# Integrated peptidogenomics decoding yak non-conventional peptides: functional mapping and biopotential mining of genetic resources

**DOI:** 10.5713/ab.25.0408

**Published:** 2025-09-30

**Authors:** Jingyun Chen, Lu Yang, Weilu Zhang, Yili Liu, Yong Wei, Li Wang, Mingfeng Jiang, Biao Li

**Affiliations:** 1 Key Laboratory of Qinghai-Tibetan Plateau Animal Genetic Resource Reservation and Utilization, Ministry of Education, Southwest Minzu University, Chengdu, China; 2 Key Laboratory of Animal Science of National Ethnic Affairs Commission of China, Southwest Minzu University, Chengdu, China; 3 Animal Science Academy of Sichuan Province, Chengdu, China

**Keywords:** Mass Spectrometry, Non-conventional Peptide, Peptidogenomics, Six-frame Translation, Yak

## Abstract

**Objective:**

Non-conventional peptides play a key role fundamental biological processes in basic plants and animals. This study investigates adaptive molecular genetic mechanisms of yaks from the perspective of non-coding peptides (NCPs).

**Methods:**

We established an integrated peptidogenomic pipeline, featuring a customized six-frame translation database using high-throughput mass spectrometry, which was utilized for the large-scale identification of NCPs in several vital organs/tissues of yaks.

**Results:**

In contrast to conventional peptides, these NCPs exhibit unique properties derived from introns, untranslated regions (UTRs), out-of-frame exons, and intergenic regions. Additionally, our findings indicate that translation events are more prevalent in unannotated transcripts than previously understood. Through transcriptome analysis and ribosome mapping analysis, 727 NCPs were identified as derived from long non-coding RNA and 944 NCPs were from circular RNA. Interestingly, the number of hydrophobic amino acids in NCPs was found to exceed that of hydrophilic amino acids in almost all tissues; in contrast to the findings for CPs, where the reverse was observed. The findings suggest a potential role in the maintenance of protein stability and minimizing the effects of oxidative stress. Furthermore, the *in vitro* antioxidant activity of the 38 candidate peptides further confirmed their physiological functions; however, specific physiological mechanisms require further investigation.

**Conclusion:**

In conclusion, this study demonstrates that a substantial portion of the yak genome can be translated into biologically functional molecules, which is crucial for functional genome research. These unique molecules will serve as basic data for future biomedical development and treatment of plateau diseases.

## INTRODUCTION

In general, peptides consisting of 2–100 amino acid residues are considered small molecules with significant biological roles [1]. However, small signal peptides or peptide hormones, ranging in length from 5 to 75 amino acids, also play crucial roles in various biological processes, such as blood pressure-lowering peptide (KVLPVP) [2] angiotensin I inhibitory peptide (GAXGLXGP) [3], and bone collagen-derived peptide (GPAGPPGPIGNV) [4]. In the past decades, research has primarily focused on conventional polypeptides (CPs) derived from the coding region of annotated coding sequences (CDSs) or traditional open reading frames (ORFs), while mRNA with protein-coding ability only accounted for about 2% of the whole genome. Recently, non-conventional peptides (NCPs, or non-coding peptides, are also referred to as microproteins by some researchers) have garnered widespread attention as newly identified endogenous peptides with important functions in various organisms [5]. These NCPs may be derived from previously unannotated CDs, such as intergenic regions (IGRs), untranslated regions (UTRs), introns, etc. Despite originating from unannotated coding regions, increasing evidence suggests that NCPs play critical roles in various cellular processes including calcium transport, muscle function, translational control, immune response and response to environmental stimuli in animals and humans [6–8]. Additionally, NCPs have been found to widely exist in plants where they play an antibacterial role [9]. However, due to the small size of these peptide fragments and limitations of genomic annotation and peptiomic technology, a large number of NCPs often cannot be further analyzed or annotated, resulting in a serious underestimation of the total number and diversity of peptides.

Given the significant physiological and pathological impacts of NCPs, there has been a growing focus on developing new strategies for their identification. The emergence of high-throughput sequencing technology and advancements in bioinformatics have enabled large-scale identification of NCPs within the entire genome. Computational methods based on sequence similarity through species comparison have been devised to identify sORFs with potential translation functions in non-CDSs [10]. However, due to their short length and low conservation, performing conservation and homology analysis is challenging, and the calculation method is not highly effective. In recent years, ribosome profiling has been extensively utilized to confirm the translation of unannotated ORFs across various species [11]. While ribosome footprinting analysis is itself an experimental approach, the assessment of the coding potential of identified regions of interest largely relies on computational bioinformatics methods [12]. This may lead to a significant number of false-positive results. Furthermore, an emerging peptidomics technology that does not require protease pre-digestion can detect endogenous peptides translated from sORFs that are smaller than 10 kDa. However, due to incomplete genomic annotations and limited mass spectrometry accuracy, many NCPs are lost during subsequent analysis processes. Recently, another distinct approach known as peptidogenomics has emerged as a leading method for identifying endogenous NCPs by integrating peptidyomics (based on high-throughput tandem mass spectrometry) with genomics. Peptidogenomics has already demonstrated successful applications in microorganisms [13], plants [9], and humans [14].

The yak (*Bos grunniens*), also referred to as the Tatar cattle, grunting cattle, hairy cattle or domestic yak, is a type of long-haired domesticated cattle that can be found on the Tibetan Plateau in the Himalayas. It is a descendant of the wild yak (*Bos mutus*). It represents a distinct bovine breed uniquely adapted to the plateau environment, displaying morphological and physiological characteristics indicative of long-term hypoxia adaptability. Therefore, the yak can serve as a valuable model for studying adaptability to altitude hypoxia, with significant research implications in the field of biomedicine. In this study, we utilized high-throughput mass spectrometry to analyze endogenous peptides in a custom peptidogenomics database generated from six-frame translation of genome sequences. This approach was employed for the identification of NCPs in key tissues related to metabolism, immunity, digestion, and reproduction in yaks. In conclusion, our findings demonstrate the widespread distribution of NCPs in the yak genome, originating not only from CDSs but also from non-CDSs, which display a distinct distribution pattern compared to CPs. Compared to conventional drugs, polypeptides offer the advantages of small molecular weight, high specificity and activity, low immunogenicity, and reduced toxic side effects. Therefore, the comprehensive identification of NCPs in key yak tissues provides valuable insights into the future biomedical functions of these latent molecules.

## MATERIALS AND METHODS

### Sample preparation

Three sexually mature adult male Maiwa yaks, approximately 5 years old, were selected. All yaks were collected from Yaks Longri Breeding Farm, Hongyuan, Aba Prefecture, Sichuan (altitude 3,500–4,000 m). They were electrocuted before bloodletting (120 volts DC, 12 seconds) and killed while unconscious by bloodletting through the carotid artery and jugular vein. Tissue samples were collected (liver, spleen, lung, muscle, testis and small intestine with 3 biological replicates per tissue), flash-frozen with liquid nitrogen and stored at −80°C.

### Peptide extraction and desalination

Weigh 3.0 g of collected yak tissue, grind it in liquid nitrogen, and transfer the resulting powder to an EP tube containing RIPA lysis and extraction buffer (Sigma-Aldrich), then incubate on ice. Take 400 μL of the upper and transfer it to a 10-kDa ultrafiltration tube, centrifuge at 8,000×g for 10 minutes at 4°C. Add 200 μL of 50 mM NH_4_HCO_3_ (Sigma-Aldrich), and centrifuge again under the same conditions. Repeat this step three times. Then, mix 100 μL of the sample with DTT solution (Sigma-Aldrich) to achieve a final DTT concentration of 10 mM. After incubating the mixture in a 56°C water bath for 1 hour, add IAM solution (Sigma-Aldrich) to reach a final IAM concentration of 55 mM. Following incubation at room temperature in the dark, desalt the peptide mixture using a C18 filter cartridge (Empore, SPE C18 Cartridge; Sigma-Aldrich). Dissolve the peptides in a sample solution containing 0.1% formic acid (Sigma-Aldrich). After centrifugation, transfer the resulting supernatant to a sample vial for LC-MS/MS analysis.

### Liquid chromatography–tandem mass spectrometry analysis

For endogenous peptide profiling, peptide segments were separated by capillary high-performance liquid chromatography, and mass spectrometry was performed with an orbitrap eclipse mass spectrometer (Thermo Fisher Scientific). The dried 5 μg peptide extract was loaded into a 75 μm×150 mm reverse-phase column (RP-C18; New Objective) and redissolved with Nano-HPLC Buffer A (2% acetonitrile and 0.1% formic acid). Linear gradient separation was performed on an Easy-nLC 1200 (Thermo Fisher Scientific) using a Nano-HPLC liquid system. Buffer B consists of 80% acetonitrile and 0.1% formic acid. The flow rate was 3,000 nl/min for 120 min. The detection methods were as follows: The mother liquor was scanned using data dependent acquisition mode (350–2,000 m/z). After higher-energy collisional dissociation (HCD) fragmentation, the 20 strongest fragments profiles (MS2 scan) were collected. The normalized collision energy was 28 eV and the dynamic exclusion duration was 25 s. We set the resolution of the measurement scan (MS1) to 60,000 at m/z 200, with a maximum injection time of 50 ms. We also set the resolution of the HCD spectrum (MS2) to 15,000 at m/z 200, with a maximum injection time of 22 ms. Peptide recognition mode and instrument operation are enabled simultaneously.

### Peptide database construction

The whole genome of yak was previously obtained by our research group and converted to FASTA format after downloading (available in the NCBI knowledge base [ https://www.ncbi.nlm.nih.gov/] and login PRJNA516576). The peptide database was constructed after the six-frame translation of genomic sequences using the sixpack function of EMBOSS:6.6.0 [15]. Whenever a stop codon was encountered, the peptides were terminated for translation, and then the next peptide was started at the next nucleotide after the previous stop codon. The ambiguous nucleotides represented by “N” in the genome sequence were replaced by random nucleotides. Other ambiguous characters were also randomly replaced according to their symbols. Location information including genomic coordinates and orientation for each putative peptide was recorded and stored in FASTA format.

### Peptide identification with PEAK studio

A customized peptide database of yaks was queried using Peaks Studio 10.6 (Bioinformatics Solutions) for peptide identification. Peptides were selected based on Mascot score (R25) and a false discovery rate (FDR<0.05). The raw data file was converted into a peptide map containing m/z values, charge states, retention times, and the intensity of all detected ions above a threshold of 8,000. To obtain quantitative information on peptides, MaxQuant software (ver. 1.3.0.5) was utilized for mass spectrometry data analysis. Bedtoolsv2.25.0 was used to retrieve mass spectrum data in order to determine the type of peptide production sequence. Peptides derived from annotated CDSsare referred to as CPs while peptides from IGRs, UTRs, different reading frames than annotated CDs, and intron regions are defined as NCPs.

### Peptide distribution on chromosomes

The peptide density on the chromosome was calculated with a sliding window of 6 Mb and a step size of 3 Mb from the R package RIdeogram:genomic Density. A hotspot region was defined as a 6 Mb region with a peptide count greater than 10. Based on the yak genome annotation [16], the physical coordinates of the translation initiation site (TSS) were extracted and the distances between the peptides and the adjacent TSSs were calculated using a Python script ( https://github.com/Peims/Calculate-thedistancebetween-peptide-and-adjac-stop-codon) to draw a frequency plot of the distance between each peptide and its TSS. To accurately estimate the number of peptides at the chromosome level, chromosome arm length was used to divide the position of CPs and NCPs.

### RNA-Seq

Sample collection is the same as before. Tissue samples from 3 adult male Maiwa yaks were collected (samples from each tissue of the three yaks were mixed to make a mixed sample). The sample processing and analysis methods were similar to those previously reported [17], and delivered to Novogene Biomedical Biotechnology for RNA-seq. After low-quality reads were filtered out from the obtained raw data, the remaining reads were mapped to the yak genome. The read count for each NCP was then calculated.

### Verification of non-conventional peptides using synthetic peptides and identification of antioxidant activity

Peptide sequences from different organs/tissues and categories were randomly selected from NCPs identified by yak peptidogenomic analysis and synthesized by TG peptide Biotechnology (Nanjing). The dried peptide powders were centrifuged and diluted with 0.1% formic acid, and each synthetic peptide was analyzed by MS using a Q Exactive mass spectrometer with the same parameters as the peptigenomic analysis.

The DPPH free radical scavenging assay kit (BC4750), ABTS free radical scavenging assay kit (BC4770), hydroxyl radical scavenging assay kit (BC1325), superoxide anion content assay kit (BC1290), and total antioxidant capacity assay kit (T-AOC, BC1310) were purchased from Sorabio, and the assays were conducted according to the manufacturers’ instructions. Each test was performed in triplicate for each sample.

### Data analysis and visualization

All antioxidant assays (DPPH, ABTS, T-AOC, hydroxyl-radical scavenging, and superoxide-anion scavenging) were evaluated using one-way ANOVA to assess the effects of different peptide concentrations. Pearson’s linear correlation coefficient was used to determine the correlation between antioxidant tests (* p<0.05 or ** p<0.01) of NCPs. In addition, unless otherwise noted, both analysis and visualization are performed using R. The data sets generated in the present investigation or analysis can be made available by the corresponding author upon reasonable request.

## RESULTS

### Peptidogenomic process for non-conventional peptides identification in animals

**Figure 1:**
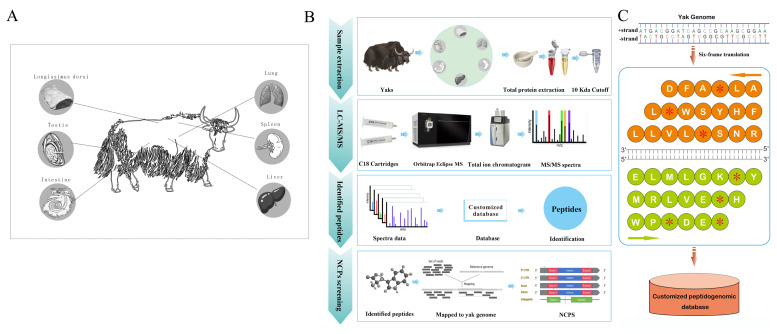
Peptidogenomic workflow for yak NCP identification. (A) Schematic representation of sample collection. (B) The peptidogenomic workflow for yak NCP identification. The samples were subjected to heat treatment in a water bath at 95°C for stabilization, and the endogenous peptides were extracted using conventional methods to minimize peptide degradation. Endogenous peptides were enriched from larger protein fragments through centrifugation with a 10-kDa molecular weight cut-off filter. Subsequently, the peptides underwent analysis using a high-resolution and high-precision mass spectrometer. MS/MS spectral data were acquired from a customized yak peptide genome database utilizing the Peaks Studio search engine. The resulting peptides were utilized for filtering out CPs, leading to the identification of NCPs. (C) Construction of customized yak peptidogenomic database. According to the previously obtained whole genome sequence of yak in FASTA format, the EMBOSS:6.6.0 software package was utilized for translation into six-frame format. The translation of genomic DNA commences with the first, second, and third nucleotides on each strand of every chromosome and concludes upon encountering a stop codon. Following the standard genetic code, triplet codons are translated into letter symbols representing amino acids, with an asterisk denoting the termination codon (n = 3). NCP, non-conventional peptide; CP, conventional polypeptide.

The direct detection of NCPs provides the most compelling evidence for confirming their existence. In order to detect NCPs in yak tissue, we developed and implemented an integrated peptidogenomic pipeline for large-scale identification. We employed the peptidogenomic approach illustrated in Figure 1A. Total proteins were extracted from 6 major organs/tissues (liver, lung, spleen, muscle, intestine and testis) following the experimental protocol. Endogenous peptides were then isolated from larger protein fragments using centrifugation with a 10 kDa cut-off filter and subsequently analyzed by LC-MS/MS (Figure 1B). To capture widely present endogenous peptides in yaks, our designed a personalized peptidogenomic database based on a six-frame translation of the yak genome sequence (Figure 1C). Subsequently, the resulting mass spectrometry data were matched with this customized peptidogenomic database using the Peaks Studio search engine to obtain prevalent endogenous peptides in yaks. This resulted in a 5.2 Gb customized peptidogenomic database containing 136 million sequences.

### Large-scale discovery of conventional polypeptides and non-conventional peptides in yak

**Figure 2:**
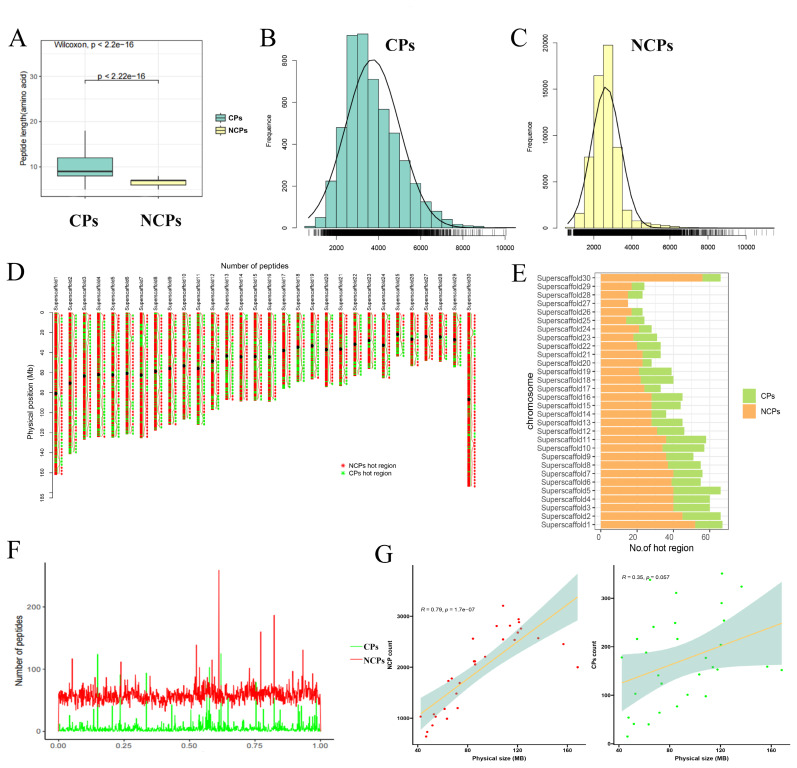
Identification of yak peptides and their localization on chromosomes. (A) Boxplot of length distribution of CPs and NCPs. Hypothesis testing with Wilcox test (* p<0.05). (B, C) Molecular weight distribution of NCPs and CPs. The rug plot above the x axis represents the distribution of observations. (D) Distribution statistics for yak genome CPs and NCPs. The blue line represents CPs, and the orange line represents NCPs. The black circle is the supposed middle of the chromosome. The * represents hotspot regions (window size = 6 Mb). (E) Statistics on the number of hot region of CPs and NCPs on chromosomes. (F) Normalized distribution of CPs and NCPs shown along the chromosomal arms. The x axis represents the normalized length of each arm with the chromosome center point to “0” and the telomere to “1”. The y axis reports the number of CPs (green) and NCPs (red). (G) Correlations between CPs or NCPs counts and chromosomal length. CP, conventional polypeptide; NCP, non-conventional peptide.

By aligning these peptides to specific genomic loci and implementing a series of stringent filtering procedures, a total of 63831 endogenous peptides from 6 different organs/tissues (liver, lung, muscle, spleen, small intestine and testis) of yak were clearly assigned to a single genomic locus. Among them, 58671 (91.92%) were NCPs and 5160 (8.08%) were CPs (Supplements 1A, 2, 3). The mean length of CPs was 9.8 amino acids, while the mean length of NCPs was 7.0 amino acids, showing a significant difference in length (Figure 2A). Additionally, the average molecular weight of NCPs was calculated as 2,643.408 Da with 99.1% of the peptides being less than 5,000 Da in weight. In contrast, CPs had the average molecular weight of 3,713.64 Da with only around 84% having a molecular weight less than or equal to 5,000 Da (Figures 2B, 2C). These results indicated that NCPs are an important component within the yak peptide population and exhibit distinct characteristics compared to CPs.

The distribution of NCPs and CPs on yak chromosomes was found to be non-uniform (Figures 2D, 2E). The majority of CPs were clustered near the chromosomal midpoint, while NCPs were evenly dispersed between the chromosomal midpoint and the telomere of each yak chromosome (Figure 2F). Additionally, 1,307 dense areas (defined in a 6MB window) were identified, with 419 CPs hotspots and 888 NCPs hotspots observed. Among these hotspots, multiple regions located on chromosomes 6, 11, 12, 23, and 25 are common to both CPs and NCPs. Furthermore, there was a strong positive correlation between the number of NCPs and chromosome length (R = 0.79; p = 1.7e-07), but no correlation was detected between the number of CPs and chromosome length (R = 0.35; p = 0.057) (Figure 2G). To assess peptide coverage on the genome, we calculated the spacing between adjacent peptides: less than 100 kb apart for 87.4% of NCPs (49,547), while only 54.5% (2,047) of CPs were within this distance range (Supplements 1B, 4). After comparing the locations of these peptides to gene models, we observed that the majority of CP and NCPs were situated within 700 bp of the standard TSS (Supplement 1D). These results reveal the widespread presence of translated NCPs across the genome and the different distribution patterns of CPs and NCPs.

As we delved into the generation mysteries of CPs and NCPs, we meticulously analyzed the nucleotide sequences of CPs and NCPs source transcripts to more accurately predict their transcription TSS. To our surprise, we observed a significant preference for non-AUG TSS in both CPs and NCPs (Supplements 2, 3). This discovery challenges the long-standing assumption that eukaryotic translation initiates at the AUG start codon. In fact, our study demonstrates a substantial frequency of non-AUG start codon usage. This finding is consistent with previous peptidomics research in other plant and animal species, which has revealed an important fact: over 90% of endogenous peptides initiate with non-AUG codons [18].

### Analysis of non-conventional peptides originating from coding or non-coding sequences

**Figure 3:**
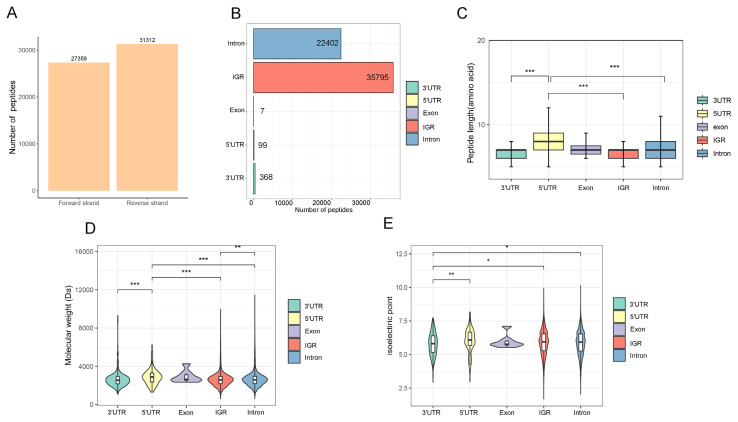
Features of yak NCPs. (A) Histogram of the number of NCPs derived from forward and reverse strands. (B) Numbers of NCPs that originated from different gene elements. (C–E) Length, molecular weight, and isoelectric point of NCPs from different gene elements. Hypothesis testing with Wilcoxon test (* p<0.05). UTR, untranslated region; IGR, intergenic region; NCP, non-conventional peptide.

The analysis of the origin of NCPs revealed that 31,312 NCPs were assigned to the reverse chain of yaks, while 27,359 NCPs were assigned to the forward chain (Figure 3A). Subsequent examination of their genomic locations showed that a majority (61.01%) originated from IGRs, with 38.18% derived from introns. Additionally, smaller percentages were found in other locations such as 3′UTR (0.63%) and 5′UTR (0.17%), with only a minimal amount detected from out-of-frame exons (0.01%) (Figure 3B). These results highlight translational evidence for these so-called non-CDSs. Furthermore, length analysis indicated that NCPs originating from the 5′UTR had the longest average length (Figure 3C). Molecular weight distribution analysis demonstrated that most NCPs (77%) had a molecular weight less than 3,000 Da. The average molecular weight of NCPs derived from the 5′ UTR and out-of-frame exons was higher compared to those derived from introns, 3′ UTR and IGRs (Figure 3D). Moreover, mean isoelectric point values of NCPs originating from the 3 ‘UTR were significantly lower than those derived from introns, IGRs and the 5′ UTR (Figure 3E). In summary, these findings indicate that identified NCPs exhibit diverse physicochemical properties based on their genetic origins which suggests potential functional differences among them.

### Verification and validation of non-conventional peptides

**Figure 4:**
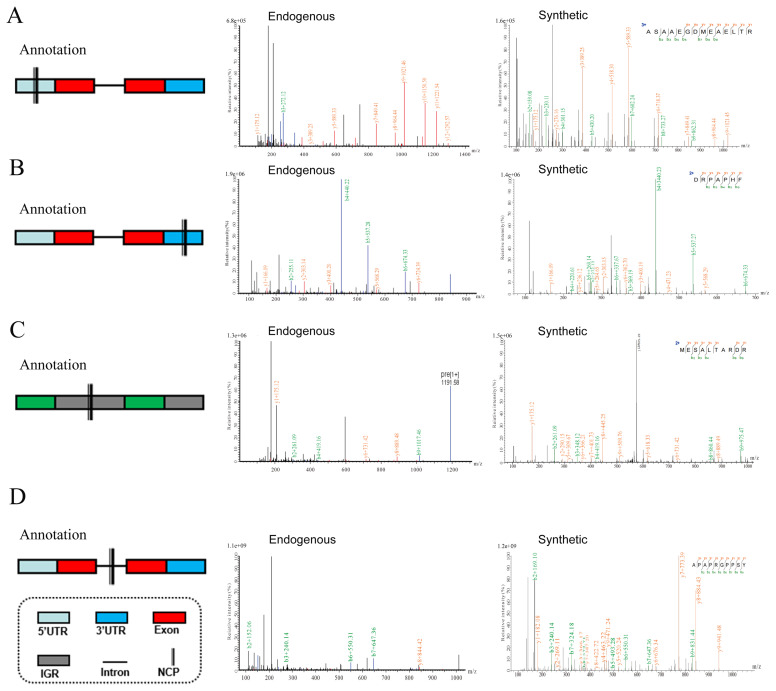
Illustrates the validation of NCPs in yaks. (A–D) The validation of NCPs was conducted by comparing the spectra of peptides identified in the peptidogenomic pipeline from 5′UTR, 3′UTR, intergene and intron NCPs (middle) with synthetic peptides (right), respectively, from genes on yak chromosomes. UTR, untranslated region; IGR, intergenic region; NCP, non-conventional peptide.

To validate the identified NCPs, we mapped these peptides to their respective genomic loci. For instance, NCP ASAAEGD MEAELTR originates from the 5′ UTR of gene XPO4 (Figure 4A), and NCP DRPAPHF is derived from the 3′ UTR of gene LPCAT3 (Figure 4B). Additionally, a substantial number of NCPs were found in IGR and introns. For example, NCP ME SALTARDR is derived from the IGR between genes CDK12 and PPP1R1B (Figure 4C), while NCP APAPRGPPSY originates from the intron of gene ATF7IP2 (Figure 4D). Subsequently, these aforementioned NCPs were synthesized and confirmed via mass spectrometry. The spectra of synthetic peptides exhibited consistency with endogenous peptide spectra obtained through peptidogenomic analysis. Furthermore, transcriptomic analyses of yak tissue were conducted to verify the potential origin of these NCPs using RNA-seq data. A comparison with all identified NCPs revealed that 727 originated from long non-coding RNAs (lncRNAs) and 944 originated from circular RNA (circRNAs; Supplement 5). While only a small percentage of NCPs (1,671, 2.8%) were supported by evidence from these databases, the results also suggest that the identified NCPs are likely derived from non-CDSs. Finally, for additional validation of the identified NCPs from a different perspective, ribosome analysis was conducted on various tissues of yak. Despite the limitations of Ribo-seq, the gene sequences obtained from the ribosome sequencing dataset were translated into amino acid sequences and matched with mass spectrometry data [19]. Additionally, the peptides identified by proteomics mass spectrometry were intersected with these sequences. Our comparative analysis showed that 2,691 NCPs detected by peptidogenomics were also identified through ribosome analysis (Supplements 1C, 6), with 1,487 NCPs originating from IGRs and 1,184 from introns.

### Analysis of tissue expression patterns of endogenous peptides

**Figure 5:**
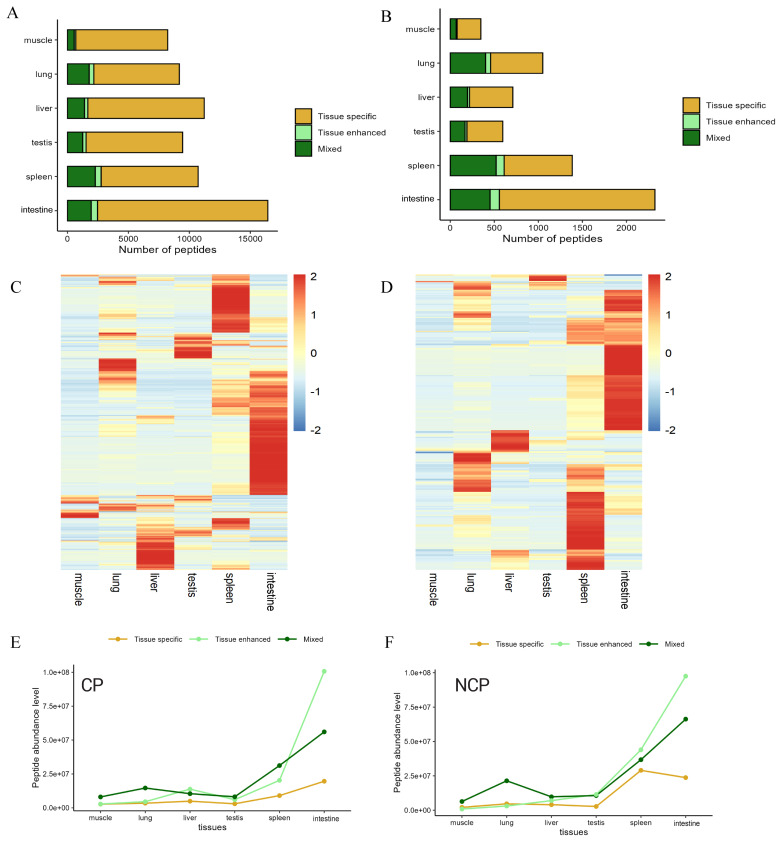
Quantitative analysis and abundance study of endogenous peptides in yak tissues. (A) The number of tissue-specific peptides, tissue-enhanced peptides, and mixed peptides in NCPs from six tissues. (B) The number of tissue-specific peptides, tissue-enhanced peptides, and mixed peptides in CPs from six tissues. (C) Heatmap of NCP abundance levels in different yak tissues. (D) Heatmap of CP abundance levels in different yak tissues. (E) Abundance levels of tissue-specific peptides, tissue-enhanced peptides, and mixed peptides in NCPs from each yak tissue. (F) Abundance levels of tissue-specific peptides, tissue-enhanced peptides, and mixed peptides in CPs from each yak tissue. Tissue-specific peptides are defined as those identified exclusively in one type of tissue. Tissue-enhanced peptides are characterized by an intra-tissue quantification level that is at least 15 times higher than that in other tissues, based on average quantification from multiple samples. Peptides that can be quantified in multiple tissues but do not meet the 15-fold difference criterion are classified as mixed. CP, conventional polypeptide; NCP, non-conventional peptide.

The expression profiles of endogenous peptides identified in yaks varied across key organs and tissues, including the liver, lung, spleen, muscle, intestine, and testis. Based on their expression specificity, these peptides were categorized into tissue-specific peptides, tissue-enhanced peptides, and mixed peptides. The majority of peptides exhibited specific expression patterns within individual tissues. Furthermore, NCPs and CPs identified a total of 951 and 145 enhanced peptides, as well as 3,765 and 718 mixed peptides, respectively; However, their distribution was not uniform across tissues (Figures 5A, 5B). Notably, the small intestine had the highest number of peptides in all categories. Both CPs and NCPs demonstrated distinct expression patterns for these endogenous peptides during immune, metabolic, and reproductive stages (Figures 5C, 5D). Additionally, compared to tissue-specific peptides, both enhanced and mixed peptides showed more dynamic expression in the spleen and intestinal tissues (Figures 5E, 5F). In NCPs, the intestine and spleen shared the largest overlap of 985 peptides, indicating a strong association. Conversely, the lungs and muscles had the smallest overlap of 55 peptides. Moreover, there were 67 overlapping peptides among the liver, lungs, spleen, intestine, and testes (Supplement 1E). These findings underscore the complexity and dynamic nature of yak endogenous peptide groups.

### Bioinformatic analysis and functional prediction

**Figure 6:**
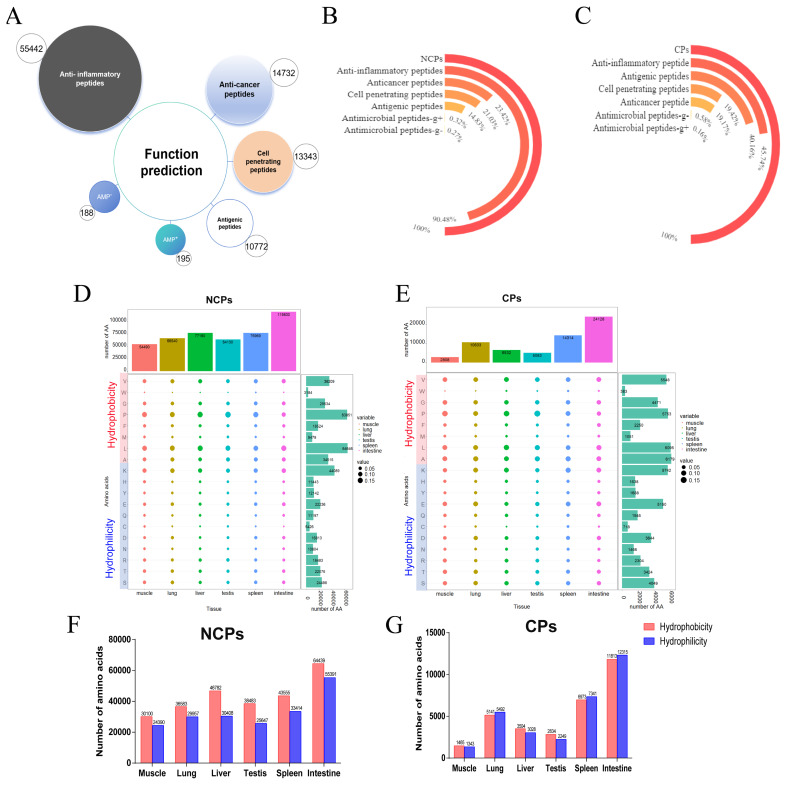
The diversity and quantity of amino acids in NCPs and CPs vary across different tissues. (A) Amino acid function prediction. (B) Proportion of NCPs with different functions. (C) Proportion of CPs with different functions. (D, E) The proportion and number of amino acids of NCPs and CPs in each tissue. (F, G) Statistical comparison of the number of hydrophobic amino acids and hydrophilic amino acids in each tissue between NCPs and CPs. NCP, non-conventional peptide; CP, conventional polypeptide.

The amino acid composition and sequence significantly influence the biological activity of polypeptides. Consequently, six distinct functional predictions were conducted for all identified endogenous peptides. The results revealed a maximum of 57,508 anti-inflammatory peptides (Figure 6A and Supplement 7), followed by 14,732 anti-cancer peptides, 13,343 cell-penetrating peptides (of which 3,178 exhibited high uptake). Furthermore, a limited number (188–195) of endogenous small peptides with antibacterial properties. Notably, it is perhaps no coincidence that 90.48% of NCPs are considered anti-inflammatory peptides (Figures 6B–6C). Further quantification of the number and proportion of amino acids in endogenous peptides identified across various tissues indicated no significant differences in amino acid distribution among different organs/tissues (Figures 6D, 6E; Supplements 8, 9). Interestingly, among the NCPs identified from six tissues, the total number of eight hydrophobic amino acids (Gly, Ala, Val, Leu, Pro, Met, Phe, and Trp) exceeded that of eleven hydrophilic amino acids (Ser, Thr, Tyr, Cys, Asn, Gln, Asp, Glu, Arg, Lys, and His). This disparity was particularly pronounced in liver, spleen, and testicular tissues (Figure 6F). In contrast, for CPs identified in lung, spleen, and intestinal tissues, the trend was reversed, with fewer hydrophobic amino acids compared to hydrophilic ones. However, there was little difference in the number of these two types of amino acids in other tissues (Figure 6G). It has been reported that a high proportion of hydrophobic amino acids is a common characteristic of anti-inflammatory and antioxidant peptides [20] were identified. Among them, the spleen-specific NCP LPWKWPWW from IGR exhibited a 61.54% sequence similarity to the antimicrobial peptide indolicidin, which was isolated from bovine neutrophil blood cells [21].

### Non-conventional peptides have the potential to maintain cellular homeostasis

In consideration of the potential physiological functions of NCPs, this study randomly selected 38 NCPs from various tissues for synthesis and assessed their antioxidant capacity using five different antioxidant detection kits: DPPH, ABTS, T-AOC, Hydroxyl radicals and superoxide anions. Multiple NCPs demonstrated the ability to scavenge at least two types of free radicals (Supplement 10). It has been reported that a higher presence of hydrophobic amino acids in low molecular weight polypeptides is crucial for maintaining protein stability and antioxidant activity; This suggests that NCPs may exert certain functions and effects through endogenous small antioxidant molecules.

## DISCUSSION

Endogenous peptides were primarily generated through protein degradation, gene coding, and gene-independent enzymatic formation *in vivo* [22]. The advent of peptiomics has enabled the large-scale identification of endogenous peptides from organs/tissues. However, peptidomic studies present formidable challenges owing to the non-specific protease digestion that occurs during sample preparation, which can lead to a lack of specificity in the resulting peptidome. Despite the widespread use of protease inhibitors in peptide extraction, numerous studies in mammals have demonstrated their limited effectiveness in preventing peptide degradation [23]. Additionally, mass spectrometry was a commonly used and effective technique for peptide detection in standard peptitomic studies, where peptides are identified by matching experimentally observed spectra with a database of predictive spectra based on annotated genes. Mass spectrometry differs from other techniques such as ribosome analysis in its ability to directly verify transcript translation [10]. Nevertheless, a significant portion of mass spectrometry fragments remain unidentified in proteomics studies due to the presence of unannotated peptides in some spectra, leading to the inability to identify NCPs. Peptigenomics represents one of the most practical methods for identifying unannotated peptides by combining proteomics with six-frame translation of the genome [24]. A personalized database constructed from whole-genome six-frame translation can effectively identify all possible peptides [25].

The yak, having undergone evolutionary adaptations to the hypoxic environment of the plateau, exhibits morphological and functional characteristics conducive to hypoxia adaptation in its body structure and organ tissue. This makes it a valuable model for studying high-altitude hypoxia adaptation. While peptigenomics has been successfully applied in identifying microorganisms, plants, and humans, its application in yaks has been limited [9,26,27]. In this study, peptiomics was combined with six-frame translation of the yak genome to establish a personalized peptidogenomic database. To our knowledge, this is the first published work on mining and analyzing yak peptidogenomic. A total of 58,671 NCPs and 5,160 CPs were identified in the customized database. This study not only demonstrates that mass spectrometry combined with peptigenomics is an effective method for detecting CPs and NCPs but also reveals translational potential in sequences previously considered untranslatable such as 5′UTR, IGRs, introns, and 3′UTR. The conventional perspective posits that ncRNAs lack coding functionality. However, recent studies have unveiled the potential of certain sORFs within ncRNAs to encode peptides or proteins. For instance, ORFs within lncRNAs, circRNAs and primary miRNAs are capable of encoding peptides or proteins, which play a pivotal role in regulating various physiological activities of cells. LINC00266-1 encodes a small peptide (RBRP) and interacts with the N6-methyladenosine (m6A) reader protein IGF2BP1, thereby enhancing mRNA stability and promoting tumor development [28]. Similarly, LINC00278 can encode micropeptide YY1BM and inhibit eEF2K transcription, thus promoting apoptosis [29]. Peptides encoded by muscle-specific lncRNA can also activate calcium pumps to enhance SERCA activity in muscles [30]. In addition to their presence in mammals, lncRNA-encoding peptides have also been identified in plants, where they play a crucial role in regulating plant cell growth and differentiation [31]. Currently identified proteins/peptides encoded by circRNA are mainly confirmed in liver cancer, colorectal cancer, gastric cancer and other cancer cells where they significantly impact cell cycle regulation, drug resistance mechanisms as well as protein translation functions [32]. In our investigation, 727 peptides were attributed to lncRNAs and 944 to cirRNAs. Despite the undetermined functionality of these NCPs, this outcome establishes a fundamental framework for the comprehensive exploration and characterization of ncRNA-encoded small peptides on a large scale. This breakthrough offers a novel perspective for genome structural and functional annotation distinct from that of protein-coding genes. Living year-round on the high plateau, yaks endure persistent hypoxia, intense radiation, extreme cold, and chronic food scarcity. These stressors impose continuous oxidative, inflammatory, DNA-damage, and metabolic burdens on the organism. To sustain normal physiological function, NCPs not only counteract the unique survival pressures of the highland environment but also maintain systemic homeostasis through multi-level regulatory networks.

By comparing the co-expression of NCPs in different organs/tissues, we identified 985 NCPs that were co-expressed in both the intestine and spleen. This finding may be associated with the role of the intestine as a significant contributor to overall immune function. Furthermore, we have identified 67 shared NCPs across five distinct organs/tissues: liver, lung, spleen, intestine, and testis. This suggests potential tissue-specific physiological functions for these peptides. This observation aligns with the diverse roles that peptides play as signaling molecules in various biological processes. For instance, glucose-dependent insulin-stimulating polypeptide (GIP) and glucagon-like peptide 1 (GLP1) are known to regulate glucose and lipid homeostasis in the liver, muscle, and adipose tissue [33,34]. They also exhibit expression in the central nervous system where they may influence appetite regulation. Additionally, GIPR and GLP1R expression has been detected in heart tissue suggesting a potential role in reducing atherosclerosis. However, it is noteworthy that no NCPs were consistently detected across all yak organs/tissues examined. This indicates potential specificity of certain NCPs within distinct functional organs/tissues.

The amino acid composition and sequence play a crucial role in determining the biological activity of polypeptides. In this study, it was observed that the number of hydrophobic amino acids in NCPs identified in 6 organs/tissues were significantly higher than that of hydrophilic amino acids, particularly in the liver and testis. Following proteolysis, highly hydrophobic peptides containing 8–10 amino acid residues are often the predominant intracellular source [35]. The length of these peptides was in line with the average size of peptides generated during protein degradation in mammalian proteasomes. Nevertheless, it is important to note that these hydrophobic-rich peptides may have pivotal physiological functions. Indeed, major histocompatibility complex (MHC) class I molecules have been consistently identified in a wide spectrum of antigenic peptides containing 8–10 residues [36]. These standard antigenic peptides exhibit high hydrophobicity and short sequences of aliphatic and aromatic amino acids, which are critical for binding to antigen pockets in MHC Class I molecules. Moreover, the hydrophobicity of peptides plays a crucial role in determining their antioxidant potential. The presence of hydrophobic amino acid residues within the peptide sequence can contribute to its ability to scavenge free radicals and protect against oxidative stress [37]. *In vitro* antioxidant assays conducted on candidate NCPs derived from yak have revealed their remarkable ability to effectively scavenge at least two types of free radicals. This suggests that these NCPs may serve as endogenous antioxidant small molecules, playing a potential role in maintaining cellular homeostasis through their antioxidative properties. The findings underscore the significance of these peptide sequences not only in immune responses but also in cellular oxidative stress management.

## CONCLUSION

In summary, this is the first study to directly and successfully identify NCPs on a large scale in the yak genome using an integrated peptidogenomic pipeline. In contrast to prior endeavors reliant on computational algorithms or ribosome profiling techniques for uncovering unannotated CDSs, our approach facilitated translation across numerous 5′UTR, 3′UTR, IGRs, and introns, thereby elucidating putative functional roles in diverse biological processes. These findings not only expand our understanding of genetic mechanisms underlying environmental adaptability in yaks but also provide valuable insights for the discovery of novel genes with potential adaptive significance.

## Data Availability

All data were deposited in the iProX repository ( https://www.iprox.cn/page/PSV023.html;?url=1748489706530orRB); Password: RWqA. Upon reasonable request, the datasets of this study can be available from the corresponding author.

## SUPPLEMENTARY MATERIAL

**Supplement 1**. (A) Pie chart showing numbers of CPs and NCPs identified. (B) Distance distribution of two neighboring CPs or NCPs. (C) The comparison of peptidogenomics and ribosome profiling sequencing results. (D) Histograms showing the distance from each CP or NCP to the neighboring TSS. (E) Comparison of NCPs identified by different organs/tissues.

**Supplement 2**. The basic information on NCPs in six different organ tissues.

**Supplement 3**. The basic information on CPs in six different organ tissues.

**Supplement 4**. CPs and NCPs with interspacing less than 100kb.

**Supplement 5**. NCPs derived from lncRNAs and circRNAs.

**Supplement 6**. Information obtained from ribosome profiling sequencing of NCPs.

**Supplement 7**. Prediction of NCP functions.

**Supplement 8**. The number of amino acids in NCPs and CPs in different organs and tissues.

**Supplement 9**. The proportion of amino acids in NCPs and CPs in different organs and tissues.

**Supplement 10**. NCPs were correlated with antioxidant activity by T-AOC, Superoxide anion, ABTS, FRAP, and Hydroxyl radical assays assays.
