# Supplementary material for: Integrated peptidogenomics decoding yak non-conventional peptides: functional mapping and biopotential mining of genetic resources
Source: Anim Biosci. 2025 Sep 30;39(5):250408. doi: 10.5713/ab.25.0408 (PMC13153706; doi:10.5713/ab.25.0408)
Supplement: Supplementary file 3 [file ab-25-0408-Supplement-3.pdf]

nnotatio	geneID	length	lectric p	ecular we	Centrom	Peptide	novo Sco	m/z	z	Mass	PTM	coded by t	TSS_Chr	TSS_star	muscle	lung	liver	testis	spleen	sintestine	Classify
CDS|exon|ID=cds-Bg		14	5.839286	5817.1	0.987323	SAASKQNKI	93	780.4203	2	1558.832	.	F	Superscaf	994093	0	0	0	0	0	BTP202209	Tissue specific
CDS|exon|ID=cds-Bg		7	5.282857	2527	0.987283	STMMSVH	89	396.6711	2	791.3306	.	K	Superscaf	997222	0	0	0	0	0	BTP202209	Tissue specific
CDS|exon|ID=cds-Bg		10	6.455	3663.7	0.987266	KTVLKSFLS	99	575.8613	2	1149.712	.	A	Superscaf	998584	0	0	0	0	0	BTP202209	Tissue specific
CDS|exon|ID=cds-Bg		7	4.37	2414.8	0.987077	TVEELYL	69	866.4449	1	865.4433	.	N	Superscaf	1013535	0	0	0	0	0	BTP202209	Tissue specific
CDS|exon|ID=cds-Bg		6	4.146667	1365.6	0.959744	LPVPVP	96	621.3958	1	620.3897	.	L	Superscaf	3156943	BTP202209	0	0	0	0	0	Tissue specific
CDS|exon|ID=cds-Bg		15	6.169333	5538.9	0.917891	A(+42.01)	94	550.6428	3	1648.912	Acetylati	T	Superscaf	6439399	0	0	0	0	0	BTP202209	Tissue specific
CDS|exon|ID=cds-Bg		12	5.9225	4345.6	0.917891	A(+42.01)	75	639.3509	2	1276.693	Acetylati	T	Superscaf	6439399	0	0	0	0	0	BTP202209	Tissue specific
CDS|exon|ID=cds-Bg		11	5.947273	3734.9	0.917891	A(+42.01)	66	575.323	2	1148.634	Acetylati	T	Superscaf	6439399	0	0	BTP202209	0	0	0	Tissue specific
CDS|exon|ID=cds-Bg		10	5.94	3423.6	0.917891	A(+42.01)	96	539.8054	2	1077.597	Acetylati	T	Superscaf	6439399	0	BTP202209	0	0	0	0	Tissue specific
CDS|exon|ID=cds-Bg		9	5.991111	3036.2	0.917891	A(+42.01)	50	466.2692	2	930.5287	Acetylati	T	Superscaf	6439399	0	0	0	0	0	BTP202209	Tissue specific
CDS|exon|ID=cds-Bg		8	5.99375	2738.9	0.917891	A(+42.01)	74	437.7591	2	873.5072	Acetylati	T	Superscaf	6439399	0	0	0	0	BTP202209	0	Tissue specific
CDS|exon|ID=cds-Bg		12	5.305833	4740.2	0.917794	KGEENLMDA	86	681.3379	2	1360.666	.	G	Superscaf	6446965	0	0	0	0	0	BTP202209	Tissue specific
CDS|exon|ID=cds-Bg		12	3.456667	3266.7	0.917781	LSEVGDTQV	51	646.8368	2	1291.666	.	I	Superscaf	6447970	0	0	0	0	0	BTP202209	Tissue specific
CDS|exon|ID=cds-Bg		14	5.195714	4911.5	0.917763	DMLEAGVLI	91	762.879	2	1523.754	.	Q	Superscaf	6449449	0	0	0	0	0	BTP202209	Tissue specific
CDS|exon|ID=cds-Bg		7	5.664286	2665	0.887962	NAFRVSP	78	395.7126	2	789.4133	.	P	Superscaf	8786652	0	BTP202209	0	0	0	0	Tissue specific
CDS|exon|ID=cds-Bg		9	5.988889	4038.5	0.87433	NGYENPTYK	96	543.2463	2	1084.482	.	P	Superscaf	9855349	0	0	0	BTP202209	0	0	Tissue specific
CDS|exon|ID=cds-Bg		9	4.365556	3038.3	0.872323	FEVVEKPQS	86	531.7745	2	1061.539	.	L	Superscaf	10013101	0	0	0	0	0	BTP202209	Tissue specific
CDS|exon|ID=cds-Bg		7	4.701429	2563.9	0.577603	D(+42.01)	94	774.3502	1	773.3443	Acetylati	T	Superscaf	33126451	0	BTP202209	BTP202209	BTP202209	BTP202209	BTP202209	Mixed
CDS|exon|ID=cds-Bg		14	4.539286	4041.8	0.472209	PPAPVVVTE	63	717.3761	2	1432.745	.	K	Superscaf	41392075	0	0	BTP202209	0	0	0	Tissue specific
CDS|exon|ID=cds-Bg		7	5.214286	2624.1	0.439262	MNMVLAH	79	408.1974	2	814.383	.	I	Superscaf	43976014	0	0	0	0	0	BTP202209	Tissue specific
CDS|exon|ID=cds-Bg		9	5.302222	4002.5	0.431943	FDPLKNQDE	91	553.2593	2	1104.509	.	N	Superscaf	44550085	0	0	0	0	BTP202209	BTP202209	Mixed
CDS|exon|ID=cds-Bg		7	5.631429	2590	0.424043	DTSLPLL	71	758.4276	1	757.4222	.	Q	Superscaf	45169878	BTP202209	0	0	0	0	0	Tissue specific
CDS|exon|ID=cds-Bg		15	6.322667	6244.7	0.421277	A(+42.01)	98	421.2166	4	1680.843	Acetylati	S	Superscaf	45386605	0	BTP202209	0	0	BTP202209	0	Mixed
CDS|exon|ID=cds-Bg		14	6.005714	5595.9	0.421277	A(+42.01)	99	509.2535	3	1524.742	Acetylati	S	Superscaf	45386605	0	0	0	BTP202209	BTP202209	0	Mixed
CDS|exon|ID=cds-Bg		13	6.033077	4985.2	0.421277	A(+42.01)	91	699.3464	2	1396.683	Acetylati	S	Superscaf	45386605	0	0	0	BTP202209	0	0	Tissue specific
CDS|exon|ID=cds-Bg		15	5.85	5837.5	0.421211	SAEDETADI	99	801.8937	2	1601.778	.	V	Superscaf	45391735	0	BTP202209	0	0	0	BTP202209	Mixed
CDS|exon|ID=cds-Bg		14	5.862143	5454.1	0.421211	AEDETADLF	99	758.3791	2	1514.746	.	V	Superscaf	45391735	0	BTP202209	0	0	0	0	Tissue specific
CDS|exon|ID=cds-Bg		13	5.563846	4985.7	0.421211	AEDETADLF	84	694.3315	2	1386.651	.	V	Superscaf	45391735	0	BTP202209	0	0	0	0	Tissue specific
CDS|exon|ID=cds-Bg		11	5.434545	4276.9	0.421211	EDETADLPT	95	608.2883	2	1214.567	.	V	Superscaf	45391735	0	0	0	0	BTP202209	0	Tissue specific
CDS|exon|ID=cds-Bg		13	5.85	5142.8	0.421211	EDETADLPT	99	722.8606	2	1443.709	.	V	Superscaf	45391735	0	BTP202209	0	0	0	BTP202209	Mixed
CDS|exon|ID=cds-Bg		12	5.525833	4674.4	0.421211	EDETADLPT	77	658.8136	2	1315.614	.	V	Superscaf	45391735	0	BTP202209	0	0	0	0	Tissue specific
CDS|exon|ID=cds-Bg		10	5.656	3851.4	0.421211	DETADLPTK	94	543.7679	2	1085.524	.	V	Superscaf	45391735	0	0	BTP202209	0	0	0	Tissue specific
CDS|exon|ID=cds-Bg		11	6.350909	4305.8	0.421211	ETADLPTKF	85	600.8263	2	1199.64	.	V	Superscaf	45391735	0	BTP202209	BTP202209	0	0	0	Mixed
CDS|exon|ID=cds-Bg		9	6.875556	3639	0.295961	S(+42.01)	86	564.8589	2	1127.707	Acetylati	Q	Superscaf	55214787	0	0	0	0	0	BTP202209	Tissue specific
CDS|exon|ID=cds-Bg		14	4.08	3775.2	0.283239	VGVATAVDC	68	688.8718	2	1375.735	.	I	Superscaf	56212249	0	0	0	0	0	BTP202209	Tissue specific
CDS|exon|ID=cds-Bg		6	6.471667	2571.8	0.267472	PESHPK	62	694.3484	1	693.3445	.	A	Superscaf	57448780	0	0	0	0	0	BTP202209	Tissue specific
CDS|exon|ID=cds-Bg		8	4.09	2124.2	0.178435	SVYVYKVL	96	485.7835	2	969.5535	.	G	Superscaf	64431634	0	BTP202209	BTP202209	0	BTP202209	0	Mixed
CDS|exon|ID=cds-Bg		6	5.193333	2178.4	0.178435	LVQGHG	71	610.3188	1	609.3234	.	F	Superscaf	64431487	BTP202209	0	0	0	0	0	Tissue specific
CDS|exon|ID=cds-Bg		14	4.860714	4429.9	0.172769	A(+42.01)	82	740.8953	2	1479.782	Acetylati	T	Superscaf	64876021	0	0	0	0	0	BTP202209	Tissue specific
CDS|exon|ID=cds-Bg		14	5.365714	4445.8	0.125553	A(+42.01)	82	657.3668	2	1312.724	Acetylati	G	Superscaf	68578966	0	0	0	0	0	BTP202209	Tissue specific
CDS|exon|ID=cds-Bg		19	4.803158	5920.3	0.125271	ASWADLVNA	88	939.4939	2	1876.98	.	G	Superscaf	68601082	0	0	0	0	0	BTP202209	Tissue specific
CDS|exon|ID=cds-Bg		9	5.903333	3319.7	0.097937	LEDKPPAPF	99	482.2593	2	962.5073	.	F	Superscaf	70744798	0	0	BTP202209	0	BTP202209	0	Mixed
CDS|exon|ID=cds-Bg		13	6.406154	4991.4	0.097935	KPLPSVPEE	98	502.2979	3	1503.877	.	F	Superscaf	70744798	0	0	0	0	BTP202209	0	Tissue specific
CDS|exon|ID=cds-Bg		14	5.266429	5594.5	0.097549	VEGEPPYLN	81	813.9073	2	1625.805	.	N	Superscaf	70775014	0	0	0	0	BTP202209	0	Tissue specific
CDS|exon|ID=cds-Bg		8	5.9025	2715.1	0.077902	LGATLRVL	71	842.5441	1	841.5385	.	A	Superscaf	72315496	0	0	BTP202209	0	0	0	Tissue specific
CDS|exon|ID=cds-Bg		10	6.276	3873.2	0.036341	LLLPGELAK	82	364.2239	3	1089.655	.	C	Superscaf	75575293	0	0	0	0	BTP202209	0	Tissue specific
CDS|exon|ID=cds-Bg		11	6.006364	4981.7	0.036774	A(+42.01)	97	682.8322	2	1363.656	Acetylati	A	Superscaf	81309574	0	0	BTP202209	0	0	0	Tissue specific
CDS|exon|ID=cds-Bg		10	6.005	4670.4	0.036774	A(+42.01)	98	647.3143	2	1292.618	Acetylati	A	Superscaf	81309574	0	0	BTP202209	0	0	0	Tissue specific
CDS|exon|ID=cds-Bg		9	6.007778	4317	0.036774	A(+42.01)	85	590.7738	2	1179.534	Acetylati	A	Superscaf	81309574	0	0	BTP202209	0	0	0	Tissue specific
CDS|exon|ID=cds-Bg		8	6.13125	3731.3	0.036774	A(+42.01)	99	510.7576	2	1019.504	Acetylati	A	Superscaf	81309574	0	0	BTP202209	0	0	0	Tissue specific
CDS|exon|ID=cds-Bg		15	5.819333	5950.5	0.037445	LEEWQSLAC	91	821.4263	2	1640.841	.	I	Superscaf	81360994	0	0	BTP202209	0	0	0	Tissue specific
CDS|exon|ID=cds-Bg		14	5.807857	5597.1	0.037445	LEEWQSLAC	98	764.8845	2	1527.757	.	I	Superscaf	81360994	0	0	BTP202209	0	0	0	Tissue specific
CDS|exon|ID=cds-Bg		5	5.462	1757	0.046132	A(+42.01)	82	560.2372	1	559.2312	Acetylati	R	Superscaf	82043476	0	BTP202209	0	0	0	0	Tissue specific
CDS|exon|ID=cds-Bg		5	4.254	1413.6	0.049246	LDAVP	68	514.2834	1	513.2798	.	R	Superscaf	82287604	0	0	BTP202209	0	0	0	Tissue specific
CDS|exon|ID=cds-Bg		5	5.704	1979.3	0.05537	PGFMC(+57	73	611.2303	1	610.2243	Carbamido	N	Superscaf	82768092	0	0	0	BTP202209	0	0	Tissue specific
CDS|exon|ID=cds-Bg		9	6.228889	3068.6	0.055382	PLLPPPPPF	99	924.5516	1	923.548	.	D	Superscaf	82768870	0	0	0	BTP202209	BTP202209	0	Tissue enhanced
CDS|exon|ID=cds-Bg		5	4.666	1710.9	0.065965	W(+42.01)	51	753.3742	1	752.3533	Acetylati	D	Superscaf	83598835	0	0	BTP202209	0	0	0	Tissue specific
CDS|exon|ID=cds-Bg		12	6.083333	4311.9	0.139073	VPPPKPATF	98	441.246	3	1320.719	.	A	Superscaf	89333646	0	BTP202209	0	0	0	0	Tissue specific
CDS|exon|ID=cds-Bg		11	6.636364	4311.9	0.139073	PPPKPATPD	99	408.2231	3	1221.651	.	A	Superscaf	89333646	0	BTP202209	0	0	BTP202209	0	Mixed
CDS|exon|ID=cds-Bg		10	6.224	3663.1	0.139073	PPPKPATPD	94	533.7819	2	1065.549	.	A	Superscaf	89333646	0	BTP202209	0	0	0	0	Tissue specific
CDS|exon|ID=cds-Bg		12	5.436667	4063.4	0.139081	VKPAETPKF	91	633.8539	2	1265.698	.	A	Superscaf	89333646	0	0	0	0	0	BTP202209	Tissue specific
CDS|exon|ID=cds-Bg		11	5.930909	4063.4	0.139081	KPAETPKPV	98	584.3188	2	1166.629	.	A	Superscaf	89333646	0	0	0	0	BTP202209	0	Tissue specific
CDS|exon|ID=cds-Bg		12	6.436667	4728.1	0.139082	AKPAETPKF	94	626.8461	2	1251.682	.	A	Superscaf	89333646	0	0	0	0	BTP202209	0	Tissue specific
CDS|exon|ID=cds-Bg		9	5.945556	3159.4	0.220925	VVLKGRPC(	97	352.8813	3	1055.627	Carbamido	L	Superscaf	95751598	0	0	0	0	0	BTP202209	Tissue specific
CDS|exon|ID=cds-Bg		5	6.622	2370.6	0.235954	N(+42.01)	58	589.2995	1	588.2867	Acetylati	S	Superscaf	96930429	0	0	0	0	0	BTP202209	Tissue specific
CDS|exon|ID=cds-Bg		8	7.05875	3343.7	0.320208	STPRPKFS	97	460.2515	2	918.4922	.	R	Superscaf	1.04E+08	0	0	0	0	BTP202209	0	Tissue specific
CDS|exon|ID=cds-Bg		17	5.901765	6172.5	0.341443	HAVSEGTKA	99	598.65	3	1792.932	.	P	Superscaf	1.05E+08	0	BTP202209	BTP202209	BTP202209	BTP202209	BTP202209	Mixed
CDS|exon|ID=cds-Bg		12	6.154167	5656.3	0.353286	A(+42.01)	98	495.9259	3	1484.758	Acetylati	A	Superscaf	1.06E+08	0	BTP202209	0	BTP202209	0	BTP202209	Mixed
CDS|exon|ID=cds-Bg		11	5.828182	5187.9	0.353286	A(+42.01)	94	679.3376	2	1356.663	Acetylati	A	Superscaf	1.06E+08	0	BTP202209	0	0	0	BTP202209	Mixed
CDS|exon|ID=cds-Bg		10	5.813	4834.5	0.353286	A(+42.01)	82	622.7961	2	1243.579	Acetylati	A	Superscaf	1.06E+08	0	0	0	BTP202209	0	0	Tissue specific
CDS|exon|ID=cds-Bg		5	7.772	2508.9	0.476887	FGRWR	51	361.2018	2	720.382	.	F	Superscaf	1.16E+08	0	0	0	BTP202209	0	0	Tissue specific
CDS|exon|ID=cds-Bg		12	6.324167	4859.3	0.496997	AYSMAKYLF	80	681.3273	2	1360.645	.	N	Superscaf	1.17E+08	0	0	0	0	0	BTP202209	Tissue specific
CDS|exon|ID=cds-Bg		9	6.157778	3854.4	0.506734	LYLGERTYY	89	589.2963	2	1176.582	.	P	Superscaf	1.18E+08	0	0	0	0	0	BTP202209	Tissue specific
CDS|exon|ID=cds-Bg		7	6.254286	3041.5	0.506734	LGERTYY	98	451.2227	2	900.4341	.	P	Superscaf	1.18E+08	0	0	0	0	0	BTP202209	Tissue specific
CDS|exon|ID=cds-Bg		8	3.64	2717.1	0.50685	VGDEVNWY	69	981.4272	1	980.424	.	H	Superscaf	1.18E+08	0	0	0	0	0	BTP202209	Tissue specific
CDS|exon|ID=cds-Bg		15	4.889333	5488.3	0.50685	LMGMGNEVI	99	843.3898	2	1684.77	.	H	Superscaf	1.18E+08	0	0	0	0	0	BTP202209	Tissue specific
CDS|exon|ID=cds-Bg		5	7.016	2110.2	0.508485	F(+42.01)	64	627.3313	1	626.3176	Acetylati	S	Superscaf	1.18E+08	0	0	0	0	0	BTP202209	Tissue specific
CDS|exon|ID=cds-Bg		8	5.585	2597.9	0.544194	VTGHPGAP	62	368.1926	2	734.3711	.	F	Superscaf	1.21E+08	0	0	0	0	0	BTP202209	Tissue specific
CDS|exon|ID=cds-Bg		10	6.204	4223.6	0.59153	ELKLEKTPS	86	586.8286	2	1171.645	.	E	Superscaf	1.25E+08	0	0	0	0	BTP202209	0	Tissue specific
CDS|exon|ID=cds-Bg		12	4.770833	4299.9	0.608374	M(+42.01)	97	701.8627	2	1401.717	Acetylati	P	Superscaf	1.26E+08	0	0	0	0	0	BTP202209	Tissue specific
CDS|exon|ID=cds-Bg		6	7.951667	2599.6	0.624163	PKKTYK	77	382.7405	2	763.4592	.	I	Superscaf	1.27E+08	0	0	0	0	BTP202209	0	Tissue specific
CDS|exon|ID=cds-Bg		7	4.701429	2290.6	0.640515	PVDFTGY	92	798.3645	1	797.3596	.	E	Superscaf	1.29E+08	0	0	0	0	0	BTP202209	Tissue specific
CDS|exon|ID=cds-Bg		9	5.393333	3285.6	0.640515	PVDFTGYWK	99	556.773	2	1111.534	.	E	Superscaf	1.29E+08	0	0	0	0	0	BTP202209	Tissue specific
CDS|exon|ID=cds-Bg		8	4.85	2817.2	0.640515	PVDFTGYW	76	984.4415	1	983.4388	.	E	Superscaf	1.29E+08	0	0	0	BTP202209	0	0	Tissue specific
CDS|exon|ID=cds-Bg		21	5.580476	9507	0.641395	T(+42.01)	86	1297.533	2	2593.049	Acetylati	P	Superscaf	1.29E+08	0	0	0	0	0	BTP202209	Tissue specific
CDS|exon|ID=cds-Bg		16	5.345625	6024.9	0.642495	SVDLHPTEF	63	929.9523	2	1857.897	.	P	Superscaf	1.29E+08	0	0	0	0	0	BTP202209	Tissue specific
CDS|exon|ID=cds-Bg		8	5.38125	3223.8	0.642784	VSTDPEHR	71	470.7278	2	939.441	.	T	Superscaf	1.29E+08	0	0	0	0	0	BTP202209	Tissue specific
CDS|exon|ID=cds-Bg		11	5.709091	4343.7	0.642832	ENLFPGLKE	85	632.8295	2	1263.65	.	L	Superscaf	1.29E+08	0	0	0	0	0	BTP202209	Tissue specific
CDS|exon|ID=cds-Bg		10	5.732	3956.3	0.642832	ENLFPGLKE	86	559.2955	2	1116.582	.	L	Superscaf	1.29E+08	0	0	0	0	0	BTP202209	Tissue specific
CDS|exon|ID=cds-Bg		9	5.7	3645	0.642832	ENLFPGLKE	92	523.778	2	1045.544	.	L	Superscaf	1.29E+08	0	0	0	0	BTP202209	BTP202209	Mixed
CDS|exon|ID=cds-Bg		8	6.01	3219.5	0.642832	ENLFPGLK	97	459.2563	2	916.5018	.	L	Superscaf	1.29E+08	0	0	0	0	0	BTP202209	Tissue specific
CDS|exon|ID=cds-Bg		17	4.842941	6008.6	0.642833	VVEEWVKET	95	679.6835	3	2036.037	.	L	Superscaf	1.29E+08	0	0	0	0	0	BTP202209	Tissue specific
CDS|exon|ID=cds-Bg		10	3.941	3288.7	0.642833	VVEEWVKET	99	628.3179	2	1254.625	.	L	Superscaf	1.29E+08	0	0	0	0	0	BTP202209	Tissue specific
CDS|exon|ID=cds-Bg		7	3.152857	1846	0.642833	VVEEWVK	98	444.7422	2	887.4752	.	L	Superscaf	1.29E+08	0	0	0	0	0	BTP202209	Tissue specific
CDS|exon|ID=cds-Bg		6	6.253333	1998.3	0.652619	PPPAPP	99	575.3169	1	574.3115	.	A	Superscaf	1.3E+08	0	0	0	BTP202209	0	0	Tissue specific
CDS|exon|ID=cds-Bg		11	6.422727	4912.4	0.653459	QLERDFSSM	97	456.9015	3	1367.687	.	Q	Superscaf	1.3E+08	0	0	0	0	0	BTP202209	Tissue specific
CDS|exon|ID=cds-Bg		6	5.47	2316.5	0.67447	FPWVNK	69	395.7175	2	789.4174	.	F	Superscaf	1.31E+08	0	0	0	0	0	BTP202209	Tissue specific
CDS|exon|ID=cds-Bg		7	3.837143	2353.8	0.72015	QTVPDVN	83	772.3943	1	771.3763	.	L	Superscaf	1.35E+08	0	0	0	0	BTP202209	0	Tissue specific
CDS|exon|ID=cds-Bg		6	6.106667	1962.1	0.721212	APPAAL	82	539.3174	1	538.3115	.	T	Superscaf	1.35E+08	0	0	BTP202209	0	0	0	Tissue specific
CDS|exon|ID=cds-Bg		7	3.354286	1883.3	0.758857	TVVDEVR	99	409.2223	2	816.4341	.	G	Superscaf	1.38E+08	0	0	0	BTP202209	0	0	Tissue specific
CDS|exon|ID=cds-Bg		5	5.376	1827	0.761917	EALSL	80	532.2953	1	531.2904	.	A	Superscaf	1.38E+08	0	0	0	BTP202209	0	0	Tissue specific
CDS|exon|ID=cds-Bg		6	5.488333	2607.8	0.776224	YEDYSK	62	402.6739	2	803.3337	.	L	Superscaf	1.39E+08	0	0	0	0	0	BTP202209	Tissue specific
CDS|exon|ID=cds-Bg		10	5.527	3420.6	0.794218	RSSWVFVAA	84	575.8193	2	1149.629	.	K	Superscaf	1.41E+08	0	0	0	0	0	BTP202209	Tissue specific
CDS|exon|ID=cds-Bg		6	5.128333	1885.2	0.800437	T(+42.01)	53	366.1992	2	730.3901	Acetylati	G	Superscaf	1.41E+08	0	0	0	BTP202209	0	0	Tissue specific
CDS|exon|ID=cds-Bg		5	4.742	2552	0.80224	C(+57.02)	52	701.1552	1	700.1615	Carbamido	G	Superscaf	1.41E+08	BTP202209	0	0	BTP202209	0	0	Mixed
CDS|exon|ID=cds-Bg		12	5.221667	4009.2	0.813597	KDVLDEKPA	80	452.2676	3	1353.787	.	Y	Superscaf	1.42E+08	0	0	BTP202209	0	0	0	Tissue specific
CDS|exon|ID=cds-Bg		11	4.810909	3540.8	0.813597	DVLDEKPAV	88	613.849	2	1225.692	.	Y	Superscaf	1.42E+08	0	0	0	0	0	BTP202209	Tissue specific
CDS|exon|ID=cds-Bg		7	4.667143	2307.6	0.826745	DVERVLK	69	429.7544	2	857.497	.	R	Superscaf	1.43E+08	BTP202209	0	BTP202209	0	0	0	Tissue enhanced
CDS|exon|ID=cds-Bg		8	5.91625	2930.2	0.826787	KVLELSGR	89	451.2762	2	900.5392	.	V	Superscaf	1.43E+08	0	0	BTP202209	0	0	0	Tissue specific
CDS|exon|ID=cds-Bg		9	5.594444	3355.7	0.82873	A(+42.01)	75	536.8014	2	1071.592	Acetylati	K	Superscaf	1.43E+08	0	0	0	BTP202209	0	0	Tissue specific
CDS|exon|ID=cds-Bg		15	6.074	5525	0.82873	A(+42.01)	97	794.4347	2	1586.863	Acetylati	K	Superscaf	1.43E+08	0	0	0	0	0	BTP202209	Tissue specific
CDS|exon|ID=cds-Bg		10	5.741	3964.5	0.828956	SGNQSVRLF	76	535.799	2	1069.588	.	S	Superscaf	1.43E+08	0	0	0	0	0	BTP202209	Tissue specific
CDS|exon|ID=cds-Bg		11	4.812727	3571.8	0.829003	VVHDGFEGI	98	391.2065	3	1170.603	.	L	Superscaf	1.43E+08	0	0	0	0	0	BTP202209	Tissue specific
CDS|exon|ID=cds-Bg		13	5.263846	4001.1	0.829067	KVVAFSPVT	89	482.6273	3	1444.865	.	V	Superscaf	1.43E+08	0	0	0	0	0	BTP202209	Tissue specific
CDS|exon|ID=cds-Bg		12	4.890833	3532.7	0.829067	VVAFSPVTE	98	439.9294	3	1316.77	.	V	Superscaf	1.43E+08	0	0	0	0	0	BTP202209	Tissue specific
CDS|exon|ID=cds-Bg		11	4.45	3064.3	0.829067	VVAFSPVTE	74	595.3372	2	1188.675	.	V	Superscaf	1.43E+08	0	0	0	0	0	BTP202209	Tissue specific
CDS|exon|ID=cds-Bg		10	4.895	3551.1	0.829074	VSGELEHVT	99	563.7943	2	1125.578	.	A	Superscaf	1.43E+08	0	0	0	0	0	BTP202209	Tissue specific
CDS|exon|ID=cds-Bg		9	4.243333	2902.3	0.829074	VSGELEHVT	84	485.7437	2	969.4767	.	A	Superscaf	1.43E+08	0	0	0	0	0	BTP202209	Tissue specific
CDS|exon|ID=cds-Bg		9	5.438889	3551.1	0.829074	SGELEHVTF	92	514.2623	2	1026.509	.	A	Superscaf	1.43E+08	0	0	0	0	0	BTP202209	Tissue specific
CDS|exon|ID=cds-Bg		13	4.129231	4282	0.840814	MLVDQVPQV	62	749.3721	2	1496.733	.	E	Superscaf	1.44E+08	0	0	0	0	0	BTP202209	Tissue specific
CDS|exon|ID=cds-Bg		11	5.277273	4542.2	0.840834	QPPLVQLHE	98	616.3231	2	1230.636	.	R	Superscaf	1.44E+08	0	0	BTP202209	0	0	0	Tissue specific
CDS|exon|ID=cds-Bg		15	5.366667	5286.9	0.845789	VLDTSESVA	94	845.9673	2	1689.93	.	A	Superscaf	1.45E+08	0	0	0	0	0	BTP202209	Tissue specific
CDS|exon|ID=cds-Bg		8	4.46875	2417.5	0.84581	SSVDAVKY	97	434.7228	2	867.4338	.	A	Superscaf	1.45E+08	0	0	0	0	0	BTP202209	Tissue specific
CDS|exon|ID=cds-Bg		15	6.043333	5792.4	0.845989	KLQWMGGGT	72	798.3943	2	1594.781	.	L	Superscaf	1.45E+08	0	0	0	0	0	BTP202209	Tissue specific
CDS|exon|ID=cds-Bg		12	4.0575	3773.3	0.846007	PSQDVRVAV	93	680.8614	2	1359.715	.	E	Superscaf	1.45E+08	0	0	0	0	0	BTP202209	Tissue specific
CDS|exon|ID=cds-Bg		13	6.500769	5633.4	0.846008	SGTGQQRPE	81	457.5739	3	1369.706	.	E	Superscaf	1.45E+08	0	0	0	0	0	BTP202209	Tissue specific
CDS|exon|ID=cds-Bg		12	4.443333	4021.7	0.846009	TVLANTVDS	80	664.8025	2	1327.597	Oxidation	E	Superscaf	1.45E+08	0	0	0	0	0	BTP202209	Tissue specific
CDS|exon|ID=cds-Bg		13	4.748462	4720.4	0.846009	FNDATDVMI	89	716.3051	2	1430.602	.	E	Superscaf	1.45E+08	0	0	0	0	0	BTP202209	Tissue specific
CDS|exon|ID=cds-Bg		17	4.970588	6154.1	0.846009	FNDATDVMI	95	967.9465	2	1933.888	.	E	Superscaf	1.45E+08	0	0	0	0	0	BTP202209	Tissue specific
CDS|exon|ID=cds-Bg		16	4.93875	5766.7	0.846009	FNDATDVMI	94	894.4158	2	1786.82	.	E	Superscaf	1.45E+08	0	0	0	0	0	BTP202209	Tissue specific

CDS|exon|ID=cds-Bg	16	4.93875	5766.7	0.846009	NDATDVMDA	91	894.4136	2	1786.82	.	E	Superscaf	1.45E+08	0	0	0	0	0	BTP20220	Tissue specific
CDS|exon|ID=cds-Bg	11	4.621818	3736.3	0.846009	DATDVMDAL	62	585.7523	2	1169.491	.	E	Superscaf	1.45E+08	0	0	0	0	0	BTP20220	Tissue specific
CDS|exon|ID=cds-Bg	15	4.907333	5170	0.846009	DATDVMDAL	93	837.3916	2	1672.777	.	E	Superscaf	1.45E+08	0	0	0	0	0	BTP20220	Tissue specific
CDS|exon|ID=cds-Bg	10	5.512	3638.2	0.846009	MDALGYVTF	92	586.7897	2	1171.57	.	E	Superscaf	1.45E+08	0	0	0	0	BTP20220	0	Tissue specific
CDS|exon|ID=cds-Bg	12	6.619167	5155.4	0.84601	FYREASSNA	81	457.9037	3	1370.694	.	E	Superscaf	1.45E+08	0	0	0	0	0	BTP20220	Tissue specific
CDS|exon|ID=cds-Bg	12	4.243333	3847.4	0.846011	AVVVGRQVN	90	652.8548	2	1303.7	.	E	Superscaf	1.45E+08	0	0	0	0	0	BTP20220	Tissue specific
CDS|exon|ID=cds-Bg	8	3.55	1868.1	0.846011	AVVVGRQV	81	414.258	2	826.5024	.	E	Superscaf	1.45E+08	0	BTP20220	0	0	0	0	Tissue specific
CDS|exon|ID=cds-Bg	8	4.8725	2165.4	0.846012	VRVLVTGK	99	436.2878	2	870.5651	.	E	Superscaf	1.45E+08	0	0	0	0	0	BTP20220	Tissue specific
CDS|exon|ID=cds-Bg	12	4.7425	4297.8	0.846012	AAEYDVVFG	82	696.8268	2	1391.647	.	E	Superscaf	1.45E+08	0	0	0	0	0	BTP20220	Tissue specific
CDS|exon|ID=cds-Bg	10	6.279	4106.7	0.846013	RVPSYQALI	82	401.5708	3	1201.693	.	E	Superscaf	1.45E+08	0	BTP20220	0	0	0	0	Tissue specific
CDS|exon|ID=cds-Bg	8	5.73125	2811.1	0.846013	ALLRGVFY	81	469.775	2	937.5385	.	E	Superscaf	1.45E+08	0	BTP20220	0	0	BTP20220	BTP20220	Tissue enhanced
CDS|exon|ID=cds-Bg	10	5.547	3653	0.846013	GVFYQTVSR	83	592.8218	2	1183.635	.	E	Superscaf	1.45E+08	0	0	BTP20220	0	BTP20220	0	Mixed
CDS|exon|ID=cds-Bg	14	5.031429	4729.2	0.846013	GVFYQTVSR	99	791.9304	2	1581.852	.	E	Superscaf	1.45E+08	0	BTP20220	0	0	BTP20220	0	Mixed
CDS|exon|ID=cds-Bg	12	5.124167	3964.3	0.846013	GVFYQTVSR	98	677.8762	2	1353.741	.	E	Superscaf	1.45E+08	0	BTP20220	BTP20220	0	BTP20220	0	Mixed
CDS|exon|ID=cds-Bg	9	5.081111	3184.6	0.846013	GVFYQTVSR	89	528.7763	2	1055.54	.	E	Superscaf	1.45E+08	0	BTP20220	0	0	0	0	Tissue specific
CDS|exon|ID=cds-Bg	13	4.959231	4431.9	0.846013	VFYQTVSRK	91	763.4189	2	1524.83	.	E	Superscaf	1.45E+08	0	BTP20220	0	0	0	0	Tissue specific
CDS|exon|ID=cds-Bg	12	5.3725	4431.9	0.846013	FYQTVSRKV	89	713.8875	2	1425.762	.	E	Superscaf	1.45E+08	0	BTP20220	0	0	0	0	Tissue specific
CDS|exon|ID=cds-Bg	11	5.362727	4044.5	0.846013	YQTVSRKVA	97	640.3522	2	1278.693	.	E	Superscaf	1.45E+08	0	BTP20220	BTP20220	0	BTP20220	0	Mixed
CDS|exon|ID=cds-Bg	10	5.333	3585	0.846013	QTVSRKVAL	99	558.8208	2	1115.63	.	E	Superscaf	1.45E+08	0	BTP20220	BTP20220	BTP20220	BTP20220	BTP20220	Mixed
CDS|exon|ID=cds-Bg	9	5.297778	2974.3	0.846013	TVSRKVALD	99	494.7921	2	987.5713	.	E	Superscaf	1.45E+08	0	BTP20220	BTP20220	0	BTP20220	BTP20220	Mixed
CDS|exon|ID=cds-Bg	8	5.14375	2576.8	0.846013	VSRKVALD	93	444.2683	2	886.5236	.	E	Superscaf	1.45E+08	0	BTP20220	0	BTP20220	BTP20220	0	Mixed
CDS|exon|ID=cds-Bg	8	4.07125	2047.1	0.847725	VGKGVVNF	71	410.2374	2	818.465	.	G	Superscaf	1.45E+08	0	0	0	0	BTP20220	BTP20220	Tissue enhanced
CDS|exon|ID=cds-Bg	8	5.695	2827.8	0.847846	SSFKEAVK	99	448.2464	2	894.481	.	V	Superscaf	1.45E+08	0	0	0	0	0	BTP20220	Tissue specific
CDS|exon|ID=cds-Bg	8	4.88625	2740.9	0.84785	SDLVAEKF	99	454.7375	2	907.465	.	V	Superscaf	1.45E+08	0	0	0	0	0	BTP20220	Tissue specific
CDS|exon|ID=cds-Bg	13	5.559231	5544.3	0.847926	LLDGSERLG	86	739.3634	2	1476.721	.	D	Superscaf	1.45E+08	0	0	0	0	0	BTP20220	Tissue specific
CDS|exon|ID=cds-Bg	15	3.634	3829.2	0.847931	VVAVGSDVI	98	752.395	2	1502.783	.	D	Superscaf	1.45E+08	0	0	0	0	0	BTP20220	Tissue specific
CDS|exon|ID=cds-Bg	8	6.1725	3002.3	0.853041	LTKEGLLL	58	443.7824	2	885.5535	.	V	Superscaf	1.45E+08	0	0	0	0	0	BTP20220	Tissue specific
CDS|exon|ID=cds-Bg	5	5.922	1869.1	0.92786	AC(+57.02	53	501.216	1	500.2053	Carbamidc	E	Superscaf	1.51E+08	0	0	0	BTP20220	0	0	Tissue specific
CDS|exon|ID=cds-Bg	7	5.852857	2719.3	0.956213	MLTVRPQ	53	422.7401	2	843.4637	.	V	Superscaf	1.53E+08	0	BTP20220	0	0	0	0	Tissue specific
CDS|exon|ID=cds-Bg	11	5.597273	3797.9	0.975565	AAVSLLGEE	99	572.8304	2	1143.65	.	E	Superscaf	1261645	0	BTP20220	0	0	0	BTP20220	Mixed
CDS|exon|ID=cds-Bg	6	8.083333	2629.8	0.951235	LKKLRP	76	377.7673	2	753.5225	.	N	Superscaf	2518155	0	0	0	0	0	BTP20220	Tissue specific
CDS|exon|ID=cds-Bg	6	4.948333	2413.7	0.934959	VENC(+57.	72	746.3527	1	745.3428	Carbamidc	P	Superscaf	3358584	BTP20220	0	0	0	0	0	Tissue specific
CDS|exon|ID=cds-Bg	6	4.111667	1676.8	0.894694	KYDVVP	58	720.3901	1	719.3854	.	M	Superscaf	5437830	0	0	BTP20220	0	0	0	Tissue specific
CDS|exon|ID=cds-Bg	9	5.513333	3379.9	0.845931	VGSENPPLF	82	484.7602	2	967.5087	.	R	Superscaf	7955701	0	0	BTP20220	0	0	0	Tissue specific
CDS|exon|ID=cds-Bg	12	5.315833	4349.6	0.845587	SVAKVLQHA	52	612.8384	2	1223.662	.	D	Superscaf	7973293	0	0	BTP20220	0	0	0	Tissue specific
CDS|exon|ID=cds-Bg	17	4.755882	6563.5	0.845388	EGAVDPNDF	68	913.4299	2	1824.849	.	G	Superscaf	7983649	0	0	0	0	0	BTP20220	Tissue specific
CDS|exon|ID=cds-Bg	13	5.786923	5425	0.845169	VEDAQLPLE	97	518.6252	3	1552.857	.	I	Superscaf	7994977	0	0	BTP20220	0	0	0	Tissue specific
CDS|exon|ID=cds-Bg	12	5.3725	4776.2	0.845169	VEDAQLPLE	74	466.5911	3	1396.756	.	I	Superscaf	7994977	0	0	BTP20220	0	0	0	Tissue specific
CDS|exon|ID=cds-Bg	7	4.898571	2092.4	0.834022	GGPVTEP	56	656.3373	1	655.3177	.	V	Superscaf	8570590	0	0	BTP20220	0	0	0	Tissue specific
CDS|exon|ID=cds-Bg	8	4.9325	2672.1	0.799589	EVC(+57.0	63	433.7075	2	865.4003	Carbamidc	G	Superscaf	10348672	0	0	0	0	BTP20220	0	Tissue specific
CDS|exon|ID=cds-Bg	8	4.395	2370.7	0.791632	APPDHVVL	83	424.2364	2	846.4599	.	I	Superscaf	10757113	BTP20220	0	0	0	0	0	Tissue specific
CDS|exon|ID=cds-Bg	11	5.692727	4643.6	0.791574	TTEPTNVRE	78	428.8772	3	1283.611	.	I	Superscaf	10757113	BTP20220	0	0	0	0	0	Tissue specific
CDS|exon|ID=cds-Bg	8	4.96	3198.7	0.791494	VPFEDTHH	92	491.2245	2	980.4352	.	I	Superscaf	10757113	BTP20220	0	0	0	0	0	Tissue specific
CDS|exon|ID=cds-Bg	5	4.296	1559.8	0.760057	YPVEP	98	604.2963	1	603.2904	.	L	Superscaf	12390052	BTP20220	BTP20220	BTP20220	BTP20220	BTP20220	BTP20220	Mixed
CDS|exon|ID=cds-Bg	9	5.158889	3712.4	0.758097	M(+42.01)	98	555.7643	2	1109.518	Acetylati	D	Superscaf	12491257	0	0	0	0	0	BTP20220	Tissue specific
CDS|exon|ID=cds-Bg	13	5.794615	5980.7	0.741051	KLEELELDE	96	553.295	3	1656.868	.	S	Superscaf	13371343	0	0	0	0	0	BTP20220	Tissue specific
CDS|exon|ID=cds-Bg	11	5.077273	5043.9	0.741051	LEELELDEG	88	701.347	2	1400.678	.	S	Superscaf	13371343	0	0	0	0	0	BTP20220	Tissue specific
CDS|exon|ID=cds-Bg	11	4.750909	4060.4	0.741049	VGELKDDDF	96	647.8089	2	1293.609	.	S	Superscaf	13371343	0	0	0	0	0	BTP20220	Tissue specific
CDS|exon|ID=cds-Bg	7	5.091429	2014.4	0.727169	PPPVVRL	57	389.2555	2	776.4908	.	I	Superscaf	14088238	0	0	0	BTP20220	0	0	Tissue specific
CDS|exon|ID=cds-Bg	7	6.88	2533.6	0.659217	K(+42.01)	56	799.5031	1	798.5076	Acetylati	P	Superscaf	17597298	0	BTP20220	0	0	0	0	Tissue specific
CDS|exon|ID=cds-Bg	7	6.444286	2954.2	0.629244	LFQPPKY	63	446.7485	2	891.4854	.	F	Superscaf	19145028	0	0	BTP20220	0	0	0	Tissue specific
CDS|exon|ID=cds-Bg	7	6.124286	2341.6	0.598166	PSPPGAP	79	622.3148	1	621.3122	.	L	Superscaf	20749510	0	0	0	0	BTP20220	0	Tissue specific
CDS|exon|ID=cds-Bg	8	6.22375	3002.3	0.594357	A(+42.01)	92	471.7614	2	941.5117	Acetylati	A	Superscaf	20946190	0	0	0	0	BTP20220	0	Tissue specific
CDS|exon|ID=cds-Bg	7	6.258571	2648.9	0.594357	A(+42.01)	94	415.2199	2	828.4276	Acetylati	A	Superscaf	20946190	0	0	BTP20220	0	BTP20220	BTP20220	Mixed
CDS|exon|ID=cds-Bg	9	4.57	2804.1	0.594312	VLERVNAVK	99	514.3147	2	1026.619	.	L	Superscaf	20948605	0	0	0	0	0	BTP20220	Tissue specific
CDS|exon|ID=cds-Bg	10	5.586	3644.9	0.594308	FSERGDAVA	87	540.2759	2	1078.541	.	Q	Superscaf	20948932	0	0	0	0	0	BTP20220	Tissue specific
CDS|exon|ID=cds-Bg	5	5.904	1674.7	0.59297	LAAAF	97	492.2802	1	491.2744	.	E	Superscaf	21017905	0	0	0	BTP20220	0	0	Tissue specific
CDS|exon|ID=cds-Bg	6	4.301667	2591	0.592307	EC(+57.02	51	758.2192	1	757.2225	Carbamidc	K	Superscaf	21052347	0	0	BTP20220	0	0	0	Tissue specific
CDS|exon|ID=cds-Bg	7	5.49	2723	0.584838	NPALEAF	94	761.3824	1	760.3755	.	P	Superscaf	21437989	BTP20220	0	0	0	0	0	Tissue specific
CDS|exon|ID=cds-Bg	8	5.30875	2418.6	0.583185	APGWGAVP	51	377.6924	2	753.3809	.	H	Superscaf	21523003	BTP20220	0	0	0	0	0	Tissue specific
CDS|exon|ID=cds-Bg	8	7.59125	3340.3	0.579957	GPSHKGKK	88	419.7482	2	837.4821	.	I	Superscaf	21689884	0	0	0	0	0	BTP20220	Tissue specific
CDS|exon|ID=cds-Bg	14	5.055714	5170.8	0.579617	A(+42.01)	96	792.4092	2	1582.809	Acetylati	K	Superscaf	21707359	0	0	0	BTP20220	BTP20220	BTP20220	Mixed
CDS|exon|ID=cds-Bg	5	5.562	1488.6	0.57868	V(+42.01)	50	587.3251	1	586.3149	Acetylati	S	Superscaf	21756159	0	0	0	BTP20220	0	0	Tissue specific
CDS|exon|ID=cds-Bg	8	7.21625	3191.6	0.576082	RAGGGLPR	96	392.2316	2	782.4511	.	G	Superscaf	21890110	0	BTP20220	0	0	0	0	Tissue specific
CDS|exon|ID=cds-Bg	6	5.7	1960.1	0.521255	KTVGYP	55	664.3769	1	663.3591	.	T	Superscaf	24721318	0	0	BTP20220	0	0	0	Tissue specific
CDS|exon|ID=cds-Bg	7	8.59	3306.7	0.503182	KTRKPRP	50	441.7912	2	881.5558	.	Q	Superscaf	25654584	BTP20220	0	0	0	0	0	Tissue specific
CDS|exon|ID=cds-Bg	7	5.284286	2166.5	0.502576	GSTLVPT	54	674.3826	1	673.3646	.	R	Superscaf	25685824	BTP20220	0	0	0	0	0	Tissue specific
CDS|exon|ID=cds-Bg	11	5.940909	4549.2	0.498711	A(+42.01)	76	622.8128	2	1243.616	Acetylati	G	Superscaf	25885372	0	0	0	BTP20220	0	0	Tissue specific
CDS|exon|ID=cds-Bg	9	6.603333	3177.4	0.496752	AAPGAAARF	95	391.2176	2	780.4242	.	V	Superscaf	25986316	0	0	0	0	0	BTP20220	Tissue specific
CDS|exon|ID=cds-Bg	6	6.045	1964.1	0.496712	LLAAGP	54	541.3431	1	540.3271	.	G	Superscaf	25988542	0	0	0	0	BTP20220	0	Tissue specific
CDS|exon|ID=cds-Bg	8	5.18875	3020.5	0.495299	THSVETPY	83	467.2177	2	932.424	.	P	Superscaf	26061613	0	0	0	0	0	BTP20220	Tissue specific
CDS|exon|ID=cds-Bg	15	5.917333	5677.3	0.49521	SSESGTLSS	96	700.8174	2	1399.622	.	L	Superscaf	26066071	0	0	BTP20220	0	0	0	Tissue specific
CDS|exon|ID=cds-Bg	13	5.953846	4910.5	0.49521	ESGTLSSGF	97	613.7858	2	1225.557	.	L	Superscaf	26066071	0	0	BTP20220	0	0	BTP20220	Mixed
CDS|exon|ID=cds-Bg	9	5.923333	3467	0.495209	SGPPGHTME	98	456.6967	2	911.3807	.	L	Superscaf	26066071	BTP20220	0	0	0	0	0	Tissue specific
CDS|exon|ID=cds-Bg	7	5.408571	2364.6	0.491062	KVGVRNL	50	393.2561	2	784.4919	.	K	Superscaf	26280441	0	0	BTP20220	0	0	0	Tissue specific
CDS|exon|ID=cds-Bg	5	4.92	1339.5	0.482047	LPPAV	67	496.3123	1	495.3057	.	I	Superscaf	26745853	BTP20220	0	0	0	0	0	Tissue specific
CDS|exon|ID=cds-Bg	12	6.194167	4802.2	0.340505	KGPLLEEQA	91	663.8822	2	1325.755	.	L	Superscaf	34054765	0	0	0	0	0	BTP20220	Tissue specific
CDS|exon|ID=cds-Bg	11	5.165455	5286.2	0.31817	DNC(+57.0	95	669.77	2	1337.531	Carbamidc	C	Superscaf	35207986	0	0	0	0	BTP20220	0	Tissue specific
CDS|exon|ID=cds-Bg	5	5.496	1668.8	0.304924	G(+42.01)	56	472.2106	1	471.1965	Acetylati	P	Superscaf	35891878	BTP20220	0	0	0	0	0	Tissue specific
CDS|exon|ID=cds-Bg	9	6.752222	3473.7	0.303432	AAHAAAAPF	53	418.2291	2	834.446	.	P	Superscaf	35968684	0	0	0	0	0	BTP20220	Tissue specific
CDS|exon|ID=cds-Bg	7	5.567143	3025.3	0.30248	AFNADFH	85	411.1812	2	820.3504	.	M	Superscaf	36018133	0	0	0	BTP20220	0	0	Tissue specific
CDS|exon|ID=cds-Bg	7	6.521429	2500.8	0.296878	A(+42.01)	53	405.7502	2	809.4759	Acetylati	E	Superscaf	36307546	0	0	BTP20220	0	0	0	Tissue specific
CDS|exon|ID=cds-Bg	7	6.258571	2569.8	0.295759	HAGGLPL	53	664.3714	1	663.3704	.	F	Superscaf	36365173	0	0	0	0	0	BTP20220	Tissue specific
CDS|exon|ID=cds-Bg	8	2.9875	1301.4	0.293964	GVGLVVVL	76	755.4999	1	754.4952	.	L	Superscaf	36457690	0	0	0	0	BTP20220	0	Tissue specific
CDS|exon|ID=cds-Bg	7	2.56	948	0.293964	GVGLVVV	69	642.4172	1	641.4112	.	L	Superscaf	36457690	0	BTP20220	0	0	BTP20220	0	Mixed
CDS|exon|ID=cds-Bg	8	5.845	3089.4	0.286968	EATLGFGH	59	416.2008	2	830.3923	.	S	Superscaf	36819346	BTP20220	0	0	0	0	0	Tissue specific
CDS|exon|ID=cds-Bg	6	6.718333	3057.6	0.285496	NQRPWP	60	399.21	2	796.398	.	N	Superscaf	36895369	0	0	BTP20220	0	0	0	Tissue specific
CDS|exon|ID=cds-Bg	5	3.63	1633.9	0.264522	EPVDY	99	622.2718	1	621.2646	.	T	Superscaf	37978425	BTP20220	0	0	0	0	0	Tissue specific
CDS|exon|ID=cds-Bg	9	6.044444	3175.4	0.171177	MLGLDAAGK	88	438.2347	2	874.4582	.	C	Superscaf	42798319	0	0	0	0	0	BTP20220	Tissue specific
CDS|exon|ID=cds-Bg	6	6.006667	1968.1	0.133031	FGPGAP	83	545.2716	1	544.2645	.	Q	Superscaf	44768242	0	0	0	0	BTP20220	0	Tissue specific
CDS|exon|ID=cds-Bg	16	4.72	6037.8	0.131848	LDEDELLGN	92	908.4643	2	1814.915	.	L	Superscaf	44829459	0	0	0	0	0	BTP20220	Tissue specific
CDS|exon|ID=cds-Bg	14	4.755	5272.9	0.131848	EDELLGNLS	91	794.4086	2	1586.804	.	L	Superscaf	44829459	0	0	0	0	0	BTP20220	Tissue specific
CDS|exon|ID=cds-Bg	6	3.525	1429.6	0.131822	VELLVG	56	629.3849	1	628.3796	.	V	Superscaf	44830726	0	0	0	0	0	BTP20220	Tissue specific
CDS|exon|ID=cds-Bg	13	5.529231	4538.8	0.11406	TAWLDGKHV	99	729.3964	2	1456.783	.	K	Superscaf	45747523	0	0	0	0	0	BTP20220	Tissue specific
CDS|exon|ID=cds-Bg	8	4.71625	2537.7	0.11406	LDGKHVVF	72	457.7572	2	913.5021	.	K	Superscaf	45747523	0	BTP20220	0	0	0	0	Tissue specific
CDS|exon|ID=cds-Bg	8	5.63625	3241.7	0.067092	AWDPLLPQ	52	470.2514	2	938.4861	.	I	Superscaf	48172927	BTP20220	0	0	0	0	0	Tissue specific
CDS|exon|ID=cds-Bg	9	5.61	3301.5	0.036412	GSVKAYTNF	76	493.7503	2	985.4869	.	P	Superscaf	49757446	0	BTP20220	0	0	0	0	Tissue specific
CDS|exon|ID=cds-Bg	7	5.641429	2169.3	0.035965	LMVALAK	97	373.2344	2	744.4568	.	T	Superscaf	49780498	0	BTP20220	0	0	0	BTP20220	Tissue enhanced
CDS|exon|ID=cds-Bg	8	5.285	2508.6	0.065611	LGDSGVGK	98	366.6962	2	731.3813	.	P	Superscaf	55025689	0	0	0	0	0	BTP20220	Tissue specific
CDS|exon|ID=cds-Bg	6	6.9	2774.1	0.161412	KGEPNR	73	350.6929	2	699.3663	.	Q	Superscaf	59972820	0	0	0	0	0	BTP20220	Tissue specific
CDS|exon|ID=cds-Bg	6	4.343333	2147.5	0.168855	LDPEVH	80	355.1791	2	708.3442	.	P	Superscaf	60356995	BTP20220	0	0	0	0	0	Tissue specific
CDS|exon|ID=cds-Bg	9	5.623333	3179.6	0.182098	LVLANMLMK	97	516.7986	2	1031.587	.	N	Superscaf	61040674	0	0	0	0	0	BTP20220	Tissue specific
CDS|exon|ID=cds-Bg	10	5.398	3679.1	0.203603	A(+42.01)	98	578.3021	2	1154.593	Acetylati	A	Superscaf	62151271	0	BTP20220	0	BTP20220	0	0	Mixed
CDS|exon|ID=cds-Bg	9	4.802222	3030.3	0.203603	A(+42.01)	96	999.4997	1	998.492	Acetylati	A	Superscaf	62151271	0	0	0	BTP20220	0	0	Tissue specific
CDS|exon|ID=cds-Bg	6	6.518333	2526.7	0.221808	APYKLN	96	353.2052	2	704.3857	.	S	Superscaf	63091420	BTP20220	0	0	0	0	0	Tissue specific
CDS|exon|ID=cds-Bg	9	4.972222	3755.4	0.259415	TTEFEPC(+	91	573.2242	2	1144.438	Carbamidc	D	Superscaf	65033329	0	0	BTP20220	0	0	0	Tissue specific
CDS|exon|ID=cds-Bg	8	5.55375	3053.4	0.259457	KWLSMNVL	72	495.7734	2	989.5368	.	Q	Superscaf	65035474	0	0	BTP20220	0	0	0	Tissue specific
CDS|exon|ID=cds-Bg	10	4.714	3462.1	0.259457	MLDEKRVMV	97	618.3083	2	1234.605	.	Q	Superscaf	65035474	0	0	BTP20220	0	0	0	Tissue specific
CDS|exon|ID=cds-Bg	8	4.42625	2737.2	0.259457	DEKRVMVD	96	496.2463	2	990.4804	.	Q	Superscaf	65035474	0	0	BTP20220	0	0	0	Tissue specific
CDS|exon|ID=cds-Bg	9	5.125556	3241.6	0.265487	EDVQPPGLK	72	491.7625	2	981.5131	.	L	Superscaf	65346814	0	0	BTP20220	0	0	0	Tissue specific
CDS|exon|ID=cds-Bg	8	5.52625	2661.8	0.301312	AAVQRKAV	57	421.7632	2	841.5134	.	T	Superscaf	67196635	BTP20220	0	0	0	0	0	Tissue specific
CDS|exon|ID=cds-Bg	7	6.945714	2923.1	0.302392	NLMALKK	91	409.2507	2	816.4891	.	S	Superscaf	67252591	0	0	0	0	0	BTP20220	Tissue specific
CDS|exon|ID=cds-Bg	9	6.277778	3246.7	0.306195	APPMPGGPF	87	430.7063	2	859.4011	.	I	Superscaf	67448284	0	0	0	BTP20220	0	0	Tissue specific
CDS|exon|ID=cds-Bg	11	5.4	3783.1	0.308026	MVFPFESGK	97	643.3157	2	1284.621	.	N	Superscaf	67543378	0	0	0	0	0	BTP20220	Tissue specific
CDS|exon|ID=cds-Bg	16	3.769375	4672.3	0.315949	SDALAVEDE	79	840.9362	2	1679.862	.	D	Superscaf	67952380	0	BTP20220	0	0	0	0	Tissue specific
CDS|exon|ID=cds-Bg	14	3.69	3877.4	0.315949	ALAVEDEQV	66	739.9071	2	1477.803	.	D	Superscaf	67952380	0	BTP20220	0	0	0	0	Tissue specific
CDS|exon|ID=cds-Bg	10	2.766	2548	0.315949	VEDEQVVPV	73	555.7855	2	1109.56	.	D	Superscaf	67952380	0	0	0	0	0	BTP20220	Tissue specific
CDS|exon|ID=cds-Bg	8	3.055	2122.5	0.315949	DEQVVPVP	81	882.4521	1	881.4494	.	D	Superscaf	67952380	0	0	0	0	BTP20220	0	Tissue specific
CDS|exon|ID=cds-Bg	8	4.01125	1966.2	0.315952	VEPVPVTK	98	434.7597	2	867.5065	.	D	Superscaf	67952380	0	BTP20220	0	0	0	0	Tissue specific
CDS|exon|ID=cds-Bg	10	5.889	3528.7	0.316015	VESLTAPSK	96	530.3042	2	1058.597	.	N	Superscaf	67955938	0	BTP20220	0	0	0	0	Tissue specific
CDS|exon|ID=cds-Bg	10	5.36	4646.3	0.345127	AC(+57.02	89	690.786	2	1379.564	Carbamidc	Q	Superscaf	69459232	0	0	0	0	BTP20220	0	Tissue specific
CDS|exon|ID=cds-Bg	16	5.67125	6874.8	0.345127	C(+57.02)	80	906.4025	2	1810.798	Carbamidc	Q	Superscaf	69459232	0	0	0	0	0	BTP20220	Tissue specific
CDS|exon|ID=cds-Bg	13	5.384615	5467.3	0.345127	EHGLQPDGG	65	705.7996	2	1409.588	.	Q	Superscaf	69459232	0	0	BTP20220	0	0	0	Tissue specific
CDS|exon|ID=cds-Bg	10	5.338	3799.2	0.345146	FVDWC(+57	90	628.7897	2	1255.57	Carbamidc	L	Superscaf	69460141	0	0	0	0	BTP20220	0	Tissue specific
CDS|exon|ID=cds-Bg	9	5.322222	3411.8	0.345146	VDWC(+57.	94	555.2557	2	1108.501	Carbamidc	L	Superscaf	69460141	0	0	0	0	0	BTP20220	Tissue specific
CDS|exon|ID=cds-Bg	6	5.688333	2394.6	0.371835	LAC(+57.0	60	730.3206	1	729.3156	Carbamidc	G	Superscaf	70838526	0	0	0	0	0	BTP20220	Tissue specific
CDS|exon|ID=cds-Bg	10	5.423	3975.4	0.428501	EDTSSLFDK	82	577.7812	2	1153.55	.	S	Superscaf	73764466	0	BTP20220	0	0	0	0	Tissue specific
CDS|exon|ID=cds-Bg	11	3.787273	2668.8	0.459638	C(+57.02)	97	530.7745	2	1059.538	Carbamidc	V	Superscaf	75372217	0	0	0	0	0	BTP20220	Tissue specific
CDS|exon|ID=cds-Bg	10	3.664	2083.1	0.459638	VVVGDGAVG	98	450.7585	2	899.5076	.	V	Superscaf	75372217	0	0	0	0	BTP20220	BTP20220	Mixed
CDS|exon|ID=cds-Bg	12	4.015833	3066.3	0.459638	VVVGDGAVG	89	581.2984	2	1160.586	Carbamidc	V	Superscaf	75372217	0	BTP20220	0	0	BTP20220	0	Tissue enhanced
CDS|exon|ID=cds-Bg	9	4.071111	2083.1	0.459638	VVGDGAVGK	98	401.2256	2	800.4392	.	V	Superscaf	75372217	0	0	0	0	0	BTP20220	Tissue specific
CDS|exon|ID=cds-Bg	11	4.380909	3066.3	0.459638	VVGDGAVGK	99	531.7641	2	1061.518	Carbamidc	V	Superscaf	75372217	0	0	0	0	BTP20220	0	Tissue specific

CDS|exon|ID=cds-B	10	4.819	3066.3	0.459638	VGDGAVGKT	99	482.2301	2	962.4491	Carbamido	V	Superscaf	75372217	0	0	0	0	BTP20220	0	Tissue specific
CDS|exon|ID=cds-B	8	6.02875	3114.5	0.476417	LDFPTMKF	99	499.7526	2	997.4943	.	Y	Superscaf	76238683	0	0	0	0	0	BTP20220	Tissue specific
CDS|exon|ID=cds-B	11	6.409091	4432.8	0.481039	AGKLARGDI	90	551.3032	2	1100.594	.	N	Superscaf	76477423	0	0	BTP20220	0	0	0	Tissue specific
CDS|exon|ID=cds-B	9	4.815556	2301.6	0.481193	TPVPGGVGF	98	780.4214	1	779.4177	.	P	Superscaf	76485415	0	0	0	0	BTP20220	0	Tissue specific
CDS|exon|ID=cds-B	13	5.311538	4855.3	0.481214	TEEVELYGE	96	748.8743	2	1495.741	.	F	Superscaf	76486453	0	0	0	0	BTP20220	BTP20220	Mixed
CDS|exon|ID=cds-B	8	5.465	3498.9	0.481229	AHLHQNVF	80	483.2501	2	964.4879	.	R	Superscaf	76487290	0	0	BTP20220	0	0	0	Tissue specific
CDS|exon|ID=cds-B	13	5.646923	4657	0.481557	AEAVQRAAA	69	614.8154	2	1227.621	.	L	Superscaf	76503970	0	0	0	0	0	BTP20220	Tissue specific
CDS|exon|ID=cds-B	7	5.727143	3148.6	0.483141	RFEELNA	74	439.7214	2	877.4293	.	L	Superscaf	76585108	0	0	0	BTP20220	0	0	Tissue specific
CDS|exon|ID=cds-B	5	5.498	1724.9	0.536902	ELGAP	72	486.2568	1	485.2485	.	W	Superscaf	79362133	0	0	0	0	BTP20220	0	Tissue specific
CDS|exon|ID=cds-B	7	6.318571	3024.5	0.537132	APPEQLR	82	405.7325	2	809.4395	.	W	Superscaf	79373839	0	0	0	0	0	BTP20220	Tissue specific
CDS|exon|ID=cds-B	11	5.863636	4760.1	0.539763	MDEEDKAFK	99	456.8859	3	1367.639	.	T	Superscaf	79509790	0	BTP20220	0	0	0	0	Tissue specific
CDS|exon|ID=cds-B	10	5.875	4388.6	0.539763	DEEDKAFKG	97	619.3038	2	1236.599	.	T	Superscaf	79509790	0	BTP20220	0	0	0	0	Tissue specific
CDS|exon|ID=cds-B	7	6.288571	2663	0.565315	TGLTDKP	77	366.2049	2	730.3861	.	Y	Superscaf	80829223	0	0	0	BTP20220	0	0	Tissue specific
CDS|exon|ID=cds-B	5	6.88	1999	0.576973	DKLGK	68	560.339	1	559.3329	.	D	Superscaf	81431341	0	0	0	0	0	BTP20220	Tissue specific
CDS|exon|ID=cds-B	11	5.33	3826.3	0.631095	VRMNVLADA	81	410.5717	3	1228.696	.	E	Superscaf	84226060	0	0	BTP20220	0	0	0	Tissue specific
CDS|exon|ID=cds-B	7	5.104286	2337.6	0.636328	V(+42.01)	82	407.2272	2	812.4432	Acetylati	R	Superscaf	84496300	0	0	0	BTP20220	0	0	Tissue specific
CDS|exon|ID=cds-B	7	5.611429	3052.4	0.645142	NYEDLPK	88	439.716	2	877.4181	.	E	Superscaf	84951331	0	0	0	0	0	BTP20220	Tissue specific
CDS|exon|ID=cds-B	7	3.971429	2209.6	0.64958	VQRVVLN	76	414.257	2	826.5025	.	D	Superscaf	85180600	0	0	0	0	BTP20220	0	Tissue specific
CDS|exon|ID=cds-B	9	5.288889	3104.4	0.649742	MKGDFVTSF	74	516.2449	2	1030.479	.	V	Superscaf	85188823	0	0	0	0	0	BTP20220	Tissue specific
CDS|exon|ID=cds-B	9	5.527778	2675.1	0.664627	GPPPVLPPF	72	435.7561	2	869.501	.	T	Superscaf	85957159	0	0	BTP20220	0	0	0	Tissue specific
CDS|exon|ID=cds-B	9	5.566667	2960.1	0.666899	LGFMSAFVK	96	500.2698	2	998.5259	.	K	Superscaf	86074804	0	BTP20220	BTP20220	0	BTP20220	BTP20220	Mixed
CDS|exon|ID=cds-B	8	5.515	2606.7	0.666899	GFMSAFVK	93	443.726	2	885.4418	.	K	Superscaf	86074804	0	0	0	0	BTP20220	0	Tissue specific
CDS|exon|ID=cds-B	6	6.17	2144.1	0.694567	KGWSVK	56	352.7123	2	703.4017	.	A	Superscaf	87503644	0	0	BTP20220	0	0	0	Tissue specific
CDS|exon|ID=cds-B	8	6.80375	2990.5	0.714035	PGRPGTPP	65	389.7063	2	777.4133	.	A	Superscaf	88508887	0	0	0	BTP20220	0	0	Tissue specific
CDS|exon|ID=cds-B	7	6.79	2726.9	0.715651	LHLGKGP	63	721.4381	1	720.4282	.	V	Superscaf	88592071	0	0	0	0	BTP20220	0	Tissue specific
CDS|exon|ID=cds-B	7	6.03	2453.7	0.716198	VPPHLKP	75	394.2425	2	786.4752	.	A	Superscaf	88620343	0	0	BTP20220	0	0	0	Tissue specific
CDS|exon|ID=cds-B	6	5.45	2219.3	0.717646	KSGVQY	66	681.3564	1	680.3493	.	P	Superscaf	88695346	0	0	0	0	0	BTP20220	Tissue specific
CDS|exon|ID=cds-B	8	6.57875	2753.9	0.717679	PAGPPALK	93	375.7276	2	749.4435	.	S	Superscaf	88696981	0	0	0	0	BTP20220	0	Tissue specific
CDS|exon|ID=cds-B	8	4.2775	2432.7	0.79326	DLVVSLAH	56	427.2458	2	852.4705	.	A	Superscaf	92599426	BTP20220	0	0	0	0	0	Tissue specific
CDS|exon|ID=cds-B	10	4.693	3441.7	0.902875	VGAVDADKE	99	520.2593	2	1038.509	.	N	Superscaf	98259787	0	0	0	0	0	BTP20220	Tissue specific
CDS|exon|ID=cds-B	9	4.547778	3637.1	0.90288	DNFDKNVLD	64	540.2521	2	1078.493	.	N	Superscaf	98259787	0	0	0	BTP20220	0	0	Tissue specific
CDS|exon|ID=cds-B	9	5.017778	3363.8	0.902883	VNQVLASRY	67	525.2889	2	1048.566	.	N	Superscaf	98259787	0	0	0	BTP20220	0	0	Tissue specific
CDS|exon|ID=cds-B	7	5.535714	2355.4	0.930174	EAAFAAG	54	636.2922	1	635.2914	.	V	Superscaf	99669775	0	0	0	BTP20220	0	0	Tissue specific
CDS|exon|ID=cds-B	5	4.998	1570.7	0.934834	KDVLP	75	571.3438	1	570.3377	.	L	Superscaf	99910506	0	0	BTP20220	0	0	0	Tissue specific
CDS|exon|ID=cds-B	14	5.659286	4937.3	0.969256	HAVSEGTKA	78	497.9298	3	1490.773	.	S	Superscaf	1.02E+08	0	0	0	0	BTP20220	BTP20220	Mixed
CDS|exon|ID=cds-B	12	6.485	4432.6	0.969257	GTKAVTKYT	93	424.2372	3	1269.693	.	S	Superscaf	1.02E+08	0	0	BTP20220	0	BTP20220	BTP20220	Mixed
CDS|exon|ID=cds-B	15	5.871333	5918.6	0.993315	VLTPEEQLA	95	585.0101	3	1752.014	.	L	Superscaf	1.03E+08	0	0	0	0	0	BTP20220	Tissue specific
CDS|exon|ID=cds-B	12	5.1325	4448	0.993315	VLTPEEQLA	87	678.3705	2	1354.734	.	L	Superscaf	1.03E+08	0	0	0	0	0	BTP20220	Tissue specific
CDS|exon|ID=cds-B	8	4.99125	2051.1	0.993388	VVPGGGLK	98	363.7289	2	725.4435	.	D	Superscaf	1.03E+08	0	BTP20220	BTP20220	0	0	BTP20220	Mixed
CDS|exon|ID=cds-B	7	5.464286	2373.7	0.996193	V(+42.01)	58	392.2211	2	782.4286	Acetylati	V	Superscaf	1.03E+08	0	0	0	0	BTP20220	0	Tissue specific
CDS|exon|ID=cds-B	6	6.74	2689.2	0.964853	Q(+42.01)	71	379.1871	2	756.3588	Acetylati	E	Superscaf	1905810	0	0	0	BTP20220	0	0	Tissue specific
CDS|exon|ID=cds-B	6	4.421667	1867	0.95838	EHVKLV	59	362.7192	2	723.4279	.	K	Superscaf	2256328	0	BTP20220	0	0	0	0	Tissue specific
CDS|exon|ID=cds-B	7	5.542857	3002.4	0.957822	LPAQQEL	53	798.4343	1	797.4283	.	R	Superscaf	2286331	0	0	0	0	0	BTP20220	Tissue specific
CDS|exon|ID=cds-B	8	6.3225	3138.6	0.936936	A(+42.01)	87	428.7112	2	855.4086	Acetylati	A	Superscaf	3418834	0	BTP20220	0	0	0	0	Tissue specific
CDS|exon|ID=cds-B	12	6.293333	4301.5	0.925013	SAPSTGGVK	97	583.3173	2	1164.625	.	G	Superscaf	4065295	0	0	0	0	0	BTP20220	Tissue specific
CDS|exon|ID=cds-B	8	6.23875	3465.9	0.92501	DFKTDLRF	99	521.2701	2	1040.529	.	G	Superscaf	4065295	0	0	0	0	0	BTP20220	Tissue specific
CDS|exon|ID=cds-B	6	4.426667	1933.1	0.925009	SEAYLV	71	681.3445	1	680.3381	.	G	Superscaf	4065295	0	BTP20220	0	0	BTP20220	0	Mixed
CDS|exon|ID=cds-B	9	6.692222	3595.2	0.921427	ARQPPPPPF	69	478.7677	2	955.5239	.	A	Superscaf	4260024	0	0	0	BTP20220	0	0	Tissue specific
CDS|exon|ID=cds-B	7	5.878571	2815.2	0.917769	EEGARLL	98	394.2178	2	786.4235	.	V	Superscaf	4457725	0	0	BTP20220	0	0	0	Tissue specific
CDS|exon|ID=cds-B	9	5.884444	3406.8	0.913029	PDPAAHLPF	85	482.7462	2	963.4814	.	I	Superscaf	4715044	0	0	0	0	BTP20220	0	Tissue specific
CDS|exon|ID=cds-B	6	6.185	2737.1	0.887848	RLAEQF	76	382.207	2	762.4024	.	R	Superscaf	6080286	0	0	0	0	0	BTP20220	Tissue specific
CDS|exon|ID=cds-B	12	6.2875	4963.1	0.887101	LKEKEELES	96	473.2668	3	1416.782	.	P	Superscaf	6120244	0	0	0	0	0	BTP20220	Tissue specific
CDS|exon|ID=cds-B	5	6.098	1839.1	0.868705	PPSST	50	488.2283	1	487.2278	.	Q	Superscaf	7118098	BTP20220	0	0	0	0	0	Tissue specific
CDS|exon|ID=cds-B	8	5.1875	2889.5	0.859616	EM(+15.99	85	460.7098	2	919.4069	Oxidation	K	Superscaf	7610734	0	BTP20220	0	0	0	0	Tissue specific
CDS|exon|ID=cds-B	11	4.795455	3198.6	0.859593	LVTYVPVTT	94	634.3672	2	1266.722	.	H	Superscaf	7611787	0	0	0	0	0	BTP20220	Tissue specific
CDS|exon|ID=cds-B	10	4.677	2845.2	0.859593	VTYVPVTTF	94	577.8243	2	1153.638	.	H	Superscaf	7611787	0	0	0	0	0	BTP20220	Tissue specific
CDS|exon|ID=cds-B	6	5.203333	2365.6	0.838537	E(+42.01)	53	358.6576	2	715.3024	Acetylati	P	Superscaf	8753841	BTP20220	0	0	0	0	0	Tissue specific
CDS|exon|ID=cds-B	5	5.562	1708.9	0.835155	E(+42.01)	55	512.236	1	511.2278	Acetylati	G	Superscaf	8937168	BTP20220	0	0	0	0	0	Tissue specific
CDS|exon|ID=cds-B	9	6.054444	3060.6	0.800557	VPPPPATLF	65	474.2872	2	946.5599	.	D	Superscaf	10812601	0	0	0	BTP20220	0	0	Tissue specific
CDS|exon|ID=cds-B	6	6.173333	2588.9	0.783781	TYFPHF	87	406.1917	2	810.3701	.	V	Superscaf	11722383	0	BTP20220	0	0	0	BTP20220	Tissue enhanced
CDS|exon|ID=cds-B	7	5.822857	2345.6	0.782182	ASRLVAP	78	713.4298	1	712.4232	.	L	Superscaf	11808685	0	BTP20220	0	0	0	0	Tissue specific
CDS|exon|ID=cds-B	7	5.064286	2044.3	0.782137	L(+42.01)	53	389.6944	2	777.3731	Acetylati	T	Superscaf	11811235	0	0	0	BTP20220	0	0	Tissue specific
CDS|exon|ID=cds-B	9	5.357778	3486.8	0.780971	LLEQVAQLK	89	521.3165	2	1040.623	.	P	Superscaf	11874568	0	0	0	0	0	BTP20220	Tissue specific
CDS|exon|ID=cds-B	8	5.4525	2829.2	0.775509	E(+42.01)	68	471.7828	2	941.5546	Acetylati	F	Superscaf	12170838	0	0	0	BTP20220	0	0	Tissue specific
CDS|exon|ID=cds-B	9	6.015556	3482.8	0.760953	DLSVKDKPF	87	353.2038	3	1056.593	.	H	Superscaf	12959782	0	BTP20220	0	0	0	0	Tissue specific
CDS|exon|ID=cds-B	7	6.97	3320.9	0.720009	QTLLPRH	77	432.754	2	863.4977	.	A	Superscaf	15179353	0	0	0	0	0	BTP20220	Tissue specific
CDS|exon|ID=cds-B	6	4.351667	1923.1	0.607685	YVALDF	96	727.3629	1	726.3588	.	N	Superscaf	21269182	0	0	0	0	BTP20220	BTP20220	Tissue enhanced
CDS|exon|ID=cds-B	21	4.980952	6466.1	0.582416	V(+42.01)	88	735.7323	3	2204.184	Acetylati	R	Superscaf	22639186	0	0	0	0	0	BTP20220	Tissue specific
CDS|exon|ID=cds-B	8	4.30875	2128.4	0.582416	V(+42.01)	94	460.7625	2	919.5127	Acetylati	R	Superscaf	22639186	0	0	BTP20220	0	0	0	Tissue specific
CDS|exon|ID=cds-B	9	5.307778	3498	0.581486	TTQPGVQFY	60	1040.502	1	1039.497	.	L	Superscaf	22689538	0	0	0	0	0	BTP20220	Tissue specific
CDS|exon|ID=cds-B	8	6.77125	3516	0.535073	GFAPYERR	98	498.2558	2	994.4984	.	K	Superscaf	25205758	0	0	0	0	BTP20220	0	Tissue specific
CDS|exon|ID=cds-B	7	6.047143	2431.8	0.516681	P(+42.01)	62	377.6925	2	753.3731	Acetylati	T	Superscaf	26203060	0	0	BTP20220	0	0	0	Tissue specific
CDS|exon|ID=cds-B	9	4.672222	3151.5	0.51388	SSHVTNVSF	71	489.2363	2	976.4614	.	A	Superscaf	26354902	0	0	0	0	0	BTP20220	Tissue specific
CDS|exon|ID=cds-B	7	7.204286	2896.1	0.490355	SSTTKTK	64	376.7167	2	751.4076	.	S	Superscaf	27630429	BTP20220	0	0	0	0	0	Tissue specific
CDS|exon|ID=cds-B	6	6.84	2603	0.488969	NAPTRA	50	629.3382	1	628.3293	.	S	Superscaf	27705445	0	0	0	0	0	BTP20220	Tissue specific
CDS|exon|ID=cds-B	7	6.404286	2777.2	0.481625	LTTPKTE	56	395.2196	2	788.428	.	S	Superscaf	28103787	0	0	0	0	BTP20220	0	Tissue specific
CDS|exon|ID=cds-B	9	7.115556	3426.8	0.468301	KPPPRPAAF	66	465.7814	2	929.5446	.	L	Superscaf	28825621	0	0	0	0	BTP20220	0	Tissue specific
CDS|exon|ID=cds-B	11	6.120909	4193.7	0.442655	PLAPMASHS	98	579.7823	2	1157.554	.	T	Superscaf	30216184	0	BTP20220	0	0	0	0	Tissue specific
CDS|exon|ID=cds-B	11	5.996364	4430.1	0.440646	PDDPMSEGK	83	410.1906	3	1227.555	.	C	Superscaf	30325177	0	0	0	BTP20220	0	0	Tissue specific
CDS|exon|ID=cds-B	8	6.0775	3696.4	0.438894	EDYDRLRP	86	532.2599	2	1062.509	.	E	Superscaf	30420301	0	0	0	0	BTP20220	0	Tissue specific
CDS|exon|ID=cds-B	7	5.654286	2545.9	0.428441	E(+42.01)	53	406.6994	2	811.3752	Acetylati	L	Superscaf	30986869	0	0	BTP20220	0	0	0	Tissue specific
CDS|exon|ID=cds-B	8	5.09625	2772.3	0.287364	T(+42.01)	85	913.4139	1	912.4077	Acetylati	E	Superscaf	38635396	0	BTP20220	0	0	0	0	Tissue specific
CDS|exon|ID=cds-B	13	5.691538	4480.7	0.285469	GFDLPAVEA	87	673.8671	2	1345.724	.	D	Superscaf	38738188	0	0	0	0	0	BTP20220	Tissue specific
CDS|exon|ID=cds-B	12	5.354167	4012.3	0.285469	GFDLPAVEA	83	609.8193	2	1217.629	.	D	Superscaf	38738188	0	0	0	0	0	BTP20220	Tissue specific
CDS|exon|ID=cds-B	11	5.96	4872.5	0.285406	RLEEASLLF	78	448.2413	3	1341.704	.	C	Superscaf	38741194	0	BTP20220	0	0	0	0	Tissue specific
CDS|exon|ID=cds-B	10	5.981	4388	0.285105	ELLEPLNEF	67	414.2335	3	1239.682	.	L	Superscaf	38757559	0	0	0	0	0	BTP20220	Tissue specific
CDS|exon|ID=cds-B	9	5.601111	3569.9	0.285069	AETVHQLSK	94	506.7727	2	1011.535	.	D	Superscaf	38759635	0	0	0	0	0	BTP20220	Tissue specific
CDS|exon|ID=cds-B	5	5.586	1970.1	0.285041	KDLAE	53	575.2986	1	574.2962	.	E	Superscaf	38761237	0	BTP20220	0	0	0	0	Tissue specific
CDS|exon|ID=cds-B	9	4.91	2971.2	0.284729	HSEVPVSLK	97	498.278	2	994.5447	.	G	Superscaf	38778193	0	0	0	0	0	BTP20220	Tissue specific
CDS|exon|ID=cds-B	10	5.921	3749.1	0.27326	A(+42.01)	68	521.7784	2	1041.545	Acetylati	I	Superscaf	39399757	0	0	0	0	BTP20220	0	Tissue specific
CDS|exon|ID=cds-B	13	5.216923	4734.1	0.273225	VLDSAFLEC	84	467.903	3	1400.694	.	P	Superscaf	39401650	0	0	0	0	0	BTP20220	Tissue specific
CDS|exon|ID=cds-B	8	5.9425	3474.9	0.273025	DLC(+57.0	59	537.7783	2	1073.544	Carbamido	R	Superscaf	39413121	0	0	0	0	BTP20220	0	Tissue specific
CDS|exon|ID=cds-B	6	5.198333	2483.7	0.160913	NDHFVK	98	380.192	2	758.3711	.	S	Superscaf	45490885	0	0	0	0	0	BTP20220	Tissue specific
CDS|exon|ID=cds-B	8	5.65875	3053.4	0.157528	A(+42.01)	99	462.7474	2	923.4825	Acetylati	Q	Superscaf	45674101	0	BTP20220	0	0	BTP20220	BTP20220	Mixed
CDS|exon|ID=cds-B	7	5.612857	2700	0.157528	A(+42.01)	98	406.2057	2	810.3984	Acetylati	Q	Superscaf	45674101	0	0	0	0	BTP20220	0	Tissue specific
CDS|exon|ID=cds-B	7	5.792857	2318.5	0.113631	PAVWPPK	55	397.7264	2	793.4486	.	N	Superscaf	48054298	0	0	0	0	BTP20220	0	Tissue specific
CDS|exon|ID=cds-B	11	5.981818	4597.3	0.109508	G(+42.01)	86	611.7641	2	1221.52	Acetylati	V	Superscaf	48277789	0	0	0	0	BTP20220	0	Tissue specific
CDS|exon|ID=cds-B	8	6.1675	3574.9	0.089702	M(+42.01)	99	1016.449	1	1015.443	Acetylati	P	Superscaf	49351615	0	0	BTP20220	0	0	0	Tissue specific
CDS|exon|ID=cds-B	10	5.499	4185.6	0.089702	M(+15.99)	96	630.2876	2	1258.565	Oxidation	P	Superscaf	49351615	0	0	BTP20220	0	0	0	Tissue specific
CDS|exon|ID=cds-B	20	5.5835	8639.6	0.089701	M(+42.01)	95	829.0399	3	2484.109	Acetylati	P	Superscaf	49351615	0	0	0	0	0	BTP20220	Tissue specific
CDS|exon|ID=cds-B	9	5.482222	3574.9	0.089702	M(+42.01)	80	558.2615	2	1114.512	Acetylati	P	Superscaf	49351615	0	0	BTP20220	0	0	0	Tissue specific
CDS|exon|ID=cds-B	10	4.761	4094.8	0.089657	TLGEEC(+5	59	622.741	2	1243.474	Carbamido	C	Superscaf	49353979	0	0	0	0	0	BTP20220	Tissue specific
CDS|exon|ID=cds-B	10	5.034	3065.2	0.089599	GDVVFKRVS	88	378.8911	3	1133.656	.	T	Superscaf	49357201	0	0	0	0	0	BTP20220	Tissue specific
CDS|exon|ID=cds-B	10	4.892	3357.6	0.075249	VDEAADALI	98	522.7806	2	1043.55	.	I	Superscaf	50135185	0	0	0	0	0	BTP20220	Tissue specific
CDS|exon|ID=cds-B	7	5.924286	2560.8	0.067541	KAVSRDP	65	386.7127	2	771.4239	.	A	Superscaf	50552944	0	0	0	BTP20220	0	0	Tissue specific
CDS|exon|ID=cds-B	5	3.592	1074.2	0.058238	LSVPV	82	514.3209	1	513.3162	.	S	Superscaf	51057385	0	BTP20220	0	0	BTP20220	0	Mixed
CDS|exon|ID=cds-B	10	6.231	4007.5	0.042361	QGKTRLVGF	94	535.3073	2	1068.604	.	V	Superscaf	51918055	0	0	BTP20220	0	0	0	Tissue specific
CDS|exon|ID=cds-B	8	6.73625	3548.9	0.042033	EFEKRKML	91	540.7936	2	1079.58	.	E	Superscaf	51936082	0	0	BTP20220	0	0	0	Tissue specific
CDS|exon|ID=cds-B	6	6.06	2540.8	0.140831	A(+42.01)	61	389.196	2	776.3738	Acetylati	C	Superscaf	61850049	0	BTP20220	0	0	0	0	Tissue specific
CDS|exon|ID=cds-B	8	5.73	2963.1	0.169825	DLLKANVK	95	450.7777	2	899.544	.	L	Superscaf	63421816	0	0	0	0	0	BTP20220	Tissue specific
CDS|exon|ID=cds-B	8	5.5	3180.6	0.175744	NSGLLDTF	91	866.422	1	865.4182	.	K	Superscaf	63742693	0	0	0	0	0	BTP20220	Tissue specific
CDS|exon|ID=cds-B	8	4.7525	2610	0.175783	SDLLLVMS	60	877.4666	1	876.4626	.	P	Superscaf	63744778	0	0	BTP20220	0	0	0	Tissue specific
CDS|exon|ID=cds-B	13	4.609231	4785.5	0.198904	M(+15.99)	87	783.353	2	1564.697	Oxidation	V	Superscaf	64998388	0	0	BTP20220	0	BTP20220	0	Mixed
CDS|exon|ID=cds-B	11	4.685455	3777.3	0.198904	M(+42.01)	90	660.8212	2	1319.633	Acetylati	V	Superscaf	64998388	0	0	0	0	BTP20220	0	Tissue specific
CDS|exon|ID=cds-B	7	5.517143	2825.2	0.199357	AELLFNT	62	807.4199	1	806.4174	.	L	Superscaf	65022913	0	BTP20220	0	0	0	0	Tissue specific
CDS|exon|ID=cds-B	7	6.174286	2983.3	0.258402	PASSHPQ	55	723.3412	1	722.3347	.	S	Superscaf	68224117	0	0	0	BTP20220	0	0	Tissue specific
CDS|exon|ID=cds-B	11	5.946364	3994.2	0.266977	LFSKMAC(+	99	634.3459	2	1266.683	Carbamido	M	Superscaf	68688982	0	0	0	0	0	BTP20220	Tissue specific
CDS|exon|ID=cds-B	8	6.4125	3467.9	0.266997	HYPSTHGL	97	456.2211	2	910.4297	.	N	Superscaf	68689957	0	0	0	0	0	BTP20220	Tissue specific
CDS|exon|ID=cds-B	9	6.425556	3865.4	0.266997	HYPSTHGLT	91	506.7437	2	1011.477	.	N	Superscaf	68689957	0	0	0	0	0	BTP20220	Tissue specific
CDS|exon|ID=cds-B	10	6.349	4324.9	0.266997	HYPSTHGLT	96	588.2764	2	1174.541	.	N	Superscaf	68689957	0	0	0	0	0	BTP20220	Tissue specific
CDS|exon|ID=cds-B	11	6.365455	4722.4	0.266997	HYPSTHGLT	87	638.799	2	1275.588	.	N	Superscaf	68689957	0	0	0	0	0	BTP20220	Tissue specific
CDS|exon|ID=cds-B	18	6.026667	6913.8	0.266997	HYPSTHGLT	92	1028.547	2	2055.09	.	N	Superscaf	68689957	0	0	0	0	0	BTP20220	Tissue specific
CDS|exon|ID=cds-B	14	5.731429	5318	0.266998	TYTVLPSRV	68	795.4503	2	1588.894	.	N	Superscaf	68689957	0	0	0	0	0	BTP20220	Tissue specific
CDS|exon|ID=cds-B	10	5.718	3445.9	0.266998	TYTVLPSRV	88	582.34	2	1162.671	.	N	Superscaf	68689957	0	0	0	0	0	BTP20220	Tissue specific
CDS|exon|ID=cds-B	8	5.62375	2588.9	0.266998	TVLPSRVK	98	450.2857	2	898.56	.	N	Superscaf	68689957	0	0	0	0	0	BTP20220	Tissue specific
CDS|exon|ID=cds-B	16	6.093125	6537.1	0.282907	AASGFNAAE	98	550.611	3	1648.817	.	D	Superscaf	69552580	0	0	0	0	0	BTP20220	Tissue specific
CDS|exon|ID=cds-B	10	4.996	3731.2	0.283067	VLYDVQELF	97	421.573	3	1261.703	.	A	Superscaf	69561259	0	0	0	0	0	BTP20220	Tissue specific
CDS|exon|ID=cds-B	11	4.863636	3845.4	0.28808	M(+42.01)	56	568.2487	2	1134.486	Acetylati	P	Superscaf	69833086	0	0	0	0	0	BTP20220	Tissue specific
CDS|exon|ID=cds-B	8	5.94625	2769.2	0.288085	ASRPPVTL	84	420.7489	2	839.4865	.	P	Superscaf	69833086	0	0	0	0	BTP20220	0	Tissue specific
CDS|exon|ID=cds-B	11	4.344545	3819.3	0.288088	VPQSVTEC(	95	637.8221	2	1273.634	Carbamido	P	Superscaf	69833086	0	0	0	0	BTP20220	0	Tissue specific
CDS|exon|ID=cds-B	9	6.202222	4109.6	0.291543	SSRALNEFE	63	530.7602	2	1059.51	.	K	Superscaf	70020637	0	0	0	0	0	BTP20220	Tissue specific
CDS|exon|ID=cds-B	6	5.998333	2407.8	0.354812	RFAVDR	98	382.2127	2	762.4136	.	A	Superscaf	73450735	0	0	BTP20220	0	0	0	Tissue specific

CDS|exon|ID=cds-Bg	11	5.854545	4475.2	0.377929	TLTSFERDA	89	659.309	2	1316.607	.	I	Superscaf	74704210	0	0	0	0	0	BTP202209 Tissue specific
CDS|exon|ID=cds-Bg	8	5.19625	2970.3	0.378042	FSPVDKMQ	98	476.2319	2	950.4531	.	Q	Superscaf	74710324	0	0	0	0	0	BTP202209 Tissue specific
CDS|exon|ID=cds-Bg	10	3.089	2325.5	0.378073	VDEVGVDVA	97	515.7728	2	1029.534	.	G	Superscaf	74711980	0	0	0	0	0	BTP202209 Tissue specific
CDS|exon|ID=cds-Bg	10	5.204	3980.6	0.378109	TPC(+57.0	94	577.2842	2	1152.56	Carbamido	L	Superscaf	74713885	0	0	BTP202209	BTP202209	0	0 Mixed
CDS|exon|ID=cds-Bg	14	5.811429	6217.8	0.378109	LVDHANNPN	92	563.288	3	1686.848	.	L	Superscaf	74713885	0	0	BTP202209	0	0	0 Tissue specific
CDS|exon|ID=cds-Bg	11	5.824545	4794.2	0.378109	VDHANNPNK	96	428.5538	3	1282.642	.	L	Superscaf	74713885	BTP202209	0	0	0	0	0 Tissue specific
CDS|exon|ID=cds-Bg	11	6.582727	5452.9	0.378109	HANNPNKKF	87	454.2288	3	1359.669	.	L	Superscaf	74713885	0	0	BTP202209	0	0	0 Tissue specific
CDS|exon|ID=cds-Bg	9	6.533333	4521.9	0.378109	NNPNKKFYG	99	576.7914	2	1151.572	.	L	Superscaf	74713885	0	0	BTP202209	BTP202209	0	0 Mixed
CDS|exon|ID=cds-Bg	7	5.805714	2601	0.38111	QGPVLLR	80	391.7505	2	781.481	.	D	Superscaf	74876734	0	0	0	BTP202209	0	0 Tissue specific
CDS|exon|ID=cds-Bg	7	6.488571	2548.6	0.384496	PAASSLK	61	673.3831	1	672.3806	.	F	Superscaf	75059377	0	0	0	0	0	BTP202209 Tissue specific
CDS|exon|ID=cds-Bg	7	5.45	2696.9	0.407691	A(+42.01)	74	777.3711	1	776.3704	Acetylati	F	Superscaf	76318062	0	0	0	0	0	BTP202209 Tissue specific
CDS|exon|ID=cds-Bg	7	4.75	2152.5	0.409361	VPAELLM(+	66	788.4369	1	787.415	Oxidation	R	Superscaf	76408180	0	BTP202209	0	0	0	0 Tissue specific
CDS|exon|ID=cds-Bg	7	7.137143	3033.3	0.409439	A(+42.01)	99	409.219	2	816.4276	Acetylati	L	Superscaf	76412566	0	0	0	BTP202209	0	0 Tissue specific
CDS|exon|ID=cds-Bg	9	6.08	3301.5	0.463664	LLKAEPLAF	74	501.303	2	1000.596	.	R	Superscaf	79350769	0	0	0	0	0	BTP202209 Tissue specific
CDS|exon|ID=cds-Bg	8	5.02875	2687.9	0.468894	AALGHVDS	84	769.3816	1	768.3766	.	A	Superscaf	79635487	0	0	BTP202209	0	0	0 Tissue specific
CDS|exon|ID=cds-Bg	11	5.898182	3548.7	0.469533	VPAAAAAAA	74	478.2627	2	954.5246	.	P	Superscaf	79670634	0	0	0	BTP202209	0	0 Tissue specific
CDS|exon|ID=cds-Bg	7	6.277143	2657	0.479888	VSMTFKR	99	434.7378	2	867.4636	.	R	Superscaf	80231881	0	0	0	BTP202209	0	0 Tissue specific
CDS|exon|ID=cds-Bg	9	6.135556	3435.8	0.536854	YAEATPMLK	87	512.2593	2	1022.511	.	A	Superscaf	83320282	0	0	0	0	0	BTP202209 Tissue specific
CDS|exon|ID=cds-Bg	7	5.951429	2833	0.553941	DSGYKLY	62	423.2023	2	844.3967	.	S	Superscaf	84246789	BTP202209	0	0	0	0	0 Tissue specific
CDS|exon|ID=cds-Bg	6	5.891667	2094.3	0.596746	KGMHVP	51	668.3435	1	667.3475	.	P	Superscaf	86567104	0	0	0	0	BTP202209	0 Tissue specific
CDS|exon|ID=cds-Bg	7	6.535714	2832.1	0.615759	KPFLPQP	72	413.7411	2	825.4749	.	Q	Superscaf	87598183	0	0	0	BTP202209	0	0 Tissue specific
CDS|exon|ID=cds-Bg	7	4.97	2582.9	0.62107	VGNQLPF	65	774.4111	1	773.4072	.	S	Superscaf	87885928	0	0	0	0	0	BTP202209 Tissue specific
CDS|exon|ID=cds-Bg	7	4.73	2196.5	0.622145	DLTGLSV	71	704.3815	1	703.3752	.	K	Superscaf	87944490	0	BTP202209	0	0	0	0 Tissue specific
CDS|exon|ID=cds-Bg	14	4.348571	5187.9	0.62678	LSDVELDDL	89	816.8928	2	1631.778	.	P	Superscaf	88195642	0	0	0	0	0	BTP202209 Tissue specific
CDS|exon|ID=cds-Bg	10	5.414	4260.8	0.645389	LDTLNEDSY	98	599.2826	2	1196.556	.	V	Superscaf	89204557	0	0	0	0	0	BTP202209 Tissue specific
CDS|exon|ID=cds-Bg	9	5.426667	3123.3	0.761354	M(+42.01)	99	496.739	2	991.4644	Acetylati	V	Superscaf	95491639	0	BTP202209	0	0	BTP202209	BTP202209 Mixed
CDS|exon|ID=cds-Bg	6	6.575	2889.2	0.766538	KC(+57.02	69	436.7056	2	871.401	Carbamido	Y	Superscaf	95772696	0	0	0	BTP202209	0	0 Tissue specific
CDS|exon|ID=cds-Bg	5	4.902	1479.7	0.792553	VGTFT	75	524.2697	1	523.2642	.	C	Superscaf	97183347	0	0	BTP202209	0	0	0 Tissue specific
CDS|exon|ID=cds-Bg	8	7.1025	3723.3	0.79359	A(+42.01)	80	473.7554	2	945.4992	Acetylati	V	Superscaf	97239277	0	0	0	BTP202209	0	0 Tissue specific
CDS|exon|ID=cds-Bg	5	4.668	1600.8	0.797031	LNVGL	72	515.3163	1	514.3115	.	S	Superscaf	97425936	0	0	0	0	0	BTP202209 Tissue specific
CDS|exon|ID=cds-Bg	9	7.44	4186.8	0.847305	RSSSSRLLF	99	354.5431	3	1060.61	.	G	Superscaf	1E+08	0	0	BTP202209	0	BTP202209	0 Mixed
CDS|exon|ID=cds-Bg	9	4.206667	2911.5	0.847351	TVRVVNTDY	67	533.7786	2	1065.545	.	P	Superscaf	1E+08	0	0	0	0	BTP202209	0 Tissue specific
CDS|exon|ID=cds-Bg	7	4.645714	2575	0.850058	ETVHELL	83	420.726	2	839.4388	.	I	Superscaf	1E+08	BTP202209	BTP202209	BTP202209	BTP202209	BTP202209	BTP202209 Mixed
CDS|exon|ID=cds-Bg	5	3.724	986.1	0.852495	A(+42.01)	58	524.3173	1	523.3005	Acetylati	W	Superscaf	1E+08	0	0	BTP202209	0	0	0 Tissue specific
CDS|exon|ID=cds-Bg	8	5.3625	3046.3	0.853619	DLAALEDK	75	437.7286	2	873.4443	.	S	Superscaf	1E+08	0	0	0	BTP202209	0	0 Tissue specific
CDS|exon|ID=cds-Bg	9	6.195556	4354	0.853638	ALLDRHQEH	85	373.527	3	1117.563	.	G	Superscaf	1E+08	0	BTP202209	0	0	0	0 Tissue specific
CDS|exon|ID=cds-Bg	19	5.235263	7114.8	0.853699	LLNEDLGDS	99	691.3728	3	2071.105	.	S	Superscaf	1E+08	0	0	0	0	0	BTP202209 Tissue specific
CDS|exon|ID=cds-Bg	6	3.671667	1513.8	0.85372	TVEVPL	77	657.3784	1	656.3745	.	L	Superscaf	1E+08	0	0	0	0	0	BTP202209 Tissue specific
CDS|exon|ID=cds-Bg	7	7.327143	3012.5	0.854077	A(+42.01)	79	415.7181	2	829.4229	Acetylati	E	Superscaf	1.01E+08	BTP202209	0	0	0	0	0 Tissue specific
CDS|exon|ID=cds-Bg	9	4.99	3238.5	0.854148	KLLVSSEDY	85	527.274	2	1052.539	.	T	Superscaf	1.01E+08	0	0	0	0	0	BTP202209 Tissue specific
CDS|exon|ID=cds-Bg	9	5.583333	3834.2	0.854195	KHEAFETDF	61	562.2554	2	1122.498	.	S	Superscaf	1.01E+08	0	0	0	0	0	BTP202209 Tissue specific
CDS|exon|ID=cds-Bg	8	5.06375	3365.8	0.854195	HEAFETDF	65	498.2071	2	994.4032	.	S	Superscaf	1.01E+08	0	0	0	0	0	BTP202209 Tissue specific
CDS|exon|ID=cds-Bg	11	5.558182	3964.2	0.854263	VEDLFLTFA	99	655.8694	2	1309.728	.	A	Superscaf	1.01E+08	0	0	0	0	0	BTP202209 Tissue specific
CDS|exon|ID=cds-Bg	10	5.14	3495.8	0.854263	VEDLFLTFA	99	591.8209	2	1181.633	.	A	Superscaf	1.01E+08	0	0	0	0	0	BTP202209 Tissue specific
CDS|exon|ID=cds-Bg	12	4.764167	4451.1	0.854361	DYVEFTRSL	92	745.3685	2	1488.725	.	S	Superscaf	1.01E+08	0	BTP202209	0	0	BTP202209	BTP202209 Mixed
CDS|exon|ID=cds-Bg	8	5.0375	3113.6	0.854361	DYVEFTRS	94	508.7368	2	1015.461	.	S	Superscaf	1.01E+08	0	BTP202209	0	0	0	0 Tissue specific
CDS|exon|ID=cds-Bg	7	6.235714	2673	0.854568	ADRLGGL	78	351.1995	2	700.3868	.	S	Superscaf	1.01E+08	0	0	BTP202209	0	0	0 Tissue specific
CDS|exon|ID=cds-Bg	12	6.178333	4222.2	0.855134	S(+42.01)	94	635.8692	2	1269.729	Acetylati	A	Superscaf	1.01E+08	0	0	0	BTP202209	0	0 Tissue specific
CDS|exon|ID=cds-Bg	9	6.133333	2974.9	0.855134	S(+42.01)	88	450.7592	2	899.5076	Acetylati	A	Superscaf	1.01E+08	0	0	0	BTP202209	0	0 Tissue specific
CDS|exon|ID=cds-Bg	7	6.55	2988.5	0.874943	NPRLM(+15	54	395.6902	2	789.3803	Oxidation	P	Superscaf	1.02E+08	0	0	0	BTP202209	0	0 Tissue specific
CDS|exon|ID=cds-Bg	5	7.052	2182.3	0.875634	HGPKY	76	601.2977	1	600.3019	.	K	Superscaf	1.02E+08	0	BTP202209	0	0	0	0 Tissue specific
CDS|exon|ID=cds-Bg	5	6.456	2133.2	0.883918	SGNFK	61	552.2791	1	551.2703	.	L	Superscaf	1.02E+08	0	0	0	0	0	BTP202209 Tissue specific
CDS|exon|ID=cds-Bg	10	6.054	4580.2	0.886291	EYHRLQNKV	97	644.3427	2	1286.673	.	F	Superscaf	1.02E+08	0	0	BTP202209	0	0	0 Tissue specific
CDS|exon|ID=cds-Bg	13	4.785385	4309.8	0.887399	A(+42.01)	65	664.8278	2	1327.644	Acetylati	M	Superscaf	1.02E+08	0	0	0	0	0	BTP202209 Tissue specific
CDS|exon|ID=cds-Bg	5	5.698	1894.2	0.892087	ARVPN	63	556.3228	1	555.3129	.	P	Superscaf	1.03E+08	0	0	0	BTP202209	0	0 Tissue specific
CDS|exon|ID=cds-Bg	10	6.18	3997.5	0.894006	STLEGRPSA	82	540.7662	2	1079.525	.	L	Superscaf	1.03E+08	0	0	0	0	BTP202209	BTP202209 Mixed
CDS|exon|ID=cds-Bg	6	5.768333	1777.9	0.895419	KPVGPP	54	594.3586	1	593.3536	.	G	Superscaf	1.03E+08	0	0	BTP202209	0	0	0 Tissue specific
CDS|exon|ID=cds-Bg	8	4.6625	2163.3	0.896578	APGVPDVK	50	391.7193	2	781.4333	.	G	Superscaf	1.03E+08	0	BTP202209	0	0	0	0 Tissue specific
CDS|exon|ID=cds-Bg	7	5.942857	2303.6	0.900558	A(+42.01)	80	357.198	2	712.3868	Acetylati	T	Superscaf	1.03E+08	0	0	0	BTP202209	0	0 Tissue specific
CDS|exon|ID=cds-Bg	10	6.625	3960.7	0.913774	LPPPPPRPF	76	541.3093	2	1080.608	.	A	Superscaf	1.04E+08	0	0	0	BTP202209	0	0 Tissue specific
CDS|exon|ID=cds-Bg	10	5.551	4044.7	0.924825	M(+42.01)	79	639.8101	2	1277.611	Acetylati	A	Superscaf	1.04E+08	0	0	0	BTP202209	0	0 Tissue specific
CDS|exon|ID=cds-Bg	7	5.872857	2345.7	0.925278	RGVPFPP	78	385.219	2	768.4282	.	V	Superscaf	1.04E+08	0	0	BTP202209	0	0	0 Tissue specific
CDS|exon|ID=cds-Bg	8	7.015	3295.6	0.931903	SSLPPKSR	59	436.2464	2	870.4922	.	T	Superscaf	1.05E+08	0	BTP202209	0	0	0	0 Tissue specific
CDS|exon|ID=cds-Bg	8	6.69625	3052.2	0.932769	GHSGPAPK	72	375.7022	2	749.382	.	T	Superscaf	1.05E+08	0	0	0	0	0	BTP202209 Tissue specific
CDS|exon|ID=cds-Bg	15	6.09	4559.3	0.949007	KAKGKKVAF	98	746.4872	2	1490.966	.	C	Superscaf	1.06E+08	0	0	0	0	BTP202209	0 Tissue specific
CDS|exon|ID=cds-Bg	11	5.443636	3013.9	0.949007	KVAPAPAVV	89	369.912	3	1106.718	.	C	Superscaf	1.06E+08	0	BTP202209	0	0	0	BTP202209 Mixed
CDS|exon|ID=cds-Bg	10	5.014	2545.5	0.949007	KVAPAPAVV	98	490.3182	2	978.6226	.	C	Superscaf	1.06E+08	0	BTP202209	0	0	BTP202209	BTP202209 Mixed
CDS|exon|ID=cds-Bg	10	5.014	2545.5	0.949007	VAPAPAVVK	98	490.3181	2	978.6226	.	C	Superscaf	1.06E+08	0	BTP202209	0	0	0	0 Tissue specific
CDS|exon|ID=cds-Bg	9	4.488889	2077.1	0.949007	VAPAPAVVK	96	426.2704	2	850.5276	.	C	Superscaf	1.06E+08	0	BTP202209	0	0	BTP202209	BTP202209 Mixed
CDS|exon|ID=cds-Bg	8	5.515	2234.2	0.949007	PAPAVVKK	78	405.2631	2	808.517	.	C	Superscaf	1.06E+08	0	0	0	0	BTP202209	0 Tissue specific
CDS|exon|ID=cds-Bg	6	6.211667	2775.2	0.950353	ESRLLQ	51	373.2113	2	744.413	.	G	Superscaf	1.06E+08	0	0	0	BTP202209	0	0 Tissue specific
CDS|exon|ID=cds-Bg	7	7.348571	2752	0.963112	PAAKPPR	69	368.7203	2	735.4391	.	A	Superscaf	1.06E+08	0	0	BTP202209	0	0	0 Tissue specific
CDS|exon|ID=cds-Bg	7	5.95	2844.2	0.976014	KPQVELR	63	435.2681	2	868.513	.	H	Superscaf	1.07E+08	0	0	0	BTP202209	0	0 Tissue specific
CDS|exon|ID=cds-Bg	5	4.916	1395.6	0.985002	VPPSP	55	496.2707	1	495.2693	.	Y	Superscaf	1.08E+08	0	0	0	BTP202209	0	0 Tissue specific
CDS|exon|ID=cds-Bg	10	5.677	4515.2	0.985842	HGDSDLQLE	98	585.2804	2	1168.547	.	F	Superscaf	1.08E+08	0	BTP202209	0	0	0	0 Tissue specific
CDS|exon|ID=cds-Bg	6	6.405	2211.4	0.985844	G(+42.01)	89	381.2139	2	760.4232	Acetylati	S	Superscaf	1.08E+08	0	0	0	BTP202209	0	0 Tissue specific
CDS|exon|ID=cds-Bg	9	5.078889	2694.9	0.98586	TFSVVPSPK	93	481.2704	2	960.528	.	A	Superscaf	1.08E+08	0	BTP202209	0	0	0	0 Tissue specific
CDS|exon|ID=cds-Bg	8	4.8975	2297.4	0.98586	FSVVPSPK	99	430.7468	2	859.4803	.	A	Superscaf	1.08E+08	0	BTP202209	0	0	0	BTP202209 Mixed
CDS|exon|ID=cds-Bg	9	4.353333	2297.4	0.98586	FSVVPSPKV	95	480.2796	2	958.5487	.	A	Superscaf	1.08E+08	0	0	0	BTP202209	0	0 Tissue specific
CDS|exon|ID=cds-Bg	8	4.58375	2321.5	0.98586	VPSPKVSD	93	414.7252	2	827.4388	.	A	Superscaf	1.08E+08	0	0	0	BTP202209	0	0 Tissue specific
CDS|exon|ID=cds-Bg	15	4.252667	4538.1	0.98586	VPSPKVSDT	81	815.9163	2	1629.825	.	A	Superscaf	1.08E+08	0	0	0	BTP202209	0	0 Tissue specific
CDS|exon|ID=cds-Bg	14	4.17	3941.4	0.98586	VPSPKVSDT	84	758.8956	2	1515.782	.	A	Superscaf	1.08E+08	0	0	0	0	BTP202209	BTP202209 Tissue enhanced
CDS|exon|ID=cds-Bg	7	6.058571	2684.1	0.985865	VPFPRLH	72	433.254	2	864.4969	.	A	Superscaf	1.08E+08	0	0	0	BTP202209	0	0 Tissue specific
CDS|exon|ID=cds-Bg	9	6.001111	3176.6	0.985866	MPGFAPLTS	76	920.4513	1	919.4473	.	A	Superscaf	1.08E+08	0	0	0	BTP202209	0	0 Tissue specific
CDS|exon|ID=cds-Bg	9	6.395556	4144.7	0.985866	LTSRGSQQY	60	520.2599	2	1038.509	.	A	Superscaf	1.08E+08	0	0	0	0	0	BTP202209 Tissue specific
CDS|exon|ID=cds-Bg	8	6.705	2878.2	0.973888	GPARAGPP	73	361.6998	2	721.3871	.	S	Superscaf	1228140	0	0	BTP202209	0	0	0 Tissue specific
CDS|exon|ID=cds-Bg	6	6.708333	2035.1	0.965709	KGGGPP	62	512.282	1	511.2754	.	N	Superscaf	1612429	0	0	BTP202209	0	BTP202209	0 Mixed
CDS|exon|ID=cds-Bg	6	6.381667	2441.9	0.890884	DLLRPP	75	355.7119	2	709.4122	.	L	Superscaf	5131075	0	0	BTP202209	0	0	0 Tissue specific
CDS|exon|ID=cds-Bg	7	5.327143	2275.5	0.758437	PVSPKDP	58	739.3843	1	738.3912	.	Q	Superscaf	11359294	0	0	0	0	BTP202209	0 Tissue specific
CDS|exon|ID=cds-Bg	9	6.126667	4197.8	0.755527	YEEHLERMK	96	412.2001	3	1233.581	.	T	Superscaf	11495836	0	BTP202209	0	0	0	0 Tissue specific
CDS|exon|ID=cds-Bg	7	6.328571	3039.2	0.737797	LNAFAKQ	57	791.4382	1	790.4337	.	R	Superscaf	12329800	0	0	0	BTP202209	0	0 Tissue specific
CDS|exon|ID=cds-Bg	6	6.796667	2528.9	0.725501	R(+42.01)	68	413.7467	2	825.4821	Acetylati	S	Superscaf	12908121	0	0	0	0	BTP202209	0 Tissue specific
CDS|exon|ID=cds-Bg	5	3.65	988.1	0.716432	LVPVG	60	484.3122	1	483.3057	.	I	Superscaf	13334242	BTP202209	0	0	0	0	0 Tissue specific
CDS|exon|ID=cds-Bg	19	5.938421	7538.3	0.677363	ASDTHC(+5	80	979.91	2	1957.811	Carbamido	D	Superscaf	15171616	0	0	0	0	0	BTP202209 Tissue specific
CDS|exon|ID=cds-Bg	12	6.0875	4531.9	0.677363	YGDSAAKGT	95	615.7833	2	1229.556	.	D	Superscaf	15171616	0	0	0	0	BTP202209	0 Tissue specific
CDS|exon|ID=cds-Bg	16	5.6375	5841.3	0.677363	YGDSAAKGT	61	838.3991	2	1674.789	.	D	Superscaf	15171616	0	0	0	0	BTP202209	0 Tissue specific
CDS|exon|ID=cds-Bg	6	3.545	1413.6	0.652846	ADVVLP	56	613.3536	1	612.3483	.	V	Superscaf	16324402	0	BTP202209	0	0	0	0 Tissue specific
CDS|exon|ID=cds-Bg	5	6.748	1797.8	0.647254	LLKAA	55	515.3572	1	514.3478	.	N	Superscaf	16587882	0	BTP202209	0	0	0	0 Tissue specific
CDS|exon|ID=cds-Bg	8	5.5725	3353.9	0.6219	LLNNPELP	64	455.2603	2	908.4967	.	C	Superscaf	17779801	BTP202209	0	BTP202209	0	BTP202209	0 Mixed
CDS|exon|ID=cds-Bg	7	5.312857	1958.2	0.508495	APGVPPP	79	634.3556	1	633.3486	.	N	Superscaf	23112481	0	0	BTP202209	0	0	0 Tissue specific
CDS|exon|ID=cds-Bg	8	5.01125	2399.7	0.506536	EPVGAPLP	51	390.2182	2	778.4225	.	A	Superscaf	23204641	0	0	0	BTP202209	0	0 Tissue specific
CDS|exon|ID=cds-Bg	13	5.885385	4943.5	0.487404	LGLASALRF	90	681.3537	2	1360.699	.	M	Superscaf	24104293	0	0	0	0	0	BTP202209 Tissue specific
CDS|exon|ID=cds-Bg	13	6.066154	5134.7	0.487093	STTGGETEE	98	690.8435	2	1379.678	.	T	Superscaf	24118972	0	BTP202209	0	0	0	0 Tissue specific
CDS|exon|ID=cds-Bg	12	5.76	4666.3	0.487093	STTGGETEE	98	626.7972	2	1251.583	.	T	Superscaf	24118972	0	BTP202209	0	0	0	0 Tissue specific
CDS|exon|ID=cds-Bg	12	6.098333	4751.3	0.487093	TTGGETEET	99	647.3289	2	1292.646	.	T	Superscaf	24118972	0	BTP202209	0	0	0	0 Tissue specific
CDS|exon|ID=cds-Bg	7	4.425714	1869.9	0.406359	V(+42.01)	60	771.4583	1	770.4538	Acetylati	L	Superscaf	27915127	0	0	BTP202209	0	0	0 Tissue specific
CDS|exon|ID=cds-Bg	10	6.833	3790.1	0.396411	LLRGLPGSG	90	499.3091	2	996.608	.	F	Superscaf	28382056	0	0	0	0	0	BTP202209 Tissue specific
CDS|exon|ID=cds-Bg	12	6.31	4644.8	0.369116	KVDQPPEAK	75	455.5992	3	1363.782	.	E	Superscaf	29666755	0	0	0	0	0	BTP202209 Tissue specific
CDS|exon|ID=cds-Bg	8	6.7	2920.3	0.361087	G(+42.01)	88	403.733	2	805.4446	Acetylati	E	Superscaf	30044439	0	0	0	BTP202209	0	0 Tissue specific
CDS|exon|ID=cds-Bg	13	6.406923	5198.3	0.358002	SAKEKGKFE	99	490.2521	3	1467.739	.	Q	Superscaf	30189331	0	0	0	0	0	BTP202209 Tissue specific
CDS|exon|ID=cds-Bg	12	6.4675	4814.9	0.358002	AKEKGKFED	99	461.2411	3	1380.707	.	Q	Superscaf	30189331	0	0	0	0	BTP202209	BTP202209 Mixed
CDS|exon|ID=cds-Bg	10	6.984	4254.7	0.358	KDPNAPKRF	95	373.8788	3	1118.62	.	Q	Superscaf	30189331	0	0	0	0	BTP202209	BTP202209 Mixed
CDS|exon|ID=cds-Bg	8	6.83875	3349.8	0.321213	LGAAERLR	91	443.2651	2	884.5192	.	H	Superscaf	31919248	0	0	BTP202209	0	0	0 Tissue specific
CDS|exon|ID=cds-Bg	10	6.397	4262.9	0.321213	AAERLRFTE	64	603.3351	2	1204.656	.	H	Superscaf	31919248	0	0	BTP202209	0	0	0 Tissue specific
CDS|exon|ID=cds-Bg	8	5.26125	3100.5	0.321209	ADLLGEQP	75	842.4232	1	841.4181	.	H	Superscaf	31919248	0	0	BTP202209	0	0	0 Tissue specific
CDS|exon|ID=cds-Bg	7	5.287143	2434.7	0.31642	LFSHVLP	84	812.4635	1	811.4592	.	I	Superscaf	32144761	0	0	0	0	0	BTP202209 Tissue specific
CDS|exon|ID=cds-Bg	8	5.86125	2516.7	0.312526	AAPKTVLP	54	398.7542	2	795.4854	.	K	Superscaf	32327845	0	0	0	BTP202209	0	0 Tissue specific
CDS|exon|ID=cds-Bg	11	6.845455	5277.9	0.238248	RLAQLKHQL	67	444.6001	3	1330.783	.	L	Superscaf	35820640	0	0	BTP202209	0	0	0 Tissue specific
CDS|exon|ID=cds-Bg	7	5.39	2702.1	0.234665	Q(+42.01)	51	412.225	2	822.4236	Acetylati	L	Superscaf	35989282	0	0	0	0	0	BTP202209 Tissue specific
CDS|exon|ID=cds-Bg	7	6.428571	2774.1	0.228065	QKATVPR	51	400.2467	2	798.4711	.	V	Superscaf	36299569	0	0	0	0	0	BTP202209 Tissue specific
CDS|exon|ID=cds-Bg	7	4.67	2196.5	0.163646	LVLLDPF	77	816.4827	1	815.4792	.	I	Superscaf	39328612	0	0	0	0	0	BTP202209 Tissue specific
CDS|exon|ID=cds-Bg	10	5.66	4651.3	0.132379	RQHEEPESF	68	653.2953	2	1304.579	.	K	Superscaf	40798819	0	0	0	0	0	BTP202209 Tissue specific
CDS|exon|ID=cds-Bg	9	5.68	4263.9	0.132379	RQHEEPESF	80	579.7604	2	1157.51	.	K	Superscaf	40798819	0	0	0	0	0	BTP202209 Tissue specific
CDS|exon|ID=cds-Bg	5	3.714	1020.1	0.031239	A(+42.01)	57	502.2858	1	501.2798	Acetylati	P	Superscaf	48493210	0	BTP202209	0	0	0	0 Tissue specific
CDS|exon|ID=cds-Bg	13	5.631538	5084.4	0.066066	VFGQSGAGN	94	668.3234	2	1334.637	.	S	Superscaf	50130805	0	0	0	0	0	BTP202209 Tissue specific
CDS|exon|ID=cds-Bg	8	5.22125	3615.1	0.066068	SC(+57.02	71	493.6867	2	985.3633	Carbamido	S	Superscaf	50130805	0	0	0	0	BTP202209	BTP202209 Mixed
CDS|exon|ID=cds-Bg	8	4.2125	1910	0.066071	VVPSPKVS	70	406.7463	2	811.4803	.	S	Superscaf	50130805	0	0	0	0	0	BTP202209 Tissue specific
CDS|exon|ID=cds-Bg	7	4.017143	2392.7	0.066072	SVHQLVE	66	406.2182	2	810.4235	.	S	Superscaf	50130805	0	BTP202209	0	0	0	0 Tissue specific
CDS|exon|ID=cds-Bg	9	5.422222	4273	0.080351	M(+42.01)	98	588.2253	2	1174.438	Acetylati	K	Superscaf	50802592	0	BTP202209	0	0	0	0 Tissue specific
CDS|exon|ID=cds-Bg	13	6.281538	6413.5	0.080351	M(+42.01)	83	579.9178	3	1736.736	Acetylati	K	Superscaf	50802592	0	BTP202209	0	0	0	0 Tissue specific
CDS|exon|ID=cds-Bg	12	5.908333	5764.7	0.080351	M(+42.01)	98	527.8845	3	1580.635	Acetylati	K	Superscaf	50802592	0	BTP202209	0	0	0	0 Tissue specific
CDS|exon|ID=cds-Bg	11	5.467273	5115.9	0.080351	M(+42.01)	75	713.2727	2	1424.534	Acetylati	K	Superscaf	50802592	0	BTP202209	0	0	0	0 Tissue specific

CDS|exon|ID=cds-Bg	11	5.605455	4584.3	0.081361	TTELDDFPT	96	640.8027	2	1279.593	.	C	Superscaf	50850151	0	BTP202209	0	0	0	0	Tissue specific
CDS|exon|ID=cds-Bg	10	5.513	4186.8	0.081362	TELDDFPTN	97	590.2783	2	1178.545	.	C	Superscaf	50850151	0	BTP202209	0	0	0	0	Tissue specific
CDS|exon|ID=cds-Bg	8	5.6725	3363.8	0.081362	LDDFPTNK	98	475.2334	2	948.4553	.	C	Superscaf	50850151	0	BTP202209	0	0	0	0	Tissue specific
CDS|exon|ID=cds-Bg	12	6.035833	4725.2	0.081418	KEPAPSGAE	84	428.5571	3	1282.652	.	C	Superscaf	50852752	0	BTP202209	0	0	0	0	Tissue specific
CDS|exon|ID=cds-Bg	6	5.688333	2572	0.110748	N(+42.01)	64	723.2413	1	722.2364	Acetylati	S	Superscaf	52232109	0	0	BTP202209	0	0	0	Tissue specific
CDS|exon|ID=cds-Bg	14	6.270714	5060.6	0.120518	STPASTSTT	98	703.3796	2	1404.746	.	Q	Superscaf	52691470	0	BTP202209	0	0	0	0	Tissue specific
CDS|exon|ID=cds-Bg	13	6.003846	4592.2	0.120518	STPASTSTT	78	639.3322	2	1276.651	.	Q	Superscaf	52691470	0	BTP202209	0	0	0	0	Tissue specific
CDS|exon|ID=cds-Bg	20	6.74	7292.3	0.164436	PEPAKSAPA	96	505.8026	4	2019.184	.	P	Superscaf	54756598	0	BTP202209	0	0	BTP202209	0	Mixed
CDS|exon|ID=cds-Bg	6	5.57	1903.9	0.176034	A(+42.01)	98	706.4123	1	705.4061	Acetylati	T	Superscaf	55301836	0	BTP202209	BTP202209	BTP202209	BTP202209	BTP202209	Mixed
CDS|exon|ID=cds-Bg	13	6.146923	4666.7	0.17605	AMEAVAAQC	99	434.9106	3	1301.712	.	L	Superscaf	55302541	0	BTP202209	0	0	BTP202209	BTP202209	Mixed
CDS|exon|ID=cds-Bg	12	6.1575	4355.4	0.17605	MEAVAAQGK	70	411.2312	3	1230.675	.	L	Superscaf	55302541	0	0	0	0	0	BTP202209	Tissue specific
CDS|exon|ID=cds-Bg	7	6.5	3065.5	0.178039	EHSGPLR	62	398.2082	2	794.4035	.	R	Superscaf	55396255	0	0	0	0	BTP202209	0	Tissue specific
CDS|exon|ID=cds-Bg	12	5.303333	4513.9	0.19393	DSTVC(+57	84	661.8137	2	1321.618	Carbamido	R	Superscaf	56143165	0	0	0	0	0	BTP202209	Tissue specific
CDS|exon|ID=cds-Bg	9	5.041111	3261.6	0.193932	AAGHDC(+5	72	973.4147	1	972.4123	Carbamido	R	Superscaf	56143165	0	0	0	0	BTP202209	0	Tissue specific
CDS|exon|ID=cds-Bg	8	4.91875	2950.3	0.193932	AGHDC(+57	76	451.6937	2	901.3752	Carbamido	R	Superscaf	56143165	0	0	0	0	BTP202209	0	Tissue specific
CDS|exon|ID=cds-Bg	10	6.099	3912.1	0.193939	SLESALKDL	99	552.3168	2	1102.623	.	R	Superscaf	56143165	0	0	0	0	0	BTP202209	Tissue specific
CDS|exon|ID=cds-Bg	8	5.65875	3090.3	0.193939	SLESALKD	81	431.7285	2	861.4443	.	R	Superscaf	56143165	0	0	0	0	BTP202209	0	Tissue specific
CDS|exon|ID=cds-Bg	5	5.788	2861.3	0.217984	HYQC(+57.	55	767.2599	1	766.2527	Carbamido	N	Superscaf	57274744	BTP202209	0	0	0	0	0	Tissue specific
CDS|exon|ID=cds-Bg	6	5.15	1676.9	0.440602	A(+42.01)	63	635.3827	1	634.369	Acetylati	T	Superscaf	67743249	0	0	0	0	BTP202209	0	Tissue specific
CDS|exon|ID=cds-Bg	5	4.384	1798	0.453573	SSHVD	56	544.2391	1	543.2289	.	P	Superscaf	68353149	0	BTP202209	BTP202209	0	BTP202209	0	Mixed
CDS|exon|ID=cds-Bg	6	3.893333	1323.6	0.524627	VRVVPP	54	666.4243	1	665.4224	.	L	Superscaf	71694373	0	0	BTP202209	0	0	0	Tissue specific
CDS|exon|ID=cds-Bg	5	7.87	2247.3	0.531712	KHKPL	51	622.3995	1	621.3962	.	T	Superscaf	72027529	0	BTP202209	0	0	0	0	Tissue specific
CDS|exon|ID=cds-Bg	6	6.748333	2151.3	0.536978	LGGKPT	63	572.338	1	571.3329	.	L	Superscaf	72275256	0	0	0	0	0	BTP202209	Tissue specific
CDS|exon|ID=cds-Bg	15	5.436667	5496.8	0.696806	GVNADVEKE	93	546.3027	3	1635.894	.	E	Superscaf	79790911	0	0	0	0	0	BTP202209	Tissue specific
CDS|exon|ID=cds-Bg	14	5.129286	5028.4	0.696806	GVNADVEKE	97	754.9034	2	1507.799	.	E	Superscaf	79790911	0	0	0	0	0	BTP202209	Tissue specific
CDS|exon|ID=cds-Bg	17	5.283529	7205.9	0.697286	SNPSENEEK	87	958.9601	2	1915.912	.	F	Superscaf	79813543	0	0	0	0	0	BTP202209	Tissue specific
CDS|exon|ID=cds-Bg	7	6.874286	2813.3	0.724154	ATRSTPP	51	365.1931	2	728.3817	.	R	Superscaf	81077020	BTP202209	0	0	0	0	0	Tissue specific
CDS|exon|ID=cds-Bg	6	6.143333	1918.1	0.731248	APPGGP	65	495.252	1	494.2489	.	T	Superscaf	81410497	0	BTP202209	0	0	0	0	Tissue specific
CDS|exon|ID=cds-Bg	7	5.204286	2108.5	0.731464	VM(+15.99	67	400.7062	2	799.3938	Oxidation	G	Superscaf	81420610	0	0	0	BTP202209	0	0	Tissue specific
CDS|exon|ID=cds-Bg	9	4.567778	2727.1	0.733431	MREYKVVVL	64	379.5532	3	1135.642	.	V	Superscaf	81513205	0	0	BTP202209	0	0	0	Tissue specific
CDS|exon|ID=cds-Bg	12	4.585	2862.8	0.733431	KVVVLGSGC	98	550.3433	2	1098.676	.	V	Superscaf	81513205	0	0	0	0	0	BTP202209	Tissue specific
CDS|exon|ID=cds-Bg	11	4.116364	2394.4	0.733431	VVVLGSGGV	97	486.2958	2	970.5811	.	V	Superscaf	81513205	0	0	0	0	0	BTP202209	Tissue specific
CDS|exon|ID=cds-Bg	10	5.575	4201.5	0.742046	A(+42.01)	87	538.7521	2	1075.493	Acetylati	G	Superscaf	81918169	0	0	0	0	BTP202209	BTP202209	Mixed
CDS|exon|ID=cds-Bg	11	5.582727	4661	0.742046	A(+42.01)	59	620.2834	2	1238.557	Acetylati	G	Superscaf	81918169	0	0	0	0	0	BTP202209	Tissue specific
CDS|exon|ID=cds-Bg	12	5.548333	4269.4	0.749883	VLDYVKEGC	89	458.2565	3	1371.751	.	E	Superscaf	82286806	0	BTP202209	0	0	0	0	Tissue specific
CDS|exon|ID=cds-Bg	11	5.325455	3821.2	0.770032	FYLPGLAPV	80	619.3326	2	1236.654	.	S	Superscaf	83234137	0	0	0	0	0	BTP202209	Tissue specific
CDS|exon|ID=cds-Bg	14	5.192143	4670.9	0.783315	SSAGTVEFL	85	734.3848	2	1466.762	.	V	Superscaf	83858845	0	0	0	0	0	BTP202209	Tissue specific
CDS|exon|ID=cds-Bg	8	5.625	3069.2	0.783315	LVDSKKNF	92	475.7664	2	949.5233	.	V	Superscaf	83858845	0	0	0	0	0	BTP202209	Tissue specific
CDS|exon|ID=cds-Bg	9	5.628889	3528.7	0.783315	LVDSKKNFY	99	557.2982	2	1112.587	.	V	Superscaf	83858845	0	0	0	0	0	BTP202209	Tissue specific
CDS|exon|ID=cds-Bg	11	5.556364	4029.5	0.823494	A(+42.01)	63	611.7826	2	1221.555	Acetylati	R	Superscaf	85748209	0	0	0	0	0	BTP202209	Tissue specific
CDS|exon|ID=cds-Bg	6	4.9	1891.1	0.826006	YVLFPL	75	376.2223	2	750.4316	.	L	Superscaf	85866526	BTP202209	0	0	0	0	0	Tissue specific
CDS|exon|ID=cds-Bg	5	4.918	1283.4	0.832561	A(+42.01)	65	482.2576	1	481.2536	Acetylati	A	Superscaf	86174761	0	0	0	BTP202209	0	0	Tissue specific
CDS|exon|ID=cds-Bg	7	5.642857	2535.8	0.906672	YELAAPP	69	760.3805	1	759.3802	.	S	Superscaf	89659729	0	0	0	0	0	BTP202209	Tissue specific
CDS|exon|ID=cds-Bg	15	6.120667	6050.4	0.91257	DAKGDKAKV	92	421.9782	4	1683.89	.	R	Superscaf	89937004	0	0	BTP202209	BTP202209	BTP202209	0	Mixed
CDS|exon|ID=cds-Bg	17	7.085882	6643	0.912572	ARLSAKPAP	86	363.2216	5	1811.078	.	R	Superscaf	89937004	0	BTP202209	0	0	0	0	Tissue specific
CDS|exon|ID=cds-Bg	16	7.1525	6331.7	0.912572	RLSAKPAPP	95	436.0167	4	1740.041	.	R	Superscaf	89937004	0	BTP202209	0	0	0	0	Tissue specific
CDS|exon|ID=cds-Bg	15	6.912	5682.9	0.912572	LSAKPAPPK	95	528.9871	3	1583.94	.	R	Superscaf	89937004	0	BTP202209	0	0	0	0	Tissue specific
CDS|exon|ID=cds-Bg	14	6.978571	5329.5	0.912572	SAKPAPPKP	96	491.2913	3	1470.856	.	R	Superscaf	89937004	0	BTP202209	0	0	BTP202209	BTP202209	Mixed
CDS|exon|ID=cds-Bg	7	6.168571	2479.5	0.923478	APNGVKK	54	357.2231	2	712.4232	.	K	Superscaf	90450132	0	0	0	0	0	BTP202209	Tissue specific
CDS|exon|ID=cds-Bg	8	5.84875	2842.1	0.947241	A(+42.01)	59	413.2327	2	824.4504	Acetylati	E	Superscaf	91566538	0	0	0	BTP202209	0	0	Tissue specific
CDS|exon|ID=cds-Bg	7	6.634286	2621.1	0.957233	A(+42.01)	82	417.7499	2	833.4871	Acetylati	E	Superscaf	92037310	0	BTP202209	0	0	0	0	Tissue specific
CDS|exon|ID=cds-Bg	11	5.668182	4091.5	0.957919	PVGMNKLWS	87	626.3109	2	1250.612	.	P	Superscaf	92069437	0	0	0	0	0	BTP202209	Tissue specific
CDS|exon|ID=cds-Bg	6	8.051667	2605.8	0.966237	KPM(+15.9	51	373.7252	2	745.4269	Oxidation	M	Superscaf	92460888	0	0	0	BTP202209	0	0	Tissue specific
CDS|exon|ID=cds-Bg	7	4.895714	1898	0.973861	T(+42.01)	80	743.428	1	742.4225	Acetylati	C	Superscaf	92819245	0	BTP202209	0	0	0	BTP202209	Mixed
CDS|exon|ID=cds-Bg	7	6.44	2885.2	0.937147	KTGPDHL	56	384.2126	2	766.3973	.	G	Superscaf	2648455	0	0	0	BTP202209	0	0	Tissue specific
CDS|exon|ID=cds-Bg	7	3.971429	1889.3	0.82794	TVTAMDV	92	736.3511	1	735.3473	.	S	Superscaf	7250377	0	0	0	0	0	BTP202209	Tissue specific
CDS|exon|ID=cds-Bg	8	3.475	1889.3	0.82794	TVTAMDVV	87	835.4193	1	834.4157	.	S	Superscaf	7250377	0	0	0	0	0	BTP202209	Tissue specific
CDS|exon|ID=cds-Bg	9	3.717778	2348.8	0.82794	TVTAMDVVY	82	998.4827	1	997.4791	.	S	Superscaf	7250377	0	0	0	0	0	BTP202209	Tissue specific
CDS|exon|ID=cds-Bg	9	4.682222	2686.9	0.827939	AMDVVYALK	96	505.2722	2	1008.531	.	S	Superscaf	7250377	0	BTP202209	0	0	BTP202209	0	Mixed
CDS|exon|ID=cds-Bg	8	4.84375	2989.4	0.739277	DLSGELDL	54	431.2123	2	860.4127	.	V	Superscaf	10986961	0	0	0	BTP202209	0	0	Tissue specific
CDS|exon|ID=cds-Bg	13	6.463846	5341.7	0.713458	ALALKHANT	99	480.6028	3	1438.793	.	S	Superscaf	12074731	0	0	0	0	BTP202209	0	Tissue specific
CDS|exon|ID=cds-Bg	5	6.098	1866.1	0.575873	LPWAP	63	583.3192	1	582.3166	.	V	Superscaf	17872771	BTP202209	0	0	0	0	0	Tissue specific
CDS|exon|ID=cds-Bg	7	3.698571	1621.8	0.55821	LAVVHVP	60	367.7349	2	733.4487	.	I	Superscaf	18617014	0	0	0	BTP202209	0	0	Tissue specific
CDS|exon|ID=cds-Bg	7	4.631429	2019.3	0.554665	TAPVLHV	51	368.7191	2	735.4279	.	G	Superscaf	18766662	BTP202209	0	0	0	0	0	Tissue specific
CDS|exon|ID=cds-Bg	11	4.939091	3527.9	0.513139	GSFVEKTVM	84	629.3108	2	1256.611	.	C	Superscaf	20516479	0	0	0	0	0	BTP202209	Tissue specific
CDS|exon|ID=cds-Bg	8	5.715	3195.5	0.513085	STDAGFHF	91	441.1912	2	880.3715	.	N	Superscaf	20518744	0	0	0	0	0	BTP202209	Tissue specific
CDS|exon|ID=cds-Bg	9	6.48	3923.1	0.512873	FEKEKMNAK	87	562.7902	2	1123.57	.	C	Superscaf	20527462	0	0	0	0	0	BTP202209	Tissue specific
CDS|exon|ID=cds-Bg	10	4.621	2892.2	0.512736	SAVTTVVNP	75	508.292	2	1014.571	.	G	Superscaf	20533507	0	BTP202209	0	0	0	BTP202209	Mixed
CDS|exon|ID=cds-Bg	14	5.057143	4542.9	0.512735	SAVTTVVNP	97	746.9022	2	1491.793	.	G	Superscaf	20533507	0	BTP202209	0	0	0	BTP202209	Mixed
CDS|exon|ID=cds-Bg	12	4.590833	3777.2	0.512735	SAVTTVVNP	86	654.3427	2	1306.677	.	G	Superscaf	20533507	0	0	0	0	BTP202209	0	Tissue specific
CDS|exon|ID=cds-Bg	8	4.31375	2197.5	0.512736	VTTVVNPK	95	429.2567	2	856.5018	.	G	Superscaf	20533507	0	BTP202209	0	0	0	0	Tissue specific
CDS|exon|ID=cds-Bg	10	4.339	3082.5	0.512735	VTTVVNPKY	94	575.308	2	1148.608	.	G	Superscaf	20533507	0	BTP202209	0	0	0	0	Tissue specific
CDS|exon|ID=cds-Bg	9	4.821111	3082.5	0.512735	TTVVNPKYE	98	525.7763	2	1049.539	.	G	Superscaf	20533507	0	BTP202209	0	0	0	0	Tissue specific
CDS|exon|ID=cds-Bg	8	4.6075	2685	0.512735	TVVNPKYE	66	475.2514	2	948.4916	.	G	Superscaf	20533507	0	BTP202209	0	0	0	0	Tissue specific
CDS|exon|ID=cds-Bg	9	5.115556	3053.2	0.512735	VVNPKYEGK	99	517.2868	2	1032.56	.	G	Superscaf	20533507	BTP202209	BTP202209	BTP202209	BTP202209	BTP202209	0	Mixed
CDS|exon|ID=cds-Bg	7	4.332857	2287.5	0.512735	VVNPKYE	98	424.7288	2	847.4439	.	G	Superscaf	20533507	0	BTP202209	0	BTP202209	BTP202209	0	Mixed
CDS|exon|ID=cds-Bg	6	5.656667	2766.1	0.461091	C(+57.02)	56	402.1789	2	802.332	Carbamido	H	Superscaf	22709970	0	0	0	BTP202209	0	0	Tissue specific
CDS|exon|ID=cds-Bg	10	5.248	3930.3	0.451919	KLTSDDVKE	99	581.7991	2	1161.588	.	A	Superscaf	23096248	0	0	0	0	0	BTP202209	Tissue specific
CDS|exon|ID=cds-Bg	8	5.395	2830.1	0.38574	GPPLVEKQ	95	434.249	2	866.4861	.	D	Superscaf	25884352	0	0	0	0	BTP202209	0	Tissue specific
CDS|exon|ID=cds-Bg	8	6.13	2797.2	0.382088	LSTLGPPP	64	391.2281	2	780.4381	.	V	Superscaf	26039394	BTP202209	0	0	0	0	0	Tissue specific
CDS|exon|ID=cds-Bg	7	5.888571	3002.4	0.371067	SQLLQPL	78	798.4692	1	797.4647	.	K	Superscaf	26503192	0	0	0	BTP202209	0	0	Tissue specific
CDS|exon|ID=cds-Bg	12	6.3675	4450	0.235458	M(+42.01)	89	603.7814	2	1205.55	Acetylati	K	Superscaf	32217913	0	BTP202209	0	0	0	0	Tissue specific
CDS|exon|ID=cds-Bg	7	5.995714	2385.7	0.235458	M(+42.01)	95	708.3003	1	707.2949	Acetylati	K	Superscaf	32217913	0	BTP202209	0	BTP202209	0	0	Mixed
CDS|exon|ID=cds-Bg	10	6.498	3695.1	0.235458	FGGPGTASR	99	473.7393	2	945.4668	.	K	Superscaf	32217913	0	0	0	0	BTP202209	0	Tissue specific
CDS|exon|ID=cds-Bg	8	5.99	2708.9	0.235458	FGGPGTAS	99	693.3187	1	692.3129	.	K	Superscaf	32217913	0	0	0	BTP202209	0	0	Tissue specific
CDS|exon|ID=cds-Bg	7	6.034286	2325.5	0.235458	FGGPGTA	85	606.288	1	605.2809	.	K	Superscaf	32217913	0	BTP202209	0	BTP202209	0	0	Mixed
CDS|exon|ID=cds-Bg	12	6.449167	4472	0.235458	GGPGTASRP	92	537.7607	2	1073.51	.	K	Superscaf	32217913	0	0	0	BTP202209	0	0	Tissue specific
CDS|exon|ID=cds-Bg	8	6.69125	3010.4	0.235458	GPGTASRP	85	371.6945	2	741.3769	.	K	Superscaf	32217913	0	0	0	0	BTP202209	0	Tissue specific
CDS|exon|ID=cds-Bg	13	6.332308	5218.1	0.235457	PSSTRSYVT	90	481.5789	3	1441.716	.	K	Superscaf	32217913	0	BTP202209	0	0	0	0	Tissue specific
CDS|exon|ID=cds-Bg	8	5.92125	3067.6	0.235457	SYVTTSTR	99	457.7317	2	913.4505	.	K	Superscaf	32217913	0	BTP202209	0	BTP202209	0	0	Mixed
CDS|exon|ID=cds-Bg	22	6.428636	8820.2	0.235456	SYVTTSTRT	67	802.4167	3	2404.235	.	K	Superscaf	32217913	0	BTP202209	0	0	0	0	Tissue specific
CDS|exon|ID=cds-Bg	13	5.937692	4958.7	0.235457	SYVTTSTRT	92	718.3574	2	1434.699	.	K	Superscaf	32217913	0	BTP202209	0	0	0	0	Tissue specific
CDS|exon|ID=cds-Bg	8	6.0275	3081.7	0.235457	YVTTSTRT	88	464.7399	2	927.4662	.	K	Superscaf	32217913	BTP202209	BTP202209	0	0	BTP202209	0	Mixed
CDS|exon|ID=cds-Bg	12	5.959167	4575.3	0.235457	YVTTSTRTY	91	674.8391	2	1347.667	.	K	Superscaf	32217913	0	BTP202209	0	BTP202209	0	0	Mixed
CDS|exon|ID=cds-Bg	10	5.956	3924.6	0.235457	YVTTSTRTY	90	589.7865	2	1177.562	.	K	Superscaf	32217913	0	BTP202209	0	0	0	0	Tissue specific
CDS|exon|ID=cds-Bg	9	5.988889	3465.1	0.235457	VTTSTRTYS	97	508.2561	2	1014.498	.	K	Superscaf	32217913	BTP202209	BTP202209	0	BTP202209	0	0	Mixed
CDS|exon|ID=cds-Bg	8	6.0275	3081.7	0.235457	VTTSTRTY	95	464.7397	2	927.4662	.	K	Superscaf	32217913	0	BTP202209	0	0	BTP202209	0	Mixed
CDS|exon|ID=cds-Bg	10	6.585	4115.8	0.235457	TTSTRTYSL	70	543.7738	2	1085.535	.	K	Superscaf	32217913	0	BTP202209	0	0	0	0	Tissue specific
CDS|exon|ID=cds-Bg	11	6.462727	4322.9	0.235457	TYSLGSALR	94	583.3129	2	1164.614	.	K	Superscaf	32217913	0	BTP202209	0	0	0	0	Tissue specific
CDS|exon|ID=cds-Bg	8	5.9375	2939.2	0.235457	TYSLGSAL	97	811.4185	1	810.4123	.	K	Superscaf	32217913	0	BTP202209	0	0	0	0	Tissue specific
CDS|exon|ID=cds-Bg	10	6.456	3925.4	0.235457	YSLGSALRP	95	532.7888	2	1063.566	.	K	Superscaf	32217913	0	BTP202209	0	0	0	0	Tissue specific
CDS|exon|ID=cds-Bg	11	6.462727	4322.9	0.235456	YSLGSALRP	78	583.314	2	1164.614	.	K	Superscaf	32217913	0	BTP202209	0	0	0	0	Tissue specific
CDS|exon|ID=cds-Bg	9	6.544444	3465.9	0.235457	SLGSALRPT	98	451.2582	2	900.5029	.	K	Superscaf	32217913	0	BTP202209	0	0	0	0	Tissue specific
CDS|exon|ID=cds-Bg	11	6.464545	4246.8	0.235456	SLGSALRPT	85	545.2975	2	1088.583	.	K	Superscaf	32217913	0	BTP202209	0	BTP202209	0	0	Mixed
CDS|exon|ID=cds-Bg	10	6.543	3863.4	0.235456	LGSALRPTT	89	501.7812	2	1001.551	.	K	Superscaf	32217913	0	0	0	BTP202209	0	0	Tissue specific
CDS|exon|ID=cds-Bg	10	7.021	4158.8	0.235456	GSALRPTTS	59	523.2905	2	1044.568	.	K	Superscaf	32217913	0	0	0	BTP202209	0	0	Tissue specific
CDS|exon|ID=cds-Bg	8	6.72125	3126.6	0.235456	GSALRPTT	98	401.7228	2	801.4344	.	K	Superscaf	32217913	0	0	0	0	BTP202209	0	Tissue specific
CDS|exon|ID=cds-Bg	9	7.137778	3861.5	0.235456	SALRPTTSR	99	494.7792	2	987.5461	.	K	Superscaf	32217913	0	0	0	BTP202209	0	0	Tissue specific
CDS|exon|ID=cds-Bg	11	6.977273	4612.4	0.235456	SALRPTTSR	68	401.5654	3	1201.678	.	K	Superscaf	32217913	0	BTP202209	0	BTP202209	0	0	Mixed
CDS|exon|ID=cds-Bg	12	6.8675	5071.9	0.235456	SALRPTTSR	99	455.9194	3	1364.741	.	K	Superscaf	32217913	0	BTP202209	BTP202209	BTP202209	0	0	Mixed
CDS|exon|ID=cds-Bg	13	6.841538	5469.4	0.235456	SALRPTTSR	96	489.6031	3	1465.789	.	K	Superscaf	32217913	0	BTP202209	0	0	0	0	Tissue specific
CDS|exon|ID=cds-Bg	10	7.077	4259	0.235456	SALRPTTSR	99	363.8716	3	1088.594	.	K	Superscaf	32217913	BTP202209	BTP202209	BTP202209	BTP202209	BTP202209	0	Mixed
CDS|exon|ID=cds-Bg	8	6.685	3212.7	0.235456	SALRPTTS	97	832.4496	1	831.445	.	K	Superscaf	32217913	0	0	0	BTP202209	0	0	Tissue specific
CDS|exon|ID=cds-Bg	11	6.975455	4688.5	0.235456	ALRPTTSRT	98	426.9098	3	1277.709	.	K	Superscaf	32217913	0	BTP202209	0	BTP202209	0	0	Mixed
CDS|exon|ID=cds-Bg	9	7.232222	3875.6	0.235456	ALRPTTSRT	93	501.7859	2	1001.562	.	K	Superscaf	32217913	0	0	0	0	BTP202209	0	Tissue specific
CDS|exon|ID=cds-Bg	7	6.828571	2829.3	0.235456	ALRPTTS	63	373.213	2	744.413	.	K	Superscaf	32217913	0	0	0	BTP202209	0	0	Tissue specific
CDS|exon|ID=cds-Bg	10	7.071	4377.2	0.235456	LRPTTSRTL	91	604.3425	2	1206.672	.	K	Superscaf	32217913	0	BTP202209	BTP202209	0	0	0	Mixed
CDS|exon|ID=cds-Bg	11	7.021818	4774.7	0.235456	LRPTTSRTL	66	654.8662	2	1307.72	.	K	Superscaf	32217913	0	BTP202209	0	0	0	0	Tissue specific
CDS|exon|ID=cds-Bg	7	6.81	3037.6	0.235456	TTSRTLY	91	421.2237	2	840.4341	.	K	Superscaf	32217913	0	BTP202209	0	0	0	0	Tissue specific
CDS|exon|ID=cds-Bg	8	6.775	3435.1	0.235456	TTSRTLYT	83	471.7472	2	941.4818	.	K	Superscaf	32217913	0	BTP202209	0	0	0	0	Tissue specific
CDS|exon|ID=cds-Bg	9	5.272222	3015.3	0.235456	YTSSPGGVY	86	930.4158	1	929.413	.	K	Superscaf	32217913	0	0	0	0	BTP202209	0	Tissue specific
CDS|exon|ID=cds-Bg	9	5.312222	2867.1	0.235455	TSSPGGVYA	98	838.3926	1	837.3868	.	K	Superscaf	32217913	0	BTP202209	0	0	BTP202209	0	Mixed
CDS|exon|ID=cds-Bg	16	5.8275	5640.3	0.235455	TSSPGGVYA	82	798.4088	2	1594.806	.	K	Superscaf	32217913	0	0	BTP202209	0	0	0	Tissue specific
CDS|exon|ID=cds-Bg	15	5.498667	4991.5	0.235455	TSSPGGVYA	95	720.3593	2	1438.705	.	K	Superscaf	32217913	0	BTP202209	0	0	0	0	Tissue specific
CDS|exon|ID=cds-Bg	13	5.881538	4680.2	0.235455	TSSPGGVYA	92	635.3052	2	1268.6	.	K	Superscaf	32217913	0	0	0	BTP202209	BTP202209	0	Mixed
CDS|exon|ID=cds-Bg	11	5.918182	3913.4	0.235455	TSSPGGVYA	99	548.2743	2	1094.536	.	K	Superscaf	32217913	BTP202209	BTP202209	0	BTP202209	BTP202209	0	Mixed
CDS|exon|ID=cds-Bg	8	5.22375	2555.8	0.235456	TSSPGGVY	99	767.3562	1	766.3497	.	K	Superscaf	32217913	BTP202209	BTP202209	0	BTP202209	0	0	Mixed
CDS|exon|ID=cds-Bg	8	5.16	2469.6	0.235455	SSPGGVYA	69	737.3458	1	736.3392	.	K	Superscaf	32217913	0	BTP202209	0	BTP202209	0	0	Mixed
CDS|exon|ID=cds-Bg	16	5.793125	5596.2	0.235455	SSPGGVYAT	50	804.4261	2	1606.843	.	K	Superscaf	32217913	0	BTP202209	0	0	0	0	Tissue specific
CDS|exon|ID=cds-Bg	12	5.8275	4282.7	0.235455	SSPGGVYAT	92	584.7816	2	1167.552	.	K	Superscaf	32217913	0	BTP202209	0	BTP202209	0	0	Mixed
CDS|exon|ID=cds-Bg	11	5.840909	3899.3	0.235455	SSPGGVYAT	86	541.2656	2	1080.52	.	K	Superscaf	32217913	BTP202209	BTP202209	0	0	BTP202209	0	Mixed
CDS|exon|ID=cds-Bg	10	5.857	3515.9	0.235455	SSPGGVYAT	99	497.7507	2	993.4879	.	K	Superscaf	32217913	BTP202209	BTP202209	0	BTP202209	BTP202209	0	Mixed

CDS|exon|ID=cds-Bg	7	5.085714	2086.2	0.235455	SPGGVYA	98	650.3123	1	649.3071	.	K	Superscaf	32217913	0	0	0	BTP202209	0	0	Tissue specific
CDS|exon|ID=cds-Bg	9	5.876667	3132.5	0.235455	SPGGVYATR	97	454.2333	2	906.4559	.	K	Superscaf	32217913	0	0	0	0	BTP202209	0	Tissue specific
CDS|exon|ID=cds-Bg	8	5.90125	2749.1	0.235455	PGGVYATR	91	410.7128	2	819.4239	.	K	Superscaf	32217913	0	0	0	BTP202209	0	0	Tissue specific
CDS|exon|ID=cds-Bg	10	6.819	4086.7	0.235455	ATRSSAVRL	94	372.8902	3	1115.652	.	K	Superscaf	32217913	BTP202209	BTP202209	BTP202209	BTP202209	0	0	Mixed
CDS|exon|ID=cds-Bg	10	6.218	3615.2	0.235454	RLRSGVPGV	99	366.2277	3	1095.662	.	K	Superscaf	32217913	0	BTP202209	0	0	BTP202209	0	Mixed
CDS|exon|ID=cds-Bg	10	5.229	3281.7	0.235454	SGVPGVRLL	86	513.3073	2	1024.603	.	K	Superscaf	32217913	0	0	0	0	BTP202209	0	Tissue specific
CDS|exon|ID=cds-Bg	8	5.0825	2317.6	0.235454	SGVPGVRL	97	392.736	2	783.4603	.	K	Superscaf	32217913	0	0	0	BTP202209	BTP202209	0	Mixed
CDS|exon|ID=cds-Bg	10	4.929	3395.9	0.235454	VPGVRLLQD	99	542.3105	2	1082.608	.	K	Superscaf	32217913	0	0	0	BTP202209	0	0	Tissue specific
CDS|exon|ID=cds-Bg	12	4.355	3807.4	0.235454	VPGVRLLQD	77	649.3564	2	1296.704	.	K	Superscaf	32217913	0	0	0	0	BTP202209	0	Tissue specific
CDS|exon|ID=cds-Bg	11	4.750909	3807.4	0.235454	PGVRLLQDS	57	599.8245	2	1197.635	.	K	Superscaf	32217913	0	0	0	0	BTP202209	0	Tissue specific
CDS|exon|ID=cds-Bg	8	5.37375	3058.5	0.235454	GVRLLQDS	66	444.249	2	886.4872	.	K	Superscaf	32217913	0	0	0	BTP202209	0	0	Tissue specific
CDS|exon|ID=cds-Bg	9	5.052222	3560.1	0.235454	RLLQDSVDF	75	546.7868	2	1091.561	.	K	Superscaf	32217913	0	BTP202209	0	0	0	0	Tissue specific
CDS|exon|ID=cds-Bg	8	4.33875	2911.3	0.235454	LLQDSVDF	97	936.4659	1	935.46	.	K	Superscaf	32217913	0	BTP202209	0	0	0	0	Tissue specific
CDS|exon|ID=cds-Bg	7	4.871429	2667	0.235452	TNEKVEL	71	416.7227	2	831.4338	.	K	Superscaf	32217913	0	0	0	0	BTP202209	0	Tissue specific
CDS|exon|ID=cds-Bg	7	4.805714	2365.6	0.235363	DVSKPDL	84	387.2036	2	772.3967	.	T	Superscaf	32222032	0	0	0	0	BTP202209	BTP202209	Mixed
CDS|exon|ID=cds-Bg	8	5.02125	2763.1	0.235363	DVSKPDLT	97	874.4494	1	873.4443	.	T	Superscaf	32222032	BTP202209	0	0	BTP202209	BTP202209	BTP202209	Mixed
CDS|exon|ID=cds-Bg	7	5.281429	3042.3	0.235361	EAEEWYK	99	477.7114	2	953.413	.	T	Superscaf	32222032	0	0	0	0	BTP202209	BTP202209	Mixed
CDS|exon|ID=cds-Bg	8	6.14625	3468.6	0.235361	AEEWYKSK	97	520.7545	2	1039.497	.	T	Superscaf	32222032	0	0	0	0	BTP202209	0	Tissue specific
CDS|exon|ID=cds-Bg	8	5.35125	3652.4	0.235332	MREMEENF	65	543.2207	2	1084.432	.	H	Superscaf	32223385	0	0	0	0	0	BTP202209	Tissue specific
CDS|exon|ID=cds-Bg	16	5.7075	6541.5	0.235304	ETNLDSLPL	93	912.9721	2	1823.938	.	K	Superscaf	32224576	0	0	BTP202209	0	0	0	Tissue specific
CDS|exon|ID=cds-Bg	17	5.755882	6939	0.235304	ETNLDSLPL	95	963.4967	2	1924.985	.	K	Superscaf	32224576	0	0	0	BTP202209	0	0	Tissue specific
CDS|exon|ID=cds-Bg	15	5.370667	5892.7	0.235304	ETNLDSLPL	97	834.924	2	1667.837	.	K	Superscaf	32224576	0	BTP202209	BTP202209	0	BTP202209	BTP202209	Mixed
CDS|exon|ID=cds-Bg	14	5.058571	5424.3	0.235304	ETNLDSLPL	97	770.8759	2	1539.742	.	K	Superscaf	32224576	0	0	0	0	0	BTP202209	Tissue specific
CDS|exon|ID=cds-Bg	13	5.858462	5121.8	0.235304	LDSLPLVDT	95	494.2749	3	1479.804	.	K	Superscaf	32224576	0	BTP202209	BTP202209	0	BTP202209	0	Mixed
CDS|exon|ID=cds-Bg	14	5.906429	5519.3	0.235304	LDSLPLVDT	91	791.4302	2	1580.852	.	K	Superscaf	32224576	0	0	0	BTP202209	0	0	Tissue specific
CDS|exon|ID=cds-Bg	12	5.45	4473	0.235304	LDSLPLVDT	96	662.8578	2	1323.703	.	K	Superscaf	32224576	0	BTP202209	BTP202209	BTP202209	BTP202209	BTP202209	Mixed
CDS|exon|ID=cds-Bg	11	5.06	4004.6	0.235304	LDSLPLVDT	92	598.8107	2	1195.608	.	K	Superscaf	32224576	0	BTP202209	0	0	0	BTP202209	Mixed
CDS|exon|ID=cds-Bg	12	5.848333	4768.4	0.235304	DSLPLVDTH	96	456.5787	3	1366.721	.	K	Superscaf	32224576	0	0	0	0	BTP202209	0	Tissue specific
CDS|exon|ID=cds-Bg	11	5.401818	4119.6	0.235304	DSLPLVDTH	97	606.3157	2	1210.619	.	K	Superscaf	32224576	0	BTP202209	BTP202209	BTP202209	BTP202209	BTP202209	Mixed
CDS|exon|ID=cds-Bg	10	4.968	3651.2	0.235304	DSLPLVDTH	94	542.2682	2	1082.524	.	K	Superscaf	32224576	0	0	0	BTP202209	BTP202209	BTP202209	Mixed
CDS|exon|ID=cds-Bg	9	4.888889	3267.8	0.235304	DSLPLVDTH	97	996.5004	1	995.4924	.	K	Superscaf	32224576	0	0	BTP202209	BTP202209	BTP202209	0	Mixed
CDS|exon|ID=cds-Bg	8	4.55125	2648.1	0.235304	DSLPLVDT	95	859.4371	1	858.4334	.	K	Superscaf	32224576	0	0	0	0	BTP202209	0	Tissue specific
CDS|exon|ID=cds-Bg	11	6.11	4356.9	0.235304	SLPLVDTHS	94	626.8535	2	1251.694	.	K	Superscaf	32224576	0	BTP202209	BTP202209	0	BTP202209	0	Mixed
CDS|exon|ID=cds-Bg	10	5.645	3708.1	0.235304	SLPLVDTHS	97	548.8022	2	1095.592	.	K	Superscaf	32224576	0	BTP202209	BTP202209	0	0	0	Mixed
CDS|exon|ID=cds-Bg	8	5.12875	2856.3	0.235304	SLPLVDTH	99	881.4716	1	880.4654	.	K	Superscaf	32224576	0	0	0	BTP202209	0	0	Tissue specific
CDS|exon|ID=cds-Bg	9	5.641111	3324.7	0.235304	LPLVDTHSK	95	505.2852	2	1008.56	.	K	Superscaf	32224576	0	0	0	BTP202209	BTP202209	0	Tissue enhanced
CDS|exon|ID=cds-Bg	8	5.12875	2856.3	0.235304	LPLVDTHS	99	441.2386	2	880.4654	.	K	Superscaf	32224576	0	0	0	BTP202209	0	0	Tissue specific
CDS|exon|ID=cds-Bg	7	5.05	2472.9	0.235304	LPLVDTH	99	794.4392	1	793.4334	.	K	Superscaf	32224576	BTP202209	0	0	BTP202209	0	0	Mixed
CDS|exon|ID=cds-Bg	9	6.172222	3620.1	0.235304	PLVDTHSKK	98	351.5321	3	1051.577	.	K	Superscaf	32224576	0	0	0	BTP202209	BTP202209	0	Mixed
CDS|exon|ID=cds-Bg	7	6.032857	3151.7	0.165715	D(+42.01)	82	462.2545	2	922.4872	Acetylati	G	Superscaf	35157055	0	0	BTP202209	0	0	0	Tissue specific
CDS|exon|ID=cds-Bg	6	5.355	3047.6	0.115803	C(+57.02)	54	507.6439	2	1013.275	Carbamido	R	Superscaf	37260445	BTP202209	0	0	0	0	0	Tissue specific
CDS|exon|ID=cds-Bg	7	5.122857	2456.7	0.091913	MADGVNK	76	367.6814	2	733.3428	.	P	Superscaf	38266951	0	0	0	BTP202209	0	0	Tissue specific
CDS|exon|ID=cds-Bg	12	4.275833	3985.5	0.085815	A(+42.01)	99	666.8123	2	1331.614	Acetylati	A	Superscaf	38524165	0	BTP202209	0	0	BTP202209	BTP202209	Mixed
CDS|exon|ID=cds-Bg	8	4.03875	2325.5	0.085815	A(+42.01)	70	788.3868	1	787.3712	Acetylati	A	Superscaf	38524165	0	0	0	0	0	BTP202209	Tissue specific
CDS|exon|ID=cds-Bg	13	6.04	5375	0.08581	KHFVGMLPE	69	404.9545	4	1615.796	Carbamido	A	Superscaf	38524165	0	0	0	0	0	BTP202209	Tissue specific
CDS|exon|ID=cds-Bg	14	6.012857	5834.5	0.08581	KHFVGMLPE	76	445.7216	4	1778.86	Carbamido	A	Superscaf	38524165	0	BTP202209	0	0	0	0	Tissue specific
CDS|exon|ID=cds-Bg	11	5.579091	4359	0.08581	VGMLPEKDC	86	456.551	3	1366.637	Carbamido	A	Superscaf	38524165	0	0	0	0	0	BTP202209	Tissue specific
CDS|exon|ID=cds-Bg	10	5.73	3909.2	0.085809	ALYDASFET	95	572.7784	2	1143.545	.	A	Superscaf	38524165	0	0	0	0	0	BTP202209	Tissue specific
CDS|exon|ID=cds-Bg	15	5.808	6375	0.085809	YDASFETKE	98	916.4339	2	1830.864	.	A	Superscaf	38524165	0	0	0	0	BTP202209	0	Tissue specific
CDS|exon|ID=cds-Bg	14	5.795714	6021.6	0.085809	YDASFETKE	92	573.5986	3	1717.78	.	A	Superscaf	38524165	0	0	0	0	BTP202209	0	Tissue specific
CDS|exon|ID=cds-Bg	12	6.225	5170.6	0.085809	YDASFETKE	94	487.5705	3	1459.694	.	A	Superscaf	38524165	0	0	0	0	0	BTP202209	Tissue specific
CDS|exon|ID=cds-Bg	11	5.905455	4702.2	0.085809	YDASFETKE	99	666.8035	2	1331.599	.	A	Superscaf	38524165	0	0	0	0	0	BTP202209	Tissue specific
CDS|exon|ID=cds-Bg	8	5.6625	3244.5	0.085809	YDASFETK	99	480.7172	2	959.4236	.	A	Superscaf	38524165	0	0	0	0	0	BTP202209	Tissue specific
CDS|exon|ID=cds-Bg	9	5.738889	3967.6	0.085702	GPEDLNRAC	96	516.2313	2	1030.45	Carbamido	T	Superscaf	38528848	0	0	0	0	BTP202209	0	Tissue specific
CDS|exon|ID=cds-Bg	7	5.445714	2298.6	0.085505	PGVLPHL	51	366.7226	2	731.433	.	M	Superscaf	38537044	0	0	0	BTP202209	0	0	Tissue specific
CDS|exon|ID=cds-Bg	9	6.837778	4146	0.08345	M(+42.01)	87	395.5256	3	1183.559	Acetylati	K	Superscaf	38623717	0	0	0	BTP202209	0	0	Tissue specific
CDS|exon|ID=cds-Bg	8	5.5175	2796.9	0.071105	KLAVSSAC(	91	418.2188	2	834.4269	Carbamido	T	Superscaf	39143977	0	0	0	0	0	BTP202209	Tissue specific
CDS|exon|ID=cds-Bg	5	6.266	1937	0.032939	H(+42.01)	58	498.2216	1	497.2234	Acetylati	N	Superscaf	40752145	0	0	0	BTP202209	0	0	Tissue specific
CDS|exon|ID=cds-Bg	7	4.931429	2094.4	0.007609	VVPSRFP	63	401.2336	2	800.4544	.	A	Superscaf	41819722	BTP202209	0	0	0	0	0	Tissue specific
CDS|exon|ID=cds-Bg	8	6.41375	3304.6	0.012903	WDSKKVTR	98	510.2836	2	1018.556	.	G	Superscaf	42684136	0	0	0	BTP202209	0	0	Tissue specific
CDS|exon|ID=cds-Bg	10	6.825	4299.9	0.027888	A(+42.01)	74	395.5505	3	1183.631	Acetylati	S	Superscaf	43315588	0	BTP202209	0	0	0	0	Tissue specific
CDS|exon|ID=cds-Bg	9	6.387778	3651.1	0.027888	A(+42.01)	87	514.7698	2	1027.53	Acetylati	S	Superscaf	43315588	0	0	0	0	0	BTP202209	Tissue specific
CDS|exon|ID=cds-Bg	9	6.472222	3800.1	0.028449	LTDLSQLKK	99	523.3142	2	1044.618	.	A	Superscaf	43339276	0	0	0	0	0	BTP202209	Tissue specific
CDS|exon|ID=cds-Bg	9	5.602222	3798.5	0.031687	VLHMDRNPY	94	382.1882	3	1143.55	.	E	Superscaf	43475746	0	0	0	0	0	BTP202209	Tissue specific
CDS|exon|ID=cds-Bg	10	5.589	3794.5	0.031821	MLLFTEVTR	94	636.8337	2	1271.658	.	D	Superscaf	43481365	0	0	0	0	0	BTP202209	Tissue specific
CDS|exon|ID=cds-Bg	12	5.380833	4575.3	0.031821	LLFTEVTRY	83	758.9066	2	1515.797	.	D	Superscaf	43481365	0	0	0	0	0	BTP202209	Tissue specific
CDS|exon|ID=cds-Bg	8	6.21875	3103.4	0.031964	TGHALALY	76	423.2284	2	844.4443	.	Q	Superscaf	43487188	0	BTP202209	0	0	BTP202209	BTP202209	Mixed
CDS|exon|ID=cds-Bg	16	6.451875	6960.8	0.032107	RMMGSEFDF	82	512.996	4	2047.964	.	V	Superscaf	43493365	0	0	0	0	0	BTP202209	Tissue specific
CDS|exon|ID=cds-Bg	10	5.891	4025.4	0.04226	ALGSQRVKE	93	587.3199	2	1172.63	.	L	Superscaf	43921087	0	0	BTP202209	0	0	0	Tissue specific
CDS|exon|ID=cds-Bg	8	5.17375	3253.6	0.042685	KNLQLDYV	58	496.772	2	991.5338	.	Y	Superscaf	43939162	0	0	BTP202209	0	0	0	Tissue specific
CDS|exon|ID=cds-Bg	8	6.32375	3411.6	0.04278	KLLDFC(+5	88	505.7698	2	1009.527	Carbamido	L	Superscaf	43943047	0	0	BTP202209	0	0	0	Tissue specific
CDS|exon|ID=cds-Bg	8	6.46125	3361.7	0.042782	YGALGSQR	94	426.221	2	850.4297	.	L	Superscaf	43943047	0	0	BTP202209	0	0	0	Tissue specific
CDS|exon|ID=cds-Bg	7	4.347143	2234.6	0.042803	LLEDPVL	99	798.4579	1	797.4534	.	S	Superscaf	43944157	0	0	BTP202209	0	0	0	Tissue specific
CDS|exon|ID=cds-Bg	11	5.877273	4255.6	0.044329	YAGLGSQRV	97	604.3229	2	1206.636	.	K	Superscaf	44008345	0	0	BTP202209	0	0	0	Tissue specific
CDS|exon|ID=cds-Bg	10	6.143	3830.1	0.044329	YAGLGSQRV	98	539.8016	2	1077.593	.	K	Superscaf	44008345	0	0	BTP202209	0	0	0	Tissue specific
CDS|exon|ID=cds-Bg	10	5.899	3796.1	0.044329	AGLGSQRVK	97	522.7922	2	1043.572	.	K	Superscaf	44008345	0	0	BTP202209	0	0	0	Tissue specific
CDS|exon|ID=cds-Bg	9	6.196667	3370.6	0.044329	AGLGSQRVK	97	458.2705	2	914.5297	.	K	Superscaf	44008345	0	0	BTP202209	0	0	0	Tissue specific
CDS|exon|ID=cds-Bg	10	5.886	4011.4	0.044329	GLGSQRVKE	96	580.3124	2	1158.615	.	K	Superscaf	44008345	0	0	BTP202209	0	0	0	Tissue specific
CDS|exon|ID=cds-Bg	7	5.848571	3063.4	0.044329	SQRVKEW	97	466.7498	2	931.4875	.	K	Superscaf	44008345	0	0	BTP202209	0	0	0	Tissue specific
CDS|exon|ID=cds-Bg	9	5.802222	3143.4	0.051369	KQTPALVAL	55	470.7943	2	939.5753	.	K	Superscaf	44305081	0	BTP202209	0	0	0	0	Tissue specific
CDS|exon|ID=cds-Bg	7	6.062857	2672.8	0.052032	LKYKPVC(+	94	454.2556	2	906.4997	Carbamido	L	Superscaf	44333014	0	0	BTP202209	0	0	0	Tissue specific
CDS|exon|ID=cds-Bg	8	6.515	3388.5	0.054089	M(+42.01)	95	557.7819	2	1113.553	Acetylati	R	Superscaf	44419708	0	0	BTP202209	0	0	0	Tissue specific
CDS|exon|ID=cds-Bg	10	6.181	4396	0.054101	LNDGLWHMK	97	607.8014	2	1213.591	.	R	Superscaf	44419708	0	0	0	0	BTP202209	0	Tissue specific
CDS|exon|ID=cds-Bg	12	6.434167	5323.9	0.054101	LNDGLWHMK	99	502.5892	3	1504.75	.	R	Superscaf	44419708	0	0	0	0	BTP202209	BTP202209	Mixed
CDS|exon|ID=cds-Bg	8	5.13875	2825.2	0.082981	LLPFPVNY	74	481.7698	2	961.5273	.	S	Superscaf	45637090	0	0	0	0	0	BTP202209	Tissue specific
CDS|exon|ID=cds-Bg	6	5.63	1785.9	0.083358	V(+42.01)	60	644.3383	1	643.3363	Acetylati	M	Superscaf	45653337	0	0	0	BTP202209	0	0	Tissue specific
CDS|exon|ID=cds-Bg	11	4.47	3501.8	0.259316	LDLFANVVF	89	418.9105	3	1253.713	.	W	Superscaf	53068099	0	0	0	0	0	BTP202209	Tissue specific
CDS|exon|ID=cds-Bg	10	6.072	3734	0.262906	TVGKSSKML	86	539.7983	2	1077.585	.	K	Superscaf	53219332	0	BTP202209	BTP202209	BTP202209	BTP202209	0	Mixed
CDS|exon|ID=cds-Bg	7	6.811429	2667.2	0.263029	RPPMGPP	65	376.1967	2	750.3846	.	W	Superscaf	53224558	0	0	0	BTP202209	0	0	Tissue specific
CDS|exon|ID=cds-Bg	7	5.511429	2463.7	0.264281	LGSDALL	67	688.3858	1	687.3803	.	I	Superscaf	53277064	0	0	BTP202209	0	0	0	Tissue specific
CDS|exon|ID=cds-Bg	7	4.06	1753	0.270757	VSSPVVR	96	372.2229	2	742.4337	.	V	Superscaf	53550336	0	0	0	0	0	BTP202209	Tissue specific
CDS|exon|ID=cds-Bg	7	5.662857	2482.6	0.29094	QVATASK	56	352.7041	2	703.3864	.	E	Superscaf	54400753	0	0	0	0	0	BTP202209	Tissue specific
CDS|exon|ID=cds-Bg	8	6.005	3089.3	0.296052	LGLDNAGK	99	394.2178	2	786.4235	.	P	Superscaf	54616156	0	0	0	0	0	BTP202209	Tissue specific
CDS|exon|ID=cds-Bg	7	4.682857	2106.5	0.298654	PVGRVET	94	379.212	2	756.413	.	C	Superscaf	54725605	0	0	0	0	BTP202209	0	Tissue specific
CDS|exon|ID=cds-Bg	7	6.251429	3301.9	0.298675	DQRWLLT	58	466.252	2	930.4923	.	W	Superscaf	54726721	0	0	0	0	BTP202209	0	Tissue specific
CDS|exon|ID=cds-Bg	5	4.318	1397.6	0.30278	DVAPP	57	498.254	1	497.2485	.	R	Superscaf	54899554	0	0	BTP202209	0	0	0	Tissue specific
CDS|exon|ID=cds-Bg	7	6.481429	2857.1	0.306	AGTEHKP	52	370.1931	2	738.366	.	V	Superscaf	55035591	BTP202209	0	0	0	0	0	Tissue specific
CDS|exon|ID=cds-Bg	9	5.304444	3294.9	0.30657	VDTLEPSPF	86	507.2695	2	1012.519	.	C	Superscaf	55059322	0	BTP202209	0	0	0	0	Tissue specific
CDS|exon|ID=cds-Bg	9	3.377778	2312.6	0.316778	VLVVPEEAY	60	509.7752	2	1017.538	.	E	Superscaf	55489639	0	0	0	0	0	BTP202209	Tissue specific
CDS|exon|ID=cds-Bg	7	6.031429	2485.8	0.356938	L(+42.01)	61	752.4204	1	751.4116	Acetylati	P	Superscaf	57182061	0	0	0	0	BTP202209	0	Tissue specific
CDS|exon|ID=cds-Bg	8	6.915	3346.5	0.358672	KLASQKTL	77	444.7786	2	887.544	.	S	Superscaf	57255085	BTP202209	0	0	0	0	0	Tissue specific
CDS|exon|ID=cds-Bg	8	5.35875	2800.1	0.38	SHSLLLLV	86	441.2754	2	880.5382	.	G	Superscaf	58153911	0	BTP202209	0	0	0	0	Tissue specific
CDS|exon|ID=cds-Bg	7	5.704286	2400.4	0.382106	KDLFVAK	75	410.7488	2	819.4854	.	K	Superscaf	58242639	0	0	BTP202209	0	0	0	Tissue specific
CDS|exon|ID=cds-Bg	8	6.19875	3365.9	0.387763	QRDLGLLP	51	456.2665	2	910.5236	.	Q	Superscaf	58480975	0	0	0	0	0	BTP202209	Tissue specific
CDS|exon|ID=cds-Bg	6	6.376667	2493.9	0.405673	TGERPF	97	353.6784	2	705.3445	.	C	Superscaf	59235658	0	BTP202209	0	0	0	0	Tissue specific
CDS|exon|ID=cds-Bg	6	8.466667	3333.8	0.424897	PQHRRK	52	411.252	2	820.478	.	Q	Superscaf	60045772	0	0	0	0	0	BTP202209	Tissue specific
CDS|exon|ID=cds-Bg	7	6.707143	2707.3	0.426915	RPVTRPP	91	411.7486	2	821.4871	.	T	Superscaf	60130657	0	0	0	BTP202209	BTP202209	0	Tissue enhanced
CDS|exon|ID=cds-Bg	6	6.806667	2868.2	0.42722	PHKC(+57.	51	403.191	2	804.3588	Carbamido	A	Superscaf	60143680	0	0	BTP202209	0	0	0	Tissue specific
CDS|exon|ID=cds-Bg	8	6.07125	2668.9	0.436292	SLGPAAPP	51	355.1967	2	708.3806	.	Q	Superscaf	60525847	0	0	BTP202209	0	0	0	Tissue specific
CDS|exon|ID=cds-Bg	13	5.223846	4816.3	0.450663	HLVSPEALI	86	742.3882	2	1482.772	.	G	Superscaf	61131526	0	0	0	0	0	BTP202209	Tissue specific
CDS|exon|ID=cds-Bg	7	5.345714	2018.3	0.451155	PGVTAPP	57	638.3517	1	637.3435	.	R	Superscaf	61152181	0	0	0	0	BTP202209	0	Tissue specific
CDS|exon|ID=cds-Bg	8	5.7275	2739.1	0.451518	LPGLPPAD	63	779.4281	1	778.4225	.	L	Superscaf	61167625	0	0	BTP202209	0	0	0	Tissue specific
CDS|exon|ID=cds-Bg	10	6.564	4813.5	0.45327	M(+42.01)	97	632.2722	2	1262.532	Acetylati	V	Superscaf	61241796	0	BTP202209	0	0	0	0	Tissue specific
CDS|exon|ID=cds-Bg	17	5.485882	6720.6	0.464398	SYEESNPKI	94	947.4437	2	1892.875	.	W	Superscaf	61710334	0	0	0	0	0	BTP202209	Tissue specific
CDS|exon|ID=cds-Bg	7	5.167143	2633.1	0.465219	VPTLPQN	76	768.4263	1	767.4177	.	A	Superscaf	61744630	0	0	BTP202209	0	0	0	Tissue specific
CDS|exon|ID=cds-Bg	7	6.258571	3243.7	0.488515	KNDLDLR	63	437.2368	2	872.4716	.	Q	Superscaf	62726781	0	0	0	0	BTP202209	0	Tissue specific
CDS|exon|ID=cds-Bg	9	6.077778	3293.7	0.49012	EAAGVRGLF	65	464.7688	2	927.525	.	A	Superscaf	62794036	0	BTP202209	BTP202209	BTP202209	BTP202209	0	Tissue enhanced
CDS|exon|ID=cds-Bg	6	5.561667	2187.3	0.493732	APKVYQ	54	353.2048	2	704.3857	.	L	Superscaf	62946565	0	0	BTP202209	0	0	0	Tissue specific
CDS|exon|ID=cds-Bg	12	5.934167	5045.6	0.499059	YARDETEFY	78	497.9068	3	1490.704	.	R	Superscaf	63170908	0	0	0	0	BTP202209	0	Tissue specific
CDS|exon|ID=cds-Bg	7	5.405714	3155.7	0.499059	RDETEFY	72	480.208	2	958.4032	.	R	Superscaf	63170908	0	0	0	0	BTP202209	0	Tissue specific
CDS|exon|ID=cds-Bg	7	6.524286	3257.7	0.51242	LQGSQRL	75	801.455	1	800.4504	.	G	Superscaf	63733981	0	0	BTP202209	0	0	0	Tissue specific
CDS|exon|ID=cds-Bg	11	5.483636	4420	0.528845	A(+42.01)	95	679.3668	2	1356.725	Acetylati	P	Superscaf	64426246	0	0	BTP202209	BTP202209	BTP202209	0	Mixed
CDS|exon|ID=cds-Bg	9	5.41	3455.9	0.528845	A(+42.01)	95	558.7957	2	1115.582	Acetylati	P	Superscaf	64426246	0	0	0	BTP202209	BTP202209	0	Tissue enhanced
CDS|exon|ID=cds-Bg	8	4.74125	2807.1	0.528845	A(+42.01)	96	480.7491	2	959.4811	Acetylati	P	Superscaf	64426246	0	0	0	0	BTP202209	BTP202209	Tissue enhanced
CDS|exon|ID=cds-Bg	7	5.688571	2429.7	0.53376	EAAPLLP	51	710.4159	1	709.401	.	Y	Superscaf	64633294	BTP202209	0	0	0	0	0	Tissue specific
CDS|exon|ID=cds-Bg	8	5.02625	2725	0.53666	FGDEPVTK	99	446.7228	2	891.4338	.	E	Superscaf	64755445	0	0	0	0	0	BTP202209	Tissue specific
CDS|exon|ID=cds-Bg	5	5.08	2385.7	0.537214	SNSEN	61	550.216	1	549.2031	.	G	Superscaf	64778973	0	0	BTP202209	0	0	0	Tissue specific
CDS|exon|ID=cds-Bg	6	6.566667	2420.6	0.548612	KNSPGP	65	599.3177	1	598.3074	.	I	Superscaf	65259279	0	0	0	0	BTP202209	0	Tissue specific
CDS|exon|ID=cds-Bg	8	6.55	3049.2	0.549655	M(+42.01)	98	466.2661	2	930.5208	Acetylati	E	Superscaf	65303041	0	0	0	BTP202209	0	0	Tissue specific
CDS|exon|ID=cds-Bg	8	6.7825	3000.5	0.550905	PPPPPLAR	87	422.7541	2	843.4966	.	A	Superscaf	65355844	0	0	BTP202209	0	0	0	Tissue specific
CDS|exon|ID=cds-Bg	12	5.535833	4409.9	0.562043	T(+42.01)	98	667.3263	2	1332.641	Acetylati	G	Superscaf	65824945	0	BTP202209	0	0	0	0	Tissue specific
CDS|exon|ID=cds-Bg	9	6.613333	3213.6	0.563133	KPSPPPPPF	80	457.2653	2	912.5068	.	V	Superscaf	65870935	0	0	BTP202209	0	0	0	Tissue specific

CDS|exon|ID=cds-Bg	7	6.442857	3149.5	0.570465	PGAHQLH	74	380.2028	2	758.3824	.	E	Superscaf	66180049	BTP202209	0	0	0	0	0	Tissue specific
CDS|exon|ID=cds-Bg	8	6.08125	3054.4	0.570465	LTMFGEKL	94	469.753	2	937.4943	.	I	Superscaf	66180001	0	BTP202209	0	0	0	BTP202209	Mixed
CDS|exon|ID=cds-Bg	9	5.152222	3618.9	0.570492	KGNFNYVEF	66	559.2673	2	1116.524	.	K	Superscaf	66180835	0	0	0	0	0	BTP202209	Tissue specific
CDS|exon|ID=cds-Bg	7	6.444286	3077.5	0.574923	C(+57.02)	62	396.1737	2	790.3214	Carbamido	G	Superscaf	66367990	BTP202209	0	0	0	0	0	Tissue specific
CDS|exon|ID=cds-Bg	9	6.76	3784.3	0.582196	LTPTHYLTK	99	537.3009	2	1072.592	.	G	Superscaf	66674449	0	0	0	0	0	BTP202209	Tissue specific
CDS|exon|ID=cds-Bg	7	5.327143	2366.5	0.582437	YHAVAAL	52	744.3974	1	743.3966	.	I	Superscaf	66684574	0	0	0	0	0	BTP202209	Tissue specific
CDS|exon|ID=cds-Bg	7	4.997143	2959.3	0.586971	QAFLEDY	89	885.3966	1	884.3915	.	A	Superscaf	66875503	0	0	0	0	0	BTP202209	Tissue specific
CDS|exon|ID=cds-Bg	7	4.267143	2212.6	0.59304	M(+42.01)	99	818.3949	1	817.3892	Acetylati	P	Superscaf	67131424	0	BTP202209	0	BTP202209	0	BTP202209	Mixed
CDS|exon|ID=cds-Bg	10	5.133	3576.9	0.607309	LLFGADVHY	99	561.282	2	1120.555	.	G	Superscaf	67732723	0	0	0	0	0	BTP202209	Tissue specific
CDS|exon|ID=cds-Bg	7	5.095714	2363.7	0.608069	MLDVMFK	97	442.2223	2	882.4343	.	D	Superscaf	67764442	0	0	0	0	0	BTP202209	Tissue specific
CDS|exon|ID=cds-Bg	14	5.43	5626.2	0.613205	TSLFQEVGI	85	785.3805	2	1568.751	Carbamido	M	Superscaf	67981045	0	BTP202209	0	0	0	0	Tissue specific
CDS|exon|ID=cds-Bg	11	5.421818	4273.7	0.613205	TSLFQEVGI	98	618.334	2	1234.656	.	M	Superscaf	67981045	0	0	0	0	0	BTP202209	Tissue specific
CDS|exon|ID=cds-Bg	9	4.315556	2730.9	0.668051	VVEFDFLGK	99	527.2815	2	1052.554	.	F	Superscaf	70292257	0	0	0	0	0	BTP202209	Tissue specific
CDS|exon|ID=cds-Bg	7	6.79	3024.3	0.723619	G(+42.01)	69	409.7306	2	817.4446	Acetylati	G	Superscaf	72633952	0	0	BTP202209	0	0	0	Tissue specific
CDS|exon|ID=cds-Bg	6	3.895	1919.1	0.72462	ESADVF	52	667.291	1	666.2861	.	S	Superscaf	72676314	0	0	BTP202209	0	0	0	Tissue specific
CDS|exon|ID=cds-Bg	7	4.868571	2610.9	0.732985	GDTQEKV	78	776.3817	1	775.3712	.	C	Superscaf	73028722	0	0	BTP202209	0	0	0	Tissue specific
CDS|exon|ID=cds-Bg	8	4.46375	2110.4	0.747976	MALVSPVL	58	415.2544	2	828.4779	.	L	Superscaf	73660788	BTP202209	0	0	0	0	0	Tissue specific
CDS|exon|ID=cds-Bg	8	7.17125	3091.4	0.749823	RPPAGPLK	95	418.2598	2	834.5075	.	F	Superscaf	73737976	0	0	BTP202209	0	0	0	Tissue specific
CDS|exon|ID=cds-Bg	12	5.918333	4670	0.751741	T(+42.01)	96	473.9214	3	1418.744	Acetylati	G	Superscaf	73819183	0	BTP202209	0	0	BTP202209	BTP202209	Mixed
CDS|exon|ID=cds-Bg	10	5.526	3890.3	0.751741	T(+42.01)	95	610.811	2	1219.612	Acetylati	G	Superscaf	73819183	0	0	0	0	BTP202209	0	Tissue specific
CDS|exon|ID=cds-Bg	12	5.8725	4625.9	0.751741	M(+42.01)	72	477.9321	3	1430.78	Acetylati	G	Superscaf	73819183	0	BTP202209	0	0	0	0	Tissue specific
CDS|exon|ID=cds-Bg	11	5.862727	4272.5	0.751741	M(+42.01)	99	440.2374	3	1317.696	Acetylati	G	Superscaf	73819183	0	0	0	0	BTP202209	BTP202209	Mixed
CDS|exon|ID=cds-Bg	10	5.475	3804.1	0.751741	M(+42.01)	79	595.8052	2	1189.601	Acetylati	G	Superscaf	73819183	0	0	BTP202209	0	0	0	Tissue specific
CDS|exon|ID=cds-Bg	9	5.414444	3492.8	0.751741	M(+42.01)	99	560.2871	2	1118.564	Acetylati	G	Superscaf	73819183	0	0	0	0	BTP202209	BTP202209	Mixed
CDS|exon|ID=cds-Bg	7	6.544286	2830	0.751801	LKMKGDY	89	427.7241	2	853.4368	.	E	Superscaf	73821682	0	0	0	0	0	BTP202209	Tissue specific
CDS|exon|ID=cds-Bg	19	4.676316	7049.9	0.753272	LDEDSLTKG	96	745.3735	3	2233.1	.	V	Superscaf	73883647	0	0	0	0	BTP202209	0	Tissue specific
CDS|exon|ID=cds-Bg	6	5.708333	2574.1	0.759148	C(+57.02)	60	378.1317	2	754.2448	Carbamido	I	Superscaf	74130823	BTP202209	0	0	0	0	0	Tissue specific
CDS|exon|ID=cds-Bg	6	4.453333	1788.9	0.780306	A(+42.01)	58	691.3528	1	690.3588	Acetylati	T	Superscaf	75022795	0	0	0	0	0	BTP202209	Tissue specific
CDS|exon|ID=cds-Bg	7	5.648571	2692.9	0.786895	G(+42.01)	59	803.4303	1	802.4072	Acetylati	R	Superscaf	75300421	0	0	0	BTP202209	0	0	Tissue specific
CDS|exon|ID=cds-Bg	8	3.7525	1640.7	0.791621	AAALVVVL	70	755.5005	1	754.4952	.	A	Superscaf	75499000	0	BTP202209	0	0	0	0	Tissue specific
CDS|exon|ID=cds-Bg	8	7.1075	3053.2	0.799852	KRLAAAPA	53	399.2462	2	796.4919	.	L	Superscaf	75846574	BTP202209	0	0	0	0	0	Tissue specific
CDS|exon|ID=cds-Bg	10	6.423	4419	0.845981	LFERLTLKH	65	424.5731	3	1270.703	.	L	Superscaf	77790223	0	0	0	0	BTP202209	0	Tissue specific
CDS|exon|ID=cds-Bg	14	5.327143	5850.8	0.846374	M(+42.01)	64	835.3832	2	1668.755	Acetylati	V	Superscaf	77806954	0	0	0	0	0	BTP202209	Tissue specific
CDS|exon|ID=cds-Bg	7	5.898571	2972.2	0.84955	KNEFLLF	73	455.7519	2	909.496	.	N	Superscaf	77940930	0	0	0	0	BTP202209	0	Tissue specific
CDS|exon|ID=cds-Bg	7	7.11	2844.9	0.858537	QPKKAAP	73	739.4454	1	738.4388	.	E	Superscaf	78319546	0	BTP202209	0	0	0	0	Tissue specific
CDS|exon|ID=cds-Bg	6	5.041667	1652.8	0.86493	GVPLAL	62	569.3646	1	568.3584	.	R	Superscaf	78588625	BTP202209	0	0	0	0	0	Tissue specific
CDS|exon|ID=cds-Bg	8	5.58625	3228.6	0.87014	TQHKVLEL	67	484.2815	2	966.5498	.	T	Superscaf	78807433	0	BTP202209	0	0	0	0	Tissue specific
CDS|exon|ID=cds-Bg	8	6.705	2878.2	0.909153	A(+42.01)	52	382.7102	2	763.3976	Acetylati	T	Superscaf	80452600	0	0	0	0	BTP202209	0	Tissue specific
CDS|exon|ID=cds-Bg	6	5.995	2600.8	0.946213	K(+42.01)	94	765.3774	1	764.3704	Acetylati	F	Superscaf	82014465	0	0	0	0	BTP202209	BTP202209	Mixed
CDS|exon|ID=cds-Bg	9	6.645556	3127.5	0.930609	G(+42.01)	77	925.5114	1	924.5068	Acetylati	E	Superscaf	2977219	0	0	0	BTP202209	0	0	Tissue specific
CDS|exon|ID=cds-Bg	6	6.045	1964.1	0.922677	A(+42.01)	65	583.3428	1	582.3377	Acetylati	G	Superscaf	3318079	0	0	0	0	0	BTP202209	Tissue specific
CDS|exon|ID=cds-Bg	6	6.566667	2494.8	0.921153	P(+42.01)	59	394.223	2	786.431	Acetylati	A	Superscaf	3383472	0	0	BTP202209	0	0	0	Tissue specific
CDS|exon|ID=cds-Bg	12	5.543333	4783.5	0.920164	LLLWSQRMV	93	747.8872	2	1493.77	.	G	Superscaf	3425692	0	0	0	0	0	BTP202209	Tissue specific
CDS|exon|ID=cds-Bg	12	5.601667	5112.8	0.920067	SDVREAEEQ	94	487.2535	3	1458.743	.	P	Superscaf	3429898	0	0	0	0	0	BTP202209	Tissue specific
CDS|exon|ID=cds-Bg	11	4.376364	4185.6	0.919934	EEVAVDAQG	86	622.8047	2	1243.605	.	A	Superscaf	3435481	0	0	0	0	0	BTP202209	Tissue specific
CDS|exon|ID=cds-Bg	10	5.115	4127.5	0.919918	LQAEEVAQG	88	572.3014	2	1142.593	.	A	Superscaf	3435481	0	0	0	0	0	BTP202209	Tissue specific
CDS|exon|ID=cds-Bg	8	5.72375	3301.6	0.91986	EAELLQLK	74	472.276	2	942.5386	.	A	Superscaf	3435481	0	0	0	0	0	BTP202209	Tissue specific
CDS|exon|ID=cds-Bg	9	5.818889	3474.8	0.919838	VGPELHHKI	99	515.2936	2	1028.577	.	Q	Superscaf	3439045	0	0	0	BTP202209	0	0	Tissue specific
CDS|exon|ID=cds-Bg	11	5.62	3941.4	0.919837	LLSAERAVT	91	590.3197	2	1178.629	.	Q	Superscaf	3439045	0	0	0	0	0	BTP202209	Tissue specific
CDS|exon|ID=cds-Bg	15	5.698	5437.8	0.919814	LLSAEKAVT	82	827.9349	2	1653.861	.	Q	Superscaf	3439045	0	0	0	0	0	BTP202209	Tissue specific
CDS|exon|ID=cds-Bg	10	6.539	3974.4	0.91976	LLPLSDARK	94	375.9025	3	1124.692	.	Q	Superscaf	3439045	0	0	0	0	0	BTP202209	Tissue specific
CDS|exon|ID=cds-Bg	9	5.488889	3149.3	0.919751	VGPEFKDKI	82	516.7888	2	1031.565	.	Q	Superscaf	3439045	0	0	0	BTP202209	0	0	Tissue specific
CDS|exon|ID=cds-Bg	9	6.733333	3589.7	0.919725	MSAAQALKK	99	474.2688	2	946.527	.	Q	Superscaf	3439045	0	0	0	0	0	BTP202209	Tissue specific
CDS|exon|ID=cds-Bg	8	5.85125	3036.3	0.919721	KLRDVSAY	84	476.2641	2	950.5185	.	Q	Superscaf	3439045	0	0	0	0	0	BTP202209	Tissue specific
CDS|exon|ID=cds-Bg	8	5.5125	2873.1	0.919716	FDATGSGF	94	801.3401	1	800.334	.	Q	Superscaf	3439045	0	BTP202209	0	0	0	0	Tissue specific
CDS|exon|ID=cds-Bg	8	5.575	2725.1	0.917833	A(+42.01)	84	447.7547	2	893.497	Acetylati	T	Superscaf	3525904	0	BTP202209	0	0	0	BTP202209	Tissue enhanced
CDS|exon|ID=cds-Bg	14	5.894286	5932.7	0.917381	LPPLTPEQG	91	796.4348	2	1590.862	.	A	Superscaf	3545146	0	0	0	0	0	BTP202209	Tissue specific
CDS|exon|ID=cds-Bg	12	5.2325	5335.1	0.917263	DQERFDNSD	52	698.8008	2	1395.59	.	L	Superscaf	3549769	0	0	0	0	0	BTP202209	Tissue specific
CDS|exon|ID=cds-Bg	7	5.141429	1938	0.916824	LAGAAVL	90	614.3854	1	613.3799	.	V	Superscaf	3569128	BTP202209	BTP202209	BTP202209	BTP202209	BTP202209	BTP202209	Mixed
CDS|exon|ID=cds-Bg	7	6.93	2707.2	0.91608	PRATPPP	67	368.2086	2	734.4075	.	S	Superscaf	3600652	0	0	BTP202209	0	0	0	Tissue specific
CDS|exon|ID=cds-Bg	5	6.648	1862	0.916058	G(+42.01)	74	621.3044	1	620.2992	Acetylati	R	Superscaf	3599215	0	0	0	0	0	BTP202209	Tissue specific
CDS|exon|ID=cds-Bg	9	6.49	3860.4	0.914006	RQVEPPSRK	75	366.2104	3	1095.615	.	G	Superscaf	3689974	0	0	0	0	BTP202209	0	Tissue specific
CDS|exon|ID=cds-Bg	10	6.93	3498.3	0.914003	KAKKPALVA	95	351.9082	3	1052.707	.	G	Superscaf	3689974	0	0	0	0	0	BTP202209	Tissue specific
CDS|exon|ID=cds-Bg	9	6.617778	3029.9	0.914003	KAKKPALVA	92	463.3117	2	924.612	.	G	Superscaf	3689974	0	0	0	0	0	BTP202209	Tissue specific
CDS|exon|ID=cds-Bg	8	6.6925	2718.6	0.914003	KAKKPALV	92	427.7937	2	853.5749	.	G	Superscaf	3689974	0	0	0	0	0	BTP202209	Tissue specific
CDS|exon|ID=cds-Bg	8	6.5075	3048.3	0.913927	ALAFPRAF	90	446.754	2	891.4966	.	M	Superscaf	3693406	0	0	0	0	0	BTP202209	Tissue specific
CDS|exon|ID=cds-Bg	6	5.025	1841.1	0.910025	SLLVTL	71	645.4153	1	644.4109	.	I	Superscaf	3861015	0	0	BTP202209	BTP202209	BTP202209	BTP202209	Mixed
CDS|exon|ID=cds-Bg	7	5.654286	2600.8	0.898018	TVAYLQK	59	411.7381	2	821.4647	.	S	Superscaf	4376262	0	0	0	0	0	BTP202209	Tissue specific
CDS|exon|ID=cds-Bg	18	4.857222	6388.1	0.752909	S(+42.01)	88	704.0483	3	2109.132	Acetylati	V	Superscaf	10602700	0	0	0	0	0	BTP202209	Tissue specific
CDS|exon|ID=cds-Bg	7	6.207143	3019.3	0.744832	Q(+42.01)	63	422.7345	2	843.4562	Acetylati	C	Superscaf	10949656	0	0	BTP202209	0	0	0	Tissue specific
CDS|exon|ID=cds-Bg	6	7.266667	2670.9	0.744378	RLFKYL	61	420.2556	2	838.5065	.	R	Superscaf	10969275	0	BTP202209	0	0	0	0	Tissue specific
CDS|exon|ID=cds-Bg	7	6.258571	2556.8	0.694608	KPGELPP	53	369.2119	2	736.4119	.	K	Superscaf	13104679	0	0	0	BTP202209	0	0	Tissue specific
CDS|exon|ID=cds-Bg	8	6.84	3204.6	0.618186	LPPKHTLP	69	451.7747	2	901.5385	.	R	Superscaf	16384321	0	0	BTP202209	0	0	0	Tissue specific
CDS|exon|ID=cds-Bg	7	5.455714	2404.7	0.57906	VVRKPLN	99	413.267	2	824.5232	.	I	Superscaf	18063022	0	0	0	0	0	BTP202209	Tissue specific
CDS|exon|ID=cds-Bg	6	7.416667	2476.8	0.574363	RLKPGM	75	701.3987	1	700.4054	.	V	Superscaf	18264844	0	0	0	0	BTP202209	BTP202209	Tissue enhanced
CDS|exon|ID=cds-Bg	8	6.555	2892.1	0.534632	YGPPGTGK	93	388.6998	2	775.3864	.	C	Superscaf	19969771	0	0	0	0	0	BTP202209	Tissue specific
CDS|exon|ID=cds-Bg	9	5.447778	3205.3	0.472169	SSDLEAKVK	99	488.7655	2	975.5236	.	M	Superscaf	22650237	0	BTP202209	0	0	0	0	Tissue specific
CDS|exon|ID=cds-Bg	8	5.2075	2663	0.465422	LDVRKVST	65	459.2789	2	916.5342	.	G	Superscaf	22939621	0	0	0	BTP202209	0	0	Tissue specific
CDS|exon|ID=cds-Bg	7	5.604286	2881.3	0.464611	M(+42.01)	75	453.1794	2	904.3459	Acetylati	M	Superscaf	22974618	BTP202209	0	0	0	0	0	Tissue specific
CDS|exon|ID=cds-Bg	5	6.366	2017.3	0.398325	P(+42.01)	55	634.3906	1	633.3849	Acetylati	L	Superscaf	25819047	0	0	0	BTP202209	BTP202209	0	Tissue enhanced
CDS|exon|ID=cds-Bg	8	5.665	3305.7	0.377919	LEEANLMK	98	474.2449	2	946.4794	.	V	Superscaf	26694553	0	0	0	0	0	BTP202209	Tissue specific
CDS|exon|ID=cds-Bg	9	5.142222	3275.5	0.342303	SLEDLKVDK	99	523.7891	2	1045.565	.	K	Superscaf	28222969	BTP202209	BTP202209	0	0	0	0	Tissue enhanced
CDS|exon|ID=cds-Bg	9	3.976667	2528.7	0.309301	VGVDQFLVK	99	502.79	2	1003.57	.	Q	Superscaf	29639017	0	0	0	0	0	BTP202209	Tissue specific
CDS|exon|ID=cds-Bg	6	3.345	1706.9	0.309301	VGVDQF	99	664.3278	1	663.3228	.	Q	Superscaf	29639017	0	0	0	0	0	BTP202209	Tissue specific
CDS|exon|ID=cds-Bg	7	6.718571	2612.9	0.309289	R(+42.01)	61	370.2196	2	738.4388	Acetylati	L	Superscaf	29639668	0	0	0	BTP202209	0	0	Tissue specific
CDS|exon|ID=cds-Bg	7	6.365714	2750.8	0.309094	A(+42.01)	99	459.2402	2	916.4694	Acetylati	G	Superscaf	29648014	0	0	0	0	0	BTP202209	Tissue specific
CDS|exon|ID=cds-Bg	6	5.621667	1741.7	0.302224	LVGAAK	52	558.3588	1	557.3537	.	D	Superscaf	29942263	0	0	0	0	0	BTP202209	Tissue specific
CDS|exon|ID=cds-Bg	7	6.515714	2524.7	0.198575	KAAPFM(+1	55	389.2055	2	776.3891	Oxidation	G	Superscaf	34390612	BTP202209	0	0	0	0	0	Tissue specific
CDS|exon|ID=cds-Bg	7	6.844286	2697.1	0.193041	PAARLPT	54	363.2146	2	724.4232	.	G	Superscaf	34628005	BTP202209	0	0	0	0	0	Tissue specific
CDS|exon|ID=cds-Bg	5	5.354	1877.4	0.170491	M(+42.01)	54	712.2411	1	711.2278	Acetylati	L	Superscaf	35595757	0	0	0	0	BTP202209	0	Tissue specific
CDS|exon|ID=cds-Bg	6	7.585	2718.9	0.069263	RHGFGK	63	351.1957	2	700.3769	.	L	Superscaf	39939679	0	0	0	0	BTP202209	0	Tissue specific
CDS|exon|ID=cds-Bg	8	5.675	3715.3	0.015341	D(+42.01)	56	508.2231	2	1014.444	Acetylati	T	Superscaf	43570269	BTP202209	0	0	0	0	0	Tissue specific
CDS|exon|ID=cds-Bg	7	5.92	3294.9	0.130773	VEEHWRR	67	506.2529	2	1010.505	.	P	Superscaf	48523606	0	0	0	0	0	BTP202209	Tissue specific
CDS|exon|ID=cds-Bg	6	4.046667	1817	0.355085	KPVVEC(+5	54	731.3648	1	730.3683	Carbamido	N	Superscaf	58149357	0	BTP202209	0	0	0	0	Tissue specific
CDS|exon|ID=cds-Bg	8	4.8925	2171.2	0.409512	VGFPSVGK	98	395.7236	2	789.4385	.	G	Superscaf	60484621	0	0	0	0	0	BTP202209	Tissue specific
CDS|exon|ID=cds-Bg	7	4.737143	1985	0.517781	AAKVGVN	80	658.3866	1	657.3809	.	T	Superscaf	65130787	BTP202209	BTP202209	BTP202209	0	BTP202209	0	Mixed
CDS|exon|ID=cds-Bg	9	4.287778	3009.3	0.521648	SDFEAVEAL	71	980.4564	1	979.4498	.	E	Superscaf	65296753	0	BTP202209	0	0	0	0	Tissue specific
CDS|exon|ID=cds-Bg	6	4.623333	1941.3	0.529551	V(+42.01)	51	401.2375	2	800.4578	Acetylati	K	Superscaf	65636130	BTP202209	0	0	BTP202209	0	0	Mixed
CDS|exon|ID=cds-Bg	16	5.673125	6788.7	0.559498	LAEQAERYD	87	951.4099	2	1900.812	Carbamido	S	Superscaf	66920962	0	0	0	0	0	BTP202209	Tissue specific
CDS|exon|ID=cds-Bg	7	4.864286	2419.6	0.560078	ALNFSVF	73	797.4153	1	796.4119	.	G	Superscaf	66945799	0	0	0	BTP202209	BTP202209	BTP202209	Mixed
CDS|exon|ID=cds-Bg	13	4.241538	4276.7	0.565272	VDEAVAVLG	77	675.853	2	1349.694	.	L	Superscaf	67168771	0	0	0	0	0	BTP202209	Tissue specific
CDS|exon|ID=cds-Bg	6	4.576667	2016.2	0.580907	KDVDPF	67	720.355	1	719.3489	.	I	Superscaf	67839625	0	BTP202209	0	0	0	0	Tissue specific
CDS|exon|ID=cds-Bg	8	6.16	3199.6	0.608034	M(+15.99)	88	478.2302	2	954.4481	Oxidation	A	Superscaf	69003469	0	BTP202209	0	0	0	0	Tissue specific
CDS|exon|ID=cds-Bg	6	7.461667	2714	0.61712	Q(+42.01)	50	369.7138	2	737.4184	Acetylati	A	Superscaf	69393723	0	0	0	BTP202209	0	0	Tissue specific
CDS|exon|ID=cds-Bg	16	4.134375	5760.5	0.636489	DDEVPDLVE	89	911.4047	2	1820.795	.	S	Superscaf	70224427	0	0	BTP202209	0	0	0	Tissue specific
CDS|exon|ID=cds-Bg	8	4.45	2610	0.708323	VDSAMELL	59	877.4302	1	876.4263	.	Y	Superscaf	73307179	0	0	BTP202209	0	0	0	Tissue specific
CDS|exon|ID=cds-Bg	6	3.99	1873.2	0.712011	M(+42.01)	58	360.1658	2	718.3207	Acetylati	M	Superscaf	73465666	0	0	0	BTP202209	0	0	Tissue specific
CDS|exon|ID=cds-Bg	7	6.564286	3093.6	0.726242	RPPPEHF	89	440.2268	2	878.4398	.	F	Superscaf	74074150	0	0	0	BTP202209	0	0	Tissue specific
CDS|exon|ID=cds-Bg	6	6.136667	1960.2	0.802859	PGGPLP	51	537.3011	1	536.2958	.	N	Superscaf	77364094	0	0	BTP202209	0	0	0	Tissue specific
CDS|exon|ID=cds-Bg	8	5.43625	2343.6	0.857175	VPAAATPP	67	723.3965	1	722.3962	.	A	Superscaf	79695114	0	0	BTP202209	0	0	0	Tissue specific
CDS|exon|ID=cds-Bg	6	7.301667	3402	0.860485	GQQRC(+57	54	804.377	1	803.382	Carbamido	N	Superscaf	79837086	0	0	BTP202209	0	0	0	Tissue specific
CDS|exon|ID=cds-Bg	7	4.682857	2692.1	0.882962	LDELDSL	82	804.3976	1	803.3912	.	E	Superscaf	80801458	0	BTP202209	0	0	0	0	Tissue specific
CDS|exon|ID=cds-Bg	7	6.914286	3124.6	0.885556	REASLLR	69	422.7519	2	843.4926	.	G	Superscaf	80913015	0	0	BTP202209	0	0	0	Tissue specific
CDS|exon|ID=cds-Bg	11	7.157273	4993.5	0.977611	DLEKRKEPK	57	465.939	3	1394.799	.	K	Superscaf	84863020	0	0	0	0	0	BTP202209	Tissue specific
CDS|exon|ID=cds-Bg	9	4.765556	2622.8	0.979312	SDALTGVVK	79	445.2517	2	888.4916	.	V	Superscaf	84935863	0	0	0	0	0	BTP202209	Tissue specific
CDS|exon|ID=cds-Bg	9	5.041111	3257.4	0.980034	VDDFDAFKK	99	542.7675	2	1083.524	.	T	Superscaf	84966874	0	0	0	0	0	BTP202209	Tissue specific
CDS|exon|ID=cds-Bg	9	5.28	3122.6	0.916142	AGELPDVPF	97	477.2545	2	952.4977	.	Q	Superscaf	3569859	0	0	0	0	0	BTP202209	Tissue specific
CDS|exon|ID=cds-Bg	7	5.364286	2177.3	0.864746	VPAGEPK	50	697.3872	1	696.3806	.	I	Superscaf	5755588	0	0	0	0	0	BTP202209	Tissue specific
CDS|exon|ID=cds-Bg	7	4.998571	2895.4	0.842038	DLM(+15.9	64	419.1851	2	836.3586	Oxidation	V	Superscaf	6721801	0	0	BTP202209	0	0	0	Tissue specific
CDS|exon|ID=cds-Bg	7	4.904286	2483.8	0.623774	C(+57.02)	72	366.6931	2	731.3636	Carbamido	L	Superscaf	16009855	0	0	0	0	BTP202209	0	Tissue specific
CDS|exon|ID=cds-Bg	11	6.321818	4022.3	0.574226	LGSLSSLPA	86	537.311	2	1072.613	.	C	Superscaf	18118228	0	0	0	0	0	BTP202209	Tissue specific
CDS|exon|ID=cds-Bg	6	4.85	2118.5	0.559511	YLVM(+15.	67	376.6862	2	751.3575	Oxidation	R	Superscaf	18744615	0	0	0	BTP202209	0	0	Tissue specific
CDS|exon|ID=cds-Bg	12	4.365833	3869.6	0.509476	NMTVRVPDA	61	652.3174	2	1302.624	.	D	Superscaf	20873650	0	0	BTP202209	0	0	0	Tissue specific
CDS|exon|ID=cds-Bg	8	7.3325	3289.8	0.473082	AAGRRTPP	74	413.2362	2	824.4617	.	A	Superscaf	22422445	0	0	BTP202209	0	0	0	Tissue specific
CDS|exon|ID=cds-Bg	15	6.851333	5646.7	0.412716	PEPAKSAPA	97	373.9662	4	1491.841	.	R	Superscaf	24991204	0	0	BTP202209	0	BTP202209	BTP202209	Mixed
CDS|exon|ID=cds-Bg	27	6.157778	10074.8	0.412709	RLLLPGELA	98	577.7275	5	2883.618	.	R	Superscaf	24991204	0	0	0	0	BTP202209	0	Tissue specific
CDS|exon|ID=cds-Bg	10	5.682	3477.7	0.41271	LPGELAKHA	99	517.8012	2	1033.592	.	R	Superscaf	24991204	0	0	0	0	BTP202209	0	Tissue specific
CDS|exon|ID=cds-Bg	9	5.936667	3149.3	0.412709	SEGTKAVTK	80	460.7537	2	919.4974	.	R	Superscaf	24991204	0	0	0	0	0	BTP202209	Tissue specific
CDS|exon|ID=cds-Bg	9	6.121111	3671.1	0.404476	A(+42.01)	92	489.7169	2	977.4236	Acetylati	R	Superscaf	25341787	0	0	0	BTP202209	0	0	Tissue specific
CDS|exon|ID=cds-Bg	12	6.346667	4980.5	0.335281	A(+42.01)	96	669.8059	2	1337.603	Acetylati	L	Superscaf	28286437	0	0	0	0	0	BTP202209	Tissue specific
CDS|exon|ID=cds-Bg	16	6.076875	6443	0.335281	A(+42.01)	92	594.2921	3	1779.857	Acetylati	L	Superscaf	28286437	0	BTP202209	0	0	0	BTP202209	Tissue enhanced
CDS|exon|ID=cds-Bg	15	5.832667	5974.6	0.335281	A(+42.01)	92	826.8853	2	1651.763	Acetylati	L	Superscaf	28286437	0	0	0	0	0	BTP202209	Tissue specific

CDS|exon|ID=cds-Bg	14	5.843571	5591.2	0.335281	A(+42.01)	92	783.37	2	1564.731	Acetylati	L	Superscaf	28286437	0	0	0	0	0	BTP202209	Tissue specific
CDS|exon|ID=cds-Bg	13	5.858462	4980.5	0.335281	A(+42.01)	92	719.3395	2	1436.672	Acetylati	L	Superscaf	28286437	0	0	0	0	0	BTP202209	Tissue specific
CDS|exon|ID=cds-Bg	11	6.630909	4555	0.335281	A(+42.01)	94	605.2872	2	1208.561	Acetylati	L	Superscaf	28286437	0	BTP202209	0	0	0	BTP202209	Mixed
CDS|exon|ID=cds-Bg	9	6.277778	3522.8	0.335281	A(+42.01)	99	966.4294	1	965.4277	Acetylati	L	Superscaf	28286437	0	0	0	0	0	BTP202209	Tissue specific
CDS|exon|ID=cds-Bg	14	6.128571	5535	0.335281	KGPSYGMSF	96	518.5941	3	1552.767	.	L	Superscaf	28286437	0	0	0	0	BTP202209	BTP202209	Mixed
CDS|exon|ID=cds-Bg	13	5.850769	5066.6	0.335281	KGPSYGMSF	87	713.3405	2	1424.672	.	L	Superscaf	28286437	0	0	0	0	0	BTP202209	Tissue specific
CDS|exon|ID=cds-Bg	12	5.865	4683.2	0.335281	KGPSYGMSF	99	669.8248	2	1337.64	.	L	Superscaf	28286437	0	0	0	0	BTP202209	0	Tissue specific
CDS|exon|ID=cds-Bg	9	6.834444	3647	0.335281	KGPSYGMSF	98	491.7413	2	981.4702	.	L	Superscaf	28286437	0	0	0	0	BTP202209	BTP202209	Mixed
CDS|exon|ID=cds-Bg	7	6.438571	2614.8	0.335281	KGPSYGM	99	739.3411	1	738.337	.	L	Superscaf	28286437	0	0	0	0	BTP202209	0	Tissue specific
CDS|exon|ID=cds-Bg	14	5.010714	5716.4	0.33528	EKKYDEELE	95	904.9462	2	1807.884	.	L	Superscaf	28286437	0	0	0	0	BTP202209	0	Tissue specific
CDS|exon|ID=cds-Bg	10	5.702	3975.7	0.335279	C(+57.02)	91	564.7621	2	1127.514	Carbamidc	L	Superscaf	28286437	0	0	0	0	BTP202209	0	Tissue specific
CDS|exon|ID=cds-Bg	9	5.817778	3163.3	0.335274	YPDGSKPVK	99	495.7644	2	989.5181	.	H	Superscaf	28286638	0	0	0	0	BTP202209	0	Tissue specific
CDS|exon|ID=cds-Bg	16	5.348125	5581.1	0.335273	YPDGSKPVK	72	855.9352	2	1709.862	.	H	Superscaf	28286638	0	0	0	0	BTP202209	0	Tissue specific
CDS|exon|ID=cds-Bg	11	5.03	3726	0.335273	DGSKPVKVF	95	585.3102	2	1168.609	.	H	Superscaf	28286638	0	0	0	0	BTP202209	0	Tissue specific
CDS|exon|ID=cds-Bg	10	5.331	3308.6	0.335273	KPVKVPENF	57	368.8836	3	1103.634	.	H	Superscaf	28286638	0	0	0	0	BTP202209	0	Tissue specific
CDS|exon|ID=cds-Bg	8	5.08875	2633.8	0.335273	KPVKVPEN	96	455.77	2	909.5283	.	H	Superscaf	28286638	0	0	0	0	BTP202209	0	Tissue specific
CDS|exon|ID=cds-Bg	20	5.4505	7700.3	0.335272	KQMEQVAQF	79	762.0562	3	2283.157	.	H	Superscaf	28286638	0	0	0	0	BTP202209	BTP202209	Tissue enhanced
CDS|exon|ID=cds-Bg	11	5.716364	4618	0.335272	KQMEQVAQF	97	675.3641	2	1348.717	.	H	Superscaf	28286638	0	0	0	0	BTP202209	BTP202209	Tissue enhanced
CDS|exon|ID=cds-Bg	9	5.24	3796.2	0.335272	KQMEQVAQF	59	554.7747	2	1107.538	.	H	Superscaf	28286638	0	0	0	0	0	BTP202209	Tissue specific
CDS|exon|ID=cds-Bg	19	5.224737	7231.9	0.335272	QMEQVAQFL	96	1078.536	2	2155.062	.	H	Superscaf	28286638	0	0	0	0	0	BTP202209	Tissue specific
CDS|exon|ID=cds-Bg	10	5.314	4149.6	0.335272	QMEQVAQFL	97	611.3156	2	1220.622	.	H	Superscaf	28286638	0	0	0	0	0	BTP202209	Tissue specific
CDS|exon|ID=cds-Bg	9	4.822222	3681.2	0.335272	QMEQVAQFL	55	547.2686	2	1092.527	.	H	Superscaf	28286638	0	0	0	0	0	BTP202209	Tissue specific
CDS|exon|ID=cds-Bg	8	4.6775	3327.8	0.335272	QMEQVAQF	57	490.7273	2	979.4433	.	H	Superscaf	28286638	0	0	0	0	0	BTP202209	Tissue specific
CDS|exon|ID=cds-Bg	15	5.266667	5213.5	0.335272	VAQFLKAAE	97	820.4339	2	1638.862	.	H	Superscaf	28286638	0	0	0	0	0	BTP202209	Tissue specific
CDS|exon|ID=cds-Bg	12	5.610833	4291.5	0.335272	FLKAAEDYG	99	671.3534	2	1340.698	.	H	Superscaf	28286638	0	0	0	0	BTP202209	BTP202209	Tissue enhanced
CDS|exon|ID=cds-Bg	16	5.50375	5859.4	0.335271	FLKAAEDYG	97	918.4438	2	1834.881	.	H	Superscaf	28286638	0	0	0	0	0	BTP202209	Tissue specific
CDS|exon|ID=cds-Bg	8	5.63625	3128.3	0.335272	FLKAAEDY	99	478.7374	2	955.465	.	H	Superscaf	28286638	0	0	0	0	0	BTP202209	Tissue specific
CDS|exon|ID=cds-Bg	11	5.622727	3904.1	0.335272	LKAAEDYGV	99	597.8189	2	1193.629	.	H	Superscaf	28286638	0	0	0	0	BTP202209	BTP202209	Tissue enhanced
CDS|exon|ID=cds-Bg	15	5.505333	5472	0.335271	LKAAEDYGV	97	844.9097	2	1687.813	.	H	Superscaf	28286638	0	0	0	0	0	BTP202209	Tissue specific
CDS|exon|ID=cds-Bg	14	5.507143	5084.6	0.335271	LKAAEDYGV	99	771.3763	2	1540.744	.	H	Superscaf	28286638	0	0	0	0	0	BTP202209	Tissue specific
CDS|exon|ID=cds-Bg	10	5.587	3550.7	0.335272	KAAEDYGVT	99	541.2773	2	1080.545	.	H	Superscaf	28286638	0	0	0	BTP202209	BTP202209	BTP202209	Mixed
CDS|exon|ID=cds-Bg	13	5.470769	4731.2	0.335271	KAAEDYGVT	98	714.8347	2	1427.66	.	H	Superscaf	28286638	0	0	0	0	BTP202209	0	Tissue specific
CDS|exon|ID=cds-Bg	9	5.125556	3082.3	0.335272	AAEDYGVTK	99	477.2306	2	952.4501	.	H	Superscaf	28286638	0	0	0	0	BTP202209	BTP202209	Mixed
CDS|exon|ID=cds-Bg	13	5.143077	4650.2	0.335271	AAEDYGVTK	91	724.3206	2	1446.634	.	H	Superscaf	28286638	0	0	0	0	0	BTP202209	Tissue specific
CDS|exon|ID=cds-Bg	12	5.115	4262.8	0.335271	AAEDYGVTK	96	650.7885	2	1299.565	.	H	Superscaf	28286638	0	0	0	0	BTP202209	BTP202209	Mixed
CDS|exon|ID=cds-Bg	8	5.01375	2771	0.335272	AEDYGVTK	99	441.7124	2	881.413	.	H	Superscaf	28286638	0	0	0	0	BTP202209	0	Tissue specific
CDS|exon|ID=cds-Bg	11	5.032727	3951.5	0.335271	AEDYGVTKT	93	615.2693	2	1228.528	.	H	Superscaf	28286638	0	0	0	0	BTP202209	0	Tissue specific
CDS|exon|ID=cds-Bg	9	5.402222	3341.8	0.335271	GVTKTDMFQ	58	513.7477	2	1025.485	.	H	Superscaf	28286638	0	0	0	0	0	BTP202209	Tissue specific
CDS|exon|ID=cds-Bg	8	5.37125	2731.1	0.335271	GVTKTDMF	79	449.7187	2	897.4266	.	H	Superscaf	28286638	0	0	0	0	BTP202209	BTP202209	Mixed
CDS|exon|ID=cds-Bg	10	4.734	3728.4	0.335271	TDMFQTVDL	81	608.7795	2	1215.548	.	H	Superscaf	28286638	0	0	0	0	0	BTP202209	Tissue specific
CDS|exon|ID=cds-Bg	9	4.651111	3341	0.335271	TDMFQTVDL	78	535.2452	2	1068.48	.	H	Superscaf	28286638	0	0	0	0	0	BTP202209	Tissue specific
CDS|exon|ID=cds-Bg	13	5.814615	4595.1	0.335263	TLMALGSLA	98	659.8711	2	1317.733	.	G	Superscaf	28287118	0	0	0	0	BTP202209	0	Tissue specific
CDS|exon|ID=cds-Bg	11	5.734545	3844.2	0.335263	MALGSLAVT	99	552.8063	2	1103.601	.	G	Superscaf	28287118	0	0	0	0	BTP202209	0	Tissue specific
CDS|exon|ID=cds-Bg	10	5.767	3247.5	0.335263	MALGSLAVT	96	495.7845	2	989.5579	.	G	Superscaf	28287118	0	0	0	0	BTP202209	0	Tissue specific
CDS|exon|ID=cds-Bg	10	5.733	3472.7	0.335263	ALGSLAVTK	99	487.2852	2	972.5604	.	G	Superscaf	28287118	0	0	0	0	BTP202209	0	Tissue specific
CDS|exon|ID=cds-Bg	9	5.768889	2876	0.335263	ALGSLAVTK	99	430.2646	2	858.5175	.	G	Superscaf	28287118	0	0	0	0	BTP202209	0	Tissue specific
CDS|exon|ID=cds-Bg	9	5.701111	3161.4	0.335263	LGSLAVTKN	99	451.7686	2	901.5233	.	G	Superscaf	28287118	0	0	BTP202209	0	BTP202209	0	Tissue enhanced
CDS|exon|ID=cds-Bg	13	6.189231	5833.6	0.335262	DGHYRGDPN	88	541.575	3	1621.71	.	G	Superscaf	28287118	0	0	0	0	BTP202209	0	Tissue specific
CDS|exon|ID=cds-Bg	11	5.906364	4993.7	0.335262	DGHYRGDPN	59	682.2917	2	1362.574	.	G	Superscaf	28287118	0	0	0	0	BTP202209	0	Tissue specific
CDS|exon|ID=cds-Bg	10	5.949	4606.3	0.335262	DGHYRGDPN	87	608.7581	2	1215.506	.	G	Superscaf	28287118	0	0	0	0	BTP202209	0	Tissue specific
CDS|exon|ID=cds-Bg	9	6.28	4194.8	0.335262	GHYRGDPNW	86	551.2451	2	1100.479	.	G	Superscaf	28287118	0	0	0	0	BTP202209	0	Tissue specific
CDS|exon|ID=cds-Bg	10	6.393	4505.1	0.335262	YRGDPNWFM	76	438.5399	3	1312.602	.	G	Superscaf	28287118	0	0	0	0	BTP202209	0	Tissue specific
CDS|exon|ID=cds-Bg	8	6.055	3665.2	0.335262	YRGDPNWF	72	527.739	2	1053.467	.	G	Superscaf	28287118	0	0	0	0	BTP202209	0	Tissue specific
CDS|exon|ID=cds-Bg	7	6.137143	3277.8	0.335262	YRGDPNW	81	454.2048	2	906.3984	.	G	Superscaf	28287118	0	0	0	0	BTP202209	0	Tissue specific
CDS|exon|ID=cds-Bg	7	5.934286	3099.5	0.335262	DPNWFMK	98	469.2146	2	936.4164	.	G	Superscaf	28287118	0	0	0	0	BTP202209	0	Tissue specific
CDS|exon|ID=cds-Bg	14	6.027857	6678.5	0.335253	AQEHKREFT	80	865.9229	2	1729.838	.	Q	Superscaf	28287571	0	0	0	0	0	BTP202209	Tissue specific
CDS|exon|ID=cds-Bg	10	6.143	4720.3	0.335253	AQEHKREFT	95	637.8076	2	1273.605	.	Q	Superscaf	28287571	0	0	0	0	0	BTP202209	Tissue specific
CDS|exon|ID=cds-Bg	15	6.11	6947.7	0.335253	EHKREFTES	94	923.4541	2	1844.902	.	Q	Superscaf	28287571	0	0	0	0	0	BTP202209	Tissue specific
CDS|exon|ID=cds-Bg	13	5.841538	6182	0.335253	EHKREFTES	71	830.897	2	1659.785	.	Q	Superscaf	28287571	0	0	0	0	BTP202209	BTP202209	Mixed
CDS|exon|ID=cds-Bg	14	6.316429	6522.2	0.335252	KREFTESQL	89	572.9586	3	1715.859	.	Q	Superscaf	28287571	0	0	0	0	BTP202209	0	Tissue specific
CDS|exon|ID=cds-Bg	13	6.218462	5902.5	0.335253	KREFTESQL	86	527.2725	3	1578.8	.	Q	Superscaf	28287571	0	0	0	0	0	BTP202209	Tissue specific
CDS|exon|ID=cds-Bg	11	5.920909	5136.8	0.335253	KREFTESQL	71	697.8471	2	1393.684	.	Q	Superscaf	28287571	0	0	0	0	0	BTP202209	Tissue specific
CDS|exon|ID=cds-Bg	12	5.925	5434.1	0.335253	REFTESQLQ	94	726.3572	2	1450.705	.	Q	Superscaf	28287571	0	0	0	0	0	BTP202209	Tissue specific
CDS|exon|ID=cds-Bg	12	5.660833	5405	0.335252	EFTESQLQE	99	716.8359	2	1431.663	.	Q	Superscaf	28287571	0	0	0	0	BTP202209	BTP202209	Mixed
CDS|exon|ID=cds-Bg	13	5.225385	5405	0.335252	EFTESQLQE	96	766.3709	2	1530.731	.	Q	Superscaf	28287571	0	0	0	0	BTP202209	0	Tissue specific
CDS|exon|ID=cds-Bg	11	5.485455	4785.3	0.335253	EFTESQLQE	99	648.3063	2	1294.604	.	Q	Superscaf	28287571	0	0	0	0	BTP202209	0	Tissue specific
CDS|exon|ID=cds-Bg	11	5.882727	4979.5	0.335252	FTESQLQEG	99	652.3152	2	1302.62	.	Q	Superscaf	28287571	0	0	0	0	BTP202209	BTP202209	Tissue enhanced
CDS|exon|ID=cds-Bg	12	5.3925	4979.5	0.335252	FTESQLQEG	98	701.8495	2	1401.689	.	Q	Superscaf	28287571	0	0	0	0	BTP202209	0	Tissue specific
CDS|exon|ID=cds-Bg	10	5.712	4359.8	0.335253	FTESQLQEG	99	583.7864	2	1165.562	.	Q	Superscaf	28287571	0	0	0	0	BTP202209	BTP202209	Mixed
CDS|exon|ID=cds-Bg	8	5.17625	3594.1	0.335253	FTESQLQE	63	491.2274	2	980.4451	.	Q	Superscaf	28287571	0	0	0	0	BTP202209	0	Tissue specific
CDS|exon|ID=cds-Bg	10	5.923	4592.1	0.335252	TESQLQEGK	99	578.7816	2	1155.552	.	Q	Superscaf	28287571	0	0	0	0	BTP202209	BTP202209	Mixed
CDS|exon|ID=cds-Bg	11	5.384545	4592.1	0.335252	TESQLQEGK	98	628.3163	2	1254.62	.	Q	Superscaf	28287571	0	0	0	0	BTP202209	BTP202209	Mixed
CDS|exon|ID=cds-Bg	9	5.737778	3972.4	0.335253	TESQLQEGK	94	510.2516	2	1018.493	.	Q	Superscaf	28287571	0	0	0	0	0	BTP202209	Tissue specific
CDS|exon|ID=cds-Bg	10	5.27	4194.6	0.335252	ESQLQEGKF	96	577.7917	2	1153.573	.	Q	Superscaf	28287571	0	0	0	0	BTP202209	0	Tissue specific
CDS|exon|ID=cds-Bg	9	7.074444	3755.4	0.335251	GMTGYGRPF	92	497.748	2	993.4814	.	Q	Superscaf	28287571	0	BTP202209	0	0	0	0	Tissue specific
CDS|exon|ID=cds-Bg	7	6.705714	2809.3	0.335251	MTGYGRP	99	391.1856	2	780.3588	.	Q	Superscaf	28287571	BTP202209	0	0	0	0	0	Tissue specific
CDS|exon|ID=cds-Bg	8	7.2	3697.3	0.335251	TGYGRPRQ	74	467.7446	2	933.478	.	Q	Superscaf	28287571	0	0	0	0	0	BTP202209	Tissue specific
CDS|exon|ID=cds-Bg	11	5.371818	3608.8	0.320692	VSEGTKAVT	96	591.821	2	1181.629	.	P	Superscaf	28906873	0	BTP202209	0	0	0	BTP202209	Mixed
CDS|exon|ID=cds-Bg	7	6.89	2765	0.314241	APPLHPK	50	380.2218	2	758.4438	.	T	Superscaf	29181559	0	BTP202209	0	0	0	0	Tissue specific
CDS|exon|ID=cds-Bg	6	5.568333	1990.1	0.305631	KLVTSF	50	694.4199	1	693.4061	.	R	Superscaf	29548368	0	0	0	0	BTP202209	0	Tissue specific
CDS|exon|ID=cds-Bg	7	4.607143	2443.7	0.305611	VPSWEFS	65	851.3912	1	850.3861	.	G	Superscaf	29549070	0	BTP202209	0	0	0	0	Tissue specific
CDS|exon|ID=cds-Bg	7	6.962857	2851.2	0.304917	TPPHLKP	78	395.2334	2	788.4545	.	K	Superscaf	29577769	BTP202209	0	0	0	0	0	Tissue specific
CDS|exon|ID=cds-Bg	10	5.131	3092.3	0.303369	VLLAAAVC(	98	523.3047	2	1044.6	Carbamidc	Y	Superscaf	29644411	0	0	0	0	0	BTP202209	Tissue specific
CDS|exon|ID=cds-Bg	7	5.188571	2673	0.303365	VYQPMEK	91	447.7197	2	893.4317	.	Y	Superscaf	29644411	0	0	0	0	0	BTP202209	Tissue specific
CDS|exon|ID=cds-Bg	14	5.498571	5068.2	0.303319	AKGKEVDNF	92	530.9654	3	1589.878	.	L	Superscaf	29646487	0	0	0	0	0	BTP202209	Tissue specific
CDS|exon|ID=cds-Bg	10	4.55	3466.7	0.303319	GKEVDNFVD	99	575.7888	2	1149.567	.	L	Superscaf	29646487	0	0	0	0	0	BTP202209	Tissue specific
CDS|exon|ID=cds-Bg	11	5.022727	3991.2	0.303319	KEVDNFVDK	99	667.8665	2	1333.724	.	L	Superscaf	29646487	0	0	0	0	0	BTP202209	Tissue specific
CDS|exon|ID=cds-Bg	10	4.551	3522.8	0.303319	EVDNFVDKL	99	603.8196	2	1205.629	.	L	Superscaf	29646487	0	0	0	0	0	BTP202209	Tissue specific
CDS|exon|ID=cds-Bg	9	3.974444	3054.4	0.303319	EVDNFVDKL	94	539.7722	2	1077.534	.	L	Superscaf	29646487	0	0	0	0	0	BTP202209	Tissue specific
CDS|exon|ID=cds-Bg	8	4.06875	2628.9	0.303319	VDNFVDKL	99	475.2504	2	948.4916	.	L	Superscaf	29646487	0	0	0	0	0	BTP202209	Tissue specific
CDS|exon|ID=cds-Bg	9	3.972222	2510.7	0.30312	VNSDVGVLK	98	465.7644	2	929.5182	.	T	Superscaf	29654950	0	0	0	0	0	BTP202209	Tissue specific
CDS|exon|ID=cds-Bg	7	5.871429	2303.7	0.297226	MGVPARP	61	364.1977	2	726.3846	.	G	Superscaf	29905492	0	0	BTP202209	0	0	0	Tissue specific
CDS|exon|ID=cds-Bg	8	6.245	3066.5	0.294941	LDRLLAGP	81	427.7626	2	853.5021	.	G	Superscaf	30003321	0	0	0	0	0	BTP202209	Tissue specific
CDS|exon|ID=cds-Bg	8	5.7775	3191.5	0.294863	LGEHAYPF	69	467.2256	2	932.4391	.	G	Superscaf	30006588	0	BTP202209	0	0	0	BTP202209	Tissue enhanced
CDS|exon|ID=cds-Bg	8	5.0075	2149.2	0.293515	VPAKVPSA	55	768.4557	1	767.4541	.	Y	Superscaf	30063798	0	0	BTP202209	0	0	0	Tissue specific
CDS|exon|ID=cds-Bg	12	4.940833	3688.8	0.293163	DAVVFHPKF	89	448.585	3	1342.74	.	T	Superscaf	30078667	0	0	0	0	0	BTP202209	Tissue specific
CDS|exon|ID=cds-Bg	9	6.237778	2984.4	0.290431	P(+42.01)	83	441.7385	2	881.4647	Acetylati	G	Superscaf	30194722	0	BTP202209	0	0	0	0	Tissue specific
CDS|exon|ID=cds-Bg	6	6.381667	2532	0.287223	PPYDPR	73	372.6848	2	743.3602	.	H	Superscaf	30331665	0	0	0	BTP202209	0	0	Tissue specific
CDS|exon|ID=cds-Bg	5	5.858	1885	0.286857	KHYVP	51	643.3649	1	642.3489	.	R	Superscaf	30347277	0	BTP202209	0	0	0	0	Tissue specific
CDS|exon|ID=cds-Bg	6	6.528333	2275.4	0.247453	YAM(+15.9	98	356.6867	2	711.3625	Oxidatior	R	Superscaf	32023648	0	0	0	0	0	BTP202209	Tissue specific
CDS|exon|ID=cds-Bg	12	6.0425	4669.1	0.247452	YAMLAKTGV	94	464.2347	3	1389.686	.	R	Superscaf	32023648	0	0	0	0	0	BTP202209	Tissue specific
CDS|exon|ID=cds-Bg	12	5.1875	4203.9	0.200763	YVDTAVRHV	87	481.2784	3	1440.82	.	L	Superscaf	34010191	0	0	0	0	BTP202209	0	Tissue specific
CDS|exon|ID=cds-Bg	8	5.595	2636.7	0.17217	LSVSKASL	63	402.7439	2	803.4752	.	R	Superscaf	35227450	0	BTP202209	0	0	0	0	Tissue specific
CDS|exon|ID=cds-Bg	7	5.355714	2505.9	0.167462	PVPDRSF	64	409.2072	2	816.413	.	A	Superscaf	35427328	0	0	BTP202209	0	0	0	Tissue specific
CDS|exon|ID=cds-Bg	6	6.376667	2690.9	0.165595	YGC(+57.0	72	413.6957	2	825.3843	Carbamidc	F	Superscaf	35507331	0	0	0	0	BTP202209	0	Tissue specific
CDS|exon|ID=cds-Bg	7	5.47	2448.5	0.164274	K(+42.01)	63	824.4261	1	823.4228	Acetylati	R	Superscaf	35563447	0	0	0	0	0	BTP202209	Tissue specific
CDS|exon|ID=cds-Bg	9	5.317778	2815	0.091576	THAVLVALK	89	476.3009	2	950.5912	.	G	Superscaf	38657032	0	0	0	0	0	BTP202209	Tissue specific
CDS|exon|ID=cds-Bg	11	6.436364	4994.7	0.091352	LEGLEERPG	86	677.8726	2	1353.736	.	F	Superscaf	38666539	0	0	0	0	0	BTP202209	Tissue specific
CDS|exon|ID=cds-Bg	10	6.106	4526.3	0.091352	LEGLEERPG	98	409.5527	3	1225.641	.	F	Superscaf	38666539	0	0	0	0	0	BTP202209	Tissue specific
CDS|exon|ID=cds-Bg	8	4.735	3087.5	0.091144	T(+42.01)	93	485.2025	2	968.3909	Acetylati	P	Superscaf	38675458	0	BTP202209	0	0	BTP202209	BTP202209	Mixed
CDS|exon|ID=cds-Bg	8	5.595	2726.8	0.090925	SSSPAVKY	85	419.7175	2	837.4232	.	N	Superscaf	38684728	0	0	0	0	0	BTP202209	Tissue specific
CDS|exon|ID=cds-Bg	9	5.825556	3914.2	0.090601	LEEEKLSQK	98	552.2983	2	1102.587	.	S	Superscaf	38698387	0	0	0	0	0	BTP202209	Tissue specific
CDS|exon|ID=cds-Bg	12	6.09	4732	0.090593	EAADPLASK	99	628.8418	2	1255.677	.	R	Superscaf	38698636	0	0	0	0	0	BTP202209	Tissue specific
CDS|exon|ID=cds-Bg	10	6.384	3995.2	0.090593	ADPLASKLN	93	528.8039	2	1055.597	.	R	Superscaf	38698636	0	0	0	0	0	BTP202209	Tissue specific
CDS|exon|ID=cds-Bg	6	4.051667	2286.6	0.090536	VHVNQY	60	380.1909	2	758.3712	.	Q	Superscaf	38701288	0	0	0	0	0	BTP202209	Tissue specific
CDS|exon|ID=cds-Bg	9	5.525556	3056.4	0.090512	LVEKPSPLT	99	492.2908	2	982.5699	.	P	Superscaf	38702359	0	0	0	0	0	BTP202209	Tissue specific
CDS|exon|ID=cds-Bg	8	5.46875	2703	0.090512	VEKPSPLT	97	435.748	2	869.4858	.	P	Superscaf	38702359	0	0	0	0	0	BTP202209	Tissue specific
CDS|exon|ID=cds-Bg	13	6.144615	5300.7	0.08801	A(+42.01)	61	687.8212	2	1373.632	Acetylati	L	Superscaf	38808613	0	0	0	BTP202209	0	0	Tissue specific
CDS|exon|ID=cds-Bg	14	6.11	5760.2	0.08801	A(+42.01)	96	769.3546	2	1536.696	Acetylati	L	Superscaf	38808613	0	BTP202209	0	0	0	BTP202209	Mixed
CDS|exon|ID=cds-Bg	12	5.845	4832.3	0.088011	A(+42.01)	68	623.7759	2	1245.537	Acetylati	L	Superscaf	38808613	0	BTP202209	0	0	0	0	Tissue specific
CDS|exon|ID=cds-Bg	11	6.083636	4406.8	0.088011	A(+42.01)	91	559.2532	2	1116.495	Acetylati	L	Superscaf	38808613	0	0	BTP202209	0	BTP202209	0	Mixed
CDS|exon|ID=cds-Bg	11	4.142727	2308.3	0.08801	LVVVGGGGV	98	471.2913	2	940.5706	.	L	Superscaf	38808613	0	0	0	0	0	BTP202209	Tissue specific
CDS|exon|ID=cds-Bg	10	3.959	1954.9	0.08801	VVVGGGGVG	99	414.7487	2	827.4865	.	L	Superscaf	38808613	0	0	0	0	0	BTP202209	Tissue specific
CDS|exon|ID=cds-Bg	9	4.398889	1954.9	0.08801	VVGGGGVGK	97	365.2157	2	728.418	.	L	Superscaf	38808613	0	0	0	0	0	BTP202209	Tissue specific
CDS|exon|ID=cds-Bg	6	7.321667	2579.7	0.004891	K(+42.01)	68	350.7003	2	699.3915	Acetylati	F	Superscaf	42345868	0	0	0	BTP202209	0	0	Tissue specific
CDS|exon|ID=cds-Bg	5	4.788	1873.2	0.00489	ALDEM	56	578.243	1	577.2418	.	F	Superscaf	42345868	0	0	0	BTP202209	0	0	Tissue specific
CDS|exon|ID=cds-Bg	8	6.29875	3245.5	0.004759	KEELLKLT	68	487.3009	2	972.5855	.	T	Superscaf	42351421	0	BTP202209	0	0	0	0	Tissue specific
CDS|exon|ID=cds-Bg	11	5.334545	3663	0.000785	PGPVESELG	99	555.2943	2	1108.576	.	T	Superscaf	42520351	0	0	0	0	BTP202209	0	Tissue specific
CDS|exon|ID=cds-Bg	9	6.272222	3202.5	0.003305	PGAGLAHPF	50	408.7157	2	815.429	.	Q	Superscaf	42694657	0	0	0	BTP202209	0	0	Tissue specific
CDS|exon|ID=cds-Bg	7	6.132857	2286.2	0.019958	ALFGVKK	86	381.7458	2	761.4799	.	D	Superscaf	43403406	0	0	0	0	BTP202209	BTP202209	Tissue enhanced
CDS|exon|ID=cds-Bg	9	5.861111	3542.8	0.022626	A(+42.01)	53	844.373	1	843.3723	Acetylati	K	Superscaf	43516738	0	0	0	BTP202209	0	0	Tissue specific
CDS|exon|ID=cds-Bg	6	4.45	1787	0.048524	VLDGPF	73	647.3359	1	646.3326	.	C	Superscaf	44619123	0	0	0	0	0	BTP202209	Tissue specific

CDS|exon|ID=cds-Bg	9	5.043333	3691.1	0.106352	FHDSDLAEF	74	540.7333	2	1079.456	.	F	Superscaf	47079751	0	0	0	0	0	BTP20220	Tissue specific
CDS|exon|ID=cds-Bg	8	4.76	2478.8	0.106361	ALPYVHTV	75	450.2511	2	898.4912	.	G	Superscaf	47079961	0	0	0	0	BTP20220	0	Tissue specific
CDS|exon|ID=cds-Bg	6	4.485	1857.2	0.109234	LMLLEV	79	717.4202	1	716.4142	.	V	Superscaf	47202561	0	BTP20220	0	0	0	0	Tissue specific
CDS|exon|ID=cds-Bg	13	5.567692	4907.5	0.110084	RAPELGHGV	95	646.8195	2	1291.627	.	L	Superscaf	47238538	0	0	BTP20220	0	0	0	Tissue specific
CDS|exon|ID=cds-Bg	12	5.135	4258.7	0.110084	APELGHGVE	96	568.7679	2	1135.526	.	L	Superscaf	47238538	BTP20220	0	0	BTP20220	0	0	Mixed
CDS|exon|ID=cds-Bg	18	5.878333	7545.3	0.110294	YKDVEELSK	99	537.7863	4	2147.122	.	M	Superscaf	47247022	0	0	BTP20220	0	0	0	Tissue specific
CDS|exon|ID=cds-Bg	17	5.891176	7085.8	0.110294	KDVEELSKT	99	497.0211	4	1984.059	.	M	Superscaf	47247022	0	0	BTP20220	0	0	0	Tissue specific
CDS|exon|ID=cds-Bg	16	5.586875	6437	0.110294	KDVEELSKT	95	914.9841	2	1827.958	.	M	Superscaf	47247022	0	0	BTP20220	0	0	0	Tissue specific
CDS|exon|ID=cds-Bg	8	6.02	2791.2	0.124558	TGPGLLSM	95	775.3999	1	774.3946	.	S	Superscaf	47854345	0	BTP20220	BTP20220	BTP20220	0	BTP20220	Mixed
CDS|exon|ID=cds-Bg	7	6.755714	2827	0.140418	KASPLLH	59	383.2401	2	764.4545	.	G	Superscaf	48529542	BTP20220	0	0	0	0	0	Tissue specific
CDS|exon|ID=cds-Bg	6	7.231667	2721.3	0.142837	PDRPPR	57	369.2056	2	736.398	.	N	Superscaf	48632197	0	0	BTP20220	0	0	BTP20220	Tissue enhanced
CDS|exon|ID=cds-Bg	10	5.225	3721.3	0.148462	LVVYPWTQR	92	654.8542	2	1307.703	.	L	Superscaf	48871516	0	0	0	0	0	BTP20220	Tissue specific
CDS|exon|ID=cds-Bg	9	5.141111	3367.9	0.148462	VVYPWTQRF	96	598.3149	2	1194.619	.	L	Superscaf	48871516	0	0	0	0	BTP20220	0	Tissue specific
CDS|exon|ID=cds-Bg	7	4.29	2331.7	0.148462	VVYPWTQ	99	892.4535	1	891.449	.	L	Superscaf	48871516	0	0	0	0	BTP20220	0	Tissue specific
CDS|exon|ID=cds-Bg	7	5.827143	2980.5	0.148462	VYPWTQR	94	475.2463	2	948.4818	.	L	Superscaf	48871516	0	0	0	0	BTP20220	0	Tissue specific
CDS|exon|ID=cds-Bg	9	5.833333	3375.7	0.148706	MLTAEEKAA	99	482.2438	2	962.4742	.	W	Superscaf	48881872	0	BTP20220	0	0	BTP20220	0	Mixed
CDS|exon|ID=cds-Bg	10	5.25	3375.7	0.148706	MLTAEEKAA	98	531.778	2	1061.543	.	W	Superscaf	48881872	0	BTP20220	BTP20220	0	BTP20220	0	Mixed
CDS|exon|ID=cds-Bg	13	5.425385	4471.9	0.148706	MLTAEEKAA	99	691.3538	2	1380.696	.	W	Superscaf	48881872	0	BTP20220	BTP20220	0	BTP20220	BTP20220	Mixed
CDS|exon|ID=cds-Bg	16	5.758125	5764.2	0.148706	MLTAEEKAA	98	584.9684	3	1751.892	.	W	Superscaf	48881872	0	0	0	0	0	BTP20220	Tissue specific
CDS|exon|ID=cds-Bg	18	5.659444	6232.6	0.148706	M(+42.01)	99	674.6929	3	2021.065	Acetylati	W	Superscaf	48881872	0	0	0	0	BTP20220	BTP20220	Mixed
CDS|exon|ID=cds-Bg	21	5.145714	7069.6	0.148706	MLTAEEKAA	96	775.0682	3	2322.193	.	W	Superscaf	48881872	0	0	0	0	BTP20220	0	Tissue specific
CDS|exon|ID=cds-Bg	12	5.420833	4084.5	0.148706	MLTAEEKAA	92	617.8201	2	1233.627	.	W	Superscaf	48881872	0	BTP20220	BTP20220	0	BTP20220	BTP20220	Mixed
CDS|exon|ID=cds-Bg	11	5.366364	3773.2	0.148706	MLTAEEKAA	99	582.3015	2	1162.59	.	W	Superscaf	48881872	0	BTP20220	BTP20220	0	BTP20220	BTP20220	Mixed
CDS|exon|ID=cds-Bg	8	5.81	3064.4	0.148706	MLTAEEKA	99	446.7251	2	891.4371	.	W	Superscaf	48881872	0	BTP20220	0	0	0	0	Tissue specific
CDS|exon|ID=cds-Bg	12	5.398333	4100.4	0.148706	LTAEEKAAV	99	625.8341	2	1249.655	.	W	Superscaf	48881872	0	BTP20220	0	0	BTP20220	BTP20220	Mixed
CDS|exon|ID=cds-Bg	17	5.654118	5861.1	0.148706	LTAEEKAAV	99	617.0101	3	1848.014	.	W	Superscaf	48881872	0	0	0	0	BTP20220	0	Tissue specific
CDS|exon|ID=cds-Bg	11	5.390909	3713	0.148706	LTAEEKAAV	80	552.2985	2	1102.587	.	W	Superscaf	48881872	0	0	0	0	0	BTP20220	Tissue specific
CDS|exon|ID=cds-Bg	10	5.328	3401.7	0.148706	LTAEEKAAV	99	516.7814	2	1031.55	.	W	Superscaf	48881872	0	BTP20220	0	0	BTP20220	BTP20220	Mixed
CDS|exon|ID=cds-Bg	11	5.345455	3747	0.148706	TAEEKAAVT	91	569.292	2	1136.571	.	W	Superscaf	48881872	0	BTP20220	0	0	BTP20220	BTP20220	Mixed
CDS|exon|ID=cds-Bg	16	5.63375	5507.7	0.148706	TAEEKAAVT	95	579.3138	3	1734.93	.	W	Superscaf	48881872	0	0	0	0	BTP20220	0	Tissue specific
CDS|exon|ID=cds-Bg	10	5.227	3349.5	0.148706	AEEKAAVTA	89	518.7676	2	1035.524	.	W	Superscaf	48881872	0	0	0	0	BTP20220	BTP20220	Mixed
CDS|exon|ID=cds-Bg	9	5.138889	3038.2	0.148706	EEKAAVTAF	73	483.249	2	964.4865	.	W	Superscaf	48881872	0	0	0	0	BTP20220	BTP20220	Mixed
CDS|exon|ID=cds-Bg	8	5.37875	2612.7	0.148706	EKAAVTAF	96	836.4499	1	835.4439	.	W	Superscaf	48881872	0	0	0	0	BTP20220	BTP20220	Mixed
CDS|exon|ID=cds-Bg	7	5.687143	2187.2	0.148706	KAAVTAF	99	707.4061	1	706.4014	.	W	Superscaf	48881872	0	0	0	0	BTP20220	0	Tissue specific
CDS|exon|ID=cds-Bg	7	6.12	2459.4	0.148707	AFWGKVK	99	418.2435	2	834.4752	.	W	Superscaf	48881872	0	0	0	0	BTP20220	0	Tissue specific
CDS|exon|ID=cds-Bg	10	4.903	3296.4	0.148707	AFWGKVKVD	98	589.8105	2	1177.613	.	W	Superscaf	48881872	0	0	0	0	BTP20220	0	Tissue specific
CDS|exon|ID=cds-Bg	8	4.69125	2597.7	0.148707	WGKVKVDE	99	480.7596	2	959.5076	.	W	Superscaf	48881872	0	0	0	0	BTP20220	0	Tissue specific
CDS|exon|ID=cds-Bg	16	5.08875	5228.6	0.148707	WGKVKVDEV	97	850.4568	2	1698.905	.	W	Superscaf	48881872	0	0	0	0	BTP20220	BTP20220	Mixed
CDS|exon|ID=cds-Bg	14	4.620714	4282.5	0.148707	WGKVKVDEV	64	743.8962	2	1485.783	.	W	Superscaf	48881872	0	0	0	0	0	BTP20220	Tissue specific
CDS|exon|ID=cds-Bg	13	4.516154	3929.1	0.148707	WGKVKVDEV	89	687.3538	2	1372.699	.	W	Superscaf	48881872	0	0	0	0	BTP20220	0	Tissue specific
CDS|exon|ID=cds-Bg	9	4.17	2597.7	0.148707	WGKVKVDEV	89	530.2939	2	1058.576	.	W	Superscaf	48881872	0	BTP20220	0	0	BTP20220	BTP20220	Mixed
CDS|exon|ID=cds-Bg	7	4.901429	2172.2	0.148707	WGKVKVD	93	416.2373	2	830.465	.	W	Superscaf	48881872	0	0	0	0	BTP20220	0	Tissue specific
CDS|exon|ID=cds-Bg	20	5.175	7285.9	0.148712	FESFGDLSS	91	719.3464	3	2155.025	.	N	Superscaf	48882211	0	0	0	0	0	BTP20220	Tissue specific
CDS|exon|ID=cds-Bg	17	5.192941	6430.1	0.148712	ESFGDLSSA	91	891.4017	2	1780.794	.	N	Superscaf	48882211	0	0	0	0	0	BTP20220	Tissue specific
CDS|exon|ID=cds-Bg	7	4.997143	2641.9	0.148712	ESFGDLS	93	754.3234	1	753.3181	.	N	Superscaf	48882211	0	0	0	0	BTP20220	0	Tissue specific
CDS|exon|ID=cds-Bg	16	5.31625	6004.6	0.148712	SFGDLSSAD	93	826.8801	2	1651.751	.	N	Superscaf	48882211	0	0	0	0	0	BTP20220	Tissue specific
CDS|exon|ID=cds-Bg	15	5.292	5621.2	0.148712	FGDLSSADA	93	783.3654	2	1564.719	.	N	Superscaf	48882211	0	0	0	0	0	BTP20220	Tissue specific
CDS|exon|ID=cds-Bg	14	5.278571	5233.8	0.148712	GDLSSADAV	91	709.8295	2	1417.651	.	N	Superscaf	48882211	0	0	0	0	0	BTP20220	Tissue specific
CDS|exon|ID=cds-Bg	16	5.2275	5702.2	0.148712	GDLSSADAV	93	823.4106	2	1644.814	.	N	Superscaf	48882211	0	0	0	0	0	BTP20220	Tissue specific
CDS|exon|ID=cds-Bg	15	6.019333	5781	0.148713	NNPKVKAHG	93	545.647	3	1633.926	.	N	Superscaf	48882211	0	0	0	0	BTP20220	0	Tissue specific
CDS|exon|ID=cds-Bg	10	5.917	3700.8	0.148713	AHGKKVLDS	95	551.3041	2	1100.598	.	N	Superscaf	48882211	0	0	0	0	0	BTP20220	Tissue specific
CDS|exon|ID=cds-Bg	11	5.672727	4121.4	0.148713	KVLDSFSNG	99	613.3146	2	1224.617	.	N	Superscaf	48882211	0	0	0	0	0	BTP20220	Tissue specific
CDS|exon|ID=cds-Bg	10	5.266	3653	0.148713	VLDSFSNGM	98	549.267	2	1096.522	.	N	Superscaf	48882211	0	0	0	0	0	BTP20220	Tissue specific
CDS|exon|ID=cds-Bg	14	5.582143	5802.5	0.148714	LDSFSNGMK	99	796.3733	2	1590.735	.	N	Superscaf	48882211	0	BTP20220	0	0	0	0	Tissue specific
CDS|exon|ID=cds-Bg	10	6.025	4272.7	0.148714	LDSFSNGMK	99	568.2623	2	1134.513	.	N	Superscaf	48882211	0	BTP20220	0	0	0	0	Tissue specific
CDS|exon|ID=cds-Bg	15	6.118667	6128.7	0.148714	SNGMKHLDD	89	817.4019	2	1632.793	.	N	Superscaf	48882211	0	BTP20220	0	0	BTP20220	0	Mixed
CDS|exon|ID=cds-Bg	10	5.804	4266.8	0.148714	SNGMKHLDD	86	565.2668	2	1128.523	.	N	Superscaf	48882211	0	BTP20220	0	0	0	0	Tissue specific
CDS|exon|ID=cds-Bg	11	6.27	4479.8	0.148714	KHLDDLKGT	93	622.8337	2	1243.656	.	N	Superscaf	48882211	0	0	0	0	BTP20220	0	Tissue specific
CDS|exon|ID=cds-Bg	10	5.923	4011.4	0.148714	HLDDLKGTF	95	558.7861	2	1115.561	.	N	Superscaf	48882211	0	0	0	0	BTP20220	0	Tissue specific
CDS|exon|ID=cds-Bg	9	5.912222	3700.1	0.148714	HLDDLKGTF	77	523.269	2	1044.524	.	N	Superscaf	48882211	0	0	0	0	0	BTP20220	Tissue specific
CDS|exon|ID=cds-Bg	8	5.96625	3312.7	0.148714	HLDDLKGT	98	449.7333	2	897.4556	.	N	Superscaf	48882211	0	0	0	0	BTP20220	0	Tissue specific
CDS|exon|ID=cds-Bg	9	5.737778	3391.7	0.148714	LDDLKGTFA	93	490.2576	2	978.5022	.	N	Superscaf	48882211	0	BTP20220	0	0	BTP20220	BTP20220	Mixed
CDS|exon|ID=cds-Bg	8	5.7075	3038.3	0.148714	DDLKGTFA	96	866.4221	1	865.4181	.	N	Superscaf	48882211	0	BTP20220	0	0	BTP20220	0	Mixed
CDS|exon|ID=cds-Bg	15	6.128	6127.6	0.148714	KGTFAALSE	93	563.9591	3	1688.856	Carbamido	N	Superscaf	48882211	0	BTP20220	0	0	0	0	Tissue specific
CDS|exon|ID=cds-Bg	7	6.534286	2526.6	0.148714	KGTFAAL	66	354.2069	2	706.4014	.	N	Superscaf	48882211	0	BTP20220	0	0	0	0	Tissue specific
CDS|exon|ID=cds-Bg	10	5.818	4265.7	0.148715	ALSELHC(+	99	593.2995	2	1184.586	Carbamido	N	Superscaf	48882211	0	BTP20220	BTP20220	BTP20220	BTP20220	0	Mixed
CDS|exon|ID=cds-Bg	13	5.539231	5685.3	0.148715	HC(+57.02	67	410.4465	4	1637.762	Carbamido	N	Superscaf	48882211	0	0	0	0	BTP20220	0	Tissue specific
CDS|exon|ID=cds-Bg	7	4.424286	2778.2	0.148716	HVDPENF	69	429.192	2	856.3715	.	N	Superscaf	48882211	0	BTP20220	0	0	0	0	Tissue specific
CDS|exon|ID=cds-Bg	7	4.731429	2626.9	0.148716	VDPENFK	99	424.7095	2	847.4075	.	N	Superscaf	48882211	0	0	0	0	BTP20220	0	Tissue specific
CDS|exon|ID=cds-Bg	12	6.089167	4634.1	0.148738	ARHFGKEFT	70	467.9252	3	1400.756	.	S	Superscaf	48883297	0	BTP20220	0	0	0	0	Tissue specific
CDS|exon|ID=cds-Bg	13	5.293846	4775.2	0.148738	FGKEFTPVL	75	749.8793	2	1497.75	.	S	Superscaf	48883297	0	0	0	0	0	BTP20220	Tissue specific
CDS|exon|ID=cds-Bg	8	5.3625	2980.3	0.148738	KEFTPVLQ	77	481.2699	2	960.528	.	S	Superscaf	48883297	0	0	0	0	BTP20220	0	Tissue specific
CDS|exon|ID=cds-Bg	10	5.432	3888.3	0.148738	TPVLQADFQ	98	573.808	2	1145.608	.	S	Superscaf	48883297	0	0	0	0	BTP20220	0	Tissue specific
CDS|exon|ID=cds-Bg	20	5.397	7925.7	0.148739	QADFQKVVN	96	746.7196	3	2237.145	.	S	Superscaf	48883297	0	0	0	0	BTP20220	0	Tissue specific
CDS|exon|ID=cds-Bg	9	4.582222	3083.3	0.148739	ADFQKVVNG	79	489.2536	2	976.4977	.	S	Superscaf	48883297	0	0	0	0	BTP20220	0	Tissue specific
CDS|exon|ID=cds-Bg	17	5.237647	6235.8	0.148739	ADFQKVVNG	93	603.993	3	1808.965	.	S	Superscaf	48883297	0	0	0	0	BTP20220	0	Tissue specific
CDS|exon|ID=cds-Bg	19	5.383684	7315	0.148739	ADFQKVVNG	96	528.2773	4	2109.087	.	S	Superscaf	48883297	0	0	0	0	0	BTP20220	Tissue specific
CDS|exon|ID=cds-Bg	8	4.40875	2786	0.148739	ADFQKVVN	92	460.7443	2	919.4763	.	S	Superscaf	48883297	0	BTP20220	0	0	BTP20220	0	Mixed
CDS|exon|ID=cds-Bg	16	5.48875	6204.8	0.148739	QKVVNGVAN	97	592.9901	3	1775.954	.	S	Superscaf	48883297	0	0	0	0	BTP20220	0	Tissue specific
CDS|exon|ID=cds-Bg	13	4.908462	4476.8	0.148739	QKVVNGVAN	93	660.8692	2	1319.731	.	S	Superscaf	48883297	0	0	0	0	BTP20220	BTP20220	Tissue enhanced
CDS|exon|ID=cds-Bg	15	5.478	5594.1	0.148739	KVVNGVANA	92	550.3048	3	1647.896	.	S	Superscaf	48883297	0	BTP20220	0	0	BTP20220	BTP20220	Mixed
CDS|exon|ID=cds-Bg	12	4.846667	3866.1	0.148739	KVVNGVANA	99	596.8419	2	1191.672	.	S	Superscaf	48883297	0	0	0	0	0	BTP20220	Tissue specific
CDS|exon|ID=cds-Bg	14	5.173571	5125.7	0.148739	VVNGVANAL	66	507.6071	3	1519.801	.	S	Superscaf	48883297	0	BTP20220	0	0	0	0	Tissue specific
CDS|exon|ID=cds-Bg	13	5.571538	5125.7	0.148739	VNGVANALA	98	474.5842	3	1420.732	.	S	Superscaf	48883297	0	BTP20220	0	0	BTP20220	0	Mixed
CDS|exon|ID=cds-Bg	10	4.842	3397.7	0.148739	VNGVANALA	77	483.2605	2	964.509	.	S	Superscaf	48883297	0	BTP20220	0	0	0	0	Tissue specific
CDS|exon|ID=cds-Bg	12	6.035833	5125.7	0.148739	NGVANALAH	98	441.561	3	1321.664	.	S	Superscaf	48883297	0	BTP20220	0	0	BTP20220	0	Mixed
CDS|exon|ID=cds-Bg	9	5.38	3397.7	0.148739	NGVANALAH	58	433.7268	2	865.4406	.	S	Superscaf	48883297	0	BTP20220	0	0	0	0	Tissue specific
CDS|exon|ID=cds-Bg	9	5.974444	3449.8	0.148739	GVANALAHR	99	454.7547	2	907.4988	.	S	Superscaf	48883297	0	0	0	0	BTP20220	0	Tissue specific
CDS|exon|ID=cds-Bg	11	6.092727	4529	0.148739	GVANALAHR	99	604.8163	2	1207.621	.	S	Superscaf	48883297	0	BTP20220	0	0	BTP20220	0	Mixed
CDS|exon|ID=cds-Bg	10	5.943	3909.3	0.148739	GVANALAHR	92	536.287	2	1070.562	.	S	Superscaf	48883297	0	0	0	0	BTP20220	0	Tissue specific
CDS|exon|ID=cds-Bg	8	5.37625	2801	0.148739	GVANALAH	90	376.7051	2	751.3976	.	S	Superscaf	48883297	0	BTP20220	0	0	0	0	Tissue specific
CDS|exon|ID=cds-Bg	8	5.975	3152.5	0.148739	VANALAHR	99	426.244	2	850.4773	.	S	Superscaf	48883297	0	0	0	0	BTP20220	0	Tissue specific
CDS|exon|ID=cds-Bg	10	6.105	4231.7	0.148739	VANALAHRY	99	384.5396	3	1150.6	.	S	Superscaf	48883297	0	BTP20220	BTP20220	0	BTP20220	0	Mixed
CDS|exon|ID=cds-Bg	9	6.783333	4231.7	0.14874	ANALAHRYH	99	526.7715	2	1051.531	.	S	Superscaf	48883297	0	BTP20220	0	0	BTP20220	BTP20220	Mixed
CDS|exon|ID=cds-Bg	8	6.6825	3612	0.14874	ANALAHRY	90	458.2404	2	914.4722	.	S	Superscaf	48883297	0	0	0	0	BTP20220	0	Tissue specific
CDS|exon|ID=cds-Bg	8	6.87875	3920.4	0.14874	NALAHRYH	91	491.2537	2	980.494	.	S	Superscaf	48883297	0	BTP20220	BTP20220	BTP20220	BTP20220	BTP20220	Mixed
CDS|exon|ID=cds-Bg	8	5.7725	3601	0.149097	SELHC(+57	99	501.239	2	1000.465	Carbamido	S	Superscaf	48898483	0	BTP20220	BTP20220	BTP20220	BTP20220	BTP20220	Mixed
CDS|exon|ID=cds-Bg	13	5.076154	5608.4	0.149894	ELHC(+57.	91	803.3672	2	1604.725	Carbamido	K	Superscaf	48932437	0	0	0	0	BTP20220	0	Tissue specific
CDS|exon|ID=cds-Bg	12	5.189167	5216.9	0.149894	HC(+57.02	98	755.8382	2	1509.667	Carbamido	K	Superscaf	48932437	0	0	0	0	BTP20220	0	Tissue specific
CDS|exon|ID=cds-Bg	11	5.162727	4829.5	0.149894	HC(+57.02	99	682.3036	2	1362.599	Carbamido	K	Superscaf	48932437	0	0	0	0	BTP20220	0	Tissue specific
CDS|exon|ID=cds-Bg	10	4.92	4209.8	0.149894	C(+57.02)	97	613.7744	2	1225.54	Carbamido	K	Superscaf	48932437	0	0	0	0	BTP20220	0	Tissue specific
CDS|exon|ID=cds-Bg	7	6.137143	2590.7	0.156143	LSEAKAP	64	358.2083	2	714.3912	.	G	Superscaf	49198510	BTP20220	0	0	0	0	0	Tissue specific
CDS|exon|ID=cds-Bg	7	6.388571	2402.6	0.15647	KGRGVLP	51	363.7285	2	725.4548	.	L	Superscaf	49212385	BTP20220	0	0	0	0	0	Tissue specific
CDS|exon|ID=cds-Bg	7	6.557143	2805.1	0.186753	LSAFSRP	71	389.2134	2	776.418	.	K	Superscaf	50501107	0	0	0	BTP20220	0	0	Tissue specific
CDS|exon|ID=cds-Bg	7	6.484286	2737.9	0.202213	L(+42.01)	80	458.7665	2	915.5218	Acetylati	W	Superscaf	51158986	0	0	BTP20220	0	0	0	Tissue specific
CDS|exon|ID=cds-Bg	8	4.7025	2705.2	0.220245	VDGRTVNL	62	437.2424	2	872.4716	.	A	Superscaf	51925993	0	0	0	0	BTP20220	0	Tissue specific
CDS|exon|ID=cds-Bg	6	7.075	2801.4	0.233489	LERMRL	94	409.2375	2	816.464	.	E	Superscaf	52490088	0	0	0	BTP20220	0	0	Tissue specific
CDS|exon|ID=cds-Bg	6	5.633333	2118.4	0.240668	LLAPPE	86	639.367	1	638.3639	.	G	Superscaf	52795279	0	0	0	0	0	BTP20220	Tissue specific
CDS|exon|ID=cds-Bg	6	7.48	2845.2	0.254725	RPWKWP	88	435.2356	2	868.4708	.	P	Superscaf	53393377	0	0	0	0	BTP20220	0	Tissue specific
CDS|exon|ID=cds-Bg	6	4.76	1729.2	0.270038	M(+15.99)	68	403.7051	2	805.3826	Oxidation	R	Superscaf	54045232	0	0	0	BTP20220	0	0	Tissue specific
CDS|exon|ID=cds-Bg	7	6.28	2471.9	0.286643	T(+42.01)	78	738.3944	1	737.3959	Acetylati	S	Superscaf	54751989	0	0	0	0	0	BTP20220	Tissue specific
CDS|exon|ID=cds-Bg	6	6.21	2283.6	0.288866	KDPTGM	63	648.2972	1	647.2949	.	D	Superscaf	54846196	0	BTP20220	BTP20220	0	0	0	Mixed
CDS|exon|ID=cds-Bg	10	5.631	4083.7	0.297181	LTRELAEDC	95	583.7864	2	1165.562	.	F	Superscaf	55200259	0	0	0	0	0	BTP20220	Tissue specific
CDS|exon|ID=cds-Bg	9	5.897778	2944.9	0.300649	AEVKKPAAA	98	442.762	2	883.5126	.	G	Superscaf	55347577	0	0	0	BTP20220	BTP20220	BTP20220	Mixed
CDS|exon|ID=cds-Bg	13	6.130769	4803.2	0.300651	AATLAERSA	75	639.3439	2	1276.678	.	G	Superscaf	55347577	0	0	0	0	0	BTP20220	Tissue specific
CDS|exon|ID=cds-Bg	10	5.146	3832.4	0.300655	LRDDEVHAG	51	562.7865	2	1123.562	.	G	Superscaf	55347577	0	0	0	BTP20220	0	0	Tissue specific
CDS|exon|ID=cds-Bg	12	4.655833	4259.8	0.300655	DDEVHAGLG	65	634.3085	2	1266.609	.	G	Superscaf	55347577	0	0	0	0	0	BTP20220	Tissue specific
CDS|exon|ID=cds-Bg	11	4.535455	3906.4	0.300655	DDEVHAGLG	62	577.7683	2	1153.525	.	G	Superscaf	55347577	0	0	0	BTP20220	0	BTP20220	Mixed
CDS|exon|ID=cds-Bg	11	5.773636	3954.4	0.300661	TTDGKLPEV	96	594.8255	2	1187.64	.	G	Superscaf	55347577	0	0	0	0	BTP20220	BTP20220	Mixed
CDS|exon|ID=cds-Bg	10	5.698	3556.9	0.300661	TDGKLPEVT	91	544.3002	2	1086.592	.	G	Superscaf	55347577	0	0	0	0	0	BTP20220	Tissue specific
CDS|exon|ID=cds-Bg	8	5.025	3118.6	0.300704	PHHVEPLE	76	479.2427	2	956.4716	.	V	Superscaf	55350031	0	0	0	BTP20220	0	0	Tissue specific
CDS|exon|ID=cds-Bg	10	5.601	4057.6	0.300705	HVEPLERLE	90	417.2329	3	1248.683	.	V	Superscaf	55350031	0	0	0	0	0	BTP20220	Tissue specific
CDS|exon|ID=cds-Bg	7	4.051429	1454.5	0.32728	VPKAVVP	56	355.2326	2	708.4534	.	R	Superscaf	56481013	0	0	0	0	BTP20220	0	Tissue specific
CDS|exon|ID=cds-Bg	7	7.208571	2920.2	0.396212	GPKSFTR	72	396.7286	2	791.429	.	G	Superscaf	59414365	0	0	BTP20220	0	0	0	Tissue specific
CDS|exon|ID=cds-Bg	11	6.485455	4252.8	0.478676	A(+42.01)	96	532.7415	2	1063.472	Acetylati	Q	Superscaf	62923600	0	0	0	BTP20220	0	0	Tissue specific
CDS|exon|ID=cds-Bg	7	5.724286	2813.1	0.492058	YGEDLTK	99	413.2011	2	824.3915	.	D	Superscaf	63493057	0	0	BTP20220	0	0	0	Tissue specific
CDS|exon|ID=cds-Bg	8	5.93375	3064.1	0.541842	SKAKVFEH	99	473.2599	2	944.5079	.	V	Superscaf	65611471	0	0	0	0	0	BTP20220	Tissue specific
CDS|exon|ID=cds-Bg	10	5.684	4115.7	0.541886	SLRPESLHG	50	389.2141	3	1164.625	.	F	Superscaf	65613403	0	0	BTP20220	0	0	0	Tissue specific
CDS|exon|ID=cds-Bg	8	4.9225	2406.6	0.542017	PVGKLVLN	95	420.2694	2	838.5276	.	V	Superscaf	65619031	0	0	BTP20220	0	0	0	Tissue specific
CDS|exon|ID=cds-Bg	7	6.375714	2976.4	0.542103	RARVANY	73	425.2355	2	848.4617	.	L	Superscaf	65622646	0	0	0	0	0	BTP20220	Tissue specific
CDS|exon|ID=cds-Bg	15	6.275333	6784.7	0.542165	SAPEHQPSA	96	566.2751	3	1695.808	.	I	Superscaf	65625187	0	0	BTP20220	0	0	0	Tissue specific
CDS|exon|ID=cds-Bg	15	6.139333	6289.9	0.542321	TYVQHGSHL	92	561.9556	3	1682.849	.	P	Superscaf	65631955	0	0	BTP20220	0	0	0	Tissue specific

CDS|exon|ID=cds-B	11	5.668182	4435.9	0.542321	TYVQHGSHL	83	600.2922	2	1198.573	.	P	Superscaf	65631955	0	0	BTP202209	0	0	0	Tissue specific
CDS|exon|ID=cds-B	9	5.627778	3741.2	0.542321	TYVQHGSHL	99	521.2584	2	1040.504	.	P	Superscaf	65631955	0	0	BTP202209	0	0	0	Tissue specific
CDS|exon|ID=cds-B	14	6.111429	5892.4	0.542321	YVQHGSHLS	88	528.2731	3	1581.801	.	P	Superscaf	65631955	0	0	BTP202209	0	0	0	Tissue specific
CDS|exon|ID=cds-B	11	6.052727	4687.2	0.542321	YVQHGSHLS	99	418.8815	3	1253.627	.	P	Superscaf	65631955	0	0	BTP202209	0	0	0	Tissue specific
CDS|exon|ID=cds-B	10	5.582	4038.4	0.542321	YVQHGSHLS	83	549.7682	2	1097.525	.	P	Superscaf	65631955	0	0	BTP202209	0	0	0	Tissue specific
CDS|exon|ID=cds-B	9	5.533333	3727.1	0.542321	YVQHGSHLS	90	514.2499	2	1026.488	.	P	Superscaf	65631955	0	0	BTP202209	0	0	0	Tissue specific
CDS|exon|ID=cds-B	8	5.515	3343.7	0.542321	YVQHGSHL	99	470.734	2	939.4562	.	P	Superscaf	65631955	0	0	BTP202209	0	0	0	Tissue specific
CDS|exon|ID=cds-B	13	6.587692	5772.3	0.542321	HGSHLSARE	81	710.3743	2	1418.738	.	P	Superscaf	65631955	0	0	BTP202209	0	0	0	Tissue specific
CDS|exon|ID=cds-B	10	5.882	4524.4	0.559478	MTADETRNL	88	589.7771	2	1177.54	.	L	Superscaf	66361822	0	BTP202209	0	0	0	0	Tissue specific
CDS|exon|ID=cds-B	8	4.8725	2535.8	0.586079	APSLPVEF	84	859.4536	1	858.4487	.	F	Superscaf	67493866	0	0	0	0	BTP202209	0	Tissue specific
CDS|exon|ID=cds-B	7	4.415714	2398.8	0.740572	PTVEELY	91	850.4156	1	849.412	.	G	Superscaf	74068189	0	0	0	0	BTP202209	BTP202209	Mixed
CDS|exon|ID=cds-B	7	5.14	2179.3	0.790244	VPAAGAQ	52	613.3376	1	612.3231	.	D	Superscaf	76181839	0	0	0	0	0	BTP202209	Tissue specific
CDS|exon|ID=cds-B	7	5.74	2463.8	0.800083	V(+42.01)	63	437.2626	2	872.512	Acetylati	S	Superscaf	76600774	0	BTP202209	0	0	0	0	Tissue specific
CDS|exon|ID=cds-B	6	6.313333	2230.4	0.810084	LHAGPA	76	565.3071	1	564.3019	.	E	Superscaf	77026309	0	0	0	0	0	BTP202209	Tissue specific
CDS|exon|ID=cds-B	6	5.785	2216.4	0.817752	MVHYGK	51	367.6829	2	733.3581	.	I	Superscaf	77352762	BTP202209	0	0	0	0	0	Tissue specific
CDS|exon|ID=cds-B	12	6.2875	4675.9	0.833937	KLEAGDTKF	93	643.8495	2	1285.688	.	W	Superscaf	78041281	0	0	0	0	0	BTP202209	Tissue specific
CDS|exon|ID=cds-B	8	3.9775	1961.2	0.91602	VLGPVHLV	61	417.2639	2	832.517	.	E	Superscaf	81534325	0	0	BTP202209	0	0	0	Tissue specific
CDS|exon|ID=cds-B	7	6.118571	2215.4	0.916138	G(+42.01)	50	594.2968	1	593.2809	Acetylati	W	Superscaf	81539308	0	0	0	0	0	BTP202209	Tissue specific
CDS|exon|ID=cds-B	17	5.094118	6621.3	0.92477	VGPGNLQEC	83	865.8935	2	1729.775	.	K	Superscaf	81906718	0	BTP202209	0	0	0	BTP202209	Mixed
CDS|exon|ID=cds-B	11	5.341818	4325.8	0.928097	VGDADLERG	90	639.3267	2	1276.641	.	I	Superscaf	82048291	0	BTP202209	0	0	0	0	Tissue specific
CDS|exon|ID=cds-B	11	5.975455	3962.5	0.928132	VGQAGTATL	98	535.8003	2	1069.588	.	F	Superscaf	82049632	0	BTP202209	0	0	0	0	Tissue specific
CDS|exon|ID=cds-B	13	4.996154	4979.8	0.928311	TEDPTNDTV	92	739.8346	2	1477.657	.	I	Superscaf	82057417	0	BTP202209	0	0	0	0	Tissue specific
CDS|exon|ID=cds-B	12	4.868333	4582.3	0.928311	EDPTNDTVD	97	689.3102	2	1376.61	.	I	Superscaf	82057417	0	BTP202209	0	0	0	0	Tissue specific
CDS|exon|ID=cds-B	7	4.855714	2413.7	0.928311	DTVDFPK	98	411.2036	2	820.3967	.	I	Superscaf	82057417	0	0	0	0	BTP202209	0	Tissue specific
CDS|exon|ID=cds-B	8	5.25875	3107.6	0.928387	YDDSTLPL	85	923.4349	1	922.4283	.	S	Superscaf	82060576	0	BTP202209	0	0	0	0	Tissue specific
CDS|exon|ID=cds-B	7	5.201429	2648.1	0.928388	DDSTLPL	75	760.371	1	759.3651	.	S	Superscaf	82060576	0	BTP202209	0	0	0	0	Tissue specific
CDS|exon|ID=cds-B	8	6.2775	3396.7	0.950113	KEQDKPPP	56	469.752	2	937.4869	.	E	Superscaf	82985259	0	0	0	BTP202209	0	0	Tissue specific
CDS|exon|ID=cds-B	5	7.556	1938.9	0.977747	K(+42.01)	94	598.3906	1	597.3849	Acetylati	Y	Superscaf	84161038	0	0	0	0	0	BTP202209	Tissue specific
CDS|exon|ID=cds-B	6	6.165	2325.5	0.982294	Y(+42.01)	66	732.3528	1	731.3489	Acetylati	Q	Superscaf	84354604	0	0	0	0	BTP202209	0	Tissue specific
CDS|exon|ID=cds-B	8	6.77625	3034.5	0.985808	LPAARTPP	83	411.7442	2	821.4759	.	P	Superscaf	84503740	0	0	BTP202209	0	0	0	Tissue specific
CDS|exon|ID=cds-B	10	6.257	4320.9	0.987788	NFAAREMAF	79	572.2744	2	1142.529	.	A	Superscaf	84588358	0	0	0	0	0	BTP202209	Tissue specific
CDS|exon|ID=cds-B	13	4.789231	4345.9	0.893756	VDQRVLEKL	79	499.2976	3	1494.877	.	H	Superscaf	4571521	0	0	0	0	0	BTP202209	Tissue specific
CDS|exon|ID=cds-B	12	5.0875	4952.6	0.893674	HDFSSDLEH	91	473.8794	3	1418.621	.	L	Superscaf	4575631	0	0	0	0	0	BTP202209	Tissue specific
CDS|exon|ID=cds-B	11	4.86	4332.9	0.893674	DFSSDLEHV	95	641.7862	2	1281.563	.	L	Superscaf	4575631	0	0	0	0	0	BTP202209	Tissue specific
CDS|exon|ID=cds-B	9	5.001111	3534	0.893674	SSDLEHVPH	99	510.7388	2	1019.467	.	L	Superscaf	4575631	0	0	0	0	0	BTP202209	Tissue specific
CDS|exon|ID=cds-B	8	4.91625	3150.6	0.893674	SDLEHVPH	99	467.2231	2	932.4352	.	L	Superscaf	4575631	0	0	0	0	0	BTP202209	Tissue specific
CDS|exon|ID=cds-B	7	5.43	2856.2	0.893363	HESAPEL	62	782.3704	1	781.3606	.	E	Superscaf	4589026	0	0	0	0	0	BTP202209	Tissue specific
CDS|exon|ID=cds-B	11	5.952727	4842.7	0.889147	T(+42.01)	55	626.7914	2	1251.573	Acetylati	S	Superscaf	4770394	0	0	0	0	BTP202209	0	Tissue specific
CDS|exon|ID=cds-B	9	6.043333	3862.6	0.889147	T(+42.01)	66	526.2542	2	1050.498	Acetylati	S	Superscaf	4770394	0	0	0	0	0	BTP202209	Tissue specific
CDS|exon|ID=cds-B	7	5.73	3001.3	0.854234	QEGSALH	64	741.3577	1	740.3453	.	G	Superscaf	6273156	0	0	0	0	0	BTP202209	Tissue specific
CDS|exon|ID=cds-B	7	4.201429	1758.9	0.851465	VFGVSPL	52	718.4125	1	717.4061	.	D	Superscaf	6392307	0	BTP202209	0	0	0	0	Tissue specific
CDS|exon|ID=cds-B	5	4.13	1463.6	0.813149	VGLEF	77	564.3	1	563.2955	.	N	Superscaf	8041107	0	0	0	0	0	BTP202209	Tissue specific
CDS|exon|ID=cds-B	6	5.983333	2314.6	0.766846	YPPYSP	87	723.3328	1	722.3275	.	R	Superscaf	10033675	0	0	BTP202209	0	0	0	Tissue specific
CDS|exon|ID=cds-B	6	3.545	1491.8	0.67807	VTAMDV	95	635.3051	1	634.2996	.	N	Superscaf	13853947	0	0	0	0	0	BTP202209	Tissue specific
CDS|exon|ID=cds-B	6	3.545	1491.8	0.67807	TAMDVV	88	635.3047	1	634.2996	.	N	Superscaf	13853947	0	0	0	0	0	BTP202209	Tissue specific
CDS|exon|ID=cds-B	6	7.075	2801.4	0.620596	ERLMLR	90	409.238	2	816.464	.	Q	Superscaf	16327869	0	0	BTP202209	0	0	0	Tissue specific
CDS|exon|ID=cds-B	7	5.867143	3212.7	0.619266	Q(+42.01)	85	448.2399	2	894.4672	Acetylati	L	Superscaf	16384660	0	0	BTP202209	0	0	BTP202209	Mixed
CDS|exon|ID=cds-B	10	3.795	3098.6	0.337953	VGDPVYQMV	58	568.2742	2	1134.538	.	I	Superscaf	28490998	0	0	BTP202209	0	0	0	Tissue specific
CDS|exon|ID=cds-B	10	5.292	3795.1	0.294978	ENDLKGAAV	92	527.27	2	1052.525	.	H	Superscaf	30339307	0	0	0	0	0	BTP202209	Tissue specific
CDS|exon|ID=cds-B	7	6.087143	2556.8	0.294942	PPAAAQP	59	651.3435	1	650.3387	.	H	Superscaf	30339307	0	0	0	0	0	BTP202209	Tissue specific
CDS|exon|ID=cds-B	15	4.924667	4918.1	0.294005	SAADEKAVL	91	765.8983	2	1529.794	.	F	Superscaf	30382360	0	0	0	0	0	BTP202209	Tissue specific
CDS|exon|ID=cds-B	9	6.161111	3066.5	0.293904	GPPPFPGTF	95	866.4371	1	865.4333	.	F	Superscaf	30386728	0	0	BTP202209	0	0	0	Tissue specific
CDS|exon|ID=cds-B	10	7.127	4955.6	0.292636	T(+42.01)	77	459.9093	3	1376.71	Acetylati	Y	Superscaf	30441253	0	0	0	0	BTP202209	0	Tissue specific
CDS|exon|ID=cds-B	6	6.555	2299.4	0.27236	YSLGPK	73	664.3645	1	663.3591	.	A	Superscaf	31314090	0	0	BTP202209	BTP202209	0	0	Mixed
CDS|exon|ID=cds-B	9	6.261111	3588.7	0.271545	QKLVKGHAY	96	522.3015	2	1042.592	.	D	Superscaf	31348858	0	0	0	0	0	BTP202209	Tissue specific
CDS|exon|ID=cds-B	7	5.848571	2452.6	0.243943	LKDVTLK	98	408.762	2	815.5117	.	P	Superscaf	32536669	0	0	0	0	0	BTP202209	Tissue specific
CDS|exon|ID=cds-B	8	5.33875	3333.8	0.231112	FVGLQERQ	90	488.7637	2	975.5137	.	T	Superscaf	33088774	0	BTP202209	0	0	0	BTP202209	Tissue enhanced
CDS|exon|ID=cds-B	7	7.194286	2973.2	0.230946	APWSGRK	71	401.2224	2	800.4293	.	F	Superscaf	33096130	0	BTP202209	0	0	0	0	Tissue specific
CDS|exon|ID=cds-B	16	5.87375	5761.1	0.217337	LPGELAKHA	92	804.4391	2	1606.868	.	R	Superscaf	33681589	0	0	0	0	BTP202209	0	Tissue specific
CDS|exon|ID=cds-B	6	4.958333	1686.8	0.193252	LVAGPF	62	603.3481	1	602.3428	.	I	Superscaf	34718329	0	0	0	0	0	BTP202209	Tissue specific
CDS|exon|ID=cds-B	12	6.02	4593.7	0.148889	KLKVSELKE	93	481.9628	3	1442.871	.	A	Superscaf	36627391	0	0	0	0	0	BTP202209	Tissue specific
CDS|exon|ID=cds-B	11	6.116364	4420.7	0.148889	VSELKEELK	97	453.6028	3	1357.793	.	A	Superscaf	36627391	0	0	BTP202209	0	0	0	Tissue specific
CDS|exon|ID=cds-B	10	5.652	3771.9	0.148889	VSELKEELK	83	601.8508	2	1201.692	.	A	Superscaf	36627391	0	0	0	0	0	BTP202209	Tissue specific
CDS|exon|ID=cds-B	7	4.254286	2358.6	0.148768	EVELSYA	72	810.3859	1	809.3807	.	Q	Superscaf	36632680	0	0	0	BTP202209	0	0	Tissue specific
CDS|exon|ID=cds-B	10	4.374	3865.4	0.148765	VLC(+57.0	64	624.7762	2	1247.543	Carbamid	Q	Superscaf	36632680	0	0	0	0	0	BTP202209	Tissue specific
CDS|exon|ID=cds-B	9	4.86	3865.4	0.148765	LC(+57.02	63	575.2419	2	1148.474	Carbamid	Q	Superscaf	36632680	0	0	0	0	0	BTP202209	Tissue specific
CDS|exon|ID=cds-B	15	5.186	5607.1	0.148741	KAVVVC(+5	97	612.3193	3	1833.941	Carbamid	K	Superscaf	36633748	0	BTP202209	0	0	0	0	Tissue specific
CDS|exon|ID=cds-B	7	3.868571	1702.8	0.148742	AVVVC(+57	99	386.7215	2	771.4313	Carbamid	K	Superscaf	36633748	0	0	0	0	BTP202209	0	Tissue specific
CDS|exon|ID=cds-B	13	4.771538	4827.4	0.148741	VVVC(+57.	98	409.7081	4	1634.809	Carbamid	K	Superscaf	36633748	0	BTP202209	0	0	0	0	Tissue specific
CDS|exon|ID=cds-B	11	4.147273	3567.9	0.148741	VVVC(+57.	92	451.2218	3	1350.649	Carbamid	K	Superscaf	36633748	0	0	0	BTP202209	0	0	Tissue specific
CDS|exon|ID=cds-B	12	5.169167	4827.4	0.148741	VVC(+57.0	99	512.9202	3	1535.74	Carbamid	K	Superscaf	36633748	0	BTP202209	0	0	BTP202209	0	Mixed
CDS|exon|ID=cds-B	11	4.660909	4178.6	0.148741	VVC(+57.0	98	690.8237	2	1379.639	Carbamid	K	Superscaf	36633748	0	0	0	0	BTP202209	0	Tissue specific
CDS|exon|ID=cds-B	11	5.639091	4827.4	0.148741	VC(+57.02	97	479.8959	3	1436.672	Carbamid	K	Superscaf	36633748	0	0	0	0	BTP202209	0	Tissue specific
CDS|exon|ID=cds-B	14	5.695	5323.6	0.14874	KKAEVEGKD	99	517.6089	3	1549.81	.	K	Superscaf	36633748	0	0	BTP202209	0	BTP202209	0	Mixed
CDS|exon|ID=cds-B	13	5.67	5012.3	0.14874	KKAEVEGKD	99	493.9301	3	1478.773	.	K	Superscaf	36633748	0	0	BTP202209	0	0	0	Tissue specific
CDS|exon|ID=cds-B	12	5.020833	4386.8	0.14874	AEVEGKDLP	99	647.816	2	1293.62	.	K	Superscaf	36633748	0	BTP202209	0	0	BTP202209	0	Mixed
CDS|exon|ID=cds-B	15	5.064667	5208.6	0.14874	AEVEGKDLP	99	545.6292	3	1633.867	.	K	Superscaf	36633748	0	BTP202209	0	0	0	BTP202209	Mixed
CDS|exon|ID=cds-B	14	4.730714	4740.2	0.14874	AEVEGKDLP	95	753.8912	2	1505.773	.	K	Superscaf	36633748	0	BTP202209	0	0	0	0	Tissue specific
CDS|exon|ID=cds-B	11	4.93	4075.5	0.14874	EVEGKDLPE	99	612.2971	2	1222.583	.	K	Superscaf	36633748	0	BTP202209	BTP202209	0	BTP202209	BTP202209	Mixed
CDS|exon|ID=cds-B	14	4.996429	4897.3	0.14874	EVEGKDLPE	99	521.9498	3	1562.83	.	K	Superscaf	36633748	0	BTP202209	0	0	0	BTP202209	Mixed
CDS|exon|ID=cds-B	13	4.631538	4428.9	0.14874	EVEGKDLPE	95	718.3718	2	1434.735	.	K	Superscaf	36633748	0	0	0	0	0	BTP202209	Tissue specific
CDS|exon|ID=cds-B	10	5.101	3650	0.14874	VEGKDLPEH	99	547.7769	2	1093.54	.	K	Superscaf	36633748	0	BTP202209	BTP202209	BTP202209	BTP202209	BTP202209	Mixed
CDS|exon|ID=cds-B	13	5.133077	4471.8	0.14874	VEGKDLPEH	99	478.9354	3	1433.788	.	K	Superscaf	36633748	0	BTP202209	0	0	BTP202209	0	Tissue enhanced
CDS|exon|ID=cds-B	12	4.749167	4003.4	0.14874	VEGKDLPEH	83	436.2378	3	1305.693	.	K	Superscaf	36633748	0	BTP202209	0	0	0	0	Tissue specific
CDS|exon|ID=cds-B	9	4.998889	3338.7	0.14874	VEGKDLPEH	99	512.259	2	1022.503	.	K	Superscaf	36633748	0	BTP202209	BTP202209	BTP202209	BTP202209	BTP202209	Mixed
CDS|exon|ID=cds-B	9	5.667778	3650	0.14874	EGKDLPEHA	95	498.2418	2	994.4719	.	K	Superscaf	36633748	0	0	0	0	BTP202209	0	Tissue specific
CDS|exon|ID=cds-B	8	5.97375	3224.5	0.14874	GKDLPEHA	95	433.7209	2	865.4293	.	K	Superscaf	36633748	0	0	BTP202209	0	BTP202209	0	Mixed
CDS|exon|ID=cds-B	9	5.311111	3280.6	0.14874	KDLPEHAVL	89	511.2859	2	1020.56	.	K	Superscaf	36633748	0	0	0	BTP202209	0	0	Tissue specific
CDS|exon|ID=cds-B	6	5.346667	2458.8	0.14874	DLPEHA	96	681.3165	1	680.3129	.	K	Superscaf	36633748	0	0	BTP202209	0	0	0	Tissue specific
CDS|exon|ID=cds-B	10	5.244	4099.4	0.148732	VELQKEEAG	99	601.3209	2	1200.635	.	V	Superscaf	36634150	0	0	0	0	BTP202209	BTP202209	Mixed
CDS|exon|ID=cds-B	14	6.735714	5800.9	0.148731	YKEESKKAL	97	558.9834	3	1673.935	.	V	Superscaf	36634150	0	0	0	0	BTP202209	0	Tissue specific
CDS|exon|ID=cds-B	13	6.636923	5455.8	0.14873	ALPPEKKQN	94	466.5947	3	1396.767	.	V	Superscaf	36634150	0	0	0	0	0	BTP202209	Tissue specific
CDS|exon|ID=cds-B	6	7.448333	2722.8	0.143071	YHLLKK	99	401.251	2	800.4908	.	M	Superscaf	36877843	0	0	0	0	0	BTP202209	Tissue specific
CDS|exon|ID=cds-B	8	7.3725	3551	0.140402	RAETLKGR	94	465.7712	2	929.5406	.	G	Superscaf	36992446	0	BTP202209	0	0	0	0	Tissue specific
CDS|exon|ID=cds-B	10	5.981	4333.8	0.106858	A(+42.01)	94	614.8287	2	1227.646	Acetylati	L	Superscaf	38436154	0	BTP202209	0	0	BTP202209	BTP202209	Mixed
CDS|exon|ID=cds-B	9	5.563333	3865.4	0.106858	A(+42.01)	91	550.782	2	1099.551	Acetylati	L	Superscaf	38436154	0	BTP202209	0	0	0	0	Tissue specific
CDS|exon|ID=cds-B	9	4.89	3289.5	0.082104	SGYVQQVKY	97	536.2753	2	1070.54	.	S	Superscaf	39501505	0	0	0	0	0	BTP202209	Tissue specific
CDS|exon|ID=cds-B	19	5.655263	7363.1	0.081928	LTAEQFDEW	99	1116.569	2	2231.13	.	L	Superscaf	39509005	0	0	0	0	0	BTP202209	Tissue specific
CDS|exon|ID=cds-B	13	5.441538	5123.6	0.081928	LTAEQFDEW	99	530.9421	3	1589.809	.	L	Superscaf	39509005	0	0	0	0	0	BTP202209	Tissue specific
CDS|exon|ID=cds-B	10	6.314	4570	0.06156	M(+15.99)	94	630.8324	2	1259.654	Oxidatio	S	Superscaf	40385635	0	0	0	BTP202209	0	0	Tissue specific
CDS|exon|ID=cds-B	8	6.43875	3605.9	0.06156	M(+42.01)	99	502.2646	2	1002.517	Acetylati	S	Superscaf	40385635	BTP202209	BTP202209	0	0	BTP202209	BTP202209	Mixed
CDS|exon|ID=cds-B	8	6.8775	3348.5	0.046937	YGKLLQPK	99	473.7874	2	945.5647	.	R	Superscaf	41014900	0	0	0	0	0	BTP202209	Tissue specific
CDS|exon|ID=cds-B	8	4.17375	2413.6	0.009502	SDFEVVPK	99	460.739	2	919.465	.	D	Superscaf	43442509	0	BTP202209	0	0	0	0	Tissue specific
CDS|exon|ID=cds-B	9	6.278889	3420.5	0.04853	LSAKFGGQA	77	439.7389	2	877.4658	.	T	Superscaf	45122386	0	0	0	0	0	BTP202209	Tissue specific
CDS|exon|ID=cds-B	5	5.832	2138.6	0.066855	M(+15.99)	78	689.2715	1	688.256	Oxidatio	G	Superscaf	45911980	0	0	0	0	0	BTP202209	Tissue specific
CDS|exon|ID=cds-B	11	5.702727	3621	0.067426	PAPTTDAVA	98	534.2892	2	1066.566	.	Q	Superscaf	45935608	0	BTP202209	0	0	0	0	Tissue specific
CDS|exon|ID=cds-B	9	4.793333	2986.4	0.072539	LALDVVANF	83	485.7866	2	969.5607	.	G	Superscaf	46156258	0	0	0	BTP202209	0	0	Tissue specific
CDS|exon|ID=cds-B	7	4.781429	1922.1	0.074425	FVGTVM(+1	73	399.2171	2	796.4153	Oxidatio	F	Superscaf	46237836	0	0	0	0	0	BTP202209	Tissue specific
CDS|exon|ID=cds-B	8	6.6075	3342.6	0.091408	SSSKLTLH	98	436.744	2	871.4763	.	E	Superscaf	46968553	0	0	0	0	BTP202209	BTP202209	Mixed
CDS|exon|ID=cds-B	8	5.565	3073.4	0.091441	SDVHRAAF	94	451.7251	2	901.4406	.	P	Superscaf	46969729	0	0	0	0	0	BTP202209	Tissue specific
CDS|exon|ID=cds-B	10	5.937	3735.8	0.115638	ASFEVEKTK	99	389.5501	3	1165.634	.	N	Superscaf	48011356	BTP202209	0	0	BTP202209	0	0	Mixed
CDS|exon|ID=cds-B	9	5.927778	3424.5	0.115639	SFEVEKTKK	93	365.872	3	1094.597	.	N	Superscaf	48011356	BTP202209	0	0	0	0	0	Tissue specific
CDS|exon|ID=cds-B	10	6.4	4821.6	0.137821	ELRDNDKTF	97	437.2199	3	1308.642	.	S	Superscaf	48965965	0	0	0	0	0	BTP202209	Tissue specific
CDS|exon|ID=cds-B	10	4.922	2801.9	0.137899	AGAVEKGVF	55	470.775	2	939.5389	.	L	Superscaf	48969346	0	0	0	0	0	BTP202209	Tissue specific
CDS|exon|ID=cds-B	8	6.92125	3486.8	0.138014	SFRNPLAK	69	466.7674	2	931.5239	.	H	Superscaf	48974260	0	0	0	0	BTP202209	0	Tissue specific
CDS|exon|ID=cds-B	6	6.826667	2882.4	0.148674	P(+42.01)	68	382.7107	2	763.3976	Acetylati	T	Superscaf	49432420	0	0	BTP202209	BTP202209	0	0	Tissue enhanced
CDS|exon|ID=cds-B	7	4.527143	1993.2	0.190414	HFVAVPP	62	383.7121	2	765.4173	.	G	Superscaf	51229321	0	0	0	BTP202209	0	0	Tissue specific
CDS|exon|ID=cds-B	7	6.031429	2317.5	0.192864	A(+42.01)	65	640.3267	1	639.3228	Acetylati	P	Superscaf	51334699	0	0	0	0	0	BTP202209	Tissue specific
CDS|exon|ID=cds-B	12	5.114167	4455.9	0.198065	VVANSKESY	98	465.58	3	1393.72	.	W	Superscaf	51558562	0	0	0	BTP202209	0	0	Tissue specific
CDS|exon|ID=cds-B	8	6.2425	3547.9	0.198066	SKESYELR	88	506.2631	2	1010.503	.	W	Superscaf	51558562	0	0	0	0	0	BTP202209	Tissue specific
CDS|exon|ID=cds-B	10	4.871	3747.2	0.199347	VDPDYWEKL	96	639.3217	2	1276.634	.	R	Superscaf	51613693	0	0	0	0	BTP202209	0	Tissue specific
CDS|exon|ID=cds-B	8	4.59375	3040.4	0.199347	VDPDYWEK	97	526.2382	2	1050.466	.	R	Superscaf	51613693	0	0	0	0	0	BTP202209	Tissue specific
CDS|exon|ID=cds-B	8	6.7825	3000.5	0.23112	PPPPLAPR	90	422.7541	2	843.4966	.	P	Superscaf	52981015	0	BTP202209	0	0	0	0	Tissue specific
CDS|exon|ID=cds-B	9	5.213333	2538.8	0.270557	VPPPVPSKF	82	459.2756	2	916.5381	.	N	Superscaf	54678822	0	0	BTP202209	0	0	0	Tissue specific
CDS|exon|ID=cds-B	7	5.911429	2299.6	0.278012	APVALPR	51	362.2267	2	722.4438	.	H	Superscaf	54999013	0	0	0	BTP202209	0	0	Tissue specific
CDS|exon|ID=cds-B	7	6.925714	3334	0.282882	DLPRPRN	80	434.2438	2	866.4722	.	P	Superscaf	55208751	BTP202209	0	0	0	0	0	Tissue specific
CDS|exon|ID=cds-B	10	5.297	3529.1	0.285239	TGHTGDVMS	51	509.2356	2	1016.46	.	P	Superscaf	55310119	0	0	0	0	0	BTP202209	Tissue specific
CDS|exon|ID=cds-B	9	6.188889	3584.1	0.28524	SLAPDTRLF	71	510.2778	2	1018.545	.	P	Superscaf	55310119	0	0	0	0	0	BTP202209	Tissue specific
CDS|exon|ID=cds-B	7	7.158571	2986.3	0.289529	RGPAC(+57	72	393.2089	2	784.4014	Carbamid	E	Superscaf	55494846	0	BTP202209	0	0	0	0	Tissue specific
CDS|exon|ID=cds-B	14	5.496429	5751.5	0.293435	MDPMNEFSA	98	798.8517	2	1595.696	.	P	Superscaf	55662763	0	0	0	0	0	BTP202209	Tissue specific
CDS|exon|ID=cds-B	9	4.813333	3248.6	0.293437	YLEPEEVLK	97	560.3	2	1118.586	.	P	Superscaf	55662763	0	0	0	0	0	BTP202209	Tissue specific
CDS|exon|ID=cds-B	8	4.7075	2789.1	0.293437	LEPEEVLK	96	478.7668	2	955.5226	.	P	Superscaf	55662763	0	0	0	0	0	BTP202209	Tissue specific

CDS|exon|ID=cds-Bg	13	5.234615	4814.2	0.293438	YSEFFTGSK	93	778.3663	2	1554.724	.	P	Superscaf	55662763	0	0	0	0	0	BTP20220	Tissue specific
CDS|exon|ID=cds-Bg	9	5.334444	3158.4	0.293438	FTGSKLVDY	99	515.2648	2	1028.518	.	P	Superscaf	55662763	0	0	0	0	0	BTP20220	Tissue specific
CDS|exon|ID=cds-Bg	8	5.31625	2771	0.293438	TGSKLVDY	98	441.73	2	881.4494	.	P	Superscaf	55662763	0	0	0	0	0	BTP20220	Tissue specific
CDS|exon|ID=cds-Bg	8	5.24625	3201.6	0.293439	ARSVHEEF	87	487.7366	2	973.4617	.	P	Superscaf	55662763	0	0	0	0	0	BTP20220	Tissue specific
CDS|exon|ID=cds-Bg	8	6.3	2699.2	0.296347	PPPPPPPP	99	795.4377	1	794.4326	.	A	Superscaf	55787974	0	0	0	BTP20220	0	0	Tissue specific
CDS|exon|ID=cds-Bg	5	5.518	1783.1	0.296869	PPMEA	76	544.2411	1	543.2363	.	P	Superscaf	55810240	0	0	0	0	BTP20220	BTP20220	Mixed
CDS|exon|ID=cds-Bg	7	5.09	2686	0.298413	LPEAYDF	97	854.3918	1	853.3857	.	P	Superscaf	55875193	BTP20220	0	0	0	0	0	Tissue specific
CDS|exon|ID=cds-Bg	7	5.1	2024.1	0.301679	LASAVLA	96	644.3954	1	643.3904	.	G	Superscaf	56017624	0	0	0	BTP20220	0	0	Tissue specific
CDS|exon|ID=cds-Bg	5	6.216	1723	0.308964	LGPPT	61	484.2746	1	483.2693	.	C	Superscaf	56331091	0	0	0	0	BTP20220	0	Tissue specific
CDS|exon|ID=cds-Bg	9	4.398889	2453.7	0.309159	C(+57.02)	66	493.787	2	985.563	Carbamido	P	Superscaf	56339482	0	0	BTP20220	0	0	0	Tissue specific
CDS|exon|ID=cds-Bg	8	6.11875	2554.7	0.319721	APPAAAGP	80	651.3435	1	650.3387	.	P	Superscaf	56794075	0	0	0	0	0	BTP20220	Tissue specific
CDS|exon|ID=cds-Bg	6	5.278333	2648	0.38089	EDHLAW	62	385.6753	2	769.3395	.	P	Superscaf	59426359	0	0	0	0	BTP20220	BTP20220	Tissue enhanced
CDS|exon|ID=cds-Bg	17	5.438235	5996.7	0.380936	GMLDPAEKD	66	894.925	2	1787.843	.	L	Superscaf	59428327	0	BTP20220	0	0	0	0	Tissue specific
CDS|exon|ID=cds-Bg	10	6.496	3565.6	0.380995	KLFPKGVFT	89	388.9076	3	1163.707	.	N	Superscaf	59430970	0	0	0	0	0	BTP20220	Tissue specific
CDS|exon|ID=cds-Bg	6	6.815	2656.1	0.380996	YLRYTP	91	406.718	2	811.4228	.	N	Superscaf	59430970	0	BTP20220	0	0	0	0	Tissue specific
CDS|exon|ID=cds-Bg	8	6.96375	3007.1	0.441828	GLGFKTPK	98	424.2544	2	846.4963	.	K	Superscaf	62048782	0	BTP20220	0	0	0	0	Tissue specific
CDS|exon|ID=cds-Bg	14	6.592143	5584.7	0.441835	VTKAAGTKK	99	791.4568	2	1580.904	.	K	Superscaf	62048782	0	0	BTP20220	0	0	0	Tissue specific
CDS|exon|ID=cds-Bg	12	7.146667	5187.2	0.441836	KAAGTKKQP	99	691.3985	2	1380.788	.	K	Superscaf	62048782	0	0	BTP20220	0	0	0	Tissue specific
CDS|exon|ID=cds-Bg	6	6.105	2534.8	0.451589	LENLPK	57	357.2105	2	712.4119	.	I	Superscaf	62469054	0	BTP20220	0	0	0	0	Tissue specific
CDS|exon|ID=cds-Bg	7	6.454286	2865.2	0.492292	D(+42.01)	82	423.225	2	844.4443	Acetylati	G	Superscaf	64220473	0	BTP20220	0	0	0	0	Tissue specific
CDS|exon|ID=cds-Bg	14	5.870714	5988.8	0.524467	A(+42.01)	69	807.366	2	1612.723	Acetylati	G	Superscaf	65605276	0	BTP20220	0	0	0	0	Tissue specific
CDS|exon|ID=cds-Bg	8	5.665	3347.7	0.5252	FDEQPLKL	90	495.2676	2	988.5229	.	T	Superscaf	65636782	0	BTP20220	0	0	0	0	Tissue specific
CDS|exon|ID=cds-Bg	7	5.62	2994.3	0.5252	FDEQPLK	98	438.7252	2	875.4388	.	T	Superscaf	65636782	0	BTP20220	0	0	0	0	Tissue specific
CDS|exon|ID=cds-Bg	6	4.073333	1825.1	0.547242	LGPVED	59	629.3132	1	628.3068	.	Q	Superscaf	66585139	0	0	0	BTP20220	0	0	Tissue specific
CDS|exon|ID=cds-Bg	7	5.274286	2109.2	0.549384	GPVDGGK	58	629.324	1	628.318	.	I	Superscaf	66677533	0	0	0	0	BTP20220	0	Tissue specific
CDS|exon|ID=cds-Bg	7	5.078571	2514.7	0.589372	G(+42.01)	75	834.4333	1	833.4283	Acetylati	I	Superscaf	68398504	0	0	BTP20220	0	0	0	Tissue specific
CDS|exon|ID=cds-Bg	11	4.451818	3020.3	0.589981	VFDPVPSGV	91	573.311	2	1144.613	.	K	Superscaf	68424568	0	0	0	0	0	BTP20220	Tissue specific
CDS|exon|ID=cds-Bg	9	6.663333	3595	0.590221	YGDGPRPPK	90	493.7557	2	985.4981	.	L	Superscaf	68434990	0	BTP20220	0	0	0	BTP20220	Mixed
CDS|exon|ID=cds-Bg	8	6.27875	3126.6	0.590221	YGDGPRPP	98	429.7078	2	857.4031	.	L	Superscaf	68434990	0	0	BTP20220	0	BTP20220	BTP20220	Mixed
CDS|exon|ID=cds-Bg	14	5.082143	3657.7	0.590226	VGGGGGVGG	99	991.4565	1	990.4518	.	L	Superscaf	68434990	0	BTP20220	0	0	0	0	Tissue specific
CDS|exon|ID=cds-Bg	8	5.5825	2926.1	0.59504	LGKQVLSY	79	454.2641	2	906.5175	.	P	Superscaf	68642368	0	0	0	0	0	BTP20220	Tissue specific
CDS|exon|ID=cds-Bg	8	6.0275	3081.7	0.684474	VTTYRTTS	67	464.7399	2	927.4662	.	N	Superscaf	72491319	0	0	0	BTP20220	0	0	Tissue specific
CDS|exon|ID=cds-Bg	6	6.106667	1962.1	0.712371	A(+42.01)	68	581.3279	1	580.322	Acetylati	L	Superscaf	73691701	0	BTP20220	0	0	0	0	Tissue specific
CDS|exon|ID=cds-Bg	7	5.375714	2480.6	0.76721	D(+42.01)	84	443.7667	2	885.5171	Acetylati	R	Superscaf	76051782	0	0	BTP20220	0	0	0	Tissue specific
CDS|exon|ID=cds-Bg	9	5.921111	4199	0.813168	RDFEGRDNM	85	570.2456	2	1138.482	.	G	Superscaf	78029799	0	0	0	0	0	BTP20220	Tissue specific
CDS|exon|ID=cds-Bg	7	5.14	1881.9	0.817464	VGAAALG	53	558.3226	1	557.3173	.	E	Superscaf	78214246	0	0	BTP20220	0	0	0	Tissue specific
CDS|exon|ID=cds-Bg	8	4.435	2548	0.858664	RVVADTEL	87	451.7491	2	901.4869	.	R	Superscaf	79987036	0	BTP20220	0	0	0	0	Tissue specific
CDS|exon|ID=cds-Bg	6	5.866667	2328.6	0.862677	C(+57.02)	50	608.2725	1	607.2635	Carbamido	V	Superscaf	80160261	0	0	0	0	0	BTP20220	Tissue specific
CDS|exon|ID=cds-Bg	7	6.884286	2807.1	0.890489	KHLLPPP	64	801.4847	1	800.4908	.	L	Superscaf	81357016	0	0	0	BTP20220	0	0	Tissue specific
CDS|exon|ID=cds-Bg	11	5.048182	4395	0.975387	LSPEEMEEL	99	667.3113	2	1332.612	.	K	Superscaf	85010188	0	0	0	0	0	BTP20220	Tissue specific
CDS|exon|ID=cds-Bg	9	6.067778	3895.2	0.97539	LSFC(+57.	98	571.2797	2	1140.549	Carbamido	K	Superscaf	85010188	0	0	0	0	0	BTP20220	Tissue specific
CDS|exon|ID=cds-Bg	10	6.975	3931.2	0.975944	DAGKKPLGP	95	519.804	2	1037.598	.	E	Superscaf	85034536	0	0	0	0	BTP20220	BTP20220	Mixed
CDS|exon|ID=cds-Bg	10	6.388	4247.6	0.975946	SAERPKEEK	71	593.8231	2	1185.635	.	E	Superscaf	85034536	0	0	0	0	BTP20220	0	Tissue specific
CDS|exon|ID=cds-Bg	8	6.5225	3552.9	0.975947	ERPKEEKL	75	514.79	2	1027.566	.	E	Superscaf	85034536	0	0	0	0	BTP20220	0	Tissue specific
CDS|exon|ID=cds-Bg	7	4.212857	1840	0.975948	VRAVVDK	79	393.743	2	785.4759	.	E	Superscaf	85034536	0	0	0	0	0	BTP20220	Tissue specific
CDS|exon|ID=cds-Bg	17	5.765294	5831.1	0.975948	AVVDKKEAC	96	431.4913	4	1721.942	.	E	Superscaf	85034536	0	0	0	0	BTP20220	0	Tissue specific
CDS|exon|ID=cds-Bg	18	6.38	7630.3	0.975948	EAGKEGPAA	93	499.7585	4	1995.013	.	E	Superscaf	85034536	0	0	0	0	BTP20220	0	Tissue specific
CDS|exon|ID=cds-Bg	18	6.397222	7070.3	0.97595	KGEQPEAAS	92	460.9978	4	1839.969	.	E	Superscaf	85034536	0	0	0	0	BTP20220	0	Tissue specific
CDS|exon|ID=cds-Bg	13	6.361538	5277.8	0.975965	LLKENTTLL	84	753.4474	2	1504.887	.	E	Superscaf	85034536	0	0	0	0	0	BTP20220	Tissue specific
CDS|exon|ID=cds-Bg	10	6.509	4167.6	0.975965	LLKENTTLL	99	391.5782	3	1171.718	.	E	Superscaf	85034536	0	0	0	0	0	BTP20220	Tissue specific
CDS|exon|ID=cds-Bg	8	6.59875	2912.1	0.976139	AASGAAPR	89	350.6852	2	699.3663	.	N	Superscaf	85042903	0	0	0	BTP20220	0	0	Tissue specific
CDS|exon|ID=cds-Bg	7	5.187143	2174.4	0.981686	G(+42.01)	55	369.2119	2	736.398	Acetylati	G	Superscaf	85281277	0	0	0	BTP20220	0	0	Tissue specific
CDS|exon|ID=cds-Bg	14	6.170714	5837.2	0.984193	PNWGGGKKC	99	788.3776	2	1574.745	Carbamido	L	Superscaf	85389316	0	BTP20220	0	0	BTP20220	BTP20220	Mixed
CDS|exon|ID=cds-Bg	15	6.194667	6234.7	0.984193	PNWGGGKKC	95	838.901	2	1675.792	Carbamido	L	Superscaf	85389316	0	0	0	0	BTP20220	BTP20220	Mixed
CDS|exon|ID=cds-Bg	18	5.781111	7081.6	0.984193	PNWGGGKKC	80	1043.5	2	2084.992	Carbamido	L	Superscaf	85389316	0	0	0	0	BTP20220	0	Tissue specific
CDS|exon|ID=cds-Bg	13	5.896154	5368.8	0.984193	PNWGGGKKC	99	724.3291	2	1446.65	Carbamido	L	Superscaf	85389316	0	0	0	0	BTP20220	BTP20220	Mixed
CDS|exon|ID=cds-Bg	12	5.916667	4758.1	0.984193	PNWGGGKKC	99	660.3008	2	1318.591	Carbamido	L	Superscaf	85389316	0	0	0	0	BTP20220	0	Tissue specific
CDS|exon|ID=cds-Bg	9	6.667778	3875.1	0.984193	PNWGGGKKC	99	502.24	2	1002.471	Carbamido	L	Superscaf	85389316	0	0	0	0	BTP20220	BTP20220	Tissue enhanced
CDS|exon|ID=cds-Bg	8	6.87375	3289.4	0.984193	P(+42.01)	96	443.2324	2	884.4504	Acetylati	L	Superscaf	85389316	0	BTP20220	0	0	BTP20220	BTP20220	Mixed
CDS|exon|ID=cds-Bg	7	6.464286	2821	0.984193	PNWGGGK	99	358.1781	2	714.3449	.	L	Superscaf	85389316	0	0	0	0	BTP20220	BTP20220	Mixed
CDS|exon|ID=cds-Bg	12	6.223333	4903.1	0.984193	WGGGKKC(+	99	455.5549	3	1363.649	Carbamido	L	Superscaf	85389316	0	0	0	0	BTP20220	0	Tissue specific
CDS|exon|ID=cds-Bg	13	6.246923	5300.6	0.984193	WGGGKKC(+	91	489.2383	3	1464.697	Carbamido	L	Superscaf	85389316	0	0	0	0	BTP20220	0	Tissue specific
CDS|exon|ID=cds-Bg	11	5.903636	4434.7	0.984193	WGGGKKC(+	83	618.7862	2	1235.554	Carbamido	L	Superscaf	85389316	0	0	0	0	BTP20220	0	Tissue specific
CDS|exon|ID=cds-Bg	10	5.929	3824	0.984193	WGGGKKC(+	83	554.7537	2	1107.495	Carbamido	L	Superscaf	85389316	0	0	0	0	BTP20220	0	Tissue specific
CDS|exon|ID=cds-Bg	9	5.296667	3413.7	0.984194	KC(+57.02	92	540.2679	2	1078.526	Carbamido	L	Superscaf	85389316	0	0	0	0	0	BTP20220	Tissue specific
CDS|exon|ID=cds-Bg	10	5.333	3873.2	0.984194	KC(+57.02	78	621.7998	2	1241.59	Carbamido	L	Superscaf	85389316	0	0	0	0	0	BTP20220	Tissue specific
CDS|exon|ID=cds-Bg	8	4.82125	2819.1	0.984194	GVC(+57.(	86	477.7382	2	953.4641	Carbamido	L	Superscaf	85389316	0	0	0	0	BTP20220	0	Tissue specific
CDS|exon|ID=cds-Bg	14	5.140714	5711.2	0.984195	FAEEVQC(+	97	827.8606	2	1653.709	Carbamido	L	Superscaf	85389316	0	0	0	0	0	BTP20220	Tissue specific
CDS|exon|ID=cds-Bg	17	5.214706	7033.7	0.984195	AEEVQC(+5	86	672.2899	3	2013.856	Carbamido	L	Superscaf	85389316	0	0	0	0	0	BTP20220	Tissue specific
CDS|exon|ID=cds-Bg	16	5.166875	6680.3	0.984195	AEEVQC(+5	90	634.5957	3	1900.772	Carbamido	L	Superscaf	85389316	0	0	0	0	0	BTP20220	Tissue specific
CDS|exon|ID=cds-Bg	15	5.146	6292.9	0.984195	AEEVQC(+5	97	877.8555	2	1753.704	Carbamido	L	Superscaf	85389316	0	0	0	0	BTP20220	0	Tissue specific
CDS|exon|ID=cds-Bg	14	5.155	5707.2	0.984195	AEEVQC(+5	94	797.8417	2	1593.673	Carbamido	L	Superscaf	85389316	0	0	0	0	BTP20220	0	Tissue specific
CDS|exon|ID=cds-Bg	13	5.114615	5323.8	0.984195	AEEVQC(+5	99	754.3253	2	1506.641	Carbamido	L	Superscaf	85389316	0	0	0	0	BTP20220	BTP20220	Mixed
CDS|exon|ID=cds-Bg	12	4.729167	4855.4	0.984195	AEEVQC(+5	94	690.2773	2	1378.546	Carbamido	L	Superscaf	85389316	0	0	0	0	0	BTP20220	Tissue specific
CDS|exon|ID=cds-Bg	15	5.11	6369	0.984195	EEVQC(+57	89	915.871	2	1829.735	Carbamido	L	Superscaf	85389316	0	0	0	0	BTP20220	BTP20220	Mixed
CDS|exon|ID=cds-Bg	14	5.083571	5981.6	0.984195	EEVQC(+57	97	842.3376	2	1682.667	Carbamido	L	Superscaf	85389316	0	0	0	0	BTP20220	0	Tissue specific
CDS|exon|ID=cds-Bg	12	5.039167	5012.5	0.984195	EEVQC(+57	95	479.5408	3	1435.604	Carbamido	L	Superscaf	85389316	0	BTP20220	0	0	BTP20220	BTP20220	Mixed
CDS|exon|ID=cds-Bg	11	4.611818	4544.1	0.984195	EEVQC(+57	92	654.7589	2	1307.509	Carbamido	L	Superscaf	85389316	0	0	0	0	0	BTP20220	Tissue specific
CDS|exon|ID=cds-Bg	14	5.245	5943.5	0.984195	EVQC(+57.	89	851.3497	2	1700.692	Carbamido	L	Superscaf	85389316	0	0	0	0	BTP20220	0	Tissue specific
CDS|exon|ID=cds-Bg	13	5.226923	5556.1	0.984195	EVQC(+57.	92	777.8148	2	1553.624	Carbamido	L	Superscaf	85389316	0	0	0	0	BTP20220	0	Tissue specific
CDS|exon|ID=cds-Bg	11	5.204545	4587	0.984195	EVQC(+57.	89	654.2849	2	1306.561	Carbamido	L	Superscaf	85389316	0	0	0	0	0	BTP20220	Tissue specific
CDS|exon|ID=cds-Bg	13	5.400769	5518	0.984195	VQC(+57.(	94	786.8297	2	1571.65	Carbamido	L	Superscaf	85389316	0	0	0	0	BTP20220	0	Tissue specific
CDS|exon|ID=cds-Bg	12	5.394167	5130.6	0.984195	VQC(+57.(	96	713.2953	2	1424.581	Carbamido	L	Superscaf	85389316	0	0	0	0	BTP20220	0	Tissue specific
CDS|exon|ID=cds-Bg	10	5.403	4161.5	0.984195	VQC(+57.(	91	589.7633	2	1177.519	Carbamido	L	Superscaf	85389316	0	0	0	0	0	BTP20220	Tissue specific
CDS|exon|ID=cds-Bg	13	5.989231	4956.4	0.984291	GYGQGAGTL	86	642.7976	2	1283.582	.	P	Superscaf	85393705	0	BTP20220	0	0	0	0	Tissue specific
CDS|exon|ID=cds-Bg	17	5.806471	6416	0.984291	YGQGAGTLS	76	835.8848	2	1669.762	.	P	Superscaf	85393705	0	0	0	0	BTP20220	0	Tissue specific
CDS|exon|ID=cds-Bg	12	5.990833	4659.1	0.984291	YGQGAGTLS	58	614.2838	2	1226.56	.	P	Superscaf	85393705	0	0	0	0	BTP20220	0	Tissue specific
CDS|exon|ID=cds-Bg	9	5.936667	3407.7	0.984291	YGQGAGTLS	94	853.4012	1	852.3977	.	P	Superscaf	85393705	0	0	0	0	0	BTP20220	Tissue specific
CDS|exon|ID=cds-Bg	13	5.85	5515.3	0.984313	VGGSERC(+	82	732.3239	2	1462.64	Carbamido	P	Superscaf	85394557	0	0	0	0	0	BTP20220	Tissue specific
CDS|exon|ID=cds-Bg	14	5.432143	5515.3	0.984313	VGGSERC(+	91	781.8585	2	1561.709	Carbamido	P	Superscaf	85394557	0	0	0	0	0	BTP20220	Tissue specific
CDS|exon|ID=cds-Bg	12	5.835833	5204	0.984313	VGGSERC(+	98	696.8063	2	1391.603	Carbamido	P	Superscaf	85394557	0	0	0	0	BTP20220	BTP20220	Mixed
CDS|exon|ID=cds-Bg	11	5.852727	4593.3	0.984313	VGGSERC(+	84	632.778	2	1263.545	Carbamido	P	Superscaf	85394557	0	0	0	0	BTP20220	BTP20220	Mixed
CDS|exon|ID=cds-Bg	10	5.87	4209.9	0.984313	VGGSERC(+	94	589.2618	2	1176.513	Carbamido	P	Superscaf	85394557	0	0	0	0	BTP20220	BTP20220	Mixed
CDS|exon|ID=cds-Bg	14	5.84	5988.8	0.984314	GSERC(+57	81	820.8641	2	1639.72	Carbamido	P	Superscaf	85394557	0	0	0	0	BTP20220	0	Tissue specific
CDS|exon|ID=cds-Bg	10	5.303	3867.1	0.984314	C(+57.02)	97	563.763	2	1125.513	Carbamido	P	Superscaf	85394557	0	BTP20220	0	0	0	0	Tissue specific
CDS|exon|ID=cds-Bg	10	6.845	4722.1	0.984381	SC(+57.02	89	425.1898	3	1272.553	Carbamido	E	Superscaf	85397407	0	0	0	0	BTP20220	BTP20220	Mixed
CDS|exon|ID=cds-Bg	8	6.5925	3956.4	0.984381	SC(+57.02	99	544.7234	2	1087.436	Carbamido	E	Superscaf	85397407	0	0	0	0	BTP20220	BTP20220	Mixed
CDS|exon|ID=cds-Bg	7	6.817143	3370.7	0.984381	SC(+57.02	98	464.7084	2	927.4055	Carbamido	E	Superscaf	85397407	0	0	0	0	BTP20220	BTP20220	Mixed
CDS|exon|ID=cds-Bg	9	5.85	3501.9	0.984381	LESTTLADK	94	489.2608	2	976.5077	.	E	Superscaf	85397407	0	0	0	0	0	BTP20220	Tissue specific
CDS|exon|ID=cds-Bg	7	6.951429	2866.9	0.984392	AKNFGPK	61	381.2178	2	760.4232	.	P	Superscaf	85398016	0	0	0	0	0	BTP20220	Tissue specific
CDS|exon|ID=cds-Bg	11	5.861818	3848	0.984392	GFGFGQGAG	86	981.4742	1	980.4716	.	P	Superscaf	85398016	0	0	0	0	0	BTP20220	Tissue specific
CDS|exon|ID=cds-Bg	13	5.543846	4467.7	0.984392	GFGFGQGAG	97	609.3044	2	1216.599	.	P	Superscaf	85398016	0	0	0	0	0	BTP20220	Tissue specific
CDS|exon|ID=cds-Bg	9	5.831111	3183.3	0.984392	GFGFGQGAG	95	797.3546	1	796.3503	.	P	Superscaf	85398016	0	0	0	0	BTP20220	0	Tissue specific
CDS|exon|ID=cds-Bg	8	5.81375	2886	0.984392	GFGFGQGA	97	740.3336	1	739.3289	.	P	Superscaf	85398016	0	0	0	0	0	BTP20220	Tissue specific
CDS|exon|ID=cds-Bg	7	5.784286	2574.7	0.984392	GFGFGQG	88	669.2969	1	668.2918	.	P	Superscaf	85398016	0	0	0	0	0	BTP20220	Tissue specific
CDS|exon|ID=cds-Bg	14	5.357143	4979.3	0.984392	FGFGQGAGA	99	688.832	2	1375.652	.	P	Superscaf	85398016	0	BTP20220	0	0	0	BTP20220	Tissue enhanced
CDS|exon|ID=cds-Bg	9	5.892222	3163.3	0.984392	GFGQGAGAL	65	777.3859	1	776.3817	.	P	Superscaf	85398016	0	0	0	0	0	BTP20220	Tissue specific
CDS|exon|ID=cds-Bg	11	5.510909	3783	0.984392	GFGQGAGAL	86	507.2603	2	1012.509	.	P	Superscaf	85398016	0	0	0	0	0	BTP20220	Tissue specific
CDS|exon|ID=cds-Bg	12	5.295833	4294.6	0.984392	FGQGAGALV	95	586.7872	2	1171.562	.	P	Superscaf	85398016	0	BTP20220	0	0	0	BTP20220	Mixed
CDS|exon|ID=cds-Bg	10	5.465	3485.7	0.984392	FGQGAGALV	94	478.7494	2	955.4875	.	P	Superscaf	85398016	0	0	0	0	0	BTP20220	Tissue specific
CDS|exon|ID=cds-Bg	10	5.21	3609.9	0.984392	QGAGALVHS	99	484.743	2	967.4723	.	P	Superscaf	85398016	0	BTP20220	0	0	0	BTP20220	Mixed
CDS|exon|ID=cds-Bg	8	5.4	2801	0.984392	QGAGALVH	87	376.7053	2	751.3976	.	P	Superscaf	85398016	0	BTP20220	0	0	0	BTP20220	Mixed
CDS|exon|ID=cds-Bg	12	5.985	4468.9	0.985347	VEAMSGMEC	99	441.556	3	1321.648	.	D	Superscaf	85439164	BTP20220	0	0	0	0	0	Tissue specific
CDS|exon|ID=cds-Bg	10	6.092	4016.4	0.985348	FDAAKSPTT	99	533.2632	2	1064.514	.	D	Superscaf	85439164	BTP20220	0	0	0	0	0	Tissue specific
CDS|exon|ID=cds-Bg	14	6.244286	4981.2	0.985777	RVPAGSEKP	84	452.2513	3	1353.736	.	G	Superscaf	85457416	0	0	0	0	0	BTP20220	Tissue specific
CDS|exon|ID=cds-Bg	6	7.57	2440.7	0.992223	K(+42.01)	77	354.218	2	706.4126	Acetylati	R	Superscaf	286410	0	0	BTP20220	0	0	0	Tissue specific
CDS|exon|ID=cds-Bg	19	5.111579	6688.4	0.914741	EDGSDPASC	89	918.4036	2	1834.797	.	R	Superscaf	3138649	0	BTP20220	0	0	BTP20220	BTP20220	Tissue enhanced
CDS|exon|ID=cds-Bg	18	5.216667	6262.9	0.914741	DGSDPASGD	88	853.8813	2	1705.754	.	R	Superscaf	3138649	0	0	0	0	BTP20220	0	Tissue specific
CDS|exon|ID=cds-Bg	11	5.09	3985.4	0.914742	DGSDPASGD	85	1080.446	1	1079.441	.	R	Superscaf	3138649	0	0	0	0	BTP20220	0	Tissue specific
CDS|exon|ID=cds-Bg	9	5.227778	3276.6	0.914742	SDPASGDFL	76	908.3965	1	907.3923	.	R	Superscaf	3138649	0	0	0	0	BTP20220	0	Tissue specific
CDS|exon|ID=cds-Bg	14	5.450714	4759.2	0.914741	PASGDFLAE	97	666.8279	2	1331.647	.	R	Superscaf	3138649	0	0	0	0	BTP20220	0	Tissue specific
CDS|exon|ID=cds-Bg	12	5.560833	5284.7	0.914662	KVDLEDYKN	98	754.3891	2	1506.768	.	V	Superscaf	3141568	0	0	0	0	0	BTP20220	Tissue specific
CDS|exon|ID=cds-Bg	8	5.57	2831.3	0.914659	TGPVPREF	87	451.7375	2	901.4658	.	V	Superscaf	3141568	0	0	0	0	0	BTP20220	Tissue specific
CDS|exon|ID=cds-Bg	8	7.13375	3121.4	0.914655	APGSPRKP	82	405.2351	2	808.4555	.	V	Superscaf	3141568	0	0	0	0	BTP20220	0	Tissue specific
CDS|exon|ID=cds-Bg	8	6.26875	3507.1	0.914653	WNPGRPEP	84	476.7335	2	951.4562	.	V	Superscaf	3141568	0	0	0	0	BTP20220	0	Tissue specific
CDS|exon|ID=cds-Bg	13	6.178462	5193.9	0.914652	PGRPEPGSA	83	663.312	2	1324.616	.	V	Superscaf	3141568	0	0	0	0	BTP20220	0	Tissue specific
CDS|exon|ID=cds-Bg	9	6.28	3375.8	0.914652	PGRPEPGSA	94	434.2183	2	866.4246	.	V	Superscaf	3141568	0	0	BTP20220	0	BTP20220	0	Mixed
CDS|exon|ID=cds-Bg	16	6.459375	6477.4	0.914652	GRPEPGSAG	96	818.3994	2	1634.791	.	V	Superscaf	3141568	0	0	0	0	BTP20220	0	Tissue specific
CDS|exon|ID=cds-Bg	12	6.425	4768.4	0.914652	PGSAGTWNP	91	598.7916	2	1195.574	.	V	Superscaf	3141568	0	0	0	0	BTP20220	0	Tissue specific
CDS|exon|ID=cds-Bg	16	6.01125	6577.2	0.914642	SSGSSSFRP	99	783.3304	2	1564.65	.	V	Superscaf	3141568	0	0	BTP20220	0	0	0	Tissue specific
CDS|exon|ID=cds-Bg	14	5.485714	5534.1	0.91464	REEGSVSSG	70	770.8632	2	1539.717	.	V	Superscaf	3141568	0	0	0	0	0	BTP20220	Tissue specific
CDS|exon|ID=cds-Bg	8	6.3775	2780	0.914639	TGKLVTTK	82	424.2641	2	846.5175	.	V	Superscaf	3141568	0	0	0	0	0	BTP20220	Tissue specific

CDS|exon|ID=cds-Bg	13	5.903077	5147	0.914636	TVTNADGRT	99	697.3472	2	1392.685	.	V	Superscaf	3141568	0	0	0	0	0	BTP202209	Tissue specific
CDS|exon|ID=cds-Bg	9	6.085556	4018.5	0.914633	LDDFFLRNK	98	389.8758	3	1166.608	.	V	Superscaf	3141568	0	0	0	0	0	BTP202209	Tissue specific
CDS|exon|ID=cds-Bg	8	5.62875	3550.1	0.914633	LDDFFLRN	91	520.2623	2	1038.513	.	V	Superscaf	3141568	0	0	0	0	0	BTP202209	Tissue specific
CDS|exon|ID=cds-Bg	8	6.09875	3665.1	0.914633	DDFFLRNK	92	527.7689	2	1053.524	.	V	Superscaf	3141568	0	BTP202209	0	0	0	BTP202209	Mixed
CDS|exon|ID=cds-Bg	17	5.281765	5909.3	0.91463	LEPDVAALC	98	817.3982	2	1632.784	.	V	Superscaf	3141568	0	BTP202209	0	0	BTP202209	0	Mixed
CDS|exon|ID=cds-Bg	10	5.175	3578.1	0.914629	FVSSSTTVN	99	549.2816	2	1096.551	.	V	Superscaf	3141568	0	BTP202209	0	0	0	0	Tissue specific
CDS|exon|ID=cds-Bg	21	5.56619	8453.3	0.914626	MEDEAESLE	99	764.6848	3	2291.037	.	V	Superscaf	3141568	0	BTP202209	0	0	0	0	Tissue specific
CDS|exon|ID=cds-Bg	14	4.974286	5376.9	0.914627	EDEAESLED	75	769.8447	2	1537.678	.	V	Superscaf	3141568	0	BTP202209	0	0	BTP202209	0	Mixed
CDS|exon|ID=cds-Bg	13	4.897692	5079.6	0.914627	EDEAESLED	97	741.3342	2	1480.657	.	V	Superscaf	3141568	0	BTP202209	0	0	BTP202209	0	Mixed
CDS|exon|ID=cds-Bg	12	5.0375	4654.1	0.914627	DEAESLEDL	93	676.8116	2	1351.614	.	V	Superscaf	3141568	0	0	0	0	BTP202209	0	Tissue specific
CDS|exon|ID=cds-Bg	9	5.36	3505.8	0.914627	ESLEDLGFK	85	519.2604	2	1036.508	.	V	Superscaf	3141568	0	BTP202209	BTP202209	0	0	0	Tissue enhanced
CDS|exon|ID=cds-Bg	8	4.8125	3037.4	0.914627	ESLEDLGF	77	909.4188	1	908.4127	.	V	Superscaf	3141568	0	0	0	0	BTP202209	0	Tissue specific
CDS|exon|ID=cds-Bg	9	5.665556	3377.6	0.914627	SLEDLGFKG	92	483.2499	2	964.4865	.	V	Superscaf	3141568	0	BTP202209	0	0	0	0	Tissue specific
CDS|exon|ID=cds-Bg	8	5.6275	3080.3	0.914627	SLEDLGFK	92	454.7389	2	907.465	.	V	Superscaf	3141568	0	BTP202209	0	0	0	0	Tissue specific
CDS|exon|ID=cds-Bg	14	6.200714	5699.1	0.914626	LEDLGFKGA	91	500.9312	3	1499.773	.	V	Superscaf	3141568	0	BTP202209	0	0	0	0	Tissue specific
CDS|exon|ID=cds-Bg	8	5.66375	2994.2	0.914627	LEDLGFKG	96	439.7337	2	877.4545	.	V	Superscaf	3141568	0	BTP202209	BTP202209	0	BTP202209	0	Mixed
CDS|exon|ID=cds-Bg	10	5.891	3869.1	0.914627	EDLGFKGAF	88	515.7501	2	1029.488	.	V	Superscaf	3141568	0	0	BTP202209	0	0	0	Tissue specific
CDS|exon|ID=cds-Bg	17	6.479412	7042.4	0.914626	EDLGFKGAF	99	890.9554	2	1779.902	.	V	Superscaf	3141568	0	0	BTP202209	0	0	0	Tissue specific
CDS|exon|ID=cds-Bg	14	6.2	5643	0.914626	EDLGFKGAF	99	722.8601	2	1443.711	.	V	Superscaf	3141568	0	0	BTP202209	0	0	0	Tissue specific
CDS|exon|ID=cds-Bg	13	6.217692	5345.7	0.914626	EDLGFKGAF	81	463.2357	3	1386.689	.	V	Superscaf	3141568	0	BTP202209	BTP202209	0	BTP202209	0	Mixed
CDS|exon|ID=cds-Bg	7	4.922857	2630	0.884987	GVHDLPQ	51	765.3865	1	764.3817	.	P	Superscaf	4234075	0	0	0	BTP202209	0	0	Tissue specific
CDS|exon|ID=cds-Bg	7	3.632857	1759	0.882426	FVVEVGR	96	403.2309	2	804.4493	.	K	Superscaf	4328353	0	BTP202209	0	0	0	0	Tissue specific
CDS|exon|ID=cds-Bg	8	5.32125	3045.5	0.831693	LEELSLTL	69	917.5177	1	916.5117	.	K	Superscaf	6195970	0	BTP202209	0	0	0	0	Tissue specific
CDS|exon|ID=cds-Bg	7	4.07	2035.3	0.826371	DLVVLGH	97	752.4265	1	751.4228	.	G	Superscaf	6391957	0	0	0	0	0	BTP202209	Tissue specific
CDS|exon|ID=cds-Bg	9	5.082222	2532.6	0.802906	AAPSVPVSK	83	428.2495	2	854.4861	.	D	Superscaf	7255609	0	0	0	0	0	BTP202209	Tissue specific
CDS|exon|ID=cds-Bg	9	6.352222	4071.5	0.71776	A(+42.01)	85	402.8658	3	1205.583	Acetylati	R	Superscaf	10390689	0	0	0	0	0	BTP202209	Tissue specific
CDS|exon|ID=cds-Bg	6	6.513333	2343.4	0.686116	YSASPK	66	652.3285	1	651.3228	.	H	Superscaf	11555451	0	0	0	0	BTP202209	0	Tissue specific
CDS|exon|ID=cds-Bg	7	5.478571	2929.4	0.676883	EELRLGE	81	423.2255	2	844.429	.	G	Superscaf	11895091	0	0	0	0	BTP202209	0	Tissue specific
CDS|exon|ID=cds-Bg	7	6.764286	2636.9	0.649044	AATTLTK	52	353.2097	2	704.4069	.	E	Superscaf	12920173	0	0	0	0	0	BTP202209	Tissue specific
CDS|exon|ID=cds-Bg	7	5.425714	2805.2	0.595225	LSNLLLD	63	394.2304	2	786.4487	.	F	Superscaf	14901564	0	0	BTP202209	BTP202209	0	0	Tissue enhanced
CDS|exon|ID=cds-Bg	7	7.44	2878.3	0.591835	RRAGPGP	53	355.7131	2	709.3983	.	P	Superscaf	15026059	BTP202209	0	0	0	0	0	Tissue specific
CDS|exon|ID=cds-Bg	9	5.594444	3989.4	0.516964	ENFYDKTSY	57	583.7522	2	1165.493	.	I	Superscaf	17782582	0	0	0	BTP202209	0	0	Tissue specific
CDS|exon|ID=cds-Bg	9	6.242222	3611.9	0.49212	GYDHSPPGK	98	479.2236	2	956.4352	.	A	Superscaf	18696898	0	0	0	0	0	BTP202209	Tissue specific
CDS|exon|ID=cds-Bg	7	4.454286	2225.5	0.039307	A(+42.01)	56	799.3608	1	798.3694	Acetylati	L	Superscaf	35366995	0	0	0	0	0	BTP202209	Tissue specific
CDS|exon|ID=cds-Bg	8	6.12	3755.4	0.022831	NSLLQTRD	75	473.7532	2	945.488	.	Y	Superscaf	35973754	0	0	0	BTP202209	0	0	Tissue specific
CDS|exon|ID=cds-Bg	7	4.774286	2463.9	0.019804	VTPELLN	84	785.4402	1	784.433	.	L	Superscaf	36085296	0	BTP202209	0	0	0	0	Tissue specific
CDS|exon|ID=cds-Bg	7	5.237143	2899.5	0.228285	R(+42.01)	59	472.7365	2	943.4723	Acetylati	K	Superscaf	45218461	0	0	0	0	0	BTP202209	Tissue specific
CDS|exon|ID=cds-Bg	7	3.575714	1528.6	0.286544	KDVVVAP	59	727.4435	1	726.4276	.	P	Superscaf	47362663	0	0	0	0	BTP202209	0	Tissue specific
CDS|exon|ID=cds-Bg	7	6.402857	2813	0.335196	PGELAKH	98	376.2072	2	750.4024	.	K	Superscaf	49154164	0	0	0	0	BTP202209	0	Tissue specific
CDS|exon|ID=cds-Bg	8	4.21125	2618	0.413034	A(+42.01)	53	464.2456	2	926.4597	Acetylati	A	Superscaf	52019857	0	0	0	0	BTP202209	0	Tissue specific
CDS|exon|ID=cds-Bg	9	4.292222	2909.2	0.457264	A(+42.01)	65	978.4404	1	977.4341	Acetylati	R	Superscaf	53648167	0	BTP202209	0	0	0	0	Tissue specific
CDS|exon|ID=cds-Bg	7	6.758571	3213.6	0.46075	RELLQKL	61	450.2856	2	898.56	.	I	Superscaf	53776390	0	0	BTP202209	0	0	0	Tissue specific
CDS|exon|ID=cds-Bg	12	6.109167	4036.1	0.465854	KVPAVPETL	98	446.6203	3	1336.844	.	M	Superscaf	53964418	0	0	0	0	0	BTP202209	Tissue specific
CDS|exon|ID=cds-Bg	11	5.779091	3567.7	0.465854	KVPAVPETL	89	403.9221	3	1208.749	.	M	Superscaf	53964418	0	0	0	0	0	BTP202209	Tissue specific
CDS|exon|ID=cds-Bg	10	5.383	3099.3	0.465854	KVPAVPETL	98	541.333	2	1080.654	.	M	Superscaf	53964418	0	0	0	0	0	BTP202209	Tissue specific
CDS|exon|ID=cds-Bg	7	5.118571	2533.9	0.571347	LDGLDLL	50	758.4252	1	757.4221	.	R	Superscaf	57848047	0	0	0	0	0	BTP202209	Tissue specific
CDS|exon|ID=cds-Bg	7	5.575714	2179.4	0.699907	KVRVLAT	90	393.7612	2	785.5123	.	V	Superscaf	62580988	0	0	0	0	0	BTP202209	Tissue specific
CDS|exon|ID=cds-Bg	6	5.463333	2396.6	0.704506	KVPSQN	53	672.3574	1	671.3602	.	I	Superscaf	62750290	0	0	BTP202209	0	0	0	Tissue specific
CDS|exon|ID=cds-Bg	7	5.13	2112.3	0.705283	YAPGVLL	76	732.425	1	731.4218	.	I	Superscaf	62779002	0	0	0	0	0	BTP202209	Tissue specific
CDS|exon|ID=cds-Bg	5	6.114	1636.8	0.709951	LAPGP	91	454.265	1	453.2587	.	L	Superscaf	62950663	BTP202209	0	0	0	0	0	Tissue specific
CDS|exon|ID=cds-Bg	7	7.155714	2948.2	0.717768	YSPGLRK	53	410.7441	2	819.4603	.	E	Superscaf	63238578	0	0	0	0	0	BTP202209	Tissue specific
CDS|exon|ID=cds-Bg	7	6.278571	2937.5	0.718124	PPMPNHP	83	395.1879	2	788.364	.	P	Superscaf	63251497	0	0	0	BTP202209	0	0	Tissue specific
CDS|exon|ID=cds-Bg	5	6.514	2134.3	0.718445	D(+42.01)	53	595.2888	1	594.2762	Acetylati	L	Superscaf	63263461	0	BTP202209	0	0	0	0	Tissue specific
CDS|exon|ID=cds-Bg	5	5.322	1805.9	0.718607	NFKLV	74	620.3748	1	619.3693	.	L	Superscaf	63269487	0	0	BTP202209	BTP202209	0	0	Mixed
CDS|exon|ID=cds-Bg	7	6.058571	2516.8	0.720071	GPPPSWG	90	697.3291	1	696.3231	.	L	Superscaf	63323233	0	0	BTP202209	0	0	0	Tissue specific
CDS|exon|ID=cds-Bg	6	7.4	2908.3	0.726593	TKYNRP	81	389.7126	2	777.4133	.	L	Superscaf	63563523	0	0	0	BTP202209	0	0	Tissue specific
CDS|exon|ID=cds-Bg	10	5.568	4774.6	0.729946	QTERYNEET	83	658.7891	2	1315.568	.	W	Superscaf	63686599	0	0	0	0	0	BTP202209	Tissue specific
CDS|exon|ID=cds-Bg	9	6.303333	3803.2	0.73003	LQPHPGLEK	69	509.7867	2	1017.561	.	V	Superscaf	63689845	0	0	0	0	0	BTP202209	Tissue specific
CDS|exon|ID=cds-Bg	10	5.591	3637.9	0.731102	SGAGLDYLA	78	1029.485	1	1028.481	.	G	Superscaf	63729145	0	0	0	0	0	BTP202209	Tissue specific
CDS|exon|ID=cds-Bg	9	4.926667	3172.6	0.766368	DGNVFTTGF	83	957.428	1	956.424	.	A	Superscaf	65027611	0	0	0	0	0	BTP202209	Tissue specific
CDS|exon|ID=cds-Bg	7	6.671429	2703.8	0.767377	PGKKSDP	72	364.7015	2	727.3864	.	D	Superscaf	65064927	BTP202209	0	BTP202209	0	0	0	Mixed
CDS|exon|ID=cds-Bg	6	7.476667	2890.4	0.813548	R(+42.01)	53	432.709	2	863.4072	Acetylati	T	Superscaf	66764347	0	0	0	0	BTP202209	0	Tissue specific
CDS|exon|ID=cds-Bg	7	6.758571	2784.1	0.817706	RAAPAWP	55	384.707	2	767.4078	.	S	Superscaf	66917656	0	0	BTP202209	0	0	0	Tissue specific
CDS|exon|ID=cds-Bg	8	6.77	3068.5	0.887505	AAPTLTAR	70	400.7338	2	799.4552	.	S	Superscaf	69487228	BTP202209	0	0	0	0	0	Tissue specific
CDS|exon|ID=cds-Bg	7	3.75	1709	0.911062	VPVGREV	76	378.2227	2	754.4337	.	L	Superscaf	70354381	0	0	0	0	BTP202209	0	Tissue specific
CDS|exon|ID=cds-Bg	16	5.39	5983.6	0.914475	HSQQTADGS	75	808.8943	2	1615.78	.	L	Superscaf	70479802	0	0	0	0	0	BTP202209	Tissue specific
CDS|exon|ID=cds-Bg	10	5.738	4391.1	0.914514	FDREDEASF	81	407.8533	3	1220.542	.	P	Superscaf	70481563	0	0	0	0	BTP202209	BTP202209	Tissue enhanced
CDS|exon|ID=cds-Bg	9	6.032222	3287.4	0.914515	SGSLAALEK	98	438.2415	2	874.476	.	P	Superscaf	70481563	0	0	0	0	0	BTP202209	Tissue specific
CDS|exon|ID=cds-Bg	10	5.48	3839.2	0.914548	SSSWSDGMA	86	1074.414	1	1073.412	.	H	Superscaf	70482862	0	0	0	0	BTP202209	0	Tissue specific
CDS|exon|ID=cds-Bg	12	5.166667	4703.2	0.914549	C(+57.02)	51	726.3401	2	1450.67	Carbamido	H	Superscaf	70482862	0	0	0	0	0	BTP202209	Tissue specific
CDS|exon|ID=cds-Bg	11	5.138182	4315.8	0.914549	C(+57.02)	64	652.8058	2	1303.602	Carbamido	H	Superscaf	70482862	0	0	0	0	0	BTP202209	Tissue specific
CDS|exon|ID=cds-Bg	7	6.528571	2574.7	0.918203	L(+42.01)	82	371.2151	2	740.4068	Acetylati	S	Superscaf	70617430	0	0	0	0	0	BTP202209	Tissue specific
CDS|exon|ID=cds-Bg	8	6.43375	3289.5	0.929656	LKMKGDYY	95	509.255	2	1016.5	.	R	Superscaf	71038720	0	0	0	0	0	BTP202209	Tissue specific
CDS|exon|ID=cds-Bg	8	6.09875	2771.1	0.936025	ALAPLLLT	78	811.526	1	810.5215	.	R	Superscaf	71273476	0	0	0	0	BTP202209	0	Tissue specific
CDS|exon|ID=cds-Bg	6	5.67	1914	0.938512	KPVYAP	57	674.3845	1	673.3799	.	V	Superscaf	71365143	0	0	BTP202209	0	0	0	Tissue specific
CDS|exon|ID=cds-Bg	7	7.09	2875	0.947404	KWLPSKP	66	428.2501	2	854.5014	.	Q	Superscaf	71692509	0	0	0	BTP202209	0	0	Tissue specific
CDS|exon|ID=cds-Bg	14	6.265	5667.1	0.947407	LSLTSTDWK	99	786.8954	2	1571.783	.	G	Superscaf	71692222	0	0	0	0	0	BTP202209	Tissue specific
CDS|exon|ID=cds-Bg	11	6.4	4526.9	0.947407	LSLTSTDWK	99	633.3375	2	1264.666	.	G	Superscaf	71692222	0	0	0	0	0	BTP202209	Tissue specific
CDS|exon|ID=cds-Bg	10	6.442	4173.5	0.947407	SLTSTDWKS	98	576.7968	2	1151.582	.	G	Superscaf	71692222	0	0	0	0	0	BTP202209	Tissue specific
CDS|exon|ID=cds-Bg	9	6.526667	3790.1	0.947407	LTSTDWKSK	98	533.2821	2	1064.55	.	G	Superscaf	71692222	0	0	0	0	0	BTP202209	Tissue specific
CDS|exon|ID=cds-Bg	14	5.462857	4987.5	0.947408	HDGSTVTKT	96	495.9217	3	1484.747	.	G	Superscaf	71692222	0	0	BTP202209	0	0	0	Tissue specific
CDS|exon|ID=cds-Bg	12	5.493333	3956.3	0.947409	GSTVTKTVK	96	617.3363	2	1232.661	.	G	Superscaf	71692222	0	0	BTP202209	0	0	0	Tissue specific
CDS|exon|ID=cds-Bg	10	5.427	3275.6	0.947409	TVTKTVKPS	99	545.3093	2	1088.608	.	G	Superscaf	71692222	0	0	BTP202209	0	0	0	Tissue specific
CDS|exon|ID=cds-Bg	11	5.312727	3847.2	0.947409	VTKTVKPSE	75	618.3174	2	1234.623	Carbamido	G	Superscaf	71692222	0	BTP202209	0	0	0	0	Tissue specific
CDS|exon|ID=cds-Bg	13	5.054615	4781.4	0.947687	SC(+57.02	72	717.8276	2	1433.646	Carbamido	L	Superscaf	71702593	0	0	0	0	0	BTP202209	Tissue specific
CDS|exon|ID=cds-Bg	14	6.225	5566.9	0.947856	LSLTGSDWK	86	764.882	2	1527.757	.	L	Superscaf	71708848	0	0	0	0	0	BTP202209	Tissue specific
CDS|exon|ID=cds-Bg	11	6.349091	4426.7	0.947856	LSLTGSDWK	92	407.8859	3	1220.64	.	L	Superscaf	71708848	0	0	0	0	0	BTP202209	Tissue specific
CDS|exon|ID=cds-Bg	8	6.8125	3636	0.95112	RSYSKLLC(	88	513.7721	2	1025.533	Carbamido	T	Superscaf	71828887	0	0	BTP202209	0	0	0	Tissue specific
CDS|exon|ID=cds-Bg	8	6.8975	3478.1	0.951121	GLLTERLR	94	479.2949	2	956.5767	.	T	Superscaf	71828887	0	0	BTP202209	0	0	0	Tissue specific
CDS|exon|ID=cds-Bg	10	6.082	4221.7	0.952039	SQPADPTLK	80	542.7829	2	1083.556	.	S	Superscaf	71862829	0	0	BTP202209	0	0	0	Tissue specific
CDS|exon|ID=cds-Bg	8	5.115	2977.5	0.952109	FELRTVDL	79	496.7736	2	991.5338	.	P	Superscaf	71865535	0	0	BTP202209	0	0	0	Tissue specific
CDS|exon|ID=cds-Bg	11	6.639091	4505	0.952249	ADTAMKERL	79	420.5627	3	1258.67	.	K	Superscaf	71870530	0	0	BTP202209	0	0	0	Tissue specific
CDS|exon|ID=cds-Bg	9	7.115556	3782.2	0.952249	TAMKERLKF	98	537.3095	2	1072.606	.	K	Superscaf	71870530	0	0	BTP202209	0	0	0	Tissue specific
CDS|exon|ID=cds-Bg	7	5.662857	2662.9	0.971542	ALDLLDK	97	394.2298	2	786.4487	.	V	Superscaf	72580900	0	0	0	0	0	BTP202209	Tissue specific
CDS|exon|ID=cds-Bg	15	5.985333	6115.6	0.971569	KFDMELDDL	75	462.9976	4	1847.97	.	E	Superscaf	72581809	0	0	0	0	0	BTP202209	Tissue specific
CDS|exon|ID=cds-Bg	9	6.92	4084.6	0.971571	ARFQPGYRS	65	541.278	2	1080.546	.	E	Superscaf	72581809	0	0	0	BTP202209	0	0	Tissue specific
CDS|exon|ID=cds-Bg	6	6.25	1943	0.975258	K(+42.01)	51	359.1971	2	716.3891	Acetylati	N	Superscaf	72717921	0	0	0	0	BTP202209	0	Tissue specific
CDS|exon|ID=cds-Bg	6	6.673333	2888.3	0.977636	ARQLLQ	66	364.7224	2	727.434	.	W	Superscaf	72805261	0	0	BTP202209	0	0	0	Tissue specific
CDS|exon|ID=cds-Bg	7	3.571429	1925.3	0.980942	VDLEPTV	75	772.4086	1	771.4014	.	I	Superscaf	72926869	0	BTP202209	0	0	0	0	Tissue specific
CDS|exon|ID=cds-Bg	13	4.23	4300.7	0.981035	AALEKDYEE	94	719.3379	2	1436.667	.	S	Superscaf	72930265	0	0	0	BTP202209	0	0	Tissue specific
CDS|exon|ID=cds-Bg	9	6.157778	3002.4	0.984307	GPPGPPSPF	62	802.4114	1	801.402	.	Q	Superscaf	73050919	0	0	BTP202209	0	0	0	Tissue specific
CDS|exon|ID=cds-Bg	6	4.031667	1652.8	0.986526	EMVFVK	67	376.7063	2	751.3939	.	P	Superscaf	73132911	0	0	0	BTP202209	0	0	Tissue specific
CDS|exon|ID=cds-Bg	13	4.515385	3735.8	0.995076	SKVVDGLEK	98	488.6179	3	1462.839	.	G	Superscaf	73447195	0	0	0	0	0	BTP202209	Tissue specific
CDS|exon|ID=cds-Bg	9	6.968889	3434.7	0.995077	KGLSGPARF	95	441.7593	2	881.5082	.	G	Superscaf	73447195	0	0	0	0	0	BTP202209	Tissue specific
CDS|exon|ID=cds-Bg	10	4.625	4101.6	0.997323	LEEDEEELF	99	640.7952	2	1279.582	.	W	Superscaf	73529995	0	0	0	0	0	BTP202209	Tissue specific
CDS|exon|ID=cds-Bg	10	5.671	4619.3	0.997359	ENDLPEWKE	87	658.3123	2	1314.62	.	K	Superscaf	73531399	0	0	0	0	BTP202209	0	Tissue specific
CDS|exon|ID=cds-Bg	13	6.397692	5029.2	0.99736	GTGDVKLLK	99	484.9543	3	1451.846	.	K	Superscaf	73531399	0	0	0	0	BTP202209	0	Tissue specific
CDS|exon|ID=cds-Bg	9	5.698889	3641	0.950743	A(+42.01)	96	514.7651	2	1027.519	Acetylati	A	Superscaf	1655986	0	0	0	0	0	BTP202209	Tissue specific
CDS|exon|ID=cds-Bg	8	6.705	2878.2	0.949774	GPAARGPP	65	361.7026	2	721.3871	.	M	Superscaf	1688530	0	0	0	BTP202209	0	0	Tissue specific
CDS|exon|ID=cds-Bg	8	4.48625	2758.2	0.945306	DTDLFVPY	76	969.4529	1	968.4491	.	V	Superscaf	1838695	0	0	0	0	0	BTP202209	Tissue specific
CDS|exon|ID=cds-Bg	6	5.638333	2020.2	0.911499	EAPAGP	86	541.2614	1	540.2543	.	R	Superscaf	2975440	0	0	BTP202209	0	0	0	Tissue specific
CDS|exon|ID=cds-Bg	6	5.895	2717.2	0.863657	RLEPEW	60	415.2131	2	828.413	.	T	Superscaf	4583887	0	BTP202209	0	0	0	0	Tissue specific
CDS|exon|ID=cds-Bg	7	5.822857	2345.6	0.840097	R(+42.01)	69	378.2227	2	754.4337	Acetylati	Q	Superscaf	5375716	0	0	0	0	BTP202209	0	Tissue specific
CDS|exon|ID=cds-Bg	9	6.268889	3010.5	0.805463	APPPPPPPF	99	866.4758	1	865.4697	.	G	Superscaf	6540598	0	BTP202209	0	BTP202209	0	0	Mixed
CDS|exon|ID=cds-Bg	8	4.92	3133.5	0.698617	ESPFVHNS	61	458.7104	2	915.4086	.	T	Superscaf	10132810	BTP202209	0	0	0	0	0	Tissue specific
CDS|exon|ID=cds-Bg	10	4.835	3508.9	0.698616	VHNSVYTAL	61	575.7951	2	1149.582	.	T	Superscaf	10132810	0	0	0	0	0	BTP202209	Tissue specific
CDS|exon|ID=cds-Bg	9	4.763333	3121.5	0.698616	VHNSVYTAL	71	502.2628	2	1002.513	.	T	Superscaf	10132810	0	0	0	0	0	BTP202209	Tissue specific
CDS|exon|ID=cds-Bg	7	5.402857	2926.2	0.681526	A(+42.01)	63	437.7261	2	873.4378	Acetylati	H	Superscaf	10707550	0	BTP202209	0	BTP202209	0	0	Tissue enhanced
CDS|exon|ID=cds-Bg	6	6.838333	2315.6	0.68139	RPSAGP	50	584.3204	1	583.3078	.	M	Superscaf	10712187	0	0	0	0	BTP202209	0	Tissue specific
CDS|exon|ID=cds-Bg	9	6.014444	3554.1	0.649441	ALPSYVDRF	71	538.7927	2	1075.577	.	I	Superscaf	11786254	0	0	0	0	0	BTP202209	Tissue specific
CDS|exon|ID=cds-Bg	11	4.866364	3710.1	0.64944	DYPLPDVAF	82	627.3271	2	1252.645	.	I	Superscaf	11786254	0	0	0	0	0	BTP202209	Tissue specific
CDS|exon|ID=cds-Bg	12	5.436667	4844.3	0.649386	TFEEEWVAK	99	748.3677	2	1494.735	.	N	Superscaf	11787997	0	0	0	0	0	BTP202209	Tissue specific
CDS|exon|ID=cds-Bg	13	6.359231	5926.2	0.649383	SAKWDYDKN	99	566.6125	3	1696.821	.	N	Superscaf	11787997	0	0	0	0	BTP202209	BTP202209	Tissue enhanced
CDS|exon|ID=cds-Bg	10	6.123	4763.1	0.649383	WDYDKNEWK	99	706.3322	2	1410.657	.	N	Superscaf	11787997	0	0	0	0	0	BTP202209	Tissue specific
CDS|exon|ID=cds-Bg	9	6.148889	4236.5	0.649383	DYDKNEWKK	99	409.1989	3	1224.577	.	N	Superscaf	11787997	BTP202209	0	0	0	0	0	Tissue specific
CDS|exon|ID=cds-Bg	8	4.7575	2577.8	0.613311	KFLVPDHV	77	477.7723	2	953.5334	.	V	Superscaf	13001005	0	0	0	BTP202209	0	0	Tissue specific
CDS|exon|ID=cds-Bg	7	5.037143	2433.7	0.607652	RAVEAAE	69	373.1932	2	744.3766	.	T	Superscaf	13190557	BTP202209	0	0	0	0	0	Tissue specific
CDS|exon|ID=cds-Bg	6	5.953333	2548	0.592021	HQGVMR	61	364.1807	2	726.3596	.	T	Superscaf	13716903	BTP202209	0	0	0	BTP202209	0	Mixed
CDS|exon|ID=cds-Bg	7	6.085714	2832.1	0.592019	LNLEGPK	95	385.7227	2	769.4333	.	T	Superscaf	13717053	0	0	0	0	0	BTP202209	Tissue specific
CDS|exon|ID=cds-Bg	15	6.062667	5666.1	0.58709	EPPKKETAS	95	532.9559	3	1595.852	.	P	Superscaf	13882600	0	BTP202209	0	0	0	0	Tissue specific
CDS|exon|ID=cds-Bg	6	5.891667	1932.2	0.58695	R(+42.01)	90	638.358	1	637.3547	Acetylati	K	Superscaf	13887319	0	0	0	0	BTP202209	0	Tissue specific
CDS|exon|ID=cds-Bg	7	6.207143	2337.7	0.581899	LPPGPPP	70	674.3859	1	673.3799	.	G	Superscaf	14057439	0	0	BTP202209	0	BTP202209	0	Mixed

CDS|exon|ID=cds-Bg	9	5.497778	3788.2	0.58015	DDHFLFDKF	72	567.2664	2	1132.519	.	G	Superscaf	14116089	BTP202209	0	0	0	0	0	Tissue specific
CDS|exon|ID=cds-Bg	12	5.489167	4126.5	0.580149	FLFDKPVSF	82	694.9107	2	1387.812	.	G	Superscaf	14116089	0	0	0	0	0	BTP202209	Tissue specific
CDS|exon|ID=cds-Bg	7	6.828571	2829.3	0.574975	SALRTPT	89	373.2117	2	744.413	.	P	Superscaf	14289988	0	0	0	0	0	BTP202209	Tissue specific
CDS|exon|ID=cds-Bg	7	5.97	2309.4	0.574973	A(+42.01)	64	688.3646	1	687.3591	Acetylati	R	Superscaf	14289982	0	0	0	0	0	BTP202209	Tissue specific
CDS|exon|ID=cds-Bg	6	3.778333	1861.1	0.569293	C(+57.02)	55	689.3647	1	688.3578	Carbamido	K	Superscaf	14481081	BTP202209	0	0	0	0	0	Tissue specific
CDS|exon|ID=cds-Bg	10	6.882	4076.5	0.568595	HTKVRAGRC	79	376.883	3	1127.631	.	P	Superscaf	14504371	0	0	0	0	0	BTP202209	Tissue specific
CDS|exon|ID=cds-Bg	9	5.826667	3874.3	0.568585	ANVQRLKEY	92	560.8065	2	1119.604	.	L	Superscaf	14504719	0	0	0	0	0	BTP202209	Tissue specific
CDS|exon|ID=cds-Bg	9	6.911111	3940.5	0.568549	MARANARLF	91	350.525	3	1048.56	.	V	Superscaf	14505961	0	0	BTP202209	0	0	0	Tissue specific
CDS|exon|ID=cds-Bg	6	5.786667	2734.1	0.566157	M(+15.99)	51	400.6437	2	799.2663	Oxidatior	G	Superscaf	14586495	0	0	0	0	BTP202209	0	Tissue specific
CDS|exon|ID=cds-Bg	7	5.257143	2542	0.562356	DPLPPLD	60	766.3949	1	765.3908	.	D	Superscaf	14714227	0	0	0	BTP202209	0	0	Tissue specific
CDS|exon|ID=cds-Bg	7	6.054286	3122.6	0.561914	LFRPDNF	85	454.7336	2	907.4551	.	P	Superscaf	14729014	0	BTP202209	0	0	0	BTP202209	Mixed
CDS|exon|ID=cds-Bg	9	4.8	2719	0.561884	PSPKVSDTV	94	465.2478	2	928.4865	.	L	Superscaf	14729725	0	0	0	BTP202209	0	0	Tissue specific
CDS|exon|ID=cds-Bg	12	4.34	3604	0.561884	SPKVSDTVV	68	660.836	2	1319.661	.	L	Superscaf	14729725	0	0	0	0	BTP202209	0	Tissue specific
CDS|exon|ID=cds-Bg	8	3.0875	1955.3	0.561884	VSDTVVEP	71	845.4227	1	844.4178	.	L	Superscaf	14729725	0	0	0	0	BTP202209	0	Tissue specific
CDS|exon|ID=cds-Bg	9	3.373333	2414.8	0.561884	VSDTVVEPY	77	1008.484	1	1007.481	.	L	Superscaf	14729725	0	0	0	0	0	BTP202209	Tissue specific
CDS|exon|ID=cds-Bg	8	3.795	2414.8	0.561884	SDTVVEPY	88	909.4188	1	908.4127	.	L	Superscaf	14729725	0	BTP202209	0	0	0	BTP202209	Mixed
CDS|exon|ID=cds-Bg	7	3.525714	2031.4	0.561884	DTVVEPY	74	822.3865	1	821.3807	.	L	Superscaf	14729725	0	BTP202209	0	0	BTP202209	BTP202209	Mixed
CDS|exon|ID=cds-Bg	11	5.903636	4535.2	0.56188	ATPTYGDLN	93	601.2937	2	1200.577	.	L	Superscaf	14729725	0	0	0	BTP202209	0	0	Tissue specific
CDS|exon|ID=cds-Bg	9	6.394444	3896.4	0.561878	LRFPGQLNA	65	508.2863	2	1014.561	.	L	Superscaf	14729725	0	0	BTP202209	0	0	0	Tissue specific
CDS|exon|ID=cds-Bg	6	5.765	2082.5	0.561877	MVPFPR	58	373.7038	2	745.3945	.	L	Superscaf	14729725	0	BTP202209	BTP202209	BTP202209	BTP202209	0	Mixed
CDS|exon|ID=cds-Bg	9	6.903333	4323.9	0.561875	RGSQQYRAL	89	539.7906	2	1077.568	.	L	Superscaf	14729725	0	0	0	BTP202209	0	0	Tissue specific
CDS|exon|ID=cds-Bg	8	5.07	2576	0.561874	ALTVPELT	84	843.4789	1	842.4749	.	L	Superscaf	14729725	0	0	0	BTP202209	0	0	Tissue specific
CDS|exon|ID=cds-Bg	13	5.370769	5082.8	0.561874	TVPELTQQM	96	754.374	2	1506.739	.	L	Superscaf	14729725	0	BTP202209	0	0	0	BTP202209	Mixed
CDS|exon|ID=cds-Bg	7	4.131429	2630	0.561862	NDLVSEY	80	839.3744	1	838.3708	.	L	Superscaf	14729725	0	0	0	0	0	BTP202209	Tissue specific
CDS|exon|ID=cds-Bg	10	4.968	4536.1	0.517066	EELHNQEVG	92	627.3055	2	1252.605	.	E	Superscaf	16236382	0	0	0	0	0	BTP202209	Tissue specific
CDS|exon|ID=cds-Bg	8	6.28875	3155.6	0.461219	HTPLYGLP	54	449.238	2	896.4756	.	A	Superscaf	18114678	0	BTP202209	0	0	0	0	Tissue specific
gene|mRNA ID=gene-B	10	5.658	3860.4	0.443334	HDEGYGPPF	61	533.2342	2	1064.456	.	L	Superscaf	18716208	0	BTP202209	0	0	0	0	Tissue specific
CDS|exon|ID=cds-Bg	13	4.75	5031.7	0.443285	FFNAPLEVT	72	778.864	2	1555.72	.	K	Superscaf	18717870	0	0	0	0	0	BTP202209	Tissue specific
CDS|exon|ID=cds-Bg	12	4.689167	4644.3	0.443285	FNAPLEVTE	80	705.3317	2	1408.651	.	K	Superscaf	18717870	0	0	0	0	0	BTP202209	Tissue specific
CDS|exon|ID=cds-Bg	11	5.054545	3525.7	0.442619	KVGSSGDVA	99	535.283	2	1068.556	.	T	Superscaf	18740352	0	0	0	0	0	BTP202209	Tissue specific
CDS|exon|ID=cds-Bg	5	6.04	1936.2	0.432911	Q(+42.01)	79	553.3022	1	552.2908	Acetylati	S	Superscaf	19066132	0	0	0	0	0	BTP202209	Tissue specific
CDS|exon|ID=cds-Bg	6	6.015	1982.1	0.43218	GPAAFP	51	559.2965	1	558.2802	.	E	Superscaf	19090969	0	0	0	0	BTP202209	0	Tissue specific
CDS|exon|ID=cds-Bg	5	4.23	1727	0.392049	LQPVE	68	585.3225	1	584.317	.	A	Superscaf	20440000	0	0	0	BTP202209	0	0	Tissue specific
CDS|exon|ID=cds-Bg	7	5.714286	2475.8	0.313019	ALGETPL	78	700.3851	1	699.3803	.	A	Superscaf	23097390	BTP202209	0	0	0	0	0	Tissue specific
CDS|exon|ID=cds-Bg	7	6.392857	3042.3	0.28786	Y(+42.01)	61	455.7154	2	909.4232	Acetylati	K	Superscaf	23942941	0	0	0	0	BTP202209	0	Tissue specific
CDS|exon|ID=cds-Bg	11	5.991818	3911.1	0.28747	LGGTDDPVK	99	579.3278	2	1156.645	.	Q	Superscaf	23956261	0	0	0	0	0	BTP202209	Tissue specific
CDS|exon|ID=cds-Bg	9	3.873333	2313.5	0.287469	FGVPSVNVA	51	889.4743	1	888.4705	.	Q	Superscaf	23956261	0	0	BTP202209	0	0	0	Tissue specific
CDS|exon|ID=cds-Bg	7	4.99	2104.2	0.287444	SVFGAPF	84	724.365	1	723.3591	.	N	Superscaf	23957107	0	0	0	0	0	BTP202209	Tissue specific
CDS|exon|ID=cds-Bg	5	8.156	2528.9	0.250262	K(+42.01)	51	364.2202	2	726.4136	Acetylati	H	Superscaf	25207306	0	0	0	BTP202209	0	0	Tissue specific
CDS|exon|ID=cds-Bg	9	4.615556	3495	0.249438	M(+42.01)	74	556.7257	2	1111.438	Acetylati	K	Superscaf	25235005	0	BTP202209	0	0	0	0	Tissue specific
CDS|exon|ID=cds-Bg	8	6.74625	2918.3	0.214406	AAPGPRPP	93	381.7154	2	761.4184	.	A	Superscaf	26412853	0	0	0	0	BTP202209	0	Tissue specific
CDS|exon|ID=cds-Bg	7	6.07	2561	0.030421	MGLPWPP	59	399.2009	2	796.3942	.	L	Superscaf	34644364	BTP202209	0	BTP202209	BTP202209	0	0	Mixed
CDS|exon|ID=cds-Bg	7	3.891429	1710.9	0.034547	D(+42.01)	50	356.7003	2	711.3803	Acetylati	T	Superscaf	34782931	0	0	0	BTP202209	0	0	Tissue specific
CDS|exon|ID=cds-Bg	13	6.102308	5732.6	0.051802	TNDMFRLAL	72	768.3729	2	1534.735	.	T	Superscaf	35363149	0	0	0	0	BTP202209	0	Tissue specific
CDS|exon|ID=cds-Bg	8	6.64875	3568	0.051803	RLALHNFG	63	464.2598	2	926.5086	.	T	Superscaf	35363149	0	0	0	0	0	BTP202209	Tissue specific
CDS|exon|ID=cds-Bg	6	4.17	1501.8	0.053599	VLTVTL	85	645.4166	1	644.4109	.	I	Superscaf	35423667	0	0	0	BTP202209	0	0	Tissue specific
CDS|exon|ID=cds-Bg	9	5.362222	3539.8	0.077729	KLDYFGEEA	72	536.2512	2	1070.492	.	S	Superscaf	36233959	0	0	0	0	0	BTP202209	Tissue specific
CDS|exon|ID=cds-Bg	10	5.392	3999.3	0.077729	KLDYFGEEA	90	617.7838	2	1233.555	.	S	Superscaf	36233959	0	BTP202209	0	0	0	BTP202209	Mixed
CDS|exon|ID=cds-Bg	8	6.43625	3061.4	0.082049	A(+42.01)	89	487.7728	2	973.5345	Acetylati	D	Superscaf	36379939	0	0	0	0	0	BTP202209	Tissue specific
CDS|exon|ID=cds-Bg	14	4.979286	4508.8	0.08692	A(+42.01)	83	659.3168	2	1316.621	Acetylati	K	Superscaf	36543790	0	BTP202209	0	0	BTP202209	0	Mixed
CDS|exon|ID=cds-Bg	11	4.988182	3175.3	0.08692	A(+42.01)	94	502.2545	2	1002.498	Acetylati	K	Superscaf	36543790	0	0	0	0	0	BTP202209	Tissue specific
CDS|exon|ID=cds-Bg	9	6.2	3757.2	0.087203	SSDAREMLK	94	518.7565	2	1035.502	.	H	Superscaf	36553405	0	0	0	0	0	BTP202209	Tissue specific
CDS|exon|ID=cds-Bg	8	5.95875	2580.9	0.136252	A(+42.01)	72	403.7337	2	805.4446	Acetylati	R	Superscaf	38202280	0	0	0	BTP202209	0	0	Tissue specific
CDS|exon|ID=cds-Bg	8	5.96625	3494.7	0.160376	A(+42.01)	57	468.7228	2	935.4348	Acetylati	D	Superscaf	39013576	0	0	0	0	0	BTP202209	Tissue specific
CDS|exon|ID=cds-Bg	5	6.566	2263.4	0.178337	QFPYK	54	682.3633	1	681.3486	.	S	Superscaf	39617485	0	0	BTP202209	0	0	0	Tissue specific
CDS|exon|ID=cds-Bg	7	6.807143	2768.9	0.20438	KPHASAP	54	707.3793	1	706.3762	.	K	Superscaf	40492852	0	0	BTP202209	0	0	0	Tissue specific
CDS|exon|ID=cds-Bg	6	7.145	2551.6	0.218448	K(+42.01)	64	364.7361	2	727.4592	Acetylati	L	Superscaf	40966066	0	0	BTP202209	0	0	0	Tissue specific
CDS|exon|ID=cds-Bg	8	5.91625	3004.3	0.218862	QGFPLGSP	54	802.4062	1	801.402	.	P	Superscaf	40980135	0	0	BTP202209	0	0	0	Tissue specific
CDS|exon|ID=cds-Bg	11	5.950909	4944.5	0.281879	A(+42.01)	97	678.8531	2	1355.704	Acetylati	P	Superscaf	43098421	0	BTP202209	0	BTP202209	0	0	Mixed
CDS|exon|ID=cds-Bg	7	6.228571	2885.3	0.297465	LPELRSS	72	401.227	2	800.4392	.	G	Superscaf	43622877	BTP202209	0	0	0	0	0	Tissue specific
CDS|exon|ID=cds-Bg	8	5.0075	2149.2	0.33051	AAVPSPKV	56	768.4598	1	767.4541	.	H	Superscaf	44733793	0	BTP202209	0	0	0	0	Tissue specific
CDS|exon|ID=cds-Bg	5	7.194	2434.8	0.333966	HLYLR	98	351.2078	2	700.402	.	I	Superscaf	44850096	0	0	0	BTP202209	0	0	Tissue specific
CDS|exon|ID=cds-Bg	13	6.787692	5675	0.334696	A(+42.01)	97	510.2985	3	1527.877	Acetylati	G	Superscaf	44874391	0	0	BTP202209	0	BTP202209	0	Mixed
CDS|exon|ID=cds-Bg	12	6.541667	5206.6	0.334696	A(+42.01)	90	700.8972	2	1399.782	Acetylati	G	Superscaf	44874391	0	BTP202209	0	0	0	0	Tissue specific
CDS|exon|ID=cds-Bg	11	6.592727	4853.2	0.334696	A(+42.01)	97	644.3571	2	1286.698	Acetylati	G	Superscaf	44874391	0	BTP202209	0	0	BTP202209	BTP202209	Tissue enhanced
CDS|exon|ID=cds-Bg	10	6.278	4384.8	0.334696	A(+42.01)	69	580.3079	2	1158.603	Acetylati	G	Superscaf	44874391	0	0	BTP202209	0	0	BTP202209	Mixed
CDS|exon|ID=cds-Bg	12	6.851667	5363.7	0.334696	A(+42.01)	96	486.619	3	1456.84	Acetylati	G	Superscaf	44874391	0	0	BTP202209	0	0	0	Tissue specific
CDS|exon|ID=cds-Bg	10	6.65	4541.9	0.334696	A(+42.01)	95	608.8345	2	1215.661	Acetylati	G	Superscaf	44874391	0	0	0	0	0	BTP202209	Tissue specific
CDS|exon|ID=cds-Bg	9	5.442222	3389.8	0.334989	SSEGPRVWF	87	532.7595	2	1063.509	.	C	Superscaf	44884360	0	0	0	0	0	BTP202209	Tissue specific
CDS|exon|ID=cds-Bg	8	5.4125	3006.4	0.334989	SEGPRVWF	69	489.2448	2	976.4766	.	C	Superscaf	44884360	0	0	0	0	BTP202209	BTP202209	Tissue enhanced
CDS|exon|ID=cds-Bg	7	5.374286	2623	0.334989	EGPRVWF	92	445.7279	2	889.4446	.	C	Superscaf	44884360	0	0	0	0	0	BTP202209	Tissue specific
CDS|exon|ID=cds-Bg	12	5.7325	4680.3	0.33499	TLATLNPES	54	1292.668	1	1291.666	.	C	Superscaf	44884360	0	0	0	0	0	BTP202209	Tissue specific
CDS|exon|ID=cds-Bg	10	6.179	4573.1	0.3354	DQYLHRFAA	84	642.3092	2	1282.609	.	C	Superscaf	44897950	0	0	0	0	0	BTP202209	Tissue specific
CDS|exon|ID=cds-Bg	7	6.787143	3091.4	0.3354	LHRFAAY	96	439.2355	2	876.4606	.	C	Superscaf	44897950	0	0	0	0	0	BTP202209	Tissue specific
CDS|exon|ID=cds-Bg	11	6.510909	4549.1	0.335493	LAQTEALMF	96	609.3337	2	1216.66	.	C	Superscaf	44901334	0	0	0	0	0	BTP202209	Tissue specific
CDS|exon|ID=cds-Bg	7	5.707143	2652.8	0.359541	EKGPAEF	56	389.1966	2	776.3704	.	E	Superscaf	45710130	0	0	0	0	0	BTP202209	Tissue specific
CDS|exon|ID=cds-Bg	6	6.14	2247.7	0.360781	W(+42.01)	96	766.3605	1	765.352	Acetylati	V	Superscaf	45751519	0	0	BTP202209	0	0	0	Tissue specific
CDS|exon|ID=cds-Bg	8	6.3225	3090.6	0.365876	DPSRPGPP	99	411.7081	2	821.4031	.	G	Superscaf	45922744	0	0	0	BTP202209	0	0	Tissue specific
CDS|exon|ID=cds-Bg	5	6.188	2002.1	0.36698	APEKY	56	607.3131	1	606.3013	.	H	Superscaf	45959914	0	0	0	BTP202209	0	0	Tissue specific
CDS|exon|ID=cds-Bg	7	2.514286	1559.8	0.372523	DVSVVDL	76	746.3924	1	745.3857	.	E	Superscaf	46146190	BTP202209	0	0	0	0	0	Tissue specific
CDS|exon|ID=cds-Bg	8	5.43125	2337.7	0.379553	VGPLPPPP	96	773.453	1	772.4483	.	P	Superscaf	46382548	0	0	0	BTP202209	0	0	Tissue specific
CDS|exon|ID=cds-Bg	6	6.106667	1962.1	0.381473	A(+42.01)	61	581.3281	1	580.322	Acetylati	W	Superscaf	46447228	0	BTP202209	0	0	0	0	Tissue specific
CDS|exon|ID=cds-Bg	7	6.678571	2676.9	0.38427	RAYAAGP	62	705.3591	1	704.3605	.	L	Superscaf	46541311	0	BTP202209	0	0	0	0	Tissue specific
CDS|exon|ID=cds-Bg	6	6.38	2385.8	0.389337	LPPGDR	59	654.3511	1	653.3497	.	G	Superscaf	46711657	0	0	0	0	0	BTP202209	Tissue specific
CDS|exon|ID=cds-Bg	7	5.931429	3109.4	0.409184	GPKFDQN	50	403.1939	2	804.3766	.	G	Superscaf	47378974	0	0	BTP202209	0	0	0	Tissue specific
CDS|exon|ID=cds-Bg	6	5.14	2411.8	0.419323	V(+42.01)	90	410.2205	2	818.4286	Acetylati	D	Superscaf	47719858	0	0	BTP202209	0	0	0	Tissue specific
CDS|exon|ID=cds-Bg	7	5.778571	2451.9	0.419429	PPPPLPD	71	366.6983	2	731.3853	.	V	Superscaf	47723386	0	0	BTP202209	0	0	BTP202209	Mixed
CDS|exon|ID=cds-Bg	8	4.915	3320.7	0.438617	V(+42.01)	96	480.2132	2	958.4144	Acetylati	S	Superscaf	48368482	0	BTP202209	0	BTP202209	BTP202209	0	Mixed
CDS|exon|ID=cds-Bg	10	5.066	4163.6	0.438617	V(+42.01)	78	605.2611	2	1208.51	Acetylati	S	Superscaf	48368482	0	BTP202209	0	0	0	0	Tissue specific
CDS|exon|ID=cds-Bg	8	4.6375	3256.6	0.440097	KNVNVQNF	66	481.7549	2	961.4981	.	P	Superscaf	48418189	0	0	0	0	BTP202209	0	Tissue specific
CDS|exon|ID=cds-Bg	16	4.6675	6030.8	0.440193	DDPVTNLNN	99	888.4236	2	1774.837	.	R	Superscaf	48421423	0	0	0	0	BTP202209	BTP202209	Mixed
CDS|exon|ID=cds-Bg	9	6.423333	3849	0.440218	YHAFSGAQK	95	504.7465	2	1007.482	.	P	Superscaf	48422161	0	0	0	0	BTP202209	BTP202209	Tissue enhanced
CDS|exon|ID=cds-Bg	12	5.794167	5028.4	0.440547	KHEAFESDL	82	452.2157	3	1353.631	.	P	Superscaf	48433357	0	0	0	0	0	BTP202209	Tissue specific
CDS|exon|ID=cds-Bg	17	5.178824	7267.2	0.440547	HEAFESDLA	89	661.3013	3	1980.893	.	P	Superscaf	48433357	0	0	0	0	0	BTP202209	Tissue specific
CDS|exon|ID=cds-Bg	7	6.312857	2991.2	0.440627	LHLEYAK	85	437.2415	2	872.4756	.	P	Superscaf	48435949	0	0	0	0	0	BTP202209	Tissue specific
CDS|exon|ID=cds-Bg	7	7.191429	2775.9	0.440628	AKRAAPF	93	380.7255	2	759.4391	.	P	Superscaf	48435949	0	0	0	0	0	BTP202209	Tissue specific
CDS|exon|ID=cds-Bg	8	6.09875	3937.4	0.44068	FDNKHTNY	91	519.7339	2	1037.457	.	S	Superscaf	48437569	0	0	0	0	0	BTP202209	Tissue specific
CDS|exon|ID=cds-Bg	13	5.737692	5015.4	0.440775	KSFSTALYC	83	709.3455	2	1416.677	.	W	Superscaf	48440602	0	BTP202209	0	0	BTP202209	0	Mixed
CDS|exon|ID=cds-Bg	7	5.065714	2684	0.440775	LYGESDL	97	796.3701	1	795.3651	.	W	Superscaf	48440602	0	BTP202209	0	0	0	0	Tissue specific
CDS|exon|ID=cds-Bg	14	5.976429	5114.8	0.445218	LPPTPASKF	94	722.8867	2	1443.761	.	R	Superscaf	48590242	0	0	BTP202209	0	0	0	Tissue specific
CDS|exon|ID=cds-Bg	7	6.404286	2869.1	0.453373	AELLKHP	97	404.2375	2	806.465	.	A	Superscaf	48864580	0	0	0	BTP202209	0	0	Tissue specific
CDS|exon|ID=cds-Bg	7	5.81	2986.3	0.459898	VNPHLQK	97	418.2416	2	834.4712	.	L	Superscaf	49084042	0	0	0	0	0	BTP202209	Tissue specific
CDS|exon|ID=cds-Bg	7	6.567143	3094.3	0.46167	KFFHPGN	51	423.713	2	845.4184	.	F	Superscaf	49143978	BTP202209	0	0	BTP202209	0	0	Tissue enhanced
CDS|exon|ID=cds-Bg	5	4.23	1429.6	0.468319	LVEGL	53	530.3164	1	529.3112	.	C	Superscaf	49367217	0	0	0	0	BTP202209	BTP202209	Tissue enhanced
CDS|exon|ID=cds-Bg	6	4.006667	1287.3	0.468804	AAVVLA	82	543.3492	1	542.3428	.	C	Superscaf	49383373	0	BTP202209	0	0	0	0	Tissue specific
CDS|exon|ID=cds-Bg	7	6.467143	2926.2	0.469401	YLGTNLK	82	404.7349	2	807.449	.	R	Superscaf	49403676	0	0	0	0	0	BTP202209	Tissue specific
CDS|exon|ID=cds-Bg	9	3.971111	2572.7	0.470903	VVGSEFVQK	93	496.7742	2	991.5338	.	S	Superscaf	49453987	0	0	0	0	0	BTP202209	Tissue specific
CDS|exon|ID=cds-Bg	10	4.14	3032.2	0.470903	VVGSEFVQK	70	578.3033	2	1154.597	.	S	Superscaf	49453987	0	BTP202209	0	0	0	0	Tissue specific
CDS|exon|ID=cds-Bg	8	4.3325	3071.4	0.470912	FDQNVNVK	82	482.2462	2	962.4821	.	A	Superscaf	49454278	0	0	0	BTP202209	0	0	Tissue specific
CDS|exon|ID=cds-Bg	8	5.6425	3146.6	0.477855	VGNAC(+57	99	451.7466	2	901.4803	Carbamido	V	Superscaf	49687540	0	BTP202209	BTP202209	0	BTP202209	0	Mixed
CDS|exon|ID=cds-Bg	7	6.448571	3146.6	0.477855	GNAC(+57.	87	402.2125	2	802.4119	Carbamido	V	Superscaf	49687540	0	BTP202209	BTP202209	BTP202209	BTP202209	0	Mixed
CDS|exon|ID=cds-Bg	6	6.103333	2534.8	0.48097	D(+42.01)	83	755.4385	1	754.4225	Acetylati	G	Superscaf	49792572	0	0	0	BTP202209	0	0	Tissue specific
CDS|exon|ID=cds-Bg	7	5.838571	2989.5	0.502731	RDYPLEL	51	453.2383	2	904.4654	.	A	Superscaf	50523958	0	0	0	0	0	BTP202209	Tissue specific
CDS|exon|ID=cds-Bg	9	5.357778	2861.3	0.524059	TSPMLLVAL	78	944.5457	1	943.5412	.	P	Superscaf	51241204	0	0	0	BTP202209	0	0	Tissue specific
CDS|exon|ID=cds-Bg	8	6.33875	2917.3	0.564244	G(+42.01)	51	407.2169	2	812.418	Acetylati	Q	Superscaf	52591969	0	0	BTP202209	0	0	0	Tissue specific
CDS|exon|ID=cds-Bg	7	6.442857	2600.7	0.568632	KFGGLYP	68	391.2225	2	780.417	.	A	Superscaf	52739934	BTP202209	0	0	0	0	0	Tissue specific
CDS|exon|ID=cds-Bg	12	6.248333	5365.1	0.568687	HSGSQRPEG	91	648.8169	2	1295.622	.	L	Superscaf	52741522	0	BTP202209	0	0	0	0	Tissue specific
CDS|exon|ID=cds-Bg	7	6.53	2694.9	0.581165	PSTSFPK	75	382.2022	2	762.3912	.	K	Superscaf	53161189	0	BTP202209	0	0	0	0	Tissue specific
CDS|exon|ID=cds-Bg	11	5.84	3980.3	0.584781	EEASAGPMA	82	544.2569	2	1086.502	.	F	Superscaf	53282827	0	BTP202209	0	0	0	0	Tissue specific
CDS|exon|ID=cds-Bg	16	5.764375	6224.6	0.5848	KATQVGEKF	98	836.9246	2	1671.843	.	V	Superscaf	53283418	0	0	0	0	BTP202209	0	Tissue specific
CDS|exon|ID=cds-Bg	10	6.44	4101.6	0.584844	VQELRKRGA	78	577.3411	2	1152.673	.	T	Superscaf	53284834	0	0	0	0	0	BTP202209	Tissue specific
CDS|exon|ID=cds-Bg	7	6.664286	2400.5	0.59018	PAAGPPK	87	637.3646	1	636.3594	.	S	Superscaf	53464177	0	0	0	0	BTP202209	0	Tissue specific
CDS|exon|ID=cds-Bg	5	3.636	1445.6	0.590733	EDVAG	56	490.2207	1	489.2071	.	G	Superscaf	53483073	BTP202209	0	0	BTP202209	0	0	Mixed
CDS|exon|ID=cds-Bg	9	6.417778	3452.1	0.593597	PPPPEPPLF	91	500.2834	2	998.5549	.	D	Superscaf	53579484	0	BTP202209	0	0	0	0	Tissue specific
CDS|exon|ID=cds-Bg	7	5.222857	2480.7	0.596902	PVDLSKW	99	422.73	2	843.449	.	M	Superscaf	53690245	0	0	0	0	0	BTP202209	Tissue specific
CDS|exon|ID=cds-Bg	14	6.209286	5361.8	0.596915	RKKYELGAF	64	775.4077	2	1548.808	Carbamido	M	Superscaf	53690245	0	0	0	0	0	BTP202209	Tissue specific
CDS|exon|ID=cds-Bg	12	5.535833	4244.6	0.596915	KYELGAPVA	82	633.3111	2	1264.612	Carbamido	M	Superscaf	53690245	0	0	0	0	0	BTP202209	Tissue specific
CDS|exon|ID=cds-Bg	13	5.545385	4704.1	0.596915	KYELGAPVA	85	714.8417	2	1427.676	Carbamido	M	Superscaf	53690245	0	0	0	0	0	BTP202209	Tissue specific
CDS|exon|ID=cds-Bg	14	5.394286	5439.9	0.596917	DDYVPKLYE	92	827.9128	2	1653.825	.	M	Superscaf	53690245	0	0	0	0	0	BTP202209	Tissue specific
CDS|exon|ID=cds-Bg	8	5.985	3351.6	0.596917	LYEQLSGK	99	469.2542	2	936.4916	.	M	Superscaf	53690245	0	BTP202209	0	0	BTP202209	BTP202209	Mixed
CDS|exon|ID=cds-Bg	10	6.202	3309.8	0.606642	GPGLPPPPF	81	463.2583	2	924.5068	.	S	Superscaf	54017191	0	0	0	BTP202209	BTP202209	0	Mixed
CDS|exon|ID=cds-Bg	7	5.385714	2092.4	0.609162	TPAATVP	53	656.3587	1	655.3541	.	H	Superscaf	54102349	0	0	0	0	BTP202209	0	Tissue specific

CDS|exon|ID=cds-B	9	6.196667	3371.7	0.620789	STADTPALK	84	452.2432	2	902.4709	.	V	Superscaf	54492379	0	BTP202209	0	0	0	Tissue specific
CDS|exon|ID=cds-B	8	5.39625	2311.6	0.626104	LPGPAVPP	79	747.438	1	746.4326	.	P	Superscaf	54672336	0	0	BTP202209	0	0	0 Tissue specific
CDS|exon|ID=cds-B	8	5.49125	3327.8	0.629795	LLPLEEHY	98	507.2673	2	1012.523	.	P	Superscaf	54796117	0	BTP202209	0	0	BTP202209	BTP202209 Tissue enhanced
CDS|exon|ID=cds-B	10	5.276	3835.4	0.629799	LLEQEVPGS	95	564.3028	2	1126.598	.	P	Superscaf	54796117	0	0	0	0	0	BTP202209 Tissue specific
CDS|exon|ID=cds-B	19	5.031579	7025.2	0.629799	LLEQEVPGS	86	1042.497	2	2082.989	Carbamido	P	Superscaf	54796117	0	0	0	0	0	BTP202209 Tissue specific
CDS|exon|ID=cds-B	10	5.05	3355.8	0.629802	LVVDPDKPF	96	395.8906	3	1184.655	.	P	Superscaf	54796117	0	0	0	0	0	BTP202209 Tissue specific
CDS|exon|ID=cds-B	9	4.415556	2707	0.629802	LVVDPDKPF	85	515.2819	2	1028.554	.	P	Superscaf	54796117	0	0	0	0	0	BTP202209 Tissue specific
CDS|exon|ID=cds-B	9	4.946667	3002.4	0.629802	VVDPDKPFF	96	536.7902	2	1071.571	.	P	Superscaf	54796117	0	0	0	0	BTP202209	BTP202209 Mixed
CDS|exon|ID=cds-B	8	4.22	2353.6	0.629802	VVDPDKPF	95	458.7423	2	915.4702	.	P	Superscaf	54796117	0	0	0	0	0	BTP202209 Tissue specific
CDS|exon|ID=cds-B	9	6.028889	3470.8	0.629802	VDPDKPFRK	94	551.3032	2	1100.598	.	P	Superscaf	54796117	0	0	0	0	BTP202209	0 Tissue specific
CDS|exon|ID=cds-B	8	5.565	3002.4	0.629802	VDPDKPFR	98	487.2567	2	972.5028	.	P	Superscaf	54796117	0	0	0	0	BTP202209	BTP202209 Mixed
CDS|exon|ID=cds-B	7	4.822857	2353.6	0.629802	VDPDKPF	94	409.2067	2	816.4017	.	P	Superscaf	54796117	0	0	0	0	BTP202209	BTP202209 Mixed
CDS|exon|ID=cds-B	7	6.36	3002.4	0.629802	DPDKPFR	95	437.7225	2	873.4344	.	P	Superscaf	54796117	0	0	0	0	BTP202209	0 Tissue specific
CDS|exon|ID=cds-B	11	5.267273	4782.5	0.629966	LFELEEQDL	88	719.8616	2	1437.714	.	L	Superscaf	54801655	0	0	0	BTP202209	0	0 Tissue specific
CDS|exon|ID=cds-B	7	6.258571	2983.2	0.630226	AHDFTKF	66	433.2139	2	864.413	.	S	Superscaf	54810604	0	BTP202209	0	0	0	0 Tissue specific
CDS|exon|ID=cds-B	7	7.215714	3018.1	0.630226	FHSLKPK	99	428.754	2	855.4966	.	S	Superscaf	54810604	0	0	0	0	0	BTP202209 Tissue specific
CDS|exon|ID=cds-B	11	5.011818	4393.9	0.630238	DDEEFALAS	86	623.7812	2	1245.551	.	S	Superscaf	54810604	0	BTP202209	0	0	0	0 Tissue specific
CDS|exon|ID=cds-B	12	6.3325	5017.8	0.630239	LEGHGLPTN	96	435.2411	3	1302.704	.	S	Superscaf	54810604	0	BTP202209	0	0	0	BTP202209 Mixed
CDS|exon|ID=cds-B	11	6.364545	4664.4	0.63024	EGHGLPTNL	66	397.5462	3	1189.62	.	S	Superscaf	54810604	0	BTP202209	0	0	0	0 Tissue specific
CDS|exon|ID=cds-B	7	6.172857	2621.1	0.638034	N(+42.01)	76	379.1981	2	756.3806	Acetylati	E	Superscaf	55073337	0	0	0	BTP202209	0	0 Tissue specific
CDS|exon|ID=cds-B	19	5.863684	6800.7	0.643948	SPPSAASPV	90	886.4307	2	1770.854	.	L	Superscaf	55272001	0	0	0	BTP202209	0	0 Tissue specific
CDS|exon|ID=cds-B	16	5.820625	5742.5	0.643949	AASPVNTPT	95	745.8624	2	1489.716	.	L	Superscaf	55272001	0	0	0	BTP202209	0	0 Tissue specific
CDS|exon|ID=cds-B	11	5.964545	4392	0.659744	T(+42.01)	83	634.8644	2	1267.718	Acetylati	Y	Superscaf	55803181	0	0	0	0	0	BTP202209 Tissue specific
CDS|exon|ID=cds-B	9	5.956667	3727.3	0.659744	T(+42.01)	66	542.8039	2	1083.596	Acetylati	Y	Superscaf	55803181	0	0	0	BTP202209	0	0 Tissue specific
CDS|exon|ID=cds-B	8	5.95375	3373.9	0.659744	T(+42.01)	85	486.262	2	970.5124	Acetylati	Y	Superscaf	55803181	0	0	0	0	0	BTP202209 Tissue specific
CDS|exon|ID=cds-B	5	6.048	1765	0.661237	LPSLP	81	526.3214	1	525.3162	.	G	Superscaf	55853272	0	0	0	BTP202209	0	0 Tissue specific
CDS|exon|ID=cds-B	6	7.455	2756.1	0.664098	QPLRPK	93	369.7333	2	737.4548	.	S	Superscaf	55949655	0	0	0	0	0	BTP202209 Tissue specific
CDS|exon|ID=cds-B	7	5.755714	2209.3	0.668052	KAAPTVS	56	673.3839	1	672.3806	.	L	Superscaf	56082214	0	0	0	0	0	BTP202209 Tissue specific
CDS|exon|ID=cds-B	11	6.598182	4732.4	0.668783	A(+42.01)	80	618.2928	2	1234.573	Acetylati	A	Superscaf	56106901	BTP202209	0	0	0	0	0 Tissue specific
CDS|exon|ID=cds-B	9	5.721111	3306.7	0.669766	LGRLAHEVC	63	476.2703	2	950.5297	.	F	Superscaf	56140141	0	0	0	0	0	BTP202209 Tissue specific
CDS|exon|ID=cds-B	10	5.738	3833.3	0.669766	LGRLAHEVC	61	379.8749	3	1136.609	.	F	Superscaf	56140141	0	0	0	0	0	BTP202209 Tissue specific
CDS|exon|ID=cds-B	12	6.404167	4603.9	0.669998	KRVLLGETC	62	453.2762	3	1356.809	.	T	Superscaf	56147893	0	BTP202209	0	0	0	0 Tissue specific
CDS|exon|ID=cds-B	14	6.439286	5475.1	0.669998	RVLLGETGK	91	399.7435	4	1594.952	.	T	Superscaf	56147893	0	0	0	0	0	BTP202209 Tissue specific
CDS|exon|ID=cds-B	11	6.100909	4135.5	0.669998	RVLLGETGK	95	410.5771	3	1228.714	.	T	Superscaf	56147893	0	BTP202209	0	0	0	0 Tissue specific
CDS|exon|ID=cds-B	13	6.106923	4826.3	0.669998	VLLGETGKE	94	480.6229	3	1438.851	.	T	Superscaf	56147893	0	BTP202209	0	0	0	BTP202209 Mixed
CDS|exon|ID=cds-B	8	5.42375	2592.8	0.669998	VLLGETGK	99	408.7441	2	815.4752	.	T	Superscaf	56147893	0	BTP202209	0	0	0	0 Tissue specific
CDS|exon|ID=cds-B	11	6.673636	4472.9	0.669998	LGETGKEKL	96	409.9051	3	1226.698	.	T	Superscaf	56147893	0	0	0	0	0	BTP202209 Tissue specific
CDS|exon|ID=cds-B	10	6.743	4119.5	0.669998	GETGKEKLF	78	372.2105	3	1113.614	.	T	Superscaf	56147893	0	0	0	0	BTP202209	BTP202209 Mixed
CDS|exon|ID=cds-B	11	5.47	3718.1	0.670036	TVRFNVLKV	74	435.6044	3	1303.798	.	P	Superscaf	56149138	0	0	0	0	0	BTP202209 Tissue specific
CDS|exon|ID=cds-B	7	6.744286	2452.7	0.673141	G(+42.01)	98	366.215	2	730.4013	Acetylati	G	Superscaf	56251627	0	0	0	BTP202209	0	0 Tissue specific
CDS|exon|ID=cds-B	13	4.956923	4346.9	0.674034	TVKVEDLTF	55	742.3853	2	1482.761	.	D	Superscaf	56283418	0	0	0	0	0	BTP202209 Tissue specific
CDS|exon|ID=cds-B	6	5.341667	2446.7	0.674565	E(+42.01)	53	356.1688	2	710.3235	Acetylati	V	Superscaf	56301295	0	0	0	BTP202209	0	0 Tissue specific
CDS|exon|ID=cds-B	12	5.925833	4177.6	0.675535	PAVSKGDGM	67	396.5444	3	1186.613	.	A	Superscaf	56333878	0	BTP202209	0	0	0	0 Tissue specific
CDS|exon|ID=cds-B	6	5.825	2372.6	0.67844	C(+57.02)	57	596.2318	1	595.2272	Carbamido	K	Superscaf	56431675	BTP202209	0	0	0	0	0 Tissue specific
CDS|exon|ID=cds-B	7	5.317143	2536.8	0.678829	VAEPNTK	59	758.4061	1	757.397	.	D	Superscaf	56444284	0	0	0	0	0	BTP202209 Tissue specific
CDS|exon|ID=cds-B	11	5.183636	3155.4	0.683836	A(+42.01)	86	563.8313	2	1125.651	Acetylati	P	Superscaf	56613184	0	BTP202209	0	0	0	0 Tissue specific
CDS|exon|ID=cds-B	10	4.728	2687	0.683836	A(+42.01)	99	499.7844	2	997.5556	Acetylati	P	Superscaf	56613184	0	BTP202209	0	0	0	0 Tissue specific
CDS|exon|ID=cds-B	9	4.057778	2038.2	0.683836	A(+42.01)	61	421.7334	2	841.4545	Acetylati	P	Superscaf	56613184	0	BTP202209	0	0	0	0 Tissue specific
CDS|exon|ID=cds-B	8	3.81875	1740.9	0.683836	A(+42.01)	62	785.4368	1	784.433	Acetylati	P	Superscaf	56613184	0	BTP202209	0	0	0	0 Tissue specific
CDS|exon|ID=cds-B	9	5.486667	3253.4	0.68476	ATDKSFVEK	99	512.7672	2	1023.524	.	L	Superscaf	56644180	0	0	0	0	0	BTP202209 Tissue specific
CDS|exon|ID=cds-B	8	6.53	3488.2	0.686008	FTTRTVRQ	80	504.7823	2	1007.551	.	G	Superscaf	56686057	0	BTP202209	0	0	0	0 Tissue specific
CDS|exon|ID=cds-B	10	5.772	3875.6	0.686008	FTTRTVRQV	86	418.9023	3	1253.688	.	G	Superscaf	56686057	0	BTP202209	0	0	0	0 Tissue specific
CDS|exon|ID=cds-B	7	7.075714	2971.5	0.688388	HRLPPPP	58	407.2435	2	812.4656	.	L	Superscaf	56766031	BTP202209	0	BTP202209	0	BTP202209	0 Tissue enhanced
CDS|exon|ID=cds-B	11	5.971818	3896.2	0.728029	TPSEKKTPS	98	565.8043	2	1129.598	.	R	Superscaf	58098916	0	0	0	BTP202209	0	0 Tissue specific
CDS|exon|ID=cds-B	11	6.137273	3639	0.732817	PPAPAPAPS	68	479.7513	2	957.4919	.	A	Superscaf	58259791	0	0	BTP202209	0	0	0 Tissue specific
CDS|exon|ID=cds-B	14	5.370714	6532.4	0.734404	S(+42.01)	97	906.902	2	1811.796	Acetylati	P	Superscaf	58313311	0	0	0	0	0	BTP202209 Tissue specific
CDS|exon|ID=cds-B	12	4.8825	3917.5	0.735107	VDLDSLVSF	91	627.8345	2	1253.662	.	G	Superscaf	58336729	0	0	BTP202209	0	0	0 Tissue specific
CDS|exon|ID=cds-B	7	6.247143	2707.7	0.742514	EDAAKAK	72	732.377	1	731.3813	.	S	Superscaf	58586029	0	BTP202209	0	0	0	0 Tissue specific
CDS|exon|ID=cds-B	7	7.641429	3002	0.746836	KAQKKPP	50	398.76	2	795.4966	.	G	Superscaf	58731376	0	0	BTP202209	0	0	0 Tissue specific
CDS|exon|ID=cds-B	6	4.295	2603.1	0.759885	D(+42.01)	58	755.2336	1	754.2405	Acetylati	N	Superscaf	59169835	0	BTP202209	0	0	0	0 Tissue specific
CDS|exon|ID=cds-B	7	4.751429	2373.8	0.79065	LPPVNPD	86	751.3938	1	750.3912	.	I	Superscaf	60204454	0	0	BTP202209	0	0	0 Tissue specific
CDS|exon|ID=cds-B	6	6.018333	2273.3	0.801962	ESGKAF	55	638.3093	1	637.3071	.	M	Superscaf	60583333	0	0	0	BTP202209	0	0 Tissue specific
CDS|exon|ID=cds-B	10	5.374	3183.4	0.86752	A(+42.01)	95	556.3431	2	1110.676	Acetylati	G	Superscaf	62788474	0	0	0	0	0	BTP202209 Tissue specific
CDS|exon|ID=cds-B	9	5.207778	3217.4	0.8677	LAEKEKVDL	96	522.7987	2	1043.586	.	S	Superscaf	62794504	0	0	0	0	0	BTP202209 Tissue specific
CDS|exon|ID=cds-B	11	5.255455	3685	0.867888	AGDLGVDLT	97	538.2827	2	1074.556	.	R	Superscaf	62801056	0	0	0	0	0	BTP202209 Tissue specific
CDS|exon|ID=cds-B	10	4.421	3190.6	0.86789	VETTEDLVA	98	1104.574	1	1103.571	.	R	Superscaf	62801056	0	0	0	0	0	BTP202209 Tissue specific
CDS|exon|ID=cds-B	5	4.872	1560.6	0.909734	SVGDK	51	505.2582	1	504.2544	.	E	Superscaf	64208316	0	0	0	0	0	BTP202209 Tissue specific
CDS|exon|ID=cds-B	7	4.695714	2080.3	0.909821	SGVDLPG	52	644.3199	1	643.3177	.	A	Superscaf	64211080	0	0	0	0	0	BTP202209 Tissue specific
CDS|exon|ID=cds-B	7	6.777143	2893.1	0.912214	KGGHLYT	51	388.2162	2	774.4024	.	A	Superscaf	64291662	0	0	0	0	0	BTP202209 Tissue specific
CDS|exon|ID=cds-B	8	5.995	3045.5	0.929101	HGPEPPLP	65	422.2251	2	842.4286	.	G	Superscaf	64859260	0	0	BTP202209	0	0	0 Tissue specific
CDS|exon|ID=cds-B	8	3.85625	1662.9	0.942492	LPGVVVPP	50	777.4816	1	776.4796	.	G	Superscaf	65309044	0	0	BTP202209	0	0	0 Tissue specific
CDS|exon|ID=cds-B	7	6.73	3277.7	0.9811	KTDLRFQ	97	454.2515	2	906.4923	.	G	Superscaf	66607441	0	0	0	0	BTP202209	BTP202209 Tissue enhanced
CDS|exon|ID=cds-B	11	4.776364	4161.8	0.981102	LVGLFEDTN	64	640.8134	2	1279.612	Carbamido	G	Superscaf	66607441	0	BTP202209	0	0	BTP202209	0 Mixed
CDS|exon|ID=cds-B	10	4.656	3808.4	0.981102	VGLFEDTNL	99	1167.531	1	1166.528	Carbamido	G	Superscaf	66607441	0	0	0	0	BTP202209	BTP202209 Mixed
CDS|exon|ID=cds-B	7	4.307143	2272.6	0.981102	VGLFEDT	99	780.3748	1	779.3701	.	G	Superscaf	66607441	0	0	0	0	BTP202209	0 Tissue specific
CDS|exon|ID=cds-B	7	5.212857	2319.7	0.986849	TVGNPLP	80	697.3854	1	696.3806	.	I	Superscaf	66800689	0	0	0	0	BTP202209	0 Tissue specific
CDS|exon|ID=cds-B	11	5.07	3755.2	0.995316	T(+42.01)	67	594.2808	2	1186.551	Acetylati	A	Superscaf	67085584	0	0	0	0	BTP202209	0 Tissue specific
CDS|exon|ID=cds-B	10	4.975	3443.9	0.995316	T(+42.01)	84	558.7618	2	1115.513	Acetylati	A	Superscaf	67085584	0	0	0	0	0	BTP202209 Tissue specific
CDS|exon|ID=cds-B	12	6.305833	4682.8	0.995414	AVKEKYAKY	86	482.9399	3	1445.803	.	V	Superscaf	67088857	0	0	0	0	0	BTP202209 Tissue specific
CDS|exon|ID=cds-B	12	6.125	5141.7	0.995456	KDELERVAK	98	482.2655	3	1443.779	.	A	Superscaf	67090324	0	0	0	0	0	BTP202209 Tissue specific
CDS|exon|ID=cds-B	9	5.738889	3512.8	0.995456	KDELERVAK	99	363.2068	3	1086.603	.	A	Superscaf	67090324	0	0	0	0	0	BTP202209 Tissue specific
CDS|exon|ID=cds-B	11	5.796364	4673.3	0.995456	DELERVAKS	99	439.567	3	1315.684	.	A	Superscaf	67090324	0	0	0	0	0	BTP202209 Tissue specific
CDS|exon|ID=cds-B	8	5.23875	3044.4	0.995456	DELERVAK	99	480.26	2	958.5083	.	A	Superscaf	67090324	0	0	0	0	0	BTP202209 Tissue specific
CDS|exon|ID=cds-B	8	7.16875	3147.5	0.996987	KPGSPPPR	59	418.2445	2	834.4711	.	R	Superscaf	67141150	0	BTP202209	0	0	0	0 Tissue specific
CDS|exon|ID=cds-B	8	6.07375	3355.7	0.693639	ATQAMEKY	77	471.2217	2	940.4324	.	D	Superscaf	9885205	0	0	0	0	0	BTP202209 Tissue specific
CDS|exon|ID=cds-B	7	5.27	2563.9	0.653482	EGSPTEG	65	676.2844	1	675.2711	.	R	Superscaf	11181298	0	0	0	BTP202209	0	0 Tissue specific
CDS|exon|ID=cds-B	6	6.053333	2217.5	0.653458	KVEPPR	67	363.2216	2	724.4232	.	R	Superscaf	11181298	0	0	0	BTP202209	0	0 Tissue specific
CDS|exon|ID=cds-B	8	4.2725	2796.2	0.639274	VTDNAVYH	98	459.7181	2	917.4243	.	F	Superscaf	11639791	0	0	0	0	0	BTP202209 Tissue specific
CDS|exon|ID=cds-B	8	5.70125	2920.2	0.638359	QLPLVKPY	77	479.2896	2	956.5695	.	H	Superscaf	11669428	0	0	0	0	0	BTP202209 Tissue specific
CDS|exon|ID=cds-B	9	7.153333	4343.7	0.63833	LFKGNNRWK	96	388.2194	3	1161.641	.	N	Superscaf	11670235	0	0	0	0	0	BTP202209 Tissue specific
CDS|exon|ID=cds-B	10	4.877	4067.7	0.638315	DTELAEELI	82	580.7854	2	1159.561	.	D	Superscaf	11670817	0	0	0	0	0	BTP202209 Tissue specific
CDS|exon|ID=cds-B	11	4.969091	4594.3	0.638315	DTELAEELI	91	673.8248	2	1345.64	.	D	Superscaf	11670817	0	0	0	0	0	BTP202209 Tissue specific
CDS|exon|ID=cds-B	12	5.728333	4619	0.638158	VDKLDASES	99	454.2511	3	1359.736	.	V	Superscaf	11675932	0	0	0	0	0	BTP202209 Tissue specific
CDS|exon|ID=cds-B	11	5.363636	4150.6	0.638158	VDKLDASES	97	616.8253	2	1231.641	.	V	Superscaf	11675932	0	0	0	0	0	BTP202209 Tissue specific
CDS|exon|ID=cds-B	8	6.25875	2659.1	0.624977	GPPPPPPP	90	378.2062	2	754.4013	.	Q	Superscaf	12101319	0	0	BTP202209	BTP202209	0	0 Mixed
CDS|exon|ID=cds-B	6	4.925	1912.1	0.556032	GVLGLQ	87	586.3532	1	585.3486	.	L	Superscaf	14326122	0	0	0	0	0	BTP202209 Tissue specific
CDS|exon|ID=cds-B	8	6.69	2846.2	0.515236	LPPPKPPP	72	421.7603	2	841.5061	.	R	Superscaf	15642085	0	0	BTP202209	0	0	0 Tissue specific
CDS|exon|ID=cds-B	7	6.061429	2584.8	0.510075	MGGEFPK	67	383.1861	2	764.3527	.	I	Superscaf	15808765	BTP202209	0	0	BTP202209	0	0 Tissue enhanced
CDS|exon|ID=cds-B	7	5.061429	2675.9	0.510053	LSEEVHK	59	841.4401	1	840.4341	.	I	Superscaf	15808765	0	0	0	0	0	BTP202209 Tissue specific
CDS|exon|ID=cds-B	7	6.055714	2592.7	0.499057	SLDALAK	97	359.2097	2	716.4068	.	G	Superscaf	16164451	0	BTP202209	0	0	0	BTP202209 Tissue enhanced
CDS|exon|ID=cds-B	13	5.280769	5198.6	0.431292	A(+42.01)	93	756.8442	2	1511.678	Acetylati	G	Superscaf	18350659	0	BTP202209	0	0	0	BTP202209 Mixed
CDS|exon|ID=cds-B	8	6.10875	2638.9	0.372588	LPPALAGP	50	735.4364	1	734.4326	.	P	Superscaf	20245375	0	0	0	0	BTP202209	0 Tissue specific
CDS|exon|ID=cds-B	9	5.83	3369.5	0.368091	A(+42.01)	78	913.4027	1	912.3977	Acetylati	A	Superscaf	20390440	0	0	0	0	BTP202209	BTP202209 Mixed
CDS|exon|ID=cds-B	6	5.516667	1792	0.35336	V(+42.01)	61	369.235	2	736.4595	Acetylati	S	Superscaf	20865733	0	0	BTP202209	0	0	0 Tissue specific
CDS|exon|ID=cds-B	7	7.028571	2979.4	0.34121	ALTARHP	88	383.2203	2	764.4293	.	R	Superscaf	21257833	0	0	0	0	0	BTP202209 Tissue specific
CDS|exon|ID=cds-B	8	7.335	3354	0.340678	RPLPRPLP	56	473.3014	2	944.5919	.	K	Superscaf	21275229	0	BTP202209	0	0	0	0 Tissue specific
CDS|exon|ID=cds-B	5	3.548	2072.4	0.333116	EVQQE	53	632.2959	1	631.2813	.	L	Superscaf	21518965	0	0	BTP202209	0	0	0 Tissue specific
CDS|exon|ID=cds-B	5	6.178	1620.8	0.323111	P(+42.01)	67	480.2493	1	479.238	Acetylati	A	Superscaf	21841738	0	0	BTP202209	BTP202209	BTP202209	0 Mixed
CDS|exon|ID=cds-B	5	4.912	1809.1	0.308368	DDAPP	55	514.2053	1	513.207	.	G	Superscaf	22317604	0	0	BTP202209	0	0	0 Tissue specific
CDS|exon|ID=cds-B	8	4.73625	2431.8	0.3019	VVGRELLL	59	449.788	2	897.5647	.	R	Superscaf	22526257	0	0	BTP202209	0	0	0 Tissue specific
CDS|exon|ID=cds-B	6	4.206667	1584.7	0.273133	EVVPKL	52	684.4262	1	683.4218	.	F	Superscaf	23454457	0	0	0	0	0	BTP202209 Tissue specific
CDS|exon|ID=cds-B	9	6.158889	3746.2	0.260636	ALTYPLHWF	96	574.2974	2	1146.586	.	T	Superscaf	23857699	0	0	0	0	0	BTP202209 Tissue specific
CDS|exon|ID=cds-B	10	5.844	3705.1	0.260548	PDLKLGPPS	90	526.7829	2	1051.555	.	P	Superscaf	23860315	0	0	BTP202209	0	0	0 Tissue specific
CDS|exon|ID=cds-B	9	4.51	3612.2	0.260117	VPQQQEHTV	85	533.2689	2	1064.525	.	F	Superscaf	23874615	BTP202209	0	0	0	0	0 Tissue specific
CDS|exon|ID=cds-B	9	5.585556	3695	0.2536	LKFLDQFAD	90	1096.564	1	1095.56	.	L	Superscaf	24084397	0	0	0	0	BTP202209	BTP202209 Mixed
CDS|exon|ID=cds-B	8	5.53625	3341.6	0.2536	KFLDQFAD	72	492.2437	2	982.476	.	L	Superscaf	24084397	0	0	0	0	BTP202209	0 Tissue specific
CDS|exon|ID=cds-B	7	7.278571	3000.4	0.253297	FRLLGAR	74	416.7585	2	831.5079	.	F	Superscaf	24095250	0	0	0	BTP202209	0	0 Tissue specific
CDS|exon|ID=cds-B	6	5.52	2181.4	0.245388	G(+42.01)	54	644.2628	1	643.2602	Acetylati	I	Superscaf	24349962	0	BTP202209	0	0	0	0 Tissue specific
CDS|exon|ID=cds-B	15	5.058667	5821.4	0.238108	VMELESKLN	91	898.4388	2	1794.871	.	N	Superscaf	24584644	0	0	0	0	0	BTP202209 Tissue specific
CDS|exon|ID=cds-B	7	4.33	2402.8	0.237157	V(+42.01)	60	427.2037	2	852.4089	Acetylati	A	Superscaf	24614830	0	0	0	BTP202209	0	0 Tissue specific
CDS|exon|ID=cds-B	7	5.48	2691	0.22944	AALLDNL	70	729.4097	1	728.4068	.	T	Superscaf	24864163	0	0	0	0	0	BTP202209 Tissue specific
CDS|exon|ID=cds-B	7	6.342857	2508.7	0.216574	GGKYVPR	97	388.7227	2	775.434	.	D	Superscaf	25279764	0	0	0	0	0	BTP202209 Tissue specific
CDS|exon|ID=cds-B	5	7.326	2326.7	0.203115	RHSPP	54	593.3168	1	592.3081	.	G	Superscaf	25713946	0	0	0	0	BTP202209	0 Tissue specific
CDS|exon|ID=cds-B	6	6.973333	2600.8	0.202931	KPHWAP	62	368.1962	2	734.3864	.	N	Superscaf	25719859	0	0	0	BTP202209	0	0 Tissue specific
CDS|exon|ID=cds-B	7	6.804286	2701.2	0.201812	ARTGM(+15	54	373.1913	2	744.3588	Oxidation	K	Superscaf	25756674	0	0	0	BTP202209	0	0 Tissue specific
CDS|exon|ID=cds-B	11	6.034545	4212.8	0.161172	ASSREVPRF	66	400.2222	3	1197.647	.	A	Superscaf	27067467	BTP202209	0	0	0	0	0 Tissue specific
CDS|exon|ID=cds-B	7	6.145714	2781.3	0.161172	SREVPRP	99	420.7374	2	839.4613	.	A	Superscaf	27067467	BTP202209	0	0	0	0	0 Tissue specific
CDS|exon|ID=cds-B	11	4.502727	3235.4	0.158726	VVEVTKDGS	99	574.8094	2	1147.608	.	S	Superscaf	27146221	0	0	0	0	0	BTP202209 Tissue specific
CDS|exon|ID=cds-B	13	5.556923	5697.4	0.158714	QATGESDWK	88	757.8202	2	1513.632	.	V	Superscaf	27146554	0	0	0	0	0	BTP202209 Tissue specific
CDS|exon|ID=cds-B	9	5.505556	3986.5	0.158707	STALEHQEY	88	539.2448	2	1076.478	.	G	Superscaf	27146839	0	0	0	0	BTP202209	BTP202209 Tissue enhanced
CDS|exon|ID=cds-B	13	5.82	4787.9	0.158705	SFLEDLKGF	98	499.9467	3	1496.824	.	G	Superscaf	27146839	0	0	0	0	BTP202209	0 Tissue specific
CDS|exon|ID=cds-B	11	5.863636	4017.1	0.158705	LEDLKGFVK	99	421.9141	3	1262.723	.	G	Superscaf	27146839	0	BTP202209	0	0	BTP202209	BTP202209 Mixed

CDS|exon|ID=cds-Bg	9	5.453333	3165.3	0.158705	LEDLKGFVK	99	524.8033	2	1047.596	.	G	Superscaf	27146839	0	0	0	0	0	BTP202209	Tissue specific
CDS|exon|ID=cds-Bg	7	4.645714	2634	0.151721	TVFLQYE	53	450.2281	2	898.4436	.	A	Superscaf	27372280	0	0	BTP202209	0	0	0	Tissue specific
CDS|exon|ID=cds-Bg	13	5.408462	4236.4	0.148545	KDSPSVWAA	95	671.3583	2	1340.709	.	I	Superscaf	27474457	0	0	0	0	0	BTP202209	Tissue specific
CDS|exon|ID=cds-Bg	15	5.488	5021.3	0.148545	KDSPSVWAA	79	795.4153	2	1588.825	.	I	Superscaf	27474457	0	0	0	0	0	BTP202209	Tissue specific
CDS|exon|ID=cds-Bg	17	5.160588	5618	0.148545	KDSPSVWAA	88	901.9658	2	1801.936	.	I	Superscaf	27474457	0	0	0	0	BTP202209	0	Tissue specific
CDS|exon|ID=cds-Bg	16	5.145	5021.3	0.148545	KDSPSVWAA	78	844.9492	2	1687.893	.	I	Superscaf	27474457	0	0	0	0	BTP202209	0	Tissue specific
CDS|exon|ID=cds-Bg	14	5.488571	4633.9	0.148545	KDSPSVWAA	79	481.5911	3	1441.757	.	I	Superscaf	27474457	0	0	0	0	BTP202209	0	Tissue specific
CDS|exon|ID=cds-Bg	8	5.285	2822	0.148545	KDSPSVWA	51	445.2227	2	888.4341	.	I	Superscaf	27474457	0	0	0	0	BTP202209	0	Tissue specific
CDS|exon|ID=cds-Bg	8	5.7575	2510.6	0.148545	AAVPGKTF	79	395.7254	2	789.4385	.	I	Superscaf	27474457	0	0	0	0	BTP202209	0	Tissue specific
CDS|exon|ID=cds-Bg	13	6.417692	6261.2	0.148473	C(+57.02)	89	850.8828	2	1699.756	Carbamido	H	Superscaf	27477046	0	0	0	0	0	BTP202209	Tissue specific
CDS|exon|ID=cds-Bg	9	6.824444	4221.9	0.148473	YEMASHLRR	68	388.1957	3	1161.571	.	H	Superscaf	27477046	0	0	0	0	BTP202209	0	Tissue specific
CDS|exon|ID=cds-Bg	8	5.65375	2785.1	0.146934	LLLDAPLA	50	825.5057	1	824.5007	.	A	Superscaf	27526681	0	0	0	0	BTP202209	0	Tissue specific
CDS|exon|ID=cds-Bg	9	6.191111	3945.3	0.133317	NALKNPFGN	56	487.7555	2	973.4981	.	L	Superscaf	27966130	0	0	BTP202209	0	0	0	Tissue specific
CDS|exon|ID=cds-Bg	10	5.335	4017.7	0.130843	EDLRLPEGD	92	578.7958	2	1155.577	.	L	Superscaf	28045963	0	0	0	0	BTP202209	0	Tissue specific
CDS|exon|ID=cds-Bg	11	5.985455	4357.9	0.130843	DLRLPEGDL	93	404.8894	3	1211.651	.	L	Superscaf	28045963	0	0	0	0	0	BTP202209	Tissue specific
CDS|exon|ID=cds-Bg	9	6.321111	3593	0.130843	RLPEGDLGK	94	492.7754	2	983.54	.	L	Superscaf	28045963	0	0	0	0	0	BTP202209	Tissue specific
CDS|exon|ID=cds-Bg	5	4.91	1325.5	0.129183	LPVGP	98	482.2968	1	481.29	.	L	Superscaf	28099561	BTP202209	0	0	0	0	0	Tissue specific
CDS|exon|ID=cds-Bg	6	5.753333	2094.4	0.128211	RPPSVF	56	351.6984	2	701.386	.	I	Superscaf	28131000	BTP202209	0	0	0	0	0	Tissue specific
CDS|exon|ID=cds-Bg	8	6.28125	3497.9	0.12189	KLQMEAPH	89	477.2439	2	952.48	.	Y	Superscaf	28334809	0	0	0	0	0	BTP202209	Tissue specific
CDS|exon|ID=cds-Bg	14	5.295714	4709.2	0.121868	SATMPSDVL	95	753.395	2	1504.781	.	V	Superscaf	28335610	0	0	0	0	0	BTP202209	Tissue specific
CDS|exon|ID=cds-Bg	13	4.953846	4240.8	0.121868	SATMPSDVL	93	689.347	2	1376.686	.	V	Superscaf	28335610	0	0	0	0	0	BTP202209	Tissue specific
CDS|exon|ID=cds-Bg	9	4.873333	2908.1	0.121868	SDVLEVTKK	86	509.7897	2	1017.571	.	V	Superscaf	28335610	0	0	0	0	BTP202209	BTP202209	Tissue enhanced
CDS|exon|ID=cds-Bg	8	4.265	2439.7	0.121868	SDVLEVTK	98	445.7437	2	889.4756	.	V	Superscaf	28335610	0	0	0	0	0	BTP202209	Tissue specific
CDS|exon|ID=cds-Bg	10	6.161	3885.3	0.097835	A(+42.01)	98	533.7616	2	1065.509	Acetylati	G	Superscaf	29111877	0	BTP202209	0	0	0	0	Tissue specific
CDS|exon|ID=cds-Bg	9	5.568889	3779.6	0.082329	M(+42.01)	83	415.5548	3	1243.646	Acetylati	V	Superscaf	29611848	0	BTP202209	BTP202209	BTP202209	0	0	Mixed
CDS|exon|ID=cds-Bg	5	3.604	976	0.050576	VVAAL	68	472.3107	1	471.3057	.	L	Superscaf	30635983	0	0	0	BTP202209	0	0	Tissue specific
CDS|exon|ID=cds-Bg	9	6.586667	3303.7	0.050555	PGRASGLPF	52	426.2333	2	850.4661	.	A	Superscaf	30636709	0	0	0	BTP202209	0	0	Tissue specific
CDS|exon|ID=cds-Bg	8	4.93875	2345.6	0.029061	VPGADGLP	54	725.3784	1	724.3755	.	R	Superscaf	31330264	0	0	0	0	BTP202209	0	Tissue specific
CDS|exon|ID=cds-Bg	8	5.24125	2295.7	0.071246	GPVRPPVP	53	409.7405	2	817.481	.	G	Superscaf	34567042	BTP202209	0	0	0	0	0	Tissue specific
CDS|exon|ID=cds-Bg	7	4.615714	2304.6	0.09024	M(+42.01)	80	854.3943	1	853.3892	Acetylati	W	Superscaf	35179381	0	BTP202209	0	0	BTP202209	0	Mixed
CDS|exon|ID=cds-Bg	9	5.022222	2867.3	0.096203	M(+42.01)	95	496.741	2	991.4684	Acetylati	G	Superscaf	35372302	0	BTP202209	0	0	BTP202209	BTP202209	Mixed
CDS|exon|ID=cds-Bg	9	5.336667	2560.6	0.10474	AAALGAAVL	63	756.4589	1	755.4541	.	R	Superscaf	35647843	0	0	0	0	BTP202209	0	Tissue specific
CDS|exon|ID=cds-Bg	8	5.7525	2552.7	0.121699	FGPVALTK	99	416.7476	2	831.4854	.	P	Superscaf	36194737	0	0	0	0	0	BTP202209	Tissue specific
CDS|exon|ID=cds-Bg	16	5.736875	5420	0.123196	M(+42.01)	95	797.8901	2	1593.771	Acetylati	A	Superscaf	36243157	0	BTP202209	0	0	0	0	Tissue specific
CDS|exon|ID=cds-Bg	9	3.641111	2744.2	0.135325	M(+42.01)	70	548.746	2	1095.48	Acetylati	S	Superscaf	36634315	0	BTP202209	0	0	0	0	Tissue specific
CDS|exon|ID=cds-Bg	14	5.798571	5160.6	0.139115	YNAPTSHVT	84	510.2748	3	1527.804	.	Y	Superscaf	36756970	0	BTP202209	0	0	0	0	Tissue specific
CDS|exon|ID=cds-Bg	7	5.422857	2459.8	0.151664	V(+42.01)	56	407.226	2	812.4392	Acetylati	V	Superscaf	37161811	BTP202209	0	0	0	0	0	Tissue specific
CDS|exon|ID=cds-Bg	9	4.536667	2921.3	0.155813	ALEEGVVRY	91	518.2748	2	1034.54	.	L	Superscaf	37295833	0	0	0	0	0	BTP202209	Tissue specific
CDS|exon|ID=cds-Bg	10	6.534	3691.8	0.159813	EPAKSAPAF	97	498.2785	2	994.5447	.	F	Superscaf	37424902	0	BTP202209	0	0	BTP202209	0	Mixed
CDS|exon|ID=cds-Bg	12	5.225833	4344.7	0.159822	PGELAKHAV	70	626.8156	2	1251.609	.	F	Superscaf	37424902	0	0	0	0	BTP202209	BTP202209	Mixed
CDS|exon|ID=cds-Bg	15	5.782	5137.4	0.161934	SVPAAAGGD	94	685.8553	2	1369.695	.	K	Superscaf	37492816	0	0	0	0	BTP202209	0	Tissue specific
CDS|exon|ID=cds-Bg	5	5.044	1653.8	0.173888	VGLSH	83	512.2812	1	511.2755	.	V	Superscaf	37878937	0	BTP202209	0	0	0	BTP202209	Tissue enhanced
CDS|exon|ID=cds-Bg	12	5.563333	4999.5	0.194882	EAGALELAF	80	620.2992	2	1238.589	.	L	Superscaf	38556439	0	0	0	0	0	BTP202209	Tissue specific
CDS|exon|ID=cds-Bg	7	5.751429	2436.6	0.228957	Q(+42.01)	78	378.721	2	755.4177	Acetylati	P	Superscaf	39656154	0	0	0	0	BTP202209	BTP202209	Tissue enhanced
CDS|exon|ID=cds-Bg	5	4.854	1299.4	0.234119	A(+42.01)	63	498.291	1	497.2849	Acetylati	A	Superscaf	39822691	0	0	BTP202209	0	0	0	Tissue specific
CDS|exon|ID=cds-Bg	10	6.445	5087.9	0.253283	M(+42.01)	96	676.7681	2	1351.525	Acetylati	L	Superscaf	40440952	0	BTP202209	0	0	0	0	Tissue specific
CDS|exon|ID=cds-Bg	15	5.416	5895.7	0.25379	YPKQSFTM(	70	872.3984	2	1742.782	Oxidation	T	Superscaf	40457392	0	0	BTP202209	0	0	0	Tissue specific
CDS|exon|ID=cds-Bg	13	5.783846	5072.9	0.253791	TMVADTPEN	98	496.5999	3	1486.781	.	T	Superscaf	40457392	0	BTP202209	0	0	0	0	Tissue specific
CDS|exon|ID=cds-Bg	13	6.184615	6107.9	0.253792	RLKQQSELQ	92	533.9682	3	1598.885	.	T	Superscaf	40457392	0	0	BTP202209	0	0	0	Tissue specific
CDS|exon|ID=cds-Bg	11	6.467273	4891	0.254014	YKEEFEKNK	95	467.2426	3	1398.714	.	S	Superscaf	40464616	0	0	0	0	BTP202209	0	Tissue specific
CDS|exon|ID=cds-Bg	8	5.71125	3656.9	0.254014	YKEEFEKN	97	543.7577	2	1085.503	.	S	Superscaf	40464616	0	0	BTP202209	0	0	0	Tissue specific
CDS|exon|ID=cds-Bg	12	6.410833	5116.2	0.254014	KEEFEKNKG	99	480.9207	3	1439.741	.	S	Superscaf	40464616	0	BTP202209	0	0	BTP202209	0	Mixed
CDS|exon|ID=cds-Bg	9	6.75	3796.8	0.254014	FEKNKGKGF	92	527.7856	2	1053.561	.	S	Superscaf	40464616	0	0	0	0	BTP202209	0	Tissue specific
CDS|exon|ID=cds-Bg	10	4.235	3230.7	0.254015	SVVADTPEL	91	529.769	2	1057.529	.	S	Superscaf	40464616	0	0	0	0	BTP202209	0	Tissue specific
CDS|exon|ID=cds-Bg	9	4.077778	2620	0.254015	SVVADTPEL	69	930.4769	1	929.4705	.	S	Superscaf	40464616	0	BTP202209	0	0	0	0	Tissue specific
CDS|exon|ID=cds-Bg	9	4.074444	2847.3	0.254015	VVADTPELQ	51	486.2538	2	970.4971	.	S	Superscaf	40464616	0	0	0	0	BTP202209	0	Tissue specific
CDS|exon|ID=cds-Bg	8	3.8775	2236.6	0.254015	VVADTPEL	98	843.4445	1	842.4385	.	S	Superscaf	40464616	0	BTP202209	0	0	0	BTP202209	Mixed
CDS|exon|ID=cds-Bg	8	5.92875	3496.1	0.254015	ADTPELQR	98	465.2365	2	928.4614	.	S	Superscaf	40464616	0	0	0	0	BTP202209	0	Tissue specific
CDS|exon|ID=cds-Bg	10	5.636	3943.4	0.271455	ALETALGGQ	80	510.2449	2	1018.475	Carbamido	D	Superscaf	41027023	BTP202209	0	0	0	0	0	Tissue specific
CDS|exon|ID=cds-Bg	11	5.280909	4339	0.271455	ETALGGQC(	99	603.2812	2	1204.551	Carbamido	D	Superscaf	41027023	BTP202209	0	0	0	0	0	Tissue specific
CDS|exon|ID=cds-Bg	8	5.29	3204.7	0.271455	LGGQC(+57	96	452.7182	2	903.4232	Carbamido	D	Superscaf	41027023	BTP202209	0	0	0	0	0	Tissue specific
CDS|exon|ID=cds-Bg	8	3.82	1749	0.271457	LPPVVPVS	88	807.4966	1	806.4902	.	D	Superscaf	41027023	BTP202209	0	0	0	0	0	Tissue specific
CDS|exon|ID=cds-Bg	8	4.29	1864	0.271457	PPVVPVSK	97	411.7568	2	821.501	.	D	Superscaf	41027023	BTP202209	0	0	0	0	0	Tissue specific
CDS|exon|ID=cds-Bg	7	6.61	2812.1	0.284176	Q(+42.01)	58	396.7213	2	791.4177	Acetylati	H	Superscaf	41437810	BTP202209	0	0	0	0	0	Tissue specific
CDS|exon|ID=cds-Bg	7	4.788571	2092.3	0.288377	LPAAEVL	53	712.4209	1	711.4167	.	A	Superscaf	41573209	0	0	BTP202209	0	0	0	Tissue specific
CDS|exon|ID=cds-Bg	7	6.27	3311.9	0.325833	C(+57.02)	54	929.3478	1	928.3565	Carbamido	V	Superscaf	42782119	0	0	0	0	0	BTP202209	Tissue specific
CDS|exon|ID=cds-Bg	7	6.988571	2892.3	0.327973	EPKPPPR	69	410.7425	2	819.4603	.	E	Superscaf	42851104	0	BTP202209	0	0	0	0	Tissue specific
CDS|exon|ID=cds-Bg	8	7.055	2925.7	0.329808	KLVKAFKK	99	481.3295	2	960.6484	.	P	Superscaf	42910228	0	0	0	0	0	BTP202209	Tissue specific
CDS|exon|ID=cds-Bg	14	4.980714	5776.6	0.33683	LQDEETVAT	92	832.392	2	1662.777	Carbamido	I	Superscaf	43136770	0	0	0	0	0	BTP202209	Tissue specific
CDS|exon|ID=cds-Bg	15	5.084	6174.1	0.33683	LQDEETVAT	94	882.9193	2	1763.825	Carbamido	I	Superscaf	43136770	0	BTP202209	0	0	BTP202209	0	Mixed
CDS|exon|ID=cds-Bg	7	3.605714	1584.7	0.33684	VVLVEPK	98	392.251	2	782.4902	.	I	Superscaf	43136770	0	0	0	0	0	BTP202209	Tissue specific
CDS|exon|ID=cds-Bg	5	7.058	2162.3	0.348605	SLHPK	51	581.338	1	580.3333	.	L	Superscaf	43516786	0	0	0	0	0	BTP202209	Tissue specific
CDS|exon|ID=cds-Bg	10	6.261	3696.9	0.35169	SGTGFKFLF	78	550.7899	2	1099.57	.	L	Superscaf	43615942	0	0	0	0	0	BTP202209	Tissue specific
CDS|exon|ID=cds-Bg	10	5.054	3379.7	0.35214	A(+42.01)	83	554.7659	2	1107.524	Acetylati	L	Superscaf	43630507	0	BTP202209	0	0	0	0	Tissue specific
CDS|exon|ID=cds-Bg	12	4.5875	3458.6	0.35262	VVGVADKDL	96	622.3647	2	1242.718	.	L	Superscaf	43645804	0	0	BTP202209	0	0	0	Tissue specific
CDS|exon|ID=cds-Bg	13	5.438462	4607.8	0.359406	A(+42.01)	94	504.9697	3	1511.892	Acetylati	P	Superscaf	43865374	0	BTP202209	0	0	BTP202209	BTP202209	Mixed
CDS|exon|ID=cds-Bg	11	4.998182	3786	0.359406	A(+42.01)	97	636.3618	2	1270.713	Acetylati	P	Superscaf	43865374	0	0	0	0	BTP202209	BTP202209	Tissue enhanced
CDS|exon|ID=cds-Bg	10	4.524	3317.6	0.359406	A(+42.01)	66	572.3142	2	1142.618	Acetylati	P	Superscaf	43865374	0	0	0	0	0	BTP202209	Tissue specific
CDS|exon|ID=cds-Bg	14	5.597857	5429.3	0.359838	ADLSREELT	96	546.6127	3	1636.824	.	K	Superscaf	43879108	0	0	0	0	0	BTP202209	Tissue specific
CDS|exon|ID=cds-Bg	8	6.01375	3366.7	0.359993	SHGGFFTN	65	433.6918	2	865.3718	.	D	Superscaf	43884157	0	0	0	0	0	BTP202209	Tissue specific
CDS|exon|ID=cds-Bg	7	4.567143	2295.4	0.365373	SFVKVYN	92	428.7299	2	855.449	.	F	Superscaf	44057869	0	0	0	0	BTP202209	BTP202209	Mixed
CDS|exon|ID=cds-Bg	8	7.3125	3610.7	0.365399	KTGKNKWF	91	504.7831	2	1007.555	.	E	Superscaf	44058712	0	0	0	0	0	BTP202209	Tissue specific
CDS|exon|ID=cds-Bg	6	7.181667	2856.1	0.365399	FQKLRF	98	419.7493	2	837.4861	.	E	Superscaf	44058712	0	BTP202209	0	0	BTP202209	BTP202209	Mixed
CDS|exon|ID=cds-Bg	6	7.268333	2538.7	0.410688	SGFLRK	77	354.2125	2	706.4126	.	T	Superscaf	45520326	0	BTP202209	0	0	0	0	Tissue specific
CDS|exon|ID=cds-Bg	16	5.415625	5472.1	0.418296	A(+42.01)	97	823.9156	2	1645.82	Acetylati	L	Superscaf	45765595	0	BTP202209	0	0	0	0	Tissue specific
CDS|exon|ID=cds-Bg	14	5.312143	4781.3	0.418296	A(+42.01)	68	718.8456	2	1435.683	Acetylati	L	Superscaf	45765595	0	0	BTP202209	BTP202209	BTP202209	BTP202209	Mixed
CDS|exon|ID=cds-Bg	9	6.844444	3580.7	0.460778	AATKYTSSK	99	478.755	2	955.4974	.	I	Superscaf	47136103	0	BTP202209	0	BTP202209	BTP202209	0	Mixed
CDS|exon|ID=cds-Bg	8	5.29625	3218.7	0.466392	QC(+57.02	87	459.7259	2	917.4389	Carbamido	R	Superscaf	47317516	BTP202209	0	0	0	0	0	Tissue specific
CDS|exon|ID=cds-Bg	10	5.779	3722.3	0.466447	GTVMGAELR	93	357.5184	3	1069.534	.	D	Superscaf	47319253	BTP202209	0	0	0	0	0	Tissue specific
CDS|exon|ID=cds-Bg	8	5.69625	3023.4	0.466447	AELRHVLA	78	454.7689	2	907.5239	.	D	Superscaf	47319253	BTP202209	0	0	0	0	0	Tissue specific
CDS|exon|ID=cds-Bg	7	5.65	2712.1	0.466447	AELRHVL	98	419.2498	2	836.4868	.	D	Superscaf	47319253	BTP202209	0	0	0	0	0	Tissue specific
CDS|exon|ID=cds-Bg	5	6.126	1827.9	0.476728	KGGDL	54	489.2593	1	488.2594	.	K	Superscaf	47651137	BTP202209	0	0	0	0	0	Tissue specific
CDS|exon|ID=cds-Bg	9	5.501111	2654.9	0.491513	VPATAAAPF	92	794.4399	1	793.4333	.	F	Superscaf	48128257	0	0	0	0	BTP202209	0	Tissue specific
CDS|exon|ID=cds-Bg	9	5.483333	3074.4	0.513726	EGPFVGSGF	91	453.226	2	904.4402	.	R	Superscaf	48844831	0	0	0	BTP202209	0	0	Tissue specific
CDS|exon|ID=cds-Bg	11	5.293636	4166.7	0.513732	GPQFGSEVE	77	609.8068	2	1217.604	.	R	Superscaf	48844831	0	0	0	BTP202209	0	0	Tissue specific
CDS|exon|ID=cds-Bg	5	4.054	2001.3	0.520723	DSDSD	52	538.1619	1	537.1555	.	I	Superscaf	49070722	0	0	BTP202209	0	0	0	Tissue specific
CDS|exon|ID=cds-Bg	7	4.051429	2270.6	0.522517	ERFEVSV	57	433.2318	2	864.4341	.	T	Superscaf	49128649	BTP202209	0	0	0	0	0	Tissue specific
CDS|exon|ID=cds-Bg	7	6.301429	2609.9	0.523837	PGGHSPP	53	648.3159	1	647.3027	.	W	Superscaf	49171312	0	0	0	0	BTP202209	0	Tissue specific
CDS|exon|ID=cds-Bg	8	6.16375	3136.5	0.544038	KHPVGRLD	52	461.2754	2	920.5192	.	S	Superscaf	49823170	0	0	0	0	BTP202209	0	Tissue specific
CDS|exon|ID=cds-Bg	7	7.028571	3153.4	0.563768	HPPAHYK	93	425.2205	2	848.4293	.	V	Superscaf	50459731	0	0	BTP202209	0	0	0	Tissue specific
CDS|exon|ID=cds-Bg	9	5.402222	3580.1	0.581997	FLEMTDDKL	98	556.2692	2	1110.527	.	R	Superscaf	51047935	0	0	0	0	0	BTP202209	Tissue specific
CDS|exon|ID=cds-Bg	11	6.530909	4671.4	0.608205	M(+42.01)	62	643.3525	2	1284.697	Acetylati	A	Superscaf	51893524	0	0	BTP202209	0	0	0	Tissue specific
CDS|exon|ID=cds-Bg	6	6.588333	2464.7	0.608236	KNFPPP	59	350.1982	2	698.3751	.	T	Superscaf	51894948	0	0	BTP202209	BTP202209	0	0	Mixed
CDS|exon|ID=cds-Bg	6	6.16	1946.1	0.608984	PAAAPP	57	523.2859	1	522.2802	.	R	Superscaf	51918793	0	0	0	BTP202209	0	0	Tissue specific
CDS|exon|ID=cds-Bg	15	5.428667	4866.1	0.612163	DSGFGGGAC	93	648.2946	2	1294.579	.	W	Superscaf	52021321	0	0	0	0	0	BTP202209	Tissue specific
CDS|exon|ID=cds-Bg	9	6.462222	3496.6	0.612212	FAEFGTLKK	93	520.7902	2	1039.57	.	S	Superscaf	52022779	0	0	0	0	0	BTP202209	Tissue specific
CDS|exon|ID=cds-Bg	16	4.975625	6834.7	0.612934	ENEEDEHSV	76	943.4096	2	1884.813	.	S	Superscaf	52046080	0	0	0	0	0	BTP202209	Tissue specific
CDS|exon|ID=cds-Bg	11	5.322727	3957.5	0.612966	MVGSYGPRA	94	598.2719	2	1194.534	.	R	Superscaf	52047322	0	0	0	0	0	BTP202209	Tissue specific
CDS|exon|ID=cds-Bg	14	5.207857	5229.9	0.612966	MVGSYGPRA	93	817.8585	2	1633.708	.	R	Superscaf	52047322	0	0	0	0	0	BTP202209	Tissue specific
CDS|exon|ID=cds-Bg	12	5.350833	4417	0.612966	MVGSYGPRA	88	679.8035	2	1357.597	.	R	Superscaf	52047322	0	0	0	0	BTP202209	BTP202209	Mixed
CDS|exon|ID=cds-Bg	11	5.614545	4240.8	0.612968	EFLTPMEEA	97	646.3135	2	1290.617	.	R	Superscaf	52047322	0	0	0	0	0	BTP202209	Tissue specific
CDS|exon|ID=cds-Bg	9	5.895556	3427.9	0.612968	LTPMEEAPK	86	508.2601	2	1014.506	.	R	Superscaf	52047322	0	0	0	0	0	BTP202209	Tissue specific
CDS|exon|ID=cds-Bg	13	4.736923	4737.3	0.613199	APDEEDHVL	94	501.2597	3	1500.757	.	Q	Superscaf	52054609	0	0	0	0	0	BTP202209	Tissue specific
CDS|exon|ID=cds-Bg	12	4.32	4268.9	0.613199	APDEEDHVL	99	458.5596	3	1372.662	.	Q	Superscaf	52054609	0	0	0	0	0	BTP202209	Tissue specific
CDS|exon|ID=cds-Bg	11	4.478182	4088.6	0.613199	DEEDHVLVL	99	445.23	3	1332.667	.	Q	Superscaf	52054609	0	0	0	0	0	BTP202209	Tissue specific
CDS|exon|ID=cds-Bg	10	3.952	3620.2	0.613199	DEEDHVLVL	99	603.2915	2	1204.572	.	Q	Superscaf	52054609	0	0	BTP202209	0	0	BTP202209	Mixed
CDS|exon|ID=cds-Bg	16	5.248125	5820.3	0.6132	VLVLHKGNF	98	578.6471	3	1732.926	.	Q	Superscaf	52054609	0	0	0	0	0	BTP202209	Tissue specific
CDS|exon|ID=cds-Bg	12	6.18	4962.2	0.6132	KGNFDEALA	96	434.2249	3	1299.657	.	Q	Superscaf	52054609	0	0	0	0	0	BTP202209	Tissue specific
CDS|exon|ID=cds-Bg	13	6.14	5421.7	0.6132	KGNFDEALA	95	488.5789	3	1462.721	.	Q	Superscaf	52054609	0	0	0	0	0	BTP202209	Tissue specific
CDS|exon|ID=cds-Bg	11	5.856364	4493.8	0.6132	KGNFDEALA	99	586.7856	2	1171.562	.	Q	Superscaf	52054609	0	0	0	0	0	BTP202209	Tissue specific
CDS|exon|ID=cds-Bg	12	6.06	4944.3	0.61324	FKNGDTASF	92	678.8223	2	1355.636	.	V	Superscaf	52055998	0	0	0	0	0	BTP202209	Tissue specific
CDS|exon|ID=cds-Bg	11	6.112727	4556.9	0.61324	KNGDTASPK	99	605.2888	2	1208.567	.	V	Superscaf	52055998	0	0	0	0	0	BTP202209	Tissue specific
CDS|exon|ID=cds-Bg	13	5.375385	4689.9	0.613294	YQLDKDGVV	98	518.2933	3	1551.866	.	G	Superscaf	52057690	0	0	0	0	0	BTP202209	Tissue specific
CDS|exon|ID=cds-Bg	12	5.351667	4230.4	0.613294	QLDKDGVVL	97	463.9398	3	1388.803	.	G	Superscaf	52057690	0	0	0	0	0	BTP202209	Tissue specific
CDS|exon|ID=cds-Bg	11	4.952727	3762	0.613294	QLDKDGVVL	95	631.358	2	1260.708	.	G	Superscaf	52057690	0	0	0	0	0	BTP202209	Tissue specific
CDS|exon|ID=cds-Bg	10	4.474	3293.6	0.613294	QLDKDGVVL	84	1133.615	1	1132.613	.	G	Superscaf	52057690	0	0	0	0	0	BTP202209	Tissue specific
CDS|exon|ID=cds-Bg	9	4.362222	2906.2	0.613294	QLDKDGVVL	84	493.7774	2	985.5444	.	G	Superscaf	52057690	0	0	0	0	0	BTP202209	Tissue specific
CDS|exon|ID=cds-Bg	9	4.343333	2682.9	0.613295	LDKDGVVLF	72	503.2826	2	1004.554	.	G	Superscaf	52057690	0	0	0	0	0	BTP202209	Tissue specific
CDS|exon|ID=cds-Bg	12	5.69	4867.3	0.613429	YKPESDELT	98	705.3408	2	1408.672	.	S	Superscaf	52062040	0	0	0	0	0	BTP202209	Tissue specific
CDS|exon|ID=cds-Bg	10	4.526	3570.8	0.613438	DEKKNVFVE	63	627.8197	2	1253.629	.	P	Superscaf	52062487	0	0	0	0	0	BTP202209	Tissue specific
CDS|exon|ID=cds-Bg	12	4.546667	4102.6	0.613444	MDSTANEVE	99	647.3013	2	1292.592	.	V	Superscaf	52062613	0	0	0	0	0	BTP202209	Tissue specific
CDS|exon|ID=cds-Bg	20	5.093	7049.8	0.613444	MDSTANEVE	98	551.5294	4	2202.099	.	V	Superscaf	52062613	0	0	0	0	0	BTP202209	Tissue specific

CDS|exon|ID=cds-Bg	12	4.0675	3731.1	0.613444	DSTANEVEA	90	631.3165	2	1260.62	.	V	Superscaf	52062613	0	0	0	BTP202209	0	0	Tissue specific
CDS|exon|ID=cds-Bg	9	3.736667	2538.7	0.613445	ANEVEAVKV	98	479.7626	2	957.5131	.	V	Superscaf	52062613	BTP202209	0	BTP202209	BTP202209	0	0	Mixed
CDS|exon|ID=cds-Bg	8	5.9125	2947.2	0.613445	VHSFPTLK	96	464.7643	2	927.5178	.	V	Superscaf	52062613	0	0	0	0	0	BTP202209	Tissue specific
CDS|exon|ID=cds-Bg	11	6.524545	4752.2	0.61345	NGERTLDGF	72	422.225	3	1263.657	.	G	Superscaf	52062889	0	0	0	0	BTP202209	BTP202209	Tissue enhanced
CDS|exon|ID=cds-Bg	10	6.636	4155.5	0.61345	GERTLDGFK	89	384.2101	3	1149.614	.	G	Superscaf	52062889	0	0	0	0	0	BTP202209	Tissue specific
CDS|exon|ID=cds-Bg	10	4.926	3872.2	0.613477	DDQKAVKDE	76	580.7921	2	1159.572	.	S	Superscaf	52063714	0	0	BTP202209	0	0	0	Tissue specific
CDS|exon|ID=cds-Bg	10	5.848	3725.1	0.620178	AGFAGDDAP	95	488.7268	2	975.441	.	I	Superscaf	52280023	0	BTP202209	0	BTP202209	0	BTP202209	Tissue enhanced
CDS|exon|ID=cds-Bg	9	5.828889	3413.8	0.620179	GFAGDDAPR	96	453.2082	2	904.4039	.	I	Superscaf	52280023	BTP202209	0	0	0	0	BTP202209	Tissue enhanced
CDS|exon|ID=cds-Bg	8	5.81125	3116.5	0.620179	FAGDDAPR	99	424.6982	2	847.3824	.	I	Superscaf	52280023	BTP202209	0	0	0	0	BTP202209	Mixed
CDS|exon|ID=cds-Bg	12	5.374167	4148.6	0.620179	AGDDAPRAV	85	601.7914	2	1201.573	.	I	Superscaf	52280023	0	0	0	0	0	BTP202209	Tissue specific
CDS|exon|ID=cds-Bg	11	5.346364	3765.2	0.620179	AGDDAPRAV	82	558.2764	2	1114.541	.	I	Superscaf	52280023	0	0	0	0	BTP202209	0	Tissue specific
CDS|exon|ID=cds-Bg	10	5.25	3540	0.620179	DDAPRAVFF	82	537.7618	2	1073.514	.	I	Superscaf	52280023	0	0	0	0	0	BTP202209	Tissue specific
CDS|exon|ID=cds-Bg	23	5.32	8755.7	0.62018	RHQGVMVGM	50	836.3959	3	2506.169	.	I	Superscaf	52280023	0	0	0	0	0	BTP202209	Tissue specific
CDS|exon|ID=cds-Bg	12	5.733333	4593.2	0.62018	RHQGVMVGM	85	443.2268	3	1326.665	.	I	Superscaf	52280023	0	0	0	0	0	BTP202209	Tissue specific
CDS|exon|ID=cds-Bg	13	5.13	4739.3	0.62018	HQGVMVGMG	83	687.3162	2	1372.623	.	I	Superscaf	52280023	0	0	0	0	0	BTP202209	Tissue specific
CDS|exon|ID=cds-Bg	12	5.084167	4355.9	0.62018	HQGVMVGMG	97	643.8021	2	1285.591	.	I	Superscaf	52280023	0	BTP202209	0	0	0	BTP202209	Mixed
CDS|exon|ID=cds-Bg	14	5.167857	5198.8	0.62018	HQGVMVGMG	98	768.8474	2	1535.686	.	I	Superscaf	52280023	0	0	0	0	0	BTP202209	Tissue specific
CDS|exon|ID=cds-Bg	22	5.072727	8106.9	0.62018	HQGVMVGMG	93	784.3642	3	2350.068	.	I	Superscaf	52280023	0	BTP202209	0	0	BTP202209	BTP202209	Mixed
CDS|exon|ID=cds-Bg	11	5.276364	3944.4	0.62018	HQGVMVGMG	99	586.2906	2	1170.564	.	I	Superscaf	52280023	0	BTP202209	0	0	BTP202209	BTP202209	Tissue enhanced
CDS|exon|ID=cds-Bg	10	4.83	3476	0.62018	HQGVMVGMG	70	522.2401	2	1042.469	.	I	Superscaf	52280023	0	0	0	0	0	BTP202209	Tissue specific
CDS|exon|ID=cds-Bg	10	5.045	3324.7	0.62018	QGVMVGMGQ	97	517.757	2	1033.505	.	I	Superscaf	52280023	0	0	0	0	0	BTP202209	Tissue specific
CDS|exon|ID=cds-Bg	8	4.85375	2416.7	0.620181	VMVGMGQK	99	425.2176	2	848.4249	.	I	Superscaf	52280023	0	0	0	0	0	BTP202209	Tissue specific
CDS|exon|ID=cds-Bg	18	5.411111	6856.5	0.620181	VGMGQKDSY	99	652.3123	3	1953.921	.	I	Superscaf	52280023	0	0	0	0	BTP202209	0	Tissue specific
CDS|exon|ID=cds-Bg	17	5.096471	6207.7	0.620181	VGMGQKDSY	96	600.278	3	1797.82	.	I	Superscaf	52280023	0	0	0	0	BTP202209	BTP202209	Mixed
CDS|exon|ID=cds-Bg	16	5.415	6207.7	0.620181	GMGQKDSYV	95	850.3809	2	1698.752	.	I	Superscaf	52280023	0	0	0	0	0	BTP202209	Tissue specific
CDS|exon|ID=cds-Bg	17	5.729412	6856.5	0.620181	M(+15.99)	88	624.621	3	1870.848	Oxidation	I	Superscaf	52280023	0	0	0	0	BTP202209	0	Tissue specific
CDS|exon|ID=cds-Bg	16	5.714375	6559.2	0.620181	MGQKDSYVG	98	600.2817	3	1797.832	.	I	Superscaf	52280023	0	0	0	0	BTP202209	0	Tissue specific
CDS|exon|ID=cds-Bg	15	5.378	5910.4	0.620181	MGQKDSYVG	94	821.8701	2	1641.731	.	I	Superscaf	52280023	0	BTP202209	0	0	BTP202209	BTP202209	Mixed
CDS|exon|ID=cds-Bg	14	5.066429	5442	0.620181	MGQKDSYVG	78	757.8222	2	1513.636	.	I	Superscaf	52280023	0	0	0	0	BTP202209	0	Tissue specific
CDS|exon|ID=cds-Bg	13	5.019231	5058.6	0.620181	MGQKDSYVG	82	714.3066	2	1426.604	.	I	Superscaf	52280023	0	0	0	0	BTP202209	0	Tissue specific
CDS|exon|ID=cds-Bg	10	5.036	3711.1	0.620181	MGQKDSYVG	77	550.2382	2	1098.465	.	I	Superscaf	52280023	0	0	0	0	BTP202209	0	Tissue specific
CDS|exon|ID=cds-Bg	9	5.265556	3299.6	0.620181	MGQKDSYVG	81	492.7248	2	983.4382	.	I	Superscaf	52280023	0	0	0	0	BTP202209	0	Tissue specific
CDS|exon|ID=cds-Bg	16	5.728125	6485	0.620181	GQKDSYVGD	97	862.9114	2	1723.813	.	I	Superscaf	52280023	0	0	0	0	BTP202209	0	Tissue specific
CDS|exon|ID=cds-Bg	14	5.693571	5890.4	0.620181	QKDSYVGDE	98	537.5961	3	1609.77	.	I	Superscaf	52280023	0	0	0	0	BTP202209	0	Tissue specific
CDS|exon|ID=cds-Bg	13	5.303846	5241.6	0.620181	QKDSYVGDE	99	727.8391	2	1453.669	.	I	Superscaf	52280023	0	0	0	0	BTP202209	0	Tissue specific
CDS|exon|ID=cds-Bg	14	5.716429	5577	0.620181	KDSYVGDEA	96	513.9167	3	1538.732	.	I	Superscaf	52280023	0	0	0	0	BTP202209	0	Tissue specific
CDS|exon|ID=cds-Bg	13	5.696923	5279.7	0.620181	KDSYVGDEA	99	741.8599	2	1481.711	.	I	Superscaf	52280023	0	0	0	0	BTP202209	0	Tissue specific
CDS|exon|ID=cds-Bg	12	5.275	4630.9	0.620181	KDSYVGDEA	99	663.81	2	1325.61	.	I	Superscaf	52280023	0	0	0	0	BTP202209	BTP202209	Mixed
CDS|exon|ID=cds-Bg	13	5.406923	5108.6	0.620181	DSYVGDEAQ	97	706.3234	2	1410.638	.	I	Superscaf	52280023	0	0	0	0	BTP202209	0	Tissue specific
CDS|exon|ID=cds-Bg	12	5.36	4811.3	0.620181	DSYVGDEAQ	99	677.813	2	1353.616	.	I	Superscaf	52280023	0	0	0	0	BTP202209	0	Tissue specific
CDS|exon|ID=cds-Bg	11	4.869091	4162.5	0.620181	DSYVGDEAQ	92	599.763	2	1197.515	.	I	Superscaf	52280023	0	BTP202209	0	0	0	BTP202209	Tissue enhanced
CDS|exon|ID=cds-Bg	10	5.059	3751	0.620182	SYVGDEAQS	97	542.2492	2	1082.488	.	I	Superscaf	52280023	0	0	0	0	0	BTP202209	Tissue specific
CDS|exon|ID=cds-Bg	10	5.567	4016.4	0.620182	YVGDEAQSK	99	576.7832	2	1151.557	.	I	Superscaf	52280023	0	0	0	0	BTP202209	0	Tissue specific
CDS|exon|ID=cds-Bg	6	6.456667	3010.4	0.620185	WHHTFY	85	445.6991	2	889.3871	.	I	Superscaf	52280023	0	0	0	0	0	BTP202209	Tissue specific
CDS|exon|ID=cds-Bg	9	6.468889	4508.2	0.620185	HHTFYNELR	81	406.1995	3	1215.578	.	I	Superscaf	52280023	BTP202209	0	0	0	0	0	Tissue specific
CDS|exon|ID=cds-Bg	8	6.32875	3888.5	0.620185	HTFYNELR	88	540.2645	2	1078.52	.	I	Superscaf	52280023	0	0	0	0	BTP202209	0	Tissue specific
CDS|exon|ID=cds-Bg	16	5.07	6034.9	0.620185	FYNELRVAP	76	642.6725	3	1925.005	.	I	Superscaf	52280023	0	0	0	0	BTP202209	0	Tissue specific
CDS|exon|ID=cds-Bg	13	4.898462	4940.7	0.620185	YNELRVAPE	72	776.889	2	1551.768	.	I	Superscaf	52280023	0	0	0	0	BTP202209	0	Tissue specific
CDS|exon|ID=cds-Bg	14	4.998571	5188	0.620185	NELRVAPEE	80	539.296	3	1614.873	.	I	Superscaf	52280023	0	0	0	0	0	BTP202209	Tissue specific
CDS|exon|ID=cds-Bg	13	4.923077	4834.6	0.620185	NELRVAPEE	93	501.6029	3	1501.789	.	I	Superscaf	52280023	0	BTP202209	0	0	BTP202209	BTP202209	Mixed
CDS|exon|ID=cds-Bg	12	4.835	4481.2	0.620185	NELRVAPEE	66	463.9084	3	1388.705	.	I	Superscaf	52280023	0	BTP202209	0	0	0	0	Tissue specific
CDS|exon|ID=cds-Bg	13	4.966923	4591.3	0.620185	ELRVAPEEH	65	751.4175	2	1500.83	.	I	Superscaf	52280023	0	0	0	0	0	BTP202209	Tissue specific
CDS|exon|ID=cds-Bg	12	4.8825	4237.9	0.620185	ELRVAPEEH	88	463.5878	3	1387.746	.	I	Superscaf	52280023	0	0	0	0	0	BTP202209	Tissue specific
CDS|exon|ID=cds-Bg	11	4.782727	3884.5	0.620185	ELRVAPEEH	87	638.3364	2	1274.662	.	I	Superscaf	52280023	0	0	0	0	BTP202209	BTP202209	Mixed
CDS|exon|ID=cds-Bg	11	5.033636	3812.4	0.620185	LRVAPEEHP	79	420.5732	3	1258.703	.	I	Superscaf	52280023	0	0	0	0	0	BTP202209	Tissue specific
CDS|exon|ID=cds-Bg	11	5.033636	3812.4	0.620186	RVAPEEHPV	79	630.3565	2	1258.703	.	I	Superscaf	52280023	0	0	0	0	BTP202209	0	Tissue specific
CDS|exon|ID=cds-Bg	10	4.939	3459	0.620186	RVAPEEHPV	92	573.8145	2	1145.619	.	I	Superscaf	52280023	0	0	0	0	BTP202209	BTP202209	Mixed
CDS|exon|ID=cds-Bg	9	4.823333	3105.6	0.620186	RVAPEEHPV	97	517.2731	2	1032.535	.	I	Superscaf	52280023	0	0	0	0	BTP202209	0	Tissue specific
CDS|exon|ID=cds-Bg	10	4.461	3163.6	0.620186	VAPEEHPVL	99	552.3075	2	1102.602	.	I	Superscaf	52280023	0	BTP202209	0	0	BTP202209	BTP202209	Mixed
CDS|exon|ID=cds-Bg	18	5.228333	6391.2	0.620186	VAPEEHPVL	96	652.0244	3	1953.057	.	I	Superscaf	52280023	0	0	0	0	BTP202209	BTP202209	Mixed
CDS|exon|ID=cds-Bg	9	4.292222	2810.2	0.620186	VAPEEHPVL	97	495.7645	2	989.5181	.	I	Superscaf	52280023	0	0	0	0	BTP202209	BTP202209	Mixed
CDS|exon|ID=cds-Bg	9	4.956667	3163.6	0.620186	APEEHPVLL	98	502.7721	2	1003.534	.	I	Superscaf	52280023	0	0	0	BTP202209	BTP202209	0	Mixed
CDS|exon|ID=cds-Bg	10	5.114	3561.1	0.620186	APEEHPVLL	64	553.2968	2	1104.582	.	I	Superscaf	52280023	0	0	0	0	BTP202209	0	Tissue specific
CDS|exon|ID=cds-Bg	8	4.82875	2810.2	0.620186	APEEHPVL	85	446.2305	2	890.4498	.	I	Superscaf	52280023	0	0	0	0	BTP202209	0	Tissue specific
CDS|exon|ID=cds-Bg	8	4.82375	2852.3	0.620186	PEEHPVLL	83	467.2523	2	932.4967	.	I	Superscaf	52280023	0	0	0	0	BTP202209	0	Tissue specific
CDS|exon|ID=cds-Bg	15	5.452667	5742.5	0.620186	EEHPVLLTE	93	843.9534	2	1685.899	.	I	Superscaf	52280023	0	0	0	0	BTP202209	0	Tissue specific
CDS|exon|ID=cds-Bg	10	4.806	3649.2	0.620186	EEHPVLLTE	77	569.2914	2	1136.571	.	I	Superscaf	52280023	0	BTP202209	0	0	0	0	Tissue specific
CDS|exon|ID=cds-Bg	14	5.612143	5317	0.620186	EHPVLLTEA	72	519.9565	3	1556.856	.	I	Superscaf	52280023	0	0	0	0	0	BTP202209	Tissue specific
CDS|exon|ID=cds-Bg	10	6.146	3934.4	0.620186	LLTEAPLNP	96	548.3222	2	1094.634	.	I	Superscaf	52280023	0	0	0	0	BTP202209	BTP202209	Tissue enhanced
CDS|exon|ID=cds-Bg	11	6.082727	4489	0.620186	LTEAPLNPK	82	584.3187	2	1166.629	.	I	Superscaf	52280023	0	0	0	0	BTP202209	0	Tissue specific
CDS|exon|ID=cds-Bg	9	6.164444	3581	0.620186	LTEAPLNPK	88	491.7799	2	981.5494	.	I	Superscaf	52280023	0	BTP202209	0	0	BTP202209	BTP202209	Mixed
CDS|exon|ID=cds-Bg	14	6.457143	6049.8	0.620187	TEAPLNPKA	91	799.9163	2	1597.825	.	I	Superscaf	52280023	0	0	0	0	BTP202209	0	Tissue specific
CDS|exon|ID=cds-Bg	13	6.511538	5678.3	0.620187	TEAPLNPKA	95	489.934	3	1466.784	.	I	Superscaf	52280023	0	0	0	0	0	BTP202209	Tissue specific
CDS|exon|ID=cds-Bg	11	6.517273	4784.4	0.620187	TEAPLNPKA	96	605.8278	2	1209.647	.	I	Superscaf	52280023	0	0	0	0	0	BTP202209	Tissue specific
CDS|exon|ID=cds-Bg	10	6.093	4135.6	0.620187	TEAPLNPKA	96	527.7786	2	1053.545	.	I	Superscaf	52280023	0	0	0	0	BTP202209	0	Tissue specific
CDS|exon|ID=cds-Bg	9	6.168889	3538.9	0.620187	TEAPLNPKA	98	940.5061	1	939.5025	.	I	Superscaf	52280023	0	0	0	0	BTP202209	BTP202209	Mixed
CDS|exon|ID=cds-Bg	8	6.1875	3227.6	0.620187	TEAPLNPK	99	435.239	2	868.4654	.	I	Superscaf	52280023	BTP202209	BTP202209	0	BTP202209	BTP202209	BTP202209	Mixed
CDS|exon|ID=cds-Bg	13	6.451538	5652.3	0.620187	EAPLNPKAN	95	749.3933	2	1496.777	.	I	Superscaf	52280023	0	0	0	0	BTP202209	0	Tissue specific
CDS|exon|ID=cds-Bg	12	6.51	5280.8	0.620187	EAPLNPKAN	98	683.8732	2	1365.736	.	I	Superscaf	52280023	0	0	0	0	BTP202209	0	Tissue specific
CDS|exon|ID=cds-Bg	10	6.516	4386.9	0.620187	EAPLNPKAN	97	555.304	2	1108.599	.	I	Superscaf	52280023	0	0	0	0	BTP202209	0	Tissue specific
CDS|exon|ID=cds-Bg	9	6.044444	3738.1	0.620187	EAPLNPKAN	95	477.2543	2	952.4977	.	I	Superscaf	52280023	0	0	0	0	BTP202209	0	Tissue specific
CDS|exon|ID=cds-Bg	12	6.720833	5226.8	0.620187	APLNPKANF	89	456.9172	3	1367.734	.	I	Superscaf	52280023	0	0	0	0	BTP202209	0	Tissue specific
CDS|exon|ID=cds-Bg	11	6.809091	4855.3	0.620187	APLNPKANF	87	619.3519	2	1236.694	.	I	Superscaf	52280023	0	0	0	0	BTP202209	0	Tissue specific
CDS|exon|ID=cds-Bg	9	6.882222	3961.4	0.620187	APLNPKANF	89	490.7835	2	979.5563	.	I	Superscaf	52280023	0	0	0	0	BTP202209	0	Tissue specific
CDS|exon|ID=cds-Bg	8	6.3975	3312.6	0.620187	APLNPKAN	97	412.7334	2	823.4551	.	I	Superscaf	52280023	0	0	0	0	BTP202209	0	Tissue specific
CDS|exon|ID=cds-Bg	7	5.641429	2853.3	0.620197	ETFNTPA	73	779.3533	1	778.3497	.	N	Superscaf	52280464	0	0	0	0	BTP202209	0	Tissue specific
CDS|exon|ID=cds-Bg	9	5.747778	3190.5	0.620198	VLSLYASGF	80	483.2736	2	964.5341	.	N	Superscaf	52280464	BTP202209	0	0	0	0	BTP202209	Tissue enhanced
CDS|exon|ID=cds-Bg	11	6.080909	4598.4	0.620202	RLDLAGRDL	67	622.8314	2	1243.652	.	N	Superscaf	52280464	0	0	0	0	0	BTP202209	Tissue specific
CDS|exon|ID=cds-Bg	12	6.045833	5057.9	0.620202	RLDLAGRDL	64	704.3625	2	1406.715	.	N	Superscaf	52280464	0	0	0	0	BTP202209	BTP202209	Tissue enhanced
CDS|exon|ID=cds-Bg	10	5.613	3949.6	0.620202	LDLAGRDLT	94	544.7808	2	1087.551	.	N	Superscaf	52280464	0	0	0	0	0	BTP202209	Tissue specific
CDS|exon|ID=cds-Bg	12	5.6475	4762.5	0.620202	LDLAGRDLT	85	682.8536	2	1363.698	.	N	Superscaf	52280464	0	0	0	0	0	BTP202209	Tissue specific
CDS|exon|ID=cds-Bg	11	5.617273	4409.1	0.620202	LDLAGRDLT	97	626.3119	2	1250.614	.	N	Superscaf	52280464	0	0	0	0	BTP202209	BTP202209	Mixed
CDS|exon|ID=cds-Bg	9	5.572222	3596.2	0.620202	DLAGRDLTD	95	488.2383	2	974.4669	.	N	Superscaf	52280464	0	0	0	0	0	BTP202209	Tissue specific
CDS|exon|ID=cds-Bg	13	5.944615	5249	0.620202	DLAGRDLTD	95	755.879	2	1509.75	.	N	Superscaf	52280464	0	0	0	0	0	BTP202209	Tissue specific
CDS|exon|ID=cds-Bg	10	5.581	4055.7	0.620202	DLAGRDLTD	85	569.7706	2	1137.53	.	N	Superscaf	52280464	0	0	0	0	BTP202209	BTP202209	Mixed
CDS|exon|ID=cds-Bg	8	5.8975	3184.7	0.620202	DLAGRDLT	77	430.7255	2	859.4399	.	N	Superscaf	52280464	0	0	0	0	0	BTP202209	Tissue specific
CDS|exon|ID=cds-Bg	8	5.8975	3184.7	0.620202	LAGRDLTD	86	430.7256	2	859.4399	.	N	Superscaf	52280464	0	0	0	0	0	BTP202209	Tissue specific
CDS|exon|ID=cds-Bg	9	5.871111	3644.2	0.620202	LAGRDLTDY	96	512.2567	2	1022.503	.	N	Superscaf	52280464	0	0	0	0	0	BTP202209	Tissue specific
CDS|exon|ID=cds-Bg	11	6.211818	4484.1	0.620202	AGRDLTDYL	86	428.2188	3	1281.639	.	N	Superscaf	52280464	0	0	0	0	0	BTP202209	Tissue specific
CDS|exon|ID=cds-Bg	8	5.6975	3226.7	0.620202	DLTDYLMK	97	998.4849	1	997.479	.	N	Superscaf	52280464	0	0	0	0	0	BTP202209	Tissue specific
CDS|exon|ID=cds-Bg	23	5.269565	8807.7	0.620205	LC(+57.02	98	1275.587	2	2549.167	Carbamido	N	Superscaf	52280464	0	0	0	0	0	BTP202209	Tissue specific
CDS|exon|ID=cds-Bg	8	4.63875	2862.2	0.620205	LC(+57.02	92	1000.477	1	999.4735	Carbamido	N	Superscaf	52280464	0	0	0	0	0	BTP202209	Tissue specific
CDS|exon|ID=cds-Bg	22	5.237273	8454.3	0.620205	C(+57.02)	95	1219.045	2	2436.082	Carbamido	N	Superscaf	52280464	0	0	0	0	0	BTP202209	Tissue specific
CDS|exon|ID=cds-Bg	7	4.447143	2508.8	0.620205	C(+57.02)	94	887.3937	1	886.3895	Carbamido	N	Superscaf	52280464	0	0	0	0	0	BTP202209	Tissue specific
CDS|exon|ID=cds-Bg	20	5.227	7409.1	0.620206	VALDFEQEM	92	705.334	3	2112.989	.	N	Superscaf	52280464	0	0	0	0	BTP202209	BTP202209	Tissue enhanced
CDS|exon|ID=cds-Bg	19	5.502105	7409.1	0.620206	ALDFEQEMA	98	1007.964	2	2013.92	.	N	Superscaf	52280464	0	0	0	0	BTP202209	0	Tissue specific
CDS|exon|ID=cds-Bg	18	5.473333	7097.8	0.620206	LDFEQEMAT	91	972.4479	2	1942.883	.	N	Superscaf	52280464	0	BTP202209	0	0	BTP202209	BTP202209	Mixed
CDS|exon|ID=cds-Bg	17	5.443529	6744.4	0.620206	DFEQEMATA	96	915.9053	2	1829.799	.	N	Superscaf	52280464	0	BTP202209	0	0	BTP202209	BTP202209	Mixed
CDS|exon|ID=cds-Bg	14	5.257143	5497.1	0.620206	DFEQEMATA	76	730.7931	2	1459.577	.	N	Superscaf	52280464	0	0	0	0	BTP202209	0	Tissue specific
CDS|exon|ID=cds-Bg	16	5.598125	6332.9	0.620206	FEQEMATAA	96	858.3906	2	1714.772	.	N	Superscaf	52280464	0	0	0	0	BTP202209	BTP202209	Tissue enhanced
CDS|exon|ID=cds-Bg	15	5.606	5945.5	0.620206	EQEMATAAS	93	784.8583	2	1567.704	.	N	Superscaf	52280464	0	BTP202209	0	0	BTP202209	BTP202209	Mixed
CDS|exon|ID=cds-Bg	14	5.776429	5520	0.620206	QEMATAASS	95	720.3344	2	1438.661	.	N	Superscaf	52280464	0	0	0	0	0	BTP202209	Tissue specific
CDS|exon|ID=cds-Bg	13	5.786154	4909.3	0.620206	EM(+15.99	97	664.3035	2	1326.597	Oxidation	N	Superscaf	52280464	0	0	0	0	BTP202209	BTP202209	Mixed
CDS|exon|ID=cds-Bg	12	6	4483.8	0.620206	MATAASSSS	97	591.7875	2	1181.56	.	N	Superscaf	52280464	0	0	0	0	BTP202209	BTP202209	Mixed
CDS|exon|ID=cds-Bg	11	6.022727	4112.3	0.620207	ATAASSSSL	99	526.2648	2	1050.519	.	N	Superscaf	52280464	0	0	0	0	0	BTP202209	Tissue specific
CDS|exon|ID=cds-Bg	13	5.968462	4955.2	0.620207	ATAASSSSL	98	651.3118	2	1300.615	.	N	Superscaf	52280464	0	0	0	0	0	BTP202209	Tissue specific
CDS|exon|ID=cds-Bg	10	6.023	3801	0.620207	TAASSSSLE	99	490.7465	2	979.4822	.	N	Superscaf	52280464	0	0	0	0	0	BTP202209	Tissue specific
CDS|exon|ID=cds-Bg	9	5.966667	3403.5	0.620207	AASSSSLEK	99	879.4573	1	878.4345	.	N	Superscaf	52280464	0	0	0	0	0	BTP202209	Tissue specific
CDS|exon|ID=cds-Bg	11	5.912727	4246.4	0.620207	AASSSSLEK	99	565.27	2	1128.53	.	N	Superscaf	52280464	0	0	0	0	0	BTP202209	Tissue specific
CDS|exon|ID=cds-Bg	14	5.529286	5676.2	0.620207	SSSLEKSYE	86	770.3589	2	1538.71	.	N	Superscaf	52280464	0	0	0	0	0	BTP202209	Tissue specific
CDS|exon|ID=cds-Bg	8	5.17875	3278.7	0.620208	SYELPDGQ	96	908.3958	1	907.3923	.	N	Superscaf	52280464	0	0	0	0	0	BTP202209	Tissue specific
CDS|exon|ID=cds-Bg	9	6.306667	4123.8	0.620209	ERFRC(+57	82	589.2903	2	1176.571	Carbamido	N	Superscaf	52280464	0	0	0	0	0	BTP202209	Tissue specific
CDS|exon|ID=cds-Bg	7	6.394286	3459.1	0.620209	ERFRC(+57	98	497.2298	2	992.4498	Carbamido	N	Superscaf	52280464	0	0	0	0	BTP202209	0	Tissue specific
CDS|exon|ID=cds-Bg	9	6.505556	3331.7	0.620218	TALAPSTMK	96	460.2422	2	918.4844	.	P	Superscaf	52281139	0	0	0	0	0	BTP202209	Tissue specific
CDS|exon|ID=cds-Bg	8	6.5025	2934.2	0.620218	ALAPSTMK	99	409.7238	2	817.4368	.	P	Superscaf	52281139	0	0	0	0	0	BTP202209	Tissue specific
CDS|exon|ID=cds-Bg	9	5.81	4024.6	0.620224	SLSTFQQMW	71	564.2615	2	1126.512	.	G	Superscaf	52281403	0	0	0	0	BTP202209	0	Tissue specific
CDS|exon|ID=cds-Bg	6	5.58	2302.7	0.646436	T(+42.01)	63	385.1525	2	768.3	Acetylati	S	Superscaf	53127459	BTP202209	0	0	0	0	0	Tissue specific
CDS|exon|ID=cds-Bg	7	5.42	2445.8	0.650287	LLEVALR	91	407.2616	2	812.512	.	A	Superscaf	53251552	0	0	BTP202209	0	0	0	Tissue specific
CDS|exon|ID=cds-Bg	14	6.662143	5301.8	0.657664	A(+42.01)	97	683.3419	2	1364.672	Acetylati	P	Superscaf	53489524	0	BTP202209	0	0	BTP202209	0	Mixed
CDS|exon|ID=cds-Bg	7	6.77	2906.4	0.665618	RPPNAPP	71	374.7068	2	747.4027	.	G	Superscaf	53745793	0	0	0	BTP202209	0	0	Tissue specific
CDS|exon|ID=cds-Bg	10	4.386	2647.9	0.6867	VGAVVPTHG	87	492.2668	2	982.5236	.	P	Superscaf	54426427	0	0	0	0	0	BTP202209	Tissue specific
CDS|exon|ID=cds-Bg	5	3.922	2203.6	0.697817	C(+57.02)	60	611.1584	1	610.1541	Carbamido	I	Superscaf	54785227	0	0	0	BTP202209	0	0	Tissue specific

CDS	exon	ID=cds-Bg	5	4.548	1369.6	0.707635	VSPVR	70	557.3392	1	556.3333	.	A	Superscaf	55102167	0	0	0	BTP202209	0	0	Tissue specific
CDS	exon	ID=cds-Bg	6	6.285	2571	0.7079	RVAHM(+15	57	383.698	2	765.3704	Oxidatior	R	Superscaf	55110738	0	0	0	0	BTP202209	0	Tissue specific
CDS	exon	ID=cds-Bg	13	5.503077	4925.5	0.748666	FSFPVDNLF	93	748.8777	2	1495.746	.	E	Superscaf	56425984	0	0	0	0	0	BTP202209	Tissue specific
CDS	exon	ID=cds-Bg	8	5.3975	2705	0.755233	LDPSLVTK	98	436.7573	2	871.5015	.	G	Superscaf	56637910	0	0	0	0	0	BTP202209	Tissue specific
CDS	exon	ID=cds-Bg	14	4.498571	4780.4	0.75703	LHLVNFVEF	62	807.4344	2	1612.861	.	N	Superscaf	56695978	0	0	0	0	0	BTP202209	Tissue specific
CDS	exon	ID=cds-Bg	10	6.369	3733	0.757426	KLFPLLATA	78	568.8372	2	1135.664	.	F	Superscaf	56708719	0	0	0	0	0	BTP202209	Tissue specific
CDS	exon	ID=cds-Bg	16	6.140625	6471	0.761943	A(+42.01)	90	841.4414	2	1680.879	Acetylati	A	Superscaf	56854474	0	BTP202209	BTP202209	0	BTP202209	0	Mixed
CDS	exon	ID=cds-Bg	14	5.819286	5510.9	0.761943	A(+42.01)	95	727.8762	2	1453.741	Acetylati	A	Superscaf	56854474	0	0	BTP202209	0	BTP202209	0	Mixed
CDS	exon	ID=cds-Bg	13	5.866923	4848.2	0.762148	LSQAADGAK	98	459.2604	3	1374.762	.	A	Superscaf	56860669	0	BTP202209	BTP202209	0	BTP202209	BTP202209	Mixed
CDS	exon	ID=cds-Bg	12	5.899167	4460.8	0.762148	LSQAADGAK	94	614.8536	2	1227.694	.	A	Superscaf	56860669	0	BTP202209	0	0	BTP202209	0	Mixed
CDS	exon	ID=cds-Bg	12	5.8575	4494.8	0.762148	SQAADGAKV	88	631.8452	2	1261.678	.	A	Superscaf	56860669	0	BTP202209	BTP202209	0	0	0	Mixed
CDS	exon	ID=cds-Bg	11	5.873636	4111.4	0.762148	QAADGAKVL	94	392.5539	3	1174.646	.	A	Superscaf	56860669	0	0	BTP202209	0	0	0	Tissue specific
CDS	exon	ID=cds-Bg	9	5.882222	3189.4	0.762149	ADGAKVLRF	91	488.7814	2	975.5501	.	A	Superscaf	56860669	0	0	BTP202209	0	0	0	Tissue specific
CDS	exon	ID=cds-Bg	9	6.426667	3583	0.776749	RPPGGGSNF	87	444.7188	2	887.4249	.	H	Superscaf	57332278	0	0	0	BTP202209	0	0	Tissue specific
CDS	exon	ID=cds-Bg	10	5.571	4243.1	0.77675	DEPTEQPVF	83	613.8071	2	1225.605	.	H	Superscaf	57332278	0	0	0	BTP202209	0	0	Tissue specific
CDS	exon	ID=cds-Bg	9	5.753333	3812.2	0.776752	EENPPSWAK	93	529.2509	2	1056.488	.	H	Superscaf	57332278	0	BTP202209	0	0	0	0	Tissue specific
CDS	exon	ID=cds-Bg	10	5.209	3534.7	0.779576	AGLVDDFEK	99	561.2934	2	1120.576	.	L	Superscaf	57423463	0	0	0	0	0	BTP202209	Tissue specific
CDS	exon	ID=cds-Bg	18	5.285556	7414.4	0.781596	NHLLFSDYF	85	1066.022	2	2130.038	.	A	Superscaf	57488485	0	0	0	0	0	BTP202209	Tissue specific
CDS	exon	ID=cds-Bg	6	6.801667	2389.8	0.82682	P(+42.01)	61	358.677	2	715.3323	Acetylati	L	Superscaf	58947715	0	0	0	0	0	BTP202209	Tissue specific
CDS	exon	ID=cds-Bg	21	5.095714	7770.4	0.829182	AGELDGESV	89	1033.952	2	2065.892	.	P	Superscaf	59024038	0	BTP202209	0	0	0	0	Tissue specific
CDS	exon	ID=cds-Bg	11	5.123636	3861.4	0.829201	EGEGGPDVF	76	550.254	2	1098.494	.	P	Superscaf	59024038	0	BTP202209	0	0	0	0	Tissue specific
CDS	exon	ID=cds-Bg	6	5.913333	2631.9	0.931035	DLKEHL	97	377.7072	2	753.4021	.	E	Superscaf	62310739	0	0	BTP202209	0	0	0	Tissue specific
CDS	exon	ID=cds-Bg	5	6.09	1908.2	0.936983	LPLWP	65	625.3685	1	624.3635	.	T	Superscaf	62502376	0	0	0	BTP202209	0	0	Tissue specific
CDS	exon	ID=cds-Bg	5	5.908	2629	0.942303	C(+57.02)	66	733.2507	1	732.2472	Carbamidc	P	Superscaf	62674408	0	BTP202209	0	0	0	0	Tissue specific
CDS	exon	ID=cds-Bg	7	5.057143	2328.6	0.960016	HGVAPPE	56	353.6849	2	705.3445	.	R	Superscaf	63245659	BTP202209	0	0	0	0	0	Tissue specific
CDS	exon	ID=cds-Bg	9	6.703333	3356	0.961199	ARM(+15.9	61	488.254	2	974.5007	Oxidatior	R	Superscaf	63284167	0	BTP202209	0	0	0	0	Tissue specific
CDS	exon	ID=cds-Bg	10	5.602	3957.2	0.929036	FVC(+57.0	92	643.3049	2	1284.599	Carbamidc	Q	Superscaf	4847500	0	0	0	0	0	BTP202209	Tissue specific
CDS	exon	ID=cds-Bg	9	5.615556	3569.8	0.929036	VC(+57.02	94	569.7705	2	1137.531	Carbamidc	Q	Superscaf	4847500	0	0	0	0	0	BTP202209	Tissue specific
CDS	exon	ID=cds-Bg	8	5.6325	3182.4	0.929036	VC(+57.02	96	496.2368	2	990.4626	Carbamidc	Q	Superscaf	4847500	0	0	0	0	0	BTP202209	Tissue specific
CDS	exon	ID=cds-Bg	12	6.844167	5163.7	0.929011	LSELAARRA	87	670.3906	2	1338.773	.	P	Superscaf	4849303	0	0	0	0	0	BTP202209	Tissue specific
CDS	exon	ID=cds-Bg	7	7.404286	3272.9	0.921285	RVHRLLR	80	475.3106	2	948.6093	.	N	Superscaf	5377057	0	0	BTP202209	0	0	0	Tissue specific
CDS	exon	ID=cds-Bg	12	5.884167	4029.2	0.921207	A(+42.01)	99	589.2972	2	1176.581	Acetylati	A	Superscaf	5382496	BTP202209	0	0	0	0	0	Tissue specific
CDS	exon	ID=cds-Bg	10	5.49	3263.5	0.921207	A(+42.01)	96	496.7391	2	991.4644	Acetylati	A	Superscaf	5382496	BTP202209	0	0	0	0	0	Tissue specific
CDS	exon	ID=cds-Bg	8	5.03375	3492.1	0.920386	ENFTERVQ	69	511.7483	2	1021.483	.	D	Superscaf	5438518	BTP202209	0	0	0	0	0	Tissue specific
CDS	exon	ID=cds-Bg	11	6.191818	4490.7	0.911277	FKSLGSNDL	84	419.226	3	1254.661	.	H	Superscaf	6060727	0	0	0	0	0	BTP202209	Tissue specific
CDS	exon	ID=cds-Bg	9	5.876667	3634.9	0.911277	SLGSNDLKF	85	490.7544	2	979.4974	.	H	Superscaf	6060727	0	0	0	0	0	BTP202209	Tissue specific
CDS	exon	ID=cds-Bg	12	5.9025	5498.1	0.867132	SHDC(+57.	95	466.2133	3	1395.62	Carbamidc	V	Superscaf	9076348	0	BTP202209	0	0	0	0	Tissue specific
CDS	exon	ID=cds-Bg	14	4.721429	5366	0.857855	VEAVEQNTL	91	824.4286	2	1646.852	.	D	Superscaf	9710289	0	0	0	0	BTP202209	BTP202209	Tissue enhanced
CDS	exon	ID=cds-Bg	12	4.198333	4544.2	0.857855	VEAVEQNTL	54	703.8406	2	1405.673	.	D	Superscaf	9710289	0	0	0	0	0	BTP202209	Tissue specific
CDS	exon	ID=cds-Bg	13	5.084615	5366	0.857855	EAVEQNTLQ	95	774.8944	2	1547.783	.	D	Superscaf	9710289	0	0	0	0	BTP202209	0	Tissue specific
gene	mRNA	ID=gene-B	11	5.169091	4629.2	0.857855	VEQNTLQEF	99	674.8564	2	1347.703	.	D	Superscaf	9710289	0	0	0	0	BTP202209	0	Tissue specific
CDS	exon	ID=cds-Bg	8	5.99875	3593	0.857855	NTLQEFLK	97	496.7716	2	991.5338	.	D	Superscaf	9710289	0	0	0	0	0	BTP202209	Tissue specific
CDS	exon	ID=cds-Bg	12	5.379167	4819.6	0.799632	LTMDLLESC	72	744.8373	2	1487.668	Carbamidc	Y	Superscaf	13687522	0	0	0	0	0	BTP202209	Tissue specific
CDS	exon	ID=cds-Bg	10	5.064	3397.6	0.799632	VYKQSVTSS	83	535.2794	2	1068.545	.	Y	Superscaf	13687522	0	BTP202209	0	0	0	0	Tissue specific
CDS	exon	ID=cds-Bg	13	5.493077	4883.6	0.732728	GVAVREERF	58	511.2827	3	1530.827	.	A	Superscaf	18257767	BTP202209	0	0	0	0	0	Tissue specific
CDS	exon	ID=cds-Bg	10	5.942	4275	0.732728	VREERFEAL	85	435.5735	3	1303.7	.	A	Superscaf	18257767	BTP202209	0	0	0	0	0	Tissue specific
CDS	exon	ID=cds-Bg	13	5.97	5848.7	0.731568	ELLKQTEGE	96	523.933	3	1568.779	.	K	Superscaf	18337018	BTP202209	0	0	0	0	0	Tissue specific
CDS	exon	ID=cds-Bg	12	5.570833	5199.9	0.731568	ELLKQTEGE	97	471.8994	3	1412.678	.	K	Superscaf	18337018	BTP202209	0	0	0	0	0	Tissue specific
CDS	exon	ID=cds-Bg	13	4.986923	5217.9	0.731442	YEETQVEAK	94	539.9336	3	1616.779	.	F	Superscaf	18345583	BTP202209	0	0	0	0	0	Tissue specific
CDS	exon	ID=cds-Bg	8	6.705	2997.1	0.731378	KEPAAPPK	83	419.2411	2	836.4755	.	S	Superscaf	18350089	0	0	0	0	BTP202209	0	Tissue specific
CDS	exon	ID=cds-Bg	7	6.271429	2528.7	0.731378	EPAAPPK	71	355.1924	2	708.3806	.	S	Superscaf	18350089	0	0	0	0	BTP202209	0	Tissue specific
CDS	exon	ID=cds-Bg	7	5.942857	2374.5	0.731189	PEVPKKP	80	397.741	2	793.4697	.	N	Superscaf	18363034	BTP202209	0	0	0	0	0	Tissue specific
CDS	exon	ID=cds-Bg	7	5.042857	2037.1	0.731089	PEVPKKV	99	398.7488	2	795.4854	.	N	Superscaf	18369919	BTP202209	0	0	0	0	0	Tissue specific
CDS	exon	ID=cds-Bg	7	5.482857	1949	0.73093	VPKVPPK	81	382.7544	2	763.4955	.	P	Superscaf	18380752	BTP202209	0	0	0	0	0	Tissue specific
CDS	exon	ID=cds-Bg	8	5.13	2322.3	0.73089	AAVPEKKV	96	421.2596	2	840.5068	.	Q	Superscaf	18383452	BTP202209	0	0	0	0	0	Tissue specific
CDS	exon	ID=cds-Bg	6	4.458333	1801	0.730652	LDFAVP	77	661.3527	1	660.3483	.	S	Superscaf	18399625	0	0	0	0	0	BTP202209	Tissue specific
CDS	exon	ID=cds-Bg	7	6.358571	2949.2	0.729714	G(+42.01)	70	437.2257	2	872.4392	Acetylati	W	Superscaf	18463881	0	0	BTP202209	0	0	0	Tissue specific
CDS	exon	ID=cds-Bg	8	5.4125	3271.7	0.729162	QVAQEALR	98	457.7554	2	913.4981	.	K	Superscaf	18498733	BTP202209	0	0	0	0	0	Tissue specific
CDS	exon	ID=cds-Bg	6	7.516667	2618.8	0.728354	KKPLM(+15	59	385.2295	2	768.4316	Oxidatior	L	Superscaf	18556854	BTP202209	0	0	0	0	0	Tissue specific
CDS	exon	ID=cds-Bg	6	5.833333	2046.4	0.690232	LLLPVR	83	355.7487	2	709.485	.	G	Superscaf	21161112	0	BTP202209	BTP202209	0	0	0	Mixed
CDS	exon	ID=cds-Bg	10	5.903	3672.1	0.666317	HLFLTSKPM	55	586.8268	2	1171.642	.	I	Superscaf	22794634	0	0	0	0	0	BTP202209	Tissue specific
CDS	exon	ID=cds-Bg	11	5.880909	4131.6	0.666317	HLFLTSKPM	95	668.3579	2	1334.706	.	I	Superscaf	22794634	0	0	0	0	0	BTP202209	Tissue specific
CDS	exon	ID=cds-Bg	10	5.232	4049.8	0.636381	MTPEDFVQF	90	643.2959	2	1284.581	.	Y	Superscaf	24839671	0	0	0	0	0	BTP202209	Tissue specific
CDS	exon	ID=cds-Bg	6	5.92	2209.4	0.620575	QGGLGL	91	544.3073	1	543.3016	.	M	Superscaf	25919047	0	0	0	0	BTP202209	0	Tissue specific
CDS	exon	ID=cds-Bg	7	7.88	2909.1	0.605506	PRAKKPP	71	397.2527	2	792.4969	.	V	Superscaf	26948982	0	0	0	BTP202209	0	0	Tissue specific
CDS	exon	ID=cds-Bg	8	6.85125	2993.1	0.587821	M(+15.99)	59	425.2542	2	848.4789	Oxidatior	K	Superscaf	28156810	0	0	0	0	0	BTP202209	Tissue specific
CDS	exon	ID=cds-Bg	7	4.49	2142.4	0.581874	A(+42.01)	53	418.2239	2	834.4235	Acetylati	P	Superscaf	28563231	BTP202209	0	0	0	0	0	Tissue specific
CDS	exon	ID=cds-Bg	7	6.568571	2684.9	0.549894	T(+42.01)	72	426.2234	2	850.4225	Acetylati	N	Superscaf	30747876	BTP202209	0	0	0	0	0	Tissue specific
CDS	exon	ID=cds-Bg	8	6.405	3153.2	0.549671	KSFGDLSK	93	441.2389	2	880.4654	.	H	Superscaf	30762976	0	0	0	0	0	BTP202209	Tissue specific
CDS	exon	ID=cds-Bg	7	5.242857	2048.3	0.534759	FPPPPVA	80	724.4021	1	723.3955	.	L	Superscaf	31780102	0	0	BTP202209	0	0	0	Tissue specific
CDS	exon	ID=cds-Bg	7	5.25	2226.7	0.492576	T(+42.01)	64	424.744	2	847.4725	Acetylati	A	Superscaf	34663362	0	0	0	BTP202209	0	0	Tissue specific
CDS	exon	ID=cds-Bg	8	6.82875	3288.7	0.417991	LAPHALLR	95	445.7805	2	889.5497	.	Q	Superscaf	39758119	0	0	0	0	BTP202209	BTP202209	Mixed
CDS	exon	ID=cds-Bg	7	6.267143	2639.9	0.417992	LAPHALL	96	734.4548	1	733.4486	.	Q	Superscaf	39758119	0	BTP202209	0	BTP202209	BTP202209	BTP202209	Mixed
CDS	exon	ID=cds-Bg	6	6.853333	2401.8	0.358815	LPPFPR	99	363.7168	2	725.4224	.	L	Superscaf	43800912	0	0	0	BTP202209	0	0	Tissue specific
CDS	exon	ID=cds-Bg	10	5.25	4009.4	0.351832	HSELSELNV	90	578.3014	2	1154.593	.	A	Superscaf	44277784	0	0	0	0	0	BTP202209	Tissue specific
CDS	exon	ID=cds-Bg	8	5.7475	3616.2	0.346028	YNDSPELR	89	497.2342	2	992.4563	.	K	Superscaf	44674342	BTP202209	0	0	0	0	0	Tissue specific
CDS	exon	ID=cds-Bg	8	3.125	1925.3	0.344186	VDTPEVVL	73	871.4757	1	870.4698	.	L	Superscaf	44800060	BTP202209	0	0	0	0	0	Tissue specific
CDS	exon	ID=cds-Bg	13	5.853077	4477.8	0.343326	VTPALLHVK	81	480.9555	3	1439.85	.	K	Superscaf	44858911	BTP202209	0	0	0	0	0	Tissue specific
CDS	exon	ID=cds-Bg	7	5.397143	2899.3	0.168685	FLDNLYP	71	881.437	1	880.433	.	F	Superscaf	56789196	0	0	0	0	0	BTP202209	Tissue specific
CDS	exon	ID=cds-Bg	8	6.00125	3644.2	0.095576	G(+42.01)	67	495.7386	2	989.46	Acetylati	P	Superscaf	61783408	0	0	0	0	BTP202209	0	Tissue specific
CDS	exon	ID=cds-Bg	7	7.117143	2958.2	0.094256	KPSSSLR	61	387.7313	2	773.4395	.	I	Superscaf	61873405	0	0	0	BTP202209	0	0	Tissue specific
CDS	exon	ID=cds-Bg	7	5.267143	2548.8	0.086996	K(+42.01)	86	868.512	1	867.5065	Acetylati	L	Superscaf	62369080	BTP202209	BTP202209	BTP202209	0	0	0	Mixed
CDS	exon	ID=cds-Bg	9	4.156667	2239.4	0.073447	GTGVVELVK	61	451.2701	2	900.528	.	E	Superscaf	63294934	0	BTP202209	0	0	0	0	Tissue specific
CDS	exon	ID=cds-Bg	5	5.942	1728.9	0.046246	LPGLF	99	546.3264	1	545.3213	.	L	Superscaf	65153281	0	0	0	BTP202209	0	0	Tissue specific
CDS	exon	ID=cds-Bg	5	5.068	1971.1	0.046597	FFESF	98	676.2973	1	675.2904	.	V	Superscaf	71495479	0	BTP202209	0	0	0	0	Tissue specific
CDS	exon	ID=cds-Bg	8	4.90875	2463.8	0.066818	MAAVLEPL	72	843.4606	1	842.4572	.	A	Superscaf	72876904	0	0	0	0	0	BTP202209	Tissue specific
CDS	exon	ID=cds-Bg	6	6.185	2457.7	0.081166	LTKELY	96	383.7193	2	765.4272	.	K	Superscaf	73856278	0	0	0	0	0	BTP202209	Tissue specific
CDS	exon	ID=cds-Bg	12	6.3825	4717.8	0.081258	SKEDATKGK	99	664.8428	2	1327.677	.	P	Superscaf	73862989	0	0	0	0	0	BTP202209	Tissue specific
CDS	exon	ID=cds-Bg	17	4.200588	5171.7	0.17401	MDHEAESVL	61	920.478	2	1838.945	.	N	Superscaf	80199493	0	0	0	0	0	BTP202209	Tissue specific
CDS	exon	ID=cds-Bg	14	5.705	5767.1	0.174677	YEEKLEASE	98	576.9591	3	1727.862	.	F	Superscaf	80244988	0	BTP202209	0	0	0	0	Tissue specific
CDS	exon	ID=cds-Bg	10	4.042	2777.1	0.174729	SEVPLVDVT	63	543.8033	2	1085.597	.	S	Superscaf	80248513	0	0	0	0	0	BTP202209	Tissue specific
CDS	exon	ID=cds-Bg	9	3.86	2393.7	0.174729	EVPLVDVTK	89	500.2877	2	998.5648	.	S	Superscaf	80248513	0	0	0	0	0	BTP202209	Tissue specific
CDS	exon	ID=cds-Bg	8	6.49875	2785.9	0.247153	AAGPLLKL	56	782.5101	1	781.5062	.	N	Superscaf	85196005	0	0	BTP202209	0	0	0	Tissue specific
CDS	exon	ID=cds-Bg	12	6.571667	4854.2	0.26339	A(+42.01)	68	718.9027	2	1435.798	Acetylati	T	Superscaf	86305165	0	0	0	0	0	BTP202209	Tissue specific
CDS	exon	ID=cds-Bg	11	6.670909	4466.8	0.26339	A(+42.01)	67	645.3689	2	1288.729	Acetylati	T	Superscaf	86305165	0	0	0	0	0	BTP202209	Tissue specific
CDS	exon	ID=cds-Bg	10	6.74	4113.4	0.26339	A(+42.01)	51	588.8265	2	1175.645	Acetylati	T	Superscaf	86305165	0	0	0	0	BTP202209	0	Tissue specific
CDS	exon	ID=cds-Bg	16	4.934375	4579.7	0.263391	VLQATVVAV	99	750.9379	2	1499.867	.	T	Superscaf	86305165	0	0	0	0	0	BTP202209	Tissue specific
CDS	exon	ID=cds-Bg	9	6.14	3393.9	0.263602	A(+42.01)	61	507.7865	2	1013.562	Acetylati	S	Superscaf	86319481	0	BTP202209	0	0	0	0	Tissue specific
CDS	exon	ID=cds-Bg	13	6.772308	5426	0.306791	KTPEAPAHF	79	365.2003	4	1456.779	.	N	Superscaf	89269903	0	0	BTP202209	0	0	0	Tissue specific
CDS	exon	ID=cds-Bg	9	6.078889	3573.9	0.306791	EAPAHPEKF	76	488.2459	2	974.4821	.	N	Superscaf	89269903	0	0	BTP202209	0	0	0	Tissue specific
CDS	exon	ID=cds-Bg	9	5.287778	3222.6	0.307942	M(+42.01)	91	1008.47	1	1007.463	Acetylati	A	Superscaf	89348596	0	BTP202209	0	0	0	BTP202209	Mixed
CDS	exon	ID=cds-Bg	10	5.325	3682.1	0.307942	M(+15.99)	73	594.2667	2	1186.522	Oxidatior	A	Superscaf	89348596	0	BTP202209	0	0	0	0	Tissue specific
CDS	exon	ID=cds-Bg	8	5.26375	2835.2	0.307942	M(+42.01)	99	861.4017	1	860.395	Acetylati	A	Superscaf	89348596	0	BTP202209	BTP202209	0	BTP202209	BTP202209	Mixed
CDS	exon	ID=cds-Bg	12	6.334167	4662.1	0.308414	LTFVTSSRA	90	439.9125	3	1316.72	.	N	Superscaf	89380717	0	0	0	0	0	BTP202209	Tissue specific
CDS	exon	ID=cds-Bg	12	5.301667	4815.3	0.308679	NNAGAFLDE	53	649.305	2	1296.599	.	S	Superscaf	89398831	0	0	0	0	0	BTP202209	Tissue specific
CDS	exon	ID=cds-Bg	8	5.7175	2492.7	0.308722	KGLAMVPL	62	414.753	2	827.4939	.	V	Superscaf	89401855	0	0	0	0	0	BTP202209	Tissue specific
CDS	exon	ID=cds-Bg	7	5.53	2670.9	0.345535	WGELAGY	86	795.3643	1	794.3599	.	G	Superscaf	91916737	0	0	0	0	0	BTP202209	Tissue specific
CDS	exon	ID=cds-Bg	17	5.395882	6329.9	0.387006	GFGDLKSPA	88	897.4526	2	1792.899	.	P	Superscaf	94749643	0	0	0	0	0	BTP202209	Tissue specific
CDS	exon	ID=cds-Bg	13	6.290769	5249.8	0.407517	ALPTQPAEE	94	461.2524	3	1380.736	.	F	Superscaf	96150811	0	0	0	0	BTP202209	0	Tissue specific
CDS	exon	ID=cds-Bg	8	4.79875	2765.1	0.409777	LELFANVP	73	451.7555	2	901.4909	.	V	Superscaf	96305436	0	0	0	BTP202209	0	0	Tissue specific
CDS	exon	ID=cds-Bg	7	7.235714	2631.7	0.417988	KTPAAPK	60	356.7246	2	711.4279	.	A	Superscaf	96866796	0	0	0	0	0	BTP202209	Tissue specific
CDS	exon	ID=cds-Bg	8	6.04125	3468.9	0.432061	NQPLPDLK	73	462.7606	2	923.5076	.	V	Superscaf	97827289	0	BTP202209	0	0	0	0	Tissue specific
CDS	exon	ID=cds-Bg	12	5.533333	4637.9	0.432312	EKAQAKVGS	96	630.3318	2	1258.652	.	V	Superscaf	97844581	0	0	BTP202209	0	0	0	Tissue specific
CDS	exon	ID=cds-Bg	11	6.064545	4367.7	0.443928	EDGTKMKGY	99	631.7872	2	1261.565	.	A	Superscaf	98638258	0	0	BTP202209	0	0	0	Tissue specific
CDS	exon	ID=cds-Bg	8	5.2975	2792	0.443929	SFGHPSSV	75	409.1944	2	816.3766	.	A	Superscaf	98638258	0	0	BTP202209	0	0	0	Tissue specific
CDS	exon	ID=cds-Bg	9	3.561111	2188.3	0.443929	SSVAGEVVF	78	894.4535	1	893.4494	.	A	Superscaf	98638258	0	0	BTP202209	0	0	0	Tissue specific
CDS	exon	ID=cds-Bg	10	4.458	3066.4	0.443929	AGEVVFNTC	67	503.7627	2	1005.513	.	A	Superscaf	98638258	0	0	BTP202209	0	0	0	Tissue specific
CDS	exon	ID=cds-Bg	10	5.874	3813.2	0.44402	PEALTDPAY	98	552.7802	2	1103.55	.	I	Superscaf	98644471	0	0	BTP202209	0	0	BTP202209	Tissue enhanced
CDS	exon	ID=cds-Bg	10	5.841	3773.1	0.44402	EALTDPAYK	95	532.7653	2	1063.519	.	I	Superscaf	98644471	0	0	BTP202209	0	0	0	Tissue specific
CDS	exon	ID=cds-Bg	8	6.14625	3036.3	0.44402	LTDPAYKG	90	432.7252	2	863.4388	.	I	Superscaf	98644471	0	0	BTP202209	0	0	0	Tissue specific
CDS	exon	ID=cds-Bg	7	6.171429	2739	0.44402	LTDPAYK	99	404.214	2	806.4174	.	I	Superscaf	98644471	0	0	BTP202209	0	0	BTP202209	Tissue enhanced
CDS	exon	ID=cds-Bg	7	6.35	3032.4	0.444233	DRKEPLF	87	452.7464	2	903.4814	.	I	Superscaf	98659060	0	0	BTP202209	0	0	0	Tissue specific
CDS	exon	ID=cds-Bg	11	5.778182	3818	0.444233	TGLAAGAQV	98	539.7958	2	1077.582	.	I	Superscaf	98659060	0	0	BTP202209	0	0	0	Tissue specific
CDS	exon	ID=cds-Bg	9	5.673333	3123.2	0.444233	LAAGAQVYK	99	460.7622	2	919.5127	.	I	Superscaf	98659060	0	0	BTP202209	0	0	0	Tissue specific
CDS	exon	ID=cds-Bg	12	5.69	4207.5	0.444234	AAGAQVYKM	61	614.2953	2	1226.579	.	I	Superscaf	98659060	0	0	BTP202209	0	0	0	Tissue specific
CDS	exon	ID=cds-Bg	9	5.647778	3141.3	0.444234	AAGAQVYKM	98	477.7383	2	953.4641	Oxidatior	I	Superscaf	98659060	0	0	BTP202209	0	0	0	Tissue specific
CDS	exon	ID=cds-Bg	8	5.60125	2830	0.444234	AGAQVYKM	97	434.222	2	866.432	.	I	Superscaf	98659060	0	0	BTP202209	0	0	0	Tissue specific
CDS	exon	ID=cds-Bg	13	6.08	4948.4	0.444252	YALDSTLPA	99	709.8678	2	1417.724	.	C	Superscaf	98660275	0	0	BTP202209	0	0	0	Tissue specific
CDS	exon	ID=cds-Bg	13	6.104615	4842.3	0.444252	ALDSTLPAC	78	684.8774	2	1367.745	.	C	Superscaf	98660275	0	0	0	0	0	BTP202209	Tissue specific
CDS	exon	ID=cds-Bg	12	6.115	4488.9	0.444252	ALDSTLPAC	93	628.3358	2	1254.661	.	C	Superscaf	98660275	0	0	BTP202209	0	0	0	Tissue specific
CDS	exon	ID=cds-Bg	11	6.123636	4177.6	0.444252	LDSTLPAGW	99	592.8179	2	1183.624	.	C	Superscaf	98660275	0	0	BTP202209	0	0	0	Tissue specific
CDS	exon	ID=cds-Bg	14	5.589286	5161.7	0.444252	DSTLPAGWK	92	772.9067	2	1543.804	.	C	Superscaf	98660275	0	0	BTP202209	0	0	0	Tissue specific

CDS|exon|ID=cds-Bg	10	6.138	3824.2	0.444252	DSTLPAGWK	95	1071.543	1	1070.54	.	C	Superscaf	98660275	0	0	BTP202209	0	0	0	Tissue specific
CDS|exon|ID=cds-Bg	9	6.12	3486.8	0.444252	DSTLPAGWK	72	487.749	2	973.4869	.	C	Superscaf	98660275	0	0	BTP202209	0	0	0	Tissue specific
CDS|exon|ID=cds-Bg	9	6.49	3412.7	0.444252	STLPAGWKP	85	478.7622	2	955.5127	.	C	Superscaf	98660275	0	0	BTP202209	0	0	0	Tissue specific
CDS|exon|ID=cds-Bg	8	6.59125	3029.3	0.444252	TLPAGWKP	87	869.4839	1	868.4807	.	C	Superscaf	98660275	0	0	BTP202209	0	0	0	Tissue specific
CDS|exon|ID=cds-Bg	10	4.284	3950.6	0.444253	LFVNVNDQT	74	582.2866	2	1162.562	.	C	Superscaf	98660275	0	0	BTP202209	0	0	0	Tissue specific
CDS|exon|ID=cds-Bg	9	4.095556	3597.2	0.444253	FVNVNDQTN	68	525.7454	2	1049.478	.	C	Superscaf	98660275	0	0	BTP202209	0	0	0	Tissue specific
CDS|exon|ID=cds-Bg	11	5.148182	3848.4	0.444263	FHPEVGPGF	83	576.7682	2	1151.525	.	G	Superscaf	98661079	0	0	BTP202209	0	0	0	Tissue specific
CDS|exon|ID=cds-Bg	10	5.68	3814.2	0.445149	PNNLAVPLY	99	564.8221	2	1127.634	.	L	Superscaf	98721574	0	0	BTP202209	0	0	0	Tissue specific
CDS|exon|ID=cds-Bg	9	5.393333	4074.6	0.44565	NNTKFVHDN	91	544.7585	2	1087.505	.	M	Superscaf	98755777	0	0	BTP202209	0	0	0	Tissue specific
CDS|exon|ID=cds-Bg	10	6.196	3505.4	0.445662	KLFAEAVKK	96	368.8961	3	1103.67	.	T	Superscaf	98756668	0	0	BTP202209	0	0	0	Tissue specific
CDS|exon|ID=cds-Bg	8	5.775	2725.7	0.445662	LFAEAVKK	99	453.2747	2	904.5381	.	T	Superscaf	98756668	0	0	BTP202209	0	0	0	Tissue specific
CDS|exon|ID=cds-Bg	7	5.208571	2257.3	0.445662	LFAEAVK	99	389.228	2	776.4432	.	T	Superscaf	98756668	0	0	BTP202209	0	0	0	Tissue specific
CDS|exon|ID=cds-Bg	14	6.215714	6431.2	0.445663	RNVDSKSLF	85	453.982	4	1811.907	.	T	Superscaf	98756668	0	0	BTP202209	0	0	0	Tissue specific
CDS|exon|ID=cds-Bg	8	5.39	3007.2	0.445663	VDSKSLFH	99	466.7437	2	931.4763	.	T	Superscaf	98756668	0	0	BTP202209	0	0	0	Tissue specific
CDS|exon|ID=cds-Bg	11	5.926364	4726.2	0.445663	VDSKSLFHY	98	460.5728	3	1378.699	.	T	Superscaf	98756668	0	0	BTP202209	0	0	0	Tissue specific
CDS|exon|ID=cds-Bg	12	5.904167	5185.7	0.445663	VDSKSLFHY	98	771.8864	2	1541.763	.	T	Superscaf	98756668	0	0	BTP202209	0	0	0	Tissue specific
CDS|exon|ID=cds-Bg	10	5.954	4115.5	0.445663	VDSKSLFHY	99	417.8861	3	1250.641	.	T	Superscaf	98756668	0	0	BTP202209	0	0	0	Tissue specific
CDS|exon|ID=cds-Bg	9	5.42	3466.7	0.445663	VDSKSLFHY	97	548.2737	2	1094.54	.	T	Superscaf	98756668	0	0	BTP202209	0	0	0	Tissue specific
CDS|exon|ID=cds-Bg	9	7.084444	4223.7	0.445663	RQYRAGKEL	77	374.2111	3	1119.615	.	T	Superscaf	98756668	0	0	BTP202209	0	0	0	Tissue specific
CDS|exon|ID=cds-Bg	8	6.625	3574.9	0.445663	QYRAGKEL	97	482.7629	2	963.5137	.	T	Superscaf	98756668	0	0	BTP202209	0	0	0	Tissue specific
CDS|exon|ID=cds-Bg	6	2.368333	1308.5	0.456937	VGVVEC(+5	57	662.33	1	661.3105	Carbamido	E	Superscaf	99526666	0	BTP202209	0	0	0	0	Tissue specific
CDS|exon|ID=cds-Bg	7	2.56	1543.8	0.461596	VSDVDVP	78	730.3602	1	729.3544	.	Q	Superscaf	99845023	0	0	0	0	BTP202209	0	Tissue specific
CDS|exon|ID=cds-Bg	8	6.915	3976.6	0.517133	TNLRLFHH	90	519.2841	2	1036.557	.	E	Superscaf	1.04E+08	0	0	0	0	BTP202209	0	Tissue specific
CDS|exon|ID=cds-Bg	8	6.1375	3564.9	0.533791	YTC(+57.0	95	511.7066	2	1021.4	Carbamido	Q	Superscaf	1.05E+08	0	BTP202209	0	0	0	BTP202209	Mixed
CDS|exon|ID=cds-Bg	11	5.721818	4453.9	0.5599	LYDLENLPA	96	631.8327	2	1261.655	.	I	Superscaf	1.07E+08	0	0	0	0	0	BTP202209	Tissue specific
CDS|exon|ID=cds-Bg	14	6.535714	5276.5	0.562326	WLALGGKAF	99	488.2712	3	1461.798	.	A	Superscaf	1.07E+08	0	0	0	0	0	BTP202209	Tissue specific
CDS|exon|ID=cds-Bg	10	6.353	3715.9	0.562326	WLALGGKAF	95	538.2987	2	1074.586	.	A	Superscaf	1.07E+08	0	0	0	0	0	BTP202209	Tissue specific
CDS|exon|ID=cds-Bg	8	3.68	2667.1	0.562339	LLDVWDQV	86	494.2591	2	986.5073	.	W	Superscaf	1.07E+08	0	0	0	0	0	BTP202209	Tissue specific
CDS|exon|ID=cds-Bg	9	4.981111	3209.7	0.567224	VGPQMHVHL	90	509.2665	2	1016.523	.	T	Superscaf	1.07E+08	0	0	0	BTP202209	0	0	Tissue specific
CDS|exon|ID=cds-Bg	7	5.087143	2439.7	0.573733	YPAPQVS	81	761.3817	1	760.3755	.	A	Superscaf	1.08E+08	0	0	0	BTP202209	0	0	Tissue specific
CDS|exon|ID=cds-Bg	7	5.815714	3005.3	0.573743	LDHKFDL	98	444.2344	2	886.4548	.	A	Superscaf	1.08E+08	0	BTP202209	BTP202209	0	0	BTP202209	Mixed
CDS|exon|ID=cds-Bg	11	5.65	4406.8	0.575678	AYSSSQRVS	95	617.7864	2	1233.563	.	P	Superscaf	1.08E+08	0	0	0	0	0	BTP202209	Tissue specific
CDS|exon|ID=cds-Bg	9	5.607778	3636	0.575678	SSSQRVSSY	94	500.7367	2	999.4621	.	P	Superscaf	1.08E+08	0	0	0	0	BTP202209	BTP202209	Tissue enhanced
CDS|exon|ID=cds-Bg	8	5.59875	3252.6	0.575678	SSQRVSSY	99	457.2205	2	912.4301	.	P	Superscaf	1.08E+08	0	0	0	0	0	BTP202209	Tissue specific
CDS|exon|ID=cds-Bg	9	7.052222	3709.2	0.575679	RRTFGGAPS	77	474.7523	2	947.4937	.	P	Superscaf	1.08E+08	0	0	0	0	0	BTP202209	Tissue specific
CDS|exon|ID=cds-Bg	13	5.931538	4463.8	0.575679	FGGAPSFPL	76	623.8242	2	1245.639	.	P	Superscaf	1.08E+08	0	0	0	0	0	BTP202209	Tissue specific
CDS|exon|ID=cds-Bg	10	5.915	3389.6	0.575679	FGGAPSFPL	95	949.4742	1	948.4705	.	P	Superscaf	1.08E+08	0	0	0	0	BTP202209	0	Tissue specific
CDS|exon|ID=cds-Bg	9	5.908889	3092.3	0.575679	FGGAPSFPL	84	892.4524	1	891.449	.	P	Superscaf	1.08E+08	0	0	0	0	BTP202209	BTP202209	Mixed
CDS|exon|ID=cds-Bg	8	5.9	2738.9	0.575679	FGGAPSFP	80	779.3702	1	778.3649	.	P	Superscaf	1.08E+08	0	0	0	0	BTP202209	0	Tissue specific
CDS|exon|ID=cds-Bg	8	5.9625	2704.9	0.575679	GAPSFPLG	97	745.3868	1	744.3806	.	P	Superscaf	1.08E+08	BTP202209	0	0	0	BTP202209	BTP202209	Mixed
CDS|exon|ID=cds-Bg	10	5.969	3481.8	0.575679	APSFPLGSF	66	493.2697	2	984.528	.	P	Superscaf	1.08E+08	0	0	0	0	BTP202209	0	Tissue specific
CDS|exon|ID=cds-Bg	11	5.83	3849.3	0.575679	GSPLSSPVF	93	572.3095	2	1142.608	.	P	Superscaf	1.08E+08	0	0	0	0	0	BTP202209	Tissue specific
CDS|exon|ID=cds-Bg	10	5.816	3552	0.575679	SPLSSPVFF	98	543.7982	2	1085.587	.	P	Superscaf	1.08E+08	0	0	0	0	BTP202209	0	Tissue specific
CDS|exon|ID=cds-Bg	13	6.146923	4637	0.57568	SSPVFPRAG	94	675.8596	2	1349.709	.	P	Superscaf	1.08E+08	0	0	0	0	BTP202209	BTP202209	Tissue enhanced
CDS|exon|ID=cds-Bg	13	6.169231	4550.9	0.57568	SPVFPRAGF	85	660.8542	2	1319.699	.	P	Superscaf	1.08E+08	0	0	0	0	BTP202209	0	Tissue specific
CDS|exon|ID=cds-Bg	12	6.185833	4253.6	0.57568	SPVFPRAGF	94	421.8981	3	1262.677	.	P	Superscaf	1.08E+08	0	0	0	0	0	BTP202209	Tissue specific
CDS|exon|ID=cds-Bg	11	6.231818	3870.2	0.57568	PVFPRAGFG	96	588.8276	2	1175.645	.	P	Superscaf	1.08E+08	0	0	0	0	0	BTP202209	Tissue specific
CDS|exon|ID=cds-Bg	8	5.7475	2666.9	0.57568	VFPRAGFG	84	425.7302	2	849.4496	.	P	Superscaf	1.08E+08	0	0	0	0	0	BTP202209	Tissue specific
CDS|exon|ID=cds-Bg	15	6.091333	5419.8	0.57568	AGFGTKGSS	97	476.9066	3	1427.7	.	P	Superscaf	1.08E+08	0	BTP202209	0	0	0	BTP202209	Mixed
CDS|exon|ID=cds-Bg	18	5.704444	6490	0.57568	AGFGTKGSS	96	909.9492	2	1817.891	.	P	Superscaf	1.08E+08	0	0	0	0	BTP202209	BTP202209	Mixed
CDS|exon|ID=cds-Bg	13	6.106154	4811.2	0.57568	FGTKGSSSS	99	650.8253	2	1299.642	.	P	Superscaf	1.08E+08	0	0	0	0	0	BTP202209	Tissue specific
CDS|exon|ID=cds-Bg	16	5.668125	5881.4	0.57568	FGTKGSSSS	90	845.92	2	1689.832	.	P	Superscaf	1.08E+08	0	0	0	0	BTP202209	0	Tissue specific
CDS|exon|ID=cds-Bg	15	5.669333	5270.7	0.57568	FGTKGSSSS	89	781.8917	2	1561.774	.	P	Superscaf	1.08E+08	0	0	0	0	0	BTP202209	Tissue specific
CDS|exon|ID=cds-Bg	15	5.680667	5494	0.57568	GTKGSSSSV	93	772.3863	2	1542.764	.	P	Superscaf	1.08E+08	0	0	0	0	BTP202209	0	Tissue specific
CDS|exon|ID=cds-Bg	14	5.66	5196.7	0.57568	TKGSSSSVT	99	743.8763	2	1485.742	.	P	Superscaf	1.08E+08	0	0	0	0	BTP202209	0	Tissue specific
CDS|exon|ID=cds-Bg	14	4.903571	4714.2	0.57568	GSSSSVTSR	86	722.3549	2	1442.7	.	P	Superscaf	1.08E+08	0	0	0	0	0	BTP202209	Tissue specific
CDS|exon|ID=cds-Bg	11	5.181818	4033.5	0.57568	SSSSVTSRV	77	600.7963	2	1199.578	.	P	Superscaf	1.08E+08	BTP202209	0	0	0	0	BTP202209	Tissue enhanced
CDS|exon|ID=cds-Bg	10	5.132	3650.1	0.57568	SSSVTSRVY	94	557.275	2	1112.546	.	P	Superscaf	1.08E+08	0	0	0	0	0	BTP202209	Tissue specific
CDS|exon|ID=cds-Bg	9	5.071111	3266.7	0.57568	SSVTSRVYQ	86	513.7639	2	1025.514	.	P	Superscaf	1.08E+08	BTP202209	0	0	0	BTP202209	BTP202209	Mixed
CDS|exon|ID=cds-Bg	8	4.995	2883.3	0.57568	SVTSRVYQ	85	470.2477	2	938.4821	.	P	Superscaf	1.08E+08	BTP202209	0	0	0	BTP202209	BTP202209	Mixed
CDS|exon|ID=cds-Bg	16	6.181875	5888.5	0.575681	QVSRTSGGA	94	496.2725	3	1485.801	.	P	Superscaf	1.08E+08	0	0	0	0	0	BTP202209	Tissue specific
CDS|exon|ID=cds-Bg	13	5.857692	4575	0.575681	QVSRTSGGA	85	573.7941	2	1145.579	.	P	Superscaf	1.08E+08	0	0	0	0	BTP202209	0	Tissue specific
CDS|exon|ID=cds-Bg	17	6.174118	5972.5	0.575681	VSRTSGGAG	93	758.9107	2	1515.812	.	P	Superscaf	1.08E+08	0	0	0	0	0	BTP202209	Tissue specific
CDS|exon|ID=cds-Bg	15	6.217333	5277.8	0.575681	VSRTSGGAG	81	453.5862	3	1357.743	.	P	Superscaf	1.08E+08	0	0	0	0	BTP202209	BTP202209	Mixed
CDS|exon|ID=cds-Bg	12	5.875	3964.3	0.575681	VSRTSGGAG	81	509.7658	2	1017.52	.	P	Superscaf	1.08E+08	0	0	0	0	BTP202209	BTP202209	Mixed
CDS|exon|ID=cds-Bg	14	6.661429	5277.8	0.575681	SRTSGGAGG	97	630.3417	2	1258.674	.	P	Superscaf	1.08E+08	0	0	0	0	0	BTP202209	Tissue specific
CDS|exon|ID=cds-Bg	13	6.736923	4894.4	0.575681	RTSGGAGGL	97	586.8259	2	1171.642	.	P	Superscaf	1.08E+08	0	0	0	0	0	BTP202209	Tissue specific
CDS|exon|ID=cds-Bg	14	6.322857	4940.3	0.575681	TSGGAGGLG	89	587.8105	2	1173.61	.	P	Superscaf	1.08E+08	0	0	0	0	0	BTP202209	Tissue specific
CDS|exon|ID=cds-Bg	13	6.372308	4556.9	0.575681	TSGGAGGLG	98	544.2954	2	1086.578	.	P	Superscaf	1.08E+08	BTP202209	0	0	0	BTP202209	BTP202209	Mixed
CDS|exon|ID=cds-Bg	12	6.401667	4245.6	0.575681	TSGGAGGLG	98	508.7771	2	1015.541	.	P	Superscaf	1.08E+08	BTP202209	0	0	0	0	BTP202209	Tissue enhanced
CDS|exon|ID=cds-Bg	11	6.005455	3596.8	0.575681	TSGGAGGLG	96	860.4418	1	859.4399	.	P	Superscaf	1.08E+08	0	0	0	0	BTP202209	0	Tissue specific
CDS|exon|ID=cds-Bg	10	6.008	3243.4	0.575681	TSGGAGGLG	95	747.3602	1	746.3558	.	P	Superscaf	1.08E+08	0	0	0	0	BTP202209	0	Tissue specific
CDS|exon|ID=cds-Bg	12	6.359167	4159.4	0.575681	SGGAGGLGA	97	493.7714	2	985.5305	.	P	Superscaf	1.08E+08	BTP202209	0	0	0	BTP202209	BTP202209	Mixed
CDS|exon|ID=cds-Bg	11	6.39	3848.1	0.575681	SGGAGGLGA	99	458.2521	2	914.4933	.	P	Superscaf	1.08E+08	0	0	0	0	BTP202209	BTP202209	Mixed
CDS|exon|ID=cds-Bg	10	5.953	3199.3	0.575681	SGGAGGLGA	85	759.3953	1	758.3922	.	P	Superscaf	1.08E+08	0	0	0	0	BTP202209	BTP202209	Tissue enhanced
CDS|exon|ID=cds-Bg	11	6.767273	4410.1	0.575681	RASRLGSTR	86	400.5685	3	1198.69	.	P	Superscaf	1.08E+08	0	0	0	0	0	BTP202209	Tissue specific
CDS|exon|ID=cds-Bg	12	6.253333	4528.1	0.575681	ASRLGSTRV	81	406.5564	3	1216.652	.	P	Superscaf	1.08E+08	0	0	0	0	BTP202209	0	Tissue specific
CDS|exon|ID=cds-Bg	13	6.207692	4987.6	0.575681	ASRLGSTRV	97	690.8621	2	1379.716	.	P	Superscaf	1.08E+08	0	0	0	0	0	BTP202209	Tissue specific
CDS|exon|ID=cds-Bg	11	6.274545	4216.8	0.575681	SRLGSTRVP	84	573.8125	2	1145.615	.	P	Superscaf	1.08E+08	0	0	0	0	0	BTP202209	Tissue specific
CDS|exon|ID=cds-Bg	10	6.334	3833.4	0.575681	SRLGSTRVP	91	530.2969	2	1058.583	.	P	Superscaf	1.08E+08	0	0	0	0	0	BTP202209	Tissue specific
CDS|exon|ID=cds-Bg	10	6.334	3833.4	0.575681	RLGSTRVPS	95	530.2975	2	1058.583	.	P	Superscaf	1.08E+08	0	0	0	0	0	BTP202209	Tissue specific
CDS|exon|ID=cds-Bg	11	6.272727	4292.9	0.575681	RLGSTRVPS	89	611.8292	2	1221.647	.	P	Superscaf	1.08E+08	0	0	0	0	0	BTP202209	Tissue specific
CDS|exon|ID=cds-Bg	8	6.4975	3066.6	0.575681	RLGSTRVP	89	443.2639	2	884.5192	.	P	Superscaf	1.08E+08	0	0	0	0	0	BTP202209	Tissue specific
CDS|exon|ID=cds-Bg	9	5.842222	3184.6	0.575681	LGSTRVPSS	96	452.2465	2	902.4821	.	P	Superscaf	1.08E+08	0	0	0	0	0	BTP202209	Tissue specific
CDS|exon|ID=cds-Bg	8	5.8625	2801.2	0.575681	LGSTRVPS	87	408.7302	2	815.4501	.	P	Superscaf	1.08E+08	0	0	0	0	0	BTP202209	Tissue specific
CDS|exon|ID=cds-Bg	9	5.806667	3290.7	0.575681	GSTRVPSSY	95	477.2363	2	952.4614	.	P	Superscaf	1.08E+08	0	0	0	0	BTP202209	BTP202209	Mixed
CDS|exon|ID=cds-Bg	8	5.78625	2993.4	0.575682	STRVPSSY	97	448.7266	2	895.4399	.	P	Superscaf	1.08E+08	BTP202209	0	0	0	0	BTP202209	Mixed
CDS|exon|ID=cds-Bg	14	5.48	5059.7	0.575682	TRVPSSYGA	71	732.8676	2	1463.726	.	P	Superscaf	1.08E+08	0	0	0	0	BTP202209	0	Tissue specific
CDS|exon|ID=cds-Bg	14	5.405	5049.6	0.575682	RVPSSYGAG	88	755.8778	2	1509.746	.	P	Superscaf	1.08E+08	0	0	0	0	BTP202209	0	Tissue specific
CDS|exon|ID=cds-Bg	13	5.399231	4662.2	0.575682	RVPSSYGAG	74	682.3435	2	1362.678	.	P	Superscaf	1.08E+08	0	0	0	0	0	BTP202209	Tissue specific
CDS|exon|ID=cds-Bg	12	4.9525	4013.4	0.575682	VPSSYGAGE	96	1207.58	1	1206.577	.	P	Superscaf	1.08E+08	0	0	0	0	0	BTP202209	Tissue specific
CDS|exon|ID=cds-Bg	11	5.132727	3601.9	0.575682	VPSSYGAGE	83	546.7799	2	1091.55	.	P	Superscaf	1.08E+08	0	0	0	0	BTP202209	0	Tissue specific
CDS|exon|ID=cds-Bg	12	5.409167	4400.8	0.575682	PSSYGAGEL	81	628.2933	2	1254.577	.	P	Superscaf	1.08E+08	0	0	0	0	BTP202209	BTP202209	Mixed
CDS|exon|ID=cds-Bg	11	5.402727	4013.4	0.575682	PSSYGAGEL	84	554.7596	2	1107.508	.	P	Superscaf	1.08E+08	0	0	0	0	BTP202209	0	Tissue specific
CDS|exon|ID=cds-Bg	10	5.646	3601.9	0.575682	PSSYGAGEL	99	993.4847	1	992.4814	.	P	Superscaf	1.08E+08	0	0	0	0	BTP202209	0	Tissue specific
CDS|exon|ID=cds-Bg	9	5.608889	3248.5	0.575682	PSSYGAGEL	98	880.4012	1	879.3974	.	P	Superscaf	1.08E+08	0	0	0	0	BTP202209	BTP202209	Mixed
CDS|exon|ID=cds-Bg	11	5.328182	4063.4	0.575682	SSYGAGELL	93	579.7668	2	1157.524	.	P	Superscaf	1.08E+08	0	0	0	0	0	BTP202209	Tissue specific
CDS|exon|ID=cds-Bg	10	5.313	3676	0.575682	SSYGAGELL	98	1011.46	1	1010.456	.	P	Superscaf	1.08E+08	0	0	0	0	BTP202209	BTP202209	Mixed
CDS|exon|ID=cds-Bg	9	5.573333	3264.5	0.575682	SSYGAGELL	95	896.4337	1	895.4286	.	P	Superscaf	1.08E+08	0	0	0	0	BTP202209	0	Tissue specific
CDS|exon|ID=cds-Bg	10	5.293	3680	0.575682	SYGAGELLD	98	1071.497	1	1070.492	.	P	Superscaf	1.08E+08	0	0	0	0	BTP202209	BTP202209	Mixed
CDS|exon|ID=cds-Bg	9	5.25	3296.6	0.575682	YGAGELLDF	89	984.4673	1	983.46	.	P	Superscaf	1.08E+08	0	BTP202209	0	0	BTP202209	BTP202209	Mixed
CDS|exon|ID=cds-Bg	8	5.22125	2909.2	0.575682	YGAGELLD	90	837.3987	1	836.3915	.	P	Superscaf	1.08E+08	BTP202209	0	0	0	BTP202209	BTP202209	Mixed
CDS|exon|ID=cds-Bg	8	5.19875	2837.1	0.575682	GAGELLDF	75	821.4008	1	820.3967	.	P	Superscaf	1.08E+08	0	0	0	0	BTP202209	BTP202209	Mixed
CDS|exon|ID=cds-Bg	11	4.764545	4144.6	0.575682	SLADAVNQE	53	603.8004	2	1205.593	.	P	Superscaf	1.08E+08	0	0	0	0	BTP202209	BTP202209	Mixed
CDS|exon|ID=cds-Bg	10	4.643	3791.2	0.575682	SLADAVNQE	65	547.2617	2	1092.509	.	P	Superscaf	1.08E+08	0	BTP202209	0	0	BTP202209	BTP202209	Mixed
CDS|exon|ID=cds-Bg	9	4.55	3403.8	0.575682	SLADAVNQE	73	946.4441	1	945.4403	.	P	Superscaf	1.08E+08	0	0	0	0	0	BTP202209	Tissue specific
CDS|exon|ID=cds-Bg	8	4.71625	2978.3	0.575682	SLADAVNQ	93	817.4047	1	816.3977	.	P	Superscaf	1.08E+08	0	BTP202209	0	0	0	BTP202209	Tissue enhanced
CDS|exon|ID=cds-Bg	9	4.527778	3407.8	0.575682	LADAVNQEF	74	1006.48	1	1005.477	.	P	Superscaf	1.08E+08	0	0	0	0	0	BTP202209	Tissue specific
CDS|exon|ID=cds-Bg	8	4.34625	3054.4	0.575682	ADAVNQEF	51	447.2016	2	892.3926	.	P	Superscaf	1.08E+08	0	0	0	0	BTP202209	BTP202209	Mixed
CDS|exon|ID=cds-Bg	7	4.107143	2743.1	0.575682	DAVNQEF	80	822.3597	1	821.3555	.	P	Superscaf	1.08E+08	0	0	0	0	BTP202209	0	Tissue specific
CDS|exon|ID=cds-Bg	13	5.058462	5580.4	0.575683	VELQELNDF	74	805.8917	2	1609.774	.	P	Superscaf	1.08E+08	0	0	0	0	0	BTP202209	Tissue specific
CDS|exon|ID=cds-Bg	11	5.476364	4579	0.575701	LKEEAENNL	61	601.3043	2	1200.599	.	S	Superscaf	1.08E+08	0	0	0	0	0	BTP202209	Tissue specific
CDS|exon|ID=cds-Bg	11	5.430909	4613	0.575701	KEEAENNLA	72	618.2965	2	1234.583	.	S	Superscaf	1.08E+08	0	0	0	0	0	BTP202209	Tissue specific
CDS|exon|ID=cds-Bg	10	5	4144.6	0.575701	EEAENNLAA	60	554.249	2	1106.488	.	S	Superscaf	1.08E+08	0	0	0	0	0	BTP202209	Tissue specific
CDS|exon|ID=cds-Bg	10	5.807	4277.5	0.575709	SEAEEWYKS	94	628.7938	2	1255.572	.	K	Superscaf	1.08E+08	0	0	0	0	BTP202209	0	Tissue specific
CDS|exon|ID=cds-Bg	8	5.33125	3425.7	0.575709	SEAEEWYK	95	521.2277	2	1040.445	.	K	Superscaf	1.08E+08	0	0	0	0	0	BTP202209	Tissue specific
CDS|exon|ID=cds-Bg	19	5.892632	8153	0.575712	VSDLTQAAN	99	686.3596	3	2056.066	.	V	Superscaf	1.08E+08	0	0	0	0	0	BTP202209	Tissue specific
CDS|exon|ID=cds-Bg	17	5.658824	7373.3	0.575712	VSDLTQAAN	90	619.9832	3	1856.934	.	V	Superscaf	1.08E+08	0	0	0	0	0	BTP202209	Tissue specific
CDS|exon|ID=cds-Bg	16	5.659375	6762.6	0.575712	VSDLTQAAN	97	865.4424	2	1728.876	.	V	Superscaf	1.08E+08	0	0	0	0	0	BTP202209	Tissue specific
CDS|exon|ID=cds-Bg	10	5.4	3844.2	0.575712	VSDLTQAAN	95	523.7761	2	1045.54	.	V	Superscaf	1.08E+08	0	0	0	0	0	BTP202209	Tissue specific
CDS|exon|ID=cds-Bg	14	5.842857	6376.3	0.575712	NDALRQAKG	63	863.8949	2	1725.782	.	V	Superscaf	1.08E+08	0	0	0	0	0	BTP202209	Tissue specific
CDS|exon|ID=cds-Bg	12	5.318333	4954.7	0.575765	ETSPEQRGS	98	678.3105	2	1354.611	.	K	Superscaf	1.08E+08	0	0	0	0	BTP202209	0	Tissue specific
CDS|exon|ID=cds-Bg	15	5.988667	6289	0.575765	ETSPEQRGS	98	571.6222	3	1711.849	.	K	Superscaf	1.08E+08	0	0	0	0	BTP202209	0	Tissue specific
CDS|exon|ID=cds-Bg	14	5.720714	5820.6	0.575765	ETSPEQRGS	87	528.9231	3	1583.754	.	K	Superscaf	1.08E+08	0	0	0	0	BTP202209	BTP202209	Mixed
CDS|exon|ID=cds-Bg	13	5.913077	5395.1	0.575765	TSPEQRGSE	95	485.9089	3	1454.711	.	K	Superscaf	1.08E+08	0	0	0	0	0	BTP202209	Tissue specific
CDS|exon|ID=cds-Bg	12	5.594167	4926.7	0.575765	TSPEQRGSE	86	664.3129	2	1326.617	.	K	Superscaf	1.08E+08	0	0	0	0	0	BTP202209	Tissue specific
CDS|exon|ID=cds-Bg	11	4.473636	4395	0.575769	VVSEATQQG	99	628.2971	2	1254.584	.	L	Superscaf	1.08E+08	0	0	0	0	BTP202209	0	Tissue specific
CDS|exon|ID=cds-Bg	10	4.599	3969.5	0.575769	VVSEATQQG	97	563.7758	2	1125.542	.	L	Superscaf	1.08E+08	0	0	0	0	BTP202209	0	Tissue specific
CDS|exon|ID=cds-Bg	6	5.778333	2143.4	0.577764	WTVTLK	74	747.4406	1	746.4327	.	P	Superscaf	1.08E+08	0	0	BTP202209	0	0	BTP202209	Mixed
CDS|exon|ID=cds-Bg	8	6.24625	3122.6	0.607753	LLDRPLAL	62	455.7948	2	909.5647	.	C	Superscaf	1.1E+08	0	BTP202209	BTP202209	0	0	BTP202209	Mixed
CDS|exon|ID=cds-Bg	6	5.091667	1692.9	0.620548	SGPVPP	76	553.2969	1	552.2907	.	A	Superscaf	1.11E+08	0	0	BTP202209	0	0	0	Tissue specific
CDS|exon|ID=cds-Bg	8	5.375	3058.5	0.640684	VGRNLSEL	56	444.2596	2	886.4872	.	V	Superscaf	1.12E+08	0	BTP202209	0	0	0	0	Tissue specific

CDS|exon|ID=cds-Bg	9	5.943333	3171.4	0.697759	STPEVKPLK	97	499.796	2	997.5807	.	L	Superscaf	1.16E+08	0	0	0	0	BTP202209	0	Tissue specific
CDS|exon|ID=cds-Bg	8	6.17875	3209.4	0.700086	KDFDTALK	99	469.2511	2	936.4916	.	S	Superscaf	1.16E+08	0	0	0	0	0	BTP202209	Tissue specific
CDS|exon|ID=cds-Bg	13	6.262308	5479.8	0.700086	DFDTALKHY	85	517.9299	3	1550.773	.	S	Superscaf	1.16E+08	0	0	0	0	0	BTP202209	Tissue specific
CDS|exon|ID=cds-Bg	12	6.139167	4600.9	0.700095	AAALEAMKD	98	656.3295	2	1310.654	.	E	Superscaf	1.16E+08	0	0	0	0	0	BTP202209	Tissue specific
CDS|exon|ID=cds-Bg	11	5.473636	4613.1	0.744497	HLGAFEESL	63	640.2977	2	1278.588	.	R	Superscaf	1.19E+08	0	0	0	0	0	BTP202209	Tissue specific
CDS|exon|ID=cds-Bg	7	3.96	1785	0.746361	GVTAELV	66	688.3857	1	687.3803	.	I	Superscaf	1.19E+08	0	0	0	0	BTP202209	0	Tissue specific
CDS|exon|ID=cds-Bg	7	3.14	1628.8	0.749229	VDVVDPK	69	386.2141	2	770.4174	.	E	Superscaf	1.19E+08	0	0	0	0	0	BTP202209	Tissue specific
CDS|exon|ID=cds-Bg	11	5.233636	3915	0.752599	KDLKEKKEV	97	672.8881	2	1343.766	.	V	Superscaf	1.2E+08	0	0	BTP202209	0	0	0	Tissue specific
CDS|exon|ID=cds-Bg	16	5.151875	6151.7	0.752599	DLKEKKEVV	99	624.9921	3	1871.959	.	V	Superscaf	1.2E+08	0	BTP202209	0	0	0	0	Tissue specific
CDS|exon|ID=cds-Bg	10	4.783	3446.6	0.752599	DLKEKKEVV	93	608.8405	2	1215.671	.	V	Superscaf	1.2E+08	0	0	BTP202209	0	BTP202209	0	Mixed
CDS|exon|ID=cds-Bg	15	5.297333	5740.2	0.752599	LKEKKEVVE	97	586.6503	3	1756.932	.	V	Superscaf	1.2E+08	0	BTP202209	0	0	0	0	Tissue specific
CDS|exon|ID=cds-Bg	9	4.984444	3035.1	0.752599	LKEKKEVVE	86	551.3276	2	1100.644	.	V	Superscaf	1.2E+08	0	0	BTP202209	0	0	0	Tissue specific
CDS|exon|ID=cds-Bg	14	5.248571	5386.8	0.752599	KEKKEVVEE	98	548.9554	3	1643.848	.	V	Superscaf	1.2E+08	0	BTP202209	0	0	0	0	Tissue specific
CDS|exon|ID=cds-Bg	16	4.859375	6006.7	0.752599	KEVVEEAEN	92	871.4198	2	1740.828	.	V	Superscaf	1.2E+08	0	BTP202209	0	0	0	0	Tissue specific
CDS|exon|ID=cds-Bg	11	4.616364	4024.5	0.752599	KEVVEEAEN	93	420.5442	3	1258.615	.	V	Superscaf	1.2E+08	0	BTP202209	0	0	0	0	Tissue specific
CDS|exon|ID=cds-Bg	10	4.104	3556.1	0.752599	EVVEEAENG	97	566.267	2	1130.52	.	V	Superscaf	1.2E+08	0	BTP202209	0	0	0	0	Tissue specific
CDS|exon|ID=cds-Bg	14	4.627857	5112.8	0.752599	VVEEAENGK	88	742.8512	2	1483.69	.	V	Superscaf	1.2E+08	0	BTP202209	0	0	0	0	Tissue specific
CDS|exon|ID=cds-Bg	11	5.422727	4458.2	0.771009	HTQTTPLVE	69	676.327	2	1350.646	.	E	Superscaf	1.21E+08	0	0	0	0	0	BTP202209	Tissue specific
CDS|exon|ID=cds-Bg	11	5.411818	3378.6	0.792007	KGPELPLVP	85	588.8688	2	1175.728	.	V	Superscaf	1.22E+08	0	0	0	0	0	BTP202209	Tissue specific
CDS|exon|ID=cds-Bg	10	6.285	4183.5	0.843705	RDDGKEALK	92	393.5424	3	1177.609	.	L	Superscaf	1.26E+08	0	BTP202209	0	0	0	0	Tissue specific
CDS|exon|ID=cds-Bg	9	5.787778	3534.7	0.843705	DDGKEALKP	96	511.7607	2	1021.508	.	L	Superscaf	1.26E+08	0	BTP202209	0	0	0	0	Tissue specific
CDS|exon|ID=cds-Bg	8	5.47	3223.6	0.843706	YTDPSYFF	67	1039.437	1	1038.433	.	L	Superscaf	1.26E+08	0	0	0	0	BTP202209	0	Tissue specific
CDS|exon|ID=cds-Bg	8	6.4575	3043.2	0.855788	QAAKLLPG	51	399.2474	2	796.4807	.	P	Superscaf	1.27E+08	BTP202209	0	0	0	0	0	Tissue specific
CDS|exon|ID=cds-Bg	14	5.977143	5113.4	0.862876	ESVPEFPLS	80	528.2975	3	1581.877	.	S	Superscaf	1.27E+08	0	0	0	0	BTP202209	0	Tissue specific
CDS|exon|ID=cds-Bg	13	6.189231	4687.9	0.862876	SVPEFPLSP	81	485.2839	3	1452.834	.	S	Superscaf	1.27E+08	0	BTP202209	0	0	0	0	Tissue specific
CDS|exon|ID=cds-Bg	12	5.09	3618.8	0.891486	A(+42.01)	84	603.8193	2	1205.629	Acetylati	P	Superscaf	1.29E+08	0	0	0	0	0	BTP202209	Tissue specific
CDS|exon|ID=cds-Bg	9	4.677778	2413.6	0.891486	VLVTGGAGY	93	836.4481	1	835.444	.	P	Superscaf	1.29E+08	0	0	0	0	0	BTP202209	Tissue specific
CDS|exon|ID=cds-Bg	7	4.614286	2164.3	0.89151	HVVDLAK	99	391.231	2	780.4493	.	G	Superscaf	1.29E+08	0	0	0	0	0	BTP202209	Tissue specific
CDS|exon|ID=cds-Bg	8	6.265	3246.7	0.897397	ALLERTGY	92	461.7513	2	921.4919	.	A	Superscaf	1.3E+08	0	0	0	0	0	BTP202209	Tissue specific
CDS|exon|ID=cds-Bg	10	6.066	3982.6	0.897398	TLDVTTGQF	96	559.8099	2	1117.609	.	A	Superscaf	1.3E+08	0	0	0	0	0	BTP202209	Tissue specific
CDS|exon|ID=cds-Bg	9	5.517778	3847.4	0.897733	EDYYYHPPF	84	590.7495	2	1179.487	.	S	Superscaf	1.3E+08	0	BTP202209	0	0	0	0	Tissue specific
CDS|exon|ID=cds-Bg	7	4.935714	2958.3	0.897735	DYYGYDY	92	958.3436	1	957.3392	.	S	Superscaf	1.3E+08	0	BTP202209	0	0	0	0	Tissue specific
CDS|exon|ID=cds-Bg	6	3.896667	2389	0.901125	DDDMDM	51	741.1958	1	740.1993	.	E	Superscaf	1.3E+08	0	0	0	BTP202209	0	0	Tissue specific
CDS|exon|ID=cds-Bg	10	5.75	4060.6	0.919583	VTSPQGFQF	98	583.7996	2	1165.588	.	E	Superscaf	1.31E+08	0	0	0	0	0	BTP202209	Tissue specific
CDS|exon|ID=cds-Bg	9	5.193333	3411.8	0.919583	VTSPQGFQF	58	505.749	2	1009.487	.	E	Superscaf	1.31E+08	0	0	0	0	0	BTP202209	Tissue specific
CDS|exon|ID=cds-Bg	9	5.885556	3162.6	0.919695	FTRVPGGPY	98	497.2596	2	992.5079	.	C	Superscaf	1.31E+08	0	0	0	0	0	BTP202209	Tissue specific
CDS|exon|ID=cds-Bg	11	5.853636	4679.1	0.919809	GNFAHLGHE	93	608.2784	2	1214.547	.	H	Superscaf	1.31E+08	0	0	0	0	0	BTP202209	Tissue specific
CDS|exon|ID=cds-Bg	12	5.8375	5138.6	0.919809	GNFAHLGHE	70	689.8094	2	1377.61	.	H	Superscaf	1.31E+08	0	0	0	0	0	BTP202209	Tissue specific
CDS|exon|ID=cds-Bg	8	5.94125	3397.7	0.919809	AHLGHESF	94	449.212	2	896.4141	.	H	Superscaf	1.31E+08	0	0	0	0	0	BTP202209	Tissue specific
CDS|exon|ID=cds-Bg	9	5.91	3857.2	0.919809	AHLGHESFY	67	530.7441	2	1059.477	.	H	Superscaf	1.31E+08	0	0	0	0	0	BTP202209	Tissue specific
CDS|exon|ID=cds-Bg	8	5.61125	3570.1	0.91981	YWQLPETY	78	1099.507	1	1098.502	.	H	Superscaf	1.31E+08	0	0	0	0	0	BTP202209	Tissue specific
CDS|exon|ID=cds-Bg	8	5.40125	2269.5	0.920095	AAPGPVPP	74	353.2041	2	704.3857	.	G	Superscaf	1.31E+08	0	0	0	0	BTP202209	0	Tissue specific
CDS|exon|ID=cds-Bg	5	3.584	1245.4	0.920171	V(+42.01)	50	541.2929	1	540.2908	Acetylati	V	Superscaf	1.31E+08	BTP202209	0	0	0	0	0	Tissue specific
CDS|exon|ID=cds-Bg	6	6.025	2590	0.920342	FTQTPY	92	756.3547	1	755.349	.	D	Superscaf	1.31E+08	0	BTP202209	0	0	0	0	Tissue specific
CDS|exon|ID=cds-Bg	11	5.18	3688.9	0.920393	SGGKSGPVE	89	540.2513	2	1078.493	.	A	Superscaf	1.31E+08	0	0	0	0	0	BTP202209	Tissue specific
CDS|exon|ID=cds-Bg	12	4.803333	3800.2	0.920394	VSLAMVGGE	96	630.3242	2	1258.638	.	A	Superscaf	1.31E+08	0	0	0	0	0	BTP202209	Tissue specific
CDS|exon|ID=cds-Bg	9	5.58	3495.8	0.920461	DAPGQYGAY	57	941.3953	1	940.3926	.	S	Superscaf	1.31E+08	0	0	0	0	BTP202209	0	Tissue specific
CDS|exon|ID=cds-Bg	7	6.265714	2806.9	0.926954	C(+57.02)	64	401.6891	2	801.348	Carbamido	H	Superscaf	1.32E+08	BTP202209	0	0	0	0	0	Tissue specific
CDS|exon|ID=cds-Bg	16	5.655	6015.7	0.933436	A(+42.01)	95	853.441	2	1704.868	Acetylati	M	Superscaf	1.32E+08	0	BTP202209	0	0	0	0	Tissue specific
CDS|exon|ID=cds-Bg	14	5.585714	5324.9	0.933436	A(+42.01)	98	748.3705	2	1494.731	Acetylati	M	Superscaf	1.32E+08	0	BTP202209	BTP202209	0	0	0	Tissue enhanced
CDS|exon|ID=cds-Bg	13	5.552308	5013.6	0.933436	A(+42.01)	99	712.8531	2	1423.694	Acetylati	M	Superscaf	1.32E+08	0	BTP202209	BTP202209	BTP202209	BTP202209	BTP202209	Mixed
CDS|exon|ID=cds-Bg	12	5.203333	4545.2	0.933436	A(+42.01)	96	648.8062	2	1295.599	Acetylati	M	Superscaf	1.32E+08	0	BTP202209	BTP202209	BTP202209	BTP202209	BTP202209	Mixed
CDS|exon|ID=cds-Bg	11	5.103636	4207.8	0.933436	A(+42.01)	66	600.2791	2	1198.547	Acetylati	M	Superscaf	1.32E+08	0	0	BTP202209	0	BTP202209	0	Mixed
CDS|exon|ID=cds-Bg	11	6.791818	4587.2	0.933507	QTPMASSPF	87	400.5448	3	1198.613	.	S	Superscaf	1.32E+08	0	BTP202209	0	0	0	0	Tissue specific
CDS|exon|ID=cds-Bg	7	6.514286	3238.8	0.933553	SLELERR	63	451.7548	2	901.4981	.	A	Superscaf	1.32E+08	0	BTP202209	0	0	0	0	Tissue specific
CDS|exon|ID=cds-Bg	10	4.422	2841.9	0.933585	ASGSFVVVQ	79	511.2868	2	1020.56	.	V	Superscaf	1.32E+08	0	BTP202209	0	0	0	0	Tissue specific
CDS|exon|ID=cds-Bg	9	4.244444	2530.6	0.933585	SGSFVVVQK	99	475.7683	2	949.5233	.	V	Superscaf	1.32E+08	0	BTP202209	0	0	0	0	Tissue specific
CDS|exon|ID=cds-Bg	8	4.2725	2590.9	0.933596	VDPEPQVK	93	456.2435	2	910.476	.	F	Superscaf	1.32E+08	0	0	0	0	BTP202209	0	Tissue specific
CDS|exon|ID=cds-Bg	10	6.247	4336.9	0.933597	TRLC(+57.	61	595.7944	2	1189.576	Carbamido	F	Superscaf	1.32E+08	0	0	0	0	BTP202209	0	Tissue specific
CDS|exon|ID=cds-Bg	11	6.193636	4796.4	0.933597	TRLC(+57.	85	677.3251	2	1352.639	Carbamido	F	Superscaf	1.32E+08	0	0	0	0	BTP202209	0	Tissue specific
CDS|exon|ID=cds-Bg	7	6.792857	3216.7	0.933597	TRLC(+57.	78	452.2373	2	902.4644	Carbamido	F	Superscaf	1.32E+08	0	0	0	0	0	BTP202209	Tissue specific
CDS|exon|ID=cds-Bg	15	6.681333	5723.1	0.933869	TPVKSPGKA	99	393.2269	4	1568.882	.	L	Superscaf	1.32E+08	0	BTP202209	0	0	0	0	Tissue specific
CDS|exon|ID=cds-Bg	10	6.64	3584.5	0.933872	KEVKLPGKG	97	542.3459	2	1082.681	.	L	Superscaf	1.32E+08	0	0	0	0	BTP202209	BTP202209	Mixed
CDS|exon|ID=cds-Bg	9	6.295556	3116.1	0.933872	EVKLPGKGK	99	478.2993	2	954.5862	.	L	Superscaf	1.32E+08	0	BTP202209	0	0	BTP202209	BTP202209	Mixed
CDS|exon|ID=cds-Bg	8	5.865	2647.7	0.933872	EVKLPGKG	91	414.2522	2	826.4912	.	L	Superscaf	1.32E+08	0	BTP202209	0	0	BTP202209	BTP202209	Mixed
CDS|exon|ID=cds-Bg	8	5.08375	3284.7	0.935064	GEFLYDNL	69	970.448	1	969.4443	.	R	Superscaf	1.32E+08	0	0	0	BTP202209	0	0	Tissue specific
CDS|exon|ID=cds-Bg	9	5.334444	3165.4	0.935242	LSDGKWVPF	92	524.775	2	1047.539	.	V	Superscaf	1.32E+08	0	0	0	0	0	BTP202209	Tissue specific
CDS|exon|ID=cds-Bg	7	2.514286	1559.8	0.942851	DVSVVLD	64	746.3923	1	745.3857	.	R	Superscaf	1.33E+08	BTP202209	0	0	0	0	0	Tissue specific
CDS|exon|ID=cds-Bg	9	5.451111	3388.6	0.954789	KNDLVEALK	98	515.2982	2	1028.587	.	H	Superscaf	1.34E+08	0	0	0	0	0	BTP202209	Tissue specific
CDS|exon|ID=cds-Bg	11	5.951818	4648.1	0.95479	NDLVEALKF	79	438.5881	3	1312.746	.	H	Superscaf	1.34E+08	0	BTP202209	0	0	0	0	Tissue specific
CDS|exon|ID=cds-Bg	8	4.915	2920.2	0.95479	NDLVEALK	97	451.253	2	900.4916	.	H	Superscaf	1.34E+08	0	BTP202209	0	0	BTP202209	BTP202209	Tissue enhanced
CDS|exon|ID=cds-Bg	16	6.26375	6245.1	0.95522	GTSAPSMTS	84	865.4023	2	1728.796	.	R	Superscaf	1.34E+08	0	0	0	0	0	BTP202209	Tissue specific
CDS|exon|ID=cds-Bg	16	5.78	7220.3	0.955234	DQAWRLVAH	70	683.3227	3	2046.948	Carbamido	R	Superscaf	1.34E+08	0	0	0	0	0	BTP202209	Tissue specific
CDS|exon|ID=cds-Bg	12	5.995833	5360.2	0.955234	RLVAHDC(+	87	516.588	3	1546.746	Carbamido	R	Superscaf	1.34E+08	0	0	BTP202209	0	0	BTP202209	Mixed
CDS|exon|ID=cds-Bg	11	5.562727	4711.4	0.955234	LVAHDC(+5	97	464.5538	3	1390.645	Carbamido	R	Superscaf	1.34E+08	0	0	0	BTP202209	0	0	Tissue specific
CDS|exon|ID=cds-Bg	10	5.521	4358	0.955234	VAHDC(+57	84	426.8596	3	1277.561	Carbamido	R	Superscaf	1.34E+08	0	0	BTP202209	BTP202209	0	0	Mixed
CDS|exon|ID=cds-Bg	8	6.14875	4046.7	0.955234	HDC(+57.0	99	554.7338	2	1107.456	Carbamido	R	Superscaf	1.34E+08	0	0	BTP202209	BTP202209	BTP202209	0	Mixed
CDS|exon|ID=cds-Bg	7	5.942857	3427	0.955234	DC(+57.02	97	486.2037	2	970.3967	Carbamido	R	Superscaf	1.34E+08	0	0	BTP202209	0	0	0	Tissue specific
CDS|exon|ID=cds-Bg	8	6.08	3825.5	0.96144	DWRYPYMQ	78	579.7539	2	1157.496	.	L	Superscaf	1.34E+08	0	0	BTP202209	0	0	0	Tissue specific
CDS|exon|ID=cds-Bg	7	4.25	1686.8	0.967487	VGAPVLF	60	702.4167	1	701.4112	.	I	Superscaf	1.34E+08	0	0	BTP202209	0	0	0	Tissue specific
CDS|exon|ID=cds-Bg	7	6.094286	2877.1	0.968576	LGGHAFQ	56	365.1858	2	728.3605	.	P	Superscaf	1.34E+08	0	0	BTP202209	0	0	0	Tissue specific
CDS|exon|ID=cds-Bg	6	6.218333	2473.7	0.971521	NHAAGP	58	566.2761	1	565.2609	.	G	Superscaf	1.35E+08	0	0	BTP202209	0	0	0	Tissue specific
CDS|exon|ID=cds-Bg	6	4.176667	2054.4	0.983919	Q(+42.01)	50	373.2016	2	744.3766	Acetylati	A	Superscaf	1.36E+08	0	0	0	BTP202209	0	0	Tissue specific
CDS|exon|ID=cds-Bg	12	6.315	4890.3	0.987597	MMATYKEKG	94	679.8233	2	1357.637	.	P	Superscaf	1.36E+08	0	0	0	0	0	BTP202209	Tissue specific
CDS|exon|ID=cds-Bg	11	6.366364	4518.8	0.987597	MATYKEKQA	95	614.3042	2	1226.596	.	P	Superscaf	1.36E+08	0	0	BTP202209	0	0	0	Tissue specific
CDS|exon|ID=cds-Bg	7	6.02	2685	0.994686	M(+42.01)	98	851.3999	1	850.3929	Acetylati	A	Superscaf	1.36E+08	BTP202209	0	0	0	0	0	Tissue specific
CDS|exon|ID=cds-Bg	5	4.296	1559.8	0.911203	PVYEP	72	604.2963	1	603.2904	.	S	Superscaf	3194944	0	BTP202209	0	0	0	0	Tissue specific
CDS|exon|ID=cds-Bg	9	5.532222	2633	0.879147	PGPVAPPPF	78	828.4601	1	827.4541	.	P	Superscaf	4348237	0	0	0	BTP202209	0	0	Tissue specific
CDS|exon|ID=cds-Bg	11	5.449091	4594.9	0.871229	VYEDKSGKG	97	672.8224	2	1343.636	.	K	Superscaf	4633177	0	0	0	0	0	BTP202209	Tissue specific
CDS|exon|ID=cds-Bg	6	3.988333	1371.5	0.848939	LVVGSP	98	571.3424	1	570.3377	.	V	Superscaf	5435200	0	0	0	BTP202209	0	0	Tissue specific
CDS|exon|ID=cds-Bg	16	6.380625	6314.7	0.840963	RKVSSAEGA	90	581.6533	3	1741.943	.	L	Superscaf	5722075	0	0	BTP202209	0	0	0	Tissue specific
CDS|exon|ID=cds-Bg	15	6.088667	5665.9	0.840964	RKVSSAEGA	96	397.4667	4	1585.842	.	L	Superscaf	5722075	0	BTP202209	0	0	0	BTP202209	Mixed
CDS|exon|ID=cds-Bg	15	6.088667	5665.9	0.840963	KVSSAEGAA	98	397.4667	4	1585.842	.	L	Superscaf	5722075	0	BTP202209	0	0	0	0	Tissue specific
CDS|exon|ID=cds-Bg	14	5.755	5017.1	0.840964	KVSSAEGAA	99	477.587	3	1429.741	.	L	Superscaf	5722075	0	BTP202209	0	0	BTP202209	BTP202209	Mixed
CDS|exon|ID=cds-Bg	15	6.156667	5846.3	0.840963	VSSAEGAAK	91	538.9564	3	1613.848	.	L	Superscaf	5722075	0	BTP202209	0	0	0	0	Tissue specific
CDS|exon|ID=cds-Bg	14	5.827857	5197.5	0.840963	VSSAEGAAK	99	486.9215	3	1457.747	.	L	Superscaf	5722075	0	0	0	0	BTP202209	BTP202209	Mixed
CDS|exon|ID=cds-Bg	13	5.448462	4548.7	0.840964	VSSAEGAAK	99	651.8275	2	1301.646	.	L	Superscaf	5722075	0	0	0	0	0	BTP202209	Tissue specific
CDS|exon|ID=cds-Bg	13	6.276154	5197.5	0.840963	SSAEGAAKE	98	453.8989	3	1358.679	.	L	Superscaf	5722075	0	0	0	0	BTP202209	0	Tissue specific
CDS|exon|ID=cds-Bg	18	6.655	6723	0.840962	SARLSAKPA	82	470.5311	4	1878.105	.	L	Superscaf	5722075	0	0	0	0	0	BTP202209	Tissue specific
CDS|exon|ID=cds-Bg	16	6.755625	6028.3	0.840962	RLSAKPAPA	96	431.0154	4	1720.036	.	L	Superscaf	5722075	0	BTP202209	0	0	0	BTP202209	Mixed
CDS|exon|ID=cds-Bg	15	6.488667	5379.5	0.840962	LSAKPAPAK	98	391.9904	4	1563.935	.	L	Superscaf	5722075	0	BTP202209	0	0	0	BTP202209	Mixed
CDS|exon|ID=cds-Bg	14	6.525	5026.1	0.840962	SAKPAPAKV	93	484.6234	3	1450.851	.	L	Superscaf	5722075	0	BTP202209	0	0	BTP202209	0	Mixed
CDS|exon|ID=cds-Bg	13	6.59	4642.7	0.840962	AKPAPAKVE	99	455.6123	3	1363.819	.	L	Superscaf	5722075	0	0	0	0	0	BTP202209	Tissue specific
CDS|exon|ID=cds-Bg	18	6.672778	6599.6	0.840961	PAPAKVETK	99	622.0366	3	1863.094	.	L	Superscaf	5722075	0	0	0	0	BTP202209	0	Tissue specific
CDS|exon|ID=cds-Bg	15	4.901333	5811.5	0.840959	AEVANQETK	71	822.3895	2	1642.769	.	L	Superscaf	5722075	0	BTP202209	0	0	0	0	Tissue specific
CDS|exon|ID=cds-Bg	7	6.31	2703.9	0.809357	RVQLAAK	56	393.2466	2	784.4919	.	P	Superscaf	6859345	0	BTP202209	0	0	0	0	Tissue specific
CDS|exon|ID=cds-Bg	6	6.083333	2054.4	0.776791	LTGPGM	57	575.281	1	574.2785	.	N	Superscaf	8031300	BTP202209	0	0	0	0	0	Tissue specific
CDS|exon|ID=cds-Bg	7	5.361429	2344.6	0.567667	LLVALLH	99	389.7604	2	777.5112	.	A	Superscaf	15555670	0	0	0	0	0	BTP202209	Tissue specific
CDS|exon|ID=cds-Bg	13	4.921538	4407.8	0.393189	VVDNGSGMC	98	671.2991	2	1340.585	Carbamido	R	Superscaf	21833476	0	BTP202209	0	0	BTP202209	BTP202209	Mixed
CDS|exon|ID=cds-Bg	10	4.651	3411.8	0.39319	VVDNGSGMC	99	533.7344	2	1065.458	Carbamido	R	Superscaf	21833476	0	0	0	0	BTP202209	BTP202209	Mixed
CDS|exon|ID=cds-Bg	11	5.816364	4407.8	0.393189	DNGSGMC(+	93	572.23	2	1142.449	Carbamido	R	Superscaf	21833476	0	0	0	0	BTP202209	0	Tissue specific
CDS|exon|ID=cds-Bg	8	6.20375	3102.3	0.393189	SGMC(+57.	89	429.1837	2	856.3571	Carbamido	R	Superscaf	21833476	0	0	0	0	BTP202209	0	Tissue specific
CDS|exon|ID=cds-Bg	9	4.72	3609.2	0.393184	VTNWDDMEK	95	569.2466	2	1136.481	.	R	Superscaf	21833476	0	BTP202209	0	0	BTP202209	BTP202209	Mixed
CDS|exon|ID=cds-Bg	8	5.41875	3132.6	0.393183	YNELRVAP	97	481.2585	2	960.5029	.	R	Superscaf	21833476	BTP202209	0	0	0	BTP202209	0	Mixed
CDS|exon|ID=cds-Bg	11	4.591818	3553.1	0.393178	DSGDGVTHT	92	542.7472	2	1083.483	.	R	Superscaf	21833476	0	0	0	0	BTP202209	0	Tissue specific
CDS|exon|ID=cds-Bg	10	4.754	3141.6	0.393178	SGDGVTHTV	90	485.2333	2	968.4563	.	R	Superscaf	21833476	0	0	0	0	BTP202209	0	Tissue specific
CDS|exon|ID=cds-Bg	9	5.871111	3644.2	0.393175	AGRDLTDYL	82	512.257	2	1022.503	.	R	Superscaf	21833476	0	0	0	0	0	BTP202209	Tissue specific
CDS|exon|ID=cds-Bg	8	5.8575	3290.8	0.393176	AGRDLTDY	94	910.4239	1	909.4192	.	R	Superscaf	21833476	0	0	0	0	BTP202209	BTP202209	Mixed
CDS|exon|ID=cds-Bg	8	5.8525	3332.9	0.393175	GRDLTDYL	65	476.7393	2	951.4661	.	R	Superscaf	21833476	0	0	0	0	BTP202209	BTP202209	Mixed
CDS|exon|ID=cds-Bg	7	5.834286	2979.5	0.393176	GRDLTDY	80	420.1965	2	838.3821	.	R	Superscaf	21833476	0	0	0	0	BTP202209	BTP202209	Mixed
CDS|exon|ID=cds-Bg	7	6.087143	2815.2	0.393175	LTDYLMK	99	442.2317	2	882.4521	.	R	Superscaf	21833476	0	0	0	0	0	BTP202209	Tissue specific
CDS|exon|ID=cds-Bg	8	6.3475	3770.4	0.39317	ERFRC(+57	95	532.7487	2	1063.487	Carbamido	R	Superscaf	21833476	0	0	0	0	BTP202209	BTP202209	Mixed
CDS|exon|ID=cds-Bg	8	4.81875	2883.3	0.393166	DLYANTVL	81	908.4686	1	907.4651	.	R	Superscaf	21833476	0	0	0	0	0	BTP202209	Tissue specific
CDS|exon|ID=cds-Bg	12	5.35	4176.6	0.389485	ESVPEFPLS	79	663.8484	2	1325.687	.	S	Superscaf	21966754	0	0	0	0	0	BTP202209	Tissue specific
CDS|exon|ID=cds-Bg	9	5.062222	2899.3	0.389486	VPEFPLSPP	78	982.5176	1	981.5171	.	S	Superscaf	21966754	0	0	0	0	BTP202209	0	Tissue specific
CDS|exon|ID=cds-Bg	8	6.125	3030.3	0.389485	EFPLSPPK	86	457.7516	2	913.4908	.	S	Superscaf	21966754	0	0	0	0	0	BTP202209	Tissue specific
CDS|exon|ID=cds-Bg	7	5.6	2565.8	0.3478	E(+42.01)	55	776.346	1	775.3388	Acetylati	E	Superscaf	23466742	0	BTP202209	0	BTP202209	0	0	Mixed
CDS|exon|ID=cds-Bg	8	5.38625	3191.6	0.206567	TSSQVHYP	76	459.7183	2	917.4243	.	G	Superscaf	28548451	0	0	0	0	0	BTP202209	Tissue specific
CDS|exon|ID=cds-Bg	9	4.668889	3256.5	0.121932	SFEVEKELF	91	564.2841	2	1126.555	.	Q	Superscaf	31593849	0	0	0	0	0	BTP202209	Tissue specific
CDS|exon|ID=cds-Bg	11	4.964545	4588.2	0.090042	VGENAEFEF	89	671.3042	2	1340.6	.	R	Superscaf	32740930	0	0	0	0	0	BTP202209	Tissue specific
CDS|exon|ID=cds-Bg	6	6.236667	2018.3	0.024586	P(+42.01)	63	581.3041	1	580.2856	Acetylati	S	Superscaf	35096143	0	0	0	0	BTP202209	0	Tissue specific
CDS|exon|ID=cds-Bg	7	6.254286	2306.3	0.032722	K(+42.01)	79	384.7328	2	767.4541	Acetylati	S	Superscaf	37158238	0	0	0	0	BTP202209	0	Tissue specific

CDS|exon|ID=cds-Bg	7	6.602857	3324.5	0.045756	C(+57.02)	79	482.2125	2	962.428	Carbamido	I	Superscaf	37627341	0	BTP202209	0	0	0	0	Tissue specific
CDS|exon|ID=cds-Bg	8	5.25875	2335.5	0.106784	AGLAPVPF	85	771.4373	1	770.4326	.	Q	Superscaf	39822961	0	0	0	0	0	BTP202209	Tissue specific
CDS|exon|ID=cds-Bg	7	6.314286	2874.1	0.107073	LSSFMWK	96	449.7271	2	897.4418	.	E	Superscaf	39833488	0	0	0	0	0	BTP202209	Tissue specific
CDS|exon|ID=cds-Bg	7	4.251429	1833	0.151685	YFVVPAP	92	396.7211	2	791.4218	.	P	Superscaf	41438620	BTP202209	0	0	0	0	0	Tissue specific
CDS|exon|ID=cds-Bg	8	5.62125	2885.9	0.519502	VSSFDKSK	55	449.2385	2	896.4603	.	S	Superscaf	54672850	0	0	0	0	BTP202209	0	Tissue specific
CDS|exon|ID=cds-Bg	8	5.58	3102.3	0.850156	FGFKEDPF	92	493.7317	2	985.4545	.	R	Superscaf	66570373	0	0	0	0	0	BTP202209	Tissue specific
CDS|exon|ID=cds-Bg	8	4.635	2735	0.861292	GDLVEELK	97	451.7427	2	901.4756	.	R	Superscaf	66970894	0	0	0	0	0	BTP202209	Tissue specific
CDS|exon|ID=cds-Bg	6	6.935	2311.7	0.983954	PGRLPP	61	636.3802	1	635.3754	.	E	Superscaf	71384254	0	0	BTP202209	0	0	0	Tissue specific
CDS|exon|ID=cds-Bg	14	6.986429	5815.3	0.985899	A(+42.01)	81	516.9536	3	1547.842	Acetylati	L	Superscaf	71454307	0	BTP202209	0	0	0	0	Tissue specific
CDS|exon|ID=cds-Bg	7	6.824286	2898.2	0.985899	A(+42.01)	98	406.7155	2	811.4188	Acetylati	L	Superscaf	71454307	0	0	0	0	BTP202209	0	Tissue specific
CDS|exon|ID=cds-Bg	17	6.842941	6540.7	0.9859	KTPPKSPGE	87	438.2495	4	1748.978	.	L	Superscaf	71454307	0	0	0	0	BTP202209	0	Tissue specific
CDS|exon|ID=cds-Bg	14	6.753571	5449.7	0.9859	KTPPKSPGE	62	370.7084	4	1478.809	.	L	Superscaf	71454307	0	0	0	0	0	BTP202209	Tissue specific
CDS|exon|ID=cds-Bg	12	6.305	5037.5	0.986049	TDTSKFTGS	96	669.3169	2	1336.626	.	D	Superscaf	71459674	0	0	0	0	BTP202209	0	Tissue specific
CDS|exon|ID=cds-Bg	11	4.651818	3539.8	0.986051	VDESGYVPC	98	607.2897	2	1212.566	.	D	Superscaf	71459674	0	BTP202209	0	0	BTP202209	0	Mixed
CDS|exon|ID=cds-Bg	10	5.117	3539.8	0.986052	DESGYVPGY	91	557.7542	2	1113.498	.	D	Superscaf	71459674	0	BTP202209	0	0	0	0	Tissue specific
CDS|exon|ID=cds-Bg	12	5.8225	4629.9	0.986052	AGTYDQKVG	98	626.3176	2	1250.626	.	D	Superscaf	71459674	0	0	0	0	0	BTP202209	Tissue specific
CDS|exon|ID=cds-Bg	11	5.804545	4318.6	0.986053	GTYDQKVQC	94	590.7985	2	1179.588	.	D	Superscaf	71459674	0	0	0	0	0	BTP202209	Tissue specific
CDS|exon|ID=cds-Bg	10	5.788	4021.3	0.986053	TYDQKVQGC	59	562.2889	2	1122.567	.	D	Superscaf	71459674	0	0	0	0	0	BTP202209	Tissue specific
CDS|exon|ID=cds-Bg	12	6.08	4604.8	0.929331	LGKHEKFPE	95	712.9138	2	1423.819	.	D	Superscaf	2509635	0	0	0	0	0	BTP202209	Tissue specific
CDS|exon|ID=cds-Bg	9	5.696667	3485.7	0.929331	HEKFPEVLK	99	563.8138	2	1125.618	.	D	Superscaf	2509635	0	0	0	0	0	BTP202209	Tissue specific
CDS|exon|ID=cds-Bg	8	5.19125	3017.3	0.929331	HEKFPEVL	75	499.7667	2	997.5233	.	D	Superscaf	2509635	0	0	0	0	0	BTP202209	Tissue specific
CDS|exon|ID=cds-Bg	9	6.176667	3796.1	0.879771	E(+42.01)	89	542.2828	2	1082.561	Acetylati	L	Superscaf	4269463	0	BTP202209	0	0	0	BTP202209	Tissue enhanced
CDS|exon|ID=cds-Bg	7	7.021429	2945.1	0.879771	KNPLPSK	99	392.2384	2	782.465	.	L	Superscaf	4269463	0	0	0	0	BTP202209	0	Tissue specific
CDS|exon|ID=cds-Bg	7	5.412857	2286.5	0.758162	AAPVLHL	60	360.7175	2	719.433	.	A	Superscaf	8587762	0	0	BTP202209	0	0	0	Tissue specific
CDS|exon|ID=cds-Bg	7	5.625714	2654.8	0.739759	EFLDAGK	96	390.2041	2	778.386	.	I	Superscaf	9241627	0	0	0	0	0	BTP202209	Tissue specific
CDS|exon|ID=cds-Bg	7	4.398571	1640.8	0.688426	GTVPVGA	63	600.3337	1	599.3279	.	E	Superscaf	11064493	0	BTP202209	0	0	0	0	Tissue specific
CDS|exon|ID=cds-Bg	8	3.88625	2425.8	0.588287	VEVVQRLF	80	495.2908	2	988.5706	.	Y	Superscaf	14620648	0	0	BTP202209	0	0	0	Tissue specific
CDS|exon|ID=cds-Bg	7	3.522857	1530.6	0.392266	VVDGVLK	75	365.2278	2	728.4432	.	V	Superscaf	21581641	BTP202209	0	0	0	0	BTP202209	Mixed
CDS|exon|ID=cds-Bg	7	6.891429	3130.8	0.372582	RMEALMR	90	453.7276	2	905.4575	.	F	Superscaf	22280740	0	0	0	0	BTP202209	0	Tissue specific
CDS|exon|ID=cds-Bg	7	5.941429	2665	0.372412	LWLPLFL	51	451.2889	2	900.5473	.	C	Superscaf	22286536	BTP202209	0	0	0	0	0	Tissue specific
CDS|exon|ID=cds-Bg	8	6.93625	3212.7	0.350491	GPRGHPPP	66	407.7168	2	813.4245	.	D	Superscaf	23065303	0	0	0	BTP202209	0	0	Tissue specific
CDS|exon|ID=cds-Bg	13	5.21	4159.6	0.276208	QPPTVVPGC	97	639.8543	2	1277.698	.	A	Superscaf	25702711	0	0	BTP202209	0	0	0	Tissue specific
CDS|exon|ID=cds-Bg	12	5.173333	3548.9	0.276208	PPTVVPGGI	99	575.8247	2	1149.639	.	A	Superscaf	25702711	0	0	0	0	BTP202209	0	Tissue specific
CDS|exon|ID=cds-Bg	8	7	2975	0.263124	KAGASTPK	52	380.2212	2	758.4286	.	W	Superscaf	26167831	0	BTP202209	0	0	0	0	Tissue specific
CDS|exon|ID=cds-Bg	8	5.86625	2426.6	0.22178	GPPVAPKP	93	381.7275	2	761.4435	.	N	Superscaf	27635728	0	0	0	0	BTP202209	0	Tissue specific
CDS|exon|ID=cds-Bg	7	5.65	2471.8	0.219806	PDPSLPA	54	696.354	1	695.3489	.	E	Superscaf	27706129	0	BTP202209	0	0	0	0	Tissue specific
CDS|exon|ID=cds-Bg	7	6.292857	3050.4	0.212713	G(+42.01)	91	454.7182	2	907.4399	Acetylati	M	Superscaf	27958293	0	0	0	0	0	BTP202209	Tissue specific
CDS|exon|ID=cds-Bg	8	5.15875	2431.8	0.204922	VATSRPLV	76	421.7578	2	841.5021	.	D	Superscaf	28234573	BTP202209	0	0	0	0	0	Tissue specific
CDS|exon|ID=cds-Bg	6	5.088333	1976.3	0.163395	LPPVPQ	89	650.3844	1	649.3799	.	E	Superscaf	29709238	0	0	0	BTP202209	0	0	Tissue specific
CDS|exon|ID=cds-Bg	9	6.343333	3694.1	0.11845	PNFAGTWKM	83	526.255	2	1050.496	.	A	Superscaf	31305442	0	BTP202209	0	0	0	0	Tissue specific
CDS|exon|ID=cds-Bg	5	3.76	1032.2	0.094422	T(+42.01)	83	514.2858	1	513.2798	Acetylati	V	Superscaf	32158863	0	0	BTP202209	0	BTP202209	0	Tissue enhanced
CDS|exon|ID=cds-Bg	11	6.426364	5267.7	0.081442	KHYAMQEAK	73	454.5569	3	1360.656	.	K	Superscaf	32619643	0	0	0	0	0	BTP202209	Tissue specific
CDS|exon|ID=cds-Bg	7	5.912857	2876.3	0.046471	N(+42.01)	94	444.2499	2	886.4872	Acetylati	N	Superscaf	33861481	0	0	BTP202209	0	0	0	Tissue specific
CDS|exon|ID=cds-Bg	8	5.10375	2843.4	0.035353	VEPTQPTP	94	868.4371	1	867.4338	.	V	Superscaf	34256332	0	0	0	0	BTP202209	0	Tissue specific
CDS|exon|ID=cds-Bg	7	6.771429	2610.9	0.011336	A(+42.01)	71	369.2229	2	736.4232	Acetylati	L	Superscaf	35108401	0	0	0	BTP202209	0	0	Tissue specific
CDS|exon|ID=cds-Bg	6	4.31	1907.2	0.00823	M(+15.99)	63	769.3423	1	768.3364	Oxidation	T	Superscaf	35219778	BTP202209	0	0	0	0	0	Tissue specific
CDS|exon|ID=cds-Bg	17	5.936471	6757.5	0.174899	LEESSGMEN	95	880.4137	2	1758.821	.	D	Superscaf	41722942	0	0	0	0	0	BTP202209	Tissue specific
CDS|exon|ID=cds-Bg	9	6.205556	3446.7	0.220325	T(+42.01)	86	509.2813	2	1016.55	Acetylati	N	Superscaf	43335931	BTP202209	0	0	0	0	0	Tissue specific
CDS|exon|ID=cds-Bg	10	5.456	3746.2	0.295087	AHLVALAEF	96	547.7999	2	1093.588	.	T	Superscaf	45990757	0	0	BTP202209	BTP202209	0	0	Mixed
CDS|exon|ID=cds-Bg	9	5.393333	3434.9	0.295087	HLVALAERD	84	512.2812	2	1022.551	.	T	Superscaf	45990757	0	BTP202209	0	0	BTP202209	0	Mixed
CDS|exon|ID=cds-Bg	8	5.11875	2815.2	0.295087	LVALAERD	96	443.7517	2	885.4919	.	T	Superscaf	45990757	0	0	0	0	BTP202209	BTP202209	Mixed
CDS|exon|ID=cds-Bg	12	6.565	5095.4	0.361479	KTC(+57.0	88	445.5738	3	1333.702	Carbamido	H	Superscaf	48348825	0	BTP202209	0	0	0	0	Tissue specific
CDS|exon|ID=cds-Bg	15	5.635333	5696.2	0.396872	A(+42.01)	81	575.9989	3	1724.979	Acetylati	S	Superscaf	49605604	0	BTP202209	0	0	0	BTP202209	Tissue enhanced
CDS|exon|ID=cds-Bg	10	6.152	3343.8	0.400266	GPPFPPPPF	64	480.2521	2	958.4912	.	N	Superscaf	49726039	0	0	BTP202209	0	0	0	Tissue specific
CDS|exon|ID=cds-Bg	9	6.172222	3046.5	0.400266	GPPFPPPPF	78	451.7414	2	901.4697	.	N	Superscaf	49726039	0	0	BTP202209	0	0	0	Tissue specific
CDS|exon|ID=cds-Bg	7	5.484286	2303.6	0.554111	VPEPTKP	72	384.218	2	766.4225	.	S	Superscaf	55189680	BTP202209	0	0	0	0	0	Tissue specific
CDS|exon|ID=cds-Bg	10	5.391	3419.7	0.570904	FLFDKPVSF	97	581.8265	2	1161.643	.	P	Superscaf	55785847	0	0	0	0	0	BTP202209	Tissue specific
CDS|exon|ID=cds-Bg	9	5.381111	3032.3	0.570904	LFDKPVSPI	94	508.2926	2	1014.575	.	P	Superscaf	55785847	0	0	0	0	0	BTP202209	Tissue specific
CDS|exon|ID=cds-Bg	8	5.30625	2678.9	0.570904	FDKPVSPL	96	451.751	2	901.4909	.	P	Superscaf	55785847	0	0	0	0	0	BTP202209	Tissue specific
CDS|exon|ID=cds-Bg	7	5.21	2325.5	0.570904	FDKPVSP	88	395.2091	2	788.4068	.	P	Superscaf	55785847	0	0	0	0	BTP202209	BTP202209	Mixed
CDS|exon|ID=cds-Bg	14	4.775	5589.5	0.572166	SDVLELTDI	90	820.3785	2	1638.737	.	A	Superscaf	55830184	0	0	0	0	0	BTP202209	Tissue specific
CDS|exon|ID=cds-Bg	9	5.444444	3797	0.572533	KSEEEFEKF	84	391.5191	3	1171.54	.	D	Superscaf	55843666	0	0	0	0	0	BTP202209	Tissue specific
CDS|exon|ID=cds-Bg	14	5.875714	5766.1	0.572577	FKDLFSEAH	99	849.4254	2	1696.846	.	I	Superscaf	55845217	0	0	0	0	0	BTP202209	Tissue specific
CDS|exon|ID=cds-Bg	9	5.795556	3748	0.572577	FKDLFSEAH	90	547.268	2	1092.524	.	I	Superscaf	55845217	0	0	0	0	0	BTP202209	Tissue specific
CDS|exon|ID=cds-Bg	8	5.835	3360.6	0.572577	KDLFSEAH	94	473.7322	2	945.4556	.	I	Superscaf	55845217	0	0	0	0	0	BTP202209	Tissue specific
CDS|exon|ID=cds-Bg	12	5.825833	5165.8	0.572672	MTEDNKDLI	98	464.5646	3	1390.676	.	K	Superscaf	55848610	0	0	0	0	0	BTP202209	Tissue specific
CDS|exon|ID=cds-Bg	10	5.139	4012.3	0.572674	YDVDYEKNA	99	622.7909	2	1243.572	.	K	Superscaf	55848610	0	0	0	0	0	BTP202209	Tissue specific
CDS|exon|ID=cds-Bg	8	6.955	3166.1	0.57269	KFLDAGKK	98	453.7722	2	905.5334	.	I	Superscaf	55849243	0	0	0	0	0	BTP202209	Tissue specific
CDS|exon|ID=cds-Bg	10	6.495	4058.2	0.57269	FLDAGKKLH	99	392.5555	3	1174.65	.	I	Superscaf	55849243	0	0	0	0	0	BTP202209	Tissue specific
CDS|exon|ID=cds-Bg	9	6.607778	3670.8	0.57269	FLDAGKKLH	99	514.7963	2	1027.581	.	I	Superscaf	55849243	0	0	0	0	0	BTP202209	Tissue specific
CDS|exon|ID=cds-Bg	10	6.701	3952.1	0.57269	KLHFAVASR	90	578.8484	2	1155.688	.	I	Superscaf	55849243	0	0	0	0	0	BTP202209	Tissue specific
CDS|exon|ID=cds-Bg	12	6.585	4737	0.57269	KLHFAVASR	82	351.9568	4	1403.804	.	I	Superscaf	55849243	0	0	0	0	0	BTP202209	Tissue specific
CDS|exon|ID=cds-Bg	9	6.363333	3483.7	0.57269	LHFAVASRK	99	514.8019	2	1027.593	.	I	Superscaf	55849243	0	0	0	0	0	BTP202209	Tissue specific
CDS|exon|ID=cds-Bg	11	6.298182	4268.6	0.57269	LHFAVASRK	88	638.8585	2	1275.709	.	I	Superscaf	55849243	0	0	0	0	0	BTP202209	Tissue specific
CDS|exon|ID=cds-Bg	8	5.94125	3015.3	0.57269	LHFAVASR	83	450.7532	2	899.4977	.	I	Superscaf	55849243	0	0	0	0	0	BTP202209	Tissue specific
CDS|exon|ID=cds-Bg	8	6.41125	3130.3	0.57269	HFAVASRK	95	458.2593	2	914.5086	.	I	Superscaf	55849243	0	0	0	0	0	BTP202209	Tissue specific
CDS|exon|ID=cds-Bg	10	5.835	4217.6	0.572691	KTFSHELSI	98	605.7881	2	1209.567	.	I	Superscaf	55849243	0	0	0	0	0	BTP202209	Tissue specific
CDS|exon|ID=cds-Bg	9	5.874444	3830.2	0.572691	KTFSHELSI	76	532.2566	2	1062.498	.	I	Superscaf	55849243	0	0	0	0	0	BTP202209	Tissue specific
CDS|exon|ID=cds-Bg	9	5.401111	3749.2	0.572691	TFSHELSDF	95	541.7415	2	1081.472	.	I	Superscaf	55849243	0	0	0	0	0	BTP202209	Tissue specific
CDS|exon|ID=cds-Bg	8	5.39125	3361.8	0.572691	TFSHELSD	95	468.2076	2	934.4032	.	I	Superscaf	55849243	0	0	0	0	0	BTP202209	Tissue specific
CDS|exon|ID=cds-Bg	8	5.26	3351.7	0.572691	FSHELSDF	75	491.2181	2	980.424	.	I	Superscaf	55849243	0	0	0	0	0	BTP202209	Tissue specific
CDS|exon|ID=cds-Bg	9	5.476667	3776.3	0.572718	ALERFLEDY	95	578.2859	2	1154.561	.	S	Superscaf	55850200	0	0	0	0	0	BTP202209	Tissue specific
CDS|exon|ID=cds-Bg	10	5.338	4164.6	0.572719	LEDYFDGNI	90	607.2892	2	1212.566	.	S	Superscaf	55850200	0	0	0	0	0	BTP202209	Tissue specific
CDS|exon|ID=cds-Bg	10	6.769	4444.8	0.572719	FDGNLKRYI	90	418.5697	3	1252.693	.	S	Superscaf	55850200	0	0	0	0	0	BTP202209	Tissue specific
CDS|exon|ID=cds-Bg	7	5.417143	2592	0.572756	PSPYEVR	92	424.2176	2	846.4235	.	Q	Superscaf	55851574	0	0	0	BTP202209	0	0	Tissue specific
CDS|exon|ID=cds-Bg	13	6.99	5894.1	0.572767	FSPANKKQN	96	388.2158	4	1548.841	.	S	Superscaf	55851967	0	0	0	0	0	BTP202209	Tissue specific
CDS|exon|ID=cds-Bg	11	6.860909	4966.2	0.572767	FSPANKKQN	95	629.847	2	1257.683	.	S	Superscaf	55851967	0	0	0	0	0	BTP202209	Tissue specific
CDS|exon|ID=cds-Bg	15	4.614667	6087.9	0.634191	YEDELSEVI	84	904.4099	2	1806.816	.	L	Superscaf	58033078	0	0	0	0	0	BTP202209	Tissue specific
CDS|exon|ID=cds-Bg	13	4.661538	5065.7	0.634191	YEDELSEVI	96	782.8697	2	1563.731	.	L	Superscaf	58033078	0	0	0	0	0	BTP202209	Tissue specific
CDS|exon|ID=cds-Bg	18	4.935556	7171	0.634191	EDELSEVLE	94	1035.493	2	2068.98	.	L	Superscaf	58033078	0	0	0	0	0	BTP202209	Tissue specific
CDS|exon|ID=cds-Bg	12	4.578333	4606.2	0.634191	EDELSEVLE	88	701.3384	2	1400.667	.	L	Superscaf	58033078	0	0	0	0	0	BTP202209	Tissue specific
CDS|exon|ID=cds-Bg	17	5.036471	6745.5	0.634191	DELSEVLEK	93	970.9712	2	1939.938	.	L	Superscaf	58033078	0	0	0	0	0	BTP202209	Tissue specific
CDS|exon|ID=cds-Bg	11	4.701818	4180.7	0.634191	DELSEVLEK	72	636.8176	2	1271.625	.	L	Superscaf	58033078	0	0	0	0	0	BTP202209	Tissue specific
CDS|exon|ID=cds-Bg	14	5.246429	5555.1	0.634191	SEVLEKPNI	92	792.3959	2	1582.784	.	L	Superscaf	58033078	0	0	0	0	0	BTP202209	Tissue specific
CDS|exon|ID=cds-Bg	13	5.213077	5171.7	0.634191	EVLEKPNDG	95	748.8804	2	1495.752	.	L	Superscaf	58033078	0	0	0	0	0	BTP202209	Tissue specific
CDS|exon|ID=cds-Bg	12	5.379167	4746.2	0.634191	VLEKPNDQA	92	456.5754	3	1366.709	.	L	Superscaf	58033078	0	0	0	0	0	BTP202209	Tissue specific
CDS|exon|ID=cds-Bg	21	5.684286	8153.1	0.634244	SDEDSDGDF	98	694.6482	3	2080.93	.	L	Superscaf	58035091	0	0	0	0	0	BTP202209	Tissue specific
CDS|exon|ID=cds-Bg	15	5.758667	6146.8	0.634248	EDSEGPSQC	97	787.355	2	1572.701	.	L	Superscaf	58035091	0	0	0	0	0	BTP202209	Tissue specific
CDS|exon|ID=cds-Bg	14	5.94	5721.3	0.634248	DSEGPSQGF	99	722.8334	2	1443.659	.	L	Superscaf	58035091	0	0	0	0	0	BTP202209	Tissue specific
CDS|exon|ID=cds-Bg	12	6.209167	4926.4	0.634249	EGPSQGPAS	96	621.8055	2	1241.6	.	L	Superscaf	58035091	0	0	0	0	0	BTP202209	Tissue specific
CDS|exon|ID=cds-Bg	12	6.499167	4519.7	0.634275	MGAEEAKPF	82	414.887	3	1241.644	.	A	Superscaf	58036132	0	0	0	0	0	BTP202209	Tissue specific
CDS|exon|ID=cds-Bg	9	5.533333	3610	0.680612	SSEAFPTNF	91	999.4374	1	998.4345	.	G	Superscaf	59681317	0	0	0	0	0	BTP202209	Tissue specific
CDS|exon|ID=cds-Bg	10	5.485	3570.9	0.680762	LGKVTNPSE	92	508.2727	2	1014.535	.	M	Superscaf	59686897	0	0	0	0	0	BTP202209	Tissue specific
CDS|exon|ID=cds-Bg	9	5.768889	3393	0.681412	MTLGLETPY	83	1024.498	1	1023.495	.	G	Superscaf	59710078	0	0	0	0	0	BTP202209	Tissue specific
CDS|exon|ID=cds-Bg	12	5.575833	4503.1	0.681514	TSLPLHPLN	84	662.8635	2	1323.719	.	K	Superscaf	59713411	0	0	0	0	0	BTP202209	Tissue specific
CDS|exon|ID=cds-Bg	9	5.413333	3368.8	0.681514	PLHPLNVSF	53	512.2833	2	1022.555	.	K	Superscaf	59713411	0	0	0	BTP202209	0	0	Tissue specific
CDS|exon|ID=cds-Bg	8	3.83125	2181.4	0.681926	LPENVVVK	84	449.2725	2	896.5331	.	K	Superscaf	59728378	0	0	0	0	0	BTP202209	Tissue specific
CDS|exon|ID=cds-Bg	8	5.03625	2701	0.681935	LSVGNAMF	58	838.4102	1	837.4055	.	K	Superscaf	59728378	0	0	0	0	0	BTP202209	Tissue specific
CDS|exon|ID=cds-Bg	7	4.901429	2347.6	0.681935	SVGNAMF	55	725.325	1	724.3214	.	K	Superscaf	59728378	0	0	0	0	0	BTP202209	Tissue specific
CDS|exon|ID=cds-Bg	11	5.269091	4257.6	0.681935	VQEELKLLI	98	454.5928	3	1360.76	.	K	Superscaf	59728378	0	0	0	0	0	BTP202209	Tissue specific
CDS|exon|ID=cds-Bg	16	5.926875	6524.2	0.681937	SSEAFPTNF	90	883.9179	2	1765.827	.	K	Superscaf	59728378	0	0	0	0	0	BTP202209	Tissue specific
CDS|exon|ID=cds-Bg	9	5.435556	3507.2	0.681995	MTLDLETPY	83	541.755	2	1081.5	.	S	Superscaf	59730712	0	0	0	0	BTP202209	BTP202209	Tissue enhanced
CDS|exon|ID=cds-Bg	13	6.037692	5147.5	0.68277	VSEELKLLI	98	535.6484	3	1603.93	.	K	Superscaf	59758024	0	0	0	0	0	BTP202209	Tissue specific
CDS|exon|ID=cds-Bg	10	5.891	3655.9	0.682927	LGKVTNPKE	94	528.8046	2	1055.597	.	K	Superscaf	59763484	0	0	0	0	0	BTP202209	Tissue specific
CDS|exon|ID=cds-Bg	8	6.20875	2919.1	0.682927	LGKVTNPK	85	428.7641	2	855.5178	.	K	Superscaf	59763484	0	0	0	0	0	BTP202209	Tissue specific
CDS|exon|ID=cds-Bg	7	5.754286	2647	0.684755	AATEPWP	63	771.3586	1	770.3599	.	S	Superscaf	59828976	0	0	0	0	BTP202209	0	Tissue specific
CDS|exon|ID=cds-Bg	6	4.888333	1752.8	0.733195	KAHVVL	56	666.425	1	665.4224	.	T	Superscaf	61549060	0	0	BTP202209	0	0	0	Tissue specific
CDS|exon|ID=cds-Bg	9	5.896667	3815.2	0.736807	ASDEFLKEF	96	547.7748	2	1093.54	.	A	Superscaf	61677169	0	0	0	BTP202209	0	0	Tissue specific
CDS|exon|ID=cds-Bg	9	6.206667	2958.3	0.817277	AAAPPPPPF	59	407.724	2	813.4384	.	S	Superscaf	64535044	0	0	0	0	BTP202209	0	Tissue specific
CDS|exon|ID=cds-Bg	7	3.697143	1565.7	0.823819	G(+42.01)	73	720.3901	1	719.3966	Acetylati	G	Superscaf	64767262	0	0	0	BTP202209	0	0	Tissue specific
CDS|exon|ID=cds-Bg	6	7.073333	2727.2	0.845921	R(+42.01)	50	365.1787	2	728.3565	Acetylati	V	Superscaf	65552086	0	0	0	0	0	BTP202209	Tissue specific
CDS|exon|ID=cds-Bg	19	5.319474	6909.5	0.892348	LLEDGGEAF	99	963.486	2	1924.963	.	L	Superscaf	67200793	0	0	0	0	0	BTP202209	Tissue specific
CDS|exon|ID=cds-Bg	18	5.282778	6556.1	0.892348	LEDGGEAPA	95	906.9443	2	1811.879	.	L	Superscaf	67200793	0	0	0	0	0	BTP202209	Tissue specific
CDS|exon|ID=cds-Bg	17	5.241765	6202.7	0.892348	EDGGEAPAA	88	850.3993	2	1698.795	.	L	Superscaf	67200793	0	0	0	0	BTP202209	0	Tissue specific
CDS|exon|ID=cds-Bg	9	6.1	4028.6	0.892649	WLNLERALY	72	589.3203	2	1176.629	.	M	Superscaf	67211563	0	0	0	0	0	BTP202209	Tissue specific
CDS|exon|ID=cds-Bg	9	4.848889	3134.6	0.892828	VQRLAETVF	66	531.7997	2	1061.587	.	A	Superscaf	67216870	0	0	0	0	0	BTP202209	Tissue specific
CDS|exon|ID=cds-Bg	13	4.841538	4597.1	0.893308	SLLSDVFPC	82	731.3712	2	1460.73	.	T	Superscaf	67234951	0	0	0	0	0	BTP202209	Tissue specific
CDS|exon|ID=cds-Bg	20	4.153	7418.5	0.893346	DGDVDPEWV	92	1137.007	2	2272.013	.	P	Superscaf	67236343	0	0	0	0	0	BTP202209	Tissue specific
CDS|exon|ID=cds-Bg	9	5.732222	3068.3	0.893412	FTSSGLVTK	97	470.2588	2	938.5073	.	K	Superscaf	67238656	0	0	0	0	0	BTP202209	Tissue specific
CDS|exon|ID=cds-Bg	8	4.835	2664	0.893466	TAAMVEFY	82	931.412	1	930.4157	.	K	Superscaf	67240366	0	0	0	0	0	BTP202209	Tissue specific
CDS|exon|ID=cds-Bg	9	4.07	3147.6	0.893587	VLFNEVLDF	98	543.2825	2	1084.555	.	D	Superscaf	67244869	0	0	0	0	0	BTP202209	Tissue specific
CDS|exon|ID=cds-Bg	7	6.274286	2992.3	0.893806	LDDAFRK	98	432.731	2	863.4501	.	H	Superscaf	67252537	0	0	0	0	0	BTP202209	Tissue specific

CDS|exon|ID=cds-B	8	6.8175	3026.6	0.905587	PPPLRPPP	97	435.7634	2	869.5123	.	R	Superscaf	67670494	0	BTP202209	0	0	0	0	Tissue specific
CDS|exon|ID=cds-B	7	6.094286	2525.8	0.919772	AARGHVP	51	354.2024	2	706.3875	.	D	Superscaf	68174635	0	0	0	BTP202209	0	0	Tissue specific
CDS|exon|ID=cds-B	11	4.702727	3200.7	0.924718	AMGPLVLTE	62	571.8265	2	1141.642	.	R	Superscaf	68350390	0	0	0	0	0	BTP202209	Tissue specific
CDS|exon|ID=cds-B	12	4.563333	3786	0.924756	SVPKVEAVK	98	636.8414	2	1271.672	.	K	Superscaf	68351788	0	0	BTP202209	0	0	0	Tissue specific
CDS|exon|ID=cds-B	9	6.564444	3347.7	0.929705	LTFATKTPC	87	468.2611	2	934.5124	.	R	Superscaf	68527201	0	0	0	0	0	BTP202209	Tissue specific
CDS|exon|ID=cds-B	7	5.875714	3158.7	0.943602	A(+42.01)	98	437.7157	2	873.4192	Acetylati	Q	Superscaf	69021019	0	0	0	BTP202209	0	0	Tissue specific
CDS|exon|ID=cds-B	7	3.855714	1758.9	0.949153	FVPVEAG	54	718.3705	1	717.3697	.	P	Superscaf	69218185	0	0	0	BTP202209	0	0	Tissue specific
CDS|exon|ID=cds-B	6	7.723333	3022.4	0.97374	KQHPPR	71	381.7204	2	761.4296	.	H	Superscaf	70091329	0	0	BTP202209	0	0	0	Tissue specific
CDS|exon|ID=cds-B	10	6.676	4417.7	0.978071	A(+42.01)	98	618.7869	2	1235.564	Acetylati	P	Superscaf	70244932	0	0	0	0	BTP202209	BTP202209	Mixed
CDS|exon|ID=cds-B	11	6.069091	4417.7	0.978071	A(+42.01)	98	445.8837	3	1334.632	Acetylati	P	Superscaf	70244932	0	BTP202209	0	0	0	0	Tissue specific
CDS|exon|ID=cds-B	13	5.992308	5264.6	0.978071	A(+42.01)	90	823.3873	2	1644.764	Acetylati	P	Superscaf	70244932	0	BTP202209	0	0	BTP202209	0	Mixed
CDS|exon|ID=cds-B	12	6.035	4877.2	0.978071	A(+42.01)	88	500.2384	3	1497.696	Acetylati	P	Superscaf	70244932	0	BTP202209	0	0	0	BTP202209	Mixed
CDS|exon|ID=cds-B	11	6.52	5118.6	0.978157	HDGKPFC(+	98	461.5404	3	1381.602	Carbamido	H	Superscaf	70248226	0	BTP202209	0	0	0	0	Tissue specific
CDS|exon|ID=cds-B	8	6.35375	3755.1	0.978158	FC(+57.02	99	541.7302	2	1081.447	Carbamido	H	Superscaf	70248226	0	BTP202209	0	0	0	0	Tissue specific
CDS|exon|ID=cds-B	10	6.153	4227.5	0.978167	YEKPSAEKF	86	392.867	3	1175.582	.	H	Superscaf	70248226	0	BTP202209	0	0	BTP202209	0	Mixed
CDS|exon|ID=cds-B	15	6.812667	6167.8	0.978169	TEERKASGF	99	399.9656	4	1595.838	.	H	Superscaf	70248226	0	BTP202209	0	0	BTP202209	0	Mixed
CDS|exon|ID=cds-B	12	6.285833	5648.6	0.978173	TGEPNMC(+	97	732.312	2	1462.612	Carbamido	V	Superscaf	70248703	0	BTP202209	0	0	BTP202209	BTP202209	Tissue enhanced
CDS|exon|ID=cds-B	9	5.875556	3105.2	0.978176	AEKVTSLGK	92	466.7725	2	931.5338	.	V	Superscaf	70248703	0	0	0	0	0	BTP202209	Tissue specific
CDS|exon|ID=cds-B	11	6.164545	5333	0.978193	HDGQPYC(+	87	466.86	3	1397.561	Carbamido	H	Superscaf	70249504	0	BTP202209	0	0	0	0	Tissue specific
CDS|exon|ID=cds-B	13	6.110769	6089.8	0.978193	HDGQPYC(+	70	540.2225	3	1617.645	Carbamido	H	Superscaf	70249504	0	BTP202209	0	0	0	0	Tissue specific
CDS|exon|ID=cds-B	7	6.427143	3515.9	0.978194	YC(+57.02	96	514.2092	2	1026.405	Carbamido	H	Superscaf	70249504	0	BTP202209	0	0	0	0	Tissue specific
CDS|exon|ID=cds-B	11	5.867273	4538.9	0.978198	YDKDPEGKA	98	624.298	2	1246.583	.	G	Superscaf	70249075	0	BTP202209	0	0	0	0	Tissue specific
CDS|exon|ID=cds-B	14	6.64	6782.5	0.978427	DWHRPC(+5	89	625.2889	3	1872.855	Carbamido	A	Superscaf	70257598	0	0	0	0	0	BTP202209	Tissue specific
CDS|exon|ID=cds-B	7	6.358571	3483.1	0.978427	DWHRPC(+5	97	492.2275	2	982.4443	Carbamido	A	Superscaf	70257598	0	0	0	0	0	BTP202209	Tissue specific
CDS|exon|ID=cds-B	11	6.165455	5333	0.978431	HEGKPYC(+	86	466.8593	3	1397.561	Carbamido	G	Superscaf	70257904	0	BTP202209	0	0	0	BTP202209	Mixed
CDS|exon|ID=cds-B	8	5.835	3955.5	0.978432	YC(+57.02	98	542.7008	2	1083.39	Carbamido	G	Superscaf	70257904	0	BTP202209	0	0	0	0	Tissue specific
CDS|exon|ID=cds-B	7	5.808571	3644.2	0.978432	YC(+57.02	99	507.1831	2	1012.353	Carbamido	G	Superscaf	70257904	0	BTP202209	0	0	0	0	Tissue specific
CDS|exon|ID=cds-B	14	5.625	5279.9	0.970528	A(+42.01)	61	733.8993	2	1465.789	Acetylati	Q	Superscaf	905482	0	0	0	0	BTP202209	BTP202209	Tissue enhanced
CDS|exon|ID=cds-B	15	5.627333	5739.4	0.970528	A(+42.01)	82	815.4303	2	1628.852	Acetylati	Q	Superscaf	905482	0	0	0	0	BTP202209	0	Tissue specific
CDS|exon|ID=cds-B	13	5.594615	4968.6	0.970528	A(+42.01)	79	698.3807	2	1394.752	Acetylati	Q	Superscaf	905482	0	0	BTP202209	0	0	0	Tissue specific
CDS|exon|ID=cds-B	12	5.7925	4543.1	0.970528	A(+42.01)	64	633.8595	2	1265.709	Acetylati	Q	Superscaf	905482	0	0	BTP202209	0	0	0	Tissue specific
CDS|exon|ID=cds-B	7	6.725714	2626.9	0.970187	ARLLAAP	79	356.2258	2	710.4438	.	R	Superscaf	916285	0	0	0	BTP202209	0	0	Tissue specific
CDS|exon|ID=cds-B	7	5.81	3133.6	0.970151	DEGWWRG	97	453.1969	2	904.3828	.	A	Superscaf	917374	0	0	0	0	BTP202209	0	Tissue specific
CDS|exon|ID=cds-B	6	3.04	932	0.970039	V(+42.01)	76	569.327	1	568.322	Acetylati	R	Superscaf	921000	0	0	0	BTP202209	0	0	Tissue specific
CDS|exon|ID=cds-B	6	4.313333	2768.3	0.912447	C(+57.02)	56	396.1289	2	790.244	Carbamido	A	Superscaf	2691139	BTP202209	0	0	0	0	0	Tissue specific
CDS|exon|ID=cds-B	13	6.220769	5238.5	0.786096	A(+42.01)	99	698.8359	2	1395.663	Acetylati	G	Superscaf	6574759	0	0	0	BTP202209	0	0	Tissue specific
CDS|exon|ID=cds-B	12	6.265833	4855.1	0.786096	A(+42.01)	99	655.3201	2	1308.631	Acetylati	G	Superscaf	6574759	0	0	0	BTP202209	0	0	Tissue specific
CDS|exon|ID=cds-B	11	6.319091	4471.7	0.786097	A(+42.01)	85	408.2059	3	1221.599	Acetylati	G	Superscaf	6574759	0	BTP202209	0	0	0	0	Tissue specific
CDS|exon|ID=cds-B	10	5.977	4003.3	0.786097	A(+42.01)	99	547.7576	2	1093.504	Acetylati	G	Superscaf	6574759	0	0	0	BTP202209	BTP202209	0	Mixed
CDS|exon|ID=cds-B	9	5.797778	3383.6	0.786097	A(+42.01)	85	479.2291	2	956.4451	Acetylati	G	Superscaf	6574759	0	BTP202209	0	BTP202209	0	0	Mixed
CDS|exon|ID=cds-B	7	4.34	2365.7	0.776422	A(+42.01)	82	871.4189	1	870.4123	Acetylati	C	Superscaf	6872296	0	BTP202209	0	0	BTP202209	BTP202209	Mixed
CDS|exon|ID=cds-B	8	5.6375	2678.8	0.768128	A(+42.01)	90	436.2355	2	870.4633	Acetylati	T	Superscaf	7127284	0	0	0	0	BTP202209	0	Tissue specific
CDS|exon|ID=cds-B	8	6.46	3779.5	0.764181	M(+42.01)	76	529.7548	2	1057.498	Acetylati	S	Superscaf	7248238	0	0	0	BTP202209	0	0	Tissue specific
CDS|exon|ID=cds-B	7	7.248571	3011.3	0.750071	KPARQGP	50	377.2177	2	752.4293	.	D	Superscaf	7682361	BTP202209	0	0	0	0	0	Tissue specific
CDS|exon|ID=cds-B	12	5.18	3910.2	0.721541	TSEVDLAKF	99	650.3771	2	1298.745	.	P	Superscaf	8559058	0	0	0	0	0	BTP202209	Tissue specific
CDS|exon|ID=cds-B	7	6.588571	3019.5	0.593806	GPPLRHE	58	403.2149	2	804.4242	.	R	Superscaf	12485679	0	0	0	BTP202209	0	0	Tissue specific
CDS|exon|ID=cds-B	6	7.946667	2889.4	0.550755	R(+42.01)	51	381.2117	2	760.4092	Acetylati	P	Superscaf	13808967	0	0	0	0	BTP202209	0	Tissue specific
CDS|exon|ID=cds-B	12	4.870833	3755.3	0.550754	VFRRFVEVC	75	478.9471	3	1433.825	.	V	Superscaf	13808929	0	0	0	0	0	BTP202209	Tissue specific
CDS|exon|ID=cds-B	13	4.931538	4214.8	0.550754	VFRRFVEVC	87	533.302	3	1596.889	.	V	Superscaf	13808929	0	0	0	0	0	BTP202209	Tissue specific
CDS|exon|ID=cds-B	11	5.33	3827.4	0.550754	RRFVEVGRV	89	451.2569	3	1350.752	.	V	Superscaf	13808929	0	BTP202209	0	0	0	0	Tissue specific
CDS|exon|ID=cds-B	9	4.123333	2529.8	0.550754	FVEVGRVAY	80	520.278	2	1038.55	.	V	Superscaf	13808929	0	0	0	0	BTP202209	0	Tissue specific
CDS|exon|ID=cds-B	9	5.861111	3102.2	0.550753	VSFGPHAGK	99	450.2388	2	898.4661	.	V	Superscaf	13808929	0	0	0	0	0	BTP202209	Tissue specific
CDS|exon|ID=cds-B	7	5.291429	2336.5	0.550754	VSFGPHA	94	357.6793	2	713.3497	.	V	Superscaf	13808929	0	0	0	0	0	BTP202209	Tissue specific
CDS|exon|ID=cds-B	8	5.89	3586.1	0.550732	KC(+57.02	77	521.738	2	1041.462	Carbamido	A	Superscaf	13809571	0	BTP202209	0	0	0	BTP202209	Mixed
CDS|exon|ID=cds-B	8	7.10875	3501.8	0.550686	WAATRWAK	99	495.2672	2	988.5242	.	F	Superscaf	13810960	0	0	0	0	0	BTP202209	Tissue specific
CDS|exon|ID=cds-B	8	6.2325	3556.1	0.550681	MTDFDRYK	98	538.2457	2	1074.481	.	T	Superscaf	13811179	0	0	0	0	0	BTP202209	Tissue specific
CDS|exon|ID=cds-B	13	7.122308	5199.2	0.550671	KLQKAALLK	99	465.9714	3	1394.897	.	G	Superscaf	13811350	0	0	0	0	0	BTP202209	Tissue specific
CDS|exon|ID=cds-B	9	7.205556	3698.7	0.550672	KLQKAALLK	99	506.8462	2	1011.68	.	G	Superscaf	13811350	0	0	0	0	0	BTP202209	Tissue specific
CDS|exon|ID=cds-B	12	6.904167	4730.8	0.550671	LQKAALLKA	91	423.2725	3	1266.802	.	G	Superscaf	13811350	0	0	0	0	0	BTP202209	Tissue specific
CDS|exon|ID=cds-B	19	6.181579	5932.6	0.550669	AAKGAAAVA	86	417.0083	4	1664.01	.	G	Superscaf	13811350	0	0	0	0	0	BTP202209	Tissue specific
CDS|exon|ID=cds-B	12	5.66	3453.3	0.550669	AVAAAAAKV	98	534.3301	2	1066.65	.	G	Superscaf	13811350	0	0	0	0	0	BTP202209	Tissue specific
CDS|exon|ID=cds-B	12	5.97	3610.4	0.550669	VAAAAAKVF	97	375.5747	3	1123.708	.	G	Superscaf	13811350	0	0	0	0	0	BTP202209	Tissue specific
CDS|exon|ID=cds-B	11	5.627273	3142	0.550669	VAAAAAKVF	98	498.8124	2	995.6127	.	G	Superscaf	13811350	0	0	0	0	0	BTP202209	Tissue specific
CDS|exon|ID=cds-B	11	6.512727	3610.4	0.550669	AAAAAKVPA	96	513.3254	2	1024.639	.	G	Superscaf	13811350	0	0	0	0	0	BTP202209	Tissue specific
CDS|exon|ID=cds-B	9	6.208889	2830.7	0.550669	AAAAKVPAK	90	413.7587	2	825.5072	.	G	Superscaf	13811350	0	0	0	0	0	BTP202209	Tissue specific
CDS|exon|ID=cds-B	10	5.243	3429.7	0.533292	AAVMVHQLS	98	542.3004	2	1082.591	.	V	Superscaf	14345692	0	0	0	0	0	BTP202209	Tissue specific
CDS|exon|ID=cds-B	7	5.917143	2989.4	0.533123	NAVRLHY	83	436.7385	2	871.4664	.	I	Superscaf	14350603	0	0	0	0	0	BTP202209	Tissue specific
CDS|exon|ID=cds-B	17	5.762353	6671.3	0.533002	AAAVLFRMS	82	984.9945	2	1967.978	.	L	Superscaf	14354506	0	0	0	0	0	BTP202209	Tissue specific
CDS|exon|ID=cds-B	8	4.55875	2501.9	0.532934	PVDGLPDL	66	825.4329	1	824.4279	.	K	Superscaf	14356468	0	0	0	0	0	BTP202209	Tissue specific
CDS|exon|ID=cds-B	8	4.5175	2461.8	0.532934	VDGLPDLG	87	785.4017	1	784.3967	.	K	Superscaf	14356468	0	0	0	0	0	BTP202209	Tissue specific
CDS|exon|ID=cds-B	7	6.297143	2276.3	0.513713	LPVKPAK	54	752.5137	1	751.4955	.	G	Superscaf	14947426	BTP202209	0	0	0	0	0	Tissue specific
CDS|exon|ID=cds-B	7	5.672857	2125.3	0.452807	GVLKPGM	86	701.3982	1	700.3942	.	K	Superscaf	16819213	0	0	0	0	BTP202209	0	Tissue specific
CDS|exon|ID=cds-B	7	4.572857	2346.5	0.452803	VGFNVKN	99	389.2162	2	776.4181	.	K	Superscaf	16819213	0	0	BTP202209	0	0	0	Tissue specific
CDS|exon|ID=cds-B	7	4.912857	2705.2	0.452798	PVLDC(+57	95	421.1952	2	840.38	Carbamido	K	Superscaf	16819213	0	0	0	0	BTP202209	0	Tissue specific
CDS|exon|ID=cds-B	11	6.074545	4127.3	0.452795	DGPKFLKSC	93	567.7908	2	1133.572	.	K	Superscaf	16819213	0	0	BTP202209	BTP202209	0	0	Mixed
CDS|exon|ID=cds-B	14	4.759286	5406.1	0.437809	QWALDDVEE	95	838.8938	2	1675.776	.	P	Superscaf	17280589	0	0	0	0	0	BTP202209	Tissue specific
CDS|exon|ID=cds-B	10	6.192	4190.5	0.437808	DLHFEGMFK	96	417.8759	3	1250.612	.	P	Superscaf	17280589	0	0	0	0	0	BTP202209	Tissue specific
CDS|exon|ID=cds-B	7	5.701429	2632.9	0.240052	LDLDGPK	99	379.2062	2	756.4017	.	S	Superscaf	23359090	0	0	0	0	0	BTP202209	Tissue specific
CDS|exon|ID=cds-B	15	5.9	6846.6	0.120211	LNLQEYQSK	99	604.3029	3	1809.893	.	V	Superscaf	34433131	0	0	BTP202209	0	0	0	Tissue specific
CDS|exon|ID=cds-B	12	6.203333	5455	0.120211	LNLQEYQSK	99	747.9004	2	1493.791	.	V	Superscaf	34433131	0	0	BTP202209	0	0	0	Tissue specific
CDS|exon|ID=cds-B	10	6.271	4730.1	0.120211	LNLQEYQSK	99	625.8387	2	1249.667	.	V	Superscaf	34433131	0	0	0	0	0	BTP202209	Tissue specific
CDS|exon|ID=cds-B	9	5.885556	4261.7	0.120211	LNLQEYQSK	95	561.7916	2	1121.572	.	V	Superscaf	34433131	0	BTP202209	0	0	BTP202209	BTP202209	Tissue enhanced
CDS|exon|ID=cds-B	13	5.625385	4997.5	0.120211	KLMSDNGVK	94	507.9433	3	1520.813	.	V	Superscaf	34433131	0	0	0	0	0	BTP202209	Tissue specific
CDS|exon|ID=cds-B	14	5.189286	5022.4	0.120213	FVADTANEA	99	725.3619	2	1448.715	.	V	Superscaf	34433131	0	0	0	0	0	BTP202209	Tissue specific
CDS|exon|ID=cds-B	13	5.166923	4635	0.120213	VADTANEAL	99	651.8279	2	1301.646	.	V	Superscaf	34433131	0	0	0	0	0	BTP202209	Tissue specific
CDS|exon|ID=cds-B	10	5.689	3473.6	0.122555	QTPKEGVKV	73	371.8909	3	1112.655	.	L	Superscaf	34505113	0	0	0	0	0	BTP202209	Tissue specific
CDS|exon|ID=cds-B	7	6.251429	2292.3	0.213615	KKLLPVA	54	384.7704	2	767.5269	.	C	Superscaf	37304227	0	0	0	BTP202209	0	0	Tissue specific
CDS|exon|ID=cds-B	5	6.116	2143.3	0.240585	YGSSH	64	550.2165	1	549.2183	.	N	Superscaf	38133291	BTP202209	0	0	0	0	0	Tissue specific
CDS|exon|ID=cds-B	7	5.798571	2131.3	0.40525	G(+42.01)	66	375.2288	2	748.4483	Acetylati	V	Superscaf	43194640	0	BTP202209	0	0	0	0	Tissue specific
CDS|exon|ID=cds-B	8	5.625	3296.9	0.409634	M(+42.01)	98	491.2308	2	980.4498	Acetylati	H	Superscaf	43329373	0	0	0	0	0	BTP202209	Tissue specific
CDS|exon|ID=cds-B	10	5.068	3680.3	0.409634	M(+42.01)	92	584.2806	2	1166.55	Acetylati	H	Superscaf	43329373	0	0	BTP202209	0	0	0	Tissue specific
CDS|exon|ID=cds-B	15	5.677333	5611.3	0.550086	LLMLSGVGN	95	534.6249	3	1600.861	.	V	Superscaf	47646553	0	0	0	0	0	BTP202209	Tissue specific
CDS|exon|ID=cds-B	13	6.147692	6058.7	0.568587	M(+42.01)	88	553.2698	3	1656.793	Acetylati	C	Superscaf	48215251	0	0	BTP202209	0	0	0	Tissue specific
CDS|exon|ID=cds-B	11	6.208182	5094.6	0.568587	M(+42.01)	73	708.8313	2	1415.65	Acetylati	C	Superscaf	48215251	0	0	0	0	BTP202209	0	Tissue specific
CDS|exon|ID=cds-B	9	5.877778	4015.5	0.568587	M(+42.01)	99	580.7554	2	1159.497	Acetylati	C	Superscaf	48215251	0	BTP202209	BTP202209	BTP202209	BTP202209	BTP202209	Mixed
CDS|exon|ID=cds-B	8	6.3825	3123.2	0.569193	AYGQALAK	98	411.2283	2	820.4443	.	L	Superscaf	48233785	0	0	0	0	0	BTP202209	Tissue specific
CDS|exon|ID=cds-B	12	6.475833	4813.1	0.57994	KKRPPVKEE	93	367.4711	4	1465.862	.	L	Superscaf	48564223	0	0	0	0	BTP202209	0	Tissue specific
CDS|exon|ID=cds-B	11	6.179091	4344.7	0.57994	KRPPVKEED	99	446.9278	3	1337.767	.	L	Superscaf	48564223	0	0	0	0	BTP202209	0	Tissue specific
CDS|exon|ID=cds-B	11	5.836364	4173.6	0.57994	RPPVKEEDL	72	634.3528	2	1266.693	.	L	Superscaf	48564223	0	BTP202209	0	0	0	0	Tissue specific
CDS|exon|ID=cds-B	10	5.823	3876.3	0.57994	RPPVKEEDL	99	404.2298	3	1209.672	.	L	Superscaf	48564223	BTP202209	BTP202209	0	0	BTP202209	0	Mixed
CDS|exon|ID=cds-B	9	5.274444	3227.5	0.57994	PPVKEEDLK	99	352.1964	3	1053.571	.	L	Superscaf	48564223	0	0	0	0	BTP202209	0	Tissue specific
CDS|exon|ID=cds-B	16	6.05	5641.9	0.579951	TGAETVFEK	97	815.9396	2	1629.873	.	V	Superscaf	48564466	0	0	0	0	BTP202209	0	Tissue specific
CDS|exon|ID=cds-B	15	5.804	5173.5	0.579951	TGAETVFEK	98	751.8958	2	1501.778	.	V	Superscaf	48564466	0	BTP202209	0	0	BTP202209	BTP202209	Mixed
CDS|exon|ID=cds-B	12	5.730833	4227.5	0.579951	TGAETVFEK	84	426.5624	3	1276.666	.	V	Superscaf	48564466	BTP202209	BTP202209	0	0	BTP202209	0	Mixed
CDS|exon|ID=cds-B	11	5.704545	3916.2	0.579951	TGAETVFEK	99	603.8209	2	1205.629	.	V	Superscaf	48564466	BTP202209	BTP202209	0	0	BTP202209	BTP202209	Mixed
CDS|exon|ID=cds-B	10	5.301	3447.8	0.579951	TGAETVFEK	96	539.7738	2	1077.534	.	V	Superscaf	48564466	BTP202209	0	0	0	BTP202209	0	Mixed
CDS|exon|ID=cds-B	10	5.622	3518.7	0.579951	GAETVFEKF	99	553.296	2	1104.582	.	V	Superscaf	48564466	0	0	0	0	0	BTP202209	Tissue specific
CDS|exon|ID=cds-B	14	6.021429	4947.1	0.579951	AETVFEKPK	89	491.6075	3	1471.804	.	V	Superscaf	48564466	0	BTP202209	0	0	0	0	Tissue specific
CDS|exon|ID=cds-B	13	5.735385	4478.7	0.579951	AETVFEKPK	99	672.8605	2	1343.709	.	V	Superscaf	48564466	0	BTP202209	0	0	0	0	Tissue specific
CDS|exon|ID=cds-B	9	5.583333	3221.4	0.579951	AETVFEKPK	90	524.788	2	1047.56	.	V	Superscaf	48564466	0	BTP202209	0	0	0	BTP202209	Mixed
CDS|exon|ID=cds-B	8	5.06375	2753	0.579951	AETVFEKP	90	460.7385	2	919.465	.	V	Superscaf	48564466	0	0	0	0	BTP202209	0	Tissue specific
CDS|exon|ID=cds-B	12	5.711667	4167.4	0.579951	ETVFEKPKA	98	637.3412	2	1272.671	.	V	Superscaf	48564466	0	BTP202209	0	0	0	0	Tissue specific
CDS|exon|ID=cds-B	8	5.52875	2910.1	0.579951	ETVFEKPK	99	489.2674	2	976.5229	.	V	Superscaf	48564466	0	BTP202209	0	0	0	BTP202209	Mixed
CDS|exon|ID=cds-B	11	6.23	3812.8	0.579952	VFEKPKAGF	99	391.2316	3	1170.676	.	V	Superscaf	48564466	BTP202209	0	0	0	0	0	Tissue specific
CDS|exon|ID=cds-B	10	6.853	3812.8	0.579952	FEKPKAGPA	96	358.2093	3	1071.608	.	V	Superscaf	48564466	0	BTP202209	0	0	0	0	Tissue specific
CDS|exon|ID=cds-B	15	5.417333	5797.5	0.58016	ELVYEELLA	98	592.6454	3	1774.925	.	G	Superscaf	48571042	0	0	0	0	0	BTP202209	Tissue specific
CDS|exon|ID=cds-B	14	4.835714	4945.6	0.580253	YQEVANPLM	60	785.3804	2	1568.754	.	L	Superscaf	48573892	0	0	0	0	BTP202209	BTP202209	Mixed
CDS|exon|ID=cds-B	7	5.502857	2462.6	0.591792	EKEKPVP	96	413.7357	2	825.4596	.	R	Superscaf	48928842	0	0	0	BTP202209	0	0	Tissue specific
CDS|exon|ID=cds-B	13	5.88	5438.9	0.599809	LYDEVHPKG	99	789.4222	2	1576.836	.	P	Superscaf	49175056	0	0	0	0	BTP202209	0	Tissue specific
CDS|exon|ID=cds-B	12	5.558333	4970.5	0.599809	LYDEVHPKG	95	725.3751	2	1448.741	.	P	Superscaf	49175056	0	0	0	0	0	BTP202209	Tissue specific
CDS|exon|ID=cds-B	11	5.516364	4659.2	0.599809	LYDEVHPKG	97	460.2396	3	1377.704	.	P	Superscaf	49175056	0	0	0	0	BTP202209	BTP202209	Mixed
CDS|exon|ID=cds-B	10	5.47	4305.8	0.599809	LYDEVHPKG	96	422.5454	3	1264.62	.	P	Superscaf	49175056	0	0	0	0	0	BTP202209	Tissue specific
CDS|exon|ID=cds-B	8	5.1825	3075.4	0.599809	LYDEVHPK	99	500.757	2	999.5025	.	P	Superscaf	49175056	0	0	0	0	BTP202209	0	Tissue specific
CDS|exon|ID=cds-B	8	7.0875	3201.6	0.60859	KMPTGGSR	79	417.2163	2	832.4225	.	R	Superscaf	49444807	0	0	0	0	BTP202209	0	Tissue specific
CDS|exon|ID=cds-B	16	6.759375	5760.8	0.608594	ASTKAKPPS	94	489.614	3	1465.825	.	R	Superscaf	49444807	0	0	0	0	0	BTP202209	Tissue specific
CDS|exon|ID=cds-B	15	6.808667	5449.5	0.608595	STKAKPPSA	99	465.9359	3	1394.788	.	R	Superscaf	49444807	0	BTP202209	0	0	0	0	Tissue specific
CDS|exon|ID=cds-B	19	6.653158	6934.1	0.608595	AKPPSAAAF	99	578.3263	3	1731.963	.	R	Superscaf	49444807	0	0	BTP202209	0	BTP202209	0	Mixed
CDS|exon|ID=cds-B	12	6.681667	4200.2	0.608595	AKPPSAAAF	99	540.3129	2	1078.614	.	R	Superscaf	49444807	0	0	BTP202209	0	BTP202209	BTP202209	Mixed
CDS|exon|ID=cds-B	11	6.741818	3888.9	0.608595	KPPSAAAPA	83	504.7935	2	1007.576	.	R	Superscaf	49444807	0	0	0	0	0	BTP202209	Tissue specific
CDS|exon|ID=cds-B	10	6.442	3420.5	0.608595	PPSAAAPAA	96	440.7465	2	879.4814	.	R	Superscaf	49444807	0	0	0	0	BTP202209	0	Tissue specific
CDS|exon|ID=cds-B	17	6.347647	6170.4	0.608595	AAAPAAKGA	96	517.2817	3	1548.826	.	R	Superscaf	49444807	0	BTP202209	0	BTP202209	0	0	Mixed
CDS|exon|ID=cds-B	14	6.597857	5096.2	0.608595	AAAPAAKGA	99	626.8517	2	1251.693	.	R	Superscaf	49444807	0	0	BTP202209	BTP202209	BTP202209	0	Mixed

CDS|exon|ID=cds-Bg	12	6.460833	4587.8	0.608596	AKGAQAPTK	99	584.8182	2	1167.625	.	R	Superscaf	49444807	0	0	0	BTP20220	0	0	Tissue specific
CDS|exon|ID=cds-Bg	8	6.54875	3438.8	0.609239	AAAGQPQR	75	399.7223	2	797.4144	.	A	Superscaf	49464463	BTP20220	0	0	0	0	0	Tissue specific
CDS|exon|ID=cds-Bg	6	2.405	1152.3	0.655706	LVDVFV	50	691.3989	1	690.3952	.	G	Superscaf	50893234	0	0	0	0	0	BTP20220	Tissue specific
CDS|exon|ID=cds-Bg	7	5.768571	2459.8	0.658598	LSPSVLR	62	386.2397	2	770.465	.	A	Superscaf	50981941	0	0	0	BTP20220	0	0	Tissue specific
CDS|exon|ID=cds-Bg	8	4.46375	1928	0.660289	VFGGAGVP	85	703.3761	1	702.3701	.	V	Superscaf	51034162	BTP20220	0	0	0	0	0	Tissue specific
CDS|exon|ID=cds-Bg	8	6.5975	3138.6	0.662999	FPPLSPLR	74	463.7805	2	925.5385	.	R	Superscaf	51117486	0	0	BTP20220	0	0	0	Tissue specific
CDS|exon|ID=cds-Bg	9	5.304444	3294.9	0.667635	REVLPTPSD	53	507.2658	2	1012.519	.	L	Superscaf	51259729	0	BTP20220	0	0	0	0	Tissue specific
CDS|exon|ID=cds-Bg	11	4.469091	3234.7	0.67203	VVASVPRAW	85	604.3081	2	1206.607	.	A	Superscaf	51394756	0	0	0	0	0	BTP20220	Tissue specific
CDS|exon|ID=cds-Bg	10	5.632	3646	0.672794	A(+42.01)	69	1079.558	1	1078.555	Acetylati	C	Superscaf	51418471	0	0	0	0	BTP20220	0	Tissue specific
CDS|exon|ID=cds-Bg	8	5.9175	2892.4	0.672996	PGVRNPPP	70	417.2333	2	832.4555	.	C	Superscaf	51424824	0	0	0	BTP20220	0	0	Tissue specific
CDS|exon|ID=cds-Bg	7	6.712857	2971.2	0.675571	C(+57.02)	73	411.7055	2	821.3854	Carbamido	S	Superscaf	51503860	0	BTP20220	0	0	0	0	Tissue specific
CDS|exon|ID=cds-Bg	9	7.395556	4449.2	0.679508	MTNRLLQRK	85	580.337	2	1158.666	.	R	Superscaf	51624859	0	0	0	0	0	BTP20220	Tissue specific
CDS|exon|ID=cds-Bg	8	6.05125	3152.5	0.684581	TATYAQAL	67	838.4274	1	837.4232	.	S	Superscaf	51780763	0	0	0	0	0	BTP20220	Tissue specific
CDS|exon|ID=cds-Bg	9	6.405556	4102.7	0.684679	STREHTAYY	73	564.2577	2	1126.504	.	A	Superscaf	51783655	0	0	0	0	0	BTP20220	Tissue specific
CDS|exon|ID=cds-Bg	8	6.08625	3436.8	0.684691	NYLHATAF	96	468.7304	2	935.4501	.	P	Superscaf	51784015	0	0	0	0	BTP20220	BTP20220	Tissue enhanced
CDS|exon|ID=cds-Bg	9	5.866667	3744.3	0.684711	LSRADLTEY	82	534.2702	2	1066.53	.	P	Superscaf	51784807	0	0	0	0	0	BTP20220	Tissue specific
CDS|exon|ID=cds-Bg	7	5.844286	2572.1	0.690104	PTPDTLP	63	740.3904	1	739.3752	.	A	Superscaf	51950227	0	0	0	0	0	BTP20220	Tissue specific
CDS|exon|ID=cds-Bg	17	4.343529	5320.9	0.702268	TLEAEAFDD	95	890.4327	2	1778.857	.	E	Superscaf	52324336	0	BTP20220	0	0	0	0	Tissue specific
CDS|exon|ID=cds-Bg	14	4.150714	4144.5	0.702268	AEAFDDVVG	98	718.8474	2	1435.683	.	E	Superscaf	52324336	0	BTP20220	0	0	0	0	Tissue specific
CDS|exon|ID=cds-Bg	13	4.006923	3833.2	0.702268	EAFDDVVGE	82	683.3314	2	1364.646	.	E	Superscaf	52324336	0	BTP20220	0	0	0	0	Tissue specific
CDS|exon|ID=cds-Bg	12	4.0725	3407.7	0.702268	AFDDVVGET	91	618.8082	2	1235.603	.	E	Superscaf	52324336	0	BTP20220	0	0	0	BTP20220	Mixed
CDS|exon|ID=cds-Bg	11	3.895455	3096.4	0.702268	FDDVVGETV	99	583.2893	2	1164.566	.	E	Superscaf	52324336	0	BTP20220	0	0	0	BTP20220	Mixed
CDS|exon|ID=cds-Bg	10	3.737	2709	0.702268	DDVVGETVG	97	509.755	2	1017.498	.	E	Superscaf	52324336	0	BTP20220	0	0	BTP20220	BTP20220	Mixed
CDS|exon|ID=cds-Bg	12	3.905833	3518	0.702268	DDVVGETVG	88	617.7914	2	1233.573	.	E	Superscaf	52324336	BTP20220	0	0	BTP20220	0	0	Mixed
CDS|exon|ID=cds-Bg	13	4.040769	3977.5	0.702268	DDVVGETVG	92	699.3243	2	1396.636	.	E	Superscaf	52324336	0	0	0	BTP20220	0	0	Tissue specific
CDS|exon|ID=cds-Bg	9	3.822222	2297.5	0.702268	DVVGETVGK	92	452.2421	2	902.4709	.	E	Superscaf	52324336	0	BTP20220	0	0	BTP20220	0	Mixed
CDS|exon|ID=cds-Bg	10	4.093	2695	0.702268	VVGETVGKT	99	502.7653	2	1003.519	.	E	Superscaf	52324336	BTP20220	0	0	0	0	0	Tissue specific
CDS|exon|ID=cds-Bg	11	4.235455	3154.5	0.702268	VVGETVGKT	90	584.2979	2	1166.582	.	E	Superscaf	52324336	0	BTP20220	0	0	0	0	Tissue specific
CDS|exon|ID=cds-Bg	10	4.659	3154.5	0.702269	VGETVGKTD	99	534.7633	2	1067.513	.	E	Superscaf	52324336	0	BTP20220	0	0	0	0	Tissue specific
CDS|exon|ID=cds-Bg	7	5.462857	3267.8	0.703788	WPENTNF	58	907.3928	1	906.3871	.	M	Superscaf	52371052	BTP20220	0	0	0	0	0	Tissue specific
CDS|exon|ID=cds-Bg	7	5.225714	3152.7	0.703788	C(+57.02)	89	475.1796	2	948.347	Carbamido	M	Superscaf	52371052	0	BTP20220	0	0	0	0	Tissue specific
CDS|exon|ID=cds-Bg	22	4.784091	6689.3	0.703978	EAEMVLAKD	81	748.0707	3	2241.193	.	V	Superscaf	52376941	0	BTP20220	0	0	0	0	Tissue specific
CDS|exon|ID=cds-Bg	14	5.315714	5102.7	0.70398	DMESPTKPD	87	780.3893	2	1558.755	.	V	Superscaf	52376941	0	BTP20220	0	0	BTP20220	BTP20220	Mixed
CDS|exon|ID=cds-Bg	12	5.475	4319.7	0.70398	ESPTKPDEA	80	657.3494	2	1312.688	.	V	Superscaf	52376941	0	BTP20220	0	0	BTP20220	0	Mixed
CDS|exon|ID=cds-Bg	15	4.393333	4607.1	0.703983	KDVVLPVQT	85	552.6431	3	1654.914	.	V	Superscaf	52376941	0	0	0	0	BTP20220	0	Tissue specific
CDS|exon|ID=cds-Bg	13	4.091538	3727.2	0.703983	VVLPVQTEE	93	706.9014	2	1411.792	.	V	Superscaf	52376941	0	BTP20220	0	0	0	BTP20220	Mixed
CDS|exon|ID=cds-Bg	12	4.4325	3727.2	0.703983	VLPVQTEET	98	657.3663	2	1312.724	.	V	Superscaf	52376941	0	BTP20220	0	0	BTP20220	BTP20220	Mixed
CDS|exon|ID=cds-Bg	8	5.17625	2945.4	0.703993	ETEVALSR	76	452.735	2	903.4661	.	V	Superscaf	52376941	0	0	0	BTP20220	0	0	Tissue specific
CDS|exon|ID=cds-Bg	8	4.76	2674.2	0.704001	LPPETEVT	95	885.4548	1	884.4491	.	V	Superscaf	52376941	0	BTP20220	0	0	0	0	Tissue specific
CDS|exon|ID=cds-Bg	11	3.68	2688.9	0.704005	SEAEVVPVP	93	577.3242	2	1152.639	.	V	Superscaf	52376941	0	0	0	0	0	BTP20220	Tissue specific
CDS|exon|ID=cds-Bg	10	3.48	2305.5	0.704005	EAEVVPVPV	95	533.808	2	1065.607	.	V	Superscaf	52376941	0	0	0	0	0	BTP20220	Tissue specific
CDS|exon|ID=cds-Bg	7	3.191429	1143.2	0.704005	VVPVPVK	90	369.2481	2	736.4847	.	V	Superscaf	52376941	0	0	0	0	0	BTP20220	Tissue specific
CDS|exon|ID=cds-Bg	10	6.622	4005.2	0.70478	KELPPSPEK	81	384.8904	3	1151.655	.	K	Superscaf	52401607	0	0	0	0	BTP20220	0	Tissue specific
CDS|exon|ID=cds-Bg	10	6.489	4293.9	0.704808	TQPTSLPKG	79	550.7989	2	1099.587	.	K	Superscaf	52402495	0	BTP20220	0	0	0	0	Tissue specific
CDS|exon|ID=cds-Bg	8	6.58875	3285.7	0.704808	TQPTSLPK	86	436.247	2	870.4811	.	K	Superscaf	52402495	0	BTP20220	0	0	0	BTP20220	Mixed
CDS|exon|ID=cds-Bg	10	5.801	3167.3	0.70481	SGSVPAAPP	99	455.7523	2	909.4919	.	K	Superscaf	52402495	0	BTP20220	0	0	0	0	Tissue specific
CDS|exon|ID=cds-Bg	8	5.795	2486.6	0.70481	SVPAAPPK	97	383.7251	2	765.4384	.	K	Superscaf	52402495	0	BTP20220	0	0	0	0	Tissue specific
CDS|exon|ID=cds-Bg	5	6.17	1662.9	0.704812	PPGLP	58	480.2816	1	479.2744	.	A	Superscaf	52402639	0	BTP20220	0	0	0	0	Tissue specific
CDS|exon|ID=cds-Bg	18	6.218333	6168.4	0.704912	RVSPSKPAS	93	441.7573	4	1763.005	.	Q	Superscaf	52405741	0	0	0	0	BTP20220	0	Tissue specific
CDS|exon|ID=cds-Bg	17	5.951176	5519.6	0.704912	VSPSKPASA	99	402.7324	4	1606.904	.	Q	Superscaf	52405741	0	BTP20220	0	0	0	0	Tissue specific
CDS|exon|ID=cds-Bg	14	6.370714	4798.8	0.704912	SKPASAPAV	98	442.2568	3	1323.751	.	Q	Superscaf	52405741	0	BTP20220	0	0	0	0	Tissue specific
CDS|exon|ID=cds-Bg	12	6.1475	3947	0.704913	PASAPAVKP	82	370.5474	3	1108.624	.	Q	Superscaf	52405741	0	BTP20220	0	0	0	0	Tissue specific
CDS|exon|ID=cds-Bg	8	5.86625	2474.6	0.704914	AVPKAPAT	68	377.7259	2	753.4385	.	Q	Superscaf	52405741	0	BTP20220	0	0	0	0	Tissue specific
CDS|exon|ID=cds-Bg	15	6.254667	5725.3	0.704939	ADLSRPKST	96	526.6207	3	1576.842	.	A	Superscaf	52406614	0	BTP20220	0	0	0	0	Tissue specific
CDS|exon|ID=cds-Bg	7	6.778571	2914.2	0.704939	A(+42.01)	93	414.7253	2	827.4501	Acetylati	A	Superscaf	52406614	0	0	0	0	0	BTP20220	Tissue specific
CDS|exon|ID=cds-Bg	8	5.985	2758	0.704944	TPPVDKKP	99	441.2567	2	880.5018	.	A	Superscaf	52406614	0	0	0	BTP20220	0	0	Tissue specific
CDS|exon|ID=cds-Bg	13	6.296923	5219.8	0.704995	TEAAAPARK	99	676.3511	2	1350.689	.	P	Superscaf	52407970	0	BTP20220	0	0	0	0	Tissue specific
CDS|exon|ID=cds-Bg	17	5.033529	5335.7	0.705224	VGSLDNVGH	97	530.9575	3	1589.853	.	P	Superscaf	52415125	0	BTP20220	0	0	0	BTP20220	Mixed
CDS|exon|ID=cds-Bg	5	6.308	1922.1	0.712885	KPPLE	53	583.3549	1	582.3377	.	K	Superscaf	52650876	BTP20220	0	0	0	0	0	Tissue specific
CDS|exon|ID=cds-Bg	6	5.931667	2192.4	0.724441	LGGTFY	59	657.3214	1	656.317	.	S	Superscaf	53006050	0	0	0	0	0	BTP20220	Tissue specific
CDS|exon|ID=cds-Bg	10	6.117	3852.2	0.801964	TAEKALPLC	98	507.2838	2	1012.555	.	D	Superscaf	55388593	0	0	BTP20220	0	0	0	Tissue specific
CDS|exon|ID=cds-Bg	17	6.020588	6422.1	0.817164	A(+42.01)	93	895.9456	2	1789.882	Acetylati	R	Superscaf	55856170	0	0	0	0	0	BTP20220	Tissue specific
CDS|exon|ID=cds-Bg	8	6.9575	3247.6	0.850414	MLPSGFRK	90	468.2584	2	934.5058	.	Q	Superscaf	56878108	0	0	0	0	0	BTP20220	Tissue specific
CDS|exon|ID=cds-Bg	17	4.970588	6359.1	0.850415	FLVHNVKEL	97	696.0414	3	2085.111	Carbamido	Q	Superscaf	56878108	0	0	0	0	0	BTP20220	Tissue specific
CDS|exon|ID=cds-Bg	11	4.238182	3630	0.850415	FLVHNVKEL	77	663.8724	2	1325.734	.	Q	Superscaf	56878108	0	0	0	0	0	BTP20220	Tissue specific
CDS|exon|ID=cds-Bg	10	4.114	3242.6	0.850415	LVHNVKELE	60	590.3393	2	1178.666	.	Q	Superscaf	56878108	0	0	0	0	BTP20220	BTP20220	Mixed
CDS|exon|ID=cds-Bg	7	7.472857	3319.7	0.850862	KGQPHRP	88	410.2315	2	818.4511	.	S	Superscaf	56891737	0	0	BTP20220	0	0	0	Tissue specific
CDS|exon|ID=cds-Bg	9	5.894444	3126.7	0.904888	P(+42.01)	66	484.7632	2	967.5014	Acetylati	R	Superscaf	58552522	0	0	0	BTP20220	0	0	Tissue specific
CDS|exon|ID=cds-Bg	7	5.241429	2269.4	0.933263	LGPVEKF	97	395.2281	2	788.4432	.	H	Superscaf	59424745	0	0	0	0	0	BTP20220	Tissue specific
CDS|exon|ID=cds-Bg	7	6.38	3213.6	0.935364	AKDERPQ	89	422.2278	2	842.4246	.	I	Superscaf	59489688	0	0	0	0	0	BTP20220	Tissue specific
CDS|exon|ID=cds-Bg	6	5.676667	2534.9	0.94099	AQVQLR	73	357.7209	2	713.4184	.	A	Superscaf	59662240	0	0	0	0	BTP20220	0	Tissue specific
CDS|exon|ID=cds-Bg	15	5.762	6216.7	0.941187	LNVLEQKQK	83	844.9664	2	1687.926	.	L	Superscaf	59668258	0	0	0	0	0	BTP20220	Tissue specific
CDS|exon|ID=cds-Bg	9	5.707778	3887.2	0.941187	LNVLEQKQK	97	550.3248	2	1098.64	.	L	Superscaf	59668258	0	0	0	0	0	BTP20220	Tissue specific
CDS|exon|ID=cds-Bg	12	5.928333	4749.2	0.941201	TLEPSEKPF	94	702.3715	2	1402.734	.	A	Superscaf	59668657	0	0	0	0	0	BTP20220	Tissue specific
CDS|exon|ID=cds-Bg	11	5.873636	4351.7	0.941202	LEPSEKPFD	94	651.848	2	1301.687	.	A	Superscaf	59668657	0	0	0	0	0	BTP20220	Tissue specific
CDS|exon|ID=cds-Bg	8	6.13875	3235.4	0.941202	SEKPFDLK	98	482.2587	2	962.5073	.	A	Superscaf	59668657	0	0	0	0	0	BTP20220	Tissue specific
CDS|exon|ID=cds-Bg	12	6.271667	4435.7	0.945793	AGSGSSPRA	98	547.7626	2	1093.515	.	Q	Superscaf	59809543	0	0	0	0	0	BTP20220	Tissue specific
CDS|exon|ID=cds-Bg	14	5.876429	5583.3	0.945794	LLALEPELE	83	525.6335	3	1573.883	.	Q	Superscaf	59809543	0	0	0	0	0	BTP20220	Tissue specific
CDS|exon|ID=cds-Bg	16	5.305625	6357.2	0.946025	DYQRQPDSC	92	914.4307	2	1826.855	.	A	Superscaf	59816914	0	0	0	0	0	BTP20220	Tissue specific
CDS|exon|ID=cds-Bg	9	6.087778	3538	0.946072	ALKMRLVDH	70	361.5412	3	1081.607	.	R	Superscaf	59818240	0	0	0	0	0	BTP20220	Tissue specific
CDS|exon|ID=cds-Bg	11	4.729091	4010.4	0.94868	ANYYQVLGV	54	613.3123	2	1224.614	.	D	Superscaf	59898130	0	0	0	BTP20220	0	0	Tissue specific
CDS|exon|ID=cds-Bg	9	4.483333	3088.4	0.94868	A(+42.01)	50	534.769	2	1067.529	Acetylati	D	Superscaf	59898130	0	0	0	BTP20220	0	0	Tissue specific
CDS|exon|ID=cds-Bg	6	7.493333	2608.9	0.950314	KGYTRP	72	361.204	2	720.3918	.	D	Superscaf	59949072	BTP20220	0	0	0	0	0	Tissue specific
CDS|exon|ID=cds-Bg	7	5.922857	2515.8	0.832053	M(+15.99)	68	756.3553	1	755.3524	Oxidation	V	Superscaf	4561348	0	0	0	0	0	BTP20220	Tissue specific
CDS|exon|ID=cds-Bg	5	4.45	1219.3	0.828652	VKLVT	92	559.3804	1	558.3741	.	C	Superscaf	4653691	0	0	BTP20220	0	0	0	Tissue specific
CDS|exon|ID=cds-Bg	8	5.33125	2846.1	0.809091	KELANLVP	55	442.2706	2	882.5175	.	R	Superscaf	5185095	BTP20220	0	0	0	0	0	Tissue specific
CDS|exon|ID=cds-Bg	9	5.412222	3360.8	0.74144	LDSPVSRSY	66	512.2578	2	1022.503	.	D	Superscaf	7022395	0	BTP20220	0	0	0	0	Tissue specific
CDS|exon|ID=cds-Bg	8	4.3725	2351.6	0.71459	GEVKPEVT	59	429.7303	2	857.4494	.	I	Superscaf	7751670	0	0	0	0	0	BTP20220	Tissue specific
CDS|exon|ID=cds-Bg	8	6.07	2612.8	0.673037	SPAAPGGP	54	653.3336	1	652.318	.	D	Superscaf	8880124	0	0	0	0	BTP20220	0	Tissue specific
CDS|exon|ID=cds-Bg	8	6.155	2889.3	0.656379	AGVPPHRP	79	415.731	2	829.4558	.	L	Superscaf	9332062	0	0	BTP20220	0	0	0	Tissue specific
CDS|exon|ID=cds-Bg	16	6.07875	6650	0.63778	SSQPLASKG	95	568.2908	3	1701.853	.	R	Superscaf	9837718	0	0	0	0	0	BTP20220	Tissue specific
CDS|exon|ID=cds-Bg	10	5.703	3580.9	0.637713	KEPSEVPTP	87	556.3026	2	1110.592	.	L	Superscaf	9839569	0	BTP20220	0	0	0	0	Tissue specific
CDS|exon|ID=cds-Bg	5	4.61	2754.3	0.597356	C(+57.02)	50	774.1719	1	773.1601	Carbamido	P	Superscaf	10935589	0	0	0	BTP20220	0	0	Tissue specific
CDS|exon|ID=cds-Bg	8	5.19625	2693	0.597283	SLGNATVL	58	774.4314	1	773.4283	.	P	Superscaf	10937437	0	0	0	BTP20220	0	0	Tissue specific
CDS|exon|ID=cds-Bg	7	6.03	2628.7	0.389905	YEGGKGS	52	697.3177	1	696.3079	.	E	Superscaf	16569865	BTP20220	0	0	0	0	0	Tissue specific
CDS|exon|ID=cds-Bg	5	4.91	1325.5	0.365448	P(+42.01)	60	524.3058	1	523.3005	Acetylati	A	Superscaf	17234316	BTP20220	0	0	0	BTP20220	0	Tissue enhanced
CDS|exon|ID=cds-Bg	9	4.404444	2959.3	0.341485	E(+42.01)	61	514.7787	2	1027.544	Acetylati	M	Superscaf	17884969	0	BTP20220	0	0	0	0	Tissue specific
CDS|exon|ID=cds-Bg	6	3.533333	1443.6	0.318942	ALEVVL	87	643.4005	1	642.3952	.	A	Superscaf	18497388	0	0	0	BTP20220	0	0	Tissue specific
CDS|exon|ID=cds-Bg	15	4.32	5523.3	0.291126	PEEVHHGEE	78	869.8771	2	1737.748	.	M	Superscaf	19252777	0	0	0	0	0	BTP20220	Tissue specific
CDS|exon|ID=cds-Bg	14	4.198571	5212	0.291126	PEEVHHGEE	81	834.3624	2	1666.711	.	M	Superscaf	19252777	0	BTP20220	0	0	BTP20220	BTP20220	Mixed
CDS|exon|ID=cds-Bg	14	5.551429	5727.3	0.291068	KSLTNDWED	99	552.6155	3	1654.832	.	L	Superscaf	19254082	0	0	0	0	0	BTP20220	Tissue specific
CDS|exon|ID=cds-Bg	10	5.598	4594.2	0.291069	KSLTNDWED	86	622.7807	2	1243.547	.	L	Superscaf	19254082	0	0	0	0	0	BTP20220	Tissue specific
CDS|exon|ID=cds-Bg	13	5.229231	5258.9	0.291068	SLTNDWEDH	98	764.3724	2	1526.737	.	L	Superscaf	19254082	0	0	0	0	0	BTP20220	Tissue specific
CDS|exon|ID=cds-Bg	10	4.979	4124.6	0.291068	NDWEDHLAV	87	613.7914	2	1225.573	.	L	Superscaf	19254082	0	0	0	0	BTP20220	0	Tissue specific
CDS|exon|ID=cds-Bg	9	4.931111	3527.9	0.291068	DWEDHLAVK	99	556.7693	2	1111.53	.	L	Superscaf	19254082	0	0	0	0	BTP20220	0	Tissue specific
CDS|exon|ID=cds-Bg	11	5.401818	4619	0.291016	C(+57.02)	98	676.3333	2	1350.66	Carbamido	R	Superscaf	19255597	0	0	0	0	0	BTP20220	Tissue specific
CDS|exon|ID=cds-Bg	11	5.636364	4033.4	0.290992	LFETALLSS	85	592.8109	2	1183.613	.	G	Superscaf	19256113	0	0	0	0	0	BTP20220	Tissue specific
CDS|exon|ID=cds-Bg	10	6.009	4784.6	0.29099	LEDPQTHSN	92	598.7828	2	1195.558	.	G	Superscaf	19256113	0	0	0	0	BTP20220	BTP20220	Mixed
CDS|exon|ID=cds-Bg	10	5.57	3544.7	0.184268	FEAEPVSKP	98	566.3037	2	1130.597	.	F	Superscaf	22154971	0	0	0	0	0	BTP20220	Tissue specific
CDS|exon|ID=cds-Bg	6	6.135	2494.7	0.147989	WLSKEP	97	380.2033	2	758.3962	.	S	Superscaf	23140363	0	0	0	0	BTP20220	0	Tissue specific
CDS|exon|ID=cds-Bg	12	5.953333	4503	0.048802	TLDAATAHP	99	614.3201	2	1226.629	.	S	Superscaf	28485148	0	0	0	0	0	BTP20220	Tissue specific
CDS|exon|ID=cds-Bg	11	5.996364	4115.6	0.051217	TLDAATAHP	89	540.7858	2	1079.561	.	S	Superscaf	28550737	0	0	0	0	0	BTP20220	Tissue specific
CDS|exon|ID=cds-Bg	7	6.294286	2806.2	0.066905	TGLFTLH	81	394.7185	2	787.4229	.	V	Superscaf	28976716	0	BTP20220	0	0	0	0	Tissue specific
CDS|exon|ID=cds-Bg	14	4.980714	5059.8	0.066928	LLLPEVGPE	81	765.8868	2	1529.761	.	L	Superscaf	28977307	0	BTP20220	0	0	0	0	Tissue specific
CDS|exon|ID=cds-Bg	15	5.866	6341.2	0.06693	SC(+57.02	97	413.1913	4	1648.738	Carbamido	L	Superscaf	28977307	0	BTP20220	0	0	0	0	Tissue specific
CDS|exon|ID=cds-Bg	14	5.516429	5692.4	0.06693	SC(+57.02	90	747.3251	2	1492.637	Carbamido	L	Superscaf	28977307	0	BTP20220	0	0	0	0	Tissue specific
CDS|exon|ID=cds-Bg	13	5.503846	5309	0.06693	SC(+57.02	99	703.8087	2	1405.605	Carbamido	L	Superscaf	28977307	0	BTP20220	0	0	0	0	Tissue specific
CDS|exon|ID=cds-Bg	12	5.004167	4257.8	0.070542	LGEVTLEEA	93	633.3207	2	1264.63	.	L	Superscaf	29075437	0	0	0	0	0	BTP20220	Tissue specific
CDS|exon|ID=cds-Bg	11	5.331818	4046.3	0.071293	YWKGFDFSV	68	696.8326	2	1391.655	.	T	Superscaf	29095972	0	0	0	0	0	BTP20220	Tissue specific
CDS|exon|ID=cds-Bg	7	5.657143	2946.3	0.071955	KNPELLD	56	828.442	1	827.4388	.	E	Superscaf	29113915	0	0	0	0	0	BTP20220	Tissue specific
CDS|exon|ID=cds-Bg	15	5.008667	4989.3	0.079745	TVKVPLKVG	91	552.3167	3	1653.93	.	H	Superscaf	29325235	0	0	0	0	0	BTP20220	Tissue specific
CDS|exon|ID=cds-Bg	7	4.735714	2391.7	0.080172	PELQLVA	79	769.443	1	768.4381	.	K	Superscaf	29337402	0	0	BTP20220	BTP20220	0	0	Mixed
CDS|exon|ID=cds-Bg	10	4.474	3887.4	0.080874	A(+42.01)	74	616.2935	2	1230.577	Acetylati	D	Superscaf	29356159	0	0	0	0	0	BTP20220	Tissue specific
CDS|exon|ID=cds-Bg	12	4.54	4355.8	0.080874	A(+42.01)	94	729.8765	2	1457.74	Acetylati	D	Superscaf	29356159	0	0	0	0	BTP20220	0	Tissue specific
CDS|exon|ID=cds-Bg	8	5.9275	3417.8	0.08101	ADC(+57.0	96	465.7386	2	929.4641	Carbamido	K	Superscaf	29359651	0	0	0	0	BTP20220	0	Tissue specific
CDS|exon|ID=cds-Bg	10	5.805	3719.8	0.081062	AYEQVAKAL	78	374.2161	3	1119.629	.	V	Superscaf	29361226	0	BTP20220	0	0	BTP20220	BTP20220	Mixed
CDS|exon|ID=cds-Bg	9	5.367778	3251.4	0.081062	AYEQVAKAL	99	496.7728	2	991.5338	.	V	Superscaf	29361226	0	0	0	0	0	BTP20220	Tissue specific
CDS|exon|ID=cds-Bg	8	5.79625	2949	0.081063	EQVAKALK	97	443.7697	2	885.5283	.	V	Superscaf	29361226	0	0	0	0	0	BTP20220	Tissue specific
CDS|exon|ID=cds-Bg	8	5.28375	2628.9	0.083928	PQAAVSPP	53	766.4069	1	765.402	.	Q	Superscaf	29439073	0	BTP20220	0	0	0	0	Tissue specific
CDS|exon|ID=cds-Bg	8	5.40125	2901.1	0.089034	SDSGLFGF	91	829.3699	1	828.3654	.	A	Superscaf	29576872	0	0	0	0	0	BTP20220	Tissue specific
CDS|exon|ID=cds-Bg	7	5.361429	2517.7	0.089034	DSGLFGF	79	742.3376	1	741.3333	.	A	Superscaf	29576872	0	0	0	0	0	BTP20220	Tissue specific
CDS|exon|ID=cds-Bg	8	5.39875	2977.2	0.089034	DSGLFGFY	93	905.4004	1	904.3967	.	A	Superscaf	29576872	0	0	0	0	0	BTP20220	Tissue specific

CDS|exon|ID=cds-Bg	9	6.101111	3670.1	0.089046	SGNLGHTPF	84	465.2257	2	928.4402	.	A	Superscaf	29576872	0	0	0	0	0	BTP202209	Tissue specific
CDS|exon|ID=cds-Bg	8	6.6	3532.9	0.090215	GSHSLRYF	92	483.7426	2	965.4719	.	D	Superscaf	29609887	0	BTP202209	0	0	0	BTP202209	Tissue enhanced
CDS|exon|ID=cds-Bg	6	3.951667	1445.7	0.090374	VSLMVP	51	645.3763	1	644.3568	.	D	Superscaf	29614132	0	0	BTP202209	0	0	0	Tissue specific
CDS|exon|ID=cds-Bg	12	6.013333	5310.1	0.106843	DWRQSPGEA	87	687.3118	2	1372.612	.	P	Superscaf	30060763	0	0	0	0	BTP202209	0	Tissue specific
CDS|exon|ID=cds-Bg	6	5.388333	2120.6	0.107784	EPPM(+15.	54	372.6864	2	743.3636	Oxidation	V	Superscaf	30087070	0	BTP202209	0	0	0	0	Tissue specific
CDS|exon|ID=cds-Bg	6	6.735	2733.2	0.10779	P(+42.01)	76	401.2157	2	800.418	Acetylati	H	Superscaf	30087297	BTP202209	0	0	0	0	0	Tissue specific
CDS|exon|ID=cds-Bg	8	4.545	2692.1	0.130504	T(+42.01)	75	889.4164	1	888.4077	Acetylati	V	Superscaf	30704119	0	0	0	0	BTP202209	0	Tissue specific
CDS|exon|ID=cds-Bg	8	6.95375	3231.5	0.180548	MKASASLR	92	432.2404	2	862.4694	.	V	Superscaf	32063263	0	0	0	BTP202209	0	0	Tissue specific
CDS|exon|ID=cds-Bg	6	5.958333	2221.4	0.19102	PAAANL	50	556.3078	1	555.3016	.	Y	Superscaf	32347815	0	BTP202209	0	0	0	0	Tissue specific
CDS|exon|ID=cds-Bg	7	5.135714	2070.2	0.203991	AGAPVYL	84	690.3802	1	689.3748	.	I	Superscaf	32699878	0	0	BTP202209	0	BTP202209	0	Tissue enhanced
CDS|exon|ID=cds-Bg	7	4.697143	2214.4	0.203992	LAAVLEY	94	778.4336	1	777.4272	.	I	Superscaf	32699878	0	BTP202209	0	0	BTP202209	BTP202209	Mixed
CDS|exon|ID=cds-Bg	10	4.865	3348.7	0.203992	AAVLEYLTA	79	540.2836	2	1078.555	.	I	Superscaf	32699878	0	BTP202209	0	0	BTP202209	BTP202209	Mixed
CDS|exon|ID=cds-Bg	9	5.047778	2923.2	0.203992	AAVLEYLTA	76	475.7626	2	949.512	.	I	Superscaf	32699878	0	BTP202209	0	0	BTP202209	BTP202209	Mixed
CDS|exon|ID=cds-Bg	8	4.92625	2611.9	0.203992	AVLEYLTA	74	879.4774	1	878.4749	.	I	Superscaf	32699878	0	0	0	0	BTP202209	0	Tissue specific
CDS|exon|ID=cds-Bg	8	4.57625	2726.1	0.203992	VLEYLTAE	92	937.4843	1	936.4804	.	I	Superscaf	32699878	0	0	0	0	BTP202209	0	Tissue specific
CDS|exon|ID=cds-Bg	18	6.272778	6411.6	0.204543	PAETAAPAF	95	607.3409	3	1819.02	.	V	Superscaf	32714998	0	0	0	0	BTP202209	0	Tissue specific
CDS|exon|ID=cds-Bg	17	6.068824	5943.2	0.204543	PAETAAPAF	82	846.4704	2	1690.925	.	V	Superscaf	32714998	0	0	0	0	0	BTP202209	Tissue specific
CDS|exon|ID=cds-Bg	12	5.474167	3974.3	0.204543	PAETAAPAF	99	590.8132	2	1179.614	.	V	Superscaf	32714998	0	BTP202209	0	0	0	0	Tissue specific
CDS|exon|ID=cds-Bg	13	6.238462	4471.5	0.204543	AAPAPVEKS	98	647.3767	2	1292.745	.	V	Superscaf	32714998	0	0	0	0	BTP202209	0	Tissue specific
CDS|exon|ID=cds-Bg	11	6.278182	3848.9	0.204543	PAPVEKSPA	95	576.3524	2	1150.671	.	V	Superscaf	32714998	0	0	0	0	BTP202209	0	Tissue specific
CDS|exon|ID=cds-Bg	11	6.654545	3936.9	0.204549	SLAALKKAL	82	528.8407	2	1055.67	.	V	Superscaf	32714998	0	0	BTP202209	0	BTP202209	0	Tissue enhanced
CDS|exon|ID=cds-Bg	10	6.718	3625.6	0.204549	SLAALKKAL	89	493.3227	2	984.6331	.	V	Superscaf	32714998	0	0	BTP202209	0	BTP202209	0	Tissue enhanced
CDS|exon|ID=cds-Bg	9	6.795556	3314.3	0.204549	SLAALKKAL	85	457.8051	2	913.596	.	V	Superscaf	32714998	0	BTP202209	0	0	BTP202209	0	Tissue enhanced
CDS|exon|ID=cds-Bg	10	6.752	3553.5	0.204549	LAALKKALA	98	485.3244	2	968.6382	.	V	Superscaf	32714998	0	0	0	0	BTP202209	0	Tissue specific
CDS|exon|ID=cds-Bg	9	6.833333	3242.2	0.204549	LAALKKALA	97	449.8065	2	897.6011	.	V	Superscaf	32714998	0	0	BTP202209	BTP202209	BTP202209	0	Mixed
CDS|exon|ID=cds-Bg	8	6.935	2930.9	0.204549	LAALKKAL	86	414.2881	2	826.564	.	V	Superscaf	32714998	0	0	0	0	BTP202209	0	Tissue specific
CDS|exon|ID=cds-Bg	9	6.837778	3200.1	0.204549	AALKKALAA	98	428.7827	2	855.5541	.	V	Superscaf	32714998	0	0	0	0	BTP202209	0	Tissue specific
CDS|exon|ID=cds-Bg	8	6.94	2888.8	0.204549	AALKKALA	84	393.2641	2	784.517	.	V	Superscaf	32714998	0	BTP202209	0	0	BTP202209	0	Tissue enhanced
CDS|exon|ID=cds-Bg	17	6.304706	6216.5	0.204552	KGTLVQTKC	99	556.3071	3	1665.905	.	V	Superscaf	32714998	0	0	BTP202209	0	0	0	Tissue specific
CDS|exon|ID=cds-Bg	14	6.162857	4977.3	0.204552	KGTLVQTKC	95	652.8599	2	1303.71	.	V	Superscaf	32714998	0	0	BTP202209	0	0	0	Tissue specific
CDS|exon|ID=cds-Bg	12	6.219167	4296.6	0.204552	KGTLVQTKC	99	580.8336	2	1159.656	.	V	Superscaf	32714998	0	0	BTP202209	0	BTP202209	0	Tissue enhanced
CDS|exon|ID=cds-Bg	20	6.4155	7635	0.204552	GTLVQTKGT	93	674.7141	3	2021.127	.	V	Superscaf	32714998	0	0	0	0	0	BTP202209	Tissue specific
CDS|exon|ID=cds-Bg	14	6.067143	5053.3	0.204553	LVQTKGTGA	92	460.9195	3	1379.741	.	V	Superscaf	32714998	0	0	BTP202209	0	0	0	Tissue specific
CDS|exon|ID=cds-Bg	13	6.073846	4699.9	0.204553	VQTKGTGAS	90	423.2246	3	1266.657	.	V	Superscaf	32714998	0	0	0	0	BTP202209	BTP202209	Mixed
CDS|exon|ID=cds-Bg	13	6.762308	5110.2	0.204553	GTGASGSFK	99	647.8571	2	1293.704	.	V	Superscaf	32714998	0	0	0	0	0	BTP202209	Tissue specific
CDS|exon|ID=cds-Bg	12	6.514167	4641.8	0.204553	GTGASGSFK	99	583.8091	2	1165.609	.	V	Superscaf	32714998	0	0	0	0	BTP202209	BTP202209	Mixed
CDS|exon|ID=cds-Bg	11	6.220909	4173.4	0.204553	GTGASGSFK	96	519.7639	2	1037.514	.	V	Superscaf	32714998	0	BTP202209	0	0	0	BTP202209	Mixed
CDS|exon|ID=cds-Bg	10	6.302	3576.7	0.204553	GTGASGSFK	93	462.7422	2	923.4712	.	V	Superscaf	32714998	0	BTP202209	0	0	0	BTP202209	Mixed
CDS|exon|ID=cds-Bg	9	6.338889	3279.4	0.204553	TGASGSFKL	99	434.2312	2	866.4498	.	V	Superscaf	32714998	0	BTP202209	0	0	0	BTP202209	Mixed
CDS|exon|ID=cds-Bg	11	6.855455	4415.4	0.204553	GASGSFKLN	97	568.8225	2	1135.635	.	V	Superscaf	32714998	0	0	0	0	BTP202209	0	Tissue specific
CDS|exon|ID=cds-Bg	9	7.046667	3806.8	0.204554	SGSFKLNKK	97	504.794	2	1007.576	.	V	Superscaf	32714998	0	0	0	0	BTP202209	0	Tissue specific
CDS|exon|ID=cds-Bg	15	7.14	5451.2	0.204562	KAAKSPAKP	99	388.003	4	1547.988	.	V	Superscaf	32714998	0	0	0	0	0	BTP202209	Tissue specific
CDS|exon|ID=cds-Bg	14	6.954286	4982.8	0.204562	KAAKSPAKP	96	474.303	3	1419.893	.	V	Superscaf	32714998	0	0	0	0	0	BTP202209	Tissue specific
CDS|exon|ID=cds-Bg	11	6.870909	3891.8	0.204563	KSPAKPKAV	96	575.8668	2	1149.723	.	V	Superscaf	32714998	0	0	0	0	0	BTP202209	Tissue specific
CDS|exon|ID=cds-Bg	16	7.0875	5788.6	0.204563	SPAKPKAVK	98	549.3523	3	1645.04	.	V	Superscaf	32714998	0	0	0	0	BTP202209	0	Tissue specific
CDS|exon|ID=cds-Bg	11	6.870909	3891.8	0.204563	SPAKPKAVK	99	384.2472	3	1149.723	.	V	Superscaf	32714998	0	0	0	0	BTP202209	BTP202209	Mixed
CDS|exon|ID=cds-Bg	10	6.584	3423.4	0.204563	SPAKPKAVK	70	511.8199	2	1021.628	.	V	Superscaf	32714998	0	0	0	0	BTP202209	0	Tissue specific
CDS|exon|ID=cds-Bg	23	6.027391	8442.1	0.204621	RLLLPGELA	90	497.0891	5	2480.411	.	L	Superscaf	32716852	0	BTP202209	0	0	0	0	Tissue specific
CDS|exon|ID=cds-Bg	9	5.823333	3576.9	0.205019	KGNYSERVC	77	505.2552	2	1008.499	.	V	Superscaf	32727856	0	0	0	0	BTP202209	0	Tissue specific
CDS|exon|ID=cds-Bg	9	4.946667	3027.3	0.20502	YLAAVLEYL	86	1054.579	1	1053.575	.	V	Superscaf	32727856	0	0	0	0	BTP202209	0	Tissue specific
CDS|exon|ID=cds-Bg	8	4.8175	2673.9	0.20502	YLAAVLEY	83	941.496	1	940.4905	.	V	Superscaf	32727856	0	BTP202209	0	0	BTP202209	0	Mixed
CDS|exon|ID=cds-Bg	12	6.5075	4895.2	0.205027	VLLPKKTES	99	472.9473	3	1415.825	.	V	Superscaf	32727856	0	0	0	0	BTP202209	0	Tissue specific
CDS|exon|ID=cds-Bg	14	6.888571	5828.4	0.205357	ALKRQGRTL	84	762.4216	2	1522.837	.	R	Superscaf	32736853	0	0	0	0	0	BTP202209	Tissue specific
CDS|exon|ID=cds-Bg	11	6.790909	4695.3	0.205358	RQGRTLYGF	89	606.3167	2	1210.621	.	R	Superscaf	32736853	0	BTP202209	0	0	0	0	Tissue specific
CDS|exon|ID=cds-Bg	8	5.24625	2381.5	0.205843	AGAPVYLA	99	761.4175	1	760.4119	.	I	Superscaf	32750212	0	BTP202209	0	0	0	0	Tissue specific
CDS|exon|ID=cds-Bg	20	5.835	7116.7	0.218816	RLLLPGELA	87	523.0572	4	2088.205	.	P	Superscaf	33102472	0	0	0	0	BTP202209	0	Tissue specific
CDS|exon|ID=cds-Bg	22	6.044091	7982.6	0.218816	RLLLPGELA	97	464.4753	5	2317.348	.	P	Superscaf	33102472	0	0	0	0	BTP202209	0	Tissue specific
CDS|exon|ID=cds-Bg	27	6.17037	10002.7	0.218816	RLLLPGELA	97	717.9103	4	2867.623	.	P	Superscaf	33102472	0	0	0	0	BTP202209	0	Tissue specific
CDS|exon|ID=cds-Bg	18	6.148889	6805.4	0.218816	RLLLPGELA	90	480.5306	4	1918.1	.	P	Superscaf	33102472	0	0	0	0	BTP202209	0	Tissue specific
CDS|exon|ID=cds-Bg	18	6.133889	6568.8	0.218817	KHAVSEGTK	99	636.0146	3	1905.032	.	P	Superscaf	33102472	0	0	0	0	BTP202209	0	Tissue specific
CDS|exon|ID=cds-Bg	17	5.921765	6100.4	0.218817	HAVSEGTKA	99	593.3182	3	1776.937	.	P	Superscaf	33102472	0	BTP202209	0	0	BTP202209	BTP202209	Mixed
CDS|exon|ID=cds-Bg	16	5.683125	5632	0.218817	HAVSEGTKA	79	550.6196	3	1648.842	.	P	Superscaf	33102472	0	0	0	0	0	BTP202209	Tissue specific
CDS|exon|ID=cds-Bg	14	6.218571	5169.4	0.218818	SEGTKAVTK	99	490.931	3	1469.773	.	P	Superscaf	33102472	0	BTP202209	0	0	BTP202209	0	Mixed
CDS|exon|ID=cds-Bg	9	6.213333	3197.3	0.218818	AVTKYTSAK	85	484.773	2	967.5338	.	P	Superscaf	33102472	0	0	0	BTP202209	BTP202209	BTP202209	Mixed
CDS|exon|ID=cds-Bg	9	5.33	3269.5	0.219131	KVLKQVHPD	76	532.3146	2	1062.619	.	E	Superscaf	33111058	0	0	0	0	0	BTP202209	Tissue specific
CDS|exon|ID=cds-Bg	11	6.574545	4594.1	0.231386	TYTEHAKRK	90	667.3624	2	1332.715	.	V	Superscaf	33443719	0	0	0	0	BTP202209	0	Tissue specific
CDS|exon|ID=cds-Bg	12	5.688333	3800	0.232584	PVAPAAPAF	99	559.8136	2	1117.613	.	T	Superscaf	33476617	0	BTP202209	0	0	0	0	Tissue specific
CDS|exon|ID=cds-Bg	18	6.128333	5940.1	0.232584	PVAPAAPAF	98	600.6993	3	1799.067	.	T	Superscaf	33476617	0	BTP202209	0	0	BTP202209	0	Mixed
CDS|exon|ID=cds-Bg	17	5.915882	5471.7	0.232584	PVAPAAPAF	92	836.4969	2	1670.972	.	T	Superscaf	33476617	0	BTP202209	0	0	BTP202209	0	Mixed
CDS|exon|ID=cds-Bg	16	5.676875	5003.3	0.232584	PVAPAAPAF	95	772.4464	2	1542.877	.	T	Superscaf	33476617	0	BTP202209	BTP202209	0	BTP202209	BTP202209	Mixed
CDS|exon|ID=cds-Bg	14	6.037857	4665.9	0.232584	APAAPAPAE	99	674.3849	2	1346.756	.	T	Superscaf	33476617	0	0	0	0	BTP202209	0	Tissue specific
CDS|exon|ID=cds-Bg	9	6.215556	3151.3	0.232584	PAAPAPAEK	99	426.2282	2	850.4548	.	T	Superscaf	33476617	0	BTP202209	0	0	0	0	Tissue specific
CDS|exon|ID=cds-Bg	11	6.251818	3886.2	0.232584	PAAPAPAEK	86	525.2835	2	1048.555	.	T	Superscaf	33476617	0	0	0	0	BTP202209	0	Tissue specific
CDS|exon|ID=cds-Bg	14	6.303571	4823	0.232584	PAAPAPAEK	96	468.9431	3	1403.814	.	T	Superscaf	33476617	0	0	0	0	BTP202209	BTP202209	Tissue enhanced
CDS|exon|ID=cds-Bg	13	6.039231	4354.6	0.232584	PAAPAPAEK	99	638.861	2	1275.719	.	T	Superscaf	33476617	0	0	0	0	BTP202209	0	Tissue specific
CDS|exon|ID=cds-Bg	14	6.549286	4954	0.232584	AAPAPAEKT	98	479.2917	3	1434.856	.	T	Superscaf	33476617	0	BTP202209	0	0	0	0	Tissue specific
CDS|exon|ID=cds-Bg	13	6.303846	4485.6	0.232584	AAPAPAEKT	97	436.593	3	1306.761	.	T	Superscaf	33476617	0	0	0	0	0	BTP202209	Tissue specific
CDS|exon|ID=cds-Bg	12	6.0175	4017.2	0.232584	AAPAPAEKT	99	590.3387	2	1178.666	.	T	Superscaf	33476617	0	0	0	0	BTP202209	0	Tissue specific
CDS|exon|ID=cds-Bg	12	6.3275	4174.3	0.232585	APAPAEKTF	99	412.9138	3	1235.724	.	T	Superscaf	33476617	0	0	0	BTP202209	0	0	Tissue specific
CDS|exon|ID=cds-Bg	11	6.355455	3863	0.232585	PAPAEKTPV	97	389.2341	3	1164.687	.	T	Superscaf	33476617	0	0	0	0	BTP202209	0	Tissue specific
CDS|exon|ID=cds-Bg	10	6.017	3394.6	0.232585	PAPAEKTPV	94	519.2982	2	1036.592	.	T	Superscaf	33476617	0	0	0	0	0	BTP202209	Tissue specific
CDS|exon|ID=cds-Bg	14	6.762143	4885.9	0.2326	TPKKVKKPA	59	684.4199	2	1366.83	.	T	Superscaf	33476617	0	0	0	0	0	BTP202209	Tissue specific
CDS|exon|ID=cds-Bg	12	6.753333	4569.7	0.238277	PEPSKSAPA	98	412.9013	3	1235.687	.	H	Superscaf	33631228	0	0	0	0	BTP202209	0	Tissue specific
CDS|exon|ID=cds-Bg	11	6.481818	4101.3	0.238277	PEPSKSAPA	98	370.2041	3	1107.592	.	H	Superscaf	33631228	0	BTP202209	0	0	BTP202209	BTP202209	Mixed
CDS|exon|ID=cds-Bg	10	6.156	3632.9	0.238277	PEPSKSAPA	99	490.7542	2	979.4974	.	H	Superscaf	33631228	0	0	0	0	BTP202209	BTP202209	Mixed
CDS|exon|ID=cds-Bg	10	6.076	3807.1	0.238528	VLLPKKTES	90	384.5627	3	1150.671	.	N	Superscaf	33637714	0	0	0	BTP202209	0	0	Tissue specific
CDS|exon|ID=cds-Bg	9	6.751111	3807.1	0.238528	LLPKKTESH	97	351.54	3	1051.603	.	N	Superscaf	33637714	0	0	BTP202209	BTP202209	BTP202209	0	Mixed
CDS|exon|ID=cds-Bg	12	6.321667	4229.4	0.238568	SAPATGGVK	92	383.8822	3	1148.63	.	V	Superscaf	33638986	0	0	0	0	BTP202209	BTP202209	Mixed
CDS|exon|ID=cds-Bg	10	6.416	3534.7	0.238568	PATGGVKKF	98	496.2858	2	990.561	.	V	Superscaf	33638986	0	0	0	0	0	BTP202209	Tissue specific
CDS|exon|ID=cds-Bg	9	6.442222	3454	0.238569	RYRPGTVAL	65	516.7992	2	1031.588	.	V	Superscaf	33638986	0	0	0	0	0	BTP202209	Tissue specific
CDS|exon|ID=cds-Bg	11	5.862727	4355.8	0.238573	KTDLRFQSS	87	626.3356	2	1250.662	.	V	Superscaf	33638986	0	0	0	0	BTP202209	0	Tissue specific
CDS|exon|ID=cds-Bg	14	4.995	5395	0.238574	QSSAVMALQ	67	747.8284	2	1493.649	Carbamido	V	Superscaf	33638986	0	0	0	0	0	BTP202209	Tissue specific
CDS|exon|ID=cds-Bg	15	5.039333	5854.5	0.238574	QSSAVMALQ	67	829.3611	2	1656.712	Carbamido	V	Superscaf	33638986	0	0	0	0	0	BTP202209	Tissue specific
CDS|exon|ID=cds-Bg	14	4.995714	5243.8	0.238574	SSAVMALQE	70	765.3324	2	1528.654	Carbamido	V	Superscaf	33638986	0	0	0	0	BTP202209	0	Tissue specific
CDS|exon|ID=cds-Bg	9	6.408889	3701.8	0.240648	A(+42.01)	84	495.2646	2	988.5189	Acetylati	P	Superscaf	33695611	0	0	0	0	0	BTP202209	Tissue specific
CDS|exon|ID=cds-Bg	7	6.172857	2546.7	0.248158	LPGELAK	99	364.2201	2	726.4276	.	V	Superscaf	33899284	0	0	0	0	BTP202209	BTP202209	Mixed
CDS|exon|ID=cds-Bg	18	6.115	6640.9	0.248159	KHAVSEGTK	99	641.3497	3	1921.027	.	V	Superscaf	33899284	0	BTP202209	BTP202209	BTP202209	BTP202209	0	Mixed
CDS|exon|ID=cds-Bg	12	5.556667	4909.7	0.515992	TMQVSSLQR	92	711.8528	2	1421.697	.	L	Superscaf	41173861	0	BTP202209	0	0	0	0	Tissue specific
CDS|exon|ID=cds-Bg	9	5.416667	3530	0.515992	VSSLQREPF	93	531.7817	2	1061.551	.	L	Superscaf	41173861	0	BTP202209	0	0	0	BTP202209	Mixed
CDS|exon|ID=cds-Bg	8	6.09375	3530	0.515992	SSLQREPF	98	482.2474	2	962.4821	.	L	Superscaf	41173861	0	BTP202209	0	0	0	BTP202209	Mixed
CDS|exon|ID=cds-Bg	16	6.184375	6206.6	0.516773	KLKKPPTDE	94	628.3641	3	1882.077	.	N	Superscaf	41195062	0	0	0	0	BTP202209	BTP202209	Mixed
CDS|exon|ID=cds-Bg	12	6.621667	4915.2	0.516773	KLKKPPTDE	96	475.9468	3	1424.824	.	N	Superscaf	41195062	0	0	0	0	BTP202209	BTP202209	Mixed
CDS|exon|ID=cds-Bg	11	5.535455	4241.6	0.516773	TDEELKETV	88	440.5718	3	1318.698	.	N	Superscaf	41195062	0	BTP202209	0	0	0	0	Tissue specific
CDS|exon|ID=cds-Bg	11	5.884545	4022.3	0.81071	A(+42.01)	99	586.3302	2	1170.65	Acetylati	G	Superscaf	49178323	0	0	0	0	BTP202209	BTP202209	Mixed
CDS|exon|ID=cds-Bg	20	4.7985	7255.3	0.81491	VDC(+57.0	88	1086.464	2	2170.926	Carbamido	T	Superscaf	49292248	0	0	0	0	0	BTP202209	Tissue specific
CDS|exon|ID=cds-Bg	16	4.469375	5763.6	0.81491	VDC(+57.0	93	877.374	2	1752.741	Carbamido	T	Superscaf	49292248	0	0	0	0	0	BTP202209	Tissue specific
CDS|exon|ID=cds-Bg	11	5.448182	4035.4	0.815459	ALGLEHSET	95	592.3162	2	1182.625	.	I	Superscaf	49307266	0	0	0	0	0	BTP202209	Tissue specific
CDS|exon|ID=cds-Bg	8	5.245	3073.4	0.81546	LEHSETVK	95	471.7465	2	941.4818	.	I	Superscaf	49307266	0	0	0	0	0	BTP202209	Tissue specific
CDS|exon|ID=cds-Bg	9	5.591111	3583	0.815701	VLRQAKDEL	88	536.309	2	1070.608	.	I	Superscaf	49313803	0	0	0	0	0	BTP202209	Tissue specific
CDS|exon|ID=cds-Bg	8	5.37375	2841.2	0.858664	EPGALLEP	62	413.2191	2	824.4279	.	Q	Superscaf	50480779	0	0	0	0	0	BTP202209	Tissue specific
CDS|exon|ID=cds-Bg	6	5.971667	2146.3	0.8588	ATGSYG	54	555.2432	1	554.2336	.	G	Superscaf	50484397	0	0	BTP202209	0	0	0	Tissue specific
CDS|exon|ID=cds-Bg	13	5.936923	5186.8	0.886297	A(+42.01)	85	746.3967	2	1490.784	Acetylati	T	Superscaf	51231244	0	0	BTP202209	0	0	0	Tissue specific
CDS|exon|ID=cds-Bg	19	4.645263	6264	0.926555	GHYTEGAEL	99	696.3607	3	2086.069	.	P	Superscaf	52324519	0	0	0	0	0	BTP202209	Tissue specific
CDS|exon|ID=cds-Bg	18	4.362222	5795.6	0.926555	GHYTEGAEL	88	653.6633	3	1957.974	.	P	Superscaf	52324519	0	0	0	0	0	BTP202209	Tissue specific
CDS|exon|ID=cds-Bg	18	4.571667	5966.7	0.926555	HYTEGAELV	89	677.3548	3	2029.048	.	P	Superscaf	52324519	0	0	0	0	0	BTP202209	Tissue specific
CDS|exon|ID=cds-Bg	17	4.267647	5498.3	0.926555	HYTEGAELV	70	634.6569	3	1900.953	.	P	Superscaf	52324519	0	0	0	0	0	BTP202209	Tissue specific
CDS|exon|ID=cds-Bg	9	4.396667	3081.5	0.926555	YTEGAELVD	97	996.4487	1	995.4447	.	P	Superscaf	52324519	0	0	0	BTP202209	0	0	Tissue specific
CDS|exon|ID=cds-Bg	17	4.394118	5347	0.926555	YTEGAELVD	99	946.9984	2	1891.989	.	P	Superscaf	52324519	0	0	0	0	0	BTP202209	Tissue specific
CDS|exon|ID=cds-Bg	16	4.06	4878.6	0.926555	YTEGAELVD	99	882.9514	2	1763.894	.	P	Superscaf	52324519	0	0	0	0	0	BTP202209	Tissue specific
CDS|exon|ID=cds-Bg	16	4.315	4887.5	0.926555	TEGAELVDS	97	865.4669	2	1728.926	.	P	Superscaf	52324519	0	0	0	0	0	BTP202209	Tissue specific
CDS|exon|ID=cds-Bg	11	4.142727	3358.8	0.926555	TEGAELVDS	76	566.7881	2	1131.566	.	P	Superscaf	52324519	0	0	0	0	0	BTP202209	Tissue specific
CDS|exon|ID=cds-Bg	16	4.108125	4915.5	0.926555	EGAELVDSV	84	586.6455	3	1756.921	.	P	Superscaf	52324519	0	0	0	0	BTP202209	0	Tissue specific
CDS|exon|ID=cds-Bg	15	4.167333	4490	0.926555	EGAELVDSV	94	543.6315	3	1627.878	.	P	Superscaf	52324519	0	0	0	0	BTP202209	BTP202209	Mixed
CDS|exon|ID=cds-Bg	14	3.769286	4021.6	0.926555	EGAELVDSV	95	750.8953	2	1499.783	.	P	Superscaf	52324519	0	0	0	0	0	BTP202209	Tissue specific
CDS|exon|ID=cds-Bg	13	3.231538	3372.8	0.926555	EGAELVDSV	73	672.8458	2	1343.682	.	P	Superscaf	52324519	0	0	0	0	0	BTP202209	Tissue specific
CDS|exon|ID=cds-Bg	10	3.904	2961.3	0.926555	EGAELVDSV	77	516.2644	2	1030.518	.	P	Superscaf	52324519	0	0	0	0	0	BTP202209	Tissue specific
CDS|exon|ID=cds-Bg	13	4.101538	3767.2	0.926556	AELVDSVLD	90	721.9131	2	1441.814	.	P	Superscaf	52324519	0	0	0	0	0	BTP202209	Tissue specific
CDS|exon|ID=cds-Bg	11	2.983636	2650	0.926556	AELVDSVLD	55	579.8146	2	1157.618	.	P	Superscaf	52324519	0	0	0	0	0	BTP202209	Tissue specific
CDS|exon|ID=cds-Bg	9	4.998889	4040.6	0.926557	ESC(+57.0	66	558.2083	2	1114.406	Carbamido	P	Superscaf	52324519	0	0	0	0	BTP202209	0	Tissue specific
CDS|exon|ID=cds-Bg	8	5.3575	3272.7	0.926564	NATLSVHQ	98	869.4424	1	868.4402	.	P	Superscaf	52324519	0	0	0	0	0	BTP202209	Tissue specific
CDS|exon|ID=cds-Bg	8	5.42875	3029.4	0.926564	ATLSVHQL	91	434.747	2	867.4814	.	P	Superscaf	52324519	0	0	0	BTP202209	0	0	Tissue specific
CDS|exon|ID=cds-Bg	10	4.665	3454.9	0.926564	ATLSVHQLV	86	548.8016	2	1095.592	.	P	Superscaf	52324519	0	0	0	BTP202209	0	0	Tissue specific

CDS|exon|ID=cds-Bg	13	6.243846	5443.2	0.926567	KLTTPTYGI	70	491.5954	3	1471.767	.	P	Superscaf	52324519	0	BTP20220	0	BTP20220	0	0	Tissue enhanced
CDS|exon|ID=cds-Bg	9	6.115556	3920.5	0.92657	PGQLNADLF	62	492.2641	2	982.5196	.	P	Superscaf	52324519	0	0	0	BTP20220	0	0	Tissue specific
CDS|exon|ID=cds-Bg	9	5.875556	3454.8	0.926571	ADLRKLAVN	75	500.2995	2	998.5872	.	P	Superscaf	52324519	0	0	BTP20220	0	BTP20220	0	Mixed
CDS|exon|ID=cds-Bg	8	5.93375	2858.1	0.926571	ADLRKLAV	60	443.2762	2	884.5443	.	P	Superscaf	52324519	0	0	BTP20220	0	0	0	Tissue specific
CDS|exon|ID=cds-Bg	8	5.8575	3143.5	0.926571	DLRKLAVN	73	464.7808	2	927.5501	.	P	Superscaf	52324519	0	0	0	0	BTP20220	0	Tissue specific
CDS|exon|ID=cds-Bg	13	5.465385	4704.4	0.926571	LAVNMVPFF	83	514.2845	3	1539.838	.	P	Superscaf	52324519	0	0	0	0	0	BTP20220	Tissue specific
CDS|exon|ID=cds-Bg	12	5.464167	4317	0.926571	LAVNMVPFF	73	697.3909	2	1392.77	.	P	Superscaf	52324519	0	0	0	0	0	BTP20220	Tissue specific
CDS|exon|ID=cds-Bg	10	5.2	3343.9	0.926571	LAVNMVPFF	84	572.3187	2	1142.627	.	P	Superscaf	52324519	0	0	0	0	BTP20220	BTP20220	Tissue enhanced
CDS|exon|ID=cds-Bg	13	5.344615	5154.9	0.926575	TVPELTQQM	89	762.3755	2	1522.734	.	P	Superscaf	52324519	0	0	0	0	0	BTP20220	Tissue specific
CDS|exon|ID=cds-Bg	15	4.944667	6093.9	0.926579	SMKEVDEQM	89	896.929	2	1791.85	.	P	Superscaf	52324519	0	BTP20220	0	0	0	0	Tissue specific
CDS|exon|ID=cds-Bg	13	4.826154	5339	0.926579	KEVDEQMLN	97	787.8945	2	1573.777	.	P	Superscaf	52324519	0	BTP20220	0	0	0	BTP20220	Mixed
CDS|exon|ID=cds-Bg	12	4.416667	4870.6	0.926579	KEVDEQMLN	53	723.8491	2	1445.682	.	P	Superscaf	52324519	0	0	0	0	0	BTP20220	Tissue specific
CDS|exon|ID=cds-Bg	12	4.416667	4870.6	0.926579	EVDEQMLNV	98	723.8472	2	1445.682	.	P	Superscaf	52324519	0	BTP20220	0	0	0	BTP20220	Tissue enhanced
CDS|exon|ID=cds-Bg	11	4.525455	4445.1	0.926579	VDEQMLNVG	99	659.3255	2	1316.639	.	P	Superscaf	52324519	0	BTP20220	0	0	0	0	Tissue specific
CDS|exon|ID=cds-Bg	10	5.471	4360.9	0.92658	MLNVQNKNS	96	567.7798	2	1133.55	.	P	Superscaf	52324519	0	0	0	0	0	BTP20220	Tissue specific
CDS|exon|ID=cds-Bg	11	5.488182	4820.4	0.92658	MLNVQNKNS	69	649.3109	2	1296.613	.	P	Superscaf	52324519	0	0	0	0	BTP20220	BTP20220	Mixed
CDS|exon|ID=cds-Bg	10	5.553	4159.8	0.926587	LHWYTGEGM	70	604.7534	2	1207.497	.	P	Superscaf	52324519	0	0	0	BTP20220	0	0	Tissue specific
CDS|exon|ID=cds-Bg	15	4.938667	5055.9	0.927327	VDLEPGTMD	94	780.3682	2	1558.73	.	S	Superscaf	52345522	0	0	0	0	BTP20220	0	Tissue specific
CDS|exon|ID=cds-Bg	10	4.537	3389	0.927817	VDLEPGTM(	75	540.2301	2	1078.449	Oxidation	S	Superscaf	52358824	0	0	0	0	BTP20220	BTP20220	Mixed
CDS|exon|ID=cds-Bg	12	4.6775	4037.8	0.927817	VDLEPGTMD	98	659.8161	2	1317.624	.	S	Superscaf	52358824	0	0	0	0	0	BTP20220	Tissue specific
CDS|exon|ID=cds-Bg	11	4.124545	3389	0.927817	VDLEPGTMD	70	581.7675	2	1161.522	.	S	Superscaf	52358824	0	BTP20220	0	0	0	BTP20220	Mixed
CDS|exon|ID=cds-Bg	8	4.59	2594.1	0.927817	VDLEPGTM	85	861.3992	1	860.395	.	S	Superscaf	52358824	0	0	0	BTP20220	BTP20220	BTP20220	Mixed
CDS|exon|ID=cds-Bg	9	5.242222	3272.9	0.927817	EPGTMDSVF	98	496.2287	2	990.444	.	S	Superscaf	52358824	0	0	0	BTP20220	0	0	Tissue specific
CDS|exon|ID=cds-Bg	8	5.495	2847.4	0.927817	PGTMDSVR	98	431.7067	2	861.4014	.	S	Superscaf	52358824	0	0	0	BTP20220	0	0	Tissue specific
CDS|exon|ID=cds-Bg	10	5.446	3757.2	0.927818	DSVRSGPF	77	525.252	2	1048.494	.	S	Superscaf	52358824	0	0	0	0	BTP20220	0	Tissue specific
CDS|exon|ID=cds-Bg	11	4.007273	3030.4	0.927848	LVDSVLDVV	98	621.8721	2	1241.734	.	Y	Superscaf	52359733	0	0	0	0	BTP20220	BTP20220	Mixed
CDS|exon|ID=cds-Bg	11	3.756364	3102.5	0.927848	VDSVLDVVF	86	629.8507	2	1257.693	.	Y	Superscaf	52359733	0	0	0	0	BTP20220	0	Tissue specific
CDS|exon|ID=cds-Bg	10	3.81	2677	0.927848	VDSVLDVVF	98	377.2234	3	1128.65	.	Y	Superscaf	52359733	0	BTP20220	0	0	BTP20220	BTP20220	Mixed
CDS|exon|ID=cds-Bg	9	4.233333	2677	0.927848	DSVLDVVRK	99	515.7964	2	1029.582	.	Y	Superscaf	52359733	0	0	0	0	BTP20220	0	Tissue specific
CDS|exon|ID=cds-Bg	9	4.261111	2691	0.927848	SVLDVVRKE	85	522.8043	2	1043.597	.	Y	Superscaf	52359733	0	0	0	BTP20220	0	0	Tissue specific
CDS|exon|ID=cds-Bg	8	4.39125	2265.5	0.927848	SVLDVVRK	91	458.2834	2	914.5549	.	Y	Superscaf	52359733	0	BTP20220	0	0	BTP20220	BTP20220	Mixed
CDS|exon|ID=cds-Bg	14	4.580714	4312.9	0.927854	MPSPKVSDT	79	774.8819	2	1547.754	.	Y	Superscaf	52359733	0	0	0	0	BTP20220	0	Tissue specific
CDS|exon|ID=cds-Bg	26	6.033077	9929.7	0.95055	A(+42.01)	91	869.4191	3	2605.249	Acetylati	R	Superscaf	52975927	0	0	0	0	0	BTP20220	Tissue specific
CDS|exon|ID=cds-Bg	22	6.141818	8796.6	0.95055	A(+42.01)	98	732.3256	3	2193.964	Acetylati	R	Superscaf	52975927	0	0	0	0	BTP20220	BTP20220	Mixed
CDS|exon|ID=cds-Bg	12	5.2625	5082.8	0.963792	WLMLQKDEF	81	775.8615	2	1549.712	.	G	Superscaf	53335939	0	0	0	0	0	BTP20220	Tissue specific
CDS|exon|ID=cds-Bg	17	5.934706	6344.9	0.893666	AAPGAVDAM	93	556.9526	3	1667.841	.	M	Superscaf	3370912	0	0	BTP20220	0	0	0	Tissue specific
CDS|exon|ID=cds-Bg	9	5.288889	3040.3	0.842165	KM(+15.99	54	520.279	2	1038.542	Oxidation	A	Superscaf	5004448	0	0	0	0	0	BTP20220	Tissue specific
CDS|exon|ID=cds-Bg	9	5.48	3177.2	0.835408	A(+42.01)	99	495.757	2	989.5029	Acetylati	E	Superscaf	5218714	0	0	0	0	0	BTP20220	Tissue specific
CDS|exon|ID=cds-Bg	10	5.498	3636.7	0.835408	A(+42.01)	99	577.2892	2	1152.566	Acetylati	E	Superscaf	5218714	0	BTP20220	BTP20220	0	BTP20220	BTP20220	Tissue enhanced
CDS|exon|ID=cds-Bg	11	5.512727	4096.2	0.835408	A(+42.01)	84	658.8214	2	1315.63	Acetylati	E	Superscaf	5218714	0	BTP20220	BTP20220	0	BTP20220	BTP20220	Mixed
CDS|exon|ID=cds-Bg	12	5.5975	4493.7	0.835408	A(+42.01)	98	709.3461	2	1416.677	Acetylati	E	Superscaf	5218714	0	0	BTP20220	0	0	0	Tissue specific
CDS|exon|ID=cds-Bg	9	5.751111	3405.6	0.835405	YKVYDLTKF	91	588.8168	2	1175.623	.	E	Superscaf	5218714	0	0	0	0	0	BTP20220	Tissue specific
CDS|exon|ID=cds-Bg	8	5.7625	2946.1	0.835405	KVYDLTKF	99	507.2844	2	1012.559	.	E	Superscaf	5218714	0	0	0	0	0	BTP20220	Tissue specific
CDS|exon|ID=cds-Bg	7	5.86	2320.4	0.28927	GDVPKKP	69	370.7188	2	739.4228	.	P	Superscaf	22535188	BTP20220	0	0	0	0	0	Tissue specific
CDS|exon|ID=cds-Bg	8	5.88375	2570.8	0.273374	A(+42.01)	58	398.7366	2	795.4603	Acetylati	G	Superscaf	23039188	0	0	0	BTP20220	0	0	Tissue specific
CDS|exon|ID=cds-Bg	16	5.550625	6505.2	0.080193	FQSLNQYLF	87	940.9664	2	1879.926	.	G	Superscaf	34249762	0	0	0	0	0	BTP20220	Tissue specific
CDS|exon|ID=cds-Bg	12	5.614167	5162.6	0.145905	DLEDKKVNG	95	481.258	3	1440.757	.	D	Superscaf	36333394	0	0	0	0	0	BTP20220	Tissue specific
CDS|exon|ID=cds-Bg	9	5.145556	3615.1	0.202001	LLQSVNQLI	65	514.3093	2	1026.607	.	H	Superscaf	38112073	0	0	0	0	0	BTP20220	Tissue specific
CDS|exon|ID=cds-Bg	11	6.037273	4210.8	0.216708	TMFLTMFGE	82	659.3307	2	1316.651	.	Q	Superscaf	38578270	0	0	0	0	BTP20220	BTP20220	Mixed
CDS|exon|ID=cds-Bg	10	6.043	3857.4	0.216708	TMFLTMFGE	97	602.7884	2	1203.567	.	Q	Superscaf	38578270	0	0	0	0	0	BTP20220	Tissue specific
CDS|exon|ID=cds-Bg	12	6.155833	4891.3	0.358412	A(+42.01)	97	630.2842	2	1258.561	Acetylati	V	Superscaf	43071286	0	0	0	0	BTP20220	BTP20220	Mixed
CDS|exon|ID=cds-Bg	11	6.201818	4280.6	0.358412	A(+42.01)	97	566.257	2	1130.503	Acetylati	V	Superscaf	43071286	0	0	0	BTP20220	0	0	Tissue specific
CDS|exon|ID=cds-Bg	9	6.378889	3235.4	0.358412	A(+42.01)	76	433.2079	2	864.4011	Acetylati	V	Superscaf	43071286	0	0	0	0	0	BTP20220	Tissue specific
CDS|exon|ID=cds-Bg	10	6.357	4040.5	0.387103	SGPFGQLFF	71	553.2897	2	1104.572	.	D	Superscaf	43981003	0	0	0	0	BTP20220	0	Tissue specific
CDS|exon|ID=cds-Bg	6	5.841667	2313.5	0.387103	SGPFGQ	96	592.2699	1	591.2653	.	D	Superscaf	43981003	0	0	0	0	BTP20220	BTP20220	Mixed
CDS|exon|ID=cds-Bg	11	6.024545	4665.3	0.387103	GPFGQLFRF	82	624.3133	2	1246.609	.	D	Superscaf	43981003	0	0	0	0	BTP20220	0	Tissue specific
CDS|exon|ID=cds-Bg	7	5.832857	2670.9	0.387103	GPFGQLF	66	765.391	1	764.3857	.	D	Superscaf	43981003	0	0	0	BTP20220	0	0	Tissue specific
CDS|exon|ID=cds-Bg	7	6.781429	2858.1	0.387273	ADLRKLA	85	393.744	2	785.4759	.	W	Superscaf	43986040	0	0	BTP20220	0	BTP20220	0	Mixed
CDS|exon|ID=cds-Bg	9	5.246667	3554.8	0.409729	LVFAFEKQG	97	555.3002	2	1108.592	.	D	Superscaf	44698525	0	0	0	0	0	BTP20220	Tissue specific
CDS|exon|ID=cds-Bg	8	5.155	3201.4	0.409729	VFAFEKQQ	94	498.7604	2	995.5076	.	D	Superscaf	44698525	0	BTP20220	0	0	0	0	Tissue specific
CDS|exon|ID=cds-Bg	6	4.95	1896.1	0.467682	VERVLK	73	372.241	2	742.4701	.	R	Superscaf	46536121	0	0	0	0	BTP20220	0	Tissue specific
CDS|exon|ID=cds-Bg	17	5.774706	6694.3	0.558566	M(+42.01)	98	835.8484	2	1669.685	Acetylati	V	Superscaf	49417807	0	BTP20220	0	0	0	0	Tissue specific
CDS|exon|ID=cds-Bg	11	6.958182	4560.9	0.62502	LHTKEKGKF	98	427.916	3	1280.728	.	V	Superscaf	51524770	0	BTP20220	0	BTP20220	0	0	Mixed
CDS|exon|ID=cds-Bg	14	4.732143	5291.9	0.629133	AFDLKC(+5	68	844.3724	2	1686.738	Carbamidc	V	Superscaf	51655201	0	0	0	0	0	BTP20220	Tissue specific
CDS|exon|ID=cds-Bg	7	6.435714	2520.9	0.745436	KGMPTVR	51	394.7336	2	787.4374	.	Q	Superscaf	55342921	0	0	0	0	0	BTP20220	Tissue specific
CDS|exon|ID=cds-Bg	11	5.478182	3526.7	0.746617	LLALGDSGV	92	515.3016	2	1028.587	.	R	Superscaf	55380286	0	0	0	0	0	BTP20220	Tissue specific
CDS|exon|ID=cds-Bg	14	5.628571	4709.1	0.746617	LLALGDSGV	94	689.879	2	1377.75	.	R	Superscaf	55380286	0	0	0	0	0	BTP20220	Tissue specific
CDS|exon|ID=cds-Bg	9	5.887778	3982.6	0.746685	LWDTAGQEF	94	538.2605	2	1074.509	.	M	Superscaf	55382446	0	0	0	0	0	BTP20220	Tissue specific
CDS|exon|ID=cds-Bg	13	6.235385	4307.9	0.82373	APAPPPAPF	84	601.8292	2	1201.649	.	H	Superscaf	57825232	0	0	0	0	BTP20220	0	Tissue specific
CDS|exon|ID=cds-Bg	5	6.972	2022.3	0.829942	ARFPP	64	587.3279	1	586.3227	.	F	Superscaf	58022317	0	0	BTP20220	BTP20220	0	0	Mixed
CDS|exon|ID=cds-Bg	8	6.23	2647	0.863033	A(+42.01)	81	393.2112	2	784.4119	Acetylati	V	Superscaf	59071225	0	0	0	BTP20220	0	0	Tissue specific
CDS|exon|ID=cds-Bg	6	7.846667	2866.3	0.924264	RPREKP	79	391.7283	2	781.4558	.	I	Superscaf	61013160	0	0	0	BTP20220	0	0	Tissue specific
CDS|exon|ID=cds-Bg	7	5.498571	3317.9	0.945979	T(+42.01)	59	857.4016	1	856.3926	Acetylati	P	Superscaf	61701636	0	0	0	0	0	BTP20220	Tissue specific
CDS|exon|ID=cds-Bg	7	6.645714	2590.8	0.957148	FGRAAGP	55	675.3544	1	674.35	.	G	Superscaf	62055655	0	BTP20220	0	0	0	0	Tissue specific
CDS|exon|ID=cds-Bg	11	6.029091	4454	0.983281	M(+42.01)	80	632.8182	2	1263.628	Acetylati	L	Superscaf	62884237	0	0	0	0	0	BTP20220	Tissue specific
CDS|exon|ID=cds-Bg	8	6.0775	2682.9	0.841621	SLAAAPPP	72	723.3981	1	722.3962	.	R	Superscaf	3350508	0	0	0	0	BTP20220	0	Tissue specific
CDS|exon|ID=cds-Bg	9	5.956667	3198.5	0.835982	A(+42.01)	73	456.2342	2	910.4582	Acetylati	V	Superscaf	3469585	0	0	0	0	0	BTP20220	Tissue specific
CDS|exon|ID=cds-Bg	12	4.389167	4039.5	0.835438	DYVFPENGI	54	693.8293	2	1385.65	.	R	Superscaf	3481024	0	0	0	0	0	BTP20220	Tissue specific
CDS|exon|ID=cds-Bg	12	5.715	4584.9	0.835241	FVEDLRKEF	90	480.2593	3	1437.762	.	N	Superscaf	3485155	0	0	0	0	0	BTP20220	Tissue specific
CDS|exon|ID=cds-Bg	8	5.2925	2982.3	0.728215	LEKLVTNF	55	482.277	2	962.5436	.	F	Superscaf	5749329	0	0	0	0	0	BTP20220	Tissue specific
CDS|exon|ID=cds-Bg	7	6.084286	2315.5	0.716342	P(+42.01)	71	638.3213	1	637.3071	Acetylati	G	Superscaf	6000097	0	0	0	BTP20220	0	0	Tissue specific
CDS|exon|ID=cds-Bg	5	6.188	1912	0.705097	EKLAL	55	573.3589	1	572.3533	.	A	Superscaf	6238087	0	0	0	BTP20220	0	0	Tissue specific
CDS|exon|ID=cds-Bg	7	3.885714	1753	0.666277	LDGPVVL	83	712.4209	1	711.4167	.	K	Superscaf	7059352	0	0	BTP20220	0	0	0	Tissue specific
CDS|exon|ID=cds-Bg	8	5.08125	2878.3	0.54179	TPFPHVDF	98	480.2325	2	958.4548	.	M	Superscaf	9692857	0	0	0	0	0	BTP20220	Tissue specific
CDS|exon|ID=cds-Bg	5	5.168	1607.8	0.500811	VGPLH	93	522.3026	1	521.2961	.	E	Superscaf	10559440	BTP20220	0	0	0	0	0	Tissue specific
CDS|exon|ID=cds-Bg	5	4.966	1399.6	0.39692	LVTAP	80	500.3075	1	499.3006	.	A	Superscaf	12757438	BTP20220	0	0	0	0	0	Tissue specific
CDS|exon|ID=cds-Bg	16	5.88375	7176.1	0.334231	HDPEKLTQE	99	497.5121	4	1986.028	.	F	Superscaf	14083504	0	0	BTP20220	0	0	0	Tissue specific
CDS|exon|ID=cds-Bg	6	8.763333	2728.8	0.27813	KPKKPR	50	377.2513	2	752.502	.	K	Superscaf	15270456	0	0	0	BTP20220	0	0	Tissue specific
CDS|exon|ID=cds-Bg	8	5.4775	3303.9	0.260717	HTPFMDEL	94	989.4359	1	988.4324	.	L	Superscaf	15638632	0	0	BTP20220	0	BTP20220	0	Mixed
CDS|exon|ID=cds-Bg	13	3.930769	3493.8	0.238536	YVLDDAVFV	92	739.4152	2	1476.823	.	L	Superscaf	16107730	0	0	0	0	0	BTP20220	Tissue specific
CDS|exon|ID=cds-Bg	12	3.786667	3034.3	0.238536	VLDDAVFVI	71	657.884	2	1313.76	.	L	Superscaf	16107730	0	0	0	0	BTP20220	BTP20220	Tissue enhanced
CDS|exon|ID=cds-Bg	6	6.725	2063.1	0.194587	KPAGAP	72	540.3038	1	539.3067	.	C	Superscaf	17037625	0	0	BTP20220	0	0	0	Tissue specific
CDS|exon|ID=cds-Bg	16	5.5925	6308.9	0.187365	HDVKFPLDS	66	600.9752	3	1799.905	.	V	Superscaf	17189950	0	0	0	0	0	BTP20220	Tissue specific
CDS|exon|ID=cds-Bg	8	6.385	2963.3	0.184653	ATAHPPPA	60	381.2013	2	760.3868	.	G	Superscaf	17247876	0	BTP20220	0	0	0	0	Tissue specific
CDS|exon|ID=cds-Bg	10	5.691	4104.6	0.18434	M(+42.01)	99	626.3157	2	1250.622	Acetylati	K	Superscaf	17254378	0	0	0	0	0	BTP20220	Tissue specific
CDS|exon|ID=cds-Bg	8	5.22	3039.5	0.18434	M(+42.01)	78	1009.491	1	1008.484	Acetylati	K	Superscaf	17254378	0	0	0	0	0	BTP20220	Tissue specific
CDS|exon|ID=cds-Bg	18	5.36	6379.2	0.174442	A(+42.01)	91	904.917	2	1807.822	Acetylati	A	Superscaf	17463655	0	BTP20220	0	0	BTP20220	0	Mixed
CDS|exon|ID=cds-Bg	9	6.082222	4153.6	0.158045	DWEKLERKE	93	411.5457	3	1231.62	.	E	Superscaf	17810491	0	0	0	0	BTP20220	0	Tissue specific
CDS|exon|ID=cds-Bg	7	6.127143	2652.8	0.019618	LEKAGYP	51	777.4109	1	776.4068	.	E	Superscaf	21569064	0	0	0	0	BTP20220	0	Tissue specific
CDS|exon|ID=cds-Bg	10	5.769	3490.7	0.035702	AVEAKKTYV	97	388.8953	3	1163.666	.	L	Superscaf	21909067	0	0	BTP20220	0	0	0	Tissue specific
CDS|exon|ID=cds-Bg	8	4.1025	2312.6	0.035788	LLPVESVY	77	919.511	1	918.5062	.	Q	Superscaf	21910897	0	0	0	0	BTP20220	BTP20220	Tissue enhanced
CDS|exon|ID=cds-Bg	9	5.345556	3902.6	0.063784	LEELLTTQC	80	553.7738	2	1105.533	Carbamidc	G	Superscaf	22503157	BTP20220	0	0	0	0	0	Tissue specific
CDS|exon|ID=cds-Bg	8	5.26625	3549.2	0.063785	EELLTTQC(	95	993.4584	1	992.4484	Carbamidc	G	Superscaf	22503157	BTP20220	0	0	0	0	0	Tissue specific
CDS|exon|ID=cds-Bg	10	4.137	3076.5	0.063792	FPPDVGGNV	97	508.7371	2	1015.461	.	Y	Superscaf	22503346	BTP20220	0	0	0	0	0	Tissue specific
CDS|exon|ID=cds-Bg	11	4.275455	3536	0.063792	FPPDVGGNV	93	590.2689	2	1178.524	.	Y	Superscaf	22503346	BTP20220	0	0	0	0	0	Tissue specific
CDS|exon|ID=cds-Bg	9	4.57	3127.4	0.063793	VGGNVDYKN	68	483.2376	2	964.4614	.	Y	Superscaf	22503346	BTP20220	0	0	0	0	0	Tissue specific
CDS|exon|ID=cds-Bg	7	3.711429	2062.3	0.063793	VGGNVDY	83	723.3302	1	722.3235	.	Y	Superscaf	22503346	BTP20220	0	0	0	0	0	Tissue specific
CDS|exon|ID=cds-Bg	5	5.332	1718.8	0.065209	EFGAG	59	480.1999	1	479.2016	.	K	Superscaf	22532764	BTP20220	0	0	BTP20220	0	0	Mixed
CDS|exon|ID=cds-Bg	13	6.398462	5299.9	0.097615	TDRETGKLK	77	469.2456	3	1404.721	.	C	Superscaf	23218804	0	0	0	0	BTP20220	0	Tissue specific
CDS|exon|ID=cds-Bg	16	6.4225	5796.1	0.097675	KAPKPDGPC	96	472.5714	3	1414.695	.	G	Superscaf	23220025	0	0	BTP20220	0	0	0	Tissue specific
CDS|exon|ID=cds-Bg	15	6.344667	5176.4	0.097675	KAPKPDGPC	95	639.824	2	1277.636	.	G	Superscaf	23220025	0	0	BTP20220	0	0	0	Tissue specific
CDS|exon|ID=cds-Bg	9	5.816667	3734.2	0.098603	PGLLHQELS	99	497.2714	2	992.5291	.	R	Superscaf	23239741	BTP20220	0	0	0	0	0	Tissue specific
CDS|exon|ID=cds-Bg	11	5.571818	4642.3	0.098606	VTAEC(+57	99	662.281	2	1322.55	Carbamidc	R	Superscaf	23239741	BTP20220	0	0	0	0	0	Tissue specific
CDS|exon|ID=cds-Bg	7	6.225714	2574.9	0.103693	G(+42.01)	72	407.2018	2	812.3738	Acetylati	P	Superscaf	23347306	0	0	0	BTP20220	0	0	Tissue specific
CDS|exon|ID=cds-Bg	13	6.115385	5480.5	0.108518	LSDLETTTS	99	744.36	2	1486.709	.	R	Superscaf	23449486	0	0	0	0	0	BTP20220	Tissue specific
CDS|exon|ID=cds-Bg	12	6.126667	5127.1	0.108518	SDLETTTSH	93	687.8202	2	1373.625	.	R	Superscaf	23449486	0	BTP20220	0	0	BTP20220	0	Tissue enhanced
CDS|exon|ID=cds-Bg	11	6.167273	4743.7	0.108518	DLETTTSHM	98	644.3027	2	1286.593	.	R	Superscaf	23449486	0	0	0	0	0	BTP20220	Tissue specific
CDS|exon|ID=cds-Bg	14	5.84	4941.3	0.118991	A(+42.01)	88	503.9622	3	1508.867	Acetylati	A	Superscaf	23670886	0	BTP20220	0	0	BTP20220	0	Mixed
CDS|exon|ID=cds-Bg	13	5.461538	4292.5	0.118991	A(+42.01)	70	677.3887	2	1352.766	Acetylati	A	Superscaf	23670886	0	0	BTP20220	0	0	0	Tissue specific
CDS|exon|ID=cds-Bg	9	6.193333	3244.4	0.118991	A(+42.01)	99	492.2969	2	982.5811	Acetylati	A	Superscaf	23670886	0	BTP20220	0	BTP20220	BTP20220	BTP20220	Mixed
CDS|exon|ID=cds-Bg	8	4.6825	2853.2	0.356059	EEFPDKVT	60	482.7332	2	963.4549	.	N	Superscaf	28686012	BTP20220	0	0	0	0	0	Tissue specific
CDS|exon|ID=cds-Bg	9	5.177778	3040.3	0.393199	MAAVQSFLA	68	469.2424	2	936.4739	.	G	Superscaf	29471404	0	0	0	0	0	BTP20220	Tissue specific
CDS|exon|ID=cds-Bg	6	7.331667	2449.5	0.400732	KWAPPK	59	363.7118	2	725.4224	.	L	Superscaf	29630923	0	BTP20220	0	0	0	0	Tissue specific
CDS|exon|ID=cds-Bg	9	6.623333	3175.5	0.406504	GAPARGGPF	53	390.2139	2	778.4085	.	T	Superscaf	29752888	0	0	0	BTP20220	0	0	Tissue specific
CDS|exon|ID=cds-Bg	12	6.116667	5320.9	0.425185	ASEPAEHEG	88	697.8513	2	1393.695	.	D	Superscaf	30147985	0	0	0	0	0	BTP20220	Tissue specific
CDS|exon|ID=cds-Bg	7	5.662857	3066.4	0.425324	EAAHEHL	51	403.7003	2	805.3718	.	D	Superscaf	30151024	BTP20220	0	0	0	0	0	Tissue specific
CDS|exon|ID=cds-Bg	8	4.96875	2053	0.4274	AKVAVLGA	98	728.4645	1	727.4592	.	N	Superscaf	30195115	0	0	0	BTP20220	0	0	Tissue specific
CDS|exon|ID=cds-Bg	11	5.814545	4266.9	0.427403	NSPLVSRLT	86	631.8564	2	1261.703	.	N	Superscaf	30195115	0	0	0	0	0	BTP20220	Tissue specific
CDS|exon|ID=cds-Bg	9	5.813333	3454	0.427403	NSPLVSRLT	66	493.7817	2	985.5556	.	N	Superscaf	30195115	0	0	BTP20220	0	BTP20220	0	Mixed
CDS|exon|ID=cds-Bg	8	4.92375	2799.1	0.427819	VSPFEEKM	99	483.7321	2	965.4528	.	Q	Superscaf	30203743	0	0	0	0	0	BTP20220	Tissue specific
CDS|exon|ID=cds-Bg	10	5.827	3909.1	0.427822	KGEEFVKNM	98	605.3167	2	1208.622	.	Q	Superscaf	30203743	0	0	0	0	0	BTP20220	Tissue specific
CDS|exon|ID=cds-Bg	9	5.392222	3440.7	0.427822	GEEFVKNMK	99	541.2698	2	1080.527	.	Q	Superscaf	30203743	0	0	0	0	0	BTP20220	Tissue specific

CDS|exon|ID=cds-Bg	12	6.409167	4795.5	0.431547	A(+42.01)	89	481.6111	3	1441.815	Acetylati	R	Superscaf	30282718	0	0	BTP202209	0	BTP202209	0	Mixed
CDS|exon|ID=cds-Bg	11	6.449091	4498.2	0.431547	A(+42.01)	94	462.6044	3	1384.794	Acetylati	R	Superscaf	30282718	BTP202209	BTP202209	BTP202209	0	BTP202209	BTP202209	Mixed
CDS|exon|ID=cds-Bg	10	6.018	3849.4	0.431547	A(+42.01)	85	615.351	2	1228.693	Acetylati	R	Superscaf	30282718	0	BTP202209	BTP202209	BTP202209	0	0	Mixed
CDS|exon|ID=cds-Bg	9	6.022222	3496	0.431547	A(+42.01)	91	558.8088	2	1115.609	Acetylati	R	Superscaf	30282718	0	0	0	0	BTP202209	0	Tissue specific
CDS|exon|ID=cds-Bg	8	6.0275	3142.6	0.431547	A(+42.01)	92	502.269	2	1002.525	Acetylati	R	Superscaf	30282718	0	BTP202209	BTP202209	0	BTP202209	BTP202209	Mixed
CDS|exon|ID=cds-Bg	7	6.077143	2759.2	0.431547	A(+42.01)	99	458.7522	2	915.4926	Acetylati	R	Superscaf	30282718	0	BTP202209	0	BTP202209	BTP202209	BTP202209	Mixed
CDS|exon|ID=cds-Bg	9	5.802222	4046.7	0.431549	WDPFRDWYF	84	641.2857	2	1280.562	.	R	Superscaf	30282718	0	0	0	0	0	BTP202209	Tissue specific
CDS|exon|ID=cds-Bg	7	5.718571	3182.7	0.431549	DPFRDWY	90	499.7204	2	997.4293	.	R	Superscaf	30282718	0	0	0	0	BTP202209	0	Tissue specific
CDS|exon|ID=cds-Bg	7	6.048571	3110.5	0.43155	RLFDQAF	71	448.7347	2	895.4551	.	R	Superscaf	30282718	0	BTP202209	0	0	0	0	Tissue specific
CDS|exon|ID=cds-Bg	10	5.91	4092.7	0.431551	FGLPRLPEE	75	622.3263	2	1242.64	.	R	Superscaf	30282718	0	0	0	0	0	BTP202209	Tissue specific
CDS|exon|ID=cds-Bg	8	5.95625	3408	0.431551	LPRLPEEW	72	520.2814	2	1038.55	.	R	Superscaf	30282718	0	0	0	0	BTP202209	0	Tissue specific
CDS|exon|ID=cds-Bg	12	5.958333	4618.1	0.431553	SHSGWPGYV	75	443.8941	3	1328.663	.	R	Superscaf	30282718	0	BTP202209	BTP202209	0	0	0	Mixed
CDS|exon|ID=cds-Bg	9	5.98	3570	0.431553	HSGWPGYVF	74	529.7607	2	1057.509	.	R	Superscaf	30282718	0	0	BTP202209	0	0	0	Tissue specific
CDS|exon|ID=cds-Bg	10	5.823	3615	0.431553	SGWPGYVRA	69	553.2916	2	1104.572	.	R	Superscaf	30282718	0	0	BTP202209	0	0	0	Tissue specific
CDS|exon|ID=cds-Bg	8	5.77875	2950.3	0.431553	SGWPGYVR	87	461.2306	2	920.4504	.	R	Superscaf	30282718	0	0	BTP202209	0	0	0	Tissue specific
CDS|exon|ID=cds-Bg	7	5.067143	2301.5	0.431553	SGWPGYV	86	765.3531	1	764.3493	.	R	Superscaf	30282718	0	0	0	BTP202209	0	0	Tissue specific
CDS|exon|ID=cds-Bg	9	6.148889	3741.2	0.431555	EGPAYNRAL	92	495.7542	2	989.493	.	R	Superscaf	30282718	0	BTP202209	0	BTP202209	0	0	Mixed
CDS|exon|ID=cds-Bg	8	7.03	3906.5	0.431556	NRALSRQL	63	479.2811	2	956.5515	.	R	Superscaf	30282718	0	0	0	BTP202209	0	BTP202209	Mixed
CDS|exon|ID=cds-Bg	14	4.888571	5087.7	0.43156	LDVNHFAPE	93	806.418	2	1610.83	.	R	Superscaf	30282718	0	0	0	0	0	BTP202209	Tissue specific
CDS|exon|ID=cds-Bg	10	4.619	3868.4	0.43156	DVNHFAPEE	77	585.7739	2	1169.535	.	R	Superscaf	30282718	0	BTP202209	0	0	0	0	Tissue specific
CDS|exon|ID=cds-Bg	13	4.804615	4734.3	0.43156	DVNHFAPEE	88	749.8773	2	1497.746	.	R	Superscaf	30282718	0	0	0	0	0	BTP202209	Tissue specific
CDS|exon|ID=cds-Bg	11	5.51	4123.6	0.43156	HFAPEELTV	63	636.3322	2	1270.656	.	R	Superscaf	30282718	0	BTP202209	0	0	0	0	Tissue specific
CDS|exon|ID=cds-Bg	9	5.165556	3106.4	0.43156	FAPEELTVK	98	517.28	2	1032.549	.	R	Superscaf	30282718	0	0	0	0	0	BTP202209	Tissue specific
CDS|exon|ID=cds-Bg	11	5.705455	3972.3	0.43156	FAPEELTVK	91	631.8511	2	1261.692	.	R	Superscaf	30282718	0	0	0	0	0	BTP202209	Tissue specific
CDS|exon|ID=cds-Bg	9	5.282222	3116.5	0.431561	APEELTVKT	95	494.2715	2	986.5284	.	R	Superscaf	30282718	0	0	0	BTP202209	0	0	Tissue specific
CDS|exon|ID=cds-Bg	8	5.6675	2931.3	0.431607	SLSPEGTL	70	803.4111	1	802.4072	.	T	Superscaf	30283732	0	BTP202209	0	0	0	0	Tissue specific
CDS|exon|ID=cds-Bg	16	5.589375	5562.2	0.431607	SLSPEGTLT	96	819.9474	2	1637.888	.	T	Superscaf	30283732	0	0	0	0	0	BTP202209	Tissue specific
CDS|exon|ID=cds-Bg	15	5.583333	5178.8	0.431607	LSPEGTLTV	96	776.4312	2	1550.856	.	T	Superscaf	30283732	0	0	0	0	0	BTP202209	Tissue specific
CDS|exon|ID=cds-Bg	8	5.51125	2630.9	0.431608	TVEAPLPK	98	427.7506	2	853.4909	.	T	Superscaf	30283732	0	0	0	0	0	BTP202209	Tissue specific
CDS|exon|ID=cds-Bg	9	6.468889	3510.8	0.431608	APLPKSATQ	90	456.7596	2	911.5076	.	T	Superscaf	30283732	0	0	0	BTP202209	0	0	Tissue specific
CDS|exon|ID=cds-Bg	9	6.4	3556.8	0.431609	LPKSATQSA	70	451.7499	2	901.4869	.	T	Superscaf	30283732	0	BTP202209	0	0	0	0	Tissue specific
CDS|exon|ID=cds-Bg	20	6.1245	8528.3	0.431611	QARAQLGGP	93	524.5176	4	2094.045	.	T	Superscaf	30283732	BTP202209	BTP202209	0	0	BTP202209	BTP202209	Mixed
CDS|exon|ID=cds-Bg	13	6.405385	5280.7	0.431611	QARAQLGGP	98	641.844	2	1281.679	.	T	Superscaf	30283732	0	0	0	0	0	BTP202209	Tissue specific
CDS|exon|ID=cds-Bg	18	6.156667	7606.3	0.431611	RAQLGGPEA	91	632.6548	3	1894.95	.	T	Superscaf	30283732	0	0	0	0	0	BTP202209	Tissue specific
CDS|exon|ID=cds-Bg	17	5.885882	6957.5	0.431611	AQLGGPEAG	99	580.6229	3	1738.849	.	T	Superscaf	30283732	BTP202209	0	0	0	BTP202209	0	Mixed
CDS|exon|ID=cds-Bg	16	5.8775	6646.2	0.431612	QLGGPEAGK	99	556.9436	3	1667.812	.	T	Superscaf	30283732	0	BTP202209	0	BTP202209	0	BTP202209	Mixed
CDS|exon|ID=cds-Bg	15	5.892667	6035.5	0.431612	LGGPEAGKS	99	514.2576	3	1539.753	.	T	Superscaf	30283732	BTP202209	BTP202209	BTP202209	BTP202209	BTP202209	BTP202209	Mixed
CDS|exon|ID=cds-Bg	14	5.886429	5682.1	0.431612	GGPEAGKSE	99	714.339	2	1426.669	.	T	Superscaf	30283732	0	0	0	0	BTP202209	BTP202209	Mixed
CDS|exon|ID=cds-Bg	6	6.361667	2603	0.502742	PLEAWR	61	386.2139	2	770.4075	.	Q	Superscaf	31788972	0	0	BTP202209	0	0	0	Tissue specific
CDS|exon|ID=cds-Bg	7	6.475714	3095.7	0.503769	HLLM(+15.	69	449.2298	2	896.4538	Oxidatior	S	Superscaf	31810662	0	0	0	0	0	BTP202209	Tissue specific
CDS|exon|ID=cds-Bg	5	6.222	2199.4	0.574933	G(+42.01)	80	604.2968	1	603.2864	Acetylati	T	Superscaf	33316113	0	BTP202209	0	0	0	0	Tissue specific
CDS|exon|ID=cds-Bg	8	6.68375	3467.8	0.606935	HTALKLLQ	95	462.2861	2	922.56	.	L	Superscaf	33992545	0	BTP202209	0	0	BTP202209	BTP202209	Mixed
CDS|exon|ID=cds-Bg	8	5.31	3609.2	0.643022	TNWDDMEK	94	519.7136	2	1037.412	.	A	Superscaf	34756159	0	BTP202209	0	0	BTP202209	BTP202209	Mixed
CDS|exon|ID=cds-Bg	7	5.135714	3211.7	0.643022	NWDDMEK	98	469.1876	2	936.3647	.	A	Superscaf	34756159	0	0	0	0	BTP202209	BTP202209	Tissue enhanced
CDS|exon|ID=cds-Bg	6	5.113333	1968.3	0.648697	PTLLVW	62	364.7173	2	727.4268	.	L	Superscaf	34876647	0	0	0	BTP202209	0	0	Tissue specific
CDS|exon|ID=cds-Bg	6	6.933333	2374.5	0.649637	KEKPPP	58	695.4149	1	694.4013	.	R	Superscaf	34896324	0	0	BTP202209	0	0	0	Tissue specific
CDS|exon|ID=cds-Bg	6	6.37	2550.1	0.67873	LEM(+15.9	94	778.4069	1	777.4055	Oxidatior	E	Superscaf	35511427	0	0	BTP202209	0	0	0	Tissue specific
CDS|exon|ID=cds-Bg	8	5.755	2755	0.679338	SPGVVKHR	65	440.2541	2	878.5086	.	G	Superscaf	35524540	0	0	0	0	BTP202209	0	Tissue specific
CDS|exon|ID=cds-Bg	5	4.304	1427.6	0.708945	A(+42.01)	79	570.3113	1	569.306	Acetylati	F	Superscaf	36150529	0	0	0	0	0	BTP202209	Tissue specific
CDS|exon|ID=cds-Bg	7	5.774286	2309.8	0.725702	R(+42.01)	71	431.765	2	861.5184	Acetylati	K	Superscaf	36505201	0	0	0	BTP202209	0	0	Tissue specific
CDS|exon|ID=cds-Bg	7	3.884286	1696.9	0.747975	LGGVDVP	64	656.3589	1	655.3541	.	N	Superscaf	36975994	0	0	0	0	0	BTP202209	Tissue specific
CDS|exon|ID=cds-Bg	7	5.551429	2567.8	0.78176	ALALEYL	84	792.4482	1	791.4429	.	G	Superscaf	37691089	0	BTP202209	0	0	BTP202209	0	Mixed
CDS|exon|ID=cds-Bg	13	5.351538	4224.3	0.823234	VLSAADKGN	99	622.352	2	1242.693	.	K	Superscaf	38568517	0	BTP202209	BTP202209	BTP202209	BTP202209	BTP202209	Mixed
CDS|exon|ID=gene-B	16	5.698125	5516.6	0.823234	VLSAADKGN	98	404.4785	4	1613.889	.	K	Superscaf	38568517	0	BTP202209	0	0	BTP202209	BTP202209	Mixed
CDS|exon|ID=cds-Bg	15	5.428667	5048.2	0.823234	VLSAADKGN	99	743.9031	2	1485.794	.	K	Superscaf	38568517	0	BTP202209	0	0	BTP202209	BTP202209	Mixed
CDS|exon|ID=cds-Bg	12	5.295833	3913	0.823234	VLSAADKGN	99	586.8334	2	1171.656	.	K	Superscaf	38568517	0	BTP202209	BTP202209	BTP202209	0	0	Mixed
CDS|exon|ID=cds-Bg	11	5.23	3601.7	0.823234	VLSAADKGN	99	551.3142	2	1100.619	.	K	Superscaf	38568517	BTP202209	BTP202209	BTP202209	BTP202209	BTP202209	BTP202209	Mixed
CDS|exon|ID=cds-Bg	10	4.779	3133.3	0.823234	VLSAADKGN	99	487.2685	2	972.524	.	K	Superscaf	38568517	0	BTP202209	0	0	BTP202209	0	Mixed
CDS|exon|ID=cds-Bg	15	6.078	5516.6	0.823235	LSAADKGNV	98	505.9457	3	1514.821	.	K	Superscaf	38568517	0	0	0	0	BTP202209	0	Tissue specific
CDS|exon|ID=cds-Bg	10	5.753	3601.7	0.823235	LSAADKGNV	99	501.7811	2	1001.551	.	K	Superscaf	38568517	0	BTP202209	BTP202209	0	BTP202209	BTP202209	Mixed
CDS|exon|ID=cds-Bg	11	5.780909	3870.9	0.823235	SAADKGNVK	87	516.2737	2	1030.541	.	K	Superscaf	38568517	0	BTP202209	0	0	0	0	Tissue specific
CDS|exon|ID=cds-Bg	14	6.085	5163.2	0.823235	SAADKGNVK	99	468.2513	3	1401.736	.	K	Superscaf	38568517	0	0	0	0	BTP202209	BTP202209	Mixed
CDS|exon|ID=cds-Bg	9	5.727778	3248.3	0.823235	SAADKGNVK	99	445.239	2	888.4665	.	K	Superscaf	38568517	0	BTP202209	BTP202209	0	BTP202209	BTP202209	Mixed
CDS|exon|ID=cds-Bg	8	5.73375	2864.9	0.823235	AADKGNVK	99	401.7226	2	801.4344	.	K	Superscaf	38568517	0	BTP202209	0	0	0	0	Tissue specific
CDS|exon|ID=cds-Bg	12	6.124167	4468.5	0.823235	ADKGNVKAA	97	622.8387	2	1243.667	.	K	Superscaf	38568517	0	0	0	0	BTP202209	0	Tissue specific
CDS|exon|ID=cds-Bg	10	5.778	3702.8	0.823235	ADKGNVKAA	98	530.2808	2	1058.551	.	K	Superscaf	38568517	0	BTP202209	0	0	0	0	Tissue specific
CDS|exon|ID=cds-Bg	9	5.488889	3197.6	0.823258	LLSHSLLVT	71	982.5886	1	981.5859	.	R	Superscaf	38569066	0	0	0	0	BTP202209	0	Tissue specific
CDS|exon|ID=cds-Bg	9	5.488889	3197.6	0.823258	LSHSLLVTL	64	491.7984	2	981.5859	.	R	Superscaf	38569066	0	0	0	0	BTP202209	0	Tissue specific
CDS|exon|ID=cds-Bg	9	5.493333	3155.5	0.823258	SHSLLVTLA	93	470.7764	2	939.5389	.	R	Superscaf	38569066	0	BTP202209	BTP202209	0	BTP202209	0	Mixed
CDS|exon|ID=cds-Bg	17	5.570588	6074.8	0.823259	SLLVTLASH	61	884.973	2	1767.941	.	R	Superscaf	38569066	0	0	0	0	0	BTP202209	Tissue specific
CDS|exon|ID=cds-Bg	19	5.383684	6694.5	0.823259	SLLVTLASH	97	669.0273	3	2004.068	.	R	Superscaf	38569066	0	0	0	0	BTP202209	0	Tissue specific
CDS|exon|ID=cds-Bg	16	5.666875	5915.6	0.823259	TLASHLPSD	86	555.2838	3	1662.836	.	R	Superscaf	38569066	0	0	0	0	BTP202209	0	Tissue specific
CDS|exon|ID=cds-Bg	15	5.643333	5604.3	0.823259	TLASHLPSD	93	531.6053	3	1591.799	.	R	Superscaf	38569066	0	0	0	0	BTP202209	0	Tissue specific
CDS|exon|ID=cds-Bg	14	5.58	5206.8	0.823259	LASHLPSDF	81	497.9227	3	1490.752	.	R	Superscaf	38569066	0	0	0	0	BTP202209	0	Tissue specific
CDS|exon|ID=cds-Bg	8	5.7125	3187.5	0.823259	ASHLPSDF	69	437.2078	2	872.4028	.	R	Superscaf	38569066	0	BTP202209	0	0	0	0	Tissue specific
CDS|exon|ID=cds-Bg	19	5.684737	7168.8	0.823259	ASHLPSDFT	75	680.6755	3	2039.011	.	R	Superscaf	38569066	0	0	0	0	BTP202209	0	Tissue specific
CDS|exon|ID=cds-Bg	18	5.696111	6781.4	0.823259	ASHLPSDFT	93	631.6531	3	1891.943	.	R	Superscaf	38569066	0	0	0	0	BTP202209	0	Tissue specific
CDS|exon|ID=cds-Bg	14	5.582857	5164.7	0.823259	ASHLPSDFT	88	483.9084	3	1448.705	.	R	Superscaf	38569066	0	BTP202209	BTP202209	0	BTP202209	0	Mixed
CDS|exon|ID=cds-Bg	13	5.549231	4853.4	0.823259	ASHLPSDFT	99	689.8401	2	1377.668	.	R	Superscaf	38569066	0	BTP202209	BTP202209	0	BTP202209	BTP202209	Mixed
CDS|exon|ID=cds-Bg	18	5.666111	6857.5	0.82326	SHLPSDFTP	96	656.9949	3	1967.974	.	R	Superscaf	38569066	0	0	0	0	BTP202209	0	Tissue specific
CDS|exon|ID=cds-Bg	13	5.549231	4853.4	0.82326	SHLPSDFTP	98	689.8398	2	1377.668	.	R	Superscaf	38569066	0	BTP202209	0	0	BTP202209	0	Mixed
CDS|exon|ID=cds-Bg	12	5.51	4542.1	0.82326	SHLPSDFTP	97	654.3214	2	1306.631	.	R	Superscaf	38569066	0	BTP202209	BTP202209	0	BTP202209	0	Mixed
CDS|exon|ID=cds-Bg	17	5.665294	6474.1	0.82326	HLPSDFTPA	94	627.9848	3	1880.942	.	R	Superscaf	38569066	0	0	0	0	BTP202209	0	Tissue specific
CDS|exon|ID=cds-Bg	16	5.676875	6086.7	0.82326	HLPSDFTPA	99	578.963	3	1733.874	.	R	Superscaf	38569066	0	0	0	0	BTP202209	0	Tissue specific
CDS|exon|ID=cds-Bg	15	5.549333	5467	0.82326	LPSDFTPAV	99	533.2764	3	1596.815	.	R	Superscaf	38569066	0	0	0	0	BTP202209	0	Tissue specific
CDS|exon|ID=cds-Bg	13	5.425385	4587.1	0.82326	LPSDFTPAV	90	677.8515	2	1353.693	.	R	Superscaf	38569066	0	0	0	0	BTP202209	0	Tissue specific
CDS|exon|ID=cds-Bg	10	5.285	3539	0.82326	LPSDFTPAV	99	542.2756	2	1082.54	.	R	Superscaf	38569066	0	0	0	0	BTP202209	0	Tissue specific
CDS|exon|ID=cds-Bg	9	5.207778	3185.6	0.82326	PSDFTPAVH	96	485.7347	2	969.4556	.	R	Superscaf	38569066	0	BTP202209	0	0	0	0	Tissue specific
CDS|exon|ID=cds-Bg	14	5.46	5163.6	0.82326	SDFTPAVHA	66	767.8783	2	1533.746	.	R	Superscaf	38569066	0	0	0	0	BTP202209	0	Tissue specific
CDS|exon|ID=cds-Bg	13	5.458462	4776.2	0.82326	SDFTPAVHA	97	694.3448	2	1386.678	.	R	Superscaf	38569066	0	0	0	0	BTP202209	0	Tissue specific
CDS|exon|ID=cds-Bg	8	5.07125	2848.2	0.82326	SDFTPAVH	99	437.2071	2	872.4028	.	R	Superscaf	38569066	0	0	0	0	BTP202209	0	Tissue specific
CDS|exon|ID=cds-Bg	12	5.44	4392.8	0.82326	DFTPAVHAS	98	650.8285	2	1299.646	.	R	Superscaf	38569066	0	0	0	0	BTP202209	0	Tissue specific
CDS|exon|ID=cds-Bg	7	4.984286	2464.8	0.82326	DFTPAVH	71	786.3788	1	785.3708	.	R	Superscaf	38569066	0	0	0	0	0	BTP202209	Tissue specific
CDS|exon|ID=cds-Bg	7	5.42	2364.6	0.82326	FTPAVHA	68	371.6968	2	741.3809	.	R	Superscaf	38569066	0	BTP202209	0	0	0	0	Tissue specific
CDS|exon|ID=cds-Bg	8	5.4325	2935.1	0.823261	VHASLDKF	96	458.7462	2	915.4814	.	R	Superscaf	38569066	0	0	0	0	0	BTP202209	Tissue specific
CDS|exon|ID=cds-Bg	7	5.425714	2547.7	0.823261	VHASLDK	82	385.2105	2	768.413	.	R	Superscaf	38569066	0	0	0	0	0	BTP202209	Tissue specific
CDS|exon|ID=cds-Bg	9	5.92	3576.8	0.823261	ASLDKFLAN	98	489.7656	2	977.5181	.	R	Superscaf	38569066	0	BTP202209	0	0	BTP202209	0	Mixed
CDS|exon|ID=cds-Bg	10	5.328	3576.8	0.823261	ASLDKFLAN	83	539.2988	2	1076.587	.	R	Superscaf	38569066	0	0	0	0	BTP202209	0	Tissue specific
CDS|exon|ID=cds-Bg	8	5.98375	2980.1	0.823261	ASLDKFLA	93	432.7447	2	863.4752	.	R	Superscaf	38569066	0	BTP202209	0	0	BTP202209	0	Mixed
gene|mRNA ID=gene-B	8	5.9075	3265.5	0.823261	SLDKFLAN	57	454.2476	2	906.481	.	R	Superscaf	38569066	0	0	0	0	BTP202209	0	Tissue specific
CDS|exon|ID=cds-Bg	7	5.94	2882.1	0.823262	LDKFLAN	86	410.7299	2	819.449	.	R	Superscaf	38569066	0	0	0	0	BTP202209	0	Tissue specific
CDS|exon|ID=cds-Bg	8	5.1975	2882.1	0.823262	LDKFLANV	94	460.2643	2	918.5175	.	R	Superscaf	38569066	0	0	0	0	BTP202209	0	Tissue specific
CDS|exon|ID=cds-Bg	7	5.731429	2459.7	0.823407	TVLTSKY	99	406.2304	2	810.4487	.	R	Superscaf	38572111	0	BTP202209	BTP202209	0	BTP202209	0	Mixed
CDS|exon|ID=cds-Bg	7	5.594286	2596	0.827209	AMSLTEL	70	764.3828	1	763.3786	.	I	Superscaf	38652795	0	0	0	0	0	BTP202209	Tissue specific
CDS|exon|ID=cds-Bg	12	6.200833	4300.6	0.831628	GAPQAGLRA	94	590.8595	2	1179.709	.	T	Superscaf	38746120	0	0	BTP202209	0	0	0	Tissue specific
CDS|exon|ID=cds-Bg	9	6.416667	3892.4	0.83163	ASPLQRLGN	96	478.2678	2	954.5247	.	T	Superscaf	38746120	0	0	BTP202209	0	0	0	Tissue specific
CDS|exon|ID=cds-Bg	12	5.471667	3827.1	0.831632	ASPLASFVT	79	567.3118	2	1132.613	.	T	Superscaf	38746120	0	0	BTP202209	0	0	0	Tissue specific
CDS|exon|ID=cds-Bg	8	4.56625	2046.3	0.849618	GPSLVPVP	59	383.2327	2	764.4432	.	G	Superscaf	39126646	0	0	0	0	0	BTP202209	Tissue specific
CDS|exon|ID=cds-Bg	8	6.775	2978.4	0.854025	GAAPPTRP	80	383.7182	2	765.4133	.	R	Superscaf	39219469	0	0	BTP202209	0	0	0	Tissue specific
CDS|exon|ID=cds-Bg	8	4.9875	2910.2	0.860107	EVHGPSYF	98	935.4264	1	934.4185	.	G	Superscaf	39348565	0	BTP202209	0	0	0	0	Tissue specific
CDS|exon|ID=cds-Bg	9	6.176667	4271	0.860108	RVQLREQHL	58	393.5616	3	1177.668	.	G	Superscaf	39348565	0	0	0	0	0	BTP202209	Tissue specific
CDS|exon|ID=cds-Bg	8	6.20125	3917.6	0.860108	RVQLREQH	69	355.8668	3	1064.584	.	G	Superscaf	39348565	0	0	0	0	0	BTP202209	Tissue specific
CDS|exon|ID=cds-Bg	8	5.60375	3622.2	0.860108	VQLREQHL	94	511.7895	2	1021.567	.	G	Superscaf	39348565	0	BTP202209	0	0	0	0	Tissue specific
CDS|exon|ID=cds-Bg	10	5.338	3849.5	0.874218	DESGPVQTF	88	543.2626	2	1084.515	.	G	Superscaf	39646993	0	0	0	0	BTP202209	0	Tissue specific
CDS|exon|ID=cds-Bg	11	6.712727	4194.2	0.888348	A(+42.01)	99	381.8823	3	1142.629	Acetylati	G	Superscaf	39945940	0	0	0	BTP202209	0	0	Tissue specific
CDS|exon|ID=cds-Bg	7	5.428571	2359.7	0.894046	APLDRGV	85	364.2069	2	726.4024	.	Q	Superscaf	40066228	0	0	0	BTP202209	0	0	Tissue specific
CDS|exon|ID=cds-Bg	7	6.59	2945.5	0.894076	MC(+57.02	80	422.7103	2	843.4095	Carbamido	G	Superscaf	40066891	0	0	BTP202209	0	0	0	Tissue specific
CDS|exon|ID=cds-Bg	7	4.86	2122.4	0.894242	T(+42.01)	55	728.3783	1	727.3752	Acetylati	P	Superscaf	40070479	0	BTP202209	0	0	0	0	Tissue specific
CDS|exon|ID=cds-Bg	7	5.835714	3084.6	0.896305	EPLGRDQ	62	814.4182	1	813.3981	.	A	Superscaf	40114150	0	0	0	0	BTP202209	0	Tissue specific
CDS|exon|ID=cds-Bg	7	4.831429	2374.8	0.898295	D(+42.01)	76	413.2068	2	824.4141	Acetylati	V	Superscaf	40156189	0	0	0	0	0	BTP202209	Tissue specific
CDS|exon|ID=cds-Bg	15	5.13	5824.5	0.984267	S(+42.01)	91	838.3815	2	1674.747	Acetylati	L	Superscaf	41974996	0	0	0	0	0	BTP202209	Tissue specific
CDS|exon|ID=cds-Bg	9	6.236667	3546.1	0.706279	GPPHQGPPM	52	459.2171	2	916.4225	.	R	Superscaf	7599985	0	0	0	BTP202209	0	0	Tissue specific
CDS|exon|ID=cds-Bg	8	6.2975	3174.6	0.706279	GPPHQGPP	93	393.6966	2	785.382	.	R	Superscaf	7599985	0	0	0	BTP202209	0	0	Tissue specific
CDS|exon|ID=cds-Bg	10	4.063	3200.6	0.634621	VLEEEVLDF	95	585.8142	2	1169.618	.	R	Superscaf	9454366	0	0	0	0	0	BTP202209	Tissue specific
CDS|exon|ID=cds-Bg	6	7.576667	2441.4	0.557021	VFRKKK	56	403.2789	2	804.5333	.	K	Superscaf	11462620	0	0	0	BTP202209	0	0	Tissue specific
CDS|exon|ID=cds-Bg	8	4.5475	2666	0.466476	PLEEAVLY	63	467.248	2	932.4855	.	W	Superscaf	13805326	0	0	0	0	0	BTP202209	Tissue specific
CDS|exon|ID=cds-Bg	7	6.254286	2377.8	0.333177	PPLPPPP	74	357.7111	2	713.4112	.	T	Superscaf	17254986	BTP202209	0	0	BTP202209	0	0	Mixed
CDS|exon|ID=cds-Bg	7	5.788571	2455.8	0.331265	PVTFRFG	58	823.4321	1	822.4388	.	D	Superscaf	17304375	0	0	BTP202209	0	0	0	Tissue specific
CDS|exon|ID=cds-Bg	6	7.085	2833.1	0.282612	DGFKHR	87	380.1982	2	758.3823	.	K	Superscaf	18563179	BTP202209	0	0	0	0	0	Tissue specific
CDS|exon|ID=cds-Bg	11	6.567273	4346.2	0.275781	M(+15.99)	91	642.8386	2	1283.67	Oxidatior	Q	Superscaf	18739357	0	0	0	0	BTP202209	0	Tissue specific
CDS|exon|ID=cds-Bg	6	3.586667	1427.6	0.158964	EVALPV	67	627.3696	1	626.3639	.	E	Superscaf	21762894	0	BTP202209	0	BTP202209	0	0	Mixed
CDS|exon|ID=cds-Bg	7	6.057143	3024.5	0.155479	M(+42.01)	99	431.6958	2	861.3803	Acetylati	P	Superscaf	21852871	0	0	0	0	BTP202209	0	Tissue specific

CDS|exon|ID=cds-Bg	6	6.726667	2535.9	0.122074	LYLPSR	97	374.72	2	747.4279	.	P	Superscaf	22717428	0	BTP202209	BTP202209	BTP202209	BTP202209	0	Mixed
CDS|exon|ID=cds-Bg	10	5.478	3319.8	0.121905	A(+42.01)	59	548.3148	2	1094.62	Acetylati	R	Superscaf	22721521	0	0	0	0	0	BTP202209	Tissue specific
CDS|exon|ID=cds-Bg	9	5.913333	2876.2	0.121663	PPPSVPPPK	91	458.2668	2	914.5225	.	T	Superscaf	22728013	0	0	0	0	0	BTP202209	Tissue specific
CDS|exon|ID=cds-Bg	7	4.577143	2500.9	0.113256	EDVLLPH	92	411.7196	2	821.4283	.	E	Superscaf	22945465	0	0	0	0	0	BTP202209	Tissue specific
CDS|exon|ID=cds-Bg	8	4.75875	3055.5	0.103799	M(+42.01)	88	969.3826	1	968.3797	Acetylati	W	Superscaf	23190187	0	0	0	0	0	BTP202209	Tissue specific
CDS|exon|ID=cds-Bg	9	5.827778	3602.1	0.078334	A(+42.01)	93	540.2446	2	1078.475	Acetylati	S	Superscaf	23849170	0	BTP202209	0	0	0	0	Tissue specific
CDS|exon|ID=cds-Bg	11	5.32	4646.2	0.045511	QNSPEAYDY	71	628.7732	2	1255.536	.	S	Superscaf	24698368	0	0	BTP202209	0	0	0	Tissue specific
CDS|exon|ID=cds-Bg	13	5.140769	4650.2	0.167202	SFEDDTVPL	88	724.3679	2	1446.724	.	N	Superscaf	30202543	0	BTP202209	0	0	0	0	Tissue specific
CDS|exon|ID=cds-Bg	14	5.177857	5109.7	0.167202	SFEDDTVPL	93	805.8998	2	1609.788	.	N	Superscaf	30202543	0	BTP202209	0	0	0	0	Tissue specific
CDS|exon|ID=cds-Bg	10	6.18	3681.4	0.186459	MQPPPPPPP	89	1054.543	1	1053.532	.	A	Superscaf	30700912	0	0	0	BTP202209	0	0	Tissue specific
CDS|exon|ID=cds-Bg	12	4.555	3845.3	0.196017	VVLPLDEMA	88	695.866	2	1389.721	.	F	Superscaf	30948376	0	0	0	0	0	BTP202209	Tissue specific
CDS|exon|ID=cds-Bg	5	4.682	1720	0.296352	EHPVP	50	578.3037	1	577.286	.	G	Superscaf	33544761	0	0	0	0	BTP202209	0	Tissue specific
CDS|exon|ID=cds-Bg	7	5.37	2346.7	0.303418	SGM(+15.9	54	370.671	2	739.3323	Oxidation	T	Superscaf	33727444	0	BTP202209	0	0	0	0	Tissue specific
CDS|exon|ID=cds-Bg	8	6.93375	3107.2	0.305945	PSFLKATK	56	446.2686	2	890.5225	.	V	Superscaf	33792910	0	0	0	0	BTP202209	0	Tissue specific
CDS|exon|ID=cds-Bg	5	5.692	1722.9	0.435444	K(+42.01)	82	653.3481	1	652.3432	Acetylati	T	Superscaf	37143889	0	0	0	0	0	BTP202209	Tissue specific
CDS|exon|ID=cds-Bg	12	5.2825	5315.1	0.525815	LLQNQYEPV	84	733.3716	2	1464.736	.	C	Superscaf	39482179	0	0	0	0	0	BTP202209	Tissue specific
CDS|exon|ID=cds-Bg	7	6.398571	3055.4	0.525817	KQLWMPF	85	949.4945	1	948.4891	.	C	Superscaf	39482179	0	0	0	0	0	BTP202209	Tissue specific
CDS|exon|ID=cds-Bg	18	5.941111	6807	0.526529	AAVATSAKG	90	592.3132	3	1773.922	.	N	Superscaf	39500773	0	0	0	0	0	BTP202209	Tissue specific
CDS|exon|ID=cds-Bg	16	5.69875	6027.3	0.526529	AAVATSAKG	97	788.3981	2	1574.79	.	N	Superscaf	39500773	0	0	0	0	0	BTP202209	Tissue specific
CDS|exon|ID=cds-Bg	14	5.488571	5046.6	0.621608	TSSSFLYPV	84	768.3641	2	1534.719	.	F	Superscaf	41960881	0	0	0	0	0	BTP202209	Tissue specific
CDS|exon|ID=cds-Bg	9	5.566667	4033.5	0.635808	QGLQHQVAH	90	509.2631	2	1016.515	.	P	Superscaf	42328567	0	0	0	0	0	BTP202209	Tissue specific
CDS|exon|ID=cds-Bg	13	6.161538	5305.1	0.63581	MATQLEAAR	99	740.892	2	1479.776	.	P	Superscaf	42328567	0	0	0	0	0	BTP202209	Tissue specific
CDS|exon|ID=cds-Bg	10	5.558	3796.1	0.639849	LDPSPEVQK	91	570.8145	2	1139.619	.	F	Superscaf	42433045	0	0	0	0	0	BTP202209	Tissue specific
CDS|exon|ID=cds-Bg	7	6.275714	3128.4	0.688805	LLQHADK	98	412.7337	2	823.4552	.	P	Superscaf	43699819	0	0	0	0	0	BTP202209	Tissue specific
CDS|exon|ID=cds-Bg	18	6.044444	6609.1	0.728625	A(+42.01)	90	856.4135	2	1710.817	Acetylati	T	Superscaf	44730157	0	0	0	BTP202209	0	0	Tissue specific
CDS|exon|ID=cds-Bg	6	5.548333	2849.3	0.732104	C(+57.02)	53	758.2171	1	757.2194	Carbamido	Q	Superscaf	44820337	0	0	0	BTP202209	0	0	Tissue specific
CDS|exon|ID=cds-Bg	6	6.263333	2189.4	0.816695	KGDPPP	55	610.3223	1	609.3122	.	F	Superscaf	47009224	0	0	BTP202209	0	0	0	Tissue specific
CDS|exon|ID=cds-Bg	12	4.358333	3973.3	0.889435	EAEAVPEVS	69	675.8237	2	1349.635	.	S	Superscaf	48891436	0	0	0	BTP202209	0	0	Tissue specific
CDS|exon|ID=cds-Bg	8	6.74625	2918.3	0.955009	APPRAGPP	85	381.7152	2	761.4184	.	L	Superscaf	50587492	0	0	0	BTP202209	0	0	Tissue specific
CDS|exon|ID=cds-Bg	8	7.68	4153	0.975728	RELELRRR	67	564.3394	2	1126.668	.	T	Superscaf	51123640	0	0	0	0	0	BTP202209	Tissue specific
CDS|exon|ID=cds-Bg	7	6.242857	3014.6	0.937211	M(+42.01)	78	421.6929	2	841.3752	Acetylati	Q	Superscaf	1466587	0	0	0	BTP202209	0	0	Tissue specific
CDS|exon|ID=cds-Bg	10	6.223	3613.9	0.930488	LPAAAPSPA	99	466.2527	2	930.4922	.	P	Superscaf	1623487	BTP202209	0	0	0	0	0	Tissue specific
CDS|exon|ID=cds-Bg	7	4.282857	2893.4	0.626859	EDDLDWL	95	905.3854	1	904.3814	.	V	Superscaf	8716024	0	0	0	BTP202209	0	0	Tissue specific
CDS|exon|ID=cds-Bg	9	5.834444	3006.4	0.512728	MTAKTLVLL	64	495.3051	2	988.5991	.	R	Superscaf	11381677	0	0	BTP202209	0	BTP202209	0	Mixed
CDS|exon|ID=cds-Bg	8	5.81625	2653	0.512728	MTAKTLVL	78	438.7629	2	875.515	.	R	Superscaf	11381677	0	0	BTP202209	0	BTP202209	0	Mixed
CDS|exon|ID=cds-Bg	9	5.834444	3006.4	0.512727	TAKTLVLLM	83	495.3048	2	988.5991	.	R	Superscaf	11381677	0	0	0	0	BTP202209	0	Tissue specific
CDS|exon|ID=cds-Bg	9	5.934444	3733.3	0.292992	TGTVDC(+5	96	561.7698	2	1121.529	Carbamido	M	Superscaf	16514710	0	0	0	0	0	BTP202209	Tissue specific
CDS|exon|ID=cds-Bg	6	4.401667	1873.1	0.022302	D(+42.01)	64	719.3621	1	718.3537	Acetylati	M	Superscaf	22837902	0	BTP202209	0	0	0	0	Tissue specific
CDS|exon|ID=cds-Bg	7	7.297143	2900.2	0.105624	RPPPKAY	56	414.7383	2	827.4653	.	G	Superscaf	25825156	0	0	0	BTP202209	0	0	Tissue specific
CDS|exon|ID=cds-Bg	11	6.174545	5123.8	0.460987	SHGQRTFGE	82	648.2844	2	1294.569	.	L	Superscaf	34126849	0	0	0	0	0	BTP202209	Tissue specific
CDS|exon|ID=cds-Bg	18	5.675556	5472.7	0.471318	A(+42.01)	50	718.3691	2	1434.71	Acetylati	K	Superscaf	34368262	0	BTP202209	0	0	0	0	Tissue specific
CDS|exon|ID=cds-Bg	9	6.201111	3001.2	0.567733	APVPKKLLM	64	498.8163	2	995.6201	.	L	Superscaf	36620386	0	0	BTP202209	0	0	0	Tissue specific
CDS|exon|ID=cds-Bg	8	3.04125	1787.9	0.764088	VVDQVVGK	97	422.2488	2	842.4861	.	G	Superscaf	41207020	0	0	0	0	0	BTP202209	Tissue specific
CDS|exon|ID=cds-Bg	7	6.52	2857.9	0.764475	KESPKFF	59	441.7403	2	881.4647	.	I	Superscaf	41216127	0	BTP202209	0	0	0	0	Tissue specific
CDS|exon|ID=cds-Bg	5	5.936	1743	0.972886	G(+42.01)	56	562.2487	1	561.2468	Acetylati	C	Superscaf	46084300	0	0	0	0	BTP202209	0	Tissue specific
CDS|exon|ID=cds-Bg	6	3.986667	1413.6	0.91497	L(+42.01)	60	356.2527	2	710.4942	Acetylati	T	Superscaf	2022280	0	0	0	BTP202209	0	0	Tissue specific
CDS|exon|ID=cds-Bg	5	6.676	1946	0.907247	ALLYK	65	607.377	1	606.3741	.	C	Superscaf	2205394	0	0	0	0	0	BTP202209	Tissue specific
CDS|exon|ID=cds-Bg	6	4.075	1881.2	0.706701	D(+42.01)	50	727.3837	1	726.3799	Acetylati	R	Superscaf	6976249	0	0	0	0	0	BTP202209	Tissue specific
CDS|exon|ID=cds-Bg	6	6.56	2642	0.601272	LGGYC(+57	52	363.1778	2	724.3326	Carbamido	T	Superscaf	9483868	BTP202209	0	0	0	0	0	Tissue specific
CDS|exon|ID=cds-Bg	8	6.5375	3249.4	0.595215	KYSPELLK	55	977.5613	1	976.5593	.	R	Superscaf	9627883	0	0	0	0	BTP202209	0	Tissue specific
CDS|exon|ID=cds-Bg	10	6.419	4623.2	0.574228	EGLLLWC(+	96	651.8503	2	1301.691	Carbamido	V	Superscaf	10127143	0	0	0	0	0	BTP202209	Tissue specific
CDS|exon|ID=cds-Bg	8	7.0725	3826.4	0.573683	NTLQTKLR	93	487.2916	2	972.5716	.	L	Superscaf	10139929	0	0	0	0	BTP202209	0	Tissue specific
CDS|exon|ID=cds-Bg	11	5.435455	4560	0.573493	HEAFESDLA	96	613.7733	2	1225.536	.	L	Superscaf	10144462	0	0	0	0	0	BTP202209	Tissue specific
CDS|exon|ID=cds-Bg	9	4.95	2851.1	0.572694	G(+42.01)	90	920.432	1	919.4286	Acetylati	F	Superscaf	10163554	0	0	0	0	0	BTP202209	Tissue specific
CDS|exon|ID=cds-Bg	10	5.284	3762.1	0.572693	SSALYGESD	95	1041.469	1	1040.466	.	F	Superscaf	10163554	0	0	0	0	0	BTP202209	Tissue specific
CDS|exon|ID=cds-Bg	9	5.24	3378.7	0.572693	SALYGESDL	95	954.4396	1	953.4341	.	F	Superscaf	10163554	BTP202209	0	0	0	0	0	Tissue specific
CDS|exon|ID=cds-Bg	8	5.185	2995.3	0.572693	ALYGESDL	99	867.4077	1	866.4022	.	F	Superscaf	10163554	BTP202209	BTP202209	0	0	BTP202209	0	Mixed
CDS|exon|ID=cds-Bg	8	6.45875	3565.2	0.387735	PVRQTAQR	66	478.2689	2	954.5359	.	T	Superscaf	14562976	BTP202209	0	0	0	0	0	Tissue specific
CDS|exon|ID=cds-Bg	7	6.045714	2345.5	0.14815	PAASALP	52	626.3586	1	625.3435	.	H	Superscaf	20261668	0	0	0	0	BTP202209	0	Tissue specific
CDS|exon|ID=cds-Bg	7	4.192857	2019.3	0.057169	PVTVAC(+5	51	397.1937	2	792.384	Carbamido	D	Superscaf	25145299	0	0	0	0	0	BTP202209	Tissue specific
CDS|exon|ID=cds-Bg	11	5.455455	4299.8	0.070346	DNQGGYGSV	88	555.2496	2	1108.49	.	T	Superscaf	25458724	0	BTP202209	0	0	0	0	Tissue specific
CDS|exon|ID=cds-Bg	6	5.546667	2715.2	0.070529	T(+42.01)	55	454.1592	2	906.3074	Acetylati	E	Superscaf	25463095	0	0	0	BTP202209	0	0	Tissue specific
CDS|exon|ID=cds-Bg	10	6.249	4254.6	0.082481	A(+42.01)	68	543.2714	2	1084.53	Acetylati	Q	Superscaf	25747381	0	0	BTP202209	0	0	0	Tissue specific
CDS|exon|ID=cds-Bg	18	5.403333	6303.7	0.089701	PGKLLSDAD	87	600.6501	3	1798.931	.	V	Superscaf	25919137	0	BTP202209	0	0	0	0	Tissue specific
CDS|exon|ID=cds-Bg	12	4.0625	3829.3	0.08979	VETSEVDVN	98	652.3212	2	1302.63	.	K	Superscaf	25921144	0	BTP202209	0	0	0	0	Tissue specific
CDS|exon|ID=cds-Bg	7	5.372857	2768	0.183036	N(+42.01)	54	875.4574	1	874.4396	Acetylati	T	Superscaf	28139199	0	0	0	0	BTP202209	0	Tissue specific
CDS|exon|ID=cds-Bg	8	5.33375	2345.6	0.276807	APPVPPFG	98	781.4215	1	780.417	.	D	Superscaf	30369520	0	0	BTP202209	0	0	0	Tissue specific
CDS|exon|ID=cds-Bg	9	7.026667	4132.6	0.278454	RFPNKPHLS	77	365.8721	3	1094.598	.	V	Superscaf	30408595	0	0	BTP202209	0	0	0	Tissue specific
CDS|exon|ID=cds-Bg	7	6.037143	2335.8	0.291017	VPPPRPP	98	380.227	2	758.4438	.	L	Superscaf	30707500	0	0	BTP202209	BTP202209	0	0	Tissue enhanced
CDS|exon|ID=cds-Bg	8	4.5375	3177.7	0.291158	DDYEPELL	94	993.4372	1	992.4338	.	H	Superscaf	30710761	0	0	0	0	BTP202209	0	Tissue specific
CDS|exon|ID=cds-Bg	7	6.187143	2822.1	0.291648	K(+42.01)	63	888.4775	1	887.4753	Acetylati	I	Superscaf	30722539	0	0	0	0	0	BTP202209	Tissue specific
CDS|exon|ID=cds-Bg	11	6.321818	4290.4	0.292434	A(+42.01)	98	413.8896	3	1238.651	Acetylati	G	Superscaf	30741241	0	BTP202209	0	0	0	0	Tissue specific
CDS|exon|ID=cds-Bg	10	5.98	3822	0.292434	A(+42.01)	98	556.2854	2	1110.556	Acetylati	G	Superscaf	30741241	0	BTP202209	0	0	BTP202209	BTP202209	Mixed
CDS|exon|ID=cds-Bg	6	4.76	2036.2	0.519126	QGFLVF	68	710.3835	1	709.3799	.	M	Superscaf	36133287	0	0	0	BTP202209	0	0	Tissue specific
CDS|exon|ID=cds-Bg	9	6.276667	3666	0.76956	DSALSTHKF	83	478.2451	2	954.4771	.	H	Superscaf	42089854	BTP202209	0	0	0	0	0	Tissue specific
CDS|exon|ID=cds-Bg	8	5.12625	2797	0.769772	SASPVYQA	93	822.397	1	821.3919	.	G	Superscaf	42094855	BTP202209	0	0	0	0	0	Tissue specific
CDS|exon|ID=cds-Bg	11	7.047273	5428.2	0.769776	RSSNLQSRS	83	446.5727	3	1336.696	.	G	Superscaf	42094855	BTP202209	0	0	0	0	0	Tissue specific
CDS|exon|ID=cds-Bg	14	6.612857	6492.4	0.770412	ERFETERNS	71	442.4778	4	1765.886	.	W	Superscaf	42110137	BTP202209	0	0	0	0	0	Tissue specific
CDS|exon|ID=cds-Bg	11	6.485455	5325.3	0.770412	ERFETERNS	94	474.2352	3	1419.686	.	W	Superscaf	42110137	BTP202209	0	0	0	0	0	Tissue specific
CDS|exon|ID=cds-Bg	10	6.058	4676.5	0.770412	ERFETERNS	99	632.7983	2	1263.584	.	W	Superscaf	42110137	BTP202209	0	0	0	0	0	Tissue specific
CDS|exon|ID=cds-Bg	11	6.647273	5030.7	0.770412	ETERNSPRF	99	445.5646	3	1333.674	.	W	Superscaf	42110137	BTP202209	0	0	0	0	0	Tissue specific
CDS|exon|ID=cds-Bg	10	6.338	4562.3	0.770412	ETERNSPRF	88	603.7964	2	1205.579	.	W	Superscaf	42110137	BTP202209	0	0	0	0	0	Tissue specific
CDS|exon|ID=cds-Bg	9	6.373333	4251	0.770412	ETERNSPRF	99	379.1871	3	1134.542	.	W	Superscaf	42110137	BTP202209	0	0	0	0	0	Tissue specific
CDS|exon|ID=cds-Bg	8	6.7675	3825.5	0.770412	TERNSPRF	90	503.7569	2	1005.499	.	W	Superscaf	42110137	BTP202209	0	0	0	0	0	Tissue specific
CDS|exon|ID=cds-Bg	9	7.347778	4135.6	0.770413	RNSPRFAKL	81	363.5484	3	1087.625	.	W	Superscaf	42110137	BTP202209	0	0	0	0	0	Tissue specific
CDS|exon|ID=cds-Bg	9	7.347778	4135.6	0.770413	NSPRFAKLF	91	363.5529	3	1087.625	.	W	Superscaf	42110137	BTP202209	0	0	0	0	0	Tissue specific
CDS|exon|ID=cds-Bg	8	6.85875	4045.6	0.770414	RNWHHGLS	71	503.7508	2	1005.489	.	W	Superscaf	42110137	BTP202209	0	0	0	0	0	Tissue specific
CDS|exon|ID=cds-Bg	10	5.648	3320.6	0.770832	VATSPAPAA	97	461.2429	2	920.4716	.	F	Superscaf	42119929	BTP202209	0	0	0	0	0	Tissue specific
CDS|exon|ID=cds-Bg	10	6.265	3866.3	0.770832	TSPAPAAHT	99	508.2454	2	1014.477	.	F	Superscaf	42119929	BTP202209	0	0	0	0	0	Tissue specific
CDS|exon|ID=cds-Bg	9	6.235556	3468.8	0.770832	SPAPAAHTY	97	457.7212	2	913.4294	.	F	Superscaf	42119929	BTP202209	0	0	0	0	0	Tissue specific
CDS|exon|ID=cds-Bg	12	6.158333	4453.9	0.771072	SGGPAESAS	79	556.7695	2	1111.526	.	I	Superscaf	42125683	BTP202209	0	0	0	0	0	Tissue specific
CDS|exon|ID=cds-Bg	11	6.201818	4070.5	0.771072	GGPAESASF	79	513.2542	2	1024.494	.	I	Superscaf	42125683	BTP202209	0	0	0	0	0	Tissue specific
CDS|exon|ID=cds-Bg	12	6.254167	4243.6	0.771074	FAPGKTTTT	93	619.3408	2	1236.671	.	I	Superscaf	42125683	0	0	0	0	0	BTP202209	Tissue specific
CDS|exon|ID=cds-Bg	11	6.647273	4736.9	0.779911	A(+42.01)	89	419.9089	3	1256.709	Acetylati	C	Superscaf	42336025	0	0	0	0	BTP202209	0	Tissue specific
CDS|exon|ID=cds-Bg	10	6.747	4126.2	0.779911	A(+42.01)	99	377.2233	3	1128.65	Acetylati	C	Superscaf	42336025	0	BTP202209	0	0	BTP202209	BTP202209	Mixed
CDS|exon|ID=cds-Bg	9	6.414444	3657.8	0.779911	A(+42.01)	99	501.2842	2	1000.555	Acetylati	C	Superscaf	42336025	0	BTP202209	0	BTP202209	BTP202209	BTP202209	Mixed
CDS|exon|ID=cds-Bg	6	5.59	2565	0.793671	F(+42.01)	62	407.1374	2	812.2656	Acetylati	S	Superscaf	42663403	BTP202209	0	0	0	0	0	Tissue specific
CDS|exon|ID=cds-Bg	8	6.43125	2980.1	0.952231	PAAFTSSK	50	404.7145	2	807.4127	.	V	Superscaf	46434517	0	0	0	0	BTP202209	0	Tissue specific
CDS|exon|ID=cds-Bg	8	4.845	2864.3	0.957816	DRVYVHPF	81	516.7651	2	1031.519	.	L	Superscaf	46567618	0	0	0	0	0	BTP202209	Tissue specific
CDS|exon|ID=cds-Bg	5	6.062	1753.1	0.946477	PLLM(+15.	53	586.3341	1	585.3196	Oxidation	N	Superscaf	1416363	BTP202209	0	0	0	0	0	Tissue specific
CDS|exon|ID=cds-Bg	6	4.08	1415.6	0.906987	GVTSVP	64	559.2993	1	558.3013	.	N	Superscaf	2461431	0	0	0	0	BTP202209	0	Tissue specific
CDS|exon|ID=cds-Bg	14	5.909286	5323.9	0.898961	GHVDSGKST	99	466.2307	3	1395.674	.	L	Superscaf	2673610	0	0	BTP202209	BTP202209	BTP202209	0	Mixed
CDS|exon|ID=cds-Bg	13	5.904615	5026.6	0.898961	HVDSGKSTT	73	447.2238	3	1338.653	.	L	Superscaf	2673610	0	BTP202209	0	BTP202209	0	0	Mixed
CDS|exon|ID=cds-Bg	8	6.2325	3215.4	0.782848	ELSELKPK	85	472.2738	2	942.5386	.	M	Superscaf	5746171	0	0	0	0	0	BTP202209	Tissue specific
CDS|exon|ID=cds-Bg	5	6.05	1742.9	0.674117	GPAPY	65	504.2435	1	503.238	.	L	Superscaf	8624058	0	0	0	BTP202209	0	0	Tissue specific
CDS|exon|ID=cds-Bg	8	5.5675	2786.9	0.674106	GELAKHAV	97	412.7331	2	823.4551	.	N	Superscaf	8623447	0	0	0	0	BTP202209	0	Tissue specific
CDS|exon|ID=cds-Bg	11	5.457273	4645.2	0.671912	EVYDDFLHY	77	495.5752	3	1483.71	.	P	Superscaf	8681584	0	0	0	0	0	BTP202209	Tissue specific
CDS|exon|ID=cds-Bg	12	5.5575	5161.9	0.67191	DPFNPFELT	78	701.3236	2	1400.636	.	P	Superscaf	8681584	0	0	0	0	0	BTP202209	Tissue specific
CDS|exon|ID=cds-Bg	11	5.515455	4850.6	0.67191	DPFNPFELT	98	665.8038	2	1329.599	.	P	Superscaf	8681584	0	0	0	0	0	BTP202209	Tissue specific
CDS|exon|ID=cds-Bg	10	5.77	4439.1	0.67191	PFNPFELTN	97	608.29	2	1214.572	.	P	Superscaf	8681584	0	0	0	0	0	BTP202209	Tissue specific
CDS|exon|ID=cds-Bg	9	5.711111	4101.7	0.67191	FNPFELTNH	98	559.7648	2	1117.519	.	P	Superscaf	8681584	0	0	0	0	0	BTP202209	Tissue specific
CDS|exon|ID=cds-Bg	8	5.74	3714.3	0.67191	NPFELTNH	98	486.2316	2	970.4508	.	P	Superscaf	8681584	0	0	0	0	0	BTP202209	Tissue specific
CDS|exon|ID=cds-Bg	13	6.295385	5316.7	0.589373	SQPGEKKLT	96	651.834	2	1301.658	.	S	Superscaf	10866010	0	0	BTP202209	0	0	0	Tissue specific
CDS|exon|ID=cds-Bg	5	5.518	1784.9	0.480472	YTVYK	96	673.3531	1	672.3483	.	L	Superscaf	13747806	0	0	0	0	0	BTP202209	Tissue specific
CDS|exon|ID=cds-Bg	12	6.735	5350.3	0.271818	M(+42.01)	54	713.3376	2	1424.665	Acetylati	G	Superscaf	19269151	0	0	0	0	0	BTP202209	Tissue specific
CDS|exon|ID=cds-Bg	8	7.1425	3643.4	0.271819	M(+42.01)	86	489.7317	2	977.4535	Acetylati	G	Superscaf	19269151	0	0	0	BTP202209	0	0	Tissue specific
CDS|exon|ID=cds-Bg	6	4.481667	2066.4	0.270068	LELVNP	56	684.3909	1	683.3854	.	K	Superscaf	19315713	0	BTP202209	0	0	BTP202209	0	Mixed
CDS|exon|ID=cds-Bg	12	5.736667	4737.1	0.029419	FGGLTHQVE	99	453.5726	3	1357.699	.	N	Superscaf	27240457	BTP202209	0	0	0	0	0	Tissue specific
CDS|exon|ID=cds-Bg	7	6.224286	2689	0.120666	KYDPPPP	77	407.2108	2	812.4068	.	G	Superscaf	29655198	BTP202209	0	BTP202209	0	0	0	Mixed
CDS|exon|ID=cds-Bg	8	7.07625	3923.6	0.155058	FNYRTTRF	84	552.7806	2	1103.551	.	G	Superscaf	30565174	0	0	0	0	0	BTP202209	Tissue specific
CDS|exon|ID=cds-Bg	15	5.678667	6187	0.181035	ADDPLLALI	68	820.432	2	1638.858	.	N	Superscaf	31252318	0	0	0	0	BTP202209	0	Tissue specific
CDS|exon|ID=cds-Bg	8	4.94125	2825.1	0.280798	VLELLDKY	88	496.7852	2	991.559	.	S	Superscaf	33892237	0	0	0	BTP202209	0	0	Tissue specific
CDS|exon|ID=cds-Bg	10	6.771	4325.7	0.280801	LKMKGDYFF	99	440.8952	3	1319.67	.	S	Superscaf	33892237	0	0	0	0	0	BTP202209	Tissue specific
CDS|exon|ID=cds-Bg	8	6.41125	3217.4	0.280801	LKMKGDYF	83	501.2585	2	1000.505	.	S	Superscaf	33892237	0	BTP202209	0	0	0	BTP202209	Tissue enhanced
CDS|exon|ID=cds-Bg	13	6.390769	5821.6	0.467534	SHRDFVTPG	91	389.7064	4	1554.802	.	Q	Superscaf	38833810	0	0	0	BTP202209	0	0	Tissue specific
CDS|exon|ID=cds-Bg	10	5.905	4169.7	0.467534	DFVTPGQQK	96	588.3099	2	1174.609	.	Q	Superscaf	38833810	0	0	0	BTP202209	0	0	Tissue specific
CDS|exon|ID=cds-Bg	9	5.721111	3340.8	0.474509	MRYVASYLL	68	558.2979	2	1114.585	.	C	Superscaf	39018577	0	0	BTP202209	0	BTP202209	0	Mixed
CDS|exon|ID=cds-Bg	8	5.68875	2987.4	0.474509	MRYVASYL	71	501.7567	2	1001.5	.	C	Superscaf	39018577	0	BTP202209	BTP202209	0	BTP202209	0	Mixed
CDS|exon|ID=cds-Bg	8	5.125	2631.8	0.474509	YVASYLLA	71	899.4824	1	898.48	.	C	Superscaf	39018577	0	0	BTP202209	0	0	0	Tissue specific
CDS|exon|ID=cds-Bg	5	6.004	1670.9	0.563873	MGPAL	86	488.2493	1	487.2465	.	A	Superscaf	41383282	0	0	0	0	0	BTP202209	Tissue specific
CDS|exon|ID=cds-Bg	8	3.13125	1925.3	0.606604	M(+42.01)	99	468.7509	2	935.4899	Acetylati	P	Superscaf	42514096	0	0	0	0	0	BTP202209	Tissue specific
CDS|exon|ID=cds-Bg	9	5.736667	3315.7	0.607258	VNLARLALF	90	508.8142	2	1015.618	.	A	Superscaf	42531331	0	0	0	0	0	BTP202209	Tissue specific

CDS|exon|ID=cds-Bg	5	4.246	1455.7	0.612057	LPLVD	98	556.3308	1	555.3268	.	G	Superscaf	42658422	BTP202209	0	0	BTP202209	0	0	Mixed
CDS|exon|ID=cds-Bg	7	6.154286	3059.4	0.620242	KWPQPLE	57	449.2471	2	896.4756	.	S	Superscaf	42875016	0	0	BTP202209	0	0	0	Tissue specific
CDS|exon|ID=cds-Bg	9	6.496667	4064.6	0.663298	EDFREAKPF	84	383.1991	3	1146.578	.	I	Superscaf	44013868	0	BTP202209	0	0	0	0	Tissue specific
CDS|exon|ID=cds-Bg	15	6.230667	5808.4	0.673684	LSLQRTGGG	99	659.3279	2	1316.643	.	P	Superscaf	44288650	0	BTP202209	0	0	BTP202209	0	Mixed
CDS|exon|ID=cds-Bg	9	5.823333	3700.1	0.673743	QLTGGHEAF	86	480.2317	2	958.4508	.	P	Superscaf	44290579	0	0	0	0	BTP202209	0	Tissue specific
CDS|exon|ID=cds-Bg	11	5.546364	4280.6	0.676977	EKEPGNKVN	82	599.8232	2	1197.635	.	V	Superscaf	44376286	0	0	0	BTP202209	0	0	Tissue specific
CDS|exon|ID=cds-Bg	10	5.499	3969.3	0.676977	EKEPGNKVN	84	564.3046	2	1126.598	.	V	Superscaf	44376286	0	0	0	BTP202209	0	0	Tissue specific
CDS|exon|ID=cds-Bg	9	5.008889	3279.5	0.677007	FVEQADALK	92	510.7694	2	1019.529	.	K	Superscaf	44377012	0	0	0	0	0	BTP202209	Tissue specific
CDS|exon|ID=cds-Bg	9	3.635556	3170.7	0.67701	NDVFVTEEW	62	569.754	2	1137.498	.	K	Superscaf	44377012	0	0	BTP202209	BTP202209	0	0	Mixed
CDS|exon|ID=cds-Bg	7	5.741429	2680.1	0.683898	EAPRPVH	92	403.2186	2	804.4242	.	P	Superscaf	44559460	0	BTP202209	0	0	0	0	Tissue specific
CDS|exon|ID=cds-Bg	5	6.05	1950.2	0.69927	AQPLP	73	525.3126	1	524.2958	.	L	Superscaf	44966068	0	0	0	BTP202209	0	0	Tissue specific
CDS|exon|ID=cds-Bg	9	6.16	2946.3	0.706131	PGAPGLPPF	59	401.7331	2	801.4384	.	V	Superscaf	45147787	0	0	BTP202209	0	0	0	Tissue specific
CDS|exon|ID=cds-Bg	10	5.089	3690	0.706679	TGVSAQVQK	86	523.286	2	1044.556	.	F	Superscaf	45162133	BTP202209	0	0	0	0	0	Tissue specific
CDS|exon|ID=cds-Bg	12	6.2	5116.5	0.70668	AQVQKQRAK	98	678.3906	2	1354.768	.	F	Superscaf	45162133	BTP202209	0	0	0	0	0	Tissue specific
CDS|exon|ID=cds-Bg	10	5.941	4165.6	0.707322	YGGRESGC(	85	560.2279	2	1118.445	Carbamido	Y	Superscaf	45179143	0	0	0	0	BTP202209	0	Tissue specific
CDS|exon|ID=cds-Bg	10	3.639	2466.6	0.711874	A(+42.01)	83	542.3223	2	1082.634	Acetylati	G	Superscaf	45299743	0	0	0	0	BTP202209	BTP202209	Mixed
CDS|exon|ID=cds-Bg	9	3.378889	2113.2	0.711874	A(+42.01)	96	485.782	2	969.5495	Acetylati	G	Superscaf	45299743	0	BTP202209	0	0	BTP202209	BTP202209	Mixed
CDS|exon|ID=cds-Bg	15	4.661333	5445.3	0.712075	VDDDSLGEF	98	825.882	2	1649.753	.	T	Superscaf	45304852	0	BTP202209	0	0	0	0	Tissue specific
CDS|exon|ID=cds-Bg	16	5.583125	6287.3	0.713522	M(+42.01)	89	836.3463	2	1670.684	Acetylati	Q	Superscaf	45343297	0	BTP202209	0	0	0	0	Tissue specific
CDS|exon|ID=cds-Bg	8	5.1925	2398.6	0.722461	M(+42.01)	83	776.3387	1	775.3323	Acetylati	G	Superscaf	45579922	0	BTP202209	0	0	0	0	Tissue specific
CDS|exon|ID=cds-Bg	6	5.026667	2026.3	0.731955	TVALLQ	55	644.3961	1	643.3904	.	T	Superscaf	45830488	0	0	0	0	0	BTP202209	Tissue specific
CDS|exon|ID=cds-Bg	11	4.956364	3292.4	0.732723	VVLGQFLVI	99	415.2742	3	1242.806	.	S	Superscaf	45851422	0	0	0	0	0	BTP202209	Tissue specific
CDS|exon|ID=cds-Bg	9	6.078889	3573.9	0.734338	A(+42.01)	75	509.2537	2	1016.493	Acetylati	W	Superscaf	45894100	0	BTP202209	0	BTP202209	0	BTP202209	Mixed
CDS|exon|ID=cds-Bg	10	5.855	3774.1	0.741599	VGNKSDLTT	99	531.7912	2	1061.572	.	E	Superscaf	46086283	0	0	0	0	0	BTP202209	Tissue specific
CDS|exon|ID=cds-Bg	12	5.440833	4356.7	0.741599	KSDLTTKKV	99	674.3757	2	1346.741	.	E	Superscaf	46086283	0	0	BTP202209	0	0	0	Tissue specific
CDS|exon|ID=cds-Bg	15	5.624667	5463	0.741599	SDLTTKKVV	95	540.9632	3	1619.873	.	E	Superscaf	46086283	0	0	0	0	0	BTP202209	Tissue specific
CDS|exon|ID=cds-Bg	15	5.394	5976.6	0.741607	LETSAKNAT	63	811.9012	2	1621.795	.	G	Superscaf	46086499	0	0	0	0	0	BTP202209	Tissue specific
CDS|exon|ID=cds-Bg	7	5.194286	2712.1	0.746799	LEDLATY	78	824.4031	1	823.3963	.	D	Superscaf	46222447	0	BTP202209	0	0	0	BTP202209	Mixed
CDS|exon|ID=cds-Bg	13	5.296923	4910.3	0.749524	FEWLVASAE	89	767.4165	2	1532.824	.	K	Superscaf	46295794	0	0	0	0	0	BTP202209	Tissue specific
CDS|exon|ID=cds-Bg	11	5.12	3789.5	0.751206	M(+42.01)	93	624.2944	2	1246.576	Acetylati	R	Superscaf	46340527	BTP202209	0	0	0	0	0	Tissue specific
CDS|exon|ID=cds-Bg	8	6.295	3347.6	0.782343	QGPLKASC(	65	430.7166	2	859.4222	Carbamido	Q	Superscaf	47164396	0	0	0	BTP202209	0	0	Tissue specific
CDS|exon|ID=cds-Bg	8	5.3975	3256.6	0.782407	SFADYNLL	83	942.4546	1	941.4494	.	A	Superscaf	47166073	0	BTP202209	BTP202209	BTP202209	0	0	Mixed
CDS|exon|ID=cds-Bg	8	5.205	2509.7	0.78241	PLLSAYVA	70	833.4741	1	832.4694	.	A	Superscaf	47166073	0	0	0	BTP202209	0	0	Tissue specific
CDS|exon|ID=cds-Bg	11	4.362727	4159.6	0.783481	DQGQSWKEE	89	652.8091	2	1303.605	.	G	Superscaf	47194525	0	0	BTP202209	BTP202209	0	0	Mixed
CDS|exon|ID=cds-Bg	10	6.286	4252.6	0.783482	QSWLQGPLK	82	564.3118	2	1126.614	.	G	Superscaf	47194525	0	0	BTP202209	BTP202209	0	0	Tissue enhanced
CDS|exon|ID=cds-Bg	12	4.808333	4658.3	0.783513	VNDGVEDLF	95	489.9	3	1466.682	Carbamido	I	Superscaf	47195296	0	0	0	0	0	BTP202209	Tissue specific
CDS|exon|ID=cds-Bg	9	6.56	3779.1	0.783547	ALPQHLKPF	83	525.8066	2	1049.602	.	Y	Superscaf	47196328	0	0	0	0	0	BTP202209	Tissue specific
CDS|exon|ID=cds-Bg	7	5.692857	2681	0.783559	AYVARLN	97	403.7289	2	805.4446	.	V	Superscaf	47196601	0	0	BTP202209	0	0	0	Tissue specific
CDS|exon|ID=cds-Bg	12	6.865833	4881.3	0.78356	VARLNSRPK	95	676.9251	2	1351.841	.	V	Superscaf	47196601	0	0	0	0	0	BTP202209	Tissue specific
CDS|exon|ID=cds-Bg	10	6.663	4101.6	0.78356	VARLNSRPK	86	385.2422	3	1152.709	.	V	Superscaf	47196601	0	0	0	0	0	BTP202209	Tissue specific
CDS|exon|ID=cds-Bg	10	5.713	4113.6	0.783561	AFLASLEHM	95	566.7747	2	1131.538	.	V	Superscaf	47196601	0	0	BTP202209	0	0	0	Tissue specific
CDS|exon|ID=cds-Bg	12	6.3475	4818.2	0.862008	TLKEKELET	92	458.2588	3	1371.761	.	L	Superscaf	49272334	0	0	0	0	0	BTP202209	Tissue specific
CDS|exon|ID=cds-Bg	14	4.647143	4763.3	0.862613	VSEGVYEST	69	731.3295	2	1460.642	.	P	Superscaf	49288447	0	BTP202209	0	0	0	0	Tissue specific
CDS|exon|ID=cds-Bg	15	4.714667	5222.8	0.862613	VSEGVYEST	91	812.8582	2	1623.705	.	P	Superscaf	49288447	0	BTP202209	0	0	0	0	Tissue specific
CDS|exon|ID=cds-Bg	10	5.585	4116.6	0.862613	YESTEAPGH	75	577.2428	2	1152.472	.	P	Superscaf	49288447	0	BTP202209	0	0	0	0	Tissue specific
CDS|exon|ID=cds-Bg	7	5.72	2683.1	0.923987	P(+42.01)	84	763.37	1	762.3548	Acetylati	P	Superscaf	50911543	0	0	0	BTP202209	0	0	Tissue specific
CDS|exon|ID=cds-Bg	8	5.51375	2959.2	0.932298	GYFLPAEF	79	943.455	1	942.4487	.	G	Superscaf	51132723	0	0	0	0	BTP202209	0	Tissue specific
CDS|exon|ID=cds-Bg	9	5.206667	2972.4	0.944094	SPGGVTVQF	98	450.7469	2	899.4825	.	I	Superscaf	51444454	0	0	0	BTP202209	0	0	Tissue specific
CDS|exon|ID=cds-Bg	9	5.063333	3150.4	0.944191	SPEDYALKV	98	511.2635	2	1020.513	.	P	Superscaf	51447280	0	0	0	BTP202209	0	0	Tissue specific
CDS|exon|ID=cds-Bg	8	4.71125	2431.8	0.957117	RVDAVLLL	56	449.7887	2	897.5647	.	S	Superscaf	51789286	0	0	BTP202209	0	0	0	Tissue specific
CDS|exon|ID=cds-Bg	9	6.197778	3426.7	0.969759	GQSVRLLAK	98	486.3015	2	970.5923	.	P	Superscaf	52123891	0	0	0	0	0	BTP202209	Tissue specific
CDS|exon|ID=cds-Bg	9	6.08	3635	0.969842	SGDTKWLPY	84	533.7616	2	1065.513	.	W	Superscaf	52125889	0	0	0	0	0	BTP202209	Tissue specific
CDS|exon|ID=cds-Bg	9	4.491111	2884.2	0.969942	YGEGHVVTF	95	504.741	2	1007.471	.	A	Superscaf	52128577	0	0	0	0	0	BTP202209	Tissue specific
CDS|exon|ID=cds-Bg	8	4.3675	2496.8	0.969942	YGEGHVVT	78	431.207	2	860.4028	.	A	Superscaf	52128577	0	0	0	0	0	BTP202209	Tissue specific
CDS|exon|ID=cds-Bg	13	4.966154	5519.3	0.969943	DGQRFVFDG	69	803.8298	2	1605.652	Carbamido	A	Superscaf	52128577	0	0	0	0	0	BTP202209	Tissue specific
CDS|exon|ID=cds-Bg	8	6.345	3618	0.969969	KFHATTQE	61	481.2324	2	960.4665	.	C	Superscaf	52129740	BTP202209	0	0	0	0	0	Tissue specific
CDS|exon|ID=cds-Bg	9	5.322222	3474.8	0.969978	VNSWKESPL	93	530.2756	2	1058.54	.	H	Superscaf	52129630	0	0	0	0	0	BTP202209	Tissue specific
CDS|exon|ID=cds-Bg	10	5.979	3735.3	0.970071	SAPVPTATG	96	514.2786	2	1026.546	.	G	Superscaf	52132084	0	0	0	0	0	BTP202209	Tissue specific
CDS|exon|ID=cds-Bg	11	6.683636	4192.9	0.970157	TTTPTATPL	97	566.3147	2	1130.618	.	N	Superscaf	52133032	0	0	0	0	0	BTP202209	Tissue specific
CDS|exon|ID=cds-Bg	9	6.717778	3397.9	0.970158	TPTATPLTK	96	465.2669	2	928.5229	.	N	Superscaf	52133032	0	0	0	0	0	BTP202209	Tissue specific
CDS|exon|ID=cds-Bg	11	6.612727	4166.9	0.970207	TM(+15.99	97	589.3081	2	1176.606	Oxidation	N	Superscaf	52133032	0	0	0	0	0	BTP202209	Tissue specific
CDS|exon|ID=cds-Bg	11	6.627273	4238.9	0.97033	TTTSTATPL	99	561.3043	2	1120.598	.	N	Superscaf	52133032	0	0	0	0	0	BTP202209	Tissue specific
CDS|exon|ID=cds-Bg	11	7.118182	4488.3	0.970337	TTTPTATPF	98	587.8231	2	1173.635	.	N	Superscaf	52133032	0	0	0	0	0	BTP202209	Tissue specific
CDS|exon|ID=cds-Bg	10	6.306	3869.4	0.970379	ATPLTETST	99	524.7777	2	1047.545	.	N	Superscaf	52133032	0	0	0	0	0	BTP202209	Tissue specific
CDS|exon|ID=cds-Bg	15	6.422	5810.9	0.970412	TTTETTTPT	98	782.4066	2	1562.804	.	N	Superscaf	52133032	0	0	0	0	0	BTP202209	Tissue specific
CDS|exon|ID=cds-Bg	15	6.380667	5856.9	0.970477	TTTETTTST	99	777.3966	2	1552.783	.	N	Superscaf	52133032	0	0	0	0	0	BTP202209	Tissue specific
CDS|exon|ID=cds-Bg	11	6.069091	3735.3	0.970478	TTAPTVPPL	92	563.3271	2	1124.644	.	N	Superscaf	52133032	0	0	0	0	0	BTP202209	Tissue specific
CDS|exon|ID=cds-Bg	8	5.495	2471.9	0.970479	TAPTVPPL	60	795.4568	1	794.4538	.	N	Superscaf	52133032	0	0	0	0	0	BTP202209	Tissue specific
CDS|exon|ID=cds-Bg	10	5.553	3348.9	0.970489	TTVLPTGTS	53	512.2699	2	1022.528	.	N	Superscaf	52133032	0	0	0	0	0	BTP202209	Tissue specific
CDS|exon|ID=cds-Bg	11	6.408182	4767	0.995034	HLDNYKKSS	74	617.3118	2	1232.615	.	L	Superscaf	52792621	0	0	0	0	0	BTP202209	Tissue specific
CDS|exon|ID=cds-Bg	18	5.424444	6615.1	0.97029	VGDPKHSSF	93	890.4307	2	1778.855	.	A	Superscaf	1824127	0	0	0	0	0	BTP202209	Tissue specific
CDS|exon|ID=cds-Bg	10	5.339	3730	0.97029	VQGGSADNL	83	494.7545	2	987.4985	.	A	Superscaf	1824127	0	0	0	0	0	BTP202209	Tissue specific
CDS|exon|ID=cds-Bg	9	6.187778	3430.6	0.958885	FDPEKGGKF	99	487.7488	2	973.4868	.	E	Superscaf	2524507	0	0	0	0	BTP202209	0	Tissue specific
CDS|exon|ID=cds-Bg	7	5.088571	2539.8	0.95319	EGALLDF	81	764.379	1	763.3752	.	R	Superscaf	2874474	0	0	0	0	0	BTP202209	Tissue specific
CDS|exon|ID=cds-Bg	9	4.263333	2821.2	0.926964	NVSPVELPF	79	501.2673	2	1000.523	.	E	Superscaf	4484413	0	0	0	0	0	BTP202209	Tissue specific
CDS|exon|ID=cds-Bg	7	6.771429	2900.2	0.845289	RDLSGPK	95	772.4167	1	771.4239	.	A	Superscaf	9499722	0	0	0	0	0	BTP202209	Tissue specific
CDS|exon|ID=cds-Bg	16	5.355625	5782.1	0.843516	FQTVADYGK	90	604.6537	3	1810.947	.	E	Superscaf	9608410	0	0	0	0	0	BTP202209	Tissue specific
CDS|exon|ID=cds-Bg	14	5.325714	4784	0.843516	TVADYGKDL	99	512.9467	3	1535.82	.	E	Superscaf	9608410	0	BTP202209	0	0	0	BTP202209	Mixed
CDS|exon|ID=cds-Bg	13	5.233077	4386.5	0.843516	VADYGKDLV	93	479.263	3	1434.772	.	E	Superscaf	9608410	0	0	0	0	0	BTP202209	Tissue specific
CDS|exon|ID=cds-Bg	11	5.637273	4075.2	0.843516	DYGKDLVEK	84	422.5616	3	1264.666	.	E	Superscaf	9608410	0	BTP202209	0	0	0	BTP202209	Mixed
CDS|exon|ID=cds-Bg	10	5.227	3606.8	0.843516	DYGKDLVEK	86	569.2916	2	1136.571	.	E	Superscaf	9608410	0	BTP202209	0	0	0	0	Tissue specific
CDS|exon|ID=cds-Bg	9	5.138889	3295.5	0.843516	DYGKDLVEK	99	533.7733	2	1065.534	.	E	Superscaf	9608410	BTP202209	BTP202209	BTP202209	0	0	BTP202209	Mixed
CDS|exon|ID=cds-Bg	8	5.41	2884	0.843516	YGKDLVEK	95	476.2592	2	950.5073	.	E	Superscaf	9608410	0	BTP202209	BTP202209	0	0	BTP202209	Mixed
CDS|exon|ID=cds-Bg	8	5.74375	3182.5	0.843508	EELTPFFK	76	505.7621	2	1009.512	.	G	Superscaf	9608743	0	BTP202209	0	0	0	0	Tissue specific
CDS|exon|ID=cds-Bg	7	6.042857	2570.9	0.843107	LPVERPK	70	419.7595	2	837.5072	.	A	Superscaf	9633795	0	0	0	0	BTP202209	0	Tissue specific
CDS|exon|ID=cds-Bg	7	5.508571	2785.1	0.832935	KPEHVNP	62	410.723	2	819.4239	.	L	Superscaf	10258317	0	0	BTP202209	0	0	0	Tissue specific
CDS|exon|ID=cds-Bg	8	6.62125	3269.6	0.826015	MLTKFETK	98	499.2722	2	996.5314	.	R	Superscaf	10683160	0	0	0	BTP202209	0	BTP202209	Tissue enhanced
CDS|exon|ID=cds-Bg	9	6.352222	3490.7	0.826015	FETKSARVK	93	355.8718	3	1064.598	.	R	Superscaf	10683160	0	0	0	0	0	BTP202209	Tissue specific
CDS|exon|ID=cds-Bg	10	5.083	3290.6	0.825762	FGTTDAVVK	99	537.7809	2	1073.551	.	H	Superscaf	10698565	0	0	0	0	0	BTP202209	Tissue specific
CDS|exon|ID=cds-Bg	9	4.804444	2670.9	0.825762	FGTTDAVVK	96	469.2516	2	936.4916	.	H	Superscaf	10698565	0	0	0	0	0	BTP202209	Tissue specific
CDS|exon|ID=cds-Bg	9	5.038889	2903.2	0.825762	GTTDAVVKH	92	464.247	2	926.4821	.	H	Superscaf	10698565	0	0	0	0	0	BTP202209	Tissue specific
CDS|exon|ID=cds-Bg	9	5.66	4247.7	0.825438	LDDKNC(+5	99	604.2719	2	1206.534	Carbamido	I	Superscaf	10718581	0	0	0	0	0	BTP202209	Tissue specific
CDS|exon|ID=cds-Bg	8	4.92	2815.3	0.825432	GDVSERVR	97	459.242	2	916.4726	.	L	Superscaf	10718863	0	0	0	0	0	BTP202209	Tissue specific
CDS|exon|ID=cds-Bg	14	5.04	4761.2	0.825384	VEATEGLGD	99	687.8408	2	1373.668	.	N	Superscaf	10721836	0	0	0	0	0	BTP202209	Tissue specific
CDS|exon|ID=cds-Bg	6	4.666667	1512.6	0.824286	K(+42.01)	68	710.4784	1	709.4738	Acetylati	T	Superscaf	10789383	0	0	0	0	BTP202209	0	Tissue specific
CDS|exon|ID=cds-Bg	7	5.088571	2030.1	0.822263	LAGGAVY	63	650.348	1	649.3435	.	P	Superscaf	10913485	0	0	0	0	0	BTP202209	Tissue specific
CDS|exon|ID=cds-Bg	10	6.377	3996.4	0.819257	A(+42.01)	62	524.2636	2	1046.514	Acetylati	P	Superscaf	11098072	0	BTP202209	0	0	0	0	Tissue specific
CDS|exon|ID=cds-Bg	9	7.011111	3737.2	0.819257	RGPAYGLSF	86	488.7696	2	975.525	.	P	Superscaf	11098072	0	BTP202209	0	0	0	0	Tissue specific
CDS|exon|ID=cds-Bg	11	5.075455	3572.8	0.803552	GSESVKDTF	98	573.8022	2	1145.593	.	C	Superscaf	12062203	0	0	0	0	BTP202209	0	Tissue specific
CDS|exon|ID=cds-Bg	12	5.849167	5116.6	0.746072	RQKEYKC(+	77	514.9283	3	1541.766	Carbamido	S	Superscaf	15591271	0	0	BTP202209	0	0	0	Tissue specific
CDS|exon|ID=cds-Bg	10	5.565	4002.3	0.745971	STANKYQVF	92	602.801	2	1203.592	.	G	Superscaf	15598042	0	0	0	0	0	BTP202209	Tissue specific
CDS|exon|ID=cds-Bg	9	5.574444	3614.9	0.745971	STANKYQVF	94	529.2676	2	1056.524	.	G	Superscaf	15598042	0	0	0	0	0	BTP202209	Tissue specific
CDS|exon|ID=cds-Bg	8	5.58625	3227.5	0.745971	STANKYQV	83	455.733	2	909.4556	.	G	Superscaf	15598042	0	0	0	0	0	BTP202209	Tissue specific
CDS|exon|ID=cds-Bg	9	4.996667	3250.8	0.745946	EGERPLPVE	81	513.2647	2	1024.519	.	K	Superscaf	15599446	0	0	BTP202209	0	0	0	Tissue specific
CDS|exon|ID=cds-Bg	8	6.0125	2999.6	0.739498	M(+42.01)	99	491.7495	2	981.4888	Acetylati	L	Superscaf	15995401	BTP202209	0	BTP202209	0	BTP202209	0	Mixed
CDS|exon|ID=cds-Bg	10	5.408	3353	0.739498	M(+42.01)	54	597.8263	2	1193.641	Acetylati	L	Superscaf	15995401	0	BTP202209	BTP202209	0	BTP202209	0	Mixed
CDS|exon|ID=cds-Bg	9	5.344444	2999.6	0.739498	M(+42.01)	98	541.2842	2	1080.557	Acetylati	L	Superscaf	15995401	0	0	0	0	BTP202209	0	Tissue specific
CDS|exon|ID=cds-Bg	10	4.945	3498.9	0.739382	AGEMLSVAE	96	522.2426	2	1042.475	.	S	Superscaf	16002598	0	0	0	0	0	BTP202209	Tissue specific
CDS|exon|ID=cds-Bg	17	5.758824	6545.2	0.739284	ALTEKSKAM	82	969.981	2	1937.956	.	C	Superscaf	16008481	0	0	0	0	0	BTP202209	Tissue specific
CDS|exon|ID=cds-Bg	7	3.251429	1993.3	0.739111	HVVEVPQ	73	404.2218	2	806.4286	.	G	Superscaf	16019284	0	0	0	BTP202209	0	0	Tissue specific
CDS|exon|ID=cds-Bg	8	6.7175	2906.2	0.737519	A(+42.01)	84	396.7178	2	791.429	Acetylati	D	Superscaf	16117087	0	0	0	BTP202209	0	0	Tissue specific
CDS|exon|ID=cds-Bg	10	6.259	3978.3	0.735086	EEGARLLAS	93	537.2993	2	1072.588	.	L	Superscaf	16266412	0	0	0	0	0	BTP202209	Tissue specific
CDS|exon|ID=cds-Bg	8	5.89625	3126.5	0.735086	EEGARLLA	69	429.7367	2	857.4606	.	L	Superscaf	16266412	0	BTP202209	BTP202209	0	0	0	Mixed
CDS|exon|ID=cds-Bg	6	7.35	2731.1	0.733124	KPC(+57.(	63	385.7147	2	769.4268	Carbamido	E	Superscaf	16387008	0	0	BTP202209	0	0	0	Tissue specific
CDS|exon|ID=cds-Bg	8	5.61625	2882.1	0.72857	P(+42.01)	50	509.295	2	1016.57	Acetylati	S	Superscaf	16666632	0	0	0	0	0	BTP202209	Tissue specific
CDS|exon|ID=cds-Bg	5	6.28	2304.7	0.722051	Y(+42.01)	72	743.3374	1	742.3286	Acetylati	S	Superscaf	17066928	0	BTP202209	0	0	0	0	Tissue specific
CDS|exon|ID=cds-Bg	6	5.538333	2184.5	0.721744	DPLTGF	67	649.3193	1	648.3119	.	A	Superscaf	17085691	BTP202209	0	0	0	0	0	Tissue specific
CDS|exon|ID=cds-Bg	12	5.8625	4322.7	0.719297	MLSPLVKDW	95	462.2579	3	1383.758	.	D	Superscaf	17236278	0	0	0	0	0	BTP202209	Tissue specific
CDS|exon|ID=cds-Bg	10	5.39	4102.5	0.715147	EC(+57.02	81	588.7593	2	1175.506	Carbamido	K	Superscaf	17490577	0	0	0	0	BTP202209	0	Tissue specific
CDS|exon|ID=cds-Bg	9	5.296667	3446.7	0.702293	KLENAVQGS	91	473.253	2	944.4927	.	P	Superscaf	18279829	0	BTP202209	0	0	0	0	Tissue specific
CDS|exon|ID=cds-Bg	12	4.586667	3947.3	0.702171	A(+42.01)	80	683.8383	2	1365.666	Acetylati	G	Superscaf	18287554	0	0	0	0	BTP202209	0	Tissue specific
CDS|exon|ID=cds-Bg	13	4.233846	3947.3	0.702171	A(+42.01)	76	733.3739	2	1464.735	Acetylati	G	Superscaf	18287554	0	0	0	0	BTP202209	0	Tissue specific
CDS|exon|ID=cds-Bg	8	6.165	3799.3	0.702169	MLQKELNH	81	506.764	2	1011.517	.	G	Superscaf	18287554	0	0	0	0	0	BTP202209	Tissue specific
CDS|exon|ID=cds-Bg	16	5.1825	5591.2	0.701361	A(+42.01)	99	931.9668	2	1861.929	Acetylati	P	Superscaf	18337225	0	0	0	0	0	BTP202209	Tissue specific
CDS|exon|ID=cds-Bg	10	5.189	3431.7	0.701361	A(+42.01)	99	580.8124	2	1159.613	Acetylati	P	Superscaf	18337225	0	BTP202209	0	0	BTP202209	0	Mixed
CDS|exon|ID=cds-Bg	9	5.096667	3120.4	0.701361	YPLEKALDV	93	524.2881	2	1046.565	.	P	Superscaf	18337225	0	0	0	0	BTP202209	0	Tissue specific
CDS|exon|ID=cds-Bg	8	5.02625	2660.9	0.701361	PLEKALDV	99	442.7574	2	883.5015	.	P	Superscaf	18337225	0	BTP202209	0	0	BTP202209	0	Mixed
CDS|exon|ID=cds-Bg	13	5.844615	5721.4	0.701341	NEFFEGFPD	83	805.8815	2	1609.752	.	L	Superscaf	18338329	0	0	0	0	BTP202209	0	Tissue specific
CDS|exon|ID=cds-Bg	8	5.84875	3571	0.699484	MKENFPNF	75	513.7372	2	1025.464	.	L	Superscaf	18452386	0	0	0	0	0	BTP202209	Tissue specific
CDS|exon|ID=cds-Bg	9	4.237778	2207.4	0.696652	VVLVSRVLK	85	506.8453	2	1011.68	.	P	Superscaf	18626332	0	0	0	0	BTP202209	0	Tissue specific
CDS|exon|ID=cds-Bg	5	5.732	2157.5	0.671011	C(+57.02)	71	604.219	1	603.2145	Carbamido	C	Superscaf	20200950	0	0	BTP202209	0	0	0	Tissue specific
CDS|exon|ID=cds-Bg	6	6.523333	2359.5	0.670161	LLYKPF	51	780.4551	1	779.4581	.	K	Superscaf	20253087	0	0	0	0	0	BTP202209	Tissue specific

CDS|exon|ID=cds-Bg	11	5.789091	4178.7	0.669358	A(+42.01)	97	623.8154	2	1245.62	Acetylati	I	Superscaf	20302330	0	0	BTP202209	0	BTP202209	0 Mixed
CDS|exon|ID=cds-Bg	10	5.292	3529.9	0.669358	A(+42.01)	75	545.7649	2	1089.519	Acetylati	I	Superscaf	20302330	0	0	BTP202209	0	0	0 Tissue specific
CDS|exon|ID=cds-Bg	9	6.097778	3635	0.669356	NTELGAFTK	95	490.7544	2	979.4974	.	I	Superscaf	20302330	0	0	0	0	0	BTP202209 Tissue specific
CDS|exon|ID=cds-Bg	9	5.913333	3259.8	0.66934	PGVLDRMMK	88	523.7761	2	1045.541	.	K	Superscaf	20303428	0	0	0	0	0	BTP202209 Tissue specific
CDS|exon|ID=cds-Bg	9	5.464444	3856.5	0.668751	PSQMEHAME	77	530.2141	2	1058.416	.	V	Superscaf	20339608	0	BTP202209	0	0	0	0 Tissue specific
CDS|exon|ID=cds-Bg	13	5.591538	5384.4	0.668751	PSQMEHAME	83	785.3108	2	1568.613	.	V	Superscaf	20339608	0	0	0	0	0	BTP202209 Tissue specific
CDS|exon|ID=cds-Bg	16	5.768125	6789	0.668751	PSQMEHAME	90	652.2676	3	1953.788	.	V	Superscaf	20339608	0	0	0	0	0	BTP202209 Tissue specific
CDS|exon|ID=cds-Bg	12	5.600833	4997	0.668751	PSQMEHAME	83	711.7765	2	1421.545	.	V	Superscaf	20339608	0	0	0	0	0	BTP202209 Tissue specific
CDS|exon|ID=cds-Bg	11	5.587273	4625.5	0.668751	PSQMEHAME	99	646.2583	2	1290.504	.	V	Superscaf	20339608	0	BTP202209	0	BTP202209	0	BTP202209 Mixed
CDS|exon|ID=cds-Bg	10	5.571	4254	0.668751	PSQMEHAME	98	580.738	2	1159.464	.	V	Superscaf	20339608	0	BTP202209	0	0	0	0 Tissue specific
CDS|exon|ID=cds-Bg	14	5.96	5627.9	0.668692	NDYFVVHMK	99	574.6423	3	1720.908	.	S	Superscaf	20342992	0	BTP202209	0	0	0	0 Tissue specific
CDS|exon|ID=cds-Bg	13	5.669231	5159.5	0.668692	NDYFVVHMK	99	531.9448	3	1592.813	.	S	Superscaf	20342992	0	BTP202209	0	0	0	0 Tissue specific
CDS|exon|ID=cds-Bg	12	5.33	4691.1	0.668692	NDYFVVHMK	84	733.3622	2	1464.718	.	S	Superscaf	20342992	0	0	0	0	BTP202209	0 Tissue specific
CDS|exon|ID=cds-Bg	11	6.309091	4160.2	0.668692	FVVHMKQKC	99	443.9308	3	1328.775	.	S	Superscaf	20342992	0	0	0	0	0	BTP202209 Tissue specific
CDS|exon|ID=cds-Bg	10	5.966	3691.8	0.668692	FVVHMKQKC	96	601.3442	2	1200.68	.	S	Superscaf	20342992	0	0	0	0	0	BTP202209 Tissue specific
CDS|exon|ID=cds-Bg	6	4.725	2282.5	0.666817	GFDDFF	79	747.2967	1	746.2911	.	G	Superscaf	20458494	BTP202209	BTP202209	BTP202209	0	0	0 Mixed
CDS|exon|ID=cds-Bg	6	5.568333	1787.9	0.663818	VGM(+15.9	58	620.3486	1	619.3363	Oxidatior	P	Superscaf	20642368	0	0	0	0	0	BTP202209 Tissue specific
CDS|exon|ID=cds-Bg	10	6.433	4244.6	0.661436	T(+42.01)	94	575.2681	2	1148.525	Acetylati	C	Superscaf	20788702	0	BTP202209	0	0	0	0 Tissue specific
CDS|exon|ID=cds-Bg	9	6.484444	3947.3	0.661436	T(+42.01)	98	546.7582	2	1091.504	Acetylati	C	Superscaf	20788702	0	BTP202209	0	0	0	0 Tissue specific
CDS|exon|ID=cds-Bg	8	6.205	3632.1	0.661427	HLYDPKMQ	96	516.2521	2	1030.491	.	C	Superscaf	20788702	0	BTP202209	0	0	0	0 Tissue specific
CDS|exon|ID=cds-Bg	10	6.161	4273.8	0.661427	YDPKMQTDK	99	611.7902	2	1221.57	.	C	Superscaf	20788702	0	0	0	0	BTP202209	0 Tissue specific
CDS|exon|ID=cds-Bg	7	5.05	2722	0.661423	VYDPKYC(+	67	472.713	2	943.4109	Carbamido	C	Superscaf	20788702	BTP202209	0	0	0	BTP202209	0 Tissue enhanced
CDS|exon|ID=cds-Bg	8	6.18375	3524	0.65756	RHEKVQTL	67	505.7889	2	1009.567	.	D	Superscaf	21026587	0	0	0	0	0	BTP202209 Tissue specific
CDS|exon|ID=cds-Bg	11	5.69	4099.8	0.657533	SLDGTRLYV	71	613.3241	2	1224.635	.	P	Superscaf	21028390	0	BTP202209	0	0	0	0 Tissue specific
CDS|exon|ID=cds-Bg	15	6.134	6784.9	0.65236	M(+42.01)	95	449.4541	4	1793.79	Acetylati	G	Superscaf	21345997	0	BTP202209	0	0	0	0 Tissue specific
CDS|exon|ID=cds-Bg	9	5.907778	4020.6	0.648825	LHNLEHSMC	99	519.2446	2	1036.476	.	N	Superscaf	21563080	0	BTP202209	0	BTP202209	0	0 Mixed
CDS|exon|ID=cds-Bg	7	5.692857	2910.3	0.648825	MHSYELG	75	418.6832	2	835.3535	.	N	Superscaf	21563080	0	BTP202209	0	0	BTP202209	0 Mixed
CDS|exon|ID=cds-Bg	12	5.88	5045.9	0.647223	TTHPVTNTT	99	678.8389	2	1355.668	.	S	Superscaf	21661339	0	0	0	0	0	BTP202209 Tissue specific
CDS|exon|ID=cds-Bg	11	4.286364	3542	0.64722	VELDPEVEC	94	608.3086	2	1214.603	.	S	Superscaf	21661339	0	0	0	0	0	BTP202209 Tissue specific
CDS|exon|ID=cds-Bg	13	6.531538	5641	0.646909	KNKQLPSAC	99	698.8777	2	1395.747	.	Q	Superscaf	21680734	0	0	0	0	BTP202209	0 Tissue specific
CDS|exon|ID=cds-Bg	8	4.74625	2807.1	0.641844	ANVELSSL	84	832.4388	1	831.4338	.	V	Superscaf	21991720	0	0	0	BTP202209	0	0 Tissue specific
CDS|exon|ID=cds-Bg	9	6.31	3328.9	0.6374	PPPLMPPHC	85	471.7452	2	941.4793	.	S	Superscaf	22264447	0	0	BTP202209	0	0	BTP202209 Mixed
CDS|exon|ID=cds-Bg	8	6.3525	3031.6	0.6374	PPPLMPPH	98	443.2357	2	884.4578	.	S	Superscaf	22264447	0	BTP202209	0	0	0	0 Tissue specific
CDS|exon|ID=cds-Bg	7	6.207143	2638.1	0.63714	HPTGVRP	74	382.2116	2	762.4136	.	S	Superscaf	22280641	0	0	0	0	BTP202209	0 Tissue specific
CDS|exon|ID=cds-Bg	10	6.95	4563.1	0.636763	LLRKGNYAE	99	407.2338	3	1218.683	.	P	Superscaf	22303552	0	0	0	BTP202209	0	0 Tissue specific
CDS|exon|ID=cds-Bg	9	5.861111	3504.8	0.636763	KGNYAERVC	96	497.2581	2	992.5039	.	P	Superscaf	22303552	0	0	BTP202209	BTP202209	BTP202209	0 Mixed
CDS|exon|ID=cds-Bg	8	5.37625	3036.4	0.636763	GNYAERVG	90	865.4128	1	864.4089	.	P	Superscaf	22303552	0	0	0	0	BTP202209	0 Tissue specific
CDS|exon|ID=cds-Bg	15	6.528	5975.3	0.636759	VLLPKKTES	97	559.3319	3	1674.978	.	P	Superscaf	22303552	0	0	0	0	BTP202209	0 Tissue specific
CDS|exon|ID=cds-Bg	14	6.994286	5975.3	0.636759	LLPKKTESH	99	526.3094	3	1575.91	.	P	Superscaf	22303552	0	0	BTP202209	0	BTP202209	0 Mixed
CDS|exon|ID=cds-Bg	13	7.116154	5378.6	0.636759	LLPKKTESH	87	488.2943	3	1461.867	.	P	Superscaf	22303552	0	0	0	0	BTP202209	0 Tissue specific
CDS|exon|ID=cds-Bg	10	5.882	3646.2	0.636206	FPVGRVHRL	86	398.5785	3	1192.719	.	K	Superscaf	22337848	0	0	0	0	BTP202209	0 Tissue specific
CDS|exon|ID=cds-Bg	9	4.921111	3045.4	0.636205	YM(+15.99	60	544.7682	2	1087.526	Oxidatior	K	Superscaf	22337848	0	0	0	0	BTP202209	0 Tissue specific
CDS|exon|ID=cds-Bg	8	4.78875	2692	0.636205	YMAAVLEY	87	959.4539	1	958.447	.	K	Superscaf	22337848	0	BTP202209	0	0	BTP202209	0 Mixed
CDS|exon|ID=cds-Bg	8	4.82875	2585.9	0.636205	MAAVLEYL	81	455.2399	2	908.4677	.	K	Superscaf	22337848	0	0	0	0	BTP202209	0 Tissue specific
CDS|exon|ID=cds-Bg	9	5.017778	2983.4	0.636205	MAAVLEYLT	54	505.7629	2	1009.515	.	K	Superscaf	22337848	0	0	0	0	BTP202209	0 Tissue specific
CDS|exon|ID=cds-Bg	7	4.664286	2232.5	0.636205	MAAVLEY	88	796.3899	1	795.3837	.	K	Superscaf	22337848	0	BTP202209	0	0	BTP202209	BTP202209 Mixed
CDS|exon|ID=cds-Bg	9	5.676667	4147.8	0.624773	ADFENTC(+	95	550.2257	2	1098.44	Carbamido	E	Superscaf	23039899	0	BTP202209	0	BTP202209	0	0 Mixed
CDS|exon|ID=cds-Bg	14	4.597143	5016.6	0.623777	A(+42.01)	83	765.3396	2	1528.668	Acetylati	P	Superscaf	23101048	0	BTP202209	0	0	BTP202209	BTP202209 Mixed
CDS|exon|ID=cds-Bg	13	4.529231	4629.2	0.623778	A(+42.01)	89	691.8057	2	1381.6	Acetylati	P	Superscaf	23101048	0	0	0	0	BTP202209	BTP202209 Mixed
CDS|exon|ID=cds-Bg	12	4.659167	4217.7	0.623778	A(+42.01)	77	634.2927	2	1266.573	Acetylati	P	Superscaf	23101048	0	BTP202209	0	0	0	0 Tissue specific
CDS|exon|ID=cds-Bg	8	4.34125	2886.3	0.623778	A(+42.01)	60	477.2313	2	952.4501	Acetylati	P	Superscaf	23101048	0	BTP202209	0	BTP202209	0	0 Mixed
CDS|exon|ID=cds-Bg	8	5.1925	3161.6	0.606307	DYGYPFDT	61	977.3896	1	976.3814	.	K	Superscaf	24173734	0	0	BTP202209	0	0	0 Tissue specific
CDS|exon|ID=cds-Bg	11	4.464545	3235.4	0.606247	PFLEDSVKV	94	420.9101	3	1259.712	.	P	Superscaf	24177262	0	0	BTP202209	0	0	0 Tissue specific
CDS|exon|ID=cds-Bg	10	4.274	3107.3	0.606247	LEDSVKVVK	96	565.8215	2	1129.634	.	P	Superscaf	24177262	0	0	BTP202209	0	0	0 Tissue specific
CDS|exon|ID=cds-Bg	9	4.147778	2510.6	0.606247	LEDSVKVVK	99	508.802	2	1015.591	.	P	Superscaf	24177262	0	0	BTP202209	0	0	0 Tissue specific
CDS|exon|ID=cds-Bg	7	6.027143	2647	0.601439	A(+42.01)	93	727.3956	1	726.3912	Acetylati	S	Superscaf	24472666	0	0	0	0	0	BTP202209 Tissue specific
CDS|exon|ID=cds-Bg	14	5.784286	5054.3	0.594248	LVLC(+57.	97	536.3036	3	1605.895	Carbamido	S	Superscaf	24914266	0	0	0	0	0	BTP202209 Tissue specific
CDS|exon|ID=cds-Bg	10	5.376	3533.8	0.594248	LVLC(+57.	99	574.3102	2	1146.611	Carbamido	S	Superscaf	24914266	0	0	0	0	0	BTP202209 Tissue specific
CDS|exon|ID=cds-Bg	9	5.308889	3180.4	0.594248	VLC(+57.0	99	517.768	2	1033.527	Carbamido	S	Superscaf	24914266	0	0	0	0	0	BTP202209 Tissue specific
CDS|exon|ID=cds-Bg	14	5.375714	6192.9	0.594247	LEDLHSEFD	99	561.923	3	1682.754	.	S	Superscaf	24914266	0	0	0	0	0	BTP202209 Tissue specific
CDS|exon|ID=cds-Bg	13	5.329231	5839.5	0.594247	EDLHSEFDE	96	524.2285	3	1569.669	.	S	Superscaf	24914266	0	0	0	0	0	BTP202209 Tissue specific
CDS|exon|ID=cds-Bg	8	5.66375	2572.9	0.594247	KVPTVSRP	93	442.2697	2	882.5287	.	S	Superscaf	24914266	0	0	0	0	0	BTP202209 Tissue specific
CDS|exon|ID=cds-Bg	10	5.002	3851.4	0.594218	EEVLQRGEA	84	572.3003	2	1142.593	.	Q	Superscaf	24916066	0	0	0	0	0	BTP202209 Tissue specific
CDS|exon|ID=cds-Bg	11	5.277273	3287.5	0.586711	FSTVPAVPL	96	565.3319	2	1128.654	.	L	Superscaf	25377046	0	0	0	0	0	BTP202209 Tissue specific
CDS|exon|ID=cds-Bg	10	5.257	2900.1	0.586711	STVPAVPLA	93	491.7986	2	981.5859	.	L	Superscaf	25377046	0	0	0	0	0	BTP202209 Tissue specific
CDS|exon|ID=cds-Bg	9	5.21	2516.7	0.586711	TVPAVPLAK	75	448.2825	2	894.5538	.	L	Superscaf	25377046	0	0	BTP202209	0	0	0 Tissue specific
CDS|exon|ID=cds-Bg	10	5.342	2914.2	0.586711	TVPAVPLAK	80	498.8056	2	995.6015	.	L	Superscaf	25377046	0	0	BTP202209	0	0	0 Tissue specific
CDS|exon|ID=cds-Bg	9	5.21	2516.7	0.586711	VPAVPLAKT	97	448.2826	2	894.5538	.	L	Superscaf	25377046	0	0	BTP202209	0	0	0 Tissue specific
CDS|exon|ID=cds-Bg	9	5.848889	3280.3	0.586558	KDVDKAFLK	99	532.3087	2	1062.607	.	I	Superscaf	25386358	0	0	BTP202209	0	0	0 Tissue specific
CDS|exon|ID=cds-Bg	8	5.85	2724.8	0.586535	GSGLAASF	74	709.3492	1	708.3442	.	C	Superscaf	25387855	0	0	0	0	0	BTP202209 Tissue specific
CDS|exon|ID=cds-Bg	6	5.961667	1998.1	0.561359	LPGAAF	50	575.3107	1	574.3115	.	T	Superscaf	26933829	0	0	0	0	BTP202209	0 Tissue specific
CDS|exon|ID=cds-Bg	7	5.682857	2237.4	0.549775	KLVFLPP	66	407.2679	2	812.516	.	S	Superscaf	27645228	BTP202209	0	0	0	0	0 Tissue specific
CDS|exon|ID=cds-Bg	7	5.37	2461.8	0.536021	SLGEVRL	93	387.2278	2	772.4443	.	D	Superscaf	28489414	0	0	0	0	0	BTP202209 Tissue specific
CDS|exon|ID=cds-Bg	7	6.061429	2700.2	0.521414	T(+42.01)	53	435.6957	2	869.3841	Acetylati	R	Superscaf	29386516	BTP202209	0	0	0	0	0 Tissue specific
CDS|exon|ID=cds-Bg	7	5.405714	3155.7	0.489685	RDTEEFY	66	480.2073	2	958.4032	.	H	Superscaf	31334626	0	0	0	0	BTP202209	0 Tissue specific
CDS|exon|ID=cds-Bg	10	5.552	3794.3	0.489541	LGPAWEVGN	98	549.7865	2	1097.562	.	L	Superscaf	31343509	0	0	0	0	0	BTP202209 Tissue specific
CDS|exon|ID=cds-Bg	9	4.973333	3145.5	0.489541	LGPAWEVGN	80	471.7348	2	941.4606	.	L	Superscaf	31343509	0	0	0	0	0	BTP202209 Tissue specific
CDS|exon|ID=cds-Bg	8	4.91875	2548.8	0.489541	LGPAWEVG	85	828.4205	1	827.4177	.	L	Superscaf	31343509	0	0	0	0	0	BTP202209 Tissue specific
CDS|exon|ID=cds-Bg	6	7.51	2655.2	0.488038	M(+42.01)	61	405.1991	2	808.3936	Acetylati	T	Superscaf	31436055	BTP202209	0	0	0	0	0 Tissue specific
CDS|exon|ID=cds-Bg	7	6.058571	2814	0.469336	TGNVC(+57	63	403.7155	2	805.4116	Carbamido	K	Superscaf	32584312	0	0	0	0	BTP202209	0 Tissue specific
CDS|exon|ID=cds-Bg	7	4.052857	1809.1	0.451014	E(+42.01)	68	754.3868	1	753.3909	Acetylati	N	Superscaf	33709264	0	0	BTP202209	0	0	0 Tissue specific
CDS|exon|ID=cds-Bg	7	6.135714	3039.4	0.430969	FSHWTPF	77	461.2146	2	920.4181	.	T	Superscaf	34940298	0	BTP202209	0	0	0	0 Tissue specific
CDS|exon|ID=cds-Bg	9	5.922222	3781.4	0.421979	DGWRLPETS	89	530.7545	2	1059.499	.	K	Superscaf	35492005	0	0	0	0	0	BTP202209 Tissue specific
CDS|exon|ID=cds-Bg	8	5.56625	2654.8	0.421887	A(+42.01)	92	864.4463	1	863.4423	Acetylati	P	Superscaf	35497738	0	0	0	BTP202209	BTP202209	0 Mixed
CDS|exon|ID=cds-Bg	7	6.395714	3065.3	0.421847	PGQLKQF	87	409.2301	2	816.4493	.	W	Superscaf	35500180	0	0	0	BTP202209	0	0 Tissue specific
CDS|exon|ID=cds-Bg	9	5.677778	3645	0.421844	LDEFPNLKA	77	523.7769	2	1045.544	.	L	Superscaf	35500402	0	0	0	BTP202209	0	0 Tissue specific
CDS|exon|ID=cds-Bg	14	6.044286	6005.8	0.421838	MQSDRFLKM	85	569.9585	3	1706.86	.	G	Superscaf	35500687	0	0	0	0	0	BTP202209 Tissue specific
CDS|exon|ID=cds-Bg	7	6.464286	2890.1	0.415896	KWGFTLY	84	457.7508	2	913.4698	.	I	Superscaf	35865687	0	0	0	0	0	BTP202209 Tissue specific
CDS|exon|ID=cds-Bg	14	5.662143	5365.9	0.414617	VDALDTASH	92	480.2351	3	1437.685	.	G	Superscaf	35944114	BTP202209	0	0	0	0	0 Tissue specific
CDS|exon|ID=cds-Bg	7	6.477143	3023.4	0.413984	PHAADSR	50	377.1912	2	752.3565	.	R	Superscaf	35982916	BTP202209	0	0	0	0	0 Tissue specific
CDS|exon|ID=cds-Bg	7	5.438571	2549.8	0.398657	EGMGAFY	55	774.3098	1	773.3054	.	I	Superscaf	36924103	0	0	0	0	0	BTP202209 Tissue specific
CDS|exon|ID=cds-Bg	15	5.055333	5457.1	0.398367	VVYENMKQT	99	869.4615	2	1736.913	.	P	Superscaf	36941806	0	BTP202209	0	0	BTP202209	BTP202209 Mixed
CDS|exon|ID=cds-Bg	9	7.164444	3894.4	0.376485	GDGRTFPKF	85	517.2788	2	1032.546	.	M	Superscaf	38285371	0	0	BTP202209	0	0	0 Tissue specific
CDS|exon|ID=cds-Bg	9	5.242222	3619.2	0.376485	RGQTC(+57	65	560.269	2	1118.529	Carbamido	M	Superscaf	38285371	0	0	0	0	0	BTP202209 Tissue specific
CDS|exon|ID=cds-Bg	9	6.207778	3490.8	0.376484	TGMLEDGKK	98	489.748	2	977.4852	.	M	Superscaf	38285371	0	0	0	0	BTP202209	0 Tissue specific
CDS|exon|ID=cds-Bg	8	6.19625	3091.4	0.376482	MSVGQRAK	97	438.7382	2	875.4647	.	M	Superscaf	38285371	0	0	0	0	0	BTP202209 Tissue specific
CDS|exon|ID=cds-Bg	9	6.258889	3470.8	0.376482	SVGQRAKLT	93	480.2839	2	958.556	.	M	Superscaf	38285371	0	0	0	0	BTP202209	0 Tissue specific
CDS|exon|ID=cds-Bg	13	5.08	4737.2	0.26946	LAPDYDALD	98	702.851	2	1403.693	.	F	Superscaf	44856808	0	0	0	0	0	BTP202209 Tissue specific
CDS|exon|ID=cds-Bg	11	5.689091	4682.2	0.267548	KLYYNLDDT	83	689.8267	2	1377.645	.	K	Superscaf	44974516	0	0	0	0	0	BTP202209 Tissue specific
CDS|exon|ID=cds-Bg	14	5.725	5566.2	0.267117	SYLENPAFM	91	827.4296	2	1652.848	.	A	Superscaf	45001027	0	0	BTP202209	0	0	0 Tissue specific
CDS|exon|ID=cds-Bg	8	7.31	3700.8	0.265129	KSLQKPQK	65	478.8014	2	955.5814	.	W	Superscaf	45123013	0	0	0	0	0	BTP202209 Tissue specific
CDS|exon|ID=cds-Bg	7	6.717143	3104.4	0.177633	T(+42.01)	84	421.7109	2	841.4083	Acetylati	C	Superscaf	50495638	BTP202209	0	0	0	0	0 Tissue specific
CDS|exon|ID=cds-Bg	7	6.471429	2596.7	0.177633	KGPSYGL	60	361.1965	2	720.3806	.	C	Superscaf	50495638	0	0	0	0	BTP202209	0 Tissue specific
CDS|exon|ID=cds-Bg	10	5.742	3763.9	0.177632	YGLSAEVKN	99	554.802	2	1107.592	.	C	Superscaf	50495638	0	0	0	0	0	BTP202209 Tissue specific
CDS|exon|ID=cds-Bg	8	6.3125	3196.4	0.149887	HLASPEGK	87	419.7232	2	837.4344	.	G	Superscaf	52199272	0	0	0	0	0	BTP202209 Tissue specific
CDS|exon|ID=cds-Bg	8	5.92	3291.7	0.149887	TLVFEQRK	94	510.7939	2	1019.576	.	G	Superscaf	52199272	0	0	0	0	0	BTP202209 Tissue specific
CDS|exon|ID=cds-Bg	7	6.107143	3360	0.09827	LNELRMQ	69	452.2375	2	902.4644	.	V	Superscaf	55368853	0	0	0	0	0	BTP202209 Tissue specific
CDS|exon|ID=cds-Bg	8	6.0975	3086.3	0.077615	KEKHTLVL	57	484.2993	2	966.5862	.	I	Superscaf	56636890	BTP202209	0	0	0	0	0 Tissue specific
CDS|exon|ID=cds-Bg	12	5.636667	4537	0.074725	KFDTPVDKL	73	700.8911	2	1399.771	.	Y	Superscaf	56814430	0	BTP202209	0	0	0	0 Tissue specific
CDS|exon|ID=cds-Bg	6	7.286667	2334.4	0.027187	LLPKKL	51	356.2533	2	710.5054	.	F	Superscaf	59733250	0	0	BTP202209	0	0	0 Tissue specific
CDS|exon|ID=cds-Bg	18	5.733333	6322.1	0.016557	A(+42.01)	93	480.2637	4	1917.033	Acetylati	E	Superscaf	60385681	0	0	BTP202209	0	0	0 Tissue specific
CDS|exon|ID=cds-Bg	10	5.481	3887.2	0.012995	A(+42.01)	86	582.774	2	1163.535	Acetylati	D	Superscaf	60604747	0	BTP202209	0	0	0	0 Tissue specific
CDS|exon|ID=cds-Bg	6	5.44	2471.8	0.16328	LANTEF	82	694.3397	1	693.3333	.	L	Superscaf	71428645	0	BTP202209	0	0	BTP202209	0 Mixed
CDS|exon|ID=cds-Bg	8	7.05	3458.9	0.168784	A(+42.01)	99	473.7597	2	945.5066	Acetylati	Q	Superscaf	71766721	BTP202209	BTP202209	BTP202209	BTP202209	BTP202209	0 Mixed
CDS|exon|ID=cds-Bg	7	6.52	2810.1	0.168784	A(+42.01)	99	395.7089	2	789.4055	Acetylati	Q	Superscaf	71766721	0	0	BTP202209	0	0	0 Tissue specific
CDS|exon|ID=cds-Bg	9	5.198889	3175.5	0.168873	ALAADHTVY	96	480.7412	2	959.4713	.	S	Superscaf	71772154	0	0	0	0	0	BTP202209 Tissue specific
CDS|exon|ID=cds-Bg	7	6.975714	3362.7	0.171568	KLLHQPH	55	436.7636	2	871.5028	.	M	Superscaf	71937700	0	0	0	0	0	BTP202209 Tissue specific
CDS|exon|ID=cds-Bg	15	5.323333	5854.7	0.297417	PGHLQEGFC	81	557.6061	3	1669.8	Carbamido	E	Superscaf	79664764	0	BTP202209	0	0	0	0 Tissue specific
CDS|exon|ID=cds-Bg	8	5.77	3328.7	0.297417	PGHLQEGF	75	442.7161	2	883.4188	.	E	Superscaf	79664764	0	BTP202209	0	0	0	0 Tissue specific
CDS|exon|ID=cds-Bg	8	5.5875	3807.4	0.297417	NRFDQLFD	52	527.7503	2	1053.488	.	E	Superscaf	79664764	BTP202209	0	0	0	0	0 Tissue specific
CDS|exon|ID=cds-Bg	16	4.93375	6128.7	0.297418	DDESDPFEV	95	903.9219	2	1805.832	.	E	Superscaf	79664764	0	0	0	0	0	BTP202209 Tissue specific
CDS|exon|ID=cds-Bg	15	4.613333	5660.3	0.297418	DDESDPFEV	72	839.8716	2	1677.737	.	E	Superscaf	79664764	0	0	0	0	0	BTP202209 Tissue specific
CDS|exon|ID=cds-Bg	11	4.411818	4015.5	0.297418	DDESDPFEV	97	647.2951	2	1292.577	.	E	Superscaf	79664764	0	BTP202209	0	0	0	BTP202209 Tissue enhanced
CDS|exon|ID=cds-Bg	10	4.556	3604	0.297418	DESDPFEVL	92	589.7819	2	1177.55	.	E	Superscaf	79664764	0	BTP202209	0	0	0	0 Tissue specific
CDS|exon|ID=cds-Bg	9	3.98	3135.6	0.297418	DESDPFEVL	60	525.7314	2	1049.455	.	E	Superscaf	79664764	0	0	0	0	0	BTP202209 Tissue specific
CDS|exon|ID=cds-Bg	9	4.732222	3192.5	0.297418	ESDPFEVLK	83	532.2661	2	1062.523	.	E	Superscaf	79664764	0	BTP202209	0	0	0	BTP202209 Tissue enhanced
CDS|exon|ID=cds-Bg	14	5.295	4459.7	0.29742	KNPLPPSVC	86	493.2947	3	1476.867	.	E	Superscaf	79664764	0	BTP202209	0	0	0	0 Tissue specific
CDS|exon|ID=cds-Bg	11	5.7	4135.5	0.297421	KEETQPPVA	94	620.3484	2	1238.687	.	E	Superscaf	79664764	0	0	0	0	0	BTP202209 Tissue specific
CDS|exon|ID=cds-Bg	10	5.296	3667.1	0.297421	KEETQPPVA	68	556.3011	2	1110.592	.	E	Superscaf	79664764	0	0	0	0	0	BTP202209 Tissue specific
CDS|exon|ID=cds-Bg	11	5.7	4135.5	0.297421	EETQPPVAL	84	620.3501	2	1238.687	.	E	Superscaf	79664764	0	BTP202209	0	0	BTP202209	0 Mixed
CDS|exon|ID=cds-Bg	10	5.296	3667.1	0.297421	EETQPPVAL	95	556.3024	2	1110.592	.	E	Superscaf	79664764	0	BTP202209	0	0	0	0 Tissue specific
CDS|exon|ID=cds-Bg	8	5.815	2816.1	0.297421	TQPPVALK	70	427.2646	2	852.5069	.	E	Superscaf	79664764	0	0	BTP202209	0	0	0 Tissue specific

CDS|exon|ID=cds-Bg	8	5.8625	3291.5	0.297484	FEKPLEEK	99	510.272	2	1018.533	.	F	Superscaf	79669177	0	0	0	0	BTP202209	0	Tissue specific
CDS|exon|ID=cds-Bg	13	4.344615	4721.2	0.297551	ENEVEEVKE	95	758.3606	2	1514.71	.	F	Superscaf	79673419	0	0	0	0	0	BTP202209	Tissue specific
CDS|exon|ID=cds-Bg	11	4.384545	3698.2	0.297644	DVDDPEAFF	82	594.7721	2	1187.535	.	F	Superscaf	79679140	0	0	0	0	0	BTP202209	Tissue specific
CDS|exon|ID=cds-Bg	9	5.028889	3286.7	0.297645	DDPEAFPAL	80	487.7262	2	973.4392	.	F	Superscaf	79679140	0	BTP202209	0	0	0	0	Tissue specific
CDS|exon|ID=cds-Bg	6	4.663333	1400.4	0.345138	PVGKVG	56	556.344	1	555.338	.	E	Superscaf	82595353	0	0	BTP202209	0	BTP202209	0	Mixed
CDS|exon|ID=cds-Bg	7	4.905714	1886.1	0.36475	A(+42.01)	65	394.2206	2	786.431	Acetylati	L	Superscaf	83799655	0	0	BTP202209	0	0	0	Tissue specific
CDS|exon|ID=cds-Bg	7	5.66	2953.4	0.516772	LDDFLFR	80	463.2411	2	924.4705	.	I	Superscaf	93134203	0	BTP202209	0	0	0	0	Tissue specific
CDS|exon|ID=cds-Bg	8	5.6875	3441	0.54489	M(+42.01)	95	551.7446	2	1101.48	Acetylati	K	Superscaf	94860778	0	0	0	BTP202209	0	0	Tissue specific
CDS|exon|ID=cds-Bg	6	6.936667	2457.9	0.560726	PYRPPP	63	363.6993	2	725.386	.	S	Superscaf	95833147	0	0	0	BTP202209	0	0	Tissue specific
CDS|exon|ID=cds-Bg	7	5.345714	2592.9	0.560969	QLEKTVP	53	407.7316	2	813.4596	.	L	Superscaf	95848152	0	BTP202209	0	0	0	0	Tissue specific
CDS|exon|ID=cds-Bg	9	5.351111	3374.7	0.573417	LGLDLWQVK	97	536.312	2	1070.612	.	G	Superscaf	96612280	0	0	0	0	0	BTP202209	Tissue specific
CDS|exon|ID=cds-Bg	8	6.83625	3260.6	0.618483	APAASHPR	51	403.7126	2	805.4194	.	G	Superscaf	99379588	0	0	0	0	0	BTP202209	Tissue specific
CDS|exon|ID=cds-Bg	13	4.995385	4582	0.636414	SSAVMALQE	53	647.3007	2	1292.592	.	P	Superscaf	1E+08	0	0	0	0	0	BTP202209	Tissue specific
CDS|exon|ID=cds-Bg	14	5.042857	5041.5	0.636414	SSAVMALQE	61	728.8311	2	1455.655	.	P	Superscaf	1E+08	0	0	0	0	BTP202209	0	Tissue specific
CDS|exon|ID=cds-Bg	6	2.903333	982	0.644425	G(+42.01)	62	619.342	1	618.3377	Acetylati	S	Superscaf	1.01E+08	0	0	0	BTP202209	0	0	Tissue specific
CDS|exon|ID=cds-Bg	8	6.53625	2657.7	0.65636	KGGGPAAP	72	654.3445	1	653.3496	.	I	Superscaf	1.02E+08	0	0	0	0	0	BTP202209	Tissue specific
CDS|exon|ID=cds-Bg	8	7.12	2977.2	0.659686	LPPKPPKP	75	437.28	2	872.5483	.	R	Superscaf	1.02E+08	0	0	BTP202209	0	0	0	Tissue specific
CDS|exon|ID=cds-Bg	9	6.314444	3930.2	0.66835	SDRAEEFKK	88	370.523	3	1108.551	.	E	Superscaf	1.02E+08	0	0	0	0	0	BTP202209	Tissue specific
CDS|exon|ID=cds-Bg	7	7.068571	3279.8	0.713087	RDKRPDL	96	450.2495	2	898.4984	.	D	Superscaf	1.05E+08	0	BTP202209	0	0	0	0	Tissue specific
CDS|exon|ID=cds-Bg	5	5.426	1785.9	0.733939	NKSVP	55	544.3101	1	543.3016	.	S	Superscaf	1.06E+08	0	0	BTP202209	0	0	0	Tissue specific
CDS|exon|ID=cds-Bg	7	6.995714	3064.6	0.734757	GRELPLR	86	420.7553	2	839.4977	.	P	Superscaf	1.07E+08	BTP202209	0	0	0	0	BTP202209	Mixed
CDS|exon|ID=cds-Bg	14	5.830714	5344.9	0.764745	M(+42.01)	51	759.8311	2	1517.653	Acetylati	E	Superscaf	1.08E+08	0	0	0	0	0	BTP202209	Tissue specific
CDS|exon|ID=cds-Bg	7	6.614286	2578.8	0.767339	FTLGPPK	54	380.222	2	758.4326	.	N	Superscaf	1.09E+08	0	BTP202209	0	0	0	0	Tissue specific
CDS|exon|ID=cds-Bg	9	6.553333	3381.7	0.767339	APARTAGSS	55	409.2041	2	816.4089	.	A	Superscaf	1.09E+08	0	0	0	0	0	BTP202209	Tissue specific
CDS|exon|ID=cds-Bg	6	5.591667	2440.7	0.768305	LVTQQK	75	358.718	2	715.4229	.	E	Superscaf	1.09E+08	0	0	BTP202209	0	0	0	Tissue specific
CDS|exon|ID=cds-Bg	9	5.486667	3062.4	0.774847	SGVLDTLTK	96	467.2644	2	932.5178	.	P	Superscaf	1.09E+08	0	0	0	0	0	BTP202209	Tissue specific
CDS|exon|ID=cds-Bg	8	4.605	2839.1	0.788937	VEFEELLK	98	503.7776	2	1005.538	.	K	Superscaf	1.1E+08	0	0	0	0	0	BTP202209	Tissue specific
CDS|exon|ID=cds-Bg	8	5.39125	2401.7	0.791925	A(+42.01)	81	823.4905	1	822.485	Acetylati	P	Superscaf	1.1E+08	0	0	0	0	BTP202209	0	Tissue specific
CDS|exon|ID=cds-Bg	7	6.084286	2776	0.813489	KNGGEPL	63	714.3783	1	713.3708	.	P	Superscaf	1.11E+08	0	0	0	0	0	BTP202209	Tissue specific
CDS|exon|ID=cds-Bg	7	6.61	2562.7	0.814371	APATSLK	52	687.4005	1	686.3962	.	L	Superscaf	1.11E+08	0	0	0	0	0	BTP202209	Tissue specific
CDS|exon|ID=cds-Bg	8	6.04125	2715	0.818997	FGLAPLPP	51	406.2387	2	810.4639	.	I	Superscaf	1.12E+08	0	BTP202209	0	0	0	0	Tissue specific
CDS|exon|ID=cds-Bg	5	8.086	2840.4	0.822349	RHC(+57.0	81	363.1786	2	724.3551	Carbamido	Q	Superscaf	1.12E+08	0	BTP202209	0	0	0	0	Tissue specific
CDS|exon|ID=cds-Bg	11	6.751818	4432.9	0.830999	STPGGHPKP	96	556.2845	2	1110.557	.	A	Superscaf	1.12E+08	0	0	0	0	0	BTP202209	Tissue specific
CDS|exon|ID=cds-Bg	10	4.421	3662.1	0.831023	VSNELLEEA	81	575.7831	2	1149.555	.	C	Superscaf	1.12E+08	0	0	0	0	0	BTP202209	Tissue specific
CDS|exon|ID=cds-Bg	15	5.863333	5324.8	0.831105	FGQGGAGPV	89	671.3343	2	1340.658	.	R	Superscaf	1.12E+08	0	BTP202209	0	0	0	BTP202209	Mixed
CDS|exon|ID=cds-Bg	13	5.884615	4640.1	0.831105	QGGAGPVGG	96	569.2896	2	1136.569	.	R	Superscaf	1.12E+08	0	BTP202209	0	0	0	0	Tissue specific
CDS|exon|ID=cds-Bg	10	5.891	3434.8	0.831105	AGPVGGQGP	96	448.2397	2	894.4671	.	R	Superscaf	1.12E+08	0	0	0	0	BTP202209	0	Tissue specific
CDS|exon|ID=cds-Bg	11	6.321818	4890.4	0.831107	EEYEGPNKK	99	449.5601	3	1345.663	.	R	Superscaf	1.12E+08	0	BTP202209	0	0	BTP202209	BTP202209	Mixed
CDS|exon|ID=cds-Bg	9	7.011111	4039.4	0.831107	YEGPNKKPP	85	363.5318	3	1087.577	.	R	Superscaf	1.12E+08	0	0	0	0	0	BTP202209	Tissue specific
CDS|exon|ID=cds-Bg	8	6.0675	2896.2	0.849446	APPPQALG	53	750.4135	1	749.4072	.	S	Superscaf	1.14E+08	BTP202209	0	0	0	0	0	Tissue specific
CDS|exon|ID=cds-Bg	7	6.895714	3049.5	0.855742	LSPLLHR	96	418.2638	2	834.5076	.	N	Superscaf	1.14E+08	BTP202209	0	0	0	0	0	Tissue specific
CDS|exon|ID=cds-Bg	9	5.726667	3415.6	0.861155	A(+42.01)	80	495.2604	2	988.5076	Acetylati	V	Superscaf	1.14E+08	0	BTP202209	0	0	0	0	Tissue specific
CDS|exon|ID=cds-Bg	7	3.482857	1911.2	0.862926	LLEVEVL	85	814.4886	1	813.4847	.	Q	Superscaf	1.14E+08	0	0	0	BTP202209	0	0	Tissue specific
CDS|exon|ID=cds-Bg	7	4.365714	1580.7	0.863488	PVVGAPG	52	596.3397	1	595.3329	.	R	Superscaf	1.14E+08	0	BTP202209	0	0	0	0	Tissue specific
CDS|exon|ID=cds-Bg	12	5.970833	4716.9	0.928578	A(+42.01)	92	707.3639	2	1412.719	Acetylati	D	Superscaf	1.18E+08	0	BTP202209	0	0	0	0	Tissue specific
CDS|exon|ID=cds-Bg	9	6.302222	3598.6	0.928578	A(+42.01)	96	536.7675	2	1071.524	Acetylati	D	Superscaf	1.18E+08	0	0	0	0	BTP202209	BTP202209	Tissue enhanced
CDS|exon|ID=cds-Bg	16	6.165	6052.4	0.933915	PSKGPLQSV	90	439.76	4	1755.015	.	S	Superscaf	1.19E+08	0	0	0	0	0	BTP202209	Tissue specific
CDS|exon|ID=cds-Bg	10	5.504	3759.2	0.933918	TLQYKLLEP	73	602.3507	2	1202.691	.	S	Superscaf	1.19E+08	0	0	0	0	BTP202209	0	Tissue specific
CDS|exon|ID=cds-Bg	14	6.023571	5225.7	0.933918	KLLEPVLLL	97	552.3444	3	1654.018	.	S	Superscaf	1.19E+08	0	0	0	0	0	BTP202209	Tissue specific
CDS|exon|ID=cds-Bg	13	6.065385	4838.3	0.933918	KLLEPVLLL	86	503.3227	3	1506.95	.	S	Superscaf	1.19E+08	0	0	0	0	0	BTP202209	Tissue specific
CDS|exon|ID=cds-Bg	12	5.366667	4821.4	0.939211	VEERPEKDF	98	502.9186	3	1505.736	.	R	Superscaf	1.19E+08	0	BTP202209	0	0	0	0	Tissue specific
CDS|exon|ID=cds-Bg	11	5.854545	4821.4	0.939211	EERPEKDFT	95	469.8956	3	1406.668	.	R	Superscaf	1.19E+08	0	BTP202209	0	0	0	0	Tissue specific
CDS|exon|ID=cds-Bg	13	5.487692	4575.8	0.939458	KSPVPAELK	92	480.2794	3	1437.819	.	P	Superscaf	1.19E+08	0	BTP202209	0	0	0	0	Tissue specific
CDS|exon|ID=cds-Bg	7	4.937143	2788.1	0.963105	D(+42.01)	58	756.277	1	755.2722	Acetylati	G	Superscaf	1.21E+08	0	0	0	0	BTP202209	0	Tissue specific
CDS|exon|ID=cds-Bg	7	4.757143	2152.4	0.978122	PAVELFP	70	386.7147	2	771.4166	.	D	Superscaf	1.21E+08	0	0	0	0	BTP202209	0	Tissue specific
CDS|exon|ID=cds-Bg	5	4.81	1407.5	0.97844	TAFAV	52	508.2747	1	507.2693	.	P	Superscaf	1.21E+08	0	0	0	BTP202209	0	0	Tissue specific
CDS|exon|ID=cds-Bg	9	5.95	4128.7	0.988952	EDFKEQTRL	96	583.2953	2	1164.577	.	L	Superscaf	1.22E+08	0	BTP202209	0	0	0	0	Tissue specific
CDS|exon|ID=cds-Bg	11	4.577273	4556.4	0.988972	MQDLQEVTQ	68	660.3076	2	1318.607	.	P	Superscaf	1.22E+08	0	0	0	0	0	BTP202209	Tissue specific
CDS|exon|ID=cds-Bg	12	4.694167	4245.7	0.989002	GDGDSGVHG	96	582.7528	2	1163.496	.	G	Superscaf	1.22E+08	0	0	0	0	BTP202209	BTP202209	Mixed
CDS|exon|ID=cds-Bg	11	4.578182	3948.4	0.989002	DGDSGVHGG	98	554.2429	2	1106.474	.	G	Superscaf	1.22E+08	0	0	0	0	BTP202209	BTP202209	Mixed
CDS|exon|ID=cds-Bg	11	5.173636	3781	0.953499	T(+42.01)	88	607.2886	2	1212.566	Acetylati	I	Superscaf	3911605	0	0	0	0	0	BTP202209	Tissue specific
CDS|exon|ID=cds-Bg	9	5.384444	2982.1	0.953499	T(+42.01)	99	476.2411	2	950.4709	Acetylati	I	Superscaf	3911605	0	0	0	0	BTP202209	BTP202209	Tissue enhanced
CDS|exon|ID=cds-Bg	8	4.61125	2922.3	0.953483	C(+57.02)	77	1060.477	1	1059.474	Carbamido	G	Superscaf	3912712	0	0	0	0	0	BTP202209	Tissue specific
CDS|exon|ID=cds-Bg	7	5.727143	2560.9	0.773769	NSGPVGR	60	686.3596	1	685.3507	.	H	Superscaf	19030561	0	0	0	0	BTP202209	BTP202209	Tissue enhanced
CDS|exon|ID=cds-Bg	7	5.72	3209.5	0.753602	A(+42.01)	94	474.697	2	947.3807	Acetylati	P	Superscaf	20727073	0	BTP202209	0	0	BTP202209	0	Mixed
CDS|exon|ID=cds-Bg	17	6.290588	8109.9	0.753602	A(+42.01)	80	737.672	3	2210	Acetylati	P	Superscaf	20727073	0	0	0	0	0	BTP202209	Tissue specific
CDS|exon|ID=cds-Bg	16	6.075	7641.5	0.753602	A(+42.01)	99	1041.957	2	2081.905	Acetylati	P	Superscaf	20727073	0	BTP202209	0	0	BTP202209	0	Mixed
CDS|exon|ID=cds-Bg	14	5.820714	6875.8	0.753602	A(+42.01)	90	633.2681	3	1896.788	Acetylati	P	Superscaf	20727073	0	0	0	0	BTP202209	BTP202209	Mixed
CDS|exon|ID=cds-Bg	12	5.821667	5911.7	0.753602	A(+42.01)	70	828.8276	2	1655.646	Acetylati	P	Superscaf	20727073	0	0	0	0	0	BTP202209	Tissue specific
CDS|exon|ID=cds-Bg	11	5.859091	5315	0.753602	A(+42.01)	95	514.8736	3	1541.603	Acetylati	P	Superscaf	20727073	0	BTP202209	0	0	BTP202209	0	Tissue enhanced
CDS|exon|ID=cds-Bg	10	6.148	4903.5	0.753602	A(+42.01)	99	714.2937	2	1426.576	Acetylati	P	Superscaf	20727073	0	BTP202209	0	BTP202209	BTP202209	BTP202209	Mixed
CDS|exon|ID=cds-Bg	9	5.635556	4254.7	0.753602	A(+42.01)	99	636.2432	2	1270.475	Acetylati	P	Superscaf	20727073	0	BTP202209	0	0	BTP202209	BTP202209	Mixed
CDS|exon|ID=cds-Bg	8	5.7125	3669	0.753602	A(+42.01)	99	556.2281	2	1110.444	Acetylati	P	Superscaf	20727073	0	BTP202209	0	0	BTP202209	0	Mixed
CDS|exon|ID=cds-Bg	13	6.016923	6436.3	0.753602	FDC(+57.0	95	571.5798	3	1711.72	Carbamido	P	Superscaf	20727073	0	BTP202209	0	0	0	0	Tissue specific
CDS|exon|ID=cds-Bg	9	5.653333	4706.5	0.753602	FDC(+57.0	94	643.7344	2	1285.46	Carbamido	P	Superscaf	20727073	0	0	0	0	BTP202209	0	Tissue specific
CDS|exon|ID=cds-Bg	7	6.071429	3698.3	0.753602	FDC(+57.0	98	529.2021	2	1056.391	Carbamido	P	Superscaf	20727073	0	BTP202209	0	0	0	0	Tissue specific
CDS|exon|ID=cds-Bg	12	6.061667	6048.9	0.753602	DC(+57.02	80	522.5562	3	1564.651	Carbamido	P	Superscaf	20727073	0	BTP202209	0	0	0	0	Tissue specific
CDS|exon|ID=cds-Bg	11	6.046364	4745	0.753601	DNLQGKKYV	99	440.9128	3	1319.72	.	P	Superscaf	20727073	0	BTP202209	0	0	0	0	Tissue specific
CDS|exon|ID=cds-Bg	14	5.970714	6767.5	0.753601	DGHHC(+57	99	471.69	4	1882.737	Carbamido	P	Superscaf	20727073	0	0	0	0	BTP202209	0	Tissue specific
CDS|exon|ID=cds-Bg	10	6.381	5073.8	0.753598	HDTC(+57.	98	685.7711	2	1369.533	Carbamido	P	Superscaf	20727073	0	0	0	0	BTP202209	0	Tissue specific
CDS|exon|ID=cds-Bg	8	6.13125	4019.7	0.753598	HDTC(+57.	99	541.7084	2	1081.407	Carbamido	P	Superscaf	20727073	0	0	0	0	0	BTP202209	Tissue specific
CDS|exon|ID=cds-Bg	9	6.681111	4326.9	0.753598	RC(+57.02	66	581.2946	2	1160.579	Carbamido	P	Superscaf	20727073	0	0	0	0	0	BTP202209	Tissue specific
CDS|exon|ID=cds-Bg	8	6.76875	3973.5	0.753598	RC(+57.02	62	524.7531	2	1047.495	Carbamido	P	Superscaf	20727073	0	0	0	0	0	BTP202209	Tissue specific
CDS|exon|ID=cds-Bg	10	5.107	3693	0.753597	ASETFVAKL	91	541.257	2	1080.509	.	P	Superscaf	20727073	0	0	0	0	BTP202209	0	Tissue specific
CDS|exon|ID=cds-Bg	8	5.33625	2684.8	0.753597	ASETFVAK	94	426.7238	2	851.4388	.	P	Superscaf	20727073	0	0	0	0	BTP202209	BTP202209	Mixed
CDS|exon|ID=cds-Bg	9	7.09	3902.1	0.753597	PKC(+57.0	95	544.2894	2	1086.568	Carbamido	P	Superscaf	20727073	0	0	0	0	BTP202209	0	Tissue specific
CDS|exon|ID=cds-Bg	17	5.998824	7790.6	0.753589	KGTVWHKDC	85	719.318	3	2154.94	Carbamido	I	Superscaf	20728003	0	0	0	0	BTP202209	0	Tissue specific
CDS|exon|ID=cds-Bg	14	5.695714	6627.4	0.753589	VWHKDC(+5	98	623.9305	3	1868.776	Carbamido	I	Superscaf	20728003	0	0	0	0	BTP202209	0	Tissue specific
CDS|exon|ID=cds-Bg	11	5.393636	4962.6	0.753589	VWHKDC(+5	75	727.3007	2	1452.592	Carbamido	I	Superscaf	20728003	0	0	0	0	BTP202209	0	Tissue specific
CDS|exon|ID=cds-Bg	13	6.133846	6627.4	0.753589	WHKDC(+57	98	590.9073	3	1769.707	Carbamido	I	Superscaf	20728003	0	0	0	0	BTP202209	0	Tissue specific
CDS|exon|ID=cds-Bg	10	5.933	4962.6	0.753589	WHKDC(+57	79	677.7662	2	1353.523	Carbamido	I	Superscaf	20728003	0	0	0	0	BTP202209	0	Tissue specific
CDS|exon|ID=cds-Bg	8	6.03	3982.5	0.753589	WHKDC(+57	80	577.2292	2	1152.448	Carbamido	I	Superscaf	20728003	0	0	0	0	BTP202209	0	Tissue specific
CDS|exon|ID=cds-Bg	22	5.475455	9398.3	0.753587	GEDFYC(+5	72	690.7982	4	2759.171	Carbamido	I	Superscaf	20728003	0	0	0	0	BTP202209	0	Tissue specific
CDS|exon|ID=cds-Bg	14	5.137143	5762.4	0.753588	GEDFYC(+5	93	881.859	2	1761.713	Carbamido	I	Superscaf	20728003	0	0	0	0	BTP202209	0	Tissue specific
CDS|exon|ID=cds-Bg	13	5.110769	5375	0.753588	GEDFYC(+5	96	808.326	2	1614.644	Carbamido	I	Superscaf	20728003	0	0	0	0	BTP202209	BTP202209	Mixed
CDS|exon|ID=cds-Bg	12	4.725	4906.6	0.753588	GEDFYC(+5	89	744.2783	2	1486.549	Carbamido	I	Superscaf	20728003	0	0	0	0	0	BTP202209	Tissue specific
CDS|exon|ID=cds-Bg	12	5.039167	5077.7	0.753588	EDFYC(+57	97	779.8159	2	1557.623	Carbamido	I	Superscaf	20728003	0	0	0	0	BTP202209	BTP202209	Mixed
CDS|exon|ID=cds-Bg	9	5.422222	3853.3	0.753588	YC(+57.02	90	584.2484	2	1166.485	Carbamido	I	Superscaf	20728003	0	0	0	0	BTP202209	0	Tissue specific
CDS|exon|ID=cds-Bg	8	4.8825	3384.9	0.753588	YC(+57.02	83	520.2021	2	1038.39	Carbamido	I	Superscaf	20728003	0	BTP202209	0	0	BTP202209	BTP202209	Mixed
CDS|exon|ID=cds-Bg	7	4.72	3073.6	0.753588	YC(+57.02	89	484.6822	2	967.3528	Carbamido	I	Superscaf	20728003	0	0	0	0	BTP202209	BTP202209	Mixed
CDS|exon|ID=cds-Bg	12	4.943333	4750.3	0.753581	HAEC(+57.	99	749.3171	2	1496.621	Carbamido	A	Superscaf	20728801	0	0	0	0	0	BTP202209	Tissue specific
CDS|exon|ID=cds-Bg	12	5.445833	4461.8	0.753581	VC(+57.02	90	675.8459	2	1349.679	Carbamido	A	Superscaf	20728801	BTP202209	0	0	0	0	0	Tissue specific
CDS|exon|ID=cds-Bg	8	5.21625	2889.1	0.753581	VC(+57.02	94	491.2451	2	980.4783	Carbamido	A	Superscaf	20728801	0	0	0	0	0	BTP202209	Tissue specific
CDS|exon|ID=cds-Bg	13	5.691538	5233.8	0.753581	KLAGQRFTA	90	731.8821	2	1461.758	.	A	Superscaf	20728801	0	0	0	0	0	BTP202209	Tissue specific
CDS|exon|ID=cds-Bg	14	4.58	5586.3	0.753581	TAVEDQYYC	95	907.3702	2	1812.733	Carbamido	A	Superscaf	20728801	0	0	0	0	BTP202209	0	Tissue specific
CDS|exon|ID=cds-Bg	12	4.2975	4877.5	0.753581	VEDQYYC(+	89	821.3267	2	1640.649	Carbamido	A	Superscaf	20728801	0	0	0	0	BTP202209	0	Tissue specific
CDS|exon|ID=cds-Bg	14	5.437143	5662	0.753581	YYC(+57.0	95	619.9567	3	1856.859	Carbamido	A	Superscaf	20728801	0	0	0	0	0	BTP202209	Tissue specific
CDS|exon|ID=cds-Bg	13	5.106154	5193.6	0.753581	YYC(+57.0	99	865.3841	2	1728.764	Carbamido	A	Superscaf	20728801	0	0	0	0	0	BTP202209	Tissue specific
CDS|exon|ID=cds-Bg	9	5.015556	4026.5	0.753581	YYC(+57.0	90	642.7517	2	1283.495	Carbamido	A	Superscaf	20728801	0	0	0	0	BTP202209	0	Tissue specific
CDS|exon|ID=cds-Bg	8	4.96625	3429.8	0.753581	YYC(+57.0	97	585.7313	2	1169.452	Carbamido	A	Superscaf	20728801	0	0	0	0	BTP202209	BTP202209	Mixed
CDS|exon|ID=cds-Bg	12	5.06	4734.1	0.753581	YC(+57.02	80	522.9054	3	1565.701	Carbamido	A	Superscaf	20728801	0	0	0	0	BTP202209	BTP202209	Mixed
CDS|exon|ID=cds-Bg	8	4.935	3567	0.753581	YC(+57.02	85	561.2225	2	1120.432	Carbamido	A	Superscaf	20728801	0	BTP202209	0	0	BTP202209	0	Mixed
CDS|exon|ID=cds-Bg	7	4.867143	2970.3	0.753581	YC(+57.02	99	504.1996	2	1006.389	Carbamido	A	Superscaf	20728801	0	0	0	0	BTP202209	BTP202209	Mixed
CDS|exon|ID=cds-Bg	14	6.087143	6849.5	0.753566	EGQSWHDYC	89	627.9285	3	1880.772	Carbamido	M	Superscaf	20730034	0	0	0	0	BTP202209	0	Tissue specific
CDS|exon|ID=cds-Bg	13	5.806154	6381.1	0.753566	EGQSWHDYC	82	877.3455	2	1752.677	Carbamido	M	Superscaf	20730034	0	0	0	0	BTP202209	0	Tissue specific
CDS|exon|ID=cds-Bg	11	6.398182	5516	0.753566	SWHDYC(+5	96	523.2224	3	1566.65	Carbamido	M	Superscaf	20730034	0	0	0	0	BTP202209	0	Tissue specific
CDS|exon|ID=cds-Bg	10	6.064	5047.6	0.753566	SWHDYC(+5	96	480.5236	3	1438.555	Carbamido	M	Superscaf	20730034	0	0	0	0	BTP202209	0	Tissue specific
CDS|exon|ID=cds-Bg	10	6.47	5132.6	0.753566	WHDYC(+57	93	494.211	3	1479.618	Carbamido	M	Superscaf	20730034	0	0	0	0	BTP202209	0	Tissue specific
CDS|exon|ID=cds-Bg	9	6.106667	4664.2	0.753566	WHDYC(+57	98	451.5134	3	1351.523	Carbamido	M	Superscaf	20730034	0	0	0	0	BTP202209	0	Tissue specific
CDS|exon|ID=cds-Bg	9	6.534444	4606	0.753566	HDYC(+57.	87	432.1853	3	1293.538	Carbamido	M	Superscaf	20730034	0	0	0	0	BTP202209	0	Tissue specific
CDS|exon|ID=cds-Bg	8	6.13375	4137.6	0.753566	HDYC(+57.	99	583.7271	2	1165.443	Carbamido	M	Superscaf	20730034	0	0	0	0	BTP202209	0	Tissue specific
CDS|exon|ID=cds-Bg	8	5.47875	3690.2	0.753565	RFVFHQEQ	78	545.7734	2	1089.536	.	M	Superscaf	20730034	0	0	0	0	0	BTP202209	Tissue specific
CDS|exon|ID=cds-Bg	15	5.204	6281.9	0.753565	VFHQEQVYC	95	636.9598	3	1907.866	Carbamido	M	Superscaf	20730034	0	0	0	0	BTP202209	0	Tissue specific
CDS|exon|ID=cds-Bg	14	4.88	5813.5	0.753565	VFHQEQVYC	98	890.8894	2	1779.771	Carbamido	M	Superscaf	20730034	0	0	0	0	BTP202209	0	Tissue specific
CDS|exon|ID=cds-Bg	13	4.506154	5345.1	0.753565	VFHQEQVYC	66	826.8413	2	1651.676	Carbamido	M	Superscaf	20730034	0	0	0	0	0	BTP202209	Tissue specific
CDS|exon|ID=cds-Bg	15	5.602667	6635.3	0.753565	FHQEQVYC(	92	961.9459	2	1921.881	Carbamido	M	Superscaf	20730034	0	0	0	0	BTP202209	0	Tissue specific
CDS|exon|ID=cds-Bg	14	5.575714	6281.9	0.753565	FHQEQVYC(	95	603.938	3	1808.797	Carbamido	M	Superscaf	20730034	0	0	0	0	BTP202209	0	Tissue specific
CDS|exon|ID=cds-Bg	13	5.255385	5813.5	0.753565	FHQEQVYC(	99	841.3556	2	1680.702	Carbamido	M	Superscaf	20730034	0	0	0	0	BTP202209	BTP202209	Mixed
CDS|exon|ID=cds-Bg	14	5.611429	6247.9	0.753565	HQEQVYC(+	89	592.6106	3	1774.813	Carbamido	M	Superscaf	20730034	0	BTP202209	0	0	0	0	Tissue specific
CDS|exon|ID=cds-Bg	13	5.583077	5894.5	0.753565	HQEQVYC(+	84	554.9149	3	1661.729	Carbamido	M	Superscaf	20730034	0	0	0	0	BTP202209	0	Tissue specific
CDS|exon|ID=cds-Bg	12	5.236667	5426.1	0.753565	HQEQVYC(+	94	767.8225	2	1533.634	Carbamido	M	Superscaf	20730034	0	BTP202209	0	0	BTP202209	BTP202209	Mixed
CDS|exon|ID=cds-Bg	13	5.459231	5628.2	0.753565	QEQVYC(+5	84	546.9241	3	1637.754	Carbamido	M	Superscaf	20730034	BTP202209	BTP202209	0	0	0	0	Mixed

CDS|exon|ID=cds-Bg	12	5.415833	5274.8	0.753565	QEQVYC(+5	98	763.3393	2	1524.67	Carbamido	M	Superscaf	20730034	0	0	0	0	BTP202209	0	Tissue specific
CDS|exon|ID=cds-Bg	11	5.022727	4806.4	0.753565	QEQVYC(+5	87	699.2921	2	1396.575	Carbamido	M	Superscaf	20730034	0	0	0	0	BTP202209	0	Tissue specific
CDS|exon|ID=cds-Bg	11	5.394545	4664.1	0.753565	EQVYC(+57	93	699.3109	2	1396.612	Carbamido	M	Superscaf	20730034	0	0	0	0	BTP202209	0	Tissue specific
CDS|exon|ID=cds-Bg	10	5.612	4238.6	0.753565	QVYC(+57.	90	423.5296	3	1267.569	Carbamido	M	Superscaf	20730034	BTP202209	0	0	0	0	0	Tissue specific
CDS|exon|ID=cds-Bg	9	5.153333	3770.2	0.753565	QVYC(+57.	97	570.7435	2	1139.474	Carbamido	M	Superscaf	20730034	0	0	0	0	BTP202209	0	Tissue specific
CDS|exon|ID=cds-Bg	10	5.645	3981.3	0.753565	VYC(+57.0	99	627.3027	2	1252.595	Carbamido	M	Superscaf	20730034	0	0	0	0	BTP202209	BTP202209	Mixed
CDS|exon|ID=cds-Bg	9	5.607778	3627.9	0.753565	VYC(+57.0	99	570.7601	2	1139.51	Carbamido	M	Superscaf	20730034	0	0	0	0	BTP202209	BTP202209	Mixed
CDS|exon|ID=cds-Bg	8	5.09125	3159.5	0.753565	VYC(+57.0	99	506.7129	2	1011.415	Carbamido	M	Superscaf	20730034	0	0	0	0	BTP202209	BTP202209	Mixed
CDS|exon|ID=cds-Bg	9	6.272222	3981.3	0.753565	YC(+57.02	97	385.515	3	1153.526	Carbamido	M	Superscaf	20730034	0	BTP202209	0	0	0	0	Tissue specific
CDS|exon|ID=cds-Bg	8	6.30875	3627.9	0.753565	YC(+57.02	98	521.2261	2	1040.442	Carbamido	M	Superscaf	20730034	0	0	0	0	BTP202209	BTP202209	Mixed
CDS|exon|ID=cds-Bg	7	5.818571	3159.5	0.753565	YC(+57.02	99	457.1789	2	912.347	Carbamido	M	Superscaf	20730034	0	0	0	0	BTP202209	BTP202209	Mixed
CDS|exon|ID=cds-Bg	14	6.245	6584.5	0.715735	EHNTEKPEF	79	574.9496	3	1721.833	.	L	Superscaf	23912377	0	0	0	0	BTP202209	0	Tissue specific
CDS|exon|ID=cds-Bg	7	5.281429	2745.9	0.698599	KDWYDVK	95	477.2393	2	952.4654	.	N	Superscaf	25353907	0	BTP202209	0	0	0	0	Tissue specific
CDS|exon|ID=cds-Bg	9	3.544444	2636.2	0.620065	M(+42.01)	99	566.2878	2	1130.564	Acetylati	T	Superscaf	31959736	0	0	BTP202209	0	0	0	Tissue specific
CDS|exon|ID=cds-Bg	6	5.618333	1797.8	0.62006	G(+42.01)	54	600.3324	1	599.3278	Acetylati	R	Superscaf	31960783	0	0	BTP202209	0	0	0	Tissue specific
CDS|exon|ID=cds-Bg	11	5.851818	3867	0.576295	A(+42.01)	97	563.3154	2	1124.619	Acetylati	T	Superscaf	35642092	0	0	0	BTP202209	0	0	Tissue specific
CDS|exon|ID=cds-Bg	10	5.463	3398.6	0.576295	A(+42.01)	93	499.267	2	996.524	Acetylati	T	Superscaf	35642092	0	0	0	BTP202209	0	0	Tissue specific
CDS|exon|ID=cds-Bg	9	5.712222	2973.1	0.576295	A(+42.01)	99	868.486	1	867.4814	Acetylati	T	Superscaf	35642092	0	0	0	BTP202209	0	0	Tissue specific
CDS|exon|ID=cds-Bg	10	5.64	4011.6	0.564168	DKEVPHPRE	85	602.3091	2	1202.604	.	L	Superscaf	36662377	0	0	BTP202209	0	0	0	Tissue specific
CDS|exon|ID=cds-Bg	7	6.737143	2542.7	0.54608	RAAAAAP	76	627.365	1	626.35	.	A	Superscaf	38183716	0	0	0	0	BTP202209	0	Tissue specific
CDS|exon|ID=cds-Bg	14	6.196429	5271.8	0.54083	T(+42.01)	88	696.3299	2	1390.651	Acetylati	S	Superscaf	38625367	0	0	0	0	BTP202209	0	Tissue specific
CDS|exon|ID=cds-Bg	9	5.814444	3913.3	0.534056	DDFKGLQHL	93	536.7731	2	1071.535	.	L	Superscaf	39195388	0	0	0	0	0	BTP202209	Tissue specific
CDS|exon|ID=cds-Bg	10	5.799	4372.8	0.534056	DDFKGLQHL	99	618.304	2	1234.598	.	L	Superscaf	39195388	0	0	0	0	0	BTP202209	Tissue specific
CDS|exon|ID=cds-Bg	8	6.1575	2870.1	0.53403	PAGLPDLK	71	405.7382	2	809.4647	.	P	Superscaf	39197521	0	0	0	0	BTP202209	0	Tissue specific
CDS|exon|ID=cds-Bg	6	4.626667	1853.2	0.528385	LPLVDT	99	657.3798	1	656.3745	.	E	Superscaf	39671896	BTP202209	0	0	BTP202209	0	0	Mixed
CDS|exon|ID=cds-Bg	9	6.104444	2994.4	0.528384	PAPGMALPF	87	425.7326	2	849.4418	.	P	Superscaf	39672505	0	0	BTP202209	0	0	0	Tissue specific
CDS|exon|ID=cds-Bg	14	4.997857	5224.8	0.525828	VDSDMYDLF	95	813.3711	2	1624.729	.	P	Superscaf	39887533	0	BTP202209	0	0	0	0	Tissue specific
CDS|exon|ID=cds-Bg	10	4.802	3608.1	0.525829	VDSDMYDLF	99	591.7697	2	1181.527	.	P	Superscaf	39887533	0	BTP202209	0	0	0	BTP202209	Tissue enhanced
CDS|exon|ID=cds-Bg	9	5.88	3540.8	0.525828	DLPKKEDAL	81	514.7823	2	1027.555	.	P	Superscaf	39887533	0	0	0	0	BTP202209	0	Tissue specific
CDS|exon|ID=cds-Bg	8	5.97125	3511	0.525825	SDADPFHR	67	472.714	2	943.4147	.	G	Superscaf	39887629	0	BTP202209	0	0	0	0	Tissue specific
CDS|exon|ID=cds-Bg	10	5.607	3866.6	0.524986	MLLYTEVTF	96	644.8314	2	1287.653	.	L	Superscaf	39958327	0	0	0	0	0	BTP202209	Tissue specific
CDS|exon|ID=cds-Bg	10	4.452	3114.3	0.524986	LDFKVVEGS	70	570.7994	2	1139.586	.	L	Superscaf	39958327	0	0	0	0	0	BTP202209	Tissue specific
CDS|exon|ID=cds-Bg	23	5.548696	7972.7	0.432265	M(+42.01)	98	1035.958	2	2069.907	Acetylati	Q	Superscaf	47758156	0	BTP202209	0	0	0	0	Tissue specific
CDS|exon|ID=cds-Bg	8	4.69875	2759.2	0.374013	PELRQVSV	62	464.267	2	926.5185	.	P	Superscaf	52658368	0	0	BTP202209	0	0	0	Tissue specific
CDS|exon|ID=cds-Bg	5	5.218	1889.1	0.292013	L(+42.01)	75	636.3214	1	635.3167	Acetylati	F	Superscaf	59556342	0	0	0	0	BTP202209	0	Tissue specific
CDS|exon|ID=cds-Bg	8	5.37125	2897.3	0.280093	EPPSGPEL	86	825.3958	1	824.3915	.	M	Superscaf	60558697	0	0	0	BTP202209	0	0	Tissue specific
CDS|exon|ID=cds-Bg	14	5.065714	4409	0.268104	LYTLVTYVF	93	822.9624	2	1643.918	.	M	Superscaf	61567180	0	0	0	0	0	BTP202209	Tissue specific
CDS|exon|ID=cds-Bg	12	4.94	3596.1	0.268104	TLVTYVPVT	88	684.889	2	1367.77	.	M	Superscaf	61567180	0	0	0	0	0	BTP202209	Tissue specific
CDS|exon|ID=cds-Bg	10	4.406	2740.3	0.268104	TLVTYVPVT	63	1093.61	1	1092.607	.	M	Superscaf	61567180	0	0	0	0	BTP202209	BTP202209	Mixed
CDS|exon|ID=cds-Bg	12	5.2675	4045.2	0.231162	SAFLDKVVG	79	652.8793	2	1303.75	.	N	Superscaf	64674991	0	0	0	0	0	BTP202209	Tissue specific
CDS|exon|ID=cds-Bg	6	2.958333	1022.1	0.230539	G(+42.01)	55	659.3755	1	658.369	Acetylati	R	Superscaf	64727565	BTP202209	0	0	0	0	0	Tissue specific
CDS|exon|ID=cds-Bg	13	4.858462	4563	0.045024	LLQAQGVEV	76	692.3752	2	1382.741	.	I	Superscaf	80332438	0	0	BTP202209	0	0	0	Tissue specific
CDS|exon|ID=cds-Bg	10	4.555	3245.5	0.045024	AQGVEVPSK	94	515.2633	2	1028.514	.	I	Superscaf	80332438	0	0	BTP202209	0	0	0	Tissue specific
CDS|exon|ID=cds-Bg	7	6.937143	2967.1	0.072039	MSLAQKK	99	403.2321	2	804.4528	.	E	Superscaf	90179887	0	0	0	0	0	BTP202209	Tissue specific
CDS|exon|ID=cds-Bg	5	6.39	1891	0.081128	APAAH	53	466.2319	1	465.2336	.	G	Superscaf	90944773	BTP202209	0	0	0	0	0	Tissue specific
CDS|exon|ID=cds-Bg	8	4.40125	2724	0.091235	A(+42.01)	53	478.7627	2	955.5127	Acetylati	I	Superscaf	91795282	0	0	0	0	0	BTP202209	Tissue specific
CDS|exon|ID=cds-Bg	12	6.415	4426.7	0.091962	LPAPGPSPS	84	398.5546	3	1192.645	.	V	Superscaf	91856380	0	0	0	0	BTP202209	0	Tissue specific
CDS|exon|ID=cds-Bg	11	6.454545	4073.3	0.091962	PAPGPSPSD	85	360.8595	3	1079.561	.	V	Superscaf	91856380	0	0	0	0	BTP202209	0	Tissue specific
CDS|exon|ID=cds-Bg	13	6.314615	5556.4	0.093714	T(+42.01)	94	725.3429	2	1448.674	Acetylati	G	Superscaf	92003791	0	BTP202209	0	BTP202209	0	0	Tissue enhanced
CDS|exon|ID=cds-Bg	11	6.191818	4482.1	0.093714	T(+42.01)	93	582.7715	2	1163.531	Acetylati	G	Superscaf	92003791	0	BTP202209	0	0	0	0	Tissue specific
CDS|exon|ID=cds-Bg	8	5.805	3118.4	0.094932	EELAPYPK	53	473.7469	2	945.4807	.	G	Superscaf	92106052	0	BTP202209	0	0	0	0	Tissue specific
CDS|exon|ID=cds-Bg	8	5.925	3103.6	0.130694	DPLHLLPP	66	901.5209	1	900.5069	.	C	Superscaf	95114593	0	0	0	BTP202209	0	0	Tissue specific
CDS|exon|ID=cds-Bg	7	5.504286	2675.1	0.155456	SSVQRVR	72	416.2445	2	830.4722	.	G	Superscaf	97197481	0	0	0	BTP202209	0	0	Tissue specific
CDS|exon|ID=cds-Bg	5	5.342	1843.1	0.155784	LLFDP	93	604.333	1	603.3268	.	L	Superscaf	97225279	0	BTP202209	0	0	BTP202209	BTP202209	Mixed
CDS|exon|ID=cds-Bg	8	4.94	2401.7	0.167222	DPGALVPL	59	781.4432	1	780.4381	.	G	Superscaf	98187307	0	0	0	0	BTP202209	0	Tissue specific
CDS|exon|ID=cds-Bg	7	4.861429	2178.5	0.179564	LETALPV	79	742.4318	1	741.4272	.	L	Superscaf	99225637	0	0	0	0	0	BTP202209	Tissue specific
CDS|exon|ID=cds-Bg	8	5.065	2221.4	0.198073	VPKSVTGP	85	392.731	2	783.449	.	V	Superscaf	1.01E+08	BTP202209	0	0	0	0	0	Tissue specific
CDS|exon|ID=cds-Bg	9	5.786667	4469	0.288061	DDHNWWQGK	92	593.2528	2	1184.5	.	I	Superscaf	1.08E+08	0	0	0	0	0	BTP202209	Tissue specific
CDS|exon|ID=cds-Bg	12	5.85	4294.5	0.288271	EEVVKLPAF	93	481.959	3	1442.861	.	F	Superscaf	1.08E+08	0	0	0	0	0	BTP202209	Tissue specific
CDS|exon|ID=cds-Bg	10	6.142	4027.4	0.296221	SAEFLLHML	99	594.8227	2	1187.637	.	P	Superscaf	1.09E+08	0	0	0	0	0	BTP202209	Tissue specific
CDS|exon|ID=cds-Bg	6	7.276667	2552.7	0.368803	A(+42.01)	64	382.2211	2	762.4388	Acetylati	D	Superscaf	1.15E+08	0	BTP202209	0	0	0	0	Tissue specific
CDS|exon|ID=cds-Bg	10	4.725	3433.9	0.390646	VTSLFQVLF	77	566.322	2	1130.634	.	R	Superscaf	1.17E+08	0	0	0	0	0	BTP202209	Tissue specific
CDS|exon|ID=cds-Bg	7	4.558571	2667	0.401432	ESVGNLQ	53	746.3722	1	745.3606	.	E	Superscaf	1.18E+08	0	0	0	0	0	BTP202209	Tissue specific
CDS|exon|ID=cds-Bg	14	4.673571	4111.4	0.40254	VTQPVVAKE	85	728.9213	2	1455.83	.	K	Superscaf	1.18E+08	BTP202209	0	0	0	0	0	Tissue specific
CDS|exon|ID=cds-Bg	13	6.16	5912.8	0.406641	NLSTLEDLN	81	530.6133	3	1588.821	.	K	Superscaf	1.18E+08	0	0	0	0	0	BTP202209	Tissue specific
CDS|exon|ID=cds-Bg	9	6.107778	4076.7	0.410029	QDSPLSQPF	96	514.2614	2	1026.509	.	G	Superscaf	1.19E+08	BTP202209	0	0	0	0	0	Tissue specific
CDS|exon|ID=cds-Bg	10	6.205	3379.9	0.414998	GPSPPPPPF	87	939.4902	1	938.4861	.	N	Superscaf	1.19E+08	0	0	0	BTP202209	0	0	Tissue specific
CDS|exon|ID=cds-Bg	6	5.885	1974.3	0.417694	GVPRLP	67	638.3959	1	637.3911	.	A	Superscaf	1.19E+08	0	0	0	BTP202209	0	0	Tissue specific
CDS|exon|ID=cds-Bg	10	4.893	3301.7	0.497634	AGDPVANVF	80	523.274	2	1044.535	.	G	Superscaf	1.26E+08	0	0	0	0	0	BTP202209	Tissue specific
CDS|exon|ID=cds-Bg	10	6.951	4213.8	0.540255	FRPGAGQPF	82	361.5326	3	1081.578	.	A	Superscaf	1.3E+08	BTP202209	0	0	0	0	0	Tissue specific
CDS|exon|ID=cds-Bg	8	5.79	2896	0.540335	SELKYVPK	98	482.2781	2	962.5436	.	G	Superscaf	1.3E+08	BTP202209	0	0	0	0	0	Tissue specific
CDS|exon|ID=cds-Bg	11	6.21	4329.7	0.557781	FDPEFTAKT	96	640.8269	2	1279.645	.	G	Superscaf	1.31E+08	0	0	0	0	0	BTP202209	Tissue specific
CDS|exon|ID=cds-Bg	9	5.66	3738.2	0.558114	C(+57.02)	77	577.7739	2	1153.537	Carbamido	L	Superscaf	1.31E+08	0	0	0	0	BTP202209	0	Tissue specific
CDS|exon|ID=cds-Bg	11	7.249091	4304.7	0.563843	RPKKPPPPS	72	586.3486	2	1170.687	.	R	Superscaf	1.32E+08	0	0	0	0	BTP202209	0	Tissue specific
CDS|exon|ID=cds-Bg	12	5.5425	4665.1	0.624684	EAC(+57.0	91	657.798	2	1313.586	Carbamido	K	Superscaf	1.37E+08	0	0	0	BTP202209	0	0	Tissue specific
CDS|exon|ID=cds-Bg	7	6.515714	2532.7	0.664967	LLKLLGL	81	385.2796	2	768.5473	.	R	Superscaf	1.4E+08	0	BTP202209	0	0	0	0	Tissue specific
CDS|exon|ID=cds-Bg	8	5.34375	3219.6	0.72041	VQQTLLEK	98	479.7814	2	957.5495	.	S	Superscaf	1.45E+08	BTP202209	0	0	0	0	0	Tissue specific
CDS|exon|ID=cds-Bg	7	6.212857	2961.4	0.825417	QGAMHTL	57	379.184	2	756.3589	.	A	Superscaf	1.54E+08	BTP202209	0	0	0	0	0	Tissue specific
CDS|exon|ID=cds-Bg	6	5.02	1988.2	0.833083	M(+42.01)	97	734.3717	1	733.368	Acetylati	F	Superscaf	1.54E+08	0	BTP202209	0	0	0	0	Tissue specific
CDS|exon|ID=cds-Bg	5	6.752	1811.8	0.885437	P(+42.01)	52	515.2905	1	514.2751	Acetylati	G	Superscaf	1.59E+08	0	0	0	0	0	BTP202209	Tissue specific
CDS|exon|ID=cds-Bg	7	6.68	2699	0.992991	LTTTKGF	51	384.2224	2	766.4225	.	L	Superscaf	421482	0	0	0	0	0	BTP202209	Tissue specific
CDS|exon|ID=cds-Bg	7	5.921429	2752.8	0.874263	KSVKEFH	55	437.7408	2	873.4708	.	Q	Superscaf	7558678	BTP202209	0	0	0	0	0	Tissue specific
CDS|exon|ID=cds-Bg	5	5.868	2166.4	0.874195	FTAYQ	54	629.2903	1	628.2856	.	S	Superscaf	7563084	0	0	0	0	0	BTP202209	Tissue specific
CDS|exon|ID=cds-Bg	7	5.814286	2766.3	0.825685	ETVHPPR	60	418.2299	2	834.4348	.	Y	Superscaf	10479048	0	BTP202209	0	0	0	0	Tissue specific
CDS|exon|ID=cds-Bg	8	6.2175	2619	0.803486	PPGPPGPP	86	358.1952	2	714.3701	.	I	Superscaf	11813356	0	0	0	BTP202209	0	0	Tissue specific
CDS|exon|ID=cds-Bg	9	4.8	3464.8	0.803485	EFGFDGDFY	81	1096.423	1	1095.419	.	I	Superscaf	11813356	0	0	0	0	0	BTP202209	Tissue specific
CDS|exon|ID=cds-Bg	9	5.884444	4066.6	0.801738	EDLLSENRK	86	552.2858	2	1102.562	.	V	Superscaf	11918491	0	0	0	0	0	BTP202209	Tissue specific
CDS|exon|ID=cds-Bg	12	5.731667	4184.5	0.786138	LLLPGELAK	84	420.9266	3	1259.76	.	S	Superscaf	12855892	0	BTP202209	0	0	BTP202209	0	Mixed
CDS|exon|ID=cds-Bg	11	5.709091	3831.1	0.786138	LLPGELAKH	98	383.2312	3	1146.676	.	S	Superscaf	12855892	0	0	0	0	BTP202209	0	Tissue specific
CDS|exon|ID=cds-Bg	11	5.681818	3861.1	0.786138	LPGELAKHA	95	561.3168	2	1120.624	.	S	Superscaf	12855892	0	0	0	0	BTP202209	0	Tissue specific
CDS|exon|ID=cds-Bg	8	5.16625	2994.6	0.745006	HYVDPTTM	54	482.2134	2	962.4167	.	G	Superscaf	15328960	0	0	0	0	0	BTP202209	Tissue specific
CDS|exon|ID=cds-Bg	8	5.99125	3231.7	0.745005	LVYHLRPY	98	354.2009	3	1059.586	.	G	Superscaf	15328960	0	0	0	0	0	BTP202209	Tissue specific
CDS|exon|ID=cds-Bg	6	7.14	2551.6	0.739629	K(+42.01)	62	672.3636	1	671.3602	Acetylati	I	Superscaf	15652234	0	0	0	0	0	BTP202209	Tissue specific
CDS|exon|ID=cds-Bg	7	5.64	2533.9	0.727404	LPPDPGY	70	758.3801	1	757.3646	.	R	Superscaf	16387158	0	0	0	BTP202209	0	0	Tissue specific
CDS|exon|ID=cds-Bg	7	6.168571	2735	0.658888	KYEPPLL	61	430.2479	2	858.485	.	I	Superscaf	20506029	0	0	BTP202209	0	0	0	Tissue specific
CDS|exon|ID=cds-Bg	13	6.316154	5297.9	0.634034	TLLQKLDFF	98	527.9597	3	1580.864	.	Y	Superscaf	21999889	0	0	0	0	0	BTP202209	Tissue specific
CDS|exon|ID=cds-Bg	7	5.75	2384.4	0.474162	FADKVPK	87	402.7328	2	803.4541	.	T	Superscaf	31610698	0	0	0	0	BTP202209	0	Tissue specific
CDS|exon|ID=cds-Bg	9	6.475556	3498.6	0.474161	TGEKGFGYK	99	493.7487	2	985.4869	.	T	Superscaf	31610698	0	0	BTP202209	0	0	0	Tissue specific
CDS|exon|ID=cds-Bg	11	6.019091	4881.8	0.446655	MDMSPLRPG	90	676.3074	2	1350.606	.	S	Superscaf	33264355	0	0	0	0	0	BTP202209	Tissue specific
CDS|exon|ID=cds-Bg	7	7.352857	3041.3	0.30383	KDPLRLK	53	435.2854	2	868.5494	.	S	Superscaf	41849566	0	BTP202209	0	0	0	0	Tissue specific
CDS|exon|ID=cds-Bg	8	5.80375	3323.7	0.258228	DYLHATAY	90	477.2195	2	952.429	.	D	Superscaf	44591629	0	0	0	0	0	BTP202209	Tissue specific
CDS|exon|ID=cds-Bg	5	4.792	1733	0.243791	T(+42.01)	89	633.3215	1	632.317	Acetylati	L	Superscaf	45459762	0	0	0	0	BTP202209	BTP202209	Mixed
CDS|exon|ID=cds-Bg	6	6.431667	2393.6	0.222535	KMFYLL	61	407.7328	2	813.4459	.	D	Superscaf	46737504	BTP202209	0	0	0	0	0	Tissue specific
CDS|exon|ID=cds-Bg	7	6.541429	2584.9	0.215516	A(+42.01)	84	404.2086	2	806.403	Acetylati	T	Superscaf	47159392	0	0	0	0	BTP202209	0	Tissue specific
CDS|exon|ID=cds-Bg	7	6.395714	3087.3	0.193005	K(+42.01)	66	456.2177	2	910.4218	Acetylati	K	Superscaf	48512676	BTP202209	0	0	0	0	0	Tissue specific
CDS|exon|ID=cds-Bg	7	6.378571	3046.5	0.190437	M(+42.01)	99	480.761	2	959.511	Acetylati	I	Superscaf	48667018	0	0	0	0	0	BTP202209	Tissue specific
CDS|exon|ID=cds-Bg	6	7.59	2633	0.186833	T(+42.01)	79	366.2001	2	730.3973	Acetylati	I	Superscaf	48883741	0	0	0	0	BTP202209	0	Tissue specific
CDS|exon|ID=cds-Bg	8	5.8175	2588.7	0.151732	A(+42.01)	74	379.7102	2	757.4082	Acetylati	R	Superscaf	50993922	0	0	0	BTP202209	0	0	Tissue specific
CDS|exon|ID=cds-Bg	7	7.007143	3168.9	0.123409	M(+42.01)	79	465.7437	2	929.4753	Acetylati	P	Superscaf	52696360	0	BTP202209	0	0	0	0	Tissue specific
CDS|exon|ID=cds-Bg	6	4.951667	2200.7	0.100287	P(+42.01)	55	370.1128	2	738.22	Acetylati	N	Superscaf	54086350	0	0	BTP202209	0	0	0	Tissue specific
CDS|exon|ID=cds-Bg	11	5.223636	4238.8	0.087023	EDEEVKATF	85	660.8246	2	1319.636	.	A	Superscaf	54883678	BTP202209	0	0	0	0	0	Tissue specific
CDS|exon|ID=cds-Bg	10	5.424	3813.3	0.087023	DEEVKATFF	66	596.303	2	1190.593	.	A	Superscaf	54883678	BTP202209	0	0	0	0	0	Tissue specific
CDS|exon|ID=cds-Bg	7	5.48	2755.1	0.087021	ADTFGLN	93	737.3478	1	736.3392	.	A	Superscaf	54883678	BTP202209	0	0	0	0	0	Tissue specific
CDS|exon|ID=cds-Bg	9	5.524444	3000.3	0.086371	SVEKPLGPT	82	464.2603	2	926.5073	.	K	Superscaf	54920962	BTP202209	0	0	0	0	0	Tissue specific
CDS|exon|ID=cds-Bg	8	5.505	2616.9	0.086371	VEKPLGPT	95	420.7442	2	839.4752	.	K	Superscaf	54920962	BTP202209	0	0	0	0	0	Tissue specific
CDS|exon|ID=cds-Bg	6	6.8	2616.8	0.057324	KDVKRH	63	391.7336	2	781.4559	.	R	Superscaf	56669080	BTP202209	0	0	0	0	0	Tissue specific
CDS|exon|ID=cds-Bg	6	7.245	2442.5	0.054263	FTPSKK	87	354.2058	2	706.4014	.	I	Superscaf	56853115	0	BTP202209	0	0	0	0	Tissue specific
CDS|exon|ID=cds-Bg	7	6.128571	2891.4	0.017657	V(+42.01)	70	436.7336	2	871.4664	Acetylati	V	Superscaf	61176628	0	0	BTP202209	0	0	0	Tissue specific
CDS|exon|ID=cds-Bg	13	5.597692	4579	0.031725	VDTPSGTNS	97	595.7844	2	1189.557	.	Y	Superscaf	62022265	0	0	0	BTP202209	0	0	Tissue specific
CDS|exon|ID=cds-Bg	5	6.192	1969.2	0.049068	MGGSH	50	488.1987	1	487.1849	.	V	Superscaf	63064909	BTP202209	0	0	0	0	0	Tissue specific
CDS|exon|ID=cds-Bg	11	4.980909	4126.6	0.066773	M(+42.01)	99	642.3217	2	1282.634	Acetylati	H	Superscaf	64129306	0	0	0	0	0	BTP202209	Tissue specific
CDS|exon|ID=cds-Bg	7	6.962857	2851.2	0.10194	KPTHPLP	76	395.2332	2	788.4545	.	P	Superscaf	66243349	0	0	0	BTP202209	0	0	Tissue specific
CDS|exon|ID=cds-Bg	6	4.465	1758.9	0.123326	D(+42.01)	60	661.3174	1	660.3118	Acetylati	H	Superscaf	67528303	0	BTP202209	0	0	0	0	Tissue specific
CDS|exon|ID=cds-Bg	8	5.715	2582.7	0.144592	ASKTPVGF	98	403.7225	2	805.4333	.	N	Superscaf	68807389	0	BTP202209	0	0	0	BTP202209	Mixed
CDS|exon|ID=cds-Bg	9	3.647778	2630	0.145953	MVGGVEEEF	67	996.4291	1	995.4269	.	T	Superscaf	68889124	0	0	0	0	0	BTP202209	Tissue specific
CDS|exon|ID=cds-Bg	6	5.665	1823.9	0.217244	V(+42.01)	70	626.3607	1	625.3435	Acetylati	Q	Superscaf	73174927	0	0	0	0	0	BTP202209	Tissue specific
CDS|exon|ID=cds-Bg	9	5.202222	3698.4	0.250605	M(+42.01)	78	560.2539	2	1118.492	Acetylati	T	Superscaf	75180337	0	0	0	0	BTP202209	0	Tissue specific
CDS|exon|ID=cds-Bg	10	5.102	4345.1	0.251482	DDEMPSHDI	92	598.2361	2	1194.461	.	N	Superscaf	75232609	0	0	0	0	BTP202209	0	Tissue specific
CDS|exon|ID=cds-Bg	9	5.465556	2970.2	0.252199	SESPVGPKF	97	449.2354	2	896.4603	.	M	Superscaf	75276133	0	0	0	0	BTP202209	0	Tissue specific
CDS|exon|ID=cds-Bg	8	5.43875	2586.8	0.252199	ESPVGPKP	99	405.7206	2	809.4283	.	M	Superscaf	75276133	0	0	BTP202209	0	BTP202209	0	Mixed
CDS|exon|ID=cds-Bg	9	5.56	2984.3	0.252199	ESPVGPKPT	92	456.2437	2	910.476	.	M	Superscaf	75276133	0	BTP202209	0	0	0	0	Tissue specific
CDS|exon|ID=cds-Bg	11	5.44	4506	0.252225	AEHQWVETS	88	656.3191	2	1310.626	.	L	Superscaf	75277714	0	BTP202209	0	0	0	0	Tissue specific

CDS|exon|ID=cds-Bg	10	5.382	4194.7	0.252225	EHQWVETSF	73	414.2005	3	1239.588	.	L	Superscaf	75277714	0	BTP202209	0	0	0	0	Tissue specific
CDS|exon|ID=cds-Bg	12	5.529167	4753.4	0.252227	EPPEALGPF	85	618.2972	2	1234.583	.	L	Superscaf	75277714	0	BTP202209	0	0	0	0	Tissue specific
CDS|exon|ID=cds-Bg	7	5.917143	2257.5	0.271358	P(+42.01)	60	723.4121	1	722.4075	Acetylati	R	Superscaf	76427710	0	0	BTP202209	0	0	0	Tissue specific
CDS|exon|ID=cds-Bg	7	5.918571	2696	0.278179	AAPEPLH	73	367.6998	2	733.3759	.	G	Superscaf	76837963	BTP202209	0	0	0	0	0	Tissue specific
CDS|exon|ID=cds-Bg	7	6.112857	2913.2	0.30425	A(+42.01)	83	871.3929	1	870.3871	Acetylati	K	Superscaf	78405190	0	BTP202209	BTP202209	BTP202209	0	0	Mixed
CDS|exon|ID=cds-Bg	8	5.7475	2673	0.378085	KGTVMTFL	72	448.7478	2	895.4837	.	S	Superscaf	82843528	0	0	0	0	0	BTP202209	Tissue specific
CDS|exon|ID=cds-Bg	16	5.3875	6092.8	0.401192	PGGLLLGDE	85	807.3904	2	1612.773	.	R	Superscaf	84232891	0	0	BTP202209	0	0	0	Tissue specific
CDS|exon|ID=cds-Bg	10	5.436	3437.8	0.401192	PGGLLLGDE	65	471.2495	2	940.4865	.	R	Superscaf	84232891	0	BTP202209	0	BTP202209	0	0	Mixed
CDS|exon|ID=cds-Bg	9	7.028889	3711.9	0.401195	KLAPEFAKF	93	353.881	3	1058.624	.	R	Superscaf	84232891	0	BTP202209	0	0	0	0	Tissue specific
CDS|exon|ID=cds-Bg	7	6.108571	2594.7	0.401195	KLAPEFA	70	388.2192	2	774.4276	.	R	Superscaf	84232891	0	BTP202209	0	0	0	0	Tissue specific
CDS|exon|ID=cds-Bg	8	4.30375	2916.3	0.401196	DSVEDHLA	73	443.2037	2	884.3876	.	R	Superscaf	84232891	0	0	0	0	0	BTP202209	Tissue specific
CDS|exon|ID=cds-Bg	9	4.48	3442.9	0.401196	DSVEDHLAW	88	536.2399	2	1070.467	.	R	Superscaf	84232891	0	BTP202209	0	0	BTP202209	BTP202209	Mixed
CDS|exon|ID=cds-Bg	8	4.66875	3031.4	0.401196	SVEDHLAW	98	478.7266	2	955.4399	.	R	Superscaf	84232891	0	BTP202209	0	0	0	0	Tissue specific
CDS|exon|ID=cds-Bg	7	4.524286	2648	0.401196	VEDHLAW	83	435.21	2	868.4079	.	R	Superscaf	84232891	0	BTP202209	0	0	0	0	Tissue specific
CDS|exon|ID=cds-Bg	11	7.160909	4663.9	0.401203	KELPSGKKY	89	440.265	3	1317.777	.	L	Superscaf	84233509	0	BTP202209	0	0	0	0	Tissue specific
CDS|exon|ID=cds-Bg	8	6.53625	3193.3	0.401203	ELPSGKKY	96	461.2549	2	920.4967	.	L	Superscaf	84233509	0	BTP202209	0	0	0	0	Tissue specific
CDS|exon|ID=cds-Bg	7	6.74	3144.7	0.401203	LRYTPQP	78	437.7415	2	873.4708	.	L	Superscaf	84233509	0	0	BTP202209	0	0	BTP202209	Mixed
CDS|exon|ID=cds-Bg	5	6.952	1978.2	0.442836	LRAAL	66	543.3594	1	542.354	.	H	Superscaf	86736525	0	0	0	BTP202209	0	0	Tissue specific
CDS|exon|ID=cds-Bg	8	5.7775	2789.1	0.475799	SSPVAFPR	91	430.7332	2	859.4551	.	K	Superscaf	88718094	0	0	0	0	0	BTP202209	Tissue specific
CDS|exon|ID=cds-Bg	5	5.664	1574.8	0.527202	M(+42.01)	73	617.325	1	616.3254	Acetylati	T	Superscaf	91808217	0	0	0	0	BTP202209	0	Tissue specific
CDS|exon|ID=cds-Bg	21	5.595238	7279.9	0.533211	A(+42.01)	92	950.4568	2	1898.908	Acetylati	R	Superscaf	92169313	0	0	BTP202209	0	0	0	Tissue specific
CDS|exon|ID=cds-Bg	20	5.5765	6982.6	0.533211	A(+42.01)	99	921.9483	2	1841.887	Acetylati	R	Superscaf	92169313	0	0	BTP202209	0	0	0	Tissue specific
CDS|exon|ID=cds-Bg	14	6.430714	6197.6	0.533948	RLAEC(+57	95	412.7157	4	1646.841	Carbamido	K	Superscaf	92213554	0	0	0	0	BTP202209	0	Tissue specific
CDS|exon|ID=cds-Bg	20	5.3235	7375.2	0.53856	LPGLVQEGE	80	1096.557	2	2191.105	.	I	Superscaf	92490790	0	0	0	0	BTP202209	BTP202209	Mixed
CDS|exon|ID=cds-Bg	9	5.822222	3549.9	0.538561	SEEATLFTK	99	513.2605	2	1024.508	.	I	Superscaf	92490790	0	0	0	0	0	BTP202209	Tissue specific
CDS|exon|ID=cds-Bg	8	6.19375	3396.8	0.539982	TAERSAYY	78	480.7231	2	959.4348	.	V	Superscaf	92576266	0	0	0	0	0	BTP202209	Tissue specific
CDS|exon|ID=cds-Bg	13	6.460769	5431.5	0.539982	LSAEEAAKG	99	487.2735	3	1458.804	.	V	Superscaf	92576266	0	0	0	0	0	BTP202209	Tissue specific
CDS|exon|ID=cds-Bg	11	6.457273	4537.6	0.539982	LSAEEAAKG	96	401.5616	3	1201.667	.	V	Superscaf	92576266	0	0	0	0	0	BTP202209	Tissue specific
CDS|exon|ID=cds-Bg	7	4.88	2702.1	0.540176	LLNVEHL	61	419.2439	2	836.4756	.	G	Superscaf	92587861	0	0	0	BTP202209	0	0	Tissue specific
CDS|exon|ID=cds-Bg	14	5.273571	5605.1	0.547417	DYDHDAFLC	99	790.8431	2	1579.679	.	D	Superscaf	93023197	0	0	0	0	BTP202209	0	Tissue specific
CDS|exon|ID=cds-Bg	7	4.062857	2056.3	0.547499	VTVDELK	90	402.228	2	802.4436	.	P	Superscaf	93028192	0	0	0	0	0	BTP202209	Tissue specific
CDS|exon|ID=cds-Bg	11	5.33	4588.2	0.54757	TAFLHPEEY	87	692.8037	2	1383.598	.	F	Superscaf	93032341	0	0	0	0	0	BTP202209	Tissue specific
CDS|exon|ID=cds-Bg	9	5.555556	3717.2	0.54757	TAFLHPEEY	96	553.759	2	1105.508	.	F	Superscaf	93032341	0	0	0	0	0	BTP202209	Tissue specific
CDS|exon|ID=cds-Bg	8	5.075	3492	0.54757	LHPEEYDY	81	533.2278	2	1064.445	.	F	Superscaf	93032341	0	0	0	0	0	BTP202209	Tissue specific
CDS|exon|ID=cds-Bg	13	5.133846	5414.2	0.547606	ADEPEWVKT	87	808.8794	2	1615.748	.	E	Superscaf	93034615	0	0	0	0	BTP202209	0	Tissue specific
CDS|exon|ID=cds-Bg	9	4.876667	3303.7	0.547606	ADEPEWVKT	96	537.7567	2	1073.503	.	E	Superscaf	93034615	0	0	BTP202209	0	0	0	Tissue specific
CDS|exon|ID=cds-Bg	12	5.06	5102.9	0.547606	DEPEWVKTE	96	773.3601	2	1544.711	.	E	Superscaf	93034615	0	0	0	0	BTP202209	0	Tissue specific
CDS|exon|ID=cds-Bg	18	4.972778	6679.5	0.547637	VGSQATDFC	95	659.9786	3	1976.923	.	D	Superscaf	93036499	0	0	0	0	BTP202209	0	Tissue specific
CDS|exon|ID=cds-Bg	11	5.153636	4267.8	0.547637	FGEALVRHD	96	660.3223	2	1318.631	.	D	Superscaf	93036499	0	0	BTP202209	0	0	0	Tissue specific
CDS|exon|ID=cds-Bg	7	5.347143	2423.7	0.547637	FGEALVR	89	396.2217	2	790.4337	.	D	Superscaf	93036499	0	0	BTP202209	0	0	0	Tissue specific
CDS|exon|ID=cds-Bg	6	5.003333	2492.9	0.547637	VRHDEF	84	401.6954	2	801.3769	.	D	Superscaf	93036499	0	0	0	0	BTP202209	0	Tissue specific
CDS|exon|ID=cds-Bg	10	4.483	3218.6	0.54856	PGFGLGDEV	60	503.2272	2	1004.445	.	P	Superscaf	93091960	0	0	0	0	0	BTP202209	Tissue specific
CDS|exon|ID=cds-Bg	8	4.07	2583.9	0.54856	FGLGDEVD	96	851.3766	1	850.3708	.	P	Superscaf	93091960	0	0	0	0	0	BTP202209	Tissue specific
CDS|exon|ID=cds-Bg	8	6.17	3971.5	0.548644	NRDWQDGK	76	509.7373	2	1017.463	.	A	Superscaf	93096853	0	0	0	0	0	BTP202209	Tissue specific
CDS|exon|ID=cds-Bg	12	6.854167	4385.5	0.548676	AKLKPGAPV	82	417.9326	3	1250.782	.	L	Superscaf	93098734	0	0	0	0	0	BTP202209	Tissue specific
CDS|exon|ID=cds-Bg	13	4.929231	4987.8	0.548823	VTDNGDGTY	89	744.3323	2	1486.669	.	A	Superscaf	93107701	0	0	0	0	0	BTP202209	Tissue specific
CDS|exon|ID=cds-Bg	8	6.38875	3470	0.548947	GDFLGRER	84	475.2438	2	948.4777	.	R	Superscaf	93114961	0	0	0	0	BTP202209	0	Tissue specific
CDS|exon|ID=cds-Bg	12	5.225	4419.1	0.548953	SVRFVPQEM	82	461.8971	3	1382.677	.	H	Superscaf	93115549	0	0	0	0	0	BTP202209	Tissue specific
CDS|exon|ID=cds-Bg	12	4.365	3552	0.548965	VVPVASLSD	98	614.8281	2	1227.646	.	A	Superscaf	93116236	0	0	0	0	0	BTP202209	Tissue specific
CDS|exon|ID=cds-Bg	8	4	2042.3	0.549001	VLVETVTK	98	444.7718	2	887.5328	.	G	Superscaf	93118435	0	0	0	0	0	BTP202209	Tissue specific
CDS|exon|ID=cds-Bg	9	5.054444	3368	0.549007	MGNRVYNVT	75	527.2594	2	1052.507	.	A	Superscaf	93118831	0	0	0	0	0	BTP202209	Tissue specific
CDS|exon|ID=cds-Bg	11	5.476364	3963.3	0.555074	EESGKPGAF	84	556.2708	2	1110.531	.	R	Superscaf	93483499	0	0	0	0	0	BTP202209	Tissue specific
CDS|exon|ID=cds-Bg	8	6.58375	3953.8	0.645365	M(+15.99)	97	394.8481	3	1181.525	Oxidation	T	Superscaf	98911516	0	BTP202209	0	0	BTP202209	0	Mixed
CDS|exon|ID=cds-Bg	7	5.987143	3305	0.645365	M(+15.99)	96	513.7183	2	1025.424	Oxidation	T	Superscaf	98911516	0	BTP202209	0	0	BTP202209	BTP202209	Mixed
CDS|exon|ID=cds-Bg	11	6.604545	5153	0.645366	KREEMRLEA	99	362.442	4	1445.741	.	T	Superscaf	98911516	0	BTP202209	0	0	0	BTP202209	Mixed
CDS|exon|ID=cds-Bg	19	5.526316	7750.9	0.646525	TTNTQAEGD	87	1009.484	2	2016.96	.	R	Superscaf	98980933	0	0	0	0	0	BTP202209	Tissue specific
CDS|exon|ID=cds-Bg	18	5.470556	7353.4	0.646525	TNTQAEGDD	89	958.9606	2	1915.912	.	R	Superscaf	98980933	0	0	0	0	0	BTP202209	Tissue specific
CDS|exon|ID=cds-Bg	13	5.256154	5039.7	0.646525	AEGDDEAAL	73	701.3423	2	1400.678	.	R	Superscaf	98980933	0	0	0	0	BTP202209	0	Tissue specific
CDS|exon|ID=cds-Bg	13	5.256154	5039.7	0.646525	EGDDEAALL	86	701.3406	2	1400.678	.	R	Superscaf	98980933	0	0	0	0	0	BTP202209	Tissue specific
CDS|exon|ID=cds-Bg	11	5.120909	4375	0.646525	EGDDEAALL	96	609.2852	2	1216.557	.	R	Superscaf	98980933	0	BTP202209	0	0	0	0	Tissue specific
CDS|exon|ID=cds-Bg	12	5.425833	4614.2	0.646525	GDDEAALLE	78	636.8246	2	1271.636	.	R	Superscaf	98980933	0	0	0	0	0	BTP202209	Tissue specific
CDS|exon|ID=cds-Bg	17	5.921176	7842.2	0.646527	RM(+15.99	91	670.9624	3	2009.871	Oxidation	R	Superscaf	98980933	0	BTP202209	0	0	0	0	Tissue specific
CDS|exon|ID=cds-Bg	14	5.988571	6513.1	0.646529	SHQKNDWME	77	587.2712	3	1758.8	.	R	Superscaf	98980933	0	0	0	0	0	BTP202209	Tissue specific
CDS|exon|ID=cds-Bg	11	5.901818	4899.3	0.646529	KNDWMEAEE	92	469.8892	3	1406.65	.	R	Superscaf	98980933	0	0	0	0	0	BTP202209	Tissue specific
CDS|exon|ID=cds-Bg	10	5.518	4430.9	0.646529	NDWMEAEEK	97	640.2832	2	1278.555	.	R	Superscaf	98980933	0	BTP202209	0	0	0	BTP202209	Tissue enhanced
CDS|exon|ID=cds-Bg	9	5.53	3834.2	0.646529	DWMEAEEKK	85	583.2624	2	1164.512	.	R	Superscaf	98980933	0	0	0	0	BTP202209	BTP202209	Mixed
CDS|exon|ID=cds-Bg	8	2.39375	1730.9	0.646531	VEVVVDEK	95	458.7501	2	915.4913	.	R	Superscaf	98980933	0	0	0	0	0	BTP202209	Tissue specific
CDS|exon|ID=cds-Bg	17	5.732353	6241.4	0.646539	KGEKVEEKT	98	611.0003	3	1829.985	.	R	Superscaf	98980933	0	0	0	0	0	BTP202209	Tissue specific
CDS|exon|ID=cds-Bg	13	5.676923	4856.2	0.64654	KVQEDKPHT	99	492.942	3	1475.81	.	R	Superscaf	98980933	0	BTP202209	0	0	0	BTP202209	Tissue enhanced
CDS|exon|ID=cds-Bg	12	5.338333	4387.8	0.64654	VQEDKPHTA	99	674.8624	2	1347.715	.	R	Superscaf	98980933	0	0	0	0	BTP202209	BTP202209	Mixed
CDS|exon|ID=cds-Bg	10	6.426	4246.3	0.646562	SQEDKAAFK	84	576.3046	2	1150.598	.	I	Superscaf	98983375	0	0	0	0	0	BTP202209	Tissue specific
CDS|exon|ID=cds-Bg	12	5.905833	4376.5	0.646664	FLDAKKGFT	98	461.5926	3	1381.761	.	L	Superscaf	98989552	0	0	0	0	0	BTP202209	Tissue specific
CDS|exon|ID=cds-Bg	11	5.944545	3989.1	0.646664	LDAKKGFTE	99	618.3516	2	1234.692	.	L	Superscaf	98989552	0	0	0	0	0	BTP202209	Tissue specific
CDS|exon|ID=cds-Bg	10	5.941	3635.7	0.646664	DAKKGFTEV	99	374.875	3	1121.608	.	L	Superscaf	98989552	0	0	0	0	0	BTP202209	Tissue specific
CDS|exon|ID=cds-Bg	17	5.052941	5942.3	0.646741	VSEAKEAEC	98	860.4197	2	1718.832	.	A	Superscaf	98994211	0	0	0	0	BTP202209	BTP202209	Mixed
CDS|exon|ID=cds-Bg	12	5.103333	4353.7	0.646742	EAEGTSQVE	94	603.2834	2	1204.557	.	A	Superscaf	98994211	0	0	0	0	0	BTP202209	Tissue specific
CDS|exon|ID=cds-Bg	11	5.897273	4357.9	0.646742	VEAGKRLEE	95	433.9171	3	1298.731	.	A	Superscaf	98994211	0	BTP202209	0	0	0	0	Tissue specific
CDS|exon|ID=cds-Bg	8	7.68	4153	0.646742	RLEELRRR	70	376.5616	3	1126.668	.	A	Superscaf	98994211	0	0	0	0	0	BTP202209	Tissue specific
CDS|exon|ID=cds-Bg	15	6.091333	6611.2	0.646743	RGETESEEF	89	460.2358	4	1836.922	.	A	Superscaf	98994211	0	0	0	0	0	BTP202209	Tissue specific
CDS|exon|ID=cds-Bg	13	5.741538	5665.1	0.646743	ETESEEFEK	99	542.2723	3	1623.799	.	A	Superscaf	98994211	0	0	0	0	0	BTP202209	Tissue specific
CDS|exon|ID=cds-Bg	11	5.386364	4586	0.646743	ETESEEFEK	97	684.8286	2	1367.646	.	A	Superscaf	98994211	0	0	0	0	0	BTP202209	Tissue specific
CDS|exon|ID=cds-Bg	12	5.951667	5239.6	0.646743	TESEEFEKL	98	499.2581	3	1494.757	.	A	Superscaf	98994211	0	0	0	0	0	BTP202209	Tissue specific
CDS|exon|ID=cds-Bg	13	5.664615	5543	0.646743	QQEAALELE	93	764.9008	2	1527.815	.	A	Superscaf	98994211	0	0	0	0	0	BTP202209	Tissue specific
CDS|exon|ID=cds-Bg	9	6.313333	3741.9	0.646744	LELEELKKK	99	565.3431	2	1128.675	.	A	Superscaf	98994211	0	0	0	0	0	BTP202209	Tissue specific
CDS|exon|ID=cds-Bg	10	5.475	4251.7	0.646744	VLEEEEQRK	98	644.3477	2	1286.683	.	A	Superscaf	98994211	0	0	0	0	0	BTP202209	Tissue specific
CDS|exon|ID=cds-Bg	16	6.216875	6806.5	0.64679	RQKMPEDGL	87	476.9915	4	1903.946	.	E	Superscaf	98997145	0	0	0	0	0	BTP202209	Tissue specific
CDS|exon|ID=cds-Bg	12	5.9175	4838.1	0.646791	EDGLSEDKK	99	464.905	3	1391.693	.	E	Superscaf	98997145	0	BTP202209	0	0	BTP202209	BTP202209	Mixed
CDS|exon|ID=cds-Bg	11	5.57	4369.7	0.646791	EDGLSEDKK	96	632.8055	2	1263.598	.	E	Superscaf	98997145	0	BTP202209	0	0	0	BTP202209	Mixed
CDS|exon|ID=cds-Bg	10	7.407	4306.4	0.646791	KKPFKC(+5	94	427.5749	3	1279.711	Carbamido	E	Superscaf	98997145	0	0	0	0	BTP202209	0	Tissue specific
CDS|exon|ID=cds-Bg	14	5.307143	4634.8	0.646916	SGVKSTHQA	99	466.9277	3	1397.763	.	W	Superscaf	99004672	0	BTP202209	0	0	BTP202209	BTP202209	Mixed
CDS|exon|ID=cds-Bg	12	5.220833	3954.1	0.646916	VKSTHQAAV	99	418.9087	3	1253.709	.	W	Superscaf	99004672	0	0	0	0	0	BTP202209	Tissue specific
CDS|exon|ID=cds-Bg	10	5.291	3485.7	0.646916	STHQAAVVS	99	514.2783	2	1026.546	.	W	Superscaf	99004672	0	0	0	0	0	BTP202209	Tissue specific
CDS|exon|ID=cds-Bg	7	5.702857	3292.8	0.646916	DSRLEQY	88	455.7149	2	909.4192	.	W	Superscaf	99004672	0	0	0	0	BTP202209	0	Tissue specific
CDS|exon|ID=cds-Bg	10	5.879	4225.8	0.646968	NEWLTKTPE	68	587.7886	2	1173.567	.	P	Superscaf	99007843	0	BTP202209	0	0	0	0	Tissue specific
CDS|exon|ID=cds-Bg	12	6.481667	4484.8	0.646968	TPEGSKSPA	98	598.3179	2	1194.625	.	P	Superscaf	99007843	0	0	0	0	BTP202209	0	Tissue specific
CDS|exon|ID=cds-Bg	11	6.1	4257.8	0.647072	DLRPGDVSC	98	400.5538	3	1198.642	.	D	Superscaf	99014152	0	BTP202209	BTP202209	0	0	0	Mixed
CDS|exon|ID=cds-Bg	9	5.43	3647.9	0.647073	LWEKQSVDK	84	378.2038	3	1131.592	.	D	Superscaf	99014152	0	0	BTP202209	0	0	0	Tissue specific
CDS|exon|ID=cds-Bg	16	5.228125	5632.1	0.647073	LWEKQSVDK	96	923.0059	2	1844.004	.	D	Superscaf	99014152	0	0	0	0	0	BTP202209	Tissue specific
CDS|exon|ID=cds-Bg	8	5.36125	3294.5	0.647073	WEKQSVDK	95	510.26	2	1018.508	.	D	Superscaf	99014152	0	0	0	0	BTP202209	BTP202209	Tissue enhanced
CDS|exon|ID=cds-Bg	14	5.547857	5278.7	0.647073	WEKQSVDKV	98	816.9301	2	1631.852	.	D	Superscaf	99014152	0	0	0	0	BTP202209	BTP202209	Mixed
CDS|exon|ID=cds-Bg	15	5.178	5278.7	0.647073	WEKQSVDKV	95	577.9795	3	1730.92	.	D	Superscaf	99014152	0	BTP202209	0	0	0	BTP202209	Tissue enhanced
CDS|exon|ID=cds-Bg	10	4.942	3692	0.647073	WEKQSVDKV	92	610.3171	2	1218.625	.	D	Superscaf	99014152	0	0	0	0	0	BTP202209	Tissue specific
CDS|exon|ID=cds-Bg	12	5.17	4283.7	0.647073	EKQSVDKVT	92	659.8445	2	1317.678	.	D	Superscaf	99014152	0	0	0	0	BTP202209	0	Tissue specific
CDS|exon|ID=cds-Bg	10	4.749	2864.1	0.647073	VDKVTSPTK	99	537.3125	2	1072.613	.	D	Superscaf	99014152	0	BTP202209	0	0	0	BTP202209	Mixed
CDS|exon|ID=cds-Bg	9	5.276667	2864.1	0.647073	DKVTSPTKV	83	487.7782	2	973.5444	.	D	Superscaf	99014152	0	BTP202209	0	0	0	0	Tissue specific
CDS|exon|ID=cds-Bg	11	5.861818	4183.4	0.660372	DVDAKKKPF	99	622.817	2	1243.623	Carbamido	Y	Superscaf	99813625	0	0	0	0	0	BTP202209	Tissue specific
CDS|exon|ID=cds-Bg	7	7.228571	3473	0.672834	T(+42.01)	83	451.7567	2	901.4981	Acetylati	N	Superscaf	1.01E+08	0	0	0	0	BTP202209	0	Tissue specific
CDS|exon|ID=cds-Bg	6	3.541667	2594.1	0.682895	EVDC(+57.	66	421.6247	2	841.2405	Carbamido	S	Superscaf	1.01E+08	0	BTP202209	0	0	0	0	Tissue specific
CDS|exon|ID=cds-Bg	6	4.558333	2322.7	0.692175	EELLDL	79	731.3809	1	730.3749	.	A	Superscaf	1.02E+08	0	0	0	0	BTP202209	0	Tissue specific
CDS|exon|ID=cds-Bg	8	5.7075	2616.8	0.699626	VSSPPLLK	99	420.762	2	839.5116	.	I	Superscaf	1.02E+08	0	0	BTP202209	0	0	0	Tissue specific
CDS|exon|ID=cds-Bg	7	6.345714	2933.4	0.715667	HLQPPPP	52	393.2255	2	784.4232	.	G	Superscaf	1.03E+08	0	0	BTP202209	0	0	0	Tissue specific
CDS|exon|ID=cds-Bg	8	6.5475	2797.9	0.743605	APSAPAPK	85	369.7142	2	737.4072	.	A	Superscaf	1.05E+08	0	0	0	0	BTP202209	0	Tissue specific
CDS|exon|ID=cds-Bg	6	6.683333	2617	0.747932	NLSRGP	63	643.3633	1	642.3449	.	S	Superscaf	1.05E+08	0	0	0	0	BTP202209	0	Tissue specific
CDS|exon|ID=cds-Bg	14	6.289286	5520.2	0.779453	QPPAMLPHK	89	540.9708	3	1619.897	.	S	Superscaf	1.07E+08	0	0	BTP202209	0	0	0	Tissue specific
CDS|exon|ID=cds-Bg	11	6.345455	4234.7	0.779453	AMLPHKAVY	92	433.5839	3	1297.733	.	S	Superscaf	1.07E+08	0	0	BTP202209	0	0	0	Tissue specific
CDS|exon|ID=cds-Bg	9	5.895556	3232.5	0.779453	AMLPHKAVY	93	515.2775	2	1028.548	.	S	Superscaf	1.07E+08	0	0	BTP202209	0	0	0	Tissue specific
CDS|exon|ID=cds-Bg	7	4.951429	1791.9	0.781574	PVVAPKP	98	354.2247	2	706.4377	.	G	Superscaf	1.07E+08	0	0	0	0	BTP202209	0	Tissue specific
CDS|exon|ID=cds-Bg	11	6.577273	4230.6	0.781575	APKPKVNPF	94	417.5815	3	1249.73	.	G	Superscaf	1.07E+08	0	0	0	0	BTP202209	0	Tissue specific
CDS|exon|ID=cds-Bg	11	5.461818	4500.2	0.781591	LLDDMTKND	86	654.8098	2	1307.607	.	P	Superscaf	1.07E+08	0	BTP202209	0	0	0	0	Tissue specific
CDS|exon|ID=cds-Bg	10	5.41	4146.8	0.781591	LDDMTKNDF	89	598.2673	2	1194.523	.	P	Superscaf	1.07E+08	0	BTP202209	0	0	0	0	Tissue specific
CDS|exon|ID=cds-Bg	11	5.807273	4573.1	0.781591	DDMTKNDPF	66	641.2914	2	1280.571	.	P	Superscaf	1.07E+08	0	BTP202209	0	0	0	0	Tissue specific
CDS|exon|ID=cds-Bg	10	5.786	4261.8	0.781591	DDMTKNDPF	82	404.1841	3	1209.533	.	P	Superscaf	1.07E+08	0	0	0	0	BTP202209	0	Tissue specific
CDS|exon|ID=cds-Bg	9	5.346667	3793.4	0.781591	DDMTKNDPF	94	541.7258	2	1081.439	.	P	Superscaf	1.07E+08	0	BTP202209	0	0	0	0	Tissue specific
CDS|exon|ID=cds-Bg	9	4.86	2532	0.781594	VPPPVTTPF	61	954.5276	1	953.5222	.	P	Superscaf	1.07E+08	0	BTP202209	0	0	0	0	Tissue specific
CDS|exon|ID=cds-Bg	8	5.4675	2532	0.781594	PPPVTTPF	78	855.4519	1	854.4538	.	P	Superscaf	1.07E+08	0	BTP202209	0	0	0	0	Tissue specific
CDS|exon|ID=cds-Bg	14	6.703571	5469.1	0.781598	TQPRGPLAF	83	708.8992	2	1415.788	.	P	Superscaf	1.07E+08	0	0	0	0	BTP202209	0	Tissue specific
CDS|exon|ID=cds-Bg	8	6.22125	2779.9	0.781598	KFSPVTPK	96	452.2675	2	902.5225	.	P	Superscaf	1.07E+08	0	BTP202209	0	0	0	0	Tissue specific
CDS|exon|ID=cds-Bg	16	6.285625	6313.8	0.781599	FSPGAPGGF	93	774.8783	2	1547.748	.	P	Superscaf	1.07E+08	0	0	0	0	0	BTP202209	Tissue specific
CDS|exon|ID=cds-Bg	19	5.538421	7352.2	0.781671	APGPLTLKE	97	708.3883	3	2122.152	.	P	Superscaf	1.07E+08	0	0	0	0	BTP202209	0	Tissue specific
CDS|exon|ID=cds-Bg	8	6.2525	2970.3	0.781672	PGPLTLKE	99	427.7515	2	853.4909	.	P	Superscaf	1.07E+08	0	0	0	0	BTP202209	0	Tissue specific
CDS|exon|ID=cds-Bg	19	5.536316	7394.3	0.781672	PGPLTLKEV	90	722.4045	3	2164.199	.	P	Superscaf	1.07E+08	0	0	0	0	BTP202209	0	Tissue specific
CDS|exon|ID=cds-Bg	18	5.511667	7040.9	0.781672	PGPLTLKEV	99	684.7091	3	2051.115	.	P	Superscaf	1.07E+08	0	0	0	0	BTP202209	0	Tissue specific

CDS|exon|ID=cds-Bg	10	4.919	4070.6	0.781672	VEELEQLTG	80	608.8212	2	1215.635	.	P	Superscaf	1.07E+08	0	0	0	0	BTP202209	BTP202209	Tissue enhanced
CDS|exon|ID=cds-Bg	7	5.547143	2567.8	0.781678	SLEGAPY	69	736.3459	1	735.3439	.	P	Superscaf	1.07E+08	0	0	0	0	0	BTP202209	Tissue specific
CDS|exon|ID=cds-Bg	7	7.592857	2776.7	0.785144	LSKKGPK	52	379.2428	2	756.4857	.	T	Superscaf	1.07E+08	0	0	0	0	0	BTP202209	Tissue specific
CDS|exon|ID=cds-Bg	8	5.8275	3394.9	0.868438	LADTERFY	89	507.7469	2	1013.482	.	F	Superscaf	1.12E+08	0	0	0	0	0	BTP202209	Tissue specific
CDS|exon|ID=cds-Bg	7	5.995714	2652.9	0.874226	A(+42.01)	55	733.3495	1	732.3442	Acetylati	G	Superscaf	1.13E+08	0	0	0	0	BTP202209	0	Tissue specific
CDS|exon|ID=cds-Bg	6	5.931667	2108.3	0.878126	LSGSLP	55	573.3224	1	572.317	.	W	Superscaf	1.13E+08	0	0	BTP202209	0	0	0	Tissue specific
CDS|exon|ID=cds-Bg	6	6.001667	2080.4	0.880918	LAPLML	62	657.3981	1	656.3931	.	C	Superscaf	1.13E+08	0	0	BTP202209	0	0	0	Tissue specific
CDS|exon|ID=cds-Bg	8	5.825	2831.2	0.890739	GSTRVPSS	51	395.7045	2	789.3981	.	S	Superscaf	1.14E+08	0	0	0	0	0	BTP202209	Tissue specific
CDS|exon|ID=cds-Bg	9	5.563333	2659.1	0.897483	VPGPPPPPF	69	427.7408	2	853.4697	.	P	Superscaf	1.14E+08	0	0	0	BTP202209	BTP202209	0	Mixed
CDS|exon|ID=cds-Bg	7	6.92	3282.4	0.900015	A(+42.01)	99	460.7628	2	919.5127	Acetylati	Y	Superscaf	1.14E+08	0	BTP202209	BTP202209	BTP202209	BTP202209	0	Mixed
CDS|exon|ID=cds-Bg	9	5.746667	3992.6	0.920386	YLRAEPEDF	77	565.2654	2	1128.52	.	W	Superscaf	1.15E+08	0	0	0	0	0	BTP202209	Tissue specific
CDS|exon|ID=cds-Bg	11	5.714545	4839.5	0.920386	YLRAEPEDF	79	480.5566	3	1438.652	.	W	Superscaf	1.15E+08	0	0	0	0	0	BTP202209	Tissue specific
CDS|exon|ID=cds-Bg	10	5.738	4452.1	0.920386	YLRAEPEDF	94	431.5334	3	1291.583	.	W	Superscaf	1.15E+08	0	0	0	0	0	BTP202209	Tissue specific
CDS|exon|ID=cds-Bg	7	6.685714	2592.8	0.925562	LAATLTK	94	359.2286	2	716.4432	.	Y	Superscaf	1.16E+08	0	BTP202209	0	0	0	0	Tissue specific
CDS|exon|ID=cds-Bg	12	5.198333	4832.5	0.925563	LTC(+57.0	81	471.5451	3	1411.619	Carbamido	D	Superscaf	1.16E+08	0	0	0	0	0	BTP202209	Tissue specific
CDS|exon|ID=cds-Bg	7	5.494286	2142	0.942469	KVVKAEK	65	401.262	2	800.512	.	V	Superscaf	1.17E+08	0	0	0	0	0	BTP202209	Tissue specific
CDS|exon|ID=cds-Bg	11	5.873636	4406.7	0.967474	VEDELKAEK	89	672.8757	2	1343.741	.	P	Superscaf	1.18E+08	0	0	0	0	BTP202209	0	Tissue specific
CDS|exon|ID=cds-Bg	10	6.619	4229.6	0.975338	FEGDFKTRF	98	408.8829	3	1223.63	.	A	Superscaf	1.19E+08	0	BTP202209	0	0	0	0	Tissue specific
CDS|exon|ID=cds-Bg	5	6.298	1921.1	0.978373	HGGLL	62	496.2867	1	495.2805	.	P	Superscaf	1.19E+08	0	0	BTP202209	0	0	0	Tissue specific
CDS|exon|ID=cds-Bg	7	3.355714	2109.4	0.978373	VDYYEVL	90	900.4323	1	899.4276	.	N	Superscaf	1.19E+08	0	0	0	BTP202209	0	0	Tissue specific
CDS|exon|ID=cds-Bg	6	7.408333	2730	0.963387	Q(+42.01)	72	377.7343	2	753.4497	Acetylati	Q	Superscaf	2212810	0	BTP202209	0	0	0	0	Tissue specific
CDS|exon|ID=cds-Bg	7	7.228571	2992	0.963064	KKPEWGK	53	436.7594	2	871.4915	.	G	Superscaf	2232543	0	0	BTP202209	0	0	0	Tissue specific
CDS|exon|ID=cds-Bg	6	3.961667	1333.4	0.952152	VPVAGF	67	589.3338	1	588.3271	.	W	Superscaf	2890969	BTP202209	0	0	0	0	0	Tissue specific
CDS|exon|ID=cds-Bg	6	5.526667	2511.9	0.857244	ETLFQP	78	734.3704	1	733.3646	.	S	Superscaf	8628027	BTP202209	0	0	0	0	0	Tissue specific
CDS|exon|ID=cds-Bg	11	6.135455	4416.7	0.807386	SSGGGEYKT	99	585.2627	2	1168.515	.	G	Superscaf	11640838	0	0	0	0	0	BTP202209	Tissue specific
CDS|exon|ID=cds-Bg	9	4.811111	2807.9	0.804489	VVGKEDSLK	99	487.7778	2	973.5444	.	S	Superscaf	11816302	0	0	BTP202209	0	0	0	Tissue specific
CDS|exon|ID=cds-Bg	9	5.915556	2983	0.804485	AFRKAVFVK	79	355.8895	3	1064.649	.	T	Superscaf	11816596	0	0	BTP202209	0	0	0	Tissue specific
CDS|exon|ID=cds-Bg	7	4.87	2307.6	0.688656	VASRLVQ	51	386.7363	2	771.4603	.	D	Superscaf	18817344	0	BTP202209	0	0	0	0	Tissue specific
CDS|exon|ID=cds-Bg	6	5.795	2506.8	0.663311	YPLEHA	94	365.1805	2	728.3493	.	L	Superscaf	20349064	0	0	0	BTP202209	0	0	Tissue specific
CDS|exon|ID=cds-Bg	7	5.781429	2835.2	0.639489	KPC(+57.0	54	431.2173	2	860.4174	Carbamido	G	Superscaf	21788916	0	0	0	BTP202209	0	0	Tissue specific
CDS|exon|ID=cds-Bg	24	5.980833	8719.2	0.616025	LPGELAKHA	99	626.3419	4	2501.349	.	V	Superscaf	23206573	0	0	0	0	BTP202209	0	Tissue specific
CDS|exon|ID=cds-Bg	23	5.98087	8365.8	0.616025	PGELAKHAV	97	598.0728	4	2388.265	.	V	Superscaf	23206573	0	0	0	0	BTP202209	0	Tissue specific
CDS|exon|ID=cds-Bg	22	5.966364	8028.4	0.616025	GELAKHAVS	94	573.808	4	2291.212	.	V	Superscaf	23206573	0	0	0	0	BTP202209	BTP202209	Tissue enhanced
CDS|exon|ID=cds-Bg	15	5.660667	5320.7	0.616025	HAVSEGTKA	97	526.9403	3	1577.805	.	V	Superscaf	23206573	0	0	0	0	BTP202209	BTP202209	Mixed
CDS|exon|ID=cds-Bg	12	5.941667	4389.7	0.616025	SEGTKAVTK	98	636.3256	2	1270.64	.	V	Superscaf	23206573	0	0	0	0	BTP202209	0	Tissue specific
CDS|exon|ID=cds-Bg	7	7.248571	3059.3	0.582074	KAAQATR	62	373.2253	2	744.4242	.	P	Superscaf	25259064	0	0	BTP202209	0	0	0	Tissue specific
CDS|exon|ID=cds-Bg	7	7.207143	2800	0.575649	LSGAPRK	59	364.7275	2	727.434	.	P	Superscaf	25647214	0	0	0	0	BTP202209	0	Tissue specific
CDS|exon|ID=cds-Bg	7	6.795714	3136.5	0.57315	E(+42.01)	94	469.7501	2	937.4869	Acetylati	T	Superscaf	25798368	0	BTP202209	0	0	0	0	Tissue specific
CDS|exon|ID=cds-Bg	9	6.406667	4276.9	0.562465	M(+42.01)	91	390.5161	3	1168.53	Acetylati	G	Superscaf	26443864	BTP202209	0	0	0	0	0	Tissue specific
CDS|exon|ID=cds-Bg	8	4.72625	2464.7	0.559393	K(+42.01)	58	485.804	2	969.5971	Acetylati	K	Superscaf	26629800	0	0	BTP202209	0	0	0	Tissue specific
CDS|exon|ID=cds-Bg	5	5.038	2591.1	0.559319	C(+57.02)	63	363.1109	2	724.2156	Carbamido	I	Superscaf	26634309	BTP202209	0	0	0	0	0	Tissue specific
CDS|exon|ID=cds-Bg	8	6.73	2830.2	0.557914	P(+42.01)	78	434.7484	2	867.4854	Acetylati	K	Superscaf	26719114	0	0	0	BTP202209	0	0	Tissue specific
CDS|exon|ID=cds-Bg	7	3.895714	1799.1	0.543651	LMAVPEV	55	379.7069	2	757.4044	.	L	Superscaf	27581128	0	0	0	BTP202209	0	0	Tissue specific
CDS|exon|ID=cds-Bg	7	5.275714	2165.3	0.54204	DVPGGLK	78	685.3851	1	684.3806	.	S	Superscaf	27678684	0	0	0	0	0	BTP202209	Tissue specific
CDS|exon|ID=cds-Bg	12	5.591667	5183	0.540221	LLNELERLQ	88	706.8889	2	1411.767	.	R	Superscaf	27788362	0	BTP202209	0	0	BTP202209	BTP202209	Mixed
CDS|exon|ID=cds-Bg	14	5.997857	5249.7	0.537035	VTQKSYKVS	99	517.9543	3	1550.842	.	K	Superscaf	27980947	0	BTP202209	0	0	0	0	Tissue specific
CDS|exon|ID=cds-Bg	10	6.205	3773.1	0.537035	SYKVSTSAF	97	548.2927	2	1094.572	.	K	Superscaf	27980947	0	BTP202209	0	0	0	0	Tissue specific
CDS|exon|ID=cds-Bg	9	6.263333	3389.7	0.537035	YKVSTSAPF	81	504.7766	2	1007.54	.	K	Superscaf	27980947	0	BTP202209	0	0	0	0	Tissue specific
CDS|exon|ID=cds-Bg	11	6.75	4648.2	0.537034	TSAPRSFSS	82	591.7953	2	1181.579	.	K	Superscaf	27980947	0	0	BTP202209	0	0	0	Tissue specific
CDS|exon|ID=cds-Bg	9	5.631111	3391.7	0.537034	SSAFSRVGN	94	462.7292	2	923.446	.	K	Superscaf	27980947	0	0	BTP202209	0	0	0	Tissue specific
CDS|exon|ID=cds-Bg	7	5.981429	2353.5	0.537032	YGGAPGL	99	634.3184	1	633.3122	.	K	Superscaf	27980947	0	BTP202209	0	0	0	0	Tissue specific
CDS|exon|ID=cds-Bg	10	5.797	4102.5	0.536949	YEELQTLAG	99	576.2994	2	1150.587	.	G	Superscaf	27986188	0	0	0	0	0	BTP202209	Tissue specific
CDS|exon|ID=cds-Bg	9	5.812222	3643	0.536949	EELQTLAGK	88	494.7677	2	987.5236	.	G	Superscaf	27986188	0	0	0	0	0	BTP202209	Tissue specific
CDS|exon|ID=cds-Bg	12	6.1975	4870.3	0.536933	LAELEAALF	97	649.8722	2	1297.735	.	H	Superscaf	27986986	0	0	0	0	0	BTP202209	Tissue specific
CDS|exon|ID=cds-Bg	9	5.853333	3539.9	0.53692	SSYGTPGFN	99	929.3972	1	928.3926	.	P	Superscaf	27988012	0	0	BTP202209	0	0	0	Tissue specific
CDS|exon|ID=cds-Bg	8	5.90875	2943.2	0.53692	SSYGTPGF	78	815.3552	1	814.3497	.	P	Superscaf	27988012	0	BTP202209	0	0	0	0	Tissue specific
CDS|exon|ID=cds-Bg	10	5.313	2880.8	0.536919	TSSKAVVVK	99	523.831	2	1045.649	.	P	Superscaf	27988012	0	0	0	0	0	BTP202209	Tissue specific
CDS|exon|ID=cds-Bg	9	4.821111	2412.4	0.536919	TSSKAVVVK	99	459.7831	2	917.5546	.	P	Superscaf	27988012	0	0	0	0	0	BTP202209	Tissue specific
CDS|exon|ID=cds-Bg	8	4.6075	2014.9	0.536919	SSKAVVVK	73	409.2585	2	816.5069	.	P	Superscaf	27988012	0	0	0	0	0	BTP202209	Tissue specific
CDS|exon|ID=cds-Bg	14	4.949286	4723	0.536918	DGKLVSESS	89	488.5903	3	1462.752	.	P	Superscaf	27988012	0	BTP202209	BTP202209	0	0	0	Mixed
CDS|exon|ID=cds-Bg	13	4.580769	4254.6	0.536918	DGKLVSESS	63	668.3327	2	1334.657	.	P	Superscaf	27988012	0	0	0	0	0	BTP202209	Tissue specific
CDS|exon|ID=cds-Bg	13	5.101538	4311.5	0.536918	GKLVSESSD	99	674.8683	2	1347.725	.	P	Superscaf	27988012	0	BTP202209	0	0	0	0	Tissue specific
CDS|exon|ID=cds-Bg	12	5.029167	4014.2	0.536918	KLVSESSDV	96	646.3565	2	1290.703	.	P	Superscaf	27988012	0	0	BTP202209	0	0	0	Tissue specific
CDS|exon|ID=cds-Bg	11	4.600909	3545.8	0.536918	LVSESSDVL	98	582.3101	2	1162.608	.	P	Superscaf	27988012	0	BTP202209	BTP202209	0	0	BTP202209	Tissue enhanced
CDS|exon|ID=cds-Bg	10	4.463	3192.4	0.536918	VSESSDVLS	99	525.769	2	1049.524	.	P	Superscaf	27988012	0	BTP202209	BTP202209	0	0	BTP202209	Mixed
CDS|exon|ID=cds-Bg	9	4.958889	3192.4	0.536918	SESSDVLSK	98	476.2351	2	950.4556	.	P	Superscaf	27988012	0	BTP202209	0	0	0	0	Tissue specific
CDS|exon|ID=cds-Bg	12	5.113333	4145.4	0.536013	TAAENEFVV	89	450.2524	3	1347.74	.	K	Superscaf	28042738	0	0	0	0	0	BTP202209	Tissue specific
CDS|exon|ID=cds-Bg	9	5.453333	3420.7	0.535688	MNKVELEAK	99	531.285	2	1060.559	.	G	Superscaf	28062349	0	0	0	0	0	BTP202209	Tissue specific
CDS|exon|ID=cds-Bg	8	5.50375	3312.8	0.533874	LLEGEESR	99	466.736	2	931.4611	.	V	Superscaf	28171867	0	0	0	0	0	BTP202209	Tissue specific
CDS|exon|ID=cds-Bg	12	5.113333	4145.4	0.527624	ATAENEFVV	96	674.8755	2	1347.74	.	R	Superscaf	28549747	0	0	0	0	0	BTP202209	Tissue specific
CDS|exon|ID=cds-Bg	8	5.29875	2831.1	0.527591	LAGLEEAL	66	815.4475	1	814.4436	.	L	Superscaf	28551712	0	0	0	BTP202209	0	0	Tissue specific
CDS|exon|ID=cds-Bg	10	5.177	3837.4	0.492166	ADSLPDSVF	95	537.2629	2	1072.515	.	N	Superscaf	30692791	0	0	0	0	0	BTP202209	Tissue specific
CDS|exon|ID=cds-Bg	11	6.232727	4924.5	0.491599	DEGFGHRPH	92	647.7974	2	1293.585	.	I	Superscaf	30727027	0	0	BTP202209	0	0	0	Tissue specific
CDS|exon|ID=cds-Bg	7	4.887143	2998.4	0.490664	C(+57.02)	50	946.4617	1	945.4379	Carbamido	G	Superscaf	30783649	0	0	0	BTP202209	0	0	Tissue specific
CDS|exon|ID=cds-Bg	7	6.16	2423.8	0.488561	TAAPTMG	55	648.2963	1	647.2949	.	M	Superscaf	30910795	0	0	0	0	BTP202209	0	Tissue specific
CDS|exon|ID=cds-Bg	15	6.051333	6501.2	0.486374	EEKSQMASI	99	826.3984	2	1650.788	.	F	Superscaf	31043067	0	0	BTP202209	0	0	0	Tissue specific
CDS|exon|ID=cds-Bg	6	5.12	2206.5	0.483837	EEALPL	53	671.3582	1	670.3537	.	T	Superscaf	31196002	0	0	0	0	BTP202209	0	Tissue specific
CDS|exon|ID=cds-Bg	9	6.542222	3377.7	0.476317	TGAGKHVPF	98	461.762	2	921.5144	.	K	Superscaf	31650535	0	0	0	0	0	BTP202209	Tissue specific
CDS|exon|ID=cds-Bg	8	4.77	2943.4	0.476295	VDWC(+57.	92	981.4116	1	980.4062	Carbamido	K	Superscaf	31651426	0	BTP202209	0	0	BTP202209	BTP202209	Mixed
CDS|exon|ID=cds-Bg	7	5.451429	2943.4	0.476295	DWC(+57.0	78	882.3414	1	881.3378	Carbamido	K	Superscaf	31651426	0	0	0	0	BTP202209	0	Tissue specific
CDS|exon|ID=cds-Bg	16	5.25875	5419.1	0.476293	QPPTVVPGC	92	554.6478	3	1660.926	.	K	Superscaf	31651426	0	0	BTP202209	0	0	0	Tissue specific
CDS|exon|ID=cds-Bg	8	5.365	3078.4	0.476291	EDMAALEK	77	453.7141	2	905.4164	.	K	Superscaf	31651426	0	BTP202209	0	0	0	0	Tissue specific
CDS|exon|ID=cds-Bg	12	5.954167	4641.1	0.475534	LMERLSVDY	98	480.26	3	1437.765	.	Q	Superscaf	31698031	0	0	0	0	0	BTP202209	Tissue specific
CDS|exon|ID=cds-Bg	9	5.713333	3225.3	0.475534	LSVDYGKKS	87	498.7695	2	995.5287	.	Q	Superscaf	31698031	0	0	0	BTP202209	0	0	Tissue specific
CDS|exon|ID=cds-Bg	12	5.821667	4506.6	0.475534	SVDYGKKSK	99	467.5849	3	1399.735	.	Q	Superscaf	31698031	0	BTP202209	0	0	0	0	Tissue specific
CDS|exon|ID=cds-Bg	12	6.243333	5107.7	0.475522	ARLDHKFDL	83	740.3726	2	1478.734	.	Q	Superscaf	31698031	0	0	0	BTP202209	0	0	Tissue specific
CDS|exon|ID=cds-Bg	9	6.387778	3965.4	0.475522	ARLDHKFDL	72	372.2032	3	1113.593	.	Q	Superscaf	31698031	0	0	BTP202209	0	0	0	Tissue specific
CDS|exon|ID=cds-Bg	6	5.956667	2493.8	0.475522	RAFVHW	90	408.2177	2	814.4238	.	Q	Superscaf	31698031	0	0	0	0	0	BTP202209	Tissue specific
CDS|exon|ID=cds-Bg	16	5.405625	6383.1	0.475521	EGEFSEARE	96	604.6068	3	1810.804	.	Q	Superscaf	31698031	0	0	0	0	0	BTP202209	Tissue specific
CDS|exon|ID=cds-Bg	6	5.91	2160.3	0.474102	L(+42.01)	92	723.3691	1	722.3639	Acetylati	L	Superscaf	31784671	0	0	0	0	0	BTP202209	Tissue specific
CDS|exon|ID=cds-Bg	10	6.339	4107.4	0.471606	A(+42.01)	93	382.2015	3	1143.588	Acetylati	M	Superscaf	31935514	0	0	0	BTP202209	0	0	Tissue specific
CDS|exon|ID=cds-Bg	8	6.2075	3047.1	0.471606	A(+42.01)	99	437.2362	2	872.4603	Acetylati	M	Superscaf	31935514	0	BTP202209	0	0	0	0	Tissue specific
CDS|exon|ID=cds-Bg	5	6.922	2526.8	0.450149	K(+42.01)	72	735.3757	1	734.3711	Acetylati	M	Superscaf	33232300	0	0	0	0	0	BTP202209	Tissue specific
CDS|exon|ID=cds-Bg	11	5.900909	4266.7	0.376686	YLLSQSSPA	87	1175.626	1	1174.623	.	Q	Superscaf	37672294	0	0	0	0	0	BTP202209	Tissue specific
CDS|exon|ID=cds-Bg	14	5.996429	6329.9	0.376621	LYEEVHPKG	98	872.4464	2	1742.885	.	F	Superscaf	37676209	0	0	0	0	0	BTP202209	Tissue specific
CDS|exon|ID=cds-Bg	12	5.713333	5250.8	0.376622	LYEEVHPKG	97	744.371	2	1486.732	.	F	Superscaf	37676209	0	0	0	0	0	BTP202209	Tissue specific
CDS|exon|ID=cds-Bg	8	5.21375	3089.4	0.376622	LYEEVHPK	92	507.7639	2	1013.518	.	F	Superscaf	37676209	0	0	0	0	BTP202209	BTP202209	Mixed
CDS|exon|ID=cds-Bg	7	4.302857	2286.5	0.369126	AEAYEVL	60	794.3933	1	793.3857	.	E	Superscaf	38128888	0	BTP202209	0	BTP202209	0	0	Mixed
CDS|exon|ID=cds-Bg	8	6.53375	3370.9	0.311608	TSAC(+57.	69	459.2397	2	916.48	Carbamido	W	Superscaf	41605519	0	BTP202209	0	0	0	0	Tissue specific
CDS|exon|ID=cds-Bg	6	4.695	2438.8	0.173804	YQPDHV	78	758.3428	1	757.3395	.	S	Superscaf	49933972	0	BTP202209	0	0	0	0	Tissue specific
CDS|exon|ID=cds-Bg	8	6.64	2944.2	0.171463	KAATSPTP	76	386.7159	2	771.4127	.	L	Superscaf	50075818	0	0	0	BTP202209	0	0	Tissue specific
CDS|exon|ID=cds-Bg	7	5.62	2394.5	0.167366	LVGAKLQ	73	364.7358	2	727.4592	.	L	Superscaf	50323408	0	0	BTP202209	0	0	0	Tissue specific
CDS|exon|ID=cds-Bg	5	7.328	2214.5	0.143009	R(+42.01)	56	579.3007	1	578.2925	Acetylati	R	Superscaf	51795457	0	0	0	0	0	BTP202209	Tissue specific
CDS|exon|ID=cds-Bg	5	7.488	2011	0.057893	K(+42.01)	54	614.3849	1	613.3799	Acetylati	N	Superscaf	56939907	0	0	0	0	0	BTP202209	Tissue specific
CDS|exon|ID=cds-Bg	14	4.251429	4958.4	0.042143	VEDSKDVNV	97	805.3856	2	1608.763	.	L	Superscaf	57891664	0	0	0	BTP202209	0	0	Tissue specific
CDS|exon|ID=cds-Bg	11	4.848182	4121.4	0.042143	SKDVNVNFE	97	633.8181	2	1265.625	.	L	Superscaf	57891664	0	0	0	BTP202209	0	0	Tissue specific
CDS|exon|ID=cds-Bg	10	4.765	3738	0.042143	KDVNVNFEK	71	590.3052	2	1178.593	.	L	Superscaf	57891664	0	0	0	BTP202209	0	0	Tissue specific
CDS|exon|ID=cds-Bg	9	5.351111	3696.1	0.04214	SC(+57.02	96	956.3745	1	955.3705	Carbamido	R	Superscaf	57891919	0	0	0	BTP202209	0	0	Tissue specific
CDS|exon|ID=cds-Bg	7	5.351429	2727	0.04214	LGGSDNF	87	709.3125	1	708.3079	.	R	Superscaf	57891919	0	0	0	BTP202209	0	0	Tissue specific
CDS|exon|ID=cds-Bg	9	6.087778	3815.1	0.04214	GGSDNFKHL	99	487.7366	2	973.4617	.	R	Superscaf	57891919	0	0	0	BTP202209	0	0	Tissue specific
CDS|exon|ID=cds-Bg	8	3.05125	2350.8	0.042079	DVDLPEVD	90	901.4106	1	900.4076	.	M	Superscaf	57895612	0	0	0	0	0	BTP202209	Tissue specific
CDS|exon|ID=cds-Bg	7	6.844286	2649.1	0.041917	LPRPPGP	96	367.2206	2	732.4282	.	A	Superscaf	57905293	0	0	0	BTP202209	0	0	Tissue specific
CDS|exon|ID=cds-Bg	11	5.705455	4207.7	0.0419	DGTEGLVRC	99	580.3042	2	1158.599	.	E	Superscaf	57906313	0	0	0	0	0	BTP202209	Tissue specific
CDS|exon|ID=cds-Bg	9	4.358889	2995.4	0.041889	AEAPEFVEM	71	1022.444	1	1021.443	.	I	Superscaf	57907081	0	0	0	0	0	BTP202209	Tissue specific
CDS|exon|ID=cds-Bg	8	4.15125	2684.1	0.041889	EAPEFVEM	75	951.4089	1	950.4055	.	I	Superscaf	57907081	0	0	0	0	0	BTP202209	Tissue specific
CDS|exon|ID=cds-Bg	12	5.009167	4897.6	0.04187	FRDQEGQDV	73	733.8644	2	1465.72	.	L	Superscaf	57908158	0	0	0	0	0	BTP202209	Tissue specific
CDS|exon|ID=cds-Bg	9	5.291111	3155.6	0.041855	AHLDATTVL	75	470.7562	2	939.5025	.	A	Superscaf	57909151	0	0	0	0	0	BTP202209	Tissue specific
CDS|exon|ID=cds-Bg	8	5.13875	2748	0.041853	DVARGVQK	76	436.7495	2	871.4875	.	A	Superscaf	57909151	0	0	0	0	0	BTP202209	Tissue specific
CDS|exon|ID=cds-Bg	12	5.179167	4168.5	0.041844	QVAEVFTGH	97	643.3458	2	1284.683	.	D	Superscaf	57909745	0	0	0	0	0	BTP202209	Tissue specific
CDS|exon|ID=cds-Bg	9	4.532222	2792.1	0.041844	VAEVFTGHL	84	486.7594	2	971.5076	.	D	Superscaf	57909745	0	0	0	0	0	BTP202209	Tissue specific
CDS|exon|ID=cds-Bg	8	5.09875	2792.1	0.041844	AEVFTGHL	81	437.225	2	872.4392	.	D	Superscaf	57909745	0	0	0	0	0	BTP202209	Tissue specific
CDS|exon|ID=cds-Bg	9	5.178889	3954.5	0.041833	EYDHLPEQA	57	551.2443	2	1100.477	.	Y	Superscaf	57910453	0	0	0	0	0	BTP202209	Tissue specific
CDS|exon|ID=cds-Bg	7	5.338571	3217.7	0.041833	YDHLPEQ	55	451.2045	2	900.3977	.	Y	Superscaf	57910453	0	0	0	0	BTP202209	0	Tissue specific
CDS|exon|ID=cds-Bg	14	5.388571	5226.5	0.041832	EAVAKADKL	93	499.9221	3	1496.747	.	Y	Superscaf	57910453	0	BTP202209	BTP202209	BTP202209	0	0	Mixed
CDS|exon|ID=cds-Bg	13	5.555385	4801	0.041832	AVAKADKLA	99	456.908	3	1367.704	.	Y	Superscaf	57910453	BTP202209	BTP202209	BTP202209	BTP202209	0	0	Mixed
CDS|exon|ID=cds-Bg	12	5.516667	4489.7	0.041832	VAKADKLAE	99	433.2287	3	1296.667	.	Y	Superscaf	57910453	0	BTP202209	BTP202209	0	BTP202209	0	Mixed
CDS|exon|ID=cds-Bg	9	5.604444	3710	0.041832	ADKLAEEHS	95	500.2395	2	998.4669	.	Y	Superscaf	57910453	0	0	0	0	0	BTP202209	Tissue specific
CDS|exon|ID=cds-Bg	6	7.388333	3151.5	0.041292	HRSKDH	64	390.2066	2	778.3834	.	W	Superscaf	57943266	0	0	0	0	0	BTP202209	Tissue specific
CDS|exon|ID=cds-Bg	5	6.112	1580.7	0.039138	GPAGP	66	398.2064	1	397.1961	.	C	Superscaf	58073317	0	0	0	0	BTP202209	0	Tissue specific
CDS|exon|ID=cds-Bg	10	5.046	3105.2	0.036	VEEAAKVTC	97	516.2881	2	1030.566	.	R	Superscaf	58262905	0	0	0	0	0	BTP202209	Tissue specific
CDS|exon|ID=cds-Bg	8	5.59125	2611.9	0.03595	A(+42.01)	50	396.2056	2	790.3973	Acetylati	V	Superscaf	58265686	BTP202209	0	0	0	0	0	Tissue specific
CDS|exon|ID=cds-Bg	11	5.868182	4220.6	0.031111	A(+42.01)	97	437.58	3	1309.724	Acetylati	A	Superscaf	58558471	0	0	0	0	BTP202209	BTP202209	Mixed

CDS|exon|ID=cds-Bg	10	5.481	3752.2	0.031111	A(+42.01)	99	591.8195	2	1181.629	Acetylati	A	Superscaf	58558471	0	0	0	BTP202209	0	0	Tissue specific
CDS|exon|ID=cds-Bg	9	5.425556	3398.8	0.031111	A(+42.01)	99	535.2789	2	1068.545	Acetylati	A	Superscaf	58558471	0	0	0	0	BTP202209	BTP202209	Tissue enhanced
CDS|exon|ID=cds-Bg	11	5.64	3850.3	0.029252	LLVTLPAAL	99	605.861	2	1209.712	.	S	Superscaf	58670737	0	0	0	0	0	BTP202209	Tissue specific
CDS|exon|ID=cds-Bg	10	5.606	3496.9	0.029252	LVTLPAALF	95	549.3195	2	1096.628	.	S	Superscaf	58670737	0	0	0	0	0	BTP202209	Tissue specific
CDS|exon|ID=cds-Bg	6	5.616667	2434.8	0.029077	MGGGC(+57	51	641.1882	1	640.1768	Carbamido	R	Superscaf	58681432	0	0	0	BTP202209	0	0	Tissue specific
CDS|exon|ID=cds-Bg	10	4.763	3130.3	0.011937	KGYLVSVDG	63	550.7806	2	1099.555	.	N	Superscaf	61160146	0	0	0	0	0	BTP202209	Tissue specific
CDS|exon|ID=cds-Bg	9	6.385556	3660.1	0.012572	A(+42.01)	99	504.2833	2	1006.556	Acetylati	Q	Superscaf	61198444	0	0	BTP202209	0	0	0	Tissue specific
CDS|exon|ID=cds-Bg	7	6.895714	2881.2	0.012572	A(+42.01)	98	383.2204	2	764.4293	Acetylati	Q	Superscaf	61198444	0	0	BTP202209	0	0	0	Tissue specific
CDS|exon|ID=cds-Bg	8	6.78375	3546	0.012993	Q(+42.01)	70	446.2235	2	890.4359	Acetylati	S	Superscaf	61224073	0	0	0	0	BTP202209	0	Tissue specific
CDS|exon|ID=cds-Bg	10	4.513	3414.9	0.052758	PEFLEDPSV	81	573.2873	2	1144.565	.	G	Superscaf	63627484	0	0	0	0	0	BTP202209	Tissue specific
CDS|exon|ID=cds-Bg	16	5.63	5996.5	0.052758	PEFLEDPSV	99	625.0129	3	1872.024	.	G	Superscaf	63627484	0	0	0	0	BTP202209	BTP202209	Mixed
CDS|exon|ID=cds-Bg	15	5.356	5528.1	0.052758	PEFLEDPSV	97	582.3156	3	1743.929	.	G	Superscaf	63627484	0	0	0	0	BTP202209	BTP202209	Mixed
CDS|exon|ID=cds-Bg	14	5.311429	5174.7	0.052758	PEFLEDPSV	94	544.6211	3	1630.845	.	G	Superscaf	63627484	0	BTP202209	0	0	BTP202209	0	Mixed
CDS|exon|ID=cds-Bg	12	5.116667	4280.8	0.052758	PEFLEDPSV	99	687.8594	2	1373.708	.	G	Superscaf	63627484	0	0	BTP202209	0	BTP202209	0	Mixed
CDS|exon|ID=cds-Bg	11	4.696364	3812.4	0.052758	PEFLEDPSV	54	623.8194	2	1245.613	.	G	Superscaf	63627484	0	0	0	0	BTP202209	0	Tissue specific
CDS|exon|ID=cds-Bg	10	5.188	3517.9	0.052758	FLEDPSVLT	92	574.8115	2	1147.613	.	G	Superscaf	63627484	0	0	0	0	BTP202209	0	Tissue specific
CDS|exon|ID=cds-Bg	9	5.155556	3130.5	0.052758	LEDPSVLTK	98	501.277	2	1000.544	.	G	Superscaf	63627484	0	0	0	0	BTP202209	0	Tissue specific
CDS|exon|ID=cds-Bg	11	5.396364	4024.4	0.052758	EDPSVLTKE	88	629.8444	2	1257.682	.	G	Superscaf	63627484	0	0	0	0	BTP202209	0	Tissue specific
CDS|exon|ID=cds-Bg	8	5.0525	2777.1	0.052758	EDPSVLTK	94	444.7358	2	887.46	.	G	Superscaf	63627484	0	0	BTP202209	0	0	0	Tissue specific
CDS|exon|ID=cds-Bg	12	6.938333	5568.5	0.05276	LQHLTARNF	59	354.7104	4	1414.816	.	G	Superscaf	63627484	0	0	0	0	0	BTP202209	Tissue specific
CDS|exon|ID=cds-Bg	11	4.870909	4367.9	0.052882	SNEDLLDQL	96	637.3324	2	1272.656	.	E	Superscaf	63634909	0	0	0	0	BTP202209	0	Tissue specific
CDS|exon|ID=cds-Bg	9	4.721111	3387.8	0.052882	EDLLDQLVK	98	536.7956	2	1071.581	.	E	Superscaf	63634909	0	0	0	0	0	BTP202209	Tissue specific
CDS|exon|ID=cds-Bg	7	6.571429	3529.1	0.052956	NTAQLQR	95	415.73	2	829.4406	.	S	Superscaf	63639540	0	0	0	BTP202209	0	0	Tissue specific
CDS|exon|ID=cds-Bg	9	5.815556	3598.1	0.053154	GRPLELSDF	74	517.2673	2	1032.524	.	V	Superscaf	63651388	0	0	0	0	BTP202209	0	Tissue specific
CDS|exon|ID=cds-Bg	10	6.575	4147.6	0.053807	ANTLRDAAF	98	528.7917	2	1055.572	.	E	Superscaf	63690799	0	0	0	0	0	BTP202209	Tissue specific
CDS|exon|ID=cds-Bg	8	5.97125	3711.2	0.091283	ELERLQKE	96	522.7866	2	1043.561	.	H	Superscaf	65955802	0	0	0	0	BTP202209	0	Tissue specific
CDS|exon|ID=cds-Bg	12	4.4275	3092.4	0.09605	FVALPGVGV	89	595.3354	2	1188.658	.	K	Superscaf	66243853	0	BTP202209	0	BTP202209	0	0	Mixed
CDS|exon|ID=cds-Bg	9	3.968889	1984.1	0.09605	FVALPGVGV	61	858.5061	1	857.501	.	K	Superscaf	66243853	0	BTP202209	BTP202209	BTP202209	0	0	Mixed
CDS|exon|ID=cds-Bg	8	4.45625	2040.3	0.09605	PGVGVSML	51	759.4059	1	758.3997	.	K	Superscaf	66243853	0	BTP202209	0	BTP202209	0	0	Mixed
CDS|exon|ID=cds-Bg	18	4.174444	6180.1	0.134024	DDEVDVDGT	97	989.4445	2	1976.881	.	E	Superscaf	68539090	0	0	BTP202209	BTP202209	BTP202209	0	Mixed
CDS|exon|ID=cds-Bg	16	3.66875	5147.9	0.134024	DDEVDVDGT	81	867.8802	2	1733.748	.	E	Superscaf	68539090	0	BTP202209	0	0	0	BTP202209	Mixed
CDS|exon|ID=cds-Bg	15	4.398667	4931.6	0.134024	VDVDGTVEE	99	809.8986	2	1617.784	.	E	Superscaf	68539090	0	BTP202209	0	BTP202209	BTP202209	0	Mixed
CDS|exon|ID=cds-Bg	14	3.944286	4282.8	0.134024	VDVDGTVEE	98	731.8475	2	1461.683	.	E	Superscaf	68539090	0	0	0	BTP202209	0	0	Tissue specific
CDS|exon|ID=cds-Bg	13	3.810769	3899.4	0.134024	VDVDGTVEE	99	688.3324	2	1374.651	.	E	Superscaf	68539090	0	BTP202209	0	0	0	BTP202209	Mixed
CDS|exon|ID=cds-Bg	13	4.846923	4520.1	0.134024	VDGTVEEDL	95	468.9021	3	1403.689	.	E	Superscaf	68539090	0	0	0	BTP202209	BTP202209	0	Tissue enhanced
CDS|exon|ID=cds-Bg	11	4.233636	3487.9	0.134024	VDGTVEEDL	97	581.2817	2	1160.556	.	E	Superscaf	68539090	0	0	0	0	BTP202209	BTP202209	Mixed
CDS|exon|ID=cds-Bg	11	5.458182	4108.6	0.134025	GTVEEDLGK	97	595.8	2	1189.594	.	E	Superscaf	68539090	0	0	0	BTP202209	0	BTP202209	Tissue enhanced
CDS|exon|ID=cds-Bg	17	5.169412	6508.3	0.134053	SLTDENALA	96	902.4484	2	1802.89	.	I	Superscaf	68540839	0	0	0	0	0	BTP202209	Tissue specific
CDS|exon|ID=cds-Bg	15	5.081333	5771.5	0.134053	TDENALAGN	93	802.3904	2	1602.774	.	I	Superscaf	68540839	0	0	0	0	0	BTP202209	Tissue specific
CDS|exon|ID=cds-Bg	14	4.977857	5374	0.134053	DENALAGNE	89	751.8686	2	1501.726	.	I	Superscaf	68540839	0	0	0	0	0	BTP202209	Tissue specific
CDS|exon|ID=cds-Bg	9	5.121111	3275.6	0.134053	AGNEELTVK	99	480.7513	2	959.4924	.	I	Superscaf	68540839	0	0	0	0	0	BTP202209	Tissue specific
CDS|exon|ID=cds-Bg	15	4.878	4891.7	0.134072	VTDTGVGMT	98	817.9207	2	1633.835	.	C	Superscaf	68542000	0	0	0	0	0	BTP202209	Tissue specific
CDS|exon|ID=cds-Bg	14	4.530714	4423.3	0.134072	VTDTGVGMT	95	753.8732	2	1505.74	.	C	Superscaf	68542000	0	0	0	0	0	BTP202209	Tissue specific
CDS|exon|ID=cds-Bg	11	4.93	3644.4	0.134072	VTDTGVGMT	96	583.2776	2	1164.544	.	C	Superscaf	68542000	0	0	0	0	0	BTP202209	Tissue specific
CDS|exon|ID=cds-Bg	12	4.741667	4025.8	0.134072	DTGVGMTRE	73	653.8173	2	1305.624	.	C	Superscaf	68542000	0	0	0	0	0	BTP202209	Tissue specific
CDS|exon|ID=cds-Bg	10	4.74	3216.8	0.134072	GVGMTREEL	81	545.7792	2	1089.549	.	C	Superscaf	68542000	0	0	0	0	0	BTP202209	Tissue specific
CDS|exon|ID=cds-Bg	9	5.965556	3693	0.134073	SGTSEFLNK	96	491.7438	2	981.4767	.	C	Superscaf	68542000	0	0	0	0	BTP202209	BTP202209	Mixed
CDS|exon|ID=cds-Bg	7	5.505714	2627.9	0.134073	SGTSEFL	85	740.3436	1	739.3388	.	C	Superscaf	68542000	0	0	0	0	0	BTP202209	Tissue specific
CDS|exon|ID=cds-Bg	8	6.00125	3309.6	0.134073	GTSEFLNK	88	448.2277	2	894.4446	.	C	Superscaf	68542000	0	0	0	0	0	BTP202209	Tissue specific
CDS|exon|ID=cds-Bg	9	4.721111	3238.5	0.13416	VLKEEASDY	94	527.2622	2	1052.502	.	M	Superscaf	68547319	0	0	0	0	0	BTP202209	Tissue specific
CDS|exon|ID=cds-Bg	10	4.344	3535	0.13419	VEKTVWDWE	88	652.8274	2	1303.645	.	L	Superscaf	68549155	0	0	0	0	0	BTP202209	Tissue specific
CDS|exon|ID=cds-Bg	8	4.28	2756.1	0.13419	VEKTVWDW	92	531.764	2	1061.518	.	L	Superscaf	68549155	0	0	0	0	0	BTP202209	Tissue specific
CDS|exon|ID=cds-Bg	9	5.19	3110.4	0.134221	TAEGEVTFK	99	491.2465	2	980.4814	.	E	Superscaf	68551012	0	0	0	0	0	BTP202209	Tissue specific
CDS|exon|ID=cds-Bg	8	5.0225	2712.9	0.134221	AEGEVTFK	97	440.723	2	879.4338	.	E	Superscaf	68551012	0	0	0	0	0	BTP202209	Tissue specific
CDS|exon|ID=cds-Bg	8	5.7925	3320.8	0.134222	PRGLFDEY	68	498.7415	2	995.4712	.	E	Superscaf	68551012	0	0	BTP202209	0	0	BTP202209	Mixed
CDS|exon|ID=cds-Bg	8	4.41125	2847.3	0.134228	SDDLPLNV	92	872.4324	1	871.4287	.	R	Superscaf	68551408	0	0	0	BTP202209	0	BTP202209	Mixed
CDS|exon|ID=cds-Bg	10	5.761	4553	0.134236	DEKYNDTFW	93	673.3035	2	1344.599	.	V	Superscaf	68551933	0	0	0	0	0	BTP202209	Tissue specific
CDS|exon|ID=cds-Bg	6	6.003333	2788.1	0.134237	NDTFWK	96	405.691	2	809.3708	.	V	Superscaf	68551933	0	0	0	0	0	BTP202209	Tissue specific
CDS|exon|ID=cds-Bg	12	5.981667	4635	0.134244	ESSPFVERL	90	716.9094	2	1431.809	.	A	Superscaf	68552470	0	0	0	0	0	BTP202209	Tissue specific
CDS|exon|ID=cds-Bg	8	4.235	2810.3	0.134245	LTEPVDEY	93	965.4434	1	964.4389	.	A	Superscaf	68552470	0	0	0	0	0	BTP202209	Tissue specific
CDS|exon|ID=cds-Bg	11	5.635455	4214.5	0.134246	RFQNVAKEG	83	425.9086	3	1274.71	.	A	Superscaf	68552470	0	0	0	0	0	BTP202209	Tissue specific
CDS|exon|ID=cds-Bg	10	4.64	3559.9	0.134247	EAVEKEFEF	77	595.7983	2	1189.587	.	A	Superscaf	68552470	0	0	0	0	BTP202209	0	Tissue specific
CDS|exon|ID=cds-Bg	8	5.59125	3147.6	0.134257	LTESPC(+5	83	890.4243	1	889.4215	Carbamido	L	Superscaf	68553196	0	0	0	0	0	BTP202209	Tissue specific
CDS|exon|ID=cds-Bg	14	3.733571	4411	0.134287	EDEDDKTVS	70	767.8572	2	1533.705	.	L	Superscaf	68555059	0	0	0	0	0	BTP202209	Tissue specific
CDS|exon|ID=cds-Bg	15	4.532667	5431.3	0.151212	A(+42.01)	91	910.9185	2	1819.826	Acetylati	P	Superscaf	69577954	0	0	0	0	0	BTP202209	Tissue specific
CDS|exon|ID=cds-Bg	5	2.926	1148.3	0.174104	VSLDV	65	532.2959	1	531.2904	.	V	Superscaf	70961467	0	BTP202209	0	0	0	0	Tissue specific
CDS|exon|ID=cds-Bg	8	6.75	2790.8	0.177986	KVPLTGKK	76	435.7909	2	869.5698	.	D	Superscaf	71195890	0	0	0	0	0	BTP202209	Tissue specific
CDS|exon|ID=cds-Bg	9	6.384444	3511	0.183283	TQTLRPGVK	75	500.2988	2	998.5873	.	F	Superscaf	71515342	0	0	0	0	0	BTP202209	Tissue specific
CDS|exon|ID=cds-Bg	5	4.36	1453.7	0.191463	EVPLP	82	554.3166	1	553.3112	.	R	Superscaf	72010704	0	0	BTP202209	BTP202209	0	0	Mixed
CDS|exon|ID=cds-Bg	7	5.37	2461.8	0.200115	LVELSGR	79	773.4482	1	772.4443	.	G	Superscaf	72533569	0	0	0	0	0	BTP202209	Tissue specific
CDS|exon|ID=cds-Bg	9	5.48	3496	0.232873	M(+42.01)	69	548.2603	2	1094.511	Acetylati	F	Superscaf	74513443	0	0	0	0	0	BTP202209	Tissue specific
CDS|exon|ID=cds-Bg	12	5.944167	5440.3	0.234209	DHDPLQAPE	96	476.5707	3	1426.695	.	I	Superscaf	74593819	0	0	0	0	BTP202209	0	Tissue specific
CDS|exon|ID=cds-Bg	6	6.28	2673.1	0.23636	APQDLR	62	350.1974	2	698.3711	.	G	Superscaf	74724115	0	0	0	0	BTP202209	0	Tissue specific
CDS|exon|ID=cds-Bg	9	3.163333	2440.8	0.245069	TVFQVVVEF	78	529.2872	2	1056.56	.	L	Superscaf	75250411	0	BTP202209	0	0	0	0	Tissue specific
CDS|exon|ID=cds-Bg	8	6.67375	3260.3	0.373015	ALKDKLEK	99	472.7911	2	943.5702	.	E	Superscaf	82983567	0	0	0	0	BTP202209	BTP202209	Mixed
CDS|exon|ID=cds-Bg	10	4.934	3712.3	0.391599	PLSEVRDLD	80	595.8044	2	1189.598	.	W	Superscaf	84106624	0	BTP202209	0	0	0	0	Tissue specific
CDS|exon|ID=cds-Bg	7	6.167143	2492.5	0.396388	V(+42.01)	82	449.7624	2	897.496	Acetylati	Q	Superscaf	84396195	0	0	0	0	0	BTP202209	Tissue specific
CDS|exon|ID=cds-Bg	17	4.851176	4767.9	0.417938	T(+42.01)	71	838.9614	2	1675.915	Acetylati	G	Superscaf	85698583	0	0	BTP202209	0	0	0	Tissue specific
CDS|exon|ID=cds-Bg	12	4.613333	2790.7	0.417938	KLVVVGAGG	99	542.3461	2	1082.681	.	G	Superscaf	85698583	0	0	0	0	0	BTP202209	Tissue specific
CDS|exon|ID=cds-Bg	11	4.147273	2322.3	0.417938	LVVVGAGGV	96	478.2986	2	954.5862	.	G	Superscaf	85698583	0	0	0	0	0	BTP202209	Tissue specific
CDS|exon|ID=cds-Bg	10	3.964	1968.9	0.417938	VVVGAGGVG	99	421.7567	2	841.5021	.	G	Superscaf	85698583	0	0	0	0	0	BTP202209	Tissue specific
CDS|exon|ID=cds-Bg	9	4.404444	1968.9	0.417938	VVGAGGVGK	98	372.2226	2	742.4337	.	G	Superscaf	85698583	0	0	0	0	0	BTP202209	Tissue specific
CDS|exon|ID=cds-Bg	7	6.141429	2602.8	0.421237	KDLASPP	62	727.4065	1	726.3912	.	I	Superscaf	85897918	0	0	0	BTP202209	0	0	Tissue specific
CDS|exon|ID=cds-Bg	7	4.455714	2565	0.467369	M(+42.01)	74	436.6935	2	871.3746	Acetylati	S	Superscaf	88686127	0	BTP202209	0	0	0	0	Tissue specific
CDS|exon|ID=cds-Bg	12	6	4764.9	0.48037	KLKDEEVAG	99	476.9503	3	1427.835	.	R	Superscaf	89471734	0	0	0	0	0	BTP202209	Tissue specific
CDS|exon|ID=cds-Bg	17	4.496471	6598.6	0.586621	T(+42.01)	88	1005.424	2	2008.838	Acetylati	I	Superscaf	95893588	0	BTP202209	0	0	0	0	Tissue specific
CDS|exon|ID=cds-Bg	11	5.032727	4173.7	0.586621	T(+42.01)	88	654.3073	2	1306.604	Acetylati	I	Superscaf	95893588	0	0	0	0	0	BTP202209	Tissue specific
CDS|exon|ID=cds-Bg	9	5.435556	3322.7	0.586621	T(+42.01)	86	525.2643	2	1048.519	Acetylati	I	Superscaf	95893588	0	0	0	BTP202209	BTP202209	0	Mixed
CDS|exon|ID=cds-Bg	12	5.574167	4175	0.586637	PTAPNVTVT	81	635.3601	2	1268.709	.	N	Superscaf	95894533	0	0	0	0	BTP202209	0	Tissue specific
CDS|exon|ID=cds-Bg	8	4.22375	2429.6	0.586671	VLKEGVEY	86	468.7537	2	935.4963	.	E	Superscaf	95896582	0	0	0	0	0	BTP202209	Tissue specific
CDS|exon|ID=cds-Bg	8	5.76125	2795.2	0.586744	MVGSYGPR	90	433.712	2	865.4116	.	A	Superscaf	95900992	0	0	0	0	0	BTP202209	Tissue specific
CDS|exon|ID=cds-Bg	12	6.531667	4099.6	0.589178	KPPGPPPGF	84	567.8178	2	1133.623	.	K	Superscaf	96048034	0	0	0	BTP202209	0	0	Tissue specific
CDS|exon|ID=cds-Bg	9	6.35	3704.1	0.59303	TSSSRLSFS	93	486.2425	2	970.472	.	F	Superscaf	96280837	0	0	0	0	BTP202209	0	Tissue specific
CDS|exon|ID=cds-Bg	9	4.575556	3047.4	0.652978	VHKSVMEEL	77	536.2761	2	1070.543	.	R	Superscaf	99904126	0	0	0	0	0	BTP202209	Tissue specific
CDS|exon|ID=cds-Bg	9	6.3	3612.8	0.673436	STQFGGYAK	98	479.7335	2	957.4556	.	K	Superscaf	1.01E+08	0	0	0	0	BTP202209	0	Tissue specific
CDS|exon|ID=cds-Bg	7	5.737143	2646.9	0.673844	KELDLGP	69	771.4218	1	770.4174	.	L	Superscaf	1.01E+08	0	0	BTP202209	0	0	0	Tissue specific
CDS|exon|ID=cds-Bg	10	5.408	3969.5	0.683199	SLC(+57.0	77	566.7899	2	1131.571	Carbamido	I	Superscaf	1.02E+08	0	0	0	0	0	BTP202209	Tissue specific
CDS|exon|ID=cds-Bg	8	4.865	2732.2	0.683298	FVELTMPY	74	500.2446	2	998.4783	.	T	Superscaf	1.02E+08	0	0	0	0	0	BTP202209	Tissue specific
CDS|exon|ID=cds-Bg	7	4.777143	2344.8	0.683298	VELTMPY	82	852.4142	1	851.4099	.	T	Superscaf	1.02E+08	0	0	0	0	0	BTP202209	Tissue specific
CDS|exon|ID=cds-Bg	6	5.531667	2417.6	0.683582	KDEFPF	85	782.3685	1	781.3646	.	G	Superscaf	1.02E+08	0	0	0	0	0	BTP202209	Tissue specific
CDS|exon|ID=cds-Bg	7	6.104286	2701.1	0.717181	T(+42.01)	57	725.3594	1	724.3392	Acetylati	G	Superscaf	1.04E+08	0	0	BTP202209	0	0	0	Tissue specific
CDS|exon|ID=cds-Bg	7	6.744286	2452.7	0.724577	KPPGPPP	99	689.3955	1	688.3907	.	R	Superscaf	1.04E+08	0	0	0	BTP202209	0	0	Tissue specific
CDS|exon|ID=cds-Bg	7	5.371429	2191.3	0.731089	AAPEVPK	98	356.2048	2	710.3962	.	P	Superscaf	1.05E+08	BTP202209	0	0	0	0	0	Tissue specific
CDS|exon|ID=cds-Bg	10	4.329	3468.9	0.735657	VLSGVHELE	85	555.7917	2	1109.572	.	G	Superscaf	1.05E+08	0	0	0	0	BTP202209	0	Tissue specific
CDS|exon|ID=cds-Bg	7	6.421429	3088.5	0.74218	KDYREPP	50	452.7209	2	903.445	.	H	Superscaf	1.05E+08	0	0	BTP202209	0	0	0	Tissue specific
CDS|exon|ID=cds-Bg	7	5.314286	2014.3	0.751778	LPPPVAP	89	690.416	1	689.4112	.	K	Superscaf	1.06E+08	0	0	BTP202209	0	0	0	Tissue specific
CDS|exon|ID=cds-Bg	12	4.705	4731.3	0.786544	NADEFENMV	95	698.8035	2	1395.598	.	C	Superscaf	1.08E+08	0	0	0	0	0	BTP202209	Tissue specific
CDS|exon|ID=cds-Bg	7	4.564286	2036.3	0.791488	GVRVELA	59	372.2227	2	742.4337	.	R	Superscaf	1.08E+08	0	0	0	BTP202209	0	0	Tissue specific
CDS|exon|ID=cds-Bg	13	5.393077	4710.2	0.792021	VDAGSGSPE	95	618.2847	2	1234.558	.	S	Superscaf	1.08E+08	0	BTP202209	0	0	0	0	Tissue specific
CDS|exon|ID=cds-Bg	6	4.265	1923.1	0.792375	D(+42.01)	59	769.3734	1	768.3694	Acetylati	G	Superscaf	1.08E+08	0	0	0	0	BTP202209	0	Tissue specific
CDS|exon|ID=cds-Bg	9	6.547778	3892.4	0.814033	APALQTAQF	66	478.2682	2	954.5247	.	A	Superscaf	1.1E+08	BTP202209	0	0	0	0	0	Tissue specific
CDS|exon|ID=cds-Bg	9	6.015556	3124.6	0.815748	AVPRASPTT	84	450.2496	2	898.4872	.	G	Superscaf	1.1E+08	BTP202209	0	0	0	0	0	Tissue specific
CDS|exon|ID=cds-Bg	8	6.015	2813.3	0.815748	VPRASPTT	99	414.7314	2	827.4501	.	G	Superscaf	1.1E+08	BTP202209	0	0	0	0	0	Tissue specific
CDS|exon|ID=cds-Bg	7	6.122857	2793.1	0.820005	SLRFDGA	98	383.1961	2	764.3817	.	P	Superscaf	1.1E+08	0	0	0	BTP202209	0	0	Tissue specific
CDS|exon|ID=cds-Bg	11	4.758182	3080.5	0.820077	PPTVVPGGD	68	1022.548	1	1021.544	.	S	Superscaf	1.1E+08	0	0	0	0	BTP202209	BTP202209	Mixed
CDS|exon|ID=cds-Bg	9	4.772222	2476.6	0.820077	VVPGGDLAK	99	428.2488	2	854.4861	.	S	Superscaf	1.1E+08	0	0	0	0	BTP202209	0	Tissue specific
CDS|exon|ID=cds-Bg	13	6.512308	5576.1	0.820079	ARLDHKFDL	76	402.7131	4	1606.829	.	S	Superscaf	1.1E+08	0	0	0	0	0	BTP202209	Tissue specific
CDS|exon|ID=cds-Bg	8	6.6125	3335.7	0.820079	DLMYAKRA	86	484.2542	2	966.4957	.	S	Superscaf	1.1E+08	0	0	0	BTP202209	0	0	Tissue specific
CDS|exon|ID=cds-Bg	10	4.327	3422.9	0.820079	VHWYVGEGM	76	603.7648	2	1205.518	.	S	Superscaf	1.1E+08	0	0	BTP202209	BTP202209	0	0	Mixed
CDS|exon|ID=cds-Bg	9	4.664444	2726.9	0.820676	YDSLVVGKL	99	497.2841	2	992.5542	.	A	Superscaf	1.1E+08	0	BTP202209	0	0	0	0	Tissue specific
CDS|exon|ID=cds-Bg	9	5.735556	3254.7	0.820701	LGPSLLTEL	92	942.548	1	941.5433	.	Q	Superscaf	1.1E+08	0	0	0	BTP202209	0	0	Tissue specific
CDS|exon|ID=cds-Bg	9	5.341111	3561	0.821901	AGQHDLVPE	78	487.2448	2	972.4777	.	P	Superscaf	1.1E+08	0	0	0	0	BTP202209	0	Tissue specific
CDS|exon|ID=cds-Bg	8	6.54625	3605	0.822102	EFKFPNRL	86	525.788	2	1049.566	.	V	Superscaf	1.1E+08	0	0	0	0	0	BTP202209	Tissue specific
CDS|exon|ID=cds-Bg	7	6.627143	3251.6	0.822102	EFKFPNR	94	469.2463	2	936.4817	.	V	Superscaf	1.1E+08	0	0	0	0	BTP202209	0	Tissue specific
CDS|exon|ID=cds-Bg	8	5.97625	2910	0.822103	LSAGGDFK	99	397.7044	2	793.397	.	V	Superscaf	1.1E+08	0	0	0	0	0	BTP202209	Tissue specific
CDS|exon|ID=cds-Bg	8	6.6525	3163.6	0.822774	QPGGGPRP	57	383.2009	2	764.3929	.	E	Superscaf	1.1E+08	0	0	0	BTP202209	0	0	Tissue specific
CDS|exon|ID=cds-Bg	15	6.698667	6175.8	0.823831	T(+42.01)	98	800.9158	2	1599.822	Acetylati	A	Superscaf	1.1E+08	0	BTP202209	0	0	0	0	Tissue specific
CDS|exon|ID=cds-Bg	14	6.747143	5864.5	0.823831	T(+42.01)	99	765.3979	2	1528.784	Acetylati	A	Superscaf	1.1E+08	0	BTP202209	0	0	0	0	Tissue specific
CDS|exon|ID=cds-Bg	13	6.438462	5215.7	0.823831	T(+42.01)	99	687.3469	2	1372.683	Acetylati	A	Superscaf	1.1E+08	0	BTP202209	0	0	BTP202209	BTP202209	Mixed
CDS|exon|ID=cds-Bg	12	6.163333	4747.3	0.823831	T(+42.01)	94	623.2993	2	1244.588	Acetylati	A	Superscaf	1.1E+08	0	0	0	0	0	BTP202209	Tissue specific
CDS|exon|ID=cds-Bg	12	6.430833	4818.2	0.823831	ENSTSTPAA	99	615.817	2	1229.625	.	A	Superscaf	1.1E+08	0	0	0	0	0	BTP202209	Tissue specific
CDS|exon|ID=cds-Bg	11	6.237273	4712.2	0.823832	KSTDHPKYS	88	654.7961	2	1307.582	.	A	Superscaf	1.1E+08	0	0	0	0	0	BTP202209	Tissue specific

CDS|exon|ID=cds-Bg	12	6.1175	4392.9	0.823834	RLVTTGVLK	67	448.6165	3	1342.83	.	A	Superscaf	1.1E+08	0	BTP20220	0	0	0	0	Tissue specific
CDS|exon|ID=cds-Bg	11	5.643636	3587.8	0.823835	TGVLKQTKG	98	544.3267	2	1086.64	.	A	Superscaf	1.1E+08	0	BTP20220	BTP20220	0	0	0	Mixed
CDS|exon|ID=cds-Bg	15	6.666667	6219.7	0.823835	ASGSFRLAK	97	412.9733	4	1647.869	.	A	Superscaf	1.1E+08	0	BTP20220	0	0	0	0	Tissue specific
CDS|exon|ID=cds-Bg	9	6.814444	3544.7	0.823835	ASGSFRLAK	93	468.7657	2	935.5188	.	A	Superscaf	1.1E+08	0	BTP20220	0	0	BTP20220	BTP20220	Mixed
CDS|exon|ID=cds-Bg	8	6.44875	3076.3	0.823835	ASGSFRLA	98	404.7184	2	807.4239	.	A	Superscaf	1.1E+08	0	BTP20220	0	0	0	0	Tissue specific
CDS|exon|ID=cds-Bg	8	6.91375	3233.4	0.823835	SGSFRLAK	99	433.2467	2	864.4817	.	A	Superscaf	1.1E+08	0	0	0	0	BTP20220	0	Tissue specific
CDS|exon|ID=cds-Bg	10	5.585	3757.1	0.823836	SDEPKRSVA	79	568.3004	2	1134.567	.	A	Superscaf	1.1E+08	0	BTP20220	0	0	0	0	Tissue specific
CDS|exon|ID=cds-Bg	7	3.895714	1799.1	0.82604	APVMVEL	92	758.4088	1	757.4044	.	S	Superscaf	1.1E+08	0	0	0	BTP20220	0	0	Tissue specific
CDS|exon|ID=cds-Bg	6	5.616667	2479.9	0.828773	Q(+42.01)	65	379.6806	2	757.3541	Acetylati	P	Superscaf	1.11E+08	0	0	0	BTP20220	0	0	Tissue specific
CDS|exon|ID=cds-Bg	19	5.922632	6534.9	0.835314	A(+42.01)	98	587.9818	3	1760.931	Acetylati	T	Superscaf	1.11E+08	0	0	0	0	0	BTP20220	Tissue specific
CDS|exon|ID=cds-Bg	16	5.656875	5375.7	0.835314	A(+42.01)	85	712.3534	2	1422.699	Acetylati	T	Superscaf	1.11E+08	0	0	0	0	0	BTP20220	Tissue specific
CDS|exon|ID=cds-Bg	11	6.058182	3490.6	0.835314	A(+42.01)	84	454.7435	2	907.4763	Acetylati	T	Superscaf	1.11E+08	0	0	0	0	0	BTP20220	Tissue specific
CDS|exon|ID=cds-Bg	14	5.887857	4978.4	0.835314	GPGAPLSAD	98	682.8701	2	1363.735	.	T	Superscaf	1.11E+08	0	0	0	0	0	BTP20220	Tissue specific
CDS|exon|ID=cds-Bg	7	5.744286	2660.9	0.835315	ADELLPK	73	393.2214	2	784.433	.	T	Superscaf	1.11E+08	0	0	0	0	BTP20220	BTP20220	Tissue enhanced
CDS|exon|ID=cds-Bg	7	5.63	2666.9	0.838929	G(+42.01)	88	833.403	1	832.3967	Acetylati	G	Superscaf	1.11E+08	BTP20220	0	0	0	0	0	Tissue specific
CDS|exon|ID=cds-Bg	11	4.775455	3760	0.839041	AHGSVVKSE	99	596.2841	2	1190.557	.	I	Superscaf	1.11E+08	0	BTP20220	BTP20220	0	BTP20220	BTP20220	Mixed
CDS|exon|ID=cds-Bg	10	4.651	3448.7	0.839041	HGSVVKSED	70	560.7661	2	1119.52	.	I	Superscaf	1.11E+08	0	BTP20220	0	0	BTP20220	BTP20220	Mixed
CDS|exon|ID=cds-Bg	9	4.324444	2829	0.839041	GSVVKSEDY	72	492.2363	2	982.4607	.	I	Superscaf	1.11E+08	0	0	0	0	0	BTP20220	Tissue specific
CDS|exon|ID=cds-Bg	8	6.2175	2619	0.848415	GPPPPPPG	91	715.3749	1	714.3701	.	R	Superscaf	1.12E+08	0	BTP20220	0	BTP20220	0	0	Mixed
CDS|exon|ID=cds-Bg	8	8.04	3837.1	0.859476	KRAQSKGR	69	465.7793	2	929.5519	.	L	Superscaf	1.12E+08	0	0	0	0	BTP20220	0	Tissue specific
CDS|exon|ID=cds-Bg	11	4.93	3544	0.865991	A(+42.01)	73	538.7296	2	1075.449	Acetylati	R	Superscaf	1.13E+08	0	0	0	0	0	BTP20220	Tissue specific
CDS|exon|ID=cds-Bg	18	5.530556	6295.9	0.865991	A(+42.01)	94	846.3794	2	1690.747	Acetylati	R	Superscaf	1.13E+08	0	0	0	BTP20220	0	0	Tissue specific
CDS|exon|ID=cds-Bg	13	5.09	4538.2	0.865991	A(+42.01)	88	646.2764	2	1290.54	Acetylati	R	Superscaf	1.13E+08	BTP20220	BTP20220	0	BTP20220	0	BTP20220	Mixed
CDS|exon|ID=cds-Bg	10	4.826	3246.7	0.865991	A(+42.01)	72	510.2204	2	1018.428	Acetylati	R	Superscaf	1.13E+08	0	BTP20220	0	0	0	0	Tissue specific
CDS|exon|ID=cds-Bg	9	4.731111	2863.3	0.865991	A(+42.01)	78	466.7043	2	931.3957	Acetylati	R	Superscaf	1.13E+08	BTP20220	0	0	0	0	0	Tissue specific
CDS|exon|ID=cds-Bg	7	5.784286	2448.7	0.868542	VTPQPGK	63	363.7095	2	725.4072	.	P	Superscaf	1.13E+08	0	0	0	0	0	BTP20220	Tissue specific
CDS|exon|ID=cds-Bg	7	4.844286	2778.2	0.87226	HYLPNVD	50	429.2098	2	856.4079	.	L	Superscaf	1.13E+08	0	0	0	BTP20220	0	0	Tissue specific
CDS|exon|ID=cds-Bg	9	6.352222	3434.6	0.873241	NFLATAGAK	98	446.7465	2	891.4814	.	D	Superscaf	1.13E+08	0	0	0	0	0	BTP20220	Tissue specific
CDS|exon|ID=cds-Bg	7	5.13	2112.3	0.873299	AYPGVLL	67	732.425	1	731.4218	.	G	Superscaf	1.13E+08	0	0	0	0	0	BTP20220	Tissue specific
CDS|exon|ID=cds-Bg	7	5.838571	2818.2	0.873364	FTPDLAH	53	800.4045	1	799.3864	.	N	Superscaf	1.13E+08	0	0	0	0	0	BTP20220	Tissue specific
CDS|exon|ID=cds-Bg	14	6.905714	6248.9	0.873472	RAGSALNRM	72	401.2263	4	1600.883	.	A	Superscaf	1.13E+08	0	0	0	0	0	BTP20220	Tissue specific
CDS|exon|ID=cds-Bg	7	4.924286	2686.1	0.878289	QDHPVLL	79	821.4493	1	820.4443	.	I	Superscaf	1.14E+08	0	0	BTP20220	0	0	0	Tissue specific
CDS|exon|ID=cds-Bg	7	7.24	3112.5	0.880387	TLRFYTK	56	464.7589	2	927.5178	.	V	Superscaf	1.14E+08	0	0	0	0	BTP20220	0	Tissue specific
CDS|exon|ID=cds-Bg	7	5.314286	2719	0.887696	EAPDDLK	51	394.1999	2	786.3759	.	G	Superscaf	1.14E+08	0	0	0	0	0	BTP20220	Tissue specific
CDS|exon|ID=cds-Bg	6	5.575	1914.1	0.889145	V(+42.01)	72	366.7	2	731.3887	Acetylati	T	Superscaf	1.14E+08	0	0	0	0	BTP20220	0	Tissue specific
CDS|exon|ID=cds-Bg	10	5.864	3399.8	0.910647	LGGPTSVRG	91	495.7702	2	989.5294	.	Q	Superscaf	1.15E+08	0	0	0	0	0	BTP20220	Tissue specific
CDS|exon|ID=cds-Bg	7	4.87	2307.6	0.914862	VAVSLQR	85	386.7358	2	771.4603	.	G	Superscaf	1.16E+08	0	0	0	0	0	BTP20220	Tissue specific
CDS|exon|ID=cds-Bg	5	5.934	2239.6	0.926697	A(+42.01)	51	558.2535	1	557.2446	Acetylati	A	Superscaf	1.16E+08	0	0	0	0	0	BTP20220	Tissue specific
CDS|exon|ID=cds-Bg	7	5.24	2524.8	0.928873	M(+42.01)	88	422.7144	2	843.416	Acetylati	Q	Superscaf	1.17E+08	0	BTP20220	0	0	0	0	Tissue specific
CDS|exon|ID=cds-Bg	9	5.888889	3339.5	0.941881	KAAAVHLLQ	93	475.7916	2	949.5709	.	P	Superscaf	1.17E+08	0	0	BTP20220	0	0	0	Tissue specific
CDS|exon|ID=cds-Bg	8	5.4075	2871.1	0.941882	AAAVHLLQ	98	411.7436	2	821.4759	.	P	Superscaf	1.17E+08	0	0	0	0	0	BTP20220	Tissue specific
CDS|exon|ID=cds-Bg	8	7.37375	3281.9	0.945052	RGPPPPPR	76	437.2547	2	872.498	.	W	Superscaf	1.18E+08	0	0	0	0	0	BTP20220	Tissue specific
CDS|exon|ID=cds-Bg	8	6.66625	2950.3	0.946488	PAALLRGP	55	397.7522	2	793.481	.	V	Superscaf	1.18E+08	BTP20220	0	0	0	0	0	Tissue specific
CDS|exon|ID=cds-Bg	7	3.878571	1797	0.983701	SLEVGVP	55	700.3865	1	699.3803	.	A	Superscaf	1.2E+08	BTP20220	0	0	0	0	0	Tissue specific
CDS|exon|ID=cds-Bg	5	2.52	674.8	0.987188	VVPVP	97	510.3281	1	509.3213	.	A	Superscaf	1.2E+08	BTP20220	BTP20220	BTP20220	BTP20220	BTP20220	BTP20220	Mixed
CDS|exon|ID=cds-Bg	7	4.097143	1596.8	0.997705	GVVGVLR	75	350.2234	2	698.4439	.	V	Superscaf	1.21E+08	0	BTP20220	0	0	0	0	Tissue specific
CDS|exon|ID=cds-Bg	8	6.7	2920.3	0.991011	G(+42.01)	76	403.733	2	805.4446	Acetylati	Y	Superscaf	529341	0	0	0	BTP20220	0	0	Tissue specific
CDS|exon|ID=cds-Bg	14	4.47	4582.9	0.965599	KVVKFPDED	97	558.9692	3	1673.888	.	S	Superscaf	2023324	0	BTP20220	0	0	0	BTP20220	Mixed
CDS|exon|ID=cds-Bg	13	4.064615	4114.5	0.965599	VVKFPDEDE	97	516.2701	3	1545.793	.	S	Superscaf	2023324	0	BTP20220	0	0	0	BTP20220	Mixed
CDS|exon|ID=cds-Bg	12	4.403333	4114.5	0.965599	VKFPDEDEV	98	724.3658	2	1446.724	.	S	Superscaf	2023324	0	0	0	0	BTP20220	0	Tissue specific
CDS|exon|ID=cds-Bg	11	4.803636	4114.5	0.965599	KFPDEDEVL	96	674.8328	2	1347.656	.	S	Superscaf	2023324	0	0	0	0	BTP20220	0	Tissue specific
CDS|exon|ID=cds-Bg	15	5.848667	6398.2	0.965597	SNQTGPAWA	87	819.8574	2	1637.707	.	S	Superscaf	2023324	0	0	0	0	BTP20220	0	Tissue specific
CDS|exon|ID=cds-Bg	11	5.932727	3789	0.960007	VLSKGHAAP	86	553.3375	2	1104.666	.	H	Superscaf	2352481	0	0	0	0	0	BTP20220	Tissue specific
CDS|exon|ID=cds-Bg	8	6.19	3043.3	0.960007	GHAAPLLY	62	421.2306	2	840.4493	.	H	Superscaf	2352481	0	0	0	0	BTP20220	BTP20220	Tissue enhanced
CDS|exon|ID=cds-Bg	13	5.688462	5195.6	0.93564	AGELKWGTD	92	503.9105	3	1508.715	.	A	Superscaf	3786385	0	0	0	0	0	BTP20220	Tissue specific
CDS|exon|ID=cds-Bg	12	5.705833	4808.2	0.93564	AGELKWGTD	93	681.8273	2	1361.646	.	A	Superscaf	3786385	0	0	0	0	0	BTP20220	Tissue specific
CDS|exon|ID=cds-Bg	7	5.707143	2461.8	0.850426	T(+42.01)	77	728.3812	1	727.3752	Acetylati	H	Superscaf	8799670	0	0	BTP20220	0	0	0	Tissue specific
CDS|exon|ID=cds-Bg	8	4.955	2831.3	0.822872	QPVLLTEL	53	456.7766	2	911.5328	.	E	Superscaf	10420105	0	0	0	BTP20220	0	0	Tissue specific
CDS|exon|ID=cds-Bg	8	5.72	2885.3	0.815299	SDAPPLTL	78	813.4318	1	812.428	.	L	Superscaf	10866097	BTP20220	0	BTP20220	BTP20220	0	0	Mixed
CDS|exon|ID=cds-Bg	9	5.366667	3622	0.815298	SVNSHFMKD	89	532.7438	2	1063.476	.	L	Superscaf	10866097	0	0	0	BTP20220	0	0	Tissue specific
CDS|exon|ID=cds-Bg	9	6.564444	3906.6	0.800812	A(+42.01)	95	543.7455	2	1085.481	Acetylati	Q	Superscaf	11718577	0	0	0	0	0	BTP20220	Tissue specific
CDS|exon|ID=cds-Bg	13	5.958462	5600.6	0.800812	A(+42.01)	80	797.8353	2	1593.662	Acetylati	Q	Superscaf	11718577	0	0	0	0	0	BTP20220	Tissue specific
CDS|exon|ID=cds-Bg	7	6.251429	2590.8	0.735895	KGTLEAP	55	715.4083	1	714.3912	.	K	Superscaf	15537742	0	BTP20220	0	0	0	0	Tissue specific
CDS|exon|ID=cds-Bg	9	7.325556	3869.9	0.735011	KLHFSPKSK	98	536.3163	2	1070.624	.	V	Superscaf	15589621	0	0	0	0	0	BTP20220	Tissue specific
CDS|exon|ID=cds-Bg	5	6.436	1903.1	0.708941	HGPPA	56	478.2388	1	477.2336	.	R	Superscaf	17123641	0	0	BTP20220	0	0	0	Tissue specific
CDS|exon|ID=cds-Bg	10	5.929	3767.2	0.703851	TDAETLPAL	86	529.7869	2	1057.565	.	D	Superscaf	17423053	0	0	0	0	0	BTP20220	Tissue specific
CDS|exon|ID=cds-Bg	7	5.517143	2739	0.676629	YEEVARK	95	447.7359	2	893.4606	.	S	Superscaf	19024159	0	0	0	0	0	BTP20220	Tissue specific
CDS|exon|ID=cds-Bg	10	6.054	3898.2	0.580488	ALATDQGSK	96	502.2732	2	1002.535	.	F	Superscaf	24680737	0	0	0	BTP20220	0	0	Tissue specific
CDS|exon|ID=cds-Bg	6	8.43	2948.5	0.577363	RRALPR	58	384.7488	2	767.4878	.	I	Superscaf	24864415	0	0	0	BTP20220	0	0	Tissue specific
CDS|exon|ID=cds-Bg	10	4.911	3007.5	0.572107	VTRLPVVLG	75	364.2277	3	1089.666	.	M	Superscaf	25173883	0	0	0	0	0	BTP20220	Tissue specific
CDS|exon|ID=cds-Bg	8	4.44375	2090.5	0.572107	VTRLPVVL	70	448.7988	2	895.5854	.	M	Superscaf	25173883	0	0	BTP20220	0	0	0	Tissue specific
CDS|exon|ID=cds-Bg	11	4.632727	3184.7	0.572106	SVGEGVTTV	88	551.2975	2	1100.583	.	M	Superscaf	25173883	0	0	BTP20220	0	0	0	Tissue specific
CDS|exon|ID=cds-Bg	10	4.528	2801.3	0.572106	VGEGVTTVR	98	507.7811	2	1013.551	.	M	Superscaf	25173883	0	0	BTP20220	0	0	0	Tissue specific
CDS|exon|ID=cds-Bg	8	4.91375	2504	0.572106	EGVTTVRP	93	429.7358	2	857.4606	.	M	Superscaf	25173883	0	0	BTP20220	0	0	0	Tissue specific
CDS|exon|ID=cds-Bg	14	5.510714	4784.1	0.571894	ESVPKLVTD	91	531.627	3	1591.864	.	K	Superscaf	25186351	0	0	BTP20220	0	0	0	Tissue specific
CDS|exon|ID=cds-Bg	10	4.59	3164.5	0.571894	ESVPKLVTD	73	567.8047	2	1133.597	.	K	Superscaf	25186351	0	0	BTP20220	0	0	BTP20220	Tissue enhanced
CDS|exon|ID=cds-Bg	9	4.491111	2777.1	0.571894	ESVPKLVTD	91	494.2697	2	986.5284	.	K	Superscaf	25186351	0	0	BTP20220	0	0	BTP20220	Mixed
CDS|exon|ID=cds-Bg	8	4.65	2351.6	0.571894	SVPKLVTD	99	858.4907	1	857.4858	.	K	Superscaf	25186351	0	0	BTP20220	0	0	BTP20220	Mixed
CDS|exon|ID=cds-Bg	8	4.625	2355.6	0.571894	VPKLVTDF	99	459.7666	2	917.5222	.	K	Superscaf	25186351	0	0	0	0	0	BTP20220	Tissue specific
CDS|exon|ID=cds-Bg	8	5.30875	2701	0.571894	LVTDFMAK	99	462.7443	2	923.4786	.	K	Superscaf	25186351	0	0	0	0	0	BTP20220	Tissue specific
CDS|exon|ID=cds-Bg	7	5.212857	2347.6	0.571894	VTDFM(+15	98	414.2005	2	826.3895	Oxidation	K	Superscaf	25186351	0	0	0	0	0	BTP20220	Tissue specific
CDS|exon|ID=cds-Bg	8	6.46375	3203.4	0.571894	TDFMAKKF	99	494.2503	2	986.4895	.	K	Superscaf	25186351	0	0	BTP20220	0	0	0	Tissue specific
CDS|exon|ID=cds-Bg	7	6.454286	2805.9	0.571894	DFMAKKF	99	443.7264	2	885.4418	.	K	Superscaf	25186351	0	0	BTP20220	0	0	0	Tissue specific
CDS|exon|ID=cds-Bg	9	6.36	3747.9	0.571894	AKKFSLDQL	94	525.3013	2	1048.592	.	K	Superscaf	25186351	0	0	BTP20220	0	0	0	Tissue specific
CDS|exon|ID=cds-Bg	8	6.4025	3436.6	0.571894	KKFSLDQL	79	489.7827	2	977.5546	.	K	Superscaf	25186351	0	0	BTP20220	0	0	0	Tissue specific
CDS|exon|ID=cds-Bg	8	5.6675	2955.3	0.571893	T(+42.01)	79	489.7777	2	977.5546	Acetylati	K	Superscaf	25186351	0	0	BTP20220	0	0	BTP20220	Mixed
CDS|exon|ID=cds-Bg	9	5.716667	3857.4	0.571893	NEGFDLLRS	85	525.7633	2	1049.514	.	K	Superscaf	25186351	0	0	BTP20220	0	0	0	Tissue specific
CDS|exon|ID=cds-Bg	8	5.755	3260.7	0.571893	EGFDLLRS	86	468.7414	2	935.4712	.	K	Superscaf	25186351	0	0	BTP20220	0	0	0	Tissue specific
CDS|exon|ID=cds-Bg	14	4.468571	4836.5	0.568773	EEVEVAPPK	91	545.2753	3	1632.811	.	H	Superscaf	25369846	0	0	BTP20220	0	0	0	Tissue specific
CDS|exon|ID=cds-Bg	9	4.224444	2731	0.568773	EEVEVAPPK	99	499.2621	2	996.5128	.	H	Superscaf	25369846	0	0	0	0	0	BTP20220	Tissue specific
CDS|exon|ID=cds-Bg	13	4.564615	4411	0.568773	EVEVAPPKD	98	502.2622	3	1503.768	.	H	Superscaf	25369846	0	0	BTP20220	0	0	0	Tissue specific
CDS|exon|ID=cds-Bg	8	4.35	2305.5	0.568773	EVEVAPPK	99	868.4753	1	867.4702	.	H	Superscaf	25369846	0	0	0	0	0	BTP20220	Tissue specific
CDS|exon|ID=cds-Bg	7	4.511429	1880	0.568773	VEVAPPK	97	370.2194	2	738.4276	.	H	Superscaf	25369846	0	0	BTP20220	0	0	BTP20220	Mixed
CDS|exon|ID=cds-Bg	11	5.101818	3985.5	0.568773	EVAPPKDHE	95	638.8346	2	1275.657	.	H	Superscaf	25369846	0	0	BTP20220	0	0	0	Tissue specific
CDS|exon|ID=cds-Bg	10	5.29	3560	0.568773	VAPPKDHEV	98	574.313	2	1146.615	.	H	Superscaf	25369846	0	0	BTP20220	0	0	0	Tissue specific
CDS|exon|ID=cds-Bg	7	3.684286	2241.6	0.567515	VDEFVTH	98	423.7018	2	845.3919	.	F	Superscaf	25444036	0	0	0	0	0	BTP20220	Tissue specific
CDS|exon|ID=cds-Bg	8	7.1825	3063.3	0.509768	AAPARPPK	53	404.2382	2	806.4762	.	G	Superscaf	28841347	0	BTP20220	0	0	0	0	Tissue specific
CDS|exon|ID=cds-Bg	5	6.738	1783.8	0.389096	K(+42.01)	64	543.3483	1	542.3428	Acetylati	R	Superscaf	35940886	0	0	BTP20220	0	0	0	Tissue specific
CDS|exon|ID=cds-Bg	9	7.277778	3936.3	0.386597	C(+57.02)	86	366.8856	3	1097.638	Carbamido	A	Superscaf	36087460	BTP20220	0	0	0	0	0	Tissue specific
CDS|exon|ID=cds-Bg	7	6.501429	2818.1	0.380335	KFNLPPP	65	406.7426	2	811.4592	.	D	Superscaf	36456292	0	0	0	0	BTP20220	0	Tissue specific
CDS|exon|ID=cds-Bg	9	5.966667	3877.3	0.371037	LFRFSQDSA	66	535.7649	2	1069.519	.	M	Superscaf	37003141	0	BTP20220	0	0	0	0	Tissue specific
CDS|exon|ID=cds-Bg	8	5.28375	3208.5	0.367007	KESELLDF	76	490.749	2	979.4862	.	F	Superscaf	37240369	0	0	0	0	BTP20220	0	Tissue specific
CDS|exon|ID=cds-Bg	7	5.957143	3209.5	0.314565	LQKEFLQ	53	453.2572	2	904.5018	.	N	Superscaf	40325739	0	0	BTP20220	0	0	0	Tissue specific
CDS|exon|ID=cds-Bg	6	7.151667	2650.1	0.153423	H(+42.01)	70	387.7291	2	773.4548	Acetylati	G	Superscaf	49806558	0	0	0	BTP20220	0	0	Tissue specific
CDS|exon|ID=cds-Bg	8	6.57375	2941.1	0.143254	WAAPAPPK	89	419.2316	2	836.4544	.	G	Superscaf	50404210	0	0	0	0	0	BTP20220	Tissue specific
CDS|exon|ID=cds-Bg	7	6.09	3126.7	0.079137	A(+42.01)	99	477.7107	2	953.4099	Acetylati	F	Superscaf	54176407	0	0	0	BTP20220	0	0	Tissue specific
CDS|exon|ID=cds-Bg	10	5.472	4184.8	0.079131	LGDLLETC(	98	602.7845	2	1203.559	Carbamido	F	Superscaf	54176407	0	0	0	0	0	BTP20220	Tissue specific
CDS|exon|ID=cds-Bg	7	4.581429	2674	0.057143	DLDGFEF	59	842.3518	1	841.3494	.	V	Superscaf	55470433	0	0	0	0	0	BTP20220	Tissue specific
CDS|exon|ID=cds-Bg	6	6.618333	2327.5	0.040003	KLSTFP	56	692.4036	1	691.3904	.	K	Superscaf	56478897	0	0	0	0	BTP20220	0	Tissue specific
CDS|exon|ID=cds-Bg	10	4.692	3234.6	0.030462	VHTSVHGVG	99	515.2631	2	1028.515	.	V	Superscaf	57040015	0	0	0	0	0	BTP20220	Tissue specific
CDS|exon|ID=cds-Bg	8	5.46625	2932	0.030462	SFVQSAFK	95	457.2399	2	912.4705	.	V	Superscaf	57040015	0	0	0	0	0	BTP20220	Tissue specific
CDS|exon|ID=cds-Bg	13	4.044615	4522.1	0.030187	ELDELVSAV	91	758.8582	2	1515.709	.	S	Superscaf	57056242	0	0	0	0	0	BTP20220	Tissue specific
CDS|exon|ID=cds-Bg	9	6.407778	4017.3	0.030187	KYNLQPKGE	98	538.7885	2	1075.566	.	S	Superscaf	57056242	0	0	0	0	0	BTP20220	Tissue specific
CDS|exon|ID=cds-Bg	13	6.03	4890.3	0.014148	VLAESDPGF	96	483.2665	3	1446.783	.	K	Superscaf	57999817	0	0	0	0	0	BTP20220	Tissue specific
CDS|exon|ID=cds-Bg	8	5.08625	3165.6	0.014068	YGMLFDEY	60	1037.424	1	1036.421	.	M	Superscaf	58004524	0	0	0	0	0	BTP20220	Tissue specific
CDS|exon|ID=cds-Bg	7	5.004286	2706.1	0.014068	GMLFDEY	88	874.3602	1	873.3578	.	M	Superscaf	58004524	0	0	0	0	0	BTP20220	Tissue specific
CDS|exon|ID=cds-Bg	8	5.6425	2949.4	0.013848	ASMLTELL	78	439.2368	2	876.4626	.	K	Superscaf	58017499	0	0	0	0	0	BTP20220	Tissue specific
CDS|exon|ID=cds-Bg	7	5.594286	2596	0.013849	ASMLTEL	80	764.3828	1	763.3786	.	K	Superscaf	58017499	0	0	0	0	0	BTP20220	Tissue specific
CDS|exon|ID=cds-Bg	7	6.055714	2547.8	0.006786	LTAHTVK	61	385.2353	2	768.4494	.	A	Superscaf	58433038	0	0	0	0	0	BTP20220	Tissue specific
CDS|exon|ID=cds-Bg	7	6.455714	2717.9	0.020246	RDKKSVP	65	415.2431	2	828.4817	.	R	Superscaf	60023476	0	0	0	0	0	BTP20220	Tissue specific
CDS|exon|ID=cds-Bg	12	5.225	5084.8	0.026561	C(+57.02)	98	706.8121	2	1411.615	Carbamido	R	Superscaf	60394840	0	BTP20220	0	0	0	0	Tissue specific
CDS|exon|ID=cds-Bg	8	7.18	3575.1	0.114434	PRSGQARP	82	434.7409	2	867.4675	.	L	Superscaf	65564566	BTP20220	0	0	0	0	0	Tissue specific
CDS|exon|ID=cds-Bg	7	7.175714	3027.3	0.218539	KAANRLP	61	385.2323	2	768.4606	.	E	Superscaf	71689171	BTP20220	0	0	0	0	0	Tissue specific
CDS|exon|ID=cds-Bg	13	5.460769	4781.2	0.220424	TKEVYELLD	96	739.8845	2	1477.766	.	C	Superscaf	71800345	0	0	0	0	0	BTP20220	Tissue specific
CDS|exon|ID=cds-Bg	12	5.371667	4383.7	0.220424	KEVYELLDS	99	689.3644	2	1376.719	.	C	Superscaf	71800345	0	0	0	0	0	BTP20220	Tissue specific
CDS|exon|ID=cds-Bg	8	5.73	3030.3	0.220425	ELLDSPGK	98	858.4553	1	857.4494	.	C	Superscaf	71800345	0	0	0	0	0	BTP20220	Tissue specific
CDS|exon|ID=cds-Bg	10	5.182	3383.7	0.220425	ELLDSPGKV	75	535.8063	2	1069.602	.	C	Superscaf	71800345	0	0	0	0	BTP20220	0	Tissue specific
CDS|exon|ID=cds-Bg	10	5.767	3642.9	0.220543	KFYPPKVEM	84	643.3329	2	1284.658	.	L	Superscaf	71807167	0	0	0	0	0	BTP20220	Tissue specific
CDS|exon|ID=cds-Bg	9	5.798889	3255.5	0.220543	KFYPPKVEM	68	569.8005	2	1137.589	.	L	Superscaf	71807167	0	0	0	0	0	BTP20220	Tissue specific
CDS|exon|ID=cds-Bg	6	5.22	1995.2	0.222262	GVHLPF	53	669.3658	1	668.3646	.	P	Superscaf	71908599	0	0	0	0	BTP20220	0	Tissue specific
CDS|exon|ID=cds-Bg	6	5.073333	2310.6	0.416851	N(+42.01)	51	392.728	2	783.4279	Acetylati	Y	Superscaf	83356567	0	0	BTP20220	0	0	0	Tissue specific
CDS|exon|ID=cds-Bg	10	4.565	3511	0.458209	PENDPNVVF	74	554.7834	2	1107.556	.	V	Superscaf	85789075	0	0	0	BTP20220	0	0	Tissue specific

CDS|exon|ID=cds-Bg	7	5.81	2924.1	0.463156	EDFEKPK	77	446.7291	2	891.4338	.	D	Superscaf	86080576	0	BTP202209	0	0	0	0	Tissue specific
CDS|exon|ID=cds-Bg	8	5.88875	2986.3	0.479687	D(+42.01)	52	472.2733	2	942.5134	Acetylati	I	Superscaf	87053410	0	0	0	0	BTP202209	0	Tissue specific
CDS|exon|ID=cds-Bg	8	5.21625	2569.9	0.499333	TFLLLVPF	66	475.2907	2	948.5684	.	P	Superscaf	88209288	BTP202209	0	0	0	0	0	Tissue specific
CDS|exon|ID=cds-Bg	10	6.041	4424.9	0.504589	RFKDLGEEH	95	639.3143	2	1276.62	.	S	Superscaf	88518403	0	0	0	0	0	BTP202209	Tissue specific
CDS|exon|ID=cds-Bg	9	6.103333	4037.5	0.504589	RFKDLGEEH	81	377.5226	3	1129.552	.	S	Superscaf	88518403	0	0	0	0	0	BTP202209	Tissue specific
CDS|exon|ID=cds-Bg	9	5.516667	3776.1	0.504589	FKDLGEEHF	98	561.265	2	1120.519	.	S	Superscaf	88518403	0	0	0	0	0	BTP202209	Tissue specific
CDS|exon|ID=cds-Bg	8	5.52125	3388.7	0.504589	KDLGEEHF	99	487.7308	2	973.4505	.	S	Superscaf	88518403	0	0	0	0	0	BTP202209	Tissue specific
CDS|exon|ID=cds-Bg	12	5.11	4187.5	0.504605	VKLVNELTE	99	695.8977	2	1389.787	.	C	Superscaf	88519297	0	0	0	0	0	BTP202209	Tissue specific
CDS|exon|ID=cds-Bg	9	5.066667	3365.7	0.504605	VNELTEFAK	99	525.7748	2	1049.539	.	C	Superscaf	88519297	0	0	0	0	0	BTP202209	Tissue specific
CDS|exon|ID=cds-Bg	7	4.262857	2586	0.504605	VNELTEF	60	426.209	2	850.4072	.	C	Superscaf	88519297	0	0	0	0	0	BTP202209	Tissue specific
CDS|exon|ID=cds-Bg	15	5.517333	6002.5	0.504605	AKTC(+57.	99	554.9096	3	1661.714	Carbamido	C	Superscaf	88519297	0	0	0	0	0	BTP202209	Tissue specific
CDS|exon|ID=cds-Bg	13	5.153846	5222.8	0.504605	TC(+57.02	99	732.295	2	1462.582	Carbamido	C	Superscaf	88519297	0	0	0	0	0	BTP202209	Tissue specific
CDS|exon|ID=cds-Bg	12	5.991667	4939.4	0.504637	LSHKDDSPD	95	451.2316	3	1350.678	.	S	Superscaf	88521199	0	0	0	0	0	BTP202209	Tissue specific
CDS|exon|ID=cds-Bg	9	5.432222	3467.9	0.504638	DDSPDLPKL	96	500.2517	2	998.492	.	S	Superscaf	88521199	0	0	BTP202209	0	0	0	Tissue specific
CDS|exon|ID=cds-Bg	10	6.851	4233.2	0.504639	KADEKKFWG	97	412.8942	3	1235.666	.	S	Superscaf	88521199	0	0	0	0	0	BTP202209	Tissue specific
CDS|exon|ID=cds-Bg	9	4.465556	2769	0.504703	AEFVEVTKL	99	518.2878	2	1034.565	.	W	Superscaf	88525132	0	0	0	0	0	BTP202209	Tissue specific
CDS|exon|ID=cds-Bg	12	6.075833	5236.7	0.504721	LKEC(+57.	98	766.8909	2	1531.774	Carbamido	A	Superscaf	88526206	0	0	0	0	0	BTP202209	Tissue specific
CDS|exon|ID=cds-Bg	10	5.301	3313.6	0.504792	LVEVSRSLG	99	363.2191	3	1086.64	.	L	Superscaf	88530361	0	0	0	0	0	BTP202209	Tissue specific
CDS|exon|ID=cds-Bg	13	6.057692	5601.5	0.504793	VGTRC(+57	67	527.2475	3	1578.724	Carbamido	L	Superscaf	88530361	0	0	0	0	0	BTP202209	Tissue specific
CDS|exon|ID=cds-Bg	7	5.555714	2744.9	0.504806	AFDEKLF	96	435.2206	2	868.433	.	L	Superscaf	88531123	0	0	0	0	0	BTP202209	Tissue specific
CDS|exon|ID=cds-Bg	10	5.849	4149.5	0.504806	AFDEKLFTF	99	627.8086	2	1253.608	.	L	Superscaf	88531123	0	0	0	0	0	BTP202209	Tissue specific
CDS|exon|ID=cds-Bg	9	5.655556	3529.8	0.504806	AFDEKLFTF	99	559.2792	2	1116.549	.	L	Superscaf	88531123	0	0	0	0	0	BTP202209	Tissue specific
CDS|exon|ID=cds-Bg	8	5.6775	3142.4	0.504806	AFDEKLFT	98	485.7457	2	969.4807	.	L	Superscaf	88531123	0	0	0	0	0	BTP202209	Tissue specific
CDS|exon|ID=cds-Bg	8	5.61	3218.5	0.504806	FDEKLFTF	98	523.7609	2	1045.512	.	L	Superscaf	88531123	0	0	0	0	0	BTP202209	Tissue specific
CDS|exon|ID=cds-Bg	7	5.628571	2831.1	0.504806	FDEKLFT	95	899.4445	1	898.4436	.	L	Superscaf	88531123	0	0	0	0	0	BTP202209	Tissue specific
CDS|exon|ID=cds-Bg	13	6.880769	5708	0.504829	LKHKPKATE	91	517.3058	3	1548.899	.	N	Superscaf	88532497	0	0	0	0	0	BTP202209	Tissue specific
CDS|exon|ID=cds-Bg	13	5.134615	5170.9	0.50483	ATEEQLKTV	79	770.3693	2	1538.729	.	N	Superscaf	88532497	0	0	0	0	0	BTP202209	Tissue specific
CDS|exon|ID=cds-Bg	12	5.105833	4783.5	0.50483	ATEEQLKTV	97	696.8344	2	1391.66	.	N	Superscaf	88532497	0	0	0	0	0	BTP202209	Tissue specific
CDS|exon|ID=cds-Bg	8	5.26375	3000.4	0.50483	LKTVMENF	94	981.5036	1	980.5001	.	N	Superscaf	88532497	0	0	0	0	0	BTP202209	Tissue specific
CDS|exon|ID=cds-Bg	7	5.232857	2613	0.50483	LKTVMEN	60	417.7211	2	833.4317	.	N	Superscaf	88532497	0	0	0	0	0	BTP202209	Tissue specific
CDS|exon|ID=cds-Bg	7	6.12	2638.8	0.505889	EGGPLYK	74	382.2016	2	762.3912	.	T	Superscaf	88594989	0	BTP202209	0	0	0	0	Tissue specific
CDS|exon|ID=cds-Bg	7	5.387143	3125.6	0.512579	D(+42.01)	68	426.1725	2	850.3457	Acetylati	L	Superscaf	88988113	BTP202209	0	0	0	0	0	Tissue specific
CDS|exon|ID=cds-Bg	8	5.23625	3232.6	0.541128	HNSANVPF	53	443.2129	2	884.4141	.	M	Superscaf	90668020	0	0	0	0	0	BTP202209	Tissue specific
CDS|exon|ID=cds-Bg	13	6.493077	5724.3	0.551204	ERGLQKGQE	89	486.594	3	1456.763	.	E	Superscaf	91260700	0	0	BTP202209	0	0	0	Tissue specific
CDS|exon|ID=cds-Bg	9	4.642222	2719	0.551207	DVGLGDGLG	92	802.3934	1	801.3868	.	E	Superscaf	91260700	BTP202209	0	0	0	0	0	Tissue specific
CDS|exon|ID=cds-Bg	15	5.786667	6697.5	0.562148	EFLGELQKK	99	631.9822	3	1892.93	.	K	Superscaf	91904686	0	0	0	0	BTP202209	0	Tissue specific
CDS|exon|ID=cds-Bg	12	5.024167	5183.7	0.562184	ELEEEVNNF	94	753.8736	2	1505.736	.	T	Superscaf	91906786	0	0	0	0	BTP202209	0	Tissue specific
CDS|exon|ID=cds-Bg	10	7.054	4267.3	0.562185	KKKAAAQLL	81	549.8515	2	1097.692	.	T	Superscaf	91906786	0	0	BTP202209	0	0	0	Tissue specific
CDS|exon|ID=cds-Bg	10	5.308	3306.6	0.574701	ASVLSLPYF	75	547.2986	2	1092.585	.	K	Superscaf	92643307	0	0	0	0	0	BTP202209	Tissue specific
CDS|exon|ID=cds-Bg	7	4.354286	2010.3	0.591487	VTLAVPQ	55	727.4429	1	726.4276	.	M	Superscaf	93630868	0	0	0	0	BTP202209	0	Tissue specific
CDS|exon|ID=cds-Bg	5	4.296	1469.7	0.614811	ELVLP	67	570.3478	1	569.3425	.	D	Superscaf	95003080	0	0	BTP202209	0	0	0	Tissue specific
CDS|exon|ID=cds-Bg	9	5.634444	3476.9	0.653754	SELYLPSST	71	996.4835	1	995.4811	.	P	Superscaf	97293916	0	0	0	0	0	BTP202209	Tissue specific
CDS|exon|ID=cds-Bg	6	7.646667	2560.9	0.690518	ATTKPR	71	673.3979	1	672.3918	.	C	Superscaf	99456868	0	BTP202209	0	0	0	0	Tissue specific
CDS|exon|ID=cds-Bg	7	6.768571	2944.2	0.70734	K(+42.01)	50	429.7548	2	857.497	Acetylati	G	Superscaf	1E+08	0	BTP202209	0	0	0	0	Tissue specific
CDS|exon|ID=cds-Bg	7	4.88	2060.3	0.735046	APAPEVP	68	680.3585	1	679.3541	.	L	Superscaf	1.02E+08	0	0	0	0	BTP202209	0	Tissue specific
CDS|exon|ID=cds-Bg	7	6.154286	2565.6	0.735136	VGSKQPK	75	372.2171	2	742.4337	.	P	Superscaf	1.02E+08	0	0	0	0	0	BTP202209	Tissue specific
CDS|exon|ID=cds-Bg	11	5.650909	4740.2	0.740542	FNEKDGQVE	90	450.5635	3	1348.674	.	A	Superscaf	1.02E+08	0	0	BTP202209	0	0	0	Tissue specific
CDS|exon|ID=cds-Bg	8	6.695	3010.4	0.750601	A(+42.01)	57	420.7488	2	839.4865	Acetylati	R	Superscaf	1.03E+08	0	0	0	0	BTP202209	0	Tissue specific
CDS|exon|ID=cds-Bg	8	4.135	2752.2	0.757242	EDDLPVYL	68	963.4644	1	962.4597	.	P	Superscaf	1.03E+08	0	BTP202209	0	0	0	0	Tissue specific
CDS|exon|ID=cds-Bg	8	5.9	3233.7	0.758119	LGTDGTHY	88	432.1967	2	862.3821	.	W	Superscaf	1.03E+08	0	0	0	0	0	BTP202209	Tissue specific
CDS|exon|ID=cds-Bg	10	4.699	3250.3	0.782737	ESVKKVQVE	97	391.8981	3	1172.677	.	L	Superscaf	1.05E+08	0	0	0	BTP202209	0	0	Tissue specific
CDS|exon|ID=cds-Bg	5	6.85	1884	0.793999	K(+42.01)	96	587.3746	1	586.369	Acetylati	F	Superscaf	1.06E+08	0	0	BTP202209	0	0	0	Tissue specific
CDS|exon|ID=cds-Bg	14	6.737857	5771	0.894498	SQPSGLQAS	96	472.9303	3	1415.773	.	T	Superscaf	1.11E+08	0	0	0	0	0	BTP202209	Tissue specific
CDS|exon|ID=cds-Bg	14	5.399286	5139.4	0.911103	FAENPKGAD	97	508.2532	3	1521.746	.	P	Superscaf	1.12E+08	0	0	0	0	0	BTP202209	Tissue specific
CDS|exon|ID=cds-Bg	12	5.340833	4440.7	0.911103	ENPKGADEF	91	652.8262	2	1303.641	.	P	Superscaf	1.12E+08	0	0	0	0	0	BTP202209	Tissue specific
CDS|exon|ID=cds-Bg	10	6.579	4793.6	0.911107	ANSTTRQDF	75	598.2926	2	1194.574	.	P	Superscaf	1.12E+08	0	0	BTP202209	BTP202209	0	0	Mixed
CDS|exon|ID=cds-Bg	7	5.96	2718.8	0.911115	SGEGFYK	98	394.1831	2	786.3548	.	P	Superscaf	1.12E+08	0	BTP202209	0	0	0	0	Tissue specific
CDS|exon|ID=cds-Bg	9	6.346667	3646.7	0.911115	SGEGFYKYK	97	539.7621	2	1077.513	.	P	Superscaf	1.12E+08	0	BTP202209	0	0	BTP202209	BTP202209	Mixed
CDS|exon|ID=cds-Bg	8	6.43	3263.3	0.911115	GEGFYKYK	88	496.2476	2	990.481	.	P	Superscaf	1.12E+08	0	BTP202209	BTP202209	BTP202209	0	0	Mixed
CDS|exon|ID=cds-Bg	9	5.67	2949.3	0.943124	PGAPATHVF	58	423.7191	2	845.4395	.	R	Superscaf	1.14E+08	BTP202209	0	0	0	0	0	Tissue specific
CDS|exon|ID=cds-Bg	7	5.99	2630.9	0.945084	AALPLLQ	60	725.4541	1	724.4483	.	A	Superscaf	1.14E+08	0	BTP202209	0	0	0	0	Tissue specific
CDS|exon|ID=cds-Bg	7	7.494286	2932.4	0.954708	PAARPRP	88	382.728	2	763.4453	.	P	Superscaf	1.15E+08	0	0	0	0	BTP202209	0	Tissue specific
CDS|exon|ID=cds-Bg	7	4.734286	2038.2	0.95887	ELVGLGA	65	658.3748	1	657.3697	.	S	Superscaf	1.15E+08	0	0	0	0	0	BTP202209	Tissue specific
CDS|exon|ID=cds-Bg	7	5.828571	2848.2	0.973339	LTAFHEL	57	415.7264	2	829.4334	.	I	Superscaf	1.16E+08	0	0	0	0	0	BTP202209	Tissue specific
CDS|exon|ID=cds-Bg	7	6.937143	2673.2	0.987711	PPPPPRP	98	379.2203	2	756.4282	.	Y	Superscaf	1.17E+08	0	0	BTP202209	BTP202209	BTP202209	BTP202209	Mixed
CDS|exon|ID=cds-Bg	10	4.646	3655.1	0.990767	ALDVHESEF	69	572.2677	2	1142.524	.	N	Superscaf	1.17E+08	0	BTP202209	0	0	0	0	Tissue specific
CDS|exon|ID=cds-Bg	8	6.46875	2733.8	0.995118	APGGFGPK	57	730.3802	1	729.3809	.	A	Superscaf	1.17E+08	0	0	0	0	BTP202209	0	Tissue specific
CDS|exon|ID=cds-Bg	10	4.296	3017.6	0.814374	LTELPVMVE	67	572.3168	2	1142.626	.	T	Superscaf	11249041	0	0	0	0	0	BTP202209	Tissue specific
CDS|exon|ID=cds-Bg	7	6.022857	2355.8	0.809669	VGPPTRP	75	362.209	2	722.4075	.	G	Superscaf	11534119	0	0	BTP202209	0	0	0	Tissue specific
CDS|exon|ID=cds-Bg	8	5.3125	2689	0.801326	PAPQVSTA	90	770.401	1	769.397	.	T	Superscaf	12039484	0	0	0	0	BTP202209	0	Tissue specific
CDS|exon|ID=cds-Bg	14	4.967857	4389.9	0.801281	M(+42.01)	88	748.8575	2	1495.705	Acetylati	E	Superscaf	12042487	0	0	0	0	0	BTP202209	Tissue specific
CDS|exon|ID=cds-Bg	10	5.433	3534.1	0.801281	M(+42.01)	55	512.2081	2	1022.405	Acetylati	E	Superscaf	12042487	0	0	0	0	BTP202209	0	Tissue specific
CDS|exon|ID=cds-Bg	13	4.907692	4018.4	0.801281	M(+42.01)	97	683.3373	2	1364.665	Acetylati	E	Superscaf	12042487	0	0	0	0	0	BTP202209	Tissue specific
CDS|exon|ID=cds-Bg	12	4.995	4208.8	0.801252	VEMDWVLKH	97	706.3533	2	1410.697	.	A	Superscaf	12044224	0	0	0	0	BTP202209	0	Tissue specific
CDS|exon|ID=cds-Bg	8	4.19375	2556.9	0.801252	VEMDWVLK	99	510.2629	2	1018.516	.	A	Superscaf	12044224	0	0	0	0	0	BTP202209	Tissue specific
CDS|exon|ID=cds-Bg	18	5.276667	7531.6	0.799943	SHGSHETDE	93	1083.459	2	2164.908	.	V	Superscaf	12123031	0	0	0	0	0	BTP202209	Tissue specific
CDS|exon|ID=cds-Bg	7	5.758571	2402.6	0.793223	VPAQKPP	58	736.4278	1	735.4279	.	M	Superscaf	12530871	0	0	BTP202209	0	0	0	Tissue specific
CDS|exon|ID=cds-Bg	15	5.241333	5178.7	0.78664	GNYSERVGA	71	776.8878	2	1551.768	.	V	Superscaf	12929575	0	0	0	0	0	BTP202209	Tissue specific
CDS|exon|ID=cds-Bg	14	5.188571	4825.3	0.78664	GNYSERVGA	68	720.3461	2	1438.684	.	V	Superscaf	12929575	0	0	0	0	0	BTP202209	Tissue specific
CDS|exon|ID=cds-Bg	10	4.994	3012.3	0.78664	SERVGAGAF	63	471.752	2	941.493	.	V	Superscaf	12929575	0	0	0	0	0	BTP202209	Tissue specific
CDS|exon|ID=cds-Bg	7	4.697143	2214.4	0.78664	YLAAVLE	86	778.4308	1	777.4272	.	V	Superscaf	12929575	0	0	0	0	BTP202209	0	Tissue specific
CDS|exon|ID=cds-Bg	7	4.77	2300.6	0.78664	AVLEYLT	79	808.4432	1	807.4378	.	V	Superscaf	12929575	0	BTP202209	BTP202209	0	BTP202209	0	Mixed
CDS|exon|ID=cds-Bg	8	6.48	2886	0.786361	TGGVKKPH	63	412.247	2	822.4711	.	A	Superscaf	12946195	0	0	0	0	0	BTP202209	Tissue specific
CDS|exon|ID=cds-Bg	5	6.682	2199.4	0.781195	KNYPP	80	618.3225	1	617.3173	.	E	Superscaf	13259823	0	0	0	BTP202209	0	0	Tissue specific
CDS|exon|ID=cds-Bg	22	5.000455	7580.2	0.758091	PGPASGGSG	62	1128.524	2	2255.038	.	E	Superscaf	14659813	0	0	0	0	0	BTP202209	Tissue specific
CDS|exon|ID=cds-Bg	21	4.938571	7242.8	0.758091	GPASGGSGE	75	1079.995	2	2157.985	.	E	Superscaf	14659813	0	0	0	0	0	BTP202209	Tissue specific
CDS|exon|ID=cds-Bg	7	7.307143	2996.4	0.757203	RASGLLR	71	386.741	2	771.4715	.	W	Superscaf	14713591	0	0	BTP202209	0	0	0	Tissue specific
CDS|exon|ID=cds-Bg	8	5.845	2642.9	0.755949	A(+42.01)	67	868.5596	1	867.5541	Acetylati	I	Superscaf	14789608	0	0	BTP202209	0	BTP202209	0	Mixed
CDS|exon|ID=cds-Bg	7	5.177143	2630	0.750744	M(+42.01)	83	840.3409	1	839.3371	Acetylati	D	Superscaf	15105067	0	BTP202209	0	0	BTP202209	BTP202209	Mixed
CDS|exon|ID=cds-Bg	11	4.277273	3770.4	0.750126	LDDEEYVVF	92	646.3102	2	1290.609	.	K	Superscaf	15142369	0	BTP202209	0	0	0	0	Tissue specific
CDS|exon|ID=cds-Bg	10	4.107	3417	0.750126	DDEEYVVPG	90	589.7686	2	1177.525	.	K	Superscaf	15142369	0	BTP202209	0	0	BTP202209	0	Mixed
CDS|exon|ID=cds-Bg	6	6.79	2506.9	0.747431	PMAWAR	99	366.1852	2	730.3585	.	V	Superscaf	15305779	0	0	BTP202209	BTP202209	0	0	Mixed
CDS|exon|ID=cds-Bg	8	6.1	2681	0.747323	GPGPPLPS	94	721.3855	1	720.3806	.	Q	Superscaf	15312025	0	0	0	BTP202209	0	0	Tissue specific
CDS|exon|ID=cds-Bg	7	7.068571	2633.6	0.746717	LPKAASK	50	357.7318	2	713.4435	.	P	Superscaf	15345493	0	0	0	BTP202209	0	0	Tissue specific
CDS|exon|ID=cds-Bg	7	5.715714	2135.2	0.745132	SGPKVAP	89	655.3714	1	654.3701	.	T	Superscaf	15445066	0	0	0	0	BTP202209	0	Tissue specific
CDS|exon|ID=cds-Bg	7	7.118571	3148.2	0.740479	KEKPNSK	76	415.7395	2	829.4658	.	L	Superscaf	15727179	0	BTP202209	0	0	0	0	Tissue specific
CDS|exon|ID=cds-Bg	5	6.188	1634.8	0.731632	PPAAP	56	452.2507	1	451.243	.	W	Superscaf	16263235	BTP202209	0	0	0	BTP202209	BTP202209	Mixed
CDS|exon|ID=cds-Bg	5	6.936	2230.7	0.727036	TLM(+15.9	52	699.3533	1	698.3422	Oxidation	R	Superscaf	16541827	0	0	0	0	0	BTP202209	Tissue specific
CDS|exon|ID=cds-Bg	9	5.348889	3549.9	0.71953	EDPFKSDPF	86	541.2448	2	1080.476	.	N	Superscaf	16996549	0	BTP202209	0	0	0	0	Tissue specific
CDS|exon|ID=cds-Bg	8	5.24125	2883.3	0.715328	HRQGLLVV	58	461.2832	2	920.5555	.	A	Superscaf	17251374	0	0	0	0	0	BTP202209	Tissue specific
CDS|exon|ID=cds-Bg	8	6.10125	2737.1	0.646736	P(+42.01)	70	410.2325	2	818.4538	Acetylati	D	Superscaf	21408025	0	0	0	BTP202209	0	0	Tissue specific
CDS|exon|ID=cds-Bg	5	6.35	1571.5	0.643888	K(+42.01)	94	570.3596	1	569.3537	Acetylati	H	Superscaf	21580645	0	0	0	0	BTP202209	0	Tissue specific
CDS|exon|ID=cds-Bg	9	6.11	4055.6	0.640839	LTLHGLQQY	62	536.7909	2	1071.571	.	T	Superscaf	21765268	0	0	0	0	0	BTP202209	Tissue specific
CDS|exon|ID=cds-Bg	8	5.965	3787.4	0.636742	HMETGNHY	87	494.6992	2	987.3869	.	P	Superscaf	22013635	0	0	0	0	0	BTP202209	Tissue specific
CDS|exon|ID=cds-Bg	8	5.05125	3035.3	0.632019	KDVHFDSL	50	480.7517	2	959.4712	.	E	Superscaf	22299939	BTP202209	0	0	0	0	0	Tissue specific
CDS|exon|ID=cds-Bg	6	5.15	1676.9	0.622315	VPALPP	58	593.3661	1	592.3584	.	G	Superscaf	22887661	BTP202209	0	0	0	0	0	Tissue specific
CDS|exon|ID=cds-Bg	8	4.795	2523.8	0.622221	LDSGVPFL	52	847.4637	1	846.4487	.	P	Superscaf	22893661	0	0	BTP202209	0	0	0	Tissue specific
CDS|exon|ID=cds-Bg	11	4.838182	3449.9	0.619804	A(+42.01)	99	591.3282	2	1180.645	Acetylati	H	Superscaf	23039986	0	0	0	0	0	BTP202209	Tissue specific
CDS|exon|ID=cds-Bg	10	4.246	2801.1	0.619804	A(+42.01)	90	513.2776	2	1024.544	Acetylati	H	Superscaf	23039986	0	0	0	0	0	BTP202209	Tissue specific
CDS|exon|ID=cds-Bg	12	5.4575	4627.1	0.618989	LAFVLYNDG	92	702.8795	2	1403.745	.	G	Superscaf	23089513	0	0	BTP202209	0	0	0	Tissue specific
CDS|exon|ID=cds-Bg	9	5.334444	3575	0.618989	VLYNDQPPK	99	537.2838	2	1072.555	.	G	Superscaf	23089513	0	BTP202209	0	0	BTP202209	BTP202209	Mixed
CDS|exon|ID=cds-Bg	9	5.042222	3179.4	0.618104	A(+42.01)	76	522.241	2	1042.469	Acetylati	P	Superscaf	23143138	0	BTP202209	0	0	BTP202209	0	Mixed
CDS|exon|ID=cds-Bg	9	5.408889	3175.4	0.618104	VGKPAPEFG	64	486.759	2	971.5076	.	P	Superscaf	23143138	0	0	0	0	0	BTP202209	Tissue specific
CDS|exon|ID=cds-Bg	7	5.335714	2724	0.618101	FFYPLDF	98	948.4471	1	947.4429	.	R	Superscaf	23143336	0	0	0	0	0	BTP202209	Tissue specific
CDS|exon|ID=cds-Bg	12	5.664167	4582.1	0.618088	LADVTRKLS	97	684.3586	2	1366.709	.	G	Superscaf	23144086	0	0	0	0	0	BTP202209	Tissue specific
CDS|exon|ID=cds-Bg	10	5.597	3917.4	0.618088	DVTRKLSSD	90	592.2999	2	1182.588	.	G	Superscaf	23144086	0	0	0	0	0	BTP202209	Tissue specific
CDS|exon|ID=cds-Bg	12	5.345	5015.4	0.618068	VDDSKEYFS	94	490.2274	3	1467.663	.	A	Superscaf	23145163	BTP202209	0	0	0	0	0	Tissue specific
CDS|exon|ID=cds-Bg	10	5.114	3799	0.618068	VDDSKEYFS	93	609.2863	2	1216.561	.	A	Superscaf	23145163	0	0	BTP202209	0	0	0	Tissue specific
CDS|exon|ID=cds-Bg	9	4.605556	2381.5	0.616173	SALVPAVGF	88	860.4841	1	859.4803	.	P	Superscaf	23260075	0	0	0	0	0	BTP202209	Tissue specific
CDS|exon|ID=cds-Bg	9	5.794444	3355.8	0.616153	LLLPVRQAF	96	528.8299	2	1055.649	.	I	Superscaf	23261212	0	0	0	0	0	BTP202209	Tissue specific
CDS|exon|ID=cds-Bg	10	5.781	3815.3	0.616153	LLLPVRQAF	87	610.3614	2	1218.712	.	I	Superscaf	23261212	0	0	0	0	0	BTP202209	Tissue specific
CDS|exon|ID=cds-Bg	7	4.411429	2113.3	0.609294	G(+42.01)	78	408.7142	2	815.4177	Acetylati	F	Superscaf	23677051	0	0	BTP202209	0	0	0	Tissue specific
CDS|exon|ID=cds-Bg	7	3.098571	1119.1	0.609266	V(+42.01)	59	755.4993	1	754.4952	Acetylati	L	Superscaf	23678752	0	0	0	0	BTP202209	0	Tissue specific
CDS|exon|ID=cds-Bg	12	5.714167	4788.2	0.589647	A(+42.01)	89	720.8531	2	1439.693	Acetylati	E	Superscaf	24867526	0	0	0	0	BTP202209	BTP202209	Mixed
CDS|exon|ID=cds-Bg	7	5.745714	2692.2	0.58048	PPVDRPH	60	409.2183	2	816.4242	.	Q	Superscaf	25423246	BTP202209	0	0	0	0	0	Tissue specific
CDS|exon|ID=cds-Bg	6	7.236667	2598.8	0.580033	RKLLFF	60	412.2594	2	822.5115	.	I	Superscaf	25450458	0	0	0	BTP202209	0	0	Tissue specific
CDS|exon|ID=cds-Bg	7	5.725714	2846.2	0.569732	AAVLQQR	70	393.2339	2	784.4555	.	D	Superscaf	26074468	0	0	0	BTP202209	0	0	Tissue specific
CDS|exon|ID=cds-Bg	8	5.1575	2375.7	0.569419	TGVSRAVP	80	393.7266	2	785.4395	.	V	Superscaf	26093110	0	0	0	0	BTP202209	0	Tissue specific
CDS|exon|ID=cds-Bg	7	4.29	1760.9	0.568394	ATVVLAF	52	720.4347	1	719.4218	.	H	Superscaf	26155444	0	0	0	0	BTP202209	0	Tissue specific
CDS|exon|ID=cds-Bg	16	6.040625	7094.7	0.566745	KEEAEKPEE	83	474.4849	4	1893.918	.	N	Superscaf	26255437	0	0	0	0	0	BTP202209	Tissue specific
CDS|exon|ID=cds-Bg	18	5.954444	6975.5	0.565635	S(+42.01)	64	644.9885	3	1931.949	Acetylati	V	Superscaf	26322574	0	0	0	0	BTP202209	0	Tissue specific
CDS|exon|ID=cds-Bg	14	6.176429	5358.7	0.565635	RGPAYGLSA	92	497.2739	3	1488.805	.	V	Superscaf	26322574	0	0	0	0	0	BTP202209	Tissue specific

CDS|exon|ID=cds-Bg	12	5.943333	4293.6	0.565635	RGPAYGLSA	85	624.3379	2	1246.667	.	V	Superscaf	26322574	0	0	0	0	0	BTP20220	Tissue specific
CDS|exon|ID=cds-Bg	8	6.5425	3088.4	0.565635	RGPAYGLS	86	820.4279	1	819.4239	.	V	Superscaf	26322574	0	0	0	0	BTP20220	0	Tissue specific
CDS|exon|ID=cds-Bg	7	6.665714	2705	0.565635	RGPAYGL	85	733.396	1	732.3918	.	V	Superscaf	26322574	0	0	0	0	BTP20220	BTP20220	Mixed
CDS|exon|ID=cds-Bg	13	5.823846	4709.9	0.565635	GPAYGLSAE	98	667.3575	2	1332.704	.	V	Superscaf	26322574	0	0	0	0	0	BTP20220	Tissue specific
CDS|exon|ID=cds-Bg	11	5.767273	4075.2	0.565635	AYGLSAEVK	95	590.3199	2	1178.629	.	V	Superscaf	26322574	0	0	0	0	0	BTP20220	Tissue specific
CDS|exon|ID=cds-Bg	8	5.72375	3007.1	0.565635	LSAEVKNK	99	444.7596	2	887.5076	.	V	Superscaf	26322574	0	0	0	0	0	BTP20220	Tissue specific
CDS|exon|ID=cds-Bg	7	5.42	3416	0.565601	EQELREW	77	495.238	2	988.4614	.	G	Superscaf	26324788	0	0	0	0	BTP20220	0	Tissue specific
CDS|exon|ID=cds-Bg	9	6.533333	3387.4	0.565542	KLQPGSVKK	89	492.8118	2	983.6127	.	G	Superscaf	26328232	0	0	0	0	BTP20220	0	Tissue specific
CDS|exon|ID=cds-Bg	14	5.688571	5190.5	0.565541	KLQPGSVKK	85	505.6212	3	1513.846	.	G	Superscaf	26328232	0	0	0	0	BTP20220	0	Tissue specific
CDS|exon|ID=cds-Bg	12	5.619167	4409.6	0.565542	KLQPGSVKK	88	442.9278	3	1325.767	.	G	Superscaf	26328232	0	0	0	0	BTP20220	0	Tissue specific
CDS|exon|ID=cds-Bg	8	6.1325	2919	0.565542	KLQPGSVK	94	856.5206	1	855.5178	.	G	Superscaf	26328232	0	0	0	0	BTP20220	0	Tissue specific
CDS|exon|ID=cds-Bg	8	6.1325	2919	0.565542	LQPGSVKK	95	428.7642	2	855.5178	.	G	Superscaf	26328232	0	0	0	0	0	BTP20220	Tissue specific
CDS|exon|ID=cds-Bg	9	5.042222	4156.8	0.565541	VNESTQNWH	98	557.7471	2	1113.484	.	G	Superscaf	26328232	0	0	0	0	0	BTP20220	Tissue specific
CDS|exon|ID=cds-Bg	7	5.461429	2593.8	0.565538	YGVKPHD	99	408.2039	2	814.3973	.	P	Superscaf	26328688	0	0	0	0	BTP20220	0	Tissue specific
CDS|exon|ID=cds-Bg	10	5.477	4796.6	0.565537	DLFENTNHT	80	609.771	2	1217.531	.	P	Superscaf	26328688	0	0	0	0	BTP20220	0	Tissue specific
CDS|exon|ID=cds-Bg	13	5.713077	4370.4	0.565533	AKTKGNKVN	97	448.2755	3	1341.809	.	M	Superscaf	26328985	0	0	0	0	0	BTP20220	Tissue specific
CDS|exon|ID=cds-Bg	11	5.319091	3590.7	0.565533	TKGNKVNVG	99	572.3442	2	1142.677	.	M	Superscaf	26328985	0	0	0	0	0	BTP20220	Tissue specific
CDS|exon|ID=cds-Bg	9	4.693333	2724.8	0.565533	GNKVNVGVK	99	457.7726	2	913.5345	.	M	Superscaf	26328985	0	0	0	0	0	BTP20220	Tissue specific
CDS|exon|ID=cds-Bg	7	4.408571	1830.8	0.565533	KVNVGVK	98	372.2408	2	742.4701	.	M	Superscaf	26328985	0	0	0	0	0	BTP20220	Tissue specific
CDS|exon|ID=cds-Bg	10	4.922	3466.6	0.565532	VGVKYAEKG	88	575.8044	2	1149.603	.	M	Superscaf	26328985	0	0	0	0	BTP20220	0	Tissue specific
CDS|exon|ID=cds-Bg	12	6.735	5663.5	0.565532	RFEPEKLRE	73	510.941	3	1529.806	.	M	Superscaf	26328985	0	0	0	0	BTP20220	0	Tissue specific
CDS|exon|ID=cds-Bg	11	6.855455	5066.8	0.565532	RFEPEKLRE	78	354.9463	4	1415.763	.	M	Superscaf	26328985	0	0	0	0	BTP20220	0	Tissue specific
CDS|exon|ID=cds-Bg	8	6.9325	3695.2	0.565532	RFEPEKLR	82	358.8713	3	1073.598	.	M	Superscaf	26328985	0	0	0	0	BTP20220	BTP20220	Mixed
CDS|exon|ID=cds-Bg	7	6.385714	3046.4	0.565532	RFEPEKL	68	459.7544	2	917.497	.	M	Superscaf	26328985	0	0	0	0	BTP20220	BTP20220	Mixed
CDS|exon|ID=cds-Bg	10	6.465	4418	0.565532	FEPEKLREG	94	420.8929	3	1259.662	.	M	Superscaf	26328985	0	0	0	0	0	BTP20220	Tissue specific
CDS|exon|ID=cds-Bg	8	5.99	3471.9	0.565532	FEPEKLRE	94	524.2753	2	1046.54	.	M	Superscaf	26328985	0	0	0	0	BTP20220	0	Tissue specific
CDS|exon|ID=cds-Bg	10	6.458	4627.3	0.565532	EPEKLREGR	88	614.3229	2	1226.637	.	M	Superscaf	26328985	0	0	0	0	BTP20220	BTP20220	Mixed
CDS|exon|ID=cds-Bg	9	6.574444	4030.6	0.565532	EPEKLREGR	92	371.8698	3	1112.594	.	M	Superscaf	26328985	0	0	0	0	BTP20220	0	Tissue specific
CDS|exon|ID=cds-Bg	9	7.365556	4345	0.565523	TRRHLYDPK	67	395.8859	3	1184.641	.	M	Superscaf	26329597	0	0	0	0	0	BTP20220	Tissue specific
CDS|exon|ID=cds-Bg	7	7.145714	3479.1	0.565523	RRHLYDP	74	478.7556	2	955.4988	.	M	Superscaf	26329597	0	0	0	0	0	BTP20220	Tissue specific
CDS|exon|ID=cds-Bg	8	6.27375	3300.6	0.565523	HLYDPKLG	78	471.7551	2	941.497	.	M	Superscaf	26329597	0	BTP20220	0	0	0	0	Tissue specific
CDS|exon|ID=cds-Bg	13	5.59	5460.2	0.565523	YDPKLGTDQ	75	745.3584	2	1488.71	.	M	Superscaf	26329597	0	0	0	0	0	BTP20220	Tissue specific
CDS|exon|ID=cds-Bg	10	5.807	4084.6	0.565523	YDPKLGTDQ	95	567.2748	2	1132.54	.	M	Superscaf	26329597	0	0	0	0	BTP20220	0	Tissue specific
CDS|exon|ID=cds-Bg	8	5.765	3136.5	0.565523	YDPKLGTD	93	454.7198	2	907.4286	.	M	Superscaf	26329597	0	0	0	0	BTP20220	BTP20220	Mixed
CDS|exon|ID=cds-Bg	12	5.584167	5000.7	0.565523	DPKLGTDQP	79	663.8275	2	1325.646	.	M	Superscaf	26329597	0	0	0	0	BTP20220	0	Tissue specific
CDS|exon|ID=cds-Bg	9	5.823333	3625.1	0.565523	DPKLGTDQP	68	970.4803	1	969.4767	.	M	Superscaf	26329597	0	0	0	0	BTP20220	0	Tissue specific
CDS|exon|ID=cds-Bg	13	5.891538	5298	0.565522	PKLGTDQPL	62	692.3566	2	1382.704	.	M	Superscaf	26329597	0	0	0	0	BTP20220	0	Tissue specific
CDS|exon|ID=cds-Bg	11	5.821818	4589.2	0.565523	PKLGTDQPL	79	606.3143	2	1210.619	.	M	Superscaf	26329597	0	0	0	0	BTP20220	0	Tissue specific
CDS|exon|ID=cds-Bg	10	5.839	3978.5	0.565523	PKLGTDQPL	53	542.2858	2	1082.561	.	M	Superscaf	26329597	0	0	0	0	BTP20220	0	Tissue specific
CDS|exon|ID=cds-Bg	8	6.18	3213.6	0.565523	PKLGTDQP	76	428.231	2	854.4498	.	M	Superscaf	26329597	0	0	0	0	BTP20220	0	Tissue specific
CDS|exon|ID=cds-Bg	9	5.333333	3783.4	0.565523	LGTDQPLDQ	79	986.4749	1	985.4716	.	M	Superscaf	26329597	0	0	0	0	0	BTP20220	Tissue specific
CDS|exon|ID=cds-Bg	8	5.29375	3172.7	0.565523	LGTDQPLD	78	858.4147	1	857.413	.	M	Superscaf	26329597	0	0	0	0	0	BTP20220	Tissue specific
CDS|exon|ID=cds-Bg	10	5.457	4138.8	0.565522	GTDQPLDQA	60	523.2421	2	1044.472	.	M	Superscaf	26329597	0	0	0	0	0	BTP20220	Tissue specific
CDS|exon|ID=cds-Bg	10	6.448	3800.2	0.56552	QAGMTAPGT	97	481.2403	2	960.4698	.	P	Superscaf	26329816	0	0	0	0	BTP20220	0	Tissue specific
CDS|exon|ID=cds-Bg	9	6.536667	3189.5	0.56552	AGMTAPGTK	98	417.2111	2	832.4113	.	P	Superscaf	26329816	0	0	0	0	BTP20220	BTP20220	Mixed
CDS|exon|ID=cds-Bg	12	5.333333	4909.7	0.565519	FEPGLGM(+	95	704.7764	2	1407.544	Oxidation	P	Superscaf	26329816	0	0	0	0	BTP20220	0	Tissue specific
CDS|exon|ID=cds-Bg	10	5.199	4124.8	0.565519	EPGLGMEHC	91	572.7208	2	1143.432	Carbamid	P	Superscaf	26329816	0	0	0	0	BTP20220	0	Tissue specific
CDS|exon|ID=cds-Bg	11	5.32	4522.3	0.565519	EPGLGMEHC	99	623.245	2	1244.48	Carbamid	P	Superscaf	26329816	0	0	0	0	BTP20220	0	Tissue specific
CDS|exon|ID=cds-Bg	9	5.446667	3713.3	0.565519	EPGLGMEHC	93	515.208	2	1028.406	Carbamid	P	Superscaf	26329816	0	0	0	0	BTP20220	0	Tissue specific
CDS|exon|ID=cds-Bg	9	5.418889	3699.3	0.565519	PGLGMEHC(	84	508.2	2	1014.39	Carbamid	P	Superscaf	26329816	0	0	0	0	BTP20220	0	Tissue specific
CDS|exon|ID=cds-Bg	10	5.53	4096.8	0.565519	PGLGMEHC(	99	558.7238	2	1115.438	Carbamid	P	Superscaf	26329816	0	0	0	0	BTP20220	BTP20220	Tissue enhanced
CDS|exon|ID=cds-Bg	8	5.725	3287.8	0.565519	PGLGMEHC(	98	450.6865	2	899.363	Carbamid	P	Superscaf	26329816	0	0	0	0	BTP20220	0	Tissue specific
CDS|exon|ID=cds-Bg	8	5.37875	3462.1	0.565519	LGMEHC(+5	98	481.6876	2	961.3633	Carbamid	P	Superscaf	26329816	0	0	0	0	BTP20220	0	Tissue specific
CDS|exon|ID=cds-Bg	13	6.529231	5435.1	0.565518	MGSNKGASG	88	662.8067	2	1323.602	.	P	Superscaf	26329816	0	0	0	0	0	BTP20220	Tissue specific
CDS|exon|ID=cds-Bg	11	6.157273	4245.7	0.565518	KGASQRGMT	75	599.303	2	1196.597	.	P	Superscaf	26329816	0	0	0	0	0	BTP20220	Tissue specific
CDS|exon|ID=cds-Bg	9	5.438889	3578.1	0.565518	YGLPRQVYD	60	555.7832	2	1109.551	.	P	Superscaf	26329816	0	0	0	0	BTP20220	0	Tissue specific
CDS|exon|ID=cds-Bg	11	5.821818	4672.3	0.565517	LPRQVYDPK	68	480.2408	3	1437.708	Carbamid	P	Superscaf	26329816	0	0	0	0	BTP20220	0	Tissue specific
CDS|exon|ID=cds-Bg	7	5.904286	3075.4	0.565517	YDPKYC(+5	94	479.7187	2	957.4266	Carbamid	P	Superscaf	26329816	0	0	0	0	BTP20220	0	Tissue specific
CDS|exon|ID=cds-Bg	6	5.891667	2722	0.565517	YDPKYC(+5	89	423.1776	2	844.3425	Carbamid	P	Superscaf	26329816	0	BTP20220	0	0	BTP20220	0	Tissue enhanced
CDS|exon|ID=cds-Bg	15	5.514667	6263.2	0.565517	C(+57.02)	90	863.8873	2	1725.767	Carbamid	P	Superscaf	26329816	0	0	0	0	BTP20220	0	Tissue specific
CDS|exon|ID=cds-Bg	14	5.522143	5666.5	0.565517	C(+57.02)	72	806.8655	2	1611.724	Carbamid	P	Superscaf	26329816	0	0	0	0	BTP20220	0	Tissue specific
CDS|exon|ID=cds-Bg	13	5.560769	5080.8	0.565517	LTPEYPELG	78	726.8506	2	1451.693	.	P	Superscaf	26329816	0	0	0	0	0	BTP20220	Tissue specific
CDS|exon|ID=cds-Bg	8	5.3975	3033.5	0.565517	TPEYPELG	93	905.4213	1	904.4178	.	P	Superscaf	26329816	0	0	0	0	BTP20220	0	Tissue specific
CDS|exon|ID=cds-Bg	7	5.315714	2736.2	0.565517	TPEYPEL	90	848.3993	1	847.3963	.	P	Superscaf	26329816	0	0	0	0	BTP20220	0	Tissue specific
CDS|exon|ID=cds-Bg	12	5.4325	4926.6	0.565517	PEYPELGEP	96	676.8069	2	1351.605	.	P	Superscaf	26329816	0	0	0	0	BTP20220	0	Tissue specific
CDS|exon|ID=cds-Bg	11	5.434545	4329.9	0.565517	PEYPELGEP	94	619.788	2	1237.562	.	P	Superscaf	26329816	0	BTP20220	0	0	BTP20220	0	Tissue enhanced
CDS|exon|ID=cds-Bg	11	5.353636	4589.2	0.565517	EYPELGEPA	83	628.2811	2	1254.552	.	P	Superscaf	26329816	0	0	0	0	BTP20220	BTP20220	Mixed
CDS|exon|ID=cds-Bg	10	5.348	3992.5	0.565517	EYPELGEPA	87	571.259	2	1140.509	.	P	Superscaf	26329816	0	0	0	0	BTP20220	BTP20220	Mixed
CDS|exon|ID=cds-Bg	10	5.567	4163.7	0.565517	YPELGEPAH	81	563.7596	2	1125.509	.	P	Superscaf	26329816	0	0	0	0	BTP20220	0	Tissue specific
CDS|exon|ID=cds-Bg	9	5.584444	3567	0.565517	YPELGEPAH	63	506.7412	2	1011.466	.	P	Superscaf	26329816	0	0	0	0	BTP20220	0	Tissue specific
CDS|exon|ID=cds-Bg	11	5.926364	4943.6	0.565517	ELGEPAHNH	75	619.2865	2	1236.564	.	P	Superscaf	26329816	0	0	0	0	0	BTP20220	Tissue specific
CDS|exon|ID=cds-Bg	12	6.2475	5734.5	0.565517	LGEPAHNHH	95	453.8802	3	1358.623	.	P	Superscaf	26329816	0	0	0	0	0	BTP20220	Tissue specific
CDS|exon|ID=cds-Bg	15	5.586667	5509.2	0.564289	SSMAEVDAA	90	772.3497	2	1542.692	.	H	Superscaf	26404054	0	0	0	0	0	BTP20220	Tissue specific
CDS|exon|ID=cds-Bg	17	4.726471	6029.7	0.564285	FVTFDDHDP	98	672.6824	3	2015.036	.	H	Superscaf	26404054	0	0	0	0	BTP20220	0	Tissue specific
CDS|exon|ID=cds-Bg	8	4.35125	2976.5	0.564285	VTFDDHDP	70	473.1982	2	944.3876	.	H	Superscaf	26404054	0	0	0	0	BTP20220	0	Tissue specific
CDS|exon|ID=cds-Bg	16	4.679375	5642.3	0.564285	VTFDDHDPV	98	623.6605	3	1867.968	.	H	Superscaf	26404054	0	0	0	0	BTP20220	BTP20220	Mixed
CDS|exon|ID=cds-Bg	15	4.342	5173.9	0.564285	VTFDDHDPV	87	870.9402	2	1739.873	.	H	Superscaf	26404054	0	0	0	0	0	BTP20220	Tissue specific
CDS|exon|ID=cds-Bg	14	4.248571	4563.2	0.564285	VTFDDHDPV	93	806.9101	2	1611.815	.	H	Superscaf	26404054	0	0	0	0	0	BTP20220	Tissue specific
CDS|exon|ID=cds-Bg	11	4.32	3856.4	0.564285	VTFDDHDPV	99	644.2944	2	1286.578	.	H	Superscaf	26404054	0	0	0	0	BTP20220	BTP20220	Mixed
CDS|exon|ID=cds-Bg	14	4.652143	5173.9	0.564285	TFDDHDPVD	87	821.4059	2	1640.804	.	H	Superscaf	26404054	0	0	0	0	0	BTP20220	Tissue specific
CDS|exon|ID=cds-Bg	14	4.881429	5244.8	0.564285	FDDHDPVDK	92	556.9548	3	1667.852	.	H	Superscaf	26404054	0	0	0	0	0	BTP20220	Tissue specific
CDS|exon|ID=cds-Bg	13	4.507692	4776.4	0.564285	FDDHDPVDK	91	770.8823	2	1539.757	.	H	Superscaf	26404054	0	0	0	0	0	BTP20220	Tissue specific
CDS|exon|ID=cds-Bg	12	4.4125	4165.7	0.564285	FDDHDPVDK	78	706.8538	2	1411.698	.	H	Superscaf	26404054	0	0	0	0	0	BTP20220	Tissue specific
CDS|exon|ID=cds-Bg	11	4.27	3812.3	0.564285	FDDHDPVDK	83	650.3115	2	1298.614	.	H	Superscaf	26404054	0	0	0	0	0	BTP20220	Tissue specific
CDS|exon|ID=cds-Bg	13	4.835385	4857.4	0.564285	DDHDPVDKL	97	507.9335	3	1520.783	.	H	Superscaf	26404054	0	0	0	0	BTP20220	BTP20220	Mixed
CDS|exon|ID=cds-Bg	12	4.426667	4389	0.564285	DDHDPVDKL	89	697.3492	2	1392.689	.	H	Superscaf	26404054	0	0	0	0	BTP20220	BTP20220	Mixed
CDS|exon|ID=cds-Bg	11	4.315455	3778.3	0.564285	DDHDPVDKL	94	633.3192	2	1264.63	.	H	Superscaf	26404054	0	0	0	0	0	BTP20220	Tissue specific
CDS|exon|ID=cds-Bg	10	4.149	3424.9	0.564285	DDHDPVDKL	97	576.7782	2	1151.546	.	H	Superscaf	26404054	0	0	0	BTP20220	BTP20220	BTP20220	Mixed
CDS|exon|ID=cds-Bg	12	4.990833	4445.9	0.564285	DHDPVDKLV	99	469.5909	3	1405.757	.	H	Superscaf	26404054	0	0	0	0	0	BTP20220	Tissue specific
CDS|exon|ID=cds-Bg	11	4.559091	3977.5	0.564285	DHDPVDKLV	93	639.8346	2	1277.662	.	H	Superscaf	26404054	0	0	0	0	0	BTP20220	Tissue specific
CDS|exon|ID=cds-Bg	10	4.45	3366.8	0.564285	DHDPVDKLV	92	575.8055	2	1149.603	.	H	Superscaf	26404054	0	0	0	0	0	BTP20220	Tissue specific
CDS|exon|ID=cds-Bg	9	4.28	3013.4	0.564285	DHDPVDKLV	97	519.265	2	1036.519	.	H	Superscaf	26404054	0	0	0	0	0	BTP20220	Tissue specific
CDS|exon|ID=cds-Bg	9	4.614444	2955.3	0.564285	HDPVDKLVL	92	518.2939	2	1034.576	.	H	Superscaf	26404054	0	0	0	0	0	BTP20220	Tissue specific
CDS|exon|ID=cds-Bg	8	4.44375	2601.9	0.564285	HDPVDKLV	88	461.7519	2	921.4919	.	H	Superscaf	26404054	0	0	0	0	0	BTP20220	Tissue specific
CDS|exon|ID=cds-Bg	8	4.2425	2335.6	0.564285	DPVDKLVL	80	449.7634	2	897.5171	.	H	Superscaf	26404054	0	0	0	0	BTP20220	0	Tissue specific
CDS|exon|ID=cds-Bg	9	5.151111	3003.2	0.564285	PVDKLVLQK	99	520.3275	2	1038.644	.	H	Superscaf	26404054	0	0	0	0	0	BTP20220	Tissue specific
CDS|exon|ID=cds-Bg	8	5.0075	2665.8	0.564285	VDKLVLQK	99	471.8013	2	941.5909	.	H	Superscaf	26404054	0	0	0	0	BTP20220	BTP20220	Mixed
CDS|exon|ID=cds-Bg	12	5.9325	4783.4	0.558311	M(+42.01)	99	740.3745	2	1478.733	Acetylati	V	Superscaf	26766625	0	BTP20220	0	0	0	0	Tissue specific
CDS|exon|ID=cds-Bg	5	5.26	2032.3	0.557095	WLLDF	93	693.3607	1	692.3533	.	K	Superscaf	26840430	0	0	0	0	BTP20220	0	Tissue specific
CDS|exon|ID=cds-Bg	7	7.217143	3452.1	0.556093	RGPPNC(+5	58	428.7136	2	855.4133	Carbamid	A	Superscaf	26900752	0	BTP20220	0	0	0	0	Tissue specific
CDS|exon|ID=cds-Bg	9	5.996667	3600.8	0.552753	A(+42.01)	91	494.7682	2	987.5236	Acetylati	R	Superscaf	27103471	0	BTP20220	0	0	0	0	Tissue specific
CDS|exon|ID=cds-Bg	7	4.61	2499.8	0.55077	NAVLEYL	98	821.4363	1	820.433	.	A	Superscaf	27223776	0	0	0	0	BTP20220	BTP20220	Mixed
CDS|exon|ID=cds-Bg	7	5.714286	2177.3	0.549652	L(+42.01)	78	795.5322	1	794.5266	Acetylati	A	Superscaf	27291124	0	BTP20220	0	0	0	BTP20220	Tissue enhanced
CDS|exon|ID=cds-Bg	17	5.064118	5327.7	0.54621	A(+42.01)	93	829.406	2	1656.803	Acetylati	R	Superscaf	27499819	0	0	0	BTP20220	0	0	Tissue specific
CDS|exon|ID=cds-Bg	16	5.02125	4956.2	0.54621	A(+42.01)	99	763.8871	2	1525.763	Acetylati	R	Superscaf	27499819	0	BTP20220	0	0	0	BTP20220	Mixed
CDS|exon|ID=cds-Bg	13	4.794615	4036.3	0.54621	VEAAAEVAA	96	643.3364	2	1284.656	.	R	Superscaf	27499819	0	0	0	0	BTP20220	BTP20220	Mixed
CDS|exon|ID=cds-Bg	7	6.114286	3152.6	0.546063	GNRFEPY	91	441.7084	2	881.4031	.	L	Superscaf	27508846	0	BTP20220	0	0	0	0	Tissue specific
CDS|exon|ID=cds-Bg	13	7.123077	6127	0.546063	RFEPYANPT	89	425.2259	4	1696.88	.	L	Superscaf	27508846	0	0	BTP20220	0	0	0	Tissue specific
CDS|exon|ID=cds-Bg	9	5.316667	3136.5	0.545752	EGPFGGGME	99	880.3484	1	879.3433	.	N	Superscaf	27527749	0	BTP20220	0	0	0	0	Tissue specific
CDS|exon|ID=cds-Bg	11	5.632727	3651.1	0.545601	VPAGMGAGL	83	529.2758	2	1056.539	.	T	Superscaf	27536425	0	0	0	BTP20220	0	0	Tissue specific
CDS|exon|ID=cds-Bg	11	5.474545	4503.2	0.545556	ESPEVAERA	97	652.3038	2	1302.599	Carbamid	A	Superscaf	27539515	0	0	0	0	BTP20220	0	Tissue specific
CDS|exon|ID=cds-Bg	8	5.88	2808	0.542611	GGPGGGNF	97	662.2877	1	661.282	.	R	Superscaf	27717322	0	BTP20220	0	BTP20220	0	0	Mixed
CDS|exon|ID=cds-Bg	7	4.761429	2464.9	0.535332	RVFTVDH	70	437.231	2	872.4504	.	R	Superscaf	28158940	0	0	0	0	0	BTP20220	Tissue specific
CDS|exon|ID=cds-Bg	10	5.163	3205.5	0.535331	KLLPVGQTV	69	551.3345	2	1100.659	.	R	Superscaf	28158940	0	0	0	0	0	BTP20220	Tissue specific
CDS|exon|ID=cds-Bg	9	4.693333	3286.5	0.535297	SAEFEVKEY	89	551.2561	2	1100.502	.	S	Superscaf	28161208	0	0	0	0	0	BTP20220	Tissue specific
CDS|exon|ID=cds-Bg	11	4.859091	3951.4	0.535291	LYGEQVDGT	66	1199.55	1	1198.551	.	F	Superscaf	28161646	0	0	0	0	0	BTP20220	Tissue specific
CDS|exon|ID=cds-Bg	7	3.768571	2178.6	0.535078	VTVMQDF	99	839.3939	1	838.3895	.	D	Superscaf	28174522	0	0	0	0	0	BTP20220	Tissue specific
CDS|exon|ID=cds-Bg	20	4.611	7228.2	0.535023	EEVPAADLS	97	1080.493	2	2158.975	.	G	Superscaf	28177879	0	0	0	0	0	BTP20220	Tissue specific
CDS|exon|ID=cds-Bg	17	5.045882	6377.2	0.535023	PAADLSDQV	90	901.9183	2	1801.822	.	G	Superscaf	28177879	0	0	0	0	BTP20220	0	Tissue specific
CDS|exon|ID=cds-Bg	10	5.411	4375.9	0.535008	LDSTDQWEK	99	634.7905	2	1267.572	.	V	Superscaf	28178722	0	0	0	0	0	BTP20220	Tissue specific
CDS|exon|ID=cds-Bg	15	6.408667	6693.3	0.534997	GYTQQLAFR	93	874.4374	2	1746.869	.	A	Superscaf	28179463	0	0	0	0	0	BTP20220	Tissue specific
CDS|exon|ID=cds-Bg	11	6.644545	5155.7	0.534997	GYTQQLAFR	98	670.3545	2	1338.704	.	A	Superscaf	28179463	0	0	0	0	0	BTP20220	Tissue specific
CDS|exon|ID=cds-Bg	10	6.667	4317.6	0.534997	LAFRQKSSA	94	585.8151	2	1169.619	.	A	Superscaf	28179463	0	0	0	0	0	BTP20220	Tissue specific
CDS|exon|ID=cds-Bg	16	6.395	6191.7	0.534907	ALALLGKLE	97	582.3466	3	1744.025	.	S	Superscaf	28184893	0	0	0	0	0	BTP20220	Tissue specific
CDS|exon|ID=cds-Bg	15	6.456	5804.3	0.534907	ALALLGKLE	91	400.2438	4	1596.956	.	S	Superscaf	28184893	0	0	0	0	0	BTP20220	Tissue specific
CDS|exon|ID=cds-Bg	9	6.942222	3464.7	0.534878	ALLALLARK	93	484.8328	2	967.6542	.	T	Superscaf	28186591	0	0	0	0	0	BTP20220	Tissue specific
CDS|exon|ID=cds-Bg	17	5.362941	6743.4	0.534854	MVFQALAQY	98	673.3401	3	2017.009	.	Q	Superscaf	28188166	0	0	0	0	0	BTP20220	Tissue specific
CDS|exon|ID=cds-Bg	9	5.134444	3415.8	0.534855	MVFQALAQY	63	535.7682	2	1069.527	.	Q	Superscaf	28188166	0	0	0	0	0	BTP20220	Tissue specific
CDS|exon|ID=cds-Bg	10	5.326	4282.8	0.53195	VHLQAGQC(	62	542.2522	2	1082.493	Carbamid	P	Superscaf	28364026	0	0	BTP20220	0	BTP20220	0	Mixed
CDS|exon|ID=cds-Bg	8	5.235	3388.8	0.53195	VHLQAGQC(	91	456.7203	2	911.4283	Carbamid	P	Superscaf	28364026	0	0	0	BTP20220	0	0	Tissue specific
CDS|exon|ID=cds-Bg	16	5.670625	6815.9	0.53194	DPTGTYHGD	94	902.4109	2	1802.807	.	F	Superscaf	28364746	0	0	0	0	BTP20220	0	Tissue specific
CDS|exon|ID=cds-Bg	7	5.464286	2853.2	0.530892	NALLEYL	67	835.453	1	834.4487	.	N	Superscaf	28428429	0	0	0	0	0	BTP20220	Tissue specific

CDS|exon|ID=cds-B	9	6.645556	3127.5	0.528368	GPPPPPPPK	89	442.2548	2	882.4963	.	L	Superscaf	28580977	0	BTP202209	0	0	0	0	Tissue specific
CDS|exon|ID=cds-B	11	6.645455	4061.5	0.528233	EPPPPKKPF	92	590.8383	2	1179.665	.	V	Superscaf	28589293	0	0	BTP202209	0	0	0	Tissue specific
CDS|exon|ID=cds-B	24	5.284583	8287.1	0.523763	MGLTAALVK	89	868.4583	3	2602.365	.	A	Superscaf	28860208	0	0	0	0	0	BTP202209	Tissue specific
CDS|exon|ID=cds-B	9	5.776667	2864.1	0.523764	MGLTAALVK	99	452.2682	2	902.5259	.	A	Superscaf	28860208	0	0	0	0	0	BTP202209	Tissue specific
CDS|exon|ID=cds-B	8	4.55125	2540.7	0.523763	QVFAEPKV	90	459.2559	2	916.5018	.	A	Superscaf	28860208	0	0	0	0	0	BTP202209	Tissue specific
CDS|exon|ID=cds-B	10	4.528	3178.5	0.521712	YLDVPVDLS	94	574.8097	2	1147.613	.	P	Superscaf	28984435	0	0	0	0	0	BTP202209	Tissue specific
CDS|exon|ID=cds-B	8	5.505	2672.9	0.521626	AAALAVER	98	400.7336	2	799.4551	.	P	Superscaf	28989685	0	0	0	BTP202209	0	0	Tissue specific
CDS|exon|ID=cds-B	9	4.417778	2640	0.507703	VSEPTTVAF	66	950.4796	1	949.4756	.	E	Superscaf	29833375	0	0	0	0	0	BTP202209	Tissue specific
CDS|exon|ID=cds-B	9	5.518889	3351.9	0.505246	VQTGMDTTK	91	490.7392	2	979.4645	.	E	Superscaf	29982055	0	0	0	0	0	BTP202209	Tissue specific
CDS|exon|ID=cds-B	13	6.059231	4655.3	0.505246	GMDTTKTVL	95	676.8522	2	1351.702	.	E	Superscaf	29982055	0	0	0	0	BTP202209	0	Tissue specific
CDS|exon|ID=cds-B	12	6.066667	4358	0.505246	MDTTKTVLT	94	648.3441	2	1294.68	.	E	Superscaf	29982055	0	0	0	0	BTP202209	0	Tissue specific
CDS|exon|ID=cds-B	13	5.888462	4795.5	0.505245	DTTKTVLTG	98	690.8619	2	1379.714	.	E	Superscaf	29982055	0	0	0	0	BTP202209	0	Tissue specific
CDS|exon|ID=cds-B	11	6.095455	3986.5	0.505246	DTTKTVLTG	98	582.8251	2	1163.64	.	E	Superscaf	29982055	0	0	0	0	BTP202209	0	Tissue specific
CDS|exon|ID=cds-B	15	5.559333	5037.5	0.505239	GVAKGAVQT	79	732.3778	2	1462.745	.	E	Superscaf	29982055	0	0	0	0	BTP202209	0	Tissue specific
CDS|exon|ID=cds-B	13	5.849231	4709.3	0.505231	DTTKTVLTG	97	675.857	2	1349.704	.	E	Superscaf	29982055	0	0	0	0	BTP202209	0	Tissue specific
CDS|exon|ID=cds-B	11	4.07	3260.7	0.503371	SYVEVLVPL	91	622.3495	2	1242.686	.	Q	Superscaf	30095890	0	BTP202209	0	0	0	BTP202209	Mixed
CDS|exon|ID=cds-B	10	3.909	2877.3	0.503371	YVEVLVPLF	69	578.8335	2	1155.654	.	Q	Superscaf	30095890	0	BTP202209	0	0	0	0	Tissue specific
CDS|exon|ID=cds-B	9	5.32	3828.1	0.503192	KFEVYEKNN	99	585.7905	2	1169.572	.	Y	Superscaf	30107001	0	0	0	0	0	BTP202209	Tissue specific
CDS|exon|ID=cds-B	9	6.477778	3239.5	0.502014	GGGSGARLL	63	394.2305	2	786.4348	.	G	Superscaf	30178282	0	0	BTP202209	0	0	0	Tissue specific
CDS|exon|ID=cds-B	13	5.716154	5800.3	0.500433	KLEELELDE	85	543.9592	3	1628.862	.	S	Superscaf	30273952	0	0	0	0	0	BTP202209	Tissue specific
CDS|exon|ID=cds-B	12	5.380833	5331.9	0.500433	LEELELDEG	99	751.3867	2	1500.767	.	S	Superscaf	30273952	0	0	0	0	BTP202209	BTP202209	Mixed
CDS|exon|ID=cds-B	8	6.7	2920.3	0.499332	LAGGPPRP	74	382.7285	2	763.434	.	A	Superscaf	30340795	0	0	0	0	0	BTP202209	Tissue specific
CDS|exon|ID=cds-B	5	5.438	2007.3	0.493748	EPYTF	52	656.3012	1	655.2853	.	L	Superscaf	30679141	0	0	0	BTP202209	0	0	Tissue specific
CDS|exon|ID=cds-B	7	6.658571	2442.6	0.487967	KPPLAGP	65	679.4091	1	678.4064	.	K	Superscaf	31029690	0	0	BTP202209	0	0	0	Tissue specific
CDS|exon|ID=cds-B	7	6.757143	2880.3	0.487506	PGQPARP	71	361.7049	2	721.3871	.	T	Superscaf	31057540	0	0	BTP202209	0	0	0	Tissue specific
CDS|exon|ID=cds-B	7	6.675714	2945.1	0.484934	EKNPAKP	56	392.2244	2	782.4286	.	R	Superscaf	31213372	0	0	0	0	0	BTP202209	Tissue specific
CDS|exon|ID=cds-B	14	5.828571	4887.3	0.480091	SSSGSSVST	76	618.7932	2	1235.574	.	P	Superscaf	31506811	0	BTP202209	0	0	0	0	Tissue specific
CDS|exon|ID=cds-B	25	5.5236	8915.7	0.480089	VEEPPGTGS	97	1148.002	2	2293.993	.	P	Superscaf	31506811	0	BTP202209	0	0	BTP202209	BTP202209	Mixed
CDS|exon|ID=cds-B	9	6.687778	3520.8	0.480085	KFTPKYVLF	87	384.5682	3	1150.686	.	P	Superscaf	31506811	0	BTP202209	0	0	BTP202209	0	Mixed
CDS|exon|ID=cds-B	8	6.30875	3684.3	0.476013	M(+42.01)	93	515.2313	2	1028.45	Acetylati	Q	Superscaf	31753996	0	BTP202209	0	0	0	0	Tissue specific
CDS|exon|ID=cds-B	12	5.710833	4344.6	0.475986	EAPAQPAPE	93	632.8454	2	1263.682	.	V	Superscaf	31755469	0	0	0	0	0	BTP202209	Tissue specific
CDS|exon|ID=cds-B	10	5.496	3438.7	0.475368	AGELNPVAF	97	498.2773	2	994.5447	.	P	Superscaf	31792963	0	0	0	0	0	BTP202209	Tissue specific
CDS|exon|ID=cds-B	10	5.53	3770	0.475325	ADSEKLPVG	83	557.8099	2	1113.603	.	A	Superscaf	31795714	0	0	0	0	0	BTP202209	Tissue specific
CDS|exon|ID=cds-B	10	4.439	3315.6	0.475324	EKEDVPLVE	94	593.319	2	1184.629	.	A	Superscaf	31795714	0	BTP202209	0	0	0	0	Tissue specific
CDS|exon|ID=cds-B	10	5.091	3358.5	0.475324	KEDVPLVEK	86	395.5668	3	1183.681	.	A	Superscaf	31795714	0	BTP202209	0	0	0	0	Tissue specific
CDS|exon|ID=cds-B	9	4.574444	2890.1	0.475324	EDVPLVEKK	90	528.8011	2	1055.586	.	A	Superscaf	31795714	0	BTP202209	0	0	0	0	Tissue specific
CDS|exon|ID=cds-B	8	3.92875	2421.7	0.475324	EDVPLVEK	84	464.7514	2	927.4913	.	A	Superscaf	31795714	0	BTP202209	0	0	0	0	Tissue specific
CDS|exon|ID=cds-B	8	5.8725	3246.7	0.473225	A(+42.01)	99	482.7394	2	963.4661	Acetylati	L	Superscaf	31922698	0	BTP202209	0	0	0	BTP202209	Tissue enhanced
CDS|exon|ID=cds-B	9	5.818889	3207.5	0.473012	SLDLVTMKK	94	517.7972	2	1033.584	.	G	Superscaf	31935547	0	0	0	0	0	BTP202209	Tissue specific
CDS|exon|ID=cds-B	9	5.744444	3835.2	0.472972	SLQETYEAK	78	534.7615	2	1067.513	.	H	Superscaf	31938220	0	0	0	0	BTP202209	BTP202209	Mixed
CDS|exon|ID=cds-B	7	5.742857	2882.2	0.457028	FGLLNGN	96	734.3818	1	733.3759	.	R	Superscaf	32904591	0	BTP202209	0	0	BTP202209	BTP202209	Mixed
CDS|exon|ID=cds-B	7	5.971429	2395.7	0.379247	PFGMALP	70	732.3734	1	731.3676	.	S	Superscaf	37618111	0	0	0	BTP202209	0	0	Tissue specific
CDS|exon|ID=cds-B	9	4.782222	3374.9	0.378808	DERVDLLSF	69	547.2778	2	1092.545	.	P	Superscaf	37644724	0	0	0	0	0	BTP202209	Tissue specific
CDS|exon|ID=cds-B	12	5.31	4127.5	0.378807	TGSTQVGKG	59	594.8303	2	1187.651	.	P	Superscaf	37644724	0	0	0	0	0	BTP202209	Tissue specific
CDS|exon|ID=cds-B	7	5.647143	2634	0.296101	MRYVASY	81	445.2144	2	888.4164	.	M	Superscaf	42656776	0	BTP202209	BTP202209	BTP202209	BTP202209	BTP202209	Tissue enhanced
CDS|exon|ID=cds-B	7	7.955714	3596.9	0.262215	R(+42.01)	59	463.761	2	925.5093	Acetylati	E	Superscaf	44710410	0	BTP202209	0	0	0	0	Tissue specific
CDS|exon|ID=cds-B	9	5.706667	3212.9	0.204428	M(+15.99)	75	523.2531	2	1044.491	Oxidation	N	Superscaf	48212296	BTP202209	0	0	0	0	0	Tissue specific
CDS|exon|ID=cds-B	15	5.668667	6560.5	0.203417	A(+42.01)	96	893.924	2	1785.838	Acetylati	G	Superscaf	48273493	0	0	0	0	BTP202209	0	Tissue specific
CDS|exon|ID=cds-B	7	4.455714	1928.1	0.18801	TVVGLDK	55	366.2213	2	730.4225	.	E	Superscaf	49207135	0	0	BTP202209	0	0	0	Tissue specific
CDS|exon|ID=cds-B	7	5.421429	2883.2	0.187711	NEASLYL	58	809.4019	1	808.3967	.	H	Superscaf	49225189	0	0	0	0	BTP202209	0	Tissue specific
CDS|exon|ID=cds-B	7	5.97	2783.2	0.185761	EGAPERP	88	378.1928	2	754.3609	.	F	Superscaf	49343640	0	0	0	0	BTP202209	0	Tissue specific
CDS|exon|ID=cds-B	9	5.705556	3685.2	0.180862	ELPMDPKSN	91	515.7457	2	1029.48	.	S	Superscaf	49640362	0	0	0	0	BTP202209	0	Tissue specific
CDS|exon|ID=cds-B	13	5.681538	4734.8	0.180861	KEEEPVKKV	99	792.4718	2	1582.929	.	S	Superscaf	49640362	0	0	BTP202209	0	0	0	Tissue specific
CDS|exon|ID=cds-B	12	5.343333	4266.4	0.180861	KEEEPVKKV	99	485.9501	3	1454.835	.	S	Superscaf	49640362	0	0	BTP202209	0	0	0	Tissue specific
CDS|exon|ID=cds-B	12	5.343333	4266.4	0.180861	EEEPVKKVL	77	485.9506	3	1454.835	.	S	Superscaf	49640362	0	0	BTP202209	0	0	0	Tissue specific
CDS|exon|ID=cds-B	7	6.642857	3012.6	0.156269	P(+42.01)	78	420.7036	2	839.3959	Acetylati	L	Superscaf	51130833	0	0	0	BTP202209	0	0	Tissue specific
CDS|exon|ID=cds-B	7	4.721429	2138.4	0.127234	VAPFDLP	85	758.4064	1	757.401	.	G	Superscaf	52890405	0	BTP202209	0	BTP202209	0	0	Mixed
CDS|exon|ID=cds-B	18	6.117778	7912.6	0.117945	KSSGNPLND	93	511.518	4	2042.051	.	R	Superscaf	53453164	0	0	0	BTP202209	0	0	Tissue specific
CDS|exon|ID=cds-B	16	5.91875	7060.8	0.117945	SGNPLNDKD	97	609.9803	3	1826.923	.	R	Superscaf	53453164	0	0	0	BTP202209	0	0	Tissue specific
CDS|exon|ID=cds-B	14	5.932143	6380.1	0.117945	NPLNDKDKD	97	561.9606	3	1682.87	.	R	Superscaf	53453164	0	0	0	BTP202209	0	0	Tissue specific
CDS|exon|ID=cds-B	9	5.271111	2977.5	0.115225	YTSVTPVLF	98	518.293	2	1034.576	.	V	Superscaf	53618023	0	0	0	0	BTP202209	BTP202209	Mixed
CDS|exon|ID=cds-B	10	5.903	3480.1	0.115119	LDPLPPPPF	93	520.293	2	1038.575	.	R	Superscaf	53624479	0	0	0	BTP202209	BTP202209	0	Tissue enhanced
CDS|exon|ID=cds-B	9	5.34	3163.4	0.112479	VGPDFQKGL	97	480.7596	2	959.5076	.	P	Superscaf	53784271	0	0	0	BTP202209	0	0	Tissue specific
CDS|exon|ID=cds-B	12	5.790833	5258.9	0.112435	YDTKLGTDK	99	715.3143	2	1428.619	Carbamido	V	Superscaf	53787031	0	0	0	0	BTP202209	0	Tissue specific
CDS|exon|ID=cds-B	9	6.232222	3665	0.112435	YDTKLGTDK	99	520.7648	2	1039.519	.	V	Superscaf	53787031	0	0	0	0	BTP202209	0	Tissue specific
CDS|exon|ID=cds-B	9	4.615556	3432	0.110558	M(+42.01)	54	536.7518	2	1071.491	Acetylati	L	Superscaf	53900809	0	BTP202209	0	0	0	0	Tissue specific
CDS|exon|ID=cds-B	9	5.006667	3038.2	0.10969	A(+42.01)	74	504.2537	2	1006.497	Acetylati	V	Superscaf	53953423	0	0	0	0	BTP202209	0	Tissue specific
CDS|exon|ID=cds-B	11	5.995455	4394.8	0.10968	SGGYGGSRD	88	591.2449	2	1180.479	.	G	Superscaf	53953825	0	0	0	BTP202209	0	0	Tissue specific
CDS|exon|ID=cds-B	9	6.161111	3066.5	0.107507	TPPGGFPPF	97	866.4379	1	865.4333	.	A	Superscaf	54085696	0	0	0	BTP202209	0	0	Tissue specific
CDS|exon|ID=cds-B	8	6.02125	2988.3	0.107506	PQGYGAPP	71	786.3749	1	785.3708	.	A	Superscaf	54085696	0	0	0	BTP202209	0	0	Tissue specific
CDS|exon|ID=cds-B	9	5.821111	3343.9	0.107495	GPPPPPPDG	91	451.222	2	900.4341	.	A	Superscaf	54086476	0	0	0	BTP202209	0	0	Tissue specific
CDS|exon|ID=cds-B	12	6.738333	5260.7	0.107408	A(+42.01)	95	391.2243	4	1560.874	Acetylati	G	Superscaf	54091771	0	0	0	BTP202209	0	0	Tissue specific
CDS|exon|ID=cds-B	10	5.197	3977.6	0.107407	FTYRGVDLD	86	607.2936	2	1212.577	.	G	Superscaf	54091771	0	0	0	0	0	BTP202209	Tissue specific
CDS|exon|ID=cds-B	7	5.744286	2971.3	0.107003	AQALDHL	65	384.206	2	766.3973	.	F	Superscaf	54115807	BTP202209	0	0	0	0	0	Tissue specific
CDS|exon|ID=cds-B	8	7.075	3642.2	0.106979	EALPGHRR	60	468.2623	2	934.5096	.	P	Superscaf	54117691	BTP202209	0	0	0	0	0	Tissue specific
CDS|exon|ID=cds-B	6	4.703333	1516.6	0.102822	KGLVVT	89	616.4008	1	615.3956	.	R	Superscaf	54369652	0	0	0	BTP202209	0	0	Tissue specific
CDS|exon|ID=cds-B	6	6.108333	2518.7	0.100653	M(+42.01)	98	413.1935	2	824.3738	Acetylati	V	Superscaf	54501145	0	BTP202209	0	0	0	0	Tissue specific
CDS|exon|ID=cds-B	5	6.626	2083.2	0.096467	ANLLK	61	558.359	1	557.3537	.	Y	Superscaf	54754887	0	0	0	0	0	BTP202209	Tissue specific
CDS|exon|ID=cds-B	8	4.9125	2926.3	0.094457	VDMGHSSY	88	448.1827	2	894.3542	.	M	Superscaf	54876526	0	0	0	0	0	BTP202209	Tissue specific
CDS|exon|ID=cds-B	8	6.53	3415.6	0.094234	NHAAPFSK	95	436.2225	2	870.4348	.	K	Superscaf	54890113	0	0	0	0	0	BTP202209	Tissue specific
CDS|exon|ID=cds-B	10	5.915	3865.3	0.094233	VLTFYRKEF	85	650.3548	2	1298.702	.	K	Superscaf	54890113	0	0	0	0	0	BTP202209	Tissue specific
CDS|exon|ID=cds-B	12	5.2225	3742.2	0.068894	GPPVASHPV	97	573.2982	2	1144.588	.	F	Superscaf	56425546	0	0	0	0	0	BTP202209	Tissue specific
CDS|exon|ID=cds-B	9	4.341111	2129.3	0.067548	VVTVVTTKK	99	487.8148	2	973.6172	.	K	Superscaf	56507305	0	BTP202209	0	0	0	0	Tissue specific
CDS|exon|ID=cds-B	7	6.618571	2668.9	0.051337	GPGVHRK	72	375.7202	2	749.4296	.	Q	Superscaf	57489601	0	0	0	0	BTP202209	0	Tissue specific
CDS|exon|ID=cds-B	6	8.32	3036.6	0.047524	RRSLLR	61	400.7631	2	799.514	.	P	Superscaf	57720592	0	0	BTP202209	0	0	0	Tissue specific
CDS|exon|ID=cds-B	10	5.136	3242.6	0.043812	EPGALSTSV	75	973.516	1	972.5128	.	R	Superscaf	57946563	0	0	0	BTP202209	0	0	Tissue specific
CDS|exon|ID=cds-B	10	6.539	4702.3	0.01674	M(+42.01)	89	705.8337	2	1409.655	Acetylati	R	Superscaf	59586148	BTP202209	0	0	0	0	0	Tissue specific
CDS|exon|ID=cds-B	8	6.54	3695.2	0.01674	M(+15.99)	96	571.7676	2	1141.523	Oxidation	R	Superscaf	59586148	BTP202209	0	0	0	0	0	Tissue specific
CDS|exon|ID=cds-B	7	6.082857	3226.8	0.01674	M(+42.01)	97	499.7226	2	997.4327	Acetylati	R	Superscaf	59586148	BTP202209	0	0	0	0	0	Tissue specific
CDS|exon|ID=cds-B	14	5.74	5787.5	0.016737	TEQRYSASS	92	513.9056	3	1538.696	.	R	Superscaf	59586148	BTP202209	0	0	0	0	0	Tissue specific
CDS|exon|ID=cds-B	10	5.42	3705	0.016737	YSASSTVSS	99	513.23	2	1024.446	.	R	Superscaf	59586148	BTP202209	0	0	0	0	0	Tissue specific
CDS|exon|ID=cds-B	9	5.813333	3771.2	0.016506	EGEFQRLAA	91	510.7588	2	1019.504	.	S	Superscaf	59600431	BTP202209	0	0	0	0	0	Tissue specific
CDS|exon|ID=cds-B	8	5.19125	2632.9	0.010795	QGLPGLVS	85	770.4385	1	769.4334	.	D	Superscaf	59946762	0	0	BTP202209	0	0	0	Tissue specific
CDS|exon|ID=cds-B	11	5.451818	3959.6	0.00422	TLAVERLLE	81	627.3736	2	1252.739	.	L	Superscaf	60344980	0	0	0	0	0	BTP202209	Tissue specific
CDS|exon|ID=cds-B	14	4.776429	4603	0.004199	LKEELVAAV	90	533.6389	3	1597.904	.	V	Superscaf	60346171	0	0	0	0	0	BTP202209	Tissue specific
CDS|exon|ID=cds-B	9	5.76	4324.9	0.003963	DDWEEKRHF	98	421.1904	3	1260.552	.	T	Superscaf	60840481	0	BTP202209	0	0	0	0	Tissue specific
CDS|exon|ID=cds-B	8	5.76375	3230.7	0.003964	FDDRGPSL	95	453.7183	2	905.4243	.	T	Superscaf	60840481	0	BTP202209	0	0	0	0	Tissue specific
CDS|exon|ID=cds-B	9	5.286667	3585.1	0.003964	VLDYDHGSF	91	354.5044	3	1060.494	.	T	Superscaf	60840481	0	BTP202209	0	0	0	0	Tissue specific
CDS|exon|ID=cds-B	9	4.915556	3431.9	0.004087	QLLQLVEPF	71	543.8109	2	1085.612	.	E	Superscaf	60848443	0	0	0	0	0	BTP202209	Tissue specific
CDS|exon|ID=cds-B	8	4.82375	2821.2	0.004087	LLQLVEPF	71	479.7816	2	957.5535	.	E	Superscaf	60848443	0	0	0	0	0	BTP202209	Tissue specific
CDS|exon|ID=cds-B	10	5.338	3451.6	0.004108	SGYSDSAVL	89	513.7566	2	1025.503	.	F	Superscaf	60849640	0	0	0	0	0	BTP202209	Tissue specific
CDS|exon|ID=cds-B	18	5.692778	6323.4	0.004148	GDVASDGKK	87	620.332	3	1857.98	.	T	Superscaf	60851863	0	0	0	0	BTP202209	0	Tissue specific
CDS|exon|ID=cds-B	16	4.2075	5800.6	0.004173	VTLDEVGDE	93	903.4115	2	1804.821	.	Q	Superscaf	60853723	0	0	0	0	0	BTP202209	Tissue specific
CDS|exon|ID=cds-B	12	5.760833	5386	0.004174	DEEDSELQK	98	497.2541	3	1488.742	.	Q	Superscaf	60853723	0	BTP202209	0	0	BTP202209	0	Mixed
CDS|exon|ID=cds-B	11	5.399091	4917.6	0.004174	DEEDSELQK	94	681.3266	2	1360.647	.	Q	Superscaf	60853723	0	0	0	0	BTP202209	0	Tissue specific
CDS|exon|ID=cds-B	8	5.05625	2384.4	0.00463	KEFVPGVK	85	452.2692	2	902.5225	.	P	Superscaf	60881278	0	0	0	BTP202209	0	0	Tissue specific
CDS|exon|ID=cds-B	7	5.838571	2627.9	0.006072	Y(+42.01)	65	765.3184	1	764.3163	Acetylati	S	Superscaf	60968338	0	0	0	0	0	BTP202209	Tissue specific
CDS|exon|ID=cds-B	6	5.693333	2509	0.020533	YMM(+15.9	51	402.639	2	803.2653	Oxidation	L	Superscaf	61845219	BTP202209	0	0	0	0	0	Tissue specific
CDS|exon|ID=cds-B	8	6.235	3419.9	0.023378	EDFRKLLM	68	526.2822	2	1050.553	.	K	Superscaf	62017537	0	0	BTP202209	0	0	0	Tissue specific
CDS|exon|ID=cds-B	8	5.99625	3062.2	0.033488	HGVYDKPK	60	472.2551	2	942.4922	.	K	Superscaf	62630170	0	BTP202209	0	0	0	0	Tissue specific
CDS|exon|ID=cds-B	6	5.471667	2407.7	0.033898	LLLEAQ	81	686.4048	1	685.401	.	T	Superscaf	62654947	0	0	0	0	0	BTP202209	Tissue specific
CDS|exon|ID=cds-B	6	7.416667	2536.7	0.035009	PAAYRK	59	353.209	2	704.3969	.	P	Superscaf	62722171	0	0	0	0	0	BTP202209	Tissue specific
CDS|exon|ID=cds-B	8	5.885	3009.3	0.04352	EAAGPLLH	78	807.4337	1	806.4286	.	E	Superscaf	63238102	BTP202209	0	0	0	0	0	Tissue specific
CDS|exon|ID=cds-B	7	5.691429	2443.7	0.043651	P(+42.01)	57	355.6662	2	709.3282	Acetylati	F	Superscaf	63245968	0	0	0	0	0	BTP202209	Tissue specific
CDS|exon|ID=cds-B	6	3.4	1553.8	0.04426	MADVVY	57	697.3217	1	696.3152	.	R	Superscaf	63282883	0	BTP202209	0	0	0	0	Tissue specific
CDS|exon|ID=cds-B	7	6.058571	2419.7	0.114341	TGGPLLS	86	644.3592	1	643.3541	.	F	Superscaf	67529863	0	0	0	BTP202209	BTP202209	0	Mixed
CDS|exon|ID=cds-B	7	7.242857	2874.9	0.121039	KLKLPEK	55	428.2857	2	854.5589	.	K	Superscaf	67935706	0	0	0	0	0	BTP202209	Tissue specific
CDS|exon|ID=cds-B	7	7.164286	2830	0.167313	KGSSAPR	62	351.6942	2	701.382	.	W	Superscaf	70739965	0	0	0	0	BTP202209	0	Tissue specific
CDS|exon|ID=cds-B	14	5.222857	5009.5	0.18844	DGGEHVVSS	99	489.9098	3	1466.711	.	K	Superscaf	72020011	0	BTP202209	0	0	0	0	Tissue specific
CDS|exon|ID=cds-B	17	5.982941	6262.6	0.188442	SSQGAKEKF	86	571.6431	3	1711.91	.	K	Superscaf	72020011	0	BTP202209	0	0	0	0	Tissue specific
CDS|exon|ID=cds-B	12	6.059167	4276.5	0.188442	KEKPVGELG	92	428.2493	3	1281.729	.	K	Superscaf	72020011	0	BTP202209	0	0	0	0	Tissue specific
CDS|exon|ID=cds-B	16	5.814375	6831.7	0.193057	M(+42.01)	98	644.3043	3	1929.892	Acetylati	E	Superscaf	72299776	0	BTP202209	0	0	0	0	Tissue specific
CDS|exon|ID=cds-B	13	5.699231	5584.4	0.193057	M(+42.01)	99	780.8475	2	1559.671	Acetylati	E	Superscaf	72299776	0	BTP202209	0	0	0	0	Tissue specific
CDS|exon|ID=cds-B	12	5.541667	4964.7	0.193057	M(+42.01)	99	712.3118	2	1422.612	Acetylati	E	Superscaf	72299776	0	BTP202209	0	0	0	0	Tissue specific
CDS|exon|ID=cds-B	17	6.641176	7155.5	0.19308	AKPKPKPNG	98	598.6664	3	1792.98	.	E	Superscaf	72299776	0	BTP202209	0	0	0	0	Tissue specific
CDS|exon|ID=cds-B	8	6.2625	3212.7	0.193491	TSGLELLR	76	444.7603	2	887.5076	.	N	Superscaf	72326356	BTP202209	0	0	0	0	0	Tissue specific
CDS|exon|ID=cds-B	7	7.195714	3355.9	0.193491	LRPSFNR	95	445.2518	2	888.493	.	N	Superscaf	72326356	BTP202209	0	0	0	0	0	Tissue specific
CDS|exon|ID=cds-B	7	4.91	2593.8	0.204054	A(+42.01)	98	860.3438	1	859.3388	Acetylati	Q	Superscaf	72967065	0	BTP202209	0	0	0	0	Tissue specific
CDS|exon|ID=cds-B	9	5.064444	3226.4	0.20917	SVANKFYVF	91	532.7802	2	1063.545	.	C	Superscaf	73276675	0	BTP202209	0	0	0	0	Tissue specific
CDS|exon|ID=cds-B	12	4.404167	4166.7	0.20921	VLEETAPED	98	679.3445	2	1356.677	.	I	Superscaf	73278970	0	BTP202209	0	0	0	BTP202209	Tissue enhanced
CDS|exon|ID=cds-B	11	3.819091	3435.8	0.209263	VVFDDSEPV	98	631.8145	2	1261.619	.	S	Superscaf	73282258	0	0	0	0	0	BTP202209	Tissue specific
CDS|exon|ID=cds-B	6	5.57	1934.1	0.247149	G(+42.01)	60	736.3675	1	735.3625	Acetylati	V	Superscaf	75578391	0	0	0	0	0	BTP202209	Tissue specific

CDS|exon|ID=cds-Bg	8	5.2125	2574.8	0.257636	G(+42.01)	74	754.3704	1	753.3657	Acetylati	P	Superscaf	76213231	0	0	0	0	0	BTP202209	Tissue specific
CDS|exon|ID=cds-Bg	13	5.403077	4369.9	0.257638	A(+42.01)	66	646.3433	2	1290.682	Acetylati	P	Superscaf	76213231	0	0	0	0	0	BTP202209	Tissue specific
CDS|exon|ID=cds-Bg	8	4.96	2789.2	0.257638	A(+42.01)	55	912.499	1	911.4964	Acetylati	P	Superscaf	76213231	0	0	BTP202209	0	0	0	Tissue specific
CDS|exon|ID=cds-Bg	7	7.287143	2984.1	0.308203	K(+42.01)	68	432.7677	2	863.5229	Acetylati	V	Superscaf	79278103	0	BTP202209	0	0	0	0	Tissue specific
CDS|exon|ID=cds-Bg	9	4.717778	2592.6	0.373934	SVYVYKVLK	98	549.8303	2	1097.648	.	V	Superscaf	83261425	0	BTP202209	BTP202209	0	BTP202209	BTP202209	Mixed
CDS|exon|ID=cds-Bg	8	4.5975	2209.2	0.373934	VYVYKVLK	99	506.3126	2	1010.616	.	V	Superscaf	83261425	0	0	0	0	BTP202209	0	Tissue specific
CDS|exon|ID=cds-Bg	8	5.155	3295.7	0.411429	LDSNAVHH	83	446.7153	2	891.4199	.	E	Superscaf	85533661	0	0	0	0	0	BTP202209	Tissue specific
CDS|exon|ID=cds-Bg	6	4.136667	1343.5	0.496616	AVGTVP	73	543.3114	1	542.3064	.	R	Superscaf	90696124	0	0	0	0	0	BTP202209	Tissue specific
CDS|exon|ID=cds-Bg	7	4.904286	2378.7	0.499384	M(+42.01)	98	798.3783	1	797.3741	Acetylati	G	Superscaf	90863941	0	0	0	0	0	BTP202209	Tissue specific
CDS|exon|ID=cds-Bg	6	6.445	2693.1	0.499846	EEM(+15.9	52	411.2086	2	820.4113	Oxidation	F	Superscaf	90891913	0	0	0	0	0	BTP202209	Tissue specific
CDS|exon|ID=cds-Bg	9	5.226667	3294.8	0.511846	TGVDNPGHP	97	447.2085	2	892.4039	.	R	Superscaf	91619122	BTP202209	0	0	0	0	0	Tissue specific
CDS|exon|ID=cds-Bg	7	4.934286	2600	0.511846	VDNPGHP	94	368.1732	2	734.3347	.	R	Superscaf	91619122	0	0	0	0	BTP202209	BTP202209	Mixed
CDS|exon|ID=cds-Bg	8	5.0575	2966.5	0.598031	LYDLVMTH	99	496.2477	2	990.4844	.	S	Superscaf	96841765	0	0	0	0	0	BTP202209	Tissue specific
CDS|exon|ID=cds-Bg	7	4.925714	2613.1	0.598031	YDLVMTH	98	439.7053	2	877.4004	.	S	Superscaf	96841765	0	0	0	0	BTP202209	BTP202209	Mixed
CDS|exon|ID=cds-Bg	10	5.727	4010.5	0.598031	ALEWPSLTA	77	558.2886	2	1114.566	.	S	Superscaf	96841765	0	0	0	0	0	BTP202209	Tissue specific
CDS|exon|ID=cds-Bg	8	5.34375	2613	0.604728	WLVSPPPP	83	446.7482	2	891.4854	.	V	Superscaf	97247773	0	0	0	BTP202209	0	0	Tissue specific
CDS|exon|ID=cds-Bg	6	6.183333	2193.2	0.740533	KYYVPK	67	399.2303	2	796.4483	.	L	Superscaf	1.05E+08	0	BTP202209	0	0	0	0	Tissue specific
CDS|exon|ID=cds-Bg	11	5.953636	3965.2	0.755416	TQAGEKAPV	99	563.3165	2	1124.619	.	K	Superscaf	1.06E+08	0	BTP202209	0	0	0	0	Tissue specific
CDS|exon|ID=cds-Bg	13	6.444615	5426.8	0.756163	SEQPPSEKS	67	481.5868	3	1441.741	.	P	Superscaf	1.06E+08	0	BTP202209	0	0	0	0	Tissue specific
CDS|exon|ID=cds-Bg	13	5.118462	4335.6	0.7566	DDAVEALAC	91	623.319	2	1244.625	.	K	Superscaf	1.06E+08	0	BTP202209	0	0	0	0	Tissue specific
CDS|exon|ID=cds-Bg	7	6.42	2976.2	0.949838	KTGNYGY	50	401.6896	2	801.3657	.	N	Superscaf	1.18E+08	BTP202209	0	0	0	0	0	Tissue specific
CDS|exon|ID=cds-Bg	7	5.968571	2805.2	0.992512	QSLLTLL	54	787.491	1	786.4851	.	A	Superscaf	1.21E+08	0	BTP202209	BTP202209	BTP202209	0	0	Mixed
CDS|exon|ID=cds-Bg	7	5.837143	2149.3	0.986691	KGPPAVT	57	669.3907	1	668.3857	.	S	Superscaf	758392	0	0	BTP202209	0	0	0	Tissue specific
CDS|exon|ID=cds-Bg	8	5.92625	3182.3	0.980712	FEVKLKHY	91	355.2007	3	1062.586	.	V	Superscaf	1098928	0	0	0	0	0	BTP202209	Tissue specific
CDS|exon|ID=cds-Bg	12	7.128333	5175.9	0.980593	SPRPPAGHP	91	422.5569	3	1264.654	.	N	Superscaf	1105648	0	0	0	0	BTP202209	0	Tissue specific
CDS|exon|ID=cds-Bg	11	6.798182	4527.1	0.980593	SPRPPAGHP	97	370.523	3	1108.553	.	N	Superscaf	1105648	0	0	0	0	BTP202209	0	Tissue specific
CDS|exon|ID=cds-Bg	12	4.75	4035.5	0.980425	ALSPGLVES	63	615.3174	2	1228.619	.	Q	Superscaf	1115122	0	BTP202209	0	0	0	0	Tissue specific
CDS|exon|ID=cds-Bg	7	5.2	2355.5	0.973341	LSEVFPK	65	410.2331	2	818.4538	.	Q	Superscaf	1519176	0	0	BTP202209	0	0	0	Tissue specific
CDS|exon|ID=cds-Bg	9	6.504444	3805.1	0.900329	FYASRPESK	96	362.1842	3	1083.535	.	I	Superscaf	5679493	0	0	0	0	0	BTP202209	Tissue specific
CDS|exon|ID=cds-Bg	7	6.528571	2406.4	0.84821	A(+42.01)	57	629.3271	1	628.318	Acetylati	P	Superscaf	8649601	0	0	BTP202209	0	0	0	Tissue specific
CDS|exon|ID=cds-Bg	8	5.6675	2954.3	0.713467	DPAAAPNP	62	752.3629	1	751.35	.	Q	Superscaf	16327897	0	BTP202209	0	0	0	0	Tissue specific
CDS|exon|ID=cds-Bg	8	5.6275	2777.9	0.695349	QLAKSLGV	95	408.251	2	814.4912	.	F	Superscaf	17359891	0	0	BTP202209	0	0	0	Tissue specific
CDS|exon|ID=cds-Bg	9	5.648889	3124.3	0.680937	PGELAKHAV	99	461.2596	2	920.5079	.	K	Superscaf	18181303	0	0	0	0	BTP202209	0	Tissue specific
CDS|exon|ID=cds-Bg	11	5.754545	4346.9	0.615014	A(+42.01)	81	621.2858	2	1240.561	Acetylati	K	Superscaf	21938164	0	0	BTP202209	0	0	0	Tissue specific
CDS|exon|ID=cds-Bg	16	6.324375	6689.4	0.615014	A(+42.01)	92	965.4752	2	1928.942	Acetylati	K	Superscaf	21938164	0	0	0	0	0	BTP202209	Tissue specific
CDS|exon|ID=cds-Bg	15	6.096667	6221	0.615014	A(+42.01)	76	901.4283	2	1800.847	Acetylati	K	Superscaf	21938164	0	BTP202209	0	0	0	BTP202209	Mixed
CDS|exon|ID=cds-Bg	7	6.394286	3000.3	0.615013	APPHFHF	96	426.7094	2	851.4078	.	K	Superscaf	21938164	0	0	BTP202209	0	0	0	Tissue specific
CDS|exon|ID=cds-Bg	11	5.962727	4705.1	0.615003	LC(+57.02	83	670.8201	2	1339.627	Carbamido	L	Superscaf	21938707	0	0	0	0	0	BTP202209	Tissue specific
CDS|exon|ID=cds-Bg	8	6.42125	3340.5	0.615003	MAQFDAKK	94	469.7408	2	937.4691	.	L	Superscaf	21938707	0	0	0	0	0	BTP202209	Tissue specific
CDS|exon|ID=cds-Bg	7	5.947143	2872.1	0.615003	MAQFDAK	93	405.6927	2	809.3741	.	L	Superscaf	21938707	0	0	0	0	0	BTP202209	Tissue specific
CDS|exon|ID=cds-Bg	21	5.804286	7439.9	0.589114	LLPGELAKH	95	738.0793	3	2211.226	.	Y	Superscaf	23413615	0	0	0	0	0	BTP202209	Tissue specific
CDS|exon|ID=cds-Bg	9	6.175556	3269.4	0.589114	AVTKYTSSK	99	492.7694	2	983.5287	.	Y	Superscaf	23413615	0	0	0	BTP202209	BTP202209	BTP202209	Mixed
CDS|exon|ID=cds-Bg	6	6.785	2482.6	0.577718	KDPPYK	72	747.4124	1	746.3962	.	L	Superscaf	24063484	0	BTP202209	0	0	0	0	Tissue specific
CDS|exon|ID=cds-Bg	9	4.318889	2733	0.557264	A(+42.01)	96	478.2565	2	954.5022	Acetylati	N	Superscaf	25228996	0	0	BTP202209	0	0	0	Tissue specific
CDS|exon|ID=cds-Bg	9	6.3	3713.2	0.492875	T(+42.01)	96	545.7806	2	1089.545	Acetylati	N	Superscaf	28898239	0	BTP202209	0	0	BTP202209	BTP202209	Mixed
CDS|exon|ID=cds-Bg	5	6.738	2081.2	0.330493	AQLKP	80	556.3433	1	555.338	.	S	Superscaf	38151481	0	0	BTP202209	0	0	0	Tissue specific
CDS|exon|ID=cds-Bg	7	7.348571	3000.4	0.328524	RAYGGPR	80	388.7104	2	775.4089	.	R	Superscaf	38263486	0	0	0	BTP202209	0	0	Tissue specific
CDS|exon|ID=cds-Bg	7	3.418571	1371.5	0.28961	V(+42.01)	78	712.4209	1	711.4167	Acetylati	T	Superscaf	40481191	0	0	BTP202209	0	0	0	Tissue specific
CDS|exon|ID=cds-Bg	7	4.33	1753.1	0.225217	VPPVLLM(+	58	392.7415	2	783.4564	Oxidation	R	Superscaf	44150455	0	0	0	BTP202209	0	0	Tissue specific
CDS|exon|ID=cds-Bg	9	6.196667	3389.8	0.218383	ATTLLEAMK	99	489.2679	2	976.5263	.	V	Superscaf	44539894	0	0	0	0	0	BTP202209	Tissue specific
CDS|exon|ID=cds-Bg	12	5.62	4892.5	0.216441	DYEGLEPKT	92	739.8342	2	1477.661	.	E	Superscaf	44650594	0	0	0	0	0	BTP202209	Tissue specific
CDS|exon|ID=cds-Bg	11	4.76	3234.3	0.21644	AVGSHVYSV	99	559.3011	2	1116.593	.	E	Superscaf	44650594	0	0	0	0	0	BTP202209	Tissue specific
CDS|exon|ID=cds-Bg	10	4.776	3202.4	0.216439	VYSVAKTDS	85	558.7795	2	1115.55	.	E	Superscaf	44650594	0	0	0	0	0	BTP202209	Tissue specific
CDS|exon|ID=cds-Bg	8	5.2625	2742.9	0.216439	SVAKTDSF	74	427.7147	2	853.4182	.	E	Superscaf	44650594	0	0	0	0	0	BTP202209	Tissue specific
CDS|exon|ID=cds-Bg	13	4.586923	4074.5	0.216439	EYVDPVDGT	92	725.8721	2	1449.735	.	E	Superscaf	44650594	0	0	0	0	0	BTP202209	Tissue specific
CDS|exon|ID=cds-Bg	8	6.5675	3544.2	0.204785	ESEPPRPR	80	484.2493	2	966.4882	.	L	Superscaf	45314653	0	0	BTP202209	0	0	0	Tissue specific
CDS|exon|ID=cds-Bg	7	6.437143	3094.7	0.201479	RPPEPQT	91	412.7151	2	823.4188	.	P	Superscaf	45503299	0	0	BTP202209	BTP202209	0	0	Mixed
CDS|exon|ID=cds-Bg	8	6.64375	2982.4	0.149168	A(+42.01)	68	434.7379	2	867.4636	Acetylati	A	Superscaf	48484093	0	0	0	0	BTP202209	0	Tissue specific
CDS|exon|ID=cds-Bg	9	4.345556	2398.5	0.147004	VGGKQVVPF	67	465.772	2	929.5334	.	Y	Superscaf	48607420	0	0	0	0	0	BTP202209	Tissue specific
CDS|exon|ID=cds-Bg	10	5.354	3622.9	0.132168	A(+42.01)	63	591.2956	2	1180.58	Acetylati	K	Superscaf	49452880	0	0	0	0	BTP202209	0	Tissue specific
CDS|exon|ID=cds-Bg	9	5.28	3311.6	0.132168	A(+42.01)	93	547.7802	2	1093.548	Acetylati	K	Superscaf	49452880	BTP202209	BTP202209	0	0	BTP202209	BTP202209	Mixed
CDS|exon|ID=cds-Bg	8	5.23375	2700.9	0.132169	A(+42.01)	99	966.495	1	965.4892	Acetylati	K	Superscaf	49452880	0	BTP202209	BTP202209	BTP202209	BTP202209	BTP202209	Mixed
CDS|exon|ID=cds-Bg	6	4.358333	1879.1	0.132169	A(+42.01)	99	725.3154	1	724.3102	Acetylati	K	Superscaf	49452880	BTP202209	BTP202209	0	BTP202209	BTP202209	BTP202209	Mixed
CDS|exon|ID=cds-Bg	7	5.121429	2389.6	0.132169	MVSEFLK	91	427.2269	2	852.4415	.	K	Superscaf	49452880	0	BTP202209	0	0	BTP202209	BTP202209	Tissue enhanced
CDS|exon|ID=cds-Bg	7	6.915714	3457.8	0.079822	KHGQERF	65	451.2412	2	900.4565	.	S	Superscaf	52435762	BTP202209	0	0	0	0	0	Tissue specific
CDS|exon|ID=cds-Bg	7	7.187143	3144.6	0.075839	LTC(+57.0	74	444.2629	2	886.5059	Carbamido	A	Superscaf	52662796	0	0	BTP202209	0	0	0	Tissue specific
CDS|exon|ID=cds-Bg	7	4.097143	1715.7	0.062477	EVVLVKK	89	407.773	2	813.5323	.	R	Superscaf	53424121	0	BTP202209	0	0	0	BTP202209	Tissue enhanced
CDS|exon|ID=cds-Bg	9	5.035556	3134.5	0.052395	MLEDSVSPK	97	503.2477	2	1004.485	.	L	Superscaf	53999196	0	0	0	0	0	BTP202209	Tissue specific
CDS|exon|ID=cds-Bg	9	5.478889	3231.4	0.052395	LEDSVSPKK	98	501.7751	2	1001.539	.	L	Superscaf	53999196	0	0	0	0	BTP202209	BTP202209	Mixed
CDS|exon|ID=cds-Bg	8	4.94625	2763	0.052395	LEDSVSPK	94	437.7272	2	873.4443	.	L	Superscaf	53999196	0	0	0	0	BTP202209	BTP202209	Tissue enhanced
CDS|exon|ID=cds-Bg	9	4.204444	2825.4	0.046589	M(+42.01)	59	524.244	2	1046.478	Acetylati	N	Superscaf	54329404	0	0	BTP202209	0	BTP202209	BTP202209	Mixed
CDS|exon|ID=cds-Bg	5	6.866	2326.4	0.010875	KQELK	57	645.3918	1	644.3857	.	V	Superscaf	56364775	0	BTP202209	0	0	0	0	Tissue specific
CDS|exon|ID=cds-Bg	6	6.053333	2217.5	0.01611	PVERPK	76	363.2176	2	724.4232	.	P	Superscaf	57902488	0	0	0	0	0	BTP202209	Tissue specific
CDS|exon|ID=cds-Bg	6	6.466667	2707.9	0.0482	ASKPQN	67	644.3389	1	643.3289	.	L	Superscaf	59731108	0	0	0	BTP202209	0	0	Tissue specific
CDS|exon|ID=cds-Bg	7	6.891429	2689.2	0.050447	RPLPPPP	99	773.453	1	772.4595	.	P	Superscaf	59859666	0	0	0	BTP202209	0	0	Tissue specific
CDS|exon|ID=cds-Bg	7	5.134286	2523.8	0.062612	A(+42.01)	62	790.361	1	789.3544	Acetylati	L	Superscaf	60552217	0	BTP202209	0	0	0	0	Tissue specific
CDS|exon|ID=cds-Bg	17	5.287059	5663.2	0.062612	A(+42.01)	67	812.4014	2	1622.794	Acetylati	L	Superscaf	60552217	0	0	0	0	BTP202209	BTP202209	Mixed
CDS|exon|ID=cds-Bg	10	4.826	3172.5	0.062612	A(+42.01)	50	1057.517	1	1056.513	Acetylati	L	Superscaf	60552217	0	0	0	0	BTP202209	0	Tissue specific
CDS|exon|ID=cds-Bg	8	4.4925	2523.8	0.062612	A(+42.01)	73	889.4285	1	888.4229	Acetylati	L	Superscaf	60552217	0	0	0	0	0	BTP202209	Tissue specific
CDS|exon|ID=cds-Bg	6	4.995	2226.5	0.062612	A(+42.01)	95	733.3388	1	732.333	Acetylati	L	Superscaf	60552217	0	BTP202209	0	0	BTP202209	BTP202209	Mixed
CDS|exon|ID=cds-Bg	12	5.864167	4554	0.062615	LDGGAPGSG	78	546.756	2	1091.5	.	L	Superscaf	60552217	0	0	BTP202209	0	0	0	Tissue specific
CDS|exon|ID=cds-Bg	7	5.842857	3140.5	0.062915	LC(+57.02	79	447.2118	2	892.4113	Carbamido	L	Superscaf	60569563	0	BTP202209	0	0	0	BTP202209	Tissue enhanced
CDS|exon|ID=cds-Bg	8	5.9075	3054.3	0.062916	LKQAPLVH	99	453.2807	2	904.5494	.	L	Superscaf	60569563	0	0	BTP202209	0	0	0	Tissue specific
CDS|exon|ID=cds-Bg	8	4.2425	2436.8	0.081401	MSVGVDHL	93	429.2116	2	856.4113	.	C	Superscaf	61623034	0	0	BTP202209	0	0	0	Tissue specific
CDS|exon|ID=cds-Bg	11	6.865455	4638.1	0.092473	KDPNAPKRP	95	402.8896	3	1205.652	.	S	Superscaf	62253772	0	0	0	0	0	BTP202209	Tissue specific
CDS|exon|ID=cds-Bg	10	4.732	3638.1	0.109642	TAEFVNLEP	78	591.803	2	1181.597	.	R	Superscaf	63232249	0	0	0	0	0	BTP202209	Tissue specific
CDS|exon|ID=cds-Bg	10	6.275	4216.7	0.109697	AEKLPNLTH	62	568.3275	2	1134.64	.	Q	Superscaf	63235486	0	0	BTP202209	0	0	0	Tissue specific
CDS|exon|ID=cds-Bg	8	6.0475	3066.3	0.109868	L(+42.01)	89	468.7502	2	935.4964	Acetylati	A	Superscaf	63245307	0	0	0	0	0	BTP202209	Tissue specific
CDS|exon|ID=cds-Bg	10	6.489	4617	0.11019	SELEYKFNP	97	657.3431	2	1312.678	.	R	Superscaf	63263560	0	0	0	0	0	BTP202209	Tissue specific
CDS|exon|ID=cds-Bg	9	6.654444	3785.2	0.11019	ALERPYTSK	79	532.7896	2	1063.566	.	R	Superscaf	63263560	0	0	0	0	0	BTP202209	Tissue specific
CDS|exon|ID=cds-Bg	10	6.493	4711	0.110192	SHAQYKELG	96	411.2169	3	1230.636	.	R	Superscaf	63263560	0	0	0	0	0	BTP202209	Tissue specific
CDS|exon|ID=cds-Bg	10	5.518	3929.3	0.110322	LHELNVPFF	99	622.3428	2	1242.676	.	M	Superscaf	63271132	0	0	0	0	0	BTP202209	Tissue specific
CDS|exon|ID=cds-Bg	9	5.466667	3575.9	0.110322	HELNVPFFK	97	565.8024	2	1129.592	.	M	Superscaf	63271132	0	0	0	0	0	BTP202209	Tissue specific
CDS|exon|ID=cds-Bg	7	4.854286	2720.1	0.110322	HELNVPF	78	428.2196	2	854.4286	.	M	Superscaf	63271132	0	0	0	0	BTP202209	BTP202209	Tissue enhanced
CDS|exon|ID=cds-Bg	11	4.279091	2863.1	0.124613	VGVPLPSSV	75	544.8011	2	1087.591	.	D	Superscaf	64085329	0	0	0	0	0	BTP202209	Tissue specific
CDS|exon|ID=cds-Bg	15	5.461333	5776.4	0.124617	YTEPSSQVS	89	821.8835	2	1641.763	.	D	Superscaf	64085329	0	0	0	0	0	BTP202209	Tissue specific
CDS|exon|ID=cds-Bg	9	5.96	2997.1	0.133904	P(+42.01)	79	489.7839	2	977.5545	Acetylati	Q	Superscaf	64615035	0	BTP202209	0	0	BTP202209	0	Mixed
CDS|exon|ID=cds-Bg	8	6.95125	3033.1	0.146813	K(+42.01)	66	402.2226	2	802.4185	Acetylati	L	Superscaf	65350354	BTP202209	0	0	0	0	0	Tissue specific
CDS|exon|ID=cds-Bg	13	5.430769	4182.7	0.184074	AAPELPVPT	91	624.8433	2	1247.676	.	S	Superscaf	67473562	0	0	0	0	0	BTP202209	Tissue specific
CDS|exon|ID=cds-Bg	5	6.11	2057.2	0.215541	KDGLW	59	618.3219	1	617.3173	.	R	Superscaf	69267171	0	0	0	0	BTP202209	0	Tissue specific
CDS|exon|ID=cds-Bg	8	7.08	3296.6	0.221906	KRWLLAGP	51	470.7871	2	939.5654	.	G	Superscaf	69629686	0	BTP202209	0	0	0	0	Tissue specific
CDS|exon|ID=cds-Bg	6	5.946667	2427.8	0.228765	M(+42.01)	73	748.3323	1	747.3261	Acetylati	T	Superscaf	70020430	0	BTP202209	0	0	0	0	Tissue specific
CDS|exon|ID=cds-Bg	11	6.532727	4373.9	0.228992	A(+42.01)	68	587.8009	2	1173.589	Acetylati	G	Superscaf	70033315	0	BTP202209	0	0	0	0	Tissue specific
CDS|exon|ID=cds-Bg	9	5.593333	3076.3	0.228992	A(+42.01)	70	862.3936	1	861.3868	Acetylati	G	Superscaf	70033315	0	BTP202209	0	0	0	0	Tissue specific
CDS|exon|ID=cds-Bg	8	5.54625	2779	0.228992	A(+42.01)	87	805.3708	1	804.3654	Acetylati	G	Superscaf	70033315	0	BTP202209	0	0	0	0	Tissue specific
CDS|exon|ID=cds-Bg	8	4.5075	2873.2	0.229416	EDSEVFKM	98	492.7196	2	983.4269	.	P	Superscaf	70057552	0	BTP202209	0	0	0	0	Tissue specific
CDS|exon|ID=cds-Bg	11	6.440909	5175.8	0.229453	PPKLHTC(+	99	461.8867	3	1382.644	Carbamido	D	Superscaf	70059523	0	0	0	0	BTP202209	0	Tissue specific
CDS|exon|ID=cds-Bg	13	6.389231	5956.7	0.229453	PPKLHTC(+	94	786.3672	2	1570.723	Carbamido	D	Superscaf	70059523	0	0	0	0	BTP202209	0	Tissue specific
CDS|exon|ID=cds-Bg	13	5.541538	5533.2	0.229473	WVGDELYC(	99	554.9263	3	1661.762	Carbamido	W	Superscaf	70060783	0	0	0	0	BTP202209	0	Tissue specific
CDS|exon|ID=cds-Bg	11	5.023636	4573.1	0.229473	WVGDELYC(	88	718.3173	2	1434.624	Carbamido	W	Superscaf	70060783	0	0	0	0	BTP202209	0	Tissue specific
CDS|exon|ID=cds-Bg	10	4.937	4046.5	0.229473	VGDELYC(+	81	625.2781	2	1248.544	Carbamido	W	Superscaf	70060783	0	BTP202209	0	0	BTP202209	0	Mixed
CDS|exon|ID=cds-Bg	9	4.642222	3426.8	0.229473	VGDELYC(+	99	556.7491	2	1111.486	Carbamido	W	Superscaf	70060783	0	0	0	0	BTP202209	0	Tissue specific
CDS|exon|ID=cds-Bg	10	6.193	3385.9	0.229728	GPPPGLLTP	72	473.2725	2	944.5331	.	S	Superscaf	70075186	0	0	BTP202209	0	0	0	Tissue specific
CDS|exon|ID=cds-Bg	11	5.991818	3813.1	0.229747	KSPAPPLLP	87	565.3386	2	1128.666	.	A	Superscaf	70076434	0	0	0	BTP202209	0	0	Tissue specific
CDS|exon|ID=cds-Bg	8	6.37875	3769.3	0.229752	FQTSHTLH	99	485.7395	2	969.4669	.	V	Superscaf	70076761	0	0	0	BTP202209	0	0	Tissue specific
CDS|exon|ID=cds-Bg	7	4.644286	2513.8	0.247858	QAVLEYL	75	835.4546	1	834.4487	.	K	Superscaf	71108272	0	BTP202209	0	0	BTP202209	0	Mixed
CDS|exon|ID=cds-Bg	9	5.452222	2797.2	0.251169	LPPVTLALL	71	468.8087	2	935.6055	.	P	Superscaf	71297098	0	0	0	0	0	BTP202209	Tissue specific
CDS|exon|ID=cds-Bg	6	6.121667	2227.3	0.25161	FAGEPK	66	648.3309	1	647.3278	.	E	Superscaf	71322444	0	0	0	0	0	BTP202209	Tissue specific
CDS|exon|ID=cds-Bg	7	6.91	3281.4	0.311507	WSKNWAK	95	460.2419	2	918.4712	.	L	Superscaf	74735434	0	0	0	0	0	BTP202209	Tissue specific
CDS|exon|ID=cds-Bg	13	6.181538	5351	0.311679	TSPAKQQAP	88	697.379	2	1392.747	.	M	Superscaf	74745232	0	BTP202209	0	0	0	0	Tissue specific
CDS|exon|ID=cds-Bg	7	5.824286	2585	0.311679	APPVRNL	87	383.7316	2	765.4497	.	M	Superscaf	74745232	0	BTP202209	0	0	0	0	Tissue specific
CDS|exon|ID=cds-Bg	7	5.965714	3248.6	0.31168	NLHQSGF	50	401.6951	2	801.377	.	M	Superscaf	74745232	0	BTP202209	0	0	0	0	Tissue specific
CDS|exon|ID=cds-Bg	7	5.928571	2591	0.324025	RDVMLPK	70	429.7407	2	857.4793	.	R	Superscaf	75448885	0	BTP202209	0	0	0	0	Tissue specific
CDS|exon|ID=cds-Bg	8	5.22	2865	0.326534	AYLEKVFY	92	516.7725	2	1031.533	.	A	Superscaf	75591577	0	0	BTP202209	0	0	0	Tissue specific
CDS|exon|ID=cds-Bg	6	5.035	1694.9	0.341301	VGPLLL	82	611.4113	1	610.4054	.	P	Superscaf	76433691	0	0	BTP202209	0	0	0	Tissue specific
CDS|exon|ID=cds-Bg	7	6.115714	2898	0.343224	DFSLEKK	92	433.7337	2	865.4545	.	M	Superscaf	76542829	0	0	0	0	0	BTP202209	Tissue specific
CDS|exon|ID=cds-Bg	9	4.766667	2474.5	0.406482	KYFPVVKLV	96	546.8418	2	1091.674	.	D	Superscaf	80147593	0	0	0	0	0	BTP202209	Tissue specific
CDS|exon|ID=cds-Bg	6	5.445	2307.6	0.461971	EDVKLR	71	380.2169	2	758.4286	.	N	Superscaf	83309632	0	0	0	0	0	BTP202209	Tissue specific
CDS|exon|ID=cds-Bg	7	5.318571	2438.7	0.466222	SGVHLTF	52	380.6989	2	759.3915	.	V	Superscaf	83552010	BTP202209	0	0	0	0	0	Tissue specific
CDS|exon|ID=cds-Bg	7	7.765714	3188.4	0.485209	QTKGKGR	63	387.7356	2	773.4507	.	S	Superscaf	84632122	BTP202209	0	0	0	0	0	Tissue specific
CDS|exon|ID=cds-Bg	7	4.408571	1809.1	0.504656	VPPYVPP	81	768.4272	1	767.4218	.	V	Superscaf	85741921	0	0	BTP202209	0	0	0	Tissue specific
CDS|exon|ID=cds-Bg	10	7.676	3898	0.507161	KPPKPPKPP	62	371.9084	3	1112.707	.	Q	Superscaf	85884580	0	BTP202209	0	0	0	0	Tissue specific

CDS|exon|ID=cds-B8	9	5.675556	3475.9	0.544751	YEETRGVLK	98	547.7943	2	1093.577	.	S	Superscaf	88026673	0	0	0	0	0	BTP202209	Tissue specific
CDS|exon|ID=cds-B8	7	6.63	3163.5	0.573422	KMTYYHF	53	495.24	2	988.4477	.	D	Superscaf	89660635	0	0	0	0	BTP202209	0	Tissue specific
CDS|exon|ID=cds-B8	7	5.354286	2040.4	0.597908	LPPPVPP	70	358.7191	2	715.4268	.	F	Superscaf	91055563	0	0	BTP202209	0	0	0	Tissue specific
CDS|exon|ID=cds-B8	9	5.686667	3713.1	0.601137	ESLLLDLKQ	87	529.8086	2	1057.602	.	C	Superscaf	91239244	BTP202209	0	0	0	0	0	Tissue specific
CDS|exon|ID=cds-B8	7	7.598571	2734.6	0.685927	KSKPGAK	51	358.2247	2	714.4388	.	V	Superscaf	96071566	0	0	0	0	BTP202209	0	Tissue specific
CDS|exon|ID=cds-B8	8	6.6025	3367	0.748991	DRHPPPPP	54	456.7412	2	911.4613	.	S	Superscaf	99665119	0	0	0	0	BTP202209	0	Tissue specific
CDS|exon|ID=cds-B8	7	5.795714	2814.3	0.7644	M(+42.01)	64	419.691	2	837.3691	Acetylati	A	Superscaf	1.01E+08	0	BTP202209	0	0	0	0	Tissue specific
CDS|exon|ID=cds-B8	11	5.744545	3970.4	0.764407	DGEVTLTTI	99	602.8416	2	1203.671	.	A	Superscaf	1.01E+08	0	BTP202209	0	0	0	0	Tissue specific
CDS|exon|ID=cds-B8	10	5.345	3502	0.764407	DGEVTLTTI	94	538.7949	2	1075.576	.	A	Superscaf	1.01E+08	0	BTP202209	0	0	0	0	Tissue specific
CDS|exon|ID=cds-B8	13	5.213077	5836.6	0.764409	LFEDDESEK	95	563.2517	3	1686.737	.	A	Superscaf	1.01E+08	0	BTP202209	0	0	0	0	Tissue specific
CDS|exon|ID=cds-B8	12	4.750833	5187.8	0.764409	LFEDDESEK	92	766.3252	2	1530.636	.	A	Superscaf	1.01E+08	0	BTP202209	0	0	0	0	Tissue specific
CDS|exon|ID=cds-B8	11	5.902727	4954.4	0.764412	LDEEHLESH	91	455.5643	3	1363.673	.	A	Superscaf	1.01E+08	0	BTP202209	0	0	0	0	Tissue specific
CDS|exon|ID=cds-B8	8	6.54375	3415.9	0.764417	GQSTPRLF	72	453.2445	2	904.4766	.	A	Superscaf	1.01E+08	0	BTP202209	0	0	0	0	Tissue specific
CDS|exon|ID=cds-B8	16	4.986875	6234.2	0.764423	LTQEELDSG	81	902.45	2	1802.89	.	A	Superscaf	1.01E+08	0	BTP202209	0	0	0	0	Tissue specific
CDS|exon|ID=cds-B8	13	4.740769	4872.6	0.764423	EELDSGLDE	94	731.356	2	1460.7	.	A	Superscaf	1.01E+08	0	BTP202209	0	0	0	0	Tissue specific
CDS|exon|ID=cds-B8	8	4.0975	2782.2	0.764428	SVDDPLEY	90	937.413	1	936.4076	.	A	Superscaf	1.01E+08	0	BTP202209	0	0	0	0	Tissue specific
CDS|exon|ID=cds-B8	12	4.774167	4090.6	0.764435	TQESDVMVG	82	669.3235	2	1336.633	.	A	Superscaf	1.01E+08	0	BTP202209	0	0	0	0	Tissue specific
CDS|exon|ID=cds-B8	10	4.511	3082.4	0.764435	ESDVMVGPF	80	554.7701	2	1107.527	.	A	Superscaf	1.01E+08	0	BTP202209	0	0	0	0	Tissue specific
CDS|exon|ID=cds-B8	9	4.654444	2656.9	0.764435	SDVMVGPFK	93	490.2483	2	978.4844	.	A	Superscaf	1.01E+08	0	BTP202209	0	0	0	0	Tissue specific
CDS|exon|ID=cds-B8	9	6.307778	3771.4	0.801055	TNTLETPTK	96	502.7647	2	1003.519	.	E	Superscaf	1.03E+08	0	0	0	0	0	BTP202209	Tissue specific
CDS|exon|ID=cds-B8	11	5.627273	4155.6	0.801352	LDEQPAMVT	85	630.3341	2	1258.659	.	P	Superscaf	1.03E+08	0	0	0	0	0	BTP202209	Tissue specific
CDS|exon|ID=cds-B8	10	5.216	3687.2	0.801352	LDEQPAMVT	99	566.2876	2	1130.564	.	P	Superscaf	1.03E+08	0	0	0	0	0	BTP202209	Tissue specific
CDS|exon|ID=cds-B8	7	6.231429	3380.8	0.801666	NQMNSKL	92	417.7093	2	833.4066	.	C	Superscaf	1.03E+08	0	0	BTP202209	0	0	0	Tissue specific
CDS|exon|ID=cds-B8	6	5.618333	2415.7	0.816348	KFEMEP	54	390.6911	2	779.3524	.	S	Superscaf	1.04E+08	0	BTP202209	0	0	0	0	Tissue specific
CDS|exon|ID=cds-B8	6	7.618333	2826.3	0.873258	L(+42.01)	62	420.7403	2	839.4766	Acetylati	R	Superscaf	1.07E+08	0	0	0	BTP202209	0	0	Tissue specific
CDS|exon|ID=cds-B8	6	1.49	708.8	0.956161	DVGVVV	64	587.3379	1	586.3326	.	P	Superscaf	1.11E+08	0	0	0	0	0	BTP202209	Tissue specific
CDS|exon|ID=cds-B8	9	4.614444	3017.3	0.957888	VVEHPEFLK	84	366.5365	3	1096.592	.	T	Superscaf	1.12E+08	0	BTP202209	0	0	BTP202209	BTP202209	Mixed
CDS|exon|ID=cds-B8	9	4.082222	3036.5	0.958024	EVVVQRLFQ	88	559.3206	2	1116.629	.	S	Superscaf	1.12E+08	0	0	BTP202209	0	0	0	Tissue specific
CDS|exon|ID=cds-B8	9	6.048889	3335.5	0.958154	AALKTASDF	67	462.2439	2	922.476	.	A	Superscaf	1.12E+08	0	0	0	0	0	BTP202209	Tissue specific
CDS|exon|ID=cds-B8	8	4.64875	2707.1	0.95816	ANVERVPF	96	466.2518	2	930.4922	.	E	Superscaf	1.12E+08	0	0	0	0	BTP202209	BTP202209	Tissue enhanced
CDS|exon|ID=cds-B8	7	4.452857	2395.8	0.95816	NVERVPF	96	430.7332	2	859.4551	.	E	Superscaf	1.12E+08	0	0	0	0	0	BTP202209	Tissue specific
CDS|exon|ID=cds-B8	16	6.054375	6416.2	0.958161	DAATLHTST	87	533.5774	3	1597.716	Oxidation	E	Superscaf	1.12E+08	0	0	0	BTP202209	0	0	Tissue specific
CDS|exon|ID=cds-B8	11	5.965455	4265.9	0.958161	DAATLHTST	84	559.7584	2	1117.507	.	E	Superscaf	1.12E+08	0	0	0	0	0	BTP202209	Tissue specific
CDS|exon|ID=cds-B8	8	5.78375	2562.7	0.958274	VLKTPSAA	94	393.7382	2	785.4647	.	A	Superscaf	1.12E+08	0	0	0	0	0	BTP202209	Tissue specific
CDS|exon|ID=cds-B8	9	5.77	3022.2	0.958274	VLKTPSAAY	89	475.2694	2	948.528	.	A	Superscaf	1.12E+08	0	0	0	0	0	BTP202209	Tissue specific
CDS|exon|ID=cds-B8	9	5.004444	3586.2	0.958309	PGELMQEDL	60	516.2418	2	1030.464	.	I	Superscaf	1.12E+08	0	BTP202209	0	0	0	0	Tissue specific
CDS|exon|ID=cds-B8	13	4.631538	4967.9	0.958309	ATDDVMLLD	89	761.837	2	1521.666	.	I	Superscaf	1.12E+08	0	0	0	0	0	BTP202209	Tissue specific
CDS|exon|ID=cds-B8	16	5.08875	6440.4	0.958407	LGWDDNYWS	84	961.437	2	1920.864	.	R	Superscaf	1.12E+08	0	0	0	0	BTP202209	0	Tissue specific
CDS|exon|ID=cds-B8	15	5.026667	6129.1	0.958407	LGWDDNYWS	79	925.9163	2	1849.827	.	R	Superscaf	1.12E+08	0	0	0	0	0	BTP202209	Tissue specific
CDS|exon|ID=cds-B8	12	4.796667	4951.8	0.958407	DDNYWSVDP	94	747.8258	2	1493.642	.	R	Superscaf	1.12E+08	0	0	0	0	BTP202209	0	Tissue specific
CDS|exon|ID=cds-B8	15	5.320667	5450.1	0.958407	WSVDPLDRA	85	813.9241	2	1625.841	.	R	Superscaf	1.12E+08	0	0	0	0	0	BTP202209	Tissue specific
CDS|exon|ID=cds-B8	13	5.213077	4827.5	0.958407	WSVDPLDRA	88	742.8892	2	1483.767	.	R	Superscaf	1.12E+08	0	0	0	0	0	BTP202209	Tissue specific
CDS|exon|ID=cds-B8	12	5.149167	4474.1	0.958407	WSVDPLDRA	87	686.345	2	1370.683	.	R	Superscaf	1.12E+08	0	0	0	0	0	BTP202209	Tissue specific
CDS|exon|ID=cds-B8	10	5.255	3737.3	0.958407	WSVDPLDRA	86	586.3065	2	1170.603	.	R	Superscaf	1.12E+08	0	0	0	0	0	BTP202209	Tissue specific
CDS|exon|ID=cds-B8	8	5.06875	3072.6	0.958407	WSVDPLDR	94	494.2454	2	986.4821	.	R	Superscaf	1.12E+08	0	0	0	0	BTP202209	0	Tissue specific
CDS|exon|ID=cds-B8	9	5.184444	3210.7	0.958407	SVDPLDRAI	83	493.2683	2	984.524	.	R	Superscaf	1.12E+08	0	0	0	0	0	BTP202209	Tissue specific
CDS|exon|ID=cds-B8	7	7.722857	2933	0.963281	PAARFKK	52	409.261	2	816.4969	.	V	Superscaf	1.12E+08	0	0	0	0	0	BTP202209	Tissue specific
CDS|exon|ID=cds-B8	7	4.69	2471.8	0.98628	EVGLTFQ	52	397.2057	2	792.4017	.	S	Superscaf	1.13E+08	BTP202209	0	0	0	0	0	Tissue specific
CDS|exon|ID=cds-B8	8	6.33625	3066.2	0.912714	KLYSSSGP	66	419.7183	2	837.4232	.	E	Superscaf	4730946	BTP202209	0	0	0	0	0	Tissue specific
CDS|exon|ID=cds-B8	10	6.771	4026.7	0.781716	PPTRPTDKP	82	561.3169	2	1120.624	.	F	Superscaf	11830714	0	0	0	0	BTP202209	0	Tissue specific
CDS|exon|ID=cds-B8	9	7.318889	4000.7	0.781716	TRPTDKPLP	89	361.8789	3	1082.62	.	F	Superscaf	11830714	0	0	0	0	BTP202209	0	Tissue specific
CDS|exon|ID=cds-B8	8	6.88875	3351.9	0.781716	TRPTDKPL	76	464.2646	2	926.5185	.	F	Superscaf	11830714	0	0	0	0	0	BTP202209	Tissue specific
CDS|exon|ID=cds-B8	11	4.475455	3321.7	0.781713	FAPVNVTTE	99	602.8304	2	1203.65	.	F	Superscaf	11830714	0	BTP202209	BTP202209	0	BTP202209	BTP202209	Mixed
CDS|exon|ID=cds-B8	14	4.152143	4130.6	0.781713	FAPVNVTTE	91	760.4015	2	1518.793	.	F	Superscaf	11830714	0	0	BTP202209	0	0	0	Tissue specific
CDS|exon|ID=cds-B8	11	4.493636	3317.7	0.781713	APVNVTTEV	95	572.8134	2	1143.614	.	F	Superscaf	11830714	0	0	0	BTP202209	0	0	Tissue specific
CDS|exon|ID=cds-B8	10	4.375	2934.3	0.781713	APVNVTTEV	99	529.2953	2	1056.582	.	F	Superscaf	11830714	0	0	0	BTP202209	BTP202209	BTP202209	Mixed
CDS|exon|ID=cds-B8	13	4.128462	4085.7	0.781713	VNVTTEVKS	94	736.8745	2	1471.734	.	F	Superscaf	11830714	0	0	BTP202209	0	0	0	Tissue specific
CDS|exon|ID=cds-B8	16	5.23875	5659.2	0.78171	VAGDSKNDP	76	803.3617	2	1604.714	.	F	Superscaf	11830714	0	0	0	0	BTP202209	0	Tissue specific
CDS|exon|ID=cds-B8	6	5.601667	2094.4	0.781711	PPMEAA	99	615.2779	1	614.2734	.	F	Superscaf	11830714	0	0	0	0	BTP202209	BTP202209	Mixed
CDS|exon|ID=cds-B8	7	5.551429	2579.9	0.781706	SDYPPLG	79	748.3506	1	747.3439	.	F	Superscaf	11830714	0	BTP202209	BTP202209	0	0	BTP202209	Mixed
CDS|exon|ID=cds-B8	6	7.463333	2562.8	0.694295	AYRPPK	80	366.2132	2	730.4125	.	L	Superscaf	16568899	0	0	0	0	0	BTP202209	Tissue specific
CDS|exon|ID=cds-B8	8	5.535	2741.1	0.683837	ELLARAVP	70	434.7655	2	867.5178	.	D	Superscaf	17135602	0	0	0	BTP202209	0	0	Tissue specific
CDS|exon|ID=cds-B8	8	4.9475	2283.4	0.680988	VSGVSLTK	77	395.7355	2	789.4596	.	V	Superscaf	17290135	0	0	0	0	BTP202209	0	Tissue specific
CDS|exon|ID=cds-B8	7	4.541429	2223.3	0.639884	V(+42.01)	58	441.7392	2	881.4647	Acetylati	T	Superscaf	19517839	0	BTP202209	0	0	0	0	Tissue specific
CDS|exon|ID=cds-B8	9	5.643333	3850.4	0.580632	MGHFDLLNY	84	555.2561	2	1108.501	.	R	Superscaf	22729500	0	0	0	0	0	BTP202209	Tissue specific
CDS|exon|ID=cds-B8	8	5.63	3478.9	0.580632	GHFDLLNY	63	489.7354	2	977.4606	.	R	Superscaf	22729500	0	0	0	0	0	BTP202209	Tissue specific
CDS|exon|ID=cds-B8	7	4.171429	2106.4	0.562836	VNPTVFF	98	823.4326	1	822.4276	.	Y	Superscaf	23693689	0	BTP202209	BTP202209	BTP202209	BTP202209	BTP202209	Mixed
CDS|exon|ID=cds-B8	9	5.494444	3163.3	0.562835	ELFADKVPK	99	523.7955	2	1045.581	.	Y	Superscaf	23693689	0	0	0	0	BTP202209	BTP202209	Tissue enhanced
CDS|exon|ID=cds-B8	14	5.436429	5281.7	0.562835	ELFADKVPK	94	804.9135	2	1607.819	.	Y	Superscaf	23693689	0	0	0	0	0	BTP202209	Tissue specific
CDS|exon|ID=cds-B8	13	5.433077	4894.3	0.562835	ELFADKVPK	73	731.3811	2	1460.751	.	Y	Superscaf	23693689	0	0	BTP202209	0	BTP202209	BTP202209	Mixed
CDS|exon|ID=cds-B8	10	5.598	3560.8	0.562835	ELFADKVPK	86	574.3196	2	1146.628	.	Y	Superscaf	23693689	0	0	0	0	BTP202209	0	Tissue specific
CDS|exon|ID=cds-B8	11	5.584545	4115.4	0.562835	FADKVPKTA	93	610.3171	2	1218.625	.	Y	Superscaf	23693689	0	0	BTP202209	0	0	0	Tissue specific
CDS|exon|ID=cds-B8	8	5.8475	2781.9	0.562835	FADKVPKT	84	453.251	2	904.5018	.	Y	Superscaf	23693689	0	0	0	0	BTP202209	0	Tissue specific
CDS|exon|ID=cds-B8	15	4.714667	5448.2	0.557138	VAHFTFQPD	65	888.415	2	1774.82	.	A	Superscaf	24002536	0	0	0	0	0	BTP202209	Tissue specific
CDS|exon|ID=cds-B8	9	6.17	3269.8	0.526345	A(+42.01)	84	463.2422	2	924.4705	Acetylati	P	Superscaf	25671424	0	BTP202209	0	0	0	0	Tissue specific
CDS|exon|ID=cds-B8	8	4.35125	2299.5	0.215779	VGGVPSNF	85	776.391	1	775.3864	.	G	Superscaf	42503929	0	0	0	0	0	BTP202209	Tissue specific
CDS|exon|ID=cds-B8	8	6.5	3505.9	0.215751	TQSRAASY	78	442.2154	2	882.4195	.	S	Superscaf	42505576	0	0	0	0	0	BTP202209	Tissue specific
CDS|exon|ID=cds-B8	7	3.487143	2412.8	0.214662	EDEFPEV	84	864.3602	1	863.3549	.	S	Superscaf	42564577	0	0	0	0	BTP202209	0	Tissue specific
CDS|exon|ID=cds-B8	7	4.431429	2236.6	0.200164	T(+42.01)	76	786.3856	1	785.3807	Acetylati	G	Superscaf	43350513	0	0	0	BTP202209	0	0	Tissue specific
CDS|exon|ID=cds-B8	7	5.045714	2202.4	0.175868	G(+42.01)	57	752.381	1	751.3752	Acetylati	C	Superscaf	44667217	0	0	0	0	0	BTP202209	Tissue specific
CDS|exon|ID=cds-B8	10	5.879	3905.6	0.150083	A(+42.01)	68	587.314	2	1172.615	Acetylati	L	Superscaf	46064218	0	BTP202209	BTP202209	0	BTP202209	0	Mixed
CDS|exon|ID=cds-B8	9	5.867778	3552.2	0.150083	A(+42.01)	99	530.772	2	1059.531	Acetylati	L	Superscaf	46064218	0	BTP202209	0	BTP202209	0	0	Tissue enhanced
CDS|exon|ID=cds-B8	8	5.25625	2903.4	0.150083	A(+42.01)	96	452.7214	2	903.4297	Acetylati	L	Superscaf	46064218	0	BTP202209	0	0	0	BTP202209	Tissue enhanced
CDS|exon|ID=cds-B8	9	4.51	2131.3	0.149236	VVVPPLPGK	90	453.2932	2	904.5745	.	V	Superscaf	46110649	0	BTP202209	0	0	0	0	Tissue specific
CDS|exon|ID=cds-B8	7	6.428571	2476.7	0.121353	ALKGVRT	70	372.7387	2	743.4653	.	P	Superscaf	47621833	0	0	BTP202209	0	0	0	Tissue specific
CDS|exon|ID=cds-B8	7	3.9	1845.1	0.081607	DTVAFVP	71	748.3842	1	747.3803	.	Q	Superscaf	49775905	0	0	BTP202209	0	0	0	Tissue specific
CDS|exon|ID=cds-B8	12	4.9375	4057.4	0.081601	ALPGDNVGF	98	615.8254	2	1229.64	.	Q	Superscaf	49775905	0	0	0	0	0	BTP202209	Tissue specific
CDS|exon|ID=cds-B8	11	4.839091	3746.1	0.081601	LPGDNVGFN	88	580.3076	2	1158.603	.	Q	Superscaf	49775905	0	0	0	BTP202209	0	0	Tissue specific
CDS|exon|ID=cds-B8	7	5.187143	2154.4	0.076937	A(+42.01)	67	760.3851	1	759.3803	Acetylati	A	Superscaf	50029177	0	0	0	BTP202209	0	0	Tissue specific
CDS|exon|ID=cds-B8	7	4.742857	2469.8	0.0718	EATFQVP	60	396.1978	2	790.3861	.	S	Superscaf	50307598	BTP202209	0	0	0	0	0	Tissue specific
CDS|exon|ID=cds-B8	8	4.49125	2516.9	0.00167	GPPVNVPQ	57	404.2208	2	806.4286	.	S	Superscaf	54108364	0	BTP202209	0	0	0	BTP202209	Mixed
CDS|exon|ID=cds-B8	7	4.707143	2168.4	0.191128	FPGVESP	56	732.353	1	731.3489	.	P	Superscaf	64558063	BTP202209	0	0	0	0	0	Tissue specific
CDS|exon|ID=cds-B8	8	5.9425	3291.6	0.195225	KDSNPLLF	67	933.4984	1	932.4967	.	S	Superscaf	64780227	0	0	0	0	0	BTP202209	Tissue specific
CDS|exon|ID=cds-B8	7	6.991429	3081.6	0.202459	T(+42.01)	65	455.2587	2	908.4868	Acetylati	K	Superscaf	65171971	0	0	0	BTP202209	0	0	Tissue specific
CDS|exon|ID=cds-B8	5	6.814	1902	0.227302	KTAFP	52	563.3167	1	562.3115	.	R	Superscaf	66518605	0	BTP202209	0	0	0	0	Tissue specific
CDS|exon|ID=cds-B8	8	6.35125	2975.5	0.255591	GPPHMPPP	79	415.2036	2	828.3952	.	S	Superscaf	68051914	0	0	BTP202209	0	0	0	Tissue specific
CDS|exon|ID=cds-B8	9	6.371111	3373	0.255591	GPPHMPPPT	78	465.7288	2	929.4429	.	S	Superscaf	68051914	0	BTP202209	0	0	0	0	Tissue specific
CDS|exon|ID=cds-B8	7	5.167143	2375.5	0.330897	DVAPYFK	85	420.2227	2	838.4225	.	V	Superscaf	72133318	0	0	BTP202209	0	0	0	Tissue specific
CDS|exon|ID=cds-B8	11	5.423636	4300.9	0.36568	DLGEEPLEF	83	600.2976	2	1198.583	.	V	Superscaf	74018353	0	BTP202209	0	0	0	0	Tissue specific
CDS|exon|ID=cds-B8	10	4.87	3996.5	0.388211	LVEALC(+5	94	585.2827	2	1168.555	Carbamido	F	Superscaf	75239695	0	0	0	0	0	BTP202209	Tissue specific
CDS|exon|ID=cds-B8	6	4.305	2134.5	0.448637	LMFDVQ	93	752.3604	1	751.3575	.	L	Superscaf	78515007	0	0	0	0	0	BTP202209	Tissue specific
CDS|exon|ID=cds-B8	7	6.094286	2525.8	0.460015	AAPGVHR	54	354.1962	2	706.3875	.	V	Superscaf	79131463	BTP202209	0	0	0	0	0	Tissue specific
CDS|exon|ID=cds-B8	9	4.974444	3250.8	0.484032	A(+42.01)	85	534.2716	2	1066.53	Acetylati	P	Superscaf	80433127	0	0	0	0	0	BTP202209	Tissue specific
CDS|exon|ID=cds-B8	9	4.517778	3246.7	0.494619	M(+42.01)	54	563.7787	2	1125.549	Acetylati	K	Superscaf	81006997	0	0	0	0	0	BTP202209	Tissue specific
CDS|exon|ID=cds-B8	8	4.335	2893.3	0.494619	M(+42.01)	99	507.2384	2	1012.465	Acetylati	K	Superscaf	81006997	0	0	0	0	0	BTP202209	Tissue specific
CDS|exon|ID=cds-B8	11	4.999091	4105.6	0.494792	SGELLLQGV	65	584.3017	2	1166.593	.	T	Superscaf	81016267	0	BTP202209	0	0	0	BTP202209	Mixed
CDS|exon|ID=cds-B8	7	6.245714	2693	0.551474	TTGDPSK	67	353.1694	2	704.334	.	N	Superscaf	84088557	0	BTP202209	0	0	0	0	Tissue specific
CDS|exon|ID=cds-B8	7	7.851429	3125.4	0.556708	RAASKLR	87	401.2547	2	800.498	.	V	Superscaf	84372205	0	0	BTP202209	0	0	0	Tissue specific
CDS|exon|ID=cds-B8	8	6.09375	3587	0.570525	STPQQLEK	89	465.7455	2	929.4818	.	D	Superscaf	85121059	0	0	0	0	0	BTP202209	Tissue specific
CDS|exon|ID=cds-B8	6	6.99	2939.4	0.58063	RLQHLL	83	390.2407	2	778.4813	.	K	Superscaf	85668685	0	0	0	0	0	BTP202209	Tissue specific
CDS|exon|ID=cds-B8	7	6.631429	2677.7	0.586178	D(+42.01)	76	372.6986	2	743.3813	Acetylati	E	Superscaf	85969536	0	0	0	0	BTP202209	0	Tissue specific
CDS|exon|ID=cds-B8	16	5.79625	5552.8	0.596212	AVSEGTKAV	99	552.9634	3	1655.873	.	H	Superscaf	86512951	0	BTP202209	0	0	BTP202209	BTP202209	Mixed
CDS|exon|ID=cds-B8	9	5.411111	3404.6	0.679687	DKLQDGSVK	98	495.2647	2	988.5189	.	L	Superscaf	91037500	0	0	0	0	0	BTP202209	Tissue specific
CDS|exon|ID=cds-B8	11	6.25	4092.6	0.700371	TPTPLGEGK	96	544.2842	2	1086.556	.	L	Superscaf	92158582	0	0	0	0	BTP202209	0	Tissue specific
CDS|exon|ID=cds-B8	9	6.282222	3311.7	0.700371	TPTPLGEGK	95	450.2439	2	898.476	.	L	Superscaf	92158582	0	0	0	0	BTP202209	0	Tissue specific
CDS|exon|ID=cds-B8	11	5.058182	3372.7	0.700841	GTPVFVHAG	84	564.7942	2	1127.576	.	T	Superscaf	92183971	0	0	0	0	BTP202209	0	Tissue specific
CDS|exon|ID=cds-B8	8	6.73625	3080.7	0.702672	MPGLPTRP	85	434.7379	2	867.4636	.	M	Superscaf	92283334	BTP202209	0	0	0	0	0	Tissue specific
CDS|exon|ID=cds-B8	14	5.625	5600.1	0.70714	QPPEPQAND	55	518.9341	3	1553.784	.	D	Superscaf	92525236	0	0	0	BTP202209	0	0	Tissue specific
CDS|exon|ID=cds-B8	12	5.28	4521	0.70714	PPEPQANDV	84	649.8199	2	1297.63	.	D	Superscaf	92525236	0	0	0	BTP202209	0	0	Tissue specific
CDS|exon|ID=cds-B8	15	5.460667	5996.5	0.707166	RLSESDKED	90	437.9709	4	1747.859	.	D	Superscaf	92525236	0	BTP202209	0	0	0	0	Tissue specific
CDS|exon|ID=cds-B8	14	5.496429	5390.9	0.707171	EPGPEAAAA	91	668.7894	2	1335.569	.	D	Superscaf	92525236	0	0	0	BTP202209	0	0	Tissue specific
CDS|exon|ID=cds-B8	15	5.714	5890.5	0.707178	DAVEREKTE	92	556.9546	3	1667.848	.	D	Superscaf	92525236	0	0	0	0	BTP202209	0	Tissue specific
CDS|exon|ID=cds-B8	11	3.587273	3262.6	0.707179	VDVDVSEEL	98	610.3037	2	1218.598	.	D	Superscaf	92525236	0	0	0	0	BTP202209	0	Tissue specific
CDS|exon|ID=cds-B8	14	4.777857	5202.7	0.70719	VEEAEEGSG	93	759.3682	2	1516.726	.	D	Superscaf	92525236	0	BTP202209	0	0	0	0	Tissue specific
CDS|exon|ID=cds-B8	13	5.059231	5321.2	0.707191	VQDTETETL	95	761.3649	2	1520.721	.	D	Superscaf	92525236	0	BTP202209	0	0	0	0	Tissue specific
CDS|exon|ID=cds-B8	11	3.764545	3304.8	0.707202	TEVVLEEDV	94	631.3275	2	1260.645	.	D	Superscaf	92525236	0	BTP202209	0	0	0	0	Tissue specific
CDS|exon|ID=cds-B8	9	3.517778	2481.8	0.707202	VVLEEDVTK	99	516.2823	2	1030.555	.	D	Superscaf	92525236	0	0	0	0	BTP202209	0	Tissue specific
CDS|exon|ID=cds-B8	8	4.71	2558.7	0.707203	DVTKKVEF	81	483.2664	2	964.5229	.	D	Superscaf	92525236	0	0	0	BTP202209	0	0	Tissue specific
CDS|exon|ID=cds-B8	7	6.155714	2751.8	0.707492	KDDASGK	54	360.6862	2	719.345	.	M	Superscaf	92544568	BTP202209	0	0	0	0	0	Tissue specific
CDS|exon|ID=cds-B8	8	5.3175	3019.4	0.790886	HFFNPVPL	59	485.7589	2	969.5072	.	E	Superscaf	97063888	0	0	0	0	0	BTP202209	Tissue specific
CDS|exon|ID=cds-B8	8	5.62	2852.2	0.790886	FNPVPLMK	53	473.2639	2	944.5153	.	E	Superscaf	97063888	0	0	0	0	0	BTP202209	Tissue specific
CDS|exon|ID=cds-B8	8	6.05625	2896.1	0.819625	GFAKPGME	81	418.7011	2	835.3898	.	D	Superscaf	98622076	0	BTP202209	0	0	0	0	Tissue specific

CDS|exon|ID=cds-Bg	6	4.866667	2563.9	0.819669	FDWLYE	57	872.3788	1	871.3752	.	L	Superscaf	98624407	0	0	0	0	BTP202209	0	Tissue specific
CDS|exon|ID=cds-Bg	6	4.67	1526.6	0.828285	VSPKVP	67	626.3846	1	625.3799	.	A	Superscaf	99089941	0	0	0	0	BTP202209	0	Tissue specific
CDS|exon|ID=cds-Bg	10	5.513	3305.8	0.853093	TLPEVKVRF	97	568.3395	2	1134.676	.	P	Superscaf	1E+08	0	0	BTP202209	0	0	0	Tissue specific
CDS|exon|ID=cds-Bg	10	4.033	3781.4	0.853096	DDSDEDLLF	78	579.2501	2	1156.488	.	P	Superscaf	1E+08	0	BTP202209	0	0	0	0	Tissue specific
CDS|exon|ID=cds-Bg	11	6.17	4346.5	0.892119	AKEKGKFED	90	627.311	2	1252.612	.	S	Superscaf	1.03E+08	0	0	0	0	0	BTP202209	Tissue specific
CDS|exon|ID=cds-Bg	10	7.144	4255.3	0.892121	KKFKDPNAF	90	391.5626	3	1171.671	.	S	Superscaf	1.03E+08	0	0	0	0	0	BTP202209	Tissue specific
CDS|exon|ID=cds-Bg	12	7.088333	5110.5	0.892121	KFKDPNAPK	98	465.5997	3	1393.783	.	S	Superscaf	1.03E+08	0	0	0	0	0	BTP202209	Tissue specific
CDS|exon|ID=cds-Bg	9	6.426667	4049.5	0.892122	LFC(+57.(	86	400.5319	3	1198.58	Carbamido	S	Superscaf	1.03E+08	0	0	0	0	0	BTP202209	Tissue specific
CDS|exon|ID=cds-Bg	8	6.4825	3696.1	0.892122	FC(+57.02	78	543.7533	2	1085.496	Carbamido	S	Superscaf	1.03E+08	0	0	0	0	0	BTP202209	Tissue specific
CDS|exon|ID=cds-Bg	16	4.909375	5353	0.955014	A(+42.01)	96	829.9117	2	1657.816	Acetylati	R	Superscaf	1.06E+08	0	0	0	BTP202209	0	0	Tissue specific
CDS|exon|ID=cds-Bg	15	6.086	6274.3	0.964756	LDSDRTPST	99	541.5921	3	1621.754	.	T	Superscaf	1.06E+08	0	BTP202209	0	0	0	0	Tissue specific
CDS|exon|ID=cds-Bg	7	5.681429	2505.8	0.965543	YEPAGPP	58	730.3351	1	729.3333	.	K	Superscaf	1.07E+08	0	0	0	0	BTP202209	0	Tissue specific
CDS|exon|ID=cds-Bg	11	5.565455	4793.5	0.965594	LTELENELN	95	652.3397	2	1302.667	.	P	Superscaf	1.07E+08	0	BTP202209	0	0	0	0	Tissue specific
CDS|exon|ID=cds-Bg	7	4.334286	1914	0.971043	SDGVLKV	87	359.2093	2	716.4068	.	K	Superscaf	1.07E+08	0	0	0	BTP202209	0	0	Tissue specific
