# Supplementary material for: Integrated peptidogenomics decoding yak non-conventional peptides: functional mapping and biopotential mining of genetic resources
Source: Anim Biosci. 2025 Sep 30;39(5):250408. doi: 10.5713/ab.25.0408 (PMC13153706; doi:10.5713/ab.25.0408)
Supplement: Supplementary file 5 [file ab-25-0408-Supplement-5.pdf]

NCPs from lncRNA	NCPs from circRNA
AAAANL	AAAAARVGL
AAAPPP	AAFLLN
AAAVLG	AAFQPL
AADLGLKK	AALAPP
AAGFSGRP	AALATL
AALLPK	AALSVP
AANAEV	AAPLLP
AAPLLP	AAPPFT
AAPMEL	AAPPPP
AAPPPP	AAPRPP
AARSLL	AAPSAF
AASTARHLYLR	AAPVVP
ACGLLG	AASLGL
AEKLLR	AATSTF
AEVLEL	AAVPVA
AGLQFPVGR	ACSLLL
AGNASKDLKVK	ADEDFVDPW
AHRLQL	ADETETPAETTHNLK
ALALAF	AEEVVVVA
ALERLK	AEEVVVVAK
ALGELP	AEEVVVVAKF
ALGLSV	AELRAL
ALGSLA	AELTAL
ALLKKP	AERRVP
ALLTPQ	AEVLEL
ALPLGRK	AFGLSL
ALPLGRKK	AFLGAPP
ALPPPP	AFPLTS
ALRLHL	AFSCSR
ALSLLQ	AGCLKY
APAAWP	AGSLLK
APFPLL	AHVPEQ
APGGLPL	AKLLLR
APGTPRP	AKRLAK
APGVAP	ALASFR
APLGPL	ALEGLL
APLPEKT	ALGTAF
APLPNL	ALKKRE
APPEVPK	ALKKVR
APPFLL	ALKLLK
APPPPP	ALPPFS
APPPTPR	ALPPPP
APPTGV	ALQDLL
APSFLP	ALRLHL
APSFPL	ALSLLQ
APWALQ	ALSLPF
ARPPPP	ALVKKLK
ASFLRK	AMELLK
ASNNELVRTK	AMLSGVT
ATKLLR	ANHLVL

ATLLLL
ATPPPP
AVKFQR
AYHLQK
AYSLLL
CCYCCW
CGPLLL
CHVLTH
CLHMAS
CVSLLL
DGLLSL
DGLVRL
DHVPLAL
DLGFLT
DLGLLP
DLGLPL
DLSLPL
DPPAPPVD
DSAMSN
DSLGFL
DVGLGL
DWDLRK
EAAPGP
EAVLNR
EAVTLP
EGFKDK
ELGVLLP
ELKEKL
ELLLSC
ELLPVP
ELSQRL
ELVLDR
EMDEEDKAFKQK
EMDEEDKAFKQKQKEEQKK
EPGPLP
EPLKPK
ESGKAG
ESNLLQ
ESPLVL
ETRELAK
EVDLNL
EVHFTR
FGEGLL
FKPSDF
FLFAFC
FLLEAH
FLLLKK
FLLLLF
FLLVQL
FLPALAA
FLSLLF
ANQVLK
APAPEVR
APLYRAL
APPPPP
APSFLP
APSFPL
AQERLL
ARLHKP
ARPPPP
ASHSLL
ASLLAA
ASLSTF
ASPQAP
ASSHKGKK
ATALLK
ATLLLL
ATLSRSL
AVELGKNQAKF
AVELNPK
AVEPLT
AVQLSK
AVWRQP
CALHFL
CCSPAP
CLLMLK
CNPDLR
DAYVRP
DDEGTLGPLPQVD
DDFGFF
DFSTVP
DGEWQL
DGLLKF
DHVFGK
DKELFL
DLELLR
DLENKFKPF
DLGLLP
DLGTLA
DLLRLFEY
DLPPPY
DLSLLP
DLVLVK
DLWKEK
DMPDLR
DNELLP
DPRGLYG
DPVTTK
DQLGADATKEKPK
DRLRLP
DRPLNP
DRVVNL

FPDLLR
FPEFQL
FPGDLP
FPGKDT
FPGLSA
FPLGSP
FPLLLN
FPSLVG
FPSSNL
FPSSPP
FREGTTPKPK
FSKVPF
FTGAAG
FTKKKT
FTKLML
FTKRTT
FTLKKK
FVDLLH
FVLLFV
FVRKKK
FVRPSL
GCFVVF
GETPLM
GFWKAP
GLFLEN
GLGGGP
GLGGLT
GLGLPLK
GLPPNP
GLSVYF
GPEPAP
GPGTLL
GPLLLF
GPPLSP
GPPPPP
GPSLPW
GPVLLA
GQEPGVK
GQVLEY
GRVGATAAV
GSLMVP
GSLTLLL
GSLVSKK
GSVSEKP
GVGALL
GVPTVG
GVTLPL
HGAPKP
HLLGLLL
HLLLPP
HLLLSF

DSLLLP
DTEDWRPR
DVGLGL
EALEKF
EALNYL
EANGSG
EAPGAP
EAVLVK
EDTEDWRP
EDTEDWRPR
EDVLLK
EEDLRK
EEVALP
EEVEVAPPKAHE
EEVEVAPPKAHEVR
EEVLLR
EFLDML
EGFDLL
EGFDLLR
EGSMSE
EKGHLLP
EKLLPR
ELAAAP
ELEAPL
ELGQGLR
ELLAYL
ELLKLE
ELLNKP
ELPFYQ
ELPFYQK
ELRVTQ
EMLRLP
ENEQLL
ENLRLK
EPAAPP
EPGPLP
ERTVFV
ERVKEF
ERWLLT
ESLLYL
ESLVRK
ESPLLL
ESSLYR
ETKKLT
ETYLFD
EVAGLK
EVAPPKAHEVR
EVEVAPP
EVEVAPPKAHE
EVEVAPPKAHEV
EVEVAPPKAHEVR

HLLPLP	EVFPVL
HLLPPPP	EVGVLR
HLPLLP	EVLLLH
HLSSSS	EVPLLT
HPAAPK	EVQTNELRAEK
HTLPAL	EVSLLP
KAAQRR	EWAPPVQ
KALKTSK	FAPVVAPKPK
KALTLL	FCLKLK
KAPLPP	FDDLPK
KASAAP	FDLWKEK
KASKKPK	FDPLNK
KAWLVGP	FDSTPA
KCSKPQ	FGDYNKELHK
KDFLVP	FGDYNKELHKAGY
KDFQAL	FGEGLL
KDPLEK	FGELAL
KEELLK	FGLDLK
KELKLP	FGVPAKK
KELLDQ	FHSSPR
KFLEGG	FKLLSH
KFLLTA	FKVSLP
KFVEAF	FLFLLY
KFVLSR	FLGEAAP
KGAAAP	FLGPFF
KGKRAH	FLLLLF
KGLLGH	FLLPKSV
KGPLTQ	FLLSDH
KGPVSY	FLLTKK
KHLSPP	FLLVVK
KKKTTK	FLRLNF
KKLEEL	FLSLLF
KKLKLR	FNPLLK
KKPNKP	FPLLLL
KKPPKK	FPPPPP
KLAPPP	FRLLQR
KLELLC	FRSLFL
KLHFGR	FTLLLF
KLKKLP	FVEKTP
KLKPPR	FVLLFV
KLLAFC	GDLAGK
KLLDFC	GDLLSL
KLLLHK	GFPPPFP
KLLLKK	GFSTLYP
KLLLSH	GHSLLQ
KLLVVH	GLDASLF
KLVDLF	GLFLEN
KMGNVKK	GLGVLPF
KNKKST	GLSPSPVAPQMF
KNMEVP	GPAAVR
KPCQAAP	GPGALP

KPEYKN
KPFFSL
KPLTKK
KPLVPPP
KPVERT
KQHYVL
KQTFPP
KQTLKP
KRFLAK
KRRRER
KSEGLL
KSFLMT
KSLDCLP
KSLENW
KSLKPGK
KSLVLT
KSMPPK
KSPALGF
KSSAWAP
KSSLPR
KSSSPTP
KSTEASK
KSVKKE
KTENLP
KTKLLL
KTPLLS
KTPWAP
KTTKKK
KTYLNF
KTYPYL
KVELCSF
KVLELQ
KVLLLLL
KVMEAR
KVPSLP
KVRAVF
KVVHVR
KWRLPR
LAAPRL
LAGLSV
LALFKK
LAPLPP
LAPPER
LAPPLL
LAVFKK
LAWGLL
LCKAGF
LCPDGLK
LCSALL
LDKSFF
LDLLVLG
GPGPAL
GPLLVP
GPPAEP
GPPAPP
GPPPPP
GPPPPYH
GPSALLR
GPSLPP
GQHLVL
GRTPDL
GSHKER
GSLLDF
GSLLLK
GTLLEL
GVFLGL
GVLPFK
GVPSVPK
GVVLAKK
GVVLSA
GWLEPL
HESFLK
HEYLSMEL
HFLLPF
HGSNLE
HGSPPPP
HGYFPP
HLLKAPH
HLLLPLLL
HLLLSF
HLLPPL
HLPLPL
HLPPPP
HLPPPPP
HLRQVGVVGK
HLVFLL
HQVLYR
HSGLDFK
HSLQLPP
HVAHLK
HVLPLEK
KAELLR
KAFYKS
KALLFL
KASAAP
KAVPSL
KDALLA
KDELLM
KDGALL
KDLVTL
KDQSLK
KEELLKT

LDSLPL
LDVGNF
LEELKAK
LEELKK
LEEQFQQGK
LEEVLK
LEEVVR
LELDTLK
LELHLK
LELLEL
LELLLP
LEPLLK
LEPSLR
LEVLPR
LFLRLP
LFLVPF
LFTKSK
LFVKKK
LGEGFL
LGEPGAV
LGGSLL
LGHLLT
LGLAGA
LGLKVF
LGPALP
LGPSALP
LGSKNKL
LGVVSVK
LHWEHR
LKALTK
LKLSLK
LKPVER
LLALQPL
LLDLKK
LLEPVT
LLERPH
LLETSL
LLFPEL
LLGFFF
LLHFVV
LLKLKSL
LLKVKLR
LLLENY
LLLKVV
LLLLEL
LLLLGN
LLLLLLL
LLLLLLLL
LLLLLLLLL
LLLLLPS
LLLMLN
KEFLLSM
KEHLQK
KEKYKLK
KELPLH
KEPLKD
KEPLKK
KEPPHK
KEPRPP
KETSSLY
KEVKAL
KEVLLL
KFAPVVAPKPK
KFFFKK
KFFLPP
KFKGRP
KFPRGL
KGAAAP
KGAFFH
KGGLLS
KGLEDR
KGVWGNK
KKAVLQ
KKFAPVVAPKPK
KKKTHK
KKKTTK
KKLEEL
KKLLKK
KKNGAPR
KKPNKP
KKSSKK
KLAVWGNK
KLGLRP
KLHFST
KLKTEL
KLLLPR
KLLPLPA
KLPGVGV
KLPKMP
KLPPPPP
KLQPLG
KLRLFR
KLTELR
KLTYLE
KLVVLGSGGVGK
KMAVLK
KMSRAP
KMYVAH
KNFCVP
KNLEVLNF
KNNDLL
KNQSLAR

LLLNTF
LLLSPN
LLLTLL
LLMLLL
LLMLLN
LLNVSK
LLPFLL
LLPPPPP
LLQHLL
LLQNSV
LLVFSD
LLVPSP
LLVTLA
LLVVVD
LPAAEC
LPAAGP
LPEFPR
LPELNG
LPELPR
LPFAFL
LPFLLL
LPFPPP
LPGLPP
LPGLSA
LPGLSAP
LPHLPR
LPKEPL
LPLAAPR
LPLAPD
LPLLHP
LPLLLLP
LPLPGP
LPLPPP
LPLPPPP
LPLVAA
LPPGAP
LPPGSLL
LPPHLR
LPPKYKK
LPPLGP
LPPLPHL
LPPLPP
LPPLPPP
LPPPPP
LPPPPPP
LPPPPPPP
LPPPPPPRP
LPPSLL
LPPTPP
LPPVAL
LPPVPP
KNVLQR
KPELSH
KPFFHY
KPGEFR
KPGGVK
KPGLPH
KPLTPR
KPPPRP
KPRLLL
KPSGGP
KPTLLK
KPVCPK
KQLADETLLK
KQPFPK
KQPMLF
KQTLKP
KRKKKH
KRLNAK
KSDGEW
KSFFPK
KSGELL
KSGTLL
KSMFPN
KTHFGP
KTLASPK
KTLLFTL
KTTLQF
KVKLDP
KVPGPP
KVPSLP
KVRAVF
KVYLLF
KWTPPK
KYGLSP
KYGLSPSPVAP
KYPPLP
LAAVLE
LAGGLL
LAGLSV
LAHEELL
LAKLLR
LALAGP
LAMVASSHKGK
LAMVASSHKGKK
LAMVASSHKGKKDT
LAPKEPR
LAPSVLF
LAVWGNK
LCSALL
LDAVVFT
LDHLTR

LPQGTP
LPRPPPP
LPRSNR
LPRTPP
LPSLGP
LPTRPC
LPVAGV
LQELLK
LQFLLG
LQPSRL
LRDNLT
LRGVFY
LSAGPL
LSALGW
LSANLL
LSDLLP
LSDLVFK
LSDRPPP
LSFCLF
LSFPTT
LSGFKTYY
LSGGLL
LSGGLN
LSGSPF
LSKKKV
LSLERL
LSLVTL
LSPGVV
LSPLPF
LSPVFG
LSQRPR
LSRLLK
LSSAGL
LSSALG
LSSLSPM
LSVGRP
LSVVLFLL
LSYLPL
LSYNLR
LTAEVL
LTAEVLEL
LTAEVLELAGN
LTDSLYP
LTGLLL
LTLGLR
LTPLPP
LTTKFT
LTVLPF
LVGGLL
LVLHAV
LVNLLK
LDLDVL
LDLESF
LDLLEC
LDLLLK
LDSLLPP
LDVQPK
LEADLP
LEAFGNAK
LEALEL
LEELYL
LEFLKK
LEKLLK
LEKLLKK
LELEQL
LELLEL
LELLKK
LELPNP
LERLYL
LERVHL
LETAVNL
LEVGLLA
LEVLEK
LEVLNF
LFALAK
LFGKKQ
LFLRLP
LGAAVL
LGADATKEKPK
LGAPEV
LGAPPV
LGARDL
LGDNVL
LGETPL
LGFLEL
LGKLAL
LGNFFSPK
LGNFFSPKVVSL
LGPAPK
LGPVEL
LGTTVL
LGVLSK
LGWTHC
LHNLSF
LKAKFPP
LKALTK
LKELAK
LKGVVT
LKKLLG
LKKRPP
LKLLEH
LKLLLC

LVVVDF
LYESSF
LYSDLK
LYVEVH
MDDTLF
MDEEDKAFKQKQK
MDGLVP
MDLKALL
MEGAEEKKK
MEPTFP
MEVKPPP
MFLEAT
MKVELC
MKVELCS
MLGASL
MLKPLR
MLLKVK
MLPPPP
MMHLLR
NASNNELVRTK
NCVPEK
NEALLEF
NGPSWL
NLLEFK
NLLVFL
NNFLVL
NNPKKP
NPSKYR
NQNLLL
NRSLLL
NSKALLR
NSSLPQR
NTLRAH
NTVLLL
PAAAAGP
PAAETP
PAAGLR
PAFLLL
PALPGL
PARLLQ
PASFLP
PASTSL
PDPPLP
PEYPFVEEY
PFASHL
PFASHLL
PFVTLK
PFWSAK
PGGLLLG
PGPAPSGTNVGSSGR
PGPYPHPP
LKLSLK
LKLVLP
LKVLLP
LLDFLK
LLDMLL
LLDPVL
LLEKPK
LLESLP
LLGLQM
LLKEPK
LLKKSL
LLKKSLL
LLKLKSL
LLKSLYR
LLLDEL
LLLDFP
LLLDPF
LLLFLPK
LLLHRP
LLLKKS
LLLLLLL
LLLLLLLL
LLLLLPS
LLLPQM
LLLRPV
LLLSRGK
LLLSSGK
LLLTLL
LLMLLL
LLNQDL
LLNVSK
LLPCLK
LLPFLE
LLPHPP
LLPLFL
LLPSPF
LLPTVF
LLQHLL
LLQLLH
LLRNPF
LLRPATP
LLRVENL
LLSGGL
LLSKKL
LLSLKY
LLVLML
LLVPFHS
LLVPSP
LLVTLA
LLVTLLH
LMKLLT

PGQPPP
PGVPGP
PGVPVH
PGVTVKDVN
PGVTVKDVNQQEF
PGVTVKDVNQQEFV
PGVTVKDVNQQEFVR
PGVTVKDVNQQEFVRALA
PGVTVKDVNQQEFVRALAA
PGVTVKDVNQQEFVRALAAF
PGVTVKDVNQQEFVRALAAFLKK
PHKKKS
PKSALP
PLFLLL
PLGLPP
PLLFSN
PLLFVT
PLLPPP
PLLPPPP
PLLSFL
PLPPPP
PLRTPP
PLVVLLY
PPAPPP
PPDLPP
PPHPVR
PPLPPP
PPPGPP
PPPPLA
PPPPLPA
PPPPPHV
PPPPPP
PPPPPPP
PPPPPPR
PPPPPRPP
PPPPRPP
PPSHTLK
PPSPPQ
PPVPLP
PPYLLL
PQKKKR
PRGLPP
PRPPPAPP
PRTVAP
PSLVFG
PSRLLY
PSRPPP
PSVPPG
PTARPP
PVLGGP
PVRLLPP
LMMKKT
LMPPPP
LNFSVF
LNLLEKDY
LNLLEKDYF
LPAAGP
LPAAPL
LPAGGL
LPAPGL
LPGLSE
LPGVAC
LPGVHVL
LPGVLP
LPKLPKL
LPKPEL
LPLAVP
LPLENK
LPLPGP
LPLPPP
LPPDAP
LPPEEE
LPPFLQ
LPPHRP
LPPLPLPP
LPPLPPP
LPPLYK
LPPPPHP
LPPPPP
LPPPPPP
LPPPPPPP
LPPPPPPPP
LPQPDL
LPSPLL
LPSRPLE
LPSRSL
LPTNLF
LPWQSK
LQAQMK
LQELLK
LQETLP
LQFLLG
LQKVLQ
LQLLFL
LQLLPQ
LQPGTP
LRALDK
LRKAEL
LRLVKL
LRPPLL
LRQVGVVGK
LSAAFL

PVTLLL
PVTRPP
QAPVYL
QAVLSL
QDLKGTN
QGLALF
QGPAAG
QKGGLE
QNTSGK
QPAPPR
QPPPPP
QRALHL
QRGVVVL
QRLLRL
QSLKGK
QSLLPL
QSLVHRK
QSPFPL
QSSYAP
QSVQPK
QTCLLL
QTHHLP
RAALVP
RAAQLR
RAKALR
RALGPR
RAPPPP
RAPTPP
RDLPNP
RDSRKL
REKMAP
RFLKRQ
RGLLTL
RGRPRP
RGYMPR
RHTLVQ
RHVFLL
RKGPLL
RLLLYK
RLLPNR
RLPPPP
RNSLLR
RPALPP
RPDRPP
RPPVFK
RQKLAL
RQWYESH
RQWYESHY
RRLLEP
RRPATL
RSFRLL
LSAFDNF
LSAGPL
LSALPF
LSAVGL
LSFPTT
LSGQRL
LSGSAL
LSGSPF
LSKHPV
LSKKKV
LSLALA
LSLEEL
LSLEFY
LSLPEAL
LSLVTL
LSPASL
LSPGAL
LSPLVLRK
LSPSLPR
LSSAGL
LSSSLLD
LSSTAF
LSSTDLR
LSSWRP
LSTLAL
LSYLPL
LTAELL
LTEKLL
LTELGTVDPK
LTELGTVDPKN
LTGLAA
LTGLLL
LTGRTWKG
LTLGLR
LTPLPK
LTSKYR
LTSYLK
LTTVLP
LTVLPQ
LTVPSR
LVDLEK
LVEAPP
LVEELR
LVETLK
LVFPER
LVGLNL
LVGVSP
LVHELR
LVHLYP
LVLDQV
LVLGGRLV

RSLTAAH
RSNLLR
RTHPPR
RVLPPR
SALRLGE
SAPGLL
SAVPKLL
SDLRLL
SDVSLTVPK
SELLVRR
sequence
SGPVLK
SGPVLL
SGRPLP
SHNGKHR
SHPFFP
SHSLLRP
SKHTRPP
SKPAAP
SKYLFT
SLAALK
SLDLLP
SLDPVP
SLFLLF
SLGLLL
SLGLVK
SLGTLP
SLGVVP
SLLERL
SLLPLP
SLLTSF
SLNSTF
SLPELT
SLSKGPFP
SLTVVPK
SLYPAPGA
SLYTFP
SNFNHKQLE
SNNELVRTK
SPLKEK
SPLLLR
SPLVPF
SPVFPR
SPVKPP
SPVVLVT
SPVYQA
SQTSSK
SSHHPF
SSKSSS
SSLLQKF
SVLLPR
LVLLDL
LVLLLPL
LVLSAL
LVPFAT
LVPVPQ
LVVLGSGGVGK
LVVVLV
LVVVVL
LVYRTH
LWTYKK
LYNLFL
LYNLFLK
LYPSRL
LYSGER
MEESVNQMQPLNEK
MFLLLF
MKPLLL
MLAAQL
MLAAQLL
MLLLNL
MLPPSP
MMTCCP
MPPLGL
MRDLYMKNGQGF
MREYKLVVL
MSPKLL
MVASSHKGK
MVASSHKGKK
MVESVRTPY
MYKPVDPY
NAFLKK
NEGFDLL
NFSVFY
NKVAPV
NLQLQP
NLVPCT
NPLLLG
NPLVVL
NPMLLL
NPRRPP
NQLLPK
NQSVLL
NSRRKP
NTAVLL
NVVLLR
NWFMKK
PAAPAP
PAASPK
PAAVVE
PALPGL
PASFLP

SVPLLL
SWTLKK
TAKNLYL
TAKNLYLG
TANLLL
TDYYLT
TFLPPP
TGGAGY
TKKLLL
TLATLP
TLFQRQ
TLLSPP
TNSLPK
TPFLTR
TPKPPR
TPPPLP
TPPPPP
TPPVPLT
TPSLLC
TPTLRLY
TPVLLL
TRAASTARHL
TRDPLV
TSCKMP
TSCLLF
TSPHPP
TTAKNLYL
TTAKNLYLG
TTKKKR
TTTASL
TVGDLLK
TVGGLL
TVLLPL
TVLNKK
TVLTQKL
TVPPLK
TVPSFK
TVSLLT
TVVALL
VALSLK
VAPPRP
VASYLL
VAVLGA
VAYVLK
VDGGFL
VDLLLL
VEDKLR
VEGPLL
VELLLK
VEPGGLP
VFQFLNAK
PAVLGF
PFLEYT
PFLVGK
PFPPPP
PGAGSQ
PGAVPP
PGEAPP
PGPAEP
PGPEAP
PGVLQP
PHLNPR
PLFLLL
PLKMLL
PLLAFK
PLLHLP
PLLMSL
PLLPPP
PLLSFL
PLLVEF
PLMLLL
PLPPPP
PLPPPPP
PLPPPPPP
PLPVAL
PLSAPP
PLSCLKL
PMLLLTG
PPAPPPPP
PPFTPP
PPHPPPP
PPKAHEVR
PPLLPDP
PPLMPV
PPLPLP
PPLPPL
PPLPPP
PPLPPPPP
PPPKLH
PPPLAP
PPPLPLR
PPPPAL
PPPPAPL
PPPPGP
PPPPGTH
PPPPHPP
PPPPLP
PPPPPLP
PPPPPP
PPPPPPP
PPPPPPPPP
PPPPPPR

VGASLVA
VGDLGL
VGGAGP
VGGLLP
VGLMVG
VGPGTF
VGPGVK
VGPLLK
VGVAPPG
VHEVTK
VKGSPLL
VKPVKVSAPR
VLALLF
VLEGKELE
VLEGKELEFY
VLLHVL
VLLLFA
VLLMAF
VLPELY
VLPSRVK
VLQPVP
VLRVFL
VLVFNT
VLYKKT
VPAGFL
VPGGLL
VPLAPP
VPPERK
VPPLLP
VPPPPP
VPPPPPP
VPVTGK
VPWGLP
VRALAAF
VRKVLQ
VSALPV
VSAVPL
VSCPLSQ
VSGPLL
VSLLPR
VSLPGP
VSLRKN
VSPLLL
VSRRPP
VSSLLP
VSTALG
VTLGNF
VTLSPL
VTVVLL
VVFFLT
VVLFFP
PPYLLL
PRLTPP
PRPPVPP
PSLRPP
PSPPPQ
PSRPPP
PTLLEH
PTPPFP
PTVLSA
PVATAA
PVDLEA
PVGLAA
PVGLLG
PVLPLQ
QALLLP
QALTVH
QALTVK
QAVLSL
QFLLPK
QKEELK
QKLKPK
QLEVLF
QLFGGF
QLLLPK
QLSNKL
QMSLLL
QPCLKK
QPGFFL
QPPPPP
QPVFLL
QQMKKK
QSLLPL
QTAGFP
QTLLEK
QTNPML
QVKEGN
RAALVP
RAHVRP
RAPPPGV
RDGKVK
RDPGSF
RDVLKY
REDSWLK
RHTLVQ
RKAPPPP
RKPKFN
RLALYL
RLEELR
RLGWSL
RLKKKP
RLLLLM

VYLFLP
VYVVFS
WAALLR
WPGTLR
YHGPPR
YHKYSL
YLLPRS
YLLVEF
YNGPGY
YQSEEK
YRAALC
YRTLPL
YTRAASTARHLYL
YVALSR

RLLLSD
RLLLYK
RLLPPL
RLPVVLG
RLSRPA
RNSLGR
RNSLLR
RPAAAGP
RPPKGF
RPPLPR
RPPVTL
RPPVTLR
RPSLPL
RQTVLR
RRPATL
RTLLPR
RTSLSF
RVFSSS
RVHRLL
RVKPVK
RYLSPR
SAALRLP
SAAPLL
SALLEL
SARLLR
SDQLGADATKEKPK
SDYPPL
sequence
SFCRLP
SFCRPL
SFLGLSL
SFYPHK
SGGLLL
SGNGLK
SGPVLK
SGPVLL
SGTVRLK
SHPFFP
SKKQPC
SLAFTP
SLENFQ
SLERLL
SLGLLL
SLGSAL
SLLAGL
SLLKNC
SLLPLP
SLLQYE
SLNSTF
SLPLVD
SLPPLK

SLRPPR
SLTLAL
SLTSPL
SLYTFP
SNSPRF
SNSRPF
SNVRLR
SPLFLHV
SPPPPPPPP
SPPSLR
SPTSLF
SRLLQE
SSGMKL
SSKSSS
SSLRSY
SSNPVY
SSPFKV
SSSKPP
SSSLEK
SSSSAK
SSYMPR
STFNDLQDLREQ
SVFRPL
SVGSLF
SVLLPR
SVNPMLL
SVNPMLLLT
SVSTQQPPK
TAALLK
TAMRDLY
TAMRDLYMK
TDTPELQR
TEAAKPVVTK
TEDWRPR
TEVGSASEVKK
TEVSSLPLVK
TGLNYL
TGNHPR
TGVVPG
TKFRPM
TLAAPL
TLASVP
TLDAGKF
TLGPPF
TLGSKFR
TLGTLQ
TLKTSR
TLLSPP
TLRLLM
TLSLLF
TLVTSF

TPLLVR
TPPPPP
TPVTLKQ
TRLSSF
TRPAVR
TSLAKP
TSPGPL
TSVALP
TTEVGSASEVK
TTEVGSASEVKK
TTEVGSASEVKKESD
TTEVGSASEVKKESDQ
TTGLVL
TVDLPL
TVFPNF
TVGLLL
TVKFPR
TVLLLP
TVLLPL
TVLSAL
TVPELT
TVSLLT
VAFMLP
VAGLTA
VAGVLP
VAGVLPF
VAKSFR
VALSLY
VAPPKAHEVR
VAPQMF
VAPQMFGN
VATRWY
VDEKLK
VDLLLL
VDPLSF
VDTRKF
VEAGRP
VEAPGR
VERVPF
VEVAPPKAHE
VEVKLR
VEVLLL
VFPLHSF
VFSLGK
VGAGSL
VGENPK
VGGLLP
VGKEDSSSTEFVEK
VGKLFEDTK
VGLMLK
VGNPLL

VGPEGF
VGPGLP
VGPPPP
VGSYLK
VGVLLL
VLAPKR
VLEMDPLPSSKPFQ
VLKTLR
VLLLDL
VLLLML
VLPELY
VLPLEK
VLPPPP
VLVAQHDAY
VLVGPS
VLWELN
VLYFPL
VLYKKT
VNPMLL
VNPMLLL
VNPMLLLT
VNPMLLLTG
VPAGELH
VPASLP
VPFPLR
VPFSLL
VPGVGN
VPPEKPP
VPQEKQ
VPSLKK
VPTFPF
VPVLPE
VSAALL
VSELLL
VSGPLR
VSGPTF
VSLFPF
VSLLPR
VSPPPP
VSPSPL
VSSFKLE
VSVVDL
VSVVLGH
VTAVDL
VTDTPEL
VTLAGL
VTLSAL
VTLVTL
VTTVPVK
VTVASQ
VTVVLL

VVAPLP
VVDLVR
VVGHGD
VVHLLF
VVLFPF
VVLGQF
VVLGSGGVGK
VVLLPL
VVLPKEFPVYL
VVPVPQ
VYKVLK
VYPLPL
VYSRPK
WKEVAQDCTK
WLPVVR
WRTVKL
WSGPPA
YDGKKPDTL
YDGKKPDTLGPN
YDLADQLHA
YGDGFF
YGVSFF
YLLDLL
YLLKRS
YPPLLL
YTDLLRLFEY
YVLASR
