# Supplementary material for: Integrated peptidogenomics decoding yak non-conventional peptides: functional mapping and biopotential mining of genetic resources
Source: Anim Biosci. 2025 Sep 30;39(5):250408. doi: 10.5713/ab.25.0408 (PMC13153706; doi:10.5713/ab.25.0408)
Supplement: Supplementary file 6 [file ab-25-0408-Supplement-6.pdf]

ncps from Ribo-seq
AAAAGK
AAAALP
AAAASLL
AAAFEEQENETVVVK
AAAGKP
AAALAGY
AAALQAV
AAAPPP
AAAVFEYPGN
AADQAAPQ
AAEDDEDDDVDTK
AAEDDEDDDVDTKKQK
AAEGLKPVGNAQPSGF
AAEVLL
AAGKGKLDVQ
AAGKGKLDVQF
AAHTVP
AALDSLSL
AALDSLSLF
AALEYL
AALFGR
AALGAQL
AALGAQLL
AALGAQLLSEW
AALKRGP
AALLAH
AALLPK
AALPLP
AAPAPVP
AAPGLP
AAPLLP
AAPLVVP
AAPPPPR
AAPPVP
AAPRPP
AAPTVTR
AAQALAL
AAQALALLR
AARPAK
AARPPPR
AASAAQRELVAQGK
AASCLLL
AASGPPPP
AASSYG
AATFLK

AAVAVPL
AAVFEYPGN
AAVPAV
AAVPVA
AAVQLGNY
AAVSGVLP
AAVSGVLPQVPEPR
AAVSLR
AAWSSK
AAYLYEVSQLKD
ACGLLG
ACGLVA
ADDDRPSWR
ADELAK
ADEWLMK
ADFDTYDDRA
ADFDTYDDRAY
ADFDTYDDRAYS
ADFDTYDDRAYSS
ADFDTYDDRAYSSF
ADMVQSQEAPK
ADMVQSQEAPKQE
ADTPENLRLK
ADTVRPEFY
ADVDLNVAAPK
ADVFAP
AEAAKPTATKTEATK
AEALGPLE
AEASLPSPK
AEAVKALVKPK
AEDDEDDDVDTK
AEEFGNETWGVTK
AEEMNEK
AEEMNEKQVYDA
AEFTRL
AEFTRLQ
AEGPDMDVNLPK
AELCPL
AELLEL
AELLRA
AEMEDLVSSK
AEPSLR
AERRVP
AETPKPL
AETPKPLG
AETPKPLGN

AEVKRF
AEVLEL
AEVLKVLGNPK
AFDCVDKMVY
AFELLH
AFELLHL
AFEVTADF
AFFLFC
AFLADPSA
AFLADPSAF
AFLHWY
AFLLSF
AFSKEKMEKTKVR
AFSPEEMKQLDALNK
AFTLLY
AGAAKPKKPAGAA
AGALHY
AGGGLP
AGLQFPVGR
AGPKVPPEPKP
AGPSLV
AGSLLK
AHLLPF
AHLVPK
AKALESPERPF
AKEWGY
AKLLPQ
AKPLTD
AKPLTDQ
AKPLTDQEKRRQ
AKVAVL
ALAAAGYDVEK
ALALAF
ALASFR
ALASLMT
ALASLMTYK
ALDFENEMATAASSSSLEK
ALDMARGM
ALDMARGMAF
ALDTSEGVTQDRFSQ
ALEAKAAAVEKQ
ALEFVA
ALEFVAR
ALEFVARNY
ALEFVARNYF
ALEFVARNYFV

ALEFVARNYFVK
ALEKLR
ALELDPNLY
ALERYP
ALESPERPF
ALETKF
ALKRQGRTLY
ALKRQGRTLYG
ALLDNM
ALLKFPL
ALLLTT
ALLMGL
ALLRGVF
ALLSSGF
ALMGCL
ALNFSV
ALPGLA
ALPGLP
ALPLHAL
ALPPVG
ALQDLL
ALRLTEY
ALRTDY
ALSARFEPF
ALSLLQ
ALVCDNGSGLCK
ALVCDNGSGLCKA
ALVCDNGSGLCKAG
ALVCDNGSGLCKAGF
ALYDATYETKESKKEDL
AMLVGKDLKVD
ANFPLLK
ANLLTGPK
ANNVLSGGTTM
APAFPQ
APEEHPTLL
APGEQPK
APGPGPTDASKVLAK
APGQGP
APGSSP
APKPPL
APKSPAKAKAVKPK
APLNPK
APLQVKVQGPK
APPLVP
APPPAAPP

APPPTPR
APSRKFF
APSVANVGSHCDL
APTDLF
APVAGP
APVVAPKPK
APYSRPK
AQADWAAK
AQGVVP
AQKDQLSEDEK
AQKDQLSEDEKF
AQKDQLSEDEKFLFVDK
AQLLLP
AQPPRL
AQPRPL
AQQAADKY
ARFEPF
ASATRF
ASAWLT
ASAWLTK
ASGVAV
ASGVAVSD
ASKERSGVSL
ASKLLR
ASLLAA
ASLSTF
ASPGAP
ASPLPGL
ASPQAP
ASSGTTDVTNR
ASSHKGKK
ASSSSK
ASSSSLEK
ASTRLFQ
ASTVELLEEGKK
ASTVELLEEGKKK
ASTVELLEEGKKKF
ATDKSFVEKL
ATEMVEVGAEEEEGGAER
ATEVSK
ATGGKYVPR
ATKLSRTKEEL
ATLRNTNPNFVR
ATLSVH
ATLSVHQ
ATPMTPGAK

ATSPQKSPSVPK
ATVLLE
AVAVVKKGTDF
AVDRLNY
AVERLL
AVFEYPGN
AVGELL
AVLGLDL
AVRLLLPGELAK
AVSEGTKAVTK
AVSEGTKAVTKY
AVVEGL
AVVLMGK
AYEQVAK
AYLASA
CCCCPC
CCCCSF
CFWFVTK
CGSAHC
CPAGPP
CPETLFQPS
CPVGCAKCAQGCVCKGASD
CPVGCAKCAQGCVCKGASDK
CSSAAL
CSVGPGYGGK
CTEQGSHPKFQKPK
CVCKGASDKCSCCA
CVQQLKEFEGK
CYVALDFENEMATAASSSSLEK
DAFFKDVDY
DAPFVF
DATYETKESKKEDL
DAVPGALDY
DAVTYTEHAKRK
DCDGDAQ
DDDHLKDLSL
DDDRGPWRN
DDDRPSWR
DDEAGKLQGSGVSSSSK
DDEAGKLQGSGVSSSSKK
DDEFTHL
DDEFTHLY
DDELNW
DDEPKFM
DDFKKAF
DDFKKAFSKEK

DDPRVEF
DDPRVEFL
DDPVDLR
DDRGPWRN
DDSFLGK
DDSRPGPWRP
DDSSKPVHL
DDSSKPVHLTA
DDTQFVRF
DDVPVPNSPF
DELEHLTK
DFAEYQPELFEK
DFAEYQPELFEKF
DFAEYQPELFEKFQNMRAQHPY
DFDLHLK
DFDLHLKGPK
DFDLHLKGPKVK
DFDLNLK
DFDVSVPK
DFENEMATAA
DFENEMATAAS
DFENEMATAASSSSLEK
DFEVVPK
DFNLKGPK
DGAEAKAAEARAAE
DGLVRL
DGPVPRP
DGSPVPSSPF
DHVFGK
DKLVTL
DKTLLRDEKK
DLAEDAPWK
DLAEDAPWKK
DLAGRDL
DLALKYFQK
DLASRDVVSR
DLDAFKNFS
DLDFNLKGPK
DLDLNLK
DLDLNLKGPK
DLDLNLKGPKVK
DLDVTGPK
DLEERLK
DLEKTYSAK
DLEPGTM
DLGEELEALK

DLGGGTFDV
DLGPPVSGPPL
DLHLKAPK
DLHLKGPK
DLHLKGPKVK
DLHLKTPK
DLHVKGPK
DLKGPKM
DLKGPKMK
DLKGPKV
DLLRLFEY
DLNLKGPK
DLNLKGPKV
DLNLKGPKVK
DLPFVVPR
DLPRPP
DLRKLAV
DLRLQDVTVL
DLSLPK
DLTDYL
DLVEALK
DLWLEREVF
DLYANNVL
DLYVPR
DMDLKGPK
DMHVNMPK
DNGSGLCKAGF
DNVTSLLNASSDKF
DNYPQSVPR
DPGGPV
DPLNDNVTSL
DPQVHR
DPRVEF
DPTVYF
DPTVYFK
DPTVYFKEQ
DPTVYFKEQF
DPTVYFKEQFL
DQEFTVK
DQLGADATKEKPK
DQRVMEHF
DRHLLP
DRHVPY
DRHVPYG
DRKPVL
DRPAPHF

DSAQGSDVSLTVPK
DSDPSYGQQGHPNTWK
DSEGHLY
DSFQEVLRF
DSGDGVTHNVP
DSLGFL
DSLLLP
DSPLGL
DSSLVF
DTALPL
DTEDWRPR
DTPENLRL
DTPENLRLK
DTPENLRLKQQSE
DTPKPL
DTSSPK
DVDFHVQGPK
DVDFHVQGPKVK
DVDKVAF
DVDLHLK
DVDLHVKGPK
DVDLHVKGPKVK
DVDLNLK
DVDLNLKGPK
DVDLNLKGPKVK
DVDMDLKGPK
DVDMDLKGPKMK
DVDVHGPDW
DVDVHGPDWH
DVDVNLPK
DVDVSLPK
DVDVSVPK
DVELNLK
DVELNLKGPK
DVGDSF
DVGELL
DVHGPDWH
DVKVKKPDLDVTGPK
DVLFLK
DVNLPK
DVQLPEVSVK
DVSLHLKGPK
DVSLPK
DVSVPK
DWHRPC
DYDAFLG

DYDDMSPR
DYDVTVPK
DYEVSVK
DYQPCPTGPN
EAESCDCLQGF
EAGGLF
EAKLLR
EALHNHYTQKSTSKSAG
EALLEL
EALLQVEDERK
EALRSL
EALSPL
EAPGAP
EAPLNPK
EAQDDLVKTREEL
EAQSKE
EATGGKYVPR
EATWVVDVK
EDELQKTKERQQK
EDEVKLP
EDGLLL
EDLEPFKMTPF
EDLGFL
EDLLLP
EDRKDGSCGVAY
EDRRPTL
EDTEDWRP
EDTEDWRPR
EDVPLV
EECWFPK
EEEDSTALVCDNGSGLCK
EEEDSTALVCDNGSGLCKA
EEEDSTALVCDNGSGLCKAGF
EEEDSTALVCDNGSGLCKAGFAGDDAPR
EEEFKTHHELKE
EEEFKTHHELKEY
EEEKSGKWEGL
EEELAA
EEELVKVREK
EEEPVQPA
EEEPVQPAVL
EEEPVQPAVLQ
EEEWDPEYTPK
EEEWDPEYTPKS
EEFGNETWGVTK
EEHPTL

EEHPTLL
EEHPTLLTEAPLNPK
EEHPVL
EEHPVLL
EEKKEEVTGTLR
EELEEESFSER
EELNREVAGHTE
EELVRM
EEPCLLK
EEPLFPF
EEPVQPAVL
EEVTGTLR
EFLASA
EFTRLQ
EFTVETR
EFVARNY
EFVEMKAK
EGEAEVDTSSPK
EGEDFSETLTRA
EGEDFSETLTRAK
EGEESRLESGMQNM
EGEWEESDVKLK
EGGDLPL
EGGELDLKGPK
EGGEVALEGGKVK
EGGEVALEGGKVKGK
EGLALSR
EGLSEASLTSALVEGK
EGPDMAVDLPK
EGPDMDVNLPK
EGPDVDVSLP
EGPDVDVSLPK
EGPDVSLKGPK
EGPEGKLK
EGPEGKLKGPK
EGPEVDVNLP
EGPEVDVNLPK
EGPEVDVSLPK
EGPFKPDHY
EGPFKPDHYRY
EGPPAFNPPVP
EGQVEVLK
EGSKAG
EGSLGLR
EGTKAVTKYTSSK
EHFLPM

EHFLPMLQ
EHFLPMLQTVAK
EHPTLL
EHPVLL
EHVTEGFENMPAAF
EKKDRVTDALN
EKPPDP
ELAAAP
ELAAPQ
ELDMNLPK
ELEAPL
ELGEFL
ELGVPL
ELHLQGF
ELKLQT
ELLKLE
ELNYFAK
ELRVAPEEHPTL
ELRVAPEEHPTLL
ELVPVA
ELVSDANQHVK
EMHFKGPK
EMTLAEGVK
ENEMATAASSSSLEK
ENEMATAASSSSLEKSY
ENFAVLGL
ENFAVLGLDL
ENFLEK
ENLEKTRHTLEK
ENLEKTRHTLEKR
ENLRLK
EPLLPR
EPPAGP
EPPAPLR
EPPGAP
EPPLLPK
EPPLLPKL
EPPLLPKLR
EPPVLP
EPSVPR
EPTPPR
EQAGPG
ERFRCP
ERFRCPET
ERPGPP
ERSGVSLAALKK

ERTDRP
ERTFQK
ESDFGVSLK
ESETLPEK
ESGKAG
ESPCAL
ESPERPF
ESSLPSASKTK
ESSLPSASKTKK
ETALLSSGF
ETELFY
ETFNVPAM
ETFNVPAMY
ETGEHLVH
ETGEHLVHVK
ETGEHLVHVKK
ETLFQPS
ETLRDLAQGTK
ETPKLP
ETPKPL
ETPKPLGN
ETSSVFVDSLTK
ETTTPTPL
EVANSTANLVK
EVDLNL
EVDLNLK
EVDLNLKGPK
EVDLNLKGPKVK
EVDVKVK
EVDVNLP
EVDVNLPK
EVDVSLPK
EVELTVSTK
EVELTVSTKKE
EVEMLVA
EVEVLK
EVFTGHL
EVGVPF
EVQTNELRAEK
EVSLLP
EVVLVK
EVVQEL
EYDVTVPK
FAPVVAPKP
FAPVVAPKPK
FCHKPCY

FCVVVNPYK
FDCHYC
FDDEPKF
FDDEPKFM
FDLHLK
FDLHLKGPK
FDRLDY
FDRMPPG
FDVSVPK
FEGGEVALEGGKVK
FEKLSK
FENEMATAASSSSLEK
FENLCK
FEWLKNLNHVSY
FFYSDQNVDSRDPVQL
FGAGGAF
FGCLDSVMENSK
FGDSLV
FGDSLVTR
FGDYNKELHK
FGDYNKELHKAGY
FGEGLL
FGFGQQ
FGGAGVGK
FGKTVPK
FGMTPSKGVLF
FGPGGP
FHAVEPY
FKDVDY
FKEQFL
FKGEGPEVDVNLPK
FKGEGPEVDVSLPK
FKQFFK
FLDAGKK
FLDVLR
FLGELL
FLKVSLGK
FLLVKK
FLNPRLY
FLSLLF
FLTPDSFEQK
FLVKEK
FLVLTK
FLVPPF
FMPGFAPL
FNDLSL

FNDLSLA
FNDLSLAK
FNDLSLAKAL
FNPFEL
FPGGPG
FPLKLT
FPLLLN
FPPERP
FPPPLP
FPSLVG
FPTSLK
FQYLSK
FRCPEA
FRCPET
FRCPETL
FRDGPPL
FRDQEGQDVLL
FRTVGQ
FRTVGQLYKEQLGK
FSPVTPK
FSVPGLKAEGPDMAVDLPK
FSVTSF
FTDKKTHL
FTDKKTHLY
FTFSKPK
FTHRLL
FTLKSK
FTMELAK
FTPDHV
FTPDHVVY
FTTLDH
FTTLDHY
FVDHVRT
FVDKNF
FVLTAAH
FVPLVK
FYEQFSK
FYNELR
FYPLDF
GAAAAP
GCVVGTKKRVLTLRK
GDDSRPGPWRP
GDFDVSVPK
GDGALF
GDGVNDAPALK
GDLLEL

GDPNKPSGF
GDSVSKLPK
GDVDVSLPK
GDVDVSVPK
GDVDVSVPKL
GDVDVTCPK
GDVKLAAQTHVSK
GDVVFK
GDYDVTVPK
GDYEVSVK
GEAADLL
GEAADLLR
GEDLLP
GEHLVHVKK
GELDLKGPK
GELLDF
GEVALEGGKVK
GEVTNDFVMLK
GFAPLTS
GFGFGQ
GFKGEGPEVD
GFKGEGPEVDVNLPK
GGAPGL
GGAVLE
GGELDLKGPK
GGPGAP
GGSGGGSFGDSLVTR
GHPPPP
GHYEVTGSDDEAGKLQGSGVSSSSK
GHYEVTGSDDEAGKLQGSGVSSSSKK
GKADGAEAKAAEAR
GKAFSY
GKLSQL
GLALEC
GLDASLF
GLGGGP
GMANLLTGPK
GMFRTVGQ
GNDEVR
GNERFRCPET
GNLPEFGEAVATASK
GPAAPP
GPALPPP
GPDAVPGALDY
GPEAPP
GPEPAP

GPGALP
GPGLQA
GPKAGG
GPKFKMPEM
GPKVKGDVDVS
GPPAEP
GPPAGP
GPPEAP
GPPGLP
GPPLRPLP
GPPLRPP
GPPPAPPK
GPPPLPR
GPPPPPPPP
GPPPPPR
GPPSLR
GPPVSEL
GPPVSGPPL
GPPVTW
GPTDASKVLAK
GQARAP
GQRAAP
GQVLEY
GRGVLR
GRLPPR
GRPAAGPP
GRPTRPP
GRTPDL
GSAAGPG
GSDDEAGKLQGSGVSSSSK
GSDDEAGKLQGSGVSSSSKK
GSGGGSFGDSLVTR
GSGLFP
GSHSLRY
GSHSLRYFY
GSHSLRYFYT
GSLDVSAPK
GSLLLK
GSLPGL
GSPEASVSGSKGDL
GSPEASVSGSKGDLK
GSPEASVSGSKGDLKSSK
GSSGPK
GTEAAHVQLK
GTFGGLGSKSK
GTGGLGL

GTLLEL
GTLVQTK
GTPGAP
GTVPSP
GVAAAL
GVAGSPEASVSGSK
GVDVLL
GVGALL
GVGLVV
GVGPVP
GVHELVK
GVKGEGPDVDVSLPK
GVKLGL
GVLAAA
GVLSLK
GVPGVRL
GVPPPP
GVPTVP
GVPVPGSPF
GVRVKETADF
GVSSSSK
GVVNRAY
GWLEPL
GWPGYVR
GWRPGSGFT
GYVVVKPKL
HAAPGP
HALLRL
HAVSEGTKAVT
HAVSEGTKAVTK
HAVSEGTKAVTKY
HAVSEGTKAVTKYTSS
HCLALLK
HEEPPAPAPGYDVHGR
HELSDF
HEMPPH
HETLYQK
HETSSVFVDSLTK
HFVGYPTNSDFELK
HGLEVL
HGQDYLVGNKL
HHLLGK
HLKPLGPGSEK
HLLLPLLL
HLLPPL
HLLPPP

HLLPPT
HLPLLP
HLPLPL
HLPPPR
HLYDPK
HQGVMV
HQGVMVG
HRFPLE
HSAPGPGPTDASK
HSAPGPGPTDASKVLAK
HSFYNEL
HSFYNELR
HSSLRR
HSSVDYADPDK
HSTTEVTQPR
HTGPAA
HTTLTM
HVGSRLY
HVGSRLYSVS
HVGSRLYSVSY
HVHEGPQEEGTEL
HVLGLQ
HVLTRM
HYEVTGSDDEAGKLQGSGVSSSSK
HYEVTGSDDEAGKLQGSGVSSSSKK
KAAAAP
KAAPPP
KAAVSH
KADEWLMK
KAEAGP
KAGGFLMKK
KALLFL
KAPKSPAKAKAVKPK
KAPLRF
KASYSGVSL
KAVPSL
KAVTKYTSSK
KCAQGCVCKGASDK
KDDLLL
KDLEEL
KDLFRK
KDLLPV
KDTPAPRLLK
KDVDEAYMNK
KEAAQR
KEAQDDLVKTR

KECLEK
KEELLK
KEESKK
KEEVLK
KEEVTGTLR
KEEVTGTLRK
KEGGGP
KEGGVT
KEGLLA
KEKLCYV
KEKLCYVA
KELNYFA
KELNYFAK
KELVLT
KEPVSSPGEEKEGEHADENK
KERSGVSL
KESYSVY
KETKDLYAPKPSSK
KETLLEMF
KEVLLP
KEYLPQ
KFAPVVAPKPK
KFHHTF
KFLDAGK
KFLPSELRDEH
KGAAAP
KGAVGP
KGDFDVSVPK
KGDVDVSLPK
KGDVDVSVPK
KGEGPEVDVNLPK
KGEGPEVDVSLPK
KGGSLL
KGGSPLNTGK
KGKKDNYPQSVPR
KGKVRK
KGKYVVL
KGMFRTVGQ
KGMFRTVGQLY
KGPDVQLPEVSVKTPK
KGPKVKGDVDVSVPK
KGPSLDVEGPDAKL
KGPSVKGEYDVTVPK
KGQHVPGSPF
KGTLVQTK
KHFVGYPTNSDFELK

KKDLEKTY
KKDLEKTYSAK
KKFPVF
KKFYEQF
KKLEEL
KKLEGDLK
KKTFYK
KKVLGY
KLACVTK
KLAELK
KLALAK
KLAPPLVTLL
KLAPPP
KLEGDLK
KLEGPDVSLKGPK
KLEVQLQEL
KLEVQLQELQSK
KLEVVR
KLGLRP
KLGTRLVPAER
KLGYTQ
KLLKTE
KLLLSH
KLLPRL
KLNVSTK
KLPKFN
KLPRLL
KLQLEK
KLRKAEL
KLRSPK
KLRTLEL
KLSELLRY
KLTYLE
KLVPHQ
KLVPHQL
KLVPHQLA
KLVPHQLAH
KLVPLK
KLVPRLL
KLVWVPSEK
KLVWVPSEKQ
KLVWVPSEKQG
KLVWVPSEKQGF
KMAVLK
KMQLAK
KMVVLSL

KNLNHVSY
KNVFVEF
KPFLSY
KPGAGV
KPGSFP
KPGSPPR
KPLLKY
KPPAAP
KPPAPL
KPPHLK
KPRLLL
KQFGVAPL
KQLEDGRTL
KQSAGH
KQSYGVVNRAY
KRVLAL
KSFSTAL
KSFTPDHV
KSLVPF
KSPLVL
KSTLLE
KSYHYR
KTAPGP
KTDGWK
KTGPKDR
KTPWYQ
KTYGVSF
KTYLQK
KVDLEK
KVEKEF
KVETFSGVYK
KVETFSGVYKK
KVEVPK
KVFLSR
KVFSRS
KVGGAP
KVKPLLQ
KVLKQVHPDTG
KVLLLLLL
KVLQRNCAA
KVLQRNCAAY
KVNFEDDSRGK
KVPVHDVTD
KVPVHDVTDASK
KVPVHDVTDASKVK
KVSLGV

KVSPLTFGRKK
ADVDLNVAAPK
ADVFAP
AEAAKPTATKTEATK
AEALGPLE
AEASLPSPK
AEAVKALVKPK
AEDDEDDDVDTK
AEEFGNETWGVTK
AEEMNEK
AEEMNEKQVVYDA
AEFTRL
AEFTRLQ
AEGPDMDVNLPK
AELCPL
AELLEL
AELLRA
AEMEDLVSSK
AEPSLR
AERRVP
AETPKPL
AETPKPLG
AETPKPLGN
AEVKRF
AEVLEL
AEVLKVLGNPK
AFDCVDKMVY
AFELLH
AFELLHL
AFEVTADF
AFFLFC
AFLADPSA
AFLADPSAF
AFLHWY
AFLLSF
AFSKEKMEKTKVR
AFSPEEMKQLDALNK
AFTLLY
AGAAKPKKPAGAA
AGALHY
AGGGLP
AGLQFPVGR
AGPKVPPEPKP
AGPSLV
AGSLLK
AHLLPF

LCYVAL
LCYVALD
LCYVALDFENEMATAASSSSLEK
LDEECWFPK
LDEECWFPKATDK
LDEELK
LDELSVR
LDFEHFLPM
LDFENEMATAASSSSLEK
LDFNLKGPK
LDGLVK
LDGVLK
LDKELK
LDKPLLK
LDLDLF
LDLLEH
LDLLLK
LDLNLK
LDLPPL
LDLSDPSLDMK
LDLWQVK
LDNLEVKKK
LDNRLP
LDPSAL
LDPSFFEK
LDSGDGVTH
LDSRLK
LDVAPL
LDVEDVK
LDVLELAEKF
LDVLTF
LDVSAPK
LDVSPQ
LDVSSLRPF
LDVTGPK
LDVTPL
LDVVRK
LEAALGEAK
LEAALGEAKK
LEAAML
LEAFGNAK
LEDLGFK
LEDLKVDK
LEDLPL
LEELPL
LEEPSPAPPSSDSPHADS

LEERLR
LEEVLK
LEEVVEKL
LEFGRGF
LEFLKK
LEFVAR
LEGDLKGPK
LEGEAEVDTSSPK
LEGEAEVDTSSPKGK
LEGGELDLKGPK
LEGLAL
LEGPDVSLK
LEGPEGKL
LEGPEGKLK
LEGPEGKLKGPK
LEGTVTGPK
LEGVAL
LEKLLK
LELAQYREVAA
LELAQYREVAAF
LELFTEL
LELLDH
LELLDHVL
LELLDHVLL
LEPSLR
LERLYL
LEVLHA
LEVLNF
LEVLSQK
LEYAKR
LFEELARASSH
LFHLGKS
LFKLGLAPQ
LFNHTM
LFNHTMF
LGAAVL
LGADATKEKPK
LGASLK
LGDVLR
LGEFAK
LGERQSY
LGFLEL
LGGDPL
LGGHLS
LGGPLL
LGGSLL

LGGVGL
LGHLLT
LGKQPR
LGLDLRTAAY
LGNFFSPK
LGNFFSPKVVSL
LGNKVDSY
LGNKVLVC
LGPVEL
LGPVPA
LGSAADF
LGSLEGEAEVDTSSPK
LGSVLR
LGTSTFT
LGVLRL
LHATAF
LHLKPLGPGSEK
LHPPPP
LHPRPP
LHRFGAK
LHSNVAHY
LHSRLLERAAK
LHTVQPK
LKDKGEYTLVVK
LKDKTEF
LKDTPAPRLLKT
LKEFEGK
LKELAK
LKGLGH
LKGPKVK
LKLEEF
LKLLQL
LKLRNWQ
LKSGAP
LKSLVSK
LKSLVSKGTLVQTK
LKTYKSQAS
LKVSLGK
LLATEQEDAAVAK
LLDEECWFPK
LLDVTPL
LLEGEESRL
LLEGEESRLE
LLEGEESRLESGMQ
LLEGEESRLESGMQN
LLEGEESRLESGMQNM

LLEKVR
LLELAK
LLEMEQ
LLEPSL
LLEQPL
LLESLP
LLESVNH
LLETSL
LLFPLE
LLGCGF
LLGDAF
LLGELLKDHSK
LLGFFF
LLGFVP
LLGGGP
LLGGPL
LLGLGPG
LLGLQF
LLGSPVG
LLHMLK
LLHVPK
LLKDKGEY
LLKDKGEYTLVVK
LLKKPP
LLKKSL
LLKLGY
LLKSLYR
LLLAAK
LLLDDF
LLLDVTPL
LLLEVG
LLLGAGESGK
LLLLCGGEDD
LLLLGAGESGK
LLLLLLL
LLLLLLLL
LLLLLLLLL
LLLLSLVV
LLLPGELAK
LLLPGELAKHA
LLLRPV
LLMLLL
LLNVHSSK
LLPEGLA
LLPGEL
LLPGELA

LLPGELAK
LLPGELAKH
LLPGELAKHA
LLPGELAKHAVSEGTK
LLPGELAKHAVSEGTKAVTKYTSSK
LLPGPV
LLPKKMDEY
LLPLFL
LLQAELL
LLQHLL
LLQHVRVPH
LLQLLH
LLQNSV
LLQNSVK
LLQNSVKQ
LLQNSVKQY
LLQTQPR
LLQVKEL
LLQVQH
LLQVTR
LLRGVFY
LLSKKL
LLSLKY
LLSPVHR
LLSWEVP
LLVAAP
LLVHRR
LLVTLA
LMFPGLP
LMHWPY
LMKLLT
LMKSKL
LMLGPSVFDVFRQY
LMQLEFGRGF
LMTTLR
LMTTLRNTTPN
LMVVGESGLGK
LNASSDKF
LNASSDKFM
LNDNVTSL
LNDPNKEALAK
LNFSVF
LNLKGPK
LNLLGF
LNYFAK
LPAGGL

LPAPPP
LPEELL
LPELLK
LPGGLL
LPGRLP
LPLHPK
LPPGLG
LPPLHK
LPPLSPR
LPPPPP
LPPPPPP
LPPPPPPP
LPPPPPPPP
LPPPPPPR
LPPPPRPP
LPPSLL
LPPVAL
LPRLLF
LPSRPLP
LPVLQLV
LQAQMK
LQELLK
LQFNWK
LQGPVG
LQGVPG
LQKVFERY
LQLEYL
LQLKPR
LQLLFL
LQLLPQ
LQLLVLP
LQNLQLQPGKA
LQNLQLQPGKAK
LQSVVL
LQVKVQGPK
LQVQHASK
LQVTRQEEEMQA
LQVVAVKAPGFGDN
LRALDK
LRDNLTL
LREAVLK
LRELHLNNNK
LRELPLR
LRELSF
LRGDPG
LRGLTL

LRGVFY
LRHFPL
LRKAEL
LRLTEY
LRLVDGKVVSE
LRLVDGKVVSETSDT
LRLVDGKVVSETSDTK
LRPPLL
LRSLAR
LRVAPEE
LRVAPEEHPT
LRVAPEEHPTL
LRVAPEEHPTLL
LSAAFL
LSAALEY
LSAALK
LSAAVESTSKKPQGAGK
LSAGPL
LSALGW
LSALPF
LSAPPP
LSARFEPF
LSEAYFRK
LSELLRY
LSELLRYY
LSGGLL
LSGGTTM
LSGQVP
LSGSAL
LSHVPV
LSKAEFL
LSKEELL
LSLAGL
LSLDPNL
LSLEEL
LSLQLT
LSMAHC
LSNFKK
LSPALPV
LSPAVP
LSPLDL
LSPLPF
LSPPLR
LSPTGVP
LSQFPK
LSQFPKAK

LSRLLK
LSRPPLP
LSRTKEEL
LSSTAF
LSVGVP
LSVPGHR
LSVRDK
LSYLPL
LTAELL
LTAEVL
LTAEVLEL
LTAVDF
LTEKNF
LTERGY
LTGLAA
LTGPDL
LTLGLR
LTLLAF
LTMDASGPK
LTPDSFEQK
LTPTHYL
LTPVLSAK
LTRSLK
LTSALVEGK
LTVDPY
LTVLDF
LTVLLM
LTVLSP
LVALPQ
LVAPGP
LVAPPG
LVCDNGSGLCK
LVCDNGSGLCKA
LVCDNGSGLCKAG
LVCDNGSGLCKAGF
LVDGKVVS
LVDGKVVSE
LVDGKVVSET
LVDGKVVSETSD
LVDGKVVSETSDT
LVDGKVVSETSDTK
LVDGKVVSETSDTKV
LVDLDMVLK
LVDLEK
LVEGKSMEGDAGTATSR
LVELVK

LVFKGDAY
LVGAAA
LVGGLY
LVGKDLKVD
LVGVGG
LVKRLTDADAM
LVKRLTDADAMKY
LVLEQL
LVLEQLRC
LVLSAL
LVMDPK
LVPGPEKEN
LVPHQLA
LVPHQLAH
LVSTLVPL
LVTFAL
LVVDLL
LVVLGH
LVVMQPEKFQSK
LVVVLV
LVVVVL
LVWVPSEK
LYASKF
LYETALLSSGF
LYEVSQL
LYEVSQLKD
LYGSAGPPPTNEEEAADKDEL
LYKEQLA
LYKEQLAK
LYKEQLG
LYKEQLGK
LYLLPAGTFVGGY
LYPSLR
LYSKLRVDRNSWQ
MACVLAK
MAMLQF
MAPLQVKVQGPK
MATLPQ
MDATANDVTSDRY
MDDREDLVYQAK
MDPLNDNVTSL
MDPLNDNVTSLLNASSDK
MDPLNDNVTSLLNASSDKF
MDYDAFLG
MEEEVRL
MEEEVRLK

MEEKKEEVTGTLR
MEGPLS
MELMMD
MELTAH
MESALTA
MESLLQL
METPSQR
MEVMNL
MEYYLH
MGFLPR
MGFVDDQQTFF
MGQKSSF
MLGPEGGEGFVVKLR
MLLLEV
MLNVQNK
MLPPPP
MLQDVLDLRK
MNFSGK
MNPVVS
MNPVVSQPG
MNPVVSQPGY
MNPVVSQPGYS
MPDVDLNLK
MPPSLP
MRLDLAGRDLTDY
MRVVPVP
MSCMDNKDGSCSVEYVPY
MSPPGA
MTESSLPSASK
MTESSLPSASKTK
MTESSLPSASKTKK
MVASSHKGK
MVASSHKGKK
MVGLDAAGK
MVHVTF
MVPVLL
MYDNRESQDTSF
MYDTPAEVAEAF
NAGGVTVSY
NAPDVEVQGK
NASAWLT
NASAWLTK
NASAWLTKNMDPLNDNVTSLLNASSDK
NASAWLTKNMDPLNDNVTSLLNASSDKF
NATLSVH
NAYLELGGLGERVLG

NDHSMAF
NDLFER
NDLSLAK
NEAGVTFT
NEATGGKYVPR
NELRVAP
NELRVAPEE
NELRVAPEEHPT
NELRVAPEEHPTL
NELRVAPEEHPTLL
NEPLLK
NETAHKPDLS
NFLTKR
NFSVFY
NGDTHLGGEDFDQRVMEHF
NGGELL
NGGELLD
NGNLPEFGEAVATASK
NHAVSLARGEVGF
NKELKTDF
NKVAVL
NKVLHF
NLLKPMGK
NLQLQP
NLQVSLTSK
NLRLMLK
NLVALK
NMAHNAEHEACDL
NMDPLNDNVTSLLNASSDKF
NNYTFL
NPGLLL
NPGLLLG
NPVAQADWAAK
NPVVSQPG
NQLTSNPENTVFDAK
NRLRQENM
NRTVSKVDDF
NSDPTDEFF
NSTKPR
NTLQTKL
NTMRVVPVP
NTPAMY
NTPAMYV
NVELPK
NVHSSKDEVQNAVR
NVPAMY

NVVDVFHAV
NVVLLA
NYEVVF
NYLHATA
PAAAAGP
PAAAAK
PAAADK
PAAAGPPR
PAAAPPAL
PAAFVP
PAALPL
PAAPAAPAPAEKTPV
PAAPAAPAPAEKTPVK
PAAPAAPAPAEKTPVKK
PAAPDFR
PAAPGF
PAASPK
PAATFAK
PAATPP
PAGPGP
PALSLP
PAPAGP
PAPATTF
PAPPAPR
PAPPPPPP
PARLLQ
PASLPVEF
PASSVP
PAVGPA
PCCDAL
PDGMVGL
PDLDFNLK
PDLDLNLK
PDVDLNLK
PDVDLNLKGPK
PEAPGP
PEEHPTLL
PEFLKA
PEPAKSAPAP
PEPAKSAPAPK
PEPAKSAPAPKK
PEVDVNLPK
PEVVDTCSLASPASVCQTK
PFLSLR
PFPPPP
PFSLLR

PGALDY
PGASGQ
PGEAPP
PGELAK
PGELAKHAVSEGTKAVTKY
PGEPAP
PGFKGEGPEVD
PGFKGEGPEVDVNLPK
PGFPLS
PGGLLL
PGGLLLG
PGGLQA
PGGLVL
PGLEVL
PGLLGVP
PGLPVG
PGPAEP
PGPAVPP
PGPEAP
PGPLLP
PGPLVG
PGPPPPPP
PGPRLPP
PGVGLPGLK
PHALLR
PHLALLR
PHYTTF
PHYVSF
PHYVTF
PHYVTFDGLYY
PKEYVK
PLEKVNPLGGAV
PLGGSDTAK
PLLALR
PLLGPL
PLLMQAW
PLLPPP
PLNDNVTS
PLNDNVTSL
PLNDNVTSLLNASSDKF
PLNDNVTSLLNASSDKFM
PLPPPP
PLPPPPPP
PLPVAL
PLSGRP
PLSPLFP

PLSPLK
PNKPSGF
PPAFNPPVP
PPAGAR
PPAPSPP
PPGKLP
PPGPALP
PPGPPPGP
PPHVPR
PPKEELSK
PPLLPDP
PPLPLP
PPLPPL
PPLPPPPP
PPLPPPT
PPLSYK
PPPAAPP
PPPAPPK
PPPEPPR
PPPFVP
PPPLAP
PPPLLPP
PPPLPA
PPPLPPGP
PPPLRPP
PPPPAL
PPPPAPL
PPPPHPGP
PPPPLA
PPPPLAP
PPPPLP
PPPPLPA
PPPPPLA
PPPPPLP
PPPPPP
PPPPPPP
PPPPPPR
PPPPSPR
PPPPVYEPVNY
PPPPWP
PPPRHP
PPPSPPR
PPPVMPR
PPPVYEPVNY
PPSLPPP
PPVPAP

PPVPLP
PPYLLL
PQAAPRP
PRELLT
PRVEFL
PSAPPL
PSFGAP
PSFKGQGPDVDVNLPK
PSFLLK
PSGTLY
PSPRPP
PSRPPP
PSRSLL
PSSLPH
PTAAALAY
PTAAGF
PTDEFF
PTGTYHGDSDL
PTSSLF
PTVLEK
PVAAEPF
PVAQADW
PVAQADWAAK
PVARSW
PVASPSGDAR
PVDLEA
PVFNPATEEKL
PVFPTELAF
PVKADP
PVPGSK
PVPGSPF
PVPKSPF
PVQLNL
PVSWESF
PVTPLK
PVTVTRTTVT
PVTVTRTTVTT
PVTVTRTTVTTT
PVVAPKPK
PVVSQPG
PVVVGV
PWTGPK
PYSQKAK
QAAGGGW
QAAPGPP
QAKRPK

QALEELR
QALLLP
QAMCRGY
QAMCRGYLA
QAMCRGYLAR
QAMVPR
QAPHPR
QAVLSL
QAVLSLY
QDGPVP
QDLLAEKVL
QDLLPR
QDPLVH
QDSVDF
QEPEPKSF
QGLALF
QGPDVDVNLPK
QGPLLE
QGVLTL
QKLKPK
QLELQK
QLKDKTEF
QLLAFH
QNMRAQHPY
QPAQPP
QPFLHQ
QPPAPW
QPPPPR
QPPPRP
QPSLVHR
QPTAPP
QRGVLGL
QRGVVVL
QRLRLQ
QSARLL
QSARLLR
QSPLSPR
QSSLPK
QSYGVVNR
QTCLLL
QTNLGL
QTPEVDVKVK
QTTMVRTAK
QVGLGP
RAAQLR
RAEFTVETR

RAGVLAH
RALLEVY
RAPGTEAAHVQLK
RASLLR
RASLVAP
RDKEKL
RDLAAF
RDLTDY
RDLTDYL
RDNSTMGYMAAKK
RDPAAAPATGNK
RDSFQEVLR
RDVLDF
RDWQSGR
RDYDDMSPR
REQEVTMLK
RFPPPP
RFYKSF
RGELVK
RGGVLR
RGLRSY
RHEMPPH
RKTAAT
RKVLRK
RLALPR
RLALYK
RLAMLK
RLAPVPGSGFL
RLDAVK
RLDLAGR
RLGRPP
RLKTKENLEKTR
RLLLPGE
RLLLPGEL
RLLLPGELA
RLLLPGELAK
RLLLPGELAKH
RLLLPGELAKHAV
RLLLVK
RLLPPL
RLQDYEEKTRK
RLSRPA
RLTDADAMK
RLTDADAMKY
RLVDGKVVSE
RLVDGKVVSETSDT

RLVDGKVVSETSDTK
RLVDGKVVSETSDTKVL
RLVDGKVVSETSDTKVLR
RLVFAK
RLVQAF
RLVSNHSLH
RLVTRF
RLYNLDVF
RMNKLGTRLVPAER
RNLLEGQD
RNLLEGQDA
RNLLEGQDAY
RNLLEGQDAYF
RNLLEGQDAYFNDLSLAK
RNSLLR
RNYFVK
RPAAAGP
RPCLCR
RPPEPL
RPPEPLT
RPPLPR
RPPPAAP
RPPRPP
RPSLPL
RPSPVVV
RQDLLAK
RQGRTLY
RQLVTLF
RQMSLLL
RRDSFQEVLR
RRDSFQEVLRF
RRELPPDQAEY
RSLGVGY
RSLKTYGVSF
RSLRSY
RSVHEEF
RTLGFN
RTLLPR
RTSLSF
RTVGQLYKEQLGK
RVAPEEHPT
RVAPEEHPTL
RVDDFKKAFSKEK
RVGNVQELSELSEQ
RVGTLR
RVHLLF

RVLDPF
RVPFLEF
RVPFSLL
RVPHNAAVQVY
RVRAVPTGDASK
RVTAPK
RVVTAF
RVYTLR
RWAASPK
RYLLEK
RYQLQR
SAAPLL
SAASVYAGAGGSGSR
SAAVESTSKKPQGAGK
SAEFTVDAK
SAEVSVPK
SAHVPPR
SAKEVANSTANLVK
SALRPTT
SAPGPGPTDASK
SAPGPGPTDASKVLAK
SARFEPF
SAWLTK
SCLLLH
SCLLLLPQ
SCLRSLGY
SCVGVFK
SDAGLGDTKMK
SDDEAGKLQGSGVSSSSK
SDDEAGKLQGSGVSSSSKK
SDDPRVEF
SDDPRVEFL
SDFGVSLK
SDLDLNLK
SDPSYGQQGHPNTWK
SDQLGADATKEKPK
SDQNVDSRDPVQL
SDVEEDGDTVSQEEDDRKPK
SDVSLTVPK
SEASLTSALVEGK
SEEEPVQPAVL
SEGTKAVTKYTSS
SEGTKAVTKYTSSK
SELLRY
SELLRYY
SEPFHR

sequence
SETLPEK
SETLTRAK
SETSAFMPVLKVVLTQANKLGV
SFCRLP
SFCRPL
SFGGKLVT
SFGGKLVTF
SFKGQGPDVDVNLPK
SFKPLR
SFQVDTSK
SFSTQSTFSNYR
SFTPDHVVY
SFTPDHVVYA
SFYNEL
SGDGVTHNVP
SGDVQRF
SGETAKGDYPLEAVRM
SGETAKGDYPLEAVRMQ
SGHSLGTPEPAPR
SGKHVVF
SGKLDAF
SGLLEC
SGLVLL
SGMFLT
SGMNVARLNF
SGNGLK
SGNQAVRLPL
SGPKPHV
SGPKPHVL
SGPKVEGGVKGGQVG
SGPKVEGGVKGGQVGL
SGPPVSEL
SGRGPF
SGSFKL
SGSSASSVTVTR
SGVPVPP
SGVSLAALK
SGVSLAALKK
SGVSLF
SGVYKKLTGKDVN
SHELSDF
SHRKFSAPR
SHRLSAF
SHSLLLV
SHTLPDF

SKERSGVSL
SKFKMPK
SKSVNVKPLVT
SLAALK
SLAALKK
SLAFTP
SLAGLL
SLAGPP
SLASTF
SLDVSAPK
SLEETLHT
SLEETLHTVDL
SLEGEAEVDTSSPK
SLEGEAEVDTSSPKGK
SLERLL
SLEVPF
SLFLLF
SLGKGDFDVSVPK
SLGSAL
SLGSLEGEAEVDTSSPKG
SLGSPF
SLGVLPL
SLHETSSVFVDSLT
SLHETSSVFVDSLTK
SLKESETLPEK
SLKMPK
SLKVDVK
SLLADF
SLLAGL
SLLPLP
SLLTPL
SLLVQTK
SLLVQTKRR
SLNDLE
SLPAGW
SLPVRF
SLQPVAVELK
SLREPPPP
SLRKVDSLKK
SLRSPY
SLSTFQ
SLSVPL
SLTELR
SLTLAL
SLTSALVEGK
SLTVPKV

SLVSKGTL
SLVSKGTLVQTK
SLYASGR
SLYTLLR
SMEGDAGTATSR
SMPDVDLNLKGPKVK
SNDVFPEFQL
SNDVFPEFQLK
SNDVFPEFQLKTY
SNFNHKQLE
SNKGQTLVVQ
SNKGQTLVVQF
SNVRLR
SNWLSK
SPEPSK
SPLVFR
SPPDLP
SPPDVLK
SPPPPPL
SPPVSTPVTGHK
SQFPKAK
SSADLKDFSSHQVNEF
SSADLKDFSSHQVNEFLAQ
SSEDPN
SSEEMC
SSEGPRVW
SSFYHAF
SSGGGPGTLKAY
SSGSSASSVTVTR
SSHQVNEF
SSLPSASK
SSLPSASKTK
SSLRKVDSLK
SSLRKVDSLKK
SSPFKV
SSPFKVSP
SSPFKVSPL
SSPGGVY
SSPNVY
SSSKPP
SSSLEK
SSSLEKSY
STAASRPPVTLR
STALSL
STALVCDNGSGLCK
STALYGESDL

STDVSVDEVK
STDVSVDEVKALASLMT
STDVSVDEVKALASLMTY
STDVSVDEVKALASLMTYK
STELLL
STKPAETPKPLG
STKPAETPKPLGN
STLLAL
STPGVKGEGPDVDVSLPK
STTATPL
STVAQLVK
SVARAGQKGY
SVDARALTQTGGPHVK
SVDSVFVL
SVEGQLEF
SVEYVPY
SVFVDSLTK
SVGGSGGGSFGDSLVTR
SVGPGYGGK
SVGVSPSLDLSK
SVLLPR
SVMGPK
SVNLVAL
SVPGLK
SVQSSGHRVRPVS
SVSLDF
SVSRVGSAAQT
SVVPSPK
SVYSDF
SVYVYK
SVYVYKVLKQ
SWTLKK
SYGFDGRGLKAENGQFEEF
SYGVVNR
TAAAVF
TADELVFF
TAFLGY
TAGLPK
TALVCDNGSGLCK
TALVCDNGSGLCKAGF
TALYGESDL
TAPQPGKY
TAPVAAEPF
TARRAF
TATPLTK
TATPSP

TAVAKPK
TAVRLLLPGELAK
TAVSVP
TAVTVP
TAVYKVPPF
TAVYKVPPFTFHVK
TCNVAHPASSTK
TCVPSVSSK
TCVVGHEALPH
TDAAVSF
TDAPQPDVK
TDASKVLAK
TDASKVLAKGVGLSKAY
TDKKTHLY
TDTEKPF
TDVDVSLPK
TEAAKPVVTK
TEDWRPR
TEEDVKFY
TEELNREVAGHTE
TEESDAVLVDK
TELYHY
TEPKSLPK
TESSLPSASK
TESSLPSASKTK
TESSLPSASKTKK
TETTTPTPLTK
TEVGSASEVKK
TFAVADEEDF
TFGADVVSK
TFGGLG
TFNVPAMY
TFPLAEKVK
TFPLAEKVKAF
TFPLAEKVKAFLADPSAF
TGAGAP
TGAPVPG
TGDGVNDAPALK
TGDGVNDAPALKK
TGEHLVH
TGEHLVHVK
TGEHLVHVKK
TGEKEQLAQAVARVK
TGGAGY
TGGKYVPR
TGGLLL

TGLKAHEPT
TGLKAHEPTY
TGLKAHEPTYF
TGLLLLSVQK
TGLLLLSVQKR
TGLTTL
TGPLSPSKDCGSPK
TGPVFP
TGVVPG
THGFLV
THGGGP
TKDLDKANY
TKENLEKTRHTLEK
TKVRTRENLER
TKYTSSK
TLAAPL
TLAAVGAASK
TLEALYR
TLEKNF
TLELRVAY
TLELVEETVQAMDVE
TLGEEKF
TLGERQSY
TLGGAL
TLGLLL
TLGPPF
TLGSKFR
TLLFQPVAELKK
TLLGAP
TLLRDEK
TLLRDEKK
TLNELL
TLNNKFASF
TLPDVDMDLKGPKMK
TLPSVF
TLRLVDGKVVSETSD
TLRLVDGKVVSETSDT
TLRLVDGKVVSETSDTK
TLSHPQQMALLDQTK
TLSLLF
TLVRLR
TMELAKKGF
TMELTAH
TMFLTMF
TMTTTETTTPTPLTK
TNEKLQQL

TNEKLQQLF
TNEKLQQLFNH
TNEKLQQLFNHT
TNEKLQQLFNHTM
TNEKLQQLFNHTMF
TPGVKGEGPDVDVSLPK
TPTATPLT
TPTPLTK
TQSLPK
TRLKTKENLEKTR
TRLLPR
TRLSSF
TRLVPAER
TRSLFVN
TRVSPYSDYEGRKDVS
TSALSL
TSALVEGKSMEGDAGTATSR
TSALWR
TSFTEK
TSQDARFY
TSQFVSLVMF
TSRKKVLL
TSRKKVLLK
TSSPVSR
TSTATPLTK
TSTSVP
TTAHGK
TTETTTPTPL
TTETTTPTPLT
TTETTTPTPLTK
TTEVGSASEVK
TTEVGSASEVKK
TTEVGSASEVKKESD
TTEVGSASEVKKESDQ
TTGLVM
TTGSPK
TTLDHY
TTLRLVDGKVVSETSDTK
TTPTATPL
TTPTVTPL
TTPVVSGK
TTQEQLAKEKDTVQK
TTTETTTPTPLTK
TTTETTTPTPLTKTTTK
TTTLAF
TTTLRLVDGKVVSETSDTK

TTTPTGTPLTK
TTTPTVT
TTTPTVTLLTK
TTYLRK
TVDLPL
TVGGPAP
TVGQLYK
TVGQLYKEQLGK
TVKNLP
TVKQSAEDVKK
TVLLLP
TVLLPL
TVLLSR
TVLSAL
TVLSLA
TVLVVW
TVPLPF
TVPLYE
TVPPSA
TVSLLT
TVTLPV
TVTPLTK
TVYFKEQ
TVYGLPR
TYETLKHEKPPQ
TYFTDKKTHLY
TYGVSF
TYGVSFF
TYVTPR
VADTPENLRL
VADTPENLRLK
VAEQTPLTALY
VAKVEPAVSSVVN
VALDFENEMATAASSSSLEK
VALDFENEMATAASSSSLEKSY
VAPEEHPTL
VAPEEHPTLL
VAPEEHPTLLTEAPLNPK
VAQADWAAK
VAQVPQ
VARNYFVK
VASLHLPSFDAH
VAVVLL
VCDNGSGLC
VCDNGSGLCK
VCDNGSGLCKA

VCDNGSGLCKAG
VCDNGSGLCKAGF
VCDNGSGLCKAGFAGDDAPR
VCTDCGTNLK
VCTDCGTNLKQ
VDAASLKGLNNLA
VDAASLKGLNNLAK
VDDFKKAF
VDDFKKAFSK
VDDFKKAFSKEK
VDDFLANEAK
VDDFLANEAKGTK
VDDLNL
VDDSKTVTDM
VDDSKTVTDML
VDDTQFVRF
VDELLK
VDGKVVSE
VDGKVVSET
VDGKVVSETS
VDGKVVSETSD
VDGKVVSETSDT
VDGKVVSETSDTK
VDGKVVSETSDTKV
VDGKVVSETSDTKVLR
VDHVRTSF
VDKPLR
VDLHVKGPK
VDLNLK
VDLNLKGPK
VDLNLKGPKVK
VDLSLPK
VDMDLKGPK
VDMDLKGPKMK
VDPLDF
VDPLSF
VDQEELVK
VDSLKKAF
VDSLKKAFSR
VDSSLPR
VDSTTCLFPVEEK
VDVDKDTLQHK
VDVDVPDVDVK
VDVDVPDVDVKGPDWHLK
VDVHGPDWHLK
VDVLNPGGPQ

VDVMLPK
VDVNLPK
VDVPDVDVK
VDVPVGEEL
VDVQLK
VDVSGPK
VDVSLPK
VDVSVPK
VDVSVPKL
VDVTCPK
VDVTSREGAKD
VDYADPDK
VDYKADEWL
VDYVNCGHVTAY
VEEHETLL
VEEHETLLL
VEELDPV
VEETVQAMDVE
VEEWQLR
VEGKSMEGDAGTATSR
VEHGSVALPA
VEHLAF
VEKLCTEQGSHPK
VELDQLV
VELNLKGPKVK
VENGKKVTVGK
VEPAVSSVVN
VEPLFP
VEPVEAVAPK
VERLLEPL
VERVPF
VEVGAEEEEGGAER
VEVLLL
VEVVLL
VFCVEKL
VFEDPF
VFEYPGN
VFLLNL
VFVDSLTK
VFVWVGK
VGAEEEEGGAER
VGDANPALQK
VGDDPAVWQLK
VGETEDSSSQMTPK
VGGHLEF
VGGLEF

VGGPVT
VGGSGGGSFGDSLVTR
VGGTVP
VGHDNPEVQF
VGHVFPK
VGKDLKVD
VGLDAAGK
VGLDAAGKTT
VGLDQMAK
VGLEHLR
VGLGLQ
VGLPASGK
VGLVPLKA
VGLVTVRA
VGLVTVRAY
VGMGQKDSY
VGNAKLADTPKPLS
VGNPLL
VGPGLP
VGPGYG
VGPGYGGK
VGPLLF
VGPVGP
VGPVLS
VGRALAH
VGRDVVQK
VGSPLQ
VGSRLYS
VGSRLYSVS
VGVAAH
VGVNGLDVSSLRPF
VGVNLPK
VGVVLA
VGVVQY
VHAKSWTLK
VHAKSWTLKK
VHEGPQEEGTEL
VHLGLQ
VHQVLY
VHQVLYR
VHQVLYRAL
VHQVLYRALVS
VHSSEETVSGAK
VHSSEETVSGAKK
VHTVSY
VHYGEVTNDF

VHYGEVTNDFVMLK
VKGDVDVSLPK
VKGDVDVSVPK
VKMTNY
VKPAETPKPLG
VKPLVT
VKVQGPK
VLAKGVGL
VLAKGVGLSK
VLAKGVGLSKAY
VLAMLQ
VLAPGP
VLAPPG
VLAPPQEELSK
VLCGTCGQPADK
VLDFEHFLPM
VLDFEHFLPMLQTVAK
VLDLDLF
VLDLEL
VLDSGDGVTH
VLDSGDGVTHN
VLDVVRK
VLEDSDLKK
VLEGLQYPFAVTSFGK
VLELDNVK
VLELQR
VLELSGR
VLEVAKDFPEY
VLEVAQH
VLFQGL
VLGLQF
VLHLPR
VLLALLH
VLLDVCPL
VLLENF
VLLHVL
VLLLDL
VLLLML
VLLPEKF
VLLPPR
VLLSAL
VLLTEA
VLLVGY
VLLVPGPEK
VLLVPGPEKEN
VLNDHSMAF

VLPLLQ
VLPPAP
VLPQVPEPR
VLPSMT
VLPSRVK
VLPVPQ
VLQRNCAAY
VLSEGEAQRL
VLSLYA
VLSRAFF
VLSVLAL
VLTADTLK
VLTADTLKLY
VLTAED
VLTPEL
VLVLHK
VLVTLGEK
VLYGDEEVPR
VMLSAGQK
VMVTNVTSLLK
VNCGHVTAY
VNDAPALK
VNDLFER
VNVGGALEY
VNVPKVK
VPASLP
VPFTPF
VPGGALG
VPGGDL
VPGLKAEGPDMAVDLPK
VPPDLW
VPPEPKPY
VPPFTF
VPPFTFH
VPPFTFHV
VPPFTFHVKK
VPPGVH
VPPLPR
VPVTGK
VQDTWHK
VQEFKK
VQMMTAK
VQQLDTLRQK
VQSAKEVANSTAN
VQSAKEVANSTANLVK
VQSGAGEGWEESDVKLK

VQSQEAPKQEAPAK
VQSSGHRVRPVS
VQTPEVDVK
VQTPEVDVKVK
VQVLPF
VQVTVPGVK
VRLLLPGELAK
VRMVLNG
VRPVSSAASVYAGAGGSGSR
VRVPQKNS
VSAALL
VSAQFPK
VSAVPL
VSEGTKAVTK
VSEGTKAVTKYTSSK
VSELTLVRVK
VSKGTLVQTK
VSKVMLSAGQK
VSKVMLSAGQKR
VSLAALKK
VSLGAL
VSLLLK
VSLLPR
VSLVAL
VSPLTF
VSPLTFGRK
VSPPPT
VSPSPL
VSPTWL
VSSFYHA
VSSFYHAF
VSTDYL
VSTPVTGHK
VSVNPY
VSVPNP
VTAEVVLAH
VTAMDVV
VTAMDVVY
VTEVENGGSLGSK
VTGGLLA
VTGSDDEAGKLQGSGVSSSSK
VTGSDDEAGKLQGSGVSSSSKK
VTLAGL
VTLDPL
VTLDPY
VTLSAL

VTLSLP
VTLSPL
VTPLTK
VTPVSP
VTRLAV
VTSVLP
VTSVPL
VTVPGVK
VTVRVPQKNS
VTVVLL
VVAPKPK
VVAVKAPGFGDN
VVAVLL
VVFEGNHY
VVGESGLGK
VVGETEDSSSQMTPK
VVLDNP
VVLLPL
VVPGSPS
VVPSPK
VVPVASPSGDAR
VVPVPQ
VVSLVAL
VVVAGY
VVVCPK
VVVFGNDKL
VVVHKETELEEGE
VVVKPKL
VVVNPYK
VVVSPVG
VVVSPVGAK
VVVSPVGAKGSAPATSSTQGK
VVVVVPNEEDWK
VVVVVPNEEDWKK
VYEPVNY
VYFKEQF
VYGPGVAK
VYKEKL
VYKVLK
VYKVLKQ
VYKVLKQVHPD
VYPLDF
VYVLAPPQEELSK
WAAVVK
WAKLFS
WCAGGP

WDDMEK
WDPEYTPK
WDPEYTPKSK
WEESDVKLK
WEPLAL
WEVDEMK
WEVQPATF
WGLLLP
WHHSFY
WHHSFYNELR
WKDVDR
WLNSLL
WLPVKK
WNELLSREVAK
WNPGRP
WPAPPRP
WPPEEF
WSGPPA
WVETSPK
WVLTAAH
WVPSEK
YAEAVARAK
YAGRRR
YALSARFEPF
YASQGGK
YCFHCK
YDAMAQENLQK
YDDMSPR
YDPLPR
YDVTAPK
YDVTVPK
YEGEDFSETLTRAK
YETALLSSGF
YETLKHEKPPQ
YEVSQL
YEVSQLKD
YEVTGSDDEAGKLQ
YEVTGSDDEAGKLQGSGVSSSSK
YEVTGSDDEAGKLQGSGVSSSSKK
YFEPLTLAAVGAASK
YFNDLSLAK
YGESDL
YGHETPL
YGVVNR
YKEQLGK
YKMKGDYHR

YLDKLEPSK
YLHATAF
YLKNRP
YLLVEF
YLTVDPY
YLYEVSQLKD
YNEATGGKYVPR
YNELRV
YNELRVAPEE
YNELRVAPEEHPT
YNELRVAPEEHPTL
YNELRVAPEEHPTLL
YPPPPPPPPPSRK
YQEGDLEKCQRAP
YREVAAF
YRSLGSVQ
YSDQNVDSRDPVQLNL
YSLGSAL
YSNRLY
YSTFGE
YTDLLRLFEY
YVALDFENEMATAASSSSLEK
YVEELERGPAA
YVPAPLR
