# Supplementary material for: Integrated peptidogenomics decoding yak non-conventional peptides: functional mapping and biopotential mining of genetic resources
Source: Anim Biosci. 2025 Sep 30;39(5):250408. doi: 10.5713/ab.25.0408 (PMC13153706; doi:10.5713/ab.25.0408)
Supplement: Supplementary file 7 [file ab-25-0408-Supplement-7.pdf]

| Peptide type | Total | AMP_g+ | AMP_g- | Anticancer peptides_Label | Anti-inflammatory peptides_Label | Allergic peptides_Label |
|---|---|---|---|---|---|---|
| CP | 5160 | 7 | 30 | 989 | 2358 | 2072 |
| NCP | 58671 | 188 | 158 | 13743 | 53084 | 8700 |
