# Supplementary material for: Integrated peptidogenomics decoding yak non-conventional peptides: functional mapping and biopotential mining of genetic resources
Source: Anim Biosci. 2025 Sep 30;39(5):250408. doi: 10.5713/ab.25.0408 (PMC13153706; doi:10.5713/ab.25.0408)
Supplement: Supplementary file 8 [file ab-25-0408-Supplement-8.pdf]

CP_counter

Aseq	muscle	lung	liver	testis	spleen	sintestine
V	240	907	595	431	1171	2204
G	220	735	455	375	1058	1628
W	8	37	39	20	118	161
P	343	973	793	784	1332	1528
F	97	337	186	121	444	1065
M	53	176	97	91	189	445
L	244	918	719	499	1228	2487
A	260	1058	620	513	1433	2295
Total	1465	5141	3504	2834	6973	11813
K	181	979	580	327	1398	2277
H	86	255	179	122	393	603
Y	69	292	156	106	395	670
E	192	943	381	300	1203	2131
Q	88	277	243	138	410	789
C	41	113	30	41	273	220
D	114	643	308	257	837	1485
N	62	210	153	133	303	605
R	156	388	299	257	477	727
T	171	642	290	254	750	1317
S	183	750	409	314	902	1491
Total	1343	5492	3028	2249	7341	12315

NCP_counter

Aseq	muscle	lung	liver	testis	spleen	sintestine
V	4020	5085	6309	4555	5873	10367
G	3430	4278	4836	4008	5173	7809
W	380	414	492	407	614	877
P	7606	9003	12937	12216	11569	10520
F	2372	2516	3113	2297	2842	5384

M	1533	1325	1456	1535	1336	2294
L	6987	8856	12283	8563	9882	18075
A	3772	5106	5356	4902	6266	9113
Total	30100	36583	46782	38483	43555	64439
K	5216	6948	6874	4992	7332	12727
H	1941	1425	1821	1663	1815	2778
Y	1275	1678	1776	1732	1915	3766
E	2306	3676	2680	2464	4105	7005
Q	1452	1461	1993	1517	1656	3118
C	1081	716	801	1087	842	899
D	1817	2820	2147	1813	3170	5046
N	1290	1461	1867	1398	1709	3079
R	2270	2636	3583	3186	2872	3936
T	2696	3226	3236	2737	3754	6427
S	3046	3910	3630	3058	4244	6610
Total	24390	29957	30408	25647	33414	55391
