# Supplementary material for: Integrated peptidogenomics decoding yak non-conventional peptides: functional mapping and biopotential mining of genetic resources
Source: Anim Biosci. 2025 Sep 30;39(5):250408. doi: 10.5713/ab.25.0408 (PMC13153706; doi:10.5713/ab.25.0408)
Supplement: Supplementary file 9 [file ab-25-0408-Supplement-9.pdf]

Amino acid ratio of NCPs

Aseq	muscle	lung	liver	testis	spleen	sintestine
A	0.069224	0.076736	0.069387	0.076438	0.081409	0.076049
C	0.019839	0.01076	0.010377	0.01695	0.010939	0.007502
D	0.033346	0.042381	0.027814	0.028271	0.041185	0.04211
E	0.04232	0.055245	0.03472	0.038422	0.053333	0.058458
F	0.043531	0.037812	0.040329	0.035818	0.036924	0.04493
G	0.062947	0.064292	0.062651	0.062498	0.067209	0.065167
H	0.035621	0.021416	0.023591	0.025932	0.023581	0.023183
K	0.095724	0.104418	0.089053	0.077842	0.095259	0.106209
L	0.128225	0.133093	0.159127	0.133526	0.128389	0.150839
M	0.028134	0.019913	0.018863	0.023936	0.017358	0.019144
N	0.023674	0.021957	0.024187	0.021799	0.022204	0.025695
P	0.139585	0.135302	0.167599	0.190488	0.150307	0.087791
Q	0.026647	0.021957	0.025819	0.023655	0.021515	0.02602
R	0.041659	0.039615	0.046418	0.04968	0.037314	0.032847
S	0.0559	0.058762	0.047027	0.047684	0.055139	0.055161
T	0.049477	0.048482	0.041923	0.042679	0.048773	0.053634
V	0.073775	0.07642	0.081733	0.071028	0.076303	0.086514
W	0.006974	0.006222	0.006374	0.006346	0.007977	0.007319
Y	0.023399	0.025218	0.023008	0.027008	0.02488	0.031428

Amino acid ratio of CPs

Aseq	muscle	lung	liver	testis	spleen	sintestine
A	0.092593	0.099502	0.094917	0.100925	0.100112	0.095118
C	0.014601	0.010627	0.004593	0.008066	0.019072	0.009118
D	0.040598	0.060472	0.047152	0.050561	0.058474	0.061547
E	0.068376	0.088686	0.058328	0.05902	0.084044	0.088321
F	0.034544	0.031694	0.028475	0.023805	0.031019	0.04414
G	0.078348	0.069124	0.069657	0.073775	0.073914	0.067473
H	0.030627	0.023982	0.027404	0.024002	0.027456	0.024992
K	0.064459	0.092072	0.088794	0.064332	0.097667	0.094372
L	0.086895	0.086335	0.110073	0.09817	0.08579	0.103075
M	0.018875	0.016552	0.01485	0.017903	0.013204	0.018443
N	0.02208	0.01975	0.023423	0.026166	0.021168	0.025075
P	0.122151	0.091508	0.121402	0.15424	0.093056	0.063329
Q	0.031339	0.026051	0.037201	0.027149	0.028643	0.032701
R	0.055556	0.03649	0.045775	0.050561	0.033324	0.030131
S	0.065171	0.070535	0.062615	0.061775	0.063015	0.061795
T	0.060897	0.060378	0.044397	0.04997	0.052396	0.054584
V	0.08547	0.0853	0.09109	0.084792	0.081808	0.091346
W	0.002849	0.00348	0.005971	0.003935	0.008244	0.006673
Y	0.024573	0.027462	0.023882	0.020854	0.027595	0.027769
