# Supplementary material for: Integrated peptidogenomics decoding yak non-conventional peptides: functional mapping and biopotential mining of genetic resources
Source: Anim Biosci. 2025 Sep 30;39(5):250408. doi: 10.5713/ab.25.0408 (PMC13153706; doi:10.5713/ab.25.0408)
Supplement: Supplementary file 10 [file ab-25-0408-Supplement-10.pdf]

Peptide	Chain length/MW	Genomics location	Tissue	T-AOC（μmol/ml）					
				15 mg/ml	10 mg/ml	7.5 mg/ml	4 mg/ml	2 mg/ml	1 mg/ml
PDPAKSAPAPK	11/4015.2	Intron	Liver	0.111±0.0353	0.1013±0.0357	0.0539±0.0173	0.0795±0.0319	0.0653±0.0275	0.065±0.0413
ASAAEGDMEAELTR	14/5385.1	5'UTR	Testis	0.1246±0.0119	0.0938±0.0062	0.0875±0.0112	0.0786±0.0099	0.0774±0.0113	0.0664±0.007
VEEVETPNSTPPR	13/4712.6	Intron	Testis	0.0755±0.0095	0.0824±0.0108	0.0767±0.0075	0.0758±0.0066	0.1237±0.0097	0.0765±0.007
AASTARHLYLR	11/4798.4	IGR	Testis	0.1225±0.0134	0.1268±0.0528	0.0839±0.0121	0.0778±0.0089	0.0837±0.0094	0.087±0.012
ADPELQLVAR	10/3763.3	Intron	Lung/Testis/Spleen	0.0859±0.0111	0.0643±0.0072	0.0853±0.0134	0.0725±0.0089	0.085±0.011	0.0845±0.0135
ATPGKAVVATSGK	13/3981.1	IGR	Lung/Testis	0.1288±0.0188	0.0639±0.0076	0.056±0.0068	0.0689±0.0099	0.0729±0.0095	0.0835±0.012
APAPRGPPSY	10/3761.2	Intron	Testis	0.8294±0.0201	0.6495±0.0357	0.5768±0.0525	0.2705±0.0693	0.1443±0.0349	0.0827±0.0086
KYAPPPPPAP	10/3574.9	Intron	Testis	1.3848±0.0725	0.6021±0.0528	0.3835±0.0685	0.125±0.0392	0.0845±0.0206	0.0813±0.0043
LLQDHRLLR	9/4353.1	Intron	Testis	0.3209±0.0695	0.2404±0.0607	0.2091±0.0191	0.1194±0.0325	0.0997±0.0201	0.0577±0.0124
RAFLEKVR	8/3243.6	Intron	Testis	0.2284±0.0523	0.1915±0.0209	0.1633±0.0342	0.0981±0.0181	0.0721±0.0015	0.0669±0.0009
MESALTARDR	10/4263	IGR	Testis	0.1368±0.0522	0.2003±0.0342	0.1662±0.0204	0.1378±0.015	0.0787±0.0118	0.1006±0.0391
GFVKVVKNK	9/2686.6	Intron	Testis/Intestine	0.3667±0.0401	0.2147±0.0522	0.1458±0.0357	0.0943±0.019	0.0851±0.0091	0.075±0.0178
AAKSQPSAPKTK	12/4788.9	IGR	Testis/Liver	0.1098±0.035	0.0678±0.0198	0.0806±0.018	0.0838±0.0042	0.0782±0.0151	0.0824±0.0057
MVGHFGVQEDR	11/4069.7	IGR	Testis	0.1528±0.0109	0.1269±0.0186	0.1121±0.0206	0.118±0.0066	0.0956±0.0153	0.056±0.0091
AAMLDTVVFK	10/3012.3	Intron	Testis/Spleen	0.1651±0.0092	0.0866±0.0099	0.0755±0.0113	0.0863±0.009	0.0677±0.0101	0.075±0.0077
HGQEVR	6/2602	IGR	Muscle	0.0808±0.0076	0.0769±0.0188	0.0722±0.0007	0.0701±0.023	0.0736±0.0208	0.0688±0.019
APEEHPTLLTEAPLNPK	17/6788.7	Intron	Spleen	0.1774±0.0094	0.1361±0.0093	0.126±0.0099	0.0951±0.0058	0.085±0.0097	0.0776±0.0078
DRPAPHF	7/3053.5	3'UTR	ung/Testis/Spleen/Liver	0.0872±0.0103	0.0766±0.0114	0.0868±0.011	0.0639±0.009	0.0839±0.0106	0.075±0.0099
DAALKQPPAPR	11/4438.9	IGR	Intestine	0.0863±0.0108	0.0775±0.0085	0.074±0.0076	0.0746±0.0116	0.0836±0.0072	0.0864±0.0129
GDYDVTVPK	9/2783.1	Intron	Lung/Intestine	0.2252±0.0112	0.1541±0.0116	0.134±0.0108	0.0954±0.0069	0.0863±0.0108	0.0792±0.0091
AAGVNVEPFWPGLFAK	16/5348.7	Intron	Intestine	0.4436±0.0376	0.2793±0.0261	0.2075±0.0262	0.135±0.024	0.106±0.0273	0.1046±0.0274
LTDCVVMRDPASK	13/4680.4	Intron	Intestine	11.6487±0.0437	10.7881±0.0639	4.0834±0.044	1.8158±0.0554	0.9774±0.0622	0.3047±0.0572
ELLFKEGVMVAK	12/3862.1	IGR	Intestine	9.0707±0.0473	8.8646±0.051	5.3944±0.0507	1.7351±0.0782	0.7071±0.055	0.4492±0.0403
YLDLSFNQMTKLPSGLP'	23/8754.8	Intron	Intestine	8.0344±0.0544	4.7445±0.0461	1.7184±0.0758	0.7894±0.0593	0.4408±0.0532	0.2829±0.0727
TALVCDNGSGLCKAGF	16/6037.6	Intron	Intestine	12.056±0.0587	10.9952±0.0481	3.5258±0.0419	1.9913±0.0666	0.7517±0.0589	0.4419±0.0554
WHHSFYNELR	10/5020.7	Intron	Intestine	0.5304±0.042	0.3288±0.0609	0.2672±0.0444	0.2162±0.0779	0.2408±0.0814	0.1478±0.0481
TLAAVGAASK	10/3145.2	Intron	Intestine	10.6432±0.0526	9.5691±0.0591	6.5083±0.0435	2.8353±0.0515	1.0607±0.0526	0.6319±0.0548
ANVSTVLTSKYR	12/4399.9	Intron	estis/Spleen/Liver/Intes	0.4209±0.0651	0.2662±0.0474	0.2061±0.0487	0.2159±0.0442	0.1877±0.0521	0.2272±0.0578
PAGMYPVAGPK	11/3528.8	IGR	Spleen	12.3291±0.0493	10.8285±0.0578	5.4009±0.052	2.4981±0.0531	0.9803±0.0411	0.2818±0.0487
MEEEECGLGKSCAR	14/6004.8	Intron	Lung	12.7123±0.0609	8.6176±0.0462	7.5132±0.0605	3.0574±0.0533	0.4212±0.0441	0.3152±0.0588
MEQASTMAEPR	11/4594.4	Intron	Lung	0.1522±0.0597	0.1249±0.0475	0.0497±0.0508	0.1244±0.0511	0.1069±0.0385	0.189±0.0537
RKEGMAAFVEKRK	13/5232.6	Intron	Liver	9.5807±0.0375	7.1293±0.0499	3.1563±0.0446	1.035±0.0564	0.4304±0.0476	0.3953±0.0532
ASRPLLR	7/3036.5	5'UTR	Lung	9.5212±0.0543	7.1564±0.0611	3.2051±0.0542	1.0214±0.0583	0.4649±0.0429	0.3156±0.0573
FGYSNRVVDL	10/3538	IGR	Muscle	0.5705±0.0596	0.3038±0.0524	0.3312±0.057	0.2991±0.048	0.2996±0.0523	0.3226±0.058
ALEQAMQAAR	10/4265.8	Intron	Testis	11.6236±0.0535	6.8947±0.0508	4.1969±0.0611	1.3371±0.0669	0.7796±0.0544	0.5805±0.0531
AEQVTKSVLF	10/3337.6	Intron	estis/Spleen/Liver/Intes	0.2283±0.0608	0.3289±0.0591	0.2129±0.0626	0.2644±0.0664	0.2025±0.0586	0.2264±0.0672
MEVKPPPGRP	10/3561.1	IGR	Liver/Testis/Spleen	11.0561±0.0712	8.9706±0.072	5.1827±0.0581	2.4674±0.0512	1.0983±0.0518	0.6061±0.0753
VVDLMVHMASKE	12/3716.2	IGR	le/Testis/Spleen/Intest	11.3815±0.0382	10.0286±0.0506	6.1802±0.058	2.0499±0.0579	0.9606±0.0605	0.5799±0.0601

Peptide	Chain length/MW	Genomics location	Tissue	ABTS(%)					
				15 mg/ml	10 mg/ml	7.5 mg/ml	4 mg/ml	2 mg/ml	1 mg/ml
PDPAKSAPAPK	11/4015.2	Intron	Liver	17.2736±1.9227	16.0131±0.8337	13.9989±0.93	17.5604±1.9145	15.813±1.9154	15.4995±1.4409
ASAAEGDMEAELTR	14/5385.1	5'UTR	Testis	35.721±1.076	24.7232±1.1244	20.1547±1.0686	17.8938±0.8966	14.4324±1.3887	13.8722±1.178
VEEVETPNSTPPR	13/4712.6	Intron	Testis	12.6851±1.114	16.0598±1.1722	17.0468±1.3106	13.7388±0.8958	16.3665±1.2973	15.0527±1.7342
AASTARHLYLR	11/4798.4	IGR	Testis	23.3293±0.9901	18.0672±1.507	17.8738±0.8522	16.4866±1.3396	14.1257±1.8011	14.8926±1.1384
ADPELQLVAR	10/3763.3	Intron	Lung/Testis/Spleen	16.0998±1.2693	12.6651±0.8432	13.1919±1.3213	10.6176±1.4583	11.598±1.3067	13.5054±0.8865
ATPGKAVVATSGK	13/3981.1	IGR	Lung/Testis	21.622±0.9605	31.5259±1.1214	10.1574±1.247	16.76±1.188	18.5207±1.7312	17.2002±1.204
APAPRGPPSY	10/3761.2	Intron	Testis	56.3092±1.4206	41.9901±0.5302	27.9312±1.3459	21.5886±2.6595	11.9648±1.9209	12.3649±1.3733
KYAPPPPPAP	10/3574.9	Intron	Testis	51.9541±1.9712	50.5135±1.7599	43.8976±1.3595	22.0488±1.5422	16.4132±1.1736	10.3375±1.2486
LLQDHRLLR	9/4353.1	Intron	Testis	31.766±1.4781	41.0431±1.9625	18.0412±28.5597	14.079±1.0412	14.4191±1.2286	11.8581±1.6838
RAFLEKVR	8/3243.6	Intron	Testis	9.6572±1.1832	24.263±1.9177	20.2014±1.2459	12.9919±1.7968	14.3524±1.9813	8.37±1.204
MESALTARDR	10/4263	IGR	Testis	25.7703±1.281	23.9629±1.3063	23.0626±1.2308	18.2806±1.0599	11.4913±1.5846	12.6251±0.921
GFVKVVKNK	9/2686.6	Intron	Testis/Intestine	14.3657±1.8445	33.6068±1.4337	22.5223±1.9	14.9526±1.2105	16.0798±1.8052	12.3049±0.923
AAKSQPSAPKTK	12/4788.9	IGR	Testis/Liver	12.2582±1.0459	9.4038±1.7398	12.3383±1.8438	9.8506±1.1032	12.2249±1.4633	8.8502±1.9787
MVGHFGVQEDR	11/4069.7	IGR	Testis	31.786±1.3428	18.2873±1.2247	20.0347±1.9649	14.6592±0.7708	14.3391±1.945	9.5238±0.8673
AAMLDTVVFK	10/3012.3	Intron	Testis/Spleen	45.5182±1.6266	37.1482±1.8216	31.4726±0.6758	22.389±1.4567	10.5175±1.4906	15.8797±1.7816
HGQEVR	6/2602	IGR	Muscle	10.4308±1.3644	10.0907±1.8431	13.4921±1.1032	9.4038±1.1274	9.5238±1.382	9.6238±1.3237
APEEHPTLLTEAPLNPK	17/6788.7	Intron	Spleen	33.38±0.9161	33.32±1.3706	24.0696±1.4017	21.1885±1.6681	14.239±0.9276	10.104±1.5094
DRPAPHF	7/3053.5	3'UTR	ung/Testis/Spleen/Liver	11.4846±1.0239	12.3583±1.9465	9.7439±1.9924	8.3167±1.9191	10.0107±1.9409	8.4834±1.9836
DAALKQPPAPR	11/4438.9	IGR	Intestine	10.3775±1.9831	9.9106±1.8008	8.1232±1.8562	10.6109±1.834	11.8581±1.9407	10.044±1.9824
GDYDVTVPK	9/2783.1	Intron	Lung/Intestine	34.4004±1.7645	30.9657±1.8416	28.7848±1.1087	10.2174±1.4002	9.4504±1.1308	8.6901±1.0893
AAGVNVEPFWPGLFAK	16/5348.7	Intron	Intestine	15.3237±0.8264	12.957±0.9048	12.574±1.1815	11.6627±1.1151	13.2689±1.7442	12.5937±0.909
LTDCVVMRDPASK	13/4680.4	Intron	Intestine	97.9846±0.9589	96.3466±1.4919	70.1881±1.1528	64.3937±1.0789	54.0065±1.3913	50.2051±1.0998
ELLFKEGVMVAK	12/3862.1	IGR	Intestine	96.5217±0.8834	85.2832±1.0427	69.0474±1.1591	52.199±0.9994	41.6301±1.3396	35.9556±1.2139
YLDLSFNQMTKLPSGLP'	23/8754.8	Intron	Intestine	96.9712±0.9501	88.2937±0.9766	62.6503±1.2105	50.7839±1.123	39.884±0.8581	33.0088±1.208
TALVCDNGSGLCKAGF	16/6037.6	Intron	Intestine	98.2918±0.9155	77.8335±0.9823	66.8619±1.0443	51.2503±1.2703	43.2372±1.5681	34.8463±1.3287
WHHSFYNELR	10/5020.7	Intron	Intestine	-0.7614±1.0723	19.0492±1.3201	17.6247±1.1499	19.0099±1.0625	18.1487±1.1293	17.4323±1.5047
TLAAVGAASK	10/3145.2	Intron	Intestine	95.3669±1.5373	93.6854±1.0034	84.3883±1.4	82.5509±1.0877	70.0705±1.0988	59.8014±1.1908
ANVSTVLTSKYR	12/4399.9	Intron	estis/Spleen/Liver/Intes	25.6855±1.1158	15.9418±1.1935	9.401±1.7951	7.4286±1.4822	7.0395±1.4012	4.744±1.2946
PAGMYPVAGPK	11/3528.8	IGR	Spleen	98.1766±0.8924	95.1169±1.9531	73.7413±1.4073	57.5326±1.8268	36.3087±1.5542	30.6501±1.9593
MEEEECGLGKSCAR	14/6004.8	Intron	Lung	98.2173±1.5885	97.5261±1.3232	86.5911±1.3837	69.8753±1.4987	62.4284±1.0752	57.7265±1.5429
MEQASTMAEPR	11/4594.4	Intron	Lung	5.7246±1.0872	10.1778±1.5253	7.3977±0.939	9.4754±1.7727	5.8061±1.3873	7.0325±1.0037
RKEGMAAFVEKRK	13/5232.6	Intron	Liver	98.192±1.5266	93.9368±1.7166	85.4448±1.5803	63.8107±1.6075	51.114±1.145	45.1562±1.1979
ASRPLLR	7/3036.5	5'UTR	Lung	82.6913±1.4021	39.2097±1.4749	28.5205±1.4937	17.1176±1.3737	12.9608±1.8326	12.7121±1.4708
FGYSNRVVDL	10/3538	IGR	Muscle	22.8984±1.4106	14.617±1.8577	11.4099±1.3427	12.9594±1.3781	12.5913±1.449	11.2539±1.3869
ALEQAMQAAR	10/4265.8	Intron	Testis	46.9333±1.4154	43.0083±1.29	20.1211±1.8701	27.0763±1.3158	21.2997±1.6907	20.8024±1.8212
AEQVTKSVLF	10/3337.6	Intron	estis/Spleen/Liver/Intes	16.0008±0.8805	15.2506±1.1887	13.9217±0.983	14.2392±1.4601	11.5223±1.5564	13.7924±1.4738
MEVKPPPGRP	10/3561.1	IGR	Liver/Testis/Spleen	97.6849±1.3316	69.9975±1.2849	56.5225±1.6899	42.452±1.4619	32.7546±1.1184	28.5556±1.4298
VVDLMVHMASKE	12/3716.2	IGR	le/Testis/Spleen/Intest	90.7479±1.5506	64.7013±1.4208	60.7004±1.8223	52.6424±1.4076	37.257±1.2285	30.8342±1.8477

Peptide	Chain length/MW	Genomics location	Tissue	Hydroxyl radical (%)						
				15 mg/ml	10 mg/ml	7.5 mg/ml	4 mg/ml	2 mg/ml	1 mg/ml	0.5 mg/ml
PDPAKSAPAPK	11/4015.2	Intron	Liver	14.6868±1.1221	14.6121±1.534	9.096±1.9556	5.3605±1.8894	7.3279±1.9289	3.2873±1.6066	414±1.7565
ASAAEGDMEAELTR	14/5385.1	5'UTR	Testis	31.422±1.1142	25.8623±1.6167	21.7408±1.3573	13.149±1.7942	5.1675±1.384	3.1316±1.3168	044±1.4584
VEEVETPNSTPPR	13/4712.6	Intron	Testis	5.1177±1.3218	5.2173±1.4528	5.3916±1.3675	9.5194±1.8122	5.678±1.6736	1.7246±1.4035	705±1.2237
AASTARHLYLR	11/4798.4	IGR	Testis	-3.7106±1.9717	-5.7527±1.9272	-5.8648±1.6262	-6.4874±1.5674	-4.4951±1.7237	-3.5674±1.8889	308±1.7021
ADPELQLVAR	10/3763.3	Intron	Lung/Testis/Spleen	18.8021±1.8132	14.3008±1.3907	12.6572±1.8457	7.3528±1.7935	10.777±1.9614	1.544±1.4579	556±1.8024
ATPGKAVVATSGK	13/3981.1	IGR	Lung/Testis	29.6635±1.6668	25.8264±1.7459	23.6915±1.8967	14.6596±1.8766	11.4128±1.6825	5.913±1.907	867±1.8766
APAPRGPPSY	10/3761.2	Intron	Testis	-0.3148±1.67	-2.6958±1.9387	-5.8245±1.9106	-6.0213±1.5718	-5.8146±1.8077	-3.6993±1.7993	1582±1.6137
KYAPPPPPAP	10/3574.9	Intron	Testis	8.9237±1.8313	3.05±1.7013	2.2235±1.7522	-3.2566±1.7641	-2.9418±1.9499	-0.2263±1.7867	377±1.7654
LLQDHRLLR	9/4353.1	Intron	Testis	27.0366±1.4496	14.8367±1.7363	16.8044±1.945	11.1865±1.6762	8.4809±1.8829	8.1956±1.832	303±1.8351
RAFLEKVR	8/3243.6	Intron	Testis	17.198±1.7033	14.7973±1.9038	9.0417±1.9695	7.989±1.7701	5.7163±1.7468	8.0087±1.8258	745±1.7762
MESALTARDR	10/4263	IGR	Testis	44.0575±1.5077	34.7403±1.8364	28.5321±1.9853	15.0039±1.8196	10.9307±1.7627	5.7753±1.601	959±1.9606
GFVKVVKNK	9/2686.6	Intron	Testis/Intestine	18.0441±1.8836	11.0882±1.8912	8.4908±1.6982	6.405±1.7961	5.185±1.8379	5.608±1.7017	719±1.8364
AAKSQPSAPKTK	12/4788.9	IGR	Testis/Liver	17.4734±1.5793	9.1598±1.8789	8.8253±1.7947	8.412±1.8744	9.1696±1.9095	8.4514±1.712	048±1.9637
MVGHFGVQEDR	11/4069.7	IGR	Testis	29.3192±1.7713	19.8642±1.6881	16.8733±1.5309	14.8367±1.6344	5.5293±1.8186	5.795±1.9787	574±1.9156
AAMLDTVVFK	10/3012.3	Intron	Testis/Spleen	30.2538±1.9616	10.8127±1.8579	11.2062±1.7237	17.4341±1.6752	5.7458±1.7607	3.4632±1.8073	7253±1.957
APEEHPTLLTEAPLNPK	17/6788.7	Intron	Spleen	38.351±1.8386	26.584±1.8615	29.4864±1.9285	17.4538±1.7262	14.4235±1.7928	11.2161±1.685	107±1.6987
DRPAPHF	7/3053.5	3'UTR	ung/Testis/Spleen/Liver	37.6525±1.8799	29.7422±1.7627	22.9044±1.6647	14.5612±1.8176	11.9146±1.8027	17.0799±1.636	795±1.6205
DAALKQPPAPR	11/4438.9	IGR	Intestine	17.7292±1.5907	17.1881±1.6635	11.2751±1.7066	11.1275±1.8925	19.8445±1.8542	8.8056±1.6572	064±1.7617
GDYDVTVPK	9/2783.1	Intron	Lung/Intestine	5.9524±1.7515	2.7745±1.6824	3.5518±1.4113	0.1279±1.9197	5.3916±1.7562	3.109±1.8766	109±1.7517
AAGVNVEPFWPGLFAK	16/5348.7	Intron	Intestine	6.6866±1.4048	2.846±1.5455	2.2281±1.596	2.3615±1.4006	0.9162±1.6806	0.4634±1.1808	755±1.3608
LTDCVVMRDPASK	13/4680.4	Intron	Intestine	12.1374±1.0797	8.5486±1.4411	4.7483±1.6863	2.6831±1.5711	2.171±1.2218	2.3699±1.8165	137±1.4698
ELLFKEGVMVAK	12/3862.1	IGR	Intestine	10.8784±1.9394	6.5786±1.701	4.4288±1.7854	4.6086±1.9086	0.9226±1.3489	0.6348±1.6902	391±1.2688
YLDLSFNQMTKLPSGLP'	23/8754.8	Intron	Intestine	11.528±1.2549	3.1507±1.7652	2.645±1.47	2.1922±1.5572	0.5015±1.4206	2.4419±1.715	735±1.9258
TALVCDNGSGLCKAGF	16/6037.6	Intron	Intestine	7.9625±1.7151	5.9015±1.3521	2.8291±1.3824	2.1435±1.7818	1.3458±0.8977	-1.1236±1.1852	845±1.5714
WHHSFYNELR	10/5020.7	Intron	Intestine	5.4148±1.4764	0.6792±1.7266	-1.6632±1.6932	-1.2802±1.4977	-0.967±1.6669	0.6285±1.582	566±1.1565
TLAAVGAASK	10/3145.2	Intron	Intestine	6.2422±1.4872	7.971±1.4629	2.8693±1.495	2.3043±1.3747	1.0072±1.5899	0.3682±1.5066	0072±1.7807
ANVSTVLTSKYR	12/4399.9	Intron	estis/Spleen/Liver/Intes	-4.5494±1.1818	-3.6945±1.5409	-1.733±1.7616	-0.711±1.2707	15.9525±1.3563	17.9352±1.3047	1713±1.3052
PAGMYPVAGPK	11/3528.8	IGR	Spleen	21.543±1.2713	18.3055±1.5842	15.9864±1.0874	17.2348±1.1168	0.1587±1.2018	2.1964±1.4971	0136±1.545
MEEEECGLGKSCAR	14/6004.8	Intron	Lung	12.6177±1.1649	10.1801±1.0997	6.6485±1.4478	4.4796±1.0319	2.9053±1.2381	2.5477±1.5633	393±1.4334

MEQASTMAEPR	11/4594.4	Intron	Lung	6.0369±1.2501	4.5261±1.471	2.7085±1.5232	3.8321±1.768	2.4249±1.4193	2.4355±1.598	2239±1.3484
RKEGMAAFVEKRK	13/5232.6	Intron	Liver	38.6276±1.5099	31.2173±1.2261	6.6887±1.448	4.7737±1.5512	5.1757±1.6926	4.067±1.3889	4122±1.4656
ASRPLLR	7/3036.5	5'UTR	Lung	6.5765±1.2839	6.3565±1.7752	15.0638±1.907	15.1082±1.7976	8.7285±1.5435	4.5473±1.3849	0901±1.1757
FGYSNRVVDL	10/3538	IGR	Muscle	7.9011±1.6413	-0.8506±1.6061	-0.4888±1.695	0.7406±1.5723	0.7596±1.5456	0.8125±1.1427	5032±1.3197
ALEQAMQAAR	10/4265.8	Intron	Testis	11.9004±1.0517	9.7928±1.229	6.5257±1.9141	3.8194±1.4285	2.4207±1.1443	3.1084±1.7984	2235±1.4305
AEQVTKSVLF	10/3337.6	Intron	estis/Spleen/Liver/Intes	4.4648±1.6709	2.1604±1.7427	-0.8739±1.6417	1.8896±1.5354	1.3754±1.1557	0.2074±1.0151	019±1.2058
MEVKPPPGRP	10/3561.1	IGR	Liver/Testis/Spleen	9.795±1.174	8.6989±1.2484	4.0966±1.415	2.2049±1.3685	0.9078±1.307	0.4676±1.808	402±1.1938
VVDLMVHMASKE	12/3716.2	IGR	le/Testis/Spleen/Intest	10.3769±1.5257	1.039±1.44	6.1258±1.5413	2.7466±1.5916	2.2747±1.3165	0.7046±1.5274	2061±1.3255

Peptide	Chain length/MW	Genomics location	Tissue	DPPH(%)						
				15 mg/ml	10 mg/ml	7.5 mg/ml	4 mg/ml	2 mg/ml	1 mg/ml	0.5 mg/ml
PDPAKSAPAPK	11/4015.2	Intron	Liver	44.311±1.4791	41.3933±1.0859	40.6411±0.9882	39.8478±0.8212	40.3838±1.0748	40.9806±1.14	2325±0.9082
ASAAEGDMEAELTR	14/5385.1	5'UTR	Testis	49.6176±1.3937	40.6143±1.4928	40.8144±1.0455	39.2439±1.0136	41.4898±1.4436	40.7±1.2023	2039±1.8922
VEEVETPNSTPPR	13/4712.6	Intron	Testis	28.9808±1.5306	34.3661±0.9419	36.7549±1.2119	39.712±1.4833	39.3761±0.8235	39.6494±1.0703	8269±1.1822
AASTARHLYLR	11/4798.4	IGR	Testis	48.651±0.848	42.2152±0.8541	41.6792±1.1678	39.2421±1.5105	39.192±0.9241	41.8811±1.182	1146±1.1976
ADPELQLVAR	10/3763.3	Intron	Lung/Testis/Spleen	44.9203±1.562	43.859±1.0721	42.8048±1.7807	42.7941±1.6591	42.4868±1.0348	43.0317±1.7567	5338±1.3992
ATPGKAVVATSGK	13/3981.1	IGR	Lung/Testis	46.2318±1.5143	50.8969±0.9395	30.2298±1.2008	14.2689±1.4783	44.9418±0.8332	46.3408±2.5236	0509±1.1236
APAPRGPPSY	10/3761.2	Intron	Testis	57.1362±0.8665	52.857±0.8024	40.3909±1.2129	27.3603±1.5556	39.8478±1.119	37.55±1.1634	5107±1.2768
KYAPPPPPAP	10/3574.9	Intron	Testis	64.0652±1.4439	51.4723±1.1611	41.0127±1.7815	41.6363±1.6026	39.93±1.5844	37.7001±0.8046	8357±1.6574
LLQDHRLLR	9/4353.1	Intron	Testis	58.3726±1.5089	50.268±0.9139	37.5661±1.214	35.0951±1.4894	38.3523±0.8244	38.1968±1.0557	9514±1.1232
RAFLEKVR	8/3243.6	Intron	Testis	55.5961±0.9562	50.2519±0.8646	44.5165±1.1775	40.784±1.5266	41.3057±0.9649	38.1021±1.2039	6834±1.2379
MESALTARDR	10/4263	IGR	Testis	63.4738±1.6398	57.599±1.6743	39.4011±1.2116	34.8056±1.4893	44.1949±1.4681	41.7739±1.6496	2068±0.7102
GFVKVVKNK	9/2686.6	Intron	Testis/Intestine	56.3125±1.2302	47.3485±1.0437	46.925±1.7643	42.9442±1.049	46.4801±0.8783	42.4868±1.4961	9252±1.2989
AAKSQPSAPKTK	12/4788.9	IGR	Testis/Liver	46.0531±1.6472	43.2176±1.1649	42.6619±1.7128	39.8353±1.4155	41.0806±1.6269	41.8954±1.5078	4713±1.3533
MVGHFGVQEDR	11/4069.7	IGR	Testis	47.6129±1.3682	42.6101±1.486	41.2432±1.7962	42.1562±1.4114	40.8805±1.8037	41.4683±1.677	1235±1.5883
AAMLDTVVFK	10/3012.3	Intron	Testis/Spleen	75.9434±1.5201	66.3486±1.5658	64.3439±0.9944	35.3541±1.7426	47.5307±1.322	41.027±1.3826	1092±1.3193
HGQEVR	6/2602	IGR	Muscle	47.8309±1.2425	40.9841±1.0535	40.5196±1.796	42.2491±1.043	38.7007±0.8608	39.9389±1.5011	1837±1.2949
APEEHPTLLTEAPLNPK	17/6788.7	Intron	Spleen	46.7696±1.3367	41.5237±0.9415	40.5392±1.5402	41.9811±0.9983	42.9424±0.9809	39.6548±1.7492	0532±1.1389
DRPAPHF	7/3053.5	3'UTR	ung/Testis/Spleen/Liver	49.3997±1.7897	46.4766±1.142	41.1074±1.0816	39.4797±1.5062	40.0229±1.3189	40.5928±1.5348	8603±1.3233
DAALKQPPAPR	11/4438.9	IGR	Intestine	45.2062±1.2458	39.9657±1.0718	41.7542±1.7677	42.1598±1.06	38.7257±0.8817	41.4683±1.4886	2295±1.301
GDYDVTVPK	9/2783.1	Intron	Lung/Intestine	48.2883±1.5581	43.4069±1.3654	41.7596±1.2875	42.8316±1.2879	42.8388±1.7283	42.7887±1.892	0883±1.767
AAGVNVEPFWPGLFAK	16/5348.7	Intron	Intestine	43.4568±1.4049	41.8789±4.9246	46.0804±2.7614	46.6604±1.701	45.1912±1.517	42.4371±3.9659	0493±1.2879
LTDCVVMRDPASK	13/4680.4	Intron	Intestine	42.6771±4.6069	44.521±0.5263	43.2997±2.7314	43.1486±1.802	44.2317±3.7447	40.5348±1.2682	4791±4.3586
ELLFKEGVMVAK	12/3862.1	IGR	Intestine	39.1081±1.4637	43.5013±2.2004	44.7027±3.38	44.6843±2.3257	44.4459±3.8849	45.2376±0.9703	3562±1.2837
YLDLSFNQMTKLPSGLP	23/8754.8	Intron	Intestine	44.5798±1.3866	45.501±2.9088	45.0098±2.4915	44.1863±3.2425	43.5011±3.9071	44.782±3.294	5812±1.994
TALVCDNGSGLCKAGF	16/6037.6	Intron	Intestine	44.8739±2.9431	41.0184±3.2	43.6412±1.8551	44.4452±1.6273	42.7294±2.4312	41.8128±1.7808	8207±0.5677
WHHSFYNELR	10/5020.7	Intron	Intestine	40.9752±2.9496	44.8211±3.3288	42.7126±2.636	43.509±2.7634	44.5815±0.7194	43.9356±0.7365	3366±1.5153
TLAAVGAASK	10/3145.2	Intron	Intestine	41.0962±3.02	40.8787±0.5477	43.2084±3.6203	44.9039±2.5913	43.5072±3.3719	41.0937±2.2702	3318±1.5065
ANVSTVLTSKYR	12/4399.9	Intron	estis/Spleen/Liver/Intes	42.2409±4.5361	41.4526±4.1575	43.2249±3.6589	43.4025±2.8132	42.902±2.4687	44.7706±3.2283	0912±2.5161
PAGMYPVAGPK	11/3528.8	IGR	Spleen	42.7367±3.8836	42.2055±1.8852	45.2312±2.3763	42.1427±4.648	42.2091±4.4441	41.339±4.4726	5187±0.7613
MEEEECGLGKSCAR	14/6004.8	Intron	Lung	43.628±4.2832	45.4202±3.0426	42.3519±2.3447	44.4047±2.0831	42.0383±3.1877	46.6854±1.1357	9172±0.5765
MEQASTMAEPR	11/4594.4	Intron	Lung	43.5086±2.0615	41.5904±2.9428	44.2056±3.9607	43.6891±3.4682	42.6118±3.1167	43.2274±1.4596	1084±4.9588
RKEGMAAFVEKRK	13/5232.6	Intron	Liver	44.4915±4.8959	42.3401±2.6322	42.3088±3.3356	42.9556±1.0355	44.1965±2.5249	42.4982±3.9281	5835±4.718
ASRPLLR	7/3036.5	5'UTR	Lung	42.4661±2.9387	43.9839±3.0963	45.3439±4.5402	42.8854±2.6585	42.7714±3.3007	44.9648±2.0822	5552±4.503
FGYSNRVVDL	10/3538	IGR	Muscle	43.983±4.2019	41.9858±1.6825	43.5142±3.846	40.4776±3.5472	44.2444±3.9181	46.604±1.2874	0377±2.7318
ALEQAMQAAR	10/4265.8	Intron	Testis	41.8813±5.8117	39.398±1.1049	43.2912±1.9079	43.8511±0.9631	43.5338±2.6071	41.6134±2.4969	4337±1.2682
AEQVTKSVLF	10/3337.6	Intron	estis/Spleen/Liver/Intes	41.4626±1.9991	39.8692±1.8329	44.5128±2.4604	44.3739±5.3817	42.4941±3.1185	44.7472±0.6393	7795±1.6725
MEVKPPPGRP	10/3561.1	IGR	Liver/Testis/Spleen	40.4638±2.7926	43.257±3.5782	45.8959±3.3409	42.6948±3.7559	44.6709±2.4108	43.7655±4.8783	0453±3.6537
VVDLMVHMASKE	12/3716.2	IGR	le/Testis/Spleen/Intest	44.759±4.5571	44.8937±1.2762	39.621±2.4926	42.0042±4.0501	43.0948±3.7945	42.1879±3.2477	5223±2.6782

Peptide	Chain length/MW	Genomics location	Tissue	Superoxide anion (%)							
				10 (mg/ml)	5 (mg/ml)	2 (mg/ml)	1 (mg/ml)	0.5 (mg/ml)	0.25 (mg/ml)	125 (mg/m	625 (mg/ml)
PDPAKSAPAPK	11/4015.2	Intron	Liver	91.0423±0.1678	87.5082±1.8273	80.8174±0.8955	65.0553±1.8333	50.282±2.2358	26.7231±0.7766	0708±1.3	933±0.8252
ASAAEGDMEAELTR	14/5385.1	5'UTR	Testis	87.2848±1.6531	78.8471±0.5751	63.422±1.1665	55.0136±0.702	41.9944±1.9502	25.1373±1.9631	5786±2.9	778±0.9065
VEEVETPNSTPPR	13/4712.6	Intron	Testis	48.3538±1.531	43.8091±0.9052	32.9451±1.8953	30.4329±2.1504	29.7883±1.7263	28.4461±1.0329	2394±3.0	0419±0.7124
AASTARHLYLR	11/4798.4	IGR	Testis	85.5343±0.3968	81.5645±0.5792	71.3689±0.3076	61.6861±1.9697	50.0293±0.7544	38.9878±1.3849	1154±1.7	609±0.2961
ADPELQLVAR	10/3763.3	Intron	Lung/Testis/Spleen	83.6336±0.208	78.3527±0.3658	71.5191±0.2983	63.389±0.235	53.7757±0.739	45.7958±0.5335	.558±2.4	0001±1.5335
ATPGKAVVATSGK	13/3981.1	IGR	Lung/Testis	17.1098±0.2793	14.6598±0.6075	12.283±0.4302	10.4519±0.6264	9.9795±0.4642	12.1915±0.2853	5279±1.5	1828±1.2181
APAPRGPPSY	10/3761.2	Intron	Testis	87.9038±0.2707	81.4803±0.3124	75.39±0.7538	65.0114±1.7035	49.6814±0.4934	32.3775±1.6759	2341±1.2	696±0.6667
KYAPPPPPAP	10/3574.9	Intron	Testis	21.8487±0.5079	22.8704±0.1689	22.4749±0.901	21.8999±0.5722	21.2481±0.256	21.7974±0.3863	5557±0.3	281±0.4769
LLQDHRLLR	9/4353.1	Intron	Testis	32.9854±0.8068	33.1539±0.2983	33.0843±0.445	32.546±0.7455	32.7217±0.7688	33.0037±0.9715	5348±0.6	9531±3.1091
RAFLEKVR	8/3243.6	Intron	Testis	9.7781±0.7461	10.148±1.929	9.9062±1.6873	11.0672±1.0345	9.4228±1.04	9.1445±0.2683	1074±0.1	375±1.9072
MESALTARDR	10/4263	IGR	Testis	10.3164±1.7006	11.3015±0.4264	10.0051±1.9655	9.4338±1.7155	9.4411±1.2152	9.2434±1.9336	0163±0.53	.646±1.548
GFVKVVKNK	9/2686.6	Intron	Testis/Intestine	9.4448±1.2814	9.2324±1.1565	10.1406±1.6579	9.8477±1.3556	9.6426±1.7212	9.7451±1.7363	6753±1.1	4922±1.4742
AAKSQPSAPKTK	12/4788.9	IGR	Testis/Liver	10.2468±1.5976	10.1809±0.8136	9.1518±0.7913	10.8108±0.5426	9.9575±0.4343	10.7925±0.7795	375±0.7	8358±1.0274
MVGHFGVQEDR	11/4069.7	IGR	Testis	10.3164±2.0354	9.855±1.1542	9.573±1.4132	9.7122±1.4303	10.8218±0.679	10.3604±1.7345	7156±0.6	8328±1.5321
AAMLDTVVFK	10/3012.3	Intron	Testis/Spleen	10.7522±0.8436	9.6096±1.1207	11.2356±0.6149	10.0051±1.8897	10.0234±0.4073	10.1443±1.7176	586±0.5	5676±1.0412
HGQEVR	6/2602	IGR	Muscle	10.0308±0.8393	10.0564±1.409	10.1809±2.0359	9.2251±0.7866	9.5876±1.6706	9.3276±1.1129	115±0.3	4336±1.2637
APEEHPTLLTEAPLNPK	17/6788.7	Intron	Spleen	10.7156±1.1261	8.445±0.8585	11.1953±1.4653	9.4009±1.1527	10.0601±1.3806	9.4778±1.5854	0161±1.4	012±1.6427
DRPAPHF	7/3053.5	3'UTR	ung/Testis/Spleen/Liver	8.6684±0.7952	10.2285±1.3321	8.9248±1.7285	9.2763±0.7319	9.0713±1.3067	10.6387±1.0551	573±1.56	733±2.0887
DAALKQPPAPR	11/4438.9	IGR	Intestine	10.0344±1.2436	9.866±0.0549	8.8405±1.2601	8.434±0.5111	9.6755±1.1497	10.9573±1.2888	0392±1.5	3583±1.0513
GDYDVTVPK	9/2783.1	Intron	Lung/Intestine	10.5508±1.0187	8.6611±0.7258	9.2983±2.1597	10.2761±1.1786	10.6131±1.0186	10.8621±1.7292	7376±2.2	9646±1.2312
AAGVNVEPFWPGLFAK	16/5348.7	Intron	Intestine	13.4329±1.1984	7.1486±1.2415	4.8378±1.3423	3.0012±1.2878	3.7391±1.154	3.4589±1.1988	3435±1.2	2325±1.2359
LTDCVVMRDPASK	13/4680.4	Intron	Intestine	95.8434±1.1099	96.0943±1.1618	91.4854±1.2252	76.0858±1.6462	51.3495±1.3418	24.4433±1.2991	0751±1.10	4484±1.4291
ELLFKEGVMVAK	12/3862.1	IGR	Intestine	94.8308±1.0256	94.1478±1.3161	85.8273±1.1024	70.1531±1.0036	35.6131±1.2547	13.4256±1.2451	445±1.14	547±1.1928
YLDLSFNQMTKLPSGLP	23/8754.8	Intron	Intestine	95.9276±1.0669	92.9045±1.3866	76.3843±1.0488	50.1556±1.5275	15.7273±1.1068	3.5944±1.0551	796±1.2	2875±1.3149
TALVCDNGSGLCKAGF	16/6037.6	Intron	Intestine	95.2684±1.1058	84.3935±0.9088	40.1322±1.419	25.9723±1.1589	37.4533±1.2006	32.2347±1.1903	2347±1.0	6386±1.1715
WHHSFYNELR	10/5020.7	Intron	Intestine	50.976±1.0899	42.2746±1.1612	31.7366±1.5116	29.3965±1.3569	29.1621±1.1795	27.0984±1.2806	082±1.0	0687±1.0386
TLAAVGAASK	10/3145.2	Intron	Intestine	95.7335±1.1218	94.7942±1.0137	95.7171±1.5548	87.12±1.5446	76.0181±1.542	54.0394±1.2155	0921±1.2	1629±1.4018
ANVSTVLTSKYR	12/4399.9	Intron	estis/Spleen/Liver/Intes	49.8517±1.337	43.17±1.3084	40.0773±1.1329	38.8578±1.3651	36.7904±1.2601	35.4464±0.9174	6257±1.2	4023±1.0694
PAGMYPVAGPK	11/3528.8	IGR	Spleen	96.5887±1.095	93.8567±1.0764	84.1024±1.1978	62.9678±1.5569	41.0404±1.0897	37.6108±1.2696	8417±1.2	3973±1.3659
MEEEECGLGKSCAR	14/6004.8	Intron	Lung	96.1016±1.0644	95.0597±1.296	87.3068±1.1959	74.8773±1.0298	52.3511±1.1533	35.419±1.202	7046±1.2	2798±1.3316
MEQASTMAEPR	11/4594.4	Intron	Lung	35.2944±1.087	52.1918±1.167	38.7662±1.0794	35.7174±0.9237	31.6744±1.0503	31.2587±1.0957	383±1.02	3758±1.0114
RKEGMAAFVEKRK	13/5232.6	Intron	Liver	95.7372±0.9408	94.9535±1.0195	86.4847±1.0495	71.964±1.0086	43.192±1.096	39.039±1.0642	5636±1.0	9241±0.9302
ASRPLLR	7/3036.5	5'UTR	Lung	32.5606±1.0841	35.5618±1.032	31.4583±1.0956	37.7463±1.0275	32.6485±0.9194	30.5043±1.036	2512±1.0	4748±1.0432
FGYSNRVVDL	10/3538	IGR	Muscle	46.3158±1.0132	40.9251±0.9998	35.657±1.0121	33.1832±1.0424	35.6716±0.9645	35.6698±1.0835	526±1.02	5718±1.0751
ALEQAMQAAR	10/4265.8	Intron	Testis	94.9901±0.8924	95.3582±1.0411	86.5323±1.041	74.1467±0.9235	48.5461±1.0849	37.5558±0.9576	177±1.12	9181±0.9918
AEQVTKSVLF	10/3337.6	Intron	estis/Spleen/Liver/Intes	43.712±1.0604	24.7308±0.9335	30.1234±0.9408	31.9692±1.0037	32.9818±0.9965	35.4373±1.0438	6387±0.9	3549±1.1904
MEVKPPPGRP	10/3561.1	IGR	Liver/Testis/Spleen	94.9828±0.9093	93.3952±0.91	86.2961±1.0776	72.1655±1.1295	48.6944±0.9663	39.4419±1.0288	9221±1.0	779±0.9691
VVDLMVHMASKE	12/3716.2	IGR	le/Testis/Spleen/Intest	94.9645±0.7685	95.642±0.8237	92.6848±0.9358	85.0674±1.1616	69.6788±0.8491	48.3557±1.1936	4144±0.9	8415±1.0971
