# Supplementary material for: Integrated peptidogenomics decoding yak non-conventional peptides: functional mapping and biopotential mining of genetic resources
Source: Anim Biosci. 2025 Sep 30;39(5):250408. doi: 10.5713/ab.25.0408 (PMC13153706; doi:10.5713/ab.25.0408)
Supplement: Supplementary file 2 [file ab-25-0408-Supplement-2.pdf]

sequenc	ref sequ	strand	Chr	DNA st	DNA er	TSS	prot sta	prot enc	CDS	exon	gene	three pr	five prir	mRNA	flag1	flag2	annotati	geneID	length	isoelectric p	molecula	Centrom	Peptide	Denovo	m/z	z	Mass	PTM	Amino a	TSS Ch	TSS sta	muscle	lung	liver	testis	spleen	sintest	Classify
RLASLDR	Superscaf	-	Superscaf	20251	20271	-461	461	467	-	-	-	-	-	-	NCP	IGR	-	.	7	6.878571429	3110.6	0.999741526	RLASLDR	70	415.7498	2	829.477	.	C	Superscaf	20433	0	BTP202209	0	0	0	0	Tissue specific
KDAATTK	Superscaf	+	Superscaf	222040	222060	2	2	8	-	-	-	-	-	-	NCP	IGR	-	ID=gene-E	7	6.792857143	2765.9	0.997168526	KDAATTK	59	734.3906	1	733.397	.	T	Superscaf	222037	0	BTP202209	0	0	0	0	Tissue specific
VLLHVLPV	Superscaf	-	Superscaf	277906	277929	-4	4	11	-	-	gene	-	-	mRNA	NCP	Intron	gene|mRN	ID=gene-E	8	3.97875	2017.3	0.996456144	VLLHVLPV	68	445.3005	2	888.5797	.	A	Superscaf	277929	0	0	0	0	BTP202209	0	Tissue specific
KAGALVLS	Superscaf	+	Superscaf	281608	281631	32	32	39	-	-	gene	-	-	mRNA	NCP	Intron	gene|mRN	ID=gene-E	8	5.67375	2478.5	0.99640894	K(+42.01)	71	800.485	1	799.4803	Acetylati	I	Superscaf	281515	0	0	0	0	0	BTP202209	Tissue specific
SGPLNFTV	Superscaf	-	Superscaf	386014	386037	-24	24	31	-	-	-	-	-	-	NCP	IGR	-	ID=gene-E	8	5.16875	2753.1	0.995077665	SGPLNFTV	73	834.4341	1	833.4283	.	E	Superscaf	386196	0	0	0	0	BTP202209	BTP202209	Tissue specific
FAKLV	Superscaf	-	Superscaf	469936	469950	-77	77	81	-	-	gene	-	-	mRNA	NCP	Intron	gene|mRN	ID=gene-E	5	5.444	1520.5	0.994007695	FAKLV	97	577.3701	1	576.3635	.	A	Superscaf	469959	0	0	0	0	BTP202209	0	Tissue specific
RNPPPR	Superscaf	-	Superscaf	473764	473781	-83	83	88	-	-	gene	-	-	mRNA	NCP	Intron	gene|mRN	ID=gene-E	6	7.638333333	2906.5	0.993958847	RNPPPR	54	368.7189	2	735.414	.	N	Superscaf	474174	0	0	BTP202209	0	0	0	Tissue specific
KTAEDGKK	Superscaf	+	Superscaf	626794	626817	44	44	51	-	-	-	-	-	-	NCP	IGR	-	ID=gene-E	8	6.74125	3248.3	0.992007494	KTAEDGKK	79	438.7421	2	875.4712	.	E	Superscaf	626665	0	0	0	0	0	BTP202209	Tissue specific
TRELLP	Superscaf	-	Superscaf	857071	857088	-18	18	23	-	-	-	-	-	-	NCP	IGR	-	ID=gene-E	6	6.461666667	2516	0.989071322	T(+42.01)	86	770.4421	1	769.4334	Acetylati	F	Superscaf	857118	0	0	0	0	0	BTP202209	Tissue specific
KEDPKQPP	Superscaf	-	Superscaf	859639	859662	-7	7	14	-	-	-	-	-	-	NCP	IGR	-	ID=gene-E	8	6.2775	3396.7	0.989038501	KEDPKQPP	70	469.7523	2	937.4869	.	R	Superscaf	859752	0	BTP202209	0	0	0	0	Tissue specific
QRLLANYK	Superscaf	-	Superscaf	924916	924939	-48	48	55	-	-	-	-	-	-	NCP	IGR	-	ID=gene-E	8	6.9	3802.2	0.988206158	QRLLANYK	56	503.3044	2	1004.577	.	V	Superscaf	925020	0	0	0	0	0	BTP202209	Tissue specific
AAPVDK	Superscaf	-	Superscaf	952297	952314	-1	1	6	-	-	-	-	-	-	NCP	IGR	-	ID=gene-E	6	5.175	1839.9	0.987857101	A(+42.01)	96	642.3438	1	641.3384	Acetylati	A	Superscaf	952320	0	0	0	0	0	BTP202209	Tissue specific
QATHNVP	Superscaf	+	Superscaf	1043128	1043148	9	9	15	-	-	gene	-	-	mRNA	NCP	Intron	gene|mRN	ID=gene-E	7	5.357142857	2873.3	0.986698882	QATHNVP	62	383.6939	2	765.377	.	A	Superscaf	1043104	0	BTP202209	0	0	0	0	Tissue specific
LSPAVLP	Superscaf	+	Superscaf	1046977	1046997	16	16	22	-	-	gene	-	-	mRNA	NCP	Intron	gene|mRN	ID=gene-E	7	5.18	2076.3	0.986649803	LSPAVLP	89	696.4261	1	695.4218	.	V	Superscaf	1046932	0	0	0	0	0	BTP202209	Tissue specific
LSTLKY	Superscaf	+	Superscaf	1145782	1145799	29	29	34	-	-	gene	-	-	mRNA	NCP	Intron	gene|mRN	ID=gene-E	6	6.595	2415.6	0.985389985	LSTLKY	83	724.421	1	723.4167	.	H	Superscaf	1145698	0	0	0	BTP202209	0	0	Tissue specific
LVELPF	Superscaf	-	Superscaf	1151215	1151232	-3	3	8	-	-	gene	-	-	mRNA	NCP	Intron	gene|mRN	ID=gene-E	6	4.493333333	1857.1	0.985320709	LVELPF	60	717.415	1	716.4108	.	L	Superscaf	1151241	0	0	0	0	BTP202209	0	Tissue specific
MMDFLM	Superscaf	+	Superscaf	1152712	1152729	29	29	34	-	-	gene	-	-	mRNA	NCP	Intron	gene|mRN	ID=gene-E	6	5.28	2266.8	0.985301621	M(+15.99)	71	410.1653	2	818.3013	Oxidation	K	Superscaf	1152628	0	0	0	0	0	BTP202209	Tissue specific
LLTVPF	Superscaf	-	Superscaf	1223890	1223907	-22	22	27	-	-	gene	-	-	mRNA	NCP	Intron	gene|mRN	ID=gene-E	6	5.045	1829.1	0.984394034	LLTVPF	89	689.4205	1	688.416	.	R	Superscaf	1223940	0	0	BTP202209	0	0	0	Tissue specific
RGTFLR	Superscaf	-	Superscaf	1229926	1229943	-39	39	44	-	-	gene	-	-	mRNA	NCP	Intron	gene|mRN	ID=gene-E	6	7.58	2733.2	0.98431707	RGTFLR	63	375.2228	2	748.4344	.	C	Superscaf	1230120	0	0	0	BTP202209	0	0	Tissue specific
LLPGELAK	Superscaf	-	Superscaf	1242058	1242081	-128	128	135	-	-	gene	-	-	mRNA	NCP	Intron	gene|mRN	ID=gene-E	8	6.14875	2900.1	0.984162299	LLPGELAK	99	420.7621	2	839.5116	.	P	Superscaf	1242132	0	0	0	BTP202209	0	BTP202209	Tissue enhanced
TVAPDPK	Superscaf	-	Superscaf	1328413	1328433	-12	12	18	-	-	gene	-	-	mRNA	NCP	Intron	gene|mRN	ID=gene-E	7	5.408571429	2263.5	0.98306123	TVAPDPK	62	364.1966	2	726.3912	.	Q	Superscaf	1328442	0	0	BTP202209	0	0	0	Tissue specific
KYPPPPLP	Superscaf	+	Superscaf	1345075	1345098	2	2	9	-	-	gene	-	-	mRNA	NCP	Intron	gene|mRN	ID=gene-E	8	6.61	2968.3	0.982848735	KYPPPPLP	67	454.7651	2	907.5167	.	S	Superscaf	1345072	0	0	0	BTP202209	0	0	Tissue specific
VTLMAELK	Superscaf	+	Superscaf	1346827	1346850	26	26	33	-	-	gene	-	-	mRNA	NCP	Intron	gene|mRN	ID=gene-E	8	5.4025	2681	0.982826395	VTLM(+15.	63	460.7679	2	919.5048	Oxidation	N	Superscaf	1346752	0	0	0	0	0	BTP202209	Tissue specific
KAYFPK	Superscaf	-	Superscaf	1413856	1413873	-93	93	98	-	-	gene	-	-	mRNA	NCP	Intron	gene|mRN	ID=gene-E	6	7.156666667	2432.4	0.981971789	KAYFPK	52	377.2151	2	752.4221	.	R	Superscaf	1414029	0	0	BTP202209	0	0	0	Tissue specific
KFPSLVH	Superscaf	-	Superscaf	1416535	1416555	-2	2	8	-	-	gene	-	-	mRNA	NCP	Intron	gene|mRN	ID=gene-E	7	5.824285714	2549.7	0.981937591	KFPSLVH	89	414.2343	2	826.4701	.	F	Superscaf	1416561	0	0	0	0	BTP202209	0	Tissue specific
FPEFLQ	Superscaf	+	Superscaf	1480342	1480359	7	7	12	-	-	-	-	-	-	NCP	IGR	-	ID=gene-E	6	5.351666667	2501.8	0.98112403	FPEFLQ	99	780.3896	1	779.3854	.	L	Superscaf	1480324	0	0	BTP202209	0	0	0	Tissue specific
SPAKPEVQ	Superscaf	+	Superscaf	1533526	1533549	24	24	31	-	-	-	-	-	-	NCP	IGR	-	ID=gene-E	8	5.36375	2874.1	0.980445808	SPAKPEVQ	88	855.4537	1	854.4498	.	P	Superscaf	1533457	0	0	0	0	0	BTP202209	Tissue specific
VAVTPSLP	Superscaf	-	Superscaf	1595281	1595304	-2	2	9	-	-	gene	-	-	mRNA	NCP	Intron	gene|mRN	ID=gene-E	8	4.60125	2120.4	0.979658373	V(+42.01)	70	413.2332	2	824.4644	Acetylati	G	Superscaf	1595337	0	BTP202209	0	0	0	0	Tissue specific
LVGGLL	Superscaf	-	Superscaf	1619254	1619271	-9	9	14	-	-	gene	-	-	mRNA	NCP	Intron	gene|mRN	ID=gene-E	6	4.98	1654.8	0.979352771	LVGGLL	75	571.3809	1	570.3741	.	K	Superscaf	1619310	0	0	BTP202209	0	0	0	Tissue specific
KPSVVT	Superscaf	-	Superscaf	1825294	1825311	-14	14	19	-	-	-	-	-	-	NCP	IGR	-	ID=gene-E	6	4.708333333	1586.7	0.976725568	KPSVVT	50	630.3805	1	629.3748	.	K	Superscaf	1825311	0	0	0	0	0	BTP202209	Tissue specific
KKSHVVLS	Superscaf	+	Superscaf	1846012	1846035	2	2	9	-	-	-	-	-	-	NCP	IGR	-	ID=gene-E	8	5.55125	2676.7	0.976461317	KKSHVVLS	59	449.2776	2	896.5443	.	I	Superscaf	1846009	0	0	BTP202209	0	0	0	Tissue specific
VALEYL	Superscaf	+	Superscaf	1850623	1850640	10	10	15	-	-	-	-	-	-	NCP	IGR	-	ID=gene-E	6	4.476666667	1903.1	0.976402599	VALEYL	76	707.3938	1	706.3901	.	E	Superscaf	1850596	0	BTP202209	0	0	0	0	Tissue specific
LPGVLP	Superscaf	+	Superscaf	1897498	1897515	168	168	173	-	-	-	-	-	-	NCP	IGR	-	ID=gene-E	6	5.088333333	1678.9	0.975804899	LPGVLP	59	595.3789	1	594.3741	.	L	Superscaf	1896997	0	BTP202209	0	0	0	0	Tissue specific
SHMDLR	Superscaf	+	Superscaf	1990618	1990635	1	1	6	-	-	-	-	-	-	NCP	IGR	-	ID=gene-E	6	6.455	2788.3	0.974617531	SHM(+15.9	64	774.3411	1	773.349	Oxidation	S	Superscaf	1990618	0	0	0	0	0	BTP202209	Tissue specific
DDDPSEK	Superscaf	+	Superscaf	2145937	2145957	7	7	13	-	-	gene	-	-	mRNA	NCP	Intron	gene|mRN	ID=gene-E	7	4.835714286	2849.2	0.972637029	D(+42.01)	74	424.1636	2	846.3243	Acetylati	V	Superscaf	2145919	BTP202209	0	0	0	0	0	Tissue specific
VGNSLL	Superscaf	+	Superscaf	2149750	2149767	23	23	28	-	-	gene	-	-	mRNA	NCP	Intron	gene|mRN	ID=gene-E	6	4.836666667	1984.2	0.972588448	VGNSLL	58	602.3498	1	601.3435	.	M	Superscaf	2149684	0	0	BTP202209	0	0	0	Tissue specific
RPHLFL	Superscaf	-	Superscaf	2185783	2185800	-95	95	100	-	-	gene	-	-	mRNA	NCP	Intron	gene|mRN	ID=gene-E	6	7.015	2700.1	0.972128994	RPHLFL	50	391.7442	2	781.4598	.	D	Superscaf	2185806	0	0	BTP202209	0	0	0	Tissue specific
KEAPKR	Superscaf	+	Superscaf	2187910	2187927	8	8	13	-	-	gene	-	-	mRNA	NCP	Intron	gene|mRN	ID=gene-E	6	7.63	2659.8	0.972101872	K(+42.01)	96	385.7283	2	769.4446	Acetylati	E	Superscaf	2187889	0	0	BTP202209	BTP202209	0	0	Tissue specific
LLMGYHLL	Superscaf	+	Superscaf	2226142	2226165	29	29	36	-	-	gene	-	-	mRNA	NCP	Intron	gene|mRN	ID=gene-E	8	6.11125	3161.6	0.971614302	LLM(+15.9	55	488.2712	2	974.5259	Oxidation	T	Superscaf	2226058	0	0	BTP202209	0	0	0	Tissue specific
VSSLPVEV	Superscaf	-	Superscaf	2229766	2229792	-22	22	30	-	-	gene	-	-	mRNA	NCP	Intron	gene|mRN	ID=gene-E	9	3.71	2280.6	0.971568054	V(+42.01)	60	972.522	1	971.5175	Acetylati	P	Superscaf	2229840	0	0	BTP202209	0	0	0	Tissue specific
KLRSPK	Superscaf	+	Superscaf	2233696	2233713	6	6	11	-	-	gene	-	-	mRNA	NCP	Intron	gene|mRN	ID=gene-E	6	8.033333333	2659.8	0.971518058	KLRSPK	69	364.7408	2	727.4704	.	S	Superscaf	2233681	0	BTP202209	0	0	0	0	Tissue specific
CSFRCAKC	Superscaf	-	Superscaf	2253868	2253891	-50	50	57	-	-	gene	-	-	mRNA	NCP	Intron	gene|mRN	ID=gene-E	8	6.5925	3956.4	0.971260769	C(+57.02)	98	544.7232	2	1087.436	Carbamido	G	Superscaf	2253981	0	0	0	0	0	BTP202209	Tissue specific
STVYVP	Superscaf	+	Superscaf	2257840	2257857	20	20	25	-	-	gene	-	-	mRNA	NCP	Intron	gene|mRN	ID=gene-E	6	4.028333333	1577.8	0.971210199	STVYVP	63	665.3451	1	664.3432	.	P	Superscaf	2257783	0	0	0	BTP202209	0	0	Tissue specific
SCPSRVPT	Superscaf	-	Superscaf	2321227	2321250	-17	17	24	-	-	-	-	-	-	NCP	IGR	-	ID=gene-E	8	5.78375	3073.6	0.970401879	S(+42.01)	60	473.2252	2	944.4385	Acetylati	R	Superscaf	2321418	0	0	0	BTP202209	0	0	Tissue specific
AAVSL	Superscaf	-	Superscaf	2369263	2369277	-3	3	7	-	-	gene	-	-	mRNA	NCP	Intron	gene|mRN	ID=gene-E	5	4.74	1359.4	0.969789489	A(+42.01)	75	502.2854	1	501.2798	Acetylati	P	Superscaf	2369781	0	BTP202209	BTP202209	BTP202209	BTP202209	BTP202209	Tissue specific
FVKHLM	Superscaf	-	Superscaf	2379031	2379048	-8	8	13	-	-	gene	-	-	mRNA	NCP	Intron	gene|mRN	ID=gene-E	6	5.756666667	2200.4	0.9696649	FVKHLM(+1	99	395.7167	2	789.4207	Oxidation	P	Superscaf	2379081	0	0	0	BTP202209	0	0	Tissue specific
SVGSVLTK	Superscaf	-	Superscaf	2509084	2509107	-22	22	29	-	-	gene	-	-	mRNA	NCP	Intron	gene|mRN	ID=gene-E	8	4.9475	2283.4	0.968006525	SVGSVLTK	99	395.7361	2	789.4596	.	N	Superscaf	2509122	0	0	0	BTP202209	0	0	Tissue enhanced
KPGKM	Superscaf	-	Superscaf	2514631	2514645	-35	35	39	-	-	gene	-	-	mRNA	NCP	Intron	gene|mRN	ID=gene-E	5	7.5	1943	0.967935911	KPGKM	61	560.3266	1	559.3152	.	V	Superscaf	2514711	0	0	BTP202209	0	0	0	Mixed
VPELQ	Superscaf	-	Superscaf	2533522	2533536	-26	26	30	-	-	-	-	-	-	NCP	IGR	-	ID=gene-E	5	4.23	1727	0.967695033	VPELQ	72	585.3243	1	584.317	.	G	Superscaf	2533653	0	0	BTP202209	0	0	0	Tissue specific
SFKPLR	Superscaf	+	Superscaf	2756608	2756625	8	8	13	-	-	-	-	-	-	NCP	IGR	-	ID=gene-E	6	7.323333333	2578.8	0.964850438	SFKPLR	60	374.2348	2	746.4438	.	Y	Superscaf	2756587	0	0	0	0	0	BTP202209	Tissue specific
TGGGGVGK	Superscaf	+	Superscaf	2759659	2759682	60	60	67	-	-	-	-	-	-	NCP	IGR	-	ID=gene-E	8	5.765	2352.4	0.964811458	TGGGGVGK	94	632.3416	1	631.3289	.	A	Superscaf	2759482	0	0	0	0	BTP202209	0	Tissue specific
AGPKAFSP	Superscaf	-	Superscaf	2830351	2830374	-3	3	10	-	-	-	-	-	-	NCP	IGR	-	ID=gene-E	8	6.43875	2833.9	0.963910069	AGPKAFSP	59	387.7184	2	773.4072	.	E	Superscaf	2830419	0	BTP202209	0	0	0	0	Tissue specific
TLYGN	Superscaf	-	Superscaf	2844619	2844633	-30	30	34	-	-	-	-	-	-	NCP	IGR	-	ID=gene-E	5	5.91	2104.4	0.963728253	T(+42.01)	70	609.2987	1	608.2806	Acetylati	Q	Superscaf	2844744	0	0	0	BTP202209	0	0	Tissue specific
SMPDP	Superscaf	+	Superscaf	2960470	2960484	24	24	28	-	-	gene	-	-	mRNA	NCP	Intron	gene|mRN	ID=gene-E	5	5.4	1841.2	0.962251044	SM(+15.99	52	562.2103	1	561.2104	Oxidation	S	Superscaf	2960401	0	0	0	0	0	BTP202209	Tissue specific
AELAFR	Superscaf	+	Superscaf	2978104	2978121	122	122	127	-	-	gene	-	-	mRNA	NCP	Intron	gene|mRN	ID=gene-E	6	6.246666667	2437.7	0.962026156	AELAFR	86	353.6974	2	705.3809	.	G	Superscaf	2977741	BTP202209	0	0	0	0	0	Tissue specific
REQVVR	Superscaf	-	Superscaf	3000997	3001014	-12	12	17	-	-	-	-	-	-	NCP	IGR	-	ID=gene-E	6	5.065	2333.8	0.961734248	REQVVR	93	393.7309	2	785.4507	.	L	Superscaf	3001089	0	0	BTP202209	0	0	0	Tissue enhanced
LPLGEL	Superscaf	+	Superscaf	3072838	3072855	26	26	31	-	-	-	-	-	-	NCP	IGR	-	ID=gene-E	6	5.571666667	2120.4	0.960818208	LPLGEL	70	641.3848	1	640.3796	.	V	Superscaf	3072763	0	BTP202209	BTP202209	BTP202209	0	0	Tissue specific
KRGPPPP	Superscaf	-	Superscaf	3116683	3116703	-1	1	7	-	-	gene	-	-	mRNA	NCP	Intron	gene|mRN	ID=gene-E	7	7.381428571	2764.1	0.960259105	K(+42.01)	69	395.7309	2	789.4497	Acetylati	K	Superscaf	3116721	0	BTP202209	0	BTP202209	0	0	Tissue specific
QPATPLM	Superscaf	+	Superscaf	3261823	3261843	24	24	30	-	-	-	-	-	-	NCP	IGR	-	ID=gene-E	7	6.075714286	2719.2	0.958408433	QPATPLM	59	379.2028	2	756.384	.	D	Superscaf	3261754	0	0	0	BTP202209	0	0	Tissue specific
AETPKP	Superscaf	+	Superscaf	3341359	3341376	16	16	21	-	-	-	-	-	-	NCP	IGR	-	ID=gene-E	6	6.351666667	2277.5	0.957394313	AETPKP	97	642.3419	1	641.3384	.	A	Superscaf	3341314	0	BTP202209	0	0	0	0	Tissue specific
VGPPFR	Superscaf	-	Superscaf	3488581	3488598	-21	21	26	-	-	-	-	-	-	NCP	IGR	-	ID=gene-E	6	5.801666667	2008.3	0.955517094	V(+42.01)	58	357.6998	2	713.386	Acetylati	E	Superscaf	3488604	0	0	0	0	0	BTP202209	Tissue specific
EPATEYL	Superscaf	+	Superscaf	3489112	3489132	28	28	34	-	-	-	-	-	-	NCP	IGR	-	ID=gene-E	7	5.275714286	2710.1	0.955510285	EPATEYL	62	411.7018	2	821.3807	.	Y	Superscaf	3489031	0	0	0	0	0	BTP202209	Mixed
VLSAAD	Superscaf	-	Superscaf	3568057	3568074	-36	36	41	-	-	-	-	-	-	NCP	IGR	-	ID=gene-E	6	4.445	1770.9	0.9545037	VLSAAD	99	575.3011	1	574.2962	.	I	Superscaf	3568203	0	0	BTP202209	0	0	0	Tissue specific
LHPTPAVH	Superscaf	-	Superscaf	3606586	3606609	-9	9	16	-	-	-	-	-	-	NCP	IGR	-	ID=gene-E	8	5.78875	2976.4	0.954012342	LHPTPAVH	75	436.2488	2	870.4712	.	L	Superscaf	3606642	0	0	0	0	0	BTP202209	Tissue specific
GVMKLLT	Superscaf	+	Superscaf	3607780	3607800	9	9	15	-	-	-	-	-	-	NCP	IGR	-	ID=gene-E	7	5.707142857	2241.5	0.953997156	GVMKLLT	88	761.4557	1	760.4517	.	H	Superscaf	3607756	0	0	0	0	0	BTP202209	Mixed
SLAVGLF	Superscaf	-	Superscaf	3635371	3635391	-19	19	25	-	-	gene	-	-	mRNA	NCP	Intron	gene|mRN	ID=gene-E	7	5.015714286	2086.2	0.953645345	SLAVGLF	56	353.7088	2	705.4061	.	G	Superscaf	3635403	0	0	0	0	0	BTP202209	Tissue specific
TGAPGLL	Superscaf	+	Superscaf	3639523	3639543	25	25	31	-	-	gene	-	-	mRNA	NCP	Intron	gene|mRN	ID=gene-E	7	6.107142857	2347.6	0.953592403	T(+42.01)	89	670.3762	1	669.3698	Acetylati	L	Superscaf	3639451	BTP202209	0	0	0	0	0	Tissue specific
WHPEKPP	Superscaf	-	Superscaf	3716863	3716883	-12	12	18	-	-	gene	-	-	mRNA	NCP	Intron	gene|mRN	ID=gene-E	7	6.477142857	3052.4	0.952606245	W(+42.01)	69	466.7313	2	931.4551	Acetylati	A	Superscaf	3717243	0	0	0	BTP202209	0	0	Tissue specific
AAPEPPR	Superscaf	-	Superscaf	3724879	3724899	-2	2	8	-	-	gene	-	-	mRNA	NCP	Intron	gene|mRN	ID=gene-E	7	6.417142857	2709.1	0.952504034	AAPEPPR	67	369.2005	2	736.3867	.	R	Superscaf	3725007	0	BTP202209	0	0	BTP202209	0	Tissue specific
RGRPPPPPS	Superscaf	-	Superscaf	3866008	3866037	-15	15	24	-	-	gene	-	-	mRNA	NCP	Intron	gene|mRN	ID=gene-E	10	7.543	4314.1	0.950704391	RGRPPPPPS	60	372.883	3	1115.631	.	Q	Superscaf	3866112	BTP202209	0	0	0	0	0	Tissue specific
LERMVLSSL	Superscaf	+	Superscaf	3975208	3975234	78	78	86	-	-	-	-	-	-	NCP	IGR	-	ID=gene-E	9	5.447777778	3272.8	0.949312027	LERM(+15.	57	532.297	2	1062.574	Oxidation	L	Superscaf	3974977	BTP202209	0	0	0	0	0	Tissue specific
KDYVT	Superscaf	+	Superscaf	4010113	4010127	11	11	15	-	-	gene	-	-	mRNA	NCP	Intron	gene|mRN	ID=gene-E	5	4.98	1736.9	0.948867108	KDYVT	53	625.3152	1	624.3119	.	S	Superscaf	4010083	0	0	0	BTP202209	0	0	Tissue specific
FYTVPGK	Superscaf	+	Superscaf	4072786	4072806	1	1	7	-	-	-	-	-	-	NCP	IGR	-	ID=gene-E	7	5.668571429	2347.5	0.948067892	FYTVPGK	69	406.2234	2	810.4276	.	F	Superscaf	4072786	0	0	0	0	BTP202209	0	Tissue specific
LPLSLLK	Superscaf	-	Superscaf	4121623	4121643	-3	3	9	-	-	-	-	-	-	NCP	IGR	-	ID=gene-E	7	6.52	2602.8	0.947445174	LPLSLLK	97	392.2696	2	782.5266	.	F	Superscaf	4121697	BTP202209	0	0	0	0	0	Tissue specific
AYHVQPRP	Superscaf	-	Superscaf	4153261	4153284	-3	3	10	-	-	-	-	-	-	NCP	IGR	-	ID=gene-E	8	6.035	3324.8	0.947041722	AYHVQPRP	86	484.2564	2	966.5035	.	K	Superscaf	4153572	0	0	0	0	BTP202209	0	Tissue specific
NDLLDF	Superscaf	+	Superscaf	4185571	4185588	9	9	14	-	-	gene	-	-	mRNA	NCP	Intron	gene|mRN	ID=gene-E	6	4.798333333	2513.9	0.946629815	NDLLDF	73	736.349	1	735.3439	.	N	Superscaf	4185547	0	BTP202209	BTP202209	0	0	0	Tissue specific
ELGVKHL	Superscaf	+	Superscaf	4188418	4188438	101	101	107	-	-	-	-	-	-	NCP	IGR	-	ID=gene-E	7	5.497142857	2517.7	0.946593475	ELGVKHL	50	398.2384	2	794.465	.	Y	Superscaf	4188118	0	0	0	0	BTP202209	BTP202209	Tissue specific
NGFSSFLK	Superscaf	-	Superscaf	4196065	4196088	-5	5	12	-	-	-	-	-	-	NCP	IGR	-	ID=gene-E	8	6.1775	3257.4	0.946495931	NGFSSFLK	58	450.2422	2	898.4548	.	I	Superscaf	4196112	0	0	BTP202209	0	0	0	Tissue specific
RGPMPSP	Superscaf	-	Superscaf	4209331	4209351	-26	26	32	-	-	gene	-	-	mRNA	NCP	Intron	gene|mRN	ID=gene-E	7	6.722857143	2713.2	0.946326815	RGPMPSP	67	371.188	2	740.364	.	Q	Superscaf	4209357	0	BTP202209	0	0	0	0	Tissue specific
SLSAPAPAF	Superscaf	-	Superscaf	4469140	4469169	-16	16	25	-	-	-	-	-	-	NCP	IGR	-	ID=gene-E	10	6.027	3363.6	0.943013891	SLSAPAPAF	69	867.4548	1	866.4498	.	T	Superscaf	4469349	BTP202209	0	0	0	0	0	Tissue specific
KSLFK	Superscaf	+	Superscaf	4534081	4534095	1	1	5	-	-	-	-	-	-	NCP	IGR	-	ID=gene-E	5	7.324	2061	0.942186023	KSLFK	66	622.398	1	621.3849	.	K	Superscaf	4534081	BTP202209	0	0	0	0	0	Tissue specific
PAAALHL	Superscaf	-	Superscaf	4601905	4601925	-7	7	13	-	-	-	-	-	-	NCP	IGR	-	ID=gene-E	7	6.272857143	2597.8	0.941321127	PAAALHL	58	692.408	1	691.4017	.	P	Superscaf	4601973	0	0	0	0	0	BTP202209	Tissue specific
TFKFPVG	Superscaf	+	Superscaf	4613392	4613412	12	12	18	-	-	-	-	-	-	NCP	IGR	-	ID=gene-E	7	5.642857143	2275.4	0.941174657	T(+42.01)	68	419.2278	2	836.4432	Acetylati	F	Superscaf	4613359	0	0	0	BTP202209	0	0	Tissue specific
RANAK	Superscaf	+	Superscaf	4620682	4620696	12	12	16	-	-	-	-	-	-	NCP	IGR	-	ID=gene-E	5	7.59	2336.5	0.941081779	R(+42.01)	64	601.3442	1	600.3344	Acetylati	V	Superscaf	4620649	0	BTP202209	0	0	0	0	Tissue specific
VDLNLK	Superscaf	+	Superscaf	4730143	4730160	118	118	123	-	-	gene	-	-	mRNA	NCP	Intron	gene|mRN	ID=gene-E	6	5.013333333	2183.4	0.93968601	VDLNLK	97	351.212	2	700.4119	.	S	Superscaf	4729792	0	BTP202209	0	0	0	0	Tissue specific
LPGVP	Superscaf	+	Superscaf	4755067	4755081	8	8	12	-	-	-	-	-	-	NCP	IGR	-	ID=gene-E	5	4.91	1325.5	0.939368244	LPGVP	76	482.2963	1	481.29	.	N	Superscaf	4755046	0	0	0	BTP202209	0	0	Mixed
KKKKDAPGF	Superscaf	-	Superscaf	4770682	4770708	-3	3	11	-	-	-	-	-	-	NCP	IGR	-	ID=gene-E	9	7.886666667	3879.9	0.939168985	KKKKDAPGF	59	514.3154	2	1026.63	.	L	Superscaf	4770810	0	BTP202209	0	0	0	0	Tissue specific
LSYVYK	Superscaf	+	Superscaf	4995934	4995951	38	38	43	-	-	-	-	-	-	NCP	IGR	-	ID=gene-E	6	5.453333333	2124.2	0.936296925	LSYVYK	82	386.7147	2	771.4167	.	T	Superscaf	4995823	0	0	0	0	0	BTP202209	Tissue enhanced
EPTAP	Superscaf	+	Superscaf	5048116	5048130	4	4	8	-	-	-	-	-	-	NCP	IGR	-	ID=gene-E	5	5.674	1809.1	0.935631593	EPTAP	51	514.246	1	513.2435	.	R	Superscaf	5048107	0	0	0	0	0	BTP202209	Tissue specific
KLPGDVP	Superscaf	+	Superscaf	5280226	5280246	10	10	16	-	-	-	-	-	-	NCP	IGR	-	ID=gene-E	7	5.322857143	2205.4	0.932671896	KLPGDVP	62	363.2073	2	724.4119	.	F	Superscaf	5280199	BTP202209	0	0	0	0	0	Tissue specific
LHALFP	Superscaf	-	Superscaf	5301085	5301102	-5	5	10	-	-	-	-	-	-	NCP	IGR	-	ID=gene-E	6	6.225	2362.6	0.932405963	LHALFP	56	697.4008	1	696.3959	.	V	Superscaf	5301150	0	0	BTP202209	0	0	0	Tissue specific
KGAQVAK	Superscaf	-	Superscaf	5303749	5303769	-11	11	17	-	-	-	-	-	-	NCP	IGR	-	ID=gene-E	7	6.162857143	2467.4	0.932371956	KGAQVAK	52	351.2183	2	700.4232	.	V	Superscaf	5303958	0	0	BTP202209	0	0	0	Tissue specific
EAPGKKPR	Superscaf	+	Superscaf	5438113	5438136	52	52	59	-	-	-	-	-	-	NCP	IGR	-	ID=gene-E	8	7.25625	3294.5	0.93065865	EAPGKKPR	57	441.7692	2	881.5082	.	S	Superscaf	5437960	0	0	0	BTP202209	0	0	Tissue specific
KGATK	Superscaf	-	Superscaf	5489356	5489370	-5	5	9	-	-	-	-	-	-	NCP	IGR	-	ID=gene-E	5	7.6	1942.9	0.930005368	KGATK	68	504.3196	1	503.3067	.	R	Superscaf	5489394	0	BTP202209	BTP202209	BTP202209	BTP202209	BTP202209	Tissue specific
PGVGPP	Superscaf	-	Superscaf	5522377	5522394	-48	48	53	-	-	-	-	-	-	NCP	IGR	-	ID=gene-E	6	5.14	1606.8	0.929584281	PGVGPP	70	523.2858	1	522.2802	.	L	Superscaf	5522442	0	BTP202209	0	0	0	0	Tissue specific
VYLTH	Superscaf	+	Superscaf	5526538	5526552	34	34	38	-	-	-	-	-	-	NCP	IGR	-	ID=gene-E	5	5.152	1830.1	0.929531263	VYLTH	97	632.3384	1	631.3329	.	I	Superscaf	5526439	0	BTP202209	0	0	BTP202209	0	Tissue specific
NPNNARPL	Superscaf	-	Superscaf	5551069	5551092	-5	5	12	-	-	-	-	-	-	NCP	IGR	-	ID=gene-E	8	6.44875	3778.4	0.929218355	NPNNARPL	50	448.2422	2	894.4672	.	Q	Superscaf	5551131	0	0	0	BTP202209	0	0	Tissue specific
KPKKGPK	Superscaf	-	Superscaf	5578552	5578572	-26	26	32	-	-	-	-	-	-	NCP	IGR	-	ID=gene-E	7	8.218571429	2845.7	0.928867959	KPKKGPK	59	391.7585	2	781.5173	.	P	Superscaf	5578650	0	BTP202209	0	0	0	0	Tissue specific
RLVLEPF	Superscaf	+	Superscaf	5644777	5644797	40	40	46	-	-	-	-	-	-	NCP	IGR	-	ID=gene-E	7	5.388571429	2505.9	0.928023528	RLVLEPF	68	437.2619	2	872.512	.	K	Superscaf	5644660	0	0	BTP202209	0	0	0	Tissue specific
KDAAK	Superscaf	+	Superscaf	5753827	5753841	30	30	34	-	-	gene	-	-	mRNA	NCP	Intron	gene|mRN	ID=gene-E	5	6.898	1970.9	0.926633114	KDAAK	52	532.3002	1	531.3016	.	F	Superscaf	5753740	BTP202209	0	0	0	0	0	Tissue specific
QTSLPDL	Superscaf	-	Superscaf	5817073	5817093	-1	1	7	-	-	gene	-	-	mRNA	NCP	Intron	gene|mRN	ID=gene-E	7	5.584285714	2847.3	0.925826592	Q(+42.01)	59	815.4122	1	814.4072	Acetylati	Q	Superscaf	5817117	0	0	BTP202209	0	0	0	Tissue specific
LNLPVLLE	Superscaf	+	Superscaf	5823790	5823813	36	36	43	-	-	gene	-	-	mRNA	NCP	Intron	gene|mRN	ID=gene-E	8	4.85625	2773.2	0.925740906	LNLPVLLE	85	910.5569	1	909.5535	.	R	Superscaf	5823685	0	0	0	0	0	BTP202209	Tissue specific
KKYKQ	Superscaf	+	Superscaf	5871487	5871501	2	2	6	-	-	gene	-	-	mRNA	NCP	Intron	gene|mRN	ID=gene-E	5	8.106	2475.4	0.925132839	KKYKQ	65	694.411	1	693.4173	.	K	Superscaf	5871484	0	0	0	0	BTP202209	0	Tissue specific
FPPPYMK	Superscaf	+	Superscaf	5879518	5879538	3	3	9	-	-	gene	-	-	mRNA	NCP	Intron	gene|mRN	ID=gene-E	7	6.504285714	2699	0.925030359	FPPPYMK	51	440.2226	2	878.436	.	R	Superscaf	5879512	0	BTP202209	0	0	0	0	Tissue specific
HVGLAQY	Superscaf	-	Superscaf	5907511	5907531	-102	102	108	-	-	-	-	-	-	NCP	IGR	-	ID=gene-E	7	5.267142857	2651.9	0.924673422	HVGLAQY	69	394.2079	2	786.4024	.	H	Superscaf	5907984	BTP202209	0	0	0	0	0	Tissue specific
EVHRAYF	Superscaf	-	Superscaf	5907655	5907675	-150	150	156	-	-	-	-	-	-	NCP	IGR	-	ID=gene-E	7	5.532857143	2852.2	0.924671586	EVHRAYF	85	461.2311	2	920.4504	.	H	Superscaf	5907984	0	BTP202209	0	0	0	0	Tissue specific
DLRLQDVT	Superscaf	-	Superscaf	5912341	5912370	-152	152	161	-	-	-	-	-	-	NCP	IGR	-	ID=gene-E	10	4.682	3540.2	0.92461172	DLRLQDVT	60	586.3373	2	1170.661	.	A	Superscaf	5912571	0	0	0	0	0	BTP202209	Tissue specific
RLYTEPP	Superscaf	-	Superscaf	5925607	5925627	-8	8	14	-	-	-	-	-	-	NCP	IGR	-	ID=gene-E	7	6.392857143	2959.5	0.924442681	RLYTEPP	56	438.2269	2	874.4548	.	R	Superscaf	5925777	0	0	0	0	0	BTP202209	Tissue specific
LLDNSRNV	Superscaf	-	Superscaf	5973706	5973729	-30	30	37	-	-	-	-	-	-	NCP	IGR	-	ID=gene-E	8	5.27375	3343.9	0.923829335	LLDNSRNV	80	465.7523	2	929.493	.	I	Superscaf	5974329	0	0	BTP202209	0	0	0	Tissue specific
PGKSSR	Superscaf	+	Superscaf	6053977	6053994	11	11	16	-	-	-	-	-	-	NCP	IGR	-	ID=gene-E	6	7.355	2518.7	0.922805881	PGKSSR	51	631.3527	1	630.3449	.	P	Superscaf	6053947	0	BTP202209	0	BTP202209	0	0	Tissue specific
FDELKKVKK	Superscaf	-	Superscaf	6130228	6130254	-178	178	186	-	-	-	-	-	-	NCP	IGR	-	ID=gene-E	9	6.29	3451.4	0.921833494	FDELKKVKK	96	567.8453	2	1133.681	.	Q	Superscaf	6130314	0	BTP202209	BTP202209	BTP202209	0	0	Tissue specific
QMKKKL	Superscaf	-	Superscaf	6160522	6160539	-14	14	19	-	-	gene	-	-	mRNA	NCP	Intron	gene|mRN	ID=gene-E	6	7.766666667	2740.8	0.921447332	QMKKKL	50	388.2521	2	774.4786	.	H	Superscaf	6160614	0	BTP202209	0	0	0	0	Tissue specific
QQKHTK	Superscaf	-	Superscaf	6232327	6232344	-3	3	8	-	-	-	-	-	-	NCP	IGR	-	ID=gene-E	6	7.483333333	3175.4	0.920531751	QQKHTK	53	385.2194	2	768.4243	.	K	Superscaf	6232350	0	BTP202209	0	0	0	0	Tissue specific
SGTLFDN	Superscaf	+	Superscaf	6339715	6339735	3	3	9	-	-	gene	-	-	mRNA	NCP	Intron	gene|mRN	ID=gene-E	7	5.431428571	2827.2	0.919162415	SGTLFDN	82	753.3385	1	752.334	.	P	Superscaf	6339709	0	BTP202209	0	0	0	0	Tissue specific
FVPGDNK	Superscaf	-	Superscaf	6406651	6406671	-1	1	7	-	-	-	-	-	-	NCP	IGR	-	ID=gene-E	7	5.124285714	2498.7	0.918308918	FVPGDNK	82	388.6989	2	775.3864	.	F	Superscaf	6406782	0	BTP202209	0	0	0	0	Mixed
KGGGTAPK	Superscaf	+	Superscaf	6478084	6478107	7	7	14	-	-	gene	-	-	mRNA	NCP	Intron	gene|mRN	ID=gene-E	8	7.03	2874.9	0.917398042	KGGGTAPK	56	358.2075	2	714.4024	.	G	Superscaf	6478066	0	BTP202209	0	0	0	0	Tissue specific
LPPPPLPPK	Superscaf	-	Superscaf	6486406	6486435	-27	27	36	-	-	-	-	-	-	NCP	IGR	-	ID=gene-E	10	6.516	3659.1	0.917291852	LPPPPLPPK	71	559.8331	2	1117.654	.	A	Superscaf	6486447	0	0	0	BTP202209	0	0	Tissue specific
VAAPRSAPF	Superscaf	-	Superscaf	6638257	6638283	-138	138	146	-	-	-	-	-	-	NCP	IGR	-	ID=gene-E	9	5.933333333	2978.3	0.915355647	VAAPRSAPF	97	433.2462	2	864.4817	.	S	Superscaf	6638676	0	0	0	BTP202209	0	0	Tissue specific
TTKHGPP	Superscaf	-	Superscaf	6786148	6786168	-37	37	43	-	-	gene	-	-	mRNA	NCP	Intron	gene|mRN	ID=gene-E	7	6.994285714	2855.2	0.913469974	T(+42.01)	58	390.205	2	778.3973	Acetylati	K	Superscaf	6786180	0	0	0	0	BTP202209	0	Tissue specific
VPSLKK	Superscaf	-	Superscaf	6856903	6856920	-25	25	30	-	-	-	-	-	-	NCP	IGR	-	ID=gene-E	6	6.24	2011	0.912567819	VPSLKK	68	671.4427	1	670.4377	.	Y	Superscaf	6856953	0	0	0	BTP202209	0	0	Tissue specific
ATQVEPL	Superscaf	-	Superscaf	6881563	6881583	-6	6	12	-	-	-	-	-	-	NCP	IGR	-	ID=gene-E	7	4.814285714	2435.8	0.912253343	A(+42.01)	57	799.4166	1	798.4123	Acetylati	A	Superscaf	6881619	0	BTP202209	0	0	0	0	Tissue specific
KESGQ	Superscaf	+	Superscaf	6951826	6951840	23	23	27	-	-	-	-	-	-	NCP	IGR	-	ID=gene-E	5	6.052	2185.3	0.9113575	KESGQ	71	548.271	1	547.2602	.	K	Superscaf	6951760	0	0	0	BTP202209	0	0	Tissue specific
VPGLVLR	Superscaf	-	Superscaf	7023364	7023384	-27	27	33	-	-	-	-	-	-	NCP	IGR	-	ID=gene-E	7	4.998571429	1990.3	0.910445247	VPGLVLR	69	377.251	2	752.4908	.	Q	Superscaf	7023390	0	0	0	0	0	BTP202209	Tissue specific
CNDDCP	Superscaf	+	Superscaf	7075015	7075032	31	31	36	-	-	-	-	-	-	NCP	IGR	-	ID=gene-E	6	4.615	2928.5	0.909786686	C(+57.02)	58	390.6111	2	779.2214	Carbamido	R	Superscaf	7074925	0	0	0	0	BTP202209	0	Tissue specific
VPAAL	Superscaf	+	Superscaf	7186975	7186989	37	37	41	-	-	-	-	-	-	NCP	IGR	-	ID=gene-E	5	4.864	1313.4	0.908359129	V(+42.01)	55	512.3043	1	511.3006	Acetylati	K	Superscaf	7186867	0	0	0	0	BTP202209	0	Tissue specific
YNLQNYE	Superscaf	+	Superscaf	7192357	7192377	6	6	12	-	-	-	-	-	-	NCP	IGR	-	ID=gene-E	7	5.284285714	3502	0.908290427	YNLQNYE	50	472.2178	2	942.4083	.	T	Superscaf	7192342	0	0	0	0	0	BTP202209	Tissue specific
GDSSSC	Superscaf	-	Superscaf	7208515	7208532	-15	15	20	-	-	-	-	-	-	NCP	IGR	-	ID=gene-E	6	5.166666667	2444.7	0.908084436	GDSSSC(+5	56	612.1827	1	611.1857	Carbamido	R	Superscaf	7208574	0	0	BTP202209	0	0	0	Tissue specific
ATAPKF	Superscaf	-	Superscaf	7356667	7356684	-8	8	13	-	-	-	-	-	-	NCP	IGR	-	ID=gene-E	6	6.681666667	2213.3	0.906195358	ATAPKF	70	634.3556	1	633.3486	.	F	Superscaf	7356819	0	0	0	0	0	BTP202209	Tissue specific
QWGKEGP	Superscaf	-	Superscaf	7394473	7394493	-22	22	28	-	-	-	-	-	-	NCP	IGR	-	ID=gene-E	7	6.105714286	2963.2	0.905713258	QWGKEGP	57	401.1975	2	800.3817	.	S	Superscaf	7394502	0	0	0	0	0	BTP202209	Tissue specific
FWGLP	Superscaf	+	Superscaf	7442488	7442502	14	14	18	-	-	-	-	-	-	NCP	IGR	-	ID=gene-E	5	5.924	1902.1	0.905101098	FWGLP	58	619.3307	1	618.3166	.	G	Superscaf	7442449	0	BTP202209	0	0	0	0	Tissue specific
VSYVY	Superscaf	+	Superscaf	7476319	7476333	4	4	8	-	-	-	-	-	-	NCP	IGR	-	ID=gene-E	5	3.4	1302.4	0.904669721	VSYVY	87	630.3121	1	629.3061	.	L	Superscaf	7476310	0	0	0	BTP202209	0	0	Mixed
VPFPFT	Superscaf	-	Superscaf	7653118	7653135	-7	7	12	-	-	-	-	-	-	NCP	IGR	-	ID=gene-E	6	5.015	1847.1	0.902415329	VPFPFT	77	707.3763	1	706.369	.	L	Superscaf	7653228	0	0	0	0	0	BTP202209	Tissue specific
AKKTYDLLF	Superscaf	-	Superscaf	7718137	7718166	-20	20	29	-	-	-	-	-	-	NCP	IGR	-	ID=gene-E	10	6.784	4079.2	0.901586123	A(+42.01)	98	423.5775	3	1267.718	Acetylati	E	Superscaf	7718733	0	0	BTP202209	0	0	0	Tissue specific
ANLTLF	Superscaf	+	Superscaf	7765402	7765419	9	9	14	-	-	gene	-	-	mRNA	NCP	Intron	gene|mRN	ID=gene-E	6	5.9	2399.7	0.900983602	ANLTLF	57	678.3807	1	677.3748	.	K	Superscaf	7765378	0	0	BTP202209	0	0	0	Tissue specific
DSHGP	Superscaf	-	Superscaf	7770412	7770426	-33	33	37	-	-	gene	-	-	mRNA	NCP	Intron	gene|mRN	ID=gene-E	5	5.702	2049.3	0.900919759	DSHGP	56	512.2089	1	511.2027	.	T	Superscaf	7770528	0	0	BTP202209	0	0	0	Tissue specific
PWTKYQ	Superscaf	-	Superscaf	7826401	7826418	-5	5	10	-	-	gene	-	-	mRNA	NCP	Intron	gene|mRN	ID=gene-E	6	6.628333333	2800.1	0.900205808	PWTKYQ	61	411.7094	2	821.4072	.	L	Superscaf	7826490	0	0	0	BTP202209	0	0	Tissue specific
VAFVEL	Superscaf	-	Superscaf	7848523	7848540	-3	3	8	-	-	gene	-	-	mRNA	NCP	Intron	gene|mRN	ID=gene-E	6	3.45	1477.6	0.899923732	V(+42.01)	60	360.2006	2	718.3901	Acetylati	L	Superscaf	7848573	0	0	0	BTP202209	0	0	Tissue specific
ALPLVL	Superscaf	-	Superscaf	7853182	7853199	-1	1	6	-	-	gene	-	-	mRNA	NCP	Intron	gene|mRN	ID=gene-E	6	5.043333333	1708.9	0.899864325	A(+42.01)	85	667.437	1	666.4316	Acetylati	A	Superscaf	7853526	0	0	0	BTP202209	0	0	Tissue specific
KPFNQNM	Superscaf	+	Superscaf	7864243	7864263	1	1	7	-	-	gene	-	-	mRNA	NCP	Intron	gene|mRN	ID=gene-E	7	6.248571429	3368.8	0.899723248	KPFNQNM	50	439.7148	2	877.4116	.	K	Superscaf	7864243	0	0	0	0	0	BTP202209	Tissue specific
TASVGPLN	Superscaf	-	Superscaf	7873378	7873401	-16	16	23	-	-	gene	-	-	mRNA	NCP	Intron	gene|mRN	ID=gene-E	8	5.23625	2677	0.89960673	T(+42.01)	80	800.4133	1	799.4076	Acetylati	R	Superscaf	7873464	0	BTP202209	0	0	0	0	Tissue specific
MDPSGVK	Superscaf	+	Superscaf	7899166	7899186	19	19	25	-	-	-	-	-	-	NCP	IGR	-	ID=gene-E	7	5.201428571	2269.5	0.899277947	M(+42.01)	99	775.3631	1	774.3582	Acetylati	S	Superscaf	7899112	0	0	0	0	0	BTP202209	Tissue specific
YGDGFF	Superscaf	+	Superscaf	8003035	8003052	2	2	7	-	-	-	-	-	-	NCP	IGR	-	ID=gene-E	6	5.255	2240.4	0.897953558	Y(+42.01)	66	747.2961	1	746.2911	Acetylati	R	Superscaf	8003032	0	0	0	BTP202209	BTP202209	0	Tissue specific
EGPPARP	Superscaf	-	Superscaf	8062561	8062581	-20	20	26	-	-	-	-	-	-	NCP	IGR	-	ID=gene-E	7	6.41	2695.1	0.897194507	EGPPARP	88	362.1905	2	722.3711	.	S	Superscaf	8062761	0	BTP202209	0	0	0	0	Tissue specific
PAAGRHVGF	Superscaf	-	Superscaf	8063419	8063445	-1	1	9	-	-	-	-	-	-	NCP	IGR	-	ID=gene-E	9	6.103333333	3160.5	0.897183491	PAAGRHVGF	70	431.2375	2	860.4617	.	P	Superscaf	8063451	0	BTP202209	0	0	0	0	Tissue specific
KKPPPK	Superscaf	-	Superscaf	8152609	8152626	-22	22	27	-	-	-	-	-	-	NCP	IGR	-	ID=gene-E	6	8.02	2417.4	0.896046349	K(+42.01)	56	368.7373	2	735.4643	Acetylati	S	Superscaf	8152638	0	0	BTP202209	0	0	0	Tissue specific
TVFQA	Superscaf	-	Superscaf	8161924	8161938	-33	33	37	-	-	-	-	-	-	NCP	IGR	-	ID=gene-E	5	4.736	1706.9	0.895927612	T(+42.01)	74	607.3062	1	606.3013	Acetylati	R	Superscaf	8161965	0	0	BTP202209	0	0	0	Tissue specific
KTEPDK	Superscaf	-	Superscaf	8187346	8187363	-16	16	21	-	-	-	-	-	-	NCP	IGR	-	ID=gene-E	6	6.416666667	2508.7	0.89560342	KTEPDK	68	359.1924	2	716.3704	.	T	Superscaf	8187384	0	0	0	0	BTP202209	BTP202209	Tissue specific
LEQAFKR	Superscaf	+	Superscaf	8215966	8215986	20	20	26	-	-	-	-	-	-	NCP	IGR	-	ID=gene-E	7	6.692857143	3205.5	0.895238449	L(+42.01)	98	467.2545	2	932.5079	Acetylati	S	Superscaf	8215909	BTP202209	0	0	0	0	0	Tissue specific
EKLFQPK	Superscaf	-	Superscaf	8368915	8368935	-2	2	8	-	-	-	-	-	-	NCP	IGR	-	ID=gene-E	7	6.587142857	3051.2	0.893288206	E(+42.01)	54	466.2685	2	930.5175	Acetylati	G	Superscaf	8369004	0	0	BTP202209	0	0	0	Tissue specific
AEENK	Superscaf	-	Superscaf	8428711	8428725	-14	14	18	-	-	-	-	-	-	NCP	IGR	-	ID=gene-E	5	5.522	2227.4	0.892525827	A(+42.01)	85	632.2919	1	631.2813	Acetylati	V	Superscaf	8428752	0	0	BTP202209	0	0	0	Tissue specific
AARTLMPK	Superscaf	-	Superscaf	8459914	8459937	-10	10	17	-	-	-	-	-	-	NCP	IGR	-	ID=gene-E	8	7.1375	3199.6	0.892127845	AARTLM(+1	67	452.254	2	902.5007	Oxidation	T	Superscaf	8460153	0	0	BTP202209	0	0	0	Tissue specific
MHELPVQ	Superscaf	+	Superscaf	8485873	8485893	58	58	64	-	-	-	-	-	-	NCP	IGR	-	ID=gene-E	7	4.927142857	2718.2	0.891796881	M(+15.99)	77	456.2104	2	910.4218	Oxidation	L	Superscaf	8485702	0	0	BTP202209	0	0	0	Tissue specific
HPAACH	Superscaf	-	Superscaf	8494144	8494161	-6	6	11	-	-	-	-	-	-	NCP	IGR	-	ID=gene-E	6	6.423333333	2785.1	0.891691456	H(+42.01)	53	734.313	1	733.2966	Acetylati	C	Superscaf	8494164	0	0	BTP202209	0	0	0	Tissue specific
LEESHE	Superscaf	+	Superscaf	8511898	8511915	5	5	10	-	-	-	-	-	-	NCP	IGR	-	ID=gene-E	6	4.818333333	2633	0.891465076	LEESHE	55	372.1666	2	742.3133	.	V	Superscaf	8511886	0	0	0	0	0	BTP202209	Tissue specific
PSGKK	Superscaf	+	Superscaf	8592079	8592093	5	5	9	-	-	-	-	-	-	NCP	IGR	-	ID=gene-E	5	7.486	1954.9	0.890442731	PSGKK	85	516.3121	1	515.3067	.	R	Superscaf	8592067	0	0	0	0	0	BTP202209	Tissue specific
CSRPCL	Superscaf	+	Superscaf	8657680	8657697	22	22	27	-	-	-	-	-	-	NCP	IGR	-	ID=gene-E	6	6.46	2894.4	0.889606219	C(+57.02)	99	396.6766	2	791.3418	Carbamido	V	Superscaf	8657617	0	BTP202209	0	0	0	0	Tissue specific
GKPPL	Superscaf	-	Superscaf	8673562	8673576	-31	31	35	-	-	-	-	-	-	NCP	IGR	-	ID=gene-E	5	6.858	1793.9	0.889403746	G(+42.01)	78	553.3342	1	552.3271	Acetylati	P	Superscaf	8673624	BTP202209	0	0	0	0	0	Tissue specific
FEKPAK	Superscaf	+	Superscaf	8684914	8684931	1	1	6	-	-	-	-	-	-	NCP	IGR	-	ID=gene-E	6	6.75	2398.4	0.889258959	FEKPAK	97	360.2067	2	718.4013	.	F	Superscaf	8684914	0	0	0	0	0	BTP202209	Tissue specific
KQGALTF	Superscaf	+	Superscaf	8794894	8794914	4	4	10	-	-	-	-	-	-	NCP	IGR	-	ID=gene-E	7	6.481428571	2826	0.887856573	KQGALTF	56	382.7174	2	763.4228	.	K	Superscaf	8794885	0	BTP202209	0	0	0	0	Tissue specific
KGLALQ	Superscaf	+	Superscaf	8796280	8796297	9	9	14	-	-	-	-	-	-	NCP	IGR	-	ID=gene-E	6	6.556666667	2394.5	0.887838938	KGLALQ	91	629.3969	1	628.3908	.	V	Superscaf	8796256	0	0	0	BTP202209	0	0	Tissue specific
QKNSK	Superscaf	-	Superscaf	8870845	8870859	-30	30	34	-	-	-	-	-	-	NCP	IGR	-	ID=gene-E	5	7.244	2527.6	0.886888203	QKNSK	55	604.3335	1	603.334	.	S	Superscaf	8870868	0	0	0	0	0	BTP202209	Tissue specific
LDKEDQQK	Superscaf	-	Superscaf	8940742	8940765	-57	57	64	-	-	-	-	-	-	NCP	IGR	-	ID=gene-E	8	5.74	3760.1	0.885996835	LDKEDQQK	84	502.256	2	1002.498	.	A	Superscaf	8940819	0	0	0	BTP202209	0	0	Tissue specific
KGEVL	Superscaf	+	Superscaf	9129331	9129345	23	23	27	-	-	-	-	-	-	NCP	IGR	-	ID=gene-E	5	4.982	1544.6	0.883592263	K(+42.01)	55	587.3367	1	586.3326	Acetylati	F	Superscaf	9129265	0	0	BTP202209	0	0	0	Tissue specific
VPKSH	Superscaf	-	Superscaf	9240931	9240945	-78	78	82	-	-	gene	-	-	mRNA	NCP	Intron	gene|mRN	ID=gene-E	5	5.862	1808.9	0.882169258	VPKSH	53	567.3243	1	566.3176	.	G	Superscaf	9241005	0	0	BTP202209	0	0	0	Tissue specific
TDDLK	Superscaf	-	Superscaf	9312070	9312084	-5	5	9	-	-	-	-	-	-	NCP	IGR	-	ID=gene-E	5	5.638	2042.3	0.881262169	T(+42.01)	62	633.2982	1	632.3017	Acetylati	E	Superscaf	9312126	0	0	0	0	0	BTP202209	Tissue specific
VTDAKQP	Superscaf	-	Superscaf	9314923	9314943	-1	1	7	-	-	-	-	-	-	NCP	IGR	-	ID=gene-E	7	5.315714286	2536.8	0.881225714	VTDAKQP	59	379.7128	2	757.397	.	V	Superscaf	9315069	0	0	0	BTP202209	0	0	Tissue specific
RAELLR	Superscaf	-	Superscaf	9336364	9336381	-4	4	9	-	-	-	-	-	-	NCP	IGR	-	ID=gene-E	6	7.12	2741.2	0.880952359	RAELLR	80	379.236	2	756.4606	.	Q	Superscaf	9336552	0	0	0	BTP202209	0	0	Tissue specific
LQGAFPVG	Superscaf	-	Superscaf	9357967	9357990	-95	95	102	-	-	-	-	-	-	NCP	IGR	-	ID=gene-E	8	5.17125	2594.8	0.880676824	LQGAFPVG	71	788.4297	1	787.4228	.	H	Superscaf	9358032	0	0	0	BTP202209	0	0	Tissue specific
SVLPEC	Superscaf	-	Superscaf	9391174	9391191	-7	7	12	-	-	-	-	-	-	NCP	IGR	-	ID=gene-E	6	4.366666667	2085.4	0.88025348	SVLPEC(+5	55	704.3408	1	703.321	Carbamido	N	Superscaf	9391359	0	0	BTP202209	0	0	0	Tissue specific
KRPKVPP	Superscaf	-	Superscaf	9430723	9430743	-5	5	11	-	-	-	-	-	-	NCP	IGR	-	ID=gene-E	7	7.02	2597.8	0.879749155	KRPKVPP	53	411.2686	2	820.5283	.	D	Superscaf	9430830	0	BTP202209	0	0	0	0	Tissue specific
AAKVTPA	Superscaf	-	Superscaf	9456127	9456147	-17	17	23	-	-	-	-	-	-	NCP	IGR	-	ID=gene-E	7	5.804285714	2137.2	0.87942523	AAKVTPA	58	657.4031	1	656.3857	.	P	Superscaf	9456327	BTP202209	0	0	BTP202209	0	0	Tissue specific
DDDGEY	Superscaf	-	Superscaf	9527434	9527451	-36	36	41	-	-	gene	-	-	mRNA	NCP	Intron	gene|mRN	ID=gene-E	6	3.96	2416.8	0.878516037	D(+42.01)	53	755.2332	1	754.2293	Acetylati	C	Superscaf	9527451	0	0	BTP202209	0	0	0	Tissue specific
RKLLKTEL	Superscaf	-	Superscaf	9545299	9545322	-6	6	13	-	-	gene	-	-	mRNA	NCP	Intron	gene|mRN	ID=gene-E	8	7.24125	3468.8	0.878288165	RKLLKTEL	89	500.8214	2	999.644	.	E	Superscaf	9545364	BTP202209	0	0	0	0	0	Tissue specific
KLLKTEL	Superscaf	-	Superscaf	9545302	9545322	-7	7	13	-	-	gene	-	-	mRNA	NCP	Intron	gene|mRN	ID=gene-E	7	6.738571429	2820	0.878288165	KLLKTEL	98	422.7774	2	843.5429	.	E	Superscaf	9545364	0	0	0	0	0	BTP202209	Tissue specific
SDFRYS	Superscaf	+	Superscaf	9559555	9559572	4	4	9	-	-	gene	-	-	mRNA	NCP	Intron	gene|mRN	ID=gene-E	6	6.038333333	2674	0.878106464	SDFRYS	64	774.3443	1	773.3344	.	L	Superscaf	9559546	0	0	0	0	0	BTP202209	Tissue specific
NLVEPPR	Superscaf	+	Superscaf	9594700	9594720	27	27	33	-	-	gene	-	-	mRNA	NCP	Intron	gene|mRN	ID=gene-E	7	5.424285714	2699.2	0.877658294	NLVEPPR	85	412.7401	2	823.4551	.	F	Superscaf	9594622	0	0	0	0	0	BTP202209	Tissue specific
ADVFAP	Superscaf	-	Superscaf	9602446	9602463	-31	31	36	-	-	gene	-	-	mRNA	NCP	Intron	gene|mRN	ID=gene-E	6	4.465	1758.9	0.877559563	A(+42.01)	52	661.3185	1	660.3118	Acetylati	A	Superscaf	9602493	0	0	0	BTP202209	0	0	Tissue specific
TVSLL	Superscaf	-	Superscaf	9691072	9691086	-1	1	5	-	-	gene	-	-	mRNA	NCP	Intron	gene|mRN	ID=gene-E	5	4.834	1487.7	0.876429537	TVSLL	82	532.3325	1	531.3268	.	T	Superscaf	9691113	0	0	0	BTP202209	0	0	Tissue specific
YPPMKLP	Superscaf	+	Superscaf	9732190	9732210	21	21	27	-	-	gene	-	-	mRNA	NCP	Intron	gene|mRN	ID=gene-E	7	6.575714286	2665	0.875905167	YPPM(+15.	77	431.2333	2	860.4466	Oxidation	L	Superscaf	9732130	BTP202209	0	0	0	0	0	Tissue specific
KPACMLR	Superscaf	+	Superscaf	9766306	9766326	11	11	17	-	-	gene	-	-	mRNA	NCP	Intron	gene|mRN	ID=gene-E	7	7.081428571	3076.5	0.875470156	KPAC(+57.	57	446.222	2	890.4466	Carbamido	T	Superscaf	9766276	0	0	BTP202209	0	0	0	Tissue specific
FPSSPP	Superscaf	-	Superscaf	9776587	9776604	-20	20	25	-	-	gene	-	-	mRNA	NCP	Intron	gene|mRN	ID=gene-E	6	5.956666667	2166.4	0.875339102	FPSSPP	76	631.3069	1	630.3013	.	Q	Superscaf	9776631	0	0	0	0	0	BTP202209	Tissue specific
DGTQG	Superscaf	-	Superscaf	9873592	9873606	-6	6	10	-	-	-	-	-	-	NCP	IGR	-	ID=gene-E	5	5.418	2014.3	0.874102235	DGTQG	60	477.2006	1	476.1867	.	E	Superscaf	9873702	0	BTP202209	0	0	0	0	Tissue specific
KKPPTL	Superscaf	-	Superscaf	9880849	9880866	-11	11	16	-	-	-	-	-	-	NCP	IGR	-	ID=gene-E	6	7.431666667	2362.5	0.874009663	K(+42.01)	93	363.2294	2	724.4483	Acetylati	L	Superscaf	9880911	0	BTP202209	0	0	0	BTP202209	Tissue specific
ATLGVVLP	Superscaf	+	Superscaf	10004782	10004805	59	59	66	-	-	-	-	-	-	NCP	IGR	-	ID=gene-E	8	4.5975	2050.3	0.872429324	A(+42.01)	81	406.2541	2	810.4851	Acetylati	L	Superscaf	10004608	0	0	0	0	0	BTP202209	Tissue specific
FRGWR	Superscaf	+	Superscaf	10007035	10007049	23	23	27	-	-	-	-	-	-	NCP	IGR	-	ID=gene-E	5	7.772	2508.9	0.872400711	FRGWR	54	361.2036	2	720.382	.	S	Superscaf	10006969	0	0	0	0	0	BTP202209	Tissue specific
RAPKSSLA	Superscaf	-	Superscaf	10095205	10095228	-8	8	15	-	-	gene	-	-	mRNA	NCP	Intron	gene|mRN	ID=gene-E	8	7.0225	3197.4	0.871276346	R(+42.01)	56	436.2581	2	870.4922	Acetylati	V	Superscaf	10095237	0	0	0	0	0	BTP202209	Tissue specific
KPSQLAK	Superscaf	+	Superscaf	10165159	10165179	4	4	10	-	-	-	-	-	-	NCP	IGR	-	ID=gene-E	7	7.015714286	2933	0.870384405	KPSQLAK	54	771.4578	1	770.465	.	Q	Superscaf	10165150	0	0	0	BTP202209	0	0	Tissue specific
LDLECFGL	Superscaf	+	Superscaf	10296094	10296117	23	23	30	-	-	-	-	-	-	NCP	IGR	-	ID=gene-E	8	5.075	3167.6	0.868714822	LDLEC(+57	58	483.7334	2	965.4528	Carbamido	R	Superscaf	10296028	0	0	BTP202209	0	0	0	Tissue specific
TNKVP	Superscaf	+	Superscaf	10382617	10382631	9	9	13	-	-	-	-	-	-	NCP	IGR	-	ID=gene-E	5	5.596	1800	0.867611687	T(+42.01)	76	600.3326	1	599.3279	Acetylati	I	Superscaf	10382593	0	0	BTP202209	BTP202209	0	0	Tissue specific
ESFVVEVK	Superscaf	+	Superscaf	10464610	10464633	36	36	43	-	-	-	-	-	-	NCP	IGR	-	ID=gene-E	8	3.4175	2090.2	0.866566085	ESFVVEVK	82	468.756	2	935.4963	.	M	Superscaf	10464505	0	0	BTP202209	BTP202209	0	0	Tissue specific
ALPPP	Superscaf	-	Superscaf	10526617	10526631	-59	59	63	-	-	-	-	-	-	NCP	IGR	-	ID=gene-E	5	6.18	1676.9	0.865775552	A(+42.01)	78	536.3054	1	535.3006	Acetylati	V	Superscaf	10526733	0	0	BTP202209	0	0	0	Tissue specific
KVFYPP	Superscaf	-	Superscaf	10660501	10660518	-6	6	11	-	-	-	-	-	-	NCP	IGR	-	ID=gene-E	6	5.58	1990.1	0.864068367	KVFYPP	61	375.7097	2	749.4112	.	R	Superscaf	10660641	0	BTP202209	0	0	0	0	Mixed
DVSER	Superscaf	-	Superscaf	10778110	10778124	-8	8	12	-	-	-	-	-	-	NCP	IGR	-	ID=gene-E	5	4.526	1869.2	0.86256878	D(+42.01)	60	647.3029	1	646.2922	Acetylati	V	Superscaf	10778241	0	BTP202209	0	0	0	0	Tissue specific
CCCEASP	Superscaf	-	Superscaf	10779316	10779336	-7	7	13	-	-	-	-	-	-	NCP	IGR	-	ID=gene-E	7	5.182857143	3214.7	0.862553325	C(+57.02)	52	442.1385	2	882.267	Carbamido	V	Superscaf	10779354	0	0	0	0	BTP202209	0	Tissue specific
NKGDFPP	Superscaf	+	Superscaf	10787743	10787763	17	17	23	-	-	-	-	-	-	NCP	IGR	-	ID=gene-E	7	6.024285714	2836.1	0.862445873	NKGDFPP	63	774.3683	1	773.3708	.	I	Superscaf	10787695	0	0	0	BTP202209	0	0	Tissue specific
PSFLLK	Superscaf	+	Superscaf	10855045	10855062	24	24	29	-	-	gene	-	-	mRNA	NCP	Intron	gene|mRN	ID=gene-E	6	6.526666667	2283.4	0.861587748	P(+42.01)	54	373.7227	2	745.4374	Acetylati	K	Superscaf	10854976	0	0	0	0	BTP202209	0	Tissue specific
VGNLPF	Superscaf	-	Superscaf	10928041	10928058	-3	3	8	-	-	-	-	-	-	NCP	IGR	-	ID=gene-E	6	4.856666667	1972.2	0.86065698	VGNLPF	76	646.3533	1	645.3486	.	M	Superscaf	10928076	0	0	0	0	0	BTP202209	Tissue specific
MDDDPP	Superscaf	-	Superscaf	10942753	10942770	-11	11	16	-	-	-	-	-	-	NCP	IGR	-	ID=gene-E	6	4.543333333	2280.8	0.860469388	M(+15.99)	53	705.2297	1	704.2323	Oxidation	G	Superscaf	10942797	0	BTP202209	0	0	0	0	Tissue enhanced
VHLMEPK	Superscaf	+	Superscaf	11036059	11036079	4	4	10	-	-	-	-	-	-	NCP	IGR	-	ID=gene-E	7	5.511428571	2575.9	0.859279611	VHLM(+15.	88	435.2281	2	868.4477	Oxidation	D	Superscaf	11036050	0	0	0	BTP202209	0	0	Tissue specific
SKPAAP	Superscaf	-	Superscaf	11133292	11133309	-23	23	28	-	-	-	-	-	-	NCP	IGR	-	ID=gene-E	6	6.676666667	2149.2	0.858039837	SKPAAP	53	570.3339	1	569.3173	.	D	Superscaf	11133330	0	0	0	0	0	BTP202209	Tissue specific
PLGVLAP	Superscaf	-	Superscaf	11245507	11245527	-6	6	12	-	-	-	-	-	-	NCP	IGR	-	ID=gene-E	7	5.221428571	1990.2	0.856608952	PLGVLAP	67	666.4248	1	665.4112	.	G	Superscaf	11245692	0	BTP202209	0	0	0	0	Tissue specific
KGPDYSK	Superscaf	+	Superscaf	11299198	11299218	3	3	9	-	-	-	-	-	-	NCP	IGR	-	ID=gene-E	7	6.58	2825.9	0.855924341	KGPDYSK	56	397.7005	2	793.397	.	C	Superscaf	11299192	0	0	BTP202209	0	0	0	Tissue specific
LPEKPF	Superscaf	+	Superscaf	11399026	11399043	25	25	30	-	-	-	-	-	-	NCP	IGR	-	ID=gene-E	6	6.17	2309.5	0.854651478	LPEKPF	54	730.4078	1	729.4061	.	I	Superscaf	11398954	0	0	0	0	0	BTP202209	Tissue specific
KEQEVVDKV	Superscaf	-	Superscaf	11484187	11484219	-18	18	28	-	-	-	-	-	-	NCP	IGR	-	ID=gene-E	11	3.955454545	3607	0.853565404	KEQEVVDKV	96	675.3312	2	1348.654	Oxidation	R	Superscaf	11484318	0	BTP202209	0	0	0	BTP202209	Tissue specific
TTACHEFFE	Superscaf	-	Superscaf	11484286	11484318	-51	51	61	-	-	-	-	-	-	NCP	IGR	-	ID=gene-E	11	5.445454545	4982.7	0.853564142	TTAC(+57.	76	704.2833	2	1406.556	Carbamido	R	Superscaf	11484318	BTP202209	0	0	0	0	0	Tissue specific
HEFFEHE	Superscaf	-	Superscaf	11484298	11484318	-55	55	61	-	-	-	-	-	-	NCP	IGR	-	ID=gene-E	7	5.114285714	3290.7	0.853564142	HEFFEHE	94	487.702	2	973.3929	.	R	Superscaf	11484318	0	0	0	0	0	BTP202209	Mixed
WLLLGT	Superscaf	-	Superscaf	11520607	11520624	-71	71	76	-	-	-	-	-	-	NCP	IGR	-	ID=gene-E	6	6.055	2281.6	0.853101206	W(+42.01)	56	744.4297	1	743.4218	Acetylati	G	Superscaf	11520843	0	0	0	0	BTP202209	0	Tissue specific
LEVMVPK	Superscaf	-	Superscaf	11641390	11641410	-35	35	41	-	-	-	-	-	-	NCP	IGR	-	ID=gene-E	7	4.427142857	1956.2	0.851561071	LEVMVPK	78	408.2388	2	814.4622	.	N	Superscaf	11641440	0	0	0	BTP202209	0	0	Tissue specific
TVQHH	Superscaf	-	Superscaf	11709988	11710002	-30	30	34	-	-	-	-	-	-	NCP	IGR	-	ID=gene-E	5	5.472	2247.6	0.850686458	TVQHH	52	621.3214	1	620.303	.	L	Superscaf	11710008	0	BTP202209	0	0	0	0	Tissue specific
SAANPML	Superscaf	-	Superscaf	11721238	11721258	-3	3	9	-	-	-	-	-	-	NCP	IGR	-	ID=gene-E	7	5.88	2665	0.850542934	SAANPML	75	703.3425	1	702.337	.	S	Superscaf	11721288	0	BTP202209	0	0	0	0	Tissue specific
VGLVPLKA	Superscaf	-	Superscaf	11898403	11898426	-41	41	48	-	-	-	-	-	-	NCP	IGR	-	ID=gene-E	8	4.99875	2121.2	0.848283875	VGLVPLKA	72	398.7667	2	795.5218	.	A	Superscaf	11898705	0	BTP202209	0	0	0	0	Tissue specific
GFRHLDSA	Superscaf	-	Superscaf	12090925	12090948	-16	16	23	-	-	-	-	-	-	NCP	IGR	-	ID=gene-E	8	6.30625	3412.8	0.845829038	GFRHLDSA	98	451.726	2	901.4406	.	K	Superscaf	12091137	0	BTP202209	0	0	0	BTP202209	Tissue specific
ALAACPK	Superscaf	-	Superscaf	12169537	12169557	-12	12	18	-	-	-	-	-	-	NCP	IGR	-	ID=gene-E	7	6.442857143	2678.8	0.8448267	ALAAC(+57	66	365.7017	2	729.3843	Carbamido	M	Superscaf	12169626	0	0	0	0	0	BTP202209	Tissue specific

ALTVS	Superscaf	-	Superscaf	12172438	12172452	-58	58	62	-	-	-	-	-	-	NCP	IGR	-	ID=gene-E	5	4.842	1445.6	0.844789786	A(+42.01)	64	532.295	1	531.2904	Acetylati	S	Superscaf	12172569	0	0	0	0	BTP20220	BTP20220	Tissue specific
LPHGPPP	Superscaf	-	Superscaf	12207688	12207708	-40	40	46	-	-	-	-	-	-	NCP	IGR	-	ID=gene-E	7	6.391428571	2620	0.844340239	LPHGPPP	69	357.6981	2	713.386	.	A	Superscaf	12207723	0	0	0	0	0	BTP20220	Tissue specific
SLVRGPR	Superscaf	-	Superscaf	12277297	12277317	-24	24	30	-	-	-	-	-	-	NCP	IGR	-	ID=gene-E	7	6.492857143	2669.1	0.843452658	SLVRGPR	99	392.7419	2	783.4715	.	R	Superscaf	12277323	0	0	0	0	0	BTP20220	Mixed
FSYGPN	Superscaf	-	Superscaf	12544699	12544716	-158	158	163	-	-	-	-	-	-	NCP	IGR	-	ID=gene-E	6	5.75	2461.7	0.84004307	F(+42.01)	81	726.307	1	725.3021	Acetylati	I	Superscaf	12544809	0	BTP20220	0	0	0	BTP20220	Tissue specific
KRLGETR	Superscaf	-	Superscaf	12692320	12692340	-32	32	38	-	-	gene	-	-	mRNA	NCP	Intron	gene|mRN	ID=gene-E	7	7.565714286	3239.7	0.838160725	KRLGETR	53	430.2515	2	858.5035	.	Q	Superscaf	12692412	0	0	0	0	BTP20220	0	Tissue specific
GLLPP	Superscaf	-	Superscaf	12712204	12712218	-8	8	12	-	-	-	-	-	-	NCP	IGR	-	ID=gene-E	5	6.106	1678.9	0.837907262	GLLPP	57	496.3134	1	495.3057	.	I	Superscaf	12712488	0	0	0	0	BTP20220	0	Tissue specific
TVEEELLK	Superscaf	+	Superscaf	12796810	12796833	30	30	37	-	-	-	-	-	-	NCP	IGR	-	ID=gene-E	8	4.73625	2849.2	0.836828341	T(+42.01)	51	501.764	2	1001.528	Acetylati	C	Superscaf	12796723	0	0	0	0	BTP20220	0	Tissue specific
SGGHVL	Superscaf	+	Superscaf	12853858	12853875	1	1	6	-	-	-	-	-	-	NCP	IGR	-	ID=gene-E	6	5.198333333	1951.1	0.836101002	SGGHVL	64	569.3026	1	568.2969	.	S	Superscaf	12853858	0	0	0	0	BTP20220	BTP20220	Tissue specific
AGTALKRLM	Superscaf	-	Superscaf	13090936	13090962	-54	54	62	-	-	-	-	-	-	NCP	IGR	-	ID=gene-E	9	6.972222222	3512.9	0.833077919	A(+42.01)	99	501.7895	2	1001.569	Acetylati	P	Superscaf	13091106	0	0	0	0	BTP20220	0	Tissue specific
AGTALKRL	Superscaf	-	Superscaf	13090936	13090959	-54	54	61	-	-	-	-	-	-	NCP	IGR	-	ID=gene-E	8	7.125	3141.4	0.833077957	A(+42.01)	82	436.2711	2	870.5287	Acetylati	P	Superscaf	13091106	0	BTP20220	0	0	0	0	Tissue specific
AEALTAW	Superscaf	-	Superscaf	13148725	13148745	-66	66	72	-	-	-	-	-	-	NCP	IGR	-	ID=gene-E	7	5.668571429	2636.9	0.832341132	AEALTAW	69	761.3806	1	760.3755	.	K	Superscaf	13148793	0	0	0	0	0	BTP20220	Tissue specific
APLPVQ	Superscaf	-	Superscaf	13182559	13182576	-23	23	28	-	-	-	-	-	-	NCP	IGR	-	ID=gene-E	6	5.041666667	1950.2	0.831909755	A(+42.01)	59	666.3885	1	665.3748	Acetylati	H	Superscaf	13182612	0	0	0	0	BTP20220	0	Tissue specific
SALEFMR	Superscaf	-	Superscaf	13252345	13252365	-3	3	9	-	-	-	-	-	-	NCP	IGR	-	ID=gene-E	7	6.127142857	2881.3	0.831019879	S(+42.01)	59	448.222	2	894.4269	Acetylati	K	Superscaf	13252578	0	0	BTP20220	0	0	0	Tissue specific
ALHVP	Superscaf	-	Superscaf	13312621	13312635	-3	3	7	-	-	-	-	-	-	NCP	IGR	-	ID=gene-E	5	5.178	1621.8	0.83025138	A(+42.01)	56	578.3353	1	577.3224	Acetylati	G	Superscaf	13312665	0	BTP20220	0	0	0	0	Tissue specific
ACMKHH	Superscaf	-	Superscaf	13356808	13356825	-3	3	8	-	-	-	-	-	-	NCP	IGR	-	ID=gene-E	6	6.951666667	2976.3	0.829687916	A(+42.01)	60	421.1823	2	840.3371	Acetylati	R	Superscaf	13356906	0	0	0	0	0	BTP20220	Tissue specific
QPRFNSR	Superscaf	-	Superscaf	13371778	13371798	-8	8	14	-	-	-	-	-	-	NCP	IGR	-	ID=gene-E	7	7.148571429	3613.2	0.829496996	QPRFNSR	77	452.7393	2	903.4675	.	D	Superscaf	13371849	BTP20220	0	0	0	0	0	Tissue specific
PFPVPTLR	Superscaf	-	Superscaf	13410331	13410354	-19	19	26	-	-	-	-	-	-	NCP	IGR	-	ID=gene-E	8	5.95625	2799.3	0.829005371	P(+42.01)	81	484.7837	2	967.5491	Acetylati	P	Superscaf	13410399	0	0	0	0	0	BTP20220	Tissue specific
VSPVLGPP	Superscaf	-	Superscaf	13441525	13441548	-8	8	15	-	-	-	-	-	-	NCP	IGR	-	ID=gene-E	8	4.56625	2046.3	0.828607618	VSPVLGPP	69	383.2278	2	764.4432	.	S	Superscaf	13441608	0	0	0	0	0	BTP20220	Tissue specific
KKQWVR	Superscaf	-	Superscaf	13469560	13469577	-27	27	32	-	-	-	-	-	-	NCP	IGR	-	ID=gene-E	6	6.963333333	2722.9	0.828250222	KKQWVR	50	422.7643	2	843.5079	.	I	Superscaf	13469586	0	0	0	BTP20220	0	0	Tissue specific
LPVCL	Superscaf	-	Superscaf	13522924	13522938	-19	19	23	-	-	-	-	-	-	NCP	IGR	-	ID=gene-E	5	4.656	1629.9	0.827569819	LPVC(+57.	98	601.3353	1	600.3305	Carbamido	S	Superscaf	13523079	0	BTP20220	0	0	BTP20220	BTP20220	Tissue specific
QNLEP	Superscaf	-	Superscaf	13551724	13551738	-6	6	10	-	-	-	-	-	-	NCP	IGR	-	ID=gene-E	5	5.312	2323.7	0.827202592	QNLEP	68	600.2977	1	599.2914	.	K	Superscaf	13551897	0	0	0	BTP20220	0	0	Tissue specific
DKFSM	Superscaf	-	Superscaf	13552396	13552410	-18	18	22	-	-	-	-	-	-	NCP	IGR	-	ID=gene-E	5	5.924	2022.2	0.827194023	D(+42.01)	77	669.2899	1	668.2839	Acetylati	N	Superscaf	13552455	0	0	0	0	BTP20220	0	Tissue specific
KGVRY	Superscaf	+	Superscaf	13564336	13564350	25	25	29	-	-	-	-	-	-	NCP	IGR	-	ID=gene-E	5	6.426	1874	0.827041777	KGVRY	58	622.3625	1	621.3598	.	P	Superscaf	13564264	0	0	BTP20220	0	0	0	Tissue specific
KKFPVFKAI	Superscaf	-	Superscaf	13899493	13899525	-22	22	32	-	-	-	-	-	-	NCP	IGR	-	ID=gene-E	11	6.107272727	3995	0.82276798	KKFPVFKAI	81	451.9339	3	1352.786	.	A	Superscaf	13899687	0	0	0	0	0	BTP20220	Tissue specific
ALEFKSQLV	Superscaf	-	Superscaf	13899514	13899549	-29	29	40	-	-	-	-	-	-	NCP	IGR	-	ID=gene-E	12	5.79	4370.5	0.822767674	ALEFKSQLV	99	645.8727	2	1289.734	.	A	Superscaf	13899687	0	BTP20220	0	0	0	BTP20220	Tissue specific
VDEDDFVH	Superscaf	-	Superscaf	13899994	13900017	-29	29	36	-	-	-	-	-	-	NCP	IGR	-	ID=gene-E	8	3.15	2667.1	0.822761707	VDEDDFVH	99	488.2045	2	974.3981	.	P	Superscaf	13900113	0	BTP20220	0	0	0	0	Tissue specific
FESLPHEN	Superscaf	-	Superscaf	13900027	13900050	-40	40	47	-	-	-	-	-	-	NCP	IGR	-	ID=gene-E	8	5.36	3529	0.822761286	FESLPHEN	87	486.7232	2	971.4348	.	P	Superscaf	13900113	0	0	0	0	BTP20220	0	Tissue specific
KPVALTSYG	Superscaf	-	Superscaf	13900051	13900110	-48	48	67	-	-	-	-	-	-	NCP	IGR	-	ID=gene-E	20	6.096	8397.4	0.822760521	KPVALTSYG	93	774.3975	3	2320.181	.	P	Superscaf	13900113	0	BTP20220	0	0	0	0	Tissue specific
TRCLVH	Superscaf	-	Superscaf	13902502	13902519	-27	27	32	-	-	-	-	-	-	NCP	IGR	-	ID=gene-E	6	5.98	2605.1	0.822729804	T(+42.01)	61	414.2117	2	826.412	Acetylati	S	Superscaf	13902669	0	0	0	0	0	BTP20220	Mixed
ESLPHEN	Superscaf	-	Superscaf	13927492	13927512	-122	122	128	-	-	-	-	-	-	NCP	IGR	-	ID=gene-E	7	5.342857143	3141.6	0.82241112	ESLPHEN	68	413.1886	2	824.3664	.	A	Superscaf	13927575	0	0	0	0	0	BTP20220	Tissue specific
KPVALTSY	Superscaf	-	Superscaf	13927513	13927536	-129	129	136	-	-	-	-	-	-	NCP	IGR	-	ID=gene-E	8	5.73875	2710.9	0.822410814	KPVALTSY	76	439.7506	2	877.4909	.	A	Superscaf	13927575	0	0	0	0	BTP20220	BTP20220	Tissue specific
SYQTNKSRH	Superscaf	-	Superscaf	13927531	13927575	-135	135	149	-	-	-	-	-	-	NCP	IGR	-	ID=gene-E	15	6.169333333	7002.9	0.822410316	SYQTNKSRH	82	944.9441	2	1887.875	.	A	Superscaf	13927575	0	0	0	0	0	BTP20220	Tissue specific
QTNKSRHDE	Superscaf	-	Superscaf	13927537	13927572	-137	137	148	-	-	-	-	-	-	NCP	IGR	-	ID=gene-E	12	6.31	5772.6	0.822410355	QTNKSRHDE	97	746.3601	2	1490.711	.	A	Superscaf	13927575	0	0	0	0	BTP20220	0	Tissue specific
KLFGP	Superscaf	-	Superscaf	13968280	13968294	-3	3	7	-	-	-	-	-	-	NCP	IGR	-	ID=gene-E	5	6.694	1843.9	0.821891111	K(+42.01)	97	603.3481	1	602.3428	Acetylati	I	Superscaf	13968345	0	0	0	0	0	BTP20220	Tissue specific
LHYLKELK	Superscaf	+	Superscaf	13990483	13990506	70	70	77	-	-	-	-	-	-	NCP	IGR	-	ID=gene-E	8	6.73625	3501.7	0.821607887	LHYLKELK	72	522.312	2	1042.617	.	Y	Superscaf	13990276	0	0	0	0	0	BTP20220	Tissue specific
KLYVPP	Superscaf	+	Superscaf	13994737	13994754	3	3	8	-	-	-	-	-	-	NCP	IGR	-	ID=gene-E	6	5.663333333	1956.1	0.821553721	KLYVPP	72	358.7193	2	715.4268	.	E	Superscaf	13994731	0	0	0	0	0	BTP20220	Tissue specific
TGGPLPL	Superscaf	-	Superscaf	13997080	13997100	-44	44	50	-	-	-	-	-	-	NCP	IGR	-	ID=gene-E	7	6.147142857	2373.7	0.821523807	T(+42.01)	60	696.3907	1	695.3854	Acetylati	R	Superscaf	13997163	0	0	0	0	0	BTP20220	Tissue specific
MHVPLR	Superscaf	-	Superscaf	14013058	14013075	-57	57	62	-	-	-	-	-	-	NCP	IGR	-	ID=gene-E	6	6.063333333	2330.8	0.821320111	M(+15.99)	53	384.7139	2	767.4112	Oxidation	R	Superscaf	14013132	0	0	BTP20220	0	0	0	Tissue specific
TSDKGNVK	Superscaf	-	Superscaf	14023354	14023377	-44	44	51	-	-	-	-	-	-	NCP	IGR	-	ID=gene-E	8	5.755	3023.2	0.821188751	T(+42.01)	82	445.7403	2	889.4505	Acetylati	G	Superscaf	14023521	0	0	0	0	BTP20220	0	Tissue specific
PALPK	Superscaf	-	Superscaf	14424961	14424975	-44	44	48	-	-	-	-	-	-	NCP	IGR	-	ID=gene-E	5	6.868	1807.9	0.816067999	PALPK	68	525.3376	1	524.3322	.	G	Superscaf	14425149	0	0	BTP20220	0	0	0	Tissue specific
SLSAPLTVI	Superscaf	-	Superscaf	14522473	14522520	-92	92	107	-	-	-	-	-	-	NCP	IGR	-	ID=gene-E	16	5.20875	5636.5	0.814824208	SLSAPLTVI	84	862.9432	2	1723.881	Carbamido	V	Superscaf	14522745	0	0	0	0	0	BTP20220	Tissue specific
GVTLPL	Superscaf	-	Superscaf	14567239	14567256	-108	108	113	-	-	-	-	-	-	NCP	IGR	-	ID=gene-E	6	5.126666667	1739	0.814253782	G(+42.01)	65	641.3854	1	640.3796	Acetylati	I	Superscaf	14567289	0	0	BTP20220	0	0	0	Mixed
MHFVVPR	Superscaf	+	Superscaf	14693986	14694006	5	5	11	-	-	gene	-	-	mRNA	NCP	Intron	gene|mRN	ID=gene-E	7	5.125714286	2364.8	0.8126376	M(+15.99)	79	472.2397	2	942.4745	Oxidation	G	Superscaf	14693974	0	0	0	BTP20220	0	0	Tissue specific
SAKTPVGF	Superscaf	+	Superscaf	14695222	14695245	11	11	18	-	-	gene	-	-	mRNA	NCP	Intron	gene|mRN	ID=gene-E	8	5.715	2582.7	0.812621802	SAKTPVGF	78	403.7221	2	805.4333	.	K	Superscaf	14695192	0	0	0	0	BTP20220	0	Tissue specific
KQNPEYSN	Superscaf	-	Superscaf	14761174	14761197	-38	38	45	-	-	gene	-	-	mRNA	NCP	Intron	gene|mRN	ID=gene-E	8	5.88375	3878.3	0.811780852	K(+42.01)	50	511.2333	2	1020.451	Acetylati	A	Superscaf	14761260	0	0	BTP20220	BTP20220	0	0	Tissue specific
LALVLF	Superscaf	+	Superscaf	14850442	14850459	88	88	93	-	-	gene	-	-	mRNA	NCP	Intron	gene|mRN	ID=gene-E	6	4.906666667	1758.9	0.810642677	LALVLF	94	675.4416	1	674.4366	.	S	Superscaf	14850181	0	0	BTP20220	0	BTP20220	0	Tissue specific
APVQPL	Superscaf	-	Superscaf	14896363	14896380	-13	13	18	-	-	gene	-	-	mRNA	NCP	Intron	gene|mRN	ID=gene-E	6	5.041666667	1950.2	0.810057141	A(+42.01)	63	666.3885	1	665.3748	Acetylati	N	Superscaf	14896392	0	0	0	0	BTP20220	0	Tissue specific
TCTYLP	Superscaf	+	Superscaf	15065617	15065634	18	18	23	-	-	-	-	-	-	NCP	IGR	-	ID=gene-E	6	6.003333333	2531	0.807898994	T(+42.01)	57	398.6796	2	795.3473	Acetylati	F	Superscaf	15065566	BTP20220	0	0	0	0	0	Tissue specific
TAAVY	Superscaf	-	Superscaf	15071902	15071916	-12	12	16	-	-	-	-	-	-	NCP	IGR	-	ID=gene-E	5	4.846	1479.6	0.807818892	TAAVY	60	524.2704	1	523.2642	.	A	Superscaf	15072066	0	0	0	BTP20220	0	0	Tissue specific
LPWPKL	Superscaf	-	Superscaf	15080107	15080124	-32	32	37	-	-	-	-	-	-	NCP	IGR	-	ID=gene-E	6	6.698333333	2376.6	0.807714232	LPWPKL	70	377.2345	2	752.4584	.	A	Superscaf	15080148	0	0	0	0	0	BTP20220	Tissue specific
KFTEGH	Superscaf	+	Superscaf	15157048	15157065	16	16	21	-	-	-	-	-	-	NCP	IGR	-	ID=gene-E	6	6.421666667	2595.8	0.806733162	KFTEGH	58	359.6841	2	717.3445	.	K	Superscaf	15157003	BTP20220	0	0	0	0	0	Tissue specific
SLGVTPVQ	Superscaf	-	Superscaf	15215611	15215634	-22	22	29	-	-	-	-	-	-	NCP	IGR	-	ID=gene-E	8	4.51375	2379.7	0.805986352	SLGVTPVQ	80	400.7279	2	799.444	.	R	Superscaf	15215694	0	0	0	0	0	BTP20220	Tissue specific
YNELARV	Superscaf	-	Superscaf	15255412	15255432	-42	42	48	-	-	-	-	-	-	NCP	IGR	-	ID=gene-E	7	5.292857143	2795.2	0.805478891	YNELARV	74	432.7319	2	863.4501	.	R	Superscaf	15255543	0	0	0	0	0	BTP20220	Tissue specific
ATTLM	Superscaf	+	Superscaf	15297853	15297867	14	14	18	-	-	-	-	-	-	NCP	IGR	-	ID=gene-E	5	6.162	1831.2	0.804937804	ATTLM(+15	54	552.264	1	551.2625	Oxidation	V	Superscaf	15297814	0	0	0	0	BTP20220	0	Tissue specific
DLLGHPKT	Superscaf	+	Superscaf	15319615	15319638	3	3	10	-	-	-	-	-	-	NCP	IGR	-	ID=gene-E	8	6.3825	3238.6	0.804660204	DLLGHPKT	94	440.7468	2	879.4814	.	P	Superscaf	15319609	0	0	0	0	BTP20220	0	Tissue specific
KTPGY	Superscaf	-	Superscaf	15344908	15344922	-28	28	32	-	-	-	-	-	-	NCP	IGR	-	ID=gene-E	5	6.84	1960.1	0.804337809	KTPGY	54	565.3002	1	564.2907	.	R	Superscaf	15345027	0	0	0	0	BTP20220	0	Tissue specific
FPSGAV	Superscaf	-	Superscaf	15360715	15360732	-11	11	16	-	-	-	-	-	-	NCP	IGR	-	ID=gene-E	6	4.908333333	1716.8	0.804136216	FPSGAV	52	577.2888	1	576.2907	.	N	Superscaf	15360990	0	0	0	0	BTP20220	0	Tissue specific
SGPLM	Superscaf	-	Superscaf	15375019	15375033	-36	36	40	-	-	-	-	-	-	NCP	IGR	-	ID=gene-E	5	5.936	1743	0.803953865	SGPLM	78	504.2482	1	503.2414	.	G	Superscaf	15375132	0	BTP20220	0	0	BTP20220	0	Tissue specific
LSENDLPK	Superscaf	+	Superscaf	15460246	15460269	11	11	18	-	-	-	-	-	-	NCP	IGR	-	ID=gene-E	8	5.66	3329.7	0.802867026	LSENDLPK	58	458.2421	2	914.4709	.	K	Superscaf	15460216	0	0	BTP20220	0	0	0	Tissue specific
RPVFARVR	Superscaf	-	Superscaf	15494593	15494616	-96	96	103	-	-	-	-	-	-	NCP	IGR	-	ID=gene-E	8	6.26	2982.5	0.80242907	RPVFARVR	73	500.8157	2	999.6089	.	G	Superscaf	15494904	0	BTP20220	0	0	0	0	Tissue specific
TVDLPL	Superscaf	-	Superscaf	15521971	15521988	-11	11	16	-	-	-	-	-	-	NCP	IGR	-	ID=gene-E	6	4.626666667	1853.2	0.802080051	TVDLPL	50	657.3784	1	656.3745	.	G	Superscaf	15522141	BTP20220	0	0	0	0	0	Tissue specific
TVVSF	Superscaf	-	Superscaf	15539533	15539547	-49	49	53	-	-	-	-	-	-	NCP	IGR	-	ID=gene-E	5	3.538	1168.3	0.801856157	T(+42.01)	91	594.3129	1	593.3061	Acetylati	G	Superscaf	15539670	0	0	0	0	BTP20220	BTP20220	Tissue specific
KGWFSLAP	Superscaf	-	Superscaf	15540445	15540468	-19	19	26	-	-	-	-	-	-	NCP	IGR	-	ID=gene-E	8	6.3825	3065.2	0.801844413	KGWFSLAP	62	453.2444	2	904.4807	.	D	Superscaf	15540588	0	0	0	0	BTP20220	0	Tissue specific
TVPLDVT	Superscaf	-	Superscaf	15615202	15615222	-346	346	352	-	-	-	-	-	-	NCP	IGR	-	ID=gene-E	7	4.044285714	1897.3	0.80089123	TVPLDVT	72	744.4113	1	743.4065	.	R	Superscaf	15615306	0	0	0	0	BTP20220	BTP20220	Tissue specific
PKTEC	Superscaf	+	Superscaf	15633001	15633015	20	20	24	-	-	-	-	-	-	NCP	IGR	-	ID=gene-E	5	6.162	2214.5	0.800664352	P(+42.01)	57	676.3091	1	675.2897	Acetylati	K	Superscaf	15632944	0	BTP20220	0	0	0	0	Tissue specific
KDVLR	Superscaf	+	Superscaf	15647545	15647559	15	15	19	-	-	-	-	-	-	NCP	IGR	-	ID=gene-E	5	5.89	1882.1	0.800478902	K(+42.01)	95	672.401	1	671.3966	Acetylati	T	Superscaf	15647503	BTP20220	0	0	0	0	0	Tissue specific
KDPEV	Superscaf	+	Superscaf	15668971	15668985	4	4	8	-	-	-	-	-	-	NCP	IGR	-	ID=gene-E	5	4.446	1642.8	0.800205701	KDPEV	67	587.3026	1	586.2962	.	L	Superscaf	15668962	0	0	0	0	BTP20220	0	Tissue specific
LGDVLRGL	Superscaf	-	Superscaf	15678067	15678090	-15	15	22	-	-	-	-	-	-	NCP	IGR	-	ID=gene-E	8	5.45125	2715.1	0.800089603	LGDVLRGL	86	421.7563	2	841.5021	.	K	Superscaf	15678537	0	0	0	BTP20220	0	0	Tissue specific
PGVAH	Superscaf	-	Superscaf	15684430	15684444	-5	5	9	-	-	-	-	-	-	NCP	IGR	-	ID=gene-E	5	5.176	1565.7	0.800008584	PGVAH	59	480.2552	1	479.2492	.	V	Superscaf	15684489	0	0	0	0	BTP20220	0	Tissue specific
KGRPRPP	Superscaf	-	Superscaf	15703507	15703527	-6	6	12	-	-	-	-	-	-	NCP	IGR	-	ID=gene-E	7	8.018571429	3075.5	0.799765258	KGRPRPP	95	404.2463	2	806.4875	.	E	Superscaf	15703710	0	BTP20220	0	0	0	0	Tissue specific
QEGLLLAR	Superscaf	-	Superscaf	15703852	15703875	-35	35	42	-	-	-	-	-	-	NCP	IGR	-	ID=gene-E	8	6.195	3353.8	0.79976082	QEGLLLAR	74	450.2683	2	898.5236	.	S	Superscaf	15703965	0	0	0	0	BTP20220	0	Tissue specific
LKGLGH	Superscaf	+	Superscaf	15930037	15930054	28	28	33	-	-	-	-	-	-	NCP	IGR	-	ID=gene-E	6	6.871666667	2389.5	0.796876825	LKGLGH	76	624.3783	1	623.3755	.	G	Superscaf	15929956	0	0	0	0	BTP20220	0	Tissue specific
WAPEL	Superscaf	+	Superscaf	15960382	15960396	2	2	6	-	-	-	-	-	-	NCP	IGR	-	ID=gene-E	5	5.482	1954.2	0.796489936	WAPEL	90	615.3134	1	614.3064	.	V	Superscaf	15960379	0	0	0	0	BTP20220	0	Tissue specific
PVAAMRG	Superscaf	+	Superscaf	16006843	16006863	138	138	144	-	-	-	-	-	-	NCP	IGR	-	ID=gene-E	7	5.831428571	2277.6	0.795897438	PVAAM(+15	55	359.1846	2	716.364	Oxidation	K	Superscaf	16006432	0	0	0	0	BTP20220	BTP20220	Tissue specific
LGPKSNP	Superscaf	-	Superscaf	16023544	16023564	-4	4	10	-	-	-	-	-	-	NCP	IGR	-	ID=gene-E	7	6.482857143	2774	0.795684485	L(+42.01)	66	754.4095	1	753.4021	Acetylati	T	Superscaf	16023567	0	BTP20220	0	0	0	0	Tissue specific
KVMLGW	Superscaf	-	Superscaf	16039267	16039284	-5	5	10	-	-	-	-	-	-	NCP	IGR	-	ID=gene-E	6	5.555	2017.2	0.79548404	KVM(+15.9	71	375.201	2	748.3942	Oxidation	L	Superscaf	16039416	0	0	BTP20220	0	0	0	Tissue specific
TLPSPYS	Superscaf	-	Superscaf	16059499	16059519	-12	12	18	-	-	-	-	-	-	NCP	IGR	-	ID=gene-E	7	6.018571429	2652	0.795226025	TLPSPYS	52	764.3806	1	763.3752	.	V	Superscaf	16059576	0	BTP20220	0	0	0	0	Tissue specific
DPVDD	Superscaf	-	Superscaf	16195348	16195362	-8	8	12	-	-	-	-	-	-	NCP	IGR	-	ID=gene-E	5	3.042	1571.9	0.793493899	D(+42.01)	56	602.2406	1	601.2231	Acetylati	V	Superscaf	16195374	0	BTP20220	0	0	0	0	Tissue specific
HNALVLY	Superscaf	+	Superscaf	16203973	16203993	11	11	17	-	-	-	-	-	-	NCP	IGR	-	ID=gene-E	7	5.234285714	2694	0.793383845	H(+42.01)	65	436.2356	2	870.46	Acetylati	I	Superscaf	16203943	0	0	BTP20220	0	0	0	Tissue specific
KDSPLQEL	Superscaf	-	Superscaf	16350355	16350378	-21	21	28	-	-	-	-	-	-	NCP	IGR	-	ID=gene-E	8	5.69	3343.7	0.791517299	KDSPLQEL	59	465.2491	2	928.4865	.	R	Superscaf	16350420	0	0	0	0	BTP20220	0	Tissue specific
LAGSLLGK	Superscaf	+	Superscaf	16591438	16591461	8	8	15	-	-	-	-	-	-	NCP	IGR	-	ID=gene-E	8	6.415	2817.9	0.788443264	LAGSLLGK	98	379.74	2	757.4698	.	L	Superscaf	16591417	BTP20220	0	0	0	0	BTP20220	Tissue specific
EHQSMLF	Superscaf	-	Superscaf	16637017	16637037	-14	14	20	-	-	-	-	-	-	NCP	IGR	-	ID=gene-E	7	5.621428571	3151.6	0.787862127	EHQSM(+15	54	454.202	2	906.3906	Oxidation	H	Superscaf	16637067	BTP20220	0	0	0	0	0	Tissue specific
HVVLLH	Superscaf	+	Superscaf	16642543	16642560	4	4	9	-	-	-	-	-	-	NCP	IGR	-	ID=gene-E	6	4.523333333	1946.2	0.787791704	HVVLLH	83	359.2227	2	716.4333	.	A	Superscaf	16642534	BTP20220	0	0	0	0	0	Tissue specific
FPESL	Superscaf	+	Superscaf	16710592	16710606	32	32	36	-	-	-	-	-	-	NCP	IGR	-	ID=gene-E	5	5.332	1887.1	0.786924053	FPESL	57	592.2949	1	591.2904	.	G	Superscaf	16710499	0	0	0	BTP20220	0	0	Tissue specific
LYGSEKF	Superscaf	-	Superscaf	16755514	16755534	-9	9	15	-	-	-	-	-	-	NCP	IGR	-	ID=gene-E	7	5.961428571	2774.9	0.786351179	LYGSEKF	51	843.419	1	842.4174	.	A	Superscaf	16755735	0	0	0	0	0	BTP20220	Tissue specific
EKPFEPK	Superscaf	-	Superscaf	16762792	16762812	-34	34	40	-	-	-	-	-	-	NCP	IGR	-	ID=gene-E	7	6.285714286	2850	0.786258377	EKPFEPK	66	437.73	2	873.4595	.	Y	Superscaf	16762827	BTP20220	0	0	0	0	0	Tissue specific
KDVVGP	Superscaf	-	Superscaf	16798348	16798365	-34	34	39	-	-	-	-	-	-	NCP	IGR	-	ID=gene-E	6	4.163333333	1514.6	0.785805043	KDVVGP	67	614.3463	1	613.3435	.	R	Superscaf	16798401	BTP20220	0	0	0	0	0	Tissue specific
DLKSSKFKA	Superscaf	-	Superscaf	16954927	16954953	-27	27	35	-	-	-	-	-	-	NCP	IGR	-	ID=gene-E	9	6.781111111	3635.6	0.783808399	DLKSSKFKA	99	512.295	2	1022.576	.	S	Superscaf	16954956	0	0	0	0	0	BTP20220	Tissue specific
VPVPPKY	Superscaf	-	Superscaf	17004844	17004864	-8	8	14	-	-	-	-	-	-	NCP	IGR	-	ID=gene-E	7	4.9	1940.1	0.783171987	VPVPPKY	62	799.4688	1	798.4639	.	F	Superscaf	17004864	0	0	0	BTP20220	BTP20220	BTP20220	Tissue specific
QGDVLDF	Superscaf	-	Superscaf	17110309	17110329	-13	13	19	-	-	-	-	-	-	NCP	IGR	-	ID=gene-E	7	4.145714286	2471.8	0.781827209	QGDVLDF	75	793.3699	1	792.3654	.	Q	Superscaf	17110365	0	0	0	0	0	BTP20220	Tissue specific
PVVCSHVL	Superscaf	-	Superscaf	17198566	17198589	-13	13	20	-	-	-	-	-	-	NCP	IGR	-	ID=gene-E	8	3.82125	2279.6	0.780701811	PVVC(+57.	73	455.752	2	909.4742	Carbamido	S	Superscaf	17198691	0	0	0	0	0	BTP20220	Tissue specific
AAVPAV	Superscaf	-	Superscaf	17393506	17393523	-52	52	57	-	-	-	-	-	-	NCP	IGR	-	ID=gene-E	6	4.06	1271.3	0.778216219	A(+42.01)	55	569.3273	1	568.322	Acetylati	P	Superscaf	17393556	0	0	BTP20220	0	0	0	Tissue specific
KPAALSYS	Superscaf	-	Superscaf	17472943	17472966	-2	2	9	-	-	-	-	-	-	NCP	IGR	-	ID=gene-E	8	6.385	3008.1	0.777203246	KPAALSYS	50	418.7248	2	835.4439	.	T	Superscaf	17473014	0	0	0	0	BTP20220	0	Tissue specific
LSPKRP	Superscaf	-	Superscaf	17543656	17543673	-9	9	14	-	-	-	-	-	-	NCP	IGR	-	ID=gene-E	6	7.46	2528.8	0.776301665	LSPKRP	62	697.4333	1	696.4282	.	R	Superscaf	17543676	0	0	0	0	BTP20220	0	Tissue specific
HARLAY	Superscaf	-	Superscaf	17637040	17637057	-13	13	18	-	-	-	-	-	-	NCP	IGR	-	ID=gene-E	6	7.005	2704	0.775110931	HARLAY	61	365.6964	2	729.3922	.	V	Superscaf	17637072	0	0	0	0	0	BTP20220	Tissue specific
PVAGP	Superscaf	-	Superscaf	17664970	17664984	-32	32	36	-	-	-	-	-	-	NCP	IGR	-	ID=gene-E	5	4.918	1283.4	0.774754836	P(+42.01)	57	482.2704	1	481.2536	Acetylati	D	Superscaf	17665026	0	0	0	0	BTP20220	0	Tissue specific
PLGAPF	Superscaf	-	Superscaf	17714011	17714028	-24	24	29	-	-	-	-	-	-	NCP	IGR	-	ID=gene-E	6	6.008333333	2024.2	0.774129479	PLGAPF	52	601.3275	1	600.3271	.	G	Superscaf	17714082	0	0	0	0	0	BTP20220	Tissue specific
VFLPALQV	Superscaf	-	Superscaf	17795101	17795124	-8	8	15	-	-	-	-	-	-	NCP	IGR	-	ID=gene-E	8	4.42625	2353.6	0.773095428	VFLPALQV	80	886.537	1	885.5323	.	G	Superscaf	17795220	0	0	0	0	BTP20220	0	Tissue specific
KELGSVPK	Superscaf	-	Superscaf	17815420	17815443	-13	13	20	-	-	-	-	-	-	NCP	IGR	-	ID=gene-E	8	5.82875	2733.8	0.772836342	KELGSVPK	54	429.2523	2	856.5018	.	P	Superscaf	17815479	0	0	0	0	0	BTP20220	Tissue specific
LLGVGH	Superscaf	-	Superscaf	17878858	17878875	-4	4	9	-	-	gene	-	-	mRNA	NCP	Intron	gene|mRN	ID=gene-E	6	5.248333333	1921.1	0.772027524	LLGVGH	99	595.3546	1	594.3489	.	S	Superscaf	17878878	0	0	0	0	BTP20220	0	Tissue specific
KYPFF	Superscaf	-	Superscaf	17896930	17896944	-4	4	8	-	-	gene	-	-	mRNA	NCP	Intron	gene|mRN	ID=gene-E	5	6.532	2040.1	0.771797128	KYPFF	66	351.1823	2	700.3584	.	I	Superscaf	17896959	0	0	0	BTP20220	0	0	Tissue specific
GKFSQK	Superscaf	-	Superscaf	17897149	17897166	-10	10	15	-	-	gene	-	-	mRNA	NCP	Intron	gene|mRN	ID=gene-E	6	7.043333333	2615.6	0.771794297	G(+42.01)	53	368.7069	2	735.3915	Acetylati	D	Superscaf	17897184	0	BTP20220	0	0	BTP20220	0	Tissue specific
TREHVVP	Superscaf	-	Superscaf	17898844	17898864	-13	13	19	-	-	gene	-	-	mRNA	NCP	Intron	gene|mRN	ID=gene-E	7	4.914285714	2428.9	0.771772646	TREHVVP	65	419.2361	2	836.4504	.	S	Superscaf	17899047	0	0	0	BTP20220	0	0	Tissue specific
ELLMEQ	Superscaf	-	Superscaf	18001411	18001428	-13	13	18	-	-	gene	-	-	mRNA	NCP	Intron	gene|mRN	ID=gene-E	6	4.966666667	2540	0.770464858	ELLMEQ	59	381.6871	2	761.3629	.	T	Superscaf	18001440	0	0	BTP20220	0	0	0	Tissue specific
PAMYV	Superscaf	+	Superscaf	18017077	18017091	9	9	13	-	-	gene	-	-	mRNA	NCP	Intron	gene|mRN	ID=gene-E	5	4.746	1479.7	0.77026514	PAMYV	98	580.2777	1	579.2726	.	V	Superscaf	18017053	0	BTP20220	0	0	0	0	Tissue specific
TEDPKVP	Superscaf	-	Superscaf	18183832	18183852	-38	38	44	-	-	-	-	-	-	NCP	IGR	-	ID=gene-E	7	5.008571429	2377.7	0.768138781	TEDPKVP	65	393.2117	2	784.3967	.	K	Superscaf	18183855	0	0	0	0	BTP20220	0	Tissue specific
VLLVF	Superscaf	-	Superscaf	18240454	18240468	-9	9	13	-	-	-	-	-	-	NCP	IGR	-	ID=gene-E	5	3.488	1094.2	0.767416873	VLLVF	81	590.3887	1	589.3839	.	L	Superscaf	18240735	0	0	0	BTP20220	0	0	Tissue specific
RPTGKF	Superscaf	-	Superscaf	18342271	18342288	-68	68	73	-	-	-	-	-	-	NCP	IGR	-	ID=gene-E	6	7.463333333	2536.8	0.766118573	RPTGKF	66	353.2108	2	704.3969	.	K	Superscaf	18342300	BTP20220	0	0	0	0	0	Tissue specific
LLHLEL	Superscaf	-	Superscaf	18443851	18443868	-31	31	36	-	-	-	-	-	-	NCP	IGR	-	ID=gene-E	6	5.788333333	2458.8	0.764823332	LLHLEL	53	737.4541	1	736.4483	.	H	Superscaf	18443949	0	0	0	0	BTP20220	0	Tissue specific
VVNPLLA	Superscaf	-	Superscaf	18509959	18509979	-37	37	43	-	-	-	-	-	-	NCP	IGR	-	ID=gene-E	7	4.241428571	1952.2	0.763980355	VVNPLLA	64	725.4536	1	724.4483	.	G	Superscaf	18510042	0	0	0	BTP20220	0	0	Tissue specific
QSLPPGLAV	Superscaf	-	Superscaf	18671818	18671856	-115	115	127	-	-	-	-	-	-	NCP	IGR	-	ID=gene-E	13	5.889230769	4700	0.761916271	QSLPPGLAV	84	460.6119	3	1378.818	.	R	Superscaf	18671856	0	0	0	BTP20220	0	0	Mixed
SLPPGLAVK	Superscaf	-	Superscaf	18671821	18671856	-116	116	127	-	-	-	-	-	-	NCP	IGR	-	ID=gene-E	12	5.909166667	4089.3	0.761916271	SLPPGLAVK	89	417.9258	3	1250.76	.	R	Superscaf	18671856	0	0	0	0	BTP20220	0	Tissue specific
LPPGLAVKE	Superscaf	-	Superscaf	18671824	18671856	-117	117	127	-	-	-	-	-	-	NCP	IGR	-	ID=gene-E	11	5.93	3705.9	0.761916271	LPPGLAVKE	97	388.9158	3	1163.728	.	R	Superscaf	18671856	0	0	0	0	BTP20220	0	Tissue specific
LSGGLL	Superscaf	-	Superscaf	18729970	18729987	-18	18	23	-	-	-	-	-	-	NCP	IGR	-	ID=gene-E	6	5.926666667	2038.2	0.761175046	LSGGLL	77	559.3438	1	558.3377	.	T	Superscaf	18730008	0	0	0	0	0	BTP20220	Mixed
CMEPP	Superscaf	+	Superscaf	18741379	18741393	16	16	20	-	-	gene	-	-	mRNA	NCP	Intron	gene|mRN	ID=gene-E	5	5.318	2057.5	0.761029608	C(+57.02)	53	649.2201	1	648.2247	Carbamido	L	Superscaf	18741334	0	0	0	0	BTP20220	0	Tissue specific
NVVVF	Superscaf	-	Superscaf	18757225	18757239	-2	2	6	-	-	-	-	-	-	NCP	IGR	-	ID=gene-E	5	2.178	984.1	0.760827557	NVVVF	58	577.3327	1	576.3271	.	I	Superscaf	18757272	0	0	0	0	0	BTP20220	Tissue specific
VGVLSA	Superscaf	-	Superscaf	18813175	18813192	-32	32	37	-	-	-	-	-	-	NCP	IGR	-	ID=gene-E	6	3.941666667	1345.4	0.760114104	V(+42.01)	89	587.3383	1	586.3326	Acetylati	H	Superscaf	18813195	BTP20220	0	0	0	0	0	Tissue specific
YGLGSY	Superscaf	+	Superscaf	19036069	19036086	3	3	8	-	-	-	-	-	-	NCP	IGR	-	ID=gene-E	6	5.82	2250.4	0.757271995	YGLGSY	98	659.2999	1	658.2962	.	V	Superscaf	19036063	0	0	0	BTP20220	0	0	Tissue specific
ATKLEN	Superscaf	+	Superscaf	19078429	19078446	27	27	32	-	-	-	-	-	-	NCP	IGR	-	ID=gene-E	6	6.15	2552.8	0.756731866	ATKLEN	69	675.3782	1	674.3599	.	A	Superscaf	19078351	0	0	0	0	BTP20220	0	Tissue specific
GKKGL	Superscaf	-	Superscaf	19156348	19156362	-44	44	48	-	-	-	-	-	-	NCP	IGR	-	ID=gene-E	5	7.48	1884.8	0.755738363	GKKGL	55	502.3367	1	501.3275	.	A	Superscaf	19156467	BTP20220	0	0	0	0	0	Tissue specific
TAEHKPP	Superscaf	-	Superscaf	19165072	19165092	-4	4	10	-	-	-	-	-	-	NCP	IGR	-	ID=gene-E	7	6.528571429	2897.2	0.755627048	TAEHKPP	52	779.4039	1	778.3973	.	N	Superscaf	19165311	BTP20220	0	0	0	0	0	Tissue specific
ENLDFL	Superscaf	+	Superscaf	19185094	19185111	16	16	21	-	-	-	-	-	-	NCP	IGR	-	ID=gene-E	6	4.84	2527.9	0.755371786	ENLDFL	62	750.3645	1	749.3596	.	F	Superscaf	19185049	0	0	0	BTP20220	0	0	Tissue specific
VDAYEVFL	Superscaf	+	Superscaf	19323811	19323834	24	24	31	-	-	-	-	-	-	NCP	IGR	-	ID=gene-E	8	3.66625	2348.6	0.753602938	VDAYEVFL	61	955.4754	1	954.4698	.	Y	Superscaf	19323742	0	0	0	0	0	BTP20220	Tissue specific
KETFP	Superscaf	-	Superscaf	19572061	19572075	-5	5	9	-	-	-	-	-	-	NCP	IGR	-	ID=gene-E	5	6.254	2016.2	0.750437631	KETFP	63	621.3157	1	620.317	.	L	Superscaf	19572075	BTP20220	0	0	0	0	0	Tissue specific
KKGAFYG	Superscaf	+	Superscaf	19594579	19594599	12	12	18	-	-	-	-	-	-	NCP	IGR	-	ID=gene-E	7	6.94	2689.6	0.750150429	KKGAFYG	50	385.711	2	769.4122	.	V	Superscaf	19594546	0	BTP20220	BTP20220	0	0	BTP20220	Tissue specific
TSLPP	Superscaf	-	Superscaf	19623832	19623846	-3	3	7	-	-	-	-	-	-	NCP	IGR	-	ID=gene-E	5	6.158	1809.1	0.749777502	TSLPP	80	514.2854	1	513.2798	.	L	Superscaf	19623885	0	0	0	0	0	BTP20220	Tissue specific
LGNMLAK	Superscaf	+	Superscaf	19661608	19661628	24	24	30	-	-	-	-	-	-	NCP	IGR	-	ID=gene-E	7	6.407142857	2752	0.749295746	LGNMLAK	87	373.7137	2	745.4156	.	R	Superscaf	19661539	0	0	0	0	0	BTP20220	Tissue specific
KSYKADK	Superscaf	+	Superscaf	19830529	19830549	13	13	19	-	-	-	-	-	-	NCP	IGR	-	ID=gene-E	7	7.078571429	2970.9	0.747141845	K(+42.01)	59	441.2416	2	880.4654	Acetylati	Q	Superscaf	19830493	0	BTP20220	0	0	0	0	Tissue specific
YTLPK	Superscaf	-	Superscaf	19909417	19909431	-2	2	6	-	-	-	-	-	-	NCP	IGR	-	ID=gene-E	5	6.842	2016.2	0.746136025	YTLPK	95	621.3585	1	620.3533	.	D	Superscaf	19909431	0	BTP20220	BTP20220	BTP20220	0	BTP20220	Tissue specific
AAHWY	Superscaf	+	Superscaf	19943230	19943244	1	1	5	-	-	-	-	-	-	NCP	IGR	-	ID=gene-E	5	6.236	2228.4	0.745704877	AAHWY	98	647.2928	1	646.2863	.	A	Superscaf	19943230	0	BTP20220	0	BTP20220	0	0	Tissue specific
PDPVN	Superscaf	+	Superscaf	20147749	20147763	4	4	8	-	-	-	-	-	-	NCP	IGR	-	ID=gene-E	5	4.196	1683	0.743097068	PDPVN	55	541.2602	1	540.2543	.	R	Superscaf	20147740	0	BTP20220	0	0	0	0	Tissue specific
KAHLTPH	Superscaf	+	Superscaf	20188732	20188752	20	20	26	-	-	-	-	-	-	NCP	IGR	-	ID=gene-E	7	7.107142857	3107.4	0.74257442	K(+42.01)	63	423.2268	2	844.4555	Acetylati	N	Superscaf	20188675	0	0	BTP20220	0	0	0	Tissue specific
LKLLTELK	Superscaf	+	Superscaf	20195947	20195970	4	4	11	-	-	-	-	-	-	NCP	IGR	-	ID=gene-E	8	6.64375	3173.4	0.742482383	LKLLTELK	97	479.319	2	956.627	.	L	Superscaf	20195938	0	0	0	0	0	BTP20220	Tissue specific
NCMDS	Superscaf	-	Superscaf	20255629	20255643	-8	8	12	-	-	-	-	-	-	NCP	IGR	-	ID=gene-E	5	4.966	2348.8	0.741721496	N(+42.01)	51	684.2014	1	683.1891	Acetylati	T	Superscaf	20255694	0	0	0	0	0	BTP20220	Tissue specific
VELAGRTPK	Superscaf	+	Superscaf	20263696	20263722	45	45	53	-	-	-	-	-	-	NCP	IGR	-	ID=gene-E	9	6.057777778	3239.6	0.741618481	VELAGRTPK	86	485.786	2	969.5607	.	C	Superscaf	20263564	0	BTP20220	0	0	0	0	Tissue specific
KSTPMGK	Superscaf	-	Superscaf	20374795	20374815	-27	27	33	-	-	-	-	-	-	NCP	IGR	-	ID=gene-E	7	7.101428571	2723.9	0.740201941	KSTPM(+15	64	382.7047	2	763.3898	Oxidation	C	Superscaf	20374932	0	0	BTP20220	0	0	0	Tissue specific
LPPGAP	Superscaf	-	Superscaf	20412610	20412627	-36	36	41	-	-	-	-	-	-	NCP	IGR	-	ID=gene-E	6	6.145	1974.2	0.739719803	LPPGAP	55	551.3149	1	550.3115	.	S	Superscaf	20412723	0	0	BTP20220	0	0	0	Tissue specific
EGPDALK	Superscaf	-	Superscaf	20422366	20422386	-1	1	7	-	-	-	-	-	-	NCP	IGR	-	ID=gene-E	7	5.742857143	2604.8	0.739595366	EGPDALK	91	365.1915	2	728.3704	.	E	Superscaf	20422386	0	0	BTP20220	0	0	0	Tissue specific
KTTNPL	Superscaf	+	Superscaf	20564008	20564025	13	13	18	-	-	gene	-	-	mRNA	NCP	Intron	gene|mRN	ID=gene-E	6	6.748333333	2550.9	0.737789336	KTTNPL	52	673.389	1	672.3806	.	G	Superscaf	20563972	0	BTP20220	0	0	0	0	Tissue specific
KNYVPL	Superscaf	+	Superscaf	20594629	20594646	11	11	16	-	-	gene	-	-	mRNA	NCP	Intron	gene|mRN	ID=gene-E	6	5.515	2215.4	0.737398889	KNYVPL	72	733.4225	1	732.417	.	H	Superscaf	20594599	0	0	BTP20220	0	0	0	Tissue specific
HTLWF	Superscaf	+	Superscaf	20631397	20631411	5	5	9	-	-	gene	-	-	mRNA	NCP	Intron	gene|mRN	ID=gene-E	5	6.294	2284.6	0.736930101	HTLWF	52	352.1845	2	702.3489	.	H	Superscaf	20631385	0	0	0	0	0	BTP20220	Tissue specific
FEKLR	Superscaf	+	Superscaf	20647327	20647341	17	17	21	-	-	-	-	-	-	NCP	IGR	-	ID=gene-E	5	7.036	2283.5	0.736726978	F(+42.01)	67	367.7172	2	733.4122	Acetylati	Q	Superscaf	20647279	0	0	0	BTP20220	0	0	Tissue specific
DYMMSH	Superscaf	+	Superscaf	20656609	20656626	12	12	17	-	-	-	-	-	-	NCP	IGR	-	ID=gene-E	6	5.566666667	2617.1	0.736608586	DYM(+15.9	50	815.2825	1	814.2626	Oxidation	V	Superscaf	20656576	0	BTP20220	0	0	0	0	Tissue specific
TTVVLT	Superscaf	+	Superscaf	20680072	20680089	43	43	48	-	-	-	-	-	-	NCP	IGR	-	ID=gene-E	6	4.261666667	1545.9	0.736309411	TTVVLT	51	633.3731	1	632.3745	.	V	Superscaf	20679946	0	0	0	0	0	BTP20220	Tissue specific
KGACAKK	Superscaf	-	Superscaf	20709028	20709048	-21	21	27	-	-	-	-	-	-	NCP	IGR	-	ID=gene-E	7	7.464285714	2910.8	0.735940156	KGAC(+57.	66	381.7206	2	761.4218	Carbamido	P	Superscaf	20709075	BTP20220	0	0	BTP20220	0	0	Tissue specific
LPPLHK	Superscaf	-	Superscaf	20843839	20843856	-9	9	14	-	-	-	-	-	-	NCP	IGR	-	ID=gene-E	6	6.981666667	2469.7	0.734221227	LPPLHK	82	352.7256	2	703.438	.	V	Superscaf	20844057	0	0	BTP20220	0	0	0	Tissue specific
AAGVPAMAE	Superscaf	-	Superscaf	20852608	20852652	-24	24	38	-	-	-	-	-	-	NCP	IGR	-	ID=gene-E	15	5.107333333	5098.7	0.73410907	A(+42.01)	78	785.8969	2	1569.782	Acetylati	P	Superscaf	20852787	0	0	BTP20220	0	0	0	Tissue specific
AESVERLQG	Superscaf	-	Superscaf	20852629	20852655	-31	31	39	-	-	-	-	-	-	NCP	IGR	-	ID=gene-E	9	5.131111111	3769.3	0.734109032	A(+42.01)	79	551.2755	2	1100.546	Acetylati	P	Superscaf	20852787	0	0	0	0	0	BTP20220	Tissue specific
AESVERLQ	Superscaf	-	Superscaf	20852629	20852652	-31	31	38	-	-	-	-	-	-	NCP	IGR	-	ID=gene-E	8	5.06625	3158.6	0.73410907	A(+42.01)	85	487.2508	2	972.4876	Acetylati	P	Superscaf	20852787	BTP20220	0	0	0	0	0	Mixed
LPLAH	Superscaf	+	Superscaf	21026329	21026343	10	10	14	-	-	-	-	-	-	NCP	IGR	-	ID=gene-E	5	6.374	1975.2	0.731894346	LPLAH	65	550.3326	1	549.3275	.	C	Superscaf	21026302	0	BTP20220	0	0	0	0	Tissue specific
GPKFLDK	Superscaf	+	Superscaf	21027475	21027495	4	4	10	-	-	-	-	-	-	NCP	IGR	-	ID=gene-E	7	6.597142857	2723.8	0.731879657	G(+42.01)	54	423.7343	2	845.4647	Acetylati	G	Superscaf	21027466	0	0	0	0	0	BTP20220	Tissue specific
DPEGFYK	Superscaf	+	Superscaf	21061834	21061854	14	14	20	-	-	-	-	-	-	NCP	IGR	-	ID=gene-E	7	5.62	2787	0.731441548	DPEGFYK	59	428.1996	2	854.381	.	M	Superscaf	21061795	0	0	BTP20220	0	0	0	Tissue specific
LAAKAAGY	Superscaf	-	Superscaf	21106060	21106083	-18	18	25	-	-	-	-	-	-	NCP	IGR	-	ID=gene-E	8	6.42875	2823.8	0.730877586	LAAKAAGY	54	382.7167	2	763.4228	.	C	Superscaf	21106152	0	BTP20220	0	0	0	0	Tissue specific
EESSCM	Superscaf	-	Superscaf	21171160	21171177	-7	7	12	-	-	-	-	-	-	NCP	IGR	-	ID=gene-E	6	4.761666667	2575	0.730047577	EESSC(+57	56	758.2202	1	757.2259	Carbamido	G	Superscaf	21171297	0	0	0	BTP20220	0	0	Tissue specific
GVDDPTFGY	Superscaf	-	Superscaf	21193300	21193326	-34	34	42	-	-	gene	-	-	mRNA	NCP	Intron	gene|mRN	ID=gene-E	9	4.65	2999.4	0.729765156	GVDDPTFGY	83	970.4136	1	969.408	.	K	Superscaf	21193548	0	0	0	0	BTP20220	0	Tissue specific
RDKNMNR	Superscaf	+	Superscaf	21226813	21226833	12	12	18	-	-	gene	-	-	mRNA	NCP	Intron	gene|mRN	ID=gene-E	7	7.257142857	3742.4	0.72933791	RDKNMNR	76	467.2353	2	932.461	.	T	Superscaf	21226780	0	0	0	0	BTP20220	BTP20220	Tissue specific
LYLPL	Superscaf	-	Superscaf	21241132	21241146	-8	8	12	-	-	gene	-	-	mRNA	NCP	Intron	gene|mRN	ID=gene-E	5	5.98	1857.1	0.729155406	LYLPL	76	618.3845	1	617.3788	.	T	Superscaf	21241185	0	0	0	0	0	BTP20220	Tissue specific
TGPKTLPP	Superscaf	-	Superscaf	21257236	21257259	-32	32	39	-	-	gene	-	-	mRNA	NCP	Intron	gene|mRN	ID=gene-E	8	6.70625	2926.3	0.72894995	T(+42.01)	52	426.749	2	851.4753	Acetylati	V	Superscaf	21257268	0	BTP20220	0	0	0	0	Tissue specific
LSQPF	Superscaf	+	Superscaf	21309979	21309993	2	2	6	-	-	gene	-	-	mRNA	NCP	Intron	gene|mRN	ID=gene-E	5	5.818	2072.3	0.728277542	LSQPF	73	591.3107	1	590.3064	.	A	Superscaf	21309976	0	BTP20220	0	0	0	0	Tissue specific
GVDLFTGK	Superscaf	+	Superscaf	21338866	21338889	10	10	17	-	-	gene	-	-	mRNA	NCP	Intron	gene|mRN	ID=gene-E	8	5.33	2612.8	0.727909091	GVDLFTGK	83	836.4458	1	835.4439	.	S	Superscaf	21338839	0	0	0	BTP20220	0	0	Tissue specific
ARPPFS	Superscaf	+	Superscaf	21345343	21345360	11	11	16	-	-	gene	-	-	mRNA	NCP	Intron	gene|mRN	ID=gene-E	6	6.756666667	2405.7	0.727826579	ARPPFS	82	674.3599	1	673.3547	.	T	Superscaf	21345313	0	BTP20220	0	0	0	0	Tissue specific
QENWLSK	Superscaf	-	Superscaf	21471538	21471558	-37	37	43	-	-	-	-	-	-	NCP	IGR	-	ID=gene-E	7	5.938571429	3364.7	0.726217436	QENWLSK	61	452.7229	2	903.445	.	D	Superscaf	21471633	0	BTP20220	0	0	0	0	Tissue specific
DAPVLSVK	Superscaf	-	Superscaf	21494806	21494829	-42	42	49	-	-	-	-	-	-	NCP	IGR	-	ID=gene-E	8	4.58625	2265.4	0.725920709	DAPVLSVK	61	414.7465	2	827.4752	.	S	Superscaf	21494871	0	BTP20220	BTP20220	0	BTP20220	0	Tissue specific
LSLQGEVL	Superscaf	-	Superscaf	21652786	21652809	-3	3	10	-	-	gene	-	-	mRNA	NCP	Intron	gene|mRN	ID=gene-E	8	4.8075	2777.1	0.723906315	LSLQGEVL	56	429.7482	2	857.4858	.	Q	Superscaf	21652884	BTP20220	0	0	0	BTP20220	0	Tissue specific
LGRPPR	Superscaf	+	Superscaf	21815572	21815589	8	8	13	-	-	gene	-	-	mRNA	NCP	Intron	gene|mRN	ID=gene-E	6	7.678333333	2623.1	0.721830717	L(+42.01)	55	369.2255	2	736.4344	Acetylati	E	Superscaf	21815551	BTP20220	0	0	0	BTP20220	0	Tissue specific
AETEERSLI	Superscaf	-	Superscaf	21863980	21864012	-32	32	42	-	-	gene	-	-	mRNA	NCP	Intron	gene|mRN	ID=gene-E	11	5.317272727	4766.5	0.721213279	A(+42.01)	84	676.7982	2	1351.589	Acetylati	A	Superscaf	21864315	0	BTP20220	0	0	0	0	Tissue specific
EDPSDNWEE	Superscaf	-	Superscaf	21873562	21873618	-35	35	53	-	-	gene	-	-	mRNA	NCP	Intron	gene|mRN	ID=gene-E	19	4.927894737	6918.6	0.721090793	EDPSDNWEE	92	938.3842	2	1874.755	.	G	Superscaf	21873699	0	0	0	BTP20220	0	0	Tissue specific
SGVYRPPG	Superscaf	-	Superscaf	21874231	21874254	-21	21	28	-	-	gene	-	-	mRNA	NCP	Intron	gene|mRN	ID=gene-E	8	5.83	2761.1	0.721082683	SGVYRPPG	84	416.7186	2	831.4239	.	V	Superscaf	21874386	BTP20220	0	0	0	0	0	Tissue specific
ATLMEVPP	Superscaf	-	Superscaf	21919414	21919437	-18	18	25	-	-	gene	-	-	mRNA	NCP	Intron	gene|mRN	ID=gene-E	8	5.0125	2534	0.720506558	ATLM(+15.	65	437.2154	2	872.4313	Oxidation	K	Superscaf	21919440	0	0	BTP20220	0	0	0	Tissue specific
KLNLGLP	Superscaf	-	Superscaf	21966418	21966438	-13	13	19	-	-	gene	-	-	mRNA	NCP	Intron	gene|mRN	ID=gene-E	7	6.48	2760	0.719907251	K(+42.01)	61	398.7544	2	795.4854	Acetylati	V	Superscaf	21966492	0	BTP20220	0	0	0	0	Tissue specific
LFVKSK	Superscaf	-	Superscaf	22053139	22053156	-5	5	10	-	-	-	-	-	-	NCP	IGR	-	ID=gene-E	6	6.103333333	2061	0.718801515	LFVKSK	53	361.2366	2	720.4534	.	R	Superscaf	22053180	0	BTP20220	0	0	0	0	Tissue specific
EGDPP	Superscaf	-	Superscaf	22062856	22062870	-34	34	38	-	-	-	-	-	-	NCP	IGR	-	ID=gene-E	5	4.952	1809.1	0.718677652	EGDPP	57	514.2053	1	513.207	.	L	Superscaf	22062918	BTP20220	0	0	0	0	0	Tissue specific
MLGMLNK	Superscaf	+	Superscaf	22064113	22064133	46	46	52	-	-	-	-	-	-	NCP	IGR	-	ID=gene-E	7	6.368571429	2812.2	0.718661548	M(+42.01)	96	848.4355	1	847.4296	Acetylati	A	Superscaf	22063978	0	0	0	0	BTP20220	BTP20220	Tissue specific
LCQLCAGK	Superscaf	-	Superscaf	22078768	22078791	-23	23	30	-	-	-	-	-	-	NCP	IGR	-	ID=gene-E	8	6.1725	3565.9	0.718474644	LC(+57.02	68	475.2313	2	948.4521	Carbamido	A	Superscaf	22078854	0	BTP20220	0	0	0	0	Tissue specific
FGYSGAFK	Superscaf	-	Superscaf	22078831	22078854	-44	44	51	-	-	-	-	-	-	NCP	IGR	-	ID=gene-E	8	6.25	2992	0.718473841	FGYSGAFK	99	438.7148	2	875.4177	.	A	Superscaf	22078854	0	0	BTP20220	0	0	0	Tissue specific
VPPELLL	Superscaf	-	Superscaf	22105051	22105071	-11	11	17	-	-	-	-	-	-	NCP	IGR	-	ID=gene-E	7	4.822857143	2160.5	0.71813955	VPPELLL	63	780.4845	1	779.4792	.	T	Superscaf	22105125	0	BTP20220	0	0	0	0	Tissue specific
VGVFTK	Superscaf	+	Superscaf	22135861	22135878	54	54	59	-	-	gene	-	-	mRNA	NCP	Intron	gene|mRN	ID=gene-E	6	4.62	1550.6	0.717746731	V(+42.01)	69	692.4041	1	691.3904	Acetylati	D	Superscaf	22135702	0	0	0	BTP20220	0	0	Tissue specific
YAVAVVKKC	Superscaf	-	Superscaf	22141630	22141665	-42	42	53	-	-	gene	-	-	mRNA	NCP	Intron	gene|mRN	ID=gene-E	12	4.844166667	3512.6	0.717672942	YAVAVVKKC	89	649.36	2	1296.708	.	M	Superscaf	22141806	0	0	0	0	0	BTP20220	Tissue specific
YAVAVVKK	Superscaf	-	Superscaf	22141630	22141653	-42	42	49	-	-	gene	-	-	mRNA	NCP	Intron	gene|mRN	ID=gene-E	8	4.6475	2018.9	0.717673095	YAVAVVKK	99	439.2777	2	876.5433	.	M	Superscaf	22141806	BTP20220	0	0	0	0	0	Tissue specific
AVAVVKKGT	Superscaf	-	Superscaf	22141633	22141665	-43	43	53	-	-	gene	-	-	mRNA	NCP	Intron	gene|mRN	ID=gene-E	11	4.77	3053.1	0.717672942	AVAVVKKGT	87	567.8293	2	1133.645	.	M	Superscaf	22141806	BTP20220	0	0	0	0	0	Tissue enhanced
AVVKKGTDF	Superscaf	-	Superscaf	22141639	22141665	-45	45	53	-	-	gene	-	-	mRNA	NCP	Intron	gene|mRN	ID=gene-E	9	5.161111111	2741.8	0.717672942	AVVKKGTDF	84	482.7752	2	963.5389	.	M	Superscaf	22141806	BTP20220	0	0	0	0	0	Tissue specific
KLNELRGK	Superscaf	-	Superscaf	22141666	22141689	-54	54	61	-	-	gene	-	-	mRNA	NCP	Intron	gene|mRN	ID=gene-E	8	7.1	3611.9	0.717672636	KLNELRGK	98	479.2939	2	956.5767	.	M	Superscaf	22141806	BTP20220	0	0	0	0	0	Tissue specific
LAMVPSHAV	Superscaf	-	Superscaf	22144225	22144260	-51	51	62	-	-	gene	-	-	mRNA	NCP	Intron	gene|mRN	ID=gene-E	12	5.01	3648.1	0.717639853	LAMVPSHAV	89	417.571	3	1249.697	.	G	Superscaf	22144491	BTP20220	0	0	0	0	0	Tissue specific
LGYEYVTAL	Superscaf	-	Superscaf	22145134	22145160	-74	74	82	-	-	gene	-	-	mRNA	NCP	Intron	gene|mRN	ID=gene-E	9	5.002222222	3057.4	0.717628377	LGYEYVTAL	79	514.7673	2	1027.523	.	K	Superscaf	22145211	0	0	0	0	BTP20220	0	Tissue specific
LLEACTFHK	Superscaf	-	Superscaf	22169206	22169235	-20	20	29	-	-	gene	-	-	mRNA	NCP	Intron	gene|mRN	ID=gene-E	10	6.186	4239.7	0.717321398	LLEAC(+57	74	608.311	2	1214.612	Carbamido	E	Superscaf	22169235	BTP20220	0	0	0	BTP20220	0	Tissue specific
ACTFHKP	Superscaf	-	Superscaf	22169215	22169235	-23	23	29	-	-	gene	-	-	mRNA	NCP	Intron	gene|mRN	ID=gene-E	7	6.668571429	3107.4	0.717321398	AC(+57.02	99	430.707	2	859.4011	Carbamido	E	Superscaf	22169235	0	0	BTP20220	0	0	0	Tissue specific
VSRPGDVS	Superscaf	-	Superscaf	22385509	22385532	-8	8	15	-	-	-	-	-	-	NCP	IGR	-	ID=gene-E	8	4.67	2461.8	0.714563408	V(+42.01)	50	429.7174	2	857.4243	Acetylati	R	Superscaf	22385589	0	0	0	0	BTP20220	0	Tissue specific
TPYPR	Superscaf	-	Superscaf	22417054	22417068	-42	42	46	-	-	-	-	-	-	NCP	IGR	-	ID=gene-E	5	7.11	2180.6	0.714161294	TPYPR	62	633.335	1	632.3282	.	N	Superscaf	22417074	BTP20220	0	0	0	0	0	Tissue specific
CSVKLH	Superscaf	+	Superscaf	22495597	22495614	15	15	20	-	-	-	-	-	-	NCP	IGR	-	ID=gene-E	6	5.668333333	2410.6	0.713159759	C(+57.02)	69	372.2002	2	742.3796	Carbamido	A	Superscaf	22495555	BTP20220	0	0	0	0	0	Tissue specific
EGHPFLFL	Superscaf	+	Superscaf	22503031	22503054	13	13	20	-	-	-	-	-	-	NCP	IGR	-	ID=gene-E	8	5.75	3161.5	0.713064892	EGHPFLFL	58	480.2517	2	958.4912	.	I	Superscaf	22502995	BTP20220	0	0	0	0	BTP20220	Mixed
VPKLKLM	Superscaf	+	Superscaf	22532911	22532931	26	26	32	-	-	-	-	-	-	NCP	IGR	-	ID=gene-E	7	6.212857143	2352.5	0.712683932	VPKLKLM	83	414.7703	2	827.5303	.	D	Superscaf	22532836	BTP20220	BTP20220	BTP20220	0	0	BTP20220	Tissue specific
MNFGELK	Superscaf	+	Superscaf	22599472	22599492	18	18	24	-	-	-	-	-	-	NCP	IGR	-	ID=gene-E	7	5.935714286	2900.2	0.711835217	MNFGELK	75	838.412	1	837.4055	.	I	Superscaf	22599421	0	0	0	0	BTP20220	0	Tissue specific
SARGP	Superscaf	+	Superscaf	22696987	22697001	39	39	43	-	-	-	-	-	-	NCP	IGR	-	ID=gene-E	5	6.946	1978.2	0.710591885	SARGP	75	487.2613	1	486.255	.	K	Superscaf	22696873	0	0	0	0	BTP20220	0	Tissue specific
VQEGPPK	Superscaf	-	Superscaf	22842127	22842147	-23	23	29	-	-	-	-	-	-	NCP	IGR	-	ID=gene-E	7	5.311428571	2476.7	0.708741137	VQEGPPK	51	754.4037	1	753.402	.	P	Superscaf	22842240	0	0	BTP20220	0	0	0	Tissue specific
DDFKKAF	Superscaf	-	Superscaf	22850323	22850343	-16	16	22	-	-	-	-	-	-	NCP	IGR	-	ID=gene-E	7	6.057142857	2845.9	0.70863663	DDFKKAF	99	435.7207	2	869.4283	.	I	Superscaf	22850349	0	0	0	0	0	BTP20220	Tissue specific
FSPLTPLT	Superscaf	-	Superscaf	23192842	23192865	-11	11	18	-	-	-	-	-	-	NCP	IGR	-	ID=gene-E	8	6.0975	2947.4	0.704269153	FSPLTPLT	56	875.4838	1	874.48	.	T	Superscaf	23192907	0	0	0	BTP20220	0	0	Tissue specific
KYRLLLH	Superscaf	+	Superscaf	23421760	23421780	14	14	20	-	-	-	-	-	-	NCP	IGR	-	ID=gene-E	7	7.384285714	3256.6	0.701350271	KYRLLLH	68	471.8017	2	941.5811	.	H	Superscaf	23421721	0	0	0	0	BTP20220	0	Tissue specific
LLPGVK	Superscaf	-	Superscaf	23530834	23530851	-85	85	90	-	-	-	-	-	-	NCP	IGR	-	ID=gene-E	6	5.661666667	1809.9	0.699959513	LLPGVK	59	626.4204	1	625.4163	.	L	Superscaf	23530998	0	0	0	0	BTP20220	0	Tissue specific
MELAAKY	Superscaf	+	Superscaf	23582176	23582196	48	48	54	-	-	-	-	-	-	NCP	IGR	-	ID=gene-E	7	6.055714286	2700.9	0.699304816	M(+42.01)	57	867.4282	1	866.4208	Acetylati	L	Superscaf	23582035	0	0	0	0	BTP20220	0	Tissue specific
AVGSLLA	Superscaf	-	Superscaf	23616661	23616681	-9	9	15	-	-	-	-	-	-	NCP	IGR	-	ID=gene-E	7	5.092857143	2010.1	0.6988651	AVGSLLA	53	630.3813	1	629.3748	.	Q	Superscaf	23616810	0	0	0	0	BTP20220	0	Tissue specific
SGPKPF	Superscaf	+	Superscaf	23767411	23767428	36	36	41	-	-	-	-	-	-	NCP	IGR	-	ID=gene-E	6	6.578333333	2211.3	0.696942934	SGPKPF	91	632.3434	1	631.3329	.	N	Superscaf	23767306	0	0	0	0	BTP20220	0	Mixed
SGPVLL	Superscaf	-	Superscaf	23789533	23789550	-7	7	12	-	-	-	-	-	-	NCP	IGR	-	ID=gene-E	6	4.985	1724.9	0.696660858	SGPVLL	85	585.3596	1	584.3533	.	Q	Superscaf	23789562	0	BTP20220	0	0	0	0	Tissue specific
TGVVPG	Superscaf	-	Superscaf	23799565	23799582	-2	2	7	-	-	gene	-	-	mRNA	NCP	Intron	gene|mRN	ID=gene-E	6	4.128333333	1329.5	0.69653294	T(+42.01)	59	571.2986	1	570.3013	Acetylati	S	Superscaf	23799585	0	0	0	BTP20220	0	0	Tissue specific
KPLGLKK	Superscaf	+	Superscaf	24012586	24012606	31	31	37	-	-	-	-	-	-	NCP	IGR	-	ID=gene-E	7	7.635714286	2746.7	0.693816684	K(+42.01)	85	413.281	2	824.5483	Acetylati	I	Superscaf	24012496	0	0	0	BTP20220	0	0	Tissue specific
KGACFLSF	Superscaf	+	Superscaf	24148069	24148092	1	1	8	-	-	-	-	-	-	NCP	IGR	-	ID=gene-E	8	6.17125	3174.3	0.69208911	KGAC(+57.	68	465.2297	2	928.4476	Carbamido	K	Superscaf	24148069	BTP20220	0	0	0	0	0	Tissue specific
VVAQRVP	Superscaf	-	Superscaf	24200845	24200865	-4	4	10	-	-	-	-	-	-	NCP	IGR	-	ID=gene-E	7	4.104285714	1908.2	0.691416204	VVAQRVP	76	384.738	2	767.4653	.	S	Superscaf	24200883	0	0	BTP20220	0	0	0	Tissue specific
ADGGSFP	Superscaf	-	Superscaf	24241855	24241875	-8	8	14	-	-	gene	-	-	mRNA	NCP	Intron	gene|mRN	ID=gene-E	7	5.484285714	2425.6	0.690893288	A(+42.01)	84	692.2878	1	691.2813	Acetylati	P	Superscaf	24241971	BTP20220	0	0	0	0	0	Tissue specific
LDRSHL	Superscaf	+	Superscaf	24489439	24489456	18	18	23	-	-	-	-	-	-	NCP	IGR	-	ID=gene-E	6	6.493333333	2770.2	0.687736398	LDRSHL	92	370.7048	2	739.3976	.	E	Superscaf	24489388	0	0	0	BTP20220	0	0	Tissue specific
ELLAGP	Superscaf	-	Superscaf	24508807	24508824	-22	22	27	-	-	-	-	-	-	NCP	IGR	-	ID=gene-E	6	5.578333333	2078.3	0.687489437	ELLAGP	75	599.3494	1	598.3326	.	T	Superscaf	24508824	0	0	0	BTP20220	0	0	Tissue specific
MHKKAGK	Superscaf	+	Superscaf	24510781	24510801	2	2	8	-	-	-	-	-	-	NCP	IGR	-	ID=gene-E	7	7.792857143	3005	0.687464229	M(+15.99)	73	408.2296	2	814.4483	Oxidation	R	Superscaf	24510778	0	0	0	BTP20220	0	0	Tissue specific
PLLTLFP	Superscaf	-	Superscaf	24650818	24650838	-1	1	7	-	-	-	-	-	-	NCP	IGR	-	ID=gene-E	7	6.078571429	2519.9	0.685678625	PLLTLFP	68	800.488	1	799.4843	.	P	Superscaf	24650853	0	0	BTP20220	BTP20220	0	0	Tissue specific
GHNLF	Superscaf	-	Superscaf	24725353	24725367	-34	34	38	-	-	-	-	-	-	NCP	IGR	-	ID=gene-E	5	6.086	2254.5	0.68472831	G(+42.01)	71	629.2916	1	628.2969	Acetylati	F	Superscaf	24725388	0	0	0	BTP20220	0	0	Tissue specific
CFCDC	Superscaf	-	Superscaf	24779797	24779811	-10	10	14	-	-	-	-	-	-	NCP	IGR	-	ID=gene-E	5	4.702	2556	0.684034098	C(+57.02)	51	761.2139	1	760.1979	Carbamido	C	Superscaf	24779895	0	BTP20220	0	0	0	0	Tissue specific
YSRVPPP	Superscaf	-	Superscaf	24819793	24819813	-22	22	28	-	-	-	-	-	-	NCP	IGR	-	ID=gene-E	7	5.857142857	2503.9	0.683524035	YSRVPPP	64	408.2316	2	814.4337	.	K	Superscaf	24819942	BTP20220	0	0	0	0	0	Tissue specific
LLYAFAEAT	Superscaf	-	Superscaf	24823687	24823722	-21	21	32	-	-	-	-	-	-	NCP	IGR	-	ID=gene-E	12	5.579166667	4116.4	0.683474192	LLYAFAEAT	79	661.8684	2	1321.728	.	L	Superscaf	24823863	0	0	BTP20220	0	0	0	Tissue specific

PDPLSR	Superscaf	+	Superscaf	24883606	24883623	38	38	43	-	-	-	-	-	-	NCP	IGR	-	ID=gene-E	6	6.331666667	2471.9	0.682710398	PDPLSR	69	684.3654	1	683.3602	.	L	Superscaf	24883495	0	0	BTP20220	0	0	0	Tissue specific
VHDSKLK	Superscaf	-	Superscaf	25207168	25207188	-32	32	38	-	-	-	-	-	-	NCP	IGR	-	ID=gene-E	7	5.957142857	2704.8	0.678584639	VHDSKLK	64	413.7476	2	825.4708	.	M	Superscaf	25207293	0	BTP20220	0	0	0	BTP20220	Tissue specific
KENPKALH	Superscaf	-	Superscaf	25377058	25377081	-14	14	21	-	-	-	-	-	-	NCP	IGR	-	ID=gene-E	8	6.75	3580.8	0.676418344	KENPKALH	63	468.7712	2	935.5189	.	A	Superscaf	25377108	BTP20220	0	0	0	0	0	Tissue specific
EDNLAAK	Superscaf	+	Superscaf	25393657	25393677	2	2	8	-	-	-	-	-	-	NCP	IGR	-	ID=gene-E	7	5.622857143	2878.1	0.676206729	EDNLAAK	65	380.6994	2	759.3763	.	H	Superscaf	25393654	0	0	0	BTP20220	0	0	Tissue specific
KDFLYPF	Superscaf	+	Superscaf	25413883	25413903	2	2	8	-	-	-	-	-	-	NCP	IGR	-	ID=gene-E	7	5.944285714	2805	0.675948829	KDFLYPF	57	929.4722	1	928.4694	.	G	Superscaf	25413880	BTP20220	0	0	0	0	0	Tissue specific
VDMATVV	Superscaf	-	Superscaf	25450177	25450197	-224	224	230	-	-	gene	-	-	mRNA	NCP	Intron	gene|mRN	ID=gene-E	7	3.038571429	1491.8	0.675486046	VDMATVV	65	734.3724	1	733.368	.	L	Superscaf	25450752	0	0	0	0	BTP20220	0	Tissue specific
QELLALNR	Superscaf	-	Superscaf	25452658	25452681	-31	31	38	-	-	gene	-	-	mRNA	NCP	Intron	gene|mRN	ID=gene-E	8	6.125	3653.2	0.675454373	Q(+42.01)	57	499.7832	2	997.5556	Acetylati	L	Superscaf	25452693	0	0	0	0	0	BTP20220	Tissue specific
KPTRP	Superscaf	+	Superscaf	25510528	25510542	1	1	5	-	-	gene	-	-	mRNA	NCP	Intron	gene|mRN	ID=gene-E	5	7.926	2189.5	0.674716591	K(+42.01)	64	640.3748	1	639.3704	Acetylati	K	Superscaf	25510528	0	0	0	0	BTP20220	0	Mixed
AEEVVVVAK	Superscaf	-	Superscaf	25513300	25513329	-3	3	12	-	-	gene	-	-	mRNA	NCP	Intron	gene|mRN	ID=gene-E	10	3.37	2329.4	0.674681054	A(+42.01)	98	566.815	2	1131.617	Acetylati	K	Superscaf	25513539	BTP20220	0	0	0	0	0	Tissue enhanced
AEEVVVVAK	Superscaf	-	Superscaf	25513300	25513326	-3	3	11	-	-	gene	-	-	mRNA	NCP	Intron	gene|mRN	ID=gene-E	9	3.135555556	1942	0.674681092	A(+42.01)	99	493.2805	2	984.5491	Acetylati	K	Superscaf	25513539	0	0	BTP20220	0	0	0	Mixed
SGDVAKK	Superscaf	-	Superscaf	25542073	25542093	-1	1	7	-	-	gene	-	-	mRNA	NCP	Intron	gene|mRN	ID=gene-E	7	5.731428571	2340.3	0.674314286	SGDVAKK	94	352.6936	2	703.3864	.	S	Superscaf	25542162	0	BTP20220	0	0	0	0	Tissue specific
MDFNVKK	Superscaf	+	Superscaf	25544305	25544325	4	4	10	-	-	gene	-	-	mRNA	NCP	Intron	gene|mRN	ID=gene-E	7	5.584285714	2703.9	0.674285826	M(+42.01)	74	462.2358	2	922.4582	Acetylati	S	Superscaf	25544296	0	BTP20220	0	0	0	BTP20220	Tissue specific
SLLKKKP	Superscaf	+	Superscaf	25573345	25573365	13	13	19	-	-	gene	-	-	mRNA	NCP	Intron	gene|mRN	ID=gene-E	7	7.594285714	2832.8	0.673915538	SLLKKKP	55	407.2803	2	812.5483	.	D	Superscaf	25573309	0	BTP20220	0	0	0	0	Tissue specific
NRVLVLL	Superscaf	+	Superscaf	25589554	25589574	9	9	15	-	-	gene	-	-	mRNA	NCP	Intron	gene|mRN	ID=gene-E	7	4.872857143	2305.7	0.673708858	N(+42.01)	58	434.7836	2	867.5541	Acetylati	N	Superscaf	25589530	0	0	BTP20220	0	0	0	Tissue specific
EALVEL	Superscaf	-	Superscaf	25676152	25676169	-4	4	9	-	-	gene	-	-	mRNA	NCP	Intron	gene|mRN	ID=gene-E	6	4.07	1869.1	0.672604691	EALVEL	59	673.374	1	672.3694	.	A	Superscaf	25676178	0	0	0	BTP20220	0	0	Tissue specific
DDCMC	Superscaf	-	Superscaf	25692742	25692756	-3	3	7	-	-	gene	-	-	mRNA	NCP	Intron	gene|mRN	ID=gene-E	5	4.346	2365.9	0.672393191	DDC(+57.(	50	716.1663	1	715.1612	Carbamido	G	Superscaf	25692780	0	0	BTP20220	BTP20220	0	0	Tissue specific
RSLKTGY	Superscaf	+	Superscaf	25851532	25851552	20	20	26	-	-	gene	-	-	mRNA	NCP	Intron	gene|mRN	ID=gene-E	7	7.188571429	3008.3	0.670368392	RSLKTGY	56	412.7331	2	823.4552	.	T	Superscaf	25851475	0	0	0	0	0	BTP20220	Tissue specific
KNHPVEYF	Superscaf	-	Superscaf	25906108	25906131	-32	32	39	-	-	-	-	-	-	NCP	IGR	-	ID=gene-E	8	5.425	3294.6	0.669672459	KNHPVEYF	53	517.2598	2	1032.503	.	G	Superscaf	25906155	0	0	0	0	BTP20220	0	Tissue specific
NVFLVA	Superscaf	-	Superscaf	25910410	25910427	-30	30	35	-	-	-	-	-	-	NCP	IGR	-	ID=gene-E	6	3.815	1648.8	0.669617681	NVFLVA	70	662.3842	1	661.3799	.	L	Superscaf	25910448	0	0	0	0	0	BTP20220	Tissue specific
LASMPLN	Superscaf	+	Superscaf	25960036	25960056	10	10	16	-	-	-	-	-	-	NCP	IGR	-	ID=gene-E	7	5.874285714	2707.1	0.668984864	L(+42.01)	55	787.3993	1	786.3946	Acetylati	V	Superscaf	25960009	0	0	0	0	BTP20220	0	Tissue specific
LSSWRP	Superscaf	-	Superscaf	26015917	26015934	-18	18	23	-	-	-	-	-	-	NCP	IGR	-	ID=gene-E	6	6.715	2633	0.668272367	LSSWRP	53	745.3966	1	744.3918	.	C	Superscaf	26015946	BTP20220	0	0	0	0	0	Tissue specific
HGRLNPY	Superscaf	-	Superscaf	26084938	26084958	-25	25	31	-	-	-	-	-	-	NCP	IGR	-	ID=gene-E	7	6.81	3312.8	0.667392246	HGRLNPY	88	428.7233	2	855.4351	.	S	Superscaf	26085000	0	0	0	0	0	BTP20220	Tissue specific
YTGLK	Superscaf	-	Superscaf	26183917	26183931	-10	10	14	-	-	-	-	-	-	NCP	IGR	-	ID=gene-E	5	6.776	1976.1	0.666130247	Y(+42.01)	65	623.347	1	622.3326	Acetylati	F	Superscaf	26183952	BTP20220	0	0	0	0	0	Tissue specific
NVLYL	Superscaf	+	Superscaf	26206231	26206245	12	12	16	-	-	-	-	-	-	NCP	IGR	-	ID=gene-E	5	4.606	1763	0.665845723	NVLYL	78	621.3584	1	620.3533	.	F	Superscaf	26206198	0	0	0	0	0	BTP20220	Tissue specific
LSGKPKK	Superscaf	+	Superscaf	26254384	26254404	4	4	10	-	-	-	-	-	-	NCP	IGR	-	ID=gene-E	7	7.592857143	2776.7	0.66523165	LSGKPKK	55	379.2489	2	756.4857	.	N	Superscaf	26254375	0	0	0	0	0	BTP20220	Tissue specific
LFSLLGD	Superscaf	-	Superscaf	26279368	26279388	-70	70	76	-	-	-	-	-	-	NCP	IGR	-	ID=gene-E	7	5.434285714	2539.8	0.664913081	LFSLLGD	53	382.7136	2	763.4116	.	V	Superscaf	26279478	0	0	0	0	0	BTP20220	Tissue specific
ALLPAGP	Superscaf	-	Superscaf	26370889	26370909	-61	61	67	-	-	-	-	-	-	NCP	IGR	-	ID=gene-E	7	6.081428571	2301.5	0.663746102	A(+42.01)	65	680.4033	1	679.3904	Acetylati	L	Superscaf	26370918	0	0	0	0	0	BTP20220	Tissue specific
NSRHVLN	Superscaf	-	Superscaf	26395069	26395089	-1	1	7	-	-	-	-	-	-	NCP	IGR	-	ID=gene-E	7	5.832857143	3198.7	0.663437784	NSRHVLN	69	420.227	2	838.4409	.	N	Superscaf	26395113	0	0	0	0	BTP20220	0	Tissue specific
LAALK	Superscaf	+	Superscaf	26395291	26395305	26	26	30	-	-	-	-	-	-	NCP	IGR	-	ID=gene-E	5	6.748	1797.8	0.66343503	LAALK	99	515.3533	1	514.3478	.	Q	Superscaf	26395216	0	0	0	0	0	BTP20220	Tissue specific
APWAVTK	Superscaf	-	Superscaf	26412145	26412165	-4	4	10	-	-	-	-	-	-	NCP	IGR	-	ID=gene-E	7	5.785714286	2352.5	0.663220049	APWAVTK	74	386.7221	2	771.4279	.	S	Superscaf	26412246	0	0	0	0	BTP20220	0	Tissue specific
ELATLLLE	Superscaf	+	Superscaf	26466625	26466648	4	4	11	-	-	-	-	-	-	NCP	IGR	-	ID=gene-E	8	5.36375	2973.4	0.66252534	ELATLLLE	64	451.2626	2	900.5168	.	A	Superscaf	26466616	0	0	0	0	0	BTP20220	Tissue specific
APFPGQLP	Superscaf	-	Superscaf	26495590	26495613	-53	53	60	-	-	-	-	-	-	NCP	IGR	-	ID=gene-E	8	6	2972.3	0.662156009	APFPGQLP	77	413.7303	2	825.4384	.	D	Superscaf	26495619	0	0	0	0	0	BTP20220	Tissue specific
TLFDQ	Superscaf	+	Superscaf	26538307	26538321	5	5	9	-	-	-	-	-	-	NCP	IGR	-	ID=gene-E	5	5.322	2160.5	0.661611441	TLFDQ	66	623.3098	1	622.2962	.	L	Superscaf	26538295	0	0	0	0	BTP20220	0	Tissue specific
LALNYSVF	Superscaf	+	Superscaf	26607361	26607384	9	9	16	-	-	-	-	-	-	NCP	IGR	-	ID=gene-E	8	5.02625	2845.1	0.660730823	LALNYSVF	78	926.4927	1	925.4909	.	E	Superscaf	26607337	0	0	0	0	BTP20220	0	Tissue specific
EGKGFGYK	Superscaf	-	Superscaf	26643331	26643354	-5	5	12	-	-	-	-	-	-	NCP	IGR	-	ID=gene-E	8	6.46875	3101.1	0.660272172	EGKGFGYK	58	443.2263	2	884.4391	.	I	Superscaf	26643372	0	0	0	0	BTP20220	0	Tissue specific
DNFGKY	Superscaf	+	Superscaf	26659456	26659473	1	1	6	-	-	-	-	-	-	NCP	IGR	-	ID=gene-E	6	5.871666667	2620.8	0.660066639	DNFGKY	68	743.3468	1	742.3286	.	D	Superscaf	26659456	0	0	0	0	BTP20220	BTP20220	Tissue specific
FPPLVGPT	Superscaf	-	Superscaf	26746447	26746470	-115	115	122	-	-	-	-	-	-	NCP	IGR	-	ID=gene-E	8	5.3575	2447.8	0.658957346	FPPLVGPT	63	414.2344	2	826.4589	.	L	Superscaf	26746479	0	BTP20220	0	0	BTP20220	BTP20220	Tissue specific
LDAELLTK	Superscaf	-	Superscaf	26794306	26794329	-9	9	16	-	-	-	-	-	-	NCP	IGR	-	ID=gene-E	8	5.8025	3074.4	0.658347099	LDAELLTK	98	451.7618	2	901.512	.	L	Superscaf	26794395	0	BTP20220	0	0	BTP20220	0	Tissue specific
AASPP	Superscaf	-	Superscaf	26931331	26931345	-1	1	5	-	-	-	-	-	-	NCP	IGR	-	ID=gene-E	5	6.064	1680.8	0.656600016	AASPP	65	442.2329	1	441.2223	.	A	Superscaf	26931423	0	BTP20220	0	0	0	0	Tissue specific
LVGRP	Superscaf	-	Superscaf	26948359	26948373	-11	11	15	-	-	-	-	-	-	NCP	IGR	-	ID=gene-E	5	5.802	1636.9	0.656382893	LVGRP	83	541.3442	1	540.3384	.	I	Superscaf	26948373	0	0	0	0	0	BTP20220	Tissue specific
FSLGGF	Superscaf	-	Superscaf	26953042	26953059	-5	5	10	-	-	-	-	-	-	NCP	IGR	-	ID=gene-E	6	5.76	2106.2	0.656323142	F(+42.01)	61	669.3213	1	668.317	Acetylati	P	Superscaf	26953122	0	0	0	BTP20220	0	0	Tissue specific
FGEGLL	Superscaf	-	Superscaf	27177721	27177738	-101	101	106	-	-	-	-	-	-	NCP	IGR	-	ID=gene-E	6	5.433333333	2114.3	0.653458273	FGEGLL	99	635.3385	1	634.3326	.	Y	Superscaf	27178134	0	0	0	0	0	BTP20220	Mixed
KPRMKAK	Superscaf	+	Superscaf	27405502	27405522	14	14	20	-	-	-	-	-	-	NCP	IGR	-	ID=gene-E	7	8.292857143	3074.2	0.650553813	KPRM(+15.	54	437.7698	2	873.5218	Oxidation	S	Superscaf	27405463	0	BTP20220	0	0	0	0	Tissue specific
APKRSFF	Superscaf	+	Superscaf	27593740	27593760	3	3	9	-	-	-	-	-	-	NCP	IGR	-	ID=gene-E	7	7.065714286	2924.1	0.648153601	APKRSFF	80	426.7397	2	851.4653	.	K	Superscaf	27593734	0	0	0	0	BTP20220	BTP20220	Tissue specific
FMSAFVK	Superscaf	+	Superscaf	27610231	27610251	49	49	55	-	-	-	-	-	-	NCP	IGR	-	ID=gene-E	7	5.45	2309.4	0.647943326	FMSAFVK	93	415.2152	2	828.4203	.	F	Superscaf	27610087	0	BTP20220	0	0	0	0	Tissue enhanced
NDSRSP	Superscaf	+	Superscaf	27626359	27626376	15	15	20	-	-	-	-	-	-	NCP	IGR	-	ID=gene-E	6	6.133333333	2761.2	0.647737717	N(+42.01)	53	359.1662	2	716.3089	Acetylati	H	Superscaf	27626317	0	BTP20220	0	0	0	0	Tissue specific
LLPLQR	Superscaf	-	Superscaf	27763207	27763224	-16	16	21	-	-	-	-	-	-	NCP	IGR	-	ID=gene-E	6	6.775	2657.1	0.645992776	LLPLQR	99	370.2437	2	738.4752	.	A	Superscaf	27763233	0	BTP20220	0	0	0	0	Mixed
KAKLKKDF	Superscaf	+	Superscaf	27881482	27881505	3	3	10	-	-	-	-	-	-	NCP	IGR	-	ID=gene-E	8	7.42625	3337.2	0.644484582	KAKLKKDF	59	489.3095	2	976.6069	.	I	Superscaf	27881476	0	0	0	0	BTP20220	0	Tissue specific
KGLLSHSL	Superscaf	-	Superscaf	28008250	28008273	-52	52	59	-	-	-	-	-	-	NCP	IGR	-	ID=gene-E	8	6.575	3212.4	0.642868171	KGLLSHSL	67	427.7568	2	853.5021	.	K	Superscaf	28008285	BTP20220	0	0	0	0	0	Tissue specific
GATKVRP	Superscaf	-	Superscaf	28092049	28092069	-1	1	7	-	-	-	-	-	-	NCP	IGR	-	ID=gene-E	7	6.474285714	2460.7	0.641799693	GATKVRP	56	364.7278	2	727.434	.	G	Superscaf	28092219	BTP20220	0	0	0	0	0	Tissue specific
QKAAKPR	Superscaf	-	Superscaf	28099420	28099440	-90	90	96	-	-	-	-	-	-	NCP	IGR	-	ID=gene-E	7	7.747142857	3156.3	0.641705706	QKAAKPR	53	399.7553	2	797.4871	.	Q	Superscaf	28099440	BTP20220	0	0	0	0	0	Tissue specific
KEGFTVP	Superscaf	-	Superscaf	28172518	28172538	-17	17	23	-	-	-	-	-	-	NCP	IGR	-	ID=gene-E	7	5.32	2313.5	0.640773637	KEGFTVP	50	389.2092	2	776.4068	.	P	Superscaf	28172577	0	0	0	BTP20220	0	0	Tissue specific
YDKAY	Superscaf	+	Superscaf	28297933	28297947	2	2	6	-	-	-	-	-	-	NCP	IGR	-	ID=gene-E	5	6.01	2110.2	0.639174555	YDKAY	51	659.2967	1	658.2962	.	T	Superscaf	28297930	0	BTP20220	BTP20220	0	0	0	Tissue specific
LAGLLLKA	Superscaf	-	Superscaf	28348513	28348536	-41	41	48	-	-	-	-	-	-	NCP	IGR	-	ID=gene-E	8	6.45875	2801.9	0.638529497	L(+42.01)	51	420.7779	2	839.548	Acetylati	L	Superscaf	28348590	0	BTP20220	0	0	0	0	Tissue specific
RLKLPF	Superscaf	+	Superscaf	28424632	28424649	5	5	10	-	-	gene	-	-	mRNA	NCP	Intron	gene|mRN	ID=gene-E	6	7.373333333	2548.8	0.637558985	RLKLPF	57	387.2534	2	772.4959	.	E	Superscaf	28424620	0	0	0	BTP20220	0	0	Tissue specific
PQQHADK	Superscaf	+	Superscaf	28487803	28487823	20	20	26	-	-	gene	-	-	mRNA	NCP	Intron	gene|mRN	ID=gene-E	7	6.274285714	3369.7	0.636753457	PQQHADK	74	412.2081	2	822.3984	.	Q	Superscaf	28487746	0	BTP20220	0	0	0	0	Tissue specific
VEDLP	Superscaf	-	Superscaf	28620739	28620753	-4	4	8	-	-	gene	-	-	mRNA	NCP	Intron	gene|mRN	ID=gene-E	5	3.694	1527.8	0.635058474	VEDLP	75	572.2912	1	571.2853	.	K	Superscaf	28620786	0	0	0	0	0	BTP20220	Tissue specific
KTMLTK	Superscaf	+	Superscaf	28641397	28641414	12	12	17	-	-	gene	-	-	mRNA	NCP	Intron	gene|mRN	ID=gene-E	6	7.378333333	2456.7	0.634795027	KTMLTK	60	361.2153	2	720.4204	.	I	Superscaf	28641364	BTP20220	0	0	0	0	0	Tissue specific
PLLAA	Superscaf	-	Superscaf	28671940	28671954	-30	30	34	-	-	gene	-	-	mRNA	NCP	Intron	gene|mRN	ID=gene-E	5	6.06	1666.8	0.634405613	PLLAA	99	484.3113	1	483.3057	.	P	Superscaf	28672011	0	0	0	0	0	BTP20220	Tissue specific
MFPLPQK	Superscaf	+	Superscaf	28710136	28710156	50	50	56	-	-	gene	-	-	mRNA	NCP	Intron	gene|mRN	ID=gene-E	7	6.457142857	2866.2	0.633918502	M(+15.99)	80	876.4565	1	875.4575	Oxidation	T	Superscaf	28709989	0	BTP20220	0	0	0	0	Tissue specific
QFKFR	Superscaf	+	Superscaf	28812688	28812702	11	11	15	-	-	-	-	-	-	NCP	IGR	-	ID=gene-E	5	7.422	2502.7	0.632610944	QFKFR	90	363.2101	2	724.402	.	I	Superscaf	28812658	0	0	0	BTP20220	0	0	Tissue specific
LPDRF	Superscaf	+	Superscaf	28833955	28833969	52	52	56	-	-	-	-	-	-	NCP	IGR	-	ID=gene-E	5	6.298	2138.5	0.63233977	LPDRF	54	647.3514	1	646.3438	.	L	Superscaf	28833802	0	BTP20220	BTP20220	BTP20220	BTP20220	BTP20220	Tissue specific
LVLELRPG	Superscaf	-	Superscaf	28855135	28855158	-58	58	65	-	-	-	-	-	-	NCP	IGR	-	ID=gene-E	8	5.52375	2769.2	0.63206959	LVLELRPG	71	448.7814	2	895.5491	.	A	Superscaf	28855173	0	BTP20220	0	0	0	0	Tissue specific
LMCCC	Superscaf	-	Superscaf	28936489	28936503	-5	5	9	-	-	-	-	-	-	NCP	IGR	-	ID=gene-E	5	5.358	2482	0.631032365	LM(+15.99	55	759.2186	1	758.222	Oxidation	F	Superscaf	28936629	0	0	0	0	BTP20220	0	Tissue specific
KKFPVF	Superscaf	-	Superscaf	28993675	28993692	-13	13	18	-	-	-	-	-	-	NCP	IGR	-	ID=gene-E	6	6.123333333	2049	0.630303151	KKFPVF	85	383.2355	2	764.4584	.	Y	Superscaf	28993692	0	0	BTP20220	0	0	BTP20220	Mixed
EDDLRK	Superscaf	+	Superscaf	29056171	29056188	28	28	33	-	-	-	-	-	-	NCP	IGR	-	ID=gene-E	6	5.94	2719.1	0.629506269	EDDLRK	83	388.1988	2	774.3871	.	I	Superscaf	29056090	0	BTP20220	0	0	0	BTP20220	Tissue specific
VNLEF	Superscaf	-	Superscaf	29099167	29099181	-12	12	16	-	-	-	-	-	-	NCP	IGR	-	ID=gene-E	5	4.018	1763	0.628958067	VNLEF	95	621.321	1	620.317	.	R	Superscaf	29099196	BTP20220	0	0	0	0	0	Tissue specific
LTVEK	Superscaf	+	Superscaf	29185000	29185014	3	3	7	-	-	-	-	-	-	NCP	IGR	-	ID=gene-E	5	5.094	1644.8	0.627863616	LTVEK	70	589.3438	1	588.3483	.	V	Superscaf	29184994	0	0	0	0	0	BTP20220	Tissue specific
AQPKY	Superscaf	-	Superscaf	29245582	29245596	-8	8	12	-	-	-	-	-	-	NCP	IGR	-	ID=gene-E	5	6.674	2187.3	0.627091138	AQPKY	60	606.3333	1	605.3173	.	C	Superscaf	29245611	0	0	BTP20220	BTP20220	0	0	Tissue specific
YHMVYM	Superscaf	-	Superscaf	29266204	29266221	-28	28	33	-	-	-	-	-	-	NCP	IGR	-	ID=gene-E	6	5.068333333	2281.7	0.62682815	YHMVYM	69	422.1794	2	842.3455	.	K	Superscaf	29266245	0	0	0	0	BTP20220	0	Tissue specific
VTAAPLY	Superscaf	-	Superscaf	29356867	29356887	-31	31	37	-	-	-	-	-	-	NCP	IGR	-	ID=gene-E	7	5.215714286	2170.4	0.625672073	VTAAPLY	70	734.4061	1	733.401	.	Y	Superscaf	29356902	0	0	0	BTP20220	0	0	Tissue specific
VPLLLDQH	Superscaf	+	Superscaf	29585293	29585316	21	21	28	-	-	-	-	-	-	NCP	IGR	-	ID=gene-E	8	5.05625	3039.5	0.622759389	VPLLLDQH	97	467.7701	2	933.5283	.	Q	Superscaf	29585233	0	0	0	BTP20220	0	0	Tissue specific
TYGAPR	Superscaf	-	Superscaf	29674393	29674410	-5	5	10	-	-	-	-	-	-	NCP	IGR	-	ID=gene-E	6	6.873333333	2451.8	0.621623356	T(+42.01)	56	353.6742	2	705.3445	Acetylati	N	Superscaf	29674446	0	0	0	0	0	BTP20220	Tissue specific
FPKQYVL	Superscaf	+	Superscaf	29801866	29801886	22	22	28	-	-	-	-	-	-	NCP	IGR	-	ID=gene-E	7	5.544285714	2616.8	0.619997918	FPKQYVL	59	447.755	2	893.501	.	L	Superscaf	29801803	0	0	0	0	0	BTP20220	Tissue specific
KLVPHSKP	Superscaf	-	Superscaf	29813335	29813358	-16	16	23	-	-	-	-	-	-	NCP	IGR	-	ID=gene-E	8	6.41625	2968.1	0.619851639	KLVPHSKP	74	453.2812	2	904.5494	.	K	Superscaf	29813382	0	0	0	0	0	BTP20220	Tissue specific
FALDGL	Superscaf	-	Superscaf	29841376	29841393	-67	67	72	-	-	-	-	-	-	NCP	IGR	-	ID=gene-E	6	5.4	2114.3	0.619494166	FALDGL	68	635.3403	1	634.3326	.	N	Superscaf	29841453	0	BTP20220	BTP20220	0	BTP20220	BTP20220	Tissue specific
TVEELLY	Superscaf	-	Superscaf	29945926	29945946	-33	33	39	-	-	-	-	-	-	NCP	IGR	-	ID=gene-E	7	4.37	2414.8	0.618161017	TVEELLY	75	866.4449	1	865.4433	.	F	Superscaf	29945952	0	0	0	0	BTP20220	BTP20220	Tissue specific
VPSLDL	Superscaf	-	Superscaf	29968258	29968275	-16	16	21	-	-	-	-	-	-	NCP	IGR	-	ID=gene-E	6	4.485	1839.1	0.617876301	VPSLDL	53	643.3636	1	642.3588	.	G	Superscaf	29968446	0	BTP20220	BTP20220	BTP20220	0	BTP20220	Tissue specific
SLYVYKVL	Superscaf	+	Superscaf	30014086	30014109	8	8	15	-	-	-	-	-	-	NCP	IGR	-	ID=gene-E	8	4.8375	2477.6	0.617291875	SLYVYKVL	72	492.79	2	983.5692	.	R	Superscaf	30014065	0	BTP20220	BTP20220	BTP20220	0	0	Tissue specific
EVLNQPM	Superscaf	-	Superscaf	30093580	30093600	-7	7	13	-	-	-	-	-	-	NCP	IGR	-	ID=gene-E	7	4.615714286	2695.2	0.61627829	EVLNQPM	75	415.7061	2	829.4004	.	L	Superscaf	30093624	0	0	BTP20220	BTP20220	BTP20220	BTP20220	Tissue enhanced
ATFKLLPF	Superscaf	+	Superscaf	30103588	30103611	17	17	24	-	-	-	-	-	-	NCP	IGR	-	ID=gene-E	8	6.43875	2996.2	0.61615064	A(+42.01)	54	489.7834	2	977.5585	Acetylati	N	Superscaf	30103540	0	BTP20220	0	0	0	BTP20220	Tissue specific
AAPYHRLP	Superscaf	+	Superscaf	30132598	30132621	3	3	10	-	-	-	-	-	-	NCP	IGR	-	ID=gene-E	8	6.82875	3378.8	0.615780735	AAPYHRLP	73	462.7543	2	923.4977	.	E	Superscaf	30132592	0	0	0	0	0	BTP20220	Tissue specific
KENLPEAK	Superscaf	+	Superscaf	30202516	30202539	9	9	16	-	-	-	-	-	-	NCP	IGR	-	ID=gene-E	8	6.20375	3386.6	0.614889215	KENLPEAK	50	464.7574	2	927.5025	.	T	Superscaf	30202492	0	0	0	0	0	BTP20220	Tissue specific
KHFGPSP	Superscaf	-	Superscaf	30281923	30281943	-1	1	7	-	-	-	-	-	-	NCP	IGR	-	ID=gene-E	7	6.722857143	2831	0.613876739	KHFGPSP	66	385.2037	2	768.3918	.	K	Superscaf	30281970	0	0	BTP20220	BTP20220	BTP20220	BTP20220	Tissue specific
KSDGLGLK	Superscaf	-	Superscaf	30303556	30303579	-3	3	10	-	-	-	-	-	-	NCP	IGR	-	ID=gene-E	8	6.50375	3033.1	0.61360086	K(+42.01)	68	430.2424	2	858.4811	Acetylati	G	Superscaf	30303630	0	0	0	0	0	BTP20220	Tissue specific
PPSLFTGL	Superscaf	-	Superscaf	30569638	30569661	-80	80	87	-	-	-	-	-	-	NCP	IGR	-	ID=gene-E	8	6.0275	2847.2	0.610208064	P(+42.01)	54	437.2425	2	872.4644	Acetylati	R	Superscaf	30569667	0	0	0	0	0	BTP20220	Tissue specific
MHEKPPP	Superscaf	-	Superscaf	30681244	30681264	-28	28	34	-	-	-	-	-	-	NCP	IGR	-	ID=gene-E	7	6.457142857	2897.3	0.608785021	M(+42.01)	63	439.2183	2	876.4163	Acetylati	F	Superscaf	30681318	0	0	0	0	0	BTP20220	Tissue specific
YQLVK	Superscaf	+	Superscaf	30803737	30803751	24	24	28	-	-	-	-	-	-	NCP	IGR	-	ID=gene-E	5	5.406	1892	0.607223196	YQLVK	66	650.3846	1	649.3799	.	N	Superscaf	30803668	0	BTP20220	0	0	BTP20220	0	Tissue specific
VGLVGK	Superscaf	+	Superscaf	30871996	30872013	23	23	28	-	-	-	-	-	-	NCP	IGR	-	ID=gene-E	6	4.61	1416.4	0.606352792	VGLVGK	96	572.3755	1	571.3693	.	R	Superscaf	30871930	0	BTP20220	0	0	0	0	Tissue specific
GVAHT	Superscaf	-	Superscaf	31068208	31068222	-70	70	74	-	-	-	-	-	-	NCP	IGR	-	ID=gene-E	5	5.222	1625.8	0.603850942	G(+42.01)	66	526.2682	1	525.2547	Acetylati	K	Superscaf	31068258	0	0	0	0	0	BTP20220	Tissue specific
YPVTR	Superscaf	-	Superscaf	31151350	31151364	-45	45	49	-	-	-	-	-	-	NCP	IGR	-	ID=gene-E	5	5.85	1843.2	0.602790804	YPVTR	56	635.3486	1	634.3438	.	S	Superscaf	31151403	0	0	0	BTP20220	0	0	Tissue specific
LDDSDVLP	Superscaf	-	Superscaf	31160785	31160808	-34	34	41	-	-	-	-	-	-	NCP	IGR	-	ID=gene-E	8	4.10625	2662.1	0.602670384	LDDSDVLP	69	873.417	1	872.4127	.	G	Superscaf	31160856	BTP20220	BTP20220	0	0	0	0	Tissue specific
PFEVP	Superscaf	+	Superscaf	31307695	31307709	21	21	25	-	-	-	-	-	-	NCP	IGR	-	ID=gene-E	5	4.26	1487.7	0.600797258	PFEVP	62	588.3007	1	587.2955	.	S	Superscaf	31307635	BTP20220	0	0	0	0	0	Tissue specific
GVLRL	Superscaf	-	Superscaf	31311580	31311594	-2	2	6	-	-	-	-	-	-	NCP	IGR	-	ID=gene-E	5	5.738	1652.9	0.60074772	GVLRL	63	557.3745	1	556.3697	.	K	Superscaf	31311633	0	BTP20220	BTP20220	BTP20220	BTP20220	0	Tissue specific
PFLEYT	Superscaf	-	Superscaf	31356004	31356021	-24	24	29	-	-	-	-	-	-	NCP	IGR	-	ID=gene-E	6	5.528333333	2360.7	0.600181234	PFLEYT	57	769.374	1	768.3694	.	R	Superscaf	31356063	0	BTP20220	0	0	0	0	Tissue specific
NSPLYK	Superscaf	-	Superscaf	31586965	31586982	-8	8	13	-	-	-	-	-	-	NCP	IGR	-	ID=gene-E	6	6.461666667	2598.8	0.597236264	N(+42.01)	81	382.2014	2	762.3912	Acetylati	S	Superscaf	31586997	0	0	BTP20220	0	0	0	Tissue specific
AVLKYCR	Superscaf	-	Superscaf	31631629	31631649	-25	25	31	-	-	-	-	-	-	NCP	IGR	-	ID=gene-E	7	6.168571429	2827.1	0.596666718	A(+42.01)	70	476.2666	2	950.5007	Acetylati	S	Superscaf	31631679	0	BTP20220	0	0	0	0	Tissue specific
SYPAAA	Superscaf	+	Superscaf	31717147	31717164	72	72	77	-	-	-	-	-	-	NCP	IGR	-	ID=gene-E	6	5.95	2114.2	0.595576321	SYPAAA	52	579.2678	1	578.27	.	A	Superscaf	31716934	0	0	BTP20220	0	0	0	Tissue specific
NPLKV	Superscaf	-	Superscaf	31729732	31729746	-31	31	35	-	-	-	-	-	-	NCP	IGR	-	ID=gene-E	5	5.486	1755.9	0.595415889	NPLKV	98	570.3594	1	569.3537	.	R	Superscaf	31729842	0	BTP20220	0	0	BTP20220	0	Tissue specific
DDDLEE	Superscaf	-	Superscaf	31809139	31809156	-26	26	31	-	-	-	-	-	-	NCP	IGR	-	ID=gene-E	6	3.555	2438.9	0.594403337	D(+42.01)	79	777.2882	1	776.2712	Acetylati	G	Superscaf	31809270	0	0	0	0	BTP20220	0	Tissue specific
AQMDLWK	Superscaf	-	Superscaf	31888063	31888083	-27	27	33	-	-	-	-	-	-	NCP	IGR	-	ID=gene-E	7	6	3053.4	0.593396943	AQMDLWK	50	446.2238	2	890.432	.	A	Superscaf	31888110	0	0	0	0	BTP20220	0	Tissue specific
KFLVAW	Superscaf	+	Superscaf	32301292	32301309	23	23	28	-	-	-	-	-	-	NCP	IGR	-	ID=gene-E	6	5.518333333	2047.1	0.588127923	KFLVAW	65	763.456	1	762.4428	.	C	Superscaf	32301226	0	0	0	0	0	BTP20220	Tissue specific
GCVLQKA	Superscaf	+	Superscaf	32421127	32421147	33	33	39	-	-	-	-	-	-	NCP	IGR	-	ID=gene-E	7	5.482857143	2626.8	0.586599876	G(+42.01)	87	409.2141	2	816.4164	Acetylati	L	Superscaf	32421031	0	BTP20220	0	0	0	0	Tissue specific
QDDKLV	Superscaf	-	Superscaf	32491315	32491332	-27	27	32	-	-	-	-	-	-	NCP	IGR	-	ID=gene-E	6	4.551666667	2255.5	0.585704951	QDDKLV	55	359.1953	2	716.3704	.	I	Superscaf	32491332	0	0	0	0	0	BTP20220	Tissue specific
CVFLLQ	Superscaf	-	Superscaf	32527273	32527290	-1	1	6	-	-	-	-	-	-	NCP	IGR	-	ID=gene-E	6	4.685	2290.6	0.585246453	C(+57.02)	51	779.4091	1	778.4047	Carbamido	C	Superscaf	32527314	0	BTP20220	0	0	0	0	Tissue specific
KELLKK	Superscaf	-	Superscaf	32601937	32601954	-44	44	49	-	-	-	-	-	-	NCP	IGR	-	ID=gene-E	6	7.4	2537.5	0.584294416	K(+42.01)	97	400.7635	2	799.5167	Acetylati	K	Superscaf	32601969	0	BTP20220	0	0	0	0	Tissue specific
NYLLKWN	Superscaf	+	Superscaf	32625964	32625984	2	2	8	-	-	-	-	-	-	NCP	IGR	-	ID=gene-E	7	6.295714286	3354.7	0.583988011	NYLLKWN	58	950.5063	1	949.5021	.	E	Superscaf	32625961	0	BTP20220	0	0	0	BTP20220	Tissue specific
GPQDLPAP	Superscaf	+	Superscaf	32699572	32699595	29	29	36	-	-	-	-	-	-	NCP	IGR	-	ID=gene-E	8	5.68625	2996.4	0.583049402	GPQDLPAP	53	397.7031	2	793.397	.	E	Superscaf	32699488	0	0	BTP20220	0	0	BTP20220	Tissue specific
MPKLPR	Superscaf	+	Superscaf	32723410	32723427	12	12	17	-	-	-	-	-	-	NCP	IGR	-	ID=gene-E	6	7.471666667	2516.9	0.582745521	MPKLPR	86	371.2244	2	740.4367	.	F	Superscaf	32723377	0	BTP20220	0	0	0	0	Tissue specific
VSVTDKF	Superscaf	-	Superscaf	32748610	32748630	-9	9	15	-	-	-	-	-	-	NCP	IGR	-	ID=gene-E	7	4.342857143	2048.2	0.582424159	V(+42.01)	71	837.4316	1	836.428	Acetylati	N	Superscaf	32748738	0	BTP20220	0	0	0	0	Tissue specific
TPQVVAKK	Superscaf	+	Superscaf	32758048	32758071	23	23	30	-	-	-	-	-	-	NCP	IGR	-	ID=gene-E	8	5.4975	2593.7	0.582303778	TPQVVAKK	62	435.7725	2	869.5334	.	R	Superscaf	32757982	0	BTP20220	0	0	0	0	Tissue specific
RLVPY	Superscaf	-	Superscaf	32895040	32895054	-44	44	48	-	-	-	-	-	-	NCP	IGR	-	ID=gene-E	5	5.74	1799.1	0.580557116	RLVPY	66	647.3851	1	646.3802	.	I	Superscaf	32895210	0	0	BTP20220	0	0	0	Tissue specific
VVPNDLA	Superscaf	-	Superscaf	33108976	33108996	-1	1	7	-	-	gene	-	-	mRNA	NCP	Intron	gene|mRN	ID=gene-E	7	3.811428571	2010.3	0.577829154	VVPNDLA	80	727.3967	1	726.3912	.	V	Superscaf	33109014	0	0	0	0	0	BTP20220	Tissue specific
PLLKKL	Superscaf	-	Superscaf	33318259	33318276	-13	13	18	-	-	gene	-	-	mRNA	NCP	Intron	gene|mRN	ID=gene-E	6	7.286666667	2334.4	0.575160637	PLLKKL	67	356.2633	2	710.5054	.	V	Superscaf	33318438	BTP20220	0	0	0	0	0	Tissue specific
MDCGP	Superscaf	+	Superscaf	33407362	33407376	9	9	13	-	-	gene	-	-	mRNA	NCP	Intron	gene|mRN	ID=gene-E	5	5.202	2003.4	0.574024528	M(+15.99)	50	637.1887	1	636.1884	Oxidation	S	Superscaf	33407338	BTP20220	0	BTP20220	0	0	0	Tissue specific
FPFTSGQ	Superscaf	-	Superscaf	33455014	33455034	-49	49	55	-	-	gene	-	-	mRNA	NCP	Intron	gene|mRN	ID=gene-E	7	5.87	2801.1	0.573416844	FPFTSGQ	53	392.1872	2	782.3599	.	L	Superscaf	33455055	BTP20220	0	0	0	0	0	Tissue specific
VKEFSPK	Superscaf	+	Superscaf	33989608	33989628	17	17	23	-	-	-	-	-	-	NCP	IGR	-	ID=gene-E	7	5.737142857	2470.5	0.566600267	VKEFSPK	98	417.7382	2	833.4647	.	M	Superscaf	33989560	BTP20220	0	0	0	0	0	Tissue specific
ASVWK	Superscaf	-	Superscaf	34061827	34061841	-1	1	5	-	-	-	-	-	-	NCP	IGR	-	ID=gene-E	5	5.466	1689.7	0.565679484	A(+42.01)	86	632.3362	1	631.3329	Acetylati	A	Superscaf	34061868	0	0	0	0	0	BTP20220	Tissue specific
KEHPLME	Superscaf	-	Superscaf	34105531	34105551	-12	12	18	-	-	-	-	-	-	NCP	IGR	-	ID=gene-E	7	5.971428571	3001.4	0.56512214	KEHPLM(+1	69	450.2183	2	898.4218	Oxidation	M	Superscaf	34105593	0	0	0	BTP20220	0	0	Tissue specific
LPKGVMY	Superscaf	+	Superscaf	34140187	34140207	11	11	17	-	-	-	-	-	-	NCP	IGR	-	ID=gene-E	7	5.628571429	2287.5	0.564680244	L(+42.01)	61	425.2369	2	848.4466	Acetylati	A	Superscaf	34140157	0	0	0	BTP20220	0	0	Tissue specific
QVTFT	Superscaf	+	Superscaf	34213138	34213152	22	22	26	-	-	-	-	-	-	NCP	IGR	-	ID=gene-E	5	4.838	1793.1	0.563750126	QVTFT	69	595.3078	1	594.3013	.	C	Superscaf	34213075	0	0	0	0	0	BTP20220	Tissue specific
CMDAC	Superscaf	-	Superscaf	34358611	34358625	-4	4	8	-	-	-	-	-	-	NCP	IGR	-	ID=gene-E	5	4.956	2265.7	0.561895209	C(+57.02)	57	656.1805	1	655.1764	Carbamido	N	Superscaf	34358655	0	0	0	BTP20220	0	0	Tissue specific
KFLLT	Superscaf	-	Superscaf	34541266	34541280	-15	15	19	-	-	-	-	-	-	NCP	IGR	-	ID=gene-E	5	6.742	1960.1	0.559566185	KFLLT	85	621.396	1	620.3897	.	R	Superscaf	34541280	BTP20220	0	0	0	0	0	Tissue specific
MSLKNK	Superscaf	-	Superscaf	34609756	34609773	-67	67	72	-	-	-	-	-	-	NCP	IGR	-	ID=gene-E	6	7.05	2641.8	0.558692835	M(+15.99)	94	368.7056	2	735.3949	Oxidation	V	Superscaf	34609878	0	BTP20220	0	0	0	BTP20220	Tissue specific
ELEEYKL	Superscaf	-	Superscaf	34746319	34746339	-1	1	7	-	-	-	-	-	-	NCP	IGR	-	ID=gene-E	7	5.288571429	2911.2	0.55695149	ELEEYKL	50	462.2375	2	922.4647	.	E	Superscaf	34746339	0	0	BTP20220	0	0	0	Tissue specific
QSMVPR	Superscaf	-	Superscaf	34878526	34878543	-5	5	10	-	-	-	-	-	-	NCP	IGR	-	ID=gene-E	6	5.69	2351.8	0.555265765	Q(+42.01)	54	380.1915	2	758.3745	Acetylati	Q	Superscaf	34878657	0	BTP20220	BTP20220	0	BTP20220	0	Mixed
VPHPQ	Superscaf	+	Superscaf	34881832	34881846	47	47	51	-	-	-	-	-	-	NCP	IGR	-	ID=gene-E	5	5.168	1905.2	0.555223649	VPHPQ	57	577.3087	1	576.302	.	F	Superscaf	34881694	0	BTP20220	BTP20220	BTP20220	0	0	Tissue specific
KNLPP	Superscaf	-	Superscaf	34910491	34910505	-29	29	33	-	-	-	-	-	-	NCP	IGR	-	ID=gene-E	5	6.746	2093.3	0.554858219	KNLPP	97	568.3433	1	567.338	.	A	Superscaf	34910604	0	BTP20220	0	0	BTP20220	0	Tissue specific
VDHLV	Superscaf	-	Superscaf	35009824	35009838	-17	17	21	-	-	-	-	-	-	NCP	IGR	-	ID=gene-E	5	3.308	1384.6	0.55359163	V(+42.01)	51	624.3351	1	623.3279	Acetylati	T	Superscaf	35009889	0	BTP20220	0	0	0	0	Tissue specific
AKNLEGY	Superscaf	-	Superscaf	35041780	35041800	-5	5	11	-	-	-	-	-	-	NCP	IGR	-	ID=gene-E	7	6	2912.1	0.553184085	A(+42.01)	57	418.7096	2	835.4075	Acetylati	R	Superscaf	35041803	0	0	0	BTP20220	0	0	Tissue specific
KPRREP	Superscaf	-	Superscaf	35164453	35164470	-6	6	11	-	-	-	-	-	-	NCP	IGR	-	ID=gene-E	6	7.846666667	2866.3	0.551619927	KPRREP	60	391.7333	2	781.4558	.	E	Superscaf	35164503	0	0	0	BTP20220	0	0	Tissue specific
YLDLLL	Superscaf	-	Superscaf	35215903	35215920	-11	11	16	-	-	-	-	-	-	NCP	IGR	-	ID=gene-E	6	5.425	2284.6	0.550963891	YLDLLL	74	749.4425	1	748.4371	.	Y	Superscaf	35215926	0	0	0	0	0	BTP20220	Tissue specific
MYLTF	Superscaf	+	Superscaf	35238178	35238192	49	49	53	-	-	-	-	-	-	NCP	IGR	-	ID=gene-E	5	5.88	1969.3	0.550679902	MYLTF	96	674.3193	1	673.3145	.	N	Superscaf	35238034	0	0	BTP20220	0	0	0	Tissue specific
ADKASFLL	Superscaf	-	Superscaf	35314753	35314776	-23	23	30	-	-	-	-	-	-	NCP	IGR	-	ID=gene-E	8	5.98375	2980.1	0.549703384	ADKASFLL	64	432.7436	2	863.4752	.	I	Superscaf	35314785	0	BTP20220	0	0	0	0	Tissue specific
LMLKL	Superscaf	-	Superscaf	35481820	35481834	-40	40	44	-	-	gene	-	-	mRNA	NCP	Intron	gene|mRN	ID=gene-E	5	6.686	1900.1	0.547573237	LM(+15.99	75	633.3949	1	632.3931	Oxidation	R	Superscaf	35481855	0	0	BTP20220	0	0	0	Tissue specific
FLEPDVP	Superscaf	+	Superscaf	35482408	35482428	24	24	30	-	-	gene	-	-	mRNA	NCP	Intron	gene|mRN	ID=gene-E	7	4.321428571	2252.6	0.547565663	FLEPDVP	61	816.4083	1	815.4065	.	V	Superscaf	35482339	BTP20220	BTP20220	0	0	0	0	Tissue specific
TMKEVAR	Superscaf	-	Superscaf	35491378	35491398	-4	4	10	-	-	gene	-	-	mRNA	NCP	Intron	gene|mRN	ID=gene-E	7	6.002857143	2623	0.547451287	T(+42.01)	75	446.7394	2	891.4484	Acetylati	H	Superscaf	35491464	0	BTP20220	0	0	0	0	Tissue specific
KWEKKR	Superscaf	+	Superscaf	35704501	35704518	15	15	20	-	-	-	-	-	-	NCP	IGR	-	ID=gene-E	6	8.181666667	3006.1	0.544733807	KWEKKR	57	437.7611	2	873.5184	.	N	Superscaf	35704459	0	0	0	BTP20220	0	0	Tissue specific
LPKAHLN	Superscaf	+	Superscaf	35912959	35912979	18	18	24	-	-	-	-	-	-	NCP	IGR	-	ID=gene-E	7	6.717142857	3040.3	0.542075733	LPKAHLN	65	396.7386	2	791.4653	.	L	Superscaf	35912908	BTP20220	0	0	0	0	0	Tissue specific
VSRTYKNV	Superscaf	-	Superscaf	36091519	36091542	-11	11	18	-	-	-	-	-	-	NCP	IGR	-	ID=gene-E	8	5.4725	2954.3	0.539798887	VSRTYKNV	96	483.7711	2	965.5294	.	Q	Superscaf	36091572	0	0	0	BTP20220	0	0	Tissue specific
PLPPLPPP	Superscaf	-	Superscaf	36380593	36380619	-51	51	59	-	-	-	-	-	-	NCP	IGR	-	ID=gene-E	9	6.228888889	3068.6	0.536112883	PLPPLPPP	74	924.5516	1	923.548	.	I	Superscaf	36380655	0	0	0	BTP20220	0	0	Mixed
HKTLGAH	Superscaf	+	Superscaf	36426100	36426120	21	21	27	-	-	-	-	-	-	NCP	IGR	-	ID=gene-E	7	7.06	3067.3	0.535532702	H(+42.01)	58	403.2268	2	804.4243	Acetylati	E	Superscaf	36426040	0	0	BTP20220	0	0	0	Tissue specific
VLLLML	Superscaf	-	Superscaf	36507718	36507735	-18	18	23	-	-	-	-	-	-	NCP	IGR	-	ID=gene-E	6	4.945	1785.1	0.534492034	VLLLM(+1	67	717.4514	1	716.4506	Oxidation	C	Superscaf	36507741	0	0	0	BTP20220	0	0	Tissue specific
KSMVLF	Superscaf	+	Superscaf	36508936	36508953	1	1	6	-	-	-	-	-	-	NCP	IGR	-	ID=gene-E	6	5.438333333	1964.1	0.534476504	KSMVLF	87	724.4087	1	723.3989	.	K	Superscaf	36508936	0	0	0	0	0	BTP20220	Tissue specific
RTCTPLM	Superscaf	+	Superscaf	36510436	36510456	51	51	57	-	-	-	-	-	-	NCP	IGR	-	ID=gene-E	7	6.695714286	3091.8	0.534457339	R(+42.01)	69	460.7263	2	919.4255	Acetylati	W	Superscaf	36510286	0	0	0	0	0	BTP20220	Tissue specific
EPKGKSW	Superscaf	+	Superscaf	36676270	36676290	12	12	18	-	-	-	-	-	-	NCP	IGR	-	ID=gene-E	7	6.648571429	2907	0.532342799	EPKGKSW	67	416.217	2	830.4286	.	S	Superscaf	36676237	0	0	0	0	0	BTP20220	Tissue specific
VGLMLK	Superscaf	+	Superscaf	36694477	36694494	38	38	43	-	-	-	-	-	-	NCP	IGR	-	ID=gene-E	6	5.57	1844	0.532110681	VGLMLK	88	660.4088	1	659.404	.	R	Superscaf	36694366	0	BTP20220	BTP20220	BTP20220	BTP20220	BTP20220	Mixed
KQPHKAP	Superscaf	-	Superscaf	36695212	36695232	-6	6	12	-	-	-	-	-	-	NCP	IGR	-	ID=gene-E	7	7.334285714	3153.3	0.532101271	KQPHKAP	50	403.235	2	804.4606	.	K	Superscaf	36695250	0	BTP20220	0	0	0	0	Tissue specific
LHLVPPNP	Superscaf	+	Superscaf	36720955	36720978	33	33	40	-	-	-	-	-	-	NCP	IGR	-	ID=gene-E	8	5.4825	2935.4	0.531772985	L(+42.01)	78	464.7686	2	927.5178	Acetylati	D	Superscaf	36720859	0	BTP20220	BTP20220	BTP20220	BTP20220	BTP20220	Tissue specific
PQHLVLF	Superscaf	-	Superscaf	36781054	36781074	-60	60	66	-	-	gene	-	-	mRNA	NCP	Intron	gene|mRN	ID=gene-E	7	5.282857143	2662	0.531006705	PQHLVLF	65	427.2486	2	852.4858	.	G	Superscaf	36781269	0	BTP20220	BTP20220	BTP20220	BTP20220	BTP20220	Tissue specific
QNYSYPG	Superscaf	+	Superscaf	36977008	36977028	2	2	8	-	-	-	-	-	-	NCP	IGR	-	ID=gene-E	7	5.761428571	3144.5	0.528508107	Q(+42.01)	72	870.3638	1	869.3555	Acetylati	A	Superscaf	36977005	0	BTP20220	0	BTP20220	0	BTP20220	Tissue specific
KGTALVLT	Superscaf	-	Superscaf	37096768	37096791	-33	33	40	-	-	-	-	-	-	NCP	IGR	-	ID=gene-E	8	5.84375	2578.8	0.526981016	KGTALVLT	50	401.7473	2	801.496	.	Y	Superscaf	37096836	0	BTP20220	0	0	0	0	Tissue specific
GLNKCR	Superscaf	+	Superscaf	37189216	37189233	67	67	72	-	-	-	-	-	-	NCP	IGR	-	ID=gene-E	6	7.146666667	2950.3	0.525802294	G(+42.01)	51	395.2085	2	788.3963	Acetylati	L	Superscaf	37189018	0	BTP20220	0	0	0	0	Tissue specific
YEQSTGY	Superscaf	+	Superscaf	37268308	37268328	28	28	34	-	-	-	-	-	-	NCP	IGR	-	ID=gene-E	7	5.481428571	3033.4	0.524793758	YEQSTGY	57	424.1774	2	846.3395	.	T	Superscaf	37268227	0	BTP20220	0	0	0	0	Tissue specific
VGQPLTK	Superscaf	+	Superscaf	37323829	37323849	19	19	25	-	-	-	-	-	-	NCP	IGR	-	ID=gene-E	7	5.738571429	2464.7	0.524085813	VGQPLTK	68	371.7205	2	741.4385	.	G	Superscaf	37323775	0	BTP20220	0	0	0	0	Tissue specific
SAKPALPK	Superscaf	-	Superscaf	37357258	37357299	-78	78	91	-	-	-	-	-	-	NCP	IGR	-	ID=gene-E	14	7.028571429	5525.9	0.523659294	SAKPALPK	88	379.7296	4	1514.893	.	R	Superscaf	37357443	0	BTP20220	0	0	0	0	Tissue specific
AKPALPKP	Superscaf	-	Superscaf	37357261	37357296	-79	79	90	-	-	-	-	-	-	NCP	IGR	-	ID=gene-E	12	6.915	4674.1	0.523659332	AKPALPKP	98	650.8876	2	1299.766	.	R	Superscaf	37357443	0	0	BTP20220	0	0	BTP20220	Tissue specific
KPALPKPE	Superscaf	-	Superscaf	37357264	37357296	-80	80	90	-	-	-	-	-	-	NCP	IGR	-	ID=gene-E	11	6.996363636	4362.8	0.523659332	KPALPKPE	95	615.3695	2	1228.729	.	R	Superscaf	37357443	0	BTP20220	0	0	0	0	Tissue specific
PALPKPEP	Superscaf	-	Superscaf	37357267	37357296	-81	81	90	-	-	-	-	-	-	NCP	IGR	-	ID=gene-E	10	6.722	3894.4	0.523659332	PALPKPEP	95	367.884	3	1100.634	.	R	Superscaf	37357443	0	0	BTP20220	0	0	0	Tissue specific
ALPKPEPR	Superscaf	-	Superscaf	37357270	37357299	-82	82	91	-	-	-	-	-	-	NCP	IGR	-	ID=gene-E	10	7.066	4025.4	0.523659294	ALPKPEPR	92	566.8441	2	1131.676	.	R	Superscaf	37357443	0	BTP20220	BTP20220	0	0	0	Tissue specific
ALPKPEPR	Superscaf	-	Superscaf	37357270	37357296	-82	82	90	-	-	-	-	-	-	NCP	IGR	-	ID=gene-E	9	6.768888889	3557	0.523659332	ALPKPEPR	99	502.7963	2	1003.581	.	R	Superscaf	37357443	0	BTP20220	0	BTP20220	0	0	Tissue specific
LPKPEPRP	Superscaf	-	Superscaf	37357273	37357296	-83	83	90	-	-	-	-	-	-	NCP	IGR	-	ID=gene-E	8	6.8625	3245.7	0.523659332	LPKPEPRP	98	467.2779	2	932.5443	.	R	Superscaf	37357443	0	0	0	BTP20220	0	0	Tissue specific
KKWDL	Superscaf	-	Superscaf	37522423	37522437	-33	33	37	-	-	-	-	-	-	NCP	IGR	-	ID=gene-E	5	6.864	2228.3	0.521553629	KKWDL	97	689.3885	1	688.3908	.	S	Superscaf	37522641	0	BTP20220	0	0	0	0	Tissue specific
KECPTAP	Superscaf	-	Superscaf	37679215	37679235	-1	1	7	-	-	gene	-	-	mRNA	NCP	Intron	gene|mRN	ID=gene-E	7	6.161428571	2863.2	0.519554307	KEC(+57.(	53	401.6894	2	801.3691	Carbamido	K	Superscaf	37679235	0	0	0	0	BTP20220	0	Tissue specific
GCDLDLRK	Superscaf	-	Superscaf	37850986	37851009	-19	19	26	-	-	-	-	-	-	NCP	IGR	-	ID=gene-E	8	6.17375	3530	0.517364027	GC(+57.02	98	488.7457	2	975.4807	Carbamido	I	Superscaf	37851054	0	0	0	0	0	BTP20220	Tissue specific
GCDLDLR	Superscaf	-	Superscaf	37850986	37851006	-19	19	25	-	-	-	-	-	-	NCP	IGR	-	ID=gene-E	7	5.664285714	3061.6	0.517364065	GC(+57.02	90	424.6977	2	847.3858	Carbamido	I	Superscaf	37851054	0	0	0	BTP20220	0	0	Tissue enhanced
ERVGLEL	Superscaf	+	Superscaf	37930720	37930740	10	10	16	-	-	-	-	-	-	NCP	IGR	-	ID=gene-E	7	5.018571429	2503.9	0.516347382	ERVGLEL	73	408.2333	2	814.4548	.	V	Superscaf	37930693	0	0	0	0	0	BTP20220	Tissue specific
KNSLLETL	Superscaf	-	Superscaf	38046931	38046954	-57	57	64	-	-	-	-	-	-	NCP	IGR	-	ID=gene-E	8	6.065	3331.7	0.514865544	KNSLLETL	50	459.2669	2	916.5229	.	F	Superscaf	38047062	0	0	0	0	BTP20220	0	Tissue specific
GVFLN	Superscaf	+	Superscaf	38139844	38139858	21	21	25	-	-	-	-	-	-	NCP	IGR	-	ID=gene-E	5	4.568	1634.8	0.513680931	GVFLN	58	549.3027	1	548.2958	.	R	Superscaf	38139784	0	0	0	0	0	BTP20220	Tissue specific
NQSFLVL	Superscaf	-	Superscaf	38171776	38171796	-17	17	23	-	-	-	-	-	-	NCP	IGR	-	ID=gene-E	7	4.882857143	2685	0.513273691	NQSFLVL	61	820.4528	1	819.449	.	P	Superscaf	38171829	0	0	0	0	0	0	Tissue specific
KLPPPPYK	Superscaf	-	Superscaf	38240512	38240535	-14	14	21	-	-	-	-	-	-	NCP	IGR	-	ID=gene-E	8	7.04	3099.3	0.512397204	K(+42.01)	66	491.2904	2	980.5695	Acetylati	G	Superscaf	38240553	0	0	BTP20220	0	0	0	Tissue specific
HLLRPPP	Superscaf	-	Superscaf	38379979	38379999	-8	8	14	-	-	-	-	-	-	NCP	IGR	-	ID=gene-E	7	7.03	2987.5	0.510618907	H(+42.01)	85	436.2643	2	870.5076	Acetylati	V	Superscaf	38380035	BTP20220	BTP20220	0	0	0	0	Tissue specific
KPSPR	Superscaf	-	Superscaf	38383615	38383629	-26	26	30	-	-	-	-	-	-	NCP	IGR	-	ID=gene-E	5	7.756	2175.4	0.510572621	KPSPR	50	584.3518	1	583.3442	.	A	Superscaf	38383632	0	BTP20220	0	0	0	0	Tissue enhanced
SKKVF	Superscaf	-	Superscaf	38388004	38388018	-17	17	21	-	-	-	-	-	-	NCP	IGR	-	ID=gene-E	5	6.128	1707.6	0.510516657	SKKVF	55	608.3832	1	607.3693	.	Y	Superscaf	38388036	0	0	0	BTP20220	0	0	Tissue specific
DPGEF	Superscaf	-	Superscaf	38415229	38415243	-5	5	9	-	-	-	-	-	-	NCP	IGR	-	ID=gene-E	5	4.788	1859.1	0.510169513	DPGEF	56	564.2281	1	563.2227	.	S	Superscaf	38415249	0	0	BTP20220	0	0	0	Tissue specific
KQWLHSP	Superscaf	-	Superscaf	38459446	38459466	-20	20	26	-	-	-	-	-	-	NCP	IGR	-	ID=gene-E	7	6.69	3299.6	0.509605628	KQWLHSP	55	448.2347	2	894.4712	.	H	Superscaf	38459505	0	0	0	0	BTP20220	0	Tissue specific
CAAEHME	Superscaf	-	Superscaf	38649799	38649819	-52	52	58	-	-	-	-	-	-	NCP	IGR	-	ID=gene-E	7	5.262857143	3050.5	0.507178448	C(+57.02)	54	432.1487	2	862.2949	Carbamido	A	Superscaf	38649819	BTP20220	0	0	0	0	0	Tissue specific
EMCCQ	Superscaf	-	Superscaf	38682880	38682894	-41	41	45	-	-	-	-	-	-	NCP	IGR	-	ID=gene-E	5	4.932	2579.1	0.506756711	E(+42.01)	55	769.2177	1	768.2241	Acetylati	T	Superscaf	38682894	0	0	0	0	BTP20220	BTP20220	Tissue specific
FGNLL	Superscaf	-	Superscaf	38703457	38703471	-7	7	11	-	-	-	-	-	-	NCP	IGR	-	ID=gene-E	5	5.764	1988.2	0.506494335	FGNLL	67	563.3093	1	562.3115	.	T	Superscaf	38703510	0	0	BTP20220	BTP20220	0	0	Mixed
TVTLKY	Superscaf	+	Superscaf	38750959	38750976	7	7	12	-	-	-	-	-	-	NCP	IGR	-	ID=gene-E	6	5.74	2076.3	0.505888601	TVTLKY	97	362.7139	2	723.4167	.	L	Superscaf	38750941	0	0	0	0	0	BTP20220	Tissue specific
VAYVLK	Superscaf	+	Superscaf	38891074	38891091	10	10	15	-	-	-	-	-	-	NCP	IGR	-	ID=gene-E	6	4.566666667	1592.6	0.504102003	V(+42.01)	57	367.7309	2	733.4374	Acetylati	R	Superscaf	38891047	BTP20220	0	0	0	0	0	Tissue specific
KDGMMKK	Superscaf	-	Superscaf	38935624	38935644	-20	20	26	-	-	-	-	-	-	NCP	IGR	-	ID=gene-E	7	7.094285714	2857	0.50353391	KDGMM(+1	55	427.2127	2	852.4197	Oxidation	H	Superscaf	38935734	0	0	0	0	0	BTP20220	Tissue specific
MHSLVHR	Superscaf	+	Superscaf	38942917	38942937	5	5	11	-	-	-	-	-	-	NCP	IGR	-	ID=gene-E	7	6.192857143	2996.5	0.503440918	M(+15.99)	98	448.2391	2	894.4494	Oxidation	N	Superscaf	38942905	0	0	0	BTP20220	0	0	Tissue specific
CTTLYKLL	Superscaf	-	Superscaf	39003196	39003219	-1	1	8	-	-	-	-	-	-	NCP	IGR	-	ID=gene-E	8	6.4275	3368.8	0.502672266	C(+57.02)	56	506.2841	2	1010.547	Carbamido	C	Superscaf	39003264	0	0	0	0	0	BTP20220	Tissue enhanced
KENHVFQ	Superscaf	-	Superscaf	39059938	39059958	-25	25	31	-	-	-	-	-	-	NCP	IGR	-	ID=gene-E	7	5.298571429	3108.4	0.50194879	KENHVFQ	52	901.4594	1	900.4454	.	K	Superscaf	39059970	0	0	0	0	BTP20220	0	Tissue specific
ALLTK	Superscaf	+	Superscaf	39082360	39082374	12	12	16	-	-	-	-	-	-	NCP	IGR	-	ID=gene-E	5	6.85	1884	0.501662965	ALLTK	93	545.3638	1	544.3585	.	G	Superscaf	39082327	0	0	0	BTP20220	BTP20220	0	Tissue specific
GSPMLQ	Superscaf	+	Superscaf	39148450	39148467	2	2	7	-	-	-	-	-	-	NCP	IGR	-	ID=gene-E	6	5.888333333	2353.7	0.500820217	GSPMLQ	94	632.3063	1	631.2999	.	L	Superscaf	39148447	0	0	0	0	0	BTP20220	Tissue specific
KFGLG	Superscaf	+	Superscaf	39262393	39262407	84	84	88	-	-	-	-	-	-	NCP	IGR	-	ID=gene-E	5	6.628	1803.8	0.499367375	K(+42.01)	82	563.3166	1	562.3115	Acetylati	N	Superscaf	39262144	0	0	0	0	0	BTP20220	Tissue specific
DSCCAC	Superscaf	-	Superscaf	39370225	39370242	-4	4	9	-	-	-	-	-	-	NCP	IGR	-	ID=gene-E	6	4.955	2863.3	0.497992377	DSC(+57.(	78	772.1918	1	771.1986	Carbamido	G	Superscaf	39370269	0	0	BTP20220	0	0	0	Tissue specific
TVHVYVR	Superscaf	-	Superscaf	39435247	39435267	-4	4	10	-	-	-	-	-	-	NCP	IGR	-	ID=gene-E	7	4.362857143	2125.5	0.497163247	TVHVYVR	64	437.2475	2	872.4868	.	L	Superscaf	39435282	BTP20220	0	0	0	0	0	Tissue specific
AHRLQL	Superscaf	-	Superscaf	39474631	39474648	-8	8	13	-	-	-	-	-	-	NCP	IGR	-	ID=gene-E	6	6.996666667	2897.3	0.496661102	A(+42.01)	96	390.2284	2	778.4449	Acetylati	D	Superscaf	39474705	0	0	BTP20220	0	0	0	Tissue enhanced
ATKKAQR	Superscaf	-	Superscaf	39524977	39524997	-1	1	7	-	-	-	-	-	-	NCP	IGR	-	ID=gene-E	7	7.78	3216.4	0.496019105	ATKKAQR	52	401.7535	2	801.4821	.	A	Superscaf	39525018	0	BTP20220	0	0	0	0	Tissue specific
KVLVVHHS	Superscaf	+	Superscaf	39579625	39579651	21	21	29	-	-	-	-	-	-	NCP	IGR	-	ID=gene-E	9	4.673333333	2832	0.495322215	KVLVVHHS	62	533.3199	2	1064.613	.	L	Superscaf	39579565	0	0	0	0	0	BTP20220	Tissue specific
MKGKY	Superscaf	+	Superscaf	39589450	39589464	18	18	22	-	-	-	-	-	-	NCP	IGR	-	ID=gene-E	5	7.372	2065.1	0.49519709	M(+15.99)	54	684.3429	1	683.3312	Oxidation	S	Superscaf	39589399	BTP20220	0	0	0	0	0	Tissue specific
KTHVAPK	Superscaf	-	Superscaf	39606415	39606435	-79	79	85	-	-	-	-	-	-	NCP	IGR	-	ID=gene-E	7	6.56	2602.7	0.494980694	KTHVAPK	60	390.7441	2	779.4653	.	R	Superscaf	39606495	0	BTP20220	0	0	0	0	Tissue specific
NGHSLLLP	Superscaf	-	Superscaf	39840970	39840993	-25	25	32	-	-	-	-	-	-	NCP	IGR	-	ID=gene-E	8	6.11125	3294.7	0.491989859	NGHSLLLP	62	425.7412	2	849.4708	.	S	Superscaf	39841116	0	0	BTP20220	0	0	0	Tissue specific
NPYKAR	Superscaf	-	Superscaf	39893638	39893655	-38	38	43	-	-	-	-	-	-	NCP	IGR	-	ID=gene-E	6	7.315	2822.1	0.491318369	NPYKAR	71	374.7079	2	747.4028	.	S	Superscaf	39893718	0	0	BTP20220	0	0	0	Tissue specific
LLVCCLPV	Superscaf	-	Superscaf	40077382	40077405	-14	14	21	-	-	-	-	-	-	NCP	IGR	-	ID=gene-E	8	4.285	2569	0.488975384	LLVC(+57.	70	487.2627	2	972.5136	Carbamido	A	Superscaf	40077438	0	0	BTP20220	0	0	0	Tissue specific
GLHETTF	Superscaf	-	Superscaf	40088125	40088145	-17	17	23	-	-	-	-	-	-	NCP	IGR	-	ID=gene-E	7	5.9	2878.3	0.488838439	GLHETTF	68	402.6974	2	803.3813	.	V	Superscaf	40088151	0	0	BTP20220	0	0	0	Mixed
MDLEALKK	Superscaf	+	Superscaf	40120654	40120677	8	8	15	-	-	-	-	-	-	NCP	IGR	-	ID=gene-E	8	6.175	3163.4	0.488423625	MDLEALKK	99	474.2631	2	946.5157	.	R	Superscaf	40120633	0	0	0	BTP20220	0	0	Tissue specific
LVLAL	Superscaf	-	Superscaf	40127023	40127037	-2	2	6	-	-	-	-	-	-	NCP	IGR	-	ID=gene-E	5	4.792	1371.5	0.488342529	LVLAL	97	528.3739	1	527.3683	.	I	Superscaf	40127073	0	BTP20220	0	0	BTP20220	BTP20220	Tissue specific
VTPRVQ	Superscaf	-	Superscaf	40231804	40231821	-10	10	15	-	-	-	-	-	-	NCP	IGR	-	ID=gene-E	6	4.873333333	1994.4	0.487006434	VTPRVQ	89	350.2102	2	698.4075	.	P	Superscaf	40231860	0	0	0	0	BTP20220	BTP20220	Tissue specific
VSVLPF	Superscaf	+	Superscaf	40566148	40566165	39	39	44	-	-	-	-	-	-	NCP	IGR	-	ID=gene-E	6	3.906666667	1461.6	0.482743234	V(+42.01)	68	352.2035	2	702.3952	Acetylati	T	Superscaf	40566034	0	0	BTP20220	0	0	0	Tissue specific
YELPNPT	Superscaf	-	Superscaf	40590700	40590720	-5	5	11	-	-	-	-	-	-	NCP	IGR	-	ID=gene-E	7	5.628571429	2907.4	0.482430134	YELPNPT	67	833.4029	1	832.3967	.	K	Superscaf	40590723	0	BTP20220	0	0	0	0	Tissue specific
YVTLF	Superscaf	+	Superscaf	40635022	40635036	5	5	9	-	-	-	-	-	-	NCP	IGR	-	ID=gene-E	5	4.73	1597.8	0.481865064	YVTLF	76	642.3469	1	641.3425	.	V	Superscaf	40635010	0	BTP20220	0	0	0	0	Tissue specific
DMLLRY	Superscaf	-	Superscaf	40681120	40681137	-28	28	33	-	-	-	-	-	-	NCP	IGR	-	ID=gene-E	6	6.183333333	2598.1	0.481277233	DM(+15.99	58	826.4088	1	825.4055	Oxidation	V	Superscaf	40681284	0	0	0	0	0	BTP20220	Tissue specific
AEEVVVVA	Superscaf	-	Superscaf	40712542	40712565	-83	83	90	-	-	-	-	-	-	NCP	IGR	-	ID=gene-E	8	2.31	1473.6	0.480876496	A(+42.01)	51	857.4604	1	856.4542	Acetylati	N	Superscaf	40712649	0	0	0	0	0	BTP20220	Tissue specific
REEKLK	Superscaf	+	Superscaf	40725622	40725639	8	8	13	-	-	-	-	-	-	NCP	IGR	-	ID=gene-E	6	7.11	2790	0.48070979	REEKLK	97	401.7347	2	801.4708	.	C	Superscaf	40725601	0	BTP20220	0	0	0	0	Tissue specific
KRLGVFF	Superscaf	-	Superscaf	40940698	40940718	-37	37	43	-	-	-	-	-	-	NCP	IGR	-	ID=gene-E	7	6.201428571	2542.7	0.477967331	KRLGVFF	53	433.7651	2	865.5174	.	N	Superscaf	40940805	0	0	BTP20220	0	0	0	Tissue specific
SVNLTKLL	Superscaf	-	Superscaf	41035882	41035905	-20	20	27	-	-	gene	-	-	mRNA	NCP	Intron	gene|mRN	ID=gene-E	8	5.6625	2906.2	0.476753607	SVNLTKLL	50	444.2776	2	886.5488	.	M	Superscaf	41035914	0	0	BTP20220	0	0	0	Tissue specific
KPMSPVH	Superscaf	+	Superscaf	41063602	41063622	47	47	53	-	-	gene	-	-	mRNA	NCP	Intron	gene|mRN	ID=gene-E	7	5.908571429	2517.8	0.476400189	KPM(+15.9	54	406.2073	2	810.4058	Oxidation	S	Superscaf	41063464	0	0	0	0	BTP20220	0	Tissue specific
KDFKV	Superscaf	+	Superscaf	41128654	41128668	10	10	14	-	-	-	-	-	-	NCP	IGR	-	ID=gene-E	5	5.586	1735.7	0.475570791	KDFKV	81	636.3694	1	635.3642	.	V	Superscaf	41128627	0	0	BTP20220	0	0	0	Tissue specific
PLLLK	Superscaf	-	Superscaf	41136916	41136930	-5	5	9	-	-	-	-	-	-	NCP	IGR	-	ID=gene-E	5	6.796	1866	0.475465443	PLLLK	73	583.4174	1	582.4105	.	G	Superscaf	41137014	0	0	BTP20220	0	0	0	Tissue specific
VVGLK	Superscaf	-	Superscaf	41179078	41179092	-71	71	75	-	-	-	-	-	-	NCP	IGR	-	ID=gene-E	5	4.338	1119.1	0.474927838	VVGLK	67	515.3534	1	514.3479	.	V	Superscaf	41179263	0	BTP20220	0	0	0	0	Tissue enhanced
RKVLQF	Superscaf	-	Superscaf	41531863	41531880	-25	25	30	-	-	gene	-	-	mRNA	NCP	Intron	gene|mRN	ID=gene-E	6	6.268333333	2468.7	0.470429459	R(+42.01)	59	416.755	2	831.4966	Acetylati	T	Superscaf	41531988	0	BTP20220	0	0	0	0	Tissue specific

KSYLVF	Superscaf	-	Superscaf	41678386	41678403	-50	50	55	-	-	-	-	-	-	NCP	IGR	-	ID=gene-E	6	5.423333333	2052.1	0.468561153	KSYLVF	51	378.7152	2	755.4218	.	F	Superscaf	41678406	0	0	0	0	BTP202209	0	Tissue specific
MLFLYP	Superscaf	+	Superscaf	42149092	42149109	10	10	15	-	-	-	-	-	-	NCP	IGR	-	ID=gene-E	6	5.858333333	2262.6	0.462559209	MLFLYP	63	392.2141	2	782.4037	.	S	Superscaf	42149065	0	0	0	BTP202209	0	0	Tissue specific
TLVRLR	Superscaf	-	Superscaf	42151420	42151437	-40	40	45	-	-	-	-	-	-	NCP	IGR	-	ID=gene-E	6	6.668333333	2401.9	0.462529524	TLVRLR	97	379.2545	2	756.497	.	T	Superscaf	42151458	0	BTP202209	0	0	0	0	Tissue specific
KKGHVPP	Superscaf	+	Superscaf	42189298	42189318	3	3	9	-	-	gene	-	-	mRNA	NCP	Intron	gene|mRN	ID=gene-E	7	6.52	2528.6	0.462046506	KKGHVPP	66	381.7338	2	761.4548	.	S	Superscaf	42189292	0	0	BTP202209	0	0	0	Tissue specific
KMNGML	Superscaf	-	Superscaf	42281497	42281514	-16	16	21	-	-	-	-	-	-	NCP	IGR	-	ID=gene-E	6	6.433333333	2458.8	0.46087092	KMNGML	61	693.3406	1	692.335	.	A	Superscaf	42281550	0	0	BTP202209	0	0	0	Tissue specific
LPPLPKRP	Superscaf	-	Superscaf	42328759	42328782	-96	96	103	-	-	gene	-	-	mRNA	NCP	Intron	gene|mRN	ID=gene-E	8	7.2075	3173.6	0.460268209	LPPLPKRP	83	459.2978	2	916.5858	.	N	Superscaf	42328920	0	0	BTP202209	0	0	0	Tissue specific
AEHLTSHKY	Superscaf	-	Superscaf	42340084	42340110	-6	6	14	-	-	gene	-	-	mRNA	NCP	Intron	gene|mRN	ID=gene-E	9	6.445555556	4038.4	0.460123766	AEHLTSHKY	79	543.271	2	1084.53	.	I	Superscaf	42340122	0	0	0	0	BTP202209	0	Tissue specific
KKEESH	Superscaf	+	Superscaf	42345679	42345696	5	5	10	-	-	gene	-	-	mRNA	NCP	Intron	gene|mRN	ID=gene-E	6	6.531666667	2790.9	0.460052539	KKEESH	61	379.2015	2	756.3766	.	L	Superscaf	42345667	0	BTP202209	0	0	0	0	Tissue specific
NSLAALKK	Superscaf	-	Superscaf	42365404	42365427	-6	6	13	-	-	-	-	-	-	NCP	IGR	-	ID=gene-E	8	6.82125	3246.3	0.459800951	N(+42.01)	97	443.7634	2	885.5283	Acetylati	V	Superscaf	42365433	0	0	0	0	0	BTP202209	Tissue specific
AAVSGRGV	Superscaf	+	Superscaf	42480349	42480372	21	21	28	-	-	-	-	-	-	NCP	IGR	-	ID=gene-E	8	5.0525	2249.4	0.458335294	A(+42.01)	71	379.7097	2	757.4082	Acetylati	T	Superscaf	42480289	BTP202209	0	0	0	0	0	Tissue specific
RAKYP	Superscaf	-	Superscaf	42556642	42556656	-2	2	6	-	-	gene	-	-	mRNA	NCP	Intron	gene|mRN	ID=gene-E	5	7.696	2225.4	0.457362601	RAKYP	67	634.3654	1	633.3598	.	P	Superscaf	42556695	0	0	0	0	BTP202209	0	Tissue specific
CMMCEM	Superscaf	-	Superscaf	42565669	42565686	-18	18	23	-	-	gene	-	-	mRNA	NCP	Intron	gene|mRN	ID=gene-E	6	5.085	2711.4	0.45724746	C(+57.02)	54	455.1088	2	908.2207	Carbamido	W	Superscaf	42565719	0	0	0	BTP202209	0	0	Tissue specific
TVYGSPLC	Superscaf	+	Superscaf	42590947	42590970	12	12	19	-	-	-	-	-	-	NCP	IGR	-	ID=gene-E	8	5.1425	2814.2	0.456925065	T(+42.01)	52	469.7182	2	937.4215	Acetylati	I	Superscaf	42590914	0	BTP202209	0	0	0	0	Tissue specific
TVGAP	Superscaf	+	Superscaf	42599887	42599901	13	13	17	-	-	-	-	-	-	NCP	IGR	-	ID=gene-E	5	4.964	1343.5	0.456811187	T(+42.01)	50	486.252	1	485.2486	Acetylati	T	Superscaf	42599851	0	0	0	0	0	BTP202209	Tissue specific
YTVAK	Superscaf	+	Superscaf	42658084	42658098	14	14	18	-	-	-	-	-	-	NCP	IGR	-	ID=gene-E	5	5.59	1636.7	0.45606912	YTVAK	62	581.3279	1	580.322	.	T	Superscaf	42658045	BTP202209	0	0	0	0	0	Tissue specific
QTLMF	Superscaf	+	Superscaf	42682516	42682530	1	1	5	-	-	-	-	-	-	NCP	IGR	-	ID=gene-E	5	5.878	2120.5	0.455757589	QTLMF	63	639.3143	1	638.3098	.	Q	Superscaf	42682516	0	0	0	0	0	BTP202209	Tissue specific
SDFGVSKL	Superscaf	+	Superscaf	42802465	42802488	23	23	30	-	-	-	-	-	-	NCP	IGR	-	ID=gene-E	8	5.1875	2684.8	0.454228012	SDFGVSKL	68	426.7261	2	851.4388	.	V	Superscaf	42802399	BTP202209	0	0	0	0	0	Tissue specific
SGPKPVH	Superscaf	-	Superscaf	42809971	42809991	-65	65	71	-	-	-	-	-	-	NCP	IGR	-	ID=gene-E	7	5.94	2443.6	0.454132341	SGPKPVH	84	361.2008	2	720.3918	.	K	Superscaf	42809994	0	0	0	0	BTP202209	0	Tissue specific
LKLLEH	Superscaf	+	Superscaf	42897379	42897396	38	38	43	-	-	-	-	-	-	NCP	IGR	-	ID=gene-E	6	6.415	2573.8	0.453017845	LKLLEH	97	376.7354	2	751.4592	.	K	Superscaf	42897268	BTP202209	0	0	0	0	0	Tissue specific
MYTMWHK	Superscaf	-	Superscaf	43064848	43064868	-24	24	30	-	-	-	-	-	-	NCP	IGR	-	ID=gene-E	7	6.701428571	3214.7	0.45088242	M(+15.99)	71	527.7345	2	1053.441	Oxidation	G	Superscaf	43064886	0	0	0	0	0	BTP202209	Tissue specific
ALQVELLP	Superscaf	-	Superscaf	43117822	43117845	-59	59	66	-	-	-	-	-	-	NCP	IGR	-	ID=gene-E	8	4.89125	2745.1	0.450206913	A(+42.01)	70	462.7725	2	923.5328	Acetylati	S	Superscaf	43118010	BTP202209	0	0	0	0	0	Tissue specific
KPGPVVY	Superscaf	+	Superscaf	43176181	43176201	11	11	17	-	-	-	-	-	-	NCP	IGR	-	ID=gene-E	7	4.852857143	1900	0.449462819	KPGPVVY	54	380.2264	2	758.4326	.	C	Superscaf	43176151	0	0	0	BTP202209	0	BTP202209	Tissue specific
SPGTLY	Superscaf	-	Superscaf	43275250	43275267	-29	29	34	-	-	-	-	-	-	NCP	IGR	-	ID=gene-E	6	6.02	2228.5	0.448199635	SPGTLY	75	637.317	1	636.3119	.	V	Superscaf	43275294	0	0	0	0	0	BTP202209	Tissue specific
SSRMEK	Superscaf	-	Superscaf	43351453	43351470	-25	25	30	-	-	gene	-	-	mRNA	NCP	Intron	gene|mRN	ID=gene-E	6	6.805	2681	0.447227975	SSRMEK	51	737.348	1	736.3538	.	L	Superscaf	43351848	0	0	BTP202209	0	0	0	Tissue specific
KKCYPPP	Superscaf	+	Superscaf	43365301	43365321	47	47	53	-	-	gene	-	-	mRNA	NCP	Intron	gene|mRN	ID=gene-E	7	7.008571429	2994.2	0.447051362	KKC(+57.(	50	445.2386	2	888.4527	Carbamido	P	Superscaf	43365163	0	0	BTP202209	0	0	0	Tissue specific
KPFLP	Superscaf	+	Superscaf	43416727	43416741	13	13	17	-	-	gene	-	-	mRNA	NCP	Intron	gene|mRN	ID=gene-E	5	6.76	1884	0.446395708	K(+42.01)	72	643.3787	1	642.3741	Acetylati	L	Superscaf	43416691	0	0	BTP202209	0	0	0	Tissue specific
LLLLPPK	Superscaf	+	Superscaf	43583734	43583754	45	45	51	-	-	gene	-	-	mRNA	NCP	Intron	gene|mRN	ID=gene-E	7	6.608571429	2556.8	0.444266135	LLLLPPK	69	397.2794	2	792.5473	.	P	Superscaf	43583602	0	0	0	0	BTP202209	0	Tissue specific
LLCSPLLM	Superscaf	+	Superscaf	43702129	43702152	21	21	28	-	-	gene	-	-	mRNA	NCP	Intron	gene|mRN	ID=gene-E	8	5.83375	3091.6	0.442756449	LLC(+57.(	72	473.7628	2	945.5027	Carbamido	E	Superscaf	43702069	0	0	0	0	BTP202209	BTP202209	Tissue specific
PSLQLVSVL	Superscaf	+	Superscaf	43717876	43717902	7	7	15	-	-	gene	-	-	mRNA	NCP	Intron	gene|mRN	ID=gene-E	9	4.583333333	2775.1	0.442555622	P(+42.01)	54	499.3068	2	996.5855	Acetylati	S	Superscaf	43717858	BTP202209	0	0	0	0	0	Tissue specific
LPAPSPPQ(	Superscaf	+	Superscaf	43737184	43737210	22	22	30	-	-	gene	-	-	mRNA	NCP	Intron	gene|mRN	ID=gene-E	9	5.95	3594.1	0.442309427	LPAPSPPQ(	62	483.7368	2	965.4641	Carbamido	N	Superscaf	43737121	0	0	0	0	0	BTP202209	Tissue specific
LSKLLR	Superscaf	-	Superscaf	43783378	43783395	-10	10	15	-	-	gene	-	-	mRNA	NCP	Intron	gene|mRN	ID=gene-E	6	7.353333333	2560.8	0.441720525	LSKLLR	95	365.2456	2	728.4908	.	P	Superscaf	43783476	0	0	0	BTP202209	BTP202209	0	Tissue specific
MVKVP	Superscaf	-	Superscaf	43923562	43923576	-46	46	50	-	-	-	-	-	-	NCP	IGR	-	ID=gene-E	5	4.358	1177.3	0.439933085	M(+15.99)	62	589.3416	1	588.3305	Oxidation	P	Superscaf	43923582	0	0	BTP202209	0	0	0	Mixed
QERKK	Superscaf	+	Superscaf	44005225	44005239	3	3	7	-	-	gene	-	-	mRNA	NCP	Intron	gene|mRN	ID=gene-E	5	7.822	2621.8	0.438891805	Q(+42.01)	66	365.7095	2	729.4133	Acetylati	Q	Superscaf	44005219	0	0	0	BTP202209	0	0	Tissue specific
KYGHAF	Superscaf	-	Superscaf	44143126	44143143	-57	57	62	-	-	-	-	-	-	NCP	IGR	-	ID=gene-E	6	6.743333333	2543.6	0.437133399	KYGHAF	57	722.3478	1	721.3547	.	I	Superscaf	44143221	BTP202209	0	0	0	0	0	Tissue specific
DVKNLLTK	Superscaf	-	Superscaf	44194777	44194800	-1	1	8	-	-	gene	-	-	mRNA	NCP	Intron	gene|mRN	ID=gene-E	8	5.79375	3049.3	0.436474724	DVKNLLTK	77	465.7834	2	929.5546	.	D	Superscaf	44195046	BTP202209	BTP202209	0	0	0	BTP202209	Mixed
HGPAP	Superscaf	+	Superscaf	44305069	44305083	10	10	14	-	-	-	-	-	-	NCP	IGR	-	ID=gene-E	5	6.436	1903.1	0.435068512	HGPAP	54	478.2396	1	477.2336	.	R	Superscaf	44305042	0	0	0	0	BTP202209	0	Mixed
ALKWVK	Superscaf	-	Superscaf	44367295	44367312	-8	8	13	-	-	-	-	-	-	NCP	IGR	-	ID=gene-E	6	6.228333333	2128.1	0.434275034	ALKWVK	80	372.7388	2	743.4694	.	V	Superscaf	44367327	BTP202209	0	BTP202209	0	0	0	Tissue specific
DLDLR	Superscaf	-	Superscaf	44560348	44560362	-27	27	31	-	-	gene	-	-	mRNA	NCP	Intron	gene|mRN	ID=gene-E	5	5.732	2178.6	0.431813465	DLDLR	89	631.3387	1	630.3337	.	M	Superscaf	44560362	0	0	0	0	BTP202209	0	Tissue enhanced
REPASPK	Superscaf	+	Superscaf	44565613	44565633	5	5	11	-	exon	gene	three_pri	-	mRNA	NCP	3UTR	exon|gene	ID=exon-E	7	6.86	2912.2	0.431746254	REPASPK	63	392.712	2	783.4239	.	N	Superscaf	44565601	0	BTP202209	0	0	0	0	Tissue specific
SLLEELTL	Superscaf	-	Superscaf	44575168	44575191	-2	2	9	-	-	gene	-	-	mRNA	NCP	Intron	gene|mRN	ID=gene-E	8	5.32125	3045.5	0.431624381	SLLEELTL	84	917.5167	1	916.5117	.	A	Superscaf	44575191	0	0	BTP202209	0	0	0	Tissue specific
TVDGLPF	Superscaf	+	Superscaf	44646823	44646843	30	30	36	-	-	gene	-	-	mRNA	NCP	Intron	gene|mRN	ID=gene-E	7	4.747142857	2184.5	0.43071075	TVDGLPF	61	748.3843	1	747.3803	.	H	Superscaf	44646736	0	0	0	BTP202209	0	0	Tissue specific
NPLLSK	Superscaf	-	Superscaf	44665027	44665044	-12	12	17	-	-	gene	-	-	mRNA	NCP	Intron	gene|mRN	ID=gene-E	6	6.515	2492.7	0.430478671	N(+42.01)	83	357.2115	2	712.4119	Acetylati	N	Superscaf	44665044	0	0	BTP202209	0	0	0	Tissue specific
SNELLMF	Superscaf	-	Superscaf	44784871	44784891	-11	11	17	-	-	gene	-	-	mRNA	NCP	Intron	gene|mRN	ID=gene-E	7	5.357142857	2871.3	0.428950509	SNELLMF	78	853.4092	1	852.4052	.	N	Superscaf	44784957	0	0	0	BTP202209	0	0	Tissue specific
AGLLKKTT(	Superscaf	+	Superscaf	44816998	44817027	68	68	77	-	-	gene	-	-	mRNA	NCP	Intron	gene|mRN	ID=gene-E	10	6.844	3697.9	0.428540744	A(+42.01)	98	522.3252	2	1042.639	Acetylati	L	Superscaf	44816797	BTP202209	0	0	0	0	0	Tissue specific
AGLLKKTT(	Superscaf	+	Superscaf	44816998	44817033	68	68	79	-	-	gene	-	-	mRNA	NCP	Intron	gene|mRN	ID=gene-E	12	6.200833333	3995.2	0.428540668	A(+42.01)	90	600.3687	2	1198.729	Acetylati	L	Superscaf	44816797	BTP202209	0	0	0	0	0	Tissue specific
AGLLKKTT(	Superscaf	+	Superscaf	44816998	44817024	68	68	76	-	-	gene	-	-	mRNA	NCP	Intron	gene|mRN	ID=gene-E	9	6.94	3344.5	0.428540783	A(+42.01)	90	465.7829	2	929.5546	Acetylati	L	Superscaf	44816797	BTP202209	0	0	0	0	0	Tissue specific
VCLDP	Superscaf	-	Superscaf	44920501	44920515	-4	4	8	-	-	-	-	-	-	NCP	IGR	-	ID=gene-E	5	4.054	1688	0.427221175	VC(+57.02	93	603.2783	1	602.2734	Carbamido	W	Superscaf	44920533	0	0	BTP202209	0	0	0	Tissue specific
FLQRQS	Superscaf	-	Superscaf	45039601	45039618	-6	6	11	-	-	-	-	-	-	NCP	IGR	-	ID=gene-E	6	6.533333333	2994.4	0.4257025	FLQRQS	93	389.7127	2	777.4133	.	S	Superscaf	45039621	0	0	BTP202209	0	0	0	Tissue specific
EVKVLR	Superscaf	-	Superscaf	45076741	45076758	-9	9	14	-	-	-	-	-	-	NCP	IGR	-	ID=gene-E	6	4.95	1896.1	0.42522893	EVKVLR	83	372.2393	2	742.4701	.	K	Superscaf	45076785	0	0	0	0	0	BTP202209	Tissue specific
LDFLPF	Superscaf	-	Superscaf	45171310	45171327	-10	10	15	-	-	gene	-	-	mRNA	NCP	Intron	gene|mRN	ID=gene-E	6	5.365	2230.5	0.424023086	LDFLPF	70	376.2103	2	750.3952	.	T	Superscaf	45171327	BTP202209	0	0	0	0	0	Tissue specific
TVKQVP	Superscaf	+	Superscaf	45383851	45383868	10	10	15	-	-	-	-	-	-	NCP	IGR	-	ID=gene-E	6	4.703333333	1814	0.421312988	T(+42.01)	73	713.4154	1	712.4119	Acetylati	L	Superscaf	45383824	0	0	0	BTP202209	0	0	Tissue specific
DDEPK	Superscaf	+	Superscaf	45384877	45384891	38	38	42	-	-	-	-	-	-	NCP	IGR	-	ID=gene-E	5	5.04	2054.3	0.421299944	DDEPK	56	603.2603	1	602.2548	.	T	Superscaf	45384766	0	0	BTP202209	0	BTP202209	0	Tissue specific
TQPGLK	Superscaf	-	Superscaf	45704761	45704778	-35	35	40	-	-	-	-	-	-	NCP	IGR	-	ID=gene-E	6	6.695	2464.7	0.417221084	TQPGLK	73	643.3754	1	642.3701	.	Q	Superscaf	45704862	0	0	BTP202209	0	0	0	Tissue specific
NQDLLR	Superscaf	-	Superscaf	45720883	45720900	-26	26	31	-	-	-	-	-	-	NCP	IGR	-	ID=gene-E	6	6.125	2974.5	0.417015513	N(+42.01)	65	400.7123	2	799.4188	Acetylati	P	Superscaf	45720918	0	BTP202209	0	0	0	0	Tissue specific
HGVLLF	Superscaf	+	Superscaf	45757585	45757602	5	5	10	-	-	-	-	-	-	NCP	IGR	-	ID=gene-E	6	5.166666667	2011.2	0.416547528	HGVLLF	59	685.4005	1	684.3959	.	W	Superscaf	45757573	BTP202209	0	0	0	0	0	Tissue specific
GKLLY	Superscaf	+	Superscaf	45854092	45854106	13	13	17	-	-	gene	-	-	mRNA	NCP	Intron	gene|mRN	ID=gene-E	5	6.666	1932	0.415317011	G(+42.01)	75	635.374	1	634.369	Acetylati	V	Superscaf	45854056	0	0	BTP202209	BTP202209	0	0	Tissue specific
KELGY	Superscaf	-	Superscaf	45875920	45875934	-23	23	27	-	-	gene	-	-	mRNA	NCP	Intron	gene|mRN	ID=gene-E	5	6.114	2004.1	0.415038684	KELGY	57	609.3217	1	608.317	.	H	Superscaf	45875967	0	0	0	BTP202209	0	0	Tissue specific
FGVSST	Superscaf	-	Superscaf	45904606	45904623	-3	3	8	-	-	-	-	-	-	NCP	IGR	-	ID=gene-E	6	4.89	1849	0.414672872	FGVSST	59	597.2901	1	596.2806	.	I	Superscaf	45904671	0	BTP202209	0	0	0	0	Tissue specific
LLQQPEPK	Superscaf	-	Superscaf	45941005	45941028	-10	10	17	-	-	-	-	-	-	NCP	IGR	-	ID=gene-E	8	6.1025	3496.9	0.414208674	LLQQPEPK	89	476.7751	2	951.5389	.	E	Superscaf	45941088	0	0	BTP202209	0	0	0	Tissue specific
GPPRPSF	Superscaf	+	Superscaf	45970771	45970791	11	11	17	-	-	-	-	-	-	NCP	IGR	-	ID=gene-E	7	6.684285714	2729.1	0.413829168	GPPRPSF	71	379.2021	2	756.3918	.	I	Superscaf	45970741	0	0	0	BTP202209	0	0	Tissue specific
AGPLTV	Superscaf	+	Superscaf	46041397	46041414	28	28	33	-	-	-	-	-	-	NCP	IGR	-	ID=gene-E	6	5.133333333	1696.9	0.412928658	A(+42.01)	99	599.3375	1	598.3326	Acetylati	S	Superscaf	46041316	0	0	BTP202209	0	0	0	Tissue specific
MNPKLEW	Superscaf	-	Superscaf	46125028	46125048	-4	4	10	-	-	-	-	-	-	NCP	IGR	-	ID=gene-E	7	6.041428571	3079.5	0.411862246	MNPKLEW	51	459.2252	2	916.4477	.	R	Superscaf	46125102	0	0	0	BTP202209	0	0	Tissue specific
VLASRVAP	Superscaf	-	Superscaf	46164172	46164195	-7	7	14	-	-	-	-	-	-	NCP	IGR	-	ID=gene-E	8	5.095	2345.6	0.411363085	VLASRVAP	81	406.7523	2	811.4916	.	D	Superscaf	46164249	0	BTP202209	0	BTP202209	0	0	Tissue specific
NLALPP	Superscaf	-	Superscaf	46223044	46223061	-20	20	25	-	-	-	-	-	-	NCP	IGR	-	ID=gene-E	6	5.998333333	2289.6	0.410612488	N(+42.01)	56	666.3927	1	665.3748	Acetylati	R	Superscaf	46223166	0	0	0	BTP202209	0	0	Tissue enhanced
QTLSSTEV	Superscaf	+	Superscaf	46242211	46242234	18	18	25	-	-	gene	-	-	mRNA	NCP	Intron	gene|mRN	ID=gene-E	8	4.90875	2951.4	0.410368014	QTLSSTEV	77	864.4285	1	863.4236	.	D	Superscaf	46242160	0	BTP202209	0	0	0	0	Tissue specific
PTQFK	Superscaf	+	Superscaf	46377676	46377690	18	18	22	-	-	-	-	-	-	NCP	IGR	-	ID=gene-E	5	6.74	2201.4	0.408640823	PTQFK	54	620.3382	1	619.3329	.	D	Superscaf	46377625	0	0	0	BTP202209	0	0	Tissue specific
VGGVP	Superscaf	+	Superscaf	46619272	46619286	15	15	19	-	-	-	-	-	-	NCP	IGR	-	ID=gene-E	5	3.648	932	0.405560247	VGGVP	57	428.2421	1	427.2431	.	L	Superscaf	46619230	0	0	BTP202209	0	0	0	Tissue specific
KDAAMK	Superscaf	-	Superscaf	46846483	46846500	-14	14	19	-	-	-	-	-	-	NCP	IGR	-	ID=gene-E	6	6.706666667	2342.4	0.402663054	KDAAM(+1!	57	679.353	1	678.337	Oxidation	I	Superscaf	46846524	0	0	0	BTP202209	0	0	Tissue specific
LAPFP	Superscaf	-	Superscaf	46880206	46880220	-1	1	5	-	-	-	-	-	-	NCP	IGR	-	ID=gene-E	5	6.016	1726.9	0.402233093	LAPFP	82	544.323	1	543.3057	.	L	Superscaf	46880244	0	0	0	0	BTP202209	0	Tissue specific
TLLLNTK	Superscaf	+	Superscaf	46955998	46956018	14	14	20	-	-	-	-	-	-	NCP	IGR	-	ID=gene-E	7	6.592857143	2920.3	0.401266597	TLLLNTK	76	401.7537	2	801.496	.	R	Superscaf	46955959	BTP202209	0	0	0	0	0	Tissue specific
LSLSGGTT	Superscaf	-	Superscaf	47055544	47055567	-49	49	56	-	-	-	-	-	-	NCP	IGR	-	ID=gene-E	8	6.04	2863.2	0.399997253	LSLSGGTT	84	735.3856	1	734.381	.	K	Superscaf	47055606	0	0	0	0	0	BTP202209	Tissue specific
LPPPLATT	Superscaf	+	Superscaf	47072542	47072565	26	26	33	-	-	-	-	-	-	NCP	IGR	-	ID=gene-E	8	6.2425	2825.3	0.399780513	LPPPLATT	56	405.2499	2	808.4694	.	V	Superscaf	47072467	0	BTP202209	0	0	0	0	Tissue specific
SLTLPSPLV	Superscaf	+	Superscaf	47120653	47120682	19	19	28	-	-	-	-	-	-	NCP	IGR	-	ID=gene-E	10	5.496	3296.8	0.399166976	SLTLPSPLV	84	514.3007	2	1026.596	.	L	Superscaf	47120599	0	BTP202209	0	0	0	0	Tissue specific
KQTKTPLTF	Superscaf	+	Superscaf	47174491	47174517	7	7	15	-	-	-	-	-	-	NCP	IGR	-	ID=gene-E	9	7.033333333	3768.2	0.398480529	K(+42.01)	56	528.3011	2	1054.602	Acetylati	V	Superscaf	47174473	BTP202209	0	0	0	0	0	Tissue specific
GVNPLL	Superscaf	-	Superscaf	47246545	47246562	-12	12	17	-	-	-	-	-	-	NCP	IGR	-	ID=gene-E	6	4.94	1938.2	0.397561888	GVNPLL	55	612.3693	1	611.3643	.	Y	Superscaf	47246637	0	0	0	BTP202209	0	0	Tissue specific
FGLDLK	Superscaf	+	Superscaf	47548384	47548401	7	7	12	-	-	-	-	-	-	NCP	IGR	-	ID=gene-E	6	6.02	2271.4	0.393713156	FGLDLK	56	692.3964	1	691.3904	.	K	Superscaf	47548366	0	0	BTP202209	0	0	0	Tissue specific
DLKAVDK	Superscaf	+	Superscaf	47635093	47635113	4	4	10	-	-	-	-	-	-	NCP	IGR	-	ID=gene-E	7	5.345714286	2424.5	0.392607497	D(+42.01)	97	415.7402	2	829.4545	Acetylati	R	Superscaf	47635084	BTP202209	0	0	0	0	0	Tissue specific
KLADNPP	Superscaf	-	Superscaf	47644636	47644656	-2	2	8	-	-	-	-	-	-	NCP	IGR	-	ID=gene-E	7	6.102857143	2816.1	0.392485815	K(+42.01)	56	398.7094	2	795.4127	Acetylati	I	Superscaf	47644701	0	0	0	0	0	BTP202209	Tissue specific
AAADYKK	Superscaf	+	Superscaf	47655745	47655765	4	4	10	-	-	-	-	-	-	NCP	IGR	-	ID=gene-E	7	6.595714286	2741.7	0.392344164	A(+42.01)	77	404.7157	2	807.4127	Acetylati	S	Superscaf	47655736	0	0	0	0	BTP202209	BTP202209	Tissue specific
YPMRMT	Superscaf	+	Superscaf	47683372	47683389	14	14	19	-	-	-	-	-	-	NCP	IGR	-	ID=gene-E	6	6.791666667	2586.2	0.391991932	YPM(+15.9	54	407.6852	2	813.3513	Oxidation	V	Superscaf	47683333	0	0	BTP202209	0	0	0	Tissue specific
MGVVTLK	Superscaf	-	Superscaf	47901076	47901096	-12	12	18	-	-	-	-	-	-	NCP	IGR	-	ID=gene-E	7	4.852857143	1888.1	0.389215963	MGVVTLK	68	747.4408	1	746.436	.	V	Superscaf	47901213	0	BTP202209	0	0	BTP202209	BTP202209	Tissue specific
LSYLPL	Superscaf	+	Superscaf	47915740	47915757	20	20	25	-	-	-	-	-	-	NCP	IGR	-	ID=gene-E	6	5.93	2240.5	0.389029022	LSYLPL	60	705.4174	1	704.4108	.	L	Superscaf	47915683	0	0	BTP202209	0	0	0	Tissue specific
KCAQSKR	Superscaf	-	Superscaf	47982988	47983008	-10	10	16	-	-	-	-	-	-	NCP	IGR	-	ID=gene-E	7	7.515714286	3476.7	0.388171508	KC(+57.02	63	439.2456	2	876.46	Carbamido	E	Superscaf	47983011	0	0	0	0	BTP202209	0	Tissue specific
TALDSVL	Superscaf	-	Superscaf	48029860	48029880	-4	4	10	-	-	-	-	-	-	NCP	IGR	-	ID=gene-E	7	4.737142857	2210.5	0.387573846	T(+42.01)	62	760.4073	1	759.4014	Acetylati	D	Superscaf	48029898	0	0	BTP202209	0	0	0	Tissue specific
LDSYLK	Superscaf	-	Superscaf	48137866	48137883	-8	8	13	-	-	-	-	-	-	NCP	IGR	-	ID=gene-E	6	6.001666667	2429.6	0.386196706	LDSYLK	61	738.3989	1	737.3959	.	K	Superscaf	48137895	BTP202209	0	0	0	0	0	Tissue specific
LAGATGPL	Superscaf	+	Superscaf	48351952	48351975	12	12	19	-	-	-	-	-	-	NCP	IGR	-	ID=gene-E	8	6.09625	2658.9	0.383466832	LAGATGPL	51	699.4005	1	698.3962	.	S	Superscaf	48351919	0	0	0	0	0	BTP202209	Tissue specific
YVLPPK	Superscaf	+	Superscaf	48466108	48466125	1	1	6	-	-	-	-	-	-	NCP	IGR	-	ID=gene-E	6	5.663333333	1956.1	0.382011312	YVLPPK	54	358.7161	2	715.4268	.	Y	Superscaf	48466108	0	0	0	BTP202209	0	0	Tissue specific
KMMLLQ	Superscaf	+	Superscaf	48630307	48630324	1	1	6	-	-	-	-	-	-	NCP	IGR	-	ID=gene-E	6	6.475	2528.9	0.37991762	KMM(+15.9	67	779.4091	1	778.4081	Oxidation	K	Superscaf	48630307	BTP202209	0	0	0	0	0	Tissue specific
YEKPLE	Superscaf	-	Superscaf	48679459	48679476	-20	20	25	-	-	-	-	-	-	NCP	IGR	-	ID=gene-E	6	5.686666667	2469.7	0.379290886	YEKPLE	88	389.7014	2	777.3908	.	V	Superscaf	48679629	0	0	BTP202209	0	0	0	Tissue specific
QRHTVSPP	Superscaf	-	Superscaf	48704476	48704499	-11	11	18	-	-	-	-	-	-	NCP	IGR	-	ID=gene-E	8	6.10125	3334.9	0.378971819	QRHTVSPP	59	461.247	2	920.4828	.	P	Superscaf	48704724	0	0	0	0	BTP202209	BTP202209	Tissue specific
LTMTF	Superscaf	+	Superscaf	48759142	48759156	28	28	32	-	-	-	-	-	-	NCP	IGR	-	ID=gene-E	5	6.054	1907.3	0.378274891	LTM(+15.9	61	628.3105	1	627.2938	Oxidation	G	Superscaf	48759061	0	0	0	0	BTP202209	BTP202209	Tissue specific
LSYVYKVL	Superscaf	-	Superscaf	48772867	48772890	-1	1	8	-	-	-	-	-	-	NCP	IGR	-	ID=gene-E	8	4.8375	2477.6	0.37809977	LSYVYKVL	65	492.7907	2	983.5692	.	L	Superscaf	48772911	0	BTP202209	BTP202209	BTP202209	0	0	Mixed
KNMGLP	Superscaf	+	Superscaf	48822790	48822807	5	5	10	-	-	-	-	-	-	NCP	IGR	-	ID=gene-E	6	6.525	2424.7	0.377463281	KNM(+15.9	57	675.3517	1	674.3422	Oxidation	N	Superscaf	48822778	0	0	0	0	BTP202209	0	Tissue specific
HSSLLSHV	Superscaf	-	Superscaf	48905023	48905046	-31	31	38	-	-	-	-	-	-	NCP	IGR	-	ID=gene-E	8	5.5225	3096.4	0.376414656	HSSLLSHV	81	440.2307	2	878.461	.	K	Superscaf	48905061	BTP202209	0	0	0	0	0	Tissue specific
EAFNLLHL	Superscaf	-	Superscaf	48914968	48914991	-15	15	22	-	-	-	-	-	-	NCP	IGR	-	ID=gene-E	8	5.7075	3400.8	0.376287848	EAFNLLHL	97	478.7627	2	955.5127	.	A	Superscaf	48914997	0	0	0	0	0	BTP202209	Tissue specific
LASLKQSR	Superscaf	+	Superscaf	48917869	48917892	5	5	12	-	-	-	-	-	-	NCP	IGR	-	ID=gene-E	8	6.93625	3512.8	0.376250858	LASLKQSR	55	451.7752	2	901.5345	.	R	Superscaf	48917857	0	0	BTP202209	0	0	0	Tissue specific
KLHAPGR	Superscaf	-	Superscaf	48917959	48917979	-13	13	19	-	-	-	-	-	-	NCP	IGR	-	ID=gene-E	7	7.48	3036.3	0.376249748	KLHAPGR	69	389.7393	2	777.4609	.	G	Superscaf	48917982	0	0	0	0	0	BTP202209	Tissue specific
THCLLR	Superscaf	+	Superscaf	48925270	48925287	36	36	41	-	-	-	-	-	-	NCP	IGR	-	ID=gene-E	6	6.976666667	2958.5	0.376156565	THC(+57.(	61	400.2121	2	798.4171	Carbamido	L	Superscaf	48925165	0	0	0	0	0	BTP202209	Tissue specific
YSDVP	Superscaf	+	Superscaf	48967648	48967662	3	3	7	-	-	-	-	-	-	NCP	IGR	-	ID=gene-E	5	4.122	1591.8	0.375616243	YSDVP	50	580.2589	1	579.254	.	R	Superscaf	48967642	0	BTP202209	0	0	BTP202209	BTP202209	Tissue specific
KRHLEK	Superscaf	+	Superscaf	49126324	49126341	26	26	31	-	-	-	-	-	-	NCP	IGR	-	ID=gene-E	6	7.838333333	2984.2	0.373592937	KRHLEK	61	810.5057	1	809.4871	.	C	Superscaf	49126249	0	0	0	0	0	BTP202209	Tissue specific
EVELE	Superscaf	-	Superscaf	49276603	49276617	-13	13	17	-	-	-	-	-	-	NCP	IGR	-	ID=gene-E	5	3.128	1629.9	0.371676777	EVELE	75	618.2966	1	617.2908	.	K	Superscaf	49276782	0	0	0	0	BTP202209	0	Tissue specific
LGPLKPF	Superscaf	-	Superscaf	49323121	49323141	-16	16	22	-	-	-	-	-	-	NCP	IGR	-	ID=gene-E	7	6.535714286	2534.7	0.371083552	L(+42.01)	70	407.2521	2	812.4796	Acetylati	L	Superscaf	49323150	0	0	0	0	BTP202209	0	Tissue specific
TDVQPLEK	Superscaf	-	Superscaf	49326973	49326996	-22	22	29	-	-	-	-	-	-	NCP	IGR	-	ID=gene-E	8	5.04875	3004.4	0.371034397	T(+42.01)	66	486.2472	2	970.4971	Acetylati	D	Superscaf	49327077	BTP202209	0	0	0	0	0	Tissue specific
SSVESLGK	Superscaf	-	Superscaf	49333492	49333515	-16	16	23	-	-	-	-	-	-	NCP	IGR	-	ID=gene-E	8	5.24375	2694.8	0.370951273	SSVESLGK	92	403.7112	2	805.4182	.	V	Superscaf	49333530	0	0	BTP202209	0	0	0	Tissue specific
HLLELPF	Superscaf	-	Superscaf	49436929	49436949	-20	20	26	-	-	-	-	-	-	NCP	IGR	-	ID=gene-E	7	5.79	2830.2	0.369632393	HLLELPF	55	868.4919	1	867.4854	.	N	Superscaf	49436997	0	BTP202209	0	0	0	0	Tissue specific
SELRF	Superscaf	-	Superscaf	49507642	49507656	-4	4	8	-	-	-	-	-	-	NCP	IGR	-	ID=gene-E	5	6.224	2198.5	0.368730812	SELRF	63	651.3435	1	650.3387	.	H	Superscaf	49507704	0	0	0	0	BTP202209	BTP202209	Tissue specific
DSVDF	Superscaf	+	Superscaf	49515742	49515756	36	36	40	-	-	-	-	-	-	NCP	IGR	-	ID=gene-E	5	3.42	1593.8	0.368627529	DSVDF	76	582.239	1	581.2333	.	N	Superscaf	49515637	0	BTP202209	0	0	0	0	Tissue specific
AVSCFA	Superscaf	-	Superscaf	49585408	49585425	-37	37	42	-	-	gene	-	-	mRNA	NCP	Intron	gene|mRN	ID=gene-E	6	4.703333333	1979.1	0.367739184	AVSC(+57.	63	654.2877	1	653.2843	Carbamido	Y	Superscaf	49585467	0	BTP202209	0	0	BTP202209	BTP202209	Tissue specific
HTLGPN	Superscaf	-	Superscaf	49587766	49587783	-49	49	54	-	-	gene	-	-	mRNA	NCP	Intron	gene|mRN	ID=gene-E	6	6.296666667	2602	0.367709117	HTLGPN	56	638.33	1	637.3184	.	I	Superscaf	49587795	0	0	0	0	BTP202209	0	Tissue specific
FMELQGY	Superscaf	-	Superscaf	49606981	49607001	-14	14	20	-	-	-	-	-	-	NCP	IGR	-	ID=gene-E	7	5.387142857	2905.3	0.36746407	FM(+15.99	75	903.3941	1	902.3844	Oxidation	I	Superscaf	49607055	0	0	0	0	BTP202209	0	Tissue specific
RRPKTK	Superscaf	+	Superscaf	49747423	49747440	19	19	24	-	-	gene	-	-	mRNA	NCP	Intron	gene|mRN	ID=gene-E	6	8.971666667	2969.3	0.36567334	RRPKTK	67	393.2593	2	784.5031	.	S	Superscaf	49747369	0	0	BTP202209	0	0	0	Tissue specific
DHMLPHQ	Superscaf	-	Superscaf	49808773	49808793	-19	19	25	-	-	gene	-	-	mRNA	NCP	Intron	gene|mRN	ID=gene-E	7	5.975714286	3323.9	0.364891032	DHMLPHQ	85	439.2029	2	876.3912	.	A	Superscaf	49808805	0	0	0	0	BTP202209	0	Tissue specific
KTLTPR	Superscaf	+	Superscaf	49834390	49834407	3	3	8	-	-	gene	-	-	mRNA	NCP	Intron	gene|mRN	ID=gene-E	6	7.64	2603	0.364564429	KTLTPR	73	358.2253	2	714.4388	.	N	Superscaf	49834384	0	0	0	0	0	BTP202209	Tissue specific
VTDVPQ	Superscaf	-	Superscaf	49866349	49866366	-1	1	6	-	-	gene	-	-	mRNA	NCP	Intron	gene|mRN	ID=gene-E	6	3.575	1757.1	0.364156922	VTDVPQ	51	658.3527	1	657.3333	.	V	Superscaf	49866450	BTP202209	0	0	0	0	0	Tissue specific
LDLAHC	Superscaf	+	Superscaf	49880851	49880868	3	3	8	-	-	gene	-	-	mRNA	NCP	Intron	gene|mRN	ID=gene-E	6	5.593333333	2635	0.363972008	LDLAHC(+5	58	728.326	1	727.3323	Carbamido	N	Superscaf	49880845	0	0	0	0	BTP202209	0	Tissue specific
LPKYPPP	Superscaf	-	Superscaf	49938511	49938531	-27	27	33	-	-	gene	-	-	mRNA	NCP	Intron	gene|mRN	ID=gene-E	7	6.654285714	2630.9	0.36323675	LPKYPPP	76	406.2363	2	810.4639	.	S	Superscaf	49938546	0	BTP202209	0	0	0	0	Tissue specific
KDGEPLVT	Superscaf	+	Superscaf	50002711	50002734	14	14	21	-	-	gene	-	-	mRNA	NCP	Intron	gene|mRN	ID=gene-E	8	5.08875	2691	0.362418102	KDGEPLVT	65	429.7308	2	857.4494	.	C	Superscaf	50002672	0	0	0	0	BTP202209	0	Tissue specific
LFKYH	Superscaf	+	Superscaf	50061994	50062008	1	1	5	-	-	-	-	-	-	NCP	IGR	-	ID=gene-E	5	6.89	2288.4	0.361662302	LFKYH	67	354.196	2	706.3802	.	L	Superscaf	50061994	0	0	BTP202209	0	0	0	Tissue specific
LPLPYRST	Superscaf	-	Superscaf	50065651	50065674	-5	5	12	-	-	-	-	-	-	NCP	IGR	-	ID=gene-E	8	6.64875	3270.8	0.361615557	LPLPYRST	68	473.7706	2	945.5283	.	A	Superscaf	50065674	0	BTP202209	0	0	0	0	Tissue specific
FGPGP	Superscaf	+	Superscaf	50145883	50145897	11	11	15	-	-	-	-	-	-	NCP	IGR	-	ID=gene-E	5	6.004	1656.8	0.360592639	FGPGP	84	474.2333	1	473.2274	.	I	Superscaf	50145853	BTP202209	0	0	0	BTP202209	0	Tissue specific
LDQATLSL	Superscaf	-	Superscaf	50247217	50247240	-11	11	18	-	-	-	-	-	-	NCP	IGR	-	ID=gene-E	8	5.59875	3174.6	0.35930042	LDQATLSL	65	860.4696	1	859.4651	.	A	Superscaf	50247339	0	BTP202209	0	0	0	0	Tissue specific
TCKPR	Superscaf	-	Superscaf	50293642	50293656	-40	40	44	-	-	-	-	-	-	NCP	IGR	-	ID=gene-E	5	7.67	2437.8	0.358708572	T(+42.01)	51	352.1884	2	702.3483	Acetylati	N	Superscaf	50293761	0	0	0	0	BTP202209	0	Tissue specific
KYHSATTL	Superscaf	-	Superscaf	50543497	50543520	-8	8	15	-	-	-	-	-	-	NCP	IGR	-	ID=gene-E	8	6.71625	3390.7	0.355522571	KYHSATTL	50	460.738	2	919.4763	.	K	Superscaf	50543601	0	BTP202209	BTP202209	BTP202209	0	0	Tissue specific
PKAAQ	Superscaf	+	Superscaf	50654689	50654703	10	10	14	-	-	-	-	-	-	NCP	IGR	-	ID=gene-E	5	6.746	2039.1	0.354104884	PKAAQ	58	514.2974	1	513.2911	.	L	Superscaf	50654662	0	0	0	BTP202209	0	0	Tissue specific
VHVLF	Superscaf	+	Superscaf	50725039	50725053	9	9	13	-	-	-	-	-	-	NCP	IGR	-	ID=gene-E	5	3.81	1360.5	0.353207855	VHVLF	79	614.3634	1	613.3588	.	I	Superscaf	50725015	0	BTP202209	0	0	0	0	Tissue specific
SPDVH	Superscaf	+	Superscaf	50748148	50748162	1	1	5	-	-	-	-	-	-	NCP	IGR	-	ID=gene-E	5	4.508	1752	0.352913193	SPDVH	66	554.2604	1	553.2496	.	S	Superscaf	50748148	0	0	BTP202209	0	0	0	Tissue specific
GPPSRY	Superscaf	-	Superscaf	50764849	50764866	-94	94	99	-	-	-	-	-	-	NCP	IGR	-	ID=gene-E	6	6.778333333	2463.8	0.352700202	GPPSRY	52	676.3337	1	675.334	.	V	Superscaf	50765103	0	0	0	BTP202209	0	0	Tissue specific
AAGPK	Superscaf	-	Superscaf	50783485	50783499	-27	27	31	-	-	-	-	-	-	NCP	IGR	-	ID=gene-E	5	6.81	1725.7	0.352462613	AAGPK	81	443.2543	1	442.254	.	L	Superscaf	50783628	BTP202209	0	0	0	0	0	Mixed
VNPLR	Superscaf	-	Superscaf	50827996	50828010	-55	55	59	-	-	-	-	-	-	NCP	IGR	-	ID=gene-E	5	5.69	1936.3	0.351895056	VNPLR	59	598.3652	1	597.3598	.	M	Superscaf	50828088	0	0	0	0	0	BTP202209	Tissue specific
YQRMLVVLI	Superscaf	-	Superscaf	50844202	50844228	-201	201	209	-	-	-	-	-	-	NCP	IGR	-	ID=gene-E	9	5.084444444	3150.7	0.351688262	YQRMLVVLI	68	567.8361	2	1133.663	.	S	Superscaf	50844330	0	0	0	0	0	BTP202209	Tissue specific
ALLLLCGGE	Superscaf	-	Superscaf	50844676	50844708	-48	48	58	-	-	-	-	-	-	NCP	IGR	-	ID=gene-E	11	5.096363636	4153.7	0.351682141	ALLLLC(+5	74	588.2815	2	1174.554	Carbamido	R	Superscaf	50844708	0	BTP202209	0	0	0	0	Tissue specific
LLLLCGGEI	Superscaf	-	Superscaf	50844679	50844708	-49	49	58	-	-	-	-	-	-	NCP	IGR	-	ID=gene-E	10	5.004	3842.4	0.351682141	LLLLC(+57	85	1104.521	1	1103.517	Carbamido	R	Superscaf	50844708	BTP202209	0	0	0	0	0	Mixed
LLLCGGEDI	Superscaf	-	Superscaf	50844682	50844708	-50	50	58	-	-	-	-	-	-	NCP	IGR	-	ID=gene-E	9	4.895555556	3489	0.351682141	LLLC(+57.	96	991.4324	1	990.4328	Carbamido	R	Superscaf	50844708	0	BTP202209	0	0	0	0	Tissue specific
YVGLGY	Superscaf	-	Superscaf	50957059	50957076	-58	58	63	-	-	-	-	-	-	NCP	IGR	-	ID=gene-E	6	4.873333333	1867	0.350249343	YVGLGY	67	671.3396	1	670.3326	.	G	Superscaf	50957091	0	0	0	0	BTP202209	0	Tissue specific
PSLPVLP	Superscaf	-	Superscaf	51024865	51024885	-53	53	59	-	-	-	-	-	-	NCP	IGR	-	ID=gene-E	7	5.22	2102.4	0.349384715	PSLPVLP	95	722.4441	1	721.4374	.	A	Superscaf	51024924	0	0	0	0	0	BTP202209	Tissue specific
YTPQP	Superscaf	-	Superscaf	51030172	51030186	-10	10	14	-	-	-	-	-	-	NCP	IGR	-	ID=gene-E	5	6.088	2142.5	0.349317122	YTPQP	99	605.2924	1	604.2856	.	T	Superscaf	51030375	BTP202209	0	0	0	0	0	Mixed
GPKRVLLF	Superscaf	-	Superscaf	51232486	51232509	-6	6	13	-	-	-	-	-	-	NCP	IGR	-	ID=gene-E	8	6.27625	2846.1	0.346737314	GPKRVLLF	57	465.2985	2	928.5858	.	H	Superscaf	51232614	0	0	0	BTP202209	0	0	Tissue enhanced
KNLKLE	Superscaf	-	Superscaf	51273127	51273144	-12	12	17	-	-	-	-	-	-	NCP	IGR	-	ID=gene-E	6	6.678333333	2665.8	0.346219179	KNLKLE	64	372.7279	2	743.4541	.	D	Superscaf	51273147	0	0	0	0	0	BTP202209	Tissue enhanced
GNRYP	Superscaf	-	Superscaf	51344752	51344766	-34	34	38	-	-	-	-	-	-	NCP	IGR	-	ID=gene-E	5	6.82	2339.7	0.345305931	GNRYP	68	606.2894	1	605.2921	.	I	Superscaf	51344769	0	0	0	BTP202209	0	0	Tissue specific
VLEKSPGK	Superscaf	-	Superscaf	51511510	51511533	-20	20	27	-	-	-	-	-	-	NCP	IGR	-	ID=gene-E	8	5.82875	2733.8	0.343179495	VLEKSPGK	97	429.2562	2	856.5018	.	F	Superscaf	51511533	BTP202209	0	0	0	0	0	Tissue specific
LNTRY	Superscaf	+	Superscaf	51536332	51536346	16	16	20	-	-	-	-	-	-	NCP	IGR	-	ID=gene-E	5	6.868	2455.9	0.342863106	LNTRY	66	666.3538	1	665.3497	.	M	Superscaf	51536287	0	0	0	0	0	BTP202209	Tissue specific
VTKHGPQ	Superscaf	-	Superscaf	51557398	51557418	-1	1	7	-	-	-	-	-	-	NCP	IGR	-	ID=gene-E	7	5.968571429	2731	0.342594418	VTKHGPQ	61	766.4136	1	765.4133	.	V	Superscaf	51557418	0	0	0	0	0	BTP202209	Tissue specific
LNPVVP	Superscaf	+	Superscaf	51613351	51613368	11	11	16	-	-	gene	-	-	mRNA	NCP	Intron	gene|mRN	ID=gene-E	6	3.998333333	1624.9	0.341881003	LNPVVP	51	638.397	1	637.3799	.	S	Superscaf	51613321	0	BTP202209	0	0	0	0	Tissue specific
TTYLRK	Superscaf	-	Superscaf	51711817	51711834	-20	20	25	-	-	gene	-	-	mRNA	NCP	Intron	gene|mRN	ID=gene-E	6	7.533333333	2725.1	0.340625469	TTYLRK	94	391.2299	2	780.4494	.	L	Superscaf	51711840	0	0	BTP202209	0	0	0	Tissue specific
LGPTFSP	Superscaf	-	Superscaf	51763783	51763803	-124	124	130	-	-	gene	-	-	mRNA	NCP	Intron	gene|mRN	ID=gene-E	7	6.034285714	2493.8	0.339962815	L(+42.01)	50	760.3851	1	759.3803	Acetylati	K	Superscaf	51763815	0	0	0	0	0	BTP202209	Tissue specific
EALEFK	Superscaf	-	Superscaf	51859360	51859377	-34	34	39	-	-	gene	-	-	mRNA	NCP	Intron	gene|mRN	ID=gene-E	6	5.61	2371.5	0.338744157	EALEFK	72	736.3842	1	735.3802	.	M	Superscaf	51859425	BTP202209	0	0	0	0	0	Tissue specific
YDGLL	Superscaf	+	Superscaf	51892183	51892197	13	13	17	-	-	-	-	-	-	NCP	IGR	-	ID=gene-E	5	5.312	1875.1	0.338325671	YDGLL	75	580.2957	1	579.2904	.	Y	Superscaf	51892147	0	0	BTP202209	0	0	0	Tissue specific
WLPPPP	Superscaf	+	Superscaf	52095244	52095261	1	1	6	-	-	gene	-	-	mRNA	NCP	Intron	gene|mRN	ID=gene-E	6	6.178333333	2229.6	0.335736414	W(+42.01)	58	374.7032	2	747.3955	Acetylati	W	Superscaf	52095244	0	0	0	0	BTP202209	BTP202209	Tissue specific
KLVDKEF	Superscaf	-	Superscaf	52104340	52104360	-1	1	7	-	-	gene	-	-	mRNA	NCP	Intron	gene|mRN	ID=gene-E	7	5.304285714	2514.6	0.335620393	KLVDKEF	62	439.7507	2	877.4909	.	K	Superscaf	52104381	0	0	BTP202209	0	0	0	Tissue specific
LDQVEVVK	Superscaf	+	Superscaf	52252219	52252242	38	38	45	-	-	-	-	-	-	NCP	IGR	-	ID=gene-E	8	3.445	2269.5	0.333734758	LDQVEVVK	61	465.2692	2	928.5229	.	V	Superscaf	52252108	0	0	BTP202209	0	0	0	Tissue specific
VGFSTK	Superscaf	+	Superscaf	52310650	52310667	4	4	9	-	-	-	-	-	-	NCP	IGR	-	ID=gene-E	6	5.566666667	1934	0.332989785	V(+42.01)	66	680.358	1	679.3541	Acetylati	M	Superscaf	52310641	0	BTP202209	0	0	0	0	Tissue specific
LEKNWALP	Superscaf	-	Superscaf	52394479	52394502	-18	18	25	-	-	gene	-	-	mRNA	NCP	Intron	gene|mRN	ID=gene-E	8	6.0675	3372.7	0.33192081	LEKNWALP	66	485.7807	2	969.5283	.	F	Superscaf	52394595	0	0	0	BTP202209	0	0	Tissue specific
QFFKFGM	Superscaf	-	Superscaf	52489096	52489116	-18	18	24	-	-	-	-	-	-	NCP	IGR	-	ID=gene-E	7	6.221428571	2910.1	0.330714392	Q(+42.01)	51	473.7207	2	945.4418	Acetylati	I	Superscaf	52489146	0	0	0	0	0	BTP202209	Tissue specific
MGSLFDL	Superscaf	+	Superscaf	52544602	52544622	23	23	29	-	-	-	-	-	-	NCP	IGR	-	ID=gene-E	7	5.401428571	2557.9	0.330006638	M(+42.01)	55	824.3826	1	823.3786	Acetylati	N	Superscaf	52544536	0	0	0	0	BTP202209	0	Tissue specific
PATGLQF	Superscaf	-	Superscaf	52761346	52761366	-5	5	11	-	-	-	-	-	-	NCP	IGR	-	ID=gene-E	7	5.99	2695	0.327242948	P(+42.01)	77	775.3973	1	774.3912	Acetylati	L	Superscaf	52761390	0	BTP202209	BTP202209	BTP202209	0	0	Tissue specific
MEDHLSLH	Superscaf	+	Superscaf	52797616	52797639	10	10	17	-	-	gene	-	-	mRNA	NCP	Intron	gene|mRN	ID=gene-E	8	5.595	3538.1	0.326780434	M(+15.99)	76	520.2283	2	1038.444	Oxidation	K	Superscaf	52797589	0	BTP202209	0	0	BTP202209	0	Tissue specific
MMGLYT	Superscaf	-	Superscaf	52986991	52987008	-22	22	27	-	-	-	-	-	-	NCP	IGR	-	ID=gene-E	6	5.94	2250.7	0.324365801	M(+15.99)	53	789.3052	1	788.3085	Oxidation	I	Superscaf	52987011	0	BTP202209	0	0	0	0	Tissue specific
DFPELKP	Superscaf	-	Superscaf	52994812	52994832	-17	17	23	-	-	-	-	-	-	NCP	IGR	-	ID=gene-E	7	5.712857143	2721	0.324266037	DFPELKP	67	423.2227	2	844.433	.	P	Superscaf	52994898	0	0	0	0	BTP202209	0	Tissue specific
KSPGYGL	Superscaf	+	Superscaf	52997104	52997124	4	4	10	-	-	-	-	-	-	NCP	IGR	-	ID=gene-E	7	6.471428571	2596.7	0.324236812	KSPGYGL	59	361.196	2	720.3806	.	E	Superscaf	52997095	0	0	BTP202209	0	0	0	Tissue specific
KLLNESGK	Superscaf	-	Superscaf	53139052	53139075	-81	81	88	-	-	-	-	-	-	NCP	IGR	-	ID=gene-E	8	6.465	3346.5	0.322426803	KLLNESGK	63	444.7589	2	887.5076	.	T	Superscaf	53139099	0	0	BTP202209	0	0	0	Tissue specific
DMPDLRK	Superscaf	-	Superscaf	53380864	53380884	-25	25	31	-	-	-	-	-	-	NCP	IGR	-	ID=gene-E	7	6.352857143	3002.5	0.319343511	D(+42.01)	74	458.7297	2	915.4484	Acetylati	F	Superscaf	53380893	0	0	0	0	0	BTP202209	Tissue specific
MTNYQP	Superscaf	+	Superscaf	53434933	53434950	13	13	18	-	-	gene	-	-	mRNA	NCP	Intron	gene|mRN	ID=gene-E	6	5.883333333	2773.3	0.318654119	M(+15.99)	58	406.1613	2	810.3218	Oxidation	T	Superscaf	53434897	0	0	0	0	0	BTP202209	Tissue specific
PWTAH	Superscaf	-	Superscaf	53608090	53608104	-47	47	51	-	-	-	-	-	-	NCP	IGR	-	ID=gene-E	5	6.466	2192.5	0.316446242	P(+42.01)	61	653.315	1	652.2969	Acetylati	H	Superscaf	53608326	0	0	0	0	0	BTP202209	Tissue specific
KPEPPGK	Superscaf	-	Superscaf	53639224	53639244	-32	32	38	-	-	-	-	-	-	NCP	IGR	-	ID=gene-E	7	6.795714286	2671.8	0.316049178	KPEPPGK	59	376.7133	2	751.4228	.	P	Superscaf	53639280	0	BTP202209	0	0	0	0	Tissue specific
NFPFEL	Superscaf	+	Superscaf	53721937	53721954	34	34	39	-	-	-	-	-	-	NCP	IGR	-	ID=gene-E	6	5.311666667	2487.8	0.314994548	NFPFEL	86	766.3807	1	765.3697	.	E	Superscaf	53721838	0	0	0	0	BTP202209	0	Tissue specific
TLEVMPY	Superscaf	-	Superscaf	53943712	53943732	-11	11	17	-	-	-	-	-	-	NCP	IGR	-	ID=gene-E	7	4.777142857	2344.8	0.31216667	TLEVMPY	83	852.4121	1	851.4099	.	T	Superscaf	53943762	0	BTP202209	0	0	0	0	Tissue specific
SSGVAQLVK	Superscaf	+	Superscaf	53990836	53990862	14	14	22	-	-	-	-	-	-	NCP	IGR	-	ID=gene-E	9	4.968888889	2807.9	0.311565718	SSGVAQLVK	84	888.5109	1	887.5076	.	N	Superscaf	53990797	0	0	0	0	BTP202209	0	Tissue specific
RHCDDD	Superscaf	-	Superscaf	54007009	54007026	-16	16	21	-	-	-	-	-	-	NCP	IGR	-	ID=gene-E	6	5.38	3088.7	0.311359612	RHC(+57.(	52	409.1457	2	816.282	Carbamido	L	Superscaf	54007065	0	0	0	BTP202209	0	0	Tissue specific
ESLRVAE	Superscaf	+	Superscaf	54099727	54099747	11	11	17	-	-	-	-	-	-	NCP	IGR	-	ID=gene-E	7	4.982857143	2547.9	0.310177332	E(+42.01)	55	423.226	2	844.429	Acetylati	M	Superscaf	54099697	0	0	0	0	BTP202209	0	Tissue specific
DTPELLH	Superscaf	-	Superscaf	54142438	54142458	-7	7	13	-	-	-	-	-	-	NCP	IGR	-	ID=gene-E	7	5.51	2898.4	0.309632726	DTPELLH	92	412.7105	2	823.4075	.	A	Superscaf	54142551	BTP202209	0	0	0	0	0	Tissue specific
FLRYP	Superscaf	-	Superscaf	54430999	54431013	-17	17	21	-	-	-	-	-	-	NCP	IGR	-	ID=gene-E	5	6.836	2186.5	0.305953379	FLRYP	54	695.3852	1	694.3802	.	V	Superscaf	54431073	0	0	0	0	BTP202209	0	Tissue specific
PDDGMM	Superscaf	+	Superscaf	54527704	54527721	1	1	6	-	-	-	-	-	-	NCP	IGR	-	ID=gene-E	6	4.951666667	2200.7	0.304720261	P(+42.01)	50	370.1112	2	738.22	Acetylati	P	Superscaf	54527704	0	0	0	0	0	BTP202209	Mixed
RMPVVVK	Superscaf	+	Superscaf	54541249	54541269	29	29	35	-	-	-	-	-	-	NCP	IGR	-	ID=gene-E	7	4.65	1826.1	0.304547511	R(+42.01)	77	443.7622	2	885.5106	Acetylati	I	Superscaf	54541165	0	0	0	0	BTP202209	0	Tissue specific
VAFEKHY	Superscaf	-	Superscaf	54563143	54563163	-1	1	7	-	-	-	-	-	-	NCP	IGR	-	ID=gene-E	7	5.387142857	2671.8	0.304268342	VAFEKHY	54	447.2324	2	892.4443	.	V	Superscaf	54563211	0	BTP202209	0	0	BTP202209	0	Tissue specific
KSLDTF	Superscaf	+	Superscaf	54594448	54594465	3	3	8	-	-	-	-	-	-	NCP	IGR	-	ID=gene-E	6	6.063333333	2401.6	0.303869212	K(+42.01)	75	752.3777	1	751.3752	Acetylati	W	Superscaf	54594442	0	BTP202209	BTP202209	BTP202209	BTP202209	BTP202209	Tissue specific
KAHFRYK	Superscaf	+	Superscaf	54616336	54616356	28	28	34	-	-	-	-	-	-	NCP	IGR	-	ID=gene-E	7	7.855714286	3363.5	0.303590081	KAHFRYK	54	475.2723	2	948.5294	.	G	Superscaf	54616255	0	BTP202209	0	0	0	0	Tissue specific
GLLTL	Superscaf	-	Superscaf	54687568	54687582	-8	8	12	-	-	-	-	-	-	NCP	IGR	-	ID=gene-E	5	6.088	1755	0.302681882	GLLTL	97	516.337	1	515.3319	.	A	Superscaf	54687582	0	0	BTP202209	0	0	0	Tissue specific
RNLLYK	Superscaf	+	Superscaf	54835558	54835575	6	6	11	-	-	-	-	-	-	NCP	IGR	-	ID=gene-E	6	7.255	2880.2	0.300794832	RNLLYK	70	403.7461	2	805.481	.	T	Superscaf	54835543	0	0	BTP202209	0	0	0	Tissue specific
GPGPYK	Superscaf	-	Superscaf	54836431	54836448	-14	14	19	-	-	-	-	-	-	NCP	IGR	-	ID=gene-E	6	6.656666667	2197.3	0.300783701	GPGPYK	53	618.3134	1	617.3173	.	G	Superscaf	54836481	0	0	0	0	0	BTP202209	Tissue specific
TAAKYL	Superscaf	+	Superscaf	54875737	54875754	1	1	6	-	-	-	-	-	-	NCP	IGR	-	ID=gene-E	6	6.658333333	2301.4	0.300282512	TAAKYL	62	666.3805	1	665.3748	.	T	Superscaf	54875737	0	0	0	0	0	BTP202209	Tissue specific
HEPTLL	Superscaf	+	Superscaf	55221637	55221654	8	8	13	-	-	gene	-	-	mRNA	NCP	Intron	gene|mRN	ID=gene-E	6	5.933333333	2486.9	0.295871962	HEPTLL	74	355.1966	2	708.3806	.	K	Superscaf	55221616	0	0	0	BTP202209	0	0	Tissue specific
TVEYTKF	Superscaf	+	Superscaf	55273510	55273530	47	47	53	-	-	gene	-	-	mRNA	NCP	Intron	gene|mRN	ID=gene-E	7	5.308571429	2535.8	0.295210494	TVEYTKF	57	887.4547	1	886.4436	.	I	Superscaf	55273372	0	0	BTP202209	0	0	0	Tissue specific
ERMFLSF	Superscaf	-	Superscaf	55364740	55364760	-15	15	21	-	-	-	-	-	-	NCP	IGR	-	ID=gene-E	7	6.05	2957.4	0.294047226	ERMFLSF	69	465.2296	2	928.4476	.	L	Superscaf	55364781	0	0	0	BTP202209	0	0	Tissue specific
EAAVPL	Superscaf	-	Superscaf	55398661	55398678	-44	44	49	-	-	-	-	-	-	NCP	IGR	-	ID=gene-E	6	4.59	1738.9	0.293614739	EAAVPL	72	599.3391	1	598.3326	.	Y	Superscaf	55398693	0	0	BTP202209	0	0	0	Tissue specific
WLFALKK	Superscaf	-	Superscaf	55400950	55400970	-8	8	14	-	-	-	-	-	-	NCP	IGR	-	ID=gene-E	7	6.975714286	2868.9	0.293585514	W(+42.01)	87	474.2889	2	946.564	Acetylati	I	Superscaf	55400976	BTP202209	0	0	0	0	0	Tissue specific
AAVVDC	Superscaf	-	Superscaf	55679803	55679820	-37	37	42	-	-	-	-	-	-	NCP	IGR	-	ID=gene-E	6	3.338333333	1619.8	0.290029914	A(+42.01)	50	676.3091	1	675.2897	Acetylati	N	Superscaf	55679904	BTP202209	0	0	0	0	0	Tissue specific
KFPWSEL	Superscaf	+	Superscaf	55760737	55760757	3	3	9	-	-	-	-	-	-	NCP	IGR	-	ID=gene-E	7	6.041428571	2882.1	0.288997892	K(+42.01)	93	948.4822	1	947.4752	Acetylati	L	Superscaf	55760731	0	0	0	BTP202209	0	0	Tissue specific
HLSNEPK	Superscaf	-	Superscaf	55807711	55807731	-1	1	7	-	-	gene	-	-	mRNA	NCP	Intron	gene|mRN	ID=gene-E	7	6.274285714	3184.5	0.288398929	HLSNEPK	60	412.7111	2	823.4188	.	H	Superscaf	55807737	0	0	0	BTP202209	0	0	Tissue specific
LNHSMK	Superscaf	+	Superscaf	55814500	55814517	2	2	7	-	-	gene	-	-	mRNA	NCP	Intron	gene|mRN	ID=gene-E	6	6.691666667	2793.1	0.288312401	LNHSMK	53	365.1915	2	728.364	.	N	Superscaf	55814497	0	0	BTP202209	0	0	0	Tissue specific
VGELLRKN	Superscaf	+	Superscaf	55856695	55856718	1	1	8	-	-	gene	-	-	mRNA	NCP	Intron	gene|mRN	ID=gene-E	8	5.8825	3143.5	0.287774299	VGELLRKN	71	464.7816	2	927.5501	.	V	Superscaf	55856695	0	0	BTP202209	BTP202209	0	BTP202209	Tissue specific
FEPEKL	Superscaf	+	Superscaf	55905076	55905093	24	24	29	-	-	gene	-	-	mRNA	NCP	Intron	gene|mRN	ID=gene-E	6	5.656666667	2397.6	0.287157472	FEPEKL	94	381.7037	2	761.3959	.	E	Superscaf	55905007	0	0	0	0	0	BTP202209	Mixed
VEVLTF	Superscaf	+	Superscaf	55962268	55962285	35	35	40	-	-	-	-	-	-	NCP	IGR	-	ID=gene-E	6	3.535	1563.8	0.28642822	VEVLTF	81	707.3912	1	706.3901	.	V	Superscaf	55962166	0	0	0	0	BTP202209	0	Tissue specific
DHPLK	Superscaf	+	Superscaf	55969525	55969539	6	6	10	-	-	-	-	-	-	NCP	IGR	-	ID=gene-E	5	6.516	2190.4	0.286335725	DHPLK	65	609.3242	1	608.3282	.	G	Superscaf	55969510	0	0	0	0	0	BTP202209	Tissue specific
KNPGLLRP	Superscaf	+	Superscaf	56025847	56025870	12	12	19	-	-	-	-	-	-	NCP	IGR	-	ID=gene-E	8	7.055	3392.8	0.285617451	KNPGLLRP	70	447.778	2	893.5446	.	I	Superscaf	56025814	BTP202209	0	0	0	0	0	Tissue specific
GKVLFP	Superscaf	+	Superscaf	56106928	56106945	12	12	17	-	-	gene	-	-	mRNA	NCP	Intron	gene|mRN	ID=gene-E	6	5.578333333	1843.9	0.284583669	G(+42.01)	66	702.4152	1	701.4112	Acetylati	Q	Superscaf	56106895	0	0	0	0	BTP202209	0	Tissue specific
EAAPALSPK	Superscaf	-	Superscaf	56146456	56146482	-4	4	12	-	-	gene	-	-	mRNA	NCP	Intron	gene|mRN	ID=gene-E	9	6.142222222	3239.4	0.284079535	EAAPALSPK	51	442.2393	2	882.481	.	R	Superscaf	56146536	0	0	0	0	0	BTP202209	Tissue specific
MQLTDF	Superscaf	+	Superscaf	56225479	56225496	8	8	13	-	-	-	-	-	-	NCP	IGR	-	ID=gene-E	6	5.393333333	2532	0.283072032	MQLTDF	99	754.3433	1	753.3367	.	P	Superscaf	56225458	0	BTP202209	0	0	0	0	Mixed
PGLVPLH	Superscaf	+	Superscaf	56228365	56228385	39	39	45	-	-	-	-	-	-	NCP	IGR	-	ID=gene-E	7	5.445714286	2298.6	0.283035195	PGLVPLH	52	366.7225	2	731.433	.	C	Superscaf	56228251	0	BTP202209	0	0	0	0	Tissue specific
TGYGMPR	Superscaf	+	Superscaf	56240917	56240937	46	46	52	-	-	-	-	-	-	NCP	IGR	-	ID=gene-E	7	6.705714286	2809.3	0.282875145	TGYGMPR	99	391.1854	2	780.3588	.	C	Superscaf	56240782	0	0	0	BTP202209	0	0	Tissue specific
KHLLN	Superscaf	-	Superscaf	56394739	56394753	-2	2	6	-	-	gene	-	-	mRNA	NCP	Intron	gene|mRN	ID=gene-E	5	6.94	2391.6	0.280913846	KHLLN	65	624.3773	1	623.3755	.	V	Superscaf	56394792	BTP202209	0	0	0	0	0	Tissue specific
PAGLAF	Superscaf	+	Superscaf	56494858	56494875	2	2	7	-	-	-	-	-	-	NCP	IGR	-	ID=gene-E	6	5.961666667	1998.1	0.279637196	PAGLAF	69	575.3161	1	574.3115	.	R	Superscaf	56494855	0	BTP202209	0	0	0	BTP202209	Tissue specific
EVEKVFK	Superscaf	+	Superscaf	56598073	56598093	9	9	15	-	exon	gene	three_pri	-	mRNA	NCP	3UTR	exon|gene	ID=exon-E	7	4.485714286	2175.2	0.27832107	E(+42.01)	97	460.7616	2	919.5015	Acetylati	T	Superscaf	56598049	0	BTP202209	BTP202209	0	BTP202209	0	Tissue specific
SCVGVFK	Superscaf	-	Superscaf	56614123	56614143	-32	32	38	-	-	-	-	-	-	NCP	IGR	-	ID=gene-E	7	4.555714286	2122.2	0.278116417	SC(+57.02	98	398.7037	2	795.3949	Carbamido	C	Superscaf	56614197	0	BTP202209	0	0	0	0	Tissue specific
KYVLPKSK	Superscaf	-	Superscaf	56639959	56639982	-5	5	12	-	-	-	-	-	-	NCP	IGR	-	ID=gene-E	8	6.605	2938.9	0.277786946	KYVLPKSK	83	481.8034	2	961.596	.	N	Superscaf	56639991	0	0	0	0	BTP202209	0	Tissue specific
LGAAPKSL	Superscaf	-	Superscaf	56681932	56681955	-13	13	20	-	-	gene	-	-	mRNA	NCP	Intron	gene|mRN	ID=gene-E	8	6.46125	2815.9	0.27725175	LGAAPKSL	61	378.7337	2	755.4541	.	M	Superscaf	56682012	0	0	0	0	BTP202209	0	Tissue specific
KSTSVFP	Superscaf	-	Superscaf	56712070	56712090	-1	1	7	-	-	gene	-	-	mRNA	NCP	Intron	gene|mRN	ID=gene-E	7	5.63	2357.5	0.276867501	KSTSVFP	67	383.2162	2	764.4068	.	K	Superscaf	56712099	0	BTP202209	0	0	BTP202209	BTP202209	Tissue specific
RGVVP	Superscaf	+	Superscaf	56854696	56854710	14	14	18	-	-	gene	-	-	mRNA	NCP	Intron	gene|mRN	ID=gene-E	5	4.606	1283.5	0.275048962	RGVVP	53	527.3243	1	526.3227	.	K	Superscaf	56854657	0	BTP202209	BTP202209	BTP202209	BTP202209	BTP202209	Tissue specific
GTGPLF	Superscaf	-	Superscaf	57022438	57022455	-2	2	7	-	-	gene	-	-	mRNA	NCP	Intron	gene|mRN	ID=gene-E	6	6.038333333	2070.3	0.272910055	G(+42.01)	67	633.3159	1	632.317	Acetylati	F	Superscaf	57022467	0	0	0	BTP202209	0	0	Tissue specific
PPSLHK	Superscaf	+	Superscaf	57041359	57041376	1	1	6	-	-	gene	-	-	mRNA	NCP	Intron	gene|mRN	ID=gene-E	6	6.931666667	2499.7	0.272668795	PPSLHK	62	678.3914	1	677.386	.	P	Superscaf	57041359	BTP202209	0	0	0	0	0	Tissue specific
LVTLANNR	Superscaf	+	Superscaf	57144004	57144027	3	3	10	-	-	-	-	-	-	NCP	IGR	-	ID=gene-E	8	5.76125	3257.8	0.271359898	LVTLANNR	51	450.7653	2	899.5189	.	N	Superscaf	57143998	BTP202209	0	0	0	0	0	Tissue specific
VELLV	Superscaf	+	Superscaf	57147970	57147984	17	17	21	-	-	-	-	-	-	NCP	IGR	-	ID=gene-E	5	3.036	1132.3	0.271309442	VELLV	58	572.3636	1	571.3581	.	H	Superscaf	57147922	0	0	0	BTP202209	0	0	Tissue specific
SVNLVAL	Superscaf	-	Superscaf	57198529	57198549	-1	1	7	-	-	gene	-	-	mRNA	NCP	Intron	gene|mRN	ID=gene-E	7	4.152857143	1998.2	0.270664691	SVNLVAL	69	715.4336	1	714.4276	.	S	Superscaf	57198582	BTP202209	0	0	0	0	0	Mixed
GVPSAF	Superscaf	+	Superscaf	57221656	57221673	5	5	10	-	-	-	-	-	-	NCP	IGR	-	ID=gene-E	6	4.908333333	1716.8	0.270369838	GVPSAF	60	577.2961	1	576.2907	.	S	Superscaf	57221644	0	0	0	0	BTP202209	0	Tissue specific
LPGLPP	Superscaf	+	Superscaf	57347146	57347163	18	18	23	-	-	gene	-	-	mRNA	NCP	Intron	gene|mRN	ID=gene-E	6	6.138333333	2016.3	0.268769723	LPGLPP	54	593.3652	1	592.3584	.	E	Superscaf	57347095	0	0	0	0	0	BTP202209	Tissue specific
VSPTWL	Superscaf	-	Superscaf	57352813	57352830	-41	41	46	-	-	gene	-	-	mRNA	NCP	Intron	gene|mRN	ID=gene-E	6	5.063333333	1998.3	0.268697463	VSPTWL	95	702.3784	1	701.3748	.	S	Superscaf	57352905	0	BTP202209	0	0	0	0	Tissue specific

KDPSAVP	Superscaf	-	Superscaf	57395830	57395850	-28	28	34	-	-	gene	-	-	mRNA	NCP	Intron	gene|mRN	ID=gene-E	7	5.287142857	2249.4	0.268148918	KDPSAVP	67	713.3826	1	712.3755	.	L	Superscaf	57395901	0	0	BTP202209	0	0	0	Tissue specific
DFGPK	Superscaf	-	Superscaf	57405358	57405372	-20	20	24	-	-	-	-	-	-	NCP	IGR	-	ID=gene-E	5	6.092	1902	0.268027503	DFGPK	98	563.2797	1	562.2751	.	L	Superscaf	57405456	0	BTP202209	0	0	BTP202209	0	Mixed
RTDLEKPF	Superscaf	+	Superscaf	57437320	57437343	2	2	9	-	-	gene	-	-	mRNA	NCP	Intron	gene|mRN	ID=gene-E	8	6.3725	3429.9	0.267619843	RTDLEKPF	68	503.2691	2	1004.529	.	A	Superscaf	57437317	0	BTP202209	BTP202209	BTP202209	0	0	Tissue specific
MTKLNGN	Superscaf	-	Superscaf	57446170	57446190	-4	4	10	-	-	gene	-	-	mRNA	NCP	Intron	gene|mRN	ID=gene-E	7	6.398571429	3081.5	0.267507035	MTKLNGN	70	777.3862	1	776.3851	.	V	Superscaf	57446211	0	0	BTP202209	0	BTP202209	0	Tissue specific
KATVEPR	Superscaf	-	Superscaf	57467902	57467922	-22	22	28	-	-	-	-	-	-	NCP	IGR	-	ID=gene-E	7	6.081428571	2588.9	0.267229932	KATVEPR	61	400.7337	2	799.4551	.	F	Superscaf	57467958	0	0	0	BTP202209	0	0	Tissue specific
LGALQRPK	Superscaf	-	Superscaf	57499684	57499707	-28	28	35	-	-	gene	-	-	mRNA	NCP	Intron	gene|mRN	ID=gene-E	8	7.05	3380.7	0.266824643	LGALQRPK	78	441.7779	2	881.5446	.	W	Superscaf	57499749	0	0	0	0	BTP202209	0	Tissue specific
SSSKPP	Superscaf	+	Superscaf	57623704	57623721	11	11	16	-	-	gene	-	-	mRNA	NCP	Intron	gene|mRN	ID=gene-E	6	6.563333333	2293.4	0.265243348	SSSKPP	52	602.3113	1	601.3071	.	S	Superscaf	57623674	0	0	0	0	0	BTP202209	Tissue specific
DGKAPQK	Superscaf	-	Superscaf	57714655	57714675	-9	9	15	-	-	gene	-	-	mRNA	NCP	Intron	gene|mRN	ID=gene-E	7	6.627142857	2905	0.264083599	DGKAPQK	69	372.1995	2	742.3973	.	C	Superscaf	57714693	0	BTP202209	0	0	BTP202209	BTP202209	Tissue specific
VLLVKSSM	Superscaf	+	Superscaf	57912067	57912090	5	5	12	-	-	gene	-	-	mRNA	NCP	Intron	gene|mRN	ID=gene-E	8	4.85125	2313.5	0.261566372	VLLVKSSM(	80	446.7646	2	891.5099	Oxidation	K	Superscaf	57912055	BTP202209	BTP202209	BTP202209	BTP202209	BTP202209	BTP202209	Tissue specific
KDFGP	Superscaf	-	Superscaf	57943939	57943953	-3	3	7	-	-	-	-	-	-	NCP	IGR	-	ID=gene-E	5	6.092	1902	0.261160089	KDFGP	58	563.2729	1	562.2751	.	D	Superscaf	57943956	BTP202209	0	0	0	0	0	Tissue specific
VGNPLL	Superscaf	-	Superscaf	57963973	57963990	-3	3	8	-	-	-	-	-	-	NCP	IGR	-	ID=gene-E	6	4.94	1938.2	0.260904598	VGNPLL	62	612.3693	1	611.3643	.	T	Superscaf	57964011	0	0	0	0	BTP202209	0	Tissue specific
RLPLQDV	Superscaf	+	Superscaf	57996655	57996675	100	100	106	-	-	gene	-	-	mRNA	NCP	Intron	gene|mRN	ID=gene-E	7	5.377142857	2715.2	0.260487834	RLPLQDV	74	420.7489	2	839.4865	.	L	Superscaf	57996358	0	0	0	0	0	BTP202209	Tissue specific
DGPKFLK	Superscaf	+	Superscaf	57997075	57997095	240	240	246	-	-	gene	-	-	mRNA	NCP	Intron	gene|mRN	ID=gene-E	7	6.597142857	2723.8	0.260482478	DGPKFLK	99	402.7334	2	803.4541	.	L	Superscaf	57996358	0	0	0	BTP202209	0	0	Tissue enhanced
VKLLFA	Superscaf	+	Superscaf	58059919	58059936	3	3	8	-	-	gene	-	-	mRNA	NCP	Intron	gene|mRN	ID=gene-E	6	5.533333333	1873.9	0.259681196	V(+42.01)	54	732.4628	1	731.4581	Acetylati	H	Superscaf	58059913	BTP202209	0	0	0	0	0	Tissue specific
PPAYKR	Superscaf	-	Superscaf	58070536	58070553	-32	32	37	-	-	gene	-	-	mRNA	NCP	Intron	gene|mRN	ID=gene-E	6	7.463333333	2562.8	0.25954582	PPAYKR	93	366.2128	2	730.4125	.	S	Superscaf	58070556	BTP202209	0	0	0	0	0	Tissue specific
ALPNLL	Superscaf	-	Superscaf	58084660	58084677	-4	4	9	-	-	gene	-	-	mRNA	NCP	Intron	gene|mRN	ID=gene-E	6	5.945	2305.6	0.259365725	ALPNLL	64	640.401	1	639.3956	.	S	Superscaf	58084713	0	0	BTP202209	0	0	0	Tissue specific
QGPDAPPK	Superscaf	+	Superscaf	58090036	58090059	7	7	14	-	-	gene	-	-	mRNA	NCP	Intron	gene|mRN	ID=gene-E	8	6.15625	3111.4	0.2592971	QGPDAPPK	62	405.2056	2	808.4079	.	A	Superscaf	58090018	0	BTP202209	BTP202209	0	BTP202209	BTP202209	Tissue specific
LSGLHFK	Superscaf	+	Superscaf	58214545	58214565	3	3	9	-	-	-	-	-	-	NCP	IGR	-	ID=gene-E	7	6.631428571	2863	0.257709531	LSGLHFK	70	401.2374	2	800.4545	.	F	Superscaf	58214539	BTP202209	0	0	0	0	0	Tissue specific
ELNARK	Superscaf	+	Superscaf	58223602	58223619	54	54	59	-	-	-	-	-	-	NCP	IGR	-	ID=gene-E	6	6.855	2804.1	0.257594084	E(+42.01)	53	386.7253	2	771.4239	Acetylati	K	Superscaf	58223443	BTP202209	0	0	0	0	0	Tissue specific
KEMTVETL	Superscaf	+	Superscaf	58276741	58276764	7	7	14	-	-	gene	-	-	mRNA	NCP	Intron	gene|mRN	ID=gene-E	8	5.12125	2839.3	0.256916435	KEM(+15.9	66	483.7464	2	965.4739	Oxidation	I	Superscaf	58276723	0	0	0	0	BTP202209	BTP202209	Tissue specific
GSYDN	Superscaf	+	Superscaf	58318768	58318782	16	16	20	-	-	-	-	-	-	NCP	IGR	-	ID=gene-E	5	5.138	2148.4	0.256380666	GSYDN	56	555.2023	1	554.1973	.	F	Superscaf	58318723	BTP202209	0	0	0	0	0	Tissue specific
FLVHVDK	Superscaf	+	Superscaf	58380787	58380807	14	14	20	-	-	gene	-	-	mRNA	NCP	Intron	gene|mRN	ID=gene-E	7	4.537142857	2240.4	0.255589789	FLVHVDK	77	429.2526	2	856.4807	.	L	Superscaf	58380748	0	0	0	BTP202209	0	0	Tissue specific
GVKYPEL	Superscaf	-	Superscaf	58432987	58433007	-37	37	43	-	-	gene	-	-	mRNA	NCP	Intron	gene|mRN	ID=gene-E	7	5.267142857	2341.5	0.25492419	GVKYPEL	50	805.441	1	804.4381	.	V	Superscaf	58433007	0	0	0	0	0	BTP202209	Tissue specific
AFRLW	Superscaf	-	Superscaf	58476448	58476462	-11	11	15	-	-	gene	-	-	mRNA	NCP	Intron	gene|mRN	ID=gene-E	5	6.826	2227.5	0.254370098	AFRLW	54	692.395	1	691.3806	.	R	Superscaf	58476465	0	0	0	0	BTP202209	0	Tissue specific
KELFFPPP	Superscaf	+	Superscaf	58549768	58549791	27	27	34	-	-	-	-	-	-	NCP	IGR	-	ID=gene-E	8	6.1	3034.3	0.253435084	KELFFPPP	55	487.7797	2	973.5272	.	Y	Superscaf	58549690	0	0	0	BTP202209	0	0	Tissue specific
CPLSLFPK	Superscaf	+	Superscaf	58713235	58713258	23	23	30	-	-	-	-	-	-	NCP	IGR	-	ID=gene-E	8	6.31	3206.5	0.251350726	C(+57.02)	54	481.2709	2	960.5103	Carbamido	Y	Superscaf	58713169	0	0	0	BTP202209	0	0	Tissue specific
KECCH	Superscaf	+	Superscaf	58920754	58920768	37	37	41	-	-	-	-	-	-	NCP	IGR	-	ID=gene-E	5	6.118	2685	0.248704779	KEC(+57.(	51	367.1344	2	732.2683	Carbamido	L	Superscaf	58920646	0	0	0	0	0	BTP202209	Tissue specific
DTVVVEK	Superscaf	-	Superscaf	59313913	59313933	-12	12	18	-	-	-	-	-	-	NCP	IGR	-	ID=gene-E	7	3.208571429	1702.9	0.243691555	DTVVVEK	57	789.4309	1	788.428	.	S	Superscaf	59314053	0	0	0	0	BTP202209	0	Tissue specific
LLKLSY	Superscaf	+	Superscaf	59436019	59436036	7	7	12	-	-	-	-	-	-	NCP	IGR	-	ID=gene-E	6	6.503333333	2371.5	0.242134627	LLKLSY	90	368.7325	2	735.4531	.	R	Superscaf	59436001	0	0	0	0	BTP202209	0	Tissue specific
SGVVP	Superscaf	+	Superscaf	59457166	59457180	6	6	10	-	-	-	-	-	-	NCP	IGR	-	ID=gene-E	5	3.59	1018.1	0.241865021	SGVVP	61	458.2541	1	457.2536	.	L	Superscaf	59457151	0	0	0	0	0	BTP202209	Tissue specific
TGFPTNF	Superscaf	+	Superscaf	59534350	59534370	1	1	7	-	-	-	-	-	-	NCP	IGR	-	ID=gene-E	7	5.957142857	2801.2	0.240880776	T(+42.01)	88	825.3752	1	824.3705	Acetylati	T	Superscaf	59534350	BTP202209	0	0	0	0	0	Tissue specific
KKYCFP	Superscaf	+	Superscaf	59560195	59560212	12	12	17	-	-	-	-	-	-	NCP	IGR	-	ID=gene-E	6	6.99	2706.8	0.240551266	KKYC(+57.	61	421.7213	2	841.4156	Carbamido	T	Superscaf	59560162	0	BTP202209	0	0	0	0	Tissue specific
EVVPQ	Superscaf	+	Superscaf	59582782	59582796	9	9	13	-	-	-	-	-	-	NCP	IGR	-	ID=gene-E	5	3.034	1373.6	0.240263299	EVVPQ	64	571.3062	1	570.3013	.	L	Superscaf	59582758	0	0	0	0	0	BTP202209	Tissue specific
GVAMVA	Superscaf	+	Superscaf	59618620	59618637	10	10	15	-	-	-	-	-	-	NCP	IGR	-	ID=gene-E	6	3.96	1291.4	0.239806292	GVAMVA	59	547.2874	1	546.2836	.	H	Superscaf	59618593	0	0	0	0	BTP202209	0	Tissue specific
TTSAG	Superscaf	-	Superscaf	59647675	59647689	-22	22	26	-	-	-	-	-	-	NCP	IGR	-	ID=gene-E	5	6.146	1787	0.239435852	TTSAG	63	436.2098	1	435.1965	.	T	Superscaf	59647791	0	0	0	0	BTP202209	0	Mixed
NSSGPPPPF	Superscaf	-	Superscaf	59657545	59657571	-6	6	14	-	-	-	-	-	-	NCP	IGR	-	ID=gene-E	9	6.026666667	3347.8	0.239309847	N(+42.01)	65	446.2188	2	890.4134	Acetylati	K	Superscaf	59657601	BTP202209	0	0	0	0	0	Tissue specific
QALKVFN	Superscaf	+	Superscaf	59670055	59670075	15	15	21	-	-	-	-	-	-	NCP	IGR	-	ID=gene-E	7	5.468571429	2727.9	0.239150409	QALKVFN	73	819.4694	1	818.465	.	R	Superscaf	59670013	0	0	0	0	0	BTP202209	Tissue specific
FPVGEVVP	Superscaf	+	Superscaf	59760064	59760087	16	16	23	-	-	gene	-	-	mRNA	NCP	Intron	gene|mRN	ID=gene-E	8	3.40875	1785	0.238002672	FPVGEVVP	66	843.4587	1	842.4538	.	K	Superscaf	59760019	0	BTP202209	0	0	0	0	Tissue specific
LNGPLLSKI	Superscaf	-	Superscaf	59845669	59845695	-1	1	9	-	-	gene	-	-	mRNA	NCP	Intron	gene|mRN	ID=gene-E	9	6.335555556	3496.8	0.236911089	LNGPLLSKI	77	477.8013	2	953.5909	.	L	Superscaf	59845698	0	0	BTP202209	0	BTP202209	0	Tissue specific
LLHQRSPL	Superscaf	+	Superscaf	59892721	59892744	2	2	9	-	-	-	-	-	-	NCP	IGR	-	ID=gene-E	8	6.74	3660.2	0.23631117	LLHQRSPL	58	482.2945	2	962.5661	.	F	Superscaf	59892718	0	BTP202209	0	0	0	0	Tissue specific
KVTLLL	Superscaf	+	Superscaf	59951461	59951478	12	12	17	-	-	gene	-	-	mRNA	NCP	Intron	gene|mRN	ID=gene-E	6	5.701666667	1926.1	0.235562256	K(+42.01)	55	728.489	1	727.4844	Acetylati	R	Superscaf	59951428	0	BTP202209	0	0	0	0	Tissue specific
LKFDRP	Superscaf	+	Superscaf	59968765	59968782	8	8	13	-	-	gene	-	-	mRNA	NCP	Intron	gene|mRN	ID=gene-E	6	6.871666667	2606.9	0.235341614	LKFDRP	83	388.2267	2	774.4388	.	V	Superscaf	59968744	0	BTP202209	0	BTP202209	0	BTP202209	Tissue specific
KAPAKPPP	Superscaf	-	Superscaf	59969788	59969811	-17	17	24	-	-	gene	-	-	mRNA	NCP	Intron	gene|mRN	ID=gene-E	8	7.09	2909	0.235328493	KAPAKPPP	66	403.2491	2	804.4857	.	R	Superscaf	59969814	0	0	0	BTP202209	0	0	Tissue enhanced
APAKPPPK	Superscaf	-	Superscaf	59969791	59969814	-18	18	25	-	-	gene	-	-	mRNA	NCP	Intron	gene|mRN	ID=gene-E	8	7.09	2909	0.235328455	APAKPPPK	66	403.2491	2	804.4857	.	R	Superscaf	59969814	0	0	0	BTP202209	0	0	Tissue specific
PPGPPYK	Superscaf	+	Superscaf	60128971	60128991	1	1	7	-	-	gene	-	-	mRNA	NCP	Intron	gene|mRN	ID=gene-E	7	6.652857143	2574.8	0.233298799	PPGPPYK	56	378.2065	2	754.4013	.	P	Superscaf	60128971	0	0	BTP202209	0	0	0	Tissue enhanced
SPRMKG	Superscaf	-	Superscaf	60230617	60230634	-10	10	15	-	-	gene	-	-	mRNA	NCP	Intron	gene|mRN	ID=gene-E	6	7.366666667	2506.8	0.232002755	SPRM(+15.	52	691.3633	1	690.3483	Oxidation	Y	Superscaf	60230958	0	0	0	0	BTP202209	0	Tissue specific
MGHRVDY	Superscaf	-	Superscaf	60237793	60237813	-44	44	50	-	-	gene	-	-	mRNA	NCP	Intron	gene|mRN	ID=gene-E	7	5.528571429	2808.3	0.231911216	MGHRVDY	89	439.2018	2	876.3912	.	M	Superscaf	60237846	BTP202209	0	0	0	0	0	Tissue specific
TTRESVVDY	Superscaf	-	Superscaf	60238897	60238923	-30	30	38	-	-	gene	-	-	mRNA	NCP	Intron	gene|mRN	ID=gene-E	9	4.594444444	3123.7	0.231897062	TTRESVVDY	73	535.2602	2	1068.509	.	L	Superscaf	60238956	0	0	0	BTP202209	0	0	Tissue specific
TTATMPK	Superscaf	-	Superscaf	60382234	60382254	-22	22	28	-	-	-	-	-	-	NCP	IGR	-	ID=gene-E	7	6.771428571	2681.1	0.230069457	TTATMPK	76	375.1998	2	748.3789	.	I	Superscaf	60382335	BTP202209	0	0	0	0	0	Tissue specific
KFQKGT	Superscaf	+	Superscaf	60395491	60395508	9	9	14	-	-	-	-	-	-	NCP	IGR	-	ID=gene-E	6	7.185	2629.7	0.229900456	K(+42.01)	62	375.7103	2	749.4072	Acetylati	M	Superscaf	60395467	0	0	0	BTP202209	0	0	Tissue specific
KMTFP	Superscaf	+	Superscaf	60473602	60473616	1	1	5	-	-	-	-	-	-	NCP	IGR	-	ID=gene-E	5	6.76	1962.2	0.228904506	KMTFP	88	623.3199	1	622.3149	.	K	Superscaf	60473602	BTP202209	0	0	0	0	0	Tissue specific
GMMADM	Superscaf	-	Superscaf	60683734	60683751	-8	8	13	-	-	-	-	-	-	NCP	IGR	-	ID=gene-E	6	5.368333333	2134.6	0.226225087	GM(+15.99	51	352.1058	2	702.2023	Oxidation	V	Superscaf	60683754	0	0	BTP202209	0	0	0	Tissue specific
RNPSF	Superscaf	-	Superscaf	60705430	60705444	-14	14	18	-	-	-	-	-	-	NCP	IGR	-	ID=gene-E	5	6.726	2353.7	0.225948481	RNPSF	62	620.3141	1	619.3078	.	R	Superscaf	60705444	0	0	BTP202209	0	0	0	Tissue specific
NGDLPF	Superscaf	-	Superscaf	60723052	60723069	-18	18	23	-	-	-	-	-	-	NCP	IGR	-	ID=gene-E	6	5.351666667	2383.7	0.225723745	N(+42.01)	65	704.313	1	703.3177	Acetylati	Q	Superscaf	60723111	0	0	BTP202209	0	0	0	Tissue specific
LGRTL	Superscaf	+	Superscaf	60739717	60739731	14	14	18	-	-	-	-	-	-	NCP	IGR	-	ID=gene-E	5	7.044	2050.4	0.225511289	LGRTL	92	559.3544	1	558.3489	.	A	Superscaf	60739678	BTP202209	0	0	0	0	0	Tissue specific
MGPPKY	Superscaf	+	Superscaf	60765568	60765585	30	30	35	-	-	-	-	-	-	NCP	IGR	-	ID=gene-E	6	6.62	2271.5	0.225181626	M(+42.01)	60	367.6791	2	733.3469	Acetylati	K	Superscaf	60765481	BTP202209	0	0	0	0	0	Tissue specific
FSHVNPLQ	Superscaf	+	Superscaf	60836299	60836322	3	3	10	-	-	-	-	-	-	NCP	IGR	-	ID=gene-E	8	5.26125	3288.7	0.224279663	FSHVNPLQ	51	471.2439	2	940.4766	.	L	Superscaf	60836293	0	0	0	0	0	BTP202209	Tissue specific
KPGCLPLT	Superscaf	-	Superscaf	60902752	60902775	-65	65	72	-	-	-	-	-	-	NCP	IGR	-	ID=gene-E	8	6.4775	3130.5	0.223432325	KPGC(+57.	50	443.2441	2	884.4789	Carbamido	P	Superscaf	60902808	0	0	BTP202209	0	0	0	Tissue specific
SPGGADAF	Superscaf	-	Superscaf	61119736	61119759	-5	5	12	-	-	-	-	-	-	NCP	IGR	-	ID=gene-E	8	5.55125	2736.9	0.220665575	SPGGADAF	53	721.3134	1	720.3078	.	S	Superscaf	61119789	0	BTP202209	0	0	0	0	Tissue specific
LLMAPPG	Superscaf	-	Superscaf	61137832	61137852	-1	1	7	-	-	-	-	-	-	NCP	IGR	-	ID=gene-E	7	6.042857143	2361.7	0.220434872	LLM(+15.9	64	357.6927	2	713.3782	Oxidation	L	Superscaf	61137864	0	0	0	0	0	BTP202209	Tissue specific
KDFKEL	Superscaf	+	Superscaf	61211101	61211118	15	15	20	-	-	-	-	-	-	NCP	IGR	-	ID=gene-E	6	6.188333333	2514.6	0.219500661	KDFKEL	67	779.4265	1	778.4225	.	S	Superscaf	61211059	0	BTP202209	0	0	0	0	Tissue specific
KTTVPF	Superscaf	-	Superscaf	61240861	61240878	-19	19	24	-	-	-	-	-	-	NCP	IGR	-	ID=gene-E	6	5.763333333	1988.2	0.219121193	KTTVPF	55	692.4036	1	691.3904	.	S	Superscaf	61240941	0	0	0	0	0	BTP202209	Tissue specific
SSLRFP	Superscaf	-	Superscaf	61332994	61333011	-7	7	12	-	-	-	-	-	-	NCP	IGR	-	ID=gene-E	6	6.646666667	2493.8	0.217946411	SSLRFP	82	353.6961	2	705.3809	.	K	Superscaf	61333014	0	BTP202209	0	0	BTP202209	BTP202209	Tissue specific
KTLSLPL	Superscaf	+	Superscaf	61475107	61475127	10	10	16	-	-	-	-	-	-	NCP	IGR	-	ID=gene-E	7	6.598571429	2646.9	0.216134298	KTLSLPL	61	386.2455	2	770.4902	.	V	Superscaf	61475080	0	0	0	0	0	BTP202209	Tissue specific
KTETQEKNF	Superscaf	-	Superscaf	61483783	61483821	-48	48	60	-	-	-	-	-	-	NCP	IGR	-	ID=gene-E	13	6.464615385	5670.2	0.216023442	KTETQEKNF	99	500.6054	3	1498.799	.	K	Superscaf	61483857	0	0	BTP202209	0	0	0	Tissue specific
KTETQEKNF	Superscaf	-	Superscaf	61483783	61483818	-48	48	59	-	-	-	-	-	-	NCP	IGR	-	ID=gene-E	12	6.191666667	5201.8	0.21602348	KTETQEKNF	55	686.3581	2	1370.704	.	K	Superscaf	61483857	0	0	BTP202209	0	0	0	Tissue specific
TETQEKNPI	Superscaf	-	Superscaf	61483786	61483824	-49	49	61	-	-	-	-	-	-	NCP	IGR	-	ID=gene-E	13	5.963076923	5627.3	0.216023403	TETQEKNPI	90	750.8773	2	1499.747	.	K	Superscaf	61483857	0	BTP202209	BTP202209	BTP202209	BTP202209	BTP202209	Tissue specific
TETQEKNPI	Superscaf	-	Superscaf	61483786	61483821	-49	49	60	-	-	-	-	-	-	NCP	IGR	-	ID=gene-E	12	6.191666667	5201.8	0.216023442	TETQEKNPI	99	686.3571	2	1370.704	.	K	Superscaf	61483857	0	0	BTP202209	0	0	0	Mixed
KNPLPSKET	Superscaf	-	Superscaf	61483801	61483827	-54	54	62	-	-	-	-	-	-	NCP	IGR	-	ID=gene-E	9	6.544444444	3768.1	0.216023365	KNPLPSKET	99	507.2844	2	1012.555	.	K	Superscaf	61483857	0	0	0	0	0	BTP202209	Mixed
TKSTFQQ	Superscaf	-	Superscaf	61556236	61556256	-6	6	12	-	-	-	-	-	-	NCP	IGR	-	ID=gene-E	7	6.465714286	3255.6	0.215099827	T(+42.01)	83	881.4344	1	880.429	Acetylati	L	Superscaf	61556274	0	BTP202209	0	0	0	0	Tissue specific
SLNDLE	Superscaf	+	Superscaf	61699447	61699464	19	19	24	-	-	-	-	-	-	NCP	IGR	-	ID=gene-E	6	4.873333333	2523.9	0.213273791	SLNDLE	72	690.3442	1	689.3232	.	W	Superscaf	61699393	0	0	0	0	BTP202209	0	Tissue specific
CECCC	Superscaf	-	Superscaf	61859173	61859187	-4	4	8	-	-	-	-	-	-	NCP	IGR	-	ID=gene-E	5	4.66	2768.3	0.211237172	C(+57.02)	51	788.1782	1	787.1758	Carbamido	D	Superscaf	61859217	0	BTP202209	0	0	0	0	Mixed
EVSLLP	Superscaf	+	Superscaf	62164864	62164881	4	4	9	-	-	-	-	-	-	NCP	IGR	-	ID=gene-E	6	4.526666667	1853.1	0.207339286	EVSLLP	64	657.3768	1	656.3745	.	C	Superscaf	62164855	0	0	BTP202209	0	0	0	Tissue specific
KSTAAPPPF	Superscaf	-	Superscaf	62174755	62174781	-2	2	10	-	-	-	-	-	-	NCP	IGR	-	ID=gene-E	9	6.576666667	3221.5	0.207213052	KSTAAPPPF	81	433.2351	2	864.4705	.	L	Superscaf	62174922	0	0	BTP202209	0	0	0	Tissue specific
AVFGL	Superscaf	+	Superscaf	62354581	62354595	10	10	14	-	-	-	-	-	-	NCP	IGR	-	ID=gene-E	5	4.69	1349.4	0.204920254	AVFGL	50	506.2978	1	505.29	.	N	Superscaf	62354554	0	0	0	0	BTP202209	0	Tissue specific
NEPLFTVS	Superscaf	+	Superscaf	62371000	62371023	12	12	19	-	-	-	-	-	-	NCP	IGR	-	ID=gene-E	8	4.825	2881.3	0.204710782	NEPLFTVS	70	453.7338	2	905.4494	.	I	Superscaf	62370967	0	0	BTP202209	BTP202209	0	0	Tissue specific
GPPQHTT	Superscaf	-	Superscaf	62376718	62376738	-10	10	16	-	-	-	-	-	-	NCP	IGR	-	ID=gene-E	7	6.41	2997.5	0.20463791	GPPQHTT	55	369.183	2	736.3504	.	G	Superscaf	62376759	0	BTP202209	BTP202209	BTP202209	BTP202209	BTP202209	Tissue specific
MDMDDD	Superscaf	-	Superscaf	62514949	62514966	-4	4	9	-	-	-	-	-	-	NCP	IGR	-	ID=gene-E	6	3.896666667	2389	0.202875373	M(+15.99)	50	815.2122	1	814.1997	Oxidation	I	Superscaf	62515065	0	0	0	BTP202209	0	0	Tissue specific
KGPGVF	Superscaf	-	Superscaf	62538910	62538927	-1	1	6	-	-	-	-	-	-	NCP	IGR	-	ID=gene-E	6	5.576666667	1787.8	0.202569848	KGPGVF	52	604.3517	1	603.338	.	K	Superscaf	62538945	0	0	BTP202209	0	0	0	Tissue specific
LSLLRVVSS	Superscaf	-	Superscaf	62597962	62597988	-10	10	18	-	-	-	-	-	-	NCP	IGR	-	ID=gene-E	9	5.082222222	2859.2	0.201816764	LSLLRVVSS	96	487.3035	2	972.5967	.	D	Superscaf	62598012	0	0	BTP202209	0	0	0	Tissue specific
LKKDLEK	Superscaf	+	Superscaf	62662234	62662254	23	23	29	-	-	-	-	-	-	NCP	IGR	-	ID=gene-E	7	6.767142857	2949	0.200997312	LKKDLEK	73	437.2725	2	872.5331	.	R	Superscaf	62662168	0	0	0	BTP202209	0	0	Tissue specific
KKPLL	Superscaf	+	Superscaf	62681758	62681772	5	5	9	-	-	-	-	-	-	NCP	IGR	-	ID=gene-E	5	7.548	1981	0.200748439	KKPLL	80	598.417	1	597.4213	.	F	Superscaf	62681746	0	0	BTP202209	0	0	0	Tissue specific
KAFSMLLR	Superscaf	-	Superscaf	62710201	62710224	-9	9	16	-	-	-	-	-	-	NCP	IGR	-	ID=gene-E	8	6.92375	3277.6	0.20038565	KAFSM(+15	59	491.283	2	980.5477	Oxidation	V	Superscaf	62710239	0	BTP202209	0	0	0	0	Tissue specific
VSSEDTLLF	Superscaf	-	Superscaf	62825620	62825646	-22	22	30	-	-	-	-	-	-	NCP	IGR	-	ID=gene-E	9	4.704444444	3045.5	0.198913911	V(+42.01)	51	1002.499	1	1001.492	Acetylati	C	Superscaf	62825658	0	BTP202209	0	0	0	0	Tissue specific
HLLPPT	Superscaf	-	Superscaf	62856580	62856597	-15	15	20	-	-	-	-	-	-	NCP	IGR	-	ID=gene-E	6	6.446666667	2398.8	0.198519256	H(+42.01)	63	360.2116	2	718.4014	Acetylati	K	Superscaf	62856744	0	0	0	BTP202209	0	0	Tissue specific
TSPCCNP	Superscaf	-	Superscaf	62861830	62861850	-39	39	45	-	-	-	-	-	-	NCP	IGR	-	ID=gene-E	7	5.751428571	3223.8	0.198452276	T(+42.01)	50	877.32	1	876.3106	Acetylati	M	Superscaf	62861940	BTP202209	0	0	0	0	0	Tissue specific
TTKPGLRK	Superscaf	+	Superscaf	62935045	62935068	6	6	13	-	-	-	-	-	-	NCP	IGR	-	ID=gene-E	8	7.69375	3368.7	0.197518677	TTKPGLRK	60	450.7783	2	899.5552	.	E	Superscaf	62935030	0	0	0	BTP202209	0	0	Tissue specific
TGVVGTLK	Superscaf	+	Superscaf	62964355	62964378	13	13	20	-	-	-	-	-	-	NCP	IGR	-	ID=gene-E	8	5.09	2211.4	0.197144947	TGVVGTLK	90	387.7384	2	773.4647	.	R	Superscaf	62964319	0	0	BTP202209	0	0	0	Tissue specific
PSDTWF	Superscaf	-	Superscaf	63026146	63026163	-24	24	29	-	-	-	-	-	-	NCP	IGR	-	ID=gene-E	6	5.475	2443.8	0.19635713	PSDTWF	59	752.3188	1	751.3177	.	R	Superscaf	63026307	0	0	BTP202209	0	0	0	Tissue specific
LAAVL	Superscaf	-	Superscaf	63041182	63041196	-88	88	92	-	-	-	-	-	-	NCP	IGR	-	ID=gene-E	5	4.8	1329.4	0.196165445	LAAVL	96	486.3284	1	485.3213	.	I	Superscaf	63041271	0	0	0	BTP202209	0	0	Mixed
KEPFP	Superscaf	-	Superscaf	63083173	63083187	-2	2	6	-	-	-	-	-	-	NCP	IGR	-	ID=gene-E	5	6.208	1956.1	0.195630021	K(+42.01)	80	659.338	1	658.3326	Acetylati	S	Superscaf	63083211	0	0	0	0	BTP202209	0	Tissue specific
LYYHR	Superscaf	+	Superscaf	63085708	63085722	27	27	31	-	-	-	-	-	-	NCP	IGR	-	ID=gene-E	5	7.13	2540.9	0.195597697	LYYHR	99	376.1967	2	750.3813	.	G	Superscaf	63085630	0	0	0	0	BTP202209	0	Tissue specific
VNEKF	Superscaf	-	Superscaf	63094138	63094152	-2	2	6	-	-	-	-	-	-	NCP	IGR	-	ID=gene-E	5	4.77	1878	0.195490206	VNEKF	61	636.3273	1	635.3278	.	P	Superscaf	63094179	BTP202209	0	0	0	0	0	Tissue specific
PNWNGKK	Superscaf	+	Superscaf	63320212	63320232	26	26	32	-	-	-	-	-	-	NCP	IGR	-	ID=gene-E	7	6.922857143	3291.5	0.192607474	PNWNGKK	83	422.2261	2	842.4399	.	N	Superscaf	63320137	0	0	0	0	0	BTP202209	Mixed
RYFKTPP	Superscaf	-	Superscaf	63433429	63433449	-33	33	39	-	-	gene	-	-	mRNA	NCP	Intron	gene|mRN	ID=gene-E	7	7.252857143	3036.4	0.191163851	R(+42.01)	67	475.7556	2	949.5021	Acetylati	R	Superscaf	63433488	0	0	BTP202209	0	0	0	Tissue specific
LVTHPA	Superscaf	+	Superscaf	63502771	63502788	3	3	8	-	-	-	-	-	-	NCP	IGR	-	ID=gene-E	6	5.403333333	2019.3	0.190279713	LVTHPA	59	637.3636	1	636.3595	.	F	Superscaf	63502765	0	0	BTP202209	0	0	0	Tissue specific
CPPLKVSF	Superscaf	+	Superscaf	63615982	63616005	15	15	22	-	-	gene	-	-	mRNA	NCP	Intron	gene|mRN	ID=gene-E	8	5.5625	2853.1	0.18883609	C(+57.02)	92	474.2566	2	946.4946	Carbamido	S	Superscaf	63615940	BTP202209	0	0	0	0	0	Tissue specific
LLAFAN	Superscaf	-	Superscaf	63638920	63638937	-22	22	27	-	exon	gene	three_pri	-	mRNA	NCP	3UTR	exon|gene	ID=exon-E	6	5.815	2313.5	0.188543685	LLAFAN	64	648.3787	1	647.3643	.	S	Superscaf	63638988	0	0	BTP202209	0	0	0	Tissue specific
MFSWLK	Superscaf	-	Superscaf	63757681	63757698	-27	27	32	-	-	-	-	-	-	NCP	IGR	-	ID=gene-E	6	6.42	2490.7	0.187029371	M(+42.01)	78	853.4269	1	852.4203	Acetylati	I	Superscaf	63757728	0	0	0	BTP202209	0	0	Mixed
KCNTLK	Superscaf	-	Superscaf	63778663	63778680	-14	14	19	-	exon	gene	three_pri	-	mRNA	NCP	3UTR	exon|gene	ID=exon-E	6	7.07	2870.1	0.186761831	KC(+57.02	75	382.2126	2	762.4058	Carbamido	F	Superscaf	63778707	0	0	0	0	0	BTP202209	Tissue specific
MGSPFPK	Superscaf	+	Superscaf	63778753	63778773	31	31	37	-	exon	gene	three_pri	-	mRNA	NCP	3UTR	exon|gene	ID=exon-E	7	6.46	2582.8	0.186760645	MGSPFPK	82	382.2007	2	762.3734	.	S	Superscaf	63778663	0	0	0	0	0	BTP202209	Mixed
KPRSEMLV	Superscaf	+	Superscaf	63784321	63784344	12	12	19	-	-	-	-	-	-	NCP	IGR	-	ID=gene-E	8	5.92875	2988.4	0.186689609	KPRSEMLV	53	480.2685	2	958.527	.	L	Superscaf	63784288	0	0	0	0	0	BTP202209	Tissue enhanced
KLNLGTVP	Superscaf	+	Superscaf	63816445	63816468	4	4	11	-	-	gene	-	-	mRNA	NCP	Intron	gene|mRN	ID=gene-E	8	5.73875	2804.1	0.186279998	KLNLGTVP	68	421.2586	2	840.5069	.	L	Superscaf	63816436	0	0	0	0	0	BTP202209	Tissue specific
KNSTL	Superscaf	-	Superscaf	64063423	64063437	-5	5	9	-	-	-	-	-	-	NCP	IGR	-	ID=gene-E	5	6.668	2199.4	0.183130911	KNSTL	51	562.3204	1	561.3122	.	L	Superscaf	64063464	0	0	0	0	0	BTP202209	Tissue specific
VGGPF	Superscaf	+	Superscaf	64074778	64074792	3	3	7	-	-	gene	-	-	mRNA	NCP	Intron	gene|mRN	ID=gene-E	5	4.744	1319.4	0.182986124	VGGPF	59	476.2491	1	475.243	.	W	Superscaf	64074772	0	0	BTP202209	0	0	0	Tissue specific
KVVYPK	Superscaf	+	Superscaf	64171969	64171986	28	28	33	-	-	gene	-	-	mRNA	NCP	Intron	gene|mRN	ID=gene-E	6	5.24	1733.7	0.181746809	KVVYPK	54	367.2301	2	732.4534	.	K	Superscaf	64171888	BTP202209	0	0	0	0	0	Tissue specific
FFLPQ	Superscaf	+	Superscaf	64345426	64345440	3	3	7	-	-	-	-	-	-	NCP	IGR	-	ID=gene-E	5	5.778	2076.3	0.179535107	FFLPQ	56	651.3575	1	650.3428	.	F	Superscaf	64345420	0	0	0	0	0	BTP202209	Tissue specific
TGPPVGGK	Superscaf	+	Superscaf	64377037	64377060	4	4	11	-	-	-	-	-	-	NCP	IGR	-	ID=gene-E	8	5.8475	2432.6	0.179131923	TGPPVGGK	98	356.7017	2	711.3915	.	S	Superscaf	64377028	0	0	0	0	BTP202209	0	Tissue specific
KPGRKTP	Superscaf	+	Superscaf	64407532	64407552	46	46	52	-	-	-	-	-	-	NCP	IGR	-	ID=gene-E	7	7.905714286	2955.2	0.178743121	KPGRKTP	73	392.2435	2	782.4763	.	D	Superscaf	64407397	0	0	0	0	0	BTP202209	Tissue specific
LVTKLPK	Superscaf	-	Superscaf	64504696	64504716	-14	14	20	-	-	-	-	-	-	NCP	IGR	-	ID=gene-E	7	6.324285714	2378.5	0.177504188	LVTKLPK	81	399.7751	2	797.5374	.	I	Superscaf	64504758	0	0	BTP202209	0	0	0	Tissue specific
VVLDNP	Superscaf	-	Superscaf	64533712	64533729	-6	6	11	-	-	gene	-	-	mRNA	NCP	Intron	gene|mRN	ID=gene-E	6	3.443333333	1699	0.177134245	VVLDNP	80	656.3593	1	655.3541	.	S	Superscaf	64533930	0	0	0	0	BTP202209	0	Mixed
AYLKNRCL	Superscaf	+	Superscaf	64872028	64872051	6	6	13	-	-	gene	-	-	mRNA	NCP	Intron	gene|mRN	ID=gene-E	8	6.82125	3777.2	0.172820322	AYLKNRC(+	60	519.2833	2	1036.549	Carbamido	G	Superscaf	64872013	0	0	0	0	BTP202209	0	Tissue specific
KPTLFK	Superscaf	+	Superscaf	64902874	64902891	54	54	59	-	-	gene	-	-	mRNA	NCP	Intron	gene|mRN	ID=gene-E	6	7.295	2412.5	0.172427083	KPTLFK	51	733.4692	1	732.4534	.	P	Superscaf	64902715	0	0	BTP202209	0	0	0	Tissue specific
RKLEPEH	Superscaf	-	Superscaf	65198443	65198463	-4	4	10	-	-	-	-	-	-	NCP	IGR	-	ID=gene-E	7	6.687142857	3278.7	0.168658262	R(+42.01)	65	475.7551	2	949.4981	Acetylati	R	Superscaf	65198520	0	BTP202209	0	0	0	0	Tissue specific
EPHQKAK	Superscaf	-	Superscaf	65222845	65222865	-3	3	9	-	-	gene	-	-	mRNA	NCP	Intron	gene|mRN	ID=gene-E	7	6.894285714	3241.4	0.168347113	E(+42.01)	58	440.2386	2	878.461	Acetylati	A	Superscaf	65222880	0	0	0	0	0	BTP202209	Tissue specific
KLPEL	Superscaf	+	Superscaf	65257255	65257269	4	4	8	-	-	gene	-	-	mRNA	NCP	Intron	gene|mRN	ID=gene-E	5	6.244	1938.1	0.16790843	K(+42.01)	86	641.3857	1	640.3796	Acetylati	F	Superscaf	65257246	0	0	BTP202209	0	0	0	Tissue specific
LSYDLP	Superscaf	-	Superscaf	65307811	65307828	-6	6	11	-	-	gene	-	-	mRNA	NCP	Intron	gene|mRN	ID=gene-E	6	5.428333333	2298.6	0.167263755	LSYDLP	53	707.3574	1	706.3537	.	P	Superscaf	65307951	0	0	0	BTP202209	0	0	Tissue specific
SVVRKHY	Superscaf	+	Superscaf	65478589	65478609	23	23	29	-	-	-	-	-	-	NCP	IGR	-	ID=gene-E	7	5.632857143	2579.8	0.165086137	S(+42.01)	56	465.7665	2	929.5083	Acetylati	S	Superscaf	65478523	BTP202209	0	0	0	0	0	Tissue specific
SAKKLVLK	Superscaf	+	Superscaf	65610379	65610402	46	46	53	-	-	gene	-	-	mRNA	NCP	Intron	gene|mRN	ID=gene-E	8	6.61	2806.7	0.163405652	SAKKLVLK	92	443.8068	2	885.6011	.	I	Superscaf	65610244	0	0	BTP202209	0	0	0	Tissue specific
LKVGSLEK	Superscaf	+	Superscaf	65645224	65645247	7	7	14	-	-	gene	-	-	mRNA	NCP	Intron	gene|mRN	ID=gene-E	8	5.78875	2749.8	0.162961345	LKVGSLEK	82	437.2725	2	872.5331	.	Q	Superscaf	65645206	0	BTP202209	BTP202209	0	BTP202209	0	Tissue specific
ANSAALFY	Superscaf	-	Superscaf	65754451	65754474	-1	1	8	-	-	gene	-	-	mRNA	NCP	Intron	gene|mRN	ID=gene-E	8	5.78375	3114.3	0.161568598	ANSAALFY	67	856.4156	1	855.4127	.	A	Superscaf	65754486	0	0	BTP202209	0	0	0	Tissue specific
RAGLKLS	Superscaf	+	Superscaf	65767426	65767446	74	74	80	-	-	gene	-	-	mRNA	NCP	Intron	gene|mRN	ID=gene-E	7	7.161428571	2816	0.161403193	RAGLKLS	62	372.7387	2	743.4653	.	F	Superscaf	65767207	0	BTP202209	0	0	0	0	Tissue specific
RVAPPK	Superscaf	+	Superscaf	65871955	65871972	7	7	12	-	-	-	-	-	-	NCP	IGR	-	ID=gene-E	6	6.52	2103.3	0.160070388	R(+42.01)	69	355.2262	2	708.4282	Acetylati	A	Superscaf	65871937	0	0	0	0	0	BTP202209	Tissue specific
LGVPTVRF	Superscaf	-	Superscaf	65958325	65958348	-54	54	61	-	-	gene	-	-	mRNA	NCP	Intron	gene|mRN	ID=gene-E	8	5.1275	2421.8	0.158969013	LGVPTVRF	75	444.7664	2	887.5229	.	H	Superscaf	65958465	0	BTP202209	0	0	0	0	Tissue specific
MNANLRP	Superscaf	-	Superscaf	65994352	65994372	-8	8	14	-	-	-	-	-	-	NCP	IGR	-	ID=gene-E	7	6.518571429	3215.8	0.158509673	MNANLRP	54	408.2208	2	814.4119	.	T	Superscaf	65994384	0	BTP202209	0	0	0	0	Tissue specific
LLVLML	Superscaf	-	Superscaf	66180745	66180762	-3	3	8	-	-	gene	-	-	mRNA	NCP	Intron	gene|mRN	ID=gene-E	6	4.945	1785.1	0.156133025	LLVLM(+15	57	717.4514	1	716.4506	Oxidation	N	Superscaf	66180831	0	0	0	0	0	BTP202209	Tissue specific
PGAGKSGK	Superscaf	-	Superscaf	66194752	66194775	-23	23	30	-	-	-	-	-	-	NCP	IGR	-	ID=gene-E	8	6.92375	2860.8	0.155954346	PGAGKSGK	59	351.2027	2	700.3868	.	F	Superscaf	66194784	0	0	0	BTP202209	0	0	Tissue specific
TQSLPK	Superscaf	-	Superscaf	66231205	66231222	-26	26	31	-	-	gene	-	-	mRNA	NCP	Intron	gene|mRN	ID=gene-E	6	6.646666667	2550.8	0.155489613	T(+42.01)	74	715.3972	1	714.3912	Acetylati	E	Superscaf	66231225	0	BTP202209	0	0	0	0	Tissue specific
EQQVKGK	Superscaf	-	Superscaf	66238303	66238323	-15	15	21	-	-	gene	-	-	mRNA	NCP	Intron	gene|mRN	ID=gene-E	7	5.71	2881	0.155399068	EQQVKGK	65	408.7292	2	815.4501	.	L	Superscaf	66238332	0	BTP202209	0	0	0	0	Tissue specific
KNFKPQNF	Superscaf	+	Superscaf	66446914	66446937	23	23	30	-	-	-	-	-	-	NCP	IGR	-	ID=gene-E	8	6.65125	3853.1	0.152739044	K(+42.01)	51	532.7769	2	1063.545	Acetylati	A	Superscaf	66446848	0	BTP202209	0	0	BTP202209	BTP202209	Tissue specific
KFYLPNP	Superscaf	+	Superscaf	66553657	66553677	17	17	23	-	-	gene	-	-	mRNA	NCP	Intron	gene|mRN	ID=gene-E	7	6.41	2940.2	0.151378008	KFYLPNP	62	439.7492	2	877.4697	.	Q	Superscaf	66553609	0	0	0	0	0	BTP202209	Tissue specific
DGAVKPPP	Superscaf	+	Superscaf	66823147	66823170	6	6	13	-	-	-	-	-	-	NCP	IGR	-	ID=gene-E	8	5.45	2500.7	0.147941719	DGAVKPPP	81	390.7154	2	779.4177	.	I	Superscaf	66823132	0	0	0	0	0	BTP202209	Tissue specific
GLFGPP	Superscaf	+	Superscaf	66863428	66863445	1	1	6	-	-	-	-	-	-	NCP	IGR	-	ID=gene-E	6	6	2010.2	0.147428175	GLFGPP	71	587.3291	1	586.3115	.	G	Superscaf	66863428	0	0	0	0	BTP202209	0	Tissue specific
VAPPPVFP	Superscaf	+	Superscaf	66887020	66887043	13	13	20	-	-	gene	-	-	mRNA	NCP	Intron	gene|mRN	ID=gene-E	8	4.5875	2048.3	0.147127278	V(+42.01)	71	433.2435	2	864.4745	Acetylati	C	Superscaf	66886984	0	0	BTP202209	0	0	0	Tissue specific
DADDC	Superscaf	+	Superscaf	67089913	67089927	2	2	6	-	-	-	-	-	-	NCP	IGR	-	ID=gene-E	5	3.99	2131.5	0.144540316	DADDC(+57	51	595.1644	1	594.1591	Carbamido	L	Superscaf	67089910	0	0	0	0	BTP202209	0	Tissue specific
FLNPRLY	Superscaf	-	Superscaf	67266715	67266735	-21	21	27	-	-	gene	-	-	mRNA	NCP	Intron	gene|mRN	ID=gene-E	7	6.51	3136.6	0.142285848	FLNPRLY	78	461.7602	2	921.5072	.	Q	Superscaf	67266825	0	0	0	0	0	BTP202209	Tissue specific
AALELSGR	Superscaf	+	Superscaf	67286398	67286421	7	7	14	-	-	-	-	-	-	NCP	IGR	-	ID=gene-E	8	6.20375	3084.4	0.142034833	AALELSGR	97	408.7302	2	815.4501	.	K	Superscaf	67286380	0	0	0	BTP202209	0	0	Tissue specific
AGHPPVK	Superscaf	+	Superscaf	67298887	67298907	24	24	30	-	-	-	-	-	-	NCP	IGR	-	ID=gene-E	7	5.988571429	2371.5	0.141875625	AGHPPVK	55	353.2106	2	704.3969	.	D	Superscaf	67298818	0	BTP202209	0	0	0	0	Tissue specific
ACKYA	Superscaf	-	Superscaf	67487410	67487424	-4	4	8	-	-	gene	-	-	mRNA	NCP	Intron	gene|mRN	ID=gene-E	5	6.492	2136.2	0.139471856	A(+42.01)	81	654.2877	1	653.2843	Acetylati	Q	Superscaf	67487544	0	BTP202209	0	0	BTP202209	BTP202209	Tissue specific
LTGTLQ	Superscaf	-	Superscaf	67492258	67492275	15	15	20	-	-	gene	-	-	mRNA	NCP	Intron	gene|mRN	ID=gene-E	6	6.106666667	2409.8	0.139410001	LTGTLQ	64	632.3541	1	631.3541	.	K	Superscaf	67492216	0	BTP202209	0	0	0	BTP202209	Tissue specific
TLQAQ	Superscaf	+	Superscaf	67513756	67513770	56	56	60	-	-	gene	-	-	mRNA	NCP	Intron	gene|mRN	ID=gene-E	5	5.966	2283.6	0.139135919	TLQAQ	54	560.3044	1	559.2966	.	G	Superscaf	67513591	0	0	0	0	BTP202209	0	Tissue enhanced
TQPPPR	Superscaf	+	Superscaf	67730818	67730835	18	18	23	-	-	gene	-	-	mRNA	NCP	Intron	gene|mRN	ID=gene-E	6	6.973333333	2669.2	0.136368136	T(+42.01)	61	369.2043	2	736.3868	Acetylati	E	Superscaf	67730767	0	BTP202209	0	0	0	0	Tissue specific
LGGPL	Superscaf	-	Superscaf	67785631	67785645	-49	49	53	-	-	-	-	-	-	NCP	IGR	-	ID=gene-E	5	6.04	1638.8	0.135669257	LGGPL	61	456.2802	1	455.2744	.	I	Superscaf	67785690	BTP202209	0	0	0	0	0	Mixed
LAPPLL	Superscaf	+	Superscaf	67827223	67827240	19	19	24	-	-	gene	-	-	mRNA	NCP	Intron	gene|mRN	ID=gene-E	6	6.093333333	2046.3	0.135138882	LAPPLL	53	623.4224	1	622.4053	.	H	Superscaf	67827169	0	BTP202209	0	0	0	0	Tissue specific
KPFLSPM	Superscaf	+	Superscaf	67909396	67909416	3	3	9	-	-	gene	-	-	mRNA	NCP	Intron	gene|mRN	ID=gene-E	7	6.461428571	2638.9	0.134091061	KPFLSPM	51	410.2223	2	818.436	.	N	Superscaf	67909390	0	BTP202209	0	0	0	BTP202209	Tissue specific
PEMAAP	Superscaf	-	Superscaf	67950502	67950519	-19	19	24	-	-	gene	-	-	mRNA	NCP	Intron	gene|mRN	ID=gene-E	6	5.601666667	2094.4	0.133566959	PEMAAP	70	615.2779	1	614.2734	.	L	Superscaf	67950573	0	0	0	0	BTP202209	0	Tissue specific
AVNLSR	Superscaf	+	Superscaf	68046031	68046048	9	9	14	-	-	gene	-	-	mRNA	NCP	Intron	gene|mRN	ID=gene-E	6	5.641666667	2293.6	0.132348874	A(+42.01)	95	351.1997	2	700.3868	Acetylati	S	Superscaf	68046007	0	0	0	0	0	BTP202209	Tissue specific
HLPGSPF	Superscaf	-	Superscaf	68046262	68046282	-42	42	48	-	-	gene	-	-	mRNA	NCP	Intron	gene|mRN	ID=gene-E	7	6.185714286	2716	0.13234589	HLPGSPF	50	377.6961	2	753.3809	.	L	Superscaf	68046327	0	BTP202209	0	0	0	0	Tissue specific
TVTFE	Superscaf	-	Superscaf	68069971	68069985	-16	16	20	-	-	gene	-	-	mRNA	NCP	Intron	gene|mRN	ID=gene-E	5	4.352	1607.9	0.132043655	T(+42.01)	62	638.3083	1	637.2959	Acetylati	T	Superscaf	68070042	0	0	0	0	0	BTP202209	Tissue specific
ATAKPF	Superscaf	-	Superscaf	68108827	68108844	-2	2	7	-	-	gene	-	-	mRNA	NCP	Intron	gene|mRN	ID=gene-E	6	6.681666667	2213.3	0.131548166	ATAKPF	62	634.3544	1	633.3486	.	I	Superscaf	68108877	0	BTP202209	0	0	0	0	Tissue specific
RVGLVK	Superscaf	+	Superscaf	68334073	68334090	27	27	32	-	-	gene	-	-	mRNA	NCP	Intron	gene|mRN	ID=gene-E	6	5.408333333	1767.9	0.128676068	R(+42.01)	67	357.236	2	712.4595	Acetylati	S	Superscaf	68333995	0	BTP202209	BTP202209	0	0	0	Tissue specific
DELVFF	Superscaf	-	Superscaf	68429713	68429730	-8	8	13	-	-	gene	-	-	mRNA	NCP	Intron	gene|mRN	ID=gene-E	6	3.855	1965.2	0.127456568	DELVFF	66	769.3734	1	768.3694	.	S	Superscaf	68429745	0	0	BTP202209	0	0	0	Tissue specific
EHLSYL	Superscaf	-	Superscaf	68499313	68499330	-1	1	6	-	-	gene	-	-	mRNA	NCP	Intron	gene|mRN	ID=gene-E	6	5.685	2594.9	0.126569102	EHLSYL	68	381.1951	2	760.3755	.	E	Superscaf	68499462	BTP202209	0	0	0	0	0	Tissue specific
PAPKLEVP	Superscaf	+	Superscaf	68526544	68526567	8	8	15	-	-	gene	-	-	mRNA	NCP	Intron	gene|mRN	ID=gene-E	8	5.4825	2570.8	0.126221805	PAPKLEVP	76	425.7548	2	849.496	.	V	Superscaf	68526523	0	0	0	0	BTP202209	0	Tissue specific
QTDSRKL	Superscaf	+	Superscaf	68539576	68539596	3	3	9	-	-	-	-	-	-	NCP	IGR	-	ID=gene-E	7	6.758571429	3273.7	0.126055673	Q(+42.01)	52	445.2394	2	888.4665	Acetylati	P	Superscaf	68539570	0	0	BTP202209	0	0	0	Tissue specific
KNWPPYK	Superscaf	-	Superscaf	68632060	68632080	-3	3	9	-	-	-	-	-	-	NCP	IGR	-	ID=gene-E	7	7.005714286	3194.4	0.124876415	KNWPPYK	53	466.7524	2	931.4916	.	Y	Superscaf	68632092	0	BTP202209	0	0	0	0	Tissue specific
WAGKPLF	Superscaf	+	Superscaf	68664367	68664387	9	9	15	-	-	gene	-	-	mRNA	NCP	Intron	gene|mRN	ID=gene-E	7	6.482857143	2681.8	0.12446447	WAGKPLF	90	818.4553	1	817.4486	.	E	Superscaf	68664343	0	0	0	0	0	BTP202209	Tissue specific
APVPPGTP	Superscaf	+	Superscaf	68666665	68666688	20	20	27	-	-	gene	-	-	mRNA	NCP	Intron	gene|mRN	ID=gene-E	8	5.465	2355.7	0.12443513	APVPPGTP	55	368.202	2	734.3962	.	T	Superscaf	68666608	0	0	0	0	BTP202209	0	Tissue specific
LSTLPVT	Superscaf	-	Superscaf	68769298	68769318	-17	17	23	-	-	-	-	-	-	NCP	IGR	-	ID=gene-E	7	5.285714286	2222.6	0.123126501	L(+42.01)	55	772.4419	1	771.4378	Acetylati	S	Superscaf	68769372	0	0	0	0	BTP202209	0	Tissue specific
KPFFPYLK	Superscaf	-	Superscaf	68787799	68787822	-3	3	10	-	-	gene	-	-	mRNA	NCP	Intron	gene|mRN	ID=gene-E	8	6.835	3199.3	0.122890558	KPFFPYLK	54	520.3043	2	1038.59	.	V	Superscaf	68787828	0	0	0	0	0	BTP202209	Tissue specific
TLNELL	Superscaf	+	Superscaf	68791669	68791686	2	2	7	-	-	gene	-	-	mRNA	NCP	Intron	gene|mRN	ID=gene-E	6	5.516666667	2479.9	0.122841288	TLNELL	60	702.4014	1	701.3959	.	M	Superscaf	68791666	0	0	0	0	BTP202209	0	Mixed
QPTLLR	Superscaf	+	Superscaf	68793724	68793741	63	63	68	-	-	gene	-	-	mRNA	NCP	Intron	gene|mRN	ID=gene-E	6	6.866666667	2701.2	0.122815085	QPTLLR	80	364.2249	2	726.4388	.	F	Superscaf	68793538	0	0	0	0	BTP202209	0	Tissue specific
EHVVLK	Superscaf	-	Superscaf	68797024	68797041	-29	29	34	-	-	gene	-	-	mRNA	NCP	Intron	gene|mRN	ID=gene-E	6	4.421666667	1867	0.122773007	EHVVLK	73	362.72	2	723.4279	.	A	Superscaf	68797059	BTP202209	0	0	0	BTP202209	0	Tissue specific
FLQSAPSK	Superscaf	+	Superscaf	68821306	68821329	9	9	16	-	-	gene	-	-	mRNA	NCP	Intron	gene|mRN	ID=gene-E	8	6.31625	3235.4	0.122463312	FLQSAPSK	98	877.4743	1	876.4705	.	R	Superscaf	68821282	BTP202209	0	0	0	0	0	Tissue specific
YESCLY	Superscaf	-	Superscaf	68895091	68895108	-5	5	10	-	-	gene	-	-	mRNA	NCP	Intron	gene|mRN	ID=gene-E	6	5.203333333	2667	0.12152256	YESC(+57.	53	417.6702	2	833.3265	Carbamido	I	Superscaf	68895177	0	0	0	0	BTP202209	0	Tissue specific
MLKVALD	Superscaf	+	Superscaf	68899252	68899272	38	38	44	-	-	gene	-	-	mRNA	NCP	Intron	gene|mRN	ID=gene-E	7	5.205714286	2269.5	0.121469465	M(+42.01)	68	416.2372	2	830.4572	Acetylati	S	Superscaf	68899141	0	BTP202209	BTP202209	BTP202209	BTP202209	BTP202209	Tissue specific
RGPYH	Superscaf	-	Superscaf	68925820	68925834	-57	57	61	-	-	gene	-	-	mRNA	NCP	Intron	gene|mRN	ID=gene-E	5	7.256	2362.7	0.121130775	RGPYH	57	629.3214	1	628.3081	.	S	Superscaf	68925882	0	0	0	0	0	BTP202209	Tissue specific
QFVHRK	Superscaf	+	Superscaf	68991256	68991273	15	15	20	-	-	gene	-	-	mRNA	NCP	Intron	gene|mRN	ID=gene-E	6	6.536666667	2735	0.120296366	QFVHRK	78	407.7355	2	813.4609	.	R	Superscaf	68991214	0	0	0	0	BTP202209	0	Tissue specific
TPVGVP	Superscaf	+	Superscaf	69021460	69021477	11	11	16	-	-	gene	-	-	mRNA	NCP	Intron	gene|mRN	ID=gene-E	6	4.183333333	1369.6	0.119911237	TPVGVP	68	569.3274	1	568.322	.	L	Superscaf	69021430	0	0	0	0	0	BTP202209	Tissue specific
VPFGVPK	Superscaf	+	Superscaf	69040816	69040836	12	12	18	-	-	gene	-	-	mRNA	NCP	Intron	gene|mRN	ID=gene-E	7	4.827142857	1827.9	0.119664391	VPFGVPK	54	372.2215	2	742.4377	.	E	Superscaf	69040783	0	0	0	BTP202209	0	0	Tissue specific
CLHMASK	Superscaf	-	Superscaf	69047116	69047136	-19	19	25	-	-	gene	-	-	mRNA	NCP	Intron	gene|mRN	ID=gene-E	7	6.54	3093.4	0.11958406	C(+57.02)	90	423.7009	2	845.3888	Carbamido	H	Superscaf	69047142	0	0	0	BTP202209	0	0	Tissue specific
RSVGGPVR	Superscaf	-	Superscaf	69360172	69360195	-25	25	32	-	-	gene	-	-	mRNA	NCP	Intron	gene|mRN	ID=gene-E	8	5.68	2613	0.115592263	RSVGGPVR	90	414.2444	2	826.4773	.	C	Superscaf	69360222	0	0	0	BTP202209	0	0	Tissue specific
KAFKTSSK	Superscaf	+	Superscaf	69361081	69361104	70	70	77	-	-	gene	-	-	mRNA	NCP	Intron	gene|mRN	ID=gene-E	8	7.32625	3268.2	0.115580673	KAFKTSSK	71	448.7617	2	895.5127	.	R	Superscaf	69360874	0	0	0	0	0	BTP202209	Tissue specific
TSLTK	Superscaf	+	Superscaf	69449758	69449772	9	9	13	-	-	gene	-	-	mRNA	NCP	Intron	gene|mRN	ID=gene-E	5	6.892	2000.2	0.114450072	T(+42.01)	87	591.3319	1	590.3276	Acetylati	L	Superscaf	69449734	0	0	0	0	0	BTP202209	Tissue specific
HVPKKKD	Superscaf	+	Superscaf	69529978	69529998	43	43	49	-	-	-	-	-	-	NCP	IGR	-	ID=gene-E	7	6.582857143	2773.8	0.113427115	HVPKKKD	82	426.2555	2	850.5024	.	P	Superscaf	69529852	BTP202209	0	0	0	0	0	Tissue specific
KSYAHPP	Superscaf	-	Superscaf	69580333	69580353	-4	4	10	-	-	gene	-	-	mRNA	NCP	Intron	gene|mRN	ID=gene-E	7	6.755714286	2917.1	0.112785042	KSYAHPP	68	400.2087	2	798.4024	.	Y	Superscaf	69580608	0	0	0	0	BTP202209	0	Tissue specific
LGVLT	Superscaf	-	Superscaf	69779935	69779949	-18	18	22	-	-	-	-	-	-	NCP	IGR	-	ID=gene-E	5	4.892	1401.6	0.110240005	LGVLT	54	502.3228	1	501.3162	.	I	Superscaf	69780018	0	0	0	BTP202209	0	0	Tissue specific
LNPHPR	Superscaf	+	Superscaf	69791263	69791280	23	23	28	-	-	gene	-	-	mRNA	NCP	Intron	gene|mRN	ID=gene-E	6	7.056666667	2893.4	0.110095524	LNPHPR	71	367.2075	2	732.4031	.	N	Superscaf	69791197	BTP202209	0	0	0	0	0	Tissue specific
RGPPLFK	Superscaf	-	Superscaf	69828274	69828294	-24	24	30	-	-	gene	-	-	mRNA	NCP	Intron	gene|mRN	ID=gene-E	7	7.218571429	2830.1	0.109623561	RGPPLFK	52	407.7523	2	813.4861	.	H	Superscaf	69828486	0	0	0	0	BTP202209	0	Tissue specific
RLESPPP	Superscaf	-	Superscaf	69890020	69890040	-50	50	56	-	-	-	-	-	-	NCP	IGR	-	ID=gene-E	7	6.362857143	2823.3	0.108836241	RLESPPP	70	398.223	2	794.4286	.	L	Superscaf	69890139	0	0	BTP202209	0	0	BTP202209	Tissue specific
EVKYTPY	Superscaf	-	Superscaf	69916144	69916164	-16	16	22	-	-	-	-	-	-	NCP	IGR	-	ID=gene-E	7	5.301428571	2547.8	0.108503136	EVKYTPY	64	450.2278	2	898.4436	.	K	Superscaf	69916248	0	0	0	0	0	BTP202209	Tissue specific
TNPALP	Superscaf	+	Superscaf	69927376	69927393	5	5	10	-	-	gene	-	-	mRNA	NCP	Intron	gene|mRN	ID=gene-E	6	6.09	2333.7	0.108359955	TNPALP	56	612.3341	1	611.3279	.	A	Superscaf	69927364	0	0	0	0	0	BTP202209	Tissue specific
LRLLDH	Superscaf	+	Superscaf	69974374	69974391	25	25	30	-	-	-	-	-	-	NCP	IGR	-	ID=gene-E	6	6.543333333	2740.2	0.107760687	LRLLDH	99	383.7314	2	765.4497	.	L	Superscaf	69974302	0	0	BTP202209	0	0	0	Tissue specific
KVPDLAVG	Superscaf	-	Superscaf	70084825	70084848	-1	1	8	-	-	-	-	-	-	NCP	IGR	-	ID=gene-E	8	4.6225	2179.3	0.106352256	K(+42.01)	61	840.4795	1	839.4752	Acetylati	K	Superscaf	70084863	0	BTP202209	BTP202209	BTP202209	0	0	Tissue specific
PGGFVP	Superscaf	+	Superscaf	70231189	70231206	25	25	30	-	-	-	-	-	-	NCP	IGR	-	ID=gene-E	6	5.003333333	1656.8	0.104486054	PGGFVP	65	573.3122	1	572.2958	.	R	Superscaf	70231117	0	0	0	BTP202209	0	0	Tissue specific
KPESVPP	Superscaf	+	Superscaf	70273048	70273068	38	38	44	-	exon	gene	three_pri	-	mRNA	NCP	3UTR	exon|gene	ID=exon-E	7	5.362857143	2289.5	0.103952274	KPESVPP	54	377.2048	2	752.4068	.	E	Superscaf	70272937	0	BTP202209	0	0	0	0	Mixed
VGMKVVK	Superscaf	-	Superscaf	70291903	70291923	-24	24	30	-	-	gene	-	-	mRNA	NCP	Intron	gene|mRN	ID=gene-E	7	4.457142857	1605.6	0.103711855	VGMKVVK	50	380.7369	2	759.4677	.	F	Superscaf	70291932	0	0	0	0	0	BTP202209	Tissue specific
VSPRHP	Superscaf	-	Superscaf	70310161	70310178	-16	16	21	-	-	gene	-	-	mRNA	NCP	Intron	gene|mRN	ID=gene-E	6	6.105	2326.7	0.103479087	V(+42.01)	69	367.7016	2	733.3871	Acetylati	V	Superscaf	70310361	0	0	BTP202209	0	0	BTP202209	Tissue specific
KGYLTGR	Superscaf	+	Superscaf	70316464	70316484	12	12	18	-	-	gene	-	-	mRNA	NCP	Intron	gene|mRN	ID=gene-E	7	7.23	2922.2	0.103398679	KGYLTGR	71	397.7305	2	793.4446	.	M	Superscaf	70316431	0	0	0	0	0	BTP202209	Tissue specific
MPALL	Superscaf	+	Superscaf	70355857	70355871	32	32	36	-	-	gene	-	-	mRNA	NCP	Intron	gene|mRN	ID=gene-E	5	6.006	1727	0.102896458	MPALL	51	544.3101	1	543.309	.	Y	Superscaf	70355764	0	0	0	0	0	BTP202209	Tissue specific
KPMGP	Superscaf	+	Superscaf	70389601	70389615	2	2	6	-	-	gene	-	-	mRNA	NCP	Intron	gene|mRN	ID=gene-E	5	6.812	1812	0.10246619	KPM(+15.9	51	545.2758	1	544.2679	Oxidation	S	Superscaf	70389598	0	BTP202209	0	0	0	0	Mixed
VSPDLRGP	Superscaf	+	Superscaf	70624663	70624686	6	6	13	-	-	gene	-	-	mRNA	NCP	Intron	gene|mRN	ID=gene-E	8	5.495	2769.2	0.099468814	V(+42.01)	55	441.7419	2	881.4606	Acetylati	A	Superscaf	70624648	0	BTP202209	0	0	0	0	Tissue specific
KPHGVPK	Superscaf	+	Superscaf	70707691	70707711	19	19	25	-	-	gene	-	-	mRNA	NCP	Intron	gene|mRN	ID=gene-E	7	6.52	2528.6	0.098410167	KPHGVPK	56	381.7332	2	761.4548	.	S	Superscaf	70707637	0	0	0	0	0	BTP202209	Tissue specific
FAFLFC	Superscaf	-	Superscaf	70744042	70744059	-10	10	15	-	-	gene	-	-	mRNA	NCP	Intron	gene|mRN	ID=gene-E	6	5.576666667	2412.6	0.097946696	FAFLFC(+5	91	804.3735	1	803.3676	Carbamido	G	Superscaf	70744110	0	0	0	0	0	BTP202209	Mixed
VLAFSM	Superscaf	+	Superscaf	70763962	70763979	6	6	11	-	-	gene	-	-	mRNA	NCP	Intron	gene|mRN	ID=gene-E	6	4.818333333	1807	0.097692697	V(+42.01)	57	363.1747	2	724.3466	Acetylati	M	Superscaf	70763947	BTP202209	0	0	0	0	0	Tissue specific
TLASVP	Superscaf	+	Superscaf	70775608	70775625	1	1	6	-	-	gene	-	-	mRNA	NCP	Intron	gene|mRN	ID=gene-E	6	5.085	1783	0.097544199	T(+42.01)	89	629.348	1	628.3432	Acetylati	T	Superscaf	70775608	0	0	0	BTP202209	0	0	Tissue specific
RQHKFK	Superscaf	-	Superscaf	70909783	70909800	-6	6	11	-	-	-	-	-	-	NCP	IGR	-	ID=gene-E	6	8.16	3203.4	0.095833342	RQHKFK	58	422.2496	2	842.4875	.	C	Superscaf	70909800	0	0	0	BTP202209	0	0	Tissue specific
EVRVQP	Superscaf	+	Superscaf	70927342	70927359	15	15	20	-	-	gene	-	-	mRNA	NCP	Intron	gene|mRN	ID=gene-E	6	4.321666667	2022.4	0.095609448	EVRVQP	52	364.2079	2	726.4024	.	K	Superscaf	70927300	0	0	0	0	0	BTP202209	Tissue specific
YVLTGR	Superscaf	-	Superscaf	70997407	70997424	-17	17	22	-	-	gene	-	-	mRNA	NCP	Intron	gene|mRN	ID=gene-E	6	5.816666667	2156.5	0.094716054	YVLTGR	71	354.7048	2	707.3966	.	T	Superscaf	70997436	0	BTP202209	BTP202209	BTP202209	BTP202209	BTP202209	Tissue enhanced
SWKETL	Superscaf	-	Superscaf	71041159	71041176	-16	16	21	-	-	gene	-	-	mRNA	NCP	Intron	gene|mRN	ID=gene-E	6	6.173333333	2554.8	0.094158174	S(+42.01)	60	403.2135	2	804.4018	Acetylati	N	Superscaf	71041203	0	BTP202209	BTP202209	BTP202209	BTP202209	BTP202209	Tissue specific
FYAPEL	Superscaf	-	Superscaf	71178340	71178357	-18	18	23	-	-	gene	-	-	mRNA	NCP	Intron	gene|mRN	ID=gene-E	6	5.443333333	2274.5	0.092408988	FYAPEL	69	739.3626	1	738.3588	.	R	Superscaf	71178360	0	BTP202209	0	0	0	0	Tissue specific
KKPMAAP	Superscaf	-	Superscaf	71206417	71206437	-12	12	18	-	-	gene	-	-	mRNA	NCP	Intron	gene|mRN	ID=gene-E	7	7.124285714	2605.7	0.092050941	KKPM(+15.	52	379.718	2	757.4156	Oxidation	K	Superscaf	71206512	0	0	0	0	BTP202209	0	Tissue specific
AELTVEVR	Superscaf	-	Superscaf	71306455	71306478	-47	47	54	-	-	-	-	-	-	NCP	IGR	-	ID=gene-E	8	4.46625	2562	0.090775324	A(+42.01)	98	479.7631	2	957.5131	Acetylati	G	Superscaf	71306736	0	0	BTP202209	0	0	0	Tissue enhanced
ENAHKDFKK	Superscaf	-	Superscaf	71351659	71351694	-14	14	25	-	-	-	-	-	-	NCP	IGR	-	ID=gene-E	12	5.991666667	4765.9	0.090198778	ENAHKDFKK	89	448.5725	3	1342.699	.	V	Superscaf	71351748	0	BTP202209	0	0	0	0	Tissue specific
SLLADF	Superscaf	-	Superscaf	71385778	71385795	-7	7	12	-	-	-	-	-	-	NCP	IGR	-	ID=gene-E	6	5.351666667	2200.4	0.089763958	SLLADF	68	665.3474	1	664.3431	.	N	Superscaf	71385828	0	BTP202209	0	0	0	0	Tissue specific
SPSPPHLK	Superscaf	+	Superscaf	71395669	71395692	13	13	20	-	-	-	-	-	-	NCP	IGR	-	ID=gene-E	8	6.69625	3220.5	0.089637762	SPSPPHLK	62	431.7501	2	861.4708	.	V	Superscaf	71395633	0	0	0	0	BTP202209	0	Tissue specific
LPAHKL	Superscaf	+	Superscaf	71443516	71443533	17	17	22	-	-	gene	-	-	mRNA	NCP	Intron	gene|mRN	ID=gene-E	6	6.935	2443.6	0.089027744	LPAHKL	99	678.4271	1	677.4224	.	D	Superscaf	71443468	BTP202209	0	0	0	BTP202209	0	Tissue specific
LLSLKY	Superscaf	-	Superscaf	71454790	71454807	-11	11	16	-	-	-	-	-	-	NCP	IGR	-	ID=gene-E	6	6.503333333	2371.5	0.08888399	LLSLKY	97	368.7322	2	735.4531	.	L	Superscaf	71454813	0	0	0	0	BTP202209	0	Tissue specific
KLSEYGP	Superscaf	-	Superscaf	71476513	71476533	-1	1	7	-	-	-	-	-	-	NCP	IGR	-	ID=gene-E	7	6.078571429	2724.9	0.088606963	KLSEYGP	57	397.2145	2	792.4017	.	K	Superscaf	71476617	0	0	BTP202209	0	0	0	Tissue specific
ATYLQY	Superscaf	+	Superscaf	71516392	71516409	4	4	9	-	-	gene	-	-	mRNA	NCP	Intron	gene|mRN	ID=gene-E	6	5.916666667	2591.9	0.088098507	ATYLQY	60	758.3662	1	757.3646	.	S	Superscaf	71516383	BTP202209	0	0	0	0	0	Tissue specific
KDDLNL	Superscaf	+	Superscaf	71555998	71556015	26	26	31	-	-	gene	-	-	mRNA	NCP	Intron	gene|mRN	ID=gene-E	6	5.508333333	2594.9	0.087593493	KDDLNL	67	717.3749	1	716.3704	.	R	Superscaf	71555923	0	0	0	0	0	BTP202209	Tissue specific
KLHRTP	Superscaf	+	Superscaf	71593951	71593968	2	2	7	-	-	-	-	-	-	NCP	IGR	-	ID=gene-E	6	7.816666667	2825.2	0.087109556	KLHRTP	75	376.2269	2	750.45	.	L	Superscaf	71593948	BTP202209	0	0	0	BTP202209	0	Tissue specific
LSELP	Superscaf	+	Superscaf	71619007	71619021	30	30	34	-	-	gene	-	-	mRNA	NCP	Intron	gene|mRN	ID=gene-E	5	5.432	1853.1	0.086790107	L(+42.01)	58	600.3223	1	599.3167	Acetylati	N	Superscaf	71618920	0	0	0	BTP202209	0	0	Mixed
LEAGSGLP	Superscaf	-	Superscaf	71681704	71681727	-16	16	23	-	-	gene	-	-	mRNA	NCP	Intron	gene|mRN	ID=gene-E	8	5.64	2759	0.085990547	LEAGSGLP	62	743.3864	1	742.3861	.	H	Superscaf	71681829	0	0	0	0	BTP202209	0	Tissue specific
LGETLP	Superscaf	-	Superscaf	71710054	71710071	-6	6	11	-	-	gene	-	-	mRNA	NCP	Intron	gene|mRN	ID=gene-E	6	5.663333333	2164.5	0.085629134	LGETLP	76	629.3492	1	628.3432	.	A	Superscaf	71710077	0	0	0	0	BTP202209	0	Tissue specific

KEPPFER	Superscaf+	Superscaf	71782003	71782023	57	57	63	-	-	gene	-	-	mRNA	NCP	Intron	gene|mRN	ID=gene-	7	6.431428571	3030.4	0.084711678	KEPPFER	62	451.7361	2	901.4657	.	N	Superscaf	71781835	0	0	BTP202209	0	0	0	Tissue specific
LYGGLFL	Superscaf-	Superscaf	71799181	71799201	-33	33	39	-	-	gene	-	-	mRNA	NCP	Intron	gene|mRN	ID=gene-	7	5.86	2501.7	0.084492642	LYGGLFL	50	782.4396	1	781.4374	.	T	Superscaf	71799219	0	0	0	BTP202209	0	0	Tissue specific
WGVLW	Superscaf-	Superscaf	71980066	71980080	-68	68	72	-	-	-	-	-	-	NCP	IGR	-	ID=gene-	5	4.746	1703.9	0.082186265	WGVLW	66	660.3452	1	659.3431	.	G	Superscaf	71980092	0	0	BTP202209	0	0	0	Mixed
GPHEAPK	Superscaf-	Superscaf	72070879	72070899	-18	18	24	-	-	-	-	-	-	NCP	IGR	-	ID=gene-	7	6.448571429	2797	0.081028237	GPHEAPK	53	368.1896	2	734.3711	.	A	Superscaf	72070923	0	0	0	BTP202209	0	0	Tissue specific
LDVKAPK	Superscaf-	Superscaf	72154255	72154275	-5	5	11	-	-	-	-	-	-	NCP	IGR	-	ID=gene-	7	5.821428571	2350.4	0.079965115	LDVKAPK	94	385.7415	2	769.4697	.	T	Superscaf	72154311	0	0	0	0	0	BTP202209	Tissue specific
KDGGDPK	Superscaf+	Superscaf	72169273	72169293	6	6	12	-	-	-	-	-	-	NCP	IGR	-	ID=gene-	7	6.237142857	2691.8	0.079773621	KDGGDPK	51	716.3708	1	715.35	.	R	Superscaf	72169258	0	BTP202209	0	0	0	0	Tissue specific
TAPVNFK	Superscaf+	Superscaf	72270649	72270669	39	39	45	-	-	-	-	-	-	NCP	IGR	-	ID=gene-	7	5.64	2498.7	0.078480982	T(+42.01)	95	409.7234	2	817.4334	Acetylati	V	Superscaf	72270535	BTP202209	BTP202209	BTP202209	0	BTP202209	0	Tissue enhanced
DRPLNP	Superscaf-	Superscaf	72313270	72313287	-7	7	12	-	-	-	-	-	-	NCP	IGR	-	ID=gene-	6	6.286666667	2685.2	0.077937562	DRPLNP	74	356.1915	2	710.3711	.	K	Superscaf	72313323	BTP202209	BTP202209	BTP202209	0	BTP202209	BTP202209	Tissue specific
MEMLCAA	Superscaf+	Superscaf	72386539	72386559	8	8	14	-	-	gene	-	-	mRNA	NCP	Intron	gene|mRN	ID=gene-	7	5.394285714	2730.2	0.077003275	M(+42.01)	59	434.1659	2	866.3336	Acetylati	L	Superscaf	72386518	0	0	BTP202209	0	0	0	Tissue specific
PKMTFLH	Superscaf-	Superscaf	72408142	72408162	-62	62	68	-	-	-	-	-	-	NCP	IGR	-	ID=gene-	7	6.767142857	2935.3	0.076727817	PKMTFLH	54	437.2426	2	872.4578	.	N	Superscaf	72408195	0	0	BTP202209	0	0	0	Tissue specific
LDGLQFF	Superscaf+	Superscaf	72497332	72497352	9	9	15	-	-	-	-	-	-	NCP	IGR	-	ID=gene-	7	5.358571429	2801.1	0.07559056	LDGLQFF	78	839.426	1	838.4225	.	C	Superscaf	72497308	0	0	BTP202209	0	0	0	Tissue specific
VGEVLVTK	Superscaf+	Superscaf	72528544	72528567	28	28	35	-	-	gene	-	-	mRNA	NCP	Intron	gene|mRN	ID=gene-	8	3.93	1942.1	0.07519254	VGEVLVTK	99	422.7586	2	843.5065	.	L	Superscaf	72528463	0	BTP202209	0	0	0	0	Tissue enhanced
WVLTSKY	Superscaf-	Superscaf	72591025	72591045	-9	9	15	-	-	gene	-	-	mRNA	NCP	Intron	gene|mRN	ID=gene-	7	5.64	2588.8	0.074395887	WVLTSKY	98	448.7464	2	895.4803	.	Y	Superscaf	72591150	BTP202209	0	0	0	0	0	Tissue specific
KPFHGPP	Superscaf+	Superscaf	72613249	72613269	15	15	21	-	-	gene	-	-	mRNA	NCP	Intron	gene|mRN	ID=gene-	7	6.811428571	2785	0.07411251	KPFHGPP	61	390.2139	2	778.4125	.	K	Superscaf	72613207	0	0	BTP202209	0	0	0	Tissue specific
FGGWK	Superscaf-	Superscaf	72618967	72618981	-4	4	8	-	-	gene	-	-	mRNA	NCP	Intron	gene|mRN	ID=gene-	5	6.61	1977	0.074039676	FGGWK	94	594.3014	1	593.2961	.	H	Superscaf	72619014	0	0	0	BTP202209	0	0	Tissue specific
MSDFPL	Superscaf+	Superscaf	72753643	72753660	32	32	37	-	-	-	-	-	-	NCP	IGR	-	ID=gene-	6	5.36	2244.6	0.072322392	M(+42.01)	54	376.1741	2	750.3258	Acetylati	V	Superscaf	72753550	BTP202209	0	0	0	0	0	Tissue specific
LPPPPPSS	Superscaf+	Superscaf	72952882	72952905	29	29	36	-	-	-	-	-	-	NCP	IGR	-	ID=gene-	8	6.105	2807.2	0.069781831	L(+42.01)	73	833.4483	1	832.433	Acetylati	N	Superscaf	72952798	0	BTP202209	0	0	0	0	Tissue specific
YYPRLH	Superscaf-	Superscaf	73063096	73063113	-18	18	23	-	-	-	-	-	-	NCP	IGR	-	ID=gene-	6	6.991666667	2878.3	0.068376575	YYPRLH	55	424.7264	2	847.434	.	E	Superscaf	73063179	0	0	0	0	0	BTP202209	Tissue specific
RDAHF	Superscaf-	Superscaf	73068916	73068930	-56	56	60	-	-	-	-	-	-	NCP	IGR	-	ID=gene-	5	6.564	2378.7	0.068302403	R(+42.01)	65	687.3337	1	686.3136	Acetylati	P	Superscaf	73068936	BTP202209	0	0	0	0	0	Tissue specific
HDGSFLK	Superscaf+	Superscaf	73088077	73088097	3	3	9	-	-	-	-	-	-	NCP	IGR	-	ID=gene-	7	6.201428571	2921.1	0.068058006	HDGSFLK	71	402.2045	2	802.3973	.	K	Superscaf	73088071	BTP202209	BTP202209	BTP202209	0	BTP202209	BTP202209	Tissue specific
RYTVKP	Superscaf+	Superscaf	73302988	73303005	8	8	13	-	-	gene	-	-	mRNA	NCP	Intron	gene|mRN	ID=gene-	6	6.498333333	2311.6	0.065317727	RYTVKP	71	382.226	2	762.4388	.	F	Superscaf	73302967	0	0	0	0	BTP202209	BTP202209	Tissue specific
GPVHPAK	Superscaf+	Superscaf	73307113	73307133	9	9	15	-	-	gene	-	-	mRNA	NCP	Intron	gene|mRN	ID=gene-	7	5.988571429	2371.5	0.065265091	GPVHPAK	52	353.2097	2	704.3969	.	G	Superscaf	73307089	BTP202209	BTP202209	BTP202209	0	BTP202209	BTP202209	Tissue specific
YGGKSKK	Superscaf+	Superscaf	73394350	73394370	2	2	8	-	-	gene	-	-	mRNA	NCP	Intron	gene|mRN	ID=gene-	7	7.5	2842.7	0.064152737	YGGKSKK	54	384.2234	2	766.4337	.	T	Superscaf	73394347	0	0	0	0	0	BTP202209	Tissue specific
TAKRPP	Superscaf-	Superscaf	73410886	73410903	-13	13	18	-	-	gene	-	-	mRNA	NCP	Intron	gene|mRN	ID=gene-	6	7.608333333	2500.8	0.063941926	T(+42.01)	69	356.21	2	710.4075	Acetylati	S	Superscaf	73411041	BTP202209	0	0	0	0	0	Tissue specific
QKFDLR	Superscaf-	Superscaf	73423066	73423083	-11	11	16	-	-	gene	-	-	mRNA	NCP	Intron	gene|mRN	ID=gene-	6	6.763333333	2880.2	0.063786619	QKFDLR	64	403.7279	2	805.4446	.	Y	Superscaf	73423119	BTP202209	0	0	0	0	0	Tissue specific
DTVRNKTK	Superscaf-	Superscaf	73424803	73424826	-5	5	12	-	-	gene	-	-	mRNA	NCP	Intron	gene|mRN	ID=gene-	8	6.46	3388.8	0.063764394	DTVRNKTK	73	481.2693	2	960.5352	.	P	Superscaf	73424886	0	0	BTP202209	0	0	BTP202209	Tissue specific
KKYCGK	Superscaf+	Superscaf	73578949	73578966	8	8	13	-	-	gene	-	-	mRNA	NCP	Intron	gene|mRN	ID=gene-	6	7.645	2747.7	0.061798964	KKYC(+57.	51	392.2159	2	782.4109	Carbamido	I	Superscaf	73578928	0	0	0	0	0	BTP202209	Tissue specific
KAACHPK	Superscaf+	Superscaf	73782718	73782738	1	1	7	-	-	-	-	-	-	NCP	IGR	-	ID=gene-	7	7.204285714	3102.2	0.05920068	KAAC(+57.	50	406.2106	2	810.417	Carbamido	K	Superscaf	73782718	0	BTP202209	0	0	0	BTP202209	Tissue specific
WGVKK	Superscaf-	Superscaf	73807699	73807713	-12	12	16	-	-	-	-	-	-	NCP	IGR	-	ID=gene-	5	6.268	1760.7	0.058882225	WGVKK	89	617.3745	1	616.3697	.	S	Superscaf	73807731	0	0	BTP202209	0	0	0	Tissue specific
LSPEELQK	Superscaf+	Superscaf	73865974	73865997	49	49	56	-	-	-	-	-	-	NCP	IGR	-	ID=gene-	8	5.72125	3357.7	0.058139049	LSPEELQK	78	472.2564	2	942.5022	.	P	Superscaf	73865830	0	0	BTP202209	0	BTP202209	0	Tissue specific
KPFQGK	Superscaf+	Superscaf	73903435	73903452	8	8	13	-	-	-	-	-	-	NCP	IGR	-	ID=gene-	6	7.146666667	2569.6	0.057661463	KPFQGK	64	352.7124	2	703.4017	.	L	Superscaf	73903414	0	0	BTP202209	0	0	0	Tissue specific
VVHRKCF	Superscaf-	Superscaf	73954375	73954395	-2	2	8	-	-	gene	-	-	mRNA	NCP	Intron	gene|mRN	ID=gene-	7	5.512857143	2710	0.057011892	V(+42.01)	99	494.263	2	986.512	Acetylati	K	Superscaf	73954425	0	0	0	0	0	BTP202209	Tissue specific
KTFLAQC	Superscaf+	Superscaf	73966144	73966164	4	4	10	-	-	gene	-	-	mRNA	NCP	Intron	gene|mRN	ID=gene-	7	6.345714286	3114.4	0.056861826	K(+42.01)	77	909.4646	1	908.4426	Acetylati	I	Superscaf	73966135	0	0	0	0	0	BTP202209	Tissue specific
LYCLY	Superscaf+	Superscaf	74095018	74095032	28	28	32	-	-	-	-	-	-	NCP	IGR	-	ID=gene-	5	5.66	2211.5	0.055218637	LYC(+57.(	76	731.3401	1	730.336	Carbamido	R	Superscaf	74094937	0	0	0	0	0	BTP202209	Tissue specific
KVTPVTR	Superscaf-	Superscaf	74112799	74112819	-4	4	10	-	-	-	-	-	-	NCP	IGR	-	ID=gene-	7	5.694285714	2249.6	0.054991836	K(+42.01)	61	421.7566	2	841.5021	Acetylati	G	Superscaf	74112948	0	0	BTP202209	0	0	0	Tissue specific
KYLPPP	Superscaf+	Superscaf	74161213	74161230	9	9	14	-	-	gene	-	-	mRNA	NCP	Intron	gene|mRN	ID=gene-	6	6.713333333	2293.5	0.054374551	KYLPPP	65	357.7121	2	713.4112	.	L	Superscaf	74161189	0	0	0	0	BTP202209	0	Tissue enhanced
DLVLK	Superscaf-	Superscaf	74164969	74164983	-30	30	34	-	-	gene	-	-	mRNA	NCP	Intron	gene|mRN	ID=gene-	5	4.934	1586.7	0.054326696	DLVLK	96	587.3747	1	586.369	.	V	Superscaf	74165025	0	0	0	0	0	BTP202209	Tissue specific
RAGKHKQ	Superscaf-	Superscaf	74226652	74226672	-2	2	8	-	-	gene	-	-	mRNA	NCP	Intron	gene|mRN	ID=gene-	7	7.924285714	3424.6	0.053540104	RAGKHKQ	50	412.7458	2	823.4777	.	S	Superscaf	74226681	0	0	0	0	0	BTP202209	Tissue specific
TSTAPGKQ	Superscaf-	Superscaf	74244946	74244969	-13	13	20	-	-	gene	-	-	mRNA	NCP	Intron	gene|mRN	ID=gene-	8	6.5525	3203.5	0.0533068	T(+42.01)	57	416.206	2	830.4134	Acetylati	K	Superscaf	74244999	0	0	0	0	0	BTP202209	Tissue specific
FTKVKM	Superscaf-	Superscaf	74251645	74251662	-14	14	19	-	-	gene	-	-	mRNA	NCP	Intron	gene|mRN	ID=gene-	6	6.206666667	2093.2	0.053221458	FTKVKM	51	377.2184	2	752.4255	.	R	Superscaf	74251710	0	0	0	0	0	BTP202209	Tissue specific
LPQLGVSLI	Superscaf-	Superscaf	74253589	74253615	-8	8	16	-	-	gene	-	-	mRNA	NCP	Intron	gene|mRN	ID=gene-	9	5.315555556	3026.4	0.053196555	LPQLGVSLI	50	462.2798	2	922.5488	.	K	Superscaf	74253627	BTP202209	0	0	0	0	0	Tissue specific
LEVAKPP	Superscaf-	Superscaf	74253823	74253843	-51	51	57	-	-	gene	-	-	mRNA	NCP	Intron	gene|mRN	ID=gene-	7	5.365714286	2233.4	0.053193648	LEVAKPP	53	377.2333	2	752.4432	.	V	Superscaf	74253876	BTP202209	0	0	0	0	0	Tissue specific
KSQEFFH	Superscaf+	Superscaf	74303950	74303970	17	17	23	-	-	-	-	-	-	NCP	IGR	-	ID=gene-	7	6.12	3282.5	0.052554481	KSQEFFH	50	461.7193	2	921.4344	.	Y	Superscaf	74303902	0	0	0	0	0	BTP202209	Tissue specific
ADLGE	Superscaf-	Superscaf	74411248	74411262	-1	1	5	-	-	-	-	-	-	NCP	IGR	-	ID=gene-	5	4.832	1799	0.051186407	ADLGE	50	504.2269	1	503.2227	.	A	Superscaf	74411436	0	0	0	0	BTP202209	0	Tissue specific
AATVVAPP(	Superscaf-	Superscaf	74411401	74411439	-4	4	16	-	-	-	-	-	-	NCP	IGR	-	ID=gene-	13	5.143076923	3662.9	0.051184151	A(+42.01)	61	583.8223	2	1165.634	Acetylati	E	Superscaf	74411667	0	BTP202209	BTP202209	0	BTP202209	0	Tissue specific
AATVVAPP(	Superscaf-	Superscaf	74411401	74411436	-4	4	15	-	-	-	-	-	-	NCP	IGR	-	ID=gene-	12	5.098333333	3279.5	0.051184189	A(+42.01)	66	540.3068	2	1078.602	Acetylati	E	Superscaf	74411667	0	BTP202209	BTP202209	BTP202209	BTP202209	BTP202209	Tissue specific
AATVVAPP(	Superscaf-	Superscaf	74411401	74411433	-4	4	14	-	-	-	-	-	-	NCP	IGR	-	ID=gene-	11	5.014545455	2968.2	0.051184227	A(+42.01)	55	504.7874	2	1007.565	Acetylati	E	Superscaf	74411667	0	0	0	0	BTP202209	0	Mixed
AATVVAPP(	Superscaf-	Superscaf	74411401	74411430	-4	4	13	-	-	-	-	-	-	NCP	IGR	-	ID=gene-	10	4.918	2614.8	0.051184265	A(+42.01)	52	448.2466	2	894.481	Acetylati	E	Superscaf	74411667	0	0	0	0	BTP202209	0	Tissue specific
AATVVAPP(	Superscaf-	Superscaf	74411401	74411427	-4	4	12	-	-	-	-	-	-	NCP	IGR	-	ID=gene-	9	4.795555556	2303.5	0.051184304	A(+42.01)	80	824.4476	1	823.4439	Acetylati	E	Superscaf	74411667	0	BTP202209	0	0	0	0	Tissue specific
AATVVAPP	Superscaf-	Superscaf	74411401	74411424	-4	4	11	-	-	-	-	-	-	NCP	IGR	-	ID=gene-	8	4.64875	2006.2	0.051184342	A(+42.01)	84	767.4282	1	766.4225	Acetylati	E	Superscaf	74411667	0	0	0	0	BTP202209	0	Mixed
ESGKVSSG	Superscaf+	Superscaf	74420566	74420589	8	8	15	-	-	-	-	-	-	NCP	IGR	-	ID=gene-	8	5.2425	2638.7	0.051067479	ESGKVSSG	62	750.3602	1	749.3555	.	R	Superscaf	74420545	0	0	BTP202209	0	0	0	Tissue specific
SSGSLALR	Superscaf-	Superscaf	74437072	74437095	-12	12	19	-	-	-	-	-	-	NCP	IGR	-	ID=gene-	8	6.46875	3114.4	0.050857012	SSGSLALR	94	395.7236	2	789.4344	.	G	Superscaf	74437140	BTP202209	0	0	0	0	0	Tissue specific
LKVFEPKLN	Superscaf-	Superscaf	74453044	74453070	-20	20	28	-	-	-	-	-	-	NCP	IGR	-	ID=gene-	9	5.761111111	3390.6	0.050653316	LKVFEPKLN	87	544.327	2	1086.644	.	L	Superscaf	74453139	0	BTP202209	0	0	0	0	Tissue specific
LKVFEPKLN	Superscaf-	Superscaf	74453044	74453073	-20	20	29	-	-	-	-	-	-	NCP	IGR	-	ID=gene-	10	5.751	3850.1	0.050653278	LKVFEPKLN	99	625.8575	2	1249.707	.	L	Superscaf	74453139	0	0	0	0	BTP202209	0	Tissue specific
LKVFEPKL	Superscaf-	Superscaf	74453044	74453067	-20	20	27	-	-	-	-	-	-	NCP	IGR	-	ID=gene-	8	5.805	2793.9	0.050653354	LKVFEPKL	72	487.3061	2	972.6008	.	L	Superscaf	74453139	0	BTP202209	0	0	0	0	Tissue specific
LKVFEPK	Superscaf-	Superscaf	74453044	74453064	-20	20	26	-	-	-	-	-	-	NCP	IGR	-	ID=gene-	7	5.78	2440.5	0.050653393	LKVFEPK	91	430.7643	2	859.5167	.	L	Superscaf	74453139	0	0	0	0	0	BTP202209	Tissue specific
KVFEPKLN	Superscaf-	Superscaf	74453047	74453070	-21	21	28	-	-	-	-	-	-	NCP	IGR	-	ID=gene-	8	5.73375	3037.2	0.050653316	KVFEPKLN	98	487.7858	2	973.5596	.	L	Superscaf	74453139	0	0	0	0	0	BTP202209	Tissue specific
VFEPKLN	Superscaf-	Superscaf	74453050	74453070	-22	22	28	-	-	-	-	-	-	NCP	IGR	-	ID=gene-	7	5.161428571	2568.8	0.050653316	VFEPKLN	99	423.7381	2	845.4647	.	L	Superscaf	74453139	0	0	0	0	0	BTP202209	Tissue specific
NHGLF	Superscaf+	Superscaf	74474944	74474958	8	8	12	-	-	gene	-	-	mRNA	NCP	Intron	gene|mRN	ID=gene-	5	6.086	2254.5	0.050374224	N(+42.01)	58	629.2916	1	628.2969	Acetylati	R	Superscaf	74474923	0	0	0	0	0	BTP202209	Tissue specific
PLSFSY	Superscaf-	Superscaf	74483578	74483595	-47	47	52	-	-	gene	-	-	mRNA	NCP	Intron	gene|mRN	ID=gene-	6	5.796666667	2304.5	0.050264094	PLSFSY	52	713.353	1	712.3431	.	C	Superscaf	74483979	0	0	BTP202209	0	0	0	Tissue specific
NMAHNAEHE	Superscaf-	Superscaf	74538196	74538234	-42	42	54	-	-	gene	-	-	mRNA	NCP	Intron	gene|mRN	ID=gene-	13	5.401538462	5939.8	0.049567395	NMAHNAEHE	94	756.3001	2	1510.593	Carbamido	R	Superscaf	74538318	0	0	BTP202209	0	0	0	Tissue specific
GPAAHVY	Superscaf-	Superscaf	74554105	74554125	-27	27	33	-	-	gene	-	-	mRNA	NCP	Intron	gene|mRN	ID=gene-	7	5.365714286	2336.5	0.04936477	GPAAHVY	53	714.3608	1	713.3497	.	Q	Superscaf	74554386	0	0	0	0	BTP202209	0	Tissue specific
FGFGFL	Superscaf-	Superscaf	74555971	74555988	-27	27	32	-	-	gene	-	-	mRNA	NCP	Intron	gene|mRN	ID=gene-	6	5.726666667	2110.2	0.049341015	FGFGFL	77	687.3477	1	686.3428	.	K	Superscaf	74556048	0	0	0	0	BTP202209	0	Tissue specific
MLQDVLDLF	Superscaf-	Superscaf	74560900	74560929	-26	26	35	-	-	gene	-	-	mRNA	NCP	Intron	gene|mRN	ID=gene-	10	5.578	3982.6	0.049278012	MLQDVLDLF	96	615.8447	2	1229.68	.	P	Superscaf	74560968	0	BTP202209	BTP202209	0	0	0	Tissue specific
EMEERSG	Superscaf-	Superscaf	74603683	74603703	-124	124	130	-	-	gene	-	-	mRNA	NCP	Intron	gene|mRN	ID=gene-	7	5.402857143	2977.5	0.048732604	EMEERSG	51	837.3408	1	836.3334	.	G	Superscaf	74603742	0	0	BTP202209	0	0	0	Tissue specific
LHPAARP	Superscaf-	Superscaf	74669353	74669373	-1	1	7	-	-	gene	-	-	mRNA	NCP	Intron	gene|mRN	ID=gene-	7	6.995714286	2919.3	0.047895249	LHPAARP	63	381.2237	2	760.4344	.	L	Superscaf	74669379	0	0	0	0	BTP202209	0	Tissue specific
YPSLKVLL	Superscaf+	Superscaf	74732164	74732187	8	8	15	-	-	-	-	-	-	NCP	IGR	-	ID=gene-	8	5.665	2708.9	0.047094312	YPSLKVLL	51	932.5773	1	931.5742	.	I	Superscaf	74732143	0	0	0	0	BTP202209	0	Tissue specific
SLPFFQRT	Superscaf+	Superscaf	74769703	74769726	2	2	9	-	-	-	-	-	-	NCP	IGR	-	ID=gene-	8	6.4825	3506	0.046615654	SLPFFQRT	66	498.2677	2	994.5236	.	P	Superscaf	74769700	0	0	0	0	BTP202209	0	Tissue specific
KMFGEKL	Superscaf+	Superscaf	74780449	74780469	49	49	55	-	-	-	-	-	-	NCP	IGR	-	ID=gene-	7	6.554285714	2771.9	0.046478671	KMFGEKL	99	426.7335	2	851.4575	.	I	Superscaf	74780305	0	BTP202209	BTP202209	0	0	0	Tissue specific
RAAPF	Superscaf-	Superscaf	74848351	74848365	-18	18	22	-	-	-	-	-	-	NCP	IGR	-	ID=gene-	5	6.916	1996.2	0.045612933	RAAPF	70	561.3124	1	560.3071	.	L	Superscaf	74848374	0	0	0	0	0	BTP202209	Tissue specific
TGLRPTT	Superscaf-	Superscaf	74848870	74848890	-33	33	39	-	-	-	-	-	-	NCP	IGR	-	ID=gene-	7	6.942857143	2829.4	0.045606239	TGLRPTT	70	745.4178	1	744.413	.	I	Superscaf	74849148	0	0	0	0	0	BTP202209	Mixed
NCTPK	Superscaf+	Superscaf	74900011	74900025	2	2	6	-	-	-	-	-	-	NCP	IGR	-	ID=gene-	5	6.6	2385.7	0.044954219	N(+42.01)	73	661.2986	1	660.2901	Acetylati	P	Superscaf	74900008	0	BTP202209	0	0	BTP202209	BTP202209	Tissue specific
KYKAPTK	Superscaf+	Superscaf	75050398	75050418	39	39	45	-	-	-	-	-	-	NCP	IGR	-	ID=gene-	7	7.675714286	2910.9	0.043036567	KYKAPTK	80	418.2603	2	834.4963	.	R	Superscaf	75050284	0	BTP202209	0	0	0	0	Tissue specific
MKPLRNLL	Superscaf+	Superscaf	75085318	75085341	18	18	25	-	-	-	-	-	-	NCP	IGR	-	ID=gene-	8	6.9875	3483	0.042591266	M(+42.01)	76	513.8089	2	1025.606	Acetylati	K	Superscaf	75085267	0	0	0	0	0	BTP202209	Tissue specific
WRYSSWK	Superscaf-	Superscaf	75177271	75177291	-14	14	20	-	-	-	-	-	-	NCP	IGR	-	ID=gene-	7	7.042857143	3396.7	0.041418817	WRYSSWK	54	506.7622	2	1011.493	.	F	Superscaf	75177309	0	0	BTP202209	0	0	0	Tissue specific
VPTVFVG	Superscaf-	Superscaf	75252469	75252489	-5	5	11	-	-	-	-	-	-	NCP	IGR	-	ID=gene-	7	3.468571429	1419.6	0.040459972	VPTVFVG	69	718.4109	1	717.4061	.	G	Superscaf	75252681	0	BTP202209	0	0	0	0	Tissue specific
FLTVPF	Superscaf-	Superscaf	75303241	75303258	-43	43	48	-	-	-	-	-	-	NCP	IGR	-	ID=gene-	6	4.961666667	1863.1	0.039812619	FLTVPF	52	362.2068	2	722.4003	.	V	Superscaf	75303324	0	0	BTP202209	0	0	0	Tissue specific
RYELQR	Superscaf-	Superscaf	75402382	75402399	-18	18	23	-	-	gene	-	-	mRNA	NCP	Intron	gene|mRN	ID=gene-	6	7.005	3146.7	0.038548478	RYELQR	91	432.7366	2	863.4613	.	I	Superscaf	75402405	0	BTP202209	0	0	0	0	Tissue specific
LTEWG	Superscaf+	Superscaf	75415159	75415173	38	38	42	-	-	gene	-	-	mRNA	NCP	Intron	gene|mRN	ID=gene-	5	5.518	2000.3	0.038385598	LTEWG	71	605.2844	1	604.2856	.	G	Superscaf	75415048	0	BTP202209	0	0	0	0	Tissue specific
HLGPSFP	Superscaf-	Superscaf	75419422	75419442	-10	10	16	-	-	-	-	-	-	NCP	IGR	-	ID=gene-	7	6.185714286	2716	0.038331164	HLGPSFP	84	754.3853	1	753.3809	.	F	Superscaf	75419481	0	0	0	0	BTP202209	0	Tissue specific
NVLEY	Superscaf+	Superscaf	75423784	75423798	5	5	9	-	-	-	-	-	-	NCP	IGR	-	ID=gene-	5	4.054	1835.1	0.038275621	NVLEY	99	637.3175	1	636.3119	.	S	Superscaf	75423772	0	0	0	0	BTP202209	0	Mixed
GVTRVY	Superscaf-	Superscaf	75461473	75461490	-3	3	8	-	-	-	-	-	-	NCP	IGR	-	ID=gene-	6	4.82	1803.1	0.037795012	G(+42.01)	73	736.3962	1	735.3915	Acetylati	L	Superscaf	75461988	0	0	0	0	BTP202209	BTP202209	Mixed
TMMDPP	Superscaf-	Superscaf	75697960	75697977	-1	1	6	-	-	-	-	-	-	NCP	IGR	-	ID=gene-	6	5.6	2226.8	0.034779581	T(+42.01)	57	383.1403	2	764.2721	Acetylati	T	Superscaf	75698010	0	0	0	0	BTP202209	0	Tissue specific
LTMPLTLL	Superscaf+	Superscaf	75708721	75708744	7	7	14	-	-	-	-	-	-	NCP	IGR	-	ID=gene-	8	6.12875	2917.5	0.034642291	LTM(+15.9	55	459.2794	2	916.5303	Oxidation	D	Superscaf	75708703	0	0	0	0	0	BTP202209	Tissue specific
TKGPRK	Superscaf-	Superscaf	75726652	75726669	-34	34	39	-	-	-	-	-	-	NCP	IGR	-	ID=gene-	6	8.173333333	2617.8	0.034413731	T(+42.01)	55	364.7285	2	727.434	Acetylati	D	Superscaf	75726810	0	0	0	0	0	BTP202209	Tissue specific
ANPLL	Superscaf-	Superscaf	75765388	75765402	-9	9	13	-	-	-	-	-	-	NCP	IGR	-	ID=gene-	5	5.938	1952.2	0.033919849	ANPLL	91	527.3169	1	526.3115	.	P	Superscaf	75765438	0	0	0	0	BTP202209	0	Tissue specific
FYWLLR	Superscaf-	Superscaf	76070752	76070769	-15	15	20	-	-	gene	-	-	mRNA	NCP	Intron	gene|mRN	ID=gene-	6	6.625	2729.1	0.030026132	FYWLLR	95	449.2519	2	896.4908	.	W	Superscaf	76070778	BTP202209	0	0	0	0	0	Tissue specific
KPLENQP	Superscaf+	Superscaf	76077799	76077819	33	33	39	-	-	gene	-	-	mRNA	NCP	Intron	gene|mRN	ID=gene-	7	6.085714286	3129.5	0.029936238	KPLENQP	51	825.4333	1	824.4392	.	A	Superscaf	76077703	0	0	0	0	0	BTP202209	Tissue specific
LPLPNC	Superscaf-	Superscaf	76111777	76111794	-13	13	18	-	-	-	-	-	-	NCP	IGR	-	ID=gene-	6	5.831666667	2564	0.029503025	LPLPNC(+5	68	357.1895	2	712.3578	Carbamido	I	Superscaf	76111797	0	0	0	0	0	BTP202209	Tissue specific
TLSLETGF	Superscaf-	Superscaf	76223311	76223334	-30	30	37	-	-	-	-	-	-	NCP	IGR	-	ID=gene-	8	5.67125	2995.4	0.028080785	TLSLETGF	92	867.4426	1	866.4385	.	S	Superscaf	76223358	0	0	0	0	BTP202209	0	Tissue specific
PTVKMY	Superscaf-	Superscaf	76248436	76248453	-30	30	35	-	-	gene	-	-	mRNA	NCP	Intron	gene|mRN	ID=gene-	6	5.663333333	2034.3	0.027760494	PTVKMY	57	369.6989	2	737.3782	.	N	Superscaf	76248453	0	0	0	0	BTP202209	0	Tissue specific
DPGGY	Superscaf+	Superscaf	76257295	76257309	18	18	22	-	-	gene	-	-	mRNA	NCP	Intron	gene|mRN	ID=gene-	5	5.374	1803	0.027647572	D(+42.01)	62	550.2166	1	549.2071	Acetylati	H	Superscaf	76257244	0	BTP202209	0	0	0	0	Tissue specific
LGPPMLK	Superscaf-	Superscaf	76267597	76267617	-1	1	7	-	-	-	-	-	-	NCP	IGR	-	ID=gene-	7	6.574285714	2518.8	0.027516135	L(+42.01)	75	407.2253	2	812.4466	Acetylati	L	Superscaf	76267752	0	BTP202209	BTP202209	BTP202209	0	0	Tissue enhanced
HSRDP	Superscaf+	Superscaf	76284829	76284843	10	10	14	-	-	-	-	-	-	NCP	IGR	-	ID=gene-	5	6.66	2400.8	0.027296487	HSRDP	56	611.2945	1	610.2823	.	I	Superscaf	76284802	BTP202209	0	0	0	0	0	Tissue specific
VNFDP	Superscaf-	Superscaf	76306591	76306605	-38	38	42	-	-	-	-	-	-	NCP	IGR	-	ID=gene-	5	4.032	1733	0.027019001	VNFDP	78	591.275	1	590.27	.	A	Superscaf	76306659	0	0	0	0	0	BTP202209	Tissue specific
PLSQGATR	Superscaf+	Superscaf	76313956	76313979	15	15	22	-	-	-	-	-	-	NCP	IGR	-	ID=gene-	8	6.61125	3339.8	0.026924976	PLSQGATR	71	415.2312	2	828.4454	.	S	Superscaf	76313914	0	0	0	0	0	BTP202209	Tissue specific
AEGAFVLL	Superscaf-	Superscaf	76315153	76315176	-18	18	25	-	-	-	-	-	-	NCP	IGR	-	ID=gene-	8	4.83375	2439.6	0.026909713	AEGAFVLL	86	819.4576	1	818.4538	.	S	Superscaf	76315188	0	0	BTP202209	0	0	0	Tissue specific
TGGPDN	Superscaf-	Superscaf	76319362	76319379	-17	17	22	-	-	-	-	-	-	NCP	IGR	-	ID=gene-	6	5.525	2337.7	0.026856121	T(+42.01)	58	602.2406	1	601.2344	Acetylati	L	Superscaf	76319409	0	0	0	0	0	0	Tissue specific
KPTLGQQ	Superscaf+	Superscaf	76346995	76347015	3	3	9	-	-	-	-	-	-	NCP	IGR	-	ID=gene-	7	6.545714286	3075.4	0.026503736	KPTLGQQ	54	386.2197	2	770.4286	.	K	Superscaf	76346989	BTP202209	0	0	0	0	0	Tissue specific
KAQVALD	Superscaf+	Superscaf	76434691	76434711	42	42	48	-	exon	gene	three_pri	-	mRNA	NCP	3UTR	exon|gen	ID=exon-	7	5.197142857	2466.6	0.025385529	KAQVALD	63	372.7212	2	743.4177	.	F	Superscaf	76434568	0	0	BTP202209	0	0	0	Mixed
LGFLEL	Superscaf-	Superscaf	76435312	76435329	-1	1	6	-	exon	gene	three_pri	-	mRNA	NCP	3UTR	exon|gen	ID=exon-	6	5.435	2170.4	0.025377649	LGFLEL	85	691.3995	1	690.3952	.	L	Superscaf	76435329	0	0	0	0	0	BTP202209	Tissue specific
VGDALSVS	Superscaf-	Superscaf	76548301	76548324	-6	6	13	-	-	gene	-	-	mRNA	NCP	Intron	gene|mRN	ID=gene-	8	4.0375	2140.3	0.023936857	VGDALSVS	83	747.3868	1	746.381	.	G	Superscaf	76548408	0	0	0	0	BTP202209	0	Tissue specific
VAWNGKK	Superscaf+	Superscaf	76561369	76561389	10	10	16	-	-	gene	-	-	mRNA	NCP	Intron	gene|mRN	ID=gene-	7	6.11	2668.7	0.023770265	V(+42.01)	54	422.7449	2	843.4603	Acetylati	R	Superscaf	76561342	0	0	0	0	0	BTP202209	Tissue specific
KVGTRVP	Superscaf-	Superscaf	76629679	76629699	-5	5	11	-	-	gene	-	-	mRNA	NCP	Intron	gene|mRN	ID=gene-	7	5.614285714	2149.4	0.022899249	K(+42.01)	60	399.7445	2	797.4759	Acetylati	M	Superscaf	76629780	0	0	BTP202209	0	0	0	Tissue specific
QAVVL	Superscaf-	Superscaf	76652551	76652565	-16	16	20	-	-	gene	-	-	mRNA	NCP	Intron	gene|mRN	ID=gene-	5	3.53	1275.4	0.022607686	QAVVL	72	529.3333	1	528.3271	.	Q	Superscaf	76652580	0	0	0	0	BTP202209	0	Tissue specific
LRMCGAF	Superscaf+	Superscaf	76661350	76661370	18	18	24	-	-	gene	-	-	mRNA	NCP	Intron	gene|mRN	ID=gene-	7	6.425714286	2955.4	0.022495414	LRM(+15.9	52	435.6983	2	869.3888	Oxidation	H	Superscaf	76661299	0	0	BTP202209	0	0	0	Tissue specific
LLLLPPQ	Superscaf+	Superscaf	76684648	76684668	15	15	21	-	-	gene	-	-	mRNA	NCP	Intron	gene|mRN	ID=gene-	7	6.024285714	2699.1	0.022198342	LLLLPPQ	51	397.2699	2	792.5109	.	T	Superscaf	76684606	0	0	0	0	BTP202209	0	Tissue specific
KDWVSL	Superscaf+	Superscaf	76704199	76704216	1	1	6	-	-	gene	-	-	mRNA	NCP	Intron	gene|mRN	ID=gene-	6	5.043333333	2143.3	0.021949087	KDWVSL	66	374.2026	2	746.3963	.	K	Superscaf	76704199	0	0	BTP202209	0	0	0	Tissue specific
DLNFPK	Superscaf-	Superscaf	76831924	76831941	-28	28	33	-	-	-	-	-	-	NCP	IGR	-	ID=gene-	6	5.98	2554.8	0.020320473	DLNFPK	52	367.197	2	732.3806	.	D	Superscaf	76831956	0	0	0	0	BTP202209	0	Tissue specific
KQKVNGF	Superscaf+	Superscaf	76836709	76836729	31	31	37	-	-	-	-	-	-	NCP	IGR	-	ID=gene-	7	5.998571429	2828.9	0.020259421	KQKVNGF	56	410.74	2	819.4603	.	L	Superscaf	76836619	0	0	0	0	0	BTP202209	Tissue specific
DFFEDTELY	Superscaf-	Superscaf	76878895	76878927	-25	25	35	-	-	gene	-	-	mRNA	NCP	Intron	gene|mRN	ID=gene-	11	5.637272727	4776.4	0.019721357	DFFEDTELY	92	731.8435	2	1461.678	.	P	Superscaf	76879029	0	BTP202209	0	0	0	0	Tissue specific
FFAYTLPK	Superscaf-	Superscaf	76882927	76882950	-288	288	295	-	-	gene	-	-	mRNA	NCP	Intron	gene|mRN	ID=gene-	8	6.39875	3102.3	0.01967006	FFAYTLPK	87	493.7708	2	985.5273	.	Y	Superscaf	76882950	0	0	BTP202209	0	0	0	Tissue enhanced
NSENKSGN(	Superscaf-	Superscaf	76889419	76889457	-45	45	57	-	-	gene	-	-	mRNA	NCP	Intron	gene|mRN	ID=gene-	13	5.33	5559.1	0.01958709	NSENKSGN(	57	750.3584	2	1498.705	.	I	Superscaf	76889601	0	BTP202209	BTP202209	BTP202209	BTP202209	0	Tissue specific
NTTVEEMPN	Superscaf-	Superscaf	76950709	76950738	-45	45	54	-	-	gene	-	-	mRNA	NCP	Intron	gene|mRN	ID=gene-	10	4.812	3919.8	0.018805699	NTTVEEMPN	76	583.2453	2	1164.479	.	C	Superscaf	76950753	0	BTP202209	BTP202209	BTP202209	BTP202209	BTP202209	Tissue specific
NTTVEEMPN	Superscaf-	Superscaf	76950709	76950750	-45	45	58	-	-	gene	-	-	mRNA	NCP	Intron	gene|mRN	ID=gene-	14	4.506428571	4996	0.018805546	NTTVEEMPN	76	782.3508	2	1562.696	.	C	Superscaf	76950753	0	BTP202209	0	0	BTP202209	0	Mixed
NTTVEEMPN	Superscaf-	Superscaf	76950709	76950747	-45	45	57	-	-	gene	-	-	mRNA	NCP	Intron	gene|mRN	ID=gene-	13	4.393076923	4642.6	0.018805585	NTTVEEMPN	86	725.8098	2	1449.612	.	C	Superscaf	76950753	0	0	BTP202209	0	0	0	Mixed
NTTVEEMPN	Superscaf-	Superscaf	76950709	76950744	-45	45	56	-	-	gene	-	-	mRNA	NCP	Intron	gene|mRN	ID=gene-	12	4.511666667	4231.1	0.018805623	NTTVEEMPN	68	668.2976	2	1334.585	.	C	Superscaf	76950753	BTP202209	0	0	0	0	0	Tissue specific
TVEEMPMNV	Superscaf-	Superscaf	76950715	76950747	-47	47	57	-	-	gene	-	-	mRNA	NCP	Intron	gene|mRN	ID=gene-	11	4.106363636	3648.4	0.018805585	TVEEMPMNV	56	618.2651	2	1234.521	.	C	Superscaf	76950753	0	0	BTP202209	0	0	0	Tissue specific
LKDVKVSLY	Superscaf-	Superscaf	77002390	77002416	-112	112	120	-	-	gene	-	-	mRNA	NCP	Intron	gene|mRN	ID=gene-	9	5.083333333	2898	0.018146756	LKDVKVSLY	77	532.8202	2	1063.628	.	T	Superscaf	77002623	0	BTP202209	0	0	BTP202209	0	Tissue specific
DDFLHFK	Superscaf-	Superscaf	77185531	77185551	-14	14	20	-	-	-	-	-	-	NCP	IGR	-	ID=gene-	7	5.744285714	3039.3	0.015811613	DDFLHFK	70	461.2238	2	920.4391	.	S	Superscaf	77185650	0	0	0	0	0	BTP202209	Tissue specific
NGAPTVKF	Superscaf-	Superscaf	77196739	77196762	-18	18	25	-	-	-	-	-	-	NCP	IGR	-	ID=gene-	8	5.68125	2796	0.015668662	NGAPTVKF	72	417.228	2	832.4443	.	C	Superscaf	77196861	0	0	0	0	BTP202209	0	Tissue specific
CLGPK	Superscaf-	Superscaf	77380852	77380866	-6	6	10	-	-	gene	-	-	mRNA	NCP	Intron	gene|mRN	ID=gene-	5	6.602	2042.2	0.013321163	C(+57.02)	83	574.3019	1	573.2944	Carbamido	S	Superscaf	77380959	0	0	0	0	0	BTP202209	Tissue specific
HLYLSR	Superscaf-	Superscaf	77389243	77389260	-11	11	16	-	-	-	-	-	-	NCP	IGR	-	ID=gene-	6	6.941666667	2818.2	0.013214131	H(+42.01)	63	415.7378	2	829.4446	Acetylati	L	Superscaf	77389266	0	BTP202209	0	0	0	0	Tissue specific
LLFTT	Superscaf+	Superscaf	77458162	77458176	25	25	29	-	-	gene	-	-	mRNA	NCP	Intron	gene|mRN	ID=gene-	5	6.1	1889.2	0.012335388	LLFTT	59	594.3594	1	593.3425	.	A	Superscaf	77458090	0	0	0	0	0	0	Tissue specific
PEAGK	Superscaf+	Superscaf	77528296	77528310	6	6	10	-	-	gene	-	-	mRNA	NCP	Intron	gene|mRN	ID=gene-	5	6.25	1839.9	0.011441113	P(+42.01)	74	543.2798	1	542.27	Acetylati	L	Superscaf	77528281	0	0	BTP202209	0	0	0	Tissue specific
TFVPKF	Superscaf-	Superscaf	77617981	77617998	-7	7	12	-	-	-	-	-	-	NCP	IGR	-	ID=gene-	6	5.588333333	1978.1	0.010297507	T(+42.01)	63	390.7177	2	779.4218	Acetylati	W	Superscaf	77618001	0	0	0	0	BTP202209	BTP202209	Tissue specific
KETVQL	Superscaf+	Superscaf	77671912	77671929	8	8	13	-	-	-	-	-	-	NCP	IGR	-	ID=gene-	6	5.186666667	2255.5	0.009609836	KETVQL	50	359.205	2	716.4069	.	I	Superscaf	77671891	0	0	BTP202209	0	0	0	Tissue specific
LTVDKK	Superscaf+	Superscaf	77697835	77697852	14	14	19	-	-	-	-	-	-	NCP	IGR	-	ID=gene-	6	5.826666667	2099.2	0.009279293	LTVDKK	74	352.2147	2	702.4276	.	F	Superscaf	77697796	0	0	0	0	0	BTP202209	Tissue specific
LGVSVPVL	Superscaf+	Superscaf	77702320	77702343	15	15	22	-	-	-	-	-	-	NCP	IGR	-	ID=gene-	8	3.73875	1724.9	0.009222029	LGVSVPVL	52	392.2513	2	782.4902	.	S	Superscaf	77702278	0	0	BTP202209	BTP202209	0	0	Tissue specific
QGHDVLT	Superscaf-	Superscaf	77832382	77832402	-125	125	131	-	-	-	-	-	-	NCP	IGR	-	ID=gene-	7	4.955714286	2690.1	0.007563654	QGHDVLT	53	769.38	1	768.3766	.	S	Superscaf	77832756	0	BTP202209	0	0	0	0	Tissue specific
LELKVE	Superscaf+	Superscaf	77900038	77900055	85	85	90	-	-	gene	-	-	mRNA	NCP	Intron	gene|mRN	ID=gene-	6	4.69	2026.2	0.006701015	LELKVE	90	365.7192	2	729.4272	.	D	Superscaf	77899786	0	0	0	0	BTP202209	0	Tissue specific
LGSVLR	Superscaf-	Superscaf	78028210	78028227	-7	7	12	-	-	gene	-	-	mRNA	NCP	Intron	gene|mRN	ID=gene-	6	5.728333333	2036.3	0.005066701	LGSVLR	72	644.4069	1	643.4017	.	E	Superscaf	78028299	0	0	0	0	BTP202209	0	Tissue specific
DPYMM	Superscaf-	Superscaf	78355813	78355827	-6	6	10	-	-	gene	-	-	mRNA	NCP	Intron	gene|mRN	ID=gene-	5	5.286	1951.4	0.000889493	D(+42.01)	50	730.239	1	729.235	Acetylati	K	Superscaf	78355836	0	0	0	0	0	BTP202209	Tissue specific
ETEYTVK	Superscaf-	Superscaf	78424759	78424779	-8	8	14	-	-	gene	-	-	mRNA	NCP	Intron	gene|mRN	ID=gene-	7	4.985714286	2573.9	1.03E-05	ETEYTVK	66	435.2208	2	868.4178	.	D	Superscaf	78424806	0	0	0	0	BTP202209	0	Tissue specific
TFGPGV	Superscaf-	Superscaf	78441301	78441318	-8	8	13	-	-	-	-	-	-	NCP	IGR	-	ID=gene-	6	5.041666667	1716.9	0.000200381	TFGPGV	51	577.3073	1	576.2907	.	F	Superscaf	78441354	0	0	0	0	BTP202209	0	Tissue specific
SHPSWLPPK	Superscaf-	Superscaf	78472606	78472635	-27	27	36	-	-	-	-	-	-	NCP	IGR	-	ID=gene-	10	6.514	4130.5	0.000599549	S(+42.01)	54	589.3013	2	1176.593	Acetylati	S	Superscaf	78472845	0	0	0	0	BTP202209	0	Tissue specific
SHPSWLPPK	Superscaf-	Superscaf	78472606	78472650	-27	27	41	-	-	-	-	-	-	NCP	IGR	-	ID=gene-	15	6.212666667	5955.6	0.000599549	S(+42.01)	68	844.9325	2	1687.857	Acetylati	S	Superscaf	78472845	0	0	BTP202209	0	0	0	Tissue specific
SVSTQQPPK	Superscaf-	Superscaf	78472705	78472734	-60	60	69	-	-	-	-	-	-	NCP	IGR	-	ID=gene-	10	6.127	3997.3	0.000600812	SVSTQQPPK	89	550.3063	2	1098.603	.	S	Superscaf	78472845	0	0	0	0	0	BTP202209	Tissue specific
SVSTQQPPK	Superscaf-	Superscaf	78472705	78472731	-60	60	68	-	-	-	-	-	-	NCP	IGR	-	ID=gene-	9	5.725555556	3528.9	0.000600812	SVSTQQPPK	85	486.2598	2	970.5084	.	S	Superscaf	78472845	0	0	0	0	BTP202209	0	Tissue specific
KKFAPVVAI	Superscaf-	Superscaf	78472729	78472764	-68	68	79	-	-	-	-	-	-	NCP	IGR	-	ID=gene-	12	6.281666667	3895.8	0.000601118	KKFAPVVAI	80	437.2822	3	1308.828	.	S	Superscaf	78472845	0	0	BTP202209	0	0	0	Tissue specific
KFAPVVAPK	Superscaf-	Superscaf	78472732	78472764	-69	69	79	-	-	-	-	-	-	NCP	IGR	-	ID=gene-	11	5.967272727	3427.4	0.000601156	KFAPVVAPK	96	394.5839	3	1180.733	.	S	Superscaf	78472845	BTP202209	BTP202209	BTP202209	BTP202209	BTP202209	BTP202209	Tissue enhanced
FAPVVAPKI	Superscaf-	Superscaf	78472735	78472764	-70	70	79	-	-	-	-	-	-	NCP	IGR	-	ID=gene-	10	5.59	2959	0.000601194	FAPVVAPKI	98	527.3251	2	1052.638	.	S	Superscaf	78472845	0	0	0	0	BTP202209	0	Tissue enhanced
FAPVVAPKI	Superscaf-	Superscaf	78472735	78472761	-70	70	78	-	-	-	-	-	-	NCP	IGR	-	ID=gene-	9	5.128888889	2490.6	0.000601194	FAPVVAPKI	97	463.2768	2	924.5432	.	S	Superscaf	78472845	BTP202209	0	0	BTP202209	0	0	Tissue specific
APVVAPKPK	Superscaf-	Superscaf	78472738	78472764	-71	71	79	-	-	-	-	-	-	NCP	IGR	-	ID=gene-	9	5.602222222	2571.6	0.000601232	APVVAPKPK	95	453.7906	2	905.5698	.	S	Superscaf	78472845	0	0	BTP202209	0	BTP202209	0	Mixed
APVVAPKP	Superscaf-	Superscaf	78472738	78472761	-71	71	78	-	-	-	-	-	-	NCP	IGR	-	ID=gene-	8	5.085	2103.2	0.000601232	APVVAPKP	98	389.7436	2	777.4749	.	S	Superscaf	78472845	BTP202209	0	0	0	0	0	Tissue specific
PVVAPKPK	Superscaf-	Superscaf	78472741	78472764	-72	72	79	-	-	-	-	-	-	NCP	IGR	-	ID=gene-	8	5.55	2260.3	0.000601271	PVVAPKPK	99	418.2727	2	834.5327	.	S	Superscaf	78472845	0	0	0	0	0	BTP202209	Tissue specific
VVAPKPK	Superscaf-	Superscaf	78472744	78472764	-73	73	79	-	-	-	-	-	-	NCP	IGR	-	ID=gene-	7	5.442857143	1922.9	0.000601309	VVAPKPK	99	369.7451	2	737.4799	.	S	Superscaf	78472845	0	0	0	0	0	BTP202209	Tissue specific
FPPPPPLDI	Superscaf-	Superscaf	78545548	78545577	-32	32	41	-	-	-	-	-	-	NCP	IGR	-	ID=gene-	10	5.431	3847.5	0.001529628	FPPPPPLDI	94	554.7679	2	1107.524	.	L	Superscaf	78545646	BTP202209	0	0	0	0	0	Tissue specific
PPPPPLDDN	Superscaf-	Superscaf	78545551	78545577	-33	33	41	-	-	-	-	-	-	NCP	IGR	-	ID=gene-	9	5.425555556	3460.1	0.001529667	PPPPPLDDN	62	481.2341	2	960.4552	.	L	Superscaf	78545646	0	0	BTP202209	0	0	0	Tissue specific
AATFLK	Superscaf+	Superscaf	78573553	78573570	25	25	30	-	-	gene	-	-	mRNA	NCP	Intron	gene|mRN	ID=gene-	6	6.628333333	2229.3	0.001886718	A(+42.01)	70	692.4049	1	691.3904	Acetylati	A	Superscaf	78573481	BTP202209	0	0	BTP202209	0	0	Tissue specific
SPYKPRPP	Superscaf-	Superscaf	78589561	78589584	-39	39	46	-	-	gene	-	-	mRNA	NCP	Intron	gene|mRN	ID=gene-	8	7.13	3309.7	0.002090836	SPYKPRPP	99	471.2627	2	940.513	.	I	Superscaf	78589608	BTP202209	0	0	0	0	0	Mixed
YKPRPP	Superscaf-	Superscaf	78589567	78589584	-41	41	46	-	-	gene	-	-	mRNA	NCP	Intron	gene|mRN	ID=gene-	6	7.51	2588.9	0.002090912	YKPRPP	97	379.2191	2	756.4282	.	I	Superscaf	78589608	BTP202209	0	0	0	0	0	Tissue specific
KAMGKPK	Superscaf+	Superscaf	78603796	78603816	49	49	55	-	-	gene	-	-	mRNA	NCP	Intron	gene|mRN	ID=gene-	7	7.608571429	2722.7	0.002272345	KAM(+15.9	54	388.224	2	774.4422	Oxidation	R	Superscaf	78603652	0	0	0	0	BTP202209	0	Tissue specific
KVEVPK	Superscaf+	Superscaf	78605770	78605787	2	2	7	-	-	gene	-	-	mRNA	NCP	Intron	gene|mRN	ID=gene-	6	4.833333333	1699.7	0.002297516	KVEVPK	99	350.2222	2	698.4326	.	G	Superscaf	78605767	BTP202209	0	0	0	0	0	Tissue specific
LDFPP	Superscaf-	Superscaf	78625420	78625434	-25	25	29	-	-	-	-	-	-	NCP	IGR	-	ID=gene-	5	5.406	1827.1	0.002548071	LDFPP	85	588.3003	1	587.2955	.	M	Superscaf	78625659	BTP202209	0	0	0	0	0	Tissue specific
ASPPVSTPV	Superscaf-	Superscaf	78688801	78688839	-7	7	19	-	-	-	-	-	-	NCP	IGR	-	ID=gene-	13	5.587692308	4270.7	0.003356239	ASPPVSTPV	91	639.3434	2	1276.678	.	G	Superscaf	78689490	BTP202209	0	0	0	0	0	Tissue specific
SPPVSTPVT	Superscaf-	Superscaf	78688804	78688839	-8	8	19	-	-	-	-	-	-	NCP	IGR	-	ID=gene-	12	5.551666667	3959.4	0.003356277	SPPVSTPVT	97	402.8862	3	1205.64	.	G	Superscaf	78689490	0	BTP202209	0	0	0	0	Mixed
PPVSTPVT(	Superscaf-	Superscaf	78688807	78688842	-9	9	20	-	-	-	-	-	-	NCP	IGR	-	ID=gene-	12	5.975	4224.8	0.003356315	PPVSTPVT(	87	425.9088	3	1274.71	.	G	Superscaf	78689490	0	0	0	0	BTP202209	0	Tissue specific
PPVSTPVT(	Superscaf-	Superscaf	78688807	78688839	-9	9	19	-	-	-	-	-	-	NCP	IGR	-	ID=gene-	11	5.54	3576	0.003356315	PPVSTPVT(	93	560.3096	2	1118.608	.	G	Superscaf	78689490	0	0	BTP202209	0	0	0	Tissue specific
VSTPVTGHI	Superscaf-	Superscaf	78688813	78688839	-11	11	19	-	-	-	-	-	-	NCP	IGR	-	ID=gene-	9	5.371111111	2901.2	0.003356392	VSTPVTGHI	97	463.2572	2	924.5029	.	G	Superscaf	78689490	0	0	0	0	BTP202209	0	Mixed
PVPMSGQGI	Superscaf-	Superscaf	78689122	78689163	-114	114	127	-	-	-	-	-	-	NCP	IGR	-	ID=gene-	14	5.963571429	5076.9	0.003360332	PVPMSGQGI	94	704.3529	2	1406.698	.	G	Superscaf	78689490	0	0	BTP202209	0	0	0	Tissue specific
NQGRYYEPY	Superscaf-	Superscaf	78689233	78689259	-151	151	159	-	-	-	-	-	-	NCP	IGR	-	ID=gene-	9	6.032222222	4294.9	0.003361747	NQGRYYEPY	53	595.2672	2	1188.52	.	G	Superscaf	78689490	0	0	0	0	BTP202209	0	Tissue specific
CSVGPGYG(	Superscaf-	Superscaf	78689260	78689289	-160	160	169	-	-	-	-	-	-	NCP	IGR	-	ID=gene-	10	5.628	3423.6	0.003362092	C(+57.02)	93	491.2249	2	980.4385	Carbamido	G	Superscaf	78689490	0	BTP202209	0	0	0	0	Tissue specific
SVGPGYGGK	Superscaf-	Superscaf	78689263	78689289	-161	161	169	-	-	-	-	-	-	NCP	IGR	-	ID=gene-	9	5.695555556	2837.9	0.00336213	SVGPGYGGK	90	411.2094	2	820.4079	.	G	Superscaf	78689490	0	0	0	0	0	BTP202209	Tissue specific
VGPGYGGKN	Superscaf-	Superscaf	78689266	78689292	-162	162	170	-	-	-	-	-	-	NCP	IGR	-	ID=gene-	9	5.665555556	3051.2	0.003362168	VGPGYGGKN	98	424.7151	2	847.4188	.	G	Superscaf	78689490	0	0	0	0	BTP202209	0	Tissue specific
VGPGYGGK	Superscaf-	Superscaf	78689266	78689289	-162	162	169	-	-	-	-	-	-	NCP	IGR	-	ID=gene-	8	5.6975	2454.5	0.003362168	VGPGYGGK	99	367.6934	2	733.3759	.	G	Superscaf	78689490	0	0	0	0	BTP202209	0	Tissue specific
DSDPSYGQG	Superscaf-	Superscaf	78689293	78689340	-171	171	186	-	-	-	-	-	-	NCP	IGR	-	ID=gene-	16	5.8725	7149	0.003362512	DSDPSYGQG	93	908.8928	2	1815.781	.	G	Superscaf	78689490	0	BTP202209	0	0	0	0	Tissue specific
DSDPSYGQG	Superscaf-	Superscaf	78689293	78689328	-171	171	182	-	-	-	-	-	-	NCP	IGR	-	ID=gene-	12	5.5325	5159.8	0.003362512	DSDPSYGQG	80	644.2632	2	1286.516	.	G	Superscaf	78689490	0	0	0	0	0	BTP202209	Tissue specific
SDPSYGQG(	Superscaf-	Superscaf	78689296	78689340	-172	172	186	-	-	-	-	-	-	NCP	IGR	-	ID=gene-	15	6.066	6737.5	0.003362551	SDPSYGQG(	94	851.3793	2	1700.754	.	G	Superscaf	78689490	0	0	0	0	0	BTP202209	Tissue specific
PAGMYPVA(	Superscaf-	Superscaf	78689383	78689415	-201	201	211	-	-	-	-	-	-	NCP	IGR	-	ID=gene-	11	5.820909091	3528.8	0.00336366	PAGMYPVA(	97	544.2827	2	1086.553	.	G	Superscaf	78689490	0	BTP202209	0	0	0	0	Tissue specific
CCGPP	Superscaf-	Superscaf	78694261	78694275	-25	25	29	-	-	-	-	-	-	NCP	IGR	-	ID=gene-	5	5.722	2143.5	0.003425859	C(+57.02)	69	590.1967	1	589.1989	Carbamido	T	Superscaf	78694362	0	0	0	0	BTP202209	0	Tissue specific
DVDVSLPK	Superscaf-	Superscaf	78730261	78730284	-8	8	15	-	-	-	-	-	-	NCP	IGR	-	ID=gene-	8	4.205	2365.6	0.003884893	DVDVSLPK	98	872.4717	1	871.4651	.	L	Superscaf	78730284	0	BTP202209	0	0	BTP202209	BTP202209	Mixed
PNLKNRDF	Superscaf+	Superscaf	78742918	78742941	12	12	19	-	-	-	-	-	-	NCP	IGR	-	ID=gene-	8	6.50625	3800.3	0.004046282	PNLKNRDF	90	502.2679	2	1002.525	.	V	Superscaf	78742885	0	0	0	0	0	BTP202209	Tissue specific
MNKLVFY	Superscaf+	Superscaf	78753205	78753225	1	1	7	-	-	-	-	-	-	NCP	IGR	-	ID=gene-	7	5.431428571	2636.9	0.00417745	MNKLVFY	53	457.7362	2	913.4731	.	M	Superscaf	78753205	0	0	0	0	0	BTP202209	Tissue specific
FRPEDELEI	Superscaf-	Superscaf	78786235	78786270	-55	55	66	-	-	-	-	-	-	NCP	IGR	-	ID=gene-	12	5.915833333	5254	0.004598614	FRPEDELEI	80	505.2581	3	1512.757	.	S	Superscaf	78786369	0	0	0	0	0	BTP202209	Tissue specific
EDELEHLTK	Superscaf-	Superscaf	78786244	78786270	-58	58	66	-	-	-	-	-	-	NCP	IGR	-	ID=gene-	9	5.383333333	3880.4	0.004598729	EDELEHLTK	99	557.2737	2	1112.535	.	S	Superscaf	78786369	0	BTP202209	BTP202209	0	0	0	Mixed
EDELEHLT	Superscaf-	Superscaf	78786244	78786267	-58	58	65	-	-	-	-	-	-	NCP	IGR	-	ID=gene-	8	4.83875	3412	0.004598729	EDELEHLT	87	493.2275	2	984.44	.	S	Superscaf	78786369	0	BTP202209	0	0	0	0	Tissue specific
DELEHLTK	Superscaf-	Superscaf	78786247	78786270	-59	59	66	-	-	-	-	-	-	NCP	IGR	-	ID=gene-	8	5.65375	3454.9	0.004598767	DELEHLTK	99	492.7519	2	983.4924	.	S	Superscaf	78786369	0	BTP202209	0	0	0	0	Tissue specific
KGGSER	Superscaf+	Superscaf	78823501	78823518	5	5	10	-	-	-	-	-	-	NCP	IGR	-	ID=gene-	6	6.89	2520.7	0.005073791	KGGSER	57	633.335	1	632.3242	.	I	Superscaf	78823489	0	BTP202209	0	0	0	0	Tissue specific
QEAYLLK	Superscaf-	Superscaf	78850411	78850431	-7	7	13	-	-	-	-	-	-	NCP	IGR	-	ID=gene-	7	6.035714286	2982.2	0.005416918	QEAYLLK	64	432.7454	2	863.4752	.	W	Superscaf	78850524	0	BTP202209	0	0	BTP202209	0	Tissue specific
NAVFP	Superscaf-	Superscaf	78880291	78880305	-1	1	5	-	-	-	-	-	-	NCP	IGR	-	ID=gene-	5	4.642	1632.8	0.005797917	N(+42.01)	77	589.2975	1	588.2907	Acetylati	N	Superscaf	78880356	0	0	0	0	BTP202209	0	Mixed
PPFKN	Superscaf+	Superscaf	78907477	78907491	4	4	8	-	-	-	-	-	-	NCP	IGR	-	ID=gene-	5	6.646	2127.3	0.006144564	P(+42.01)	65	644.3383	1	643.3329	Acetylati	V	Superscaf	78907468	0	0	BTP202209	0	BTP202209	0	Tissue specific
SLFTVLAT	Superscaf-	Superscaf	78982612	78982635	-9	9	16	-	-	-	-	-	-	NCP	IGR	-	ID=gene-	8	5.275	2583.9	0.007102606	SLFTVLAT	53	851.4832	1	850.48	.	S	Superscaf	78982635	0	0	BTP202209	0	0	0	Tissue specific
KLPLR	Superscaf+	Superscaf	78998863	78998877	4	4	8	-	-	-	-	-	-	NCP	IGR	-	ID=gene-	5	7.752	2161.4	0.007309821	KLPLR	61	626.4224	1	625.4275	.	L	Superscaf	78998854	0	0	0	0	BTP202209	0	Tissue specific
KNKEC	Superscaf-	Superscaf	79245649	79245663	-18	18	22	-	-	-	-	-	-	NCP	IGR	-	ID=gene-	5	6.626	2544.7	0.010456575	KNKEC(+57	58	678.3258	1	677.3167	Carbamido	C	Superscaf	79245669	0	0	0	0	BTP202209	0	Mixed
EAYLV	Superscaf-	Superscaf	79364713	79364727	-1	1	5	-	-	-	-	-	-	NCP	IGR	-	ID=gene-	5	4.176	1549.7	0.011974753	EAYLV	63	594.3118	1	593.306	.	E	Superscaf	79364757	0	0	0	0	BTP202209	0	Tissue specific
KEHAAPPN	Superscaf+	Superscaf	79419145	79419168	27	27	34	-	-	-	-	-	-	NCP	IGR	-	ID=gene-	8	6.325	3407.7	0.012668812	KEHAAPPN	52	863.4529	1	862.4297	.	S	Superscaf	79419067	0	0	0	0	BTP202209	0	Tissue specific
EKTKCPV	Superscaf-	Superscaf	79431217	79431237	-2	2	8	-	-	-	-	-	-	NCP	IGR	-	ID=gene-	7	5.792857143	2682.9	0.012822741	E(+42.01)	59	452.2332	2	902.4531	Acetylati	G	Superscaf	79431240	0	0	0	0	0	BTP202209	Tissue specific
SVTLF	Superscaf+	Superscaf	79455928	79455942	8	8	12	-	-	gene	-	-	mRNA	NCP	Intron	gene|mRN	ID=gene-	5	4.734	1521.7	0.01313783	SVTLF	94	566.3165	1	565.3112	.	C	Superscaf	79455907	0	BTP202209	0	0	0	0	Tissue specific
LPTVEC	Superscaf+	Superscaf	79467601	79467618	18	18	23	-	-	gene	-	-	mRNA	NCP	Intron	gene|mRN	ID=gene-	6	4.508333333	2099.5	0.013286672	LPTVEC(+5	54	718.3304	1	717.3367	Carbamido	E	Superscaf	79467550	0	0	0	0	BTP202209	0	Tissue specific
QLLLEYL	Superscaf+	Superscaf	79536520	79536540	4	4	10	-	-	-	-	-	-	NCP	IGR	-	ID=gene-	7	5.492857143	2909.3	0.014165454	QLLLEYL	50	891.515	1	890.5113	.	Y	Superscaf	79536511	0	BTP202209	0	0	BTP202209	BTP202209	Tissue specific
PLFGSPP	Superscaf-	Superscaf	79588594	79588614	-33	33	39	-	-	-	-	-	-	NCP	IGR	-	ID=gene-	7	6.001428571	2433.7	0.014829446	PLFGSPP	53	357.6935	2	713.3748	.	V	Superscaf	79588773	BTP202209	0	0	0	0	0	Tissue specific
LLAKVPGQ	Superscaf-	Superscaf	79609237	79609260	-3	3	10	-	-	-	-	-	-	NCP	IGR	-	ID=gene-	8	5.705	2731.9	0.015092664	LLAKVPGQ	70	413.2645	2	824.512	.	S	Superscaf	79609281	0	0	0	0	0	BTP202209	Tissue specific
QQAKGGKP	Superscaf+	Superscaf	79682260	79682283	11	11	18	-	-	gene	-	-	mRNA	NCP	Intron	gene|mRN	ID=gene-	8	6.88	3401.5	0.016023776	QQAKGGKP	61	407.2357	2	812.4504	.	K	Superscaf	79682230	0	0	0	0	BTP202209	0	Tissue specific
SDLPFEL	Superscaf-	Superscaf	79853944	79853964	-3	3	9	-	-	-	-	-	-	NCP	IGR	-	ID=gene-	7	5.087142857	2652	0.018212908	SDLPFEL	84	820.4065	1	819.4014	.	K	Superscaf	79853964	0	BTP202209	0	0	0	0	Tissue specific
CCMVN	Superscaf-	Superscaf	79900918	79900932	-9	9	13	-	-	gene	-	-	mRNA	NCP	Intron	gene|mRN	ID=gene-	5	4.24	2139.6	0.018811871	C(+57.02)	64	363.1312	2	724.2343	Carbamido	C	Superscaf	79901109	0	0	0	0	0	BTP202209	Tissue specific
QDDAAPR	Superscaf-	Superscaf	79997092	79997112	-30	30	36	-	-	-	-	-	-	NCP	IGR	-	ID=gene-	7	5.812857143	3042.5	0.02003818	QDDAAPR	77	386.681	2	771.3511	.	A	Superscaf	79997145	0	0	0	0	0	BTP202209	Tissue specific
MLGPL	Superscaf-	Superscaf	80098999	80099013	-1	1	5	-	-	gene	-	-	mRNA	NCP	Intron	gene|mRN	ID=gene-	5	5.996	1713	0.02133759	MLGPL	70	530.2937	1	529.2934	.	M	Superscaf	80099034	0	0	0	0	0	BTP202209	Tissue specific
RAKPAVP	Superscaf+	Superscaf	80172946	80172966	34	34	40	-	-	-	-	-	-	NCP	IGR	-	ID=gene-	7	6.448571429	2414.6	0.022280484	RAKPAVP	54	369.7285	2	737.4548	.	E	Superscaf	80172847	0	0	0	0	BTP202209	0	Tissue specific
APDFA	Superscaf-	Superscaf	80196952	80196966	-13	13	17	-	-	-	-	-	-	NCP	IGR	-	ID=gene-	5	5.358	1758.9	0.022586583	A(+42.01)	63	562.2531	1	561.2435	Acetylati	H	Superscaf	80196987	0	BTP202209	0	0	BTP202209	BTP202209	Tissue specific
LEAFLF	Superscaf-	Superscaf	80271430	80271447	-1	1	6	-	-	-	-	-	-	NCP	IGR	-	ID=gene-	6	5.36	2218.4	0.023536247	LEAFLF	57	739.3997	1	738.3952	.	L	Superscaf	80271477	BTP202209	BTP202209	0	0	0	0	Tissue specific
HKLLGH	Superscaf+	Superscaf	80349166	80349183	20	20	25	-	-	-	-	-	-	NCP	IGR	-	ID=gene-	6	7.141666667	2711.9	0.024527455	HKLLGH	59	704.4286	1	703.4129	.	C	Superscaf	80349109	0	BTP202209	0	0	0	BTP202209	Tissue specific
MDDGH	Superscaf+	Superscaf	80470333	80470347	13	13	17	-	-	gene	-	-	mRNA	NCP	Intron	gene|mRN	ID=gene-	5	5.05	2111.5	0.026072448	M(+15.99)	70	590.1962	1	589.1802	Oxidation	Y	Superscaf	80470297	0	BTP202209	0	0	0	0	Tissue specific
CDMMDF	Superscaf-	Superscaf	80486119	80486136	-10	10	15	-	-	gene	-	-	mRNA	NCP	Intron	gene|mRN	ID=gene-	6	4.656666667	2539.1	0.026273734	C(+57.02)	52	446.6315	2	891.2449	Carbamido	V	Superscaf	80486169	0	BTP202209	0	0	0	0	Tissue specific
KELTTPK	Superscaf+	Superscaf	80521939	80521959	18	18	24	-	-	gene	-	-	mRNA	NCP	Intron	gene|mRN	ID=gene-	7	6.862857143	2848.1	0.026730473	KELTTPK	63	408.744	2	815.4752	.	G	Superscaf	80521888	0	0	0	0	BTP202209	0	Tissue specific
KGVVP	Superscaf+	Superscaf	80619421	80619435	39	39	43	-	-	gene	-	-	mRNA	NCP	Intron	gene|mRN	ID=gene-	5	4.402	1103.1	0.02797346	KGVVP	81	499.322	1	498.3166	.	V	Superscaf	80619307	0	0	0	0	BTP202209	0	Tissue specific
KNYKYC	Superscaf+	Superscaf	80852422	80852439	3	3	8	-	-	gene	-	-	mRNA	NCP	Intron	gene|mRN	ID=gene-	6	6.871666667	3038.2	0.030944442	KNYKYC(+5	51	438.1994	2	874.4007	Carbamido	H	Superscaf	80852416	BTP202209	0	0	0	0	0	Tissue specific
NSPLGF	Superscaf+	Superscaf	80901277	80901294	11	11	16	-	-	gene	-	-	mRNA	NCP	Intron	gene|mRN	ID=gene-	6	5.803333333	2355.6	0.031567389	NSPLGF	86	634.3189	1	633.3122	.	Y	Superscaf	80901247	0	0	0	0	0	BTP202209	Mixed
LEPLVPVK	Superscaf+	Superscaf	80937244	80937267	68	68	75	-	-	gene	-	-	mRNA	NCP	Intron	gene|mRN	ID=gene-	8	4.69	2275.5	0.032026002	LEPLVPVK	86	447.7849	2	893.5585	.	L	Superscaf	80937043	0	BTP202209	0	0	0	0	Tissue specific
KHHRAWP	Superscaf+	Superscaf	81010213	81010233	4	4	10	-	-	-	-	-	-	NCP	IGR	-	ID=gene-	7	7.698571429	3531.9	0.032956426	KHHRAWP	59	466.2556	2	930.4937	.	N	Superscaf	81010204	0	0	0	0	BTP202209	0	Tissue specific
PPYVP	Superscaf-	Superscaf	81015391	81015405	-6	6	10	-	-	-	-	-	-	NCP	IGR	-	ID=gene-	5	4.912	1471.7	0.03302245	PPYVP	67	572.3043	1	571.3006	.	K	Superscaf	81015510	0	BTP202209	0	0	0	0	Tissue specific
ALKERK	Superscaf-	Superscaf	81091957	81091974	-10	10	15	-	-	gene	-	-	mRNA	NCP	Intron	gene|mRN	ID=gene-	6	7.576666667	2675.8	0.033998739	ALKERK	65	372.7388	2	743.4653	.	T	Superscaf	81092001	0	0	0	0	0	BTP202209	Tissue specific
AKFAH	Superscaf-	Superscaf	81109816	81109830	-20	20	24	-	-	gene	-	-	mRNA	NCP	Intron	gene|mRN	ID=gene-	5	6.97	2098.1	0.034226458	A(+42.01)	59	615.322	1	614.3176	Acetylati	L	Superscaf	81109905	0	0	BTP202209	0	0	0	Tissue specific
AAAALY	Superscaf-	Superscaf	81135472	81135489	-5	5	10	-	-	gene	-	-	mRNA	NCP	Intron	gene|mRN	ID=gene-	6	5.953333333	2058.1	0.034553596	A(+42.01)	75	621.3303	1	620.317	Acetylati	S	Superscaf	81135840	0	BTP202209	0	0	0	0	Tissue specific
AAPPSLP	Superscaf+	Superscaf	81192703	81192723	8	8	14	-	-	-	-	-	-	NCP	IGR	-	ID=gene-	7	6.085714286	2371.6	0.035283345	A(+42.01)	54	694.3749	1	693.3697	Acetylati	V	Superscaf	81192682	0	BTP202209	0	0	0	0	Tissue specific

MLCLVCKK	Superscaf	+	Superscaf	81200305	81200328	29	29	36	-	-	-	-	-	-	NCP	IGR	-	ID=gene-E	8	5.90375	3186.5	0.035380278	M(+42.01)	91	547.277	2	1092.549	Acetylati	H	Superscaf	81200221	0	0	BTP202209	0	0	0	Tissue specific
LPPVSGPG	Superscaf	+	Superscaf	81299749	81299772	110	110	117	-	-	-	-	-	-	NCP	IGR	-	ID=gene-E	8	5.3125	2343.6	0.036648282	LPPVSGPG	52	723.4121	1	722.3962	.	E	Superscaf	81299422	0	0	0	0	BTP202209	0	Tissue specific
AMLKRQP	Superscaf	+	Superscaf	81406360	81406380	5	5	11	-	-	-	-	-	-	NCP	IGR	-	ID=gene-E	7	7.171428571	3101.5	0.038007673	A(+42.01)	52	451.2425	2	900.4851	Acetylati	I	Superscaf	81406348	0	BTP202209	BTP202209	0	BTP202209	0	Tissue specific
GSLSYDTLF	Superscaf	-	Superscaf	81444271	81444297	-18	18	26	-	-	gene	-	-	mRNA	NCP	Intron	gene|mRNA	ID=gene-E	9	5.638888889	3376.8	0.038491074	GSLSYDTLF	56	952.4697	1	951.4549	.	P	Superscaf	81444348	0	BTP202209	0	BTP202209	0	0	Tissue specific
ELTALAPS	Superscaf	+	Superscaf	81539386	81539409	8	8	15	-	-	-	-	-	-	NCP	IGR	-	ID=gene-E	8	5.71625	2873.2	0.039703879	ELTALAPS	72	801.4308	1	800.428	.	T	Superscaf	81539365	0	0	0	0	0	BTP202209	Tissue specific
KPAAYK	Superscaf	-	Superscaf	81579235	81579252	-28	28	33	-	-	gene	-	-	mRNA	NCP	Intron	gene|mRNA	ID=gene-E	6	7.246666667	2356.3	0.040211992	K(+42.01)	77	360.2069	2	718.4014	Acetylati	G	Superscaf	81579267	0	0	0	BTP202209	BTP202209	0	Tissue specific
LLPGLF	Superscaf	+	Superscaf	81792394	81792411	58	58	63	-	-	gene	-	-	mRNA	NCP	Intron	gene|mRNA	ID=gene-E	6	5.948333333	2082.3	0.042929969	LLPGLF	99	659.41	1	658.4053	.	N	Superscaf	81792223	0	0	0	BTP202209	0	0	Tissue specific
SDGVLK	Superscaf	+	Superscaf	81802642	81802659	8	8	13	-	-	gene	-	-	mRNA	NCP	Intron	gene|mRNA	ID=gene-E	6	5.056666667	1914	0.043060641	SDGVLK	98	618.3434	1	617.3384	.	K	Superscaf	81802621	0	0	0	BTP202209	0	0	Tissue specific
KYLVPPP	Superscaf	+	Superscaf	81861673	81861693	1	1	7	-	-	-	-	-	-	NCP	IGR	-	ID=gene-E	7	5.754285714	2293.5	0.043813342	KYLVPPP	77	407.2447	2	812.4796	.	K	Superscaf	81861673	0	0	0	0	0	BTP202209	Mixed
PPVPY	Superscaf	+	Superscaf	81962878	81962892	21	21	25	-	-	-	-	-	-	NCP	IGR	-	ID=gene-E	5	4.912	1471.7	0.045103801	PPVPY	81	572.3058	1	571.3006	.	R	Superscaf	81962818	0	0	0	BTP202209	0	0	Mixed
RLPPPPP	Superscaf	+	Superscaf	81975661	81975681	31	31	37	-	-	-	-	-	-	NCP	IGR	-	ID=gene-E	7	6.891428571	2689.2	0.045266796	RLPPPPP	79	773.4525	1	772.4595	.	Y	Superscaf	81975571	0	0	0	0	0	BTP202209	Tissue specific
TLPGATRP	Superscaf	+	Superscaf	81991840	81991863	42	42	49	-	-	-	-	-	-	NCP	IGR	-	ID=gene-E	8	6.79875	3080.6	0.045473093	T(+42.01)	69	427.7321	2	853.4658	Acetylati	C	Superscaf	81991717	0	0	0	0	BTP202209	0	Tissue specific
LPVPPPPP	Superscaf	+	Superscaf	82036261	82036284	16	16	23	-	-	-	-	-	-	NCP	IGR	-	ID=gene-E	8	5.4725	2377.8	0.046039503	LPVPPPPP	72	407.2436	2	812.4796	.	G	Superscaf	82036216	0	0	0	0	BTP202209	0	Tissue specific
ESHLPGSPF	Superscaf	-	Superscaf	82049938	82049964	-6	6	14	-	-	gene	-	-	mRNA	NCP	Intron	gene|mRNA	ID=gene-E	9	5.8	3524.9	0.046213898	ESHLPGSPF	61	485.7329	2	969.4556	.	M	Superscaf	82049970	0	0	0	0	BTP202209	0	Tissue specific
TPLSAP	Superscaf	+	Superscaf	82108141	82108158	32	32	37	-	-	-	-	-	-	NCP	IGR	-	ID=gene-E	6	6.135	2120.4	0.046956041	TPLSAP	56	585.3226	1	584.317	.	M	Superscaf	82108048	0	BTP202209	0	0	0	0	Tissue specific
RTGSLVLR	Superscaf	+	Superscaf	82135450	82135473	9	9	16	-	-	-	-	-	-	NCP	IGR	-	ID=gene-E	8	6.4575	3082.6	0.047304256	RTGSLVLR	69	451.2818	2	900.5505	.	R	Superscaf	82135426	0	0	BTP202209	0	BTP202209	0	Tissue specific
LFEDPY	Superscaf	-	Superscaf	82160314	82160331	-5	5	10	-	-	-	-	-	-	NCP	IGR	-	ID=gene-E	6	4.935	2374.7	0.047621295	LFEDPY	76	783.3542	1	782.3486	.	P	Superscaf	82160367	0	BTP202209	BTP202209	BTP202209	BTP202209	BTP202209	Tissue specific
KPPAFTK	Superscaf	+	Superscaf	82187509	82187529	3	3	9	-	-	-	-	-	-	NCP	IGR	-	ID=gene-E	7	7.158571429	2707.8	0.047968057	KPPAFTK	63	394.7363	2	787.4592	.	A	Superscaf	82187503	0	0	0	0	BTP202209	0	Tissue specific
DPPPREPP	Superscaf	+	Superscaf	82225429	82225452	34	34	41	-	-	-	-	-	-	NCP	IGR	-	ID=gene-E	8	6.05625	3172.8	0.048451573	DPPPREPP	60	452.7332	2	903.445	.	I	Superscaf	82225330	0	0	0	BTP202209	0	0	Tissue specific
GPVGVP	Superscaf	+	Superscaf	82329553	82329570	6	6	11	-	-	gene	-	-	mRNA	NCP	Intron	gene|mRNA	ID=gene-E	6	4.09	1269.4	0.049779252	GPVGVP	76	525.308	1	524.2958	.	K	Superscaf	82329538	0	BTP202209	0	0	0	0	Tissue specific
TSFKEMK	Superscaf	+	Superscaf	82356475	82356495	15	15	21	-	-	gene	-	-	mRNA	NCP	Intron	gene|mRNA	ID=gene-E	7	6.591428571	2902.1	0.050122533	TSFKEMK	96	435.7231	2	869.4317	.	E	Superscaf	82356433	0	BTP202209	BTP202209	BTP202209	BTP202209	BTP202209	Tissue specific
KMVGDLC	Superscaf	+	Superscaf	82465264	82465284	3	3	9	-	-	gene	-	-	mRNA	NCP	Intron	gene|mRNA	ID=gene-E	7	5.061428571	2487.8	0.051509695	KMVGDLC(+	62	411.6952	2	821.3776	Carbamido	E	Superscaf	82465258	0	BTP202209	BTP202209	BTP202209	BTP202209	BTP202209	Mixed
CLGKP	Superscaf	-	Superscaf	82522288	82522302	-8	8	12	-	-	-	-	-	-	NCP	IGR	-	ID=gene-E	5	6.602	2042.2	0.052236804	C(+57.02)	75	574.3019	1	573.2944	Carbamido	L	Superscaf	82522914	0	BTP202209	0	0	BTP202209	BTP202209	Tissue specific
NSPRMLK	Superscaf	+	Superscaf	82607119	82607139	6	6	12	-	-	gene	-	-	mRNA	NCP	Intron	gene|mRNA	ID=gene-E	7	7.088571429	3159.6	0.05331848	NSPRMLK	99	423.2358	2	844.4589	.	Q	Superscaf	82607104	0	0	0	0	BTP202209	0	Tissue specific
KPAAAK	Superscaf	+	Superscaf	82640992	82641009	43	43	48	-	-	-	-	-	-	NCP	IGR	-	ID=gene-E	6	7.306666667	2208.1	0.053750392	KPAAAK	66	585.3729	1	584.3646	.	S	Superscaf	82640866	0	0	0	0	0	BTP202209	Tissue specific
KGLKFLP	Superscaf	-	Superscaf	82648150	82648170	-3	3	9	-	-	-	-	-	-	NCP	IGR	-	ID=gene-E	7	7.027142857	2665.7	0.053841663	K(+42.01)	65	844.5273	1	843.5218	Acetylati	R	Superscaf	82648188	0	0	0	0	0	BTP202209	Tissue specific
KMLYF	Superscaf	-	Superscaf	82699678	82699692	-4	4	8	-	-	gene	-	-	mRNA	NCP	Intron	gene|mRNA	ID=gene-E	5	6.522	2040.2	0.054498694	KMLYF	57	701.3785	1	700.3618	.	S	Superscaf	82699812	0	0	0	0	0	BTP202209	Tissue specific
TVLKVHA	Superscaf	-	Superscaf	82770715	82770735	-16	16	22	-	-	gene	-	-	mRNA	NCP	Intron	gene|mRNA	ID=gene-E	7	5.122857143	2150.3	0.055404482	T(+42.01)	81	405.2465	2	808.4807	Acetylati	L	Superscaf	82770753	0	0	0	0	0	BTP202209	Tissue specific
ANLVTR	Superscaf	-	Superscaf	82808161	82808178	-8	8	13	-	-	gene	-	-	mRNA	NCP	Intron	gene|mRNA	ID=gene-E	6	5.783333333	2307.7	0.055881954	A(+42.01)	69	358.2072	2	714.4024	Acetylati	R	Superscaf	82808307	0	0	0	0	0	BTP202209	Tissue specific
TGALDLK	Superscaf	+	Superscaf	82918078	82918098	9	9	15	-	-	-	-	-	-	NCP	IGR	-	ID=gene-E	7	6.17	2592.8	0.057283499	T(+42.01)	72	759.4211	1	758.4174	Acetylati	R	Superscaf	82918054	0	0	0	0	0	BTP202209	Tissue specific
KDGLELLL	Superscaf	+	Superscaf	82988200	82988223	3	3	10	-	-	gene	-	-	mRNA	NCP	Intron	gene|mRNA	ID=gene-E	8	5.7275	3016.3	0.058177621	KDGLELLL	62	450.772	2	899.5328	.	S	Superscaf	82988194	BTP202209	0	0	0	0	0	Tissue specific
CTECCC	Superscaf	-	Superscaf	83264083	83264100	-13	13	18	-	-	gene	-	-	mRNA	NCP	Intron	gene|mRNA	ID=gene-E	6	4.971666667	3165.8	0.061695389	C(+57.02)	52	889.2186	1	888.2234	Carbamido	L	Superscaf	83264103	0	0	BTP202209	0	BTP202209	0	Tissue specific
VTPPYLV	Superscaf	+	Superscaf	83349412	83349432	4	4	10	-	-	gene	-	-	mRNA	NCP	Intron	gene|mRNA	ID=gene-E	7	4.395714286	1885.2	0.062783414	VTPPYLV	75	394.733	2	787.4479	.	P	Superscaf	83349403	0	0	0	0	0	BTP202209	Tissue specific
PPLPS	Superscaf	+	Superscaf	83371450	83371464	10	10	14	-	-	gene	-	-	mRNA	NCP	Intron	gene|mRNA	ID=gene-E	5	6.112	1749	0.063064419	PPLPS	77	510.2901	1	509.2849	.	K	Superscaf	83371423	0	0	0	BTP202209	0	0	Tissue specific
LSGNGKLL	Superscaf	+	Superscaf	83409028	83409051	2	2	9	-	-	-	-	-	-	NCP	IGR	-	ID=gene-E	8	6.33875	3103.3	0.063543574	LSGNGKLL	59	401.2489	2	800.4756	.	R	Superscaf	83409025	0	0	0	BTP202209	0	0	Tissue specific
NPPTFLSQ	Superscaf	-	Superscaf	83459659	83459682	-27	27	34	-	-	-	-	-	-	NCP	IGR	-	ID=gene-E	8	5.91625	3403.9	0.064189167	NPPTFLSQ	63	452.2365	2	902.4498	.	E	Superscaf	83459715	0	0	0	BTP202209	0	0	Tissue specific
ALLME	Superscaf	+	Superscaf	83518456	83518470	45	45	49	-	-	-	-	-	-	NCP	IGR	-	ID=gene-E	5	5.39	1815.1	0.064938884	ALLME	54	576.3016	1	575.2989	.	D	Superscaf	83518324	BTP202209	0	0	0	0	0	Tissue specific
EAELGLFG	Superscaf	+	Superscaf	83560288	83560311	2	2	9	-	-	gene	-	-	mRNA	NCP	Intron	gene|mRNA	ID=gene-E	8	5.23	2851.1	0.065472281	EAELGLFG	70	835.4189	1	834.4123	.	R	Superscaf	83560285	0	0	0	0	0	BTP202209	Tissue specific
YNLTELK	Superscaf	-	Superscaf	83565412	83565432	-3	3	9	-	-	gene	-	-	mRNA	NCP	Intron	gene|mRNA	ID=gene-E	7	6.074285714	3054.4	0.065537617	YNLTELK	75	440.7433	2	879.4702	.	E	Superscaf	83565441	0	0	BTP202209	0	0	0	Tissue specific
KLAEC	Superscaf	+	Superscaf	83696854	83696868	2	2	6	-	-	-	-	-	-	NCP	IGR	-	ID=gene-E	5	5.996	2144.3	0.067213626	KLAEC(+57	57	620.3193	1	619.2999	Carbamido	H	Superscaf	83696851	0	0	0	0	0	BTP202209	Mixed
MDGLVP	Superscaf	-	Superscaf	83757787	83757804	-21	21	26	-	-	-	-	-	-	NCP	IGR	-	ID=gene-E	6	4.495	1771.1	0.067990579	M(+42.01)	96	673.3208	1	672.3152	Acetylati	I	Superscaf	83757921	0	BTP202209	0	0	0	0	Mixed
VFEPKL	Superscaf	+	Superscaf	83768032	83768049	31	31	36	-	-	-	-	-	-	NCP	IGR	-	ID=gene-E	6	5.12	1972.1	0.068121212	VFEPKL	79	366.717	2	731.4218	.	K	Superscaf	83767942	0	0	0	0	BTP202209	0	Tissue specific
QPSGLPG	Superscaf	-	Superscaf	83838499	83838519	-100	100	106	-	-	-	-	-	-	NCP	IGR	-	ID=gene-E	7	5.978571429	2616.9	0.069019733	Q(+42.01)	79	697.3495	1	696.3442	Acetylati	G	Superscaf	83838519	0	0	0	0	BTP202209	0	Tissue specific
LSSGYG	Superscaf	+	Superscaf	83871433	83871450	7	7	12	-	-	-	-	-	-	NCP	IGR	-	ID=gene-E	6	5.823333333	2174.3	0.069439672	LSSGYG	63	583.2738	1	582.265	.	E	Superscaf	83871415	0	0	0	0	BTP202209	0	Mixed
LPWGGGKK	Superscaf	-	Superscaf	83917288	83917311	-24	24	31	-	-	-	-	-	-	NCP	IGR	-	ID=gene-E	8	6.945	3046.1	0.070024367	LPWGGGKK	84	421.7554	2	841.481	.	H	Superscaf	83917359	0	0	0	0	0	BTP202209	Tissue specific
LPGGNHH	Superscaf	-	Superscaf	83926681	83926701	-26	26	32	-	-	-	-	-	-	NCP	IGR	-	ID=gene-E	7	6.401428571	3121.5	0.070144136	LPGGNHH	78	731.3585	1	730.3511	.	E	Superscaf	83926704	0	0	0	0	BTP202209	0	Tissue specific
LQKPRLL	Superscaf	+	Superscaf	83965498	83965518	1	1	7	-	-	-	-	-	-	NCP	IGR	-	ID=gene-E	7	7.198571429	3125.5	0.07063909	LQKPRLL	90	434.2896	2	866.5701	.	L	Superscaf	83965498	0	0	0	0	0	BTP202209	Tissue specific
LLSHSL	Superscaf	+	Superscaf	84064897	84064914	15	15	20	-	-	-	-	-	-	NCP	IGR	-	ID=gene-E	6	6.148333333	2446.7	0.07190652	LLSHSL	93	669.3911	1	668.3857	.	E	Superscaf	84064855	0	0	0	0	0	BTP202209	Tissue specific
PAPPPPPPF	Superscaf	-	Superscaf	84100222	84100251	-416	416	425	-	-	-	-	-	-	NCP	IGR	-	ID=gene-E	10	6.272	3347.9	0.072356947	PAPPPPPPF	90	482.2664	2	962.5225	.	L	Superscaf	84101646	0	0	0	0	0	BTP202209	Mixed
PRPPPSPPF	Superscaf	-	Superscaf	84100846	84100878	-664	664	674	-	-	-	-	-	-	NCP	IGR	-	ID=gene-E	11	6.649090909	4068.8	0.072364904	PRPPPSPPF	77	379.2203	3	1134.618	.	C	Superscaf	84102240	0	BTP202209	0	0	0	0	Tissue specific
PPPPRSPP	Superscaf	-	Superscaf	84101329	84101352	-273	273	280	-	-	-	-	-	-	NCP	IGR	-	ID=gene-E	8	6.78	3056.6	0.072371063	PPPPRSPP	80	422.7366	2	843.4603	.	H	Superscaf	84101457	0	BTP202209	0	0	0	0	Tissue specific
LPVGVRPS	Superscaf	-	Superscaf	84226855	84226878	-13	13	20	-	-	-	-	-	-	NCP	IGR	-	ID=gene-E	8	5.12375	2357.7	0.073971637	LPVGVRPS	67	412.7517	2	823.4916	.	C	Superscaf	84226881	0	0	0	0	0	BTP202209	Tissue specific
YETPK	Superscaf	-	Superscaf	84291550	84291564	-10	10	14	-	-	-	-	-	-	NCP	IGR	-	ID=gene-E	5	6.29	2088.3	0.074796559	YETPK	60	637.3123	1	636.3119	.	D	Superscaf	84291654	0	0	0	0	BTP202209	0	Tissue specific
AAHWC	Superscaf	+	Superscaf	84401029	84401043	10	10	14	-	-	-	-	-	-	NCP	IGR	-	ID=gene-E	5	6.108	2354.6	0.07619252	AAHWC(+57	59	644.2619	1	643.2537	Carbamido	I	Superscaf	84401002	0	BTP202209	0	0	0	0	Tissue specific
LPAGGL	Superscaf	-	Superscaf	84404236	84404253	-8	8	13	-	-	-	-	-	-	NCP	IGR	-	ID=gene-E	6	6.036666667	1950.1	0.076233412	LPAGGL	67	527.318	1	526.3115	.	L	Superscaf	84404259	0	0	0	0	BTP202209	0	Tissue specific
VLATAAQ	Superscaf	-	Superscaf	84426073	84426093	-6	6	12	-	-	-	-	-	-	NCP	IGR	-	ID=gene-E	7	5.174285714	2295.5	0.076511854	VLATAAQ	62	673.3746	1	672.3806	.	E	Superscaf	84426156	0	0	0	0	BTP202209	0	Tissue specific
LSTGFKTH	Superscaf	-	Superscaf	84496576	84496599	-3	3	10	-	-	-	-	-	-	NCP	IGR	-	ID=gene-E	8	6.6875	3304.6	0.077410834	LSTGFKTH	58	445.7367	2	889.4658	.	V	Superscaf	84496617	0	0	0	0	BTP202209	0	Tissue specific
KDGNPLPS	Superscaf	-	Superscaf	84517519	84517542	-4	4	11	-	-	-	-	-	-	NCP	IGR	-	ID=gene-E	8	6.04375	3185.5	0.077677877	KDGNPLPS	53	414.2156	2	826.4185	.	S	Superscaf	84517644	BTP202209	0	0	0	0	0	Tissue specific
TCPGF	Superscaf	-	Superscaf	84681844	84681858	-34	34	38	-	-	-	-	-	-	NCP	IGR	-	ID=gene-E	5	5.86	2005.3	0.079773175	TC(+57.02	59	581.2379	1	580.2315	Carbamido	D	Superscaf	84682032	0	0	0	BTP202209	0	0	Tissue specific
LPPGPPGP	Superscaf	-	Superscaf	84725362	84725385	-23	23	30	-	-	-	-	-	-	NCP	IGR	-	ID=gene-E	8	6.1775	2635	0.08032807	LPPGPPGP	76	731.4013	1	730.4013	.	H	Superscaf	84725394	BTP202209	0	0	0	0	0	Tissue specific
AASSLGPK	Superscaf	+	Superscaf	84736918	84736941	112	112	119	-	-	-	-	-	-	NCP	IGR	-	ID=gene-E	8	6.42375	2845.9	0.08047542	AASSLGPK	56	365.7049	2	729.402	.	L	Superscaf	84736585	0	BTP202209	0	BTP202209	BTP202209	0	Tissue specific
VPTGKHF	Superscaf	-	Superscaf	84867820	84867840	-22	22	28	-	-	-	-	-	-	NCP	IGR	-	ID=gene-E	7	5.944285714	2507.7	0.082144544	VPTGKHF	70	393.2189	2	784.4232	.	V	Superscaf	84867867	0	BTP202209	BTP202209	BTP202209	0	0	Tissue specific
DGCEELKK	Superscaf	-	Superscaf	84870199	84870222	-17	17	24	-	-	-	-	-	-	NCP	IGR	-	ID=gene-E	8	5.7325	3435.7	0.082174878	D(+42.01)	73	510.7397	2	1019.459	Acetylati	T	Superscaf	84870225	0	BTP202209	0	BTP202209	BTP202209	BTP202209	Tissue specific
LNKGM	Superscaf	+	Superscaf	84928948	84928962	6	6	10	-	-	-	-	-	-	NCP	IGR	-	ID=gene-E	5	6.57	2087.3	0.082923984	L(+42.01)	57	620.3188	1	619.3	Acetylati	E	Superscaf	84928933	0	0	0	BTP202209	0	0	Tissue specific
MLVCVDGL	Superscaf	+	Superscaf	84939139	84939162	11	11	18	-	-	-	-	-	-	NCP	IGR	-	ID=gene-E	8	3.95875	2372.8	0.083053928	M(+15.99)	71	482.7208	2	963.4406	Oxidation	R	Superscaf	84939109	0	0	0	BTP202209	0	0	Tissue specific
SPNVYN	Superscaf	+	Superscaf	84968623	84968640	43	43	48	-	-	-	-	-	-	NCP	IGR	-	ID=gene-E	6	4.743333333	2373.7	0.083429877	SPNVYN	73	693.3194	1	692.3129	.	N	Superscaf	84968497	0	0	0	0	BTP202209	0	Tissue specific
VTFLQYE	Superscaf	-	Superscaf	84986560	84986580	-4	4	10	-	-	-	-	-	-	NCP	IGR	-	ID=gene-E	7	4.645714286	2634	0.083658591	VTFLQYE	60	899.447	1	898.4436	.	H	Superscaf	84986691	0	0	0	BTP202209	0	0	Tissue specific
LALELGHK	Superscaf	-	Superscaf	85005184	85005207	-4	4	11	-	-	-	-	-	-	NCP	IGR	-	ID=gene-E	8	6.31	3182.4	0.083896064	LALELGHK	59	440.7654	2	879.5178	.	S	Superscaf	85005207	0	0	0	0	BTP202209	0	Tissue specific
VPAHPP	Superscaf	+	Superscaf	85020031	85020048	26	26	31	-	-	-	-	-	-	NCP	IGR	-	ID=gene-E	6	5.418333333	1943.2	0.084085377	VPAHPP	50	617.3308	1	616.3333	.	F	Superscaf	85019956	0	BTP202209	0	0	0	0	Tissue specific
KGGGGAGP	Superscaf	-	Superscaf	85048150	85048173	-39	39	46	-	-	-	-	-	-	NCP	IGR	-	ID=gene-E	8	6.48875	2603.6	0.084443921	K(+42.01)	58	642.3227	1	641.3133	Acetylati	M	Superscaf	85048218	0	0	0	0	BTP202209	0	Tissue specific
EFGTNLK	Superscaf	+	Superscaf	85173043	85173063	7	7	13	-	-	-	-	-	-	NCP	IGR	-	ID=gene-E	7	6.047142857	2926.2	0.086036424	EFGTNLK	92	404.7115	2	807.4127	.	R	Superscaf	85173025	0	0	0	0	BTP202209	0	Tissue specific
PGPPPPP	Superscaf	-	Superscaf	85430113	85430133	-37	37	43	-	-	-	-	-	-	NCP	IGR	-	ID=gene-E	7	6.252857143	2321.7	0.089314309	PGPPPPP	62	658.3634	1	657.3486	.	A	Superscaf	85430178	0	0	0	BTP202209	0	0	Tissue enhanced
TWLLT	Superscaf	+	Superscaf	85675225	85675239	1	1	5	-	-	-	-	-	-	NCP	IGR	-	ID=gene-E	5	6.182	2028.4	0.092439717	TWLLT	63	633.3613	1	632.3533	.	T	Superscaf	85675225	0	0	BTP202209	0	0	0	Tissue specific
APKSRFF	Superscaf	+	Superscaf	85682281	85682301	43	43	49	-	-	-	-	-	-	NCP	IGR	-	ID=gene-E	7	7.065714286	2924.1	0.092529688	APKSRFF	88	426.7397	2	851.4653	.	L	Superscaf	85682155	0	0	0	0	BTP202209	0	Tissue specific
APVMVLE	Superscaf	-	Superscaf	85859593	85859613	-15	15	21	-	-	-	-	-	-	NCP	IGR	-	ID=gene-E	7	3.895714286	1799.1	0.094790583	APVMVLE	81	758.4088	1	757.4044	.	L	Superscaf	85859664	0	BTP202209	BTP202209	BTP202209	BTP202209	BTP202209	Tissue specific
PALTLKSAF	Superscaf	-	Superscaf	85931116	85931142	-74	74	82	-	-	-	-	-	-	NCP	IGR	-	ID=gene-E	9	6.505555556	3253.5	0.095702568	PALTLKSAF	78	449.2775	2	896.5331	.	G	Superscaf	85931370	0	0	0	0	0	BTP202209	Tissue specific
ALLKCGR	Superscaf	-	Superscaf	85932142	85932162	-18	18	24	-	-	-	-	-	-	NCP	IGR	-	ID=gene-E	7	7.067142857	3018.3	0.095715651	ALLKC(+57	79	409.2413	2	816.464	Carbamido	L	Superscaf	85932432	0	BTP202209	0	0	0	0	Tissue specific
EPFSLR	Superscaf	-	Superscaf	86007484	86007501	-61	61	66	-	-	-	-	-	-	NCP	IGR	-	ID=gene-E	6	6.236666667	2535.9	0.096676332	EPFSLR	56	374.7028	2	747.3915	.	R	Superscaf	86007537	0	BTP202209	0	0	0	0	Tissue specific
DTLQFTSF	Superscaf	-	Superscaf	86016955	86016978	-2	2	9	-	-	-	-	-	-	NCP	IGR	-	ID=gene-E	8	5.5375	3328.8	0.096797096	D(+42.01)	53	500.7371	2	999.4549	Acetylati	K	Superscaf	86017041	0	0	0	0	0	BTP202209	Tissue specific
KNGLHPP	Superscaf	-	Superscaf	86154757	86154777	-3	3	9	-	-	-	-	-	-	NCP	IGR	-	ID=gene-E	7	6.755714286	3010.3	0.098554201	KNGLHPP	54	381.7101	2	761.4184	.	N	Superscaf	86154933	0	0	0	0	BTP202209	0	Tissue specific
EKSNGLQ	Superscaf	+	Superscaf	86185231	86185251	7	7	13	-	-	-	-	-	-	NCP	IGR	-	ID=gene-E	7	5.95	3135.4	0.098942774	EKSNGLQ	56	388.1999	2	774.3871	.	S	Superscaf	86185213	0	0	BTP202209	0	0	0	Tissue specific
KAMSVDS	Superscaf	-	Superscaf	86208631	86208651	-29	29	35	-	-	-	-	-	-	NCP	IGR	-	ID=gene-E	7	5.12	2329.5	0.099241146	KAM(+15.9	72	753.348	1	752.3375	Oxidation	N	Superscaf	86208681	0	BTP202209	0	0	0	0	Tissue specific
KSVKVPPKE	Superscaf	-	Superscaf	86386609	86386635	-19	19	27	-	-	-	-	-	-	NCP	IGR	-	ID=gene-E	9	5.635555556	2888.9	0.101510533	KSVKVPPKE	54	506.3168	2	1010.612	.	H	Superscaf	86386641	0	BTP202209	0	0	0	0	Tissue specific
SLKAVQVP	Superscaf	+	Superscaf	86400928	86400951	8	8	15	-	-	-	-	-	-	NCP	IGR	-	ID=gene-E	8	4.92125	2464.6	0.101693113	SLKAVQVP	70	421.2587	2	840.5069	.	K	Superscaf	86400907	0	0	0	BTP202209	0	0	Tissue specific
FLRSMF	Superscaf	+	Superscaf	86476273	86476290	6	6	11	-	-	-	-	-	-	NCP	IGR	-	ID=gene-E	6	6.521666667	2531.9	0.102653833	FLRSM(+15	55	816.4083	1	815.4	Oxidation	T	Superscaf	86476258	0	0	0	0	0	BTP202209	Tissue specific
TMMCMP	Superscaf	-	Superscaf	86574490	86574507	-24	24	29	-	-	gene	-	-	mRNA	NCP	Intron	gene|mRNA	ID=gene-E	6	5.85	2435.1	0.103906192	T(+42.01)	65	406.6432	2	811.2737	Acetylati	A	Superscaf	86574564	0	0	0	0	BTP202209	0	Tissue specific
GVVAVGPP	Superscaf	-	Superscaf	86655745	86655768	-13	13	20	-	-	gene	-	-	mRNA	NCP	Intron	gene|mRNA	ID=gene-E	8	3.82	1580.7	0.10494227	G(+42.01)	50	369.2115	2	736.4119	Acetylati	L	Superscaf	86655792	0	0	0	BTP202209	0	0	Tissue specific
KEKGPGR	Superscaf	+	Superscaf	86746462	86746482	5	5	11	-	exon	gene	three_pri	-	mRNA	NCP	3UTR	exon|gene	ID=exon-E	7	7.385714286	2943.1	0.106098997	KEKGPGR	63	386.2224	2	770.4398	.	P	Superscaf	86746450	0	0	0	BTP202209	0	0	Tissue specific
KELTPPK	Superscaf	+	Superscaf	86754565	86754585	8	8	14	-	-	gene	-	-	mRNA	NCP	Intron	gene|mRNA	ID=gene-E	7	6.83	2788	0.106202318	KELTPPK	51	406.7513	2	811.4803	.	R	Superscaf	86754544	0	BTP202209	0	BTP202209	0	0	Tissue specific
AAPLLP	Superscaf	-	Superscaf	86803999	86804016	-81	81	86	-	-	gene	-	-	mRNA	NCP	Intron	gene|mRNA	ID=gene-E	6	6.1	2004.2	0.106832648	A(+42.01)	77	623.3748	1	622.369	Acetylati	E	Superscaf	86804406	0	0	BTP202209	0	0	0	Mixed
MHDLRL	Superscaf	+	Superscaf	86843191	86843208	34	34	39	-	-	gene	-	-	mRNA	NCP	Intron	gene|mRNA	ID=gene-E	6	6.505	2758.3	0.107332383	M(+15.99)	72	400.7105	2	799.4011	Oxidation	Q	Superscaf	86843092	0	BTP202209	0	0	BTP202209	BTP202209	Tissue specific
FKTEDF	Superscaf	-	Superscaf	87039856	87039873	-5	5	10	-	-	-	-	-	-	NCP	IGR	-	ID=gene-E	6	5.57	2477.7	0.109840046	FKTEDF	54	786.3616	1	785.3596	.	K	Superscaf	87039888	0	0	0	0	0	BTP202209	Tissue specific
LLFVPR	Superscaf	+	Superscaf	87042664	87042681	16	16	21	-	-	-	-	-	-	NCP	IGR	-	ID=gene-E	6	5.75	2080.4	0.109875851	LLFVPR	98	372.7406	2	743.4694	.	F	Superscaf	87042619	0	0	0	0	BTP202209	0	Tissue specific
QALPGVR	Superscaf	-	Superscaf	87051070	87051090	-10	10	16	-	-	-	-	-	-	NCP	IGR	-	ID=gene-E	7	5.811428571	2558.9	0.109983035	QALPGVR	86	370.7239	2	739.434	.	K	Superscaf	87051096	0	0	0	BTP202209	0	0	Mixed
KELVTL	Superscaf	-	Superscaf	87129886	87129903	-19	19	24	-	-	gene	-	-	mRNA	NCP	Intron	gene|mRNA	ID=gene-E	6	5.241666667	1998.2	0.110988014	KELVTL	79	702.4387	1	701.4323	.	S	Superscaf	87129939	0	BTP202209	0	0	0	0	Tissue specific
RPALPP	Superscaf	+	Superscaf	87180397	87180414	12	12	17	-	-	-	-	-	-	NCP	IGR	-	ID=gene-E	6	6.943333333	2325.7	0.111632076	RPALPP	58	650.3929	1	649.3911	.	R	Superscaf	87180364	0	0	0	0	0	BTP202209	Tissue specific
VVSGPV	Superscaf	-	Superscaf	87247051	87247068	-16	16	21	-	-	-	-	-	-	NCP	IGR	-	ID=gene-E	6	2.991666667	1018.1	0.112481978	VVSGPV	92	557.3272	1	556.322	.	F	Superscaf	87247278	0	0	0	0	0	BTP202209	Tissue specific
KEEWNF	Superscaf	-	Superscaf	87261583	87261600	-1	1	6	-	-	gene	-	-	mRNA	NCP	Intron	gene|mRNA	ID=gene-E	6	5.493333333	2830.1	0.112667274	K(+42.01)	78	894.3955	1	893.3919	Acetylati	K	Superscaf	87261699	0	0	0	0	0	BTP202209	Tissue specific
TAVKHEQEY	Superscaf	-	Superscaf	87320056	87320082	-172	172	180	-	-	-	-	-	-	NCP	IGR	-	ID=gene-E	9	5.292222222	3718.1	0.11341286	TAVKHEQEY	94	552.7672	2	1103.525	.	K	Superscaf	87320235	0	BTP202209	0	0	0	0	Tissue specific
LTMHVTK	Superscaf	-	Superscaf	87339961	87339981	-13	13	19	-	-	-	-	-	-	NCP	IGR	-	ID=gene-E	7	6.017142857	2608	0.113666667	LTMHVTK	50	415.2284	2	828.4528	.	Q	Superscaf	87340008	0	BTP202209	0	0	BTP202209	BTP202209	Tissue specific
LFVLY	Superscaf	+	Superscaf	87355534	87355548	9	9	13	-	-	-	-	-	-	NCP	IGR	-	ID=gene-E	5	4.62	1553.7	0.113865238	LFVLY	73	654.3841	1	653.3788	.	A	Superscaf	87355510	0	0	BTP202209	0	0	0	Tissue specific
KPSCCMK	Superscaf	+	Superscaf	87417739	87417759	15	15	21	-	-	gene	-	-	mRNA	NCP	Intron	gene|mRNA	ID=gene-E	7	6.75	3200.5	0.11465841	KPSC(+57.	64	455.6962	2	909.3871	Carbamido	E	Superscaf	87417697	0	0	BTP202209	BTP202209	BTP202209	BTP202209	Tissue specific
LLPFL	Superscaf	-	Superscaf	87426106	87426120	-4	4	8	-	exon	gene	three_pri	-	mRNA	NCP	3UTR	exon|gene	ID=exon-E	5	5.944	1785	0.114765097	LLPFL	69	602.3895	1	601.3839	.	S	Superscaf	87426162	BTP202209	BTP202209	BTP202209	BTP202209	BTP202209	BTP202209	Tissue specific
AALSYL	Superscaf	-	Superscaf	87464515	87464532	-25	25	30	-	-	-	-	-	-	NCP	IGR	-	ID=gene-E	6	5.89	2172.3	0.115254848	AALSYL	75	637.3529	1	636.3483	.	A	Superscaf	87464571	0	0	BTP202209	0	0	0	Tissue specific
SLKRGFPF	Superscaf	+	Superscaf	87553930	87553953	38	38	45	-	-	gene	-	-	mRNA	NCP	Intron	gene|mRNA	ID=gene-E	8	6.92375	3263.5	0.116394973	SLKRGFPF	53	476.2701	2	950.5338	.	Y	Superscaf	87553819	0	0	0	0	0	BTP202209	Tissue specific
MLWGLRL	Superscaf	-	Superscaf	87567100	87567120	-29	29	35	-	-	gene	-	-	mRNA	NCP	Intron	gene|mRNA	ID=gene-E	7	6.615714286	2904.4	0.116562903	MLWGLRL	61	444.7568	2	887.5051	.	F	Superscaf	87567126	0	0	0	0	0	BTP202209	Tissue specific
KSDGEGNS	Superscaf	+	Superscaf	87658657	87658680	4	4	11	-	-	-	-	-	-	NCP	IGR	-	ID=gene-E	8	5.58	3263.5	0.117730341	K(+42.01)	66	835.3566	1	834.3355	Acetylati	N	Superscaf	87658648	0	0	0	0	0	BTP202209	Tissue specific
MPGVP	Superscaf	-	Superscaf	87853054	87853068	-25	25	29	-	-	-	-	-	-	NCP	IGR	-	ID=gene-E	5	4.864	1343.6	0.120209086	MPGVP	71	500.2546	1	499.2465	.	G	Superscaf	87853197	0	BTP202209	0	0	BTP202209	BTP202209	Mixed
PGPGLP	Superscaf	-	Superscaf	87908347	87908364	-122	122	127	-	-	-	-	-	-	NCP	IGR	-	ID=gene-E	6	6.136666667	1960.2	0.120914124	PGPGLP	64	537.301	1	536.2958	.	N	Superscaf	87908403	0	0	0	BTP202209	0	0	Tissue specific
VFLRPF	Superscaf	-	Superscaf	87925708	87925725	-10	10	15	-	-	-	-	-	-	NCP	IGR	-	ID=gene-E	6	5.666666667	2114.4	0.121135493	VFLRPF	96	389.7324	2	777.4537	.	L	Superscaf	87925749	0	0	0	0	0	BTP202209	Tissue specific
DEGLRLR	Superscaf	-	Superscaf	87999007	87999027	-7	7	13	-	-	-	-	-	-	NCP	IGR	-	ID=gene-E	7	6.52	3138.7	0.122070124	DEGLRLR	74	429.7408	2	857.4719	.	A	Superscaf	87999078	BTP202209	0	0	0	0	0	Tissue specific
KELPL	Superscaf	+	Superscaf	88014115	88014129	8	8	12	-	-	-	-	-	-	NCP	IGR	-	ID=gene-E	5	6.244	1938.1	0.122262765	K(+42.01)	75	641.3854	1	640.3796	Acetylati	R	Superscaf	88014094	0	0	BTP202209	0	0	0	Tissue specific
VDMLVPK	Superscaf	+	Superscaf	88066102	88066122	2	2	8	-	-	-	-	-	-	NCP	IGR	-	ID=gene-E	7	4.391428571	1942.2	0.122925648	VDM(+15.9	60	409.2273	2	816.4415	Oxidation	K	Superscaf	88066099	0	0	BTP202209	0	0	0	Tissue specific
SDPLLSK	Superscaf	-	Superscaf	88080061	88080081	-7	7	13	-	-	-	-	-	-	NCP	IGR	-	ID=gene-E	7	6.047142857	2690.9	0.123103639	SDPLLSK	88	380.2155	2	758.4174	.	S	Superscaf	88080081	0	0	0	0	0	BTP202209	Tissue specific
LTKSAWR	Superscaf	+	Superscaf	88107619	88107639	3	3	9	-	-	-	-	-	-	NCP	IGR	-	ID=gene-E	7	7.228571429	3089.4	0.123455029	LTKSAWR	95	431.2496	2	860.4868	.	Q	Superscaf	88107613	0	0	BTP202209	0	0	0	Tissue specific
KHGEYGKK	Superscaf	+	Superscaf	88166497	88166520	2	2	9	-	-	-	-	-	-	NCP	IGR	-	ID=gene-E	8	7.20375	3504.5	0.124205779	K(+42.01)	54	494.7676	2	987.5137	Acetylati	S	Superscaf	88166494	0	0	BTP202209	BTP202209	0	0	Tissue specific
KQETALL	Superscaf	-	Superscaf	88172581	88172601	-3	3	9	-	-	-	-	-	-	NCP	IGR	-	ID=gene-E	7	6.16	2920.2	0.124283356	KQETALL	66	401.7361	2	801.4596	.	I	Superscaf	88172673	0	0	0	0	BTP202209	0	Tissue specific
QYAVLKN	Superscaf	-	Superscaf	88336276	88336296	-1	1	7	-	-	-	-	-	-	NCP	IGR	-	ID=gene-E	7	5.494285714	2800	0.126370621	QYAVLKN	52	418.2365	2	834.4599	.	Q	Superscaf	88336350	0	BTP202209	0	0	0	0	Tissue specific
EWLVFH	Superscaf	-	Superscaf	88339252	88339269	-9	9	14	-	-	-	-	-	-	NCP	IGR	-	ID=gene-E	6	4.693333333	2312.6	0.126408568	EWLVFH	83	830.4176	1	829.4122	.	P	Superscaf	88339380	0	0	0	0	0	BTP202209	Tissue specific
SFDKA	Superscaf	-	Superscaf	88410649	88410663	-24	24	28	-	-	-	-	-	-	NCP	IGR	-	ID=gene-E	5	5.978	1962	0.127318947	SFDKA	68	567.2751	1	566.27	.	K	Superscaf	88410669	BTP202209	0	0	0	0	0	Tissue specific
KWAPLR	Superscaf	-	Superscaf	88488025	88488042	-12	12	17	-	-	-	-	-	-	NCP	IGR	-	ID=gene-E	6	7.448333333	2645.9	0.128305563	KWAPLR	52	385.7406	2	769.4598	.	H	Superscaf	88488111	BTP202209	0	0	0	0	0	Tissue specific
HDFDTPK	Superscaf	-	Superscaf	88511965	88511985	-3	3	9	-	-	-	-	-	-	NCP	IGR	-	ID=gene-E	7	5.94	3033.4	0.128610821	HDFDTPK	63	430.1936	2	858.3871	.	N	Superscaf	88511985	0	0	0	0	0	BTP202209	Tissue specific
QGFLVL	Superscaf	-	Superscaf	88621708	88621725	-13	13	18	-	-	-	-	-	-	NCP	IGR	-	ID=gene-E	6	4.843333333	2002.2	0.130010147	QGFLVL	78	676.4	1	675.3956	.	R	Superscaf	88621743	BTP202209	0	0	0	0	0	Tissue specific
VEAGRP	Superscaf	-	Superscaf	88675243	88675260	-26	26	31	-	-	-	-	-	-	NCP	IGR	-	ID=gene-E	6	5.378333333	2020.3	0.130692769	VEAGRP	61	628.3438	1	627.334	.	H	Superscaf	88675290	BTP202209	0	0	0	0	0	Tissue specific
MDVLF	Superscaf	+	Superscaf	88723348	88723362	15	15	19	-	-	-	-	-	-	NCP	IGR	-	ID=gene-E	5	4.036	1523.8	0.131306153	M(+42.01)	50	666.3141	1	665.3094	Acetylati	I	Superscaf	88723306	0	BTP202209	0	0	0	0	Tissue specific
LPGLK	Superscaf	-	Superscaf	88747015	88747029	-21	21	25	-	-	-	-	-	-	NCP	IGR	-	ID=gene-E	5	6.794	1809.9	0.13160793	LPGLK	99	527.3543	1	526.3478	.	G	Superscaf	88747038	0	BTP202209	0	0	BTP202209	BTP202209	Tissue specific
APAAWP	Superscaf	-	Superscaf	88791328	88791345	-8	8	13	-	-	-	-	-	-	NCP	IGR	-	ID=gene-E	6	6.091666667	2135.3	0.132172962	APAAWP	59	612.312	1	611.3067	.	P	Superscaf	88791471	0	0	0	0	BTP202209	BTP202209	Tissue specific
LRLLGP	Superscaf	-	Superscaf	88819756	88819773	-76	76	81	-	-	-	-	-	-	NCP	IGR	-	ID=gene-E	6	6.828333333	2343.7	0.132535446	LRLLGP	57	668.4429	1	667.438	.	Y	Superscaf	88819992	0	BTP202209	BTP202209	0	0	0	Tissue specific
CCADF	Superscaf	+	Superscaf	88996348	88996362	23	23	27	-	-	-	-	-	-	NCP	IGR	-	ID=gene-E	5	4.902	2281.6	0.13478716	C(+57.02)	51	357.6193	2	713.2149	Carbamido	V	Superscaf	88996282	0	BTP202209	0	0	0	BTP202209	Tissue specific
WDHHGLS	Superscaf	-	Superscaf	89141578	89141598	-3	3	9	-	-	-	-	-	-	NCP	IGR	-	ID=gene-E	7	5.952857143	3211.6	0.136638979	WDHHGLS	92	426.1926	2	850.3722	.	M	Superscaf	89141667	0	0	0	BTP202209	0	0	Tissue specific
GDVKLAAQT	Superscaf	-	Superscaf	89227873	89227911	-11	11	23	-	-	gene	-	-	mRNA	NCP	Intron	gene|mRNA	ID=gene-E	13	5.53	4632.9	0.137739321	G(+42.01)	92	698.3829	2	1394.752	Acetylati	P	Superscaf	89228211	0	0	0	0	BTP202209	0	Tissue specific
LTQKPM	Superscaf	-	Superscaf	89303161	89303178	-13	13	18	-	-	gene	-	-	mRNA	NCP	Intron	gene|mRNA	ID=gene-E	6	6.658333333	2538.9	0.138699314	LTQKPM(+1	68	733.396	1	732.384	Oxidation	K	Superscaf	89303277	0	BTP202209	0	0	BTP202209	0	Tissue specific
LGSVTP	Superscaf	-	Superscaf	89309335	89309352	-6	6	11	-	-	gene	-	-	mRNA	NCP	Intron	gene|mRNA	ID=gene-E	6	5.076666667	1769	0.138778039	L(+42.01)	66	615.3311	1	614.3275	Acetylati	P	Superscaf	89309358	0	0	0	0	BTP202209	0	Tissue specific
ESVPTPK	Superscaf	-	Superscaf	89328766	89328786	-45	45	51	-	-	gene	-	-	mRNA	NCP	Intron	gene|mRNA	ID=gene-E	7	5.395714286	2349.6	0.139025802	ESVPTPK	85	379.2071	2	756.4017	.	E	Superscaf	89328852	0	BTP202209	0	0	BTP202209	BTP202209	Tissue specific
RQVKPKTLS	Superscaf	-	Superscaf	89332378	89332419	-199	199	212	-	-	gene	-	-	mRNA	NCP	Intron	gene|mRNA	ID=gene-E	14	6.467142857	6061.7	0.139071859	RQVKPKTLS	94	432.7486	4	1726.969	.	A	Superscaf	89333646	0	0	0	0	BTP202209	0	Tissue specific
LSEEERKVE	Superscaf	-	Superscaf	89332399	89332437	-206	206	218	-	-	gene	-	-	mRNA	NCP	Intron	gene|mRNA	ID=gene-E	13	5.613846154	5606.3	0.139072126	LSEEERKVE	99	768.8881	2	1535.769	.	A	Superscaf	89333646	0	0	BTP202209	0	0	0	Tissue specific
SEEERKVHG	Superscaf	-	Superscaf	89332402	89332446	-207	207	221	-	-	gene	-	-	mRNA	NCP	Intron	gene|mRNA	ID=gene-E	15	5.03	6051.8	0.139072165	SEEERKVHG	64	892.9276	2	1783.849	.	A	Superscaf	89333646	0	0	0	0	BTP202209	0	Tissue specific
AAEGLKPVG	Superscaf	-	Superscaf	89332606	89332653	-275	275	290	-	-	gene	-	-	mRNA	NCP	Intron	gene|mRNA	ID=gene-E	16	5.608125	5726.1	0.139074766	AAEGLKPVG	86	771.8973	2	1541.784	.	A	Superscaf	89333646	0	BTP202209	0	0	0	0	Tissue enhanced
AAEGLKPVG	Superscaf	-	Superscaf	89332606	89332650	-275	275	289	-	-	gene	-	-	mRNA	NCP	Intron	gene|mRNA	ID=gene-E	15	5.616666667	5338.7	0.139074766	AAEGLKPVG	68	698.3619	2	1394.715	.	A	Superscaf	89333646	0	0	0	0	BTP202209	0	Tissue specific
AAEGLKPVG	Superscaf	-	Superscaf	89332606	89332635	-275	275	284	-	-	gene	-	-	mRNA	NCP	Intron	gene|mRNA	ID=gene-E	10	5.463	3398.6	0.139074766	AAEGLKPVG	97	478.2621	2	954.5134	.	A	Superscaf	89333646	0	0	BTP202209	0	0	0	Mixed
AEGLKPVGN	Superscaf	-	Superscaf	89332609	89332653	-276	276	290	-	-	gene	-	-	mRNA	NCP	Intron	gene|mRNA	ID=gene-E	15	5.580666667	5414.8	0.139074804	AEGLKPVGN	69	736.3778	2	1470.747	.	A	Superscaf	89333646	0	BTP202209	0	0	0	0	Tissue specific
AEGLKPVGN	Superscaf	-	Superscaf	89332609	89332635	-276	276	284	-	-	gene	-	-	mRNA	NCP	Intron	gene|mRNA	ID=gene-E	9	5.401111111	3087.3	0.139074804	AEGLKPVGN	98	442.7431	2	883.4763	.	A	Superscaf	89333646	0	0	BTP202209	0	BTP202209	BTP202209	Tissue specific
EGLKPVGN	Superscaf	-	Superscaf	89332612	89332635	-277	277	284	-	-	gene	-	-	mRNA	NCP	Intron	gene|mRNA	ID=gene-E	8	5.32375	2776	0.139074842	EGLKPVGN	95	407.2253	2	812.4392	.	A	Superscaf	89333646	0	BTP202209	0	0	0	0	Mixed
VGNAKLADT	Superscaf	-	Superscaf	89332663	89332704	-294	294	307	-	-	gene	-	-	mRNA	NCP	Intron	gene|mRNA	ID=gene-E	14	5.902857143	5027.4	0.139075493	VGNAKLADT	92	705.8999	2	1409.788	.	A	Superscaf	89333646	0	BTP202209	0	0	0	BTP202209	Tissue specific
LADTPKPLS	Superscaf	-	Superscaf	89332678	89332704	-299	299	307	-	-	gene	-	-	mRNA	NCP	Intron	gene|mRNA	ID=gene-E	9	6.166666667	3353.7	0.139075684	LADTPKPLS	81	471.2669	2	940.5229	.	A	Superscaf	89333646	0	0	0	BTP202209	0	0	Tissue specific
TPKPLSSTK	Superscaf	-	Superscaf	89332687	89332731	-302	302	316	-	-	gene	-	-	mRNA	NCP	Intron	gene|mRNA	ID=gene-E	15	6.706	5804.3	0.139075799	TPKPLSSTK	96	527.9644	3	1580.877	.	A	Superscaf	89333646	0	BTP202209	BTP202209	BTP202209	0	0	Tissue specific
SSTKPAETF	Superscaf	-	Superscaf	89332702	89332743	-307	307	320	-	-	gene	-	-	mRNA	NCP	Intron	gene|mRNA	ID=gene-E	14	6.385714286	5495	0.13907599	SSTKPAETF	93	713.8777	2	1425.746	.	A	Superscaf	89333646	BTP202209	0	0	0	0	0	Tissue specific
STKPAETPK	Superscaf	-	Superscaf	89332705	89332743	-308	308	320	-	-	gene	-	-	mRNA	NCP	Intron	gene|mRNA	ID=gene-E	13	6.44	5111.6	0.139076028	STKPAETPK	73	670.3622	2	1338.714	.	A	Superscaf	89333646	0	BTP202209	0	0	0	0	Mixed
STKPAETPK	Superscaf	-	Superscaf	89332705	89332740	-308	308	319	-	-	gene	-	-	mRNA	NCP	Intron	gene|mRNA	ID=gene-E	12	6.525833333	4514.9	0.139076028	STKPAETPK	86	613.3417	2	1224.671	.	A	Superscaf	89333646	0	0	0	0	BTP202209	0	Mixed
STKPAETPK	Superscaf	-	Superscaf	89332705	89332734	-308	308	317	-	-	gene	-	-	mRNA	NCP	Intron	gene|mRNA	ID=gene-E	10	6.636	3864.2	0.139076028	STKPAETPK	99	528.2885	2	1054.566	.	A	Superscaf	89333646	0	0	0	0	BTP202209	BTP202209	Mixed
TKPAETPKE	Superscaf	-	Superscaf	89332708	89332743	-309	309	320	-	-	gene	-	-	mRNA	NCP	Intron	gene|mRNA	ID=gene-E	12	6.503333333	4728.2	0.139076066	TKPAETPKE	92	626.8461	2	1251.682	.	A	Superscaf	89333646	0	0	0	0	BTP202209	0	Tissue specific
KPAETPKPL	Superscaf	-	Superscaf	89332711	89332743	-310	310	320	-	-	gene	-	-	mRNA	NCP	Intron	gene|mRNA	ID=gene-E	11	6.500909091	4330.7	0.139076105	KPAETPKPL	98	576.3221	2	1150.635	.	A	Superscaf	89333646	0	BTP202209	0	0	0	0	Tissue specific
KPAETPKPL	Superscaf	-	Superscaf	89332711	89332740	-310	310	319	-	-	gene	-	-	mRNA	NCP	Intron	gene|mRNA	ID=gene-E	10	6.61	3734	0.139076105	KPAETPKPL	86	519.3016	2	1036.592	.	A	Superscaf	89333646	0	BTP202209	0	0	0	0	Mixed
KPAETPKP	Superscaf	-	Superscaf	89332711	89332734	-310	310	317	-	-	gene	-	-	mRNA	NCP	Intron	gene|mRNA	ID=gene-E	8	6.76875	3083.3	0.139076105	KPAETPKP	99	434.2487	2	866.4861	.	A	Superscaf	89333646	0	0	0	BTP202209	0	0	Tissue specific
AETPKPLGN	Superscaf	-	Superscaf	89332717	89332743	-312	312	320	-	-	gene	-	-	mRNA	NCP	Intron	gene|mRNA	ID=gene-E	9	6.163333333	3524.9	0.139076181	AETPKPLGN	87	463.7502	2	925.4869	.	A	Superscaf	89333646	0	0	0	0	0	BTP202209	Mixed
AETPKPLG	Superscaf	-	Superscaf	89332717	89332740	-312	312	319	-	-	gene	-	-	mRNA	NCP	Intron	gene|mRNA	ID=gene-E	8	6.2575	2928.2	0.139076181	AETPKPLG	97	812.4506	1	811.4439	.	A	Superscaf	89333646	0	BTP202209	0	0	0	0	Tissue enhanced
ETPKPLGN	Superscaf	-	Superscaf	89332720	89332743	-313	313	320	-	-	gene	-	-	mRNA	NCP	Intron	gene|mRNA	ID=gene-E	8	6.18125	3213.6	0.139076219	ETPKPLGN	97	428.231	2	854.4498	.	A	Superscaf	89333646	BTP202209	BTP202209	0	0	BTP202209	0	Mixed
VKPAETPKE	Superscaf	-	Superscaf	89332744	89332776	-321	321	331	-	-	gene	-	-	mRNA	NCP	Intron	gene|mRNA	ID=gene-E	11	6.009090909	3734	0.139076525	VKPAETPKE	87	568.8364	2	1135.66	.	A	Superscaf	89333646	0	0	0	0	0	BTP202209	Tissue enhanced
VKPAETPKE	Superscaf	-	Superscaf	89332744	89332770	-321	321	329	-	-	gene	-	-	mRNA	NCP	Intron	gene|mRNA	ID=gene-E	9	6.016666667	3083.3	0.139076525	VKPAETPKE	93	483.7829	2	965.5545	.	A	Superscaf	89333646	BTP202209	0	0	0	0	0	Tissue specific
VKPAETPKE	Superscaf	-	Superscaf	89332852	89332887	-357	357	368	-	-	gene	-	-	mRNA	NCP	Intron	gene|mRNA	ID=gene-E	12	5.959166667	4330.7	0.139077902	VKPAETPKE	94	625.8563	2	1249.703	.	A	Superscaf	89333646	0	0	0	0	BTP202209	0	Tissue specific
KPTETPKPL	Superscaf	-	Superscaf	89332891	89332920	-370	370	379	-	-	gene	-	-	mRNA	NCP	Intron	gene|mRNA	ID=gene-E	10	6.661	3820.2	0.1390784	KPTETPKPL	96	534.3066	2	1066.602	.	A	Superscaf	89333646	0	0	0	0	0	BTP202209	Tissue specific
KPTETPKP	Superscaf	-	Superscaf	89332891	89332914	-370	370	377	-	-	gene	-	-	mRNA	NCP	Intron	gene|mRNA	ID=gene-E	8	6.8325	3169.5	0.1390784	KPTETPKP	93	449.2547	2	896.4967	.	A	Superscaf	89333646	0	BTP202209	0	0	0	0	Tissue specific
TETPKPLG	Superscaf	-	Superscaf	89332897	89332920	-372	372	379	-	-	gene	-	-	mRNA	NCP	Intron	gene|mRNA	ID=gene-E	8	6.32125	3014.4	0.139078476	TETPKPLG	92	842.4585	1	841.4545	.	A	Superscaf	89333646	BTP202209	0	0	0	0	0	Tissue specific
SNVKPAETP	Superscaf	-	Superscaf	89333170	89333199	-463	463	472	-	-	gene	-	-	mRNA	NCP	Intron	gene|mRNA	ID=gene-E	10	5.894	3726	0.139081957	SNVKPAETP	97	535.7938	2	1069.577	.	A	Superscaf	89333646	0	BTP202209	0	0	0	0	Tissue specific
AKPAETSKP	Superscaf	-	Superscaf	89333212	89333247	-477	477	488	-	-	gene	-	-	mRNA	NCP	Intron	gene|mRNA	ID=gene-E	12	6.159166667	4802.2	0.139082493	AKPAETSKP	99	635.8333	2	1269.657	.	A	Superscaf	89333646	0	BTP202209	0	0	0	0	Tissue specific
PAETSKPLF	Superscaf	-	Superscaf	89333218	89333247	-479	479	488	-	-	gene	-	-	mRNA	NCP	Intron	gene|mRNA	ID=gene-E	10	5.815	4022.5	0.139082569	PAETSKPLF	99	536.2676	2	1070.524	.	A	Superscaf	89333646	BTP202209	BTP202209	0	0	0	0	Tissue specific
EAPKPLGN	Superscaf	-	Superscaf	89333260	89333283	-493	493	500	-	-	gene	-	-	mRNA	NCP	Intron	gene|mRNA	ID=gene-E	8	6.1175	3127.4	0.139083105	EAPKPLGN	66	413.226	2	824.4391	.	A	Superscaf	89333646	0	BTP202209	0	0	0	0	Tissue specific
RTVTVNTEG	Superscaf	-	Superscaf	89388721	89388750	-61	61	70	-	-	-	-	-	-	NCP	IGR	-	ID=gene-E	10	5.437	3942.6	0.139790285	RTVTVNTEG	54	392.5496	3	1174.631	.	S	Superscaf	89388789	0	BTP202209	0	0	0	0	Tissue specific
RTVTVNTEG	Superscaf	-	Superscaf	89388721	89388765	-61	61	75	-	-	-	-	-	-	NCP	IGR	-	ID=gene-E	15	4.944	5584.4	0.139790285	RTVTVNTEG	63	893.9486	2	1785.89	.	S	Superscaf	89388789	0	BTP202209	0	0	0	0	Tissue specific
TGSPTSPLN	Superscaf	-	Superscaf	89415070	89415105	-128	128	139	-	-	-	-	-	-	NCP	IGR	-	ID=gene-E	12	6.113333333	4689.2	0.140126259	TGSPTSPLN	83	601.3065	2	1200.599	.	T	Superscaf	89415207	0	BTP202209	0	0	0	0	Tissue specific
TGSPTSPL	Superscaf	-	Superscaf	89415070	89415093	-128	128	135	-	-	-	-	-	-	NCP	IGR	-	ID=gene-E	8	6.12125	2887.3	0.140126259	TGSPTSPL	79	759.3846	1	758.381	.	T	Superscaf	89415207	0	0	BTP202209	0	0	0	Mixed
GSPTSPLN	Superscaf	-	Superscaf	89415073	89415096	-129	129	136	-	-	-	-	-	-	NCP	IGR	-	ID=gene-E	8	5.98125	3086.5	0.140126298	GSPTSPLN	90	772.3827	1	771.3763	.	T	Superscaf	89415207	0	0	0	0	0	BTP202209	Tissue specific
SPTSPLNAE	Superscaf	-	Superscaf	89415076	89415105	-130	130	139	-	-	-	-	-	-	NCP	IGR	-	ID=gene-E	10	6.086	3994.4	0.140126336	SPTSPLNAE	86	522.2706	2	1042.53	.	T	Superscaf	89415207	0	0	0	0	BTP202209	0	Tissue specific
LEAVAEEKE	Superscaf	-	Superscaf	89416606	89416656	-42	42	58	-	-	-	-	-	-	NCP	IGR	-	ID=gene-E	17	5.524117647	6182.5	0.140145845	LEAVAEEKE	96	493.7678	4	1971.046	.	I	Superscaf	89416818	BTP202209	0	0	0	0	0	Tissue specific
EAVAEEKPE	Superscaf	-	Superscaf	89416609	89416656	-43	43	58	-	-	-	-	-	-	NCP	IGR	-	ID=gene-E	16	5.495625	5829.1	0.140145883	EAVAEEKPE	93	929.9835	2	1857.962	.	I	Superscaf	89416818	0	0	0	0	0	BTP202209	Tissue specific
EAVAEEKPE	Superscaf	-	Superscaf	89416609	89416647	-43	43	55	-	-	-	-	-	-	NCP	IGR	-	ID=gene-E	13	5.156153846	4589.9	0.140145883	EAVAEEKPE	94	499.5945	3	1495.767	.	I	Superscaf	89416818	BTP202209	0	0	0	0	0	Mixed
AVAEEKPHV	Superscaf	-	Superscaf	89416612	89416647	-44	44	55	-	-	-	-	-	-	NCP	IGR	-	ID=gene-E	12	5.3175	4164.4	0.140145921	AVAEEKPHV	98	456.5806	3	1366.724	.	I	Superscaf	89416818	0	BTP202209	0	0	0	0	Tissue specific
VAEEKPHVK	Superscaf	-	Superscaf	89416615	89416647	-45	45	55	-	-	-	-	-	-	NCP	IGR	-	ID=gene-E	11	5.253636364	3853.1	0.14014596	VAEEKPHVK	97	432.9014	3	1295.687	.	I	Superscaf	89416818	0	BTP202209	0	0	0	0	Mixed
AEEKPHVKF	Superscaf	-	Superscaf	89416618	89416647	-46	46	55	-	-	-	-	-	-	NCP	IGR	-	ID=gene-E	10	5.779	3853.1	0.140145998	AEEKPHVKF	96	399.8787	3	1196.619	.	I	Superscaf	89416818	0	BTP202209	0	0	0	0	Tissue specific
EEKPHVKPY	Superscaf	-	Superscaf	89416621	89416647	-47	47	55	-	-	-	-	-	-	NCP	IGR	-	ID=gene-E	9	5.752222222	3541.8	0.140146036	EEKPHVKPY	99	563.7965	2	1125.582	.	I	Superscaf	89416818	0	0	0	0	0	BTP202209	Mixed
EKPHVKPY	Superscaf	-	Superscaf	89416624	89416647	-48	48	55	-	-	-	-	-	-	NCP	IGR	-	ID=gene-E	8	6.06875	3116.3	0.140146074	EKPHVKPY	96	499.2675	2	996.5392	.	I	Superscaf	89416818	0	BTP202209	0	0	0	0	Tissue specific
LKELVTNF	Superscaf	+	Superscaf	89454220	89454243	28	28	35	-	-	-	-	-	-	NCP	IGR	-	ID=gene-E	8	5.2925	2982.3	0.140625459	LKELVTNF	62	482.2762	2	962.5436	.	R	Superscaf	89454139	BTP202209	0	0	0	0	0	Tissue specific
VFYFT	Superscaf	+	Superscaf	89455966	89455980	124	124	128	-	-	-	-	-	-	NCP	IGR	-	ID=gene-E	5	4.63	1631.8	0.140647722	VFYFT	91	676.332	1	675.3268	.	H	Superscaf	89455597	0	0	0	0	0	BTP202209	Tissue specific
PVMKFK	Superscaf	-	Superscaf	89462467	89462484	-31	31	36	-	-	-	-	-	-	NCP	IGR	-	ID=gene-E	6	6.168333333	2033.1	0.140730616	PVM(+15.9	61	383.22	2	764.4255	Oxidation	G	Superscaf	89462541	0	0	0	BTP202209	0	0	Tissue specific
LVGTVFP	Superscaf	-	Superscaf	89473924	89473944	-33	33	39	-	-	-	-	-	-	NCP	IGR	-	ID=gene-E	7	4.322857143	1773	0.140876703	LVGTVFP	60	366.7189	2	731.4218	.	K	Superscaf	89473944	0	BTP202209	0	0	0	0	Tissue specific
TVMSY	Superscaf	+	Superscaf	89513677	89513691	4	4	8	-	-	-	-	-	-	NCP	IGR	-	ID=gene-E	5	4.724	1611.9	0.141383591	T(+42.01)	74	642.2783	1	641.2731	Acetylati	P	Superscaf	89513668	0	BTP202209	0	0	BTP202209	0	Tissue specific
YMMSCP	Superscaf	-	Superscaf	89519446	89519463	-27	27	32	-	-	-	-	-	-	NCP	IGR	-	ID=gene-E	6	5.693333333	2509	0.141457151	YMM(+15.9	58	402.639	2	803.2653	Oxidation	H	Superscaf	89519466	0	BTP202209	0	0	0	0	Tissue specific
EDSDC	Superscaf	-	Superscaf	89520790	89520804	-11	11	15	-	-	-	-	-	-	NCP	IGR	-	ID=gene-E	5	3.972	2217.6	0.141474289	E(+42.01)	51	667.178	1	666.1803	Acetylati	H	Superscaf	89520861	0	BTP202209	0	0	BTP202209	BTP202209	Tissue specific
ATDQEKR	Superscaf	-	Superscaf	89556664	89556684	-40	40	46	-	-	-	-	-	-	NCP	IGR	-	ID=gene-E	7	6.412857143	3273.7	0.141931716	ATDQEKR	94	424.2187	2	846.4195	.	V	Superscaf	89556861	0	BTP202209	0	0	0	0	Tissue specific
EGTLHFK	Superscaf	-	Superscaf	89559778	89559798	-17	17	23	-	-	-	-	-	-	NCP	IGR	-	ID=gene-E	7	6.358571429	2949.2	0.141971422	EGTLHFK	60	416.214	2	830.4286	.	K	Superscaf	89559891	0	0	0	0	0	BTP202209	Tissue specific
KRTPP	Superscaf	-	Superscaf	89685604	89685618	-24	24	28	-	-	-	-	-	-	NCP	IGR	-	ID=gene-E	5	7.926	2189.5	0.143575822	K(+42.01)	52	640.3761	1	639.3704	Acetylati	K	Superscaf	89685957	0	0	0	BTP202209	0	0	Tissue specific
VPPGPLQP	Superscaf	-	Superscaf	89691310	89691333	-39	39	46	-	-	-	-	-	-	NCP	IGR	-	ID=gene-E	8	5.35	2611	0.143648579	VPPGPLQP	61	402.7335	2	803.4541	.	P	Superscaf	89691366	0	0	0	0	0	BTP202209	Tissue specific
GPVLVPR	Superscaf	-	Superscaf	89715529	89715549	-83	83	89	-	-	-	-	-	-	NCP	IGR	-	ID=gene-E	7	5.044285714	1974.3	0.143957394	GPVLVPR	69	369.2418	2	736.4595	.	G	Superscaf	89715591	0	0	0	0	0	BTP202209	Tissue specific
MGYPMQL	Superscaf	+	Superscaf	89839879	89839899	5	5	11	-	-	-	-	-	-	NCP	IGR	-	ID=gene-E	7	5.865714286	2801.3	0.145542974	M(+42.01)	88	881.3868	1	880.3823	Acetylati	M	Superscaf	89839867	0	0	BTP202209	0	0	0	Tissue specific
WAKPENL	Superscaf	+	Superscaf	89841610	89841630	13	13	19	-	-	-	-	-	-	NCP	IGR	-	ID=gene-E	7	6.08	3019.3	0.145565046	WAKPENL	88	429.2322	2	856.4443	.	I	Superscaf	89841574	0	0	BTP202209	0	0	0	Tissue specific
KLTFLPQ	Superscaf	-	Superscaf	89845516	89845536	-8	8	14	-	-	-	-	-	-	NCP	IGR	-	ID=gene-E	7	6.522857143	2908.2	0.145614851	KLTFLPQ	82	423.7548	2	845.501	.	I	Superscaf	89845548	0	0	0	BTP202209	0	BTP202209	Tissue specific
LTAVTK	Superscaf	+	Superscaf	89884444	89884461	9	9	14	-	-	-	-	-	-	NCP	IGR	-	ID=gene-E	6	5.8	1928.1	0.146111219	L(+42.01)	59	674.4012	1	673.4011	Acetylati	S	Superscaf	89884420	0	0	0	0	0	BTP202209	Tissue specific
REHEPK	Superscaf	-	Superscaf	89908090	89908107	-11	11	16	-	-	-	-	-	-	NCP	IGR	-	ID=gene-E	6	6.805	2925.3	0.146412728	REHEPK	60	398.213	2	794.4034	.	S	Superscaf	89908164	0	0	0	0	0	BTP202209	Tissue specific
TKKFL	Superscaf	-	Superscaf	89949997	89950011	-3	3	7	-	-	-	-	-	-	NCP	IGR	-	ID=gene-E	5	7.494	2075.1	0.146947082	TKKFL	59	636.415	1	635.4006	.	N	Superscaf	89950062	0	0	BTP202209	0	0	0	Tissue specific
KKLKLR	Superscaf	-	Superscaf	89972575	89972592	-33	33	38	-	-	-	-	-	-	NCP	IGR	-	ID=gene-E	6	8.656666667	2760.8	0.147234973	KKLKLR	50	393.2698	2	784.5646	.	L	Superscaf	89972622	0	0	0	0	0	BTP202209	Tissue specific
KLVVDYC	Superscaf	+	Superscaf	89995789	89995809	16	16	22	-	-	-	-	-	-	NCP	IGR	-	ID=gene-E	7	4.195714286	2278.5	0.147530973	KLVVDYC(+	54	448.73	2	895.4473	Carbamido	E	Superscaf	89995744	BTP202209	0	0	0	0	0	Tissue specific
APGHPVK	Superscaf	-	Superscaf	90111325	90111345	-45	45	51	-	-	-	-	-	-	NCP	IGR	-	ID=gene-E	7	5.988571429	2371.5	0.149004166	APGHPVK	79	353.2106	2	704.3969	.	M	Superscaf	90111381	0	BTP202209	0	0	BTP202209	0	Tissue specific
NAFRAPS	Superscaf	+	Superscaf	90266770	90266790	20	20	26	-	-	-	-	-	-	NCP	IGR	-	ID=gene-E	7	6.524285714	2976.3	0.150986236	NAFRAPS	95	381.6975	2	761.382	.	Q	Superscaf	90266713	0	0	0	0	0	BTP202209	Tissue specific
PLPPKY	Superscaf	-	Superscaf	90340276	90340293	-32	32	37	-	-	-	-	-	-	NCP	IGR	-	ID=gene-E	6	6.713333333	2293.5	0.151923506	PLPPKY	55	714.4172	1	713.4112	.	W	Superscaf	90340365	0	BTP202209	0	0	0	0	Tissue specific
VTFEPFQ	Superscaf	-	Superscaf	90420217	90420237	-39	39	45	-	-	-	-	-	-	NCP	IGR	-	ID=gene-E	7	4.665714286	2545.9	0.152942829	V(+42.01)	90	909.4313	1	908.428	Acetylati	W	Superscaf	90420327	0	0	BTP202209	0	0	0	Tissue specific
VAGGNLY	Superscaf	+	Superscaf	90476455	90476475	14	14	20	-	-	-	-	-	-	NCP	IGR	-	ID=gene-E	7	5.001428571	2315.5	0.153659917	VAGGNLY	63	693.3531	1	692.3493	.	C	Superscaf	90476416	0	0	BTP202209	0	0	0	Tissue specific
TTPGKENFF	Superscaf	-	Superscaf	90486367	90486399	-13	13	23	-	-	-	-	-	-	NCP	IGR	-	ID=gene-E	11	6.878181818	4778.3	0.153786304	T(+42.01)	96	444.9127	3	1331.72	Acetylati	A	Superscaf	90486570	0	0	BTP202209	0	0	0	Tissue specific
VTGLDLPK	Superscaf	-	Superscaf	90561988	90562011	-3	3	10	-	-	-	-	-	-	NCP	IGR	-	ID=gene-E	8	5.43375	2618.9	0.154750543	VTGLDLPK	72	421.7507	2	841.4909	.	N	Superscaf	90562245	0	0	BTP202209	0	0	0	Tissue specific
KSMNVP	Superscaf	-	Superscaf	90593395	90593412	-22	22	27	-	-	-	-	-	-	NCP	IGR	-	ID=gene-E	6	5.48	2157.4	0.155151012	KSMNVP	76	675.3362	1	674.3422	.	V	Superscaf	90593424	0	0	0	0	0	BTP202209	Tissue specific
GFPDVP	Superscaf	-	Superscaf	90676000	90676017	-18	18	23	-	-	gene	-	-	mRNA	NCP	Intron	gene|mRNA	ID=gene-E	6	4.503333333	1771	0.156204303	G(+42.01)	70	673.3199	1	672.3118	Acetylati	V	Superscaf	90676059	0	0	0	0	0	BTP202209	Tissue specific
EDVLLK	Superscaf	-	Superscaf	90704575	90704592	-12	12	17	-	-	gene	-	-	mRNA	NCP	Intron	gene|mRNA	ID=gene-E	6	4.648333333	2012.2	0.156568661	EDVLLK	78	358.7122	2	715.4116	.	H	Superscaf	90704622	0	0	0	0	0	BTP202209	Tissue specific
LPLPNF	Superscaf	-	Superscaf	90836470	90836487	-21	21	26	-	-	gene	-	-	mRNA	NCP	Intron	gene|mRNA	ID=gene-E	6	5.908333333	2365.7	0.158250446	LPLPNF	99	700.4	1	699.3955	.	G	Superscaf	90836547	0	0	BTP202209	0	0	0	Tissue specific
MMMCEC	Superscaf	-	Superscaf	91055638	91055655	-1	1	6	-	-	gene	-	-	mRNA	NCP	Intron	gene|mRNA	ID=gene-E	6	5.085	2711.4	0.161045045	M(+42.01)	52	468.1282	2	934.2363	Acetylati	M	Superscaf	91055703	0	0	0	BTP202209	0	0	Mixed
AGGLALF	Superscaf	-	Superscaf	91121734	91121754	-1	1	7	-	-	gene	-	-	mRNA	NCP	Intron	gene|mRNA	ID=gene-E	7	5.917142857	2311.4	0.161887831	AGGLALF	75	648.3693	1	647.3642	.	A	Superscaf	91121847	0	0	BTP202209	0	0	0	Tissue specific
YRLTK	Superscaf	-	Superscaf	91232524	91232538	-8	8	12	-	-	gene	-	-	mRNA	NCP	Intron	gene|mRNA	ID=gene-E	5	7.734	2327.6	0.163300508	Y(+42.01)	56	361.7116	2	721.4122	Acetylati	R	Superscaf	91232559	0	0	0	0	0	BTP202209	Tissue specific

MWKSVVGHI	Superscaf	-	Superscaf	91259650	91259679	-39	39	48	-	-	gene	-	-	mRNA	NCP	Intron	gene|mRN	ID=gene-E	10	4.359	3078.4	0.16364639	M(+42.01)	97	600.2966	2	1198.581	Acetylati	G	Superscaf	91259763	BTP20220	0	0	0	0	0	Tissue specific
DYKGEVEKI	Superscaf	-	Superscaf	91271518	91271556	-6	6	18	-	-	gene	-	-	mRNA	NCP	Intron	gene|mRN	ID=gene-E	13	5.823846154	5435.8	0.163797718	DYKGEVEKI	98	516.9252	3	1547.758	.	S	Superscaf	91271724	0	0	0	BTP20220	0	0	Tissue specific
EEPAAPAPI	Superscaf	-	Superscaf	91287205	91287237	-68	68	78	-	-	gene	-	-	mRNA	NCP	Intron	gene|mRN	ID=gene-E	11	5.947272727	3956.3	0.163997742	EEPAAPAPI	87	560.3044	2	1118.597	.	P	Superscaf	91287336	0	0	BTP20220	0	0	0	Tissue specific
FGFVSF	Superscaf	-	Superscaf	91293544	91293561	-53	53	58	-	-	gene	-	-	mRNA	NCP	Intron	gene|mRN	ID=gene-E	6	4.681666667	1842.9	0.16407857	FGFVSF	63	352.1823	2	702.3377	.	P	Superscaf	91293675	0	0	0	0	0	BTP20220	Tissue specific
GVPLT	Superscaf	+	Superscaf	91468735	91468749	5	5	9	-	-	-	-	-	-	NCP	IGR	-	ID=gene-E	5	4.956	1385.6	0.16631242	GVPLT	82	486.2914	1	485.2849	.	E	Superscaf	91468723	0	0	0	0	0	BTP20220	Tissue specific
PSVYPSFP	Superscaf	-	Superscaf	91868422	91868445	-18	18	25	-	-	-	-	-	-	NCP	IGR	-	ID=gene-E	8	5.175	2625.9	0.171408805	PSVYPSFP	66	447.222	2	892.433	.	S	Superscaf	91868574	0	0	0	0	0	BTP20220	Tissue specific
FSMMPKAK	Superscaf	+	Superscaf	91892383	91892406	17	17	24	-	-	-	-	-	-	NCP	IGR	-	ID=gene-E	8	6.8075	3099.3	0.171714331	FSMMPKAK	64	470.2382	2	938.4717	.	R	Superscaf	91892335	0	0	0	0	0	BTP20220	Tissue specific
NPLFAPP	Superscaf	-	Superscaf	91921612	91921632	-39	39	45	-	-	-	-	-	-	NCP	IGR	-	ID=gene-E	7	5.97	2661	0.172087028	NPLFAPP	69	378.2105	2	754.4013	.	R	Superscaf	91921683	0	0	0	0	BTP20220	0	Tissue specific
MNESQK	Superscaf	+	Superscaf	91926382	91926399	5	5	10	-	-	-	-	-	-	NCP	IGR	-	ID=gene-E	6	5.908333333	2856.2	0.17214785	MNESQK	55	368.6669	2	735.3221	.	A	Superscaf	91926370	0	0	BTP20220	0	0	0	Tissue specific
FPAAPQ	Superscaf	-	Superscaf	92062441	92062458	-32	32	37	-	-	-	-	-	-	NCP	IGR	-	ID=gene-E	6	5.961666667	2295.5	0.17388273	FPAAPQ	74	630.324	1	629.3173	.	S	Superscaf	92062509	0	0	0	0	0	BTP20220	Tissue specific
FVTPSVP	Superscaf	-	Superscaf	92066752	92066772	-95	95	101	-	-	-	-	-	-	NCP	IGR	-	ID=gene-E	7	4.327142857	1843.1	0.173937699	FVTPSVP	67	746.4174	1	745.401	.	R	Superscaf	92066805	0	0	0	BTP20220	0	0	Tissue specific
LEFPT	Superscaf	-	Superscaf	92074927	92074941	-17	17	21	-	-	-	-	-	-	NCP	IGR	-	ID=gene-E	5	5.502	1901.2	0.174041938	LEFPT	59	606.312	1	605.306	.	V	Superscaf	92074944	0	BTP20220	0	0	0	0	Tissue specific
KCWEPLK	Superscaf	-	Superscaf	92075806	92075826	-77	77	83	-	-	-	-	-	-	NCP	IGR	-	ID=gene-E	7	6.555714286	3165.4	0.174053146	KC(+57.0	98	480.7506	2	959.4899	Carbamido	V	Superscaf	92075868	0	0	0	0	0	BTP20220	Tissue specific
LSLNNAPK	Superscaf	+	Superscaf	92083018	92083041	48	48	55	-	-	-	-	-	-	NCP	IGR	-	ID=gene-E	8	6.315	3400.7	0.174145106	LSLNNAPK	64	428.7518	2	855.4814	.	I	Superscaf	92082877	BTP20220	0	0	0	0	0	Tissue specific
SSLRSY	Superscaf	+	Superscaf	92103313	92103330	8	8	13	-	-	-	-	-	-	NCP	IGR	-	ID=gene-E	6	6.573333333	2611.9	0.174403887	SSLRSY	50	712.3613	1	711.3552	.	L	Superscaf	92103292	0	0	0	0	0	BTP20220	Tissue specific
KSASVLV	Superscaf	-	Superscaf	92292079	92292099	-46	46	52	-	-	-	-	-	-	NCP	IGR	-	ID=gene-E	7	4.728571429	1899.9	0.176810831	KSASVLV	68	352.2202	2	702.4276	.	S	Superscaf	92292105	BTP20220	0	0	0	0	0	Tissue specific
LLVDPL	Superscaf	+	Superscaf	92406418	92406435	12	12	17	-	-	-	-	-	-	NCP	IGR	-	ID=gene-E	6	4.535	1809.1	0.17826876	LLVDPL	88	669.4156	1	668.4108	.	S	Superscaf	92406385	0	0	0	BTP20220	0	0	Tissue specific
LLAGAKEK	Superscaf	+	Superscaf	92534644	92534667	10	10	17	-	-	-	-	-	-	NCP	IGR	-	ID=gene-E	8	6.58375	2989	0.179903763	LLAGAKEK	97	415.259	2	828.5068	.	G	Superscaf	92534617	0	0	0	BTP20220	0	0	Tissue specific
AYTNF	Superscaf	+	Superscaf	92581609	92581623	5	5	9	-	-	-	-	-	-	NCP	IGR	-	ID=gene-E	5	5.82	2152.4	0.18050261	AYTNF	89	615.2754	1	614.27	.	P	Superscaf	92581597	0	BTP20220	0	BTP20220	0	0	Tissue specific
SSLKLEER	Superscaf	-	Superscaf	92613931	92613954	-4	4	11	-	-	-	-	-	-	NCP	IGR	-	ID=gene-E	8	6.2825	3441.8	0.180914746	SSLKLEER	90	481.2704	2	960.524	.	F	Superscaf	92614041	0	0	0	0	0	BTP20220	Tissue specific
ETLPVAY	Superscaf	-	Superscaf	92624038	92624058	-4	4	10	-	-	-	-	-	-	NCP	IGR	-	ID=gene-E	7	4.815714286	2284.6	0.18104362	ETLPVAY	55	792.4135	1	791.4065	.	E	Superscaf	92624142	BTP20220	0	0	0	0	0	Tissue specific
KEVLMPK	Superscaf	+	Superscaf	92708287	92708307	15	15	21	-	-	-	-	-	-	NCP	IGR	-	ID=gene-E	7	5.818571429	2424.6	0.182117874	KEVLMPK	66	422.7554	2	843.4888	.	L	Superscaf	92708245	0	0	BTP20220	0	0	0	Tissue specific
EDGRR	Superscaf	-	Superscaf	92794648	92794662	-29	29	33	-	-	-	-	-	-	NCP	IGR	-	ID=gene-E	5	6.736	2431.9	0.183219058	EDGRR	54	632.3064	1	631.3038	.	S	Superscaf	92794782	0	0	0	0	BTP20220	0	Tissue specific
LLLLYDR	Superscaf	+	Superscaf	92797552	92797572	18	18	24	-	-	-	-	-	-	NCP	IGR	-	ID=gene-E	7	6.187142857	2933.4	0.183256087	LLLLYDR	60	453.2826	2	904.5381	.	K	Superscaf	92797501	0	0	BTP20220	0	0	0	Tissue specific
VLAPEL	Superscaf	-	Superscaf	92895193	92895210	-75	75	80	-	-	-	-	-	-	NCP	IGR	-	ID=gene-E	6	4.583333333	1781	0.184501101	VLAPEL	89	641.3851	1	640.3796	.	A	Superscaf	92895423	0	0	0	0	BTP20220	0	Tissue specific
VGPVP	Superscaf	+	Superscaf	92899594	92899608	9	9	13	-	-	-	-	-	-	NCP	IGR	-	ID=gene-E	5	3.714	972.1	0.184557218	VGPVP	93	468.2806	1	467.2744	.	Q	Superscaf	92899570	0	0	0	0	BTP20220	0	Mixed
QMVQTK	Superscaf	-	Superscaf	93124153	93124170	-9	9	14	-	-	-	-	-	-	NCP	IGR	-	ID=gene-E	6	5.553333333	2458.8	0.187420557	Q(+42.01)	80	396.6935	2	791.3848	Acetylati	R	Superscaf	93124371	0	0	BTP20220	0	0	0	Tissue specific
MTLPL	Superscaf	+	Superscaf	93197098	93197112	161	161	165	-	-	-	-	-	-	NCP	IGR	-	ID=gene-E	5	6.108	1813.2	0.188350674	M(+42.01)	57	616.3331	1	615.3302	Acetylati	K	Superscaf	93196618	0	0	BTP20220	0	0	0	Tissue specific
APLLGAH	Superscaf	-	Superscaf	93311830	93311850	-7	7	13	-	-	-	-	-	-	NCP	IGR	-	ID=gene-E	7	6.265714286	2583.8	0.189813615	A(+42.01)	59	720.4005	1	719.3966	Acetylati	K	Superscaf	93311949	0	0	0	0	0	BTP20220	Tissue specific
KQKNLLE	Superscaf	-	Superscaf	93312916	93312936	-2	2	8	-	-	-	-	-	-	NCP	IGR	-	ID=gene-E	7	6.531428571	3276.5	0.189827463	KQKNLLE	50	436.7648	2	871.5127	.	N	Superscaf	93313233	0	0	BTP20220	0	0	0	Tissue specific
LMVGRTHNI	Superscaf	-	Superscaf	93339550	93339576	-13	13	21	-	-	-	-	-	-	NCP	IGR	-	ID=gene-E	9	5.996666667	3638.3	0.190167071	LMVGRTHNI	63	520.7848	2	1039.56	.	F	Superscaf	93339663	0	0	0	0	BTP20220	0	Tissue specific
AFEFLA	Superscaf	-	Superscaf	93373765	93373782	-6	6	11	-	-	-	-	-	-	NCP	IGR	-	ID=gene-E	6	5.366666667	2176.3	0.190603345	AFEFLA	83	697.3521	1	696.3482	.	L	Superscaf	93374001	0	0	0	0	BTP20220	0	Tissue specific
LDLKER	Superscaf	+	Superscaf	93375034	93375051	4	4	9	-	-	-	-	-	-	NCP	IGR	-	ID=gene-E	6	6.441666667	2661	0.190619526	LDLKER	58	387.222	2	772.4443	.	K	Superscaf	93375025	BTP20220	0	0	0	0	0	Tissue specific
LGPKKST	Superscaf	-	Superscaf	93412831	93412851	-3	3	9	-	-	-	-	-	-	NCP	IGR	-	ID=gene-E	7	7.134285714	2705.8	0.191101473	L(+42.01)	57	772.4572	1	771.449	Acetylati	K	Superscaf	93413103	0	0	0	BTP20220	0	0	Tissue specific
KFHKK	Superscaf	+	Superscaf	93507949	93507963	3	3	7	-	-	-	-	-	-	NCP	IGR	-	ID=gene-E	5	8.458	2412.3	0.192314317	K(+42.01)	58	365.224	2	728.4333	Acetylati	F	Superscaf	93507943	0	0	BTP20220	0	0	0	Tissue specific
AAAFLDK	Superscaf	+	Superscaf	93567184	93567204	3	3	9	-	-	-	-	-	-	NCP	IGR	-	ID=gene-E	7	6.032857143	2554.6	0.193069619	AAAFLDK	53	735.4001	1	734.3962	.	Q	Superscaf	93567178	0	0	0	0	0	BTP20220	Tissue specific
KGGDPLK	Superscaf	-	Superscaf	93708598	93708618	-21	21	27	-	-	-	-	-	-	NCP	IGR	-	ID=gene-E	7	6.667142857	2633.7	0.19487278	KGGDPLK	69	357.7146	2	713.4072	.	R	Superscaf	93708618	0	BTP20220	0	0	BTP20220	BTP20220	Tissue specific
VGLPNGKS	Superscaf	+	Superscaf	93733474	93733497	2	2	9	-	-	-	-	-	-	NCP	IGR	-	ID=gene-E	8	5.63125	2733.9	0.195189973	V(+42.01)	56	813.4492	1	812.4392	Acetylati	A	Superscaf	93733471	0	BTP20220	0	0	BTP20220	0	Tissue specific
KYWALFQ	Superscaf	-	Superscaf	93770413	93770433	-2	2	8	-	-	-	-	-	-	NCP	IGR	-	ID=gene-E	7	6.345714286	3117.3	0.19566098	KYWALFQ	54	478.2636	2	954.4963	.	N	Superscaf	93770442	0	BTP20220	0	0	0	0	Tissue specific
HVVTP	Superscaf	-	Superscaf	93869314	93869328	-8	8	12	-	-	-	-	-	-	NCP	IGR	-	ID=gene-E	5	4.084	1354.6	0.196922061	HVVTP	67	552.3131	1	551.3068	.	V	Superscaf	93869352	0	BTP20220	0	0	0	0	Tissue specific
VTLSP	Superscaf	+	Superscaf	93883498	93883512	11	11	15	-	-	-	-	-	-	NCP	IGR	-	ID=gene-E	5	4.898	1471.7	0.19710292	VTLSP	51	516.3019	1	515.2955	.	G	Superscaf	93883468	0	BTP20220	0	0	BTP20220	0	Tissue specific
AAAASQGGF	Superscaf	-	Superscaf	94051780	94051833	-3	3	20	-	-	gene	-	-	mRNA	NCP	Intron	gene|mRN	ID=gene-E	18	6.183888889	6344.6	0.199248674	A(+42.01)	99	709.3212	2	1416.634	Acetylati	K	Superscaf	94052454	0	0	0	0	BTP20220	0	Mixed
AAAASQGGF	Superscaf	-	Superscaf	94051780	94051830	-3	3	19	-	-	gene	-	-	mRNA	NCP	Intron	gene|mRN	ID=gene-E	17	6.196470588	6047.3	0.199248674	A(+42.01)	99	680.8117	2	1359.613	Acetylati	K	Superscaf	94052454	0	BTP20220	0	0	0	BTP20220	Tissue specific
AAAASQGGF	Superscaf	-	Superscaf	94051780	94051827	-3	3	18	-	-	gene	-	-	mRNA	NCP	Intron	gene|mRN	ID=gene-E	16	6.22875	5663.9	0.199248674	A(+42.01)	99	637.2955	2	1272.581	Acetylati	K	Superscaf	94052454	0	0	BTP20220	0	0	0	Tissue specific
TVALTPLP	Superscaf	-	Superscaf	94243726	94243749	-53	53	60	-	-	-	-	-	-	NCP	IGR	-	ID=gene-E	8	5.455	2487.9	0.201696166	T(+42.01)	82	427.2544	2	852.4957	Acetylati	T	Superscaf	94244172	0	BTP20220	0	0	BTP20220	BTP20220	Tissue specific
KGLVGL	Superscaf	-	Superscaf	94251313	94251330	-11	11	16	-	-	-	-	-	-	NCP	IGR	-	ID=gene-E	6	5.606666667	1769.8	0.201792907	K(+42.01)	79	628.4006	1	627.3956	Acetylati	K	Superscaf	94251393	0	0	0	0	BTP20220	0	Tissue specific
VTRSPYK	Superscaf	-	Superscaf	94317190	94317210	-13	13	19	-	-	-	-	-	-	NCP	IGR	-	ID=gene-E	7	6.381428571	2695	0.202632901	V(+42.01)	62	446.7497	2	891.4814	Acetylati	T	Superscaf	94317213	BTP20220	0	0	0	0	0	Tissue specific
MGPEA	Superscaf	-	Superscaf	94369159	94369173	-23	23	27	-	-	gene	-	-	mRNA	NCP	Intron	gene|mRN	ID=gene-E	5	5.452	1743	0.203295555	MGPEA	52	504.2155	1	503.205	.	F	Superscaf	94369188	0	0	0	BTP20220	0	0	Tissue specific
VMLLQHS	Superscaf	+	Superscaf	94533664	94533684	2	2	8	-	-	gene	-	-	mRNA	NCP	Intron	gene|mRN	ID=gene-E	7	5.232857143	2692.1	0.205393148	VM(+15.99	89	843.4546	1	842.432	Oxidation	N	Superscaf	94533661	0	0	0	0	0	BTP20220	Tissue specific
MEPTFP	Superscaf	-	Superscaf	94671082	94671099	-26	26	31	-	-	gene	-	-	mRNA	NCP	Intron	gene|mRN	ID=gene-E	6	5.596666667	2256.7	0.207145357	M(+42.01)	99	763.3314	1	762.3258	Acetylati	R	Superscaf	94671222	0	0	0	0	BTP20220	0	Mixed
VEKEKKEV	Superscaf	-	Superscaf	94689778	94689801	-7	7	14	-	-	gene	-	-	mRNA	NCP	Intron	gene|mRN	ID=gene-E	8	4.86	2681.7	0.207383748	VEKEKKEV	80	494.7861	2	987.56	.	E	Superscaf	94689807	0	0	0	0	BTP20220	0	Tissue specific
PPPPKSR	Superscaf	+	Superscaf	94772398	94772418	17	17	23	-	-	-	-	-	-	NCP	IGR	-	ID=gene-E	7	7.34	2850.2	0.208437231	PPPPKSR	57	389.7263	2	777.4496	.	K	Superscaf	94772350	0	0	0	BTP20220	0	0	Tissue specific
KNEDF	Superscaf	-	Superscaf	94834336	94834350	-2	2	6	-	-	-	-	-	-	NCP	IGR	-	ID=gene-E	5	5.364	2289.5	0.209226999	KNEDF	64	652.2977	1	651.2864	.	R	Superscaf	94834368	0	BTP20220	0	0	BTP20220	BTP20220	Tissue specific
GPALDY	Superscaf	+	Superscaf	94861111	94861128	28	28	33	-	-	gene	-	-	mRNA	NCP	Intron	gene|mRN	ID=gene-E	6	5.483333333	2170.4	0.209568405	GPALDY	58	635.2999	1	634.2962	.	V	Superscaf	94861030	0	0	0	BTP20220	0	0	Tissue specific
EVLNPFL	Superscaf	-	Superscaf	94864576	94864596	-7	7	13	-	-	-	-	-	-	NCP	IGR	-	ID=gene-E	7	4.624285714	2453.8	0.209612587	EVLNPFL	77	831.4574	1	830.4538	.	V	Superscaf	94864623	0	0	0	0	BTP20220	0	Tissue specific
VGEYL	Superscaf	-	Superscaf	94864966	94864980	-4	4	8	-	-	-	-	-	-	NCP	IGR	-	ID=gene-E	5	4.166	1535.7	0.20961756	VGEYL	89	580.2977	1	579.2904	.	Q	Superscaf	94864989	0	BTP20220	0	0	0	BTP20220	Mixed
VHLAC	Superscaf	+	Superscaf	94897708	94897722	24	24	28	-	-	-	-	-	-	NCP	IGR	-	ID=gene-E	5	4.922	1870.1	0.210035052	VHLAC(+57	86	599.2953	1	598.2897	Carbamido	G	Superscaf	94897639	0	0	BTP20220	0	0	0	Tissue specific
YLCCFGY	Superscaf	-	Superscaf	94970197	94970217	-7	7	13	-	-	gene	-	-	mRNA	NCP	Intron	gene|mRN	ID=gene-E	7	5.541428571	3128.5	0.210959355	YLC(+57.0	57	491.6953	2	981.3724	Carbamido	S	Superscaf	94970232	0	0	BTP20220	0	0	0	Tissue specific
MTLFLEYL	Superscaf	-	Superscaf	95238370	95238393	-15	15	22	-	-	-	-	-	-	NCP	IGR	-	ID=gene-E	8	5.5725	3101.6	0.214378813	M(+42.01)	81	536.2846	2	1070.536	Acetylati	K	Superscaf	95238582	BTP20220	0	0	0	0	0	Tissue specific
EVWEK	Superscaf	+	Superscaf	95309701	95309715	10	10	14	-	-	-	-	-	-	NCP	IGR	-	ID=gene-E	5	4.414	1846	0.21528835	EVWEK	92	690.3434	1	689.3384	.	E	Superscaf	95309674	0	BTP20220	0	0	0	0	Tissue specific
ATPPKGY	Superscaf	-	Superscaf	95496265	95496285	-24	24	30	-	-	gene	-	-	mRNA	NCP	Intron	gene|mRN	ID=gene-E	7	6.645714286	2608.8	0.217667216	ATPPKGY	63	367.1961	2	732.3806	.	K	Superscaf	95496288	0	0	BTP20220	0	0	0	Tissue specific
KQAAGLPR	Superscaf	-	Superscaf	95501110	95501133	-15	15	22	-	-	gene	-	-	mRNA	NCP	Intron	gene|mRN	ID=gene-E	8	7.055	3338.6	0.217728995	KQAAGLPR	52	420.756	2	839.4977	.	R	Superscaf	95501142	0	0	BTP20220	0	0	0	Tissue specific
VAALF	Superscaf	+	Superscaf	95517643	95517657	3	3	7	-	-	gene	-	-	mRNA	NCP	Intron	gene|mRN	ID=gene-E	5	4.7	1363.4	0.217939806	VAALF	63	520.3115	1	519.3057	.	V	Superscaf	95517637	0	0	0	0	BTP20220	0	Tissue specific
CKRWLVL	Superscaf	+	Superscaf	95526127	95526147	4	4	10	-	-	gene	-	-	mRNA	NCP	Intron	gene|mRN	ID=gene-E	7	6.195714286	2936.3	0.218047985	C(+57.02)	51	487.7834	2	973.5531	Carbamido	L	Superscaf	95526118	0	0	BTP20220	0	0	0	Tissue specific
GPPLVG	Superscaf	+	Superscaf	95566606	95566623	31	31	36	-	-	gene	-	-	mRNA	NCP	Intron	gene|mRN	ID=gene-E	6	5.086666667	1622.8	0.21856413	GPPLVG	51	539.3174	1	538.3115	.	C	Superscaf	95566516	0	0	BTP20220	0	0	0	Mixed
AVRPPYK	Superscaf	-	Superscaf	95586979	95586999	-51	51	57	-	-	gene	-	-	mRNA	NCP	Intron	gene|mRN	ID=gene-E	7	6.397142857	2562.8	0.218823905	A(+42.01)	63	436.7536	2	871.4915	Acetylati	V	Superscaf	95587119	0	BTP20220	BTP20220	BTP20220	BTP20220	0	Tissue specific
KAGRELL	Superscaf	-	Superscaf	95587126	95587146	-2	2	8	-	-	gene	-	-	mRNA	NCP	Intron	gene|mRN	ID=gene-E	7	6.81	2858.1	0.21882578	KAGRELL	61	393.7439	2	785.4759	.	D	Superscaf	95587233	0	0	BTP20220	BTP20220	0	0	Tissue specific
AAKRPTT	Superscaf	+	Superscaf	95594854	95594874	72	72	78	-	-	gene	-	-	mRNA	NCP	Intron	gene|mRN	ID=gene-E	7	7.414285714	2872.2	0.218924319	AAKRPTT	85	372.7145	2	743.429	.	I	Superscaf	95594641	BTP20220	0	0	0	0	0	Tissue specific
CFTPGF	Superscaf	-	Superscaf	95667469	95667486	-27	27	32	-	-	gene	-	-	mRNA	NCP	Intron	gene|mRN	ID=gene-E	6	5.796666667	2392.7	0.219850228	C(+57.02)	75	728.306	1	727.2999	Carbamido	E	Superscaf	95667489	0	0	BTP20220	0	0	0	Tissue specific
YELLLK	Superscaf	+	Superscaf	95764843	95764860	4	4	9	-	-	gene	-	-	mRNA	NCP	Intron	gene|mRN	ID=gene-E	6	6.093333333	2413.6	0.221091839	YELLLK	99	389.737	2	777.4636	.	M	Superscaf	95764834	0	0	BTP20220	0	0	0	Tissue specific
DFDLPK	Superscaf	+	Superscaf	95857564	95857581	44	44	49	-	-	-	-	-	-	NCP	IGR	-	ID=gene-E	6	5.573333333	2369.6	0.222274119	DFDLPK	74	367.6881	2	733.3646	.	T	Superscaf	95857435	BTP20220	0	0	0	0	0	Tissue specific
DRPEPL	Superscaf	-	Superscaf	95949418	95949435	-14	14	19	-	-	-	-	-	-	NCP	IGR	-	ID=gene-E	6	5.921666667	2514	0.223445343	D(+42.01)	53	768.3885	1	767.3813	Acetylati	S	Superscaf	95949480	0	BTP20220	0	0	0	0	Tissue specific
LTTGP	Superscaf	+	Superscaf	95955607	95955621	88	88	92	-	-	-	-	-	-	NCP	IGR	-	ID=gene-E	5	6.262	1783.1	0.223524259	LTTGP	54	488.2805	1	487.2642	.	L	Superscaf	95955346	0	0	0	BTP20220	0	0	Tissue specific
ATDFSLPR	Superscaf	-	Superscaf	96024934	96024957	-26	26	33	-	-	-	-	-	-	NCP	IGR	-	ID=gene-E	8	6.215	3230.7	0.224408244	ATDFSLPR	83	453.7372	2	905.4606	.	D	Superscaf	96024972	0	0	BTP20220	0	0	0	Tissue specific
ASLLAA	Superscaf	-	Superscaf	96094336	96094353	-2	2	7	-	-	-	-	-	-	NCP	IGR	-	ID=gene-E	6	5.95	2024.1	0.225293184	ASLLAA	69	545.327	1	544.322	.	S	Superscaf	96094395	0	0	BTP20220	0	0	0	Tissue specific
YLVEPF	Superscaf	+	Superscaf	96199894	96199911	60	60	65	-	-	-	-	-	-	NCP	IGR	-	ID=gene-E	6	4.44	1963.2	0.226639148	YLVEPF	77	767.3979	1	766.3901	.	Y	Superscaf	96199717	0	0	0	BTP20220	0	0	Mixed
KQYLQPP	Superscaf	+	Superscaf	96215200	96215220	45	45	51	-	-	gene	-	-	mRNA	NCP	Intron	gene|mRN	ID=gene-E	7	6.468571429	3177.5	0.226834314	KQYLQPP	51	437.2474	2	872.4756	.	T	Superscaf	96215068	0	0	0	0	0	BTP20220	Tissue specific
WGKVK	Superscaf	+	Superscaf	96233425	96233439	12	12	16	-	-	-	-	-	-	NCP	IGR	-	ID=gene-E	5	6.268	1760.7	0.2270667	WGKVK	95	617.3754	1	616.3697	.	L	Superscaf	96233392	0	0	0	BTP20220	0	0	Tissue specific
MQTLKC	Superscaf	-	Superscaf	96272122	96272139	-11	11	16	-	-	-	-	-	-	NCP	IGR	-	ID=gene-E	6	6.445	2787.2	0.227560123	M(+42.01)	92	411.6941	2	821.3776	Acetylati	D	Superscaf	96272169	0	0	BTP20220	BTP20220	0	0	Tissue specific
QAGLAYSL	Superscaf	-	Superscaf	96280840	96280863	-55	55	62	-	-	gene	-	-	mRNA	NCP	Intron	gene|mRN	ID=gene-E	8	5.87	3080.3	0.227671286	QAGLAYSL	60	822.4323	1	821.4283	.	R	Superscaf	96280887	0	0	BTP20220	0	0	0	Tissue specific
KSKSTVP	Superscaf	-	Superscaf	96308779	96308799	-17	17	23	-	-	gene	-	-	mRNA	NCP	Intron	gene|mRN	ID=gene-E	7	6.238571429	2438.5	0.228027534	KSKSTVP	56	373.7236	2	745.4334	.	T	Superscaf	96308886	0	0	BTP20220	0	0	0	Tissue specific
VGAVPF	Superscaf	-	Superscaf	96352636	96352653	-17	17	22	-	-	gene	-	-	mRNA	NCP	Intron	gene|mRN	ID=gene-E	6	3.961666667	1333.4	0.228586752	VGAVPF	63	589.3339	1	588.3271	.	P	Superscaf	96352680	0	0	BTP20220	0	0	0	Tissue specific
VALSLK	Superscaf	-	Superscaf	96400807	96400824	-12	12	17	-	-	gene	-	-	mRNA	NCP	Intron	gene|mRN	ID=gene-E	6	5.566666667	1869.9	0.229200978	V(+42.01)	99	672.4274	1	671.4218	Acetylati	L	Superscaf	96401109	0	BTP20220	0	0	0	0	Mixed
TPLNL	Superscaf	+	Superscaf	96582370	96582384	35	35	39	-	-	-	-	-	-	NCP	IGR	-	ID=gene-E	5	6.04	2038.4	0.231516077	TPLNL	96	557.3287	1	556.3221	.	P	Superscaf	96582268	0	BTP20220	0	0	0	0	Tissue specific
KDTALPPPI	Superscaf	-	Superscaf	96640390	96640416	-51	51	59	-	-	gene	-	-	mRNA	NCP	Intron	gene|mRN	ID=gene-E	9	6.271111111	3291.7	0.232255886	KDTALPPPI	75	468.2671	2	934.5123	.	Y	Superscaf	96640482	0	0	0	0	0	BTP20220	Tissue specific
KTTPVGPR	Superscaf	-	Superscaf	96800098	96800121	-12	12	19	-	-	gene	-	-	mRNA	NCP	Intron	gene|mRN	ID=gene-E	8	6.51625	2884.3	0.234292314	KTTPVGPR	55	428.2601	2	854.4974	.	G	Superscaf	96800130	0	0	0	0	0	BTP20220	Tissue specific
KGPPQHR	Superscaf	+	Superscaf	96879055	96879075	2	2	8	-	-	-	-	-	-	NCP	IGR	-	ID=gene-E	7	7.472857143	3319.7	0.23529909	KGPPQHR	51	410.2312	2	818.4511	.	K	Superscaf	96879052	BTP20220	0	0	0	0	0	Tissue specific
LSFLL	Superscaf	+	Superscaf	97079071	97079085	41	41	45	-	-	-	-	-	-	NCP	IGR	-	ID=gene-E	5	5.82	1831	0.237849482	LSFLL	77	592.3685	1	591.3632	.	S	Superscaf	97078951	0	0	0	0	BTP20220	0	Tissue specific
AAKSNMK	Superscaf	+	Superscaf	97198822	97198842	34	34	40	-	-	gene	-	-	mRNA	NCP	Intron	gene|mRN	ID=gene-E	7	6.908571429	2911	0.23937642	AAKSNMK	82	375.2056	2	748.3901	.	F	Superscaf	97198723	0	0	0	0	BTP20220	0	Tissue specific
KPRLLK	Superscaf	-	Superscaf	97202218	97202235	-11	11	16	-	-	gene	-	-	mRNA	NCP	Intron	gene|mRN	ID=gene-E	6	8.083333333	2629.8	0.239419722	K(+42.01)	69	398.7725	2	795.533	Acetylati	I	Superscaf	97202394	0	0	0	BTP20220	0	0	Tissue specific
DYYLR	Superscaf	-	Superscaf	97213969	97213983	-90	90	94	-	-	-	-	-	-	NCP	IGR	-	ID=gene-E	5	6.206	2332.7	0.239569558	DYYLR	71	365.1805	2	728.3493	.	K	Superscaf	97213983	0	0	0	0	0	BTP20220	Tissue specific
LDVKLF	Superscaf	+	Superscaf	97418593	97418610	9	9	14	-	-	-	-	-	-	NCP	IGR	-	ID=gene-E	6	5.025	1974.1	0.242178707	LDVKLF	83	367.7248	2	733.4374	.	N	Superscaf	97418569	0	BTP20220	0	0	0	0	Tissue specific
SLAEPDV	Superscaf	-	Superscaf	97448605	97448625	-1	1	7	-	-	-	-	-	-	NCP	IGR	-	ID=gene-E	7	4.31	2222.5	0.242561388	SLAEPDV	52	730.3561	1	729.3544	.	S	Superscaf	97448760	0	0	0	0	BTP20220	0	Tissue specific
LVAKFCK	Superscaf	-	Superscaf	97485568	97485588	-4	4	10	-	-	gene	-	-	mRNA	NCP	Intron	gene|mRN	ID=gene-E	7	5.997142857	2574.6	0.243032701	L(+42.01)	53	454.2558	2	906.4997	Acetylati	S	Superscaf	97485684	0	BTP20220	0	0	0	0	Tissue specific
FGEHLL	Superscaf	+	Superscaf	97548766	97548783	9	9	14	-	-	-	-	-	-	NCP	IGR	-	ID=gene-E	6	5.703333333	2436.7	0.243838535	FGEHLL	93	358.1907	2	714.3701	.	R	Superscaf	97548742	0	BTP20220	0	0	0	0	Tissue specific
MVLVEALK	Superscaf	+	Superscaf	97552537	97552560	8	8	15	-	-	-	-	-	-	NCP	IGR	-	ID=gene-E	8	4.58625	2283.5	0.243886619	MVLVEALK	83	451.7714	2	901.5306	.	E	Superscaf	97552516	0	0	BTP20220	0	0	0	Tissue specific
TEKFLT	Superscaf	+	Superscaf	97845367	97845384	3	3	8	-	-	gene	-	-	mRNA	NCP	Intron	gene|mRN	ID=gene-E	6	6.246666667	2429.7	0.247620477	TEKFLT	58	738.3937	1	737.3959	.	T	Superscaf	97845361	0	0	0	BTP20220	0	0	Tissue specific
KTLSPLLK	Superscaf	-	Superscaf	97872088	97872111	-7	7	14	-	-	gene	-	-	mRNA	NCP	Intron	gene|mRN	ID=gene-E	8	6.99125	3115.3	0.247961195	KTLSPLLK	50	450.2947	2	898.5851	.	S	Superscaf	97872267	0	0	0	BTP20220	0	0	Tissue specific
NQNLLL	Superscaf	-	Superscaf	98011567	98011584	-12	12	17	-	-	-	-	-	-	NCP	IGR	-	ID=gene-E	6	5.735	2864.3	0.249739683	NQNLLL	61	357.7126	2	713.4072	.	K	Superscaf	98011623	0	BTP20220	0	0	0	0	Tissue specific
KNKDPPCL	Superscaf	+	Superscaf	98054659	98054682	5	5	12	-	-	-	-	-	-	NCP	IGR	-	ID=gene-E	8	6.4325	3558.9	0.250289147	KNKDPPC(+	60	486.2522	2	970.4905	Carbamido	G	Superscaf	98054647	0	0	0	BTP20220	0	0	Tissue specific
YELLTAK	Superscaf	+	Superscaf	98147908	98147928	5	5	11	-	-	-	-	-	-	NCP	IGR	-	ID=gene-E	7	6.161428571	2769	0.251478159	YELLTAK	81	419.2377	2	836.4644	.	L	Superscaf	98147896	0	BTP20220	0	0	0	0	Tissue specific
WDFDKSK	Superscaf	-	Superscaf	98176327	98176347	-27	27	33	-	-	-	-	-	-	NCP	IGR	-	ID=gene-E	7	6.067142857	3057.2	0.251840528	W(+42.01)	83	484.2261	2	966.4446	Acetylati	C	Superscaf	98176386	0	0	BTP20220	0	0	0	Tissue specific
LERYCE	Superscaf	-	Superscaf	98238955	98238972	-15	15	20	-	-	-	-	-	-	NCP	IGR	-	ID=gene-E	6	5.643333333	2898.4	0.252639094	LERYC(+57	61	435.1982	2	868.3749	Carbamido	V	Superscaf	98239020	BTP20220	0	0	0	0	0	Tissue specific
WSFSEAR	Superscaf	-	Superscaf	98302069	98302089	-5	5	11	-	-	gene	-	-	mRNA	NCP	Intron	gene|mRN	ID=gene-E	7	6.104285714	3066.4	0.253443857	W(+42.01)	57	462.7054	2	923.4137	Acetylati	T	Superscaf	98302089	0	BTP20220	0	0	0	0	Tissue specific
NYALGP	Superscaf	+	Superscaf	98303146	98303163	25	25	30	-	-	gene	-	-	mRNA	NCP	Intron	gene|mRN	ID=gene-E	6	5.89	2355.6	0.25345759	NYALGP	53	634.3187	1	633.3122	.	S	Superscaf	98303074	0	0	0	0	BTP20220	0	Tissue specific
LTHLE	Superscaf	+	Superscaf	98335165	98335179	12	12	16	-	-	gene	-	-	mRNA	NCP	Intron	gene|mRN	ID=gene-E	5	5.86	2149.5	0.253865862	LTHLE	64	612.3333	1	611.3279	.	N	Superscaf	98335132	0	BTP20220	0	0	0	0	Tissue specific
KKDFRWQ	Superscaf	-	Superscaf	98366170	98366190	-8	8	14	-	-	-	-	-	-	NCP	IGR	-	ID=gene-E	7	7.175714286	3521.8	0.254261205	KKDFRWQ	58	504.2665	2	1006.535	.	R	Superscaf	98366220	0	BTP20220	0	0	0	0	Tissue specific
SLDLYK	Superscaf	-	Superscaf	98405944	98405961	-9	9	14	-	-	-	-	-	-	NCP	IGR	-	ID=gene-E	6	6.001666667	2429.6	0.254768361	SLDLYK	82	369.7038	2	737.3959	.	S	Superscaf	98406033	0	0	0	BTP20220	0	0	Tissue specific
EQALLR	Superscaf	-	Superscaf	98413708	98413725	-23	23	28	-	-	-	-	-	-	NCP	IGR	-	ID=gene-E	6	6.268333333	2703.1	0.254867359	EQALLR	74	365.2174	2	728.418	.	M	Superscaf	98413809	0	0	0	0	0	BTP20220	Tissue specific
LGPPPPHPI	Superscaf	-	Superscaf	98430391	98430417	-20	20	28	-	-	-	-	-	-	NCP	IGR	-	ID=gene-E	9	6.371111111	3294.8	0.255080083	L(+42.01)	56	475.7671	2	949.5021	Acetylati	W	Superscaf	98430489	0	BTP20220	0	0	BTP20220	0	Tissue specific
KDANFH	Superscaf	+	Superscaf	98524219	98524236	26	26	31	-	-	-	-	-	-	NCP	IGR	-	ID=gene-E	6	6.201666667	2795	0.256276478	KDANFH	55	731.3378	1	730.3398	.	N	Superscaf	98524144	0	BTP20220	0	0	0	BTP20220	Tissue specific
NYVNK	Superscaf	-	Superscaf	98589559	98589573	-20	20	24	-	-	gene	-	-	mRNA	NCP	Intron	gene|mRN	ID=gene-E	5	5.244	2121.3	0.257109625	NYVNK	54	637.3281	1	636.3231	.	A	Superscaf	98589729	0	0	0	BTP20220	0	0	Tissue specific
AFSLYK	Superscaf	+	Superscaf	98598583	98598600	28	28	33	-	-	gene	-	-	mRNA	NCP	Intron	gene|mRN	ID=gene-E	6	6.426666667	2363.4	0.257224689	AFSLYK	99	364.7005	2	727.3904	.	D	Superscaf	98598502	0	0	0	BTP20220	0	0	Tissue specific
TGLSPL	Superscaf	-	Superscaf	98715766	98715783	-13	13	18	-	-	-	-	-	-	NCP	IGR	-	ID=gene-E	6	6.073333333	2122.4	0.258718883	T(+42.01)	50	629.3486	1	628.3432	Acetylati	I	Superscaf	98715906	0	0	0	BTP20220	0	0	Tissue specific
KPFLEPYK	Superscaf	+	Superscaf	98925907	98925930	3	3	10	-	-	gene	-	-	mRNA	NCP	Intron	gene|mRN	ID=gene-E	8	6.5525	3237.4	0.261398378	KPFLEPYK	55	511.2823	2	1020.564	.	S	Superscaf	98925901	0	0	0	BTP20220	0	0	Tissue specific
EVVAF	Superscaf	-	Superscaf	99027661	99027675	-33	33	37	-	-	gene	-	-	mRNA	NCP	Intron	gene|mRN	ID=gene-E	5	2.944	1124.2	0.262695838	EVVAF	69	564.301	1	563.2955	.	I	Superscaf	99027723	0	0	BTP20220	0	0	0	Mixed
MDMKR	Superscaf	+	Superscaf	99033325	99033339	7	7	11	-	-	gene	-	-	mRNA	NCP	Intron	gene|mRN	ID=gene-E	5	6.994	2271.7	0.262768059	M(+42.01)	99	361.6683	2	721.3251	Acetylati	M	Superscaf	99033307	0	BTP20220	BTP20220	BTP20220	BTP20220	BTP20220	Tissue specific
CEARW	Superscaf	-	Superscaf	99078841	99078855	-10	10	14	-	-	gene	-	-	mRNA	NCP	Intron	gene|mRN	ID=gene-E	5	6.182	2497.9	0.263348431	C(+57.02)	55	361.1622	2	720.3013	Carbamido	I	Superscaf	99078987	0	BTP20220	BTP20220	0	0	0	Tissue specific
KMGLR	Superscaf	+	Superscaf	99079822	99079836	30	30	34	-	-	gene	-	-	mRNA	NCP	Intron	gene|mRN	ID=gene-E	5	7.64	2139.4	0.263360939	KMGLR	60	604.3508	1	603.3527	.	R	Superscaf	99079735	0	0	BTP20220	0	0	0	Tissue specific
NFKLLS	Superscaf	+	Superscaf	99199645	99199662	1	1	6	-	-	-	-	-	-	NCP	IGR	-	ID=gene-E	6	6.378333333	2542.7	0.264888795	NFKLLS	53	721.4225	1	720.417	.	N	Superscaf	99199645	0	BTP20220	BTP20220	BTP20220	0	0	Mixed
AYLFE	Superscaf	+	Superscaf	99239563	99239577	14	14	18	-	-	-	-	-	-	NCP	IGR	-	ID=gene-E	5	5.272	1937.1	0.265397787	A(+42.01)	69	684.3218	1	683.3166	Acetylati	N	Superscaf	99239524	0	0	0	0	0	BTP20220	Tissue specific
SEDLLKF	Superscaf	-	Superscaf	99263317	99263337	-13	13	19	-	-	-	-	-	-	NCP	IGR	-	ID=gene-E	7	5.578571429	2783	0.265700673	SEDLLKF	94	426.2284	2	850.4436	.	S	Superscaf	99263403	0	0	0	0	BTP20220	0	Tissue specific
KHNTQ	Superscaf	+	Superscaf	99284797	99284811	3	3	7	-	-	gene	-	-	mRNA	NCP	Intron	gene|mRN	ID=gene-E	5	6.984	2693	0.265974563	KHNTQ	54	627.3313	1	626.3136	.	M	Superscaf	99284791	0	BTP20220	BTP20220	0	BTP20220	BTP20220	Tissue specific
QVAVPP	Superscaf	+	Superscaf	99538993	99539010	8	8	13	-	-	-	-	-	-	NCP	IGR	-	ID=gene-E	6	4.045	1596.8	0.269215802	Q(+42.01)	56	652.3738	1	651.3592	Acetylati	E	Superscaf	99538972	0	0	BTP20220	0	0	0	Tissue specific
QDVCALLL	Superscaf	-	Superscaf	99851167	99851190	-5	5	12	-	-	-	-	-	-	NCP	IGR	-	ID=gene-E	8	4.7	2979.4	0.273196314	QDVC(+57.	62	466.2407	2	930.4844	Carbamido	F	Superscaf	99851196	0	0	0	0	0	BTP20220	Tissue specific
QPVREK	Superscaf	+	Superscaf	99876838	99876855	1	1	6	-	-	-	-	-	-	NCP	IGR	-	ID=gene-E	6	5.945	2490.8	0.273523643	QPVREK	70	378.7209	2	755.429	.	Q	Superscaf	99876838	BTP20220	BTP20220	BTP20220	BTP20220	BTP20220	BTP20220	Tissue specific
LLFGP	Superscaf	-	Superscaf	99922342	99922356	-4	4	8	-	-	-	-	-	-	NCP	IGR	-	ID=gene-E	5	5.942	1728.9	0.274103862	LLFGP	99	546.3274	1	545.3213	.	Y	Superscaf	99922413	0	0	BTP20220	0	0	0	Tissue specific
SNSPRF	Superscaf	-	Superscaf	1E+08	1E+08	-11	11	16	-	-	-	-	-	-	NCP	IGR	-	ID=gene-E	6	6.551666667	2737.1	0.27510513	SNSPRF	62	354.1764	2	706.3398	.	G	Superscaf	1E+08	0	0	0	0	0	BTP20220	Tissue specific
KEEELVEV	Superscaf	-	Superscaf	1E+08	1E+08	-6	6	13	-	-	-	-	-	-	NCP	IGR	-	ID=gene-E	8	3.575	2523.8	0.275143421	KEEELVEV	73	487.7539	2	973.4968	.	R	Superscaf	1E+08	0	0	0	0	BTP20220	0	Tissue specific
DYTRHFT	Superscaf	-	Superscaf	1E+08	1E+08	-24	24	30	-	-	-	-	-	-	NCP	IGR	-	ID=gene-E	7	6.502857143	3321.9	0.276272261	DYTRHFT	96	470.2184	2	938.4246	.	L	Superscaf	1E+08	0	0	BTP20220	0	0	0	Tissue specific
ESYKNPRR	Superscaf	-	Superscaf	1E+08	1E+08	-22	22	29	-	-	-	-	-	-	NCP	IGR	-	ID=gene-E	8	7.19125	3968.5	0.278122589	ESYKNPRR	55	525.2723	2	1048.541	.	T	Superscaf	1E+08	BTP20220	0	0	0	0	0	Tissue specific
LCFTVPL	Superscaf	-	Superscaf	1E+08	1E+08	-44	44	50	-	-	-	-	-	-	NCP	IGR	-	ID=gene-E	7	5.041428571	2414.8	0.278594973	LC(+57.0	55	425.2298	2	848.4466	Carbamido	I	Superscaf	1E+08	0	0	0	0	0	BTP20220	Tissue specific
KAHFRP	Superscaf	+	Superscaf	1E+08	1E+08	37	37	42	-	-	-	-	-	-	NCP	IGR	-	ID=gene-E	6	7.648333333	2773	0.278784783	KAHFRP	72	378.2132	2	754.4238	.	I	Superscaf	1E+08	0	BTP20220	BTP20220	0	BTP20220	BTP20220	Tissue specific
LLGLQF	Superscaf	+	Superscaf	1E+08	1E+08	86	86	91	-	-	-	-	-	-	NCP	IGR	-	ID=gene-E	6	5.84	2355.6	0.280761001	LLGLQF	76	690.4166	1	689.4112	.	K	Superscaf	1E+08	0	BTP20220	0	0	BTP20220	BTP20220	Tissue specific
TTWGP	Superscaf	-	Superscaf	1.01E+08	1.01E+08	-33	33	37	-	-	-	-	-	-	NCP	IGR	-	ID=gene-E	5	6.244	1956.3	0.281519975	T(+42.01)	53	603.2673	1	602.27	Acetylati	V	Superscaf	1.01E+08	0	BTP20220	0	0	0	0	Tissue specific
KPHLTSK	Superscaf	-	Superscaf	1.01E+08	1.01E+08	-19	19	25	-	-	-	-	-	-	NCP	IGR	-	ID=gene-E	7	7.365714286	3028.2	0.281996911	KPHLTSK	50	405.7502	2	809.4759	.	V	Superscaf	1.01E+08	0	0	0	0	BTP20220	0	Tissue specific
GLMKP	Superscaf	+	Superscaf	1.01E+08	1.01E+08	27	27	31	-	-	-	-	-	-	NCP	IGR	-	ID=gene-E	5	6.748	1828	0.283664658	GLM(+15.9	58	561.3125	1	560.2992	Oxidation	Y	Superscaf	1.01E+08	0	0	0	0	BTP20220	0	Tissue specific
KLDLSNRP	Superscaf	+	Superscaf	1.01E+08	1.01E+08	16	16	23	-	-	-	-	-	-	NCP	IGR	-	ID=gene-E	8	6.6025	3553	0.284188798	KLDLSNRP	98	471.7656	2	941.5294	.	C	Superscaf	1.01E+08	0	0	0	BTP20220	0	0	Tissue specific
ARYLLF	Superscaf	-	Superscaf	1.01E+08	1.01E+08	-14	14	19	-	-	-	-	-	-	NCP	IGR	-	ID=gene-E	6	6.646666667	2513.8	0.284219936	ARYLLF	60	391.7389	2	781.4486	.	I	Superscaf	1.01E+08	BTP20220	0	0	0	0	0	Tissue specific
VCLVA	Superscaf	-	Superscaf	1.01E+08	1.01E+08	-32	32	36	-	-	-	-	-	-	NCP	IGR	-	ID=gene-E	5	3.404	1250.4	0.284265303	VC(+57.0	54	561.3035	1	560.2992	Carbamido	G	Superscaf	1.01E+08	0	0	BTP20220	0	BTP20220	0	Tissue specific
KPDVPSH	Superscaf	-	Superscaf	1.01E+08	1.01E+08	-42	42	48	-	-	-	-	-	-	NCP	IGR	-	ID=gene-E	7	5.511428571	2557.8	0.286050907	KPDVPSH	64	390.2046	2	778.3973	.	R	Superscaf	1.01E+08	0	0	BTP20220	0	0	0	Tissue specific
STPMFAK	Superscaf	+	Superscaf	1.01E+08	1.01E+08	2	2	8	-	-	-	-	-	-	NCP	IGR	-	ID=gene-E	7	6.5	2656.9	0.288624366	STPMFAK	55	391.2065	2	780.384	.	P	Superscaf	1.01E+08	0	0	BTP20220	0	0	0	Tissue specific
VLSVEL	Superscaf	+	Superscaf	1.01E+08	1.01E+08	6	6	11	-	-	-	-	-	-	NCP	IGR	-	ID=gene-E	6	3.476666667	1515.7	0.289243373	VLSVEL	68	659.396	1	658.3901	.	N	Superscaf	1.01E+08	0	BTP20220	0	0	0	0	Mixed
TPLTRLY	Superscaf	+	Superscaf	1.01E+08	1.01E+08	13	13	19	-	-	-	-	-	-	NCP	IGR	-	ID=gene-E	7	6.82	2947.5	0.291202376	TPLTRLY	79	863.4951	1	862.4912	.	K	Superscaf	1.01E+08	0	0	0	0	BTP20220	0	Tissue specific
MCCMP	Superscaf	-	Superscaf	1.01E+08	1.01E+08	-6	6	10	-	-	-	-	-	-	NCP	IGR	-	ID=gene-E	5	5.568	2251.8	0.291514902	M(+42.01)	53	378.614	2	755.2111	Acetylati	I	Superscaf	1.01E+08	0	0	0	0	0	BTP20220	Tissue specific
QSPPTFYQ	Superscaf	-	Superscaf	1.01E+08	1.01E+08	-11	11	18	-	-	-	-	-	-	NCP	IGR	-	ID=gene-E	8	5.90625	3524	0.291650508	Q(+42.01)	64	505.226	2	1008.455	Acetylati	P	Superscaf	1.01E+08	0	BTP20220	0	0	0	BTP20220	Tissue specific
LDAGVSRL	Superscaf	-	Superscaf	1.01E+08	1.01E+08	-58	58	65	-	-	-	-	-	-	NCP	IGR	-	ID=gene-E	8	5.42	2759.1	0.2925133	LDAGVSRL	55	415.7328	2	829.4658	.	S	Superscaf	1.01E+08	0	BTP20220	0	BTP20220	0	0	Tissue specific
HLLLPVNF	Superscaf	+	Superscaf	1.01E+08	1.01E+08	3	3	10	-	-	gene	-	-	mRNA	NCP	Intron	gene|mRN	ID=gene-E	8	5.34	3001.4	0.293849127	HLLLPVNF	77	476.788	2	951.5541	.	I	Superscaf	1.01E+08	0	BTP20220	BTP20220	BTP20220	BTP20220	BTP20220	Tissue specific
QVTPGKK	Superscaf	-	Superscaf	1.01E+08	1.01E+08	-35	35	41	-	-	gene	-	-	mRNA	NCP	Intron	gene|mRN	ID=gene-E	7	6.275714286	2579.7	0.294141723	Q(+42.01)	61	400.2407	2	798.46	Acetylati	H	Superscaf	1.01E+08	0	0	0	0	BTP20220	0	Tissue specific
KYVDY	Superscaf	-	Superscaf	1.02E+08	1.02E+08	-25	25	29	-	-	gene	-	-	mRNA	NCP	Intron	gene|mRN	ID=gene-E	5	4.806	1798.9	0.294880997	K(+42.01)	75	729.3422	1	728.3381	Acetylati	H	Superscaf	1.02E+08	0	0	BTP20220	0	0	0	Tissue specific
MDRRKK	Superscaf	+	Superscaf	1.02E+08	1.02E+08	4	4	9	-	-	gene	-	-	mRNA	NCP	Intron	gene|mRN	ID=gene-E	6	8.286666667	3017.4	0.295024981	M(+42.01)	66	438.255	2	874.4807	Acetylati	L	Superscaf	1.02E+08	0	0	0	0	BTP20220	0	Tissue specific
NPGHFP	Superscaf	-	Superscaf	1.02E+08	1.02E+08	-20	20	25	-	-	gene	-	-	mRNA	NCP	Intron	gene|mRN	ID=gene-E	6	6.175	2575.9	0.298698246	NPGHFP	55	668.3143	1	667.3078	.	N	Superscaf	1.02E+08	0	0	0	0	0	BTP20220	Tissue specific
RVFLPQ	Superscaf	+	Superscaf	1.02E+08	1.02E+08	43	43	48	-	-	-	-	-	-	NCP	IGR	-	ID=gene-E	6	5.695	2337.7	0.30039759	RVFLPQ	56	759.4595	1	758.4439	.	C	Superscaf	1.02E+08	0	0	0	0	0	BTP20220	Tissue specific
KTMLE	Superscaf	+	Superscaf	1.02E+08	1.02E+08	1	1	5	-	-	-	-	-	-	NCP	IGR	-	ID=gene-E	5	6.244	2016.3	0.301001793	KTM(+15.9	61	637.3171	1	636.3152	Oxidation	K	Superscaf	1.02E+08	0	0	BTP20220	0	0	0	Tissue specific
LPDMP	Superscaf	+	Superscaf	1.02E+08	1.02E+08	52	52	56	-	-	-	-	-	-	NCP	IGR	-	ID=gene-E	5	5.46	1811.2	0.301388873	LPDMP	72	572.2739	1	571.2676	.	S	Superscaf	1.02E+08	0	0	BTP20220	0	0	0	Tissue specific
SLLYQE	Superscaf	+	Superscaf	1.02E+08	1.02E+08	37	37	42	-	-	-	-	-	-	NCP	IGR	-	ID=gene-E	6	5.361666667	2585.9	0.304538152	SLLYQE	84	752.3802	1	751.3752	.	E	Superscaf	1.02E+08	0	0	0	BTP20220	0	0	Tissue specific
AELKVEGK	Superscaf	-	Superscaf	1.02E+08	1.02E+08	-1	1	8	-	-	-	-	-	-	NCP	IGR	-	ID=gene-E	8	5.48625	2749.8	0.304587766	A(+42.01)	78	458.2542	2	914.5073	Acetylati	A	Superscaf	1.02E+08	0	0	0	0	0	BTP20220	Tissue specific
KGPVLLH	Superscaf	-	Superscaf	1.02E+08	1.02E+08	-13	13	19	-	-	-	-	-	-	NCP	IGR	-	ID=gene-E	7	5.937142857	2429.6	0.305389698	KGPVLLH	64	382.2429	2	762.4752	.	K	Superscaf	1.02E+08	0	0	BTP20220	0	0	0	Tissue specific
LLLKSLP	Superscaf	+	Superscaf	1.02E+08	1.02E+08	28	28	34	-	-	-	-	-	-	NCP	IGR	-	ID=gene-E	7	6.52	2602.8	0.305852748	L(+42.01)	76	413.2733	2	824.5371	Acetylati	R	Superscaf	1.02E+08	0	0	0	0	BTP20220	BTP20220	Tissue specific
GKQDLLSF	Superscaf	-	Superscaf	1.02E+08	1.02E+08	-12	12	19	-	-	-	-	-	-	NCP	IGR	-	ID=gene-E	8	5.93125	3265.5	0.306295142	G(+42.01)	59	475.2508	2	948.4916	Acetylati	T	Superscaf	1.02E+08	0	0	0	0	0	BTP20220	Tissue specific
CSYEGP	Superscaf	-	Superscaf	1.02E+08	1.02E+08	-8	8	13	-	-	gene	-	-	mRNA	NCP	Intron	gene|mRN	ID=gene-E	6	5.308333333	2488.8	0.30687976	C(+57.02)	57	712.2485	1	711.2534	Carbamido	P	Superscaf	1.02E+08	0	0	0	0	0	BTP20220	Mixed
EFTLL	Superscaf	-	Superscaf	1.03E+08	1.03E+08	-28	28	32	-	-	gene	-	-	mRNA	NCP	Intron	gene|mRN	ID=gene-E	5	5.438	1917.2	0.307189952	EFTLL	81	622.3438	1	621.3373	.	G	Superscaf	1.03E+08	0	0	0	0	BTP20220	0	Tissue specific
ENFRAL	Superscaf	+	Superscaf	1.03E+08	1.03E+08	3	3	8	-	-	gene	-	-	mRNA	NCP	Intron	gene|mRN	ID=gene-E	6	6.145	2723.1	0.307912012	ENFRAL	73	375.1992	2	748.3868	.	P	Superscaf	1.03E+08	0	BTP20220	0	0	0	0	Mixed
RNALNVQ	Superscaf	+	Superscaf	1.03E+08	1.03E+08	19	19	25	-	-	-	-	-	-	NCP	IGR	-	ID=gene-E	7	5.604285714	3117.6	0.311538533	RNALNVQ	60	407.7294	2	813.4457	.	L	Superscaf	1.03E+08	0	BTP20220	0	0	0	BTP20220	Tissue specific
VYVYK	Superscaf	-	Superscaf	1.03E+08	1.03E+08	-16	16	20	-	-	-	-	-	-	NCP	IGR	-	ID=gene-E	5	4.212	1387.4	0.315251581	VYVYK	96	671.3743	1	670.369	.	K	Superscaf	1.03E+08	0	BTP20220	0	0	0	BTP20220	Tissue specific
KPMLPALR	Superscaf	-	Superscaf	1.03E+08	1.03E+08	-4	4	11	-	-	-	-	-	-	NCP	IGR	-	ID=gene-E	8	7.10375	3181.6	0.315681849	KPMLPALR	52	463.2864	2	924.5579	.	P	Superscaf	1.03E+08	0	BTP20220	0	0	0	BTP20220	Tissue specific
YVSVY	Superscaf	-	Superscaf	1.03E+08	1.03E+08	-9	9	13	-	-	-	-	-	-	NCP	IGR	-	ID=gene-E	5	3.4	1302.4	0.318875921	YVSVY	73	630.3107	1	629.3061	.	G	Superscaf	1.03E+08	0	0	0	0	BTP20220	0	Tissue specific
GLSGLL	Superscaf	-	Superscaf	1.04E+08	1.04E+08	-19	19	24	-	-	-	-	-	-	NCP	IGR	-	ID=gene-E	6	5.926666667	2038.2	0.31997022	G(+42.01)	64	601.354	1	600.3483	Acetylati	L	Superscaf	1.04E+08	0	BTP20220	0	0	BTP20220	0	Tissue specific
KQADLLH	Superscaf	-	Superscaf	1.04E+08	1.04E+08	-1	1	7	-	-	-	-	-	-	NCP	IGR	-	ID=gene-E	7	6.275714286	3128.4	0.320460277	KQADLLH	67	412.7354	2	823.4552	.	K	Superscaf	1.04E+08	BTP20220	0	0	0	0	0	Tissue specific
KSLEF	Superscaf	+	Superscaf	1.04E+08	1.04E+08	7	7	11	-	-	-	-	-	-	NCP	IGR	-	ID=gene-E	5	6.02	2018.1	0.321764048	KSLEF	63	623.3369	1	622.3326	.	E	Superscaf	1.04E+08	0	0	0	0	0	BTP20220	Tissue specific
CLCAF	Superscaf	-	Superscaf	1.04E+08	1.04E+08	-23	23	27	-	-	-	-	-	-	NCP	IGR	-	ID=gene-E	5	5.504	2223.5	0.322628217	C(+57.02)	53	712.2747	1	711.272	Carbamido	G	Superscaf	1.04E+08	BTP20220	0	0	0	0	0	Tissue specific
VPLPRVFW	Superscaf	-	Superscaf	1.04E+08	1.04E+08	-23	23	30	-	-	-	-	-	-	NCP	IGR	-	ID=gene-E	8	5.08875	2591	0.323405897	V(+42.01)	57	352.5373	3	1054.596	Acetylati	L	Superscaf	1.04E+08	0	0	0	0	0	BTP20220	Tissue specific
LVPVAEGY	Superscaf	-	Superscaf	1.04E+08	1.04E+08	-22	22	29	-	-	-	-	-	-	NCP	IGR	-	ID=gene-E	8	4.14375	2184.4	0.324761233	LVPVAEGY	85	847.4525	1	846.4487	.	P	Superscaf	1.04E+08	0	BTP20220	0	BTP20220	0	0	Tissue specific
LSLKDL	Superscaf	+	Superscaf	1.04E+08	1.04E+08	16	16	21	-	-	-	-	-	-	NCP	IGR	-	ID=gene-E	6	6.055	2323.5	0.325453724	LSLKDL	50	688.4214	1	687.4167	.	I	Superscaf	1.04E+08	0	0	BTP20220	0	0	0	Tissue specific
NPLEVRK	Superscaf	-	Superscaf	1.04E+08	1.04E+08	-6	6	12	-	-	-	-	-	-	NCP	IGR	-	ID=gene-E	7	5.915714286	2830.2	0.32574303	NPLEVRK	81	428.255	2	854.4974	.	V	Superscaf	1.04E+08	0	0	0	0	BTP20220	BTP20220	Tissue specific
QKAQLSY	Superscaf	+	Superscaf	1.04E+08	1.04E+08	7	7	13	-	-	gene	-	-	mRNA	NCP	Intron	gene|mRN	ID=gene-E	7	6.34	3197.4	0.329250814	Q(+42.01)	64	879.4439	1	878.4498	Acetylati	Y	Superscaf	1.04E+08	0	BTP20220	0	0	0	BTP20220	Tissue specific
LEEAEVLPI	Superscaf	-	Superscaf	1.04E+08	1.04E+08	-85	85	93	-	-	gene	-	-	mRNA	NCP	Intron	gene|mRN	ID=gene-E	9	4.435555556	2985.4	0.32925594	LEEAEVLPI	54	506.7806	2	1011.549	.	C	Superscaf	1.04E+08	0	0	0	BTP20220	0	0	Mixed
LEEAEVLP	Superscaf	-	Superscaf	1.04E+08	1.04E+08	-85	85	92	-	-	gene	-	-	mRNA	NCP	Intron	gene|mRN	ID=gene-E	8	4.2425	2632	0.32925594	LEEAEVLP	79	450.2393	2	898.4647	.	C	Superscaf	1.04E+08	0	0	0	0	BTP20220	0	Tissue specific
KVVSGPK	Superscaf	+	Superscaf	1.04E+08	1.04E+08	20	20	26	-	-	gene	-	-	mRNA	NCP	Intron	gene|mRN	ID=gene-E	7	5.347142857	1954.9	0.329453668	KVVSGPK	94	357.7286	2	713.4435	.	L	Superscaf	1.04E+08	0	0	0	0	BTP20220	0	Tissue specific
VNLPK	Superscaf	-	Superscaf	1.04E+08	1.04E+08	-7	7	11	-	-	-	-	-	-	NCP	IGR	-	ID=gene-E	5	5.486	1755.9	0.330549114	VNLPK	95	570.3594	1	569.3537	.	G	Superscaf	1.04E+08	0	0	0	0	0	BTP20220	Mixed
KPTTVAP	Superscaf	-	Superscaf	1.04E+08	1.04E+08	-77	77	83	-	-	-	-	-	-	NCP	IGR	-	ID=gene-E	7	5.917142857	2249.5	0.330667583	KPTTVAP	52	713.428	1	712.4119	.	S	Superscaf	1.04E+08	0	0	0	0	BTP20220	0	Tissue enhanced
KESDLTKGI	Superscaf	+	Superscaf	1.04E+08	1.04E+08	36	36	44	-	-	-	-	-	-	NCP	IGR	-	ID=gene-E	9	6.201111111	3558.8	0.331677993	KESDLTKGI	63	495.7719	2	989.5393	.	R	Superscaf	1.04E+08	0	0	0	BTP20220	0	0	Tissue specific
FRSLFL	Superscaf	-	Superscaf	1.05E+08	1.05E+08	-19	19	24	-	-	-	-	-	-	NCP	IGR	-	ID=gene-E	6	6.56	2513.8	0.333692808	FRSLFL	50	391.7388	2	781.4486	.	L	Superscaf	1.05E+08	0	BTP20220	0	0	BTP20220	0	Tissue specific
KLYPPPP	Superscaf	+	Superscaf	1.05E+08	1.05E+08	8	8	14	-	-	-	-	-	-	NCP	IGR	-	ID=gene-E	7	6.654285714	2630.9	0.335368358	KLYPPPP	92	406.2393	2	810.4639	.	K	Superscaf	1.05E+08	0	BTP20220	0	0	0	0	Tissue enhanced
RLALGGYL	Superscaf	-	Superscaf	1.05E+08	1.05E+08	-48	48	55	-	-	-	-	-	-	NCP	IGR	-	ID=gene-E	8	6.54	3074.4	0.335487898	RLALGGYL	56	431.7609	2	861.5072	.	Y	Superscaf	1.05E+08	0	0	0	BTP20220	0	0	Tissue specific
KKVPAAP	Superscaf	-	Superscaf	1.05E+08	1.05E+08	-55	55	61	-	-	-	-	-	-	NCP	IGR	-	ID=gene-E	7	6.302857143	2234.2	0.335661604	KKVPAAP	50	355.73	2	709.4486	.	Q	Superscaf	1.05E+08	BTP20220	0	0	0	0	0	Tissue enhanced
MTMKYP	Superscaf	-	Superscaf	1.05E+08	1.05E+08	-17	17	22	-	-	-	-	-	-	NCP	IGR	-	ID=gene-E	6	6.621666667	2405.8	0.339613694	M(+42.01)	84	406.6866	2	811.3608	Acetylati	R	Superscaf	1.05E+08	0	0	0	BTP20220	0	0	Tissue specific
RLVSPQTT	Superscaf	+	Superscaf	1.05E+08	1.05E+08	28	28	35	-	-	-	-	-	-	NCP	IGR	-	ID=gene-E	8	5.92875	3128.7	0.341481006	RLVSPQTT	53	451.2512	2	900.5029	.	L	Superscaf	1.05E+08	0	0	BTP20220	0	0	0	Tissue specific
TKGGSS	Superscaf	+	Superscaf	1.05E+08	1.05E+08	12	12	17	-	-	-	-	-	-	NCP	IGR	-	ID=gene-E	6	6.595	2227.3	0.341831019	TKGGSS	51	536.2695	1	535.2602	.	P	Superscaf	1.05E+08	0	0	0	0	BTP20220	BTP20220	Tissue specific
KYFFVP	Superscaf	-	Superscaf	1.05E+08	1.05E+08	-4	4	9	-	-	gene	-	-	mRNA	NCP	Intron	gene|mRN	ID=gene-E	6	5.443333333	2040.1	0.343164513	KYFFVP	67	400.718	2	799.4268	.	Y	Superscaf	1.05E+08	0	0	0	BTP20220	0	0	Tissue specific
PGAKPGAK	Superscaf	-	Superscaf	1.05E+08	1.05E+08	-23	23	30	-	-	gene	-	-	mRNA	NCP	Intron	gene|mRN	ID=gene-E	8	7.0075	2828.8	0.343185934	PGAKPGAK	71	363.2172	2	724.4232	.	H	Superscaf	1.05E+08	BTP20220	0	0	0	0	0	Tissue specific
HLAKDSGFI	Superscaf	+	Superscaf	1.05E+08	1.05E+08	136	136	148	-	-	-	-	-	-	NCP	IGR	-	ID=gene-E	13	5.598461538	4631.8	0.343374291	HLAKDSGFI	79	470.2592	3	1407.762	.	A	Superscaf	1.05E+08	0	0	0	BTP20220	0	0	Tissue specific
PLPPPPYK	Superscaf	+	Superscaf	1.05E+08	1.05E+08	5	5	12	-	-	-	-	-	-	NCP	IGR	-	ID=gene-E	8	6.61	2968.3	0.344280654	PLPPPPYK	80	454.7638	2	907.5167	.	L	Superscaf	1.05E+08	0	0	BTP20220	0	0	0	Tissue specific
NTLAFVLL	Superscaf	-	Superscaf	1.06E+08	1.06E+08	-1	1	8	-	-	gene	-	-	mRNA	NCP	Intron	gene|mRN	ID=gene-E	8	5.1725	2753.1	0.345756524	N(+42.01)	55	466.774	2	931.5378	Acetylati	N	Superscaf	1.06E+08	0	0	0	0	0	BTP20220	Tissue specific
DGLLSL	Superscaf	+	Superscaf	1.06E+08	1.06E+08	4	4	9	-	-	gene	-	-	mRNA	NCP	Intron	gene|mRN	ID=gene-E	6	5.426666667	2152.4	0.34601431	DGLLSL	75	617.3478	1	616.3432	.	D	Superscaf	1.06E+08	0	BTP20220	BTP20220	0	BTP20220	BTP20220	Tissue specific
KVAAAAPK	Superscaf	+	Superscaf	1.06E+08	1.06E+08	1	1	8	-	-	-	-	-	-	NCP	IGR	-	ID=gene-E	8	6.2325	2519.4	0.346341333	KVAAAAPK	72	378.2479	2	754.4701	.	K	Superscaf	1.06E+08	0	0	BTP20220	0	BTP20220	0	Tissue specific
ELKKVF	Superscaf	+	Superscaf	1.06E+08	1.06E+08	22	22	27	-	exon	gene	three_pri	-	mRNA	NCP	3UTR	exon|gene	ID=exon-E	6	5.693333333	2103.1	0.346617748	ELKKVF	93	382.2377	2	762.4639	.	L	Superscaf	1.06E+08	0	0	BTP20220	BTP20220	0	0	Mixed
YEVPPF	Superscaf	+	Superscaf	1.06E+08	1.06E+08	7	7	12	-	-	gene	-	-	mRNA	NCP	Intron	gene|mRN	ID=gene-E	6	4.493333333	1947.2	0.347060907	YEVPPF	79	751.3651	1	750.3588	.	F	Superscaf	1.06E+08	0	0	0	0	BTP20220	BTP20220	Mixed
PPPSLAAP	Superscaf	+	Superscaf	1.06E+08	1.06E+08	12	12	19	-	-	gene	-	-	mRNA	NCP	Intron	gene|mRN	ID=gene-E	8	6.1125	2709	0.347254619	PPPSLAAP	83	375.2163	2	748.4119	.	E	Superscaf	1.06E+08	0	0	0	0	0	BTP20220	Tissue specific
KSYSDKGF	Superscaf	+	Superscaf	1.06E+08	1.06E+08	1	1	8	-	-	gene	-	-	mRNA	NCP	Intron	gene|mRN	ID=gene-E	8	6.365	3259.3	0.349037774	KSYSDKGF	52	466.2271	2	930.4446	.	K	Superscaf	1.06E+08	0	BTP20220	0	0	0	0	Tissue specific
EGLLLLPK	Superscaf	+	Superscaf	1.06E+08	1.06E+08	11	11	18	-	-	gene	-	-	mRNA	NCP	Intron	gene|mRN	ID=gene-E	8	6.14375	2942.2	0.35011287	EGLLLLPK	85	441.7847	2	881.5585	.	K	Superscaf	1.06E+08	0	0	0	0	0	BTP20220	Tissue specific
YEGKSLR	Superscaf	+	Superscaf	1.06E+08	1.06E+08	19	19	25	-	-	gene	-	-	mRNA	NCP	Intron	gene|mRN	ID=gene-E	7	6.715714286	3036.3	0.350158506	Y(+42.01)	89	447.7424	2	893.4606	Acetylati	P	Superscaf	1.06E+08	0	0	0	0	0	BTP20220	Tissue specific
PNYKLP	Superscaf	+	Superscaf	1.06E+08	1.06E+08	20	20	25	-	-	gene	-	-	mRNA	NCP	Intron	gene|mRN	ID=gene-E	6	6.565	2552.8	0.350338868	PNYKLP	51	366.2016	2	730.4014	.	L	Superscaf	1.06E+08	0	0	BTP20220	0	0	0	Tissue specific
FDGVKLR	Superscaf	+	Superscaf	1.06E+08	1.06E+08	36	36	42	-	-	-	-	-	-	NCP	IGR	-	ID=gene-E	7	5.842857143	2566.8	0.351024167	FDGVKLR	91	417.7437	2	833.4759	.	Y	Superscaf	1.06E+08	0	0	0	0	BTP20220	0	Tissue specific
FGPLKYY	Superscaf	-	Superscaf	1.06E+08	1.06E+08	-66	66	72	-	-	-	-	-	-	NCP	IGR	-	ID=gene-E	7	6.398571429	2762.9	0.351598801	FGPLKYY	96	444.2354	2	886.4589	.	L	Superscaf	1.06E+08	0	0	0	0	BTP20220	0	Tissue specific
LLLRAF	Superscaf	+	Superscaf	1.06E+08	1.06E+08	38	38	43	-	-	-	-	-	-	NCP	IGR	-	ID=gene-E	6	6.7	2407.7	0.352690269	LLLRAF	91	366.7402	2	731.4694	.	H	Superscaf	1.06E+08	0	0	0	0	BTP20220	0	Tissue specific
AVPPKGM	Superscaf	-	Superscaf	1.06E+08	1.06E+08	-3	3	9	-	-	gene	-	-	mRNA	NCP	Intron	gene|mRN	ID=gene-E	7	5.725714286	2123.3	0.353325725	A(+42.01)	61	741.3868	1	740.3891	Acetylati	K	Superscaf	1.06E+08	0	0	0	0	BTP20220	0	Mixed
KGTTFLRY	Superscaf	-	Superscaf	1.06E+08	1.06E+08	-5	5	12	-	-	-	-	-	-	NCP	IGR	-	ID=gene-E	8	7.08125	3409.8	0.354363562	KGTTFLRY	90	493.2751	2	984.5392	.	A	Superscaf	1.06E+08	0	0	0	0	BTP20220	0	Tissue specific
ADDLRFK	Superscaf	+	Superscaf	1.07E+08	1.07E+08	12	12	18	-	-	-	-	-	-	NCP	IGR	-	ID=gene-E	7	6.274285714	2992.3	0.35809172	A(+42.01)	79	453.7363	2	905.4606	Acetylati	M	Superscaf	1.07E+08	0	0	0	0	0	BTP20220	Tissue specific
KNFMPK	Superscaf	-	Superscaf	1.07E+08	1.07E+08	-1	1	6	-	-	gene	-	-	mRNA	NCP	Intron	gene|mRN	ID=gene-E	6	7.07	2629.8	0.35941259	KNFM(+15.	64	390.7057	2	779.4	Oxidation	K	Superscaf	1.07E+08	0	0	0	0	0	BTP20220	Tissue specific
NLLEFK	Superscaf	+	Superscaf	1.07E+08	1.07E+08	19	19	24	-	-	-	-	-	-	NCP	IGR	-	ID=gene-E	6	5.968333333	2584.8	0.360694327	NLLEFK	95	382.2194	2	762.4276	.	H	Superscaf	1.07E+08	0	0	0	0	0	BTP20220	Tissue specific
GVAVLLEN	Superscaf	+	Superscaf	1.07E+08	1.07E+08	62	62	69	-	-	gene	-	-	mRNA	NCP	Intron	gene|mRN	ID=gene-E	8	4.0725	2337.6	0.362144148	GVAVLLEN	78	814.4634	1	813.4596	.	N	Superscaf	1.07E+08	0	BTP20220	BTP20220	BTP20220	BTP20220	0	Tissue specific
QSAKALVY	Superscaf	+	Superscaf	1.07E+08	1.07E+08	10	10	17	-	-	gene	-	-	mRNA	NCP	Intron	gene|mRN	ID=gene-E	8	5.59375	2898	0.362185193	QSAKALVY	74	440.2491	2	878.4861	.	N	Superscaf	1.07E+08	0	0	0	0	BTP20220	0	Tissue specific
LNVLPF	Superscaf	-	Superscaf	1.07E+08	1.07E+08	-14	14	19	-	-	-	-	-	-	NCP	IGR	-	ID=gene-E	6	4.858333333	2028.3	0.363278153	LNVLPF	78	702.4158	1	701.4112	.	V	Superscaf	1.07E+08	0	BTP20220	BTP20220	BTP20220	BTP20220	0	Tissue specific
LLVAVAF	Superscaf	+	Superscaf	1.07E+08	1.07E+08	35	35	41	-	-	-	-	-	-	NCP	IGR	-	ID=gene-E	7	4.211428571	1716.8	0.363758876	LLVAVAF	56	732.462	1	731.4581	.	S	Superscaf	1.07E+08	0	0	0	BTP20220	0	0	Tissue specific

FVLAP	Superscaf	-	Superscaf	1.07E+08	1.07E+08	-35	35	39	-	-	-	-	-	-	NCP	IGR	-	ID=gene-E	5	4.756	1389.5	0.366475056	FVLAP	89	546.3264	1	545.3213	.	T	Superscaf	1.07E+08	BTP20220	BTP20220	0	0	0	0	Tissue specific
KMATGHF	Superscaf	-	Superscaf	1.07E+08	1.07E+08	-5	5	11	-	-	-	-	-	-	NCP	IGR	-	ID=gene-E	7	6.725714286	2853.1	0.36716177	KM(+15.99	51	404.1985	2	806.3745	Oxidation	V	Superscaf	1.07E+08	0	BTP20220	0	0	0	0	Tissue specific
KSLQP	Superscaf	+	Superscaf	1.07E+08	1.07E+08	14	14	18	-	-	gene	-	-	mRNA	NCP	Intron	gene|mRNA	ID=gene-E	5	6.67	2153.3	0.368086571	KSLQP	59	572.3398	1	571.3329	.	T	Superscaf	1.07E+08	0	0	BTP20220	0	0	0	Tissue specific
AERPVR	Superscaf	-	Superscaf	1.07E+08	1.07E+08	-11	11	16	-	-	gene	-	-	mRNA	NCP	Intron	gene|mRNA	ID=gene-E	6	6.176666667	2371.8	0.370167677	A(+42.01)	83	769.4278	1	768.4242	Acetylati	P	Superscaf	1.07E+08	0	0	0	BTP20220	0	0	Tissue specific
TLPMMLP	Superscaf	-	Superscaf	1.07E+08	1.07E+08	-2	2	8	-	-	-	-	-	-	NCP	IGR	-	ID=gene-E	7	6.084285714	2522.1	0.370577327	T(+42.01)	72	422.7203	2	843.4235	Acetylati	L	Superscaf	1.07E+08	0	BTP20220	0	0	BTP20220	0	Tissue specific
KQGVPPR	Superscaf	+	Superscaf	1.08E+08	1.08E+08	1	1	7	-	-	-	-	-	-	NCP	IGR	-	ID=gene-E	7	6.388571429	2700	0.370880595	KQGVPPR	56	391.2361	2	780.4606	.	K	Superscaf	1.08E+08	0	BTP20220	BTP20220	0	0	0	Tissue specific
PLLMFPPP	Superscaf	-	Superscaf	1.08E+08	1.08E+08	-2	2	9	-	-	-	-	-	-	NCP	IGR	-	ID=gene-E	8	6.04875	2815.3	0.371871573	PLLMFPPP	59	456.2646	2	910.4986	.	Q	Superscaf	1.08E+08	0	0	0	0	BTP20220	0	Tissue specific
PCGGM	Superscaf	-	Superscaf	1.08E+08	1.08E+08	-36	36	40	-	-	-	-	-	-	NCP	IGR	-	ID=gene-E	5	5.802	1889.2	0.372714933	PC(+57.02	53	521.1847	1	520.1774	Carbamido	Q	Superscaf	1.08E+08	0	BTP20220	0	0	0	0	Tissue specific
NSFLKLFW	Superscaf	-	Superscaf	1.08E+08	1.08E+08	-1	1	8	-	-	-	-	-	-	NCP	IGR	-	ID=gene-E	8	6.205	3456.7	0.37283409	N(+42.01)	54	548.8052	2	1095.575	Acetylati	N	Superscaf	1.08E+08	0	0	0	BTP20220	0	0	Tissue specific
DFLSF	Superscaf	+	Superscaf	1.08E+08	1.08E+08	5	5	9	-	-	-	-	-	-	NCP	IGR	-	ID=gene-E	5	5.118	1923.1	0.372840937	DFLSF	88	628.2955	1	627.2904	.	L	Superscaf	1.08E+08	0	0	0	BTP20220	0	0	Tissue specific
VTEPHHLK	Superscaf	+	Superscaf	1.08E+08	1.08E+08	17	17	24	-	-	-	-	-	-	NCP	IGR	-	ID=gene-E	8	5.86875	3221.6	0.373189268	V(+42.01)	60	501.7666	2	1001.529	Acetylati	V	Superscaf	1.08E+08	0	0	0	0	0	BTP20220	Tissue specific
KGNQRFK	Superscaf	-	Superscaf	1.08E+08	1.08E+08	-12	12	18	-	-	-	-	-	-	NCP	IGR	-	ID=gene-E	7	7.535714286	3477.7	0.374085531	KGNQRFK	64	439.2467	2	876.493	.	N	Superscaf	1.08E+08	0	0	0	0	0	BTP20220	Tissue specific
RFGLTAH	Superscaf	+	Superscaf	1.08E+08	1.08E+08	11	11	17	-	-	-	-	-	-	NCP	IGR	-	ID=gene-E	7	6.904285714	3015.4	0.376922514	RFGLTAH	71	401.2245	2	800.4293	.	I	Superscaf	1.08E+08	0	0	0	0	0	BTP20220	Tissue specific
YGPMK	Superscaf	+	Superscaf	1.08E+08	1.08E+08	32	32	36	-	-	gene	-	-	mRNA	NCP	Intron	gene|mRNA	ID=gene-E	5	6.684	1934.1	0.381088246	YGPMK	97	595.2899	1	594.2836	.	W	Superscaf	1.08E+08	0	0	0	0	0	BTP20220	Tissue specific
KYCLR	Superscaf	-	Superscaf	1.08E+08	1.08E+08	-14	14	18	-	-	gene	-	-	mRNA	NCP	Intron	gene|mRNA	ID=gene-E	5	7.432	2515.8	0.382551684	K(+42.01)	59	391.2067	2	780.3952	Acetylati	R	Superscaf	1.08E+08	0	0	0	BTP20220	0	0	Tissue specific
NGFSLP	Superscaf	-	Superscaf	1.09E+08	1.09E+08	-6	6	11	-	-	gene	-	-	mRNA	NCP	Intron	gene|mRNA	ID=gene-E	6	5.803333333	2355.6	0.384887707	NGFSLP	60	634.3315	1	633.3122	.	E	Superscaf	1.09E+08	0	BTP20220	0	0	BTP20220	BTP20220	Tissue specific
TPAVSKY	Superscaf	-	Superscaf	1.09E+08	1.09E+08	-12	12	18	-	-	-	-	-	-	NCP	IGR	-	ID=gene-E	7	5.704285714	2357.5	0.385493173	T(+42.01)	75	807.4214	1	806.4174	Acetylati	A	Superscaf	1.09E+08	0	BTP20220	BTP20220	BTP20220	BTP20220	BTP20220	Tissue specific
RVKRRLFG	Superscaf	+	Superscaf	1.09E+08	1.09E+08	21	21	28	-	-	-	-	-	-	NCP	IGR	-	ID=gene-E	8	7.43125	3452.9	0.386017887	RVKRRLFG	62	516.3334	2	1030.651	.	N	Superscaf	1.09E+08	0	0	0	0	BTP20220	0	Tissue specific
PPPVLLFLI	Superscaf	+	Superscaf	1.09E+08	1.09E+08	22	22	30	-	-	-	-	-	-	NCP	IGR	-	ID=gene-E	9	5.366666667	2813.2	0.388027729	PPPVLLFLI	57	504.8307	2	1007.642	.	L	Superscaf	1.09E+08	0	0	0	0	0	BTP20220	Tissue specific
VVRTLK	Superscaf	-	Superscaf	1.09E+08	1.09E+08	-55	55	60	-	-	-	-	-	-	NCP	IGR	-	ID=gene-E	6	5.501666667	1868.1	0.388301275	V(+42.01)	69	379.2471	2	756.4858	Acetylati	L	Superscaf	1.09E+08	0	0	0	0	0	BTP20220	Tissue specific
KLLRPL	Superscaf	-	Superscaf	1.09E+08	1.09E+08	-2	2	7	-	-	-	-	-	-	NCP	IGR	-	ID=gene-E	6	7.456666667	2514.8	0.38900111	K(+42.01)	78	391.2694	2	780.5222	Acetylati	F	Superscaf	1.09E+08	0	0	0	0	0	BTP20220	Tissue enhanced
LLALKY	Superscaf	+	Superscaf	1.09E+08	1.09E+08	11	11	16	-	-	-	-	-	-	NCP	IGR	-	ID=gene-E	6	6.56	2299.4	0.389425984	LLALKY	98	360.7351	2	719.4581	.	N	Superscaf	1.09E+08	0	0	0	BTP20220	0	0	Tissue specific
ASKVLPR	Superscaf	-	Superscaf	1.09E+08	1.09E+08	-13	13	19	-	-	-	-	-	-	NCP	IGR	-	ID=gene-E	7	6.354285714	2502.7	0.390259551	ASKVLPR	91	385.7465	2	769.481	.	I	Superscaf	1.09E+08	0	0	0	0	BTP20220	BTP20220	Tissue specific
ERVWY	Superscaf	-	Superscaf	1.09E+08	1.09E+08	-32	32	36	-	-	-	-	-	-	NCP	IGR	-	ID=gene-E	5	5.106	2060.4	0.39034979	ERVWY	61	376.6887	2	751.3653	.	I	Superscaf	1.09E+08	0	0	0	0	0	BTP20220	Tissue specific
LLEGEERRN	Superscaf	+	Superscaf	1.09E+08	1.09E+08	40	40	48	-	-	gene	-	-	mRNA	NCP	Intron	gene|mRNA	ID=gene-E	9	6.095555556	3949.7	0.392273448	LLEGEERRN	67	566.7936	2	1131.571	.	Y	Superscaf	1.09E+08	0	0	0	0	0	BTP20220	Tissue specific
RLSEPYK	Superscaf	+	Superscaf	1.09E+08	1.09E+08	20	20	26	-	-	gene	-	-	mRNA	NCP	Intron	gene|mRNA	ID=gene-E	7	6.762857143	3076.4	0.394868634	RLSEPYK	59	446.7491	2	891.4814	.	S	Superscaf	1.09E+08	0	BTP20220	0	0	0	0	Tissue specific
CGKSP	Superscaf	+	Superscaf	1.09E+08	1.09E+08	12	12	16	-	-	-	-	-	-	NCP	IGR	-	ID=gene-E	5	6.542	2072.2	0.396226303	C(+57.02)	56	548.2482	1	547.2424	Carbamido	A	Superscaf	1.09E+08	0	0	0	BTP20220	0	0	Tissue specific
KELDKLP	Superscaf	+	Superscaf	1.1E+08	1.1E+08	23	23	29	-	-	-	-	-	-	NCP	IGR	-	ID=gene-E	7	6.275714286	2818	0.397938232	KELDKLP	61	421.7516	2	841.4909	.	K	Superscaf	1.1E+08	0	0	0	0	0	BTP20220	Tissue specific
KPKTTATK	Superscaf	+	Superscaf	1.1E+08	1.1E+08	32	32	39	-	-	-	-	-	-	NCP	IGR	-	ID=gene-E	8	7.64125	3246.4	0.398827074	KPKTTATK	55	437.7704	2	873.5283	.	N	Superscaf	1.1E+08	0	0	BTP20220	BTP20220	0	0	Tissue specific
LDELPL	Superscaf	+	Superscaf	1.1E+08	1.1E+08	19	19	24	-	-	-	-	-	-	NCP	IGR	-	ID=gene-E	6	5.071666667	2234.6	0.398955183	LDELPL	69	699.3883	1	698.385	.	I	Superscaf	1.1E+08	0	0	0	0	0	BTP20220	Mixed
LQVPL	Superscaf	+	Superscaf	1.1E+08	1.1E+08	64	64	68	-	-	gene	-	-	mRNA	NCP	Intron	gene|mRNA	ID=gene-E	5	4.782	1654.9	0.399544621	LQVPL	58	569.364	1	568.3585	.	E	Superscaf	1.1E+08	0	0	BTP20220	0	0	0	Tissue specific
KEVTQKP	Superscaf	+	Superscaf	1.1E+08	1.1E+08	1	1	7	-	-	gene	-	-	mRNA	NCP	Intron	gene|mRNA	ID=gene-E	7	5.882857143	2707.9	0.399754705	KEVTQKP	50	415.2366	2	828.4705	.	K	Superscaf	1.1E+08	0	0	0	0	0	BTP20220	Tissue specific
TQYFR	Superscaf	+	Superscaf	1.1E+08	1.1E+08	6	6	10	-	-	gene	-	-	mRNA	NCP	Intron	gene|mRNA	ID=gene-E	5	6.816	2503.9	0.401686511	T(+42.01)	70	378.6926	2	755.3602	Acetylati	R	Superscaf	1.1E+08	0	0	BTP20220	0	0	0	Tissue specific
SAAYVY	Superscaf	-	Superscaf	1.1E+08	1.1E+08	-17	17	22	-	-	gene	-	-	mRNA	NCP	Intron	gene|mRNA	ID=gene-E	6	4.84	1925	0.401772121	SAAYVY	74	673.317	1	672.3119	.	R	Superscaf	1.1E+08	BTP20220	0	0	0	0	0	Tissue specific
GVGVL	Superscaf	-	Superscaf	1.1E+08	1.1E+08	-15	15	19	-	-	gene	-	-	mRNA	NCP	Intron	gene|mRNA	ID=gene-E	5	3.584	948	0.401920771	GVGVL	63	444.2796	1	443.2744	.	S	Superscaf	1.1E+08	0	0	0	0	0	BTP20220	Tissue specific
EVLLK	Superscaf	-	Superscaf	1.1E+08	1.1E+08	-13	13	17	-	-	-	-	-	-	NCP	IGR	-	ID=gene-E	5	4.984	1600.7	0.404669007	EVLLK	77	601.3896	1	600.3846	.	S	Superscaf	1.1E+08	BTP20220	0	0	0	0	0	Tissue specific
LKLADF	Superscaf	-	Superscaf	1.1E+08	1.1E+08	-35	35	40	-	-	-	-	-	-	NCP	IGR	-	ID=gene-E	6	6.028333333	2285.4	0.405863375	LKLADF	87	353.7089	2	705.4061	.	E	Superscaf	1.1E+08	0	0	0	0	BTP20220	0	Tissue specific
VGVLCGKP	Superscaf	-	Superscaf	1.1E+08	1.1E+08	-11	11	18	-	-	gene	-	-	mRNA	NCP	Intron	gene|mRNA	ID=gene-E	8	4.8725	2339.5	0.408946017	VGVLC(+57	55	415.2341	2	828.4528	Carbamido	I	Superscaf	1.1E+08	0	0	0	0	0	BTP20220	Tissue specific
LAQALSPK	Superscaf	+	Superscaf	1.11E+08	1.11E+08	68	68	75	-	-	gene	-	-	mRNA	NCP	Intron	gene|mRNA	ID=gene-E	8	6.42125	3129.3	0.409080628	LAQALSPK	89	414.2509	2	826.4912	.	S	Superscaf	1.11E+08	0	0	0	0	BTP20220	0	Tissue specific
DLPVYF	Superscaf	-	Superscaf	1.11E+08	1.11E+08	-28	28	33	-	-	gene	-	-	mRNA	NCP	Intron	gene|mRNA	ID=gene-E	6	4.398333333	1949.2	0.409911633	DLPVYF	87	753.3783	1	752.3745	.	G	Superscaf	1.11E+08	0	0	0	BTP20220	0	0	Tissue specific
QNTPTEK	Superscaf	-	Superscaf	1.11E+08	1.11E+08	-3	3	9	-	-	gene	-	-	mRNA	NCP	Intron	gene|mRNA	ID=gene-E	7	6.197142857	3233.7	0.413412684	Q(+42.01)	86	430.2068	2	858.4083	Acetylati	Q	Superscaf	1.11E+08	0	0	0	BTP20220	0	0	Tissue specific
FFKPK	Superscaf	+	Superscaf	1.11E+08	1.11E+08	19	19	23	-	-	-	-	-	-	NCP	IGR	-	ID=gene-E	5	7.348	2049	0.414258224	F(+42.01)	51	354.7101	2	707.4006	Acetylati	P	Superscaf	1.11E+08	0	0	BTP20220	BTP20220	0	0	Tissue specific
LLSVTR	Superscaf	+	Superscaf	1.11E+08	1.11E+08	24	24	29	-	-	-	-	-	-	NCP	IGR	-	ID=gene-E	6	5.821666667	2136.5	0.415642824	L(+42.01)	71	365.7266	2	729.4385	Acetylati	K	Superscaf	1.11E+08	0	0	0	0	0	BTP20220	Tissue specific
GVHVLT	Superscaf	+	Superscaf	1.11E+08	1.11E+08	2	2	7	-	-	-	-	-	-	NCP	IGR	-	ID=gene-E	6	4.345	1667.9	0.417876176	GVHVLT	53	625.3644	1	624.3595	.	W	Superscaf	1.11E+08	0	0	0	0	0	BTP20220	Tissue specific
WGMKVL	Superscaf	+	Superscaf	1.11E+08	1.11E+08	55	55	60	-	-	-	-	-	-	NCP	IGR	-	ID=gene-E	6	5.555	2017.2	0.420070549	WGM(+15.9	56	375.2016	2	748.3942	Oxidation	V	Superscaf	1.11E+08	0	0	0	BTP20220	0	0	Tissue specific
VFQFLNAK	Superscaf	-	Superscaf	1.11E+08	1.11E+08	-2	2	9	-	-	-	-	-	-	NCP	IGR	-	ID=gene-E	8	5.47	3115.3	0.420458701	VFQFLNAK	99	483.772	2	965.5334	.	Q	Superscaf	1.11E+08	0	0	0	0	0	BTP20220	Tissue specific
VKPVKVSAF	Superscaf	-	Superscaf	1.11E+08	1.11E+08	-18	18	27	-	-	-	-	-	-	NCP	IGR	-	ID=gene-E	10	5.454	2955.1	0.420518031	VKPVKVSAF	85	360.9005	3	1079.681	.	H	Superscaf	1.11E+08	0	0	BTP20220	0	0	0	Tissue specific
PQRVVL	Superscaf	-	Superscaf	1.11E+08	1.11E+08	-4	4	9	-	-	-	-	-	-	NCP	IGR	-	ID=gene-E	6	4.781666667	1950.3	0.421295902	P(+42.01)	52	753.4595	1	752.4545	Acetylati	F	Superscaf	1.11E+08	0	0	BTP20220	0	0	0	Tissue specific
LVLRLQQ	Superscaf	-	Superscaf	1.12E+08	1.12E+08	-27	27	33	-	-	-	-	-	-	NCP	IGR	-	ID=gene-E	7	5.714285714	2930.4	0.421945703	LVLRLQQ	67	435.2803	2	868.5494	.	L	Superscaf	1.12E+08	0	0	BTP20220	0	0	0	Tissue specific
LKGVVT	Superscaf	-	Superscaf	1.12E+08	1.12E+08	-1	1	6	-	-	-	-	-	-	NCP	IGR	-	ID=gene-E	6	4.703333333	1516.6	0.42237402	LKGVVT	58	616.4008	1	615.3956	.	L	Superscaf	1.12E+08	0	0	0	BTP20220	0	0	Tissue specific
SLDGLLQ	Superscaf	-	Superscaf	1.12E+08	1.12E+08	-50	50	56	-	-	gene	-	-	mRNA	NCP	Intron	gene|mRNA	ID=gene-E	7	5.458571429	2763.1	0.422657817	SLDGLLQ	66	745.4045	1	744.4017	.	S	Superscaf	1.12E+08	0	0	0	BTP20220	0	0	Tissue specific
LLAARRL	Superscaf	-	Superscaf	1.12E+08	1.12E+08	-10	10	16	-	-	gene	-	-	mRNA	NCP	Intron	gene|mRNA	ID=gene-E	7	7.357142857	2980.4	0.422659653	LLAARRL	63	406.7754	2	811.5392	.	Q	Superscaf	1.12E+08	0	0	0	BTP20220	0	0	Tissue specific
EEMMMAF	Superscaf	-	Superscaf	1.12E+08	1.12E+08	-38	38	44	-	-	gene	-	-	mRNA	NCP	Intron	gene|mRNA	ID=gene-E	7	5.027142857	2664.2	0.422826665	EEMMMAF	51	888.3264	1	887.3228	.	I	Superscaf	1.12E+08	0	0	0	BTP20220	0	0	Tissue specific
KPPCLDK	Superscaf	-	Superscaf	1.12E+08	1.12E+08	-5	5	11	-	-	gene	-	-	mRNA	NCP	Intron	gene|mRNA	ID=gene-E	7	6.578571429	2962.2	0.424057335	K(+42.01)	59	450.2334	2	898.4582	Acetylati	G	Superscaf	1.12E+08	0	BTP20220	0	0	0	0	Tissue specific
SAAHHGR	Superscaf	-	Superscaf	1.12E+08	1.12E+08	-52	52	58	-	-	gene	-	-	mRNA	NCP	Intron	gene|mRNA	ID=gene-E	7	7.09	3191.5	0.424803189	SAAHHGR	60	368.182	2	734.3572	.	L	Superscaf	1.12E+08	0	0	0	BTP20220	0	0	Tissue specific
VLGYSCST	Superscaf	+	Superscaf	1.12E+08	1.12E+08	10	10	17	-	-	-	-	-	-	NCP	IGR	-	ID=gene-E	8	5.065	2860.2	0.425844545	VLGYSC(+5	60	443.7062	2	885.3902	Carbamido	N	Superscaf	1.12E+08	0	0	0	BTP20220	0	0	Tissue specific
LNTEF	Superscaf	+	Superscaf	1.12E+08	1.12E+08	2	2	6	-	-	-	-	-	-	NCP	IGR	-	ID=gene-E	5	5.324	2160.5	0.425982408	LNTEF	97	623.3027	1	622.2962	.	A	Superscaf	1.12E+08	0	0	0	0	0	BTP20220	Tissue specific
KATSLGLK	Superscaf	-	Superscaf	1.12E+08	1.12E+08	-3	3	10	-	-	-	-	-	-	NCP	IGR	-	ID=gene-E	8	6.955	3033.1	0.426378822	K(+42.01)	71	430.2652	2	858.5175	Acetylati	F	Superscaf	1.12E+08	0	0	BTP20220	0	BTP20220	0	Tissue specific
KPMFPPP	Superscaf	-	Superscaf	1.12E+08	1.12E+08	-30	30	36	-	-	-	-	-	-	NCP	IGR	-	ID=gene-E	7	6.595714286	2576.9	0.42704205	KPMFPPP	63	407.2278	2	812.4255	.	N	Superscaf	1.12E+08	0	0	0	BTP20220	0	0	Tissue specific
LFKCGK	Superscaf	-	Superscaf	1.12E+08	1.12E+08	-2	2	7	-	-	-	-	-	-	NCP	IGR	-	ID=gene-E	6	6.988333333	2560.6	0.427068712	LFKC(+57.	97	376.7083	2	751.405	Carbamido	I	Superscaf	1.12E+08	0	0	0	BTP20220	0	0	Tissue specific
KLNVTDF	Superscaf	-	Superscaf	1.12E+08	1.12E+08	-22	22	28	-	-	-	-	-	-	NCP	IGR	-	ID=gene-E	7	5.158571429	2614.9	0.428283163	K(+42.01)	81	878.46	1	877.4545	Acetylati	D	Superscaf	1.12E+08	0	0	0	BTP20220	0	0	Tissue specific
VLDLDLF	Superscaf	-	Superscaf	1.12E+08	1.12E+08	-8	8	14	-	-	-	-	-	-	NCP	IGR	-	ID=gene-E	7	4.194285714	2270.6	0.429009775	VLDLDLF	60	834.4586	1	833.4534	.	P	Superscaf	1.12E+08	0	0	BTP20220	0	0	0	Tissue specific
KANLYL	Superscaf	+	Superscaf	1.12E+08	1.12E+08	4	4	9	-	-	-	-	-	-	NCP	IGR	-	ID=gene-E	6	6.465	2542.7	0.429992413	KANLYL	66	721.4236	1	720.417	.	Q	Superscaf	1.12E+08	0	0	BTP20220	0	0	0	Tissue specific
KFVKLA	Superscaf	+	Superscaf	1.13E+08	1.13E+08	8	8	13	-	-	gene	-	-	mRNA	NCP	Intron	gene|mRNA	ID=gene-E	6	6.16	1988.9	0.437225793	KFVKLA	70	353.235	2	704.4584	.	F	Superscaf	1.13E+08	0	0	0	0	BTP20220	0	Tissue specific
AASKPVEA	Superscaf	-	Superscaf	1.13E+08	1.13E+08	-18	18	26	-	-	-	-	-	-	NCP	IGR	-	ID=gene-E	9	5.446666667	2859.9	0.438047272	A(+42.01)	94	443.2356	2	884.4603	Acetylati	L	Superscaf	1.13E+08	0	0	0	0	0	BTP20220	Tissue specific
WKEVAQDC	Superscaf	-	Superscaf	1.13E+08	1.13E+08	-8	8	17	-	-	-	-	-	-	NCP	IGR	-	ID=gene-E	10	5.478	4205.6	0.438230541	WKEVAQDC(	99	632.8011	2	1263.592	Carbamido	Y	Superscaf	1.13E+08	0	0	0	0	BTP20220	0	Tissue specific
AVELNPK	Superscaf	-	Superscaf	1.13E+08	1.13E+08	-18	18	24	-	-	-	-	-	-	NCP	IGR	-	ID=gene-E	7	5.238571429	2492.7	0.438230924	AVELNPK	93	385.7223	2	769.4333	.	Y	Superscaf	1.13E+08	BTP20220	0	0	0	0	0	Tissue specific
KKFVPP	Superscaf	-	Superscaf	1.13E+08	1.13E+08	-37	37	42	-	-	-	-	-	-	NCP	IGR	-	ID=gene-E	6	6.26	1999	0.43837835	KKFVPP	61	358.2297	2	714.4428	.	I	Superscaf	1.13E+08	BTP20220	0	0	0	0	0	Tissue specific
YSLCDAAHA	Superscaf	-	Superscaf	1.13E+08	1.13E+08	-35	35	49	-	-	-	-	-	-	NCP	IGR	-	ID=gene-E	15	5.474666667	5960.5	0.438515678	YSLC(+57.	86	832.39	2	1662.767	Carbamido	F	Superscaf	1.13E+08	0	0	BTP20220	0	0	0	Tissue specific
SLCDAAHAG	Superscaf	-	Superscaf	1.13E+08	1.13E+08	-36	36	49	-	-	-	-	-	-	NCP	IGR	-	ID=gene-E	14	5.461428571	5501	0.438515716	SLC(+57.(	97	750.8561	2	1499.704	Carbamido	F	Superscaf	1.13E+08	0	BTP20220	0	0	0	0	Tissue specific
YGLKPPTL	Superscaf	-	Superscaf	1.13E+08	1.13E+08	-51	51	58	-	-	-	-	-	-	NCP	IGR	-	ID=gene-E	8	6.5575	3004.3	0.43851629	YGLKPPTL	93	444.7606	2	887.5116	.	F	Superscaf	1.13E+08	0	0	0	0	0	BTP20220	Tissue specific
YGLKPPT	Superscaf	-	Superscaf	1.13E+08	1.13E+08	-51	51	57	-	-	-	-	-	-	NCP	IGR	-	ID=gene-E	7	6.64	2650.9	0.43851629	YGLKPPT	78	388.2193	2	774.4276	.	F	Superscaf	1.13E+08	0	0	0	0	0	BTP20220	Tissue specific
DKLGTKL	Superscaf	-	Superscaf	1.13E+08	1.13E+08	-59	59	65	-	-	-	-	-	-	NCP	IGR	-	ID=gene-E	7	6.701428571	2749.9	0.43930908	DKLGTKL	98	387.7388	2	773.4647	.	R	Superscaf	1.13E+08	0	0	0	0	0	BTP20220	Tissue specific
LALLEPL	Superscaf	-	Superscaf	1.13E+08	1.13E+08	-29	29	35	-	-	-	-	-	-	NCP	IGR	-	ID=gene-E	7	5.637142857	2487.8	0.440000155	LALLEPL	70	768.4841	1	767.4792	.	Q	Superscaf	1.13E+08	0	0	0	0	0	BTP20220	Tissue specific
KMVYVLT	Superscaf	+	Superscaf	1.13E+08	1.13E+08	12	12	18	-	-	-	-	-	-	NCP	IGR	-	ID=gene-E	7	4.808571429	2050.3	0.440363289	KM(+15.99	52	435.2369	2	868.4728	Oxidation	S	Superscaf	1.13E+08	0	0	0	0	0	BTP20220	Tissue specific
LDGLLEK	Superscaf	-	Superscaf	1.13E+08	1.13E+08	-15	15	21	-	-	-	-	-	-	NCP	IGR	-	ID=gene-E	7	5.691428571	2662.9	0.440956017	LDGLLEK	51	787.4527	1	786.4487	.	G	Superscaf	1.13E+08	0	BTP20220	0	0	0	0	Tissue specific
VGLMKL	Superscaf	+	Superscaf	1.13E+08	1.13E+08	17	17	22	-	-	-	-	-	-	NCP	IGR	-	ID=gene-E	6	5.57	1844	0.441521546	VGLMKL	88	660.409	1	659.404	.	I	Superscaf	1.13E+08	BTP20220	0	0	0	0	0	Tissue specific
EVADFL	Superscaf	+	Superscaf	1.13E+08	1.13E+08	52	52	57	-	-	-	-	-	-	NCP	IGR	-	ID=gene-E	6	3.945	1889.1	0.441737598	EVADFL	56	693.3435	1	692.3381	.	S	Superscaf	1.13E+08	BTP20220	0	0	0	0	0	Tissue specific
PDSVKKP	Superscaf	-	Superscaf	1.13E+08	1.13E+08	-9	9	15	-	-	-	-	-	-	NCP	IGR	-	ID=gene-E	7	5.818571429	2406.5	0.441878751	PDSVKKP	79	385.7242	2	769.4333	.	G	Superscaf	1.13E+08	0	0	0	0	BTP20220	BTP20220	Mixed
LLAPQ	Superscaf	-	Superscaf	1.13E+08	1.13E+08	-83	83	87	-	-	-	-	-	-	NCP	IGR	-	ID=gene-E	5	5.986	1966.2	0.441894052	LLAPQ	68	541.3429	1	540.3271	.	G	Superscaf	1.13E+08	0	0	BTP20220	0	0	0	Tissue specific
TLDYVGW	Superscaf	-	Superscaf	1.13E+08	1.13E+08	-7	7	13	-	-	-	-	-	-	NCP	IGR	-	ID=gene-E	7	4.714285714	2445.8	0.44197817	TLDYVGW	60	853.4177	1	852.4017	.	V	Superscaf	1.13E+08	0	0	0	0	0	BTP20220	Tissue specific
FFPYK	Superscaf	-	Superscaf	1.13E+08	1.13E+08	-15	15	19	-	-	-	-	-	-	NCP	IGR	-	ID=gene-E	5	6.532	2040.1	0.442132329	FFPYK	99	701.36	1	700.3584	.	Y	Superscaf	1.13E+08	0	0	0	0	BTP20220	0	Tissue specific
METPTNR	Superscaf	+	Superscaf	1.13E+08	1.13E+08	25	25	31	-	-	-	-	-	-	NCP	IGR	-	ID=gene-E	7	6.357142857	3174.9	0.442137991	M(+42.01)	78	445.704	2	889.3964	Acetylati	E	Superscaf	1.13E+08	0	0	0	BTP20220	0	0	Tissue specific
KDPTRE	Superscaf	-	Superscaf	1.13E+08	1.13E+08	-39	39	44	-	-	-	-	-	-	NCP	IGR	-	ID=gene-E	6	6.586666667	2689.1	0.444139722	KDPTRE	65	373.2022	2	744.3766	.	R	Superscaf	1.13E+08	0	0	0	BTP20220	0	0	Tissue specific
VSLQFK	Superscaf	-	Superscaf	1.13E+08	1.13E+08	-39	39	44	-	-	-	-	-	-	NCP	IGR	-	ID=gene-E	6	5.421666667	2203.3	0.44491197	VSLQFK	62	361.2211	2	720.417	.	T	Superscaf	1.13E+08	0	0	0	0	BTP20220	0	Tissue specific
HPAKKQ	Superscaf	+	Superscaf	1.13E+08	1.13E+08	8	8	13	-	-	-	-	-	-	NCP	IGR	-	ID=gene-E	6	7.506666667	2815.9	0.445762484	H(+42.01)	63	375.7205	2	749.4184	Acetylati	I	Superscaf	1.13E+08	0	BTP20220	0	0	0	0	Tissue specific
KDVEHKN	Superscaf	-	Superscaf	1.13E+08	1.13E+08	-11	11	17	-	-	-	-	-	-	NCP	IGR	-	ID=gene-E	7	5.524285714	2990.2	0.446033467	KDVEHKN	65	435.223	2	868.4402	.	W	Superscaf	1.13E+08	0	0	BTP20220	0	0	0	Tissue specific
GPVKVKC	Superscaf	+	Superscaf	1.13E+08	1.13E+08	24	24	30	-	-	-	-	-	-	NCP	IGR	-	ID=gene-E	7	5.252857143	2157.2	0.446858159	GPVKVKC(+	68	394.2246	2	786.4422	Carbamido	I	Superscaf	1.13E+08	0	0	0	0	0	BTP20220	Tissue specific
TVPNAER	Superscaf	-	Superscaf	1.14E+08	1.14E+08	-1	1	7	-	-	-	-	-	-	NCP	IGR	-	ID=gene-E	7	5.462857143	2717.2	0.448188439	TVPNAER	52	393.7147	2	785.4031	.	T	Superscaf	1.14E+08	0	0	BTP20220	0	0	0	Tissue specific
VVTATLLE	Superscaf	-	Superscaf	1.14E+08	1.14E+08	-1	1	8	-	-	-	-	-	-	NCP	IGR	-	ID=gene-E	8	4.2825	2238.6	0.448514736	V(+42.01)	70	887.5048	1	886.5012	Acetylati	V	Superscaf	1.14E+08	BTP20220	0	0	0	0	0	Tissue specific
LPLPPPKY	Superscaf	+	Superscaf	1.14E+08	1.14E+08	27	27	34	-	-	-	-	-	-	NCP	IGR	-	ID=gene-E	8	6.57	2984.3	0.448597821	LPLPPPKY	99	924.5524	1	923.548	.	L	Superscaf	1.14E+08	BTP20220	0	0	0	0	0	Tissue enhanced
PPVLLHV	Superscaf	-	Superscaf	1.14E+08	1.14E+08	-31	31	37	-	-	gene	-	-	mRNA	NCP	Intron	gene|mRNA	ID=gene-E	7	4.592857143	2001.3	0.451761138	P(+42.01)	97	408.7567	2	815.4905	Acetylati	Y	Superscaf	1.14E+08	0	0	BTP20220	0	0	0	Tissue specific
ALGHTAPP	Superscaf	+	Superscaf	1.14E+08	1.14E+08	28	28	35	-	-	gene	-	-	mRNA	NCP	Intron	gene|mRNA	ID=gene-E	8	6.33875	2965.3	0.451948615	ALGHTAPP	60	382.2125	2	762.4024	.	K	Superscaf	1.14E+08	0	0	BTP20220	0	0	0	Tissue specific
RKKSQF	Superscaf	+	Superscaf	1.14E+08	1.14E+08	18	18	23	-	-	gene	-	-	mRNA	NCP	Intron	gene|mRNA	ID=gene-E	6	7.841666667	2967.1	0.452209576	RKKSQF	52	397.2424	2	792.4606	.	T	Superscaf	1.14E+08	0	0	BTP20220	0	0	0	Tissue specific
GLVAKLQ	Superscaf	+	Superscaf	1.14E+08	1.14E+08	1	1	7	-	-	-	-	-	-	NCP	IGR	-	ID=gene-E	7	5.62	2394.5	0.455872858	GLVAKLQ	61	364.7357	2	727.4592	.	G	Superscaf	1.14E+08	0	0	0	0	BTP20220	0	Tissue specific
FGGFGQQ	Superscaf	-	Superscaf	1.14E+08	1.14E+08	-17	17	23	-	-	-	-	-	-	NCP	IGR	-	ID=gene-E	7	5.738571429	2888.1	0.456615613	FGGFGQQ	94	740.3333	1	739.3289	.	A	Superscaf	1.14E+08	0	0	BTP20220	0	0	0	Tissue specific
LSYPPR	Superscaf	+	Superscaf	1.14E+08	1.14E+08	5	5	10	-	-	-	-	-	-	NCP	IGR	-	ID=gene-E	6	6.78	2519.9	0.45717288	LSYPPR	72	366.7094	2	731.3966	.	G	Superscaf	1.14E+08	0	BTP20220	0	0	0	0	Tissue specific
EVSLFP	Superscaf	-	Superscaf	1.14E+08	1.14E+08	-2	2	7	-	-	-	-	-	-	NCP	IGR	-	ID=gene-E	6	4.443333333	1887.1	0.457738295	EVSLFP	72	691.3639	1	690.3588	.	K	Superscaf	1.14E+08	0	0	0	0	0	BTP20220	Tissue specific
FSPPRF	Superscaf	-	Superscaf	1.14E+08	1.14E+08	-24	24	29	-	-	-	-	-	-	NCP	IGR	-	ID=gene-E	6	6.666666667	2481.8	0.458905771	FSPPRF	78	375.6978	2	749.386	.	G	Superscaf	1.14E+08	0	0	0	BTP20220	0	0	Tissue specific
CMLAV	Superscaf	-	Superscaf	1.14E+08	1.14E+08	-27	27	31	-	-	-	-	-	-	NCP	IGR	-	ID=gene-E	5	4.554	1621.9	0.45898702	C(+57.02)	51	593.2769	1	592.2713	Carbamido	H	Superscaf	1.14E+08	0	0	0	BTP20220	0	0	Tissue specific
KRLEVVTK	Superscaf	+	Superscaf	1.14E+08	1.14E+08	1	1	8	-	-	-	-	-	-	NCP	IGR	-	ID=gene-E	8	5.74625	2762	0.459446015	KRLEVVTK	66	486.812	2	971.6127	.	K	Superscaf	1.14E+08	0	0	0	BTP20220	0	0	Tissue specific
FFVASQF	Superscaf	-	Superscaf	1.15E+08	1.15E+08	-1	1	7	-	-	gene	-	-	mRNA	NCP	Intron	gene|mRNA	ID=gene-E	7	4.827142857	2467.6	0.460109396	FFVASQF	52	845.4159	1	844.4119	.	F	Superscaf	1.15E+08	0	0	0	0	BTP20220	0	Tissue specific
LPPATEM	Superscaf	-	Superscaf	1.15E+08	1.15E+08	-10	10	16	-	-	gene	-	-	mRNA	NCP	Intron	gene|mRNA	ID=gene-E	7	5.728571429	2534	0.461497553	LPPATEM	75	758.3801	1	757.368	.	A	Superscaf	1.15E+08	0	0	BTP20220	0	0	0	Tissue specific
ESNHPK	Superscaf	-	Superscaf	1.15E+08	1.15E+08	-24	24	29	-	-	gene	-	-	mRNA	NCP	Intron	gene|mRNA	ID=gene-E	6	6.323333333	2831.1	0.461561091	ESNHPK	63	711.3383	1	710.3347	.	L	Superscaf	1.15E+08	BTP20220	0	0	0	0	0	Tissue specific
EAVEPVH	Superscaf	-	Superscaf	1.15E+08	1.15E+08	-1	1	7	-	-	gene	-	-	mRNA	NCP	Intron	gene|mRNA	ID=gene-E	7	3.764285714	2119.4	0.46184703	EAVEPVH	50	390.6973	2	779.3813	.	E	Superscaf	1.15E+08	BTP20220	0	0	0	0	0	Tissue specific
LLLEVG	Superscaf	-	Superscaf	1.15E+08	1.15E+08	-10	10	15	-	-	gene	-	-	mRNA	NCP	Intron	gene|mRNA	ID=gene-E	6	4.521666667	1783	0.462412101	LLLEVG	65	643.4005	1	642.3952	.	M	Superscaf	1.15E+08	0	0	0	0	0	BTP20220	Tissue specific
KLNLSH	Superscaf	+	Superscaf	1.15E+08	1.15E+08	11	11	16	-	-	-	-	-	-	NCP	IGR	-	ID=gene-E	6	6.73	2775	0.463819767	KLNLSH	55	356.2094	2	710.4075	.	L	Superscaf	1.15E+08	BTP20220	0	BTP20220	0	0	0	Tissue specific
MEHYK	Superscaf	+	Superscaf	1.15E+08	1.15E+08	13	13	17	-	-	-	-	-	-	NCP	IGR	-	ID=gene-E	5	6.392	2344.6	0.464357907	M(+42.01)	57	749.3376	1	748.3214	Acetylati	L	Superscaf	1.15E+08	0	0	0	0	BTP20220	0	Tissue specific
EAVRVYST	Superscaf	+	Superscaf	1.15E+08	1.15E+08	2	2	9	-	-	-	-	-	-	NCP	IGR	-	ID=gene-E	8	4.73375	2626	0.464795634	EAVRVYST	74	462.7417	2	923.4713	.	T	Superscaf	1.15E+08	BTP20220	0	0	0	0	0	Tissue enhanced
LGLEVL	Superscaf	+	Superscaf	1.15E+08	1.15E+08	9	9	14	-	-	-	-	-	-	NCP	IGR	-	ID=gene-E	6	4.521666667	1783	0.465130117	LGLEVL	75	643.4005	1	642.3952	.	Q	Superscaf	1.15E+08	BTP20220	0	0	0	0	0	Tissue specific
SKLQPK	Superscaf	-	Superscaf	1.15E+08	1.15E+08	-15	15	20	-	-	-	-	-	-	NCP	IGR	-	ID=gene-E	6	7.181666667	2621.7	0.469858051	S(+42.01)	53	371.7267	2	741.4385	Acetylati	S	Superscaf	1.15E+08	BTP20220	0	0	0	0	BTP20220	Tissue specific
VGDASHFE	Superscaf	-	Superscaf	1.15E+08	1.15E+08	-4	4	11	-	-	-	-	-	-	NCP	IGR	-	ID=gene-E	8	4.61625	2836.1	0.469968717	V(+42.01)	55	452.1988	2	902.377	Acetylati	H	Superscaf	1.15E+08	BTP20220	0	0	0	0	0	Tissue specific
GHAAKPL	Superscaf	-	Superscaf	1.15E+08	1.15E+08	-11	11	17	-	-	gene	-	-	mRNA	NCP	Intron	gene|mRNA	ID=gene-E	7	6.802857143	2698.8	0.470350786	G(+42.01)	55	368.2167	2	734.4075	Acetylati	P	Superscaf	1.15E+08	0	BTP20220	0	0	0	0	Tissue specific
EPDYQPR	Superscaf	-	Superscaf	1.15E+08	1.15E+08	-2	2	8	-	-	gene	-	-	mRNA	NCP	Intron	gene|mRNA	ID=gene-E	7	5.837142857	3230.8	0.470451697	EPDYQPR	73	452.7178	2	903.4086	.	K	Superscaf	1.15E+08	BTP20220	0	0	0	0	0	Tissue specific
DGAALLKE	Superscaf	+	Superscaf	1.15E+08	1.15E+08	12	12	19	-	-	gene	-	-	mRNA	NCP	Intron	gene|mRNA	ID=gene-E	8	5.7375	2932.1	0.470767627	DGAALLKE	76	408.731	2	815.4388	.	P	Superscaf	1.15E+08	0	0	BTP20220	0	0	0	Tissue specific
KSLVEYLQ	Superscaf	-	Superscaf	1.15E+08	1.15E+08	-32	32	39	-	-	gene	-	-	mRNA	NCP	Intron	gene|mRNA	ID=gene-E	8	5.23875	3054.3	0.471141013	KSLVEYLQ	62	490.2752	2	978.5386	.	S	Superscaf	1.15E+08	0	0	0	0	0	BTP20220	Tissue specific
AGDDKN	Superscaf	+	Superscaf	1.15E+08	1.15E+08	53	53	58	-	-	gene	-	-	mRNA	NCP	Intron	gene|mRNA	ID=gene-E	6	5.513333333	2496.7	0.471245749	AGDDKN	50	619.2695	1	618.2609	.	P	Superscaf	1.15E+08	0	0	0	0	BTP20220	0	Tissue specific
TSTVPDFK	Superscaf	-	Superscaf	1.15E+08	1.15E+08	-6	6	13	-	-	gene	-	-	mRNA	NCP	Intron	gene|mRNA	ID=gene-E	8	5.40375	2783.1	0.471401706	T(+42.01)	56	468.7305	2	935.46	Acetylati	Q	Superscaf	1.15E+08	0	BTP20220	0	0	BTP20220	BTP20220	Tissue specific
KVEGL	Superscaf	-	Superscaf	1.15E+08	1.15E+08	-37	37	41	-	-	gene	-	-	mRNA	NCP	Intron	gene|mRNA	ID=gene-E	5	4.982	1544.6	0.472124607	K(+42.01)	96	587.3389	1	586.3326	Acetylati	M	Superscaf	1.15E+08	0	BTP20220	0	0	0	0	Mixed
LQVGELK	Superscaf	-	Superscaf	1.16E+08	1.16E+08	-6	6	12	-	-	-	-	-	-	NCP	IGR	-	ID=gene-E	7	5.22	2508.7	0.474744122	LQVGELK	87	393.7379	2	785.4647	.	D	Superscaf	1.16E+08	0	BTP20220	BTP20220	0	BTP20220	0	Tissue specific
PGVPVH	Superscaf	+	Superscaf	1.16E+08	1.16E+08	35	35	40	-	-	-	-	-	-	NCP	IGR	-	ID=gene-E	6	4.36	1591.8	0.476254229	P(+42.01)	68	647.3482	1	646.3438	Acetylati	I	Superscaf	1.16E+08	0	0	0	0	BTP20220	0	Tissue specific
QTWEKQS	Superscaf	-	Superscaf	1.16E+08	1.16E+08	-2	2	8	-	-	-	-	-	-	NCP	IGR	-	ID=gene-E	7	6.051428571	3422.8	0.476280585	QTWEKQS	76	453.7218	2	905.4243	.	L	Superscaf	1.16E+08	0	BTP20220	0	0	BTP20220	0	Tissue specific
ELMSY	Superscaf	-	Superscaf	1.16E+08	1.16E+08	-19	19	23	-	-	-	-	-	-	NCP	IGR	-	ID=gene-E	5	5.258	1993.3	0.476286667	ELMSY	85	642.2795	1	641.2731	.	T	Superscaf	1.16E+08	0	0	0	0	0	BTP20220	Mixed
KNNLLC	Superscaf	-	Superscaf	1.16E+08	1.16E+08	-21	21	26	-	-	gene	-	-	mRNA	NCP	Intron	gene|mRNA	ID=gene-E	6	6.256666667	2954.3	0.481754335	KNNLLC(+5	68	381.2008	2	760.3901	Carbamido	I	Superscaf	1.16E+08	0	0	0	0	BTP20220	0	Tissue specific
AHLEHT	Superscaf	+	Superscaf	1.16E+08	1.16E+08	8	8	13	-	-	-	-	-	-	NCP	IGR	-	ID=gene-E	6	6.155	2727.1	0.482357046	AHLEHT	53	707.3353	1	706.3398	.	I	Superscaf	1.16E+08	0	0	0	0	BTP20220	0	Tissue specific
KTLLFVH	Superscaf	+	Superscaf	1.16E+08	1.16E+08	38	38	44	-	-	-	-	-	-	NCP	IGR	-	ID=gene-E	7	5.9	2579.8	0.482600258	KTLLFVH	60	429.2631	2	856.517	.	N	Superscaf	1.16E+08	0	0	0	0	0	BTP20220	Tissue specific
MMKPPPP	Superscaf	+	Superscaf	1.16E+08	1.16E+08	17	17	23	-	-	-	-	-	-	NCP	IGR	-	ID=gene-E	7	6.634285714	2561	0.483529457	M(+42.01)	71	420.2187	2	838.4081	Acetylati	D	Superscaf	1.16E+08	0	0	0	0	0	BTP20220	Tissue specific
MEHGVGY	Superscaf	+	Superscaf	1.17E+08	1.17E+08	5	5	11	-	-	gene	-	-	mRNA	NCP	Intron	gene|mRNA	ID=gene-E	7	4.88	2470.8	0.486230909	M(+42.01)	99	834.3424	1	833.3378	Acetylati	L	Superscaf	1.17E+08	0	0	0	BTP20220	0	0	Tissue specific
ERDSWLK	Superscaf	+	Superscaf	1.17E+08	1.17E+08	15	15	21	-	-	-	-	-	-	NCP	IGR	-	ID=gene-E	7	6.32	3217.6	0.486526489	ERDSWLK	66	467.2426	2	932.4716	.	D	Superscaf	1.17E+08	BTP20220	0	0	0	0	0	Tissue enhanced
RNSHLV	Superscaf	+	Superscaf	1.17E+08	1.17E+08	13	13	18	-	exon	gene	three_pri	-	mRNA	NCP	3UTR	exon|gene	ID=exon-E	6	5.903333333	2602	0.487924515	RNSHLV	52	363.2054	2	724.398	.	A	Superscaf	1.17E+08	0	0	0	BTP20220	0	0	Tissue specific
KNEEPG	Superscaf	+	Superscaf	1.17E+08	1.17E+08	17	17	22	-	-	gene	-	-	mRNA	NCP	Intron	gene|mRNA	ID=gene-E	6	5.643333333	2550.8	0.488158201	KNEEPG	56	673.3168	1	672.3079	.	C	Superscaf	1.17E+08	0	BTP20220	0	0	0	0	Mixed
LGRVVL	Superscaf	-	Superscaf	1.17E+08	1.17E+08	-4	4	9	-	-	-	-	-	-	NCP	IGR	-	ID=gene-E	6	4.781666667	1652.9	0.489869747	LGRVVL	56	656.4443	1	655.438	.	H	Superscaf	1.17E+08	0	0	0	0	0	BTP20220	Tissue specific
LSNHAY	Superscaf	-	Superscaf	1.17E+08	1.17E+08	-4	4	9	-	-	gene	-	-	mRNA	NCP	Intron	gene|mRNA	ID=gene-E	6	6.056666667	2724	0.490292518	LSNHAY	96	704.3329	1	703.3289	.	L	Superscaf	1.17E+08	0	0	0	BTP20220	0	0	Tissue specific
TNPPPP	Superscaf	-	Superscaf	1.17E+08	1.17E+08	-5	5	10	-	-	-	-	-	-	NCP	IGR	-	ID=gene-E	6	6.19	2343.8	0.49090659	TNPPPP	61	622.3198	1	621.3122	.	R	Superscaf	1.17E+08	0	0	BTP20220	BTP20220	0	0	Tissue specific
LVLVAK	Superscaf	-	Superscaf	1.17E+08	1.17E+08	-29	29	34	-	-	-	-	-	-	NCP	IGR	-	ID=gene-E	6	4.62	1486.5	0.492139976	LVLVAK	90	642.4518	1	641.4476	.	C	Superscaf	1.17E+08	0	BTP20220	0	0	0	0	Tissue specific
RDDLRK	Superscaf	-	Superscaf	1.17E+08	1.17E+08	-38	38	43	-	-	-	-	-	-	NCP	IGR	-	ID=gene-E	6	7.196666667	2942.4	0.493449752	RDDLRK	78	401.7227	2	801.4457	.	H	Superscaf	1.17E+08	0	0	0	0	0	BTP20220	Tissue specific
EAELRAPV	Superscaf	+	Superscaf	1.17E+08	1.17E+08	87	87	94	-	-	-	-	-	-	NCP	IGR	-	ID=gene-E	8	5.19	2813.2	0.493790394	EAELRAPV	64	442.7386	2	883.4763	.	L	Superscaf	1.17E+08	0	0	0	BTP20220	0	0	Tissue specific
KCHGVLGK	Superscaf	+	Superscaf	1.17E+08	1.17E+08	6	6	13	-	-	-	-	-	-	NCP	IGR	-	ID=gene-E	8	6.25125	3090.2	0.494894791	KC(+57.02	58	449.7417	2	897.4854	Carbamido	R	Superscaf	1.17E+08	0	0	0	0	0	BTP20220	Tissue specific
DFMDGLK	Superscaf	-	Superscaf	1.17E+08	1.17E+08	-3	3	9	-	-	-	-	-	-	NCP	IGR	-	ID=gene-E	7	5.551428571	2701	0.495880069	DFMDGLK	94	413.192	2	824.3738	.	I	Superscaf	1.17E+08	0	0	0	0	0	BTP20220	Tissue specific
TAERVAP	Superscaf	+	Superscaf	1.17E+08	1.17E+08	27	27	33	-	-	gene	-	-	mRNA	NCP	Intron	gene|mRNA	ID=gene-E	7	5.55	2431.8	0.497259313	T(+42.01)	50	393.2049	2	784.4079	Acetylati	D	Superscaf	1.17E+08	0	0	0	0	0	BTP20220	Tissue specific
KGYRL	Superscaf	+	Superscaf	1.17E+08	1.17E+08	6	6	10	-	-	gene	-	-	mRNA	NCP	Intron	gene|mRNA	ID=gene-E	5	7.622	2227.4	0.497757938	KGYRL	57	636.3834	1	635.3755	.	P	Superscaf	1.17E+08	0	0	0	0	0	BTP20220	Tissue specific
KSTAAGPP	Superscaf	+	Superscaf	1.18E+08	1.18E+08	4	4	11	-	-	-	-	-	-	NCP	IGR	-	ID=gene-E	8	6.57	2844	0.498586418	KSTAAGPP	63	364.6978	2	727.3864	.	G	Superscaf	1.18E+08	0	0	0	BTP20220	0	0	Tissue specific
PPFPHWP	Superscaf	+	Superscaf	1.18E+08	1.18E+08	10	10	16	-	-	gene	-	-	mRNA	NCP	Intron	gene|mRNA	ID=gene-E	7	6.308571429	2883.3	0.498919562	PPFPHWP	57	439.2245	2	876.4282	.	T	Superscaf	1.18E+08	BTP20220	0	0	0	BTP20220	BTP20220	Tissue specific
VTGGGLL	Superscaf	+	Superscaf	1.18E+08	1.18E+08	25	25	31	-	-	gene	-	-	mRNA	NCP	Intron	gene|mRNA	ID=gene-E	7	5.2	1996.2	0.500461648	VTGGGLL	56	616.3646	1	615.3592	.	H	Superscaf	1.18E+08	0	0	0	0	BTP20220	0	Tissue specific
NPGSLAVP	Superscaf	+	Superscaf	1.18E+08	1.18E+08	12	12	19	-	-	gene	-	-	mRNA	NCP	Intron	gene|mRNA	ID=gene-E	8	5.2075	2616.9	0.500717521	N(+42.01)	50	398.7119	2	795.4127	Acetylati	L	Superscaf	1.18E+08	0	0	0	0	BTP20220	BTP20220	Tissue specific
GLPPYPK	Superscaf	+	Superscaf	1.18E+08	1.18E+08	17	17	23	-	-	gene	-	-	mRNA	NCP	Intron	gene|mRNA	ID=gene-E	7	6.607142857	2590.8	0.500753708	G(+42.01)	73	407.2257	2	812.4432	Acetylati	V	Superscaf	1.18E+08	0	0	BTP20220	0	0	0	Mixed
RGVVVL	Superscaf	+	Superscaf	1.18E+08	1.18E+08	22	22	27	-	-	gene	-	-	mRNA	NCP	Intron	gene|mRNA	ID=gene-E	6	3.785	1299.5	0.50117732	RGVVVL	53	642.4275	1	641.4224	.	N	Superscaf	1.18E+08	0	0	0	0	0	BTP20220	Tissue specific
KGVAKLY	Superscaf	+	Superscaf	1.18E+08	1.18E+08	4	4	10	-	-	gene	-	-	mRNA	NCP	Intron	gene|mRNA	ID=gene-E	7	6.158571429	2358.3	0.502811557	KGVAKLY	71	389.7427	2	777.4749	.	P	Superscaf	1.18E+08	0	0	BTP20220	0	0	0	Tissue specific
LLLFLPK	Superscaf	+	Superscaf	1.18E+08	1.18E+08	53	53	59	-	-	gene	-	-	mRNA	NCP	Intron	gene|mRNA	ID=gene-E	7	6.491428571	2606.8	0.50570064	LLLFLPK	97	422.2868	2	842.5629	.	Q	Superscaf	1.18E+08	0	0	0	BTP20220	0	0	Tissue specific
ATMMDF	Superscaf	+	Superscaf	1.18E+08	1.18E+08	19	19	24	-	-	gene	-	-	mRNA	NCP	Intron	gene|mRNA	ID=gene-E	6	5.416666667	2250.7	0.505863826	ATM(+15.9	59	366.1389	2	730.2666	Oxidation	V	Superscaf	1.18E+08	0	0	BTP20220	0	0	0	Tissue specific
KASSSQK	Superscaf	-	Superscaf	1.18E+08	1.18E+08	-19	19	25	-	-	gene	-	-	mRNA	NCP	Intron	gene|mRNA	ID=gene-E	7	6.884285714	3009	0.508148705	KASSSQK	65	368.201	2	734.3923	.	V	Superscaf	1.18E+08	0	BTP20220	0	0	0	0	Tissue specific
NSVSAAPK	Superscaf	-	Superscaf	1.18E+08	1.18E+08	-20	20	27	-	-	-	-	-	-	NCP	IGR	-	ID=gene-E	8	5.60625	2791.9	0.51023861	N(+42.01)	82	408.2094	2	814.4185	Acetylati	K	Superscaf	1.18E+08	BTP20220	0	0	0	0	0	Tissue specific
LVLAMQ	Superscaf	+	Superscaf	1.18E+08	1.18E+08	42	42	47	-	-	-	-	-	-	NCP	IGR	-	ID=gene-E	6	4.896666667	2000.3	0.510425819	LVLAM(+15	65	690.3901	1	689.3782	Oxidation	R	Superscaf	1.18E+08	0	0	0	BTP20220	0	0	Tissue specific
LYSSSML	Superscaf	+	Superscaf	1.19E+08	1.19E+08	39	39	45	-	-	gene	-	-	mRNA	NCP	Intron	gene|mRNA	ID=gene-E	7	5.772857143	2688	0.511542419	LYSSSM(+1	83	408.6925	2	815.3735	Oxidation	S	Superscaf	1.19E+08	0	0	0	BTP20220	0	0	Tissue specific
KNYSLR	Superscaf	+	Superscaf	1.19E+08	1.19E+08	5	5	10	-	-	-	-	-	-	NCP	IGR	-	ID=gene-E	6	7.205	2910.2	0.511761608	KNYSLR	55	390.7277	2	779.429	.	E	Superscaf	1.19E+08	0	0	0	0	BTP20220	0	Tissue specific
PTHVK	Superscaf	-	Superscaf	1.19E+08	1.19E+08	-19	19	23	-	-	-	-	-	-	NCP	IGR	-	ID=gene-E	5	6.032	1823	0.512478772	PTHVK	65	581.338	1	580.3333	.	G	Superscaf	1.19E+08	0	0	0	BTP20220	0	0	Tissue specific
LLPTL	Superscaf	+	Superscaf	1.19E+08	1.19E+08	46	46	50	-	-	-	-	-	-	NCP	IGR	-	ID=gene-E	5	6.154	1795.1	0.512958003	LLPTL	98	556.3698	1	555.3632	.	T	Superscaf	1.19E+08	0	0	0	0	BTP20220	0	Tissue specific
KNQEF	Superscaf	-	Superscaf	1.19E+08	1.19E+08	-28	28	32	-	-	-	-	-	-	NCP	IGR	-	ID=gene-E	5	5.9	2488.7	0.513581448	K(+42.01)	71	707.3339	1	706.3286	Acetylati	D	Superscaf	1.19E+08	0	0	BTP20220	0	0	0	Tissue specific
DWSFY	Superscaf	-	Superscaf	1.19E+08	1.19E+08	-18	18	22	-	-	-	-	-	-	NCP	IGR	-	ID=gene-E	5	5.136	2168.4	0.515094729	DWSFY	96	717.2852	1	716.2806	.	A	Superscaf	1.19E+08	0	0	BTP20220	0	0	0	Tissue specific
AVRSF	Superscaf	-	Superscaf	1.19E+08	1.19E+08	-35	35	39	-	-	-	-	-	-	NCP	IGR	-	ID=gene-E	5	5.588	1730.9	0.515331553	A(+42.01)	60	621.333	1	620.3282	Acetylati	K	Superscaf	1.19E+08	0	0	BTP20220	0	0	0	Tissue specific
DSKPVNL	Superscaf	+	Superscaf	1.19E+08	1.19E+08	16	16	22	-	-	-	-	-	-	NCP	IGR	-	ID=gene-E	7	5.154285714	2550.8	0.515563365	DSKPVNL	87	772.4162	1	771.4127	.	C	Superscaf	1.19E+08	0	0	BTP20220	0	0	0	Tissue specific
LLLSKKL	Superscaf	-	Superscaf	1.19E+08	1.19E+08	-17	17	23	-	-	-	-	-	-	NCP	IGR	-	ID=gene-E	7	7.011428571	2733.8	0.518208662	LLLSKKL	63	407.7903	2	813.5687	.	L	Superscaf	1.19E+08	0	0	BTP20220	0	0	0	Tissue specific
ETHLVH	Superscaf	-	Superscaf	1.19E+08	1.19E+08	-6	6	11	-	-	-	-	-	-	NCP	IGR	-	ID=gene-E	6	5.151666667	2415.8	0.518599032	ETHLVH	64	735.3761	1	734.3712	.	F	Superscaf	1.19E+08	0	0	0	0	0	BTP20220	Tissue specific
HSLKL	Superscaf	+	Superscaf	1.19E+08	1.19E+08	15	15	19	-	-	-	-	-	-	NCP	IGR	-	ID=gene-E	5	6.994	2178.3	0.519108789	HSLKL	50	597.3696	1	596.3646	.	R	Superscaf	1.19E+08	0	0	0	0	BTP20220	0	Tissue specific
VVFLQ	Superscaf	-	Superscaf	1.19E+08	1.19E+08	-17	17	21	-	-	-	-	-	-	NCP	IGR	-	ID=gene-E	5	3.422	1351.5	0.519229286	VVFLQ	98	605.3637	1	604.3585	.	Y	Superscaf	1.19E+08	0	0	0	0	BTP20220	BTP20220	Tissue specific
VLLKEY	Superscaf	+	Superscaf	1.19E+08	1.19E+08	7	7	12	-	-	-	-	-	-	NCP	IGR	-	ID=gene-E	6	5.096666667	2060.2	0.519920973	VLLKEY	98	382.7289	2	763.4479	.	W	Superscaf	1.19E+08	BTP20220	0	0	0	0	0	Mixed
PVVHPP	Superscaf	+	Superscaf	1.19E+08	1.19E+08	54	54	59	-	-	-	-	-	-	NCP	IGR	-	ID=gene-E	6	4.415	1631.9	0.519938684	PVVHPP	51	645.3763	1	644.3646	.	H	Superscaf	1.19E+08	0	BTP20220	0	0	0	0	Tissue specific
LVCNGPS	Superscaf	-	Superscaf	1.19E+08	1.19E+08	-36	36	42	-	-	-	-	-	-	NCP	IGR	-	ID=gene-E	7	4.908571429	2553.9	0.522885682	LVC(+57.(	51	373.6771	2	745.3428	Carbamido	M	Superscaf	1.19E+08	0	0	BTP20220	0	0	0	Tissue specific
NNDTH	Superscaf	+	Superscaf	1.19E+08	1.19E+08	3	3	7	-	-	-	-	-	-	NCP	IGR	-	ID=gene-E	5	5.582	2622.1	0.523120745	N(+42.01)	59	642.2354	1	641.2405	Acetylati	F	Superscaf	1.19E+08	0	BTP20220	0	0	0	0	Tissue specific
KYFQSK	Superscaf	+	Superscaf	1.2E+08	1.2E+08	35	35	40	-	-	-	-	-	-	NCP	IGR	-	ID=gene-E	6	6.991666667	2777.8	0.523845483	KYFQSK	62	400.7175	2	799.4228	.	L	Superscaf	1.2E+08	0	0	0	0	0	BTP20220	Tissue specific
LSEVVVVK	Superscaf	+	Superscaf	1.2E+08	1.2E+08	1	1	8	-	-	-	-	-	-	NCP	IGR	-	ID=gene-E	8	3.0775	1630.7	0.52452076	LSEVVVVK	94	436.774	2	871.5378	.	L	Superscaf	1.2E+08	0	0	BTP20220	0	0	0	Tissue specific
VEEKKC	Superscaf	-	Superscaf	1.2E+08	1.2E+08	-5	5	10	-	-	-	-	-	-	NCP	IGR	-	ID=gene-E	6	5.156666667	2373.5	0.524746682	VEEKKC(+5	53	792.3789	1	791.3847	Carbamido	L	Superscaf	1.2E+08	0	0	0	0	0	BTP20220	Tissue specific
FQFKLR	Superscaf	-	Superscaf	1.2E+08	1.2E+08	-11	11	16	-	-	-	-	-	-	NCP	IGR	-	ID=gene-E	6	7.181666667	2856.1	0.524849352	FQFKLR	95	419.7483	2	837.4861	.	Q	Superscaf	1.2E+08	0	0	0	0	0	BTP20220	Tissue specific
LTLLATPL	Superscaf	-	Superscaf	1.2E+08	1.2E+08	-6	6	13	-	-	-	-	-	-	NCP	IGR	-	ID=gene-E	8	6.1625	2857.3	0.52927398	LTLLATPL	62	841.5348	1	840.532	.	S	Superscaf	1.2E+08	0	0	0	BTP20220	0	0	Tissue specific
KEVAAHK	Superscaf	-	Superscaf	1.2E+08	1.2E+08	-4	4	10	-	-	-	-	-	-	NCP	IGR	-	ID=gene-E	7	6.047142857	2604.6	0.529907102	KEVAAHK	59	391.725	2	781.4446	.	K	Superscaf	1.2E+08	BTP20220	0	0	0	0	0	Tissue specific
VNTLLRPDC	Superscaf	+	Superscaf	1.2E+08	1.2E+08	6	6	17	-	-	-	-	-	-	NCP	IGR	-	ID=gene-E	12	6.05	4758.3	0.530367475	VNTLLRPDC	92	685.3906	2	1368.773	.	H	Superscaf	1.2E+08	BTP20220	0	0	0	0	0	Tissue specific
ARLLCEF	Superscaf	+	Superscaf	1.2E+08	1.2E+08	36	36	42	-	-	-	-	-	-	NCP	IGR	-	ID=gene-E	7	6.065714286	3065.5	0.530406569	ARLLC(+57	70	454.7356	2	907.4586	Carbamido	S	Superscaf	1.2E+08	BTP20220	0	0	0	0	0	Tissue specific
KAYAAPK	Superscaf	+	Superscaf	1.2E+08	1.2E+08	13	13	19	-	-	gene	-	-	mRNA	NCP	Intron	gene|mRNA	ID=gene-E	7	7.071428571	2667.6	0.531155636	KAYAAPK	56	374.7206	2	747.4279	.	G	Superscaf	1.2E+08	BTP20220	0	0	0	0	0	Tissue specific
TLHTAPPK	Superscaf	+	Superscaf	1.2E+08	1.2E+08	85	85	92	-	-	-	-	-	-	NCP	IGR	-	ID=gene-E	8	6.87375	3222.6	0.532938332	TLHTAPPK	65	432.745	2	863.4865	.	P	Superscaf	1.2E+08	0	0	0	BTP20220	0	0	Tissue specific
SVAVFCR	Superscaf	+	Superscaf	1.2E+08	1.2E+08	19	19	25	-	-	-	-	-	-	NCP	IGR	-	ID=gene-E	7	4.708571429	2316.6	0.53461591	SVAVFC(+5	94	419.7132	2	837.4167	Carbamido	I	Superscaf	1.2E+08	BTP20220	0	0	0	0	0	Tissue specific
LPLSADNK	Superscaf	+	Superscaf	1.2E+08	1.2E+08	4	4	11	-	-	-	-	-	-	NCP	IGR	-	ID=gene-E	8	6.01	3215.5	0.535486085	LPLSADNK	71	429.2388	2	856.4654	.	T	Superscaf	1.2E+08	0	BTP20220	0	0	0	0	Tissue specific
LTTKPSLK	Superscaf	-	Superscaf	1.2E+08	1.2E+08	-3	3	10	-	-	-	-	-	-	NCP	IGR	-	ID=gene-E	8	7.06	3159.4	0.536241119	LTTKPSLK	57	444.2805	2	886.5488	.	H	Superscaf	1.2E+08	0	0	0	BTP20220	0	0	Tissue specific
SELLVRR	Superscaf	-	Superscaf	1.21E+08	1.21E+08	-30	30	36	-	-	-	-	-	-	NCP	IGR	-	ID=gene-E	7	6.054285714	2813.3	0.536644266	S(+42.01)	70	457.7737	2	913.5345	Acetylati	I	Superscaf	1.21E+08	BTP20220	0	0	0	0	0	Tissue specific
GHVNN	Superscaf	-	Superscaf	1.21E+08	1.21E+08	-16	16	20	-	-	-	-	-	-	NCP	IGR	-	ID=gene-E	5	4.876	2110.4	0.538224719	GHVNN	50	540.2573	1	539.2452	.	T	Superscaf	1.21E+08	0	BTP20220	BTP20220	0	0	0	Tissue specific
FTKALSS	Superscaf	+	Superscaf	1.21E+08	1.21E+08	34	34	40	-	-	-	-	-	-	NCP	IGR	-	ID=gene-E	7	6.444285714	2684.8	0.541022416	FTKALSS	52	377.2161	2	752.4068	.	T	Superscaf	1.21E+08	0	BTP20220	BTP20220	BTP20220	0	0	Mixed
PLLMQ	Superscaf	+	Superscaf	1.21E+08	1.21E+08	3	3	7	-	-	-	-	-	-	NCP	IGR	-	ID=gene-E	5	5.932	2026.4	0.541785254	PLLMQ	99	601.3352	1	600.3305	.	R	Superscaf	1.21E+08	0	0	BTP20220	0	0	0	Tissue specific
RMLALK	Superscaf	+	Superscaf	1.21E+08	1.21E+08	3	3	8	-	-	-	-	-	-	NCP	IGR	-	ID=gene-E	6	7.371666667	2506.8	0.544471023	RMLALK	76	366.232	2	730.4523	.	Y	Superscaf	1.21E+08	0	0	BTP20220	0	0	0	Tissue specific
KKSSKHK	Superscaf	+	Superscaf	1.21E+08	1.21E+08	9	9	15	-	-	-	-	-	-	NCP	IGR	-	ID=gene-E	7	8.272857143	3260.1	0.544848463	KKSSKHK	50	421.7687	2	841.5134	.	K	Superscaf	1.21E+08	0	0	BTP20220	0	0	0	Tissue specific
TEMHK	Superscaf	-	Superscaf	1.21E+08	1.21E+08	-7	7	11	-	-	-	-	-	-	NCP	IGR	-	ID=gene-E	5	6.566	2282.6	0.545114894	T(+42.01)	52	703.3044	1	702.3007	Acetylati	L	Superscaf	1.21E+08	0	0	0	BTP20220	0	0	Tissue specific
SLPEV	Superscaf	-	Superscaf	1.21E+08	1.21E+08	-6	6	10	-	-	-	-	-	-	NCP	IGR	-	ID=gene-E	5	4.236	1499.7	0.545294835	SLPEV	59	544.298	1	543.2904	.	Q	Superscaf	1.21E+08	0	0	BTP20220	0	0	0	Tissue specific
KGGGKPNL	Superscaf	-	Superscaf	1.21E+08	1.21E+08	-4	4	11	-	-	-	-	-	-	NCP	IGR	-	ID=gene-E	8	6.885	3116.2	0.545691976	KGGGKPNL	50	385.7234	2	769.4446	.	R	Superscaf	1.21E+08	0	0	BTP20220	BTP20220	0	0	Tissue specific
KPTTYC	Superscaf	-	Superscaf	1.21E+08	1.21E+08	-35	35	40	-	-	-	-	-	-	NCP	IGR	-	ID=gene-E	6	6.63	2646	0.546939592	KPTTYC(+5	69	769.3521	1	768.3476	Carbamido	W	Superscaf	1.21E+08	0	0	BTP20220	BTP20220	0	BTP20220	Tissue specific
LRNLPLQ	Superscaf	-	Superscaf	1.21E+08	1.21E+08	-10	10	16	-	-	-	-	-	-	NCP	IGR	-	ID=gene-E	7	6.58	3253.8	0.547176836	LRNLPLQ	64	427.2648	2	852.5181	.	H	Superscaf	1.21E+08	0	0	BTP20220	0	0	0	Tissue specific
KETTPSEK	Superscaf	-	Superscaf	1.21E+08	1.21E+08	-3	3	10	-	-	gene	-	-	mRNA	NCP	Intron	gene|mRNA	ID=gene-E	8	6.37	3303.6	0.548073979	KETTPSEK	58	460.2451	2	918.4658	.	G	Superscaf	1.21E+08	0	0	BTP20220	0	0	BTP20220	Tissue specific
MEPLTF	Superscaf	+	Superscaf	1.21E+08	1.21E+08	14	14	19	-	-	gene	-	-	mRNA	NCP	Intron	gene|mRNA	ID=gene-E	6	5.543333333	2272.7	0.548266965	M(+42.01)	72	779.3612	1	778.3571	Acetylati	T	Superscaf	1.21E+08	0	0	BTP20220	BTP20220	0	0	Tissue specific
QKVESK	Superscaf	-	Superscaf	1.22E+08	1.22E+08	-9	9	14	-	-	gene	-	-	mRNA	NCP	Intron	gene|mRNA	ID=gene-E	6	5.671666667	2356.4	0.55032401	Q(+42.01)	52	380.7184	2	759.4127	Acetylati	N	Superscaf	1.22E+08	0	0	BTP20220	0	0	0	Tissue specific
LVEWT	Superscaf	-	Superscaf	1.22E+08	1.22E+08	-16	16	20	-	-	-	-	-	-	NCP	IGR	-	ID=gene-E	5	4.324	1703	0.551522854	LVEWT	76	647.3359	1	646.3326	.	Y	Superscaf	1.22E+08	0	0	0	BTP20220	0	0	Tissue specific
NAYKL	Superscaf	+	Superscaf	1.22E+08	1.22E+08	4	4	8	-	-	-	-	-	-	NCP	IGR	-	ID=gene-E	5	6.562	2189.3	0.554729397	NAYKL	53	608.3461	1	607.3329	.	N	Superscaf	1.22E+08	0	0	0	0	BTP20220	0	Tissue specific
GFTPGLG	Superscaf	+	Superscaf	1.22E+08	1.22E+08	26	26	32	-	-	-	-	-	-	NCP	IGR	-	ID=gene-E	7	6.028571429	2367.6	0.555166473	GFTPGLG	67	648.3323	1	647.3278	.	T	Superscaf	1.22E+08	0	0	0	0	0	BTP20220	Tissue specific
RSVGPGVR	Superscaf	-	Superscaf	1.22E+08	1.22E+08	-35	35	42	-	-	-	-	-	-	NCP	IGR	-	ID=gene-E	8	5.68	2613	0.556095749	RSVGPGVR	79	414.2444	2	826.4773	.	G	Superscaf	1.22E+08	0	0	BTP20220	0	0	0	Tissue specific
QLLAF	Superscaf	-	Superscaf	1.22E+08	1.22E+08	-13	13	17	-	-	gene	-	-	mRNA	NCP	Intron	gene|mRNA	ID=gene-E	5	5.822	2016.2	0.557161817	QLLAF	87	591.3474	1	590.3428	.	L	Superscaf	1.22E+08	0	0	0	0	BTP20220	0	Tissue specific
LVLLLPL	Superscaf	-	Superscaf	1.22E+08	1.22E+08	-8	8	14	-	-	-	-	-	-	NCP	IGR	-	ID=gene-E	7	5.171428571	2104.4	0.558989537	L(+42.01)	58	411.7919	2	821.5626	Acetylati	V	Superscaf	1.22E+08	0	BTP20220	0	0	0	0	Tissue enhanced
GKDEPQR	Superscaf	-	Superscaf	1.22E+08	1.22E+08	-14	14	20	-	-	-	-	-	-	NCP	IGR	-	ID=gene-E	7	6.372857143	3199.6	0.560752992	G(+42.01)	95	436.2192	2	870.4195	Acetylati	L	Superscaf	1.22E+08	BTP20220	0	0	0	0	0	Tissue specific
ATLRVLEP	Superscaf	+	Superscaf	1.22E+08	1.22E+08	12	12	19	-	-	-	-	-	-	NCP	IGR	-	ID=gene-E	8	5.59875	2827.3	0.56103897	A(+42.01)	60	470.7758	2	939.5389	Acetylati	L	Superscaf	1.22E+08	0	0	BTP20220	0	0	0	Tissue specific
NLRSHL	Superscaf	-	Superscaf	1.22E+08	1.22E+08	-7	7	12	-	-	-	-	-	-	NCP	IGR	-	ID=gene-E	6	6.9	2955.4	0.561239759	NLRSHL	96	370.2127	2	738.4136	.	K	Superscaf	1.22E+08	0	BTP20220	0	0	0	0	Tissue specific
TQCFR	Superscaf	-	Superscaf	1.22E+08	1.22E+08	-3	3	7	-	-	-	-	-	-	NCP	IGR	-	ID=gene-E	5	6.688	2630.1	0.561483353	TQC(+57.(	72	356.1685	2	710.317	Carbamido	I	Superscaf	1.22E+08	0	BTP20220	0	0	0	0	Tissue specific
KPVALT	Superscaf	+	Superscaf	1.23E+08	1.23E+08	30	30	35	-	-	-	-	-	-	NCP	IGR	-	ID=gene-E	6	5.761666667	1868	0.565522354	KPVALT	98	628.4005	1	627.3956	.	P	Superscaf	1.23E+08	0	0	0	BTP20220	BTP20220	0	Tissue specific
LPELNGKL1	Superscaf	+	Superscaf	1.23E+08	1.23E+08	4	4	12	-	-	-	-	-	-	NCP	IGR	-	ID=gene-E	9	6.123333333	3583	0.565544617	LPELNGKL1	50	492.7893	2	983.5651	.	W	Superscaf	1.23E+08	0	0	BTP20220	BTP20220	BTP20220	BTP20220	Tissue specific
KSDSFGF	Superscaf	-	Superscaf	1.23E+08	1.23E+08	-7	7	13	-	-	-	-	-	-	NCP	IGR	-	ID=gene-E	7	5.857142857	2718.8	0.567333242	K(+42.01)	75	829.3698	1	828.3654	Acetylati	H	Superscaf	1.23E+08	0	0	BTP20220	BTP20220	0	0	Tissue specific
EEDRLK	Superscaf	-	Superscaf	1.23E+08	1.23E+08	-5	5	10	-	-	-	-	-	-	NCP	IGR	-	ID=gene-E	6	5.981666667	2733.1	0.567589498	E(+42.01)	67	416.213	2	830.4134	Acetylati	D	Superscaf	1.23E+08	0	BTP20220	0	0	0	0	Tissue specific
KTYLNF	Superscaf	-	Superscaf	1.23E+08	1.23E+08	-41	41	46	-	-	-	-	-	-	NCP	IGR	-	ID=gene-E	6	6.466666667	2662.9	0.568721323	KTYLNF	57	393.2121	2	784.4119	.	L	Superscaf	1.23E+08	0	0	0	0	BTP20220	0	Mixed
LFSYVLT	Superscaf	+	Superscaf	1.23E+08	1.23E+08	28	28	34	-	-	-	-	-	-	NCP	IGR	-	ID=gene-E	7	5.044285714	2334.6	0.569144361	LFSYVLT	65	842.4604	1	841.4586	.	I	Superscaf	1.23E+08	0	0	0	0	0	BTP20220	Tissue specific
GVLGVGH	Superscaf	+	Superscaf	1.23E+08	1.23E+08	38	38	44	-	-	-	-	-	-	NCP	IGR	-	ID=gene-E	7	4.497142857	1865	0.569403485	GVLGVGH	80	638.3602	1	637.3547	.	D	Superscaf	1.23E+08	0	0	0	0	0	BTP20220	Tissue specific
NHLMEPK	Superscaf	+	Superscaf	1.23E+08	1.23E+08	25	25	31	-	-	-	-	-	-	NCP	IGR	-	ID=gene-E	7	6.284285714	3172.6	0.569674277	NHLM(+15.	73	442.7227	2	883.4222	Oxidation	V	Superscaf	1.23E+08	0	0	0	BTP20220	0	0	Tissue enhanced
DKVHL	Superscaf	+	Superscaf	1.23E+08	1.23E+08	10	10	14	-	-	-	-	-	-	NCP	IGR	-	ID=gene-E	5	5.256	1853	0.570113152	D(+42.01)	73	653.3594	1	652.3544	Acetylati	E	Superscaf	1.23E+08	BTP20220	0	0	0	0	0	Tissue specific

LGKFTEV	Superscaf+	Superscaf	1.23E+08	1.23E+08	2	2	8	-	-	-	-	-	-	NCP	IGR	-	ID=gene-E	7	5.274285714	2329.5	0.571494078	LGKFTEV	99	397.2244	2	792.4381	.	H	Superscaf	1.23E+08	BTP20220	0	0	0	0	0	Tissue specific
SAKKLLV	Superscaf+	Superscaf	1.23E+08	1.23E+08	4	4	10	-	-	-	-	-	-	NCP	IGR	-	ID=gene-E	7	6.162857143	2338.3	0.573349264	SAKKLLV	98	379.7592	2	757.5062	.	R	Superscaf	1.23E+08	0	BTP20220	BTP20220	BTP20220	BTP20220	BTP20220	Tissue specific
CENND	Superscaf+	Superscaf	1.24E+08	1.24E+08	2	2	6	-	-	-	-	-	-	NCP	IGR	-	ID=gene-E	5	4.406	2616.1	0.574913881	C(+57.02)	54	651.2119	1	650.1966	Carbamidc	K	Superscaf	1.24E+08	0	0	0	0	BTP20220	0	Tissue specific
KTAAYP	Superscaf+	Superscaf	1.24E+08	1.24E+08	7	7	12	-	-	-	-	-	-	NCP	IGR	-	ID=gene-E	6	6.711666667	2285.4	0.575249167	KTAAYP	55	650.3551	1	649.3435	.	T	Superscaf	1.24E+08	0	0	BTP20220	0	0	0	Tissue specific
FLLPLEF	Superscaf-	Superscaf	1.24E+08	1.24E+08	-7	7	13	-	-	-	-	-	-	NCP	IGR	-	ID=gene-E	7	5.488571429	2597.9	0.576259309	FLLPLEF	52	878.499	1	877.4949	.	T	Superscaf	1.24E+08	0	0	0	0	BTP20220	0	Tissue specific
NAPLF	Superscaf-	Superscaf	1.24E+08	1.24E+08	-62	62	66	-	-	-	-	-	-	NCP	IGR	-	ID=gene-E	5	5.838	1986.2	0.576266424	N(+42.01)	69	603.313	1	602.3064	Acetylati	T	Superscaf	1.24E+08	0	0	0	BTP20220	0	0	Tissue specific
PPLPFD	Superscaf+	Superscaf	1.24E+08	1.24E+08	1	1	6	-	-	-	-	-	-	NCP	IGR	-	ID=gene-E	6	5.555	2164.5	0.576335317	PPLPFD	87	685.3535	1	684.3482	.	P	Superscaf	1.24E+08	0	0	0	0	0	BTP20220	Tissue specific
NPPLLQLLI	Superscaf+	Superscaf	1.24E+08	1.24E+08	33	33	41	-	-	-	-	-	-	NCP	IGR	-	ID=gene-E	9	5.951111111	3649.2	0.576634148	NPPLLQLLI	50	510.8242	2	1019.638	.	P	Superscaf	1.24E+08	0	BTP20220	0	0	0	BTP20220	Tissue specific
LLHQLPTH	Superscaf+	Superscaf	1.24E+08	1.24E+08	62	62	69	-	-	-	-	-	-	NCP	IGR	-	ID=gene-E	8	6.45	3645.2	0.576911061	LLHQLPTH	55	479.7685	2	957.5396	.	L	Superscaf	1.24E+08	0	0	0	0	0	BTP20220	Tissue specific
NPPVT	Superscaf+	Superscaf	1.24E+08	1.24E+08	63	63	67	-	-	-	-	-	-	NCP	IGR	-	ID=gene-E	5	4.908	1669	0.576919285	N(+42.01)	66	569.2917	1	568.2856	Acetylati	G	Superscaf	1.24E+08	0	BTP20220	0	0	0	BTP20220	Tissue specific
TVSFGR	Superscaf-	Superscaf	1.24E+08	1.24E+08	-3	3	8	-	-	-	-	-	-	NCP	IGR	-	ID=gene-E	6	5.736666667	2114.4	0.577712189	T(+42.01)	54	354.6864	2	707.3602	Acetylati	M	Superscaf	1.24E+08	0	BTP20220	0	BTP20220	0	BTP20220	Tissue specific
LAKAANK	Superscaf+	Superscaf	1.24E+08	1.24E+08	2	2	8	-	-	-	-	-	-	NCP	IGR	-	ID=gene-E	7	6.99	2820.8	0.581838062	L(+42.01)	68	379.2308	2	756.4493	Acetylati	K	Superscaf	1.24E+08	0	0	0	0	0	BTP20220	Tissue specific
RVPLFE	Superscaf-	Superscaf	1.24E+08	1.24E+08	-15	15	20	-	-	-	-	-	-	NCP	IGR	-	ID=gene-E	6	5.29	2152.5	0.582365951	RVPLFE	53	760.4332	1	759.4279	.	N	Superscaf	1.24E+08	0	0	0	BTP20220	0	0	Tissue specific
SSLAKVTL	Superscaf+	Superscaf	1.24E+08	1.24E+08	8	8	15	-	-	-	-	-	-	NCP	IGR	-	ID=gene-E	8	5.70125	2650.8	0.582589577	SSLAKVTL	55	409.7512	2	817.4909	.	G	Superscaf	1.24E+08	0	0	BTP20220	0	0	0	Tissue specific
KTPAWVK	Superscaf-	Superscaf	1.24E+08	1.24E+08	-3	3	9	-	-	gene	-	-	mRNA	NCP	Intron	gene|mRN	ID=gene-E	7	6.317142857	2509.6	0.583477617	KTPAWVK	56	415.247	2	828.4858	.	R	Superscaf	1.24E+08	0	0	0	0	0	BTP20220	Tissue specific
PFFSLPR	Superscaf-	Superscaf	1.24E+08	1.24E+08	-5	5	11	-	-	gene	-	-	mRNA	NCP	Intron	gene|mRN	ID=gene-E	7	6.568571429	2835.2	0.583613605	P(+42.01)	76	453.2449	2	904.4807	Acetylati	N	Superscaf	1.24E+08	0	0	0	BTP20220	0	0	Tissue specific
PFVVLL	Superscaf-	Superscaf	1.24E+08	1.24E+08	-24	24	29	-	-	gene	-	-	mRNA	NCP	Intron	gene|mRN	ID=gene-E	6	3.956666667	1431.6	0.584068546	PFVVLL	71	687.4449	1	686.4366	.	F	Superscaf	1.24E+08	0	0	0	BTP20220	0	0	Tissue enhanced
QCVKTK	Superscaf-	Superscaf	1.24E+08	1.24E+08	-52	52	57	-	-	gene	-	-	mRNA	NCP	Intron	gene|mRN	ID=gene-E	6	6.113333333	2530.7	0.584692105	Q(+42.01)	57	403.2179	2	804.4164	Acetylati	Y	Superscaf	1.24E+08	0	0	0	BTP20220	0	0	Tissue specific
AVVAPCN	Superscaf+	Superscaf	1.24E+08	1.24E+08	8	8	14	-	-	gene	-	-	mRNA	NCP	Intron	gene|mRN	ID=gene-E	7	4.11	2142.4	0.587313457	AVVAPC(+5	54	365.6842	2	729.348	Carbamidc	L	Superscaf	1.24E+08	0	0	0	0	0	BTP20220	Tissue specific
VDTLP	Superscaf+	Superscaf	1.25E+08	1.25E+08	41	41	45	-	-	gene	-	-	mRNA	NCP	Intron	gene|mRN	ID=gene-E	5	4.356	1499.8	0.589940163	VDTLP	60	544.2963	1	543.2904	.	P	Superscaf	1.25E+08	0	0	0	0	0	BTP20220	Tissue specific
NVLKVTK	Superscaf-	Superscaf	1.25E+08	1.25E+08	-28	28	34	-	-	gene	-	-	mRNA	NCP	Intron	gene|mRN	ID=gene-E	7	5.342857143	2284.4	0.59087839	NVLKVTK	69	401.2616	2	800.512	.	M	Superscaf	1.25E+08	0	BTP20220	0	0	0	0	Tissue specific
KNEYA	Superscaf-	Superscaf	1.25E+08	1.25E+08	-4	4	8	-	-	gene	-	-	mRNA	NCP	Intron	gene|mRN	ID=gene-E	5	6.01	2261.4	0.591277711	KNEYA	54	624.297	1	623.2914	.	W	Superscaf	1.25E+08	0	BTP20220	0	0	0	0	Tissue specific
LSLSMEGV	Superscaf+	Superscaf	1.25E+08	1.25E+08	25	25	32	-	-	-	-	-	-	NCP	IGR	-	ID=gene-E	8	4.7825	2567.9	0.592472576	LSLSM(+15	50	426.2118	2	850.4106	Oxidation	H	Superscaf	1.25E+08	BTP20220	0	0	0	0	0	Tissue specific
KAAEVT	Superscaf+	Superscaf	1.25E+08	1.25E+08	2	2	7	-	-	-	-	-	-	NCP	IGR	-	ID=gene-E	6	5.255	1914	0.592639053	KAAEVT	61	618.3441	1	617.3384	.	W	Superscaf	1.25E+08	0	0	0	0	BTP20220	0	Tissue specific
LLMARL	Superscaf+	Superscaf	1.25E+08	1.25E+08	30	30	35	-	-	-	-	-	-	NCP	IGR	-	ID=gene-E	6	6.745	2391.8	0.59398035	LLMARL	76	358.7297	2	715.4415	.	G	Superscaf	1.25E+08	0	0	0	0	BTP20220	0	Tissue specific
SFKVEKK	Superscaf+	Superscaf	1.25E+08	1.25E+08	29	29	35	-	-	-	-	-	-	NCP	IGR	-	ID=gene-E	7	6.228571429	2601.5	0.59428423	SFKVEKK	79	433.2561	2	864.5068	.	T	Superscaf	1.25E+08	0	0	0	0	0	BTP20220	Tissue specific
ESGKQEC	Superscaf-	Superscaf	1.25E+08	1.25E+08	-31	31	37	-	-	gene	-	-	mRNA	NCP	Intron	gene|mRN	ID=gene-E	7	5.5	3196.5	0.59453383	ESGKQEC(+	54	837.3416	1	836.3334	Carbamidc	L	Superscaf	1.25E+08	0	0	0	0	0	BTP20220	Tissue specific
PGSQKTK	Superscaf+	Superscaf	1.25E+08	1.25E+08	36	36	42	-	-	gene	-	-	mRNA	NCP	Intron	gene|mRN	ID=gene-E	7	7.087142857	2963.1	0.595098021	P(+42.01)	87	394.2184	2	786.4235	Acetylati	S	Superscaf	1.25E+08	0	0	0	0	BTP20220	0	Tissue specific
KDSVH	Superscaf-	Superscaf	1.25E+08	1.25E+08	-6	6	10	-	-	gene	-	-	mRNA	NCP	Intron	gene|mRN	ID=gene-E	5	5.196	1883	0.595687076	KDSVH	69	585.3053	1	584.2918	.	C	Superscaf	1.25E+08	0	0	BTP20220	0	0	0	Tissue specific
KAVFGQ	Superscaf+	Superscaf	1.25E+08	1.25E+08	3	3	8	-	-	gene	-	-	mRNA	NCP	Intron	gene|mRN	ID=gene-E	6	5.476666667	2075.1	0.599286399	KAVFGQ	61	649.3645	1	648.3595	.	E	Superscaf	1.25E+08	0	0	0	BTP20220	0	0	Tissue specific
ERKSFLFL	Superscaf+	Superscaf	1.25E+08	1.25E+08	1	1	8	-	-	-	-	-	-	NCP	IGR	-	ID=gene-E	8	6.54	3407.7	0.599342975	ERKSFLFL	52	520.2988	2	1038.586	.	E	Superscaf	1.25E+08	0	0	0	0	0	BTP20220	Tissue specific
TKNPQ	Superscaf+	Superscaf	1.25E+08	1.25E+08	11	11	15	-	-	gene	-	-	mRNA	NCP	Intron	gene|mRN	ID=gene-E	5	6.726	2410.7	0.599874459	T(+42.01)	58	629.3275	1	628.3181	Acetylati	S	Superscaf	1.25E+08	0	0	0	0	BTP20220	0	Tissue specific
VLSST	Superscaf+	Superscaf	1.26E+08	1.26E+08	13	13	17	-	-	-	-	-	-	NCP	IGR	-	ID=gene-E	5	4.774	1517.7	0.600358358	V(+42.01)	58	548.2889	1	547.2853	Acetylati	Q	Superscaf	1.26E+08	0	BTP20220	0	0	0	0	Tissue specific
PPPATHT	Superscaf-	Superscaf	1.26E+08	1.26E+08	-22	22	28	-	-	gene	-	-	mRNA	NCP	Intron	gene|mRN	ID=gene-E	7	6.51	2738.2	0.603799773	PPPATHT	64	360.6868	2	719.3602	.	W	Superscaf	1.26E+08	0	0	BTP20220	0	0	0	Tissue specific
KPNDK	Superscaf-	Superscaf	1.26E+08	1.26E+08	-35	35	39	-	-	gene	-	-	mRNA	NCP	Intron	gene|mRN	ID=gene-E	5	6.832	2282.4	0.606279091	KPNDK	64	601.3323	1	600.3231	.	I	Superscaf	1.26E+08	BTP20220	0	0	0	0	0	Tissue specific
SVYVY	Superscaf-	Superscaf	1.26E+08	1.26E+08	-3	3	7	-	-	gene	-	-	mRNA	NCP	Intron	gene|mRN	ID=gene-E	5	3.4	1302.4	0.606583737	SVYVY	95	630.3121	1	629.3061	.	G	Superscaf	1.26E+08	0	0	BTP20220	0	0	0	Mixed
LLSDDLLFI	Superscaf+	Superscaf	1.26E+08	1.26E+08	9	9	17	-	-	gene	-	-	mRNA	NCP	Intron	gene|mRN	ID=gene-E	9	5.222222222	3360.8	0.606589666	LLSDDLLFI	71	524.7984	2	1047.585	.	N	Superscaf	1.26E+08	0	BTP20220	0	0	0	0	Tissue specific
SCFGPK	Superscaf+	Superscaf	1.26E+08	1.26E+08	6	6	11	-	-	gene	-	-	mRNA	NCP	Intron	gene|mRN	ID=gene-E	6	6.365	2459.6	0.606729901	SC(+57.02	91	695.3151	1	694.3109	Carbamidc	L	Superscaf	1.26E+08	0	0	0	BTP20220	0	0	Tissue specific
FPLSLQPLI	Superscaf+	Superscaf	1.26E+08	1.26E+08	16	16	24	-	-	gene	-	-	mRNA	NCP	Intron	gene|mRN	ID=gene-E	9	5.925555556	3469.9	0.607913825	FPLSLQPLI	67	1027.619	1	1026.611	.	W	Superscaf	1.26E+08	0	0	0	BTP20220	0	0	Tissue specific
RGVFSAY	Superscaf-	Superscaf	1.26E+08	1.26E+08	-29	29	35	-	-	gene	-	-	mRNA	NCP	Intron	gene|mRN	ID=gene-E	7	5.652857143	2487.7	0.608168207	RGVFSAY	51	400.215	2	798.4024	.	M	Superscaf	1.26E+08	BTP20220	0	0	0	0	0	Tissue specific
SGDLMF	Superscaf-	Superscaf	1.26E+08	1.26E+08	-2	2	7	-	-	gene	-	-	mRNA	NCP	Intron	gene|mRN	ID=gene-E	6	5.305	2204.5	0.608235952	SGDLMF	55	669.2885	1	668.2839	.	N	Superscaf	1.26E+08	0	0	BTP20220	0	0	0	Tissue specific
SLVSVVR	Superscaf-	Superscaf	1.26E+08	1.26E+08	-50	50	56	-	-	-	-	-	-	NCP	IGR	-	ID=gene-E	7	4.014285714	1769	0.610441304	SLVSVVR	80	380.238	2	758.465	.	A	Superscaf	1.26E+08	0	0	0	BTP20220	0	0	Tissue specific
HVVPEVR	Superscaf-	Superscaf	1.26E+08	1.26E+08	-17	17	23	-	-	-	-	-	-	NCP	IGR	-	ID=gene-E	7	3.981428571	2031.4	0.610486098	H(+42.01)	73	439.2554	2	876.4818	Acetylati	M	Superscaf	1.26E+08	BTP20220	0	0	0	0	0	Tissue specific
KVVGFER	Superscaf+	Superscaf	1.26E+08	1.26E+08	18	18	24	-	-	-	-	-	-	NCP	IGR	-	ID=gene-E	7	5.024285714	2227.4	0.610700008	KVVGFER	60	417.7483	2	833.4759	.	V	Superscaf	1.26E+08	0	0	0	BTP20220	0	0	Tissue specific
RAYLFL	Superscaf+	Superscaf	1.26E+08	1.26E+08	2	2	7	-	-	gene	-	-	mRNA	NCP	Intron	gene|mRN	ID=gene-E	6	6.646666667	2513.8	0.610847167	RAYLFL	60	391.7394	2	781.4486	.	P	Superscaf	1.26E+08	BTP20220	0	0	0	0	0	Tissue specific
NSSLPQR	Superscaf+	Superscaf	1.26E+08	1.26E+08	8	8	14	-	-	gene	-	-	mRNA	NCP	Intron	gene|mRN	ID=gene-E	7	6.494285714	3313.8	0.611486295	NSSLPQR	54	401.2147	2	800.4141	.	Q	Superscaf	1.26E+08	0	0	0	0	BTP20220	BTP20220	Tissue specific
MMVTKK	Superscaf+	Superscaf	1.26E+08	1.26E+08	14	14	19	-	-	-	-	-	-	NCP	IGR	-	ID=gene-E	6	6.251666667	2077.3	0.611573205	M(+15.99)	83	385.2048	2	768.3874	Oxidation	D	Superscaf	1.26E+08	0	0	0	0	0	BTP20220	Tissue specific
CVGKPNP	Superscaf+	Superscaf	1.26E+08	1.26E+08	16	16	22	-	-	-	-	-	-	NCP	IGR	-	ID=gene-E	7	5.534285714	2622.9	0.612194699	C(+57.02)	59	771.3807	1	770.3745	Carbamidc	Q	Superscaf	1.26E+08	0	0	0	BTP20220	0	0	Tissue specific
KYMSKK	Superscaf-	Superscaf	1.26E+08	1.26E+08	-23	23	28	-	-	gene	-	-	mRNA	NCP	Intron	gene|mRN	ID=gene-E	6	7.718333333	2619.6	0.612423298	KYM(+15.9	72	400.7179	2	799.4262	Oxidation	I	Superscaf	1.26E+08	0	0	0	BTP20220	0	0	Tissue specific
YGGGKGGY	Superscaf-	Superscaf	1.26E+08	1.26E+08	-28	28	35	-	-	gene	-	-	mRNA	NCP	Intron	gene|mRN	ID=gene-E	8	6.36375	2873.9	0.612455162	YGGGKGGY	74	379.6757	2	757.3395	.	E	Superscaf	1.26E+08	0	0	0	0	0	BTP20220	Tissue specific
LTGDW	Superscaf+	Superscaf	1.26E+08	1.26E+08	25	25	29	-	-	gene	-	-	mRNA	NCP	Intron	gene|mRN	ID=gene-E	5	5.468	1986.3	0.612659509	LTGDW	99	591.275	1	590.27	.	L	Superscaf	1.26E+08	BTP20220	0	0	0	0	0	Tissue specific
EKSLVR	Superscaf+	Superscaf	1.26E+08	1.26E+08	15	15	20	-	-	gene	-	-	mRNA	NCP	Intron	gene|mRN	ID=gene-E	6	5.896666667	2279.5	0.612907731	EKSLVR	95	366.223	2	730.4337	.	H	Superscaf	1.26E+08	0	0	0	BTP20220	0	0	Tissue specific
TAGFY	Superscaf+	Superscaf	1.27E+08	1.27E+08	19	19	23	-	-	-	-	-	-	NCP	IGR	-	ID=gene-E	5	5.932	1853	0.614539482	TAGFY	66	558.2535	1	557.2485	.	I	Superscaf	1.27E+08	0	0	0	0	BTP20220	0	Tissue specific
APQLR	Superscaf+	Superscaf	1.27E+08	1.27E+08	9	9	13	-	-	gene	-	-	mRNA	NCP	Intron	gene|mRN	ID=gene-E	5	6.942	2261.6	0.615098968	A(+42.01)	63	626.3613	1	625.3547	Acetylati	K	Superscaf	1.27E+08	BTP20220	BTP20220	BTP20220	0	BTP20220	BTP20220	Mixed
RVSSFKLE	Superscaf+	Superscaf	1.27E+08	1.27E+08	17	17	24	-	-	-	-	-	-	NCP	IGR	-	ID=gene-E	8	5.8175	3050.3	0.615567144	RVSSFKLE	95	483.267	2	964.5341	.	Y	Superscaf	1.27E+08	0	0	BTP20220	0	0	0	Tissue specific
VCDLP	Superscaf-	Superscaf	1.27E+08	1.27E+08	-1	1	5	-	-	-	-	-	-	NCP	IGR	-	ID=gene-E	5	4.054	1688	0.619724499	VC(+57.02	89	603.2783	1	602.2734	Carbamidc	V	Superscaf	1.27E+08	0	0	0	0	0	BTP20220	Tissue specific
LVVLL	Superscaf-	Superscaf	1.27E+08	1.27E+08	-36	36	40	-	-	-	-	-	-	NCP	IGR	-	ID=gene-E	5	3.588	1060.2	0.623194642	LVVLL	90	556.405	1	555.3996	.	M	Superscaf	1.27E+08	BTP20220	0	0	0	0	0	Mixed
KQKPEP	Superscaf-	Superscaf	1.28E+08	1.28E+08	-56	56	61	-	-	gene	-	-	mRNA	NCP	Intron	gene|mRN	ID=gene-E	6	6.825	2647.8	0.626892045	KQKPEP	51	363.7105	2	725.4072	.	V	Superscaf	1.28E+08	0	0	0	BTP20220	0	0	Tissue specific
KDFGKK	Superscaf-	Superscaf	1.28E+08	1.28E+08	-4	4	9	-	-	gene	-	-	mRNA	NCP	Intron	gene|mRN	ID=gene-E	6	7.273333333	2501.4	0.627420967	KDFGKK	81	361.712	2	721.4122	.	K	Superscaf	1.28E+08	0	0	BTP20220	0	0	0	Tissue specific
GDCPP	Superscaf-	Superscaf	1.28E+08	1.28E+08	-17	17	21	-	-	-	-	-	-	NCP	IGR	-	ID=gene-E	5	5.312	1969.3	0.628882607	GDC(+57.(	69	545.2084	1	544.1951	Carbamidc	R	Superscaf	1.28E+08	0	0	BTP20220	0	0	0	Tissue specific
ALELD	Superscaf+	Superscaf	1.28E+08	1.28E+08	11	11	15	-	-	-	-	-	-	NCP	IGR	-	ID=gene-E	5	4.834	1855.1	0.629219653	ALELD	95	560.2903	1	559.2853	.	W	Superscaf	1.28E+08	0	0	BTP20220	0	BTP20220	BTP20220	Tissue specific
VTHEYTV	Superscaf+	Superscaf	1.28E+08	1.28E+08	19	19	25	-	-	-	-	-	-	NCP	IGR	-	ID=gene-E	7	4.218571429	2299.7	0.629552797	VTHEYTV	75	424.71	2	847.4076	.	I	Superscaf	1.28E+08	BTP20220	BTP20220	BTP20220	BTP20220	BTP20220	BTP20220	Tissue specific
KRWKY	Superscaf-	Superscaf	1.28E+08	1.28E+08	-34	34	38	-	-	-	-	-	-	NCP	IGR	-	ID=gene-E	5	8.358	2571.7	0.630144415	KRWKY	66	390.7254	2	779.4442	.	K	Superscaf	1.28E+08	0	BTP20220	0	0	0	0	Tissue specific
FSLDQLLHT	Superscaf-	Superscaf	1.28E+08	1.28E+08	-12	12	20	-	-	-	-	-	-	NCP	IGR	-	ID=gene-E	9	5.76	3870.4	0.630388238	FSLDQLLHT	87	537.2833	2	1072.555	.	D	Superscaf	1.28E+08	0	0	0	0	0	BTP20220	Mixed
SLDQLLHT	Superscaf-	Superscaf	1.28E+08	1.28E+08	-13	13	20	-	-	-	-	-	-	NCP	IGR	-	ID=gene-E	8	5.795	3483	0.630388277	SLDQLLHT	80	463.7488	2	925.4869	.	D	Superscaf	1.28E+08	0	BTP20220	0	0	0	0	Tissue specific
AVSVTP	Superscaf+	Superscaf	1.28E+08	1.28E+08	32	32	37	-	-	-	-	-	-	NCP	IGR	-	ID=gene-E	6	4.088333333	1429.6	0.631580464	A(+42.01)	78	615.3335	1	614.3275	Acetylati	F	Superscaf	1.28E+08	0	BTP20220	0	0	BTP20220	BTP20220	Mixed
KDPQPPK	Superscaf+	Superscaf	1.28E+08	1.28E+08	4	4	10	-	-	-	-	-	-	NCP	IGR	-	ID=gene-E	7	6.714285714	2971.2	0.632475389	KDPQPPK	54	405.2354	2	808.4443	.	D	Superscaf	1.28E+08	0	0	BTP20220	0	0	0	Tissue specific
LLTVDL	Superscaf-	Superscaf	1.28E+08	1.28E+08	-83	83	88	-	-	-	-	-	-	NCP	IGR	-	ID=gene-E	6	4.573333333	1869.2	0.633464989	L(+42.01)	55	358.2204	2	714.4163	Acetylati	V	Superscaf	1.28E+08	0	0	BTP20220	0	0	0	Tissue specific
KLLPLK	Superscaf-	Superscaf	1.28E+08	1.28E+08	-5	5	10	-	-	-	-	-	-	NCP	IGR	-	ID=gene-E	6	7.286666667	2334.4	0.633809265	K(+42.01)	97	377.2629	2	752.516	Acetylati	N	Superscaf	1.28E+08	BTP20220	0	0	0	0	0	Tissue specific
RTLVPAER	Superscaf+	Superscaf	1.28E+08	1.28E+08	3	3	10	-	-	-	-	-	-	NCP	IGR	-	ID=gene-E	8	6.19625	3122.7	0.633975244	RTLVPAER	98	471.2789	2	940.5453	.	V	Superscaf	1.28E+08	0	0	BTP20220	0	0	0	Tissue specific
ERLVE	Superscaf+	Superscaf	1.28E+08	1.28E+08	14	14	18	-	-	-	-	-	-	NCP	IGR	-	ID=gene-E	5	4.636	1853.2	0.635557227	ERLVE	93	645.3544	1	644.3493	.	F	Superscaf	1.28E+08	0	0	0	BTP20220	0	0	Tissue specific
KMALP	Superscaf+	Superscaf	1.28E+08	1.28E+08	3	3	7	-	-	-	-	-	-	NCP	IGR	-	ID=gene-E	5	6.758	1842	0.636582327	KM(+15.99	53	575.3281	1	574.3149	Oxidation	G	Superscaf	1.28E+08	0	BTP20220	0	0	0	0	Tissue specific
NAFLVL	Superscaf+	Superscaf	1.28E+08	1.28E+08	36	36	41	-	-	-	-	-	-	NCP	IGR	-	ID=gene-E	6	4.811666667	2002.2	0.638483492	NAFLVL	74	676.4	1	675.3956	.	L	Superscaf	1.28E+08	0	0	BTP20220	0	0	0	Tissue specific
SLNSTF	Superscaf-	Superscaf	1.29E+08	1.29E+08	-3	3	8	-	-	gene	-	-	mRNA	NCP	Intron	gene|mRN	ID=gene-E	6	5.793333333	2501.8	0.640192896	SLNSTF	78	668.3226	1	667.3177	.	W	Superscaf	1.29E+08	0	BTP20220	BTP20220	BTP20220	BTP20220	0	Tissue specific
GHMPPDG	Superscaf+	Superscaf	1.29E+08	1.29E+08	36	36	42	-	-	gene	-	-	mRNA	NCP	Intron	gene|mRN	ID=gene-E	7	5.835714286	2672.1	0.640588353	GHMPPDG	57	355.6497	2	709.2853	.	L	Superscaf	1.29E+08	0	0	0	BTP20220	0	0	Tissue specific
DVGVYK	Superscaf-	Superscaf	1.29E+08	1.29E+08	-4	4	9	-	-	gene	-	-	mRNA	NCP	Intron	gene|mRN	ID=gene-E	6	4.056666667	1636.7	0.640683335	DVGVYK	97	680.358	1	679.3541	.	E	Superscaf	1.29E+08	0	0	BTP20220	0	0	0	Tissue specific
KDHYPP	Superscaf-	Superscaf	1.29E+08	1.29E+08	-32	32	37	-	-	gene	-	-	mRNA	NCP	Intron	gene|mRN	ID=gene-E	6	6.426666667	2633.9	0.640700013	KDHYPP	61	756.3676	1	755.3602	.	A	Superscaf	1.29E+08	0	0	0	0	0	BTP20220	Tissue specific
AAPENKP	Superscaf-	Superscaf	1.29E+08	1.29E+08	-28	28	34	-	-	-	-	-	-	NCP	IGR	-	ID=gene-E	7	6.144285714	2788	0.642066863	A(+42.01)	58	768.3972	1	767.3813	Acetylati	F	Superscaf	1.29E+08	BTP20220	0	0	0	0	0	Tissue specific
PAKYNPS	Superscaf+	Superscaf	1.29E+08	1.29E+08	3	3	9	-	-	-	-	-	-	NCP	IGR	-	ID=gene-E	7	6.444285714	2894.1	0.642338458	P(+42.01)	69	409.7044	2	817.397	Acetylati	Y	Superscaf	1.29E+08	0	0	0	0	BTP20220	0	Tissue specific
LTYSPP	Superscaf+	Superscaf	1.29E+08	1.29E+08	15	15	20	-	-	gene	-	-	mRNA	NCP	Intron	gene|mRN	ID=gene-E	6	6.075	2268.6	0.64428874	LTYSPP	61	677.3481	1	676.3432	.	T	Superscaf	1.29E+08	0	0	0	BTP20220	0	0	Tissue specific
PVVGQ	Superscaf+	Superscaf	1.29E+08	1.29E+08	3	3	7	-	-	-	-	-	-	NCP	IGR	-	ID=gene-E	5	3.584	1245.4	0.645233661	PVVGQ	57	499.2788	1	498.2802	.	F	Superscaf	1.29E+08	BTP20220	0	0	0	0	0	Tissue specific
LASESLLK	Superscaf-	Superscaf	1.29E+08	1.29E+08	-2	2	9	-	-	-	-	-	-	NCP	IGR	-	ID=gene-E	8	6.035	3032.2	0.645490261	LASESLLK	96	430.7561	2	859.5015	.	S	Superscaf	1.29E+08	0	0	0	0	0	BTP20220	Tissue specific
FVDVLPF	Superscaf-	Superscaf	1.29E+08	1.29E+08	-29	29	35	-	-	-	-	-	-	NCP	IGR	-	ID=gene-E	7	3.744285714	1877.1	0.646037774	FVDVLPF	69	836.4531	1	835.4479	.	I	Superscaf	1.29E+08	0	0	0	0	0	BTP20220	Tissue specific
TDTMLEK	Superscaf-	Superscaf	1.29E+08	1.29E+08	-5	5	11	-	-	gene	-	-	mRNA	NCP	Intron	gene|mRN	ID=gene-E	7	5.817142857	2825.3	0.646561876	TDTMLEK	51	837.412	1	836.395	.	H	Superscaf	1.29E+08	0	BTP20220	0	0	0	0	Tissue specific
RLRLQK	Superscaf+	Superscaf	1.29E+08	1.29E+08	6	6	11	-	-	-	-	-	-	NCP	IGR	-	ID=gene-E	6	8.145	3083.5	0.647244383	RLRLQK	95	407.2731	2	812.5344	.	A	Superscaf	1.29E+08	0	0	BTP20220	BTP20220	0	0	Tissue specific
NPLPAWF	Superscaf-	Superscaf	1.29E+08	1.29E+08	-24	24	30	-	-	gene	-	-	mRNA	NCP	Intron	gene|mRN	ID=gene-E	7	5.911428571	2850.2	0.647524623	NPLPAWF	52	422.7184	2	843.4279	.	N	Superscaf	1.29E+08	0	0	0	0	BTP20220	0	Tissue specific
PMSPK	Superscaf+	Superscaf	1.29E+08	1.29E+08	10	10	14	-	-	-	-	-	-	NCP	IGR	-	ID=gene-E	5	6.754	1898.1	0.647713209	PMSPK	56	559.2972	1	558.2836	.	G	Superscaf	1.29E+08	0	0	BTP20220	0	BTP20220	0	Tissue specific
EPLKSQ	Superscaf+	Superscaf	1.29E+08	1.29E+08	31	31	36	-	-	-	-	-	-	NCP	IGR	-	ID=gene-E	6	6.095	2578.8	0.647944562	EPLKSQ	50	701.39	1	700.3755	.	E	Superscaf	1.29E+08	0	0	BTP20220	0	0	0	Tissue specific
YDLPP	Superscaf+	Superscaf	1.29E+08	1.29E+08	29	29	33	-	-	-	-	-	-	NCP	IGR	-	ID=gene-E	5	5.442	1899.2	0.648830536	YDLPP	82	604.2964	1	603.2904	.	M	Superscaf	1.29E+08	0	0	BTP20220	0	0	0	Tissue specific
GPMDC	Superscaf-	Superscaf	1.29E+08	1.29E+08	-19	19	23	-	-	-	-	-	-	NCP	IGR	-	ID=gene-E	5	5.202	2003.4	0.649398017	GPM(+15.9	52	595.1831	1	594.1778	Oxidation	I	Superscaf	1.29E+08	0	BTP20220	0	0	0	0	Tissue specific
GSNPLR	Superscaf-	Superscaf	1.29E+08	1.29E+08	-15	15	20	-	-	gene	-	-	mRNA	NCP	Intron	gene|mRN	ID=gene-E	6	6.683333333	2617	0.650146089	GSNPLR	55	643.3575	1	642.3449	.	N	Superscaf	1.29E+08	0	0	0	0	0	BTP20220	Tissue specific
KGLSPF	Superscaf+	Superscaf	1.3E+08	1.3E+08	5	5	10	-	-	gene	-	-	mRNA	NCP	Intron	gene|mRN	ID=gene-E	6	6.525	2227.3	0.651433067	K(+42.01)	77	690.3786	1	689.3748	Acetylati	N	Superscaf	1.3E+08	0	0	0	0	0	BTP20220	Tissue specific
LYVTSP	Superscaf+	Superscaf	1.3E+08	1.3E+08	15	15	20	-	-	gene	-	-	mRNA	NCP	Intron	gene|mRN	ID=gene-E	6	5.025	1931.2	0.65283641	L(+42.01)	50	361.198	2	720.3694	Acetylati	Q	Superscaf	1.3E+08	0	BTP20220	0	0	0	0	Tissue specific
ERPVSK	Superscaf+	Superscaf	1.3E+08	1.3E+08	28	28	33	-	-	gene	-	-	mRNA	NCP	Intron	gene|mRN	ID=gene-E	6	5.95	2263.5	0.656188619	ERPVSK	63	358.2102	2	714.4024	.	V	Superscaf	1.3E+08	0	0	0	0	0	BTP20220	Tissue specific
RMPFFKY	Superscaf-	Superscaf	1.3E+08	1.3E+08	-77	77	83	-	-	gene	-	-	mRNA	NCP	Intron	gene|mRN	ID=gene-E	7	7.024285714	3060.4	0.657185564	RMPFFKY	50	494.7498	2	987.5	.	I	Superscaf	1.3E+08	0	0	0	0	BTP20220	0	Tissue specific
EVNVNR	Superscaf+	Superscaf	1.3E+08	1.3E+08	7	7	12	-	-	gene	-	-	mRNA	NCP	Intron	gene|mRN	ID=gene-E	6	4.133333333	2267.7	0.657864208	EVNVNR	99	365.6947	2	729.377	.	S	Superscaf	1.3E+08	0	0	0	0	0	BTP20220	Tissue specific
KELVMPK	Superscaf+	Superscaf	1.3E+08	1.3E+08	53	53	59	-	-	gene	-	-	mRNA	NCP	Intron	gene|mRN	ID=gene-E	7	5.818571429	2424.6	0.659752979	KELVMPK	71	422.7554	2	843.4888	.	R	Superscaf	1.3E+08	0	0	0	0	0	BTP20220	Tissue specific
KQRLGQP	Superscaf+	Superscaf	1.3E+08	1.3E+08	33	33	39	-	-	-	-	-	-	NCP	IGR	-	ID=gene-E	7	7.15	3326.7	0.660683211	KQRLGQP	61	413.7413	2	825.4821	.	N	Superscaf	1.3E+08	0	BTP20220	0	0	0	BTP20220	Tissue specific
WNPGRP	Superscaf-	Superscaf	1.3E+08	1.3E+08	-34	34	39	-	-	-	-	-	-	NCP	IGR	-	ID=gene-E	6	6.771666667	2744.2	0.662741749	WNPGRP	79	363.6869	2	725.3609	.	S	Superscaf	1.3E+08	0	0	0	0	BTP20220	0	Mixed
FVVGF	Superscaf+	Superscaf	1.3E+08	1.3E+08	7	7	11	-	-	-	-	-	-	NCP	IGR	-	ID=gene-E	5	3.386	1072.1	0.662889557	FVVGF	65	568.3231	1	567.3057	.	M	Superscaf	1.3E+08	0	0	0	0	BTP20220	0	Tissue specific
AERLRFP	Superscaf-	Superscaf	1.31E+08	1.31E+08	-15	15	21	-	-	-	-	-	-	NCP	IGR	-	ID=gene-E	7	6.931428571	3112.6	0.665110325	A(+42.01)	70	465.7601	2	929.5082	Acetylati	L	Superscaf	1.31E+08	0	0	0	0	0	BTP20220	Tissue specific
KYELP	Superscaf+	Superscaf	1.31E+08	1.31E+08	2	2	6	-	-	-	-	-	-	NCP	IGR	-	ID=gene-E	5	6.18	2044.2	0.665152174	KYELP	73	649.3544	1	648.3483	.	S	Superscaf	1.31E+08	0	0	BTP20220	0	0	0	Tissue specific
LEMRL	Superscaf+	Superscaf	1.31E+08	1.31E+08	12	12	16	-	-	-	-	-	-	NCP	IGR	-	ID=gene-E	5	6.338	2152.6	0.667640673	LEMRL	57	661.3694	1	660.3629	.	T	Superscaf	1.31E+08	0	BTP20220	0	0	0	0	Tissue specific
FLKHGF	Superscaf+	Superscaf	1.31E+08	1.31E+08	3	3	8	-	-	-	-	-	-	NCP	IGR	-	ID=gene-E	6	6.706666667	2513.6	0.669429298	FLKHGF	55	374.7129	2	747.4067	.	T	Superscaf	1.31E+08	0	0	0	BTP20220	0	0	Tissue specific
KRLCC	Superscaf+	Superscaf	1.31E+08	1.31E+08	32	32	36	-	-	-	-	-	-	NCP	IGR	-	ID=gene-E	5	7.304	2642	0.671027845	KRLC(+57.	76	368.676	2	735.352	Carbamidc	I	Superscaf	1.31E+08	0	0	0	0	0	BTP20220	Tissue specific
VSAKGPVN	Superscaf+	Superscaf	1.31E+08	1.31E+08	86	86	93	-	-	-	-	-	-	NCP	IGR	-	ID=gene-E	8	4.89	2394.5	0.673416734	VSAKGPVN	62	386.2207	2	770.4286	.	A	Superscaf	1.31E+08	0	0	0	BTP20220	0	0	Tissue specific
FVFLVLGW	Superscaf-	Superscaf	1.31E+08	1.31E+08	-10	10	17	-	-	-	-	-	-	NCP	IGR	-	ID=gene-E	8	4.3475	2305.5	0.674355038	FVFLVLGW	67	490.7929	2	979.5531	.	T	Superscaf	1.31E+08	0	0	BTP20220	0	0	0	Tissue specific
EGLLAL	Superscaf-	Superscaf	1.31E+08	1.31E+08	-11	11	16	-	-	-	-	-	-	NCP	IGR	-	ID=gene-E	6	5.525	2094.3	0.67515391	EGLLAL	51	615.3693	1	614.3639	.	L	Superscaf	1.31E+08	0	0	0	0	BTP20220	BTP20220	Tissue specific
KGPSSR	Superscaf+	Superscaf	1.31E+08	1.31E+08	27	27	32	-	-	-	-	-	-	NCP	IGR	-	ID=gene-E	6	7.355	2518.7	0.675276548	KGPSSR	50	631.353	1	630.3449	.	T	Superscaf	1.31E+08	0	0	0	0	BTP20220	0	Tissue specific
VVVDLVAM	Superscaf+	Superscaf	1.31E+08	1.31E+08	56	56	63	-	-	-	-	-	-	NCP	IGR	-	ID=gene-E	8	2.59	1447.7	0.676033648	VVVDLVAM(	53	431.2417	2	860.4677	Oxidation	K	Superscaf	1.31E+08	0	BTP20220	0	0	0	0	Tissue specific
PVKVLFW	Superscaf-	Superscaf	1.31E+08	1.31E+08	-31	31	37	-	-	-	-	-	-	NCP	IGR	-	ID=gene-E	7	4.77	2073.2	0.676283668	P(+42.01)	50	465.7776	2	929.5374	Acetylati	K	Superscaf	1.31E+08	0	0	BTP20220	0	0	0	Tissue specific
KLNLQNK	Superscaf+	Superscaf	1.32E+08	1.32E+08	3	3	9	-	-	-	-	-	-	NCP	IGR	-	ID=gene-E	7	6.844285714	3447.7	0.676912162	KLNLQNK	63	429.268	2	856.513	.	K	Superscaf	1.32E+08	0	BTP20220	0	0	0	0	Tissue specific
VVPLQY	Superscaf-	Superscaf	1.32E+08	1.32E+08	-2	2	7	-	-	gene	-	-	mRNA	NCP	Intron	gene|mRN	ID=gene-E	6	3.931666667	1761	0.678020461	V(+42.01)	59	760.4237	1	759.4167	Acetylati	S	Superscaf	1.32E+08	0	BTP20220	0	0	0	0	Tissue specific
KQELPY	Superscaf+	Superscaf	1.32E+08	1.32E+08	36	36	41	-	-	gene	-	-	mRNA	NCP	Intron	gene|mRN	ID=gene-E	6	6.091666667	2654.9	0.680204659	KQELPY	67	777.4104	1	776.4068	.	E	Superscaf	1.32E+08	0	0	0	0	0	BTP20220	Tissue specific
KLATTVP	Superscaf+	Superscaf	1.32E+08	1.32E+08	39	39	45	-	-	gene	-	-	mRNA	NCP	Intron	gene|mRN	ID=gene-E	7	5.871428571	2265.5	0.680249874	KLATTVP	52	365.2268	2	728.4432	.	S	Superscaf	1.32E+08	0	0	0	BTP20220	0	0	Tissue specific
KRDLLR	Superscaf+	Superscaf	1.32E+08	1.32E+08	14	14	19	-	-	gene	-	-	mRNA	NCP	Intron	gene|mRN	ID=gene-E	6	7.698333333	2884.3	0.68333068	KRDLLR	60	400.7646	2	799.5028	.	L	Superscaf	1.32E+08	0	BTP20220	0	0	0	0	Tissue enhanced
KPEARFQ	Superscaf+	Superscaf	1.32E+08	1.32E+08	16	16	22	-	-	gene	-	-	mRNA	NCP	Intron	gene|mRN	ID=gene-E	7	6.738571429	3189.5	0.68405844	KPEARFQ	56	438.2342	2	874.4661	.	Q	Superscaf	1.32E+08	0	0	0	0	0	BTP20220	Tissue specific
DQLLTH	Superscaf+	Superscaf	1.32E+08	1.32E+08	69	69	74	-	-	gene	-	-	mRNA	NCP	Intron	gene|mRN	ID=gene-E	6	5.783333333	2746.2	0.68407114	DQLLTH	92	363.6908	2	725.3708	.	E	Superscaf	1.32E+08	0	0	0	0	0	BTP20220	Tissue specific
RQLLLLC	Superscaf+	Superscaf	1.32E+08	1.32E+08	20	20	26	-	exon	gene	three_pri	-	mRNA	NCP	3UTR	exon|gene	ID=exon-E	7	6.478571429	3258.8	0.685868487	RQLLLLC(+	55	458.2842	2	914.5372	Carbamidc	R	Superscaf	1.32E+08	0	0	0	0	BTP20220	0	Tissue specific
DKLTP	Superscaf+	Superscaf	1.32E+08	1.32E+08	12	12	16	-	-	gene	-	-	mRNA	NCP	Intron	gene|mRN	ID=gene-E	5	6.304	1968.2	0.687241763	D(+42.01)	58	615.3424	1	614.3275	Acetylati	V	Superscaf	1.32E+08	BTP20220	0	0	0	0	0	Tissue specific
VFPSLVG	Superscaf-	Superscaf	1.32E+08	1.32E+08	-13	13	19	-	-	gene	-	-	mRNA	NCP	Intron	gene|mRN	ID=gene-E	7	4.201428571	1758.9	0.68759193	VFPSLVG	85	718.4109	1	717.4061	.	K	Superscaf	1.32E+08	0	0	0	0	0	BTP20220	Mixed
KTVKP	Superscaf-	Superscaf	1.32E+08	1.32E+08	-20	20	24	-	-	gene	-	-	mRNA	NCP	Intron	gene|mRN	ID=gene-E	5	6.462	1671.7	0.687658948	K(+42.01)	63	614.3848	1	613.3799	Acetylati	V	Superscaf	1.32E+08	0	0	BTP20220	0	0	0	Tissue specific
MQFLTM	Superscaf-	Superscaf	1.32E+08	1.32E+08	-6	6	11	-	-	gene	-	-	mRNA	NCP	Intron	gene|mRN	ID=gene-E	6	5.856666667	2492	0.688566152	M(+15.99)	65	786.3498	1	785.3452	Oxidation	K	Superscaf	1.32E+08	0	0	BTP20220	0	0	0	Tissue specific
LFAKPP	Superscaf+	Superscaf	1.33E+08	1.33E+08	39	39	44	-	-	-	-	-	-	NCP	IGR	-	ID=gene-E	6	6.636666667	2195.3	0.691737235	LFAKPP	59	672.402	1	671.4006	.	S	Superscaf	1.33E+08	0	0	BTP20220	0	0	0	Tissue specific
EVHHLLL	Superscaf+	Superscaf	1.33E+08	1.33E+08	2	2	8	-	-	-	-	-	-	NCP	IGR	-	ID=gene-E	7	5.191428571	2725.1	0.691866453	EVHHLLL	67	430.753	2	859.4916	.	N	Superscaf	1.33E+08	0	BTP20220	0	0	0	0	Mixed
KVMVFGPS	Superscaf+	Superscaf	1.33E+08	1.33E+08	5	5	12	-	-	-	-	-	-	NCP	IGR	-	ID=gene-E	8	4.865	2245.4	0.692223887	KVM(+15.9	52	440.7368	2	879.4524	Oxidation	Q	Superscaf	1.33E+08	0	BTP20220	0	BTP20220	0	BTP20220	Tissue specific
HELSELLC	Superscaf+	Superscaf	1.33E+08	1.33E+08	29	29	36	-	-	-	-	-	-	NCP	IGR	-	ID=gene-E	8	5.33375	3500	0.692532167	HELSELLC(	68	500.7411	2	999.4695	Carbamidc	F	Superscaf	1.33E+08	0	0	0	BTP20220	0	0	Tissue specific
KEPPGFPK	Superscaf-	Superscaf	1.33E+08	1.33E+08	-8	8	15	-	-	-	-	-	-	NCP	IGR	-	ID=gene-E	8	6.63125	3059.2	0.692974369	KEPPGFPK	62	450.2599	2	898.4912	.	G	Superscaf	1.33E+08	0	0	0	0	0	BTP20220	Tissue specific
TNVVPVP	Superscaf+	Superscaf	1.33E+08	1.33E+08	11	11	17	-	-	-	-	-	-	NCP	IGR	-	ID=gene-E	7	3.505714286	1669	0.695168589	T(+42.01)	73	767.4261	1	766.4225	Acetylati	K	Superscaf	1.33E+08	BTP20220	0	0	0	0	0	Tissue specific
LESGLLT	Superscaf+	Superscaf	1.33E+08	1.33E+08	18	18	24	-	-	-	-	-	-	NCP	IGR	-	ID=gene-E	7	5.62	2563.9	0.696206083	LESGLLT	62	732.4125	1	731.4065	.	S	Superscaf	1.33E+08	0	0	0	0	0	BTP20220	Tissue specific
SPLKLR	Superscaf-	Superscaf	1.33E+08	1.33E+08	-5	5	10	-	-	-	-	-	-	NCP	IGR	-	ID=gene-E	6	7.406666667	2544.8	0.697471256	SPLKLR	92	357.2358	2	712.4595	.	T	Superscaf	1.33E+08	0	0	0	0	0	BTP20220	Tissue specific
SFLYL	Superscaf-	Superscaf	1.33E+08	1.33E+08	-6	6	10	-	-	-	-	-	-	NCP	IGR	-	ID=gene-E	5	5.756	1937.1	0.697632798	SFLYL	69	642.3495	1	641.3425	.	K	Superscaf	1.33E+08	0	0	0	0	0	BTP20220	Mixed
DVKSF	Superscaf-	Superscaf	1.33E+08	1.33E+08	-4	4	8	-	-	-	-	-	-	NCP	IGR	-	ID=gene-E	5	4.774	1650.7	0.69781951	DVKSF	57	595.3185	1	594.3013	.	E	Superscaf	1.33E+08	0	0	0	0	0	BTP20220	Tissue specific
KEGLN	Superscaf+	Superscaf	1.33E+08	1.33E+08	10	10	14	-	-	-	-	-	-	NCP	IGR	-	ID=gene-E	5	6.064	2141.3	0.698008517	KEGLN	66	560.306	1	559.2966	.	N	Superscaf	1.33E+08	0	0	0	0	0	BTP20220	Tissue specific
MGKLVFH	Superscaf+	Superscaf	1.33E+08	1.33E+08	4	4	10	-	-	gene	-	-	mRNA	NCP	Intron	gene|mRN	ID=gene-E	7	5.787142857	2497.7	0.698338563	M(+15.99)	70	424.2259	2	846.4422	Oxidation	L	Superscaf	1.33E+08	0	0	0	0	BTP20220	0	Tissue specific
TVMQAAH	Superscaf+	Superscaf	1.33E+08	1.33E+08	20	20	26	-	-	gene	-	-	mRNA	NCP	Intron	gene|mRN	ID=gene-E	7	5.365714286	2622	0.699282604	T(+42.01)	50	815.3777	1	814.3644	Acetylati	S	Superscaf	1.33E+08	0	0	0	0	BTP20220	0	Tissue specific
SSSFRNLG	Superscaf+	Superscaf	1.33E+08	1.33E+08	18	18	25	-	-	-	-	-	-	NCP	IGR	-	ID=gene-E	8	6.33	3433.8	0.701372011	SSSFRNLG	88	434.2186	2	866.4246	.	S	Superscaf	1.33E+08	0	0	0	0	0	BTP20220	Tissue specific
LRLWN	Superscaf+	Superscaf	1.33E+08	1.33E+08	1	1	5	-	-	gene	-	-	mRNA	NCP	Intron	gene|mRN	ID=gene-E	5	6.804	2478.9	0.701710472	LRLWN	88	351.2067	2	700.402	.	L	Superscaf	1.33E+08	0	0	0	0	0	BTP20220	Mixed
RKGGK	Superscaf+	Superscaf	1.33E+08	1.33E+08	82	82	86	-	-	gene	-	-	mRNA	NCP	Intron	gene|mRN	ID=gene-E	5	8.436	2180.2	0.702077164	R(+42.01)	57	587.3738	1	586.355	Acetylati	G	Superscaf	1.33E+08	0	0	0	0	0	BTP20220	Tissue specific
ALKLLKGS	Superscaf+	Superscaf	1.33E+08	1.33E+08	18	18	25	-	-	gene	-	-	mRNA	NCP	Intron	gene|mRN	ID=gene-E	8	6.88625	2989	0.70209805	ALKLLKGS	54	829.5479	1	828.5433	.	L	Superscaf	1.33E+08	0	0	0	BTP20220	0	0	Tissue specific
HQGVMV	Superscaf-	Superscaf	1.34E+08	1.34E+08	4	4	9	-	-	-	-	-	-	NCP	IGR	-	ID=gene-E	6	4.16	1899.2	0.702527208	HQGVMV	61	670.3311	1	669.3268	.	D	Superscaf	1.34E+08	0	0	0	0	0	BTP20220	Tissue specific
VGDLLAY	Superscaf-	Superscaf	1.34E+08	1.34E+08	-3	3	9	-	-	-	-	-	-	NCP	IGR	-	ID=gene-E	7	4.654285714	2186.4	0.703383115	VGDLLAY	73	750.3996	1	749.3959	.	T	Superscaf	1.34E+08	BTP20220	0	0	0	0	0	Tissue specific
DPPFKP	Superscaf+	Superscaf	1.34E+08	1.34E+08	22	22	27	-	-	-	-	-	-	NCP	IGR	-	ID=gene-E	6	6.181666667	2279.5	0.704011877	DPPFKP	90	350.6842	2	699.3591	.	F	Superscaf	1.34E+08	0	0	0	0	0	BTP20220	Tissue specific
VSVPNP	Superscaf+	Superscaf	1.34E+08	1.34E+08	12	12	17	-	-	-	-	-	-	NCP	IGR	-	ID=gene-E	6	3.948333333	1654.9	0.704869849	V(+42.01)	64	654.3414	1	653.3384	Acetylati	G	Superscaf	1.34E+08	0	0	0	BTP20220	0	0	Tissue specific
SCNPPRP	Superscaf+	Superscaf	1.34E+08	1.34E+08	45	45	51	-	-	-	-	-	-	NCP	IGR	-	ID=gene-E	7	6.538571429	3226.8	0.705077448	SC(+57.02	55	414.1931	2	826.3755	Carbamidc	K	Superscaf	1.34E+08	0	0	0	0	BTP20220	0	Tissue specific
VKKGVTP	Superscaf-	Superscaf	1.34E+08	1.34E+08	-20	20	26	-	-	-	-	-	-	NCP	IGR	-	ID=gene-E	7	5.468571429	1969	0.70680001	VKKGVTP	56	364.7424	2	727.4592	.	I	Superscaf	1.34E+08	0	0	0	0	BTP20220	0	Tissue specific
FELVAVR	Superscaf+	Superscaf	1.34E+08	1.34E+08	13	13	19	-	-	-	-	-	-	NCP	IGR	-	ID=gene-E	7	4.494285714	2126.4	0.706935693	FELVAVR	77	417.2461	2	832.4807	.	T	Superscaf	1.34E+08	BTP20220	0	0	0	0	0	Tissue specific
LQNPVL	Superscaf-	Superscaf	1.34E+08	1.34E+08	-42	42	47	-	-	gene	-	-	mRNA	NCP	Intron	gene|mRN	ID=gene-E	6	4.886666667	2251.6	0.707323194	LQNPVL	53	683.4061	1	682.4014	.	R	Superscaf	1.34E+08	0	0	BTP20220	0	0	0	Tissue specific
QPKSVPK	Superscaf-	Superscaf	1.34E+08	1.34E+08	-26	26	32	-	-	gene	-	-	mRNA	NCP	Intron	gene|mRN	ID=gene-E	7	6.201428571	2605.7	0.707661464	QPKSVPK	69	392.2441	2	782.465	.	W	Superscaf	1.34E+08	0	0	0	0	0	BTP20220	Tissue specific
EYAALLL	Superscaf-	Superscaf	1.34E+08	1.34E+08	-19	19	25	-	-	-	-	-	-	NCP	IGR	-	ID=gene-E	7	5.551428571	2567.8	0.708449013	EYAALLL	74	792.4473	1	791.4429	.	I	Superscaf	1.34E+08	0	0	0	0	0	BTP20220	Tissue specific
YKSPPNR	Superscaf+	Superscaf	1.34E+08	1.34E+08	31	31	37	-	-	gene	-	-	mRNA	NCP	Intron	gene|mRN	ID=gene-E	7	7.121428571	3231.6	0.709889308	Y(+42.01)	61	452.2449	2	902.461	Acetylati	G	Superscaf	1.34E+08	0	0	0	BTP20220	0	0	Tissue specific
WAHPAAY	Superscaf+	Superscaf	1.34E+08	1.34E+08	14	14	20	-	-	gene	-	-	mRNA	NCP	Intron	gene|mRN	ID=gene-E	7	6.214285714	2877.1	0.710027171	W(+42.01)	54	429.1971	2	856.3868	Acetylati	P	Superscaf	1.34E+08	BTP20220	0	0	0	0	0	Tissue specific
KGGYVRL	Superscaf+	Superscaf	1.34E+08	1.34E+08	4	4	10	-	-	gene	-	-	mRNA	NCP	Intron	gene|mRN	ID=gene-E	7	6.297142857	2524.7	0.711148744	KGGYVRL	57	396.7392	2	791.4653	.	K	Superscaf	1.34E+08	0	0	0	BTP20220	0	0	Tissue specific
TVQAH	Superscaf+	Superscaf	1.34E+08	1.34E+08	47	47	51	-	-	gene	-	-	mRNA	NCP	Intron	gene|mRN	ID=gene-E	5	5.158	1939.2	0.711633561	TVQAH	55	555.2817	1	554.2812	.	G	Superscaf	1.34E+08	0	0	0	0	BTP20220	0	Tissue specific
LDVGF	Superscaf+	Superscaf	1.34E+08	1.34E+08	4	4	8	-	-	gene	-	-	mRNA	NCP	Intron	gene|mRN	ID=gene-E	5	4.08	1449.6	0.711798354	LDVGF	97	550.2852	1	549.2798	.	D	Superscaf	1.34E+08	0	0	0	0	0	BTP20220	Tissue specific
GVKLP	Superscaf+	Superscaf	1.34E+08	1.34E+08	26	26	30	-	-	-	-	-	-	NCP	IGR	-	ID=gene-E	5	5.598	1456.5	0.712709306	GVKLP	59	513.3378	1	512.3322	.	I	Superscaf	1.34E+08	0	0	0	0	0	BTP20220	Tissue specific
CCCFMAP	Superscaf+	Superscaf	1.34E+08	1.34E+08	12	12	18	-	-	gene	-	-	mRNA	NCP	Intron	gene|mRN	ID=gene-E	7	5.515714286	3164.7	0.714468898	C(+57.02)	51	481.1534	2	960.2962	Carbamidc	T	Superscaf	1.34E+08	0	0	0	BTP20220	0	0	Tissue specific
MWKEK	Superscaf-	Superscaf	1.34E+08	1.34E+08	-1	1	5	-	-	gene	-	-	mRNA	NCP	Intron	gene|mRN	ID=gene-E	5	6.868	2260.4	0.714541731	M(+15.99)	51	369.1912	2	736.3578	Oxidation	M	Superscaf	1.34E+08	0	0	0	0	BTP20220	0	Tissue specific
FVRKKK	Superscaf-	Superscaf	1.34E+08	1.34E+08	-5	5	10	-	-	gene	-	-	mRNA	NCP	Intron	gene|mRN	ID=gene-E	6	7.576666667	2441.4	0.714758204	FVRKKK	51	403.2785	2	804.5333	.	I	Superscaf	1.34E+08	0	0	0	0	BTP20220	0	Tissue specific
QQLEVLRA	Superscaf+	Superscaf	1.34E+08	1.34E+08	27	27	34	-	-	gene	-	-	mRNA	NCP	Intron	gene|mRN	ID=gene-E	8	5.4075	3313.8	0.714930456	Q(+42.01)	52	499.7851	2	997.5556	Acetylati	G	Superscaf	1.34E+08	0	0	0	0	BTP20220	0	Tissue specific
KWDDLPQ	Superscaf-	Superscaf	1.35E+08	1.35E+08	-32	32	38	-	-	-	-	-	-	NCP	IGR	-	ID=gene-E	7	5.642857143	3119.5	0.715537452	K(+42.01)	72	472.2281	2	942.4447	Acetylati	V	Superscaf	1.35E+08	0	0	0	0	BTP20220	0	Tissue specific
SDGLRPLM	Superscaf+	Superscaf	1.35E+08	1.35E+08	10	10	17	-	-	gene	-	-	mRNA	NCP	Intron	gene|mRN	ID=gene-E	8	6.17375	3156.7	0.715938418	SDGLRPLM(	92	452.7291	2	903.4484	Oxidation	V	Superscaf	1.35E+08	0	0	0	0	BTP20220	BTP20220	Tissue specific
APVPPPP	Superscaf+	Superscaf	1.35E+08	1.35E+08	35	35	41	-	-	gene	-	-	mRNA	NCP	Intron	gene|mRN	ID=gene-E	7	5.36	1998.3	0.716505937	APVPPPP	81	674.3754	1	673.3799	.	K	Superscaf	1.35E+08	0	0	BTP20220	0	0	0	Tissue specific
VEKAM	Superscaf+	Superscaf	1.35E+08	1.35E+08	14	14	18	-	-	gene	-	-	mRNA	NCP	Intron	gene|mRN	ID=gene-E	5	4.946	1576.7	0.718278841	VEKAM	61	577.3065	1	576.2941	.	M	Superscaf	1.35E+08	BTP20220	0	0	0	0	0	Tissue specific
KADLMR	Superscaf-	Superscaf	1.35E+08	1.35E+08	-37	37	42	-	-	-	-	-	-	NCP	IGR	-	ID=gene-E	6	6.87	2564.9	0.719451825	KADLMR	78	367.2066	2	732.3952	.	S	Superscaf	1.35E+08	0	0	0	0	0	BTP20220	Tissue specific
VGPVGN	Superscaf-	Superscaf	1.35E+08	1.35E+08	-11	11	16	-	-	gene	-	-	mRNA	NCP	Intron	gene|mRN	ID=gene-E	6	3.941666667	1528.7	0.720144354	VGPVGN	72	542.2911	1	541.286	.	L	Superscaf	1.35E+08	0	0	0	BTP20220	0	0	Tissue specific
GVTTL	Superscaf-	Superscaf	1.35E+08	1.35E+08	-24	24	28	-	-	gene	-	-	mRNA	NCP	Intron	gene|mRN	ID=gene-E	5	5.002	1445.7	0.72054249	G(+42.01)	57	532.2947	1	531.2904	Acetylati	R	Superscaf	1.35E+08	0	0	0	0	0	BTP20220	Mixed
NFGMLTH	Superscaf-	Superscaf	1.35E+08	1.35E+08	-4	4	10	-	-	-	-	-	-	NCP	IGR	-	ID=gene-E	7	6.101428571	3023.5	0.721650483	NFGM(+15.	64	418.1912	2	834.3694	Oxidation	P	Superscaf	1.35E+08	0	BTP20220	0	0	0	0	Tissue specific
RFVKKK	Superscaf+	Superscaf	1.35E+08	1.35E+08	1	1	6	-	-	gene	-	-	mRNA	NCP	Intron	gene|mRN	ID=gene-E	6	7.576666667	2441.4	0.722640619	RFVKKK	61	403.2795	2	804.5333	.	R	Superscaf	1.35E+08	0	0	0	0	0	BTP20220	Mixed
KKKYG	Superscaf-	Superscaf	1.35E+08	1.35E+08	-25	25	29	-	-	-	-	-	-	NCP	IGR	-	ID=gene-E	5	8.17	2162	0.724685538	KKKYG	55	623.3753	1	622.3802	.	D	Superscaf	1.35E+08	0	0	0	0	BTP20220	0	Tissue specific
PPVPE	Superscaf+	Superscaf	1.35E+08	1.35E+08	7	7	11	-	-	-	-	-	-	NCP	IGR	-	ID=gene-E	5	4.424	1437.7	0.725179918	PPVPE	57	538.2852	1	537.2798	.	R	Superscaf	1.35E+08	BTP20220	0	0	BTP20220	0	0	Tissue specific
ETTHAKGK	Superscaf+	Superscaf	1.35E+08	1.35E+08	14	14	21	-	-	-	-	-	-	NCP	IGR	-	ID=gene-E	8	6.9175	3385.6	0.725438966	ETTHAKGK	77	436.234	2	870.4559	.	G	Superscaf	1.35E+08	0	0	BTP20220	0	0	0	Tissue specific
VDNPPAF	Superscaf+	Superscaf	1.35E+08	1.35E+08	13	13	19	-	-	-	-	-	-	NCP	IGR	-	ID=gene-E	7	4.64	2381.7	0.725616535	VDNPPAF	62	759.3678	1	758.3599	.	E	Superscaf	1.35E+08	0	0	0	BTP20220	0	0	Tissue specific
TVFMKSP	Superscaf-	Superscaf	1.35E+08	1.35E+08	-3	3	9	-	-	-	-	-	-	NCP	IGR	-	ID=gene-E	7	5.64	2345.6	0.725888857	TVFMKSP	55	405.2102	2	808.4153	.	L	Superscaf	1.35E+08	0	0	0	0	0	BTP20220	Tissue specific
EDLRLSPF	Superscaf+	Superscaf	1.35E+08	1.35E+08	4	4	11	-	-	-	-	-	-	NCP	IGR	-	ID=gene-E	8	5.79625	3300.8	0.7260981	EDLRLSPF	96	488.7565	2	975.5025	.	M	Superscaf	1.35E+08	BTP20220	0	0	0	0	0	Mixed
KNVHSLTN	Superscaf-	Superscaf	1.35E+08	1.35E+08	-18	18	25	-	-	-	-	-	-	NCP	IGR	-	ID=gene-E	8	5.7925	3415.8	0.726547456	KNVHSLTN	59	456.7432	2	911.4825	.	K	Superscaf	1.35E+08	0	0	BTP20220	0	0	0	Tissue specific
ALQDLL	Superscaf-	Superscaf	1.35E+08	1.35E+08	-48	48	53	-	-	-	-	-	-	NCP	IGR	-	ID=gene-E	6	5.43	2393.7	0.72719588	A(+42.01)	56	357.7109	2	713.3959	Acetylati	N	Superscaf	1.35E+08	0	0	BTP20220	0	0	0	Tissue specific
TVHSLLCY	Superscaf+	Superscaf	1.36E+08	1.36E+08	11	11	18	-	-	-	-	-	-	NCP	IGR	-	ID=gene-E	8	5.305	3152.6	0.727871692	TVHSLLC(+	56	496.7491	2	991.4797	Carbamidc	I	Superscaf	1.36E+08	0	0	BTP20220	0	0	0	Tissue specific
KMLVGW	Superscaf-	Superscaf	1.36E+08	1.36E+08	-4	4	9	-	-	-	-	-	-	NCP	IGR	-	ID=gene-E	6	5.555	2017.2	0.728589353	KM(+15.99	55	375.2013	2	748.3942	Oxidation	L	Superscaf	1.36E+08	0	0	BTP20220	0	0	0	Tissue specific
ENCCC	Superscaf+	Superscaf	1.36E+08	1.36E+08	21	21	25	-	-	-	-	-	-	NCP	IGR	-	ID=gene-E	5	4.738	2779.3	0.73067857	ENC(+57.(	65	742.1921	1	741.188	Carbamidc	S	Superscaf	1.36E+08	0	0	BTP20220	0	0	0	Tissue specific
KSPTGTVRF	Superscaf-	Superscaf	1.36E+08	1.36E+08	-1	1	9	-	-	-	-	-	-	NCP	IGR	-	ID=gene-E	9	6.332222222	3317.7	0.731629267	KSPTGTVRF	83	496.783	2	991.545	.	K	Superscaf	1.36E+08	0	0	BTP20220	0	0	0	Tissue specific
LLVGD	Superscaf+	Superscaf	1.36E+08	1.36E+08	27	27	31	-	-	gene	-	-	mRNA	NCP	Intron	gene|mRN	ID=gene-E	5	4.18	1415.6	0.733895441	LLVGD	63	516.2982	1	515.2955	.	S	Superscaf	1.36E+08	0	0	BTP20220	0	0	0	Tissue specific
WELLD	Superscaf-	Superscaf	1.36E+08	1.36E+08	-14	14	18	-	-	gene	-	-	mRNA	NCP	Intron	gene|mRN	ID=gene-E	5	4.808	2070.4	0.733907108	W(+42.01)	78	717.3383	1	716.3381	Acetylati	N	Superscaf	1.36E+08	0	0	0	0	0	BTP20220	Mixed
NKLLS	Superscaf-	Superscaf	1.36E+08	1.36E+08	-17	17	21	-	-	gene	-	-	mRNA	NCP	Intron	gene|mRN	ID=gene-E	5	6.558	2155.3	0.733930557	N(+42.01)	52	616.3659	1	615.3592	Acetylati	N	Superscaf	1.36E+08	0	0	0	0	0	BTP20220	Tissue specific
QKLLLQ	Superscaf+	Superscaf	1.36E+08	1.36E+08	2	2	7	-	-	-	-	-	-	NCP	IGR	-	ID=gene-E	6	6.496666667	2750	0.734300882	QKLLLQ	51	371.7429	2	741.4749	.	Y	Superscaf	1.36E+08	0	0	0	0	0	BTP20220	Tissue specific
APELW	Superscaf+	Superscaf	1.36E+08	1.36E+08	10	10	14	-	-	gene	-	-	mRNA	NCP	Intron	gene|mRN	ID=gene-E	5	5.482	1954.2	0.736237546	A(+42.01)	56	657.3217	1	656.317	Acetylati	E	Superscaf	1.36E+08	0	0	0	0	0	BTP20220	Tissue specific
QELEGMK	Superscaf+	Superscaf	1.36E+08	1.36E+08	4	4	10	-	-	gene	-	-	mRNA	NCP	Intron	gene|mRN	ID=gene-E	7	5.647142857	2952.3	0.73651924	QELEGMK	72	417.7037	2	833.3953	.	K	Superscaf	1.36E+08	0	0	0	0	0	BTP20220	Tissue specific
NSGSPPP	Superscaf-	Superscaf	1.36E+08	1.36E+08	-11	11	17	-	-	-	-	-	-	NCP	IGR	-	ID=gene-E	7	5.948571429	2673	0.738100459	NSGSPPP	61	655.3079	1	654.2973	.	I	Superscaf	1.36E+08	0	0	0	0	0	BTP20220	Tissue specific
KNMKVGS	Superscaf+	Superscaf	1.36E+08	1.36E+08	61	61	67	-	-	gene	-	-	mRNA	NCP	Intron	gene|mRN	ID=gene-E	7	6.041428571	2585.7	0.738852547	KNM(+15.9	56	390.2054	2	778.4007	Oxidation	E	Superscaf	1.36E+08	0	0	0	0	BTP20220	0	Tissue specific
MEQLLRN	Superscaf+	Superscaf	1.37E+08	1.37E+08	28	28	34	-	-	-	-	-	-	NCP	IGR	-	ID=gene-E	7	6.107142857	3360	0.741488511	M(+42.01)	63	473.2429	2	944.4749	Acetylati	N	Superscaf	1.37E+08	0	0	BTP20220	0	0	0	Tissue specific
MYDLHY	Superscaf-	Superscaf	1.37E+08	1.37E+08	-4	4	9	-	-	-	-	-	-	NCP	IGR	-	ID=gene-E	6	5.601666667	2675.1	0.74163938	M(+15.99)	55	429.1705	2	856.3425	Oxidation	W	Superscaf	1.37E+08	0	0	BTP20220	0	0	0	Tissue specific
FGQRQGF	Superscaf+	Superscaf	1.37E+08	1.37E+08	6	6	12	-	-	-	-	-	-	NCP	IGR	-	ID=gene-E	7	6.422857143	3239.6	0.742275792	FGQRQGF	67	420.2107	2	838.4086	.	T	Superscaf	1.37E+08	0	0	BTP20220	0	0	0	Tissue specific
ASLTTFL	Superscaf+	Superscaf	1.37E+08	1.37E+08	19	19	25	-	-	-	-	-	-	NCP	IGR	-	ID=gene-E	7	6.028571429	2583.9	0.743011968	A(+42.01)	65	794.4258	1	793.4222	Acetylati	Q	Superscaf	1.37E+08	0	0	BTP20220	BTP20220	0	0	Tissue specific
LQVTPP	Superscaf+	Superscaf	1.37E+08	1.37E+08	5	5	10	-	-	-	-	-	-	NCP	IGR	-	ID=gene-E	6	5.126666667	2036.4	0.744474029	LQVTPP	76	654.3916	1	653.3748	.	C	Superscaf	1.37E+08	0	0	BTP20220	0	0	0	Tissue specific
VKPPLPHP	Superscaf+	Superscaf	1.37E+08	1.37E+08	36	36	43	-	-	-	-	-	-	NCP	IGR	-	ID=gene-E	8	6.06375	2791.1	0.746337401	V(+42.01)	55	463.7773	2	925.5385	Acetylati	R	Superscaf	1.37E+08	0	0	0	BTP20220	0	0	Tissue specific
REVKQLYF	Superscaf+	Superscaf	1.37E+08	1.37E+08	40	40	47	-	-	-	-	-	-	NCP	IGR	-	ID=gene-E	8	5.81125	3353.7	0.747353816	R(+42.01)	66	562.7998	2	1123.603	Acetylati	T	Superscaf	1.37E+08	BTP20220	0	0	0	0	0	Tissue specific
LSLKV	Superscaf+	Superscaf	1.37E+08	1.37E+08	63	63	67	-	-	-	-	-	-	NCP	IGR	-	ID=gene-E	5	5.476	1558.6	0.747562753	L(+42.01)	73	601.3906	1	600.3846	Acetylati	I	Superscaf	1.37E+08	0	0	0	0	BTP20220	0	Mixed
LPPKYKK	Superscaf-	Superscaf	1.37E+08	1.37E+08	-17	17	23	-	-	-	-	-	-	NCP	IGR	-	ID=gene-E	7	7.637142857	2892.9	0.748091254	LPPKYKK	72	437.2797	2	872.5483	.	A	Superscaf	1.37E+08	0	0	BTP20220	0	0	0	Tissue specific
HLLFMVM	Superscaf-	Superscaf	1.37E+08	1.37E+08	-1	1	7	-	-	-	-	-	-	NCP	IGR	-	ID=gene-E	7	5.218571429	2456.9	0.748451099	HLLFMVM	53	445.7381	2	889.4554	.	H	Superscaf	1.37E+08	BTP20220	0	0	0	0	0	Tissue specific
LDLSP	Superscaf-	Superscaf	1.37E+08	1.37E+08	-4	4	8	-	-	-	-	-	-	NCP	IGR	-	ID=gene-E	5	5.382	1839.1	0.749027071	LDLSP	93	544.2954	1	543.2904	.	P	Superscaf	1.37E+08	0	0	0	0	0	BTP20220	Tissue specific
DAPFVF	Superscaf-	Superscaf	1.37E+08	1.37E+08	-7	7	12	-	-	-	-	-	-	NCP	IGR	-	ID=gene-E	6	4.375	1835	0.749247216	DAPFVF	69	695.3386	1	694.3326	.	V	Superscaf	1.37E+08	BTP20220	0	0	0	0	0	Tissue specific
KNKDRF	Superscaf-	Superscaf	1.37E+08	1.37E+08	-7	7	12	-	-	-	-	-	-	NCP	IGR	-	ID=gene-E	6	7.35	2981.2	0.749591836	KNKDRF	62	404.2201	2	806.4399	.	K	Superscaf	1.37E+08	0	0	BTP20220	0	0	0	Tissue specific
VDLFEGK	Superscaf-	Superscaf	1.37E+08	1.37E+08	-1	1	7	-	-	-	-	-	-	NCP	IGR	-	ID=gene-E	7	4.765714286	2343.5	0.750571682	VDLFEGK	99	404.2146	2	806.4174	.	V	Superscaf	1.37E+08	0	0	0	BTP20220	0	0	Mixed
LVTFAL	Superscaf-	Superscaf	1.37E+08	1.37E+08	-3	3	8	-	-	gene	-	-	mRNA	NCP	Intron	gene|mRN	ID=gene-E	6	4.998333333	1803	0.75244936	LVTFAL	86	663.4043	1	662.4003	.	L	Superscaf	1.37E+08	0	0	0	0	BTP20220	0	Tissue specific
VDDLTEF	Superscaf-	Superscaf	1.37E+08	1.37E+08	-17	17	23	-	-	-	-	-	-	NCP	IGR	-	ID=gene-E	7	3.878571429	2386.8	0.752571463	VDDLTEF	89	838.3823	1	837.3756	.	R	Superscaf	1.37E+08	0	0	0	0	BTP20220	0	Mixed
ATRFAF	Superscaf-	Superscaf	1.38E+08	1.38E+08	-7	7	12	-	-	-	-	-	-	NCP	IGR	-	ID=gene-E	6	6.715	2443.7	0.753922553	ATRFAF	52	712.3735	1	711.3704	.	N	Superscaf	1.38E+08	0	0	0	0	0	BTP20220	Tissue specific
LFGGF	Superscaf-	Superscaf	1.38E+08	1.38E+08	-25	25	29	-	-	-	-	-	-	NCP	IGR	-	ID=gene-E	5	5.776	1722.8	0.754562025	LFGGF	99	540.28	1	539.2744	.	H	Superscaf	1.38E+08	0	0	BTP20220	0	0	0	Tissue specific
KVPTKHG	Superscaf-	Superscaf	1.38E+08	1.38E+08	-22	22	28	-	-	-	-	-	-	NCP	IGR	-	ID=gene-E	7	6.552857143	2588.7	0.755793613	KVPTKHG	55	766.4556	1	765.4497	.	K	Superscaf	1.38E+08	0	0	0	BTP20220	0	0	Tissue specific
YPFLP	Superscaf-	Superscaf	1.38E+08	1.38E+08	-39	39	43	-	-	-	-	-	-	NCP	IGR	-	ID=gene-E	5	5.944	1875.1	0.757943575	YPFLP	55	636.3402	1	635.3318	.	I	Superscaf	1.38E+08	0	BTP20220	0	0	0	0	Tissue specific
KDTPPNP	Superscaf+	Superscaf	1.38E+08	1.38E+08	8	8	14	-	-	-	-	-	-	NCP	IGR	-	ID=gene-E	7	6.221428571	2886.3	0.75809957	KDTPPNP	71	768.3928	1	767.3813	.	S	Superscaf	1.38E+08	0	0	0	0	BTP20220	0	Tissue specific
NHLTMRP	Superscaf+	Superscaf	1.38E+08	1.38E+08	38	38	44	-	-	-	-	-	-	NCP	IGR	-	ID=gene-E	7	6.902857143	3325	0.758561434	NHLTM(+15	62	442.7227	2	883.4334	Oxidation	L	Superscaf	1.38E+08	0	0	0	0	0	BTP20220	Tissue specific
HALMRLD	Superscaf+	Superscaf	1.38E+08	1.38E+08	26	26	32	-	-	gene	-	-	mRNA	NCP	Intron	gene|mRN	ID=gene-E	7	6.435714286	3069.6	0.759954334	HALMRLD	84	428.2265	2	854.4432	.	S	Superscaf	1.38E+08	0	0	0	0	BTP20220	0	Tissue specific
MFLRGER	Superscaf+	Superscaf	1.38E+08	1.38E+08	14	14	20	-	-	gene	-	-	mRNA	NCP	Intron	gene|mRN	ID=gene-E	7	6.845714286	3132.7	0.760688635	M(+42.01)	84	475.7463	2	949.4803	Acetylati	V	Superscaf	1.38E+08	0	0	0	BTP20220	0	0	Tissue specific
KNVGL	Superscaf+	Superscaf	1.38E+08	1.38E+08	2	2	6	-	-	-	-	-	-	NCP	IGR	-	ID=gene-E	5	5.42	1715.8	0.761254395	K(+42.01)	74	572.3383	1	571.3329	Acetylati	D	Superscaf	1.38E+08	0	0	0	0	BTP20220	0	Tissue enhanced
KRTVTAM	Superscaf-	Superscaf	1.38E+08	1.38E+08	-1	1	7	-	-	gene	-	-	mRNA	NCP	Intron	gene|mRN	ID=gene-E	7	6.475714286	2595	0.762120133	KRTVTAM	61	403.7297	2	805.448	.	K	Superscaf	1.38E+08	0	0	0	0	0	BTP20220	Tissue specific

YGPTLL	Superscaf	+	Superscaf	1.38E+08	1.38E+08	8	8	13	-	exon	gene	three_pri	-	mRNA	NCP	3UTR	exon|gene	ID=exon-E	6	6.07	2198.5	0.764062343	YGPTLL	84	663.3693	1	662.3639	.	K	Superscaf	1.38E+08	BTP202209	0	0	0	0	0	Tissue specific
EDTLQK	Superscaf	+	Superscaf	1.38E+08	1.38E+08	7	7	12	-	-	gene	-	-	mRNA	NCP	Intron	gene|mRN	ID=gene-E	6	5.681666667	2667	0.76500998	EDTLQK	68	733.3854	1	732.3654	.	V	Superscaf	1.38E+08	0	0	0	0	BTP202209	0	Tissue specific
VLLLV	Superscaf	+	Superscaf	1.39E+08	1.39E+08	25	25	29	-	-	gene	-	-	mRNA	NCP	Intron	gene|mRN	ID=gene-E	5	3.588	1060.2	0.76700104	VLLLV	78	556.4061	1	555.3996	.	N	Superscaf	1.39E+08	0	0	BTP202209	0	0	0	Tissue specific
AVTTNK	Superscaf	-	Superscaf	1.39E+08	1.39E+08	-21	21	26	-	-	-	-	-	-	NCP	IGR	-	ID=gene-E	6	5.705	2171.4	0.767565766	AVTTNK	54	633.3582	1	632.3493	.	K	Superscaf	1.39E+08	0	0	0	0	0	BTP202209	Tissue specific
TKEQER	Superscaf	+	Superscaf	1.39E+08	1.39E+08	1	1	6	-	-	-	-	-	-	NCP	IGR	-	ID=gene-E	6	6.52	2976.4	0.769655288	T(+42.01)	60	416.7153	2	831.4086	Acetylati	T	Superscaf	1.39E+08	0	0	0	0	0	BTP202209	Tissue specific
CKGPARP	Superscaf	+	Superscaf	1.39E+08	1.39E+08	45	45	51	-	-	-	-	-	-	NCP	IGR	-	ID=gene-E	7	7.158571429	2986.3	0.771650708	C(+57.02)	80	393.2079	2	784.4014	Carbamido	Q	Superscaf	1.39E+08	0	0	0	0	0	BTP202209	Tissue specific
LATKAVE	Superscaf	-	Superscaf	1.39E+08	1.39E+08	-1	1	7	-	-	gene	-	-	mRNA	NCP	Intron	gene|mRN	ID=gene-E	7	5.358571429	2267.4	0.771775642	LATKAVE	95	366.2173	2	730.4225	.	L	Superscaf	1.39E+08	0	0	0	0	0	BTP202209	Tissue specific
SLDSGGLR	Superscaf	-	Superscaf	1.39E+08	1.39E+08	-9	9	16	-	-	-	-	-	-	NCP	IGR	-	ID=gene-E	8	6.12375	3128.5	0.772612843	SLDSGGLR	84	402.7124	2	803.4137	.	S	Superscaf	1.39E+08	0	0	BTP202209	0	0	0	Tissue specific
FLEKGK	Superscaf	-	Superscaf	1.39E+08	1.39E+08	-23	23	28	-	-	gene	-	-	mRNA	NCP	Intron	gene|mRN	ID=gene-E	6	6.688333333	2400.4	0.774259857	FLEKGK	97	361.2134	2	720.417	.	A	Superscaf	1.39E+08	0	0	0	0	0	BTP202209	Tissue specific
GGPTH	Superscaf	+	Superscaf	1.39E+08	1.39E+08	7	7	11	-	-	gene	-	-	mRNA	NCP	Intron	gene|mRN	ID=gene-E	5	6.472	1949.2	0.776118868	G(+42.01)	59	510.2337	1	509.2234	Acetylati	F	Superscaf	1.39E+08	0	0	0	0	0	BTP202209	Tissue specific
LGSLAEAY	Superscaf	-	Superscaf	1.39E+08	1.39E+08	-11	11	18	-	-	-	-	-	-	NCP	IGR	-	ID=gene-E	8	5.56625	2895.1	0.777070215	LGSLAEAY	67	412.212	2	822.4123	.	L	Superscaf	1.39E+08	0	0	0	0	0	BTP202209	Tissue specific
PPPPLP	Superscaf	+	Superscaf	1.39E+08	1.39E+08	66	66	71	-	-	-	-	-	-	NCP	IGR	-	ID=gene-E	6	6.246666667	2040.4	0.77817205	PPPPLP	98	617.3632	1	616.3584	.	I	Superscaf	1.39E+08	0	0	0	0	0	BTP202209	Tissue specific
MGHLLVK	Superscaf	-	Superscaf	1.4E+08	1.4E+08	-3	3	9	-	-	gene	-	-	mRNA	NCP	Intron	gene|mRN	ID=gene-E	7	5.858571429	2463.7	0.779160503	MGHLLVK	59	399.2374	2	796.463	.	Q	Superscaf	1.4E+08	0	0	BTP202209	0	BTP202209	0	Tissue specific
AFLEMK	Superscaf	+	Superscaf	1.4E+08	1.4E+08	2	2	7	-	-	gene	-	-	mRNA	NCP	Intron	gene|mRN	ID=gene-E	6	6.031666667	2317.5	0.779526161	AFLEMK	66	369.6942	2	737.3782	.	Q	Superscaf	1.4E+08	0	0	0	0	0	BTP202209	Tissue specific
KSVKKE	Superscaf	+	Superscaf	1.4E+08	1.4E+08	17	17	22	-	-	-	-	-	-	NCP	IGR	-	ID=gene-E	6	6.353333333	2214.1	0.780015874	KSVKKE	50	359.7249	2	717.4385	.	S	Superscaf	1.4E+08	0	0	0	0	0	BTP202209	Tissue specific
VKMLK	Superscaf	-	Superscaf	1.4E+08	1.4E+08	-1	1	5	-	-	-	-	-	-	NCP	IGR	-	ID=gene-E	5	6.242	1661.7	0.780131856	V(+42.01)	73	660.4084	1	659.404	Acetylati	V	Superscaf	1.4E+08	0	0	0	0	0	BTP202209	Tissue specific
FLQSQR	Superscaf	-	Superscaf	1.4E+08	1.4E+08	-7	7	12	-	-	-	-	-	-	NCP	IGR	-	ID=gene-E	6	6.533333333	2994.4	0.780367494	FLQSQR	81	389.7128	2	777.4133	.	C	Superscaf	1.4E+08	BTP202209	0	0	0	0	0	Tissue specific
KKLAVVHMI	Superscaf	+	Superscaf	1.4E+08	1.4E+08	4	4	13	-	-	-	-	-	-	NCP	IGR	-	ID=gene-E	10	5.377	3357.6	0.780603208	KKLAVVHMI	58	385.2231	3	1152.669	.	R	Superscaf	1.4E+08	0	0	0	BTP202209	0	0	Tissue specific
LGSPAKPV	Superscaf	+	Superscaf	1.4E+08	1.4E+08	52	52	59	-	-	-	-	-	-	NCP	IGR	-	ID=gene-E	8	5.74875	2488.6	0.781332957	LGSPAKPV	60	768.4557	1	767.4541	.	S	Superscaf	1.4E+08	0	0	0	BTP202209	0	0	Tissue specific
KAVYW	Superscaf	-	Superscaf	1.4E+08	1.4E+08	-19	19	23	-	-	-	-	-	-	NCP	IGR	-	ID=gene-E	5	5.462	1765.8	0.782259096	KAVYW	50	666.3519	1	665.3537	.	L	Superscaf	1.4E+08	0	0	0	0	BTP202209	0	Tissue specific
RGFVFP	Superscaf	+	Superscaf	1.4E+08	1.4E+08	3	3	8	-	-	-	-	-	-	NCP	IGR	-	ID=gene-E	6	5.665	2058.3	0.782499744	RGFVFP	54	361.7069	2	721.3911	.	C	Superscaf	1.4E+08	0	0	0	0	0	BTP202209	Tissue specific
DPGAAL	Superscaf	+	Superscaf	1.4E+08	1.4E+08	28	28	33	-	-	-	-	-	-	NCP	IGR	-	ID=gene-E	6	5.543333333	2022.2	0.782529926	D(+42.01)	65	585.2847	1	584.2806	Acetylati	R	Superscaf	1.4E+08	BTP202209	0	0	0	0	0	Tissue specific
LDNLPGM	Superscaf	-	Superscaf	1.4E+08	1.4E+08	-24	24	30	-	-	-	-	-	-	NCP	IGR	-	ID=gene-E	7	5.48	2721.2	0.783282971	LDNLPGM	73	759.3702	1	758.3633	.	D	Superscaf	1.4E+08	0	BTP202209	BTP202209	0	0	0	Tissue specific
PPAPF	Superscaf	+	Superscaf	1.4E+08	1.4E+08	15	15	19	-	-	-	-	-	-	NCP	IGR	-	ID=gene-E	5	6.08	1710.9	0.783337328	PPAPF	68	528.2921	1	527.2744	.	D	Superscaf	1.4E+08	0	0	BTP202209	0	0	0	Tissue specific
PDPLPY	Superscaf	-	Superscaf	1.4E+08	1.4E+08	-13	13	18	-	-	-	-	-	-	NCP	IGR	-	ID=gene-E	6	5.585	2236.6	0.789286331	PDPLPY	60	701.3456	1	700.3431	.	D	Superscaf	1.4E+08	0	0	0	0	0	BTP202209	Tissue specific
RLLFLPK	Superscaf	-	Superscaf	1.4E+08	1.4E+08	-15	15	21	-	-	-	-	-	-	NCP	IGR	-	ID=gene-E	7	7.174285714	2902.2	0.789715221	RLLFLPK	96	886.5821	1	885.58	.	N	Superscaf	1.4E+08	0	0	0	BTP202209	0	0	Tissue specific
GPPPPAPLF	Superscaf	+	Superscaf	1.4E+08	1.4E+08	54	54	62	-	exon	gene	-	five_prim	mRNA	NCP	5UTR	exon|gene	ID=exon-E	9	6.692222222	3297.8	0.791230071	G(+42.01)	86	472.2681	2	942.5286	Acetylati	P	Superscaf	1.4E+08	0	0	0	BTP202209	0	0	Tissue specific
NGLGNER	Superscaf	-	Superscaf	1.41E+08	1.41E+08	-3	3	9	-	-	-	-	-	-	NCP	IGR	-	ID=gene-E	7	6.102857143	3215.7	0.79290172	N(+42.01)	77	401.1965	2	800.3777	Acetylati	A	Superscaf	1.41E+08	0	0	BTP202209	0	0	0	Tissue specific
LLVYSV	Superscaf	-	Superscaf	1.41E+08	1.41E+08	-31	31	36	-	-	gene	-	-	mRNA	NCP	Intron	gene|mRN	ID=gene-E	6	3.883333333	1549.7	0.794651901	L(+42.01)	67	368.2168	2	734.4214	Acetylati	A	Superscaf	1.41E+08	0	0	0	0	BTP202209	0	Tissue specific
KELTDPF	Superscaf	-	Superscaf	1.41E+08	1.41E+08	-4	4	10	-	-	gene	-	-	mRNA	NCP	Intron	gene|mRN	ID=gene-E	7	5.745714286	2781.1	0.794723549	KELTDPF	66	849.4315	1	848.4279	.	L	Superscaf	1.41E+08	0	0	0	0	0	BTP202209	Tissue specific
EVEREVP	Superscaf	+	Superscaf	1.41E+08	1.41E+08	26	26	32	-	-	gene	-	-	mRNA	NCP	Intron	gene|mRN	ID=gene-E	7	3.817142857	2262.7	0.795314784	EVEREVP	83	429.2211	2	856.429	.	S	Superscaf	1.41E+08	0	0	0	0	BTP202209	0	Tissue enhanced
MERTF	Superscaf	-	Superscaf	1.41E+08	1.41E+08	-3	3	7	-	-	-	-	-	-	NCP	IGR	-	ID=gene-E	5	6.348	2230.7	0.797024685	M(+42.01)	83	725.3278	1	724.3214	Acetylati	K	Superscaf	1.41E+08	0	0	0	BTP202209	0	0	Tissue specific
VTHFRPK	Superscaf	-	Superscaf	1.41E+08	1.41E+08	-9	9	15	-	-	-	-	-	-	NCP	IGR	-	ID=gene-E	7	6.628571429	2859.2	0.797216026	VTHFRPK	71	442.7589	2	883.5028	.	D	Superscaf	1.41E+08	0	0	0	0	0	BTP202209	Tissue specific
KSHVPP	Superscaf	+	Superscaf	1.41E+08	1.41E+08	18	18	23	-	-	-	-	-	-	NCP	IGR	-	ID=gene-E	6	5.935	2146.3	0.797816251	KSHVPP	73	664.3776	1	663.3704	.	F	Superscaf	1.41E+08	0	0	BTP202209	0	0	0	Tissue specific
EPPVVKT	Superscaf	-	Superscaf	1.41E+08	1.41E+08	-1	1	7	-	-	-	-	-	-	NCP	IGR	-	ID=gene-E	7	4.584285714	1966.2	0.798505682	EPPVVKT	72	385.225	2	768.4381	.	E	Superscaf	1.41E+08	BTP202209	0	0	0	0	0	Tissue specific
QEPPGRPQ	Superscaf	+	Superscaf	1.41E+08	1.41E+08	38	38	45	-	-	-	-	-	-	NCP	IGR	-	ID=gene-E	8	6.26875	3605.2	0.799350878	QEPPGRPQ	75	454.7317	2	907.4511	.	K	Superscaf	1.41E+08	BTP202209	0	0	0	0	0	Tissue specific
ALLTQ	Superscaf	+	Superscaf	1.41E+08	1.41E+08	32	32	36	-	-	gene	-	-	mRNA	NCP	Intron	gene|mRN	ID=gene-E	5	6.032	2026.3	0.799459745	A(+42.01)	93	587.3389	1	586.3326	Acetylati	P	Superscaf	1.41E+08	BTP202209	0	0	0	0	0	Tissue specific
TPLPPR	Superscaf	+	Superscaf	1.41E+08	1.41E+08	65	65	70	-	-	gene	-	-	mRNA	NCP	Intron	gene|mRN	ID=gene-E	6	7.028333333	2411.9	0.799495129	T(+42.01)	53	361.7117	2	721.4122	Acetylati	K	Superscaf	1.41E+08	0	0	BTP202209	0	BTP202209	0	Tissue specific
APVEALLPF	Superscaf	+	Superscaf	1.41E+08	1.41E+08	88	88	96	-	-	gene	-	-	mRNA	NCP	Intron	gene|mRN	ID=gene-E	9	5.033333333	2817.1	0.800118229	APVEALLPF	53	956.5425	1	955.5378	.	E	Superscaf	1.41E+08	0	0	0	0	0	BTP202209	Tissue specific
TTYSKER	Superscaf	-	Superscaf	1.41E+08	1.41E+08	-15	15	21	-	-	gene	-	-	mRNA	NCP	Intron	gene|mRN	ID=gene-E	7	6.874285714	3180.6	0.800180428	TTYSKER	60	442.7263	2	883.4399	.	W	Superscaf	1.41E+08	0	0	BTP202209	0	0	0	Tissue specific
KHYGGCSK	Superscaf	-	Superscaf	1.41E+08	1.41E+08	-23	23	30	-	-	gene	-	-	mRNA	NCP	Intron	gene|mRN	ID=gene-E	8	6.92125	3579.7	0.800723159	KHYGGC(+5	56	468.7268	2	935.4283	Carbamido	R	Superscaf	1.41E+08	BTP202209	0	0	0	0	0	Tissue specific
AVPPCVQ	Superscaf	+	Superscaf	1.41E+08	1.41E+08	3	3	9	-	-	gene	-	-	mRNA	NCP	Intron	gene|mRN	ID=gene-E	7	4.184285714	2182.5	0.80126524	A(+42.01)	67	406.7011	2	811.3898	Acetylati	Q	Superscaf	1.41E+08	0	BTP202209	0	0	0	0	Tissue specific
LPLAPAR	Superscaf	+	Superscaf	1.41E+08	1.41E+08	43	43	49	-	-	gene	-	-	mRNA	NCP	Intron	gene|mRN	ID=gene-E	7	6.765714286	2653	0.80161116	LPLAPAR	79	369.2365	2	736.4595	.	A	Superscaf	1.41E+08	0	0	0	0	0	BTP202209	Tissue specific
VTVKKWT	Superscaf	+	Superscaf	1.41E+08	1.41E+08	97	97	103	-	-	-	-	-	-	NCP	IGR	-	ID=gene-E	7	5.49	2258.4	0.802992126	VTVKKWT	65	431.2628	2	860.512	.	E	Superscaf	1.41E+08	0	0	0	BTP202209	0	0	Tissue specific
VMFLH	Superscaf	+	Superscaf	1.42E+08	1.42E+08	21	21	25	-	-	-	-	-	-	NCP	IGR	-	ID=gene-E	5	4.96	1732	0.805298044	VMFLH	51	646.3433	1	645.3309	.	S	Superscaf	1.42E+08	BTP202209	0	0	0	0	0	Tissue specific
LSGAGLML	Superscaf	+	Superscaf	1.42E+08	1.42E+08	52	52	59	-	-	-	-	-	-	NCP	IGR	-	ID=gene-E	8	5.91625	2721	0.805401747	LSGAGLM(+	56	777.4102	1	776.4102	Oxidation	N	Superscaf	1.42E+08	BTP202209	0	0	BTP202209	0	0	Tissue specific
EARVAC	Superscaf	-	Superscaf	1.42E+08	1.42E+08	-7	7	12	-	-	gene	-	-	mRNA	NCP	Intron	gene|mRN	ID=gene-E	6	5.173333333	2282.6	0.80781332	EARVAC(+5	52	353.1707	2	704.3275	Carbamido	N	Superscaf	1.42E+08	0	0	0	0	BTP202209	0	Tissue specific
TALKFYP	Superscaf	-	Superscaf	1.42E+08	1.42E+08	-14	14	20	-	-	-	-	-	-	NCP	IGR	-	ID=gene-E	7	6.53	2714.9	0.808814358	TALKFYP	71	420.2357	2	838.4589	.	C	Superscaf	1.42E+08	0	0	BTP202209	0	0	0	Tissue specific
VADDD	Superscaf	+	Superscaf	1.42E+08	1.42E+08	12	12	16	-	-	-	-	-	-	NCP	IGR	-	ID=gene-E	5	2.986	1545.8	0.808855901	VADDD	53	534.2049	1	533.1969	.	V	Superscaf	1.42E+08	0	0	0	0	BTP202209	0	Tissue specific
FFEPQPP	Superscaf	-	Superscaf	1.42E+08	1.42E+08	-11	11	17	-	-	gene	-	-	mRNA	NCP	Intron	gene|mRN	ID=gene-E	7	5.532857143	2823.2	0.808950079	FFEPQPP	67	431.211	2	860.4068	.	N	Superscaf	1.42E+08	0	0	0	0	BTP202209	0	Tissue specific
QSARLL	Superscaf	+	Superscaf	1.42E+08	1.42E+08	10	10	15	-	-	-	-	-	-	NCP	IGR	-	ID=gene-E	6	6.678333333	2661	0.809155229	Q(+42.01)	88	365.2156	2	728.4181	Acetylati	C	Superscaf	1.42E+08	0	0	0	BTP202209	0	0	Mixed
DSLMCK	Superscaf	-	Superscaf	1.42E+08	1.42E+08	-21	21	26	-	-	-	-	-	-	NCP	IGR	-	ID=gene-E	6	5.856666667	2573.9	0.809248719	DSLMC(+57	73	377.1653	2	752.3197	Carbamido	F	Superscaf	1.42E+08	BTP202209	0	0	0	0	0	Mixed
GLAPLH	Superscaf	+	Superscaf	1.42E+08	1.42E+08	43	43	48	-	-	-	-	-	-	NCP	IGR	-	ID=gene-E	6	6.306666667	2272.5	0.810856332	G(+42.01)	59	649.3643	1	648.3595	Acetylati	G	Superscaf	1.42E+08	0	0	0	0	0	BTP202209	Tissue specific
FKFVL	Superscaf	-	Superscaf	1.42E+08	1.42E+08	-7	7	11	-	-	-	-	-	-	NCP	IGR	-	ID=gene-E	5	5.336	1596.6	0.811082827	FKFVL	99	653.4002	1	652.3948	.	K	Superscaf	1.42E+08	0	BTP202209	0	0	0	0	Tissue specific
EAVHPPR	Superscaf	+	Superscaf	1.42E+08	1.42E+08	22	22	28	-	-	gene	-	-	mRNA	NCP	Intron	gene|mRN	ID=gene-E	7	5.741428571	2680.1	0.811104402	EAVHPPR	94	403.2244	2	804.4242	.	G	Superscaf	1.42E+08	BTP202209	0	0	0	0	0	Tissue specific
NRGHDLSF	Superscaf	-	Superscaf	1.42E+08	1.42E+08	-4	4	11	-	-	gene	-	-	mRNA	NCP	Intron	gene|mRN	ID=gene-E	8	6.23	3698.2	0.811574605	NRGHDLSF	57	473.228	2	944.4464	.	G	Superscaf	1.42E+08	BTP202209	0	0	0	0	0	Tissue specific
KVALLSVP	Superscaf	+	Superscaf	1.42E+08	1.42E+08	3	3	10	-	-	gene	-	-	mRNA	NCP	Intron	gene|mRN	ID=gene-E	8	4.9625	2207.3	0.811813379	KVALLSVP	50	826.5364	1	825.5323	.	Y	Superscaf	1.42E+08	BTP202209	0	0	0	0	0	Tissue specific
DWDDMEK	Superscaf	+	Superscaf	1.42E+08	1.42E+08	22	22	28	-	-	gene	-	-	mRNA	NCP	Intron	gene|mRN	ID=gene-E	7	4.787142857	3026.5	0.812195449	DWDDMEK	99	938.3519	1	937.3488	.	R	Superscaf	1.42E+08	0	0	BTP202209	BTP202209	BTP202209	0	Mixed
KPSRPPP	Superscaf	+	Superscaf	1.42E+08	1.42E+08	21	21	27	-	-	-	-	-	-	NCP	IGR	-	ID=gene-E	7	7.34	2850.2	0.812506061	KPSRPPP	76	389.7302	2	777.4496	.	I	Superscaf	1.42E+08	BTP202209	0	0	0	0	0	Tissue specific
LSDHGPVP	Superscaf	+	Superscaf	1.42E+08	1.42E+08	4	4	11	-	-	-	-	-	-	NCP	IGR	-	ID=gene-E	8	5.09875	2740.1	0.813663592	LSDHGPVP	86	411.2114	2	820.4079	.	P	Superscaf	1.42E+08	BTP202209	0	0	0	0	0	Tissue specific
KDCAFPK	Superscaf	+	Superscaf	1.42E+08	1.42E+08	6	6	12	-	-	-	-	-	-	NCP	IGR	-	ID=gene-E	7	6.467142857	2970.1	0.814616508	KDC(+57.0	58	433.2097	2	864.4163	Carbamido	K	Superscaf	1.42E+08	0	0	BTP202209	0	0	0	Tissue specific
DLPLTVE	Superscaf	+	Superscaf	1.42E+08	1.42E+08	59	59	65	-	-	-	-	-	-	NCP	IGR	-	ID=gene-E	7	4.425714286	2278.7	0.814619606	DLPLTVE	57	786.4219	1	785.4171	.	V	Superscaf	1.42E+08	0	0	BTP202209	0	0	0	Tissue specific
TVGPSLVH	Superscaf	+	Superscaf	1.42E+08	1.42E+08	47	47	54	-	-	-	-	-	-	NCP	IGR	-	ID=gene-E	8	4.75625	2388.7	0.815924946	TVGPSLVH	89	405.2256	2	808.4443	.	G	Superscaf	1.42E+08	0	0	BTP202209	0	0	0	Mixed
AGPHKTLLL	Superscaf	-	Superscaf	1.42E+08	1.42E+08	-34	34	42	-	-	gene	-	-	mRNA	NCP	Intron	gene|mRN	ID=gene-E	9	6.37	3563.9	0.816235406	AGPHKTLLL	68	483.2793	2	964.5341	.	L	Superscaf	1.42E+08	0	0	0	BTP202209	0	0	Tissue specific
PEAPP	Superscaf	+	Superscaf	1.43E+08	1.43E+08	2	2	6	-	-	gene	-	-	mRNA	NCP	Intron	gene|mRN	ID=gene-E	5	5.628	1749	0.817231853	PEAPP	66	510.2573	1	509.2485	.	A	Superscaf	1.43E+08	0	0	0	BTP202209	0	0	Tissue specific
DSLRVAP	Superscaf	+	Superscaf	1.43E+08	1.43E+08	33	33	39	-	-	gene	-	-	mRNA	NCP	Intron	gene|mRN	ID=gene-E	7	5.387142857	2445.8	0.817312911	D(+42.01)	86	400.2175	2	798.4235	Acetylati	Q	Superscaf	1.43E+08	0	0	0	BTP202209	0	0	Mixed
PLLKSYST	Superscaf	+	Superscaf	1.43E+08	1.43E+08	7	7	14	-	-	gene	-	-	mRNA	NCP	Intron	gene|mRN	ID=gene-E	8	6.44375	3136.4	0.817482448	PLLKSYST	56	454.7549	2	907.5015	.	M	Superscaf	1.43E+08	0	0	BTP202209	0	0	0	Tissue specific
AAKVPTA	Superscaf	+	Superscaf	1.43E+08	1.43E+08	12	12	18	-	-	gene	-	-	mRNA	NCP	Intron	gene|mRN	ID=gene-E	7	5.804285714	2137.2	0.81797304	AAKVPTA	56	657.4032	1	656.3857	.	G	Superscaf	1.43E+08	0	0	0	BTP202209	0	0	Tissue specific
NAHLNP	Superscaf	+	Superscaf	1.43E+08	1.43E+08	3	3	8	-	-	gene	-	-	mRNA	NCP	Intron	gene|mRN	ID=gene-E	6	6.118333333	2815.2	0.818620393	N(+42.01)	53	707.3496	1	706.3398	Acetylati	Q	Superscaf	1.43E+08	0	0	BTP202209	0	0	0	Tissue specific
GVPPPAPV	Superscaf	+	Superscaf	1.43E+08	1.43E+08	28	28	35	-	-	-	-	-	-	NCP	IGR	-	ID=gene-E	8	4.64875	1958.2	0.818678231	GVPPPAPV	71	733.4196	1	732.417	.	T	Superscaf	1.43E+08	0	BTP202209	0	0	0	0	Tissue specific
NGGELL	Superscaf	-	Superscaf	1.43E+08	1.43E+08	-11	11	16	-	-	gene	-	-	mRNA	NCP	Intron	gene|mRN	ID=gene-E	6	5.421666667	2323.6	0.820215459	NGGELL	72	602.3129	1	601.3071	.	N	Superscaf	1.43E+08	0	0	0	0	0	BTP202209	Tissue specific
KHTCGK	Superscaf	-	Superscaf	1.43E+08	1.43E+08	-12	12	17	-	-	gene	-	-	mRNA	NCP	Intron	gene|mRN	ID=gene-E	6	7.431666667	2837	0.82051253	KHTC(+57.	68	365.6895	2	729.3592	Carbamido	V	Superscaf	1.43E+08	0	0	0	0	BTP202209	0	Tissue specific
DLHPHL	Superscaf	+	Superscaf	1.43E+08	1.43E+08	21	21	26	-	-	gene	-	-	mRNA	NCP	Intron	gene|mRN	ID=gene-E	6	6.068333333	2695.1	0.820863806	DLHPHL	98	366.1944	2	730.3762	.	S	Superscaf	1.43E+08	0	0	0	0	BTP202209	0	Mixed
FLDKVR	Superscaf	-	Superscaf	1.43E+08	1.43E+08	-2	2	7	-	-	gene	-	-	mRNA	NCP	Intron	gene|mRN	ID=gene-E	6	5.821666667	2269.5	0.821167992	FLDKVR	97	389.2326	2	776.4544	.	L	Superscaf	1.43E+08	0	0	0	0	BTP202209	0	Tissue specific
DSFTPA	Superscaf	+	Superscaf	1.43E+08	1.43E+08	17	17	22	-	-	gene	-	-	mRNA	NCP	Intron	gene|mRN	ID=gene-E	6	5.496666667	2228.5	0.821542181	DSFTPA	57	637.281	1	636.2755	.	S	Superscaf	1.43E+08	0	0	0	0	0	BTP202209	Tissue specific
DLVEPTA	Superscaf	+	Superscaf	1.43E+08	1.43E+08	4	4	10	-	-	gene	-	-	mRNA	NCP	Intron	gene|mRN	ID=gene-E	7	4.431428571	2236.6	0.822991237	DLVEPTA	69	744.3782	1	743.3701	.	D	Superscaf	1.43E+08	0	0	0	0	BTP202209	0	Tissue specific
PVATAA	Superscaf	-	Superscaf	1.43E+08	1.43E+08	-7	7	12	-	-	gene	-	-	mRNA	NCP	Intron	gene|mRN	ID=gene-E	6	5.148333333	1668.8	0.824352425	PVATAA	54	529.2969	1	528.2908	.	K	Superscaf	1.43E+08	0	0	0	0	0	BTP202209	Tissue specific
APTVE	Superscaf	-	Superscaf	1.43E+08	1.43E+08	-14	14	18	-	-	gene	-	-	mRNA	NCP	Intron	gene|mRN	ID=gene-E	5	4.414	1471.7	0.824430269	APTVE	55	516.2751	1	515.2591	.	T	Superscaf	1.43E+08	0	0	BTP202209	0	0	0	Tissue specific
EVSLP	Superscaf	-	Superscaf	1.43E+08	1.43E+08	-15	15	19	-	-	gene	-	-	mRNA	NCP	Intron	gene|mRN	ID=gene-E	5	4.236	1499.7	0.826268241	EVSLP	77	544.2963	1	543.2904	.	V	Superscaf	1.43E+08	0	0	BTP202209	0	0	0	Mixed
LMVMRRC	Superscaf	+	Superscaf	1.43E+08	1.43E+08	19	19	25	-	-	gene	-	-	mRNA	NCP	Intron	gene|mRN	ID=gene-E	7	6.288571429	2979.7	0.826359589	LMVM(+15.	58	491.2398	2	980.4718	Oxidation	I	Superscaf	1.43E+08	0	0	BTP202209	0	0	0	Tissue specific
RRPWE	Superscaf	+	Superscaf	1.43E+08	1.43E+08	24	24	28	-	-	gene	-	-	mRNA	NCP	Intron	gene|mRN	ID=gene-E	5	7.386	2587.1	0.827948802	RRPWE	91	372.1992	2	742.3875	.	N	Superscaf	1.43E+08	0	0	BTP202209	0	0	0	Tissue specific
KAVSRPT	Superscaf	+	Superscaf	1.43E+08	1.43E+08	27	27	33	-	-	-	-	-	-	NCP	IGR	-	ID=gene-E	7	6.432857143	2546.8	0.828142515	KAVSRPT	58	379.7335	2	757.4446	.	D	Superscaf	1.43E+08	0	0	BTP202209	0	0	0	Tissue specific
LPGPM	Superscaf	+	Superscaf	1.43E+08	1.43E+08	6	6	10	-	-	gene	-	-	mRNA	NCP	Intron	gene|mRN	ID=gene-E	5	6.06	1697	0.829013531	LPGPM(+15	53	530.2584	1	529.257	Oxidation	R	Superscaf	1.43E+08	0	0	0	0	0	BTP202209	Tissue specific
DVPPPP	Superscaf	+	Superscaf	1.43E+08	1.43E+08	12	12	17	-	-	-	-	-	-	NCP	IGR	-	ID=gene-E	6	4.695	1761.1	0.82936584	DVPPPP	76	621.3253	1	620.317	.	G	Superscaf	1.43E+08	0	0	0	0	0	BTP202209	Tissue specific
KHGFSPP	Superscaf	-	Superscaf	1.44E+08	1.44E+08	-7	7	13	-	-	gene	-	-	mRNA	NCP	Intron	gene|mRN	ID=gene-E	7	6.722857143	2831	0.830701246	KHGFSPP	62	385.2042	2	768.3918	.	E	Superscaf	1.44E+08	0	0	BTP202209	0	0	0	Tissue specific
KEHYTL	Superscaf	-	Superscaf	1.44E+08	1.44E+08	-54	54	59	-	-	-	-	-	-	NCP	IGR	-	ID=gene-E	6	6.453333333	2724	0.83198459	KEHYTL	68	395.7072	2	789.4021	.	S	Superscaf	1.44E+08	0	0	0	0	0	BTP202209	Tissue specific
PMLLTLL	Superscaf	+	Superscaf	1.44E+08	1.44E+08	39	39	45	-	-	-	-	-	-	NCP	IGR	-	ID=gene-E	7	6.071428571	2520	0.832090053	PM(+15.99	76	408.7521	2	815.4827	Oxidation	D	Superscaf	1.44E+08	BTP202209	0	0	0	0	0	Tissue specific
FLDVLR	Superscaf	+	Superscaf	1.44E+08	1.44E+08	27	27	32	-	-	gene	-	-	mRNA	NCP	Intron	gene|mRN	ID=gene-E	6	5.195	2154.5	0.833091665	FLDVLR	82	381.7288	2	761.4435	.	Y	Superscaf	1.44E+08	0	0	BTP202209	0	0	0	Tissue specific
KRPEYNP	Superscaf	+	Superscaf	1.44E+08	1.44E+08	123	123	129	-	-	gene	-	-	mRNA	NCP	Intron	gene|mRN	ID=gene-E	7	6.77	3273.7	0.833576366	KRPEYNP	52	452.237	2	902.461	.	P	Superscaf	1.44E+08	0	0	BTP202209	0	0	0	Tissue specific
KSPVAF	Superscaf	+	Superscaf	1.44E+08	1.44E+08	6	6	11	-	-	-	-	-	-	NCP	IGR	-	ID=gene-E	6	5.536666667	1887.9	0.833673949	KSPVAF	55	648.3773	1	647.3642	.	K	Superscaf	1.44E+08	0	0	0	0	BTP202209	0	Tissue specific
KVEGAPV	Superscaf	+	Superscaf	1.44E+08	1.44E+08	27	27	33	-	-	gene	-	-	mRNA	NCP	Intron	gene|mRN	ID=gene-E	7	4.464285714	1839.9	0.834712016	KVEGAPV	59	699.4021	1	698.3962	.	K	Superscaf	1.44E+08	0	0	0	BTP202209	0	0	Tissue specific
WGGLVNAG	Superscaf	+	Superscaf	1.44E+08	1.44E+08	5	5	12	-	-	-	-	-	-	NCP	IGR	-	ID=gene-E	8	5.15125	2679.9	0.835348467	W(+42.01)	63	815.3965	1	814.3973	Acetylati	V	Superscaf	1.44E+08	0	0	0	0	0	BTP202209	Tissue specific
GHPRALPP	Superscaf	+	Superscaf	1.44E+08	1.44E+08	23	23	30	-	-	gene	-	-	mRNA	NCP	Intron	gene|mRN	ID=gene-E	8	6.9025	3242.7	0.836449765	GHPRALPP	63	422.741	2	843.4715	.	S	Superscaf	1.44E+08	BTP202209	0	0	0	0	0	Tissue specific
KDVHEEF	Superscaf	-	Superscaf	1.44E+08	1.44E+08	-2	2	8	-	-	gene	-	-	mRNA	NCP	Intron	gene|mRN	ID=gene-E	7	4.602857143	2738	0.836792358	KDVHEEF	52	452.2178	2	902.4134	.	K	Superscaf	1.44E+08	0	0	0	BTP202209	0	0	Tissue specific
LTALPGFG	Superscaf	+	Superscaf	1.44E+08	1.44E+08	36	36	43	-	-	gene	-	-	mRNA	NCP	Intron	gene|mRN	ID=gene-E	8	6.02875	2735	0.837247681	LTALPGFG	64	775.432	1	774.4276	.	N	Superscaf	1.44E+08	0	0	0	0	BTP202209	0	Tissue specific
NFTKVWF	Superscaf	-	Superscaf	1.44E+08	1.44E+08	-20	20	26	-	-	-	-	-	-	NCP	IGR	-	ID=gene-E	7	5.504285714	2764	0.838102249	NFTKVWF	75	471.247	2	940.4807	.	A	Superscaf	1.44E+08	0	0	BTP202209	BTP202209	0	0	Tissue specific
GPFAVP	Superscaf	+	Superscaf	1.44E+08	1.44E+08	14	14	19	-	-	gene	-	-	mRNA	NCP	Intron	gene|mRN	ID=gene-E	6	5.011666667	1670.8	0.840537462	GPFAVP	50	587.3167	1	586.3115	.	P	Superscaf	1.44E+08	0	0	BTP202209	0	BTP202209	BTP202209	Tissue specific
PVGGP	Superscaf	+	Superscaf	1.44E+08	1.44E+08	159	159	163	-	-	-	-	-	-	NCP	IGR	-	ID=gene-E	5	4.908	1269.4	0.842245259	PVGGP	64	426.2333	1	425.2274	.	A	Superscaf	1.44E+08	0	0	0	BTP202209	0	0	Tissue specific
TAPWFN	Superscaf	+	Superscaf	1.45E+08	1.45E+08	40	40	45	-	-	-	-	-	-	NCP	IGR	-	ID=gene-E	6	5.938333333	2556.9	0.844282796	TAPWFN	75	368.1735	2	734.3387	.	R	Superscaf	1.45E+08	0	BTP202209	BTP202209	BTP202209	BTP202209	BTP202209	Tissue specific
RSLLVK	Superscaf	-	Superscaf	1.45E+08	1.45E+08	-8	8	13	-	-	-	-	-	-	NCP	IGR	-	ID=gene-E	6	6.356666667	2207.4	0.844332448	R(+42.01)	59	379.2486	2	756.4857	Acetylati	N	Superscaf	1.45E+08	0	0	BTP202209	BTP202209	0	0	Tissue specific
KNPKYK	Superscaf	+	Superscaf	1.45E+08	1.45E+08	3	3	8	-	-	gene	-	-	mRNA	NCP	Intron	gene|mRN	ID=gene-E	6	7.765	2798.8	0.844806515	KNPKYK	93	389.2278	2	776.4544	.	G	Superscaf	1.45E+08	0	0	BTP202209	0	0	0	Tissue specific
LSPPVLTR	Superscaf	+	Superscaf	1.45E+08	1.45E+08	15	15	22	-	-	-	-	-	-	NCP	IGR	-	ID=gene-E	8	5.94125	2811.3	0.845222514	LSPPVLTR	61	441.7703	2	881.5334	.	A	Superscaf	1.45E+08	0	0	BTP202209	0	0	0	Tissue specific
PASLPTPP	Superscaf	+	Superscaf	1.45E+08	1.45E+08	21	21	28	-	-	-	-	-	-	NCP	IGR	-	ID=gene-E	8	6.17625	2795.2	0.845380001	P(+42.01)	52	411.2193	2	820.433	Acetylati	P	Superscaf	1.45E+08	0	0	BTP202209	0	BTP202209	0	Tissue specific
LNNPVAQ	Superscaf	-	Superscaf	1.45E+08	1.45E+08	-4	4	10	-	-	gene	-	-	mRNA	NCP	Intron	gene|mRN	ID=gene-E	7	4.967142857	2806.2	0.845908961	LNNPVAQ	80	755.402	1	754.3973	.	R	Superscaf	1.45E+08	0	0	BTP202209	0	0	0	Tissue specific
RFAVVK	Superscaf	-	Superscaf	1.45E+08	1.45E+08	-13	13	18	-	-	gene	-	-	mRNA	NCP	Intron	gene|mRN	ID=gene-E	6	5.333333333	1815.9	0.847096023	R(+42.01)	52	381.2416	2	760.4595	Acetylati	T	Superscaf	1.45E+08	0	0	0	BTP202209	0	0	Tissue specific
AQKLLR	Superscaf	+	Superscaf	1.45E+08	1.45E+08	2	2	7	-	-	-	-	-	-	NCP	IGR	-	ID=gene-E	6	7.355	2746	0.84833771	AQKLLR	94	364.7357	2	727.4704	.	A	Superscaf	1.45E+08	BTP202209	0	0	0	0	0	Tissue specific
VGVAPGP	Superscaf	+	Superscaf	1.45E+08	1.45E+08	27	27	33	-	-	gene	-	-	mRNA	NCP	Intron	gene|mRN	ID=gene-E	7	4.365714286	1580.7	0.851075923	VGVAPGP	75	596.3397	1	595.3329	.	G	Superscaf	1.45E+08	0	0	0	BTP202209	0	0	Tissue specific
WGPPTSRP	Superscaf	+	Superscaf	1.45E+08	1.45E+08	51	51	58	-	-	gene	-	-	mRNA	NCP	Intron	gene|mRN	ID=gene-E	8	6.71625	3265.8	0.851346524	W(+42.01)	65	470.2381	2	938.461	Acetylati	C	Superscaf	1.45E+08	0	0	BTP202209	0	0	0	Tissue specific
KPVHDAP	Superscaf	-	Superscaf	1.45E+08	1.45E+08	-17	17	23	-	-	gene	-	-	mRNA	NCP	Intron	gene|mRN	ID=gene-E	7	5.56	2485.7	0.851438063	KPVHDAP	57	382.2075	2	762.4024	.	H	Superscaf	1.45E+08	0	0	0	BTP202209	BTP202209	0	Tissue specific
LVVGL	Superscaf	+	Superscaf	1.45E+08	1.45E+08	10	10	14	-	-	gene	-	-	mRNA	NCP	Intron	gene|mRN	ID=gene-E	5	3.586	1004.1	0.852220524	LVVGL	90	500.3423	1	499.337	.	L	Superscaf	1.45E+08	0	0	BTP202209	0	0	0	Tissue specific
VFAALPPP	Superscaf	+	Superscaf	1.45E+08	1.45E+08	43	43	50	-	-	-	-	-	-	NCP	IGR	-	ID=gene-E	8	5.3	2375.6	0.853435931	VFAALPPP	54	406.247	2	810.4639	.	N	Superscaf	1.45E+08	0	0	BTP202209	0	0	0	Tissue specific
YQEKVGK	Superscaf	-	Superscaf	1.45E+08	1.45E+08	-31	31	37	-	-	-	-	-	-	NCP	IGR	-	ID=gene-E	7	5.711428571	2729.8	0.853466571	YQEKVGK	66	426.2389	2	850.4548	.	K	Superscaf	1.45E+08	BTP202209	0	0	0	0	0	Tissue specific
DWFMAGK	Superscaf	+	Superscaf	1.45E+08	1.45E+08	24	24	30	-	-	gene	-	-	mRNA	NCP	Intron	gene|mRN	ID=gene-E	7	5.974285714	2774	0.853913709	DWFM(+15.	57	435.6862	2	869.3741	Oxidation	G	Superscaf	1.45E+08	0	0	0	0	0	BTP202209	Tissue specific
ATTSTTLP	Superscaf	+	Superscaf	1.46E+08	1.46E+08	51	51	58	-	-	gene	-	-	mRNA	NCP	Intron	gene|mRN	ID=gene-E	8	6.2625	2975.5	0.856220622	ATTSTTLP	56	791.4099	1	790.4072	.	H	Superscaf	1.46E+08	0	0	0	0	0	BTP202209	Tissue specific
KVFKPK	Superscaf	-	Superscaf	1.46E+08	1.46E+08	-11	11	16	-	-	gene	-	-	mRNA	NCP	Intron	gene|mRN	ID=gene-E	6	6.833333333	2130	0.858393956	KVFKPK	64	373.7539	2	745.485	.	I	Superscaf	1.46E+08	0	BTP202209	0	0	0	BTP202209	Tissue specific
LTVEL	Superscaf	-	Superscaf	1.46E+08	1.46E+08	-1	1	5	-	-	gene	-	-	mRNA	NCP	Intron	gene|mRN	ID=gene-E	5	4.342	1529.8	0.859368752	LTVEL	61	574.3418	1	573.3373	.	L	Superscaf	1.46E+08	0	0	0	0	BTP202209	0	Tissue specific
NVEAPSL	Superscaf	+	Superscaf	1.46E+08	1.46E+08	15	15	21	-	-	gene	-	-	mRNA	NCP	Intron	gene|mRN	ID=gene-E	7	4.658571429	2407.7	0.859517326	N(+42.01)	66	771.386	1	770.381	Acetylati	H	Superscaf	1.46E+08	0	0	0	0	BTP202209	0	Tissue specific
CDRDLR	Superscaf	+	Superscaf	1.46E+08	1.46E+08	21	21	26	-	-	gene	-	-	mRNA	NCP	Intron	gene|mRN	ID=gene-E	6	6.41	3059.7	0.86021479	C(+57.02)	61	417.6907	2	833.3813	Carbamido	R	Superscaf	1.46E+08	0	0	0	BTP202209	0	0	Tissue specific
SLDVLTHF	Superscaf	-	Superscaf	1.46E+08	1.46E+08	-10	10	17	-	-	-	-	-	-	NCP	IGR	-	ID=gene-E	8	5.02625	2906.3	0.861254961	SLDVLTHF	72	931.4841	1	930.4811	.	V	Superscaf	1.46E+08	0	0	0	0	BTP202209	0	Tissue specific
KGVMFHL	Superscaf	+	Superscaf	1.46E+08	1.46E+08	35	35	41	-	-	gene	-	-	mRNA	NCP	Intron	gene|mRN	ID=gene-E	7	5.787142857	2497.7	0.862580268	KGVMFHL	58	416.2289	2	830.4473	.	T	Superscaf	1.46E+08	BTP202209	0	0	0	0	0	Tissue specific
LPPPP	Superscaf	+	Superscaf	1.46E+08	1.46E+08	6	6	10	-	-	-	-	-	-	NCP	IGR	-	ID=gene-E	5	6.236	1703	0.864454503	LPPPP	99	520.3118	1	519.3057	.	T	Superscaf	1.46E+08	0	0	0	0	0	BTP202209	Mixed
LTVALK	Superscaf	-	Superscaf	1.46E+08	1.46E+08	-48	48	53	-	-	-	-	-	-	NCP	IGR	-	ID=gene-E	6	5.708333333	1884	0.8655068	LTVALK	70	644.4317	1	643.4268	.	F	Superscaf	1.46E+08	0	0	0	0	BTP202209	0	Tissue specific
APPLF	Superscaf	-	Superscaf	1.46E+08	1.46E+08	-53	53	57	-	-	-	-	-	-	NCP	IGR	-	ID=gene-E	5	6.016	1726.9	0.866289185	APPLF	98	544.3124	1	543.3057	.	Q	Superscaf	1.46E+08	0	0	0	BTP202209	0	0	Tissue specific
PYPGP	Superscaf	+	Superscaf	1.46E+08	1.46E+08	39	39	43	-	-	gene	-	-	mRNA	NCP	Intron	gene|mRN	ID=gene-E	5	6.106	1769	0.867643985	PYPGP	90	530.2592	1	529.2536	.	P	Superscaf	1.46E+08	0	0	BTP202209	0	0	0	Tissue specific
SFVELK	Superscaf	-	Superscaf	1.46E+08	1.46E+08	-3	3	8	-	-	gene	-	-	mRNA	NCP	Intron	gene|mRN	ID=gene-E	6	5.016666667	2018.1	0.867785559	SFVELK	94	361.7062	2	721.401	.	E	Superscaf	1.46E+08	0	0	0	0	BTP202209	0	Tissue specific
LTVDPGF	Superscaf	+	Superscaf	1.47E+08	1.47E+08	14	14	20	-	-	gene	-	-	mRNA	NCP	Intron	gene|mRN	ID=gene-E	7	4.747142857	2184.5	0.868465694	LTVDPGF	54	748.3846	1	747.3803	.	M	Superscaf	1.47E+08	0	0	0	0	0	BTP202209	Tissue specific
KTNRH	Superscaf	-	Superscaf	1.47E+08	1.47E+08	-5	5	9	-	-	gene	-	-	mRNA	NCP	Intron	gene|mRN	ID=gene-E	5	8.006	2731.1	0.869493892	KTNRH	52	655.3754	1	654.3561	.	K	Superscaf	1.47E+08	0	0	0	BTP202209	0	0	Tissue specific
QSFKEAF	Superscaf	+	Superscaf	1.47E+08	1.47E+08	82	82	88	-	-	gene	-	-	mRNA	NCP	Intron	gene|mRN	ID=gene-E	7	5.895714286	2974.1	0.869878868	Q(+42.01)	75	449.7181	2	897.4232	Acetylati	Q	Superscaf	1.47E+08	0	BTP202209	0	0	0	0	Tissue specific
LLGQL	Superscaf	-	Superscaf	1.47E+08	1.47E+08	-27	27	31	-	-	gene	-	-	mRNA	NCP	Intron	gene|mRN	ID=gene-E	5	5.912	1968.2	0.87015119	LLGQL	55	543.3492	1	542.3428	.	N	Superscaf	1.47E+08	0	0	0	0	BTP202209	0	Tissue specific
TDSPK	Superscaf	+	Superscaf	1.47E+08	1.47E+08	11	11	15	-	-	-	-	-	-	NCP	IGR	-	ID=gene-E	5	6.244	1998.2	0.87182303	TDSPK	54	547.2678	1	546.265	.	K	Superscaf	1.47E+08	0	0	0	0	0	BTP202209	Tissue specific
RNLGTGM	Superscaf	+	Superscaf	1.47E+08	1.47E+08	28	28	34	-	-	-	-	-	-	NCP	IGR	-	ID=gene-E	7	6.624285714	2962.5	0.871975773	RNLGTGM	72	374.6913	2	747.3698	.	V	Superscaf	1.47E+08	BTP202209	0	0	0	0	0	Tissue specific
RVMQKL	Superscaf	-	Superscaf	1.47E+08	1.47E+08	-37	37	42	-	-	-	-	-	-	NCP	IGR	-	ID=gene-E	6	6.313333333	2452.8	0.873452141	RVMQKL	65	387.7338	2	773.4582	.	W	Superscaf	1.47E+08	0	BTP202209	0	0	0	0	Tissue specific
VVLELKK	Superscaf	+	Superscaf	1.47E+08	1.47E+08	151	151	157	-	-	-	-	-	-	NCP	IGR	-	ID=gene-E	7	4.951428571	2069.1	0.874323617	VVLELKK	92	414.778	2	827.548	.	V	Superscaf	1.47E+08	0	BTP202209	0	0	0	0	Tissue specific
LAFLLHK	Superscaf	-	Superscaf	1.47E+08	1.47E+08	-2	2	8	-	-	-	-	-	-	NCP	IGR	-	ID=gene-E	7	6.681428571	2847	0.875410265	LAFLLHK	50	421.2707	2	840.5222	.	I	Superscaf	1.47E+08	0	0	0	0	0	BTP202209	Tissue specific
EVHNGG	Superscaf	-	Superscaf	1.47E+08	1.47E+08	-6	6	11	-	-	-	-	-	-	NCP	IGR	-	ID=gene-E	6	4.693333333	2236.5	0.875599004	EVHNGG	53	612.2781	1	611.2663	.	T	Superscaf	1.47E+08	0	0	0	0	BTP202209	0	Tissue specific
VLNVHS	Superscaf	+	Superscaf	1.47E+08	1.47E+08	56	56	61	-	-	gene	-	-	mRNA	NCP	Intron	gene|mRN	ID=gene-E	6	4.11	1953.2	0.876539985	VLNVHS	64	668.3767	1	667.3653	.	D	Superscaf	1.47E+08	0	0	0	0	0	BTP202209	Tissue specific
MTFLTFM	Superscaf	-	Superscaf	1.47E+08	1.47E+08	-35	35	41	-	-	gene	-	-	mRNA	NCP	Intron	gene|mRN	ID=gene-E	7	5.928571429	2666.2	0.87701126	MTFLTFM	65	890.4138	1	889.4078	.	E	Superscaf	1.47E+08	0	0	0	0	0	BTP202209	Mixed
RATVTRPV	Superscaf	-	Superscaf	1.47E+08	1.47E+08	-31	31	38	-	-	gene	-	-	mRNA	NCP	Intron	gene|mRN	ID=gene-E	8	5.8625	2741.3	0.877131489	RATVTRPV	70	450.2684	2	898.5349	.	R	Superscaf	1.47E+08	0	0	0	0	0	BTP202209	Tissue specific
KDGAKTK	Superscaf	+	Superscaf	1.47E+08	1.47E+08	20	20	26	-	-	gene	-	-	mRNA	NCP	Intron	gene|mRN	ID=gene-E	7	7.244285714	2822.8	0.877259674	KDGAKTK	58	374.2244	2	746.4286	.	V	Superscaf	1.47E+08	0	0	0	BTP202209	0	0	Tissue specific
EAFTRLQ	Superscaf	-	Superscaf	1.47E+08	1.47E+08	-20	20	26	-	-	gene	-	-	mRNA	NCP	Intron	gene|mRN	ID=gene-E	7	6.234285714	3134.6	0.877301829	E(+42.01)	79	906.4648	1	905.4606	Acetylati	V	Superscaf	1.47E+08	0	0	0	0	BTP202209	0	Tissue specific
ELYKVP	Superscaf	-	Superscaf	1.47E+08	1.47E+08	-62	62	67	-	-	-	-	-	-	NCP	IGR	-	ID=gene-E	6	5.15	2044.2	0.877729189	ELYKVP	55	374.7145	2	747.4167	.	V	Superscaf	1.47E+08	0	0	0	BTP202209	0	0	Tissue specific
VPKGPAAF	Superscaf	+	Superscaf	1.47E+08	1.47E+08	13	13	20	-	-	-	-	-	-	NCP	IGR	-	ID=gene-E	8	5.72875	2450.5	0.877772989	VPKGPAAF	63	393.7269	2	785.4435	.	R	Superscaf	1.47E+08	0	0	BTP202209	0	0	0	Tissue specific
TATFFL	Superscaf	-	Superscaf	1.47E+08	1.47E+08	-1	1	6	-	-	-	-	-	-	NCP	IGR	-	ID=gene-E	6	6.003333333	2234.5	0.878367093	T(+42.01)	58	741.3907	1	740.3745	Acetylati	T	Superscaf	1.47E+08	BTP202209	0	0	0	0	0	Tissue specific
LGEKWDR	Superscaf	-	Superscaf	1.47E+08	1.47E+08	-30	30	36	-	-	-	-	-	-	NCP	IGR	-	ID=gene-E	7	6.361428571	3131.5	0.878452703	LGEKWDR	99	452.2362	2	902.461	.	N	Superscaf	1.47E+08	0	0	0	0	0	BTP202209	Tissue specific
LEASYAY	Superscaf	-	Superscaf	1.47E+08	1.47E+08	-11	11	17	-	-	-	-	-	-	NCP	IGR	-	ID=gene-E	7	5.462857143	2703.9	0.878829187	LEASYAY	58	816.3779	1	815.3701	.	S	Superscaf	1.47E+08	0	0	BTP202209	0	0	0	Tissue specific
RYPHK	Superscaf	-	Superscaf	1.47E+08	1.47E+08	-25	25	29	-	-	gene	-	-	mRNA	NCP	Intron	gene|mRN	ID=gene-E	5	8.01	2533.8	0.880728172	RYPHK	95	350.6914	2	699.3816	.	T	Superscaf	1.47E+08	BTP202209	0	0	0	0	0	Tissue specific
NVDVLP	Superscaf	-	Superscaf	1.48E+08	1.48E+08	-24	24	29	-	-	gene	-	-	mRNA	NCP	Intron	gene|mRN	ID=gene-E	6	3.443333333	1699	0.882129641	NVDVLP	85	656.3587	1	655.3541	.	M	Superscaf	1.48E+08	0	BTP202209	0	0	0	0	Tissue specific
TMGPKVR	Superscaf	+	Superscaf	1.48E+08	1.48E+08	42	42	48	-	-	gene	-	-	mRNA	NCP	Intron	gene|mRN	ID=gene-E	7	6.435714286	2520.9	0.882849291	TM(+15.99	53	402.7193	2	803.4324	Oxidation	D	Superscaf	1.48E+08	0	0	0	0	0	BTP202209	Tissue specific
LSAPGGLLS	Superscaf	+	Superscaf	1.48E+08	1.48E+08	38	38	46	-	-	-	-	-	-	NCP	IGR	-	ID=gene-E	9	5.951111111	3070.3	0.883024948	LSAPGGLLS	61	814.4652	1	813.4596	.	G	Superscaf	1.48E+08	0	0	0	0	BTP202209	0	Tissue specific
VAMNLPR	Superscaf	+	Superscaf	1.48E+08	1.48E+08	8	8	14	-	-	-	-	-	-	NCP	IGR	-	ID=gene-E	7	5.745714286	2619.1	0.884428329	V(+42.01)	62	421.7363	2	841.448	Acetylati	N	Superscaf	1.48E+08	0	0	0	0	0	BTP202209	Tissue specific
KPPVPPP	Superscaf	+	Superscaf	1.48E+08	1.48E+08	22	22	28	-	-	gene	-	-	mRNA	NCP	Intron	gene|mRN	ID=gene-E	7	5.891428571	2155.4	0.885311013	KPPVPPP	57	366.2282	2	730.4377	.	K	Superscaf	1.48E+08	0	0	0	0	0	BTP202209	Tissue specific
KAGHEYK	Superscaf	-	Superscaf	1.48E+08	1.48E+08	-55	55	61	-	-	-	-	-	-	NCP	IGR	-	ID=gene-E	7	6.848571429	3050.1	0.886099174	KAGHEYK	60	416.7227	2	831.4239	.	K	Superscaf	1.48E+08	0	0	0	0	0	BTP202209	Tissue specific
VLGLQ	Superscaf	+	Superscaf	1.48E+08	1.48E+08	4	4	8	-	-	-	-	-	-	NCP	IGR	-	ID=gene-E	5	4.716	1614.8	0.886314346	VLGLQ	98	529.3329	1	528.3271	.	H	Superscaf	1.48E+08	0	0	0	BTP202209	0	0	Mixed
SLLRNSDD	Superscaf	+	Superscaf	1.48E+08	1.48E+08	6	6	13	-	-	-	-	-	-	NCP	IGR	-	ID=gene-E	8	5.67875	3542.1	0.887015482	SLLRNSDD	93	460.2249	2	918.4407	.	S	Superscaf	1.48E+08	0	0	0	0	0	BTP202209	Tissue specific
YNPLSR	Superscaf	+	Superscaf	1.48E+08	1.48E+08	6	6	11	-	-	gene	-	-	mRNA	NCP	Intron	gene|mRN	ID=gene-E	6	6.631666667	2779.2	0.887155449	YNPLSR	56	375.2001	2	748.3868	.	K	Superscaf	1.48E+08	0	0	0	BTP202209	0	0	Tissue specific
KAFASSPK	Superscaf	+	Superscaf	1.48E+08	1.48E+08	33	33	40	-	-	gene	-	-	mRNA	NCP	Intron	gene|mRN	ID=gene-E	8	6.8325	3051	0.887702924	KAFASSPK	58	418.2302	2	834.4599	.	S	Superscaf	1.48E+08	0	BTP202209	0	BTP202209	0	0	Tissue specific
TCRLFDL	Superscaf	-	Superscaf	1.48E+08	1.48E+08	-7	7	13	-	-	-	-	-	-	NCP	IGR	-	ID=gene-E	7	6.102857143	3137.7	0.88803557	TC(+57.02	76	462.7326	2	923.4535	Carbamido	N	Superscaf	1.48E+08	0	0	BTP202209	0	0	0	Tissue specific
GPPGPPVT	Superscaf	-	Superscaf	1.48E+08	1.48E+08	-62	62	69	-	-	-	-	-	-	NCP	IGR	-	ID=gene-E	8	5.45875	2341.7	0.888104119	GPPGPPVT	50	361.1965	2	720.3806	.	H	Superscaf	1.48E+08	0	0	0	0	0	BTP202209	Tissue specific
GSHKER	Superscaf	+	Superscaf	1.48E+08	1.48E+08	15	15	20	-	-	-	-	-	-	NCP	IGR	-	ID=gene-E	6	7.16	2843.1	0.888920702	G(+42.01)	82	378.1864	2	754.3722	Acetylati	C	Superscaf	1.48E+08	0	0	0	0	BTP202209	0	Tissue specific
KGWPGAK	Superscaf	+	Superscaf	1.48E+08	1.48E+08	23	23	29	-	-	-	-	-	-	NCP	IGR	-	ID=gene-E	7	7.09	2706.7	0.888938681	KGWPGAK	86	372.212	2	742.4126	.	Y	Superscaf	1.48E+08	0	0	0	0	BTP202209	0	Tissue specific
FFGNPYF	Superscaf	-	Superscaf	1.48E+08	1.48E+08	-15	15	21	-	-	gene	-	-	mRNA	NCP	Intron	gene|mRN	ID=gene-E	7	5.682857143	2853.1	0.889789692	FFGNPYF	62	446.2071	2	890.3962	.	I	Superscaf	1.48E+08	0	0	0	0	0	BTP202209	Tissue specific
EEGRPPYP	Superscaf	+	Superscaf	1.48E+08	1.48E+08	97	97	104	-	-	gene	-	-	mRNA	NCP	Intron	gene|mRN	ID=gene-E	8	5.96625	3268.8	0.88984776	EEGRPPYP	59	472.7318	2	943.4399	.	N	Superscaf	1.48E+08	0	0	0	0	0	BTP202209	Tissue specific
KMQLAK	Superscaf	-	Superscaf	1.48E+08	1.48E+08	-4	4	9	-	-	gene	-	-	mRNA	NCP	Intron	gene|mRN	ID=gene-E	6	7.146666667	2583.7	0.890260622	KMQLAK	98	359.7157	2	717.4207	.	T	Superscaf	1.48E+08	0	0	0	BTP202209	0	0	Tissue specific
TVLLF	Superscaf	+	Superscaf	1.48E+08	1.48E+08	21	21	25	-	-	-	-	-	-	NCP	IGR	-	ID=gene-E	5	4.794	1491.7	0.89137772	TVLLF	64	592.3677	1	591.3632	.	R	Superscaf	1.48E+08	0	0	0	0	BTP202209	BTP202209	Mixed
FFSLVHKR	Superscaf	+	Superscaf	1.48E+08	1.48E+08	17	17	24	-	-	gene	-	-	mRNA	NCP	Intron	gene|mRN	ID=gene-E	8	6.33875	3248.5	0.891801714	F(+42.01)	69	538.3033	2	1074.597	Acetylati	N	Superscaf	1.48E+08	0	0	0	0	BTP202209	0	Tissue specific
FVNVEF	Superscaf	-	Superscaf	1.48E+08	1.48E+08	-11	11	16	-	-	gene	-	-	mRNA	NCP	Intron	gene|mRN	ID=gene-E	6	3.265	1797	0.89205598	FVNVEF	68	754.3777	1	753.3697	.	R	Superscaf	1.48E+08	0	0	0	BTP202209	0	0	Tissue specific
KCKAAQE	Superscaf	+	Superscaf	1.49E+08	1.49E+08	23	23	29	-	-	gene	-	-	mRNA	NCP	Intron	gene|mRN	ID=gene-E	7	6.487142857	3181.3	0.893611264	KC(+57.02	72	417.7101	2	833.4065	Carbamido	N	Superscaf	1.49E+08	0	BTP202209	0	0	0	0	Mixed
LDEVLK	Superscaf	-	Superscaf	1.49E+08	1.49E+08	-49	49	54	-	-	gene	-	-	mRNA	NCP	Intron	gene|mRN	ID=gene-E	6	4.648333333	2012.2	0.894031088	LDEVLK	99	358.712	2	715.4116	.	I	Superscaf	1.49E+08	0	BTP202209	0	0	BTP202209	BTP202209	Mixed
WNDDMEK	Superscaf	+	Superscaf	1.49E+08	1.49E+08	27	27	33	-	-	gene	-	-	mRNA	NCP	Intron	gene|mRN	ID=gene-E	7	5.135714286	3211.7	0.894688654	WNDDMEK	78	469.1876	2	936.3647	.	F	Superscaf	1.49E+08	0	0	0	0	0	BTP202209	Tissue specific
FLGPFF	Superscaf	+	Superscaf	1.49E+08	1.49E+08	19	19	24	-	-	gene	-	-	mRNA	NCP	Intron	gene|mRN	ID=gene-E	6	5.781666667	2150.3	0.895372691	FLGPFF	62	364.1963	2	726.3741	.	L	Superscaf	1.49E+08	BTP202209	0	0	0	0	0	Mixed
LGKELLD	Superscaf	-	Superscaf	1.49E+08	1.49E+08	-33	33	39	-	-	gene	-	-	mRNA	NCP	Intron	gene|mRN	ID=gene-E	7	5.691428571	2662.9	0.895378735	LGKELLD	88	394.2303	2	786.4487	.	L	Superscaf	1.49E+08	BTP202209	0	0	0	0	0	Tissue specific
KAAPYGK	Superscaf	-	Superscaf	1.49E+08	1.49E+08	-20	20	26	-	-	-	-	-	-	NCP	IGR	-	ID=gene-E	7	7.064285714	2653.6	0.896961943	KAAPYGK	52	367.717	2	733.4122	.	C	Superscaf	1.49E+08	0	0	0	0	0	BTP202209	Tissue specific
LSSALDL	Superscaf	+	Superscaf	1.49E+08	1.49E+08	2	2	8	-	-	-	-	-	-	NCP	IGR	-	ID=gene-E	7	5.47	2549.8	0.897826266	LSSALDL	57	359.6965	2	717.3909	.	F	Superscaf	1.49E+08	0	0	0	0	BTP202209	0	Tissue specific
KFKGRP	Superscaf	-	Superscaf	1.49E+08	1.49E+08	-4	4	9	-	-	-	-	-	-	NCP	IGR	-	ID=gene-E	6	7.998333333	2607.7	0.898421135	K(+42.01)	58	387.7339	2	773.4548	Acetylati	N	Superscaf	1.49E+08	0	0	0	BTP202209	0	0	Tissue specific
FPGVPVGR	Superscaf	+	Superscaf	1.49E+08	1.49E+08	17	17	24	-	-	-	-	-	-	NCP	IGR	-	ID=gene-E	8	5.0975	2305.6	0.899661253	FPGVPVGR	71	414.7371	2	827.4653	.	P	Superscaf	1.49E+08	0	0	BTP202209	0	0	0	Tissue specific
LYKKKKKKF	Superscaf	-	Superscaf	1.49E+08	1.49E+08	-14	14	22	-	-	-	-	-	-	NCP	IGR	-	ID=gene-E	9	8.395555556	4010.7	0.90025532	LYKKKKKKF	52	404.2779	3	1209.796	.	Q	Superscaf	1.49E+08	0	0	0	0	0	BTP202209	Tissue specific
VYKNVR	Superscaf	-	Superscaf	1.49E+08	1.49E+08	-41	41	46	-	-	-	-	-	-	NCP	IGR	-	ID=gene-E	6	5.261666667	2173.4	0.901011655	VYKNVR	69	389.7306	2	777.4497	.	F	Superscaf	1.49E+08	0	0	0	0	0	BTP202209	Tissue specific
TVPLPNSF	Superscaf	+	Superscaf	1.49E+08	1.49E+08	21	21	28	-	-	gene	-	-	mRNA	NCP	Intron	gene|mRN	ID=gene-E	8	5.21	2793.2	0.901454278	TVPLPNSF	77	437.7351	2	873.4596	.	E	Superscaf	1.49E+08	0	0	0	0	0	BTP202209	Tissue specific
TSRTLY	Superscaf	+	Superscaf	1.49E+08	1.49E+08	4	4	9	-	-	-	-	-	-	NCP	IGR	-	ID=gene-E	6	6.856666667	2640.1	0.901719447	TSRTLY	79	370.6987	2	739.3864	.	L	Superscaf	1.49E+08	0	0	0	0	0	BTP202209	Tissue specific
MEKGDQK	Superscaf	-	Superscaf	1.49E+08	1.49E+08	-12	12	18	-	-	-	-	-	-	NCP	IGR	-	ID=gene-E	7	6.148571429	3053.3	0.902585988	M(+15.99)	50	447.2054	2	892.396	Oxidation	K	Superscaf	1.49E+08	0	BTP202209	0	0	0	0	Tissue specific
CVTKKL	Superscaf	-	Superscaf	1.49E+08	1.49E+08	-13	13	18	-	-	-	-	-	-	NCP	IGR	-	ID=gene-E	6	6.168333333	2273.4	0.903188776	C(+57.02)	62	374.7253	2	747.4313	Carbamido	L	Superscaf	1.49E+08	0	0	BTP202209	0	0	0	Tissue specific
FKKVL	Superscaf	-	Superscaf	1.49E+08	1.49E+08	-4	4	8	-	-	-	-	-	-	NCP	IGR	-	ID=gene-E	5	6.188	1677.6	0.904059372	FKKVL	52	634.4353	1	633.4213	.	E	Superscaf	1.49E+08	0	0	0	0	0	BTP202209	Tissue specific
RLNPVEF	Superscaf	-	Superscaf	1.49E+08	1.49E+08	-17	17	23	-	-	-	-	-	-	NCP	IGR	-	ID=gene-E	7	5.307142857	2749.2	0.904425528	RLNPVEF	70	437.7409	2	873.4708	.	E	Superscaf	1.49E+08	0	0	0	0	BTP202209	0	Tissue specific
APKNAHTK	Superscaf	-	Superscaf	1.49E+08	1.49E+08	-32	32	39	-	-	-	-	-	-	NCP	IGR	-	ID=gene-E	8	7.16875	3510.7	0.904660974	APKNAHTK	54	433.7458	2	865.477	.	D	Superscaf	1.49E+08	0	0	BTP202209	0	0	0	Tissue specific
KDLFT	Superscaf	-	Superscaf	1.49E+08	1.49E+08	-2	2	6	-	-	-	-	-	-	NCP	IGR	-	ID=gene-E	5	6.14	2018.2	0.904937312	K(+42.01)	65	665.3475	1	664.3432	Acetylati	I	Superscaf	1.49E+08	0	0	0	BTP202209	0	0	Tissue specific
VGKLMGK	Superscaf	-	Superscaf	1.49E+08	1.49E+08	-6	6	12	-	-	-	-	-	-	NCP	IGR	-	ID=gene-E	7	6.164285714	2256.3	0.9055077	VGKLMGK	85	366.7234	2	731.4363	.	G	Superscaf	1.49E+08	0	0	BTP202209	0	0	0	Tissue specific
LTVVQ	Superscaf	+	Superscaf	1.5E+08	1.5E+08	15	15	19	-	-	-	-	-	-	NCP	IGR	-	ID=gene-E	5	3.632	1361.6	0.906848232	LTVVQ	55	559.3552	1	558.3377	.	A	Superscaf	1.5E+08	0	0	0	0	0	BTP202209	Tissue specific
APRSPKAP	Superscaf	+	Superscaf	1.5E+08	1.5E+08	13	13	20	-	-	gene	-	-	mRNA	NCP	Intron	gene|mRN	ID=gene-E	8	7.14	3135.4	0.907144691	APRSPKAP	59	412.2417	2	822.4711	.	N	Superscaf	1.5E+08	0	0	0	0	0	BTP202209	Tissue specific
KQAAVLP	Superscaf	+	Superscaf	1.5E+08	1.5E+08	6	6	12	-	-	-	-	-	-	NCP	IGR	-	ID=gene-E	7	5.672857143	2392.5	0.908007598	KQAAVLP	64	726.4481	1	725.4435	.	S	Superscaf	1.5E+08	0	0	0	BTP202209	0	0	Tissue specific
VTLAF	Superscaf	+	Superscaf	1.5E+08	1.5E+08	142	142	146	-	-	-	-	-	-	NCP	IGR	-	ID=gene-E	5	4.802	1449.6	0.90836641	VTLAF	63	550.3335	1	549.3162	.	P	Superscaf	1.5E+08	0	0	0	0	BTP202209	BTP202209	Mixed
AESHSVK	Superscaf	+	Superscaf	1.5E+08	1.5E+08	17	17	23	-	-	gene	-	-	mRNA	NCP	Intron	gene|mRN	ID=gene-E	7	5.418571429	2591.7	0.909018659	AESHSVK	54	379.2003	2	756.3766	.	R	Superscaf	1.5E+08	0	0	0	0	0	BTP202209	Tissue specific
RGPKFL	Superscaf	+	Superscaf	1.5E+08	1.5E+08	21	21	26	-	-	gene	-	-	mRNA	NCP	Intron	gene|mRN	ID=gene-E	6	7.371666667	2492.7	0.90903637	RGPKFL	63	359.2272	2	716.4333	.	E	Superscaf	1.5E+08	0	BTP202209	0	0	0	BTP202209	Tissue specific
APELRPT	Superscaf	-	Superscaf	1.5E+08	1.5E+08	-19	19	25	-	-	gene	-	-	mRNA	NCP	Intron	gene|mRN	ID=gene-E	7	6.444285714	2811.3	0.909214743	APELRPT	76	392.22	2	782.4286	.	F	Superscaf	1.5E+08	0	0	0	0	0	BTP202209	Tissue specific
AMLLNY	Superscaf	+	Superscaf	1.5E+08	1.5E+08	19	19	24	-	-	gene	-	-	mRNA	NCP	Intron	gene|mRN	ID=gene-E	6	5.8	2445.8	0.911234071	AM(+15.99	72	740.3619	1	739.3575	Oxidation	V	Superscaf	1.5E+08	0	0	0	0	0	BTP202209	Tissue specific
GYSDN	Superscaf	-	Superscaf	1.5E+08	1.5E+08	-13	13	17	-	-	gene	-	-	mRNA	NCP	Intron	gene|mRN	ID=gene-E	5	5.138	2148.4	0.912095486	GYSDN	61	555.2023	1	554.1973	.	E	Superscaf	1.5E+08	0	0	0	0	0	BTP202209	Tissue specific
LPEELL	Superscaf	-	Superscaf	1.5E+08	1.5E+08	-42	42	47	-	-	-	-	-	-	NCP	IGR	-	ID=gene-E	6	5.113333333	2248.6	0.913409203	LPEELL	71	713.4064	1	712.4007	.	L	Superscaf	1.5E+08	0	0	0	BTP202209	0	0	Tissue specific
RAPPERK	Superscaf	-	Superscaf	1.5E+08	1.5E+08	-13	13	19	-	-	-	-	-	-	NCP	IGR	-	ID=gene-E	7	7.585714286	3177.6	0.913635239	RAPPERK	98	427.2467	2	852.4929	.	L	Superscaf	1.5E+08	0	0	0	0	0	BTP202209	Mixed
KLALGAGK	Superscaf	+	Superscaf	1.5E+08	1.5E+08	15	15	22	-	-	-	-	-	-	NCP	IGR	-	ID=gene-E	8	6.9275	2860.8	0.914809983	K(+42.01)	77	400.2545	2	798.4963	Acetylati	A	Superscaf	1.5E+08	0	0	0	0	0	BTP202209	Tissue specific
LTAFWTK	Superscaf	+	Superscaf	1.5E+08	1.5E+08	4	4	10	-	-	gene	-	-	mRNA	NCP	Intron	gene|mRN	ID=gene-E	7	6.595714286	2842.1	0.915369354	LTAFWTK	55	433.738	2	865.4698	.	F	Superscaf	1.5E+08	0	0	0	0	0	BTP202209	Tissue specific
FLVLVVLH	Superscaf	+	Superscaf	1.5E+08	1.5E+08	12	12	19	-	-	gene	-	-	mRNA	NCP	Intron	gene|mRN	ID=gene-E	8	3.87625	2067.3	0.91650619	FLVLVVLH	56	470.3128	2	938.5953	.	L	Superscaf	1.5E+08	0	0	0	0	0	BTP202209	Tissue specific
TVYGLPR	Superscaf	-	Superscaf	1.5E+08	1.5E+08	-9	9	15	-	-	gene	-	-	mRNA	NCP	Intron	gene|mRN	ID=gene-E	7	5.885714286	2493.9	0.918632626	TVYGLPR	73	403.233	2	804.4493	.	S	Superscaf	1.5E+08	0	0	BTP202209	0	0	0	Tissue specific
VAKKPK	Superscaf	-	Superscaf	1.51E+08	1.51E+08	-24	24	29	-	-	-	-	-	-	NCP	IGR	-	ID=gene-E	6	6.923333333	2053.9	0.919025138	V(+42.01)	63	356.7385	2	711.4643	Acetylati	Y	Superscaf	1.51E+08	0	BTP202209	0	0	0	0	Tissue specific
LFKGGKK	Superscaf	-	Superscaf	1.51E+08	1.51E+08	-72	72	78	-	-	-	-	-	-	NCP	IGR	-	ID=gene-E	7	7.517142857	2740.6	0.919115644	LFKGGKK	67	389.251	2	776.4908	.	V	Superscaf	1.51E+08	0	0	0	0	0	BTP202209	Tissue specific
AQMKLF	Superscaf	-	Superscaf	1.51E+08	1.51E+08	-10	10	15	-	-	gene	-	-	mRNA	NCP	Intron	gene|mRN	ID=gene-E	6	6.436666667	2502.7	0.919829557	A(+42.01)	69	795.4166	1	794.3997	Acetylati	H	Superscaf	1.51E+08	0	0	BTP202209	0	0	0	Tissue specific
LFSRSL	Superscaf	-	Superscaf	1.51E+08	1.51E+08	-101	101	106	-	-	gene	-	-	mRNA	NCP	Intron	gene|mRN	ID=gene-E	6	6.593333333	2509.8	0.920439613	LFSRSL	72	361.7122	2	721.4122	.	I	Superscaf	1.51E+08	0	0	0	0	0	BTP202209	Tissue specific
KFVGFGP	Superscaf	-	Superscaf	1.51E+08	1.51E+08	-3	3	9	-	-	gene	-	-	mRNA	NCP	Intron	gene|mRN	ID=gene-E	7	5.562857143	2175.2	0.921430552	KFVGFGP	60	376.2165	2	750.4064	.	Y	Superscaf	1.51E+08	0	0	BTP202209	0	0	0	Tissue specific
VEVLLL	Superscaf	-	Superscaf	1.51E+08	1.51E+08	-20	20	25	-	-	gene	-	-	mRNA	NCP	Intron	gene|mRN	ID=gene-E	6	3.526666667	1485.7	0.924130666	VEVLLL	81	685.4465	1	684.4421	.	E	Superscaf	1.51E+08	0	0	0	0	0	BTP202209	Tissue specific
VHAKSWT	Superscaf	-	Superscaf	1.51E+08	1.51E+08	-4	4	10	-	-	-	-	-	-	NCP	IGR	-	ID=gene-E	7	5.921428571	2706.9	0.92523426	VHAKSWT	90	414.7202	2	827.429	.	W	Superscaf	1.51E+08	0	0	0	0	0	BTP202209	Tissue specific
KGFMP	Superscaf	-	Superscaf	1.51E+08	1.51E+08	-34	34	38	-	-	-	-	-	-	NCP	IGR	-	ID=gene-E	5	6.648	1862	0.9270554	K(+42.01)	76	621.3049	1	620.2992	Acetylati	T	Superscaf	1.51E+08	0	0	0	0	0	BTP202209	Mixed
PLMHPLK	Superscaf	+	Superscaf	1.51E+08	1.51E+08	2	2	8	-	-	-	-	-	-	NCP	IGR	-	ID=gene-E	7	6.805714286	2841.2	0.927118326	PLMHPLK	57	418.2423	2	834.4786	.	D	Superscaf	1.51E+08	0	BTP202209	BTP202209	BTP202209	0	0	Tissue specific
LLTGPELK	Superscaf	-	Superscaf	1.51E+08	1.51E+08	-9	9	16	-	-	gene	-	-	mRNA	NCP	Intron	gene|mRN	ID=gene-E	8	6.2125	2986.3	0.928916897	LLTGPELK	99	435.7669	2	869.5222	.	I	Superscaf	1.51E+08	0	0	0	BTP202209	0	0	Tissue specific
KSATVYD	Superscaf	-	Superscaf	1.51E+08	1.51E+08	-159	159	165	-	-	gene	-	-	mRNA	NCP	Intron	gene|mRN	ID=gene-E	7	5.228571429	2431.6	0.930350192	KSATVYD	57	392.1921	2	782.381	.	V	Superscaf	1.51E+08	BTP202209	0	0	0	0	0	Tissue specific
TGLPALV	Superscaf	+	Superscaf	1.51E+08	1.51E+08	33	33	39	-	-	-	-	-	-	NCP	IGR	-	ID=gene-E	7	5.254285714	2050.3	0.931151627	TGLPALV	81	670.4108	1	669.4061	.	Q	Superscaf	1.51E+08	0	0	0	0	0	BTP202209	Tissue specific
PPPKAHTK	Superscaf	+	Superscaf	1.52E+08	1.52E+08	2	2	9	-	-	gene	-	-	mRNA	NCP	Intron	gene|mRN	ID=gene-E	8	7.315	3277.5	0.932397483	PPPKAHTK	78	438.2575	2	874.5024	.	P	Superscaf	1.52E+08	0	BTP202209	0	BTP202209	0	0	Tissue specific
EHFFEKH	Superscaf	+	Superscaf	1.52E+08	1.52E+08	21	21	27	-	-	-	-	-	-	NCP	IGR	-	ID=gene-E	7	6.045714286	3333.6	0.933167857	EHFFEKH	84	487.2357	2	972.4453	.	N	Superscaf	1.52E+08	0	0	0	BTP202209	0	0	Tissue specific

PACHNP	Superscaf	+	Superscaf	1.52E+08	1.52E+08	18	18	23	-	-	-	-	-	-	NCP	IGR	-	ID=gene-E	6	6.106666667	2788.2	0.933186639	PAC(+57.	57	695.3021	1	694.2857	Carbamidc	D	Superscaf	1.52E+08	0	0	BTP20220	0	0	0	Tissue specific
GLTAPGP	Superscaf	+	Superscaf	1.52E+08	1.52E+08	81	81	87	-	-	gene	-	-	mRNA	NCP	Intron	gene|mRN	ID=gene-E	7	6.152857143	2331.6	0.934316092	GLTAPGP	95	612.334	1	611.3278	.	S	Superscaf	1.52E+08	0	0	0	0	BTP20220	0	Tissue specific
EPKPPALGF	Superscaf	-	Superscaf	1.52E+08	1.52E+08	-4	4	12	-	-	-	-	-	-	NCP	IGR	-	ID=gene-E	9	6.236666667	3205.5	0.934815597	EPKPPALGF	60	453.2601	2	904.5018	.	P	Superscaf	1.52E+08	0	0	0	BTP20220	0	0	Tissue specific
KYSRHLLE	Superscaf	+	Superscaf	1.52E+08	1.52E+08	19	19	26	-	-	gene	-	-	mRNA	NCP	Intron	gene|mRN	ID=gene-E	8	6.82625	3712.1	0.935263232	KYSRHLLE	54	523.2883	2	1044.572	.	T	Superscaf	1.52E+08	0	0	0	0	0	BTP20220	Tissue specific
LDSRLE	Superscaf	-	Superscaf	1.52E+08	1.52E+08	-33	33	38	-	-	gene	-	-	mRNA	NCP	Intron	gene|mRN	ID=gene-E	6	5.765	2576	0.935264035	LDSRLE	80	366.6968	2	731.3813	.	L	Superscaf	1.52E+08	0	0	0	BTP20220	0	0	Tissue specific
KTMAHR	Superscaf	+	Superscaf	1.52E+08	1.52E+08	20	20	25	-	-	gene	-	-	mRNA	NCP	Intron	gene|mRN	ID=gene-E	6	7.731666667	2817.2	0.935289473	KTM(+15.	55	380.2066	2	758.3857	Oxidatior	P	Superscaf	1.52E+08	0	0	0	0	BTP20220	0	Tissue specific
YLLVEF	Superscaf	-	Superscaf	1.52E+08	1.52E+08	-17	17	22	-	-	gene	-	-	mRNA	NCP	Intron	gene|mRN	ID=gene-E	6	4.386666667	1979.2	0.935734315	YLLVEF	89	783.4233	1	782.4214	.	N	Superscaf	1.52E+08	0	0	0	BTP20220	0	0	Tissue specific
KALNP	Superscaf	+	Superscaf	1.52E+08	1.52E+08	20	20	24	-	-	gene	-	-	mRNA	NCP	Intron	gene|mRN	ID=gene-E	5	6.69	2067.2	0.936987784	KALNP	52	542.3289	1	541.3224	.	V	Superscaf	1.52E+08	0	0	0	BTP20220	0	0	Tissue specific
PPKPEPKP	Superscaf	-	Superscaf	1.52E+08	1.52E+08	-1	1	8	-	-	-	-	-	-	NCP	IGR	-	ID=gene-E	8	6.775	3049.3	0.939384246	PPKPEPKP	97	445.2593	2	888.5068	.	P	Superscaf	1.52E+08	0	BTP20220	BTP20220	0	0	BTP20220	Tissue enhanced
NGAVLKK	Superscaf	-	Superscaf	1.52E+08	1.52E+08	-14	14	20	-	-	-	-	-	-	NCP	IGR	-	ID=gene-E	7	6.122857143	2495.5	0.93952823	NGAVLKK	72	365.2371	2	728.4545	.	T	Superscaf	1.52E+08	0	0	0	0	BTP20220	BTP20220	Tissue specific
PPKPEPKPK	Superscaf	-	Superscaf	1.52E+08	1.52E+08	-1	1	9	-	-	-	-	-	-	NCP	IGR	-	ID=gene-E	9	7.104444444	3517.7	0.939677837	PPKPEPKPK	98	509.3073	2	1016.602	.	P	Superscaf	1.52E+08	0	0	0	0	BTP20220	0	Tissue enhanced
LPLNPF	Superscaf	+	Superscaf	1.52E+08	1.52E+08	80	80	85	-	-	-	-	-	-	NCP	IGR	-	ID=gene-E	6	5.908333333	2365.7	0.939960066	LPLNPF	64	700.4	1	699.3955	.	V	Superscaf	1.52E+08	0	0	0	0	0	BTP20220	Tissue specific
KNKPFK	Superscaf	+	Superscaf	1.52E+08	1.52E+08	41	41	46	-	-	gene	-	-	mRNA	NCP	Intron	gene|mRN	ID=gene-E	6	7.735	2726.7	0.941466003	KNKPFK	87	381.2307	2	760.4595	.	L	Superscaf	1.52E+08	0	0	0	0	0	BTP20220	Tissue specific
EVPAACQ	Superscaf	+	Superscaf	1.52E+08	1.52E+08	2	2	8	-	-	-	-	-	-	NCP	IGR	-	ID=gene-E	7	4.604285714	2581.9	0.943513103	EVPAAC(+5	55	774.3416	1	773.3378	Carbamidc	G	Superscaf	1.52E+08	0	BTP20220	0	0	BTP20220	BTP20220	Tissue specific
ACPTTYM	Superscaf	+	Superscaf	1.53E+08	1.53E+08	105	105	111	-	-	gene	-	-	mRNA	NCP	Intron	gene|mRN	ID=gene-E	7	5.972857143	2860.4	0.945483353	AC(+57.02	52	430.1685	2	858.3252	Carbamidc	A	Superscaf	1.53E+08	0	0	0	0	0	BTP20220	Tissue specific
GGPVR	Superscaf	+	Superscaf	1.53E+08	1.53E+08	134	134	138	-	-	-	-	-	-	NCP	IGR	-	ID=gene-E	5	5.8	1580.8	0.945891855	GGPVR	58	485.2769	1	484.2758	.	R	Superscaf	1.53E+08	0	0	0	0	0	BTP20220	Tissue specific
ELRRF	Superscaf	-	Superscaf	1.53E+08	1.53E+08	-26	26	30	-	-	gene	-	-	mRNA	NCP	Intron	gene|mRN	ID=gene-E	5	7.24	2463.9	0.947071342	ELRRF	73	360.71	2	719.4078	.	A	Superscaf	1.53E+08	0	0	0	0	0	BTP20220	Tissue specific
ALLLK	Superscaf	+	Superscaf	1.53E+08	1.53E+08	12	12	16	-	-	gene	-	-	mRNA	NCP	Intron	gene|mRN	ID=gene-E	5	6.74	1839.9	0.947218998	ALLLK	99	557.4	1	556.3948	.	Y	Superscaf	1.53E+08	0	0	0	0	0	BTP20220	Tissue specific
LTWPAAF	Superscaf	+	Superscaf	1.53E+08	1.53E+08	55	55	61	-	-	-	-	-	-	NCP	IGR	-	ID=gene-E	7	6.031428571	2624.9	0.947962097	LTWPAAF	60	805.4245	1	804.417	.	H	Superscaf	1.53E+08	0	0	0	0	0	BTP20220	Tissue specific
DTMSV	Superscaf	-	Superscaf	1.53E+08	1.53E+08	-22	22	26	-	-	-	-	-	-	NCP	IGR	-	ID=gene-E	5	4.186	1563.9	0.948657266	DTM(+15.	52	568.2242	1	567.221	Oxidatior	V	Superscaf	1.53E+08	0	0	0	0	0	BTP20220	Tissue specific
LTLPV	Superscaf	+	Superscaf	1.53E+08	1.53E+08	13	13	17	-	-	-	-	-	-	NCP	IGR	-	ID=gene-E	5	4.958	1441.7	0.94869001	LTLPV	60	542.3505	1	541.3475	.	P	Superscaf	1.53E+08	0	0	0	BTP20220	0	0	Tissue specific
VLEDLGRQ	Superscaf	+	Superscaf	1.53E+08	1.53E+08	35	35	42	-	-	gene	-	-	mRNA	NCP	Intron	gene|mRN	ID=gene-E	8	5.06625	3100.6	0.949227998	VLEDLGRQ	84	465.2553	2	928.4977	.	E	Superscaf	1.53E+08	BTP20220	0	0	0	0	0	Tissue specific
SFLRKA	Superscaf	-	Superscaf	1.53E+08	1.53E+08	-6	6	11	-	-	gene	-	-	mRNA	NCP	Intron	gene|mRN	ID=gene-E	6	7.276666667	2552.7	0.949609953	SFLRKA	86	361.2205	2	720.4282	.	I	Superscaf	1.53E+08	0	0	BTP20220	0	0	0	Tissue specific
LSLFALAP	Superscaf	+	Superscaf	1.53E+08	1.53E+08	46	46	53	-	-	gene	-	-	mRNA	NCP	Intron	gene|mRN	ID=gene-E	8	5.93	2791	0.949888969	LSLFALAP	64	416.2576	2	830.4902	.	S	Superscaf	1.53E+08	0	0	0	0	BTP20220	0	Tissue specific
AAFVDVT	Superscaf	+	Superscaf	1.53E+08	1.53E+08	17	17	23	-	-	gene	-	-	mRNA	NCP	Intron	gene|mRN	ID=gene-E	7	3.86	1819	0.950442908	AAFVDVT	54	722.368	1	721.3646	.	N	Superscaf	1.53E+08	0	BTP20220	0	0	0	0	Tissue specific
EVSVQYR	Superscaf	-	Superscaf	1.53E+08	1.53E+08	-21	21	27	-	-	gene	-	-	mRNA	NCP	Intron	gene|mRN	ID=gene-E	7	4.424285714	2527.9	0.951240556	E(+42.01)	91	461.7427	2	921.4556	Acetylati	N	Superscaf	1.53E+08	0	0	0	0	0	BTP20220	Tissue specific
LMLVA	Superscaf	+	Superscaf	1.53E+08	1.53E+08	8	8	12	-	-	gene	-	-	mRNA	NCP	Intron	gene|mRN	ID=gene-E	5	4.746	1389.6	0.952015443	LMLVA	89	546.3298	1	545.3247	.	V	Superscaf	1.53E+08	0	0	0	0	0	BTP20220	Tissue specific
KHAGQKR	Superscaf	-	Superscaf	1.53E+08	1.53E+08	-11	11	17	-	-	gene	-	-	mRNA	NCP	Intron	gene|mRN	ID=gene-E	7	7.924285714	3424.6	0.952163175	K(+42.01)	69	433.7489	2	865.4882	Acetylati	K	Superscaf	1.53E+08	0	0	0	0	0	BTP20220	Tissue specific
HRLVPPH	Superscaf	-	Superscaf	1.53E+08	1.53E+08	-22	22	28	-	-	-	-	-	-	NCP	IGR	-	ID=gene-E	7	6.36	2916.4	0.95284507	H(+42.01)	50	449.2497	2	896.498	Acetylati	Y	Superscaf	1.53E+08	BTP20220	0	0	0	0	0	Tissue specific
YGTGPFN	Superscaf	+	Superscaf	1.53E+08	1.53E+08	13	13	19	-	-	gene	-	-	mRNA	NCP	Intron	gene|mRN	ID=gene-E	7	5.902857143	2773.1	0.954648729	YGTGPFN	61	755.3342	1	754.3286	.	T	Superscaf	1.53E+08	0	0	0	0	0	BTP20220	Tissue specific
QEALEMR	Superscaf	-	Superscaf	1.53E+08	1.53E+08	-7	7	13	-	-	gene	-	-	mRNA	NCP	Intron	gene|mRN	ID=gene-E	7	5.8	3146.7	0.95550609	QEALEM(+1	97	446.7117	2	891.412	Oxidatior	R	Superscaf	1.53E+08	0	0	BTP20220	0	0	0	Tissue specific
PPPWN	Superscaf	-	Superscaf	1.53E+08	1.53E+08	-60	60	64	-	-	-	-	-	-	NCP	IGR	-	ID=gene-E	5	6.04	2135.5	0.956836561	PPPWN	87	610.2988	1	609.2911	.	R	Superscaf	1.53E+08	0	0	0	0	0	BTP20220	Tissue specific
LPPPPQK	Superscaf	-	Superscaf	1.53E+08	1.53E+08	-2	2	8	-	-	-	-	-	-	NCP	IGR	-	ID=gene-E	7	6.652857143	2782.1	0.957177126	LPPPPQK	96	388.7358	2	775.4592	.	P	Superscaf	1.53E+08	0	0	0	0	0	BTP20220	Tissue specific
AAVPYH	Superscaf	+	Superscaf	1.54E+08	1.54E+08	1	1	6	-	-	-	-	-	-	NCP	IGR	-	ID=gene-E	6	5.265	2039.2	0.957969265	AAVPYH	86	657.3342	1	656.3282	.	A	Superscaf	1.54E+08	0	0	0	0	0	BTP20220	Tissue specific
KPLTLSQPF	Superscaf	+	Superscaf	1.54E+08	1.54E+08	25	25	33	-	-	-	-	-	-	NCP	IGR	-	ID=gene-E	9	6.495555556	3579	0.959141485	KPLTLSQPF	65	490.7898	2	979.5702	.	V	Superscaf	1.54E+08	0	0	0	BTP20220	0	0	Tissue specific
ATKAVCVLK	Superscaf	-	Superscaf	1.54E+08	1.54E+08	-190	190	198	-	-	-	-	-	-	NCP	IGR	-	ID=gene-E	9	5.45	2896	0.959380259	A(+42.01)	99	516.2975	2	1030.585	Acetylati	K	Superscaf	1.54E+08	0	0	0	BTP20220	BTP20220	0	Tissue specific
ATKAVCVL	Superscaf	-	Superscaf	1.54E+08	1.54E+08	-190	190	197	-	-	-	-	-	-	NCP	IGR	-	ID=gene-E	8	4.91375	2427.6	0.959380259	A(+42.01)	64	452.2502	2	902.4895	Acetylati	K	Superscaf	1.54E+08	0	0	0	0	0	BTP20220	Tissue specific
KDPLWS	Superscaf	+	Superscaf	1.54E+08	1.54E+08	53	53	58	-	-	-	-	-	-	NCP	IGR	-	ID=gene-E	6	6.093333333	2480.7	0.959832866	KDPLWS	58	745.3763	1	744.3806	.	Q	Superscaf	1.54E+08	0	BTP20220	0	0	0	0	Tissue specific
PEVYRK	Superscaf	-	Superscaf	1.54E+08	1.54E+08	-5	5	10	-	-	-	-	-	-	NCP	IGR	-	ID=gene-E	6	5.946666667	2339.6	0.962139244	PEVYRK	66	396.2311	2	790.4337	.	P	Superscaf	1.54E+08	0	BTP20220	0	0	0	0	Tissue specific
NFPLTS	Superscaf	-	Superscaf	1.54E+08	1.54E+08	-36	36	41	-	-	gene	-	-	mRNA	NCP	Intron	gene|mRN	ID=gene-E	6	5.896666667	2455.8	0.962681439	NFPLTS	71	678.3428	1	677.3384	.	T	Superscaf	1.54E+08	0	0	0	BTP20220	0	0	Tissue specific
EMLYG	Superscaf	-	Superscaf	1.54E+08	1.54E+08	-34	34	38	-	-	gene	-	-	mRNA	NCP	Intron	gene|mRN	ID=gene-E	5	5.316	1907.2	0.962732774	EMLYG	50	612.2792	1	611.2625	.	V	Superscaf	1.54E+08	0	0	0	0	BTP20220	0	Tissue specific
LLLLSCST	Superscaf	-	Superscaf	1.54E+08	1.54E+08	-13	13	20	-	-	-	-	-	-	NCP	IGR	-	ID=gene-E	8	5.85375	3163.6	0.963235876	LLLLSC(+5	56	453.7464	2	905.4892	Carbamidc	H	Superscaf	1.54E+08	0	0	BTP20220	0	0	0	Tissue specific
VEVLKLGK	Superscaf	+	Superscaf	1.54E+08	1.54E+08	2	2	9	-	-	-	-	-	-	NCP	IGR	-	ID=gene-E	8	5.07875	2366.4	0.964040791	VEVLKLGK	99	443.2953	2	884.5695	.	I	Superscaf	1.54E+08	0	0	0	BTP20220	0	0	Tissue specific
DPPPLFD	Superscaf	+	Superscaf	1.54E+08	1.54E+08	79	79	85	-	-	-	-	-	-	NCP	IGR	-	ID=gene-E	7	5.185714286	2576	0.965560767	DPPPLFD	92	800.381	1	799.3752	.	S	Superscaf	1.54E+08	0	0	0	BTP20220	0	0	Tissue specific
RPLKDL	Superscaf	-	Superscaf	1.54E+08	1.54E+08	-40	40	45	-	-	-	-	-	-	NCP	IGR	-	ID=gene-E	6	6.955	2572.9	0.967274417	RPLKDL	56	741.4497	1	740.4544	.	G	Superscaf	1.54E+08	BTP20220	0	0	0	0	0	Tissue specific
QAHLYR	Superscaf	-	Superscaf	1.54E+08	1.54E+08	-32	32	37	-	-	-	-	-	-	NCP	IGR	-	ID=gene-E	6	6.943333333	3003.4	0.967744353	QAHLYR	97	394.2114	2	786.4136	.	R	Superscaf	1.54E+08	0	0	BTP20220	0	0	0	Tissue specific
LKVPVP	Superscaf	+	Superscaf	1.54E+08	1.54E+08	2	2	7	-	-	-	-	-	-	NCP	IGR	-	ID=gene-E	6	4.72	1496.6	0.968080557	L(+42.01)	52	694.4404	1	693.4425	Acetylati	F	Superscaf	1.54E+08	0	0	BTP20220	0	0	0	Tissue specific
KSEHPPR	Superscaf	-	Superscaf	1.54E+08	1.54E+08	-5	5	11	-	-	-	-	-	-	NCP	IGR	-	ID=gene-E	7	7.084285714	3220.6	0.968108979	KSEHPPR	66	425.7298	2	849.4457	.	L	Superscaf	1.54E+08	0	0	BTP20220	0	0	0	Tissue specific
KSTLKV	Superscaf	-	Superscaf	1.54E+08	1.54E+08	-21	21	26	-	-	-	-	-	-	NCP	IGR	-	ID=gene-E	6	6.278333333	2071.1	0.968905326	K(+42.01)	95	717.4492	1	716.4432	Acetylati	S	Superscaf	1.54E+08	0	0	0	0	0	BTP20220	Tissue specific
RWKLR	Superscaf	-	Superscaf	1.54E+08	1.54E+08	-10	10	14	-	-	-	-	-	-	NCP	IGR	-	ID=gene-E	5	8.626	2646	0.968946333	RWKLR	51	379.7482	2	757.4711	.	G	Superscaf	1.54E+08	0	0	0	0	BTP20220	0	Tissue specific
LSPPFR	Superscaf	-	Superscaf	1.54E+08	1.54E+08	-27	27	32	-	-	gene	-	-	mRNA	NCP	Intron	gene|mRN	ID=gene-E	6	6.75	2447.8	0.969645365	LSPPFR	80	358.7119	2	715.4017	.	G	Superscaf	1.54E+08	0	0	0	BTP20220	0	0	Tissue specific
KKLVVAP	Superscaf	+	Superscaf	1.54E+08	1.54E+08	19	19	25	-	-	gene	-	-	mRNA	NCP	Intron	gene|mRN	ID=gene-E	7	5.397142857	1938.9	0.969699455	KKLVVAP	58	377.7617	2	753.5112	.	F	Superscaf	1.54E+08	0	0	BTP20220	0	0	0	Tissue specific
VSFYY	Superscaf	-	Superscaf	1.55E+08	1.55E+08	-1	1	5	-	-	-	-	-	-	NCP	IGR	-	ID=gene-E	5	4.496	1689.8	0.971862308	VSFYY	88	678.3094	1	677.306	.	V	Superscaf	1.55E+08	BTP20220	0	0	0	0	0	Tissue specific
LEPKYKS	Superscaf	+	Superscaf	1.55E+08	1.55E+08	9	9	15	-	-	-	-	-	-	NCP	IGR	-	ID=gene-E	7	6.617142857	2896	0.972409323	LEPKYKS	91	432.7439	2	863.4752	.	K	Superscaf	1.55E+08	BTP20220	0	0	0	0	0	Tissue specific
TVELMFP	Superscaf	-	Superscaf	1.55E+08	1.55E+08	-21	21	27	-	-	gene	-	-	mRNA	NCP	Intron	gene|mRN	ID=gene-E	7	4.751428571	2272.7	0.972595461	T(+42.01)	50	878.4456	1	877.4255	Acetylati	D	Superscaf	1.55E+08	0	0	BTP20220	0	0	0	Tissue specific
AAAAVTRP	Superscaf	-	Superscaf	1.55E+08	1.55E+08	-6	6	13	-	-	-	-	-	-	NCP	IGR	-	ID=gene-E	8	5.95875	2628.9	0.97333661	AAAAVTRP	83	378.7204	2	755.429	.	R	Superscaf	1.55E+08	BTP20220	0	0	0	0	0	Tissue specific
PAGMRK	Superscaf	+	Superscaf	1.55E+08	1.55E+08	21	21	26	-	-	-	-	-	-	NCP	IGR	-	ID=gene-E	6	7.423333333	2434.7	0.97413246	PAGM(+15.	51	675.3542	1	674.3533	Oxidatior	T	Superscaf	1.55E+08	BTP20220	0	0	0	0	0	Tissue specific
KAPPDPK	Superscaf	+	Superscaf	1.55E+08	1.55E+08	14	14	20	-	-	-	-	-	-	NCP	IGR	-	ID=gene-E	7	6.767142857	2671.8	0.974349698	KAPPDPK	76	376.7136	2	751.4228	.	G	Superscaf	1.55E+08	0	0	BTP20220	0	0	0	Tissue enhanced
VGGDGVVV	Superscaf	+	Superscaf	1.55E+08	1.55E+08	25	25	32	-	-	-	-	-	-	NCP	IGR	-	ID=gene-E	8	2.61	1303.4	0.97491473	VGGDGVVV	67	701.3807	1	700.3755	.	P	Superscaf	1.55E+08	0	0	BTP20220	0	0	0	Tissue specific
KKHGPR	Superscaf	-	Superscaf	1.55E+08	1.55E+08	-5	5	10	-	-	-	-	-	-	NCP	IGR	-	ID=gene-E	6	8.35	2840	0.975285744	KKHGPR	58	361.7302	2	721.4347	.	E	Superscaf	1.55E+08	BTP20220	0	0	0	0	0	Tissue specific
LSVKPR	Superscaf	-	Superscaf	1.55E+08	1.55E+08	-1	1	6	-	-	gene	-	-	mRNA	NCP	Intron	gene|mRN	ID=gene-E	6	6.41	2191.4	0.976380272	LSVKPR	62	350.2281	2	698.4439	.	L	Superscaf	1.55E+08	0	0	BTP20220	0	0	0	Tissue specific
VLRNPLLA	Superscaf	-	Superscaf	1.55E+08	1.55E+08	-9	9	16	-	-	-	-	-	-	NCP	IGR	-	ID=gene-E	8	5.80375	2954.4	0.976816048	VLRNPLLA	53	448.2874	2	894.5651	.	H	Superscaf	1.55E+08	0	0	BTP20220	0	0	0	Tissue specific
KSSSYAH	Superscaf	-	Superscaf	1.55E+08	1.55E+08	-8	8	14	-	-	-	-	-	-	NCP	IGR	-	ID=gene-E	7	6.578571429	3009.1	0.976910035	KSSSYAH	84	390.1921	2	778.361	.	G	Superscaf	1.55E+08	0	0	BTP20220	0	0	0	Tissue specific
SVCGYLLL	Superscaf	-	Superscaf	1.55E+08	1.55E+08	-18	18	25	-	-	-	-	-	-	NCP	IGR	-	ID=gene-E	8	5.03375	2786.1	0.976986197	SVC(+57.	51	462.7429	2	923.4786	Carbamidc	K	Superscaf	1.55E+08	0	0	0	0	0	BTP20220	Tissue specific
KAETFGP	Superscaf	+	Superscaf	1.55E+08	1.55E+08	1	1	7	-	-	-	-	-	-	NCP	IGR	-	ID=gene-E	7	6.18	2624.8	0.978898494	KAETFGP	68	375.1925	2	748.3755	.	K	Superscaf	1.55E+08	0	0	0	0	0	BTP20220	Tissue specific
VARPPR	Superscaf	-	Superscaf	1.55E+08	1.55E+08	-3	3	8	-	-	-	-	-	-	NCP	IGR	-	ID=gene-E	6	6.69	2283.7	0.980303711	V(+42.01)	54	369.2291	2	736.4344	Acetylati	V	Superscaf	1.55E+08	0	0	BTP20220	0	0	0	Tissue specific
LLETSL	Superscaf	-	Superscaf	1.55E+08	1.55E+08	-8	8	13	-	-	-	-	-	-	NCP	IGR	-	ID=gene-E	6	5.561666667	2266.6	0.980700431	LLETSL	67	675.3903	1	674.3851	.	H	Superscaf	1.55E+08	0	0	0	BTP20220	0	0	Tissue specific
YSESFK	Superscaf	-	Superscaf	1.55E+08	1.55E+08	-31	31	36	-	-	-	-	-	-	NCP	IGR	-	ID=gene-E	6	5.91	2507.6	0.982484504	YSESFK	73	380.6833	2	759.3439	.	A	Superscaf	1.55E+08	0	0	BTP20220	0	0	0	Tissue specific
KTPPRH	Superscaf	+	Superscaf	1.56E+08	1.56E+08	14	14	19	-	-	-	-	-	-	NCP	IGR	-	ID=gene-E	6	7.87	2809.2	0.98469196	KTPPRH	52	368.2191	2	734.4188	.	N	Superscaf	1.56E+08	0	0	0	0	0	BTP20220	Tissue specific
PYLNPPE	Superscaf	+	Superscaf	1.56E+08	1.56E+08	49	49	55	-	-	gene	-	-	mRNA	NCP	Intron	gene|mRN	ID=gene-E	7	5.595714286	2847.3	0.984829785	PYLNPPE	64	415.2113	2	828.4017	.	L	Superscaf	1.56E+08	0	0	0	BTP20220	0	0	Tissue specific
TLFMMF	Superscaf	+	Superscaf	1.56E+08	1.56E+08	32	32	37	-	-	gene	-	-	mRNA	NCP	Intron	gene|mRN	ID=gene-E	6	5.828333333	2268.7	0.984856945	TLFMMF	81	789.3634	1	788.3601	.	G	Superscaf	1.56E+08	0	0	BTP20220	0	0	0	Tissue specific
NLLYPPPPF	Superscaf	-	Superscaf	1.56E+08	1.56E+08	-7	7	15	-	-	gene	-	-	mRNA	NCP	Intron	gene|mRN	ID=gene-E	9	6.058888889	3450	0.985041017	NLLYPPPPF	81	504.279	2	1006.549	.	Y	Superscaf	1.56E+08	0	0	0	0	BTP20220	0	Tissue specific
KEMTFL	Superscaf	-	Superscaf	1.56E+08	1.56E+08	-61	61	66	-	-	gene	-	-	mRNA	NCP	Intron	gene|mRN	ID=gene-E	6	6.116666667	2403.7	0.986117069	KEMTFL	53	768.3928	1	767.3887	.	G	Superscaf	1.56E+08	0	0	BTP20220	0	0	0	Tissue specific
KRSLVPSTI	Superscaf	-	Superscaf	1.56E+08	1.56E+08	-11	11	19	-	-	-	-	-	-	NCP	IGR	-	ID=gene-E	9	6.294444444	3325.7	0.988228893	KRSLVPSTI	60	500.8052	2	999.6077	.	N	Superscaf	1.56E+08	0	0	0	0	BTP20220	0	Tissue specific
SVQTCPK	Superscaf	-	Superscaf	1.56E+08	1.56E+08	-61	61	67	-	-	-	-	-	-	NCP	IGR	-	ID=gene-E	7	5.56	2783.1	0.988626378	SVQTC(+57	75	410.2079	2	818.3956	Carbamidc	R	Superscaf	1.56E+08	0	0	BTP20220	0	0	0	Tissue specific
LQEPGP	Superscaf	-	Superscaf	1.56E+08	1.56E+08	-44	44	49	-	-	gene	-	-	mRNA	NCP	Intron	gene|mRN	ID=gene-E	6	5.57	2361.7	0.989292398	LQEPGP	97	640.3401	1	639.3228	.	R	Superscaf	1.56E+08	0	0	0	0	0	BTP20220	Tissue specific
AAAAKLAL	Superscaf	+	Superscaf	1.56E+08	1.56E+08	24	24	31	-	-	gene	-	-	mRNA	NCP	Intron	gene|mRN	ID=gene-E	8	6.475	2731.7	0.989361138	A(+42.01)	74	385.7462	2	769.4697	Acetylati	E	Superscaf	1.56E+08	0	0	0	0	0	BTP20220	Tissue specific
KTSYVYSL	Superscaf	-	Superscaf	1.56E+08	1.56E+08	-29	29	36	-	-	gene	-	-	mRNA	NCP	Intron	gene|mRN	ID=gene-E	8	5.61625	2905.1	0.9895542	KTSYVYSL	63	480.7525	2	959.4964	.	T	Superscaf	1.56E+08	0	0	BTP20220	0	0	0	Tissue specific
GVLSWY	Superscaf	+	Superscaf	1.56E+08	1.56E+08	5	5	10	-	-	gene	-	-	mRNA	NCP	Intron	gene|mRN	ID=gene-E	6	4.863333333	2020.2	0.991970631	GVLSWY	91	724.364	1	723.3592	.	S	Superscaf	1.56E+08	0	0	BTP20220	BTP20220	0	0	Tissue specific
LAPPF	Superscaf	+	Superscaf	1.56E+08	1.56E+08	19	19	23	-	-	gene	-	-	mRNA	NCP	Intron	gene|mRN	ID=gene-E	5	6.016	1726.9	0.991974724	LAPPF	90	544.312	1	543.3057	.	L	Superscaf	1.56E+08	0	0	0	BTP20220	0	0	Tissue specific
PRVEF	Superscaf	+	Superscaf	1.56E+08	1.56E+08	14	14	18	-	-	gene	-	-	mRNA	NCP	Intron	gene|mRN	ID=gene-E	5	5.152	1799.1	0.992306682	PRVEF	83	647.3489	1	646.3438	.	S	Superscaf	1.56E+08	0	0	BTP20220	0	0	0	Tissue specific
VTLEVT	Superscaf	-	Superscaf	1.56E+08	1.56E+08	-10	10	15	-	-	-	-	-	-	NCP	IGR	-	ID=gene-E	6	3.71	1573.9	0.992809974	VTLEVT	80	661.3732	1	660.3694	.	L	Superscaf	1.56E+08	0	0	0	BTP20220	0	0	Tissue specific
LYDASDL	Superscaf	-	Superscaf	1.56E+08	1.56E+08	-12	12	18	-	-	gene	-	-	mRNA	NCP	Intron	gene|mRN	ID=gene-E	7	5.037142857	2684	0.993666263	LYDASDL	76	796.3719	1	795.3651	.	F	Superscaf	1.56E+08	0	0	0	0	0	BTP20220	Tissue specific
CCPCS	Superscaf	-	Superscaf	1.56E+08	1.56E+08	-31	31	35	-	-	-	-	-	-	NCP	IGR	-	ID=gene-E	5	5.408	2477.9	0.994509317	C(+57.02)	60	363.1107	2	724.1979	Carbamidc	A	Superscaf	1.56E+08	0	0	0	BTP20220	0	0	Mixed
SRSLLVD	Superscaf	-	Superscaf	1.56E+08	1.56E+08	-8	8	14	-	-	-	-	-	-	NCP	IGR	-	ID=gene-E	7	5.292857143	2533.9	0.995141751	SRSLLVD	80	395.2252	2	788.4392	.	V	Superscaf	1.56E+08	0	0	BTP20220	0	0	0	Tissue specific
VKCPKC	Superscaf	-	Superscaf	1.57E+08	1.57E+08	-2	2	7	-	-	gene	-	-	mRNA	NCP	Intron	gene|mRN	ID=gene-E	6	5.97	2445.6	0.995858686	V(+42.01)	91	417.2074	2	832.3935	Acetylati	L	Superscaf	1.57E+08	0	0	0	0	0	BTP20220	Tissue specific
GVPPLKM	Superscaf	-	Superscaf	1.57E+08	1.57E+08	-8	8	14	-	-	-	-	-	-	NCP	IGR	-	ID=gene-E	7	5.72	2165.4	0.996915267	G(+42.01)	65	392.2305	2	782.436	Acetylati	L	Superscaf	1.57E+08	0	0	0	BTP20220	0	0	Tissue specific
PGVPGLVA	Superscaf	+	Superscaf	1.57E+08	1.57E+08	27	27	34	-	-	-	-	-	-	NCP	IGR	-	ID=gene-E	8	4.5675	1934.1	0.996952946	PGVPGLVA	54	709.4227	1	708.417	.	D	Superscaf	1.57E+08	0	0	BTP20220	0	0	0	Tissue specific
LFGKKQ	Superscaf	+	Superscaf	1.57E+08	1.57E+08	9	9	14	-	-	gene	-	-	mRNA	NCP	Intron	gene|mRN	ID=gene-E	6	7.093333333	2585.6	0.997315315	LFGKKQ	95	360.7259	2	719.433	.	V	Superscaf	1.57E+08	0	0	BTP20220	0	0	0	Mixed
VTVEVW	Superscaf	-	Superscaf	1.57E+08	1.57E+08	-4	4	9	-	-	-	-	-	-	NCP	IGR	-	ID=gene-E	6	2.606666667	1349.6	0.997794852	V(+42.01)	61	774.4111	1	773.3959	Acetylati	I	Superscaf	1.57E+08	0	0	BTP20220	0	0	0	Tissue specific
SEMGSSGE	Superscaf	-	Superscaf	1.57E+08	1.57E+08	-3	3	10	-	-	-	-	-	-	NCP	IGR	-	ID=gene-E	8	5.14625	2967.3	0.99816667	S(+42.01)	62	841.2936	1	840.2807	Acetylati	S	Superscaf	1.57E+08	BTP20220	0	0	0	0	0	Tissue specific
PLFLG	Superscaf	+	Superscaf	119845	119859	279	279	283	-	-	-	-	-	-	NCP	IGR	-	.	5	5.942	1728.9	0.99767885	PLFLG	97	546.3275	1	545.3213	.	A	Superscaf	119011	0	0	0	BTP20220	0	0	Tissue specific
FLSSSGY	Superscaf	-	Superscaf	145171	145191	-41	41	47	-	-	-	-	-	-	NCP	IGR	-	.	7	5.732857143	2647.8	0.997188278	FLSSSGY	58	760.3522	1	759.3439	.	S	Superscaf	145227	0	BTP20220	BTP20220	BTP20220	BTP20220	BTP20220	Tissue specific
LDTLY	Superscaf	+	Superscaf	160927	160941	92	92	96	-	-	-	-	-	-	NCP	IGR	-	.	5	5.424	1975.3	0.996883269	LDTLY	86	624.3214	1	623.3167	.	E	Superscaf	160654	0	0	0	0	BTP20220	0	Tissue specific
ATLSVH	Superscaf	+	Superscaf	220699	220716	13	13	18	-	-	-	-	-	-	NCP	IGR	-	.	6	5.3	2065.3	0.995725686	ATLSVH	92	627.3439	1	626.3387	.	S	Superscaf	220663	0	0	0	0	0	BTP20220	Mixed
LMVLVL	Superscaf	-	Superscaf	232429	232446	-39	39	44	-	-	-	-	-	-	NCP	IGR	-	.	6	3.948333333	1431.7	0.995498526	LMVLVL	53	687.4466	1	686.4401	.	S	Superscaf	232500	0	0	BTP20220	0	0	0	Tissue specific
ESTLATRK	Superscaf	+	Superscaf	284548	284571	14	14	21	-	-	-	-	-	-	NCP	IGR	-	.	8	6.8075	3385.8	0.994489091	ESTLATRK	58	453.2552	2	904.4977	.	C	Superscaf	284509	0	0	BTP20220	0	0	0	Tissue specific
GVLAAA	Superscaf	-	Superscaf	421147	421164	-111	111	116	-	-	-	-	-	-	NCP	IGR	-	.	6	5.001666667	1584.6	0.991843875	GVLAAA	55	501.3022	1	500.2958	.	P	Superscaf	421242	0	0	BTP20220	0	0	0	Tissue specific
DVDLRK	Superscaf	-	Superscaf	451990	452007	-15	15	20	-	-	-	-	-	-	NCP	IGR	-	ID=gene-E	6	5.403333333	2293.6	0.99124658	DVDLRK	97	373.2123	2	744.413	.	I	Superscaf	452097	0	0	BTP20220	0	BTP20220	BTP20220	Tissue specific
LHAELLT	Superscaf	-	Superscaf	503143	503163	-3	3	9	-	-	-	-	-	-	NCP	IGR	-	ID=gene-E	7	5.9	2814.2	0.990255909	LHAELLT	76	398.7305	2	795.449	.	Y	Superscaf	503166	0	0	BTP20220	0	0	0	Tissue specific
QDPLFK	Superscaf	+	Superscaf	564901	564918	4	4	9	-	-	gene	-	-	mRNA	NCP	Intron	gene|mRN	ID=gene-E	6	6.02	2568.8	0.989059982	QDPLFK	75	374.2042	2	746.3962	.	E	Superscaf	564892	0	0	BTP20220	0	0	0	Tissue specific
HTGPPPP	Superscaf	-	Superscaf	568252	568272	-29	29	35	-	-	-	-	-	-	NCP	IGR	-	ID=gene-E	7	6.47	2664.1	0.98899503	HTGPPPP	72	351.6843	2	701.3497	.	P	Superscaf	568302	0	0	BTP20220	0	0	0	Tissue specific
KAGALVL	Superscaf	+	Superscaf	656179	656199	39	39	45	-	-	-	-	-	-	NCP	IGR	-	ID=gene-E	7	5.672857143	2095.1	0.987292264	K(+42.01)	83	713.4546	1	712.4483	Acetylati	Y	Superscaf	656065	0	0	0	0	BTP20220	0	Mixed
VGLPR	Superscaf	-	Superscaf	747601	747615	-13	13	17	-	-	-	-	-	-	NCP	IGR	-	ID=gene-E	5	5.802	1636.9	0.985521931	VGLPR	81	541.3442	1	540.3384	.	H	Superscaf	747621	0	BTP20220	0	0	0	0	Tissue specific
SYPYKK	Superscaf	+	Superscaf	761926	761943	10	10	15	-	-	-	-	-	-	NCP	IGR	-	ID=gene-E	6	7.13	2576.6	0.98524446	SYPYKK	90	393.2118	2	784.4119	.	L	Superscaf	761899	0	0	BTP20220	BTP20220	BTP20220	0	Tissue specific
TVFNK	Superscaf	+	Superscaf	810538	810552	50	50	54	-	-	gene	-	-	mRNA	NCP	Intron	gene|mRN	ID=gene-E	5	5.432	1850	0.984303114	T(+42.01)	62	650.3602	1	649.3435	Acetylati	S	Superscaf	810391	0	BTP20220	BTP20220	0	BTP20220	0	Tissue specific
PEFLKA	Superscaf	+	Superscaf	863758	863775	13	13	18	-	-	gene	-	-	mRNA	NCP	Intron	gene|mRN	ID=gene-E	6	6.123333333	2283.4	0.983272415	PEFLKA	56	352.7016	2	703.3904	.	K	Superscaf	863722	0	0	0	BTP20220	0	0	Mixed
PGARAGPGF	Superscaf	-	Superscaf	894469	894498	-20	20	29	-	-	gene	-	-	mRNA	NCP	Intron	gene|mRN	ID=gene-E	10	6.591	3512.9	0.982677443	PGARAGPGF	74	438.7371	2	875.4613	.	A	Superscaf	894765	0	0	BTP20220	0	0	0	Tissue specific
LSGEVLR	Superscaf	+	Superscaf	958066	958086	21	21	27	-	-	gene	-	-	mRNA	NCP	Intron	gene|mRN	ID=gene-E	7	5.37	2461.8	0.981446019	LSGEVLR	52	387.232	2	772.4443	.	K	Superscaf	958006	0	0	BTP20220	0	BTP20220	0	Tissue specific
LPKEPL	Superscaf	+	Superscaf	992563	992580	12	12	17	-	-	gene	-	-	mRNA	NCP	Intron	gene|mRN	ID=gene-E	6	6.253333333	2275.5	0.980778019	LPKEPL	83	696.4279	1	695.4218	.	E	Superscaf	992530	0	0	BTP20220	0	0	0	Tissue specific
LEYAALL	Superscaf	-	Superscaf	1016008	1016028	-38	38	44	-	-	gene	-	-	mRNA	NCP	Intron	gene|mRN	ID=gene-E	7	5.551428571	2567.8	0.980323933	LEYAALL	63	792.4459	1	791.4429	.	H	Superscaf	1016046	0	0	BTP20220	0	0	0	Tissue specific
ESARLLR	Superscaf	-	Superscaf	1039825	1039845	-34	34	40	-	-	gene	-	-	mRNA	NCP	Intron	gene|mRN	ID=gene-E	7	6.914285714	3124.6	0.9798627	ESARLLR	73	422.7614	2	843.4926	.	A	Superscaf	1039926	0	0	BTP20220	0	0	0	Tissue specific
TSGDLLSK	Superscaf	-	Superscaf	1040269	1040292	-7	7	14	-	-	gene	-	-	mRNA	NCP	Intron	gene|mRN	ID=gene-E	8	6.06625	3048.3	0.979854044	T(+42.01)	62	431.7286	2	861.4443	Acetylati	L	Superscaf	1040448	0	0	0	BTP20220	BTP20220	0	Tissue specific
RVVTAF	Superscaf	+	Superscaf	1122358	1122375	86	86	91	-	-	gene	-	-	mRNA	NCP	Intron	gene|mRN	ID=gene-E	6	4.798333333	1745	0.978264451	RVVTAF	75	692.4053	1	691.4017	.	S	Superscaf	1122103	0	0	0	BTP20220	0	0	Mixed
LENHWQL	Superscaf	+	Superscaf	1134403	1134423	34	34	40	-	-	gene	-	-	mRNA	NCP	Intron	gene|mRN	ID=gene-E	7	5.674285714	3486	0.978031134	LENHWQL	52	470.2369	2	938.461	.	T	Superscaf	1134304	0	0	0	0	0	BTP20220	Tissue specific
RGVFHH	Superscaf	-	Superscaf	1144312	1144329	-73	73	78	-	-	-	-	-	-	NCP	IGR	-	ID=gene-E	6	6.231666667	2572.9	0.977839297	RGVFHH	68	376.6999	2	751.3878	.	R	Superscaf	1144470	0	0	0	0	0	BTP20220	Tissue specific
QRVPKAP	Superscaf	-	Superscaf	1146148	1146168	-4	4	10	-	-	-	-	-	-	NCP	IGR	-	ID=gene-E	7	6.395714286	2714	0.977803684	QRVPKAP	66	398.2484	2	794.4763	.	N	Superscaf	1146189	0	0	0	0	0	BTP20220	Tissue specific
STMPF	Superscaf	+	Superscaf	1149817	1149831	8	8	12	-	-	-	-	-	-	NCP	IGR	-	ID=gene-E	5	5.948	1877.2	0.977732747	STMPF	76	582.2571	1	581.252	.	P	Superscaf	1149796	0	0	0	0	0	BTP20220	Tissue specific
GVDKAVFP	Superscaf	+	Superscaf	1173844	1173867	3	3	10	-	-	gene	-	-	mRNA	NCP	Intron	gene|mRN	ID=gene-E	8	4.56	2213.3	0.977267274	GVDKAVFP	62	416.7274	2	831.449	.	N	Superscaf	1173838	0	0	0	0	0	BTP20220	Tissue specific
TVTSAY	Superscaf	+	Superscaf	1181578	1181595	23	23	28	-	-	gene	-	-	mRNA	NCP	Intron	gene|mRN	ID=gene-E	6	5.07	1949.2	0.977117616	T(+42.01)	66	683.3221	1	682.3174	Acetylati	K	Superscaf	1181512	0	0	0	0	0	BTP20220	Tissue specific
PTVGWV	Superscaf	-	Superscaf	1194661	1194678	-53	53	58	-	-	-	-	-	-	NCP	IGR	-	ID=gene-E	6	4.115	1558.8	0.976864255	PTVGWV	56	658.3539	1	657.3486	.	W	Superscaf	1194765	0	0	0	0	0	BTP20220	Tissue specific
LDLSPST	Superscaf	+	Superscaf	1230499	1230519	26	26	32	-	-	gene	-	-	mRNA	NCP	Intron	gene|mRN	ID=gene-E	7	5.588571429	2620	0.97617017	LDLSPST	53	732.3794	1	731.3701	.	Q	Superscaf	1230424	0	0	0	0	0	BTP20220	Tissue specific
LPVEP	Superscaf	+	Superscaf	1253485	1253499	32	32	36	-	-	gene	-	-	mRNA	NCP	Intron	gene|mRN	ID=gene-E	5	4.36	1453.7	0.975725147	LPVEP	78	554.3168	1	553.3112	.	D	Superscaf	1253392	0	0	0	0	0	BTP20220	Tissue specific
PQTHHT	Superscaf	+	Superscaf	1261312	1261329	47	47	52	-	-	gene	-	-	mRNA	NCP	Intron	gene|mRN	ID=gene-E	6	6.698333333	2982.5	0.975573513	PQTHHT	61	720.3535	1	719.3351	.	V	Superscaf	1261174	0	0	0	0	0	BTP20220	Tissue specific
FNDLSL	Superscaf	+	Superscaf	1299391	1299408	5	5	10	-	-	gene	-	-	mRNA	NCP	Intron	gene|mRN	ID=gene-E	6	5.25	2485.8	0.974836088	FNDLSL	83	708.3531	1	707.349	.	G	Superscaf	1299379	0	0	0	0	0	BTP20220	Tissue specific
PSFGAPL	Superscaf	+	Superscaf	1326859	1326879	13	13	19	-	-	gene	-	-	mRNA	NCP	Intron	gene|mRN	ID=gene-E	7	5.961428571	2407.6	0.974304094	PSFGAPL	67	688.3638	1	687.3591	.	R	Superscaf	1326823	0	0	0	0	0	BTP20220	Mixed
KNRLPVAPF	Superscaf	+	Superscaf	1382749	1382775	106	106	114	-	-	gene	-	-	mRNA	NCP	Intron	gene|mRN	ID=gene-E	9	6.312222222	3390.8	0.97322163	KNRLPVAPF	66	496.3102	2	990.5974	.	I	Superscaf	1382434	0	0	0	0	0	BTP20220	Tissue specific
MLTAGAQ	Superscaf	-	Superscaf	1444723	1444743	-2	2	8	-	-	-	-	-	-	NCP	IGR	-	ID=gene-E	7	5.988571429	2653	0.972021578	MLTAGAQ	59	691.353	1	690.337	.	K	Superscaf	1444848	0	0	BTP20220	0	0	0	Tissue specific
QHDSVR	Superscaf	+	Superscaf	1479187	1479204	8	8	13	-	-	-	-	-	-	NCP	IGR	-	ID=gene-E	6	5.441666667	2674.1	0.971354217	QHDSVR	58	371.1893	2	740.3565	.	R	Superscaf	1479166	0	0	BTP20220	0	0	0	Tissue specific
KTPRGTP	Superscaf	-	Superscaf	1534708	1534728	-21	21	27	-	-	gene	-	-	mRNA	NCP	Intron	gene|mRN	ID=gene-E	7	7.447142857	2884.3	0.970278958	K(+42.01)	82	399.7263	2	797.4395	Acetylati	F	Superscaf	1534818	0	0	BTP20220	0	0	0	Tissue specific
KEGLSPAGF	Superscaf	-	Superscaf	1571254	1571280	-14	14	22	-	-	-	-	-	-	NCP	IGR	-	ID=gene-E	9	6.131111111	3211.4	0.969571103	KEGLSPAGF	51	428.226	2	854.4498	.	L	Superscaf	1571409	0	0	0	0	0	BTP20220	Tissue specific
PAAAASSK	Superscaf	-	Superscaf	1571461	1571484	-17	17	24	-	-	-	-	-	-	NCP	IGR	-	ID=gene-E	8	6.435	2817.8	0.969567153	PAAAASSK	56	351.6895	2	701.3708	.	K	Superscaf	1571625	0	0	BTP20220	BTP20220	0	0	Tissue specific
YPSDWLM	Superscaf	+	Superscaf	1577611	1577631	4	4	10	-	-	-	-	-	-	NCP	IGR	-	ID=gene-E	7	5.461428571	2843.3	0.969448112	YPSDWLM(+	63	464.1987	2	926.3844	Oxidatior	A	Superscaf	1577602	BTP20220	BTP20220	BTP20220	BTP20220	0	BTP20220	Tissue specific
TQVVLD	Superscaf	+	Superscaf	1580638	1580655	32	32	37	-	-	-	-	-	-	NCP	IGR	-	ID=gene-E	6	3.521666667	1773.1	0.96938955	T(+42.01)	71	716.3804	1	715.3752	Acetylati	V	Superscaf	1580545	0	0	BTP20220	0	0	0	Tissue specific
AFLMPK	Superscaf	+	Superscaf	1580659	1580676	75	75	80	-	-	-	-	-	-	NCP	IGR	-	ID=gene-E	6	6.545	2229.4	0.969389144	A(+42.01)	66	374.7133	2	747.3989	Acetylati	C	Superscaf	1580437	0	0	BTP20220	0	BTP20220	0	Tissue specific
ESLHELL	Superscaf	-	Superscaf	1729075	1729095	-7	7	13	-	-	-	-	-	-	NCP	IGR	-	ID=gene-E	7	5.378571429	2914.3	0.966514909	ESLHELL	63	840.4448	1	839.4388	.	S	Superscaf	1729212	0	0	BTP20220	BTP20220	0	0	Tissue specific
QSFPKAK	Superscaf	-	Superscaf	1756186	1756206	-16	16	22	-	-	gene	-	-	mRNA	NCP	Intron	gene|mRN	ID=gene-E	7	6.944285714	2967	0.965989887	QSFPKAK	79	403.2307	2	804.4493	.	C	Superscaf	1756209	0	0	0	0	BTP20220	0	Tissue specific
LPSRSQPP	Superscaf	-	Superscaf	1781068	1781091	-11	11	18	-	-	gene	-	-	mRNA	NCP	Intron	gene|mRN	ID=gene-E	8	6.58125	3391.9	0.965507972	LPSRSQPP	63	441.2401	2	880.4766	.	Q	Superscaf	1781346	0	0	BTP20220	0	0	0	Tissue specific
EVFLL	Superscaf	+	Superscaf	1847773	1847787	32	32	36	-	-	gene	-	-	mRNA	NCP	Intron	gene|mRN	ID=gene-E	5	4.132	1519.7	0.964216359	EVFLL	66	620.3648	1	619.3581	.	N	Superscaf	1847680	0	0	BTP20220	0	0	0	Tissue specific
QALVLSLY	Superscaf	-	Superscaf	1870915	1870938	-1	1	8	-	-	gene	-	-	mRNA	NCP	Intron	gene|mRN	ID=gene-E	8	5.11875	2825.1	0.963768024	QALVLSLY	71	906.5256	1	905.5222	.	Q	Superscaf	1871010	0	0	BTP20220	0	0	0	Tissue specific
NKPVERK	Superscaf	-	Superscaf	1882558	1882578	-16	16	22	-	-	gene	-	-	mRNA	NCP	Intron	gene|mRN	ID=gene-E	7	6.452857143	2945.2	0.963542608	NKPVERK	98	435.7543	2	869.5082	.	T	Superscaf	1882578	0	0	0	0	0	BTP20220	Tissue specific
SGTRVSPS	Superscaf	-	Superscaf	1940788	1940811	-30	30	37	-	-	-	-	-	-	NCP	IGR	-	ID=gene-E	8	5.825	2831.2	0.962414886	SGTRVSPS	55	395.7046	2	789.3981	.	C	Superscaf	1940811	0	BTP20220	0	0	BTP20220	BTP20220	Tissue specific
EPAMDPK	Superscaf	-	Superscaf	2003938	2003958	-45	45	51	-	-	-	-	-	-	NCP	IGR	-	ID=gene-E	7	5.757142857	2663	0.961192002	EPAM(+15.	61	402.179	2	802.3531	Oxidatior	A	Superscaf	2004225	0	BTP20220	0	0	BTP20220	0	Tissue specific
VPYALL	Superscaf	-	Superscaf	2029513	2029530	-74	74	79	-	-	-	-	-	-	NCP	IGR	-	ID=gene-E	6	4.99	1815	0.960696783	VPYALL	65	675.4049	1	674.4003	.	S	Superscaf	2029533	BTP20220	0	0	0	0	0	Tissue specific
PGMDC	Superscaf	+	Superscaf	2048434	2048448	11	11	15	-	-	-	-	-	-	NCP	IGR	-	ID=gene-E	5	5.202	2003.4	0.960330423	PGM(+15.9	55	595.1831	1	594.1778	Oxidatior	R	Superscaf	2048404	0	0	BTP20220	0	0	0	Mixed
QMLGY	Superscaf	-	Superscaf	2114185	2114199	-5	5	9	-	-	-	-	-	-	NCP	IGR	-	ID=gene-E	5	5.802	2092.4	0.959057111	QMLGY	93	611.2839	1	610.2785	.	G	Superscaf	2114211	0	0	BTP20220	0	0	0	Tissue specific
PVGMQPR	Superscaf	-	Superscaf	2252668	2252688	-6	6	12	-	-	-	-	-	-	NCP	IGR	-	ID=gene-E	7	5.818571429	2603.1	0.956375178	PVGM(+15.	50	400.7075	2	799.4011	Oxidatior	S	Superscaf	2252745	0	0	0	0	0	BTP20220	Tissue specific
EGGHG	Superscaf	-	Superscaf	2274403	2274417	-92	92	96	-	-	-	-	-	-	NCP	IGR	-	ID=gene-E	5	5.744	1937.1	0.955954381	EGGHG	61	456.1758	1	455.1765	.	W	Superscaf	2274462	0	0	BTP20220	0	0	0	Mixed
GPLQYR	Superscaf	+	Superscaf	2283784	2283801	7	7	12	-	-	-	-	-	-	NCP	IGR	-	ID=gene-E	6	6.72	2707.1	0.955772654	GPLQYR	53	367.2026	2	732.3918	.	R	Superscaf	2283766	0	0	BTP20220	0	0	0	Tissue specific
NDGMVP	Superscaf	+	Superscaf	2288437	2288454	5	5	10	-	-	-	-	-	-	NCP	IGR	-	ID=gene-E	6	4.4	2014.4	0.955682545	N(+42.01)	52	690.2677	1	689.269	Acetylati	T	Superscaf	2288425	0	0	0	0	0	BTP20220	Tissue specific
FDDNVF	Superscaf	+	Superscaf	2428909	2428926	8	8	13	-	-	-	-	-	-	NCP	IGR	-	ID=gene-E	6	3.718333333	2194.5	0.95296221	FDDNVF	57	378.6636	2	755.3126	.	G	Superscaf	2428888	0	0	BTP20220	0	0	0	Tissue specific
VEVGLLD	Superscaf	+	Superscaf	2499769	2499789	20	20	26	-	-	gene	-	-	mRNA	NCP	Intron	gene|mRN	ID=gene-E	7	3.445714286	1841.1	0.951589901	VEVGLLD	67	744.4114	1	743.4065	.	A	Superscaf	2499712	0	0	BTP20220	0	0	0	Tissue specific
GNNSL	Superscaf	+	Superscaf	2500045	2500059	4	4	8	-	-	gene	-	-	mRNA	NCP	Intron	gene|mRN	ID=gene-E	5	5.69	2227.5	0.951584672	G(+42.01)	54	546.2563	1	545.2446	Acetylati	F	Superscaf	2500036	0	0	BTP20220	0	0	BTP20220	Tissue specific
LTEGL	Superscaf	-	Superscaf	2507218	2507232	-9	9	13	-	-	gene	-	-	mRNA	NCP	Intron	gene|mRN	ID=gene-E	5	5.536	1827.1	0.951445762	LTEGL	52	532.2957	1	531.2904	.	T	Superscaf	2507403	0	0	0	0	0	BTP20220	Tissue specific
HLAAP	Superscaf	-	Superscaf	2566405	2566419	-7	7	11	-	-	gene	-	-	mRNA	NCP	Intron	gene|mRN	ID=gene-E	5	6.382	1933.1	0.950299566	HLAAP	62	508.2875	1	507.2805	.	W	Superscaf	2566698	0	0	0	0	0	BTP20220	Tissue specific
NDMSDC	Superscaf	-	Superscaf	2575672	2575689	-5	5	10	-	-	gene	-	-	mRNA	NCP	Intron	gene|mRN	ID=gene-E	6	4.633333333	2760.3	0.950120046	NDM(+15.9	56	757.2183	1	756.2054	Oxidatior	K	Superscaf	2575698	0	0	0	BTP20220	0	0	Tissue specific
WPPGFPP	Superscaf	+	Superscaf	2628028	2628048	33	33	39	-	-	-	-	-	-	NCP	IGR	-	ID=gene-E	7	6.077142857	2560.9	0.949106079	WPPGFPP	59	399.2012	2	796.3907	.	F	Superscaf	2627932	0	0	0	BTP20220	BTP20220	0	Tissue specific
KVGLPL	Superscaf	-	Superscaf	2737771	2737788	-14	14	19	-	-	-	-	-	-	NCP	IGR	-	ID=gene-E	6	5.661666667	1809.9	0.946980889	KVGLPL	74	626.4209	1	625.4163	.	K	Superscaf	2737812	BTP20220	0	0	0	0	0	Tissue specific
KDVLLEF	Superscaf	-	Superscaf	2791156	2791176	-3	3	9	-	-	-	-	-	-	NCP	IGR	-	ID=gene-E	7	4.767142857	2399.6	0.945946995	KDVLLEF	81	432.2452	2	862.48	.	T	Superscaf	2791176	0	0	0	BTP20220	0	0	Tissue specific
LAPPKSR	Superscaf	+	Superscaf	2869000	2869020	15	15	21	-	-	-	-	-	-	NCP	IGR	-	ID=gene-E	7	7.254285714	2840.1	0.944439493	LAPPKSR	61	384.744	2	767.4653	.	T	Superscaf	2868958	0	BTP20220	0	0	0	0	Tissue specific
EPTMVK	Superscaf	-	Superscaf	2870293	2870310	-15	15	20	-	-	-	-	-	-	NCP	IGR	-	ID=gene-E	6	5.256666667	2000.3	0.944414512	EPTMVK	51	352.6891	2	703.3575	.	G	Superscaf	2870337	0	BTP20220	0	0	BTP20220	BTP20220	Tissue specific
RCHVLPK	Superscaf	+	Superscaf	2895889	2895909	7	7	13	-	-	-	-	-	-	NCP	IGR	-	ID=gene-E	7	6.484285714	3013.4	0.94391877	RC(+57.02	60	455.2665	2	908.5014	Carbamidc	T	Superscaf	2895871	0	0	0	0	BTP20220	0	Tissue specific
RTTYSGL	Superscaf	+	Superscaf	2910589	2910609	36	36	42	-	-	-	-	-	-	NCP	IGR	-	ID=gene-E	7	6.73	2937.4	0.943634094	RTTYSGL	62	399.2104	2	796.4079	.	R	Superscaf	2910484	0	BTP20220	0	BTP20220	BTP20220	BTP20220	Tissue specific
VGGFHFK	Superscaf	-	Superscaf	2962882	2962902	-32	32	38	-	-	-	-	-	-	NCP	IGR	-	ID=gene-E	7	5.747142857	2457.5	0.942621405	VGGFHFK	80	396.2104	2	790.4126	.	G	Superscaf	2962944	0	0	0	0	0	BTP20220	Tissue specific
SGFNLG	Superscaf	-	Superscaf	3017350	3017367	-17	17	22	-	-	-	-	-	-	NCP	IGR	-	ID=gene-E	6	5.748333333	2315.5	0.941566654	SGFNLG	53	594.2938	1	593.2809	.	F	Superscaf	3017580	0	0	0	BTP20220	0	0	Tissue specific
VSKLSWKP	Superscaf	-	Superscaf	3031807	3031830	-14	14	21	-	-	-	-	-	-	NCP	IGR	-	ID=gene-E	8	6.12625	2921	0.941286568	V(+42.01)	51	493.7802	2	985.5596	Acetylati	R	Superscaf	3031866	0	0	0	0	0	BTP20220	Tissue specific
SPYKHR	Superscaf	-	Superscaf	3043126	3043143	-4	4	9	-	-	-	-	-	-	NCP	IGR	-	ID=gene-E	6	7.621666667	2917.2	0.941067484	SPYKHR	76	394.2079	2	786.4136	.	D	Superscaf	3043149	0	0	0	0	BTP20220	0	Tissue specific
MWTAY	Superscaf	-	Superscaf	3072505	3072519	-11	11	15	-	-	-	-	-	-	NCP	IGR	-	ID=gene-E	5	5.97	2066.4	0.940498598	M(+42.01)	53	713.2937	1	712.2891	Acetylati	E	Superscaf	3072525	0	BTP20220	0	BTP20220	BTP20220	0	Tissue specific
SGRPPVV	Superscaf	-	Superscaf	3079699	3079719	-22	22	28	-	-	-	-	-	-	NCP	IGR	-	ID=gene-E	7	5.001428571	2004.3	0.940359165	SGRPPVV	64	356.2094	2	710.4075	.	D	Superscaf	3079746	0	0	0	BTP20220	0	0	Tissue specific
MELPP	Superscaf	-	Superscaf	3091696	3091710	-1	1	5	-	-	-	-	-	-	NCP	IGR	-	ID=gene-E	5	5.51	1825.2	0.940126951	M(+42.01)	98	628.2986	1	627.2938	Acetylati	M	Superscaf	3091716	0	0	BTP20220	0	0	0	Tissue specific
LSGGAAPG	Superscaf	-	Superscaf	3097717	3097740	-13	13	20	-	-	-	-	-	-	NCP	IGR	-	ID=gene-E	8	5.98875	2588.7	0.940010176	LSGGAAPG	53	629.3251	1	628.318	.	P	Superscaf	3098238	0	0	0	BTP20220	0	0	Tissue specific
GVAVYG	Superscaf	+	Superscaf	3155902	3155919	40	40	45	-	-	-	-	-	-	NCP	IGR	-	ID=gene-E	6	3.936666667	1365.4	0.938883501	GVAVYG	55	565.2962	1	564.2907	.	E	Superscaf	3155785	0	0	0	0	0	BTP20220	Tissue specific
VVAAP	Superscaf	+	Superscaf	3358300	3358314	50	50	54	-	-	gene	-	-	mRNA	NCP	Intron	gene|mRN	ID=gene-E	5	3.668	960	0.934963985	VVAAP	94	456.2799	1	455.2744	.	F	Superscaf	3358153	0	0	BTP20220	BTP20220	0	0	Tissue specific
VSKPTLN	Superscaf	-	Superscaf	3403720	3403740	-6	6	12	-	-	gene	-	-	mRNA	NCP	Intron	gene|mRN	ID=gene-E	7	5.662857143	2536.8	0.93408428	VSKPTLN	99	379.7229	2	757.4334	.	L	Superscaf	3403758	0	0	0	0	BTP20220	0	Tissue specific
KTSTDRK	Superscaf	+	Superscaf	3421960	3421980	42	42	48	-	-	gene	-	-	mRNA	NCP	Intron	gene|mRN	ID=gene-E	7	7.421428571	3175.5	0.93373105	KTSTDRK	56	418.2349	2	834.4559	.	P	Superscaf	3421837	0	0	0	BTP20220	0	0	Tissue specific
KAGTSGLP	Superscaf	-	Superscaf	3466975	3466998	-2	2	9	-	-	gene	-	-	mRNA	NCP	Intron	gene|mRN	ID=gene-E	8	6.52375	2846	0.932859246	KAGTSGLP	53	730.4087	1	729.4021	.	L	Superscaf	3467025	0	0	0	0	0	BTP20220	Tissue specific
VSMTY	Superscaf	+	Superscaf	3485605	3485619	1	1	5	-	-	gene	-	-	mRNA	NCP	Intron	gene|mRN	ID=gene-E	5	4.724	1611.9	0.932498638	VSMTY	90	600.2672	1	599.2625	.	V	Superscaf	3485605	0	0	0	0	0	BTP20220	Mixed
VSVPGPQ	Superscaf	-	Superscaf	3517174	3517194	-29	29	35	-	-	gene	-	-	mRNA	NCP	Intron	gene|mRN	ID=gene-E	7	4.271428571	1966.2	0.931887167	V(+42.01)	62	725.3798	1	724.3755	Acetylati	K	Superscaf	3517299	BTP20220	0	0	0	0	0	Tissue specific
KGTLSVH	Superscaf	+	Superscaf	3533413	3533433	9	9	15	-	-	-	-	-	-	NCP	IGR	-	ID=gene-E	7	5.927142857	2519.7	0.931572688	KGTLSVH	88	371.2097	2	740.4181	.	Q	Superscaf	3533389	0	0	0	0	0	BTP20220	Mixed
PPPPRQPPF	Superscaf	-	Superscaf	3582874	3582900	-7	7	15	-	-	-	-	-	-	NCP	IGR	-	ID=gene-E	9	6.723333333	3621.3	0.930614726	PPPPRQPPF	97	491.7751	2	981.5396	.	H	Superscaf	3582921	0	0	0	BTP20220	0	0	Tissue specific
RKKKYAR	Superscaf	+	Superscaf	3609043	3609063	102	102	108	-	-	-	-	-	-	NCP	IGR	-	ID=gene-E	7	8.917142857	3473.6	0.930108062	RKKKYAR	59	475.311	2	948.5981	.	F	Superscaf	3608740	0	0	BTP20220	0	0	0	Tissue specific
EGPFHYK	Superscaf	-	Superscaf	3615808	3615828	-69	69	75	-	-	-	-	-	-	NCP	IGR	-	ID=gene-E	7	6.28	2995.2	0.929977053	EGPFHYK	55	439.2096	2	876.413	.	S	Superscaf	3615855	0	0	0	0	BTP20220	0	Tissue specific
LGKSEQLK	Superscaf	+	Superscaf	3694534	3694557	60	60	67	-	-	-	-	-	-	NCP	IGR	-	ID=gene-E	8	6.495	3360.5	0.928452413	LGKSEQLK	77	451.7677	2	901.5233	.	E	Superscaf	3694357	0	0	0	BTP20220	0	0	Tissue specific
EHALLA	Superscaf	+	Superscaf	3708925	3708942	10	10	15	-	-	-	-	-	-	NCP	IGR	-	ID=gene-E	6	5.801666667	2374.6	0.928173837	EHALLA	98	653.3593	1	652.3544	.	P	Superscaf	3708898	0	0	0	BTP20220	0	0	Tissue specific
SALVMD	Superscaf	-	Superscaf	3735382	3735399	-3	3	8	-	-	-	-	-	-	NCP	IGR	-	ID=gene-E	6	4.4	1831.1	0.92766148	SALVMD	51	635.3026	1	634.2996	.	V	Superscaf	3735459	0	0	0	BTP20220	0	0	Tissue specific
QVGLGP	Superscaf	-	Superscaf	3758173	3758190	-31	31	36	-	-	-	-	-	-	NCP	IGR	-	ID=gene-E	6	4.978333333	1896.1	0.927220117	Q(+42.01)	53	612.3328	1	611.3279	Acetylati	V	Superscaf	3758217	0	0	0	0	0	BTP20220	Tissue specific
NPVAGF	Superscaf	-	Superscaf	3759961	3759978	-6	6	11	-	-	-	-	-	-	NCP	IGR	-	ID=gene-E	6	4.863333333	1930.1	0.927185491	N(+42.01)	62	646.3254	1	645.3122	Acetylati	V	Superscaf	3760032	0	0	0	0	0	BTP20220	Tissue specific
VAHVYKP	Superscaf	+	Superscaf	3793033	3793053	22	22	28	-	-	-	-	-	-	NCP	IGR	-	ID=gene-E	7	5.044285714	2196.3	0.926544971	VAHVYKP	59	407.2316	2	812.4545	.	F	Superscaf	3792970	0	0	0	BTP20220	0	0	Tissue specific
GADSY	Superscaf	+	Superscaf	3821173	3821187	34	34	38	-	-	-	-	-	-	NCP	IGR	-	ID=gene-E	5	5.26	1863	0.926000137	GADSY	52	512.2083	1	511.1914	.	K	Superscaf	3821074	BTP20220	BTP20220	BTP20220	BTP20220	BTP20220	BTP20220	Tissue specific
LLLTGKFL	Superscaf	-	Superscaf	3854806	3854829	-6	6	13	-	-	-	-	-	-	NCP	IGR	-	ID=gene-E	8	6.455	2964.2	0.925348637	LLLTGKFL	83	452.7948	2	903.5793	.	W	Superscaf	3854889	0	0	0	0	0	BTP20220	Tissue specific
VTEEALLP	Superscaf	-	Superscaf	3872569	3872592	-26	26	33	-	-	-	-	-	-	NCP	IGR	-	ID=gene-E	8	4.65625	2604	0.925004645	V(+42.01)	73	913.4874	1	912.4804	Acetylati	I	Superscaf	3872784	0	0	0	0	BTP20220	0	Tissue specific
LLMVEPK	Superscaf	-	Superscaf	3904903	3904923	-16	16	22	-	-	gene	-	-	mRNA	NCP	Intron	gene|mRN	ID=gene-E	7	5.281428571	2309.6	0.924378533	LLM(+15.9	69	423.2451	2	844.4728	Oxidatior	K	Superscaf	3904923	BTP20220	0	0	0	0	0	Tissue specific
FMGQAW	Superscaf	+	Superscaf	3958264	3958281	12	12	17	-	-	gene	-	-	mRNA	NCP	Intron	gene|mRN	ID=gene-E	6	5.793333333	2504.8	0.92334522	FMGQAW	59	739.3201	1	738.3159	.	R	Superscaf	3958231	0	0	BTP20220	0	0	0	Tissue specific
KHAAPPP	Superscaf	-	Superscaf	3988120	3988140	-37	37	43	-	-	-	-	-	-	NCP	IGR	-	ID=gene-E	7	6.895714286	2722.9	0.92276698	KHAAPPP	58	717.4152	1	716.3969	.	R	Superscaf	3988185	0	BTP20220	0	0	0	0	Tissue specific
AAQGEPQVG	Superscaf	+	Superscaf	4013161	4013193	78	78	88	-	-	-	-	-	-	NCP	IGR	-	ID=gene-E	11	5.427272727	4370.7	0.922281812	A(+42.01)	78	622.8165	2	1243.62	Acetylati	H	Superscaf	4012930	0	0	BTP20220	BTP20220	0	0	Mixed
AAQGEPQVG	Superscaf	+	Superscaf	4013161	4013190	78	78	87	-	-	-	-	-	-	NCP	IGR	-	ID=gene-E	10	4.996	3902.3	0.92228187	A(+42.01)	68	558.7683	2	1115.525	Acetylati	H	Superscaf	4012930	0	0	0	0	0	BTP20220	Mixed
EPFLDPL	Superscaf	-	Superscaf	4143673	4143693	-19	19	25	-	-	-	-	-	-	NCP	IGR	-	ID=gene-E	7	5.175714286	2606	0.919754591	EPFLDPL	69	830.4234	1	829.4221	.	R	Superscaf	4143846	0	0	0	0	BTP20220	0	Tissue specific
RDSGKNP	Superscaf	-	Superscaf	4164502	4164522	-68	68	74	-	-	gene	-	-	mRNA	NCP	Intron	gene|mRN	ID=gene-E	7	6.69	3143.5	0.919351224	RDSGKNP	71	387.1939	2	772.3828	.	G	Superscaf	4164615	0	0	0	0	0	BTP20220	Tissue specific
MSLLLNTFY	Superscaf	+	Superscaf	4263766	4263792	15	15	23	-	-	-	-	-	-	NCP	IGR	-	ID=gene-E	9	5.827777778	3656.2	0.917428793	MSLLLNTFY	76	551.2839	2	1100.558	.	N	Superscaf	4263724	0	0	0	0	BTP20220	0	Tissue specific
TYLVHR	Superscaf	-	Superscaf	4279858	4279875	-5	5	10	-	-	-	-	-	-	NCP	IGR	-	ID=gene-E	6	6.086666667	2478.9	0.917117335	T(+42.01)	81	415.7325	2	829.4446	Acetylati	R	Superscaf	4279914	BTP20220	0	0	0	0	0	Tissue specific
SVYGLA	Superscaf	-	Superscaf	4336837	4336854	-25	25	30	-	-	-	-	-	-	NCP	IGR	-	ID=gene-E	6	4.885	1804.9	0.916013898	SVYGLA	52	609.3228	1	608.317	.	S	Superscaf	4336872	0	0	0	0	0	BTP20220	Tissue specific
QTYVY	Superscaf	-	Superscaf	4453069	4453083	-9	9	13	-	-	-	-	-	-	NCP	IGR	-	ID=gene-E	5	4.7	1927.2	0.913763045	QTYVY	78	673.318	1	672.3119	.	F	Superscaf	4453155	0	0	BTP20220	0	0	0	Mixed
ATEPSL	Superscaf	+	Superscaf	4468375	4468392	45	45	50	-	-	gene	-	-	mRNA	NCP	Intron	gene|mRN	ID=gene-E	6	5.621666667	2208.5	0.913466576	ATEPSL	54	617.3187	1	616.3068	.	I	Superscaf	4468243	0	0	0	BTP20220	0	0	Tissue specific
LGKRCAY	Superscaf	+	Superscaf	4548637	4548657	5	5	11	-	-	-	-	-	-	NCP	IGR	-	ID=gene-E	7	7.021428571	3124.4	0.91191219	LGKRC(+57	99	434.2275	2	866.4432	Carbamidc	T	Superscaf	4548625	0	0	BTP20220	0	0	0	Tissue specific
MPGGK	Superscaf	+	Superscaf	4548685	4548699	16	16	20	-	-	-	-	-	-	NCP	IGR	-	ID=gene-E	5	6.746	1771.9	0.911911377	M(+42.01)	53	531.2529	1	530.2523	Acetylati	A	Superscaf	4548640	0	BTP20220	0	0	0	0	Mixed
HSLLVTAL	Superscaf	+	Superscaf	4623694	4623717	7	7	14	-	-	-	-	-	-	NCP	IGR	-	ID=gene-E	8	5.47	2772.1	0.910458603	HSLLVTAL	62	427.2594	2	852.5069	.	F	Superscaf	4623676	BTP20220	0	BTP20220	0	0	0	Tissue specific
SLTLKY	Superscaf	-	Superscaf	4738210	4738227	-1	1	6	-	-	-	-	-	-	NCP	IGR	-	ID=gene-E	6	6.595	2415.6	0.908241039	SLTLKY	85	724.421	1	723.4167	.	S	Superscaf	4738239	0	0	BTP20220	0	0	BTP20220	Tissue specific
HVSPSKE	Superscaf	-	Superscaf	4745080	4745100	-1	1	7	-	-	gene	-	-	mRNA	NCP	Intron	gene|mRN	ID=gene-E	7	5.458571429	2617.8	0.908107939	HVSPSKE	74	392.2065	2	782.3923	.	H	Superscaf	4745106	0	BTP20220	BTP20220	BTP20220	BTP20220	BTP20220	Tissue specific
ARPFP	Superscaf	+	Superscaf	4753585	4753599	48	48	52	-	-	gene	-	-	mRNA	NCP	Intron	gene|mRN	ID=gene-E	5	6.972	2022.3	0.90794335	ARPFP	68	587.3279	1	586.3227	.	C	Superscaf	4753444	0	0	0	0	0	BTP20220	Tissue specific
PSVPPG	Superscaf	-	Superscaf	4882027	4882044	-1	1	6	-	-	gene	-	-	mRNA	NCP	Intron	gene|mRN	ID=gene-E	6	5.091666667	1692.9	0.905455926	P(+42.01)	63	595.3063	1	594.3013	Acetylati	P	Superscaf	4882356	0	0	0	BTP20220	0	0	Tissue specific
TPAKMPK	Superscaf	-	Superscaf	5064757	5064777	-3	3	9	-	-	-	-	-	-	NCP	IGR	-	ID=gene-E	7	7.197142857	2691.9	0.901917179	T(+42.01)	70	407.7288	2	813.4418	Acetylati	L	Superscaf	5064807	0	0	0	0	BTP20220	0	Tissue specific
VVFAPPK	Superscaf	+	Superscaf	5069071	5069091	186	186	192	-	-	-	-	-	-	NCP	IGR	-	ID=gene-E	7	4.834285714	1841.9	0.901833635	VVFAPPK	80	379.2348	2	756.4534	.	L	Superscaf	5068516	0	0	0	0	BTP20220	0	Tissue specific
KPLKVSH	Superscaf	+	Superscaf	5076649	5076669	5	5	11	-	-	-	-	-	-	NCP	IGR	-	ID=gene-E	7	6.432857143	2630.7	0.901686882	KPLKVSH	56	404.7489	2	807.4966	.	K	Superscaf	5076637	0	0	BTP20220	0	0	0	Tissue specific
SREGMLTP	Superscaf	+	Superscaf	5078821	5078844	25	25	32	-	-	-	-	-	-	NCP	IGR	-	ID=gene-E	8	6.27375	3214.8	0.901644762	SREGMLTP	65	445.7221	2	889.4327	.	S	Superscaf	5078749	0	0	BTP20220	BTP20220	0	BTP20220	Tissue specific
DEGLRPL	Superscaf	-	Superscaf	5096038	5096058	-8	8	14	-	-	-	-	-	-	NCP	IGR	-	ID=gene-E	7	5.882857143	2827.3	0.901311401	DEGLRPL	53	400.2171	2	798.4235	.	N	Superscaf	5096121	0	BTP20220	0	0	0	0	Tissue specific

NGCPPPYHT	Superscaf	+	Superscaf	5127433	5127489	2	2	20	-	-	gene	-	-	mRNA	NCP	Intron	gene|mRN	ID=gene-E	19	5.611578947	7819.9	0.900702718	NGC(+57.	93	1132.024	2	2262.042	Carbamido	L	Superscaf	5127430	0	0	0	0	BTP20220	0	Tissue specific
KPFWNK	Superscaf	-	Superscaf	5186767	5186784	-6	6	11	-	-	-	-	-	-	NCP	IGR	-	ID=gene-E	6	7.093333333	2784.9	0.899554431	KPFWNK	68	410.2352	2	818.4439	.	E	Superscaf	5186796	0	0	0	BTP20220	0	0	Tissue specific
YALPHALL	Superscaf	+	Superscaf	5197648	5197671	60	60	67	-	-	-	-	-	-	NCP	IGR	-	ID=gene-E	8	6.19125	3099.4	0.899343597	YALPHALL	73	449.2616	2	896.512	.	M	Superscaf	5197471	0	0	0	BTP20220	0	0	Tissue specific
LPHALLEVF	Superscaf	+	Superscaf	5197654	5197680	62	62	70	-	-	-	-	-	-	NCP	IGR	-	ID=gene-E	9	5.758888889	3402.9	0.899343422	LPHALLEVI	79	524.317	2	1046.624	.	M	Superscaf	5197471	0	0	BTP20220	0	0	0	Tissue specific
PGPHELL	Superscaf	+	Superscaf	5258062	5258082	15	15	21	-	-	-	-	-	-	NCP	IGR	-	ID=gene-E	7	5.905714286	2724.1	0.898173697	P(+42.01)	66	402.7159	2	803.4177	Acetylati	S	Superscaf	5258020	0	0	0	0	0	BTP20220	Tissue specific
DLLYRAL	Superscaf	-	Superscaf	5331448	5331468	-10	10	16	-	-	-	-	-	-	NCP	IGR	-	ID=gene-E	7	6.192857143	2891.3	0.896752527	DLLYRAL	61	432.2537	2	862.4912	.	R	Superscaf	5331468	0	0	0	0	BTP20220	0	Tissue specific
CGEQHYR	Superscaf	-	Superscaf	5353114	5353134	-58	58	64	-	-	-	-	-	-	NCP	IGR	-	ID=gene-E	7	6.267142857	3647.2	0.896332951	C(+57.02)	56	475.1982	2	948.3872	Carbamido	Q	Superscaf	5353155	0	BTP20220	0	0	0	0	Tissue specific
HGPPPP	Superscaf	+	Superscaf	5372188	5372205	8	8	13	-	-	-	-	-	-	NCP	IGR	-	ID=gene-E	6	6.46	2266.6	0.895963628	HGPPPP	76	601.3066	1	600.3019	.	Y	Superscaf	5372167	0	0	0	0	BTP20220	0	Mixed
GPPYHPP	Superscaf	+	Superscaf	5381188	5381208	41	41	47	-	-	-	-	-	-	NCP	IGR	-	ID=gene-E	7	6.345714286	2726.1	0.895789279	GPPYHPP	93	382.6884	2	763.3653	.	P	Superscaf	5381068	0	BTP20220	0	0	0	0	Tissue enhanced
LVLSKH	Superscaf	-	Superscaf	5398150	5398167	-124	124	129	-	-	-	-	-	-	NCP	IGR	-	ID=gene-E	6	5.828333333	2178.3	0.895460856	LVLSKH	53	696.4348	1	695.433	.	W	Superscaf	5398338	0	0	BTP20220	0	0	0	Tissue specific
EAPTSMP	Superscaf	+	Superscaf	5447743	5447763	9	9	15	-	-	-	-	-	-	NCP	IGR	-	ID=gene-E	7	5.685714286	2564	0.894500396	E(+42.01)	63	790.3271	1	789.3215	Acetylati	A	Superscaf	5447719	0	0	0	0	0	BTP20220	Tissue specific
NDFTGKK	Superscaf	-	Superscaf	5461918	5461938	-6	6	12	-	-	-	-	-	-	NCP	IGR	-	ID=gene-E	7	6.548571429	3027.2	0.894225888	NDFTGKK	83	405.21	2	808.4079	.	L	Superscaf	5461965	0	0	BTP20220	0	0	0	Tissue specific
LTYGFN	Superscaf	+	Superscaf	5490964	5490981	5	5	10	-	-	-	-	-	-	NCP	IGR	-	ID=gene-E	6	5.838333333	2491.8	0.893663451	LTYGFN	98	714.3455	1	713.3384	.	R	Superscaf	5490952	0	0	0	0	0	BTP20220	Mixed
VSMLFM	Superscaf	+	Superscaf	5508622	5508639	34	34	39	-	-	-	-	-	-	NCP	IGR	-	ID=gene-E	6	4.773333333	1867.2	0.893321492	VSMLFM	63	727.3488	1	726.3445	.	T	Superscaf	5508523	0	BTP20220	0	0	0	0	Tissue specific
YLLQDM	Superscaf	-	Superscaf	5512000	5512017	-44	44	49	-	-	-	-	-	-	NCP	IGR	-	ID=gene-E	6	5.331666667	2560	0.893256074	YLLQDM	53	391.69	2	781.368	.	F	Superscaf	5512074	0	0	0	BTP20220	0	0	Tissue specific
AEVQPKGP	Superscaf	+	Superscaf	5568538	5568561	16	16	23	-	-	-	-	-	-	NCP	IGR	-	ID=gene-E	8	5.4	2788	0.892161062	AEVQPKGP	60	413.2263	2	824.4391	.	L	Superscaf	5568493	0	0	BTP20220	0	0	0	Tissue specific
YAAPPP	Superscaf	+	Superscaf	5574535	5574552	7	7	12	-	-	-	-	-	-	NCP	IGR	-	ID=gene-E	6	6.1	2094.3	0.892045042	YAAPPP	71	615.3118	1	614.3064	.	V	Superscaf	5574517	0	BTP20220	0	0	0	0	Tissue specific
LYEYTQ	Superscaf	+	Superscaf	5584327	5584344	37	37	42	-	-	-	-	-	-	NCP	IGR	-	ID=gene-E	6	5.45	2706.1	0.891855413	LYEYTQ	63	408.6938	2	815.3701	.	F	Superscaf	5584219	0	0	0	0	0	BTP20220	Tissue specific
VPLTFWT	Superscaf	+	Superscaf	5584597	5584617	29	29	35	-	-	-	-	-	-	NCP	IGR	-	ID=gene-E	7	5.244285714	2399.8	0.891850126	V(+42.01)	56	453.239	2	904.4694	Acetylati	I	Superscaf	5584513	0	0	0	0	BTP20220	0	Tissue specific
AMNLRK	Superscaf	-	Superscaf	5596666	5596683	-62	62	67	-	-	-	-	-	-	NCP	IGR	-	ID=gene-E	6	7.276666667	2750.1	0.89161646	A(+42.01)	69	395.7142	2	789.4167	Acetylati	K	Superscaf	5597097	0	0	BTP20220	0	0	0	Tissue specific
LGEAPA	Superscaf	+	Superscaf	5711863	5711880	12	12	17	-	-	gene	-	-	mRNA	NCP	Intron	gene|mRN	ID=gene-E	6	5.585	2036.2	0.889385593	LGEAPA	89	557.2908	1	556.2856	.	R	Superscaf	5711830	0	0	0	BTP20220	0	0	Tissue specific
KYKLPPPP	Superscaf	+	Superscaf	5712304	5712327	33	33	40	-	-	gene	-	-	mRNA	NCP	Intron	gene|mRN	ID=gene-E	8	7.04	3099.3	0.889376936	KYKLPPPP	61	470.2853	2	938.5589	.	R	Superscaf	5712208	BTP20220	0	0	0	0	0	Tissue specific
KHSPAAYRK	Superscaf	+	Superscaf	5778106	5778132	15	15	23	-	-	gene	-	-	mRNA	NCP	Intron	gene|mRN	ID=gene-E	9	7.501111111	4008.2	0.888102578	KHSPAAYRK	58	353.1985	3	1056.583	.	I	Superscaf	5778064	0	0	0	0	BTP20220	0	Tissue specific
LPVKRGPPF	Superscaf	+	Superscaf	5806549	5806575	32	32	40	-	-	gene	-	-	mRNA	NCP	Intron	gene|mRN	ID=gene-E	9	6.405555556	3117.5	0.88755176	LPVKRGPPF	81	480.8016	2	959.5916	.	P	Superscaf	5806456	0	0	0	0	BTP20220	0	Tissue specific
GLSVVK	Superscaf	+	Superscaf	5814340	5814357	4	4	9	-	-	-	-	-	-	NCP	IGR	-	ID=gene-E	6	4.561666667	1502.5	0.887401056	G(+42.01)	73	644.3959	1	643.3904	Acetylati	G	Superscaf	5814331	0	0	0	0	0	BTP20220	Tissue specific
KYDDLP	Superscaf	+	Superscaf	5853157	5853174	22	22	27	-	-	-	-	-	-	NCP	IGR	-	ID=gene-E	6	5.603333333	2441.7	0.886649339	KYDDLP	60	750.355	1	749.3596	.	V	Superscaf	5853094	0	0	BTP20220	0	0	0	Tissue specific
RKLTLQY	Superscaf	+	Superscaf	5882170	5882190	7	7	13	-	-	-	-	-	-	NCP	IGR	-	ID=gene-E	7	7.185714286	3291.7	0.886087425	R(+42.01)	62	482.2776	2	962.5549	Acetylati	H	Superscaf	5882152	0	0	0	0	0	BTP20220	Tissue specific
LDVSAKP	Superscaf	+	Superscaf	5970526	5970546	14	14	20	-	-	-	-	-	-	NCP	IGR	-	ID=gene-E	7	5.241428571	2265.4	0.884376351	LDVSAKP	99	365.211	2	728.4068	.	K	Superscaf	5970487	0	0	0	0	BTP20220	0	Tissue specific
KPEGMF	Superscaf	-	Superscaf	5976880	5976897	-5	5	10	-	-	-	-	-	-	NCP	IGR	-	ID=gene-E	6	6.076666667	2287.5	0.88425336	KPEGMF	50	354.6785	2	707.3312	.	I	Superscaf	5976978	0	0	0	0	BTP20220	0	Tissue specific
HLDVKP	Superscaf	+	Superscaf	6012934	6012951	3	3	8	-	-	-	-	-	-	NCP	IGR	-	ID=gene-E	6	5.43	2190.4	0.88355515	HLDVKP	57	354.7047	2	707.3966	.	R	Superscaf	6012928	BTP20220	0	0	0	0	0	Tissue specific
KYAPPPP	Superscaf	-	Superscaf	6083527	6083547	-3	3	9	-	-	-	-	-	-	NCP	IGR	-	ID=gene-E	7	6.66	2588.8	0.882188011	KYAPPPP	80	385.2123	2	768.417	.	I	Superscaf	6083607	0	0	0	0	BTP20220	BTP20220	Tissue specific
EGSYKS	Superscaf	+	Superscaf	6146653	6146670	23	23	28	-	-	-	-	-	-	NCP	IGR	-	ID=gene-E	6	5.991666667	2417.5	0.880965591	EGSYKS	65	670.2976	1	669.2969	.	V	Superscaf	6146587	0	0	0	0	BTP20220	BTP20220	Tissue specific
VKALGPH	Superscaf	-	Superscaf	6208096	6208116	-8	8	14	-	-	-	-	-	-	NCP	IGR	-	ID=gene-E	7	5.942857143	2387.5	0.879775648	V(+42.01)	82	763.4435	1	762.4388	Acetylati	G	Superscaf	6208140	0	0	0	0	BTP20220	0	Tissue specific
LKVFKPK	Superscaf	-	Superscaf	6213496	6213516	-7	7	13	-	-	-	-	-	-	NCP	IGR	-	ID=gene-E	7	6.711428571	2483.4	0.879671073	LKVFKPK	99	430.2899	2	858.569	.	C	Superscaf	6213555	0	BTP20220	0	0	0	0	Tissue specific
HLLKAPH	Superscaf	+	Superscaf	6238594	6238614	92	92	98	-	-	-	-	-	-	NCP	IGR	-	ID=gene-E	7	7.028571429	3063.3	0.879185034	HLLKAPH	61	408.2471	2	814.4813	.	T	Superscaf	6238321	0	BTP20220	0	0	0	0	Tissue specific
LLLDRMM	Superscaf	+	Superscaf	6258808	6258828	31	31	37	-	-	-	-	-	-	NCP	IGR	-	ID=gene-E	7	6.167142857	2863.5	0.878793576	LLLDRMM	55	446.2456	2	890.4718	.	R	Superscaf	6258718	0	0	BTP20220	0	0	0	Tissue specific
ESQYPVEKI	Superscaf	+	Superscaf	6259543	6259569	9	9	17	-	-	-	-	-	-	NCP	IGR	-	ID=gene-E	9	5.05	3463.8	0.878779226	ESQYPVEKI	74	546.7897	2	1091.55	.	F	Superscaf	6259519	0	0	0	0	0	BTP20220	Tissue specific
DEDCCCC	Superscaf	-	Superscaf	6345658	6345678	-15	15	21	-	-	gene	-	-	mRNA	NCP	Intron	gene|mRN	ID=gene-E	7	4.177142857	3591.3	0.877111667	D(+42.01)	74	530.6318	2	1059.24	Acetylati	G	Superscaf	6345714	0	0	0	0	BTP20220	0	Tissue specific
TKGKLLK	Superscaf	-	Superscaf	6410815	6410835	-24	24	30	-	-	gene	-	-	mRNA	NCP	Intron	gene|mRN	ID=gene-E	7	7.668571429	2806.8	0.875849858	TKGKLLK	94	394.2719	2	786.5327	.	G	Superscaf	6410877	0	0	0	0	BTP20220	0	Tissue specific
EHYKLSA	Superscaf	-	Superscaf	6410935	6410955	-1	1	7	-	-	gene	-	-	mRNA	NCP	Intron	gene|mRN	ID=gene-E	7	6.27	3021.2	0.875847534	EHYKLSA	53	424.2177	2	846.4235	.	E	Superscaf	6411135	BTP20220	0	0	0	0	0	Tissue specific
MYLVMM	Superscaf	+	Superscaf	6480622	6480639	46	46	51	-	-	-	-	-	-	NCP	IGR	-	ID=gene-E	6	4.815	1927.4	0.874498056	M(+15.99)	56	819.3561	1	818.3377	Oxidation	K	Superscaf	6480487	0	BTP20220	0	0	0	0	Tissue specific
LPLPHHK	Superscaf	+	Superscaf	6486166	6486186	24	24	30	-	-	-	-	-	-	NCP	IGR	-	ID=gene-E	7	7.068571429	3089.4	0.874390635	LPLPHHK	68	421.258	2	840.497	.	D	Superscaf	6486097	0	0	0	0	0	BTP20220	Tissue specific
KLGYPF	Superscaf	-	Superscaf	6507850	6507867	-75	75	80	-	-	-	-	-	-	NCP	IGR	-	ID=gene-E	6	6.521666667	2303.4	0.873970768	KLGYPF	68	724.397	1	723.3955	.	R	Superscaf	6507873	0	0	0	0	BTP20220	BTP20220	Tissue specific
ATELLEL	Superscaf	-	Superscaf	6547240	6547260	-1	1	7	-	-	-	-	-	-	NCP	IGR	-	ID=gene-E	7	5.275714286	2620	0.873207896	ATELLEL	71	788.4381	1	787.4327	.	A	Superscaf	6547263	BTP20220	0	0	0	0	0	Tissue specific
LAAASFFR	Superscaf	+	Superscaf	6550531	6550554	4	4	11	-	-	-	-	-	-	NCP	IGR	-	ID=gene-E	8	6.43	3094.3	0.873144105	LAAASFFR	96	441.7422	2	881.4759	.	L	Superscaf	6550522	0	0	0	0	BTP20220	0	Tissue specific
KYLCEP	Superscaf	-	Superscaf	6562420	6562437	-1	1	6	-	-	-	-	-	-	NCP	IGR	-	ID=gene-E	6	5.986666667	2629.9	0.872913983	KYLC(+57.	57	405.1947	2	808.3789	Carbamido	K	Superscaf	6562455	0	0	0	0	0	BTP20220	Tissue specific
VSGFPYL	Superscaf	+	Superscaf	6599824	6599844	9	9	15	-	-	-	-	-	-	NCP	IGR	-	ID=gene-E	7	5.01	2218.4	0.872189571	VSGFPYL	62	391.7138	2	781.401	.	D	Superscaf	6599800	0	0	BTP20220	0	0	0	Tissue specific
ALDNLTSL	Superscaf	-	Superscaf	6605398	6605421	-23	23	30	-	-	-	-	-	-	NCP	IGR	-	ID=gene-E	8	5.56875	3160.6	0.872081569	ALDNLTSL	52	846.4547	1	845.4494	.	E	Superscaf	6605433	0	0	0	0	BTP20220	0	Tissue specific
YSTVYK	Superscaf	+	Superscaf	6661294	6661311	16	16	21	-	-	gene	-	-	mRNA	NCP	Intron	gene|mRN	ID=gene-E	6	5.545	2168.3	0.870999221	YSTVYK	98	760.3829	1	759.3803	.	K	Superscaf	6661249	0	0	0	0	BTP20220	0	Tissue specific
KDLKPV	Superscaf	+	Superscaf	6684658	6684675	10	10	15	-	-	gene	-	-	mRNA	NCP	Intron	gene|mRN	ID=gene-E	6	5.788333333	2039.1	0.870546762	KDLKPV	83	350.2261	2	698.4326	.	K	Superscaf	6684631	0	0	BTP20220	0	0	0	Tissue specific
DYHKPK	Superscaf	-	Superscaf	6739711	6739728	-45	45	50	-	-	gene	-	-	mRNA	NCP	Intron	gene|mRN	ID=gene-E	6	7	2764.9	0.869480623	DYHKPK	63	394.2095	2	786.4024	.	K	Superscaf	6739746	0	0	0	0	BTP20220	BTP20220	Tissue specific
KEHFEV	Superscaf	-	Superscaf	6766540	6766557	-4	4	9	-	-	-	-	-	-	NCP	IGR	-	ID=gene-E	6	4.875	2326.5	0.868961061	KEHFEV	53	394.7049	2	787.3864	.	T	Superscaf	6766632	0	BTP20220	0	0	0	0	Tissue specific
KHGNP	Superscaf	-	Superscaf	6950794	6950808	-22	22	26	-	-	gene	-	-	mRNA	NCP	Intron	gene|mRN	ID=gene-E	5	7.002	2319.5	0.865392917	KHGNP	60	552.2939	1	551.2816	.	N	Superscaf	6950868	0	0	BTP20220	0	0	0	Tissue specific
VGTNKEK	Superscaf	-	Superscaf	6985714	6985734	-12	12	18	-	-	gene	-	-	mRNA	NCP	Intron	gene|mRN	ID=gene-E	7	5.801428571	2653.8	0.864716551	VGTNKEK	56	388.2255	2	774.4235	.	F	Superscaf	6985746	0	0	0	0	0	BTP20220	Tissue specific
LTNNKYVNE	Superscaf	+	Superscaf	6987940	6987966	15	15	23	-	-	gene	-	-	mRNA	NCP	Intron	gene|mRN	ID=gene-E	9	5.262222222	3894.4	0.864673327	LTNNKYVNE	55	547.7798	2	1093.54	.	I	Superscaf	6987898	BTP20220	0	0	0	0	0	Tissue specific
RTPTYH	Superscaf	+	Superscaf	7112155	7112172	29	29	34	-	-	-	-	-	-	NCP	IGR	-	ID=gene-E	6	7.228333333	2860.4	0.862267994	RTPTYH	90	774.3758	1	773.382	.	L	Superscaf	7112071	0	0	0	0	BTP20220	BTP20220	Tissue specific
DSLNFF	Superscaf	-	Superscaf	7114435	7114452	-24	24	29	-	-	-	-	-	-	NCP	IGR	-	ID=gene-E	6	5.166666667	2519.8	0.86222384	DSLNFF	65	742.3376	1	741.3333	.	T	Superscaf	7114476	0	0	BTP20220	0	0	0	Tissue specific
PSFLVSFP	Superscaf	+	Superscaf	7140220	7140243	27	27	34	-	-	-	-	-	-	NCP	IGR	-	ID=gene-E	8	5.1125	2569.8	0.86172438	PSFLVSFP	61	447.2506	2	892.4694	.	K	Superscaf	7140142	BTP20220	0	0	0	0	BTP20220	Tissue specific
LPNKENP	Superscaf	+	Superscaf	7165150	7165170	5	5	11	-	-	gene	-	-	mRNA	NCP	Intron	gene|mRN	ID=gene-E	7	6.051428571	3115.5	0.861241652	LPNKENP	95	406.2183	2	810.4235	.	D	Superscaf	7165138	0	BTP20220	0	BTP20220	BTP20220	BTP20220	Tissue specific
YVKLGFP	Superscaf	-	Superscaf	7193044	7193064	-28	28	34	-	-	-	-	-	-	NCP	IGR	-	ID=gene-E	7	5.59	2303.4	0.860701466	Y(+42.01)	73	433.2467	2	864.4745	Acetylati	S	Superscaf	7193118	0	BTP20220	0	0	BTP20220	BTP20220	Tissue specific
LERLKDY	Superscaf	-	Superscaf	7207198	7207218	-32	32	38	-	-	-	-	-	-	NCP	IGR	-	ID=gene-E	7	6.33	3120.5	0.860427364	LERLKDY	85	468.7598	2	935.5076	.	E	Superscaf	7207254	0	0	0	0	BTP20220	BTP20220	Mixed
VSCKNK	Superscaf	-	Superscaf	7215577	7215594	-18	18	23	-	-	-	-	-	-	NCP	IGR	-	ID=gene-E	6	5.931666667	2502.6	0.860265157	VSC(+57.	83	368.1923	2	734.3745	Carbamido	C	Superscaf	7215612	BTP20220	0	0	0	0	0	Tissue specific
FFPPPPP	Superscaf	+	Superscaf	7284664	7284684	2	2	8	-	-	-	-	-	-	NCP	IGR	-	ID=gene-E	7	6.065714286	2461.8	0.858927183	FFPPPPP	68	399.7088	2	797.4111	.	S	Superscaf	7284661	0	0	0	0	0	BTP20220	Tissue specific
KFLQLGK	Superscaf	-	Superscaf	7305856	7305876	-26	26	32	-	-	-	-	-	-	NCP	IGR	-	ID=gene-E	7	6.934285714	2939	0.858516785	KFLQLGK	63	417.269	2	832.517	.	T	Superscaf	7306038	0	0	0	0	BTP20220	0	Tissue specific
PVSPP	Superscaf	+	Superscaf	7313278	7313292	29	29	33	-	-	-	-	-	-	NCP	IGR	-	ID=gene-E	5	4.916	1395.6	0.858373169	PVSPP	61	496.2851	1	495.2693	.	L	Superscaf	7313194	0	BTP20220	0	0	0	0	Tissue specific
YPLLLST	Superscaf	+	Superscaf	7318897	7318917	45	45	51	-	-	-	-	-	-	NCP	IGR	-	ID=gene-E	7	6.015714286	2638	0.858264238	YPLLLST	55	806.4625	1	805.4586	.	E	Superscaf	7318765	0	0	0	0	BTP20220	0	Tissue specific
WDGTF	Superscaf	+	Superscaf	7389865	7389879	80	80	84	-	-	-	-	-	-	NCP	IGR	-	ID=gene-E	5	5.368	2020.3	0.856890011	WDGTF	86	625.2599	1	624.2543	.	K	Superscaf	7389628	0	0	0	0	BTP20220	0	Tissue specific
PGVKLLV	Superscaf	+	Superscaf	7446436	7446456	110	110	116	-	-	-	-	-	-	NCP	IGR	-	ID=gene-E	7	4.852857143	1809.9	0.855794359	PGVKLLV	60	363.2493	2	724.4847	.	Q	Superscaf	7446109	0	0	0	0	BTP20220	0	Tissue specific
VGDVSSF	Superscaf	-	Superscaf	7532680	7532700	-28	28	34	-	-	gene	-	-	mRNA	NCP	Intron	gene|mRN	ID=gene-E	7	3.682857143	1863	0.854124186	VGDVSSF	75	710.3312	1	709.3282	.	L	Superscaf	7532706	0	0	0	0	0	BTP20220	Tissue specific
SGPAKYR	Superscaf	-	Superscaf	7548235	7548255	-20	20	26	-	-	gene	-	-	mRNA	NCP	Intron	gene|mRN	ID=gene-E	7	7.161428571	2906.1	0.853822953	S(+42.01)	61	410.7231	2	819.4239	Acetylati	P	Superscaf	7548255	0	BTP20220	0	0	0	BTP20220	Tissue specific
LEPTF	Superscaf	+	Superscaf	7568518	7568532	30	30	34	-	-	gene	-	-	mRNA	NCP	Intron	gene|mRN	ID=gene-E	5	5.502	1901.2	0.853430275	LEPTF	50	606.3098	1	605.306	.	R	Superscaf	7568431	0	0	0	0	0	BTP20220	Tissue specific
CCSGGY	Superscaf	+	Superscaf	7644229	7644246	1	1	6	-	-	gene	-	-	mRNA	NCP	Intron	gene|mRN	ID=gene-E	6	5.553333333	2608.9	0.851964022	C(+57.02)	68	745.2189	1	744.2207	Carbamido	C	Superscaf	7644229	0	0	BTP20220	0	0	0	Tissue specific
ASEGSRGGF	Superscaf	+	Superscaf	7698460	7698504	77	77	91	-	-	-	-	-	-	NCP	IGR	-	ID=gene-E	15	6.459333333	5611.2	0.850913279	A(+42.01)	87	697.8396	2	1393.67	Acetylati	R	Superscaf	7698232	BTP20220	0	0	0	0	0	Tissue specific
AHHRPTT	Superscaf	+	Superscaf	7710601	7710621	50	50	56	-	-	-	-	-	-	NCP	IGR	-	ID=gene-E	7	7.331428571	3331.9	0.850678626	AHHRPTT	78	410.2184	2	818.4147	.	I	Superscaf	7710454	0	0	0	0	BTP20220	0	Tissue specific
KCFTKP	Superscaf	+	Superscaf	7737706	7737723	8	8	13	-	-	-	-	-	-	NCP	IGR	-	ID=gene-E	6	7.135	2644.8	0.850153777	KC(+57.02	76	390.7057	2	779.4	Carbamido	T	Superscaf	7737685	0	0	0	0	0	BTP20220	Tissue specific
QQTLLEK	Superscaf	+	Superscaf	7737952	7737972	54	54	60	-	-	-	-	-	-	NCP	IGR	-	ID=gene-E	7	6.107142857	3219.6	0.850148955	QQTLLEK	77	430.2471	2	858.4811	.	R	Superscaf	7737793	0	BTP20220	0	0	0	0	Tissue specific
VDPKGKP	Superscaf	-	Superscaf	7738909	7738929	-3	3	9	-	-	-	-	-	-	NCP	IGR	-	ID=gene-E	7	5.86	2320.4	0.850130422	VDPKGKP	65	740.4388	1	739.4228	.	S	Superscaf	7739055	0	0	0	0	BTP20220	0	Tissue specific
RLLLF	Superscaf	-	Superscaf	7768672	7768686	-8	8	12	-	-	-	-	-	-	NCP	IGR	-	ID=gene-E	5	6.836	2096.4	0.849554158	RLLLF	73	661.4388	1	660.4323	.	Q	Superscaf	7768689	0	BTP20220	0	0	BTP20220	BTP20220	Mixed
AEEELKK	Superscaf	-	Superscaf	7810759	7810779	-52	52	58	-	-	-	-	-	-	NCP	IGR	-	ID=gene-E	7	5.877142857	2878	0.848738998	A(+42.01)	82	444.7322	2	887.46	Acetylati	F	Superscaf	7810785	0	0	BTP20220	0	0	0	Tissue specific
LATVLDF	Superscaf	-	Superscaf	7880908	7880928	-3	3	9	-	-	gene	-	-	mRNA	NCP	Intron	gene|mRN	ID=gene-E	7	4.708571429	2214.5	0.847380516	LATVLDF	93	778.4315	1	777.4272	.	L	Superscaf	7880949	0	0	0	BTP20220	0	0	Tissue specific
HKKYTSY	Superscaf	-	Superscaf	7912918	7912938	-38	38	44	-	-	gene	-	-	mRNA	NCP	Intron	gene|mRN	ID=gene-E	7	7.228571429	3256.4	0.84676062	HKKYTSY	51	463.7369	2	925.4658	.	P	Superscaf	7912947	0	0	0	BTP20220	0	0	Tissue specific
PAAMRPK	Superscaf	+	Superscaf	7973281	7973301	21	21	27	-	-	gene	-	-	mRNA	NCP	Intron	gene|mRN	ID=gene-E	7	7.27	2786.1	0.84559165	PAAMRPK	50	385.7176	2	769.4268	.	D	Superscaf	7973221	0	BTP20220	BTP20220	0	0	0	Tissue specific
EGFKP	Superscaf	+	Superscaf	8000032	8000046	10	10	14	-	-	gene	-	-	mRNA	NCP	Intron	gene|mRN	ID=gene-E	5	6.142	1916	0.845073715	EGFKP	75	577.3065	1	576.2907	.	S	Superscaf	8000005	0	0	BTP20220	0	0	0	Tissue specific
KGFTKY	Superscaf	+	Superscaf	8131528	8131545	38	38	43	-	-	-	-	-	-	NCP	IGR	-	ID=gene-E	6	7.186666667	2478.5	0.842527148	K(+42.01)	71	393.2112	2	784.4119	Acetylati	F	Superscaf	8131417	0	0	BTP20220	0	0	0	Tissue specific
YFNQLSK	Superscaf	-	Superscaf	8276065	8276085	-5	5	11	-	-	gene	-	-	mRNA	NCP	Intron	gene|mRN	ID=gene-E	7	6.228571429	3259.5	0.839728034	YFNQLSK	74	450.231	2	898.4548	.	C	Superscaf	8276091	0	0	0	BTP20220	0	0	Tissue specific
KYCGSW	Superscaf	-	Superscaf	8279410	8279427	-14	14	19	-	-	gene	-	-	mRNA	NCP	Intron	gene|mRN	ID=gene-E	6	6.326666667	2720.9	0.839663314	KYC(+57.	67	800.3325	1	799.3323	Carbamido	S	Superscaf	8279460	0	0	0	0	0	BTP20220	Tissue specific
PFLKEWL	Superscaf	-	Superscaf	8295499	8295519	2	2	8	-	-	-	-	-	-	NCP	IGR	-	ID=gene-E	7	6.084285714	2852.1	0.839351681	PFLKEWL	51	466.7639	2	931.5167	.	L	Superscaf	8295496	0	0	0	0	0	BTP20220	Tissue specific
RCLHY	Superscaf	-	Superscaf	8393995	8394009	-11	11	15	-	-	gene	-	-	mRNA	NCP	Intron	gene|mRN	ID=gene-E	5	7.002	2667.1	0.837444356	RC(+57.02	61	748.344	1	747.3486	Carbamido	E	Superscaf	8394024	0	0	0	BTP20220	0	0	Tissue specific
KSPDPPKP	Superscaf	+	Superscaf	8395534	8395557	23	23	30	-	-	gene	-	-	mRNA	NCP	Intron	gene|mRN	ID=gene-E	8	6.66625	3081.3	0.837414378	KSPDPPKP	80	433.2408	2	864.4705	.	E	Superscaf	8395468	0	0	BTP20220	0	0	0	Tissue specific
MEKPY	Superscaf	+	Superscaf	8398915	8398929	3	3	7	-	-	gene	-	-	mRNA	NCP	Intron	gene|mRN	ID=gene-E	5	6.134	2062.3	0.837349077	MEKPY	70	667.3074	1	666.3047	.	P	Superscaf	8398909	BTP20220	0	0	0	0	0	Tissue specific
KESLALFP	Superscaf	-	Superscaf	8445448	8445471	-9	9	16	-	-	gene	-	-	mRNA	NCP	Intron	gene|mRN	ID=gene-E	8	6.05	3020.2	0.83644776	KESLALFP	61	452.7584	2	903.5065	.	K	Superscaf	8445474	0	BTP20220	0	0	0	0	Tissue specific
SPLSTLTLI	Superscaf	+	Superscaf	8453044	8453070	7	7	15	-	-	gene	-	-	mRNA	NCP	Intron	gene|mRN	ID=gene-E	9	6.071111111	3312.8	0.8363006	SPLSTLTLI	57	944.563	1	943.559	.	K	Superscaf	8453026	0	BTP20220	0	0	0	0	Tissue specific
SAVRLR	Superscaf	+	Superscaf	8506729	8506746	43	43	48	-	-	gene	-	-	mRNA	NCP	Intron	gene|mRN	ID=gene-E	6	6.533333333	2345.7	0.835261128	SAVRLR	86	351.2229	2	700.4344	.	K	Superscaf	8506603	0	0	0	BTP20220	0	0	Tissue specific
TGATLAW	Superscaf	+	Superscaf	8604094	8604114	23	23	29	-	-	gene	-	-	mRNA	NCP	Intron	gene|mRN	ID=gene-E	7	6.134285714	2594.9	0.833375531	T(+42.01)	71	761.38	1	760.3755	Acetylati	I	Superscaf	8604028	0	0	0	0	BTP20220	0	Tissue specific
LSAAVTAL	Superscaf	-	Superscaf	8670307	8670330	-145	145	152	-	exon	gene	three_pri	-	mRNA	NCP	3UTR	exon|gene	ID=exon-E	8	5.27875	2421.6	0.832093214	LSAAVTAL	69	745.4425	1	744.4381	.	I	Superscaf	8670375	0	0	0	0	0	BTP20220	Tissue specific
DLRKLAV	Superscaf	-	Superscaf	8698225	8698245	-21	21	27	-	-	gene	-	-	mRNA	NCP	Intron	gene|mRN	ID=gene-E	7	5.921428571	2546.8	0.831552621	DLRKLAV	95	407.7596	2	813.5072	.	E	Superscaf	8698251	0	0	0	0	BTP20220	0	Mixed
VRELME	Superscaf	-	Superscaf	8767216	8767233	3	3	8	-	-	-	-	-	-	NCP	IGR	-	ID=gene-E	6	4.821666667	2224.7	0.830216622	VRELME	72	776.3945	1	775.3898	.	S	Superscaf	8767210	BTP20220	0	0	0	0	0	Tissue specific
EVELSAF	Superscaf	-	Superscaf	8785663	8785683	-56	56	62	-	-	-	-	-	-	NCP	IGR	-	ID=gene-E	7	4.228571429	2286.5	0.829859325	EVELSAF	87	794.3948	1	793.3857	.	S	Superscaf	8785716	0	0	0	0	0	BTP20220	Tissue specific
QVCVQ	Superscaf	+	Superscaf	8798479	8798493	43	43	47	-	-	-	-	-	-	NCP	IGR	-	ID=gene-E	5	3.264	1807.1	0.829611251	QVC(+57.	52	633.2985	1	632.2952	Carbamido	K	Superscaf	8798353	0	0	0	BTP20220	0	0	Tissue specific
KYPLTLY	Superscaf	+	Superscaf	8836180	8836200	1	1	7	-	-	-	-	-	-	NCP	IGR	-	ID=gene-E	7	6.55	2829.1	0.828881029	K(+42.01)	88	939.5154	1	938.5113	Acetylati	K	Superscaf	8836180	0	0	BTP20220	0	0	0	Mixed
MKETLL	Superscaf	-	Superscaf	8887198	8887215	-2	2	7	-	-	gene	-	-	mRNA	NCP	Intron	gene|mRN	ID=gene-E	6	6.2	2369.7	0.827893089	M(+42.01)	59	776.4236	1	775.415	Acetylati	K	Superscaf	8887218	0	0	0	0	0	BTP20220	Tissue specific
AMVSNSG	Superscaf	+	Superscaf	8887315	8887335	11	11	17	-	-	gene	-	-	mRNA	NCP	Intron	gene|mRN	ID=gene-E	7	4.93	2343.6	0.827890766	A(+42.01)	59	707.3046	1	706.2956	Acetylati	H	Superscaf	8887285	0	0	BTP20220	0	0	0	Tissue specific
LGERLF	Superscaf	+	Superscaf	8889427	8889444	16	16	21	-	-	gene	-	-	mRNA	NCP	Intron	gene|mRN	ID=gene-E	6	6.231666667	2465.8	0.827849923	LGERLF	99	367.7118	2	733.4122	.	C	Superscaf	8889382	0	0	0	0	BTP20220	0	Tissue specific
NVALSYK	Superscaf	+	Superscaf	8980645	8980665	37	37	43	-	-	-	-	-	-	NCP	IGR	-	ID=gene-E	7	5.498571429	2572.7	0.826083367	NVALSYK	56	397.7216	2	793.4334	.	S	Superscaf	8980537	0	0	BTP20220	0	0	0	Tissue specific
AAALALGR	Superscaf	+	Superscaf	9048247	9048270	44	44	51	-	-	gene	-	-	mRNA	NCP	Intron	gene|mRN	ID=gene-E	8	6.59625	2898.1	0.824774151	A(+42.01)	60	392.7356	2	783.4603	Acetylati	G	Superscaf	9048118	0	0	BTP20220	0	0	0	Tissue specific
KETAGGK	Superscaf	-	Superscaf	9064549	9064569	-64	64	70	-	-	gene	-	-	mRNA	NCP	Intron	gene|mRN	ID=gene-E	7	6.741428571	2665.7	0.82445851	KETAGGK	54	690.366	1	689.3708	.	V	Superscaf	9064614	0	0	BTP20220	0	0	0	Tissue specific
RALSGPK	Superscaf	-	Superscaf	9076861	9076881	-24	24	30	-	-	gene	-	-	mRNA	NCP	Intron	gene|mRN	ID=gene-E	7	7.207142857	2800	0.824220079	RALSGPK	78	364.7284	2	727.434	.	A	Superscaf	9076929	0	0	0	0	BTP20220	0	Tissue specific
AALLTEA	Superscaf	+	Superscaf	9083041	9083061	7	7	13	-	-	gene	-	-	mRNA	NCP	Intron	gene|mRN	ID=gene-E	7	5.681428571	2463.7	0.8241004	AALLTEA	95	688.3862	1	687.3803	.	G	Superscaf	9083023	0	0	BTP20220	0	0	0	Tissue specific
KSLKNVH	Superscaf	+	Superscaf	9141802	9141822	15	15	21	-	-	-	-	-	-	NCP	IGR	-	ID=gene-E	7	6.305714286	2890	0.822962453	K(+42.01)	60	434.2613	2	866.4974	Acetylati	I	Superscaf	9141760	0	0	0	0	0	BTP20220	Tissue specific
QHAVPL	Superscaf	+	Superscaf	9238945	9238962	27	27	32	-	-	gene	-	-	mRNA	NCP	Intron	gene|mRN	ID=gene-E	6	5.256666667	2232.5	0.821081272	QHAVPL	65	664.3738	1	663.3704	.	K	Superscaf	9238867	0	0	0	BTP20220	0	0	Tissue specific
SGDVQRF	Superscaf	-	Superscaf	9256780	9256800	-11	11	17	-	-	gene	-	-	mRNA	NCP	Intron	gene|mRN	ID=gene-E	7	5.215714286	2739.1	0.820735827	SGDVQRF	79	404.6995	2	807.3875	.	S	Superscaf	9256812	0	BTP20220	0	0	0	0	Tissue specific
TMLPLKD	Superscaf	-	Superscaf	9328888	9328908	-28	28	34	-	-	gene	-	-	mRNA	NCP	Intron	gene|mRN	ID=gene-E	7	6.178571429	2693.1	0.819339407	T(+42.01)	76	859.451	1	858.4521	Acetylati	H	Superscaf	9328953	0	0	BTP20220	0	0	0	Tissue specific
STVLKKAP	Superscaf	-	Superscaf	9336559	9336582	-3	3	10	-	-	gene	-	-	mRNA	NCP	Intron	gene|mRN	ID=gene-E	8	6.24875	2719.8	0.819190794	STVLKKAP	55	422.275	2	842.5225	.	E	Superscaf	9336672	BTP20220	0	0	0	0	0	Tissue specific
YEELVKE	Superscaf	-	Superscaf	9480745	9480765	-30	30	36	-	-	-	-	-	-	NCP	IGR	-	ID=gene-E	7	4.434285714	2557.8	0.816398593	YEELVKE	89	455.2309	2	908.4491	.	K	Superscaf	9480783	0	0	0	0	BTP20220	0	Mixed
TDMGVPK	Superscaf	-	Superscaf	9507754	9507774	-27	27	33	-	-	-	-	-	-	NCP	IGR	-	ID=gene-E	7	5.322857143	2283.6	0.815875546	TDMGVPK	74	374.1856	2	746.3633	.	P	Superscaf	9507849	BTP20220	0	0	0	0	0	Tissue specific
KNDPPPP	Superscaf	+	Superscaf	9581467	9581487	25	25	31	-	-	gene	-	-	mRNA	NCP	Intron	gene|mRN	ID=gene-E	7	6.188571429	2826.2	0.814448044	KNDPPPP	70	382.7014	2	763.3864	.	V	Superscaf	9581395	0	0	0	BTP20220	0	0	Tissue specific
TVYFPC	Superscaf	+	Superscaf	9587527	9587544	6	6	11	-	-	gene	-	-	mRNA	NCP	Intron	gene|mRN	ID=gene-E	6	4.831666667	2167.5	0.814330746	TVYFPC(+5	53	786.3502	1	785.3418	Carbamido	Q	Superscaf	9587512	BTP20220	BTP20220	0	0	0	0	Tissue specific
KFEVPLL	Superscaf	-	Superscaf	9660445	9660465	-39	39	45	-	-	gene	-	-	mRNA	NCP	Intron	gene|mRN	ID=gene-E	7	5.242857143	2325.5	0.812918582	KFEVPLL	63	845.5118	1	844.5058	.	I	Superscaf	9660939	0	0	BTP20220	0	0	0	Tissue specific
KAHPSSK	Superscaf	+	Superscaf	9689809	9689829	44	44	50	-	-	-	-	-	-	NCP	IGR	-	ID=gene-E	7	7.25	2972	0.812349928	KAHPSSK	59	377.7118	2	753.4133	.	K	Superscaf	9689680	0	0	0	BTP20220	0	0	Tissue specific
YRALFQ	Superscaf	+	Superscaf	9719059	9719076	8	8	13	-	-	-	-	-	-	NCP	IGR	-	ID=gene-E	6	6.591666667	2771.1	0.81178354	YRALFQ	68	797.4266	1	796.4232	.	E	Superscaf	9719038	0	BTP20220	0	0	0	0	Tissue specific
CHRLEWN	Superscaf	-	Superscaf	9835279	9835299	-6	6	12	-	-	gene	-	-	mRNA	NCP	Intron	gene|mRN	ID=gene-E	7	6.267142857	3756.4	0.809532803	C(+57.02)	67	507.734	2	1013.45	Carbamido	S	Superscaf	9835359	0	0	BTP20220	0	0	0	Tissue specific
KTDPPPF	Superscaf	+	Superscaf	9836530	9836550	44	44	50	-	-	gene	-	-	mRNA	NCP	Intron	gene|mRN	ID=gene-E	7	6.231428571	2677	0.809508577	KTDPPPF	59	401.2079	2	800.4068	.	D	Superscaf	9836401	BTP20220	0	0	0	0	0	Tissue specific
QPTVLN	Superscaf	+	Superscaf	9938857	9938874	5	5	10	-	-	gene	-	-	mRNA	NCP	Intron	gene|mRN	ID=gene-E	6	4.978333333	2295.7	0.807527004	Q(+42.01)	67	713.3812	1	712.3755	Acetylati	L	Superscaf	9938845	0	0	BTP20220	0	0	0	Tissue specific
MPKRLP	Superscaf	-	Superscaf	9944086	9944103	-14	14	19	-	-	gene	-	-	mRNA	NCP	Intron	gene|mRN	ID=gene-E	6	7.471666667	2516.9	0.807425741	MPKRLP	84	371.2249	2	740.4367	.	K	Superscaf	9944172	0	BTP20220	0	0	0	BTP20220	Tissue specific
GSEGKPAEI	Superscaf	+	Superscaf	9956263	9956292	68	68	77	-	-	gene	-	-	mRNA	NCP	Intron	gene|mRN	ID=gene-E	10	6.184	3767.9	0.807189692	GSEGKPAEI	75	508.2711	2	1014.535	.	I	Superscaf	9956062	0	0	0	0	0	BTP20220	Tissue specific
EGTLTFG	Superscaf	+	Superscaf	10027435	10027455	18	18	24	-	-	gene	-	-	mRNA	NCP	Intron	gene|mRN	ID=gene-E	7	5.668571429	2555.9	0.805811573	EGTLTFG	59	724.3506	1	723.3439	.	R	Superscaf	10027384	0	0	0	BTP20220	0	0	Tissue specific
TSTTPTPLT	Superscaf	-	Superscaf	10049929	10049955	-5	5	13	-	-	gene	-	-	mRNA	NCP	Intron	gene|mRN	ID=gene-E	9	6.323333333	3399.1	0.805375845	T(+42.01)	83	960.4859	1	959.4811	Acetylati	V	Superscaf	10050024	0	BTP20220	0	0	0	0	Tissue specific
YYAGAK	Superscaf	+	Superscaf	10171183	10171200	4	4	9	-	-	gene	-	-	mRNA	NCP	Intron	gene|mRN	ID=gene-E	6	6.511666667	2307.3	0.803027854	YYAGAK	69	672.3322	1	671.3278	.	I	Superscaf	10171174	0	0	0	0	0	BTP20220	Tissue specific
GLGVQ	Superscaf	-	Superscaf	10171672	10171686	-8	8	12	-	-	gene	-	-	mRNA	NCP	Intron	gene|mRN	ID=gene-E	5	4.714	1558.7	0.803018442	G(+42.01)	60	515.2858	1	514.2751	Acetylati	S	Superscaf	10171731	0	0	0	0	0	BTP20220	Tissue specific
KDELGYVF	Superscaf	-	Superscaf	10187974	10187997	-1	1	8	-	-	gene	-	-	mRNA	NCP	Intron	gene|mRN	ID=gene-E	8	4.8775	2803	0.802702569	KDELGYVF	53	485.7511	2	969.4807	.	K	Superscaf	10188012	0	0	0	BTP20220	0	0	Tissue specific
KGHHLVP	Superscaf	-	Superscaf	10210447	10210467	-41	41	47	-	-	gene	-	-	mRNA	NCP	Intron	gene|mRN	ID=gene-E	7	6.167142857	2695.9	0.802267422	KGHHLVP	53	394.2293	2	786.45	.	T	Superscaf	10210467	0	0	0	BTP20220	BTP20220	0	Tissue specific
NPLNPK	Superscaf	+	Superscaf	10288960	10288977	31	31	36	-	-	-	-	-	-	NCP	IGR	-	ID=gene-E	6	6.523333333	2690	0.800747023	NPLNPK	91	682.385	1	681.3809	.	D	Superscaf	10288870	0	0	BTP20220	0	0	0	Tissue specific
DLALKYFQK	Superscaf	-	Superscaf	10460497	10460523	-8	8	16	-	-	gene	-	-	mRNA	NCP	Intron	gene|mRN	ID=gene-E	9	6.357777778	3824	0.797424919	DLALKYFQK	88	563.3165	2	1124.623	.	P	Superscaf	10460583	0	0	BTP20220	0	BTP20220	0	Tissue specific
KELAVQP	Superscaf	-	Superscaf	10536346	10536366	-14	14	20	-	-	gene	-	-	mRNA	NCP	Intron	gene|mRN	ID=gene-E	7	5.272857143	2506.7	0.795956168	KELAVQP	65	784.4536	1	783.449	.	D	Superscaf	10536402	0	0	BTP20220	0	0	BTP20220	Tissue specific
KFWGKY	Superscaf	+	Superscaf	10543363	10543380	5	5	10	-	-	gene	-	-	mRNA	NCP	Intron	gene|mRN	ID=gene-E	6	7.08	2607.6	0.795820338	KFWGKY	97	414.7222	2	827.433	.	E	Superscaf	10543351	0	0	0	0	0	BTP20220	Tissue specific
KPSPTPPP	Superscaf	+	Superscaf	10546633	10546656	47	47	54	-	-	gene	-	-	mRNA	NCP	Intron	gene|mRN	ID=gene-E	8	6.68125	2936.3	0.795756896	KPSPTPPP	61	410.7318	2	819.449	.	C	Superscaf	10546495	0	BTP20220	BTP20220	BTP20220	BTP20220	0	Tissue specific
QSNALLRH	Superscaf	+	Superscaf	10553398	10553421	7	7	14	-	-	gene	-	-	mRNA	NCP	Intron	gene|mRN	ID=gene-E	8	6.63375	3877.4	0.795625887	QSNALLRH	96	469.7602	2	937.5093	.	K	Superscaf	10553380	0	BTP20220	BTP20220	0	BTP20220	0	Tissue specific
EKALSRL	Superscaf	+	Superscaf	10574896	10574916	42	42	48	-	-	gene	-	-	mRNA	NCP	Intron	gene|mRN	ID=gene-E	7	6.768571429	2944.2	0.795209622	E(+42.01)	72	429.7538	2	857.497	Acetylati	K	Superscaf	10574773	0	BTP20220	0	0	0	BTP20220	Tissue specific
QPVYM	Superscaf	+	Superscaf	10608487	10608501	13	13	17	-	-	gene	-	-	mRNA	NCP	Intron	gene|mRN	ID=gene-E	5	4.672	1779.1	0.794559225	QPVYM	92	637.3004	1	636.2941	.	L	Superscaf	10608451	0	0	0	BTP20220	0	0	Tissue specific
KEYEPK	Superscaf	+	Superscaf	10652893	10652910	17	17	22	-	-	-	-	-	-	NCP	IGR	-	ID=gene-E	6	6.313333333	2584.7	0.793699215	KEYEPK	50	397.207	2	792.4017	.	L	Superscaf	10652845	0	0	BTP20220	0	0	0	Tissue specific
NYCVCC	Superscaf	+	Superscaf	10669405	10669422	2	2	7	-	-	gene	-	-	mRNA	NCP	Intron	gene|mRN	ID=gene-E	6	4.355	2813.3	0.793379449	NYC(+57.	54	438.1418	2	874.2772	Carbamido	I	Superscaf	10669402	0	BTP20220	BTP20220	BTP20220	0	0	Tissue specific
APGGLPL	Superscaf	-	Superscaf	10724275	10724295	-9	9	15	-	-	gene	-	-	mRNA	NCP	Intron	gene|mRN	ID=gene-E	7	6.074285714	2287.5	0.792316797	APGGLPL	68	624.3728	1	623.3642	.	K	Superscaf	10725129	0	BTP20220	0	0	0	0	Tissue specific
LPNFC	Superscaf	+	Superscaf	10940623	10940637	6	6	10	-	-	-	-	-	-	NCP	IGR	-	ID=gene-E	5	5.638	2260.6	0.788127188	LPNFC(+57	58	650.2969	1	649.2894	Carbamido	I	Superscaf	10940608	0	0	0	BTP20220	0	0	Tissue specific
LDPSGL	Superscaf	-	Superscaf	10951903	10951920	-12	12	17	-	-	-	-	-	-	NCP	IGR	-	ID=gene-E	6	5.48	2136.4	0.787908685	LDPSGL	79	601.3284	1	600.3119	.	I	Superscaf	10951962	0	0	BTP20220	0	0	0	Tissue specific
VKQLLVG	Superscaf	+	Superscaf	10964110	10964130	21	21	27	-	-	-	-	-	-	NCP	IGR	-	ID=gene-E	7	4.76	2083.2	0.78767223	VKQLLVG	92	756.4951	1	755.4905	.	D	Superscaf	10964050	0	BTP20220	0	0	0	0	Tissue specific
VAVLLKE	Superscaf	-	Superscaf	10987798	10987818	-24	24	30	-	-	-	-	-	-	NCP	IGR	-	ID=gene-E	7	4.42	1912	0.787213496	V(+42.01)	50	407.2635	2	812.5007	Acetylati	K	Superscaf	10987884	0	BTP20220	0	0	BTP20220	0	Tissue specific
LDGVTVTG	Superscaf	-	Superscaf	11031499	11031522	-36	36	43	-	-	-	-	-	-	NCP	IGR	-	ID=gene-E	8	4.24375	2154.5	0.786367139	LDGVTVTG	68	761.4024	1	760.3967	.	L	Superscaf	11031609	0	0	0	0	BTP20220	0	Tissue specific
APLPHHK	Superscaf	+	Superscaf	11131489	11131509	11	11	17	-	-	-	-	-	-	NCP	IGR	-	ID=gene-E	7	7.074285714	3047.3	0.784430823	A(+42.01)	50	421.2426	2	840.4606	Acetylati	A	Superscaf	11131459	0	BTP20220	0	0	0	0	Tissue specific
MNPVVS	Superscaf	+	Superscaf	11146975	11146992	17	17	22	-	exon	gene	three_pri	-	mRNA	NCP	3UTR	exon|gene	ID=exon-E	6	3.856666667	1689	0.784130984	M(+42.01)	99	688.3312	1	687.3262	Acetylati	S	Superscaf	11146927	BTP20220	0	0	0	0	0	Mixed
SVSHAKPP	Superscaf	+	Superscaf	11192845	11192868	13	13	20	-	-	gene	-	-	mRNA	NCP	Intron	gene|mRN	ID=gene-E	8	5.91375	2841	0.783242564	SVSHAKPP	78	411.7239	2	821.4395	.	K	Superscaf	11192809	0	0	BTP20220	0	0	0	Tissue specific
MGGKHS	Superscaf	+	Superscaf	11211961	11211978	44	44	49	-	-	gene	-	-	mRNA	NCP	Intron	gene|mRN	ID=gene-E	6	6.783333333	2437.6	0.782872486	M(+42.01)	76	658.2856	1	657.2905	Acetylati	T	Superscaf	11211832	0	0	BTP20220	0	0	0	Tissue specific
VPGATTPP	Superscaf	+	Superscaf	11213047	11213070	10	10	17	-	-	gene	-	-	mRNA	NCP	Intron	gene|mRN	ID=gene-E	8	5.49375	2415.8	0.782851339	VPGATTPP	68	739.3843	1	738.3912	.	Q	Superscaf	11213020	0	0	0	0	0	BTP20220	Tissue specific
QTLGPLE	Superscaf	-	Superscaf	11310214	11310234	-6	6	12	-	-	-	-	-	-	NCP	IGR	-	ID=gene-E	7	5.661428571	2775.2	0.780969693	Q(+42.01)	59	799.418	1	798.4123	Acetylati	Y	Superscaf	11310252	0	0	0	0	0	BTP20220	Tissue specific
KAAAVLH	Superscaf	+	Superscaf	11312527	11312547	28	28	34	-	-	-	-	-	-	NCP	IGR	-	ID=gene-E	7	5.91	2375.4	0.7809249	KAAAVLH	78	355.2201	2	708.4282	.	Q	Superscaf	11312446	0	0	BTP20220	0	0	0	Tissue specific
KQPVHN	Superscaf	-	Superscaf	11333173	11333190	-23	23	28	-	-	-	-	-	-	NCP	IGR	-	ID=gene-E	6	5.781666667	2632.9	0.780525134	KQPVHN	59	361.7014	2	721.3871	.	S	Superscaf	11333280	0	0	0	0	0	BTP20220	Tissue specific
QFVELCS	Superscaf	-	Superscaf	11513158	11513178	-1	1	7	-	-	gene	-	-	mRNA	NCP	Intron	gene|mRN	ID=gene-E	7	4.432857143	2746.1	0.777039545	QFVELC(+5	68	441.705	2	881.3953	Carbamido	Q	Superscaf	11513229	0	BTP20220	BTP20220	0	BTP20220	0	Tissue specific
QKMVPR	Superscaf	-	Superscaf	11649211	11649228	-8	8	13	-	-	-	-	-	-	NCP	IGR	-	ID=gene-E	6	6.366666667	2436.8	0.774404845	QKMVPR	64	379.7265	2	757.4269	.	F	Superscaf	11649228	0	0	0	0	0	BTP20220	Tissue specific
QFMRDF	Superscaf	-	Superscaf	11682676	11682693	-5	5	10	-	-	gene	-	-	mRNA	NCP	Intron	gene|mRN	ID=gene-E	6	6.015	2817.3	0.773756773	QFMRDF	61	422.189	2	842.3745	.	L	Superscaf	11682714	0	0	BTP20220	0	0	0	Tissue specific
VHTKLAQ	Superscaf	-	Superscaf	11691361	11691381	-10	10	16	-	-	gene	-	-	mRNA	NCP	Intron	gene|mRN	ID=gene-E	7	5.93	2761	0.773588524	VHTKLAQ	74	398.7354	2	795.4603	.	I	Superscaf	11691525	0	BTP20220	0	0	0	BTP20220	Tissue specific
MHSDLR	Superscaf	-	Superscaf	11744578	11744595	-1	1	6	-	-	gene	-	-	mRNA	NCP	Intron	gene|mRN	ID=gene-E	6	6.455	2788.3	0.772557999	M(+15.99)	66	774.3419	1	773.349	Oxidation	M	Superscaf	11744607	0	BTP20220	0	0	0	0	Tissue specific
PGLPP	Superscaf	+	Superscaf	11935438	11935452	67	67	71	-	-	-	-	-	-	NCP	IGR	-	ID=gene-E	5	6.17	1662.9	0.768861925	PGLPP	87	480.2809	1	479.2744	.	L	Superscaf	11935240	0	BTP20220	0	0	0	0	Mixed
VKKPPAA	Superscaf	+	Superscaf	11952775	11952795	7	7	13	-	-	-	-	-	-	NCP	IGR	-	ID=gene-E	7	6.302857143	2234.2	0.768526066	VKKPPAA	51	355.729	2	709.4486	.	G	Superscaf	11952757	0	0	0	0	BTP20220	BTP20220	Tissue specific
DGYSLKY	Superscaf	-	Superscaf	11958934	11958954	-5	5	11	-	-	-	-	-	-	NCP	IGR	-	ID=gene-E	7	5.951428571	2833	0.768406793	DGYSLKY	52	423.2022	2	844.3967	.	K	Superscaf	11958990	BTP20220	0	0	0	0	0	Tissue specific
KPWSHAL	Superscaf	-	Superscaf	11960701	11960721	-17	17	23	-	-	-	-	-	-	NCP	IGR	-	ID=gene-E	7	6.742857143	3000.2	0.768372573	KPWSHAL	52	419.7342	2	837.4497	.	R	Superscaf	11960739	0	0	BTP20220	0	0	0	Tissue specific
SAPGKQER	Superscaf	-	Superscaf	11975677	11975700	-102	102	109	-	-	gene	-	-	mRNA	NCP	Intron	gene|mRN	ID=gene-E	8	6.6675	3482.8	0.768082495	SAPGKQER	63	436.738	2	871.4511	.	I	Superscaf	11975766	0	0	0	0	BTP20220	0	Tissue specific
KGVLTMM	Superscaf	-	Superscaf	12057748	12057768	-4	4	10	-	-	gene	-	-	mRNA	NCP	Intron	gene|mRN	ID=gene-E	7	5.674285714	2259.6	0.766493193	KGVLTM(+1	72	406.2059	2	810.3979	Oxidation	S	Superscaf	12057768	0	0	0	0	0	BTP20220	Tissue specific
RVHLFL	Superscaf	-	Superscaf	12107041	12107058	-5	5	10	-	-	-	-	-	-	NCP	IGR	-	ID=gene-E	6	5.965	2362.7	0.765538659	RVHLFL	50	392.7477	2	783.4755	.	E	Superscaf	12107112	0	0	0	BTP20220	0	0	Tissue specific
DYLKEK	Superscaf	+	Superscaf	12112711	12112728	6	6	11	-	-	gene	-	-	mRNA	NCP	Intron	gene|mRN	ID=gene-E	6	6.218333333	2586.7	0.765428855	DYLKEK	84	398.2083	2	794.4174	.	H	Superscaf	12112696	0	0	0	0	BTP20220	0	Tissue specific
QVGCKP	Superscaf	+	Superscaf	12122464	12122481	32	32	37	-	-	gene	-	-	mRNA	NCP	Intron	gene|mRN	ID=gene-E	6	5.446666667	2299.5	0.765239982	Q(+42.01)	64	730.3561	1	729.348	Acetylati	E	Superscaf	12122371	0	0	0	0	BTP20220	0	Tissue specific
DHKFLM	Superscaf	-	Superscaf	12226060	12226077	-1	1	6	-	-	gene	-	-	mRNA	NCP	Intron	gene|mRN	ID=gene-E	6	6.251666667	2611.9	0.763233776	DHKFLM	52	395.6977	2	789.3843	.	D	Superscaf	12226140	0	0	0	BTP20220	0	0	Tissue specific
LDRLLVK	Superscaf	-	Superscaf	12227791	12227811	-20	20	26	-	-	gene	-	-	mRNA	NCP	Intron	gene|mRN	ID=gene-E	7	5.915714286	2588.9	0.763200195	LDRLLVK	75	428.7838	2	855.5541	.	K	Superscaf	12227907	0	BTP20220	0	0	0	0	Tissue specific
RNVLSYR	Superscaf	-	Superscaf	12231205	12231225	-8	8	14	-	-	gene	-	-	mRNA	NCP	Intron	gene|mRN	ID=gene-E	7	6.321428571	3090.6	0.763134081	RNVLSYR	50	454.2568	2	906.5035	.	S	Superscaf	12231231	0	0	0	0	0	BTP20220	Tissue specific
KVYLPPP	Superscaf	+	Superscaf	12259261	12259281	1	1	7	-	-	gene	-	-	mRNA	NCP	Intron	gene|mRN	ID=gene-E	7	5.754285714	2293.5	0.762590758	KVYLPPP	59	407.2451	2	812.4796	.	K	Superscaf	12259261	0	0	0	BTP20220	BTP20220	0	Tissue specific
KPNPPKP	Superscaf	-	Superscaf	12324307	12324327	-5	5	11	-	-	-	-	-	-	NCP	IGR	-	ID=gene-E	7	7.155714286	2883.1	0.761331098	KPNPPKP	77	389.2329	2	776.4544	.	P	Superscaf	12324363	0	0	0	0	0	BTP20220	Tissue specific
NPHKPR	Superscaf	-	Superscaf	12345514	12345531	-12	12	17	-	-	-	-	-	-	NCP	IGR	-	ID=gene-E	6	7.683333333	3008.4	0.760920468	NPHKPR	51	374.713	2	747.414	.	R	Superscaf	12345555	0	0	0	0	0	BTP20220	Tissue specific
FGEGLGKG	Superscaf	+	Superscaf	12357565	12357588	3	3	10	-	-	-	-	-	-	NCP	IGR	-	ID=gene-E	8	6.0375	2823.9	0.760686976	FGEGLGKG	82	764.381	1	763.3864	.	V	Superscaf	12357559	0	0	0	0	0	BTP20220	Tissue specific
KELFHEK	Superscaf	-	Superscaf	12368734	12368754	-22	22	28	-	-	-	-	-	-	NCP	IGR	-	ID=gene-E	7	6.424285714	3148.3	0.760470739	KELFHEK	79	465.7552	2	929.497	.	G	Superscaf	12368778	0	0	0	0	0	BTP20220	Tissue specific
GRHVEF	Superscaf	+	Superscaf	12425998	12426015	22	22	27	-	exon	gene	three_pri	-	mRNA	NCP	3UTR	exon|gene	ID=exon-E	6	5.503333333	2378.7	0.759361841	G(+42.01)	57	393.6915	2	785.382	Acetylati	F	Superscaf	12425935	0	BTP20220	0	0	0	0	Tissue specific
AAAEVNR	Superscaf	+	Superscaf	12522514	12522534	14	14	20	-	exon	gene	three_pri	-	mRNA	NCP	3UTR	exon|gene	ID=exon-E	7	5.35	2604.9	0.757492686	AAAEVNR	59	730.3807	1	729.3769	.	G	Superscaf	12522475	0	0	0	BTP20220	0	0	Tissue specific
LSDMPPK	Superscaf	-	Superscaf	12531274	12531294	-17	17	23	-	-	gene	-	-	mRNA	NCP	Intron	gene|mRN	ID=gene-E	7	6.102857143	2663	0.757323043	LSDMPPK	66	394.1967	2	786.3945	.	Q	Superscaf	12531372	0	0	0	BTP20220	0	0	Tissue specific
TATAPF	Superscaf	-	Superscaf	12532357	12532374	-51	51	56	-	-	gene	-	-	mRNA	NCP	Intron	gene|mRN	ID=gene-E	6	6.146666667	2142.4	0.757302128	TATAPF	76	607.3062	1	606.3013	.	E	Superscaf	12532374	0	0	BTP20220	0	0	0	Tissue specific
TALSPKVR	Superscaf	+	Superscaf	12558721	12558744	5	5	12	-	-	gene	-	-	mRNA	NCP	Intron	gene|mRN	ID=gene-E	8	6.37625	2900.2	0.756791455	T(+42.01)	87	457.2755	2	912.5392	Acetylati	T	Superscaf	12558709	0	0	0	0	BTP20220	BTP20220	Tissue specific
AAQRLGK	Superscaf	+	Superscaf	12589246	12589266	27	27	33	-	-	gene	-	-	mRNA	NCP	Intron	gene|mRN	ID=gene-E	7	7.162857143	3001.2	0.756200376	A(+42.01)	87	393.2336	2	784.4555	Acetylati	D	Superscaf	12589168	0	0	0	BTP20220	0	0	Tissue enhanced
KMVLDLSE	Superscaf	-	Superscaf	12659074	12659097	-45	45	52	-	-	gene	-	-	mRNA	NCP	Intron	gene|mRN	ID=gene-E	8	4.915	2767.1	0.754848051	KMVLDLSE	75	467.7411	2	933.4841	.	R	Superscaf	12659136	0	0	0	0	0	BTP20220	Tissue specific
PPDLDLR	Superscaf	-	Superscaf	12758743	12758763	-13	13	19	-	-	-	-	-	-	NCP	IGR	-	ID=gene-E	7	5.894285714	2853.4	0.752917952	PPDLDLR	73	413.2247	2	824.4391	.	S	Superscaf	12758772	0	0	0	0	0	BTP20220	Tissue specific
APVEPPGK	Superscaf	+	Superscaf	12765127	12765150	56	56	63	-	-	-	-	-	-	NCP	IGR	-	ID=gene-E	8	5.48125	2514.7	0.752794263	APVEPPGK	56	397.7186	2	793.4333	.	P	Superscaf	12764962	0	0	0	0	0	BTP20220	Tissue specific
TVREPF	Superscaf	-	Superscaf	12897961	12897978	-25	25	30	-	-	gene	-	-	mRNA	NCP	Intron	gene|mRN	ID=gene-E	6	5.381666667	2196.6	0.75022196	T(+42.01)	69	790.4034	1	789.4021	Acetylati	L	Superscaf	12897993	BTP20220	0	0	0	0	0	Tissue specific
KPVVYFAK	Superscaf	-	Superscaf	12918550	12918573	-44	44	51	-	-	gene	-	-	mRNA	NCP	Intron	gene|mRN	ID=gene-E	8	5.3675	2432.4	0.749823124	K(+42.01)	61	497.2916	2	992.5695	Acetylati	A	Superscaf	12918573	0	0	BTP20220	0	0	0	Tissue specific
KAGPYDPP	Superscaf	+	Superscaf	12918634	12918657	18	18	25	-	-	gene	-	-	mRNA	NCP	Intron	gene|mRN	ID=gene-E	8	6.1575	2960.2	0.749821497	KAGPYDPP	57	422.7101	2	843.4126	.	P	Superscaf	12918583	0	0	0	0	0	BTP20220	Tissue specific
NPPALPP	Superscaf	+	Superscaf	12922882	12922902	68	68	74	-	-	gene	-	-	mRNA	NCP	Intron	gene|mRN	ID=gene-E	7	6.087142857	2611	0.74973929	NPPALPP	62	353.2065	2	704.3857	.	G	Superscaf	12922681	0	BTP20220	0	0	0	0	Tissue specific
LKVSLGK	Superscaf	+	Superscaf	12935191	12935211	8	8	14	-	-	-	-	-	-	NCP	IGR	-	ID=gene-E	7	6.155714286	2324.3	0.749500918	LKVSLGK	99	372.7509	2	743.4905	.	R	Superscaf	12935170	0	0	0	0	BTP20220	BTP20220	Tissue specific
AVVVTVRA	Superscaf	+	Superscaf	12938395	12938418	8	8	15	-	-	-	-	-	-	NCP	IGR	-	ID=gene-E	8	3.66625	1668.9	0.749438812	AVVVTVRA	57	814.5124	1	813.5072	.	E	Superscaf	12938374	0	0	0	BTP20220	0	0	Tissue specific
KSCKPLR	Superscaf	-	Superscaf	12974284	12974304	-1	1	7	-	-	-	-	-	-	NCP	IGR	-	ID=gene-E	7	7.602857143	3245.5	0.748743855	KSC(+57.	60	444.7605	2	887.5011	Carbamido	K	Superscaf	12974382	0	0	0	0	BTP20220	0	Tissue specific
HHLAQKEK	Superscaf	+	Superscaf	12977632	12977655	7	7	14	-	-	-	-	-	-	NCP	IGR	-	ID=gene-E	8	6.94125	3877.1	0.748678961	HHLAQKEK	78	495.7845	2	989.5406	.	D	Superscaf	12977614	0	BTP20220	0	0	0	BTP20220	Tissue specific
KEAVGGK	Superscaf	+	Superscaf	13010821	13010841	20	20	26	-	-	-	-	-	-	NCP	IGR	-	ID=gene-E	7	5.808571429	2268.2	0.748036292	KEAVGGK	86	688.3961	1	687.3915	.	A	Superscaf	13010764	0	0	BTP20220	0	0	0	Tissue specific
AGGAVVP	Superscaf	+	Superscaf	13028890	13028910	8	8	14	-	-	-	-	-	-	NCP	IGR	-	ID=gene-E	7	4.325714286	1554.6	0.747686373	A(+42.01)	67	612.3333	1	611.3278	Acetylati	D	Superscaf	13028869	0	0	BTP20220	0	0	0	Tissue specific
KMKPKP	Superscaf	+	Superscaf	13038460	13038477	53	53	58	-	-	gene	-	-	mRNA	NCP	Intron	gene|mRN	ID=gene-E	6	7.928333333	2451.5	0.747501102	KMKPKP	50	364.7216	2	727.4415	.	D	Superscaf	13038304	0	0	0	BTP20220	0	0	Tissue specific
LPKPAKP	Superscaf	-	Superscaf	13051006	13051026	-20	20	26	-	-	gene	-	-	mRNA	NCP	Intron	gene|mRN	ID=gene-E	7	7.197142857	2613.7	0.747258082	LPKPAKP	96	375.7456	2	749.4799	.	S	Superscaf	13051086	0	0	0	0	0	BTP20220	Tissue specific
CPPPKF	Superscaf	+	Superscaf	13071343	13071360	6	6	11	-	-	gene	-	-	mRNA	NCP	Intron	gene|mRN	ID=gene-E	6	6.523333333	2453.7	0.7468643	C(+57.02)	97	394.1958	2	786.3734	Carbamido	P	Superscaf	13071328	0	0	0	0	0	BTP20220	Tissue specific
RFTPD	Superscaf	+	Superscaf	13116169	13116183	5	5	9	-	-	-	-	-	-	NCP	IGR	-	ID=gene-E	5	6.408	2182.6	0.745996273	RFTPD	68	635.3124	1	634.3074	.	S	Superscaf	13116157	0	0	0	0	0	BTP20220	Tissue specific
GMVPGKP	Superscaf	-	Superscaf	13160401	13160421	-45	45	51	-	-	-	-	-	-	NCP	IGR	-	ID=gene-E	7	5.718571429	2109.3	0.745139574	G(+42.01)	85	372.1894	2	742.3683	Acetylati	I	Superscaf	13160445	0	0	0	0	BTP20220	BTP20220	Mixed
AKLCKAP	Superscaf	+	Superscaf	13186339	13186359	34	34	40	-	-	-	-	-	-	NCP	IGR	-	ID=gene-E	7	6.974285714	2835.9	0.744637267	AKLC(+57.	73	394.2345	2	786.4421	Carbamido	C	Superscaf	13186240	0	0	0	0	0	BTP20220	Tissue specific
CMMYCACK	Superscaf	+	Superscaf	13191397	13191420	58	58	65	-	-	-	-	-	-	NCP	IGR	-	ID=gene-E	8	5.9975	3739.3	0.744539257	C(+57.02)	50	599.1981	2	1196.379	Carbamido	M	Superscaf	13191226	0	0	0	0	0	BTP20220	Tissue specific
DAKPPPR	Superscaf	+	Superscaf	13223707	13223727	170	170	176	-	-	-	-	-	-	NCP	IGR	-	ID=gene-E	7	6.912857143	2852.2	0.743913611	D(+42.01)	93	411.7318	2	821.4395	Acetylati	D	Superscaf	13223200	0	0	0	0	BTP20220	0	Tissue specific
AAPLK	Superscaf	-	Superscaf	13233982	13233996	-10	10	14	-	-	-	-	-	-	NCP	IGR	-	ID=gene-E	5	6.812	1781.8	0.743714745	AAPLK	74	499.322	1	498.3166	.	I	Superscaf	13234002	0	0	0	BTP20220	0	0	Tissue specific
RPPGYLL	Superscaf	+	Superscaf	13240513	13240533	18	18	24	-	-	-	-	-	-	NCP	IGR	-	ID=gene-E	7	6.707142857	2787.2	0.743588151	RPPGYLL	54	408.2492	2	814.4701	.	S	Superscaf	13240462	0	0	BTP20220	0	0	0	Tissue specific
KMEGVTV	Superscaf	+	Superscaf	13289977	13289997	4	4	10	-	-	gene	-	-	mRNA	NCP	Intron	gene|mRN	ID=gene-E	7	4.458571429	1960.2	0.742630247	KMEGVTV	98	763.3983	1	762.3946	.	R	Superscaf	13289968	0	0	0	0	0	BTP20220	Tissue specific
LSNANAK	Superscaf	-	Superscaf	13333573	13333593	-30	30	36	-	-	-	-	-	-	NCP	IGR	-	ID=gene-E	7	6.322857143	3021.2	0.741785981	LSNANAK	53	359.198	2	716.3817	.	R	Superscaf	13333725	0	0	0	0	0	BTP20220	Tissue specific
LLSPEHK	Superscaf	-	Superscaf	13341916	13341936	-52	52	58	-	-	gene	-	-	mRNA	NCP	Intron	gene|mRN	ID=gene-E	7	6.355714286	2941.2	0.741624414	LLSPEHK	54	412.2345	2	822.4599	.	L	Superscaf	13342053	0	0	0	0	BTP20220	0	Tissue specific
KMVFLT	Superscaf	+	Superscaf	13454761	13454778	13	13	18	-	-	gene	-	-	mRNA	NCP	Intron	gene|mRN	ID=gene-E	6	5.58	1978.2	0.739439152	KMVFLT	65	369.7133	2	737.4146	.	D	Superscaf	13454725	0	0	0	0	BTP20220	0	Tissue specific
WAVPPGP	Superscaf	+	Superscaf	13477651	13477671	72	72	78	-	-	-	-	-	-	NCP	IGR	-	ID=gene-E	7	5.254285714	2147.4	0.738995814	W(+42.01)	66	383.2028	2	764.3857	Acetylati	L	Superscaf	13477438	0	0	0	0	BTP20220	0	Tissue specific
GRPNLYP	Superscaf	+	Superscaf	13478659	13478679	2	2	8	-	-	-	-	-	-	NCP	IGR	-	ID=gene-E	7	6.625714286	3030.5	0.738976293	GRPNLYP	54	408.7155	2	815.429	.	W	Superscaf	13478656	0	0	0	BTP20220	0	0	Tissue specific
SLSKGPFP	Superscaf	-	Superscaf	13488025	13488048	-33	33	40	-	-	-	-	-	-	NCP	IGR	-	ID=gene-E	8	6.39125	2948.1	0.738794856	SLSKGPFP	68	416.7306	2	831.449	.	L	Superscaf	13488450	0	0	0	0	0	BTP20220	Tissue specific
HCTKK	Superscaf	+	Superscaf	13503793	13503807	2	2	6	-	-	gene	-	-	mRNA	NCP	Intron	gene|mRN	ID=gene-E	5	7.724	2539.7	0.738489673	H(+42.01)	51	358.178	2	714.3483	Acetylati	D	Superscaf	13503790	0	0	0	0	0	BTP20220	Tissue specific
KGKYVLTH	Superscaf	+	Superscaf	13504402	13504425	1	1	8	-	-	gene	-	-	mRNA	NCP	Intron	gene|mRN	ID=gene-E	8	6.40125	3064.2	0.738477705	KGKYVLTH	59	473.2782	2	944.5443	.	K	Superscaf	13504402	0	0	0	BTP20220	0	0	Tissue specific

MLKPY	Superscaf	-	Superscaf	13539214	13539228	-14	14	18	-	-	-	-	-	-	NCP	IGR	-	ID=gene-E	5	6.686	1990.2	0.737803721	M(+15.99)	89	709.3589	1	708.3516	Oxidation	R	Superscaf	13539255	0	0	0	BTP20220	0	0	Tissue specific
ASDPPLTL	Superscaf	+	Superscaf	13580596	13580619	15	15	22	-	-	-	-	-	-	NCP	IGR	-	ID=gene-E	8	5.72	2885.3	0.737002156	ASDPPLTL	67	813.4353	1	812.428	.	L	Superscaf	13580554	0	0	0	0	0	BTP20220	Tissue specific
RGPAAYR	Superscaf	+	Superscaf	13585528	13585548	76	76	82	-	-	-	-	-	-	NCP	IGR	-	ID=gene-E	7	7.355714286	3014.4	0.736906703	RGPAAYR	76	395.713	2	789.4245	.	Q	Superscaf	13585303	0	0	0	BTP20220	0	0	Tissue specific
MVEKPPP	Superscaf	-	Superscaf	13621462	13621482	-6	6	12	-	-	gene	-	-	mRNA	NCP	Intron	gene|mRN	ID=gene-E	7	5.372857143	2277.6	0.736210817	M(+15.99)	76	855.4258	1	854.4208	Oxidation	S	Superscaf	13621482	0	0	0	0	0	BTP20220	Mixed
EPMAHF	Superscaf	+	Superscaf	13664359	13664376	23	23	28	-	-	gene	-	-	mRNA	NCP	Intron	gene|mRN	ID=gene-E	6	5.726666667	2452.8	0.735380146	EPM(+15.9	63	374.1583	2	746.3057	Oxidation	V	Superscaf	13664293	0	0	0	0	0	BTP20220	Tissue specific
KDGSPPP	Superscaf	+	Superscaf	13670776	13670796	2	2	8	-	-	gene	-	-	mRNA	NCP	Intron	gene|mRN	ID=gene-E	7	6.18	2572.8	0.735255818	KDGSPPP	63	697.3413	1	696.3442	.	I	Superscaf	13670773	0	0	BTP20220	BTP20220	0	0	Tissue specific
KPDRMGP	Superscaf	-	Superscaf	13672612	13672632	-13	13	19	-	-	gene	-	-	mRNA	NCP	Intron	gene|mRN	ID=gene-E	7	6.827142857	2872.3	0.735220263	KPDRM(+15	55	408.7032	2	815.3959	Oxidation	Q	Superscaf	13672665	0	0	0	BTP20220	0	0	Tissue specific
VPVAPGT	Superscaf	+	Superscaf	13677766	13677786	35	35	41	-	-	gene	-	-	mRNA	NCP	Intron	gene|mRN	ID=gene-E	7	4.445714286	1680.9	0.735120452	VPVAPGT	56	640.3647	1	639.3591	.	V	Superscaf	13677664	0	0	BTP20220	0	0	0	Tissue specific
KPAHVAV	Superscaf	+	Superscaf	13679989	13680009	11	11	17	-	-	-	-	-	-	NCP	IGR	-	ID=gene-E	7	5.095714286	2048.1	0.735077402	KPAHVAV	50	361.2192	2	720.4282	.	G	Superscaf	13679959	0	0	0	0	0	BTP20220	Tissue specific
APTLGLPP	Superscaf	+	Superscaf	13696171	13696194	45	45	52	-	-	-	-	-	-	NCP	IGR	-	ID=gene-E	8	6.1725	2725.1	0.734763969	APTLGLPP	50	383.2284	2	764.4432	.	A	Superscaf	13696039	BTP20220	0	0	0	0	0	Tissue specific
SEPLAGF	Superscaf	+	Superscaf	13719778	13719798	9	9	15	-	-	-	-	-	-	NCP	IGR	-	ID=gene-E	7	5.521428571	2495.7	0.734306861	SEPLAGF	82	720.3542	1	719.3489	.	Y	Superscaf	13719754	0	0	0	BTP20220	0	0	Tissue specific
TCNQLPLT	Superscaf	-	Superscaf	13782046	13782069	-4	4	11	-	-	-	-	-	-	NCP	IGR	-	ID=gene-E	8	5.925	3632.3	0.733100941	T(+42.01)	58	988.4892	1	987.4695	Acetylati	Q	Superscaf	13782075	0	0	0	0	0	BTP20220	Tissue specific
KVVGVGN	Superscaf	+	Superscaf	13913593	13913613	10	10	16	-	-	-	-	-	-	NCP	IGR	-	ID=gene-E	7	3.87	1659.7	0.730553503	KVVGVGN	73	672.4032	1	671.3966	.	P	Superscaf	13913566	0	0	0	0	0	BTP20220	Mixed
MQPPGPPP	Superscaf	+	Superscaf	13989988	13990011	8	8	15	-	-	gene	-	-	mRNA	NCP	Intron	gene|mRN	ID=gene-E	8	6.10875	2966.5	0.729074004	M(+42.01)	73	431.708	2	861.4055	Acetylati	E	Superscaf	13989967	0	BTP20220	0	0	0	BTP20220	Tissue specific
ANPSFPLG	Superscaf	-	Superscaf	14054410	14054433	-5	5	12	-	-	gene	-	-	mRNA	NCP	Intron	gene|mRN	ID=gene-E	8	5.8925	3004.3	0.727826429	ANPSFPLG	53	802.4077	1	801.402	.	Y	Superscaf	14054694	BTP20220	0	0	0	0	0	Tissue specific
EVGLP	Superscaf	+	Superscaf	14167228	14167242	8	8	12	-	-	-	-	-	-	NCP	IGR	-	ID=gene-E	5	4.294	1413.6	0.725641807	EVGLP	91	514.285	1	513.2798	.	N	Superscaf	14167207	0	0	0	0	0	BTP20220	Tissue specific
KVSRALD	Superscaf	+	Superscaf	14168605	14168625	41	41	47	-	-	-	-	-	-	NCP	IGR	-	ID=gene-E	7	5.878571429	2576.8	0.725615024	KVSRALD	67	394.7335	2	787.4551	.	V	Superscaf	14168485	0	0	0	BTP20220	0	0	Tissue specific
DGDTP	Superscaf	+	Superscaf	14184919	14184933	46	46	50	-	-	-	-	-	-	NCP	IGR	-	ID=gene-E	5	4.948	1855.2	0.725299208	DGDTP	66	504.1919	1	503.1863	.	L	Superscaf	14184784	BTP20220	0	0	0	0	0	Tissue specific
PKRDLP	Superscaf	+	Superscaf	14189206	14189223	15	15	20	-	-	-	-	-	-	NCP	IGR	-	ID=gene-E	6	7.008333333	2556.9	0.72521613	PKRDLP	85	363.2175	2	724.4232	.	P	Superscaf	14189164	0	0	0	BTP20220	0	0	Tissue specific
VTAPH	Superscaf	-	Superscaf	14197981	14197995	-2	2	6	-	-	-	-	-	-	NCP	IGR	-	ID=gene-E	5	5.288	1665.9	0.725046254	VTAPH	54	524.2751	1	523.2755	.	W	Superscaf	14198064	0	0	BTP20220	0	0	0	Tissue specific
KAKHGKG	Superscaf	-	Superscaf	14206393	14206413	-36	36	42	-	-	-	-	-	-	NCP	IGR	-	ID=gene-E	7	7.824285714	2930.8	0.724883234	KAKHGKG	78	363.2311	2	724.4344	.	A	Superscaf	14206422	0	0	0	0	0	BTP20220	Tissue specific
MEAKLKK	Superscaf	-	Superscaf	14323000	14323020	-9	9	15	-	-	gene	-	-	mRNA	NCP	Intron	gene|mRN	ID=gene-E	7	7.17	2866.9	0.722625061	M(+42.01)	88	445.262	2	888.5103	Acetylati	W	Superscaf	14323062	0	0	0	BTP20220	0	0	Tissue specific
LTLQDY	Superscaf	+	Superscaf	14332744	14332761	74	74	79	-	-	gene	-	-	mRNA	NCP	Intron	gene|mRN	ID=gene-E	6	5.461666667	2586	0.722436419	LTLQDY	88	752.3788	1	751.3752	.	C	Superscaf	14332525	0	0	BTP20220	0	0	0	Tissue specific
TSLAKP	Superscaf	+	Superscaf	14355484	14355501	40	40	45	-	-	gene	-	-	mRNA	NCP	Intron	gene|mRN	ID=gene-E	6	6.708333333	2251.4	0.721996044	TSLAKP	55	616.3639	1	615.3591	.	R	Superscaf	14355367	0	0	0	0	BTP20220	0	Tissue specific
WAAVVK	Superscaf	+	Superscaf	14449180	14449197	2	2	7	-	-	gene	-	-	mRNA	NCP	Intron	gene|mRN	ID=gene-E	6	4.611666667	1617.6	0.720181558	W(+42.01)	76	358.2126	2	714.4064	Acetylati	T	Superscaf	14449177	0	0	0	0	BTP20220	BTP20220	Tissue specific
VLVGDGGTC	Superscaf	-	Superscaf	14492125	14492154	-32	32	41	-	-	gene	-	-	mRNA	NCP	Intron	gene|mRN	ID=gene-E	10	4.91	2820	0.719349667	VLVGDGGTC	95	451.7487	2	901.4869	.	K	Superscaf	14492817	0	0	0	BTP20220	0	0	Tissue specific
LGVEVHPL	Superscaf	-	Superscaf	14492212	14492235	-61	61	68	-	-	gene	-	-	mRNA	NCP	Intron	gene|mRN	ID=gene-E	8	4.38	2386.7	0.719348098	LGVEVHPL	85	432.2509	2	862.4912	.	K	Superscaf	14492817	0	0	0	BTP20220	0	0	Tissue specific
GVEVHPLVF	Superscaf	-	Superscaf	14492215	14492247	-62	62	72	-	-	gene	-	-	mRNA	NCP	Intron	gene|mRN	ID=gene-E	11	4.423636364	3437.9	0.719347866	GVEVHPLVF	70	617.8295	2	1233.651	.	K	Superscaf	14492817	0	0	0	0	0	BTP20220	Tissue specific
CFSKLKK	Superscaf	+	Superscaf	14505505	14505525	24	24	30	-	-	gene	-	-	mRNA	NCP	Intron	gene|mRN	ID=gene-E	7	7.34	3115.1	0.719090728	C(+57.02)	81	455.7545	2	909.5106	Carbamido	A	Superscaf	14505436	0	0	0	0	BTP20220	0	Tissue specific
LFPPHGS	Superscaf	-	Superscaf	14509438	14509458	-10	10	16	-	-	gene	-	-	mRNA	NCP	Intron	gene|mRN	ID=gene-E	7	6.185714286	2716	0.719014563	LFPPHGS	75	754.3868	1	753.3809	.	L	Superscaf	14509575	0	0	0	0	0	BTP20220	Tissue specific
ASRALLR	Superscaf	-	Superscaf	14637505	14637525	-5	5	11	-	-	gene	-	-	mRNA	NCP	Intron	gene|mRN	ID=gene-E	7	7.314285714	3010.4	0.716534459	ASRALLR	78	393.7493	2	785.4871	.	L	Superscaf	14637585	0	BTP20220	0	0	0	0	Tissue specific
PATKY	Superscaf	-	Superscaf	14642497	14642511	-4	4	8	-	-	gene	-	-	mRNA	NCP	Intron	gene|mRN	ID=gene-E	5	6.85	1974.1	0.716437902	P(+42.01)	71	621.3208	1	620.317	Acetylati	A	Superscaf	14642541	0	0	0	BTP20220	0	0	Tissue specific
PGDGY	Superscaf	-	Superscaf	14703835	14703849	-46	46	50	-	-	gene	-	-	mRNA	NCP	Intron	gene|mRN	ID=gene-E	5	5.374	1803	0.71525005	P(+42.01)	56	550.2164	1	549.2071	Acetylati	Q	Superscaf	14703897	0	0	0	0	0	BTP20220	Tissue specific
FARSNK	Superscaf	-	Superscaf	14706871	14706888	-1	1	6	-	-	gene	-	-	mRNA	NCP	Intron	gene|mRN	ID=gene-E	6	7.181666667	2796	0.715191198	FARSNK	63	361.7034	2	721.3871	.	F	Superscaf	14707056	0	0	0	0	0	BTP20220	Tissue specific
AARAAPVV	Superscaf	+	Superscaf	14791036	14791059	21	21	28	-	-	-	-	-	-	NCP	IGR	-	ID=gene-E	8	5.1425	2231.4	0.71356117	A(+42.01)	58	398.7367	2	795.4603	Acetylati	K	Superscaf	14790976	0	BTP20220	0	0	BTP20220	BTP20220	Tissue specific
RLELLT	Superscaf	+	Superscaf	14832370	14832387	9	9	14	-	-	-	-	-	-	NCP	IGR	-	ID=gene-E	6	6.408333333	2532	0.712760825	RLELLT	68	372.7328	2	743.4541	.	Q	Superscaf	14832346	0	0	0	0	0	BTP20220	Tissue specific
SSPKEKL	Superscaf	-	Superscaf	14835331	14835351	-1	1	7	-	-	-	-	-	-	NCP	IGR	-	ID=gene-E	7	6.62	2819.9	0.712703425	SSPKEKL	76	394.7281	2	787.4439	.	S	Superscaf	14835471	0	0	0	BTP20220	0	0	Tissue specific
NGAQSQK	Superscaf	+	Superscaf	14847610	14847630	44	44	50	-	-	-	-	-	-	NCP	IGR	-	ID=gene-E	7	6.302857143	3278.5	0.712465634	NGAQSQK	77	366.6887	2	731.3562	.	G	Superscaf	14847481	BTP20220	0	0	0	0	0	Tissue specific
KPPTHLR	Superscaf	-	Superscaf	14851009	14851029	-7	7	13	-	-	-	-	-	-	NCP	IGR	-	ID=gene-E	7	7.6	3162.6	0.71239981	KPPTHLR	53	424.7573	2	847.5028	.	A	Superscaf	14851122	0	0	0	0	0	BTP20220	Tissue specific
VELLLK	Superscaf	+	Superscaf	14857357	14857374	6	6	11	-	-	-	-	-	-	NCP	IGR	-	ID=gene-E	6	5.15	1954.1	0.712276935	VELLLK	83	714.4734	1	713.4687	.	G	Superscaf	14857342	0	0	0	0	BTP20220	BTP20220	Tissue specific
VGGGSLLA	Superscaf	-	Superscaf	14992597	14992620	-27	27	34	-	-	-	-	-	-	NCP	IGR	-	ID=gene-E	8	5.19625	2293.4	0.709657805	VGGGSLLA	57	673.3844	1	672.3806	.	A	Superscaf	14992662	0	0	0	0	BTP20220	BTP20220	Tissue specific
KALLSNR	Superscaf	+	Superscaf	15005572	15005592	4	4	10	-	-	gene	-	-	mRNA	NCP	Intron	gene|mRN	ID=gene-E	7	7.081428571	3115.4	0.709406594	KALLSNR	57	401.2466	2	800.4868	.	K	Superscaf	15005563	0	0	0	0	BTP20220	BTP20220	Tissue specific
KALVQSP	Superscaf	-	Superscaf	15020248	15020268	-2	2	8	-	-	gene	-	-	mRNA	NCP	Intron	gene|mRN	ID=gene-E	7	5.624285714	2464.6	0.709122383	KALVQSP	55	742.4312	1	741.4385	.	M	Superscaf	15020268	0	0	0	0	0	BTP20220	Tissue specific
PPPSRPP	Superscaf	-	Superscaf	15021175	15021195	-1	1	7	-	-	gene	-	-	mRNA	NCP	Intron	gene|mRN	ID=gene-E	7	6.848571429	2719.2	0.709104431	PPPSRPP	97	374.2096	2	746.4075	.	P	Superscaf	15021225	0	0	0	BTP20220	0	0	Tissue specific
SETTLYPK	Superscaf	-	Superscaf	15042628	15042651	-41	41	48	-	-	gene	-	-	mRNA	NCP	Intron	gene|mRN	ID=gene-E	8	6.205	3222.6	0.708688921	SETTLYPK	75	469.7393	2	937.4756	.	A	Superscaf	15042807	0	0	0	0	0	BTP20220	Tissue specific
VGFVPKC	Superscaf	-	Superscaf	15076783	15076803	-16	16	22	-	-	gene	-	-	mRNA	NCP	Intron	gene|mRN	ID=gene-E	7	4.644285714	2076.2	0.708027544	VGFVPKC(+	56	403.716	2	805.4156	Carbamido	I	Superscaf	15076884	0	0	0	0	BTP20220	BTP20220	Tissue specific
KYAPSP	Superscaf	+	Superscaf	15085078	15085095	28	28	33	-	-	gene	-	-	mRNA	NCP	Intron	gene|mRN	ID=gene-E	6	6.616666667	2297.4	0.707866964	KYAPSP	51	662.3438	1	661.3435	.	L	Superscaf	15084997	0	BTP20220	0	0	0	BTP20220	Tissue specific
VAVESEDL	Superscaf	-	Superscaf	15133045	15133068	-4	4	11	-	-	-	-	-	-	NCP	IGR	-	ID=gene-E	8	3.38625	2310.6	0.706937935	VAVESEDL	75	861.4187	1	860.4127	.	V	Superscaf	15133071	0	0	0	BTP20220	0	0	Tissue specific
SHLLVTLA	Superscaf	-	Superscaf	15151177	15151200	-4	4	11	-	-	-	-	-	-	NCP	IGR	-	ID=gene-E	8	5.47	2772.1	0.706586796	SHLLVTLA	73	853.5114	1	852.5069	.	Q	Superscaf	15151242	0	0	BTP20220	0	0	0	Mixed
DSPLLRW	Superscaf	-	Superscaf	15260674	15260694	-4	4	10	-	-	gene	-	-	mRNA	NCP	Intron	gene|mRN	ID=gene-E	7	6.222857143	3014.5	0.704466371	DSPLLRW	66	443.7422	2	885.4708	.	N	Superscaf	15260748	0	0	BTP20220	0	0	0	Tissue specific
KAVLGLGA	Superscaf	+	Superscaf	15287956	15287979	42	42	49	-	-	gene	-	-	mRNA	NCP	Intron	gene|mRN	ID=gene-E	8	5.71	2392.4	0.703937979	KAVLGLGA	66	364.7358	2	727.4592	.	L	Superscaf	15287833	0	0	0	0	0	BTP20220	Mixed
LLDLVTPM	Superscaf	-	Superscaf	15339847	15339870	-39	39	46	-	-	-	-	-	-	NCP	IGR	-	ID=gene-E	8	4.93625	2578.1	0.702933075	LLDLVTPM	51	901.5028	1	900.499	.	V	Superscaf	15339942	0	0	BTP20220	0	0	0	Tissue specific
SAAVGAP	Superscaf	-	Superscaf	15342892	15342912	-6	6	12	-	-	-	-	-	-	NCP	IGR	-	ID=gene-E	7	5.144285714	1952	0.702874164	SAAVGAP	79	572.3024	1	571.2966	.	S	Superscaf	15343083	0	0	BTP20220	0	0	0	Tissue specific
KETKKQEK	Superscaf	+	Superscaf	15351298	15351321	9	9	16	-	-	-	-	-	-	NCP	IGR	-	ID=gene-E	8	7.1975	3732.8	0.702711318	KETKKQEK	71	509.8051	2	1017.582	.	K	Superscaf	15351274	0	0	BTP20220	0	0	0	Tissue specific
GSPMQL	Superscaf	-	Superscaf	15362221	15362238	-6	6	11	-	-	-	-	-	-	NCP	IGR	-	ID=gene-E	6	5.888333333	2353.7	0.702499903	GSPMQL	95	632.3063	1	631.2999	.	M	Superscaf	15362256	0	0	BTP20220	0	0	0	Tissue specific
PFVSLPL	Superscaf	-	Superscaf	15362548	15362568	-7	7	13	-	-	-	-	-	-	NCP	IGR	-	ID=gene-E	7	5.102857143	2152.4	0.702493513	PFVSLPL	74	772.4572	1	771.4531	.	T	Superscaf	15362601	0	0	0	0	0	BTP20220	Tissue specific
LAVSKSSK	Superscaf	+	Superscaf	15374539	15374562	5	5	12	-	-	-	-	-	-	NCP	IGR	-	ID=gene-E	8	6.065	2751.7	0.702261241	L(+42.01)	77	431.2509	2	860.4967	Acetylati	R	Superscaf	15374527	0	0	0	BTP20220	0	0	Tissue specific
GVVAPGP	Superscaf	+	Superscaf	15386602	15386622	6	6	12	-	-	-	-	-	-	NCP	IGR	-	ID=gene-E	7	4.365714286	1580.7	0.702027691	GVVAPGP	67	596.3397	1	595.3329	.	N	Superscaf	15386587	0	0	0	BTP20220	0	0	Tissue specific
HDGHLNY	Superscaf	-	Superscaf	15386737	15386757	-11	11	17	-	-	-	-	-	-	NCP	IGR	-	ID=gene-E	7	5.881428571	3357.8	0.702025076	HDGHLNY	73	428.1905	2	854.3671	.	E	Superscaf	15386811	0	0	0	BTP20220	0	0	Tissue specific
MPGERGK	Superscaf	-	Superscaf	15439210	15439230	-1	1	7	-	-	-	-	-	-	NCP	IGR	-	ID=gene-E	7	6.815714286	2846.2	0.701008901	M(+42.01)	88	408.7124	2	815.3959	Acetylati	M	Superscaf	15439386	0	0	0	0	0	BTP20220	Tissue specific
LVPTLRVP	Superscaf	+	Superscaf	15448153	15448176	18	18	25	-	-	-	-	-	-	NCP	IGR	-	ID=gene-E	8	5.23125	2427.9	0.700835656	LVPTLRVP	71	447.79	2	893.5698	.	D	Superscaf	15448102	0	0	0	0	0	BTP20220	Tissue specific
LEALVLL	Superscaf	-	Superscaf	15461344	15461364	-10	10	16	-	-	-	-	-	-	NCP	IGR	-	ID=gene-E	7	4.737142857	2150.4	0.700580261	LEALVLL	62	770.4988	1	769.4949	.	T	Superscaf	15461493	0	0	BTP20220	0	0	0	Tissue specific
SYVNAG	Superscaf	+	Superscaf	15467116	15467133	8	8	13	-	-	-	-	-	-	NCP	IGR	-	ID=gene-E	6	4.79	2048.2	0.700468541	S(+42.01)	54	652.2881	1	651.2864	Acetylati	I	Superscaf	15467095	0	0	0	0	0	BTP20220	Tissue specific
PAARPSPP	Superscaf	+	Superscaf	15477799	15477822	48	48	55	-	-	-	-	-	-	NCP	IGR	-	ID=gene-E	8	6.71	3004.4	0.700261541	PAARPSPP	53	396.728	2	791.429	.	N	Superscaf	15477658	0	0	0	0	BTP20220	BTP20220	Tissue specific
LHEGFLE	Superscaf	+	Superscaf	15500845	15500865	6	6	12	-	-	gene	-	-	mRNA	NCP	Intron	gene|mRN	ID=gene-E	7	5.348571429	2862.2	0.699815298	LHEGFLE	78	422.712	2	843.4127	.	L	Superscaf	15500830	0	0	0	0	0	BTP20220	Tissue specific
ARGPAVS	Superscaf	+	Superscaf	15508402	15508422	2	2	8	-	-	gene	-	-	mRNA	NCP	Intron	gene|mRN	ID=gene-E	7	5.821428571	2289.5	0.699668952	A(+42.01)	69	350.1899	2	698.3711	Acetylati	L	Superscaf	15508399	0	0	0	0	0	BTP20220	Tissue specific
FVEDPF	Superscaf	-	Superscaf	15514321	15514338	-33	33	38	-	-	gene	-	-	mRNA	NCP	Intron	gene|mRN	ID=gene-E	6	3.908333333	1949.2	0.699554384	FVEDPF	71	753.3426	1	752.3381	.	A	Superscaf	15514398	0	0	0	BTP20220	0	0	Tissue specific
MGMMHP	Superscaf	-	Superscaf	15567517	15567534	-33	33	38	-	-	gene	-	-	mRNA	NCP	Intron	gene|mRN	ID=gene-E	6	6.185	2368.9	0.698524208	MGM(+15.9	54	368.1292	2	734.255	Oxidation	A	Superscaf	15567672	0	BTP20220	0	0	0	0	Mixed
PEAQPGK	Superscaf	+	Superscaf	15584944	15584964	13	13	19	-	-	gene	-	-	mRNA	NCP	Intron	gene|mRN	ID=gene-E	7	6.171428571	2788	0.698186664	PEAQPGK	59	363.688	2	725.3708	.	K	Superscaf	15584908	BTP20220	0	0	0	0	0	Tissue specific
KALTY	Superscaf	+	Superscaf	15590983	15590997	76	76	80	-	-	gene	-	-	mRNA	NCP	Intron	gene|mRN	ID=gene-E	5	6.786	1990.1	0.698069831	KALTY	76	595.3437	1	594.3377	.	L	Superscaf	15590758	0	0	0	BTP20220	0	0	Tissue specific
LPVQP	Superscaf	+	Superscaf	15600832	15600846	42	42	46	-	-	gene	-	-	mRNA	NCP	Intron	gene|mRN	ID=gene-E	5	4.846	1638.9	0.697879098	LPVQP	78	553.3334	1	552.3271	.	G	Superscaf	15600709	0	BTP20220	0	0	0	0	Tissue specific
LSLLHLPL	Superscaf	-	Superscaf	15602257	15602280	-33	33	40	-	-	gene	-	-	mRNA	NCP	Intron	gene|mRN	ID=gene-E	8	6.18375	3107.5	0.697851328	L(+42.01)	81	474.2984	2	946.5851	Acetylati	T	Superscaf	15602310	0	0	0	BTP20220	0	0	Tissue specific
DLPRPLS	Superscaf	-	Superscaf	15611329	15611349	-17	17	23	-	-	gene	-	-	mRNA	NCP	Intron	gene|mRN	ID=gene-E	7	6.281428571	2825.3	0.697675701	DLPRPLS	59	399.2291	2	796.4443	.	F	Superscaf	15611367	0	0	BTP20220	0	0	0	Tissue specific
LDTPGTY	Superscaf	-	Superscaf	15634084	15634104	-3	3	9	-	-	-	-	-	-	NCP	IGR	-	ID=gene-E	7	5.705714286	2654.1	0.697235035	LDTPGTY	90	766.3594	1	765.3545	.	G	Superscaf	15634344	0	BTP20220	0	0	BTP20220	0	Tissue specific
GLHKPAA	Superscaf	-	Superscaf	15689293	15689313	-6	6	12	-	-	gene	-	-	mRNA	NCP	Intron	gene|mRN	ID=gene-E	7	6.802857143	2698.8	0.696165875	G(+42.01)	59	368.2095	2	734.4025	Acetylati	S	Superscaf	15689379	0	0	BTP20220	0	0	0	Tissue specific
KRKPPSP	Superscaf	+	Superscaf	15699073	15699093	9	9	15	-	-	gene	-	-	mRNA	NCP	Intron	gene|mRN	ID=gene-E	7	7.831428571	2981.2	0.695976479	KRKPPSP	54	405.2564	2	808.4919	.	L	Superscaf	15699049	0	0	BTP20220	0	0	0	Tissue specific
DDTGQQ	Superscaf	+	Superscaf	15723091	15723108	11	11	16	-	-	-	-	-	-	NCP	IGR	-	ID=gene-E	6	4.956666667	2739.2	0.695511412	D(+42.01)	59	705.2581	1	704.2613	Acetylati	K	Superscaf	15723061	0	0	0	BTP20220	0	0	Tissue specific
DHLKFGLL	Superscaf	+	Superscaf	15800314	15800337	8	8	15	-	-	-	-	-	-	NCP	IGR	-	ID=gene-E	8	6.21125	3244.5	0.694015821	D(+42.01)	61	492.7765	2	983.544	Acetylati	V	Superscaf	15800293	BTP20220	0	0	0	0	0	Tissue specific
CCQSGM	Superscaf	+	Superscaf	15854581	15854598	17	17	22	-	-	-	-	-	-	NCP	IGR	-	ID=gene-E	6	5.515	2834.3	0.69296502	C(+57.02)	54	758.2125	1	757.2194	Carbamido	R	Superscaf	15854533	BTP20220	0	0	0	0	0	Tissue specific
VPPVTPP	Superscaf	-	Superscaf	15873109	15873129	-2	2	8	-	-	-	-	-	-	NCP	IGR	-	ID=gene-E	7	4.532857143	1747.1	0.692606155	VPPVTPP	62	706.4108	1	705.4061	.	S	Superscaf	15873195	0	0	0	BTP20220	0	0	Tissue specific
KHRNPP	Superscaf	-	Superscaf	15931006	15931023	-11	11	16	-	-	gene	-	-	mRNA	NCP	Intron	gene|mRN	ID=gene-E	6	7.683333333	3008.4	0.691484998	KHRNPP	55	374.7128	2	747.414	.	D	Superscaf	15931023	0	BTP20220	BTP20220	BTP20220	0	0	Tissue specific
PPPPNRT	Superscaf	+	Superscaf	15985489	15985509	7	7	13	-	-	-	-	-	-	NCP	IGR	-	ID=gene-E	7	6.842857143	2992.6	0.69042984	PPPPNRT	52	389.7126	2	777.4133	.	P	Superscaf	15985471	BTP20220	0	0	0	0	0	Tissue specific
FKVSEKK	Superscaf	+	Superscaf	16012396	16012416	21	21	27	-	-	-	-	-	-	NCP	IGR	-	ID=gene-E	7	6.228571429	2601.5	0.689908768	FKVSEKK	75	433.2582	2	864.5068	.	F	Superscaf	16012336	0	0	BTP20220	0	0	0	Tissue specific
GPTLVFL	Superscaf	+	Superscaf	16035067	16035087	40	40	46	-	-	-	-	-	-	NCP	IGR	-	ID=gene-E	7	5.177142857	2126.4	0.689469729	GPTLVFL	58	746.4421	1	745.4374	.	F	Superscaf	16034950	0	0	0	0	BTP20220	0	Tissue specific
DFRKPGEK	Superscaf	+	Superscaf	16053052	16053075	7	7	14	-	-	-	-	-	-	NCP	IGR	-	ID=gene-E	8	6.7725	3444.7	0.689121379	DFRKPGEK	95	488.7625	2	975.5137	.	I	Superscaf	16053034	0	0	0	BTP20220	0	0	Tissue specific
VPPKAVP	Superscaf	+	Superscaf	16061707	16061727	11	11	17	-	-	-	-	-	-	NCP	IGR	-	ID=gene-E	7	4.951428571	1791.9	0.688953827	VPPKAVP	66	354.2254	2	706.4377	.	E	Superscaf	16061677	0	0	0	BTP20220	0	0	Tissue specific
KPYVPP	Superscaf	+	Superscaf	16077817	16077834	11	11	16	-	-	-	-	-	-	NCP	IGR	-	ID=gene-E	6	5.716666667	1940.1	0.688641904	KPYVPP	62	350.7118	2	699.3955	.	T	Superscaf	16077787	0	BTP20220	0	0	0	0	Tissue enhanced
ETGAKNLV	Superscaf	-	Superscaf	16170247	16170270	-63	63	70	-	-	gene	-	-	mRNA	NCP	Intron	gene|mRN	ID=gene-E	8	5.35875	2850.1	0.686851819	ETGAKNLV	61	416.2317	2	830.4498	.	I	Superscaf	16170315	0	0	0	BTP20220	0	0	Tissue specific
KPACNAK	Superscaf	+	Superscaf	16206625	16206645	42	42	48	-	-	-	-	-	-	NCP	IGR	-	ID=gene-E	7	6.892857143	3079.2	0.686147392	KPAC(+57.	54	394.7002	2	787.4011	Carbamido	L	Superscaf	16206502	0	0	0	0	0	BTP20220	Tissue specific
KERDSLP	Superscaf	-	Superscaf	16249831	16249851	-42	42	48	-	-	-	-	-	-	NCP	IGR	-	ID=gene-E	7	6.378571429	3028.4	0.685310679	KERDSLP	60	422.7283	2	843.445	.	R	Superscaf	16249869	0	0	0	BTP20220	0	0	Tissue specific
EVAKER	Superscaf	-	Superscaf	16265167	16265184	-26	26	31	-	-	-	-	-	-	NCP	IGR	-	ID=gene-E	6	5.493333333	2279.5	0.685013745	EVAKER	63	366.2004	2	730.3973	.	K	Superscaf	16265298	0	BTP20220	0	0	0	0	Tissue specific
ESLLLTKA	Superscaf	-	Superscaf	16307374	16307397	-10	10	17	-	-	gene	-	-	mRNA	NCP	Intron	gene|mRN	ID=gene-E	8	6.14125	3046.3	0.684196262	ESLLLTKA	79	437.7644	2	873.5171	.	I	Superscaf	16307448	0	0	0	0	BTP20220	0	Tissue specific
SVTKKPP	Superscaf	+	Superscaf	16400254	16400274	25	25	31	-	-	-	-	-	-	NCP	IGR	-	ID=gene-E	7	6.327142857	2392.5	0.682397636	SVTKKPP	68	378.7337	2	755.4541	.	V	Superscaf	16400182	0	BTP20220	0	BTP20220	BTP20220	BTP20220	Tissue specific
PPKTPNK	Superscaf	+	Superscaf	16400890	16400910	31	31	37	-	-	-	-	-	-	NCP	IGR	-	ID=gene-E	7	7.188571429	2943.2	0.68238532	P(+42.01)	72	412.2328	2	822.4599	Acetylati	M	Superscaf	16400800	0	0	0	BTP20220	0	0	Tissue specific
LEFGRGF	Superscaf	-	Superscaf	16408924	16408944	-13	13	19	-	-	-	-	-	-	NCP	IGR	-	ID=gene-E	7	6.122857143	2797.1	0.682229736	LEFGRGF	70	413.2142	2	824.418	.	T	Superscaf	16408965	0	0	0	BTP20220	0	0	Tissue specific
VVLLLLGV	Superscaf	+	Superscaf	16448110	16448133	52	52	59	-	-	-	-	-	-	NCP	IGR	-	ID=gene-E	8	3.73625	1710.9	0.681470814	VVLLLLGV	53	413.2981	2	824.5735	.	Q	Superscaf	16447957	BTP20220	0	0	0	0	0	Tissue specific
DPCPRGP	Superscaf	-	Superscaf	16457503	16457523	-11	11	17	-	-	-	-	-	-	NCP	IGR	-	ID=gene-E	7	6.231428571	2955.5	0.681288971	DPC(+57.(	99	399.6805	2	797.349	Carbamido	V	Superscaf	16457544	BTP20220	0	0	0	0	0	Tissue specific
YGQVSR	Superscaf	-	Superscaf	16469152	16469169	-12	12	17	-	-	-	-	-	-	NCP	IGR	-	ID=gene-E	6	5.62	2399.7	0.681063438	Y(+42.01)	85	376.1956	2	750.366	Acetylati	N	Superscaf	16469169	0	0	0	0	0	BTP20220	Tissue specific
TLPFDK	Superscaf	-	Superscaf	16471465	16471482	-26	26	31	-	-	-	-	-	-	NCP	IGR	-	ID=gene-E	6	6.166666667	2355.6	0.681018645	TLPFDK	92	720.3894	1	719.3854	.	K	Superscaf	16471551	0	0	0	0	BTP20220	0	Tissue specific
SLPAKAK	Superscaf	-	Superscaf	16479112	16479132	-24	24	30	-	-	-	-	-	-	NCP	IGR	-	ID=gene-E	7	7.068571429	2633.6	0.680870498	SLPAKAK	59	714.4626	1	713.4435	.	S	Superscaf	16479246	0	0	BTP20220	0	0	0	Tissue specific
LKLTELKL	Superscaf	-	Superscaf	16497658	16497681	-20	20	27	-	-	-	-	-	-	NCP	IGR	-	ID=gene-E	8	6.64375	3173.4	0.680511284	LKLTELKL	87	479.3193	2	956.627	.	C	Superscaf	16497693	0	0	BTP20220	0	0	0	Tissue specific
PAAPTY	Superscaf	-	Superscaf	16537162	16537179	-35	35	40	-	-	-	-	-	-	NCP	IGR	-	ID=gene-E	6	6.138333333	2154.4	0.679746379	PAAPTY	58	619.3131	1	618.3013	.	R	Superscaf	16537230	0	0	0	BTP20220	0	0	Tissue specific
PHAPFR	Superscaf	+	Superscaf	16543714	16543731	165	165	170	-	-	-	-	-	-	NCP	IGR	-	ID=gene-E	6	7.075	2642	0.679619495	PHAPFR	97	362.697	2	723.3816	.	H	Superscaf	16543222	0	0	0	BTP20220	0	0	Mixed
LEADPVS	Superscaf	+	Superscaf	16585465	16585485	78	78	84	-	-	-	-	-	-	NCP	IGR	-	ID=gene-E	7	4.31	2222.5	0.6788109	LEADPVS	82	730.3602	1	729.3544	.	K	Superscaf	16585234	0	0	BTP20220	0	0	0	Tissue specific
KSPDLDL	Superscaf	-	Superscaf	16667236	16667256	-14	14	20	-	-	-	-	-	-	NCP	IGR	-	ID=gene-E	7	5.66	2719	0.67722735	KSPDLDL	53	787.423	1	786.4123	.	I	Superscaf	16667289	BTP20220	0	0	0	0	0	Tissue specific
TLQALVLK	Superscaf	+	Superscaf	16676656	16676679	33	33	40	-	-	gene	-	-	mRNA	NCP	Intron	gene|mRN	ID=gene-E	8	5.735	2848.1	0.677044867	TLQALVLK	64	443.2892	2	884.5695	.	L	Superscaf	16676560	0	0	0	BTP20220	0	0	Tissue specific
ELGGLDHK	Superscaf	+	Superscaf	16684813	16684836	56	56	63	-	-	gene	-	-	mRNA	NCP	Intron	gene|mRN	ID=gene-E	8	5.9275	3226.5	0.676886901	ELGGLDHK	74	434.7281	2	867.445	.	K	Superscaf	16684648	0	0	0	0	0	BTP20220	Tissue specific
KDPPCLP	Superscaf	-	Superscaf	16734793	16734813	-75	75	81	-	-	gene	-	-	mRNA	NCP	Intron	gene|mRN	ID=gene-E	7	6.087142857	2831.2	0.675919063	KDPPC(+57	56	826.4271	1	825.4055	Carbamido	K	Superscaf	16734813	0	BTP20220	0	0	0	0	Tissue specific
LTGLPGGR	Superscaf	+	Superscaf	16790470	16790493	50	50	57	-	-	-	-	-	-	NCP	IGR	-	ID=gene-E	8	6.6825	2982.4	0.674840782	LTGLPGGR	65	385.7248	2	769.4446	.	K	Superscaf	16790323	0	0	0	0	0	BTP20220	Tissue specific
DHWQGPP	Superscaf	+	Superscaf	16816669	16816689	35	35	41	-	-	-	-	-	-	NCP	IGR	-	ID=gene-E	7	5.81	3140.6	0.674333479	DHWQGPP	93	418.6866	2	835.3613	.	V	Superscaf	16816567	0	BTP20220	0	0	0	0	Tissue specific
QFMPAK	Superscaf	+	Superscaf	16830274	16830291	17	17	22	-	-	-	-	-	-	NCP	IGR	-	ID=gene-E	6	6.49	2486.7	0.674070067	QFMPAK	81	361.1954	2	720.3629	.	G	Superscaf	16830226	0	0	0	0	0	BTP20220	Tissue specific
DVGELL	Superscaf	+	Superscaf	16845913	16845930	93	93	98	-	-	-	-	-	-	NCP	IGR	-	ID=gene-E	6	4.02	1841.1	0.673767207	DVGELL	56	645.3433	1	644.3381	.	C	Superscaf	16845637	0	0	BTP20220	0	0	0	Tissue specific
PMDMF	Superscaf	-	Superscaf	16890841	16890855	-1	1	5	-	-	-	-	-	-	NCP	IGR	-	ID=gene-E	5	5.25	1879.3	0.672897204	P(+42.01)	54	698.254	1	697.2451	Acetylati	P	Superscaf	16891071	0	0	0	BTP20220	0	0	Tissue specific
YDSPPLG	Superscaf	+	Superscaf	16903909	16903929	120	120	126	-	-	-	-	-	-	NCP	IGR	-	ID=gene-E	7	5.551428571	2579.9	0.672644018	YDSPPLG	72	748.3489	1	747.3439	.	I	Superscaf	16903552	0	BTP20220	BTP20220	0	0	0	Tissue specific
AATKLF	Superscaf	-	Superscaf	16977562	16977579	-34	34	39	-	exon	gene	three_pri	-	mRNA	NCP	3UTR	exon|gene	ID=exon-E	6	6.628333333	2229.3	0.671217736	A(+42.01)	52	692.4041	1	691.3904	Acetylati	P	Superscaf	16977651	0	0	0	0	BTP20220	0	Tissue enhanced
KPDQVPQ	Superscaf	+	Superscaf	16984537	16984557	29	29	35	-	-	gene	-	-	mRNA	NCP	Intron	gene|mRN	ID=gene-E	7	5.23	2776.1	0.671082602	KPDQVPQ	54	811.4279	1	810.4235	.	S	Superscaf	16984453	0	0	0	0	BTP20220	0	Tissue specific
GVLMVG	Superscaf	+	Superscaf	17000692	17000709	13	13	18	-	-	gene	-	-	mRNA	NCP	Intron	gene|mRN	ID=gene-E	6	3.945	1319.5	0.670769808	GVLMVG	82	575.3198	1	574.3149	.	W	Superscaf	17000656	0	0	0	BTP20220	0	0	Tissue specific
TTSFTLAK	Superscaf	-	Superscaf	17005531	17005554	-41	41	48	-	-	gene	-	-	mRNA	NCP	Intron	gene|mRN	ID=gene-E	8	6.56125	3096.4	0.670675981	TTSFTLAK	99	868.4739	1	867.4702	.	P	Superscaf	17005650	0	0	0	0	0	BTP20220	Tissue specific
NDFKLFH	Superscaf	-	Superscaf	17017603	17017623	-48	48	54	-	-	gene	-	-	mRNA	NCP	Intron	gene|mRN	ID=gene-E	7	6.092857143	3224.5	0.670442257	NDFKLFH	61	460.7322	2	919.4551	.	A	Superscaf	17017641	0	0	0	0	BTP20220	0	Tissue specific
KNKVLYP	Superscaf	-	Superscaf	17030710	17030730	-33	33	39	-	-	-	-	-	-	NCP	IGR	-	ID=gene-E	7	6.118571429	2683.8	0.670188431	K(+42.01)	76	452.2602	2	902.5225	Acetylati	L	Superscaf	17030817	0	0	0	BTP20220	0	0	Tissue specific
ATSDLPK	Superscaf	-	Superscaf	17035699	17035719	-4	4	10	-	-	-	-	-	-	NCP	IGR	-	ID=gene-E	7	6.174285714	2662.9	0.670091815	A(+42.01)	83	387.204	2	772.3967	Acetylati	T	Superscaf	17036022	0	0	BTP20220	0	0	0	Tissue specific
EWALVR	Superscaf	+	Superscaf	17091214	17091231	76	76	81	-	-	-	-	-	-	NCP	IGR	-	ID=gene-E	6	5.311666667	2265.6	0.669016788	EWALVR	62	387.2246	2	772.4232	.	I	Superscaf	17090989	0	0	0	0	0	BTP20220	Tissue specific
LLQQQM	Superscaf	+	Superscaf	17096203	17096220	37	37	42	-	-	-	-	-	-	NCP	IGR	-	ID=gene-E	6	5.776666667	2910.4	0.668920173	LLQQQM	90	760.4008	1	759.3949	.	V	Superscaf	17096095	0	0	0	BTP20220	0	0	Tissue specific
ETLFQPS	Superscaf	+	Superscaf	17177401	17177421	5	5	11	-	-	-	-	-	-	NCP	IGR	-	ID=gene-E	7	5.548571429	2895.3	0.667347661	ETLFQPS	91	821.401	1	820.3967	.	K	Superscaf	17177389	0	0	0	BTP20220	0	0	Tissue specific
VKVTLLH	Superscaf	-	Superscaf	17184088	17184108	-1	1	7	-	-	-	-	-	-	NCP	IGR	-	ID=gene-E	7	5.117142857	2192.4	0.667218162	V(+42.01)	63	851.5312	1	850.5276	Acetylati	V	Superscaf	17184165	0	0	0	BTP20220	0	0	Tissue specific
ECFKSSG	Superscaf	-	Superscaf	17276920	17276940	-7	7	13	-	-	-	-	-	-	NCP	IGR	-	ID=gene-E	7	5.827142857	2931.1	0.665420408	EC(+57.02	76	814.3328	1	813.3327	Carbamido	L	Superscaf	17277018	BTP20220	0	0	0	0	0	Tissue specific
KSGVPEK	Superscaf	-	Superscaf	17303059	17303079	-14	14	20	-	-	-	-	-	-	NCP	IGR	-	ID=gene-E	7	5.807142857	2380.4	0.664914209	KSGVPEK	51	372.7187	2	743.4177	.	E	Superscaf	17303232	0	BTP20220	0	BTP20220	BTP20220	BTP20220	Tissue specific
KHVDARP	Superscaf	+	Superscaf	17335456	17335476	5	5	11	-	-	-	-	-	-	NCP	IGR	-	ID=gene-E	7	6.197142857	2797.1	0.664286819	KHVDARP	50	411.7365	2	821.4507	.	Y	Superscaf	17335444	0	BTP20220	BTP20220	BTP20220	BTP20220	BTP20220	Tissue specific
LDELLP	Superscaf	-	Superscaf	17385610	17385627	-26	26	31	-	-	-	-	-	-	NCP	IGR	-	ID=gene-E	6	5.071666667	2234.6	0.663315611	LDELLP	72	699.3903	1	698.385	.	L	Superscaf	17385765	0	BTP20220	0	0	0	BTP20220	Tissue specific
VTPTNP	Superscaf	+	Superscaf	17394604	17394621	12	12	17	-	-	-	-	-	-	NCP	IGR	-	ID=gene-E	6	5.178333333	2066.5	0.663141436	VTPTNP	57	628.3244	1	627.3228	.	I	Superscaf	17394571	0	0	BTP20220	0	0	0	Tissue specific
KLDPTK	Superscaf	+	Superscaf	17441347	17441364	4	4	9	-	-	gene	-	-	mRNA	NCP	Intron	gene|mRN	ID=gene-E	6	6.876666667	2436.6	0.662236226	K(+42.01)	92	372.2168	2	742.4225	Acetylati	M	Superscaf	17441338	0	BTP20220	0	0	BTP20220	BTP20220	Mixed
PLGKTNP	Superscaf	+	Superscaf	17478994	17479014	18	18	24	-	-	-	-	-	-	NCP	IGR	-	ID=gene-E	7	6.604285714	2788.1	0.661507109	PLGKTNP	57	363.7095	2	725.4072	.	D	Superscaf	17478943	0	BTP20220	0	BTP20220	0	0	Tissue specific
LPDRKPP	Superscaf	-	Superscaf	17486389	17486409	-6	6	12	-	-	-	-	-	-	NCP	IGR	-	ID=gene-E	7	6.907142857	2894.3	0.6613639	LPDRKPP	75	411.7433	2	821.4759	.	G	Superscaf	17486532	0	BTP20220	BTP20220	0	BTP20220	0	Tissue specific
PGCDLR	Superscaf	-	Superscaf	17495077	17495094	-58	58	63	-	-	-	-	-	-	NCP	IGR	-	ID=gene-E	6	6.166666667	2634.1	0.661195709	P(+42.01)	67	380.1754	2	758.3381	Acetylati	L	Superscaf	17495130	0	0	BTP20220	0	0	0	Tissue specific
PPPVVPR	Superscaf	-	Superscaf	17513200	17513220	-80	80	86	-	-	-	-	-	-	NCP	IGR	-	ID=gene-E	7	5.137142857	1998.4	0.660844687	PPPVVPR	98	381.2367	2	760.4595	.	P	Superscaf	17513304	0	0	BTP20220	0	0	0	Tissue specific
LPLKPPP	Superscaf	-	Superscaf	17527885	17527905	-26	26	32	-	-	-	-	-	-	NCP	IGR	-	ID=gene-E	7	6.7	2524.8	0.660560302	LPLKPPP	97	381.2483	2	760.4847	.	R	Superscaf	17528031	0	0	0	0	0	BTP20220	Tissue specific
PVREPK	Superscaf	+	Superscaf	17543605	17543622	32	32	37	-	-	-	-	-	-	NCP	IGR	-	ID=gene-E	6	6.053333333	2217.5	0.660255931	PVREPK	71	363.2175	2	724.4232	.	E	Superscaf	17543512	0	0	0	BTP20220	0	0	Tissue specific
TVLPLLQ	Superscaf	-	Superscaf	17555017	17555037	-100	100	106	-	-	-	-	-	-	NCP	IGR	-	ID=gene-E	7	5.202857143	2405.8	0.660034872	T(+42.01)	73	825.505	1	824.5007	Acetylati	D	Superscaf	17555115	0	0	BTP20220	0	0	0	Tissue specific
KASEDPK	Superscaf	-	Superscaf	17592391	17592411	-8	8	14	-	-	gene	-	-	mRNA	NCP	Intron	gene|mRN	ID=gene-E	7	6.238571429	2805.9	0.659311099	KASEDPK	64	387.7102	2	773.3919	.	P	Superscaf	17592477	0	0	0	0	BTP20220	BTP20220	Tissue specific
GVVPNGL	Superscaf	-	Superscaf	17604325	17604345	-7	7	13	-	-	gene	-	-	mRNA	NCP	Intron	gene|mRN	ID=gene-E	7	4.232857143	1882.1	0.659079989	GVVPNGL	58	655.3754	1	654.3701	.	K	Superscaf	17604612	0	0	0	0	BTP20220	BTP20220	Tissue specific
DCHSCM	Superscaf	+	Superscaf	17680453	17680470	15	15	20	-	-	-	-	-	-	NCP	IGR	-	ID=gene-E	6	5.338333333	2957.5	0.657605778	DC(+57.02	51	413.1198	2	824.2252	Carbamido	R	Superscaf	17680411	0	0	0	0	0	BTP20220	Tissue specific
LQKAALL	Superscaf	-	Superscaf	17690014	17690034	-8	8	14	-	-	-	-	-	-	NCP	IGR	-	ID=gene-E	7	6.481428571	2761.9	0.657420564	LQKAALL	82	378.751	2	755.4905	.	G	Superscaf	17690253	0	0	0	0	0	BTP20220	Tissue specific
VLEELLF	Superscaf	-	Superscaf	17697475	17697495	-12	12	18	-	-	-	-	-	-	NCP	IGR	-	ID=gene-E	7	4.265714286	2298.6	0.657276077	VLEELLF	67	862.4882	1	861.4847	.	K	Superscaf	17697552	0	0	0	BTP20220	0	0	Tissue specific
WPEPEF	Superscaf	-	Superscaf	17714446	17714463	-20	20	25	-	-	-	-	-	-	NCP	IGR	-	ID=gene-E	6	5.068333333	2439.8	0.65694748	WPEPEF	87	804.3549	1	803.3489	.	M	Superscaf	17714484	BTP20220	0	0	0	0	0	Tissue specific
AASRRYP	Superscaf	-	Superscaf	17720881	17720901	-9	9	15	-	-	-	-	-	-	NCP	IGR	-	ID=gene-E	7	7.314285714	3100.5	0.656822804	A(+42.01)	55	431.7302	2	861.4457	Acetylati	R	Superscaf	17721069	0	0	BTP20220	0	0	0	Tissue specific
TRQGQPP	Superscaf	+	Superscaf	17774833	17774853	20	20	26	-	-	-	-	-	-	NCP	IGR	-	ID=gene-E	7	6.737142857	3239.8	0.655777987	T(+42.01)	61	413.2127	2	824.4141	Acetylati	N	Superscaf	17774776	BTP20220	0	0	0	0	0	Tissue specific
ASLVVPG	Superscaf	-	Superscaf	17775220	17775240	-39	39	45	-	-	-	-	-	-	NCP	IGR	-	ID=gene-E	7	4.278571429	1682.8	0.655770493	A(+42.01)	50	684.3871	1	683.3854	Acetylati	S	Superscaf	17775309	0	BTP20220	0	0	0	0	Tissue specific
ERSLAVP	Superscaf	-	Superscaf	17816908	17816928	-34	34	40	-	-	-	-	-	-	NCP	IGR	-	ID=gene-E	7	5.422857143	2459.8	0.654963176	ERSLAVP	73	386.2139	2	770.4286	.	S	Superscaf	17816952	0	0	0	0	0	BTP20220	Tissue specific
KFLMPK	Superscaf	-	Superscaf	17843791	17843808	-14	14	19	-	-	gene	-	-	mRNA	NCP	Intron	gene|mRN	ID=gene-E	6	7.165	2386.5	0.654442627	KFLM(+15.	50	390.2261	2	778.4411	Oxidation	Y	Superscaf	17843856	0	0	BTP20220	0	0	0	Tissue specific
LLLVVKP	Superscaf	-	Superscaf	17860810	17860830	-14	14	20	-	-	gene	-	-	mRNA	NCP	Intron	gene|mRN	ID=gene-E	7	4.854285714	1866	0.654112985	LLLVVKP	51	391.2837	2	780.5473	.	S	Superscaf	17860929	0	0	0	0	0	BTP20220	Tissue specific
RLPLGVCA	Superscaf	-	Superscaf	17915545	17915568	-48	48	55	-	-	gene	-	-	mRNA	NCP	Intron	gene|mRN	ID=gene-E	8	5.75375	2887.3	0.653052946	RLPLGVC(+	56	443.2502	2	884.4902	Carbamido	V	Superscaf	17915787	BTP20220	0	0	0	0	0	Tissue specific
TAELGSPP	Superscaf	+	Superscaf	17957776	17957799	12	12	19	-	-	gene	-	-	mRNA	NCP	Intron	gene|mRN	ID=gene-E	8	5.75	2843.2	0.652235114	T(+42.01)	91	813.3966	1	812.3916	Acetylati	D	Superscaf	17957743	0	0	0	0	0	BTP20220	Tissue specific
GSSME	Superscaf	-	Superscaf	17995174	17995188	-2	2	6	-	-	gene	-	-	mRNA	NCP	Intron	gene|mRN	ID=gene-E	5	5.26	1861.1	0.651511051	GSSME	52	510.1925	1	509.1792	.	G	Superscaf	17995260	0	BTP20220	0	0	0	0	Tissue specific
AAHVSHK	Superscaf	-	Superscaf	18105622	18105642	-1	1	7	-	-	gene	-	-	mRNA	NCP	Intron	gene|mRN	ID=gene-E	7	6.091428571	2713.8	0.649372035	AAHVSHK	71	375.2067	2	748.398	.	A	Superscaf	18105705	0	0	BTP20220	0	0	0	Tissue specific
KNYFAAP	Superscaf	-	Superscaf	18155731	18155751	-2	2	8	-	-	gene	-	-	mRNA	NCP	Intron	gene|mRN	ID=gene-E	7	6.375714286	2872	0.648401641	KNYFAAP	67	405.7128	2	809.4072	.	N	Superscaf	18155961	0	0	0	0	BTP20220	BTP20220	Tissue specific
GTWKPR	Superscaf	+	Superscaf	18223177	18223194	33	33	38	-	-	gene	-	-	mRNA	NCP	Intron	gene|mRN	ID=gene-E	6	7.531666667	2676	0.647095561	GTWKPR	69	372.7059	2	743.4078	.	C	Superscaf	18223081	0	0	0	0	0	BTP20220	Tissue specific
AAPLGLHQ	Superscaf	-	Superscaf	18223900	18223923	-1	1	8	-	-	gene	-	-	mRNA	NCP	Intron	gene|mRN	ID=gene-E	8	6.18875	3194.5	0.647081444	A(+42.01)	76	424.7337	2	847.4552	Acetylati	A	Superscaf	18224013	BTP20220	0	0	0	0	0	Tissue specific
KDVQKP	Superscaf	+	Superscaf	18291079	18291096	15	15	20	-	-	gene	-	-	mRNA	NCP	Intron	gene|mRN	ID=gene-E	6	5.733333333	2296.4	0.645780593	KDVQKP	51	357.7073	2	713.4072	.	H	Superscaf	18291037	0	0	0	BTP20220	0	0	Tissue specific
LDPTEF	Superscaf	+	Superscaf	18313234	18313251	10	10	15	-	-	gene	-	-	mRNA	NCP	Intron	gene|mRN	ID=gene-E	6	5.08	2312.7	0.645351547	LDPTEF	99	721.3404	1	720.333	.	N	Superscaf	18313207	0	0	0	0	0	BTP20220	Tissue specific
VFGGPK	Superscaf	+	Superscaf	18374125	18374142	11	11	16	-	-	gene	-	-	mRNA	NCP	Intron	gene|mRN	ID=gene-E	6	5.576666667	1787.8	0.644172352	VFGGPK	50	604.3516	1	603.338	.	M	Superscaf	18374095	0	0	BTP20220	0	0	0	Tissue specific
ANQGY	Superscaf	-	Superscaf	18388978	18388992	-40	40	44	-	-	gene	-	-	mRNA	NCP	Intron	gene|mRN	ID=gene-E	5	5.742	2275.5	0.643884771	A(+42.01)	55	594.2566	1	593.2445	Acetylati	L	Superscaf	18388992	0	0	BTP20220	0	0	0	Tissue specific
GVPPVF	Superscaf	-	Superscaf	18391339	18391356	-69	69	74	-	-	gene	-	-	mRNA	NCP	Intron	gene|mRN	ID=gene-E	6	4.008333333	1359.5	0.643838991	GVPPVF	93	615.3473	1	614.3428	.	Q	Superscaf	18392091	BTP20220	0	0	0	0	0	Tissue specific
VGPFML	Superscaf	-	Superscaf	18400504	18400521	-23	23	28	-	-	gene	-	-	mRNA	NCP	Intron	gene|mRN	ID=gene-E	6	4.913333333	1747	0.643661504	VGPFML	57	663.3477	1	662.3461	.	L	Superscaf	18400629	0	0	0	BTP20220	0	0	Tissue specific
ALAATVF	Superscaf	+	Superscaf	18400606	18400626	11	11	17	-	-	gene	-	-	mRNA	NCP	Intron	gene|mRN	ID=gene-E	7	5.15	2072.2	0.643659471	ALAATVF	66	692.4036	1	691.3904	.	L	Superscaf	18400576	0	0	0	0	BTP20220	0	Tissue specific
QTGAKFV	Superscaf	+	Superscaf	18400816	18400836	20	20	26	-	-	gene	-	-	mRNA	NCP	Intron	gene|mRN	ID=gene-E	7	5.627142857	2472.6	0.643655404	QTGAKFV	52	375.7105	2	749.4072	.	D	Superscaf	18400759	0	0	0	0	BTP20220	0	Tissue specific
CLAFKCL	Superscaf	+	Superscaf	18468151	18468171	7	7	13	-	-	gene	-	-	mRNA	NCP	Intron	gene|mRN	ID=gene-E	7	6.177142857	3045.3	0.642351417	C(+57.02)	64	456.2354	2	910.4405	Carbamido	T	Superscaf	18468133	0	0	0	BTP20220	0	0	Tissue specific
EDLVKLQ	Superscaf	-	Superscaf	18493831	18493851	-16	16	22	-	-	gene	-	-	mRNA	NCP	Intron	gene|mRN	ID=gene-E	7	4.791428571	2622.9	0.641854106	EDLVKLQ	95	422.7411	2	843.4702	.	T	Superscaf	18493863	0	0	0	0	0	BTP20220	Tissue specific
WFPFFC	Superscaf	-	Superscaf	18548431	18548448	-106	106	111	-	-	gene	-	-	mRNA	NCP	Intron	gene|mRN	ID=gene-E	6	5.608333333	2611.9	0.640796798	W(+42.01)	50	473.2081	2	944.389	Acetylati	L	Superscaf	18548481	0	BTP20220	BTP20220	0	0	0	Mixed
YAHRYH	Superscaf	+	Superscaf	18554953	18554970	4	4	9	-	-	gene	-	-	mRNA	NCP	Intron	gene|mRN	ID=gene-E	6	7.213333333	3118.5	0.640670495	Y(+42.01)	62	444.7142	2	887.4038	Acetylati	S	Superscaf	18554944	0	0	BTP20220	0	0	BTP20220	Tissue specific
LPVKHSR	Superscaf	+	Superscaf	18555436	18555456	10	10	16	-	-	gene	-	-	mRNA	NCP	Intron	gene|mRN	ID=gene-E	7	6.578571429	2811.1	0.640661084	LPVKHSR	51	418.7635	2	835.5028	.	S	Superscaf	18555409	0	BTP20220	0	0	0	0	Tissue specific
KDKHEPP	Superscaf	+	Superscaf	18567040	18567060	6	6	12	-	-	gene	-	-	mRNA	NCP	Intron	gene|mRN	ID=gene-E	7	6.551428571	3068.3	0.640436364	KDKHEPP	52	425.7185	2	849.4344	.	I	Superscaf	18567025	0	0	0	0	0	BTP20220	Tissue specific
ADDFEV	Superscaf	-	Superscaf	18585970	18585987	-48	48	53	-	-	gene	-	-	mRNA	NCP	Intron	gene|mRN	ID=gene-E	6	3.443333333	1947.2	0.64006983	A(+42.01)	68	737.298	1	736.2915	Acetylati	N	Superscaf	18586008	0	0	0	0	BTP20220	0	Tissue specific
HESFL	Superscaf	+	Superscaf	18592042	18592056	17	17	21	-	-	gene	-	-	mRNA	NCP	Intron	gene|mRN	ID=gene-E	5	5.59	2169.4	0.6399523	HESFL	80	632.3022	1	631.2966	.	E	Superscaf	18591994	0	0	BTP20220	0	0	0	Tissue specific
PSRGQARP	Superscaf	-	Superscaf	18624364	18624387	-128	128	135	-	-	gene	-	-	mRNA	NCP	Intron	gene|mRN	ID=gene-E	8	7.18	3575.1	0.639326188	PSRGQARP	64	434.7393	2	867.4675	.	A	Superscaf	18624450	0	0	0	BTP20220	0	0	Tissue specific
GKFPA	Superscaf	+	Superscaf	18658165	18658179	1	1	5	-	-	gene	-	-	mRNA	NCP	Intron	gene|mRN	ID=gene-E	5	6.702	1801.8	0.638671784	G(+42.01)	50	561.3112	1	560.2958	Acetylati	G	Superscaf	18658165	0	0	0	BTP20220	0	0	Tissue specific
ANEFLPR	Superscaf	+	Superscaf	18684208	18684228	20	20	26	-	exon	gene	three_pri	-	mRNA	NCP	3UTR	exon|gene	ID=exon-E	7	6.167142857	3060.5	0.638167327	ANEFLPR	69	423.7261	2	845.4395	.	Y	Superscaf	18684151	0	0	0	BTP20220	0	0	Tissue specific
KWGGGVT	Superscaf	+	Superscaf	18712549	18712569	6	6	12	-	-	gene	-	-	mRNA	NCP	Intron	gene|mRN	ID=gene-E	7	5.724285714	2284.4	0.637618485	KWGGGVT	57	352.6882	2	703.3653	.	E	Superscaf	18712534	0	0	0	0	0	BTP20220	Tissue specific
TSPAE	Superscaf	-	Superscaf	18823705	18823719	-77	77	81	-	-	gene	-	-	mRNA	NCP	Intron	gene|mRN	ID=gene-E	5	5.55	1855.1	0.63546599	T(+42.01)	53	546.2389	1	545.2333	Acetylati	R	Superscaf	18824187	0	0	0	BTP20220	0	0	Tissue specific
PCLKGK	Superscaf	-	Superscaf	18830368	18830385	-37	37	42	-	-	gene	-	-	mRNA	NCP	Intron	gene|mRN	ID=gene-E	6	7.125	2510.6	0.635336898	PC(+57.02	51	351.6974	2	701.3894	Carbamido	T	Superscaf	18830412	0	0	0	0	0	BTP20220	Tissue specific
KEVAFAF	Superscaf	+	Superscaf	18853273	18853293	34	34	40	-	-	gene	-	-	mRNA	NCP	Intron	gene|mRN	ID=gene-E	7	5.137142857	2291.3	0.634893269	KEVAFAF	68	406.22	2	810.4276	.	Q	Superscaf	18853174	BTP20220	0	BTP20220	0	0	0	Tissue specific
MLALKLD	Superscaf	+	Superscaf	18897034	18897054	5	5	11	-	-	gene	-	-	mRNA	NCP	Intron	gene|mRN	ID=gene-E	7	6.06	2622.9	0.634045808	M(+42.01)	72	423.2479	2	844.4728	Acetylati	G	Superscaf	18897022	0	0	0	0	0	BTP20220	Tissue specific
KDTPPP	Superscaf	+	Superscaf	18973546	18973563	2	2	7	-	-	-	-	-	-	NCP	IGR	-	ID=gene-E	6	6.356666667	2289.6	0.63256416	KDTPPP	54	654.3405	1	653.3384	.	P	Superscaf	18973543	BTP20220	0	0	0	0	0	Tissue specific
FPASTELL	Superscaf	+	Superscaf	18991552	18991575	23	23	30	-	exon	gene	-	five_prim	mRNA	NCP	5UTR	exon|gene	ID=exon-E	8	5.64875	2949.3	0.632215345	FPASTELL	52	877.465	1	876.4592	.	R	Superscaf	18991486	0	0	0	0	BTP20220	0	Tissue specific
ELLLNNL	Superscaf	+	Superscaf	19002331	19002351	8	8	14	-	-	gene	-	-	mRNA	NCP	Intron	gene|mRN	ID=gene-E	7	5.422857143	3032.5	0.632006661	ELLLNNL	76	828.4796	1	827.4753	.	S	Superscaf	19002310	0	0	0	0	BTP20220	0	Tissue specific
KLPTPLT	Superscaf	-	Superscaf	19023517	19023537	-58	58	64	-	-	-	-	-	-	NCP	IGR	-	ID=gene-E	7	6.765714286	2645	0.63159638	K(+42.01)	82	811.4895	1	810.4851	Acetylati	R	Superscaf	19023537	0	0	0	0	BTP20220	BTP20220	Tissue specific
LSGSK	Superscaf	-	Superscaf	19025497	19025511	-24	24	28	-	-	gene	-	-	mRNA	NCP	Intron	gene|mRN	ID=gene-E	5	6.61	1885.9	0.631558152	LSGSK	53	491.2845	1	490.2751	.	S	Superscaf	19025583	0	0	0	BTP20220	0	0	Tissue specific
LVVSY	Superscaf	+	Superscaf	19084282	19084296	18	18	22	-	-	gene	-	-	mRNA	NCP	Intron	gene|mRN	ID=gene-E	5	3.464	1196.3	0.630419741	LVVSY	87	580.3313	1	579.3268	.	D	Superscaf	19084231	0	BTP20220	BTP20220	0	BTP20220	BTP20220	Tissue specific
LTTFDN	Superscaf	-	Superscaf	19123513	19123530	-23	23	28	-	-	gene	-	-	mRNA	NCP	Intron	gene|mRN	ID=gene-E	6	5.483333333	2544	0.629659948	L(+42.01)	65	376.6803	2	751.3388	Acetylati	L	Superscaf	19123539	0	BTP20220	0	0	0	BTP20220	Tissue specific
LGNMALK	Superscaf	-	Superscaf	19132810	19132830	-16	16	22	-	-	-	-	-	-	NCP	IGR	-	ID=gene-E	7	6.407142857	2752	0.629479847	LGNMALK	97	373.7131	2	745.4156	.	I	Superscaf	19132899	0	0	BTP20220	0	0	0	Tissue specific
QPGSLAAP	Superscaf	+	Superscaf	19144603	19144626	2	2	9	-	-	gene	-	-	mRNA	NCP	Intron	gene|mRN	ID=gene-E	8	5.99	2942.2	0.629251409	QPGSLAAP	52	370.7036	2	739.3864	.	P	Superscaf	19144600	0	0	0	0	0	BTP20220	Tissue specific
AALLF	Superscaf	+	Superscaf	19190899	19190913	8	8	12	-	-	-	-	-	-	NCP	IGR	-	ID=gene-E	5	5.896	1716.8	0.62835503	AALLF	65	534.338	1	533.3213	.	H	Superscaf	19190878	0	BTP20220	0	0	0	0	Mixed
WDFALYE	Superscaf	+	Superscaf	19195210	19195230	9	9	15	-	-	gene	-	-	mRNA	NCP	Intron	gene|mRN	ID=gene-E	7	5.031428571	2875.2	0.628271429	WDFALYE	61	943.416	1	942.4123	.	T	Superscaf	19195186	0	0	0	BTP20220	0	0	Tissue specific
VGDSLLP	Superscaf	-	Superscaf	19211272	19211292	-4	4	10	-	-	gene	-	-	mRNA	NCP	Intron	gene|mRN	ID=gene-E	7	4.697142857	2136.4	0.627960377	V(+42.01)	50	742.3956	1	741.3909	Acetylati	V	Superscaf	19211361	0	BTP20220	0	0	0	0	Tissue specific
EPVLQ	Superscaf	-	Superscaf	19249942	19249956	-78	78	82	-	-	gene	-	-	mRNA	NCP	Intron	gene|mRN	ID=gene-E	5	4.23	1727	0.627211623	EPVLQ	95	585.3226	1	584.317	.	N	Superscaf	19250127	0	BTP20220	0	0	0	0	Tissue specific
QLLLPK	Superscaf	+	Superscaf	19250035	19250052	62	62	67	-	-	gene	-	-	mRNA	NCP	Intron	gene|mRN	ID=gene-E	6	6.605	2476.7	0.627209764	QLLLPK	55	356.2365	2	710.4691	.	W	Superscaf	19249852	0	0	0	BTP20220	0	0	Tissue specific
NPGPPPPP	Superscaf	-	Superscaf	19291507	19291530	-7	7	14	-	-	-	-	-	-	NCP	IGR	-	ID=gene-E	8	6.1475	2918.4	0.626406514	N(+42.01)	71	407.7008	2	813.402	Acetylati	F	Superscaf	19291554	0	BTP20220	0	0	0	0	Tissue specific
KARTVH	Superscaf	+	Superscaf	19355260	19355277	3	3	8	-	-	gene	-	-	mRNA	NCP	Intron	gene|mRN	ID=gene-E	6	6.773333333	2445.7	0.625172011	KARTVH	54	356.2204	2	710.4188	.	Q	Superscaf	19355254	0	BTP20220	0	0	0	0	Tissue specific
KDAAKRH	Superscaf	-	Superscaf	19366999	19367019	-12	12	18	-	-	gene	-	-	mRNA	NCP	Intron	gene|mRN	ID=gene-E	7	7.548571429	3239.4	0.624944619	KDAAKRH	87	413.2369	2	824.4617	.	L	Superscaf	19367247	0	BTP20220	0	0	0	0	Tissue specific
ESDLPLNL	Superscaf	+	Superscaf	19496650	19496673	5	5	12	-	-	gene	-	-	mRNA	NCP	Intron	gene|mRN	ID=gene-E	8	5.19	3214.7	0.622433782	ESDLPLNL	59	900.4642	1	899.46	.	V	Superscaf	19496638	0	BTP20220	0	0	0	0	Tissue specific
PGPLGVT	Superscaf	-	Superscaf	19498273	19498293	-8	8	14	-	-	gene	-	-	mRNA	NCP	Intron	gene|mRN	ID=gene-E	7	5.292857143	2020.3	0.622402409	PGPLGVT	76	640.3647	1	639.3591	.	N	Superscaf	19498302	0	0	0	0	0	BTP20220	Tissue specific
APPPTPR	Superscaf	+	Superscaf	19511848	19511868	53	53	59	-	-	-	-	-	-	NCP	IGR	-	ID=gene-E	7	6.93	2707.2	0.62213952	A(+42.01)	87	389.2158	2	776.418	Acetylati	P	Superscaf	19511692	0	0	BTP20220	0	0	0	Tissue specific
VPLGP	Superscaf	+	Superscaf	19568878	19568892	5	5	9	-	-	-	-	-	-	NCP	IGR	-	ID=gene-E	5	4.91	1325.5	0.621035212	VPLGP	83	482.2964	1	481.29	.	V	Superscaf	19568866	0	0	0	0	BTP20220	0	Tissue specific
GVGRLVK	Superscaf	-	Superscaf	19591441	19591461	-7	7	13	-	-	-	-	-	-	NCP	IGR	-	ID=gene-E	7	5.488571429	2065.2	0.620598148	GVGRLVK	81	364.7411	2	727.4704	.	E	Superscaf	19591461	0	0	0	BTP20220	0	0	Tissue specific
MKLQNL	Superscaf	-	Superscaf	19603351	19603368	-17	17	22	-	-	-	-	-	-	NCP	IGR	-	ID=gene-E	6	6.418333333	2754.1	0.620367561	MKLQNL	52	373.7141	2	745.4156	.	R	Superscaf	19603455	0	0	0	0	0	BTP20220	Tissue specific
SGFLT	Superscaf	-	Superscaf	19655059	19655073	-34	34	38	-	-	-	-	-	-	NCP	IGR	-	ID=gene-E	5	5.928	1819	0.619366259	SGFLT	74	524.2698	1	523.2642	.	S	Superscaf	19655097	0	0	0	0	0	BTP20220	Tissue specific
FGVNK	Superscaf	+	Superscaf	19685524	19685538	22	22	26	-	-	-	-	-	-	NCP	IGR	-	ID=gene-E	5	5.32	1749.8	0.618776284	FGVNK	63	564.3131	1	563.3067	.	I	Superscaf	19685461	0	0	0	0	0	BTP20220	Tissue specific
LSVDEVR	Superscaf	+	Superscaf	19686403	19686423	51	51	57	-	-	-	-	-	-	NCP	IGR	-	ID=gene-E	7	4.087142857	2222.6	0.618759145	LSVDEVR	97	409.2229	2	816.4341	.	E	Superscaf	19686253	0	0	0	BTP20220	0	0	Tissue specific
TFDWRC	Superscaf	-	Superscaf	19687951	19687968	-14	14	19	-	-	-	-	-	-	NCP	IGR	-	ID=gene-E	6	6.108333333	2957.5	0.618729225	T(+42.01)	50	926.3741	1	925.3752	Acetylati	Q	Superscaf	19687980	BTP20220	0	0	0	0	0	Tissue specific
YDPPGLR	Superscaf	+	Superscaf	19793083	19793103	14	14	20	-	-	-	-	-	-	NCP	IGR	-	ID=gene-E	7	6.277142857	2845.3	0.616693215	YDPPGLR	77	409.2114	2	816.413	.	D	Superscaf	19793044	BTP20220	0	0	0	0	0	Tissue specific
GLRQAM	Superscaf	-	Superscaf	19813165	19813182	-13	13	18	-	-	-	-	-	-	NCP	IGR	-	ID=gene-E	6	6.688333333	2593	0.616304372	G(+42.01)	59	359.1895	2	716.364	Acetylati	N	Superscaf	19813206	0	0	0	0	BTP20220	BTP20220	Tissue specific
PPRGPPPPF	Superscaf	+	Superscaf	19844968	19845003	7	7	18	-	-	gene	-	-	mRNA	NCP	Intron	gene|mRN	ID=gene-E	12	6.644166667	4320.1	0.615688137	PPRGPPPPF	84	601.8445	2	1201.661	.	P	Superscaf	19844950	0	0	BTP20220	0	0	0	Tissue specific
LDKLRL	Superscaf	-	Superscaf	19940710	19940727	-4	4	9	-	-	gene	-	-	mRNA	NCP	Intron	gene|mRN	ID=gene-E	6	6.901666667	2588.9	0.613834377	LDKLRL	91	379.2478	2	756.4857	.	S	Superscaf	19940754	0	0	0	0	BTP20220	BTP20220	Tissue specific
FPDPEK	Superscaf	-	Superscaf	19943035	19943052	-5	5	10	-	-	gene	-	-	mRNA	NCP	Intron	gene|mRN	ID=gene-E	6	5.668333333	2367.6	0.613789352	FPDPEK	72	732.3549	1	731.3489	.	H	Superscaf	19943076	BTP20220	BTP20220	0	0	0	0	Tissue specific
PPDEE	Superscaf	+	Superscaf	20037670	20037684	5	5	9	-	-	gene	-	-	mRNA	NCP	Intron	gene|mRN	ID=gene-E	5	4.402	1937.3	0.611956739	PPDEE	62	586.2463	1	585.2282	.	L	Superscaf	20037658	0	BTP20220	0	0	0	0	Tissue specific
VLPPAP	Superscaf	+	Superscaf	20103196	20103213	23	23	28	-	-	gene	-	-	mRNA	NCP	Intron	gene|mRN	ID=gene-E	6	5.15	1676.9	0.610687726	VLPPAP	76	593.3635	1	592.3584	.	R	Superscaf	20103130	0	0	0	BTP20220	0	0	Tissue specific

LAVDLLLP	Superscaf	+	Superscaf	20123725	20123748	20	20	27	-	-	-	-	-	-	NCP	IGR	-	ID=gene-E	8	4.90125	2473.8	0.610290052	LAVDLLLP	67	853.5369	1	852.532	.	L	Superscaf	20123668	0	BTP202209	0	0	0	0	Tissue specific
LSNHVLGL	Superscaf	+	Superscaf	20158384	20158407	4	4	11	-	-	-	-	-	-	NCP	IGR	-	ID=gene-E	8	5.32375	2957.3	0.609618857	LSNHVLGL	62	426.7495	2	851.4865	.	K	Superscaf	20158375	BTP202209	0	0	0	0	0	Tissue specific
RSDLRLL	Superscaf	-	Superscaf	20161021	20161041	-48	48	54	-	-	-	-	-	-	NCP	IGR	-	ID=gene-E	7	6.872857143	3152.7	0.609567848	RSDLRLL	91	436.7672	2	871.5239	.	S	Superscaf	20161047	0	0	0	0	BTP202209	0	Tissue specific
FDDLNLK	Superscaf	+	Superscaf	20208184	20208204	3	3	9	-	-	gene	-	-	mRNA	NCP	Intron	gene|mRN	ID=gene-E	7	5.504285714	2982.3	0.608654504	FDDLNLK	90	432.7256	2	863.4388	.	Y	Superscaf	20208178	0	0	0	BTP202209	0	0	Tissue specific
AAAEAPVVV	Superscaf	-	Superscaf	20213791	20213817	-49	49	57	-	-	gene	-	-	mRNA	NCP	Intron	gene|mRN	ID=gene-E	9	3.733333333	2008.1	0.608545805	A(+42.01)	65	868.4751	1	867.4702	Acetylati	G	Superscaf	20213889	0	BTP202209	0	0	0	0	Tissue specific
KPMYPR	Superscaf	+	Superscaf	20215627	20215644	45	45	50	-	-	gene	-	-	mRNA	NCP	Intron	gene|mRN	ID=gene-E	6	7.418333333	2623	0.608510424	KPMYPR	53	396.2168	2	790.416	.	N	Superscaf	20215495	0	0	0	0	BTP202209	0	Tissue specific
KDMCSPK	Superscaf	-	Superscaf	20254093	20254113	-13	13	19	-	-	gene	-	-	mRNA	NCP	Intron	gene|mRN	ID=gene-E	7	6.457142857	3026.3	0.607765446	KDM(+15.9	67	441.1996	2	880.3782	Oxidation	K	Superscaf	20254143	0	0	0	BTP202209	0	0	Mixed
AVALLSY	Superscaf	-	Superscaf	20266228	20266248	-6	6	12	-	-	gene	-	-	mRNA	NCP	Intron	gene|mRN	ID=gene-E	7	5.048571429	2172.3	0.607530443	AVALLSY	57	736.4251	1	735.4167	.	F	Superscaf	20266251	BTP202209	0	0	0	0	0	Tissue specific
KGRPHSK	Superscaf	+	Superscaf	20297326	20297346	26	26	32	-	-	gene	-	-	mRNA	NCP	Intron	gene|mRN	ID=gene-E	7	7.968571429	3223.4	0.606928209	KGRPHSK	69	405.235	2	808.4667	.	L	Superscaf	20297251	0	0	0	0	BTP202209	0	Tissue specific
VPGTCKK	Superscaf	+	Superscaf	20323543	20323563	26	26	32	-	-	gene	-	-	mRNA	NCP	Intron	gene|mRN	ID=gene-E	7	6.185714286	2554.7	0.6064205	VPGTC(+57	58	395.2123	2	788.4214	Carbamido	H	Superscaf	20323468	BTP202209	0	0	0	0	0	Tissue specific
KCPPQ	Superscaf	-	Superscaf	20352466	20352480	-26	26	30	-	-	gene	-	-	mRNA	NCP	Intron	gene|mRN	ID=gene-E	5	6.602	2339.6	0.605860502	KC(+57.02	51	629.3124	1	628.3002	Carbamido	P	Superscaf	20352492	0	0	0	0	BTP202209	0	Tissue specific
KLYFAAP	Superscaf	+	Superscaf	20412169	20412189	3	3	9	-	-	-	-	-	-	NCP	IGR	-	ID=gene-E	7	6.457142857	2628.7	0.604704197	KLYFAAP	58	405.2368	2	808.4483	.	F	Superscaf	20412163	0	0	0	0	BTP202209	0	Tissue specific
KDLADPV	Superscaf	-	Superscaf	20473621	20473641	-23	23	29	-	-	-	-	-	-	NCP	IGR	-	ID=gene-E	7	4.854285714	2293.5	0.603514138	KDLADPV	71	757.4015	1	756.4017	.	G	Superscaf	20473647	0	0	0	0	0	BTP202209	Tissue specific
PQMHTKK	Superscaf	-	Superscaf	20591350	20591370	-1	1	7	-	-	-	-	-	-	NCP	IGR	-	ID=gene-E	7	7.328571429	3273.6	0.601234236	PQMHTKK	63	435.238	2	868.4589	.	P	Superscaf	20591394	0	BTP202209	0	0	0	0	Tissue specific
MRKPEKQ	Superscaf	-	Superscaf	20620693	20620713	-7	7	13	-	-	-	-	-	-	NCP	IGR	-	ID=gene-E	7	7.308571429	3330.7	0.600665989	MRKPEKQ	71	458.7526	2	915.496	.	N	Superscaf	20620782	0	0	0	0	BTP202209	BTP202209	Tissue specific
AVPPLKF	Superscaf	-	Superscaf	20710777	20710797	-5	5	11	-	-	gene	-	-	mRNA	NCP	Intron	gene|mRN	ID=gene-E	7	5.688571429	2195.3	0.598921452	A(+42.01)	61	407.2505	2	812.4796	Acetylati	K	Superscaf	20710827	0	0	0	BTP202209	0	0	Tissue specific
EHLLTRE	Superscaf	-	Superscaf	20724598	20724618	-37	37	43	-	-	-	-	-	-	NCP	IGR	-	ID=gene-E	7	6.182857143	3223.8	0.598653799	EHLLTRE	68	449.2471	2	896.4716	.	S	Superscaf	20724663	0	0	0	0	0	BTP202209	Tissue specific
TPFQYPP	Superscaf	+	Superscaf	20798488	20798508	21	21	27	-	-	-	-	-	-	NCP	IGR	-	ID=gene-E	7	6.031428571	2867.3	0.597222869	T(+42.01)	74	891.423	1	890.4174	Acetylati	R	Superscaf	20798428	0	0	0	0	0	BTP202209	Tissue specific
LPGLELR	Superscaf	+	Superscaf	20842750	20842770	25	25	31	-	-	-	-	-	-	NCP	IGR	-	ID=gene-E	7	6.312857143	2769.2	0.596365706	LPGLELR	99	399.2462	2	796.4807	.	S	Superscaf	20842678	BTP202209	0	0	0	0	0	Tissue specific
LPPNPPLP	Superscaf	+	Superscaf	20847607	20847630	45	45	52	-	-	gene	-	-	mRNA	NCP	Intron	gene|mRN	ID=gene-E	8	6.10875	2990.5	0.596271589	LPPNPPLP	77	422.7512	2	843.4854	.	A	Superscaf	20847475	0	BTP202209	0	0	0	0	Tissue specific
KKPRMAK	Superscaf	-	Superscaf	20892109	20892129	-53	53	59	-	-	gene	-	-	mRNA	NCP	Intron	gene|mRN	ID=gene-E	7	8.292857143	3074.2	0.595409836	KKPRM(+15	54	437.7702	2	873.5218	Oxidation	R	Superscaf	20892999	0	0	0	BTP202209	0	0	Tissue specific
VSLLAYY	Superscaf	+	Superscaf	20892235	20892255	3	3	9	-	-	gene	-	-	mRNA	NCP	Intron	gene|mRN	ID=gene-E	7	4.997142857	2320.5	0.595407395	V(+42.01)	67	435.7319	2	869.4534	Acetylati	T	Superscaf	20892229	0	0	0	0	BTP202209	0	Tissue specific
SLRPESL	Superscaf	+	Superscaf	20938000	20938020	4	4	10	-	-	gene	-	-	mRNA	NCP	Intron	gene|mRN	ID=gene-E	7	6.228571429	2885.3	0.594521125	SLRPESL	95	401.2269	2	800.4392	.	S	Superscaf	20937991	0	0	BTP202209	0	BTP202209	0	Tissue specific
SLPLFGPG	Superscaf	+	Superscaf	20945785	20945808	17	17	24	-	-	-	-	-	-	NCP	IGR	-	ID=gene-E	8	5.9575	2747	0.594370306	SLPLFGPG	55	787.4304	1	786.4276	.	S	Superscaf	20945737	0	0	0	BTP202209	0	0	Tissue specific
VRTVGGL	Superscaf	+	Superscaf	20948845	20948865	42	42	48	-	-	gene	-	-	mRNA	NCP	Intron	gene|mRN	ID=gene-E	7	5.03	1994.3	0.594311105	VRTVGGL	53	351.2146	2	700.4232	.	A	Superscaf	20948722	0	0	0	BTP202209	0	0	Tissue specific
LGPLA	Superscaf	+	Superscaf	21017665	21017679	24	24	28	-	-	gene	-	-	mRNA	NCP	Intron	gene|mRN	ID=gene-E	5	6.05	1652.8	0.592978475	LGPLA	82	470.2958	1	469.29	.	L	Superscaf	21017596	0	0	0	0	0	BTP202209	Tissue specific
TVHLLPL	Superscaf	-	Superscaf	21038008	21038028	-15	15	21	-	-	gene	-	-	mRNA	NCP	Intron	gene|mRN	ID=gene-E	7	5.48	2414.8	0.592584403	T(+42.01)	71	417.756	2	833.5011	Acetylati	H	Superscaf	21038130	0	0	0	0	BTP202209	0	Tissue specific
VYPALKPR	Superscaf	-	Superscaf	21060229	21060252	-21	21	28	-	-	gene	-	-	mRNA	NCP	Intron	gene|mRN	ID=gene-E	8	6.345	2916.2	0.59215402	VYPALKPR	91	472.2891	2	942.5651	.	A	Superscaf	21060255	0	0	BTP202209	0	0	0	Tissue specific
KVLEPY	Superscaf	-	Superscaf	21111292	21111309	-6	6	11	-	-	-	-	-	-	NCP	IGR	-	ID=gene-E	6	5.15	2044.2	0.591165267	K(+42.01)	71	790.4307	1	789.4272	Acetylati	P	Superscaf	21111315	0	0	0	0	0	BTP202209	Tissue specific
VNLEYL	Superscaf	-	Superscaf	21144127	21144144	-9	9	14	-	-	-	-	-	-	NCP	IGR	-	ID=gene-E	6	4.375	2188.5	0.590529395	VNLEYL	63	750.3997	1	749.3959	.	A	Superscaf	21144198	0	0	0	0	BTP202209	BTP202209	Tissue specific
KDPSYF	Superscaf	+	Superscaf	21162052	21162069	4	4	9	-	-	-	-	-	-	NCP	IGR	-	ID=gene-E	6	5.971666667	2447.6	0.590182265	KDPSYF	50	756.3646	1	755.3489	.	S	Superscaf	21162043	0	0	0	0	0	BTP202209	Tissue specific
STTATPL	Superscaf	+	Superscaf	21175093	21175113	9	9	15	-	-	-	-	-	-	NCP	IGR	-	ID=gene-E	7	6.224285714	2578	0.58992966	STTATPL	73	690.3652	1	689.3596	.	S	Superscaf	21175069	BTP202209	0	0	0	0	0	Tissue specific
EDVNWY	Superscaf	-	Superscaf	21267760	21267777	-69	69	74	-	-	-	-	-	-	NCP	IGR	-	ID=gene-E	6	3.858333333	2419.8	0.588135159	EDVNWY	62	825.3394	1	824.334	.	H	Superscaf	21267816	0	0	0	0	BTP202209	0	Tissue specific
LPLVPAAH	Superscaf	-	Superscaf	21271147	21271170	-10	10	17	-	-	-	-	-	-	NCP	IGR	-	ID=gene-E	8	5.52375	2623.9	0.588069451	LPLVPAAH	50	817.4896	1	816.4857	.	E	Superscaf	21271176	BTP202209	0	0	0	0	0	Tissue specific
PFTMVP	Superscaf	+	Superscaf	21348835	21348852	22	22	27	-	-	gene	-	-	mRNA	NCP	Intron	gene|mRN	ID=gene-E	6	5.06	1831.2	0.586565087	PFTMVP	74	691.3522	1	690.3411	.	I	Superscaf	21348772	0	0	0	0	0	BTP202209	Mixed
LGPPFLQ	Superscaf	+	Superscaf	21478273	21478293	17	17	23	-	-	gene	-	-	mRNA	NCP	Intron	gene|mRN	ID=gene-E	7	5.951428571	2677	0.584058374	L(+42.01)	81	407.2289	2	812.4432	Acetylati	P	Superscaf	21478225	0	0	0	0	BTP202209	0	Tissue specific
SSSEFPK	Superscaf	+	Superscaf	21493681	21493701	1	1	7	-	-	-	-	-	-	NCP	IGR	-	ID=gene-E	7	5.968571429	2768.9	0.583759988	SSSEFPK	77	391.1967	2	780.3654	.	S	Superscaf	21493681	0	0	0	0	BTP202209	0	Tissue specific
SALQY	Superscaf	+	Superscaf	21579223	21579237	32	32	36	-	-	gene	-	-	mRNA	NCP	Intron	gene|mRN	ID=gene-E	5	5.798	2118.3	0.582103526	SALQY	70	581.2911	1	580.2856	.	K	Superscaf	21579130	BTP202209	0	0	0	0	0	Tissue specific
LDLTGL	Superscaf	-	Superscaf	21589540	21589557	-14	14	19	-	-	gene	-	-	mRNA	NCP	Intron	gene|mRN	ID=gene-E	6	5.568333333	2166.5	0.581903672	LDLTGL	91	631.364	1	630.3588	.	A	Superscaf	21589653	0	0	0	0	0	BTP202209	Tissue specific
EAKSRTLG	Superscaf	-	Superscaf	21633760	21633783	-25	25	32	-	-	gene	-	-	mRNA	NCP	Intron	gene|mRN	ID=gene-E	8	6.7375	3285.6	0.581047206	EAKSRTLG	68	431.2406	2	860.4716	.	Q	Superscaf	21633825	0	BTP202209	0	0	0	0	Tissue specific
KKTGMSK	Superscaf	+	Superscaf	21636310	21636330	4	4	10	-	-	gene	-	-	mRNA	NCP	Intron	gene|mRN	ID=gene-E	7	7.592857143	2854.9	0.580997881	KKTGM(+15	60	398.2306	2	794.432	Oxidation	E	Superscaf	21636301	BTP202209	0	0	0	0	0	Tissue specific
KSTLGHE	Superscaf	+	Superscaf	21755524	21755544	72	72	78	-	-	gene	-	-	mRNA	NCP	Intron	gene|mRN	ID=gene-E	7	6.387142857	2945.2	0.578689222	KSTLGHE	51	771.4053	1	770.3923	.	L	Superscaf	21755311	0	0	BTP202209	0	0	0	Tissue specific
LDFTVW	Superscaf	+	Superscaf	21780388	21780405	15	15	20	-	-	-	-	-	-	NCP	IGR	-	ID=gene-E	6	4.475	2076.4	0.578207772	LDFTVW	60	390.6966	2	779.3854	.	L	Superscaf	21780346	BTP202209	0	BTP202209	0	0	0	Tissue specific
KPQGKTT	Superscaf	-	Superscaf	21800944	21800964	-1	1	7	-	-	gene	-	-	mRNA	NCP	Intron	gene|mRN	ID=gene-E	7	7.208571429	2977.2	0.577809633	K(+42.01)	85	401.2257	2	800.4392	Acetylati	K	Superscaf	21801174	0	0	0	BTP202209	0	0	Tissue specific
APESPLR	Superscaf	+	Superscaf	21832360	21832380	1	1	7	-	exon	gene	-	five_prim	mRNA	NCP	5UTR	exon|gene	ID=exon-E	7	6.322857143	2797.2	0.577201241	APESPLR	59	385.214	2	768.413	.	A	Superscaf	21832360	0	0	BTP202209	0	0	0	Tissue specific
NGHLFY	Superscaf	-	Superscaf	21847828	21847845	-6	6	11	-	-	-	-	-	-	NCP	IGR	-	ID=gene-E	6	6.015	2714	0.576901751	NGHLFY	70	750.3571	1	749.3497	.	N	Superscaf	21847881	0	0	0	BTP202209	0	0	Tissue specific
GVPSVPK	Superscaf	-	Superscaf	21898381	21898401	-33	33	39	-	-	gene	-	-	mRNA	NCP	Intron	gene|mRN	ID=gene-E	7	4.855714286	1823.9	0.5759227	G(+42.01)	95	363.219	2	724.4119	Acetylati	F	Superscaf	21898599	0	0	0	0	0	BTP202209	Tissue specific
VPEPSAL	Superscaf	-	Superscaf	21903043	21903063	-36	36	42	-	-	-	-	-	-	NCP	IGR	-	ID=gene-E	7	4.785714286	2148.4	0.575832417	VPEPSAL	81	356.7016	2	711.3802	.	L	Superscaf	21903081	0	0	0	0	0	BTP202209	Tissue specific
RLAPPER	Superscaf	-	Superscaf	21921004	21921024	-41	41	47	-	-	gene	-	-	mRNA	NCP	Intron	gene|mRN	ID=gene-E	7	7.048571429	3062.6	0.57548459	RLAPPER	99	419.7413	2	837.4821	.	T	Superscaf	21921168	0	0	0	BTP202209	0	0	Tissue specific
EVPDPT	Superscaf	-	Superscaf	21946654	21946671	-35	35	40	-	-	gene	-	-	mRNA	NCP	Intron	gene|mRN	ID=gene-E	6	4.22	1909.3	0.574987919	EVPDPT	50	657.3067	1	656.3017	.	L	Superscaf	21946836	0	0	0	BTP202209	0	0	Tissue specific
GSLNTEFK	Superscaf	-	Superscaf	21969484	21969507	-25	25	32	-	-	gene	-	-	mRNA	NCP	Intron	gene|mRN	ID=gene-E	8	6.00125	3309.6	0.574545684	G(+42.01)	84	469.2326	2	936.4553	Acetylati	H	Superscaf	21969516	0	0	0	BTP202209	0	0	Mixed
NLKAKAL	Superscaf	+	Superscaf	21970042	21970062	12	12	18	-	-	gene	-	-	mRNA	NCP	Intron	gene|mRN	ID=gene-E	7	6.984285714	2862.9	0.574534936	NLKAKAL	56	379.2485	2	756.4857	.	R	Superscaf	21970009	0	0	0	0	BTP202209	0	Tissue specific
RETKNH	Superscaf	-	Superscaf	21972058	21972075	-10	10	15	-	-	gene	-	-	mRNA	NCP	Intron	gene|mRN	ID=gene-E	6	7.208333333	3156.6	0.574495953	R(+42.01)	56	826.4272	1	825.4093	Acetylati	G	Superscaf	21972180	BTP202209	0	0	0	0	0	Tissue specific
VLTLGNER	Superscaf	+	Superscaf	22076080	22076103	7	7	14	-	-	gene	-	-	mRNA	NCP	Intron	gene|mRN	ID=gene-E	8	5.48125	3072.6	0.572481381	VLTLGNER	99	451.2567	2	900.5029	.	S	Superscaf	22076062	0	0	0	0	BTP202209	0	Tissue specific
KFTAGRP	Superscaf	-	Superscaf	22078315	22078335	-53	53	59	-	-	gene	-	-	mRNA	NCP	Intron	gene|mRN	ID=gene-E	7	7.257142857	2848.1	0.572438157	KFTAGRP	51	388.729	2	775.434	.	L	Superscaf	22078359	0	0	BTP202209	0	0	0	Tissue specific
ELGSLNK	Superscaf	-	Superscaf	22088035	22088055	-13	13	19	-	-	-	-	-	-	NCP	IGR	-	ID=gene-E	7	5.997142857	2878.1	0.572249922	ELGSLNK	55	760.4153	1	759.4127	.	P	Superscaf	22088064	0	0	0	0	0	BTP202209	Tissue specific
AAPPERK	Superscaf	+	Superscaf	22224457	22224477	12	12	18	-	-	-	-	-	-	NCP	IGR	-	ID=gene-E	7	6.908571429	2840.1	0.569608018	A(+42.01)	84	405.7196	2	809.4395	Acetylati	R	Superscaf	22224424	BTP202209	0	0	0	0	0	Tissue specific
ETPKAVP	Superscaf	-	Superscaf	22230109	22230129	-6	6	12	-	-	-	-	-	-	NCP	IGR	-	ID=gene-E	7	5.444285714	2277.5	0.569498563	ETPKAVP	79	371.2094	2	740.4068	.	T	Superscaf	22230225	0	0	0	0	BTP202209	0	Tissue specific
ERMALL	Superscaf	-	Superscaf	22258831	22258848	-27	27	32	-	-	-	-	-	-	NCP	IGR	-	ID=gene-E	6	6.285	2463.9	0.568942401	ERM(+15.9	66	374.7034	2	747.3949	Oxidation	V	Superscaf	22258848	0	0	0	0	BTP202209	0	Tissue specific
KEEVTSALF	Superscaf	+	Superscaf	22274479	22274505	12	12	20	-	-	-	-	-	-	NCP	IGR	-	ID=gene-E	9	5.683333333	3413.8	0.568639192	KEEVTSALF	85	516.7877	2	1031.561	.	I	Superscaf	22274446	BTP202209	0	0	0	0	0	Tissue specific
KKPPKK	Superscaf	-	Superscaf	22286935	22286952	-29	29	34	-	-	-	-	-	-	NCP	IGR	-	ID=gene-E	6	8.593333333	2548.4	0.568398148	KKPPKK	58	363.2509	2	724.4959	.	H	Superscaf	22286955	0	0	0	0	0	BTP202209	Tissue enhanced
KGFHRK	Superscaf	-	Superscaf	22292350	22292367	-37	37	42	-	-	-	-	-	-	NCP	IGR	-	ID=gene-E	6	8.213333333	2890	0.568293283	K(+42.01)	79	407.7351	2	813.4609	Acetylati	L	Superscaf	22292505	0	0	0	0	0	BTP202209	Tissue specific
EGTFVRF	Superscaf	+	Superscaf	22362577	22362597	7	7	13	-	-	-	-	-	-	NCP	IGR	-	ID=gene-E	7	5.348571429	2543.9	0.566933231	EGTFVRF	64	428.2212	2	854.4286	.	G	Superscaf	22362559	0	BTP202209	0	0	0	0	Tissue enhanced
LPSLPYPF	Superscaf	-	Superscaf	22417963	22417986	-8	8	15	-	-	-	-	-	-	NCP	IGR	-	ID=gene-E	8	5.96	2949.3	0.565860586	LPSLPYPF	50	933.4899	1	932.5007	.	S	Superscaf	22417995	0	0	BTP202209	0	0	0	Tissue specific
LAMLR	Superscaf	-	Superscaf	22440730	22440744	-28	28	32	-	-	-	-	-	-	NCP	IGR	-	ID=gene-E	5	6.898	2038.4	0.565419862	LAMLR	69	603.3617	1	602.3574	.	N	Superscaf	22440780	0	0	0	BTP202209	0	0	Tissue specific
PVYPTAA	Superscaf	-	Superscaf	22451368	22451388	-6	6	12	-	-	-	-	-	-	NCP	IGR	-	ID=gene-E	7	5.261428571	2154.4	0.565213734	PVYPTAA	61	359.686	2	717.3697	.	E	Superscaf	22451394	0	0	0	0	BTP202209	0	Tissue specific
VVALP	Superscaf	-	Superscaf	22484635	22484649	-14	14	18	-	-	gene	-	-	mRNA	NCP	Intron	gene|mRN	ID=gene-E	5	3.66	1002.1	0.564569612	VVALP	52	498.3266	1	497.3213	.	Y	Superscaf	22484814	0	0	0	BTP202209	BTP202209	0	Tissue specific
WKALDF	Superscaf	-	Superscaf	22537510	22537527	-11	11	16	-	-	gene	-	-	mRNA	NCP	Intron	gene|mRN	ID=gene-E	6	6.013333333	2458.6	0.563545594	WKALDF	61	779.4198	1	778.4014	.	D	Superscaf	22537569	0	0	BTP202209	0	0	0	Tissue specific
CQTGEKL	Superscaf	-	Superscaf	22555792	22555812	-5	5	11	-	-	-	-	-	-	NCP	IGR	-	ID=gene-E	7	6.015714286	3138.5	0.563191493	C(+57.02)	72	835.4003	1	834.3906	Carbamido	T	Superscaf	22555908	0	0	0	BTP202209	0	0	Tissue specific
FCHKPCY	Superscaf	-	Superscaf	22570783	22570803	-17	17	23	-	-	-	-	-	-	NCP	IGR	-	ID=gene-E	7	6.401428571	3443.8	0.562901182	FC(+57.02	99	506.2121	2	1010.41	Carbamido	D	Superscaf	22570833	BTP202209	0	0	0	0	0	Tissue specific
MDHYDSQQT	Superscaf	-	Superscaf	22580896	22580931	-62	62	73	-	-	-	-	-	-	NCP	IGR	-	ID=gene-E	12	5.2075	5743.7	0.562705046	M(+42.01)	80	779.7892	2	1557.568	Acetylati	Q	Superscaf	22581390	BTP202209	0	0	0	0	0	Mixed
MDHYDSQQT	Superscaf	-	Superscaf	22580896	22580925	-62	62	71	-	-	-	-	-	-	NCP	IGR	-	ID=gene-E	10	5.386	4872.7	0.562705162	M(+42.01)	77	640.7437	2	1279.478	Acetylati	Q	Superscaf	22581390	0	0	0	0	0	BTP202209	Tissue specific
LGTLPVDF	Superscaf	+	Superscaf	22582339	22582362	22	22	29	-	-	-	-	-	-	NCP	IGR	-	ID=gene-E	8	4.90125	2537.9	0.562677334	LGTLPVDF	98	861.468	1	860.4644	.	W	Superscaf	22582276	0	0	BTP202209	0	0	0	Tissue specific
VWQKPTR	Superscaf	-	Superscaf	22603630	22603650	-28	28	34	-	-	-	-	-	-	NCP	IGR	-	ID=gene-E	7	6.41	2989.4	0.562265077	VWQKPTR	54	457.7634	2	913.5134	.	R	Superscaf	22603653	0	0	BTP202209	0	0	0	Tissue specific
NLSLPF	Superscaf	-	Superscaf	22611865	22611882	-42	42	47	-	-	-	-	-	-	NCP	IGR	-	ID=gene-E	6	5.805	2411.7	0.562105659	NLSLPF	57	690.378	1	689.3748	.	Q	Superscaf	22611882	0	0	0	0	BTP202209	0	Mixed
KSHPRP	Superscaf	-	Superscaf	22613452	22613469	-17	17	22	-	-	-	-	-	-	NCP	IGR	-	ID=gene-E	6	7.728333333	2795.1	0.562074926	KSHPRP	58	361.2069	2	720.4031	.	V	Superscaf	22613691	0	0	0	BTP202209	0	0	Tissue specific
ATAGSLK	Superscaf	-	Superscaf	22623898	22623918	-48	48	54	-	-	-	-	-	-	NCP	IGR	-	ID=gene-E	7	6.562857143	2522.6	0.561872574	ATAGSLK	66	647.3826	1	646.365	.	K	Superscaf	22623930	0	0	0	BTP202209	0	0	Tissue specific
DDPLTNLNT	Superscaf	-	Superscaf	22654063	22654110	-1	1	16	-	-	-	-	-	-	NCP	IGR	-	ID=gene-E	16	5.095625	6171	0.561287886	DDPLTNLNT	99	881.9254	2	1761.842	.	D	Superscaf	22654305	0	0	0	BTP202209	0	0	Mixed
DDPLTNL	Superscaf	-	Superscaf	22654063	22654083	-1	1	7	-	-	-	-	-	-	NCP	IGR	-	ID=gene-E	7	5.162857143	2861.4	0.561288408	DDPLTNL	85	394.1939	2	786.3759	.	D	Superscaf	22654305	0	0	0	0	BTP202209	0	Tissue specific
PLTNLNTAF	Superscaf	-	Superscaf	22654069	22654110	-3	3	16	-	-	-	-	-	-	NCP	IGR	-	ID=gene-E	14	5.399285714	5348	0.561287886	PLTNLNTAF	87	766.8973	2	1531.788	.	D	Superscaf	22654305	0	0	0	0	BTP202209	0	Tissue specific
VSSFYHAF	Superscaf	-	Superscaf	22655407	22655430	-56	56	63	-	-	-	-	-	-	NCP	IGR	-	ID=gene-E	8	5.19875	2932.1	0.561262323	VSSFYHAF	99	479.225	2	956.4392	.	I	Superscaf	22655790	0	0	0	0	BTP202209	0	Tissue specific
VSSFYHA	Superscaf	-	Superscaf	22655407	22655427	-56	56	62	-	-	-	-	-	-	NCP	IGR	-	ID=gene-E	7	5.158571429	2544.7	0.561262381	VSSFYHA	95	405.6916	2	809.3708	.	I	Superscaf	22655790	0	0	0	0	BTP202209	0	Tissue specific
SSFYHAF	Superscaf	-	Superscaf	22655410	22655430	-57	57	63	-	-	-	-	-	-	NCP	IGR	-	ID=gene-E	7	5.941428571	2932.1	0.561262323	SSFYHAF	98	429.6908	2	857.3708	.	I	Superscaf	22655790	0	0	0	BTP202209	BTP202209	BTP202209	Mixed
AADQAAPQ	Superscaf	-	Superscaf	22663771	22663794	-107	107	114	-	-	-	-	-	-	NCP	IGR	-	ID=gene-E	8	5.58125	3215.5	0.561100348	AADQAAPQ	64	386.1916	2	770.3558	.	Y	Superscaf	22664277	0	0	0	0	BTP202209	0	Tissue specific
LYLEYAKRA	Superscaf	-	Superscaf	22670146	22670181	-24	24	35	-	-	gene	-	-	mRNA	NCP	Intron	gene|mRN	ID=gene-E	12	6.403333333	4827.2	0.56097666	LYLEYAKRA	95	481.2637	3	1440.776	.	A	Superscaf	22670352	0	0	0	0	BTP202209	0	Tissue specific
LYLEYAKR	Superscaf	-	Superscaf	22670146	22670169	-24	24	31	-	-	gene	-	-	mRNA	NCP	Intron	gene|mRN	ID=gene-E	8	6.6275	3479.8	0.560976892	LYLEYAKR	96	528.2968	2	1054.581	.	A	Superscaf	22670352	0	0	0	0	BTP202209	0	Tissue specific
HVNMAGTNF	Superscaf	-	Superscaf	22671142	22671171	-82	82	91	-	-	gene	-	-	mRNA	NCP	Intron	gene|mRN	ID=gene-E	10	5.464	3987.6	0.560957488	HVNMAGTNF	76	552.2484	2	1102.487	.	G	Superscaf	22671303	BTP202209	0	0	0	0	0	Tissue specific
VNMAGTNPY	Superscaf	-	Superscaf	22671145	22671171	-83	83	91	-	-	gene	-	-	mRNA	NCP	Intron	gene|mRN	ID=gene-E	9	5.227777778	3367.9	0.560957488	VNMAGTNPY	93	966.4299	1	965.4277	.	G	Superscaf	22671303	0	BTP202209	0	0	0	0	Tissue specific
RRELPPDQA	Superscaf	-	Superscaf	22678075	22678107	-26	26	36	-	-	gene	-	-	mRNA	NCP	Intron	gene|mRN	ID=gene-E	11	6.076363636	4969.8	0.560823167	RRELPPDQA	66	687.3425	2	1372.674	.	G	Superscaf	22678206	0	BTP202209	0	0	0	0	Tissue specific
TGPDAVPGA	Superscaf	-	Superscaf	22678132	22678167	-45	45	56	-	-	gene	-	-	mRNA	NCP	Intron	gene|mRN	ID=gene-E	12	5.0575	3925.4	0.560822005	TGPDAVPGA	91	1175.554	1	1174.551	.	G	Superscaf	22678206	0	0	0	0	0	BTP202209	Tissue specific
GPDAVPGAL	Superscaf	-	Superscaf	22678135	22678167	-46	46	56	-	-	gene	-	-	mRNA	NCP	Intron	gene|mRN	ID=gene-E	11	4.923636364	3527.9	0.560822005	GPDAVPGAL	84	1074.508	1	1073.503	.	G	Superscaf	22678206	0	0	0	BTP202209	0	0	Tissue specific
DAVPGALDY	Superscaf	-	Superscaf	22678141	22678167	-48	48	56	-	-	gene	-	-	mRNA	NCP	Intron	gene|mRN	ID=gene-E	9	4.654444444	2893.2	0.560822005	DAVPGALDY	91	920.4324	1	919.4286	.	G	Superscaf	22678206	0	0	0	BTP202209	0	0	Tissue specific
MSFSTALYC	Superscaf	-	Superscaf	22678168	22678206	-57	57	69	-	-	gene	-	-	mRNA	NCP	Intron	gene|mRN	ID=gene-E	13	5.430769231	4918.5	0.56082125	MSFSTALYC	69	710.8152	2	1419.623	.	G	Superscaf	22678206	0	0	0	0	BTP202209	0	Tissue specific
STALYGESD	Superscaf	-	Superscaf	22678177	22678206	-60	60	69	-	-	gene	-	-	mRNA	NCP	Intron	gene|mRN	ID=gene-E	10	5.369	3776.2	0.56082125	STALYGESD	87	528.2473	2	1054.482	.	G	Superscaf	22678206	0	0	0	0	0	BTP202209	Mixed
TALYGESDI	Superscaf	-	Superscaf	22678180	22678206	-61	61	69	-	-	gene	-	-	mRNA	NCP	Intron	gene|mRN	ID=gene-E	9	5.334444444	3392.8	0.56082125	TALYGESDI	98	968.454	1	967.4498	.	G	Superscaf	22678206	0	0	0	0	0	BTP202209	Mixed
HQPPPPQG	Superscaf	-	Superscaf	22719178	22719201	-6	6	13	-	-	-	-	-	-	NCP	IGR	-	ID=gene-E	8	6.2575	3488	0.560027354	HQPPPPQG	63	429.2096	2	856.4191	.	V	Superscaf	22719282	0	0	0	BTP202209	0	0	Tissue specific
FKAPPPPP	Superscaf	-	Superscaf	22731442	22731465	-19	19	26	-	-	-	-	-	-	NCP	IGR	-	ID=gene-E	8	6.5925	2854.1	0.559789854	F(+42.01)	52	446.7485	2	891.4854	Acetylati	F	Superscaf	22731477	0	0	0	BTP202209	0	0	Mixed
HSGGPPPP	Superscaf	-	Superscaf	22744636	22744659	-23	23	30	-	-	-	-	-	-	NCP	IGR	-	ID=gene-E	8	6.30125	2947.3	0.559534343	HSGGPPPP	76	745.3726	1	744.3555	.	P	Superscaf	22744680	0	0	0	BTP202209	0	0	Tissue specific
HLLPPPP	Superscaf	-	Superscaf	22744873	22744893	-9	9	15	-	-	-	-	-	-	NCP	IGR	-	ID=gene-E	7	6.392857143	2676.1	0.559529811	H(+42.01)	72	406.7392	2	811.4592	Acetylati	I	Superscaf	22744914	0	0	0	BTP202209	0	0	Tissue enhanced
KGFVGP	Superscaf	-	Superscaf	22804789	22804806	-26	26	31	-	-	-	-	-	-	NCP	IGR	-	ID=gene-E	6	5.576666667	1787.8	0.558369556	KGFVGP	50	604.3528	1	603.338	.	P	Superscaf	22804935	0	0	0	BTP202209	0	0	Tissue specific
KNTLML	Superscaf	+	Superscaf	22892731	22892748	12	12	17	-	-	gene	-	-	mRNA	NCP	Intron	gene|mRN	ID=gene-E	6	6.565	2540.9	0.5566665	KNTLML	50	360.2102	2	718.4047	.	F	Superscaf	22892698	0	0	0	0	BTP202209	0	Tissue specific
KPLKTH	Superscaf	-	Superscaf	22919002	22919019	-2	2	7	-	-	-	-	-	-	NCP	IGR	-	ID=gene-E	6	7.646666667	2644.8	0.556157744	K(+42.01)	93	383.2329	2	764.4545	Acetylati	H	Superscaf	22919040	0	0	0	0	BTP202209	0	Tissue specific
SKLRSGLL	Superscaf	-	Superscaf	22950223	22950246	-24	24	31	-	-	gene	-	-	mRNA	NCP	Intron	gene|mRN	ID=gene-E	8	6.97125	3241.5	0.555553012	SKLRSGLL	66	437.2789	2	872.5443	.	K	Superscaf	22950285	0	0	0	BTP202209	0	0	Tissue specific
RGPRQLLS	Superscaf	-	Superscaf	22963570	22963593	-5	5	12	-	-	gene	-	-	mRNA	NCP	Intron	gene|mRN	ID=gene-E	8	7.135	3633.2	0.555294539	RGPRQLLS	60	463.7794	2	925.5457	.	N	Superscaf	22963641	0	0	0	BTP202209	0	0	Mixed
SHLLVTL	Superscaf	+	Superscaf	22964761	22964781	16	16	22	-	-	gene	-	-	mRNA	NCP	Intron	gene|mRN	ID=gene-E	7	5.391428571	2460.8	0.555271532	SHLLVTL	63	782.4749	1	781.4698	.	N	Superscaf	22964716	0	0	0	0	BTP202209	0	Mixed
SSAKPPK	Superscaf	-	Superscaf	22997152	22997172	-16	16	22	-	-	-	-	-	-	NCP	IGR	-	ID=gene-E	7	7.065714286	2689.7	0.554644259	SSAKPPK	56	357.7113	2	713.4072	.	Q	Superscaf	22997178	0	0	BTP202209	0	0	0	Tissue specific
KEYCMP	Superscaf	-	Superscaf	22999585	22999602	-5	5	10	-	-	-	-	-	-	NCP	IGR	-	ID=gene-E	6	5.948333333	2648	0.5545972	KEYC(+57.	53	422.1776	2	842.3303	Carbamido	E	Superscaf	22999662	0	0	0	0	BTP202209	0	Tissue specific
QKDNLT	Superscaf	+	Superscaf	23009722	23009739	4	4	9	-	-	-	-	-	-	NCP	IGR	-	ID=gene-E	6	6.046666667	2838.2	0.55440089	Q(+42.01)	61	760.3809	1	759.3763	Acetylati	N	Superscaf	23009713	0	BTP202209	0	0	0	0	Tissue specific
KKAMKPQ	Superscaf	-	Superscaf	23021371	23021391	-9	9	15	-	-	-	-	-	-	NCP	IGR	-	ID=gene-E	7	7.562857143	3036.1	0.554175241	KKAMKPQ	65	415.7494	2	829.4844	.	V	Superscaf	23021427	0	0	0	0	0	BTP202209	Tissue specific
TQGRMLP	Superscaf	-	Superscaf	23034043	23034063	-62	62	68	-	-	gene	-	-	mRNA	NCP	Intron	gene|mRN	ID=gene-E	7	6.705714286	3016.6	0.553929839	T(+42.01)	67	422.72	2	843.4273	Acetylati	D	Superscaf	23034327	0	0	BTP202209	0	0	0	Tissue specific
LGLPGLGW	Superscaf	-	Superscaf	23042137	23042160	-21	21	28	-	-	-	-	-	-	NCP	IGR	-	ID=gene-E	8	6.005	2816.1	0.553773036	LGLPGLGW	59	812.4528	1	811.4592	.	C	Superscaf	23042166	0	0	0	0	BTP202209	0	Tissue specific
EELRTTP	Superscaf	-	Superscaf	23044504	23044524	-25	25	31	-	-	-	-	-	-	NCP	IGR	-	ID=gene-E	7	6.077142857	2985.6	0.553727255	EELRTTP	73	423.226	2	844.429	.	K	Superscaf	23044590	BTP202209	0	0	0	0	0	Tissue specific
YYMFMC	Superscaf	+	Superscaf	23069872	23069889	8	8	13	-	-	-	-	-	-	NCP	IGR	-	ID=gene-E	6	5.553333333	2635.1	0.553236045	YYM(+15.9	55	473.6518	2	945.3071	Oxidation	R	Superscaf	23069851	0	0	0	0	0	BTP202209	Tissue specific
FDVAEK	Superscaf	+	Superscaf	23103856	23103873	25	25	30	-	-	-	-	-	-	NCP	IGR	-	ID=gene-E	6	4.571666667	2004.1	0.552577922	FDVAEK	98	354.6779	2	707.3489	.	N	Superscaf	23103784	0	0	0	0	BTP202209	0	Tissue specific
KESTLGSK	Superscaf	+	Superscaf	23107381	23107404	18	18	25	-	-	-	-	-	-	NCP	IGR	-	ID=gene-E	8	6.5675	3177.3	0.552509542	KESTLGSK	50	425.2448	2	848.4603	.	V	Superscaf	23107330	0	0	0	BTP202209	0	0	Tissue specific
TEDKSK	Superscaf	+	Superscaf	23115838	23115855	17	17	22	-	-	gene	-	-	mRNA	NCP	Intron	gene|mRN	ID=gene-E	6	6.313333333	2554.7	0.552345882	T(+42.01)	89	375.1867	2	748.3603	Acetylati	E	Superscaf	23115790	0	0	BTP202209	0	0	0	Tissue specific
VGYPHK	Superscaf	-	Superscaf	23215249	23215266	-20	20	25	-	-	-	-	-	-	NCP	IGR	-	ID=gene-E	6	5.876666667	2182.3	0.550420721	VGYPHK	88	350.6904	2	699.3704	.	S	Superscaf	23215284	0	0	0	BTP202209	0	0	Mixed
DGDMM	Superscaf	+	Superscaf	23222470	23222484	35	35	39	-	-	gene	-	-	mRNA	NCP	Intron	gene|mRN	ID=gene-E	5	4.682	1863.3	0.55028094	D(+42.01)	50	610.1813	1	609.1774	Acetylati	T	Superscaf	23222368	0	0	0	BTP202209	0	0	Tissue specific
VLESLQ	Superscaf	-	Superscaf	23292046	23292063	-79	79	84	-	-	-	-	-	-	NCP	IGR	-	ID=gene-E	6	4.418333333	2126.4	0.548933496	VLESLQ	61	688.3848	1	687.3803	.	H	Superscaf	23292156	0	0	BTP202209	0	0	0	Tissue specific
TLDDSLP	Superscaf	+	Superscaf	23334226	23334246	20	20	26	-	-	gene	-	-	mRNA	NCP	Intron	gene|mRN	ID=gene-E	7	5.201428571	2648.1	0.548116593	TLDDSLP	61	760.3693	1	759.3651	.	T	Superscaf	23334169	BTP202209	0	0	0	0	0	Mixed
LPYLQPN	Superscaf	+	Superscaf	23372926	23372946	2	2	8	-	-	gene	-	-	mRNA	NCP	Intron	gene|mRN	ID=gene-E	7	5.897142857	3048.5	0.547367142	LPYLQPN	75	422.7306	2	843.449	.	F	Superscaf	23372923	BTP202209	0	0	0	0	0	Tissue specific
KGYLHP	Superscaf	-	Superscaf	23443072	23443089	-26	26	31	-	-	gene	-	-	mRNA	NCP	Intron	gene|mRN	ID=gene-E	6	6.873333333	2535.7	0.546008775	KGYLHP	68	357.6934	2	713.386	.	K	Superscaf	23443137	0	0	BTP202209	0	0	0	Tissue specific
NNYLLLRV	Superscaf	-	Superscaf	23621719	23621742	-11	11	18	-	-	-	-	-	-	NCP	IGR	-	ID=gene-E	8	5.6475	3361.9	0.542549039	N(+42.01)	59	523.8038	2	1045.592	Acetylati	Q	Superscaf	23621769	0	BTP202209	0	0	0	0	Tissue specific
ATETVP	Superscaf	-	Superscaf	23657377	23657394	-6	6	11	-	-	-	-	-	-	NCP	IGR	-	ID=gene-E	6	4.766666667	1869.2	0.541858614	ATETVP	57	617.3114	1	616.3068	.	L	Superscaf	23657409	0	BTP202209	0	0	0	0	Tissue specific
QQLPSGK	Superscaf	+	Superscaf	23673442	23673462	1	1	7	-	-	gene	-	-	mRNA	NCP	Intron	gene|mRN	ID=gene-E	7	6.424285714	3061.3	0.541547447	Q(+42.01)	51	400.2204	2	798.4235	Acetylati	Q	Superscaf	23673442	0	0	0	0	BTP202209	0	Tissue specific
KAYGPL	Superscaf	+	Superscaf	23691550	23691567	7	7	12	-	-	gene	-	-	mRNA	NCP	Intron	gene|mRN	ID=gene-E	6	6.611666667	2227.3	0.541196831	K(+42.01)	62	690.3775	1	689.3748	Acetylati	A	Superscaf	23691532	BTP202209	0	0	0	0	0	Tissue specific
KDLQPC	Superscaf	+	Superscaf	23865967	23865984	18	18	23	-	-	gene	-	-	mRNA	NCP	Intron	gene|mRN	ID=gene-E	6	5.943333333	2767.1	0.537819128	K(+42.01)	73	802.3733	1	801.3691	Acetylati	P	Superscaf	23865916	0	0	0	0	BTP202209	0	Tissue specific
PQLVTPR	Superscaf	-	Superscaf	23926183	23926203	-9	9	15	-	-	gene	-	-	mRNA	NCP	Intron	gene|mRN	ID=gene-E	7	5.931428571	2685.2	0.536652947	P(+42.01)	75	426.7546	2	851.4865	Acetylati	H	Superscaf	23926296	0	0	0	0	0	BTP202209	Mixed
LPQSDLR	Superscaf	-	Superscaf	23950084	23950104	-7	7	13	-	-	gene	-	-	mRNA	NCP	Intron	gene|mRN	ID=gene-E	7	6.188571429	3098.6	0.536190088	LPQSDLR	86	414.7308	2	827.4501	.	V	Superscaf	23950152	0	0	0	0	0	BTP202209	Tissue specific
PAAGLPPK	Superscaf	+	Superscaf	24047188	24047211	15	15	22	-	-	gene	-	-	mRNA	NCP	Intron	gene|mRN	ID=gene-E	8	6.57875	2753.9	0.534309545	PAAGLPPK	53	375.7252	2	749.4435	.	E	Superscaf	24047146	0	0	0	BTP202209	0	0	Tissue specific
RPKEQK	Superscaf	-	Superscaf	24050401	24050418	-5	5	10	-	-	gene	-	-	mRNA	NCP	Intron	gene|mRN	ID=gene-E	6	7.568333333	2959.2	0.53424744	RPKEQK	99	393.2289	2	784.4555	.	T	Superscaf	24050451	0	0	0	0	BTP202209	0	Tissue specific
LFTMKPLL	Superscaf	-	Superscaf	24123202	24123225	-10	10	17	-	-	-	-	-	-	NCP	IGR	-	ID=gene-E	8	6.4675	3022.4	0.532837483	LFTMKPLL	94	481.7889	2	961.567	.	I	Superscaf	24123243	0	BTP202209	0	0	0	0	Tissue specific
KGKYGNK	Superscaf	-	Superscaf	24206950	24206970	-4	4	10	-	-	gene	-	-	mRNA	NCP	Intron	gene|mRN	ID=gene-E	7	7.461428571	3056	0.531215705	K(+42.01)	57	418.7366	2	835.4552	Acetylati	V	Superscaf	24206976	0	BTP202209	0	0	0	0	Tissue specific
PGAPRVR	Superscaf	-	Superscaf	24218893	24218913	-8	8	14	-	-	gene	-	-	mRNA	NCP	Intron	gene|mRN	ID=gene-E	7	6.587142857	2581	0.53098442	PGAPRVR	66	376.7289	2	751.4453	.	Y	Superscaf	24218937	0	BTP202209	0	0	0	0	Tissue specific
MELLQ	Superscaf	-	Superscaf	24253711	24253725	-28	28	32	-	-	-	-	-	-	NCP	IGR	-	ID=gene-E	5	5.316	2114.5	0.530310263	M(+42.01)	94	675.3361	1	674.3309	Acetylati	L	Superscaf	24253743	0	0	0	0	0	BTP202209	Tissue specific
FVAHKF	Superscaf	-	Superscaf	24412672	24412689	-72	72	77	-	-	gene	-	-	mRNA	NCP	Intron	gene|mRN	ID=gene-E	6	5.718333333	2174.2	0.527231817	FVAHKF	67	748.4091	1	747.4067	.	F	Superscaf	24412731	0	0	0	0	0	BTP202209	Tissue specific
PGFLVRVQ	Superscaf	+	Superscaf	24419770	24419793	5	5	12	-	-	gene	-	-	mRNA	NCP	Intron	gene|mRN	ID=gene-E	8	5.0175	2635	0.527094244	PGFLVRVQ	78	458.2732	2	914.5338	.	P	Superscaf	24419758	BTP202209	0	0	0	0	0	Tissue specific
LLSKKL	Superscaf	+	Superscaf	24567193	24567210	95	95	100	-	-	gene	-	-	mRNA	NCP	Intron	gene|mRN	ID=gene-E	6	7.183333333	2380.4	0.524239414	LLSKKL	73	351.25	2	700.4847	.	L	Superscaf	24566911	0	0	0	BTP202209	0	0	Mixed
QGLALF	Superscaf	-	Superscaf	24590656	24590673	-40	40	45	-	-	gene	-	-	mRNA	NCP	Intron	gene|mRN	ID=gene-E	6	5.846666667	2313.5	0.523785037	QGLALF	84	648.37	1	647.3643	.	S	Superscaf	24590700	0	BTP202209	0	0	0	0	Mixed
KTPFQEK	Superscaf	-	Superscaf	24616294	24616314	-4	4	10	-	-	gene	-	-	mRNA	NCP	Intron	gene|mRN	ID=gene-E	7	6.665714286	3095.3	0.523288482	KTPFQEK	56	439.2407	2	876.4705	.	K	Superscaf	24616338	0	0	BTP202209	0	0	0	Tissue specific
KGTLRAGP	Superscaf	-	Superscaf	24696985	24697008	-2	2	9	-	-	-	-	-	-	NCP	IGR	-	ID=gene-E	8	7.15875	3111.4	0.521725788	KGTLRAGP	58	400.2482	2	798.4711	.	Q	Superscaf	24697035	0	BTP202209	0	0	0	0	Tissue specific
TETNKLR	Superscaf	-	Superscaf	24700774	24700794	-32	32	38	-	-	-	-	-	-	NCP	IGR	-	ID=gene-E	7	6.881428571	3287.8	0.52165247	T(+42.01)	75	452.2567	2	902.4821	Acetylati	K	Superscaf	24700932	0	BTP202209	0	0	0	0	Tissue specific
LSAKPPK	Superscaf	-	Superscaf	24710731	24710751	-42	42	48	-	-	gene	-	-	mRNA	NCP	Intron	gene|mRN	ID=gene-E	7	7.108571429	2659.7	0.521459646	LSAKPPK	62	370.7356	2	739.4592	.	R	Superscaf	24710808	0	0	0	0	0	BTP202209	Tissue specific
TTKAGKK	Superscaf	-	Superscaf	24714694	24714714	-26	26	32	-	-	gene	-	-	mRNA	NCP	Intron	gene|mRN	ID=gene-E	7	7.752857143	2808.8	0.5213829	TTKAGKK	52	367.2311	2	732.4493	.	R	Superscaf	24714756	BTP202209	0	0	0	0	0	Tissue specific
NYLTELK	Superscaf	-	Superscaf	24734242	24734262	-16	16	22	-	-	gene	-	-	mRNA	NCP	Intron	gene|mRN	ID=gene-E	7	6.074285714	3054.4	0.521004339	NYLTELK	70	440.7441	2	879.4702	.	I	Superscaf	24734265	BTP202209	0	0	0	0	0	Tissue specific
TVLLPF	Superscaf	-	Superscaf	24797200	24797217	-11	11	16	-	-	-	-	-	-	NCP	IGR	-	ID=gene-E	6	5.045	1829.1	0.519785173	TVLLPF	55	689.4232	1	688.416	.	L	Superscaf	24797310	0	0	0	0	BTP202209	0	Tissue specific
KVKLPLQ	Superscaf	-	Superscaf	24845614	24845634	-1	1	7	-	-	gene	-	-	mRNA	NCP	Intron	gene|mRN	ID=gene-E	7	6.198571429	2591.7	0.518847546	KVKLPLQ	93	413.2809	2	824.5483	.	K	Superscaf	24845718	0	0	0	0	0	BTP202209	Mixed
LLMLK	Superscaf	-	Superscaf	24948790	24948804	-3	3	7	-	-	-	-	-	-	NCP	IGR	-	ID=gene-E	5	6.686	1900.1	0.516849589	LLMLK	99	617.4033	1	616.3982	.	K	Superscaf	24948828	BTP202209	0	0	0	0	0	Mixed
HVRGFPL	Superscaf	-	Superscaf	24978457	24978477	-16	16	22	-	-	-	-	-	-	NCP	IGR	-	ID=gene-E	7	6.011428571	2644	0.516274951	HVRGFPL	53	413.2396	2	824.4657	.	L	Superscaf	24978543	BTP202209	0	0	0	0	0	Tissue specific
FPGKDT	Superscaf	-	Superscaf	25059163	25059180	-24	24	29	-	-	gene	-	-	mRNA	NCP	Intron	gene|mRN	ID=gene-E	6	6.165	2299.5	0.514712083	FPGKDT	51	664.3299	1	663.3228	.	L	Superscaf	25059312	0	BTP202209	0	0	0	0	Tissue specific
FLDAGKK	Superscaf	-	Superscaf	25069678	25069698	-32	32	38	-	-	gene	-	-	mRNA	NCP	Intron	gene|mRN	ID=gene-E	7	6.557142857	2697.7	0.514508395	FLDAGKK	99	389.7245	2	777.4384	.	Q	Superscaf	25069869	0	0	0	BTP202209	0	0	Tissue specific
QWCYVPK	Superscaf	+	Superscaf	25090390	25090410	8	8	14	-	-	-	-	-	-	NCP	IGR	-	ID=gene-E	7	5.465714286	2988.3	0.514107293	QWC(+57.0	57	490.7353	2	979.4586	Carbamido	L	Superscaf	25090369	0	0	0	0	0	BTP202209	Tissue specific
VVSLA	Superscaf	+	Superscaf	25206412	25206426	56	56	60	-	-	gene	-	-	mRNA	NCP	Intron	gene|mRN	ID=gene-E	5	3.536	1048.1	0.511860565	VVSLA	99	488.3065	1	487.3006	.	R	Superscaf	25206247	BTP202209	0	0	0	0	0	Tissue specific
KSNLEL	Superscaf	-	Superscaf	25272973	25272990	-3	3	8	-	-	-	-	-	-	NCP	IGR	-	ID=gene-E	6	6.001666667	2580.8	0.510571509	KSNLEL	68	703.3973	1	702.3912	.	E	Superscaf	25272996	0	0	0	0	0	BTP202209	Tissue specific
EPRHHSKR	Superscaf	-	Superscaf	25301536	25301559	-5	5	12	-	-	gene	-	-	mRNA	NCP	Intron	gene|mRN	ID=gene-E	8	7.705	4151.7	0.510018251	EPRHHSKR	67	523.7834	2	1045.553	.	A	Superscaf	25301598	0	BTP202209	0	0	0	0	Tissue specific
KKYVLST	Superscaf	-	Superscaf	25306360	25306380	-3	3	9	-	-	gene	-	-	mRNA	NCP	Intron	gene|mRN	ID=gene-E	7	6.19	2530.6	0.509924889	KKYVLST	59	419.7599	2	837.496	.	S	Superscaf	25306428	0	BTP202209	0	0	0	0	Tissue specific
KVLFPAP	Superscaf	-	Superscaf	25319269	25319289	-7	7	13	-	-	gene	-	-	mRNA	NCP	Intron	gene|mRN	ID=gene-E	7	5.688571429	2195.3	0.509674897	KVLFPAP	69	386.2391	2	770.469	.	E	Superscaf	25319301	BTP202209	0	0	0	0	0	Tissue specific
MMTANR	Superscaf	-	Superscaf	25345393	25345410	-22	22	27	-	-	-	-	-	-	NCP	IGR	-	ID=gene-E	6	6.703333333	2697.3	0.509169046	MMTANR	53	362.1728	2	722.3204	.	G	Superscaf	25345563	0	0	BTP202209	0	BTP202209	0	Tissue specific
QPDVGPK	Superscaf	-	Superscaf	25427539	25427559	-19	19	25	-	-	-	-	-	-	NCP	IGR	-	ID=gene-E	7	5.275714286	2462.7	0.507578176	QPDVGPK	51	370.7072	2	739.3864	.	I	Superscaf	25427610	0	0	0	BTP202209	0	0	Tissue specific
LLCMNPM	Superscaf	-	Superscaf	25545061	25545081	-14	14	20	-	-	-	-	-	-	NCP	IGR	-	ID=gene-E	7	5.741428571	2969.6	0.505302283	LLC(+57.0	63	439.6991	2	877.386	Carbamido	P	Superscaf	25545201	BTP202209	0	0	0	0	0	Tissue specific
NLLLSVSK	Superscaf	-	Superscaf	25564027	25564050	-17	17	24	-	-	gene	-	-	mRNA	NCP	Intron	gene|mRN	ID=gene-E	8	5.55625	2892.1	0.504934935	NLLLSVSK	96	437.2726	2	872.5331	.	H	Superscaf	25564083	0	0	0	BTP202209	0	0	Tissue specific
SVMCVD	Superscaf	+	Superscaf	25666762	25666779	11	11	16	-	-	gene	-	-	mRNA	NCP	Intron	gene|mRN	ID=gene-E	6	3.236666667	1752.1	0.502945519	SVM(+15.9	61	363.6431	2	725.2724	Oxidation	S	Superscaf	25666732	0	0	0	0	BTP202209	0	Tissue specific
DPLLQK	Superscaf	-	Superscaf	25682251	25682268	-8	8	13	-	-	gene	-	-	mRNA	NCP	Intron	gene|mRN	ID=gene-E	6	6.103333333	2534.8	0.502645564	D(+42.01)	93	755.4379	1	754.4225	Acetylati	Y	Superscaf	25682292	0	BTP202209	0	0	0	0	Tissue specific
EQPKLP	Superscaf	-	Superscaf	25689769	25689786	-15	15	20	-	-	gene	-	-	mRNA	NCP	Intron	gene|mRN	ID=gene-E	6	6.198333333	2532.8	0.502499973	EQPKLP	51	711.4058	1	710.3962	.	Q	Superscaf	25689786	BTP202209	0	0	0	0	0	Tissue specific
MMGVY	Superscaf	+	Superscaf	25850056	25850070	23	23	27	-	-	gene	-	-	mRNA	NCP	Intron	gene|mRN	ID=gene-E	5	4.626	1499.8	0.499395965	M(+15.99)	54	632.2504	1	631.2346	Oxidation	I	Superscaf	25849990	0	BTP202209	0	0	0	0	Tissue specific
YLLTAVT	Superscaf	-	Superscaf	25852843	25852863	-4	4	10	-	-	gene	-	-	mRNA	NCP	Intron	gene|mRN	ID=gene-E	7	5.242857143	2272.6	0.499341877	YLLTAVT	59	390.733	2	779.4429	.	K	Superscaf	25852875	0	BTP202209	0	0	0	0	Tissue specific
LTGGLEL	Superscaf	+	Superscaf	25857352	25857372	28	28	34	-	-	gene	-	-	mRNA	NCP	Intron	gene|mRN	ID=gene-E	7	5.661428571	2477.8	0.499254557	LTGGLEL	71	702.4008	1	701.3959	.	G	Superscaf	25857271	0	0	BTP202209	0	0	0	Tissue specific
AVSAVPQ	Superscaf	-	Superscaf	25862323	25862343	-232	232	238	-	-	gene	-	-	mRNA	NCP	Intron	gene|mRN	ID=gene-E	7	4.238571429	1954.1	0.49915829	A(+42.01)	67	713.3807	1	712.3755	Acetylati	I	Superscaf	25862430	0	0	0	0	0	BTP202209	Tissue specific
LGSYNHR	Superscaf	-	Superscaf	25873663	25873683	-45	45	51	-	-	gene	-	-	mRNA	NCP	Intron	gene|mRN	ID=gene-E	7	6.721428571	3358.8	0.498938684	LGSYNHR	51	846.4233	1	845.4144	.	K	Superscaf	25873692	0	0	0	0	0	BTP202209	Tissue specific
YYGTY	Superscaf	+	Superscaf	25892884	25892898	12	12	16	-	-	gene	-	-	mRNA	NCP	Intron	gene|mRN	ID=gene-E	5	5.896	2073.3	0.498566572	Y(+42.01)	59	708.3008	1	707.2802	Acetylati	S	Superscaf	25892851	0	0	0	0	0	BTP202209	Tissue specific
GAVFLAP	Superscaf	+	Superscaf	25894108	25894128	10	10	16	-	-	gene	-	-	mRNA	NCP	Intron	gene|mRN	ID=gene-E	7	5.11	1998.1	0.498542752	GAVFLAP	57	674.3845	1	673.3799	.	G	Superscaf	25894081	0	0	0	0	0	BTP202209	Tissue specific
KGKPAAR	Superscaf	-	Superscaf	26047684	26047704	-15	15	21	-	-	-	-	-	-	NCP	IGR	-	ID=gene-E	7	7.792857143	2842.9	0.49556865	KGKPAAR	61	364.2313	2	726.45	.	I	Superscaf	26047851	0	0	0	0	BTP202209	0	Tissue specific
EDLFTGL	Superscaf	+	Superscaf	26059837	26059857	5	5	11	-	-	-	-	-	-	NCP	IGR	-	ID=gene-E	7	5.161428571	2626	0.495333299	EDLFTGL	77	794.3925	1	793.3857	.	L	Superscaf	26059825	0	0	0	0	BTP202209	0	Tissue specific
GHVANTK	Superscaf	-	Superscaf	26083702	26083722	-2	2	8	-	-	-	-	-	-	NCP	IGR	-	ID=gene-E	7	5.894285714	2690.9	0.494871137	GHVANTK	52	363.6913	2	725.382	.	C	Superscaf	26083725	0	0	0	0	BTP202209	0	Tissue specific
PGADFPK	Superscaf	+	Superscaf	26084548	26084568	38	38	44	-	-	-	-	-	-	NCP	IGR	-	ID=gene-E	7	6.111428571	2550.7	0.494854753	PGADFPK	64	731.363	1	730.3649	.	H	Superscaf	26084437	0	0	0	BTP202209	0	0	Tissue specific
KPYGAP	Superscaf	+	Superscaf	26111275	26111292	2	2	7	-	-	-	-	-	-	NCP	IGR	-	ID=gene-E	6	6.665	2211.3	0.494337225	KPYGAP	64	632.3369	1	631.3329	.	V	Superscaf	26111272	0	BTP202209	0	0	0	0	Tissue specific
KVPSLALP	Superscaf	-	Superscaf	26117332	26117355	-1	1	8	-	-	-	-	-	-	NCP	IGR	-	ID=gene-E	8	5.75	2544.7	0.494219811	KVPSLALP	55	412.7646	2	823.5167	.	K	Superscaf	26117409	BTP202209	0	0	0	0	0	Tissue specific
LLLLLPS	Superscaf	+	Superscaf	26156503	26156523	42	42	48	-	-	-	-	-	-	NCP	IGR	-	ID=gene-E	7	5.982857143	2487.8	0.493461296	L(+42.01)	61	405.7743	2	809.5262	Acetylati	L	Superscaf	26156380	0	BTP202209	0	0	0	0	Tissue specific
KETAVLL	Superscaf	-	Superscaf	26205550	26205570	-28	28	34	-	-	-	-	-	-	NCP	IGR	-	ID=gene-E	7	5.352857143	2309.5	0.492511468	KETAVLL	63	387.2402	2	772.4694	.	Q	Superscaf	26205636	0	0	0	0	BTP202209	BTP202209	Tissue specific
ESLTVGP	Superscaf	-	Superscaf	26212000	26212020	-1	1	7	-	-	-	-	-	-	NCP	IGR	-	ID=gene-E	7	4.811428571	2194.5	0.492386559	E(+42.01)	52	744.3903	1	743.3701	Acetylati	E	Superscaf	26212083	0	BTP202209	0	0	0	0	Tissue specific
YESKVY	Superscaf	-	Superscaf	26218858	26218875	-18	18	23	-	-	-	-	-	-	NCP	IGR	-	ID=gene-E	6	4.993333333	2196.3	0.492253808	YESKVY	53	788.3776	1	787.3752	.	T	Superscaf	26219136	0	0	0	0	0	BTP202209	Mixed
SLLEETLL	Superscaf	+	Superscaf	26276164	26276187	128	128	135	-	-	-	-	-	-	NCP	IGR	-	ID=gene-E	8	5.32125	3045.5	0.491143922	SLLEETLL	75	917.5154	1	916.5117	.	T	Superscaf	26275783	0	0	BTP202209	0	0	0	Tissue specific
VMGELLC	Superscaf	-	Superscaf	26293834	26293854	-17	17	23	-	-	-	-	-	-	NCP	IGR	-	ID=gene-E	7	4.56	2386.8	0.490801789	VM(+15.99	58	837.3987	1	836.3772	Oxidation	D	Superscaf	26293872	0	0	BTP202209	0	0	0	Tissue specific
FLLSDH	Superscaf	-	Superscaf	26296771	26296788	-60	60	65	-	-	-	-	-	-	NCP	IGR	-	ID=gene-E	6	5.613333333	2508.8	0.49074497	FLLSDH	85	366.1893	2	730.365	.	V	Superscaf	26296821	0	BTP202209	0	0	0	0	Tissue specific
KAFPPGP	Superscaf	+	Superscaf	26322115	26322135	8	8	14	-	-	gene	-	-	mRNA	NCP	Intron	gene|mRN	ID=gene-E	7	6.587142857	2476.6	0.490254108	KAFPPGP	79	357.1993	2	712.3907	.	I	Superscaf	26322094	0	0	0	BTP202209	0	0	Tissue specific
KHFYAR	Superscaf	+	Superscaf	26352643	26352660	6	6	11	-	-	-	-	-	-	NCP	IGR	-	ID=gene-E	6	7.541666667	2895.1	0.489662971	KHFYAR	64	411.2178	2	820.4344	.	E	Superscaf	26352628	BTP202209	0	0	0	0	0	Tissue specific
TPAGVPP	Superscaf	-	Superscaf	26377018	26377038	-53	53	59	-	-	-	-	-	-	NCP	IGR	-	ID=gene-E	7	5.345714286	2018.3	0.489190875	TPAGVPP	63	638.358	1	637.3435	.	P	Superscaf	26377071	0	0	BTP202209	BTP202209	0	0	Tissue specific
PPKEPKPP	Superscaf	-	Superscaf	26397229	26397252	-7	7	14	-	-	gene	-	-	mRNA	NCP	Intron	gene|mRN	ID=gene-E	8	6.775	3049.3	0.488799417	PPKEPKPP	58	445.2524	2	888.5068	.	S	Superscaf	26397264	0	BTP202209	0	0	0	0	Tissue specific
SLGTPEK	Superscaf	-	Superscaf	26404003	26404023	-9	9	15	-	-	-	-	-	-	NCP	IGR	-	ID=gene-E	7	6.202857143	2662.9	0.488668292	SLGTPEK	51	366.1943	2	730.3861	.	H	Superscaf	26404104	0	0	0	0	0	BTP202209	Tissue specific
KLAPARAL	Superscaf	+	Superscaf	26419921	26419944	8	8	15	-	-	-	-	-	-	NCP	IGR	-	ID=gene-E	8	7.1025	3095.3	0.488359971	K(+42.01)	51	441.2862	2	880.5494	Acetylati	H	Superscaf	26419900	0	0	BTP202209	0	0	0	Tissue specific
AEEFLVL	Superscaf	+	Superscaf	26441773	26441793	71	71	77	-	-	-	-	-	-	NCP	IGR	-	ID=gene-E	7	4.271428571	2256.5	0.48793685	AEEFLVL	70	820.4545	1	819.4378	.	I	Superscaf	26441563	0	0	0	0	BTP202209	0	Tissue specific
EAAPGLR	Superscaf	-	Superscaf	26462494	26462514	-60	60	66	-	-	-	-	-	-	NCP	IGR	-	ID=gene-E	7	6.324285714	2685	0.487535574	EAAPGLR	85	357.2	2	712.3868	.	C	Superscaf	26462784	0	0	0	0	0	BTP202209	Tissue enhanced
LPTHLRC	Superscaf	-	Superscaf	26471104	26471124	-2	2	8	-	-	-	-	-	-	NCP	IGR	-	ID=gene-E	7	6.88	3295.9	0.487368836	LPTHLRC(+	72	448.7438	2	895.4698	Carbamido	A	Superscaf	26471367	0	0	0	0	BTP202209	0	Tissue specific
PAAGPGRGF	Superscaf	-	Superscaf	26485498	26485524	-186	186	194	-	-	-	-	-	-	NCP	IGR	-	ID=gene-E	9	6.623333333	3175.5	0.48708997	PAAGPGRGF	54	390.2133	2	778.4085	.	E	Superscaf	26486064	0	0	0	0	BTP202209	0	Tissue specific
GKPLASHVS	Superscaf	-	Superscaf	26509240	26509266	-109	109	117	-	-	-	-	-	-	NCP	IGR	-	ID=gene-E	9	5.884444444	3154.3	0.48663019	G(+42.01)	56	469.2627	2	936.5029	Acetylati	G	Superscaf	26509521	0	0	BTP202209	0	0	0	Tissue specific
KGVTFSLC	Superscaf	-	Superscaf	26512675	26512698	-41	41	48	-	-	-	-	-	-	NCP	IGR	-	ID=gene-E	8	5.55	2873.1	0.486563727	KGVTFSLC(	74	911.4619	1	910.4582	Carbamido	K	Superscaf	26512704	0	BTP202209	0	0	0	0	Tissue specific
LLNLEEE	Superscaf	+	Superscaf	26542411	26542431	9	9	15	-	-	-	-	-	-	NCP	IGR	-	ID=gene-E	7	4.715714286	2933.4	0.485987928	LLNLEEE	91	859.4363	1	858.4334	.	P	Superscaf	26542387	0	BTP202209	0	0	0	0	Tissue specific
PRFGMR	Superscaf	-	Superscaf	26549848	26549865	-66	66	71	-	-	-	-	-	-	NCP	IGR	-	ID=gene-E	6	7.503333333	2691.2	0.485843963	PRFGM(+15	53	779.4038	1	778.3908	Oxidation	Q	Superscaf	26550042	0	0	0	0	0	BTP202209	Tissue specific
TGPGLDL	Superscaf	+	Superscaf	26685118	26685138	107	107	113	-	exon	gene	-	five_prim	mRNA	NCP	5UTR	exon|gene	ID=exon-E	7	5.671428571	2447.8	0.48322431	TGPGLDL	82	672.3538	1	671.349	.	G	Superscaf	26684800	0	0	BTP202209	0	0	0	Tissue specific
KQHKPP	Superscaf	-	Superscaf	26773186	26773203	-4	4	9	-	-	gene	-	-	mRNA	NCP	Intron	gene|mRN	ID=gene-E	6	7.553333333	2842	0.481518872	KQHKPP	59	367.7179	2	733.4235	.	E	Superscaf	26773215	0	0	0	BTP202209	0	0	Tissue specific
AFKRSP	Superscaf	+	Superscaf	26778076	26778093	61	61	66	-	-	gene	-	-	mRNA	NCP	Intron	gene|mRN	ID=gene-E	6	7.33	2536.7	0.481424174	AFKRSP	55	353.2042	2	704.3969	.	V	Superscaf	26777896	0	0	0	BTP202209	0	0	Tissue specific
LLYDRYN	Superscaf	-	Superscaf	26856766	26856786	-7	7	13	-	-	gene	-	-	mRNA	NCP	Intron	gene|mRN	ID=gene-E	7	6.06	3282.8	0.479900231	LLYDRYN	96	478.7546	2	955.4763	.	T	Superscaf	26856846	0	0	0	0	BTP202209	0	Tissue specific
ERFVQNL	Superscaf	-	Superscaf	26907001	26907021	-6	6	12	-	-	gene	-	-	mRNA	NCP	Intron	gene|mRN	ID=gene-E	7	5.214285714	3022.5	0.478927396	ERFVQNL	80	453.2522	2	904.4766	.	K	Superscaf	26907114	BTP202209	0	0	0	0	0	Tissue specific
AELLRA	Superscaf	-	Superscaf	26920801	26920818	-18	18	23	-	-	gene	-	-	mRNA	NCP	Intron	gene|mRN	ID=gene-E	6	6.33	2403.7	0.478660208	A(+42.01)	67	357.711	2	713.4072	Acetylati	W	Superscaf	26920866	0	0	0	0	BTP202209	0	Tissue specific
WPDGAR	Superscaf	+	Superscaf	26961622	26961639	18	18	23	-	-	gene	-	-	mRNA	NCP	Intron	gene|mRN	ID=gene-E	6	6.318333333	2532.9	0.477869682	WPDGAR	89	351.1701	2	700.3292	.	G	Superscaf	26961571	0	0	0	0	BTP202209	0	Tissue specific
EPVLNLRNT	Superscaf	+	Superscaf	26971171	26971197	14	14	22	-	-	gene	-	-	mRNA	NCP	Intron	gene|mRN	ID=gene-E	9	5.51	3709.4	0.477684585	E(+42.01)	71	549.3055	2	1096.588	Acetylati	G	Superscaf	26971132	0	0	0	0	BTP202209	BTP202209	Tissue specific
KTYFLL	Superscaf	+	Superscaf	27043276	27043293	18	18	23	-	-	-	-	-	-	NCP	IGR	-	ID=gene-E	6	6.561666667	2419.6	0.476288397	KTYFLL	52	392.7292	2	783.4531	.	E	Superscaf	27043225	BTP202209	0	0	0	0	0	Tissue specific
QELRTPL	Superscaf	-	Superscaf	27051202	27051222	-4	4	10	-	-	-	-	-	-	NCP	IGR	-	ID=gene-E	7	6.345714286	3126.7	0.476134847	QELRTPL	74	428.7463	2	855.4814	.	R	Superscaf	27051279	0	BTP202209	0	0	0	0	Mixed
LLQVKEL	Superscaf	-	Superscaf	27061069	27061089	-48	48	54	-	-	-	-	-	-	NCP	IGR	-	ID=gene-E	7	5.221428571	2564.8	0.475943766	LLQVKEL	73	421.7697	2	841.5273	.	Q	Superscaf	27061101	0	0	0	0	BTP202209	0	Tissue specific
QRSPPLGP	Superscaf	-	Superscaf	27068992	27069015	-35	35	42	-	-	-	-	-	-	NCP	IGR	-	ID=gene-E	8	6.6175	3305.8	0.475790273	QRSPPLGP	53	426.2451	2	850.4661	.	H	Superscaf	27069015	BTP202209	0	0	0	0	0	Tissue specific
NDKWP	Superscaf	-	Superscaf	27094528	27094542	-9	9	13	-	-	-	-	-	-	NCP	IGR	-	ID=gene-E	5	6.062	2340.6	0.475295926	NDKWP	64	659.3272	1	658.3074	.	E	Superscaf	27094569	0	0	0	0	BTP202209	0	Tissue specific
KDMTKK	Superscaf	-	Superscaf	27097411	27097428	-3	3	8	-	-	-	-	-	-	NCP	IGR	-	ID=gene-E	6	7.411666667	2585.7	0.475240037	KDMTKK	64	375.7194	2	749.4105	.	R	Superscaf	27097449	0	0	0	0	BTP202209	0	Tissue specific
HSPPARK	Superscaf	-	Superscaf	27167623	27167643	-4	4	10	-	-	-	-	-	-	NCP	IGR	-	ID=gene-E	7	7.484285714	3106.4	0.473880276	HSPPARK	65	396.7319	2	791.4402	.	Q	Superscaf	27167655	0	0	0	0	0	BTP202209	Tissue specific
KADVVRV	Superscaf	-	Superscaf	27209341	27209361	-9	9	15	-	-	-	-	-	-	NCP	IGR	-	ID=gene-E	7	4.212857143	1840	0.473072379	KADVVRV	63	393.7386	2	785.4759	.	I	Superscaf	27209430	0	0	0	BTP202209	0	0	Tissue specific
LPVQVFN	Superscaf	-	Superscaf	27456655	27456675	-12	12	18	-	-	-	-	-	-	NCP	IGR	-	ID=gene-E	7	4.117142857	2285.6	0.468282976	LPVQVFN	89	816.4615	1	815.4541	.	S	Superscaf	27456684	0	0	0	0	BTP202209	0	Tissue specific

KHSSAFK	Superscaf	-	Superscaf	27459850	27459870	-25	25	31	-	-	gene	-	-	mRNA	NCP	Intron	gene|mRN	ID=gene-E	7	7.132857143	3022	0.468221103	KHSSAFK	64	402.7261	2	803.429	.	K	Superscaf	27459885	0	0	0	0	BTP202209	0	Tissue specific
KYPPPPAP	Superscaf	-	Superscaf	27506491	27506514	-25	25	32	-	-	gene	-	-	mRNA	NCP	Intron	gene|mRN	ID=gene-E	8	6.615	2926.2	0.46731781	KYPPPPAP	68	433.7404	2	865.4697	.	V	Superscaf	27506535	0	0	0	0	BTP202209	0	Mixed
GEVFP	Superscaf	+	Superscaf	27603853	27603867	18	18	22	-	-	-	-	-	-	NCP	IGR	-	ID=gene-E	5	4.194	1447.6	0.465432504	G(+42.01)	63	590.2806	1	589.2747	Acetylati	C	Superscaf	27603802	0	0	0	BTP202209	0	0	Tissue specific
KAYPPPP	Superscaf	-	Superscaf	27620260	27620280	-41	41	47	-	-	-	-	-	-	NCP	IGR	-	ID=gene-E	7	6.66	2588.8	0.465114655	KAYPPPP	57	385.215	2	768.417	.	R	Superscaf	27620289	0	0	0	0	BTP202209	0	Tissue specific
LPASP	Superscaf	-	Superscaf	27745717	27745731	-17	17	21	-	-	gene	-	-	mRNA	NCP	Intron	gene|mRN	ID=gene-E	5	6.056	1722.9	0.462685212	LPASP	56	484.2849	1	483.2693	.	T	Superscaf	27745842	0	0	0	0	BTP202209	0	Tissue specific
MKMEAMSR	Superscaf	-	Superscaf	27795400	27795423	-19	19	26	-	-	-	-	-	-	NCP	IGR	-	ID=gene-E	8	6.58375	3351.9	0.461722893	M(+42.01)	85	513.2402	2	1024.45	Acetylati	R	Superscaf	27795426	0	0	0	0	BTP202209	0	Tissue specific
KLGLLY	Superscaf	+	Superscaf	27942469	27942486	20	20	25	-	-	gene	-	-	mRNA	NCP	Intron	gene|mRN	ID=gene-E	6	6.551666667	2285.4	0.458874919	K(+42.01)	86	748.4581	1	747.4531	Acetylati	N	Superscaf	27942412	BTP202209	0	0	0	0	0	Tissue specific
YVLTGP	Superscaf	-	Superscaf	27978880	27978897	-9	9	14	-	-	gene	-	-	mRNA	NCP	Intron	gene|mRN	ID=gene-E	6	5.073333333	1845.1	0.458169795	YVLTGP	57	649.3533	1	648.3483	.	E	Superscaf	27978909	0	0	0	0	BTP202209	0	Tissue specific
LYEESTPK	Superscaf	-	Superscaf	28003576	28003599	-2	2	9	-	-	gene	-	-	mRNA	NCP	Intron	gene|mRN	ID=gene-E	8	5.79125	3250.6	0.457691424	LYEESTPK	84	483.7463	2	965.4705	.	S	Superscaf	28003602	BTP202209	0	0	0	0	0	Tissue specific
KVHNNKK	Superscaf	+	Superscaf	28045297	28045317	5	5	11	-	-	-	-	-	-	NCP	IGR	-	ID=gene-E	7	6.804285714	3218.3	0.456883527	KVHNNKK	52	434.2577	2	866.5086	.	T	Superscaf	28045285	0	0	0	BTP202209	0	0	Tissue specific
APKPPL	Superscaf	+	Superscaf	28126867	28126884	14	14	19	-	-	-	-	-	-	NCP	IGR	-	ID=gene-E	6	6.773333333	2145.3	0.455303927	APKPPL	52	622.3995	1	621.3849	.	L	Superscaf	28126828	0	0	0	0	0	BTP202209	Tissue specific
KLVVTSK	Superscaf	+	Superscaf	28176427	28176447	42	42	48	-	-	-	-	-	-	NCP	IGR	-	ID=gene-E	7	5.381428571	2071.1	0.454344106	KLVVTSK	62	387.7563	2	773.501	.	N	Superscaf	28176304	0	0	0	0	BTP202209	0	Tissue specific
LMKFFR	Superscaf	-	Superscaf	28254505	28254522	-12	12	17	-	-	gene	-	-	mRNA	NCP	Intron	gene|mRN	ID=gene-E	6	7.198333333	2616.9	0.452832131	L(+42.01)	75	442.2417	2	882.4786	Acetylati	K	Superscaf	28254615	0	0	0	0	BTP202209	BTP202209	Tissue specific
STKPLYT	Superscaf	+	Superscaf	28427863	28427883	20	20	26	-	-	-	-	-	-	NCP	IGR	-	ID=gene-E	7	6.631428571	2797.1	0.449474878	STKPLYT	63	405.221	2	808.433	.	K	Superscaf	28427806	0	BTP202209	0	0	0	0	Tissue specific
KNRSPRP	Superscaf	-	Superscaf	28431895	28431915	-15	15	21	-	-	-	-	-	-	NCP	IGR	-	ID=gene-E	7	7.85	3420.9	0.449396796	KNRSPRP	67	427.7521	2	853.4882	.	D	Superscaf	28431936	0	0	BTP202209	0	0	0	Tissue specific
LRAAPP	Superscaf	+	Superscaf	28488517	28488534	45	45	50	-	-	-	-	-	-	NCP	IGR	-	ID=gene-E	6	6.896666667	2299.6	0.448300331	L(+42.01)	73	666.3906	1	665.386	Acetylati	P	Superscaf	28488385	BTP202209	0	0	0	0	0	Tissue specific
QNAAPY	Superscaf	-	Superscaf	28529434	28529451	-2	2	7	-	-	gene	-	-	mRNA	NCP	Intron	gene|mRN	ID=gene-E	6	5.843333333	2626.9	0.447507946	QNAAPY	52	663.3206	1	662.3024	.	L	Superscaf	28529454	0	0	0	0	BTP202209	0	Tissue specific
DLVLVKK	Superscaf	-	Superscaf	28546387	28546407	-67	67	73	-	-	gene	-	-	mRNA	NCP	Intron	gene|mRN	ID=gene-E	7	4.915714286	2055.1	0.447179581	DLVLVKK	76	407.7725	2	813.5323	.	S	Superscaf	28546422	0	0	0	0	BTP202209	0	Tissue specific
EAPAPPKAE	Superscaf	-	Superscaf	28648741	28648779	-14	14	26	-	-	gene	-	-	mRNA	NCP	Intron	gene|mRN	ID=gene-E	13	6.512307692	4824.9	0.445197078	EAPAPPKAE	99	436.5809	3	1306.724	.	I	Superscaf	28649187	0	0	0	0	BTP202209	0	Mixed
DLRASKP	Superscaf	+	Superscaf	28653016	28653036	13	13	19	-	-	gene	-	-	mRNA	NCP	Intron	gene|mRN	ID=gene-E	7	6.778571429	2914.2	0.445114639	DLRASKP	71	393.726	2	785.4395	.	F	Superscaf	28652980	0	0	0	0	BTP202209	0	Tissue specific
PLLKMLQ	Superscaf	-	Superscaf	28659040	28659060	-34	34	40	-	-	gene	-	-	mRNA	NCP	Intron	gene|mRN	ID=gene-E	7	6.482857143	2848.2	0.44499798	PLLKMLQ	64	421.762	2	841.5095	.	G	Superscaf	28659081	0	BTP202209	0	0	0	0	Mixed
QPTPK	Superscaf	+	Superscaf	28745302	28745316	48	48	52	-	-	-	-	-	-	NCP	IGR	-	ID=gene-E	5	6.904	2151.4	0.443327574	QPTPK	57	570.3334	1	569.3173	.	S	Superscaf	28745161	0	0	BTP202209	0	0	0	Tissue specific
HYLPPLQ	Superscaf	+	Superscaf	28776433	28776453	3	3	9	-	-	-	-	-	-	NCP	IGR	-	ID=gene-E	7	6.208571429	3071.5	0.442724585	HYLPPLQ	60	434.2366	2	866.465	.	K	Superscaf	28776427	0	0	0	0	BTP202209	0	Tissue specific
DNELLP	Superscaf	+	Superscaf	28782139	28782156	19	19	24	-	-	-	-	-	-	NCP	IGR	-	ID=gene-E	6	4.976666667	2477.9	0.442614143	DNELLP	64	700.347	1	699.3439	.	G	Superscaf	28782085	0	0	0	0	0	BTP202209	Tissue specific
FPRPKFS	Superscaf	+	Superscaf	28785739	28785759	34	34	40	-	-	-	-	-	-	NCP	IGR	-	ID=gene-E	7	7.105714286	2950.2	0.442544368	F(+42.01)	82	460.7536	2	919.4915	Acetylati	P	Superscaf	28785640	0	0	0	BTP202209	0	0	Tissue specific
KVLLMEC	Superscaf	+	Superscaf	28787566	28787586	24	24	30	-	-	-	-	-	-	NCP	IGR	-	ID=gene-E	7	5.098571429	2557.9	0.442508987	KVLLM(+15	63	454.7313	2	907.4507	Oxidation	R	Superscaf	28787497	BTP202209	0	0	0	0	0	Tissue specific
LLESTML	Superscaf	-	Superscaf	28831003	28831023	-4	4	10	-	-	-	-	-	-	NCP	IGR	-	ID=gene-E	7	5.588571429	2638.1	0.441667801	LLESTM(+1	74	411.7196	2	821.4205	Oxidation	L	Superscaf	28831074	0	BTP202209	0	0	0	0	Tissue specific
HGCFSQ	Superscaf	+	Superscaf	28849954	28849971	78	78	83	-	-	-	-	-	-	NCP	IGR	-	ID=gene-E	6	5.898333333	2884.2	0.44130086	H(+42.01)	54	777.2882	1	776.2912	Acetylati	F	Superscaf	28849723	0	0	0	BTP202209	0	0	Tissue specific
KFTVP	Superscaf	-	Superscaf	28850659	28850673	-25	25	29	-	-	-	-	-	-	NCP	IGR	-	ID=gene-E	5	5.61	1590.7	0.441287265	K(+42.01)	67	633.3575	1	632.3533	Acetylati	I	Superscaf	28850673	0	0	0	0	BTP202209	0	Tissue specific
LSLTSKY	Superscaf	+	Superscaf	28937221	28937241	4	4	10	-	-	-	-	-	-	NCP	IGR	-	ID=gene-E	7	6.464285714	2799	0.439610817	LSLTSKY	90	811.4537	1	810.4487	.	A	Superscaf	28937212	0	0	0	BTP202209	0	0	Mixed
VLLAF	Superscaf	+	Superscaf	28945054	28945068	4	4	8	-	-	-	-	-	-	NCP	IGR	-	ID=gene-E	5	4.692	1405.5	0.439459242	VLLAF	99	562.3589	1	561.3526	.	A	Superscaf	28945045	0	BTP202209	0	0	0	0	Mixed
RLHFLP	Superscaf	-	Superscaf	28992640	28992657	-17	17	22	-	-	-	-	-	-	NCP	IGR	-	ID=gene-E	6	7.015	2700.1	0.438537649	RLHFLP	67	391.7353	2	781.4598	.	H	Superscaf	28992678	0	0	0	0	BTP202209	0	Tissue specific
LHVTFPK	Superscaf	-	Superscaf	29017891	29017911	-8	8	14	-	-	-	-	-	-	NCP	IGR	-	ID=gene-E	7	5.945714286	2563.8	0.438048588	LHVTFPK	61	421.2513	2	840.4858	.	L	Superscaf	29018022	0	BTP202209	0	0	0	0	Tissue specific
SGLEMRK	Superscaf	-	Superscaf	29019262	29019282	-3	3	9	-	-	-	-	-	-	NCP	IGR	-	ID=gene-E	7	6.728571429	2948.3	0.438022038	SGLEMRK	87	410.7199	2	819.4272	.	V	Superscaf	29019282	0	0	BTP202209	0	0	0	Tissue specific
PGAGLK	Superscaf	+	Superscaf	29074036	29074053	16	16	21	-	-	-	-	-	-	NCP	IGR	-	ID=gene-E	6	6.663333333	2065.1	0.436961361	PGAGLK	71	542.3287	1	541.3224	.	G	Superscaf	29073991	0	0	BTP202209	0	0	0	Tissue specific
DGLPKYK	Superscaf	-	Superscaf	29080378	29080398	-28	28	34	-	-	-	-	-	-	NCP	IGR	-	ID=gene-E	7	6.622857143	2795.9	0.436838486	DGLPKYK	77	410.7314	2	819.449	.	S	Superscaf	29080485	0	0	0	0	BTP202209	0	Tissue specific
AHRLQLLR	Superscaf	+	Superscaf	29110360	29110383	1	1	8	-	-	-	-	-	-	NCP	IGR	-	ID=gene-E	8	7.34	3899.5	0.436257806	A(+42.01)	75	350.2142	3	1047.63	Acetylati	A	Superscaf	29110360	0	0	0	0	BTP202209	0	Tissue specific
LLKSLYR	Superscaf	-	Superscaf	29133664	29133684	-24	24	30	-	-	-	-	-	-	NCP	IGR	-	ID=gene-E	7	7.111428571	3020.3	0.435806566	LLKSLYR	89	446.7826	2	891.5541	.	L	Superscaf	29133699	BTP202209	0	0	0	0	0	Tissue specific
KMYLSFG	Superscaf	+	Superscaf	29144464	29144484	2	2	8	-	-	-	-	-	-	NCP	IGR	-	ID=gene-E	7	6.322857143	2720.9	0.435597417	KMYLSFG	59	423.2226	2	844.4153	.	L	Superscaf	29144461	0	0	0	0	BTP202209	0	Tissue specific
YFMGPK	Superscaf	+	Superscaf	29153293	29153310	5	5	10	-	-	-	-	-	-	NCP	IGR	-	ID=gene-E	6	6.483333333	2321.5	0.435426495	YFM(+15.9	69	379.6754	2	757.3469	Oxidation	S	Superscaf	29153281	0	0	0	0	BTP202209	0	Tissue specific
GSPPFKF	Superscaf	-	Superscaf	29154025	29154045	-10	10	16	-	-	-	-	-	-	NCP	IGR	-	ID=gene-E	7	6.421428571	2598.7	0.435412262	G(+42.01)	82	411.2106	2	820.4119	Acetylati	N	Superscaf	29154048	0	0	0	0	BTP202209	0	Tissue specific
TLGMKGY	Superscaf	+	Superscaf	29155276	29155296	3	3	9	-	-	-	-	-	-	NCP	IGR	-	ID=gene-E	7	6.514285714	2644.9	0.435388035	TLGMKGY	50	385.2011	2	768.384	.	F	Superscaf	29155270	0	0	0	0	BTP202209	0	Tissue specific
LSKPLPP	Superscaf	+	Superscaf	29223259	29223279	7	7	13	-	-	-	-	-	-	NCP	IGR	-	ID=gene-E	7	6.611428571	2570.8	0.434071499	LSKPLPP	55	376.2379	2	750.4639	.	D	Superscaf	29223241	BTP202209	0	0	0	0	0	Tissue specific
LSKLWDV	Superscaf	+	Superscaf	29276113	29276133	62	62	68	-	-	-	-	-	-	NCP	IGR	-	ID=gene-E	7	5.177142857	2496.7	0.433047945	LSKLWDV	83	430.746	2	859.4803	.	E	Superscaf	29275930	0	0	0	0	BTP202209	0	Tissue specific
ALPLHAL	Superscaf	-	Superscaf	29290576	29290596	-24	24	30	-	-	-	-	-	-	NCP	IGR	-	ID=gene-E	7	6.267142857	2639.9	0.43276786	ALPLHAL	80	734.4548	1	733.4486	.	T	Superscaf	29290851	0	0	BTP202209	0	0	0	Mixed
QNCAKLK	Superscaf	-	Superscaf	29403499	29403519	-15	15	21	-	-	-	-	-	-	NCP	IGR	-	ID=gene-E	7	6.794285714	3394.6	0.430581029	QNC(+57.(	68	431.2261	2	860.4538	Carbamido	L	Superscaf	29403534	BTP202209	0	0	0	0	0	Tissue specific
DEAVPLP	Superscaf	+	Superscaf	29413162	29413182	12	12	18	-	-	-	-	-	-	NCP	IGR	-	ID=gene-E	7	4.398571429	2176.5	0.430393899	DEAVPLP	72	740.3795	1	739.3752	.	A	Superscaf	29413129	0	0	0	0	0	BTP202209	Tissue specific
QALTVH	Superscaf	-	Superscaf	29415760	29415777	-38	38	43	-	-	-	-	-	-	NCP	IGR	-	ID=gene-E	6	5.295	2292.6	0.430343645	QALTVH	90	668.3704	1	667.3653	.	S	Superscaf	29415783	BTP202209	0	0	0	0	0	Tissue specific
VGVAPLT	Superscaf	-	Superscaf	29433874	29433894	-62	62	68	-	-	-	-	-	-	NCP	IGR	-	ID=gene-E	7	4.4	1696.9	0.429992797	VGVAPLT	59	656.3953	1	655.3904	.	R	Superscaf	29433909	0	0	0	0	BTP202209	0	Tissue specific
ALTVLDF	Superscaf	+	Superscaf	29463631	29463651	6	6	12	-	-	-	-	-	-	NCP	IGR	-	ID=gene-E	7	4.708571429	2214.5	0.429416533	ALTVLDF	80	778.4279	1	777.4272	.	D	Superscaf	29463616	0	0	0	0	BTP202209	0	Tissue enhanced
LATKLE	Superscaf	-	Superscaf	29469190	29469207	-8	8	13	-	-	-	-	-	-	NCP	IGR	-	ID=gene-E	6	6.245	2309.5	0.429308937	LATKLE	60	674.4058	1	673.401	.	K	Superscaf	29469240	0	0	0	BTP202209	0	0	Tissue specific
RYVPQA	Superscaf	-	Superscaf	29472772	29472789	-27	27	32	-	-	-	-	-	-	NCP	IGR	-	ID=gene-E	6	5.731666667	2367.7	0.429239569	RYVPQA	63	733.3852	1	732.3918	.	E	Superscaf	29472846	0	0	0	BTP202209	0	0	Tissue specific
RSGFKGK	Superscaf	-	Superscaf	29483227	29483247	-23	23	29	-	-	-	-	-	-	NCP	IGR	-	ID=gene-E	7	7.62	2951	0.429037043	RSGFKGK	87	390.223	2	778.4449	.	V	Superscaf	29483271	0	0	0	BTP202209	0	0	Tissue specific
PDVLSH	Superscaf	-	Superscaf	29561323	29561340	-15	15	20	-	-	-	-	-	-	NCP	IGR	-	ID=gene-E	6	4.753333333	2105.4	0.427524719	P(+42.01)	56	355.1849	2	708.3442	Acetylati	C	Superscaf	29561352	0	0	0	BTP202209	0	0	Tissue specific
KPQSKHR	Superscaf	+	Superscaf	29596906	29596926	22	22	28	-	-	gene	-	-	mRNA	NCP	Intron	gene|mRN	ID=gene-E	7	7.922857143	3536.8	0.426835573	KPQSKHR	53	440.7607	2	879.5038	.	N	Superscaf	29596843	BTP202209	0	0	0	0	0	Tissue specific
THVELL	Superscaf	-	Superscaf	29623765	29623782	-22	22	27	-	-	gene	-	-	mRNA	NCP	Intron	gene|mRN	ID=gene-E	6	4.883333333	2149.5	0.426315488	THVELL	68	356.2049	2	710.3963	.	N	Superscaf	29623791	BTP202209	0	0	0	0	0	Tissue specific
LDLLLKKK	Superscaf	+	Superscaf	29647525	29647548	4	4	11	-	-	gene	-	-	mRNA	NCP	Intron	gene|mRN	ID=gene-E	8	7.01375	3230.3	0.425855243	LDLLLKKK	99	485.8353	2	969.6586	.	A	Superscaf	29647516	0	0	0	BTP202209	0	0	Tissue specific
MDDFER	Superscaf	+	Superscaf	29647897	29647914	12	12	17	-	-	gene	-	-	mRNA	NCP	Intron	gene|mRN	ID=gene-E	6	5.191666667	2656.2	0.425848156	M(+42.01)	99	854.3333	1	853.3276	Acetylati	V	Superscaf	29647864	0	0	0	BTP202209	0	0	Mixed
KETPVP	Superscaf	-	Superscaf	29654404	29654421	-21	21	26	-	-	gene	-	-	mRNA	NCP	Intron	gene|mRN	ID=gene-E	6	5.348333333	1966.2	0.425722143	KETPVP	88	670.3752	1	669.3697	.	N	Superscaf	29654448	0	0	0	0	0	BTP202209	Mixed
PAKMQPF	Superscaf	-	Superscaf	29675197	29675217	-4	4	10	-	-	gene	-	-	mRNA	NCP	Intron	gene|mRN	ID=gene-E	7	6.462857143	2824.1	0.425319415	PAKMQPF	73	409.7155	2	817.4156	.	I	Superscaf	29675283	0	0	0	BTP202209	0	0	Tissue specific
NPMVFV	Superscaf	+	Superscaf	29714155	29714172	1	1	6	-	-	gene	-	-	mRNA	NCP	Intron	gene|mRN	ID=gene-E	6	3.823333333	1693	0.424565025	NPM(+15.9	59	722.3484	1	721.3469	Oxidation	N	Superscaf	29714155	0	0	0	BTP202209	0	0	Tissue specific
CAQDLRK	Superscaf	-	Superscaf	29785171	29785191	-71	71	77	-	-	-	-	-	-	NCP	IGR	-	ID=gene-E	7	6.591428571	3389.8	0.423189694	C(+57.02)	63	466.7274	2	931.4545	Carbamido	D	Superscaf	29785227	0	0	0	BTP202209	0	0	Tissue specific
RYLQNNF	Superscaf	-	Superscaf	29788405	29788425	-26	26	32	-	-	-	-	-	-	NCP	IGR	-	ID=gene-E	7	6.335714286	3653.2	0.423127065	RYLQNNF	72	477.736	2	953.4719	.	K	Superscaf	29788569	0	0	0	0	BTP202209	0	Tissue specific
LSPLVDTHS	Superscaf	-	Superscaf	29795896	29795922	-6	6	14	-	-	gene	-	-	mRNA	NCP	Intron	gene|mRN	ID=gene-E	9	5.19	3239.7	0.422981881	LSPLVDTHS	93	484.7545	2	967.4974	.	L	Superscaf	29795940	BTP202209	0	0	0	0	0	Mixed
LSPLVDTH	Superscaf	-	Superscaf	29795896	29795919	-6	6	13	-	-	gene	-	-	mRNA	NCP	Intron	gene|mRN	ID=gene-E	8	5.12875	2856.3	0.422981939	LSPLVDTH	91	441.2383	2	880.4654	.	L	Superscaf	29795940	BTP202209	0	0	0	0	0	Mixed
AMKSKR	Superscaf	+	Superscaf	29867770	29867787	9	9	14	-	-	gene	-	-	mRNA	NCP	Intron	gene|mRN	ID=gene-E	6	7.948333333	2651.8	0.421590167	A(+42.01)	51	389.7079	2	777.4167	Acetylati	E	Superscaf	29867746	BTP202209	0	0	0	0	0	Tissue specific
KNLEVLN	Superscaf	+	Superscaf	29880592	29880612	31	31	37	-	-	gene	-	-	mRNA	NCP	Intron	gene|mRN	ID=gene-E	7	5.105714286	2794.1	0.421341802	KNLEVLN	72	415.241	2	828.4705	.	G	Superscaf	29880502	BTP202209	0	0	0	0	0	Tissue specific
PGAPVNL	Superscaf	+	Superscaf	29938021	29938041	183	183	189	-	-	-	-	-	-	NCP	IGR	-	ID=gene-E	7	5.14	2233.5	0.420229651	PGAPVNL	52	667.3758	1	666.3701	.	G	Superscaf	29937475	0	0	0	0	0	BTP202209	Tissue specific
AFANLR	Superscaf	+	Superscaf	29951617	29951634	31	31	36	-	-	-	-	-	-	NCP	IGR	-	ID=gene-E	6	6.611666667	2608.9	0.419966413	A(+42.01)	97	367.2019	2	732.3918	Acetylati	N	Superscaf	29951527	0	0	0	BTP202209	0	0	Mixed
TSAPRSFSS	Superscaf	-	Superscaf	30009748	30009774	-42	42	50	-	-	gene	-	-	mRNA	NCP	Intron	gene|mRN	ID=gene-E	9	6.423333333	3616	0.418840493	TSAPRSFSS	84	470.2296	2	938.4457	.	S	Superscaf	30011133	0	0	0	0	BTP202209	0	Tissue specific
TSAPRSFS	Superscaf	-	Superscaf	30009748	30009771	-42	42	49	-	-	gene	-	-	mRNA	NCP	Intron	gene|mRN	ID=gene-E	8	6.51625	3232.6	0.418840551	TSAPRSFS	92	426.7128	2	851.4137	.	S	Superscaf	30011133	0	0	0	0	0	BTP202209	Tissue specific
KDVDEAYM	Superscaf	-	Superscaf	30010321	30010350	-233	233	242	-	-	gene	-	-	mRNA	NCP	Intron	gene|mRN	ID=gene-E	10	5.148	3924.3	0.418829338	KDVDEAYM	57	606.7806	2	1211.549	.	S	Superscaf	30011133	0	BTP202209	0	0	0	0	Tissue specific
MNKVELESF	Superscaf	-	Superscaf	30010342	30010398	-240	240	258	-	-	gene	-	-	mRNA	NCP	Intron	gene|mRN	ID=gene-E	19	5.289473684	7674.8	0.418828408	MNKVELESF	81	1119.057	2	2236.105	.	S	Superscaf	30011133	BTP202209	0	0	0	0	0	Tissue specific
KYEELQTL	Superscaf	-	Superscaf	30010582	30010614	-320	320	330	-	-	gene	-	-	mRNA	NCP	Intron	gene|mRN	ID=gene-E	11	6.155454545	4570.9	0.418824225	KYEELQTL	96	640.3448	2	1278.682	.	S	Superscaf	30011133	0	0	0	0	BTP202209	0	Tissue specific
LLEGEESRI	Superscaf	-	Superscaf	30010900	30010947	-426	426	441	-	-	gene	-	-	mRNA	NCP	Intron	gene|mRN	ID=gene-E	16	5.465	6722.8	0.418817777	LLEGEESRI	80	911.9146	2	1821.824	.	S	Superscaf	30011133	0	0	BTP202209	0	0	0	Tissue specific
LLEGEESRI	Superscaf	-	Superscaf	30010900	30010944	-426	426	440	-	-	gene	-	-	mRNA	NCP	Intron	gene|mRN	ID=gene-E	15	5.446	6351.3	0.418817835	LLEGEESRI	86	846.3958	2	1690.783	.	S	Superscaf	30011133	0	0	0	BTP202209	0	0	Tissue specific
LLEGEESRI	Superscaf	-	Superscaf	30010900	30010941	-426	426	439	-	-	gene	-	-	mRNA	NCP	Intron	gene|mRN	ID=gene-E	14	5.448571429	5754.6	0.418817893	LLEGEESRI	99	789.3741	2	1576.74	.	S	Superscaf	30011133	0	BTP202209	0	0	0	0	Tissue specific
LLEGEESRI	Superscaf	-	Superscaf	30010900	30010929	-426	426	435	-	-	gene	-	-	mRNA	NCP	Intron	gene|mRN	ID=gene-E	10	5.323	4091.7	0.418818125	LLEGEESRI	98	587.7982	2	1173.588	.	S	Superscaf	30011133	0	0	0	BTP202209	0	0	Tissue specific
LLEGEESRI	Superscaf	-	Superscaf	30010900	30010926	-426	426	434	-	-	gene	-	-	mRNA	NCP	Intron	gene|mRN	ID=gene-E	9	5.556666667	3666.2	0.418818183	LLEGEESRI	98	523.2779	2	1044.545	.	S	Superscaf	30011133	0	0	0	BTP202209	0	0	Tissue specific
EGEESRLES	Superscaf	-	Superscaf	30010906	30010947	-428	428	441	-	-	gene	-	-	mRNA	NCP	Intron	gene|mRN	ID=gene-E	14	5.391428571	6016	0.418817777	EGEESRLES	80	798.8315	2	1595.656	.	S	Superscaf	30011133	0	0	0	0	0	BTP202209	Tissue specific
MEVMNL	Superscaf	+	Superscaf	30018961	30018978	194	194	199	-	-	gene	-	-	mRNA	NCP	Intron	gene|mRN	ID=gene-E	6	4.351666667	2118.6	0.418662251	M(+42.01)	63	778.3475	1	777.3401	Acetylati	D	Superscaf	30018382	0	0	0	BTP202209	0	0	Tissue specific
RAAPPKP	Superscaf	-	Superscaf	30086155	30086175	-60	60	66	-	-	-	-	-	-	NCP	IGR	-	ID=gene-E	7	7.348571429	2752	0.417360936	R(+42.01)	74	389.7352	2	777.4496	Acetylati	I	Superscaf	30086286	0	0	0	BTP202209	0	0	Tissue specific
RGKTLPL	Superscaf	-	Superscaf	30090160	30090180	-45	45	51	-	-	-	-	-	-	NCP	IGR	-	ID=gene-E	7	7.322857143	2856.2	0.417283376	R(+42.01)	65	413.7589	2	825.5072	Acetylati	I	Superscaf	30090192	0	0	0	0	0	BTP202209	Tissue specific
KETQARP	Superscaf	+	Superscaf	30216166	30216186	94	94	100	-	-	-	-	-	-	NCP	IGR	-	ID=gene-E	7	6.888571429	3199.6	0.414843185	KETQARP	58	415.2237	2	828.4453	.	W	Superscaf	30215887	BTP202209	0	0	0	0	0	Tissue specific
LNLTNR	Superscaf	-	Superscaf	30224761	30224778	-9	9	14	-	-	-	-	-	-	NCP	IGR	-	ID=gene-E	6	6.678333333	2946.5	0.414676795	LNLTNR	94	365.7127	2	729.4133	.	I	Superscaf	30224811	0	0	0	BTP202209	0	0	Tissue specific
YVLGAPL	Superscaf	-	Superscaf	30246721	30246741	-9	9	15	-	-	-	-	-	-	NCP	IGR	-	ID=gene-E	7	5.13	2112.3	0.414251467	YVLGAPL	63	732.426	1	731.4218	.	H	Superscaf	30246756	0	0	0	BTP202209	0	0	Tissue specific
MEEPQR	Superscaf	+	Superscaf	30266509	30266526	6	6	11	-	-	-	-	-	-	NCP	IGR	-	ID=gene-E	6	5.816666667	2819.4	0.413868317	M(+42.01)	74	416.186	2	830.3593	Acetylati	A	Superscaf	30266494	0	0	0	0	BTP202209	0	Tissue specific
RLNSLTK	Superscaf	-	Superscaf	30268105	30268125	-12	12	18	-	-	-	-	-	-	NCP	IGR	-	ID=gene-E	7	7.154285714	3201.6	0.413837351	RLNSLTK	67	416.2539	2	830.4974	.	M	Superscaf	30268818	BTP202209	0	0	0	0	0	Tissue specific
LPLLLLP	Superscaf	+	Superscaf	30270313	30270333	35	35	41	-	-	-	-	-	-	NCP	IGR	-	ID=gene-E	7	6.071428571	2441.8	0.413794592	LPLLLLP	71	778.5413	1	777.5364	.	I	Superscaf	30270211	0	0	0	BTP202209	0	0	Tissue specific
SGVSAVP	Superscaf	+	Superscaf	30300304	30300324	4	4	10	-	-	-	-	-	-	NCP	IGR	-	ID=gene-E	7	4.235714286	1712.8	0.413213796	SGVSAVP	76	616.3365	1	615.3228	.	P	Superscaf	30300295	0	0	0	0	0	BTP202209	Tissue specific
KGSSSPR	Superscaf	-	Superscaf	30418222	30418242	-10	10	16	-	-	-	-	-	-	NCP	IGR	-	ID=gene-E	7	7.115714286	2902.1	0.410930234	KGSSSPR	53	359.6967	2	717.3769	.	W	Superscaf	30418308	0	0	0	BTP202209	0	0	Tissue specific
MELLKT	Superscaf	+	Superscaf	30483898	30483915	3	3	8	-	-	-	-	-	-	NCP	IGR	-	ID=gene-E	6	6.2	2369.7	0.409658432	M(+42.01)	68	776.4201	1	775.415	Acetylati	G	Superscaf	30483892	0	0	0	BTP202209	0	0	Tissue specific
LSPPDKP	Superscaf	+	Superscaf	30517132	30517152	19	19	25	-	-	-	-	-	-	NCP	IGR	-	ID=gene-E	7	6.181428571	2628.9	0.409014776	LSPPDKP	53	377.2139	2	752.4068	.	I	Superscaf	30517078	0	0	0	BTP202209	0	0	Tissue specific
KVGNSPF	Superscaf	-	Superscaf	30526195	30526215	-7	7	13	-	-	-	-	-	-	NCP	IGR	-	ID=gene-E	7	5.511428571	2470.6	0.408839264	KVGNSPF	71	748.397	1	747.3915	.	R	Superscaf	30526422	BTP202209	0	0	0	0	0	Tissue specific
YAASEY	Superscaf	-	Superscaf	30527020	30527037	3	3	8	-	-	-	-	-	-	NCP	IGR	-	ID=gene-E	6	5.376666667	2350.5	0.408823346	YAASEY	69	352.1514	2	702.2861	.	L	Superscaf	30527014	BTP202209	0	0	0	0	0	Mixed
KAPTDKK	Superscaf	+	Superscaf	30552304	30552324	16	16	22	-	-	gene	-	-	mRNA	NCP	Intron	gene|mRN	ID=gene-E	7	7.291428571	2862.9	0.408333646	K(+42.01)	73	415.2344	2	828.4705	Acetylati	P	Superscaf	30552259	BTP202209	0	0	0	0	0	Tissue specific
DGKPF	Superscaf	-	Superscaf	30680329	30680343	-20	20	24	-	-	-	-	-	-	NCP	IGR	-	ID=gene-E	5	6.092	1902	0.405854472	DGKPF	58	563.2806	1	562.2751	.	L	Superscaf	30680379	BTP202209	0	0	0	0	0	Tissue specific
VFVKSDR	Superscaf	+	Superscaf	30716974	30716994	27	27	33	-	-	-	-	-	-	NCP	IGR	-	ID=gene-E	7	4.947142857	2299.5	0.405144701	VFVKSDR	77	425.7433	2	849.4708	.	L	Superscaf	30716896	0	0	0	BTP202209	0	0	Tissue specific
LVPHYK	Superscaf	-	Superscaf	30729376	30729393	-15	15	20	-	-	-	-	-	-	NCP	IGR	-	ID=gene-E	6	5.878333333	2238.4	0.404904586	LVPHYK	54	378.7184	2	755.433	.	L	Superscaf	30729417	BTP202209	0	0	0	0	0	Tissue specific
PGGLL	Superscaf	-	Superscaf	30737350	30737364	-70	70	74	-	-	-	-	-	-	NCP	IGR	-	ID=gene-E	5	6.04	1638.8	0.404750222	PGGLL	99	456.2802	1	455.2744	.	S	Superscaf	30737394	BTP202209	0	BTP202209	0	0	0	Mixed
SLAFF	Superscaf	-	Superscaf	30809047	30809061	5	5	9	-	-	-	-	-	-	NCP	IGR	-	ID=gene-E	5	5.728	1822.9	0.403361761	SLAFF	51	584.3167	1	583.3006	.	E	Superscaf	30809035	0	0	0	BTP202209	0	0	Tissue specific
KRVAVFF	Superscaf	-	Superscaf	30837391	30837411	-20	20	26	-	-	-	-	-	-	NCP	IGR	-	ID=gene-E	7	5.354285714	2203.3	0.402812744	KRVAVFF	60	433.7643	2	865.5174	.	S	Superscaf	30837417	0	0	0	0	BTP202209	0	Tissue specific
TFLHLQH	Superscaf	-	Superscaf	30902032	30902052	-27	27	33	-	-	-	-	-	-	NCP	IGR	-	ID=gene-E	7	6.4	3341.8	0.401560928	TFLHLQH	83	448.2408	2	894.4712	.	S	Superscaf	30902085	0	0	0	0	BTP202209	0	Tissue specific
GPVLVQTC	Superscaf	-	Superscaf	30903712	30903735	-19	19	26	-	-	-	-	-	-	NCP	IGR	-	ID=gene-E	8	4.43125	2582	0.401528335	GPVLVQTC(	67	873.4485	1	872.4426	Carbamido	Q	Superscaf	30903858	BTP202209	0	0	0	0	0	Tissue specific
APRPQLPP	Superscaf	-	Superscaf	30906259	30906282	-34	34	41	-	-	-	-	-	-	NCP	IGR	-	ID=gene-E	8	6.70125	3273.8	0.401479011	APRPQLPP	80	438.2564	2	874.5024	.	L	Superscaf	30906303	0	0	0	BTP202209	0	0	Tissue specific
KGAATALPK	Superscaf	-	Superscaf	30984484	30984510	-4	4	12	-	-	gene	-	-	mRNA	NCP	Intron	gene|mRN	ID=gene-E	9	6.924444444	3256.3	0.399964073	KGAATALPK	53	428.7648	2	855.5178	.	D	Superscaf	30984528	0	0	BTP202209	0	0	0	Tissue specific
KQPTYFAP	Superscaf	-	Superscaf	31141315	31141338	-2	2	9	-	-	-	-	-	-	NCP	IGR	-	ID=gene-E	8	6.46	3309.6	0.396926993	K(+42.01)	56	497.2575	2	992.4967	Acetylati	Q	Superscaf	31141392	0	0	0	0	0	BTP202209	Tissue specific
ATDVSVP	Superscaf	+	Superscaf	31150309	31150329	1	1	7	-	-	-	-	-	-	NCP	IGR	-	ID=gene-E	7	3.928571429	1841.1	0.396752876	A(+42.01)	65	730.3602	1	729.3545	Acetylati	A	Superscaf	31150309	0	0	0	BTP202209	0	0	Mixed
KEFVPYPK	Superscaf	+	Superscaf	31205221	31205244	7	7	14	-	-	-	-	-	-	NCP	IGR	-	ID=gene-E	8	5.805	2884	0.39568941	KEFVPYPK	59	504.2863	2	1006.549	.	G	Superscaf	31205203	0	BTP202209	0	0	0	0	Tissue specific
LFDTPPP	Superscaf	+	Superscaf	31256026	31256046	29	29	35	-	-	-	-	-	-	NCP	IGR	-	ID=gene-E	7	5.694285714	2562	0.394705595	LFDTPPP	75	393.709	2	785.3959	.	I	Superscaf	31255942	0	0	0	0	BTP202209	0	Tissue specific
GCDKLR	Superscaf	-	Superscaf	31277839	31277856	-4	4	9	-	-	-	-	-	-	NCP	IGR	-	ID=gene-E	6	6.74	2765.1	0.39428323	G(+42.01)	82	395.7052	2	789.3803	Acetylati	G	Superscaf	31278057	0	BTP202209	0	0	0	0	Tissue specific
VAWVGSH	Superscaf	-	Superscaf	31290844	31290864	-8	8	14	-	-	-	-	-	-	NCP	IGR	-	ID=gene-E	7	4.45	2138.3	0.394031321	V(+42.01)	56	399.2007	2	796.3868	Acetylati	F	Superscaf	31290876	0	0	0	0	BTP202209	0	Tissue specific
LALGAER	Superscaf	-	Superscaf	31298722	31298742	-2	2	8	-	-	-	-	-	-	NCP	IGR	-	ID=gene-E	7	6.278571429	2701	0.393878758	LALGAER	70	365.2151	2	728.418	.	T	Superscaf	31298790	0	0	0	BTP202209	0	0	Tissue specific
WLPVKK	Superscaf	+	Superscaf	31311037	31311054	3	3	8	-	-	-	-	-	-	NCP	IGR	-	ID=gene-E	6	6.275	2154.2	0.393640328	WLPVKK	94	385.7478	2	769.485	.	K	Superscaf	31311031	0	0	0	0	0	BTP202209	Tissue specific
VNMLP	Superscaf	-	Superscaf	31331053	31331067	-5	5	9	-	-	-	-	-	-	NCP	IGR	-	ID=gene-E	5	4.688	1659	0.393252763	VNMLP	55	573.3019	1	572.2992	.	F	Superscaf	31331097	0	0	0	0	0	BTP202209	Tissue specific
MQVLNC	Superscaf	-	Superscaf	31424866	31424883	-1	1	6	-	-	-	-	-	-	NCP	IGR	-	ID=gene-E	6	4.635	2518	0.391435953	M(+42.01)	99	806.3512	1	805.3463	Acetylati	M	Superscaf	31424886	0	0	0	0	0	BTP202209	Tissue specific
NNPPPR	Superscaf	+	Superscaf	31495969	31495986	6	6	11	-	-	-	-	-	-	NCP	IGR	-	ID=gene-E	6	6.746666667	2854.4	0.390058995	N(+42.01)	55	368.6859	2	735.3664	Acetylati	N	Superscaf	31495954	BTP202209	0	0	BTP202209	0	0	Tissue specific
KKDAGPK	Superscaf	+	Superscaf	31542184	31542204	29	29	35	-	-	-	-	-	-	NCP	IGR	-	ID=gene-E	7	7.211428571	2762.7	0.389163952	KKDAGPK	60	372.2218	2	742.4337	.	C	Superscaf	31542100	0	BTP202209	0	0	0	0	Tissue specific
FPPHRT	Superscaf	-	Superscaf	31581976	31581993	-47	47	52	-	-	-	-	-	-	NCP	IGR	-	ID=gene-E	6	7.16	2728.2	0.388393412	FPPHRT	56	754.3867	1	753.3922	.	A	Superscaf	31581999	0	0	0	0	BTP202209	0	Tissue specific
MTEDDL	Superscaf	-	Superscaf	31633972	31633989	-12	12	17	-	-	-	-	-	-	NCP	IGR	-	ID=gene-E	6	4.57	2370.9	0.387386474	MTEDDL	61	362.1452	2	722.2792	.	C	Superscaf	31634028	0	0	BTP202209	0	0	0	Tissue specific
MRQLVQL	Superscaf	-	Superscaf	31690072	31690092	-22	22	28	-	-	-	-	-	-	NCP	IGR	-	ID=gene-E	7	5.681428571	2948.5	0.386300002	MRQLVQL	57	444.2593	2	886.5059	.	W	Superscaf	31690101	0	0	0	BTP202209	0	0	Mixed
KSLLF	Superscaf	+	Superscaf	31790665	31790679	32	32	36	-	-	-	-	-	-	NCP	IGR	-	ID=gene-E	5	6.572	1946	0.384352067	KSLLF	62	607.3754	1	606.3741	.	L	Superscaf	31790572	0	0	BTP202209	0	0	0	Tissue specific
KPPKGLH	Superscaf	+	Superscaf	31812727	31812747	7	7	13	-	-	-	-	-	-	NCP	IGR	-	ID=gene-E	7	7.374285714	2882	0.383924705	K(+42.01)	73	409.7462	2	817.481	Acetylati	P	Superscaf	31812709	0	0	BTP202209	0	0	0	Tissue specific
VHSPPPK	Superscaf	+	Superscaf	31900381	31900401	7	7	13	-	-	-	-	-	-	NCP	IGR	-	ID=gene-E	7	5.987142857	2483.7	0.382227226	VHSPPPK	54	381.2202	2	760.4232	.	N	Superscaf	31900363	BTP202209	0	0	0	0	0	Tissue specific
ELGAFH	Superscaf	+	Superscaf	31904716	31904733	58	58	63	-	-	-	-	-	-	NCP	IGR	-	ID=gene-E	6	5.71	2394.6	0.382143334	ELGAFH	62	673.317	1	672.3231	.	N	Superscaf	31904545	0	0	BTP202209	0	0	0	Tissue specific
PSTSPSR	Superscaf	+	Superscaf	32082433	32082453	9	9	15	-	-	-	-	-	-	NCP	IGR	-	ID=gene-E	7	6.704285714	2871.3	0.378701666	P(+42.01)	73	387.1866	2	772.3715	Acetylati	N	Superscaf	32082409	0	0	BTP202209	0	0	0	Tissue specific
LLLATKK	Superscaf	-	Superscaf	32104174	32104194	-11	11	17	-	-	-	-	-	-	NCP	IGR	-	ID=gene-E	7	7.138571429	2705.8	0.378280637	LLLATKK	61	393.7768	2	785.5374	.	N	Superscaf	32104350	0	0	BTP202209	0	0	0	Tissue specific
ATCQAY	Superscaf	-	Superscaf	32128549	32128566	-2	2	7	-	-	-	-	-	-	NCP	IGR	-	ID=gene-E	6	5.816666667	2676	0.377808657	ATC(+57.(	51	713.2933	1	712.285	Carbamido	T	Superscaf	32128722	0	0	BTP202209	0	0	0	Tissue specific
YRLPTAA	Superscaf	+	Superscaf	32133334	32133354	4	4	10	-	-	-	-	-	-	NCP	IGR	-	ID=gene-E	7	6.752857143	2819.2	0.377715934	YRLPTAA	61	396.2222	2	790.4337	.	K	Superscaf	32133325	BTP202209	0	0	0	0	0	Tissue specific
EGPDAGR	Superscaf	+	Superscaf	32133610	32133630	5	5	11	-	-	-	-	-	-	NCP	IGR	-	ID=gene-E	7	5.887142857	2729.1	0.377710589	EGPDAGR	81	351.1701	2	700.314	.	L	Superscaf	32133598	0	0	0	0	BTP202209	0	Tissue specific
PHYVSF	Superscaf	-	Superscaf	32167735	32167752	-1	1	6	-	-	-	-	-	-	NCP	IGR	-	ID=gene-E	6	5.118333333	2187.4	0.377049794	PHYVSF	96	749.3579	1	748.3544	.	P	Superscaf	32167758	BTP202209	0	0	0	0	0	Tissue specific
SLTSPL	Superscaf	-	Superscaf	32203213	32203230	-3	3	8	-	-	-	-	-	-	NCP	IGR	-	ID=gene-E	6	6.025	2208.5	0.376362738	SLTSPL	68	617.3478	1	616.3432	.	S	Superscaf	32203311	0	0	BTP202209	0	0	0	Tissue specific
KTKLLL	Superscaf	-	Superscaf	32208301	32208318	-1	1	6	-	-	-	-	-	-	NCP	IGR	-	ID=gene-E	6	7.325	2394.5	0.376264206	KTKLLL	55	358.2572	2	714.5003	.	K	Superscaf	32208348	0	0	BTP202209	0	0	0	Tissue enhanced
EALTLKNQ	Superscaf	+	Superscaf	32218837	32218860	2	2	9	-	-	gene	-	-	mRNA	NCP	Intron	gene|mRN	ID=gene-E	8	6.06625	3516.9	0.376060053	E(+42.01)	51	479.763	2	957.5131	Acetylati	T	Superscaf	32218834	0	0	0	0	BTP202209	0	Tissue specific
PPLMPK	Superscaf	-	Superscaf	32237611	32237628	-33	33	38	-	-	gene	-	-	mRNA	NCP	Intron	gene|mRN	ID=gene-E	6	6.728333333	2205.5	0.375696598	PPLM(+15.	76	698.3806	1	697.3832	Oxidation	S	Superscaf	32237757	0	0	BTP202209	0	0	0	Tissue specific
ALLEYLT	Superscaf	+	Superscaf	32269519	32269539	14	14	20	-	-	gene	-	-	mRNA	NCP	Intron	gene|mRN	ID=gene-E	7	5.624285714	2654	0.37507862	ALLEYLT	88	822.4578	1	821.4534	.	K	Superscaf	32269480	0	0	0	0	BTP202209	0	Tissue specific
LYRTTVL	Superscaf	-	Superscaf	32312440	32312460	-31	31	37	-	-	gene	-	-	mRNA	NCP	Intron	gene|mRN	ID=gene-E	7	5.92	2610.1	0.374247426	LYRTTVL	50	433.261	2	864.5069	.	Q	Superscaf	32312475	0	0	0	0	0	BTP202209	Tissue specific
KPMVRP	Superscaf	-	Superscaf	32355634	32355651	-15	15	20	-	-	gene	-	-	mRNA	NCP	Intron	gene|mRN	ID=gene-E	6	6.475	2163.5	0.373411003	KPMVRP	50	364.2245	2	726.421	.	T	Superscaf	32355678	0	0	BTP202209	0	0	0	Tissue specific
EHLKPQ	Superscaf	-	Superscaf	32378410	32378427	-39	39	44	-	-	gene	-	-	mRNA	NCP	Intron	gene|mRN	ID=gene-E	6	6.413333333	2815.1	0.37296993	EHLKPQ	61	376.2111	2	750.4024	.	A	Superscaf	32378448	0	0	0	0	BTP202209	0	Tissue specific
LSVNPMLL	Superscaf	-	Superscaf	32410273	32410296	-3	3	10	-	-	gene	-	-	mRNA	NCP	Intron	gene|mRN	ID=gene-E	8	5.135	2749.2	0.372352766	LSVNPM(+1	99	902.4988	1	901.4943	Oxidation	N	Superscaf	32410356	0	BTP202209	0	0	0	0	Tissue specific
PGLDGK	Superscaf	-	Superscaf	32422987	32423004	-30	30	35	-	-	gene	-	-	mRNA	NCP	Intron	gene|mRN	ID=gene-E	6	6.155	2165.3	0.372106667	PGLDGK	75	586.314	1	585.3122	.	R	Superscaf	32423046	0	0	0	0	0	BTP202209	Tissue specific
RGPNSLLP	Superscaf	+	Superscaf	32512546	32512569	106	106	113	-	-	-	-	-	-	NCP	IGR	-	ID=gene-E	8	6.5475	3307.8	0.37037218	RGPNSLLP	50	427.2536	2	852.4818	.	N	Superscaf	32512231	BTP202209	0	0	0	0	0	Tissue specific
WSVLGCG	Superscaf	+	Superscaf	32521516	32521536	9	9	15	-	-	gene	-	-	mRNA	NCP	Intron	gene|mRN	ID=gene-E	7	4.93	2443.7	0.370198528	WSVLGC(+5	61	778.3555	1	777.348	Carbamido	A	Superscaf	32521492	BTP202209	0	0	0	0	0	Tissue specific
RTVLLF	Superscaf	+	Superscaf	32527273	32527290	7	7	12	-	-	gene	-	-	mRNA	NCP	Intron	gene|mRN	ID=gene-E	6	5.788333333	2140.5	0.370087098	RTVLLF	65	374.7401	2	747.4643	.	Q	Superscaf	32527255	BTP202209	0	0	0	0	0	Tissue specific
RNVGVPR	Superscaf	+	Superscaf	32554009	32554029	7	7	13	-	-	-	-	-	-	NCP	IGR	-	ID=gene-E	7	5.6	2529	0.369569279	RNVGVPR	64	399.2437	2	796.4667	.	R	Superscaf	32553991	BTP202209	0	0	0	0	0	Tissue specific
LQLVL	Superscaf	+	Superscaf	32579533	32579547	16	16	20	-	-	-	-	-	-	NCP	IGR	-	ID=gene-E	5	4.718	1670.9	0.369075106	LQLVL	67	585.3949	1	584.3897	.	E	Superscaf	32579488	BTP202209	0	0	0	0	0	Tissue specific
FVDKLK	Superscaf	+	Superscaf	32602267	32602284	15	15	20	-	-	-	-	-	-	NCP	IGR	-	ID=gene-E	6	5.651666667	2089.1	0.368634789	FVDKLK	98	375.2302	2	748.4483	.	E	Superscaf	32602225	0	0	0	0	0	BTP202209	Tissue specific
KPKEKLQC	Superscaf	-	Superscaf	32615383	32615406	-7	7	14	-	-	-	-	-	-	NCP	IGR	-	ID=gene-E	8	6.92375	3717.9	0.368380672	K(+42.01)	50	536.7973	2	1071.575	Acetylati	I	Superscaf	32615406	0	0	0	0	0	BTP202209	Tissue specific
EVPLLT	Superscaf	+	Superscaf	32652070	32652087	40	40	45	-	-	-	-	-	-	NCP	IGR	-	ID=gene-E	6	4.668333333	1867.2	0.36767032	EVPLLT	82	671.3945	1	670.3901	.	S	Superscaf	32651953	BTP202209	0	0	0	0	0	Mixed
TSPTGWK	Superscaf	-	Superscaf	32693389	32693409	13	13	19	-	-	gene	-	-	mRNA	NCP	Intron	gene|mRN	ID=gene-E	7	6.662857143	2808.1	0.366870091	T(+42.01)	95	409.7043	2	817.397	Acetylati	K	Superscaf	32693353	0	0	BTP202209	0	0	0	Tissue specific
LPPPPDLPF	Superscaf	+	Superscaf	32747179	32747214	30	30	41	-	-	-	-	-	-	NCP	IGR	-	ID=gene-E	12	5.969166667	4154.9	0.365828121	LPPPPDLPF	80	617.3391	2	1232.68	.	G	Superscaf	32747092	0	BTP202209	0	0	0	0	Tissue specific
LAVVEY	Superscaf	+	Superscaf	32799808	32799825	10	10	15	-	-	-	-	-	-	NCP	IGR	-	ID=gene-E	6	3.48	1549.7	0.364809274	LAVVEY	83	693.3788	1	692.3745	.	E	Superscaf	32799781	0	0	BTP202209	0	0	0	Tissue specific
LAKKTLNL	Superscaf	-	Superscaf	32828566	32828589	-19	19	26	-	-	-	-	-	-	NCP	IGR	-	ID=gene-E	8	6.9225	3302.5	0.36425224	LAKKTLNL	86	450.7959	2	899.5804	.	K	Superscaf	32828619	0	0	0	BTP202209	0	0	Tissue specific
FPKLTGV	Superscaf	-	Superscaf	32895658	32895678	-8	8	14	-	-	-	-	-	-	NCP	IGR	-	ID=gene-E	7	5.714285714	2241.4	0.362953016	FPKLTGV	67	381.2351	2	760.4483	.	C	Superscaf	32895708	0	0	0	BTP202209	0	0	Tissue specific
QPPRMAK	Superscaf	-	Superscaf	32931616	32931636	-2	2	8	-	-	-	-	-	-	NCP	IGR	-	ID=gene-E	7	7.217142857	3085.5	0.362256665	QPPRM(+15	57	422.2335	2	842.4432	Oxidation	V	Superscaf	32931642	0	0	0	BTP202209	0	0	Tissue specific
ECCCNF	Superscaf	+	Superscaf	32933974	32933991	14	14	19	-	-	-	-	-	-	NCP	IGR	-	ID=gene-E	6	4.861666667	3166.7	0.362211059	E(+42.01)	55	466.1369	2	930.267	Acetylati	S	Superscaf	32933935	0	0	0	BTP202209	0	0	Tissue specific
KEYLNP	Superscaf	-	Superscaf	32982436	32982453	-57	57	62	-	-	-	-	-	-	NCP	IGR	-	ID=gene-E	6	6.051666667	2640.9	0.36127256	KEYLNP	51	763.3944	1	762.3912	.	P	Superscaf	32982525	0	0	0	BTP202209	0	0	Tissue specific
SVVGTTN	Superscaf	+	Superscaf	32998492	32998512	2	2	8	-	-	-	-	-	-	NCP	IGR	-	ID=gene-E	7	4.302857143	2072.4	0.360961566	SVVGTTN	51	677.3437	1	676.3392	.	R	Superscaf	32998489	0	0	0	0	BTP202209	0	Tissue specific
KGGSLLR	Superscaf	-	Superscaf	33112660	33112680	-11	11	17	-	-	-	-	-	-	NCP	IGR	-	ID=gene-E	7	7.154285714	2802	0.358750626	K(+42.01)	54	772.465	1	771.4603	Acetylati	L	Superscaf	33112716	0	0	0	BTP202209	0	0	Tissue specific
KADLGGLL	Superscaf	+	Superscaf	33228130	33228153	3	3	10	-	-	-	-	-	-	NCP	IGR	-	ID=gene-E	8	6.07625	2846	0.356514413	KADLGGLL	55	786.4692	1	785.4647	.	K	Superscaf	33228124	0	BTP202209	0	0	0	BTP202209	Tissue specific
KAVQFPP	Superscaf	-	Superscaf	33458467	33458487	-57	57	63	-	-	-	-	-	-	NCP	IGR	-	ID=gene-E	7	5.641428571	2452.6	0.35205384	KAVQFPP	62	786.4358	1	785.4435	.	P	Superscaf	33458493	BTP202209	0	0	0	0	0	Tissue specific
YGVDAP	Superscaf	+	Superscaf	33579421	33579438	4	4	9	-	-	-	-	-	-	NCP	IGR	-	ID=gene-E	6	4.486666667	1817	0.349711542	YGVDAP	76	621.3002	1	620.2806	.	I	Superscaf	33579412	BTP202209	0	0	0	0	0	Tissue specific
LVTLDY	Superscaf	-	Superscaf	33602404	33602421	-20	20	25	-	-	-	-	-	-	NCP	IGR	-	ID=gene-E	6	4.52	1975.3	0.349266461	LVTLDY	70	723.3893	1	722.3851	.	S	Superscaf	33602433	0	0	BTP202209	0	0	0	Tissue specific
KRMPPPP	Superscaf	+	Superscaf	33638389	33638409	6	6	12	-	-	-	-	-	-	NCP	IGR	-	ID=gene-E	7	7.35	2838.3	0.348569529	KRM(+15.9	52	838.4636	1	837.4531	Oxidation	P	Superscaf	33638374	0	BTP202209	0	0	0	0	Tissue specific
AETFAAK	Superscaf	-	Superscaf	33742498	33742518	-3	3	9	-	-	-	-	-	-	NCP	IGR	-	ID=gene-E	7	6.147142857	2612.7	0.346553388	AETFAAK	55	369.2002	2	736.3755	.	R	Superscaf	33742566	0	0	BTP202209	0	0	0	Tissue specific
LAPRPVH	Superscaf	-	Superscaf	33755962	33755982	-10	10	16	-	-	-	-	-	-	NCP	IGR	-	ID=gene-E	7	6.135714286	2608	0.346292649	L(+42.01)	87	416.2373	2	830.4763	Acetylati	L	Superscaf	33756000	0	0	0	0	0	BTP202209	Tissue specific
LTGAVAF	Superscaf	+	Superscaf	33790246	33790266	4	4	10	-	-	-	-	-	-	NCP	IGR	-	ID=gene-E	7	5.142857143	2058.2	0.345628716	LTGAVAF	74	678.3771	1	677.3748	.	E	Superscaf	33790237	0	0	0	BTP202209	0	0	Tissue specific
TVPLEGK	Superscaf	+	Superscaf	33808630	33808650	13	13	19	-	-	-	-	-	-	NCP	IGR	-	ID=gene-E	7	5.391428571	2279.5	0.345272698	T(+42.01)	90	393.223	2	784.433	Acetylati	K	Superscaf	33808594	0	0	0	BTP202209	0	0	Tissue specific
KYSMKK	Superscaf	+	Superscaf	33823168	33823185	2	2	7	-	-	-	-	-	-	NCP	IGR	-	ID=gene-E	6	7.718333333	2619.6	0.344991217	KYSM(+15.	74	400.7179	2	799.4262	Oxidation	T	Superscaf	33823165	0	0	0	BTP202209	0	0	Tissue specific
KLGTKMQ	Superscaf	+	Superscaf	34002766	34002786	6	6	12	-	-	-	-	-	-	NCP	IGR	-	ID=gene-E	7	7.051428571	2967.2	0.341513123	KLGTKM(+1	57	411.2268	2	820.4477	Oxidation	S	Superscaf	34002751	0	0	BTP202209	0	0	0	Tissue specific
KLSWT	Superscaf	-	Superscaf	34040320	34040334	-3	3	7	-	-	-	-	-	-	NCP	IGR	-	ID=gene-E	5	6.764	2129.3	0.340785981	KLSWT	73	634.354	1	633.3486	.	A	Superscaf	34040421	0	0	BTP202209	0	0	0	Tissue specific
CSHAAPK	Superscaf	+	Superscaf	34105039	34105059	12	12	18	-	-	gene	-	-	mRNA	NCP	Intron	gene|mRN	ID=gene-E	7	6.624285714	3017.2	0.339532537	C(+57.02)	65	385.6909	2	769.3541	Carbamido	T	Superscaf	34105006	0	0	BTP202209	0	0	0	Tissue specific
VGGLDL	Superscaf	-	Superscaf	34125034	34125051	-13	13	18	-	-	gene	-	-	mRNA	NCP	Intron	gene|mRN	ID=gene-E	6	4.478333333	1712.9	0.339145379	VGGLDL	68	573.3231	1	572.317	.	A	Superscaf	34125129	0	BTP202209	0	0	0	0	Tissue specific
DPVLRPP	Superscaf	-	Superscaf	34137109	34137129	-67	67	73	-	-	gene	-	-	mRNA	NCP	Intron	gene|mRN	ID=gene-E	7	5.515714286	2425.9	0.33891148	D(+42.01)	78	418.2352	2	834.4599	Acetylati	P	Superscaf	34137240	0	0	0	BTP202209	0	0	Tissue specific
REVLSEK	Superscaf	-	Superscaf	34194955	34194975	-14	14	20	-	-	-	-	-	-	NCP	IGR	-	ID=gene-E	7	5.514285714	2705	0.337791253	REVLSEK	52	430.7389	2	859.4763	.	Q	Superscaf	34195032	0	0	0	BTP202209	0	0	Tissue specific
LTAELLQLL	Superscaf	-	Superscaf	34273081	34273107	-25	25	33	-	-	-	-	-	-	NCP	IGR	-	ID=gene-E	9	5.702222222	3512	0.336278175	LTAELLQLL	52	507.3078	2	1012.617	.	G	Superscaf	34273242	0	BTP202209	0	0	0	0	Tissue specific
KPGSGLR	Superscaf	+	Superscaf	34296268	34296288	6	6	12	-	-	-	-	-	-	NCP	IGR	-	ID=gene-E	7	7.2	2786	0.335829259	KPGSGLR	68	357.711	2	713.4184	.	H	Superscaf	34296253	BTP202209	0	0	0	0	0	Tissue specific
SVARLAKLE	Superscaf	+	Superscaf	34304920	34304949	2	2	11	-	-	-	-	-	-	NCP	IGR	-	ID=gene-E	10	6.314	3723.9	0.335661533	SVARLAKLE	78	372.2349	3	1113.687	.	P	Superscaf	34304917	0	BTP202209	0	0	0	0	Tissue specific
LMMKKT	Superscaf	-	Superscaf	34461778	34461795	-20	20	25	-	-	gene	-	-	mRNA	NCP	Intron	gene|mRN	ID=gene-E	6	7.248333333	2430.7	0.332624104	LMMKKT	51	376.2103	2	750.4132	.	K	Superscaf	34461894	0	0	BTP202209	BTP202209	0	0	Tissue specific
PKKMPK	Superscaf	-	Superscaf	34496770	34496787	-11	11	16	-	-	gene	-	-	mRNA	NCP	Intron	gene|mRN	ID=gene-E	6	7.928333333	2451.5	0.331946461	PKKMPK	69	364.7211	2	727.4415	.	I	Superscaf	34496799	0	0	0	BTP202209	0	0	Tissue specific
PTVVGPP	Superscaf	+	Superscaf	34540066	34540086	112	112	118	-	-	gene	-	-	mRNA	NCP	Intron	gene|mRN	ID=gene-E	7	4.485714286	1707	0.331107946	PTVVGPP	58	666.3908	1	665.3748	.	T	Superscaf	34539733	0	0	BTP202209	0	BTP202209	BTP202209	Tissue specific
EAKPPLH	Superscaf	+	Superscaf	34609114	34609134	29	29	35	-	-	gene	-	-	mRNA	NCP	Intron	gene|mRN	ID=gene-E	7	6.45	2853.1	0.329770785	EAKPPLH	52	396.2222	2	790.4337	.	W	Superscaf	34609030	0	BTP202209	0	0	BTP202209	BTP202209	Tissue specific
VTLLDF	Superscaf	-	Superscaf	34631449	34631466	-16	16	21	-	-	gene	-	-	mRNA	NCP	Intron	gene|mRN	ID=gene-E	6	4.49	1903.2	0.329338311	V(+42.01)	67	749.4007	1	748.4007	Acetylati	I	Superscaf	34631697	0	BTP202209	0	0	0	0	Tissue specific
RPTAYFP	Superscaf	-	Superscaf	34644511	34644531	-65	65	71	-	-	gene	-	-	mRNA	NCP	Intron	gene|mRN	ID=gene-E	7	6.721428571	2879.3	0.329085298	RPTAYFP	70	426.2318	2	850.4337	.	K	Superscaf	34644540	0	BTP202209	BTP202209	BTP202209	0	BTP202209	Tissue specific
YVRSLD	Superscaf	+	Superscaf	34659895	34659912	28	28	33	-	-	gene	-	-	mRNA	NCP	Intron	gene|mRN	ID=gene-E	6	5.175	2256.6	0.328787435	YVRSLD	68	376.6981	2	751.3864	.	P	Superscaf	34659814	0	BTP202209	0	0	0	0	Tissue specific
METMNLP	Superscaf	-	Superscaf	34686694	34686714	-2	2	8	-	-	gene	-	-	mRNA	NCP	Intron	gene|mRN	ID=gene-E	7	5.562857143	2853.5	0.328268396	M(+42.01)	73	439.1926	2	876.3721	Acetylati	Q	Superscaf	34686720	0	BTP202209	0	0	0	0	Tissue specific
TSPLPLKP	Superscaf	-	Superscaf	34744933	34744956	-3	3	10	-	-	gene	-	-	mRNA	NCP	Intron	gene|mRN	ID=gene-E	8	6.60125	2968.3	0.327140501	TSPLPLKP	70	426.762	2	851.5116	.	V	Superscaf	34745055	0	0	BTP202209	0	0	0	Tissue specific
KAAGGLPR	Superscaf	-	Superscaf	34816996	34817019	-36	36	43	-	-	-	-	-	-	NCP	IGR	-	ID=gene-E	8	7.095	3025.2	0.325744952	KAAGGLPR	60	385.2425	2	768.4606	.	Q	Superscaf	34817019	0	BTP202209	0	0	0	0	Tissue specific
FLLVVK	Superscaf	-	Superscaf	34835566	34835583	-20	20	25	-	-	-	-	-	-	NCP	IGR	-	ID=gene-E	6	4.53	1562.6	0.325385448	F(+42.01)	58	380.7562	2	759.4894	Acetylati	E	Superscaf	34835601	BTP202209	0	0	0	0	0	Tissue specific
SVDMRS	Superscaf	-	Superscaf	34839601	34839618	-2	2	7	-	-	-	-	-	-	NCP	IGR	-	ID=gene-E	6	5.14	2198.6	0.325307307	SVDM(+15.	53	710.3142	1	709.3065	Oxidation	D	Superscaf	34839648	0	0	BTP202209	0	0	0	Tissue specific
DDFERR	Superscaf	-	Superscaf	34846828	34846845	-17	17	22	-	-	-	-	-	-	NCP	IGR	-	ID=gene-E	6	6.026666667	2933.5	0.325167351	DDFERR	86	419.1942	2	836.3776	.	V	Superscaf	34846926	BTP202209	0	BTP202209	0	0	0	Tissue specific
AAPPKATG	Superscaf	-	Superscaf	34871287	34871310	-17	17	24	-	-	-	-	-	-	NCP	IGR	-	ID=gene-E	8	6.6125	2771.9	0.32469357	AAPPKATG	56	356.7017	2	711.3915	.	V	Superscaf	34871484	0	0	BTP202209	0	0	0	Tissue specific
KSAALPK	Superscaf	-	Superscaf	34878238	34878258	-6	6	12	-	-	-	-	-	-	NCP	IGR	-	ID=gene-E	7	7.068571429	2633.6	0.324559017	KSAALPK	84	357.7287	2	713.4435	.	P	Superscaf	34878381	0	0	BTP202209	BTP202209	0	0	Tissue specific
HPKPSTP	Superscaf	-	Superscaf	34910932	34910952	-7	7	13	-	-	-	-	-	-	NCP	IGR	-	ID=gene-E	7	6.92	2881.2	0.323925876	H(+42.01)	86	403.212	2	804.413	Acetylati	I	Superscaf	34911012	BTP202209	0	BTP202209	0	0	0	Tissue specific
SLAVTNK	Superscaf	+	Superscaf	34913554	34913574	16	16	22	-	-	-	-	-	-	NCP	IGR	-	ID=gene-E	7	5.622857143	2510.7	0.323875099	SLAVTNK	81	366.7145	2	731.4177	.	K	Superscaf	34913509	0	0	BTP202209	0	0	0	Tissue specific
KPSVDKK	Superscaf	-	Superscaf	35004358	35004378	-7	7	13	-	-	-	-	-	-	NCP	IGR	-	ID=gene-E	7	6.31	2537.5	0.322116619	KPSVDKK	71	401.2408	2	800.4756	.	L	Superscaf	35004390	0	BTP202209	0	0	0	0	Tissue specific
VLPLLTQ	Superscaf	-	Superscaf	35036683	35036703	-56	56	62	-	-	-	-	-	-	NCP	IGR	-	ID=gene-E	7	5.202857143	2405.8	0.321490623	VLPLLTQ	88	783.4949	1	782.4902	.	G	Superscaf	35036742	0	BTP202209	BTP202209	BTP202209	0	BTP202209	Tissue specific
HGPSK	Superscaf	+	Superscaf	35038441	35038455	4	4	8	-	-	-	-	-	-	NCP	IGR	-	ID=gene-E	5	7.056	2106.2	0.321456695	HGPSK	56	525.2797	1	524.2707	.	V	Superscaf	35038432	0	BTP202209	0	0	0	0	Tissue specific
NVTLLAS	Superscaf	-	Superscaf	35093329	35093349	-2	2	8	-	-	-	-	-	-	NCP	IGR	-	ID=gene-E	7	5.085714286	2395.7	0.320393635	NVTLLAS	51	717.4104	1	716.4069	.	K	Superscaf	35093400	0	0	0	0	BTP202209	0	Tissue specific
LEKGLDG	Superscaf	-	Superscaf	35119477	35119497	-18	18	24	-	-	-	-	-	-	NCP	IGR	-	ID=gene-E	7	5.69	2606.8	0.319887262	LEKGLDG	79	366.1991	2	730.3861	.	I	Superscaf	35119497	0	0	0	0	BTP202209	0	Tissue specific
MEALAK	Superscaf	+	Superscaf	35167144	35167161	5	5	10	-	-	-	-	-	-	NCP	IGR	-	ID=gene-E	6	6.121666667	2241.4	0.318964216	M(+42.01)	99	704.3629	1	703.3574	Acetylati	K	Superscaf	35167132	0	0	0	0	BTP202209	0	Mixed
QTPKVHP	Superscaf	+	Superscaf	35178343	35178363	7	7	13	-	-	-	-	-	-	NCP	IGR	-	ID=gene-E	7	6.015714286	2771.1	0.318747282	Q(+42.01)	70	424.7347	2	847.4552	Acetylati	G	Superscaf	35178325	0	BTP202209	0	0	0	0	Tissue specific
YGLVDY	Superscaf	-	Superscaf	35220385	35220402	-36	36	41	-	-	gene	-	-	mRNA	NCP	Intron	gene|mRN	ID=gene-E	6	4.373333333	1981.2	0.317933168	YGLVDY	84	729.3409	1	728.3381	.	K	Superscaf	35220447	0	0	0	BTP202209	0	0	Tissue specific
NAGFVF	Superscaf	-	Superscaf	35239822	35239839	-1	1	6	-	-	gene	-	-	mRNA	NCP	Intron	gene|mRN	ID=gene-E	6	4.726666667	1980.1	0.317556758	N(+42.01)	64	696.3481	1	695.3278	Acetylati	N	Superscaf	35240085	0	0	BTP202209	BTP202209	0	0	Tissue specific
APNPAK	Superscaf	-	Superscaf	35255692	35255709	-34	34	39	-	-	-	-	-	-	NCP	IGR	-	ID=gene-E	6	6.631666667	2362.5	0.317249424	A(+42.01)	64	639.336	1	638.3387	Acetylati	I	Superscaf	35255769	0	0	0	0	0	BTP202209	Tissue specific
PVGVP	Superscaf	+	Superscaf	35365075	35365089	9	9	13	-	-	gene	-	-	mRNA	NCP	Intron	gene|mRN	ID=gene-E	5	3.714	972.1	0.315131207	PVGVP	91	468.2813	1	467.2744	.	D	Superscaf	35365051	BTP202209	0	0	0	0	0	Mixed
SAAKPLE	Superscaf	-	Superscaf	35372689	35372709	-31	31	37	-	-	gene	-	-	mRNA	NCP	Intron	gene|mRN	ID=gene-E	7	6.137142857	2590.7	0.31498364	SAAKPLE	62	358.2076	2	714.3912	.	P	Superscaf	35372829	0	0	0	BTP202209	0	0	Tissue specific
KGPKF	Superscaf	+	Superscaf	35377165	35377179	7	7	11	-	-	gene	-	-	mRNA	NCP	Intron	gene|mRN	ID=gene-E	5	7.446	1958.9	0.314897076	KGPKF	52	576.348	1	575.3431	.	E	Superscaf	35377147	0	0	0	BTP202209	0	0	Tissue specific
SLMVHGK	Superscaf	+	Superscaf	35436862	35436882	11	11	17	-	-	-	-	-	-	NCP	IGR	-	ID=gene-E	7	5.815714286	2493.7	0.313740887	SLM(+15.9	57	394.2141	2	786.4058	Oxidation	G	Superscaf	35436832	0	BTP202209	0	0	0	0	Tissue specific
KPPTVPP	Superscaf	+	Superscaf	35548888	35548908	8	8	14	-	-	-	-	-	-	NCP	IGR	-	ID=gene-E	7	5.924285714	2215.5	0.311571428	KPPTVPP	58	368.2284	2	734.4326	.	M	Superscaf	35548867	0	BTP202209	0	0	0	0	Tissue specific
KNLQAF	Superscaf	-	Superscaf	35582158	35582175	-14	14	19	-	-	gene	-	-	mRNA	NCP	Intron	gene|mRN	ID=gene-E	6	6.38	2727.9	0.31092719	KNLQAF	51	720.405	1	719.3966	.	T	Superscaf	35582193	BTP202209	0	0	0	0	0	Tissue specific
QLELQK	Superscaf	-	Superscaf	35636632	35636649	-9	9	14	-	-	gene	-	-	mRNA	NCP	Intron	gene|mRN	ID=gene-E	6	6.036666667	2822.1	0.309872264	QLELQK	75	379.7226	2	757.4334	.	R	Superscaf	35636679	0	0	0	BTP202209	0	0	Tissue specific
KAAERR	Superscaf	-	Superscaf	35660815	35660832	-26	26	31	-	-	gene	-	-	mRNA	NCP	Intron	gene|mRN	ID=gene-E	6	7.753333333	2814.1	0.309403944	KAAERR	73	365.7133	2	729.4245	.	L	Superscaf	35660868	0	0	0	0	BTP202209	0	Tissue specific
LPPPPLFFF	Superscaf	-	Superscaf	35661490	35661516	6	6	14	-	-	gene	-	-	mRNA	NCP	Intron	gene|mRN	ID=gene-E	9	6.046666667	3168.6	0.309390698	LPPPPLFFF	58	512.7983	2	1023.579	.	Y	Superscaf	35661475	BTP202209	0	0	0	0	0	Tissue specific
PLSLLKPF	Superscaf	+	Superscaf	35746117	35746140	91	91	98	-	-	gene	-	-	mRNA	NCP	Intron	gene|mRN	ID=gene-E	8	6.43	2974.2	0.307751897	PLSLLKPF	50	457.7892	2	913.5637	.	R	Superscaf	35745847	0	0	BTP202209	BTP202209	0	0	Tissue specific

SCLRSLGY	Superscaf+	Superscaf	35748331	35748354	14	14	21	-	-	gene	-	-	mRNA	NCP	Intron	gene|mRN	ID=gene-E	8	6.34125	3464.9	0.307709022	SC(+57.02	78	478.2356	2	954.4593	Carbamido	G	Superscaf	35748292	BTP20220	0	0	0	0	0	Tissue specific
KFLLVPP	Superscaf+	Superscaf	35835253	35835273	40	40	46	-	-	-	-	-	-	NCP	IGR	-	ID=gene-E	7	5.682857143	2237.4	0.306025777	KFLLVPP	72	407.2639	2	812.516	.	S	Superscaf	35835136	0	0	0	BTP20220	0	0	Mixed
NLRRFN	Superscaf+	Superscaf	35886985	35887002	76	76	81	-	-	-	-	-	-	NCP	IGR	-	ID=gene-E	6	7.3	3231.8	0.30502401	NLRRFN	62	410.232	2	818.4511	.	M	Superscaf	35886760	BTP20220	0	0	0	0	0	Tissue specific
KPVERT	Superscaf+	Superscaf	35926651	35926668	34	34	39	-	-	-	-	-	-	NCP	IGR	-	ID=gene-E	6	6.091666667	2277.6	0.304255851	KPVERT	69	365.2099	2	728.418	.	P	Superscaf	35926552	BTP20220	0	0	0	0	0	Tissue specific
NPEKPVK	Superscaf+	Superscaf	35934898	35934918	7	7	13	-	exon	gene	three_pri	-	mRNA	NCP	3UTR	exon|gene	ID=exon-E	7	5.815714286	2633.8	0.304096084	NPEKPVK	99	406.2354	2	810.4599	.	S	Superscaf	35934880	0	0	0	BTP20220	0	0	Tissue specific
LGLGGAV	Superscaf+	Superscaf	35937973	35937993	13	13	19	-	-	gene	-	-	mRNA	NCP	Intron	gene|mRN	ID=gene-E	7	5.127142857	1910	0.304036535	LGLGGAV	97	586.3541	1	585.3486	.	V	Superscaf	35937937	0	0	0	BTP20220	0	0	Tissue specific
GPEAPP	Superscaf+	Superscaf	35967772	35967789	31	31	36	-	-	gene	-	-	mRNA	NCP	Intron	gene|mRN	ID=gene-E	6	5.685	2046.3	0.303459515	GPEAPP	88	567.2755	1	566.27	.	G	Superscaf	35967682	0	0	0	BTP20220	0	0	Tissue specific
VLYRLAV	Superscaf+	Superscaf	35982403	35982423	36	36	42	-	-	-	-	-	-	NCP	IGR	-	ID=gene-E	7	4.914285714	2126.4	0.303176118	VLYRLAV	77	417.265	2	832.517	.	K	Superscaf	35982298	BTP20220	BTP20220	0	BTP20220	0	0	Tissue specific
HTPPP	Superscaf-	Superscaf	35990887	35990901	-6	6	10	-	-	gene	-	-	mRNA	NCP	Intron	gene|mRN	ID=gene-E	5	6.604	2029.4	0.303011936	HTPPP	52	548.2898	1	547.2755	.	Y	Superscaf	35990952	0	0	0	BTP20220	0	0	Tissue specific
LPPLVPK	Superscaf+	Superscaf	36010558	36010578	25	25	31	-	-	gene	-	-	mRNA	NCP	Intron	gene|mRN	ID=gene-E	7	5.8	2187.4	0.302630877	LPPLVPK	89	382.2568	2	762.5003	.	E	Superscaf	36010486	0	0	0	BTP20220	0	0	Tissue specific
LSDLSAPK	Superscaf+	Superscaf	36053323	36053346	42	42	49	-	-	gene	-	-	mRNA	NCP	Intron	gene|mRN	ID=gene-E	8	6.04375	3002.2	0.301802646	LSDLSAPK	86	415.7338	2	829.4545	.	V	Superscaf	36053200	0	0	0	BTP20220	0	0	Tissue specific
LESVVPY	Superscaf+	Superscaf	36064336	36064356	32	32	38	-	-	-	-	-	-	NCP	IGR	-	ID=gene-E	7	3.834285714	1959.2	0.30158943	LESVVPY	56	806.4261	1	805.4221	.	P	Superscaf	36064243	0	0	BTP20220	0	0	0	Tissue specific
KFVVKPP	Superscaf+	Superscaf	36078298	36078318	1	1	7	-	-	-	-	-	-	NCP	IGR	-	ID=gene-E	7	5.365714286	1999	0.301319046	KFVVKPP	71	407.7659	2	813.5112	.	K	Superscaf	36078298	BTP20220	BTP20220	0	BTP20220	0	0	Tissue specific
KPFGTGR	Superscaf-	Superscaf	36079072	36079092	-64	64	70	-	-	-	-	-	-	NCP	IGR	-	ID=gene-E	7	7.25	2834.1	0.301304057	KPFGTGR	61	381.7216	2	761.4184	.	Y	Superscaf	36079134	0	0	0	0	BTP20220	0	Tissue specific
KKLAAPG	Superscaf-	Superscaf	36089704	36089724	-38	38	44	-	-	-	-	-	-	NCP	IGR	-	ID=gene-E	7	7.11	2547.5	0.301098162	KKLAAPG	52	684.4382	1	683.433	.	G	Superscaf	36089817	0	0	0	BTP20220	0	0	Tissue specific
NPAGPRP	Superscaf-	Superscaf	36089857	36089877	-15	15	21	-	-	-	-	-	-	NCP	IGR	-	ID=gene-E	7	6.722857143	2866.3	0.301095199	NPAGPRP	71	354.6861	2	707.3715	.	D	Superscaf	36089898	0	0	BTP20220	0	0	0	Tissue specific
VDKPPSVL	Superscaf-	Superscaf	36093562	36093585	-31	31	38	-	-	-	-	-	-	NCP	IGR	-	ID=gene-E	8	4.62125	2291.5	0.301023391	VDKPPSVL	81	427.7507	2	853.4909	.	L	Superscaf	36093648	0	BTP20220	0	0	0	0	Tissue specific
VFECR	Superscaf-	Superscaf	36135796	36135810	-12	12	16	-	-	-	-	-	-	NCP	IGR	-	ID=gene-E	5	4.896	2047.4	0.300205675	VFEC(+57.	94	355.667	2	709.3217	Carbamido	A	Superscaf	36135813	0	0	BTP20220	0	0	0	Mixed
EDFHNHP	Superscaf-	Superscaf	36142996	36143016	-16	16	22	-	-	gene	-	-	mRNA	NCP	Intron	gene|mRN	ID=gene-E	7	5.508571429	3397.9	0.300066126	EDFHNHP	82	448.1881	2	894.362	.	C	Superscaf	36143085	0	0	0	BTP20220	0	0	Tissue specific
VEFPGLKE	Superscaf+	Superscaf	36186769	36186792	22	22	29	-	-	-	-	-	-	NCP	IGR	-	ID=gene-E	8	4.98875	2694.9	0.299218374	VEFPGLKE	80	459.7486	2	917.4858	.	I	Superscaf	36186706	0	BTP20220	0	0	0	0	Tissue specific
SFPLG	Superscaf-	Superscaf	36265597	36265611	-5	5	9	-	-	gene	-	-	mRNA	NCP	Intron	gene|mRN	ID=gene-E	5	5.882	1758.9	0.297691991	SFPLG	90	520.2764	1	519.2693	.	C	Superscaf	36265641	0	0	BTP20220	0	0	0	Mixed
KMTDRK	Superscaf-	Superscaf	36270646	36270663	-9	9	14	-	-	gene	-	-	mRNA	NCP	Intron	gene|mRN	ID=gene-E	6	7.581666667	2766.1	0.297594156	KMTDRK	75	389.7176	2	777.4167	.	T	Superscaf	36270750	0	0	0	BTP20220	0	0	Tissue specific
QTLVWCLHK	Superscaf+	Superscaf	36301810	36301839	83	83	92	-	exon	gene	three_pri	-	mRNA	NCP	3UTR	exon|gene	ID=exon-E	10	5.56	4340.9	0.296990412	QTLVWC(+5	83	657.3365	2	1312.66	Carbamido	A	Superscaf	36301564	0	0	0	BTP20220	BTP20220	0	Tissue specific
ADLLKEPQE	Superscaf+	Superscaf	36309076	36309102	31	31	39	-	-	gene	-	-	mRNA	NCP	Intron	gene|mRN	ID=gene-E	9	5.453333333	3697.1	0.296849759	A(+42.01)	75	542.7691	2	1083.545	Acetylati	R	Superscaf	36308986	0	BTP20220	0	0	0	0	Tissue specific
KVGPRLL	Superscaf+	Superscaf	36366238	36366258	18	18	24	-	-	gene	-	-	mRNA	NCP	Intron	gene|mRN	ID=gene-E	7	6.39	2458.7	0.295742894	KVGPRLL	57	391.7652	2	781.5174	.	D	Superscaf	36366187	0	0	0	0	BTP20220	0	Mixed
RLAEFR	Superscaf+	Superscaf	36391915	36391932	67	67	72	-	-	-	-	-	-	NCP	IGR	-	ID=gene-E	6	7.036666667	2775.2	0.2952457	RLAEFR	96	396.2278	2	790.4449	.	K	Superscaf	36391717	0	0	0	0	BTP20220	0	Tissue specific
RFAAPPPP	Superscaf+	Superscaf	36397096	36397119	14	14	21	-	-	-	-	-	-	NCP	IGR	-	ID=gene-E	8	6.685	3008.4	0.29514525	RFAAPPPP	65	426.7469	2	851.4653	.	A	Superscaf	36397057	0	0	0	0	BTP20220	0	Tissue specific
TERWW	Superscaf+	Superscaf	36408172	36408186	6	6	10	-	-	-	-	-	-	NCP	IGR	-	ID=gene-E	5	6.458	2525	0.29493093	T(+42.01)	51	819.3649	1	818.3712	Acetylati	Q	Superscaf	36408157	0	0	0	BTP20220	0	0	Tissue specific
MDALKK	Superscaf-	Superscaf	36411943	36411960	-3	3	8	-	-	-	-	-	-	NCP	IGR	-	ID=gene-E	6	6.7	2384.5	0.294857844	M(+42.01)	99	374.2064	2	746.3997	Acetylati	L	Superscaf	36411960	0	0	BTP20220	0	0	0	Mixed
NPARPPP	Superscaf+	Superscaf	36415804	36415824	6	6	12	-	-	-	-	-	-	NCP	IGR	-	ID=gene-E	7	6.77	2906.4	0.294783015	NPARPPP	69	374.713	2	747.4027	.	P	Superscaf	36415789	BTP20220	0	0	0	0	0	Tissue specific
GHSLEK	Superscaf+	Superscaf	36428875	36428892	7	7	12	-	exon	gene	three_pri	-	mRNA	NCP	3UTR	exon|gene	ID=exon-E	6	6.363333333	2547.7	0.294529945	G(+42.01)	94	712.3719	1	711.3552	Acetylati	A	Superscaf	36428857	BTP20220	0	0	0	0	0	Tissue specific
VGKLSHSL	Superscaf-	Superscaf	36753403	36753426	-15	15	22	-	-	-	-	-	-	NCP	IGR	-	ID=gene-E	8	5.8275	2859	0.288245125	VGKLSHSL	83	420.7493	2	839.4865	.	E	Superscaf	36753468	0	0	0	BTP20220	0	0	Mixed
KDYSGP	Superscaf+	Superscaf	36776266	36776283	33	33	38	-	-	-	-	-	-	NCP	IGR	-	ID=gene-E	6	6.053333333	2357.5	0.287802484	KDYSGP	55	666.311	1	665.302	.	P	Superscaf	36776170	BTP20220	0	0	0	0	0	Tissue specific
VHVVYP	Superscaf-	Superscaf	36790996	36791013	-41	41	46	-	-	gene	-	-	mRNA	NCP	Intron	gene|mRN	ID=gene-E	6	3.258333333	1416.6	0.287517227	V(+42.01)	67	378.2129	2	754.4014	Acetylati	F	Superscaf	36791016	0	0	0	0	BTP20220	0	Tissue specific
FPVRGHL	Superscaf+	Superscaf	36823789	36823809	15	15	21	-	-	-	-	-	-	NCP	IGR	-	ID=gene-E	7	6.011428571	2644	0.286882111	FPVRGHL	81	413.2405	2	824.4657	.	L	Superscaf	36823747	0	BTP20220	0	0	0	0	Tissue specific
VTLLEEL	Superscaf-	Superscaf	36840418	36840438	-1	1	7	-	-	-	-	-	-	NCP	IGR	-	ID=gene-E	7	4.415714286	2308.7	0.286560079	VTLLEEL	73	408.7378	2	815.464	.	V	Superscaf	36840444	0	0	0	0	BTP20220	0	Tissue specific
AAVAVPL	Superscaf+	Superscaf	36851254	36851274	103	103	109	-	exon	gene	-	five_prim	mRNA	NCP	5UTR	exon|gene	ID=exon-E	7	4.334285714	1624.7	0.286350232	A(+42.01)	60	682.4108	1	681.4061	Acetylati	S	Superscaf	36850948	0	0	0	BTP20220	0	0	Tissue specific
WMHMG	Superscaf-	Superscaf	36872176	36872190	-26	26	30	-	-	gene	-	-	mRNA	NCP	Intron	gene|mRN	ID=gene-E	5	6.19	2186.6	0.28594518	W(+42.01)	52	360.1292	2	718.2567	Acetylati	A	Superscaf	36872280	0	0	0	BTP20220	0	0	Tissue specific
KPPAAPQP	Superscaf+	Superscaf	36921190	36921213	28	28	35	-	-	-	-	-	-	NCP	IGR	-	ID=gene-E	8	6.57875	3051.3	0.284995816	KPPAAPQP	64	403.2391	2	804.4493	.	G	Superscaf	36921109	0	0	BTP20220	0	0	0	Tissue specific
KADFHH	Superscaf-	Superscaf	36934756	36934773	-73	73	78	-	-	gene	-	-	mRNA	NCP	Intron	gene|mRN	ID=gene-E	6	6.565	2818	0.284733218	KADFHH	62	377.6907	2	753.3558	.	T	Superscaf	36934863	0	0	0	BTP20220	0	0	Tissue specific
SLLTQKL	Superscaf+	Superscaf	36950893	36950913	20	20	26	-	-	gene	-	-	mRNA	NCP	Intron	gene|mRN	ID=gene-E	7	6.505714286	2920.2	0.284420656	SLLTQKL	75	401.7535	2	801.496	.	A	Superscaf	36950836	0	0	0	BTP20220	0	0	Tissue specific
VDLSNTT	Superscaf+	Superscaf	37146367	37146387	5	5	11	-	-	gene	-	-	mRNA	NCP	Intron	gene|mRN	ID=gene-E	7	4.728571429	2540	0.28063517	VDLSNTT	72	749.3651	1	748.3603	.	G	Superscaf	37146355	0	0	BTP20220	0	0	0	Tissue specific
VFCER	Superscaf+	Superscaf	37165510	37165524	5	5	9	-	-	gene	-	-	mRNA	NCP	Intron	gene|mRN	ID=gene-E	5	4.896	2047.4	0.280264569	VFC(+57.0	91	355.667	2	709.3217	Carbamido	V	Superscaf	37165498	0	0	BTP20220	0	0	0	Tissue specific
ADVSKEP	Superscaf+	Superscaf	37200994	37201014	32	32	38	-	-	gene	-	-	mRNA	NCP	Intron	gene|mRN	ID=gene-E	7	4.847142857	2337.5	0.279577281	ADVSKEP	64	373.1852	2	744.3654	.	S	Superscaf	37200901	0	0	0	BTP20220	0	0	Tissue specific
DVGDSF	Superscaf+	Superscaf	37223254	37223271	21	21	26	-	-	gene	-	-	mRNA	NCP	Intron	gene|mRN	ID=gene-E	6	3.845	1891.1	0.27914626	D(+42.01)	58	681.2675	1	680.2653	Acetylati	L	Superscaf	37223194	0	0	BTP20220	0	0	0	Tissue specific
AAKPLAL	Superscaf-	Superscaf	37246933	37246953	-4	4	10	-	-	gene	-	-	mRNA	NCP	Intron	gene|mRN	ID=gene-E	7	6.58	2446.5	0.278687642	AAKPLAL	57	683.443	1	682.4377	.	E	Superscaf	37246953	0	0	0	BTP20220	0	0	Tissue specific
KAADMPK	Superscaf-	Superscaf	37298521	37298541	-19	19	25	-	-	gene	-	-	mRNA	NCP	Intron	gene|mRN	ID=gene-E	7	6.648571429	2679.8	0.277688605	KAADM(+15	64	388.7097	2	775.3898	Oxidation	T	Superscaf	37298754	BTP20220	0	0	0	0	0	Tissue specific
LVELK	Superscaf-	Superscaf	37313014	37313028	-18	18	22	-	-	gene	-	-	mRNA	NCP	Intron	gene|mRN	ID=gene-E	5	4.984	1600.7	0.277408055	LVELK	67	601.39	1	600.3846	.	F	Superscaf	37313100	0	BTP20220	BTP20220	0	BTP20220	BTP20220	Tissue specific
PPNVKPP	Superscaf+	Superscaf	37395937	37395957	9	9	15	-	-	gene	-	-	mRNA	NCP	Intron	gene|mRN	ID=gene-E	7	5.764285714	2414.7	0.275802079	PPNVKPP	84	374.7201	2	747.4279	.	S	Superscaf	37395913	BTP20220	0	0	0	0	0	Tissue specific
KPTEWYK	Superscaf-	Superscaf	37402117	37402137	-12	12	18	-	-	gene	-	-	mRNA	NCP	Intron	gene|mRN	ID=gene-E	7	6.725714286	3083.3	0.275682399	KPTEWYK	74	476.2583	2	950.4861	.	Q	Superscaf	37402140	0	0	BTP20220	0	0	0	Tissue specific
YELRLSH	Superscaf+	Superscaf	37411165	37411185	30	30	36	-	-	gene	-	-	mRNA	NCP	Intron	gene|mRN	ID=gene-E	7	6.41	3243.7	0.275507178	YELRLSH	67	459.2436	2	916.4766	.	S	Superscaf	37411078	0	0	0	BTP20220	0	0	Tissue specific
QPTPTPLL	Superscaf+	Superscaf	37423972	37423995	39	39	46	-	-	-	-	-	-	NCP	IGR	-	ID=gene-E	8	6.19625	3124.7	0.275259104	Q(+42.01)	53	454.757	2	907.5015	Acetylati	L	Superscaf	37423858	BTP20220	0	0	0	0	0	Tissue specific
EDVPTLL	Superscaf+	Superscaf	37424248	37424268	21	21	27	-	-	-	-	-	-	NCP	IGR	-	ID=gene-E	7	4.425714286	2278.7	0.275253817	EDVPTLL	73	786.4224	1	785.4171	.	L	Superscaf	37424188	0	0	0	BTP20220	0	0	Tissue specific
KYLWVT	Superscaf+	Superscaf	37427083	37427100	22	22	27	-	-	-	-	-	-	NCP	IGR	-	ID=gene-E	6	5.633333333	2205.4	0.275198974	KYLWVT	53	405.2343	2	808.4483	.	S	Superscaf	37427020	0	0	0	0	BTP20220	0	Tissue specific
KFVYPP	Superscaf+	Superscaf	37466122	37466139	27	27	32	-	-	-	-	-	-	NCP	IGR	-	ID=gene-E	6	5.58	1990.1	0.274442957	KFVYPP	75	375.7148	2	749.4112	.	N	Superscaf	37466044	0	0	BTP20220	BTP20220	0	0	Tissue specific
APMTKP	Superscaf+	Superscaf	37482112	37482129	18	18	23	-	-	gene	-	-	mRNA	NCP	Intron	gene|mRN	ID=gene-E	6	6.773333333	2223.5	0.2741333	APM(+15.9	62	660.3446	1	659.3312	Oxidation	S	Superscaf	37482061	0	0	0	BTP20220	0	0	Tissue specific
APAEKPP	Superscaf-	Superscaf	37534867	37534887	-58	58	64	-	-	gene	-	-	mRNA	NCP	Intron	gene|mRN	ID=gene-E	7	6.271428571	2528.7	0.273111606	APAEKPP	69	355.201	2	708.3806	.	L	Superscaf	37534950	BTP20220	0	0	0	0	0	Tissue specific
MNYPGHG	Superscaf-	Superscaf	37613011	37613031	-34	34	40	-	-	gene	-	-	mRNA	NCP	Intron	gene|mRN	ID=gene-E	7	6.092857143	2979.4	0.271598294	M(+42.01)	98	817.3271	1	816.3224	Acetylati	K	Superscaf	37613865	0	0	0	0	BTP20220	0	Tissue specific
LLVSFFV	Superscaf+	Superscaf	37635658	37635678	12	12	18	-	-	gene	-	-	mRNA	NCP	Intron	gene|mRN	ID=gene-E	7	4.085714286	1865	0.27115972	LLVSFFV	54	412.746	2	823.4843	.	V	Superscaf	37635625	0	0	0	BTP20220	0	0	Tissue specific
GVPLK	Superscaf+	Superscaf	37660714	37660728	1	1	5	-	-	gene	-	-	mRNA	NCP	Intron	gene|mRN	ID=gene-E	5	5.598	1456.5	0.27067461	GVPLK	93	513.3384	1	512.3322	.	G	Superscaf	37660714	BTP20220	0	0	0	0	0	Mixed
VYPKPR	Superscaf+	Superscaf	37685677	37685694	8	8	13	-	-	gene	-	-	mRNA	NCP	Intron	gene|mRN	ID=gene-E	6	6.46	2251.5	0.270191126	VYPKPR	82	380.2349	2	758.4438	.	A	Superscaf	37685656	BTP20220	0	0	0	0	0	Tissue specific
FSPGTKK	Superscaf+	Superscaf	37695967	37695987	8	8	14	-	-	-	-	-	-	NCP	IGR	-	ID=gene-E	7	7.062857143	2739.8	0.269991796	F(+42.01)	52	403.7165	2	805.4334	Acetylati	R	Superscaf	37695946	BTP20220	0	0	0	0	0	Tissue specific
KGYPPPP	Superscaf+	Superscaf	37746910	37746930	1	1	7	-	-	gene	-	-	mRNA	NCP	Intron	gene|mRN	ID=gene-E	7	6.652857143	2574.8	0.26900525	KGYPPPP	73	378.2071	2	754.4013	.	K	Superscaf	37746910	0	0	0	BTP20220	0	0	Mixed
KLTLKGYKG	Superscaf-	Superscaf	37804474	37804500	-9	9	17	-	-	gene	-	-	mRNA	NCP	Intron	gene|mRN	ID=gene-E	9	7.221111111	3877	0.267890368	KLTLKGYKG	82	539.8326	2	1077.655	.	F	Superscaf	37804650	0	0	0	BTP20220	0	0	Tissue specific
YKLTSGWV	Superscaf-	Superscaf	37816288	37816311	-50	50	57	-	-	-	-	-	-	NCP	IGR	-	ID=gene-E	8	5.68125	2886.1	0.26766164	YKLTSGWV	91	477.2564	2	952.5018	.	V	Superscaf	37816311	BTP20220	0	0	0	0	0	Mixed
PVCPLK	Superscaf-	Superscaf	37860748	37860765	-12	12	17	-	exon	gene	three_pri	-	mRNA	NCP	3UTR	exon|gene	ID=exon-E	6	5.556666667	2082.3	0.266800758	PVC(+57.0	63	357.1988	2	712.3942	Carbamido	V	Superscaf	37860771	0	0	0	BTP20220	0	0	Tissue specific
VGMFLQG	Superscaf-	Superscaf	37863271	37863291	-23	23	29	-	-	gene	-	-	mRNA	NCP	Intron	gene|mRN	ID=gene-E	7	4.971428571	2317.6	0.266751841	VGMFLQG	61	751.3777	1	750.3734	.	A	Superscaf	37863354	0	0	0	BTP20220	0	0	Tissue specific
GSVLLNR	Superscaf+	Superscaf	37902271	37902291	15	15	21	-	-	gene	-	-	mRNA	NCP	Intron	gene|mRN	ID=gene-E	7	5.682857143	2633	0.265996579	G(+42.01)	66	400.7341	2	799.4552	Acetylati	R	Superscaf	37902229	0	0	BTP20220	0	0	0	Tissue specific
KSALKLY	Superscaf-	Superscaf	37927018	37927038	-7	7	13	-	-	gene	-	-	mRNA	NCP	Intron	gene|mRN	ID=gene-E	7	6.971428571	2797.8	0.265517337	KSALKLY	76	411.7569	2	821.501	.	C	Superscaf	37927053	BTP20220	0	0	0	0	0	Tissue specific
VAVHVH	Superscaf+	Superscaf	37934224	37934241	62	62	67	-	-	gene	-	-	mRNA	NCP	Intron	gene|mRN	ID=gene-E	6	3.533333333	1550.7	0.265377846	V(+42.01)	55	352.1956	2	702.3813	Acetylati	M	Superscaf	37934041	BTP20220	0	0	0	0	0	Tissue specific
AAAKPENLS	Superscaf-	Superscaf	37971376	37971405	-65	65	74	-	-	gene	-	-	mRNA	NCP	Intron	gene|mRN	ID=gene-E	10	6.037	3852.1	0.26465814	A(+42.01)	62	528.2885	2	1054.566	Acetylati	P	Superscaf	37971462	0	0	0	BTP20220	BTP20220	0	Tissue specific
AAAKPENLS	Superscaf-	Superscaf	37971376	37971414	-65	65	77	-	-	gene	-	-	mRNA	NCP	Intron	gene|mRN	ID=gene-E	13	5.227692308	4471.8	0.264657966	A(+42.01)	96	695.8859	2	1389.762	Acetylati	P	Superscaf	37971462	0	0	0	0	BTP20220	0	Tissue specific
AAAKPENLS	Superscaf-	Superscaf	37971376	37971402	-65	65	73	-	-	gene	-	-	mRNA	NCP	Intron	gene|mRN	ID=gene-E	9	6.043333333	3498.7	0.264658198	A(+42.01)	95	942.4871	1	941.4818	Acetylati	P	Superscaf	37971462	0	0	BTP20220	0	0	0	Tissue specific
AAAKPENL	Superscaf-	Superscaf	37971376	37971399	-65	65	72	-	-	gene	-	-	mRNA	NCP	Intron	gene|mRN	ID=gene-E	8	6.08875	3115.3	0.264658256	A(+42.01)	99	428.2313	2	854.4498	Acetylati	P	Superscaf	37971462	0	0	0	0	BTP20220	0	Mixed
QCCGPGP	Superscaf+	Superscaf	37987789	37987809	28	28	34	-	-	gene	-	-	mRNA	NCP	Intron	gene|mRN	ID=gene-E	7	5.747142857	3051.5	0.264340466	Q(+42.01)	50	409.1449	2	816.2895	Acetylati	G	Superscaf	37987708	0	0	0	BTP20220	0	0	Tissue specific
LGNKVLVC	Superscaf-	Superscaf	37996906	37996929	-33	33	40	-	-	gene	-	-	mRNA	NCP	Intron	gene|mRN	ID=gene-E	8	4.7625	2654.9	0.264163851	LGNKVLVC(	72	451.7587	2	901.5055	Carbamido	L	Superscaf	37996962	0	0	0	0	BTP20220	0	Tissue specific
SKSVNVKPL	Superscaf-	Superscaf	38005267	38005299	-21	21	31	-	-	gene	-	-	mRNA	NCP	Intron	gene|mRN	ID=gene-E	11	5.005454545	3388.6	0.26400176	SKSVNVKPL	98	586.3551	2	1170.697	.	R	Superscaf	38005401	0	0	0	0	BTP20220	0	Tissue specific
ERYVPR	Superscaf+	Superscaf	38023543	38023560	20	20	25	-	-	gene	-	-	mRNA	NCP	Intron	gene|mRN	ID=gene-E	6	6.116666667	2520	0.263648124	ERYVPR	84	410.2258	2	818.4399	.	L	Superscaf	38023486	0	0	BTP20220	0	0	0	Tissue specific
LGAAVLSL	Superscaf-	Superscaf	38043874	38043897	-34	34	41	-	-	-	-	-	-	NCP	IGR	-	ID=gene-E	8	5.20375	2363.5	0.263254284	LGAAVLSL	74	743.4629	1	742.4589	.	M	Superscaf	38044047	0	0	0	BTP20220	0	0	Tissue enhanced
AMLSGVT	Superscaf+	Superscaf	38061151	38061171	18	18	24	-	-	-	-	-	-	NCP	IGR	-	ID=gene-E	7	5.132857143	2114.4	0.262919761	A(+42.01)	64	720.358	1	719.3524	Acetylati	V	Superscaf	38061100	0	0	0	0	BTP20220	0	Tissue specific
EPKTPLSPE	Superscaf+	Superscaf	38063047	38063073	44	44	52	-	-	-	-	-	-	NCP	IGR	-	ID=gene-E	9	6.261111111	3377.8	0.262882928	EPKTPLSPE	60	483.2643	2	964.5229	.	E	Superscaf	38062918	BTP20220	0	0	0	0	0	Tissue specific
PVNGP	Superscaf-	Superscaf	38067817	38067831	-94	94	98	-	-	-	-	-	-	NCP	IGR	-	ID=gene-E	5	4.796	1568.8	0.262790786	P(+42.01)	53	525.2684	1	524.2595	Acetylati	R	Superscaf	38067852	BTP20220	0	0	BTP20220	BTP20220	0	Tissue specific
KDEAAAK	Superscaf+	Superscaf	38072371	38072391	2	2	8	-	-	-	-	-	-	NCP	IGR	-	ID=gene-E	7	6.247142857	2707.7	0.262702478	KDEAAAK	50	732.3761	1	731.3813	.	K	Superscaf	38072368	BTP20220	0	0	0	0	0	Tissue specific
LEKGDLQ	Superscaf+	Superscaf	38077753	38077773	3	3	9	-	-	-	-	-	-	NCP	IGR	-	ID=gene-E	7	5.644285714	2920.2	0.262598252	LEKGDLQ	72	401.7177	2	801.4232	.	L	Superscaf	38077747	BTP20220	0	0	0	0	0	Tissue specific
LYPHKVVF	Superscaf-	Superscaf	38310487	38310510	-13	13	20	-	-	gene	-	-	mRNA	NCP	Intron	gene|mRN	ID=gene-E	8	5.09375	2625.8	0.258091143	LYPHKVVF	92	501.7914	2	1001.57	.	L	Superscaf	38310519	0	BTP20220	0	0	0	0	Mixed
LLKLSYR	Superscaf+	Superscaf	38317609	38317629	14	14	20	-	-	gene	-	-	mRNA	NCP	Intron	gene|mRN	ID=gene-E	7	7.111428571	3020.3	0.257953279	LLKLSYR	88	446.7827	2	891.5541	.	I	Superscaf	38317570	0	0	0	0	BTP20220	0	Tissue specific
VADGPV	Superscaf-	Superscaf	38363821	38363838	-14	14	19	-	-	gene	-	-	mRNA	NCP	Intron	gene|mRN	ID=gene-E	6	3.543333333	1357.5	0.257058411	VADGPV	79	557.2909	1	556.2856	.	E	Superscaf	38363850	BTP20220	0	0	0	0	0	Tissue specific
HQGKGPK	Superscaf-	Superscaf	38371771	38371791	-34	34	40	-	-	gene	-	-	mRNA	NCP	Intron	gene|mRN	ID=gene-E	7	7.28	3099.2	0.256904395	HQGKGPK	60	376.2109	2	750.4136	.	W	Superscaf	38371950	BTP20220	0	0	0	0	0	Tissue specific
VDLSGPK	Superscaf+	Superscaf	38432416	38432436	3	3	9	-	-	gene	-	-	mRNA	NCP	Intron	gene|mRN	ID=gene-E	7	5.234285714	2251.4	0.255729964	VDLSGPK	98	358.2025	2	714.3912	.	H	Superscaf	38432410	0	0	0	0	BTP20220	0	Tissue specific
PLLMFCK	Superscaf-	Superscaf	38438008	38438028	-40	40	46	-	-	gene	-	-	mRNA	NCP	Intron	gene|mRN	ID=gene-E	7	6.321428571	2857.2	0.255621671	PLLMFC(+5	68	454.737	2	907.4659	Carbamido	A	Superscaf	38438031	0	0	0	BTP20220	0	0	Tissue specific
GSLGPGAYK	Superscaf+	Superscaf	38460022	38460048	13	13	21	-	-	-	-	-	-	NCP	IGR	-	ID=gene-E	9	6.365555556	3205.3	0.255195239	GSLGPGAYK	72	425.2274	2	848.4391	.	E	Superscaf	38459986	BTP20220	0	0	0	0	0	Tissue specific
SWEVY	Superscaf+	Superscaf	38472304	38472318	2	2	6	-	-	-	-	-	-	NCP	IGR	-	ID=gene-E	5	4.09	1795	0.254957622	S(+42.01)	73	725.3108	1	724.3068	Acetylati	L	Superscaf	38472301	BTP20220	0	0	0	0	0	Tissue specific
LSEAYFRK	Superscaf-	Superscaf	38515948	38515971	-68	68	75	-	-	-	-	-	-	NCP	IGR	-	ID=gene-E	8	6.5675	3437.7	0.254112253	LSEAYFRK	97	507.2721	2	1012.534	.	I	Superscaf	38516010	0	0	BTP20220	0	0	0	Tissue specific
SPPDVLK	Superscaf-	Superscaf	38528767	38528787	-41	41	47	-	-	-	-	-	-	NCP	IGR	-	ID=gene-E	7	5.281428571	2291.5	0.253864062	SPPDVLK	99	378.2164	2	754.4225	.	S	Superscaf	38528985	0	0	0	BTP20220	0	0	Tissue specific
VDVDKDTLG	Superscaf-	Superscaf	38528818	38528850	-58	58	68	-	-	-	-	-	-	NCP	IGR	-	ID=gene-E	11	4.921818182	4152.6	0.253862842	VDVDKDTLG	99	433.2277	3	1296.667	.	S	Superscaf	38528985	BTP20220	0	0	0	0	0	Tissue specific
EVYGPK	Superscaf-	Superscaf	38532226	38532243	-89	89	94	-	-	-	-	-	-	NCP	IGR	-	ID=gene-E	6	5.148333333	1988.1	0.253797134	EVYGPK	53	692.3694	1	691.3541	.	V	Superscaf	38532249	0	BTP20220	BTP20220	0	BTP20220	0	Tissue specific
FGERALK	Superscaf-	Superscaf	38554537	38554557	-9	9	15	-	-	-	-	-	-	NCP	IGR	-	ID=gene-E	7	6.738571429	2892.1	0.253365009	FGERALK	99	410.7358	2	819.4603	.	S	Superscaf	38554560	BTP20220	0	BTP20220	0	BTP20220	BTP20220	Tissue specific
AEYKKL	Superscaf+	Superscaf	38616091	38616108	9	9	14	-	-	-	-	-	-	NCP	IGR	-	ID=gene-E	6	6.726666667	2486.5	0.252173032	A(+42.01)	95	397.2306	2	792.4381	Acetylati	S	Superscaf	38616067	BTP20220	0	0	0	0	0	Tissue specific
HLLFVV	Superscaf+	Superscaf	38634556	38634573	13	13	18	-	-	-	-	-	-	NCP	IGR	-	ID=gene-E	6	4.171666667	1713.9	0.251815445	HLLFVV	63	364.2247	2	726.4428	.	Q	Superscaf	38634520	0	0	BTP20220	0	0	0	Tissue specific
NTGLLLAR	Superscaf+	Superscaf	38668486	38668509	2	2	9	-	-	-	-	-	-	NCP	IGR	-	ID=gene-E	8	6.57875	3311.8	0.251158251	N(+42.01)	77	450.2676	2	898.5236	Acetylati	H	Superscaf	38668483	0	0	0	0	BTP20220	0	Tissue specific
ASDRLSL	Superscaf+	Superscaf	38763256	38763276	47	47	53	-	-	-	-	-	-	NCP	IGR	-	ID=gene-E	7	6.152857143	2845.2	0.249323024	ASDRLSL	87	381.2102	2	760.4079	.	R	Superscaf	38763118	0	0	BTP20220	0	0	0	Tissue specific
VLLGHR	Superscaf-	Superscaf	38815369	38815386	-23	23	28	-	-	-	-	-	-	NCP	IGR	-	ID=gene-E	6	6.046666667	2272.6	0.248313879	V(+42.01)	56	368.7198	2	735.4391	Acetylati	C	Superscaf	38815575	BTP20220	0	0	0	0	0	Tissue specific
KCYEPKL	Superscaf+	Superscaf	38843056	38843076	14	14	20	-	-	-	-	-	-	NCP	IGR	-	ID=gene-E	7	6.522857143	3098.3	0.247777644	KC(+57.02	54	469.2402	2	936.4739	Carbamido	L	Superscaf	38843017	0	0	0	0	BTP20220	0	Tissue specific
ELVMPEKF	Superscaf+	Superscaf	38871076	38871099	3	3	10	-	-	-	-	-	-	NCP	IGR	-	ID=gene-E	8	4.96125	2769.1	0.247234959	ELVMPEKF	73	496.7544	2	991.5048	.	T	Superscaf	38871070	0	0	0	BTP20220	0	0	Tissue specific
MNPVVG	Superscaf+	Superscaf	38935195	38935212	2	2	7	-	-	-	-	-	-	NCP	IGR	-	ID=gene-E	6	3.905	1602.9	0.245993368	M(+42.01)	74	658.3203	1	657.3156	Acetylati	R	Superscaf	38935192	BTP20220	0	0	0	0	0	Tissue specific
VHDLTK	Superscaf+	Superscaf	38964262	38964279	1	1	6	-	-	-	-	-	-	NCP	IGR	-	ID=gene-E	6	5.468333333	2250.5	0.245430466	VHDLTK	79	712.4073	1	711.3915	.	V	Superscaf	38964262	BTP20220	0	0	0	0	0	Tissue specific
ALMFL	Superscaf+	Superscaf	39002980	39002994	6	6	10	-	-	-	-	-	-	NCP	IGR	-	ID=gene-E	5	5.842	1777	0.244680724	ALM(+15.9	52	610.333	1	609.3196	Oxidation	S	Superscaf	39002965	BTP20220	0	0	0	0	0	Tissue specific
KDTTSLC	Superscaf+	Superscaf	39008866	39008886	24	24	30	-	-	-	-	-	-	NCP	IGR	-	ID=gene-E	7	6.064285714	2997.4	0.244566621	KDTTSLC(+	60	824.3907	1	823.3746	Carbamido	Y	Superscaf	39008797	0	0	0	0	BTP20220	0	Tissue specific
KPANEC	Superscaf+	Superscaf	39031141	39031158	34	34	39	-	-	gene	-	-	mRNA	NCP	Intron	gene|mRN	ID=gene-E	6	5.951666667	2725	0.244135309	KPANEC(+5	51	718.3304	1	717.3116	Carbamido	S	Superscaf	39031042	0	0	0	0	BTP20220	0	Tissue specific
VSPLTF	Superscaf-	Superscaf	39112732	39112749	-26	26	31	-	-	-	-	-	-	NCP	IGR	-	ID=gene-E	6	4.995	1859.1	0.242555244	VSPLTF	97	663.3699	1	662.3639	.	S	Superscaf	39112785	0	0	0	BTP20220	0	0	Tissue specific
KNTLTPV	Superscaf-	Superscaf	39119308	39119328	-3	3	9	-	-	-	-	-	-	NCP	IGR	-	ID=gene-E	7	5.784285714	2550.9	0.242427837	KNTLTPV	53	772.4432	1	771.449	.	S	Superscaf	39119352	0	BTP20220	0	0	0	0	Tissue specific
LLLDVALP	Superscaf-	Superscaf	39138463	39138486	-13	13	20	-	-	-	-	-	-	NCP	IGR	-	ID=gene-E	8	4.90125	2473.8	0.24205683	LLLDVALP	74	853.5365	1	852.532	.	L	Superscaf	39138486	0	BTP20220	0	0	0	0	Tissue enhanced
DSRLTPV	Superscaf+	Superscaf	39241672	39241692	30	30	36	-	-	-	-	-	-	NCP	IGR	-	ID=gene-E	7	5.46	2532	0.240058176	DSRLTPV	58	394.2187	2	786.4235	.	S	Superscaf	39241585	0	0	BTP20220	0	0	0	Tissue specific
PKVTVLGL	Superscaf-	Superscaf	39369583	39369606	-4	4	11	-	-	-	-	-	-	NCP	IGR	-	ID=gene-E	8	5.0625	2207.4	0.237581035	PKVTVLGL	61	413.7718	2	825.5323	.	I	Superscaf	39369624	0	0	0	BTP20220	0	0	Tissue specific
DLLHPSPEE	Superscaf+	Superscaf	39423049	39423084	12	12	23	-	-	gene	-	-	mRNA	NCP	Intron	gene|mRN	ID=gene-E	12	5.913333333	5189.9	0.236545398	DLLHPSPEE	90	483.9149	3	1448.726	.	R	Superscaf	39423016	0	0	0	0	BTP20220	0	Mixed
RLVQSPNS	Superscaf+	Superscaf	39423100	39423123	29	29	36	-	-	gene	-	-	mRNA	NCP	Intron	gene|mRN	ID=gene-E	8	5.6825	3313.8	0.236544642	RLVQSPNS	51	450.7468	2	899.4825	.	R	Superscaf	39423016	0	0	0	0	BTP20220	0	Tissue specific
VESSVEVP	Superscaf-	Superscaf	39453838	39453861	-6	6	13	-	-	-	-	-	-	NCP	IGR	-	ID=gene-E	8	3.0125	1955.2	0.23594938	VESSVEVP	59	845.421	1	844.4178	.	V	Superscaf	39453930	0	BTP20220	0	0	0	0	Tissue specific
ESPPK	Superscaf-	Superscaf	39558160	39558174	-23	23	27	-	-	-	-	-	-	NCP	IGR	-	ID=gene-E	5	6.248	1952.1	0.233929289	ESPPK	64	557.3015	1	556.2856	.	K	Superscaf	39558183	0	0	0	BTP20220	0	0	Tissue specific
KPPTKGK	Superscaf+	Superscaf	39614173	39614193	37	37	43	-	-	-	-	-	-	NCP	IGR	-	ID=gene-E	7	7.76	2774.8	0.232844443	KPPTKGK	50	378.2397	2	754.4701	.	R	Superscaf	39614065	0	0	0	0	BTP20220	0	Tissue specific
HGQDNY	Superscaf+	Superscaf	39617533	39617550	4	4	9	-	-	-	-	-	-	NCP	IGR	-	ID=gene-E	6	5.541666667	2995.4	0.232779432	H(+42.01)	70	775.2904	1	774.2933	Acetylati	I	Superscaf	39617524	0	0	0	0	BTP20220	0	Tissue specific
ELGPP	Superscaf+	Superscaf	39705316	39705330	14	14	18	-	-	-	-	-	-	NCP	IGR	-	ID=gene-E	5	5.554	1751	0.231079514	ELGPP	50	512.2657	1	511.2642	.	P	Superscaf	39705277	0	0	0	0	BTP20220	0	Tissue specific
YFANPK	Superscaf-	Superscaf	39871261	39871278	-16	16	21	-	-	gene	-	-	mRNA	NCP	Intron	gene|mRN	ID=gene-E	6	6.435	2560.7	0.227865819	YFANPK	59	739.3774	1	738.3701	.	H	Superscaf	39871311	0	0	0	0	BTP20220	0	Tissue specific
LMTVGC	Superscaf-	Superscaf	40184413	40184430	-7	7	12	-	-	-	-	-	-	NCP	IGR	-	ID=gene-E	6	4.875	2005.4	0.221801419	LMTVGC(+5	99	680.3073	1	679.3033	Carbamido	R	Superscaf	40184436	0	0	0	0	BTP20220	0	Tissue specific
LAETLYK	Superscaf-	Superscaf	40194889	40194909	-45	45	51	-	-	-	-	-	-	NCP	IGR	-	ID=gene-E	7	6.161428571	2769	0.221598486	LAETLYK	80	837.4663	1	836.4644	.	P	Superscaf	40194957	0	0	0	BTP20220	0	0	Tissue specific
KPTRLT	Superscaf+	Superscaf	40204144	40204161	12	12	17	-	-	-	-	-	-	NCP	IGR	-	ID=gene-E	6	7.64	2603	0.221419315	KPTRLT	50	358.2257	2	714.4388	.	I	Superscaf	40204111	0	0	0	BTP20220	0	0	Tissue specific
EFVRPLF	Superscaf+	Superscaf	40473238	40473258	29	29	35	-	-	-	-	-	-	NCP	IGR	-	ID=gene-E	7	5.317142857	2539.9	0.21620807	EFVRPLF	54	454.2534	2	906.4963	.	H	Superscaf	40473154	0	0	0	BTP20220	BTP20220	0	Tissue specific
KKQPTKK	Superscaf-	Superscaf	40481491	40481511	-28	28	34	-	-	-	-	-	-	NCP	IGR	-	ID=gene-E	7	8.205714286	3219.2	0.216048245	KKQPTKK	66	429.2805	2	856.5494	.	K	Superscaf	40481607	0	0	0	BTP20220	0	0	Tissue specific
NTDLSGPK	Superscaf-	Superscaf	40504627	40504650	-51	51	58	-	-	-	-	-	-	NCP	IGR	-	ID=gene-E	8	6.0725	3245.6	0.215600143	NTDLSGPK	74	416.2183	2	830.4134	.	S	Superscaf	40504656	BTP20220	0	0	0	0	0	Tissue specific
QTLDVV	Superscaf+	Superscaf	40518670	40518687	1	1	6	-	-	-	-	-	-	NCP	IGR	-	ID=gene-E	6	3.521666667	1773.1	0.215328307	QTLDVV	69	674.3702	1	673.3646	.	Q	Superscaf	40518670	0	0	0	0	BTP20220	0	Mixed
EGLKLAY	Superscaf-	Superscaf	40562395	40562415	-7	7	13	-	-	-	-	-	-	NCP	IGR	-	ID=gene-E	7	6.081428571	2668.8	0.214481485	EGLKLAY	77	397.2248	2	792.4381	.	G	Superscaf	40562442	0	0	0	0	BTP20220	0	Tissue specific
THDTPLL	Superscaf+	Superscaf	40594252	40594272	8	8	14	-	-	-	-	-	-	NCP	IGR	-	ID=gene-E	7	5.982857143	2870.4	0.213864552	T(+42.01)	58	419.7176	2	837.4232	Acetylati	K	Superscaf	40594231	0	BTP20220	0	0	BTP20220	BTP20220	Tissue specific
KVMFLM	Superscaf-	Superscaf	40628530	40628547	-13	13	18	-	-	-	-	-	-	NCP	IGR	-	ID=gene-E	6	5.45	1952.2	0.213200794	KVMFLM(+1	91	392.7121	2	783.4023	Oxidation	T	Superscaf	40628562	0	0	BTP20220	BTP20220	0	0	Tissue specific
YEHNSP	Superscaf-	Superscaf	40632649	40632666	-11	11	16	-	-	-	-	-	-	NCP	IGR	-	ID=gene-E	6	5.643333333	2822.2	0.213121027	YEHNSP	64	746.3064	1	745.3031	.	P	Superscaf	40632789	0	0	0	BTP20220	0	0	Tissue specific
GPAAAAAGK	Superscaf+	Superscaf	40638550	40638576	59	59	67	-	-	-	-	-	-	NCP	IGR	-	ID=gene-E	9	6.453333333	2956.9	0.213006576	GPAAAAAGK	55	357.1952	2	712.3867	.	T	Superscaf	40638376	0	0	0	BTP20220	0	0	Tissue specific
QAKAPVP	Superscaf-	Superscaf	40671955	40671975	-50	50	56	-	-	-	-	-	-	NCP	IGR	-	ID=gene-E	7	5.718571429	2376.5	0.212359781	Q(+42.01)	65	376.7141	2	751.4228	Acetylati	K	Superscaf	40672149	0	0	0	BTP20220	0	0	Tissue specific
KPDVPP	Superscaf-	Superscaf	40696816	40696833	-419	419	424	-	-	-	-	-	-	NCP	IGR	-	ID=gene-E	6	5.268333333	1892.1	0.211878389	KPDVPP	57	652.3723	1	651.3591	.	R	Superscaf	40696890	0	0	0	0	BTP20220	0	Tissue specific
LPHQKVGL	Superscaf-	Superscaf	40719523	40719546	-75	75	82	-	-	-	-	-	-	NCP	IGR	-	ID=gene-E	8	5.90125	3040.3	0.211438537	LPHQKVGL	60	446.274	2	890.5338	.	V	Superscaf	40719561	0	0	0	0	BTP20220	0	Tissue specific
KEYNGP	Superscaf-	Superscaf	41080243	41080260	-10	10	15	-	-	-	-	-	-	NCP	IGR	-	ID=gene-E	6	6.05	2584.8	0.204453067	KEYNGP	56	354.1692	2	706.3286	.	L	Superscaf	41080338	0	BTP20220	0	0	BTP20220	0	Tissue specific
LDQVKYF	Superscaf+	Superscaf	41170057	41170077	37	37	43	-	-	gene	-	-	mRNA	NCP	Intron	gene|mRN	ID=gene-E	7	5.068571429	2690.9	0.2027137	LDQVKYF	73	456.7426	2	911.4752	.	R	Superscaf	41169949	0	0	0	0	BTP20220	BTP20220	Tissue specific
GVSVQKR	Superscaf-	Superscaf	41176291	41176311	-94	94	100	-	-	gene	-	-	mRNA	NCP	Intron	gene|mRN	ID=gene-E	7	5.4	2408.6	0.202592975	GVSVQKR	74	387.2325	2	772.4555	.	G	Superscaf	41176419	0	BTP20220	0	0	0	0	Tissue enhanced
FFKGSPR	Superscaf-	Superscaf	41182822	41182842	-16	16	22	-	-	gene	-	-	mRNA	NCP	Intron	gene|mRN	ID=gene-E	7	7.058571429	2910.1	0.202466497	FFKGSPR	75	419.7291	2	837.4496	.	Y	Superscaf	41182917	0	0	0	BTP20220	0	0	Tissue specific
KELGGSF	Superscaf+	Superscaf	41198305	41198325	7	7	13	-	-	gene	-	-	mRNA	NCP	Intron	gene|mRN	ID=gene-E	7	6.005714286	2612.7	0.202166659	K(+42.01)	57	779.3896	1	778.3861	Acetylati	Y	Superscaf	41198287	0	0	0	BTP20220	0	0	Tissue specific
KLEDKKTW	Superscaf-	Superscaf	41222329	41222352	-76	76	83	-	-	-	-	-	-	NCP	IGR	-	ID=gene-E	8	6.72625	3519.7	0.20170136	KLEDKKTW	52	524.2936	2	1046.576	.	N	Superscaf	41222694	0	0	0	BTP20220	0	0	Tissue specific
YPLPQML	Superscaf-	Superscaf	41228566	41228586	-15	15	21	-	-	-	-	-	-	NCP	IGR	-	ID=gene-E	7	5.945714286	2823.3	0.201580634	YPLPQM(+1	65	439.2249	2	876.4415	Oxidation	I	Superscaf	41228772	0	0	0	BTP20220	BTP20220	0	Tissue specific
ASSDLQVK	Superscaf-	Superscaf	41244655	41244678	-29	29	36	-	-	-	-	-	-	NCP	IGR	-	ID=gene-E	8	5.215	2922.1	0.201269002	A(+42.01)	99	445.2344	2	888.4553	Acetylati	E	Superscaf	41244891	0	0	0	BTP20220	0	0	Mixed
MLFWY	Superscaf+	Superscaf	41327974	41327988	3	3	7	-	-	-	-	-	-	NCP	IGR	-	ID=gene-E	5	5.752	2098.4	0.199655647	M(+42.01)	70	801.3643	1	800.3568	Acetylati	L	Superscaf	41327968	0	0	0	0	BTP20220	0	Tissue specific
LLKGGAK	Superscaf+	Superscaf	41358034	41358054	11	11	17	-	-	-	-	-	-	NCP	IGR	-	ID=gene-E	7	7.057142857	2549.5	0.199073399	L(+42.01)	63	364.7413	2	727.4592	Acetylati	Q	Superscaf	41358004	0	0	0	0	BTP20220	0	Tissue specific
AGVFLKK	Superscaf+	Superscaf	41362759	41362779	1	1	7	-	-	-	-	-	-	NCP	IGR	-	ID=gene-E	7	6.132857143	2286.2	0.198981896	AGVFLKK	99	381.7457	2	761.4799	.	A	Superscaf	41362759	0	0	0	0	BTP20220	0	Tissue specific
GPATKE	Superscaf-	Superscaf	41430361	41430378	-11	11	16	-	-	-	-	-	-	NCP	IGR	-	ID=gene-E	6	6.296666667	2237.4	0.197672796	GPATKE	62	602.3113	1	601.3071	.	S	Superscaf	41430432	BTP20220	0	0	0	0	0	Tissue specific
QNTMDP	Superscaf+	Superscaf	41444914	41444931	3	3	8	-	-	-	-	-	-	NCP	IGR	-	ID=gene-E	6	5.435	2725.3	0.197390967	Q(+42.01)	55	747.2984	1	746.2905	Acetylati	Q	Superscaf	41444908	0	0	BTP20220	0	0	0	Tissue specific
PLTVLPP	Superscaf+	Superscaf	41482198	41482218	20	20	26	-	-	-	-	-	-	NCP	IGR	-	ID=gene-E	7	5.341428571	2116.5	0.196668879	PLTVLPP	79	736.4584	1	735.4531	.	I	Superscaf	41482141	0	0	0	0	BTP20220	0	Tissue specific
RFFSK	Superscaf+	Superscaf	41579065	41579079	24	24	28	-	-	-	-	-	-	NCP	IGR	-	ID=gene-E	5	7.428	2275.4	0.194793101	R(+42.01)	84	363.7057	2	725.386	Acetylati	P	Superscaf	41578996	0	0	0	BTP20220	0	0	Tissue specific
LGRSPEDK	Superscaf+	Superscaf	41618173	41618196	10	10	17	-	-	-	-	-	-	NCP	IGR	-	ID=gene-E	8	6.3275	3325.7	0.194035574	LGRSPEDK	59	451.2432	2	900.4664	.	S	Superscaf	41618146	0	BTP20220	0	0	0	0	Tissue specific
QTRKLAV	Superscaf+	Superscaf	41659417	41659437	18	18	24	-	-	-	-	-	-	NCP	IGR	-	ID=gene-E	7	6.382857143	2790.1	0.193236914	Q(+42.01)	78	429.2637	2	856.5131	Acetylati	I	Superscaf	41659366	BTP20220	0	0	0	0	0	Tissue specific
KPDRTPP	Superscaf-	Superscaf	41872753	41872773	-32	32	38	-	-	-	-	-	-	NCP	IGR	-	ID=gene-E	7	6.985714286	2938.4	0.189105518	KPDRTPP	70	405.726	2	809.4395	.	R	Superscaf	41872887	0	0	0	BTP20220	0	0	Tissue specific
KVVSSGK	Superscaf+	Superscaf	41876023	41876043	11	11	17	-	-	-	-	-	-	NCP	IGR	-	ID=gene-E	7	5.258571429	2000.9	0.189042193	KVVSSGK	71	352.7209	2	703.4228	.	K	Superscaf	41875993	0	0	0	BTP20220	0	0	Tissue specific
NLHQVTK	Superscaf+	Superscaf	41901982	41902002	1	1	7	-	-	-	-	-	-	NCP	IGR	-	ID=gene-E	7	5.842857143	3046.4	0.188539479	NLHQVTK	97	420.2381	2	838.4661	.	N	Superscaf	41901982	0	0	BTP20220	0	0	0	Tissue specific
LCVSP	Superscaf+	Superscaf	41923867	41923881	14	14	18	-	-	-	-	-	-	NCP	IGR	-	ID=gene-E	5	4.596	1659.9	0.188115778	LC(+57.02	56	575.2798	1	574.2785	Carbamido	T	Superscaf	41923828	0	0	0	BTP20220	0	0	Tissue specific
TVYLLP	Superscaf-	Superscaf	42038866	42038883	-42	42	47	-	-	-	-	-	-	NCP	IGR	-	ID=gene-E	6	5.075	1901.2	0.185888686	TVYLLP	75	705.4157	1	704.4108	.	H	Superscaf	42038910	0	0	0	0	BTP20220	0	Tissue specific
DVDLR	Superscaf+	Superscaf	42123403	42123417	12	12	16	-	-	gene	-	-	mRNA	NCP	Intron	gene|mRN	ID=gene-E	5	4.536	1825.2	0.184251628	DVDLR	74	617.3223	1	616.318	.	E	Superscaf	42123370	0	BTP20220	0	0	0	0	Tissue specific
NGPSFP	Superscaf-	Superscaf	42123694	42123711	-20	20	25	-	-	gene	-	-	mRNA	NCP	Intron	gene|mRN	ID=gene-E	6	5.856666667	2339.6	0.184245935	NGPSFP	92	618.2859	1	617.2809	.	N	Superscaf	42123726	0	0	BTP20220	0	0	0	Tissue specific
QTVLLFP	Superscaf+	Superscaf	42162115	42162135	25	25	31	-	-	gene	-	-	mRNA	NCP	Intron	gene|mRN	ID=gene-E	7	5.131428571	2439.8	0.183501828	QTVLLFP	50	817.4893	1	816.4745	.	N	Superscaf	42162043	0	0	0	0	BTP20220	0	Tissue specific
APNSPVFL	Superscaf+	Superscaf	42183709	42183732	58	58	65	-	-	gene	-	-	mRNA	NCP	Intron	gene|mRN	ID=gene-E	8	5.14625	2707	0.183083588	APNSPVFL	60	844.4541	1	843.449	.	Q	Superscaf	42183538	0	0	0	0	BTP20220	BTP20220	Tissue specific
SVVVL	Superscaf-	Superscaf	42210400	42210414	-8	8	12	-	-	gene	-	-	mRNA	NCP	Intron	gene|mRN	ID=gene-E	5	2.332	736.8	0.182566873	SVVVL	75	516.3368	1	515.3319	.	I	Superscaf	42210423	0	0	BTP20220	0	0	0	Tissue specific
EFLDF	Superscaf+	Superscaf	42232291	42232305	31	31	35	-	-	gene	-	-	mRNA	NCP	Intron	gene|mRN	ID=gene-E	5	4.626	1965.2	0.182142939	EFLDF	66	670.3058	1	669.301	.	V	Superscaf	42232201	BTP20220	0	0	0	0	0	Tissue specific
KERAALE	Superscaf+	Superscaf	42260176	42260196	13	13	19	-	-	-	-	-	-	NCP	IGR	-	ID=gene-E	7	6.422857143	2944.2	0.181602811	KERAALE	58	408.7319	2	815.4501	.	W	Superscaf	42260140	0	BTP20220	0	0	0	0	Tissue specific
ESGVVTEK	Superscaf-	Superscaf	42269248	42269271	-13	13	20	-	-	-	-	-	-	NCP	IGR	-	ID=gene-E	8	4.295	2397.6	0.181427067	E(+42.01)	54	445.7261	2	889.4393	Acetylati	S	Superscaf	42269310	0	0	0	BTP20220	0	0	Tissue specific
ATEGPAVRE	Superscaf-	Superscaf	42274135	42274161	-4	4	12	-	-	-	-	-	-	NCP	IGR	-	ID=gene-E	9	6.175555556	3377.9	0.181332369	A(+42.01)	76	499.7712	2	997.5305	Acetylati	Q	Superscaf	42274440	0	0	0	BTP20220	0	0	Mixed
ATEGPAVR	Superscaf-	Superscaf	42274135	42274158	-4	4	11	-	-	-	-	-	-	NCP	IGR	-	ID=gene-E	8	5.6025	2729.1	0.181332427	A(+42.01)	97	421.7206	2	841.4294	Acetylati	Q	Superscaf	42274440	0	0	0	0	BTP20220	0	Tissue enhanced
MFLGKVK	Superscaf-	Superscaf	42350500	42350520	-10	10	16	-	-	-	-	-	-	NCP	IGR	-	ID=gene-E	7	6.094285714	2346.4	0.179853626	MFLGKVK	67	411.7456	2	821.4833	.	L	Superscaf	42350595	0	0	0	BTP20220	0	0	Tissue specific
MKLVFK	Superscaf+	Superscaf	42428449	42428466	13	13	18	-	-	-	-	-	-	NCP	IGR	-	ID=gene-E	6	6.115	2049.1	0.178344149	M(+42.01)	81	404.251	2	806.4724	Acetylati	I	Superscaf	42428413	0	0	0	BTP20220	0	0	Tissue specific
EKVLWK	Superscaf+	Superscaf	42584635	42584652	7	7	12	-	-	gene	-	-	mRNA	NCP	Intron	gene|mRN	ID=gene-E	6	5.761666667	2242.3	0.175319502	EKVLWK	90	401.7433	2	801.4749	.	I	Superscaf	42584617	0	0	0	BTP20220	0	0	Tissue specific
LTRSLK	Superscaf+	Superscaf	42591034	42591051	2	2	7	-	-	gene	-	-	mRNA	NCP	Intron	gene|mRN	ID=gene-E	6	7.445	2604.9	0.175195581	LTRSLK	53	359.2367	2	716.4545	.	L	Superscaf	42591031	BTP20220	0	0	0	0	0	Tissue specific
NMKLHM	Superscaf+	Superscaf	42593641	42593658	23	23	28	-	-	-	-	-	-	NCP	IGR	-	ID=gene-E	6	6.703333333	2781.2	0.175145094	NMKLHM	94	387.1947	2	772.3724	.	I	Superscaf	42593575	0	BTP20220	0	0	0	0	Tissue specific
DLEVRGLK	Superscaf-	Superscaf	42624151	42624174	-25	25	32	-	-	gene	-	-	mRNA	NCP	Intron	gene|mRN	ID=gene-E	8	5.5775	2958.3	0.174554131	DLEVRGLK	69	465.2726	2	928.5341	.	F	Superscaf	42624300	BTP20220	0	0	0	0	0	Tissue specific
GVPVWF	Superscaf-	Superscaf	42660646	42660663	-24	24	29	-	-	gene	-	-	mRNA	NCP	Intron	gene|mRN	ID=gene-E	6	3.94	1548.7	0.173847497	GVPVWF	51	352.689	2	703.3693	.	F	Superscaf	42660705	0	0	0	BTP20220	0	0	Tissue specific
TLEHSLK	Superscaf+	Superscaf	42893638	42893658	48	48	54	-	-	-	-	-	-	NCP	IGR	-	ID=gene-E	7	6.388571429	3001.3	0.169335392	T(+42.01)	82	435.2427	2	868.4654	Acetylati	Q	Superscaf	42893497	0	0	0	0	BTP20220	BTP20220	Tissue specific
QLVVSTQ	Superscaf-	Superscaf	42924850	42924870	-18	18	24	-	-	-	-	-	-	NCP	IGR	-	ID=gene-E	7	4.212857143	2355.7	0.168730951	QLVVSTQ	50	387.7163	2	773.4283	.	L	Superscaf	42924882	0	0	BTP20220	0	0	0	Tissue specific
KDSTFQQ	Superscaf-	Superscaf	42947545	42947565	-14	14	20	-	-	-	-	-	-	NCP	IGR	-	ID=gene-E	7	5.957142857	3269.6	0.168291447	KDSTFQQ	93	853.4045	1	852.3977	.	G	Superscaf	42947922	0	BTP20220	BTP20220	BTP20220	0	0	Tissue specific
KGTLLPGK	Superscaf-	Superscaf	43041658	43041681	-4	4	11	-	-	gene	-	-	mRNA	NCP	Intron	gene|mRN	ID=gene-E	8	7.02625	2973.1	0.166468827	KGTLLPGK	78	407.2614	2	812.512	.	Q	Superscaf	43041684	0	BTP20220	0	BTP20220	0	0	Tissue specific
ELPFYQK	Superscaf+	Superscaf	43112443	43112463	6	6	12	-	-	-	-	-	-	NCP	IGR	-	ID=gene-E	7	6.004285714	3042.3	0.165098086	ELPFYQK	97	924.4811	1	923.4752	.	N	Superscaf	43112428	0	0	BTP20220	0	0	0	Tissue specific
LSVFHPPP	Superscaf+	Superscaf	43165327	43165350	11	11	18	-	-	gene	-	-	mRNA	NCP	Intron	gene|mRN	ID=gene-E	8	5.45375	2756.1	0.164073893	LSVFHPPP	64	447.2501	2	892.4807	.	V	Superscaf	43165297	0	0	0	BTP20220	0	0	Tissue specific
YNEALRV	Superscaf+	Superscaf	43180381	43180401	49	49	55	-	-	gene	-	-	mRNA	NCP	Intron	gene|mRN	ID=gene-E	7	5.292857143	2795.2	0.163782421	YNEALRV	65	432.7317	2	863.4501	.	K	Superscaf	43180237	0	0	BTP20220	0	0	0	Tissue specific
PAAALPPP	Superscaf+	Superscaf	43376374	43376397	159	159	166	-	-	gene	-	-	mRNA	NCP	Intron	gene|mRN	ID=gene-E	8	6.155	2636.9	0.159986826	PAAALPPP	91	367.2206	2	732.417	.	N	Superscaf	43375900	0	0	0	BTP20220	0	0	Tissue specific
PTLGNPK	Superscaf-	Superscaf	43407964	43407984	-13	13	19	-	-	gene	-	-	mRNA	NCP	Intron	gene|mRN	ID=gene-E	7	6.604285714	2788.1	0.159375122	PTLGNPK	56	363.7095	2	725.4072	.	I	Superscaf	43408068	0	0	0	BTP20220	0	0	Tissue specific
LDEKDEL	Superscaf+	Superscaf	43416106	43416126	15	15	21	-	-	gene	-	-	mRNA	NCP	Intron	gene|mRN	ID=gene-E	7	4.868571429	2849.2	0.159217447	LDEKDEL	94	861.4172	1	860.4127	.	F	Superscaf	43416064	BTP20220	0	0	0	0	0	Tissue specific
YLGDVKK	Superscaf-	Superscaf	43429693	43429713	-4	4	10	-	-	gene	-	-	mRNA	NCP	Intron	gene|mRN	ID=gene-E	7	5.722857143	2458.5	0.158954326	YLGDVKK	75	411.734	2	821.4647	.	S	Superscaf	43429791	0	0	BTP20220	0	0	0	Tissue specific
QGYVLY	Superscaf+	Superscaf	43541170	43541187	5	5	10	-	-	gene	-	-	mRNA	NCP	Intron	gene|mRN	ID=gene-E	6	4.82	2180.4	0.156795556	QGYVLY	71	742.3738	1	741.3697	.	Q	Superscaf	43541158	0	0	0	0	BTP20220	BTP20220	Tissue specific
LEDGK	Superscaf+	Superscaf	43573723	43573737	31	31	35	-	-	gene	-	-	mRNA	NCP	Intron	gene|mRN	ID=gene-E	5	5.576	1956.1	0.156165204	LEDGK	61	561.2979	1	560.2806	.	P	Superscaf	43573633	0	0	BTP20220	0	0	0	Tissue specific
SALQKLK	Superscaf+	Superscaf	43601818	43601838	25	25	31	-	-	gene	-	-	mRNA	NCP	Intron	gene|mRN	ID=gene-E	7	6.97	2949	0.155621009	SALQKLK	96	394.2537	2	786.4963	.	K	Superscaf	43601746	0	0	0	0	BTP20220	0	Tissue specific
KHDQPF	Superscaf-	Superscaf	43679803	43679820	-26	26	31	-	-	gene	-	-	mRNA	NCP	Intron	gene|mRN	ID=gene-E	6	6.288333333	2835.1	0.154110835	KHDQPF	50	771.386	1	770.3711	.	F	Superscaf	43679916	0	0	0	BTP20220	0	0	Tissue specific
VPSLDLP	Superscaf-	Superscaf	43701337	43701357	-1	1	7	-	-	gene	-	-	mRNA	NCP	Intron	gene|mRN	ID=gene-E	7	4.744285714	2176.5	0.153693756	VPSLDLP	62	740.4154	1	739.4116	.	V	Superscaf	43701423	0	0	0	BTP20220	0	0	Tissue specific
ESPLYQ	Superscaf+	Superscaf	43756591	43756608	1	1	6	-	-	gene	-	-	mRNA	NCP	Intron	gene|mRN	ID=gene-E	6	5.415	2569.9	0.152623784	ESPLYQ	85	736.3513	1	735.3439	.	E	Superscaf	43756591	0	0	BTP20220	0	0	0	Tissue specific
VPDPAAP	Superscaf+	Superscaf	43852633	43852653	34	34	40	-	-	-	-	-	-	NCP	IGR	-	ID=gene-E	7	4.844285714	2046.3	0.150763807	V(+42.01)	52	354.6798	2	707.3489	Acetylati	T	Superscaf	43852534	0	0	BTP20220	0	0	0	Tissue specific
QAFDSFY	Superscaf-	Superscaf	43879963	43879983	-7	7	13	-	-	-	-	-	-	NCP	IGR	-	ID=gene-E	7	5.277142857	2951.2	0.150234543	Q(+42.01)	73	460.1941	2	918.3759	Acetylati	S	Superscaf	43879998	BTP20220	0	0	0	0	0	Tissue specific
NCHCCH	Superscaf-	Superscaf	43918570	43918587	-10	10	15	-	-	-	-	-	-	NCP	IGR	-	ID=gene-E	6	5.941666667	3593.2	0.149486951	N(+42.01)	59	929.2734	1	928.2739	Acetylati	L	Superscaf	43918596	0	0	0	0	BTP20220	0	Tissue specific
VELPF	Superscaf+	Superscaf	44036488	44036502	27	27	31	-	-	-	-	-	-	NCP	IGR	-	ID=gene-E	5	4.196	1503.7	0.147203448	VELPF	73	604.3308	1	603.3268	.	I	Superscaf	44036410	0	0	0	0	BTP20220	0	Tissue specific
AHVPEQ	Superscaf+	Superscaf	44058091	44058108	11	11	16	-	-	-	-	-	-	NCP	IGR	-	ID=gene-E	6	4.796666667	2304.6	0.146785033	AHVPEQ	95	680.3229	1	679.3289	.	T	Superscaf	44058061	0	0	0	0	BTP20220	0	Tissue specific
LVGPRLL	Superscaf-	Superscaf	44149684	44149704	-21	21	27	-	-	-	-	-	-	NCP	IGR	-	ID=gene-E	7	5.852857143	2343.7	0.145011214	LVGPRLL	50	384.2596	2	766.5065	.	F	Superscaf	44149848	0	0	0	0	BTP20220	BTP20220	Tissue specific
SVGLSPF	Superscaf-	Superscaf	44159677	44159697	-15	15	21	-	-	-	-	-	-	NCP	IGR	-	ID=gene-E	7	5.012857143	2142.3	0.144817693	SVGLSPF	72	706.3741	1	705.3697	.	S	Superscaf	44159742	0	BTP20220	0	0	BTP20220	BTP20220	Mixed
TRSSWTR	Superscaf+	Superscaf	44224645	44224665	68	68	74	-	-	-	-	-	-	NCP	IGR	-	ID=gene-E	7	7.404285714	3386	0.143559544	TRSSWTR	60	447.2341	2	892.4515	.	I	Superscaf	44224444	0	0	0	BTP20220	0	0	Tissue specific
LDVQPK	Superscaf-	Superscaf	44254048	44254065	-8	8	13	-	-	-	-	-	-	NCP	IGR	-	ID=gene-E	6	5.106666667	2181.4	0.142990193	LDVQPK	94	350.2031	2	698.3962	.	R	Superscaf	44254101	0	0	0	BTP20220	0	0	Tissue specific
APHQPVR	Superscaf-	Superscaf	44274760	44274780	-17	17	23	-	-	-	-	-	-	NCP	IGR	-	ID=gene-E	7	6.088571429	2865.3	0.142589033	APHQPVR	54	402.7282	2	803.4402	.	M	Superscaf	44274849	0	0	0	BTP20220	0	0	Tissue specific
RSLRSY	Superscaf+	Superscaf	44368699	44368716	1	1	6	-	-	-	-	-	-	NCP	IGR	-	ID=gene-E	6	7.42	2877.3	0.140769899	R(+42.01)	74	412.2227	2	822.4348	Acetylati	R	Superscaf	44368699	0	0	0	BTP20220	0	0	Tissue specific
KGDQKK	Superscaf+	Superscaf	44407399	44407416	5	5	10	-	-	gene	-	-	mRNA	NCP	Intron	gene|mRN	ID=gene-E	6	7.301666667	2724.7	0.140020448	KGDQKK	83	352.2075	2	702.4024	.	T	Superscaf	44407387	0	0	0	0	BTP20220	BTP20220	Tissue specific
DNSGGL	Superscaf-	Superscaf	44415364	44415381	-29	29	34	-	-	gene	-	-	mRNA	NCP	Intron	gene|mRN	ID=gene-E	6	5.33	2339.6	0.1398662	DNSGGL	54	562.2478	1	561.2394	.	R	Superscaf	44415501	0	0	0	BTP20220	0	0	Tissue specific
QALLLKAL	Superscaf+	Superscaf	44455267	44455290	37	37	44	-	-	gene	-	-	mRNA	NCP	Intron	gene|mRN	ID=gene-E	8	6.41875	3115.3	0.139093336	QALLLKAL	68	435.2937	2	868.5745	.	W	Superscaf	44455159	0	0	BTP20220	0	0	0	Tissue specific
FFKKP	Superscaf+	Superscaf	44522113	44522127	2	2	6	-	-	-	-	-	-	NCP	IGR	-	ID=gene-E	5	7.348	2049	0.137798992	F(+42.01)	51	354.7039	2	707.4006	Acetylati	F	Superscaf	44522110	0	0	0	BTP20220	0	0	Tissue specific
MGNPLLR	Superscaf-	Superscaf	44525398	44525418	-10	10	16	-	-	-	-	-	-	NCP	IGR	-	ID=gene-E	7	6.592857143	2958.5	0.13773526	MGNPLLR	60	400.7202	2	799.4374	.	P	Superscaf	44525454	0	0	BTP20220	0	0	0	Tissue specific
NGEGPF	Superscaf+	Superscaf	44589760	44589777	8	8	13	-	-	-	-	-	-	NCP	IGR	-	ID=gene-E	6	5.391666667	2341.6	0.136488904	N(+42.01)	72	662.2856	1	661.2708	Acetylati	S	Superscaf	44589739	0	0	0	BTP20220	0	0	Tissue specific
VGAPP	Superscaf-	Superscaf	44717668	44717682	-6	6	10	-	-	gene	-	-	mRNA	NCP	Intron	gene|mRN	ID=gene-E	5	4.918	1283.4	0.134011938	V(+42.01)	90	482.2676	1	481.2536	Acetylati	A	Superscaf	44717964	0	0	0	BTP20220	0	0	Tissue specific
KASPVFPR	Superscaf+	Superscaf	44737057	44737080	50	50	57	-	-	-	-	-	-	NCP	IGR	-	ID=gene-E	8	6.285	2874.1	0.133636282	KASPVFPR	92	451.2597	2	900.5181	.	L	Superscaf	44736910	0	0	0	0	BTP20220	0	Tissue specific
GLPPKNF	Superscaf+	Superscaf	44754304	44754324	7	7	13	-	-	-	-	-	-	NCP	IGR	-	ID=gene-E	7	6.454285714	2778	0.133302341	G(+42.01)	63	407.7339	2	813.4385	Acetylati	N	Superscaf	44754286	0	0	0	0	BTP20220	0	Tissue specific
SLLNKPK	Superscaf-	Superscaf	44809222	44809242	-7	7	13	-	-	gene	-	-	mRNA	NCP	Intron	gene|mRN	ID=gene-E	7	6.975714286	2961.1	0.132238816	S(+42.01)	99	421.2595	2	840.5069	Acetylati	T	Superscaf	44809269	0	0	0	0	BTP20220	0	Tissue specific
SLLVQTK	Superscaf+	Superscaf	44837926	44837946	104	104	110	-	-	gene	-	-	mRNA	NCP	Intron	gene|mRN	ID=gene-E	7	5.651428571	2566.8	0.131682944	SLLVQTK	96	394.7462	2	787.4803	.	G	Superscaf	44837617	0	0	BTP20220	0	0	0	Tissue specific

ALETNTHVK	Superscaf	-	Superscaf	44859169	44859201	-21	21	31	-	-	gene	-	-	mRNA	NCP	Intron	gene|mRNA	ID=gene-E	11	5.650909091	4416.9	0.131271327	ALETNTHVK	86	661.8373	2	1321.667	.	C	Superscaf	44859252	0	0	0	0	BTP20220	0	Tissue specific
KSCFRAC	Superscaf	+	Superscaf	44906209	44906229	7	7	13	-	-	-	-	-	-	NCP	IGR	-	ID=gene-E	7	6.817142857	3370.7	0.130360598	KSC(+57.	84	464.7085	2	927.4055	Carbamidc	V	Superscaf	44906191	0	0	0	0	BTP20220	0	Tissue specific
VPPGLFY	Superscaf	+	Superscaf	44918824	44918844	16	16	22	-	-	-	-	-	-	NCP	IGR	-	ID=gene-E	7	5.098571429	2172.4	0.1301163	VPPGLFY	58	792.4135	1	791.4218	.	R	Superscaf	44918779	0	BTP20220	0	0	0	0	Tissue specific
KDHPHK	Superscaf	+	Superscaf	44969608	44969625	11	11	16	-	-	-	-	-	-	NCP	IGR	-	ID=gene-E	6	7.321666667	2925.1	0.129132891	KDHPHK	57	381.1999	2	760.398	.	E	Superscaf	44969578	0	0	0	0	BTP20220	BTP20220	Tissue specific
KELDFKG	Superscaf	-	Superscaf	45042469	45042489	-3	3	9	-	-	-	-	-	-	NCP	IGR	-	ID=gene-E	7	6.157142857	2811.9	0.127721831	KELDFKG	55	418.7282	2	835.4439	.	D	Superscaf	45042513	BTP20220	0	0	0	0	0	Tissue specific
ELPEEQ	Superscaf	+	Superscaf	45071056	45071073	8	8	13	-	-	-	-	-	-	NCP	IGR	-	ID=gene-E	6	4.598333333	2578	0.127168283	ELPEEQ	60	744.3525	1	743.3337	.	P	Superscaf	45071035	BTP20220	0	0	0	0	0	Tissue specific
RGYLL	Superscaf	+	Superscaf	45082120	45082134	59	59	63	-	exon	gene	-	five_prim	mRNA	NCP	5UTR	exon|gene	ID=exon-E	5	6.87	2112.4	0.126954079	RGYLL	68	621.3699	1	620.3646	.	V	Superscaf	45081946	BTP20220	0	0	0	0	0	Tissue specific
TSKKPPPP	Superscaf	+	Superscaf	45103108	45103131	23	23	30	-	-	-	-	-	-	NCP	IGR	-	ID=gene-E	8	7.11125	3067.3	0.126547458	T(+42.01)	63	447.2665	2	892.5018	Acetylati	I	Superscaf	45103042	0	0	BTP20220	0	0	BTP20220	Tissue specific
QQELPLR	Superscaf	-	Superscaf	45127120	45127140	-27	27	33	-	-	-	-	-	-	NCP	IGR	-	ID=gene-E	7	6.22	3339.9	0.126082507	QQELPLR	76	442.2569	2	882.4923	.	L	Superscaf	45127170	0	0	0	0	0	BTP20220	Tissue specific
PPHAVGK	Superscaf	-	Superscaf	45138805	45138825	-68	68	74	-	-	-	-	-	-	NCP	IGR	-	ID=gene-E	7	5.988571429	2371.5	0.125856219	PPHAVGK	75	353.2023	2	704.3969	.	Y	Superscaf	45138867	0	BTP20220	0	0	BTP20220	BTP20220	Tissue specific
VVPKWQ	Superscaf	-	Superscaf	45146350	45146367	-66	66	71	-	-	-	-	-	-	NCP	IGR	-	ID=gene-E	6	4.596666667	1943.1	0.125710163	V(+42.01)	55	798.4652	1	797.4435	Acetylati	L	Superscaf	45146379	0	0	0	BTP20220	0	0	Tissue specific
DLGTRKP	Superscaf	+	Superscaf	45163462	45163482	64	64	70	-	-	gene	-	-	mRNA	NCP	Intron	gene|mRNA	ID=gene-E	7	6.892857143	2914.3	0.12537872	DLGTRKP	76	393.726	2	785.4395	.	E	Superscaf	45163273	0	0	0	0	BTP20220	0	Tissue specific
NVGVLPF	Superscaf	-	Superscaf	45209833	45209853	-67	67	73	-	-	-	-	-	-	NCP	IGR	-	ID=gene-E	7	4.162857143	1972.2	0.124480714	NVGVLPF	67	745.4214	1	744.417	.	G	Superscaf	45209880	0	0	BTP20220	0	0	0	Tissue specific
KGVFK	Superscaf	+	Superscaf	45234472	45234486	12	12	16	-	-	-	-	-	-	NCP	IGR	-	ID=gene-E	5	6.186	1621.5	0.12400368	KGVFK	52	578.3728	1	577.3588	.	K	Superscaf	45234439	BTP20220	0	0	0	0	0	Tissue specific
PGPRPPPP	Superscaf	+	Superscaf	45237265	45237288	78	78	85	-	-	-	-	-	-	NCP	IGR	-	ID=gene-E	8	6.81625	2970.5	0.123949417	PGPRPPPP	90	407.7301	2	813.4496	.	V	Superscaf	45237034	0	0	0	BTP20220	0	0	Mixed
KLKKKPPG	Superscaf	-	Superscaf	45275329	45275352	-2	2	9	-	-	-	-	-	-	NCP	IGR	-	ID=gene-E	8	7.93875	3199.1	0.123212282	KLKKKPPG	70	448.3064	2	894.6014	.	I	Superscaf	45275367	0	0	0	0	0	BTP20220	Tissue specific
LLLKTK	Superscaf	-	Superscaf	45326035	45326052	-49	49	54	-	-	gene	-	-	mRNA	NCP	Intron	gene|mRNA	ID=gene-E	6	7.325	2394.5	0.122230442	LLLKTK	52	358.258	2	714.5003	.	S	Superscaf	45326061	BTP20220	0	0	0	0	0	Tissue specific
TVAKL	Superscaf	-	Superscaf	45334780	45334794	-42	42	46	-	-	gene	-	-	mRNA	NCP	Intron	gene|mRNA	ID=gene-E	5	5.654	1530.6	0.122061148	T(+42.01)	51	573.3586	1	572.3533	Acetylati	L	Superscaf	45334821	0	0	0	0	BTP20220	0	Tissue specific
KTGSLLST	Superscaf	+	Superscaf	45379768	45379791	4	4	11	-	-	gene	-	-	mRNA	NCP	Intron	gene|mRNA	ID=gene-E	8	6.51125	3034.3	0.12118975	KTGSLLST	56	806.4625	1	805.4545	.	L	Superscaf	45379759	0	0	0	0	BTP20220	0	Tissue specific
TQYVYK	Superscaf	-	Superscaf	45426706	45426723	-23	23	28	-	-	gene	-	-	mRNA	NCP	Intron	gene|mRNA	ID=gene-E	6	5.54	2395.6	0.120280881	TQYVYK	80	801.4104	1	800.4069	.	V	Superscaf	45426756	0	0	0	0	BTP20220	0	Tissue specific
KANMPT	Superscaf	+	Superscaf	45452074	45452091	61	61	66	-	-	gene	-	-	mRNA	NCP	Intron	gene|mRNA	ID=gene-E	6	6.625	2482.8	0.119789612	KANMPT	56	661.3286	1	660.3265	.	L	Superscaf	45451894	0	0	0	0	0	BTP20220	Tissue specific
MLSYK	Superscaf	+	Superscaf	45560779	45560793	8	8	12	-	-	gene	-	-	mRNA	NCP	Intron	gene|mRNA	ID=gene-E	5	6.562	2036.2	0.117684525	MLSYK	52	641.3314	1	640.3254	.	L	Superscaf	45560758	0	BTP20220	0	0	0	0	Tissue specific
YHNLVE	Superscaf	-	Superscaf	45586162	45586179	-6	6	11	-	-	gene	-	-	mRNA	NCP	Intron	gene|mRNA	ID=gene-E	6	4.643333333	2454.8	0.117192908	YHNLVE	67	387.6913	2	773.3708	.	I	Superscaf	45586422	0	0	BTP20220	0	0	0	Tissue specific
STELLLR	Superscaf	+	Superscaf	45634054	45634074	2	2	8	-	-	gene	-	-	mRNA	NCP	Intron	gene|mRNA	ID=gene-E	7	6.304285714	2915.4	0.116265389	STELLLR	99	416.2494	2	830.4861	.	T	Superscaf	45634051	0	0	BTP20220	0	0	0	Mixed
EHNVTTF	Superscaf	+	Superscaf	45660730	45660750	7	7	13	-	-	gene	-	-	mRNA	NCP	Intron	gene|mRNA	ID=gene-E	7	4.965714286	2824.3	0.11574879	EHNVTTF	51	424.1989	2	846.3871	.	Y	Superscaf	45660712	0	0	0	0	0	BTP20220	Tissue specific
KAFVGP	Superscaf	-	Superscaf	45662602	45662619	-14	14	19	-	-	gene	-	-	mRNA	NCP	Intron	gene|mRNA	ID=gene-E	6	5.585	1801.8	0.115712595	KAFVGP	67	618.3583	1	617.3536	.	W	Superscaf	45662742	0	BTP20220	0	0	0	0	Tissue specific
KYVGSP	Superscaf	+	Superscaf	45709234	45709251	16	16	21	-	-	gene	-	-	mRNA	NCP	Intron	gene|mRNA	ID=gene-E	6	5.558333333	1946	0.114809535	KYVGSP	58	650.3584	1	649.3435	.	S	Superscaf	45709189	0	0	BTP20220	0	0	0	Tissue specific
PALPETK	Superscaf	+	Superscaf	45736516	45736536	10	10	16	-	exon	gene	three_pri	-	mRNA	NCP	3UTR	exon|gene	ID=exon-E	7	6.298571429	2630.9	0.114281143	PALPETK	82	378.2169	2	754.4225	.	M	Superscaf	45736489	0	0	BTP20220	0	0	0	Tissue specific
GPRLLEY	Superscaf	-	Superscaf	45747562	45747582	-11	11	17	-	-	gene	-	-	mRNA	NCP	Intron	gene|mRNA	ID=gene-E	7	6.267142857	2875.3	0.11406723	GPRLLEY	60	424.2311	2	846.4599	.	A	Superscaf	45747645	0	0	BTP20220	0	0	0	Tissue specific
KPTMHR	Superscaf	+	Superscaf	45775582	45775599	9	9	14	-	-	gene	-	-	mRNA	NCP	Intron	gene|mRNA	ID=gene-E	6	7.778333333	2843.3	0.113524662	KPTM(+15.	51	393.2154	2	784.4014	Oxidation	W	Superscaf	45775558	0	0	BTP20220	0	0	0	Tissue specific
SVDFVLY	Superscaf	-	Superscaf	45804022	45804042	-26	26	32	-	-	-	-	-	-	NCP	IGR	-	ID=gene-E	7	3.681428571	1995.2	0.112973844	SVDFVLY	73	842.4299	1	841.4221	.	M	Superscaf	45804282	0	0	0	BTP20220	0	0	Tissue specific
SPGSPP	Superscaf	-	Superscaf	45815863	45815880	-2	2	7	-	-	gene	-	-	mRNA	NCP	Intron	gene|mRNA	ID=gene-E	6	6.038333333	2076.3	0.112744593	SPGSPP	70	541.267	1	540.2543	.	L	Superscaf	45815964	0	0	BTP20220	0	0	0	Tissue specific
FGTPGLG	Superscaf	+	Superscaf	45917980	45918000	35	35	41	-	-	gene	-	-	mRNA	NCP	Intron	gene|mRNA	ID=gene-E	7	6.028571429	2367.6	0.11076697	FGTPGLG	69	648.3323	1	647.3278	.	G	Superscaf	45917878	0	0	0	BTP20220	0	0	Tissue specific
KPTLFN	Superscaf	-	Superscaf	45959839	45959856	-5	5	10	-	-	gene	-	-	mRNA	NCP	Intron	gene|mRNA	ID=gene-E	6	6.573333333	2540.8	0.109956401	KPTLFN	69	719.4039	1	718.4014	.	R	Superscaf	45959946	0	0	BTP20220	0	0	0	Tissue specific
ALDRLGF	Superscaf	-	Superscaf	46022245	46022265	-25	25	31	-	-	-	-	-	-	NCP	IGR	-	ID=gene-E	7	6.165714286	2763.1	0.108747808	ALDRLGF	58	396.2187	2	790.4337	.	Q	Superscaf	46022304	0	BTP20220	0	0	0	0	Tissue specific
AEKLLR	Superscaf	+	Superscaf	46086058	46086075	4	4	9	-	-	-	-	-	-	NCP	IGR	-	ID=gene-E	6	6.95	2560.8	0.107512084	A(+42.01)	76	386.238	2	770.465	Acetylati	G	Superscaf	46086049	0	0	BTP20220	0	0	0	Tissue specific
GKVLQY	Superscaf	-	Superscaf	46112056	46112073	-61	61	66	-	-	-	-	-	-	NCP	IGR	-	ID=gene-E	6	5.5	2189.3	0.107008616	GKVLQY	63	707.4061	1	706.4014	.	S	Superscaf	46112076	0	0	BTP20220	0	0	0	Tissue specific
PSPVVPP	Superscaf	+	Superscaf	46145392	46145412	25	25	31	-	-	gene	-	-	mRNA	NCP	Intron	gene|mRNA	ID=gene-E	7	4.411428571	1733	0.106362983	PSPVVPP	62	692.4056	1	691.3904	.	L	Superscaf	46145320	0	BTP20220	0	0	0	0	Tissue specific
LSALAAF	Superscaf	-	Superscaf	46159084	46159104	-40	40	46	-	-	gene	-	-	mRNA	NCP	Intron	gene|mRNA	ID=gene-E	7	5.882857143	2411.5	0.106097829	LSALAAF	81	692.3945	1	691.3904	.	F	Superscaf	46159167	0	0	0	BTP20220	0	BTP20220	Tissue specific
SLCSSPLL	Superscaf	-	Superscaf	46164958	46164984	-5	5	13	-	-	gene	-	-	mRNA	NCP	Intron	gene|mRNA	ID=gene-E	9	5.87	3531	0.105983958	SLC(+57.	54	977.4884	1	976.4899	Carbamidc	S	Superscaf	46164987	0	0	BTP20220	0	0	0	Tissue specific
RLSMLPH	Superscaf	-	Superscaf	46221355	46221375	-1	1	7	-	-	gene	-	-	mRNA	NCP	Intron	gene|mRNA	ID=gene-E	7	6.862857143	3067.6	0.104891909	RLSMLPH	50	427.2463	2	852.464	.	R	Superscaf	46221438	0	0	BTP20220	BTP20220	0	0	Tissue specific
KPATTVP	Superscaf	+	Superscaf	46381438	46381458	14	14	20	-	-	gene	-	-	mRNA	NCP	Intron	gene|mRNA	ID=gene-E	7	5.917142857	2249.5	0.101791794	KPATTVP	57	357.211	2	712.4119	.	D	Superscaf	46381399	0	BTP20220	0	0	BTP20220	BTP20220	Tissue specific
MEAEPK	Superscaf	-	Superscaf	46414474	46414491	-7	7	12	-	-	gene	-	-	mRNA	NCP	Intron	gene|mRNA	ID=gene-E	6	5.708333333	2339.6	0.101152087	M(+42.01)	54	746.3521	1	745.3316	Acetylati	I	Superscaf	46414491	0	0	0	BTP20220	0	0	Tissue specific
KVFVVF	Superscaf	+	Superscaf	46419103	46419120	15	15	20	-	-	gene	-	-	mRNA	NCP	Intron	gene|mRNA	ID=gene-E	6	3.45	1243.2	0.101062443	K(+42.01)	50	390.7318	2	779.4581	Acetylati	V	Superscaf	46419061	0	BTP20220	0	0	0	0	Tissue specific
GVDYPL	Superscaf	-	Superscaf	46449193	46449210	-4	4	9	-	-	-	-	-	-	NCP	IGR	-	ID=gene-E	6	4.48	1859.1	0.10047973	G(+42.01)	57	705.3429	1	704.3381	Acetylati	G	Superscaf	46449273	0	BTP20220	0	0	BTP20220	0	Tissue specific
LATALQK	Superscaf	-	Superscaf	46461490	46461510	-68	68	74	-	-	-	-	-	-	NCP	IGR	-	ID=gene-E	7	6.56	2806	0.100241533	LATALQK	98	372.7328	2	743.4541	.	V	Superscaf	46461609	0	BTP20220	0	0	BTP20220	BTP20220	Tissue specific
LLLAAK	Superscaf	+	Superscaf	46474027	46474044	51	51	56	-	-	-	-	-	-	NCP	IGR	-	ID=gene-E	6	6.62	2151.2	0.099998803	LLLAAK	92	628.4368	1	627.4319	.	V	Superscaf	46473877	0	0	0	0	BTP20220	0	Tissue specific
CCKDYAK	Superscaf	-	Superscaf	46489972	46489992	-4	4	10	-	-	-	-	-	-	NCP	IGR	-	ID=gene-E	7	6.31	3290.5	0.099689959	C(+57.02)	96	493.704	2	985.3997	Carbamidc	G	Superscaf	46490034	0	0	BTP20220	0	0	0	Tissue specific
LMLLLN	Superscaf	-	Superscaf	46538551	46538568	-17	17	22	-	-	-	-	-	-	NCP	IGR	-	ID=gene-E	6	5.846666667	2381.8	0.098749252	LMLLLN	58	716.4352	1	715.4302	.	R	Superscaf	46538625	BTP20220	0	0	0	0	0	Tissue specific
VLPVEP	Superscaf	-	Superscaf	46541710	46541727	-2	2	7	-	-	-	-	-	-	NCP	IGR	-	ID=gene-E	6	3.633333333	1453.7	0.098688076	VLPVEP	98	653.3859	1	652.3796	.	N	Superscaf	46541745	BTP20220	0	0	0	0	0	Tissue specific
RALVLLAK	Superscaf	+	Superscaf	46561114	46561137	35	35	42	-	-	-	-	-	-	NCP	IGR	-	ID=gene-E	8	6.31	2800	0.098312189	RALVLLAK	84	442.3067	2	882.6014	.	P	Superscaf	46561012	0	BTP20220	0	0	0	BTP20220	Tissue specific
ALVLLAK	Superscaf	+	Superscaf	46561117	46561137	36	36	42	-	-	-	-	-	-	NCP	IGR	-	ID=gene-E	7	5.674285714	2151.2	0.098312189	ALVLLAK	99	364.2557	2	726.5003	.	P	Superscaf	46561012	0	0	0	BTP20220	0	0	Tissue specific
NLAHRY	Superscaf	-	Superscaf	46576084	46576101	-2	2	7	-	-	-	-	-	-	NCP	IGR	-	ID=gene-E	6	6.903333333	2989.4	0.098022401	NLAHRY	98	387.2057	2	772.398	.	S	Superscaf	46576119	0	0	0	BTP20220	0	0	Tissue specific
CAVVED	Superscaf	-	Superscaf	46604230	46604247	-13	13	18	-	-	gene	-	-	mRNA	NCP	Intron	gene|mRNA	ID=gene-E	6	2.871666667	1734	0.097477334	C(+57.02)	53	692.2887	1	691.2847	Carbamidc	R	Superscaf	46604328	0	0	0	BTP20220	0	0	Tissue specific
LPPLRPKP	Superscaf	+	Superscaf	46638439	46638462	2	2	9	-	-	gene	-	-	mRNA	NCP	Intron	gene|mRNA	ID=gene-E	8	7.2075	3173.6	0.096814738	LPPLRPKP	75	459.2979	2	916.5858	.	L	Superscaf	46638436	0	0	0	0	BTP20220	0	Tissue specific
KAALTAL	Superscaf	+	Superscaf	46746538	46746558	2	2	8	-	-	gene	-	-	mRNA	NCP	Intron	gene|mRNA	ID=gene-E	7	6.612857143	2506.6	0.094721386	K(+42.01)	54	729.4473	1	728.4432	Acetylati	N	Superscaf	46746535	0	0	0	0	BTP20220	0	Tissue specific
FNGRMH	Superscaf	+	Superscaf	46775794	46775811	15	15	20	-	-	gene	-	-	mRNA	NCP	Intron	gene|mRNA	ID=gene-E	6	6.826666667	2921.4	0.094154882	FNGRMH	63	381.1859	2	760.3439	.	Y	Superscaf	46775752	BTP20220	0	0	0	0	0	Tissue specific
LLEAPVE	Superscaf	+	Superscaf	46844206	46844226	32	32	38	-	-	gene	-	-	mRNA	NCP	Intron	gene|mRNA	ID=gene-E	7	4.388571429	2206.5	0.092829979	LLEAPVE	91	770.4247	1	769.4221	.	E	Superscaf	46844113	0	BTP20220	0	0	0	0	Tissue specific
YGDNYLR	Superscaf	-	Superscaf	46973347	46973367	-8	8	14	-	-	-	-	-	-	NCP	IGR	-	ID=gene-E	7	6.058571429	3226.7	0.090329077	YGDNYLR	59	450.7172	2	899.4137	.	P	Superscaf	46973385	0	0	0	BTP20220	0	0	Tissue specific
LEAVDP	Superscaf	+	Superscaf	47064223	47064240	111	111	116	-	-	-	-	-	-	NCP	IGR	-	ID=gene-E	6	4.081666667	1839.1	0.08856926	LEAVDP	68	643.3277	1	642.3224	.	I	Superscaf	47063893	0	BTP20220	0	0	0	0	Tissue specific
KPHSPP	Superscaf	-	Superscaf	47120785	47120802	-13	13	18	-	-	-	-	-	-	NCP	IGR	-	ID=gene-E	6	6.985	2483.7	0.087473898	KPHSPP	53	662.3581	1	661.3547	.	I	Superscaf	47120844	0	0	0	BTP20220	0	0	Tissue specific
ELYKKLR	Superscaf	+	Superscaf	47126320	47126340	26	26	32	-	-	-	-	-	-	NCP	IGR	-	ID=gene-E	7	7.297142857	3177.4	0.087366651	ELYKKLR	65	475.2942	2	948.5756	.	V	Superscaf	47126245	BTP20220	0	0	0	0	0	Tissue specific
KARKLR	Superscaf	-	Superscaf	47243161	47243178	-30	30	35	-	-	gene	-	-	mRNA	NCP	Intron	gene|mRNA	ID=gene-E	6	8.833333333	2899.1	0.085104005	KARKLR	54	386.2621	2	770.5239	.	S	Superscaf	47243268	0	0	0	0	BTP20220	0	Tissue specific
VTAALMLL	Superscaf	+	Superscaf	47258698	47258721	32	32	39	-	-	gene	-	-	mRNA	NCP	Intron	gene|mRNA	ID=gene-E	8	5.2825	2451.8	0.084803004	VTAALMLL	59	416.2523	2	830.4935	.	G	Superscaf	47258605	0	0	0	0	BTP20220	0	Tissue specific
KDKVFPGK	Superscaf	-	Superscaf	47278939	47278962	-3	3	10	-	-	gene	-	-	mRNA	NCP	Intron	gene|mRNA	ID=gene-E	8	6.2425	2838.8	0.084411023	KDKVFPGK	55	459.7758	2	917.5334	.	I	Superscaf	47279019	0	0	0	0	BTP20220	0	Tissue specific
QNSFLVL	Superscaf	-	Superscaf	47283940	47283960	-10	10	16	-	-	gene	-	-	mRNA	NCP	Intron	gene|mRNA	ID=gene-E	7	4.882857143	2685	0.084314234	QNSFLVL	61	820.4545	1	819.449	.	I	Superscaf	47283987	0	BTP20220	0	0	BTP20220	0	Tissue specific
AAVTKTH	Superscaf	+	Superscaf	47285029	47285049	51	51	57	-	-	gene	-	-	mRNA	NCP	Intron	gene|mRNA	ID=gene-E	7	6.061428571	2505.7	0.084293145	A(+42.01)	52	385.2187	2	768.413	Acetylati	G	Superscaf	47284879	0	BTP20220	0	BTP20220	BTP20220	BTP20220	Tissue specific
KMLSDF	Superscaf	+	Superscaf	47296522	47296539	4	4	9	-	-	gene	-	-	mRNA	NCP	Intron	gene|mRNA	ID=gene-E	6	5.933333333	2375.6	0.084070633	KMLSDF	73	740.3563	1	739.3574	.	A	Superscaf	47296513	0	BTP20220	0	0	0	0	Mixed
EYVSQL	Superscaf	+	Superscaf	47323984	47324001	16	16	21	-	-	gene	-	-	mRNA	NCP	Intron	gene|mRNA	ID=gene-E	6	4.365	2232.5	0.083538813	EYVSQL	82	738.3654	1	737.3596	.	K	Superscaf	47323939	0	BTP20220	0	0	0	0	Tissue specific
VPPERK	Superscaf	+	Superscaf	47329498	47329515	16	16	21	-	-	gene	-	-	mRNA	NCP	Intron	gene|mRNA	ID=gene-E	6	6.053333333	2217.5	0.08343203	VPPERK	84	363.2249	2	724.4232	.	C	Superscaf	47329453	BTP20220	0	0	0	0	0	Tissue specific
KGLLGLL	Superscaf	+	Superscaf	47331406	47331426	58	58	64	-	-	gene	-	-	mRNA	NCP	Intron	gene|mRNA	ID=gene-E	7	6.514285714	2476.6	0.083395023	KGLLGLL	61	357.2429	2	712.4847	.	E	Superscaf	47331235	0	0	BTP20220	0	0	0	Tissue specific
LAGLDAR	Superscaf	+	Superscaf	47352340	47352360	2	2	8	-	-	gene	-	-	mRNA	NCP	Intron	gene|mRNA	ID=gene-E	7	6.242857143	2687	0.082989622	LAGLDAR	98	358.2072	2	714.4024	.	K	Superscaf	47352337	0	BTP20220	0	0	0	0	Tissue specific
YVVAGP	Superscaf	-	Superscaf	47425009	47425026	-23	23	28	-	-	gene	-	-	mRNA	NCP	Intron	gene|mRNA	ID=gene-E	6	3.991666667	1405.5	0.081582396	YVVAGP	72	605.3266	1	604.322	.	N	Superscaf	47425053	0	0	BTP20220	0	0	0	Tissue specific
PTVPPP	Superscaf	-	Superscaf	47470702	47470719	-8	8	13	-	-	gene	-	-	mRNA	NCP	Intron	gene|mRNA	ID=gene-E	6	5.288333333	1747.1	0.08069752	PTVPPP	96	607.3431	1	606.3377	.	G	Superscaf	47470719	0	0	BTP20220	0	0	0	Tissue specific
KVGQP	Superscaf	+	Superscaf	47488858	47488872	3	3	7	-	-	gene	-	-	mRNA	NCP	Intron	gene|mRNA	ID=gene-E	5	5.532	1713.8	0.080345975	KVGQP	53	528.3108	1	527.3068	.	H	Superscaf	47488852	0	0	BTP20220	0	0	0	Tissue specific
QGKEYPGP	Superscaf	+	Superscaf	47496628	47496651	2	2	9	-	-	gene	-	-	mRNA	NCP	Intron	gene|mRNA	ID=gene-E	8	6.10125	3233.5	0.080195329	QGKEYPGP	51	438.2173	2	874.4185	.	P	Superscaf	47496625	BTP20220	0	0	0	0	0	Tissue specific
EPPLLVD	Superscaf	-	Superscaf	47521732	47521752	-54	54	60	-	-	gene	-	-	mRNA	NCP	Intron	gene|mRNA	ID=gene-E	7	4.392857143	2218.6	0.079709231	EPPLLVD	93	782.4266	1	781.4221	.	M	Superscaf	47521824	BTP20220	0	0	0	0	0	Tissue specific
VLNAVVT	Superscaf	+	Superscaf	47531695	47531715	2	2	8	-	-	gene	-	-	mRNA	NCP	Intron	gene|mRNA	ID=gene-E	7	3.42	1658.9	0.079516291	VLNAVVT	58	715.4308	1	714.4276	.	G	Superscaf	47531692	0	0	BTP20220	0	0	0	Tissue specific
VPPHVKP	Superscaf	+	Superscaf	47570059	47570079	16	16	22	-	-	gene	-	-	mRNA	NCP	Intron	gene|mRNA	ID=gene-E	7	5.175714286	2100.3	0.078773346	VPPHVKP	83	387.2346	2	772.4595	.	S	Superscaf	47570014	0	0	BTP20220	0	0	0	Tissue specific
PEPLKTP	Superscaf	-	Superscaf	47607589	47607609	-3	3	9	-	-	gene	-	-	mRNA	NCP	Intron	gene|mRNA	ID=gene-E	7	6.338571429	2657	0.078046553	PEPLKTP	80	391.2209	2	780.4381	.	S	Superscaf	47607627	0	0	BTP20220	0	0	0	Tissue specific
RPPVFK	Superscaf	+	Superscaf	47826160	47826177	2	2	7	-	-	-	-	-	-	NCP	IGR	-	ID=gene-E	6	6.43	2179.4	0.073813836	RPPVFK	64	743.4434	1	742.449	.	K	Superscaf	47826157	0	0	BTP20220	0	0	0	Tissue specific
YTFHPP	Superscaf	+	Superscaf	47855515	47855532	4	4	9	-	-	-	-	-	-	NCP	IGR	-	ID=gene-E	6	6.31	2538.9	0.073245357	YTFHPP	75	381.1785	2	760.3544	.	K	Superscaf	47855506	0	BTP20220	0	0	0	0	Tissue specific
LGDKVEF	Superscaf	-	Superscaf	47872729	47872749	-28	28	34	-	-	-	-	-	-	NCP	IGR	-	ID=gene-E	7	4.765714286	2343.5	0.072911938	LGDKVEF	67	404.2143	2	806.4174	.	R	Superscaf	47872761	0	0	0	BTP20220	0	0	Tissue specific
ANPLPL	Superscaf	-	Superscaf	47887375	47887392	-68	68	73	-	-	-	-	-	-	NCP	IGR	-	ID=gene-E	6	5.998333333	2289.6	0.072628366	ANPLPL	75	624.3728	1	623.3643	.	C	Superscaf	47887395	0	0	0	0	BTP20220	0	Tissue specific
FNNTKK	Superscaf	+	Superscaf	47954755	47954772	14	14	19	-	-	-	-	-	-	NCP	IGR	-	ID=gene-E	6	7.051666667	2915.1	0.071323507	FNNTKK	72	376.2076	2	750.4024	.	I	Superscaf	47954716	0	0	BTP20220	0	0	0	Tissue specific
PVHKLRP	Superscaf	-	Superscaf	47997880	47997900	-8	8	14	-	-	gene	-	-	mRNA	NCP	Intron	gene|mRNA	ID=gene-E	7	6.667142857	2765.1	0.070488304	PVHKLRP	54	423.7675	2	845.5235	.	A	Superscaf	47997900	BTP20220	0	0	0	0	0	Tissue specific
ESAHVPR	Superscaf	+	Superscaf	48058051	48058071	30	30	36	-	-	gene	-	-	mRNA	NCP	Intron	gene|mRNA	ID=gene-E	7	5.652857143	2726.1	0.069323052	ESAHVPR	75	398.2026	2	794.4035	.	T	Superscaf	48057964	0	0	0	0	0	BTP20220	Tissue specific
NAVLTAH	Superscaf	+	Superscaf	48069295	48069315	9	9	15	-	-	gene	-	-	mRNA	NCP	Intron	gene|mRNA	ID=gene-E	7	5.364285714	2589.9	0.069105305	NAVLTAH	53	725.3813	1	724.3868	.	A	Superscaf	48069271	0	0	0	BTP20220	0	0	Mixed
TMFLTM	Superscaf	-	Superscaf	48102841	48102858	-5	5	10	-	-	gene	-	-	mRNA	NCP	Intron	gene|mRNA	ID=gene-E	6	6.003333333	2278.8	0.068455722	TMFLTM	90	743.3434	1	742.3394	.	L	Superscaf	48102897	0	BTP20220	0	0	0	0	Mixed
APGVHPK	Superscaf	+	Superscaf	48177745	48177765	13	13	19	-	-	gene	-	-	mRNA	NCP	Intron	gene|mRNA	ID=gene-E	7	5.988571429	2371.5	0.067005097	APGVHPK	70	353.2044	2	704.3969	.	W	Superscaf	48177709	BTP20220	0	0	0	0	0	Mixed
EDAAVTK	Superscaf	+	Superscaf	48178933	48178953	15	15	21	-	-	gene	-	-	mRNA	NCP	Intron	gene|mRNA	ID=gene-E	7	4.928571429	2325.5	0.066982091	EDAAVTK	80	733.37	1	732.3654	.	R	Superscaf	48178891	0	0	0	BTP20220	0	0	Tissue specific
NPLGKKP	Superscaf	-	Superscaf	48185989	48186009	-20	20	26	-	-	gene	-	-	mRNA	NCP	Intron	gene|mRNA	ID=gene-E	7	7.062857143	2859	0.066845447	NPLGKKP	88	377.2342	2	752.4544	.	T	Superscaf	48186036	0	0	BTP20220	0	0	0	Tissue specific
LSELDSL	Superscaf	-	Superscaf	48196408	48196428	-13	13	19	-	-	gene	-	-	mRNA	NCP	Intron	gene|mRNA	ID=gene-E	7	5.07	2664	0.066643676	LSELDSL	83	776.4008	1	775.3963	.	I	Superscaf	48196437	0	BTP20220	0	0	0	0	Tissue specific
KSWLVP	Superscaf	-	Superscaf	48205402	48205419	-15	15	20	-	-	-	-	-	-	NCP	IGR	-	ID=gene-E	6	5.598333333	2069.2	0.066469559	KSWLVP	73	365.2156	2	728.4221	.	M	Superscaf	48205515	0	BTP20220	0	0	0	0	Tissue specific
CCCYCDP	Superscaf	-	Superscaf	48236395	48236415	-12	12	18	-	-	gene	-	-	mRNA	NCP	Intron	gene|mRNA	ID=gene-E	7	5.001428571	3551.2	0.065869301	C(+57.02)	50	538.6517	2	1075.287	Carbamidc	A	Superscaf	48236421	0	0	BTP20220	0	0	0	Tissue specific
KDLLYH	Superscaf	+	Superscaf	48333388	48333405	7	7	12	-	-	-	-	-	-	NCP	IGR	-	ID=gene-E	6	6.32	2665.9	0.063991024	KDLLYH	65	394.7149	2	787.4228	.	C	Superscaf	48333370	0	0	0	BTP20220	0	0	Tissue specific
NQMVFK	Superscaf	-	Superscaf	48367762	48367779	-12	12	17	-	-	-	-	-	-	NCP	IGR	-	ID=gene-E	6	5.338333333	2434.7	0.063325348	NQM(+15.	63	782.3876	1	781.3793	Oxidation	S	Superscaf	48367812	0	0	0	0	0	BTP20220	Tissue specific
DYGLL	Superscaf	+	Superscaf	48396343	48396357	24	24	28	-	-	-	-	-	-	NCP	IGR	-	ID=gene-E	5	5.312	1875.1	0.062771916	DYGLL	98	580.2957	1	579.2904	.	Y	Superscaf	48396274	0	0	BTP20220	0	0	0	Tissue specific
LTGALLK	Superscaf	+	Superscaf	48436207	48436227	5	5	11	-	-	-	-	-	-	NCP	IGR	-	ID=gene-E	7	6.6	2534.7	0.061999806	L(+42.01)	52	757.4797	1	756.4745	Acetylati	Y	Superscaf	48436195	0	0	0	BTP20220	0	0	Tissue specific
GPAPKPDP	Superscaf	+	Superscaf	48471889	48471912	3	3	10	-	-	-	-	-	-	NCP	IGR	-	ID=gene-E	8	6.2375	2838.1	0.061308742	GPAPKPDP	51	778.4146	1	777.402	.	L	Superscaf	48471883	0	0	0	0	0	BTP20220	Tissue specific
AARDLKEK	Superscaf	-	Superscaf	48509758	48509781	-181	181	188	-	-	-	-	-	-	NCP	IGR	-	ID=gene-E	8	6.80625	3398.6	0.060575384	AARDLKEK	95	465.7704	2	929.5294	.	Q	Superscaf	48509916	BTP20220	0	0	0	0	0	Mixed
TPVKFPP	Superscaf	+	Superscaf	48512479	48512499	11	11	17	-	-	-	-	-	-	NCP	IGR	-	ID=gene-E	7	5.807142857	2265.5	0.060522748	TPVKFPP	61	393.2337	2	784.4483	.	G	Superscaf	48512449	0	BTP20220	0	0	0	0	Tissue specific
NMSLEKK	Superscaf	-	Superscaf	48536353	48536373	-20	20	26	-	-	-	-	-	-	NCP	IGR	-	ID=gene-E	7	6.502857143	3067.3	0.060060412	NM(+15.99	73	433.2238	2	864.4375	Oxidation	A	Superscaf	48536406	BTP20220	0	0	0	0	0	Tissue specific
KHGTSSK	Superscaf	+	Superscaf	48543307	48543327	4	4	10	-	-	-	-	-	-	NCP	IGR	-	ID=gene-E	7	7.275714286	3018.1	0.059925743	KHGTSSK	68	372.6983	2	743.3926	.	N	Superscaf	48543298	0	0	BTP20220	0	0	0	Tissue specific
PFVEDL	Superscaf	+	Superscaf	48650689	48650706	13	13	18	-	-	-	-	-	-	NCP	IGR	-	ID=gene-E	6	3.991666667	1915.2	0.057846276	P(+42.01)	66	761.3697	1	760.3643	Acetylati	L	Superscaf	48650653	0	0	0	BTP20220	0	0	Tissue specific
VGGGGLLS	Superscaf	-	Superscaf	48719377	48719400	-51	51	58	-	-	-	-	-	-	NCP	IGR	-	ID=gene-E	8	5.19	2279.4	0.05651597	VGGGGLLS	54	659.371	1	658.365	.	F	Superscaf	48719439	0	0	0	BTP20220	0	0	Tissue specific
SVGSPP	Superscaf	-	Superscaf	48719518	48719535	-7	7	12	-	-	-	-	-	-	NCP	IGR	-	ID=gene-E	6	4.988333333	1738.9	0.056513356	SVGSPP	51	543.2789	1	542.27	.	N	Superscaf	48719544	0	BTP20220	0	0	0	0	Tissue specific
LGPAFSK	Superscaf	+	Superscaf	48725422	48725442	19	19	25	-	-	-	-	-	-	NCP	IGR	-	ID=gene-E	7	6.452857143	2538.6	0.056398963	L(+42.01)	77	381.2101	2	760.4119	Acetylati	P	Superscaf	48725368	0	0	0	BTP20220	0	0	Tissue specific
FVGMPF	Superscaf	+	Superscaf	48777412	48777429	53	53	58	-	-	-	-	-	-	NCP	IGR	-	ID=gene-E	6	4.83	1781	0.0553922	FVGMPF	54	697.3499	1	696.3305	.	C	Superscaf	48777256	0	0	BTP20220	0	0	0	Tissue specific
SSVPTVK	Superscaf	-	Superscaf	48823939	48823959	-2	2	8	-	-	-	-	-	-	NCP	IGR	-	ID=gene-E	7	4.847142857	1970.1	0.054491115	SSVPTVK	99	359.2095	2	716.4069	.	Y	Superscaf	48823983	0	BTP20220	0	0	0	0	Tissue specific
SADRMHL	Superscaf	+	Superscaf	48837040	48837060	66	66	72	-	-	-	-	-	-	NCP	IGR	-	ID=gene-E	7	6.392857143	3099.6	0.054237405	SADRMHL	63	415.1966	2	828.3912	.	P	Superscaf	48836845	0	0	0	BTP20220	BTP20220	BTP20220	Tissue specific
EFLFD	Superscaf	-	Superscaf	48854362	48854376	-13	13	17	-	-	gene	-	-	mRNA	NCP	Intron	gene|mRNA	ID=gene-E	5	4.626	1965.2	0.053902069	EFLFD	69	670.3066	1	669.301	.	T	Superscaf	48854421	0	0	0	BTP20220	0	0	Mixed
AAQALAL	Superscaf	+	Superscaf	48913495	48913515	74	74	80	-	-	-	-	-	-	NCP	IGR	-	ID=gene-E	7	5.955714286	2562.7	0.052756802	A(+42.01)	53	699.401	1	698.3962	Acetylati	P	Superscaf	48913276	0	0	0	0	BTP20220	0	Tissue enhanced
DSEAV	Superscaf	+	Superscaf	48944146	48944160	15	15	19	-	-	-	-	-	-	NCP	IGR	-	ID=gene-E	5	3.578	1531.7	0.052163341	DSEAV	50	520.2294	1	519.2177	.	G	Superscaf	48944104	0	BTP20220	0	0	BTP20220	0	Tissue specific
MAVVKK	Superscaf	+	Superscaf	48953347	48953364	15	15	20	-	-	-	-	-	-	NCP	IGR	-	ID=gene-E	6	5.208333333	1619.6	0.0519851	M(+42.01)	96	359.2147	2	716.4255	Acetylati	H	Superscaf	48953305	0	0	0	BTP20220	0	0	Tissue specific
KDRALTH	Superscaf	+	Superscaf	48979195	48979215	9	9	15	-	-	-	-	-	-	NCP	IGR	-	ID=gene-E	7	7.084285714	3210.6	0.051484478	K(+42.01)	60	441.7411	2	881.4719	Acetylati	L	Superscaf	48979171	0	0	BTP20220	0	0	0	Tissue specific
KPSYCCH	Superscaf	+	Superscaf	48994936	48994956	40	40	46	-	-	-	-	-	-	NCP	IGR	-	ID=gene-E	7	6.43	3439.8	0.051179643	KPSYC(+57	73	476.1974	2	950.3738	Carbamidc	E	Superscaf	48994819	0	0	BTP20220	0	0	0	Tissue specific
TELLSLPY	Superscaf	+	Superscaf	49003762	49003785	52	52	59	-	-	-	-	-	-	NCP	IGR	-	ID=gene-E	8	5.66625	3063.5	0.051008663	TELLSLPY	68	935.505	1	934.5011	.	I	Superscaf	49003609	0	BTP20220	BTP20220	BTP20220	BTP20220	0	Tissue specific
GTVPSP	Superscaf	-	Superscaf	49035982	49035999	-44	44	49	-	-	-	-	-	-	NCP	IGR	-	ID=gene-E	6	5.13	1753	0.050384817	GTVPSP	72	557.2896	1	556.2856	.	F	Superscaf	49036089	0	0	BTP20220	BTP20220	0	0	Tissue specific
KPPKPKL	Superscaf	+	Superscaf	49124533	49124553	44	44	50	-	-	-	-	-	-	NCP	IGR	-	ID=gene-E	7	7.728571429	2770.8	0.048669909	KPPKPKL	54	404.2747	2	806.5378	.	S	Superscaf	49124404	0	0	0	BTP20220	0	0	Tissue specific
KVEKEF	Superscaf	+	Superscaf	49172200	49172217	43	43	48	-	-	-	-	-	-	NCP	IGR	-	ID=gene-E	6	5.233333333	2175.2	0.047746864	K(+42.01)	91	411.2222	2	820.433	Acetylati	T	Superscaf	49172074	0	0	0	BTP20220	0	0	Tissue specific
SYCRVP	Superscaf	+	Superscaf	49267951	49267968	35	35	40	-	-	gene	-	-	mRNA	NCP	Intron	gene|mRNA	ID=gene-E	6	5.57	2414.8	0.045892581	SYC(+57.	97	391.185	2	780.3588	Carbamidc	P	Superscaf	49267849	0	BTP20220	0	0	0	0	Mixed
DRTPLV	Superscaf	+	Superscaf	49291000	49291017	5	5	10	-	-	gene	-	-	mRNA	NCP	Intron	gene|mRNA	ID=gene-E	6	5.423333333	2148.6	0.045446222	DRTPLV	65	350.6971	2	699.3915	.	S	Superscaf	49290988	0	0	0	BTP20220	0	0	Tissue specific
AVRSDSVH	Superscaf	-	Superscaf	49307644	49307667	-13	13	20	-	-	-	-	-	-	NCP	IGR	-	ID=gene-E	8	4.8375	2758.1	0.045123783	A(+42.01)	55	456.7242	2	911.4461	Acetylati	R	Superscaf	49307670	0	0	0	0	0	BTP20220	Tissue specific
KNVTPFE	Superscaf	-	Superscaf	49311244	49311264	-24	24	30	-	-	-	-	-	-	NCP	IGR	-	ID=gene-E	7	5.24	2612.9	0.045054125	KNVTPFE	56	417.7202	2	833.4283	.	S	Superscaf	49311312	0	0	0	0	0	BTP20220	Tissue specific
KFVAPP	Superscaf	+	Superscaf	49372936	49372953	8	8	13	-	-	-	-	-	-	NCP	IGR	-	ID=gene-E	6	5.64	1841.9	0.043859476	KFVAPP	76	658.3859	1	657.3849	.	P	Superscaf	49372915	BTP20220	0	0	0	0	0	Tissue specific
DTPKPL	Superscaf	-	Superscaf	49423126	49423143	-8	8	13	-	-	-	-	-	-	NCP	IGR	-	ID=gene-E	6	6.303333333	2305.6	0.042887513	DTPKPL	89	670.3757	1	669.3697	.	V	Superscaf	49423362	0	0	BTP20220	0	0	0	Tissue specific
VGPAREK	Superscaf	+	Superscaf	49438900	49438920	10	10	16	-	-	-	-	-	-	NCP	IGR	-	ID=gene-E	7	6.001428571	2488.7	0.04258198	VGPAREK	79	378.7209	2	755.429	.	L	Superscaf	49438873	0	0	0	BTP20220	0	0	Tissue specific
SLDGNLH	Superscaf	+	Superscaf	49527550	49527570	8	8	14	-	-	-	-	-	-	NCP	IGR	-	ID=gene-E	7	5.654285714	3015.4	0.040865213	SLDGNLH	52	378.1888	2	754.361	.	C	Superscaf	49527529	0	BTP20220	0	0	0	0	Tissue specific
EYPKAGK	Superscaf	-	Superscaf	49651663	49651683	-11	11	17	-	-	gene	-	-	mRNA	NCP	Intron	gene|mRNA	ID=gene-E	7	6.664285714	2767.8	0.038461681	EYPKAGK	53	396.7084	2	791.4177	.	N	Superscaf	49651707	0	0	BTP20220	0	0	0	Tissue specific
KLQNKP	Superscaf	+	Superscaf	49691899	49691916	9	9	14	-	-	gene	-	-	mRNA	NCP	Intron	gene|mRNA	ID=gene-E	6	7.136666667	2835	0.037682542	KLQNKP	70	364.225	2	726.4388	.	R	Superscaf	49691875	BTP20220	0	0	0	0	0	Tissue specific
FLDEER	Superscaf	-	Superscaf	49721029	49721046	-9	9	14	-	-	-	-	-	-	NCP	IGR	-	ID=gene-E	6	5.271666667	2652.1	0.03711842	FLDEER	97	404.6949	2	807.3762	.	L	Superscaf	49721079	0	0	0	0	0	BTP20220	Tissue specific
ASVGGQPK	Superscaf	-	Superscaf	49727833	49727856	-12	12	19	-	-	-	-	-	-	NCP	IGR	-	ID=gene-E	8	5.66625	2705.8	0.03698654	ASVGGQPK	68	372.2047	2	742.3973	.	P	Superscaf	49727883	0	BTP20220	0	0	0	0	Tissue specific
LDVAGFK	Superscaf	+	Superscaf	49749448	49749468	34	34	40	-	-	gene	-	-	mRNA	NCP	Intron	gene|mRNA	ID=gene-E	7	5.165714286	2229.3	0.036568009	LDVAGFK	51	375.2125	2	748.4119	.	M	Superscaf	49749349	0	0	BTP20220	BTP20220	0	0	Tissue specific
LQAVLSL	Superscaf	+	Superscaf	49801774	49801794	1	1	7	-	-	-	-	-	-	NCP	IGR	-	ID=gene-E	7	5.041428571	2365.6	0.035554681	LQAVLSL	77	743.4637	1	742.4589	.	L	Superscaf	49801774	0	0	BTP20220	0	0	0	Tissue enhanced
PPGMGLPP	Superscaf	-	Superscaf	49871581	49871604	-15	15	22	-	-	-	-	-	-	NCP	IGR	-	ID=gene-E	8	6.10875	2669.1	0.034202763	PPGMGLPP	52	383.209	2	764.3891	.	K	Superscaf	49871625	0	BTP20220	0	0	0	0	Tissue specific
RRVLLFK	Superscaf	-	Superscaf	49910431	49910451	-6	6	12	-	-	-	-	-	-	NCP	IGR	-	ID=gene-E	7	6.957142857	2860.2	0.033450465	RRVLLFK	54	466.3153	2	930.6127	.	M	Superscaf	49910610	BTP20220	0	0	0	0	0	Tissue specific
LLFPLE	Superscaf	-	Superscaf	49994896	49994913	-2	2	7	-	-	-	-	-	-	NCP	IGR	-	ID=gene-E	6	5.49	2210.5	0.031814801	LLFPLE	57	731.4321	1	730.4265	.	I	Superscaf	49994955	0	0	0	BTP20220	0	0	Tissue specific
NTTSAVTVK	Superscaf	+	Superscaf	50004604	50004630	5	5	13	-	-	-	-	-	-	NCP	IGR	-	ID=gene-E	9	5.16	2952.3	0.031626625	NTTSAVTVK	98	920.5013	1	919.4974	.	K	Superscaf	50004592	0	0	0	0	0	BTP20220	Tissue specific
RLKELKDQ	Superscaf	+	Superscaf	50004643	50004666	18	18	25	-	-	-	-	-	-	NCP	IGR	-	ID=gene-E	8	6.755	3740.1	0.031625928	RLKELKDQ	83	515.3038	2	1028.598	.	K	Superscaf	50004592	0	0	0	BTP20220	0	0	Tissue specific
TGLRLGE	Superscaf	+	Superscaf	50024731	50024751	2	2	8	-	-	-	-	-	-	NCP	IGR	-	ID=gene-E	7	6.344285714	2773.2	0.031236968	TGLRLGE	74	745.4178	1	744.413	.	K	Superscaf	50024728	0	0	0	0	0	BTP20220	Tissue specific
TAYLVF	Superscaf	+	Superscaf	50031616	50031633	6	6	11	-	-	-	-	-	-	NCP	IGR	-	ID=gene-E	6	4.945	1909.1	0.031103694	T(+42.01)	62	378.2	2	754.3901	Acetylati	Y	Superscaf	50031601	BTP20220	0	0	0	0	0	Tissue specific
RCRASLLR	Superscaf	-	Superscaf	50035303	50035326	-37	37	44	-	-	-	-	-	-	NCP	IGR	-	ID=gene-E	8	7.62	3933.6	0.031032176	R(+42.01)	72	358.5435	3	1072.592	Acetylati	G	Superscaf	50035362	0	0	0	BTP20220	0	0	Tissue enhanced
LVAMGSL	Superscaf	+	Superscaf	50102017	50102037	34	34	40	-	-	-	-	-	-	NCP	IGR	-	ID=gene-E	7	5.054285714	2070.3	0.029740273	LVAMGSL	75	690.3905	1	689.3782	.	G	Superscaf	50101918	0	0	BTP20220	0	0	0	Tissue specific
KKHMPK	Superscaf	+	Superscaf	50130460	50130477	4	4	9	-	-	-	-	-	-	NCP	IGR	-	ID=gene-E	6	8.143333333	2733.8	0.029189513	KKHM(+15.	53	392.722	2	783.4425	Oxidation	V	Superscaf	50130451	0	0	BTP20220	0	0	0	Tissue specific
EVYVYK	Superscaf	-	Superscaf	50187913	50187930	-13	13	18	-	-	-	-	-	-	NCP	IGR	-	ID=gene-E	6	4.046666667	1812.9	0.028076897	EVYVYK	96	400.7074	2	799.4116	.	E	Superscaf	50187957	0	0	0	0	0	BTP20220	Tissue specific
KLVDEKF	Superscaf	-	Superscaf	50206591	50206611	-24	24	30	-	-	-	-	-	-	NCP	IGR	-	ID=gene-E	7	5.304285714	2514.6	0.027715127	KLVDEKF	75	439.7523	2	877.4909	.	F	Superscaf	50206638	BTP20220	0	0	0	0	0	Tissue specific
APLDRVP	Superscaf	-	Superscaf	50290168	50290188	-23	23	29	-	-	-	-	-	-	NCP	IGR	-	ID=gene-E	7	5.475714286	2399.8	0.026096602	APLDRVP	81	384.223	2	766.4337	.	S	Superscaf	50290245	0	0	0	0	0	BTP20220	Tissue specific
MYVLK	Superscaf	+	Superscaf	50304709	50304723	4	4	8	-	-	-	-	-	-	NCP	IGR	-	ID=gene-E	5	5.426	1652.8	0.025815122	MYVLK	94	653.3666	1	652.3618	.	I	Superscaf	50304700	0	0	BTP20220	0	0	0	Tissue specific
NGPSWL	Superscaf	-	Superscaf	50365573	50365590	-3	3	8	-	-	-	-	-	-	NCP	IGR	-	ID=gene-E	6	5.871666667	2494.8	0.024636391	NGPSWL	92	673.3296	1	672.3231	.	P	Superscaf	50365593	0	0	BTP20220	0	0	0	Tissue specific
HHALHY	Superscaf	-	Superscaf	50371108	50371125	-8	8	13	-	-	-	-	-	-	NCP	IGR	-	ID=gene-E	6	6.738333333	2983.3	0.024529202	HHALHY	60	389.1919	2	776.3718	.	S	Superscaf	50371131	0	0	BTP20220	0	0	0	Tissue specific
SHSLTVM	Superscaf	-	Superscaf	50386927	50386947	-14	14	20	-	-	-	-	-	-	NCP	IGR	-	ID=gene-E	7	5.315714286	2508.9	0.024222798	SHSLTVM	72	387.6937	2	773.3742	.	V	Superscaf	50387043	0	0	0	BTP20220	0	0	Tissue specific
PTVKVSR	Superscaf	-	Superscaf	50396899	50396919	-17	17	23	-	-	-	-	-	-	NCP	IGR	-	ID=gene-E	7	5.572857143	2235.5	0.024029684	PTVKVSR	80	393.7434	2	785.4759	.	Y	Superscaf	50397102	0	0	BTP20220	0	0	0	Tissue specific
[illegible]																																						

NLATLKT	Superscaf	–	Superscaf	52784821	52784841	-7	7	13	–	–	gene	–	–	mRNA	NCP	Intron	gene|mRN	ID=gene-E
MLDLL	Superscaf	+	Superscaf	52804393	52804407	40	40	44	–	–	gene	–	–	mRNA	NCP	Intron	gene|mRN	ID=gene-E
RPHQEK	Superscaf	+	Superscaf	52807066	52807083	29	29	34	–	–	gene	–	–	mRNA	NCP	Intron	gene|mRN	ID=gene-E
MATVLLPR	Superscaf	–	Superscaf	52813987	52814010	-52	52	59	–	–	gene	–	–	mRNA	NCP	Intron	gene|mRN	ID=gene-E
RDLKEKL	Superscaf	–	Superscaf	52824232	52824252	-9	9	15	–	–	gene	–	–	mRNA	NCP	Intron	gene|mRN	ID=gene-E
QTWLGGS	Superscaf	+	Superscaf	52850233	52850253	6	6	12	–	–	–	–	–	–	NCP	IGR	–	ID=gene-E
KDPMHQ	Superscaf	+	Superscaf	52881973	52881990	39	39	44	–	–	–	–	–	–	NCP	IGR	–	ID=gene-E
VQLYY	Superscaf	+	Superscaf	52886197	52886211	19	19	23	–	–	–	–	–	–	NCP	IGR	–	ID=gene-E
FSPTTTK	Superscaf	+	Superscaf	52902244	52902264	8	8	14	–	–	–	–	–	–	NCP	IGR	–	ID=gene-E
WAGKVEA	Superscaf	–	Superscaf	52919773	52919793	-11	11	17	–	–	–	–	–	–	NCP	IGR	–	ID=gene-E
KTPHGPK	Superscaf	+	Superscaf	52956109	52956129	46	46	52	–	–	–	–	–	–	NCP	IGR	–	ID=gene-E
KLEGLKFQ	Superscaf	+	Superscaf	52959967	52959990	67	67	74	–	–	–	–	–	–	NCP	IGR	–	ID=gene-E
KDWQTK	Superscaf	+	Superscaf	53009332	53009349	1	1	6	–	–	gene	–	–	mRNA	NCP	Intron	gene|mRN	ID=gene-E
GDDDL	Superscaf	+	Superscaf	53038831	53038845	1	1	5	–	–	gene	–	–	mRNA	NCP	Intron	gene|mRN	ID=gene-E
TTAFPAST	Superscaf	–	Superscaf	53113381	53113404	-11	11	18	–	–	gene	–	–	mRNA	NCP	Intron	gene|mRN	ID=gene-E
KLQDKDK	Superscaf	–	Superscaf	53119261	53119281	-31	31	37	–	–	gene	–	–	mRNA	NCP	Intron	gene|mRN	ID=gene-E
YLDRSGF	Superscaf	+	Superscaf	53200018	53200038	9	9	15	–	–	gene	–	–	mRNA	NCP	Intron	gene|mRN	ID=gene-E
NSASKPK	Superscaf	–	Superscaf	53271979	53271999	-22	22	28	–	–	gene	–	–	mRNA	NCP	Intron	gene|mRN	ID=gene-E
LPGLSQ	Superscaf	+	Superscaf	53399239	53399256	2	2	7	–	–	–	–	–	–	NCP	IGR	–	ID=gene-E
RTAVQK	Superscaf	+	Superscaf	53445760	53445777	22	22	27	–	–	–	–	–	–	NCP	IGR	–	ID=gene-E
KEVTLPK	Superscaf	–	Superscaf	53490919	53490939	-14	14	20	–	–	–	–	–	–	NCP	IGR	–	ID=gene-E
PKLGLFY	Superscaf	–	Superscaf	53530900	53530920	-23	23	29	–	–	–	–	–	–	NCP	IGR	–	ID=gene-E
LVAVY	Superscaf	–	Superscaf	53613916	53613930	-21	21	25	–	–	–	–	–	–	NCP	IGR	–	ID=gene-E
LDDLKP	Superscaf	+	Superscaf	53712997	53713014	18	18	23	–	–	gene	–	–	mRNA	NCP	Intron	gene|mRN	ID=gene-E
KYLDLR	Superscaf	+	Superscaf	53732617	53732634	14	14	19	–	–	gene	–	–	mRNA	NCP	Intron	gene|mRN	ID=gene-E
KPFVEP	Superscaf	+	Superscaf	53948632	53948649	11	11	16	–	–	gene	–	–	mRNA	NCP	Intron	gene|mRN	ID=gene-E
KQGVLQF	Superscaf	+	Superscaf	53953132	53953152	30	30	36	–	–	gene	–	–	mRNA	NCP	Intron	gene|mRN	ID=gene-E
HNEYGN	Superscaf	–	Superscaf	53955589	53955606	-3	3	8	–	–	gene	–	–	mRNA	NCP	Intron	gene|mRN	ID=gene-E
DMYEMM	Superscaf	+	Superscaf	53963743	53963760	34	34	39	–	–	gene	–	–	mRNA	NCP	Intron	gene|mRN	ID=gene-E
KVGLEPV	Superscaf	+	Superscaf	54038662	54038682	1	1	7	–	–	gene	–	–	mRNA	NCP	Intron	gene|mRN	ID=gene-E
WGQGY	Superscaf	–	Superscaf	54063814	54063828	-8	8	12	–	–	–	–	–	–	NCP	IGR	–	ID=gene-E
CTQGELK	Superscaf	+	Superscaf	54097072	54097092	10	10	16	–	–	–	–	–	–	NCP	IGR	–	ID=gene-E
VYLKV	Superscaf	–	Superscaf	54106447	54106461	-42	42	46	–	–	–	–	–	–	NCP	IGR	–	ID=gene-E
PAAKNF	Superscaf	–	Superscaf	54149251	54149268	-3	3	8	–	–	–	–	–	–	NCP	IGR	–	ID=gene-E
NVGFRYK	Superscaf	–	Superscaf	54164560	54164580	-70	70	76	–	–	–	–	–	–	NCP	IGR	–	ID=gene-E
KVDPLVSL	Superscaf	+	Superscaf	54183799	54183822	4	4	11	–	–	gene	–	–	mRNA	NCP	Intron	gene|mRN	ID=gene-E
VSLVPVKD	Superscaf	+	Superscaf	54192484	54192507	3	3	10	–	–	gene	–	–	mRNA	NCP	Intron	gene|mRN	ID=gene-E
KGVWAAR	Superscaf	–	Superscaf	54225028	54225048	-17	17	23	–	–	gene	–	–	mRNA	NCP	Intron	gene|mRN	ID=gene-E
LVFKELEK	Superscaf	+	Superscaf	54283600	54283623	2	2	9	–	–	gene	–	–	mRNA	NCP	Intron	gene|mRN	ID=gene-E
ANELEF	Superscaf	–	Superscaf	54453493	54453510	-76	76	81	–	–	gene	–	–	mRNA	NCP	Intron	gene|mRN	ID=gene-E
HLLEAK	Superscaf	+	Superscaf	54479413	54479430	6	6	11	–	–	gene	–	–	mRNA	NCP	Intron	gene|mRN	ID=gene-E
LGPHHKQ	Superscaf	–	Superscaf	54550126	54550146	-16	16	22	–	–	–	–	–	–	NCP	IGR	–	ID=gene-E
VSTRLLT	Superscaf	–	Superscaf	54552862	54552882	-3	3	9	–	–	–	–	–	–	NCP	IGR	–	ID=gene-E
ELGNYG	Superscaf	–	Superscaf	54560308	54560325	-5	5	10	–	–	–	–	–	–	NCP	IGR	–	ID=gene-E
KKNFPGP	Superscaf	–	Superscaf	54566218	54566238	-10	10	16	–	–	–	–	–	–	NCP	IGR	–	ID=gene-E
KLFDF	Superscaf	+	Superscaf	54598429	54598443	73	73	77	–	–	gene	–	–	mRNA	NCP	Intron	gene|mRN	ID=gene-E
WAAVVPK	Superscaf	–	Superscaf	54625720	54625740	-14	14	20	–	–	gene	–	–	mRNA	NCP	Intron	gene|mRN	ID=gene-E
VFKLAP	Superscaf	–	Superscaf	54651721	54651738	-1	1	6	–	–	gene	–	–	mRNA	NCP	Intron	gene|mRN	ID=gene-E
LPRLLF	Superscaf	–	Superscaf	54667519	54667536	-16	16	21	–	–	gene	–	–	mRNA	NCP	Intron	gene|mRN	ID=gene-E
EVPQHLNK	Superscaf	–	Superscaf	54672310	54672333	-2	2	9	–	–	gene	–	–	mRNA	NCP	Intron	gene|mRN	ID=gene-E
KVGDYL	Superscaf	+	Superscaf	54693307	54693324	10	10	15	–	–	gene	–	–	mRNA	NCP	Intron	gene|mRN	ID=gene-E
EPEPTLL	Superscaf	–	Superscaf	54785200	54785220	-21	21	27	–	–	gene	–	–	mRNA	NCP	Intron	gene|mRN	ID=gene-E
PVVVPSRR	Superscaf	–	Superscaf	54828448	54828471	-13	13	20	–	–	gene	–	–	mRNA	NCP	Intron	gene|mRN	ID=gene-E
HFVVK	Superscaf	+	Superscaf	54837751	54837765	3	3	7	–	–	gene	–	–	mRNA	NCP	Intron	gene|mRN	ID=gene-E
KDGKVPK	Superscaf	+	Superscaf	54860845	54860865	2	2	8	–	–	gene	–	–	mRNA	NCP	Intron	gene|mRN	ID=gene-E
KDYVYK	Superscaf	+	Superscaf	54870463	54870480	10	10	15	–	–	–	–	–	–	NCP	IGR	–	ID=gene-E
TTTVHEK	Superscaf	–	Superscaf	54899842	54899862	-2	2	8	–	–	gene	–	–	mRNA	NCP	Intron	gene|mRN	ID=gene-E
TQPVEL	Superscaf	–	Superscaf	54907261	54907278	-19	19	24	–	–	gene	–	–	mRNA	NCP	Intron	gene|mRN	ID=gene-E
VYKVNY	Superscaf	+	Superscaf	54985597	54985614	24	24	29	–	–	gene	–	–	mRNA	NCP	Intron	gene|mRN	ID=gene-E
MLPTLLVK	Superscaf	–	Superscaf	55014430	55014453	-7	7	14	–	–	gene	–	–	mRNA	NCP	Intron	gene|mRN	ID=gene-E
KRHTSSK	Superscaf	–	Superscaf	55162081	55162101	-62	62	68	–	–	–	–	–	–	NCP	IGR	–	ID=gene-E
QLAPVPF	Superscaf	+	Superscaf	55242655	55242675	22	22	28	–	–	–	–	–	–	NCP	IGR	–	ID=gene-E
QFPFPK	Superscaf	+	Superscaf	55280080	55280097	10	10	15	–	–	–	–	–	–	NCP	IGR	–	ID=gene-E
TKFGGQR	Superscaf	+	Superscaf	55415050	55415070	14	14	20	–	–	–	–	–	–	NCP	IGR	–	ID=gene-E
MCKMFP	Superscaf	+	Superscaf	55542400	55542417	32	32	37	–	–	–	–	–	–	NCP	IGR	–	ID=gene-E
MEMTVP	Superscaf	–	Superscaf	55616065	55616082	-92	92	97	–	–	gene	–	–	mRNA	NCP	Intron	gene|mRN	ID=gene-E
RTPVLTL	Superscaf	+	Superscaf	55628845	55628865	4	4	10	–	–	gene	–	–	mRNA	NCP	Intron	gene|mRN	ID=gene-E
DMALLR	Superscaf	–	Superscaf	55629538	55629555	-28	28	33	–	–	gene	–	–	mRNA	NCP	Intron	gene|mRN	ID=gene-E
KSVMLGP	Superscaf	–	Superscaf	55663399	55663419	-11	11	17	–	–	gene	–	–	mRNA	NCP	Intron	gene|mRN	ID=gene-E
VTHSLLV	Superscaf	–	Superscaf	55698208	55698228	-1	1	7	–	–	gene	–	–	mRNA	NCP	Intron	gene|mRN	ID=gene-E
LSPVARK	Superscaf	+	Superscaf	55772392	55772412	5	5	11	–	–	gene	–	–	mRNA	NCP	Intron	gene|mRN	ID=gene-E
GKNLPEN	Superscaf	–	Superscaf	55887349	55887369	-13	13	19	–	–	gene	–	–	mRNA	NCP	Intron	gene|mRN	ID=gene-E
TYGVSFF	Superscaf	–	Superscaf	55904563	55904583	-26	26	32	–	–	gene	–	–	mRNA	NCP	Intron	gene|mRN	ID=gene-E
KLVPRLLG	Superscaf	–	Superscaf	55918930	55918953	-28	28	35	–	–	gene	–	–	mRNA	NCP	Intron	gene|mRN	ID=gene-E
VDEKTKEVI	Superscaf	–	Superscaf	55918978	55919007	-44	44	53	–	–	gene	–	–	mRNA	NCP	Intron	gene|mRN	ID=gene-E
RWAASPK	Superscaf	–	Superscaf	55919032	55919052	-62	62	68	–	–	gene	–	–	mRNA	NCP	Intron	gene|mRN	ID=gene-E
EPELLGFN	Superscaf	–	Superscaf	55923745	55923768	-11	11	18	–	–	gene	–	–	mRNA	NCP	Intron	gene|mRN	ID=gene-E
KYPLT	Superscaf	–	Superscaf	55986097	55986111	-1	1	5	–	–	gene	–	–	mRNA	NCP	Intron	gene|mRN	ID=gene-E
YPHGV	Superscaf	+	Superscaf	55994512	55994526	10	10	14	–	–	gene	–	–	mRNA	NCP	Intron	gene|mRN	ID=gene-E
SVVSPP	Superscaf	–	Superscaf	56041882	56041899	-22	22	27	–	–	–	–	–	–	NCP	IGR	–	ID=gene-E
RMELVH	Superscaf	+	Superscaf	56082061	56082078	5	5	10	–	–	–	–	–	–	NCP	IGR	–	ID=gene-E
KFTKTK	Superscaf	+	Superscaf	56109565	56109582	77	77	82	–	–	–	–	–	–	NCP	IGR	–	ID=gene-E
NFALNVP	Superscaf	–	Superscaf	56114590	56114610	-59	59	65	–	–	–	–	–	–	NCP	IGR	–	ID=gene-E
ATTKKFK	Superscaf	+	Superscaf	56119045	56119065	12	12	18	–	–	–	–	–	–	NCP	IGR	–	ID=gene-E
LKTVETR	Superscaf	+	Superscaf	56192443	56192463	21	21	27	–	–	–	–	–	–	NCP	IGR	–	ID=gene-E
LGGVLLF	Superscaf	+	Superscaf	56200582	56200602	1	1	7	–	–	–	–	–	–	NCP	IGR	–	ID=gene-E
KEDDLRK	Superscaf	+	Superscaf	56245075	56245095	14	14	20	–	–	–	–	–	–	NCP	IGR	–	ID=gene-E
KLKLVSHK	Superscaf	+	Superscaf	56256778	56256801	13	13	20	–	–	–	–	–	–	NCP	IGR	–	ID=gene-E
RNCARL	Superscaf	–	Superscaf	56266897	56266914	-12	12	17	–	–	–	–	–	–	NCP	IGR	–	ID=gene-E
MGVFLQQ	Superscaf	–	Superscaf	56305996	56306016	-29	29	35	–	–	–	–	–	–	NCP	IGR	–	ID=gene-E
KMQMLK	Superscaf	–	Superscaf	56322826	56322843	-105	105	110	–	–	–	–	–	–	NCP	IGR	–	ID=gene-E
KAEDSLLA	Superscaf	–	Superscaf	56323753	56323797	-44	44	58	–	–	–	–	–	–	NCP	IGR	–	ID=gene-E
AEDSLLAAI	Superscaf	–	Superscaf	56323756	56323797	-45	45	58	–	–	–	–	–	–	NCP	IGR	–	ID=gene-E
CAELEEELK	Superscaf	–	Superscaf	56342050	56342076	-41	41	49	–	–	–	–	–	–	NCP	IGR	–	ID=gene-E
CAELEEELK	Superscaf	–	Superscaf	56342050	56342097	-41	41	56	–	–	–	–	–	–	NCP	IGR	–	ID=gene-E
CAELEEELK	Superscaf	–	Superscaf	56342050	56342094	-41	41	55	–	–	–	–	–	–	NCP	IGR	–	ID=gene-E
EEELKTVTN	Superscaf	–	Superscaf	56342062	56342097	-45	45	56	–	–	–	–	–	–	NCP	IGR	–	ID=gene-E
KTVTNNLK	Superscaf	–	Superscaf	56342074	56342097	-49	49	56	–	–	–	–	–	–	NCP	IGR	–	ID=gene-E
AEFAERSVI	Superscaf	–	Superscaf	56342950	56342979	-10	10	19	–	–	–	–	–	–	NCP	IGR	–	ID=gene-E
HAKEENLSM	Superscaf	–	Superscaf	56351524	56351553	-11	11	20	–	–	–	–	–	–	NCP	IGR	–	ID=gene-E
AKEENLSMH	Superscaf	–	Superscaf	56351527	56351556	-12	12	21	–	–	–	–	–	–	NCP	IGR	–	ID=gene-E
AKEENLSMH	Superscaf	–	Superscaf	56351527	56351553	-12	12	20	–	–	–	–	–	–	NCP	IGR	–	ID=gene-E
EENLSMHQ	Superscaf	–	Superscaf	56351533	56351556	-14	14	21	–	–	–	–	–	–	NCP	IGR	–	ID=gene-E
MHQMLDQTI	Superscaf	–	Superscaf	56351548	56351592	-19	19	33	–	–	–	–	–	–	NCP	IGR	–	ID=gene-E
HQMLDQTLI	Superscaf	–	Superscaf	56351551	56351592	-20	20	33	–	–	–	–	–	–	NCP	IGR	–	ID=gene-E
QMLDQTLLE	Superscaf	–	Superscaf	56351554	56351592	-21	21	33	–	–	–	–	–	–	NCP	IGR	–	ID=gene-E
LDQTLLELN	Superscaf	–	Superscaf	56351560	56351592	-23	23	33	–	–	–	–	–	–	NCP	IGR	–	ID=gene-E
QTLLELNNM	Superscaf	–	Superscaf	56351566	56351592	-25	25	33	–	–	–	–	–	–	NCP	IGR	–	ID=gene-E
TLLELNNM	Superscaf	–	Superscaf	56351569	56351592	-26	26	33	–	–	–	–	–	–	NCP	IGR	–	ID=gene-E
LLELNNM	Superscaf	–	Superscaf	56351572	56351592	-27	27	33	–	–	–	–	–	–	NCP	IGR	–	ID=gene-E
LELNNM	Superscaf	–	Superscaf	56351575	56351592	-28	28	33	–	–	–	–	–	–	NCP	IGR	–	ID=gene-E
AFVQRT	Superscaf	+	Superscaf	56363401	56363418	12	12	17	–	–	–	–	–	–	NCP	IGR	–	ID=gene-E
TVGDLLK	Superscaf	–	Superscaf	56439673	56439693	-37	37	43	–	–	–	–	–	–	NCP	IGR	–	ID=gene-E
FDLYPP	Superscaf	–	Superscaf	56457397	56457414	-2	2	7	–	–	–	–	–	–	NCP	IGR	–	ID=gene-E
QQAVYK	Superscaf	+	Superscaf	56533978	56533995	10	10	15	–	–	gene	–	–	mRNA	NCP	Intron	gene|mRN	ID=gene-E
FFANRP	Superscaf	–	Superscaf	56553631	56553648	-5	5	10	–	–	gene	–	–	mRNA	NCP	Intron	gene|mRN	ID=gene-E
NEGSPFL	Superscaf	+	Superscaf	56584768	56584788	16	16	22	–	–	gene	–	–	mRNA	NCP	Intron	gene|mRN	ID=gene-E
PSPLLAGK	Superscaf	–	Superscaf	56653150	56653173	-20	20	27	–	–	gene	–	–	mRNA	NCP	Intron	gene|mRN	ID=gene-E
KPPAFAK	Superscaf	–	Superscaf	56788798	56788818	-5	5	11	–	–	–	–	–	–	NCP	IGR	–	ID=gene-E
SHALALF	Superscaf	–	Superscaf	56834743	56834763	-24	24	30	–	–	–	–	–	–	NCP	IGR	–	ID=gene-E
VVAPK	Superscaf	–	Superscaf	56894479	56894493	-41	41	45	–	–	–	–	–	–	NCP	IGR	–	ID=gene-E
MDDLFLP	Superscaf	+	Superscaf	57015250	57015270	32	32	38	–	–	–	–	–	–	NCP	IGR	–	ID=gene-E
HLKVPQ	Superscaf	+	Superscaf	57063769	57063786	14	14	19	–	–	–	–	–	–	NCP	IGR	–	ID=gene-E
PTVAHVP	Superscaf	–	Superscaf	57143059	57143079	-33	33	39	–	–	–	–	–	–	NCP	IGR	–	ID=gene-E
NELATLL	Superscaf	+	Superscaf	57148348	57148368	48	48	54	–	–	–	–	–	–	NCP	IGR	–	ID=gene-E
ALGGF	Superscaf	–	Superscaf	57203851	57203865	-30	30	34	–	–	–	–	–	–	NCP	IGR	–	ID=gene-E
KVWSHH	Superscaf	+	Superscaf	57211321	57211338	16	16	21	–	–	–	–	–	–	NCP	IGR	–	ID=gene-E
TGPELKY	Superscaf	–	Superscaf	57217426	57217446	-15	15	21	–	–	–	–	–	–	NCP	IGR	–	ID=gene-E
TQWEK	Superscaf	–	Superscaf	57222280	57222294	-6	6	10	–	–	–	–	–	–	NCP	IGR	–	ID=gene-E
KGFDGDR	Superscaf	–	Superscaf	57258694	57258714	-5	5	11	–	–	–	–	–	–	NCP	IGR	–	ID=gene-E
MEDLNGK	Superscaf	–	Superscaf	57259624	57259644	-11	11	17	–	–	–	–	–	–	NCP	IGR	–	ID=gene-E
KRFATLK	Superscaf	+	Superscaf	57309436	57309456	22	22	28	–	–	–	–	–	–	NCP	IGR	–	ID=gene-E
ALDYSN	Superscaf	+	Superscaf	57338062	57338079	6	6	11	–	–	–	–	–	–	NCP	IGR	–	ID=gene-E
VGAGSFL	Superscaf	+	Superscaf	57346768	57346788	12	12	18	–	–	–	–	–	–	NCP	IGR	–	ID=gene-E
KAFASPR	Superscaf	+	Superscaf	57358978	57358998	15	15	21	–	–	–	–	–	–	NCP	IGR	–	ID=gene-E
EGGPKSH	Superscaf	–	Superscaf	57405289	57405309	-21	21	27	–	–	–	–	–	–	NCP	IGR	–	ID=gene-E
LLSAAGT	Superscaf	+	Superscaf	57538162	57538182	30	30	36	–	–	–	–	–	–	NCP	IGR	–	ID=gene-E
PSFPWTK	Superscaf	+	Superscaf	57548479	57548499	105	105	111	–	–	gene	–	–	mRNA	NCP	Intron	gene|mRN	ID=gene-E
TSSHRAAP	Superscaf	+	Superscaf	57566941	57566964	35	35	42	–	–	gene	–	–	mRNA	NCP	Intron	gene|mRN	ID=gene-E
LSAAPLVM	Superscaf	–	Superscaf	57607933	57607956	-49	49	56	–	–	–	–	–	–	NCP	IGR	–	ID=gene-E
LPAGGGKN	Superscaf	–	Superscaf	57658954	57658977	-26	26	33	–	–	–	–	–	–	NCP	IGR	–	ID=gene-E
MVPLLEL	Superscaf	–	Superscaf	57739813	57739833	-17	17	23	–	–	gene	–	–	mRNA	NCP	Intron	gene|mRN	ID=gene-E
KMKVLR	Superscaf	–	Superscaf	57797809	57797826	-21	21	26	–	–	–	–	–	–	NCP	IGR	–	ID=gene-E
EGLKKKMQ	Superscaf	+	Superscaf	57802210	57802233	3	3	10	–	–	–	–	–	–	NCP	IGR	–	ID=gene-E
SSFGWQ	Superscaf	+	Superscaf	57803953	57803970	13	13	18	–	–	–	–	–	–	NCP	IGR	–	ID=gene-E
KLNSGLK	Superscaf	–	Superscaf	57838933	57838953	-18	18	24	–	–	–	–	–	–	NCP	IGR	–	ID=gene-E
RFRCTEP	Superscaf	–	Superscaf	57846520	57846540	-5	5	11	–	–	–	–	–	–	NCP	IGR	–	ID=gene-E
KTVVP	Superscaf	–	Superscaf	57964255	57964269	-25	25	29	–	–	gene	–	–	mRNA	NCP	Intron	gene|mRN	ID=gene-E
HPKTQ	Superscaf	–	Superscaf	57965731	57965745	-27	27	31	–	–	gene	–	–	mRNA	NCP	Intron	gene|mRN	ID=gene-E
PLSSSPVFF	Superscaf	–	Superscaf	57967603	57967629	-4	4	12	–	–	gene	–	–	mRNA	NCP	Intron	gene|mRN	ID=gene-E
KPAEGV	Superscaf	–	Superscaf	57998293	57998310	-46	46	51	–	–	gene	–	–	mRNA	NCP	Intron	gene|mRN	ID=gene-E
QRVPPPP	Superscaf	+	Superscaf	57998983	57999003	17	17	23	–	–	gene	–	–	mRNA	NCP	Intron	gene|mRN	ID=gene-E
EPTFYK	Superscaf	–	Superscaf	58336912	58336929	-34	34	39	–	–	–	–	–	–	NCP	IGR	–	ID=gene-E
VAAHEVR	Superscaf	–	Superscaf	58370098	58370118	-6	6	12	–	–	gene	–	–	mRNA	NCP	Intron	gene|mRN	ID=gene-E
GLPKAGK	Superscaf	+	Superscaf	58381606	58381626	23	23	29	–	–	gene	–	–	mRNA	NCP	Intron	gene|mRN	ID=gene-E
SGPETESSF	Superscaf	–	Superscaf	58429642	58429692	-131	131	147	–	–	gene	–	–	mRNA	NCP	Intron	gene|mRN	ID=gene-E
LLVGHTPVF	Superscaf	–	Superscaf	58442950	58442979	-48	48	57	–	–	gene	–	–	mRNA	NCP	Intron	gene|mRN	ID=gene-E
TDVDLHAY	Superscaf	–	Superscaf	58442980	58443003	-58	58	65	–	–	gene	–	–	mRNA	NCP	Intron	gene|mRN	ID=gene-E
ALEKPGWEN	Superscaf	–	Superscaf	58443115	58443147	-103	103	113	–	–	gene	–	–	mRNA	NCP	Intron	gene|mRN	ID=gene-E
LNVDRVFAI	Superscaf	–	Superscaf	58446232	58446258	-63	63	71	–	exon	gene	–	five_prim	mRNA	NCP	5UTR	exon|gene	ID=exon-E
LNVDRVFAI	Superscaf	–	Superscaf	58446232	58446261	-63	63	72	–	exon	gene	–	five_prim	mRNA	NCP	5UTR	exon|gene	ID=exon-E
NVDRVFAL	Superscaf	–	Superscaf	58446235	58446258	-64	64	71	–	exon	gene	–	five_prim	mRNA	NCP	5UTR	exon|gene	ID=exon-E
SDEEKVLRF	Superscaf	–	Superscaf	58446262	58446288	-73	73	81	–	–	gene	–	–	mRNA	NCP	Intron	gene|mRN	ID=gene-E
VSFTVP	Superscaf	+	Superscaf	58505977	58505994	27	27	32	–	–	–	–	–	–	NCP	IGR	–	ID=gene-E
EMHCEC	Superscaf	+	Superscaf	58523365	58523382	5	5	10	–	–	gene	–	–	mRNA	NCP	Intron	gene|mRN	ID=gene-E
VVLFPF	Superscaf	+	Superscaf	58533868	58533885	22	22	27	–	–	gene	–	–	mRNA	NCP	Intron	gene|mRN	ID=gene-E
SLHTLYVW	Superscaf	–	Superscaf	58603138	58603161	-10	10	17	–	–	gene	–	–	mRNA	NCP	Intron	gene|mRN	ID=gene-E
LFNSLK	Superscaf	+	Superscaf	58652236	58652253	13	13	18	–	–	–	–	–	–	NCP	IGR	–	ID=gene-E
PDFVKS	Superscaf	–	Superscaf	58709782	58709799	-7	7	12	–	–	–	–	–	–	NCP	IGR	–	ID=gene-E
KMQVLYR	Superscaf	–	Superscaf	58763797	58763817	-7	7	13	–	–	–	–	–	–	NCP	IGR	–	ID=gene-E
QEKLTSK	Superscaf	–	Superscaf	58797301	58797321	-27	27	33	–	–	–	–	–	–	NCP	IGR	–	ID=gene-E
DPSVKAK	Superscaf	–	Superscaf	58847467	58847487	-14	14	20	–	–	gene	–	–	mRNA	NCP	Intron	gene|mRN	ID=gene-E
MTGLKKK	Superscaf	–	Superscaf	58998349	58998369	-28	28	34	–	–	–	–	–	–	NCP	IGR	–	ID=gene-E
LPVGPDP	Superscaf	–	Superscaf	59085373	59085393	-8	8	14	–	–	gene	–	–	mRNA	NCP	Intron	gene|mRN	ID=gene-E
AQKYLLR	Superscaf	–	Superscaf	59092420	59092440	-1	1	7	–	–	gene	–	–	mRNA	NCP	Intron	gene|mRN	ID=gene-E
LGLWYK	Superscaf	+	Superscaf	59245387	59245404	20	20	25	–	–	–	–	–	–	NCP	IGR	–	ID=gene-E
EEEGCKC	Superscaf	+	Superscaf	59266726	59266746	23	23	29	–	–	–	–	–	–	NCP	IGR	–	ID=gene-E
QMDSQK	Superscaf	+	Superscaf	59267509	59267526	17	17	22	–	–	–	–	–	–	NCP	IGR	–	ID=gene-E
VRNCML	Superscaf	–	Superscaf	59401642	59401659	-24	24	29	–	–	gene	–	–	mRNA	NCP	Intron	gene|mRN	ID=gene-E
TATTEMK	Superscaf	–	Superscaf	59418136	59418156	-2	2	8	–	–	–	–	–	–	NCP	IGR	–	ID=gene-E
YLLPKM	Superscaf	–	Superscaf	59421838	59421855	-33	33	38	–	–	–	–	–	–	NCP	IGR	–	ID=gene-E
GYKLL	Superscaf	+	Superscaf	59496139	59496153	28	28	32	–	–	–	–	–	–	NCP	IGR	–	ID=gene-E
TQAFK	Superscaf	+	Superscaf	59497471	59497485	20	20	24	–	–	–	–	–	–	NCP	IGR	–	ID=gene-E
AKPLTDQEK	Superscaf	–	Superscaf	59541004	59541039	-67	67	78	–	–	gene	–	–	mRNA	NCP	Intron	gene|mRN	ID=gene-E
AKPLTDQ	Superscaf	–	Superscaf	59541004	59541024	-67	67	73	–	–	gene	–	–	mRNA	NCP	Intron	gene|mRN	ID=gene-E
AKPLTD	Superscaf	–	Superscaf	59541004	59541021	-67	67	72	–	–	gene	–	–	mRNA	NCP	Intron	gene|mRN	ID=gene-E
PSPSVLK	Superscaf	–	Superscaf	59568535	59568555	-28	28	34	–	–	gene	–	–	mRNA	NCP	Intron	gene|mRN	ID=gene-E
SQFLEKEY	Superscaf	–	Superscaf	59573611	59573634	-14	14	21	–	–	gene	–	–	mRNA	NCP	Intron	gene|mRN	ID=gene-E
SPKQPDL	Superscaf	–	Superscaf	59577949	59577969	-40	40	46	–	exon	gene	three_pri	–	mRNA	NCP	3UTR	exon|gene	ID=exon-E
VALAPPPR	Superscaf	–	Superscaf	59638354	59638377	-126	126	133	–	–	gene	–	–	mRNA	NCP	Intron	gene|mRN	ID=gene-E
KTAVPGR	Superscaf	–	Superscaf	59648425	59648445	-8	8	14	–	–	gene	–	–	mRNA	NCP	Intron	gene|mRN	ID=gene-E
TCQLLLH	Superscaf	+	Superscaf	59689192	59689212	34	34	40	–	–	gene	–	–	mRNA	NCP	Intron	gene|mRN	ID=gene-E
ASLDKF	Superscaf	–	Superscaf	59690386	59690403	-17	17	22	–	–	gene	–	–	mRNA	NCP	Intron	gene|mRN	ID=gene-E
KFLLPK	Superscaf	–	Superscaf	59712256	59712273	-5	5	10	–	–	gene	–	–	mRNA	NCP	Intron	gene|mRN	ID=gene-E
TVPGWVP	Superscaf	–	Superscaf	59713219	59713239	-15	15	21	–	–	gene	–	–	mRNA	NCP	Intron	gene|mRN	ID=gene-E
PVALPPPL	Superscaf	+	Superscaf	59724106	59724129	11	11	18	–	–	gene	–	–	mRNA	NCP	Intron	gene|mRN	ID=gene-E
GAVAVHPP	Superscaf	+	Superscaf	59741224	59741247	31	31	38	–	–	gene	–	–	mRNA	NCP	Intron	gene|mRN	ID=gene-E
KDRTLNK	Superscaf	+	Superscaf	59744923	59744943	11	11	17	–	–	–	–	–	–	NCP	IGR	–	ID=gene-E
KSEPLLH	Superscaf	–	Superscaf	59822413	59822433	-23	23	29	–	–	–	–	–	–	NCP	IGR	–	ID=gene-E
ATSGYG	Superscaf	+	Superscaf	59833678	59833695	3	3	8	–	–	–	–	–	–	NCP	IGR	–	ID=gene-E
KHNARP	Superscaf	–	Superscaf	59838442	59838459	-18	18	23	–	–	gene	–	–	mRNA	NCP	Intron	gene|mRN	ID=gene-E
PVFPQR	Superscaf	+	Superscaf	59844208	59844225	24	24	29	–	–	gene	–	–	mRNA	NCP	Intron	gene|mRN	ID=gene-E
RVALALT	Superscaf	–	Superscaf	59874673	59874693	-26	26	32	–	–	gene	–	–	mRNA	NCP	Intron	gene|mRN	ID=gene-E
GVLKAVD	Superscaf	+	Superscaf	59915128	59915148	3	3	9	–	–	–	–	–	–	NCP	IGR	–	ID=gene-E
TGGLLL	Superscaf	+	Superscaf	59924743	59924760	7	7	12	–	–	gene	–	–	mRNA	NCP	Intron	gene|mRN	ID=gene-E
FALLMFLK	Superscaf	–	Superscaf	59947414	59947437	-1	1	8	–	–	–	–	–	–	NCP	IGR	–	ID=gene-E
KPAVVNK	Superscaf	+	Superscaf	59996584	59996604	26	26	32	–	–	gene	–	–	mRNA	NCP	Intron	gene|mRN	ID=gene-E
NPNGF	Superscaf	+	Superscaf	59997910	59997924	2	2	6	–	–	gene	–	–	mRNA	NCP	Intron	gene|mRN	ID=gene-E
CCPPPP	Superscaf	–	Superscaf	60015499	60015516	-24	24	29	–	–	gene	–	–	mRNA	NCP	Intron	gene|mRN	ID=gene-E
CCCDYS	Superscaf	+	Superscaf	60042157	60042174	20	20	25	–	–	–	–	–	–	NCP	IGR	–	ID=gene-E
TLDWVAF	Superscaf	–	Superscaf	60068419	60068439	-36	36	42	–	–	–	–	–	–	NCP	IGR	–	ID=gene-E
KLLQF	Superscaf	+	Superscaf	60105367	60105381	5	5	9	–	–	–	–	–	–	NCP	IGR	–	ID=gene-E
LPSFY	Superscaf	–	Superscaf	60115039	60115053	-32	32	36	–	–	–	–	–	–	NCP	IGR	–	ID=gene-E
LSKVVEF	Superscaf	–	Superscaf	60132784	60132804	-21	21	27	–	–	–	–	–	–	NCP	IGR	–	ID=gene-E
GKFNLL	Superscaf	–	Superscaf	60258790	60258807	-19	19	24	–	–	gene	–	–	mRNA	NCP	Intron	gene|mRN	ID=gene-E

7	6.598571429	2878.2	0.022213648	NLATLKT	98	760.454	1	759.449	.	T	Superscaf 52784922	0	0	0	0	0	BTP20220	Tissue specific
5	5.332	1843.2	0.022592673	MLDLL	62	604.3357	1	603.3302	.	G	Superscaf 52804276	0	0	0	BTP20220	0	0	Mixed
6	7.21	3110.5	0.022644438	RPHQEK	54	397.7095	2	793.4194	.	L	Superscaf 52806982	0	BTP20220	0	0	0	0	Tissue specific
8	5.915	2773.3	0.022778468	M(+42.01)	72	471.7719	2	941.5368	Acetylati	F	Superscaf 52814013	BTP20220	0	0	0	0	0	Tissue specific
7	6.912857143	3129.4	0.022976869	RDLKEKL	91	451.2753	2	900.5392	.	R	Superscaf 52824291	0	BTP20220	0	0	0	0	Tissue specific
7	5.952857143	2866.2	0.023480396	QTWLGGS	58	748.36	1	747.3552	.	L	Superscaf 52850218	0	BTP20220	0	0	0	0	Tissue specific
6	6.333333333	2819.2	0.024095062	KDPM(+15.	51	771.3362	1	770.3381	Oxidatior	C	Superscaf 52881859	BTP20220	0	0	0	0	0	Tissue specific
5	4.59	1883.1	0.024176863	VQLYY	75	685.3522	1	684.3483	.	H	Superscaf 52886143	BTP20220	0	0	0	0	0	Tissue specific
7	6.684285714	2769.1	0.024487624	FSPTTTK	72	391.2069	2	780.4017	.	S	Superscaf 52902223	0	0	0	0	0	BTP20220	Tissue specific
7	5.265714286	2340.4	0.024827085	WAGKVEA	65	380.7018	2	759.3915	.	I	Superscaf 52919814	0	0	0	0	0	BTP20220	Tissue specific
7	7.452857143	2926.1	0.025530756	KTPHGPK	52	382.7286	2	763.434	.	Q	Superscaf 52955974	BTP20220	0	0	0	0	0	Tissue specific
8	6.47	3364.5	0.025605469	KLEGLKFQ	75	481.7855	2	961.5596	.	S	Superscaf 52959769	0	0	0	0	0	BTP20220	Tissue specific
6	6.753333333	2883.1	0.026561455	KDWQTK	63	403.2131	2	804.413	.	K	Superscaf 53009332	0	BTP20220	0	0	0	0	Tissue specific
5	4.172	1885.2	0.027132723	GDDDL	72	534.2104	1	533.1969	.	G	Superscaf 53038831	0	0	BTP20220	0	0	0	Tissue specific
8	6.13625	2923.3	0.028576434	T(+42.01)	78	837.3953	1	836.3916	Acetylati	E	Superscaf 53113485	0	BTP20220	0	0	0	0	Mixed
7	6.684285714	3192.3	0.028690304	KLQDKDK	63	437.7514	2	873.4919	.	S	Superscaf 53119290	0	BTP20220	0	0	0	BTP20220	Tissue specific
7	6.071428571	2941.3	0.030254218	YLDRSGF	70	429.2097	2	856.4079	.	I	Superscaf 53199994	0	0	0	0	0	BTP20220	Tissue specific
7	6.938571429	2949	0.031647792	NSASKPK	58	366.2123	2	730.3973	.	F	Superscaf 53272080	0	0	0	BTP20220	0	0	Tissue specific
6	5.926666667	2335.6	0.034112267	LPGLSQ	65	614.3613	1	613.3435	.	T	Superscaf 53399236	0	0	0	0	BTP20220	0	Tissue specific
6	6.45	2436.7	0.035013178	RTAVQK	51	351.7177	2	701.4184	.	S	Superscaf 53445697	0	0	0	BTP20220	0	0	Tissue specific
7	5.93	2450.6	0.035887712	KEVTLPK	52	814.5124	1	813.496	.	I	Superscaf 53490945	0	BTP20220	0	0	0	0	Tissue specific
7	6.444285714	2656.8	0.036661971	PKLGLFY	60	419.2437	2	836.4796	.	N	Superscaf 53531013	0	0	0	0	0	BTP20220	Tissue specific
5	3.532	1124.2	0.038269632	LVAVY	75	564.3369	1	563.3318	.	Y	Superscaf 53613966	0	0	0	0	0	BTP20220	Tissue specific
6	5.656666667	2335.6	0.040188402	LDDLKP	89	700.3857	1	699.3802	.	S	Superscaf 53712946	0	0	BTP20220	0	0	0	Tissue specific
6	6.848333333	2695	0.040568357	K(+42.01)	71	425.2478	2	848.4756	Acetylati	N	Superscaf 53732578	0	0	0	0	0	BTP20220	Tissue specific
6	5.173333333	1956.1	0.044751633	K(+42.01)	79	758.407	1	757.401	Acetylati	S	Superscaf 53948602	0	BTP20220	0	0	0	0	Mixed
7	5.495714286	2727.9	0.044838779	KQGVLQF	57	410.2386	2	818.465	.	K	Superscaf 53953045	0	0	0	BTP20220	0	0	Tissue specific
6	5.543333333	2995.4	0.04488636	H(+42.01)	67	775.2897	1	774.2933	Acetylati	M	Superscaf 53955669	0	0	BTP20220	0	0	0	Tissue specific
6	4.85	2411	0.045044268	DM(+15.9	50	426.1377	2	850.2548	Oxidatior	G	Superscaf 53963644	0	0	0	0	0	BTP20220	Tissue specific
7	4.458571429	1882	0.046495125	KVGLEPV	79	741.447	1	740.4432	.	K	Superscaf 54038662	0	0	0	0	0	BTP20220	Tissue specific
5	5.828	2191.4	0.04698221	WGQGY	64	610.2604	1	609.2547	.	Q	Superscaf 54063828	0	0	0	0	0	BTP20220	Tissue specific
7	6.015714286	3138.5	0.047626274	C(+57.02)	59	835.3997	1	834.3906	Carbamidc	F	Superscaf 54097045	0	0	0	0	0	BTP20220	Tissue specific
5	4.276	1281.3	0.047807827	VYLKV	66	621.3955	1	620.3897	.	I	Superscaf 54106464	0	0	0	0	0	BTP20220	Tissue specific
6	6.495	2412.5	0.048636755	PAAKNF	58	647.3589	1	646.3438	.	K	Superscaf 54149349	0	0	0	0	BTP20220	0	Tissue specific
7	6.145714286	2858.1	0.048933224	N(+42.01)	55	463.2523	2	924.4818	Acetylati	K	Superscaf 54164619	0	0	0	0	0	BTP20220	Tissue specific
8	4.58125	2307.5	0.049305801	KVDPLVSL	71	435.7672	2	869.5222	.	S	Superscaf 54183790	0	0	0	0	BTP20220	0	Tissue specific
8	3.83375	1954.1	0.049473991	V(+42.01)	63	449.7638	2	897.5171	Acetylati	L	Superscaf 54192478	0	0	BTP20220	0	0	0	Tissue specific
7	6.342857143	2563.7	0.050104228	K(+42.01)	61	415.2323	2	828.4606	Acetylati	M	Superscaf 54225147	0	0	0	BTP20220	0	0	Tissue specific
8	5.42	2882	0.051238514	LVFKELEK	89	503.3004	2	1004.591	.	R	Superscaf 54283597	0	0	BTP20220	0	0	0	Tissue specific
6	4.888333333	2499.8	0.054528607	ANELEF	51	722.3317	1	721.3282	.	V	Superscaf 54453552	0	0	BTP20220	0	0	0	Tissue specific
6	6.421666667	2531.7	0.055030565	HLLEAK	81	355.711	2	709.4122	.	S	Superscaf 54479398	BTP20220	0	0	0	0	0	Tissue specific
7	6.974285714	3306.6	0.05639997	LGPHHKQ	61	408.7323	2	815.4402	.	R	Superscaf 54550395	0	0	BTP20220	0	0	0	Tissue specific
7	5.922857143	2534	0.056452954	VSTRLLT	56	395.2429	2	788.4756	.	G	Superscaf 54552882	BTP20220	0	0	0	0	0	Tissue specific
6	5.368333333	2429.7	0.056597151	ELGNYG	52	652.287	1	651.2864	.	S	Superscaf 54560352	0	0	BTP20220	0	0	0	Tissue specific
7	6.991428571	2893	0.056711602	KKNFPGP	60	394.2215	2	786.4388	.	N	Superscaf 54566238	0	0	0	0	0	BTP20220	Tissue specific
5	5.93	2008.1	0.05733539	KLFDF	65	669.3547	1	668.3533	.	K	Superscaf 54598213	0	0	0	0	0	BTP20220	Tissue specific
7	4.852857143	1955	0.057863899	WAAVVPK	55	385.7389	2	769.4486	.	A	Superscaf 54625740	0	0	0	0	0	BTP20220	Tissue specific
6	5.586666667	1857.9	0.058367426	V(+42.01)	77	358.7184	2	715.4268	Acetylati	V	Superscaf 54651831	0	0	0	0	BTP20220	0	Tissue specific
6	6.746666667	2433.8	0.058673365	LPRLLF	56	758.5017	1	757.485	.	S	Superscaf 54667539	0	0	BTP20220	0	0	0	Tissue specific
8	5.48625	3411.8	0.058766146	EVPQHLNK	67	482.762	2	963.5137	.	K	Superscaf 54672363	0	BTP20220	0	0	0	0	Tissue specific
6	5.053333333	1990.1	0.059172767	KVGDYL	80	694.3745	1	693.3697	.	S	Superscaf 54693280	0	0	0	0	0	BTP20220	Tissue specific
7	5.361428571	2630.1	0.060952336	EPEPTLL	67	399.715	2	797.4171	.	M	Superscaf 54785223	0	0	0	BTP20220	0	0	Tissue specific
8	4.975	2355.8	0.061789863	P(+42.01)	86	476.2886	2	950.5661	Acetylati	E	Superscaf 54828519	0	0	0	BTP20220	0	0	Tissue specific
5	4.562	1475.5	0.061970022	HFVVK	65	629.3696	1	628.3697	.	M	Superscaf 54837745	0	0	0	BTP20220	0	0	Tissue specific
7	6.351428571	2451.4	0.062417253	K(+42.01)	52	407.2444	2	812.4756	Acetylati	G	Superscaf 54860842	0	0	BTP20220	0	0	0	Tissue specific
6	5.628333333	2267.3	0.062603512	KDYVYK	83	815.4265	1	814.4225	.	F	Superscaf 54870436	0	0	0	0	0	BTP20220	Tissue specific
7	5.734285714	2706.1	0.063172456	TTTVHEK	72	408.2215	2	814.4185	.	R	Superscaf 54899901	0	BTP20220	0	BTP20220	BTP20220	0	Tissue specific
6	4.613333333	2124.5	0.06331613	T(+42.01)	65	728.3782	1	727.3752	Acetylati	E	Superscaf 54907320	0	0	BTP20220	0	0	0	Tissue specific
6	4.411666667	1984.1	0.064833159	VYKVNY	75	393.2115	2	784.4119	.	N	Superscaf 54985528	0	0	0	0	0	BTP20220	Tissue specific
8	5.7825	2635	0.06539153	M(+15.99)	62	486.7919	2	971.5725	Oxidatior	V	Superscaf 55014459	0	0	0	0	BTP20220	0	Tissue specific
7	7.96	3369.6	0.068250891	KRHTSSK	54	422.2516	2	842.4722	.	S	Superscaf 55162146	0	BTP20220	0	0	0	0	Tissue specific
7	5.104285714	2337.6	0.069811261	QLAPVPF	92	771.4373	1	770.4326	.	P	Superscaf 55242592	0	0	0	0	0	BTP20220	Tissue specific
6	6.491666667	2528.7	0.070536021	QFPFPK	82	382.2066	2	762.4064	.	Q	Superscaf 55280053	0	BTP20220	0	0	0	0	Tissue specific
7	7.157142857	3107.4	0.073149806	TKFGGQR	54	397.2236	2	792.4242	.	K	Superscaf 55415011	0	0	0	0	0	BTP20220	Tissue specific
6	6.34	2521.9	0.075616025	M(+42.01)	53	436.177	2	870.3438	Acetylati	D	Superscaf 55542307	0	0	0	0	0	BTP20220	Tissue specific
6	4.591666667	1903.4	0.077042597	M(+42.01)	60	383.1601	2	764.3085	Acetylati	R	Superscaf 55616139	0	0	0	0	BTP20220	0	Mixed
7	6.011428571	2488	0.077290091	R(+42.01)	70	421.2592	2	840.5069	Acetylati	I	Superscaf 55628836	0	0	BTP20220	0	0	0	Tissue specific
6	6.243333333	2449.9	0.077303511	D(+42.01)	70	380.7019	2	759.3949	Acetylati	K	Superscaf 55629579	0	0	BTP20220	0	0	0	Tissue specific
7	5.631428571	2211.4	0.077959252	KSVM(+15.	51	374.2079	2	746.3997	Oxidatior	A	Superscaf 55663440	0	0	BTP20220	0	0	0	Tissue specific
7	4.537142857	2107.4	0.078633352	VTHSLLV	58	384.7326	2	767.4542	.	V	Superscaf 55698261	0	0	BTP20220	0	0	0	Tissue specific
7	6.354285714	2502.7	0.080069975	LSPVARK	94	385.7466	2	769.481	.	G	Superscaf 55772380	0	0	BTP20220	0	0	0	Tissue specific
7	6.004285714	3075.4	0.082296195	GKNLPEN	54	771.3989	1	770.3923	.	M	Superscaf 55887486	0	BTP20220	0	0	0	0	Tissue specific
7	4.971428571	2312.5	0.082629556	TYGVSFF	72	820.3864	1	819.3803	.	E	Superscaf 55904655	BTP20220	0	0	0	0	0	Tissue specific
8	6.33875	2812.1	0.082907782	KLVPRLLG	76	448.3068	2	894.6014	.	L	Superscaf 55919076	0	0	0	0	0	BTP20220	Mixed
10	4.705	3560.9	0.082908712	VDEKTKEVI	94	594.8255	2	1187.64	.	L	Superscaf 55919076	0	0	0	BTP20220	0	0	Tissue specific
7	7.201428571	2987.2	0.082909758	RWAASPK	95	408.2281	2	814.4449	.	L	Superscaf 55919076	0	BTP20220	0	0	0	0	Tissue specific
8	5.195	3176.6	0.083001028	EPELLGFN	76	459.7234	2	917.4494	.	Q	Superscaf 55923807	0	0	0	BTP20220	0	0	Tissue specific
5	6.842	2016.2	0.084208517	K(+42.01)	95	663.3682	1	662.3639	Acetylati	K	Superscaf 55986129	0	0	BTP20220	0	0	0	Tissue specific
5	5.104	1713.9	0.084371479	YPHGV	54	572.2809	1	571.2755	.	Q	Superscaf 55994485	0	0	BTP20220	0	0	0	Tissue specific
6	3.993333333	1441.6	0.085288831	SVVSPP	70	585.3226	1	584.317	.	Q	Superscaf 56041914	0	0	BTP20220	0	0	0	Tissue specific
6	5.55	2418.9	0.086066924	RMELVH	68	392.718	2	783.4061	.	E	Superscaf 56082049	0	0	BTP20220	0	0	0	Tissue specific
6	7.96	2587.6	0.086599558	KFTKTK	51	376.7345	2	751.4592	.	K	Superscaf 56109337	0	0	BTP20220	0	0	0	Tissue specific
7	4.942857143	2582.9	0.08669687	NFALNVP	58	387.7113	2	773.4072	.	L	Superscaf 56114679	0	0	BTP20220	0	0	0	Tissue specific
7	7.682857143	2898.9	0.086783144	ATTKKFK	66	412.2599	2	822.4963	.	I	Superscaf 56119012	BTP20220	BTP20220	BTP20220	BTP20220	BTP20220	BTP20220	Tissue specific
7	6.108571429	2691.1	0.088204546	LKTVETR	98	423.7543	2	845.4971	.	H	Superscaf 56192383	0	0	BTP20220	0	0	0	Tissue specific
7	5.051428571	2042.2	0.088362163	LGGVLLF	66	718.4471	1	717.4425	.	L	Superscaf 56200582	0	BTP20220	BTP20220	0	BTP20220	0	Tissue specific
7	6.482857143	3187.5	0.0892238	KEDDLRK	63	452.2496	2	902.4821	.	P	Superscaf 56245036	0	0	BTP20220	0	BTP20220	0	Tissue specific
8	6.80625	3115.1	0.089450437	K(+42.01)	89	497.8223	2	993.6335	Acetylati	S	Superscaf 56256742	0	0	BTP20220	0	0	0	Tissue specific
6	7.325	3144.7	0.089646398	R(+42.01)	51	416.2234	2	830.4181	Acetylati	L	Superscaf 56266989	0	0	BTP20220	0	0	0	Tissue specific
7	4.925714286	2631	0.090403576	MGVFLQQ	69	822.4156	1	821.4106	.	I	Superscaf 56306022	0	0	0	BTP20220	0	0	Tissue specific
6	7.101666667	2643.9	0.090729501	KMQMLK	97	389.7172	2	777.4241	.	S	Superscaf 56323062	0	0	BTP20220	0	0	0	Tissue enhanced
15	5.708	5568.8	0.090747453	KAEDSLLA	99	751.8851	2	1501.762	.	H	Superscaf 56323833	0	0	0	0	BTP20220	0	Tissue specific
14	5.42	5100.4	0.090747511	AEDSLLAAI	95	687.8376	2	1373.668	.	H	Superscaf 56323833	0	0	0	0	0	BTP20220	Tissue specific
9	5.068888889	3774.2	0.091101786	C(+57.02)	97	560.7607	2	1119.512	Carbamidc	G	Superscaf 56342217	0	0	0	0	BTP20220	0	Tissue specific
16	5.32625	6584.4	0.091101786	C(+57.02)	99	945.9731	2	1889.94	Carbamidc	G	Superscaf 56342217	0	0	0	0	0	BTP20220	Tissue specific
15	5.032	6116	0.091101786	C(+57.02)	69	881.927	2	1761.846	Carbamidc	G	Superscaf 56342217	0	BTP20220	0	0	BTP20220	0	Tissue specific
12	5.415	4908.5	0.091102019	EEELKTVTN	90	709.3826	2	1416.746	.	G	Superscaf 56342217	0	0	0	0	BTP20220	BTP20220	Tissue specific
8	6.1675	3278.6	0.091102251	KTVTNNLK	98	459.2734	2	916.5342	.	G	Superscaf 56342217	0	BTP20220	0	0	BTP20220	0	Tissue specific
10	5.667	3759.1	0.091119216	AEFAERSVI	91	569.297	2	1136.583	.	I	Superscaf 56343054	0	0	0	0	BTP20220	0	Tissue specific
10	6.02	4575.1	0.091285257	HAKEENLSM	90	399.1876	3	1194.545	.	F	Superscaf 56351592	0	0	0	0	BTP20220	0	Tissue specific
10	5.826	4566.1	0.091285315	AKEENLSMH	99	593.7777	2	1185.545	.	F	Superscaf 56351592	0	0	0	0	0	BTP20220	Tissue specific
9	5.845555556	3955.4	0.091285315	AKEENLSMH	99	529.7484	2	1057.486	.	F	Superscaf 56351592	0	BTP20220	0	0	0	0	Tissue specific
8	5.3125	3786.4	0.091285431	EENLSMHQ	62	494.2117	2	986.4127	.	F	Superscaf 56351592	BTP20220	0	0	0	0	0	Tissue specific
15	5.573333333	6797.1	0.091285722	MHQMLDQTI	57	915.9268	2	1829.847	.	F	Superscaf 56351592	0	BTP20220	0	0	0	0	Tissue specific
14	5.560714286	6425.6	0.09128578	HQMLDQTLI	73	850.4072	2	1698.807	.	F	Superscaf 56351592	0	0	BTP20220	0	0	0	Tissue specific
13	5.404615385	5805.9	0.091285838	QMLDQTLLE	75	781.8785	2	1561.748	.	F	Superscaf 56351592	0	0	0	0	0	BTP20220	Tissue enhanced
11	5.350909091	4823.7	0.091285954	LDQTLLELN	84	652.3298	2	1302.649	.	F	Superscaf 56351592	0	0	0	0	BTP20220	BTP20220	Tissue specific
9	5.545555556	4058.8	0.09128607	QTLLELNNM	62	538.2733	2	1074.538	.	F	Superscaf 56351592	BTP20220	0	0	0	0	0	Tissue specific
8	5.5325	3448.1	0.091286128	TLLELNNM	98	947.4856	1	946.4794	.	F	Superscaf 56351592	0	0	0	0	0	BTP20220	Mixed
7	5.39	3050.6	0.091286186	LLELNNM	86	846.4359	1	845.4317	.	F	Superscaf 56351592	0	0	BTP20220	0	0	0	Tissue specific
6	5.291666667	2697.2	0.091286244	LELNNM	87	733.352	1	732.3476	.	F	Superscaf 56351592	0	0	0	0	0	BTP20220	Tissue specific
6	5.74	2355.7	0.091515263	A(+42.01)	61	382.2074	2	762.4024	Acetylati	L	Superscaf 56363368	0	0	0	0	0	BTP20220	Tissue specific
7	5.31	2281.5	0.092992322	TVGDLLK	62	373.2219	2	744.4381	.	L	Superscaf 56440113	0	0	0	BTP20220	0	0	Mixed
6	5.448333333	2286.6	0.093335559	FDLYPP	78	376.1859	2	750.3588	.	Y	Superscaf 56457462	0	0	0	0	0	BTP20220	Tissue specific
6	5.453333333	2460.6	0.094818602	QQAVYK	60	368.7032	2	735.3915	.	H	Superscaf 56533951	0	0	BTP20220	BTP20220	0	0	Tissue specific
6	6.575	2669	0.095199195	FFANRP	53	376.1962	2	750.3813	.	W	Superscaf 56553648	0	BTP20220	0	0	BTP20220	0	Tissue specific
7	5.434285714	2781.1	0.095802184	N(+42.01)	68	805.3708	1	804.3654	Acetylati	N	Superscaf 56584723	0	BTP20220	0	0	BTP20220	BTP20220	Tissue specific
8	6.49625	2842	0.097126448	P(+42.01)	72	824.4813	1	823.4803	Acetylati	L	Superscaf 56653236	0	0	0	0	BTP20220	BTP20220	Tissue specific
7	7.085714286	2621.6	0.099753363	KPPAFAK	50	379.7323	2	757.4486	.	I	Superscaf 56788935	0	0	BTP20220	BTP20220	0	0	Tissue specific
7	6.107142857	2719.9	0.100643119	SHALALF	61	379.7123	2	757.4122	.	P	Superscaf 56834838	0	0	0	BTP20220	0	0	Tissue specific
5	4.412	1117.1	0.101799946	V(+42.01)	54	555.3593	1	554.3428	Acetylati	F	Superscaf 56894529	0	0	BTP20220	0	0	0	Tissue specific
7	5.061428571	2626.1	0.104138758	M(+42.01)	62	892.4092	1	891.4048	Acetylati	K	Superscaf 57015157	0	0	0	0	0	BTP20220	Tissue specific
6	5.876666667	2389.6	0.105078361	HLKVPQ	92	361.2203	2	720.4282	.	R	Superscaf 57063730	0	BTP20220	0	0	0	0	Tissue specific
7	4.677142857	2003.3	0.106613866	PTVAHVP	62	720.3904	1	719.3966	.	S	Superscaf 57143091	0	BTP20220	0	0	BTP20220	BTP20220	Tissue specific
7	5.588571429	2791.2	0.106716291	NELATLL	58	773.439	1	772.433	.	M	Superscaf 57148207	0	BTP20220	0	0	0	0	Tissue specific
5	5.884	1646.7	0.107791144	ALGGF	95	464.2488	1	463.243	.	R	Superscaf 57203940	0	0	0	0	BTP20220	0	Tissue specific
6	6.081666667	2617.8	0.107935805	KVWSHH	51	397.2103	2	792.4031	.	I	Superscaf 57211276	0	0	BTP20220	0	0	0	Tissue specific
7	6.2	2739	0.108054033	TGPELKY	83	404.215	2	806.4174	.	K	Superscaf 57217506	0	0	0	BTP20220	0	0	Tissue specific
5	6.206	2428.7	0.108148034	T(+42.01)	74	733.3479	1	732.3442	Acetylati	A	Superscaf 57222306	0	BTP20220	0	0	0	0	Tissue specific
7	6.265714286	2922.2	0.108853216	KGFDGDR	52	397.7002	2	793.3718	.	T	Superscaf 57258888	BTP20220	0	0	0	0	0	Tissue specific
7	5.577142857	2924.3	0.108871226	M(+42.01)	72	848.3667	1	847.3746	Acetylati	A	Superscaf 57259686	0	0	0	0	0	BTP20220	Tissue specific
7	7.75	3035.2	0.109835869	KRFATLK	51	432.279	2	862.5388	.	R	Superscaf 57309373	0	0	0	BTP20220	0	0	Tissue specific
6	5.286666667	2515.8	0.11039023	A(+42.01)	61	362.6581	2	723.3075	Acetylati	M	Superscaf 57338047	0	0	0	BTP20220	0	0	Tissue specific
7	5.014285714	2030.1	0.110558828	VGAGSFL	52	650.338	1	649.3435	.	Q	Superscaf 57346735	BTP20220	0	0	0	BTP20220	0	Tissue specific
7	7.142857143	2848	0.110795283	KAFASPR	52	388.7275	2	775.434	.	A	Superscaf 57358936	0	0	0	0	0	BTP20220	Tissue specific
7	6.352857143	2829	0.111692127	EGGPKSH	66	356.1695	2	710.3347	.	H	Superscaf 57405360	0	BTP20220	0	0	0	0	Tissue specific
7	6.025714286	2407.6	0.114265302	LLSAAGT	99	632.3586	1	631.3541	.	G	Superscaf 57538075	0	0	0	0	0	BTP20220	Tissue specific
7	6.56	2838.1	0.114465097	P(+42.01)	75	452.7281	2	903.449	Acetylati	G	Superscaf 57548167	0	0	0	0	0	BTP20220	Tissue specific
8	6.8225	3392.8	0.114822626	TSSHRAAP	70	413.7148	2	825.4093	.	S	Superscaf 57566839	0	0	0	0	0	BTP20220	Tissue specific
8	5.21625	2421.7	0.115616464	LSAAPLVM	51	801.4509	1	800.4466	.	V	Superscaf 57607977	BTP20220	0	0	0	0	0	Mixed
8	6.42	2959.1	0.11660452	LPAGGGKN	52	713.3807	1	712.3868	.	T	Superscaf 57658986	0	BTP20220	0	0	0	0	Tissue specific
7	4.744285714	2194.6	0.118170409	M(+15.99)	64	436.744	2	871.4725	Oxidatior	Q	Superscaf 57739851	BTP20220	0	0	0	0	0	Tissue specific
6	6.995	2310.5	0.119293541	KMKVLR	62	387.7559	2	773.4946	.	E	Superscaf 57797871	0	0	0	0	0	BTP20220	Tissue specific
8	6.97375	3463.6	0.119378769	EGLKKKMQ	86	481.273	2	960.5426	.	S	Superscaf 57802204	0	0	0	0	0	BTP20220	Tissue specific
6	5.725	2588.8	0.119412524	SSFGWQ	91	356.1569	2	710.3024	.	N	Superscaf 57803917	0	BTP20220	0	0	BTP20220	BTP20220	Tissue specific
7	6.928571429	2921	0.120089935	KLNSGLK	75	380.2372	2	758.465	.	A	Superscaf 57838971	0	0	0	0	BTP20220	0	Tissue specific
7	6.867142857	3431.1	0.120236862	RFRC(+57.	91	483.2331	2	964.4548	Carbamidc	S	Superscaf 57846543	0	0	0	0	BTP20220	0	Tissue specific
5	4.514	1203.3	0.12251688	KTVVP	51	543.3485	1	542.3428	.	A	Superscaf 57964287	0	0	0	0	0	BTP20220	Mixed
5	7.162	2433.7	0.122545464	HPKTQ	77	610.3369	1	609.3234	.	Q	Superscaf 57965751	0	BTP20220	0	0	0	0	Tissue specific
9	5.266666667	2903.2	0.122581716	PLSSSPVFF	61	465.7479	2	929.4858	.	D	Superscaf 57967629	0	0	0	0	BTP20220	0	Tissue specific
6	5.208333333	1839.9	0.123176049	K(+42.01)	73	642.3438	1	641.3384	Acetylati	E	Superscaf 57998433	0	0	0	BTP20220	0	0	Tissue specific
7	5.944285714	2609.1	0.123189411	QRVPPPP	66	395.7302	2	789.4497	.	L	Superscaf 57998935	0	0	0	BTP20220	0	0	Tissue specific
6	6.155	2475.7	0.129733634	EPTFYK	53	392.6959	2	783.3802	.	G	Superscaf 58336956	0	0	BTP20220	0	0	0	Tissue specific
7	4.801428571	2316.6	0.130376303	V(+42.01)	94	412.2295	2	822.4348	Acetylati	D	Superscaf 58370235	0	0	0	0	0	BTP20220	Tissue specific
7	7.102857143	2533.5	0.130599164	G(+42.01)	59	356.7197	2	711.4279	Acetylati	K	Superscaf 58381540	0	0	0	BTP20220	0	0	Tissue specific
17	5.980588235	7033	0.131529413	SGPETESSF	88	622.964	3	1865.876	.	E	Superscaf 58430025	0	BTP20220	0	0	0	0	Tissue specific
10	5.142	3365.8	0.131787131	LLVGHTPVF	98	373.8798	3	1118.624	.	G	Superscaf 58443306	0	0	0	0	0	BTP20220	Tissue specific
8	4.715	2964.4	0.131787712	TDVDLHAY	97	467.217	2	932.424	.	G	Superscaf 58443306	0	0	BTP20220	0	BTP20220	0	Tissue specific
11	5.745454545	4426.8	0.131790327	ALEKPGWEN	82	624.3035	2	1246.598	.	G	Superscaf 58443306	0	0	0	0	BTP20220	0	Tissue specific
9	4.733333333	3062.5	0.13185069	LNVDRVFAI	83	523.8004	2	1045.592	.	Y	Superscaf 58446408	0	0	0	0	BTP20220	0	Tissue specific
10	4.826	3522	0.13185069	LNVDRVFAI	92	605.3326	2	1208.655	.	Y	Superscaf 58446408	BTP20220	0	0	0	0	0	Tissue specific
8	4.5775	2709.1	0.131850748	NVDRVFAL	92	467.2592	2	932.5079	.	Y	Superscaf 58446408	0	BTP20220	0	0	0	0	Tissue specific
9	5.227777778	3503.9	0.131851271	SDEEKVLRF	85	561.7914	2	1121.572	.	Y	Superscaf 58446408	0	0	0	BTP20220	0	0	Tissue specific
6	3.998333333	1505.7	0.133007692	VSFTVP	50	649.3533	1	648.3483	.	W	Superscaf 58505899	0	0	BTP20220	0	0	0	Tissue specific
6	4.97	3013.6	0.133344422	EMHC(+57.	58	433.1306	2	864.2565	Carbamidc	L	Superscaf 58523353	0	0	BTP20220	0	0	0	Tissue specific
6	3.873333333	1465.6	0.13354782	VVLFPF	84	721.426	1	720.421	.	K	Superscaf 58533805	0	BTP20220	0	0	0	0	Tissue specific
8	5.41375	3093.5	0.13488928	SLHTLYVW	61	509.7776	2	1017.528	.	G	Superscaf 58603212	BTP20220	0	0	0	0	0	Tissue specific
6	6.378333333	2542.7	0.135840096	LFNSLK	97	361.2143	2	720.417	.	I	Superscaf 58652200	0	BTP20220	0	0	0	0	Tissue specific
6	5.028333333	1988.1	0.136954513	P(+42.01)	59	734.3731	1	733.3646	Acetylati	L	Superscaf 58709880	0	0	0	0	BTP20220	0	Tissue specific
7	6.22	2912.3	0.13800055	KM(+15.9	79	477.263	2	952.5164	Oxidatior	V	Superscaf 58763844	0	0	BTP20220	0	0	0	Tissue specific
7	6.648571429	3107.3	0.138649377	QEKLTSK	58	417.2396	2	832.4654	.	P	Superscaf 58797342	0	0	0	0	0	BTP20220	Tissue specific
7	5.778571429	2380.4	0.139620876	DPSVKAK	54	372.7174	2	743.4177	.	P	Superscaf 58847526	0	0	0	0	0	BTP20220	Tissue specific
7	7.635714286	2824.9	0.142542808	MTGLKKK	57	403.2478	2	804.4891	.	A	Superscaf 58998393	0	0	0	BTP20220	0	0	Tissue specific
7	4.831428571	2074.4	0.144228086	LPVGPDP	80	694.3746	1	693.3697	.	V	Superscaf 59085573	0	0	0	BTP20220	0	0	Tissue specific
7	7.112857143	3205.5	0.144364556	AQKYLLR	54	446.2729	2	890.5338	.	A	Superscaf 59092440	0	0	BTP20220	0	0	0	Tissue specific
6	6.536666667	2458.6	0.147326865	LGLWYK	98	390.2243	2	778.4377	.	G	Superscaf 59245330	0	0	0	BTP20220	0	0	Tissue specific
7	5.058571429	3213.6	0.147740109	E(+42.01)	51	953.3307	1	952.3266	Acetylati	A	Superscaf 59266660	0	0	0	0	0	BTP20220	Tissue specific
6	5.906666667	2856.2	0.147755272	QMDSQK	50	368.6676	2	735.3221	.	M	Superscaf 59267461	0	BTP20220	BTP20220	BTP20220	BTP20220	0	Tissue specific
6	5.486666667	2556.1	0.150352848	VRNC(+57.	51	396.695	2	791.3782	Carbamidc	K	Superscaf 59401704	0	0	BTP20220	0	0	0	Tissue specific
7	6.331428571	2769.2	0.150672266	TATTEMK	50	391.189	2	780.3688	.	L	Superscaf 59418210	0	0	BTP20220	0	0	0	Tissue specific
6	6.568333333	2343.6	0.150743958	YLLPKM(+1	69	390.7216	2	779.4251	Oxidatior	P	Superscaf 59421867	BTP20220	0	0	0	0	0	Tissue specific
5	6.666	1932	0.152182847	GYKLL	55	593.3665	1	592.3584	.	L	Superscaf 59496058	0	BTP20220	0	0	0	0	Tissue specific
5	6.684	2175.3	0.152208642	T(+42.01)	61	636.3441	1	635.3279	Acetylati	L	Superscaf 59497414	0	0	BTP20220	0	0	0	Tissue specific
12	6.943333333	5692.4	0.153051687	A(+42.01)	88	504.6125	3	1510.822	Acetylati	G	Superscaf 59541765	0	0	0	0	BTP20220	0	Tissue specific
7	6.17	2890.2	0.153051687	A(+42.01)	99	814.4259	1	813.4232	Acetylati	G	Superscaf 59541765	0	0	0	0	0	BTP20220	Tissue specific
6	6.256666667	2279.5	0.153051687	A(+42.01)	81	686.3699	1	685.3646	Acetylati	G	Superscaf 59541765	0	0	BTP20220	0	0	0	Tissue specific
7	5.668571429	2263.4	0.153584844	PSPSVLK	99	364.2199	2	726.4276	.	S	Superscaf 59568807	0	0	BTP20220	0	0	0	Tissue specific
8	5.57875	3513.8	0.153683144	SQFLEKEY	79	522.2534	2	1042.497	.	P	Superscaf 59573889	0	BTP20220	0	0	0	0	Tissue specific
7	6.088571429	2902.2	0.153767152	SPKQPDL	59	392.7126	2	783.4127	.	Q	Superscaf 59578008	0	BTP20220	0	0	0	0	Tissue specific
8	5.96	2637	0.154936936	V(+42.01)	61	431.7646	2	861.5072	Acetylati	I	Superscaf 59639124	0	0	BTP20220	0	0	0	Tissue specific
7	6.474285714	2460.7	0.155131968	K(+42.01)	66	385.7274	2	769.4446	Acetylati	S	Superscaf 59648457	0	0	0	BTP20220	0	0	Tissue specific
7	6.104285714	3273.8	0.155921448	T(+42.01)	70	926.47	1	925.4692	Acetylati	I	Superscaf 59689093	0	0	0	BTP20220	0	0	Tissue specific
6	5.978333333	2315.4	0.155944571	ASLDKF	98	680.358	1	679.3541	.	T	Superscaf 59690481	0	0	BTP20220	0	0	0	Tissue specific
6	7.203333333	2368.4	0.156368098	KFLLPK	59	373.2528	2	744.4897	.	C	Superscaf 59712291	BTP20220	0	0	0	0	0	Tissue specific
7	4.427142857	1896.2	0.156386747	T(+42.01)	52	797.4165	1	796.4119	Acetylati	A	Superscaf 59713245	0	0	BTP20220	0	0	0	Tissue specific
8	5.3975	2367.7	0.156597581	P(+42.01)	52	423.2584	2	844.5058	Acetylati	C	Superscaf 59724076	0	0	BTP20220	0	0	0	Tissue specific
8	4.775	2214.4	0.156929083	GAVAVHPP	51	374.2148	2	746.4075	.	G	Superscaf 59741134	0	0	BTP20220	0	0	0	Tissue specific
7	7.304285714	3344.7	0.157000716	KDRTLNK	74	437.7565	2	873.5032	.	K	Superscaf 59744893	0	0	BTP20220	0	0	0	Tissue specific
7	6.355714286	2941.2	0.158501363	KSEPLLH	52	412.2412	2	822.4599	.	F	Superscaf 59822445	0	0	0	BTP20220	0	0	Tissue specific
6	5.971666667	2146.3	0.158719517	ATSGYG	59	555.2432	1	554.2336	.	Q	Superscaf 59833672	0	0	0	0	0	BTP20220	Mixed
6	7.636666667	2982.3	0.158811775	KHNARP	52	361.7109	2	721.3983	.	K	Superscaf 59838621	0	0	BTP20220	0	0	0	Tissue specific
6	5.748333333	2321.7	0.158923437	PVFPQR	91	372.2123	2	742.4126	.	T	Superscaf 59844139	0	BTP20220	0	0	0	0	Mixed
7	5.898571429	2375.7	0.159513413	RVALALT	63	372.2358	2	742.4701	.	K	Superscaf 59874750	0	0	0	BTP20220	0	0	Tissue specific
7	4.382857143	1841.9	0.160296851	GVLKAVD	68	701.4171	1	700.4119	.	S	Superscaf 59915122	0	0	BTP20220	BTP20220	0	0	Tissue specific
6	6.068333333	2052.3	0.160483052	TGGLLL	56	573.3583	1	572.3533	.	V	Superscaf 59924725	0	0	0	0	BTP20220	0	Tissue specific
8	6.30125	2986.2	0.160922091	FALLMFLK	69	491.7991	2	981.5721	.	F	Superscaf 59947449	0	BTP20220	0	0	0	0	Tissue specific
7	5.315714286	2182.2	0.161874301	KPAVVNK	50	378.2397	2	754.4701	.	I	Superscaf 59996509	0	0	BTP20220	0	0	0	Tissue specific
5	5.714	2215.5	0.16189998	N(+42.01)	78	590.2555	1	589.2496	Acetylati	K	Superscaf 59997907	0	0	0	BTP20220	0	0	Tissue specific
6	5.873333333	2521	0.162240603	C(+57.02)	90	385.1512	2	768.2935	Carbamidc	I	Superscaf 60015549	0	0	0	0	BTP20220	0	Tissue specific
6	4.895	3011.5	0.162756853	C(+57.02)	53	906.2552	1	905.2354	Carbamidc	Q	Superscaf 60042100	0	0	0	0	0	BTP20220	Tissue specific
7	4.695714286	2387.7	0.163265435	TLDWVAF	82	851.4253	1	850.4225	.	Q	Superscaf 60068478	0	0	0	BTP20220	0	0	Tissue specific
5	6.566	2173.3	0.163980958	K(+42.01)	76	690.4147	1	689.4112	Acetylati	S	Superscaf 60105355	0	0	0	0	BTP20220	0	Tissue specific
5	5.82	1921.1	0.164168262	LPSFY	51	626.3176	1	625.3112	.	K	Superscaf 60115056	BTP20220	0	0	0	0	0	Tissue specific
7	4.3	2018.1	0.164511906	LSKVVEF	69	411.2477	2	820.4694	.	E	Superscaf 60132837	0	BTP20220	0	0	0	0	Tissue specific
6	6.426666667	2456.6	0.166952097	G(+42.01)	55	733.4232	1	732.417	Acetylati	I	Superscaf 60258813	0	0	0	0	BTP20220	0	Tissue specific

KPHYGPK	Superscaf	-	Superscaf	60373387	60373407	-47	47	53	-	-	-	-	-	-	NCP	IGR	-	ID=gene-E	7	7.328571429	2988.1	0.169171346	KPHYGPK	51	413.7357	2	825.4497	.	R	Superscaf	60373485	0	BTP202209	0	0	BTP202209	BTP202209	Tissue specific
MHVKHR	Superscaf	-	Superscaf	60434470	60434487	-2	2	7	-	-	-	-	-	-	NCP	IGR	-	ID=gene-E	6	6.905	2728.1	0.170354259	MHVKHR	64	404.2185	2	806.4333	.	S	Superscaf	60434511	0	0	0	BTP202209	0	0	Tissue specific
VLLTKKP	Superscaf	+	Superscaf	60449491	60449511	12	12	18	-	-	-	-	-	-	NCP	IGR	-	ID=gene-E	7	6.324285714	2378.5	0.170645151	VLLTKKP	84	399.7747	2	797.5374	.	D	Superscaf	60449458	0	BTP202209	0	BTP202209	BTP202209	BTP202209	Tissue specific
LRVPPLTPG	Superscaf	-	Superscaf	60490105	60490131	-173	173	181	-	-	-	-	-	-	NCP	IGR	-	ID=gene-E	9	5.977777778	3376	0.171431668	LRVPPLTPG	51	510.821	2	1019.613	.	K	Superscaf	60490209	0	0	0	0	0	BTP202209	Tissue specific
HLVALLH	Superscaf	+	Superscaf	60500095	60500115	9	9	15	-	-	-	-	-	-	NCP	IGR	-	ID=gene-E	7	5.591428571	2610.9	0.171625131	HLVALLH	59	401.7491	2	801.4861	.	I	Superscaf	60500071	0	0	0	0	BTP202209	0	Tissue specific
LVEKVLR	Superscaf	-	Superscaf	60533104	60533124	-30	30	36	-	-	-	-	-	-	NCP	IGR	-	ID=gene-E	7	5.097142857	2249.5	0.172264373	LVEKVLR	82	428.7828	2	855.5541	.	H	Superscaf	60533184	0	0	0	0	BTP202209	0	Tissue specific
LAKAQ	Superscaf	+	Superscaf	60550858	60550872	16	16	20	-	-	-	-	-	-	NCP	IGR	-	ID=gene-E	5	6.682	2055.1	0.172608191	LAKAQ	67	530.3273	1	529.3224	.	P	Superscaf	60550813	0	BTP202209	0	0	0	0	Tissue specific
AMRAQR	Superscaf	+	Superscaf	60572464	60572481	48	48	53	-	-	-	-	-	-	NCP	IGR	-	ID=gene-E	6	7.493333333	2902.4	0.173026606	AMRAQR	92	366.6942	2	731.386	.	V	Superscaf	60572323	0	0	0	0	0	BTP202209	Tissue specific
GFKTAF	Superscaf	-	Superscaf	60616954	60616971	-6	6	11	-	-	-	-	-	-	NCP	IGR	-	ID=gene-E	6	6.536666667	2249.3	0.173888185	G(+42.01)	70	712.3613	1	711.3591	Acetylati	P	Superscaf	60617064	0	0	0	0	0	BTP202209	Tissue specific
LVRTLF	Superscaf	+	Superscaf	60647935	60647952	3	3	8	-	-	-	-	-	-	NCP	IGR	-	ID=gene-E	6	5.788333333	2140.5	0.174488153	LVRTLF	51	374.7375	2	747.4643	.	N	Superscaf	60647929	BTP202209	0	0	0	0	0	Tissue specific
KSGSKAP	Superscaf	-	Superscaf	60664579	60664599	-9	9	15	-	-	-	-	-	-	NCP	IGR	-	ID=gene-E	7	7.018571429	2649.6	0.174810475	KSGSKAP	52	674.3845	1	673.3759	.	S	Superscaf	60664617	0	0	0	BTP202209	0	0	Tissue specific
VNVPKTR	Superscaf	-	Superscaf	60679159	60679179	-34	34	40	-	-	-	-	-	-	NCP	IGR	-	ID=gene-E	7	5.534285714	2448.8	0.175092826	VNVPKTR	97	407.2499	2	812.4868	.	V	Superscaf	60679473	0	0	BTP202209	0	0	0	Mixed
LECVEPNCF	Superscaf	-	Superscaf	60679363	60679395	-102	102	112	-	-	-	-	-	-	NCP	IGR	-	ID=gene-E	11	5.486363636	4810.5	0.175096777	LEC(+57.0	96	464.551	3	1390.633	Carbamido	V	Superscaf	60679473	0	BTP202209	0	0	0	0	Mixed
LECVEPNCF	Superscaf	-	Superscaf	60679363	60679392	-102	102	111	-	-	-	-	-	-	NCP	IGR	-	ID=gene-E	10	5.061	4342.1	0.175096777	LEC(+57.0	93	632.2738	2	1262.538	Carbamido	V	Superscaf	60679473	0	0	0	0	0	BTP202209	Tissue specific
KYVPPP	Superscaf	-	Superscaf	60681208	60681225	-4	4	9	-	-	-	-	-	-	NCP	IGR	-	ID=gene-E	6	5.716666667	1940.1	0.175132507	KYVPPP	55	350.7028	2	699.3955	.	I	Superscaf	60681234	0	0	0	0	BTP202209	0	Tissue specific
KGELFR	Superscaf	-	Superscaf	60691363	60691380	-6	6	11	-	-	-	-	-	-	NCP	IGR	-	ID=gene-E	6	6.858333333	2580.8	0.175329165	KGELFR	51	375.2127	2	748.4232	.	V	Superscaf	60691473	0	BTP202209	0	0	0	0	Tissue specific
LYEYSGQ	Superscaf	+	Superscaf	60692581	60692601	8	8	14	-	-	-	-	-	-	NCP	IGR	-	ID=gene-E	7	5.402857143	2989.3	0.175352753	LYEYSGQ	56	430.1957	2	858.3759	.	K	Superscaf	60692560	0	0	0	0	0	BTP202209	Tissue specific
SYCRPV	Superscaf	+	Superscaf	60695317	60695334	3	3	8	-	-	-	-	-	-	NCP	IGR	-	ID=gene-E	6	5.57	2414.8	0.175405737	SYC(+57.0	98	391.186	2	780.3588	Carbamido	L	Superscaf	60695311	0	BTP202209	0	0	0	BTP202209	Tissue specific
AKALVK	Superscaf	+	Superscaf	60796849	60796866	3	3	8	-	-	-	-	-	-	NCP	IGR	-	ID=gene-E	6	6.25	1912.8	0.177371973	AKALVK	60	629.4316	1	628.4272	.	T	Superscaf	60796843	0	0	BTP202209	0	0	0	Tissue specific
AEELGLK	Superscaf	-	Superscaf	60827053	60827073	-9	9	15	-	-	-	-	-	-	NCP	IGR	-	ID=gene-E	7	5.732857143	2634.8	0.177956893	AEELGLK	94	380.2144	2	758.4174	.	M	Superscaf	60827079	0	0	0	0	0	BTP202209	Tissue specific
KNYRPP	Superscaf	-	Superscaf	60847018	60847035	-5	5	10	-	-	-	-	-	-	NCP	IGR	-	ID=gene-E	6	7.361666667	2848.2	0.178343529	KNYRPP	58	387.7095	2	773.4184	.	S	Superscaf	60847077	0	0	0	0	0	BTP202209	Tissue specific
KAKPL	Superscaf	+	Superscaf	60882421	60882435	29	29	33	-	-	gene	-	-	mRNA	NCP	Intron	gene|mRNA	ID=gene-E	5	7.556	1938.9	0.179029132	KAKPL	55	556.3916	1	555.3744	.	Y	Superscaf	60882337	0	0	0	BTP202209	0	0	Tissue specific
MGVTTK	Superscaf	-	Superscaf	60890344	60890361	-9	9	14	-	-	gene	-	-	mRNA	NCP	Intron	gene|mRNA	ID=gene-E	6	5.753333333	1932.2	0.179182566	MGVTTK	58	636.3319	1	635.3312	.	A	Superscaf	60890379	0	0	0	0	0	BTP202209	Tissue specific
KEYLP	Superscaf	-	Superscaf	60899158	60899172	-17	17	21	-	-	gene	-	-	mRNA	NCP	Intron	gene|mRNA	ID=gene-E	5	6.18	2044.2	0.179353255	KEYLP	67	649.3543	1	648.3483	.	L	Superscaf	60899253	0	BTP202209	0	0	0	0	Mixed
TVYYLT	Superscaf	+	Superscaf	60932980	60932997	1	1	6	-	-	gene	-	-	mRNA	NCP	Intron	gene|mRNA	ID=gene-E	6	5.06	2067.4	0.180008241	TVYYLT	55	759.3867	1	758.3851	.	T	Superscaf	60932980	0	0	0	0	BTP202209	0	Tissue specific
VGPGY	Superscaf	-	Superscaf	60933916	60933930	-42	42	46	-	-	gene	-	-	mRNA	NCP	Intron	gene|mRNA	ID=gene-E	5	4.78	1391.5	0.180026368	VGPGY	85	492.2441	1	491.238	.	H	Superscaf	60934023	0	0	BTP202209	0	0	0	Tissue specific
LNDTEM	Superscaf	-	Superscaf	60954178	60954195	-1	1	6	-	-	gene	-	-	mRNA	NCP	Intron	gene|mRNA	ID=gene-E	6	4.976666667	2556.1	0.180418755	LNDTEM(+1	52	369.6516	2	737.2902	Oxidation	L	Superscaf	60954231	0	0	BTP202209	0	0	0	Tissue specific
PLLRLLK	Superscaf	-	Superscaf	60987238	60987258	-39	39	45	-	-	gene	-	-	mRNA	NCP	Intron	gene|mRNA	ID=gene-E	7	7.245714286	2868.2	0.181058984	PLLRLLK	60	426.8028	2	851.5956	.	V	Superscaf	60987393	0	0	0	0	0	BTP202209	Tissue specific
SGDLFGF	Superscaf	-	Superscaf	60990892	60990912	-1	1	7	-	-	gene	-	-	mRNA	NCP	Intron	gene|mRNA	ID=gene-E	7	5.361428571	2517.7	0.181129746	SGDLFGF	73	742.3376	1	741.3333	.	S	Superscaf	60991014	0	0	0	0	0	BTP202209	Tissue specific
KTNETSK	Superscaf	+	Superscaf	60997525	60997545	12	12	18	-	-	gene	-	-	mRNA	NCP	Intron	gene|mRNA	ID=gene-E	7	6.692857143	3137.4	0.181258199	KTNETSK	50	404.2137	2	806.4134	.	Y	Superscaf	60997492	0	BTP202209	0	0	0	0	Tissue specific
KPEGEP	Superscaf	+	Superscaf	61116172	61116189	5	5	10	-	-	-	-	-	-	NCP	IGR	-	ID=gene-E	6	5.791666667	2291.5	0.183555878	KPEGEP	52	656.3239	1	655.3177	.	D	Superscaf	61116160	0	0	BTP202209	0	0	0	Tissue specific
MSVFHKK	Superscaf	+	Superscaf	61150741	61150761	22	22	28	-	-	gene	-	-	mRNA	NCP	Intron	gene|mRNA	ID=gene-E	7	6.282857143	2698.8	0.18422533	MSVFHKK	58	438.7499	2	875.4688	.	N	Superscaf	61150678	0	BTP202209	0	0	0	0	Tissue specific
LFLGGLS	Superscaf	+	Superscaf	61153750	61153770	21	21	27	-	-	gene	-	-	mRNA	NCP	Intron	gene|mRNA	ID=gene-E	7	5.862857143	2425.6	0.184283601	LFLGGLS	79	706.4114	1	705.4061	.	S	Superscaf	61153690	0	0	0	0	0	BTP202209	Tissue specific
VFLTAAH	Superscaf	-	Superscaf	61302622	61302642	-41	41	47	-	-	-	-	-	-	NCP	IGR	-	ID=gene-E	7	5.374285714	2380.6	0.187166608	VFLTAAH	98	758.4164	1	757.4122	.	D	Superscaf	61302657	0	0	BTP202209	0	0	0	Tissue specific
SPTVKY	Superscaf	-	Superscaf	61331893	61331910	-27	27	32	-	-	gene	-	-	mRNA	NCP	Intron	gene|mRNA	ID=gene-E	6	5.651666667	2046.2	0.18773346	S(+42.01)	71	736.3962	1	735.3803	Acetylati	W	Superscaf	61332006	0	0	0	BTP202209	0	0	Tissue specific
YCMSH	Superscaf	-	Superscaf	61379884	61379898	-5	5	9	-	-	gene	-	-	mRNA	NCP	Intron	gene|mRNA	ID=gene-E	5	5.94	2419.8	0.188662839	Y(+42.01)	50	755.2373	1	754.2415	Acetylati	T	Superscaf	61379934	0	0	0	0	0	BTP202209	Tissue specific
ATNHLK	Superscaf	-	Superscaf	61563007	61563024	-1	1	6	-	-	-	-	-	-	NCP	IGR	-	ID=gene-E	6	6.878333333	2747	0.192209139	A(+42.01)	55	363.1967	2	724.3868	Acetylati	A	Superscaf	61563078	0	0	0	0	BTP202209	0	Tissue specific
KQLFMP	Superscaf	-	Superscaf	61563085	61563102	-14	14	19	-	-	-	-	-	-	NCP	IGR	-	ID=gene-E	6	6.483333333	2528.8	0.192210649	KQLFM(+15	61	390.2086	2	778.4047	Oxidation	G	Superscaf	61563168	0	0	0	0	0	BTP202209	Tissue specific
AVPFLVVLI	Superscaf	-	Superscaf	61587898	61587924	-15	15	23	-	-	-	-	-	-	NCP	IGR	-	ID=gene-E	9	3.971111111	2096.3	0.19269117	A(+42.01)	73	506.8277	2	1011.637	Acetylati	S	Superscaf	61587963	0	0	0	0	0	BTP202209	Tissue specific
PANSSKK	Superscaf	+	Superscaf	61607353	61607373	6	6	12	-	-	gene	-	-	mRNA	NCP	Intron	gene|mRNA	ID=gene-E	7	6.938571429	2949	0.193067929	PANSSKK	62	366.2015	2	730.3973	.	Y	Superscaf	61607338	0	0	0	BTP202209	0	0	Tissue specific
LSGPPEK	Superscaf	+	Superscaf	61708603	61708623	97	97	103	-	-	gene	-	-	mRNA	NCP	Intron	gene|mRNA	ID=gene-E	7	6.17	2602.8	0.195028704	LSGPPEK	78	364.2086	2	726.3912	.	F	Superscaf	61708315	0	0	0	0	0	BTP202209	Tissue specific
LTVLEEL	Superscaf	+	Superscaf	61748884	61748904	33	33	39	-	-	gene	-	-	mRNA	NCP	Intron	gene|mRNA	ID=gene-E	7	4.415714286	2308.7	0.195808772	LTVLEEL	83	816.4669	1	815.464	.	V	Superscaf	61748788	0	0	0	0	0	BTP202209	Tissue specific
NVLVVLV	Superscaf	+	Superscaf	62025007	62025027	5	5	11	-	-	gene	-	-	mRNA	NCP	Intron	gene|mRNA	ID=gene-E	7	2.481428571	1303.5	0.20115608	NVLVVLV	54	755.5005	1	754.4952	.	S	Superscaf	62024995	0	0	0	BTP202209	0	0	Tissue specific
LVYDVL	Superscaf	-	Superscaf	62114749	62114766	-5	5	10	-	-	gene	-	-	mRNA	NCP	Intron	gene|mRNA	ID=gene-E	6	3.431666667	1577.8	0.202893995	LVYDVL	78	721.4108	1	720.4058	.	F	Superscaf	62114766	0	0	0	0	0	BTP202209	Tissue specific
FSHKLT	Superscaf	+	Superscaf	62142979	62142996	7	7	12	-	-	-	-	-	-	NCP	IGR	-	ID=gene-E	6	6.833333333	2609.8	0.203440688	FSHKLT	50	366.7075	2	731.3966	.	Q	Superscaf	62142961	0	0	0	0	0	BTP202209	Tissue specific
FDAGLL	Superscaf	-	Superscaf	62225743	62225760	-1	1	6	-	-	-	-	-	-	NCP	IGR	-	ID=gene-E	6	5.4	2114.3	0.205043468	FDAGLL	68	635.3372	1	634.3326	.	F	Superscaf	62225787	0	0	0	0	0	BTP202209	Tissue specific
WNAAPLL	Superscaf	-	Superscaf	62285776	62285796	-4	4	10	-	-	gene	-	-	mRNA	NCP	Intron	gene|mRNA	ID=gene-E	7	5.942857143	2790.1	0.206206048	W(+42.01)	52	413.729	2	825.4385	Acetylati	F	Superscaf	62285880	0	0	0	0	0	BTP202209	Tissue specific
LEVLSQK	Superscaf	+	Superscaf	62298115	62298135	68	68	74	-	-	gene	-	-	mRNA	NCP	Intron	gene|mRNA	ID=gene-E	7	5.178571429	2594.8	0.206445001	LEVLSQK	99	408.7426	2	815.4752	.	I	Superscaf	62297914	0	0	BTP202209	0	0	0	Tissue specific
GAPGFFL	Superscaf	+	Superscaf	62441026	62441046	56	56	62	-	-	-	-	-	-	NCP	IGR	-	ID=gene-E	7	5.885714286	2371.5	0.209212569	G(+42.01)	64	750.3792	1	749.3748	Acetylati	P	Superscaf	62440861	0	0	0	0	BTP202209	0	Tissue specific
DPYLVRT	Superscaf	-	Superscaf	62460589	62460609	-17	17	23	-	-	-	-	-	-	NCP	IGR	-	ID=gene-E	7	5.457142857	2608.1	0.20959142	DPYLVRT	61	432.2361	2	862.4548	.	E	Superscaf	62460609	0	0	0	0	BTP202209	0	Tissue specific
HEEPTLL	Superscaf	+	Superscaf	62521996	62522016	19	19	25	-	-	-	-	-	-	NCP	IGR	-	ID=gene-E	7	5.545714286	2912.4	0.210780607	HEEPTLL	67	419.7173	2	837.4232	.	T	Superscaf	62521942	0	0	0	0	BTP202209	0	Mixed
VPPPLP	Superscaf	+	Superscaf	62647606	62647623	19	19	24	-	-	-	-	-	-	NCP	IGR	-	ID=gene-E	6	5.196666667	1703	0.21321313	VPPPLP	54	619.3792	1	618.3741	.	W	Superscaf	62647552	BTP202209	0	0	0	0	0	Tissue specific
ETPKHEQEK	Superscaf	+	Superscaf	62690212	62690238	5	5	13	-	-	-	-	-	-	NCP	IGR	-	ID=gene-E	9	6.134444444	4178.6	0.214038224	ETPKHEQEK	70	563.2896	2	1124.546	.	K	Superscaf	62690200	0	0	BTP202209	0	0	0	Tissue specific
KPLYPPP	Superscaf	+	Superscaf	62766022	62766042	12	12	18	-	-	-	-	-	-	NCP	IGR	-	ID=gene-E	7	6.654285714	2630.9	0.215506335	KPLYPPP	75	406.2366	2	810.4639	.	K	Superscaf	62765989	0	0	0	0	BTP202209	0	Tissue specific
EDVPSE	Superscaf	-	Superscaf	62766364	62766381	-22	22	27	-	-	-	-	-	-	NCP	IGR	-	ID=gene-E	6	3.565	1983.3	0.215512959	EDVPSE	90	675.2821	1	674.2759	.	K	Superscaf	62766387	0	0	0	0	BTP202209	BTP202209	Mixed
ASVNKYK	Superscaf	+	Superscaf	62860006	62860026	23	23	29	-	-	-	-	-	-	NCP	IGR	-	ID=gene-E	7	6.035714286	2687.7	0.217326399	ASVNKYK	63	405.2351	2	808.4443	.	K	Superscaf	62859940	0	0	0	0	0	BTP202209	Tissue specific
KDVKFQ	Superscaf	+	Superscaf	62923927	62923944	1	1	6	-	-	-	-	-	-	NCP	IGR	-	ID=gene-E	6	5.596666667	2346.4	0.218564272	KDVKFQ	55	382.7229	2	763.4228	.	K	Superscaf	62923927	0	0	0	0	0	BTP202209	Tissue specific
VNSCDSF	Superscaf	-	Superscaf	62937955	62937975	-28	28	34	-	-	-	-	-	-	NCP	IGR	-	ID=gene-E	7	4.32	2748.1	0.218835934	VNSC(+57.	59	414.6621	2	827.312	Carbamido	K	Superscaf	62938035	0	0	0	0	0	BTP202209	Tissue specific
DYSPLP	Superscaf	-	Superscaf	62964274	62964291	-17	17	22	-	-	-	-	-	-	NCP	IGR	-	ID=gene-E	6	5.481666667	2282.6	0.219345619	DYSPLP	77	691.3278	1	690.3224	.	C	Superscaf	62964369	0	0	0	0	BTP202209	BTP202209	Tissue specific
GPKMHTR	Superscaf	-	Superscaf	62976316	62976336	-6	6	12	-	-	-	-	-	-	NCP	IGR	-	ID=gene-E	7	7.52	3140.6	0.219578821	GPKMHTR	59	413.7166	2	825.4279	.	F	Superscaf	62976360	0	0	0	BTP202209	0	0	Tissue specific
WSLHSTM	Superscaf	-	Superscaf	63047191	63047211	-4	4	10	-	-	-	-	-	-	NCP	IGR	-	ID=gene-E	7	6.157142857	3035.5	0.220951363	WSLHSTM	91	431.1979	2	860.3851	.	I	Superscaf	63047226	0	0	0	BTP202209	0	0	Tissue specific
KVLLAPD	Superscaf	+	Superscaf	63060907	63060927	1	1	7	-	-	-	-	-	-	NCP	IGR	-	ID=gene-E	7	5.284285714	2235.4	0.221216982	KVLLAPD	93	755.4651	1	754.4589	.	K	Superscaf	63060907	0	BTP202209	0	0	0	0	Tissue specific
VQEFKK	Superscaf	+	Superscaf	63102205	63102222	13	13	18	-	-	gene	-	-	mRNA	NCP	Intron	gene|mRNA	ID=gene-E	6	5.638333333	2360.4	0.222016746	VQEFKK	56	389.724	2	777.4385	.	G	Superscaf	63102169	0	BTP202209	0	0	0	0	Tissue specific
DSFSTPF	Superscaf	-	Superscaf	63117268	63117288	-27	27	33	-	-	-	-	-	-	NCP	IGR	-	ID=gene-E	7	5.445714286	2688	0.222308451	D(+42.01)	54	842.3545	1	841.3494	Acetylati	N	Superscaf	63117312	0	BTP202209	0	0	0	0	Tissue specific
TLQYYLT	Superscaf	-	Superscaf	63124636	63124656	-20	20	26	-	-	-	-	-	-	NCP	IGR	-	ID=gene-E	7	5.998571429	3031.5	0.222451137	TLQYYLT	55	901.4606	1	900.4593	.	S	Superscaf	63124731	0	0	0	0	0	BTP202209	Tissue specific
KMTAMVP	Superscaf	+	Superscaf	63139567	63139587	1	1	7	-	-	-	-	-	-	NCP	IGR	-	ID=gene-E	7	5.727142857	2257.6	0.222740286	KMTAM(+15	54	397.2053	2	792.3874	Oxidation	K	Superscaf	63139567	BTP202209	0	0	0	0	0	Tissue specific
RLNLVTEK	Superscaf	-	Superscaf	63182437	63182460	-17	17	24	-	-	-	-	-	-	NCP	IGR	-	ID=gene-E	8	5.9525	3243.7	0.223570493	RLNLVTEK	66	486.7934	2	971.5764	.	T	Superscaf	63182493	BTP202209	0	0	0	0	0	Tissue specific
LFRPDN	Superscaf	-	Superscaf	63257374	63257391	-4	4	9	-	-	-	-	-	-	NCP	IGR	-	ID=gene-E	6	6.15	2735.2	0.225021698	LFRPDN	99	381.1994	2	760.3868	.	V	Superscaf	63257451	0	0	BTP202209	0	0	0	Mixed
KQDLDLR	Superscaf	+	Superscaf	63281929	63281949	9	9	15	-	-	-	-	-	-	NCP	IGR	-	ID=gene-E	7	6.292857143	3257.7	0.225497222	K(+42.01)	73	465.2586	2	928.4977	Acetylati	T	Superscaf	63281905	0	BTP202209	0	0	0	0	Tissue specific
KGMDFH	Superscaf	+	Superscaf	63340426	63340443	10	10	15	-	-	-	-	-	-	NCP	IGR	-	ID=gene-E	6	6.25	2555.8	0.226630056	KGM(+15.9	62	375.6627	2	749.3167	Oxidation	I	Superscaf	63340399	0	0	0	0	0	BTP202209	Tissue specific
KYLNPLP	Superscaf	+	Superscaf	63380680	63380700	2	2	8	-	-	-	-	-	-	NCP	IGR	-	ID=gene-E	7	6.481428571	2906.2	0.227409602	KYLNPLP	51	422.7477	2	843.4854	.	W	Superscaf	63380677	BTP202209	0	0	0	0	0	Tissue specific
QAVLSYL	Superscaf	-	Superscaf	63444862	63444882	-1	1	7	-	-	-	-	-	-	NCP	IGR	-	ID=gene-E	7	4.995714286	2471.7	0.22865253	QAVLSYL	64	793.4412	1	792.4381	.	Q	Superscaf	63444933	0	BTP202209	0	0	0	0	Tissue specific
KGDKLR	Superscaf	-	Superscaf	63481141	63481158	-2	2	7	-	-	-	-	-	-	NCP	IGR	-	ID=gene-E	6	7.526666667	2647.8	0.229355097	K(+42.01)	53	379.725	2	757.4446	Acetylati	L	Superscaf	63481200	0	0	0	0	BTP202209	0	Tissue specific
MELPV	Superscaf	+	Superscaf	63536989	63537003	4	4	8	-	-	-	-	-	-	NCP	IGR	-	ID=gene-E	5	4.25	1487.8	0.230436631	M(+42.01)	69	630.3163	1	629.3094	Acetylati	V	Superscaf	63536980	0	0	0	BTP202209	0	0	Mixed
WGFTKF	Superscaf	-	Superscaf	63645154	63645171	-188	188	193	-	-	-	-	-	-	NCP	IGR	-	ID=gene-E	6	6.515	2464.6	0.232531319	WGFTKF	97	393.2007	2	784.3908	.	A	Superscaf	63645285	0	0	BTP202209	0	0	0	Tissue specific
VPYFS	Superscaf	-	Superscaf	63664153	63664167	-4	4	8	-	-	-	-	-	-	NCP	IGR	-	ID=gene-E	5	4.624	1567.7	0.232899248	VPYFS	71	612.3038	1	611.2955	.	P	Superscaf	63664335	0	0	0	0	BTP202209	BTP202209	Tissue specific
DPPKF	Superscaf	+	Superscaf	63684541	63684555	10	10	14	-	-	-	-	-	-	NCP	IGR	-	ID=gene-E	5	6.158	1942.1	0.233294075	D(+42.01)	94	645.3348	1	644.317	Acetylati	I	Superscaf	63684514	0	0	0	0	BTP202209	BTP202209	Tissue specific
PGGDLVV	Superscaf	+	Superscaf	63772687	63772707	2	2	8	-	-	-	-	-	-	NCP	IGR	-	ID=gene-E	7	3.884285714	1696.9	0.235001082	PGGDLVV	56	656.3595	1	655.3541	.	L	Superscaf	63772684	0	BTP202209	0	0	0	0	Tissue specific
NMDQLQ	Superscaf	+	Superscaf	63825910	63825927	1	1	6	-	-	-	-	-	-	NCP	IGR	-	ID=gene-E	6	5.235	2954.5	0.236031781	N(+42.01)	50	806.3505	1	805.3276	Acetylati	N	Superscaf	63825910	0	0	BTP202209	0	0	0	Tissue specific
KRCAPR	Superscaf	+	Superscaf	63870406	63870423	27	27	32	-	-	-	-	-	-	NCP	IGR	-	ID=gene-E	6	8.1	3000.4	0.236893476	KRC(+57.0	51	394.2215	2	786.4282	Carbamido	D	Superscaf	63870328	0	0	0	BTP202209	0	0	Tissue specific
KHLVDFK	Superscaf	+	Superscaf	63925393	63925413	9	9	15	-	-	-	-	-	-	NCP	IGR	-	ID=gene-E	7	5.928571429	2708.8	0.237958337	KHLVDFK	75	443.7595	2	885.5072	.	R	Superscaf	63925369	0	0	0	0	0	BTP202209	Tissue specific
TVTLNA	Superscaf	+	Superscaf	63983104	63983121	4	4	9	-	-	-	-	-	-	NCP	IGR	-	ID=gene-E	6	5.078333333	2056.4	0.239075949	TVTLNA	62	618.3429	1	617.3384	.	A	Superscaf	63983095	0	0	BTP202209	0	0	0	Tissue specific
KGAGL	Superscaf	-	Superscaf	63990025	63990039	-30	30	34	-	-	-	-	-	-	NCP	IGR	-	ID=gene-E	5	6.736	1727.7	0.239209979	KGAGL	74	445.2801	1	444.2696	.	Y	Superscaf	63990066	0	0	0	0	0	BTP202209	Tissue specific
LPCKKPP	Superscaf	-	Superscaf	64009714	64009734	-9	9	15	-	-	-	-	-	-	NCP	IGR	-	ID=gene-E	7	7.054285714	2888.1	0.23959127	LPC(+57.0	54	420.2397	2	838.4734	Carbamido	K	Superscaf	64009764	0	0	0	0	BTP202209	0	Tissue specific
LDPNPL	Superscaf	+	Superscaf	64029661	64029678	1	1	6	-	-	-	-	-	-	NCP	IGR	-	ID=gene-E	6	5.49	2389.8	0.239977557	LDPNPL	77	668.3592	1	667.3541	.	L	Superscaf	64029661	0	0	0	0	0	BTP202209	Mixed
KNLTAVT	Superscaf	+	Superscaf	64052797	64052817	7	7	13	-	-	-	-	-	-	NCP	IGR	-	ID=gene-E	7	5.744285714	2524.8	0.240425601	K(+42.01)	58	788.4484	1	787.444	Acetylati	K	Superscaf	64052779	0	0	0	BTP202209	0	0	Tissue specific
TAKNLYL	Superscaf	+	Superscaf	64060519	64060539	63	63	69	-	-	gene	-	-	mRNA	NCP	Intron	gene|mRNA	ID=gene-E	7	6.474285714	2940.2	0.240575143	TAKNLYL	76	411.7385	2	821.4647	.	I	Superscaf	64060333	0	BTP202209	0	0	0	0	Mixed
LTRLHY	Superscaf	-	Superscaf	64140400	64140417	-1	1	6	-	-	gene	-	-	mRNA	NCP	Intron	gene|mRNA	ID=gene-E	6	7.083333333	2832.3	0.242122092	LTRLHY	99	401.7303	2	801.4497	.	L	Superscaf	64140468	0	BTP202209	0	0	0	0	Tissue specific
WLPTSN	Superscaf	+	Superscaf	64153921	64153938	64	64	69	-	-	gene	-	-	mRNA	NCP	Intron	gene|mRNA	ID=gene-E	6	5.965	2595	0.242383935	WLPTSN	52	359.1844	2	716.3493	.	S	Superscaf	64153732	0	0	0	0	0	BTP202209	Tissue specific
PLYDAPK	Superscaf	-	Superscaf	64234723	64234743	-3	3	9	-	-	-	-	-	-	NCP	IGR	-	ID=gene-E	7	6.138571429	2678.9	0.243948721	PLYDAPK	55	803.4297	1	802.4225	.	K	Superscaf	64234755	0	0	BTP202209	0	0	0	Tissue specific
EAASKPK	Superscaf	+	Superscaf	64325665	64325685	16	16	22	-	-	-	-	-	-	NCP	IGR	-	ID=gene-E	7	6.674285714	2705.7	0.245709874	EAASKPK	79	365.7021	2	729.402	.	L	Superscaf	64325620	0	0	BTP202209	0	0	0	Tissue specific
AQFLVA	Superscaf	-	Superscaf	64355926	64355943	-7	7	12	-	-	-	-	-	-	NCP	IGR	-	ID=gene-E	6	4.858333333	1974.1	0.246295898	AQFLVA	74	648.3698	1	647.3643	.	S	Superscaf	64356114	0	0	0	BTP202209	0	0	Tissue specific
CLRVPVP	Superscaf	-	Superscaf	64412095	64412115	-16	16	22	-	-	-	-	-	-	NCP	IGR	-	ID=gene-E	7	4.908571429	2262.7	0.247383649	C(+57.02)	69	420.7403	2	839.4687	Carbamido	N	Superscaf	64412217	0	0	0	0	0	BTP202209	Tissue specific
SHLSLV	Superscaf	+	Superscaf	64435738	64435755	19	19	24	-	-	-	-	-	-	NCP	IGR	-	ID=gene-E	6	5.151666667	2093.3	0.247841512	SHLSLV	77	655.3762	1	654.3701	.	E	Superscaf	64435684	0	0	0	0	0	BTP202209	Tissue specific
KLVVFEP	Superscaf	+	Superscaf	64456108	64456128	19	19	25	-	-	-	-	-	-	NCP	IGR	-	ID=gene-E	7	4.388571429	1972.1	0.24823599	KLVVFEP	71	416.2555	2	830.4902	.	L	Superscaf	64456054	0	0	0	BTP202209	0	0	Tissue specific
SLERLLV	Superscaf	-	Superscaf	64463548	64463568	-19	19	25	-	-	-	-	-	-	NCP	IGR	-	ID=gene-E	7	5.371428571	2517.9	0.248380071	SLERLLV	93	415.259	2	828.5069	.	T	Superscaf	64463583	0	BTP202209	0	0	0	0	Mixed
LTVFPK	Superscaf	-	Superscaf	64470013	64470030	-5	5	10	-	-	-	-	-	-	NCP	IGR	-	ID=gene-E	6	5.671666667	1944.1	0.24850527	LTVFPK	90	352.7206	2	703.4268	.	L	Superscaf	64470159	0	0	0	0	BTP202209	0	Tissue specific
VSKPGLT	Superscaf	+	Superscaf	64475557	64475577	50	50	56	-	-	-	-	-	-	NCP	IGR	-	ID=gene-E	7	5.742857143	2237.4	0.248612633	VSKPGLT	78	351.2114	2	700.4119	.	N	Superscaf	64475410	0	0	0	BTP202209	0	0	Tissue specific
LPYPQYP	Superscaf	+	Superscaf	64544614	64544634	3	3	9	-	-	-	-	-	-	NCP	IGR	-	ID=gene-E	7	5.978571429	2895.3	0.249949969	LPYPQYP	67	439.2245	2	876.4381	.	V	Superscaf	64544608	0	0	0	0	0	BTP202209	Tissue specific
HLLHKAT	Superscaf	+	Superscaf	64564249	64564269	29	29	35	-	-	-	-	-	-	NCP	IGR	-	ID=gene-E	7	7.061428571	3123.4	0.250330214	HLLHKAT	60	410.2513	2	818.4763	.	Q	Superscaf	64564165	0	0	0	0	0	BTP202209	Tissue specific
KHWCSP	Superscaf	-	Superscaf	64595842	64595859	-25	25	30	-	-	-	-	-	-	NCP	IGR	-	ID=gene-E	6	6.703333333	2921.2	0.250942034	KHWC(+57.	54	407.6825	2	813.3592	Carbamido	K	Superscaf	64595901	0	0	0	BTP202209	0	0	Tissue specific
KDYVPK	Superscaf	-	Superscaf	64664266	64664283	-29	29	34	-	-	-	-	-	-	NCP	IGR	-	ID=gene-E	6	5.735	2145.2	0.25226711	KDYVPK	50	375.2096	2	748.4119	.	H	Superscaf	64664286	0	BTP202209	0	0	BTP202209	BTP202209	Tissue specific
FVMMVL	Superscaf	+	Superscaf	64671943	64671960	76	76	81	-	-	-	-	-	-	NCP	IGR	-	ID=gene-E	6	3.826666667	1483.8	0.252415781	FVMM(+15.	68	378.1934	2	754.3758	Oxidation	F	Superscaf	64671718	0	0	0	0	BTP202209	0	Tissue specific
ETGHF	Superscaf	-	Superscaf	64673674	64673688	-18	18	22	-	-	-	-	-	-	NCP	IGR	-	ID=gene-E	5	5.758	2127.4	0.252449303	ETGHF	68	590.2546	1	589.2496	.	I	Superscaf	64673754	0	BTP202209	0	0	BTP202209	BTP202209	Tissue specific
TLFLSAN	Superscaf	+	Superscaf	64767313	64767333	13	13	19	-	-	gene	-	-	mRNA	NCP	Intron	gene|mRNA	ID=gene-E	7	5.868571429	2783.1	0.254262685	T(+42.01)	52	404.2167	2	806.4174	Acetylati	K	Superscaf	64767277	0	BTP202209	0	BTP202209	0	0	Tissue specific
EMQRPK	Superscaf	-	Superscaf	64808209	64808226	-15	15	20	-	-	-	-	-	-	NCP	IGR	-	ID=gene-E	6	6.903333333	2862.3	0.255054664	EM(+15.99	72	402.7109	2	803.3959	Oxidation	L	Superscaf	64808268	0	BTP202209	0	0	0	0	Tissue specific
PLLYM	Superscaf	-	Superscaf	64828000	64828014	-29	29	33	-	-	gene	-	-	mRNA	NCP	Intron	gene|mRNA	ID=gene-E	5	5.934	1875.2	0.25543793	PLLYM	84	636.3416	1	635.3353	.	I	Superscaf	64828017	0	0	0	BTP202209	0	0	Tissue specific
MHQSY	Superscaf	+	Superscaf	64832599	64832613	6	6	10	-	-	-	-	-	-	NCP	IGR	-	ID=gene-E	5	6.066	2444.8	0.255526992	M(+42.01)	55	707.2755	1	706.2745	Acetylati	W	Superscaf	64832584	0	BTP202209	0	0	0	0	Tissue specific
LARLHS	Superscaf	+	Superscaf	64861123	64861140	7	7	12	-	-	gene	-	-	mRNA	NCP	Intron	gene|mRNA	ID=gene-E	6	7.001666667	2670	0.256079379	LARLHS	73	696.4016	1	695.4078	.	E	Superscaf	64861105	0	0	0	0	0	BTP202209	Tissue specific
GAPAPPPP	Superscaf	+	Superscaf	64879426	64879449	143	143	150	-	-	gene	-	-	mRNA	NCP	Intron	gene|mRNA	ID=gene-E	8	6.18875	2606.9	0.256433829	GAPAPPPP	95	703.3741	1	702.3701	.	P	Superscaf	64879000	0	0	0	0	0	BTP202209	Tissue specific
RSAPGSGSK	Superscaf	+	Superscaf	64889284	64889331	159	159	174	-	-	gene	-	-	mRNA	NCP	Intron	gene|mRNA	ID=gene-E	16	6.435625	5864	0.256624736	RSAPGSGSK	88	414.2481	4	1652.969	.	S	Superscaf	64888810	0	BTP202209	0	0	0	BTP202209	Mixed
DDDDDFDEI	Superscaf	+	Superscaf	64889386	64889439	193	193	210	-	-	gene	-	-	mRNA	NCP	Intron	gene|mRNA	ID=gene-E	18	4.317777778	6612.3	0.256626711	DDDDDFDEI	87	708.3126	3	2121.922	.	S	Superscaf	64888810	0	0	BTP202209	0	0	0	Tissue specific
DDDDDFDEI	Superscaf	+	Superscaf	64889386	64889436	193	193	209	-	-	gene	-	-	mRNA	NCP	Intron	gene|mRNA	ID=gene-E	17	3.998823529	6143.9	0.256626711	DDDDDFDEI	92	997.9168	2	1993.828	.	S	Superscaf	64888810	0	BTP202209	0	0	0	0	Tissue specific
NWAEGK	Superscaf	+	Superscaf	64913563	64913580	8	8	13	-	-	-	-	-	-	NCP	IGR	-	ID=gene-E	6	6.041666667	2625.8	0.257094915	N(+42.01)	85	746.3491	1	745.3395	Acetylati	M	Superscaf	64913542	BTP202209	0	0	0	0	0	Tissue specific
LYANSYL	Superscaf	-	Superscaf	64939075	64939095	-2	2	8	-	-	-	-	-	-	NCP	IGR	-	ID=gene-E	7	5.77	2917.2	0.257588972	LYANSYL	50	843.4198	1	842.4174	.	L	Superscaf	64939164	0	0	0	0	0	BTP202209	Tissue specific
PVSELCFR	Superscaf	+	Superscaf	64940803	64940826	24	24	31	-	-	-	-	-	-	NCP	IGR	-	ID=gene-E	8	5.305	3121.6	0.257622436	PVSELC(+5	59	504.2489	2	1006.491	Carbamido	L	Superscaf	64940734	0	0	0	0	BTP202209	0	Tissue specific
VVGMDL	Superscaf	+	Superscaf	64967818	64967835	34	34	39	-	-	gene	-	-	mRNA	NCP	Intron	gene|mRNA	ID=gene-E	6	3.445	1433.7	0.2581456	VVGM(+15.	60	649.3322	1	648.3152	Oxidation	N	Superscaf	64967719	0	0	0	0	BTP202209	BTP202209	Tissue specific
VHEVLYR	Superscaf	-	Superscaf	65040910	65040930	-10	10	16	-	-	-	-	-	-	NCP	IGR	-	ID=gene-E	7	4.744285714	2506.9	0.259561075	VHEVLYR	99	458.2537	2	914.4974	.	N	Superscaf	65040954	0	0	BTP202209	0	0	0	Tissue specific
KAQNQLGL	Superscaf	-	Superscaf	65089396	65089419	-12	12	19	-	-	-	-	-	-	NCP	IGR	-	ID=gene-E	8	6.3	3601.9	0.26050004	KAQNQLGL	56	436.253	2	870.4923	.	A	Superscaf	65089764	0	0	0	0	0	BTP202209	Tissue specific
NPLVLF	Superscaf	+	Superscaf	65106760	65106777	207	207	212	-	-	gene	-	-	mRNA	NCP	Intron	gene|mRNA	ID=gene-E	6	4.858333333	2028.3	0.260836305	NPLVLF	65	702.417	1	701.4112	.	K	Superscaf	65106142	0	0	BTP202209	0	0	0	Mixed
PKHGP	Superscaf	+	Superscaf	65153701	65153715	13	13	17	-	-	-	-	-	-	NCP	IGR	-	ID=gene-E	5	7.18	2060.2	0.261745349	PKHGP	60	535.2909	1	534.2914	.	W	Superscaf	65153665	0	0	0	0	BTP202209	BTP202209	Tissue specific
GVFLQ	Superscaf	+	Superscaf	65158525	65158539	16	16	20	-	-	-	-	-	-	NCP	IGR	-	ID=gene-E	5	4.616	1648.8	0.261838769	GVFLQ	64	563.3175	1	562.3115	.	D	Superscaf	65158480	0	BTP202209	0	0	BTP202209	BTP202209	Tissue specific
HGVKGLP	Superscaf	+	Superscaf	65229403	65229423	17	17	23	-	-	gene	-	-	mRNA	NCP	Intron	gene|mRNA	ID=gene-E	7	5.935714286	2373.5	0.263211369	HGVKGLP	62	354.2122	2	706.4126	.	G	Superscaf	65229355	0	BTP202209	0	0	0	0	Tissue specific
CCCFPHRP	Superscaf	-	Superscaf	65318851	65318874	-11	11	18	-	-	-	-	-	-	NCP	IGR	-	ID=gene-E	8	6.43625	4087.8	0.26494359	C(+57.02)	58	392.4873	3	1174.447	Carbamido	N	Superscaf	65318979	0	0	0	BTP202209	0	0	Tissue specific
RFTATKK	Superscaf	-	Superscaf	65413357	65413377	-4	4	10	-	-	-	-	-	-	NCP	IGR	-	ID=gene-E	7	7.828571429	3079.3	0.266773763	RFTATKK	68	426.251	2	850.5024	.	Y	Superscaf	65413386	0	0	0	BTP202209	0	0	Tissue specific
PGNTRPP	Superscaf	+	Superscaf	65428429	65428449	40	40	46	-	-	-	-	-	-	NCP	IGR	-	ID=gene-E	7	6.795714286	2952.5	0.267065642	PGNTRPP	59	369.6932	2	737.382	.	W	Superscaf	65428312	0	BTP202209	0	0	0	0	Tissue specific
AFSTVVP	Superscaf	-	Superscaf	65469148	65469168	-84	84	90	-	-	gene	-	-	mRNA	NCP	Intron	gene|mRNA	ID=gene-E	7	4.287142857	1817	0.267854193	AFSTVVP	63	720.3902	1	719.3854	.	V	Superscaf	65469186	0	BTP202209	0	0	0	0	Tissue specific
KPYQPP	Superscaf	+	Superscaf	65515117	65515134	5	5	10	-	-	gene	-	-	mRNA	NCP	Intron	gene|mRNA	ID=gene-E	6	6.658333333	2550.8	0.268744414	KPYQPP	68	365.1995	2	728.3857	.	T	Superscaf	65515105	0	0	0	0	0	BTP202209	Tissue specific
KLLLPPA	Superscaf	-	Superscaf	65533378	65533398	-3	3	9	-	-	gene	-	-	mRNA	NCP	Intron	gene|mRNA	ID=gene-E	7	6.614285714	2514.7	0.26909805	KLLLPPA	74	376.2573	2	750.5003	.	T	Superscaf	65533488	0	0	0	BTP202209	0	0	Tissue specific
KNKKGM	Superscaf	-	Superscaf	65570956	65570973	-22	22	27	-	-	-	-	-	-	NCP	IGR	-	ID=gene-E	6	7.725	2670.7	0.269825774	KNKKGM	51	353.2042	2	704.4003	.	G	Superscaf	65571192	0	0	0	0	0	BTP202209	Tissue specific
LDLTNLL	Superscaf	+	Superscaf	65583391	65583411	19	19	25	-	-	-	-	-	-	NCP	IGR	-	ID=gene-E	7	5.547142857	2819.3	0.270066586	LDLTNLL	52	801.4686	1	800.4644	.	V	Superscaf	65583337	0	0	0	0	0	BTP202209	Tissue specific
SGDFVHRLF	Superscaf	-	Superscaf	65655490	65655519	-3	3	12	-	-	gene	-	-	mRNA	NCP	Intron	gene|mRNA	ID=gene-E	10	5.671	3792.3	0.271462832	SGDFVHRLF	97	570.8095	2	1139.609	.	S	Superscaf	65655564	BTP202209	0	0	0	0	0	Tissue specific
DVAKL	Superscaf	-	Superscaf	65705608	65705622	-1	1	5	-	-	-	-	-	-	NCP	IGR	-	ID=gene-E	5	4.942	1544.6	0.2724334	DVAKL	80	545.3278	1	544.322	.	D	Superscaf	65705676	0	0	0	0	BTP202209	0	Tissue specific
KAPSPLR	Superscaf	-	Superscaf	65851204	65851224	-46	46	52	-	-	-	-	-	-	NCP	IGR	-	ID=gene-E	7	7.254285714	2840.1	0.275252965	KAPSPLR	72	384.733	2	767.4653	.	G	Superscaf	65851452	0	0	BTP202209	0	0	0	Tissue specific
PSVPEVL	Superscaf	+	Superscaf	65860873	65860893	15	15	21	-	-	-	-	-	-	NCP	IGR	-	ID=gene-E	7	3.925714286	1837.1	0.275440212	PSVPEVL	71	740.4154	1	739.4116	.	M	Superscaf	65860831	0	0	0	0	0	BTP202209	Tissue specific
KTESLGKP	Superscaf	-	Superscaf	65888368	65888391	-9	9	16	-	-	-	-	-	-	NCP	IGR	-	ID=gene-E	8	6.645	3131.3	0.275972671	KTESLGKP	59	430.2473	2	858.481	.	E	Superscaf	65888451	0	0	0	0	0	BTP202209	Tissue specific
VLPLEEAY	Superscaf	-	Superscaf	65904367	65904390	-86	86	93	-	-	-	-	-	-	NCP	IGR	-	ID=gene-E	8	4.5475	2666	0.276282502	VLPLEEAY	90	933.4868	1	932.4855	.	D	Superscaf	65904498	0	0	0	0	0	BTP202209	Tissue specific
VVDPKKPFF	Superscaf	-	Superscaf	65928979	65929008	-54	54	63	-	-	-	-	-	-	NCP	IGR	-	ID=gene-E	10	6.103	3527.7	0.27675913	VVDPKKPFF	93	405.2506	3	1212.734	.	H	Superscaf	65929041	0	0	0	0	0	BTP202209	Tissue specific
VVDPKKPFF	Superscaf	-	Superscaf	65928979	65929005	-54	54	62	-	-	-	-	-	-	NCP	IGR	-	ID=gene-E	9	5.698888889	3059.3	0.27675913	VVDPKKPFF	81	362.5539	3	1084.639	.	H	Superscaf	65929041	0	0	BTP202209	0	0	0	Tissue specific
VVDPKKP	Superscaf	-	Superscaf	65928979	65928999	-54	54	60	-	-	-	-	-	-	NCP	IGR	-	ID=gene-E	7	5.007142857	2023.1	0.27675913	VVDPKKP	98	391.74	2	781.4697	.	H	Superscaf	65929041	BTP202209	0	0	0	0	0	Mixed
AGDFPKVK	Superscaf	-	Superscaf	65985346	65985369	-50	50	57	-	-	-	-	-	-	NCP	IGR	-	ID=gene-E	8	5.7775	2681.7	0.277850715	AGDFPKVK	90	431.2459	2	860.4755	.	H	Superscaf	65985516	0	BTP202209	0	0	0	0	Tissue specific
GMKAPY	Superscaf	-	Superscaf	66052474	66052491	-55	55	60	-	-	-	-	-	-	NCP	IGR	-	ID=gene-E	6	6.573333333	2245.4	0.279150694	G(+42.01)	51	724.3307	1	723.3262	Acetylati	A	Superscaf	66052509	0	0	0	0	0	BTP202209	Tissue specific
KPAAPGPV	Superscaf	-	Superscaf	66071626	66071649	-41	41	48	-	-	-	-	-	-	NCP	IGR	-	ID=gene-E	8	5.83125	2400.5	0.279521586	KPAAPGPV	51	368.7202	2	735.4279	.	D	Superscaf	66071691	0	0	0	0	0	BTP202209	Tissue specific
KFFPNPP	Superscaf	-	Superscaf	66115825	66115845	-1	1	7	-	-	-	-	-	-	NCP	IGR	-	ID=gene-E	7	6.43	2852.1	0.280377529	KFFPNPP	64	423.7356	2	845.4435	.	K	Superscaf	66115854	0	0	0	0	0	BTP202209	Tissue specific
NLAKKLA	Superscaf	+	Superscaf	66195208	66195228	3	3	9	-	-	-	-	-	-	NCP	IGR	-	ID=gene-E	7	6.984285714	2862.9	0.281914834	NLAKKLA	91	379.248	2	756.4857	.	E	Superscaf	66195202	0	0	BTP202209	0	0	0	Tissue specific
PKVPHHK	Superscaf	+	Superscaf	66197362	66197382	20	20	26	-	-	-	-	-	-	NCP	IGR	-	ID=gene-E	7	6.751428571	2851	0.281956548	PKVPHHK	50	421.7577	2	841.4922	.	C	Superscaf	66197305	0	0	BTP202209	0	0	0	Tissue specific
QPAPPR	Superscaf	-	Superscaf	66199480	66199497	-134	134	139	-	-	-	-	-	-	NCP	IGR	-	ID=gene-E	6	6.888333333	2583	0.281997565	Q(+42.01)	67	354.1898	2	706.3762	Acetylati	F	Superscaf	66199569	0	0	0	0	0	BTP202209	Tissue specific
NHRAKK	Superscaf	-	Superscaf	66288352	66288369	-44	44	49	-	-	-	-	-	-	NCP	IGR	-	ID=gene-E	6	8.21	3113.3	0.283718631	NHRAKK	52	377.2325	2	752.4406	.	Q	Superscaf	66288546	0	BTP202209	0	0	0	0	Tissue specific
PFVYPP	Superscaf	-	Superscaf	66289351	66289368	-5	5	10	-	-	-	-	-	-	NCP	IGR	-	ID=gene-E	6	5.006666667	1859.1	0.283737977	PFVYPP	63	360.1912	2	718.369	.	F	Superscaf	66289464	0	BTP202209	0	0	0	0	Tissue specific
KATLRLY	Superscaf	-	Superscaf	66378508	66378528	-2	2	8	-	-	-	-	-	-	NCP	IGR	-	ID=gene-E	7	7.238571429	2992.3	0.285464563	KATLRLY	90	432.7686	2	863.5229	.	V	Superscaf	66378528	0	0	BTP202209	0	0	0	Tissue specific
WDLEY	Superscaf	-	Superscaf	66425593	66425607	-51	51	55	-	-	-	-	-	-	NCP	IGR	-	ID=gene-E	5	4.744	2176.5	0.286375395	WDLEY	85	725.312	1	724.3068	.	Y	Superscaf	66425667	0	BTP202209	0	0	BTP202209	BTP202209	Tissue specific
PPAAAMH	Superscaf	+	Superscaf	66442090	66442110	40	40	46	-	-	gene	-	-	mRNA	NCP	Intron	gene|mRNA	ID=gene-E	7	6.285714286	2599.9	0.286695871	PPAAAM(+1	54	710.3258	1	709.3217	Oxidation	R	Superscaf	66441973	0	0	0	0	0	BTP202209	Tissue specific
PPFFPPP	Superscaf	+	Superscaf	66446575	66446595	95	95	101	-	-	gene	-	-	mRNA	NCP	Intron	gene|mRNA	ID=gene-E	7	6.065714286	2461.8	0.286782726	PPFFPPP	91	798.4177	1	797.4111	.	T	Superscaf	66446293	0	BTP202209	BTP202209	BTP202209	0	0	Tissue specific
LHTDRP	Superscaf	+	Superscaf	66510517	66510534	14	14	19	-	-	-	-	-	-	NCP	IGR	-	ID=gene-E	6	6.688333333	2768.3	0.288021006	LHTDRP	52	369.7012	2	737.382	.	V	Superscaf	66510478	0	0	BTP202209	0	0	0	Tissue specific
PGKQAWT	Superscaf	+	Superscaf	66534715	66534735	4	4	10	-	-	-	-	-	-	NCP	IGR	-	ID=gene-E	7	6.585714286	2949.2	0.288489616	PGKQAWT	71	394.2094	2	786.4024	.	L	Superscaf	66534706	0	0	BTP202209	0	0	0	Tissue specific
TAKKPQH	Superscaf	-	Superscaf	66535654	66535674	-6	6	12	-	-	-	-	-	-	NCP	IGR	-	ID=gene-E	7	7.367142857	3213.4	0.288507801	TAKKPQH	75	405.2364	2	808.4555	.	T	Superscaf	66535704	0	0	0	0	0	BTP202209	Tissue specific
EPTLKDF	Superscaf	+	Superscaf	66564148	66564168	56	56	62	-	-	-	-	-	-	NCP	IGR	-	ID=gene-E	7	5.745714286	2781.1	0.289059606	EPTLKDF	71	425.2154	2	848.4279	.	S	Superscaf	66563983	0	0	0	0	0	BTP202209	Tissue specific
DTVPTPK	Superscaf	-	Superscaf	66617134	66617154	-22	22	28	-	-	-	-	-	-	NCP	IGR	-	ID=gene-E	7	5.481428571	2349.7	0.290085716	DTVPTPK	86	379.2073	2	756.4017	.	A	Superscaf	66617166	0	0	0	0	0	BTP202209	Tissue specific
EFLAGT	Superscaf	+	Superscaf	66641563	66641580	22	22	27	-	-	-	-	-	-	NCP	IGR	-	ID=gene-E	6	5.533333333	2172.4	0.2905588	EFLAGT	59	637.3175	1	636.3119	.	H	Superscaf	66641500	BTP202209	0	0	0	0	0	Tissue specific
GPVRPPTP	Superscaf	+	Superscaf	66698737	66698760	28	28	35	-	-	-	-	-	-	NCP	IGR	-	ID=gene-E	8	6.0575	2693.2	0.291666013	GPVRPPTP	79	410.7335	2	819.4603	.	C	Superscaf	66698656	0	0	0	0	0	BTP202209	Tissue specific
PMAYQL	Superscaf	+	Superscaf	66725074	66725091	45	45	50	-	-	-	-	-	-	NCP	IGR	-	ID=gene-E	6	5.893333333	2443.8	0.292176047	PMAYQL	62	722.3524	1	721.3469	.	V	Superscaf	66724942	0	0	BTP202209	0	0	0	Tissue specific
QRYFPK	Superscaf	+	Superscaf	66775996	66776013	31	31	36	-	-	gene	-	-	mRNA	NCP	Intron	gene|mRNA	ID=gene-E	6	7.265	2912.2	0.293162186	QRYFPK	51	419.7277	2	837.4497	.	Q	Superscaf	66775906	0	BTP202209	0	0	0	BTP202209	Tissue specific
LPGGVP	Superscaf	-	Superscaf	66796510	66796527	-29	29	34	-	-	gene	-	-	mRNA	NCP	Intron	gene|mRNA	ID=gene-E	6	5.086666667	1622.8	0.293559453	LPGGVP	63	539.3172	1	538.3115	.	A	Superscaf	66796611	0	BTP202209	BTP202209	0	0	0	Tissue specific
LEKQPY	Superscaf	-	Superscaf	66809767	66809784	-56	56	61	-	-	gene	-	-	mRNA	NCP	Intron	gene|mRNA	ID=gene-E	6	6.091666667	2654.9	0.293816184	LEKQPY	56	777.4107	1	776.4068	.	F	Superscaf	66809796	0	0	0	0	0	BTP202209	Tissue specific
KHDLNQK	Superscaf	+	Superscaf	66816499	66816519	7	7	13	-	-	gene	-	-	mRNA	NCP	Intron	gene|mRNA	ID=gene-E	7	6.725714286	3528.8	0.293946554	KHDLNQK	58	441.7444	2	881.4719	.	S	Superscaf	66816481	0	0	BTP202209	0	0	0	Tissue specific
MLPTAF	Superscaf	+	Superscaf	66869452	66869469	31	31	36	-	-	gene	-	-	mRNA	NCP	Intron	gene|mRNA	ID=gene-E	6	6.01	2158.5	0.294972024	M(+42.01)	57	721.3705	1	720.3516	Acetylati	K	Superscaf	66869362	0	0	BTP202209	0	0	0	Tissue specific
LVMGGGKR	Superscaf	+	Superscaf	66932473	66932496	17	17	24	-	-	gene	-	-	mRNA	NCP	Intron	gene|mRNA	ID=gene-E	8	6.2675	2734	0.296192468	LVMGGGKR	65	409.241	2	816.464	.	F	Superscaf	66932425	0	0	0	BTP202209	0	0	Tissue specific
VDVML	Superscaf	+	Superscaf	66941965	66941979	22	22	26	-	-	gene	-	-	mRNA	NCP	Intron	gene|mRNA	ID=gene-E	5	2.94	1136.4	0.296376287	VDVM(+15.	61	592.2956	1	591.2938	Oxidation	G	Superscaf	66941902	BTP202209	0	0	0	0	0	Tissue specific
RGGPLSF	Superscaf	-	Superscaf	66971803	66971823	-33	33	39	-	-	-	-	-	-	NCP	IGR	-	ID=gene-E	7	6.591428571	2705	0.29695412	RGGPLSF	67	367.2027	2	732.3918	.	G	Superscaf	66971889	BTP202209	0	0	0	0	0	Tissue specific
HYASSPR	Superscaf	+	Superscaf	67044808	67044828	3	3	9	-	-	gene	-	-	mRNA	NCP	Intron	gene|mRNA	ID=gene-E	7	6.812857143	3143.5	0.298367911	HYASSPR	74	409.1997	2	816.3878	.	Y	Superscaf	67044802	0	0	0	0	0	BTP202209	Tissue specific
KTTPVR	Superscaf	-	Superscaf	67046431	67046448	-48	48	53	-	-	gene	-	-	mRNA	NCP	Intron	gene|mRNA	ID=gene-E	6	6.643333333	2249.6	0.298399342	KTTPVR	57	351.2177	2	700.4232	.	I	Superscaf	67046559	0	0	BTP202209	0	0	0	Tissue specific
KDFPPK	Superscaf	-	Superscaf	67062526	67062543	-36	36	41	-	-	gene	-	-	mRNA	NCP	Intron	gene|mRNA	ID=gene-E	6	6.755	2410.5	0.298711032	KDFPPK	58	731.4062	1	730.4013	.	F	Superscaf	67062543	0	0	BTP202209	0	0	0	Tissue specific
FCCFGPF	Superscaf	+	Superscaf	67132969	67132989	2	2	8	-	-	gene	-	-	mRNA	NCP	Intron	gene|mRNA	ID=gene-E	7	5.535714286	2968.3	0.300075209	FC(+57.02	65	467.6849	2	933.3513	Carbamido	L	Superscaf	67132966	0	0	BTP202209	0	0	0	Tissue specific
CCQDDP	Superscaf	-	Superscaf	67164826	67164843	-50	50	55	-	-	gene	-	-	mRNA	NCP	Intron	gene|mRNA	ID=gene-E	6	4.655	2942.5	0.300692141	C(+57.02)	51	794.2484	1	793.2371	Carbamido	K	Superscaf	67164897	0	0	BTP202209	0	0	0	Tissue specific
EVADWL	Superscaf	+	Superscaf	67178974	67178991	30	30	35	-	-	gene	-	-	mRNA	NCP	Intron	gene|mRNA	ID=gene-E	6	4.013333333	2028.3	0.300966126	EVADWL	61	732.3539	1	731.349	.	A	Superscaf	67178887	0	0	0	0	0	BTP202209	Tissue specific
KELDDG	Superscaf	-	Superscaf	67186432	67186449	-6	6	11	-	-	gene	-	-	mRNA	NCP	Intron	gene|mRNA	ID=gene-E	6	5.141666667	2367.6	0.301110556	KELDDG	52	676.3088	1	675.3075	.	L	Superscaf	67186524	0	0	0	0	0	BTP202209	Tissue specific
EPAPPRP	Superscaf	-	Superscaf	67237975	67237995	-119	119	125	-	-	gene	-	-	mRNA	NCP	Intron	gene|mRNA	ID=gene-E	7	6.457142857	2735.2	0.302108721	EPAPPRP	57	382.2055	2	762.4024	.	Q	Superscaf	67238721	0	0	0	0	0	BTP202209	Tissue specific
PEHAP	Superscaf	-	Superscaf	67239769	67239783	-91	91	95	-	-	gene	-	-	mRNA	NCP	Intron	gene|mRNA	ID=gene-E	5	5.886	2031.3	0.302143463	PEHAP	58	550.272	1	549.2547	.	K	Superscaf	67239933	0	0	0	BTP202209	0	0	Tissue specific
HSRSH	Superscaf	-	Superscaf	67306624	67306638	-176	176	180	-	-	gene	-	-	mRNA	NCP	Intron	gene|mRNA	ID=gene-E	5	7.46	2655	0.303438155	HSRSH	74	623.3128	1	622.2935	.	E	Superscaf	67306641	0	0	0	0	0	BTP202209	Tissue specific
VPGVPGSP	Superscaf	-	Superscaf	67307941	67307964	-30	30	37	-	-	gene	-	-	mRNA	NCP	Intron	gene|mRNA	ID=gene-E	8	4.565	1990.2	0.303463659	VPGVPGSP	71	709.3837	1	708.3806	.	Q	Superscaf	67308063	BTP202209	0	0	0	0	0	Tissue specific
ALGGFWL	Superscaf	-	Superscaf	67351183	67351203	-9	9	15	-	-	-	-	-	-	NCP	IGR	-	ID=gene-E	7	5.898571429	2526.7	0.30430107	ALGGFWL	58	382.2128	2	762.4064	.	S	Superscaf	67351242	BTP202209	0	0	0	0	0	Tissue specific
KDLLEPF	Superscaf	+	Superscaf	67424770	67424790	2	2	8	-	-	-	-	-	-	NCP	IGR	-	ID=gene-E	7	5.667142857	2737	0.305726132	KDLLEPF	77	861.4698	1	860.4643	.	P	Superscaf	67424767	0	0	0	BTP202209	0	0	Tissue specific
ALAAKVS	Superscaf	-	Superscaf	67442335	67442355	-1	1	7	-	-	gene	-	-	mRNA	NCP	Intron	gene|mRNA	ID=gene-E	7	5.637142857	2139.1	0.30606629	A(+42.01)	56	351.2131	2	700.4119	Acetylati	A	Superscaf	67442364	0	BTP202209	0	0	0	0	Tissue specific
PPPDPPPP	Superscaf	-	Superscaf	67564681	67564704	-4	4	11	-	-	gene	-	-	mRNA	NCP	Intron	gene|mRNA	ID=gene-E	8	5.88375	2773.3	0.308435602	PPPDPPPP	85	407.2034	2	812.4068	.	I	Superscaf	67564767	0	0	0	0	0	BTP202209	Tissue specific
YHLSPPP	Superscaf	+	Superscaf	67604347	67604367	40	40	46	-	-	gene	-	-	mRNA	NCP	Intron	gene|mRNA	ID=gene-E	7	6.258571429	2828.2	0.309203761	YHLSPPP	74	405.7107	2	809.4072	.	S	Superscaf	67604230	0	0	0	0	0	BTP202209	Tissue specific
KNFFPGGP	Superscaf	-	Superscaf	67620061	67620084	-8	8	15	-	-	-	-	-	-	NCP	IGR	-	ID=gene-E	8	6.33125	3109.3	0.309508074	KNFFPGGP	61	432.2266	2	862.4337	.	F	Superscaf	67620144	0	0	BTP202209	0	0	0	Tissue specific
KSGDVAK	Superscaf	-	Superscaf	67680859	67680879	-8	8	14	-	-	-	-	-	-	NCP	IGR	-	ID=gene-E	7	5.731428571	2340.3	0.310685468	KSGDVAK	76	352.6938	2	703.3864	.	T	Superscaf	67680918	0	0	BTP202209	0	0	0	Tissue specific
CADPLR	Superscaf	-	Superscaf	67699303	67699320	-51	51	56	-	-	-	-	-	-	NCP	IGR	-	ID=gene-E	6	6.175	2648.1	0.311042648	C(+57.02)	66	366.1755	2	730.3432	Carbamido	S	Superscaf	67699467	BTP202209	0	0	0	0	0	Tissue specific
KPPAGAPP	Superscaf	-	Superscaf	67709878	67709901	-146	146	153	-	-	-	-	-	-	NCP	IGR	-	ID=gene-E	8	6.61875	2737.9	0.31124744	KPPAGAPP	57	367.7178	2	733.4122	.	P	Superscaf	67710024	0	0	0	0	BTP202209	0	Tissue specific
LDKDRL	Superscaf	-	Superscaf	67801570	67801587	-7	7	12	-	-	gene	-	-	mRNA	NCP	Intron	gene|mRNA	ID=gene-E	6	6.4	2647	0.313023118	L(+42.01)	90	401.2213	2	800.4392	Acetylati	A	Superscaf	67801653	BTP202209	0	0	0	0	0	Tissue specific
QGDPLEY	Superscaf	+	Superscaf	67854034	67854054	10	10	16	-	-	-	-	-	-	NCP	IGR	-	ID=gene-E	7	5.107142857	2895.3	0.314039118	QGDPLEY	60	821.3643	1	820.3603	.	H	Superscaf	67854007	BTP202209	0	0	0	0	0	Tissue specific
ESKTKSK	Superscaf	-	Superscaf	67858006	67858026	-3	3	9	-	-	-	-	-	-	NCP	IGR	-	ID=gene-E	7	7.19	2995	0.314116039	ESKTKSK	72	404.2267	2	806.4498	.	N	Superscaf	67858026	BTP202209	0	0	0	0	0	Tissue specific
DCDSGY	Superscaf	+	Superscaf	67895614	67895631	35	35	40	-	-	-	-	-	-	NCP	IGR	-	ID=gene-E	6	4.711666667	2548.9	0.314844343	D(+42.01)	50	758.2209	1	757.2225	Acetylati	K	Superscaf	67895512	0	0	0	BTP202209	0	0	Tissue specific
LLLENY	Superscaf	+	Superscaf	68007586	68007603	36	36	41	-	-	gene	-	-	mRNA	NCP	Intron	gene|mRNA	ID=gene-E	6	5.371666667	2541.9	0.317012756	LLLENY	90	764.412	1	763.4116	.	I	Superscaf	68007481	0	BTP202209	0	0	0	0	Tissue specific
MDMPCC	Superscaf	+	Superscaf	68025952	68025969	38	38	43	-	-	gene	-	-	mRNA	NCP	Intron	gene|mRNA	ID=gene-E	6	5.135	2663.3	0.317368426	M(+15.99)	54	436.1229	2	870.238	Oxidation	Y	Superscaf	68025841	0	0	0	0	0	BTP202209	Tissue specific
KPDVLN	Superscaf	+	Superscaf	68063767	68063784	56	56	61	-	-	-	-	-	-	NCP	IGR	-	ID=gene-E	6	5.066666667	2167.4	0.318100739	KPDVLN	51	685.3859	1	684.3806	.	E	Superscaf	68063602	0	0	0	0	BTP202209	0	Tissue specific
MRLVQF	Superscaf	+	Superscaf	68081887	68081904	23	23	28	-	-	-	-	-	-	NCP	IGR	-	ID=gene-E	6	5.603333333	2371.8	0.318451645	MRLVQF	61	397.221	2	792.4316	.	D	Superscaf	68081821	0	0	0	BTP202209	0	0	Tissue enhanced
KNSGLVR	Superscaf	+	Superscaf	68132932	68132952	7	7	13	-	-	-	-	-	-	NCP	IGR	-	ID=gene-E	7	6.22	2748	0.319440166	KNSGLVR	57	387.2289	2	772.4555	.	V	Superscaf	68132914	0	BTP202209	0	0	0	BTP202209	Tissue specific
EAGMAK	Superscaf	-	Superscaf	68137720	68137737	-4	4	9	-	-	-	-	-	-	NCP	IGR	-	ID=gene-E	6	6.12	2185.3	0.319532889	EAGMAK	57	606.2948	1	605.2843	.	T	Superscaf	68137767	0	BTP202209	0	0	0	0	Tissue specific
HQVHRK	Superscaf	+	Superscaf	68154676	68154693	12	12	17	-	-	-	-	-	-	NCP	IGR	-	ID=gene-E	6	6.888333333	2967.3	0.319861253	HQVHRK	70	402.7316	2	803.4514	.	N	Superscaf	68154643	BTP202209	0	0	0	0	0	Tissue specific
HTGGKLLG	Superscaf	+	Superscaf	68195890	68195913	9	9	16	-	-	-	-	-	-	NCP	IGR	-	ID=gene-E	8	6.71625	3084.3	0.32065939	HTGGKLLG	57	391.7278	2	781.4446	.	H	Superscaf	68195866	0	BTP202209	0	0	0	0	Tissue specific
VLLTTF	Superscaf	-	Superscaf	68202793	68202810	-3	3	8	-	-	-	-	-	-	NCP	IGR	-	ID=gene-E	6	5.083333333	1889.2	0.320793072	V(+42.01)	74	368.2126	2	734.4214	Acetylati	K	Superscaf	68202858	BTP202209	0	0	0	0	0	Tissue specific
LSPLMFP	Superscaf	-	Superscaf	68247028	68247048	-23	23	29	-	-	-	-	-	-	NCP	IGR	-	ID=gene-E	7	5.924285714	2523.9	0.321649712	LSPLM(+15	62	410.719	2	819.42	Oxidation	R	Superscaf	68247075	0	0	BTP202209	0	0	0	Tissue specific
NRSHVLN	Superscaf	+	Superscaf	68265424	68265444	23	23	29	-	-	-	-	-	-	NCP	IGR	-	ID=gene-E	7	5.832857143	3198.7	0.322005963	NRSHVLN	75	420.2271	2	838.4409	.	F	Superscaf	68265358	BTP202209	0	0	0	0	0	Tissue specific
GHQQLYW	Superscaf	+	Superscaf	68280940	68280960	66	66	72	-	-	-	-	-	-	NCP	IGR	-	ID=gene-E	7	6.055714286	3477.9	0.322306441	GHQQLYW	71	466.2232	2	930.4348	.	I	Superscaf	68280745	0	0	0	0	0	BTP202209	Tissue specific
LARALPF	Superscaf	+	Superscaf	68324389	68324409	28	28	34	-	-	-	-	-	-	NCP	IGR	-	ID=gene-E	7	6.648571429	2703	0.32314786	LARALPF	56	394.24	2	786.4752	.	Q	Superscaf	68324308	0	0	0	0	BTP202209	0	Tissue specific
KHMELR	Superscaf	+	Superscaf	68346022	68346039	51	51	56	-	-	-	-	-	-	NCP	IGR	-	ID=gene-E	6	7.173333333	2887.3	0.323566798	KHMELR	50	407.2184	2	812.4327	.	M	Superscaf	68345872	0	0	0	0	BTP202209	0	Tissue specific
KPAVP	Superscaf	+	Superscaf	68353693	68353707	3	3	7	-	-	-	-	-	-	NCP	IGR	-	ID=gene-E	5	5.672	1454.5	0.323715352	K(+42.01)	71	553.3334	1	552.3271	Acetylati	Q	Superscaf	68353687	0	0	0	0	0	BTP202209	Tissue specific
VVLKEPK	Superscaf	-	Superscaf	68370175	68370195	-6	6	12	-	-	-	-	-	-	NCP	IGR	-	ID=gene-E	7	4.997142857	2053.1	0.324034537	VVLKEPK	99	406.7645	2	811.5167	.	L	Superscaf	68370288	0	0	BTP202209	0	0	0	Tissue specific
VTLAED	Superscaf	+	Superscaf	68407429	68407446	2	2	7	-	-	-	-	-	-	NCP	IGR	-	ID=gene-E	6	4.12	1899.2	0.324755986	V(+42.01)	55	689.326	1	688.3279	Acetylati	K	Superscaf	68407426	0	0	BTP202209	0	0	0	Tissue specific
FSPYLLR	Superscaf	-	Superscaf	68582653	68582673	-24	24	30	-	-	gene	-	-	mRNA	NCP	Intron	gene|mRNA	ID=gene-E	7	6.548571429	2923.3	0.328449317	FSPYLLR	54	448.2567	2	894.4963	.	L	Superscaf	68582679	0	0	BTP202209	0	0	0	Tissue specific
KNKMVP	Superscaf	+	Superscaf	68665132	68665149	9	9	14	-	-	-	-	-	-	NCP	IGR	-	ID=gene-E	6	6.156666667	2242.4	0.329746578	KNKMVP	57	358.7105	2	715.4051	.	K	Superscaf	68665108	0	0	BTP202209	0	0	0	Tissue specific
KSHAK	Superscaf	-	Superscaf	68689552	68689566	-3	3	7	-	-	-	-	-	-	NCP	IGR	-	ID=gene-E	5	7.754	2251.2	0.330219488	KSHAK	73	570.3337	1	569.3286	.	K	Superscaf	68689599	0	BTP202209	BTP202209	0	0	0	Tissue specific
EARLLR	Superscaf	-	Superscaf	68737027	68737044	-53	53	58	-	-	-	-	-	-	NCP	IGR	-	ID=gene-E	6	7.12	2741.2	0.331138873	EARLLR	81	379.2357	2	756.4606	.	S	Superscaf	68737098	0	0	0	0	BTP202209	0	Tissue specific
EDAAMGK	Superscaf	-	Superscaf	68827273	68827293	-37	37	43	-	-	-	-	-	-	NCP	IGR	-	ID=gene-E	7	5.67	2596.8	0.332886548	EDAAM(+15	50	737.3263	1	736.3062	Oxidation	K	Superscaf	68827317	0	0	0	BTP202209	0	0	Tissue specific

TEDLRDK	Superscaf	+	Superscaf	68875885	68875905	5	5	11	-	-	-	-	-	-	NCP	IGR	-	ID=gene-E	7	6.024285714	3116.6	0.333827952	T(+42.01)	84	459.7375	2	917.4454	Acetylati	C	Superscaf	68875873	0	0	0	0	0	BTP202209	Tissue specific
YVLTKK	Superscaf	-	Superscaf	68929444	68929461	-29	29	34	-	-	-	-	-	-	NCP	IGR	-	ID=gene-E	6	6.275	2147.2	0.334865158	YVLTKK	60	376.2423	2	750.4639	.	S	Superscaf	68929470	0	0	0	BTP202209	0	0	Mixed
QVTWL	Superscaf	-	Superscaf	68957869	68957883	-77	77	81	-	-	gene	-	-	mRNA	NCP	Intron	gene|mRN	ID=gene-E	5	4.81	1888.2	0.335415627	QVTWL	65	646.3544	1	645.3486	.	C	Superscaf	68957964	0	0	BTP202209	0	0	0	Tissue specific
KLTTSTK	Superscaf	-	Superscaf	68978518	68978538	-4	4	10	-	-	gene	-	-	mRNA	NCP	Intron	gene|mRN	ID=gene-E	7	7.247142857	2866.1	0.335815509	KLTTSTK	54	389.7374	2	777.4596	.	Q	Superscaf	68978661	0	0	0	0	BTP202209	0	Tissue specific
APATLHVG	Superscaf	-	Superscaf	68994817	68994840	-33	33	40	-	-	gene	-	-	mRNA	NCP	Intron	gene|mRN	ID=gene-E	8	5.55125	2627.9	0.33613115	APATLHVG	54	765.4194	1	764.4181	.	L	Superscaf	68994849	0	0	BTP202209	0	0	0	Tissue specific
EVFMYA	Superscaf	-	Superscaf	69013108	69013125	-55	55	60	-	-	-	-	-	-	NCP	IGR	-	ID=gene-E	6	4.355	1955.2	0.336485368	EVFMYA	51	759.334	1	758.3309	.	K	Superscaf	69013134	0	BTP202209	0	0	0	0	Tissue specific
SLAALKK	Superscaf	+	Superscaf	69090751	69090771	32	32	38	-	-	-	-	-	-	NCP	IGR	-	ID=gene-E	7	7.022857143	2649.6	0.337988977	SLAALKK	99	365.7438	2	729.4749	.	A	Superscaf	69090658	0	0	0	0	0	BTP202209	Tissue enhanced
TDPEATPK	Superscaf	+	Superscaf	69161518	69161541	18	18	25	-	-	-	-	-	-	NCP	IGR	-	ID=gene-E	8	5.95125	3086.5	0.339359428	TDPEATPK	72	429.7177	2	857.413	.	N	Superscaf	69161467	0	BTP202209	0	0	BTP202209	BTP202209	Tissue specific
PFSLL	Superscaf	-	Superscaf	69162064	69162078	-32	32	36	-	-	-	-	-	-	NCP	IGR	-	ID=gene-E	5	5.884	1815	0.339370001	PFSLL	99	576.338	1	575.3318	.	H	Superscaf	69162081	0	0	0	0	BTP202209	BTP202209	Mixed
GHAAHSN	Superscaf	-	Superscaf	69177394	69177414	-3	3	9	-	-	-	-	-	-	NCP	IGR	-	ID=gene-E	7	6.325714286	3139.4	0.339666877	G(+42.01)	64	368.1617	2	734.3096	Acetylati	D	Superscaf	69177513	0	0	0	0	BTP202209	BTP202209	Tissue specific
PAEKD	Superscaf	-	Superscaf	69283243	69283257	-16	16	20	-	-	-	-	-	-	NCP	IGR	-	ID=gene-E	5	5.65	1954.1	0.341716714	P(+42.01)	52	601.2819	1	600.2755	Acetylati	S	Superscaf	69283323	0	0	0	0	BTP202209	0	Tissue specific
ECCFQ	Superscaf	+	Superscaf	69317275	69317289	17	17	21	-	-	-	-	-	-	NCP	IGR	-	ID=gene-E	5	4.878	2595	0.342375767	EC(+57.02	55	372.1286	2	742.2415	Carbamido	Q	Superscaf	69317227	0	0	0	0	BTP202209	0	Mixed
TLEALRY	Superscaf	-	Superscaf	69344842	69344862	-14	14	20	-	-	-	-	-	-	NCP	IGR	-	ID=gene-E	7	6.307142857	2949.4	0.342909621	T(+42.01)	99	454.2461	2	906.4811	Acetylati	D	Superscaf	69344949	0	BTP202209	0	0	0	0	Mixed
KNTHTLLL	Superscaf	-	Superscaf	69352555	69352578	-10	10	17	-	-	-	-	-	-	NCP	IGR	-	ID=gene-E	8	6.7175	3540	0.343058988	KNTHTLLL	76	470.2836	2	938.5549	.	N	Superscaf	69352632	0	0	0	0	0	BTP202209	Tissue specific
LSLTAVPQ	Superscaf	-	Superscaf	69400345	69400368	-66	66	73	-	-	-	-	-	-	NCP	IGR	-	ID=gene-E	8	5.2675	2747.1	0.343984474	LSLTAVPQ	54	414.743	2	827.4753	.	A	Superscaf	69400389	0	BTP202209	0	0	0	0	Tissue specific
TKFRPM	Superscaf	+	Superscaf	69439546	69439563	25	25	30	-	-	-	-	-	-	NCP	IGR	-	ID=gene-E	6	7.426666667	2611	0.344743627	T(+42.01)	67	419.2257	2	836.4214	Acetylati	R	Superscaf	69439474	0	0	0	0	BTP202209	BTP202209	Tissue specific
KERLT	Superscaf	+	Superscaf	69529825	69529839	3	3	7	-	-	-	-	-	-	NCP	IGR	-	ID=gene-E	5	7.246	2293.6	0.346491941	KERLT	67	646.3853	1	645.3809	.	I	Superscaf	69529819	0	0	0	0	0	BTP202209	Tissue specific
KLSYL	Superscaf	+	Superscaf	69614581	69614595	12	12	16	-	-	gene	-	-	mRNA	NCP	Intron	gene|mRN	ID=gene-E	5	6.608	2018.1	0.348133298	K(+42.01)	61	665.3842	1	664.3796	Acetylati	V	Superscaf	69614548	BTP202209	0	0	0	0	BTP202209	Tissue specific
YGVTGP	Superscaf	-	Superscaf	69664012	69664029	-12	12	17	-	-	-	-	-	-	NCP	IGR	-	ID=gene-E	6	5.071666667	1789	0.349090563	YGVTGP	59	593.2943	1	592.2856	.	F	Superscaf	69664119	0	BTP202209	0	0	0	0	Tissue specific
TPSKAPK	Superscaf	-	Superscaf	69671125	69671145	-24	24	30	-	-	-	-	-	-	NCP	IGR	-	ID=gene-E	7	7.187142857	2703.8	0.349228311	TPSKAPK	57	364.7225	2	727.4228	.	T	Superscaf	69671172	0	0	0	0	0	BTP202209	Tissue specific
DNPAP	Superscaf	+	Superscaf	69715096	69715110	6	6	10	-	-	gene	-	-	mRNA	NCP	Intron	gene|mRN	ID=gene-E	5	5.4	1994.3	0.350079839	D(+42.01)	57	555.243	1	554.2336	Acetylati	N	Superscaf	69715081	0	0	0	BTP202209	0	0	Tissue specific
VDLVL	Superscaf	+	Superscaf	69820912	69820926	7	7	11	-	-	-	-	-	-	NCP	IGR	-	ID=gene-E	5	2.986	1118.3	0.352129037	VDLVL	75	558.3478	1	557.3425	.	L	Superscaf	69820894	0	0	BTP202209	0	0	0	Tissue specific
PAKPSVK	Superscaf	+	Superscaf	69828319	69828339	11	11	17	-	-	gene	-	-	mRNA	NCP	Intron	gene|mRN	ID=gene-E	7	6.254285714	2306.3	0.352272479	PAKPSVK	99	363.7277	2	725.4435	.	N	Superscaf	69828289	BTP202209	0	0	0	0	0	Mixed
LVQRVVL	Superscaf	+	Superscaf	69849454	69849474	4	4	10	-	-	gene	-	-	mRNA	NCP	Intron	gene|mRN	ID=gene-E	7	4.052857143	1966.3	0.352681772	LVQRVVL	87	413.7777	2	825.5436	.	I	Superscaf	69849445	0	0	0	BTP202209	0	0	Tissue specific
PEVAADP	Superscaf	+	Superscaf	69866386	69866406	44	44	50	-	-	gene	-	-	mRNA	NCP	Intron	gene|mRN	ID=gene-E	7	4.404285714	2134.4	0.353009672	PEVAADP	59	698.3492	1	697.3282	.	G	Superscaf	69866257	0	BTP202209	0	0	0	0	Tissue specific
KPEAPPN	Superscaf	+	Superscaf	69980452	69980472	2	2	8	-	-	-	-	-	-	NCP	IGR	-	ID=gene-E	7	6.184285714	2814.1	0.355218637	KPEAPPN	56	752.381	1	751.3864	.	S	Superscaf	69980449	0	BTP202209	0	0	0	0	Tissue specific
LEGLLN	Superscaf	-	Superscaf	70014400	70014417	-14	14	19	-	-	-	-	-	-	NCP	IGR	-	ID=gene-E	6	5.423333333	2379.7	0.355876063	LEGLLN	58	658.3734	1	657.3697	.	G	Superscaf	70014456	0	0	BTP202209	0	0	0	Tissue specific
PLVTKTK	Superscaf	+	Superscaf	70359370	70359390	58	58	64	-	-	gene	-	-	mRNA	NCP	Intron	gene|mRN	ID=gene-E	7	6.402857143	2422.6	0.362556639	PLVTKTK	50	393.7635	2	785.501	.	L	Superscaf	70359199	0	0	BTP202209	0	0	0	Tissue specific
LEEELKR	Superscaf	-	Superscaf	70380688	70380708	-41	41	47	-	-	gene	-	-	mRNA	NCP	Intron	gene|mRN	ID=gene-E	7	6.017142857	3100.5	0.362969477	LEEELKR	97	458.7575	2	915.5025	.	D	Superscaf	70380720	0	BTP202209	0	0	0	0	Tissue specific
LSSTFQQ	Superscaf	-	Superscaf	70405756	70405776	-20	20	26	-	-	-	-	-	-	NCP	IGR	-	ID=gene-E	7	5.807142857	3126.5	0.363454935	LSSTFQQ	84	810.397	1	809.3919	.	E	Superscaf	70405779	BTP202209	0	0	0	0	0	Mixed
LLEEKLK	Superscaf	+	Superscaf	70447747	70447767	5	5	11	-	-	gene	-	-	mRNA	NCP	Intron	gene|mRN	ID=gene-E	7	6.265714286	2848	0.364268119	LLEEKLK	99	436.7746	2	871.5378	.	L	Superscaf	70447735	0	0	0	0	BTP202209	0	Tissue specific
KPKKYSK	Superscaf	-	Superscaf	70463764	70463784	-2	2	8	-	-	gene	-	-	mRNA	NCP	Intron	gene|mRN	ID=gene-E	7	8.085714286	3053.9	0.364578299	KPKKYSK	65	439.7787	2	877.5385	.	C	Superscaf	70463871	0	0	0	0	BTP202209	0	Tissue specific
KDPAPPP	Superscaf	+	Superscaf	70516822	70516842	36	36	42	-	-	-	-	-	-	NCP	IGR	-	ID=gene-E	7	6.275714286	2540.8	0.365605803	KDPAPPP	89	721.3872	1	720.3806	.	R	Superscaf	70516717	0	0	0	0	0	BTP202209	Tissue specific
VASKKAAP	Superscaf	+	Superscaf	70555822	70555845	2	2	9	-	-	gene	-	-	mRNA	NCP	Intron	gene|mRN	ID=gene-E	8	6.19	2591.5	0.366361065	VASKKAAP	56	386.2443	2	770.465	.	T	Superscaf	70555819	0	0	0	0	BTP202209	0	Tissue specific
KVGDLEK	Superscaf	-	Superscaf	70577782	70577802	-9	9	15	-	exon	gene	three_pri	-	mRNA	NCP	3UTR	exon|gene	ID=exon-E	7	5.374285714	2424.5	0.366786335	K(+42.01)	54	830.4499	1	829.4545	Acetylati	Y	Superscaf	70577805	0	0	0	0	0	BTP202209	Tissue specific
KDGPLK	Superscaf	+	Superscaf	70615573	70615590	2	2	7	-	-	gene	-	-	mRNA	NCP	Intron	gene|mRN	ID=gene-E	6	6.783333333	2336.4	0.367518183	KDGPLK	71	657.3923	1	656.3857	.	N	Superscaf	70615570	0	BTP202209	0	BTP202209	0	0	Tissue specific
DLKEHLH	Superscaf	-	Superscaf	70632928	70632948	-6	6	12	-	-	gene	-	-	mRNA	NCP	Intron	gene|mRN	ID=gene-E	7	6.152857143	3251.6	0.367854274	DLKEHLH	99	446.2361	2	890.461	.	M	Superscaf	70632990	0	BTP202209	0	0	BTP202209	BTP202209	Tissue specific
LDKDNLR	Superscaf	-	Superscaf	70644463	70644483	-2	2	8	-	-	gene	-	-	mRNA	NCP	Intron	gene|mRN	ID=gene-E	7	6.258571429	3243.7	0.368077657	LDKDNLR	79	437.2421	2	872.4716	.	L	Superscaf	70644537	0	0	0	BTP202209	0	0	Tissue specific
RLLLSD	Superscaf	+	Superscaf	70667974	70667991	28	28	33	-	-	gene	-	-	mRNA	NCP	Intron	gene|mRN	ID=gene-E	6	6.225	2503.9	0.368532964	R(+42.01)	51	758.4507	1	757.4333	Acetylati	C	Superscaf	70667893	0	0	0	BTP202209	0	0	Tissue specific
KPGLAY	Superscaf	-	Superscaf	70723228	70723245	-49	49	54	-	-	gene	-	-	mRNA	NCP	Intron	gene|mRN	ID=gene-E	6	6.611666667	2227.3	0.369602995	KPGLAY	64	648.3779	1	647.3642	.	V	Superscaf	70723467	0	0	BTP202209	0	0	0	Tissue specific
SYLGKP	Superscaf	-	Superscaf	70730098	70730115	-30	30	35	-	-	gene	-	-	mRNA	NCP	Intron	gene|mRN	ID=gene-E	6	6.555	2299.4	0.369736017	SYLGKP	92	664.3645	1	663.3591	.	I	Superscaf	70730130	0	BTP202209	0	0	0	0	Mixed
EVLTGL	Superscaf	-	Superscaf	70852162	70852179	-28	28	33	-	-	-	-	-	-	NCP	IGR	-	ID=gene-E	6	4.613333333	1827.1	0.372099889	EVLTGL	57	631.364	1	630.3588	.	G	Superscaf	70852290	BTP202209	0	0	0	0	0	Tissue specific
MHGTLR	Superscaf	+	Superscaf	70894582	70894599	2	2	7	-	-	-	-	-	-	NCP	IGR	-	ID=gene-E	6	7.096666667	2688.2	0.372921381	M(+15.99)	82	365.6814	2	729.3592	Oxidation	K	Superscaf	70894579	0	BTP202209	BTP202209	0	BTP202209	0	Tissue specific
QALLNKVV	Superscaf	+	Superscaf	70907500	70907523	36	36	43	-	-	-	-	-	-	NCP	IGR	-	ID=gene-E	8	4.8475	2693.9	0.373171546	QALLNKVV	65	442.78	2	883.5491	.	Q	Superscaf	70907395	0	0	0	0	BTP202209	0	Mixed
QGPLGLVSI	Superscaf	+	Superscaf	70909234	70909260	7	7	15	-	-	-	-	-	-	NCP	IGR	-	ID=gene-E	9	5.278888889	2986.3	0.373205126	QGPLGLVSI	70	883.521	1	882.5175	.	N	Superscaf	70909216	0	BTP202209	0	0	0	0	Tissue specific
LVQLGSLF	Superscaf	+	Superscaf	70956103	70956126	32	32	39	-	-	-	-	-	-	NCP	IGR	-	ID=gene-E	8	5.09	2739	0.374112776	LVQLGSLF	68	438.7614	2	875.5117	.	H	Superscaf	70956010	0	0	0	0	BTP202209	0	Tissue specific
TRSLFVN	Superscaf	+	Superscaf	70985722	70985742	35	35	41	-	-	-	-	-	-	NCP	IGR	-	ID=gene-E	7	5.691428571	2767.2	0.374686368	TRSLFVN	73	418.7333	2	835.4552	.	G	Superscaf	70985620	0	0	0	0	0	BTP202209	Mixed
VEKGPKV	Superscaf	-	Superscaf	71002837	71002857	-9	9	15	-	-	-	-	-	-	NCP	IGR	-	ID=gene-E	7	4.995714286	1997	0.375017812	VEKGPKV	98	378.7338	2	755.4541	.	I	Superscaf	71002881	0	0	0	0	0	BTP202209	Tissue specific
PPNQEEC	Superscaf	-	Superscaf	71020579	71020599	-44	44	50	-	-	-	-	-	-	NCP	IGR	-	ID=gene-E	7	5.017142857	3318.9	0.375361397	PPNQEEC(+	50	437.1757	2	872.3334	Carbamido	S	Superscaf	71020626	0	0	0	0	BTP202209	0	Tissue specific
GPVKVG	Superscaf	+	Superscaf	71066428	71066445	11	11	16	-	-	-	-	-	-	NCP	IGR	-	ID=gene-E	6	4.663333333	1400.4	0.376249294	GPVKVG	60	556.3441	1	555.338	.	G	Superscaf	71066398	0	0	0	0	0	BTP202209	Tissue specific
TGKGKLLK	Superscaf	-	Superscaf	71097667	71097690	-4	4	11	-	-	-	-	-	-	NCP	IGR	-	ID=gene-E	8	7.45625	3104.1	0.376854258	T(+42.01)	70	443.7878	2	885.5648	Acetylati	A	Superscaf	71097729	0	0	0	BTP202209	0	0	Tissue specific
RTSLFVN	Superscaf	+	Superscaf	71121079	71121099	2	2	8	-	-	-	-	-	-	NCP	IGR	-	ID=gene-E	7	5.691428571	2767.2	0.377307648	RTSLFVN	79	418.7338	2	835.4552	.	A	Superscaf	71121076	0	BTP202209	0	0	0	0	Mixed
LMLVGK	Superscaf	+	Superscaf	71128855	71128872	38	38	43	-	-	-	-	-	-	NCP	IGR	-	ID=gene-E	6	5.57	1844	0.377458235	LMLVGK	97	660.4086	1	659.404	.	H	Superscaf	71128744	0	0	0	BTP202209	0	0	Tissue specific
LLMGM	Superscaf	-	Superscaf	71168488	71168502	-14	14	18	-	-	gene	-	-	mRNA	NCP	Intron	gene|mRN	ID=gene-E	5	5.886	1747.1	0.378225755	LLMGM	53	564.2869	1	563.2811	.	M	Superscaf	71168544	0	0	0	0	BTP202209	0	Tissue specific
KLAEDR	Superscaf	+	Superscaf	71177083	71177100	4	4	9	-	-	gene	-	-	mRNA	NCP	Intron	gene|mRN	ID=gene-E	6	6.448333333	2618.9	0.378392203	KLAEDR	92	366.2114	2	730.3973	.	T	Superscaf	71177074	0	0	BTP202209	0	0	0	Tissue specific
TNFVL	Superscaf	+	Superscaf	71295319	71295333	22	22	26	-	-	-	-	-	-	NCP	IGR	-	ID=gene-E	5	4.68	1735	0.380681923	T(+42.01)	54	635.3372	1	634.3326	Acetylati	K	Superscaf	71295256	0	0	0	0	0	BTP202209	Tissue specific
KGMVVR	Superscaf	+	Superscaf	71339452	71339469	18	18	23	-	-	-	-	-	-	NCP	IGR	-	ID=gene-E	6	5.37	1786	0.381536588	KGM(+15.9	57	353.2062	2	704.4003	Oxidation	F	Superscaf	71339401	0	0	0	BTP202209	0	0	Tissue specific
KGLALVR	Superscaf	+	Superscaf	71341582	71341602	13	13	19	-	-	-	-	-	-	NCP	IGR	-	ID=gene-E	7	6.35	2432.6	0.381577837	KGLALVR	73	378.7567	2	755.5017	.	G	Superscaf	71341546	0	0	0	0	BTP202209	0	Tissue specific
KRYWSK	Superscaf	-	Superscaf	71377093	71377110	-8	8	13	-	-	-	-	-	-	NCP	IGR	-	ID=gene-E	6	7.911666667	2955.1	0.382265531	KRYWSK	60	434.2378	2	866.4763	.	N	Superscaf	71377173	0	0	0	0	0	BTP202209	Tissue specific
VGALLRGP	Superscaf	+	Superscaf	71394445	71394468	9	9	16	-	-	gene	-	-	mRNA	NCP	Intron	gene|mRN	ID=gene-E	8	5.8725	2598.9	0.382601565	VGALLRGP	53	391.7461	2	781.481	.	E	Superscaf	71394421	0	0	BTP202209	0	0	0	Tissue specific
LVAPPG	Superscaf	-	Superscaf	71411017	71411034	-16	16	21	-	-	gene	-	-	mRNA	NCP	Intron	gene|mRN	ID=gene-E	6	5.095	1636.8	0.382922492	LVAPPG	98	553.3336	1	552.3271	.	D	Superscaf	71411037	0	0	0	0	0	BTP202209	Tissue specific
YVKPEM	Superscaf	-	Superscaf	71436037	71436054	-13	13	18	-	-	gene	-	-	mRNA	NCP	Intron	gene|mRN	ID=gene-E	6	5.111666667	2062.3	0.383407022	YVKPEM(+1	56	391.6848	2	781.368	Oxidation	F	Superscaf	71436153	0	0	0	0	BTP202209	0	Tissue specific
PLFSPR	Superscaf	-	Superscaf	71596801	71596818	-32	32	37	-	-	-	-	-	-	NCP	IGR	-	ID=gene-E	6	6.75	2447.8	0.386520325	PLFSPR	50	358.7103	2	715.4017	.	V	Superscaf	71596821	0	0	0	0	BTP202209	0	Tissue specific
TARMGK	Superscaf	+	Superscaf	71662171	71662188	2	2	7	-	-	gene	-	-	mRNA	NCP	Intron	gene|mRN	ID=gene-E	6	7.461666667	2494.8	0.387786259	T(+42.01)	50	353.1961	2	704.364	Acetylati	G	Superscaf	71662168	0	0	0	BTP202209	0	0	Tissue specific
KSRGLLTL	Superscaf	+	Superscaf	71715274	71715297	16	16	23	-	-	gene	-	-	mRNA	NCP	Intron	gene|mRN	ID=gene-E	8	7.0775	3255.6	0.388814634	KSRGLLTL	50	444.2856	2	886.56	.	P	Superscaf	71715229	0	0	0	BTP202209	0	0	Tissue specific
NLEFVAR	Superscaf	+	Superscaf	71780656	71780676	14	14	20	-	-	-	-	-	-	NCP	IGR	-	ID=gene-E	7	5.267142857	2723.1	0.390080801	NLEFVAR	95	424.7336	2	847.4551	.	L	Superscaf	71780617	0	0	0	BTP202209	0	0	Tissue specific
ACLFLQ	Superscaf	+	Superscaf	71793196	71793213	2	2	7	-	-	-	-	-	-	NCP	IGR	-	ID=gene-E	6	5.688333333	2601.9	0.390323647	AC(+57.02	58	751.3765	1	750.3734	Carbamido	I	Superscaf	71793193	0	0	0	0	BTP202209	0	Tissue specific
EVFTGHL	Superscaf	-	Superscaf	71808682	71808702	-23	23	29	-	-	-	-	-	-	NCP	IGR	-	ID=gene-E	7	4.967142857	2480.8	0.390623543	EVFTGHL	77	401.7069	2	801.4021	.	Y	Superscaf	71808750	0	0	0	BTP202209	0	0	Tissue specific
TSPFLPR	Superscaf	-	Superscaf	71837374	71837394	-48	48	54	-	-	-	-	-	-	NCP	IGR	-	ID=gene-E	7	6.718571429	2845.3	0.391179183	T(+42.01)	54	430.2446	2	858.4599	Acetylati	L	Superscaf	71837400	0	0	BTP202209	0	0	0	Tissue specific
GALGQRK	Superscaf	-	Superscaf	71850388	71850408	-6	6	12	-	-	-	-	-	-	NCP	IGR	-	ID=gene-E	7	7.155714286	2987.2	0.391431208	G(+42.01)	70	386.2244	2	770.4399	Acetylati	L	Superscaf	71850408	0	0	0	BTP202209	0	0	Tissue specific
ESTLVLK	Superscaf	-	Superscaf	71872249	71872269	-10	10	16	-	-	gene	-	-	mRNA	NCP	Intron	gene|mRN	ID=gene-E	7	5.304285714	2381.6	0.391854561	ESTLVLK	54	395.237	2	788.4644	.	V	Superscaf	71872335	0	BTP202209	0	0	0	BTP202209	Tissue specific
GPLGHLKP	Superscaf	+	Superscaf	71872903	71872926	42	42	49	-	-	gene	-	-	mRNA	NCP	Intron	gene|mRN	ID=gene-E	8	6.72875	3064.3	0.391867226	G(+42.01)	64	430.7513	2	859.4916	Acetylati	E	Superscaf	71872780	0	0	0	0	0	BTP202209	Tissue specific
AAQGEPQVG	Superscaf	+	Superscaf	71874973	71875008	21	21	32	-	-	gene	-	-	mRNA	NCP	Intron	gene|mRN	ID=gene-E	12	5.473333333	4724.1	0.391907313	A(+42.01)	66	679.3571	2	1356.704	Acetylati	K	Superscaf	71874913	0	0	0	BTP202209	0	0	Tissue specific
DGFHFK	Superscaf	-	Superscaf	71890900	71890917	-13	13	18	-	-	gene	-	-	mRNA	NCP	Intron	gene|mRN	ID=gene-E	6	6.205	2571.7	0.39221575	DGFHFK	65	375.6782	2	749.3497	.	K	Superscaf	71891019	0	0	0	0	BTP202209	0	Tissue specific
PPLPP	Superscaf	-	Superscaf	72172198	72172212	-41	41	45	-	-	-	-	-	-	NCP	IGR	-	ID=gene-E	5	6.236	1703	0.397663276	PPLPP	79	520.3123	1	519.3057	.	K	Superscaf	72172392	0	0	BTP202209	0	0	0	Tissue enhanced
HWRSPPP	Superscaf	-	Superscaf	72361321	72361341	-10	10	16	-	-	-	-	-	-	NCP	IGR	-	ID=gene-E	7	6.974285714	3190.7	0.40132577	HWRSPPP	57	438.7187	2	875.4402	.	K	Superscaf	72361341	0	0	BTP202209	0	0	0	Tissue specific
KNLVFST	Superscaf	+	Superscaf	72431590	72431610	9	9	15	-	-	-	-	-	-	NCP	IGR	-	ID=gene-E	7	5.545714286	2586.8	0.402686577	KNLVFST	62	404.73	2	807.449	.	I	Superscaf	72431566	0	0	0	0	0	BTP202209	Tissue specific
LPTLAPD	Superscaf	+	Superscaf	72439228	72439248	57	57	63	-	-	gene	-	-	mRNA	NCP	Intron	gene|mRN	ID=gene-E	7	5.725714286	2501.9	0.402834492	LPTLAPD	53	726.402	1	725.3959	.	G	Superscaf	72439060	0	0	0	0	0	BTP202209	Tissue specific
AASLVFPR	Superscaf	+	Superscaf	72441517	72441540	5	5	12	-	-	gene	-	-	mRNA	NCP	Intron	gene|mRN	ID=gene-E	8	5.78	2733	0.40287882	A(+42.01)	57	451.7548	2	901.5021	Acetylati	R	Superscaf	72441505	0	0	0	BTP202209	0	0	Tissue specific
TCKLVQT	Superscaf	-	Superscaf	72554260	72554280	-13	13	19	-	-	gene	-	-	mRNA	NCP	Intron	gene|mRN	ID=gene-E	7	5.635714286	2813.2	0.405062164	T(+42.01)	71	446.2319	2	890.4532	Acetylati	R	Superscaf	72554283	0	BTP202209	0	0	0	0	Tissue specific
ECMTAK	Superscaf	-	Superscaf	72581959	72581976	-9	9	14	-	-	-	-	-	-	NCP	IGR	-	ID=gene-E	6	6.046666667	2559.9	0.405598574	EC(+57.02	54	739.3147	1	738.304	Carbamido	S	Superscaf	72582003	0	BTP202209	0	0	0	0	Tissue specific
LGPVG	Superscaf	+	Superscaf	72710203	72710217	70	70	74	-	-	-	-	-	-	NCP	IGR	-	ID=gene-E	5	4.844	1285.4	0.408082105	LGPVG	70	442.2653	1	441.2587	.	Y	Superscaf	72709996	0	0	0	BTP202209	0	0	Mixed
YCADY	Superscaf	-	Superscaf	72747775	72747789	-21	21	25	-	-	-	-	-	-	NCP	IGR	-	ID=gene-E	5	5.066	2227.5	0.408809712	Y(+42.01)	56	733.2501	1	732.2425	Acetylati	K	Superscaf	72747804	0	0	0	BTP202209	0	0	Tissue specific
PVEGVHK	Superscaf	-	Superscaf	72920863	72920883	-10	10	16	-	-	gene	-	-	mRNA	NCP	Intron	gene|mRN	ID=gene-E	7	4.688571429	2148.3	0.412161678	PVEGVHK	55	383.2208	2	764.418	.	S	Superscaf	72920883	0	0	0	0	BTP202209	0	Tissue specific
FLVGVA	Superscaf	+	Superscaf	72986242	72986259	17	17	22	-	-	gene	-	-	mRNA	NCP	Intron	gene|mRN	ID=gene-E	6	3.908333333	1349.4	0.413427787	FLVGVA	56	605.3638	1	604.3584	.	L	Superscaf	72986194	0	0	0	0	BTP202209	0	Tissue specific
TLENLKK	Superscaf	-	Superscaf	72994756	72994776	-39	39	45	-	-	gene	-	-	mRNA	NCP	Intron	gene|mRN	ID=gene-E	7	6.657142857	3063.3	0.413592666	T(+42.01)	82	444.2582	2	886.5124	Acetylati	G	Superscaf	72994785	0	0	0	0	0	BTP202209	Tissue specific
AALYLTKP	Superscaf	+	Superscaf	73002814	73002837	52	52	59	-	-	gene	-	-	mRNA	NCP	Intron	gene|mRN	ID=gene-E	8	6.52875	2992.2	0.413748715	AALYLTKP	56	438.7675	2	875.5116	.	P	Superscaf	73002661	0	0	BTP202209	BTP202209	0	0	Tissue specific
DLDQLRK	Superscaf	-	Superscaf	73008739	73008759	-19	19	25	-	-	gene	-	-	mRNA	NCP	Intron	gene|mRN	ID=gene-E	7	6.292857143	3257.7	0.413863456	DLDQLRK	85	444.2513	2	886.4872	.	R	Superscaf	73008774	0	0	0	0	BTP202209	0	Tissue specific
DDEETTALV	Superscaf	-	Superscaf	73023151	73023204	-7	7	24	-	-	-	-	-	-	NCP	IGR	-	ID=gene-E	18	4.676666667	6527.4	0.414142554	D(+42.01)	97	982.9363	2	1963.868	Acetylati	S	Superscaf	73023408	0	0	0	0	0	BTP202209	Tissue specific
VCDNGSGLV	Superscaf	-	Superscaf	73023175	73023204	-15	15	24	-	-	-	-	-	-	NCP	IGR	-	ID=gene-E	10	4.674	3393.7	0.414143019	VC(+57.02	97	524.7579	2	1047.502	Carbamido	S	Superscaf	73023408	0	BTP202209	0	0	0	0	Tissue specific
WDDMEK	Superscaf	-	Superscaf	73024516	73024533	-38	38	43	-	-	-	-	-	-	NCP	IGR	-	ID=gene-E	6	5.09	2615	0.414168989	WDDMEK	98	412.1656	2	822.3218	.	G	Superscaf	73024743	0	BTP202209	0	0	BTP202209	BTP202209	Mixed
QPLGKHL	Superscaf	-	Superscaf	73025095	73025115	-20	20	26	-	-	-	-	-	-	NCP	IGR	-	ID=gene-E	7	6.744285714	3040.3	0.414180201	QPLGKHL	70	396.7386	2	791.4653	.	G	Superscaf	73025118	0	0	BTP202209	0	0	0	Tissue specific
DLAGRDL	Superscaf	-	Superscaf	73025587	73025607	-50	50	56	-	-	-	-	-	-	NCP	IGR	-	ID=gene-E	7	5.807142857	2787.2	0.414189729	DLAGRDL	87	380.2019	2	758.3922	.	H	Superscaf	73025769	0	0	0	0	0	BTP202209	Tissue specific
RFRCPET	Superscaf	-	Superscaf	73025938	73025958	-92	92	98	-	-	-	-	-	-	NCP	IGR	-	ID=gene-E	7	6.867142857	3431.1	0.414196527	RFRC(+57.	95	483.2327	2	964.4548	Carbamido	V	Superscaf	73026039	0	0	0	0	BTP202209	BTP202209	Tissue specific
VTMELLK	Superscaf	+	Superscaf	73040455	73040475	14	14	20	-	-	-	-	-	-	NCP	IGR	-	ID=gene-E	7	5.314285714	2369.7	0.414477658	VTM(+15.9	70	425.237	2	848.4677	Oxidation	K	Superscaf	73040416	0	BTP202209	0	0	0	0	Tissue specific
LRLRYL	Superscaf	-	Superscaf	73057651	73057668	-1	1	6	-	-	-	-	-	-	NCP	IGR	-	ID=gene-E	6	7.52	2817.3	0.41481067	LRLRYL	81	417.2696	2	832.5283	.	L	Superscaf	73057716	BTP202209	0	0	0	0	0	Tissue specific
AEEFPV	Superscaf	+	Superscaf	73110550	73110567	4	4	9	-	-	gene	-	-	mRNA	NCP	Intron	gene|mRN	ID=gene-E	6	4.04	1887.1	0.415835095	A(+42.01)	88	733.3378	1	732.333	Acetylati	L	Superscaf	73110541	0	BTP202209	0	0	0	0	Tissue specific
KDLGK	Superscaf	-	Superscaf	73127362	73127376	-17	17	21	-	-	gene	-	-	mRNA	NCP	Intron	gene|mRN	ID=gene-E	5	6.88	1999	0.416160671	KDLGK	78	560.3383	1	559.3329	.	L	Superscaf	73127382	0	0	BTP202209	0	0	0	Tissue specific
LSPDSVPV	Superscaf	+	Superscaf	73168918	73168941	32	32	39	-	-	-	-	-	-	NCP	IGR	-	ID=gene-E	8	4.11375	2206.5	0.416965431	LSPDSVPV	64	813.4321	1	812.428	.	L	Superscaf	73168825	BTP202209	0	0	0	0	0	Tissue specific
LKEPLTV	Superscaf	-	Superscaf	73265974	73265994	-7	7	13	-	-	-	-	-	-	NCP	IGR	-	ID=gene-E	7	5.392857143	2335.6	0.418844985	LKEPLTV	73	400.249	2	798.485	.	M	Superscaf	73266063	0	0	BTP202209	0	0	0	Tissue specific
KFLEPQ	Superscaf	+	Superscaf	73296976	73296993	11	11	16	-	-	-	-	-	-	NCP	IGR	-	ID=gene-E	6	6.061666667	2582.8	0.41944536	KFLEPQ	58	381.2131	2	760.4119	.	L	Superscaf	73296946	BTP202209	0	0	0	0	0	Tissue specific
TVLKFLLK	Superscaf	+	Superscaf	73304467	73304490	10	10	17	-	-	-	-	-	-	NCP	IGR	-	ID=gene-E	8	6.17875	2781.9	0.419590428	T(+42.01)	83	502.3227	2	1002.648	Acetylati	Y	Superscaf	73304440	0	0	0	0	0	BTP202209	Tissue specific
HDLGHF	Superscaf	-	Superscaf	73309105	73309122	-25	25	30	-	-	-	-	-	-	NCP	IGR	-	ID=gene-E	6	5.93	2689	0.419680246	HDLGHF	76	363.1707	2	724.3293	.	H	Superscaf	73309158	0	0	0	0	0	BTP202209	Tissue specific
HEPFLH	Superscaf	+	Superscaf	73324570	73324587	24	24	29	-	-	-	-	-	-	NCP	IGR	-	ID=gene-E	6	6.026666667	2743.1	0.419979737	HEPFLH	74	390.2028	2	778.3762	.	A	Superscaf	73324501	0	BTP202209	0	0	0	0	Tissue specific
TLHFRFL	Superscaf	-	Superscaf	73337299	73337319	-61	61	67	-	-	gene	-	-	mRNA	NCP	Intron	gene|mRN	ID=gene-E	7	6.828571429	3147.6	0.420226242	TLHFRFL	97	467.268	2	932.5232	.	I	Superscaf	73337412	0	BTP202209	0	0	0	0	Tissue specific
AQLSLVY	Superscaf	+	Superscaf	73388167	73388187	22	22	28	-	-	gene	-	-	mRNA	NCP	Intron	gene|mRN	ID=gene-E	7	4.995714286	2471.7	0.421211335	AQLSLVY	73	793.4419	1	792.4381	.	L	Superscaf	73388104	0	0	0	0	0	BTP202209	Tissue specific
LMLKPPP	Superscaf	+	Superscaf	73428763	73428783	80	80	86	-	-	gene	-	-	mRNA	NCP	Intron	gene|mRN	ID=gene-E	7	6.621428571	2558.9	0.421997504	LM(+15.99	52	406.2474	2	810.4673	Oxidation	Y	Superscaf	73428526	0	0	0	BTP202209	0	0	Tissue specific
MVSLAPFP	Superscaf	+	Superscaf	73452559	73452582	4	4	11	-	-	gene	-	-	mRNA	NCP	Intron	gene|mRN	ID=gene-E	8	5.18875	2481.8	0.42245833	MVSLAPFP	50	431.2278	2	860.4466	.	L	Superscaf	73452550	0	0	0	0	0	BTP202209	Tissue specific
LSVLF	Superscaf	+	Superscaf	73487008	73487022	19	19	23	-	-	gene	-	-	mRNA	NCP	Intron	gene|mRN	ID=gene-E	5	4.624	1477.6	0.423125458	LSVLF	76	578.3528	1	577.3475	.	G	Superscaf	73486954	0	0	0	0	0	BTP202209	Tissue specific
KKMATK	Superscaf	+	Superscaf	73497823	73497840	3	3	8	-	-	gene	-	-	mRNA	NCP	Intron	gene|mRN	ID=gene-E	6	7.92	2485.5	0.423334898	KKM(+15.9	50	361.7166	2	721.4156	Oxidation	K	Superscaf	73497817	0	BTP202209	0	0	0	0	Tissue specific
LSYEPN	Superscaf	-	Superscaf	73527796	73527813	-9	9	14	-	-	gene	-	-	mRNA	NCP	Intron	gene|mRN	ID=gene-E	6	5.375	2555.9	0.423915345	LSYEPN	56	722.3303	1	721.3282	.	F	Superscaf	73527957	0	0	0	0	0	BTP202209	Tissue specific
RLPSHVTP	Superscaf	-	Superscaf	73528177	73528200	-18	18	25	-	-	gene	-	-	mRNA	NCP	Intron	gene|mRN	ID=gene-E	8	6.1425	3077.6	0.423922723	RLPSHVTP	52	453.7563	2	905.5083	.	R	Superscaf	73528293	0	0	0	0	0	BTP202209	Tissue specific
VPLQVSH	Superscaf	+	Superscaf	73528639	73528659	5	5	11	-	-	gene	-	-	mRNA	NCP	Intron	gene|mRN	ID=gene-E	7	4.457142857	2304.6	0.42393167	VPLQVSH	88	779.4349	1	778.4337	.	V	Superscaf	73528627	0	0	0	0	0	BTP202209	Tissue specific
KSFMVPR	Superscaf	-	Superscaf	73548640	73548660	-72	72	78	-	-	gene	-	-	mRNA	NCP	Intron	gene|mRN	ID=gene-E	7	6.244285714	2596.9	0.424319003	KSFMVPR	54	432.7376	2	863.4687	.	N	Superscaf	73548897	0	0	0	0	0	BTP202209	Tissue specific
RLLGPLF	Superscaf	+	Superscaf	73553149	73553169	44	44	50	-	-	gene	-	-	mRNA	NCP	Intron	gene|mRN	ID=gene-E	7	6.635714286	2731.1	0.424406323	RLLGPLF	57	408.2601	2	814.5065	.	K	Superscaf	73553020	0	0	0	0	BTP202209	0	Mixed
QSPPAATL	Superscaf	-	Superscaf	73570816	73570839	-27	27	34	-	-	-	-	-	-	NCP	IGR	-	ID=gene-E	8	6.06	3042.4	0.424748456	Q(+42.01)	57	826.4159	1	825.4232	Acetylati	S	Superscaf	73570899	0	0	0	0	0	BTP202209	Tissue specific
KSKALLY	Superscaf	-	Superscaf	73571374	73571394	-1	1	7	-	-	-	-	-	-	NCP	IGR	-	ID=gene-E	7	6.971428571	2797.8	0.424759262	KSKALLY	59	411.7568	2	821.501	.	K	Superscaf	73571439	0	0	0	0	0	BTP202209	Tissue specific
LLPRSKV	Superscaf	+	Superscaf	73591882	73591902	5	5	11	-	-	-	-	-	-	NCP	IGR	-	ID=gene-E	7	6.348571429	2544.8	0.425156414	LLPRSKV	99	406.7702	2	811.528	.	R	Superscaf	73591870	0	0	0	0	0	BTP202209	Tissue specific
ELVAEAL	Superscaf	+	Superscaf	73600009	73600029	6	6	12	-	-	-	-	-	-	NCP	IGR	-	ID=gene-E	7	4.348571429	2180.4	0.425313798	ELVAEAL	91	744.4105	1	743.4065	.	V	Superscaf	73599994	BTP202209	0	0	0	0	0	Tissue specific
APEYNK	Superscaf	+	Superscaf	73686256	73686273	4	4	9	-	-	-	-	-	-	NCP	IGR	-	ID=gene-E	6	6.058333333	2598.8	0.42698403	APEYNK	57	721.3548	1	720.3442	.	I	Superscaf	73686247	0	0	0	0	BTP202209	0	Tissue specific
MMYLLSH	Superscaf	+	Superscaf	73689298	73689318	16	16	22	-	-	-	-	-	-	NCP	IGR	-	ID=gene-E	7	6.055714286	2912.4	0.42704294	M(+42.01)	66	468.7136	2	935.4245	Acetylati	I	Superscaf	73689253	BTP202209	0	0	0	0	0	Tissue specific
NPPGVVL	Superscaf	-	Superscaf	73744417	73744437	-4	4	10	-	-	gene	-	-	mRNA	NCP	Intron	gene|mRN	ID=gene-E	7	4.28	1922.2	0.428110357	N(+42.01)	69	737.417	1	736.4119	Acetylati	V	Superscaf	73744527	0	BTP202209	0	0	0	0	Tissue specific
KEVFVK	Superscaf	-	Superscaf	73754473	73754490	-1	1	6	-	-	gene	-	-	mRNA	NCP	Intron	gene|mRN	ID=gene-E	6	4.696666667	1749.7	0.428305098	KEVFVK	70	375.2307	2	748.4483	.	K	Superscaf	73754589	0	BTP202209	0	0	BTP202209	0	Tissue specific
TSFNTPA	Superscaf	+	Superscaf	73795231	73795251	10	10	16	-	-	gene	-	-	mRNA	NCP	Intron	gene|mRN	ID=gene-E	7	5.992857143	2811.2	0.429094404	T(+42.01)	70	779.3538	1	778.3497	Acetylati	Q	Superscaf	73795204	0	BTP202209	0	0	0	0	Tissue specific
DGNVGF	Superscaf	-	Superscaf	73877869	73877886	-44	44	49	-	-	-	-	-	-	NCP	IGR	-	ID=gene-E	6	4.3	1990.2	0.430694745	DGNVGF	57	608.2654	1	607.2602	.	H	Superscaf	73877901	BTP202209	0	0	0	BTP202209	0	Tissue specific
NRLPPPP	Superscaf	+	Superscaf	73912120	73912140	21	21	27	-	-	-	-	-	-	NCP	IGR	-	ID=gene-E	7	6.764285714	2948.5	0.431358038	NRLPPPP	74	395.7313	2	789.4497	.	D	Superscaf	73912060	0	0	0	BTP202209	0	0	Tissue specific
LLNTFY	Superscaf	-	Superscaf	73933666	73933683	-10	10	15	-	-	-	-	-	-	NCP	IGR	-	ID=gene-E	6	5.84	2547.9	0.431775291	LLNTFY	96	770.4049	1	769.401	.	S	Superscaf	73933695	0	0	0	0	0	BTP202209	Tissue specific
KNSSL	Superscaf	+	Superscaf	74006833	74006847	7	7	11	-	-	-	-	-	-	NCP	IGR	-	ID=gene-E	5	6.498	2185.3	0.43319222	KNSSL	50	548.2991	1	547.2966	.	I	Superscaf	74006815	0	0	0	BTP202209	0	0	Tissue specific
KLLLPR	Superscaf	-	Superscaf	74063920	74063937	-28	28	33	-	-	gene	-	-	mRNA	NCP	Intron	gene|mRN	ID=gene-E	6	7.456666667	2514.8	0.434297748	KLLLPR	69	370.2624	2	738.5115	.	L	Superscaf	74064273	0	BTP202209	0	0	0	0	Tissue enhanced
AYSDF	Superscaf	-	Superscaf	74066305	74066319	-41	41	45	-	-	gene	-	-	mRNA	NCP	Intron	gene|mRN	ID=gene-E	5	5.162	1953.1	0.434343935	AYSDF	58	602.2419	1	601.2383	.	N	Superscaf	74066334	0	0	0	BTP202209	0	0	Tissue specific
FDRGPPL	Superscaf	-	Superscaf	74130442	74130462	-10	10	16	-	-	gene	-	-	mRNA	NCP	Intron	gene|mRN	ID=gene-E	7	6.251428571	2773.2	0.435585991	FDRGPPL	60	401.2158	2	800.418	.	H	Superscaf	74130519	0	BTP202209	0	BTP202209	BTP202209	0	Tissue specific
KEFVTVVR	Superscaf	-	Superscaf	74145100	74145123	-11	11	18	-	-	gene	-	-	mRNA	NCP	Intron	gene|mRN	ID=gene-E	8	4.46625	2327.6	0.435869853	KEFVTVVR	62	489.2877	2	976.5706	.	R	Superscaf	74145234	0	BTP202209	0	0	0	0	Tissue specific
LEFTPTN	Superscaf	+	Superscaf	74238244	74238264	43	43	49	-	-	-	-	-	-	NCP	IGR	-	ID=gene-E	7	5.635714286	2895.4	0.43767365	LEFTPTN	85	821.4018	1	820.3967	.	R	Superscaf	74238118	0	BTP202209	0	0	0	0	Tissue specific
CYPSLP	Superscaf	-	Superscaf	74256124	74256141	-34	34	39	-	-	-	-	-	-	NCP	IGR	-	ID=gene-E	6	5.823333333	2456.8	0.438019908	C(+57.02)	79	778.3354	1	777.3367	Carbamido	E	Superscaf	74256147	0	0	0	0	0	BTP202209	Tissue specific
LPPWL	Superscaf	-	Superscaf	74270563	74270577	-10	10	14	-	-	-	-	-	-	NCP	IGR	-	ID=gene-E	5	6.09	1908.2	0.438299529	LPPWL	98	625.3696	1	624.3635	.	D	Superscaf	74270619	0	0	BTP202209	0	0	0	Mixed
GGLSFRK	Superscaf	-	Superscaf	74276320	74276340	-1	1	7	-	-	-	-	-	-	NCP	IGR	-	ID=gene-E	7	7.082857143	2836	0.438411017	GGLSFRK	96	382.7289	2	763.434	.	G	Superscaf	74276361	0	BTP202209	0	0	0	0	Tissue specific
SEEFYQK	Superscaf	-	Superscaf	74317096	74317116	-4	4	10	-	-	-	-	-	-	NCP	IGR	-	ID=gene-E	7	5.521428571	3160.4	0.439200672	SEEFYQK	53	465.7229	2	929.413	.	L	Superscaf	74317302	0	BTP202209	0	0	0	0	Tissue specific
KMKVPGK	Superscaf	+	Superscaf	74485165	74485185	3	3	9	-	-	-	-	-	-	NCP	IGR	-	ID=gene-E	7	6.748571429	2411.4	0.442455441	KMKVPGK	70	394.2469	2	786.4786	.	L	Superscaf	74485159	0	0	0	0	0	BTP202209	Tissue specific
MLSVAA	Superscaf	-	Superscaf	74518759	74518776	-14	14	19	-	-	-	-	-	-	NCP	IGR	-	ID=gene-E	6	4.908333333	1730.9	0.443106012	M(+42.01)	99	633.3254	1	632.3203	Acetylati	M	Superscaf	74518803	BTP202209	0	0	0	0	0	Tissue specific
LSLDVKN	Superscaf	-	Superscaf	74563114	74563134	-14	14	20	-	-	-	-	-	-	NCP	IGR	-	ID=gene-E	7	5.108571429	2566.8	0.443964976	LSLDVKN	82	394.7281	2	787.444	.	L	Superscaf	74563200	0	0	0	0	BTP202209	0	Tissue specific
GLPPSSVT	Superscaf	+	Superscaf	74641744	74641767	19	19	26	-	-	-	-	-	-	NCP	IGR	-	ID=gene-E	8	5.305	2489.8	0.445487699	G(+42.01)	54	799.4271	1	798.4123	Acetylati	I	Superscaf	74641690	0	0	0	0	BTP202209	0	Tissue specific
MANVLFP	Superscaf	+	Superscaf	74659324	74659344	4	4	10	-	-	-	-	-	-	NCP	IGR	-	ID=gene-E	7	4.991428571	2357.7	0.445828148	M(+15.99)	54	807.4073	1	806.3997	Oxidation	Q	Superscaf	74659315	0	0	0	BTP202209	0	0	Tissue specific
VFELL	Superscaf	-	Superscaf	74668159	74668173	-17	17	21	-	-	-	-	-	-	NCP	IGR	-	ID=gene-E	5	4.132	1519.7	0.445999243	VFELL	97	620.3628	1	619.3581	.	K	Superscaf	74668254	BTP202209	0	0	0	0	0	Tissue specific
KYREN	Superscaf	-	Superscaf	74686390	74686404	-6	6	10	-	-	-	-	-	-	NCP	IGR	-	ID=gene-E	5	6.958	2598.9	0.446352299	KYREN	53	709.366	1	708.3555	.	V	Superscaf	74686431	0	0	0	BTP202209	0	0	Tissue specific
ECCSSD	Superscaf	+	Superscaf	74708041	74708058	53	53	58	-	-	-	-	-	-	NCP	IGR	-	ID=gene-E	6	4.598333333	2775.2	0.446771585	EC(+57.02	54	757.2168	1	756.2054	Carbamido	N	Superscaf	74707885	BTP202209	0	0	0	0	0	Tissue specific
LSAAAGLK	Superscaf	+	Superscaf	74768077	74768100	3	3	10	-	-	-	-	-	-	NCP	IGR	-	ID=gene-E	8	6.42625	2789.8	0.447934223	LSAAAGLK	59	365.7244	2	729.4385	.	S	Superscaf	74768071	0	0	0	0	0	BTP202209	Tissue specific
RSMPSR	Superscaf	+	Superscaf	74899411	74899428	7	7	12	-	-	gene	-	-	mRNA	NCP	Intron	gene|mRN	ID=gene-E	6	7.488333333	2773.3	0.450477594	RSM(+15.9	64	375.1929	2	748.365	Oxidation	L	Superscaf	74899393	0	0	0	BTP202209	0	0	Tissue specific
EMLLQR	Superscaf	-	Superscaf	74900485	74900502	-37	37	42	-	-	gene	-	-	mRNA	NCP	Intron	gene|mRN	ID=gene-E	6	6.223333333	2763.3	0.450498393	EMLLQR	63	395.2164	2	788.4214	.	E	Superscaf	74900544	0	0	0	0	BTP202209	0	Tissue specific
ERLFSNF	Superscaf	+	Superscaf	74960593	74960613	8	8	14	-	-	gene	-	-	mRNA	NCP	Intron	gene|mRN	ID=gene-E	7	6.001428571	3182.6	0.451662425	ERLFSNF	74	456.7314	2	911.4501	.	R	Superscaf	74960572	0	0	BTP202209	0	0	0	Tissue specific
QTDFFLR	Superscaf	+	Superscaf	74979034	74979054	21	21	27	-	-	gene	-	-	mRNA	NCP	Intron	gene|mRN	ID=gene-E	7	6.121428571	3196.7	0.452019547	QTDFFLR	57	463.743	2	925.4658	.	P	Superscaf	74978974	0	0	0	0	0	BTP202209	Tissue specific
KAPLKKP	Superscaf	-	Superscaf	75022261	75022281	-57	57	63	-	-	-	-	-	-	NCP	IGR	-	ID=gene-E	7	7.688571429	2744.7	0.452856667	KAPLKKP	55	391.2612	2	780.5221	.	W	Superscaf	75022380	0	0	0	0	BTP202209	BTP202209	Tissue specific
PVEPTKP	Superscaf	+	Superscaf	75038098	75038118	55	55	61	-	-	-	-	-	-	NCP	IGR	-	ID=gene-E	7	5.484285714	2303.6	0.453163362	PVEPTKP	80	384.2182	2	766.4225	.	T	Superscaf	75037936	0	0	0	0	0	BTP202209	Tissue specific
ERGDFV	Superscaf	+	Superscaf	75045517	75045534	1	1	6	-	-	-	-	-	-	NCP	IGR	-	ID=gene-E	6	4.733333333	2170.5	0.453307036	ERGDFV	54	722.348	1	721.3395	.	E	Superscaf	75045517	0	0	0	0	0	BTP202209	Tissue specific
DPVTYF	Superscaf	+	Superscaf	75052057	75052074	16	16	21	-	-	-	-	-	-	NCP	IGR	-	ID=gene-E	6	4.49	1993.3	0.453433687	DPVTYF	66	741.3423	1	740.3381	.	N	Superscaf	75052012	0	0	0	0	BTP202209	0	Tissue specific
YTLMVK	Superscaf	+	Superscaf	75111679	75111696	16	16	21	-	-	-	-	-	-	NCP	IGR	-	ID=gene-E	6	5.61	2050.3	0.454588307	YTLMVK	58	377.7133	2	753.4095	.	L	Superscaf	75111634	0	0	0	BTP202209	0	0	Tissue specific
QTLKPM	Superscaf	-	Superscaf	75148531	75148548	-12	12	17	-	-	gene	-	-	mRNA	NCP	Intron	gene|mRN	ID=gene-E	6	6.658333333	2538.9	0.455301971	Q(+42.01)	55	759.4035	1	758.3997	Acetylati	A	Superscaf	75148677	0	0	0	0	0	BTP202209	Tissue specific
TLAAAVK	Superscaf	-	Superscaf	75203074	75203094	-6	6	12	-	-	-	-	-	-	NCP	IGR	-	ID=gene-E	7	5.758571429	2153.2	0.456358233	TLAAAVK	68	673.4219	1	672.417	.	H	Superscaf	75203163	0	0	BTP202209	0	0	0	Tissue specific
KCGMLK	Superscaf	+	Superscaf	75248092	75248109	7	7	12	-	-	-	-	-	-	NCP	IGR	-	ID=gene-E	6	7.033333333	2544.7	0.457230037	KC(+57.02	89	736.3896	1	735.3771	Carbamido	Q	Superscaf	75248074	BTP202209	0	0	BTP202209	BTP202209	0	Tissue specific
NDPPV	Superscaf	-	Superscaf	75264703	75264717	-45	45	49	-	-	-	-	-	-	NCP	IGR	-	ID=gene-E	5	4.196	1683	0.45755172	NDPPV	52	541.2602	1	540.2543	.	P	Superscaf	75264741	0	BTP202209	0	0	0	0	Tissue specific
LNLVRQ	Superscaf	+	Superscaf	75271660	75271677	5	5	10	-	-	-	-	-	-	NCP	IGR	-	ID=gene-E	6	5.63	2563	0.457686447	LNLVRQ	80	371.7303	2	741.4497	.	A	Superscaf	75271648	0	0	BTP202209	0	0	0	Tissue specific
ATPLLSK	Superscaf	+	Superscaf	75298678	75298698	55	55	61	-	-	-	-	-	-	NCP	IGR	-	ID=gene-E	7	6.604285714	2604.8	0.458209669	ATPLLSK	57	365.2293	2	728.4432	.	Q	Superscaf	75298516	0	0	BTP202209	0	0	0	Tissue specific
PAKPVTT	Superscaf	+	Superscaf	75407407	75407427	2	2	8	-	-	gene	-	-	mRNA	NCP	Intron	gene|mRN	ID=gene-E	7	5.917142857	2249.5	0.460315279	P(+42.01)	61	378.2173	2	754.4225	Acetylati	I	Superscaf	75407404	0	0	BTP202209	0	0	0	Tissue specific
KDWFPK	Superscaf	-	Superscaf	75442687	75442704	-5	5	10	-	-	gene	-	-	mRNA	NCP	Intron	gene|mRN	ID=gene-E	6	6.686666667	2599.7	0.4609985	KDWFPK	56	410.7228	2	819.4279	.	N	Superscaf	75442734	0	0	0	BTP202209	0	0	Tissue specific
MLLSMY	Superscaf	-	Superscaf	75487009	75487026	-84	84	89	-	-	-	-	-	-	NCP	IGR	-	ID=gene-E	6	5.8	2292.7	0.461856826	M(+15.99)	50	416.1898	2	830.3554	Oxidation	F	Superscaf	75487032	0	0	0	BTP202209	0	0	Tissue specific
SRLLQE	Superscaf	+	Superscaf	75510949	75510966	12	12	17	-	-	-	-	-	-	NCP	IGR	-	ID=gene-E	6	6.211666667	2775.2	0.46232044	SRLLQE	68	373.2124	2	744.413	.	E	Superscaf	75510916	0	0	0	0	0	BTP202209	Tissue specific
MMHLVG	Superscaf	+	Superscaf	75565930	75565947	17	17	22	-	-	gene	-	-	mRNA	NCP	Intron	gene|mRN	ID=gene-E	6	5.173333333	2013.4	0.463385184	M(+15.99)	56	719.3196	1	718.3143	Oxidation	D	Superscaf	75565882	0	0	0	BTP202209	0	0	Tissue specific
SVGPVQN	Superscaf	+	Superscaf	75565972	75565992	1	1	7	-	-	gene	-	-	mRNA	NCP	Intron	gene|mRN	ID=gene-E	7	4.144285714	2225.5	0.463385998	SVGPVQN	68	700.3669	1	699.3552	.	S	Superscaf	75565972	0	0	0	BTP202209	0	0	Tissue specific
VTFDPH	Superscaf	-	Superscaf	75737839	75737856	-69	69	74	-	-	-	-	-	-	NCP	IGR	-	ID=gene-E	6	4.811666667	2153.5	0.466714318	VTFDPH	75	358.173	2	714.3337	.	Y	Superscaf	75737919	0	0	BTP202209	0	0	0	Tissue specific
FHLLF	Superscaf	+	Superscaf	75738136	75738150	69	69	73	-	-	-	-	-	-	NCP	IGR	-	ID=gene-E	5	6.102	2101.3	0.466720069	FHLLF	71	676.3792	1	675.3744	.	L	Superscaf	75737932	0	0	0	BTP202209	0	0	Tissue specific
KMPKLR	Superscaf	-	Superscaf	75839428	75839445	-27	27	32	-	-	-	-	-	-	NCP	IGR	-	ID=gene-E	6	8.045	2647.9	0.468681657	K(+42.01)	62	415.7426	2	829.4844	Acetylati	G	Superscaf	75839499	0	0	0	0	BTP202209	0	Tissue specific
HSLELST	Superscaf	+	Superscaf	75839788	75839808	24	24	30	-	-	-	-	-	-	NCP	IGR	-	ID=gene-E	7	5.808571429	2916.3	0.468688629	HSLELST	75	393.7014	2	785.3919	.	F	Superscaf	75839719	0	0	0	0	0	BTP202209	Tissue specific
TVKNLKEK	Superscaf	+	Superscaf	75910597	75910620	18	18	25	-	-	-	-	-	-	NCP	IGR	-	ID=gene-E	8	6.295	3178.3	0.470059893	TVKNLKEK	64	480.2905	2	958.5811	.	L	Superscaf	75910546	0	BTP202209	0	0	0	BTP202209	Tissue specific
FAYVYPFP	Superscaf	+	Superscaf	75926596	75926619	9	9	16	-	-	-	-	-	-	NCP	IGR	-	ID=gene-E	8	5.1125	2679.9	0.470369724	F(+42.01)	72	523.2551	2	1044.496	Acetylati	I	Superscaf	75926572	0	0	0	0	0	BTP202209	Tissue specific
GVVEL	Superscaf	+	Superscaf	76022044	76022058	24	24	28	-	-	gene	-	-	mRNA	NCP	Intron	gene|mRN	ID=gene-E	5	3.034	1076.2	0.472218139	GVVEL	54	516.3005	1	515.2955	.	N	Superscaf	76021975	0	0	0	0	0	BTP202209	Tissue specific
NTLDATP	Superscaf	-	Superscaf	76037005	76037025	-26	26	32	-	-	gene	-	-	mRNA	NCP	Intron	gene|mRN	ID=gene-E	7	5.677142857	2805.3	0.472507869	NTLDATP	81	731.37	1	730.3497	.	I	Superscaf	76037094	0	0	0	BTP202209	0	0	Tissue specific
QGDLKGPK	Superscaf	+	Superscaf	76053868	76053891	47	47	54	-	-	gene	-	-	mRNA	NCP	Intron	gene|mRN	ID=gene-E	8	6.54	3244.4	0.472834433	Q(+42.01)	85	442.7512	2	883.4763	Acetylati	T	Superscaf	76053730	0	0	0	0	BTP202209	0	Tissue specific
LTLVFMT	Superscaf	+	Superscaf	76070137	76070157	102	102	108	-	-	gene	-	-	mRNA	NCP	Intron	gene|mRN	ID=gene-E	7	5.178571429	2260.7	0.473149493	LTLVFMT	67	824.4504	1	823.4514	.	R	Superscaf	76069834	0	BTP202209	0	0	0	0	Tissue specific
KDRWPKK	Superscaf	-	Superscaf	76154800	76154820	-52	52	58	-	-	gene	-	-	mRNA	NCP	Intron	gene|mRN	ID=gene-E	7	7.877142857	3329.5	0.474789049	KDRWPKK	55	479.2784	2	956.5555	.	A	Superscaf	76154820	BTP202209	0	0	0	0	0	Tissue specific
DVGQLPLT	Superscaf	-	Superscaf	76215823	76215846	-29	29	36	-	-	gene	-	-	mRNA	NCP	Intron	gene|mRN	ID=gene-E	8	4.9225	2761.2	0.4759708	D(+42.01)	60	442.7365	2	883.4651	Acetylati	P	Superscaf	76215846	0	0	0	BTP202209	0	0	Tissue specific
PDCCAL	Superscaf	+	Superscaf	76268383	76268400	9	9	14	-	-	gene	-	-	mRNA	NCP	Intron	gene|mRN	ID=gene-E	6	5.218333333	2585	0.47698866	P(+42.01)	61	777.2947	1	776.2833	Acetylati	H	Superscaf	76268359	0	0	0	BTP202209	0	0	Tissue specific
QQLAELR	Superscaf	-	Superscaf	76296619	76296639	-18	18	24	-	-	-	-	-	-	NCP	IGR	-	ID=gene-E	7	6.18	3313.8	0.477535469	QQLAELR	55	857.4687	1	856.4766	.	D	Superscaf	76296756	0	0	0	BTP202209	0	0	Tissue specific
LTFTPARP	Superscaf	+	Superscaf	76301317	76301340	131	131	138	-	-	gene	-	-	mRNA	NCP	Intron	gene|mRN	ID=gene-E	8	6.7375	3170.7	0.477626449	LTFTPARP	84	451.7627	2	901.5021	.	Q	Superscaf	76300927	BTP202209	0	0	0	0	0	Tissue specific
QDSKLK	Superscaf	-	Superscaf	76348561	76348578	-2	2	7	-	-	gene	-	-	mRNA	NCP	Intron	gene|mRN	ID=gene-E	6	6.626666667	2695.8	0.478541361	QDSKLK	81	359.7086	2	717.4021	.	A	Superscaf	76348599	0	0	0	0	BTP202209	0	Tissue specific
LSTDNPK	Superscaf	-	Superscaf	76372786	76372806	-4	4	10	-	-	-	-	-	-	NCP	IGR	-	ID=gene-E	7	6.087142857	2948.3	0.479010495	LSTDNPK	75	387.7078	2	773.3919	.	K	Superscaf	76372839	0	0	BTP202209	BTP202209	0	BTP202209	Tissue specific
MLTVPPPP	Superscaf	+	Superscaf	76475479	76475502	33	33	40	-	-	gene	-	-	mRNA	NCP	Intron	gene|mRN	ID=gene-E	8	5.4325	2472	0.480999214	MLTVPPPP	72	426.2327	2	850.4622	.	F	Superscaf	76475383	0	0	0	0	0	BTP202209	Tissue specific
YDDMPK	Superscaf	+	Superscaf	76526284	76526301	6	6	11	-	-	-	-	-	-	NCP	IGR	-	ID=gene-E	6	5.565	2459.8	0.481983087	YDDMPK	51	384.6712	2	767.316	.	V	Superscaf	76526269	0	0	BTP202209	0	0	0	Tissue specific
PSRSLLL	Superscaf	-	Superscaf	76618324	76618344	-20	20	26	-	-	-	-	-	-	NCP	IGR	-	ID=gene-E	7	6.622857143	2813.2	0.483765504	PSRSLLL	74	785.4858	1	784.4807	.	G	Superscaf	76618551	0	BTP202209	0	0	0	0	Tissue specific
WSAPSDKEC	Superscaf	-	Superscaf	76626652	76626681	-41	41	50	-	-	-	-	-	-	NCP	IGR	-	ID=gene-E	10	5.713	4004.3	0.483926781	WSAPSDKEC	91	570.2515	2	1138.493	.	F	Superscaf	76626756	0	0	0	0	BTP202209	0	Tissue specific
LALKFLP	Superscaf	-	Superscaf	76627444	76627464	-16	16	22	-	-	-	-	-	-	NCP	IGR	-	ID=gene-E	7	6.497142857	2564.7	0.483942118	LALKFLP	87	401.2634	2	800.516	.	H	Superscaf	76627524	0	0	BTP202209	0	0	0	Tissue specific
KSEAKPR	Superscaf	+	Superscaf	76689688	76689708	62	62	68	-	-	-	-	-	-	NCP	IGR	-	ID=gene-E	7	7.351428571	3043.2	0.485147516	KSEAKPR	78	408.2386	2	814.4661	.	R	Superscaf	76689505	0	0	0	0	0	BTP202209	Tissue specific
GPGFTAAP	Superscaf	+	Superscaf	76842664	76842687	62	62	69	-	-	gene	-	-	mRNA	NCP	Intron	gene|mRN	ID=gene-E	8	6.07375	2676.9	0.488109999	GPGFTAAP	61	359.1815	2	716.3493	.	G	Superscaf	76842481	0	0	0	0	BTP202209	0	Tissue specific
KDGKPPP	Superscaf	+	Superscaf	76889626	76889646	3	3	9	-	-	gene	-	-	mRNA	NCP	Intron	gene|mRN	ID=gene-E	7	6.76	2657.8	0.48901945	K(+42.01)	71	390.7154	2	779.4177	Acetylati	F	Superscaf	76889620	0	0	0	0	BTP202209	0	Tissue specific
GAAVLSL	Superscaf	+	Superscaf	76900513	76900533	24	24	30	-	-	gene	-	-	mRNA	NCP	Intron	gene|mRN	ID=gene-E	7	5.092857143	2010.1	0.489230284	GAAVLSL	71	630.3809	1	629.3748	.	G	Superscaf	76900444	0	0	0	0	0	BTP202209	Tissue specific
SDGLVVK	Superscaf	+	Superscaf	76948252	76948272	19	19	25	-	-	-	-	-	-	NCP	IGR	-	ID=gene-E	7	4.334285714	1914	0.490154782	SDGLVVK	57	359.21	2	716.4068	.	R	Superscaf	76948198	0	0	0	0	0	BTP202209	Tissue specific
LVSAF	Superscaf	+	Superscaf	76950922	76950936	13	13	17	-	-	-	-	-	-	NCP	IGR	-	ID=gene-E	5	4.632	1435.5	0.490206488	LVSAF	50	536.3062	1	535.3006	.	D	Superscaf	76950886	0	BTP202209	0	0	0	0	Tissue specific
KHALSPR	Superscaf	-	Superscaf	76966978	76966998	-4	4	10	-	-	gene	-	-	mRNA	NCP	Intron	gene|mRN	ID=gene-E	7	7.438571429	3122.4	0.490517423	KHALSPR	59	404.7359	2	807.4715	.	P	Superscaf	76967040	0	0	0	BTP202209	0	0	Tissue specific
LPPHRP	Superscaf	+	Superscaf	77051974	77051991	1	1	6	-	-	gene	-	-	mRNA	NCP	Intron	gene|mRN	ID=gene-E	6	7.205	2634.1	0.492163428	LPPHRP	82	358.7117	2	715.4129	.	L	Superscaf	77051974	0	0	0	0	BTP202209	BTP202209	Tissue specific
DLFTGHL	Superscaf	+	Superscaf	77056732	77056752	3	3	9	-	-	gene	-	-	mRNA	NCP	Intron	gene|mRN	ID=gene-E	7	5.785714286	2820.2	0.49225557	DLFTGHL	77	401.7066	2	801.4021	.	D	Superscaf	77056726	0	0	0	BTP202209	0	0	Tissue specific
MLLLAPP	Superscaf	-	Superscaf	77100328	77100348	-26	26	32	-	-	gene	-	-	mRNA	NCP	Intron	gene|mRN	ID=gene-E	7	6.044285714	2417.8	0.493099836	MLLLAPP	51	377.7248	2	753.4459	.	N	Superscaf	77100402	0	0	0	0	0	BTP202209	Tissue specific
KPPLYK	Superscaf	+	Superscaf	77112616	77112633	1	1	6	-	-	gene	-	-	mRNA	NCP	Intron	gene|mRN	ID=gene-E	6	7.286666667	2424.5	0.493337801	KPPLYK	99	373.2324	2	744.4534	.	K	Superscaf	77112616	BTP202209	0	0	0	0	0	Tissue specific
HLVLKTQ	Superscaf	-	Superscaf	77114722	77114742	-10	10	16	-	-	gene	-	-	mRNA	NCP	Intron	gene|mRN	ID=gene-E	7	5.924285714	2803.1	0.493378585	HLVLKTQ	84	419.7594	2	837.5072	.	H	Superscaf	77114811	0	0	BTP202209	0	0	0	Tissue specific
KPAPVH	Superscaf	-	Superscaf	77155048	77155065	-15	15	20	-	-	-	-	-	-	NCP	IGR	-	ID=gene-E	6	5.991666667	2074.2	0.494159526	KPAPVH	74	648.3794	1	647.3755	.	G	Superscaf	77155080	0	0	0	0	BTP202209	0	Tissue specific
PGARAVLP	Superscaf	+	Superscaf	77158192	77158215	1	1	8	-	-	-	-	-	-	NCP	IGR	-	ID=gene-E	8	5.91875	2596.9	0.494220411	PGARAVLP	64	390.7392	2	779.4653	.	P	Superscaf	77158192	0	0	0	0	0	BTP202209	Tissue specific
FASLVHR	Superscaf	-	Superscaf	77171044	77171064	-3	3	9	-	-	-	-	-	-	NCP	IGR	-	ID=gene-E	7	5.93	2704	0.494469299	FASLVHR	86	415.2446	2	828.4606	.	F	Superscaf	77171172	BTP202209	0	0	0	0	0	Tissue specific
LTVLLM	Superscaf	+	Superscaf	77181532	77181549	16	16	21	-	-	-	-	-	-	NCP	IGR	-	ID=gene-E	6	5.036666667	1829.2	0.494672406	LTVLLM	92	689.4243	1	688.4193	.	W	Superscaf	77181487	0	0	0	0	0	BTP202209	Mixed
LPPGLSSK	Superscaf	-	Superscaf	77199916	77199939	-101	101	108	-	-	-	-	-	-	NCP	IGR	-	ID=gene-E	8	6.45375	2914.1	0.495028425	L(+42.01)	71	420.7405	2	839.4752	Acetylati	A	Superscaf	77200095	0	BTP202209	0	0	0	0	Tissue specific
MSSLVHR	Superscaf	-	Superscaf	77226049	77226069	-37	37	43	-	-	-	-	-	-	NCP	IGR	-	ID=gene-E	7	5.92	2760.2	0.495534508	M(+15.99)	83	423.2211	2	844.4225	Oxidation	G	Superscaf	77226114	0	0	0	0	0	BTP202209	Tissue specific
GKVPPP	Superscaf	-	Superscaf	77284210	77284227	-10	10	15	-	-	-	-	-	-	NCP	IGR	-	ID=gene-E	6	5.768333333	1777.9	0.496660835	G(+42.01)	52	636.3779	1	635.3642	Acetylati	A	Superscaf	77284260	0	0	0	0	0	BTP202209	Tissue specific
KGWKQLK	Superscaf	-	Superscaf	77325448	77325468	-5	5	11	-	-	-	-	-	-	NCP	IGR	-	ID=gene-E	7	7.53	3193.2	0.497459437	KGWKQLK	52	444.2774	2	886.5388	.	I	Superscaf	77325468	0	0	0	0	0	BTP202209	Tissue specific
DRPKVGL	Superscaf	+	Superscaf	77349424	77349444	1	1	7	-	-	-	-	-	-	NCP	IGR	-	ID=gene-E	7	5.96	2516.8	0.497923748	DRPKVGL	94	392.736	2	783.4603	.	D	Superscaf	77349424	0	0	BTP202209	0	0	0	Tissue specific
LSAGLDF	Superscaf	+	Superscaf	77364610	77364630	13	13	19	-	-	-	-	-	-	NCP	IGR	-	ID=gene-E	7	5.44	2497.7	0.498217835	L(+42.01)	61	764.3837	1	763.3752	Acetylati	V	Superscaf	77364574	0	0	0	0	BTP202209	0	Tissue specific
EGQYRPP	Superscaf	-	Superscaf	77365867	77365887	-29	29	35	-	-	-	-	-	-	NCP	IGR	-	ID=gene-E	7	6.265714286	3116.6	0.498242178	EGQYRPP	59	423.7158	2	845.4031	.	G	Superscaf	77365968	0	0	0	0	0	BTP202209	Tissue specific
QPSWWL	Superscaf	-	Superscaf	77380432	77380449	-8	8	13	-	-	-	-	-	-	NCP	IGR	-	ID=gene-E	6	5.898333333	2738.1	0.498524239	QPSWWL	69	816.4005	1	815.3966	.	A	Superscaf	77380635	0	0	0	BTP202209	0	0	Tissue specific
RLVSVK	Superscaf	+	Superscaf	77399086	77399103	29	29	34	-	-	-	-	-	-	NCP	IGR	-	ID=gene-E	6	5.36	1854	0.498885486	RLVSVK	58	351.2353	2	700.4595	.	Y	Superscaf	77399002	0	0	0	0	0	BTP202209	Tissue specific
VTGGKLR	Superscaf	-	Superscaf	77433376	77433396	-16	16	22	-	-	gene	-	-	mRNA	NCP	Intron	gene|mRN	ID=gene-E	7	6.421428571	2462.7	0.499549535	VTGGKLR	53	365.7313	2	729.4497	.	E	Superscaf	77433534	0	0	BTP202209	0	0	0	Tissue specific

KSSLEPMK	Superscaf	+	Superscaf	77435251	77435274	6	6	13	-	-	gene	-	-	mRNA	NCP	Intron	gene|mRN	ID=gene-	8	6.51125	3191.4	0.499585846	K(+42.01)	55	489.2473	2	976.4899	Acetylati	V	Superscaf	77435236	0	0	0	BTP20220	0	0	Tissue specific
MEFYPV	Superscaf	+	Superscaf	77461927	77461944	46	46	51	-	-	-	-	-	-	NCP	IGR	-	ID=gene-	6	4.401666667	1981.3	0.500102444	M(+15.99)	51	422.183	2	842.352	Oxidatior	G	Superscaf	77461792	0	0	0	BTP20220	0	0	Tissue specific
QVPPPP	Superscaf	-	Superscaf	77531515	77531532	-10	10	15	-	-	-	-	-	-	NCP	IGR	-	ID=gene-	6	5.141666667	1960.3	0.501450063	QVPPPP	79	634.3651	1	633.3486	.	S	Superscaf	77531544	0	0	BTP20220	0	0	0	Tissue specific
MEKVPPP	Superscaf	-	Superscaf	77575345	77575365	-3	3	9	-	-	gene	-	-	mRNA	NCP	Intron	gene|mRN	ID=gene-	7	5.372857143	2277.6	0.50229886	M(+42.01)	63	839.4314	1	838.4258	Acetylati	D	Superscaf	77575419	0	0	BTP20220	0	0	0	Tissue specific
MTHPPY	Superscaf	-	Superscaf	77596819	77596836	-38	38	43	-	-	gene	-	-	mRNA	NCP	Intron	gene|mRN	ID=gene-	6	6.355	2523	0.502714719	M(+15.99)	78	381.1699	2	760.3214	Oxidatior	P	Superscaf	77596878	0	0	BTP20220	0	0	0	Tissue specific
LPLPGP	Superscaf	-	Superscaf	77642413	77642430	-89	89	94	-	-	gene	-	-	mRNA	NCP	Intron	gene|mRN	ID=gene-	6	6.138333333	2016.3	0.503597677	LPLPGP	54	593.3652	1	592.3584	.	W	Superscaf	77642922	0	0	0	0	0	BTP20220	Tissue specific
QELLDF	Superscaf	-	Superscaf	77688289	77688306	-10	10	15	-	-	gene	-	-	mRNA	NCP	Intron	gene|mRN	ID=gene-	6	4.88	2541.9	0.504486097	QELLDF	89	764.3787	1	763.3752	.	A	Superscaf	77688321	0	0	0	0	0	BTP20220	Mixed
HGLPPPP	Superscaf	+	Superscaf	77764909	77764929	3	3	9	-	-	gene	-	-	mRNA	NCP	Intron	gene|mRN	ID=gene-	7	6.391428571	2620	0.505969895	HGLPPPP	85	357.7002	2	713.386	.	A	Superscaf	77764903	0	0	0	0	BTP20220	0	Tissue specific
VVGSVL	Superscaf	+	Superscaf	77780707	77780724	17	17	22	-	-	-	-	-	-	NCP	IGR	-	ID=gene-	6	2.938333333	1034.1	0.506275834	VVGSVL	77	573.3593	1	572.3533	.	S	Superscaf	77780659	0	0	BTP20220	0	0	0	Tissue specific
ALPYAP	Superscaf	+	Superscaf	77785378	77785395	34	34	39	-	-	-	-	-	-	NCP	IGR	-	ID=gene-	6	6.046666667	2110.3	0.506366291	ALPYAP	64	631.3544	1	630.3377	.	S	Superscaf	77785279	0	0	BTP20220	0	0	0	Tissue specific
AYAAPA	Superscaf	+	Superscaf	77854258	77854275	5	5	10	-	-	-	-	-	-	NCP	IGR	-	ID=gene-	6	6.006666667	2042.1	0.507700199	AYAAPA	56	563.2797	1	562.2751	.	F	Superscaf	77854246	BTP20220	0	0	0	BTP20220	0	Tissue specific
RYYPK	Superscaf	-	Superscaf	77877430	77877444	-10	10	14	-	-	-	-	-	-	NCP	IGR	-	ID=gene-	5	7.624	2373.6	0.50814894	RYYPK	54	363.7048	2	725.386	.	H	Superscaf	77877555	0	0	BTP20220	0	0	0	Tissue specific
LSQGKLL	Superscaf	-	Superscaf	77989285	77989305	-14	14	20	-	-	-	-	-	-	NCP	IGR	-	ID=gene-	7	6.425714286	2820	0.510315088	LSQGKLL	68	379.7408	2	757.4698	.	T	Superscaf	77989305	0	BTP20220	0	0	0	0	Tissue specific
VPAGFL	Superscaf	+	Superscaf	78029338	78029355	5	5	10	-	-	-	-	-	-	NCP	IGR	-	ID=gene-	6	4.958333333	1686.8	0.511090741	V(+42.01)	79	645.3552	1	644.3533	Acetylati	M	Superscaf	78029326	0	0	0	0	BTP20220	0	Tissue specific
LLLAGKSF	Superscaf	+	Superscaf	78032842	78032865	27	27	34	-	-	-	-	-	-	NCP	IGR	-	ID=gene-	8	6.35375	2908	0.511158598	LLLAGKSF	65	424.7628	2	847.5167	.	G	Superscaf	78032764	0	0	0	0	0	BTP20220	Mixed
AVKPFR	Superscaf	-	Superscaf	78198514	78198531	-10	10	15	-	-	-	-	-	-	NCP	IGR	-	ID=gene-	6	6.383333333	2153.3	0.514366948	A(+42.01)	64	380.2329	2	758.4438	Acetylati	K	Superscaf	78198537	0	0	BTP20220	0	0	0	Tissue specific
LTTLWF	Superscaf	+	Superscaf	78290767	78290784	42	42	47	-	-	-	-	-	-	NCP	IGR	-	ID=gene-	6	6.065	2415.8	0.51615349	LTTLWF	67	390.7153	2	779.4218	.	H	Superscaf	78290644	0	0	0	BTP20220	0	0	Tissue specific
AEGKPLH	Superscaf	+	Superscaf	78304648	78304668	6	6	12	-	-	-	-	-	-	NCP	IGR	-	ID=gene-	7	6.402857143	2813	0.516422305	A(+42.01)	62	397.2126	2	792.413	Acetylati	L	Superscaf	78304633	0	BTP20220	0	0	0	0	Tissue specific
FNFFSLP	Superscaf	-	Superscaf	78447859	78447879	-17	17	23	-	-	gene	-	-	mRNA	NCP	Intron	gene|mRN	ID=gene-	7	5.687142857	2833.1	0.519195682	FNFFSLP	55	871.4312	1	870.4276	.	Y	Superscaf	78447888	0	0	0	0	0	BTP20220	Tissue specific
HSTASPP	Superscaf	+	Superscaf	78453019	78453039	26	26	32	-	-	gene	-	-	mRNA	NCP	Intron	gene|mRN	ID=gene-	7	6.3	2770.1	0.519295609	H(+42.01)	81	738.3466	1	737.3344	Acetylati	V	Superscaf	78452944	0	0	0	0	0	BTP20220	Tissue specific
VERVDK	Superscaf	-	Superscaf	78453067	78453084	-10	10	15	-	-	gene	-	-	mRNA	NCP	Intron	gene|mRN	ID=gene-	6	4.448333333	1954.2	0.519296539	VERVDK	58	373.2159	2	744.413	.	A	Superscaf	78453171	0	0	BTP20220	0	0	0	Tissue specific
LSNGEGP	Superscaf	-	Superscaf	78470188	78470208	-16	16	22	-	-	gene	-	-	mRNA	NCP	Intron	gene|mRN	ID=gene-	7	5.504285714	2691	0.519628098	LSNGEGP	67	673.3161	1	672.3079	.	S	Superscaf	78470280	0	BTP20220	0	0	BTP20220	BTP20220	Tissue specific
FKMAPK	Superscaf	-	Superscaf	78490000	78490017	-14	14	19	-	-	gene	-	-	mRNA	NCP	Intron	gene|mRN	ID=gene-	6	7.171666667	2344.4	0.520011771	FKMAPK	52	361.2082	2	720.3992	.	M	Superscaf	78490035	0	0	0	BTP20220	0	0	Tissue specific
VPNLSSAC	Superscaf	+	Superscaf	78587713	78587736	6	6	13	-	-	gene	-	-	mRNA	NCP	Intron	gene|mRN	ID=gene-	8	5.01125	2951.3	0.521904049	VPNLSSAC(	51	424.1984	2	846.3906	Carbamidc	Q	Superscaf	78587698	0	0	0	BTP20220	0	0	Tissue specific
LAPGKPN	Superscaf	-	Superscaf	78596602	78596622	-14	14	20	-	-	gene	-	-	mRNA	NCP	Intron	gene|mRN	ID=gene-	7	6.531428571	2701.9	0.522076191	LAPGKPN	63	696.4027	1	695.3966	.	T	Superscaf	78596652	0	0	BTP20220	0	0	0	Tissue specific
YHDELF	Superscaf	+	Superscaf	78649780	78649797	4	4	9	-	-	gene	-	-	mRNA	NCP	Intron	gene|mRN	ID=gene-	6	5.15	2657	0.523106019	YHDELF	63	412.1832	2	822.3548	.	I	Superscaf	78649771	0	0	0	BTP20220	0	0	Tissue specific
KTLCYK	Superscaf	+	Superscaf	78728419	78728436	1	1	6	-	-	gene	-	-	mRNA	NCP	Intron	gene|mRN	ID=gene-	6	7.111666667	2732.9	0.524628916	KTLC(+57.	53	406.7152	2	811.4262	Carbamidc	K	Superscaf	78728419	0	BTP20220	0	0	0	0	Tissue specific
PEVNKR	Superscaf	-	Superscaf	78749704	78749721	-4	4	9	-	-	gene	-	-	mRNA	NCP	Intron	gene|mRN	ID=gene-	6	5.905	2476.8	0.525041114	P(+42.01)	70	392.7235	2	783.4239	Acetylati	T	Superscaf	78749799	0	0	0	0	0	BTP20220	Tissue specific
QMWKSK	Superscaf	+	Superscaf	78754723	78754740	23	23	28	-	-	gene	-	-	mRNA	NCP	Intron	gene|mRN	ID=gene-	6	7.075	2829	0.525138311	QM(+15.99	68	412.2087	2	822.4058	Oxidatior	I	Superscaf	78754657	BTP20220	0	0	0	0	0	Tissue specific
VYHMYR	Superscaf	-	Superscaf	78803614	78803631	-14	14	19	-	-	gene	-	-	mRNA	NCP	Intron	gene|mRN	ID=gene-	6	5.903333333	2559	0.526085118	VYHMYR	99	434.7087	2	867.4061	.	L	Superscaf	78803682	0	0	BTP20220	0	0	0	Tissue specific
LSWLKL	Superscaf	+	Superscaf	78841384	78841401	108	108	113	-	-	gene	-	-	mRNA	NCP	Intron	gene|mRN	ID=gene-	6	6.541666667	2438.6	0.526816559	LSWLKL	75	380.2393	2	758.4691	.	C	Superscaf	78841063	0	BTP20220	BTP20220	0	0	BTP20220	Tissue specific
KTPWYQ	Superscaf	-	Superscaf	78891793	78891810	-23	23	28	-	-	gene	-	-	mRNA	NCP	Intron	gene|mRN	ID=gene-	6	6.628333333	2800.1	0.527792764	KTPWYQ	77	411.7097	2	821.4072	.	K	Superscaf	78891831	0	BTP20220	0	0	0	0	Mixed
KYTVQE	Superscaf	+	Superscaf	78983083	78983100	5	5	10	-	-	gene	-	-	mRNA	NCP	Intron	gene|mRN	ID=gene-	6	5.133333333	2361.6	0.529560656	KYTVQE	60	767.4036	1	766.3861	.	K	Superscaf	78983071	0	0	0	0	BTP20220	0	Tissue specific
LVSVTRPL	Superscaf	+	Superscaf	78984790	78984813	7	7	14	-	-	gene	-	-	mRNA	NCP	Intron	gene|mRN	ID=gene-	8	5.15375	2473.9	0.529593713	LVSVTRPL	95	442.7802	2	883.5491	.	Q	Superscaf	78984772	0	0	0	0	0	BTP20220	Tissue specific
EHKGPQ	Superscaf	-	Superscaf	79163176	79163193	-8	8	13	-	-	gene	-	-	mRNA	NCP	Intron	gene|mRN	ID=gene-	6	6.411666667	2759	0.533048278	EHKGPQ	52	695.3566	1	694.3398	.	G	Superscaf	79163328	0	0	0	BTP20220	0	0	Tissue specific
KEFSSFLK	Superscaf	-	Superscaf	79191985	79192008	-36	36	43	-	-	-	-	-	-	NCP	IGR	-	ID=gene-	8	6.375	3257.3	0.533606184	KEFSSFLK	80	493.272	2	984.528	.	E	Superscaf	79192110	0	0	0	0	0	BTP20220	Tissue specific
PRNGPLQ	Superscaf	+	Superscaf	79214218	79214238	33	33	39	-	-	-	-	-	-	NCP	IGR	-	ID=gene-	7	6.624285714	3181.7	0.534036741	PRNGPLQ	64	391.2181	2	780.4242	.	K	Superscaf	79214122	0	0	0	0	BTP20220	0	Tissue specific
LGERQSY	Superscaf	-	Superscaf	79229956	79229976	-7	7	13	-	-	-	-	-	-	NCP	IGR	-	ID=gene-	7	6.131428571	3178.6	0.534341518	LGERQSY	97	426.712	2	851.4137	.	K	Superscaf	79230027	0	0	0	0	BTP20220	0	Tissue specific
PLAKDLL	Superscaf	+	Superscaf	79240699	79240719	7	7	13	-	-	-	-	-	-	NCP	IGR	-	ID=gene-	7	6.138571429	2588.8	0.534549564	PLAKDLL	94	385.2433	2	768.4745	.	P	Superscaf	79240681	0	0	0	0	0	BTP20220	Tissue specific
KNDTPL	Superscaf	+	Superscaf	79256665	79256682	21	21	26	-	-	-	-	-	-	NCP	IGR	-	ID=gene-	6	6.155	2564.9	0.534858756	KNDTPL	66	687.3684	1	686.3599	.	C	Superscaf	79256605	0	0	0	0	0	BTP20220	Tissue specific
GLMVWKQ	Superscaf	+	Superscaf	79257085	79257105	10	10	16	-	-	-	-	-	-	NCP	IGR	-	ID=gene-	7	5.568571429	2627.9	0.534866889	G(+42.01)	62	460.2412	2	918.4633	Acetylati	S	Superscaf	79257058	0	0	BTP20220	0	0	0	Tissue specific
LLVVPGP	Superscaf	+	Superscaf	79297360	79297380	17	17	23	-	-	-	-	-	-	NCP	IGR	-	ID=gene-	7	4.361428571	1678.9	0.535646842	L(+42.01)	58	368.7302	2	735.4531	Acetylati	V	Superscaf	79297312	0	0	0	0	0	BTP20220	Tissue specific
LPLPVLE	Superscaf	+	Superscaf	79428424	79428444	45	45	51	-	-	gene	-	-	mRNA	NCP	Intron	gene|mRN	ID=gene-	7	4.822857143	2160.5	0.538184985	LPLPVLE	96	780.4845	1	779.4792	.	S	Superscaf	79428292	0	0	0	0	0	BTP20220	Tissue specific
RLSGP	Superscaf	-	Superscaf	79489399	79489413	-38	38	42	-	-	-	-	-	-	NCP	IGR	-	ID=gene-	5	6.938	2020.3	0.539365807	RLSGP	89	529.3126	1	528.302	.	G	Superscaf	79489458	0	0	0	0	0	BTP20220	Tissue specific
KAAVLSL	Superscaf	+	Superscaf	79550545	79550565	3	3	9	-	-	gene	-	-	mRNA	NCP	Intron	gene|mRN	ID=gene-	7	5.631428571	2181.2	0.54054994	K(+42.01)	79	743.4626	1	742.4589	Acetylati	G	Superscaf	79550539	0	BTP20220	0	0	BTP20220	BTP20220	Tissue specific
KFFYHK	Superscaf	-	Superscaf	79553956	79553973	-1	1	6	-	-	gene	-	-	mRNA	NCP	Intron	gene|mRN	ID=gene-	6	7.281666667	2790.8	0.540615997	KFFYHK	60	435.2409	2	868.4595	.	K	Superscaf	79553982	0	0	0	0	BTP20220	BTP20220	Tissue specific
LFGRLGR	Superscaf	-	Superscaf	79590136	79590156	3	3	9	-	-	gene	-	-	mRNA	NCP	Intron	gene|mRN	ID=gene-	7	7.271428571	2986.4	0.541316647	LFGRLGR	73	409.7509	2	817.4922	.	Q	Superscaf	79590130	0	0	0	0	BTP20220	0	Tissue specific
TVFPL	Superscaf	+	Superscaf	79620172	79620186	11	11	15	-	-	-	-	-	-	NCP	IGR	-	ID=gene-	5	4.858	1475.7	0.541898314	TVFPL	70	576.3373	1	575.3318	.	A	Superscaf	79620142	0	BTP20220	0	0	0	0	Tissue specific
KLRWYLH	Superscaf	-	Superscaf	79710322	79710342	-36	36	42	-	-	-	-	-	-	NCP	IGR	-	ID=gene-	7	7.371428571	3429.8	0.54364413	KLRWYLH	58	508.2878	2	1014.576	.	V	Superscaf	79710345	0	0	BTP20220	0	0	0	Tissue specific
YVYSVN	Superscaf	+	Superscaf	79726048	79726065	8	8	13	-	-	-	-	-	-	NCP	IGR	-	ID=gene-	6	3.735	1899.1	0.543948674	YVYSVN	61	744.3525	1	743.349	.	E	Superscaf	79726027	0	0	0	0	0	BTP20220	Tissue specific
KFVHVPP	Superscaf	+	Superscaf	79780522	79780542	10	10	16	-	-	-	-	-	-	NCP	IGR	-	ID=gene-	7	5.058571429	2150.3	0.5450036	KFVHVPP	57	412.2471	2	822.4752	.	N	Superscaf	79780495	0	0	0	0	0	BTP20220	Tissue specific
RDGKVK	Superscaf	-	Superscaf	79783813	79783830	-30	30	35	-	-	-	-	-	-	NCP	IGR	-	ID=gene-	6	6.53	2294.4	0.545067332	RDGKVK	54	351.7186	2	701.4184	.	S	Superscaf	79783914	0	BTP20220	0	0	0	0	Tissue specific
LAPPPPPP	Superscaf	-	Superscaf	79789645	79789668	-53	53	60	-	-	-	-	-	-	NCP	IGR	-	ID=gene-	8	6.225	2689.1	0.545180273	LAPPPPPP	74	393.2369	2	784.4483	.	A	Superscaf	79789755	0	BTP20220	0	BTP20220	0	0	Tissue specific
AVGSLPF	Superscaf	-	Superscaf	79858036	79858056	-37	37	43	-	-	-	-	-	-	NCP	IGR	-	ID=gene-	7	5.061428571	2070.2	0.546504711	AVGSLPF	52	690.3792	1	689.3748	.	P	Superscaf	79858131	0	BTP20220	0	0	0	0	Tissue specific
QYLLPLL	Superscaf	+	Superscaf	79926946	79926966	45	45	51	-	-	-	-	-	-	NCP	IGR	-	ID=gene-	7	5.932857143	2821.2	0.547839199	QYLLPLL	57	859.5266	1	858.5215	.	G	Superscaf	79926814	0	0	0	BTP20220	0	0	Tissue specific
GLVEPY	Superscaf	+	Superscaf	80046670	80046687	28	28	33	-	-	gene	-	-	mRNA	NCP	Intron	gene|mRN	ID=gene-	6	4.521666667	1873.1	0.550157735	GLVEPY	73	677.3478	1	676.3431	.	N	Superscaf	80046589	0	0	BTP20220	0	0	0	Tissue specific
QSGSFKL	Superscaf	+	Superscaf	80083258	80083278	29	29	35	-	-	gene	-	-	mRNA	NCP	Intron	gene|mRN	ID=gene-	7	6.311428571	2884	0.550866287	QSGSFKL	94	383.7074	2	765.4021	.	K	Superscaf	80083174	0	0	0	BTP20220	0	0	Mixed
SDLRLSFP	Superscaf	+	Superscaf	80083651	80083674	2	2	9	-	-	gene	-	-	mRNA	NCP	Intron	gene|mRN	ID=gene-	8	6.10375	3258.7	0.550873897	S(+42.01)	92	976.5033	1	975.5025	Acetylati	F	Superscaf	80083648	0	0	BTP20220	0	0	0	Tissue specific
LALDDD	Superscaf	+	Superscaf	80107648	80107665	4	4	9	-	-	-	-	-	-	NCP	IGR	-	ID=gene-	6	4.481666667	2252.6	0.551338615	LALDDD	51	661.2957	1	660.2966	.	V	Superscaf	80107639	0	BTP20220	0	0	0	0	Tissue specific
ESLMALH	Superscaf	+	Superscaf	80125846	80125866	86	86	92	-	-	-	-	-	-	NCP	IGR	-	ID=gene-	7	5.745714286	2818.2	0.551691032	ESLMALH	70	400.7098	2	799.3898	.	L	Superscaf	80125591	0	0	BTP20220	0	0	0	Tissue specific
VPVQPLC	Superscaf	-	Superscaf	80150962	80150982	-7	7	13	-	-	-	-	-	-	NCP	IGR	-	ID=gene-	7	4.178571429	2224.6	0.55217742	VPVQPLC(+	83	812.4301	1	811.4262	Carbamidc	S	Superscaf	80150997	0	0	0	BTP20220	0	0	Tissue specific
SLDPDFK	Superscaf	+	Superscaf	80179474	80179494	4	4	10	-	-	-	-	-	-	NCP	IGR	-	ID=gene-	7	5.588571429	2753	0.552729574	SLDPDFK	77	411.2102	2	820.3967	.	L	Superscaf	80179465	0	0	BTP20220	0	0	0	Tissue specific
ESALLVR	Superscaf	+	Superscaf	80257666	80257686	6	6	12	-	-	-	-	-	-	NCP	IGR	-	ID=gene-	7	5.377142857	2475.8	0.554243815	ESALLVR	84	394.2306	2	786.4599	.	P	Superscaf	80257651	0	BTP20220	0	0	BTP20220	BTP20220	Tissue specific
KGDCLK	Superscaf	+	Superscaf	80272510	80272527	6	6	11	-	-	gene	-	-	mRNA	NCP	Intron	gene|mRN	ID=gene-	6	6.57	2584.7	0.554531279	KGDC(+57.	50	360.6857	2	719.3636	Carbamidc	A	Superscaf	80272495	0	0	0	0	BTP20220	BTP20220	Tissue specific
KAKLLVP	Superscaf	+	Superscaf	80312944	80312964	37	37	43	-	-	gene	-	-	mRNA	NCP	Intron	gene|mRN	ID=gene-	7	6.251428571	2292.3	0.555314311	KAKLLVP	56	384.7713	2	767.5269	.	P	Superscaf	80312836	0	0	BTP20220	0	0	0	Tissue specific
FGSLPY	Superscaf	+	Superscaf	80349370	80349387	38	38	43	-	-	gene	-	-	mRNA	NCP	Intron	gene|mRN	ID=gene-	6	5.845	2218.4	0.556019725	FGSLPY	68	683.3364	1	682.3326	.	S	Superscaf	80349259	0	0	0	BTP20220	0	0	Tissue specific
GVTALLQ	Superscaf	+	Superscaf	80358331	80358351	35	35	41	-	-	gene	-	-	mRNA	NCP	Intron	gene|mRN	ID=gene-	7	5.161428571	2323.6	0.556193261	GVTALLQ	82	701.4166	1	700.4119	.	S	Superscaf	80358229	BTP20220	0	0	0	0	0	Tissue specific
LTVDLM	Superscaf	+	Superscaf	80396566	80396583	7	7	12	-	-	gene	-	-	mRNA	NCP	Intron	gene|mRN	ID=gene-	6	4.535	1887.3	0.556933707	LTVDLM(+1	65	707.3586	1	706.3571	Oxidatior	N	Superscaf	80396548	0	0	0	BTP20220	0	0	Tissue specific
LAPGLALAL	Superscaf	+	Superscaf	80429542	80429568	54	54	62	-	-	-	-	-	-	NCP	IGR	-	ID=gene-	9	6.027777778	2982.2	0.55757231	LAPGLALAL	77	838.5368	1	837.5323	.	L	Superscaf	80429383	0	0	BTP20220	0	0	0	Tissue specific
SGGHFRP	Superscaf	+	Superscaf	80485807	80485827	1	1	7	-	-	-	-	-	-	NCP	IGR	-	ID=gene-	7	6.821428571	2971.3	0.558661919	SGGHFRP	59	379.188	2	756.3667	.	S	Superscaf	80485807	0	0	0	BTP20220	0	0	Tissue specific
DKVGNDK	Superscaf	-	Superscaf	80486953	80486973	-17	17	23	-	-	-	-	-	-	NCP	IGR	-	ID=gene-	7	5.257142857	2653.8	0.558684113	DKVGNDK	72	388.1945	2	774.3871	.	L	Superscaf	80486973	0	0	0	BTP20220	0	0	Tissue specific
EDVGLLP	Superscaf	+	Superscaf	80536201	80536221	33	33	39	-	-	-	-	-	-	NCP	IGR	-	ID=gene-	7	4.345714286	2178.5	0.559637833	EDVGLLP	85	742.3956	1	741.3908	.	F	Superscaf	80536105	0	0	0	BTP20220	0	0	Tissue specific
LHAPPPPPF	Superscaf	+	Superscaf	80539870	80539896	15	15	23	-	exon	gene	three_pri	-	mRNA	NCP	3UTR	exon|gene	ID=exon-	9	6.376666667	3308.8	0.559708886	L(+42.01)	91	482.7676	2	963.5178	Acetylati	G	Superscaf	80539828	0	0	BTP20220	0	0	0	Mixed
GRPPKAAP	Superscaf	+	Superscaf	80565094	80565117	126	126	133	-	-	-	-	-	-	NCP	IGR	-	ID=gene-	8	7.17625	3049.3	0.560197366	GRPPKAAP	60	397.2381	2	792.4606	.	G	Superscaf	80564719	0	0	0	BTP20220	0	0	Tissue specific
RPAHKL	Superscaf	+	Superscaf	80583106	80583123	32	32	37	-	-	-	-	-	-	NCP	IGR	-	ID=gene-	6	7.731666667	2739	0.56054618	RPAHKL	85	361.2202	2	720.4395	.	V	Superscaf	80583013	0	0	0	0	0	BTP20220	Tissue specific
FAPLRST	Superscaf	+	Superscaf	80586820	80586840	10	10	16	-	-	-	-	-	-	NCP	IGR	-	ID=gene-	7	6.678571429	2819.2	0.560618104	FAPLRST	72	396.2231	2	790.4337	.	C	Superscaf	80586793	0	0	0	0	0	BTP20220	Tissue specific
LDLSAPK	Superscaf	+	Superscaf	80598430	80598450	2	2	8	-	-	gene	-	-	mRNA	NCP	Intron	gene|mRN	ID=gene-	7	6.095714286	2618.8	0.56084294	LDLSAPK	93	372.2183	2	742.4225	.	P	Superscaf	80598427	0	0	0	BTP20220	0	0	Tissue enhanced
THSLDLR	Superscaf	-	Superscaf	80617579	80617599	-11	11	17	-	-	gene	-	-	mRNA	NCP	Intron	gene|mRN	ID=gene-	7	6.498571429	3167.7	0.561213773	THSLDLR	67	421.2331	2	840.4454	.	K	Superscaf	80617635	0	0	0	BTP20220	0	BTP20220	Tissue specific
LTPFQLTN	Superscaf	-	Superscaf	80690434	80690457	-12	12	19	-	-	gene	-	-	mRNA	NCP	Intron	gene|mRN	ID=gene-	8	5.9825	3434	0.562624659	LTPFQLTN	67	467.2536	2	932.4967	.	N	Superscaf	80690460	0	0	0	0	0	BTP20220	Tissue specific
YGVKP	Superscaf	-	Superscaf	80706526	80706540	-20	20	24	-	-	-	-	-	-	NCP	IGR	-	ID=gene-	5	5.534	1562.6	0.562936292	YGVKP	98	563.3165	1	562.3115	.	L	Superscaf	80706744	0	0	BTP20220	0	0	0	Tissue specific
RPGVLLRQ	Superscaf	-	Superscaf	80708611	80708634	-45	45	52	-	-	-	-	-	-	NCP	IGR	-	ID=gene-	8	6.425	3249.8	0.562976669	RPGVLLRQ	88	469.791	2	937.5821	.	D	Superscaf	80708652	0	0	0	BTP20220	0	0	Tissue specific
VNSHEFE	Superscaf	+	Superscaf	80740291	80740311	12	12	18	-	-	-	-	-	-	NCP	IGR	-	ID=gene-	7	4.371428571	2838.2	0.563590174	VNSHEFE	53	431.1927	2	860.3664	.	T	Superscaf	80740258	0	0	BTP20220	0	0	0	Tissue specific
RKPEPPR	Superscaf	-	Superscaf	80770378	80770398	-16	16	22	-	-	gene	-	-	mRNA	NCP	Intron	gene|mRN	ID=gene-	7	7.625714286	3203.7	0.564172829	R(+42.01)	87	461.2605	2	920.5192	Acetylati	K	Superscaf	80770473	0	0	BTP20220	0	0	0	Tissue specific
TPGDCK	Superscaf	+	Superscaf	80788576	80788593	7	7	12	-	-	gene	-	-	mRNA	NCP	Intron	gene|mRN	ID=gene-	6	6.088333333	2497.8	0.564525245	TPGDC(+57	53	677.2914	1	676.285	Carbamidc	W	Superscaf	80788558	0	0	0	0	0	BTP20220	Tissue specific
EKLGDHT	Superscaf	-	Superscaf	80830306	80830326	-10	10	16	-	-	gene	-	-	mRNA	NCP	Intron	gene|mRN	ID=gene-	7	6	2973.3	0.565333375	EKLGDHT	51	400.2041	2	798.3871	.	M	Superscaf	80830377	0	BTP20220	0	0	0	0	Tissue specific
SGFGDRK	Superscaf	-	Superscaf	80845435	80845455	-21	21	27	-	-	gene	-	-	mRNA	NCP	Intron	gene|mRN	ID=gene-	7	6.652857143	2894.1	0.565626358	SGFGDRK	54	383.6919	2	765.3769	.	D	Superscaf	80845638	0	0	0	0	BTP20220	0	Tissue specific
WSPPHLK	Superscaf	-	Superscaf	80862499	80862519	-24	24	30	-	-	-	-	-	-	NCP	IGR	-	ID=gene-	7	6.782857143	3026.3	0.565956814	WSPPHLK	74	432.7415	2	863.4653	.	Q	Superscaf	80862543	0	0	0	BTP20220	0	0	Tissue specific
HNFHKN	Superscaf	-	Superscaf	80865847	80865864	-4	4	9	-	-	-	-	-	-	NCP	IGR	-	ID=gene-	6	6.87	3288.6	0.56602165	HNFHKN	52	398.6991	2	795.3776	.	V	Superscaf	80865882	0	0	0	BTP20220	0	0	Tissue specific
KPLLKNP	Superscaf	-	Superscaf	80902456	80902476	-24	24	30	-	-	gene	-	-	mRNA	NCP	Intron	gene|mRN	ID=gene-	7	7.064285714	2915.1	0.566730608	KPLLKNP	77	405.2652	2	808.517	.	K	Superscaf	80902788	0	BTP20220	0	0	0	0	Tissue specific
MPLPVAP	Superscaf	+	Superscaf	80957116	80957136	17	17	23	-	-	-	-	-	-	NCP	IGR	-	ID=gene-	7	5.235714286	2048.4	0.567789136	M(+42.01)	54	383.7113	2	765.4095	Acetylati	S	Superscaf	80957068	0	0	BTP20220	0	0	0	Tissue specific
PSGVPSPR	Superscaf	+	Superscaf	80969041	80969064	10	10	17	-	-	-	-	-	-	NCP	IGR	-	ID=gene-	8	5.87375	2725.1	0.568020072	P(+42.01)	66	419.723	2	837.4344	Acetylati	A	Superscaf	80969014	0	0	BTP20220	0	0	0	Tissue specific
GVLLNKVV	Superscaf	+	Superscaf	80977375	80977398	8	8	15	-	-	gene	-	-	mRNA	NCP	Intron	gene|mRN	ID=gene-	8	4.135	2069.2	0.568181465	GVLLNKVV	93	421.2772	2	840.5433	.	K	Superscaf	80977354	0	0	0	BTP20220	0	0	Tissue specific
LVLPVPQ	Superscaf	+	Superscaf	80986618	80986638	33	33	39	-	-	gene	-	-	mRNA	NCP	Intron	gene|mRN	ID=gene-	7	4.315714286	1992.3	0.568360462	LVLPVPQ	79	765.4833	1	764.4796	.	K	Superscaf	80986522	0	0	0	0	BTP20220	0	Tissue specific
GPVMKVH	Superscaf	-	Superscaf	80993956	80993976	-8	8	14	-	-	gene	-	-	mRNA	NCP	Intron	gene|mRN	ID=gene-	7	5.05	2094.3	0.568502567	GPVM(+15.	65	392.206	2	782.4109	Oxidatior	P	Superscaf	80993985	BTP20220	0	0	0	0	0	Tissue specific
FSCPPK	Superscaf	-	Superscaf	81017671	81017688	-4	4	9	-	-	gene	-	-	mRNA	NCP	Intron	gene|mRN	ID=gene-	6	6.42	2499.7	0.568961824	FSC(+57.(	60	368.1772	2	734.3421	Carbamidc	V	Superscaf	81017721	0	0	0	BTP20220	0	0	Tissue specific
MEVLAP	Superscaf	-	Superscaf	81058849	81058866	-45	45	50	-	-	gene	-	-	mRNA	NCP	Intron	gene|mRN	ID=gene-	6	4.545	1799.1	0.569759264	M(+42.01)	70	701.3528	1	700.3466	Acetylati	E	Superscaf	81058917	0	BTP20220	0	0	0	0	Tissue specific
KAWLVGP	Superscaf	+	Superscaf	81083956	81083976	22	22	28	-	-	-	-	-	-	NCP	IGR	-	ID=gene-	7	5.7	2294.4	0.570245478	KAWLVGP	63	385.7286	2	769.4486	.	P	Superscaf	81083893	0	0	0	0	0	BTP20220	Tissue specific
KDLPF	Superscaf	-	Superscaf	81120841	81120855	-31	31	35	-	-	gene	-	-	mRNA	NCP	Intron	gene|mRN	ID=gene-	5	6.094	1958.1	0.570959781	KDLPF	71	619.3419	1	618.3377	.	M	Superscaf	81120861	0	0	0	0	BTP20220	0	Tissue specific
KDEAAKK	Superscaf	-	Superscaf	81135727	81135747	-27	27	33	-	-	gene	-	-	mRNA	NCP	Intron	gene|mRN	ID=gene-	7	6.778571429	2864.8	0.571248058	KDEAAKK	69	395.2265	2	788.4391	.	S	Superscaf	81135786	0	0	BTP20220	0	0	0	Tissue specific
LGKYGPAN	Superscaf	-	Superscaf	81137212	81137235	-58	58	65	-	-	gene	-	-	mRNA	NCP	Intron	gene|mRN	ID=gene-	8	6.38125	3121.3	0.571276816	LGKYGPAN	98	410.2288	2	818.4286	.	L	Superscaf	81137337	0	0	BTP20220	0	0	0	Tissue specific
DGLVWGSS	Superscaf	-	Superscaf	81137485	81137511	-55	55	63	-	-	gene	-	-	mRNA	NCP	Intron	gene|mRN	ID=gene-	9	5.32	3121.3	0.571282103	DGLVWGSS	89	474.7399	2	947.4713	.	R	Superscaf	81137658	0	0	BTP20220	0	0	0	Tissue specific
PLASFVTQ	Superscaf	-	Superscaf	81141826	81141849	-5	5	12	-	-	gene	-	-	mRNA	NCP	Intron	gene|mRN	ID=gene-	8	5.205	2781.1	0.57136617	PLASFVTQ	73	862.4641	1	861.4596	.	I	Superscaf	81141888	0	0	0	BTP20220	0	0	Tissue specific
PFHPPLP	Superscaf	+	Superscaf	81177655	81177675	147	147	153	-	-	gene	-	-	mRNA	NCP	Intron	gene|mRN	ID=gene-	7	6.321428571	2710.1	0.572060022	P(+42.01)	65	423.7356	2	845.4435	Acetylati	V	Superscaf	81177217	0	0	BTP20220	0	0	0	Tissue specific
PDRLVN	Superscaf	-	Superscaf	81205387	81205404	-19	19	24	-	-	gene	-	-	mRNA	NCP	Intron	gene|mRN	ID=gene-	6	5.236666667	2347.8	0.572597071	PDRLVN	64	357.1994	2	712.3868	.	H	Superscaf	81205434	0	BTP20220	0	0	0	0	Tissue specific
GDLKFNP	Superscaf	+	Superscaf	81230254	81230274	15	15	21	-	-	-	-	-	-	NCP	IGR	-	ID=gene-	7	5.978571429	2852.1	0.573078638	GDLKFNP	59	395.7074	2	789.402	.	F	Superscaf	81230212	BTP20220	0	0	0	0	0	Tissue specific
LLEGEFPQ	Superscaf	-	Superscaf	81266833	81266856	-21	21	28	-	-	gene	-	-	mRNA	NCP	Intron	gene|mRN	ID=gene-	8	5.225	3190.6	0.573787015	LLEGEFPQ	74	466.7362	2	931.465	.	F	Superscaf	81266910	BTP20220	0	0	0	0	0	Tissue specific
HGVVPLAT	Superscaf	-	Superscaf	81295549	81295572	-25	25	32	-	-	gene	-	-	mRNA	NCP	Intron	gene|mRN	ID=gene-	8	4.79875	2316.6	0.574343119	HGVVPLAT	93	793.4541	1	792.4494	.	F	Superscaf	81295602	0	0	0	BTP20220	0	0	Mixed
GVVLH	Superscaf	+	Superscaf	81307522	81307536	9	9	13	-	-	gene	-	-	mRNA	NCP	Intron	gene|mRN	ID=gene-	5	3.908	1270.4	0.574574984	GVVLH	72	524.3171	1	523.3118	.	S	Superscaf	81307498	0	0	BTP20220	BTP20220	0	0	Tissue specific
PGCGFPPP	Superscaf	-	Superscaf	81354973	81354996	-40	40	47	-	-	gene	-	-	mRNA	NCP	Intron	gene|mRN	ID=gene-	8	5.955	2917.3	0.575493905	PGC(+57.(	50	414.6875	2	827.3636	Carbamidc	L	Superscaf	81355029	0	0	BTP20220	BTP20220	0	0	Tissue specific
LGLEFAQ	Superscaf	+	Superscaf	81383821	81383841	28	28	34	-	-	-	-	-	-	NCP	IGR	-	ID=gene-	7	5.471428571	2739	0.576052566	LGLEFAQ	70	777.4117	1	776.4068	.	G	Superscaf	81383740	0	0	BTP20220	BTP20220	0	0	Tissue specific
LFKPVGN	Superscaf	-	Superscaf	81450964	81450984	-47	47	53	-	-	gene	-	-	mRNA	NCP	Intron	gene|mRN	ID=gene-	7	5.554285714	2440.6	0.577352835	LFKPVGN	59	387.7277	2	773.4435	.	L	Superscaf	81451065	0	0	BTP20220	0	0	0	Tissue specific
YLRPPSFL	Superscaf	-	Superscaf	81470581	81470604	-29	29	36	-	-	-	-	-	-	NCP	IGR	-	ID=gene-	8	6.5175	3260.7	0.577732732	YLRPPSFL	73	496.7793	2	991.5491	.	I	Superscaf	81470631	0	0	0	BTP20220	0	0	Tissue specific
VNEAPP	Superscaf	+	Superscaf	81490138	81490155	12	12	17	-	-	-	-	-	-	NCP	IGR	-	ID=gene-	6	4.541666667	2008.3	0.578111466	VNEAPP	57	626.3089	1	625.3071	.	A	Superscaf	81490105	0	0	BTP20220	0	0	0	Tissue specific
KGPGLTTP	Superscaf	+	Superscaf	81506458	81506481	26	26	33	-	-	-	-	-	-	NCP	IGR	-	ID=gene-	8	6.665	2886.2	0.578427514	KGPGLTTP	63	385.7292	2	769.4334	.	G	Superscaf	81506383	0	0	BTP20220	0	0	0	Tissue specific
TTLPELK	Superscaf	-	Superscaf	81591289	81591309	-32	32	38	-	-	gene	-	-	mRNA	NCP	Intron	gene|mRN	ID=gene-	7	6.325714286	2733.1	0.580070324	TTLPELK	98	401.2384	2	800.4644	.	C	Superscaf	81591369	0	0	BTP20220	0	0	0	Tissue specific
LGCLKL	Superscaf	+	Superscaf	81651319	81651336	14	14	19	-	-	gene	-	-	mRNA	NCP	Intron	gene|mRN	ID=gene-	6	6.445	2411.6	0.581232845	LGC(+57.(	61	352.2165	2	702.4098	Carbamidc	G	Superscaf	81651280	0	0	BTP20220	BTP20220	0	0	Tissue specific
FPVPYPP	Superscaf	+	Superscaf	81679183	81679203	29	29	35	-	-	-	-	-	-	NCP	IGR	-	ID=gene-	7	5.191428571	2196.5	0.58177245	FPVPYPP	75	408.7153	2	815.4218	.	C	Superscaf	81679099	0	0	BTP20220	BTP20220	0	0	Tissue specific
LSGVYPL	Superscaf	-	Superscaf	81688765	81688785	-21	21	27	-	-	-	-	-	-	NCP	IGR	-	ID=gene-	7	5.081428571	2184.4	0.581958012	LSGVYPL	86	374.7135	2	747.4167	.	R	Superscaf	81688785	0	0	BTP20220	0	0	0	Tissue specific
GQMKVVYF	Superscaf	-	Superscaf	81708664	81708687	-4	4	11	-	-	gene	-	-	mRNA	NCP	Intron	gene|mRN	ID=gene-	8	4.78125	2594.8	0.58234337	G(+42.01)	50	507.2694	2	1012.505	Acetylati	P	Superscaf	81708726	0	0	BTP20220	0	0	0	Tissue specific
EPRPPGL	Superscaf	+	Superscaf	81735592	81735612	7	7	13	-	-	gene	-	-	mRNA	NCP	Intron	gene|mRN	ID=gene-	7	6.404285714	2737.2	0.582864849	EPRPPGL	60	383.2194	2	764.418	.	M	Superscaf	81735574	0	0	0	0	BTP20220	0	Tissue specific
DLGFLKDL	Superscaf	-	Superscaf	81738523	81738546	-53	53	60	-	-	gene	-	-	mRNA	NCP	Intron	gene|mRN	ID=gene-	8	5.63375	3036.3	0.582921609	DLGFLKDL	63	460.7563	2	919.5015	.	S	Superscaf	81738585	0	0	0	BTP20220	0	0	Tissue specific
SPCCLVT	Superscaf	-	Superscaf	81750187	81750207	-41	41	47	-	-	gene	-	-	mRNA	NCP	Intron	gene|mRN	ID=gene-	7	4.932857143	2643.1	0.583147491	SPC(+57.(	88	836.3606	1	835.3568	Carbamidc	E	Superscaf	81750243	0	0	BTP20220	0	0	0	Tissue specific
MLYGAR	Superscaf	+	Superscaf	81771229	81771246	16	16	21	-	-	gene	-	-	mRNA	NCP	Intron	gene|mRN	ID=gene-	6	6.69	2441.8	0.583554983	M(+15.99)	62	363.6909	2	725.353	Oxidatior	R	Superscaf	81771184	0	0	BTP20220	0	0	0	Tissue specific
VAGAGLV	Superscaf	-	Superscaf	81778111	81778131	-13	13	19	-	-	gene	-	-	mRNA	NCP	Intron	gene|mRN	ID=gene-	7	4.28	1570.6	0.583688258	VAGAGLV	81	586.3549	1	585.3486	.	R	Superscaf	81778137	0	0	BTP20220	0	0	0	Tissue specific
LYKPSGK	Superscaf	-	Superscaf	81794473	81794493	-10	10	16	-	-	gene	-	-	mRNA	NCP	Intron	gene|mRN	ID=gene-	7	7.01	2767.8	0.584005119	L(+42.01)	70	417.7434	2	833.4647	Acetylati	G	Superscaf	81794550	0	0	0	BTP20220	0	0	Tissue specific
LQYKKM	Superscaf	+	Superscaf	81798718	81798735	71	71	76	-	-	gene	-	-	mRNA	NCP	Intron	gene|mRN	ID=gene-	6	7.086666667	2731.9	0.584087326	LQYKKM	53	405.7379	2	809.447	.	E	Superscaf	81798508	0	0	0	BTP20220	0	0	Tissue specific
TPATDVFP	Superscaf	+	Superscaf	81814021	81814044	3	3	10	-	-	gene	-	-	mRNA	NCP	Intron	gene|mRN	ID=gene-	8	5.01625	2580	0.584383679	T(+42.01)	67	889.4141	1	888.4229	Acetylati	Y	Superscaf	81814015	0	0	0	BTP20220	0	0	Tissue specific
VVARAGPV	Superscaf	-	Superscaf	81822622	81822645	-9	9	16	-	-	gene	-	-	mRNA	NCP	Intron	gene|mRN	ID=gene-	8	4.38375	1906.1	0.584550243	VVARAGPV	81	384.739	2	767.4653	.	T	Superscaf	81822720	0	0	0	BTP20220	0	0	Tissue specific
GPAEPLN	Superscaf	+	Superscaf	81875464	81875484	11	11	17	-	-	-	-	-	-	NCP	IGR	-	ID=gene-	7	5.6	2659	0.585573564	GPAEPLN	66	697.3469	1	696.3442	.	L	Superscaf	81875434	0	0	BTP20220	0	0	0	Tissue specific
NPALPLE	Superscaf	+	Superscaf	81885367	81885387	48	48	54	-	-	-	-	-	-	NCP	IGR	-	ID=gene-	7	5.601428571	2715.1	0.585765342	NPALPLE	60	377.2091	2	752.4068	.	N	Superscaf	81885226	0	0	BTP20220	0	0	0	Tissue specific
VPGFP	Superscaf	+	Superscaf	81977197	81977211	34	34	38	-	-	gene	-	-	mRNA	NCP	Intron	gene|mRN	ID=gene-	5	4.81	1359.5	0.587543692	VPGFP	93	516.2799	1	515.2744	.	H	Superscaf	81977098	0	0	BTP20220	0	0	0	Tissue specific
VTLVLT	Superscaf	-	Superscaf	82070833	82070850	-8	8	13	-	-	-	-	-	-	NCP	IGR	-	ID=gene-	6	4.17	1501.8	0.589357017	VTLVLT	77	645.4166	1	644.4109	.	V	Superscaf	82070874	0	BTP20220	0	0	0	0	Tissue specific
KNGCLVH	Superscaf	-	Superscaf	82114801	82114821	-10	10	16	-	-	-	-	-	-	NCP	IGR	-	ID=gene-	7	5.672857143	2921.2	0.590208487	KNGC(+57.	65	414.2127	2	826.4119	Carbamidc	I	Superscaf	82114845	BTP20220	0	BTP20220	0	0	0	Mixed
ELSDEP	Superscaf	+	Superscaf	82134796	82134813	1	1	6	-	-	gene	-	-	mRNA	NCP	Intron	gene|mRN	ID=gene-	6	4.561666667	2336.7	0.590595703	ELSDEP	65	689.3007	1	688.2915	.	E	Superscaf	82134796	0	0	0	0	0	BTP20220	Tissue specific
QNSLDF	Superscaf	-	Superscaf	82161016	82161033	2	2	7	-	-	-	-	-	-	NCP	IGR	-	ID=gene-	6	5.195	2743.1	0.591103471	QNSLDF	65	723.3324	1	722.3235	.	L	Superscaf	82161013	0	0	0	0	BTP20220	0	Tissue specific
KAADFQLH	Superscaf	-	Superscaf	82173538	82173561	-124	124	131	-	-	-	-	-	-	NCP	IGR	-	ID=gene-	8	6.18125	3473.7	0.591345968	KAADFQLH	98	465.2439	2	928.4766	.	Y	Superscaf	82173771	0	0	0	BTP20220	0	0	Tissue specific
GVPLNP	Superscaf	-	Superscaf	82211311	82211328	-92	92	97	-	-	gene	-	-	mRNA	NCP	Intron	gene|mRN	ID=gene-	6	4.993333333	1922.2	0.592077468	GVPLNP	53	596.3373	1	595.3329	.	R	Superscaf	82211385	0	0	BTP20220	BTP20220	BTP20220	0	Tissue specific
ARRNPLE	Superscaf	-	Superscaf	82233253	82233273	-75	75	81	-	-	gene	-	-	mRNA	NCP	Intron	gene|mRN	ID=gene-	7	6.921428571	3321.9	0.592502389	A(+42.01)	68	449.2469	2	896.4828	Acetylati	G	Superscaf	82233309	0	0	0	0	0	BTP20220	Tissue specific
ALLTKAVVF	Superscaf	-	Superscaf	82242223	82242258	-26	26	37	-	exon	gene	three_pri	-	mRNA	NCP	3UTR	exon|gene	ID=exon-	12	5.13	3641	0.5926761	ALLTKAVVF	84	621.8574	2	1241.705	.	S	Superscaf	82242525	0	0	BTP20220	0	0	0	Tissue specific
LTKNLALEI	Superscaf	-	Superscaf	82242742	82242768	-29	29	37	-	exon	gene	three_pri	-	mRNA	NCP	3UTR	exon|gene	ID=exon-	9	6.093333333	3613	0.59268615	LTKNLALEI	82	507.8115	2	1013.612	.	G	Superscaf	82242870	0	0	BTP20220	0	0	0	Tissue specific
AELNVRVNC	Superscaf	-	Superscaf	82242769	82242798	-38	38	47	-	exon	gene	three_pri	-	mRNA	NCP	3UTR	exon|gene	ID=exon-	10	4.78	3871.5	0.592686673	AELNVRVNC	97	594.3111	2	1186.613	Carbamidc	G	Superscaf	82242870	0	0	BTP20220	0	0	0	Tissue specific
AELNVRVN	Superscaf	-	Superscaf	82242769	82242792	-38	38	45	-	exon	gene	three_pri	-	mRNA	NCP	3UTR	exon|gene	ID=exon-	8	4.6	2932.4	0.592686673	AELNVRVN	94	457.7549	2	913.4981	.	G	Superscaf	82242870	0	0	BTP20220	0	0	0	Tissue specific
ELNVRVN	Superscaf	-	Superscaf	82242772	82242792	-39	39	45	-	exon	gene	three_pri	-	mRNA	NCP	3UTR	exon|gene	ID=exon-	7	4.397142857	2621.1	0.592686731	ELNVRVN	96	422.2361	2	842.461	.	G	Superscaf	82242870	0	0	BTP20220	0	0	0	Tissue specific
AGGSLSHL	Superscaf	-	Superscaf	82244890	82244913	-51	51	58	-	-	-	-	-	-	NCP	IGR	-	ID=gene-	8	6.10875	2999.2	0.592727748	AGGSLSHL	93	371.1964	2	740.3817	.	Y	Superscaf	82244913	0	BTP20220	BTP20220	0	0	0	Tissue specific
AGRGQARP	Superscaf	-	Superscaf	82246627	82246650	-15	15	22	-	-	-	-	-	-	NCP	IGR	-	ID=gene-	8	7.18125	3462.9	0.592761386	A(+42.01)	68	427.7321	2	853.4518	Acetylati	A	Superscaf	82246656	0	0	0	BTP20220	0	0	Tissue specific
RLVDHP	Superscaf	+	Superscaf	82253140	82253157	7	7	12	-	-	-	-	-	-	NCP	IGR	-	ID=gene-	6	5.6	2370.8	0.592887515	RLVDHP	66	368.7021	2	735.4028	.	H	Superscaf	82253122	0	0	0	0	0	BTP20220	Tissue specific
LPSLPAAGF	Superscaf	-	Superscaf	82262095	82262121	-4	4	12	-	-	-	-	-	-	NCP	IGR	-	ID=gene-	9	6.061111111	3022.3	0.593060934	LPSLPAAGF	74	411.7437	2	821.4647	.	L	Superscaf	82262280	0	0	0	BTP20220	0	0	Tissue specific
NNNLGGGL	Superscaf	-	Superscaf	82282990	82283013	-3	3	10	-	-	-	-	-	-	NCP	IGR	-	ID=gene-	8	5.7625	3388.8	0.59346558	NNNLGGGL	52	758.3781	1	757.3719	.	Q	Superscaf	82283163	0	BTP20220	0	0	BTP20220	BTP20220	Tissue specific
YPELG	Superscaf	-	Superscaf	82295089	82295103	-1	1	5	-	-	-	-	-	-	NCP	IGR	-	ID=gene-	5	5.426	1873.1	0.593699885	YPELG	82	578.2803	1	577.2747	.	Y	Superscaf	82295208	0	0	0	0	0	BTP20220	Tissue specific
ATLRVLPEA	Superscaf	-	Superscaf	82318351	82318386	-7	7	18	-	-	gene	-	-	mRNA	NCP	Intron	gene|mRN	ID=gene-	12	6.018333333	4529	0.59415037	A(+42.01)	94	669.8881	2	1337.767	Acetylati	A	Superscaf	82318524	0	0	0	0	BTP20220	0	Tissue specific
ATLRVLPEA	Superscaf	-	Superscaf	82318351	82318380	-7	7	16	-	-	gene	-	-	mRNA	NCP	Intron	gene|mRN	ID=gene-	10	5.646	3749.3	0.59415037	A(+42.01)	87	570.3231	2	1138.635	Acetylati	A	Superscaf	82318524	0	0	BTP20220	0	0	0	Tissue specific
ATLRVLPEA	Superscaf	-	Superscaf	82318351	82318377	-7	7	15	-	-	gene	-	-	mRNA	NCP	Intron	gene|mRN	ID=gene-	9	5.645555556	3138.6	0.59415037	A(+42.01)	97	506.2938	2	1010.576	Acetylati	A	Superscaf	82318524	0	0	BTP20220	0	0	0	Mixed
ATLRVLPE	Superscaf	-	Superscaf	82318351	82318374	-7	7	14	-	-	gene	-	-	mRNA	NCP	Intron	gene|mRN	ID=gene-	8	5.59875	2827.3	0.59415037	A(+42.01)	64	470.7767	2	939.5389	Acetylati	A	Superscaf	82318524	0	0	0	0	BTP20220	0	Mixed
ATLRVLP	Superscaf	-	Superscaf	82318351	82318371	-7	7	13	-	-	gene	-	-	mRNA	NCP	Intron	gene|mRN	ID=gene-	7	5.938571429	2401.8	0.59415037	A(+42.01)	95	406.2542	2	810.4963	Acetylati	A	Superscaf	82318524	0	0	0	0	0	BTP20220	Mixed
VLPEAQAK	Superscaf	-	Superscaf	82318363	82318386	-11	11	18	-	-	gene	-	-	mRNA	NCP	Intron	gene|mRN	ID=gene-	8	5.36625	2818	0.594150602	VLPEAQAK	96	428.2489	2	854.4861	.	A	Superscaf	82318524	0	0	0	0	0	BTP20220	Tissue specific
VDVFREDLC	Superscaf	-	Superscaf	82318930	82318962	-8	8	18	-	-	gene	-	-	mRNA	NCP	Intron	gene|mRN	ID=gene-	11	4.788181818	4089.7	0.594161582	VDVFREDLC	99	691.341	2	1380.671	Carbamidc	R	Superscaf	82319052	0	0	0	BTP20220	0	0	Mixed
DVFREDLC	Superscaf	-	Superscaf	82318933	82318962	-9	9	18	-	-	gene	-	-	mRNA	NCP	Intron	gene|mRN	ID=gene-	10	5.267	4089.7	0.59416164	DVFREDLC	76	641.8063	2	1281.602	Carbamidc	R	Superscaf	82319052	0	0	0	BTP20220	0	0	Tissue specific
TENLLGSYF	Superscaf	-	Superscaf	82319107	82319139	-50	50	60	-	-	gene	-	-	mRNA	NCP	Intron	gene|mRN	ID=gene-	11	5.995454545	4459.9	0.59416501	TENLLGSYF	93	634.827	2	1267.645	.	G	Superscaf	82319190	0	0	0	0	0	BTP20220	Tissue specific
FSERGDAVI	Superscaf	-	Superscaf	82320292	82320321	-50	50	59	-	-	gene	-	-	mRNA	NCP	Intron	gene|mRN	ID=gene-	10	5.637	3731.1	0.594187958	FSERGDAVI	94	555.2817	2	1108.551	.	A	Superscaf	82320558	BTP20220	0	0	0	0	0	Tissue specific
GHAAPVAA	Superscaf	-	Superscaf	82329034	82329057	-61	61	68	-	-	gene	-	-	mRNA	NCP	Intron	gene|mRN	ID=gene-	8	5.4925	2499.6	0.594357253	G(+42.01)	66	368.1896	2	734.3711	Acetylati	S	Superscaf	82329213	0	0	BTP20220	0	0	0	Tissue specific
LSALPF	Superscaf	-	Superscaf	82343836	82343853	-30	30	35	-	-	gene	-	-	mRNA	NCP	Intron	gene|mRN	ID=gene-	6	5.906666667	2126.3	0.594643904	LSALPF	76	647.3757	1	646.369	.	T	Superscaf	82343904	0	0	0	0	0	BTP20220	Tissue enhanced
GTLEFK	Superscaf	-	Superscaf	82348654	82348671	-8	8	13	-	-	gene	-	-	mRNA	NCP	Intron	gene|mRN	ID=gene-	6	6.153333333	2329.5	0.594737208	G(+42.01)	66	368.6964	2	735.3803	Acetylati	P	Superscaf	82348755	0	BTP20220	0	0	0	0	Tissue specific
LTEKPQK	Superscaf	+	Superscaf	82350205	82350225	48	48	54	-	-	gene	-	-	mRNA	NCP	Intron	gene|mRN	ID=gene-	7	6.737142857	3061.3	0.594767244	LTEKPQK	66	422.2496	2	842.4861	.	I	Superscaf	82350064	0	0	BTP20220	0	0	0	Tissue specific
PPPPRGPP	Superscaf	-	Superscaf	82355032	82355055	-19	19	26	-	-	gene	-	-	mRNA	NCP	Intron	gene|mRN	ID=gene-	8	6.81625	2970.5	0.594860722	PPPPRGPP	79	407.7307	2	813.4496	.	W	Superscaf	82355217	0	BTP20220	0	0	0	0	Tissue specific
MELMMD	Superscaf	-	Superscaf	82369573	82369590	-87	87	92	-	-	gene	-	-	mRNA	NCP	Intron	gene|mRN	ID=gene-	6	4.903333333	2304.9	0.595142318	M(+15.99)	51	843.2917	1	842.286	Oxidatior	L	Superscaf	82369689	0	BTP20220	0	0	0	0	Tissue specific
AAAAVVEF	Superscaf	-	Superscaf	82389169	82389192	-1	1	8	-	-	-	-	-	-	NCP	IGR	-	ID=gene-	8	4.0975	2058.1	0.595521808	A(+42.01)	94	819.4223	1	818.4174	Acetylati	A	Superscaf	82389216	0	0	BTP20220	0	0	0	Mixed
TVPVP	Superscaf	-	Superscaf	82429297	82429311	-14	14	18	-	-	-	-	-	-	NCP	IGR	-	ID=gene-	5	3.826	1072.3	0.596298914	T(+42.01)	99	554.3182	1	553.3112	Acetylati	A	Superscaf	82429344	BTP20220	0	0	0	0	0	Mixed
EDHLERL	Superscaf	-	Superscaf	82430068	82430088	-47	47	53	-	-	-	-	-	-	NCP	IGR	-	ID=gene-	7	5.674285714	3237.8	0.596313845	EDHLERL	80	456.2313	2	910.4508	.	P	Superscaf	82430154	0	0	0	BTP20220	0	0	Tissue specific
YMPALPR	Superscaf	-	Superscaf	82437994	82438014	-25	25	31	-	-	-	-	-	-	NCP	IGR	-	ID=gene-	7	6.681428571	2819.3	0.596467337	Y(+42.01)	52	445.2416	2	888.4527	Acetylati	D	Superscaf	82438038	0	0	BTP20220	0	0	0	Tissue specific
YGPLTL	Superscaf	-	Superscaf	82469146	82469163	-18	18	23	-	-	gene	-	-	mRNA	NCP	Intron	gene|mRN	ID=gene-	6	6.07	2198.5	0.597070616	YGPLTL	94	663.3682	1	662.3639	.	D	Superscaf	82469409	0	0	BTP20220	0	0	0	Mixed
LSPTGVP	Superscaf	-	Superscaf	82557046	82557066	-21	21	27	-	-	gene	-	-	mRNA	NCP	Intron	gene|mRN	ID=gene-	7	5.251428571	2106.4	0.598772859	LSPTGVP	60	670.3737	1	669.3697	.	E	Superscaf	82557240	0	0	0	0	0	BTP20220	Tissue specific
TAAELYP	Superscaf	+	Superscaf	82675717	82675737	18	18	24	-	-	-	-	-	-	NCP	IGR	-	ID=gene-	7	5.675714286	2595.9	0.601071003	TAAELYP	68	764.3796	1	763.3752	.	R	Superscaf	82675666	BTP20220	0	0	0	0	0	Tissue specific
KDREVAK	Superscaf	-	Superscaf	82712860	82712880	-32	32	38	-	-	-	-	-	-	NCP	IGR	-	ID=gene-	7	6.064285714	2733.9	0.601790302	KDREVAK	55	423.2373	2	844.4766	.	G	Superscaf	82712958	0	0	0	BTP20220	0	0	Tissue specific
MEGAEEKKR	Superscaf	+	Superscaf	82762336	82762362	19	19	27	-	-	-	-	-	-	NCP	IGR	-	ID=gene-	9	6.291111111	3661.8	0.602748438	M(+42.01)	98	546.2716	2	1090.533	Acetylati	L	Superscaf	82762282	0	0	0	BTP20220	0	0	Tissue specific
KNNGDP	Superscaf	+	Superscaf	82784158	82784175	30	30	35	-	-	-	-	-	-	NCP	IGR	-	ID=gene-	6	5.966666667	2708	0.603171036	KNNGDP	68	644.3015	1	643.2925	.	K	Superscaf	82784071	0	0	0	0	BTP20220	0	Tissue specific
QSPLSPR	Superscaf	-	Superscaf	82792144	82792164	-129	129	135	-	-	-	-	-	-	NCP	IGR	-	ID=gene-	7	6.621428571	3054.5	0.60332569	Q(+42.01)	75	413.7292	2	825.4344	Acetylati	V	Superscaf	82792212	BTP20220	0	0	0	0	0	Tissue specific
KGVLVDP	Superscaf	-	Superscaf	82845562	82845582	-247	247	253	-	-	gene	-	-	mRNA	NCP	Intron	gene|mRN	ID=gene-	7	4.422857143	1868	0.604360166	KGVLVDP	54	727.4335	1	726.4276	.	L	Superscaf	82845720	0	0	0	0	BTP20220	0	Tissue specific
RPGVMTK	Superscaf	+	Superscaf	82884691	82884711	5	5	11	-	-	gene	-	-	mRNA	NCP	Intron	gene|mRN	ID=gene-	7	6.435714286	2520.9	0.605117926	R(+42.01)	54	423.7356	2	845.4429	Acetylati	N	Superscaf	82884679	0	0	0	0	0	BTP20220	Tissue specific
MLGMLK	Superscaf	+	Superscaf	82900399	82900416	11	11	16	-	-	gene	-	-	mRNA	NCP	Intron	gene|mRN	ID=gene-	6	6.528333333	2215.5	0.605422122	M(+42.01)	98	734.3931	1	733.3867	Acetylati	H	Superscaf	82900369	0	0	0	0	0	BTP20220	Mixed
KNSPR	Superscaf	+	Superscaf	82921762	82921776	5	5	9	-	-	gene	-	-	mRNA	NCP	Intron	gene|mRN	ID=gene-	5	7.578	2434.7	0.60583583	KNSPR	56	601.3298	1	600.3344	.	A	Superscaf	82921750	0	BTP20220	0	0	0	0	Tissue specific
MTKAEG	Superscaf	+	Superscaf	82964344	82964361	5	5	10	-	-	gene	-	-	mRNA	NCP	Intron	gene|mRN	ID=gene-	6	6.205	2271.5	0.60666046	MTKAEG	55	636.2928	1	635.2949	.	N	Superscaf	82964332	0	0	0	0	0	BTP20220	Tissue specific
GGAVLE	Superscaf	-	Superscaf	82966114	82966131	-33	33	38	-	-	gene	-	-	mRNA	NCP	Intron	gene|mRN	ID=gene-	6	4.526666667	1684.8	0.606694737	GGAVLE	88	545.2903	1	544.2856	.	D	Superscaf	82966194	BTP20220	0	0	0	0	0	Tissue specific
RTPLAVR	Superscaf	-	Superscaf	82984915	82984935	-12	12	18	-	-	gene	-	-	mRNA	NCP	Intron	gene|mRN	ID=gene-	7	6.621428571	2697.2	0.607058831	RTPLAVR	99	406.757	2	811.5028	.	K	Superscaf	82984935	0	0	BTP20220	0	0	0	Tissue specific
KEEMY	Superscaf	-	Superscaf	83111458	83111472	-5	5	9	-	-	gene	-	-	mRNA	NCP	Intron	gene|mRN	ID=gene-	5	5.518	2150.4	0.609509421	KEEMY	54	699.2987	1	698.2945	.	L	Superscaf	83111703	0	0	0	BTP20220	0	0	Tissue specific
GVPPPPPPP	Superscaf	+	Superscaf	83174983	83175009	53	53	61	-	-	gene	-	-	mRNA	NCP	Intron	gene|mRN	ID=gene-	9	5.563333333	2659.1	0.610739626	GVPPPPPPP	61	427.7413	2	853.4697	.	N	Superscaf	83174827	BTP20220	0	0	0	0	0	Tissue enhanced
PSVPKKP	Superscaf	-	Superscaf	83181094	83181114	-25	25	31	-	-	gene	-	-	mRNA	NCP	Intron	gene|mRN	ID=gene-	7	6.294285714	2332.4	0.610857969	P(+42.01)	90	397.7421	2	793.4697	Acetylati	T	Superscaf	83181132	0	BTP20220	0	0	0	0	Tissue specific
RLLSVLNK	Superscaf	+	Superscaf	83238817	83238840	5	5	12	-	-	-	-	-	-	NCP	IGR	-	ID=gene-	8	6.19125	3157.5	0.611975814	RLLSVLNK	62	471.8065	2	941.6022	.	H	Superscaf	83238805	0	0	0	0	BTP20220	0	Tissue specific
STLFFE	Superscaf	-	Superscaf	83275972	83275989	-2	2	7	-	-	-	-	-	-	NCP	IGR	-	ID=gene-	6	5.395	2334.6	0.612695346	STLFFE	75	743.359	1	742.3537	.	L	Superscaf	83275989	0	0	0	0	BTP20220	0	Tissue specific
TTPYPK	Superscaf	+	Superscaf	83280703	83280720	9	9	14	-	-	-	-	-	-	NCP	IGR	-	ID=gene-	6	6.843333333	2397.7	0.612786965	TTPYPK	79	353.697	2	705.3697	.	D	Superscaf	83280679	0	0	0	0	0	BTP20220	Tissue specific
PSLFER	Superscaf	+	Superscaf	83315971	83315988	9	9	14	-	-	-	-	-	-	NCP	IGR	-	ID=gene-	6	6.236666667	2535.9	0.613469954	PSLFER	96	374.7019	2	747.3915	.	G	Superscaf	83315947	0	0	0	BTP20220	0	0	Tissue specific
KDVKP	Superscaf	-	Superscaf	83321569	83321583	-1	1	5	-	-	-	-	-	-	NCP	IGR	-	ID=gene-	5	5.75	1685.7	0.613578363	KDVKP	72	586.3546	1	585.3486	.	K	Superscaf	83321637	0	BTP20220	0	0	0	0	Tissue specific
KANGSLK	Superscaf	+	Superscaf	83336800	83336820	1	1	7	-	-	-	-	-	-	NCP	IGR	-	ID=gene-	7	6.934285714	2878.9	0.613873321	KANGSLK	56	359.2215	2	716.418	.	K	Superscaf	83336800	0	BTP20220	0	0	0	0	Tissue specific
LHLSFK	Superscaf	-	Superscaf	83358001	83358018	-26	26	31	-	-	-	-	-	-	NCP	IGR	-	ID=gene-	6	6.741666667	2565.7	0.614283893	LHLSFK	97	372.7224	2	743.433	.	C	Superscaf	83358069	0	0	BTP20220	0	0	0	Tissue specific
QPWKYT	Superscaf	-	Superscaf	83434642	83434659	-6	6	11	-	-	-	-	-	-	NCP	IGR	-	ID=gene-	6	6.628333333	2800.1	0.615768097	QPWKYT	53	411.7115	2	821.4072	.	S	Superscaf	83434686	0	0	BTP20220	0	0	0	Tissue specific
VSGDLKP	Superscaf	-	Superscaf	83470948	83470968	-24	24	30	-	-	-	-	-	-	NCP	IGR	-	ID=gene-	7	5.234285714	2251.4	0.616471188	VSGDLKP	57	715.3961	1	714.3912	.	A	Superscaf	83471040	0	0	0	BTP20220	0	0	Tissue specific
EFGLP	Superscaf	-	Superscaf	83475394	83475408	-37	37	41	-	-	-	-	-	-	NCP	IGR	-	ID=gene-	5	5.39	1801	0.616557287	EFGLP	65	562.2861	1	561.2798	.	V	Superscaf	83475444	0	0	0	0	BTP20220	0	Tissue specific
FHDGAR	Superscaf	+	Superscaf	83556451	83556468	57	57	62	-	-	gene	-	-	mRNA	NCP	Intron	gene|mRN	ID=gene-	6	6.465	2676	0.618127011	FHDGAR	66	351.6722	2	701.3245	.	Y	Superscaf	83556283	0	0	0	0	BTP20220	0	Tissue specific
SELLLTK	Superscaf	-	Superscaf	83558125	83558145	-38	38	44	-	-	gene	-	-	mRNA	NCP	Intron	gene|mRN	ID=gene-	7	6.158571429	2735	0.618159429	SELLLTK	99	402.247	2	802.48	.	V	Superscaf	83558148	0	0	0	BTP20220	0	0	Tissue specific
MFLLQGA	Superscaf	+	Superscaf	83569342	83569362	3	3	9	-	-	gene	-	-	mRNA	NCP	Intron	gene|mRN	ID=gene-	7	5.832857143	2685	0.618376654	MFLLQGA	97	779.4089	1	778.4047	.	D	Superscaf	83569336	0	0	0	0	BTP20220	0	Tissue specific
KQLDPAL	Superscaf	-	Superscaf	83580931	83580951	-77	77	83	-	-	gene	-	-	mRNA	NCP	Intron	gene|mRN	ID=gene-	7	6.091428571	2846.1	0.618601083	KQLDPAL	55	784.442	1	783.449	.	P	Superscaf	83580981	0	BTP20220	0	0	BTP20220	0	Tissue specific
KCPVK	Superscaf	+	Superscaf	83689081	83689095	32	32	36	-	-	-	-	-	-	NCP	IGR	-	ID=gene-	5	6.16	1859.9	0.62069548	KC(+57.02	57	631.3539	1	630.3523	Carbamidc	E	Superscaf	83688988	0	BTP20220	0	0	0	0	Tissue specific
AAFKGNK	Superscaf	+	Superscaf	83735443	83735463	2	2	8	-	-	-	-	-	-	NCP	IGR	-	ID=gene-	7	6.911428571	2840.8	0.621593312	AAFKGNK	68	368.2133	2	734.4075	.	G	Superscaf	83735440	BTP20220	0	0	0	0	0	Tissue specific
RSKVALD	Superscaf	+	Superscaf	83904955	83904975	51	51	57	-	-	gene	-	-	mRNA	NCP	Intron	gene|mRN	ID=gene-	7	5.878571429	2576.8	0.624876026	RSKVALD	76	394.7335	2	787.4551	.	A	Superscaf	83904805	BTP20220	0	BTP20220	0	BTP20220	0	Mixed
AAVPV	Superscaf	+	Superscaf	83962870	83962884	22	22	26	-	-	gene	-	-	mRNA	NCP	Intron	gene|mRN	ID=gene-	5	3.668	960	0.625997589	AAVPV	55	456.2801	1	455.2744	.	K	Superscaf	83962807	0	0	0	0	BTP20220	0	Tissue specific
QALHQPL	Superscaf	+	Superscaf	84023659	84023679	6	6	12	-	-	-	-	-	-	NCP	IGR	-	ID=gene-	7	6.167142857	3196.6	0.627174809	QALHQPL	52	806.4409	1	805.4446	.	V	Superscaf	84023644	0	BTP20220	0	0	0	0	Tissue specific
TMPPPPPP	Superscaf	+	Superscaf	84140515	84140538	12	12	19	-	-	gene	-	-	mRNA	NCP	Intron	gene|mRN	ID=gene-	8	6.26	2793.4	0.629437804	T(+42.01)	75	446.2108	2	890.4208	Acetylati	I	Superscaf	84140482	0	0	0	0	0	BTP20220	Tissue specific
MVGAP	Superscaf	+	Superscaf	84156562	84156576	19	19	23	-	-	gene	-	-	mRNA	NCP	Intron	gene|mRN	ID=gene-	5	4.808	1317.5	0.629748565	MVGAP	73	474.2425	1	473.2308	.	G	Superscaf	84156508	0	0	0	0	0	BTP20220	Tissue specific
MLAAQL	Superscaf	+	Superscaf	84174043	84174060	43	43	48	-	-	gene	-	-	mRNA	NCP	Intron	gene|mRN	ID=gene-	6	5.9	2311.6	0.630087096	M(+42.01)	78	688.3693	1	687.3625	Acetylati	M	Superscaf	84173917	0	0	0	0	0	BTP20220	Tissue specific
SGMNVARLN	Superscaf	-	Superscaf	84193687	84193716	-31	31	40	-	-	gene	-	-	mRNA	NCP	Intron	gene|mRN	ID=gene-	10	5.646	3946.5	0.630467516	SGMNVARLN	98	554.7799	2	1107.55	.	T	Superscaf	84193800	BTP20220	0	0	0	0	0	Tissue specific
VTEVENGGS	Superscaf	-	Superscaf	84198061	84198099	-59	59	71	-	-	gene	-	-	mRNA	NCP	Intron	gene|mRN	ID=gene-	13	4.874615385	4325.7	0.630552221	VTEVENGGS	69	638.8215	2	1275.631	.	W	Superscaf	84198318	0	0	0	BTP20220	0	0	Tissue specific
SGETAKGDY	Superscaf	-	Superscaf	84200512	84200562	-137	137	153	-	-	gene	-	-	mRNA	NCP	Intron	gene|mRN	ID=gene-	17	5.614117647	6510.3	0.630599686	SGETAKGDY	88	926.4445	2	1850.883	.	D	Superscaf	84200676	0	0	0	BTP20220	0	0	Tissue specific
SGETAKGDY	Superscaf	-	Superscaf	84200512	84200559	-137	137	152	-	-	gene	-	-	mRNA	NCP	Intron	gene|mRN	ID=gene-	16	5.611875	5899.6	0.630599686	SGETAKGDY	76	575.2792	3	1722.825	.	D	Superscaf	84200676	BTP20220	0	0	0	0	0	Tissue specific
LFEELARAS	Superscaf	-	Superscaf	84203578	84203610	-17	17	27	-	-	gene	-	-	mRNA	NCP	Intron	gene|mRN	ID=gene-	11	5.966363636	4603.1	0.630659062	LFEELARAS	96	630.3199	2	1258.631	.	T	Superscaf	84203706	0	0	BTP20220	0	0	0	Tissue specific
GWRPGSGFT	Superscaf	-	Superscaf	84207214	84207240	-29	29	37	-	-	gene	-	-	mRNA	NCP	Intron	gene|mRN	ID=gene-	9	6.505555556	3573	0.630729475	GWRPGSGFT	66	482.7349	2	963.4562	.	F	Superscaf	84207267	0	0	0	BTP20220	0	0	Tissue specific
SGFTNTMRV	Superscaf	-	Superscaf	84207229	84207267	-34	34	46	-	-	gene	-	-	mRNA	NCP	Intron	gene|mRN	ID=gene-	13	4.977692308	4154.9	0.630729766	SGFTNTMRV	94	702.8673	2	1403.723	.	F	Superscaf	84207267	0	0	BTP20220	0	0	0	Tissue specific

FTNTMRVVF	Superscaf	-	Superscaf	84207235	84207267	-36	36	46	-	-	gene	-	-	mRNA	NCP	Intron	gene|mRN	ID=gene-E	11	4.823636364	3474.2	0.630729882	FTNTMRVVF	96	630.8413	2	1259.67	.	F	Superscaf	84207267	0	0	0	0	BTP20220	0	Tissue specific
NTMRVVPVF	Superscaf	-	Superscaf	84207241	84207267	-38	38	46	-	-	gene	-	-	mRNA	NCP	Intron	gene|mRN	ID=gene-E	9	4.561111111	2689.3	0.630729998	NTMRVVPVF	72	506.7826	2	1011.554	.	F	Superscaf	84207267	0	0	0	0	BTP20220	0	Tissue specific
MRVVPVP	Superscaf	-	Superscaf	84207247	84207267	-40	40	46	-	-	gene	-	-	mRNA	NCP	Intron	gene|mRN	ID=gene-E	7	4.158571429	1695.1	0.630730114	MRVVPVP	74	399.2384	2	796.4629	.	F	Superscaf	84207267	0	0	0	BTP20220	0	0	Mixed
SVTPEVR	Superscaf	+	Superscaf	84227218	84227238	44	44	50	-	-	gene	-	-	mRNA	NCP	Intron	gene|mRN	ID=gene-E	7	4.641428571	2192.6	0.631116866	SVTPEVR	68	394.2149	2	786.4235	.	A	Superscaf	84227089	0	0	0	0	0	BTP20220	Tissue specific
PVGEAQL	Superscaf	-	Superscaf	84234658	84234678	-10	10	16	-	-	gene	-	-	mRNA	NCP	Intron	gene|mRN	ID=gene-E	7	4.734285714	2335.6	0.631260947	P(+42.01)	50	755.3901	1	754.3861	Acetylati	G	Superscaf	84234762	0	BTP20220	BTP20220	0	0	BTP20220	Tissue specific
GLSVELP	Superscaf	+	Superscaf	84241723	84241743	22	22	28	-	-	gene	-	-	mRNA	NCP	Intron	gene|mRN	ID=gene-E	7	4.732857143	2150.4	0.631397765	G(+42.01)	58	756.4094	1	755.4065	Acetylati	V	Superscaf	84241660	0	0	0	0	BTP20220	0	Tissue specific
TFKTLGS	Superscaf	-	Superscaf	84271972	84271992	-37	37	43	-	-	gene	-	-	mRNA	NCP	Intron	gene|mRN	ID=gene-E	7	6.558571429	2684.9	0.631983557	TFKTLGS	53	377.2169	2	752.4069	.	V	Superscaf	84272007	0	0	0	BTP20220	0	0	Tissue specific
LHWFY	Superscaf	-	Superscaf	84283102	84283116	-24	24	28	-	-	gene	-	-	mRNA	NCP	Intron	gene|mRN	ID=gene-E	5	6.12	2346.6	0.632199097	LHWFY	51	383.1888	2	764.3646	.	P	Superscaf	84283119	0	BTP20220	0	0	0	0	Tissue specific
VGLKTEL	Superscaf	+	Superscaf	84321715	84321735	2	2	8	-	-	gene	-	-	mRNA	NCP	Intron	gene|mRN	ID=gene-E	7	5.345714286	2295.5	0.632946864	VGLKTEL	94	759.457	1	758.4538	.	A	Superscaf	84321712	0	0	BTP20220	0	0	0	Tissue specific
RHDNP	Superscaf	-	Superscaf	84347629	84347643	-2	2	6	-	-	-	-	-	-	NCP	IGR	-	ID=gene-E	5	6.606	2614.1	0.633448706	R(+42.01)	53	680.3218	1	679.3038	Acetylati	R	Superscaf	84347814	0	0	0	BTP20220	0	0	Tissue specific
TAAAFDN	Superscaf	+	Superscaf	84435034	84435054	23	23	29	-	-	gene	-	-	mRNA	NCP	Intron	gene|mRN	ID=gene-E	7	5.492857143	2727	0.635141363	T(+42.01)	64	376.1603	2	750.3184	Acetylati	M	Superscaf	84434968	0	0	0	BTP20220	0	0	Tissue specific
LHPYKK	Superscaf	+	Superscaf	84441145	84441162	20	20	25	-	-	gene	-	-	mRNA	NCP	Intron	gene|mRN	ID=gene-E	6	7.501666667	2706.8	0.635259707	LHPYKK	82	393.2391	2	784.4595	.	M	Superscaf	84441088	0	0	0	0	BTP20220	0	Tissue specific
NRTPKK	Superscaf	-	Superscaf	84451819	84451836	-3	3	8	-	exon	gene	-	five_prim	mRNA	NCP	5UTR	exon|gene	ID=exon-E	6	8.08	2917.2	0.635466416	NRTPKK	62	372.2234	2	742.4449	.	I	Superscaf	84452034	0	0	0	0	BTP20220	0	Tissue specific
LLPASGK	Superscaf	+	Superscaf	84471025	84471045	12	12	18	-	-	-	-	-	-	NCP	IGR	-	ID=gene-E	7	6.524285714	2504.6	0.635838353	L(+42.01)	97	364.2253	2	726.4276	Acetylati	V	Superscaf	84470992	0	0	0	0	0	BTP20220	Tissue specific
KRHCDK	Superscaf	+	Superscaf	84485521	84485538	6	6	11	-	-	gene	-	-	mRNA	NCP	Intron	gene|mRN	ID=gene-E	6	7.636666667	3202.5	0.636119078	K(+42.01)	50	443.2268	2	884.4286	Acetylati	Y	Superscaf	84485506	0	0	0	0	BTP20220	0	Tissue specific
AALSVP	Superscaf	-	Superscaf	84561259	84561276	-47	47	52	-	-	gene	-	-	mRNA	NCP	Intron	gene|mRN	ID=gene-E	6	5	1696.8	0.637585795	A(+42.01)	77	599.339	1	598.3326	Acetylati	R	Superscaf	84561309	0	0	0	0	BTP20220	0	Tissue specific
SGVHKSR	Superscaf	-	Superscaf	84603487	84603507	-12	12	18	-	-	gene	-	-	mRNA	NCP	Intron	gene|mRN	ID=gene-E	7	6.488571429	2801	0.638403569	SGVHKSR	70	385.7142	2	769.4195	.	N	Superscaf	84603513	0	BTP20220	0	0	0	0	Tissue specific
PHALTKT	Superscaf	+	Superscaf	84604138	84604158	2	2	8	-	-	gene	-	-	mRNA	NCP	Intron	gene|mRN	ID=gene-E	7	6.955714286	2885.2	0.638416176	PHALTKT	51	384.2216	2	766.4337	.	I	Superscaf	84604135	0	0	BTP20220	0	0	0	Tissue specific
LPQPAL	Superscaf	+	Superscaf	84611077	84611094	32	32	37	-	exon	gene	three_pri	-	mRNA	NCP	3UTR	exon|gene	ID=exon-E	6	6.038333333	2303.6	0.638550554	LPQPAL	52	638.3964	1	637.3799	.	P	Superscaf	84610984	0	0	0	BTP20220	0	0	Tissue specific
TAVAGGFL	Superscaf	-	Superscaf	84621127	84621150	-11	11	18	-	-	gene	-	-	mRNA	NCP	Intron	gene|mRN	ID=gene-E	8	5.24625	2355.5	0.638745179	TAVAGGFL	67	735.3995	1	734.3962	.	H	Superscaf	84621201	0	BTP20220	0	0	0	0	Tissue specific
AVSLFRA	Superscaf	-	Superscaf	84624970	84624990	-30	30	36	-	-	gene	-	-	mRNA	NCP	Intron	gene|mRN	ID=gene-E	7	5.705714286	2395.6	0.638819602	AVSLFRA	53	763.4591	1	762.4388	.	L	Superscaf	84625026	0	0	0	0	0	BTP20220	Tissue specific
PWGGSYG	Superscaf	-	Superscaf	84654778	84654798	-1	1	7	-	-	gene	-	-	mRNA	NCP	Intron	gene|mRN	ID=gene-E	7	5.92	2598.8	0.639396854	P(+42.01)	51	383.1708	2	764.3129	Acetylati	P	Superscaf	84654822	0	0	BTP20220	0	0	0	Tissue specific
KSLRTVL	Superscaf	+	Superscaf	84789028	84789048	14	14	20	-	-	gene	-	-	mRNA	NCP	Intron	gene|mRN	ID=gene-E	7	6.381428571	2604.9	0.641996696	KSLRTVL	88	408.7674	2	815.5229	.	Y	Superscaf	84788989	0	0	0	0	BTP20220	0	Tissue specific
YGPLKN	Superscaf	-	Superscaf	84870826	84870843	-4	4	9	-	-	gene	-	-	mRNA	NCP	Intron	gene|mRN	ID=gene-E	6	6.51	2512.7	0.643580769	Y(+42.01)	84	367.1963	2	732.3806	Acetylati	A	Superscaf	84870867	0	0	0	0	0	BTP20220	Tissue specific
QEWGWAK	Superscaf	-	Superscaf	84917827	84917847	-33	33	39	-	-	-	-	-	-	NCP	IGR	-	ID=gene-E	7	6.054285714	3166.4	0.644490975	QEWGWAK	58	452.717	2	903.4239	.	L	Superscaf	84917865	0	0	0	0	0	BTP20220	Tissue specific
TPKNKK	Superscaf	-	Superscaf	84942931	84942948	-45	45	50	-	-	gene	-	-	mRNA	NCP	Intron	gene|mRN	ID=gene-E	6	7.91	2736.8	0.644977131	T(+42.01)	71	379.2343	2	756.4494	Acetylati	A	Superscaf	84943044	0	0	0	0	BTP20220	0	Tissue specific
SAPYYRQ	Superscaf	+	Superscaf	84963523	84963543	35	35	41	-	-	-	-	-	-	NCP	IGR	-	ID=gene-E	7	6.532857143	3210.6	0.645375909	SAPYYRQ	77	442.7153	2	883.4188	.	R	Superscaf	84963421	0	0	0	0	BTP20220	0	Tissue specific
KVVCKP	Superscaf	+	Superscaf	84977692	84977709	2	2	7	-	-	gene	-	-	mRNA	NCP	Intron	gene|mRN	ID=gene-E	6	5.133333333	1859.9	0.645650301	K(+42.01)	98	386.7214	2	771.4313	Acetylati	V	Superscaf	84977689	0	0	0	BTP20220	0	0	Tissue specific
KHVTPP	Superscaf	-	Superscaf	85062790	85062807	-15	15	20	-	-	gene	-	-	mRNA	NCP	Intron	gene|mRN	ID=gene-E	6	6.076666667	2160.4	0.647298281	KHVTPP	56	678.3918	1	677.386	.	R	Superscaf	85062807	0	0	0	0	0	BTP20220	Mixed
GAAGPSR	Superscaf	+	Superscaf	85086028	85086048	32	32	38	-	-	gene	-	-	mRNA	NCP	Intron	gene|mRN	ID=gene-E	7	6.674285714	2586.8	0.647748301	GAAGPSR	61	615.3323	1	614.3136	.	F	Superscaf	85085935	0	0	0	0	0	BTP20220	Tissue specific
TMAPKPK	Superscaf	-	Superscaf	85115773	85115793	-58	58	64	-	-	gene	-	-	mRNA	NCP	Intron	gene|mRN	ID=gene-E	7	7.197142857	2691.9	0.648324333	TM(+15.99	85	394.7282	2	787.4262	Oxidation	E	Superscaf	85115814	0	0	BTP20220	0	0	0	Tissue specific
TVVPPKF	Superscaf	+	Superscaf	85145440	85145460	33	33	39	-	-	-	-	-	-	NCP	IGR	-	ID=gene-E	7	4.907142857	1928.1	0.648898854	TVVPPKF	83	394.2378	2	786.4639	.	R	Superscaf	85145344	0	0	0	0	BTP20220	0	Tissue specific
AQTAKAP	Superscaf	-	Superscaf	85173955	85173975	-26	26	32	-	-	gene	-	-	mRNA	NCP	Intron	gene|mRN	ID=gene-E	7	6.611428571	2747.9	0.649451066	A(+42.01)	69	364.6995	2	727.3864	Acetylati	K	Superscaf	85174071	0	0	BTP20220	0	0	0	Tissue specific
AVLSRAF	Superscaf	-	Superscaf	85201774	85201794	-6	6	12	-	-	gene	-	-	mRNA	NCP	Intron	gene|mRN	ID=gene-E	7	5.705714286	2395.6	0.6499898	AVLSRAF	56	763.456	1	762.4388	.	R	Superscaf	85201794	0	0	0	0	0	BTP20220	Tissue specific
KNNTVVEL	Superscaf	-	Superscaf	85255213	85255236	-7	7	14	-	-	gene	-	-	mRNA	NCP	Intron	gene|mRN	ID=gene-E	8	4.53625	2838.2	0.651024682	KNNTVVEL	50	458.7565	2	915.5025	.	T	Superscaf	85255260	0	0	0	0	0	BTP20220	Tissue specific
FEMASF	Superscaf	-	Superscaf	85273213	85273230	-10	10	15	-	-	-	-	-	-	NCP	IGR	-	ID=gene-E	6	5.271666667	2266.5	0.651373265	FEM(+15.9	55	747.2979	1	746.2945	Oxidation	H	Superscaf	85273242	0	0	0	BTP20220	0	0	Tissue specific
TKFVLF	Superscaf	-	Superscaf	85378267	85378284	-27	27	32	-	-	gene	-	-	mRNA	NCP	Intron	gene|mRN	ID=gene-E	6	5.535	1994.1	0.653407706	TKFVLF	53	377.724	2	753.4425	.	S	Superscaf	85378311	0	BTP20220	0	0	BTP20220	0	Tissue specific
KGVRCR	Superscaf	-	Superscaf	85379812	85379829	-2	2	7	-	-	gene	-	-	mRNA	NCP	Intron	gene|mRN	ID=gene-E	6	7.041666667	2649	0.653437626	KGVRC(+57	53	388.2173	2	774.4283	Carbamido	Q	Superscaf	85379835	0	0	BTP20220	0	0	0	Tissue specific
LCLVCK	Superscaf	+	Superscaf	85455715	85455732	9	9	14	-	-	-	-	-	-	NCP	IGR	-	ID=gene-E	6	5.29	2346.6	0.654907539	LC(+57.02	99	792.4072	1	791.4034	Carbamido	T	Superscaf	85455691	0	0	0	0	0	BTP20220	Tissue specific
NPPGRPP	Superscaf	+	Superscaf	85544155	85544175	16	16	22	-	-	-	-	-	-	NCP	IGR	-	ID=gene-E	7	6.762857143	2892.4	0.656620239	NPPGRPP	87	367.6993	2	733.3871	.	I	Superscaf	85544110	0	0	0	0	0	BTP20220	Tissue specific
SVNPLVP	Superscaf	+	Superscaf	85629931	85629951	11	11	17	-	-	-	-	-	-	NCP	IGR	-	ID=gene-E	7	4.238571429	2008.3	0.658281349	SVNPLVP	80	363.212	2	724.4119	.	A	Superscaf	85629901	0	0	0	0	BTP20220	BTP20220	Tissue specific
PPPDKPPP	Superscaf	+	Superscaf	85634638	85634661	21	21	28	-	-	-	-	-	-	NCP	IGR	-	ID=gene-E	8	6.31375	2904.3	0.658372552	PPPDKPPP	95	422.7302	2	843.449	.	A	Superscaf	85634578	0	0	0	BTP20220	0	0	Tissue enhanced
KLGVLLAE	Superscaf	+	Superscaf	85669039	85669062	3	3	10	-	-	-	-	-	-	NCP	IGR	-	ID=gene-E	8	5.36125	2562.7	0.659038702	KLGVLLAE	59	421.7768	2	841.5273	.	K	Superscaf	85669033	0	0	BTP20220	0	0	0	Tissue specific
ADLDLTKR	Superscaf	-	Superscaf	85681540	85681563	-3	3	10	-	-	-	-	-	-	NCP	IGR	-	ID=gene-E	8	6.36875	3355.8	0.659280792	ADLDLTKR	99	466.2622	2	930.5134	.	H	Superscaf	85681569	0	0	0	BTP20220	0	0	Tissue specific
AEELTFP	Superscaf	+	Superscaf	85702468	85702488	30	30	36	-	-	gene	-	-	mRNA	NCP	Intron	gene|mRN	ID=gene-E	7	5.25	2638	0.659686077	A(+42.01)	72	424.7046	2	847.3963	Acetylati	L	Superscaf	85702381	0	0	BTP20220	0	0	0	Tissue specific
PGEPKPP	Superscaf	+	Superscaf	85851451	85851471	8	8	14	-	-	-	-	-	-	NCP	IGR	-	ID=gene-E	7	6.304285714	2540.8	0.662571233	P(+42.01)	50	382.2069	2	762.3912	Acetylati	K	Superscaf	85851430	0	0	0	BTP20220	0	0	Tissue specific
RPFCGT	Superscaf	+	Superscaf	85859146	85859163	12	12	17	-	-	-	-	-	-	NCP	IGR	-	ID=gene-E	6	6.676666667	2654.1	0.662720252	RPFC(+57.	50	369.177	2	736.3326	Carbamido	Y	Superscaf	85859113	0	0	0	BTP20220	0	0	Tissue specific
LEYLG	Superscaf	+	Superscaf	85863661	85863675	37	37	41	-	-	-	-	-	-	NCP	IGR	-	ID=gene-E	5	5.362	1889.1	0.662807688	LEYLG	75	594.3112	1	593.306	.	G	Superscaf	85863553	0	0	0	0	0	BTP20220	Tissue specific
EGVSPP	Superscaf	+	Superscaf	85928569	85928586	1	1	6	-	-	gene	-	-	mRNA	NCP	Intron	gene|mRN	ID=gene-E	6	4.578333333	1781	0.664064675	EGVSPP	69	585.2866	1	584.2806	.	E	Superscaf	85928569	0	0	0	0	0	BTP20220	Tissue specific
TKTTHGK	Superscaf	+	Superscaf	85930330	85930350	11	11	17	-	-	gene	-	-	mRNA	NCP	Intron	gene|mRN	ID=gene-E	7	7.518571429	3046.3	0.664098778	T(+42.01)	75	407.7298	2	813.4344	Acetylati	P	Superscaf	85930300	0	0	0	BTP20220	0	0	Tissue specific
GPSVMLK	Superscaf	-	Superscaf	85937659	85937679	-16	16	22	-	-	gene	-	-	mRNA	NCP	Intron	gene|mRN	ID=gene-E	7	5.631428571	2211.4	0.664240709	GPSVMLK	69	366.2067	2	730.4047	.	V	Superscaf	85937745	0	0	0	BTP20220	0	0	Tissue specific
AFLGYK	Superscaf	-	Superscaf	85989814	85989831	-39	39	44	-	-	gene	-	-	mRNA	NCP	Intron	gene|mRN	ID=gene-E	6	6.475	2277.3	0.665250726	AFLGYK	98	698.3859	1	697.3799	.	R	Superscaf	85989858	0	0	0	0	0	BTP20220	Tissue specific
EPKVHR	Superscaf	-	Superscaf	85998913	85998930	-18	18	23	-	-	gene	-	-	mRNA	NCP	Intron	gene|mRN	ID=gene-E	6	6.268333333	2499.8	0.665426934	E(+42.01)	63	404.2299	2	806.4399	Acetylati	V	Superscaf	85998957	0	0	0	0	BTP20220	0	Tissue specific
KPPPKP	Superscaf	-	Superscaf	86028031	86028048	-14	14	19	-	-	-	-	-	-	NCP	IGR	-	ID=gene-E	6	7.446666667	2286.4	0.665990824	K(+42.01)	84	353.2161	2	704.4221	Acetylati	T	Superscaf	86028051	0	0	0	0	0	BTP20220	Tissue specific
YSCDDSL	Superscaf	-	Superscaf	86044804	86044824	-4	4	10	-	-	-	-	-	-	NCP	IGR	-	ID=gene-E	7	4.851428571	2988.4	0.666315644	YSC(+57.0	53	430.1549	2	858.3065	Carbamido	G	Superscaf	86044824	0	0	0	0	BTP20220	0	Tissue specific
KEALTLL	Superscaf	+	Superscaf	86064157	86064177	7	7	13	-	-	gene	-	-	mRNA	NCP	Intron	gene|mRN	ID=gene-E	7	6.207142857	2662.9	0.666690428	KEALTLL	54	787.4899	1	786.485	.	G	Superscaf	86064139	0	0	0	0	BTP20220	0	Tissue specific
TGAGPSFP	Superscaf	-	Superscaf	86099326	86099349	-21	21	28	-	-	gene	-	-	mRNA	NCP	Intron	gene|mRN	ID=gene-E	8	6.03125	2749	0.6673715	TGAGPSFP	51	733.3495	1	732.3442	.	C	Superscaf	86099370	0	0	0	BTP20220	0	0	Tissue specific
APGAAAAGK	Superscaf	+	Superscaf	86135668	86135694	174	174	182	-	-	gene	-	-	mRNA	NCP	Intron	gene|mRN	ID=gene-E	9	6.453333333	2956.9	0.668075287	APGAAAAGK	57	357.1953	2	712.3867	.	A	Superscaf	86135149	0	0	0	0	BTP20220	0	Tissue specific
RAPPPGWP	Superscaf	+	Superscaf	86156413	86156436	10	10	17	-	-	-	-	-	-	NCP	IGR	-	ID=gene-E	8	6.73	3133.6	0.668477028	RAPPPGWP	50	439.2337	2	876.4606	.	D	Superscaf	86156386	0	0	0	0	BTP20220	0	Tissue specific
VPGAKPR	Superscaf	-	Superscaf	86193985	86194005	-3	3	9	-	-	-	-	-	-	NCP	IGR	-	ID=gene-E	7	6.441428571	2400.6	0.669204635	VPGAKPR	63	362.7216	2	723.4391	.	V	Superscaf	86194023	0	0	0	0	BTP20220	0	Tissue specific
FPSSNL	Superscaf	-	Superscaf	86233063	86233080	-28	28	33	-	-	gene	-	-	mRNA	NCP	Intron	gene|mRN	ID=gene-E	6	5.755	2441.7	0.669961407	FPSSNL	97	664.3299	1	663.3228	.	R	Superscaf	86233083	0	0	0	0	0	BTP20220	Tissue specific
LDFTGHL	Superscaf	-	Superscaf	86271379	86271399	-18	18	24	-	-	gene	-	-	mRNA	NCP	Intron	gene|mRN	ID=gene-E	7	5.785714286	2820.2	0.670703422	LDFTGHL	94	802.4055	1	801.4021	.	Y	Superscaf	86271435	0	0	0	0	0	BTP20220	Tissue specific
AAPLTK	Superscaf	-	Superscaf	86292124	86292141	-155	155	160	-	-	-	-	-	-	NCP	IGR	-	ID=gene-E	6	6.765	2179.3	0.671105163	A(+42.01)	73	642.3794	1	641.3748	Acetylati	R	Superscaf	86292243	0	0	0	BTP20220	0	0	Tissue specific
LDVTLP	Superscaf	-	Superscaf	86293561	86293578	-25	25	30	-	exon	gene	three_pri	-	mRNA	NCP	3UTR	exon|gene	ID=exon-E	6	4.626666667	1853.2	0.671132991	LDVTLP	71	657.3793	1	656.3745	.	A	Superscaf	86293605	0	0	0	0	0	BTP20220	Tissue specific
KTHFGP	Superscaf	-	Superscaf	86300686	86300703	-25	25	30	-	-	gene	-	-	mRNA	NCP	Intron	gene|mRN	ID=gene-E	6	6.935	2507.7	0.671270972	KTHFGP	50	686.3511	1	685.3547	.	E	Superscaf	86300736	0	0	0	0	0	BTP20220	Tissue specific
LFAPMK	Superscaf	+	Superscaf	86309221	86309238	7	7	12	-	-	gene	-	-	mRNA	NCP	Intron	gene|mRN	ID=gene-E	6	6.545	2229.4	0.671436258	L(+42.01)	70	374.7077	2	747.3989	Acetylati	L	Superscaf	86309203	0	0	0	0	0	BTP20220	Tissue specific
VGLFLVK	Superscaf	+	Superscaf	86381608	86381628	17	17	23	-	-	-	-	-	-	NCP	IGR	-	ID=gene-E	7	4.735714286	1859.9	0.672838081	V(+42.01)	54	817.5155	1	816.5109	Acetylati	R	Superscaf	86381560	0	0	0	0	0	BTP20220	Tissue specific
PAPGAVF	Superscaf	-	Superscaf	86395036	86395056	-16	16	22	-	-	-	-	-	-	NCP	IGR	-	ID=gene-E	7	5.155714286	1982.1	0.673098123	P(+42.01)	56	700.3636	1	699.3591	Acetylati	S	Superscaf	86395074	0	0	0	0	0	BTP20220	Tissue specific
KLLPKKTE	Superscaf	-	Superscaf	86428444	86428467	-2	2	9	-	-	gene	-	-	mRNA	NCP	Intron	gene|mRN	ID=gene-E	8	7.15375	3272.4	0.673745092	K(+42.01)	99	499.8148	2	997.6171	Acetylati	K	Superscaf	86428494	0	0	0	0	0	BTP20220	Mixed
TYSLGGTL	Superscaf	-	Superscaf	86437108	86437131	-25	25	32	-	-	gene	-	-	mRNA	NCP	Intron	gene|mRN	ID=gene-E	8	6.0375	2939.3	0.673912876	TYSLGGTL	57	811.4182	1	810.4123	.	C	Superscaf	86437152	BTP20220	0	0	0	0	0	Tissue specific
KKNSFHR	Superscaf	-	Superscaf	86510341	86510361	-1	1	7	-	-	-	-	-	-	NCP	IGR	-	ID=gene-E	7	7.771428571	3572.8	0.675331082	KKNSFHR	53	458.7645	2	915.5038	.	K	Superscaf	86510439	0	0	0	0	0	BTP20220	Tissue specific
MAVELH	Superscaf	-	Superscaf	86522863	86522880	-1	1	6	-	-	-	-	-	-	NCP	IGR	-	ID=gene-E	6	4.76	2081.4	0.675573579	M(+15.99)	76	379.1803	2	756.3476	Oxidation	M	Superscaf	86523069	0	0	BTP20220	0	0	0	Tissue specific
AVLQPSHL	Superscaf	+	Superscaf	86548813	86548836	27	27	34	-	-	gene	-	-	mRNA	NCP	Intron	gene|mRN	ID=gene-E	8	5.4	2969.3	0.676076119	A(+42.01)	61	453.7624	2	905.4971	Acetylati	Q	Superscaf	86548735	0	0	0	0	0	BTP20220	Tissue specific
NAPRVVK	Superscaf	-	Superscaf	86553538	86553558	-1	1	7	-	-	gene	-	-	mRNA	NCP	Intron	gene|mRN	ID=gene-E	7	5.461428571	2362.6	0.676167621	N(+42.01)	74	413.2496	2	824.4868	Acetylati	N	Superscaf	86553564	0	0	BTP20220	0	0	0	Tissue specific
LGPEFP	Superscaf	+	Superscaf	86568583	86568600	24	24	29	-	-	gene	-	-	mRNA	NCP	Intron	gene|mRN	ID=gene-E	6	5.541666667	2138.4	0.676458978	LGPEFP	67	659.3386	1	658.3326	.	T	Superscaf	86568514	0	0	0	0	BTP20220	0	Tissue specific
LVMMPQ	Superscaf	-	Superscaf	86569756	86569773	-13	13	18	-	-	gene	-	-	mRNA	NCP	Intron	gene|mRN	ID=gene-E	6	4.905	2044.5	0.676481694	LVM(+15.9	61	750.3614	1	749.3452	Oxidation	T	Superscaf	86569863	0	0	BTP20220	0	0	0	Tissue specific
KPFVVLP	Superscaf	-	Superscaf	86609236	86609256	-2	2	8	-	-	-	-	-	-	NCP	IGR	-	ID=gene-E	7	4.828571429	1884	0.677246251	KPFVVLP	53	400.2534	2	798.5003	.	P	Superscaf	86609298	0	0	0	0	0	BTP20220	Tissue specific
KELCKPQ	Superscaf	-	Superscaf	86647666	86647686	-29	29	35	-	-	gene	-	-	mRNA	NCP	Intron	gene|mRN	ID=gene-E	7	6.521428571	3249.5	0.677990474	KELC(+57.	51	451.7413	2	901.4691	Carbamido	T	Superscaf	86647701	0	0	0	BTP20220	0	0	Tissue specific
VDLTVFP	Superscaf	+	Superscaf	86699239	86699259	2	2	8	-	-	gene	-	-	mRNA	NCP	Intron	gene|mRN	ID=gene-E	7	3.894285714	1887.2	0.678989219	VDLTVFP	70	790.4314	1	789.4272	.	T	Superscaf	86699236	0	0	0	0	BTP20220	BTP20220	Tissue specific
ASLALLR	Superscaf	-	Superscaf	86775181	86775201	-5	5	11	-	-	-	-	-	-	NCP	IGR	-	ID=gene-E	7	6.631428571	2715	0.680459888	A(+42.01)	70	393.2458	2	784.4807	Acetylati	F	Superscaf	86775309	0	BTP20220	0	0	0	0	Tissue specific
RGTNAPG	Superscaf	-	Superscaf	86779120	86779140	-4	4	10	-	-	-	-	-	-	NCP	IGR	-	ID=gene-E	7	6.708571429	2886.3	0.680536169	RGTNAPG	50	672.3542	1	671.3351	.	L	Superscaf	86779149	0	0	0	BTP20220	0	0	Tissue specific
ELAPF	Superscaf	-	Superscaf	86790262	86790276	-1	1	5	-	-	-	-	-	-	NCP	IGR	-	ID=gene-E	5	5.4	1815	0.680751941	ELAPF	50	576.312	1	575.2955	.	E	Superscaf	86790381	0	BTP20220	0	0	0	0	Tissue specific
LLGGLA	Superscaf	+	Superscaf	86799925	86799942	2	2	7	-	-	-	-	-	-	NCP	IGR	-	ID=gene-E	6	5.983333333	1966.1	0.680939072	LLGGLA	66	543.3486	1	542.3428	.	L	Superscaf	86799922	0	0	BTP20220	BTP20220	0	0	Tissue specific
MQSLK	Superscaf	-	Superscaf	86829091	86829105	-4	4	8	-	-	gene	-	-	mRNA	NCP	Intron	gene|mRN	ID=gene-E	5	6.56	2187.4	0.681503891	M(+42.01)	97	648.3357	1	647.3312	Acetylati	W	Superscaf	86829114	0	0	BTP20220	0	0	0	Tissue specific
KPFAQK	Superscaf	-	Superscaf	86851627	86851644	-7	7	12	-	-	gene	-	-	mRNA	NCP	Intron	gene|mRN	ID=gene-E	6	7.155	2583.6	0.681940316	KPFAQK	59	359.7109	2	717.4173	.	K	Superscaf	86851668	0	0	BTP20220	BTP20220	0	0	Tissue specific
YKLPPGP	Superscaf	-	Superscaf	86861347	86861367	-11	11	17	-	-	gene	-	-	mRNA	NCP	Intron	gene|mRN	ID=gene-E	7	6.607142857	2590.8	0.68212855	YKLPPGP	52	386.2172	2	770.4326	.	R	Superscaf	86861805	0	0	BTP20220	0	0	0	Tissue specific
TGTSVPR	Superscaf	-	Superscaf	86964205	86964225	-37	37	43	-	-	gene	-	-	mRNA	NCP	Intron	gene|mRN	ID=gene-E	7	5.967142857	2461.9	0.684120465	T(+42.01)	57	380.2023	2	758.3923	Acetylati	H	Superscaf	86964273	0	0	BTP20220	0	0	0	Tissue specific
SASFSRP	Superscaf	+	Superscaf	87038116	87038136	2	2	8	-	-	gene	-	-	mRNA	NCP	Intron	gene|mRN	ID=gene-E	7	6.514285714	2835.1	0.685551801	SASFSRP	70	376.1884	2	750.366	.	T	Superscaf	87038113	0	0	0	BTP20220	0	0	Tissue specific
PPPFLG	Superscaf	-	Superscaf	87045001	87045018	-47	47	52	-	-	gene	-	-	mRNA	NCP	Intron	gene|mRN	ID=gene-E	6	6.055	2050.3	0.685685134	PPPFLG	88	627.3483	1	626.3428	.	E	Superscaf	87045027	BTP20220	0	BTP20220	BTP20220	0	0	Tissue specific
RLGTLVL	Superscaf	+	Superscaf	87116257	87116277	5	5	11	-	-	gene	-	-	mRNA	NCP	Intron	gene|mRN	ID=gene-E	7	5.885714286	2403.8	0.687065054	RLGTLVL	60	386.2616	2	770.5014	.	I	Superscaf	87116245	0	0	BTP20220	0	0	0	Tissue specific
ERPLDW	Superscaf	+	Superscaf	87180307	87180324	43	43	48	-	-	gene	-	-	mRNA	NCP	Intron	gene|mRN	ID=gene-E	6	5.853333333	2703.2	0.688305426	ERPLDW	88	408.2005	2	814.3973	.	V	Superscaf	87180181	0	0	0	0	BTP20220	0	Tissue specific
KLMKKPP	Superscaf	-	Superscaf	87195559	87195579	-5	5	11	-	-	gene	-	-	mRNA	NCP	Intron	gene|mRN	ID=gene-E	7	7.65	2804.9	0.688600791	KLMKKPP	51	421.2772	2	840.5255	.	L	Superscaf	87195600	0	0	0	0	0	BTP20220	Tissue specific
AALKP	Superscaf	-	Superscaf	87234100	87234114	-5	5	9	-	-	gene	-	-	mRNA	NCP	Intron	gene|mRN	ID=gene-E	5	6.812	1781.8	0.689347163	AALKP	95	499.321	1	498.3166	.	F	Superscaf	87234120	0	BTP20220	0	0	0	0	Tissue enhanced
MEDLLDLGE	Superscaf	+	Superscaf	87309652	87309684	7	7	17	-	-	-	-	-	-	NCP	IGR	-	ID=gene-E	11	5.092727273	4477.3	0.690810279	M(+42.01)	94	681.3141	2	1360.618	Acetylati	R	Superscaf	87309634	0	0	0	0	BTP20220	0	Tissue specific
HLLEHLE	Superscaf	-	Superscaf	87328861	87328881	-59	59	65	-	-	gene	-	-	mRNA	NCP	Intron	gene|mRN	ID=gene-E	7	5.651428571	3150.6	0.691182274	HLLEHLE	74	445.7376	2	889.4658	.	Q	Superscaf	87328962	0	0	0	BTP20220	0	0	Tissue specific
SAQKNPP	Superscaf	+	Superscaf	87345034	87345054	14	14	20	-	-	gene	-	-	mRNA	NCP	Intron	gene|mRN	ID=gene-E	7	6.442857143	3045.3	0.691495475	SAQKNPP	77	741.3907	1	740.3817	.	L	Superscaf	87344995	0	0	0	0	BTP20220	0	Tissue specific
PHQDDD	Superscaf	+	Superscaf	87345208	87345225	8	8	13	-	-	gene	-	-	mRNA	NCP	Intron	gene|mRN	ID=gene-E	6	4.741666667	2802.3	0.691498845	PHQDDD	50	363.6312	2	725.2617	.	R	Superscaf	87345187	0	0	0	BTP20220	0	0	Tissue specific
FSLLY	Superscaf	+	Superscaf	87353656	87353670	18	18	22	-	-	gene	-	-	mRNA	NCP	Intron	gene|mRN	ID=gene-E	5	5.756	1937.1	0.691662446	FSLLY	75	642.3456	1	641.3425	.	H	Superscaf	87353605	0	BTP20220	0	0	0	0	Tissue specific
APTKNAK	Superscaf	-	Superscaf	87356272	87356292	-27	27	33	-	-	gene	-	-	mRNA	NCP	Intron	gene|mRN	ID=gene-E	7	7.108571429	2891	0.691713107	APTKNAK	51	365.2169	2	728.418	.	T	Superscaf	87356409	0	0	0	0	0	BTP20220	Tissue specific
LFFVPPP	Superscaf	+	Superscaf	87366970	87366990	13	13	19	-	-	gene	-	-	mRNA	NCP	Intron	gene|mRN	ID=gene-E	7	5.12	2140.4	0.691920281	LFFVPPP	57	408.7319	2	815.4581	.	K	Superscaf	87366934	0	0	0	BTP20220	0	0	Tissue specific
SLDPAYP	Superscaf	+	Superscaf	87412447	87412467	102	102	108	-	-	gene	-	-	mRNA	NCP	Intron	gene|mRN	ID=gene-E	7	5.558571429	2593.9	0.692800973	SLDPAYP	51	762.3805	1	761.3596	.	E	Superscaf	87412144	BTP20220	0	0	0	0	0	Tissue specific
LYSSWYL	Superscaf	-	Superscaf	87447805	87447825	-24	24	30	-	-	-	-	-	-	NCP	IGR	-	ID=gene-E	7	5.79	2919.2	0.693485705	LYSSWYL	55	931.4543	1	930.4487	.	I	Superscaf	87447855	0	BTP20220	0	0	0	0	Tissue specific
FARAQR	Superscaf	+	Superscaf	87471826	87471843	12	12	17	-	-	-	-	-	-	NCP	IGR	-	ID=gene-E	6	7.448333333	2918.3	0.693950888	FARAQR	54	374.7137	2	747.414	.	H	Superscaf	87471793	0	0	0	0	0	BTP20220	Mixed
TAAKQVK	Superscaf	-	Superscaf	87545524	87545544	-16	16	22	-	-	gene	-	-	mRNA	NCP	Intron	gene|mRN	ID=gene-E	7	6.242857143	2567.6	0.695378099	TAAKQVK	59	373.2298	2	744.4493	.	T	Superscaf	87545670	0	BTP20220	0	0	BTP20220	BTP20220	Tissue specific
WEVLR	Superscaf	+	Superscaf	87552217	87552231	24	24	28	-	-	gene	-	-	mRNA	NCP	Intron	gene|mRN	ID=gene-E	5	5.17	1954.3	0.695507714	W(+42.01)	66	744.4044	1	743.3966	Acetylati	L	Superscaf	87552148	0	BTP20220	0	0	BTP20220	0	Tissue specific
PPETTAHP	Superscaf	+	Superscaf	87586966	87586989	17	17	24	-	-	-	-	-	-	NCP	IGR	-	ID=gene-E	8	6.09875	3163.7	0.696180651	PPETTAHP	59	425.214	2	848.4028	.	V	Superscaf	87586918	0	0	0	0	BTP20220	0	Tissue specific
KMTAPPR	Superscaf	+	Superscaf	87638698	87638718	24	24	30	-	-	-	-	-	-	NCP	IGR	-	ID=gene-E	7	7.342857143	2872.3	0.697182476	KMTAPPR	63	400.7282	2	799.4374	.	F	Superscaf	87638629	0	BTP20220	BTP20220	0	BTP20220	BTP20220	Tissue specific
SPPLVVP	Superscaf	-	Superscaf	87656443	87656463	-30	30	36	-	-	-	-	-	-	NCP	IGR	-	ID=gene-E	7	4.365714286	1749	0.69752612	SPPLVVP	58	708.4263	1	707.4218	.	G	Superscaf	87656526	0	0	0	0	0	BTP20220	Tissue specific
KDYGVP	Superscaf	+	Superscaf	87657865	87657882	16	16	21	-	-	-	-	-	-	NCP	IGR	-	ID=gene-E	6	5.106666667	1974.1	0.697553658	KDYGVP	50	678.3432	1	677.3384	.	L	Superscaf	87657820	0	0	0	BTP20220	0	BTP20220	Tissue specific
TEQALSAF	Superscaf	-	Superscaf	87700450	87700473	-35	35	42	-	-	-	-	-	-	NCP	IGR	-	ID=gene-E	8	5.5725	3180.5	0.698378346	T(+42.01)	55	454.7197	2	907.4287	Acetylati	A	Superscaf	87700533	0	0	0	0	0	BTP20220	Tissue specific
AALVEC	Superscaf	-	Superscaf	87714082	87714099	-62	62	67	-	-	-	-	-	-	NCP	IGR	-	ID=gene-E	6	4.376666667	1987.2	0.698642338	AALVEC(+5	58	662.3303	1	661.3105	Carbamido	D	Superscaf	87714276	0	0	BTP20220	0	0	0	Tissue specific
SELQNLK	Superscaf	-	Superscaf	87728878	87728898	-5	5	11	-	-	-	-	-	-	NCP	IGR	-	ID=gene-E	7	5.951428571	3191.5	0.698928873	SELQNLK	98	416.2317	2	830.4498	.	I	Superscaf	87728991	0	0	0	BTP20220	0	0	Tissue specific
LLFKAGP	Superscaf	+	Superscaf	87746842	87746862	15	15	21	-	-	-	-	-	-	NCP	IGR	-	ID=gene-E	7	6.495714286	2508.6	0.699276758	LLFKAGP	87	373.2316	2	744.4534	.	E	Superscaf	87746800	0	BTP20220	0	0	0	0	Tissue specific
KLDSDGLL	Superscaf	-	Superscaf	87773212	87773235	-26	26	33	-	-	gene	-	-	mRNA	NCP	Intron	gene|mRN	ID=gene-E	8	5.65875	3032.3	0.699787431	K(+42.01)	71	451.744	2	901.4756	Acetylati	N	Superscaf	87773364	0	0	BTP20220	0	0	0	Tissue specific
KLERQPGL	Superscaf	+	Superscaf	87778336	87778359	24	24	31	-	-	gene	-	-	mRNA	NCP	Intron	gene|mRN	ID=gene-E	8	6.7	3494.9	0.69988666	KLERQPGL	61	470.782	2	939.5501	.	G	Superscaf	87778267	0	BTP20220	0	0	0	0	Tissue specific
ALPHALL	Superscaf	-	Superscaf	87779392	87779412	-100	100	106	-	-	gene	-	-	mRNA	NCP	Intron	gene|mRN	ID=gene-E	7	6.267142857	2639.9	0.699907111	ALPHALL	52	734.4547	1	733.4486	.	S	Superscaf	87779541	0	0	0	0	0	BTP20220	Tissue enhanced
LLARAEVV	Superscaf	-	Superscaf	87794680	87794703	-1	1	8	-	-	gene	-	-	mRNA	NCP	Intron	gene|mRN	ID=gene-E	8	4.7475	2403.7	0.700203173	LLARAEVV	63	435.7729	2	869.5334	.	L	Superscaf	87794895	0	0	BTP20220	0	0	0	Tissue specific
KTALRSP	Superscaf	+	Superscaf	87816265	87816285	24	24	30	-	-	gene	-	-	mRNA	NCP	Intron	gene|mRN	ID=gene-E	7	7.287142857	2900.2	0.700621181	KTALRSP	73	386.7364	2	771.4603	.	K	Superscaf	87816196	0	0	0	0	0	BTP20220	Tissue specific
KPGVPGKP	Superscaf	+	Superscaf	87822226	87822249	8	8	15	-	-	gene	-	-	mRNA	NCP	Intron	gene|mRN	ID=gene-E	8	6.29	2543.6	0.70073662	KPGVPGKP	60	390.2394	2	778.4701	.	G	Superscaf	87822205	0	0	0	0	0	BTP20220	Tissue specific
KGVHPP	Superscaf	-	Superscaf	87829828	87829845	-1	1	6	-	-	gene	-	-	mRNA	NCP	Intron	gene|mRN	ID=gene-E	6	5.983333333	2060.2	0.700883838	KGVHPP	69	634.365	1	633.3598	.	K	Superscaf	87829911	0	0	0	0	0	BTP20220	Tissue specific
NLPGSFP	Superscaf	+	Superscaf	87835753	87835773	24	24	30	-	-	gene	-	-	mRNA	NCP	Intron	gene|mRN	ID=gene-E	7	5.874285714	2693	0.700998579	NLPGSFP	94	731.3686	1	730.3649	.	M	Superscaf	87835684	0	0	0	0	0	BTP20220	Tissue specific
LDDKLL	Superscaf	+	Superscaf	87867616	87867633	52	52	57	-	-	-	-	-	-	NCP	IGR	-	ID=gene-E	6	5.603333333	2351.6	0.701615628	LDDKLL	56	358.7116	2	715.4116	.	R	Superscaf	87867463	0	0	0	0	0	BTP20220	Tissue specific
GLYGPGD	Superscaf	+	Superscaf	87877642	87877662	28	28	34	-	-	-	-	-	-	NCP	IGR	-	ID=gene-E	7	5.545714286	2453.7	0.701809788	G(+42.01)	52	720.3334	1	719.3126	Acetylati	S	Superscaf	87877561	BTP20220	0	0	0	0	0	Tissue specific
PSLFPP	Superscaf	+	Superscaf	88003363	88003380	4	4	9	-	-	-	-	-	-	NCP	IGR	-	ID=gene-E	6	6.006666667	2136.4	0.70424446	P(+42.01)	66	699.3782	1	698.3639	Acetylati	I	Superscaf	88003354	0	0	0	0	0	BTP20220	Tissue specific
LHVLLST	Superscaf	-	Superscaf	88010278	88010298	-3	3	9	-	-	-	-	-	-	NCP	IGR	-	ID=gene-E	7	5.391428571	2460.8	0.704378374	LHVLLST	71	782.4745	1	781.4698	.	K	Superscaf	88010352	0	0	0	0	0	BTP20220	Tissue specific
WVLGGSL	Superscaf	-	Superscaf	88086664	88086684	-29	29	35	-	-	-	-	-	-	NCP	IGR	-	ID=gene-E	7	5.067142857	2211.4	0.70585764	WVLGGSL	71	731.4056	1	730.4014	.	L	Superscaf	88087098	0	0	0	BTP20220	0	0	Tissue specific
MQPPP	Superscaf	+	Superscaf	88089691	88089705	15	15	19	-	-	gene	-	-	mRNA	NCP	Intron	gene|mRN	ID=gene-E	5	6.06	1994.4	0.70591626	M(+42.01)	99	611.2842	1	610.2784	Acetylati	Y	Superscaf	88089649	0	0	0	0	BTP20220	0	Mixed
SPTGSLP	Superscaf	+	Superscaf	88149997	88150017	34	34	40	-	-	gene	-	-	mRNA	NCP	Intron	gene|mRN	ID=gene-E	7	6.062857143	2489.8	0.707084126	SPTGSLP	64	658.3364	1	657.3333	.	C	Superscaf	88149898	0	0	0	0	0	BTP20220	Tissue specific
TLHVSSQH	Superscaf	-	Superscaf	88164043	88164066	-144	144	151	-	-	-	-	-	-	NCP	IGR	-	ID=gene-E	8	5.5875	3367.8	0.707356137	TLHVSSQH	95	454.7313	2	907.4512	.	K	Superscaf	88164066	0	0	0	0	BTP20220	0	Tissue specific
GPPSLR	Superscaf	+	Superscaf	88166137	88166154	28	28	33	-	-	-	-	-	-	NCP	IGR	-	ID=gene-E	6	6.831666667	2357.7	0.707396688	GPPSLR	60	626.3595	1	625.3547	.	A	Superscaf	88166056	0	0	0	BTP20220	0	0	Tissue specific
VDKPLR	Superscaf	-	Superscaf	88169977	88169994	-45	45	50	-	-	-	-	-	-	NCP	IGR	-	ID=gene-E	6	5.958333333	2219.5	0.707471052	VDKPLR	56	364.2248	2	726.4388	.	A	Superscaf	88170093	0	0	0	0	0	BTP20220	Tissue specific
RVTKLLT	Superscaf	-	Superscaf	88242130	88242150	-23	23	29	-	-	-	-	-	-	NCP	IGR	-	ID=gene-E	7	6.502857143	2619	0.708868344	R(+42.01)	54	436.7802	2	871.5491	Acetylati	R	Superscaf	88242186	0	0	0	BTP20220	0	0	Tissue specific
VCVLGAGK	Superscaf	+	Superscaf	88291177	88291200	33	33	40	-	-	gene	-	-	mRNA	NCP	Intron	gene|mRN	ID=gene-E	8	4.8375	2313.4	0.709818172	VC(+57.02	76	402.2234	2	802.4371	Carbamido	L	Superscaf	88291081	0	0	BTP20220	0	0	0	Tissue specific
FPGAFLLG	Superscaf	+	Superscaf	88295857	88295880	43	43	50	-	-	gene	-	-	mRNA	NCP	Intron	gene|mRN	ID=gene-E	8	5.8975	2724.9	0.709908804	FPGAFLLG	55	821.4565	1	820.4483	.	N	Superscaf	88295731	0	0	0	0	BTP20220	0	Tissue specific
TGQLKSH	Superscaf	+	Superscaf	88302940	88302960	5	5	11	-	-	gene	-	-	mRNA	NCP	Intron	gene|mRN	ID=gene-E	7	6.734285714	3130.4	0.710045971	T(+42.01)	59	406.7137	2	811.4188	Acetylati	N	Superscaf	88302928	0	0	0	0	0	BTP20220	Tissue specific
QVEPP	Superscaf	+	Superscaf	88328761	88328775	16	16	20	-	-	-	-	-	-	NCP	IGR	-	ID=gene-E	5	4.294	1711	0.710546012	QVEPP	62	569.2906	1	568.2856	.	G	Superscaf	88328716	0	0	0	BTP20220	0	0	Tissue specific
PLQVPEPP	Superscaf	+	Superscaf	88382956	88382979	7	7	14	-	-	-	-	-	-	NCP	IGR	-	ID=gene-E	8	5.00625	2739.2	0.711595534	PLQVPEPP	74	438.7485	2	875.4752	.	L	Superscaf	88382938	BTP20220	0	0	0	0	0	Tissue specific
LYFVVL	Superscaf	-	Superscaf	88390039	88390056	-22	22	27	-	-	-	-	-	-	NCP	IGR	-	ID=gene-E	6	3.85	1553.7	0.711732701	LYFVVL	53	753.4595	1	752.4472	.	S	Superscaf	88390317	0	0	0	BTP20220	0	0	Tissue specific
PWVAEKQ	Superscaf	+	Superscaf	88410019	88410039	32	32	38	-	-	-	-	-	-	NCP	IGR	-	ID=gene-E	7	5.26	2679.9	0.712119628	P(+42.01)	65	450.2282	2	898.4548	Acetylati	K	Superscaf	88409926	0	0	0	0	0	BTP20220	Tissue specific
LTVGLVPL	Superscaf	+	Superscaf	88424230	88424253	52	52	59	-	-	-	-	-	-	NCP	IGR	-	ID=gene-E	8	4.5925	2092.4	0.712394833	LTVGLVPL	64	406.2662	2	810.5215	.	K	Superscaf	88424077	0	0	0	0	0	BTP20220	Tissue specific
LVSKASLV	Superscaf	-	Superscaf	88433770	88433793	-5	5	12	-	-	-	-	-	-	NCP	IGR	-	ID=gene-E	8	4.885	2253.3	0.712579582	LVSKASLV	83	408.7621	2	815.5117	.	W	Superscaf	88433820	0	0	0	0	0	BTP20220	Tissue specific
VPTAASPL	Superscaf	+	Superscaf	88451779	88451802	24	24	31	-	-	-	-	-	-	NCP	IGR	-	ID=gene-E	8	5.35375	2431.7	0.712928338	VPTAASPL	64	378.2175	2	754.4225	.	G	Superscaf	88451710	0	0	0	0	0	BTP20220	Tissue specific
KLDHPTP	Superscaf	+	Superscaf	88476319	88476339	22	22	28	-	-	gene	-	-	mRNA	NCP	Intron	gene|mRN	ID=gene-E	7	6.487142857	2925.3	0.713403572	KLDHPTP	50	404.2144	2	806.4286	.	V	Superscaf	88476256	BTP20220	0	0	0	0	0	Tissue specific
GPPLLSF	Superscaf	+	Superscaf	88482910	88482930	9	9	15	-	-	gene	-	-	mRNA	NCP	Intron	gene|mRN	ID=gene-E	7	5.955714286	2449.7	0.713531211	GPPLLSF	59	365.7133	2	729.4061	.	S	Superscaf	88482886	0	0	0	BTP20220	0	0	Tissue specific
LNYDAL	Superscaf	-	Superscaf	88490755	88490772	-6	6	11	-	-	gene	-	-	mRNA	NCP	Intron	gene|mRN	ID=gene-E	6	5.336666667	2485.8	0.713683135	LNYDAL	61	708.3546	1	707.349	.	H	Superscaf	88490844	0	BTP20220	0	0	0	0	Tissue specific
KETSHPPP	Superscaf	-	Superscaf	88526578	88526601	-2	2	9	-	-	gene	-	-	mRNA	NCP	Intron	gene|mRN	ID=gene-E	8	6.4575	3306.7	0.714376871	KETSHPPP	60	446.723	2	891.445	.	R	Superscaf	88526757	0	BTP20220	0	0	0	0	Tissue specific
RSPLQRF	Superscaf	-	Superscaf	88544902	88544922	-7	7	13	-	-	gene	-	-	mRNA	NCP	Intron	gene|mRN	ID=gene-E	7	7.23	3369.9	0.714731728	RSPLQRF	71	452.2687	2	902.5086	.	L	Superscaf	88544937	0	0	0	BTP20220	0	0	Tissue specific
AFKPPPPP	Superscaf	-	Superscaf	88546402	88546425	-1	1	8	-	-	gene	-	-	mRNA	NCP	Intron	gene|mRN	ID=gene-E	8	6.5925	2854.1	0.714760776	A(+42.01)	67	446.7488	2	891.4854	Acetylati	A	Superscaf	88546470	0	BTP20220	0	0	0	0	Tissue enhanced
CDMSQPP	Superscaf	+	Superscaf	88569412	88569432	35	35	41	-	-	gene	-	-	mRNA	NCP	Intron	gene|mRN	ID=gene-E	7	5.381428571	3037.6	0.715206381	C(+57.02)	50	446.6567	2	891.3102	Carbamido	A	Superscaf	88569310	0	0	0	0	BTP20220	0	Tissue specific
EGPGDN	Superscaf	+	Superscaf	88594672	88594689	3	3	8	-	-	gene	-	-	mRNA	NCP	Intron	gene|mRN	ID=gene-E	6	4.973333333	2365.7	0.715695558	E(+42.01)	57	630.2354	1	629.2292	Acetylati	Y	Superscaf	88594666	BTP20220	0	0	0	0	0	Tissue specific
KPDVPPP	Superscaf	+	Superscaf	88602637	88602657	32	32	38	-	-	-	-	-	-	NCP	IGR	-	ID=gene-E	7	5.415714286	2229.5	0.715849803	KPDVPPP	51	375.2108	2	748.4119	.	D	Superscaf	88602544	BTP20220	0	0	0	0	0	Tissue specific
KARLPLR	Superscaf	+	Superscaf	88619383	88619403	6	6	12	-	-	-	-	-	-	NCP	IGR	-	ID=gene-E	7	7.934285714	3121.5	0.716174103	KARLPLR	77	427.2909	2	852.5657	.	D	Superscaf	88619368	0	BTP20220	0	0	0	0	Tissue specific
HMALKKK	Superscaf	+	Superscaf	88624402	88624422	15	15	21	-	-	gene	-	-	mRNA	NCP	Intron	gene|mRN	ID=gene-E	7	7.794285714	3061.1	0.716271299	HM(+15.99	85	436.2603	2	870.5109	Oxidation	P	Superscaf	88624360	0	0	BTP20220	0	0	0	Tissue specific
RAGVLAH	Superscaf	+	Superscaf	88721056	88721076	25	25	31	-	-	-	-	-	-	NCP	IGR	-	ID=gene-E	7	6.048571429	2541.8	0.718143069	RAGVLAH	79	723.4277	1	722.4188	.	L	Superscaf	88720984	0	0	0	0	0	BTP20220	Tissue specific
KPHLVSH	Superscaf	-	Superscaf	88745671	88745691	-6	6	12	-	-	gene	-	-	mRNA	NCP	Intron	gene|mRN	ID=gene-E	7	6.125714286	2782	0.718619755	KPHLVSH	55	409.2404	2	816.4606	.	T	Superscaf	88745787	0	0	BTP20220	0	0	0	Tissue specific
TGVSLLF	Superscaf	+	Superscaf	88796023	88796043	55	55	61	-	-	gene	-	-	mRNA	NCP	Intron	gene|mRN	ID=gene-E	7	5.088571429	2172.4	0.719594855	TGVSLLF	50	368.7192	2	735.4167	.	T	Superscaf	88795861	0	0	0	0	0	BTP20220	Tissue specific
NKYLGY	Superscaf	+	Superscaf	88807018	88807035	13	13	18	-	-	gene	-	-	mRNA	NCP	Intron	gene|mRN	ID=gene-E	6	6.403333333	2634.8	0.719807781	NKYLGY	82	757.3748	1	756.3806	.	S	Superscaf	88806982	0	0	0	0	0	BTP20220	Tissue specific
VSVLL	Superscaf	+	Superscaf	88846630	88846644	43	43	47	-	-	-	-	-	-	NCP	IGR	-	ID=gene-E	5	3.528	1090.2	0.720574894	VSVLL	96	530.3527	1	529.3475	.	R	Superscaf	88846504	BTP20220	0	0	0	0	0	Mixed
KAATN	Superscaf	-	Superscaf	88927633	88927647	-43	43	47	-	-	-	-	-	-	NCP	IGR	-	ID=gene-E	5	6.744	2085.2	0.722143572	KAATN	70	504.2785	1	503.2704	.	F	Superscaf	88927662	0	0	0	BTP20220	0	0	Tissue specific
YHTKSR	Superscaf	-	Superscaf	88935832	88935849	-11	11	16	-	-	-	-	-	-	NCP	IGR	-	ID=gene-E	6	7.66	2977.3	0.722302351	YHTKSR	63	396.2123	2	790.4086	.	M	Superscaf	88935903	0	0	0	BTP20220	0	0	Tissue specific
EDSVLLR	Superscaf	+	Superscaf	89017897	89017917	12	12	18	-	-	gene	-	-	mRNA	NCP	Intron	gene|mRN	ID=gene-E	7	4.941428571	2576	0.723891595	EDSVLLR	99	416.2311	2	830.4498	.	Q	Superscaf	89017864	0	0	0	0	BTP20220	0	Tissue specific
KPTTTKAK	Superscaf	-	Superscaf	89076454	89076477	-3	3	10	-	-	gene	-	-	mRNA	NCP	Intron	gene|mRN	ID=gene-E	8	7.64125	3246.4	0.725025591	KPTTTKAK	65	437.7702	2	873.5283	.	W	Superscaf	89076573	0	0	0	BTP20220	0	0	Tissue specific
LSCSAGPF	Superscaf	+	Superscaf	89173081	89173104	5	5	12	-	-	gene	-	-	mRNA	NCP	Intron	gene|mRN	ID=gene-E	8	5.76625	3039.3	0.726896838	LSC(+57.0	50	838.3795	1	837.3691	Carbamido	C	Superscaf	89173069	0	BTP20220	0	0	0	0	Tissue specific
KERAAPP	Superscaf	-	Superscaf	89184439	89184459	-8	8	14	-	-	-	-	-	-	NCP	IGR	-	ID=gene-E	7	6.908571429	2840.1	0.727116793	KERAAPP	72	384.7251	2	767.429	.	L	Superscaf	89184609	0	0	0	BTP20220	0	0	Tissue specific
TSGLELL	Superscaf	-	Superscaf	89198173	89198193	-25	25	31	-	-	-	-	-	-	NCP	IGR	-	ID=gene-E	7	5.62	2563.9	0.727382761	TSGLELL	81	732.4128	1	731.4065	.	S	Superscaf	89198268	0	0	0	BTP20220	0	0	Tissue specific
GPAAVTWK	Superscaf	-	Superscaf	89213542	89213565	-19	19	26	-	-	-	-	-	-	NCP	IGR	-	ID=gene-E	8	5.80875	2649.8	0.727680392	GPAAVTWK	74	415.2312	2	828.4493	.	V	Superscaf	89213583	0	0	0	0	0	BTP20220	Tissue specific
STGSEFL	Superscaf	+	Superscaf	89217205	89217225	4	4	10	-	-	-	-	-	-	NCP	IGR	-	ID=gene-E	7	5.505714286	2627.9	0.727751329	STGSEFL	72	740.3436	1	739.3388	.	L	Superscaf	89217196	0	0	0	BTP20220	0	0	Tissue specific
TVVMP	Superscaf	+	Superscaf	89225542	89225556	39	39	43	-	-	-	-	-	-	NCP	IGR	-	ID=gene-E	5	3.716	1106.4	0.72791278	T(+42.01)	75	604.3085	1	603.2938	Acetylati	S	Superscaf	89225428	0	0	0	0	BTP20220	0	Tissue specific
QPGLGGLT	Superscaf	-	Superscaf	89248870	89248893	-29	29	36	-	-	-	-	-	-	NCP	IGR	-	ID=gene-E	8	6.04375	2944.3	0.728364543	QPGLGGLT	86	742.4067	1	741.4021	.	T	Superscaf	89248914	0	0	0	BTP20220	0	0	Tissue specific
KAATKPK	Superscaf	-	Superscaf	89266456	89266476	-17	17	23	-	-	-	-	-	-	NCP	IGR	-	ID=gene-E	7	7.727142857	2762.7	0.728705108	KAATKPK	57	372.2403	2	742.4701	.	L	Superscaf	89266725	BTP20220	0	0	0	0	0	Tissue specific
AAPKRPP	Superscaf	-	Superscaf	89271736	89271756	-4	4	10	-	-	-	-	-	-	NCP	IGR	-	ID=gene-E	7	7.348571429	2752	0.728807358	AAPKRPP	68	368.7245	2	735.4391	.	P	Superscaf	89271825	0	0	0	0	0	BTP20220	Tissue specific
KRRLDP	Superscaf	-	Superscaf	89295679	89295696	-8	8	13	-	-	-	-	-	-	NCP	IGR	-	ID=gene-E	6	7.751666667	2868.3	0.729271031	KRRLDP	56	784.4887	1	783.4715	.	I	Superscaf	89295705	0	0	0	BTP20220	0	0	Tissue specific
TGLRAGP	Superscaf	+	Superscaf	89319301	89319321	33	33	39	-	-	-	-	-	-	NCP	IGR	-	ID=gene-E	7	6.79	2643	0.729728487	T(+42.01)	67	357.1992	2	712.3868	Acetylati	T	Superscaf	89319205	BTP20220	0	0	0	0	0	Tissue specific
LGQALGR	Superscaf	-	Superscaf	89327296	89327316	-34	34	40	-	-	-	-	-	-	NCP	IGR	-	ID=gene-E	7	6.618571429	2872.2	0.729883315	LGQALGR	57	357.7214	2	713.4184	.	N	Superscaf	89327322	0	0	BTP20220	0	0	0	Tissue specific
LSVAVP	Superscaf	+	Superscaf	89338792	89338809	8	8	13	-	-	-	-	-	-	NCP	IGR	-	ID=gene-E	6	3.996666667	1385.5	0.730105943	L(+42.01)	62	627.3691	1	626.3639	Acetylati	V	Superscaf	89338771	0	0	0	0	BTP20220	0	Tissue specific
SLDAFVP	Superscaf	+	Superscaf	89342383	89342403	4	4	10	-	-	-	-	-	-	NCP	IGR	-	ID=gene-E	7	4.632857143	2184.4	0.730175485	SLDAFVP	67	748.3849	1	747.3802	.	A	Superscaf	89342374	BTP20220	0	0	0	0	0	Tissue specific
LSLTEFL	Superscaf	+	Superscaf	89401606	89401626	12	12	18	-	-	-	-	-	-	NCP	IGR	-	ID=gene-E	7	5.55	2654	0.731322378	L(+42.01)	52	432.7376	2	863.4641	Acetylati	P	Superscaf	89401573	0	0	0	0	BTP20220	0	Tissue specific
NTPMQL	Superscaf	+	Superscaf	89407570	89407587	47	47	52	-	-	-	-	-	-	NCP	IGR	-	ID=gene-E	6	5.936666667	2667.2	0.731437875	NTPMQL	99	703.3434	1	702.3371	.	E	Superscaf	89407432	0	BTP20220	0	0	0	0	Tissue specific
AALLECT	Superscaf	-	Superscaf	89424502	89424522	-143	143	149	-	-	-	-	-	-	NCP	IGR	-	ID=gene-E	7	5.538571429	2738.1	0.731765775	AALLEC(+5	56	777.3711	1	776.3738	Carbamido	L	Superscaf	89424798	0	BTP20220	0	0	0	0	Tissue specific
MELQKAK	Superscaf	-	Superscaf	89534857	89534877	-12	12	18	-	-	gene	-	-	mRNA	NCP	Intron	gene|mRN	ID=gene-E	7	6.585714286	3009.2	0.733902874	M(+15.99)	73	453.2336	2	904.4688	Oxidation	F	Superscaf	89534883	0	0	0	BTP20220	0	0	Tissue specific
DNCMSH	Superscaf	+	Superscaf	89563546	89563563	1	1	6	-	-	gene	-	-	mRNA	NCP	Intron	gene|mRN	ID=gene-E	6	5.403333333	2968.5	0.734458456	DNC(+57.0	64	390.1327	2	778.2374	Carbamido	D	Superscaf	89563546	0	0	0	0	BTP20220	BTP20220	Tissue specific
AVPPLLY	Superscaf	+	Superscaf	89574529	89574549	7	7	13	-	-	gene	-	-	mRNA	NCP	Intron	gene|mRN	ID=gene-E	7	5.177142857	2152.4	0.734671149	A(+42.01)	73	407.7416	2	813.4636	Acetylati	E	Superscaf	89574511	0	BTP20220	0	0	0	0	Tissue specific
KYTLACK	Superscaf	+	Superscaf	89597821	89597841	30	30	36	-	-	gene	-	-	mRNA	NCP	Intron	gene|mRN	ID=gene-E	7	6.955714286	3044.2	0.735122214	KYTLAC(+5	52	442.2318	2	882.4633	Carbamido	D	Superscaf	89597734	0	0	0	0	BTP20220	0	Tissue specific
KAPPLP	Superscaf	-	Superscaf	89616076	89616093	-65	65	70	-	-	gene	-	-	mRNA	NCP	Intron	gene|mRN	ID=gene-E	6	6.773333333	2145.3	0.735475735	KAPPLP	53	622.3985	1	621.3849	.	L	Superscaf	89616177	0	0	0	BTP20220	0	0	Tissue specific
NGELRHV	Superscaf	+	Superscaf	89670859	89670879	18	18	24	-	-	gene	-	-	mRNA	NCP	Intron	gene|mRN	ID=gene-E	7	5.561428571	2941.4	0.736536644	NGELRHV	91	412.7216	2	823.4301	.	R	Superscaf	89670808	0	BTP20220	0	0	0	0	Tissue specific
ANGGLGL	Superscaf	-	Superscaf	89675017	89675037	-18	18	24	-	-	gene	-	-	mRNA	NCP	Intron	gene|mRN	ID=gene-E	7	5.9	2506.7	0.736617167	ANGGLGL	69	601.3284	1	600.3231	.	A	Superscaf	89675082	0	0	0	BTP20220	0	0	Tissue specific
KSSYLMK	Superscaf	-	Superscaf	89688832	89688852	-21	21	27	-	-	gene	-	-	mRNA	NCP	Intron	gene|mRN	ID=gene-E	7	6.89	2888	0.736884704	KSSYLMK	58	856.4461	1	855.4524	.	Y	Superscaf	89689068	0	0	0	0	0	BTP20220	Tissue specific
PLVGGP	Superscaf	-	Superscaf	89689981	89689998	-4	4	9	-	-	gene	-	-	mRNA	NCP	Intron	gene|mRN	ID=gene-E	6	5.086666667	1622.8	0.736906955	PLVGGP	67	539.3167	1	538.3115	.	A	Superscaf	89690202	0	BTP20220	0	0	0	0	Tissue specific
ALVEF	Superscaf	+	Superscaf	89697988	89698002	75	75	79	-	-	gene	-	-	mRNA	NCP	Intron	gene|mRN	ID=gene-E	5	4.14	1477.6	0.737062016	ALVEF	97	578.3167	1	577.3112	.	L	Superscaf	89697766	0	0	0	BTP20220	0	0	Tissue specific
LPPPPLFR	Superscaf	+	Superscaf	89713159	89713182	19	19	26	-	-	gene	-	-	mRNA	NCP	Intron	gene|mRN	ID=gene-E	8	6.675	3092.6	0.737355812	LPPPPLFR	56	468.785	2	935.5592	.	Y	Superscaf	89713105	0	BTP20220	0	0	0	0	Mixed
KNHEPTR	Superscaf	-	Superscaf	89772940	89772960	-45	45	51	-	-	-	-	-	-	NCP	IGR	-	ID=gene-E	7	7.078571429	3494	0.738513512	KNHEPTR	56	441.233	2	880.4515	.	A	Superscaf	89773140	BTP20220	0	0	0	0	0	Tissue specific
RVVVGY	Superscaf	+	Superscaf	89817067	89817084	59	59	64	-	-	gene	-	-	mRNA	NCP	Intron	gene|mRN	ID=gene-E	6	3.731666667	1405.6	0.739368061	RVVVGY	52	692.4049	1	691.4017	.	V	Superscaf	89816893	0	0	0	BTP20220	0	0	Tissue specific
KPQFRPP	Superscaf	-	Superscaf	89836294	89836314	-8	8	14	-	-	gene	-	-	mRNA	NCP	Intron	gene|mRN	ID=gene-E	7	7.218571429	3127.5	0.739740405	KPQFRPP	50	435.2448	2	868.4919	.	T	Superscaf	89836335	0	0	BTP20220	0	0	0	Tissue specific
VCLLHKE	Superscaf	+	Superscaf	89850157	89850177	15	15	21	-	-	gene	-	-	mRNA	NCP	Intron	gene|mRN	ID=gene-E	7	5.361428571	2806.1	0.740008871	VC(+57.02	94	449.7431	2	897.4742	Carbamido	Q	Superscaf	89850115	0	0	BTP20220	0	0	0	Tissue specific
EAVLLLKK	Superscaf	-	Superscaf	89857603	89857626	-30	30	37	-	-	gene	-	-	mRNA	NCP	Intron	gene|mRN	ID=gene-E	8	5.8325	2733.8	0.740153068	EAVLLLKK	98	457.3061	2	912.6008	.	L	Superscaf	89857728	0	BTP20220	0	0	0	0	Tissue specific
KLNPYAK	Superscaf	-	Superscaf	89859034	89859054	-50	50	56	-	-	gene	-	-	mRNA	NCP	Intron	gene|mRN	ID=gene-E	7	6.978571429	2995.1	0.74018078	KLNPYAK	99	417.2462	2	832.4807	.	F	Superscaf	89859132	BTP20220	0	0	BTP20220	0	0	Tissue specific
SDQKGVQGK	Superscaf	-	Superscaf	89859223	89859294	-26	26	49	-	-	gene	-	-	mRNA	NCP	Intron	gene|mRN	ID=gene-E	24	5.66375	8566.7	0.74018444	SDQKGVQGK	93	631.8577	4	2523.413	.	F	Superscaf	89859405	0	BTP20220	0	0	0	0	Tissue specific
SDQKGVQGK	Superscaf	-	Superscaf	89859223	89859276	-26	26	43	-	-	gene	-	-	mRNA	NCP	Intron	gene|mRN	ID=gene-E	18	5.638333333	6609.8	0.74018444	SDQKGVQGK	97	963.5379	2	1925.069	.	F	Superscaf	89859405	BTP20220	0	0	0	0	0	Tissue specific
SDQKGVQGK	Superscaf	-	Superscaf	89859223	89859270	-26	26	41	-	-	gene	-	-	mRNA	NCP	Intron	gene|mRN	ID=gene-E	16	5.533125	5715.9	0.74018444	SDQKGVQGK	82	556.9828	3	1667.932	.	F	Superscaf	89859405	BTP20220	0	0	0	0	0	Tissue specific
SDQKGVQGK	Superscaf	-	Superscaf	89859223	89859267	-26	26	40	-	-	gene	-	-	mRNA	NCP	Intron	gene|mRN	ID=gene-E	15	5.252666667	5247.5	0.74018444	SDQKGVQGK	96	514.2844	3	1539.837	.	F	Superscaf	89859405	BTP20220	0	0	0	0	0	Tissue specific
KEKKAVGDK	Superscaf	-	Superscaf	89859268	89859318	-41	41	57	-	-	gene	-	-	mRNA	NCP	Intron	gene|mRN	ID=gene-E	17	6.153529412	5712.5	0.740185312	KEKKAVGDK	75	463.7979	4	1851.167	.	F	Superscaf	89859405	BTP20220	0	0	0	0	0	Tissue specific
GMEVVLL	Superscaf	-	Superscaf	89874274	89874294	-47	47	53	-	-	gene	-	-	mRNA	NCP	Intron	gene|mRN	ID=gene-E	7	3.842857143	1801.1	0.740475913	GMEVVLL	63	760.424	1	759.42	.	V	Superscaf	89874378	0	0	0	BTP20220	0	0	Tissue specific
VPGHNLP	Superscaf	-	Superscaf	89881024	89881044	-1	1	7	-	-	gene	-	-	mRNA	NCP	Intron	gene|mRN	ID=gene-E	7	5.364285714	2541.9	0.740606631	VPGHNLP	51	367.2075	2	732.3918	.	V	Superscaf	89881044	BTP20220	0	0	0	0	0	Tissue specific
MQFLT	Superscaf	-	Superscaf	89964469	89964483	-3	3	7	-	-	-	-	-	-	NCP	IGR	-	ID=gene-E	5	5.878	2120.5	0.7422226	M(+15.99)	61	655.3096	1	654.3047	Oxidation	F	Superscaf	89964483	0	0	BTP20220	0	0	0	Tissue specific
NGSSLDF	Superscaf	+	Superscaf	90003466	90003486	38	38	44	-	-	-	-	-	-	NCP	IGR	-	ID=gene-E	7	5.31	2813.1	0.742977803	NGSSLDF	68	739.3261	1	738.3184	.	G	Superscaf	90003355	0	BTP20220	0	0	0	0	Tissue specific
KRHPPPPG	Superscaf	-	Superscaf	90054160	90054183	-34	34	41	-	-	-	-	-	-	NCP	IGR	-	ID=gene-E	8	7.4075	3383.8	0.743959527	K(+42.01)	70	464.2648	2	926.5086	Acetylati	K	Superscaf	90054207	0	BTP20220	0	0	0	0	Tissue specific
LAGGAP	Superscaf	-	Superscaf	90134179	90134196	-18	18	23	-	-	-	-	-	-	NCP	IGR	-	ID=gene-E	6	6.043333333	1908	0.745509149	LAGGAP	66	485.2812	1	484.2645	.	G	Superscaf	90134250	0	0	BTP20220	0	0	0	Tissue specific
KKVGLEPL	Superscaf	-	Superscaf	90141649	90141672	-5	5	12	-	-	-	-	-	-	NCP	IGR	-	ID=gene-E	8	5.86625	2703.8	0.74565381	K(+42.01)	71	463.2878	2	924.5644	Acetylati	G	Superscaf	90141690	0	0	BTP20220	0	0	0	Tissue specific
KLTTDK	Superscaf	+	Superscaf	90143191	90143208	1	1	6	-	-	-	-	-	-	NCP	IGR	-	ID=gene-E	6	6.915	2496.7	0.745683672	KLTTDK	50	353.2089	2	704.4069	.	K	Superscaf	90143191	BTP20220	0	0	0	0	0	Tissue specific
LSGPSPQA	Superscaf	-	Superscaf	90165841	90165864	-83	83	90	-	-	-	-	-	-	NCP	IGR	-	ID=gene-E	8	5.9475	3014.3	0.746122305	LSGPSPQA	57	756.3868	1	755.3813	.	S	Superscaf	90165975	0	BTP20220	0	0	0	0	Tissue specific
PVPPPPPP	Superscaf	-	Superscaf	90204790	90204813	-7	7	14	-	-	gene	-	-	mRNA	NCP	Intron	gene|mRN	ID=gene-E	8	5.5125	2361.8	0.746876578	PVPPPPPP	94	797.4677	1	796.4483	.	A	Superscaf	90204900	0	0	0	BTP20220	0	0	Tissue specific
ESNPPLR	Superscaf	-	Superscaf	90239365	90239385	-29	29	35	-	-	-	-	-	-	NCP	IGR	-	ID=gene-E	7	6.235714286	3082.6	0.747546146	ESNPPLR	79	406.715	2	811.4188	.	I	Superscaf	90239400	0	0	BTP20220	0	0	0	Tissue specific
AAPSLSGK	Superscaf	-	Superscaf	90271105	90271128	-16	16	23	-	-	-	-	-	-	NCP	IGR	-	ID=gene-E	8	6.42375	2845.9	0.748160813	AAPSLSGK	54	365.7106	2	729.402	.	R	Superscaf	90271185	0	0	0	BTP20220	0	0	Tissue specific
GVFYQ	Superscaf	-	Superscaf	90275557	90275571	-32	32	36	-	-	-	-	-	-	NCP	IGR	-	ID=gene-E	5	4.552	1754.9	0.748247029	GVFYQ	98	613.297	1	612.2907	.	R	Superscaf	90275574	0	0	0	BTP20220	0	0	Mixed
VTEADLP	Superscaf	-	Superscaf	90356629	90356649	-73	73	79	-	-	-	-	-	-	NCP	IGR	-	ID=gene-E	7	4.431428571	2236.6	0.749817043	VTEADLP	67	744.3713	1	743.3701	.	K	Superscaf	90356811	0	0	BTP20220	0	0	0	Tissue specific

PKVPPPPPF	Superscaf	-	Superscaf	90360229	90360255	-37	37	45	-	-	-	-	-	-	NCP	IGR	-	ID=gene-E	9	5.982222222	2830.2	0.749886759	P(+42.01)	73	967.5569	1	966.5538	Acetylati	W	Superscaf	90360510	0	0	0	0	BTP20220	0	Tissue specific
MAMLQF	Superscaf	-	Superscaf	90392200	90392217	-20	20	25	-	-	-	-	-	-	NCP	IGR	-	ID=gene-E	6	5.771666667	2405.8	0.750505899	MAMLQF	81	740.345	1	739.3397	.	S	Superscaf	90392244	0	0	0	BTP20220	0	0	Tissue specific
VPAFLGF	Superscaf	+	Superscaf	90396133	90396153	1	1	7	-	-	-	-	-	-	NCP	IGR	-	ID=gene-E	7	5.032857143	2074.2	0.750582064	V(+42.01)	64	396.7211	2	791.4218	Acetylati	V	Superscaf	90396133	BTP20220	0	0	0	0	BTP20220	Tissue specific
PLVLPPPR	Superscaf	-	Superscaf	90422635	90422658	-83	83	90	-	-	-	-	-	-	NCP	IGR	-	ID=gene-E	8	5.99	2705.2	0.751095293	P(+42.01)	55	465.7945	2	929.5698	Acetylati	G	Superscaf	90422679	0	0	0	0	BTP20220	0	Tissue specific
EAPQGG	Superscaf	+	Superscaf	90502975	90502992	14	14	19	-	-	-	-	-	-	NCP	IGR	-	ID=gene-E	6	5.521666667	2279.5	0.752651132	EAPQGG	52	558.2471	1	557.2445	.	R	Superscaf	90502936	0	0	BTP20220	0	0	0	Tissue specific
FFPPPPG	Superscaf	+	Superscaf	90538957	90538977	13	13	19	-	-	-	-	-	-	NCP	IGR	-	ID=gene-E	7	6.018571429	2421.7	0.753347947	FFPPPPG	89	379.6941	2	757.3799	.	R	Superscaf	90538921	0	0	0	BTP20220	0	0	Mixed
TLRDQGF	Superscaf	+	Superscaf	90564190	90564210	62	62	68	-	-	-	-	-	-	NCP	IGR	-	ID=gene-E	7	6.191428571	3106.6	0.753836602	TLRDQGF	91	418.7106	2	835.4188	.	R	Superscaf	90564007	BTP20220	0	0	0	0	0	Tissue specific
KVGPRLK	Superscaf	-	Superscaf	90575398	90575418	-10	10	16	-	-	-	-	-	-	NCP	IGR	-	ID=gene-E	7	6.927142857	2573.7	0.754053652	KVGPRLK	88	399.2701	2	796.5283	.	Y	Superscaf	90575490	0	0	BTP20220	0	0	0	Tissue specific
MLYVVQFL	Superscaf	-	Superscaf	90576610	90576633	-26	26	33	-	-	-	-	-	-	NCP	IGR	-	ID=gene-E	8	4.3125	2535.9	0.754077123	MLYVVQFL	55	506.7747	2	1011.546	.	C	Superscaf	90576633	0	0	0	BTP20220	0	0	Tissue specific
FSNRPK	Superscaf	+	Superscaf	90613930	90613947	10	10	15	-	-	-	-	-	-	NCP	IGR	-	ID=gene-E	6	7.228333333	2822.1	0.75479985	FSNRPK	57	374.713	2	747.4028	.	L	Superscaf	90613903	0	0	0	BTP20220	0	0	Tissue specific
YAVKPY	Superscaf	+	Superscaf	90658927	90658944	2	2	7	-	-	-	-	-	-	NCP	IGR	-	ID=gene-E	6	5.563333333	2036.1	0.755671247	YAVKPY	67	370.6955	2	739.3904	.	T	Superscaf	90658924	0	0	0	BTP20220	0	0	Tissue specific
KSPGGGP	Superscaf	-	Superscaf	90795358	90795378	-1	1	7	-	-	-	-	-	-	NCP	IGR	-	ID=gene-E	7	6.561428571	2418.5	0.758313326	K(+42.01)	62	641.338	1	640.318	Acetylati	K	Superscaf	90795471	0	0	0	BTP20220	0	0	Tissue specific
KMWHLK	Superscaf	-	Superscaf	90830182	90830199	-7	7	12	-	-	-	-	-	-	NCP	IGR	-	ID=gene-E	6	7.448333333	2808	0.758987716	K(+42.01)	53	450.7362	2	899.4688	Acetylati	L	Superscaf	90830235	0	0	0	BTP20220	0	0	Tissue specific
MNNLLR	Superscaf	+	Superscaf	90858253	90858270	12	12	17	-	-	-	-	-	-	NCP	IGR	-	ID=gene-E	6	6.548333333	2920.5	0.75953133	M(+42.01)	56	401.7155	2	801.4167	Acetylati	V	Superscaf	90858220	0	0	BTP20220	0	0	0	Tissue specific
MKLAVL	Superscaf	+	Superscaf	90913417	90913434	23	23	28	-	-	-	-	-	-	NCP	IGR	-	ID=gene-E	6	5.578333333	1858	0.760599618	M(+42.01)	80	716.4344	1	715.4302	Acetylati	K	Superscaf	90913351	BTP20220	0	0	0	0	0	Tissue specific
HTYMR	Superscaf	+	Superscaf	90938704	90938718	55	55	59	-	-	gene	-	-	mRNA	NCP	Intron	gene|mRN	ID=gene-E	5	7.258	2497	0.761089318	HTYMR	81	354.1665	2	706.3221	.	V	Superscaf	90938542	BTP20220	0	0	0	0	0	Tissue specific
KRLVPPP	Superscaf	-	Superscaf	90953410	90953430	-55	55	61	-	-	gene	-	-	mRNA	NCP	Intron	gene|mRN	ID=gene-E	7	6.482857143	2482.8	0.761374109	KRLVPPP	56	403.7704	2	805.5174	.	Y	Superscaf	90953466	0	0	0	BTP20220	0	0	Tissue specific
RLGPTYPP	Superscaf	-	Superscaf	90989791	90989814	-88	88	95	-	-	-	-	-	-	NCP	IGR	-	ID=gene-E	8	6.725	3168.7	0.762078652	RLGPTYPP	66	450.7592	2	899.4865	.	P	Superscaf	90989913	BTP20220	0	0	0	0	0	Tissue specific
SLHSYPF	Superscaf	+	Superscaf	90994339	90994359	99	99	105	-	-	-	-	-	-	NCP	IGR	-	ID=gene-E	7	6.052857143	2924.2	0.762166727	SLHSYPF	51	425.7079	2	849.4021	.	G	Superscaf	90994045	0	0	0	BTP20220	0	0	Tissue specific
TLKPPLAL	Superscaf	+	Superscaf	91018243	91018266	64	64	71	-	-	-	-	-	-	NCP	IGR	-	ID=gene-E	8	6.60375	2912.2	0.762629644	TLKPPLAL	52	426.7851	2	851.548	.	A	Superscaf	91018054	0	0	0	BTP20220	0	0	Tissue specific
SPVYAGA	Superscaf	-	Superscaf	91107067	91107087	-17	17	23	-	-	gene	-	-	mRNA	NCP	Intron	gene|mRN	ID=gene-E	7	5.092857143	2100.2	0.764349781	SPVYAGA	85	664.3293	1	663.3228	.	A	Superscaf	91107132	0	0	0	BTP20220	0	0	Tissue specific
VPPPPLP	Superscaf	-	Superscaf	91134784	91134804	-135	135	141	-	-	gene	-	-	mRNA	NCP	Intron	gene|mRN	ID=gene-E	7	5.354285714	2040.4	0.764886539	VPPPPLP	98	716.4312	1	715.4268	.	M	Superscaf	91135446	BTP20220	0	0	0	0	0	Tissue specific
EPPGHKP	Superscaf	-	Superscaf	91221478	91221498	-32	32	38	-	-	-	-	-	-	NCP	IGR	-	ID=gene-E	7	6.488571429	2823.1	0.766565427	EPPGHKP	55	381.1998	2	760.3868	.	S	Superscaf	91221513	BTP20220	0	0	0	0	0	Mixed
VVDLVR	Superscaf	-	Superscaf	91238893	91238910	-25	25	30	-	-	-	-	-	-	NCP	IGR	-	ID=gene-E	6	3.285	1413.7	0.76690268	VVDLVR	95	350.7203	2	699.4279	.	I	Superscaf	91238994	0	0	0	0	BTP20220	0	Tissue specific
PLYTPVM	Superscaf	+	Superscaf	91300675	91300695	37	37	43	-	-	-	-	-	-	NCP	IGR	-	ID=gene-E	7	5.217142857	2256.7	0.76809913	PLYTPVM(+	50	836.4111	1	835.415	Oxidatior	L	Superscaf	91300567	BTP20220	0	0	0	0	0	Tissue specific
FGGFVDF	Superscaf	-	Superscaf	91310236	91310256	-1	1	7	-	-	-	-	-	-	NCP	IGR	-	ID=gene-E	7	4.478571429	2168.3	0.768284285	FGGFVDF	79	788.3594	1	787.3541	.	F	Superscaf	91310298	0	0	BTP20220	0	0	0	Tissue specific
LSSNPLLF	Superscaf	-	Superscaf	91400176	91400199	-13	13	20	-	-	-	-	-	-	NCP	IGR	-	ID=gene-E	8	5.81125	3148.5	0.770026034	LSSNPLLF	78	890.4945	1	889.4909	.	I	Superscaf	91400208	BTP20220	0	0	0	0	0	Tissue specific
KAVAVLG	Superscaf	+	Superscaf	91412554	91412574	3	3	9	-	-	-	-	-	-	NCP	IGR	-	ID=gene-E	7	4.818571429	1741.7	0.770265742	KAVAVLG	89	657.4286	1	656.4221	.	H	Superscaf	91412548	BTP20220	0	0	0	0	0	Mixed
NPQVKK	Superscaf	+	Superscaf	91513204	91513221	11	11	16	-	-	-	-	-	-	NCP	IGR	-	ID=gene-E	6	6.14	2481.6	0.772214897	NPQVKK	57	357.2203	2	712.4232	.	N	Superscaf	91513174	0	0	0	BTP20220	0	0	Tissue specific
AYHLQK	Superscaf	+	Superscaf	91580539	91580556	10	10	15	-	-	-	-	-	-	NCP	IGR	-	ID=gene-E	6	6.773333333	2823	0.773518885	AYHLQK	98	759.4114	1	758.4075	.	S	Superscaf	91580512	0	0	BTP20220	0	0	0	Tissue specific
QFSKGGP	Superscaf	+	Superscaf	91744882	91744902	4	4	10	-	-	-	-	-	-	NCP	IGR	-	ID=gene-E	7	6.398571429	2781.9	0.776701498	Q(+42.01)	69	762.3887	1	761.3708	Acetylati	L	Superscaf	91744873	0	0	BTP20220	0	0	0	Tissue specific
MNQLAK	Superscaf	+	Superscaf	91774729	91774746	1	1	6	-	-	-	-	-	-	NCP	IGR	-	ID=gene-E	6	6.425	2712	0.777279505	M(+15.99)	70	360.6955	2	719.3636	Oxidatior	M	Superscaf	91774729	BTP20220	0	0	0	0	0	Tissue specific
FKLLDN	Superscaf	+	Superscaf	91825243	91825260	5	5	10	-	-	gene	-	-	mRNA	NCP	Intron	gene|mRN	ID=gene-E	6	5.926666667	2570.8	0.778257743	FKLLDN	78	375.2116	2	748.4119	.	C	Superscaf	91825231	0	BTP20220	0	0	0	0	Tissue specific
CCLVHR	Superscaf	-	Superscaf	91831357	91831374	-10	10	15	-	-	gene	-	-	mRNA	NCP	Intron	gene|mRN	ID=gene-E	6	5.728333333	2793.3	0.778376145	C(+57.02)	75	422.6983	2	843.3843	Carbamidc	D	Superscaf	91831413	0	0	BTP20220	0	0	0	Tissue specific
NSKLLAR	Superscaf	-	Superscaf	91848739	91848759	-1	1	7	-	-	gene	-	-	mRNA	NCP	Intron	gene|mRN	ID=gene-E	7	7.081428571	3115.4	0.778712759	NSKLLAR	71	401.2544	2	800.4868	.	N	Superscaf	91848819	BTP20220	0	0	0	0	0	Tissue specific
VWFKLSS	Superscaf	+	Superscaf	91969813	91969833	22	22	28	-	-	-	-	-	-	NCP	IGR	-	ID=gene-E	7	5.492857143	2502.6	0.781057439	VWFKLSS	61	433.7385	2	865.4698	.	V	Superscaf	91969750	0	0	0	0	0	BTP20220	Tissue specific
VSVNSLKK	Superscaf	+	Superscaf	92101021	92101044	4	4	11	-	-	gene	-	-	mRNA	NCP	Intron	gene|mRN	ID=gene-E	8	5.27875	2653.7	0.78359837	VSVNSLKK	97	437.7698	2	873.5283	.	H	Superscaf	92101012	0	0	0	0	BTP20220	0	Tissue specific
VGQLY	Superscaf	-	Superscaf	92179549	92179563	-13	13	17	-	-	gene	-	-	mRNA	NCP	Intron	gene|mRN	ID=gene-E	5	4.652	1720.9	0.785119118	VGQLY	97	579.3118	1	578.3064	.	F	Superscaf	92179614	0	0	0	0	BTP20220	0	Tissue specific
KGWAGPK	Superscaf	-	Superscaf	92184754	92184774	-1	1	7	-	-	gene	-	-	mRNA	NCP	Intron	gene|mRN	ID=gene-E	7	7.09	2706.7	0.785219916	KGWAGPK	56	372.2164	2	742.4126	.	K	Superscaf	92184834	BTP20220	0	0	0	0	0	Tissue specific
SGPMQL	Superscaf	-	Superscaf	92188000	92188017	-10	10	15	-	-	gene	-	-	mRNA	NCP	Intron	gene|mRN	ID=gene-E	6	5.888333333	2353.7	0.785282777	SGPMQL	85	632.3053	1	631.2999	.	E	Superscaf	92188038	BTP20220	0	0	0	0	0	Tissue specific
NPSFPLG	Superscaf	-	Superscaf	92198668	92198688	-3	3	9	-	-	gene	-	-	mRNA	NCP	Intron	gene|mRN	ID=gene-E	7	5.874285714	2693	0.78548937	NPSFPLG	71	731.3693	1	730.3649	.	C	Superscaf	92198715	0	0	0	0	0	BTP20220	Mixed
KSSVTSP	Superscaf	+	Superscaf	92246017	92246037	29	29	35	-	-	gene	-	-	mRNA	NCP	Intron	gene|mRN	ID=gene-E	7	5.658571429	2353.5	0.786406315	KSSVTSP	63	353.1874	2	704.3704	.	L	Superscaf	92245933	BTP20220	0	0	0	0	0	Tissue specific
LAAKFLL	Superscaf	+	Superscaf	92253880	92253900	55	55	61	-	-	gene	-	-	mRNA	NCP	Intron	gene|mRN	ID=gene-E	7	6.457142857	2538.6	0.786558588	L(+42.01)	52	409.2679	2	816.5109	Acetylati	P	Superscaf	92253718	BTP20220	0	0	0	0	0	Tissue specific
GDVKNF	Superscaf	+	Superscaf	92255317	92255334	5	5	10	-	-	gene	-	-	mRNA	NCP	Intron	gene|mRN	ID=gene-E	6	4.928333333	2161.3	0.786586416	G(+42.01)	88	721.3503	1	720.3442	Acetylati	R	Superscaf	92255305	0	0	BTP20220	0	0	0	Tissue specific
FDPAWK	Superscaf	-	Superscaf	92278891	92278908	-19	19	24	-	-	-	-	-	-	NCP	IGR	-	ID=gene-E	6	6.066666667	2442.6	0.787042942	FDPAWK	80	382.189	2	762.3701	.	L	Superscaf	92278914	0	0	0	0	BTP20220	0	Tissue specific
MRFSLLR	Superscaf	-	Superscaf	92301526	92301546	-42	42	48	-	-	-	-	-	-	NCP	IGR	-	ID=gene-E	7	7.198571429	3146.7	0.787481285	MRFSLLR	76	461.7713	2	921.5218	.	N	Superscaf	92301912	0	0	0	0	0	BTP20220	Tissue specific
TAAVSPF	Superscaf	-	Superscaf	92324725	92324745	-13	13	19	-	-	gene	-	-	mRNA	NCP	Intron	gene|mRN	ID=gene-E	7	5.147142857	2128.3	0.787930549	TAAVSPF	57	692.354	1	691.3541	.	V	Superscaf	92324778	BTP20220	0	0	BTP20220	0	0	Tissue specific
LVGEAPPL	Superscaf	-	Superscaf	92329747	92329770	-4	4	11	-	-	gene	-	-	mRNA	NCP	Intron	gene|mRN	ID=gene-E	8	4.97125	2415.7	0.788027803	LVGEAPPL	54	795.4607	1	794.4538	.	T	Superscaf	92329881	0	0	BTP20220	0	0	0	Tissue specific
KPAGLDY	Superscaf	+	Superscaf	92361697	92361717	9	9	15	-	-	gene	-	-	mRNA	NCP	Intron	gene|mRN	ID=gene-E	7	6.091428571	2638.8	0.788646537	K(+42.01)	64	805.4061	1	804.4017	Acetylati	W	Superscaf	92361673	0	0	0	BTP20220	0	0	Tissue specific
FETTPF	Superscaf	+	Superscaf	92380696	92380713	1	1	6	-	-	gene	-	-	mRNA	NCP	Intron	gene|mRN	ID=gene-E	6	5.59	2332.7	0.789014465	FETTPF	64	741.3555	1	740.3381	.	F	Superscaf	92380696	0	0	0	BTP20220	0	0	Tissue specific
PLKKHP	Superscaf	-	Superscaf	92403061	92403078	-8	8	13	-	-	gene	-	-	mRNA	NCP	Intron	gene|mRN	ID=gene-E	6	7.608333333	2584.7	0.789447578	P(+42.01)	54	381.2368	2	760.4595	Acetylati	L	Superscaf	92403141	0	0	0	BTP20220	0	0	Tissue specific
KLLTDSK	Superscaf	+	Superscaf	92434828	92434848	10	10	16	-	-	gene	-	-	mRNA	NCP	Intron	gene|mRN	ID=gene-E	7	6.66	2836	0.790062768	KLLTDSK	82	402.7432	2	803.4752	.	Y	Superscaf	92434801	BTP20220	0	0	0	0	0	Tissue specific
TDTATLPV	Superscaf	+	Superscaf	92438527	92438550	65	65	72	-	-	gene	-	-	mRNA	NCP	Intron	gene|mRN	ID=gene-E	8	5.1075	2606.1	0.790134401	T(+42.01)	53	430.2272	2	858.4335	Acetylati	N	Superscaf	92438335	BTP20220	0	0	0	0	0	Tissue specific
PPPPKRS	Superscaf	-	Superscaf	92456251	92456271	-4	4	10	-	-	gene	-	-	mRNA	NCP	Intron	gene|mRN	ID=gene-E	7	7.34	2850.2	0.790477638	PPPPKRS	51	389.7383	2	777.4496	.	K	Superscaf	92456454	0	0	0	BTP20220	0	0	Tissue specific
PLLKVFW	Superscaf	-	Superscaf	92488771	92488791	-2	2	8	-	-	-	-	-	-	NCP	IGR	-	ID=gene-E	7	5.624285714	2426.6	0.79110741	PLLKVFW	50	451.7781	2	901.5425	.	I	Superscaf	92488809	0	0	BTP20220	0	0	0	Tissue specific
DLTVNH	Superscaf	+	Superscaf	92563708	92563725	63	63	68	-	-	-	-	-	-	NCP	IGR	-	ID=gene-E	6	4.746666667	2378.8	0.792558616	D(+42.01)	54	370.6863	2	739.35	Acetylati	S	Superscaf	92563522	0	0	BTP20220	0	0	0	Tissue specific
KHLPPK	Superscaf	-	Superscaf	92570119	92570136	-1	1	6	-	-	-	-	-	-	NCP	IGR	-	ID=gene-E	6	7.608333333	2584.7	0.792682769	KHLPPK	51	360.2355	2	718.449	.	K	Superscaf	92570157	BTP20220	0	BTP20220	0	0	0	Tissue specific
KFETLLK	Superscaf	-	Superscaf	92686588	92686608	-1	1	7	-	-	gene	-	-	mRNA	NCP	Intron	gene|mRN	ID=gene-E	7	6.667142857	2854	0.79493827	KFETLLK	73	439.7691	2	877.5273	.	K	Superscaf	92686755	0	0	0	BTP20220	0	0	Tissue specific
KDGPP	Superscaf	-	Superscaf	92689759	92689773	-55	55	59	-	-	gene	-	-	mRNA	NCP	Intron	gene|mRN	ID=gene-E	5	6.256	1852	0.794999678	KDGPP	62	513.2654	1	512.2594	.	R	Superscaf	92690568	BTP20220	0	0	0	0	BTP20220	Tissue specific
HALLKQDG	Superscaf	-	Superscaf	92749474	92749497	-111	111	118	-	-	gene	-	-	mRNA	NCP	Intron	gene|mRN	ID=gene-E	8	6.2375	3425.7	0.7961561	HALLKQDG	56	441.252	2	880.4766	.	S	Superscaf	92749533	BTP20220	0	0	0	0	0	Tissue specific
KTPWAP	Superscaf	+	Superscaf	92785162	92785179	17	17	22	-	-	-	-	-	-	NCP	IGR	-	ID=gene-E	6	6.796666667	2378.6	0.796847222	KTPWAP	70	350.1948	2	698.3751	.	I	Superscaf	92785114	0	0	0	0	BTP20220	0	Tissue specific
HLLYR	Superscaf	-	Superscaf	92839195	92839209	-13	13	17	-	-	gene	-	-	mRNA	NCP	Intron	gene|mRN	ID=gene-E	5	7.194	2434.8	0.797893607	HLLYR	90	351.2067	2	700.402	.	S	Superscaf	92839260	0	0	BTP20220	0	0	0	Tissue specific
LLNPFL	Superscaf	+	Superscaf	92888860	92888877	14	14	19	-	-	-	-	-	-	NCP	IGR	-	ID=gene-E	6	5.855	2381.7	0.798855403	LLNPFL	87	716.4316	1	715.4268	.	L	Superscaf	92888821	0	BTP20220	0	0	0	0	Tissue specific
QDLLEF	Superscaf	-	Superscaf	92915146	92915163	-16	16	21	-	-	-	-	-	-	NCP	IGR	-	ID=gene-E	6	4.88	2541.9	0.79936445	Q(+42.01)	54	806.4089	1	805.3857	Acetylati	P	Superscaf	92915322	BTP20220	0	0	0	0	0	Tissue specific
YELDLRK	Superscaf	+	Superscaf	92923846	92923866	22	22	28	-	-	-	-	-	-	NCP	IGR	-	ID=gene-E	7	6.33	3120.5	0.799532931	Y(+42.01)	73	489.7693	2	977.5181	Acetylati	S	Superscaf	92923783	0	0	0	0	BTP20220	0	Tissue specific
SADEYG	Superscaf	+	Superscaf	93013549	93013566	17	17	22	-	-	-	-	-	-	NCP	IGR	-	ID=gene-E	6	4.92	2288.5	0.80127009	S(+42.01)	57	683.244	1	682.2446	Acetylati	Y	Superscaf	93013501	BTP20220	0	BTP20220	BTP20220	0	0	Tissue specific
SLADAVN	Superscaf	+	Superscaf	93068761	93068781	4	4	10	-	-	-	-	-	-	NCP	IGR	-	ID=gene-E	7	4.582857143	2367.6	0.802339308	SLADAVN	68	689.3428	1	688.3392	.	D	Superscaf	93068752	BTP20220	BTP20220	BTP20220	BTP20220	BTP20220	0	Tissue specific
FVGAAP	Superscaf	+	Superscaf	93074326	93074343	1	1	6	-	-	-	-	-	-	NCP	IGR	-	ID=gene-E	6	4.965	1644.7	0.802447078	FVGAAP	88	561.2998	1	560.2958	.	F	Superscaf	93074326	0	BTP20220	0	0	0	0	Tissue specific
VGDVLLP	Superscaf	-	Superscaf	93074902	93074922	-6	6	12	-	-	-	-	-	-	NCP	IGR	-	ID=gene-E	7	3.885714286	1753	0.802458232	VGDVLLP	60	712.4225	1	711.4167	.	G	Superscaf	93074994	BTP20220	0	0	0	0	0	Tissue specific
LLLSRGK	Superscaf	+	Superscaf	93109153	93109173	31	31	37	-	-	-	-	-	-	NCP	IGR	-	ID=gene-E	7	7.155714286	2858.1	0.803121526	LLLSRGK	87	393.7566	2	785.5123	.	A	Superscaf	93109063	0	BTP20220	0	0	0	0	Tissue specific
DKPLTPT	Superscaf	-	Superscaf	93132490	93132510	-20	20	26	-	-	-	-	-	-	NCP	IGR	-	ID=gene-E	7	6.335714286	2703.1	0.803573463	D(+42.01)	74	813.4324	1	812.428	Acetylati	S	Superscaf	93132606	0	0	BTP20220	0	0	0	Tissue specific
AARPRAAP	Superscaf	-	Superscaf	93190522	93190545	-46	46	53	-	-	-	-	-	-	NCP	IGR	-	ID=gene-E	8	7.275	3217.6	0.804697291	AARPRAAP	89	405.24	2	808.4667	.	S	Superscaf	93190848	0	BTP20220	0	0	0	0	Tissue specific
GPAAYVP	Superscaf	+	Superscaf	93192220	93192240	2	2	8	-	-	-	-	-	-	NCP	IGR	-	ID=gene-E	7	5.181428571	2054.2	0.804730174	GPAAYVP	54	674.3517	1	673.3435	.	V	Superscaf	93192217	BTP20220	0	0	0	0	0	Tissue specific
LSYCPPPPF	Superscaf	-	Superscaf	93279979	93280005	-36	36	44	-	-	-	-	-	-	NCP	IGR	-	ID=gene-E	9	5.982222222	3469	0.806429687	LSYC(+57.	80	514.2484	2	1026.484	Carbamidc	P	Superscaf	93280050	0	0	0	0	0	BTP20220	Tissue specific
KPFAAVP	Superscaf	-	Superscaf	93315217	93315237	-22	22	28	-	-	-	-	-	-	NCP	IGR	-	ID=gene-E	7	5.694285714	2153.2	0.807112094	KPFAAVP	53	365.2168	2	728.4221	.	G	Superscaf	93315549	0	BTP20220	0	0	0	0	Tissue specific
DLKLCSF	Superscaf	-	Superscaf	93366001	93366021	-7	7	13	-	-	-	-	-	-	NCP	IGR	-	ID=gene-E	7	5.835714286	2943.2	0.808095561	DLKLC(+57	52	441.7209	2	881.4316	Carbamidc	H	Superscaf	93366150	0	0	0	0	BTP20220	0	Tissue specific
KPCFQE	Superscaf	+	Superscaf	93367312	93367329	6	6	11	-	-	-	-	-	-	NCP	IGR	-	ID=gene-E	6	5.901666667	2815.1	0.808120949	KPC(+57.(	57	404.6939	2	807.3585	Carbamidc	K	Superscaf	93367297	0	0	0	BTP20220	0	0	Tissue specific
TGDCVKK	Superscaf	+	Superscaf	93476848	93476868	19	19	25	-	-	-	-	-	-	NCP	IGR	-	ID=gene-E	7	5.71	2628.8	0.810242188	TGDC(+57.	51	404.2129	2	806.3956	Carbamidc	L	Superscaf	93476794	0	BTP20220	0	0	0	0	Tissue specific
KQVPSGK	Superscaf	-	Superscaf	93487417	93487437	-12	12	18	-	-	-	-	-	-	NCP	IGR	-	ID=gene-E	7	6.154285714	2565.6	0.810446863	KQVPSGK	63	743.4367	1	742.4337	.	E	Superscaf	93487485	0	0	BTP20220	0	0	0	Tissue specific
KSRPLH	Superscaf	-	Superscaf	93488848	93488865	-7	7	12	-	-	-	-	-	-	NCP	IGR	-	ID=gene-E	6	7.675	2811.1	0.810474576	KSRPLH	71	369.2219	2	736.4344	.	R	Superscaf	93488925	BTP20220	0	0	0	0	0	Tissue specific
TAPAVRLK	Superscaf	+	Superscaf	93491338	93491361	14	14	21	-	-	-	-	-	-	NCP	IGR	-	ID=gene-E	8	6.41875	2828.1	0.810522796	T(+42.01)	59	449.2711	2	896.5443	Acetylati	G	Superscaf	93491299	0	0	BTP20220	0	BTP20220	0	Tissue specific
FTPVASK	Superscaf	-	Superscaf	93557089	93557109	-13	13	19	-	-	-	-	-	-	NCP	IGR	-	ID=gene-E	7	5.678571429	2285.4	0.811796109	FTPVASK	98	375.2116	2	748.4119	.	D	Superscaf	93557343	0	0	0	0	0	BTP20220	Tissue specific
VSKATTK	Superscaf	+	Superscaf	93560443	93560463	16	16	22	-	-	-	-	-	-	NCP	IGR	-	ID=gene-E	7	6.32	2426.5	0.811861061	VSKATTK	54	367.7288	2	733.4334	.	I	Superscaf	93560398	0	BTP20220	0	0	0	0	Tissue specific
EAALPC	Superscaf	-	Superscaf	93635077	93635094	-4	4	9	-	-	-	-	-	-	NCP	IGR	-	ID=gene-E	6	5.426666667	2324.6	0.813306399	EAALPC(+5	72	660.3144	1	659.2949	Carbamidc	T	Superscaf	93635370	0	BTP20220	0	0	0	0	Tissue specific
LSDLATY	Superscaf	+	Superscaf	93674902	93674922	14	14	20	-	-	-	-	-	-	NCP	IGR	-	ID=gene-E	7	5.545714286	2670	0.814077637	L(+42.01)	72	824.4011	1	823.3964	Acetylati	N	Superscaf	93674863	0	0	0	0	BTP20220	0	Tissue specific
NRMFLT	Superscaf	+	Superscaf	93693286	93693303	13	13	18	-	-	-	-	-	-	NCP	IGR	-	ID=gene-E	6	6.651666667	2755.3	0.814433655	NRMFLT	55	391.2037	2	780.3953	.	D	Superscaf	93693250	BTP20220	0	0	0	0	0	Tissue specific
VAGTVKR	Superscaf	-	Superscaf	93693865	93693885	-33	33	39	-	-	-	-	-	-	NCP	IGR	-	ID=gene-E	7	5.574285714	2123.3	0.814444868	VAGTVKR	56	365.7249	2	729.4497	.	H	Superscaf	93694047	0	0	BTP20220	0	0	0	Tissue specific
APGLP	Superscaf	+	Superscaf	93763984	93763998	44	44	48	-	-	-	-	-	-	NCP	IGR	-	ID=gene-E	5	6.114	1636.8	0.81580277	A(+42.01)	69	496.2746	1	495.2693	Acetylati	A	Superscaf	93763855	BTP20220	0	0	0	0	0	Tissue specific
GVLVLLGT	Superscaf	-	Superscaf	93940000	93940023	-91	91	98	-	-	-	-	-	-	NCP	IGR	-	ID=gene-E	8	4.55125	2052.3	0.819211438	GVLVLLGT	60	771.4949	1	770.4902	.	Y	Superscaf	93940059	BTP20220	0	0	0	0	0	Tissue specific
KKLTEVR	Superscaf	+	Superscaf	93986716	93986736	19	19	25	-	-	-	-	-	-	NCP	IGR	-	ID=gene-E	7	6.567142857	2762	0.820116125	K(+42.01)	71	458.2829	2	914.5549	Acetylati	R	Superscaf	93986662	0	0	BTP20220	0	0	0	Tissue specific
VSNGPQ	Superscaf	-	Superscaf	94034407	94034424	-2	2	7	-	-	-	-	-	-	NCP	IGR	-	ID=gene-E	6	4.835	2225.5	0.821039693	VSNGPQ	60	601.2932	1	600.2867	.	I	Superscaf	94034427	0	0	BTP20220	0	0	0	Tissue specific
KGGWPF	Superscaf	-	Superscaf	94037908	94037925	-12	12	17	-	-	-	-	-	-	NCP	IGR	-	ID=gene-E	6	6.558333333	2314.4	0.821107493	KGGWPF	55	691.3557	1	690.3489	.	L	Superscaf	94038144	0	0	0	0	0	BTP20220	Tissue specific
KDNLLL	Superscaf	-	Superscaf	94054027	94054044	-10	10	15	-	-	-	-	-	-	NCP	IGR	-	ID=gene-E	6	6.01	2536.8	0.821419648	KDNLLL	53	358.2198	2	714.4276	.	K	Superscaf	94054149	0	0	0	0	0	BTP20220	Tissue specific
FVPGKTHVI	Superscaf	-	Superscaf	94057108	94057140	-6	6	16	-	-	-	-	-	-	NCP	IGR	-	ID=gene-E	11	5.56	3834.2	0.821479314	FVPGKTHVI	97	416.5781	3	1246.719	.	V	Superscaf	94057332	0	0	0	BTP20220	0	0	Tissue specific
ALLDRML	Superscaf	-	Superscaf	94079434	94079454	-3	3	9	-	-	-	-	-	-	NCP	IGR	-	ID=gene-E	7	6.205714286	2803.3	0.821911672	ALLDRML	84	416.2396	2	830.4684	.	M	Superscaf	94079475	BTP20220	0	0	BTP20220	0	0	Tissue specific
LNLVYKYL	Superscaf	-	Superscaf	94090852	94090875	-4	4	11	-	-	-	-	-	-	NCP	IGR	-	ID=gene-E	8	5.55125	3044.3	0.822132789	LNLVYKYL	67	1025.598	1	1024.596	.	C	Superscaf	94090959	0	0	BTP20220	BTP20220	0	0	Tissue specific
KPVFAAP	Superscaf	-	Superscaf	94178125	94178145	-64	64	70	-	-	-	-	-	-	NCP	IGR	-	ID=gene-E	7	5.694285714	2153.2	0.823822889	KPVFAAP	62	365.2111	2	728.4221	.	A	Superscaf	94178175	BTP20220	0	0	0	0	0	Tissue specific
RSLFSNF	Superscaf	+	Superscaf	94196377	94196397	18	18	24	-	-	-	-	-	-	NCP	IGR	-	ID=gene-E	7	6.352857143	3140.5	0.824176352	R(+42.01)	69	456.7321	2	911.4501	Acetylati	F	Superscaf	94196326	0	BTP20220	0	0	BTP20220	BTP20220	Tissue specific
TVLVTHK	Superscaf	+	Superscaf	94197625	94197645	8	8	14	-	-	-	-	-	-	NCP	IGR	-	ID=gene-E	7	5.195714286	2236.5	0.82420052	T(+42.01)	50	420.2457	2	838.4913	Acetylati	L	Superscaf	94197604	BTP20220	0	0	BTP20220	0	0	Tissue specific
TVLVTH	Superscaf	+	Superscaf	94197625	94197642	8	8	13	-	-	-	-	-	-	NCP	IGR	-	ID=gene-E	6	4.438333333	1768.1	0.82420052	TVLVTH	58	669.3912	1	668.3857	.	L	Superscaf	94197604	0	BTP20220	0	0	0	0	Mixed
YVLRAS	Superscaf	-	Superscaf	94238068	94238085	-32	32	37	-	-	-	-	-	-	NCP	IGR	-	ID=gene-E	6	5.683333333	2156.4	0.824983726	YVLRAS	65	354.7054	2	707.3966	.	G	Superscaf	94238172	BTP20220	0	0	0	0	0	Tissue specific
MMGLK	Superscaf	-	Superscaf	94244680	94244694	-5	5	9	-	-	gene	-	-	mRNA	NCP	Intron	gene|mRN	ID=gene-E	5	6.638	1862.1	0.825111772	M(+42.01)	79	621.3107	1	620.3026	Acetylati	L	Superscaf	94244796	0	0	0	0	0	BTP20220	Tissue specific
LTHWERK	Superscaf	+	Superscaf	94245742	94245762	14	14	20	-	-	gene	-	-	mRNA	NCP	Intron	gene|mRN	ID=gene-E	7	7.101428571	3439.9	0.825132338	LTHWERK	59	485.2619	2	968.5192	.	F	Superscaf	94245703	BTP20220	0	0	0	0	0	Tissue specific
APPPPPPP	Superscaf	-	Superscaf	94248211	94248234	-41	41	48	-	-	-	-	-	-	NCP	IGR	-	ID=gene-E	8	6.265	2673.1	0.825180152	APPPPPPP	81	385.2154	2	768.417	.	M	Superscaf	94248408	BTP20220	0	0	BTP20220	0	0	Tissue specific
QTLLHPR	Superscaf	-	Superscaf	94249300	94249320	-75	75	81	-	-	-	-	-	-	NCP	IGR	-	ID=gene-E	7	6.97	3320.9	0.825201241	QTLLHPR	81	432.7555	2	863.4977	.	S	Superscaf	94249455	0	0	0	0	BTP20220	0	Mixed
LYFAAGP	Superscaf	-	Superscaf	94263406	94263426	-8	8	14	-	-	-	-	-	-	NCP	IGR	-	ID=gene-E	7	5.918571429	2457.6	0.825474414	LYFAAGP	57	369.6911	2	737.3748	.	L	Superscaf	94263489	0	0	BTP20220	0	0	0	Tissue specific
ADPRVRQ	Superscaf	-	Superscaf	94308109	94308129	-51	51	57	-	-	-	-	-	-	NCP	IGR	-	ID=gene-E	7	6.065714286	2968.5	0.826340117	A(+42.01)	62	442.2392	2	882.4671	Acetylati	A	Superscaf	94308159	0	0	0	0	0	BTP20220	Tissue specific
SHRLSAF	Superscaf	-	Superscaf	94321825	94321845	-11	11	17	-	-	-	-	-	-	NCP	IGR	-	ID=gene-E	7	6.741428571	3087.4	0.826605737	SHRLSAF	92	409.2185	2	816.4242	.	M	Superscaf	94321929	0	0	BTP20220	0	BTP20220	0	Tissue specific
DLSMCHP	Superscaf	+	Superscaf	94383973	94383993	32	32	38	-	-	-	-	-	-	NCP	IGR	-	ID=gene-E	7	5.612857143	3062.6	0.827809275	DLSM(+15.	53	438.1744	2	874.3314	Oxidatior	L	Superscaf	94383880	0	BTP20220	0	0	0	0	Tissue specific
KPLERVK	Superscaf	+	Superscaf	94420792	94420812	5	5	11	-	-	-	-	-	-	NCP	IGR	-	ID=gene-E	7	6.534285714	2701.9	0.8285223	K(+42.01)	73	456.2857	2	910.56	Acetylati	R	Superscaf	94420780	BTP20220	0	0	0	0	0	Tissue specific
LNLVVLGH	Superscaf	+	Superscaf	94516225	94516248	3	3	10	-	-	-	-	-	-	NCP	IGR	-	ID=gene-E	8	4.61375	2573.9	0.830370424	LNLVVLGH	97	432.7667	2	863.5229	.	I	Superscaf	94516219	BTP20220	0	0	0	0	0	Tissue specific
MNFKFV	Superscaf	-	Superscaf	94578082	94578099	-13	13	18	-	-	-	-	-	-	NCP	IGR	-	ID=gene-E	6	5.31	2211.4	0.831568326	MNFKFV	53	393.2093	2	784.3942	.	F	Superscaf	94578135	0	0	0	BTP20220	0	0	Mixed
KMSLP	Superscaf	-	Superscaf	94605892	94605906	-12	12	16	-	-	-	-	-	-	NCP	IGR	-	ID=gene-E	5	6.69	1914.1	0.832106886	KM(+15.99	53	591.3186	1	590.3098	Oxidatior	T	Superscaf	94605930	0	0	BTP20220	0	0	0	Tissue specific
AESVHVPPF	Superscaf	-	Superscaf	94612129	94612155	-39	39	47	-	-	-	-	-	-	NCP	IGR	-	ID=gene-E	9	4.601111111	2752.1	0.83222767	A(+42.01)	55	487.7498	2	973.4869	Acetylati	Y	Superscaf	94612173	0	0	0	BTP20220	0	0	Tissue specific
RQLFQEK	Superscaf	-	Superscaf	94706560	94706580	-39	39	45	-	-	-	-	-	-	NCP	IGR	-	ID=gene-E	7	6.64	3504.9	0.83405639	RQLFQEK	72	474.7639	2	947.5189	.	H	Superscaf	94706580	BTP20220	0	0	0	0	0	Tissue specific
LLLEMEQ	Superscaf	+	Superscaf	94750843	94750863	8	8	14	-	-	-	-	-	-	NCP	IGR	-	ID=gene-E	7	5.111428571	2893.4	0.83491396	LLLEMEQ	93	875.4503	1	874.447	.	E	Superscaf	94750822	0	0	0	BTP20220	0	0	Tissue specific
KTSSYKP	Superscaf	-	Superscaf	94775152	94775172	-18	18	24	-	-	-	-	-	-	NCP	IGR	-	ID=gene-E	7	7.047142857	2898	0.83538472	KTSSYKP	68	405.7288	2	809.4283	.	E	Superscaf	94775214	0	0	0	BTP20220	0	0	Tissue specific
GTGLSPPK	Superscaf	-	Superscaf	94830868	94830891	-6	6	13	-	-	-	-	-	-	NCP	IGR	-	ID=gene-E	8	6.55875	2872.1	0.836463698	GTGLSPPK	50	378.7235	2	755.4177	.	K	Superscaf	94831164	0	0	0	0	BTP20220	0	Tissue specific
RYVASY	Superscaf	-	Superscaf	94932697	94932714	-16	16	21	-	-	-	-	-	-	NCP	IGR	-	ID=gene-E	6	5.63	2262.5	0.838435685	RYVASY	81	379.6936	2	757.3759	.	R	Superscaf	94932753	0	0	0	BTP20220	0	0	Tissue specific
NMDKKK	Superscaf	+	Superscaf	94961002	94961019	16	16	21	-	-	-	-	-	-	NCP	IGR	-	ID=gene-E	6	7.225	2784.9	0.83898383	N(+42.01)	54	403.2086	2	804.4164	Acetylati	K	Superscaf	94960957	0	0	BTP20220	0	0	0	Tissue specific
TGRELPP	Superscaf	-	Superscaf	94970791	94970811	-24	24	30	-	-	-	-	-	-	NCP	IGR	-	ID=gene-E	7	6.437142857	2797.3	0.839173401	TGRELPP	87	385.2154	2	768.413	.	V	Superscaf	94971069	0	BTP20220	0	0	0	0	Tissue specific
REMFL	Superscaf	-	Superscaf	95025130	95025144	-20	20	24	-	-	-	-	-	-	NCP	IGR	-	ID=gene-E	5	6.238	2186.6	0.840225712	REM(+15.9	65	356.1772	2	710.3421	Oxidatior	F	Superscaf	95025216	BTP20220	0	0	BTP20220	0	0	Tissue specific
DCDCPP	Superscaf	+	Superscaf	95055721	95055738	2	2	7	-	-	-	-	-	-	NCP	IGR	-	ID=gene-E	6	4.763333333	2669.2	0.840818128	D(+42.01)	50	805.2474	1	804.2418	Acetylati	D	Superscaf	95055718	BTP20220	0	0	0	0	0	Tissue specific
PSVEF	Superscaf	-	Superscaf	95117407	95117421	-34	34	38	-	-	-	-	-	-	NCP	IGR	-	ID=gene-E	5	4.136	1533.7	0.842012719	P(+42.01)	63	620.2909	1	619.2853	Acetylati	E	Superscaf	95117484	0	BTP20220	0	0	0	0	Tissue specific
NSPLQFR	Superscaf	+	Superscaf	95126689	95126709	15	15	21	-	-	-	-	-	-	NCP	IGR	-	ID=gene-E	7	6.465714286	3317.8	0.842192471	NSPLQFR	99	431.2402	2	860.4504	.	I	Superscaf	95126647	BTP20220	0	0	0	0	0	Tissue specific
AYVKAGP	Superscaf	-	Superscaf	95198683	95198703	-30	30	36	-	-	-	-	-	-	NCP	IGR	-	ID=gene-E	7	5.672857143	2185.2	0.843586683	A(+42.01)	52	374.2126	2	746.3962	Acetylati	Q	Superscaf	95198703	0	0	BTP20220	0	0	0	Tissue specific
NVVLLR	Superscaf	-	Superscaf	95228263	95228280	-31	31	36	-	-	-	-	-	-	NCP	IGR	-	ID=gene-E	6	4.688333333	1952.3	0.84415952	NVVLLR	74	357.2384	2	712.4595	.	S	Superscaf	95228289	0	0	0	BTP20220	0	0	Mixed
HLAAPPR	Superscaf	-	Superscaf	95258356	95258376	-75	75	81	-	-	-	-	-	-	NCP	IGR	-	ID=gene-E	7	6.995714286	2919.3	0.844742291	HLAAPPR	73	381.2237	2	760.4344	.	R	Superscaf	95258592	0	0	0	0	0	BTP20220	Tissue specific
TVYTLLR	Superscaf	-	Superscaf	95276599	95276619	-1	1	7	-	-	-	-	-	-	NCP	IGR	-	ID=gene-E	7	5.92	2610.1	0.845095579	TVYTLLR	92	433.2584	2	864.5069	.	T	Superscaf	95276640	0	BTP20220	0	0	BTP20220	BTP20220	Tissue specific
KYEFRV	Superscaf	-	Superscaf	95337466	95337483	-22	22	27	-	-	-	-	-	-	NCP	IGR	-	ID=gene-E	6	5.81	2389.6	0.84627431	KYEFRV	59	421.2287	2	840.4493	.	S	Superscaf	95337534	0	0	BTP20220	0	0	0	Tissue specific
PYFGPLP	Superscaf	+	Superscaf	95424058	95424078	3	3	9	-	-	-	-	-	-	NCP	IGR	-	ID=gene-E	7	5.998571429	2509.8	0.847951222	PYFGPLP	59	790.4159	1	789.4061	.	I	Superscaf	95424052	0	0	0	0	BTP20220	BTP20220	Tissue specific
YMAAV	Superscaf	-	Superscaf	95466916	95466930	-15	15	19	-	-	gene	-	-	mRNA	NCP	Intron	gene|mRN	ID=gene-E	5	4.69	1453.6	0.848781196	YMAAV	97	554.2621	1	553.257	.	V	Superscaf	95467005	0	BTP20220	0	0	BTP20220	BTP20220	Tissue specific
LSVPHRGP	Superscaf	-	Superscaf	95489884	95489907	-20	20	27	-	-	gene	-	-	mRNA	NCP	Intron	gene|mRN	ID=gene-E	8	6.0725	2977.4	0.849225987	LSVPHRGP	61	431.7482	2	861.4821	.	E	Superscaf	95490015	0	0	BTP20220	0	0	0	Tissue specific
AYLRDF	Superscaf	+	Superscaf	95505976	95505993	3	3	8	-	-	gene	-	-	mRNA	NCP	Intron	gene|mRN	ID=gene-E	6	6.145	2571.9	0.849537619	AYLRDF	97	392.7017	2	783.3915	.	V	Superscaf	95505970	0	0	BTP20220	0	0	0	Tissue specific
NELGGLK	Superscaf	-	Superscaf	95629702	95629722	-24	24	30	-	-	-	-	-	-	NCP	IGR	-	ID=gene-E	7	6.038571429	2792	0.851933657	NELGGLK	52	730.3965	1	729.4021	.	T	Superscaf	95629722	0	BTP20220	0	0	0	0	Tissue specific
MRLSPP	Superscaf	-	Superscaf	95712061	95712078	-45	45	50	-	-	-	-	-	-	NCP	IGR	-	ID=gene-E	6	6.795	2431.9	0.853528594	MRLSPP	61	700.3798	1	699.3738	.	G	Superscaf	95712126	0	0	BTP20220	0	0	0	Tissue specific
ERLPHF	Superscaf	-	Superscaf	95795422	95795439	-7	7	12	-	-	-	-	-	-	NCP	IGR	-	ID=gene-E	6	6.555	2772.2	0.855142936	ERLPHF	71	399.7185	2	797.4184	.	A	Superscaf	95795514	0	0	0	0	0	BTP20220	Tissue specific
ANLRMAP	Superscaf	-	Superscaf	95833726	95833746	-15	15	21	-	-	-	-	-	-	NCP	IGR	-	ID=gene-E	7	6.605714286	2930.4	0.855884719	ANLRM(+15	66	394.7025	2	787.4011	Oxidatior	T	Superscaf	95833770	0	0	0	0	BTP20220	0	Tissue specific
SFEVLGR	Superscaf	+	Superscaf	95853511	95853531	36	36	42	-	-	-	-	-	-	NCP	IGR	-	ID=gene-E	7	5.298571429	2495.8	0.856267869	SFEVLGR	89	404.2202	2	806.4286	.	L	Superscaf	95853406	BTP20220	0	0	0	0	0	Tissue specific
KGGPMPK	Superscaf	-	Superscaf	95870011	95870031	-6	6	12	-	-	-	-	-	-	NCP	IGR	-	ID=gene-E	7	7.11	2577.7	0.856587403	KGGPMPK	50	357.6971	2	713.3894	.	N	Superscaf	95870055	0	0	0	BTP20220	0	0	Tissue specific
SYNLVLY	Superscaf	+	Superscaf	96007282	96007302	4	4	10	-	-	-	-	-	-	NCP	IGR	-	ID=gene-E	7	4.91	2605.9	0.859245748	SYNLVLY	51	871.4635	1	870.4487	.	K	Superscaf	96007273	BTP20220	0	0	0	0	0	Tissue specific
QPVPPGP	Superscaf	+	Superscaf	96225394	96225414	4	4	10	-	-	-	-	-	-	NCP	IGR	-	ID=gene-E	7	5.26	2257.6	0.863469634	Q(+42.01)	52	367.196	2	732.3806	Acetylati	P	Superscaf	96225385	0	0	0	BTP20220	0	0	Tissue specific
VNVRTKP	Superscaf	-	Superscaf	96225697	96225717	-5	5	11	-	-	-	-	-	-	NCP	IGR	-	ID=gene-E	7	5.534285714	2448.8	0.863475502	VNVRTKP	79	407.2503	2	812.4868	.	V	Superscaf	96225798	0	0	BTP20220	0	0	0	Tissue specific
FTKEKT	Superscaf	+	Superscaf	96234253	96234270	30	30	35	-	-	-	-	-	-	NCP	IGR	-	ID=gene-E	6	6.873333333	2544.7	0.863641194	FTKEKT	70	377.2054	2	752.4068	.	C	Superscaf	96234166	0	0	BTP20220	0	0	0	Tissue specific
VNFKEYK	Superscaf	+	Superscaf	96240649	96240669	26	26	32	-	-	-	-	-	-	NCP	IGR	-	ID=gene-E	7	5.607142857	2805.9	0.863765057	VNFKEYK	57	464.2515	2	926.4861	.	Y	Superscaf	96240574	0	0	0	BTP20220	0	0	Tissue specific
SLFRLH	Superscaf	+	Superscaf	96394381	96394398	13	13	18	-	-	-	-	-	-	NCP	IGR	-	ID=gene-E	6	6.911666667	2746.1	0.866742181	SLFRLH	50	386.7311	2	771.4391	.	I	Superscaf	96394345	0	0	BTP20220	0	0	0	Tissue specific
PTPPP	Superscaf	+	Superscaf	96428137	96428151	12	12	16	-	-	-	-	-	-	NCP	IGR	-	ID=gene-E	5	6.346	1747.1	0.867395889	PTPPP	67	508.2747	1	507.2693	.	H	Superscaf	96428104	0	0	0	0	0	BTP20220	Tissue specific
PDAGDE	Superscaf	+	Superscaf	96473446	96473463	29	29	34	-	-	-	-	-	-	NCP	IGR	-	ID=gene-E	6	4.575	2194.5	0.868273328	PDAGDE	50	603.2185	1	602.2183	.	C	Superscaf	96473362	0	0	0	BTP20220	0	0	Tissue specific
LGKMHMK	Superscaf	+	Superscaf	96496711	96496731	12	12	18	-	-	-	-	-	-	NCP	IGR	-	ID=gene-E	7	7.217142857	2950.2	0.868723871	LGKMHM(+1	55	430.7258	2	859.4408	Oxidatior	K	Superscaf	96496678	0	0	0	0	0	BTP20220	Tissue specific
KDLKEF	Superscaf	+	Superscaf	96496930	96496947	39	39	44	-	-	-	-	-	-	NCP	IGR	-	ID=gene-E	6	6.188333333	2514.6	0.868728112	K(+42.01)	74	411.2221	2	820.433	Acetylati	D	Superscaf	96496816	0	0	0	BTP20220	0	0	Tissue specific
APVLEYL	Superscaf	+	Superscaf	96508543	96508563	2	2	8	-	-	-	-	-	-	NCP	IGR	-	ID=gene-E	7	4.737142857	2240.5	0.868953005	APVLEYL	64	804.4483	1	803.4429	.	V	Superscaf	96508540	0	0	0	BTP20220	0	0	Mixed
KRFSLRP	Superscaf	+	Superscaf	96547867	96547887	1	1	7	-	-	gene	-	-	mRNA	NCP	Intron	gene|mRN	ID=gene-E	7	7.814285714	3227.6	0.869714541	KRFSLRP	69	452.2789	2	902.545	.	K	Superscaf	96547867	0	0	BTP20220	0	0	0	Tissue specific
KDANLLY	Superscaf	-	Superscaf	96576640	96576660	-7	7	13	-	-	-	-	-	-	NCP	IGR	-	ID=gene-E	7	5.965714286	2954.2	0.87027175	KDANLLY	58	836.4478	1	835.4439	.	C	Superscaf	96576660	0	BTP20220	0	0	0	0	Tissue specific
LSTREVL	Superscaf	+	Superscaf	96637894	96637914	18	18	24	-	-	-	-	-	-	NCP	IGR	-	ID=gene-E	7	5.45	2562	0.871457975	LSTREVL	60	409.241	2	816.4705	.	T	Superscaf	96637843	0	0	0	BTP20220	0	0	Tissue specific
HTAPY	Superscaf	-	Superscaf	96644356	96644370	-3	3	7	-	-	-	-	-	-	NCP	IGR	-	ID=gene-E	5	6.42	2125.4	0.871583116	H(+42.01)	62	630.2769	1	629.2809	Acetylati	M	Superscaf	96644442	BTP20220	0	0	0	0	0	Tissue specific
RRKLLK	Superscaf	+	Superscaf	96659041	96659058	2	2	7	-	-	-	-	-	-	NCP	IGR	-	ID=gene-E	6	8.826666667	2941.2	0.871867501	RRKLLK	57	407.2872	2	812.5708	.	R	Superscaf	96659038	0	0	0	BTP20220	0	0	Tissue specific
WGAKPLF	Superscaf	-	Superscaf	96686770	96686790	-4	4	10	-	-	-	-	-	-	NCP	IGR	-	ID=gene-E	7	6.482857143	2681.8	0.872404491	WGAKPLF	84	409.7301	2	817.4486	.	Q	Superscaf	96686808	0	0	0	0	BTP20220	BTP20220	Tissue specific
VVVAPGP	Superscaf	-	Superscaf	96689470	96689490	-5	5	11	-	-	-	-	-	-	NCP	IGR	-	ID=gene-E	7	3.512857143	1283.4	0.872456779	V(+42.01)	85	680.4038	1	679.3904	Acetylati	S	Superscaf	96689490	0	BTP20220	0	0	0	0	Tissue specific
KKAPSPK	Superscaf	+	Superscaf	96727243	96727263	4	4	10	-	-	-	-	-	-	NCP	IGR	-	ID=gene-E	7	7.645714286	2774.7	0.873188278	KKAPSPK	60	378.2401	2	754.4701	.	S	Superscaf	96727234	BTP20220	0	0	0	0	0	Tissue enhanced
KGEHYGP	Superscaf	-	Superscaf	96728305	96728325	-11	11	17	-	-	-	-	-	-	NCP	IGR	-	ID=gene-E	7	6.35	2905.1	0.873208845	KGEHYGP	50	394.1895	2	786.366	.	S	Superscaf	96728400	BTP20220	0	0	0	0	0	Tissue specific
ERQFSFP	Superscaf	+	Superscaf	96823942	96823962	1	1	7	-	-	-	-	-	-	NCP	IGR	-	ID=gene-E	7	6.081428571	3180.6	0.87506092	ERQFSFP	63	455.7309	2	909.4344	.	E	Superscaf	96823942	BTP20220	0	0	0	0	0	Tissue specific
PPVAAPN	Superscaf	-	Superscaf	96853123	96853143	-18	18	24	-	-	-	-	-	-	NCP	IGR	-	ID=gene-E	7	5.192857143	2231.5	0.875626029	PPVAAPN	65	665.3486	1	664.3544	.	G	Superscaf	96853320	0	0	0	0	BTP20220	0	Tissue specific
SALGLEL	Superscaf	-	Superscaf	96947890	96947910	-7	7	13	-	-	-	-	-	-	NCP	IGR	-	ID=gene-E	7	5.547142857	2477.7	0.877461256	SALGLEL	73	702.3993	1	701.3959	.	F	Superscaf	96947922	0	0	0	0	0	BTP20220	Mixed
KVRLGGGN	Superscaf	-	Superscaf	96978550	96978573	-15	15	22	-	-	-	-	-	-	NCP	IGR	-	ID=gene-E	8	6.225	2959.2	0.878055008	KVRLGGGN	62	400.7399	2	799.4664	.	K	Superscaf	96978573	0	BTP20220	0	0	0	0	Tissue specific
KPPTVPN	Superscaf	+	Superscaf	97095343	97095363	25	25	31	-	-	-	-	-	-	NCP	IGR	-	ID=gene-E	7	5.797142857	2474.8	0.880316783	KPPTVPN	90	376.7179	2	751.4228	.	V	Superscaf	97095271	0	BTP20220	0	0	0	0	Tissue specific
QVKNKPK	Superscaf	+	Superscaf	97128937	97128957	8	8	14	-	-	gene	-	-	mRNA	NCP	Intron	gene|mRN	ID=gene-E	7	6.654285714	2950	0.880967353	QVKNKPK	81	841.5115	1	840.5181	.	N	Superscaf	97128916	0	0	BTP20220	0	0	0	Tissue specific
LKLLAPEPF	Superscaf	-	Superscaf	97180147	97180173	-4	4	12	-	-	-	-	-	-	NCP	IGR	-	ID=gene-E	9	6.697777778	3589	0.88195907	LKLLAPEPF	81	518.8279	2	1035.644	.	H	Superscaf	97180284	0	0	0	0	BTP20220	0	Tissue specific
KLLAPEPR	Superscaf	-	Superscaf	97180150	97180173	-5	5	12	-	-	-	-	-	-	NCP	IGR	-	ID=gene-E	8	6.7875	3235.6	0.881959128	KLLAPEPR	82	462.2872	2	922.5599	.	H	Superscaf	97180284	0	0	BTP20220	0	0	0	Tissue enhanced
NPVVLEPA	Superscaf	-	Superscaf	97209553	97209576	-22	22	29	-	-	-	-	-	-	NCP	IGR	-	ID=gene-E	8	4.15375	2361.7	0.882528537	NPVVLEPA	53	838.4746	1	837.4596	.	S	Superscaf	97209582	BTP20220	0	0	BTP20220	0	0	Tissue specific
TNFAP	Superscaf	+	Superscaf	97245655	97245669	4	4	8	-	-	-	-	-	-	NCP	IGR	-	ID=gene-E	5	5.948	2030.3	0.883227676	T(+42.01)	69	591.2769	1	590.27	Acetylati	N	Superscaf	97245646	BTP20220	0	0	BTP20220	0	0	Tissue specific
PTGYCLK	Superscaf	+	Superscaf	97411438	97411458	30	30	36	-	-	-	-	-	-	NCP	IGR	-	ID=gene-E	7	6.457142857	2899.2	0.886438176	PTGYC(+57	56	419.7181	2	837.4055	Carbamidc	C	Superscaf	97411351	0	0	0	0	0	BTP20220	Tissue specific
MFVLLQ	Superscaf	-	Superscaf	97436548	97436565	-70	70	75	-	-	-	-	-	-	NCP	IGR	-	ID=gene-E	6	4.806666667	2076.4	0.886924448	MFVLLQ	60	750.4194	1	749.4146	.	V	Superscaf	97436625	0	0	BTP20220	0	0	0	Tissue specific
AEEGDGN	Superscaf	+	Superscaf	97482991	97483011	11	11	17	-	-	-	-	-	-	NCP	IGR	-	ID=gene-E	7	4.682857143	2765.1	0.887823848	A(+42.01)	54	733.2501	1	732.2562	Acetylati	F	Superscaf	97482961	0	0	0	0	BTP20220	0	Tissue specific
NAPSFPL	Superscaf	-	Superscaf	97483282	97483302	-17	17	23	-	-	-	-	-	-	NCP	IGR	-	ID=gene-E	7	5.881428571	2707	0.887829483	NAPSFPL	96	745.3869	1	744.3806	.	K	Superscaf	97483326	BTP20220	0	0	0	0	0	Mixed
NEGFDLL	Superscaf	+	Superscaf	97495786	97495806	2	2	8	-	-	-	-	-	-	NCP	IGR	-	ID=gene-E	7	5.001428571	2825.2	0.888071632	NEGFDLL	98	807.3854	1	806.381	.	I	Superscaf	97495783	0	BTP20220	0	0	0	0	Tissue specific
RHLMPTR	Superscaf	+	Superscaf	97526482	97526502	3	3	9	-	-	-	-	-	-	NCP	IGR	-	ID=gene-E	7	7.667142857	3377.1	0.88866608	RHLMPTR	95	455.7602	2	909.4967	.	D	Superscaf	97526476	0	0	BTP20220	0	0	0	Tissue specific
EKPPDP	Superscaf	-	Superscaf	97567180	97567197	-26	26	31	-	-	-	-	-	-	NCP	IGR	-	ID=gene-E	6	5.805	2317.6	0.889454225	EKPPDP	67	682.3298	1	681.3333	.	S	Superscaf	97567245	BTP20220	0	0	0	0	0	Tissue specific
CTYCCC	Superscaf	-	Superscaf	97582360	97582377	-32	32	37	-	-	gene	-	-	mRNA	NCP	Intron	gene|mRN	ID=gene-E	6	5.378333333	3199.8	0.889748195	C(+57.02)	56	462.1269	2	922.2442	Carbamidc	I	Superscaf	97582377	0	0	0	0	BTP20220	0	Tissue specific
AMVSEFL	Superscaf	-	Superscaf	97651999	97652019	-35	35	41	-	-	-	-	-	-	NCP	IGR	-	ID=gene-E	7	4.59	2232.5	0.891096802	A(+42.01)	80	854.3933	1	853.3892	Acetylati	G	Superscaf	97652031	0	0	0	0	0	BTP20220	Mixed
GDDGP	Superscaf	-	Superscaf	97662382	97662396	-24	24	28	-	-	-	-	-	-	NCP	IGR	-	ID=gene-E	5	4.836	1755	0.891297875	GDDGP	61	460.1663	1	459.1601	.	R	Superscaf	97662489	0	0	0	0	BTP20220	0	Mixed
VAPVT	Superscaf	+	Superscaf	97741723	97741737	1	1	5	-	-	-	-	-	-	NCP	IGR	-	ID=gene-E	5	3.77	1046.2	0.892834367	V(+42.01)	51	528.301	1	527.2955	Acetylati	V	Superscaf	97741723	BTP20220	0	0	0	0	0	Tissue specific
NSSFTLH	Superscaf	+	Superscaf	97748080	97748100	72	72	78	-	-	-	-	-	-	NCP	IGR	-	ID=gene-E	7	6.05	3121.5	0.892957475	N(+42.01)	61	424.1981	2	846.3871	Acetylati	S	Superscaf	97747867	0	0	0	0	0	BTP20220	Tissue specific
HVVVLHP	Superscaf	-	Superscaf	97766944	97766964	-12	12	18	-	-	-	-	-	-	NCP	IGR	-	ID=gene-E	7	3.922857143	1930.2	0.893322789	HVVVLHP	72	400.7407	2	799.4705	.	L	Superscaf	97767021	0	BTP20220	0	0	0	0	Tissue specific
PVVVMLC	Superscaf	-	Superscaf	97791232	97791252	-15	15	21	-	-	-	-	-	-	NCP	IGR	-	ID=gene-E	7	3.292857143	1648	0.893793143	P(+42.01)	54	438.2157	2	874.4293	Acetylati	L	Superscaf	97791294	0	0	0	0	0	BTP20220	Tissue specific
KNKKLT	Superscaf	+	Superscaf	97796884	97796901	25	25	30	-	-	-	-	-	-	NCP	IGR	-	ID=gene-E	6	7.856666667	2752.8	0.893902597	KNKKLT	50	366.2411	2	730.4701	.	V	Superscaf	97796812	0	0	BTP20220	0	0	0	Tissue specific
PDLPLPD	Superscaf	-	Superscaf	97884832	97884852	-4	4	10	-	-	-	-	-	-	NCP	IGR	-	ID=gene-E	7	5.257142857	2542	0.89560577	PDLPLPD	66	766.3949	1	765.3908	.	C	Superscaf	97884861	0	0	BTP20220	0	0	0	Tissue specific
KDELPY	Superscaf	+	Superscaf	97916563	97916580	19	19	24	-	-	-	-	-	-	NCP	IGR	-	ID=gene-E	6	5.645	2455.7	0.896220262	KDELPY	77	764.3793	1	763.3752	.	L	Superscaf	97916509	0	0	0	0	BTP20220	0	Mixed
PDVTLER	Superscaf	-	Superscaf	97921345	97921365	-8	8	14	-	-	-	-	-	-	NCP	IGR	-	ID=gene-E	7	5.108571429	2574.1	0.896312869	P(+42.01)	76	436.226	2	870.4446	Acetylati	V	Superscaf	97921419	0	0	BTP20220	0	0	0	Tissue specific
KQPAEPP	Superscaf	-	Superscaf	97923370	97923390	-12	12	18	-	-	gene	-	-	mRNA	NCP	Intron	gene|mRN	ID=gene-E	7	6.218571429	2828.1	0.896352084	KQPAEPP	53	383.7157	2	765.402	.	V	Superscaf	97923480	0	0	BTP20220	0	0	0	Tissue specific
LEKGLDL	Superscaf	-	Superscaf	97941799	97941819	-47	47	53	-	-	gene	-	-	mRNA	NCP	Intron	gene|mRN	ID=gene-E	7	5.691428571	2662.9	0.896708974	LEKGLDL	71	394.2304	2	786.4487	.	L	Superscaf	97941846	BTP20220	0	0	0	0	0	Mixed
RLKKKP	Superscaf	+	Superscaf	97992415	97992432	14	14	19	-	-	-	-	-	-	NCP	IGR	-	ID=gene-E	6	8.71	2744.8	0.897689187	RLKKKP	95	385.2669	2	768.5333	.	G	Superscaf	97992376	BTP20220	0	0	0	0	0	Tissue specific
YDHME	Superscaf	+	Superscaf	98126896	98126910	12	12	16	-	-	-	-	-	-	NCP	IGR	-	ID=gene-E	5	5.038	2287.7	0.900293502	YDHME	72	694.2447	1	693.2428	.	L	Superscaf	98126863	0	BTP20220	0	0	0	0	Tissue specific
AVLELY	Superscaf	-	Superscaf	98142316	98142333	-22	22	27	-	-	gene	-	-	mRNA	NCP	Intron	gene|mRN	ID=gene-E	6	4.476666667	1903.1	0.900592121	AVLELY	72	707.3943	1	706.3901	.	C	Superscaf	98142339	0	0	BTP20220	0	0	0	Tissue specific
KLHQELS	Superscaf	+	Superscaf	98221102	98221122	8	8	14	-	-	-	-	-	-	NCP	IGR	-	ID=gene-E	7	6.262857143	3214.5	0.902117865	K(+42.01)	98	896.4823	1	895.4763	Acetylati	G	Superscaf	98221081	0	0	0	BTP20220	0	0	Tissue specific
AAATPDLG	Superscaf	+	Superscaf	98321635	98321658	33	33	40	-	-	-	-	-	-	NCP	IGR	-	ID=gene-E	8	5.72625	2731	0.904064754	A(+42.01)	63	379.1886	2	756.3654	Acetylati	W	Superscaf	98321539	0	BTP20220	0	0	0	0	Tissue specific
AYLSLEL	Superscaf	+	Superscaf	98420653	98420673	2	2	8	-	-	-	-	-	-	NCP	IGR	-	ID=gene-E	7	5.502857143	2639.9	0.905982305	AYLSLEL	56	808.4418	1	807.4378	.	R	Superscaf	98420650	BTP20220	0	0	0	0	0	Tissue specific
KYPQSR	Superscaf	-	Superscaf	98505388	98505405	-14	14	19	-	-	-	-	-	-	NCP	IGR	-	ID=gene-E	6	7.298333333	2908.2	0.907623255	KYPQSR	56	389.7116	2	777.4133	.	S	Superscaf	98505456	0	0	BTP20220	0	0	0	Tissue specific
KSVYVLK	Superscaf	-	Superscaf	98506645	98506665	-3	3	9	-	-	-	-	-	-	NCP	IGR	-	ID=gene-E	7	5.257142857	2133.1	0.907647598	KSVYVLK	54	418.7709	2	835.5167	.	I	Superscaf	98506836	BTP20220	0	0	0	0	0	Tissue specific
KPPRKGQ	Superscaf	-	Superscaf	98528452	98528472	-5	5	11	-	-	-	-	-	-	NCP	IGR	-	ID=gene-E	7	7.78	3168.4	0.908069905	KPPRKGQ	52	405.7523	2	809.4871	.	F	Superscaf	98528478	BTP20220	0	0	0	0	0	Tissue specific
NPGLLLG	Superscaf	+	Superscaf	98546488	98546508	25	25	31	-	-	-	-	-	-	NCP	IGR	-	ID=gene-E	7	5.941428571	2588.9	0.908419184	NPGLLLG	54	683.4075	1	682.4014	.	E	Superscaf	98546416	0	0	0	0	BTP20220	0	Mixed
KPGSLDL	Superscaf	+	Superscaf	98628745	98628765	16	16	22	-	-	-	-	-	-	NCP	IGR	-	ID=gene-E	7	6.088571429	2604.8	0.910012147	KPGSLDL	88	729.4111	1	728.4068	.	S	Superscaf	98628700	0	0	0	0	0	BTP20220	Tissue specific
SRLLQRF	Superscaf	-	Superscaf	98628874	98628894	-24	24	30	-	-	-	-	-	-	NCP	IGR	-	ID=gene-E	7	7.184285714	3385.9	0.910014645	SRLLQRF	73	460.276	2	918.5399	.	I	Superscaf	98629044	BTP20220	0	0	0	0	0	Tissue specific
SGPKPHVL	Superscaf	+	Superscaf	98634793	98634816	11	11	18	-	-	-	-	-	-	NCP	IGR	-	ID=gene-E	8	5.945	2797	0.91012927	SGPKPHVL	88	417.7436	2	833.4759	.	M	Superscaf	98634763	0	0	0	0	BTP20220	0	Tissue specific
SGPKPHV	Superscaf	+	Superscaf	98634793	98634813	11	11	17	-	-	-	-	-	-	NCP	IGR	-	ID=gene-E	7	5.94	2443.6	0.91012927	SGPKPHV	98	361.2008	2	720.3918	.	M	Superscaf	98634763	BTP20220	0	0	0	0	0	Tissue specific
KSKYPHR	Superscaf	+	Superscaf	98640187	98640207	40	40	46	-	-	-	-	-	-	NCP	IGR	-	ID=gene-E	7	7.924285714	3385.6	0.910233729	KSKYPHR	55	458.256	2	914.5086	.	N	Superscaf	98640070	0	0	0	0	0	BTP20220	Tissue specific
STFTHKTVY	Superscaf	-	Superscaf	98709994	98710020	-51	51	59	-	-	-	-	-	-	NCP	IGR	-	ID=gene-E	9	5.971111111	3510.9	0.911585588	STFTHKTVY	96	542.2753	2	1082.54	.	V	Superscaf	98710131	BTP20220	0	0	0	0	0	Tissue specific
KGGDSLK	Superscaf	+	Superscaf	98745898	98745918	59	59	65	-	-	-	-	-	-	NCP	IGR	-	ID=gene-E	7	6.578571429	2679.7	0.912280893	KGGDSLK	54	352.6964	2	703.3864	.	N	Superscaf	98745724	0	0	0	BTP20220	0	0	Tissue specific
MDPCHLSVF	Superscaf	-	Superscaf	98746180	98746215	-18	18	29	-	-	-	-	-	-	NCP	IGR	-	ID=gene-E	12	5.599166667	4692.3	0.912286354	M(+42.01)	79	734.8363	2	1467.664	Acetylati	S	Superscaf	98746854	BTP20220	0	0	0	0	0	Tissue specific
DEGAKRLT	Superscaf	+	Superscaf	98746513	98746536	9	9	16	-	-	-	-	-	-	NCP	IGR	-	ID=gene-E	8	6.39875	3313.7	0.912292803	DEGAKRLT	71	445.2332	2	888.4665	.	T	Superscaf	98746489	BTP20220	0	0	0	0	0	Tissue specific
KKTKCGK	Superscaf	+	Superscaf	98831293	98831313	4	4	10	-	-	-	-	-	-	NCP	IGR	-	ID=gene-E	7	8.068571429	3154.1	0.913934625	KKTKC(+57	68	425.2443	2	848.4902	Carbamidc	Y	Superscaf	98831284	0	0	0	BTP20220	0	0	Tissue specific
VMPGHRP	Superscaf	-	Superscaf	98831566	98831586	-12	12	18	-	-	-	-	-	-	NCP	IGR	-	ID=gene-E	7	6.095714286	2612.1	0.913939912	VM(+15.99	74	405.2065	2	808.4014	Oxidatior	D	Superscaf	98831784	0	0	0	BTP20220	0	0	Tissue specific
KSKAFPK	Superscaf	+	Superscaf	98877838	98877858	13	13	19	-	-	-	-	-	-	NCP	IGR	-	ID=gene-E	7	7.528571429	2824.7	0.914836	K(+42.01)	54	424.2558	2	846.4963	Acetylati	P	Superscaf	98877802	BTP20220	0	0	0	0	0	Tissue specific
KGVSLFPK	Superscaf	-	Superscaf	98886628	98886651	-37	37	44	-	-	-	-	-	-	NCP	IGR	-	ID=gene-E	8	6.11125	2695.7	0.915006225	KGVSLFPK	67	438.2683	2	874.5276	.	G	Superscaf	98886684	0	0	BTP20220	0	0	0	Tissue specific
KFVEAF	Superscaf	-	Superscaf	98933215	98933232	-3	3	8	-	-	-	-	-	-	NCP	IGR	-	ID=gene-E	6	4.99	1980	0.915908413	KFVEAF	53	740.4041	1	739.3904	.	L	Superscaf	98933232	BTP20220	0	0	0	0	0	Tissue specific
TLGYTPF	Superscaf	+	Superscaf	98959516	98959536	12	12	18	-	-	-	-	-	-	NCP	IGR	-	ID=gene-E	7	6.064285714	2630	0.91641775	TLGYTPF	73	798.4014	1	797.3959	.	V	Superscaf	98959483	0	0	0	0	0	BTP20220	Tissue specific

QLNMH	Superscaf	-	Superscaf	98966959	98966973	-4	4	8	-	-	-	-	-	-	NCP	IGR	-	ID=gene-E	5	6.076	2552	0.916561889	QLNM(+15.	84	658.2856	1	657.2905	Oxidatior	L	Superscaf	98967159	BTP20220	0	0	0	0	0	Tissue specific
APALHAGP	Superscaf	+	Superscaf	99030727	99030750	73	73	80	-	-	-	-	-	-	NCP	IGR	-	ID=gene-E	8	6.275	2879.1	0.917796799	APALHAGP	57	367.1995	2	732.3918	.	P	Superscaf	99030511	BTP20220	0	0	0	0	0	Tissue specific
DFLRA	Superscaf	+	Superscaf	99103609	99103623	20	20	24	-	-	-	-	-	-	NCP	IGR	-	ID=gene-E	5	6.242	2112.4	0.919208208	DFLRA	96	621.3338	1	620.3282	.	H	Superscaf	99103552	0	0	0	0	0	BTP20220	Tissue specific
FRQANF	Superscaf	+	Superscaf	99133654	99133671	13	13	18	-	-	-	-	-	-	NCP	IGR	-	ID=gene-E	6	6.466666667	2942.3	0.91979005	FRQANF	57	391.6993	2	781.3871	.	D	Superscaf	99133618	0	BTP20220	0	0	0	0	Tissue specific
ATLFGPK	Superscaf	-	Superscaf	99148819	99148839	-13	13	19	-	-	-	-	-	-	NCP	IGR	-	ID=gene-E	7	6.574285714	2552.7	0.92008373	ATLFGPK	99	367.2151	2	732.417	.	S	Superscaf	99148875	0	0	BTP20220	0	0	0	Tissue specific
LGPPPPPPI	Superscaf	-	Superscaf	99169339	99169365	-52	52	60	-	-	-	-	-	-	NCP	IGR	-	ID=gene-E	9	6.192222222	3028.5	0.920481114	LGPPPPPPI	81	442.7639	2	883.5167	.	G	Superscaf	99169731	0	0	0	0	BTP20220	0	Tissue specific
AALAFGV	Superscaf	-	Superscaf	99215575	99215595	-7	7	13	-	-	-	-	-	-	NCP	IGR	-	ID=gene-E	7	5.07	1972	0.921376505	AALAFGV	51	648.3799	1	647.3642	.	T	Superscaf	99215598	BTP20220	0	0	0	0	0	Tissue specific
LPPFE	Superscaf	-	Superscaf	99238369	99238383	-65	65	69	-	-	-	-	-	-	NCP	IGR	-	ID=gene-E	5	5.456	1841.1	0.921817926	LPPFE	99	602.3169	1	601.3111	.	S	Superscaf	99238473	0	0	BTP20220	0	0	0	Tissue specific
KGVLSHSL	Superscaf	-	Superscaf	99255331	99255354	-71	71	78	-	-	-	-	-	-	NCP	IGR	-	ID=gene-E	8	5.8275	2859	0.922146407	KGVLSHSL	63	420.749	2	839.4865	.	H	Superscaf	99255414	0	0	0	0	BTP20220	0	Tissue specific
MAPKPK	Superscaf	+	Superscaf	99386008	99386025	6	6	11	-	-	-	-	-	-	NCP	IGR	-	ID=gene-E	6	7.308333333	2294.4	0.924677055	M(+42.01)	84	357.2023	2	712.3942	Acetylati	D	Superscaf	99385993	BTP20220	0	0	0	0	0	Tissue specific
FGVPPPP	Superscaf	-	Superscaf	99391711	99391731	-8	8	14	-	-	-	-	-	-	NCP	IGR	-	ID=gene-E	7	5.235714286	2034.3	0.924787498	FGVPPPP	61	710.3906	1	709.3799	.	F	Superscaf	99391851	0	BTP20220	0	0	0	0	Mixed
AAVVD	Superscaf	-	Superscaf	99408415	99408429	-8	8	12	-	-	gene	-	-	mRNA	NCP	Intron	gene|mRN	ID=gene-E	5	3.002	1034.1	0.925110982	AAVVD	68	474.2513	1	473.2485	.	A	Superscaf	99408576	0	BTP20220	0	0	0	0	Tissue specific
SAGPLGGP	Superscaf	+	Superscaf	99419488	99419511	13	13	20	-	-	-	-	-	-	NCP	IGR	-	ID=gene-E	8	6.02375	2614.8	0.925325418	S(+42.01)	77	697.3491	1	696.3442	Acetylati	G	Superscaf	99419452	0	0	0	0	0	BTP20220	Tissue specific
SALLVPR	Superscaf	-	Superscaf	99436378	99436398	-269	269	275	-	-	-	-	-	-	NCP	IGR	-	ID=gene-E	7	5.817142857	2387.7	0.925652504	SALLVPR	66	378.2397	2	754.4701	.	N	Superscaf	99436425	0	BTP20220	BTP20220	0	0	0	Tissue specific
PPPYVPP	Superscaf	-	Superscaf	99484882	99484902	-104	104	110	-	-	-	-	-	-	NCP	IGR	-	ID=gene-E	7	5.308571429	2146.5	0.926591817	PPPYVPP	93	766.4105	1	765.4061	.	Q	Superscaf	99484914	0	BTP20220	0	0	0	0	Mixed
PPYVPPP	Superscaf	-	Superscaf	99484885	99484905	-105	105	111	-	-	-	-	-	-	NCP	IGR	-	ID=gene-E	7	5.308571429	2146.5	0.926591875	PPYVPPP	79	766.412	1	765.4061	.	Q	Superscaf	99484914	0	0	0	BTP20220	0	0	Mixed
KPGNEEK	Superscaf	+	Superscaf	99537667	99537687	37	37	43	-	-	-	-	-	-	NCP	IGR	-	ID=gene-E	7	6.228571429	3019.2	0.927614034	KPGNEEK	63	401.2059	2	800.4028	.	G	Superscaf	99537559	0	0	0	0	0	BTP20220	Tissue specific
ALVAVLG	Superscaf	-	Superscaf	99648409	99648429	-26	26	32	-	-	-	-	-	-	NCP	IGR	-	ID=gene-E	7	4.281428571	1626.7	0.929758627	ALVAVLG	51	642.4161	1	641.4112	.	N	Superscaf	99648459	BTP20220	0	0	0	0	0	Tissue specific
NPYKLR	Superscaf	+	Superscaf	99698035	99698052	4	4	9	-	-	gene	-	-	mRNA	NCP	Intron	gene|mRN	ID=gene-E	6	7.308333333	2864.2	0.930719668	NPYKLR	82	395.7315	2	789.4497	.	C	Superscaf	99698026	0	BTP20220	0	0	0	0	Tissue specific
GPVAAMM	Superscaf	+	Superscaf	99714535	99714555	18	18	24	-	-	-	-	-	-	NCP	IGR	-	ID=gene-E	7	5.115714286	2000.3	0.931039202	GPVAAMM	56	676.3095	1	675.3084	.	A	Superscaf	99714484	0	0	0	0	0	BTP20220	Tissue specific
NVDGSHM	Superscaf	-	Superscaf	99972889	99972909	-7	7	13	-	-	gene	-	-	mRNA	NCP	Intron	gene|mRN	ID=gene-E	7	4.767142857	2680.1	0.936042401	NVDGSHM(+	56	775.2897	1	774.2967	Oxidatior	S	Superscaf	99972930	0	0	BTP20220	0	0	0	Tissue specific
EQKQPQ	Superscaf	+	Superscaf	99976036	99976053	4	4	9	-	-	gene	-	-	mRNA	NCP	Intron	gene|mRN	ID=gene-E	6	6.035	3063.4	0.936103345	EQKQPQ	83	757.3704	1	756.3766	.	A	Superscaf	99976027	0	BTP20220	0	0	0	0	Tissue specific
PVKLSHK	Superscaf	-	Superscaf	1E+08	1E+08	-2	2	8	-	-	-	-	-	-	NCP	IGR	-	ID=gene-E	7	6.432857143	2630.7	0.936687801	PVKLSHK	71	404.7537	2	807.4966	.	S	Superscaf	1E+08	BTP20220	0	0	0	0	0	Tissue enhanced
SEPVCSK	Superscaf	+	Superscaf	1E+08	1E+08	4	4	10	-	-	-	-	-	-	NCP	IGR	-	ID=gene-E	7	5.091428571	2583.8	0.937498487	SEPVC(+57	50	806.3618	1	805.364	Carbamido	L	Superscaf	1E+08	BTP20220	0	0	0	0	0	Tissue specific
RMVTLK	Superscaf	-	Superscaf	1E+08	1E+08	-1	1	6	-	-	-	-	-	-	NCP	IGR	-	ID=gene-E	6	6.46	2239.6	0.937927998	RMVTLK	71	747.4401	1	746.4473	.	R	Superscaf	1E+08	BTP20220	0	0	0	0	0	Tissue specific
RRSLF	Superscaf	+	Superscaf	1E+08	1E+08	10	10	14	-	-	gene	-	-	mRNA	NCP	Intron	gene|mRN	ID=gene-E	5	7.732	2421.8	0.938902053	R(+42.01)	63	720.4253	1	719.4078	Acetylati	F	Superscaf	1E+08	0	0	BTP20220	0	0	0	Tissue specific
VLQDVKL	Superscaf	-	Superscaf	1E+08	1E+08	-3	3	9	-	-	-	-	-	-	NCP	IGR	-	ID=gene-E	7	4.331428571	2197.4	0.939668178	VLQDVKL	98	407.7546	2	813.496	.	S	Superscaf	1E+08	BTP20220	0	0	0	0	0	Tissue specific
SRELQGK	Superscaf	+	Superscaf	1E+08	1E+08	42	42	48	-	-	-	-	-	-	NCP	IGR	-	ID=gene-E	7	6.714285714	3187.5	0.939737604	S(+42.01)	50	430.2324	2	858.4559	Acetylati	V	Superscaf	1E+08	BTP20220	0	0	BTP20220	0	0	Tissue specific
QTTERGY	Superscaf	+	Superscaf	1E+08	1E+08	21	21	27	-	-	gene	-	-	mRNA	NCP	Intron	gene|mRN	ID=gene-E	7	6.331428571	3236.8	0.940249904	QTTERGY	84	427.701	2	853.393	.	Q	Superscaf	1E+08	0	0	0	0	0	BTP20220	Tissue specific
PVLST	Superscaf	-	Superscaf	1E+08	1E+08	-28	28	32	-	-	gene	-	-	mRNA	NCP	Intron	gene|mRN	ID=gene-E	5	4.898	1471.7	0.940707708	PVLST	67	516.3126	1	515.2955	.	Y	Superscaf	1E+08	BTP20220	0	0	0	0	0	Tissue specific
TQLLLGP	Superscaf	-	Superscaf	1E+08	1E+08	-7	7	13	-	-	gene	-	-	mRNA	NCP	Intron	gene|mRN	ID=gene-E	7	6.055714286	2703.1	0.941931638	T(+42.01)	57	392.2404	2	782.4538	Acetylati	S	Superscaf	1E+08	0	0	0	0	BTP20220	0	Tissue specific
KAAWAGAP	Superscaf	+	Superscaf	1E+08	1E+08	5	5	12	-	-	gene	-	-	mRNA	NCP	Intron	gene|mRN	ID=gene-E	8	6.4975	2874.9	0.942079321	KAAWAGAP	67	386.2107	2	770.4075	.	V	Superscaf	1E+08	0	BTP20220	0	0	0	0	Tissue specific
WKAFR	Superscaf	+	Superscaf	1E+08	1E+08	7	7	11	-	-	gene	-	-	mRNA	NCP	Intron	gene|mRN	ID=gene-E	5	7.578	2342.5	0.942732738	WKAFR	93	354.2069	2	706.3914	.	L	Superscaf	1E+08	0	0	0	0	BTP20220	BTP20220	Tissue specific
KDLTLRPK	Superscaf	+	Superscaf	1E+08	1E+08	9	9	16	-	-	gene	-	-	mRNA	NCP	Intron	gene|mRN	ID=gene-E	8	7.25	3438.8	0.943721782	KDLTLRPK	64	485.8036	2	969.5971	.	L	Superscaf	1E+08	0	0	0	0	BTP20220	0	Tissue specific
ADKLTR	Superscaf	-	Superscaf	1E+08	1E+08	-13	13	18	-	-	-	-	-	-	NCP	IGR	-	ID=gene-E	6	7	2590.9	0.945811125	A(+42.01)	99	373.2122	2	744.413	Acetylati	Q	Superscaf	1E+08	0	0	BTP20220	0	0	0	Tissue specific
RTVYLSE	Superscaf	+	Superscaf	1E+08	1E+08	5	5	11	-	-	gene	-	-	mRNA	NCP	Intron	gene|mRN	ID=gene-E	7	5.404285714	2668.1	0.946230179	RTVYLSE	77	434.2404	2	866.4498	.	H	Superscaf	1E+08	BTP20220	0	0	0	0	0	Tissue specific
VDFPR	Superscaf	-	Superscaf	1.01E+08	1.01E+08	-5	5	9	-	-	-	-	-	-	NCP	IGR	-	ID=gene-E	5	5.102	1785.1	0.946483831	VDFPR	50	633.3351	1	632.3282	.	Q	Superscaf	1.01E+08	BTP20220	0	0	0	0	0	Tissue specific
PQGAKP	Superscaf	+	Superscaf	1.01E+08	1.01E+08	33	33	38	-	-	-	-	-	-	NCP	IGR	-	ID=gene-E	6	6.663333333	2362.5	0.947743548	PQGAKP	68	597.3343	1	596.3282	.	T	Superscaf	1.01E+08	0	0	0	BTP20220	0	0	Tissue specific
ALGNPLR	Superscaf	+	Superscaf	1.01E+08	1.01E+08	16	16	22	-	-	-	-	-	-	NCP	IGR	-	ID=gene-E	7	6.631428571	2898.3	0.948874988	ALGNPLR	61	370.7285	2	739.434	.	D	Superscaf	1.01E+08	0	0	0	0	BTP20220	0	Tissue specific
DPPPYK	Superscaf	+	Superscaf	1.01E+08	1.01E+08	121	121	126	-	-	-	-	-	-	NCP	IGR	-	ID=gene-E	6	6.211666667	2351.6	0.95030493	DPPPYK	84	716.3605	1	715.3541	.	I	Superscaf	1.01E+08	0	0	0	0	BTP20220	0	Tissue specific
SSVASPNPF	Superscaf	+	Superscaf	1.01E+08	1.01E+08	79	79	87	-	-	-	-	-	-	NCP	IGR	-	ID=gene-E	9	5.758888889	3381.8	0.951693216	SSVASPNPF	89	457.7364	2	913.4617	.	E	Superscaf	1.01E+08	0	0	0	0	BTP20220	0	Tissue specific
QRFPPPP	Superscaf	-	Superscaf	1.01E+08	1.01E+08	-13	13	19	-	-	-	-	-	-	NCP	IGR	-	ID=gene-E	7	6.727142857	2996.5	0.951976091	QRFPPPP	57	838.4644	1	837.4496	.	E	Superscaf	1.01E+08	BTP20220	0	0	0	0	0	Tissue specific
LQDPPK	Superscaf	-	Superscaf	1.01E+08	1.01E+08	-38	38	43	-	-	-	-	-	-	NCP	IGR	-	ID=gene-E	6	6.156666667	2518.8	0.952023266	LQDPPK	84	697.3812	1	696.3806	.	V	Superscaf	1.01E+08	0	0	0	0	0	BTP20220	Tissue specific
VGGGLVT	Superscaf	+	Superscaf	1.01E+08	1.01E+08	8	8	14	-	-	-	-	-	-	NCP	IGR	-	ID=gene-E	7	4.345714286	1642.8	0.952189075	VGGGLVT	68	602.3486	1	601.3435	.	L	Superscaf	1.01E+08	0	0	0	BTP20220	0	0	Tissue specific
KPPGAAPR	Superscaf	+	Superscaf	1.01E+08	1.01E+08	32	32	39	-	-	gene	-	-	mRNA	NCP	Intron	gene|mRN	ID=gene-E	8	7.17625	3049.3	0.952499545	KPPGAAPR	63	397.2299	2	792.4606	.	P	Superscaf	1.01E+08	0	0	BTP20220	0	0	0	Tissue specific
RDLVRKL	Superscaf	-	Superscaf	1.01E+08	1.01E+08	-10	10	16	-	-	gene	-	-	mRNA	NCP	Intron	gene|mRN	ID=gene-E	7	6.598571429	2884.3	0.954504473	RDLVRKL	60	450.2857	2	898.5712	.	V	Superscaf	1.01E+08	0	0	0	0	BTP20220	0	Tissue specific
VTRLLSK	Superscaf	-	Superscaf	1.01E+08	1.01E+08	-46	46	52	-	-	gene	-	-	mRNA	NCP	Intron	gene|mRN	ID=gene-E	7	6.381428571	2604.9	0.954963091	VTRLLSK	70	408.767	2	815.5229	.	N	Superscaf	1.01E+08	0	0	0	BTP20220	0	0	Tissue specific
KMPPDAK	Superscaf	+	Superscaf	1.01E+08	1.01E+08	37	37	43	-	-	gene	-	-	mRNA	NCP	Intron	gene|mRN	ID=gene-E	7	6.688571429	2705.9	0.95500405	KM(+15.99	83	401.7066	2	801.4055	Oxidatior	D	Superscaf	1.01E+08	0	0	0	0	BTP20220	0	Tissue specific
RVSGLSPK	Superscaf	+	Superscaf	1.01E+08	1.01E+08	4	4	11	-	-	gene	-	-	mRNA	NCP	Intron	gene|mRN	ID=gene-E	8	6.26375	2872.1	0.955107172	R(+42.01)	54	443.2649	2	884.5079	Acetylati	S	Superscaf	1.01E+08	0	0	0	0	0	BTP20220	Tissue specific
QLTGSPK	Superscaf	-	Superscaf	1.01E+08	1.01E+08	-31	31	37	-	-	gene	-	-	mRNA	NCP	Intron	gene|mRN	ID=gene-E	7	6.55	2848.1	0.955333634	QLTGSPK	51	365.7069	2	729.4021	.	F	Superscaf	1.01E+08	0	0	0	0	0	BTP20220	Tissue specific
EVDVNLP	Superscaf	-	Superscaf	1.01E+08	1.01E+08	-2	2	8	-	-	gene	-	-	mRNA	NCP	Intron	gene|mRN	ID=gene-E	7	3.411428571	2124.5	0.955406197	EVDVNLP	71	785.4046	1	784.3967	.	N	Superscaf	1.01E+08	BTP20220	0	0	0	0	0	Mixed
KSHGVPP	Superscaf	+	Superscaf	1.01E+08	1.01E+08	83	83	89	-	-	gene	-	-	mRNA	NCP	Intron	gene|mRN	ID=gene-E	7	5.94	2443.6	0.955958061	KSHGVPP	56	361.1986	2	720.3918	.	R	Superscaf	1.01E+08	0	0	0	BTP20220	0	0	Tissue specific
KSLEFK	Superscaf	-	Superscaf	1.01E+08	1.01E+08	-5	5	10	-	-	gene	-	-	mRNA	NCP	Intron	gene|mRN	ID=gene-E	6	6.64	2486.5	0.956026964	KSLEFK	86	376.219	2	750.4276	.	I	Superscaf	1.01E+08	BTP20220	0	0	0	0	0	Tissue specific
GPRAPAPP	Superscaf	+	Superscaf	1.01E+08	1.01E+08	12	12	19	-	-	gene	-	-	mRNA	NCP	Intron	gene|mRN	ID=gene-E	8	6.74625	2918.3	0.956308502	GPRAPAPP	59	381.7217	2	761.4184	.	N	Superscaf	1.01E+08	0	0	BTP20220	0	0	0	Tissue specific
VYYTVPL	Superscaf	-	Superscaf	1.01E+08	1.01E+08	-36	36	42	-	-	gene	-	-	mRNA	NCP	Intron	gene|mRN	ID=gene-E	7	4.304285714	2007.3	0.956876923	VYYTVPL	80	854.46	1	853.4586	.	L	Superscaf	1.01E+08	0	BTP20220	BTP20220	0	0	0	Tissue specific
MSFSTAL	Superscaf	+	Superscaf	1.01E+08	1.01E+08	55	55	61	-	-	gene	-	-	mRNA	NCP	Intron	gene|mRN	ID=gene-E	7	5.874285714	2587.9	0.959107326	MSFSTAL	88	756.357	1	755.3524	.	G	Superscaf	1.01E+08	0	0	0	BTP20220	0	0	Mixed
LPGVGVP	Superscaf	+	Superscaf	1.01E+08	1.01E+08	22	22	28	-	-	gene	-	-	mRNA	NCP	Intron	gene|mRN	ID=gene-E	7	4.36	1622.8	0.959681964	LPGVGVP	65	638.3851	1	637.3799	.	S	Superscaf	1.01E+08	BTP20220	0	0	0	0	0	Tissue specific
VQLHYV	Superscaf	-	Superscaf	1.01E+08	1.01E+08	-31	31	36	-	-	gene	-	-	mRNA	NCP	Intron	gene|mRN	ID=gene-E	6	4.146666667	2043.3	0.95983656	VQLHYV	63	758.4185	1	757.4123	.	V	Superscaf	1.01E+08	0	0	0	0	0	BTP20220	Tissue specific
VEVTY	Superscaf	+	Superscaf	1.01E+08	1.01E+08	2	2	6	-	-	gene	-	-	mRNA	NCP	Intron	gene|mRN	ID=gene-E	5	3.082	1282.5	0.960461974	VEVTY	55	610.3068	1	609.301	.	G	Superscaf	1.01E+08	0	0	BTP20220	0	0	0	Tissue specific
SSFHL	Superscaf	-	Superscaf	1.01E+08	1.01E+08	-1	1	5	-	-	gene	-	-	mRNA	NCP	Intron	gene|mRN	ID=gene-E	5	6.082	2127.3	0.960641146	SSFHL	76	590.302	1	589.286	.	S	Superscaf	1.01E+08	0	BTP20220	0	0	0	0	Tissue specific
KFQHPP	Superscaf	-	Superscaf	1.01E+08	1.01E+08	-5	5	10	-	-	gene	-	-	mRNA	NCP	Intron	gene|mRN	ID=gene-E	6	6.843333333	2761	0.961779208	KFQHPP	69	377.2013	2	752.3969	.	L	Superscaf	1.01E+08	0	0	0	0	0	BTP20220	Tissue specific
LLRPSQEL	Superscaf	+	Superscaf	1.01E+08	1.01E+08	4	4	11	-	-	-	-	-	-	NCP	IGR	-	ID=gene-E	8	6.19375	3466	0.963121249	LLRPSQEL	64	478.2917	2	954.5498	.	K	Superscaf	1.01E+08	0	0	BTP20220	0	0	0	Tissue specific
LDAAKGVG	Superscaf	+	Superscaf	1.01E+08	1.01E+08	21	21	28	-	-	-	-	-	-	NCP	IGR	-	ID=gene-E	8	5.33375	2450.5	0.963293449	LDAAKGVG	63	365.708	2	729.402	.	S	Superscaf	1.01E+08	BTP20220	0	0	0	0	0	Tissue specific
KTGKPNW	Superscaf	+	Superscaf	1.01E+08	1.01E+08	79	79	85	-	-	gene	-	-	mRNA	NCP	Intron	gene|mRN	ID=gene-E	7	7.082857143	3092.3	0.965074878	KTGKPNW	58	415.7335	2	829.4446	.	K	Superscaf	1.01E+08	0	0	0	0	BTP20220	0	Tissue specific
TLPPRHP	Superscaf	+	Superscaf	1.01E+08	1.01E+08	68	68	74	-	-	gene	-	-	mRNA	NCP	Intron	gene|mRN	ID=gene-E	7	7.108571429	3031.6	0.965347063	TLPPRHP	75	409.2439	2	816.4606	.	L	Superscaf	1.01E+08	0	0	0	0	BTP20220	0	Tissue specific
NHGEAGK	Superscaf	-	Superscaf	1.02E+08	1.02E+08	-28	28	34	-	-	gene	-	-	mRNA	NCP	Intron	gene|mRN	ID=gene-E	7	6.274285714	3016.2	0.96768111	NHGEAGK	63	356.675	2	711.33	.	K	Superscaf	1.02E+08	0	0	0	0	0	BTP20220	Tissue specific
SLGLENR	Superscaf	+	Superscaf	1.02E+08	1.02E+08	4	4	10	-	-	gene	-	-	mRNA	NCP	Intron	gene|mRN	ID=gene-E	7	6.142857143	3058.5	0.967692672	SLGLENR	54	394.7141	2	787.4188	.	L	Superscaf	1.02E+08	0	0	0	0	BTP20220	BTP20220	Tissue specific
EEELAAL	Superscaf	+	Superscaf	1.02E+08	1.02E+08	16	16	22	-	-	-	-	-	-	NCP	IGR	-	ID=gene-E	7	4.808571429	2605.9	0.967926338	E(+42.01)	99	816.397	1	815.3912	Acetylati	K	Superscaf	1.02E+08	BTP20220	0	0	0	0	0	Mixed
FMYSAK	Superscaf	-	Superscaf	1.02E+08	1.02E+08	-10	10	15	-	-	-	-	-	-	NCP	IGR	-	ID=gene-E	6	6.388333333	2381.5	0.969305736	FMYSAK	64	746.3448	1	745.3469	.	G	Superscaf	1.02E+08	BTP20220	0	0	0	0	0	Tissue specific
LQGAQML	Superscaf	+	Superscaf	1.02E+08	1.02E+08	9	9	15	-	-	-	-	-	-	NCP	IGR	-	ID=gene-E	7	5.857142857	2908.3	0.969391312	LQGAQML	91	760.4006	1	759.3949	.	P	Superscaf	1.02E+08	0	0	0	BTP20220	0	0	Tissue specific
LLEPF	Superscaf	-	Superscaf	1.02E+08	1.02E+08	-10	10	14	-	-	-	-	-	-	NCP	IGR	-	ID=gene-E	5	5.392	1857.1	0.970616811	LLEPF	93	618.3497	1	617.3424	.	R	Superscaf	1.02E+08	0	BTP20220	0	0	0	0	Tissue specific
GNVQLSR	Superscaf	+	Superscaf	1.02E+08	1.02E+08	29	29	35	-	-	-	-	-	-	NCP	IGR	-	ID=gene-E	7	5.635714286	2890.3	0.97214424	G(+42.01)	80	408.2225	2	814.4297	Acetylati	Y	Superscaf	1.02E+08	0	BTP20220	0	0	0	0	Tissue specific
VDKDLLEPC	Superscaf	+	Superscaf	1.02E+08	1.02E+08	46	46	57	-	-	-	-	-	-	NCP	IGR	-	ID=gene-E	12	4.485833333	4027.5	0.972445124	VDKDLLEPC	74	666.3261	2	1330.644	Carbamido	E	Superscaf	1.02E+08	0	0	0	0	0	BTP20220	Tissue specific
DGPKLVRE	Superscaf	+	Superscaf	1.02E+08	1.02E+08	55	55	62	-	exon	gene	three_pri	-	mRNA	NCP	3UTR	exon|gene	ID=exon-E	8	5.6175	2942.3	0.972512749	DGPKLVRE	81	457.2562	2	912.5029	.	G	Superscaf	1.02E+08	BTP20220	0	0	0	0	0	Tissue specific
VYAGAF	Superscaf	-	Superscaf	1.02E+08	1.02E+08	-7	7	12	-	-	gene	-	-	mRNA	NCP	Intron	gene|mRN	ID=gene-E	6	4.858333333	1766.8	0.973118178	VYAGAF	77	627.3115	1	626.3064	.	N	Superscaf	1.02E+08	0	0	0	BTP20220	0	0	Tissue specific
PPAAEVP	Superscaf	+	Superscaf	1.02E+08	1.02E+08	32	32	38	-	-	gene	-	-	mRNA	NCP	Intron	gene|mRN	ID=gene-E	7	4.88	2060.3	0.973278817	PPAAEVP	60	680.3574	1	679.3541	.	I	Superscaf	1.02E+08	0	0	0	BTP20220	0	0	Tissue specific
CCCCCDL	Superscaf	+	Superscaf	1.02E+08	1.02E+08	9	9	15	-	-	gene	-	-	mRNA	NCP	Intron	gene|mRN	ID=gene-E	7	4.864285714	3693.4	0.974119829	C(+57.02)	56	545.1464	2	1088.285	Carbamido	D	Superscaf	1.02E+08	BTP20220	0	0	0	0	0	Tissue specific
SKTPPPP	Superscaf	-	Superscaf	1.02E+08	1.02E+08	-811	811	817	-	-	gene	-	-	mRNA	NCP	Intron	gene|mRN	ID=gene-E	7	6.735714286	2598.9	0.974974553	SKTPPPP	74	723.397	1	722.3962	.	I	Superscaf	1.02E+08	0	0	BTP20220	0	0	0	Tissue specific
ELKRTP	Superscaf	+	Superscaf	1.02E+08	1.02E+08	24	24	29	-	-	-	-	-	-	NCP	IGR	-	ID=gene-E	6	7.088333333	2631	0.975944482	ELKRTP	56	372.2301	2	742.4337	.	R	Superscaf	1.02E+08	BTP20220	0	0	0	0	0	Tissue specific
KEVSFQVD	Superscaf	-	Superscaf	1.02E+08	1.02E+08	-20	20	27	-	-	gene	-	-	mRNA	NCP	Intron	gene|mRN	ID=gene-E	8	4.0925	2686.9	0.977635803	KEVSFQVD	56	476.237	2	950.4709	.	D	Superscaf	1.02E+08	BTP20220	0	0	0	0	0	Tissue specific
PPPPPRGP	Superscaf	+	Superscaf	1.02E+08	1.02E+08	32	32	39	-	-	gene	-	-	mRNA	NCP	Intron	gene|mRN	ID=gene-E	8	6.81625	2970.5	0.977667698	PPPPPRGP	86	407.7307	2	813.4496	.	E	Superscaf	1.02E+08	0	BTP20220	0	0	0	0	Tissue specific
PATTPP	Superscaf	+	Superscaf	1.02E+08	1.02E+08	38	38	43	-	-	gene	-	-	mRNA	NCP	Intron	gene|mRN	ID=gene-E	6	6.33	2118.5	0.977674089	PATTPP	85	583.3049	1	582.3013	.	D	Superscaf	1.02E+08	0	0	0	0	0	BTP20220	Tissue specific
CGFFTP	Superscaf	+	Superscaf	1.02E+08	1.02E+08	49	49	54	-	-	gene	-	-	mRNA	NCP	Intron	gene|mRN	ID=gene-E	6	5.796666667	2392.7	0.977784008	C(+57.02)	63	364.6586	2	727.2999	Carbamido	L	Superscaf	1.02E+08	BTP20220	0	0	0	0	0	Tissue specific
VPLRGSY	Superscaf	-	Superscaf	1.02E+08	1.02E+08	-44	44	50	-	exon	gene	three_pri	-	mRNA	NCP	3UTR	exon|gene	ID=exon-E	7	5.764285714	2479.8	0.978307985	VPLRGSY	68	396.2221	2	790.4337	.	V	Superscaf	1.02E+08	BTP20220	0	0	0	0	0	Tissue specific
AHRLQLR	Superscaf	-	Superscaf	1.02E+08	1.02E+08	-52	52	58	-	-	gene	-	-	mRNA	NCP	Intron	gene|mRN	ID=gene-E	7	7.534285714	3546.1	0.979185018	A(+42.01)	88	468.2795	2	934.5461	Acetylati	N	Superscaf	1.02E+08	0	0	0	0	BTP20220	0	Tissue specific
VGELALK	Superscaf	-	Superscaf	1.02E+08	1.02E+08	-37	37	43	-	-	gene	-	-	mRNA	NCP	Intron	gene|mRN	ID=gene-E	7	5.272857143	2209.3	0.979581995	VGELALK	85	729.4474	1	728.4432	.	L	Superscaf	1.02E+08	BTP20220	0	0	0	0	0	Tissue specific
QLKKLLH	Superscaf	-	Superscaf	1.02E+08	1.02E+08	-47	47	53	-	-	gene	-	-	mRNA	NCP	Intron	gene|mRN	ID=gene-E	7	7.237142857	3227.4	0.979876663	QLKKLLH	98	440.2913	2	878.5701	.	F	Superscaf	1.02E+08	0	0	0	0	BTP20220	0	Tissue specific
MLVARLT	Superscaf	-	Superscaf	1.02E+08	1.02E+08	-21	21	27	-	-	gene	-	-	mRNA	NCP	Intron	gene|mRN	ID=gene-E	7	5.86	2435.9	0.980279217	MLVARLT	88	402.2426	2	802.4735	.	T	Superscaf	1.02E+08	0	0	0	BTP20220	0	0	Tissue specific
PGLGHVGP	Superscaf	-	Superscaf	1.02E+08	1.02E+08	-12	12	19	-	-	gene	-	-	mRNA	NCP	Intron	gene|mRN	ID=gene-E	8	5.51	2539.8	0.980629601	PGLGHVGP	52	367.2012	2	732.3918	.	I	Superscaf	1.02E+08	0	0	BTP20220	0	0	0	Tissue specific
GHLLKFK	Superscaf	-	Superscaf	1.02E+08	1.02E+08	-10	10	16	-	-	gene	-	-	mRNA	NCP	Intron	gene|mRN	ID=gene-E	7	7.211428571	2948	0.980883833	GHLLKFK	57	421.7678	2	841.5174	.	D	Superscaf	1.02E+08	0	0	0	0	0	BTP20220	Tissue specific
PVPLPPPP	Superscaf	+	Superscaf	1.02E+08	1.02E+08	7	7	14	-	-	gene	-	-	mRNA	NCP	Intron	gene|mRN	ID=gene-E	8	5.4725	2377.8	0.982449432	PVPLPPPP	68	407.2438	2	812.4796	.	D	Superscaf	1.02E+08	0	BTP20220	0	0	0	0	Tissue specific
DPKAVPP	Superscaf	+	Superscaf	1.02E+08	1.02E+08	3	3	9	-	-	gene	-	-	mRNA	NCP	Intron	gene|mRN	ID=gene-E	7	5.375714286	2203.4	0.982489228	D(+42.01)	69	383.2136	2	764.4068	Acetylati	T	Superscaf	1.02E+08	0	0	0	0	0	BTP20220	Tissue specific
HLPLPL	Superscaf	-	Superscaf	1.02E+08	1.02E+08	-6	6	11	-	-	gene	-	-	mRNA	NCP	Intron	gene|mRN	ID=gene-E	6	6.355	2354.7	0.982799118	HLPLPL	54	689.432	1	688.4272	.	I	Superscaf	1.02E+08	0	0	0	0	BTP20220	0	Mixed
GPKGYLK	Superscaf	+	Superscaf	1.03E+08	1.03E+08	7	7	13	-	-	gene	-	-	mRNA	NCP	Intron	gene|mRN	ID=gene-E	7	7.051428571	2681.7	0.985150943	GPKGYLK	56	381.7292	2	761.4435	.	A	Superscaf	1.03E+08	0	BTP20220	0	0	0	0	Tissue specific
NDPLEP	Superscaf	+	Superscaf	1.03E+08	1.03E+08	29	29	34	-	-	-	-	-	-	NCP	IGR	-	ID=gene-E	6	5.03	2461.9	0.988765856	N(+42.01)	70	363.6689	2	725.3232	Acetylati	P	Superscaf	1.03E+08	0	0	0	0	0	BTP20220	Tissue specific
LLLGPLY	Superscaf	+	Superscaf	1.03E+08	1.03E+08	12	12	18	-	-	gene	-	-	mRNA	NCP	Intron	gene|mRN	ID=gene-E	7	5.978571429	2507.8	0.991109025	LLLGPLY	99	788.4879	1	787.4843	.	E	Superscaf	1.03E+08	BTP20220	0	0	0	0	0	Mixed
EEAPGHRP	Superscaf	-	Superscaf	1.03E+08	1.03E+08	-37	37	44	-	-	gene	-	-	mRNA	NCP	Intron	gene|mRN	ID=gene-E	8	6.1725	3402.9	0.991999072	EEAPGHRP	99	446.716	2	891.4199	.	E	Superscaf	1.03E+08	BTP20220	0	0	0	0	0	Tissue specific
CDVDLR	Superscaf	-	Superscaf	1.03E+08	1.03E+08	-12	12	17	-	-	gene	-	-	mRNA	NCP	Intron	gene|mRN	ID=gene-E	6	4.616666667	2410.9	0.993094259	C(+57.02)	99	389.1798	2	776.3487	Carbamido	G	Superscaf	1.03E+08	0	BTP20220	0	0	0	0	Tissue enhanced
GVKYPLE	Superscaf	-	Superscaf	1.03E+08	1.03E+08	-15	15	21	-	-	gene	-	-	mRNA	NCP	Intron	gene|mRN	ID=gene-E	7	5.267142857	2341.5	0.993521969	GVKYPLE	86	805.441	1	804.4381	.	V	Superscaf	1.03E+08	0	0	0	0	0	BTP20220	Tissue specific
MGPDLLR	Superscaf	+	Superscaf	1.03E+08	1.03E+08	33	33	39	-	-	gene	-	-	mRNA	NCP	Intron	gene|mRN	ID=gene-E	7	6.244285714	2773.3	0.995198068	MGPDLLR	70	401.2233	2	800.4214	.	L	Superscaf	1.03E+08	0	0	BTP20220	0	0	0	Tissue specific
EGSPPLTL	Superscaf	+	Superscaf	1.03E+08	1.03E+08	115	115	122	-	-	-	-	-	-	NCP	IGR	-	ID=gene-E	8	5.745	2885.3	0.995478386	EGSPPLTL	62	813.4325	1	812.428	.	L	Superscaf	1.03E+08	0	0	0	0	BTP20220	BTP20220	Tissue specific
LSGAVP	Superscaf	+	Superscaf	1.03E+08	1.03E+08	13	13	18	-	-	-	-	-	-	NCP	IGR	-	ID=gene-E	6	4.991666667	1682.8	0.995768232	LSGAVP	74	543.3119	1	542.3064	.	L	Superscaf	1.03E+08	0	BTP20220	0	0	0	0	Tissue specific
SLENFQ	Superscaf	+	Superscaf	1.03E+08	1.03E+08	4	4	9	-	-	gene	-	-	mRNA	NCP	Intron	gene|mRN	ID=gene-E	6	5.236666667	2757.1	0.995800941	SLENFQ	65	737.3481	1	736.3392	.	K	Superscaf	1.03E+08	0	0	0	0	0	BTP20220	Tissue specific
KRLSSH	Superscaf	+	Superscaf	1.03E+08	1.03E+08	25	25	30	-	-	gene	-	-	mRNA	NCP	Intron	gene|mRN	ID=gene-E	6	7.571666667	2857.1	0.998911978	KRLSSH	64	364.2083	2	726.4136	.	G	Superscaf	1.03E+08	BTP20220	0	0	0	0	0	Tissue specific
GYDEEF	Superscaf	+	Superscaf	1.03E+08	1.03E+08	18	18	23	-	-	-	-	-	-	NCP	IGR	-	.	6	4.42	2406.7	0.999118281	G(+42.01)	66	801.291	1	800.2864	Acetylati	L	Superscaf	1.03E+08	0	0	0	0	0	BTP20220	Tissue specific
VGAPVP	Superscaf	-	Superscaf	27961	27978	-63	63	68	-	-	-	-	-	-	NCP	IGR	-	.	6	4.098333333	1283.4	0.999483943	VGAPVP	78	539.3164	1	538.3115	.	K	Superscaf	28014	0	0	BTP20220	0	0	0	Tissue specific
ATLAPAF	Superscaf	-	Superscaf	103060	103080	-11	11	17	-	-	-	-	-	-	NCP	IGR	-	.	7	6.05	2409.6	0.998098678	ATLAPAF	50	690.384	1	689.3748	.	G	Superscaf	103320	0	0	0	0	0	BTP20220	Tissue specific
KTPVP	Superscaf	-	Superscaf	211825	211839	-6	6	10	-	-	-	-	-	-	NCP	IGR	-	.	5	5.774	1540.7	0.996092605	KTPVP	58	541.3325	1	540.3271	.	D	Superscaf	211992	0	0	BTP20220	0	0	0	Tissue specific
YVVED	Superscaf	-	Superscaf	249586	249600	-65	65	69	-	-	-	-	-	-	NCP	IGR	-	.	5	2.37	1296.5	0.995396099	YVVED	73	624.2859	1	623.2802	.	P	Superscaf	250011	0	0	0	BTP20220	0	0	Tissue specific
NQAGY	Superscaf	+	Superscaf	379396	379410	34	34	38	-	-	-	-	-	-	NCP	IGR	-	.	5	5.742	2275.5	0.993001739	N(+42.01)	52	594.2566	1	593.2445	Acetylati	E	Superscaf	379297	0	0	0	BTP20220	0	0	Tissue specific
RLPDVN	Superscaf	-	Superscaf	432820	432837	-33	33	38	-	-	-	-	-	-	NCP	IGR	-	.	6	5.236666667	2347.8	0.992016272	RLPDVN	73	357.2004	2	712.3868	.	G	Superscaf	432942	0	0	0	BTP20220	0	0	Tissue specific
LSRQL	Superscaf	-	Superscaf	436174	436188	-26	26	30	-	-	-	-	-	-	NCP	IGR	-	.	5	6.81	2349.7	0.991954462	LSRQL	92	616.3761	1	615.3704	.	V	Superscaf	436317	0	0	0	BTP20220	0	0	Tissue specific
KARLGLP	Superscaf	-	Superscaf	514090	514110	-6	6	12	-	-	-	-	-	-	NCP	IGR	-	.	7	7.25	2770	0.990517182	K(+42.01)	59	398.7598	2	795.4966	Acetylati	Q	Superscaf	514422	0	BTP20220	0	0	0	0	Tissue specific
PLGFAF	Superscaf	-	Superscaf	534880	534897	-13	13	18	-	-	-	-	-	-	NCP	IGR	-	.	6	5.871666667	2074.2	0.990133763	PLGFAF	52	651.3525	1	650.3428	.	S	Superscaf	534993	0	0	0	0	0	BTP20220	Tissue specific
QTARLLR	Superscaf	-	Superscaf	585310	585330	-246	246	252	-	-	-	-	-	-	NCP	IGR	-	.	7	7.382857143	3323.9	0.989203521	Q(+42.01)	73	450.2742	2	898.5349	Acetylati	P	Superscaf	585762	0	0	0	0	0	BTP20220	Tissue specific
PGAGNVP	Superscaf	-	Superscaf	643753	643773	-12	12	18	-	-	-	-	-	-	NCP	IGR	-	.	7	5.138571429	2177.4	0.988125533	PGAGNVP	58	611.3229	1	610.3074	.	G	Superscaf	643773	BTP20220	0	0	0	0	0	Tissue specific
AALDNP	Superscaf	-	Superscaf	647458	647475	-133	133	138	-	-	-	-	-	-	NCP	IGR	-	.	6	5.45	2321.6	0.988057249	AALDNP	56	600.2986	1	599.2914	.	R	Superscaf	647937	0	0	0	BTP20220	0	0	Tissue specific
FLPRAAV	Superscaf	-	Superscaf	676846	676866	-45	45	51	-	-	-	-	-	-	NCP	IGR	-	.	7	5.794285714	2349.6	0.987515129	F(+42.01)	72	408.247	2	814.4701	Acetylati	Q	Superscaf	676911	BTP20220	0	0	0	0	0	Tissue specific
PVPLGPK	Superscaf	-	Superscaf	682156	682176	-112	112	118	-	-	-	-	-	-	NCP	IGR	-	.	7	5.798571429	2131.3	0.987417185	PVPLGPK	59	354.2253	2	706.4377	.	E	Superscaf	682398	0	0	0	0	BTP20220	0	Tissue specific
GVETLPV	Superscaf	-	Superscaf	683140	683160	-57	57	63	-	-	-	-	-	-	NCP	IGR	-	.	7	4	1811.1	0.987399035	GVETLPV	78	714.4032	1	713.3959	.	C	Superscaf	683163	0	0	0	0	0	BTP20220	Tissue specific
CRTPGPP	Superscaf	-	Superscaf	770176	770196	-63	63	69	-	-	-	-	-	-	NCP	IGR	-	.	7	6.74	2941.5	0.985793646	C(+57.02)	53	784.3861	1	783.3698	Carbamido	A	Superscaf	770214	0	0	0	0	0	BTP20220	Tissue specific
AGLGRPGPA	Superscaf	-	Superscaf	802126	802158	-50	50	60	-	-	-	-	-	-	NCP	IGR	-	.	11	6.940909091	4177.7	0.985204104	A(+42.01)	70	545.809	2	1089.604	Acetylati	V	Superscaf	804201	0	0	0	BTP20220	0	0	Tissue specific
PVVAVTK	Superscaf	-	Superscaf	819322	819342	-32	32	38	-	-	-	-	-	-	NCP	IGR	-	.	7	4.084285714	1514.6	0.984887143	P(+42.01)	72	378.2411	2	754.4589	Acetylati	L	Superscaf	819540	0	0	0	0	0	BTP20220	Tissue specific
MVGHFGVQE	Superscaf	-	Superscaf	824683	824715	-32	32	42	-	-	-	-	-	-	NCP	IGR	-	.	11	4.850909091	4069.7	0.984788037	MVGHFGVQE	93	637.7988	2	1273.587	.	A	Superscaf	824856	0	0	0	0	BTP20220	0	Tissue specific
PLAGPAPP	Superscaf	-	Superscaf	832600	832623	-143	143	150	-	-	-	-	-	-	NCP	IGR	-	.	8	6.14875	2622.9	0.984642173	P(+42.01)	66	381.2154	2	760.4119	Acetylati	K	Superscaf	832761	0	0	0	0	0	BTP20220	Tissue specific
KPSGVP	Superscaf	+	Superscaf	987508	987525	21	21	26	-	-	-	-	-	-	NCP	IGR	-	.	6	5.665	1823.9	0.981784988	KPSGVP	61	584.338	1	583.3329	.	N	Superscaf	987448	0	0	BTP20220	0	0	0	Tissue specific
GPPRPPP	Superscaf	-	Superscaf	997855	997875	-31	31	37	-	-	-	-	-	-	NCP	IGR	-	.	7	6.89	2633.1	0.981594081	GPPRPPP	99	359.2039	2	716.3969	.	R	Superscaf	998385	0	0	0	0	0	BTP20220	Tissue specific
SVVPSPK	Superscaf	-	Superscaf	1008004	1008024	-256	256	262	-	-	-	-	-	-	NCP	IGR	-	.	7	4.814285714	1910	0.981406882	SVVPSPK	95	357.2114	2	712.4119	.	D	Superscaf	1008132	0	0	0	0	0	BTP20220	Tissue specific
KGPPRTP	Superscaf	-	Superscaf	1017889	1017909	-39	39	45	-	-	-	-	-	-	NCP	IGR	-	.	7	7.414285714	2824.2	0.981224552	KGPPRTP	56	376.7285	2	751.434	.	T	Superscaf	1018086	0	BTP20220	0	0	0	0	Tissue specific
AESDWDTVI	Superscaf	-	Superscaf	1070929	1070970	-65	65	78	-	-	-	-	-	-	NCP	IGR	-	.	14	5.430714286	5203.8	0.980245835	A(+42.01)	99	563.9642	3	1688.873	Acetylati	T	Superscaf	1071339	0	0	BTP20220	0	0	0	Tissue specific
AESDWDTVI	Superscaf	-	Superscaf	1070929	1070967	-65	65	77	-	-	-	-	-	-	NCP	IGR	-	.	13	5.099230769	4735.4	0.980245891	A(+42.01)	92	781.3938	2	1560.778	Acetylati	T	Superscaf	1071339	0	0	0	BTP20220	0	0	Mixed
AESDWDTVI	Superscaf	-	Superscaf	1070929	1070964	-65	65	76	-	-	-	-	-	-	NCP	IGR	-	.	12	4.7125	4267	0.980245946	A(+42.01)	98	717.3473	2	1432.683	Acetylati	T	Superscaf	1071339	0	0	0	0	BTP20220	0	Mixed
PPLRFPGP	Superscaf	-	Superscaf	1087867	1087890	-6	6	13	-	-	-	-	-	-	NCP	IGR	-	.	8	6.67375	3036.5	0.979933744	PPLRFPGP	98	440.7547	2	879.4966	.	A	Superscaf	1087929	0	0	0	BTP20220	0	0	Tissue specific
LLTLCDF	Superscaf	-	Superscaf	1162198	1162218	-41	41	47	-	-	-	-	-	-	NCP	IGR	-	.	7	5.42	2842.3	0.978562756	LLTLC(+57	62	441.2253	2	880.4364	Carbamido	W	Superscaf	1162221	BTP20220	0	0	0	0	0	Tissue specific
DVEPLY	Superscaf	-	Superscaf	1163203	1163220	-26	26	31	-	-	-	-	-	-	NCP	IGR	-	.	6	4.021666667	1987.3	0.978544274	DVEPLY	50	735.3644	1	734.3486	.	A	Superscaf	1163475	0	0	0	BTP20220	0	0	Tissue specific
TGPDVLL	Superscaf	-	Superscaf	1207834	1207854	-42	42	48	-	-	-	-	-	-	NCP	IGR	-	.	7	4.818571429	2150.5	0.977720994	TGPDVLL	58	714.4109	1	713.3959	.	G	Superscaf	1207920	BTP20220	0	0	0	0	0	Tissue specific
PGFVLLR	Superscaf	-	Superscaf	1264036	1264056	-86	86	92	-	-	-	-	-	-	NCP	IGR	-	.	7	5.781428571	2377.7	0.976684342	PGFVLLR	66	401.2504	2	800.4908	.	T	Superscaf	1264107	0	0	0	BTP20220	0	0	Tissue specific
KEPRTH	Superscaf	-	Superscaf	1272415	1272432	-20	20	25	-	-	-	-	-	-	NCP	IGR	-	.	6	7.356666667	2897.3	0.976529846	KEPRTH	55	384.2179	2	766.4086	.	E	Superscaf	1272450	0	0	0	0	BTP20220	0	Tissue specific
KQEAGP	Superscaf	-	Superscaf	1284826	1284843	-33	33	38	-	-	-	-	-	-	NCP	IGR	-	.	6	6.15	2450.6	0.976300923	KQEAGP	65	629.3127	1	628.318	.	N	Superscaf	1284897	0	0	0	BTP20220	0	0	Tissue specific
KASGGAVPK	Superscaf	-	Superscaf	1310038	1310064	-3	3	11	-	-	-	-	-	-	NCP	IGR	-	.	9	6.16	2874.8	0.975835719	KASGGAVPK	61	407.7412	2	813.4708	.	Y	Superscaf	1310253	0	BTP20220	0	0	0	0	Tissue specific
PAPWS	Superscaf	-	Superscaf	1423003	1423017	-34	34	38	-	-	-	-	-	-	NCP	IGR	-	.	5	6.038	1896.1	0.973752288	PAPWS	83	557.2714	1	556.2645	.	N	Superscaf	1423077	0	0	0	BTP20220	0	0	Tissue specific
KGGHVLP	Superscaf	-	Superscaf	1469101	1469121	-77	77	83	-	-	-	-	-	-	NCP	IGR	-	.	7	5.935714286	2373.5	0.972901894	KGGHVLP	62	354.2129	2	706.4126	.	I	Superscaf	1469256	0	0	0	BTP20220	0	0	Tissue specific
KLFGGAH	Superscaf	-	Superscaf	1480468	1480488	-6	6	12	-	-	-	-	-	-	NCP	IGR	-	.	7	6.678571429	2734.8	0.972692229	KLFGGAH	60	365.2077	2	728.3969	.	S	Superscaf	1480734	0	BTP20220	0	0	0	0	Tissue specific
KDPLVTD	Superscaf	-	Superscaf	1583047	1583067	-20	20	26	-	-	-	-	-	-	NCP	IGR	-	.	7	4.927142857	2379.7	0.970800147	KDPLVTD	59	787.4177	1	786.4123	.	S	Superscaf	1583250	0	BTP20220	0	0	0	0	Tissue specific
QAAPGPP	Superscaf	-	Superscaf	1607446	1607466	-81	81	87	-	-	-	-	-	-	NCP	IGR	-	.	7	6.08	2542.8	0.970350105	Q(+42.01)	52	679.328	1	678.3337	Acetylati	H	Superscaf	1607466	0	0	0	BTP20220	0	0	Tissue specific
LPAEPV	Superscaf	-	Superscaf	1611502	1611519	-143	143	148	-	-	-	-	-	-	NCP	IGR	-	.	6	4.636666667	1765	0.970275347	LPAEPV	58	625.3547	1	624.3483	.	P	Superscaf	1611591	BTP20220	0	0	0	0	0	Tissue specific
EHVPDL	Superscaf	+	Superscaf	1642477	1642494	3	3	8	-	-	-	-	-	-	NCP	IGR	-	.	6	4.343333333	2147.5	0.96970401	EHVPDL	58	355.1776	2	708.3442	.	C	Superscaf	1642471	BTP20220	0	0	0	0	0	Tissue specific
FPGEGP	Superscaf	-	Superscaf	1663027	1663044	-49	49	54	-	-	-	-	-	-	NCP	IGR	-	.	6	5.54	2082.3	0.969324962	FPGEGP	50	603.2664	1	602.27	.	R	Superscaf	1663116	0	0	0	BTP20220	0	0	Tissue specific
KPEPAAP	Superscaf	-	Superscaf	1738558	1738578	-6	6	12	-	-	-	-	-	-	NCP	IGR	-	.	7	6.271428571	2528.7	0.967931729	KPEPAAP	57	355.1956	2	708.3806	.	S	Superscaf	1738806	0	0	0	BTP20220	0	0	Tissue specific
HLELCLK	Superscaf	+	Superscaf	1840453	1840473	16	16	22	-	-	-	-	-	-	NCP	IGR	-	ID=gene-E	7	6.215714286	3159.5	0.966052264	HLELC(+57	99	456.7498	2	911.4899	Carbamido	V	Superscaf	1840408	0	0	0	0	BTP20220	0	Tissue specific
YPAAHLL	Superscaf	+	Superscaf	1863940	1863960	93	93	99	-	-	gene	-	-	mRNA	NCP	Intron	gene|mRN	ID=gene-E	7	6.221428571	2746	0.965619044	Y(+42.01)	58	826.4476	1	825.4385	Acetylati	E	Superscaf	1863664	0	0	0	0	0	BTP20220	Tissue specific
RKNAAHP	Superscaf	+	Superscaf	1902406	1902426	82	82	88	-	-	gene	-	-	mRNA	NCP	Intron	gene|mRN	ID=gene-E	7	7.405714286	3293.6	0.964909534	RKNAAHP	65	397.2188	2	792.4354	.	T	Superscaf	1902163	0	0	0	0	BTP20220	0	Tissue specific
LSAVTNK	Superscaf	-	Superscaf	1905313	1905333	-26	26	32	-	-	gene	-	-	mRNA	NCP	Intron	gene|mRN	ID=gene-E	7	5.622857143	2510.7	0.964855914	LSAVTNK	88	366.7147	2	731.4177	.	E	Superscaf	1905426	0	0	0	0	0	BTP20220	Tissue specific
PLPMPGAP	Superscaf	-	Superscaf	1919410	1919433	-65	65	72	-	-	gene	-	-	mRNA	NCP	Intron	gene|mRN	ID=gene-E	8	6.115	2683.1	0.964595838	PLPMPGAP	55	779.4163	1	778.4047	.	K	Superscaf	1919439	BTP20220	0	0	0	0	0	Tissue specific
PFHAAF	Superscaf	+	Superscaf	1925728	1925745	121	121	126	-	-	gene	-	-	mRNA	NCP	Intron	gene|mRN	ID=gene-E	6	6.148333333	2354.5	0.964479412	PFHAAF	52	689.3487	1	688.3333	.	T	Superscaf	1925368	BTP20220	0	0	0	0	0	Tissue specific
LPPRPPP	Superscaf	-	Superscaf	1927729	1927749	-76	76	82	-	-	gene	-	-	mRNA	NCP	Intron	gene|mRN	ID=gene-E	7	6.891428571	2689.2	0.964442448	LPPRPPP	61	387.2411	2	772.4595	.	L	Superscaf	1927797	0	0	0	0	BTP20220	0	Tissue specific
VPPAPPGP	Superscaf	+	Superscaf	1942501	1942524	15	15	22	-	-	gene	-	-	mRNA	NCP	Intron	gene|mRN	ID=gene-E	8	5.43625	2295.6	0.964169922	VPPAPPGP	78	731.4025	1	730.4013	.	A	Superscaf	1942459	0	BTP20220	0	0	0	0	Tissue specific
TTGAAAP	Superscaf	-	Superscaf	1950379	1950399	-8	8	14	-	-	gene	-	-	mRNA	NCP	Intron	gene|mRN	ID=gene-E	7	6.198571429	2363.6	0.964024667	TTGAAAP	50	588.3008	1	587.2914	.	P	Superscaf	1950813	0	0	0	0	0	BTP20220	Tissue specific
DPPPFR	Superscaf	-	Superscaf	1975765	1975782	-147	147	152	-	-	gene	-	-	mRNA	NCP	Intron	gene|mRN	ID=gene-E	6	6.351666667	2459.9	0.963556474	DPPPFR	94	364.6885	2	727.3653	.	R	Superscaf	1976004	0	0	0	BTP20220	0	0	Tissue specific
GSAPAPPK	Superscaf	-	Superscaf	1978381	1978404	-172	172	179	-	-	gene	-	-	mRNA	NCP	Intron	gene|mRN	ID=gene-E	8	6.54125	2783.9	0.963508111	GSAPAPPK	76	362.6997	2	723.3915	.	T	Superscaf	1978599	0	0	0	0	BTP20220	0	Tissue specific
QAGLAARP	Superscaf	-	Superscaf	2010106	2010129	-38	38	45	-	-	gene	-	-	mRNA	NCP	Intron	gene|mRN	ID=gene-E	8	6.59	3181.5	0.96292294	QAGLAARP	62	392.2201	2	782.4399	.	A	Superscaf	2010207	0	0	0	0	0	BTP20220	Tissue specific
LTALLAK	Superscaf	+	Superscaf	2034319	2034339	4	4	10	-	-	gene	-	-	mRNA	NCP	Intron	gene|mRN	ID=gene-E	7	6.607142857	2548.7	0.962476384	LTALLAK	64	365.2517	2	728.4796	.	S	Superscaf	2034310	BTP20220	0	0	0	0	0	Tissue specific
GVWHVP	Superscaf	-	Superscaf	2055037	2055054	-58	58	63	-	-	gene	-	-	mRNA	NCP	Intron	gene|mRN	ID=gene-E	6	4.291666667	1781	0.962094293	G(+42.01)	56	736.3682	1	735.3704	Acetylati	V	Superscaf	2055054	BTP20220	0	0	0	0	0	Tissue specific
ASPLK	Superscaf	-	Superscaf	2072632	2072646	-11	11	15	-	-	gene	-	-	mRNA	NCP	Intron	gene|mRN	ID=gene-E	5	6.744	1853.9	0.961769807	A(+42.01)	62	557.3392	1	556.322	Acetylati	V	Superscaf	2072658	BTP20220	0	0	0	0	0	Tissue specific
ESPCAL	Superscaf	+	Superscaf	2097007	2097024	27	27	32	-	-	gene	-	-	mRNA	NCP	Intron	gene|mRN	ID=gene-E	6	5.37	2396.7	0.961320152	ESPC(+57.	92	676.2937	1	675.2897	Carbamido	G	Superscaf	2096929	0	0	0	BTP20220	0	0	Tissue specific
HGARPPP	Superscaf	-	Superscaf	2109544	2109564	-25	25	31	-	-	gene	-	-	mRNA	NCP	Intron	gene|mRN	ID=gene-E	7	7.034285714	2889.3	0.96108885	HGARPPP	51	366.1992	2	730.3874	.	R	Superscaf	2109825	0	0	BTP20220	0	0	0	Tissue specific
VLLPPGV	Superscaf	-	Superscaf	2142910	2142930	-160	160	166	-	-	-	-	-	-	NCP	IGR	-	ID=gene-E	7	4.361428571	1678.9	0.96047341	V(+42.01)	57	368.7375	2	735.4531	Acetylati	R	Superscaf	2142963	0	0	0	0	0	BTP20220	Tissue specific
KHAAPGA	Superscaf	-	Superscaf	2145295	2145315	-44	44	50	-	-	-	-	-	-	NCP	IGR	-	ID=gene-E	7	6.808571429	2656.7	0.960429419	K(+42.01)	50	693.3593	1	692.3605	Acetylati	R	Superscaf	2145390	0	0	0	0	0	BTP20220	Tissue specific
AVSAASRL	Superscaf	-	Superscaf	2189302	2189325	-63	63	70	-	-	gene	-	-	mRNA	NCP	Intron	gene|mRN	ID=gene-E	8	5.77	2702.9	0.959617649	AVSAASRL	73	387.7267	2	773.4395	.	Q	Superscaf	2189436	0	BTP20220	0	0	0	BTP20220	Tissue specific
KECLSLK	Superscaf	+	Superscaf	2258929	2258949	11	11	17	-	-	gene	-	-	mRNA	NCP	Intron	gene|mRN	ID=gene-E	7	6.48	3038.2	0.958333426	K(+42.01)	65	460.2438	2	918.4844	Acetylati	G	Superscaf	2258899	0	0	BTP20220	0	0	0	Tissue specific
PRLTPP	Superscaf	-	Superscaf	2301025	2301042	-148	148	153	-	-	-	-	-	-	NCP	IGR	-	ID=gene-E	6	7.028333333	2411.9	0.957557016	P(+42.01)	57	361.7169	2	721.4122	Acetylati	V	Superscaf	2301057	0	0	BTP20220	0	0	0	Tissue specific
LTTLRAL	Superscaf	-	Superscaf	2311198	2311218	-32	32	38	-	-	-	-	-	-	NCP	IGR	-	ID=gene-E	7	6.825714286	2815.3	0.957369319	LTTLRAL	97	394.2535	2	786.4963	.	L	Superscaf	2311353	0	0	0	0	0	BTP20220	Tissue specific
EGAAPGG	Superscaf	-	Superscaf	2326366	2326386	-68	68	74	-	-	-	-	-	-	NCP	IGR	-	ID=gene-E	7	5.638571429	2277.4	0.957089543	EGAAPGG	52	558.2548	1	557.2445	.	L	Superscaf	2326440	0	0	0	0	0	BTP20220	Tissue specific
LEGAGYR	Superscaf	-	Superscaf	2344399	2344419	-10	10	16	-	-	gene	-	-	mRNA	NCP	Intron	gene|mRN	ID=gene-E	7	6.225714286	2793.1	0.956756922	LEGAGYR	78	765.3746	1	764.3817	.	G	Superscaf	2344566	0	0	0	0	BTP20220	0	Mixed
VRVPVLL	Superscaf	-	Superscaf	2367646	2367666	-34	34	40	-	-	-	-	-	-	NCP	IGR	-	ID=gene-E	7	4.145714286	1693	0.956328129	V(+42.01)	57	419.2796	2	836.5483	Acetylati	E	Superscaf	2367702	BTP20220	0	0	0	0	0	Tissue specific
GDGLSVSPF	Superscaf	+	Superscaf	2372062	2372088	12	12	20	-	-	-	-	-	-	NCP	IGR	-	ID=gene-E	9	4.983333333	2801.1	0.956246564	GDGLSVSPF	76	828.4082	1	827.4025	.	T	Superscaf	2372029	0	0	0	0	BTP20220	0	Tissue specific
KPAAPRP	Superscaf	-	Superscaf	2381947	2381967	-8	8	14	-	-	-	-	-	-	NCP	IGR	-	ID=gene-E	7	7.348571429	2752	0.956064345	KPAAPRP	73	368.7213	2	735.4391	.	A	Superscaf	2382024	0	BTP20220	0	0	0	0	Tissue specific
YESSVGY	Superscaf	+	Superscaf	2384752	2384772	34	34	40	-	-	-	-	-	-	NCP	IGR	-	ID=gene-E	7	4.552857143	2408.6	0.956012607	YESSVGY	55	402.675	2	803.3337	.	I	Superscaf	2384653	0	0	0	0	0	BTP20220	Tissue specific
RPAEC	Superscaf	-	Superscaf	2390719	2390733	-157	157	161	-	-	-	-	-	-	NCP	IGR	-	ID=gene-E	5	6.264	2308.7	0.955902655	R(+42.01)	54	674.2949	1	673.2853	Acetylati	N	Superscaf	2390745	BTP20220	0	0	0	0	0	Tissue specific
KKRWRP	Superscaf	-	Superscaf	2400658	2400675	-13	13	18	-	-	-	-	-	-	NCP	IGR	-	ID=gene-E	6	8.865	3098.4	0.955719274	KKRWRP	50	435.7731	2	869.5347	.	A	Superscaf	2400738	0	0	0	0	BTP20220	0	Tissue specific
KSPEMEQK	Superscaf	+	Superscaf	2446555	2446578	1	1	8	-	-	gene	-	-	mRNA	NCP	Intron	gene|mRN	ID=gene-E	8	6.1625	3490.8	0.954872588	KSPEM(+15	57	496.7341	2	991.4644	Oxidatior	K	Superscaf	2446555	0	0	0	BTP20220	0	0	Tissue specific
EPKTNR	Superscaf	+	Superscaf	2530456	2530473	20	20	25	-	-	gene	-	-	mRNA	NCP	Intron	gene|mRN	ID=gene-E	6	6.993333333	2874.3	0.953325135	EPKTNR	61	372.7057	2	743.3926	.	P	Superscaf	2530399	0	0	0	0	0	BTP20220	Tissue specific
ATPGMP	Superscaf	-	Superscaf	2549590	2549607	-95	95	100	-	-	gene	-	-	mRNA	NCP	Intron	gene|mRN	ID=gene-E	6	6.145	2052.4	0.952972206	ATPGM(+15	60	589.2736	1	588.2578	Oxidatior	F	Superscaf	2549652	0	0	0	0	BTP20220	0	Tissue specific
RLRACPP	Superscaf	-	Superscaf	2584444	2584464	-4	4	10	-	-	-	-	-	-	NCP	IGR	-	ID=gene-E	7	7.305714286	3222.8	0.952329265	RLRAC(+57	68	869.4645	1	868.4701	Carbamido	I	Superscaf	2584749	BTP20220	0	0	0	0	0	Tissue specific
MAKLLLPT	Superscaf	-	Superscaf	2612914	2612937	-80	80	87	-	-	-	-	-	-	NCP	IGR	-	ID=gene-E	8	6.535	2946.3	0.951804077	MAKLLLPT	54	443.7822	2	885.5358	.	P	Superscaf	2613129	0	0	0	BTP20220	0	0	Tissue specific
ADALPA	Superscaf	-	Superscaf	2656171	2656188	-49	49	54	-	-	gene	-	-	mRNA	NCP	Intron	gene|mRN	ID=gene-E	6	5.551666667	2036.2	0.951006307	ADALPA	81	557.2909	1	556.2856	.	P	Superscaf	2656236	0	0	0	BTP20220	0	0	Tissue specific
QGGLQLR	Superscaf	-	Superscaf	2658415	2658435	-94	94	100	-	-	gene	-	-	mRNA	NCP	Intron	gene|mRN	ID=gene-E	7	6.565714286	3171.6	0.950964861	QGGLQLR	80	386.2269	2	770.4399	.	S	Superscaf	2658567	0	BTP20220	0	0	0	0	Tissue specific
KFAAAP	Superscaf	-	Superscaf	2666266	2666283	-200	200	205	-	-	gene	-	-	mRNA	NCP	Intron	gene|mRN	ID=gene-E	6	6.596666667	2127.1	0.950820104	KFAAAP	79	604.3516	1	603.338	.	G	Superscaf	2666298	0	0	BTP20220	0	0	BTP20220	Tissue specific
KYTPAAP	Superscaf	+	Superscaf	2690767	2690787	23	23	29	-	-	-	-	-	-	NCP	IGR	-	ID=gene-E	7	6.652857143	2622.8	0.950368125	KYTPAAP	53	374.2035	2	746.3962	.	S	Superscaf	2690701	0	0	0	0	BTP20220	0	Tissue specific
KAHGQEK	Superscaf	-	Superscaf	2707102	2707122	-47	47	53	-	-	-	-	-	-	NCP	IGR	-	ID=gene-E	7	6.847142857	3201.3	0.950066824	KAHGQEK	61	399.2098	2	796.4191	.	Q	Superscaf	2707227	0	0	0	0	BTP20220	BTP20220	Tissue specific
LAGRTPK	Superscaf	-	Superscaf	2721835	2721855	-62	62	68	-	-	-	-	-	-	NCP	IGR	-	ID=gene-E	7	7.328571429	2814.1	0.949795072	LAGRTPK	64	371.735	2	741.4497	.	D	Superscaf	2721882	0	0	0	0	BTP20220	0	Tissue specific
PPAVP	Superscaf	-	Superscaf	2727592	2727606	-21	21	25	-	-	-	-	-	-	NCP	IGR	-	ID=gene-E	5	4.984	1323.5	0.949688994	PPAVP	56	480.2796	1	479.2744	.	V	Superscaf	2727621	0	0	BTP20220	0	0	0	Tissue specific
KHTPPGP	Superscaf	-	Superscaf	2757973	2757993	-217	217	223	-	-	-	-	-	-	NCP	IGR	-	ID=gene-E	7	6.961428571	2795.1	0.949128502	KHTPPGP	55	367.2068	2	732.3918	.	A	Superscaf	2758089	0	0	0	0	0	BTP20220	Tissue specific
DALPVQ	Superscaf	+	Superscaf	2847178	2847195	57	57	62	-	-	gene	-	-	mRNA	NCP	Intron	gene|mRN	ID=gene-E	6	4.486666667	2024.3	0.947483161	DALPVQ	61	642.3439	1	641.3384	.	V	Superscaf	2847010	0	0	0	BTP20220	0	0	Tissue specific
KELPDK	Superscaf	-	Superscaf	2851297	2851314	-3	3	8	-	-	gene	-	-	mRNA	NCP	Intron	gene|mRN	ID=gene-E	6	6.325	2464.6	0.947407186	KELPDK	63	729.4135	1	728.4068	.	V	Superscaf	2851332	0	BTP20220	0	0	0	0	Tissue specific
GTLPALV	Superscaf	-	Superscaf	2851963	2851983	-3	3	9	-	-	gene	-	-	mRNA	NCP	Intron	gene|mRN	ID=gene-E	7	5.254285714	2050.3	0.947394846	GTLPALV	84	670.4108	1	669.4061	.	P	Superscaf	2851992	0	0	0	0	0	BTP20220	Tissue specific
PRKMSK	Superscaf	+	Superscaf	2857897	2857914	4	4	9	-	-	gene	-	-	mRNA	NCP	Intron	gene|mRN	ID=gene-E	6	7.995	2677.9	0.947285448	PRKM(+15.	63	381.7107	2	761.4218	Oxidatior	G	Superscaf	2857888	0	0	0	0	BTP20220	BTP20220	Tissue specific
EGTLP	Superscaf	-	Superscaf	2906215	2906229	-34	34	38	-	-	gene	-	-	mRNA	NCP	Intron	gene|mRN	ID=gene-E	5	5.6	1811.3	0.946394272	EGTLP	54	516.2659	1	515.2591	.	G	Superscaf	2906325	0	0	0	BTP20220	0	0	Tissue specific
QSAGP	Superscaf	-	Superscaf	2924677	2924691	-47	47	51	-	-	gene	-	-	mRNA	NCP	Intron	gene|mRN	ID=gene-E	5	5.924	1940.1	0.946053739	Q(+42.01)	64	501.2384	1	500.2231	Acetylati	A	Superscaf	2924877	BTP20220	0	0	0	0	0	Tissue specific
FALLDF	Superscaf	+	Superscaf	2958439	2958456	4	4	9	-	-	gene	-	-	mRNA	NCP	Intron	gene|mRN	ID=gene-E	6	5.318333333	2204.4	0.945430939	FALLDF	65	725.381	1	724.3795	.	R	Superscaf	2958430	0	BTP20220	0	0	0	0	Tissue specific
KGSSGVP	Superscaf	+	Superscaf	2994604	2994624	22	22	28	-	-	gene	-	-	mRNA	NCP	Intron	gene|mRN	ID=gene-E	7	5.62	2167.2	0.944763816	KGSSGVP	50	631.3357	1	630.3337	.	T	Superscaf	2994541	BTP20220	0	0	0	0	0	Tissue specific
TPPTRAAP	Superscaf	-	Superscaf	3010216	3010239	-81	81	88	-	-	gene	-	-	mRNA	NCP	Intron	gene|mRN	ID=gene-E	8	6.845	3078.6	0.944475796	TPPTRAAP	64	405.7261	2	809.4395	.	P	Superscaf	3010254	BTP20220	BTP20220	BTP20220	BTP20220	0	0	Tissue specific
LLLALVGQ	Superscaf	-	Superscaf	3028024	3028047	-12	12	19	-	-	gene	-	-	mRNA	NCP	Intron	gene|mRN	ID=gene-E	8	5.195	2632.9	0.944147325	LLLALVGQ	95	826.5377	1	825.5323	.	S	Superscaf	3028239	BTP20220	0	0	0	0	0	Mixed
HGAGLPQ	Superscaf	-	Superscaf	3029281	3029301	-25	25	31	-	-	gene	-	-	mRNA	NCP	Intron	gene|mRN	ID=gene-E	7	6.211428571	2827.1	0.944124195	HGAGLPQ	82	679.3483	1	678.3449	.	T	Superscaf	3029427	0	BTP20220	0	0	0	0	Tissue specific
GPPLVGT	Superscaf	-	Superscaf	3057802	3057822	-12	12	18	-	-	gene	-	-	mRNA	NCP	Intron	gene|mRN	ID=gene-E	7	5.292857143	2020.3	0.943598122	GPPLVGT	56	640.3656	1	639.3591	.	V	Superscaf	3057867	BTP20220	0	0	0	0	0	Mixed
SPVLLHQ	Superscaf	-	Superscaf	3094036	3094056	-191	191	197	-	-	gene	-	-	mRNA	NCP	Intron	gene|mRN	ID=gene-E	7	5.311428571	2658	0.942929782	SPVLLHQ	54	793.4528	1	792.4494	.	L	Superscaf	3094200	0	0	0	0	0	BTP20220	Tissue specific
VDVLNPGGF	Superscaf	-	Superscaf	3100012	3100041	-55	55	64	-	-	gene	-	-	mRNA	NCP	Intron	gene|mRN	ID=gene-E	10	4.455	3241.7	0.942819388	VDVLNPGGF	80	498.2596	2	994.5084	.	A	Superscaf	3100308	0	0	BTP20220	0	0	0	Tissue specific
KSLKGLP	Superscaf	+	Superscaf	3109411	3109431	17	17	23	-	-	gene	-	-	mRNA	NCP	Intron	gene|mRN	ID=gene-E	7	7.055714286	2661.7	0.942646188	KSLKGLP	66	371.7436	2	741.4749	.	T	Superscaf	3109363	0	BTP20220	0	0	0	0	Tissue specific
KAPQAAAPF	Superscaf	+	Superscaf	3132004	3132030	23	23	31	-	-	gene	-	-	mRNA	NCP	Intron	gene|mRN	ID=gene-E	9	6.485555556	3336.5	0.942229347	KAPQAAAPF	50	425.7441	2	849.4708	.	P	Superscaf	3131938	BTP20220	0	0	0	0	0	Tissue specific
VTGPAARP	Superscaf	-	Superscaf	3139657	3139680	-17	17	24	-	-	gene	-	-	mRNA	NCP	Intron	gene|mRN	ID=gene-E	8	5.9875	2641	0.942088242	VTGPAARP	66	384.7208	2	767.429	.	T	Superscaf	3139833	0	0	0	BTP20220	0	0	Tissue specific
SAAPPPP	Superscaf	-	Superscaf	3206431	3206451	-45	45	51	-	-	gene	-	-	mRNA	NCP	Intron	gene|mRN	ID=gene-E	7	6.131428571	2355.6	0.940856643	SAAPPPP	68	636.3446	1	635.3278	.	Q	Superscaf	3206583	0	0	0	BTP20220	0	0	Tissue specific
KATAMPK	Superscaf	+	Superscaf	3225289	3225309	6	6	12	-	-	-	-	-	-	NCP	IGR	-	ID=gene-E	7	7.157142857	2665.8	0.940508805	KATAM(+15	55	381.7129	2	761.4105	Oxidatior	G	Superscaf	3225274	BTP20220	0	0	BTP20220	0	0	Tissue specific
VLPHQLAH	Superscaf	-	Superscaf	3257593	3257616	-34	34	41	-	-	gene	-	-	mRNA	NCP	Intron	gene|mRN	ID=gene-E	8	5.63875	3205.6	0.939912899	VLPHQLAH	68	457.7624	2	913.5134	.	K	Superscaf	3257628	0	0	0	BTP20220	0	0	Tissue specific
VSVRDF	Superscaf	+	Superscaf	3285889	3285906	22	22	27	-	-	gene	-	-	mRNA	NCP	Intron	gene|mRN	ID=gene-E	6	4.148333333	1831.1	0.939391087	VSVRDF	97	361.6938	2	721.3759	.	A	Superscaf	3285826	BTP20220	0	0	0	0	0	Tissue specific
LPPPPGP	Superscaf	-	Superscaf	3298954	3298974	-97	97	103	-	-	gene	-	-	mRNA	NCP	Intron	gene|mRN	ID=gene-E	7	6.207142857	2337.7	0.939150046	L(+42.01)	99	358.7057	2	715.3904	Acetylati	D	Superscaf	3299160	0	0	BTP20220	0	0	0	Tissue specific

VPLKGRP	Superscaf	-	Superscaf	3305440	3305460	-15	15	21	-	-	gene	-	-	mRNA	NCP	Intron	gene|mRN	ID=gene-E	7	6.435714286	2442.7	0.939030411	VPLKGRP	93	383.7498	2	765.4861	.	S	Superscaf	3305508	BTP20220	0	0	0	0	0	Tissue specific
KHEARP	Superscaf	-	Superscaf	3308227	3308244	-215	215	220	-	-	gene	-	-	mRNA	NCP	Intron	gene|mRN	ID=gene-E	6	7.271666667	2811.1	0.93897906	KHEARP	60	369.2126	2	736.398	.	G	Superscaf	3308253	0	0	BTP20220	BTP20220	0	0	Tissue specific
KERLVF	Superscaf	+	Superscaf	3313939	3313956	46	46	51	-	-	gene	-	-	mRNA	NCP	Intron	gene|mRN	ID=gene-E	6	5.863333333	2283.5	0.938873701	K(+42.01)	54	417.2476	2	832.4807	Acetylati	F	Superscaf	3313804	0	0	0	BTP20220	0	0	Tissue specific
FPDRSP	Superscaf	-	Superscaf	3324340	3324357	-19	19	24	-	-	-	-	-	-	NCP	IGR	-	ID=gene-E	6	6.248333333	2505.9	0.938681853	FPDRSP	56	359.6786	2	717.3445	.	G	Superscaf	3324558	0	0	0	0	0	BTP20220	Tissue specific
QANPKR	Superscaf	-	Superscaf	3352651	3352668	-47	47	52	-	-	gene	-	-	mRNA	NCP	Intron	gene|mRN	ID=gene-E	6	7.313333333	2973.3	0.938159654	QANPKR	69	357.2011	2	712.398	.	C	Superscaf	3352827	0	0	0	0	BTP20220	0	Tissue specific
KMGAAP	Superscaf	-	Superscaf	3379111	3379128	-69	69	74	-	-	gene	-	-	mRNA	NCP	Intron	gene|mRN	ID=gene-E	6	6.633333333	2097.2	0.937671596	KM(+15.99	60	590.2856	1	589.2894	Oxidation	L	Superscaf	3379353	0	0	BTP20220	0	0	0	Tissue specific
WAGCLHGL	Superscaf	+	Superscaf	3403120	3403143	9	9	16	-	-	gene	-	-	mRNA	NCP	Intron	gene|mRN	ID=gene-E	8	6.0525	3344.7	0.937228637	W(+42.01)	84	478.2346	2	954.4382	Acetylati	F	Superscaf	3403096	BTP20220	0	0	0	0	0	Tissue specific
PPSPVVPV	Superscaf	-	Superscaf	3437488	3437511	-4	4	11	-	-	gene	-	-	mRNA	NCP	Intron	gene|mRN	ID=gene-E	8	3.86	1733	0.936594715	PPSPVVPV	55	396.2401	2	790.4589	.	E	Superscaf	3438024	0	0	0	BTP20220	0	0	Tissue specific
PQGPNPR	Superscaf	-	Superscaf	3473431	3473451	-36	36	42	-	-	-	-	-	-	NCP	IGR	-	ID=gene-E	7	6.67	3165.7	0.935931797	P(+42.01)	57	404.2132	2	806.4035	Acetylati	G	Superscaf	3473625	0	0	BTP20220	0	0	0	Tissue specific
MSRPPA	Superscaf	+	Superscaf	3557980	3557997	21	21	26	-	-	gene	-	-	mRNA	NCP	Intron	gene|mRN	ID=gene-E	6	6.801666667	2389.8	0.934372337	M(+42.01)	70	700.3448	1	699.3373	Acetylati	V	Superscaf	3557920	BTP20220	0	0	0	0	0	Tissue specific
LQLEYPV	Superscaf	+	Superscaf	3572194	3572214	5	5	11	-	-	gene	-	-	mRNA	NCP	Intron	gene|mRN	ID=gene-E	7	4.684285714	2539.9	0.934110103	LQLEYPV	52	431.2373	2	860.4644	.	G	Superscaf	3572182	BTP20220	0	0	0	0	0	Tissue specific
SPGRTLVL	Superscaf	+	Superscaf	3575698	3575721	23	23	30	-	-	gene	-	-	mRNA	NCP	Intron	gene|mRN	ID=gene-E	8	5.9	2771.2	0.934045416	SPGRTLVL	57	421.7574	2	841.5021	.	Q	Superscaf	3575632	0	0	0	0	BTP20220	0	Tissue enhanced
PGVPGP	Superscaf	-	Superscaf	3577963	3577980	-53	53	58	-	-	gene	-	-	mRNA	NCP	Intron	gene|mRN	ID=gene-E	6	5.14	1606.8	0.934003748	PGVPGP	83	523.2858	1	522.2802	.	T	Superscaf	3578004	0	BTP20220	0	0	0	0	Mixed
PPPLPP	Superscaf	-	Superscaf	3607120	3607137	-1	1	6	-	-	-	-	-	-	NCP	IGR	-	ID=gene-E	6	6.246666667	2040.4	0.933465944	PPPLPP	99	617.3632	1	616.3584	.	P	Superscaf	3607293	BTP20220	0	0	0	0	0	Tissue specific
KEPRPP	Superscaf	-	Superscaf	3621727	3621744	-38	38	43	-	-	gene	-	-	mRNA	NCP	Intron	gene|mRN	ID=gene-E	6	7.103333333	2554.9	0.933196516	KEPRPP	59	362.2098	2	722.4075	.	W	Superscaf	3621759	0	0	0	BTP20220	0	0	Tissue specific
KDLVAF	Superscaf	+	Superscaf	3629731	3629748	25	25	30	-	-	-	-	-	-	NCP	IGR	-	ID=gene-E	6	5.031666667	1932	0.933048881	KDLVAF	51	692.4061	1	691.3904	.	L	Superscaf	3629659	BTP20220	0	0	0	0	0	Tissue enhanced
PGMTDLR	Superscaf	-	Superscaf	3665143	3665163	-88	88	94	-	-	gene	-	-	mRNA	NCP	Intron	gene|mRN	ID=gene-E	7	6.322857143	2817.4	0.932395648	PGM(+15.9	50	403.2003	2	804.38	Oxidation	S	Superscaf	3665187	BTP20220	0	0	0	0	0	Tissue specific
LGATAASG	Superscaf	-	Superscaf	3667660	3667683	-99	99	106	-	-	gene	-	-	mRNA	NCP	Intron	gene|mRN	ID=gene-E	8	6.02375	2662.8	0.932349166	LGATAASG	56	647.3376	1	646.3286	.	R	Superscaf	3667773	BTP20220	0	0	0	0	0	Tissue specific
PLLYLFLP	Superscaf	+	Superscaf	3715345	3715368	13	13	20	-	-	gene	-	-	mRNA	NCP	Intron	gene|mRN	ID=gene-E	8	5.9575	2935.3	0.931469611	PLLYLFLP	67	488.2919	2	974.584	.	K	Superscaf	3715309	BTP20220	0	0	BTP20220	0	0	Tissue specific
RLGLTLK	Superscaf	+	Superscaf	3727354	3727374	13	13	19	-	-	gene	-	-	mRNA	NCP	Intron	gene|mRN	ID=gene-E	7	7.277142857	2872.2	0.931248159	RLGLTLK	76	400.7695	2	799.528	.	L	Superscaf	3727318	0	BTP20220	0	0	0	0	Tissue specific
TVSRRPLT	Superscaf	+	Superscaf	3751405	3751428	15	15	22	-	-	gene	-	-	mRNA	NCP	Intron	gene|mRN	ID=gene-E	8	6.5675	3166.8	0.93080448	T(+42.01)	57	486.291	2	970.556	Acetylati	I	Superscaf	3751363	0	0	0	0	BTP20220	0	Tissue specific
TKAAGVLP	Superscaf	-	Superscaf	3754375	3754398	-12	12	19	-	-	gene	-	-	mRNA	NCP	Intron	gene|mRN	ID=gene-E	8	5.82	2476.6	0.930749698	T(+42.01)	86	798.4712	1	797.4647	Acetylati	I	Superscaf	3754443	0	0	0	BTP20220	0	0	Tissue specific
FTKGAP	Superscaf	+	Superscaf	3775669	3775686	4	4	9	-	-	gene	-	-	mRNA	NCP	Intron	gene|mRN	ID=gene-E	6	6.673333333	2199.3	0.930357038	FTKGAP	58	620.349	1	619.3329	.	C	Superscaf	3775660	BTP20220	0	0	0	0	0	Tissue specific
KPTAGP	Superscaf	-	Superscaf	3820897	3820914	-58	58	63	-	-	gene	-	-	mRNA	NCP	Intron	gene|mRN	ID=gene-E	6	6.81	2149.3	0.929522803	KPTAGP	55	570.3248	1	569.3173	.	A	Superscaf	3820926	BTP20220	0	0	0	0	0	Tissue specific
KSLLLGSR	Superscaf	-	Superscaf	3865987	3866010	-99	99	106	-	-	gene	-	-	mRNA	NCP	Intron	gene|mRN	ID=gene-E	8	6.97125	3241.5	0.928691002	KSLLLGSR	50	437.278	2	872.5443	.	G	Superscaf	3866031	0	0	BTP20220	0	0	BTP20220	Tissue specific
KAGREQR	Superscaf	-	Superscaf	3874030	3874050	-2	2	8	-	-	gene	-	-	mRNA	NCP	Intron	gene|mRN	ID=gene-E	7	7.445714286	3410.8	0.928542703	KAGREQR	54	422.7468	2	843.4675	.	H	Superscaf	3874281	BTP20220	0	0	0	0	0	Tissue specific
MTRTRVLL	Superscaf	+	Superscaf	4032145	4032168	5	5	12	-	-	gene	-	-	mRNA	NCP	Intron	gene|mRN	ID=gene-E	8	6.53625	3170.9	0.925626198	MTRTRVLL	50	495.3064	2	988.5851	.	G	Superscaf	4032133	BTP20220	0	0	0	0	0	Tissue specific
ELLEAPV	Superscaf	-	Superscaf	4056457	4056477	-21	21	27	-	-	-	-	-	-	NCP	IGR	-	ID=gene-E	7	4.388571429	2206.5	0.925177816	ELLEAPV	84	770.4247	1	769.4221	.	S	Superscaf	4056492	0	0	BTP20220	0	0	0	Tissue specific
VTLVTLAS	Superscaf	-	Superscaf	4056577	4056600	-24	24	31	-	-	-	-	-	-	NCP	IGR	-	ID=gene-E	8	4.59	2196.5	0.925175547	VTLVTLAS	94	803.4853	1	802.48	.	H	Superscaf	4056822	BTP20220	0	BTP20220	0	0	0	Tissue specific
VTLVTLA	Superscaf	-	Superscaf	4056577	4056597	-24	24	30	-	-	-	-	-	-	NCP	IGR	-	ID=gene-E	7	4.434285714	1813.1	0.925175603	VTLVTLA	91	716.4537	1	715.448	.	H	Superscaf	4056822	0	BTP20220	0	0	0	0	Mixed
TDHPTLLT	Superscaf	+	Superscaf	4059850	4059873	64	64	71	-	-	-	-	-	-	NCP	IGR	-	ID=gene-E	8	6.05125	3267.9	0.925115176	T(+42.01)	59	470.2407	2	938.4709	Acetylati	R	Superscaf	4059661	0	0	BTP20220	0	0	0	Tissue specific
QPYLPY	Superscaf	-	Superscaf	4067260	4067277	-41	41	46	-	-	-	-	-	-	NCP	IGR	-	ID=gene-E	6	5.925	2557.9	0.924978609	QPYLPY	64	390.6985	2	779.3854	.	K	Superscaf	4067394	0	BTP20220	0	0	0	0	Tissue specific
ARPNPP	Superscaf	-	Superscaf	4079008	4079025	-45	45	50	-	-	-	-	-	-	NCP	IGR	-	ID=gene-E	6	6.848333333	2569	0.924761916	ARPNPP	81	651.3563	1	650.35	.	G	Superscaf	4079064	0	0	0	BTP20220	0	0	Mixed
KELDTF	Superscaf	+	Superscaf	4081123	4081140	3	3	8	-	-	-	-	-	-	NCP	IGR	-	ID=gene-E	6	5.653333333	2443.7	0.924722904	KELDTF	80	752.379	1	751.3752	.	G	Superscaf	4081117	BTP20220	0	0	0	0	0	Tissue specific
RGMRVP	Superscaf	+	Superscaf	4134193	4134210	27	27	32	-	-	-	-	-	-	NCP	IGR	-	ID=gene-E	6	6.59	2303.8	0.923744022	RGMRVP	89	358.206	2	714.3959	.	S	Superscaf	4134115	0	BTP20220	0	0	0	0	Tissue specific
PPPPKLE	Superscaf	-	Superscaf	4175926	4175946	-51	51	57	-	-	-	-	-	-	NCP	IGR	-	ID=gene-E	7	6.305714286	2596.9	0.922974197	PPPPKLE	55	389.2269	2	776.4432	.	T	Superscaf	4176012	BTP20220	0	BTP20220	0	0	0	Tissue specific
LVEVP	Superscaf	-	Superscaf	4187932	4187946	-80	80	84	-	-	-	-	-	-	NCP	IGR	-	ID=gene-E	5	3.1	1116.3	0.922752855	LVEVP	77	556.3339	1	555.3268	.	G	Superscaf	4187958	0	0	BTP20220	0	0	0	Tissue specific
KVPPPPPPF	Superscaf	+	Superscaf	4189504	4189530	-18	18	26	-	-	-	-	-	-	NCP	IGR	-	ID=gene-E	9	5.982222222	2830.2	0.922723638	KVPPPPPPF	88	925.5526	1	924.5432	.	L	Superscaf	4189593	0	0	BTP20220	0	0	0	Tissue specific
PGGGVPPGF	Superscaf	+	Superscaf	4190026	4190052	38	38	46	-	-	-	-	-	-	NCP	IGR	-	ID=gene-E	9	5.453333333	2538.8	0.92271401	PGGGVPPGF	54	367.6953	2	733.3759	.	E	Superscaf	4189915	0	BTP20220	0	0	0	0	Tissue specific
LGRPWYP	Superscaf	-	Superscaf	4206817	4206837	-12	12	18	-	-	gene	-	-	mRNA	NCP	Intron	gene|mRN	ID=gene-E	7	6.694285714	2960.4	0.922404409	L(+42.01)	51	465.7534	2	929.4759	Acetylati	F	Superscaf	4206879	0	0	BTP20220	0	0	0	Tissue specific
AAVSGVLPG	Superscaf	-	Superscaf	4260487	4260528	-57	57	70	-	-	gene	-	-	mRNA	NCP	Intron	gene|mRN	ID=gene-E	14	4.871428571	4353.9	0.921414072	A(+42.01)	82	731.4042	2	1460.799	Acetylati	W	Superscaf	4260768	0	0	0	0	0	BTP20220	Mixed
AAVSGVLPG	Superscaf	-	Superscaf	4260487	4260525	-57	57	69	-	-	gene	-	-	mRNA	NCP	Intron	gene|mRN	ID=gene-E	13	4.418461538	3705.1	0.921414127	A(+42.01)	61	653.3538	2	1304.698	Acetylati	W	Superscaf	4260768	0	BTP20220	0	0	0	0	Tissue specific
AAVSGVLPG	Superscaf	-	Superscaf	4260487	4260519	-57	57	67	-	-	gene	-	-	mRNA	NCP	Intron	gene|mRN	ID=gene-E	11	4.356363636	2942.2	0.921414238	A(+42.01)	80	540.3061	2	1078.602	Acetylati	W	Superscaf	4260768	0	0	BTP20220	0	0	0	Tissue specific
AAVSGVLP	Superscaf	-	Superscaf	4260487	4260510	-57	57	64	-	-	gene	-	-	mRNA	NCP	Intron	gene|mRN	ID=gene-E	8	4.49625	1994.1	0.921414404	A(+42.01)	85	755.4286	1	754.4225	Acetylati	W	Superscaf	4260768	0	BTP20220	0	0	0	0	Mixed
VLPQVPEPF	Superscaf	-	Superscaf	4260502	4260528	-62	62	70	-	-	gene	-	-	mRNA	NCP	Intron	gene|mRN	ID=gene-E	9	4.945555556	3050.6	0.921414072	VLPQVPEPF	78	517.8022	2	1033.592	.	W	Superscaf	4260768	BTP20220	0	0	0	0	0	Tissue specific
GPPSRPGP	Superscaf	+	Superscaf	4265938	4265961	15	15	22	-	-	gene	-	-	mRNA	NCP	Intron	gene|mRN	ID=gene-E	8	6.6975	2976.4	0.92131386	GPPSRPGP	66	382.7053	2	763.3976	.	P	Superscaf	4265896	0	0	BTP20220	0	0	0	Tissue specific
KPVSFC	Superscaf	+	Superscaf	4273687	4273704	24	24	29	-	-	gene	-	-	mRNA	NCP	Intron	gene|mRN	ID=gene-E	6	5.37	2162.3	0.921171039	KPVSFC(+5	64	369.1805	2	736.3578	Carbamido	T	Superscaf	4273618	0	0	BTP20220	0	0	0	Tissue specific
LVPRQPP	Superscaf	-	Superscaf	4295809	4295829	-7	7	13	-	-	gene	-	-	mRNA	NCP	Intron	gene|mRN	ID=gene-E	7	5.898571429	2625.1	0.920762941	LVPRQPP	95	403.7544	2	805.481	.	D	Superscaf	4295832	0	0	BTP20220	0	0	0	Tissue specific
VHSSEETVS	Superscaf	-	Superscaf	4308058	4308096	-45	45	57	-	-	gene	-	-	mRNA	NCP	Intron	gene|mRN	ID=gene-E	13	5.313076923	4563.8	0.920536675	VHSSEETVS	90	453.5671	3	1357.684	.	T	Superscaf	4308129	0	0	0	0	0	BTP20220	Tissue specific
VHSSEETVS	Superscaf	-	Superscaf	4308058	4308093	-45	45	56	-	-	gene	-	-	mRNA	NCP	Intron	gene|mRN	ID=gene-E	12	4.944166667	4095.4	0.92053673	VHSSEETVS	99	615.7994	2	1229.589	.	T	Superscaf	4308129	0	0	0	0	BTP20220	0	Tissue specific
VADEKLR	Superscaf	+	Superscaf	4321204	4321224	17	17	23	-	-	gene	-	-	mRNA	NCP	Intron	gene|mRN	ID=gene-E	7	5.527142857	2618.9	0.920294527	VADEKLR	77	415.7383	2	829.4658	.	E	Superscaf	4321156	0	0	BTP20220	0	0	0	Tissue specific
DPPPP	Superscaf	-	Superscaf	4348738	4348752	-30	30	34	-	-	-	-	-	-	NCP	IGR	-	ID=gene-E	5	5.634	1761.1	0.91978677	D(+42.01)	62	564.264	1	563.2591	Acetylati	P	Superscaf	4348824	0	0	BTP20220	0	0	0	Tissue specific
ALPKAVR	Superscaf	-	Superscaf	4383334	4383354	-137	137	143	-	-	gene	-	-	mRNA	NCP	Intron	gene|mRN	ID=gene-E	7	6.402857143	2430.6	0.919148532	ALPKAVR	81	377.7485	2	753.4861	.	A	Superscaf	4383456	0	0	BTP20220	0	0	0	Tissue specific
LPPPHPEN	Superscaf	+	Superscaf	4456828	4456851	77	77	84	-	-	-	-	-	-	NCP	IGR	-	ID=gene-E	8	5.925	3344.9	0.917792872	LPPPHPEN	99	450.7312	2	899.4501	.	I	Superscaf	4456600	0	0	0	BTP20220	0	0	Tissue specific
KGPPALPH	Superscaf	+	Superscaf	4464136	4464159	58	58	65	-	-	gene	-	-	mRNA	NCP	Intron	gene|mRN	ID=gene-E	8	6.775	3062.3	0.917658075	KGPPALPH	68	408.744	2	815.4653	.	Q	Superscaf	4463965	0	0	BTP20220	0	0	0	Tissue specific
GMGHDP	Superscaf	-	Superscaf	4465936	4465953	-19	19	24	-	exon	gene	-	five_prim	mRNA	NCP	5UTR	exon|gene	ID=exon-E	6	5.758333333	2334.7	0.917624984	G(+42.01)	50	671.2477	1	670.2381	Acetylati	S	Superscaf	4465953	0	0	BTP20220	0	0	0	Tissue specific
RPFGP	Superscaf	+	Superscaf	4499623	4499637	9	9	13	-	-	gene	-	-	mRNA	NCP	Intron	gene|mRN	ID=gene-E	5	6.962	2008.3	0.917003679	RPFGP	67	573.313	1	572.3071	.	E	Superscaf	4499599	BTP20220	0	0	BTP20220	0	0	Tissue specific
APPVTRD	Superscaf	+	Superscaf	4500283	4500303	43	43	49	-	-	gene	-	-	mRNA	NCP	Intron	gene|mRN	ID=gene-E	7	5.554285714	2443.9	0.916991394	A(+42.01)	93	399.2139	2	796.4079	Acetylati	A	Superscaf	4500157	0	0	0	BTP20220	0	0	Tissue specific
LSGEGLPF	Superscaf	-	Superscaf	4526122	4526145	-34	34	41	-	-	gene	-	-	mRNA	NCP	Intron	gene|mRN	ID=gene-E	8	5.5725	2835.1	0.916514736	LSGEGLPF	66	819.4211	1	818.4174	.	A	Superscaf	4526205	0	0	0	BTP20220	0	0	Tissue specific
VGGLP	Superscaf	-	Superscaf	4531312	4531326	-6	6	10	-	-	gene	-	-	mRNA	NCP	Intron	gene|mRN	ID=gene-E	5	4.844	1285.4	0.916419172	VGGLP	70	442.2648	1	441.2587	.	L	Superscaf	4531584	0	0	0	BTP20220	0	0	Tissue specific
RPVEC	Superscaf	+	Superscaf	4618561	4618575	20	20	24	-	-	gene	-	-	mRNA	NCP	Intron	gene|mRN	ID=gene-E	5	5.06	1997.4	0.914809854	RPVEC(+57	60	660.3138	1	659.3061	Carbamido	G	Superscaf	4618504	0	0	0	BTP20220	0	0	Mixed
RLGRFGY	Superscaf	-	Superscaf	4702699	4702719	-3	3	9	-	-	-	-	-	-	NCP	IGR	-	ID=gene-E	7	7.225714286	3092.5	0.913257808	RLGRFGY	76	434.741	2	867.4715	.	F	Superscaf	4702782	0	0	0	BTP20220	0	0	Mixed
VVRQLH	Superscaf	-	Superscaf	4711930	4711947	-162	162	167	-	-	-	-	-	-	NCP	IGR	-	ID=gene-E	6	4.996666667	2232.6	0.913087597	V(+42.01)	70	397.2371	2	792.4606	Acetylati	Y	Superscaf	4712289	0	0	0	BTP20220	0	0	Tissue specific
KGAMVPR	Superscaf	+	Superscaf	4753441	4753461	10	10	16	-	-	-	-	-	-	NCP	IGR	-	ID=gene-E	7	6.362857143	2434.7	0.912321866	KGAMVPR	67	379.7263	2	757.4269	.	C	Superscaf	4753414	0	0	0	0	BTP20220	0	Mixed
EPAALYQ	Superscaf	-	Superscaf	4754542	4754562	-3	3	9	-	-	-	-	-	-	NCP	IGR	-	ID=gene-E	7	5.55	2809.1	0.912301558	EPAALYQ	62	791.3877	1	790.3861	.	Q	Superscaf	4754706	0	0	0	BTP20220	0	0	Tissue specific
NAFNLK	Superscaf	+	Superscaf	4806853	4806870	34	34	39	-	-	gene	-	-	mRNA	NCP	Intron	gene|mRN	ID=gene-E	6	6.34	2713.9	0.911336731	NAFNLK	91	706.3855	1	705.3809	.	F	Superscaf	4806754	0	BTP20220	0	0	0	0	Tissue specific
LSVAGVTK	Superscaf	+	Superscaf	4876999	4877022	22	22	29	-	-	gene	-	-	mRNA	NCP	Intron	gene|mRN	ID=gene-E	8	4.99	2211.3	0.910042769	LSVAGVTK	52	387.7472	2	773.4647	.	I	Superscaf	4876936	0	0	BTP20220	BTP20220	0	0	Tissue specific
QSQTN	Superscaf	+	Superscaf	4901662	4901676	2	2	6	-	-	gene	-	-	mRNA	NCP	Intron	gene|mRN	ID=gene-E	5	5.784	2599	0.909588023	Q(+42.01)	50	619.2715	1	618.2609	Acetylati	Y	Superscaf	4901659	0	0	BTP20220	0	0	0	Tissue specific
RFLKRQ	Superscaf	-	Superscaf	4903345	4903362	-4	4	9	-	-	gene	-	-	mRNA	NCP	Intron	gene|mRN	ID=gene-E	6	8.061666667	3117.5	0.909556925	RFLKRQ	58	424.2641	2	846.5187	.	P	Superscaf	4903413	0	BTP20220	BTP20220	BTP20220	BTP20220	0	Tissue specific
QELNPVP	Superscaf	+	Superscaf	4934515	4934535	143	143	149	-	-	gene	-	-	mRNA	NCP	Intron	gene|mRN	ID=gene-E	7	4.694285714	2661.1	0.908981935	QELNPVP	51	796.4191	1	795.4127	.	E	Superscaf	4934089	0	0	0	0	0	BTP20220	Tissue specific
LALSLRFVF	Superscaf	+	Superscaf	4990783	4990809	20	20	28	-	-	-	-	-	-	NCP	IGR	-	ID=gene-E	9	5.797777778	3128.5	0.907943955	LALSLRFVF	72	508.3163	2	1014.623	.	H	Superscaf	4990726	0	0	0	0	0	BTP20220	Tissue specific
PSRKFF	Superscaf	+	Superscaf	4992010	4992027	29	29	34	-	-	-	-	-	-	NCP	IGR	-	ID=gene-E	6	7.24	2612.8	0.907921489	PSRKFF	90	391.2209	2	780.4282	.	S	Superscaf	4991926	0	0	0	BTP20220	0	0	Mixed
LELPVP	Superscaf	-	Superscaf	4999063	4999080	-43	43	48	-	-	-	-	-	-	NCP	IGR	-	ID=gene-E	6	4.63	1807.1	0.907791395	LELPVP	65	667.3989	1	666.3952	.	R	Superscaf	4999272	0	0	0	0	0	BTP20220	Mixed
VAPPSPR	Superscaf	-	Superscaf	5094175	5094195	-37	37	43	-	-	gene	-	-	mRNA	NCP	Intron	gene|mRN	ID=gene-E	7	5.908571429	2355.7	0.906036988	V(+42.01)	72	383.2228	2	764.418	Acetylati	R	Superscaf	5094219	0	0	0	0	0	BTP20220	Tissue specific
APGGKKTK	Superscaf	-	Superscaf	5167858	5167881	-17	17	24	-	-	-	-	-	-	NCP	IGR	-	ID=gene-E	8	7.50125	3046	0.904677842	APGGKKTK	71	393.7505	2	785.4759	.	V	Superscaf	5168088	0	0	BTP20220	0	0	BTP20220	Tissue specific
CGLVSP	Superscaf	-	Superscaf	5187643	5187660	-11	11	16	-	-	-	-	-	-	NCP	IGR	-	ID=gene-E	6	4.825	1957.2	0.904313016	C(+57.02)	60	632.3063	1	631.2999	Carbamido	N	Superscaf	5187666	0	0	0	0	0	BTP20220	Mixed
QKQLTQ	Superscaf	+	Superscaf	5279287	5279304	14	14	19	-	-	-	-	-	-	NCP	IGR	-	ID=gene-E	6	6.533333333	3051.4	0.902622632	QKQLTQ	64	745.4166	1	744.413	.	P	Superscaf	5279248	BTP20220	0	0	0	0	0	Tissue specific
PPKPMYG	Superscaf	-	Superscaf	5296999	5297019	-27	27	33	-	-	gene	-	-	mRNA	NCP	Intron	gene|mRN	ID=gene-E	7	6.574285714	2608.9	0.902295876	PPKPM(+15	50	403.1976	2	804.384	Oxidation	K	Superscaf	5297109	BTP20220	0	0	0	0	0	Tissue specific
GTAGPPPP	Superscaf	-	Superscaf	5314198	5314221	-89	89	96	-	-	gene	-	-	mRNA	NCP	Intron	gene|mRN	ID=gene-E	8	6.21125	2653	0.901978583	GTAGPPPP	84	693.36	1	692.3493	.	G	Superscaf	5314257	0	0	0	BTP20220	0	0	Tissue specific
DPGHPYP	Superscaf	-	Superscaf	5328253	5328273	-46	46	52	-	-	gene	-	-	mRNA	NCP	Intron	gene|mRN	ID=gene-E	7	5.87	2800.2	0.901719393	DPGHPYP	54	782.3524	1	781.3395	.	K	Superscaf	5328282	0	BTP20220	0	0	0	0	Tissue specific
AVRPALY	Superscaf	-	Superscaf	5336731	5336751	-94	94	100	-	-	gene	-	-	mRNA	NCP	Intron	gene|mRN	ID=gene-E	7	5.82	2421.7	0.901563015	A(+42.01)	70	416.2369	2	830.465	Acetylati	S	Superscaf	5336865	0	0	0	BTP20220	0	0	Tissue specific
LGLAGA	Superscaf	-	Superscaf	5346016	5346033	-124	124	129	-	-	gene	-	-	mRNA	NCP	Intron	gene|mRN	ID=gene-E	6	5.99	1924	0.901391807	LGLAGA	96	501.3025	1	500.2958	.	C	Superscaf	5346318	0	0	BTP20220	0	0	0	Tissue specific
RVGTLR	Superscaf	+	Superscaf	5405152	5405169	39	39	44	-	-	gene	-	-	mRNA	NCP	Intron	gene|mRN	ID=gene-E	6	6.666666667	2345.8	0.900301037	RVGTLR	61	351.2239	2	700.4344	.	G	Superscaf	5405038	BTP20220	0	0	0	0	0	Tissue specific
SSEEMC	Superscaf	+	Superscaf	5405926	5405943	16	16	21	-	-	gene	-	-	mRNA	NCP	Intron	gene|mRN	ID=gene-E	6	4.761666667	2575	0.900286761	SSEEM(+15	70	758.2202	1	757.2259	Oxidation	T	Superscaf	5405881	0	0	0	0	BTP20220	0	Tissue specific
LGPAVPAVI	Superscaf	-	Superscaf	5414674	5414700	-3	3	11	-	-	-	-	-	-	NCP	IGR	-	ID=gene-E	9	4.791111111	2345.6	0.900125237	LGPAVPAVI	63	824.4829	1	823.4803	.	L	Superscaf	5414724	0	0	0	0	0	BTP20220	Tissue specific
QAHVLAT	Superscaf	-	Superscaf	5419252	5419272	-127	127	133	-	-	-	-	-	-	NCP	IGR	-	ID=gene-E	7	5.398571429	2603.9	0.900040906	QAHVLAT	65	370.2135	2	738.4024	.	D	Superscaf	5419272	0	0	0	BTP20220	0	0	Tissue specific
QGLVQFK	Superscaf	+	Superscaf	5434204	5434224	36	36	42	-	-	-	-	-	-	NCP	IGR	-	ID=gene-E	7	5.495714286	2727.9	0.899765114	QGLVQFK	82	410.2389	2	818.465	.	D	Superscaf	5434099	0	BTP20220	0	0	0	0	Tissue specific
PVETLPR	Superscaf	-	Superscaf	5492359	5492379	-41	41	47	-	-	gene	-	-	mRNA	NCP	Intron	gene|mRN	ID=gene-E	7	5.584285714	2500	0.898692439	P(+42.01)	69	427.2503	2	852.4705	Acetylati	H	Superscaf	5492460	BTP20220	0	0	0	0	0	Tissue specific
SVACHDF	Superscaf	-	Superscaf	5542093	5542113	-52	52	58	-	-	gene	-	-	mRNA	NCP	Intron	gene|mRN	ID=gene-E	7	4.68	2699	0.897775089	SVAC(+57.	56	418.1676	2	834.333	Carbamido	N	Superscaf	5542152	BTP20220	0	0	0	0	0	Tissue specific
GPMLLK	Superscaf	+	Superscaf	5542540	5542557	6	6	11	-	-	gene	-	-	mRNA	NCP	Intron	gene|mRN	ID=gene-E	6	6.62	2181.4	0.8977669	G(+42.01)	67	358.7069	2	715.3939	Acetylati	L	Superscaf	5542525	0	0	0	0	0	BTP20220	Tissue specific
ELGSFLVPV	Superscaf	-	Superscaf	5557678	5557704	-2	2	10	-	-	gene	-	-	mRNA	NCP	Intron	gene|mRN	ID=gene-E	9	4.891111111	3134.5	0.897487512	ELGSFLVPV	71	488.2611	2	974.5073	.	A	Superscaf	5557716	0	0	0	BTP20220	0	0	Tissue specific
TKLVTVK	Superscaf	-	Superscaf	5572978	5572998	-2	2	8	-	-	gene	-	-	mRNA	NCP	Intron	gene|mRN	ID=gene-E	7	5.502857143	2085.2	0.897205412	T(+42.01)	97	415.7787	2	829.5273	Acetylati	V	Superscaf	5573052	0	0	0	BTP20220	0	0	Tissue specific
LPPPLPPKF	Superscaf	-	Superscaf	5582317	5582343	-27	27	35	-	-	gene	-	-	mRNA	NCP	Intron	gene|mRN	ID=gene-E	9	6.611111111	3199.6	0.897033042	LPPPLPPKF	87	478.3014	2	954.5901	.	A	Superscaf	5582529	0	0	0	0	BTP20220	0	Tissue specific
SGACKMP	Superscaf	-	Superscaf	5586232	5586252	-24	24	30	-	-	gene	-	-	mRNA	NCP	Intron	gene|mRN	ID=gene-E	7	6.354285714	2755	0.89696094	SGAC(+57.	60	375.674	2	749.32	Carbamido	Q	Superscaf	5586255	0	0	0	BTP20220	0	0	Tissue specific
KPSTPGSGF	Superscaf	+	Superscaf	5600335	5600361	12	12	20	-	exon	gene	-	five_prim	mRNA	NCP	5UTR	exon|gene	ID=exon-E	9	6.496666667	3239.5	0.896700698	KPSTPGSGF	62	414.2145	2	826.4185	.	P	Superscaf	5600302	0	0	0	0	BTP20220	BTP20220	Tissue specific
TEALGSPP	Superscaf	+	Superscaf	5601766	5601789	30	30	37	-	-	gene	-	-	mRNA	NCP	Intron	gene|mRN	ID=gene-E	8	5.75	2843.2	0.896674359	T(+42.01)	77	813.3966	1	812.3916	Acetylati	A	Superscaf	5601679	0	0	0	0	0	BTP20220	Tissue specific
PRGSAPPP	Superscaf	+	Superscaf	5605081	5605104	107	107	114	-	-	-	-	-	-	NCP	IGR	-	ID=gene-E	8	6.70375	2990.4	0.896613213	PRGSAPPP	72	389.7128	2	777.4133	.	R	Superscaf	5604763	BTP20220	0	0	0	0	0	Tissue specific
EGEAFW	Superscaf	+	Superscaf	5644348	5644365	16	16	21	-	-	-	-	-	-	NCP	IGR	-	ID=gene-E	6	4.966666667	2373.6	0.89588904	EGEAFW	62	738.3063	1	737.302	.	E	Superscaf	5644303	BTP20220	0	0	0	0	0	Tissue specific
TYLVK	Superscaf	-	Superscaf	5663806	5663820	-1	1	5	-	-	-	-	-	-	NCP	IGR	-	ID=gene-E	5	5.582	1678.8	0.89553019	TYLVK	99	623.3738	1	622.369	.	T	Superscaf	5663823	BTP20220	0	0	0	0	0	Tissue specific
LHNLVSV	Superscaf	-	Superscaf	5706400	5706420	-2	2	8	-	-	gene	-	-	mRNA	NCP	Intron	gene|mRN	ID=gene-E	7	4.377142857	2306.6	0.894744428	LHNLVSV	65	391.2301	2	780.4494	.	W	Superscaf	5706924	0	0	0	0	0	BTP20220	Tissue enhanced
SGDQPVVP	Superscaf	+	Superscaf	5755468	5755491	36	36	43	-	-	-	-	-	-	NCP	IGR	-	ID=gene-E	8	4.10875	2377.7	0.893839308	SGDQPVVP	60	798.3954	1	797.3919	.	D	Superscaf	5755363	0	BTP20220	0	0	0	0	Tissue specific
DKPPAPP	Superscaf	-	Superscaf	5770579	5770599	-21	21	27	-	-	-	-	-	-	NCP	IGR	-	ID=gene-E	7	6.275714286	2540.8	0.893560639	DKPPAPP	85	721.3872	1	720.3806	.	G	Superscaf	5771088	0	0	0	BTP20220	0	0	Tissue specific
WGSPGY	Superscaf	-	Superscaf	5784118	5784135	-18	18	23	-	-	-	-	-	-	NCP	IGR	-	ID=gene-E	6	5.911666667	2301.5	0.893310966	WGSPGY	52	666.2847	1	665.2809	.	L	Superscaf	5784198	0	0	0	BTP20220	0	0	Tissue specific
KSRHAPK	Superscaf	-	Superscaf	5809912	5809932	-48	48	54	-	-	-	-	-	-	NCP	IGR	-	ID=gene-E	7	7.975714286	3237.4	0.892835137	KSRHAPK	57	412.2475	2	822.4824	.	V	Superscaf	5810241	BTP20220	0	0	0	0	0	Mixed
AAPEPRP	Superscaf	-	Superscaf	5817886	5817906	-193	193	199	-	-	-	-	-	-	NCP	IGR	-	ID=gene-E	7	6.417142857	2709.1	0.892688056	AAPEPRP	80	369.1953	2	736.3867	.	G	Superscaf	5818014	0	0	0	0	0	BTP20220	Tissue specific
PEAPPER	Superscaf	-	Superscaf	5839396	5839416	-139	139	145	-	-	-	-	-	-	NCP	IGR	-	ID=gene-E	7	6.017142857	2823.3	0.892291301	PEAPPER	71	398.2079	2	794.3922	.	R	Superscaf	5839485	0	0	0	0	0	BTP20220	Tissue specific
SGRHGVP	Superscaf	-	Superscaf	5852209	5852229	-59	59	65	-	-	gene	-	-	mRNA	NCP	Intron	gene|mRN	ID=gene-E	7	6.038571429	2583.9	0.892054964	SGRHGVP	52	709.3697	1	708.3667	.	G	Superscaf	5852790	0	0	0	0	0	BTP20220	Tissue specific
FVEKTP	Superscaf	+	Superscaf	5912065	5912082	13	13	18	-	-	gene	-	-	mRNA	NCP	Intron	gene|mRN	ID=gene-E	6	5.211666667	2016.2	0.890950969	FVEKTP	72	360.7054	2	719.3854	.	A	Superscaf	5912029	0	BTP20220	0	0	0	0	Tissue specific
QAPFLRP	Superscaf	-	Superscaf	6039490	6039510	-54	54	60	-	-	-	-	-	-	NCP	IGR	-	ID=gene-E	7	6.641428571	2986.4	0.888600545	QAPFLRP	62	414.7418	2	827.4653	.	P	Superscaf	6039774	0	BTP20220	0	0	0	0	Tissue specific
APPLPPPPF	Superscaf	-	Superscaf	6104647	6104676	-99	99	108	-	-	gene	-	-	mRNA	NCP	Intron	gene|mRN	ID=gene-E	10	6.24	3363.9	0.88739855	APPLPPPPF	64	490.2828	2	978.5538	.	E	Superscaf	6104751	0	0	BTP20220	0	0	0	Tissue specific
LSPSVPK	Superscaf	+	Superscaf	6172105	6172125	13	13	19	-	-	gene	-	-	mRNA	NCP	Intron	gene|mRN	ID=gene-E	7	5.668571429	2263.4	0.886154446	LSPSVPK	80	364.2195	2	726.4276	.	S	Superscaf	6172069	0	0	0	0	BTP20220	BTP20220	Tissue specific
ATNKWLP	Superscaf	-	Superscaf	6178681	6178701	-2	2	8	-	-	gene	-	-	mRNA	NCP	Intron	gene|mRN	ID=gene-E	7	6.552857143	2991.3	0.886033151	ATNKWLP	65	415.2277	2	828.4494	.	G	Superscaf	6178725	0	0	0	0	0	BTP20220	Tissue specific
PPAGAR	Superscaf	-	Superscaf	6207010	6207027	-56	56	61	-	-	gene	-	-	mRNA	NCP	Intron	gene|mRN	ID=gene-E	6	6.895	2243.5	0.885510674	PPAGAR	59	568.3151	1	567.3129	.	T	Superscaf	6207102	0	0	BTP20220	0	0	0	Tissue specific
PPGRPPPP	Superscaf	-	Superscaf	6237547	6237570	-31	31	38	-	-	gene	-	-	mRNA	NCP	Intron	gene|mRN	ID=gene-E	8	6.81625	2970.5	0.884947305	PPGRPPPP	68	407.7352	2	813.4496	.	A	Superscaf	6237603	0	0	0	0	0	BTP20220	Mixed
LLLLKNVT	Superscaf	+	Superscaf	6311542	6311565	16	16	23	-	-	gene	-	-	mRNA	NCP	Intron	gene|mRN	ID=gene-E	8	5.7	2876.2	0.883582459	LLLLKNVT	55	457.3064	2	912.6008	.	T	Superscaf	6311497	0	0	0	0	BTP20220	0	Tissue specific
VYSPTFL	Superscaf	-	Superscaf	6392773	6392793	-4	4	10	-	-	gene	-	-	mRNA	NCP	Intron	gene|mRN	ID=gene-E	7	5.09	2318.6	0.882084199	VYSPTFL	76	826.4272	1	825.4272	.	T	Superscaf	6392820	0	BTP20220	0	0	0	0	Tissue specific
PPANP	Superscaf	-	Superscaf	6403498	6403512	-106	106	110	-	-	gene	-	-	mRNA	NCP	Intron	gene|mRN	ID=gene-E	5	6.066	1920.2	0.881886486	PPANP	61	495.2552	1	494.2489	.	V	Superscaf	6403584	0	0	0	0	0	BTP20220	Tissue specific
QTALLLF	Superscaf	+	Superscaf	6447832	6447852	13	13	19	-	-	gene	-	-	mRNA	NCP	Intron	gene|mRN	ID=gene-E	7	5.945714286	2767.1	0.88106863	QTALLLF	62	805.4791	1	804.4745	.	K	Superscaf	6447796	0	0	BTP20220	0	0	0	Tissue specific
RELDLH	Superscaf	-	Superscaf	6449989	6450006	-17	17	22	-	-	gene	-	-	mRNA	NCP	Intron	gene|mRN	ID=gene-E	6	6.083333333	2812.3	0.881028899	R(+42.01)	75	412.7094	2	823.4188	Acetylati	L	Superscaf	6450138	0	0	BTP20220	0	0	0	Tissue specific
PAAEFFLP	Superscaf	-	Superscaf	6467800	6467823	-19	19	26	-	-	gene	-	-	mRNA	NCP	Intron	gene|mRN	ID=gene-E	8	5.6	2851.1	0.880700262	PAAEFFLP	68	446.2261	2	890.4537	.	P	Superscaf	6467868	0	0	0	BTP20220	0	0	Tissue specific
HESPAPPP	Superscaf	-	Superscaf	6496414	6496437	-28	28	35	-	-	gene	-	-	mRNA	NCP	Intron	gene|mRN	ID=gene-E	8	5.96375	3089.5	0.880172474	HESPAPPP	58	416.2016	2	830.3922	.	H	Superscaf	6496467	0	0	0	0	BTP20220	0	Tissue specific
TVAAF	Superscaf	-	Superscaf	6502822	6502836	-12	12	16	-	-	gene	-	-	mRNA	NCP	Intron	gene|mRN	ID=gene-E	5	4.81	1407.5	0.880054443	TVAAF	53	508.2848	1	507.2693	.	S	Superscaf	6502929	BTP20220	0	0	0	0	0	Tissue specific
AVEPNK	Superscaf	-	Superscaf	6528859	6528876	-7	7	12	-	-	gene	-	-	mRNA	NCP	Intron	gene|mRN	ID=gene-E	6	5.115	2139.3	0.879574133	A(+42.01)	83	350.1865	2	698.3599	Acetylati	N	Superscaf	6528987	0	BTP20220	0	0	0	0	Tissue specific
KFPGTVP	Superscaf	-	Superscaf	6529705	6529725	-7	7	13	-	-	gene	-	-	mRNA	NCP	Intron	gene|mRN	ID=gene-E	7	5.76	2225.4	0.879558473	KFPGTVP	60	373.2132	2	744.417	.	D	Superscaf	6529725	0	0	0	BTP20220	0	0	Tissue specific
LLFTEV	Superscaf	-	Superscaf	6570247	6570264	-3	3	8	-	-	gene	-	-	mRNA	NCP	Intron	gene|mRN	ID=gene-E	6	4.531666667	1917.2	0.878810726	LLFTEV	95	721.4095	1	720.4058	.	V	Superscaf	6570348	BTP20220	0	0	0	0	0	Tissue specific
KPSPTYK	Superscaf	+	Superscaf	6590509	6590529	2	2	8	-	-	gene	-	-	mRNA	NCP	Intron	gene|mRN	ID=gene-E	7	7.135714286	2852	0.878436936	KPSPTYK	63	410.7321	2	819.449	.	K	Superscaf	6590506	BTP20220	0	0	0	0	0	Tissue specific
ERMLRL	Superscaf	+	Superscaf	6595147	6595164	4	4	9	-	-	gene	-	-	mRNA	NCP	Intron	gene|mRN	ID=gene-E	6	7.075	2801.4	0.878351443	ERMLRL	99	409.2384	2	816.464	.	F	Superscaf	6595138	0	0	BTP20220	0	0	0	Tissue specific
DGDDP	Superscaf	-	Superscaf	6620293	6620307	-18	18	22	-	-	-	-	-	-	NCP	IGR	-	ID=gene-E	5	4.236	1869.2	0.877887677	D(+42.01)	50	560.1827	1	559.1761	Acetylati	C	Superscaf	6620319	BTP20220	0	0	0	0	0	Tissue specific
VSALTVVYF	Superscaf	-	Superscaf	6676633	6676659	-36	36	44	-	-	gene	-	-	mRNA	NCP	Intron	gene|mRN	ID=gene-E	9	3.927777778	2292.5	0.876848258	VSALTVVYF	62	499.7783	2	997.5484	.	P	Superscaf	6676671	0	0	0	0	0	BTP20220	Tissue specific
WVPSPK	Superscaf	+	Superscaf	6703720	6703737	1	1	6	-	-	-	-	-	-	NCP	IGR	-	ID=gene-E	6	5.651666667	2053.2	0.876348801	WVPSPK	93	357.2095	2	712.3908	.	W	Superscaf	6703720	BTP20220	0	0	0	0	0	Mixed
ADMMFP	Superscaf	+	Superscaf	6714394	6714411	21	21	26	-	-	-	-	-	-	NCP	IGR	-	ID=gene-E	6	5.378333333	2190.6	0.876151918	ADMMFP	58	711.2817	1	710.2768	.	P	Superscaf	6714334	0	0	0	BTP20220	0	0	Tissue specific
TGGFAL	Superscaf	-	Superscaf	6748306	6748323	-1	1	6	-	-	-	-	-	-	NCP	IGR	-	ID=gene-E	6	5.991666667	2044.2	0.875526407	TGGFAL	67	565.2961	1	564.2907	.	T	Superscaf	6748353	0	BTP20220	0	0	0	0	Tissue specific
KPFGGSK	Superscaf	-	Superscaf	6785680	6785700	-17	17	23	-	-	gene	-	-	mRNA	NCP	Intron	gene|mRN	ID=gene-E	7	6.982857143	2639.6	0.874836984	KPFGGSK	51	360.7072	2	719.3966	.	E	Superscaf	6785703	0	0	0	BTP20220	0	0	Tissue specific
QAAGP	Superscaf	-	Superscaf	6791905	6791919	-65	65	69	-	-	gene	-	-	mRNA	NCP	Intron	gene|mRN	ID=gene-E	5	5.992	1868	0.874722274	QAAGP	62	443.2263	1	442.2176	.	L	Superscaf	6791922	0	0	0	BTP20220	0	0	Tissue enhanced
LVTELR	Superscaf	-	Superscaf	6814684	6814701	-27	27	32	-	-	gene	-	-	mRNA	NCP	Intron	gene|mRN	ID=gene-E	6	5.411666667	2178.6	0.874302058	L(+42.01)	97	386.73	2	771.449	Acetylati	M	Superscaf	6814713	0	0	0	0	0	BTP20220	Mixed
CYAEQ	Superscaf	+	Superscaf	6832429	6832443	2	2	6	-	-	gene	-	-	mRNA	NCP	Intron	gene|mRN	ID=gene-E	5	5.114	2392.7	0.873974804	C(+57.02)	51	670.2542	1	669.2428	Carbamido	K	Superscaf	6832426	0	0	0	0	0	BTP20220	Tissue specific
GYPGAK	Superscaf	-	Superscaf	6855193	6855210	-60	60	65	-	-	gene	-	-	mRNA	NCP	Intron	gene|mRN	ID=gene-E	6	6.61	2171.2	0.873554864	GYPGAK	61	592.3152	1	591.3016	.	F	Superscaf	6855255	BTP20220	0	0	0	0	0	Tissue specific
KFHTVPP	Superscaf	-	Superscaf	6932620	6932640	-18	18	24	-	-	-	-	-	-	NCP	IGR	-	ID=gene-E	7	5.991428571	2547.8	0.872126659	KFHTVPP	51	413.2303	2	824.4545	.	S	Superscaf	6932679	0	0	BTP20220	0	0	0	Tissue specific
RAAGPLR	Superscaf	-	Superscaf	6937180	6937200	-98	98	104	-	-	-	-	-	-	NCP	IGR	-	ID=gene-E	7	7.401428571	2908.3	0.872042549	RAAGPLR	52	370.7285	2	739.4453	.	R	Superscaf	6937395	0	0	BTP20220	0	0	0	Tissue specific
QHPELLK	Superscaf	+	Superscaf	6938392	6938412	84	84	90	-	-	-	-	-	-	NCP	IGR	-	ID=gene-E	7	6.351428571	3168.5	0.872020194	QHPELLK	83	432.7433	2	863.4865	.	R	Superscaf	6938143	0	0	0	BTP20220	0	0	Tissue specific
EVGVLR	Superscaf	-	Superscaf	6940822	6940839	-16	16	21	-	-	-	-	-	-	NCP	IGR	-	ID=gene-E	6	4.321666667	1725	0.871975428	EVGVLR	58	672.4006	1	671.3966	.	E	Superscaf	6940950	0	0	0	BTP20220	0	0	Tissue specific
LPPPMPP	Superscaf	+	Superscaf	6964081	6964101	40	40	46	-	-	-	-	-	-	NCP	IGR	-	ID=gene-E	7	6.175714286	2411.9	0.871546357	LPPPMPP	58	374.705	2	747.3989	.	S	Superscaf	6963964	BTP20220	0	0	0	0	0	Tissue specific
GPASFHSP	Superscaf	-	Superscaf	6990562	6990585	-3	3	10	-	-	-	-	-	-	NCP	IGR	-	ID=gene-E	8	6.1275	3057.3	0.871057857	GPASFHSP	51	400.1899	2	798.366	.	A	Superscaf	6990639	0	BTP20220	0	0	0	0	Tissue specific
AATRWAK	Superscaf	-	Superscaf	7077634	7077654	-67	67	73	-	-	gene	-	-	mRNA	NCP	Intron	gene|mRN	ID=gene-E	7	7.282857143	2975.2	0.869451859	AATRWAK	61	402.2272	2	802.4449	.	P	Superscaf	7077897	BTP20220	0	0	0	0	0	Tissue specific
PPPPAVGL	Superscaf	-	Superscaf	7085122	7085145	-8	8	15	-	-	gene	-	-	mRNA	NCP	Intron	gene|mRN	ID=gene-E	8	5.39625	2311.6	0.869313687	PPPPAVGL	72	374.2247	2	746.4326	.	E	Superscaf	7085175	0	BTP20220	0	0	0	0	Tissue specific
RRLPQ	Superscaf	+	Superscaf	7126504	7126518	79	79	83	-	-	-	-	-	-	NCP	IGR	-	ID=gene-E	5	7.89	2599.1	0.868550557	R(+42.01)	55	711.4369	1	710.4187	Acetylati	S	Superscaf	7126270	0	0	BTP20220	0	0	0	Tissue specific
AVSRVY	Superscaf	+	Superscaf	7169857	7169874	63	63	68	-	-	-	-	-	-	NCP	IGR	-	ID=gene-E	6	4.686666667	1803	0.867750851	A(+42.01)	66	736.3942	1	735.3915	Acetylati	H	Superscaf	7169671	0	BTP20220	0	0	0	0	Tissue specific
NGAPGGL	Superscaf	-	Superscaf	7229050	7229070	-33	33	39	-	-	-	-	-	-	NCP	IGR	-	ID=gene-E	7	5.945714286	2490.7	0.866658974	NGAPGGL	52	585.3047	1	584.2918	.	R	Superscaf	7229082	0	BTP20220	0	0	0	0	Tissue specific
PASARGAK	Superscaf	+	Superscaf	7268476	7268499	53	53	60	-	-	-	-	-	-	NCP	IGR	-	ID=gene-E	8	7.06375	3069.2	0.865931701	PASARGAK	71	379.2128	2	756.4242	.	S	Superscaf	7268320	BTP20220	0	0	0	0	0	Tissue specific
RPRHVLL	Superscaf	-	Superscaf	7291177	7291197	-2	2	8	-	-	-	-	-	-	NCP	IGR	-	ID=gene-E	7	6.767142857	2961.5	0.865513034	R(+42.01)	57	932.5773	1	931.5715	Acetylati	R	Superscaf	7291212	0	0	BTP20220	0	0	0	Tissue specific
LNADKG	Superscaf	-	Superscaf	7296424	7296441	-40	40	45	-	-	-	-	-	-	NCP	IGR	-	ID=gene-E	6	6.015	2438.6	0.865416308	LNADKG	71	617.3287	1	616.318	.	W	Superscaf	7296465	0	0	0	0	BTP20220	0	Tissue specific
PGVHKPP	Superscaf	-	Superscaf	7319527	7319547	-10	10	16	-	-	-	-	-	-	NCP	IGR	-	ID=gene-E	7	6.028571429	2397.6	0.864990115	PGVHKPP	66	366.2123	2	730.4126	.	R	Superscaf	7319565	0	0	0	BTP20220	0	0	Tissue specific
LPDFSVT	Superscaf	-	Superscaf	7370368	7370388	-30	30	36	-	-	-	-	-	-	NCP	IGR	-	ID=gene-E	7	4.705714286	2270.6	0.864052347	LPDFSVT	74	778.3937	1	777.3909	.	A	Superscaf	7370400	0	0	0	BTP20220	0	0	Tissue specific
QLEVPRP	Superscaf	-	Superscaf	7372690	7372710	-42	42	48	-	-	-	-	-	-	NCP	IGR	-	ID=gene-E	7	5.458571429	2713.2	0.864009518	QLEVPRP	86	419.7414	2	837.4708	.	S	Superscaf	7372755	0	0	BTP20220	0	0	0	Tissue specific
DEMDC	Superscaf	+	Superscaf	7396012	7396026	29	29	33	-	-	-	-	-	-	NCP	IGR	-	ID=gene-E	5	3.986	2205.7	0.863579451	D(+42.01)	52	356.095	2	710.1887	Acetylati	G	Superscaf	7395928	0	BTP20220	0	0	0	0	Tissue specific
APRTVYA	Superscaf	+	Superscaf	7405705	7405725	42	42	48	-	-	-	-	-	-	NCP	IGR	-	ID=gene-E	7	5.898571429	2465.8	0.863400552	APRTVYA	81	389.2149	2	776.418	.	D	Superscaf	7405582	BTP20220	0	0	0	0	0	Tissue specific
KDPLTK	Superscaf	+	Superscaf	7426942	7426959	19	19	24	-	-	-	-	-	-	NCP	IGR	-	ID=gene-E	6	6.876666667	2436.6	0.863008888	KDPLTK	56	351.2183	2	700.4119	.	K	Superscaf	7426888	BTP20220	0	0	0	0	0	Tissue specific
KEPVPTP	Superscaf	-	Superscaf	7450579	7450599	-44	44	50	-	-	-	-	-	-	NCP	IGR	-	ID=gene-E	7	5.484285714	2303.6	0.862572846	KEPVPTP	59	384.2227	2	766.4225	.	L	Superscaf	7450698	BTP20220	0	0	0	0	0	Tissue specific
PAARPKE	Superscaf	+	Superscaf	7455016	7455036	40	40	46	-	-	-	-	-	-	NCP	IGR	-	ID=gene-E	7	6.908571429	2840.1	0.862491005	PAARPKE	62	384.7224	2	767.429	.	L	Superscaf	7454899	BTP20220	0	0	0	0	0	Tissue specific
VGDMVVP	Superscaf	-	Superscaf	7571170	7571190	-117	117	123	-	-	gene	-	-	mRNA	NCP	Intron	gene|mRN	ID=gene-E	7	2.998571429	1417.7	0.860348531	VGDMVVP	50	716.3578	1	715.3575	.	V	Superscaf	7571913	BTP20220	0	0	0	0	0	Tissue specific
PKLPP	Superscaf	+	Superscaf	7602388	7602402	19	19	23	-	-	gene	-	-	mRNA	NCP	Intron	gene|mRN	ID=gene-E	5	6.924	1834	0.859772822	PKLPP	72	551.3528	1	550.3478	.	F	Superscaf	7602334	BTP20220	0	0	0	0	0	Mixed
ALLSSLDL	Superscaf	+	Superscaf	7677715	7677738	11	11	18	-	-	gene	-	-	mRNA	NCP	Intron	gene|mRN	ID=gene-E	8	5.53375	2903.2	0.858383241	ALLSSLDL	81	416.2401	2	830.4749	.	T	Superscaf	7677685	0	BTP20220	0	0	0	0	Tissue specific
KATPSK	Superscaf	-	Superscaf	7699351	7699368	-31	31	36	-	-	gene	-	-	mRNA	NCP	Intron	gene|mRN	ID=gene-E	6	7.335	2366.4	0.857984273	KATPSK	58	631.3766	1	630.3701	.	L	Superscaf	7699416	0	BTP20220	0	0	0	0	Tissue specific
SGPSLVH	Superscaf	+	Superscaf	7715683	7715703	4	4	10	-	-	gene	-	-	mRNA	NCP	Intron	gene|mRN	ID=gene-E	7	5.314285714	2374.6	0.857682972	SGPSLVH	95	696.3651	1	695.3602	.	P	Superscaf	7715674	0	0	0	BTP20220	0	0	Mixed
KERALDP	Superscaf	-	Superscaf	7762843	7762863	-90	90	96	-	-	-	-	-	-	NCP	IGR	-	ID=gene-E	7	6.427142857	2956.3	0.856813101	KERALDP	50	414.7313	2	827.4501	.	F	Superscaf	7763025	0	BTP20220	0	0	0	0	Tissue specific
FPPPVAP	Superscaf	-	Superscaf	7828339	7828359	-30	30	36	-	-	gene	-	-	mRNA	NCP	Intron	gene|mRN	ID=gene-E	7	5.242857143	2048.3	0.855605019	FPPPVAP	86	724.4021	1	723.3955	.	T	Superscaf	7828500	0	0	BTP20220	0	0	0	Tissue specific
AGGHRLLN	Superscaf	-	Superscaf	7831645	7831668	-56	56	63	-	-	gene	-	-	mRNA	NCP	Intron	gene|mRN	ID=gene-E	8	6.71	3477.9	0.855543985	A(+42.01)	73	440.2478	2	878.4722	Acetylati	P	Superscaf	7831791	0	0	0	BTP20220	0	0	Tissue specific
YCPSLP	Superscaf	-	Superscaf	7869295	7869312	-36	36	41	-	-	-	-	-	-	NCP	IGR	-	ID=gene-E	6	5.823333333	2456.8	0.854849637	Y(+42.01)	85	778.3342	1	777.3367	Acetylati	R	Superscaf	7869348	BTP20220	0	0	0	0	0	Tissue specific
FAFFAE	Superscaf	-	Superscaf	7910536	7910553	-23	23	28	-	-	-	-	-	-	NCP	IGR	-	ID=gene-E	6	5.283333333	2210.3	0.854088942	FAFFAE	53	366.1795	2	730.3326	.	L	Superscaf	7910652	0	BTP20220	0	0	0	0	Tissue specific
EDKPAW	Superscaf	-	Superscaf	7917592	7917609	-9	9	14	-	-	gene	-	-	mRNA	NCP	Intron	gene|mRN	ID=gene-E	6	5.69	2480.7	0.853958793	EDKPAW	50	373.1824	2	744.3442	.	R	Superscaf	7917651	0	0	0	BTP20220	0	0	Tissue specific
LAATPGGK	Superscaf	+	Superscaf	7918507	7918530	27	27	34	-	-	gene	-	-	mRNA	NCP	Intron	gene|mRN	ID=gene-E	8	6.56625	2773.9	0.853941805	LAATPGGK	64	357.7168	2	713.4072	.	H	Superscaf	7918429	0	0	0	BTP20220	0	0	Tissue specific
RLLAVPF	Superscaf	-	Superscaf	7953784	7953804	-22	22	28	-	-	gene	-	-	mRNA	NCP	Intron	gene|mRN	ID=gene-E	7	5.788571429	2391.7	0.853291172	RLLAVPF	50	815.5103	1	814.5065	.	G	Superscaf	7953813	BTP20220	0	0	0	0	0	Tissue specific
FETKTPP	Superscaf	-	Superscaf	8004949	8004969	-19	19	25	-	-	-	-	-	-	NCP	IGR	-	ID=gene-E	7	6.3	2751.1	0.852347428	FETKTPP	64	410.2138	2	818.4174	.	T	Superscaf	8005176	BTP20220	0	0	0	0	0	Tissue specific
LPPPKAPPF	Superscaf	+	Superscaf	8005753	8005779	1	1	9	-	-	-	-	-	-	NCP	IGR	-	ID=gene-E	9	6.615555556	3157.5	0.852332487	LPPPKAPPF	98	457.2775	2	912.5432	.	L	Superscaf	8005753	0	0	BTP20220	0	0	0	Tissue specific
FAFVQT	Superscaf	+	Superscaf	8103094	8103111	18	18	23	-	-	gene	-	-	mRNA	NCP	Intron	gene|mRN	ID=gene-E	6	4.86	2094.3	0.850537187	FAFVQT	51	712.3629	1	711.3591	.	S	Superscaf	8103043	0	0	0	BTP20220	0	0	Tissue specific
RDPGSLR	Superscaf	-	Superscaf	8111959	8111979	-16	16	22	-	-	gene	-	-	mRNA	NCP	Intron	gene|mRN	ID=gene-E	7	6.917142857	3080.6	0.850373616	RDPGSLR	63	400.7154	2	799.43	.	G	Superscaf	8112006	0	0	0	0	BTP20220	0	Tissue specific
KAPLPP	Superscaf	-	Superscaf	8118127	8118144	-35	35	40	-	-	gene	-	-	mRNA	NCP	Intron	gene|mRN	ID=gene-E	6	6.773333333	2145.3	0.850259902	KAPLPP	52	622.3985	1	621.3849	.	P	Superscaf	8118150	0	BTP20220	0	0	0	0	Tissue specific
GDGAP	Superscaf	-	Superscaf	8141338	8141352	-137	137	141	-	-	gene	-	-	mRNA	NCP	Intron	gene|mRN	ID=gene-E	5	5.446	1654.8	0.849831828	GDGAP	71	416.1809	1	415.1703	.	S	Superscaf	8141415	0	0	0	0	BTP20220	0	Tissue specific
SSGSHPAG	Superscaf	-	Superscaf	8185681	8185704	-127	127	134	-	-	gene	-	-	mRNA	NCP	Intron	gene|mRN	ID=gene-E	8	6.11125	3013.2	0.84901375	S(+42.01)	53	741.317	1	740.3089	Acetylati	V	Superscaf	8185830	0	0	BTP20220	0	0	0	Tissue specific
ATLPKY	Superscaf	-	Superscaf	8221720	8221737	-114	114	119	-	-	gene	-	-	mRNA	NCP	Intron	gene|mRN	ID=gene-E	6	6.705	2327.5	0.848349117	ATLPKY	54	692.4036	1	691.3904	.	K	Superscaf	8222349	0	BTP20220	0	0	0	0	Tissue specific
PPVVLRL	Superscaf	+	Superscaf	8285554	8285574	26	26	32	-	-	-	-	-	-	NCP	IGR	-	ID=gene-E	7	5.045714286	2030.4	0.847171636	PPVVLRL	67	397.2715	2	792.5222	.	P	Superscaf	8285479	0	0	0	BTP20220	0	0	Tissue specific
LDFHALPY	Superscaf	-	Superscaf	8297866	8297889	-101	101	108	-	-	-	-	-	-	NCP	IGR	-	ID=gene-E	8	5.7475	3233.6	0.846944485	LDFHALPY	73	488.2487	2	974.4861	.	E	Superscaf	8298042	0	0	BTP20220	0	0	0	Tissue specific
RLSKLKK	Superscaf	+	Superscaf	8299870	8299890	7	7	13	-	-	-	-	-	-	NCP	IGR	-	ID=gene-E	7	8.231428571	3144.2	0.846907576	R(+42.01)	68	457.8045	2	913.6072	Acetylati	M	Superscaf	8299852	0	0	0	0	BTP20220	0	Tissue specific
GPPAAE	Superscaf	+	Superscaf	8311168	8311185	39	39	44	-	-	-	-	-	-	NCP	IGR	-	ID=gene-E	6	5.638333333	2020.2	0.846699238	GPPAAE	52	541.2606	1	540.2543	.	K	Superscaf	8311054	BTP20220	0	0	0	0	0	Tissue specific
ATLPK	Superscaf	-	Superscaf	8371789	8371803	-3	3	7	-	-	gene	-	-	mRNA	NCP	Intron	gene|mRN	ID=gene-E	5	6.914	1868	0.845581132	A(+42.01)	66	571.3433	1	570.3377	Acetylati	R	Superscaf	8371815	0	0	BTP20220	0	0	0	Tissue specific
PDGMLRL	Superscaf	+	Superscaf	8402020	8402040	35	35	41	-	-	-	-	-	-	NCP	IGR	-	ID=gene-E	7	6.244285714	2773.3	0.845023407	PDGMLRL	53	401.2116	2	800.4214	.	V	Superscaf	8401918	BTP20220	0	0	0	0	0	Tissue specific
DDLDGLHL	Superscaf	+	Superscaf	8413195	8413218	4	4	11	-	-	-	-	-	-	NCP	IGR	-	ID=gene-E	8	5.05125	3211.7	0.844817228	DDLDGLHL	64	449.2186	2	896.424	.	H	Superscaf	8413186	BTP20220	0	0	0	0	0	Tissue specific
NVPRFM	Superscaf	+	Superscaf	8454595	8454612	10	10	15	-	-	gene	-	-	mRNA	NCP	Intron	gene|mRN	ID=gene-E	6	5.616666667	2341.8	0.844053711	NVPRFM(+1	72	390.2045	2	778.3796	Oxidation	A	Superscaf	8454568	BTP20220	0	0	0	0	0	Tissue specific
KVGPP	Superscaf	+	Superscaf	8595187	8595201	16	16	20	-	-	-	-	-	-	NCP	IGR	-	ID=gene-E	5	5.662	1440.5	0.841460531	KVGPP	55	497.3059	1	496.3009	.	I	Superscaf	8595142	BTP20220	0	0	0	0	0	Tissue enhanced
GPAAVP	Superscaf	-	Superscaf	8598910	8598927	-73	73	78	-	-	-	-	-	-	NCP	IGR	-	ID=gene-E	6	5.101666667	1594.7	0.841391804	GPAAVP	55	511.2953	1	510.2802	.	F	Superscaf	8599128	0	BTP20220	0	0	0	0	Tissue specific
LLGKLAL	Superscaf	-	Superscaf	8610343	8610363	-31	31	37	-	-	-	-	-	-	NCP	IGR	-	ID=gene-E	7	6.521428571	2490.6	0.841180866	LLGKLAL	99	727.5062	1	726.5003	.	G	Superscaf	8610426	BTP20220	0	0	0	0	0	Tissue specific
RGKGGPPK	Superscaf	-	Superscaf	8611861	8611884	-1	1	8	-	-	-	-	-	-	NCP	IGR	-	ID=gene-E	8	7.59375	3152.3	0.841152811	RGKGGPPK	50	398.7477	2	795.4715	.	R	Superscaf	8611992	0	0	BTP20220	0	0	0	Tissue specific
PKGGLGSL	Superscaf	+	Superscaf	8632492	8632515	2	2	9	-	-	gene	-	-	mRNA	NCP	Intron	gene|mRN	ID=gene-E	8	6.44875	2787.9	0.84077227	P(+42.01)	51	770.442	1	769.4333	Acetylati	A	Superscaf	8632489	0	0	0	0	BTP20220	0	Tissue specific
TTGLVLD	Superscaf	-	Superscaf	8660134	8660154	-28	28	34	-	-	-	-	-	-	NCP	IGR	-	ID=gene-E	7	4.851428571	2210.6	0.840262465	TTGLVLD	87	718.3943	1	717.3909	.	P	Superscaf	8660361	BTP20220	0	0	0	0	0	Tissue specific
KESAFLH	Superscaf	-	Superscaf	8676460	8676480	-5	5	11	-	-	-	-	-	-	NCP	IGR	-	ID=gene-E	7	6.244285714	2949.1	0.83996133	KESAFLH	50	416.227	2	830.4286	.	T	Superscaf	8676525	0	0	0	0	BTP20220	0	Tissue specific
TVEALER	Superscaf	-	Superscaf	8705578	8705598	-81	81	87	-	-	-	-	-	-	NCP	IGR	-	ID=gene-E	7	5.104285714	2562	0.839424245	T(+42.01)	77	430.2252	2	858.4447	Acetylati	Q	Superscaf	8705961	0	0	BTP20220	BTP20220	0	0	Tissue specific
KHLQCF	Superscaf	-	Superscaf	8790646	8790663	-18	18	23	-	-	-	-	-	-	NCP	IGR	-	ID=gene-E	6	6.576666667	3025.3	0.837855212	K(+42.01)	69	437.7206	2	873.4167	Acetylati	V	Superscaf	8790699	BTP20220	0	0	BTP20220	0	0	Tissue specific
EHARAG	Superscaf	-	Superscaf	8807671	8807688	-11	11	16	-	-	-	-	-	-	NCP	IGR	-	ID=gene-E	6	6.596666667	2613.9	0.837541184	EHARAG	73	640.3245	1	639.3088	.	S	Superscaf	8807754	BTP20220	0	0	0	0	0	Tissue specific
ELVPVA	Superscaf	+	Superscaf	8842291	8842308	17	17	22	-	-	-	-	-	-	NCP	IGR	-	ID=gene-E	6	3.586666667	1427.6	0.836902614	ELVPVA	85	627.3701	1	626.3639	.	E	Superscaf	8842243	0	BTP20220	0	0	0	0	Mixed
EGAGLSLF	Superscaf	-	Superscaf	8882989	8883012	-19	19	26	-	-	-	-	-	-	NCP	IGR	-	ID=gene-E	8	5.5375	2809	0.836151824	EGAGLSLF	52	793.4043	1	792.4017	.	K	Superscaf	8883057	0	0	BTP20220	0	0	0	Tissue specific
AHTRFSK	Superscaf	+	Superscaf	8929918	8929938	5	5	11	-	-	gene	-	-	mRNA	NCP	Intron	gene|mRN	ID=gene-E	7	7.4	3216.5	0.835286268	AHTRFSK	50	423.7379	2	845.4507	.	L	Superscaf	8929906	BTP20220	0	0	0	0	0	Tissue specific
LVVLY	Superscaf	+	Superscaf	8938060	8938074	21	21	25	-	-	gene	-	-	mRNA	NCP	Intron	gene|mRN	ID=gene-E	5	3.524	1166.3	0.835136199	LVVLY	81	606.3835	1	605.3788	.	D	Superscaf	8938000	0	0	0	0	0	BTP20220	Tissue specific
KSVTPP	Superscaf	-	Superscaf	9000316	9000333	-27	27	32	-	-	gene	-	-	mRNA	NCP	Intron	gene|mRN	ID=gene-E	6	5.758333333	1924.1	0.833987824	KSVTPP	52	628.3752	1	627.3591	.	A	Superscaf	9000354	0	BTP20220	0	0	0	0	Tissue specific
DKKKMR	Superscaf	-	Superscaf	9030913	9030930	-3	3	8	-	-	-	-	-	-	NCP	IGR	-	ID=gene-E	6	8.116666667	2837	0.833423459	DKKKMR	51	403.2452	2	804.464	.	G	Superscaf	9030933	0	BTP20220	0	0	0	0	Tissue specific
SNGLLTLL	Superscaf	+	Superscaf	9104371	9104394	34	34	41	-	-	gene	-	-	mRNA	NCP	Intron	gene|mRN	ID=gene-E	8	5.93875	3088.5	0.832068407	SNGLLTLL	57	830.4957	1	829.4909	.	L	Superscaf	9104272	0	0	0	0	0	BTP20220	Tissue specific
SPPGPT	Superscaf	-	Superscaf	9192217	9192234	-24	24	29	-	-	-	-	-	-	NCP	IGR	-	ID=gene-E	6	6.18	2090.4	0.830448188	S(+42.01)	52	597.2822	1	596.2806	Acetylati	P	Superscaf	9192261	0	0	0	0	BTP20220	0	Tissue specific
KAVFAPK	Superscaf	+	Superscaf	9245518	9245538	103	103	109	-	-	-	-	-	-	NCP	IGR	-	ID=gene-E	7	6.185714286	2284.2	0.82946499	KAVFAPK	51	380.736	2	759.4643	.	S	Superscaf	9245212	0	0	0	BTP20220	0	0	Tissue specific
VDDLNL	Superscaf	-	Superscaf	9364198	9364215	-13	13	18	-	-	-	-	-	-	NCP	IGR	-	ID=gene-E	6	3.885	2126.5	0.827275979	VDDLNL	70	688.3494	1	687.3439	.	D	Superscaf	9364263	BTP20220	0	0	0	0	0	Tissue specific
TPKPAAP	Superscaf	-	Superscaf	9369427	9369447	-38	38	44	-	-	-	-	-	-	NCP	IGR	-	ID=gene-E	7	6.744285714	2500.7	0.827179474	T(+42.01)	77	362.2118	2	722.3962	Acetylati	K	Superscaf	9369501	0	BTP20220	0	0	0	0	Tissue specific
LVVVLV	Superscaf	+	Superscaf	9386959	9386976	23	23	28	-	-	-	-	-	-	NCP	IGR	-	ID=gene-E	6	1.993333333	706.8	0.82685615	LVVVLV	66	641.4581	1	640.4523	.	F	Superscaf	9386893	0	0	0	0	0	BTP20220	Tissue specific
FDCHYC	Superscaf	+	Superscaf	9396232	9396249	28	28	33	-	-	-	-	-	-	NCP	IGR	-	ID=gene-E	6	5.29	3049.5	0.826685108	FDC(+57.(	99	451.151	2	900.2894	Carbamido	N	Superscaf	9396151	0	0	BTP20220	0	0	0	Mixed
HMASKE	Superscaf	-	Superscaf	9422953	9422970	-5	5	10	-	-	-	-	-	-	NCP	IGR	-	ID=gene-E	6	6.333333333	2579.8	0.826192236	HMASKE	98	351.6642	2	701.3167	.	M	Superscaf	9423171	0	0	0	BTP20220	0	0	Tissue specific
VLTYC	Superscaf	+	Superscaf	9448006	9448020	55	55	59	-	-	-	-	-	-	NCP	IGR	-	ID=gene-E	5	4.638	1796.1	0.825730186	VLTYC(+57	89	655.3104	1	654.3047	Carbamido	F	Superscaf	9447844	0	BTP20220	0	0	0	0	Tissue specific
VEVGLFT	Superscaf	-	Superscaf	9489283	9489303	-47	47	53	-	-	-	-	-	-	NCP	IGR	-	ID=gene-E	7	3.882857143	1861.1	0.824968717	VEVGLFT	60	764.4153	1	763.4116	.	L	Superscaf	9489306	BTP20220	0	0	0	0	0	Tissue specific
YPPDRP	Superscaf	-	Superscaf	9538414	9538431	-4	4	9	-	-	-	-	-	-	NCP	IGR	-	ID=gene-E	6	6.381666667	2532	0.824062545	YPPDRP	62	372.6858	2	743.3602	.	A	Superscaf	9538467	BTP20220	0	0	0	0	0	Tissue specific

VYRSAF	Superscaf	-	Superscaf	9580510	9580527	-25	25	30	-	-	-	-	-	-	NCP	IGR	-	ID=gene-E	6	5.6	2190.4	0.823286079	VYRSAF	85	371.6962	2	741.3809	.	P	Superscaf	9580572	0	BTP20220	0	0	0	0	Mixed
ARPLAFC	Superscaf	-	Superscaf	9697699	9697719	-5	5	11	-	-	-	-	-	-	NCP	IGR	-	ID=gene-E	7	6.511428571	2935.3	0.82112446	ARPLAFC(+	56	417.7263	2	833.4218	Carbamidc	V	Superscaf	9697719	0	0	0	BTP20220	0	0	Tissue specific
APEPRP	Superscaf	-	Superscaf	9704212	9704229	-28	28	33	-	-	-	-	-	-	NCP	IGR	-	ID=gene-E	6	6.483333333	2397.8	0.821004382	A(+42.01)	98	354.6804	2	707.3602	Acetylati	K	Superscaf	9704229	0	BTP20220	0	0	0	0	Tissue specific
LLLLTVVV	Superscaf	+	Superscaf	9709387	9709410	115	115	122	-	-	-	-	-	-	NCP	IGR	-	ID=gene-E	8	3.80625	1811.1	0.820908818	LLLLTVVV	51	435.3096	2	868.5997	.	T	Superscaf	9709045	BTP20220	0	0	0	0	0	Tissue specific
KYVPPPP	Superscaf	-	Superscaf	9712084	9712104	-8	8	14	-	-	gene	-	-	mRNA	NCP	Intron	gene|mRN	ID=gene-E	7	5.8	2277.5	0.820859127	KYVPPPP	74	399.2286	2	796.4483	.	N	Superscaf	9712104	0	0	0	BTP20220	0	0	Tissue specific
AVAPPV	Superscaf	-	Superscaf	9763072	9763089	-1	1	6	-	-	gene	-	-	mRNA	NCP	Intron	gene|mRN	ID=gene-E	6	4.106666667	1297.4	0.819918702	A(+42.01)	55	595.3428	1	594.3377	Acetylati	A	Superscaf	9763326	0	0	BTP20220	0	0	0	Tissue specific
GQKGPLQ	Superscaf	-	Superscaf	9770902	9770922	-11	11	17	-	-	gene	-	-	mRNA	NCP	Intron	gene|mRN	ID=gene-E	7	6.465714286	2975.2	0.819774222	G(+42.01)	91	769.4168	1	768.413	Acetylati	L	Superscaf	9770994	0	0	BTP20220	0	0	0	Mixed
MEVPGSL	Superscaf	-	Superscaf	9824788	9824808	-29	29	35	-	-	gene	-	-	mRNA	NCP	Intron	gene|mRN	ID=gene-E	7	4.7	2168.5	0.818780288	M(+42.01)	74	774.3687	1	773.3629	Acetylati	K	Superscaf	9824928	BTP20220	0	0	0	0	0	Tissue specific
KMTFPP	Superscaf	-	Superscaf	9858031	9858048	-6	6	11	-	-	gene	-	-	mRNA	NCP	Intron	gene|mRN	ID=gene-E	6	6.683333333	2299.6	0.818167173	KMTFPP	78	360.6855	2	719.3676	.	E	Superscaf	9858270	0	0	0	0	0	BTP20220	Tissue specific
HPALGGLL	Superscaf	-	Superscaf	9866944	9866967	-1	1	8	-	-	gene	-	-	mRNA	NCP	Intron	gene|mRN	ID=gene-E	8	6.22375	2923.2	0.818002661	HPALGGLL	60	777.4593	1	776.4545	.	H	Superscaf	9866973	0	0	BTP20220	0	0	0	Tissue specific
GPGGM	Superscaf	-	Superscaf	9894337	9894351	-18	18	22	-	-	gene	-	-	mRNA	NCP	Intron	gene|mRN	ID=gene-E	5	5.992	1600.8	0.81749756	GPGGM(+15	65	434.1657	1	433.1631	Oxidatior	N	Superscaf	9894393	BTP20220	0	0	0	0	0	Tissue specific
DCDVD	Superscaf	+	Superscaf	9947587	9947601	27	27	31	-	-	-	-	-	-	NCP	IGR	-	ID=gene-E	5	2.786	1820.2	0.816515357	D(+42.01)	67	665.2172	1	664.201	Acetylati	P	Superscaf	9947509	0	0	BTP20220	0	0	0	Tissue specific
LTVTFM	Superscaf	-	Superscaf	9957028	9957045	-18	18	23	-	-	-	-	-	-	NCP	IGR	-	ID=gene-E	6	5.045	1907.3	0.816341162	LTVTFM	65	711.3654	1	710.3673	.	P	Superscaf	9957324	0	0	0	0	0	BTP20220	Tissue specific
KPASAKK	Superscaf	-	Superscaf	9957541	9957561	-41	41	47	-	-	-	-	-	-	NCP	IGR	-	ID=gene-E	7	7.605714286	2748.6	0.816331644	KPASAKK	59	365.2329	2	728.4544	.	W	Superscaf	9957819	0	0	0	0	BTP20220	0	Tissue specific
MTKPMR	Superscaf	+	Superscaf	9959143	9959160	20	20	25	-	-	-	-	-	-	NCP	IGR	-	ID=gene-E	6	7.471666667	2595.1	0.81630215	M(+15.99)	52	419.2036	2	836.3884	Oxidatior	I	Superscaf	9959086	0	0	BTP20220	0	0	0	Tissue specific
VEDKLR	Superscaf	-	Superscaf	9981070	9981087	-37	37	42	-	-	-	-	-	-	NCP	IGR	-	ID=gene-E	6	5.445	2307.6	0.815897704	VEDKLR	81	380.2218	2	758.4286	.	E	Superscaf	9981126	0	BTP20220	0	0	0	0	Tissue specific
VFGPPF	Superscaf	-	Superscaf	10044406	10044423	-87	87	92	-	-	-	-	-	-	NCP	IGR	-	ID=gene-E	6	4.921666667	1746.9	0.814729464	VFGPPF	79	663.3482	1	662.3428	.	I	Superscaf	10044432	0	0	0	0	BTP20220	0	Tissue specific
MPCRQK	Superscaf	+	Superscaf	10074145	10074162	10	10	15	-	-	-	-	-	-	NCP	IGR	-	ID=gene-E	6	7.203333333	3022.5	0.814180925	M(+42.01)	62	431.209	2	860.3997	Acetylati	S	Superscaf	10074118	0	0	0	0	BTP20220	0	Tissue specific
KETANLG	Superscaf	+	Superscaf	10180132	10180152	24	24	30	-	-	-	-	-	-	NCP	IGR	-	ID=gene-E	7	6.124285714	2850.1	0.812225927	KETANLG	53	732.3854	1	731.3813	.	Y	Superscaf	10180063	0	0	0	0	0	BTP20220	Tissue specific
PRRCAC	Superscaf	-	Superscaf	10191139	10191156	-55	55	60	-	-	-	-	-	-	NCP	IGR	-	ID=gene-E	6	7.313333333	3117.7	0.812022957	PRRC(+57.	52	410.1927	2	818.364	Carbamidc	R	Superscaf	10191459	0	0	0	BTP20220	0	0	Tissue specific
GVLTK	Superscaf	-	Superscaf	10252930	10252944	-19	19	23	-	-	-	-	-	-	NCP	IGR	-	ID=gene-E	5	5.644	1516.6	0.810883271	GVLTK	77	517.3324	1	516.3271	.	P	Superscaf	10253073	BTP20220	BTP20220	0	0	0	0	Tissue specific
NATKLSK	Superscaf	+	Superscaf	10257289	10257309	33	33	39	-	-	-	-	-	-	NCP	IGR	-	ID=gene-E	7	7.014285714	2979.1	0.810802758	N(+42.01)	74	402.2377	2	802.4548	Acetylati	L	Superscaf	10257193	0	0	0	BTP20220	0	0	Tissue specific
PGPTPP	Superscaf	-	Superscaf	10258783	10258800	-151	151	156	-	-	-	-	-	-	NCP	IGR	-	ID=gene-E	6	6.283333333	2044.4	0.810775256	PGPTPP	78	565.2943	1	564.2907	.	N	Superscaf	10258833	0	0	0	0	BTP20220	BTP20220	Tissue specific
GVEFK	Superscaf	+	Superscaf	10325815	10325829	4	4	8	-	-	-	-	-	-	NCP	IGR	-	ID=gene-E	5	4.882	1578.6	0.809538899	GVEFK	89	579.3124	1	578.3064	.	I	Superscaf	10325806	0	BTP20220	0	0	0	0	Mixed
FPKLLT	Superscaf	+	Superscaf	10349371	10349388	30	30	35	-	-	-	-	-	-	NCP	IGR	-	ID=gene-E	6	6.668333333	2297.5	0.80910435	FPKLLT	76	718.4504	1	717.4425	.	S	Superscaf	10349284	BTP20220	0	0	0	0	0	Tissue specific
KAAFLQR	Superscaf	-	Superscaf	10356502	10356522	-1	1	7	-	-	-	-	-	-	NCP	IGR	-	ID=gene-E	7	7.092857143	3091.3	0.808972763	KAAFLQR	62	417.248	2	832.4919	.	K	Superscaf	10356528	BTP20220	0	0	0	0	0	Tissue specific
KLNVSTK	Superscaf	-	Superscaf	10370371	10370391	-16	16	22	-	-	-	-	-	-	NCP	IGR	-	ID=gene-E	7	6.154285714	2667.8	0.808716947	KLNVSTK	76	395.244	2	788.4756	.	S	Superscaf	10370415	0	0	BTP20220	0	0	0	Tissue specific
SHNGF	Superscaf	+	Superscaf	10376314	10376328	86	86	90	-	-	-	-	-	-	NCP	IGR	-	ID=gene-E	5	6.026	2284.5	0.808607439	SHNGF	54	561.2375	1	560.2343	.	T	Superscaf	10376059	0	0	0	BTP20220	0	0	Tissue specific
LSMGML	Superscaf	-	Superscaf	10467898	10467915	-10	10	15	-	-	-	-	-	-	NCP	IGR	-	ID=gene-E	6	5.851666667	2130.5	0.806918106	LSMGML	52	651.3155	1	650.3132	.	S	Superscaf	10467957	BTP20220	0	0	0	0	0	Tissue specific
LKKKVS	Superscaf	-	Superscaf	10473526	10473543	-35	35	40	-	-	-	-	-	-	NCP	IGR	-	ID=gene-E	6	6.813333333	2142	0.806814297	LKKKVS	68	351.7507	2	701.4799	.	E	Superscaf	10473588	0	0	0	0	0	BTP20220	Tissue specific
LPPPPRPNF	Superscaf	+	Superscaf	10483621	10483647	31	31	39	-	-	-	-	-	-	NCP	IGR	-	ID=gene-E	9	6.661111111	3623.3	0.806627927	LPPPPRPNF	73	492.7838	2	983.5552	.	Q	Superscaf	10483531	0	0	0	0	BTP20220	0	Tissue specific
HLSAAGR	Superscaf	+	Superscaf	10515196	10515216	107	107	113	-	-	gene	-	-	mRNA	NCP	Intron	gene|mRN	ID=gene-E	7	6.86	2925.2	0.806045634	HLSAAGR	77	356.1949	2	710.3823	.	Q	Superscaf	10514878	0	0	0	0	0	BTP20220	Tissue specific
MENFV	Superscaf	-	Superscaf	10560181	10560195	-1	1	5	-	-	gene	-	-	mRNA	NCP	Intron	gene|mRN	ID=gene-E	5	3.972	1781.1	0.805215991	M(+42.01)	73	681.2914	1	680.2839	Acetylati	M	Superscaf	10560291	0	0	BTP20220	0	0	0	Tissue specific
VSPPPT	Superscaf	+	Superscaf	10569460	10569477	19	19	24	-	-	gene	-	-	mRNA	NCP	Intron	gene|mRN	ID=gene-E	6	5.185	1793.1	0.805044783	V(+42.01)	57	639.3234	1	638.3275	Acetylati	F	Superscaf	10569406	0	0	0	BTP20220	0	0	Tissue specific
APVLDF	Superscaf	+	Superscaf	10602742	10602759	18	18	23	-	-	gene	-	-	mRNA	NCP	Intron	gene|mRN	ID=gene-E	6	4.458333333	1801	0.804430893	APVLDF	65	661.366	1	660.3483	.	A	Superscaf	10602691	0	0	0	0	BTP20220	BTP20220	Tissue specific
HLGPPPPPF	Superscaf	+	Superscaf	10604587	10604613	259	259	267	-	-	gene	-	-	mRNA	NCP	Intron	gene|mRN	ID=gene-E	9	6.371111111	3294.8	0.804396696	H(+42.01)	86	475.7607	2	949.5021	Acetylati	G	Superscaf	10603813	0	0	0	0	0	BTP20220	Mixed
PSTTTPK	Superscaf	+	Superscaf	10619872	10619892	54	54	60	-	-	-	-	-	-	NCP	IGR	-	ID=gene-E	7	6.801428571	2719.1	0.804114873	PSTTTPK	59	366.201	2	730.3861	.	A	Superscaf	10619713	0	0	0	BTP20220	0	0	Tissue specific
ALAFSPTQ	Superscaf	-	Superscaf	10700773	10700796	-49	49	56	-	-	-	-	-	-	NCP	IGR	-	ID=gene-E	8	5.9575	3092.4	0.802622589	ALAFSPTQ	62	417.7214	2	833.4283	.	S	Superscaf	10700847	0	BTP20220	0	0	0	0	Tissue specific
PGAPRYK	Superscaf	-	Superscaf	10715722	10715742	-9	9	15	-	-	-	-	-	-	NCP	IGR	-	ID=gene-E	7	7.25	2860.1	0.802346908	PGAPRYK	54	788.4376	1	787.434	.	D	Superscaf	10715778	BTP20220	0	0	0	0	0	Tissue specific
SPGHRTP	Superscaf	+	Superscaf	10741171	10741191	38	38	44	-	-	gene	-	-	mRNA	NCP	Intron	gene|mRN	ID=gene-E	7	7.018571429	3021.5	0.801877499	SPGHRTP	95	376.2025	2	750.3773	.	Y	Superscaf	10741060	BTP20220	0	0	BTP20220	0	0	Tissue specific
MLSRLLK	Superscaf	+	Superscaf	10766857	10766877	171	171	177	-	-	-	-	-	-	NCP	IGR	-	ID=gene-E	7	7.124285714	2932.3	0.801403718	M(+42.01)	75	451.7772	2	901.5419	Acetylati	P	Superscaf	10766347	BTP20220	0	0	0	0	0	Mixed
VYKEKL	Superscaf	-	Superscaf	10862818	10862835	-19	19	24	-	-	gene	-	-	mRNA	NCP	Intron	gene|mRN	ID=gene-E	6	5.723333333	2175.2	0.799633761	V(+42.01)	70	411.2352	2	820.4694	Acetylati	I	Superscaf	10862874	0	0	0	BTP20220	0	0	Tissue specific
CSQDDAV	Superscaf	+	Superscaf	10906789	10906809	4	4	10	-	-	-	-	-	-	NCP	IGR	-	ID=gene-E	7	4.044285714	2714.1	0.798822656	C(+57.02)	58	418.6563	2	835.3018	Carbamidc	G	Superscaf	10906780	BTP20220	0	0	0	0	0	Tissue specific
VFFGPHQ	Superscaf	+	Superscaf	10927153	10927173	20	20	26	-	-	-	-	-	-	NCP	IGR	-	ID=gene-E	7	5.21	2639.9	0.798447039	V(+42.01)	55	437.217	2	872.418	Acetylati	R	Superscaf	10927096	0	0	0	0	0	BTP20220	Tissue specific
SSVTSVR	Superscaf	-	Superscaf	10929685	10929705	-26	26	32	-	-	-	-	-	-	NCP	IGR	-	ID=gene-E	7	4.904285714	2196.5	0.798400336	SSVTSVR	93	368.2022	2	734.3923	.	V	Superscaf	10929759	BTP20220	0	0	0	0	0	Tissue specific
NQLTSNPEN	Superscaf	-	Superscaf	10930132	10930176	-124	124	138	-	-	-	-	-	-	NCP	IGR	-	ID=gene-E	15	5.355333333	6274.1	0.798391649	NQLTSNPEN	92	839.4021	2	1676.801	.	P	Superscaf	10930272	0	0	0	0	BTP20220	BTP20220	Tissue specific
DLGGGTFDV	Superscaf	-	Superscaf	10931353	10931379	-33	33	41	-	-	-	-	-	-	NCP	IGR	-	ID=gene-E	9	4.648888889	2853.2	0.798369459	DLGGGTFDV	77	880.4014	1	879.3974	.	G	Superscaf	10931697	BTP20220	0	0	0	0	0	Tissue specific
NGDTHLGGE	Superscaf	-	Superscaf	10931425	10931481	-57	57	75	-	-	-	-	-	-	NCP	IGR	-	ID=gene-E	19	5.235789474	7970.2	0.798367578	NGDTHLGGE	97	1102.471	2	2202.939	.	G	Superscaf	10931697	BTP20220	0	0	0	0	0	Tissue specific
DQRVMEHF	Superscaf	-	Superscaf	10931458	10931481	-68	68	75	-	-	-	-	-	-	NCP	IGR	-	ID=gene-E	8	5.1775	3475.1	0.798367578	DQRVMEHF	94	531.2434	2	1060.476	.	G	Superscaf	10931697	0	0	BTP20220	0	0	0	Tissue specific
YEGEDFSET	Superscaf	-	Superscaf	10931620	10931661	-122	122	135	-	-	-	-	-	-	NCP	IGR	-	ID=gene-E	14	5.784285714	5792.5	0.798364258	YEGEDFSET	90	823.3861	2	1644.763	.	G	Superscaf	10931697	0	BTP20220	0	0	0	0	Tissue enhanced
EGEDFSETI	Superscaf	-	Superscaf	10931623	10931661	-123	123	135	-	-	-	-	-	-	NCP	IGR	-	ID=gene-E	13	5.793846154	5333	0.798364258	EGEDFSETI	99	741.8535	2	1481.7	.	G	Superscaf	10931697	0	0	0	BTP20220	0	0	Tissue enhanced
EGEDFSETI	Superscaf	-	Superscaf	10931623	10931658	-123	123	134	-	-	-	-	-	-	NCP	IGR	-	ID=gene-E	12	5.465	4864.6	0.798364313	EGEDFSETI	95	677.8076	2	1353.605	.	G	Superscaf	10931697	0	0	BTP20220	0	0	0	Tissue specific
SETLTRAK	Superscaf	-	Superscaf	10931638	10931661	-128	128	135	-	-	-	-	-	-	NCP	IGR	-	ID=gene-E	8	6.8075	3385.8	0.798364258	SETLTRAK	98	453.2553	2	904.4977	.	G	Superscaf	10931697	0	0	0	BTP20220	0	0	Tissue specific
VLEDSDLKK	Superscaf	-	Superscaf	10931839	10931865	-13	13	21	-	-	-	-	-	-	NCP	IGR	-	ID=gene-E	9	5.142222222	3275.5	0.798360495	VLEDSDLKK	99	523.7882	2	1045.565	.	D	Superscaf	10932093	BTP20220	0	0	0	0	0	Tissue specific
VLLDVCPL	Superscaf	-	Superscaf	10932358	10932381	-28	28	35	-	-	-	-	-	-	NCP	IGR	-	ID=gene-E	8	4.02875	2394.8	0.798350977	VLLDVC(+5	92	928.5114	1	927.5099	Carbamidc	S	Superscaf	10932588	0	0	0	0	BTP20220	0	Tissue specific
LYGSAGPPF	Superscaf	-	Superscaf	10933405	10933467	-169	169	189	-	-	-	-	-	-	NCP	IGR	-	ID=gene-E	21	5.366666667	8078	0.798330946	LYGSAGPPF	96	1102.001	2	2201.996	.	L	Superscaf	10933467	0	0	BTP20220	0	0	0	Tissue specific
FWKAPK	Superscaf	-	Superscaf	10948627	10948644	-3	3	8	-	-	-	-	-	-	NCP	IGR	-	ID=gene-E	6	7.195	2499.5	0.798051004	FWKAPK	78	388.726	2	775.438	.	G	Superscaf	10948689	0	BTP20220	0	0	0	0	Tissue specific
KYLLVEF	Superscaf	+	Superscaf	10997269	10997289	1	1	7	-	-	-	-	-	-	NCP	IGR	-	ID=gene-E	7	5.151428571	2447.6	0.797153742	KYLLVEF	93	911.5198	1	910.5164	.	K	Superscaf	10997269	0	BTP20220	0	0	0	0	Tissue specific
RTRDKSK	Superscaf	+	Superscaf	11018851	11018871	5	5	11	-	-	-	-	-	-	NCP	IGR	-	ID=gene-E	7	8.025714286	3426.8	0.796755659	R(+42.01)	68	466.7678	2	931.5199	Acetylati	P	Superscaf	11018839	0	BTP20220	0	0	0	0	Tissue specific
ALRQSLSK	Superscaf	+	Superscaf	11023594	11023617	9	9	16	-	-	-	-	-	-	NCP	IGR	-	ID=gene-E	8	6.93625	3512.8	0.796668119	ALRQSLSK	97	451.772	2	901.5345	.	L	Superscaf	11023570	0	0	0	BTP20220	0	0	Tissue specific
TGPLAVP	Superscaf	-	Superscaf	11061493	11061513	-64	64	70	-	-	-	-	-	-	NCP	IGR	-	ID=gene-E	7	5.3	2034.3	0.795969123	TGPLAVP	65	654.3787	1	653.3748	.	A	Superscaf	11061756	0	0	0	0	BTP20220	0	Tissue specific
PKEYVK	Superscaf	-	Superscaf	11087716	11087733	-17	17	22	-	-	-	-	-	-	NCP	IGR	-	ID=gene-E	6	5.776666667	2159.2	0.795485492	PKEYVK	51	382.221	2	762.4276	.	E	Superscaf	11087769	0	0	0	0	BTP20220	0	Tissue specific
KVELSQLRV	Superscaf	-	Superscaf	11196265	11196321	-61	61	79	-	-	gene	-	-	mRNA	NCP	Intron	gene|mRN	ID=gene-E	19	5.721052632	6489.8	0.793482574	KVELSQLRV	93	648.0505	3	1941.137	.	C	Superscaf	11196333	0	0	BTP20220	0	0	0	Tissue specific
KYKPLDLRF	Superscaf	-	Superscaf	11199691	11199720	-22	22	31	-	-	gene	-	-	mRNA	NCP	Intron	gene|mRN	ID=gene-E	10	7.317	4306.6	0.793419879	KYKPLDLRF	97	419.9253	3	1256.761	.	V	Superscaf	11199831	BTP20220	0	0	0	0	0	Tissue specific
QPTYP	Superscaf	-	Superscaf	11201926	11201940	-36	36	40	-	-	gene	-	-	mRNA	NCP	Intron	gene|mRN	ID=gene-E	5	6.088	2142.5	0.793378931	QPTYP	60	605.2917	1	604.2856	.	P	Superscaf	11202171	BTP20220	0	0	0	0	0	Tissue specific
MGWPVQ	Superscaf	-	Superscaf	11231980	11231997	-30	30	35	-	-	gene	-	-	mRNA	NCP	Intron	gene|mRN	ID=gene-E	6	4.926666667	2143.5	0.792824526	M(+15.99)	54	733.3374	1	732.3265	Oxidatior	T	Superscaf	11231997	0	0	BTP20220	0	0	0	Tissue specific
TVEGGAP	Superscaf	+	Superscaf	11232505	11232525	5	5	11	-	-	gene	-	-	mRNA	NCP	Intron	gene|mRN	ID=gene-E	7	4.858571429	2066.3	0.792814787	TVEGGAP	60	630.3119	1	629.3021	.	Q	Superscaf	11232493	BTP20220	0	0	0	0	0	Tissue specific
RNGRPK	Superscaf	+	Superscaf	11319277	11319294	31	31	36	-	-	-	-	-	-	NCP	IGR	-	ID=gene-E	6	8.156666667	2997.4	0.791214323	RNGRPK	62	364.2229	2	726.4249	.	S	Superscaf	11319187	0	0	0	BTP20220	0	0	Tissue specific
RQAGRFF	Superscaf	+	Superscaf	11333029	11333049	67	67	73	-	-	-	-	-	-	NCP	IGR	-	ID=gene-E	7	7.16	3291.7	0.79096061	RQAGRFF	63	441.2379	2	880.4667	.	T	Superscaf	11332831	0	BTP20220	0	0	0	0	Tissue specific
TALNVP	Superscaf	-	Superscaf	11341294	11341311	-19	19	24	-	-	-	-	-	-	NCP	IGR	-	ID=gene-E	6	5.04	1996.3	0.790808216	T(+42.01)	59	656.3588	1	655.3541	Acetylati	G	Superscaf	11341383	0	0	0	BTP20220	0	0	Tissue specific
ACARPLLS	Superscaf	-	Superscaf	11462311	11462334	-7	7	14	-	-	-	-	-	-	NCP	IGR	-	ID=gene-E	8	6.47	3284.7	0.788575933	A(+42.01)	82	465.2455	2	928.48	Acetylati	T	Superscaf	11462412	0	0	0	0	BTP20220	0	Tissue specific
VSAPEYR	Superscaf	+	Superscaf	11513365	11513385	5	5	11	-	-	-	-	-	-	NCP	IGR	-	ID=gene-E	7	5.377142857	2565.9	0.787634292	VSAPEYR	51	411.204	2	820.4079	.	R	Superscaf	11513353	0	0	BTP20220	0	0	0	Tissue specific
TADFLAT	Superscaf	+	Superscaf	11515537	11515557	6	6	12	-	-	-	-	-	-	NCP	IGR	-	ID=gene-E	7	5.647142857	2569.9	0.787594229	T(+42.01)	60	780.3719	1	779.3701	Acetylati	R	Superscaf	11515522	0	0	BTP20220	BTP20220	0	0	Tissue specific
DPQGEKQ	Superscaf	+	Superscaf	11533435	11533455	12	12	18	-	-	-	-	-	-	NCP	IGR	-	ID=gene-E	7	5.642857143	3161.5	0.787264098	DPQGEKQ	65	801.3669	1	800.3664	.	Q	Superscaf	11533402	0	0	0	0	0	BTP20220	Tissue specific
ATQKRL	Superscaf	-	Superscaf	11549131	11549148	-61	61	66	-	-	-	-	-	-	NCP	IGR	-	ID=gene-E	6	7.446666667	2790.1	0.786974639	ATQKRL	66	358.7272	2	715.434	.	G	Superscaf	11549325	0	BTP20220	0	0	0	0	Tissue specific
PGAPPNP	Superscaf	+	Superscaf	11560204	11560224	5	5	11	-	-	-	-	-	-	NCP	IGR	-	ID=gene-E	7	6.085714286	2554.9	0.786770341	PGAPPNP	71	649.3175	1	648.3231	.	P	Superscaf	11560192	0	0	0	BTP20220	0	0	Tissue specific
AVSGAGP	Superscaf	-	Superscaf	11587183	11587203	-58	58	64	-	-	gene	-	-	mRNA	NCP	Intron	gene|mRN	ID=gene-E	7	5.137142857	1938	0.78627271	A(+42.01)	58	600.3008	1	599.2914	Acetylati	D	Superscaf	11587254	BTP20220	0	0	0	0	0	Tissue specific
SSNLELHSL	Superscaf	-	Superscaf	11593693	11593719	-30	30	38	-	-	gene	-	-	mRNA	NCP	Intron	gene|mRN	ID=gene-E	9	5.688888889	3852.3	0.786152522	SSNLELHSL	96	500.2582	2	998.5032	.	I	Superscaf	11593782	0	0	0	0	0	BTP20220	Mixed
KWGFTK	Superscaf	+	Superscaf	11602750	11602767	29	29	34	-	-	-	-	-	-	NCP	IGR	-	ID=gene-E	6	7.225	2545.6	0.78598563	KWGFTK	99	383.7141	2	765.4174	.	T	Superscaf	11602666	0	0	0	0	0	BTP20220	Tissue specific
SPAGLRGP	Superscaf	-	Superscaf	11610901	11610924	-72	72	79	-	-	gene	-	-	mRNA	NCP	Intron	gene|mRN	ID=gene-E	8	6.6225	2966.3	0.785835174	SPAGLRGP	76	377.713	2	753.4133	.	F	Superscaf	11610927	0	0	BTP20220	0	0	0	Tissue specific
VYKVLKQVF	Superscaf	+	Superscaf	11681335	11681367	57	57	67	-	-	gene	-	-	mRNA	NCP	Intron	gene|mRN	ID=gene-E	11	4.875454545	3729	0.784535844	VYKVLKQVF	94	663.3796	2	1324.75	.	G	Superscaf	11681167	0	0	0	0	0	BTP20220	Tissue specific
LDDLEL	Superscaf	+	Superscaf	11691085	11691102	64	64	69	-	-	gene	-	-	mRNA	NCP	Intron	gene|mRN	ID=gene-E	6	4.516666667	2308.7	0.784356281	LDDLEL	67	717.3689	1	716.3592	.	H	Superscaf	11690896	BTP20220	0	0	0	0	0	Tissue specific
KPTPP	Superscaf	-	Superscaf	11701705	11701719	-17	17	21	-	-	gene	-	-	mRNA	NCP	Intron	gene|mRN	ID=gene-E	5	7.034	1878.1	0.784160449	KPTPP	55	539.3289	1	538.3115	.	A	Superscaf	11701866	BTP20220	0	0	0	0	0	Mixed
TPPGPLQ	Superscaf	-	Superscaf	11705425	11705445	-5	5	11	-	-	gene	-	-	mRNA	NCP	Intron	gene|mRN	ID=gene-E	7	6.147142857	2671.1	0.784091723	T(+42.01)	82	376.2064	2	750.3912	Acetylati	E	Superscaf	11705499	BTP20220	0	0	0	0	0	Tissue specific
KLTTVPP	Superscaf	+	Superscaf	11722657	11722677	4	4	10	-	-	gene	-	-	mRNA	NCP	Intron	gene|mRN	ID=gene-E	7	5.911428571	2291.6	0.783773877	K(+42.01)	67	399.2373	2	796.4694	Acetylati	G	Superscaf	11722648	BTP20220	0	0	0	0	0	Tissue specific
PLGPFAV	Superscaf	+	Superscaf	11746564	11746584	48	48	54	-	-	gene	-	-	mRNA	NCP	Intron	gene|mRN	ID=gene-E	7	5.15	2024.2	0.783332909	PLGPFAV	88	700.4001	1	699.3955	.	P	Superscaf	11746423	0	0	0	BTP20220	0	0	Tissue specific
HATSR	Superscaf	-	Superscaf	11796046	11796060	-9	9	13	-	-	gene	-	-	mRNA	NCP	Intron	gene|mRN	ID=gene-E	5	7.316	2360.7	0.782420319	H(+42.01)	61	613.2969	1	612.298	Acetylati	I	Superscaf	11796177	0	0	BTP20220	0	0	0	Tissue specific
LDGLQPC	Superscaf	-	Superscaf	11812099	11812119	-82	82	88	-	-	gene	-	-	mRNA	NCP	Intron	gene|mRN	ID=gene-E	7	5.41	2949.4	0.782124109	LDGLQPC(+	60	802.3724	1	801.3691	Carbamidc	A	Superscaf	11812131	0	0	0	0	BTP20220	0	Tissue specific
APKQT	Superscaf	-	Superscaf	11889358	11889372	-14	14	18	-	-	-	-	-	-	NCP	IGR	-	ID=gene-E	5	6.848	2125.3	0.780699168	APKQT	68	544.3116	1	543.3016	.	V	Superscaf	11889630	0	0	0	0	BTP20220	BTP20220	Tissue specific
KKLLKK	Superscaf	+	Superscaf	11976727	11976744	3	3	8	-	-	gene	-	-	mRNA	NCP	Intron	gene|mRN	ID=gene-E	6	8.486666667	2580.4	0.779087582	K(+42.01)	95	400.2901	2	798.5691	Acetylati	F	Superscaf	11976721	0	0	0	0	BTP20220	BTP20220	Tissue specific
VKNPPPP	Superscaf	-	Superscaf	11994349	11994369	-22	22	28	-	-	gene	-	-	mRNA	NCP	Intron	gene|mRN	ID=gene-E	7	5.764285714	2414.7	0.778762487	VKNPPPP	78	748.4413	1	747.4279	.	L	Superscaf	11994537	BTP20220	0	0	0	0	0	Tissue specific
LLKKPP	Superscaf	+	Superscaf	12008560	12008577	2	2	7	-	-	gene	-	-	mRNA	NCP	Intron	gene|mRN	ID=gene-E	6	7.34	2318.4	0.778500418	LLKKPP	83	695.4782	1	694.4741	.	R	Superscaf	12008557	0	BTP20220	0	0	0	0	Tissue specific
VVPSPKV	Superscaf	-	Superscaf	12044677	12044697	-1	1	7	-	-	gene	-	-	mRNA	NCP	Intron	gene|mRN	ID=gene-E	7	4.002857143	1526.6	0.777834181	VVPSPKV	89	363.2305	2	724.4483	.	V	Superscaf	12044808	0	0	BTP20220	0	0	0	Tissue specific
LAPDPSR	Superscaf	+	Superscaf	12138673	12138693	14	14	20	-	-	-	-	-	-	NCP	IGR	-	ID=gene-E	7	6.287142857	2783.2	0.776100414	LAPDPSR	72	378.2101	2	754.3973	.	V	Superscaf	12138634	0	0	0	0	0	BTP20220	Tissue specific
FDPAL	Superscaf	-	Superscaf	12279556	12279570	-13	13	17	-	-	-	-	-	-	NCP	IGR	-	ID=gene-E	5	5.35	1801	0.773501921	FDPAL	51	562.2878	1	561.2798	.	Q	Superscaf	12279639	0	0	0	0	0	BTP20220	Tissue specific
KDLVT	Superscaf	-	Superscaf	12303466	12303480	-5	5	9	-	-	-	-	-	-	NCP	IGR	-	ID=gene-E	5	5.044	1630.8	0.773060899	KDLVT	76	575.3388	1	574.3326	.	T	Superscaf	12303609	0	0	BTP20220	0	0	0	Mixed
HGTPH	Superscaf	+	Superscaf	12312505	12312519	67	67	71	-	-	gene	-	-	mRNA	NCP	Intron	gene|mRN	ID=gene-E	5	6.796	2271.6	0.772894173	HGTPH	53	548.2535	1	547.2503	.	T	Superscaf	12312307	0	0	BTP20220	0	0	0	Tissue specific
TLHGSFR	Superscaf	+	Superscaf	12353347	12353367	4	4	10	-	-	gene	-	-	mRNA	NCP	Intron	gene|mRN	ID=gene-E	7	6.855714286	3087.5	0.772140727	TLHGSFR	55	409.2175	2	816.4242	.	I	Superscaf	12353338	0	0	0	BTP20220	0	0	Tissue specific
NPPLAPP	Superscaf	+	Superscaf	12408127	12408147	65	65	71	-	-	gene	-	-	mRNA	NCP	Intron	gene|mRN	ID=gene-E	7	6.087142857	2611	0.771130304	NPPLAPP	80	353.2051	2	704.3857	.	A	Superscaf	12407935	BTP20220	0	0	0	0	0	Mixed
MRGDKYR	Superscaf	+	Superscaf	12518863	12518883	7	7	13	-	-	gene	-	-	mRNA	NCP	Intron	gene|mRN	ID=gene-E	7	7.372857143	3305.8	0.769087766	M(+15.99)	57	471.2264	2	940.4549	Oxidatior	R	Superscaf	12518845	0	0	BTP20220	0	0	0	Tissue specific
AAPLHVP	Superscaf	-	Superscaf	12540589	12540609	-21	21	27	-	-	gene	-	-	mRNA	NCP	Intron	gene|mRN	ID=gene-E	7	5.458571429	2270.5	0.768687027	AAPLHVP	54	352.712	2	703.4017	.	G	Superscaf	12540810	0	0	0	BTP20220	0	0	Tissue specific
LTAPEPP	Superscaf	-	Superscaf	12549058	12549078	-16	16	22	-	-	gene	-	-	mRNA	NCP	Intron	gene|mRN	ID=gene-E	7	5.807142857	2499.9	0.768530815	LTAPEPP	58	724.3837	1	723.3802	.	A	Superscaf	12549114	0	0	BTP20220	0	0	0	Tissue specific
VLHTPH	Superscaf	-	Superscaf	12571543	12571560	-47	47	52	-	-	gene	-	-	mRNA	NCP	Intron	gene|mRN	ID=gene-E	6	5.665	2327.7	0.768116132	VLHTPH	81	352.1962	2	702.3813	.	F	Superscaf	12571842	BTP20220	0	0	0	0	0	Tissue specific
VTGLPDLR	Superscaf	-	Superscaf	12575776	12575799	-5	5	12	-	-	gene	-	-	mRNA	NCP	Intron	gene|mRN	ID=gene-E	8	5.56125	2799.3	0.768037944	VTGLPDLR	50	435.7481	2	869.497	.	E	Superscaf	12575931	BTP20220	0	0	0	0	0	Tissue specific
TQAGLK	Superscaf	-	Superscaf	12712654	12712671	-118	118	123	-	-	-	-	-	-	NCP	IGR	-	ID=gene-E	6	6.648333333	2438.6	0.765513324	TQAGLK	93	617.359	1	616.3544	.	N	Superscaf	12712815	0	0	0	0	0	BTP20220	Tissue specific
VLEMDPLPS	Superscaf	-	Superscaf	12740593	12740634	-14	14	27	-	-	gene	-	-	mRNA	NCP	Intron	gene|mRN	ID=gene-E	14	5.359285714	5160.8	0.764997543	VLEMDPLPS	95	794.4047	2	1586.801	.	H	Superscaf	12740736	0	0	BTP20220	0	0	0	Tissue specific
KKHYGR	Superscaf	-	Superscaf	13033192	13033209	-22	22	27	-	-	gene	-	-	mRNA	NCP	Intron	gene|mRN	ID=gene-E	6	8.243333333	2962.1	0.759600964	KKHYGR	53	394.7311	2	787.4453	.	G	Superscaf	13033224	0	0	BTP20220	0	0	0	Tissue specific
MQTATLP	Superscaf	+	Superscaf	13046770	13046790	10	10	16	-	-	gene	-	-	mRNA	NCP	Intron	gene|mRN	ID=gene-E	7	6.108571429	2779.3	0.75935046	M(+42.01)	63	402.2044	2	802.3895	Acetylati	T	Superscaf	13046743	0	0	BTP20220	BTP20220	0	0	Tissue specific
TTLDDKLLC	Superscaf	-	Superscaf	13068745	13068780	-31	31	42	-	-	gene	-	-	mRNA	NCP	Intron	gene|mRN	ID=gene-E	12	5.965833333	4691.2	0.758944852	T(+42.01)	99	694.3865	2	1386.761	Acetylati	A	Superscaf	13068939	BTP20220	0	0	0	0	0	Tissue specific
TTLDDKLLC	Superscaf	-	Superscaf	13068745	13068777	-31	31	41	-	-	gene	-	-	mRNA	NCP	Intron	gene|mRN	ID=gene-E	11	5.964545455	4337.8	0.758944908	T(+42.01)	98	637.8428	2	1273.677	Acetylati	A	Superscaf	13068939	0	0	0	0	0	BTP20220	Tissue specific
TTLDDKLL	Superscaf	-	Superscaf	13068745	13068768	-31	31	38	-	-	gene	-	-	mRNA	NCP	Intron	gene|mRN	ID=gene-E	8	5.835	3146.6	0.758945074	T(+42.01)	94	480.765	2	959.5175	Acetylati	A	Superscaf	13068939	0	0	0	0	0	BTP20220	Tissue specific
TVRMNTF	Superscaf	-	Superscaf	13102726	13102746	-34	34	40	-	-	gene	-	-	mRNA	NCP	Intron	gene|mRN	ID=gene-E	7	5.78	2799.4	0.758318346	TVRM(+15.	66	442.7177	2	883.4222	Oxidatior	D	Superscaf	13102818	0	0	0	BTP20220	0	0	Tissue specific
QFPKALG	Superscaf	+	Superscaf	13111801	13111821	1	1	7	-	-	-	-	-	-	NCP	IGR	-	ID=gene-E	7	6.448571429	2765.9	0.758150956	QFPKALG	71	380.7213	2	759.4279	.	Q	Superscaf	13111801	0	BTP20220	BTP20220	0	0	0	Tissue specific
SLPLVDT	Superscaf	-	Superscaf	13199344	13199364	-34	34	40	-	-	-	-	-	-	NCP	IGR	-	ID=gene-E	7	4.777142857	2236.6	0.756536215	SLPLVDT	69	744.4111	1	743.4065	.	P	Superscaf	13199466	0	BTP20220	0	0	0	0	Tissue specific
PVVPPPF	Superscaf	+	Superscaf	13258996	13259016	23	23	29	-	-	gene	-	-	mRNA	NCP	Intron	gene|mRN	ID=gene-E	7	4.382857143	1737	0.755435928	PVVPPPF	89	752.4288	1	751.4268	.	G	Superscaf	13258930	0	0	BTP20220	0	0	0	Tissue specific
DPVSPP	Superscaf	+	Superscaf	13259836	13259853	110	110	115	-	-	gene	-	-	mRNA	NCP	Intron	gene|mRN	ID=gene-E	6	4.591666667	1807.1	0.755420489	DPVSPP	66	611.2942	1	610.2962	.	T	Superscaf	13259509	BTP20220	0	0	0	0	0	Tissue specific
CVPKK	Superscaf	+	Superscaf	13340239	13340253	62	62	66	-	-	gene	-	-	mRNA	NCP	Intron	gene|mRN	ID=gene-E	5	6.16	1859.9	0.753937502	C(+57.02)	66	631.359	1	630.3523	Carbamidc	A	Superscaf	13340056	0	0	BTP20220	0	0	0	Tissue specific
AGKRASPP	Superscaf	-	Superscaf	13379086	13379109	-20	20	27	-	-	gene	-	-	mRNA	NCP	Intron	gene|mRN	ID=gene-E	8	7.09875	3095.3	0.753220798	A(+42.01)	73	413.2303	2	824.4504	Acetylati	S	Superscaf	13379316	0	0	0	0	0	BTP20220	Tissue specific
WPKVPK	Superscaf	+	Superscaf	13384477	13384494	3	3	8	-	-	gene	-	-	mRNA	NCP	Intron	gene|mRN	ID=gene-E	6	6.328333333	2138.2	0.753121471	WPKVPK	99	377.7319	2	753.4537	.	L	Superscaf	13384471	0	0	BTP20220	0	0	0	Tissue specific
LSGSFPF	Superscaf	+	Superscaf	13508875	13508895	41	41	47	-	-	gene	-	-	mRNA	NCP	Intron	gene|mRN	ID=gene-E	7	5.795714286	2529.7	0.750826881	LSGSFPF	70	754.3741	1	753.3697	.	G	Superscaf	13508755	0	0	0	0	BTP20220	0	Tissue specific
KTALYT	Superscaf	-	Superscaf	13523329	13523346	-7	7	12	-	-	gene	-	-	mRNA	NCP	Intron	gene|mRN	ID=gene-E	6	6.743333333	2387.6	0.75056033	KTALYT	52	696.3986	1	695.3854	.	V	Superscaf	13523436	0	0	0	0	BTP20220	0	Tissue specific
KEVLCSF	Superscaf	+	Superscaf	13571704	13571724	69	69	75	-	-	gene	-	-	mRNA	NCP	Intron	gene|mRN	ID=gene-E	7	5.017142857	2603.8	0.749667993	KEVLC(+57	58	441.7226	2	881.4316	Carbamidc	L	Superscaf	13571500	BTP20220	BTP20220	0	0	0	0	Tissue specific
DLQTLPK	Superscaf	+	Superscaf	13590130	13590150	19	19	25	-	-	gene	-	-	mRNA	NCP	Intron	gene|mRN	ID=gene-E	7	6.164285714	2932.3	0.749328123	DLQTLPK	98	814.4653	1	813.4596	.	V	Superscaf	13590076	0	0	BTP20220	0	0	0	Tissue specific
QGQFE	Superscaf	-	Superscaf	13674031	13674045	-11	11	15	-	-	gene	-	-	mRNA	NCP	Intron	gene|mRN	ID=gene-E	5	5.194	2331.6	0.74778067	Q(+42.01)	64	650.282	1	649.2708	Acetylati	I	Superscaf	13674045	0	0	BTP20220	0	0	0	Tissue specific
LEEVALT	Superscaf	+	Superscaf	13749229	13749249	31	31	37	-	-	gene	-	-	mRNA	NCP	Intron	gene|mRN	ID=gene-E	7	4.421428571	2266.6	0.746393524	LEEVALT	53	774.4188	1	773.4171	.	M	Superscaf	13749139	0	0	0	BTP20220	0	0	Tissue specific
LEMYRDL	Superscaf	+	Superscaf	13785241	13785261	20	20	26	-	-	gene	-	-	mRNA	NCP	Intron	gene|mRN	ID=gene-E	7	5.76	3023.6	0.745729278	LEMYRDL	52	470.2385	2	938.4531	.	W	Superscaf	13785184	0	0	0	BTP20220	0	0	Tissue specific
VGKLWN	Superscaf	-	Superscaf	13867684	13867701	-20	20	25	-	-	gene	-	-	mRNA	NCP	Intron	gene|mRN	ID=gene-E	6	5.498333333	2242.4	0.744208663	VGKLWN	85	716.408	1	715.4017	.	I	Superscaf	13867740	BTP20220	0	0	0	0	0	Mixed
DPKGKGK	Superscaf	+	Superscaf	13868998	13869018	4	4	10	-	-	gene	-	-	mRNA	NCP	Intron	gene|mRN	ID=gene-E	7	7.204285714	2748.7	0.744184371	DPKGKGK	51	365.2212	2	728.418	.	C	Superscaf	13868989	0	0	BTP20220	0	0	0	Tissue specific
ASKQLFR	Superscaf	-	Superscaf	13871494	13871514	-37	37	43	-	-	gene	-	-	mRNA	NCP	Intron	gene|mRN	ID=gene-E	7	7.044285714	3163.4	0.744138332	A(+42.01)	91	446.2558	2	890.4974	Acetylati	P	Superscaf	13871643	0	0	BTP20220	0	0	0	Mixed
HALPPT	Superscaf	-	Superscaf	13877383	13877400	-2	2	7	-	-	gene	-	-	mRNA	NCP	Intron	gene|mRN	ID=gene-E	6	6.453333333	2356.7	0.744029764	HALPPT	75	635.3386	1	634.3438	.	P	Superscaf	13877442	0	0	0	0	BTP20220	0	Mixed
KPFYHKGPF	Superscaf	-	Superscaf	13888234	13888260	-2	2	10	-	-	gene	-	-	mRNA	NCP	Intron	gene|mRN	ID=gene-E	9	7.008888889	3712.9	0.74382945	KPFYHKGPF	57	535.7936	2	1069.571	.	G	Superscaf	13888335	0	0	0	0	BTP20220	0	Tissue specific
APGALR	Superscaf	-	Superscaf	13890829	13890846	-23	23	28	-	-	gene	-	-	mRNA	NCP	Intron	gene|mRN	ID=gene-E	6	6.841666667	2259.5	0.743781751	A(+42.01)	76	626.3595	1	625.3547	Acetylati	K	Superscaf	13890864	0	0	0	BTP20220	0	0	Tissue specific
LPDTQQK	Superscaf	-	Superscaf	13899439	13899459	-4	4	10	-	-	gene	-	-	mRNA	NCP	Intron	gene|mRN	ID=gene-E	7	6.117142857	3189.6	0.743622883	LPDTQQK	71	415.222	2	828.4341	.	F	Superscaf	13899507	BTP20220	0	0	0	0	0	Tissue specific
YSAFF	Superscaf	-	Superscaf	13932190	13932204	-3	3	7	-	-	gene	-	-	mRNA	NCP	Intron	gene|mRN	ID=gene-E	5	5.664	1929	0.743018898	YSAFF	88	634.2842	1	633.2798	.	G	Superscaf	13932258	0	0	0	0	BTP20220	0	Tissue specific
KELRVPF	Superscaf	-	Superscaf	13941991	13942011	-32	32	38	-	-	gene	-	-	mRNA	NCP	Intron	gene|mRN	ID=gene-E	7	5.925714286	2620.9	0.742838006	K(+42.01)	98	465.7723	2	929.5334	Acetylati	L	Superscaf	13942206	0	0	BTP20220	0	0	0	Tissue specific
KNDLLL	Superscaf	+	Superscaf	13964563	13964580	7	7	12	-	exon	gene	three_pri	-	mRNA	NCP	3UTR	exon|gene	ID=exon-E	6	6.01	2536.8	0.742421719	KNDLLL	63	358.2148	2	714.4276	.	S	Superscaf	13964545	0	0	0	0	BTP20220	0	Tissue specific
LGFSPP	Superscaf	+	Superscaf	13996102	13996119	49	49	54	-	-	-	-	-	-	NCP	IGR	-	ID=gene-E	6	5.951666667	2096.3	0.741839978	LGFSPP	59	617.319	1	616.322	.	N	Superscaf	13995958	0	0	0	BTP20220	0	0	Tissue specific
KVSSSVLKA	Superscaf	-	Superscaf	14063248	14063274	-55	55	63	-	-	-	-	-	-	NCP	IGR	-	ID=gene-E	9	5.391111111	2751.7	0.740601296	KVSSSVLKA	95	459.7836	2	917.5546	.	Y	Superscaf	14063409	0	0	0	0	BTP20220	0	Tissue specific
VTAGL	Superscaf	-	Superscaf	14079124	14079138	-107	107	111	-	-	-	-	-	-	NCP	IGR	-	ID=gene-E	5	4.9	1359.5	0.740308683	VTAGL	50	460.2748	1	459.2693	.	G	Superscaf	14079405	0	0	0	BTP20220	0	0	Tissue specific
LLAPERP	Superscaf	+	Superscaf	14113252	14113272	2	2	8	-	-	-	-	-	-	NCP	IGR	-	ID=gene-E	7	6.365714286	2767.2	0.739679078	LLAPERP	93	398.239	2	794.465	.	L	Superscaf	14113249	0	BTP20220	BTP20220	0	0	0	Tissue specific
EFSAQR	Superscaf	+	Superscaf	14114347	14114364	6	6	11	-	-	-	-	-	-	NCP	IGR	-	ID=gene-E	6	6.135	2767.1	0.739658935	E(+42.01)	71	390.1912	2	778.361	Acetylati	R	Superscaf	14114332	BTP20220	0	0	0	0	0	Tissue specific
MGAVVVR	Superscaf	-	Superscaf	14180626	14180646	-3	3	9	-	-	-	-	-	-	NCP	IGR	-	ID=gene-E	7	4.071428571	1628.9	0.738436356	MGAVVVR	89	366.2128	2	730.416	.	L	Superscaf	14180778	0	0	BTP20220	0	0	0	Tissue specific
LNGKAPK	Superscaf	+	Superscaf	14183281	14183301	21	21	27	-	-	-	-	-	-	NCP	IGR	-	ID=gene-E	7	7.022857143	2832.9	0.738387385	LNGKAPK	64	364.2197	2	726.4388	.	N	Superscaf	14183221	0	0	0	0	BTP20220	0	Tissue specific
EPMEEP	Superscaf	-	Superscaf	14199400	14199417	-1	1	6	-	-	-	-	-	-	NCP	IGR	-	ID=gene-E	6	4.668333333	2322.8	0.738090123	EPM(+15.9	55	374.1447	2	746.2792	Oxidatior	E	Superscaf	14199459	0	0	0	0	0	BTP20220	Tissue specific
ESPQLLH	Superscaf	-	Superscaf	14206417	14206437	-3	3	9	-	-	-	-	-	-	NCP	IGR	-	ID=gene-E	7	5.771428571	3083.5	0.737960638	ESPQLLH	70	412.2196	2	822.4235	.	L	Superscaf	14206686	0	0	0	0	0	BTP20220	Tissue specific
ESRPVFL	Superscaf	-	Superscaf	14276653	14276673	-10	10	16	-	-	-	-	-	-	NCP	IGR	-	ID=gene-E	7	5.345714286	2535.9	0.736665127	ESRPVFL	83	847.4642	1	846.4599	.	N	Superscaf	14276757	0	0	0	BTP20220	0	0	Tissue specific
EGPFG	Superscaf	+	Superscaf	14403667	14403681	91	91	95	-	-	-	-	-	-	NCP	IGR	-	ID=gene-E	5	5.388	1744.9	0.73432245	EGPFG	94	506.2238	1	505.2172	.	A	Superscaf	14403397	0	0	0	BTP20220	0	0	Tissue specific
PPPVAP	Superscaf	-	Superscaf	14415406	14415423	-18	18	23	-	-	-	-	-	-	NCP	IGR	-	ID=gene-E	6	5.203333333	1660.9	0.734105868	PPPVAP	84	577.3326	1	576.3271	.	L	Superscaf	14415495	0	0	0	0	BTP20220	0	Tissue specific
KEEVTLP	Superscaf	+	Superscaf	14417350	14417370	2	2	8	-	-	-	-	-	-	NCP	IGR	-	ID=gene-E	7	4.998571429	2407.7	0.734069955	KEEVTLP	54	408.2336	2	814.4436	.	N	Superscaf	14417347	0	0	0	0	BTP20220	0	Tissue specific
TTEEDLK	Superscaf	+	Superscaf	14482219	14482239	37	37	43	-	-	-	-	-	-	NCP	IGR	-	ID=gene-E	7	5.455714286	2879.3	0.732873439	TTEEDLK	88	418.2112	2	834.397	.	R	Superscaf	14482111	0	0	0	BTP20220	0	0	Tissue specific
RLPKKT	Superscaf	+	Superscaf	14490949	14490966	7	7	12	-	-	-	-	-	-	NCP	IGR	-	ID=gene-E	6	8.175	2673.9	0.732712468	RLPKKT	70	371.744	2	741.4861	.	M	Superscaf	14490931	BTP20220	0	0	0	0	0	Mixed
PGNCCC	Superscaf	-	Superscaf	14509285	14509302	-41	41	46	-	-	-	-	-	-	NCP	IGR	-	ID=gene-E	6	5.456666667	2988.5	0.732374259	PGNC(+57.	51	767.2129	1	766.2197	Carbamidc	Q	Superscaf	14509308	0	BTP20220	0	0	0	BTP20220	Tissue specific
RLSTHDP	Superscaf	+	Superscaf	14512888	14512908	38	38	44	-	-	-	-	-	-	NCP	IGR	-	ID=gene-E	7	6.544285714	3151.7	0.732307746	RLSTHDP	55	413.2184	2	824.4141	.	G	Superscaf	14512777	0	0	0	0	0	BTP20220	Tissue specific
YAPSLGP	Superscaf	+	Superscaf	14536198	14536218	177	177	183	-	-	gene	-	-	mRNA	NCP	Intron	gene|mRN	ID=gene-E	7	5.987142857	2479.7	0.73187779	YAPSLGP	61	704.3579	1	703.3541	.	P	Superscaf	14535670	0	0	0	0	BTP20220	0	Tissue specific
LGGEAGRP	Superscaf	+	Superscaf	14593663	14593686	55	55	62	-	-	gene	-	-	mRNA	NCP	Intron	gene|mRN	ID=gene-E	8	6.27375	2968.3	0.730817786	L(+42.01)	61	798.4177	1	797.4031	Acetylati	Q	Superscaf	14593501	BTP20220	0	0	0	0	0	Tissue specific
TKSPVP	Superscaf	+	Superscaf	14609185	14609202	20	20	25	-	-	gene	-	-	mRNA	NCP	Intron	gene|mRN	ID=gene-E	6	5.758333333	1924.1	0.730531592	T(+42.01)	89	670.3751	1	669.3698	Acetylati	G	Superscaf	14609128	0	0	0	0	0	BTP20220	Tissue specific
SERPLP	Superscaf	-	Superscaf	14622316	14622333	-32	32	37	-	-	gene	-	-	mRNA	NCP	Intron	gene|mRN	ID=gene-E	6	6.373333333	2485.9	0.730289389	S(+42.01)	89	370.6985	2	739.3864	Acetylati	L	Superscaf	14622414	0	0	0	0	0	BTP20220	Tissue specific
EKALLAP	Superscaf	-	Superscaf	14631418	14631438	-17	17	23	-	-	gene	-	-	mRNA	NCP	Intron	gene|mRN	ID=gene-E	7	6.18	2560.7	0.730121446	E(+42.01)	58	783.4594	1	782.4538	Acetylati	I	Superscaf	14631441	0	0	0	0	0	BTP20220	Tissue specific
NHRPPK	Superscaf	-	Superscaf	14684527	14684544	-43	43	48	-	-	gene	-	-	mRNA	NCP	Intron	gene|mRN	ID=gene-E	6	7.683333333	3008.4	0.7291419	NHRPPK	52	374.7136	2	747.414	.	A	Superscaf	14684712	0	0	0	BTP20220	0	0	Tissue specific
RPPGSLGR	Superscaf	+	Superscaf	14694274	14694297	2	2	9	-	-	gene	-	-	mRNA	NCP	Intron	gene|mRN	ID=gene-E	8	7.215	3303.8	0.728962005	RPPGSLGR	67	420.241	2	838.4773	.	A	Superscaf	14694271	0	0	0	0	0	BTP20220	Tissue specific
LPPPPPLF	Superscaf	+	Superscaf	14700832	14700864	2	2	12	-	-	gene	-	-	mRNA	NCP	Intron	gene|mRN	ID=gene-E	11	6.104545455	3949.5	0.728840876	LPPPPPLF	89	606.8394	2	1211.659	.	A	Superscaf	14700829	0	0	0	BTP20220	0	0	Tissue specific
WRKKGP	Superscaf	-	Superscaf	14724571	14724588	-1	1	6	-	-	gene	-	-	mRNA	NCP	Intron	gene|mRN	ID=gene-E	6	8.066666667	2746.9	0.728403284	WRKKGP	50	771.4698	1	770.4551	.	W	Superscaf	14724762	0	0	0	BTP20220	0	0	Tissue specific
GPDDKLR	Superscaf	-	Superscaf	14724583	14724603	-5	5	11	-	-	gene	-	-	mRNA	NCP	Intron	gene|mRN	ID=gene-E	7	6.384285714	2928.3	0.728403007	GPDDKLR	75	400.7089	2	799.4188	.	W	Superscaf	14724762	BTP20220	0	0	0	0	0	Tissue specific
RGFVLEEV	Superscaf	-	Superscaf	14732548	14732571	-45	45	52	-	-	gene	-	-	mRNA	NCP	Intron	gene|mRN	ID=gene-E	8	4.32875	2537.9	0.728256036	RGFVLEEV	70	474.7603	2	947.5076	.	A	Superscaf	14732580	0	0	0	0	0	BTP20220	Tissue specific
ESGPPP	Superscaf	+	Superscaf	14749831	14749848	32	32	37	-	-	-	-	-	-	NCP	IGR	-	ID=gene-E	6	5.628333333	2118.4	0.72793736	ESGPPP	60	583.2699	1	582.2649	.	L	Superscaf	14749738	BTP20220	0	0	0	0	0	Tissue specific
ENHPVLL	Superscaf	+	Superscaf	14758615	14758635	7	7	13	-	-	-	-	-	-	NCP	IGR	-	ID=gene-E	7	4.925714286	2686.1	0.727775283	ENHPVLL	96	821.4493	1	820.4443	.	A	Superscaf	14758597	0	0	0	0	BTP20220	0	Tissue specific
LQVPPP	Superscaf	+	Superscaf	14770150	14770167	9	9	14	-	-	-	-	-	-	NCP	IGR	-	ID=gene-E	6	5.088333333	1976.3	0.727562574	LQVPPP	95	650.3961	1	649.3799	.	S	Superscaf	14770126	0	0	0	0	0	BTP20220	Tissue specific
KRLNAK	Superscaf	+	Superscaf	14800171	14800188	5	5	10	-	-	gene	-	-	mRNA	NCP	Intron	gene|mRN	ID=gene-E	6	7.941666667	2847	0.727008833	KRLNAK	50	365.2427	2	728.4657	.	T	Superscaf	14800159	0	0	0	0	0	BTP20220	Tissue specific
LYVFF	Superscaf	+	Superscaf	14806453	14806467	10	10	14	-	-	gene	-	-	mRNA	NCP	Intron	gene|mRN	ID=gene-E	5	4.52	1587.7	0.726893016	LYVFF	86	688.3666	1	687.3632	.	A	Superscaf	14806426	0	0	BTP20220	0	0	0	Tissue specific
LDDLSAPK	Superscaf	+	Superscaf	14865556	14865579	8	8	15	-	-	gene	-	-	mRNA	NCP	Intron	gene|mRN	ID=gene-E	8	5.705	3030.3	0.725802689	LDDLSAPK	90	858.4557	1	857.4494	.	T	Superscaf	14865535	0	0	0	0	0	BTP20220	Tissue specific
KVVRVH	Superscaf	+	Superscaf	14950603	14950620	1	1	6	-	-	-	-	-	-	NCP	IGR	-	ID=gene-E	6	4.681666667	1736.9	0.724234098	KVVRVH	79	369.2413	2	736.4708	.	K	Superscaf	14950603	0	BTP20220	0	0	0	0	Tissue specific
PPPFAGPPF	Superscaf	+	Superscaf	14968717	14968743	104	104	112	-	-	-	-	-	-	NCP	IGR	-	ID=gene-E	9	6.141111111	3020.4	0.723899817	PPPFAGPPF	63	438.7305	2	875.4541	.	A	Superscaf	14968408	0	0	0	0	BTP20220	0	Tissue specific
MLPRAE	Superscaf	-	Superscaf	15040195	15040212	-23	23	28	-	-	-	-	-	-	NCP	IGR	-	ID=gene-E	6	6.338333333	2447.9	0.722581563	M(+15.99)	63	366.6929	2	731.3636	Oxidatior	Q	Superscaf	15040371	0	0	BTP20220	0	0	0	Tissue specific
PLSLLLLF	Superscaf	-	Superscaf	15056023	15056046	-11	11	18	-	exon	gene	three_pri	-	mRNA	NCP	3UTR	exon|gene	ID=exon-E	8	5.92	2875.2	0.722289503	PLSLLLLF	65	458.294	2	914.584	.	N	Superscaf	15056175	0	0	BTP20220	0	0	0	Tissue specific
GLSTLRL	Superscaf	+	Superscaf	15080503	15080523	2	2	8	-	-	gene	-	-	mRNA	NCP	Intron	gene|mRN	ID=gene-E	7	6.697142857	2787.2	0.721838022	G(+42.01)	50	401.2494	2	800.4756	Acetylati	A	Superscaf	15080500	0	0	0	0	0	BTP20220	Tissue specific
KPKPPK	Superscaf	+	Superscaf	15123892	15123909	11	11	16	-	-	gene	-	-	mRNA	NCP	Intron	gene|mRN	ID=gene-E	6	8.02	2417.4	0.721037762	K(+42.01)	59	368.7373	2	735.4643	Acetylati	Y	Superscaf	15123862	0	0	0	BTP20220	0	0	Tissue specific
KNVVAPK	Superscaf	+	Superscaf	15177328	15177348	1	1	7	-	-	-	-	-	-	NCP	IGR	-	ID=gene-E	7	5.315714286	2182.2	0.720052074	KNVVAPK	51	378.2388	2	754.4701	.	K	Superscaf	15177328	0	BTP20220	0	0	0	0	Tissue specific
QEVVPVP	Superscaf	-	Superscaf	15233680	15233700	-49	49	55	-	-	gene	-	-	mRNA	NCP	Intron	gene|mRN	ID=gene-E	7	3.067142857	1711	0.719012654	QEVVPVP	77	767.4261	1	766.4225	.	T	Superscaf	15233727	0	0	BTP20220	0	0	0	Tissue specific
KAVKELK	Superscaf	+	Superscaf	15254521	15254541	2	2	8	-	-	gene	-	-	mRNA	NCP	Intron	gene|mRN	ID=gene-E	7	6.348571429	2495.4	0.71862824	K(+42.01)	95	429.2747	2	856.5381	Acetylati	V	Superscaf	15254518	0	0	0	BTP20220	0	0	Tissue specific
LSAVMD	Superscaf	-	Superscaf	15266569	15266586	-3	3	8	-	-	gene	-	-	mRNA	NCP	Intron	gene|mRN	ID=gene-E	6	4.4	1831.1	0.718406068	LSAVMD	55	635.3026	1	634.2996	.	S	Superscaf	15266595	BTP20220	0	0	0	0	0	Tissue specific
NFLGSKAK	Superscaf	+	Superscaf	15279676	15279699	16	16	23	-	-	gene	-	-	mRNA	NCP	Intron	gene|mRN	ID=gene-E	8	6.7525	3266.3	0.718164198	NFLGSKAK	86	432.7456	2	863.4865	.	G	Superscaf	15279631	0	0	0	BTP20220	0	0	Tissue specific
VKVGV	Superscaf	+	Superscaf	15366142	15366156	1	1	5	-	-	-	-	-	-	NCP	IGR	-	ID=gene-E	5	3.142	765.7	0.716569488	VKVGV	89	501.3383	1	500.3322	.	V	Superscaf	15366142	0	0	0	0	BTP20220	0	Tissue specific
VLTGKSVQ	Superscaf	+	Superscaf	15373234	15373257	6	6	13	-	-	-	-	-	-	NCP	IGR	-	ID=gene-E	8	4.94375	2510.7	0.71643851	VLTGKSVQ	87	416.2477	2	830.4861	.	P	Superscaf	15373219	0	0	0	0	BTP20220	BTP20220	Tissue specific
APVLGF	Superscaf	-	Superscaf	15388240	15388257	-6	6	11	-	-	-	-	-	-	NCP	IGR	-	ID=gene-E	6	4.958333333	1686.8	0.716161833	APVLGF	50	603.347	1	602.3428	.	I	Superscaf	15388284	0	0	0	0	0	BTP20220	Mixed
VPAKLP	Superscaf	+	Superscaf	15400018	15400035	10	10	15	-	-	-	-	-	-	NCP	IGR	-	ID=gene-E	6	5.723333333	1807.9	0.715944586	V(+42.01)	58	666.4254	1	665.4112	Acetylati	D	Superscaf	15399991	0	0	0	0	0	BTP20220	Tissue specific
LWTRSLY	Superscaf	-	Superscaf	15424903	15424923	-4	4	10	-	-	-	-	-	-	NCP	IGR	-	ID=gene-E	7	6.64	3122.6	0.715485524	LWTRSLY	65	469.7569	2	937.5021	.	D	Superscaf	15425034	0	0	0	BTP20220	0	0	Tissue specific
KLLYPV	Superscaf	-	Superscaf	15507667	15507684	-26	26	31	-	-	-	-	-	-	NCP	IGR	-	ID=gene-E	6	5.61	1972.1	0.713958988	KLLYPV	59	366.7354	2	731.4581	.	G	Superscaf	15507741	0	BTP20220	0	0	0	0	Tissue specific
KVLLLVP	Superscaf	+	Superscaf	15532885	15532905	27	27	33	-	-	-	-	-	-	NCP	IGR	-	ID=gene-E	7	4.854285714	1866	0.713493784	KVLLLVP	62	391.283	2	780.5473	.	P	Superscaf	15532807	0	0	0	BTP20220	0	0	Tissue specific
CLGPCAP	Superscaf	-	Superscaf	15602185	15602205	-9	9	15	-	-	-	-	-	-	NCP	IGR	-	ID=gene-E	7	5.801428571	2808.2	0.712215537	C(+57.02)	52	774.3231	1	773.32	Carbamidc	R	Superscaf	15602241	0	0	0	0	BTP20220	0	Tissue specific
QELDPK	Superscaf	-	Superscaf	15605032	15605049	-32	32	37	-	-	-	-	-	-	NCP	IGR	-	ID=gene-E	6	5.643333333	2606.9	0.712163079	QELDPK	60	365.1912	2	728.3704	.	R	Superscaf	15605064	0	0	0	0	0	BTP20220	Tissue specific
HVSPLH	Superscaf	-	Superscaf	15620134	15620151	-2	2	7	-	-	-	-	-	-	NCP	IGR	-	ID=gene-E	6	5.523333333	2313.6	0.711884521	H(+42.01)	67	366.1909	2	730.3762	Acetylati	L	Superscaf	15620262	0	0	0	0	0	BTP20220	Tissue specific
QRMSLLL	Superscaf	-	Superscaf	15622210	15622230	-6	6	12	-	-	-	-	-	-	NCP	IGR	-	ID=gene-E	7	6.54	3074.6	0.711846174	QRM(+15.9	65	438.7514	2	875.4899	Oxidatior	T	Superscaf	15622317	0	0	0	0	BTP20220	0	Tissue specific
HVLPLEK	Superscaf	-	Superscaf	15634033	15634053	-13	13	19	-	-	-	-	-	-	NCP	IGR	-	ID=gene-E	7	5.544285714	2557.8	0.711628097	HVLPLEK	99	418.2545	2	834.4963	.	L	Superscaf	15634233	0	0	0	0	BTP20220	BTP20220	Tissue specific
KLGYTQ	Superscaf	-	Superscaf	15698827	15698844	-1	1	6	-	-	-	-	-	-	NCP	IGR	-	ID=gene-E	6	6.588333333	2586.8	0.71043302	KLGYTQ	84	355.196	2	708.3806	.	K	Superscaf	15698901	0	0	0	0	0	BTP20220	Tissue specific
SLDGALLT	Superscaf	-	Superscaf	15699844	15699867	-2	2	9	-	-	-	-	-	-	NCP	IGR	-	ID=gene-E	8	5.63875	2861.2	0.710414151	SLDGALLT	66	789.4323	1	788.428	.	P	Superscaf	15699870	0	0	BTP20220	0	0	BTP20220	Tissue specific
GVPGV	Superscaf	+	Superscaf	15776356	15776370	6	6	10	-	-	gene	-	-	mRNA	NCP	Intron	gene|mRN	ID=gene-E	5	3.648	932	0.709003044	GVPGV	69	428.2493	1	427.2431	.	S	Superscaf	15776341	BTP20220	0	0	0	0	0	Mixed
GLPAYK	Superscaf	+	Superscaf	15800005	15800022	5	5	10	-	-	gene	-	-	mRNA	NCP	Intron	gene|mRN	ID=gene-E	6	6.611666667	2227.3	0.70856678	GLPAYK	70	648.3661	1	647.3642	.	N	Superscaf	15799993	0	0	0	0	0	BTP20220	Tissue specific
PLLSVC	Superscaf	+	Superscaf	15830419	15830436	4	4	9	-	-	gene	-	-	mRNA	NCP	Intron	gene|mRN	ID=gene-E	6	4.826666667	2013.3	0.70800579	P(+42.01)	54	365.6946	2	729.3731	Acetylati	F	Superscaf	15830410	0	BTP20220	0	0	BTP20220	0	Tissue specific
KNKMKPF	Superscaf	-	Superscaf	15854617	15854637	-18	18	24	-	-	gene	-	-	mRNA	NCP	Intron	gene|mRN	ID=gene-E	7	7.451428571	3098.2	0.7075594	KNKMKPF	57	446.7598	2	891.5	.	E	Superscaf	15854655	0	0	0	0	BTP20220	0	Tissue specific
MCCLEKK	Superscaf	-	Superscaf	15888520	15888540	-9	9	15	-	-	gene	-	-	mRNA	NCP	Intron	gene|mRN	ID=gene-E	7	6.352857143	3258.6	0.706934055	M(+15.99)	79	513.7173	2	1025.434	Oxidatior	K	Superscaf	15888669	0	0	0	0	BTP20220	0	Tissue specific
QLPGKAKL	Superscaf	+	Superscaf	15896842	15896865	59	59	66	-	-	-	-	-	-	NCP	IGR	-	ID=gene-E	8	6.9225	3200.3	0.7067805	QLPGKAKL	90	427.7756	2	853.5385	.	K	Superscaf	15896668	0	BTP20220	0	0	0	0	Tissue specific
VLNVHVT	Superscaf	+	Superscaf	15937153	15937173	1	1	7	-	-	-	-	-	-	NCP	IGR	-	ID=gene-E	7	3.644285714	1967.3	0.706037014	VLNVHVT	66	781.4439	1	780.4494	.	V	Superscaf	15937153	0	0	0	0	0	BTP20220	Tissue specific
QHTVEVL	Superscaf	-	Superscaf	15986671	15986691	-41	41	47	-	-	gene	-	-	mRNA	NCP	Intron	gene|mRN	ID=gene-E	7	4.138571429	2406.8	0.705123649	Q(+42.01)	61	434.2308	2	866.4498	Acetylati	A	Superscaf	15986760	BTP20220	0	0	0	0	0	Tissue specific
LPPPL	Superscaf	-	Superscaf	16003444	16003458	-2	2	6	-	-	-	-	-	-	NCP	IGR	-	ID=gene-E	5	6.172	1719	0.70481438	LPPPL	63	536.3429	1	535.337	.	S	Superscaf	16003521	0	BTP20220	0	0	0	0	Tissue specific
LEALLY	Superscaf	-	Superscaf	16007629	16007646	-4	4	9	-	-	-	-	-	-	NCP	IGR	-	ID=gene-E	6	5.473333333	2256.5	0.704737132	LEALLY	58	721.4103	1	720.4058	.	V	Superscaf	16007718	0	BTP20220	0	0	0	0	Tissue specific
PLMFPLLM	Superscaf	+	Superscaf	16067650	16067673	8	8	15	-	-	-	-	-	-	NCP	IGR	-	ID=gene-E	8	5.94	2865.4	0.703629927	PLMFPLLM(	65	489.273	2	976.5126	Oxidatior	L	Superscaf	16067629	0	0	BTP20220	0	0	0	Tissue specific
LTVGEC	Superscaf	-	Superscaf	16086067	16086084	-5	5	10	-	-	-	-	-	-	NCP	IGR	-	ID=gene-E	6	4.453333333	2059.4	0.703290334	LTVGEC(+5	55	678.3253	1	677.3054	Carbamidc	A	Superscaf	16086249	0	BTP20220	0	0	0	0	Tissue specific
KSAYFFH	Superscaf	+	Superscaf	16093567	16093587	34	34	40	-	-	-	-	-	-	NCP	IGR	-	ID=gene-E	7	6.521428571	3017.1	0.70315194	KSAYFFH	55	450.2281	2	898.4337	.	T	Superscaf	16093468	0	BTP20220	0	0	0	0	Tissue specific
LYMSSSL	Superscaf	+	Superscaf	16098343	16098363	8	8	14	-	-	-	-	-	-	NCP	IGR	-	ID=gene-E	7	5.772857143	2688	0.703063846	LYM(+15.9	70	408.6927	2	815.3735	Oxidatior	R	Superscaf	16098322	0	0	0	0	BTP20220	0	Tissue specific
QQPFDLK	Superscaf	-	Superscaf	16099633	16099653	-29	29	35	-	-	-	-	-	-	NCP	IGR	-	ID=gene-E	7	5.967142857	3179.5	0.703040052	QQPFDLK	77	438.228	2	874.4548	.	F	Superscaf	16099671	0	0	0	0	BTP20220	0	Tissue specific
DDDEPP	Superscaf	-	Superscaf	16160719	16160736	-372	372	377	-	-	-	-	-	-	NCP	IGR	-	ID=gene-E	6	4.121666667	2334.8	0.701913369	D(+42.01)	74	365.1271	2	728.2501	Acetylati	L	Superscaf	16161339	0	BTP20220	0	0	0	0	Tissue specific
FEMHPF	Superscaf	-	Superscaf	16214791	16214808	-5	5	10	-	-	gene	-	-	mRNA	NCP	Intron	gene|mRN	ID=gene-E	6	5.636666667	2528.9	0.700916005	FEMHPF	65	807.3517	1	806.3421	.	V	Superscaf	16215027	0	0	0	0	0	BTP20220	Tissue specific
HCKPK	Superscaf	+	Superscaf	16222531	16222545	5	5	9	-	-	gene	-	-	mRNA	NCP	Intron	gene|mRN	ID=gene-E	5	7.678	2479.6	0.700773295	H(+42.01)	83	356.1833	2	710.3534	Acetylati	L	Superscaf	16222519	0	0	BTP20220	0	0	0	Tissue specific
KAGEKTH	Superscaf	+	Superscaf	16297180	16297200	2	2	8	-	-	-	-	-	-	NCP	IGR	-	ID=gene-E	7	6.972857143	2988.1	0.699396275	KAGEKTH	69	385.7151	2	769.4082	.	P	Superscaf	16297177	0	0	0	0	0	BTP20220	Tissue specific
LPAGAHC	Superscaf	-	Superscaf	16340770	16340790	-25	25	31	-	-	gene	-	-	mRNA	NCP	Intron	gene|mRN	ID=gene-E	7	6.128571429	2816.1	0.698592252	LPAGAHC(+	56	725.3509	1	724.3326	Carbamidc	R	Superscaf	16340814	0	0	BTP20220	0	0	0	Tissue specific

APVLPPLP	Superscaf	-	Superscaf	16356103	16356126	-16	16	23	-	-	gene	-	-	mRNA	NCP	Intron	gene|mRN	ID=gene-E	8	5.3975	2367.7	0.698309378	A(+42.01)	66	423.2595	2	844.5058	Acetylati	F	Superscaf	16356132	0	BTP202209	0	0	0	0	Tissue specific
AMWYM	Superscaf	-	Superscaf	16370092	16370106	-21	21	25	-	-	gene	-	-	mRNA	NCP	Intron	gene|mRN	ID=gene-E	5	5.814	2040.4	0.698051515	AMWYM	50	701.2754	1	700.2713	.	K	Superscaf	16370130	0	BTP202209	0	0	0	0	Tissue specific
KSFSPLPP	Superscaf	-	Superscaf	16423108	16423131	-17	17	24	-	-	gene	-	-	mRNA	NCP	Intron	gene|mRN	ID=gene-E	8	6.4325	2988.2	0.697073463	KSFSPLPP	65	436.7476	2	871.4803	.	Q	Superscaf	16423152	0	0	BTP202209	0	0	0	Tissue specific
KFSVTH	Superscaf	-	Superscaf	16428112	16428129	-19	19	24	-	-	gene	-	-	mRNA	NCP	Intron	gene|mRN	ID=gene-E	6	5.836666667	2256.4	0.696981274	KFSVTH	78	359.6978	2	717.381	.	N	Superscaf	16428510	BTP202209	0	0	0	0	0	Tissue specific
KHLADGF	Superscaf	-	Superscaf	16445062	16445082	-8	8	14	-	exon	gene	three_pri	-	mRNA	NCP	3UTR	exon|gene	ID=exon-E	7	6.25	2849	0.696668574	KHLADGF	53	394.2076	2	786.4024	.	A	Superscaf	16445130	0	0	BTP202209	0	0	0	Tissue specific
TLRDLKY	Superscaf	+	Superscaf	16464475	16464495	24	24	30	-	-	gene	-	-	mRNA	NCP	Intron	gene|mRN	ID=gene-E	7	6.802857143	3092.5	0.696310499	TLRDLKY	94	454.7618	2	907.5127	.	C	Superscaf	16464406	0	0	0	0	BTP202209	0	Tissue specific
ALGSDVLL	Superscaf	+	Superscaf	16494679	16494702	11	11	18	-	-	-	-	-	-	NCP	IGR	-	ID=gene-E	8	4.8225	2463.7	0.695753328	ALGSDVLL	58	787.4523	1	786.4487	.	K	Superscaf	16494649	BTP202209	0	0	0	0	0	Tissue specific
GEPLR	Superscaf	-	Superscaf	16532707	16532721	-22	22	26	-	-	gene	-	-	mRNA	NCP	Intron	gene|mRN	ID=gene-E	5	6.446	2062.4	0.695052063	G(+42.01)	68	613.3297	1	612.3231	Acetylati	N	Superscaf	16532919	0	0	0	0	0	BTP202209	Tissue specific
ELSEP	Superscaf	+	Superscaf	16534495	16534509	3	3	7	-	-	gene	-	-	mRNA	NCP	Intron	gene|mRN	ID=gene-E	5	4.88	1925.2	0.695019083	ELSEP	63	574.2814	1	573.2646	.	L	Superscaf	16534489	0	0	0	0	BTP202209	0	Tissue specific
YSVPVEF	Superscaf	-	Superscaf	16640656	16640676	-17	17	23	-	-	gene	-	-	mRNA	NCP	Intron	gene|mRN	ID=gene-E	7	3.762857143	1993.2	0.693060821	YSVPVEF	82	840.4145	1	839.4065	.	L	Superscaf	16640727	0	BTP202209	BTP202209	0	0	0	Tissue specific
LPPPPLK	Superscaf	+	Superscaf	16724959	16724979	20	20	26	-	-	gene	-	-	mRNA	NCP	Intron	gene|mRN	ID=gene-E	7	6.7	2524.8	0.691505842	LPPPPLK	99	381.2474	2	760.4847	.	T	Superscaf	16724902	0	BTP202209	0	0	0	0	Tissue specific
LPDLPPPP	Superscaf	-	Superscaf	16879669	16879692	-7	7	14	-	-	gene	-	-	mRNA	NCP	Intron	gene|mRN	ID=gene-E	8	5.80375	2805.3	0.688652143	LPDLPPPP	55	845.483	1	844.4694	.	I	Superscaf	16879833	0	BTP202209	0	0	0	0	Tissue specific
DNAPVLK	Superscaf	+	Superscaf	16951744	16951764	79	79	85	-	-	gene	-	-	mRNA	NCP	Intron	gene|mRN	ID=gene-E	7	5.202857143	2478.7	0.687322767	DNAPVLK	79	378.7206	2	755.4177	.	K	Superscaf	16951510	0	0	0	0	0	BTP202209	Tissue specific
NSLLRPR	Superscaf	+	Superscaf	16983622	16983642	49	49	55	-	-	gene	-	-	mRNA	NCP	Intron	gene|mRN	ID=gene-E	7	7.267142857	3321.9	0.686734773	NSLLRPR	72	428.2646	2	854.5086	.	C	Superscaf	16983478	0	0	BTP202209	0	0	0	Tissue specific
APELLSK	Superscaf	-	Superscaf	17018908	17018928	-27	27	33	-	-	-	-	-	-	NCP	IGR	-	ID=gene-E	7	6.131428571	2632.8	0.686083919	APELLSK	55	757.4432	1	756.4381	.	N	Superscaf	17018964	0	0	0	0	BTP202209	0	Tissue specific
RAGGPRPP	Superscaf	-	Superscaf	17039665	17039688	-373	373	380	-	-	-	-	-	-	NCP	IGR	-	ID=gene-E	8	7.2975	3215.7	0.685700998	RAGGPRPP	81	404.2311	2	806.451	.	I	Superscaf	17039694	BTP202209	0	0	0	0	0	Tissue specific
KPFYSP	Superscaf	+	Superscaf	17070292	17070309	68	68	73	-	-	-	-	-	-	NCP	IGR	-	ID=gene-E	6	6.526666667	2373.5	0.685136191	KPFYSP	51	738.3954	1	737.3748	.	L	Superscaf	17070091	0	0	0	0	BTP202209	BTP202209	Tissue specific
MLSLAKF	Superscaf	+	Superscaf	17077165	17077185	6	6	12	-	-	-	-	-	-	NCP	IGR	-	ID=gene-E	7	6.375714286	2628.8	0.685009362	M(+42.01)	75	426.244	2	850.4622	Acetylati	Q	Superscaf	17077150	0	0	0	0	BTP202209	0	Tissue specific
VAGPPHL	Superscaf	-	Superscaf	17159947	17159967	-3	3	9	-	-	gene	-	-	mRNA	NCP	Intron	gene|mRN	ID=gene-E	7	5.451428571	2256.5	0.683482438	V(+42.01)	69	366.7063	2	731.3966	Acetylati	G	Superscaf	17159985	BTP202209	0	0	0	0	0	Tissue specific
DSVGPK	Superscaf	-	Superscaf	17200384	17200401	-33	33	38	-	-	gene	-	-	mRNA	NCP	Intron	gene|mRN	ID=gene-E	6	5.11	1898	0.682736629	D(+42.01)	68	644.3121	1	643.3177	Acetylati	W	Superscaf	17200527	0	0	0	0	BTP202209	0	Tissue specific
VATVSPLR	Superscaf	+	Superscaf	17271196	17271219	28	28	35	-	-	gene	-	-	mRNA	NCP	Intron	gene|mRN	ID=gene-E	8	5.15875	2431.8	0.681430383	VATVSPLR	81	421.7578	2	841.5021	.	V	Superscaf	17271115	BTP202209	0	0	0	0	0	Tissue specific
HSLLVT	Superscaf	+	Superscaf	17302828	17302845	51	51	56	-	-	gene	-	-	mRNA	NCP	Intron	gene|mRN	ID=gene-E	6	5.293333333	2107.4	0.680847037	HSLLVT	97	669.3917	1	668.3857	.	T	Superscaf	17302678	0	BTP202209	0	0	BTP202209	0	Mixed
EAFKENE	Superscaf	-	Superscaf	17413471	17413491	-7	7	13	-	-	gene	-	-	mRNA	NCP	Intron	gene|mRN	ID=gene-E	7	5.187142857	3040.3	0.678806159	EAFKENE	64	866.3976	1	865.3817	.	Y	Superscaf	17413494	0	0	0	0	BTP202209	BTP202209	Tissue specific
DGKLP	Superscaf	+	Superscaf	17444665	17444679	27	27	31	-	-	gene	-	-	mRNA	NCP	Intron	gene|mRN	ID=gene-E	5	6.192	1868	0.678230893	D(+42.01)	71	571.3076	1	570.3013	Acetylati	T	Superscaf	17444587	0	BTP202209	0	0	0	0	Tissue specific
MLDWYT	Superscaf	+	Superscaf	17508811	17508828	6	6	11	-	-	gene	-	-	mRNA	NCP	Intron	gene|mRN	ID=gene-E	6	5.463333333	2520	0.677047657	MLDWYT	53	414.683	2	827.3524	.	S	Superscaf	17508796	0	0	0	BTP202209	0	0	Tissue specific
KDSTVP	Superscaf	-	Superscaf	17557324	17557341	-19	19	24	-	-	gene	-	-	mRNA	NCP	Intron	gene|mRN	ID=gene-E	6	5.203333333	1998.2	0.676152829	KDSTVP	55	646.3404	1	645.3333	.	A	Superscaf	17557356	0	0	0	0	0	BTP202209	Tissue specific
NALQFR	Superscaf	+	Superscaf	17559877	17559894	11	11	16	-	-	gene	-	-	mRNA	NCP	Intron	gene|mRN	ID=gene-E	6	6.55	2908.3	0.676105739	NALQFR	68	374.7115	2	747.4028	.	H	Superscaf	17559847	0	0	0	0	0	BTP202209	Tissue specific
EVAAP	Superscaf	-	Superscaf	17734294	17734308	-26	26	30	-	-	gene	-	-	mRNA	NCP	Intron	gene|mRN	ID=gene-E	5	4.312	1385.5	0.672888653	EVAAP	64	486.2568	1	485.2485	.	L	Superscaf	17734353	0	0	0	0	0	BTP202209	Tissue specific
KQVTAF	Superscaf	-	Superscaf	17767855	17767872	-1	1	6	-	-	-	-	-	-	NCP	IGR	-	ID=gene-E	6	5.57	2175.3	0.672269561	KQVTAF	70	693.3903	1	692.3857	.	K	Superscaf	17768040	0	0	0	0	0	BTP202209	Tissue specific
RLVALLH	Superscaf	+	Superscaf	17948809	17948829	50	50	56	-	-	-	-	-	-	NCP	IGR	-	ID=gene-E	7	6.044285714	2640	0.668931788	RLVALLH	93	411.2641	2	820.5283	.	I	Superscaf	17948662	0	0	0	0	0	BTP202209	Tissue specific
FAPLTS	Superscaf	-	Superscaf	18052309	18052326	-11	11	16	-	-	-	-	-	-	NCP	IGR	-	ID=gene-E	6	5.998333333	2170.4	0.667022774	FAPLTS	76	635.3367	1	634.3326	.	V	Superscaf	18052488	0	BTP202209	0	0	0	0	Mixed
LLQAELL	Superscaf	+	Superscaf	18238333	18238353	49	49	55	-	-	-	-	-	-	NCP	IGR	-	ID=gene-E	7	5.544285714	2761.1	0.663591485	LLQAELL	50	400.245	2	798.485	.	G	Superscaf	18238189	0	0	0	0	0	BTP202209	Tissue specific
CAASLPPPF	Superscaf	-	Superscaf	18413263	18413289	-27	27	35	-	-	-	-	-	-	NCP	IGR	-	ID=gene-E	9	5.991111111	3294.7	0.66036477	C(+57.02)	57	455.2353	2	908.4426	Carbamido	F	Superscaf	18413388	0	0	0	0	0	BTP202209	Tissue specific
AGHSKSQ	Superscaf	-	Superscaf	18431878	18431898	-3	3	9	-	-	-	-	-	-	NCP	IGR	-	ID=gene-E	7	6.618571429	3074.2	0.660021525	A(+42.01)	51	756.3656	1	755.3562	Acetylati	L	Superscaf	18431898	0	0	BTP202209	0	0	0	Tissue specific
NEHAPQ	Superscaf	-	Superscaf	18456349	18456366	-3	3	8	-	-	-	-	-	-	NCP	IGR	-	ID=gene-E	6	5.698333333	2901.3	0.65957021	NEHAPQ	77	695.3185	1	694.3034	.	P	Superscaf	18456528	0	0	BTP202209	0	0	0	Tissue specific
PPPPVHQK	Superscaf	+	Superscaf	18486268	18486291	24	24	31	-	-	-	-	-	-	NCP	IGR	-	ID=gene-E	8	6.0225	3048.4	0.65901824	PPPPVHQK	73	450.2574	2	898.5024	.	L	Superscaf	18486199	0	0	0	0	0	BTP202209	Tissue specific
KRPLLNF	Superscaf	+	Superscaf	18498838	18498858	14	14	20	-	-	-	-	-	-	NCP	IGR	-	ID=gene-E	7	7.092857143	3145.5	0.65878644	KRPLLNF	63	444.2799	2	886.5388	.	I	Superscaf	18498799	0	0	0	BTP202209	0	0	Tissue specific
QAYGRGP	Superscaf	-	Superscaf	18543655	18543675	-7	7	13	-	-	-	-	-	-	NCP	IGR	-	ID=gene-E	7	6.618571429	2962.3	0.657959785	Q(+42.01)	62	395.689	2	789.377	Acetylati	R	Superscaf	18543705	0	0	0	0	BTP202209	0	Tissue specific
KNSLLTTH	Superscaf	+	Superscaf	18614749	18614772	4	4	11	-	-	-	-	-	-	NCP	IGR	-	ID=gene-E	8	6.68	3570	0.656648393	KNSLLTTH	52	457.2636	2	912.5029	.	A	Superscaf	18614740	BTP202209	0	0	0	0	0	Tissue specific
VMTYL	Superscaf	+	Superscaf	18626974	18626988	122	122	126	-	-	-	-	-	-	NCP	IGR	-	ID=gene-E	5	4.784	1581.9	0.656423068	VMTYL	99	626.3196	1	625.3145	.	L	Superscaf	18626611	0	0	BTP202209	0	0	0	Tissue specific
LDDPPR	Superscaf	+	Superscaf	18633376	18633393	4	4	9	-	-	-	-	-	-	NCP	IGR	-	ID=gene-E	6	5.88	2500	0.656304927	LDDPPR	73	356.6836	2	711.3551	.	R	Superscaf	18633367	0	0	BTP202209	0	0	0	Tissue specific
DGDGP	Superscaf	+	Superscaf	18637432	18637446	8	8	12	-	-	-	-	-	-	NCP	IGR	-	ID=gene-E	5	4.836	1755	0.656230169	DGDGP	58	460.1669	1	459.1601	.	V	Superscaf	18637411	0	BTP202209	0	0	0	0	Tissue specific
KPSYPYK	Superscaf	+	Superscaf	18716746	18716766	2	2	8	-	-	-	-	-	-	NCP	IGR	-	ID=gene-E	7	7.011428571	2914	0.654767102	KPSYPYK	53	441.7377	2	881.4647	.	E	Superscaf	18716743	0	0	0	0	BTP202209	0	Tissue specific
PDTASRP	Superscaf	-	Superscaf	18727957	18727977	-13	13	19	-	-	-	-	-	-	NCP	IGR	-	ID=gene-E	7	6.365714286	2827.3	0.654560314	P(+42.01)	83	393.1973	2	784.3715	Acetylati	A	Superscaf	18728064	0	BTP202209	0	0	0	0	Tissue specific
VALPYLK	Superscaf	+	Superscaf	18735388	18735408	1	1	7	-	-	-	-	-	-	NCP	IGR	-	ID=gene-E	7	5.668571429	2283.4	0.654423248	V(+42.01)	77	423.2573	2	844.5058	Acetylati	V	Superscaf	18735388	0	BTP202209	0	0	0	0	Tissue specific
GFETSVLQK	Superscaf	-	Superscaf	18743110	18743136	-18	18	26	-	-	-	-	-	-	NCP	IGR	-	ID=gene-E	9	5.361111111	3323.6	0.654280704	G(+42.01)	70	525.7758	2	1049.539	Acetylati	I	Superscaf	18743175	BTP202209	0	0	0	0	0	Tissue specific
MVQQPR	Superscaf	+	Superscaf	18799393	18799410	3	3	8	-	-	-	-	-	-	NCP	IGR	-	ID=gene-E	6	5.685	2579.1	0.653242724	MVQQPR	52	379.7094	2	757.3905	.	E	Superscaf	18799387	BTP202209	0	0	0	0	0	Tissue specific
MLSLQLK	Superscaf	-	Superscaf	18904048	18904068	-2	2	8	-	-	-	-	-	-	NCP	IGR	-	ID=gene-E	7	6.394285714	2894.2	0.651312295	MLSLQLK	70	832.4942	1	831.4888	.	A	Superscaf	18904110	0	0	0	0	BTP202209	0	Tissue specific
ELGPAY	Superscaf	-	Superscaf	18964483	18964500	-112	112	117	-	-	-	-	-	-	NCP	IGR	-	ID=gene-E	6	5.525	2184.4	0.65019762	ELGPAY	73	649.3171	1	648.3118	.	S	Superscaf	18964635	0	0	BTP202209	0	0	0	Tissue specific
LPAKLAK	Superscaf	-	Superscaf	18976693	18976713	-4	4	10	-	-	-	-	-	-	NCP	IGR	-	ID=gene-E	7	7.111428571	2603.6	0.64997235	L(+42.01)	50	391.7578	2	781.5062	Acetylati	Q	Superscaf	18976755	0	0	BTP202209	0	0	0	Tissue specific
KSLAFAF	Superscaf	-	Superscaf	18994285	18994305	-30	30	36	-	-	-	-	-	-	NCP	IGR	-	ID=gene-E	7	6.342857143	2602.6	0.649647863	KSLAFAF	56	392.2262	2	782.4326	.	A	Superscaf	18994452	0	BTP202209	0	0	0	0	Tissue specific
KTTKAVGP	Superscaf	-	Superscaf	18997588	18997611	-8	8	15	-	-	-	-	-	-	NCP	IGR	-	ID=gene-E	8	6.35375	2677.8	0.649586884	KTTKAVGP	66	401.2391	2	800.4756	.	E	Superscaf	18997737	0	0	0	BTP202209	0	0	Tissue specific
KMSFTP	Superscaf	+	Superscaf	19038292	19038309	2	2	7	-	-	-	-	-	-	NCP	IGR	-	ID=gene-E	6	6.58	2345.6	0.648836205	KM(+15.99	52	363.6832	2	725.3418	Oxidation	S	Superscaf	19038289	BTP202209	0	0	0	0	0	Tissue specific
KPDKLGK	Superscaf	-	Superscaf	19039882	19039902	-1	1	7	-	-	-	-	-	-	NCP	IGR	-	ID=gene-E	7	7.205714286	2804.8	0.648806821	KPDKLGK	56	393.2462	2	784.4807	.	K	Superscaf	19039914	0	BTP202209	0	0	0	0	Tissue specific
KYLLPP	Superscaf	+	Superscaf	19082797	19082814	13	13	18	-	-	-	-	-	-	NCP	IGR	-	ID=gene-E	6	6.66	2309.5	0.648015305	KYLLPP	66	365.7271	2	729.4424	.	I	Superscaf	19082761	0	0	0	0	BTP202209	0	Tissue enhanced
LSVAPSPP	Superscaf	-	Superscaf	19113805	19113828	-16	16	23	-	-	-	-	-	-	NCP	IGR	-	ID=gene-E	8	5.2825	2443.7	0.647443248	LSVAPSPP	65	767.4263	1	766.4225	.	I	Superscaf	19113894	0	0	0	0	BTP202209	0	Tissue specific
TVREVPT	Superscaf	-	Superscaf	19169620	19169640	-52	52	58	-	-	-	-	-	-	NCP	IGR	-	ID=gene-E	7	4.762857143	2206.7	0.646413789	T(+42.01)	67	843.4586	1	842.4498	Acetylati	C	Superscaf	19169640	0	0	0	0	BTP202209	0	Tissue specific
KLFLASNL	Superscaf	-	Superscaf	19175218	19175241	-57	57	64	-	-	-	-	-	-	NCP	IGR	-	ID=gene-E	8	6.28375	3207.4	0.646310478	K(+42.01)	67	474.2896	2	946.5488	Acetylati	K	Superscaf	19175289	0	0	0	BTP202209	0	0	Tissue specific
LDLKEF	Superscaf	+	Superscaf	19206727	19206744	6	6	11	-	-	-	-	-	-	NCP	IGR	-	ID=gene-E	6	5.561666667	2399.6	0.645729402	LDLKEF	80	764.4165	1	763.4116	.	S	Superscaf	19206712	0	BTP202209	0	0	0	0	Tissue specific
NKVAVLG	Superscaf	-	Superscaf	19255429	19255449	-21	21	27	-	-	-	-	-	-	NCP	IGR	-	ID=gene-E	7	4.731428571	2027.1	0.644831032	NKVAVLG	89	700.4338	1	699.4279	.	S	Superscaf	19255491	0	0	BTP202209	0	0	0	Mixed
KGKLY	Superscaf	+	Superscaf	19265632	19265646	9	9	13	-	-	-	-	-	-	NCP	IGR	-	ID=gene-E	5	7.418	2047	0.644642947	K(+42.01)	57	650.3847	1	649.3799	Acetylati	N	Superscaf	19265608	0	0	0	0	0	BTP202209	Tissue specific
AASSYG	Superscaf	-	Superscaf	19338370	19338387	-15	15	20	-	-	-	-	-	-	NCP	IGR	-	ID=gene-E	6	5.838333333	2146.2	0.643301231	AASSYG	64	555.2424	1	554.2336	.	T	Superscaf	19338450	0	0	0	0	0	BTP202209	Tissue specific
AGLLLPK	Superscaf	-	Superscaf	19346116	19346136	-11	11	17	-	-	-	-	-	-	NCP	IGR	-	ID=gene-E	7	6.567142857	2474.6	0.6431583	AGLLLPK	52	356.2365	2	710.4691	.	G	Superscaf	19346166	0	0	0	0	BTP202209	0	Tissue specific
ADKPDLG	Superscaf	-	Superscaf	19390093	19390113	-13	13	19	-	-	-	-	-	-	NCP	IGR	-	ID=gene-E	7	5.707142857	2590.8	0.642347139	A(+42.01)	96	379.1895	2	756.3654	Acetylati	L	Superscaf	19390128	0	0	BTP202209	0	0	0	Mixed
ADKPDL	Superscaf	-	Superscaf	19390093	19390110	-13	13	18	-	-	-	-	-	-	NCP	IGR	-	ID=gene-E	6	5.663333333	2293.5	0.642347195	A(+42.01)	99	350.6783	2	699.3439	Acetylati	L	Superscaf	19390128	0	0	0	BTP202209	0	0	Mixed
LVCEFK	Superscaf	-	Superscaf	19423303	19423320	-68	68	73	-	-	-	-	-	-	NCP	IGR	-	ID=gene-E	6	4.906666667	2220.4	0.641734632	LVC(+57.0	56	795.4018	1	794.3997	Carbamido	A	Superscaf	19423326	0	0	0	0	0	BTP202209	Tissue specific
LSLPVFR	Superscaf	-	Superscaf	19437850	19437870	-40	40	46	-	-	-	-	-	-	NCP	IGR	-	ID=gene-E	7	5.74	2463.8	0.641466256	LSLPVFR	95	416.2562	2	830.5014	.	V	Superscaf	19437948	0	0	BTP202209	0	0	0	Tissue specific
DEPWAF	Superscaf	-	Superscaf	19502833	19502850	-25	25	30	-	-	-	-	-	-	NCP	IGR	-	ID=gene-E	6	4.98	2399.7	0.640267692	DEPWAF	75	764.3228	1	763.3177	.	T	Superscaf	19502910	0	0	0	0	BTP202209	0	Mixed
GPGPAPP	Superscaf	-	Superscaf	19557376	19557396	-170	170	176	-	-	-	-	-	-	NCP	IGR	-	ID=gene-E	7	6.165714286	2255.5	0.639261585	GPGPAPP	53	592.2989	1	591.3016	.	V	Superscaf	19557891	0	0	0	0	0	BTP202209	Tissue specific
LLGGPLWG	Superscaf	+	Superscaf	19586038	19586061	21	21	28	-	-	-	-	-	-	NCP	IGR	-	ID=gene-E	8	6.005	2816.1	0.638732856	LLGGPLWG	84	812.4528	1	811.4592	.	R	Superscaf	19585978	0	BTP202209	0	0	0	0	Tissue specific
PPGPPPGP	Superscaf	-	Superscaf	19590439	19590462	-186	186	193	-	-	-	-	-	-	NCP	IGR	-	ID=gene-E	8	6.2175	2619	0.638651679	PPGPPPGP	86	358.1908	2	714.3701	.	P	Superscaf	19590747	BTP202209	0	0	0	0	0	Tissue enhanced
CDSSD	Superscaf	+	Superscaf	19694089	19694103	1	1	5	-	-	-	-	-	-	NCP	IGR	-	ID=gene-E	5	4.464	2175.5	0.636740009	C(+57.02)	58	625.174	1	624.1697	Carbamido	C	Superscaf	19694089	0	0	0	0	0	BTP202209	Tissue specific
SPFKQP	Superscaf	-	Superscaf	19737763	19737780	-55	55	60	-	-	-	-	-	-	NCP	IGR	-	ID=gene-E	6	6.525	2524.7	0.635934382	S(+42.01)	68	373.1987	2	744.3806	Acetylati	C	Superscaf	19737909	0	0	0	BTP202209	0	0	Tissue specific
VGPALRP	Superscaf	-	Superscaf	19977697	19977717	-105	105	111	-	-	-	-	-	-	NCP	IGR	-	ID=gene-E	7	5.904285714	2285.6	0.631508716	VGPALRP	57	355.2206	2	708.4282	.	V	Superscaf	19977921	BTP202209	0	0	0	0	0	Tissue specific
LYLDAGK	Superscaf	+	Superscaf	20020714	20020734	4	4	10	-	-	-	-	-	-	NCP	IGR	-	ID=gene-E	7	6.045714286	2654.8	0.630715263	L(+42.01)	83	411.2275	2	820.433	Acetylati	K	Superscaf	20020705	BTP202209	0	0	0	0	0	Tissue specific
VLAAC	Superscaf	-	Superscaf	20032039	20032053	-92	92	96	-	-	-	-	-	-	NCP	IGR	-	ID=gene-E	5	4.608	1561.7	0.630506482	VLAAC(+57	63	533.2786	1	532.2679	Carbamido	R	Superscaf	20032137	0	0	0	BTP202209	0	0	Tissue specific
MGPGMGP	Superscaf	+	Superscaf	20084332	20084352	20	20	26	-	-	-	-	-	-	NCP	IGR	-	ID=gene-E	7	6.001428571	2309.7	0.629541821	MGPGMGP	55	646.2665	1	645.2615	.	S	Superscaf	20084275	0	0	0	0	0	BTP202209	Tissue specific
KPKFPHTF	Superscaf	-	Superscaf	20106289	20106312	-8	8	15	-	-	-	-	-	-	NCP	IGR	-	ID=gene-E	8	7.145	3403.6	0.629136767	KPKFPHTF	53	501.29	2	1000.549	.	S	Superscaf	20106372	0	0	0	0	0	BTP202209	Tissue specific
LSVVTPGP	Superscaf	-	Superscaf	20120467	20120490	-15	15	22	-	-	-	-	-	-	NCP	IGR	-	ID=gene-E	8	4.595	2106.4	0.628875252	LSVVTPGP	68	385.2237	2	768.4381	.	A	Superscaf	20120826	0	0	0	BTP202209	0	0	Tissue specific
FRGGPLAK	Superscaf	+	Superscaf	20190088	20190111	59	59	66	-	-	-	-	-	-	NCP	IGR	-	ID=gene-E	8	7.0275	3101.3	0.627591084	FRGGPLAK	70	423.2533	2	844.4919	.	H	Superscaf	20189914	0	0	0	0	0	BTP202209	Tissue specific
KAPEC	Superscaf	+	Superscaf	20191240	20191254	37	37	41	-	-	-	-	-	-	NCP	IGR	-	ID=gene-E	5	6.06	2128.3	0.627570002	KAPEC(+57	57	604.2863	1	603.2686	Carbamido	I	Superscaf	20191132	BTP202209	BTP202209	BTP202209	BTP202209	0	0	Tissue specific
KDAYVRK	Superscaf	+	Superscaf	20206417	20206437	52	52	58	-	-	-	-	-	-	NCP	IGR	-	ID=gene-E	7	6.412857143	2767.9	0.627289949	KDAYVRK	51	440.2596	2	878.4974	.	F	Superscaf	20206264	BTP202209	0	0	BTP202209	0	0	Tissue specific
PPSVTFL	Superscaf	-	Superscaf	20274451	20274471	-19	19	25	-	-	-	-	-	-	NCP	IGR	-	ID=gene-E	7	5.181428571	2196.5	0.626035055	PPSVTFL	50	380.7206	2	759.4167	.	A	Superscaf	20274501	0	0	0	BTP202209	0	0	Tissue specific
KNRFR	Superscaf	+	Superscaf	20315455	20315469	33	33	37	-	-	-	-	-	-	NCP	IGR	-	ID=gene-E	5	8.43	2750.1	0.625278842	KNRFR	54	360.7232	2	719.4191	.	L	Superscaf	20315359	0	0	0	0	0	BTP202209	Tissue specific
KPEVPGP	Superscaf	-	Superscaf	20330935	20330955	-76	76	82	-	-	-	-	-	-	NCP	IGR	-	ID=gene-E	7	5.404285714	2203.4	0.624993201	KPEVPGP	54	362.2035	2	722.3962	.	P	Superscaf	20330958	0	0	0	BTP202209	0	0	Tissue specific
DPRVAPR	Superscaf	+	Superscaf	20359198	20359218	92	92	98	-	-	-	-	-	-	NCP	IGR	-	ID=gene-E	7	6.158571429	2695.2	0.624471886	DPRVAPR	53	405.7289	2	809.4507	.	Q	Superscaf	20358925	0	0	0	0	0	BTP202209	Tissue specific
KTDGP	Superscaf	+	Superscaf	20447413	20447427	27	27	31	-	-	gene	-	-	mRNA	NCP	Intron	gene|mRN	ID=gene-E	5	6.302	1912.1	0.622844861	KTDGP	56	517.2582	1	516.2544	.	N	Superscaf	20447335	0	0	0	0	BTP202209	BTP202209	Tissue specific
AAGPLAQR	Superscaf	+	Superscaf	20451571	20451594	52	52	59	-	-	gene	-	-	mRNA	NCP	Intron	gene|mRN	ID=gene-E	8	6.59	3181.5	0.622768	AAGPLAQR	60	392.2308	2	782.4399	.	K	Superscaf	20451418	0	0	0	0	BTP202209	0	Tissue specific
EAARLKP	Superscaf	+	Superscaf	20465224	20465244	26	26	32	-	-	gene	-	-	mRNA	NCP	Intron	gene|mRN	ID=gene-E	7	6.862857143	2856.1	0.622516225	EAARLKP	50	392.7356	2	783.4603	.	I	Superscaf	20465149	0	0	0	0	BTP202209	0	Tissue specific
EETPG	Superscaf	-	Superscaf	20479987	20480001	-1	1	5	-	-	gene	-	-	mRNA	NCP	Intron	gene|mRN	ID=gene-E	5	5.048	1883.2	0.62224403	EETPG	53	532.2317	1	531.2177	.	E	Superscaf	20480106	0	BTP202209	0	0	0	0	Tissue specific
DVFLKSH	Superscaf	+	Superscaf	20493445	20493465	1	1	7	-	-	gene	-	-	mRNA	NCP	Intron	gene|mRN	ID=gene-E	7	5.348571429	2623.8	0.621995685	D(+42.01)	59	444.2414	2	886.4548	Acetylati	D	Superscaf	20493445	BTP202209	0	0	0	0	0	Tissue specific
MDLEKV	Superscaf	+	Superscaf	20531011	20531028	2	2	7	-	-	gene	-	-	mRNA	NCP	Intron	gene|mRN	ID=gene-E	6	4.61	2030.3	0.621302831	M(+42.01)	99	776.3817	1	775.3786	Acetylati	H	Superscaf	20531008	0	BTP202209	0	0	BTP202209	BTP202209	Tissue specific
KDKLGDLL	Superscaf	+	Superscaf	20534350	20534373	1	1	8	-	-	gene	-	-	mRNA	NCP	Intron	gene|mRN	ID=gene-E	8	6.16625	3117.3	0.621241132	K(+42.01)	65	472.2749	2	942.5386	Acetylati	K	Superscaf	20534350	0	0	0	BTP202209	0	0	Tissue specific
KMMMVK	Superscaf	+	Superscaf	20746564	20746581	1	1	6	-	-	-	-	-	-	NCP	IGR	-	ID=gene-E	6	6.121666667	2051.3	0.617326931	KM(+15.99	51	400.1958	2	798.3802	Oxidation	K	Superscaf	20746564	0	0	0	BTP202209	0	0	Tissue specific
EPVGL	Superscaf	+	Superscaf	20747074	20747088	45	45	49	-	-	-	-	-	-	NCP	IGR	-	ID=gene-E	5	4.294	1413.6	0.61731758	EPVGL	55	514.2841	1	513.2798	.	A	Superscaf	20746942	0	0	0	0	0	BTP202209	Tissue specific
KGSYF	Superscaf	+	Superscaf	20750722	20750736	12	12	16	-	-	-	-	-	-	NCP	IGR	-	ID=gene-E	5	6.506	1996	0.617250292	KGSYF	59	601.2959	1	600.2907	.	E	Superscaf	20750689	0	BTP202209	0	0	0	0	Tissue specific
WKGPSH	Superscaf	-	Superscaf	20763979	20763996	-2	2	7	-	-	-	-	-	-	NCP	IGR	-	ID=gene-E	6	6.861666667	2632.8	0.61700571	W(+42.01)	54	753.3721	1	752.3605	Acetylati	L	Superscaf	20764110	0	0	0	0	BTP202209	0	Tissue specific
WTFFK	Superscaf	-	Superscaf	20772760	20772774	-7	7	11	-	-	-	-	-	-	NCP	IGR	-	ID=gene-E	5	6.624	2167.3	0.616843798	WTFFK	80	728.3768	1	727.3693	.	N	Superscaf	20772774	0	BTP202209	0	0	0	0	Tissue specific
LAATFPF	Superscaf	-	Superscaf	20913103	20913123	-4	4	10	-	-	-	-	-	-	NCP	IGR	-	ID=gene-E	7	5.972857143	2485.7	0.614255045	LAATFPF	63	766.4022	1	765.4061	.	T	Superscaf	20913147	0	BTP202209	0	0	0	0	Tissue specific
PAGKPAAP	Superscaf	-	Superscaf	20917201	20917224	-46	46	53	-	-	-	-	-	-	NCP	IGR	-	ID=gene-E	8	6.58375	2711.8	0.614179402	PAGKPAAP	51	354.7	2	707.3965	.	L	Superscaf	20917269	0	BTP202209	BTP202209	0	0	0	Tissue specific
PVEEK	Superscaf	-	Superscaf	20944852	20944866	-55	55	59	-	-	gene	-	-	mRNA	NCP	Intron	gene|mRN	ID=gene-E	5	4.496	1656.8	0.613669542	PVEEK	90	601.3225	1	600.3118	.	P	Superscaf	20944875	0	BTP202209	0	0	0	0	Tissue specific
KMSLVLF	Superscaf	+	Superscaf	20958250	20958270	7	7	13	-	-	gene	-	-	mRNA	NCP	Intron	gene|mRN	ID=gene-E	7	5.515714286	2317.5	0.613422303	KM(+15.99	59	427.2492	2	852.4779	Oxidation	F	Superscaf	20958232	BTP202209	0	0	BTP202209	0	0	Tissue specific
DPLVP	Superscaf	-	Superscaf	20960440	20960454	-10	10	14	-	-	gene	-	-	mRNA	NCP	Intron	gene|mRN	ID=gene-E	5	4.31	1439.7	0.613382019	DPLVP	51	540.3027	1	539.2955	.	L	Superscaf	20960457	0	BTP202209	0	0	0	0	Tissue specific
GVVGGGPP	Superscaf	-	Superscaf	20974426	20974449	-91	91	98	-	-	gene	-	-	mRNA	NCP	Intron	gene|mRN	ID=gene-E	8	4.56	1864	0.61312388	GVVGGGPP	51	639.3342	1	638.3387	.	Q	Superscaf	20974458	0	BTP202209	0	BTP202209	0	BTP202209	Tissue specific
ARFTKK	Superscaf	+	Superscaf	21050578	21050595	7	7	12	-	-	gene	-	-	mRNA	NCP	Intron	gene|mRN	ID=gene-E	6	8.045	2681.8	0.611719358	ARFTKK	50	375.736	2	749.4548	.	E	Superscaf	21050560	0	0	0	0	0	BTP202209	Tissue specific
KDVASY	Superscaf	+	Superscaf	21078364	21078381	26	26	31	-	-	gene	-	-	mRNA	NCP	Intron	gene|mRN	ID=gene-E	6	5.011666667	2034.1	0.611206842	KDVASY	54	682.3409	1	681.3333	.	Y	Superscaf	21078289	0	0	0	0	BTP202209	0	Tissue specific
KPVTEPK	Superscaf	+	Superscaf	21084811	21084831	19	19	25	-	-	gene	-	-	mRNA	NCP	Intron	gene|mRN	ID=gene-E	7	5.975714286	2434.6	0.611087871	KPVTEPK	76	399.7334	2	797.4647	.	S	Superscaf	21084757	0	0	BTP202209	0	0	0	Tissue specific
RLHGALP	Superscaf	+	Superscaf	21226138	21226158	21	21	27	-	-	-	-	-	-	NCP	IGR	-	ID=gene-E	7	6.942857143	2921.3	0.608481079	RLHGALP	84	382.2317	2	762.45	.	D	Superscaf	21226078	0	0	0	0	0	BTP202209	Tissue specific
DWHRPC	Superscaf	-	Superscaf	21273160	21273177	-12	12	17	-	-	gene	-	-	mRNA	NCP	Intron	gene|mRN	ID=gene-E	6	6.421666667	3129.7	0.607613808	DWHRPC(+5	99	435.6863	2	869.3602	Carbamido	D	Superscaf	21273246	0	BTP202209	0	BTP202209	0	0	Mixed
NWRAGGP	Superscaf	-	Superscaf	21273229	21273249	-3	3	9	-	-	gene	-	-	mRNA	NCP	Intron	gene|mRN	ID=gene-E	7	6.617142857	3015.4	0.60761248	NWRAGGP	71	379.1901	2	756.3667	.	S	Superscaf	21273330	0	BTP202209	0	0	0	0	Tissue specific
LRHVRP	Superscaf	+	Superscaf	21278746	21278763	9	9	14	-	-	gene	-	-	mRNA	NCP	Intron	gene|mRN	ID=gene-E	6	6.898333333	2608.1	0.607510773	L(+42.01)	64	410.2432	2	818.4875	Acetylati	F	Superscaf	21278722	0	BTP202209	0	0	0	0	Tissue specific
NLTVLDF	Superscaf	-	Superscaf	21287509	21287529	-46	46	52	-	-	gene	-	-	mRNA	NCP	Intron	gene|mRN	ID=gene-E	7	4.621428571	2499.9	0.607349083	NLTVLDF	63	821.438	1	820.433	.	A	Superscaf	21287619	0	0	0	BTP202209	0	0	Tissue specific
DQAVYF	Superscaf	-	Superscaf	21313954	21313971	-33	33	38	-	-	gene	-	-	mRNA	NCP	Intron	gene|mRN	ID=gene-E	6	4.296666667	2180.4	0.606861358	D(+42.01)	62	784.3503	1	783.3439	Acetylati	S	Superscaf	21314076	0	BTP202209	0	0	0	0	Tissue specific
VLGVP	Superscaf	+	Superscaf	21446797	21446811	21	21	25	-	-	gene	-	-	mRNA	NCP	Intron	gene|mRN	ID=gene-E	5	3.65	988.1	0.604411109	VLGVP	57	484.3109	1	483.3057	.	K	Superscaf	21446737	0	0	0	0	0	BTP202209	Tissue specific
SDPRLYP	Superscaf	+	Superscaf	21491215	21491235	20	20	26	-	-	-	-	-	-	NCP	IGR	-	ID=gene-E	7	6.235714286	2931.4	0.603591703	SDPRLYP	92	424.2177	2	846.4235	.	S	Superscaf	21491158	0	0	0	BTP202209	0	0	Tissue enhanced
HSSLRR	Superscaf	-	Superscaf	21518941	21518958	-3	3	8	-	-	-	-	-	-	NCP	IGR	-	ID=gene-E	6	7.741666667	3037.5	0.603080349	HSSLRR	51	378.2142	2	754.4198	.	R	Superscaf	21518967	0	BTP202209	BTP202209	BTP202209	0	BTP202209	Tissue specific
KYLPPPP	Superscaf	+	Superscaf	21645532	21645552	27	27	33	-	-	-	-	-	-	NCP	IGR	-	ID=gene-E	7	6.654285714	2630.9	0.600745308	KYLPPPP	78	406.2389	2	810.4639	.	C	Superscaf	21645454	0	0	BTP202209	0	0	0	Mixed
LFMQPK	Superscaf	+	Superscaf	21814357	21814374	14	14	19	-	-	-	-	-	-	NCP	IGR	-	ID=gene-E	6	6.483333333	2528.8	0.597631367	LFMQPK	71	382.2143	2	762.4098	.	L	Superscaf	21814318	0	BTP202209	0	0	0	0	Tissue specific
RGMKWPVD	Superscaf	-	Superscaf	21878515	21878538	-16	16	23	-	-	-	-	-	-	NCP	IGR	-	ID=gene-E	8	5.9225	3061.5	0.596447855	R(+42.01)	57	515.765	2	1029.507	Acetylati	P	Superscaf	21878592	BTP202209	0	0	0	0	0	Tissue specific
SPAAPGPV	Superscaf	-	Superscaf	21886333	21886356	-73	73	80	-	-	-	-	-	-	NCP	IGR	-	ID=gene-E	8	5.32375	2315.5	0.596303651	S(+42.01)	52	369.1882	2	736.3755	Acetylati	R	Superscaf	21886368	0	BTP202209	0	0	0	0	Tissue specific
MVHFVK	Superscaf	+	Superscaf	21987064	21987081	4	4	9	-	-	gene	-	-	mRNA	NCP	Intron	gene|mRN	ID=gene-E	6	4.76	1847	0.594445766	MVHFVK	70	380.7168	2	759.4102	.	R	Superscaf	21987055	0	0	0	BTP202209	0	0	Tissue specific
AAMAKKK	Superscaf	+	Superscaf	21990193	21990213	49	49	55	-	-	gene	-	-	mRNA	NCP	Intron	gene|mRN	ID=gene-E	7	7.575714286	2710.6	0.594387996	A(+42.01)	59	403.2323	2	804.4528	Acetylati	N	Superscaf	21990049	0	0	BTP202209	0	0	0	Tissue specific
DFNRVAL	Superscaf	-	Superscaf	22027471	22027491	-9	9	15	-	-	gene	-	-	mRNA	NCP	Intron	gene|mRN	ID=gene-E	7	5.231428571	2709.1	0.593700399	DFNRVAL	92	417.7256	2	833.4395	.	K	Superscaf	22027557	0	BTP202209	0	0	0	0	Mixed
QMFLLQ	Superscaf	+	Superscaf	22121506	22121523	59	59	64	-	-	-	-	-	-	NCP	IGR	-	ID=gene-E	6	5.748333333	2687.1	0.591965968	QM(+15.99	72	795.4042	1	794.3997	Oxidation	D	Superscaf	22121332	0	BTP202209	0	0	0	0	Tissue specific
KSTLLE	Superscaf	-	Superscaf	22212055	22212072	-38	38	43	-	-	-	-	-	-	NCP	IGR	-	ID=gene-E	6	6.188333333	2381.6	0.590295782	K(+42.01)	52	732.4125	1	731.4065	Acetylati	A	Superscaf	22212156	0	0	0	0	BTP202209	BTP202209	Tissue specific
FTHRLLA	Superscaf	-	Superscaf	22213450	22213470	-22	22	28	-	-	-	-	-	-	NCP	IGR	-	ID=gene-E	7	6.905714286	3071.5	0.590269995	FTHRLLA	96	429.2526	2	856.4919	.	G	Superscaf	22213524	0	BTP202209	0	BTP202209	0	0	Tissue specific
LAKPGAK	Superscaf	+	Superscaf	22254361	22254381	24	24	30	-	-	-	-	-	-	NCP	IGR	-	ID=gene-E	7	7.11	2547.5	0.589515387	L(+42.01)	83	363.7275	2	725.4435	Acetylati	G	Superscaf	22254292	BTP202209	0	0	0	0	0	Tissue specific
MAELVK	Superscaf	+	Superscaf	22272922	22272939	23	23	28	-	-	-	-	-	-	NCP	IGR	-	ID=gene-E	6	5.118333333	1930.1	0.589173083	M(+42.01)	79	366.6986	2	731.3887	Acetylati	I	Superscaf	22272856	BTP202209	BTP202209	0	0	0	0	Tissue specific
KCWPK	Superscaf	+	Superscaf	22285393	22285407	6	6	10	-	-	-	-	-	-	NCP	IGR	-	ID=gene-E	5	7.338	2386.5	0.588943109	KC(+57.02	51	359.6888	2	717.3632	Carbamido	G	Superscaf	22285378	0	BTP202209	0	0	0	0	Tissue specific
LSETFK	Superscaf	-	Superscaf	22290436	22290453	-6	6	11	-	-	gene	-	-	mRNA	NCP	Intron	gene|mRN	ID=gene-E	6	6.105	2415.6	0.588850035	LSETFK	83	362.6956	2	723.3803	.	I	Superscaf	22290465	0	BTP202209	0	0	0	0	Tissue specific
KEPPLYR	Superscaf	+	Superscaf	22301842	22301862	89	89	95	-	-	gene	-	-	mRNA	NCP	Intron	gene|mRN	ID=gene-E	7	6.851428571	3030.4	0.588639595	KEPPLYR	50	451.7567	2	901.5021	.	S	Superscaf	22301578	0	BTP202209	0	0	0	0	Tissue specific
TMLQRPQ	Superscaf	+	Superscaf	22371289	22371309	33	33	39	-	-	-	-	-	-	NCP	IGR	-	ID=gene-E	7	6.66	3330	0.587358637	T(+42.01)	59	466.2359	2	930.4593	Acetylati	P	Superscaf	22371193	0	0	0	0	BTP202209	0	Tissue specific
GFDLL	Superscaf	-	Superscaf	22382548	22382562	-3	3	7	-	-	-	-	-	-	NCP	IGR	-	ID=gene-E	5	5.276	1803	0.587151074	GFDLL	72	564.301	1	563.2955	.	P	Superscaf	22382610	0	BTP202209	0	0	BTP202209	0	Tissue specific
RKAPVP	Superscaf	+	Superscaf	22434802	22434819	17	17	22	-	-	-	-	-	-	NCP	IGR	-	ID=gene-E	6	6.52	2103.3	0.586187188	R(+42.01)	61	709.4348	1	708.4282	Acetylati	G	Superscaf	22434754	0	0	BTP202209	0	0	0	Tissue specific
LASPKK	Superscaf	-	Superscaf	22502701	22502718	-5	5	10	-	-	-	-	-	-	NCP	IGR	-	ID=gene-E	6	7.243333333	2322.3	0.584934783	LASPKK	57	643.4113	1	642.4064	.	L	Superscaf	22502772	0	BTP202209	0	0	0	0	Tissue specific
APEVK	Superscaf	+	Superscaf	22538137	22538151	46	46	50	-	-	gene	-	-	mRNA	NCP	Intron	gene|mRN	ID=gene-E	5	5.056	1542.6	0.584281217	APEVK	57	543.3051	1	542.3064	.	R	Superscaf	22538002	0	BTP202209	0	0	0	0	Tissue specific
LGPHKPY	Superscaf	+	Superscaf	22543108	22543128	22	22	28	-	-	gene	-	-	mRNA	NCP	Intron	gene|mRN	ID=gene-E	7	6.791428571	2873.1	0.584189416	L(+42.01)	62	427.2393	2	852.4493	Acetylati	I	Superscaf	22543045	0	0	0	BTP202209	0	0	Tissue specific
KNAQLLK	Superscaf	-	Superscaf	22575853	22575873	-1	1	7	-	-	-	-	-	-	NCP	IGR	-	ID=gene-E	7	6.931428571	3162.3	0.58358543	KNAQLLK	56	407.7595	2	813.5072	.	K	Superscaf	22575969	0	BTP202209	0	0	0	0	Tissue specific
VVPGSPS	Superscaf	-	Superscaf	22577317	22577337	-20	20	26	-	-	-	-	-	-	NCP	IGR	-	ID=gene-E	7	4.275714286	1738.9	0.583558427	VVPGSPS	85	642.3443	1	641.3384	.	K	Superscaf	22577394	0	0	0	0	BTP202209	0	Tissue specific
VGEVP	Superscaf	-	Superscaf	22624285	22624299	-11	11	15	-	-	-	-	-	-	NCP	IGR	-	ID=gene-E	5	3.098	1060.2	0.582692207	VGEVP	66	500.2702	1	499.2642	.	E	Superscaf	22624359	0	0	0	0	0	BTP202209	Tissue specific
VASRFK	Superscaf	+	Superscaf	22634908	22634925	16	16	21	-	-	-	-	-	-	NCP	IGR	-	ID=gene-E	6	6.28	2199.3	0.582496209	VASRFK	60	354.2104	2	706.4126	.	R	Superscaf	22634863	0	0	0	0	0	BTP202209	Tissue specific
AKLSHK	Superscaf	+	Superscaf	22768081	22768098	1	1	6	-	-	gene	-	-	mRNA	NCP	Intron	gene|mRN	ID=gene-E	6	7.458333333	2604.6	0.580039818	A(+42.01)	68	363.2121	2	724.4232	Acetylati	A	Superscaf	22768081	0	0	0	0	0	BTP202209	Tissue specific
KSGSKPK	Superscaf	-	Superscaf	22808947	22808967	-23	23	29	-	-	gene	-	-	mRNA	NCP	Intron	gene|mRN	ID=gene-E	7	7.55	2806.7	0.579285985	KSGSKPK	70	366.2184	2	730.4337	.	A	Superscaf	22809030	0	0	0	0	0	BTP202209	Tissue specific
HMPKKRKK	Superscaf	+	Superscaf	22828516	22828539	1	1	8	-	-	-	-	-	-	NCP	IGR	-	ID=gene-E	8	8.67	3851	0.578924977	H(+42.01)	70	365.556	3	1093.654	Acetylati	H	Superscaf	22828516	0	0	0	BTP202209	0	0	Tissue specific
TGKLNLLK	Superscaf	-	Superscaf	22867561	22867584	-10	10	17	-	-	gene	-	-	mRNA	NCP	Intron	gene|mRN	ID=gene-E	8	6.91625	3288.5	0.578204788	T(+42.01)	74	464.7937	2	927.5753	Acetylati	Y	Superscaf	22867668	0	BTP202209	0	0	0	0	Tissue specific
PVLPLTPK	Superscaf	+	Superscaf	22895065	22895088	3	3	10	-	-	gene	-	-	mRNA	NCP	Intron	gene|mRN	ID=gene-E	8	5.89125	2584.9	0.577697473	PVLPLTPK	61	432.7802	2	863.548	.	H	Superscaf	22895059	0	BTP202209	0	0	BTP202209	0	Tissue specific
TATLPQH	Superscaf	-	Superscaf	22907035	22907055	-6	6	12	-	-	-	-	-	-	NCP	IGR	-	ID=gene-E	7	6.371428571	3027.5	0.57747674	TATLPQH	87	384.2048	2	766.3973	.	S	Superscaf	22907088	0	0	0	0	BTP202209	0	Tissue specific
QFLKDK	Superscaf	+	Superscaf	22963966	22963983	7	7	12	-	-	gene	-	-	mRNA	NCP	Intron	gene|mRN	ID=gene-E	6	6.593333333	2699.8	0.576426697	QFLKDK	53	389.7242	2	777.4385	.	K	Superscaf	22963948	0	BTP202209	0	0	0	0	Tissue specific
PAKNDSP	Superscaf	-	Superscaf	23002567	23002587	-33	33	39	-	-	gene	-	-	mRNA	NCP	Intron	gene|mRN	ID=gene-E	7	6.06	2846.1	0.575714642	PAKNDSP	57	364.6895	2	727.35	.	L	Superscaf	23002725	0	0	0	BTP202209	0	0	Tissue specific

MDGPAAL	Superscaf	-	Superscaf	24640288	24640308	-2	2	8	-	-	-	-	-	-	NCP	IGR	-	ID=gene-E	7	5.572857143	2393.7	0.54550669	M(+42.01)	57	716.3252	1	715.321	Acetylati	T	Superscaf	24640383	0	BTP20220	0	0	0	0	Tissue specific
GFLLDGN	Superscaf	-	Superscaf	24641887	24641907	-17	17	23	-	-	-	-	-	-	NCP	IGR	-	ID=gene-E	7	5.394285714	2697	0.545477196	GFLLDGN	99	735.365	1	734.3599	.	F	Superscaf	24641940	0	0	0	0	BTP20220	0	Mixed
GDGRLFR	Superscaf	-	Superscaf	24734626	24734646	-12	12	18	-	-	-	-	-	-	NCP	IGR	-	ID=gene-E	7	6.841428571	3044.5	0.543766615	GDGRLFR	61	410.7232	2	819.4351	.	L	Superscaf	24734982	0	BTP20220	0	0	0	0	Tissue specific
KGGLLSH	Superscaf	+	Superscaf	24758260	24758280	7	7	13	-	-	-	-	-	-	NCP	IGR	-	ID=gene-E	7	6.701428571	2772.9	0.543330683	KGGLLSH	63	356.2094	2	710.4075	.	N	Superscaf	24758242	0	BTP20220	0	0	0	0	Tissue specific
KAMVP	Superscaf	-	Superscaf	24818359	24818373	-2	2	6	-	-	-	-	-	-	NCP	IGR	-	ID=gene-E	5	5.562	1488.6	0.54222226	KAM(+15.9	68	561.3016	1	560.2992	Oxidation	E	Superscaf	24818460	0	0	0	0	BTP20220	0	Tissue specific
APFSRK	Superscaf	-	Superscaf	24845377	24845394	-10	10	15	-	-	-	-	-	-	NCP	IGR	-	ID=gene-E	6	7.33	2536.7	0.541723855	APFSRK	67	353.2042	2	704.3969	.	V	Superscaf	24845613	0	0	0	0	BTP20220	0	Tissue specific
KEELYPK	Superscaf	-	Superscaf	24980062	24980082	-5	5	11	-	-	-	-	-	-	NCP	IGR	-	ID=gene-E	7	6.265714286	2938.1	0.539239519	KEELYPK	62	453.7544	2	905.4858	.	H	Superscaf	24980142	0	0	0	0	BTP20220	BTP20220	Tissue specific
MLYLPRS	Superscaf	-	Superscaf	25050040	25050060	-13	13	19	-	-	-	-	-	-	NCP	IGR	-	ID=gene-E	7	6.587142857	2907.4	0.537948767	MLYLPRS	73	440.2397	2	878.4684	.	D	Superscaf	25050084	0	0	0	0	0	BTP20220	Tissue specific
KYPRPP	Superscaf	-	Superscaf	25065112	25065129	-11	11	16	-	-	-	-	-	-	NCP	IGR	-	ID=gene-E	6	7.51	2588.9	0.537670818	KYPRPP	94	379.2195	2	756.4282	.	S	Superscaf	25065198	0	0	0	0	0	BTP20220	Tissue specific
EEELTVK	Superscaf	+	Superscaf	25083544	25083564	1	1	7	-	-	-	-	-	-	NCP	IGR	-	ID=gene-E	7	4.558571429	2495.8	0.537330782	E(+42.01)	93	445.2328	2	888.444	Acetylati	E	Superscaf	25083544	0	0	0	BTP20220	0	BTP20220	Tissue specific
PYVTR	Superscaf	+	Superscaf	25113406	25113420	2	2	6	-	-	-	-	-	-	NCP	IGR	-	ID=gene-E	5	5.85	1843.2	0.536780085	PYVTR	53	635.3488	1	634.3438	.	A	Superscaf	25113403	0	0	0	BTP20220	0	0	Tissue specific
RALLNYL	Superscaf	-	Superscaf	25125628	25125648	-22	22	28	-	-	-	-	-	-	NCP	IGR	-	ID=gene-E	7	6.541428571	3076.5	0.536554538	RALLNYL	56	431.7603	2	861.5072	.	S	Superscaf	25125690	0	0	0	0	0	BTP20220	Tissue specific
LGAWCPR	Superscaf	-	Superscaf	25162918	25162938	-6	6	12	-	-	-	-	-	-	NCP	IGR	-	ID=gene-E	7	6.562857143	3060.5	0.535866719	LGAWC(+57	52	430.2184	2	858.4171	Carbamido	I	Superscaf	25162965	0	0	0	0	0	BTP20220	Tissue specific
TVHLFGK	Superscaf	+	Superscaf	25297381	25297401	14	14	20	-	-	-	-	-	-	NCP	IGR	-	ID=gene-E	7	5.898571429	2523.7	0.533386534	TVHLFGK	54	401.237	2	800.4545	.	H	Superscaf	25297342	0	0	0	0	0	BTP20220	Tissue specific
KVYLGFP	Superscaf	+	Superscaf	25324375	25324395	5	5	11	-	-	-	-	-	-	NCP	IGR	-	ID=gene-E	7	5.59	2303.4	0.532888627	K(+42.01)	64	433.2433	2	864.4745	Acetylati	Y	Superscaf	25324363	0	BTP20220	0	0	BTP20220	BTP20220	Tissue specific
SKHTRPP	Superscaf	+	Superscaf	25369831	25369851	4	4	10	-	-	-	-	-	-	NCP	IGR	-	ID=gene-E	7	7.557142857	3192.6	0.532050185	SKHTRPP	52	411.7398	2	821.4507	.	P	Superscaf	25369822	0	BTP20220	0	0	0	0	Tissue specific
AVPLPPLP	Superscaf	+	Superscaf	25397710	25397733	6	6	13	-	-	-	-	-	-	NCP	IGR	-	ID=gene-E	8	5.3975	2367.7	0.531535899	A(+42.01)	74	423.2571	2	844.5058	Acetylati	S	Superscaf	25397695	0	BTP20220	0	0	0	0	Tissue specific
LDTLTPK	Superscaf	-	Superscaf	25404454	25404474	-1	1	7	-	-	-	-	-	-	NCP	IGR	-	ID=gene-E	7	6.29	2719.1	0.53141156	LDTLTPK	67	394.2303	2	786.4487	.	L	Superscaf	25404477	0	BTP20220	0	0	0	0	Tissue specific
LPPGHLP	Superscaf	-	Superscaf	25440346	25440366	-62	62	68	-	-	-	-	-	-	NCP	IGR	-	ID=gene-E	7	6.345714286	2636	0.530749528	LPPGHLP	55	365.7152	2	729.4173	.	A	Superscaf	25440501	0	0	0	0	0	BTP20220	Tissue specific
KGMTHVP	Superscaf	+	Superscaf	25454095	25454115	40	40	46	-	-	-	-	-	-	NCP	IGR	-	ID=gene-E	7	5.982857143	2491.8	0.530495926	KGMTHVP	60	385.2072	2	768.3953	.	T	Superscaf	25453978	0	0	0	BTP20220	0	0	Tissue specific
MGEGDC	Superscaf	-	Superscaf	25494721	25494738	-11	11	16	-	-	-	-	-	-	NCP	IGR	-	ID=gene-E	6	4.816666667	2388.8	0.52974663	M(+15.99)	54	684.2	1	683.1891	Oxidation	H	Superscaf	25494750	0	0	BTP20220	0	0	0	Tissue specific
KDLEHGP	Superscaf	-	Superscaf	25536901	25536921	-61	61	67	-	-	-	-	-	-	NCP	IGR	-	ID=gene-E	7	5.967142857	2913.2	0.52896856	KDLEHGP	52	795.4045	1	794.3922	.	L	Superscaf	25536951	0	BTP20220	0	0	0	0	Tissue specific
YSQALLR	Superscaf	+	Superscaf	25559329	25559349	7	7	13	-	-	-	-	-	-	NCP	IGR	-	ID=gene-E	7	6.532857143	3120.5	0.528554873	YSQALLR	82	425.7411	2	849.4708	.	N	Superscaf	25559311	0	0	BTP20220	0	0	0	Tissue specific
GSAMPK	Superscaf	-	Superscaf	25565977	25565994	-91	91	96	-	-	-	-	-	-	NCP	IGR	-	ID=gene-E	6	6.576666667	2169.3	0.528432305	GSAMPK	52	590.2909	1	589.2894	.	K	Superscaf	25566087	0	BTP20220	0	0	0	BTP20220	Tissue specific
HLYDPK	Superscaf	+	Superscaf	25580902	25580919	3	3	8	-	-	-	-	-	-	NCP	IGR	-	ID=gene-E	6	6.373333333	2649.9	0.528157012	HLYDPK	68	386.7024	2	771.3915	.	S	Superscaf	25580896	0	0	BTP20220	BTP20220	BTP20220	0	Tissue enhanced
KAVSYPK	Superscaf	+	Superscaf	25607362	25607382	20	20	26	-	-	-	-	-	-	NCP	IGR	-	ID=gene-E	7	6.162857143	2428.4	0.527668899	K(+42.01)	63	417.7411	2	833.4647	Acetylati	L	Superscaf	25607305	0	0	0	0	0	BTP20220	Tissue specific
KFYKRH	Superscaf	-	Superscaf	25623391	25623408	-17	17	22	-	-	-	-	-	-	NCP	IGR	-	ID=gene-E	6	8.161666667	3052.2	0.527373297	KFYKRH	60	439.7511	2	877.4922	.	Q	Superscaf	25623438	0	0	0	0	BTP20220	0	Tissue specific
NSLVLVSP	Superscaf	-	Superscaf	25634335	25634358	-28	28	35	-	-	-	-	-	-	NCP	IGR	-	ID=gene-E	8	4.37875	2407.7	0.527171323	NSLVLVSP	67	414.7484	2	827.4753	.	T	Superscaf	25634385	0	BTP20220	0	0	0	BTP20220	Tissue specific
FSQQHP	Superscaf	-	Superscaf	25712803	25712820	-1	1	6	-	-	-	-	-	-	NCP	IGR	-	ID=gene-E	6	6.058333333	2949.3	0.525724083	F(+42.01)	56	393.1876	2	784.3504	Acetylati	F	Superscaf	25712835	0	BTP20220	0	0	0	0	Tissue specific
KTTPVVR	Superscaf	-	Superscaf	25808059	25808079	-35	35	41	-	-	-	-	-	-	NCP	IGR	-	ID=gene-E	7	5.694285714	2249.6	0.52396702	KTTPVVR	50	400.7522	2	799.4916	.	M	Superscaf	25808130	0	0	0	0	BTP20220	0	Tissue specific
KQSTQRQ	Superscaf	-	Superscaf	25848019	25848039	-57	57	63	-	-	-	-	-	-	NCP	IGR	-	ID=gene-E	7	7.094285714	3730.2	0.523229953	KQSTQRQ	54	438.2413	2	874.4621	.	P	Superscaf	25848048	0	0	BTP20220	0	0	0	Tissue specific
VSLEKNR	Superscaf	-	Superscaf	25849900	25849920	-1	1	7	-	-	-	-	-	-	NCP	IGR	-	ID=gene-E	7	5.827142857	2876.2	0.523195258	V(+42.01)	57	444.2509	2	886.4872	Acetylati	V	Superscaf	25850085	0	0	0	0	0	BTP20220	Tissue specific
KYYKVG	Superscaf	-	Superscaf	25882774	25882791	-10	10	15	-	-	-	-	-	-	NCP	IGR	-	ID=gene-E	6	6.128333333	2153.1	0.522588948	KYYKVG	84	379.2228	2	756.417	.	C	Superscaf	25882848	0	0	0	0	BTP20220	0	Tissue specific
PLTNTHSKF	Superscaf	+	Superscaf	25906267	25906293	70	70	78	-	-	-	-	-	-	NCP	IGR	-	ID=gene-E	9	7.168888889	4202.8	0.522155451	PLTNTHSKF	89	527.2983	2	1052.573	.	Y	Superscaf	25906060	0	BTP20220	0	0	0	0	Tissue specific
VGGRELVL	Superscaf	+	Superscaf	25907152	25907175	1	1	8	-	-	-	-	-	-	NCP	IGR	-	ID=gene-E	8	4.735	2375.7	0.522139183	VGGRELVL	53	421.7573	2	841.5021	.	V	Superscaf	25907152	0	0	0	0	BTP20220	0	Tissue specific
KFQPPPP	Superscaf	+	Superscaf	25909942	25909962	5	5	11	-	-	-	-	-	-	NCP	IGR	-	ID=gene-E	7	6.581428571	2816.1	0.522087776	K(+42.01)	66	426.733	2	851.4541	Acetylati	K	Superscaf	25909930	0	0	0	0	0	BTP20220	Tissue specific
YAMPGF	Superscaf	+	Superscaf	25911856	25911873	20	20	25	-	-	-	-	-	-	NCP	IGR	-	ID=gene-E	6	5.863333333	2164.4	0.522052527	YAMPGF	82	685.3002	1	684.2941	.	H	Superscaf	25911799	0	0	0	0	BTP20220	0	Tissue specific
QPLPKPP	Superscaf	-	Superscaf	25920220	25920240	-14	14	20	-	-	-	-	-	-	NCP	IGR	-	ID=gene-E	7	6.652857143	2782.1	0.521898197	QPLPKPP	63	388.7353	2	775.4592	.	S	Superscaf	25920279	0	0	0	0	BTP20220	0	Tissue specific
PVFVL	Superscaf	-	Superscaf	25941241	25941255	-9	9	13	-	-	-	-	-	-	NCP	IGR	-	ID=gene-E	5	3.552	1078.2	0.521510573	PVFVL	68	574.359	1	573.3526	.	L	Superscaf	25941258	0	BTP20220	0	0	0	0	Tissue specific
AGKLHPR	Superscaf	-	Superscaf	25959112	25959132	-55	55	61	-	-	-	-	-	-	NCP	IGR	-	ID=gene-E	7	7.48	3036.3	0.52118083	A(+42.01)	70	410.7425	2	819.4715	Acetylati	S	Superscaf	25959222	0	0	0	0	0	BTP20220	Tissue specific
AALVD	Superscaf	+	Superscaf	26119549	26119563	14	14	18	-	-	-	-	-	-	NCP	IGR	-	ID=gene-E	5	4.198	1387.5	0.518221661	AALVD	88	488.2687	1	487.2642	.	F	Superscaf	26119510	0	0	0	0	0	BTP20220	Tissue specific
AGGLP	Superscaf	+	Superscaf	26202361	26202375	4	4	8	-	-	-	-	-	-	NCP	IGR	-	ID=gene-E	5	6.048	1596.7	0.516694185	A(+42.01)	50	456.2445	1	455.238	Acetylati	V	Superscaf	26202352	0	0	BTP20220	0	0	0	Tissue specific
LNRMAF	Superscaf	+	Superscaf	26240824	26240841	10	10	15	-	-	gene	-	-	mRNA	NCP	Intron	gene|mRN	ID=gene-E	6	6.566666667	2669.1	0.515984675	LNRM(+15.	55	384.1903	2	766.3796	Oxidation	L	Superscaf	26240797	0	0	0	0	0	BTP20220	Tissue specific
ELEKFDK	Superscaf	-	Superscaf	26287543	26287563	-171	171	177	-	-	gene	-	-	mRNA	NCP	Intron	gene|mRN	ID=gene-E	7	5.764285714	2940.1	0.515122882	ELEKFDK	94	454.7386	2	907.465	.	T	Superscaf	26288553	0	0	0	BTP20220	0	0	Mixed
LVGAPLQ	Superscaf	-	Superscaf	26294896	26294916	-54	54	60	-	-	gene	-	-	mRNA	NCP	Intron	gene|mRN	ID=gene-E	7	5.128571429	2263.5	0.514987255	LVGAPLQ	56	697.4221	1	696.417	.	F	Superscaf	26294928	0	0	0	0	BTP20220	0	Tissue specific
KEAAQR	Superscaf	+	Superscaf	26371951	26371968	45	45	50	-	-	-	-	-	-	NCP	IGR	-	ID=gene-E	6	6.901666667	2776	0.513566022	KEAAQR	53	351.6922	2	701.382	.	A	Superscaf	26371819	0	0	0	0	0	BTP20220	Tissue specific
VSKDLTK	Superscaf	+	Superscaf	26413870	26413890	3	3	9	-	-	gene	-	-	mRNA	NCP	Intron	gene|mRN	ID=gene-E	7	5.805714286	2482.6	0.512792766	VSKDLTK	76	395.7392	2	789.4596	.	I	Superscaf	26413864	0	0	0	0	0	BTP20220	Tissue specific
AGALVRK	Superscaf	+	Superscaf	26510134	26510154	11	11	17	-	-	-	-	-	-	NCP	IGR	-	ID=gene-E	7	6.355714286	2390.5	0.511017166	A(+42.01)	89	378.7393	2	755.4653	Acetylati	V	Superscaf	26510104	0	0	0	0	0	BTP20220	Tissue enhanced
FEKLRF	Superscaf	+	Superscaf	26514175	26514192	7	7	12	-	-	-	-	-	-	NCP	IGR	-	ID=gene-E	6	6.776666667	2670.9	0.510942684	F(+42.01)	87	441.2524	2	880.4807	Acetylati	D	Superscaf	26514157	0	0	0	0	0	BTP20220	Tissue specific
KERYVL	Superscaf	-	Superscaf	26543335	26543352	-38	38	43	-	-	gene	-	-	mRNA	NCP	Intron	gene|mRN	ID=gene-E	6	5.893333333	2355.6	0.510404825	KERYVL	70	404.2396	2	806.465	.	S	Superscaf	26543385	0	0	0	0	BTP20220	BTP20220	Tissue specific
TAYKAR	Superscaf	-	Superscaf	26600194	26600211	-2	2	7	-	-	gene	-	-	mRNA	NCP	Intron	gene|mRN	ID=gene-E	6	7.455	2596.8	0.509356054	TAYKAR	81	355.1966	2	708.3918	.	G	Superscaf	26600247	0	0	0	BTP20220	0	0	Tissue specific
KTSFGK	Superscaf	-	Superscaf	26821306	26821323	-10	10	15	-	-	-	-	-	-	NCP	IGR	-	ID=gene-E	6	7.19	2402.4	0.505277618	K(+42.01)	86	355.1961	2	708.3806	Acetylati	L	Superscaf	26821350	0	BTP20220	0	0	0	0	Tissue specific
RLGSPVTR	Superscaf	+	Superscaf	26833642	26833665	22	22	29	-	-	-	-	-	-	NCP	IGR	-	ID=gene-E	8	6.4975	3066.6	0.505049968	RLGSPVTR	64	443.2655	2	884.5192	.	C	Superscaf	26833579	0	0	BTP20220	BTP20220	0	0	Tissue specific
AELRALV	Superscaf	+	Superscaf	26861980	26862000	1	1	7	-	-	-	-	-	-	NCP	IGR	-	ID=gene-E	7	5.425714286	2403.7	0.504527326	A(+42.01)	97	407.2439	2	812.4756	Acetylati	A	Superscaf	26861980	0	0	0	0	BTP20220	0	Tissue enhanced
LPPPPLPPF	Superscaf	+	Superscaf	26881972	26881998	13	13	21	-	-	-	-	-	-	NCP	IGR	-	ID=gene-E	9	6.228888889	3068.6	0.50415846	LPPPPLPPF	80	924.5524	1	923.548	.	F	Superscaf	26881936	0	0	0	0	BTP20220	0	Tissue specific
MPKLDR	Superscaf	+	Superscaf	26908180	26908197	34	34	39	-	-	-	-	-	-	NCP	IGR	-	ID=gene-E	6	6.916666667	2591	0.503675217	M(+15.99)	75	409.2154	2	816.4163	Oxidation	L	Superscaf	26908081	0	0	BTP20220	0	0	0	Tissue specific
RGPPLLY	Superscaf	+	Superscaf	26911111	26911131	29	29	35	-	-	-	-	-	-	NCP	IGR	-	ID=gene-E	7	6.707142857	2787.2	0.503621099	RGPPLLY	68	408.2475	2	814.4701	.	E	Superscaf	26911027	0	0	0	0	BTP20220	BTP20220	Tissue enhanced
AMGLM	Superscaf	+	Superscaf	26988724	26988738	47	47	51	-	-	-	-	-	-	NCP	IGR	-	ID=gene-E	5	5.894	1705	0.502189629	AMGLM	98	522.2393	1	521.2342	.	S	Superscaf	26988586	0	0	0	0	BTP20220	0	Mixed
GPLTPRP	Superscaf	+	Superscaf	27055168	27055188	25	25	31	-	-	gene	-	-	mRNA	NCP	Intron	gene|mRN	ID=gene-E	7	6.877142857	2709.2	0.500963951	GPLTPRP	53	369.2123	2	736.4232	.	T	Superscaf	27055096	0	BTP20220	0	0	0	0	Tissue specific
PDPPP	Superscaf	-	Superscaf	27057241	27057255	-1	1	5	-	-	gene	-	-	mRNA	NCP	Intron	gene|mRN	ID=gene-E	5	5.634	1761.1	0.500925825	P(+42.01)	58	564.2642	1	563.2591	Acetylati	P	Superscaf	27057342	0	0	BTP20220	0	0	0	Tissue specific
ERYWPF	Superscaf	-	Superscaf	27060538	27060555	-10	10	15	-	-	gene	-	-	mRNA	NCP	Intron	gene|mRN	ID=gene-E	6	6.218333333	2785.2	0.500864956	ERYWPF	64	449.2166	2	896.418	.	F	Superscaf	27060621	BTP20220	0	0	0	0	0	Tissue specific
APRTVY	Superscaf	-	Superscaf	27093832	27093849	-7	7	12	-	-	gene	-	-	mRNA	NCP	Intron	gene|mRN	ID=gene-E	6	5.878333333	2154.5	0.500250844	APRTVY	93	353.6964	2	705.3809	.	F	Superscaf	27093861	BTP20220	0	0	0	BTP20220	0	Tissue specific
KPPDLLA	Superscaf	+	Superscaf	27106183	27106203	28	28	34	-	-	gene	-	-	mRNA	NCP	Intron	gene|mRN	ID=gene-E	7	6.184285714	2572.8	0.500022973	KPPDLLA	51	377.2299	2	752.4432	.	G	Superscaf	27106102	0	0	0	BTP20220	0	0	Tissue specific
QKAAPVP	Superscaf	-	Superscaf	27134014	27134034	-28	28	34	-	-	gene	-	-	mRNA	NCP	Intron	gene|mRN	ID=gene-E	7	5.718571429	2376.5	0.499509627	Q(+42.01)	61	376.7141	2	751.4228	Acetylati	E	Superscaf	27134037	0	BTP20220	0	0	BTP20220	0	Tissue specific
TYVTPR	Superscaf	+	Superscaf	27213550	27213567	13	13	18	-	-	gene	-	-	mRNA	NCP	Intron	gene|mRN	ID=gene-E	6	5.963333333	2240.7	0.498042632	TYVTPR	99	368.7021	2	735.3915	.	N	Superscaf	27213514	0	0	0	0	BTP20220	0	Tissue specific
MFSGLP	Superscaf	-	Superscaf	27320293	27320310	-21	21	26	-	-	gene	-	-	mRNA	NCP	Intron	gene|mRN	ID=gene-E	6	5.86	2130.4	0.496073745	M(+42.01)	69	693.3253	1	692.3203	Acetylati	D	Superscaf	27320319	0	0	0	0	BTP20220	0	Tissue specific
PLNPKGSP	Superscaf	-	Superscaf	27461548	27461571	-11	11	18	-	-	gene	-	-	mRNA	NCP	Intron	gene|mRN	ID=gene-E	8	6.46	3111.4	0.49346817	PLNPKGSP	53	405.2341	2	808.4443	.	S	Superscaf	27461583	BTP20220	0	0	0	0	0	Tissue specific
FGVGMGK	Superscaf	+	Superscaf	27478798	27478818	70	70	76	-	-	-	-	-	-	NCP	IGR	-	ID=gene-E	7	5.554285714	2119.2	0.493150047	FGVGM(+15	67	711.3414	1	710.3421	Oxidation	E	Superscaf	27478591	0	0	0	0	BTP20220	0	Tissue specific
FPGGGSL	Superscaf	+	Superscaf	27560449	27560469	46	46	52	-	-	gene	-	-	mRNA	NCP	Intron	gene|mRN	ID=gene-E	7	5.907142857	2353.5	0.491643985	FPGGGSL	50	634.3202	1	633.3122	.	I	Superscaf	27560314	0	0	0	0	BTP20220	0	Tissue specific
KGLSSGK	Superscaf	-	Superscaf	27571006	27571026	-12	12	18	-	-	gene	-	-	mRNA	NCP	Intron	gene|mRN	ID=gene-E	7	6.965714286	2651.6	0.49144926	K(+42.01)	65	359.7032	2	717.4021	Acetylati	F	Superscaf	27571083	BTP20220	0	0	0	0	0	Tissue specific
PAAPP	Superscaf	-	Superscaf	27579049	27579063	-12	12	16	-	-	gene	-	-	mRNA	NCP	Intron	gene|mRN	ID=gene-E	5	6.188	1634.8	0.491301016	P(+42.01)	87	494.254	1	493.2536	Acetylati	G	Superscaf	27579294	0	0	0	0	BTP20220	0	Tissue enhanced
NPKRPP	Superscaf	-	Superscaf	27591760	27591777	-2	2	7	-	-	gene	-	-	mRNA	NCP	Intron	gene|mRN	ID=gene-E	6	7.468333333	2726.1	0.491066505	NPKRPP	97	354.7101	2	707.4078	.	K	Superscaf	27591780	0	0	0	0	0	BTP20220	Tissue specific
GHERQY	Superscaf	+	Superscaf	27596851	27596868	3	3	8	-	-	gene	-	-	mRNA	NCP	Intron	gene|mRN	ID=gene-E	6	6.475	3061.5	0.490972601	GHERQY	53	395.1868	2	788.3565	.	E	Superscaf	27596845	0	0	0	0	BTP20220	0	Tissue specific
RVLVP	Superscaf	+	Superscaf	27619015	27619029	21	21	25	-	-	gene	-	-	mRNA	NCP	Intron	gene|mRN	ID=gene-E	5	4.608	1339.6	0.490563839	RVLVP	87	583.3924	1	582.3853	.	K	Superscaf	27618955	0	0	0	0	0	BTP20220	Mixed
FDSPKF	Superscaf	-	Superscaf	27693487	27693504	-14	14	19	-	-	-	-	-	-	NCP	IGR	-	ID=gene-E	6	5.941666667	2375.5	0.489190139	FDSPKF	50	740.3563	1	739.3541	.	N	Superscaf	27693636	0	0	BTP20220	0	0	0	Tissue specific
KNLFPSN	Superscaf	-	Superscaf	27722791	27722811	-4	4	10	-	-	-	-	-	-	NCP	IGR	-	ID=gene-E	7	6.285714286	3123.4	0.488649568	KNLFPSN	54	819.4204	1	818.4286	.	T	Superscaf	27722901	0	0	0	0	BTP20220	0	Tissue specific
YGGSSSSSS	Superscaf	+	Superscaf	27746077	27746118	42	42	55	-	-	gene	-	-	mRNA	NCP	Intron	gene|mRN	ID=gene-E	14	6.122857143	5440.8	0.488219668	YGGSSSSSS	98	669.7814	2	1337.548	.	F	Superscaf	27745954	0	0	0	0	0	BTP20220	Tissue specific
KLNLKYVC	Superscaf	-	Superscaf	27755842	27755865	-13	13	20	-	-	gene	-	-	mRNA	NCP	Intron	gene|mRN	ID=gene-E	8	5.94125	3285.5	0.488039883	K(+42.01)	69	540.3001	2	1078.585	Acetylati	D	Superscaf	27755877	0	0	0	0	0	BTP20220	Tissue specific
VTDLPF	Superscaf	+	Superscaf	27802171	27802188	4	4	9	-	-	gene	-	-	mRNA	NCP	Intron	gene|mRN	ID=gene-E	6	4.543333333	1887.2	0.48718545	VTDLPF	57	691.3557	1	690.3588	.	T	Superscaf	27802162	0	BTP20220	0	0	0	0	Tissue specific
ALTALTY	Superscaf	-	Superscaf	27846748	27846768	-2	2	8	-	-	-	-	-	-	NCP	IGR	-	ID=gene-E	7	6.102857143	2583.9	0.486363167	ALTALTY	62	752.4153	1	751.4116	.	T	Superscaf	27846816	0	BTP20220	0	0	0	0	Tissue specific
CMDDQ	Superscaf	+	Superscaf	27876742	27876756	54	54	58	-	-	gene	-	-	mRNA	NCP	Intron	gene|mRN	ID=gene-E	5	4.472	2390.9	0.485810035	C(+57.02)	54	684.2007	1	683.1891	Carbamido	E	Superscaf	27876583	0	0	0	0	0	BTP20220	Tissue specific
YNFPDPP	Superscaf	-	Superscaf	27906262	27906282	-9	9	15	-	-	gene	-	-	mRNA	NCP	Intron	gene|mRN	ID=gene-E	7	5.488571429	2867.3	0.485265424	YNFPDPP	62	425.1898	2	848.3704	.	V	Superscaf	27906336	0	0	0	0	0	BTP20220	Tissue specific
ATTRWLL	Superscaf	+	Superscaf	27913285	27913305	25	25	31	-	-	gene	-	-	mRNA	NCP	Intron	gene|mRN	ID=gene-E	7	6.812857143	2988.5	0.485135884	ATTRWLL	60	430.7542	2	859.4916	.	V	Superscaf	27913213	0	0	0	BTP20220	0	0	Tissue specific
KLFPPPPP	Superscaf	+	Superscaf	27926908	27926931	11	11	18	-	-	gene	-	-	mRNA	NCP	Intron	gene|mRN	ID=gene-E	8	6.5875	2896.2	0.484884551	KLFPPPPP	76	446.775	2	891.5218	.	I	Superscaf	27926878	0	0	0	0	BTP20220	0	Tissue specific
FVGPLK	Superscaf	+	Superscaf	28190983	28191000	37	37	42	-	-	gene	-	-	mRNA	NCP	Intron	gene|mRN	ID=gene-E	6	5.578333333	1843.9	0.480013768	FVGPLK	70	660.4036	1	659.4006	.	D	Superscaf	28190875	0	0	0	0	0	BTP20220	Tissue enhanced
VGPVPLT	Superscaf	-	Superscaf	28277089	28277109	-10	10	16	-	-	-	-	-	-	NCP	IGR	-	ID=gene-E	7	4.44	1723	0.478425478	V(+42.01)	50	724.4211	1	723.4167	Acetylati	W	Superscaf	28277244	0	BTP20220	0	0	0	0	Tissue specific
KKMEPF	Superscaf	-	Superscaf	28333735	28333752	-35	35	40	-	-	-	-	-	-	NCP	IGR	-	ID=gene-E	6	6.705	2458.6	0.477380691	KKMEPF	59	779.4249	1	778.4047	.	Q	Superscaf	28333947	0	0	0	0	BTP20220	0	Tissue specific
SRPLT	Superscaf	-	Superscaf	28344862	28344876	-4	4	8	-	-	-	-	-	-	NCP	IGR	-	ID=gene-E	5	7.05	2120.5	0.477175508	S(+42.01)	53	615.3455	1	614.3387	Acetylati	R	Superscaf	28345026	0	0	0	0	BTP20220	0	Tissue specific
KEYTH	Superscaf	-	Superscaf	28436767	28436781	-4	4	8	-	-	-	-	-	-	NCP	IGR	-	ID=gene-E	5	6.548	2370.6	0.475480309	KEYTH	67	677.3251	1	676.3181	.	E	Superscaf	28436799	0	BTP20220	0	0	0	0	Tissue specific
KHQNPPP	Superscaf	-	Superscaf	28482070	28482090	-17	17	23	-	-	-	-	-	-	NCP	IGR	-	ID=gene-E	7	6.755714286	3307.7	0.47464458	KHQNPPP	52	409.2169	2	816.4242	.	Q	Superscaf	28482147	BTP20220	0	0	0	0	0	Tissue specific
STVPP	Superscaf	-	Superscaf	28550083	28550097	-57	57	61	-	-	-	-	-	-	NCP	IGR	-	ID=gene-E	5	4.962	1455.7	0.473390183	STVPP	50	500.2678	1	499.2642	.	L	Superscaf	28550103	0	0	0	BTP20220	0	0	Tissue specific
AELRADP	Superscaf	-	Superscaf	28552687	28552707	-26	26	32	-	-	gene	-	-	mRNA	NCP	Intron	gene|mRN	ID=gene-E	7	5.895714286	2799.2	0.473342041	A(+42.01)	66	407.203	2	812.4028	Acetylati	L	Superscaf	28552806	0	BTP20220	0	0	0	BTP20220	Tissue specific
SLLEEVN	Superscaf	+	Superscaf	28562722	28562742	13	13	19	-	-	gene	-	-	mRNA	NCP	Intron	gene|mRN	ID=gene-E	7	4.212857143	2537.9	0.473156944	SLLEEVN	64	803.427	1	802.4072	.	T	Superscaf	28562686	0	0	0	0	BTP20220	BTP20220	Tissue specific
PVVPLDP	Superscaf	+	Superscaf	28568575	28568595	1	1	7	-	-	gene	-	-	mRNA	NCP	Intron	gene|mRN	ID=gene-E	7	3.978571429	1777.1	0.473048985	PVVPLDP	64	736.4305	1	735.4167	.	P	Superscaf	28568575	0	0	0	0	BTP20220	0	Tissue specific
DTAGQEDYI	Superscaf	-	Superscaf	28595908	28595946	-83	83	95	-	-	gene	-	-	mRNA	NCP	Intron	gene|mRN	ID=gene-E	13	5.827692308	5724.7	0.472544493	DTAGQEDYI	86	768.3557	2	1534.701	.	A	Superscaf	28596321	BTP20220	0	0	0	0	0	Tissue specific
SLGVLPK	Superscaf	-	Superscaf	28610137	28610157	-4	4	10	-	-	gene	-	-	mRNA	NCP	Intron	gene|mRN	ID=gene-E	7	5.664285714	2193.3	0.472282369	SLGVLPK	54	357.2354	2	712.4483	.	G	Superscaf	28610232	0	BTP20220	0	0	0	0	Tissue specific
SKLTGYQP	Superscaf	-	Superscaf	28619677	28619700	-29	29	36	-	-	gene	-	-	mRNA	NCP	Intron	gene|mRN	ID=gene-E	8	6.43875	3307.6	0.472106347	SKLTGYQP	55	447.2419	2	892.4654	.	R	Superscaf	28619838	0	0	0	0	0	BTP20220	Tissue specific
KGHVLGL	Superscaf	+	Superscaf	28648705	28648725	2	2	8	-	-	-	-	-	-	NCP	IGR	-	ID=gene-E	7	5.89	2389.5	0.471570978	KGHVLGL	79	362.2292	2	722.4439	.	G	Superscaf	28648702	0	BTP20220	0	0	0	0	Tissue enhanced
LRSDLRLL	Superscaf	-	Superscaf	28649194	28649217	-23	23	30	-	-	-	-	-	-	NCP	IGR	-	ID=gene-E	8	6.76125	3506.1	0.471561903	LRSDLRLL	92	493.3097	2	984.608	.	E	Superscaf	28649331	0	0	BTP20220	0	BTP20220	0	Tissue specific
KPPPTAP	Superscaf	-	Superscaf	28691899	28691919	-16	16	22	-	-	-	-	-	-	NCP	IGR	-	ID=gene-E	7	6.784285714	2526.8	0.47077426	KPPPTAP	57	354.2062	2	706.4013	.	T	Superscaf	28691928	0	BTP20220	0	0	0	0	Tissue specific
KPTRNV	Superscaf	+	Superscaf	28717429	28717446	36	36	41	-	-	-	-	-	-	NCP	IGR	-	ID=gene-E	6	6.456666667	2448.8	0.470303411	KPTRNV	51	714.4126	1	713.4184	.	K	Superscaf	28717324	0	BTP20220	0	0	0	0	Tissue specific
KRWAAFY	Superscaf	+	Superscaf	28757830	28757850	40	40	46	-	-	-	-	-	-	NCP	IGR	-	ID=gene-E	7	7.081428571	3113.3	0.469558155	KRWAAFY	65	471.2574	2	940.4919	.	P	Superscaf	28757713	BTP20220	0	0	0	0	0	Tissue specific
YALPPPPP	Superscaf	+	Superscaf	28758919	28758942	74	74	81	-	-	-	-	-	-	NCP	IGR	-	ID=gene-E	8	6.145	2811.2	0.469538013	Y(+42.01)	61	447.2334	2	892.4694	Acetylati	G	Superscaf	28758700	0	0	0	0	BTP20220	0	Tissue specific
LEDNP	Superscaf	+	Superscaf	28777261	28777275	54	54	58	-	-	gene	-	-	mRNA	NCP	Intron	gene|mRN	ID=gene-E	5	4.776	2124.5	0.469199858	LEDNP	50	587.2563	1	586.2598	.	F	Superscaf	28777102	0	BTP20220	0	0	0	0	Tissue specific
MELLKLG	Superscaf	+	Superscaf	28791571	28791591	9	9	15	-	-	gene	-	-	mRNA	NCP	Intron	gene|mRN	ID=gene-E	7	6.088571429	2622.9	0.468935798	M(+42.01)	50	845.4835	1	844.4728	Acetylati	F	Superscaf	28791547	0	BTP20220	0	0	0	0	Tissue specific
LARSLKT	Superscaf	+	Superscaf	28820992	28821012	2	2	8	-	-	gene	-	-	mRNA	NCP	Intron	gene|mRN	ID=gene-E	7	7.241428571	2916.2	0.468393124	LARSLKT	99	394.7519	2	787.4916	.	I	Superscaf	28820989	0	0	0	0	0	BTP20220	Mixed
KVYTAF	Superscaf	-	Superscaf	28845769	28845786	-8	8	13	-	-	-	-	-	-	NCP	IGR	-	ID=gene-E	6	5.571666667	2024.1	0.467936165	KVYTAF	51	728.3875	1	727.3904	.	N	Superscaf	28845804	0	0	0	BTP20220	BTP20220	0	Tissue specific
AAVYQA	Superscaf	-	Superscaf	28847503	28847520	-19	19	24	-	-	-	-	-	-	NCP	IGR	-	ID=gene-E	6	4.895	2004.1	0.467904181	A(+42.01)	70	664.3286	1	663.3228	Acetylati	R	Superscaf	28847529	0	BTP20220	0	0	0	0	Tissue specific
LQKQPY	Superscaf	+	Superscaf	28848565	28848582	46	46	51	-	-	-	-	-	-	NCP	IGR	-	ID=gene-E	6	6.496666667	2840.1	0.467884593	LQKQPY	79	388.7169	2	775.4228	.	P	Superscaf	28848430	0	0	0	0	0	BTP20220	Tissue specific
VSCNP	Superscaf	+	Superscaf	28849672	28849686	19	19	23	-	-	-	-	-	-	NCP	IGR	-	ID=gene-E	5	4.482	1903.2	0.467864229	V(+42.01)	67	618.2449	1	617.2479	Acetylati	K	Superscaf	28849618	0	0	0	0	0	BTP20220	Tissue specific
AAGRGWK	Superscaf	+	Superscaf	28871233	28871253	19	19	25	-	-	-	-	-	-	NCP	IGR	-	ID=gene-E	7	7.195714286	2861	0.467466423	AAGRGWK	91	373.2084	2	744.4031	.	L	Superscaf	28871179	0	0	0	0	0	BTP20220	Tissue specific
LLVLDL	Superscaf	-	Superscaf	28874089	28874106	-29	29	34	-	-	-	-	-	-	NCP	IGR	-	ID=gene-E	6	4.481666667	1825.1	0.4674138	LLVLDL	61	685.4468	1	684.4421	.	S	Superscaf	28874109	0	0	0	0	0	BTP20220	Tissue specific
TLFGNER	Superscaf	+	Superscaf	28957720	28957740	9	9	15	-	-	-	-	-	-	NCP	IGR	-	ID=gene-E	7	6.192857143	3106.6	0.465871161	TLFGNER	84	418.7146	2	835.4188	.	L	Superscaf	28957696	0	0	BTP20220	0	0	0	Tissue specific
VGFPP	Superscaf	-	Superscaf	28962316	28962330	-12	12	16	-	-	-	-	-	-	NCP	IGR	-	ID=gene-E	5	4.81	1359.5	0.465786498	VGFPP	55	516.2787	1	515.2744	.	I	Superscaf	28962351	BTP20220	0	0	0	0	0	Tissue specific
QERLHLV	Superscaf	-	Superscaf	28986214	28986234	-11	11	17	-	-	-	-	-	-	NCP	IGR	-	ID=gene-E	7	5.597142857	3011.5	0.465345586	QERLHLV	64	447.7611	2	893.5083	.	T	Superscaf	28986285	BTP20220	0	0	0	0	0	Tissue specific
CPCDAL	Superscaf	+	Superscaf	29009269	29009286	3	3	8	-	-	-	-	-	-	NCP	IGR	-	ID=gene-E	6	5.218333333	2585	0.464920389	C(+57.02)	52	777.2941	1	776.2833	Carbamido	P	Superscaf	29009263	BTP20220	0	0	0	0	0	Tissue specific
REPLTRHN	Superscaf	-	Superscaf	29071270	29071293	-1	1	8	-	-	-	-	-	-	NCP	IGR	-	ID=gene-E	8	7.06875	4027.8	0.463776663	REPLTRHN	65	511.7744	2	1021.542	.	R	Superscaf	29071338	0	0	0	0	0	BTP20220	Tissue specific
GTLLEL	Superscaf	-	Superscaf	29075137	29075154	-14	14	19	-	-	-	-	-	-	NCP	IGR	-	ID=gene-E	6	5.61	2180.5	0.463705446	G(+42.01)	85	687.3909	1	686.3851	Acetylati	F	Superscaf	29075193	0	BTP20220	0	0	0	0	Mixed
PPLLEAP	Superscaf	+	Superscaf	29107342	29107362	38	38	44	-	-	-	-	-	-	NCP	IGR	-	ID=gene-E	7	5.728571429	2455.8	0.463111366	PPLLEAP	53	368.7116	2	735.4166	.	G	Superscaf	29107231	0	BTP20220	0	0	0	BTP20220	Tissue specific
KLLFVPP	Superscaf	+	Superscaf	29107960	29107980	12	12	18	-	-	-	-	-	-	NCP	IGR	-	ID=gene-E	7	5.682857143	2237.4	0.463099967	KLLFVPP	82	407.2635	2	812.516	.	D	Superscaf	29107927	0	BTP20220	0	0	0	BTP20220	Tissue specific
MDFEDDYTF	Superscaf	+	Superscaf	29235679	29235708	31	31	40	-	-	gene	-	-	mRNA	NCP	Intron	gene|mRN	ID=gene-E	10	4.882	4279	0.460744009	M(+42.01)	98	651.2344	2	1300.455	Acetylati	L	Superscaf	29235589	0	BTP20220	0	0	0	0	Tissue enhanced
MDFEDDYTF	Superscaf	+	Superscaf	29235679	29235705	31	31	39	-	-	gene	-	-	mRNA	NCP	Intron	gene|mRN	ID=gene-E	9	4.793333333	3895.6	0.460744064	M(+42.01)	95	607.7181	2	1213.423	Acetylati	L	Superscaf	29235589	0	BTP20220	0	0	0	0	Mixed
EHGLSNY	Superscaf	-	Superscaf	29241247	29241267	-10	10	16	-	-	gene	-	-	mRNA	NCP	Intron	gene|mRN	ID=gene-E	7	5.644285714	3135.5	0.460641473	EHGLSNY	57	819.3559	1	818.3559	.	I	Superscaf	29241318	0	0	BTP20220	0	0	0	Tissue specific
ANPSFPL	Superscaf	+	Superscaf	29304478	29304498	34	34	40	-	-	gene	-	-	mRNA	NCP	Intron	gene|mRN	ID=gene-E	7	5.881428571	2707	0.45947517	ANPSFPL	73	745.3873	1	744.3806	.	T	Superscaf	29304379	0	0	0	0	BTP20220	0	Tissue specific
KPFKFVLGK	Superscaf	+	Superscaf	29306887	29306913	110	110	118	-	-	gene	-	-	mRNA	NCP	Intron	gene|mRN	ID=gene-E	9	6.492222222	3168.1	0.459430625	KPFKFVLGK	96	355.2253	3	1062.659	.	N	Superscaf	29306560	0	0	0	0	BTP20220	BTP20220	Tissue specific
FDVELLKLE	Superscaf	+	Superscaf	29307052	29307078	165	165	173	-	-	gene	-	-	mRNA	NCP	Intron	gene|mRN	ID=gene-E	9	4.73	3178.5	0.459427581	FDVELLKLE	98	553.3079	2	1104.607	.	N	Superscaf	29306560	0	BTP20220	0	0	0	0	Tissue specific
DVELLKLE	Superscaf	+	Superscaf	29307055	29307078	166	166	173	-	-	gene	-	-	mRNA	NCP	Intron	gene|mRN	ID=gene-E	8	4.63625	2791.1	0.459427581	DVELLKLE	92	479.7756	2	957.5382	.	N	Superscaf	29306560	0	0	0	0	0	BTP20220	Mixed
APTHKPK	Superscaf	-	Superscaf	29333338	29333358	-51	51	57	-	-	gene	-	-	mRNA	NCP	Intron	gene|mRN	ID=gene-E	7	7.46	2940.1	0.458942844	APTHKPK	57	389.7373	2	777.4497	.	S	Superscaf	29333370	0	BTP20220	0	0	0	0	Tissue specific
PERPGPGSA	Superscaf	+	Superscaf	29375578	29375607	49	49	58	-	-	gene	-	-	mRNA	NCP	Intron	gene|mRN	ID=gene-E	10	6.249	3673.1	0.458163556	PERPGPGSA	64	462.7292	2	923.446	.	I	Superscaf	29375434	0	0	0	0	BTP20220	0	Tissue specific
PERPGPGSA	Superscaf	+	Superscaf	29375578	29375604	49	49	57	-	-	gene	-	-	mRNA	NCP	Intron	gene|mRN	ID=gene-E	9	6.28	3375.8	0.458163611	PERPGPGSA	77	434.2183	2	866.4246	.	I	Superscaf	29375434	0	0	0	0	BTP20220	0	Tissue specific
QLYVPR	Superscaf	-	Superscaf	29379010	29379027	-7	7	12	-	-	gene	-	-	mRNA	NCP	Intron	gene|mRN	ID=gene-E	6	5.725	2409.8	0.458100474	QLYVPR	81	388.2208	2	774.4388	.	T	Superscaf	29379066	0	0	0	0	0	BTP20220	Tissue specific
LYLRPP	Superscaf	-	Superscaf	29451931	29451948	-46	46	51	-	-	-	-	-	-	NCP	IGR	-	ID=gene-E	6	6.83	2489.9	0.456755438	LYLRPP	68	379.7341	2	757.4486	.	P	Superscaf	29451966	0	0	0	0	BTP20220	BTP20220	Tissue specific
KPHVPPPPF	Superscaf	-	Superscaf	29458534	29458560	-7	7	15	-	-	-	-	-	-	NCP	IGR	-	ID=gene-E	9	6.125555556	3112.5	0.456633478	KPHVPPPPF	63	483.2911	2	964.5494	.	L	Superscaf	29458575	0	0	0	0	0	BTP20220	Tissue specific
VKDEQPR	Superscaf	+	Superscaf	29466211	29466231	17	17	23	-	-	-	-	-	-	NCP	IGR	-	ID=gene-E	7	5.52	2902.3	0.456491986	VKDEQPR	79	436.234	2	870.4559	.	T	Superscaf	29466163	0	0	0	0	BTP20220	0	Tissue specific
KCVPP	Superscaf	+	Superscaf	29466781	29466795	4	4	8	-	-	-	-	-	-	NCP	IGR	-	ID=gene-E	5	5.472	1728.9	0.456481583	KC(+57.02	60	600.3245	1	599.3101	Carbamido	R	Superscaf	29466772	0	0	0	0	0	BTP20220	Tissue specific
NQLLNVSK	Superscaf	-	Superscaf	29516506	29516529	-39	39	46	-	-	gene	-	-	mRNA	NCP	Intron	gene|mRN	ID=gene-E	8	5.48125	3362.7	0.455564234	NQLLNVSK	78	458.2663	2	914.5185	.	I	Superscaf	29516550	0	0	0	0	0	BTP20220	Tissue specific
GDNVGF	Superscaf	+	Superscaf	29564725	29564742	3	3	8	-	-	gene	-	-	mRNA	NCP	Intron	gene|mRN	ID=gene-E	6	4.3	1990.2	0.454674939	GDNVGF	84	608.2657	1	607.2602	.	R	Superscaf	29564719	0	0	0	0	0	BTP20220	Tissue specific
LTYVMVLV	Superscaf	-	Superscaf	29583346	29583369	-1	1	8	-	-	gene	-	-	mRNA	NCP	Intron	gene|mRN	ID=gene-E	8	3.7375	1935.3	0.454331362	LTYVM(+15	67	477.272	2	952.5303	Oxidation	L	Superscaf	29583411	0	BTP20220	0	0	0	0	Tissue specific
AGPSLV	Superscaf	-	Superscaf	29599618	29599635	-38	38	43	-	-	gene	-	-	mRNA	NCP	Intron	gene|mRN	ID=gene-E	6	4.991666667	1682.8	0.454031334	AGPSLV	83	543.3118	1	542.3064	.	F	Superscaf	29599743	0	0	0	0	0	BTP20220	Tissue specific
ADELAK	Superscaf	+	Superscaf	29630095	29630112	56	56	61	-	-	gene	-	-	mRNA	NCP	Intron	gene|mRN	ID=gene-E	6	5.658333333	2281.4	0.453469182	A(+42.01)	99	688.3511	1	687.3439	Acetylati	V	Superscaf	29629930	0	BTP20220	0	0	0	0	Mixed
MFKFVSP	Superscaf	-	Superscaf	29631124	29631144	-75	75	81	-	-	gene	-	-	mRNA	NCP	Intron	gene|mRN	ID=gene-E	7	5.49	2335.5	0.453450147	M(+42.01)	53	449.2373	2	896.4466	Acetylati	L	Superscaf	29631147	0	0	0	0	0	BTP20220	Tissue specific
SLAMTKH	Superscaf	-	Superscaf	29647663	29647683	-9	9	15	-	-	gene	-	-	mRNA	NCP	Intron	gene|mRN	ID=gene-E	7	6.755714286	2905.2	0.453145083	SLAMTKH	63	394.212	2	786.4058	.	G	Superscaf	29647692	BTP20220	0	0	0	0	0	Tissue specific
LAPSPAPK	Superscaf	+	Superscaf	29665087	29665110	71	71	78	-	-	gene	-	-	mRNA	NCP	Intron	gene|mRN	ID=gene-E	8	6.5425	2840	0.45282364	LAPSPAPK	98	390.7335	2	779.4541	.	V	Superscaf	29664877	0	0	BTP20220	0	0	0	Tissue enhanced
RDPSFN	Superscaf	-	Superscaf	29694250	29694267	-16	16	21	-	-	gene	-	-	mRNA	NCP	Intron	gene|mRN	ID=gene-E	6	6.1	2765.2	0.452285836	RDPSFN	78	368.1738	2	734.3347	.	L	Superscaf	29694279	0	0	0	0	BTP20220	0	Tissue specific
EMVHAK	Superscaf	+	Superscaf	29732065	29732082	87	87	92	-	-	gene	-	-	mRNA	NCP	Intron	gene|mRN	ID=gene-E	6	5.386666667	2196.4	0.451588334	EM(+15.99	62	365.6824	2	729.348	Oxidation	V	Superscaf	29731807	BTP20220	0	0	0	0	0	Tissue specific
MMKAH	Superscaf	-	Superscaf	29760379	29760393	-10	10	14	-	-	gene	-	-	mRNA	NCP	Intron	gene|mRN	ID=gene-E	5	6.97	2142.4	0.451066134	MM(+15.99	51	633.2891	1	632.2775	Oxidation	P	Superscaf	29760423	0	0	0	BTP20220	0	0	Tissue specific
MELLQDT	Superscaf	+	Superscaf	29776306	29776326	3	3	9	-	-	gene	-	-	mRNA	NCP	Intron	gene|mRN	ID=gene-E	7	5.154285714	2923.5	0.450772248	M(+42.01)	63	891.4123	1	890.4055	Acetylati	K	Superscaf	29776300	0	0	0	0	BTP20220	BTP20220	Tissue specific
KSHVEQG	Superscaf	-	Superscaf	29778523	29778543	-6	6	12	-	-	gene	-	-	mRNA	NCP	Intron	gene|mRN	ID=gene-E	7	5.407142857	2805	0.450731355	KSHVEQG	51	392.7083	2	783.3875	.	A	Superscaf	29778624	0	0	BTP20220	0	0	0	Tissue specific
RNCLLLH	Superscaf	-	Superscaf	29833765	29833785	-19	19	25	-	-	gene	-	-	mRNA	NCP	Intron	gene|mRN	ID=gene-E	7	6.674285714	3511.1	0.44971241	RNC(+57.0	82	463.2574	2	924.4963	Carbamido	R	Superscaf	29833962	0	0	0	BTP20220	0	0	Tissue specific
QGELEF	Superscaf	-	Superscaf	29847235	29847252	-20	20	25	-	-	gene	-	-	mRNA	NCP	Intron	gene|mRN	ID=gene-E	6	4.92	2499.8	0.44946401	QGELEF	56	722.3324	1	721.3282	.	I	Superscaf	29847258	0	0	0	0	BTP20220	0	Tissue specific
ESLLTY	Superscaf	-	Superscaf	29876527	29876544	-2	2	7	-	-	gene	-	-	mRNA	NCP	Intron	gene|mRN	ID=gene-E	6	5.508333333	2372.7	0.448923716	E(+42.01)	59	767.381	1	766.3749	Acetylati	F	Superscaf	29876562	0	0	0	BTP20220	0	0	Tissue specific
ENMLEK	Superscaf	-	Superscaf	29876794	29876811	-30	30	35	-	-	gene	-	-	mRNA	NCP	Intron	gene|mRN	ID=gene-E	6	5.553333333	2641	0.448918791	ENM(+15.9	64	779.3552	1	778.3531	Oxidation	P	Superscaf	29876826	0	0	0	0	0	BTP20220	Tissue specific
AAPSVTGK	Superscaf	-	Superscaf	29884447	29884470	-26	26	33	-	-	gene	-	-	mRNA	NCP	Intron	gene|mRN	ID=gene-E	8	5.7825	2506.6	0.44877752	AAPSVTGK	51	365.7096	2	729.4021	.	I	Superscaf	29884479	0	0	0	0	BTP20220	0	Tissue specific
NTLPEQ	Superscaf	+	Superscaf	29942524	29942541	45	45	50	-	-	gene	-	-	mRNA	NCP	Intron	gene|mRN	ID=gene-E	6	5.515	2721.2	0.447706393	N(+42.01)	71	743.3457	1	742.3497	Acetylati	T	Superscaf	29942392	BTP20220	0	0	0	0	0	Tissue specific
MLLLKLVQ	Superscaf	-	Superscaf	29969248	29969271	-55	55	62	-	-	gene	-	-	mRNA	NCP	Intron	gene|mRN	ID=gene-E	8	5.6325	2864.2	0.447213355	M(+42.01)	68	500.316	2	998.6198	Acetylati	A	Superscaf	29969511	0	0	0	0	BTP20220	0	Tissue specific
SAHLQW	Superscaf	+	Superscaf	30011812	30011829	11	11	16	-	-	gene	-	-	mRNA	NCP	Intron	gene|mRN	ID=gene-E	6	6.135	2805.1	0.446428368	S(+42.01)	79	783.3786	1	782.3712	Acetylati	R	Superscaf	30011782	0	BTP20220	0	0	0	0	Tissue specific
EPELT	Superscaf	+	Superscaf	30017875	30017889	35	35	39	-	-	gene	-	-	mRNA	NCP	Intron	gene|mRN	ID=gene-E	5	5.05	1939.3	0.446316591	E(+42.01)	70	630.2997	1	629.2908	Acetylati	L	Superscaf	30017773	0	0	0	0	0	BTP20220	Tissue specific
KSYSLPP	Superscaf	-	Superscaf	30097549	30097569	-3	3	9	-	-	-	-	-	-	NCP	IGR	-	ID=gene-E	7	6.477142857	2722.9	0.444846884	KSYSLPP	56	396.2244	2	790.4225	.	R	Superscaf	30097569	0	0	0	0	0	BTP20220	Tissue specific
PASELPR	Superscaf	+	Superscaf	30124432	30124452	4	4	10	-	-	-	-	-	-	NCP	IGR	-	ID=gene-E	7	6.322857143	2797.2	0.444351024	PASELPR	60	385.2139	2	768.413	.	R	Superscaf	30124423	0	0	0	0	0	BTP20220	Tissue specific
KSTPLQTP	Superscaf	+	Superscaf	30131344	30131367	11	11	18	-	-	gene	-	-	mRNA	NCP	Intron	gene|mRN	ID=gene-E	8	6.58875	3285.7	0.444223476	KSTPLQTP	60	436.2482	2	870.4811	.	R	Superscaf	30131314	0	BTP20220	0	0	0	0	Tissue specific
KSTSVGK	Superscaf	+	Superscaf	30156928	30156948	26	26	32	-	-	gene	-	-	mRNA	NCP	Intron	gene|mRN	ID=gene-E	7	6.191428571	2398.4	0.443751632	KSTSVGK	51	353.7072	2	705.4021	.	V	Superscaf	30156853	0	0	0	0	BTP20220	0	Tissue specific
YFPPVR	Superscaf	+	Superscaf	30158584	30158601	13	13	18	-	-	gene	-	-	mRNA	NCP	Intron	gene|mRN	ID=gene-E	6	5.75	2170.5	0.443721142	YFPPVR	80	389.7125	2	777.4173	.	L	Superscaf	30158548	0	0	0	BTP20220	0	0	Tissue specific
LPLEGKG	Superscaf	+	Superscaf	30208213	30208233	5	5	11	-	-	gene	-	-	mRNA	NCP	Intron	gene|mRN	ID=gene-E	7	6.165714286	2532.7	0.442805674	LPLEGKG	90	357.2124	2	712.4119	.	F	Superscaf	30208201	0	0	BTP20220	0	0	0	Tissue specific
ALPVLPPP	Superscaf	+	Superscaf	30217126	30217149	42	42	49	-	-	gene	-	-	mRNA	NCP	Intron	gene|mRN	ID=gene-E	8	5.3975	2367.7	0.442641217	A(+42.01)	65	423.2589	2	844.5058	Acetylati	A	Superscaf	30217003	0	0	0	0	BTP20220	0	Tissue specific
KNDTLP	Superscaf	+	Superscaf	30230635	30230652	3	3	8	-	-	-	-	-	-	NCP	IGR	-	ID=gene-E	6	6.155	2564.9	0.442392153	KNDTLP	61	687.371	1	686.3599	.	Y	Superscaf	30230629	0	0	0	0	BTP20220	0	Tissue enhanced
KFFPF	Superscaf	-	Superscaf	30274231	30274245	-5	5	9	-	-	-	-	-	-	NCP	IGR	-	ID=gene-E	5	6.496	1968	0.441588075	KFFPF	52	685.3712	1	684.3635	.	S	Superscaf	30274377	0	0	0	0	0	BTP20220	Tissue specific
APSCYPQ	Superscaf	+	Superscaf	30278011	30278031	1	1	7	-	-	-	-	-	-	NCP	IGR	-	ID=gene-E	7	5.804285714	3025.4	0.441518242	APSC(+57.	63	822.3514	1	821.3378	Carbamido	A	Superscaf	30278011	0	0	BTP20220	0	0	0	Tissue specific
YQGKSLR	Superscaf	+	Superscaf	30283219	30283239	7	7	13	-	-	-	-	-	-	NCP	IGR	-	ID=gene-E	7	7.062857143	3221.5	0.44142218	YQGKSLR	77	426.2389	2	850.4661	.	S	Superscaf	30283201	0	0	0	0	BTP20220	0	Tissue specific
LLKPKP	Superscaf	-	Superscaf	30347833	30347850	-29	29	34	-	-	-	-	-	-	NCP	IGR	-	ID=gene-E	6	7.34	2318.4	0.440230422	LLKPKP	72	695.4782	1	694.4741	.	L	Superscaf	30348102	0	BTP20220	0	0	0	0	Tissue specific
DLDVKGSP	Superscaf	+	Superscaf	30480394	30480417	12	12	19	-	-	-	-	-	-	NCP	IGR	-	ID=gene-E	8	4.95125	2662.9	0.437785209	DLDVKGSP	78	415.716	2	829.4181	.	N	Superscaf	30480361	0	0	0	0	BTP20220	BTP20220	Tissue specific
ALTTYG	Superscaf	+	Superscaf	30482428	30482445	41	41	46	-	-	-	-	-	-	NCP	IGR	-	ID=gene-E	6	6.115	2216.5	0.437747802	ALTTYG	51	625.3202	1	624.3119	.	S	Superscaf	30482308	BTP20220	0	0	0	0	0	Tissue specific
WWLLLVE	Superscaf	+	Superscaf	30488560	30488580	9	9	15	-	-	-	-	-	-	NCP	IGR	-	ID=gene-E	7	4.705714286	2538.9	0.437634641	W(+42.01)	70	500.7756	2	999.5429	Acetylati	L	Superscaf	30488536	0	0	BTP20220	BTP20220	0	0	Tissue specific
TVHAKPP	Superscaf	-	Superscaf	30502978	30502998	-7	7	13	-	-	-	-	-	-	NCP	IGR	-	ID=gene-E	7	6.068571429	2471.7	0.4373687	TVHAKPP	70	375.2175	2	748.4232	.	D	Superscaf	30503016	0	0	BTP20220	0	0	0	Tissue specific
HAKPPPP	Superscaf	-	Superscaf	30502984	30503004	-9	9	15	-	-	-	-	-	-	NCP	IGR	-	ID=gene-E	7	6.935714286	2749	0.437368589	H(+42.01)	53	393.2246	2	784.4232	Acetylati	D	Superscaf	30503016	0	0	BTP20220	0	0	0	Tissue specific
GKPPAVK	Superscaf	-	Superscaf	30519112	30519132	-8	8	14	-	-	-	-	-	-	NCP	IGR	-	ID=gene-E	7	6.295714286	2220.2	0.437071106	G(+42.01)	83	369.7274	2	737.4435	Acetylati	S	Superscaf	30519186	BTP20220	0	0	0	0	0	Tissue specific
FGRSFSP	Superscaf	+	Superscaf	30545746	30545766	5	5	11	-	-	-	-	-	-	NCP	IGR	-	ID=gene-E	7	6.478571429	2825.1	0.436579839	FGRSFSP	76	399.1992	2	796.3868	.	R	Superscaf	30545734	0	0	0	0	BTP20220	0	Tissue specific
NVNANY	Superscaf	-	Superscaf	30562516	30562533	-9	9	14	-	-	-	-	-	-	NCP	IGR	-	ID=gene-E	6	4.651666667	2560.9	0.43627057	N(+42.01)	57	736.3239	1	735.3188	Acetylati	K	Superscaf	30562590	0	0	0	0	0	BTP20220	Tissue specific
MTDTFAP	Superscaf	-	Superscaf	30579595	30579615	-22	22	28	-	-	-	-	-	-	NCP	IGR	-	ID=gene-E	7	5.654285714	2614.1	0.43595549	MTDTFAP	61	391.6783	2	781.3316	.	D	Superscaf	30579684	0	0	0	0	BTP20220	0	Tissue specific
VDCVTPK	Superscaf	-	Superscaf	30614296	30614316	-6	6	12	-	-	-	-	-	-	NCP	IGR	-	ID=gene-E	7	4.365714286	2200.5	0.435315426	VDC(+57.0	68	409.7064	2	817.4004	Carbamido	P	Superscaf	30614352	BTP20220	0	0	0	0	0	Tissue specific
CDMDMC	Superscaf	-	Superscaf	30645037	30645054	-25	25	30	-	-	gene	-	-	mRNA	NCP	Intron	gene|mRN	ID=gene-E	6	4.58	2737.4	0.43474846	C(+57.02)	56	432.1053	2	862.1966	Carbamido	Y	Superscaf	30645063	0	BTP20220	0	0	0	0	Tissue specific
KEPLGGP	Superscaf	-	Superscaf	30647638	30647658	-1	1	7	-	-	gene	-	-	mRNA	NCP	Intron	gene|mRN	ID=gene-E	7	6.211428571	2516.7	0.434700429	KEPLGGP	77	697.386	1	696.3806	.	K	Superscaf	30647658	0	BTP20220	0	0	0	0	Tissue specific
DLVAPPK	Superscaf	-	Superscaf	30648712	30648732	-22	22	28	-	-	gene	-	-	mRNA	NCP	Intron	gene|mRN	ID=gene-E	7	5.33	2219.4	0.434680619	DLVAPPK	70	370.2194	2	738.4276	.	V	Superscaf	30648765	0	0	0	0	BTP20220	0	Tissue specific
KDEDP	Superscaf	+	Superscaf	30659761	30659775	3	3	7	-	-	gene	-	-	mRNA	NCP	Intron	gene|mRN	ID=gene-E	5	5.04	2054.3	0.43447693	KDEDP	64	603.2684	1	602.2548	.	H	Superscaf	30659755	0	0	0	BTP20220	0	0	Tissue specific
KPSSKGPP	Superscaf	+	Superscaf	30664807	30664830	98	98	105	-	-	gene	-	-	mRNA	NCP	Intron	gene|mRN	ID=gene-E	8	6.96375	3013.1	0.43438369	KPSSKGPP	76	399.2335	2	796.4443	.	P	Superscaf	30664516	0	0	0	BTP20220	0	0	Tissue specific
HMGGPL	Superscaf	+	Superscaf	30675703	30675720	94	94	99	-	-	gene	-	-	mRNA	NCP	Intron	gene|mRN	ID=gene-E	6	6.26	2276.6	0.434182822	HM(+15.99	50	627.298	1	626.2846	Oxidation	I	Superscaf	30675424	0	BTP20220	0	0	0	0	Tissue specific
LKLLSYR	Superscaf	-	Superscaf	30724558	30724578	-43	43	49	-	-	-	-	-	-	NCP	IGR	-	ID=gene-E	7	7.111428571	3020.3	0.433281631	LKLLSYR	85	446.7822	2	891.5541	.	M	Superscaf	30724584	0	0	BTP20220	0	0	0	Tissue specific
LAPLLKDV	Superscaf	-	Superscaf	30791776	30791799	-48	48	55	-	-	gene	-	-	mRNA	NCP	Intron	gene|mRN	ID=gene-E	8	5.37125	2588.8	0.432041732	LAPLLKDV	94	434.7774	2	867.5429	.	E	Superscaf	30791835	0	0	0	0	0	BTP20220	Tissue specific
VPGGALG	Superscaf	+	Superscaf	30796567	30796587	147	147	153	-	-	gene	-	-	mRNA	NCP	Intron	gene|mRN	ID=gene-E	7	5.172857143	1894	0.431953417	VPGGALG	50	570.3239	1	569.3173	.	R	Superscaf	30796129	0	BTP20220	0	0	0	0	Tissue specific
SQGLLALR	Superscaf	+	Superscaf	30864382	30864405	17	17	24	-	-	gene	-	-	mRNA	NCP	Intron	gene|mRN	ID=gene-E	8	6.5025	3311.7	0.430702506	SQGLLALR	64	429.2678	2	856.513	.	N	Superscaf	30864334	0	0	0	0	0	BTP20220	Tissue specific
PCCDAL	Superscaf	+	Superscaf	30867052	30867069	25	25	30	-	-	gene	-	-	mRNA	NCP	Intron	gene|mRN	ID=gene-E	6	5.218333333	2585	0.430653368	P(+42.01)	56	777.2947	1	776.2833	Acetylati	P	Superscaf	30866980	BTP20220	0	0	0	0	0	Tissue specific
CGDDPK	Superscaf	-	Superscaf	30872407	30872424	-83	83	88	-	-	gene	-	-	mRNA	NCP	Intron	gene|mRN	ID=gene-E	6	5.495	2511.8	0.430554595	C(+57.02)	59	367.1457	2	732.2748	Carbamido	Y	Superscaf	30872454	BTP20220	0	0	0	0	0	Tissue specific
TVALPVDF	Superscaf	+	Superscaf	30878911	30878934	21	21	28	-	-	gene	-	-	mRNA	NCP	Intron	gene|mRN	ID=gene-E	8	4.16	2198.5	0.430434517	TVALPVDF	50	861.468	1	860.4644	.	S	Superscaf	30878851	0	0	0	0	BTP20220	0	Tissue specific
TKGVHLR	Superscaf	+	Superscaf	30903841	30903861	1	1	7	-	-	gene	-	-	mRNA	NCP	Intron	gene|mRN	ID=gene-E	7	6.652857143	2785.1	0.429974736	TKGVHLR	66	405.7491	2	809.4871	.	T	Superscaf	30903841	0	0	BTP20220	0	0	0	Tissue specific
CPGVLAK	Superscaf	-	Superscaf	30914467	30914487	-28	28	34	-	-	gene	-	-	mRNA	NCP	Intron	gene|mRN	ID=gene-E	7	5.575714286	2353.5	0.429778738	C(+57.02)	60	372.7058	2	743.4	Carbamido	I	Superscaf	30914499	0	BTP20220	0	0	0	0	Tissue specific
PPRKLLK	Superscaf	+	Superscaf	30977875	30977895	14	14	20	-	-	gene	-	-	mRNA	NCP	Intron	gene|mRN	ID=gene-E	7	7.828571429	2967.2	0.42860917	PPRKLLK	53	426.2996	2	850.5752	.	E	Superscaf	30977836	0	0	0	0	0	BTP20220	Tissue specific
KDVEEN	Superscaf	+	Superscaf	31031482	31031499	6	6	11	-	-	-	-	-	-	NCP	IGR	-	ID=gene-E	6	4.093333333	2327.6	0.427620438	KDVEEN	69	367.1764	2	732.329	.	V	Superscaf	31031467	0	0	BTP20220	0	0	0	Tissue specific
NPTRQPP	Superscaf	+	Superscaf	31230046	31230066	3	3	9	-	-	gene	-	-	mRNA	NCP	Intron	gene|mRN	ID=gene-E	7	6.75	3265.9	0.423957847	NPTRQPP	55	405.2133	2	808.4191	.	G	Superscaf	31230040	0	0	0	0	0	BTP20220	Tissue specific
MDARKK	Superscaf	+	Superscaf	31286770	31286787	21	21	26	-	-	-	-	-	-	NCP	IGR	-	ID=gene-E	6	7.496666667	2679.9	0.422911622	M(+42.01)	94	395.7087	2	789.4167	Acetylati	L	Superscaf	31286710	0	BTP20220	0	0	0	0	Tissue specific
FSVLP	Superscaf	+	Superscaf	31294171	31294185	5	5	9	-	-	-	-	-	-	NCP	IGR	-	ID=gene-E	5	4.688	1461.6	0.422775165	FSVLP	51	562.3168	1	561.3162	.	S	Superscaf	31294159	0	0	0	0	BTP20220	0	Tissue specific
GLVLEAP	Superscaf	+	Superscaf	31316359	31316379	11	11	17	-	-	gene	-	-	mRNA	NCP	Intron	gene|mRN	ID=gene-E	7	4.781428571	2078.3	0.422365794	G(+42.01)	64	740.4175	1	739.4116	Acetylati	G	Superscaf	31316329	0	0	0	0	0	BTP20220	Tissue specific
AAEVLKR	Superscaf	+	Superscaf	31322308	31322328	14	14	20	-	-	gene	-	-	mRNA	NCP	Intron	gene|mRN	ID=gene-E	7	5.962857143	2518.7	0.422256064	AAEVLKR	80	393.7439	2	785.4759	.	G	Superscaf	31322269	BTP20220	0	0	0	0	0	Mixed
MAVYPP	Superscaf	-	Superscaf	31354750	31354767	-11	11	16	-	-	-	-	-	-	NCP	IGR	-	ID=gene-E	6	5.005	1817.1	0.421657723	MAVYPP	68	677.3281	1	676.3254	.	K	Superscaf	31354776	0	0	0	0	0	BTP20220	Tissue specific
CEHAAA	Superscaf	+	Superscaf	31394563	31394580	8	8	13	-	-	-	-	-	-	NCP	IGR	-	ID=gene-E	6	5.648333333	2564.8	0.420923367	C(+57.02)	52	350.6432	2	699.2646	Carbamido	G	Superscaf	31394542	BTP20220	0	0	0	0	0	Tissue specific
KRGELKT	Superscaf	+	Superscaf	31419415	31419435	63	63	69	-	-	-	-	-	-	NCP	IGR	-	ID=gene-E	7	7.42	3059.3	0.420464914	KRGELKT	55	416.2555	2	830.4974	.	M	Superscaf	31419229	0	0	0	0	0	BTP20220	Tissue specific
DFDLPKG	Superscaf	-	Superscaf	31420612	31420632	-11	11	17	-	-	-	-	-	-	NCP	IGR	-	ID=gene-E	7	5.63	2666.9	0.420442835	DFDLPKG	85	396.1989	2	790.386	.	L	Superscaf	31420695	0	0	0	0	0	BTP20220	Tissue specific
DPLSP	Superscaf	+	Superscaf	31423516	31423530	89	89	93	-	-	-	-	-	-	NCP	IGR	-	ID=gene-E	5	5.446	1823.1	0.420389381	DPLSP	85	528.2649	1	527.2591	.	L	Superscaf	31423252	0	0	0	0	0	BTP20220	Tissue specific
LGKAVLGA	Superscaf	-	Superscaf	31477945	31477968	-1	1	8	-	-	-	-	-	-	NCP	IGR	-	ID=gene-E	8	5.71	2392.4	0.419385266	LGKAVLGA	75	364.7355	2	727.4592	.	L	Superscaf	31478046	0	0	0	BTP20220	0	0	Tissue specific
LGSHESP	Superscaf	+	Superscaf	31483474	31483494	20	20	26	-	-	-	-	-	-	NCP	IGR	-	ID=gene-E	7	5.774285714	2800.1	0.419283338	LGSHESP	55	363.6727	2	725.3344	.	C	Superscaf	31483417	0	0	BTP20220	0	0	0	Tissue specific
GPLDLLF	Superscaf	-	Superscaf	31544758	31544778	-10	10	16	-	-	gene	-	-	mRNA	NCP	Intron	gene|mRN	ID=gene-E	7	5.522857143	2493.8	0.418152948	GPLDLLF	68	774.437	1	773.4323	.	K	Superscaf	31544892	0	0	0	0	0	BTP20220	Mixed
NSARLLR	Superscaf	-	Superscaf	31549510	31549530	-1	1	7	-	-	gene	-	-	mRNA	NCP	Intron	gene|mRN	ID=gene-E	7	7.227142857	3295.8	0.418065297	NSARLLR	96	415.2526	2	828.493	.	N	Superscaf	31549530	0	0	0	BTP20220	0	0	Mixed
KQNLKE	Superscaf	+	Superscaf	31561672	31561689	4	4	9	-	-	gene	-	-	mRNA	NCP	Intron	gene|mRN	ID=gene-E	6	6.623333333	2923.1	0.417841023	KQNLKE	56	380.2198	2	758.4286	.	F	Superscaf	31561663	0	0	0	0	0	BTP20220	Tissue specific
QDRGQL	Superscaf	-	Superscaf	31619548	31619565	-43	43	48	-	-	gene	-	-	mRNA	NCP	Intron	gene|mRN	ID=gene-E	6	6.163333333	2932.4	0.416773493	QDRGQL	75	716.3791	1	715.3613	.	S	Superscaf	31619679	BTP20220	0	0	0	0	0	Tissue specific
KQKFC	Superscaf	+	Superscaf	31636081	31636095	2	2	6	-	-	gene	-	-	mRNA	NCP	Intron	gene|mRN	ID=gene-E	5	7.126	2520.6	0.416468595	K(+42.01)	50	752.3809	1	751.3687	Acetylati	S	Superscaf	31636078	0	0	0	0	BTP20220	0	Tissue specific
LLSGTSY	Superscaf	-	Superscaf	31681138	31681158	-3	3	9	-	-	gene	-	-	mRNA	NCP	Intron	gene|mRN	ID=gene-E	7	5.925714286	2627.9	0.415637403	LLSGTSY	90	740.3808	1	739.3752	.	A	Superscaf	31681161	BTP20220	0	0	0	0	0	Tissue specific
PGPVVYLR	Superscaf	+	Superscaf	31726999	31727022	29	29	36	-	-	-	-	-	-	NCP	IGR	-	ID=gene-E	8	5.12125	2433.8	0.414791436	PGPVVYLR	69	450.771	2	899.5228	.	K	Superscaf	31726915	0	0	0	0	BTP20220	BTP20220	Tissue specific

PALGT	Superscaf	+	Superscaf	31730188	31730202	20	20	24	-	-	-	-	-	-	NCP	IGR	-	ID=gene-E	5	6.16	1696.9	0.414732781	P(+42.01)	50	500.2685	1	499.2642	Acetylati	I	Superscaf	31730131	0	0	BTP20220	0	0	0	Tissue specific
KDDLGKF	Superscaf	-	Superscaf	31792000	31792020	-1	1	7	-	-	-	-	-	-	NCP	IGR	-	ID=gene-E	7	6.121428571	2797.9	0.413592541	KDDLGKF	56	411.7249	2	821.4283	.	K	Superscaf	31792110	BTP20220	0	0	0	0	0	Tissue specific
FVKSPF	Superscaf	-	Superscaf	31806124	31806141	-33	33	38	-	-	-	-	-	-	NCP	IGR	-	ID=gene-E	6	5.446666667	1964	0.413332077	FVKSPF	58	724.3973	1	723.3955	.	D	Superscaf	31806234	0	0	0	0	0	BTP20220	Tissue specific
GPEGAP	Superscaf	-	Superscaf	32033761	32033778	-59	59	64	-	-	-	-	-	-	NCP	IGR	-	ID=gene-E	6	5.63	2006.2	0.409133287	G(+42.01)	57	569.257	1	568.2493	Acetylati	E	Superscaf	32033856	BTP20220	0	0	0	0	0	Tissue specific
PLSGRAAP	Superscaf	+	Superscaf	32050393	32050416	26	26	33	-	-	-	-	-	-	NCP	IGR	-	ID=gene-E	8	6.62875	2980.3	0.408826397	PLSGRAAP	57	384.7157	2	767.429	.	G	Superscaf	32050318	0	0	0	0	0	BTP20220	Tissue specific
KGTFY	Superscaf	-	Superscaf	32051935	32051949	-19	19	23	-	-	gene	-	-	mRNA	NCP	Intron	gene|mRN	ID=gene-E	5	6.676	2010.1	0.408798121	K(+42.01)	67	657.3218	1	656.317	Acetylati	M	Superscaf	32051970	0	BTP20220	0	0	0	0	Tissue specific
KPEAFR	Superscaf	-	Superscaf	32055796	32055813	-14	14	19	-	-	gene	-	-	mRNA	NCP	Intron	gene|mRN	ID=gene-E	6	6.92	2578.8	0.408726849	KPEAFR	58	374.2076	2	746.4075	.	I	Superscaf	32055819	0	BTP20220	0	0	0	0	Tissue specific
TVGDLF	Superscaf	+	Superscaf	32061514	32061531	7	7	12	-	-	gene	-	-	mRNA	NCP	Intron	gene|mRN	ID=gene-E	6	4.488333333	1847.1	0.408621379	TVGDLF	69	651.3318	1	650.3275	.	Q	Superscaf	32061496	0	0	BTP20220	0	0	0	Tissue specific
AEVRELQ	Superscaf	-	Superscaf	32062348	32062368	-4	4	10	-	-	gene	-	-	mRNA	NCP	Intron	gene|mRN	ID=gene-E	7	4.978571429	2775.2	0.408605941	A(+42.01)	90	443.7337	2	885.4556	Acetylati	R	Superscaf	32062941	0	0	0	0	BTP20220	0	Tissue specific
SPQGVPFL	Superscaf	-	Superscaf	32067694	32067717	-5	5	12	-	-	gene	-	-	mRNA	NCP	Intron	gene|mRN	ID=gene-E	8	5.17	2707	0.408507278	SPQGVPFL	57	844.454	1	843.449	.	R	Superscaf	32067909	0	0	BTP20220	0	0	0	Tissue specific
LMPNVLL	Superscaf	+	Superscaf	32083480	32083500	5	5	11	-	-	gene	-	-	mRNA	NCP	Intron	gene|mRN	ID=gene-E	7	5.057142857	2365.8	0.408216159	LM(+15.9	67	408.2366	2	814.4623	Oxidatior	F	Superscaf	32083468	0	0	0	0	BTP20220	0	Tissue specific
KSMAPR	Superscaf	+	Superscaf	32143177	32143194	21	21	26	-	-	-	-	-	-	NCP	IGR	-	ID=gene-E	6	7.375	2520.8	0.407115096	KSM(+15.9	61	353.1954	2	704.364	Oxidatior	L	Superscaf	32143117	0	0	0	0	0	BTP20220	Tissue specific
QLLWH	Superscaf	+	Superscaf	32183377	32183391	22	22	26	-	-	gene	-	-	mRNA	NCP	Intron	gene|mRN	ID=gene-E	5	6.218	2463.8	0.406373658	Q(+42.01)	77	738.3829	1	737.386	Acetylati	R	Superscaf	32183314	0	0	BTP20220	0	0	0	Tissue specific
EPSLVHRK	Superscaf	+	Superscaf	32192827	32192850	43	43	50	-	-	gene	-	-	mRNA	NCP	Intron	gene|mRN	ID=gene-E	8	6.15875	3236.6	0.406199185	E(+42.01)	88	504.2786	2	1006.556	Acetylati	G	Superscaf	32192701	0	BTP20220	0	0	0	0	Tissue specific
KESSVLS	Superscaf	+	Superscaf	32223877	32223897	22	22	28	-	-	-	-	-	-	NCP	IGR	-	ID=gene-E	7	5.14	2397.5	0.40562652	K(+42.01)	58	791.4121	1	790.4072	Acetylati	L	Superscaf	32223814	0	BTP20220	0	0	0	0	Tissue specific
GPPSPPPPF	Superscaf	+	Superscaf	32231356	32231385	44	44	53	-	-	-	-	-	-	NCP	IGR	-	ID=gene-E	10	6.205	3379.9	0.405488403	GPPSPPPPF	73	470.2491	2	938.4861	.	G	Superscaf	32231227	0	0	BTP20220	0	0	0	Tissue specific
VTLGTFP	Superscaf	+	Superscaf	32235775	32235795	4	4	10	-	-	-	-	-	-	NCP	IGR	-	ID=gene-E	7	5.255714286	2170.5	0.40540706	VTLGTFP	69	734.4026	1	733.401	.	E	Superscaf	32235766	0	0	0	0	0	BTP20220	Tissue specific
VFEVELL	Superscaf	+	Superscaf	32302456	32302476	36	36	42	-	-	gene	-	-	mRNA	NCP	Intron	gene|mRN	ID=gene-E	7	3.411428571	1945.2	0.404177121	VFEVELL	82	848.4723	1	847.4691	.	H	Superscaf	32302351	0	0	0	0	BTP20220	0	Tissue specific
RMLKSSH	Superscaf	+	Superscaf	32303641	32303661	8	8	14	-	-	gene	-	-	mRNA	NCP	Intron	gene|mRN	ID=gene-E	7	7.311428571	3228.6	0.404155264	R(+42.01)	58	458.7394	2	915.4597	Acetylati	I	Superscaf	32303620	0	0	0	BTP20220	0	0	Tissue specific
LPEQKVL	Superscaf	-	Superscaf	32334418	32334438	-13	13	19	-	-	-	-	-	-	NCP	IGR	-	ID=gene-E	7	5.267142857	2548.8	0.403587579	LPEQKVL	71	413.7545	2	825.496	.	Y	Superscaf	32334447	0	0	BTP20220	0	0	0	Tissue specific
YRVFYM	Superscaf	-	Superscaf	32380516	32380533	-16	16	21	-	-	gene	-	-	mRNA	NCP	Intron	gene|mRN	ID=gene-E	6	5.551666667	2326.7	0.402737351	Y(+42.01)	51	460.7116	2	919.4262	Acetylati	I	Superscaf	32380536	0	0	0	BTP20220	0	0	Tissue specific
LGALLLLK	Superscaf	-	Superscaf	32450230	32450253	-31	31	38	-	-	-	-	-	-	NCP	IGR	-	ID=gene-E	8	6.45375	2844	0.401451358	L(+42.01)	54	441.8032	2	881.595	Acetylati	K	Superscaf	32450265	0	0	0	0	0	BTP20220	Tissue specific
TFHRK	Superscaf	-	Superscaf	32458852	32458866	-11	11	15	-	-	-	-	-	-	NCP	IGR	-	ID=gene-E	5	8.02	2521.8	0.40129249	T(+42.01)	78	365.6972	2	729.3922	Acetylati	Y	Superscaf	32458872	0	0	BTP20220	0	0	0	Tissue specific
KLGKSVE	Superscaf	-	Superscaf	32475526	32475546	-8	8	14	-	-	-	-	-	-	NCP	IGR	-	ID=gene-E	7	5.761428571	2396.4	0.400984825	KLGKSVE	53	760.4542	1	759.449	.	F	Superscaf	32475600	BTP20220	0	BTP20220	0	0	0	Tissue specific
KDVLEL	Superscaf	+	Superscaf	32628223	32628240	11	11	16	-	-	-	-	-	-	NCP	IGR	-	ID=gene-E	6	4.648333333	2012.2	0.398168367	KDVLEL	64	716.416	1	715.4116	.	L	Superscaf	32628193	BTP20220	0	0	0	0	0	Tissue specific
LAKVAVP	Superscaf	-	Superscaf	32652310	32652330	-76	76	82	-	-	gene	-	-	mRNA	NCP	Intron	gene|mRN	ID=gene-E	7	4.865714286	1781.8	0.397724024	L(+42.01)	53	370.2384	2	738.4639	Acetylati	F	Superscaf	32652420	0	0	0	BTP20220	0	0	Tissue specific
NLPGHGP	Superscaf	-	Superscaf	32655184	32655204	-90	90	96	-	-	-	-	-	-	NCP	IGR	-	ID=gene-E	7	6.217142857	2839.2	0.397671013	N(+42.01)	54	367.1852	2	732.3555	Acetylati	N	Superscaf	32655297	0	0	0	0	BTP20220	0	Tissue specific
KVLLCQF	Superscaf	+	Superscaf	32658220	32658240	4	4	10	-	-	-	-	-	-	NCP	IGR	-	ID=gene-E	7	5.407142857	2759	0.397615013	KVLLC(+57	52	454.2533	2	906.4997	Carbamidc	S	Superscaf	32658211	0	0	0	0	BTP20220	0	Tissue specific
KMKPPR	Superscaf	-	Superscaf	32667898	32667915	-25	25	30	-	-	-	-	-	-	NCP	IGR	-	ID=gene-E	6	8.098333333	2631.9	0.397436557	KM(+15.9	70	386.7241	2	771.4425	Oxidatior	T	Superscaf	32667948	0	0	0	0	BTP20220	0	Tissue enhanced
LDVGNF	Superscaf	+	Superscaf	32677183	32677200	4	4	9	-	-	-	-	-	-	NCP	IGR	-	ID=gene-E	6	4.301666667	2046.3	0.397265294	LDVGNF	94	664.3271	1	663.3228	.	C	Superscaf	32677174	0	0	0	BTP20220	0	0	Tissue specific
HSLLK	Superscaf	-	Superscaf	32768407	32768421	-13	13	17	-	-	-	-	-	-	NCP	IGR	-	ID=gene-E	5	6.994	2178.3	0.395582712	HSLLK	79	597.3696	1	596.3646	.	E	Superscaf	32768676	0	0	0	0	BTP20220	0	Tissue specific
RPPVVP	Superscaf	-	Superscaf	32829574	32829591	-10	10	15	-	-	-	-	-	-	NCP	IGR	-	ID=gene-E	6	4.943333333	1661	0.394454425	RPPVVP	77	664.4022	1	663.4067	.	S	Superscaf	32829618	BTP20220	0	0	0	0	0	Tissue specific
FESAENK	Superscaf	-	Superscaf	32853973	32853993	-27	27	33	-	-	-	-	-	-	NCP	IGR	-	ID=gene-E	7	5.538571429	2998.2	0.394004327	FESAENK	62	824.3657	1	823.3712	.	S	Superscaf	32854032	0	0	0	0	BTP20220	BTP20220	Tissue specific
LFPLTQV	Superscaf	+	Superscaf	32892118	32892138	22	22	28	-	-	-	-	-	-	NCP	IGR	-	ID=gene-E	7	5.131428571	2439.8	0.393300738	LFPLTQV	66	817.4777	1	816.4745	.	F	Superscaf	32892055	0	0	0	0	BTP20220	0	Tissue specific
QKKMPK	Superscaf	-	Superscaf	32895118	32895135	-8	8	13	-	-	-	-	-	-	NCP	IGR	-	ID=gene-E	6	7.82	2724.8	0.393245458	Q(+42.01)	64	409.2286	2	816.4528	Acetylati	D	Superscaf	32895159	0	0	0	BTP20220	0	0	Tissue specific
ARLPKKK	Superscaf	-	Superscaf	32932438	32932458	-2	2	8	-	-	-	-	-	-	NCP	IGR	-	ID=gene-E	7	8.325714286	3056.1	0.392557031	ARLPKKK	51	420.7871	2	839.5704	.	E	Superscaf	32932539	0	BTP20220	0	0	BTP20220	BTP20220	Tissue specific
MSFQLYW	Superscaf	+	Superscaf	33020569	33020589	3	3	9	-	-	-	-	-	-	NCP	IGR	-	ID=gene-E	7	5.727142857	3092.5	0.390931445	MSFQLYW	55	487.7259	2	973.4368	.	K	Superscaf	33020563	BTP20220	0	0	0	0	0	Tissue specific
LSQRPR	Superscaf	-	Superscaf	33092008	33092025	-112	112	117	-	-	-	-	-	-	NCP	IGR	-	ID=gene-E	6	7.521666667	2982.5	0.389613799	LSQRPR	70	378.7211	2	755.4402	.	A	Superscaf	33092094	0	0	BTP20220	0	0	0	Tissue specific
KDSGTLLK	Superscaf	-	Superscaf	33092347	33092370	-2	2	9	-	-	-	-	-	-	NCP	IGR	-	ID=gene-E	8	6.57375	3133.3	0.389607436	KDSGTLLK	75	861.5044	1	860.4967	.	G	Superscaf	33092463	0	0	0	BTP20220	0	0	Tissue specific
KAPAHYK	Superscaf	+	Superscaf	33128908	33128928	6	6	12	-	-	-	-	-	-	NCP	IGR	-	ID=gene-E	7	7.295714286	2976	0.388933119	KAPAHYK	53	407.7278	2	813.4497	.	Q	Superscaf	33128893	0	0	BTP20220	0	0	0	Tissue specific
LSYNFPAL	Superscaf	-	Superscaf	33228997	33229020	-21	21	28	-	-	-	-	-	-	NCP	IGR	-	ID=gene-E	8	5.81375	3182.5	0.387086911	LSYNFPAL	74	462.743	2	923.4752	.	S	Superscaf	33229071	0	0	BTP20220	0	0	0	Tissue specific
KNLEVTVK	Superscaf	-	Superscaf	33334642	33334665	-27	27	34	-	-	-	-	-	-	NCP	IGR	-	ID=gene-E	8	5.0775	2709.9	0.385138277	KNLEVTVK	99	465.7843	2	929.5546	.	V	Superscaf	33334806	BTP20220	0	0	0	0	0	Tissue specific
PGMLEY	Superscaf	+	Superscaf	33387763	33387780	55	55	60	-	-	-	-	-	-	NCP	IGR	-	ID=gene-E	6	5.48	2244.6	0.384158564	PGM(+15.9	65	725.3137	1	724.3102	Oxidatior	N	Superscaf	33387601	0	0	BTP20220	0	0	0	Tissue specific
KFLRNP	Superscaf	+	Superscaf	33420310	33420327	7	7	12	-	-	-	-	-	-	NCP	IGR	-	ID=gene-E	6	7.278333333	2792.1	0.383558231	KFLRNP	78	387.7355	2	773.4548	.	T	Superscaf	33420292	0	0	BTP20220	0	0	0	Tissue specific
AAALTVR	Superscaf	-	Superscaf	33459418	33459438	-19	19	25	-	-	-	-	-	-	NCP	IGR	-	ID=gene-E	7	5.904285714	2333.6	0.382836824	AAALTVR	80	351.2158	2	700.4232	.	S	Superscaf	33459498	BTP20220	0	0	0	0	0	Tissue specific
DPLENHPP	Superscaf	-	Superscaf	33491629	33491652	-1	1	8	-	-	-	-	-	-	NCP	IGR	-	ID=gene-E	8	5.50875	3419	0.382242633	D(+42.01)	74	480.725	2	959.4348	Acetylati	D	Superscaf	33491685	0	0	0	BTP20220	0	0	Tissue specific
HLDRAP	Superscaf	+	Superscaf	33564625	33564642	20	20	25	-	-	-	-	-	-	NCP	IGR	-	ID=gene-E	6	6.603333333	2682.1	0.380896324	HLDRAP	54	354.6967	2	707.3715	.	Y	Superscaf	33564568	0	BTP20220	0	0	0	0	Tissue specific
RLGVGAPV	Superscaf	-	Superscaf	33566107	33566130	-41	41	48	-	-	-	-	-	-	NCP	IGR	-	ID=gene-E	8	5.125	2245.5	0.380868878	RLGVGAPV	55	384.7382	2	767.4653	.	E	Superscaf	33566130	0	0	0	0	BTP20220	0	Tissue specific
VGAGMF	Superscaf	-	Superscaf	33604207	33604224	-4	4	9	-	-	-	-	-	-	NCP	IGR	-	ID=gene-E	6	4.865	1664.8	0.38016623	VGAGMF	86	581.2739	1	580.2679	.	A	Superscaf	33604284	0	0	0	0	0	BTP20220	Tissue specific
HSDFPK	Superscaf	+	Superscaf	33679387	33679404	3	3	8	-	-	-	-	-	-	NCP	IGR	-	ID=gene-E	6	6.293333333	2607.8	0.378779526	HSDFPK	78	730.3588	1	729.3445	.	N	Superscaf	33679381	0	0	0	0	0	BTP20220	Mixed
FTLKSK	Superscaf	-	Superscaf	33739546	33739563	-34	34	39	-	-	-	-	-	-	NCP	IGR	-	ID=gene-E	6	7.191666667	2458.5	0.377669887	FTLKSK	53	362.2208	2	722.4327	.	I	Superscaf	33739608	0	0	0	0	BTP20220	0	Mixed
MMLDLNKAF	Superscaf	-	Superscaf	33778072	33778098	-70	70	78	-	-	-	-	-	-	NCP	IGR	-	ID=gene-E	9	6.371111111	3706.1	0.376959104	MMLDLNKAF	89	355.1913	3	1062.557	.	V	Superscaf	33778227	0	0	0	0	0	BTP20220	Tissue specific
STSETMGHS	Superscaf	-	Superscaf	33778513	33778563	-35	35	51	-	-	-	-	-	-	NCP	IGR	-	ID=gene-E	17	5.994117647	6705.5	0.376950527	STSETMGHS	97	876.4012	2	1750.794	.	T	Superscaf	33778695	0	BTP20220	0	0	0	BTP20220	Mixed
STSETMGHS	Superscaf	-	Superscaf	33778513	33778560	-35	35	50	-	-	-	-	-	-	NCP	IGR	-	ID=gene-E	16	6.02625	6318.1	0.376950582	STSETMGHS	98	802.8668	2	1603.726	.	T	Superscaf	33778695	0	0	0	0	BTP20220	0	Tissue specific
SADRLAAAF	Superscaf	-	Superscaf	33778537	33778563	-43	43	51	-	-	-	-	-	-	NCP	IGR	-	ID=gene-E	9	6.105555556	3429.7	0.376950527	SADRLAAAF	94	461.2409	2	920.4716	.	T	Superscaf	33778695	0	BTP20220	0	0	0	0	Tissue specific
KAQDEGLLS	Superscaf	-	Superscaf	33778621	33778662	-71	71	84	-	-	-	-	-	-	NCP	IGR	-	ID=gene-E	14	4.711428571	4751.2	0.376948701	KAQDEGLLS	78	759.3936	2	1516.777	.	T	Superscaf	33778695	0	0	0	0	0	BTP20220	Tissue specific
VGLPPLEKF	Superscaf	-	Superscaf	33784225	33784251	-14	14	22	-	-	-	-	-	-	NCP	IGR	-	ID=gene-E	9	5.441111111	2960.2	0.376845611	VGLPPLEKF	92	500.2963	2	998.58	.	I	Superscaf	33784485	0	0	BTP20220	0	0	0	Tissue specific
FGATGCRLV	Superscaf	-	Superscaf	33784288	33784317	-35	35	44	-	-	-	-	-	-	NCP	IGR	-	ID=gene-E	10	5.748	3650.2	0.376844394	FGATGC(+5	78	556.272	2	1110.532	Carbamidc	I	Superscaf	33784485	0	0	0	0	BTP20220	BTP20220	Tissue specific
ATGCRLVM	Superscaf	-	Superscaf	33784294	33784317	-37	37	44	-	-	-	-	-	-	NCP	IGR	-	ID=gene-E	8	5.75375	2965.5	0.376844394	ATGC(+57.	74	454.2264	2	906.4415	Carbamidc	I	Superscaf	33784485	0	0	0	0	0	BTP20220	Tissue specific
CALKFFC	Superscaf	+	Superscaf	33799774	33799794	48	48	54	-	-	-	-	-	-	NCP	IGR	-	ID=gene-E	7	6.105714286	3079.3	0.376558919	C(+57.02)	57	473.2152	2	944.4248	Carbamidc	G	Superscaf	33799633	0	0	BTP20220	0	0	0	Tissue specific
NAPKSEK	Superscaf	+	Superscaf	33872902	33872922	17	17	23	-	-	gene	-	-	mRNA	NCP	Intron	gene|mRN	ID=gene-E	7	6.587142857	2991.1	0.375210064	NAPKSEK	77	387.2128	2	772.4079	.	G	Superscaf	33872854	0	0	BTP20220	0	0	0	Tissue specific
KQKPSSH	Superscaf	+	Superscaf	33875167	33875187	-1	1	7	-	-	gene	-	-	mRNA	NCP	Intron	gene|mRN	ID=gene-E	7	7.197142857	3271.4	0.375168286	KQKPSSH	55	811.4287	1	810.4348	.	K	Superscaf	33875211	0	0	BTP20220	0	0	0	Tissue specific
AFFPPRP	Superscaf	+	Superscaf	33926866	33926886	25	25	31	-	-	-	-	-	-	NCP	IGR	-	ID=gene-E	7	6.662857143	2747.1	0.374214692	A(+42.01)	78	437.2331	2	872.4544	Acetylati	P	Superscaf	33926794	BTP20220	0	0	0	0	0	Tissue specific
LLHKKM	Superscaf	-	Superscaf	33951763	33951780	-23	23	28	-	-	-	-	-	-	NCP	IGR	-	ID=gene-E	6	7.463333333	2634.8	0.37375552	L(+42.01)	55	406.2475	2	810.4786	Acetylati	N	Superscaf	33951810	0	0	0	0	0	BTP20220	Tissue enhanced
PPSGGPL	Superscaf	-	Superscaf	33957697	33957717	-43	43	49	-	-	-	-	-	-	NCP	IGR	-	ID=gene-E	7	6.071428571	2343.6	0.373646011	P(+42.01)	65	666.34	1	665.3384	Acetylati	K	Superscaf	33957741	0	0	BTP20220	0	0	0	Tissue specific
KYFLLPP	Superscaf	+	Superscaf	33959389	33959409	1	1	7	-	-	-	-	-	-	NCP	IGR	-	ID=gene-E	7	6.491428571	2696.9	0.373614802	KYFLLPP	65	439.264	2	876.5109	.	K	Superscaf	33959389	0	0	0	0	0	BTP20220	Tissue specific
GVARVQ	Superscaf	-	Superscaf	34042255	34042272	-11	11	16	-	-	-	-	-	-	NCP	IGR	-	ID=gene-E	6	4.733333333	1868.1	0.372086384	G(+42.01)	51	671.3944	1	670.3762	Acetylati	F	Superscaf	34042398	0	0	0	0	0	BTP20220	Tissue specific
EEHDMD	Superscaf	+	Superscaf	34060918	34060935	2	2	7	-	-	-	-	-	-	NCP	IGR	-	ID=gene-E	6	4.286666667	2665.2	0.371742143	EEHDM(+15	59	396.129	2	790.244	Oxidatior	K	Superscaf	34060915	0	0	0	0	0	BTP20220	Tissue specific
VTVPGGDL	Superscaf	-	Superscaf	34090342	34090365	-21	21	28	-	-	-	-	-	-	NCP	IGR	-	ID=gene-E	8	4.215	2094.4	0.371199303	VTVPGGDL	97	757.4065	1	756.4017	.	T	Superscaf	34090389	0	0	BTP20220	0	0	0	Mixed
QLNGAHRSY	Superscaf	+	Superscaf	34125289	34125315	10	10	18	-	-	-	-	-	-	NCP	IGR	-	ID=gene-E	9	6.524444444	4280.8	0.370554647	QLNGAHRSY	52	523.2587	2	1044.51	.	I	Superscaf	34125262	0	0	0	0	0	BTP20220	Tissue specific
PSARLPSP	Superscaf	-	Superscaf	34133848	34133871	-50	50	57	-	-	-	-	-	-	NCP	IGR	-	ID=gene-E	8	6.6275	3092.5	0.37039848	PSARLPSP	64	412.7398	2	823.4551	.	L	Superscaf	34134162	0	0	0	0	0	BTP20220	Tissue specific
QEFTPR	Superscaf	+	Superscaf	34140976	34140993	46	46	51	-	-	-	-	-	-	NCP	IGR	-	ID=gene-E	6	6.323333333	2807.3	0.370265464	Q(+42.01)	69	819.3861	1	818.3923	Acetylati	A	Superscaf	34140841	0	0	0	0	BTP20220	0	Tissue specific
ARASHTQ	Superscaf	-	Superscaf	34160131	34160151	-40	40	46	-	-	-	-	-	-	NCP	IGR	-	ID=gene-E	7	6.892857143	3282.7	0.369912093	ARASHTQ	60	385.6948	2	769.3831	.	A	Superscaf	34160262	0	0	0	0	0	BTP20220	Tissue specific
WAPRML	Superscaf	-	Superscaf	34182685	34182702	-160	160	165	-	-	-	-	-	-	NCP	IGR	-	ID=gene-E	6	6.783333333	2549	0.369496137	WAPRML	89	387.2074	2	772.4054	.	W	Superscaf	34182717	0	0	0	0	0	BTP20220	Tissue specific
VPGRVLL	Superscaf	-	Superscaf	34202974	34202994	-22	22	28	-	-	-	-	-	-	NCP	IGR	-	ID=gene-E	7	4.998571429	1990.3	0.369121849	VPGRVLL	71	377.2515	2	752.4908	.	L	Superscaf	34203135	0	0	0	0	0	BTP20220	Tissue specific
TKNLP	Superscaf	+	Superscaf	34218394	34218408	70	70	74	-	-	-	-	-	-	NCP	IGR	-	ID=gene-E	5	6.792	2153.4	0.368837536	T(+42.01)	86	614.3492	1	613.3435	Acetylati	A	Superscaf	34218187	0	0	0	0	0	BTP20220	Tissue specific
YGPGAP	Superscaf	-	Superscaf	34226236	34226253	-25	25	30	-	-	-	-	-	-	NCP	IGR	-	ID=gene-E	6	6.036666667	2040.2	0.368692834	YGPGAP	88	561.2667	1	560.2595	.	R	Superscaf	34226352	0	0	0	0	BTP20220	0	Tissue specific
KTVEVF	Superscaf	+	Superscaf	34276861	34276878	1	1	6	-	-	gene	-	-	mRNA	NCP	Intron	gene|mRN	ID=gene-E	6	4.161666667	1678.8	0.36775905	K(+42.01)	61	764.4144	1	763.4116	Acetylati	K	Superscaf	34276861	0	0	0	BTP20220	0	0	Tissue specific
KACYLSP	Superscaf	-	Superscaf	34319359	34319379	-65	65	71	-	-	gene	-	-	mRNA	NCP	Intron	gene|mRN	ID=gene-E	7	6.342857143	2899.1	0.366975114	KAC(+57.(	73	419.7114	2	837.4055	Carbamidc	G	Superscaf	34319448	0	BTP20220	0	0	0	0	Tissue specific
KLTVNKT	Superscaf	-	Superscaf	34320172	34320192	-3	3	9	-	-	gene	-	-	mRNA	NCP	Intron	gene|mRN	ID=gene-E	7	6.275714286	2681.9	0.366960118	KLTVNKT	53	402.2458	2	802.4913	.	T	Superscaf	34320396	0	0	BTP20220	0	0	0	Tissue specific
SGTSQNEG	Superscaf	-	Superscaf	34342111	34342134	-9	9	16	-	-	-	-	-	-	NCP	IGR	-	ID=gene-E	8	5.51375	3391.8	0.366555395	SGTSQNEG	50	390.1628	2	778.3093	.	H	Superscaf	34342200	0	0	0	BTP20220	0	0	Tissue specific
NPPSPSSPF	Superscaf	-	Superscaf	34353313	34353339	-40	40	48	-	-	gene	-	-	mRNA	NCP	Intron	gene|mRN	ID=gene-E	9	5.903333333	3483.9	0.366348718	N(+42.01)	80	971.4423	1	970.4396	Acetylati	S	Superscaf	34353423	0	0	0	0	BTP20220	0	Tissue specific
VPPPAVEPK	Superscaf	-	Superscaf	34413364	34413396	-39	39	49	-	-	-	-	-	-	NCP	IGR	-	ID=gene-E	11	5.474545455	3360.6	0.36524096	VPPPAVEPK	93	386.8991	3	1157.681	.	F	Superscaf	34413507	0	0	BTP20220	0	0	0	Tissue specific
PPPAVEPKK	Superscaf	-	Superscaf	34413367	34413396	-40	40	49	-	-	-	-	-	-	NCP	IGR	-	ID=gene-E	10	6.022	3360.6	0.36524096	PPPAVEPKK	98	530.3116	2	1058.612	.	F	Superscaf	34413507	0	0	BTP20220	0	0	0	Tissue specific
YPAPVFH	Superscaf	+	Superscaf	34417969	34417989	45	45	51	-	-	-	-	-	-	NCP	IGR	-	ID=gene-E	7	5.335714286	2452.7	0.365156241	YPAPVFH	60	830.4163	1	829.4122	.	K	Superscaf	34417837	0	0	BTP20220	0	0	0	Tissue specific
VPPLSNSV	Superscaf	-	Superscaf	34422892	34422915	-43	43	50	-	-	-	-	-	-	NCP	IGR	-	ID=gene-E	8	4.41875	2391.7	0.36506538	VPPLSNSV	53	406.7291	2	811.444	.	L	Superscaf	34423152	0	0	BTP20220	0	0	0	Tissue specific
KGYKNF	Superscaf	+	Superscaf	34450654	34450671	11	11	16	-	-	gene	-	-	mRNA	NCP	Intron	gene|mRN	ID=gene-E	6	7	2677.7	0.364553418	KGYKNF	58	378.7126	2	755.3966	.	I	Superscaf	34450624	0	0	0	BTP20220	0	0	Tissue specific
AEEFTLP	Superscaf	+	Superscaf	34476802	34476822	14	14	20	-	-	-	-	-	-	NCP	IGR	-	ID=gene-E	7	5.25	2638	0.36407106	A(+42.01)	78	424.7047	2	847.3963	Acetylati	M	Superscaf	34476763	0	0	0	BTP20220	0	0	Mixed
LNEVVTR	Superscaf	+	Superscaf	34487836	34487856	27	27	33	-	-	-	-	-	-	NCP	IGR	-	ID=gene-E	7	4.557142857	2421.9	0.363867536	LNEVVTR	99	415.7387	2	829.4658	.	K	Superscaf	34487758	0	0	BTP20220	BTP20220	BTP20220	0	Tissue specific
VPLGGP	Superscaf	+	Superscaf	34489723	34489740	27	27	32	-	-	-	-	-	-	NCP	IGR	-	ID=gene-E	6	5.086666667	1622.8	0.363832786	VPLGGP	68	539.3167	1	538.3115	.	D	Superscaf	34489645	0	0	BTP20220	BTP20220	BTP20220	0	Tissue specific
RKLVETL	Superscaf	+	Superscaf	34537987	34538007	15	15	21	-	-	-	-	-	-	NCP	IGR	-	ID=gene-E	7	6.03	2647	0.362942495	R(+42.01)	67	450.7784	2	899.544	Acetylati	H	Superscaf	34537945	0	0	BTP20220	BTP20220	0	0	Tissue specific
AALVLEDGS	Superscaf	-	Superscaf	34601758	34601805	-11	11	26	-	-	-	-	-	-	NCP	IGR	-	ID=gene-E	16	5.12375	5482.1	0.361765734	A(+42.01)	82	857.4577	2	1712.91	Acetylati	S	Superscaf	34601877	0	0	BTP20220	0	BTP20220	0	Tissue specific
GPALPDGRF	Superscaf	-	Superscaf	34619563	34619589	-21	21	29	-	-	-	-	-	-	NCP	IGR	-	ID=gene-E	9	6.194444444	3381.8	0.361437706	GPALPDGRF	71	465.2447	2	928.4766	.	G	Superscaf	34619652	0	BTP20220	0	0	0	0	Tissue specific
GPALPDGR	Superscaf	-	Superscaf	34619563	34619586	-21	21	28	-	-	-	-	-	-	NCP	IGR	-	ID=gene-E	8	6.28375	2994.4	0.361437761	GPALPDGR	92	391.7096	2	781.4082	.	G	Superscaf	34619652	0	0	0	0	0	BTP20220	Tissue specific
EETPAFF	Superscaf	+	Superscaf	34637257	34637277	2	2	8	-	-	-	-	-	-	NCP	IGR	-	ID=gene-E	7	5.178571429	2672	0.361111449	EETPAFF	59	420.6989	2	839.3701	.	L	Superscaf	34637254	0	0	0	0	0	BTP20220	Tissue specific
KPSSERP	Superscaf	-	Superscaf	34687798	34687818	-16	16	22	-	-	-	-	-	-	NCP	IGR	-	ID=gene-E	7	6.811428571	2984.3	0.360179214	KPSSERP	64	400.7113	2	799.4188	.	L	Superscaf	34688025	0	0	BTP20220	0	0	0	Tissue specific
KGSPRAGP	Superscaf	-	Superscaf	34729210	34729233	-16	16	23	-	-	-	-	-	-	NCP	IGR	-	ID=gene-E	8	7.0925	3081.3	0.35941531	KGSPRAGP	56	385.2183	2	768.4242	.	P	Superscaf	34729251	0	0	0	0	BTP20220	0	Tissue specific
ETFFKVS	Superscaf	-	Superscaf	34757449	34757469	-4	4	10	-	-	-	-	-	-	NCP	IGR	-	ID=gene-E	7	5.161428571	2449.6	0.358894494	ETFFKVS	54	429.221	2	856.433	.	P	Superscaf	34757472	BTP20220	0	0	0	0	0	Tissue specific
MAPSRK	Superscaf	-	Superscaf	34768906	34768923	-26	26	31	-	-	-	-	-	-	NCP	IGR	-	ID=gene-E	6	7.375	2520.8	0.358683223	M(+15.99)	92	374.1992	2	746.3745	Oxidatior	K	Superscaf	34769073	0	0	0	0	BTP20220	0	Tissue specific
KVRAVF	Superscaf	-	Superscaf	34773211	34773228	-10	10	15	-	-	-	-	-	-	NCP	IGR	-	ID=gene-E	6	5.333333333	1815.9	0.358603817	KVRAVF	65	360.231	2	718.449	.	S	Superscaf	34773324	0	0	0	0	BTP20220	0	Tissue specific
PSTGKGGK	Superscaf	-	Superscaf	34779178	34779201	-9	9	16	-	-	-	-	-	-	NCP	IGR	-	ID=gene-E	8	6.9875	2947	0.358493644	PSTGKGGK	55	366.201	2	730.3973	.	K	Superscaf	34779222	0	BTP20220	0	BTP20220	BTP20220	0	Tissue specific
KVMVFK	Superscaf	-	Superscaf	34881172	34881189	-1	1	6	-	-	-	-	-	-	NCP	IGR	-	ID=gene-E	6	5.118333333	1695.7	0.356612464	KVM(+15.9	54	384.2269	2	766.4411	Oxidatior	K	Superscaf	34881270	0	0	0	0	0	BTP20220	Tissue specific
STSSVFV	Superscaf	-	Superscaf	34891765	34891785	-62	62	68	-	-	-	-	-	-	NCP	IGR	-	ID=gene-E	7	4.15	1935.1	0.356417019	STSSVFV	51	363.687	2	725.3596	.	C	Superscaf	34899165	0	BTP20220	BTP20220	0	0	0	Tissue specific
ATPVKVT	Superscaf	-	Superscaf	34905544	34905564	-1703	1703	1709	-	-	-	-	-	-	NCP	IGR	-	ID=gene-E	7	5.017142857	1912.1	0.356162864	ATPVKVT	55	358.2253	2	714.4276	.	S	Superscaf	34909404	0	0	BTP20220	0	0	0	Tissue specific
QEWYEL	Superscaf	+	Superscaf	34957537	34957554	26	26	31	-	-	-	-	-	-	NCP	IGR	-	ID=gene-E	6	4.936666667	2801.2	0.355203903	QEWYEL	78	867.3861	1	866.381	.	E	Superscaf	34957462	0	0	0	0	0	BTP20220	Mixed
LVHLYP	Superscaf	+	Superscaf	34962664	34962681	14	14	19	-	-	-	-	-	-	NCP	IGR	-	ID=gene-E	6	5.251666667	2123.4	0.355109335	L(+42.01)	52	392.2285	2	782.4327	Acetylati	G	Superscaf	34962625	0	0	0	0	BTP20220	0	Mixed
MDYHGS	Superscaf	-	Superscaf	35043802	35043819	-61	61	66	-	-	-	-	-	-	NCP	IGR	-	ID=gene-E	6	5.603333333	2542.9	0.353612735	MDYHGS	50	355.1391	2	708.2537	.	V	Superscaf	35043822	BTP20220	0	0	0	0	0	Tissue specific
KLLEQEKQ	Superscaf	+	Superscaf	35094265	35094288	26	26	33	-	-	-	-	-	-	NCP	IGR	-	ID=gene-E	8	6.1475	3716	0.352681828	KLLEQEKQ	88	508.2854	2	1014.571	.	K	Superscaf	35094190	0	BTP20220	0	0	0	0	Tissue specific
TERLLAL	Superscaf	-	Superscaf	35188876	35188896	-12	12	18	-	-	-	-	-	-	NCP	IGR	-	ID=gene-E	7	6.352857143	2843.3	0.350936773	T(+42.01)	51	429.2585	2	856.5018	Acetylati	I	Superscaf	35188911	0	0	0	0	0	BTP20220	Tissue specific
VEVEL	Superscaf	-	Superscaf	35199721	35199735	-12	12	16	-	-	-	-	-	-	NCP	IGR	-	ID=gene-E	5	2.484	1204.4	0.350736846	VEVEL	91	588.3195	1	587.3166	.	Q	Superscaf	35199858	0	BTP20220	0	0	0	0	Tissue specific
ASSTLLD	Superscaf	-	Superscaf	35223196	35223216	-12	12	18	-	-	-	-	-	-	NCP	IGR	-	ID=gene-E	7	5.548571429	2593.9	0.350303737	ASSTLLD	55	706.3597	1	705.3545	.	C	Superscaf	35223219	0	0	0	0	0	BTP20220	Tissue specific
NKPSVPK	Superscaf	-	Superscaf	35298181	35298201	-2	2	8	-	-	-	-	-	-	NCP	IGR	-	ID=gene-E	7	6.167142857	2591.7	0.34892063	NKPSVPK	99	385.2314	2	768.4493	.	T	Superscaf	35298276	0	0	0	0	BTP20220	0	Tissue specific
AVSLWGF	Superscaf	+	Superscaf	35385784	35385804	27	27	33	-	-	-	-	-	-	NCP	IGR	-	ID=gene-E	7	5.002857143	2259.4	0.347304782	AVSLWGF	60	779.4219	1	778.4014	.	G	Superscaf	35385706	0	0	0	0	0	BTP20220	Tissue specific
EGLDFY	Superscaf	+	Superscaf	35429239	35429256	1	1	6	-	-	-	-	-	-	NCP	IGR	-	ID=gene-E	6	4.88	2334.6	0.346503305	EGLDFY	76	743.323	1	742.3173	.	E	Superscaf	35429239	0	0	0	BTP20220	0	0	Mixed
KANLLGK	Superscaf	-	Superscaf	35457271	35457291	-4	4	10	-	-	-	-	-	-	NCP	IGR	-	ID=gene-E	7	6.977142857	2848.9	0.345986196	KANLLGK	56	372.2466	2	742.4701	.	K	Superscaf	35457291	0	0	BTP20220	0	0	0	Tissue specific
EHAAVTK	Superscaf	-	Superscaf	35503240	35503260	-10	10	16	-	-	-	-	-	-	NCP	IGR	-	ID=gene-E	7	5.588571429	2533.7	0.345138293	EHAAVTK	80	378.2081	2	754.3973	.	L	Superscaf	35503272	0	0	0	0	BTP20220	0	Tissue specific
SGNFTPK	Superscaf	+	Superscaf	35503441	35503461	1	1	7	-	-	-	-	-	-	NCP	IGR	-	ID=gene-E	7	6.444285714	2868.1	0.345134585	SGNFTPK	77	375.6974	2	749.3708	.	S	Superscaf	35503441	0	0	0	0	0	BTP20220	Tissue specific
KGHVGPK	Superscaf	-	Superscaf	35516572	35516592	-51	51	57	-	-	-	-	-	-	NCP	IGR	-	ID=gene-E	7	6.472857143	2488.5	0.344892383	KGHVGPK	72	361.7118	2	721.4235	.	K	Superscaf	35516640	0	BTP20220	0	0	0	0	Tissue specific
EALTVLD	Superscaf	-	Superscaf	35533249	35533269	-16	16	22	-	-	-	-	-	-	NCP	IGR	-	ID=gene-E	7	4.385714286	2252.6	0.344584773	E(+42.01)	61	802.4159	1	801.412	Acetylati	D	Superscaf	35533269	0	0	0	0	0	BTP20220	Tissue specific
VEFPASP	Superscaf	-	Superscaf	35538310	35538330	-12	12	18	-	-	-	-	-	-	NCP	IGR	-	ID=gene-E	7	4.714285714	2182.4	0.344491423	VEFPASP	66	746.3688	1	745.3646	.	K	Superscaf	35538342	0	0	BTP20220	0	0	0	Tissue specific
YLAAVL	Superscaf	-	Superscaf	35557969	35557986	-4	4	9	-	-	-	-	-	-	NCP	IGR	-	ID=gene-E	6	4.943333333	1788.9	0.344128866	YLAAVL	99	649.3904	1	648.3846	.	N	Superscaf	35558046	0	0	BTP20220	0	0	0	Mixed
YEELVK	Superscaf	+	Superscaf	35565424	35565441	24	24	29	-	-	-	-	-	-	NCP	IGR	-	ID=gene-E	6	4.636666667	2132.3	0.343991357	YEELVK	99	390.7088	2	779.4065	.	K	Superscaf	35565355	0	0	0	0	0	BTP20220	Tissue specific
KPAAHAR	Superscaf	-	Superscaf	35565664	35565684	-3	3	9	-	-	-	-	-	-	NCP	IGR	-	ID=gene-E	7	7.492857143	3008.2	0.343986875	KPAAHAR	57	375.7206	2	749.4296	.	K	Superscaf	35565690	0	0	0	0	BTP20220	BTP20220	Tissue specific
VPLAALL	Superscaf	-	Superscaf	35606719	35606739	-25	25	31	-	-	-	-	-	-	NCP	IGR	-	ID=gene-E	7	5.182857143	2020.2	0.343229611	V(+42.01)	53	369.7446	2	737.4687	Acetylati	F	Superscaf	35606814	0	0	BTP20220	0	0	0	Tissue specific
QLLEC	Superscaf	+	Superscaf	35620282	35620296	22	22	26	-	-	-	-	-	-	NCP	IGR	-	ID=gene-E	5	5.17	2328.7	0.34297955	Q(+42.01)	59	704.3413	1	703.3211	Acetylati	E	Superscaf	35620219	BTP20220	0	0	0	0	0	Tissue specific
QKDPQQ	Superscaf	+	Superscaf	35693920	35693937	2	2	7	-	-	-	-	-	-	NCP	IGR	-	ID=gene-E	6	5.993333333	3049.4	0.341621234	QKDPQQ	51	372.1857	2	742.361	.	T	Superscaf	35693917	BTP20220	0	0	0	0	0	Tissue specific
THLTLP	Superscaf	+	Superscaf	35806843	35806860	13	13	18	-	-	-	-	-	-	NCP	IGR	-	ID=gene-E	6	6.485	2458.9	0.339538356	T(+42.01)	60	362.2091	2	722.3963	Acetylati	L	Superscaf	35806807	BTP20220	0	0	0	0	0	Tissue specific
FLDELVS	Superscaf	-	Superscaf	35814091	35814111	-15	15	21	-	-	-	-	-	-	NCP	IGR	-	ID=gene-E	7	4.187142857	2314.6	0.339404611	FLDELVS	68	822.4244	1	821.4171	.	V	Superscaf	35814183	0	0	BTP20220	0	0	0	Tissue specific
TRYFPL	Superscaf	+	Superscaf	35859469	35859486	4	4	9	-	-	-	-	-	-	NCP	IGR	-	ID=gene-E	6	6.785	2584	0.338567663	T(+42.01)	64	419.719	2	837.4385	Acetylati	I	Superscaf	35859460	0	0	0	0	0	BTP20220	Tissue specific
MLLEEAR	Superscaf	+	Superscaf	35876044	35876064	7	7	13	-	-	-	-	-	-	NCP	IGR	-	ID=gene-E	7	5.847142857	2889.4	0.33826188	M(+15.99)	76	439.2339	2	876.4375	Oxidatior	G	Superscaf	35876026	BTP20220	0	0	0	0	0	Tissue specific
KGFKTPK	Superscaf	-	Superscaf	35884231	35884251	-31	31	37	-	-	-	-	-	-	NCP	IGR	-	ID=gene-E	7	7.642857143	2824.8	0.33811087	K(+42.01)	99	424.2544	2	846.4963	Acetylati	A	Superscaf	35884335	0	0	0	0	BTP20220	0	Tissue specific
VGMFLGA	Superscaf	-	Superscaf	35911264	35911284	-26	26	32	-	-	-	-	-	-	NCP	IGR	-	ID=gene-E	7	5.024285714	2018.2	0.337612243	VGM(+15.9	51	710.3518	1	709.3469	Oxidatior	L	Superscaf	35911476	0	0	BTP20220	0	0	0	Tissue specific
NVASGF	Superscaf	-	Superscaf	35913106	35913123	-4	4	9	-	-	-	-	-	-	NCP	IGR	-	ID=gene-E	6	4.76	1976.1	0.337578323	NVASGF	72	594.2855	1	593.2809	.	E	Superscaf	35913189	0	0	0	0	BTP20220	0	Tissue specific
ASCLLL	Superscaf	-	Superscaf	35991733	35991750	-6	6	11	-	-	-	-	-	-	NCP	IGR	-	ID=gene-E	6	5.776666667	2340.6	0.336128039	A(+42.01)	67	718.3771	1	717.3731	Acetylati	I	Superscaf	35991834	0	BTP20220	0	0	0	0	Tissue specific
YTDLLRLFF	Superscaf	-	Superscaf	36021850	36021879	-17	17	26	-	-	-	-	-	-	NCP	IGR	-	ID=gene-E	10	5.822	4249.9	0.335572306	YTDLLRLFF	91	666.8423	2	1331.676	.	T	Superscaf	36022068	0	0	0	0	BTP20220	BTP20220	Tissue specific
TFGADVVSK	Superscaf	-	Superscaf	36030307	36030333	-48	48	56	-	-	-	-	-	-	NCP	IGR	-	ID=gene-E	9	4.71	2656.8	0.335416371	TFGADVVSK	97	462.2425	2	922.476	.	F	Superscaf	36030381	0	0	0	0	BTP20220	0	Tissue specific
KKWLPLT	Superscaf	-	Superscaf	36091588	36091608	-27	27	33	-	-	-	-	-	-	NCP	IGR	-	ID=gene-E	7	7.165714286	2905.1	0.334286146	KKWLPLT	70	443.2854	2	884.5483	.	K	Superscaf	36091692	0	0	0	0	BTP20220	0	Tissue specific
KDRLNAG	Superscaf	-	Superscaf	36104860	36104880	-1	1	7	-	-	-	-	-	-	NCP	IGR	-	ID=gene-E	7	6.692857143	3087.4	0.334041343	K(+42.01)	90	408.2213	2	814.4297	Acetylati	K	Superscaf	36104892	0	0	0	0	0	BTP20220	Mixed
HAAVKPP	Superscaf	-	Superscaf	36133123	36133143	-22	22	28	-	-	-	-	-	-	NCP	IGR	-	ID=gene-E	7	5.995714286	2385.5	0.333520029	HAAVKPP	76	360.2069	2	718.4126	.	Q	Superscaf	36133209	BTP20220	0	0	0	0	0	Tissue specific
YGAADL	Superscaf	-	Superscaf	36148729	36148746	-59	59	64	-	-	-	-	-	-	NCP	IGR	-	ID=gene-E	6	5.436666667	2144.3	0.33323223	YGAADL	60	609.2856	1	608.2806	.	S	Superscaf	36148749	0	0	0	0	BTP20220	0	Tissue specific
HSPMPQ	Superscaf	-	Superscaf	36254590	36254607	-26	26	31	-	-	-	-	-	-	NCP	IGR	-	ID=gene-E	6	6.211666667	2660.1	0.331279611	HSPM(+15.	51	712.3013	1	711.301	Oxidatior	D	Superscaf	36254613	0	0	0	0	BTP20220	0	Tissue specific
KEVDQGK	Superscaf	-	Superscaf	36272446	36272466	-6	6	12	-	-	-	-	-	-	NCP	IGR	-	ID=gene-E	7	5.327142857	2681.8	0.3309502	KEVDQGK	50	402.2099	2	802.4185	.	S	Superscaf	36272559	0	0	0	0	BTP20220	0	Tissue specific
VFLPALT	Superscaf	-	Superscaf	36315859	36315879	-7	7	13	-	-	-	-	-	-	NCP	IGR	-	ID=gene-E	7	5.184285714	2140.4	0.330149442	V(+42.01)	54	401.747	2	801.4636	Acetylati	A	Superscaf	36316020	0	0	BTP20220	0	0	0	Tissue specific
PDAPPRP	Superscaf	+	Superscaf	36382117	36382137	13	13	19	-	-	-	-	-	-	NCP	IGR	-	ID=gene-E	7	6.421428571	2721.2	0.328927306	P(+42.01)	74	396.2104	2	790.3973	Acetylati	M	Superscaf	36382081	0	0	BTP20220	0	0	0	Tissue specific
RPSPGE	Superscaf	-	Superscaf	36415627	36415644	-6	6	11	-	-	-	-	-	-	NCP	IGR	-	ID=gene-E	6	6.371666667	2429.8	0.328309265	RPSPGE	51	642.3265	1	641.3132	.	L	Superscaf	36415671	0	0	0	0	BTP20220	0	Tissue specific
AKVLGLG	Superscaf	-	Superscaf	36429682	36429702	-53	53	59	-	-	-	-	-	-	NCP	IGR	-	ID=gene-E	7	5.665714286	2081.1	0.328049964	AKVLGLG	85	657.4274	1	656.4221	.	T	Superscaf	36429981	0	0	0	0	BTP20220	0	Tissue specific
LAEAW	Superscaf	-	Superscaf	36447493	36447507	-4	4	8	-	-	-	-	-	-	NCP	IGR	-	ID=gene-E	5	5.426	1928.1	0.327721549	LAEAW	99	589.2963	1	588.2907	.	K	Superscaf	36447513	0	0	0	BTP20220	0	0	Tissue specific
ELKEELKK	Superscaf	+	Superscaf	36587575	36587598	9	9	16	-	-	gene	-	-	mRNA	NCP	Intron	gene|mRN	ID=gene-E	8	6.355	3388.5	0.325137554	ELKEELKK	77	508.8013	2	1015.591	.	I	Superscaf	36587551	BTP20220	0	0	0	0	0	Tissue specific
VGLLETL	Superscaf	-	Superscaf	36626782	36626802	-28	28	34	-	-	-	-	-	-	NCP	IGR	-	ID=gene-E	7	4.808571429	2180.5	0.324414432	VGLLETL	68	744.4494	1	743.4429	.	T	Superscaf	36626862	0	0	0	0	BTP20220	0	Tissue specific
KMKVPPP	Superscaf	-	Superscaf	36662734	36662754	-17	17	23	-	-	-	-	-	-	NCP	IGR	-	ID=gene-E	7	6.304285714	2320.5	0.323751293	KM(+15.99	63	406.7462	2	811.4626	Oxidatior	L	Superscaf	36662811	0	0	0	BTP20220	0	0	Tissue specific
LSCGSSSTS	Superscaf	-	Superscaf	36754969	36754998	-6	6	15	-	-	-	-	-	-	NCP	IGR	-	ID=gene-E	10	5.788	3904.3	0.322049842	LSC(+57.(	69	499.7263	2	997.4386	Carbamidc	A	Superscaf	36755109	0	BTP20220	0	BTP20220	0	0	Tissue specific
DCLQGF	Superscaf	+	Superscaf	36802723	36802740	20	20	25	-	-	-	-	-	-	NCP	IGR	-	ID=gene-E	6	5.178333333	2646	0.321169235	DC(+57.02	84	739.3057	1	738.3007	Carbamidc	R	Superscaf	36802666	0	0	0	0	0	BTP20220	Tissue specific
DLVTPF	Superscaf	+	Superscaf	36833515	36833532	43	43	48	-	-	-	-	-	-	NCP	IGR	-	ID=gene-E	6	4.543333333	1887.2	0.320601273	DLVTPF	57	691.3557	1	690.3588	.	F	Superscaf	36833389	BTP20220	0	0	0	0	0	Tissue specific
MKLFVK	Superscaf	-	Superscaf	36861712	36861729	-8	8	13	-	-	-	-	-	-	NCP	IGR	-	ID=gene-E	6	6.115	2049.1	0.320081176	M(+42.01)	55	404.2515	2	806.4724	Acetylati	E	Superscaf	36861762	BTP20220	0	0	0	0	0	Tissue specific
DYDMC	Superscaf	+	Superscaf	36865186	36865200	17	17	21	-	-	-	-	-	-	NCP	IGR	-	ID=gene-E	5	4.474	2239.7	0.320017153	D(+42.01)	50	761.2122	1	760.2044	Acetylati	G	Superscaf	36865138	BTP20220	0	0	0	0	0	Tissue specific
YVDYK	Superscaf	-	Superscaf	36912055	36912069	-4	4	8	-	-	-	-	-	-	NCP	IGR	-	ID=gene-E	5	4.806	1798.9	0.319152649	YVDYK	65	687.3298	1	686.3275	.	L	Superscaf	36912105	0	0	0	0	0	BTP20220	Tissue specific
ETTTPLP	Superscaf	+	Superscaf	37025353	37025373	2	2	8	-	-	-	-	-	-	NCP	IGR	-	ID=gene-E	7	5.912857143	2646.2	0.317062744	ETTTPLP	88	758.3859	1	757.3858	.	I	Superscaf	37025350	0	0	0	BTP20220	0	0	Tissue specific
GPDRLVP	Superscaf	-	Superscaf	37085929	37085949	-25	25	31	-	-	-	-	-	-	NCP	IGR	-	ID=gene-E	7	5.468571429	2385.8	0.315945413	GPDRLVP	52	377.2152	2	752.418	.	S	Superscaf	37085976	0	0	0	0	0	BTP20220	Tissue specific
VGSFKV	Superscaf	-	Superscaf	37128523	37128540	-12	12	17	-	-	-	-	-	-	NCP	IGR	-	ID=gene-E	6	4.478333333	1536.5	0.315159817	VGSFKV	78	636.3694	1	635.3643	.	L	Superscaf	37128543	0	0	BTP20220	0	0	0	Tissue specific
LSFPTTK	Superscaf	-	Superscaf	37173151	37173171	-3	3	9	-	-	-	-	-	-	NCP	IGR	-	ID=gene-E	7	6.605714286	2725	0.314336593	LSFPTTK	99	397.2242	2	792.4381	.	S	Superscaf	37173222	BTP20220	0	0	0	0	0	Tissue enhanced
FQAKKF	Superscaf	+	Superscaf	37205071	37205088	21	21	26	-	-	-	-	-	-	NCP	IGR	-	ID=gene-E	6	7.018333333	2633.6	0.31374788	FQAKKF	54	384.7289	2	767.433	.	K	Superscaf	37205011	0	0	0	0	BTP20220	0	Tissue specific
LVFVQ	Superscaf	+	Superscaf	37218349	37218363	9	9	13	-	-	-	-	-	-	NCP	IGR	-	ID=gene-E	5	3.422	1351.5	0.313503021	LVFVQ	54	605.3605	1	604.3585	.	S	Superscaf	37218325	0	BTP20220	0	0	0	0	Tissue specific
GGLSAPGA	Superscaf	+	Superscaf	37225474	37225497	46	46	53	-	-	-	-	-	-	NCP	IGR	-	ID=gene-E	8	5.98875	2588.7	0.313371434	GGLSAPGA	61	629.324	1	628.318	.	S	Superscaf	37225339	0	0	0	0	0	BTP20220	Tissue specific
KSMLPEK	Superscaf	-	Superscaf	37307554	37307574	-4	4	10	-	-	-	-	-	-	NCP	IGR	-	ID=gene-E	7	6.63	2808	0.311857514	K(+42.01)	58	445.744	2	889.4579	Acetylati	N	Superscaf	37307580	0	0	0	0	BTP20220	0	Tissue specific
KTKHPP	Superscaf	+	Superscaf	37309480	37309497	3	3	8	-	-	-	-	-	-	NCP	IGR	-	ID=gene-E	6	7.7	2628.8	0.311822044	K(+42.01)	53	375.2193	2	748.4232	Acetylati	K	Superscaf	37309474	0	BTP20220	0	0	BTP20220	BTP20220	Tissue specific
VGPLELK	Superscaf	+	Superscaf	37320439	37320459	111	111	117	-	-	-	-	-	-	NCP	IGR	-	ID=gene-E	7	5.312857143	2235.4	0.311619849	VGPLELK	93	378.2348	2	754.4589	.	T	Superscaf	37320109	BTP20220	0	0	0	0	0	Tissue specific
RVLPL	Superscaf	+	Superscaf	37343398	37343412	24	24	28	-	-	gene	-	-	mRNA	NCP	Intron	gene|mRN	ID=gene-E	5	5.804	1693	0.311196478	RVLPL	73	597.4058	1	596.4009	.	M	Superscaf	37343329	0	0	0	BTP20220	0	0	Tissue specific
KGGVLQR	Superscaf	+	Superscaf	37346140	37346160	15	15	21	-	-	gene	-	-	mRNA	NCP	Intron	gene|mRN	ID=gene-E	7	6.295714286	2675.9	0.311145791	KGGVLQR	84	379.2368	2	756.4606	.	N	Superscaf	37346098	0	0	0	0	BTP20220	0	Tissue specific
WPPEEF	Superscaf	-	Superscaf	37397110	37397127	-45	45	50	-	-	gene	-	-	mRNA	NCP	Intron	gene|mRN	ID=gene-E	6	5.068333333	2439.8	0.310205699	WPPEEF	90	804.3549	1	803.3489	.	L	Superscaf	37397187	0	0	0	0	0	BTP20220	Tissue specific
AAGTLY	Superscaf	-	Superscaf	37416823	37416840	-7	7	12	-	-	gene	-	-	mRNA	NCP	Intron	gene|mRN	ID=gene-E	6	6.03	2130.3	0.30984209	A(+42.01)	86	637.3195	1	636.3119	Acetylati	S	Superscaf	37417026	0	0	0	BTP20220	0	0	Mixed
NYPELK	Superscaf	+	Superscaf	37468648	37468665	27	27	32	-	-	gene	-	-	mRNA	NCP	Intron	gene|mRN	ID=gene-E	6	6.051666667	2640.9	0.308886172	N(+42.01)	51	805.4014	1	804.4017	Acetylati	S	Superscaf	37468570	BTP20220	0	0	0	0	0	Tissue specific
FPQTEC	Superscaf	-	Superscaf	37505407	37505424	-38	38	43	-	-	gene	-	-	mRNA	NCP	Intron	gene|mRN	ID=gene-E	6	5.366666667	2744.2	0.308208148	F(+42.01)	56	823.3192	1	822.3218	Acetylati	K	Superscaf	37505442	0	0	BTP20220	0	0	0	Tissue specific
HLAWEKK	Superscaf	-	Superscaf	37521634	37521654	-32	32	38	-	-	gene	-	-	mRNA	NCP	Intron	gene|mRN	ID=gene-E	7	6.882857143	3173.3	0.307908784	HLAWEKK	59	456.2668	2	910.5024	.	S	Superscaf	37521678	BTP20220	0	0	0	0	0	Tissue specific
GKGVSHR	Superscaf	-	Superscaf	37532746	37532766	-7	7	13	-	-	gene	-	-	mRNA	NCP	Intron	gene|mRN	ID=gene-E	7	6.53	2714.9	0.307703822	G(+42.01)	55	391.7151	2	781.4195	Acetylati	T	Superscaf	37533039	0	0	BTP20220	0	0	0	Tissue specific
TQTHNVP	Superscaf	+	Superscaf	37564444	37564464	15	15	21	-	-	gene	-	-	mRNA	NCP	Intron	gene|mRN	ID=gene-E	7	5.43	2959.5	0.307119148	T(+42.01)	77	419.705	2	837.3981	Acetylati	R	Superscaf	37564402	0	0	0	0	BTP20220	0	Mixed
PGVFMLR	Superscaf	-	Superscaf	37702432	37702452	-14	14	20	-	-	-	-	-	-	NCP	IGR	-	ID=gene-E	7	5.748571429	2395.8	0.304573944	PGVFMLR	80	410.226	2	818.4473	.	E	Superscaf	37702635	0	0	0	0	0	BTP20220	Tissue specific
NSSVGKKP	Superscaf	+	Superscaf	37708261	37708284	4	4	11	-	-	-	-	-	-	NCP	IGR	-	ID=gene-E	8	6.065	2935	0.304466372	NSSVGKKP	51	408.7383	2	815.4501	.	T	Superscaf	37708252	0	0	0	0	BTP20220	0	Tissue specific
SLVVHSR	Superscaf	-	Superscaf	37718644	37718664	-22	22	28	-	-	-	-	-	-	NCP	IGR	-	ID=gene-E	7	5.098571429	2388.7	0.304274912	SLVVHSR	59	399.242	2	796.4555	.	N	Superscaf	37718679	BTP20220	0	0	0	0	0	Tissue specific
SLDRPF	Superscaf	+	Superscaf	37730170	37730187	55	55	60	-	-	-	-	-	-	NCP	IGR	-	ID=gene-E	6	6.195	2521.9	0.304062369	SLDRPF	63	734.3905	1	733.3759	.	K	Superscaf	37730008	0	0	0	0	0	BTP20220	Tissue specific
KAFLLSQL	Superscaf	-	Superscaf	37752151	37752174	-18	18	25	-	-	-	-	-	-	NCP	IGR	-	ID=gene-E	8	6.31375	3221.4	0.303656816	KAFLLSQL	66	460.2915	2	918.5538	.	T	Superscaf	37752177	0	0	0	0	0	BTP20220	Tissue specific
LAMPV	Superscaf	-	Superscaf	37822276	37822290	-43	43	47	-	-	gene	-	-	mRNA	NCP	Intron	gene|mRN	ID=gene-E	5	4.81	1373.6	0.302363518	LAMPV	65	530.292	1	529.2934	.	A	Superscaf	37822299	0	0	0	0	0	BTP20220	Tissue specific
KPRGGLH	Superscaf	-	Superscaf	37824895	37824915	-18	18	24	-	-	gene	-	-	mRNA	NCP	Intron	gene|mRN	ID=gene-E	7	7.472857143	3022.3	0.3023151	KPRGGLH	51	382.7229	2	763.4453	.	D	Superscaf	37825086	0	0	BTP20220	0	0	0	Tissue specific
KPASLPGP	Superscaf	-	Superscaf	37840114	37840137	-31	31	38	-	-	gene	-	-	mRNA	NCP	Intron	gene|mRN	ID=gene-E	8	6.53625	2826	0.302034328	KPASLPGP	61	383.7274	2	765.4384	.	S	Superscaf	37840161	BTP20220	0	0	0	0	0	Tissue specific
AAYLVK	Superscaf	+	Superscaf	37846402	37846419	2	2	7	-	-	gene	-	-	mRNA	NCP	Intron	gene|mRN	ID=gene-E	6	5.57	1903.9	0.301918456	AAYLVK	55	664.4002	1	663.3955	.	K	Superscaf	37846399	0	0	BTP20220	0	0	0	Tissue specific
VGCVFV	Superscaf	-	Superscaf	37851520	37851537	-14	14	19	-	-	gene	-	-	mRNA	NCP	Intron	gene|mRN	ID=gene-E	6	2.745	1270.4	0.301824054	V(+42.01)	53	722.3484	1	721.3469	Acetylati	L	Superscaf	37851540	0	BTP20220	0	0	0	BTP20220	Tissue specific
LEVNSHLV	Superscaf	-	Superscaf	37948924	37948947	-28	28	35	-	-	-	-	-	-	NCP	IGR	-	ID=gene-E	8	4.2325	2732.1	0.300027315	LEVNSHLV	59	455.7573	2	909.4919	.	K	Superscaf	37948959	0	BTP20220	0	0	0	0	Tissue specific
AEVKRF	Superscaf	+	Superscaf	37977310	37977327	9	9	14	-	-	gene	-	-	mRNA	NCP	Intron	gene|mRN	ID=gene-E	6	5.87	2241.4	0.299503843	A(+42.01)	99	396.2222	2	790.4337	Acetylati	A	Superscaf	37977286	0	0	0	0	0	BTP20220	Tissue enhanced
TALPDT	Superscaf	-	Superscaf	37995286	37995303	-19	19	24	-	-	gene	-	-	mRNA	NCP	Intron	gene|mRN	ID=gene-E	6	5.721666667	2208.6	0.299172274	T(+42.01)	54	659.3268	1	658.3174	Acetylati	N	Superscaf	37995396	0	0	0	BTP20220	0	0	Tissue specific
KPTPNPH	Superscaf	-	Superscaf	38061043	38061063	-15	15	21	-	-	gene	-	-	mRNA	NCP	Intron	gene|mRN	ID=gene-E	7	6.881428571	3094.5	0.297959323	KPTPNPH	53	395.7145	2	789.4133	.	Q	Superscaf	38061063	0	0	0	BTP20220	0	0	Tissue specific
EVGVLFT	Superscaf	-	Superscaf	38065453	38065473	-54	54	60	-	-	-	-	-	-	NCP	IGR	-	ID=gene-E	7	3.882857143	1861.1	0.29787798	EVGVLFT	61	764.4153	1	763.4116	.	P	Superscaf	38065824	0	BTP20220	0	0	0	0	Tissue specific
LSARPST	Superscaf	+	Superscaf	38066944	38066964	5	5	11	-	-	-	-	-	-	NCP	IGR	-	ID=gene-E	7	6.707142857	2815.2	0.297850478	LSARPST	52	366.2048	2	730.3973	.	Q	Superscaf	38066932	0	0	0	0	BTP20220	0	Tissue specific
KPSQQPK	Superscaf	-	Superscaf	38098819	38098839	-14	14	20	-	-	gene	-	-	mRNA	NCP	Intron	gene|mRN	ID=gene-E	7	7.008571429	3216.4	0.29726254	KPSQQPK	76	406.7337	2	811.4551	.	E	Superscaf	38098926	0	0	0	0	0	BTP20220	Tissue specific
QFHPDR	Superscaf	-	Superscaf	38109148	38109165	-14	14	19	-	-	-	-	-	-	NCP	IGR	-	ID=gene-E	6	6.458333333	3015.5	0.297072076	Q(+42.01)	50	421.2026	2	840.3878	Acetylati	H	Superscaf	38109273	BTP20220	0	0	0	0	0	Tissue specific
YGGPAP	Superscaf	+	Superscaf	38199541	38199558	6	6	11	-	-	-	-	-	-	NCP	IGR	-	ID=gene-E	6	6.036666667	2040.2	0.295404767	YGGPAP	86	561.2667	1	560.2595	.	W	Superscaf	38199526	0	0	BTP20220	0	0	0	Tissue specific
LAAQFCC	Superscaf	+	Superscaf	38199640	38199660	39	39	45	-	-	-	-	-	-	NCP	IGR	-	ID=gene-E	7	5.598571429	3145.5	0.295402885	LAAQFC(+5	55	869.3505	1	868.3571	Carbamidc	W	Superscaf	38199526	0	0	0	0	0	BTP20220	Tissue specific
PVLMH	Superscaf	+	Superscaf	38246500	38246514	10	10	14	-	-	gene	-	-	mRNA	NCP	Intron	gene|mRN	ID=gene-E	5	5.124	1682	0.294538658	PVLM(+15.	51	612.3163	1	611.3101	Oxidatior	Y	Superscaf	38246473	0	0	0	0	BTP20220	0	Tissue specific
LAPDVEL	Superscaf	-	Superscaf	38247931	38247951	-21	21	27	-	-	gene	-	-	mRNA	NCP	Intron	gene|mRN	ID=gene-E	7	4.352857143	2192.5	0.294512152	LAPDVEL	69	756.4105	1	755.4065	.	R	Superscaf	38247978	0	0	0	0	0	BTP20220	Tissue specific
PFGQYPP	Superscaf	-	Superscaf	38277448	38277468	-4	4	10	-	-	gene	-	-	mRNA	NCP	Intron	gene|mRN	ID=gene-E	7	5.951428571	2767.1	0.293967708	PFGQYPP	55	403.1972	2	804.3806	.	P	Superscaf	38277540	0	0	0	0	BTP20220	0	Tissue specific
KPALPTPP	Superscaf	-	Superscaf	38299684	38299707	-4	4	11	-	-	gene	-	-	mRNA	NCP	Intron	gene|mRN	ID=gene-E	8	6.68375	2880.2	0.293557507	KPALPTPP	77	410.7515	2	819.4854	.	G	Superscaf	38299806	0	BTP20220	0	0	0	0	Tissue specific
KAYPPK	Superscaf	+	Superscaf	38346355	38346372	10	10	15	-	-	gene	-	-	mRNA	NCP	Intron	gene|mRN	ID=gene-E	6	7.293333333	2382.4	0.292696766	KAYPPK	58	352.2125	2	702.4064	.	K	Superscaf	38346328	0	0	BTP20220	0	0	0	Tissue specific
RFVLPQ	Superscaf	+	Superscaf	38366455	38366472	5	5	10	-	-	gene	-	-	mRNA	NCP	Intron	gene|mRN	ID=gene-E	6	5.695	2337.7	0.292326019	RFVLPQ	64	759.4583	1	758.4439	.	C	Superscaf	38366443	0	0	0	0	0	BTP20220	Tissue specific
FPAVPEV	Superscaf	+	Superscaf	38394718	38394738	2	2	8	-	-	gene	-	-	mRNA	NCP	Intron	gene|mRN	ID=gene-E	7	3.902857143	1799	0.291804649	FPAVPEV	66	379.706	2	757.401	.	D	Superscaf	38394715	0	0	0	0	BTP20220	0	Mixed
VTVLYT	Superscaf	+	Superscaf	38402101	38402118	1	1	6	-	-	gene	-	-	mRNA	NCP	Intron	gene|mRN	ID=gene-E	6	4.116666667	1607.9	0.291668524	VTVLYT	65	695.3938	1	694.3901	.	V	Superscaf	38402101	0	BTP20220	0	0	0	0	Tissue specific
KLEDVKF	Superscaf	-	Superscaf	38430172	38430192	-5	5	11	-	-	gene	-	-	mRNA	NCP	Intron	gene|mRN	ID=gene-E	7	5.304285714	2514.6	0.291150696	KLEDVKF	68	439.7512	2	877.4909	.	Y	Superscaf	38430201	0	0	BTP20220	0	0	0	Tissue specific
KELAVTK	Superscaf	+	Superscaf	38459950	38459970	24	24	30	-	-	-	-	-	-	NCP	IGR	-	ID=gene-E	7	5.89	2424.5	0.290601437	KELAVTK	81	788.4851	1	787.4803	.	M	Superscaf	38459881	0	BTP20220	0	0	0	0	Tissue specific
KGRLLLK	Superscaf	+	Superscaf	38467369	38467389	20	20	26	-	-	-	-	-	-	NCP	IGR	-	ID=gene-E	7	7.735714286	2943.1	0.290464593	KGRLLLK	62	414.2932	2	826.5752	.	Q	Superscaf	38467312	0	0	BTP20220	0	0	0	Tissue specific
EKLVA	Superscaf	-	Superscaf	38475421	38475435	-8	8	12	-	-	-	-	-	-	NCP	IGR	-	ID=gene-E	5	4.992	1558.6	0.290316184	EKLVA	82	559.3428	1	558.3377	.	E	Superscaf	38475489	0	0	BTP20220	0	0	0	Tissue specific
LAKYTSSK	Superscaf	+	Superscaf	38500837	38500860	3	3	10	-	-	-	-	-	-	NCP	IGR	-	ID=gene-E	8	6.87875	3225.3	0.289847217	LAKYTSSK	86	449.2542	2	896.4967	.	A	Superscaf	38500831	0	0	0	0	0	BTP20220	Tissue specific
NNMHAA	Superscaf	-	Superscaf	38507239	38507256	-7	7	12	-	-	-	-	-	-	NCP	IGR	-	ID=gene-E	6	6.033333333	2807.2	0.289729242	N(+42.01)	54	358.1489	2	714.2755	Acetylati	K	Superscaf	38507274	0	0	0	0	0	BTP20220	Tissue specific
PAPPVDR	Superscaf	+	Superscaf	38540158	38540178	10	10	16	-	-	-	-	-	-	NCP	IGR	-	ID=gene-E	7	5.521428571	2383.8	0.289121992	PAPPVDR	51	376.2029	2	750.4024	.	P	Superscaf	38540131	0	0	0	0	BTP20220	0	Tissue specific
AAAVRRP	Superscaf	+	Superscaf	38551921	38551941	5	5	11	-	-	-	-	-	-	NCP	IGR	-	ID=gene-E	7	6.554285714	2568.9	0.288905022	AAAVRRP	51	370.7294	2	739.4453	.	S	Superscaf	38551909	0	0	0	BTP20220	0	0	Tissue specific
LTVLPF	Superscaf	-	Superscaf	38567863	38567880	-66	66	71	-	-	-	-	-	-	NCP	IGR	-	ID=gene-E	6	5.045	1829.1	0.288611025	LTVLPF	57	689.4242	1	688.416	.	N	Superscaf	38567892	BTP20220	0	0	0	0	0	Tissue specific
NAKLGLL	Superscaf	-	Superscaf	38590879	38590899	-1	1	7	-	-	gene	-	-	mRNA	NCP	Intron	gene|mRN	ID=gene-E	7	6.44	2733.9	0.288186437	NAKLGLL	50	728.464	1	727.4592	.	N	Superscaf	38590914	0	0	BTP20220	0	0	0	Tissue specific
CFTKFYL	Superscaf	+	Superscaf	38603146	38603166	5	5	11	-	-	gene	-	-	mRNA	NCP	Intron	gene|mRN	ID=gene-E	7	6.27	3039.3	0.287960171	C(+57.02)	54	489.7418	2	977.4681	Carbamidc	L	Superscaf	38603134	BTP20220	0	0	0	0	0	Tissue specific
DPPPPQPP	Superscaf	-	Superscaf	38646517	38646540	-11	11	18	-	-	gene	-	-	mRNA	NCP	Intron	gene|mRN	ID=gene-E	8	5.8025	3046.6	0.287160132	DPPPPQPP	75	422.7104	2	843.4126	.	C	Superscaf	38646588	0	0	0	BTP20220	0	0	Tissue specific

AEAPGHGP	Superscaf	-	Superscaf	38648560	38648583	-54	54	61	-	-	gene	-	-	mRNA	NCP	Intron	gene|mRN	ID=gene-E	8	5.92375	2937.2	0.287122449	A(+42.01)	64	389.1788	2	776.3453	Acetylati	D	Superscaf	38648664	0	0	0	BTP202209	0	0	Tissue enhanced
WGMLPPPPF	Superscaf	+	Superscaf	38721490	38721516	20	20	28	-	-	gene	-	-	mRNA	NCP	Intron	gene|mRN	ID=gene-E	9	6.121111111	3235.8	0.285777191	WGM(+15.9	71	504.2597	2	1006.495	Oxidation	T	Superscaf	38721433	0	0	0	BTP202209	0	0	Tissue specific
LEGLGAL	Superscaf	-	Superscaf	38748163	38748183	-53	53	59	-	-	gene	-	-	mRNA	NCP	Intron	gene|mRN	ID=gene-E	7	5.588571429	2391.6	0.285285316	LEGLGAL	77	672.384	1	671.3854	.	T	Superscaf	38748711	0	BTP202209	0	0	0	0	Tissue specific
KFNHFFK	Superscaf	-	Superscaf	38803984	38804004	-20	20	26	-	-	-	-	-	-	NCP	IGR	-	ID=gene-E	7	6.988571429	3315.4	0.284255691	KFNHFFK	63	484.2551	2	966.5075	.	C	Superscaf	38804070	0	0	0	BTP202209	0	0	Tissue specific
KGVYPR	Superscaf	+	Superscaf	38964940	38964957	2	2	7	-	-	-	-	-	-	NCP	IGR	-	ID=gene-E	6	6.405	2211.4	0.281286894	KGVYPR	60	360.2108	2	718.4126	.	E	Superscaf	38964937	0	0	0	0	BTP202209	0	Tissue specific
YRLLNGN	Superscaf	+	Superscaf	39010393	39010413	29	29	35	-	-	gene	-	-	mRNA	NCP	Intron	gene|mRN	ID=gene-E	7	6.452857143	3305.8	0.280448453	YRLLNGN	78	849.4684	1	848.4504	.	V	Superscaf	39010309	0	0	0	0	0	BTP202209	Tissue specific
KVETFSGVY	Superscaf	-	Superscaf	39014299	39014331	-9	9	19	-	-	gene	-	-	mRNA	NCP	Intron	gene|mRN	ID=gene-E	11	5.614545455	3755.8	0.280376185	KVETFSGVY	86	429.2418	3	1284.708	.	S	Superscaf	39014376	0	0	0	BTP202209	0	0	Tissue specific
KVETFSGVY	Superscaf	-	Superscaf	39014299	39014328	-9	9	18	-	-	gene	-	-	mRNA	NCP	Intron	gene|mRN	ID=gene-E	10	5.202	3287.4	0.280376241	KVETFSGVY	97	579.313	2	1156.613	.	S	Superscaf	39014376	0	0	0	0	0	BTP202209	Tissue specific
ERMLYR	Superscaf	-	Superscaf	39025876	39025893	-39	39	44	-	-	gene	-	-	mRNA	NCP	Intron	gene|mRN	ID=gene-E	6	7.021666667	2907.5	0.280162923	ERMLYR	76	434.2372	2	866.4432	.	N	Superscaf	39025902	0	0	0	0	0	BTP202209	Tissue specific
LTVSLVLP	Superscaf	+	Superscaf	39034000	39034023	22	22	29	-	-	gene	-	-	mRNA	NCP	Intron	gene|mRN	ID=gene-E	8	4.55625	2178.5	0.280012964	LTVSLVLP	54	841.5348	1	840.532	.	K	Superscaf	39033937	0	BTP202209	0	0	0	0	Tissue specific
VVLAC	Superscaf	-	Superscaf	39055105	39055119	-1	1	5	-	-	gene	-	-	mRNA	NCP	Intron	gene|mRN	ID=gene-E	5	3.404	1250.4	0.279623846	VVLAC(+57	59	561.3096	1	560.2992	Carbamido	V	Superscaf	39055173	0	0	0	0	0	BTP202209	Tissue specific
KGMPPR	Superscaf	+	Superscaf	39062728	39062745	14	14	19	-	-	gene	-	-	mRNA	NCP	Intron	gene|mRN	ID=gene-E	6	7.47	2460.8	0.279483183	KGM(+15.9	52	351.1963	2	700.369	Oxidation	K	Superscaf	39062689	0	BTP202209	0	0	0	0	Tissue specific
VNATPQ	Superscaf	-	Superscaf	39070603	39070620	-2	2	7	-	-	gene	-	-	mRNA	NCP	Intron	gene|mRN	ID=gene-E	6	4.985	2253.6	0.279337928	VNATPQ	51	629.3274	1	628.3181	.	L	Superscaf	39070860	0	0	0	0	BTP202209	0	Tissue specific
DAFVRSP	Superscaf	+	Superscaf	39128398	39128418	8	8	14	-	-	gene	-	-	mRNA	NCP	Intron	gene|mRN	ID=gene-E	7	5.315714286	2479.8	0.278271837	D(+42.01)	67	417.2158	2	832.4079	Acetylati	L	Superscaf	39128377	0	BTP202209	0	0	0	0	Tissue specific
APLPL	Superscaf	-	Superscaf	39142552	39142566	-10	10	14	-	-	gene	-	-	mRNA	NCP	Intron	gene|mRN	ID=gene-E	5	6.116	1692.9	0.278010876	A(+42.01)	72	552.3365	1	551.3318	Acetylati	Y	Superscaf	39142662	BTP202209	0	0	0	0	0	Tissue specific
LKLSQKNS	Superscaf	-	Superscaf	39142675	39142698	-5	5	12	-	-	gene	-	-	mRNA	NCP	Intron	gene|mRN	ID=gene-E	8	6.7325	3617.8	0.278008441	LKLSQKNS	85	459.2727	2	916.5342	.	H	Superscaf	39142722	0	0	0	BTP202209	0	0	Tissue specific
MYDNRESQI	Superscaf	-	Superscaf	39276676	39276711	-23	23	34	-	-	-	-	-	-	NCP	IGR	-	ID=gene-E	12	5.48	5487.4	0.275536556	MYDNRESQI	78	746.8018	2	1491.594	.	E	Superscaf	39276783	0	0	0	0	0	BTP202209	Tissue specific
VDSTTCLFF	Superscaf	-	Superscaf	39282949	39282987	-17	17	29	-	-	-	-	-	-	NCP	IGR	-	ID=gene-E	13	4.666923077	4573.2	0.275420794	VDSTTC(+5	99	762.8625	2	1523.718	Carbamido	L	Superscaf	39283020	0	0	0	0	0	BTP202209	Tissue specific
TSQFVSLVW	Superscaf	-	Superscaf	39302464	39302493	-17	17	26	-	-	-	-	-	-	NCP	IGR	-	ID=gene-E	10	4.623	3274.7	0.275061004	TSQFVSLVW	69	579.7959	2	1157.579	.	N	Superscaf	39302607	0	0	BTP202209	0	0	0	Tissue specific
VVTYKP	Superscaf	+	Superscaf	39326086	39326103	30	30	35	-	-	-	-	-	-	NCP	IGR	-	ID=gene-E	6	4.705	1662.8	0.274625515	VVTYKP	52	353.7122	2	705.4061	.	G	Superscaf	39325999	0	0	0	BTP202209	0	0	Tissue specific
LEEVEPTP	Superscaf	-	Superscaf	39342469	39342492	-7	7	14	-	-	gene	-	-	mRNA	NCP	Intron	gene|mRN	ID=gene-E	8	4.34625	2702.2	0.274323218	LEEVEPTP	75	913.451	1	912.444	.	I	Superscaf	39342675	0	0	BTP202209	0	0	0	Tissue specific
KQLEDGRTI	Superscaf	+	Superscaf	39365701	39365727	23	23	31	-	-	-	-	-	-	NCP	IGR	-	ID=gene-E	9	6.311111111	3966.5	0.273894646	KQLEDGRTI	85	530.2921	2	1058.572	.	F	Superscaf	39365635	0	0	0	BTP202209	0	0	Tissue specific
PVLHLLFP	Superscaf	-	Superscaf	39367570	39367593	-35	35	42	-	exon	gene	three_pri	-	mRNA	NCP	3UTR	exon|gene	ID=exon-E	8	5.45125	2742.1	0.273860227	P(+42.01)	57	489.295	2	976.5745	Acetylati	Q	Superscaf	39367701	0	0	0	0	0	BTP202209	Tissue specific
DMCWK	Superscaf	+	Superscaf	39370636	39370650	17	17	21	-	-	gene	-	-	mRNA	NCP	Intron	gene|mRN	ID=gene-E	5	5.874	2363.7	0.27380384	DM(+15.99	59	755.2714	1	754.2778	Oxidation	N	Superscaf	39370588	0	0	BTP202209	0	0	0	Tissue specific
NEHYAK	Superscaf	+	Superscaf	39374842	39374859	41	41	46	-	-	gene	-	-	mRNA	NCP	Intron	gene|mRN	ID=gene-E	6	6.273333333	2881.1	0.273726205	NEHYAK	65	381.1861	2	760.3504	.	V	Superscaf	39374722	0	0	0	BTP202209	0	0	Tissue specific
LTAAVAL	Superscaf	-	Superscaf	39396211	39396231	-32	32	38	-	-	-	-	-	-	NCP	IGR	-	ID=gene-E	7	5.221428571	2038.2	0.273331996	LTAAVAL	51	658.4116	1	657.4061	.	R	Superscaf	39396231	0	0	BTP202209	0	0	0	Tissue specific
EVDALV	Superscaf	-	Superscaf	39409630	39409647	-5	5	10	-	-	-	-	-	-	NCP	IGR	-	ID=gene-E	6	3.031666667	1501.7	0.273084536	EVDALV	57	645.3401	1	644.3381	.	C	Superscaf	39409668	0	0	0	BTP202209	0	0	Tissue specific
EVLPLLAK	Superscaf	-	Superscaf	39429865	39429888	-14	14	21	-	-	gene	-	-	mRNA	NCP	Intron	gene|mRN	ID=gene-E	8	5.4025	2602.8	0.272711189	EVLPLLAK	87	441.7847	2	881.5585	.	S	Superscaf	39429912	0	BTP202209	0	0	0	0	Tissue specific
YLLKLK	Superscaf	+	Superscaf	39440722	39440739	6	6	11	-	-	gene	-	-	mRNA	NCP	Intron	gene|mRN	ID=gene-E	6	7.18	2456.5	0.272511041	YLLKLK	55	389.2682	2	776.516	.	N	Superscaf	39440707	0	0	0	BTP202209	0	0	Tissue specific
LDFAFGL	Superscaf	-	Superscaf	39458233	39458253	-48	48	54	-	-	gene	-	-	mRNA	NCP	Intron	gene|mRN	ID=gene-E	7	5.411428571	2501.7	0.272187993	LDFAFGL	73	782.4056	1	781.401	.	S	Superscaf	39458472	0	0	BTP202209	0	0	0	Tissue specific
DLDKLLK	Superscaf	-	Superscaf	39533614	39533634	-26	26	32	-	-	-	-	-	-	NCP	IGR	-	ID=gene-E	7	6.194285714	2820	0.270797582	DLDKLLK	97	422.76	2	843.5065	.	E	Superscaf	39533661	BTP202209	0	0	0	0	0	Tissue specific
FEPELK	Superscaf	-	Superscaf	39549193	39549210	-15	15	20	-	-	-	-	-	-	NCP	IGR	-	ID=gene-E	6	5.656666667	2397.6	0.270510281	FEPELK	95	762.3984	1	761.3959	.	S	Superscaf	39549213	BTP202209	0	0	0	0	0	Mixed
DLLKGPK	Superscaf	-	Superscaf	39550534	39550554	-5	5	11	-	-	-	-	-	-	NCP	IGR	-	ID=gene-E	7	6.668571429	2689.8	0.27048549	DLLKGPK	65	385.7408	2	769.4697	.	V	Superscaf	39550584	0	0	0	0	0	BTP202209	Tissue specific
KPAAETPK	Superscaf	-	Superscaf	39561001	39561024	-84	84	91	-	-	-	-	-	-	NCP	IGR	-	ID=gene-E	8	6.73375	3057.2	0.27029237	KPAAETPK	51	421.2448	2	840.4705	.	A	Superscaf	39561129	0	0	0	0	0	BTP202209	Tissue specific
LSSSALSH	Superscaf	-	Superscaf	39572560	39572583	-38	38	45	-	-	-	-	-	-	NCP	IGR	-	ID=gene-E	8	6.03625	3171.4	0.270079163	LSSSALSH	89	401.2068	2	800.4028	.	T	Superscaf	39572628	BTP202209	0	0	0	0	0	Tissue specific
LDVLGVTK	Superscaf	-	Superscaf	39576517	39576540	-21	21	28	-	-	-	-	-	-	NCP	IGR	-	ID=gene-E	8	4.64625	2281.5	0.270006176	LDVLGVTK	97	422.7595	2	843.5065	.	V	Superscaf	39576825	0	0	0	BTP202209	0	0	Tissue enhanced
LTSDTGEL	Superscaf	+	Superscaf	39583144	39583167	4	4	11	-	-	-	-	-	-	NCP	IGR	-	ID=gene-E	8	5.3575	3019.5	0.26988394	LTSDTGEL	52	835.4022	1	834.397	.	K	Superscaf	39583135	0	0	BTP202209	0	0	0	Tissue specific
DSVSEFLK	Superscaf	-	Superscaf	39621937	39621960	-16	16	23	-	-	gene	-	-	mRNA	NCP	Intron	gene|mRN	ID=gene-E	8	4.84375	2813	0.269168398	D(+42.01)	90	483.7332	2	965.4705	Acetylati	R	Superscaf	39621975	0	0	0	0	0	BTP202209	Tissue specific
FTQLYKP	Superscaf	-	Superscaf	39634051	39634071	-3	3	9	-	-	gene	-	-	mRNA	NCP	Intron	gene|mRN	ID=gene-E	7	6.477142857	3014.3	0.26894501	FTQLYKP	71	448.7464	2	895.4803	.	S	Superscaf	39634071	0	0	BTP202209	0	0	0	Tissue specific
GPLGDLR	Superscaf	-	Superscaf	39651202	39651222	-31	31	37	-	-	gene	-	-	mRNA	NCP	Intron	gene|mRN	ID=gene-E	7	6.275714286	2699.1	0.268628658	GPLGDLR	98	364.2074	2	726.4024	.	I	Superscaf	39651237	0	0	0	0	BTP202209	0	Tissue specific
MYMDPP	Superscaf	+	Superscaf	39740092	39740109	1	1	6	-	-	gene	-	-	mRNA	NCP	Intron	gene|mRN	ID=gene-E	6	5.455	2288.8	0.266989127	MYM(+15.9	59	385.1526	2	768.2822	Oxidation	M	Superscaf	39740092	0	0	0	BTP202209	0	0	Tissue specific
NHTKKKK	Superscaf	+	Superscaf	39835876	39835896	89	89	95	-	-	-	-	-	-	NCP	IGR	-	ID=gene-E	7	8.355714286	3487.5	0.265222324	NHTKKKK	53	442.2751	2	882.5399	.	H	Superscaf	39835612	0	BTP202209	BTP202209	0	0	BTP202209	Tissue specific
VAKTAVT	Superscaf	-	Superscaf	39888085	39888105	-6	6	12	-	-	gene	-	-	mRNA	NCP	Intron	gene|mRN	ID=gene-E	7	4.977142857	1886	0.264259323	V(+42.01)	53	731.425	1	730.4225	Acetylati	T	Superscaf	39888246	0	0	BTP202209	0	0	0	Tissue specific
PPTLGPP	Superscaf	-	Superscaf	39905995	39906015	-4	4	10	-	-	gene	-	-	mRNA	NCP	Intron	gene|mRN	ID=gene-E	7	6.24	2397.8	0.263928971	PPTLGPP	71	678.3904	1	677.3748	.	P	Superscaf	39906129	0	0	BTP202209	0	0	0	Tissue specific
EKLSYDK	Superscaf	-	Superscaf	39985201	39985221	-12	12	18	-	-	-	-	-	-	NCP	IGR	-	ID=gene-E	7	6.141428571	2970.1	0.262468008	EKLSYDK	51	441.7353	2	881.4494	.	K	Superscaf	39985230	0	0	0	0	0	BTP202209	Tissue specific
LTPLVT	Superscaf	-	Superscaf	40056319	40056336	-74	74	79	-	-	-	-	-	-	NCP	IGR	-	ID=gene-E	6	5.22	1839.2	0.261156283	LTPLVT	86	643.4006	1	642.3952	.	E	Superscaf	40056429	0	0	0	BTP202209	0	0	Tissue specific
VTASNVLK	Superscaf	-	Superscaf	40092667	40092690	-2	2	9	-	-	-	-	-	-	NCP	IGR	-	ID=gene-E	8	4.92	2510.7	0.26048573	VTASNVLK	52	416.2489	2	830.4861	.	S	Superscaf	40092741	0	BTP202209	0	BTP202209	0	0	Tissue specific
FNVDPF	Superscaf	+	Superscaf	40094794	40094811	27	27	32	-	-	-	-	-	-	NCP	IGR	-	ID=gene-E	6	4.273333333	2120.4	0.260446608	FNVDPF	64	738.345	1	737.3384	.	Y	Superscaf	40094716	0	BTP202209	0	0	0	0	Tissue specific
QDCHK	Superscaf	-	Superscaf	40101256	40101270	-22	22	26	-	-	-	-	-	-	NCP	IGR	-	ID=gene-E	5	6.194	2696	0.260327471	Q(+42.01)	50	365.1567	2	728.2912	Acetylati	D	Superscaf	40101294	BTP202209	0	0	0	0	0	Tissue specific
KTLLQPL	Superscaf	+	Superscaf	40115545	40115565	78	78	84	-	-	-	-	-	-	NCP	IGR	-	ID=gene-E	7	6.594285714	2874.2	0.260063798	KTLLQPL	63	812.5226	1	811.5167	.	F	Superscaf	40115314	0	0	BTP202209	0	0	0	Tissue specific
KLHFDP	Superscaf	-	Superscaf	40192930	40192947	-5	5	10	-	-	-	-	-	-	NCP	IGR	-	ID=gene-E	6	6.343333333	2577.8	0.258636478	KLHFDP	62	378.7042	2	755.3966	.	L	Superscaf	40193007	0	0	0	BTP202209	0	0	Tissue specific
VYQFPK	Superscaf	-	Superscaf	40285453	40285470	-20	20	25	-	-	-	-	-	-	NCP	IGR	-	ID=gene-E	6	5.471666667	2263.4	0.25692988	VYQFPK	50	391.214	2	780.417	.	L	Superscaf	40285509	0	BTP202209	0	0	0	0	Tissue specific
GDLPGLVRI	Superscaf	-	Superscaf	40289779	40289805	-54	54	62	-	-	-	-	-	-	NCP	IGR	-	ID=gene-E	9	5.545555556	3052.5	0.256849921	G(+42.01)	77	491.288	2	980.5654	Acetylati	R	Superscaf	40289955	0	0	BTP202209	0	0	0	Mixed
GDLPGLVR	Superscaf	-	Superscaf	40289779	40289802	-54	54	61	-	-	-	-	-	-	NCP	IGR	-	ID=gene-E	8	5.49125	2699.1	0.256849976	G(+42.01)	81	434.7458	2	867.4814	Acetylati	R	Superscaf	40289955	0	0	BTP202209	0	0	0	Tissue specific
GKELDSL	Superscaf	-	Superscaf	40297408	40297428	-3	3	9	-	-	-	-	-	-	NCP	IGR	-	ID=gene-E	7	5.648571429	2692.9	0.256709314	G(+42.01)	54	803.4303	1	802.4072	Acetylati	Q	Superscaf	40297431	0	0	BTP202209	0	0	0	Tissue specific
KYAKPP	Superscaf	+	Superscaf	40335574	40335591	19	19	24	-	-	-	-	-	-	NCP	IGR	-	ID=gene-E	6	7.293333333	2382.4	0.256005393	KYAKPP	63	352.2066	2	702.4064	.	V	Superscaf	40335520	0	0	BTP202209	0	0	0	Tissue specific
LDDALVT	Superscaf	+	Superscaf	40386022	40386042	30	30	36	-	-	-	-	-	-	NCP	IGR	-	ID=gene-E	7	4.35	2238.6	0.255074818	LDDALVT	70	746.3909	1	745.3857	.	N	Superscaf	40385935	BTP202209	0	0	0	0	0	Tissue specific
RELTQAR	Superscaf	+	Superscaf	40591489	40591509	14	14	20	-	-	gene	-	-	mRNA	NCP	Intron	gene|mRN	ID=gene-E	7	6.988571429	3396	0.251284956	RELTQAR	85	437.2478	2	872.4828	.	F	Superscaf	40591450	0	0	BTP202209	0	0	0	Tissue specific
LDTEFR	Superscaf	+	Superscaf	40601470	40601487	26	26	31	-	-	gene	-	-	mRNA	NCP	Intron	gene|mRN	ID=gene-E	6	5.823333333	2624.1	0.251100911	LDTEFR	95	390.6962	2	779.3813	.	M	Superscaf	40601395	0	0	0	0	BTP202209	0	Tissue specific
VPDDFP	Superscaf	+	Superscaf	40653388	40653405	12	12	17	-	-	-	-	-	-	NCP	IGR	-	ID=gene-E	6	4.003333333	1885.2	0.250143277	VPDDFP	57	689.3272	1	688.3068	.	F	Superscaf	40653355	0	0	0	BTP202209	0	0	Tissue specific
EAEVSVP	Superscaf	+	Superscaf	40659496	40659516	9	9	15	-	-	-	-	-	-	NCP	IGR	-	ID=gene-E	7	3.491428571	1883.1	0.250030559	EAEVSVP	50	730.3585	1	729.3544	.	I	Superscaf	40659472	0	0	BTP202209	0	0	0	Tissue specific
QNGKLFK	Superscaf	+	Superscaf	40725286	40725306	10	10	16	-	-	-	-	-	-	NCP	IGR	-	ID=gene-E	7	6.852857143	3182.3	0.248817055	QNGKLFK	84	417.744	2	833.4759	.	K	Superscaf	40725259	BTP202209	0	0	0	0	0	Tissue specific
LNLFEML	Superscaf	-	Superscaf	40732726	40732746	-15	15	21	-	-	-	-	-	-	NCP	IGR	-	ID=gene-E	7	5.4	2841.3	0.248679823	LNLFEML	75	879.4612	1	878.4572	.	S	Superscaf	40732917	0	0	0	0	0	BTP202209	Tissue specific
PTSTPKC	Superscaf	+	Superscaf	40765888	40765908	5	5	11	-	-	-	-	-	-	NCP	IGR	-	ID=gene-E	7	6.585714286	2907.3	0.248068146	P(+42.01)	73	416.6929	2	831.3796	Acetylati	A	Superscaf	40765876	0	0	0	0	0	BTP202209	Tissue specific
VFFNTLR	Superscaf	-	Superscaf	40801360	40801380	-8	8	14	-	-	-	-	-	-	NCP	IGR	-	ID=gene-E	7	5.662857143	2771.2	0.247413861	VFFNTLR	94	448.7482	2	895.4916	.	E	Superscaf	40801389	0	0	0	0	0	BTP202209	Tissue specific
PKQMPAP	Superscaf	-	Superscaf	40839463	40839483	-13	13	19	-	-	-	-	-	-	NCP	IGR	-	ID=gene-E	7	6.58	2774.1	0.246711047	PKQM(+15.	54	784.4001	1	783.3949	Oxidation	L	Superscaf	40839549	0	0	0	0	BTP202209	0	Tissue specific
VAALYLE	Superscaf	-	Superscaf	40967986	40968006	-42	42	48	-	-	-	-	-	-	NCP	IGR	-	ID=gene-E	7	4.697142857	2214.4	0.244340425	VAALYLE	58	778.4312	1	777.4272	.	F	Superscaf	40968006	0	0	0	0	0	BTP202209	Tissue specific
VHLSHAK	Superscaf	+	Superscaf	41169031	41169051	2	2	8	-	-	-	-	-	-	NCP	IGR	-	ID=gene-E	7	6.085714286	2755.9	0.240632127	V(+42.01)	63	417.2371	2	832.4555	Acetylati	T	Superscaf	41169028	0	0	BTP202209	0	0	0	Tissue specific
LSVPPFK	Superscaf	-	Superscaf	41290831	41290851	-19	19	25	-	-	-	-	-	-	NCP	IGR	-	ID=gene-E	7	5.64	2267.4	0.238385512	LSVPPFK	81	394.2325	2	786.4639	.	N	Superscaf	41290899	0	0	0	0	BTP202209	BTP202209	Tissue enhanced
GCKAKGK	Superscaf	-	Superscaf	41327035	41327055	-9	9	15	-	-	-	-	-	-	NCP	IGR	-	ID=gene-E	7	7.457142857	2896.8	0.237717725	GC(+57.02	83	374.712	2	747.4061	Carbamido	E	Superscaf	41327055	0	0	BTP202209	0	0	0	Tissue specific
ELRYF	Superscaf	-	Superscaf	41425165	41425179	-14	14	18	-	-	-	-	-	-	NCP	IGR	-	ID=gene-E	5	6.22	2274.6	0.235907817	ELRYF	80	364.1915	2	726.3701	.	Q	Superscaf	41425209	0	BTP202209	0	0	0	0	Tissue enhanced
LLVGG	Superscaf	+	Superscaf	41485030	41485044	17	17	21	-	-	-	-	-	-	NCP	IGR	-	ID=gene-E	5	4.78	1301.4	0.2348036	LLVGG	83	458.2966	1	457.29	.	Q	Superscaf	41484982	0	0	BTP202209	0	0	0	Mixed
EEVRLPP	Superscaf	-	Superscaf	41586328	41586348	-22	22	28	-	-	-	-	-	-	NCP	IGR	-	ID=gene-E	7	5.111428571	2528	0.232935036	EEVRLPP	96	420.2324	2	838.4548	.	E	Superscaf	41586357	0	0	0	BTP202209	0	0	Tissue specific
FPPLSLLK	Superscaf	+	Superscaf	41639422	41639445	39	39	46	-	-	-	-	-	-	NCP	IGR	-	ID=gene-E	8	6.43	2974.2	0.231955656	FPPLSLLK	84	914.5712	1	913.5637	.	K	Superscaf	41639308	0	0	0	0	BTP202209	0	Tissue specific
KGLLLGL	Superscaf	-	Superscaf	41679448	41679468	-2	2	8	-	-	-	-	-	-	NCP	IGR	-	ID=gene-E	7	6.514285714	2476.6	0.231217427	KGLLLGL	61	357.2485	2	712.4847	.	Y	Superscaf	41679516	0	0	0	0	0	BTP202209	Tissue specific
KSQDTPK	Superscaf	+	Superscaf	41708821	41708841	40	40	46	-	-	-	-	-	-	NCP	IGR	-	ID=gene-E	7	6.658571429	3077.3	0.230675639	KSQDTPK	55	402.2176	2	802.4185	.	A	Superscaf	41708704	0	0	0	0	0	BTP202209	Tissue specific
DLETPY	Superscaf	-	Superscaf	41726884	41726901	-7	7	12	-	-	-	-	-	-	NCP	IGR	-	ID=gene-E	6	5.11	2384.8	0.23034252	DLETPY	87	737.3323	1	736.3279	.	A	Superscaf	41726943	0	BTP202209	0	0	0	0	Tissue specific
KSYLSSH	Superscaf	+	Superscaf	41781460	41781480	9	9	15	-	-	-	-	-	-	NCP	IGR	-	ID=gene-E	7	6.572857143	3051.2	0.229335804	K(+42.01)	97	432.214	2	862.4185	Acetylati	K	Superscaf	41781436	BTP202209	0	0	0	0	0	Tissue specific
ARPLM	Superscaf	-	Superscaf	41800654	41800668	-7	7	11	-	-	-	-	-	-	NCP	IGR	-	ID=gene-E	5	6.962	2022.4	0.228981879	ARPLM	50	587.3287	1	586.3261	.	G	Superscaf	41800671	0	0	0	0	0	BTP202209	Tissue specific
FAFVQTC	Superscaf	+	Superscaf	41860372	41860392	11	11	17	-	-	-	-	-	-	NCP	IGR	-	ID=gene-E	7	4.882857143	2680	0.227880263	FAFVQTC(+	95	872.3933	1	871.3898	Carbamido	L	Superscaf	41860342	0	0	0	0	0	BTP202209	Tissue specific
RFPLMR	Superscaf	-	Superscaf	41877382	41877399	-40	40	45	-	-	-	-	-	-	NCP	IGR	-	ID=gene-E	6	7.505	2747.3	0.227566567	RFPLMR	97	410.2348	2	818.4585	.	I	Superscaf	41877426	0	0	0	0	0	BTP202209	Tissue specific
MCGSC	Superscaf	+	Superscaf	41907670	41907684	4	4	8	-	-	-	-	-	-	NCP	IGR	-	ID=gene-E	5	5.488	2223.6	0.227007957	M(+15.99)	51	672.1763	1	671.1713	Oxidation	N	Superscaf	41907661	0	0	BTP202209	0	0	0	Tissue enhanced
GFSTAPF	Superscaf	+	Superscaf	41944357	41944377	34	34	40	-	-	-	-	-	-	NCP	IGR	-	ID=gene-E	7	5.922857143	2501.7	0.22633115	GFSTAPF	52	726.3524	1	725.3384	.	L	Superscaf	41944258	0	0	BTP202209	0	0	0	Tissue specific
MDFSCW	Superscaf	+	Superscaf	41945482	41945499	12	12	17	-	-	-	-	-	-	NCP	IGR	-	ID=gene-E	6	5.131666667	2666.1	0.226310455	M(+15.99)	53	452.1546	2	902.2939	Oxidation	R	Superscaf	41945449	0	0	0	BTP202209	0	0	Tissue specific
EMKPFP	Superscaf	+	Superscaf	42222919	42222936	1	1	6	-	-	gene	-	-	mRNA	NCP	Intron	gene|mRN	ID=gene-E	6	6.131666667	2327.6	0.221193098	EM(+15.99	88	382.6843	2	763.3574	Oxidation	E	Superscaf	42222919	0	0	BTP202209	0	0	0	Tissue specific
SHPLGPK	Superscaf	-	Superscaf	42235687	42235707	-22	22	28	-	-	gene	-	-	mRNA	NCP	Intron	gene|mRN	ID=gene-E	7	6.794285714	2797	0.220957535	S(+42.01)	53	389.2104	2	776.4181	Acetylati	E	Superscaf	42235734	0	BTP202209	0	0	0	0	Tissue specific
ALGNLMNK	Superscaf	+	Superscaf	42244573	42244596	1	1	8	-	-	gene	-	-	mRNA	NCP	Intron	gene|mRN	ID=gene-E	8	6.2825	3348.7	0.220793576	ALGNLM(+1	57	438.7421	2	875.4535	Oxidation	A	Superscaf	42244573	0	BTP202209	0	0	0	0	Tissue specific
GVPPLK	Superscaf	-	Superscaf	42253417	42253434	-60	60	65	-	-	gene	-	-	mRNA	NCP	Intron	gene|mRN	ID=gene-E	6	5.715	1793.9	0.220630558	G(+42.01)	69	652.4005	1	651.3955	Acetylati	A	Superscaf	42253536	0	0	BTP202209	0	0	0	Tissue specific
TPVKGPQ	Superscaf	-	Superscaf	42359164	42359184	-1	1	7	-	-	-	-	-	-	NCP	IGR	-	ID=gene-E	7	5.784285714	2448.7	0.218679988	T(+42.01)	70	384.7208	2	767.4177	Acetylati	T	Superscaf	42359259	0	0	BTP202209	0	0	0	Tissue specific
HDTLLLL	Superscaf	+	Superscaf	42406276	42406296	5	5	11	-	-	-	-	-	-	NCP	IGR	-	ID=gene-E	7	5.858571429	2842.3	0.217811001	HDTLLLL	52	412.7473	2	823.4803	.	A	Superscaf	42406264	0	0	0	0	0	BTP202209	Tissue specific
VATGLCK	Superscaf	+	Superscaf	42433201	42433221	40	40	46	-	-	-	-	-	-	NCP	IGR	-	ID=gene-E	7	5.608571429	2413.6	0.217314367	VATGLC(+5	52	374.7031	2	747.3949	Carbamido	K	Superscaf	42433084	0	0	0	0	0	BTP202209	Tissue specific
KGGGLTLH	Superscaf	+	Superscaf	42434098	42434121	27	27	34	-	-	-	-	-	-	NCP	IGR	-	ID=gene-E	8	6.71625	3084.3	0.217297766	KGGGLTLH	58	391.7285	2	781.4446	.	P	Superscaf	42434020	0	0	BTP202209	0	0	0	Tissue specific
NSSVAPY	Superscaf	-	Superscaf	42519358	42519378	-25	25	31	-	-	-	-	-	-	NCP	IGR	-	ID=gene-E	7	4.964285714	2471.7	0.215725191	NSSVAPY	65	737.348	1	736.3392	.	S	Superscaf	42519384	0	0	0	0	BTP202209	BTP202209	Tissue specific
PRPLNL	Superscaf	+	Superscaf	42524821	42524838	4	4	9	-	-	-	-	-	-	NCP	IGR	-	ID=gene-E	6	6.788333333	2627.1	0.21562448	PRPLNL	61	355.2199	2	708.4282	.	T	Superscaf	42524812	0	0	0	0	0	BTP202209	Tissue specific
DDLLLK	Superscaf	-	Superscaf	42534643	42534660	-6	6	11	-	-	-	-	-	-	NCP	IGR	-	ID=gene-E	6	5.603333333	2351.6	0.215443313	DDLLLK	99	716.4177	1	715.4116	.	K	Superscaf	42534741	0	0	0	0	0	BTP202209	Mixed
HDLKNPP	Superscaf	+	Superscaf	42570514	42570534	2	2	8	-	-	-	-	-	-	NCP	IGR	-	ID=gene-E	7	6.327142857	3124.5	0.214781613	H(+42.01)	70	431.7172	2	861.4344	Acetylati	I	Superscaf	42570511	0	0	0	BTP202209	0	0	Tissue specific
GDEWQL	Superscaf	+	Superscaf	42643549	42643566	4	4	9	-	-	-	-	-	-	NCP	IGR	-	ID=gene-E	6	4.946666667	2625	0.213434529	GDEWQL	76	747.3298	1	746.3235	.	R	Superscaf	42643540	BTP202209	BTP202209	0	BTP202209	0	0	Tissue specific
RLGFTLT	Superscaf	+	Superscaf	42672106	42672126	4	4	10	-	-	-	-	-	-	NCP	IGR	-	ID=gene-E	7	6.747142857	2835.3	0.212907736	RLGFTLT	70	404.2436	2	806.465	.	A	Superscaf	42672097	BTP202209	0	0	0	0	0	Tissue specific
MYLPK	Superscaf	+	Superscaf	42683761	42683775	14	14	18	-	-	-	-	-	-	NCP	IGR	-	ID=gene-E	5	6.686	1990.2	0.212692869	M(+15.99)	67	667.3531	1	666.3411	Oxidation	V	Superscaf	42683722	0	0	0	BTP202209	0	0	Tissue specific
VTLVSVL	Superscaf	-	Superscaf	42789892	42789912	-33	33	39	-	-	-	-	-	-	NCP	IGR	-	ID=gene-E	7	3.452857143	1487.7	0.21073516	VTLVSVL	74	730.4675	1	729.4636	.	S	Superscaf	42789942	0	0	0	0	0	BTP202209	Tissue specific
EVFGK	Superscaf	+	Superscaf	42814714	42814728	12	12	16	-	-	-	-	-	-	NCP	IGR	-	ID=gene-E	5	4.882	1578.6	0.210277426	EVFGK	98	579.3123	1	578.3064	.	L	Superscaf	42814681	BTP202209	0	0	0	0	0	Tissue enhanced
VVLLPL	Superscaf	+	Superscaf	42975934	42975951	3	3	8	-	-	-	-	-	-	NCP	IGR	-	ID=gene-E	6	4.04	1397.6	0.207303649	V(+42.01)	60	695.4792	1	694.4629	Acetylati	M	Superscaf	42975928	0	0	0	0	BTP202209	0	Tissue specific
HSLLVTN	Superscaf	-	Superscaf	42989911	42989931	-40	40	46	-	-	-	-	-	-	NCP	IGR	-	ID=gene-E	7	5.31	2704.1	0.207045787	HSLLVTN	57	392.2279	2	782.4286	.	L	Superscaf	42989937	0	0	0	0	0	BTP202209	Tissue specific
AAGPVYL	Superscaf	+	Superscaf	43003195	43003215	26	26	32	-	-	gene	-	-	mRNA	NCP	Intron	gene|mRN	ID=gene-E	7	5.135714286	2070.2	0.206800762	AAGPVYL	84	690.3791	1	689.3748	.	T	Superscaf	43003120	0	0	0	0	0	BTP202209	Tissue specific
LVPVPQK	Superscaf	-	Superscaf	43024786	43024806	-3	3	9	-	-	-	-	-	-	NCP	IGR	-	ID=gene-E	7	4.852857143	2107.3	0.206402513	LVPVPQK	65	390.7516	2	779.4905	.	V	Superscaf	43024842	0	0	0	0	BTP202209	0	Tissue specific
KLHRPT	Superscaf	+	Superscaf	43060609	43060626	6	6	11	-	-	-	-	-	-	NCP	IGR	-	ID=gene-E	6	7.816666667	2825.2	0.205741809	KLHRPT	63	376.2269	2	750.45	.	S	Superscaf	43060594	0	0	0	0	0	BTP202209	Tissue specific
KLGYGP	Superscaf	+	Superscaf	43142656	43142673	1	1	6	-	-	-	-	-	-	NCP	IGR	-	ID=gene-E	6	6.603333333	2213.3	0.204228443	KLGYGP	69	634.354	1	633.3486	.	K	Superscaf	43142656	0	0	0	0	0	BTP202209	Tissue specific
AYPGHGP	Superscaf	-	Superscaf	43223560	43223580	-4	4	10	-	-	-	-	-	-	NCP	IGR	-	ID=gene-E	7	6.258571429	2659.9	0.202736104	A(+42.01)	81	740.3337	1	739.3289	Acetylati	K	Superscaf	43223589	0	0	0	0	BTP202209	0	Mixed
KGHGAPPP	Superscaf	-	Superscaf	43296586	43296609	-5	5	12	-	-	-	-	-	-	NCP	IGR	-	ID=gene-E	8	6.77375	3006.2	0.201389076	KGHGAPPP	61	380.7018	2	759.4027	.	K	Superscaf	43296654	BTP202209	0	0	0	0	0	Tissue specific
KDWPVR	Superscaf	+	Superscaf	43306486	43306503	25	25	30	-	-	-	-	-	-	NCP	IGR	-	ID=gene-E	6	5.943333333	2392.7	0.20120658	KDWPVR	55	400.73	2	799.434	.	P	Superscaf	43306414	0	0	0	0	BTP202209	0	Tissue specific
ADSYLN	Superscaf	+	Superscaf	43314520	43314537	11	11	16	-	-	-	-	-	-	NCP	IGR	-	ID=gene-E	6	5.286666667	2515.8	0.201058392	A(+42.01)	64	362.6576	2	723.3075	Acetylati	S	Superscaf	43314490	0	0	BTP202209	0	0	0	Tissue specific
QGCCC	Superscaf	+	Superscaf	43344700	43344714	7	7	11	-	-	-	-	-	-	NCP	IGR	-	ID=gene-E	5	5.336	2665.1	0.200501774	Q(+42.01)	58	726.1865	1	725.1931	Acetylati	F	Superscaf	43344682	0	0	0	0	0	BTP202209	Tissue specific
KCPYLFQ	Superscaf	-	Superscaf	43347925	43347945	-93	93	99	-	-	-	-	-	-	NCP	IGR	-	ID=gene-E	7	6.261428571	3202.5	0.200442177	KC(+57.02	61	478.2437	2	954.4633	Carbamido	V	Superscaf	43347945	0	0	BTP202209	0	0	0	Tissue specific
RSLGLAH	Superscaf	+	Superscaf	43350679	43350699	60	60	66	-	-	-	-	-	-	NCP	IGR	-	ID=gene-E	7	6.854285714	2967.3	0.20039138	RSLGLAH	88	377.2203	2	752.4293	.	F	Superscaf	43350502	0	0	BTP202209	0	0	0	Tissue specific
KAYSTAL	Superscaf	+	Superscaf	43360795	43360815	14	14	20	-	-	-	-	-	-	NCP	IGR	-	ID=gene-E	7	6.518571429	2684.8	0.200204789	KAYSTAL	52	377.209	2	752.4068	.	K	Superscaf	43360756	0	0	BTP202209	0	0	0	Tissue specific
CDKPLR	Superscaf	-	Superscaf	43384942	43384959	-32	32	37	-	-	-	-	-	-	NCP	IGR	-	ID=gene-E	6	6.795	2805.2	0.19975945	C(+57.02)	70	394.7008	2	787.4011	Carbamido	I	Superscaf	43384962	0	0	BTP202209	0	0	0	Tissue specific
TAPLYE	Superscaf	-	Superscaf	43451692	43451709	-7	7	12	-	-	-	-	-	-	NCP	IGR	-	ID=gene-E	6	5.618333333	2284.6	0.198528238	T(+42.01)	65	368.1793	2	734.3486	Acetylati	F	Superscaf	43451949	0	0	0	0	0	BTP202209	Tissue specific
KYPPKF	Superscaf	-	Superscaf	43486684	43486701	-21	21	26	-	-	-	-	-	-	NCP	IGR	-	ID=gene-E	6	7.203333333	2458.5	0.197882807	KYPPKF	53	390.2284	2	778.4377	.	I	Superscaf	43486704	0	BTP202209	0	0	0	0	Tissue specific
GSLEKNF	Superscaf	-	Superscaf	43540933	43540953	-28	28	34	-	-	-	-	-	-	NCP	IGR	-	ID=gene-E	7	5.925714286	2912.1	0.196882123	GSLEKNF	73	794.4004	1	793.397	.	N	Superscaf	43540962	0	0	0	0	0	BTP202209	Tissue specific
LLVGNKCDI	Superscaf	-	Superscaf	43553401	43553439	-24	24	36	-	-	-	-	-	-	NCP	IGR	-	ID=gene-E	13	6.122307692	5151.6	0.196651817	LLVGNKC(+	96	497.2831	3	1488.833	Carbamido	E	Superscaf	43553568	0	0	BTP202209	0	0	0	Tissue specific
LVGNKCDLT	Superscaf	-	Superscaf	43553404	43553436	-25	25	35	-	-	-	-	-	-	NCP	IGR	-	ID=gene-E	11	5.806363636	4329.8	0.196651872	LVGNKC(+5	92	416.8905	3	1247.654	Carbamido	E	Superscaf	43553568	0	BTP202209	0	0	0	0	Tissue specific
VGNKCDLT	Superscaf	-	Superscaf	43553407	43553439	-26	26	36	-	-	-	-	-	-	NCP	IGR	-	ID=gene-E	11	6.148181818	4444.8	0.196651817	VGNKC(+57	99	421.8945	3	1262.665	Carbamido	E	Superscaf	43553568	0	0	0	0	BTP202209	0	Tissue specific
VGNKCDLT	Superscaf	-	Superscaf	43553407	43553436	-26	26	35	-	-	-	-	-	-	NCP	IGR	-	ID=gene-E	10	5.789	3976.4	0.196651872	VGNKC(+57	99	568.2913	2	1134.57	Carbamido	E	Superscaf	43553568	0	BTP202209	0	0	BTP202209	0	Tissue specific
LETSAKNAT	Superscaf	-	Superscaf	43553827	43553871	-11	11	25	-	-	-	-	-	-	NCP	IGR	-	ID=gene-E	15	5.371333333	6048.7	0.196643849	LETSAKNAT	67	819.9005	2	1637.79	.	E	Superscaf	43553991	0	0	0	BTP202209	0	0	Tissue specific
LETSAKNAT	Superscaf	-	Superscaf	43553827	43553868	-11	11	24	-	-	-	-	-	-	NCP	IGR	-	ID=gene-E	14	5.363571429	5661.3	0.196643904	LETSAKNAT	82	746.3646	2	1490.721	.	E	Superscaf	43553991	0	BTP202209	0	0	0	0	Tissue specific
KRMGPGATA	Superscaf	-	Superscaf	43553896	43553937	-34	34	47	-	-	-	-	-	-	NCP	IGR	-	ID=gene-E	14	6.712857143	5240.6	0.196642631	KRMGPGATA	60	444.2328	3	1329.682	.	E	Superscaf	43553991	0	BTP202209	0	0	0	0	Tissue specific
RMGPGATAG	Superscaf	-	Superscaf	43553899	43553937	-35	35	47	-	-	-	-	-	-	NCP	IGR	-	ID=gene-E	13	6.48	4772.2	0.196642631	RMGPGATAG	97	601.7994	2	1201.587	.	E	Superscaf	43553991	0	BTP202209	0	0	BTP202209	BTP202209	Tissue specific
MGPGATAGG	Superscaf	-	Superscaf	43553902	43553949	-36	36	51	-	-	-	-	-	-	NCP	IGR	-	ID=gene-E	16	5.894375	5571.9	0.19664241	MGPGATAGG	95	492.2473	3	1473.725	.	E	Superscaf	43553991	0	BTP202209	0	0	0	0	Tissue specific
MGPGATAGG	Superscaf	-	Superscaf	43553902	43553937	-36	36	47	-	-	-	-	-	-	NCP	IGR	-	ID=gene-E	12	6.123333333	4123.4	0.196642631	MGPGATAGG	99	523.7489	2	1045.486	.	E	Superscaf	43553991	0	0	0	0	BTP202209	0	Tissue specific
KCEPRF	Superscaf	+	Superscaf	43599628	43599645	6	6	11	-	-	-	-	-	-	NCP	IGR	-	ID=gene-E	6	6.753333333	2853.2	0.195799542	KC(+57.02	50	418.6995	2	835.401	Carbamido	Q	Superscaf	43599613	0	0	0	0	BTP202209	BTP202209	Tissue specific
KPEFSR	Superscaf	-	Superscaf	43615159	43615176	-5	5	10	-	-	-	-	-	-	NCP	IGR	-	ID=gene-E	6	6.863333333	2650.9	0.195513071	KPEFSR	61	382.2053	2	762.4024	.	L	Superscaf	43615191	0	0	0	0	BTP202209	BTP202209	Tissue specific
HHLLEKL	Superscaf	+	Superscaf	43753066	43753086	19	19	25	-	-	-	-	-	-	NCP	IGR	-	ID=gene-E	7	6.582857143	3193.5	0.192969305	HHLLEKL	51	445.2688	2	888.5181	.	A	Superscaf	43753012	BTP202209	0	0	0	0	0	Tissue specific
KFTVMK	Superscaf	-	Superscaf	43854679	43854696	-25	25	30	-	-	-	-	-	-	NCP	IGR	-	ID=gene-E	6	6.206666667	2093.2	0.191095097	KFTVMK	58	377.2151	2	752.4255	.	I	Superscaf	43854702	0	0	0	0	0	BTP202209	Tissue specific
VDYSPR	Superscaf	-	Superscaf	43861033	43861050	-55	55	60	-	-	-	-	-	-	NCP	IGR	-	ID=gene-E	6	5.228333333	2240.6	0.190977897	VDYSPR	80	368.684	2	735.3551	.	Y	Superscaf	43861074	BTP202209	0	0	0	0	0	Tissue specific
CSSPLLLH	Superscaf	-	Superscaf	43950733	43950756	-61	61	68	-	-	-	-	-	-	NCP	IGR	-	ID=gene-E	8	6.02625	3369.8	0.189323259	C(+57.02)	68	463.7401	2	925.4691	Carbamido	G	Superscaf	43950903	0	0	0	0	0	BTP202209	Tissue specific
VYGSLQ	Superscaf	-	Superscaf	44006758	44006775	-2	2	7	-	-	-	-	-	-	NCP	IGR	-	ID=gene-E	6	4.823333333	2104.3	0.188289982	VYGSLQ	64	666.3557	1	665.3384	.	R	Superscaf	44006868	0	0	0	0	0	BTP202209	Tissue specific
QGPWEGK	Superscaf	-	Superscaf	44010496	44010516	-38	38	44	-	-	-	-	-	-	NCP	IGR	-	ID=gene-E	7	6.105714286	2963.2	0.188220979	QGPWEGK	79	401.1963	2	800.3817	.	G	Superscaf	44011353	0	0	0	0	0	BTP202209	Tissue specific
PLPPPP	Superscaf	-	Superscaf	44011216	44011233	-26	26	31	-	-	-	-	-	-	NCP	IGR	-	ID=gene-E	6	6.246666667	2040.4	0.188207754	PLPPPP	96	617.364	1	616.3584	.	G	Superscaf	44011260	0	0	0	0	0	BTP202209	Tissue enhanced
KGFLVGP	Superscaf	-	Superscaf	44018194	44018214	-2	2	8	-	-	-	-	-	-	NCP	IGR	-	ID=gene-E	7	5.634285714	2141.2	0.188078989	KGFLVGP	50	359.2144	2	716.4221	.	G	Superscaf	44018217	0	BTP202209	0	0	0	0	Tissue specific
LGMSRK	Superscaf	+	Superscaf	44052037	44052054	14	14	19	-	-	-	-	-	-	NCP	IGR	-	ID=gene-E	6	7.313333333	2522.8	0.187454806	LGM(+15.9	63	354.1935	2	706.3796	Oxidation	E	Superscaf	44051998	0	BTP202209	0	0	0	0	Tissue specific
KPPGVAY	Superscaf	+	Superscaf	44219818	44219838	5	5	11	-	-	-	-	-	-	NCP	IGR	-	ID=gene-E	7	5.712857143	2211.3	0.184360011	KPPGVAY	80	366.2007	2	730.4013	.	E	Superscaf	44219806	0	BTP202209	0	0	0	0	Tissue specific
METGWPK	Superscaf	-	Superscaf	44268751	44268771	-29	29	35	-	-	-	-	-	-	NCP	IGR	-	ID=gene-E	7	6.2	2824.2	0.183457436	M(+15.99)	75	453.7021	2	905.3953	Oxidation	K	Superscaf	44268903	0	BTP202209	0	0	0	0	Tissue specific
PSRPVVK	Superscaf	-	Superscaf	44312617	44312637	-5	5	11	-	-	-	-	-	-	NCP	IGR	-	ID=gene-E	7	5.54	2175.4	0.182648323	PSRPVVK	73	391.7463	2	781.481	.	E	Superscaf	44312772	0	BTP202209	0	0	0	0	Tissue specific
LAQLQEA	Superscaf	+	Superscaf	44352760	44352780	2	2	8	-	-	-	-	-	-	NCP	IGR	-	ID=gene-E	7	5.502857143	2976.3	0.18190788	LAQLQEA	73	772.4176	1	771.4127	.	L	Superscaf	44352757	0	0	0	0	0	BTP202209	Tissue specific
HGSTK	Superscaf	-	Superscaf	44357665	44357679	-17	17	21	-	-	-	-	-	-	NCP	IGR	-	ID=gene-E	5	7.102	2166.3	0.181817518	HGSTK	51	529.2832	1	528.2656	.	S	Superscaf	44357700	0	BTP202209	0	0	0	0	Tissue specific
KHATAA	Superscaf	-	Superscaf	44388355	44388372	-9	9	14	-	-	-	-	-	-	NCP	IGR	-	ID=gene-E	6	6.986666667	2419.5	0.181251382	KHATAA	51	598.3375	1	597.3234	.	R	Superscaf	44388510	0	BTP202209	0	0	0	BTP202209	Tissue specific
NYMLAK	Superscaf	+	Superscaf	44399983	44400000	34	34	39	-	-	-	-	-	-	NCP	IGR	-	ID=gene-E	6	6.426666667	2560.8	0.181036902	NYMLAK	85	739.3788	1	738.3734	.	H	Superscaf	44399884	0	BTP202209	0	0	0	0	Tissue specific
VGLFSPR	Superscaf	-	Superscaf	44524078	44524098	-17	17	23	-	-	-	-	-	-	NCP	IGR	-	ID=gene-E	7	5.738571429	2407.7	0.1787479	VGLFSPR	54	388.2287	2	774.4388	.	I	Superscaf	44524137	0	BTP202209	0	0	0	0	Tissue specific
PVLLWFL	Superscaf	-	Superscaf	44607115	44607135	-21	21	27	-	-	-	-	-	-	NCP	IGR	-	ID=gene-E	7	5.087142857	2311.6	0.177216273	P(+42.01)	51	465.2822	2	928.5422	Acetylati	Y	Superscaf	44607153	0	0	0	0	0	BTP202209	Tissue specific
DLPLTL	Superscaf	+	Superscaf	44633401	44633418	26	26	31	-	-	-	-	-	-	NCP	IGR	-	ID=gene-E	6	5.623333333	2206.6	0.17673148	DLPLTL	70	671.3945	1	670.3901	.	F	Superscaf	44633326	0	0	0	0	0	BTP202209	Tissue specific
EVFPGLK	Superscaf	-	Superscaf	44655430	44655450	-56	56	62	-	-	-	-	-	-	NCP	IGR	-	ID=gene-E	7	5.241428571	2269.4	0.176325098	EVFPGLK	88	395.2268	2	788.4432	.	W	Superscaf	44655450	0	0	0	0	0	BTP202209	Tissue specific
KDLPPPP	Superscaf	+	Superscaf	44672629	44672649	44	44	50	-	-	-	-	-	-	NCP	IGR	-	ID=gene-E	7	6.27	2582.9	0.17600786	KDLPPPP	63	763.4296	1	762.4276	.	V	Superscaf	44672500	0	BTP202209	0	0	0	0	Tissue specific
FLYPP	Superscaf	-	Superscaf	44691649	44691663	-4	4	8	-	-	-	-	-	-	NCP	IGR	-	ID=gene-E	5	5.944	1875.1	0.175657145	FLYPP	76	636.3381	1	635.3318	.	T	Superscaf	44691678	0	0	0	0	0	BTP202209	Mixed
NRAAF	Superscaf	+	Superscaf	44729980	44729994	6	6	10	-	-	-	-	-	-	NCP	IGR	-	ID=gene-E	5	6.738	2255.5	0.174950125	NRAAF	62	578.3019	1	577.2972	.	I	Superscaf	44729965	0	0	BTP202209	0	0	0	Tissue specific
TVGAYVPL	Superscaf	+	Superscaf	44739658	44739681	1	1	8	-	-	-	-	-	-	NCP	IGR	-	ID=gene-E	8	4.5575	2156.4	0.174771447	TVGAYVPL	54	410.2413	2	818.4538	.	T	Superscaf	44739658	0	0	0	0	0	BTP202209	Tissue specific
LCPGDLK	Superscaf	+	Superscaf	44743021	44743041	29	29	35	-	-	-	-	-	-	NCP	IGR	-	ID=gene-E	7	5.994285714	2807.1	0.174709471	LC(+57.02	91	401.7092	2	801.4055	Carbamido	S	Superscaf	44742937	0	0	BTP202209	0	0	0	Tissue specific
CKDLKEK	Superscaf	-	Superscaf	44749057	44749077	-21	21	27	-	-	-	-	-	-	NCP	IGR	-	ID=gene-E	7	6.63	3181.3	0.174598137	C(+57.02)	94	460.7535	2	919.4797	Carbamido	S	Superscaf	44749116	0	0	BTP202209	0	0	0	Tissue specific
TVLDL	Superscaf	-	Superscaf	44972557	44972571	-44	44	48	-	-	gene	-	-	mRNA	NCP	Intron	gene|mRN	ID=gene-E	5	4.292	1515.8	0.170475764	TVLDL	74	560.3257	1	559.3217	.	A	Superscaf	44972586	0	0	0	BTP202209	0	0	Tissue specific
RLQTVSF	Superscaf	-	Superscaf	45081874	45081894	-42	42	48	-	-	-	-	-	-	NCP	IGR	-	ID=gene-E	7	5.725714286	2781.2	0.168459289	RLQTVSF	72	425.7413	2	849.4708	.	I	Superscaf	45081936	0	0	0	0	BTP202209	0	Tissue specific
GPVSMAH	Superscaf	+	Superscaf	45100705	45100725	22	22	28	-	-	gene	-	-	mRNA	NCP	Intron	gene|mRN	ID=gene-E	7	5.33	2320.6	0.168111949	GPVSM(+15	56	357.669	2	713.3167	Oxidation	T	Superscaf	45100642	0	0	0	0	0	BTP202209	Tissue specific
DSVRLF	Superscaf	-	Superscaf	45101434	45101451	-38	38	43	-	-	gene	-	-	mRNA	NCP	Intron	gene|mRN	ID=gene-E	6	5.145	2184.5	0.168098558	DSVRLF	61	736.3968	1	735.3915	.	L	Superscaf	45101547	0	BTP202209	0	0	0	0	Tissue specific
NWKGVT	Superscaf	+	Superscaf	45104629	45104646	18	18	23	-	-	gene	-	-	mRNA	NCP	Intron	gene|mRN	ID=gene-E	6	5.59	2286.5	0.168039626	NWKGVT	76	352.6893	2	703.3653	.	F	Superscaf	45104578	0	BTP202209	0	0	0	0	Tissue specific
ADYSLN	Superscaf	-	Superscaf	45107974	45107991	-26	26	31	-	-	gene	-	-	mRNA	NCP	Intron	gene|mRN	ID=gene-E	6	5.286666667	2515.8	0.167977927	A(+42.01)	53	362.6574	2	723.3075	Acetylati	F	Superscaf	45108063	0	0	0	0	0	BTP202209	Tissue specific
VGHGVSH	Superscaf	-	Superscaf	45257398	45257418	-6	6	12	-	-	gene	-	-	mRNA	NCP	Intron	gene|mRN	ID=gene-E	7	4.685714286	2217.4	0.165221729	VGHGVSH	76	692.3418	1	691.3401	.	L	Superscaf	45257475	0	0	0	0	0	BTP202209	Tissue specific
FDHLRK	Superscaf	+	Superscaf	45305011	45305028	12	12	17	-	-	-	-	-	-	NCP	IGR	-	ID=gene-E	6	7.086666667	2889.2	0.164343557	FDHLRK	82	408.2281	2	814.4449	.	V	Superscaf	45304978	0	0	0	BTP202209	BTP202209	0	Tissue specific
VVKKLAL	Superscaf	+	Superscaf	45363079	45363099	16	16	22	-	-	gene	-	-	mRNA	NCP	Intron	gene|mRN	ID=gene-E	7	5.351428571	1954.9	0.16327243	VVKKLAL	99	385.7778	2	769.5425	.	V	Superscaf	45363034	0	BTP202209	0	0	0	0	Mixed
MMMGHP	Superscaf	+	Superscaf	45381748	45381765	32	32	37	-	-	gene	-	-	mRNA	NCP	Intron	gene|mRN	ID=gene-E	6	6.185	2368.9	0.162928134	M(+15.99)	56	368.129	2	734.255	Oxidation	V	Superscaf	45381655	0	0	0	BTP202209	0	0	Tissue specific
LSGGFLK	Superscaf	+	Superscaf	45421183	45421203	83	83	89	-	-	-	-	-	-	NCP	IGR	-	ID=gene-E	7	6.4	2540.6	0.162200696	LSGGFLK	69	721.4225	1	720.417	.	V	Superscaf	45420937	0	0	0	0	0	BTP202209	Tissue specific
ANCGL	Superscaf	+	Superscaf	45474199	45474213	29	29	33	-	-	-	-	-	-	NCP	IGR	-	ID=gene-E	5	5.68	2144.4	0.16122292	ANC(+57.0	50	534.2437	1	533.2267	Carbamido	S	Superscaf	45474115	BTP202209	0	0	0	0	0	Tissue specific
DLHYMVY	Superscaf	+	Superscaf	45490213	45490233	39	39	45	-	-	-	-	-	-	NCP	IGR	-	ID=gene-E	7	4.801428571	2675.1	0.160927429	DLHYMVY	65	470.7138	2	939.416	.	D	Superscaf	45490099	0	0	0	0	BTP202209	0	Tissue specific
VESTGVFT	Superscaf	+	Superscaf	45490384	45490407	16	16	23	-	-	-	-	-	-	NCP	IGR	-	ID=gene-E	8	4.17625	2288.6	0.16092422	VESTGVFT	63	839.4116	1	838.4072	.	D	Superscaf	45490339	0	0	0	0	BTP202209	BTP202209	Tissue specific
PELNGKLTC	Superscaf	+	Superscaf	45490762	45490791	47	47	56	-	-	gene	-	-	mRNA	NCP	Intron	gene|mRN	ID=gene-E	10	6.085	3898.4	0.160917137	PELNGKLTC	69	530.2764	2	1058.543	.	P	Superscaf	45490624	0	0	0	0	0	BTP202209	Tissue specific
TCRLEKPAK	Superscaf	+	Superscaf	45490834	45490860	71	71	79	-	-	gene	-	-	mRNA	NCP	Intron	gene|mRN	ID=gene-E	9	7.034444444	3996.4	0.160915864	TC(+57.02	96	368.2045	3	1101.596	Carbamido	P	Superscaf	45490624	0	0	BTP202209	0	BTP202209	0	Tissue specific
KAVLLKK	Superscaf	-	Superscaf	45546721	45546741	-1	1	7	-	-	-	-	-	-	NCP	IGR	-	ID=gene-E	7	6.742857143	2423.3	0.159885133	KAVLLKK	50	400.2906	2	798.5691	.	K	Superscaf	45546765	0	0	BTP202209	0	BTP202209	BTP202209	Tissue specific
KPFPVHT	Superscaf	+	Superscaf	45604633	45604653	23	23	29	-	-	-	-	-	-	NCP	IGR	-	ID=gene-E	7	5.991428571	2547.8	0.158816939	KPFPVHT	50	413.2413	2	824.4545	.	R	Superscaf	45604567	0	BTP202209	0	0	0	0	Tissue specific
PAPGKPPK	Superscaf	+	Superscaf	45609670	45609693	13	13	20	-	-	-	-	-	-	NCP	IGR	-	ID=gene-E	8	7.08375	2895	0.158723976	PAPGKPPK	60	396.2383	2	790.4701	.	I	Superscaf	45609634	0	0	0	0	0	BTP202209	Tissue specific
KEALWL	Superscaf	+	Superscaf	45640378	45640395	29	29	34	-	-	-	-	-	-	NCP	IGR	-	ID=gene-E	6	6.138333333	2438.6	0.158157674	KEALWL	73	380.2238	2	758.4327	.	V	Superscaf	45640294	0	0	0	0	BTP202209	0	Tissue specific
ERPLPAH	Superscaf	+	Superscaf	45675745	45675765	1	1	7	-	-	gene	-	-	mRNA	NCP	Intron	gene|mRN	ID=gene-E	7	6.595714286	3033.5	0.15750527	ERPLPAH	84	410.2268	2	818.4399	.	E	Superscaf	45675745	0	0	0	0	0	BTP202209	Tissue specific
VPVPP	Superscaf	-	Superscaf	45684517	45684531	-30	30	34	-	-	gene	-	-	mRNA	NCP	Intron	gene|mRN	ID=gene-E	5	3.78	1012.2	0.15734358	V(+42.01)	57	550.3223	1	549.3162	Acetylati	V	Superscaf	45684537	0	BTP202209	0	0	0	BTP202209	Tissue specific
NLSSVFVQ	Superscaf	+	Superscaf	45716812	45716835	9	9	16	-	-	gene	-	-	mRNA	NCP	Intron	gene|mRN	ID=gene-E	8	4.235	2715	0.156747729	NLSSVFVQ	54	447.2323	2	892.4654	.	S	Superscaf	45716788	0	BTP202209	0	0	0	0	Tissue specific
MQLFVK	Superscaf	+	Superscaf	45722395	45722412	5	5	10	-	-	gene	-	-	mRNA	NCP	Intron	gene|mRN	ID=gene-E	6	5.433333333	2191.4	0.156644861	MQLFVK	99	383.2191	2	764.4255	.	Y	Superscaf	45722383	0	0	0	0	0	BTP202209	Tissue enhanced
KSPGLKL	Superscaf	+	Superscaf	45734413	45734433	12	12	18	-	-	gene	-	-	mRNA	NCP	Intron	gene|mRN	ID=gene-E	7	7.055714286	2661.7	0.156423132	KSPGLKL	66	371.7433	2	741.4749	.	P	Superscaf	45734380	0	0	0	BTP202209	0	0	Tissue specific
LPTAAQPP	Superscaf	-	Superscaf	45859429	45859452	-115	115	122	-	-	gene	-	-	mRNA	NCP	Intron	gene|mRN	ID=gene-E	8	6.1375	2996.4	0.154117142	LPTAAQPP	82	397.7228	2	793.4333	.	S	Superscaf	45859572	BTP202209	0	0	0	0	0	Tissue specific
LEQLLEQL	Superscaf	-	Superscaf	45892951	45892974	-27	27	34	-	-	gene	-	-	mRNA	NCP	Intron	gene|mRN	ID=gene-E	8	5.2075	3486	0.153498825	LEQLLEQL	57	493.2802	2	984.5491	.	S	Superscaf	45893028	BTP202209	0	0	0	0	0	Tissue specific
NCKPLR	Superscaf	+	Superscaf	45923656	45923673	13	13	18	-	-	gene	-	-	mRNA	NCP	Intron	gene|mRN	ID=gene-E	6	7.201666667	2990.4	0.152932579	NC(+57.02	84	394.2079	2	786.4171	Carbamido	P	Superscaf	45923620	0	0	0	0	BTP202209	0	Tissue specific
FYLKPP	Superscaf	+	Superscaf	45923950	45923967	14	14	19	-	-	gene	-	-	mRNA	NCP	Intron	gene|mRN	ID=gene-E	6	6.576666667	2343.5	0.152927156	FYLKPP	79	382.7281	2	763.4268	.	S	Superscaf	45923911	0	0	0	0	0	BTP202209	Tissue specific
RGPMPP	Superscaf	-	Superscaf	45979789	45979806	-7	7	12	-	-	gene	-	-	mRNA	NCP	Intron	gene|mRN	ID=gene-E	6	6.896666667	2329.8	0.151897199	RGPMPP	78	654.3372	1	653.3319	.	I	Superscaf	45979863	0	0	BTP202209	BTP202209	0	0	Tissue specific
GTFLK	Superscaf	-	Superscaf	46077895	46077909	-38	38	42	-	-	gene	-	-	mRNA	NCP	Intron	gene|mRN	ID=gene-E	5	6.74	1904	0.150087678	GTFLK	54	565.3325	1	564.3271	.	A	Superscaf	46077909	0	0	0	0	0	BTP202209	Tissue specific
KSMCSP	Superscaf	-	Superscaf	46081906	46081923	-28	28	33	-	-	gene	-	-	mRNA	NCP	Intron	gene|mRN	ID=gene-E	6	6.361666667	2529.8	0.150013639	KSM(+15.9	53	363.1496	2	724.2884	Oxidation	P	Superscaf	46082001	0	0	0	0	0	BTP202209	Tissue specific
EGLVLP	Superscaf	-	Superscaf	46095481	46095498	-14	14	19	-	-	gene	-	-	mRNA	NCP	Intron	gene|mRN	ID=gene-E	6	4.575	1767	0.149763247	EGLVLP	54	627.3645	1	626.3639	.	I	Superscaf	46095513	0	0	0	0	0	BTP202209	Tissue specific
PGVYWK	Superscaf	+	Superscaf	46153822	46153839	59	59	64	-	-	gene	-	-	mRNA	NCP	Intron	gene|mRN	ID=gene-E	6	5.593333333	2089.2	0.14868714	PGVYWK	57	375.2033	2	748.3908	.	R	Superscaf	46153648	0	0	0	0	0	BTP202209	Tissue specific
TTGLTTF	Superscaf	+	Superscaf	46154974	46154994	4	4	10	-	-	gene	-	-	mRNA	NCP	Intron	gene|mRN	ID=gene-E	7	6.221428571	2628.1	0.148665836	TTGLTTF	84	740.3823	1	739.3752	.	R	Superscaf	46154965	0	0	BTP202209	0	0	0	Tissue specific
AQPLVH	Superscaf	-	Superscaf	46178632	46178649	-12	12	17	-	-	gene	-	-	mRNA	NCP	Intron	gene|mRN	ID=gene-E	6	5.256666667	2232.5	0.148229517	AQPLVH	68	664.3751	1	663.3704	.	G	Superscaf	46178751	0	0	0	0	0	BTP202209	Tissue specific
QQAKNR	Superscaf	+	Superscaf	46206661	46206678	9	9	14	-	-	gene	-	-	mRNA	NCP	Intron	gene|mRN	ID=gene-E	6	7.205	3246.6	0.147712519	QQAKNR	57	372.7112	2	743.4038	.	Y	Superscaf	46206637	BTP202209	0	0	0	0	0	Tissue specific
KDPKVTK	Superscaf	-	Superscaf	46238809	46238829	-3	3	9	-	-	-	-	-	-	NCP	IGR	-	ID=gene-E	7	6.431428571	2551.6	0.14711949	K(+42.01)	62	429.2627	2	856.5018	Acetylati	T	Superscaf	46238835	0	0	0	0	0	BTP202209	Tissue specific
ASGKPQ	Superscaf	-	Superscaf	46255846	46255863	-7	7	12	-	-	-	-	-	-	NCP	IGR	-	ID=gene-E	6	6.56	2408.5	0.146805296	A(+42.01)	62	629.3275	1	628.318	Acetylati	P	Superscaf	46255893	0	0	0	0	BTP202209	0	Tissue specific
QPSVHR	Superscaf	+	Superscaf	46281316	46281333	106	106	111	-	-	-	-	-	-	NCP	IGR	-	ID=gene-E	6	5.996666667	2600	0.146335499	Q(+42.01)	54	383.2003	2	764.3929	Acetylati	D	Superscaf	46281001	0	0	0	0	0	BTP202209	Tissue specific
MTLSVQK	Superscaf	-	Superscaf	46326259	46326279	-62	62	68	-	-	-	-	-	-	NCP	IGR	-	ID=gene-E	7	5.618571429	2584.9	0.145506465	MTLSVQK	68	403.7238	2	805.4368	.	V	Superscaf	46326294	0	0	0	0	BTP202209	0	Tissue specific
PFLFTEV	Superscaf	+	Superscaf	46337704	46337724	9	9	15	-	-	-	-	-	-	NCP	IGR	-	ID=gene-E	7	4.712857143	2288.6	0.14529536	PFLFTEV	73	852.4472	1	851.4429	.	I	Superscaf	46337680	0	BTP202209	0	0	0	0	Tissue specific
QFAEFCF	Superscaf	-	Superscaf	46346911	46346931	-22	22	28	-	-	-	-	-	-	NCP	IGR	-	ID=gene-E	7	5.192857143	3095.4	0.145125536	QFAEFC(+5	63	474.698	2	947.3847	Carbamido	I	Superscaf	46346940	0	0	0	0	BTP202209	0	Mixed
KVLFCYF	Superscaf	-	Superscaf	46440571	46440591	-52	52	58	-	-	-	-	-	-	NCP	IGR	-	ID=gene-E	7	5.337142857	2641.8	0.143397967	KVLFC(+57	61	488.7579	2	975.4888	Carbamido	Q	Superscaf	46440618	0	BTP202209	0	0	0	0	Tissue specific
ATLTVFPK	Superscaf	+	Superscaf	46478584	46478607	5	5	12	-	-	-	-	-	-	NCP	IGR	-	ID=gene-E	8	5.8225	2652.9	0.142696757	A(+42.01)	92	459.7676	2	917.5222	Acetylati	L	Superscaf	46478572	0	BTP202209	0	0	0	0	Tissue specific
LPLKDLT	Superscaf	+	Superscaf	46540246	46540266	1	1	7	-	-	-	-	-	-	NCP	IGR	-	ID=gene-E	7	6.211428571	2675	0.14155945	LPLKDLT	97	799.491	1	798.485	.	L	Superscaf	46540246	0	0	BTP202209	0	BTP202209	0	Tissue specific
DSEDC	Superscaf	+	Superscaf	46549309	46549323	13	13	17	-	-	-	-	-	-	NCP	IGR	-	ID=gene-E	5	3.972	2217.6	0.141392393	D(+42.01)	51	667.1757	1	666.1803	Acetylati	M	Superscaf	46549273	0	0	0	0	BTP202209	0	Tissue specific
VSGAALK	Superscaf	+	Superscaf	46551484	46551504	6	6	12	-	-	-	-	-	-	NCP	IGR	-	ID=gene-E	7	5.63	2125.1	0.141352164	VSGAALK	80	645.3918	1	644.3857	.	L	Superscaf	46551469	0	0	0	0	BTP202209	BTP202209	Tissue specific
VGLNVKS	Superscaf	-	Superscaf	46570942	46570962	-5	5	11	-	-	-	-	-	-	NCP	IGR	-	ID=gene-E	7	4.682857143	2099.2	0.140993259	VGLNVKS	81	716.4286	1	715.4228	.	R	Superscaf	46570989	0	0	0	BTP202209	0	0	Tissue specific
ALRLTEY	Superscaf	-	Superscaf	46580059	46580079	-23	23	29	-	-	-	-	-	-	NCP	IGR	-	ID=gene-E	7	6.307142857	2949.4	0.140825095	ALRLTEY	85	433.2413	2	864.4705	.	T	Superscaf	46580262	0	BTP202209	0	0	0	0	Tissue enhanced
SRTLSGMES	Superscaf	-	Superscaf	46580080	46580109	-30	30	39	-	-	-	-	-	-	NCP	IGR	-	ID=gene-E	10	6.091	4103.7	0.140824541	SRTLSGMES	82	1130.513	1	1129.507	.	T	Superscaf	46580262	0	0	0	0	BTP202209	0	Tissue specific
LEELVVQPI	Superscaf	-	Superscaf	46582108	46582134	-22	22	30	-	-	-	-	-	-	NCP	IGR	-	ID=gene-E	9	4.036666667	2859.3	0.14078719	LEELVVQPI	74	520.3036	2	1038.596	.	C	Superscaf	46582221	0	0	0	0	0	BTP202209	Mixed
TNGGVGK	Superscaf	+	Superscaf	46615063	46615083	3	3	9	-	-	-	-	-	-	NCP	IGR	-	ID=gene-E	7	5.655714286	2354.5	0.140179442	TNGGVGK	87	632.3414	1	631.3289	.	Y	Superscaf	46615057	0	0	BTP202209	0	0	0	Tissue specific
DKPKTY	Superscaf	+	Superscaf	46621327	46621344	5	5	10	-	-	-	-	-	-	NCP	IGR	-	ID=gene-E	6	6.823333333	2542.7	0.140063957	DKPKTY	57	751.4085	1	750.3912	.	I	Superscaf	46621315	0	0	0	0	0	BTP202209	Tissue specific
SLRPLQF	Superscaf	+	Superscaf	46641139	46641159	13	13	19	-	-	-	-	-	-	NCP	IGR	-	ID=gene-E	7	6.547142857	3074.5	0.139698467	SLRPLQF	62	430.7513	2	859.4916	.	M	Superscaf	46641103	0	0	0	0	0	BTP202209	Tissue specific
EKLGSR	Superscaf	+	Superscaf	46669660	46669677	47	47	52	-	-	-	-	-	-	NCP	IGR	-	ID=gene-E	6	6.891666667	2576.8	0.139172449	E(+42.01)	67	366.2011	2	730.3973	Acetylati	H	Superscaf	46669522	BTP202209	0	0	0	0	0	Tissue specific
LTTKLSPK	Superscaf	+	Superscaf	46790941	46790964	3	3	10	-	-	-	-	-	-	NCP	IGR	-	ID=gene-E	8	7.06	3159.4	0.136935297	LTTKLSPK	78	444.28	2	886.5488	.	Q	Superscaf	46790935	BTP202209	0	0	0	0	0	Tissue specific
CMDHSGK	Superscaf	-	Superscaf	46822159	46822179	-20	20	26	-	-	-	-	-	-	NCP	IGR	-	ID=gene-E	7	6.102857143	3137.5	0.136359532	C(+57.02)	58	834.3278	1	833.316	Carbamido	C	Superscaf	46822443	0	0	BTP202209	0	0	0	Tissue specific

TMLADHAAF	Superscaf	-	Superscaf	46872739	46872780	-7	7	20	-	-	gene	-	-	mRNA	NCP	Intron	gene|mRN	ID=gene-E	14	5.978571429	5852.7	0.135426191	T(+42.01)	95	822.4127	2	1642.814	Acetylati	S	Superscaf	46872855	BTP20220	0	0	0	0	0	Tissue specific
TMLADHAAF	Superscaf	-	Superscaf	46872739	46872765	-7	7	15	-	-	gene	-	-	mRNA	NCP	Intron	gene|mRN	ID=gene-E	9	6.404444444	3736.3	0.135426468	T(+42.01)	86	522.2479	2	1042.487	Acetylati	S	Superscaf	46872855	BTP20220	0	0	0	0	0	Tissue specific
FFPPPKY	Superscaf	-	Superscaf	46910239	46910259	-10	10	16	-	-	gene	-	-	mRNA	NCP	Intron	gene|mRN	ID=gene-E	7	6.465714286	2714.9	0.134734887	FFPPPKY	66	448.2356	2	894.4639	.	I	Superscaf	46910277	BTP20220	0	0	0	0	0	Tissue specific
FLGFL	Superscaf	-	Superscaf	47012113	47012127	-14	14	18	-	-	gene	-	-	mRNA	NCP	Intron	gene|mRN	ID=gene-E	5	5.778	1778.9	0.13285592	FLGFL	53	596.3425	1	595.337	.	L	Superscaf	47012133	0	0	0	0	BTP20220	0	Tissue specific
MHAVPY	Superscaf	-	Superscaf	47039497	47039514	-34	34	39	-	-	gene	-	-	mRNA	NCP	Intron	gene|mRN	ID=gene-E	6	5.22	2099.4	0.132350763	M(+15.99)	53	733.3372	1	732.3265	Oxidation	F	Superscaf	47039550	0	BTP20220	0	0	0	0	Tissue specific
APAEPPR	Superscaf	+	Superscaf	47041258	47041278	5	5	11	-	-	gene	-	-	mRNA	NCP	Intron	gene|mRN	ID=gene-E	7	6.417142857	2709.1	0.132318226	APAEPPR	75	369.2047	2	736.3867	.	I	Superscaf	47041246	0	0	0	0	BTP20220	0	Mixed
MLSSFLR	Superscaf	-	Superscaf	47041366	47041386	-43	43	49	-	-	gene	-	-	mRNA	NCP	Intron	gene|mRN	ID=gene-E	7	6.472857143	2881.3	0.132316234	M(+15.99)	73	435.2243	2	868.4477	Oxidation	K	Superscaf	47041554	0	0	0	0	0	BTP20220	Tissue specific
MVPPWR	Superscaf	+	Superscaf	47043544	47043561	25	25	30	-	-	gene	-	-	mRNA	NCP	Intron	gene|mRN	ID=gene-E	6	5.833333333	2221.7	0.132276116	M(+15.99)	56	401.209	2	800.4003	Oxidation	A	Superscaf	47043472	0	0	0	0	BTP20220	0	Tissue specific
ALVFVPVL	Superscaf	+	Superscaf	47107162	47107185	32	32	39	-	-	gene	-	-	mRNA	NCP	Intron	gene|mRN	ID=gene-E	8	3.72	1742.9	0.131102564	A(+42.01)	52	450.2863	2	898.5527	Acetylati	I	Superscaf	47107069	0	0	0	0	0	BTP20220	Tissue specific
VGEPPRG	Superscaf	+	Superscaf	47145718	47145738	17	17	23	-	-	gene	-	-	mRNA	NCP	Intron	gene|mRN	ID=gene-E	7	5.502857143	2343.7	0.130391449	VGEPPRG	62	356.1914	2	710.3711	.	K	Superscaf	47145670	0	0	0	0	BTP20220	BTP20220	Tissue specific
SAAGPPPP	Superscaf	+	Superscaf	47147881	47147904	55	55	62	-	-	gene	-	-	mRNA	NCP	Intron	gene|mRN	ID=gene-E	8	6.11125	2652.9	0.130351497	SAAGPPPP	73	693.3629	1	692.3493	.	V	Superscaf	47147719	0	0	0	0	0	BTP20220	Tissue specific
PPMLK	Superscaf	+	Superscaf	47148874	47148888	32	32	36	-	-	gene	-	-	mRNA	NCP	Intron	gene|mRN	ID=gene-E	5	6.814	1868.1	0.130333347	PPM(+15.9	93	601.3444	1	600.3305	Oxidation	R	Superscaf	47148781	0	0	0	0	BTP20220	0	Tissue specific
RALPHM	Superscaf	+	Superscaf	47228692	47228709	1	1	6	-	-	-	-	-	-	NCP	IGR	-	ID=gene-E	6	7.066666667	2642.1	0.12886104	R(+42.01)	53	383.7126	2	765.3956	Acetylati	R	Superscaf	47228692	0	0	0	0	0	BTP20220	Tissue specific
PALLTNRL	Superscaf	+	Superscaf	47257642	47257665	18	18	25	-	-	-	-	-	-	NCP	IGR	-	ID=gene-E	8	6.62	3351.9	0.128326943	PALLTNRL	80	449.2778	2	896.5443	.	E	Superscaf	47257591	0	0	0	0	0	BTP20220	Tissue specific
KAAHLLY	Superscaf	+	Superscaf	47298844	47298864	14	14	20	-	-	-	-	-	-	NCP	IGR	-	ID=gene-E	7	6.712857143	2877	0.127567023	KAAHLLY	59	408.2372	2	814.4701	.	Q	Superscaf	47298805	0	0	0	BTP20220	0	0	Mixed
LSEGKPL	Superscaf	+	Superscaf	47315086	47315106	42	42	48	-	-	gene	-	-	mRNA	NCP	Intron	gene|mRN	ID=gene-E	7	6.124285714	2618.8	0.127267437	LSEGKPL	63	372.2173	2	742.4225	.	L	Superscaf	47314963	0	0	0	BTP20220	0	0	Mixed
VPGVR	Superscaf	+	Superscaf	47354131	47354145	8	8	12	-	-	gene	-	-	mRNA	NCP	Intron	gene|mRN	ID=gene-E	5	4.606	1283.5	0.126547358	VPGVR	57	527.3284	1	526.3227	.	N	Superscaf	47354110	0	0	0	0	0	BTP20220	Tissue specific
FARPPD	Superscaf	-	Superscaf	47463421	47463438	-12	12	17	-	-	-	-	-	-	NCP	IGR	-	ID=gene-E	6	6.305	2433.8	0.124531436	FARPPD	61	351.6886	2	701.3496	.	V	Superscaf	47463462	0	0	0	0	BTP20220	0	Tissue specific
LGPELTP	Superscaf	-	Superscaf	47473813	47473833	-8	8	14	-	-	-	-	-	-	NCP	IGR	-	ID=gene-E	7	5.754285714	2501.9	0.124339699	LGPELTP	72	726.3986	1	725.3959	.	L	Superscaf	47473860	0	BTP20220	0	0	0	0	Tissue specific
LGLDKV	Superscaf	+	Superscaf	47476879	47476896	1	1	6	-	-	-	-	-	-	NCP	IGR	-	ID=gene-E	6	5.106666667	1884	0.124283202	LGLDKV	95	644.3975	1	643.3904	.	L	Superscaf	47476879	0	BTP20220	0	0	0	0	Tissue specific
KDYEEVGVI	Superscaf	+	Superscaf	47541691	47541717	74	74	82	-	-	gene	-	-	mRNA	NCP	Intron	gene|mRN	ID=gene-E	9	3.75	2899.2	0.123087571	KDYEEVGVI	83	527.239	2	1052.466	.	L	Superscaf	47541472	0	BTP20220	0	0	0	0	Tissue specific
NLTLTLR	Superscaf	-	Superscaf	47659873	47659893	-18	18	24	-	-	-	-	-	-	NCP	IGR	-	ID=gene-E	7	6.738571429	3100.7	0.120907801	NLTLTLR	50	415.7577	2	829.5021	.	S	Superscaf	47659899	0	BTP20220	0	0	0	0	Tissue specific
LDDVVKL	Superscaf	-	Superscaf	47689318	47689338	-1	1	7	-	-	-	-	-	-	NCP	IGR	-	ID=gene-E	7	3.948571429	1998.2	0.120364685	LDDVVKL	83	401.2379	2	800.4644	.	L	Superscaf	47689353	0	BTP20220	0	0	0	0	Tissue specific
MLLLLE	Superscaf	-	Superscaf	47749603	47749620	-3	3	8	-	-	gene	-	-	mRNA	NCP	Intron	gene|mRN	ID=gene-E	6	5.481666667	2210.6	0.119252777	MLLLLE	56	366.2194	2	730.4299	.	Y	Superscaf	47749626	0	BTP20220	BTP20220	0	0	BTP20220	Tissue specific
PYSSFLR	Superscaf	+	Superscaf	47755045	47755065	39	39	45	-	-	-	-	-	-	NCP	IGR	-	ID=gene-E	7	6.505714286	2953.3	0.119152343	PYSSFLR	66	435.2243	2	868.4443	.	T	Superscaf	47754931	0	BTP20220	0	0	0	0	Tissue specific
SQLPFLQ	Superscaf	+	Superscaf	47843692	47843712	2	2	8	-	-	gene	-	-	mRNA	NCP	Intron	gene|mRN	ID=gene-E	7	5.817142857	3036.4	0.117517239	SQLPFLQ	69	832.455	1	831.449	.	D	Superscaf	47843689	0	0	0	0	BTP20220	0	Tissue specific
EKNTEF	Superscaf	-	Superscaf	48002287	48002304	-32	32	37	-	-	gene	-	-	mRNA	NCP	Intron	gene|mRN	ID=gene-E	6	5.6	2701	0.114591991	E(+42.01)	53	809.3641	1	808.3603	Acetylati	V	Superscaf	48002304	0	0	0	0	BTP20220	BTP20220	Tissue specific
CSPAK	Superscaf	-	Superscaf	48006649	48006663	-13	13	17	-	-	-	-	-	-	NCP	IGR	-	ID=gene-E	5	6.552	2086.2	0.114511589	C(+57.02)	51	604.2717	1	603.2686	Carbamido	K	Superscaf	48006753	0	BTP20220	0	0	0	0	Tissue specific
LDAPGHR	Superscaf	-	Superscaf	48094675	48094695	-28	28	34	-	-	gene	-	-	mRNA	NCP	Intron	gene|mRN	ID=gene-E	7	6.512857143	2979.4	0.112887829	LDAPGHR	97	383.2031	2	764.3929	.	E	Superscaf	48094695	0	0	BTP20220	0	0	0	Tissue specific
SGAELLEL	Superscaf	+	Superscaf	48113434	48113457	64	64	71	-	-	-	-	-	-	NCP	IGR	-	ID=gene-E	8	5.25625	2903.2	0.112541761	SGAELLEL	75	831.444	1	830.4385	.	Y	Superscaf	48113245	0	0	BTP20220	0	0	0	Mixed
ALPPYVP	Superscaf	+	Superscaf	48149113	48149133	23	23	29	-	-	gene	-	-	mRNA	NCP	Intron	gene|mRN	ID=gene-E	7	5.222857143	2136.4	0.111883713	ALPPYVP	70	378.7153	2	755.4218	.	V	Superscaf	48149047	0	BTP20220	0	0	0	0	Tissue specific
GVHVLFL	Superscaf	+	Superscaf	48180961	48180981	27	27	33	-	-	-	-	-	-	NCP	IGR	-	ID=gene-E	7	4.428571429	2011.2	0.111296273	GVHVLFL	55	392.747	2	783.4643	.	P	Superscaf	48180883	0	BTP20220	0	0	0	0	Tissue specific
EEHALLL	Superscaf	+	Superscaf	48188020	48188040	10	10	16	-	-	-	-	-	-	NCP	IGR	-	ID=gene-E	7	5.427142857	2842.2	0.111166069	EEHALLL	62	412.7271	2	823.4439	.	L	Superscaf	48187993	0	0	0	0	BTP20220	0	Tissue specific
KFTLEQEK	Superscaf	+	Superscaf	48222862	48222885	14	14	21	-	-	-	-	-	-	NCP	IGR	-	ID=gene-E	8	6.195	3536.8	0.110523349	K(+42.01)	74	532.7875	2	1063.555	Acetylati	I	Superscaf	48222823	BTP20220	0	0	0	0	0	Tissue specific
PAPPVLPPF	Superscaf	+	Superscaf	48251635	48251661	55	55	63	-	-	-	-	-	-	NCP	IGR	-	ID=gene-E	9	5.533333333	2689.1	0.109992573	PAPPVLPPF	85	884.5215	1	883.5167	.	D	Superscaf	48251473	BTP20220	0	0	0	0	0	Tissue specific
PAAAMP	Superscaf	+	Superscaf	48296482	48296499	19	19	24	-	exon	gene	three_pri	-	mRNA	NCP	3UTR	exon|gene	ID=exon-E	6	6.068333333	1980.2	0.109165531	PAAAM(+15	58	573.2762	1	572.2628	Oxidation	H	Superscaf	48296428	0	0	0	0	BTP20220	0	Tissue specific
ALPKPEP	Superscaf	+	Superscaf	48311683	48311703	32	32	38	-	-	gene	-	-	mRNA	NCP	Intron	gene|mRN	ID=gene-E	7	6.265714286	2570.8	0.108885091	ALPKPEP	78	376.2201	2	750.4276	.	A	Superscaf	48311590	0	0	BTP20220	0	0	0	Tissue specific
KNVLGAR	Superscaf	+	Superscaf	48325564	48325584	56	56	62	-	-	gene	-	-	mRNA	NCP	Intron	gene|mRN	ID=gene-E	7	6.268571429	2675.9	0.108629054	KNVLGAR	96	379.2368	2	756.4606	.	L	Superscaf	48325399	0	0	0	0	BTP20220	0	Tissue specific
LQKGAPY	Superscaf	+	Superscaf	48361678	48361698	19	19	25	-	-	-	-	-	-	NCP	IGR	-	ID=gene-E	7	6.474285714	2838	0.107962928	LQKGAPY	73	388.7169	2	775.4228	.	G	Superscaf	48361624	0	BTP20220	0	0	0	0	Tissue specific
LTAAPEL	Superscaf	+	Superscaf	48365410	48365430	2	2	8	-	-	-	-	-	-	NCP	IGR	-	ID=gene-E	7	5.721428571	2489.8	0.10789409	LTAAPEL	65	714.402	1	713.3959	.	H	Superscaf	48365407	0	0	0	BTP20220	0	0	Tissue specific
KNTKPNL	Superscaf	-	Superscaf	48383956	48383976	-50	50	56	-	-	-	-	-	-	NCP	IGR	-	ID=gene-E	7	7.015714286	3218.5	0.107552007	KNTKPNL	74	407.7417	2	813.4708	.	P	Superscaf	48383985	BTP20220	0	0	0	BTP20220	0	Tissue specific
LMTVMP	Superscaf	+	Superscaf	48406156	48406173	19	19	24	-	-	-	-	-	-	NCP	IGR	-	ID=gene-E	6	5.051666667	1831.3	0.107142581	LMTVMP	71	691.3575	1	690.3445	.	S	Superscaf	48406102	0	BTP20220	0	0	0	0	Tissue specific
KQDAGPLP	Superscaf	+	Superscaf	48594175	48594198	6	6	13	-	-	-	-	-	-	NCP	IGR	-	ID=gene-E	8	6.11625	3127.4	0.103674438	KQDAGPLP	51	413.2257	2	824.4391	.	I	Superscaf	48594160	0	0	0	BTP20220	0	0	Tissue specific
YAAYLF	Superscaf	-	Superscaf	48599536	48599553	-17	17	22	-	-	-	-	-	-	NCP	IGR	-	ID=gene-E	6	5.803333333	2282.4	0.103575665	Y(+42.01)	65	789.3786	1	788.3745	Acetylati	Q	Superscaf	48599595	0	BTP20220	0	0	0	0	Tissue specific
LQALYTVS	Superscaf	+	Superscaf	48611971	48611994	33	33	40	-	-	-	-	-	-	NCP	IGR	-	ID=gene-E	8	5.1875	2869.2	0.103346189	LQALYTVS	68	894.4918	1	893.4858	.	I	Superscaf	48611875	0	BTP20220	0	0	0	0	Tissue specific
LLSVLTKH	Superscaf	-	Superscaf	48630262	48630285	-23	23	30	-	-	-	-	-	-	NCP	IGR	-	ID=gene-E	8	5.935	2929.2	0.10300881	LLSVLTKH	74	455.793	2	909.5648	.	G	Superscaf	48630354	0	0	0	0	BTP20220	0	Tissue specific
LDNLKGPT	Superscaf	+	Superscaf	48692347	48692370	12	12	19	-	-	-	-	-	-	NCP	IGR	-	ID=gene-E	8	6.11	3215.6	0.101863645	LDNLKGPT	70	429.2389	2	856.4654	.	A	Superscaf	48692314	0	0	BTP20220	0	0	0	Tissue specific
LLQTQPR	Superscaf	+	Superscaf	48720469	48720489	14	14	20	-	-	-	-	-	-	NCP	IGR	-	ID=gene-E	7	6.692857143	3311.9	0.101344986	LLQTQPR	81	428.2545	2	854.4974	.	K	Superscaf	48720430	0	BTP20220	0	0	0	0	Tissue specific
LSPQEEPGK	Superscaf	+	Superscaf	48756193	48756219	2	2	10	-	-	-	-	-	-	NCP	IGR	-	ID=gene-E	9	5.784444444	3639	0.100685942	LSPQEEPGK	67	492.7503	2	983.4923	.	T	Superscaf	48756190	0	0	0	BTP20220	0	0	Tissue specific
DSFAY	Superscaf	-	Superscaf	48785344	48785358	-22	22	26	-	-	gene	-	-	mRNA	NCP	Intron	gene|mRN	ID=gene-E	5	5.162	1953.1	0.10014847	DSFAY	58	602.2419	1	601.2383	.	I	Superscaf	48785370	0	0	0	BTP20220	0	0	Tissue specific
KPMLKP	Superscaf	+	Superscaf	49039360	49039377	3	3	8	-	-	-	-	-	-	NCP	IGR	-	ID=gene-E	6	7.301666667	2336.5	0.095463061	KPMLKP	58	357.2228	2	712.4305	.	P	Superscaf	49039354	0	0	0	0	BTP20220	0	Tissue specific
DHTSLPL	Superscaf	-	Superscaf	49057099	49057119	-7	7	13	-	-	-	-	-	-	NCP	IGR	-	ID=gene-E	7	5.861428571	2856.3	0.095135807	DHTSLPL	74	391.7042	2	781.397	.	N	Superscaf	49057209	0	0	0	0	0	BTP20220	Tissue specific
FLTLKGS	Superscaf	+	Superscaf	49075711	49075731	5	5	11	-	-	-	-	-	-	NCP	IGR	-	ID=gene-E	7	6.48	2640.8	0.094792507	FLTLKGS	55	383.2278	2	764.4432	.	G	Superscaf	49075699	0	0	0	0	0	BTP20220	Tissue specific
QTPKYH	Superscaf	-	Superscaf	49085785	49085802	-3	3	8	-	-	-	-	-	-	NCP	IGR	-	ID=gene-E	6	6.911666667	2893.2	0.094606746	QTPKYH	60	387.1938	2	772.3868	.	F	Superscaf	49085802	0	0	BTP20220	0	BTP20220	0	Tissue specific
ATMSPMK	Superscaf	+	Superscaf	49133587	49133607	7	7	13	-	-	-	-	-	-	NCP	IGR	-	ID=gene-E	7	6.538571429	2641	0.093724977	ATMSPM(+1	56	391.1881	2	780.351	Oxidation	F	Superscaf	49133569	0	0	BTP20220	0	BTP20220	0	Tissue specific
AGGPR	Superscaf	+	Superscaf	49148233	49148247	28	28	32	-	-	-	-	-	-	NCP	IGR	-	ID=gene-E	5	7.004	1892.1	0.093454941	AGGPR	60	457.2441	1	456.2444	.	E	Superscaf	49148152	0	0	0	0	BTP20220	0	Tissue specific
TYTLLK	Superscaf	+	Superscaf	49170250	49170267	30	30	35	-	-	-	-	-	-	NCP	IGR	-	ID=gene-E	6	6.736666667	2429.7	0.09304878	T(+42.01)	61	390.731	2	779.4429	Acetylati	I	Superscaf	49170163	BTP20220	0	0	0	0	0	Tissue specific
TPPPAPK	Superscaf	+	Superscaf	49232347	49232367	9	9	15	-	-	-	-	-	-	NCP	IGR	-	ID=gene-E	7	6.784285714	2526.8	0.091903338	T(+42.01)	69	375.2087	2	748.4119	Acetylati	D	Superscaf	49232323	0	0	BTP20220	0	0	0	Tissue specific
YLEAVLAT	Superscaf	-	Superscaf	49248538	49248561	-7	7	14	-	-	-	-	-	-	NCP	IGR	-	ID=gene-E	8	4.92625	2611.9	0.091604638	YLEAVLAT	61	879.4788	1	878.4749	.	I	Superscaf	49248615	BTP20220	0	0	0	0	0	Tissue specific
CMWEVGK	Superscaf	+	Superscaf	49264213	49264233	4	4	10	-	-	-	-	-	-	NCP	IGR	-	ID=gene-E	7	5.084285714	2675	0.091315566	C(+57.02)	62	476.198	2	950.399	Carbamido	S	Superscaf	49264204	0	BTP20220	0	0	0	0	Tissue specific
GPPPNRPP	Superscaf	+	Superscaf	49276444	49276467	25	25	32	-	-	-	-	-	-	NCP	IGR	-	ID=gene-E	8	6.705	3229.8	0.091089908	GPPPNRPP	76	416.2259	2	830.4398	.	S	Superscaf	49276372	0	0	0	BTP20220	0	0	Tissue specific
KGSSSRP	Superscaf	+	Superscaf	49317604	49317624	20	20	26	-	-	gene	-	-	mRNA	NCP	Intron	gene|mRN	ID=gene-E	7	7.115714286	2902.1	0.090330763	KGSSSRP	62	359.6946	2	717.3769	.	S	Superscaf	49317547	0	BTP20220	0	0	0	0	Tissue specific
APELEAE	Superscaf	+	Superscaf	49317949	49317969	37	37	43	-	-	gene	-	-	mRNA	NCP	Intron	gene|mRN	ID=gene-E	7	4.854285714	2589.9	0.090324399	A(+42.01)	84	800.3652	1	799.3599	Acetylati	K	Superscaf	49317841	0	BTP20220	0	0	0	0	Tissue specific
KELPLAGK	Superscaf	+	Superscaf	49337209	49337232	1	1	8	-	-	-	-	-	-	NCP	IGR	-	ID=gene-E	8	6.61875	3015.1	0.089969091	KELPLAGK	93	428.2666	2	854.5225	.	K	Superscaf	49337209	BTP20220	0	0	0	0	0	Tissue specific
AALPLKK	Superscaf	+	Superscaf	49346539	49346559	31	31	37	-	-	-	-	-	-	NCP	IGR	-	ID=gene-E	7	7.111428571	2603.6	0.089797053	AALPLKK	59	370.7545	2	739.4955	.	V	Superscaf	49346449	0	0	BTP20220	0	0	0	Tissue specific
PLATGLP	Superscaf	+	Superscaf	49348036	49348056	16	16	22	-	-	-	-	-	-	NCP	IGR	-	ID=gene-E	7	6.154285714	2387.7	0.089769441	PLATGLP	61	668.4049	1	667.3904	.	T	Superscaf	49347991	0	BTP20220	0	0	0	0	Tissue specific
ESNRGPP	Superscaf	+	Superscaf	49484521	49484541	28	28	34	-	-	gene	-	-	mRNA	NCP	Intron	gene|mRN	ID=gene-E	7	6.234285714	3026.5	0.08725196	ESNRGPP	51	378.6798	2	755.3562	.	L	Superscaf	49484440	0	0	0	0	BTP20220	0	Tissue specific
LTTVGHF	Superscaf	-	Superscaf	49506517	49506537	-4	4	10	-	-	-	-	-	-	NCP	IGR	-	ID=gene-E	7	5.44	2452.8	0.086846241	LTTVGHF	52	774.4085	1	773.4072	.	R	Superscaf	49506570	BTP20220	0	0	0	0	0	Tissue specific
KDMENF	Superscaf	-	Superscaf	49529875	49529892	-27	27	32	-	-	-	-	-	-	NCP	IGR	-	ID=gene-E	6	5.428333333	2661	0.086415455	KDMENF	75	783.33	1	782.3268	.	F	Superscaf	49529934	0	0	0	0	BTP20220	0	Tissue specific
DPFLDL	Superscaf	+	Superscaf	49650169	49650186	19	19	24	-	-	-	-	-	-	NCP	IGR	-	ID=gene-E	6	4.946666667	2254.6	0.084196619	DPFLDL	94	719.3602	1	718.3537	.	M	Superscaf	49650115	0	0	0	0	BTP20220	0	Tissue specific
KTVPGAR	Superscaf	+	Superscaf	49760479	49760499	15	15	21	-	-	gene	-	-	mRNA	NCP	Intron	gene|mRN	ID=gene-E	7	6.474285714	2460.7	0.082161883	KTVPGAR	51	364.7241	2	727.434	.	N	Superscaf	49760437	0	0	BTP20220	0	0	0	Tissue specific
FSNPPGP	Superscaf	+	Superscaf	49817746	49817766	37	37	43	-	-	gene	-	-	mRNA	NCP	Intron	gene|mRN	ID=gene-E	7	5.92	2677	0.081105586	FSNPPGP	59	358.1725	2	714.3337	.	G	Superscaf	49817638	0	0	0	BTP20220	0	0	Tissue specific
EKVLL	Superscaf	+	Superscaf	49832251	49832265	4	4	8	-	-	gene	-	-	mRNA	NCP	Intron	gene|mRN	ID=gene-E	5	4.984	1600.7	0.08083815	EKVLL	60	601.3889	1	600.3846	.	T	Superscaf	49832242	0	0	0	BTP20220	0	0	Tissue specific
LAFLTKK	Superscaf	+	Superscaf	49836451	49836471	18	18	24	-	-	gene	-	-	mRNA	NCP	Intron	gene|mRN	ID=gene-E	7	7.067142857	2739.8	0.08076057	LAFLTKK	90	410.7663	2	819.5218	.	G	Superscaf	49836400	BTP20220	0	0	0	0	0	Tissue specific
PAAEPP	Superscaf	+	Superscaf	49868956	49868973	54	54	59	-	-	gene	-	-	mRNA	NCP	Intron	gene|mRN	ID=gene-E	6	5.693333333	2060.3	0.080161067	PAAEPP	69	581.29	1	580.2856	.	V	Superscaf	49868797	BTP20220	0	0	0	0	0	Tissue specific
ETMKFER	Superscaf	-	Superscaf	49873135	49873155	-6	6	12	-	-	-	-	-	-	NCP	IGR	-	ID=gene-E	7	6.385714286	3124.6	0.08008393	ETM(+15.9	58	478.7306	2	955.4433	Oxidation	I	Superscaf	49873272	0	0	BTP20220	0	0	0	Tissue specific
PAAWHAK	Superscaf	+	Superscaf	49936612	49936632	4	4	10	-	-	-	-	-	-	NCP	IGR	-	ID=gene-E	7	6.797142857	2886	0.078913089	PAAWHAK	50	390.7057	2	779.4078	.	S	Superscaf	49936603	0	0	0	0	BTP20220	0	Tissue specific
MLRSGK	Superscaf	+	Superscaf	50006101	50006118	11	11	16	-	-	-	-	-	-	NCP	IGR	-	ID=gene-E	6	7.313333333	2522.8	0.077631412	M(+42.01)	83	367.2036	2	732.3952	Acetylati	R	Superscaf	50006071	0	0	0	0	BTP20220	BTP20220	Tissue specific
QALKLLKT	Superscaf	+	Superscaf	50020909	50020932	20	20	27	-	-	-	-	-	-	NCP	IGR	-	ID=gene-E	8	6.9525	3316.5	0.077358166	QALKLLKT	91	457.8043	2	913.596	.	S	Superscaf	50020852	0	0	BTP20220	0	0	0	Tissue specific
KPWTQ	Superscaf	+	Superscaf	50048371	50048385	9	9	13	-	-	-	-	-	-	NCP	IGR	-	ID=gene-E	5	6.822	2340.6	0.076851792	KPWTQ	75	659.358	1	658.3439	.	E	Superscaf	50048347	0	0	BTP20220	0	0	0	Tissue specific
MVLDAP	Superscaf	+	Superscaf	50055325	50055342	17	17	22	-	-	-	-	-	-	NCP	IGR	-	ID=gene-E	6	4.503333333	1785.1	0.07672347	MVLDAP	93	645.3313	1	644.3203	.	S	Superscaf	50055277	0	0	BTP20220	0	0	0	Tissue specific
VVGSGSPK	Superscaf	+	Superscaf	50062936	50062959	9	9	16	-	-	-	-	-	-	NCP	IGR	-	ID=gene-E	8	4.9175	2167.2	0.076582973	VVGSGSPK	50	365.7119	2	729.4021	.	G	Superscaf	50062912	0	0	0	0	BTP20220	0	Tissue specific
LSYSEFR	Superscaf	+	Superscaf	50073595	50073615	6	6	12	-	-	-	-	-	-	NCP	IGR	-	ID=gene-E	7	6.065714286	3041.4	0.076386422	LSYSEFR	50	451.2306	2	900.4341	.	N	Superscaf	50073580	0	0	BTP20220	0	0	0	Tissue specific
KHPREVK	Superscaf	+	Superscaf	50143732	50143752	49	49	55	-	-	gene	-	-	mRNA	NCP	Intron	gene|mRN	ID=gene-E	7	6.764285714	2968.2	0.075092737	KHPREVK	72	447.2632	2	892.5242	.	S	Superscaf	50143588	0	0	0	0	BTP20220	0	Tissue specific
ENLLTLL	Superscaf	+	Superscaf	50147458	50147478	3	3	9	-	-	-	-	-	-	NCP	IGR	-	ID=gene-E	7	5.582857143	2833.3	0.07502401	ENLLTLL	56	815.4841	1	814.48	.	N	Superscaf	50147452	0	BTP20220	0	0	BTP20220	BTP20220	Tissue specific
VLEEDLSK	Superscaf	-	Superscaf	50178958	50178981	-10	10	17	-	-	gene	-	-	mRNA	NCP	Intron	gene|mRN	ID=gene-E	8	4.59875	2821.1	0.074442934	VLEEDLSK	86	466.7448	2	931.4862	.	I	Superscaf	50179059	0	0	0	0	BTP20220	BTP20220	Tissue specific
NGSLPF	Superscaf	+	Superscaf	50208064	50208081	22	22	27	-	-	gene	-	-	mRNA	NCP	Intron	gene|mRN	ID=gene-E	6	5.803333333	2355.6	0.073906181	NGSLPF	71	634.3212	1	633.3122	.	L	Superscaf	50208001	0	BTP20220	0	0	0	0	Tissue specific
TQGPLCP	Superscaf	+	Superscaf	50248996	50249016	53	53	59	-	-	gene	-	-	mRNA	NCP	Intron	gene|mRN	ID=gene-E	7	5.964285714	2919.4	0.07315113	TQGPLC(+5	60	772.3712	1	771.3585	Carbamido	K	Superscaf	50248840	0	0	0	0	BTP20220	0	Tissue specific
PGVTVKDVN	Superscaf	+	Superscaf	50270527	50270553	14	14	22	-	-	-	-	-	-	NCP	IGR	-	ID=gene-E	9	4.102222222	2508.8	0.072753878	PGVTVKDVN	96	464.7575	2	927.5025	.	A	Superscaf	50270488	0	0	0	BTP20220	0	0	Tissue specific
TKVNLP	Superscaf	-	Superscaf	50295904	50295921	-3	3	8	-	-	-	-	-	-	NCP	IGR	-	ID=gene-E	6	5.66	2153.4	0.072285962	T(+42.01)	54	713.4148	1	712.4119	Acetylati	S	Superscaf	50296014	0	0	0	BTP20220	0	0	Tissue specific
PGRLTPP	Superscaf	+	Superscaf	50299033	50299053	28	28	34	-	-	-	-	-	-	NCP	IGR	-	ID=gene-E	7	6.877142857	2709.2	0.072228192	PGRLTPP	66	737.4276	1	736.4232	.	L	Superscaf	50298952	0	0	BTP20220	0	0	0	Tissue specific
KAEVVAP	Superscaf	+	Superscaf	50339164	50339184	63	63	69	-	-	-	-	-	-	NCP	IGR	-	ID=gene-E	7	4.471428571	1853.9	0.071487971	KAEVVAP	74	357.213	2	712.4119	.	A	Superscaf	50338978	0	0	BTP20220	0	0	0	Tissue specific
LEPGSYK	Superscaf	+	Superscaf	50341333	50341353	55	55	61	-	-	-	-	-	-	NCP	IGR	-	ID=gene-E	7	6.078571429	2724.9	0.071447964	L(+42.01)	67	418.2159	2	834.4123	Acetylati	R	Superscaf	50341171	0	0	0	BTP20220	0	0	Tissue specific
VLLAAAV	Superscaf	+	Superscaf	50391058	50391078	27	27	33	-	-	-	-	-	-	NCP	IGR	-	ID=gene-E	7	4.288571429	1640.7	0.07053078	VLLAAAV	72	656.4308	1	655.4268	.	L	Superscaf	50390980	0	0	0	0	BTP20220	BTP20220	Tissue specific
KNGSLPF	Superscaf	+	Superscaf	50398681	50398701	9	9	15	-	-	-	-	-	-	NCP	IGR	-	ID=gene-E	7	6.365714286	2824	0.070390173	KNGSLPF	62	762.4138	1	761.4072	.	T	Superscaf	50398657	0	0	0	BTP20220	0	0	Tissue specific
KLLKPEK	Superscaf	-	Superscaf	50458657	50458677	-21	21	27	-	-	gene	-	-	mRNA	NCP	Intron	gene|mRN	ID=gene-E	7	7.242857143	2874.9	0.069283909	KLLKPEK	96	428.2857	2	854.5589	.	F	Superscaf	50458689	0	0	0	0	BTP20220	0	Tissue specific
VGFDLRLPY	Superscaf	-	Superscaf	50462851	50462877	-6	6	14	-	-	-	-	-	-	NCP	IGR	-	ID=gene-E	9	5.455555556	3248.7	0.06920644	VGFDLRLPY	82	540.2958	2	1078.581	.	A	Superscaf	50462952	0	0	0	BTP20220	0	0	Tissue specific
KGMGDLK	Superscaf	+	Superscaf	50484640	50484660	4	4	10	-	-	-	-	-	-	NCP	IGR	-	ID=gene-E	7	6.588571429	2667.8	0.06880465	KGMGDLK	60	374.7018	2	747.3949	.	P	Superscaf	50484631	0	0	0	BTP20220	0	0	Tissue specific
PDDPPR	Superscaf	+	Superscaf	50531320	50531337	29	29	34	-	-	gene	-	-	mRNA	NCP	Intron	gene|mRN	ID=gene-E	6	5.933333333	2484	0.067943687	P(+42.01)	71	369.6818	2	737.3344	Acetylati	G	Superscaf	50531236	0	0	0	0	BTP20220	0	Tissue specific
TVNPPF	Superscaf	-	Superscaf	50600053	50600070	-16	16	21	-	-	-	-	-	-	NCP	IGR	-	ID=gene-E	6	5.003333333	2056.4	0.066675899	TVNPPF	63	674.3572	1	673.3435	.	H	Superscaf	50600673	0	0	0	0	BTP20220	0	Tissue specific
LFDRDL	Superscaf	+	Superscaf	50600317	50600334	6	6	11	-	-	-	-	-	-	NCP	IGR	-	ID=gene-E	6	5.69	2566	0.06667103	LFDRDL	79	389.7149	2	777.402	.	K	Superscaf	50600302	0	0	0	0	BTP20220	0	Tissue specific
PPLHKPK	Superscaf	+	Superscaf	50613121	50613141	24	24	30	-	-	-	-	-	-	NCP	IGR	-	ID=gene-E	7	7.421428571	2922.1	0.066434803	PPLHKPK	59	408.7548	2	815.5017	.	F	Superscaf	50613052	0	0	0	0	BTP20220	0	Tissue specific
LPPELP	Superscaf	+	Superscaf	50722180	50722197	9	9	14	-	-	-	-	-	-	NCP	IGR	-	ID=gene-E	6	5.68	2144.5	0.064423253	LPPELP	96	665.3849	1	664.3795	.	P	Superscaf	50722156	0	0	0	0	BTP20220	0	Tissue specific
KEMHVW	Superscaf	+	Superscaf	50734483	50734500	19	19	24	-	-	-	-	-	-	NCP	IGR	-	ID=gene-E	6	5.365	2411.7	0.064196322	KEM(+15.9	55	423.201	2	844.3901	Oxidation	M	Superscaf	50734429	0	0	BTP20220	0	0	0	Tissue specific
PAGASN	Superscaf	+	Superscaf	50790937	50790954	97	97	102	-	-	-	-	-	-	NCP	IGR	-	ID=gene-E	6	5.9	2237.4	0.063155022	PAGASN	52	516.2439	1	515.2339	.	Y	Superscaf	50790649	0	0	0	0	BTP20220	BTP20220	Tissue specific
KTFGVT	Superscaf	-	Superscaf	50835106	50835123	-106	106	111	-	-	-	-	-	-	NCP	IGR	-	ID=gene-E	6	5.708333333	1948.1	0.062340319	KTFGVT	57	652.3736	1	651.3592	.	S	Superscaf	50835159	0	0	BTP20220	0	0	0	Tissue specific
DDDSG	Superscaf	+	Superscaf	50882497	50882511	8	8	12	-	-	-	-	-	-	NCP	IGR	-	ID=gene-E	5	4.112	1915.2	0.061466242	DDDSG	54	508.1518	1	507.1449	.	L	Superscaf	50882476	0	0	BTP20220	0	0	0	Mixed
AAVSSVR	Superscaf	+	Superscaf	50888119	50888139	17	17	23	-	-	-	-	-	-	NCP	IGR	-	ID=gene-E	7	4.88	2038.2	0.061362433	A(+42.01)	89	366.1999	2	730.3973	Acetylati	I	Superscaf	50888071	0	0	BTP20220	0	0	0	Tissue specific
PHALMRL	Superscaf	+	Superscaf	50993275	50993295	24	24	30	-	-	gene	-	-	mRNA	NCP	Intron	gene|mRN	ID=gene-E	7	6.911428571	2995.5	0.059422819	PHALMRL	98	419.2399	2	836.4691	.	R	Superscaf	50993206	0	0	0	0	BTP20220	0	Tissue specific
VFQAAVPK	Superscaf	-	Superscaf	51000076	51000099	-76	76	83	-	-	-	-	-	-	NCP	IGR	-	ID=gene-E	8	4.90125	2426.5	0.059297318	VFQAAVPK	99	430.2542	2	858.4963	.	L	Superscaf	51000132	BTP20220	0	0	0	BTP20220	0	Tissue specific
VERLLEPL	Superscaf	-	Superscaf	51022078	51022101	-33	33	40	-	-	gene	-	-	mRNA	NCP	Intron	gene|mRN	ID=gene-E	8	5.18	2897.4	0.058891489	VERLLEPL	76	484.7914	2	967.5702	.	W	Superscaf	51022155	0	0	0	0	BTP20220	0	Tissue specific
KYRLLPG	Superscaf	+	Superscaf	51071989	51072009	42	42	48	-	-	gene	-	-	mRNA	NCP	Intron	gene|mRN	ID=gene-E	7	7.198571429	2918.2	0.05797093	KYRLLPG	55	423.767	2	845.5123	.	L	Superscaf	51071866	0	0	0	0	BTP20220	0	Tissue specific
AAVPWN	Superscaf	+	Superscaf	51101092	51101109	34	34	39	-	-	gene	-	-	mRNA	NCP	Intron	gene|mRN	ID=gene-E	6	4.94	2083.3	0.057434177	AAVPWN	81	657.3332	1	656.3282	.	G	Superscaf	51100993	0	0	0	0	BTP20220	0	Tissue specific
LELLTDR	Superscaf	-	Superscaf	51135475	51135495	-18	18	24	-	-	gene	-	-	mRNA	NCP	Intron	gene|mRN	ID=gene-E	7	5.917142857	2943.5	0.056799923	LELLTDR	98	430.246	2	858.4811	.	V	Superscaf	51135579	0	BTP20220	0	0	0	0	Mixed
NPPSSKP	Superscaf	-	Superscaf	51181312	51181332	-54	54	60	-	-	gene	-	-	mRNA	NCP	Intron	gene|mRN	ID=gene-E	7	6.487142857	2844.1	0.055954455	NPPSSKP	57	363.6889	2	725.3708	.	P	Superscaf	51181338	0	BTP20220	0	0	0	0	Tissue specific
ESSHAPK	Superscaf	-	Superscaf	51208591	51208611	-42	42	48	-	-	gene	-	-	mRNA	NCP	Intron	gene|mRN	ID=gene-E	7	6.318571429	2929.1	0.05545129	ESSHAPK	52	378.1935	2	754.361	.	W	Superscaf	51208731	0	BTP20220	0	0	0	0	Tissue specific
HWMLDK	Superscaf	+	Superscaf	51248371	51248388	5	5	10	-	-	gene	-	-	mRNA	NCP	Intron	gene|mRN	ID=gene-E	6	6.32	2751.1	0.054717599	HWMLDK	90	415.2044	2	828.3953	.	Q	Superscaf	51248359	0	BTP20220	0	0	BTP20220	0	Tissue specific
PLPPHMT	Superscaf	+	Superscaf	51353056	51353076	182	182	188	-	-	gene	-	-	mRNA	NCP	Intron	gene|mRN	ID=gene-E	7	6.392857143	2754.3	0.052786617	PLPPHM(+1	51	404.7115	2	807.3949	Oxidation	K	Superscaf	51352513	0	BTP20220	0	0	0	0	Tissue specific
AAVLGDA	Superscaf	-	Superscaf	51368731	51368751	-23	23	29	-	-	gene	-	-	mRNA	NCP	Intron	gene|mRN	ID=gene-E	7	4.711428571	1996.1	0.05249749	A(+42.01)	80	658.3392	1	657.3333	Acetylati	C	Superscaf	51368907	0	BTP20220	0	0	0	0	Tissue specific
LPRAGTP	Superscaf	-	Superscaf	51377632	51377652	-2	2	8	-	-	gene	-	-	mRNA	NCP	Intron	gene|mRN	ID=gene-E	7	6.837142857	2683.1	0.05233331	LPRAGTP	66	356.2102	2	710.4075	.	S	Superscaf	51377676	0	BTP20220	0	0	0	0	Tissue specific
DSLTSKY	Superscaf	-	Superscaf	51387166	51387186	-1	1	7	-	-	gene	-	-	mRNA	NCP	Intron	gene|mRN	ID=gene-E	7	6.034285714	2857.1	0.052157454	DSLTSKY	84	407.2094	2	812.3916	.	D	Superscaf	51387198	0	0	0	0	BTP20220	0	Tissue specific
LPPAAGPL	Superscaf	+	Superscaf	51422230	51422253	18	18	25	-	-	gene	-	-	mRNA	NCP	Intron	gene|mRN	ID=gene-E	8	6.10875	2638.9	0.051510639	LPPAAGPL	59	735.437	1	734.4326	.	P	Superscaf	51422179	0	0	0	BTP20220	0	0	Tissue specific
LSLPFVR	Superscaf	-	Superscaf	51497368	51497388	-11	11	17	-	-	-	-	-	-	NCP	IGR	-	ID=gene-E	7	5.74	2463.8	0.050124766	LSLPFVR	84	416.257	2	830.5014	.	I	Superscaf	51497409	0	0	BTP20220	0	0	0	Tissue specific
SETFVAK	Superscaf	-	Superscaf	51502108	51502128	-8	8	14	-	-	-	-	-	-	NCP	IGR	-	ID=gene-E	7	5.238571429	2373.5	0.050037336	SETFVAK	85	391.2057	2	780.4017	.	F	Superscaf	51502176	BTP20220	0	0	0	0	0	Tissue specific
SRYKKM	Superscaf	-	Superscaf	51563362	51563379	-5	5	10	-	-	-	-	-	-	NCP	IGR	-	ID=gene-E	6	7.888333333	2800	0.048907554	SRYKKM	58	406.7272	2	811.4374	.	F	Superscaf	51563457	0	0	BTP20220	0	0	0	Tissue enhanced
KAAPPPR	Superscaf	+	Superscaf	51567928	51567948	6	6	12	-	-	-	-	-	-	NCP	IGR	-	ID=gene-E	7	7.348571429	2752	0.048823278	KAAPPPR	77	368.7208	2	735.4391	.	V	Superscaf	51567913	0	0	BTP20220	0	0	0	Tissue specific
AVCAAY	Superscaf	+	Superscaf	51570862	51570879	24	24	29	-	-	-	-	-	-	NCP	IGR	-	ID=gene-E	6	4.79	1979.1	0.048769216	AVC(+57.0	60	654.2874	1	653.2843	Carbamido	S	Superscaf	51570793	0	BTP20220	0	0	0	0	Tissue specific
LLWHQK	Superscaf	-	Superscaf	51605434	51605451	-7	7	12	-	-	gene	-	-	mRNA	NCP	Intron	gene|mRN	ID=gene-E	6	6.805	2932.2	0.048131531	LLWHQK	54	412.7454	2	823.4705	.	I	Superscaf	51605508	0	0	0	0	BTP20220	0	Tissue specific
TPKMPP	Superscaf	+	Superscaf	51651088	51651105	64	64	69	-	-	gene	-	-	mRNA	NCP	Intron	gene|mRN	ID=gene-E	6	6.82	2249.6	0.047289438	T(+42.01)	59	728.3599	1	727.3575	Acetylati	K	Superscaf	51650899	0	BTP20220	0	0	0	0	Tissue specific
DFLNNP	Superscaf	-	Superscaf	51665722	51665739	-49	49	54	-	-	gene	-	-	mRNA	NCP	Intron	gene|mRN	ID=gene-E	6	5.258333333	2683.1	0.047019512	DFLNNP	83	719.3364	1	718.3286	.	D	Superscaf	51665805	0	BTP20220	0	0	0	0	Tissue specific
EELFKRV	Superscaf	+	Superscaf	51724177	51724197	5	5	11	-	-	gene	-	-	mRNA	NCP	Intron	gene|mRN	ID=gene-E	7	5.485714286	2709	0.045941247	EELFKRV	78	460.7622	2	919.5127	.	N	Superscaf	51724165	0	BTP20220	0	0	0	0	Tissue specific
WLPVVR	Superscaf	+	Superscaf	51748507	51748524	1	1	6	-	-	gene	-	-	mRNA	NCP	Intron	gene|mRN	ID=gene-E	6	4.821666667	1866.2	0.045492533	WLPVVR	99	385.2387	2	768.4646	.	W	Superscaf	51748507	0	0	0	0	BTP20220	0	Tissue enhanced
SPFVLGV	Superscaf	+	Superscaf	51830836	51830856	25	25	31	-	-	gene	-	-	mRNA	NCP	Intron	gene|mRN	ID=gene-E	7	4.201428571	1758.9	0.04397391	SPFVLGV	52	359.7087	2	717.4061	.	K	Superscaf	51830764	0	0	0	0	BTP20220	BTP20220	Tissue specific
TDLRKEE	Superscaf	-	Superscaf	51847339	51847359	-3	3	9	-	-	gene	-	-	mRNA	NCP	Intron	gene|mRN	ID=gene-E	7	6.06	3130.6	0.04366951	T(+42.01)	71	466.7422	2	931.4611	Acetylati	I	Superscaf	51847443	BTP20220	0	0	BTP20220	0	0	Tissue specific
AVLPMTR	Superscaf	-	Superscaf	51883384	51883404	-24	24	30	-	-	gene	-	-	mRNA	NCP	Intron	gene|mRN	ID=gene-E	7	5.905714286	2419.9	0.043004656	A(+42.01)	65	423.235	2	844.4477	Acetylati	S	Superscaf	51883404	0	0	BTP20220	0	0	0	Tissue specific
LTVRPP	Superscaf	+	Superscaf	51936193	51936210	3	3	8	-	exon	gene	three_pri	-	mRNA	NCP	3UTR	exon|gene	ID=exon-E	6	5.978333333	2074.5	0.042030643	LTVRPP	60	682.4217	1	681.4174	.	C	Superscaf	51936187	0	0	BTP20220	0	0	0	Tissue specific
ALEFVA	Superscaf	+	Superscaf	51939496	51939513	85	85	90	-	-	gene	-	-	mRNA	NCP	Intron	gene|mRN	ID=gene-E	6	4.453333333	1788.9	0.041969719	ALEFVA	85	649.3533	1	648.3483	.	H	Superscaf	51939244	0	BTP20220	0	0	0	0	Tissue specific
MLPQNA	Superscaf	+	Superscaf	51966859	51966876	9	9	14	-	-	gene	-	-	mRNA	NCP	Intron	gene|mRN	ID=gene-E	6	5.851666667	2581	0.041465005	M(+42.01)	69	715.3434	1	714.337	Acetylati	S	Superscaf	51966835	0	0	BTP20220	0	0	0	Tissue specific
GTFGLA	Superscaf	-	Superscaf	51978976	51978993	-22	22	27	-	-	gene	-	-	mRNA	NCP	Intron	gene|mRN	ID=gene-E	6	5.991666667	2044.2	0.041241506	G(+42.01)	65	607.3063	1	606.3013	Acetylati	I	Superscaf	51979041	0	0	BTP20220	0	0	0	Tissue specific
LHAGLAAP	Superscaf	-	Superscaf	52016782	52016805	-32	32	39	-	-	-	-	-	-	NCP	IGR	-	ID=gene-E	8	6.235	2895.1	0.040544059	LHAGLAAP	51	375.2168	2	748.4232	.	I	Superscaf	52016934	0	0	BTP20220	0	0	0	Tissue specific
KMSRAP	Superscaf	-	Superscaf	52077391	52077408	-13	13	18	-	-	-	-	-	-	NCP	IGR	-	ID=gene-E	6	7.375	2520.8	0.03942623	KM(+15.99	57	353.1874	2	704.364	Oxidation	S	Superscaf	52077498	0	0	0	0	BTP20220	0	Tissue specific
FPEPLK	Superscaf	-	Superscaf	52102273	52102290	-15	15	20	-	-	-	-	-	-	NCP	IGR	-	ID=gene-E	6	6.17	2309.5	0.038967279	FPEPLK	72	365.7099	2	729.4061	.	D	Superscaf	52102296	0	0	0	0	0	BTP20220	Tissue enhanced
RLHEEGP	Superscaf	-	Superscaf	52122928	52122948	-54	54	60	-	-	-	-	-	-	NCP	IGR	-	ID=gene-E	7	6.148571429	3107.6	0.038586239	RLHEEGP	56	419.2102	2	836.414	.	W	Superscaf	52122975	0	0	0	0	0	BTP20220	Tissue specific
VLPLLYK	Superscaf	+	Superscaf	52200148	52200168	26	26	32	-	-	-	-	-	-	NCP	IGR	-	ID=gene-E	7	5.662857143	2325.5	0.037161908	VLPLLYK	56	423.2722	2	844.5422	.	P	Superscaf	52200073	0	0	0	BTP20220	0	0	Tissue specific
KPQMPPP	Superscaf	+	Superscaf	52343872	52343892	11	11	17	-	-	-	-	-	-	NCP	IGR	-	ID=gene-E	7	6.62	2800.2	0.034510902	KPQM(+15.	55	405.7072	2	809.4105	Oxidation	L	Superscaf	52343842	0	0	0	BTP20220	0	0	Tissue specific
SCLLLH	Superscaf	+	Superscaf	52416334	52416351	28	28	33	-	-	-	-	-	-	NCP	IGR	-	ID=gene-E	6	6.038333333	2649	0.033174387	SC(+57.02	91	371.6986	2	741.3843	Carbamido	L	Superscaf	52416253	0	0	0	BTP20220	0	0	Tissue specific
NPVSTFHV	Superscaf	-	Superscaf	52425577	52425600	-22	22	29	-	-	-	-	-	-	NCP	IGR	-	ID=gene-E	8	4.62375	2722.1	0.033003789	N(+42.01)	57	471.7409	2	941.4607	Acetylati	C	Superscaf	52425699	0	0	BTP20220	0	0	0	Tissue specific
NYALLRL	Superscaf	-	Superscaf	52466215	52466235	-17	17	23	-	-	-	-	-	-	NCP	IGR	-	ID=gene-E	7	6.541428571	3076.5	0.032254271	NYALLRL	60	431.7657	2	861.5072	.	A	Superscaf	52466295	0	0	0	BTP20220	0	0	Tissue enhanced
KPFNPPP	Superscaf	-	Superscaf	52474342	52474362	-27	27	33	-	-	-	-	-	-	NCP	IGR	-	ID=gene-E	7	6.547142857	2802.1	0.032104368	KPFNPPP	53	398.7224	2	795.4279	.	I	Superscaf	52474452	0	BTP20220	0	0	0	0	Tissue specific
KHVWMK	Superscaf	+	Superscaf	52519450	52519467	50	50	55	-	-	-	-	-	-	NCP	IGR	-	ID=gene-E	6	6.451666667	2454.6	0.031272401	KHVWM(+15	60	422.7234	2	843.4425	Oxidation	N	Superscaf	52519303	0	0	0	0	BTP20220	0	Tissue specific
DAYVRP	Superscaf	+	Superscaf	52542112	52542129	48	48	53	-	-	-	-	-	-	NCP	IGR	-	ID=gene-E	6	5.285	2168.5	0.030854398	DAYVRP	66	360.6817	2	719.3602	.	C	Superscaf	52541971	0	0	BTP20220	0	0	0	Tissue specific
VTLNV	Superscaf	+	Superscaf	52551427	52551441	113	113	117	-	-	-	-	-	-	NCP	IGR	-	ID=gene-E	5	3.584	1347.6	0.030682637	VTLNV	80	545.3278	1	544.3221	.	V	Superscaf	52551091	0	0	0	0	BTP20220	0	Mixed
KAQLYYP	Superscaf	+	Superscaf	52579597	52579617	12	12	18	-	-	-	-	-	-	NCP	IGR	-	ID=gene-E	7	6.43	3000.2	0.030162927	KAQLYYP	58	441.7429	2	881.4647	.	E	Superscaf	52579564	0	0	0	0	BTP20220	0	Tissue specific
EFCRPL	Superscaf	-	Superscaf	52654471	52654488	-16	16	21	-	-	-	-	-	-	NCP	IGR	-	ID=gene-E	6	6.126666667	2738.2	0.028781923	EFC(+57.0	93	411.1985	2	820.3901	Carbamido	L	Superscaf	52654518	BTP20220	0	0	0	0	0	Tissue specific
APDLPKL	Superscaf	-	Superscaf	52657987	52658007	-33	33	39	-	-	-	-	-	-	NCP	IGR	-	ID=gene-E	7	6.184285714	2572.8	0.028717015	APDLPKL	83	753.4486	1	752.4432	.	Y	Superscaf	52658007	0	0	BTP20220	0	0	0	Tissue specific
VSDSLLP	Superscaf	+	Superscaf	52666225	52666245	5	5	11	-	-	-	-	-	-	NCP	IGR	-	ID=gene-E	7	4.655714286	2222.5	0.028565064	V(+42.01)	61	772.4086	1	771.4014	Acetylati	S	Superscaf	52666213	0	0	0	BTP20220	0	0	Mixed
TSMMSVH	Superscaf	+	Superscaf	52706875	52706895	13	13	19	-	-	-	-	-	-	NCP	IGR	-	ID=gene-E	7	5.282857143	2527	0.02781527	TSMMSVH	88	396.6708	2	791.3306	.	L	Superscaf	52706839	0	0	0	BTP20220	0	0	Tissue specific
AYLFD	Superscaf	-	Superscaf	52718953	52718967	-6	6	10	-	-	-	-	-	-	NCP	IGR	-	ID=gene-E	5	5.222	1923.1	0.027592601	A(+42.01)	74	670.3066	1	669.301	Acetylati	S	Superscaf	52718979	0	0	BTP20220	0	BTP20220	0	Mixed
QTYVYK	Superscaf	-	Superscaf	52791127	52791144	-22	22	27	-	-	-	-	-	-	NCP	IGR	-	ID=gene-E	6	5.54	2395.6	0.026261288	QTYVYK	80	801.4104	1	800.4069	.	T	Superscaf	52791219	0	0	0	BTP20220	0	0	Tissue specific
KVPQLELV	Superscaf	+	Superscaf	52900360	52900383	5	5	12	-	-	-	-	-	-	NCP	IGR	-	ID=gene-E	8	4.60875	2548.8	0.024246362	KVPQLELV	56	463.2879	2	924.5644	.	E	Superscaf	52900348	0	0	0	0	BTP20220	0	Tissue specific
LLGVETL	Superscaf	+	Superscaf	52975399	52975419	14	14	20	-	-	-	-	-	-	NCP	IGR	-	ID=gene-E	7	4.808571429	2180.5	0.022862314	LLGVETL	99	744.4494	1	743.4429	.	L	Superscaf	52975360	0	0	0	0	BTP20220	0	Tissue specific
KPQHDAK	Superscaf	+	Superscaf	53119264	53119284	6	6	12	-	-	-	-	-	-	NCP	IGR	-	ID=gene-E	7	6.858571429	3227.4	0.020208708	KPQHDAK	50	412.2288	2	822.4348	.	K	Superscaf	53119249	BTP20220	0	0	0	0	0	Tissue specific
VTPLK	Superscaf	-	Superscaf	53198059	53198073	-11	11	15	-	-	-	-	-	-	NCP	IGR	-	ID=gene-E	5	5.71	1556.7	0.018755435	VTPLK	69	557.3646	1	556.3585	.	K	Superscaf	53198334	0	BTP20220	0	0	0	0	Tissue specific
ALGVGL	Superscaf	+	Superscaf	53214007	53214024	12	12	17	-	-	-	-	-	-	NCP	IGR	-	ID=gene-E	6	4.986666667	1612.7	0.018461217	ALGVGL	53	529.333	1	528.3271	.	P	Superscaf	53213974	0	0	0	BTP20220	0	0	Tissue specific
MVFHHK	Superscaf	-	Superscaf	53456005	53456022	-10	10	15	-	-	-	-	-	-	NCP	IGR	-	ID=gene-E	6	6.025	2466.7	0.013997537	M(+42.01)	61	420.7114	2	839.4112	Acetylati	L	Superscaf	53456082	0	BTP20220	0	0	0	0	Tissue specific
NPSKYR	Superscaf	+	Superscaf	53538616	53538633	85	85	90	-	-	-	-	-	-	NCP	IGR	-	ID=gene-E	6	7.258333333	2894.2	0.012473767	N(+42.01)	97	403.7102	2	805.4082	Acetylati	L	Superscaf	53538364	0	0	0	0	BTP20220	0	Tissue specific
LKDYL	Superscaf	+	Superscaf	53585773	53585787	4	4	8	-	-	-	-	-	-	NCP	IGR	-	ID=gene-E	5	6.066	2046.2	0.011604006	LKDYL	94	651.3686	1	650.3639	.	S	Superscaf	53585764	0	BTP20220	0	0	0	0	Tissue specific
MNPVV	Superscaf	+	Superscaf	53613010	53613024	15	15	19	-	-	-	-	-	-	NCP	IGR	-	ID=gene-E	5	3.492	1305.6	0.011101617	M(+42.01)	99	601.3002	1	600.2941	Acetylati	H	Superscaf	53612968	0	0	0	0	BTP20220	0	Mixed
DSLQLRR	Superscaf	+	Superscaf	53738341	53738361	53	53	59	-	-	-	-	-	-	NCP	IGR	-	ID=gene-E	7	6.825714286	3410	0.008789761	DSLQLRR	62	444.2549	2	886.4984	.	L	Superscaf	53738185	0	0	0	0	0	BTP20220	Tissue specific
FLVHNVK	Superscaf	+	Superscaf	53853223	53853243	1	1	7	-	-	-	-	-	-	NCP	IGR	-	ID=gene-E	7	4.885714286	2425.6	0.00667075	FLVHNVK	97	428.7542	2	855.4966	.	F	Superscaf	53853223	0	0	0	0	BTP20220	BTP20220	Tissue specific
APPPPP	Superscaf	-	Superscaf	53949970	53949987	-1	1	6	-	-	-	-	-	-	NCP	IGR	-	ID=gene-E	6	6.253333333	1998.3	0.004886295	APPPPP	99	575.3177	1	574.3115	.	A	Superscaf	53950116	0	0	0	0	BTP20220	0	Tissue specific
[truncated: 14,928,894 more chars]
