# Supplementary material for: Integrated peptidogenomics decoding yak non-conventional peptides: functional mapping and biopotential mining of genetic resources
Source: Anim Biosci. 2025 Sep 30;39(5):250408. doi: 10.5713/ab.25.0408 (PMC13153706; doi:10.5713/ab.25.0408)
Supplement: Supplementary file 4 [file ab-25-0408-Supplement-4.pdf]

name	group	Adjacent
SAASKQNKLEQVEK;STMMSVH	CPs	3.111
STMMSVH;KTVLKSFLSK	CPs	1.317
KTVLKSFLSK;TVEELYL	CPs	14.876
TVEELYL;LPVPVP	CPs	2143.561
LPVPVP;ALHVPKAPGFAQMLK	CPs	3282.372
ALHVPKAPGFAQMLK;KGEENLMDAQVK	CPs	7.587
KGEENLMDAQVK;LSEVGDTQVVVF	CPs	1.032
LSEVGDTQVVVF;DMLEAGVLDTYLGK	CPs	1.431
DMLEAGVLDTYLGK;NAFRVSP	CPs	2337.173
NAFRVSP;NGYENPTYK	CPs	1069.078
NGYENPTYK;FEVVEKPQS	CPs	157.416
FEVVEKPQS;DDDLAAL	CPs	23113.56
DDDLAAL;PPAPVVVTETPEPT	CPs	8265.561
PPAPVVVTETPEPT;MNMVLAH	CPs	2583.915
MNMVLAH;FDPLKNQDE	CPs	574.035
FDPLKNQDE;DTSLPLL	CPs	619.556
DTSLPLL;ADGKAGEEKPEKPQR	CPs	216.913
ADGKAGEEKPEKPQR;SAEDETADLPTKPTK	CPs	5.169
SAEDETADLPTKPTK;AEDETADLPTKPT	CPs	0.003
AEDETADLPTKPT;EDETADLPTKP	CPs	0.003
EDETADLPTKP;ETADLPTKPTK	CPs	0.006
ETADLPTKPTK;SLLLRKLPF	CPs	9822.821
SLLLRKLPF;VGVATAVDGLVTQF	CPs	997.648
VGVATAVDGLVTQF;PESHPK	CPs	1236.564
PESHPK;SVYVYKVL	CPs	6982.794
SVYVYKVL;LVQGHG	CPs	0.048
LVQGHG;ADPDVLTEVPAALK	CPs	444.306
ADPDVLTEVPAALK;AAADALLGSLVTGL	CPs	3702.966
AAADALLGSLVTGL;ASWADLVNAHAVPGSGVVK	CPs	22.095
ASWADLVNAHAVPGSGVVK;LEDKPPAPP	CPs	2143.725
LEDKPPAPP;KPLPSVPEEKKPR	CPs	0.093
KPLPSVPEEKKPR;VEGEPPYLNENPLR	CPs	30.309
VEGEPPYLNENPLR;LGATLRVL	CPs	1540.818
LGATLRVL;LLLPGELAKH	CPs	3259.44
LLLPGELAKH;AEFTRLQNCLA	CPs	5734.098
AEFTRLQNCLA;LEEWQSLAGPPSSKL	CPs	52.686
LEEWQSLAGPPSSKL;AEMAP	CPs	681.222
AEMAP;LDAVP	CPs	244.269
LDAVP;PGFMC	CPs	480.284
PGFMC;PLLPPPPPP	CPs	0.901
PLLPPPPPP;WYFVP	CPs	829.953
WYFVP;VPPPKPATPDFR	CPs	5733.635
VPPPKPATPDFR;PPPKPATPDF	CPs	0.003
PPPKPATPDF;VKPAETPKPVSN	CPs	0.567
VKPAETPKPVSN;AKPAETPKPLSN	CPs	0.072
AKPAETPKPLSN;VVLKGRPCK	CPs	6418.573
VVLKGRPCK;NATNK	CPs	1178.645
NATNK;STPRPKFS	CPs	6607.696
STPRPKFS;HAVSEGTKAVTKYTSSK	CPs	1665.33
HAVSEGTKAVTKYTSSK;ADNEKLDNQRLK	CPs	928.854

ADNEKLDNQRLK;FGRWR	CPs	9693.48
FGRWR;AYSMAKYLRDSG	CPs	1577.109
AYSMAKYLRDSG;LYLGERTYY	CPs	763.65
LYLGERTYY;VGDEVNWY	CPs	9.054
VGDEVNWY;LMGMGNEVDLHSVHF	CPs	0.024
LMGMGNEVDLHSVHF;FHGPK	CPs	128.202
FHGPK;VTGHPGAP	CPs	2800.539
VTGHPGAP;ELKLEKTPSQ	CPs	3712.329
ELKLEKTPSQ;MDVNTALQEVLK	CPs	1321.002
MDVNTALQEVLK;PKKTYK	CPs	1238.294
PKKTYK;PVDFTGY	CPs	1282.378
PVDFTGY;TRDQNGTWEMESNDNFEGYMK	CPs	69.06
TRDQNGTWEMESNDNFEGYMK;SVDLHPTEPWMLASLY	CPs	86.265
SVDLHPTEPWMLASLY;VSTDPEHR	CPs	22.674
VSTDPEHR;ENLFPGLKEAF	CPs	3.759
ENLFPGLKEAF;VVEEWVKETHADLWPAK	CPs	0.033
VVEEWVKETHADLWPAK;PPPAPP	CPs	767.463
PPPAPP;QLERDFSSMKK	CPs	65.898
QLERDFSSMKK;FPWVNK	CPs	1647.792
FPWVNK;QTVPDVN	CPs	3582.492
QTVPDVN;APPAAL	CPs	83.31
APPAAL;TVVDEVR	CPs	2952.362
TVVDEVR;EALSL	CPs	239.958
EALSL;YEDYSK	CPs	1122.015
YEDYSK;RSSWVFVAAK	CPs	1411.18
RSSWVFVAAK;TLVYPP	CPs	487.728
TLVYPP;CCSDC	CPs	141.369
CCSDC;KDVLDEKPAVLK	CPs	890.739
KDVLDEKPAVLK;DVERVLK	CPs	1031.103
DVERVLK;KVLELSGR	CPs	3.285
KVLELSGR;ASVDLEKLR	CPs	152.415
ASVDLEKLR;SGNQSVRLPL	CPs	17.715
SGNQSVRLPL;VVHDGFEGLAK	CPs	3.711
VVHDGFEGLAK;KVVAFSPVTELKK	CPs	5.019
KVVAFSPVTELKK;VVAFSPVTELK	CPs	0.003
VVAFSPVTELK;VSGELEHVTR	CPs	0.513
VSGELEHVTR;MLVDQVPQVPEGW	CPs	920.694
MLVDQVPQVPEGW;QPPLVQLHEGN	CPs	1.608
QPPLVQLHEGN;VLDTSESVALRLKPY	CPs	388.554
VLDTSESVALRLKPY;SSVDAVKY	CPs	1.65
SSVDAVKY;KLQWMGGGTFTGEAL	CPs	14.064
KLQWMGGGTFTGEAL;PSQDVRVAVVQY	CPs	1.449
PSQDVRVAVVQY;SGTGQQRPERAAL	CPs	0.036
SGTGQQRPERAAL;TVLANTVDSMDF	CPs	0.057
TVLANTVDSMDF;FNDATDVMDALGY	CPs	0.036
FNDATDVMDALGY;NDATDVMDALGYVTRF	CPs	0.003
NDATDVMDALGYVTRF;DATDVMDALGY	CPs	0.003
DATDVMDALGY;MDALGYVTRF	CPs	0.015
MDALGYVTRF;FYREASSNAAKK	CPs	0.027
FYREASSNAAKK;AVVVGRQVNEPH	CPs	0.135
AVVVGRQVNEPH;VRVLVTGK	CPs	0.036

VRVLVTGK;AAEYDVVFGERH	CPs	0.024
AAEYDVVFGERH;RVPSYQALLR	CPs	0.042
RVPSYQALLR;ALLRGVFY	CPs	0.018
ALLRGVFY;GVFYQTVSRK	CPs	0.012
GVFYQTVSRK;VFYQTVSRKVALD	CPs	0.003
VFYQTVSRKVALD;VGKGVVNF	CPs	134.274
VGKGVVNF;SSFKEAVK	CPs	9.501
SSFKEAVK;SDLVAEKF	CPs	0.315
SDLVAEKF;LLDGSERLGEQNF	CPs	5.94
LLDGSERLGEQNF;VVAVGSDVDTDVLSK	CPs	0.408
VVAVGSDVDTDVLSK;LTKEGLLL	CPs	400.751
LTKEGLLL;ACPPG	CPs	5867.719
ACPPG;MLTVRPQ	CPs	2223.593
AAVSLLGEEKK;LKKLRP	CPs	1256.408
LKKLRP;VENCPK	CPs	840.453
VENCPK;KYDVVP	CPs	2079.195
KYDVVP;VGSENPPLR	CPs	2517.997
VGSENPPLR;SVAKVLQHAASN	CPs	17.724
SVAKVLQHAASN;EGAVDPNDPDKVNTLNQ	CPs	10.272
EGAVDPNDPDKVNTLNQ;VEDAQLPLEQKKR	CPs	11.322
VEDAQLPLEQKKR;GGPVTEP	CPs	575.613
GGPVTEP;EVCAPPPP	CPs	1778.049
EVCAPPPP;APPDHVVL	CPs	410.883
APPDHVVL;TTEPTNVRETH	CPs	2.991
TTEPTNVRETH;VPFEDTHH	CPs	4.14
VPFEDTHH;YPVEP	CPs	1623.351
YPVEP;MENQVLTPH	CPs	101.181
MENQVLTPH;KLEELELDEQQRK	CPs	880.23
KLEELELDEQQRK;LEELELDEQQR	CPs	0.003
LEELELDEQQR;VGELKDDDFEK	CPs	0.069
VGELKDDDFEK;PPPVVRL	CPs	716.745
PPPVVRL;KGGVLKR	CPs	3508.943
KGGVLKR;LFQPPKY	CPs	1547.715
LFQPPKY;PSPPGAP	CPs	1604.794
PSPPGAP;AKPCGVRL	CPs	196.683
AKPCGVRL;VLERVNAVK	CPs	2.304
VLERVNAVK;FSERGDAVAK	CPs	0.24
FSERGDAVAK;LAAAF	CPs	69.087
LAAAF;ECDDGY	CPs	34.229
ECDDGY;NPALEAF	CPs	385.69
NPALEAF;APGWGAVP	CPs	85.326
APGWGAVP;GPSHKGKK	CPs	166.683
GPSHKGKK;AELEEVTLDGKPLQ	CPs	17.556
AELEEVTLDGKPLQ;VPAKM	CPs	48.449
VPAKM;RAGGGLPR	CPs	134.104
RAGGGLPR;KTVGYP	CPs	2831.181
KTVGYP;KTRKPRP	CPs	933.242
KTRKPRP;GSTLVPT	CPs	31.264
GSTLVPT;ASNVTNKTDPR	CPs	199.563
ASNVTNKTDPR;AAPGAAARP	CPs	101.16
AAPGAAARP;LLAAGP	CPs	2.1

LLAAGP;THSVETPY	CPs	72.972
THSVETPY;SSESGTLSSGPPGHT	CPs	4.575
SSESGTLSSGPPGHT;SGPPGHTME	CPs	0.024
SGPPGHTME;KVGVRNL	CPs	214.193
KVGVRNL;LPPAV	CPs	465.484
LPPAV;KGPLLEEQALTK	CPs	7308.93
KGPLLEEQALTK;DNCPLEHNPDQ	CPs	1153.305
DNCPLEHNPDQ;GPEAG	CPs	684
GPEAG;AAHAAAAPR	CPs	77.058
AAHAAAAPR;AFNADFH	CPs	49.134
AFNADFH;AVPPTRK	CPs	289.311
AVPPTRK;HAGGLPL	CPs	57.774
HAGGLPL;GVGLVVVL	CPs	92.661
GVGLVVVL;EATLGFGH	CPs	361.299
EATLGFGH;NQRPWP	CPs	75.993
NQRPWP;EPVDY	CPs	1083.056
EPVDY;MLGLDAAGK	CPs	4820.119
MLGLDAAGK;FGPGAP	CPs	1969.752
FGPGAP;LDEDELLGNLSEVELK	CPs	61.112
LDEDELLGNLSEVELK;VELLVG	CPs	1.33
VELLVG;TAWLDGKHVVFGK	CPs	917.163
TAWLDGKHVVFGK;LDGKHVVF	CPs	0.009
LDGKHVVF;AWDPLLPQ	CPs	2425.32
AWDPLLPQ;GSVKAYTNF	CPs	1584.246
GSVKAYTNF;LMVALAK	CPs	23.103
LMVALAK;LGDSGVGK	CPs	5245.17
LGDSGVGK;KGEPNR	CPs	4947.005
KGEPNR;LDPEVH	CPs	384.286
LDPEVH;LVLANMLMK	CPs	683.847
LVLANMLMK;ADADKPEVLR	CPs	1110.498
ADADKPEVLR;APYKLN	CPs	940.053
APYKLN;TTEFEPCFD	CPs	1941.951
TTEFEPCFD;KWLSMNVL	CPs	2.127
KWLSMNVL;MLDEKRVMVD	CPs	0.024
MLDEKRVMVD;EDVQPPGLK	CPs	311.358
EDVQPPGLK;AAVQRKAV	CPs	1849.953
AAVQRKAV;NLMALKK	CPs	55.773
NLMALKK;APPMPGGPH	CPs	196.362
APPMPGGPH;MVFPFESGKPF	CPs	94.527
MVFPFESGKPF;SDALAVEDEQVVPVPL	CPs	409.146
SDALAVEDEQVVPVPL;VEDEQVVPVP	CPs	0.015
VEDEQVVPVP;VEPVPVTK	CPs	0.153
VEPVPVTK;VESLTAPSKK	CPs	3.228
VESLTAPSKK;ACWELYCLEH	CPs	1503.288
ACWELYCLEH;CLEHGLQPDGQMPSDK	CPs	0.018
CLEHGLQPDGQMPSDK;EHGLQPDGQMPSD	CPs	0.006
EHGLQPDGQMPSD;FVDWCPTGFK	CPs	0.945
FVDWCPTGFK;LACGFY	CPs	1378.169
LACGFY;EDTSSLFDKL	CPs	2926.114
EDTSSLFDKL;CVVVGDGAVGK	CPs	1607.85
CVVVGDGAVGK;VVVGDGAVGKTC	CPs	0.003

VVVGDGAVGKTC;VVGDGAVGK	CPs	0.003
VVGDGAVGK;VGDGAVGKTC	CPs	0.003
VGDGAVGKTC;LDFPTMKF	CPs	866.424
LDFPTMKF;AGKLARGDLSN	CPs	238.635
AGKLARGDLSN;TPVPGGVGP	CPs	7.983
TPVPGGVGP;TEEVELYGETKAK	CPs	1.053
TEEVELYGETKAK;AHLHQNVF	CPs	0.81
AHLHQNVF;AEAVQRAAAAPSS	CPs	16.932
AEAVQRAAAAPSS;RFEELNA	CPs	81.792
RFEELNA;ELGAP	CPs	2776.071
ELGAP;APPEQLR	CPs	11.898
APPEQLR;MDEEDKAFKQK	CPs	135.849
MDEEDKAFKQK;TGLTDKP	CPs	1319.448
TGLTDKP;DKLGK	CPs	601.992
DKLGK;VRMNVLADALK	CPs	2794.767
VRMNVLADALK;VAPPLQF	CPs	270.216
VAPPLQF;NYEDLPK	CPs	455.124
NYEDLPK;VQRVVLN	CPs	229.179
VQRVVLN;MKGDFVTSF	CPs	8.367
MKGDFVTSF;GPPPVLPPP	CPs	768.627
GPPPVLPPP;LGFMSAFVK	CPs	117.3
LGFMSAFVK;KGWSVK	CPs	1428.741
KGWSVK;PGRPGTPP	CPs	1005.255
PGRPGTPP;LHLGKGP	CPs	83.454
LHLGKGP;VPPHLKP	CPs	28.221
VPPHLKP;KSGVQY	CPs	74.805
KSGVQY;PAGPPALK	CPs	1.692
PAGPPALK;DLVVSLAH	CPs	3902.859
DLVVSLAH;VGAVDADKHQ	CPs	5660.247
VGAVDADKHQ;DNFDKNVLD	CPs	0.27
DNFDKNVLD;VNQVLASRY	CPs	0.168
VNQVLASRY;EAAFAAG	CPs	1409.247
EAAFAAG;KDVLP	CPs	240.626
KDVLP;HAVSEGTKAVTKYT	CPs	1777.48
HAVSEGTKAVTKYT;GTKAVTKYTSSK	CPs	0.015
GTKAVTKYTSSK;VLTPEEQLADKLRLK	CPs	1242.339
VLTPEEQLADKLRLK;VVPGGGLK	CPs	3.759
VVPGGGLK;VGREPLA	CPs	144.828
QSMRPP;EHVKLV	CPs	350.926
EHVKLV;LPAQQEL	CPs	30.267
LPAQQEL;ASRDPPAT	CPs	1132.338
ASRDPPAT;SAPSTGGVKKPH	CPs	646.395
SAPSTGGVKKPH;DFKTDLRF	CPs	0.147
DFKTDLRF;SEAYLV	CPs	0.057
SEAYLV;ARQPPPPPP	CPs	194.219
ARQPPPPPP;EEGARLL	CPs	198.31
EEGARLL;PDPAAHLPF	CPs	256.974
PDPAAHLPF;RLAEQF	CPs	1365.185
RLAEQF;LKEKEELESGKK	CPs	40.471
LKEKEELESGKK;PPSST	CPs	997.38
PPSST;EMGTPDVR	CPs	492.723

EMGTPDVR;LVTYVPVTTFK	CPs	1.26
LVTYVPVTTFK;EADPDK	CPs	1141.598
EADPDK;EGPAP	CPs	183.342
EGPAP;VPPPPATLR	CPs	1875.709
VPPPPATLR;TYFPHF	CPs	909.551
TYFPHF;ASRLVAP	CPs	86.635
ASRLVAP;LGPFVGM	CPs	2.433
LGPFVGM;LLEQVAQLK	CPs	63.222
LLEQVAQLK;EASLLLRV	CPs	296.135
EASLLLRV;DLSVKDKPR	CPs	789.163
DLSVKDKPR;QTLLPRH	CPs	2219.745
QTLLPRH;YVALDF	CPs	6089.646
YVALDF;VSVTRAVFGDLPLGAGTVEKF	CPs	1369.941
VSVTRAVFGDLPLGAGTVEKF;TTQPGVQFY	CPs	50.427
TTQPGVQFY;GFAPYERR	CPs	2516.289
GFAPYERR;PSPMPAL	CPs	997.116
PSPMPAL;SSHVTNVSF	CPs	151.86
SSHVTNVSF;SSTTKTK	CPs	1275.446
SSTTKTK;NAPTRA	CPs	75.091
NAPTRA;LTTPKTE	CPs	398.183
LTTPKTE;KPPPRPAAP	CPs	722.311
KPPPRPAAP;PLAPMASHSTF	CPs	1390.386
PLAPMASHSTF;PDDPMSEGKPR	CPs	108.957
PDDPMSEGKPR;EDYDRLRP	CPs	94.998
EDYDRLRP;ESFPPPP	CPs	566.682
ESFPPPP;TTTVATDY	CPs	7648.455
TTTVATDY;GFDLPAVEAATKK	CPs	102.759
GFDLPAVEAATKK;RLEEASLLHQF	CPs	3.423
RLEEASLLHQF;ELLEPLNERK	CPs	16.32
ELLEPLNERK;AETVHQLSK	CPs	1.926
AETVHQLSK;KDLAE	CPs	1.533
KDLAE;HSEVPVSLK	CPs	16.932
HSEVPVSLK;AGAELRAALE	CPs	621.762
AGAELRAALE;VLDSAFLEGGHEK	CPs	1.923
VLDSAFLEGGHEK;DLCFVHRK	CPs	10.883
DLCFVHRK;NDHFVK	CPs	6078.109
NDHFVK;ASEVARHL	CPs	183.51
ASEVARHL;PAVWPPK	CPs	2379.885
PAVWPPK;GDDGLCGHPRP	CPs	223.524
GDDGLCGHPRP;MNFSGKYQ	CPs	1073.781
MNFSGKYQ;TLGEECEMEF	CPs	2.406
TLGEECEMEF;GDVVFKRVSK	CPs	3.129
GDVVFKRVSK;VDEAADALLK	CPs	778.011
VDEAADALLK;KAVSRDP	CPs	417.876
KAVSRDP;LSVPV	CPs	504.366
LSVPV;QGKTRLVGPN	CPs	860.784
QGKTRLVGPN;EFEKRKML	CPs	17.802
EFEKRKML;AQMELK	CPs	9913.958
AQMELK;DLLKANVK	CPs	1571.902
DLLKANVK;NSGLLDTF	CPs	320.892
NSGLLDTF;SDLLLVMS	CPs	2.124

SDLLLVMS;MDSQGRKVVVCDN	CPs	1253.475
MDSQGRKVVVCDN;AELLFNT	CPs	24.579
AELLFNT;PASSHPQ	CPs	3201.117
PASSHPQ;LFSKMACVLAK	CPs	464.904
LFSKMACVLAK;HYPSTHGL	CPs	1.092
HYPSTHGL;TYTVLPSRVKNLAQ	CPs	0.024
TYTVLPSRVKNLAQ;TVLPSRVK	CPs	0.006
TVLPSRVK;AASGFNAAEDAQTLRK	CPs	862.515
AASGFNAAEDAQTLRK;VLYDVQELRK	CPs	8.688
VLYDVQELRK;MDAGVTESGLN	CPs	271.773
MDAGVTESGLN;ASRPPVTL	CPs	0.276
ASRPPVTL;VPQSVTECVKQ	CPs	0.174
VPQSVTECVKQ;SSRALNEFH	CPs	187.293
SSRALNEFH;RFAVDR	CPs	3430.131
RFAVDR;TLTSFERDAMF	CPs	1253.286
TLTSFERDAMF;FSPVDKMQ	CPs	6.129
FSPVDKMQ;VDEVGVDVAK	CPs	1.683
VDEVGVDVAK;TPCQLLVDHA	CPs	1.929
TPCQLLVDHA;LVDHANNPNKKFYQ	CPs	0.015
LVDHANNPNKKFYQ;VDHANNPNKKF	CPs	0.003
VDHANNPNKKF;HANNPNKKFYQ	CPs	0.006
HANNPNKKFYQ;QGPVLLR	CPs	162.693
QGPVLLR;PAASSLK	CPs	183.558
PAASSLK;AAALENF	CPs	1257.557
AAALENF;VPAELLM	CPs	90.496
VPAELLM;AMQAAKR	CPs	4.227
AMQAAKR;LLKAEPLAF	CPs	2939.805
LLKAEPLAF;AALGHVDS	CPs	283.518
AALGHVDS;VPAAAAAAASR	CPs	34.694
VPAAAAAAASR;VSMTFKR	CPs	561.388
VSMTFKR;YAEATPMLK	CPs	3088.395
YAEATPMLK;DSGYKLY	CPs	926.387
DSGYKLY;KGMHVP	CPs	2320.666
KGMHVP;KPFLPQP	CPs	1030.746
KPFLPQP;VGNQLPF	CPs	287.946
VGNQLPF;DLTGLSV	CPs	58.313
DLTGLSV;LSDVELDDLEKDEL	CPs	251.242
LSDVELDDLEKDEL;LDTLNEDSYK	CPs	1008.927
LDTLNEDSYK;MDDKASVGK	CPs	6287.028
MDDKASVGK;KCFFDR	CPs	281.054
KCFFDR;VGTFT	CPs	1410.423
VGTFT;ARNTLSSR	CPs	56.173
ARNTLSSR;LNVGL	CPs	186.557
LNVGL;RSSSSRLLR	CPs	2725.609
RSSSSRLLR;TVRVVNTDY	CPs	2.466
TVRVVNTDY;ETVHELL	CPs	146.792
ETVHELL;AVPVP	CPs	132.1
AVPVP;DLAALEDK	CPs	60.924
DLAALEDK;ALLDRHQEH	CPs	1.068
ALLDRHQEH;LLNEDLGDSLDSVEALLKK	CPs	3.321
LLNEDLGDSLDSVEALLKK;TVEVPL	CPs	1.106

TVEVPL;AARMPSR	CPs	19.372
AARMPSR;KLLVSSEDY	CPs	3.855
KLLVSSEDY;KHEAFETDF	CPs	2.52
KHEAFETDF;VEDLFLTFAKK	CPs	3.681
VEDLFLTFAKK;DYVEFTRSLFVN	CPs	5.328
DYVEFTRSLFVN;ADRLGGL	CPs	11.238
ADRLGGL;SAPAAKVSKKEL	CPs	30.681
SAPAAKVSKKEL;NPRLMGS	CPs	1073.958
NPRLMGS;HGPKY	CPs	37.475
HGPKY;SGNFK	CPs	449.091
SGNFK;EYHRLQNKVT	CPs	128.668
EYHRLQNKVT;AELVQGQSAPVGM	CPs	60.042
AELVQGQSAPVGM;ARVPN	CPs	254.142
ARVPN;STLEGRPSAY	CPs	104.079
STLEGRPSAY;KPVGPP	CPs	76.605
KPVGPP;APGVPDVK	CPs	62.808
APGVPDVK;AAGPVTR	CPs	215.798
AAGPVTR;LPPPPPRPPN	CPs	716.497
LPPPPPRPPN;MMNEVKESLR	CPs	599.124
MMNEVKESLR;RGVPFPP	CPs	24.543
RGVPFPP;SSLPPKSR	CPs	359.234
SSLPPKSR;GHSGPAPK	CPs	46.9
GHSGPAPK;KAKGKKVAPAPAVVK	CPs	880.335
KAKGKKVAPAPAVVK;KVAPAPAVVKK	CPs	0.015
KVAPAPAVVKK;VAPAPAVVK	CPs	0.003
VAPAPAVVK;PAPAVVKK	CPs	0.006
PAPAVVKK;ESRLLQ	CPs	72.995
ESRLLQ;PAAKPPR	CPs	691.669
PAAKPPR;KPQVELR	CPs	699.519
KPQVELR;VPPSP	CPs	487.242
VPPSP;HGDSDLQLER	CPs	45.555
HGDSDLQLER;GKYVPR	CPs	0.138
GKYVPR;TFSVVPSPK	CPs	0.855
TFSVVPSPK;FSVVPSPKV	CPs	0.003
FSVVPSPKV;VPSPKVSD	CPs	0.009
VPSPKVSD;VPFPRLH	CPs	0.264
VPFPRLH;MPGFAPLTS	CPs	0.027
MPGFAPLTS;LTSRGSQQY	CPs	0.018
GPARAGPP;KGGGPP	CPs	384.589
KGGGPP;DLLRPP	CPs	3518.607
DLLRPP;PVSPKDP	CPs	6228.234
PVSPKDP;YEEHLERMK	CPs	136.803
YEEHLERMK;LNAFAKQ	CPs	833.76
LNAFAKQ;RVERPK	CPs	578.237
RVERPK;LVPVG	CPs	426.454
LVPVG;ASDTHCGYGDSAAKGTTPY	CPs	1837.14
ASDTHCGYGDSAAKGTTPY;YGDSAAKGTTPYVQAF	CPs	0.021
YGDSAAKGTTPYVQAF;ADVVLP	CPs	1152.894
ADVVLP;LLKAA	CPs	262.973
LLKAA;LLNNPELP	CPs	1192.225
LLNNPELP;APGVPPP	CPs	5332.821

APGVPPP;EPVGAPLP	CPs	92.106
EPVGAPLP;LGLASALRPDGEY	CPs	899.628
LGLASALRPDGEY;STTGGETEETLKK	CPs	14.637
STTGGETEETLKK;VAAEVLK	CPs	3796.458
VAAEVLK;LLRGLPGSGK	CPs	467.796
LLRGLPGSGK;KVDQPPEAKKPK	CPs	1283.547
KVDQPPEAKKPK;GLPRPGAP	CPs	377.552
GLPRPGAP;SAKEKGKFEDMAK	CPs	145.063
SAKEKGKFEDMAK;KDPNAPKRPP	CPs	0.111
KDPNAPKRPP;LGAAERLR	CPs	1729.854
LGAAERLR;AAERLRFTEL	CPs	0.006
AAERLRFTEL;ADLLGEQP	CPs	0.195
ADLLGEQP;LFSHVLP	CPs	225.222
LFSHVLP;AAPKTVLP	CPs	183.072
AAPKTVLP;RLAQLKHQLPQ	CPs	3492.882
RLAQLKHQLPQ;QVTHLSP	CPs	168.513
QVTHLSP;QKATVPR	CPs	310.341
QKATVPR;LVLLDPF	CPs	3029.262
LVLLDPF;RQHEEPESFF	CPs	1470.306
RQHEEPESFF;AAVTV	CPs	7694.025
AAVTV;VFGQSGAGNNWAK	CPs	1637.697
VFGQSGAGNNWAK;SCDCLQGF	CPs	0.105
SCDCLQGF;VVPSPKVS	CPs	0.129
VVPSPKVS;SVHQLVE	CPs	0.057
SVHQLVE;MQDHNKDDM	CPs	671.478
MQDHNKDDM;TTELDDFPTNK	CPs	47.499
TTELDDFPTNK;KEPAPSGAELER	CPs	2.664
KEPAPSGAELER;NMCSGP	CPs	1379.255
NMCSGP;STPASTSTTTPVKK	CPs	459.409
STPASTSTTTPVKK;PEPAKSAPAPKKGSKKAVTK	CPs	2065.176
PEPAKSAPAPKKGSKKAVTK;AAYKLV	CPs	545.409
AAYKLV;AMEAVAAQGKAKK	CPs	0.72
AMEAVAAQGKAKK;EHSGPLR	CPs	93.555
EHSGPLR;DSTVCLADADKK	CPs	747.255
DSTVCLADADKK;AAGHDCFPV	CPs	0.111
AAGHDCFPV;SLESALKDLK	CPs	0.303
SLESALKDLK;HYQCC	CPs	1130.727
HYQCC;AVPPLP	CPs	10468.43
AVPPLP;SSHVD	CPs	609.951
SSHVD;VRVVPP	CPs	3341.236
VRVVPP;KHKPL	CPs	333.177
KHKPL;LGGKPT	CPs	247.664
LGGKPT;GVNADVEKHLELGKK	CPs	7515.751
GVNADVEKHLELGKK;SNPSENEEKEAQSQLVK	CPs	22.596
SNPSENEEKEAQSQLVK;ATRSTPP	CPs	1263.456
ATRSTPP;APPGGP	CPs	333.549
APPGGP;VMPPPFP	CPs	10.164
VMPPPFP;MREYKVVVL	CPs	92.508
MREYKVVVL;KVVVLGSGGVGK	CPs	0.012
KVVVLGSGGVGK;AAAAAEQQQF	CPs	405.123
AAAAAEQQQF;VLDYVKEGGHKK	CPs	368.487

VLDYVKEGGHKK;FYLPGLAPVNF	CPs	947.502
FYLPGLAPVNF;SSAGTVEFLVDSKK	CPs	624.624
SSAGTVEFLVDSKK;LVDSKKNF	CPs	0.024
LVDSKKNF;ATAATEEPFPF	CPs	1889.376
ATAATEEPFPF;YVLFPL	CPs	118.137
YVLFPL;AVPPG	CPs	308.241
AVPPG;YELAAPP	CPs	3485.016
YELAAPP;DAKGDKAKVKDEPQR	CPs	277.35
DAKGDKAKVKDEPQR;ARLSAKPAPPKPEPKPK	CPs	0.051
ARLSAKPAPPKPEPKPK;APNGVKK	CPs	512.909
APNGVKK;AAAVAQRP	CPs	1117.414
AAAVAQRP;AVPRPPR	CPs	469.857
AVPRPPR;PVGMNKLWSGY	CPs	32.271
PVGMNKLWSGY;KPMKAR	CPs	391.127
KPMKAR;TAVSVPK	CPs	358.525
KTGPDHL;TVTAMDV	CPs	4602.057
TVTAMDV;AMDVVYALK	CPs	0.009
AMDVVYALK;DLSGELDL	CPs	3736.296
DLSGELDL;ALALKHANTKPFE	CPs	1087.998
ALALKHANTKPFE;LPWAP	CPs	5797.908
LPWAP;LAVVHVP	CPs	744.336
LAVVHVP;TAPVLHV	CPs	149.411
TAPVLHV;GSFVEKTVMPY	CPs	1749.904
GSFVEKTVMPY;STDAGFHF	CPs	2.265
STDAGFHF;FEKEKMNAK	CPs	8.925
FEKEKMNAK;SAVTTVVNPK	CPs	5.799
SAVTTVVNPK;VTTVVNPKYE	CPs	0.006
VTTVVNPKYE;VVNPKYEGK	CPs	0.009
VVNPKYEGK;CQLYGY	CPs	2176.355
CQLYGY;KLTSDDVKEQ	CPs	386.455
KLTSDDVKEQ;GPPLVEKQ	CPs	2788.818
GPPLVEKQ;LSTLGPPP	CPs	153.944
LSTLGPPP;SQLLQPL	CPs	464.407
SQLLQPL;MFGGPGTASRPS	CPs	5714.601
MFGGPGTASRPS;FGGPGTASRP	CPs	0.003
FGGPGTASRP;GGPGTASRPSST	CPs	0.003
GGPGTASRPSST;GPGTASRP	CPs	0.003
GPGTASRP;PSSTRSYVTTSTR	CPs	0.021
PSSTRSYVTTSTR;SYVTTSTRTYSLGSALRPTTSR	CPs	0.015
SYVTTSTRTYSLGSALRPTTSR;YVTTSTRT	CPs	0.003
YVTTSTRT;VTTSTRTYS	CPs	0.003
VTTSTRTYS;TTSTRTYSLG	CPs	0.003
TTSTRTYSLG;TYSLGSALRPT	CPs	0.015
TYSLGSALRPT;YSLGSALRPTT	CPs	0.003
YSLGSALRPTT;SLGSALRPT	CPs	0.003
SLGSALRPT;LGSALRPTTS	CPs	0.003
LGSALRPTTS;GSALRPTTSR	CPs	0.003
GSALRPTTSR;SALRPTTSRTL	CPs	0.003
SALRPTTSRTL;ALRPTTSRTLY	CPs	0.003
ALRPTTSRTLY;LRPTTSRTLYT	CPs	0.003
LRPTTSRTLYT;TTSRTLY	CPs	0.009

TTSRTLY;YTSSPGGVY	CPs	0.018
YTSSPGGVY;TSSPGGVYA	CPs	0.003
TSSPGGVYA;SSPGGVYATRSSAVRL	CPs	0.003
SSPGGVYATRSSAVRL;SPGGVYA	CPs	0.003
SPGGVYA;PGGVYATR	CPs	0.003
PGGVYATR;ATRSSAVRLR	CPs	0.015
ATRSSAVRLR;RLRSGVPGVR	CPs	0.021
RLRSGVPGVR;SGVPGVRLLQ	CPs	0.009
SGVPGVRLLQ;VPGVRLLQDS	CPs	0.006
VPGVRLLQDS;PGVRLLQDSVD	CPs	0.003
PGVRLLQDSVD;GVRLLQDS	CPs	0.003
GVRLLQDS;RLLQDSVDF	CPs	0.006
RLLQDSVDF;TNEKVEL	CPs	0.069
TNEKVEL;DVSKPDL	CPs	3.765
DVSKPDL;EAEEWYK	CPs	0.081
EAEEWYK;AEEWYKSK	CPs	0.003
AEEWYKSK;MREMEENF	CPs	1.203
MREMEENF;ETNLDSLPLVDTHSKR	CPs	1.182
ETNLDSLPLVDTHSKR;LDSLPLVDTHSKRT	CPs	0.009
LDSLPLVDTHSKRT;DSLPLVDTHSKR	CPs	0.003
DSLPLVDTHSKR;SLPLVDTHSK	CPs	0.003
SLPLVDTHSK;LPLVDTHS	CPs	0.003
LPLVDTHS;PLVDTHSKR	CPs	0.003
PLVDTHSKR;DLRLLDH	CPs	2932.494
DLRLLDH;CMCCYY	CPs	2103.327
CMCCYY;MADGVNK	CPs	1006.719
MADGVNK;ASGVQVADEVCR	CPs	256.986
ASGVQVADEVCR;KHFVGMLPEKDCR	CPs	0.201
KHFVGMLPEKDCR;VGMLPEKDCRY	CPs	0.009
VGMLPEKDCRY;ALYDASFETK	CPs	0.033
ALYDASFETK;YDASFETKESRKEEL	CPs	0.006
YDASFETKESRKEEL;GPEDLNRAC	CPs	4.503
GPEDLNRAC;PGVLPHL	CPs	8.316
PGVLPHL;MDHMSPRLR	CPs	86.613
MDHMSPRLR;KLAVSSAC	CPs	520.215
KLAVSSAC;HSAAA	CPs	1608.336
HSAAA;VVPSRFP	CPs	1067.43
VVPSRFP;WDSKKVTR	CPs	864.387
WDSKKVTR;AKPLTDGERR	CPs	631.476
AKPLTDGERR;LTDLSQLKK	CPs	23.658
LTDLSQLKK;VLHMDRNPY	CPs	136.44
VLHMDRNPY;MLLFTEVTRY	CPs	5.628
MLLFTEVTRY;LLFTEVTRYLDF	CPs	0.003
LLFTEVTRYLDF;TGHALALY	CPs	6.021
TGHALALY;RMMGSEFDFEEMKRKK	CPs	6.03
RMMGSEFDFEEMKRKK;ALGSQRVKEW	CPs	427.866
ALGSQRVKEW;KNLQLDYV	CPs	17.892
KNLQLDYV;KLLDFCKS	CPs	4.035
KLLDFCKS;YGALGSQR	CPs	0.045
YGALGSQR;LLEDPVL	CPs	0.906
LLEDPVL;YAGLGSQRVKE	CPs	64.284

YAGLGSQRVKE;AGLGSQRVK	CPs	0.003
AGLGSQRVK;GLGSQRVKEW	CPs	0.003
GLGSQRVKEW;KQTPALVAL	CPs	296.691
KQTPALVAL;LKYKPVC	CPs	27.939
LKYKPVC;MFKKFDEK	CPs	86.691
MFKKFDEK;LNDGLWHMKT	CPs	0.507
LNDGLWHMKT;LLPFPVNY	CPs	1216.986
LLPFPVNY;VAPGKM	CPs	15.932
VAPGKM;LDLFANVVHVK	CPs	7414.924
LDLFANVVHVK;TVGKSSKMLQ	CPs	151.278
TVGKSSKMLQ;RPPMGPP	CPs	5.172
RPPMGPP;LGSDALL	CPs	52.788
LGSDALL;VSSPVVR	CPs	272.909
VSSPVVR;QVATASK	CPs	850.486
QVATASK;LGLDNAGK	CPs	215.439
LGLDNAGK;PVGRVET	CPs	109.653
PVGRVET;DQRWLLT	CPs	0.873
DQRWLLT;DVAPP	CPs	172.974
DVAPP;AGTEHKP	CPs	135.713
AGTEHKP;VDTLEPSPR	CPs	24.031
VDTLEPSPR;VLVVPEEAY	CPs	430.155
VLVVPEEAY;LSLPSPP	CPs	1692.38
LSLPSPP;KLASQKTL	CPs	73.078
KLASQKTL;SHSLLLLV	CPs	898.793
SHSLLLLV;KDLFVAK	CPs	88.737
KDLFVAK;QRDLGLLP	CPs	238.345
QRDLGLLP;TGERPF	CPs	754.755
TGERPF;PQHRRK	CPs	810.09
PQHRRK;RPVTRPP	CPs	85.053
RPVTRPP;PHKCYT	CPs	12.855
PHKCYT;SLGPAAPP	CPs	382.311
SLGPAAPP;HLVSPEALDFLDK	CPs	605.607
HLVSPEALDFLDK;PGVTAPP	CPs	20.709
PGVTAPP;LPGLPPAD	CPs	15.3
LPGLPPAD;MNPQCARCGK	CPs	73.874
MNPQCARCGK;SYEESNPKDPAAVTETR	CPs	468.883
SYEESNPKDPAAVTETR;VPTLPQN	CPs	34.626
VPTLPQN;KNDLDLR	CPs	981.71
KNDLDLR;EAAGVRGLR	CPs	67.63
EAAGVRGLR;APKVYQ	CPs	152.205
APKVYQ;YARDETEFYLGK	CPs	224.46
YARDETEFYLGK;RDETEFY	CPs	0.006
RDETEFY;LQGSQRL	CPs	563.031
LQGSQRL;AEVEETLKRLQ	CPs	692.19
AEVEETLKRLQ;EAAPLLP	CPs	207.12
EAAPLLP;FGDEPVTK	CPs	122.202
FGDEPVTK;SNSEN	CPs	23.354
SNSEN;KNSPGP	CPs	480.315
KNSPGP;MEALGKLK	CPs	43.915
MEALGKLK;PPPPPLAR	CPs	52.68
PPPPPLAR;TTETGPDSEVKK	CPs	469.353

TTETGPDSEVKK;KPSPPPPPP	CPs	45.936
KPSPPPPPP;PGAHQLH	CPs	308.976
PGAHQLH;LTMFGEKL	CPs	0.03
LTMFGEKL;KGNFNYVEF	CPs	1.143
KGNFNYVEF;CCRGAAP	CPs	186.714
CCRGAAP;LTPTHYLTK	CPs	306.492
LTPTHYLTK;YHAVAAL	CPs	10.143
YHAVAAL;QAFLEDY	CPs	191.085
QAFLEDY;MDVGELL	CPs	255.717
MDVGELL;LLFGADVHYS	CPs	601.326
LLFGADVHYS;MLDVMFK	CPs	31.995
MLDVMFK;TSLFQEVGLKNCSS	CPs	216.447
TSLFQEVGLKNCSS;VVEFDFLGK	CPs	2311.254
VVEFDFLGK;GHKLQPP	CPs	2341.665
GHKLQPP;ESADVF	CPs	42.176
ESADVF;GDTQEKV	CPs	352.519
GDTQEKV;MALVSPVL	CPs	631.73
MALVSPVL;RPPAGPLK	CPs	77.794
RPPAGPLK;TMDKSELVQKAK	CPs	80.835
TMDKSELVQKAK;MDKSELVQKAKL	CPs	0.003
MDKSELVQKAKL;LKMKGDY	CPs	2.55
LKMKGDY;LDEDSLTKQPEEVFDVLEK	CPs	61.956
LDEDSLTKQPEEVFDVLEK;CAMMNP	CPs	247.632
CAMMNP;AALEVF	CPs	891.615
AALEVF;GKELLDS	CPs	277.659
GKELLDS;AAALVVVL	CPs	199.173
AAALVVVL;KRLAAAPA	CPs	346.827
KRLAAAPA;LFERLTLKHD	CPs	1943.919
LFERLTLKHD;MELSDANLQTLTEY	CPs	16.569
MELSDANLQTLTEY;KNEFLLF	CPs	133.829
KNEFLLF;QPKKAAP	CPs	378.724
QPKKAAP;GVPLAL	CPs	269.373
GVPLAL;TQHKVLEL	CPs	219.555
TQHKVLEL;APGGAPRP	CPs	1644.054
APGGAPRP;KLQDGY	CPs	1561.736
GPKPPPPPP;AGALLP	CPs	340.425
AGALLP;PMKLLQ	CPs	65.384
PMKLLQ;LLLWSQRMVEGY	CPs	42.403
LLLWSQRMVEGY;SDVREAEEQLRK	CPs	4.194
SDVREAEEQLRK;EEVAVDAQQQK	CPs	5.676
EEVAVDAQQQK;LQAEEVAQQK	CPs	0.723
LQAEEVAQQK;EAELLQLK	CPs	2.457
EAELLQLK;VGPELHHKL	CPs	0.972
VGPELHHKL;LLSAERAVTGY	CPs	0.024
LLSAERAVTGY;LLSAEKAVTGYKDPY	CPs	0.984
LLSAEKAVTGYKDPY;LLPLSDARKL	CPs	2.337
LLPLSDARKL;VGPEFKDKL	CPs	0.366
VGPEFKDKL;MSAAQALKK	CPs	1.116
MSAAQALKK;KLRDVSAY	CPs	0.168
KLRDVSAY;FDATGSGF	CPs	0.246
FDATGSGF;AAPELVPR	CPs	80.787

AAPELVPR;LPPLTPEQQEALQK	CPs	19.386
LPPLTPEQQEALQK;DQERFDNSDLSA	CPs	5.058
DQERFDNSDLSA;LAGAAVL	CPs	18.876
LAGAAVL;PRATPPP	CPs	31.911
PRATPPP;GKFMP	CPs	0.969
GKFMP;RQVEPPSRK	CPs	88.02
RQVEPPSRK;KAKKPALVAK	CPs	0.147
KAKKPALVAK;ALAFPRAF	CPs	3.261
ALAFPRAF;SLLVTL	CPs	167.468
SLLVTL;TVAYLQK	CPs	515.241
TVAYLQK;SRELQDVDLAEVKPLVEK	CPs	6226.846
SRELQDVDLAEVKPLVEK;QGSRQVK	CPs	346.626
QGSRQVK;RLFKYL	CPs	19.481
RLFKYL;KPGELPP	CPs	2135.746
KPGELPP;LPPKHTLP	CPs	3279.381
LPPKHTLP;VVRKPLN	CPs	1678.998
VVRKPLN;RLKPGM	CPs	201.531
RLKPGM;YGPPGTGK	CPs	1704.945
YGPPGTGK;SSDLEAKVK	CPs	2680.415
SSDLEAKVK;LDVRKVST	CPs	289.528
LDVRKVST;MFMSSFN	CPs	34.826
MFMSSFN;PLLLH	CPs	2844.441
PLLLH;LEEANLMK	CPs	875.659
LEEANLMK;SLEDLKVDK	CPs	1528.323
SLEDLKVDK;VGVDQFLVK	CPs	1416.21
VGVDQFLVK;RPGALAL	CPs	0.519
RPGALAL;AYAYLFK	CPs	8.358
AYAYLFK;LVGAAK	CPs	294.81
LVGAAK;KAAPFMP	CPs	4447.761
KAAPFMP;PAARLPT	CPs	237.498
PAARLPT;MMMEP	CPs	967.653
MMMEP;RHGFGK	CPs	4343.895
RHGFGK;DTLHLSQC	CPs	3630.545
DTLHLSQC;VEEHWRR	CPs	4953.376
VEEHWRR;KPVVEC	CPs	9625.706
KPVVEC;VGFPSVGK	CPs	2335.549
VGFPSVGK;AAKVGVN	CPs	4645.998
AAKVGVN;SDFEAVEAL	CPs	165.948
SDFEAVEAL;VVCLLR	CPs	339.176
VVCLLR;LAEQAERYDDMAACMK	CPs	1285.054
LAEQAERYDDMAACMK;ALNFSVF	CPs	24.891
ALNFSVF;VDEAVAVLQAHQA	CPs	222.864
VDEAVAVLQAHQA;KDVDPF	CPs	670.938
KDVDPF;MEQPPAPK	CPs	1164.099
MEQPPAPK;QAKPPR	CPs	389.876
QAKPPR;DDEVPDLVENFDEASK	CPs	831.16
DDEVPDLVENFDEASK;VDSAMELL	CPs	3082.542
VDSAMELL;MADVEL	CPs	158.241
MADVEL;RPPPEHF	CPs	610.695
RPPPEHF;PGGPLP	CPs	3287.769
PGGPLP;VPAAATPP	CPs	2330.849

VPAAATPP;GQQRCR	CPs	142.026
GQQRCR;LDELDSL	CPs	964.51
LDELDSL;REASLLR	CPs	111.347
REASLLR;DLEKRKEPKPR	CPs	3950.236
DLEKRKEPKPR;SDALTGVVK	CPs	72.996
SDALTGVVK;VDDFDAFKK	CPs	30.96
AGELPDVPR;VPAGEPK	CPs	2187.103
VPAGEPK;DLMESLN	CPs	966.291
DLMESLN;CGLVLNG	CPs	9288.048
CGLVLNG;LGSLSSLPATK	CPs	2108.424
LGSLSSLPATK;YLVMNP	CPs	626.24
YLVMNP;NMTVRVPDAVSD	CPs	2129.137
NMTVRVPDAVSD;AAGRRTPP	CPs	1548.72
AAGRRTPP;PEPAKSAPAPKKGSK	CPs	2568.798
PEPAKSAPAPKKGSK;RLLLPGELAKHAVSEGTKAVTKYT	CPs	0.294
RLLLPGELAKHAVSEGTKAVTKYTSSK;LPGELAKHAV	CPs	0.009
LPGELAKHAV;SEGTKAVTK	CPs	0.03
SEGTKAVTK;AAGESMAQR	CPs	350.361
AAGESMAQR;ANKGPSYGMSRE	CPs	2944.503
ANKGPSYGMSRE;KGPSYGMSREVQSK	CPs	0.006
KGPSYGMSREVQSK;EKKYDEELEERLVE	CPs	0.045
EKKYDEELEERLVE;CGPDVGRPDR	CPs	0.057
CGPDVGRPDR;YPDGSKPVK	CPs	0.21
YPDGSKPVK;DGSKPVKVPEN	CPs	0.006
DGSKPVKVPEN;KPVKVPENPP	CPs	0.009
KPVKVPENPP;KQMEQVAQFLKAAEDYGVTK	CPs	0.042
KQMEQVAQFLKAAEDYGVTK;QMEQVAQFLK	CPs	0.003
QMEQVAQFLK;VAQFLKAAEDYGVTK	CPs	0.012
VAQFLKAAEDYGVTK;FLKAAEDYGVTKTDMF	CPs	0.009
FLKAAEDYGVTKTDMF;LKAAEDYGVTK	CPs	0.003
LKAAEDYGVTK;KAAEDYGVTKTDM	CPs	0.003
KAAEDYGVTKTDM;AAEDYGVTK	CPs	0.003
AAEDYGVTK;AEDYGVTKTDM	CPs	0.003
AEDYGVTKTDM;GVTKTDMFQ	CPs	0.012
GVTKTDMFQ;TDMFQTVDLF	CPs	0.012
TDMFQTVDLF;TLMALGSLAVTKN	CPs	0.336
TLMALGSLAVTKN;MALGSLAVTK	CPs	0.006
MALGSLAVTK;ALGSLAVTKN	CPs	0.003
ALGSLAVTKN;DGHYRGDPNWFMK	CPs	0.03
DGHYRGDPNWFMK;GHYRGDPNW	CPs	0.003
GHYRGDPNW;YRGDPNWFMK	CPs	0.006
YRGDPNWFMK;AQEHKREFTESQLQ	CPs	0.375
AQEHKREFTESQLQ;EHKREFTESQLQEGK	CPs	0.006
EHKREFTESQLQEGK;KREFTESQLQEGKH	CPs	0.006
KREFTESQLQEGKH;REFTESQLQEGK	CPs	0.003
REFTESQLQEGK;EFTESQLQEGKH	CPs	0.003
EFTESQLQEGKH;FTESQLQEGKHV	CPs	0.003
FTESQLQEGKHV;TESQLQEGKH	CPs	0.003
TESQLQEGKH;ESQLQEGKHV	CPs	0.003
ESQLQEGKHV;GMTGYGRPR	CPs	0.072
GMTGYGRPR;MTGYGRP	CPs	0.003

MTGYGRP;TGYGRPRQ	CPs	0.003
TGYGRPRQ;VSEGTKAVTKY	CPs	619.512
VSEGTKAVTKY;APPLHPK	CPs	274.539
APPLHPK;KLVTSF	CPs	366.425
KLVTSF;VPSWEFS	CPs	0.834
VPSWEFS;TPPHLKP	CPs	29.53
TPPHLKP;VLLAAAVCTK	CPs	65.841
VLLAAAVCTK;VYQPMEK	CPs	0.165
VYQPMEK;AKGKEVDNFVDKLK	CPs	1.971
AKGKEVDNFVDKLK;GKEVDNFVDK	CPs	0.006
GKEVDNFVDK;KEVDNFVDKLK	CPs	0.003
KEVDNFVDKLK;EVDNFVDKL	CPs	0.003
EVDNFVDKL;VNSDVGVLK	CPs	8.478
VNSDVGVLK;MGVPARP	CPs	250.782
MGVPARP;LDRLLAGP	CPs	97.271
LDRLLAGP;LGEHAYPF	CPs	3.309
LGEHAYPF;VPAKVPSA	CPs	57.36
VPAKVPSA;DAVVFHPKFVGK	CPs	14.941
DAVVFHPKFVGK;PAAPPPPPP	CPs	116.286
PAAPPPPPP;PPYDPR	CPs	136.559
PPYDPR;KHYVP	CPs	15.57
KHYVP;YAMLAK	CPs	1676.773
YAMLAK;YVDTAVRHVLLR	CPs	1986.825
YVDTAVRHVLLR;LSVSKASL	CPs	1216.782
LSVSKASL;PVPDRSF	CPs	200.325
PVPDRSF;YGCWLK	CPs	79.481
YGCWLK;KQFGVGF	CPs	56.176
KQFGVGF;THAVLVALK	CPs	3093.609
THAVLVALK;LEGLEERPQRK	CPs	9.528
LEGLEERPQRK;TAAENVCY	CPs	8.835
TAAENVCY;SSSPAVKY	CPs	9.342
SSSPAVKY;LEEEKLSQK	CPs	13.776
LEEEKLSQK;EAADPLASKLNK	CPs	0.33
EAADPLASKLNK;VHVNQY	CPs	2.421
VHVNQY;LVEKPSPLT	CPs	1.053
LVEKPSPLT;AAAGWRDGSGQEK	CPs	106.419
AAAGWRDGSGQEK;LVVVGGGGVGK	CPs	0.045
LVVVGGGGVGK;KPQGKT	CPs	3537.036
KPQGKT;ALDEM	CPs	0.042
ALDEM;KEELLKLT	CPs	5.568
KEELLKLT;PGPVESELGKP	CPs	169.125
PGPVESELGKP;PGAGLAHPP	CPs	174.045
PGAGLAHPP;ALFGVKK	CPs	708.674
ALFGVKK;AGPAASSQN	CPs	113.551
AGPAASSQN;VLDGPF	CPs	1102.082
VLDGPF;FHDSDLAEF	CPs	2460.787
FHDSDLAEF;ALPYVHTV	CPs	0.39
ALPYVHTV;LMLLEV	CPs	122.24
LMLLEV;RAPELGHGVEGGN	CPs	36.184
RAPELGHGVEGGN;YKDVEELSKTKDLPQAQR	CPs	8.919
YKDVEELSKTKDLPQAQR;KDVEELSKTKDLPQAQ	CPs	0.003

KDVEELSKTKDLPQAQ;TGPGLLSM	CPs	606.987
TGPGLLSM;KASPLLH	CPs	674.936
KASPLLH;PDRPPR	CPs	102.901
PDRPPR;LVVYPWTQRF	CPs	239.397
LVVYPWTQRF;VVYPWTQ	CPs	0.003
VVYPWTQ;VYPWTQR	CPs	0.003
VYPWTQR;MLTAEEKAA	CPs	10.359
MLTAEEKAA;LTAEEKAAVTAF	CPs	0.003
LTAEEKAAVTAF;TAEEKAAVTAFWGKVK	CPs	0.003
TAEEKAAVTAFWGKVK;AEEKAAVTAF	CPs	0.003
AEEKAAVTAF;AFWGKVK	CPs	0.024
AFWGKVK;WGKVKVDE	CPs	0.006
WGKVKVDE;FESFGDLSSADAVMNNPKVK	CPs	0.207
FESFGDLSSADAVMNNPKVK;ESFGDLSSADAVMNNPK	CPs	0.003
ESFGDLSSADAVMNNPK;GDLSSADAVMNNPKVK	CPs	0.009
GDLSSADAVMNNPKVK;NNPKVKAHGKKVLDS	CPs	0.03
NNPKVKAHGKKVLDS;AHGKKVLDSF	CPs	0.018
AHGKKVLDSF;KVLDSFSNGMK	CPs	0.012
KVLDSFSNGMK;LDSFSNGMKHLDDL	CPs	0.006
LDSFSNGMKHLDDL;SNGMKHLDDLKGTFA	CPs	0.012
SNGMKHLDDLKGTFA;HLDDLKGTF	CPs	0.015
HLDDLKGTF;LDDLKGTFA	CPs	0.003
LDDLKGTFA;KGTFAALSELHCDKL	CPs	0.012
KGTFAALSELHCDKL;HCDKLHVDPENFK	CPs	0.03
HCDKLHVDPENFK;HVDPENF	CPs	0.015
HVDPENF;VDPENFK	CPs	0.003
VDPENFK;ARHFGKEFTPVL	CPs	0.948
ARHFGKEFTPVL;FGKEFTPVLQADF	CPs	0.009
FGKEFTPVLQADF;KEFTPVLQ	CPs	0.006
KEFTPVLQ;TPVLQADFQK	CPs	0.009
TPVLQADFQK;QADFQKVVNGVANALAHRYH	CPs	0.012
QADFQKVVNGVANALAHRYH;ADFQKVVNG	CPs	0.003
ADFQKVVNG;QKVVNGVANALAHRYH	CPs	0.009
QKVVNGVANALAHRYH;KVVNGVANALAH	CPs	0.003
KVVNGVANALAH;VVNGVANALAHRYH	CPs	0.003
VVNGVANALAHRYH;VNGVANALAH	CPs	0.003
VNGVANALAH;NGVANALAHRYH	CPs	0.003
NGVANALAHRYH;GVANALAHR	CPs	0.003
GVANALAHR;VANALAHRYH	CPs	0.003
VANALAHRYH;ANALAHRY	CPs	0.003
ANALAHRY;NALAHRYH	CPs	0.003
NALAHRYH;SELHCDKL	CPs	15.198
SELHCDKL;ELHCDKLHVDPEN	CPs	33.939
ELHCDKLHVDPEN;HCDKLHVDPENF	CPs	0.006
HCDKLHVDPENF;CDKLHVDPEN	CPs	0.003
CDKLHVDPEN;LSEAKAP	CPs	265.905
LSEAKAP;KGRGVLP	CPs	13.929
KGRGVLP;LSAFSRP	CPs	1288.641
LSAFSRP;LAPLWFK	CPs	657.888
LAPLWFK;VDGRTVNL	CPs	767.337
VDGRTVNL;LERMRL	CPs	563.588

LERMRL;LLAPPE	CPs	305.494
LLAPPE;RPWKWP	CPs	598.191
RPWKWP;MVRMVP	CPs	651.618
MVRMVP;TAPTPLP	CPs	706.649
TAPTPLP;KDPTGM	CPs	94.579
KDPTGM;LTRELAEDGY	CPs	353.814
LTRELAEDGY;AEVKKPAAA	CPs	147.591
AEVKKPAAA;AATLAERSAGLAF	CPs	0.063
AATLAERSAGLAF;LRDDEVHAGL	CPs	0.186
LRDDEVHAGL;DDEVHAGLGELL	CPs	0.006
DDEVHAGLGELL;TTDGKLPEVTK	CPs	0.228
TTDGKLPEVTK;PHHVEPLE	CPs	1.866
PHHVEPLE;HVEPLERLEK	CPs	0.006
HVEPLERLEK;VPKAVVP	CPs	1130.874
VPKAVVP;GPKSFTR	CPs	2933.334
GPKSFTR;AAAGTCGTGTR	CPs	3509.205
AAAGTCGTGTR;YGEDLTK	CPs	569.466
YGEDLTK;SKAKVFEH	CPs	2118.483
SKAKVFEH;SLRPESLHQV	CPs	1.872
SLRPESLHQV;PVGKLVLN	CPs	5.58
PVGKLVLN;RARVANY	CPs	3.672
RARVANY;SAPEHQPSALEHRTH	CPs	2.649
SAPEHQPSALEHRTH;TYVQHGSHLSAREKA	CPs	6.612
TYVQHGSHLSAREKA;YVQHGSHLSAR	CPs	0.003
YVQHGSHLSAR;HGSHLSAREKANL	CPs	0.009
HGSHLSAREKANL;MTADETRNLQ	CPs	730.092
MTADETRNLQ;APSLPVEF	CPs	1131.993
APSLPVEF;PTVEELY	CPs	6574.302
PTVEELY;VPAAGAQ	CPs	2113.719
VPAAGAQ;VRPFLSL	CPs	418.707
VRPFLSL;LHAGPA	CPs	425.577
LHAGPA;MVHYGK	CPs	326.336
MVHYGK;KLEAGDTKPEAK	CPs	688.729
KLEAGDTKPEAK;VLGPVHLV	CPs	3492.963
VLGPVHLV;GPPGPGA	CPs	5.019
GPPGPGA;VGPGNLQEGESEGDSQK	CPs	367.329
VGPGNLQEGESEGDSQK;VGDADLERQKF	CPs	141.558
VGDADLERQKF;VGQAGTATLPR	CPs	1.506
VGQAGTATLPR;TEDPTNDTVDFPK	CPs	7.602
TEDPTNDTVDFPK;YDDSTLPL	CPs	3.258
YDDSTLPL;KEQDKPPP	CPs	924.536
KEQDKPPP;KPALK	CPs	1175.908
KPALK;YAPDPK	CPs	193.497
YAPDPK;LPAARTPP	CPs	149.517
LPAARTPP;NFAAREMAPH	CPs	84.294
VDQRVLEKLPSLV;HDFSSDLEHVPH	CPs	3.534
HDFSSDLEHVPH;HESAPEL	CPs	13.386
HESAPEL;TNPTEHTLPSN	CPs	181.446
TNPTEHTLPSN;QEGSALH	CPs	1502.513
QEGSALH;VFGVSPL	CPs	119.136
VFGVSPL;VGLEF	CPs	1648.935

VGLEF;YPPYSP	CPs	1992.649
YPPYSP;VTAMDV	CPs	3820.461
VTAMDV;TAMDVV	CPs	0.003
TAMDVV;ERLMLR	CPs	2473.424
ERLMLR;QNSLVHR	CPs	57.181
QNSLVHR;VGDPVYQMVQ	CPs	12106.28
VGDPVYQMVQ;ENDLKGAAVH	CPs	1849.446
ENDLKGAAVH;PPAAAQP	CPs	1.548
PPAAAQP;SAADEKAVLAAEEVK	CPs	40.317
SAADEKAVLAAEEVK;GPPPFPGTP	CPs	4.356
GPPPFPGTP;THWFHRNPLK	CPs	54.561
THWFHRNPLK;YSLGPK	CPs	872.585
YSLGPK;QKLVKGHAY	CPs	35.044
QKLVKGHAY;LKDVTLK	CPs	1187.853
LKDVTLK;FVGLQERQ	CPs	552.192
FVGLQERQ;APWSGRK	CPs	7.131
APWSGRK;LPGELAKHAVSEGTKA	CPs	585.663
LPGELAKHAVSEGTKA;LVAGPF	CPs	1036.539
LVAGPF;KLKVSELKEELK	CPs	1909.122
KLKVSELKEELK;VSELKEELKKR	CPs	0.009
VSELKEELKKR;EVELSYA	CPs	5.223
EVELSYA;VLCHNCAVEF	CPs	0.096
VLCHNCAVEF;KAVVVCPKDEDYKQR	CPs	1.017
KAVVVCPKDEDYKQR;AVVVCPK	CPs	0.003
AVVVCPK;VVVCPKDEDYKQR	CPs	0.003
VVVCPKDEDYKQR;VVCPKDEDYKQ	CPs	0.003
VVCPKDEDYKQ;VCPKDEDYKQR	CPs	0.003
VCPKDEDYKQR;KKAEVEGKDLPEHA	CPs	0.039
KKAEVEGKDLPEHA;AEVEGKDLPEHAVLK	CPs	0.006
AEVEGKDLPEHAVLK;EVEGKDLPEHA	CPs	0.003
EVEGKDLPEHA;VEGKDLPEHAVLK	CPs	0.003
VEGKDLPEHAVLK;EGKDLPEHA	CPs	0.003
EGKDLPEHA;KDLPEHAVL	CPs	0.006
KDLPEHAVL;DLPEHA	CPs	0.003
DLPEHA;VELQKEEAQK	CPs	0.339
VELQKEEAQK;YKEESKKALPPEKK	CPs	0.042
YKEESKKALPPEKK;ALPPEKKQNTGSK	CPs	0.021
ALPPEKKQNTGSK;YHLLKK	CPs	243.582
YHLLKK;RAETLKGR	CPs	114.84
RAETLKGR;AEQELEALRK	CPs	1443.543
AEQELEALRK;SGYVQQVKY	CPs	1065.318
SGYVQQVKY;LTAEQFDEWVKPKDMLGPK	CPs	7.545
LTAEQFDEWVKPKDMLGPK;MNQEKLAKLQ	CPs	876.567
MNQEKLAKLQ;YGKLLQPK	CPs	629.304
YGKLLQPK;SDFEVVPK	CPs	2428.875
SDFEVVPK;LSAKFGGQA	CPs	1679.568
LSAKFGGQA;MMTQF	CPs	788.607
MMTQF;PAPTTDAVAPK	CPs	24.582
PAPTTDAVAPK;LALDVVANR	CPs	220.008
LALDVVANR;FVGTVMK	CPs	81.179
FVGTVMK;SSSKLTLH	CPs	730.876

SSSKLTLH;SDVHRAAF	CPs	1.386
SDVHRAAF;ASFEVEKTKK	CPs	1041.351
ASFEVEKTKK;ELRDNDKTRY	CPs	954.645
ELRDNDKTRY;AGAVEKGVPL	CPs	3.348
AGAVEKGVPL;SFRNPLAK	CPs	4.932
SFRNPLAK;PQQPPR	CPs	458.748
PQQPPR;HFVAVPP	CPs	1796.277
HFVAVPP;ASPLGGP	CPs	105.426
ASPLGGP;VVANSKESYELR	CPs	223.857
VVANSKESYELR;VDPDYWEKLL	CPs	55.173
VDPDYWEKLL;PPPPLAPR	CPs	1367.334
PPPPLAPR;VPPPVPSKP	CPs	1697.177
VPPPVPSKP;APVALPR	CPs	320.83
APVALPR;DLPRPRN	CPs	209.585
DLPRPRN;TGHTGDVMSL	CPs	101.434
TGHTGDVMSL;SLAPDTRLF	CPs	0.03
SLAPDTRLF;RGPACPK	CPs	184.571
RGPACPK;MDPMNEFSALNEAK	CPs	168.115
MDPMNEFSALNEAK;YLEPEEVLK	CPs	0.078
YLEPEEVLK;YSEFFTGSKLVDY	CPs	0.027
YSEFFTGSKLVDY;ARSVHEEF	CPs	0.039
ARSVHEEF;PPPPPPPP	CPs	125.139
PPPPPPPP;PPMEA	CPs	22.479
PPMEA;LPEAYDF	CPs	66.456
LPEAYDF;LASAVLA	CPs	140.526
LASAVLA;LGPPT	CPs	313.512
LGPPT;CVLPKVTVA	CPs	8.421
CVLPKVTVA;APPAAAGP	CPs	454.5
APPAAAGP;EDHLAW	CPs	2632.431
EDHLAW;GMLDPAEKDEKGMPVTA	CPs	1.968
GMLDPAEKDEKGMPVTA;KLFPKGVFTK	CPs	2.544
KLFPKGVFTK;YLRYTP	CPs	0.051
YLRYTP;GLGFKTPK	CPs	2617.896
GLGFKTPK;VTKAAGTKKQFQKF	CPs	0.312
VTKAAGTKKQFQKF;LENLPK	CPs	419.75
LENLPK;DKHPPLP	CPs	1751.629
DKHPPLP;AGEGQRAEPEREGW	CPs	1384.668
AGEGQRAEPEREGW;FDEQPLKL	CPs	31.554
FDEQPLKL;LGPVED	CPs	948.552
LGPVED;GPVDGGK	CPs	92.175
GPVDGGK;GDVKNFL	CPs	1720.902
GDVKNFL;VFDPVPSGVTK	CPs	26.19
VFDPVPSGVTK;YGDGPRPPK	CPs	10.344
YGDGPRPPK;VGGGGGVGGGGGGF	CPs	0.207
VGGGGGVGGGGGGF;LGKQVLSY	CPs	207.162
LGKQVLSY;VTTYRTTS	CPs	3848.822
VTTYRTTS;AAAPLP	CPs	1200.505
AAAPLP;DEKLLVK	CPs	2360.024
DEKLLVK;RDFEGRDNM	CPs	1977.801
RDFEGRDNM;VGAAALG	CPs	184.864
VGAAALG;RVVADTEL	CPs	1773.021

RVVADTEL;CSTGAL	CPs	172.718
CSTGAL;KHLLPPP	CPs	1196.89
KHLLPPP;LSPEEMEELEK	CPs	3653.55
LSPEEMEELEK;LSFCEKETK	CPs	0.135
LSFCEKETK;DAGKKPLGPR	CPs	23.844
DAGKKPLGPR;SAERPKEEKL	CPs	0.114
SAERPKEEKL;VRAVVDK	CPs	0.054
VRAVVDK;AVVDKKEAGKEGPAAPR	CPs	0.006
AVVDKKEAGKEGPAAPR;EAGKEGPAAPRKDEEKQR	CPs	0.018
EAGKEGPAAPRKDEEKQR;KGEQPEAASKTAAPEKAK	CPs	0.078
KGEQPEAASKTAAPEKAK;LLKENTTLLKLGY	CPs	0.651
LLKENTTLLKLGY;AASGAAPR	CPs	7.5
AASGAAPR;GVVAHGR	CPs	238.701
GVVAHGR;PNWGGGKKCGVCQK	CPs	107.898
PNWGGGKKCGVCQK;WGGGKKCGVCQKT	CPs	0.006
WGGGKKCGVCQKT;KCGVCQKTV	CPs	0.015
KCGVCQKTV;GVCQKTVY	CPs	0.006
GVCQKTVY;FAEEVQCEGSSFHK	CPs	0.024
FAEEVQCEGSSFHK;AEEVQCEGSSFHKSCFL	CPs	0.003
AEEVQCEGSSFHKSCFL;EEVQCEGSSFHKSCF	CPs	0.003
EEVQCEGSSFHKSCF;EVQCEGSSFHKSC	CPs	0.003
EVQCEGSSFHKSC;VQCEGSSFHKSCF	CPs	0.003
VQCEGSSFHKSCF;GYGQGAGTLSMDK	CPs	4.155
GYGQGAGTLSMDK;YGQGAGTLSMDKGESLG	CPs	0.003
YGQGAGTLSMDKGESLG;VGGSERCPRCSQA	CPs	0.948
VGGSERCPRCSQA;GSERCPRCSQAVYA	CPs	0.006
GSERCPRCSQAVYA;CSQAVYAAEK	CPs	0.021
CSQAVYAAEK;SCFRCAKCGK	CPs	2.862
SCFRCAKCGK;LESTTLADK	CPs	0.033
LESTTLADK;AKNFGPK	CPs	0.444
AKNFGPK;GFGFGQGAGAL	CPs	0.021
GFGFGQGAGAL;FGFGQGAGALVHSE	CPs	0.003
FGFGQGAGALVHSE;GFGQGAGAL	CPs	0.003
GFGQGAGAL;FGQGAGALVHSE	CPs	0.003
FGQGAGALVHSE;QGAGALVH	CPs	0.006
QGAGALVH;VEAMSGMEGRKK	CPs	41.094
VEAMSGMEGRKK;FDAAKSPTTQ	CPs	0.039
FDAAKSPTTQ;RVPAGSEKPSAAGK	CPs	18.462
KAPPRP;EDGSDPASGDFLAEGGGVR	CPs	2852.371
EDGSDPASGDFLAEGGGVR;DGSDPASGDFL	CPs	0.003
DGSDPASGDFL;PASGDFLAEGGGVR	CPs	0.012
PASGDFLAEGGGVR;KVDLEDYKNQQK	CPs	2.919
KVDLEDYKNQQK;TGPVPREF	CPs	0.117
TGPVPREF;APGSPRKP	CPs	0.156
APGSPRKP;WNPGRPEP	CPs	0.084
WNPGRPEP;PGRPEPGSAGTWN	CPs	0.006
PGRPEPGSAGTWN;GRPEPGSAGTWNPGRP	CPs	0.003
GRPEPGSAGTWNPGRP;SSGSSSFRPDSSGHGN	CPs	0.354
SSGSSSFRPDSSGHGN;REEGSVSSGTKQEF	CPs	0.081
REEGSVSSGTKQEF;TGKLVTTK	CPs	0.045
TGKLVTTK;TVTNADGRTETTK	CPs	0.114

TVTNADGRTETTK;LDDFFLRNK	CPs	0.12
LDDFFLRNK;LEPDVAALGESGSSSSK	CPs	0.075
LEPDVAALGESGSSSSK;FVSSSTTVNR	CPs	0.072
FVSSSTTVNR;MEDEAESLEDLGFKGAHGTQK	CPs	0.063
MEDEAESLEDLGFKGAHGTQK;EDEAESLEDLGFKG	CPs	0.003
EDEAESLEDLGFKG;DEAESLEDLGFK	CPs	0.003
DEAESLEDLGFK;ESLEDLGF	CPs	0.009
ESLEDLGF;SLEDLGFKG	CPs	0.003
SLEDLGFKG;LEDLGFKGAHGTQK	CPs	0.003
LEDLGFKGAHGTQK;EDLGFKGAHG	CPs	0.003
EDLGFKGAHG;GVHDLPQ	CPs	1091.184
GVHDLPQ;FVVEVGR	CPs	94.269
FVVEVGR;LEELSLTL	CPs	1867.713
LEELSLTL;DLVVLGH	CPs	195.9
DLVVLGH;AAPSVPVSK	CPs	863.874
AAPSVPVSK;ARDYDHLFK	CPs	3134.612
ARDYDHLFK;YSASPK	CPs	1164.942
YSASPK;EELRLGE	CPs	339.913
EELRLGE;AATTLTK	CPs	1024.878
AATTLTK;LSNLLLD	CPs	1981.307
LSNLLLD;RRAGPGP	CPs	124.81
RRAGPGP;ENFYDKTSY	CPs	2756.292
ENFYDKTSY;GYDHSPPGK	CPs	914.631
GYDHSPPGK;AACTVVH	CPs	16670.02
AACTVVH;NSLLQTRD	CPs	606.555
NSLLQTRD;VTPELLN	CPs	111.452
VTPELLN;RDDLVER	CPs	9133.267
RDDLVER;KDVVVAP	CPs	2144.742
KDVVVAP;PGELAKH	CPs	1791.117
PGELAKH;ADPELELV	CPs	2865.531
ADPELELV;AAEVDFGDL	CPs	1628.328
AAEVDFGDL;RELLQKL	CPs	128.31
RELLQKL;KVPAVPETLKKK	CPs	187.893
KVPAVPETLKKK;LDGLDLL	CPs	3883.692
LDGLDLL;KVRVLAT	CPs	4732.839
KVRVLAT;KVPSQN	CPs	169.317
KVPSQN;YAPGVLL	CPs	28.619
YAPGVLL;LAPGP	CPs	171.829
LAPGP;YSPGLRK	CPs	287.789
YSPGLRK;PPMPNHP	CPs	13.078
PPMPNHP;DHGPK	CPs	11.841
DHGPK;NFKLV	CPs	5.954
NFKLV;GPPPSWG	CPs	53.89
GPPPSWG;TKYNRP	CPs	240.131
TKYNRP;QTERYNEETF	CPs	123.433
QTERYNEETF;LQPHPGLEK	CPs	3.066
LQPHPGLEK;SGAGLDYLAY	CPs	39.471
SGAGLDYLAY;DGNVFTTGF	CPs	1298.313
DGNVFTTGF;PGKKSDP	CPs	37.166
PGKKSDP;RSMWAR	CPs	1699.735
RSMWAR;RAAPAWP	CPs	153.081

RAAPAWP;AAPTLTAR	CPs	2569.587
AAPTLTAR;VPVGREV	CPs	867.228
VPVGREV;HSQQTADGSAVSTVTK	CPs	125.673
HSQQTADGSAVSTVTK;FDREDEASPR	CPs	1.434
FDREDEASPR;SGSLAALEK	CPs	0.03
SGSLAALEK;SSSWSDGMAF	CPs	1.215
SSSWSDGMAF;CALVHNFFPEAF	CPs	0.03
CALVHNFFPEAF;LSPKSAP	CPs	134.535
LSPKSAP;LKMKGDYY	CPs	421.62
LKMKGDYY;ALAPLLLT	CPs	234.489
ALAPLLLT;KPVYAP	CPs	91.544
KPVYAP;KWLPSKP	CPs	327.381
KWLPSKP;LSLTSTDWKSKGSY	CPs	0.073
LSLTSTDWKSKGSY;SLTSTDWKSK	CPs	0.003
SLTSTDWKSK;HDGSTVTKTVKPSE	CPs	0.054
HDGSTVTKTVKPSE;VTKTVKPSECS	CPs	0.015
VTKTVKPSECS;SCEVTHEGSTVTK	CPs	10.251
SCEVTHEGSTVTK;LSLTGSDWKSKGSY	CPs	6.213
LSLTGSDWKSKGSY;RSYSKLLC	CPs	120.168
RSYSKLLC;GLLTERLR	CPs	0.024
GLLTERLR;SQPADPTLKQ	CPs	33.798
SQPADPTLKQ;FELRTVDL	CPs	2.595
FELRTVDL;ADTAMKERLKP	CPs	5.139
ADTAMKERLKP;ALDLLDK	CPs	710.244
ALDLLDK;KFDMELDDLPKEKLK	CPs	1.011
KFDMELDDLPKEKLK;ARFQPGYRS	CPs	0.066
ARFQPGYRS;KGVMPK	CPs	135.74
KGVMPK;ARQLLQ	CPs	87.544
ARQLLQ;VDLEPTV	CPs	121.707
VDLEPTV;AALEKDYEEVGVD	CPs	3.441
AALEKDYEEVGVD;GPPGPPSPP	CPs	120.453
GPPGPPSPP;EMVFVK	CPs	81.692
EMVFVK;SKVVDGLEKVVYK	CPs	314.755
SKVVDGLEKVVYK;KGLSGPARP	CPs	0.036
KGLSGPARP;LEEDEEELFK	CPs	82.671
LEEDEEELFK;ENDLPEWKER	CPs	1.341
ENDLPEWKER;GTGDVKLLKHKEK	CPs	0.03
ADLDSPQKL;GPAARGPP	CPs	32.583
GPAARGPP;DTDLFVPY	CPs	150.21
DTDLFVPY;EAPAGP	CPs	1136.646
EAPAGP;RLEPEW	CPs	1608.528
RLEPEW;RSLAVAP	CPs	792.12
RSLAVAP;APPPPPPPP	CPs	1164.465
APPPPPPPP;ESPFVHNS	CPs	3592.329
ESPFVHNS;VHNSVYTALF	CPs	0.012
VHNSVYTALF;ANQVLCK	CPs	574.599
ANQVLCK;RPSAGP	CPs	4.592
RPSAGP;ALPSYVDRR	CPs	1074.154
ALPSYVDRR;DYPLPDVAHVK	CPs	0.027
DYPLPDVAHVK;TFEEEWVAKQTK	CPs	1.833
TFEEEWVAKQTK;SAKWDYDKNEWKK	CPs	0.075

SAKWDYDKNEWKK;KFLVPDHV	CPs	1212.813
KFLVPDHV;RAVEAAE	CPs	190.269
RAVEAAE;HQGVMR	CPs	525.563
HQGVMR;LNLEGPK	CPs	0.051
LNLEGPK;EPPKKETASTGPQVK	CPs	165.682
EPPKKETASTGPQVK;RVAGPP	CPs	4.752
RVAGPP;LPPGPPP	CPs	169.832
LPPGPPP;DDHFLFDKP	CPs	58.809
DDHFLFDKP;FLFDKPVSPLLL	CPs	0.009
FLFDKPVSPLLL;SALRTPT	CPs	173.959
SALRTPT;AAFGAPL	CPs	0.054
AAFGAPL;CQVLVA	CPs	190.976
CQVLVA;HTKVRAGRGF	CPs	23.458
HTKVRAGRGF;ANVQRLKEY	CPs	0.333
ANVQRLKEY;MARANARLF	CPs	1.209
MARANARLF;MECCGK	CPs	80.45
MECCGK;DPLPPLD	CPs	127.774
DPLPPLD;LFRPDNF	CPs	14.859
LFRPDNF;PSPKVSDTV	CPs	1.002
PSPKVSDTV;SPKVSDTVVEPY	CPs	0.003
SPKVSDTVVEPY;VSDTVVEP	CPs	0.009
VSDTVVEP;SDTVVEPY	CPs	0.003
SDTVVEPY;ATPTYGDLNHL	CPs	0.126
ATPTYGDLNHL;LRFPGQLNA	CPs	0.066
LRFPGQLNA;MVPFPR	CPs	0.051
MVPFPR;RGSQQYRAL	CPs	0.057
RGSQQYRAL;ALTVPELT	CPs	0.021
ALTVPELT;TVPELTQQMFDAK	CPs	0.006
TVPELTQQMFDAK;NDLVSEY	CPs	0.393
NDLVSEY;EELHNQEVQK	CPs	1506.111
EELHNQEVQK;HTPLYGLP	CPs	1877.705
HTPLYGLP;HDEGYGPPPP	CPs	601.302
HDEGYGPPPP;FFNAPLEVTEENF	CPs	1.65
FFNAPLEVTEENF;KVGSSGDVALH	CPs	22.38
KVGSSGDVALH;QGPLP	CPs	326.401
QGPLP;GPAAFP	CPs	24.567
GPAAFP;LQPVE	CPs	1349.274
LQPVE;ALGETPL	CPs	2657.111
ALGETPL;YSYKPNP	CPs	845.875
YSYKPNP;LGGTDDPVKKK	CPs	13.095
LGGTDDPVKKK;FGVPSVNVA	CPs	0.066
FGVPSVNVA;SVFGAPF	CPs	0.843
SVFGAPF;KERPR	CPs	1250.1
KERPR;MDALEEESF	CPs	27.711
MDALEEESF;AAPGPRPP	CPs	1177.836
AAPGPRPP;MGLPWPP	CPs	8231.496
MGLPWPP;DVVLAGP	CPs	138.693
DVVLAGP;TNDMFRLALHNFG	CPs	580.164
TNDMFRLALHNFG;VLTVTL	CPs	60.413
VLTVTL;KLDYFGEEA	CPs	811.282
KLDYFGEEA;AVWTRATK	CPs	145.254

AVWTRATK;ATVVASGGDGKEQG	CPs	163.758
ATVVASGGDGKEQG;SSDAREMLK	CPs	9.531
SSDAREMLK;AAPVGPPR	CPs	1649.088
AAPVGPPR;AQQAADYK	CPs	811.077
AQQAADYK;QFPYK	CPs	603.903
QFPYK;KPHASAP	CPs	875.604
KPHASAP;KLKNAL	CPs	472.977
KLKNAL;QGFPLGSP	CPs	13.958
QGFPLGSP;AEQELEALRKQ	CPs	2118.691
AEQELEALRKQ;LPELRSS	CPs	524.042
LPELRSS;AAVPSPKV	CPs	1110.997
AAVPSPKV;HLYLR	CPs	116.219
HLYLR;AALTQNPQFKKLK	CPs	24.547
AALTQNPQFKKLK;ALTQNPQFKK	CPs	0.003
ALTQNPQFKK;SSEGPRVWF	CPs	9.828
SSEGPRVWF;TLATLNPESSLF	CPs	0.06
TLATLNPESSLF;DQYLHRFAAY	CPs	13.767
DQYLHRFAAY;LAQTEALMRGK	CPs	3.126
LAQTEALMRGK;EKGPAEF	CPs	808.568
EKGPAEF;WPMPPP	CPs	41.65
WPMPPP;DPSRPGPP	CPs	171.309
DPSRPGPP;APEKY	CPs	37.119
APEKY;DVSVVDL	CPs	186.369
DVSVVDL;VGPLPPPP	CPs	236.358
VGPLPPPP;AAAPPL	CPs	64.551
AAAPPL;RAYAAGP	CPs	94.044
RAYAAGP;LPPGDR	CPs	170.34
LPPGDR;GPKFDQN	CPs	667.299
GPKFDQN;VDFLRQ	CPs	340.899
VDFLRQ;PPPPLPD	CPs	3.555
PPPPLPD;VDYHAANQ	CPs	645.144
VDYHAANQ;KNVNVQNF	CPs	49.74
KNVNVQNF;DDPVTNLNNAFEVAEK	CPs	3.225
DDPVTNLNNAFEVAEK;YHAFSGAQK	CPs	0.864
YHAFSGAQK;KHEAFESDLAAH	CPs	11.058
KHEAFESDLAAH;HEAFESDLAAHQDRVEQ	CPs	0.003
HEAFESDLAAHQDRVEQ;LHLEYAK	CPs	2.685
LHLEYAK;AKRAAPF	CPs	0.015
AKRAAPF;FDNKHTNY	CPs	1.767
FDNKHTNY;KSFSTALYGESDL	CPs	3.18
KSFSTALYGESDL;LPPTPASKPLPDDP	CPs	149.382
LPPTPASKPLPDDP;AELLKHP	CPs	274.197
AELLKHP;VNPHLQK	CPs	219.378
VNPHLQK;KFFHPGN	CPs	59.603
KFFHPGN;LVEGL	CPs	223.554
LVEGL;AAVVLA	CPs	16.282
AAVVLA;YLGTNLK	CPs	20.096
YLGTNLK;VVGSEFVQK	CPs	50.47
VVGSEFVQK;FDQNVNVK	CPs	0.291
FDQNVNVK;VGNACRLL	CPs	233.454
VGNACRLL;DPLLKQ	CPs	104.729

DPLLKQ;RDYPLEL	CPs	731.623
RDYPLEL;TSPMLLVAL	CPs	717.084
TSPMLLVAL;GLPPHPGP	CPs	1351.089
GLPPHPGP;KFGGLYP	CPs	147.548
KFGGLYP;HSGSQRPEQTGL	CPs	1.852
HSGSQRPEQTGL;PSTSFPK	CPs	419.529
PSTSFPK;EEASAGPMAPK	CPs	121.575
EEASAGPMAPK;KATQVGEKPAKDESAN	CPs	0.633
KATQVGEKPAKDESAN;VQELRKRGAP	CPs	1.473
VQELRKRGAP;PAAGPPK	CPs	179.409
PAAGPPK;EDVAG	CPs	18.596
EDVAG;PPPPEPPLR	CPs	96.3
PPPPEPPLR;PVDLSKW	CPs	111.073
PVDLSKW;RKKYELGAPVAGTC	CPs	0.459
RKKYELGAPVAGTC;KYELGAPVAGTCY	CPs	0.006
KYELGAPVAGTCY;DDYVPKLYEQLSGK	CPs	0.051
DDYVPKLYEQLSGK;GPGLPPPPPP	CPs	326.958
GPGLPPPPPP;TPAATVP	CPs	84.753
TPAATVP;STADTPALK	CPs	390.9
STADTPALK;LPGPAVPP	CPs	178.733
LPGPAVPP;LLPLEEHY	CPs	124.069
LLPLEEHY;LLEQEVPGSR	CPs	0.135
LLEQEVPGSR;LVVDPDKPFR	CPs	0.108
LVVDPDKPFR;VVDPDKPF	CPs	0.003
VVDPDKPF;VDPDKPFRK	CPs	0.003
VDPDKPFRK;DPDKPFR	CPs	0.003
DPDKPFR;LFELEEQDLFR	CPs	5.493
LFELEEQDLFR;AHDFTKF	CPs	8.742
AHDFTKF;FHSLKPK	CPs	0.018
FHSLKPK;DDEEFALASHL	CPs	0.393
DDEEFALASHL;LEGHGLPTNLPR	CPs	0.045
LEGHGLPTNLPR;NPPPPPP	CPs	262.073
NPPPPPP;SPPSAASPVNTPTTGPHGP	CPs	198.841
SPPSAASPVNTPTTGPHGP;AASPVNTPTTGPHGPS	CPs	0.012
AASPVNTPTTGPHGPS;TQFLPPNLLAL	CPs	531.081
TQFLPPNLLAL;LPSLP	CPs	50.172
LPSLP;QPLRPK	CPs	96.215
QPLRPK;KAAPTVS	CPs	132.91
KAAPTVS;AATRTASRACE	CPs	24.582
AATRTASRACE;LGRLAHEVG	CPs	33.057
LGRLAHEVG;KRVLLGETGKEK	CPs	7.788
KRVLLGETGKEK;RVLLGETGKEKLPR	CPs	0.003
RVLLGETGKEKLPR;VLLGETGK	CPs	0.003
VLLGETGK;LGETGKEKLPR	CPs	0.006
LGETGKEKLPR;TVRFNVLKVTK	CPs	1.266
TVRFNVLKVTK;GPPPKPP	CPs	104.4
GPPPKPP;TVKVEDLTFTSPF	CPs	30.027
TVKVEDLTFTSPF;EEHLAA	CPs	17.862
EEHLAA;PAVSKGDGMRGL	CPs	32.616
PAVSKGDGMRGL;CSAATS	CPs	97.659
CSAATS;VAEPNTK	CPs	13.074

VAEPNTK;AAVTVSVPGRK	CPs	168.363
AAVTVSVPGRK;ATDKSFVEK	CPs	31.05
ATDKSFVEK;FTTRTVRQ	CPs	41.976
FTTRTVRQ;HRLPPPP	CPs	80.013
HRLPPPP;TPSEKKTPSVG	CPs	1332.774
TPSEKKTPSVG;PPAPAPAPSGP	CPs	160.995
PPAPAPAPSGP;SDFDEFERQLNENK	CPs	53.361
SDFDEFERQLNENK;VDLDSLVSRPGP	CPs	23.628
VDLDSLVSRPGP;EDAAKAK	CPs	249.054
EDAAKAK;KAQKKPP	CPs	145.311
KAQKKPP;DDDHDP	CPs	438.72
DDDHDP;LPPVNPD	CPs	1034.373
LPPVNPD;ESGKAF	CPs	380.307
ESGKAF;AELRALVAVK	CPs	2204.157
AELRALVAVK;LAEKEKVDL	CPs	6.069
LAEKEKVDL;AGDLGVDLTSK	CPs	6.312
AGDLGVDLTSK;VETTEDLVAK	CPs	0.084
VETTEDLVAK;SVGDK	CPs	1406.861
SVGDK;SGVDLPG	CPs	2.914
SGVDLPG;KGGHLYT	CPs	80.465
KGGHLYT;HGPEPPLP	CPs	567.778
HGPEPPLP;LPGVVVPP	CPs	450.219
LPGVVVPP;KTDLRFQ	CPs	1298.055
KTDLRFQ;LVGLFEDTNLC	CPs	0.063
LVGLFEDTNLC;VGLFEDT	CPs	0.003
VGLFEDT;TVGNPLP	CPs	193.23
TVGNPLP;TEWETAAPAVA	CPs	284.655
TEWETAAPAVA;AVKEKYAKYLPH	CPs	3.306
AVKEKYAKYLPH;KDELERVAKSNR	CPs	1.425
KDELERVAKSNR;DELERVAK	CPs	0.003
DELERVAK;KPGSPPPR	CPs	51.474
ATQAMEKY;EGSPTEG	CPs	1295.802
EGSPTEG;KVEPPR	CPs	0.78
KVEPPR;VTDNAVYH	CPs	457.689
VTDNAVYH;QLPLVKPY	CPs	29.529
QLPLVKPY;LFKGNNRWK	CPs	0.936
LFKGNNRWK;DTELAEELLQ	CPs	0.459
DTELAEELLQ;VDKLDASESLRK	CPs	5.085
VDKLDASESLRK;GPPPPPPP	CPs	425.342
GPPPPPPP;GVLGLQ	CPs	2224.722
GVLGLQ;LPPPKPPP	CPs	1316.416
LPPPKPPP;MGGEFPK	CPs	166.515
MGGEFPK;LSEEVHK	CPs	0.708
LSEEVHK;SLDALAK	CPs	354.837
SLDALAK;AEQESLEFGKADF	CPs	2186.631
AEQESLEFGKADF;LPPALAGP	CPs	1894.287
LPPALAGP;AFGGAQASY	CPs	145.101
AFGGAQASY;VVKPPR	CPs	475.338
VVKPPR;ALTARHP	CPs	392.052
ALTARHP;RPLPRPLP	CPs	17.201
RPLPRPLP;EVQQE	CPs	244.003

EVQQE;PAGPP	CPs	322.836
PAGPP;DDAPP	CPs	475.71
DDAPP;VVGRELLL	CPs	208.713
VVGRELLL;EVVPKL	CPs	928.266
EVVPKL;ALTYPLHWF	CPs	403.236
ALTYPLHWF;PDLKLGPPSE	CPs	2.85
PDLKLGPPSE;VPQQQEHTV	CPs	13.925
VPQQQEHTV;LKFLDQFAD	CPs	210.28
LKFLDQFAD;FRLLGAR	CPs	9.809
FRLLGAR;GAPGDW	CPs	255.204
GAPGDW;VMELESKLNEAKEEF	CPs	234.85
VMELESKLNEAKEEF;VAVDDRH	CPs	30.717
VAVDDRH;AALLDNL	CPs	249.024
AALLDNL;GGKYVPR	CPs	415.19
GGKYVPR;RHSPP	CPs	434.278
RHSPP;KPHWAP	CPs	5.928
KPHWAP;ARTGMPP	CPs	36.119
ARTGMPP;ASSREVPRPLS	CPs	1311.357
ASSREVPRPLS;SREVPRP	CPs	0.006
SREVPRP;VVEVTKDGSSK	CPs	78.925
VVEVTKDGSSK;QATGESDWKDTNY	CPs	0.363
QATGESDWKDTNY;STALEHQEY	CPs	0.255
STALEHQEY;SFLEDLKGFVKSK	CPs	0.03
SFLEDLKGFVKSK;LEDLKGFVK	CPs	0.006
LEDLKGFVK;TVFLQYE	CPs	225.372
TVFLQYE;KDSPSVWAAVPGK	CPs	102.477
KDSPSVWAAVPGK;AAVPGKTF	CPs	0.021
AAVPGKTF;CYEMASHLRRSQY	CPs	2.313
CYEMASHLRRSQY;YEMASHLRR	CPs	0.003
YEMASHLRR;LLLDAPLA	CPs	49.659
LLLDAPLA;NALKNPFGN	CPs	439.386
NALKNPFGN;EDLRLPEGDL	CPs	79.836
EDLRLPEGDL;DLRLPEGDLGK	CPs	0.003
DLRLPEGDLGK;LPVGP	CPs	53.574
LPVGP;RPPSVF	CPs	31.382
RPPSVF;KLQMEAPH	CPs	203.95
KLQMEAPH;SATMPSDVLEVTKK	CPs	0.693
SATMPSDVLEVTKK;SDVLEVTK	CPs	0.015
SDVLEVTK;AAASGYTDLR	CPs	775.502
AAASGYTDLR;MEREVQRVR	CPs	500.358
MEREVQRVR;VVAAL	CPs	1024.591
VVAAL;PGRASGLPP	CPs	0.672
PGRASGLPP;VPGADGLP	CPs	693.591
VPGADGLP;GPVRPPVP	CPs	3236.721
GPVRPPVP;MESVALY	CPs	612.906
MESVALY;MEGTGVLPF	CPs	192.426
MEGTGVLPF;AAALGAAVL	CPs	275.469
AAALGAAVL;FGPVALTK	CPs	547.224
FGPVALTK;MESPAPSPPAGVPTSK	CPs	48.309
MESPAPSPPAGVPTSK;MELEDGVVY	CPs	391.383
MELEDGVVY;YNAPTSHVTPSVKK	CPs	122.298

YNAPTSHVTPSVKK;VASLREP	CPs	404.922
VASLREP;ALEEGVVRY	CPs	133.875
ALEEGVVRY;EPAKSAPAPK	CPs	129.069
EPAKSAPAPK;PGELAKHAVSED	CPs	0.303
PGELAKHAVSED;SVPAAAGGDKEAAAR	CPs	68.139
SVPAAAGGDKEAAAR;VGLSH	CPs	385.761
VGLSH;EAGALELAHQSN	CPs	677.433
EAGALELAHQSN;QPAAVTK	CPs	1099.535
QPAAVTK;AVLGP	CPs	166.57
AVLGP;MNPNCARCCK	CPs	618.381
MNPNCARCCK;YPKQSFTMVADTPEN	CPs	16.353
YPKQSFTMVADTPEN;TMVADTPENLRLK	CPs	0.018
TMVADTPENLRLK;RLKQQSELQSQVR	CPs	0.03
RLKQQSELQSQVR;YKEEFEKNKGK	CPs	7.176
YKEEFEKNKGK;KEEFEKNKGKGF	CPs	0.003
KEEFEKNKGKGF;SVVADTPELQ	CPs	0.036
SVVADTPELQ;VVADTPEL	CPs	0.003
VVADTPEL;ADTPELQR	CPs	0.006
ADTPELQR;ALETALGGQC	CPs	562.731
ALETALGGQC;ETALGGQCVDR	CPs	0.006
ETALGGQCVDR;LPPVVPVS	CPs	0.06
LPPVVPVS;PPVVPVSK	CPs	0.003
PPVVPVSK;QPKPSPP	CPs	410.424
QPKPSPP;LPAAEVL	CPs	135.546
LPAAEVL;CCSMSLR	CPs	1208.661
CCSMSLR;EPKPPPR	CPs	69.054
EPKPPPR;KLVKAFKK	CPs	59.199
KLVKAFKK;LQDEETVATLQECK	CPs	226.581
LQDEETVATLQECK;VVLVEPK	CPs	0.321
VVLVEPK;SLHPK	CPs	379.656
SLHPK;SGTGFKFLPF	CPs	99.534
SGTGFKFLPF;ADLGAWVTSF	CPs	14.511
ADLGAWVTSF;VVGVADKDLLKS	CPs	15.504
VVGVADKDLLKS;ASLLKVDQEVKLK	CPs	218.952
ASLLKVDQEVKLK;ADLSREELTAVMRF	CPs	13.965
ADLSREELTAVMRF;SHGGFFTN	CPs	5.001
SHGGFFTN;SFVKVYN	CPs	173.604
SFVKVYN;KTGKNKWF	CPs	0.816
KTGKNKWF;FQKLRF	CPs	0.024
FQKLRF;SGFLRK	CPs	1461.38
SGFLRK;ADESDTAVKPPAPPLP	CPs	245.482
ADESDTAVKPPAPPLP;AATKYTSSK	CPs	1370.808
AATKYTSSK;QCGDVLRA	CPs	181.176
QCGDVLRA;GTVMGAELRH	CPs	1.755
GTVMGAELRH;AELRHVLA	CPs	0.015
AELRHVLA;KGGDL	CPs	331.734
KGGDL;VPATAAAPP	CPs	477.102
VPATAAAPP;EGPFVGSGR	CPs	716.766
EGPFVGSGR;GPQFGSEVELR	CPs	0.18
GPQFGSEVELR;DSDSD	CPs	225.588
DSDSD;ERFEVSV	CPs	57.897

ERFEVSV;PGGHSPP	CPs	42.594
PGGHSPP;KHPVGRLD	CPs	651.864
KHPVGRLD;HPPAHYK	CPs	636.63
HPPAHYK;FLEMTDDKL	CPs	588.21
FLEMTDDKL;MDLRLAGRQAL	CPs	845.688
MDLRLAGRQAL;KNFPPP	CPs	1.016
KNFPPP;PAAAPP	CPs	24.109
PAAAPP;DSGFGGGAGVETGGK	CPs	102.612
DSGFGGGAGVETGGK;FAEFGTLKK	CPs	1.581
FAEFGTLKK;ENEEDEHSVNYKPPAQ	CPs	23.295
ENEEDEHSVNYKPPAQ;MVGSYGPRAEE	CPs	1.032
MVGSYGPRAEE;EFLTPMEEAPK	CPs	0.036
EFLTPMEEAPK;APDEEDHVLVLHK	CPs	7.473
APDEEDHVLVLHK;DEEDHVLVLH	CPs	0.006
DEEDHVLVLH;VLVLHKGNFDEALAAH	CPs	0.015
VLVLHKGNFDEALAAH;KGNFDEALAAHK	CPs	0.015
KGNFDEALAAHK;FKNGDTASPKEY	CPs	1.275
FKNGDTASPKEY;YQLDKDGVVLFKK	CPs	1.764
YQLDKDGVVLFKK;QLDKDGVVLFK	CPs	0.003
QLDKDGVVLFK;LDKDGVVLF	CPs	0.003
LDKDGVVLF;YKPESDELTAEK	CPs	4.338
YKPESDELTAEK;DEKKNVFVEF	CPs	0.285
DEKKNVFVEF;MDSTANEVEAVK	CPs	0.21
MDSTANEVEAVK;DSTANEVEAVKV	CPs	0.003
DSTANEVEAVKV;VHSFPTLK	CPs	0.033
VHSFPTLK;NGERTLDGFKK	CPs	0.147
NGERTLDGFKK;DDQKAVKDEL	CPs	0.879
DDQKAVKDEL;AGFAGDDAPR	CPs	216.234
AGFAGDDAPR;AGDDAPRAVFPS	CPs	0.009
AGDDAPRAVFPS;RHQGVMVGMGQKDSYVGDEAQSK	CPs	0.051
RHQGVMVGMGQKDSYVGDEAQSK;HQGVMVGMGQKDS	CPs	0.003
HQGVMVGMGQKDS;QGVMVGMGQK	CPs	0.003
QGVMVGMGQK;VGMGQKDSYVGDEAQSKR	CPs	0.012
VGMGQKDSYVGDEAQSKR;GMGQKDSYVGDEAQSK	CPs	0.003
GMGQKDSYVGDEAQSK;MGQKDSYVGDEAQSKRG	CPs	0.003
MGQKDSYVGDEAQSKRG;QKDSYVGDEAQSKR	CPs	0.006
QKDSYVGDEAQSKR;KDSYVGDEAQSKRG	CPs	0.003
KDSYVGDEAQSKRG;DSYVGDEAQSKR	CPs	0.003
DSYVGDEAQSKR;SYVGDEAQSK	CPs	0.003
SYVGDEAQSK;YVGDEAQSKR	CPs	0.003
YVGDEAQSKR;WHHTFY	CPs	0.099
WHHTFY;HHTFYNELR	CPs	0.003
HHTFYNELR;FYNELRVAPEEHPVLL	CPs	0.009
FYNELRVAPEEHPVLL;YNELRVAPEEHPV	CPs	0.003
YNELRVAPEEHPV;NELRVAPEEHPVLL	CPs	0.003
NELRVAPEEHPVLL;ELRVAPEEHPVL	CPs	0.003
ELRVAPEEHPVL;RVAPEEHPVLL	CPs	0.006
RVAPEEHPVLL;VAPEEHPVLLTEAPLNPK	CPs	0.003
VAPEEHPVLLTEAPLNPK;APEEHPVLL	CPs	0.003
APEEHPVLL;EEHPVLLTEAPLNPK	CPs	0.006
EEHPVLLTEAPLNPK;LTEAPLNPKAN	CPs	0.018

LTEAPLNPKAN;TEAPLNPKANREKM	CPs	0.003
TEAPLNPKANREKM;EAPLNPKANREK	CPs	0.003
EAPLNPKANREK;APLNPKANREKM	CPs	0.003
APLNPKANREKM;ETFNTPA	CPs	0.33
ETFNTPA;VLSLYASGR	CPs	0.042
VLSLYASGR;RLDLAGRDLTD	CPs	0.114
RLDLAGRDLTD;LDLAGRDLTDYL	CPs	0.003
LDLAGRDLTDYL;DLAGRDLTD	CPs	0.003
DLAGRDLTD;LAGRDLTDY	CPs	0.003
LAGRDLTDY;AGRDLTDYLMK	CPs	0.003
AGRDLTDYLMK;LCYVALDFEQEMATAASSSSLEK	CPs	0.105
LCYVALDFEQEMATAASSSSLEK;CYVALDF	CPs	0.003
CYVALDF;VALDFEQEMATAASSSSLEK	CPs	0.006
VALDFEQEMATAASSSSLEK;DFEQEMATAASSSS	CPs	0.009
DFEQEMATAASSSS;FEQEMATAASSSSLEK	CPs	0.003
FEQEMATAASSSSLEK;ATAASSSSLEKSY	CPs	0.015
ATAASSSSLEKSY;TAASSSSLEK	CPs	0.003
TAASSSSLEK;AASSSSLEKSY	CPs	0.003
AASSSSLEKSY;SSSLEKSYELPDGQ	CPs	0.009
SSSLEKSYELPDGQ;ERFRCPEAL	CPs	0.06
ERFRCPEAL;TALAPSTMK	CPs	0.303
TALAPSTMK;SLSTFQQMW	CPs	0.177
SLSTFQQMW;TPAYEM	CPs	845.846
TPAYEM;LLEVALR	CPs	124.237
LLEVALR;AATATMATSGSARK	CPs	238.047
AATATMATSGSARK;RPPNAPP	CPs	256.665
RPPNAPP;VGAVVPTHGF	CPs	680.274
VGAVVPTHGF;CDDSD	CPs	358.713
CDDSD;VSPVR	CPs	316.832
VSPVR;RVAHMH	CPs	8.541
RVAHMH;FSFPVDNLRASPF	CPs	1315.423
FSFPVDNLRASPF;LDPSLVTK	CPs	211.911
LDPSLVTK;LHLVNFVEPVGLNY	CPs	57.993
LHLVNFVEPVGLNY;KLFPLLATAY	CPs	12.768
KLFPLLATAY;AASEQPQAGELLAKAR	CPs	145.755
AASEQPQAGELLAKAR;LSQAADGAKVLRF	CPs	6.624
LSQAADGAKVLRF;RPPGGGSNF	CPs	471.129
RPPGGGSNF;DEPTEQPVRR	CPs	0.039
DEPTEQPVRR;EENPPSWAK	CPs	0.063
EENPPSWAK;AGLVDDFEKK	CPs	91.146
AGLVDDFEKK;NHLLFSDYREPLVEEAAQ	CPs	65.157
NHLLFSDYREPLVEEAAQ;PSMAPR	CPs	1459.302
PSMAPR;AGELDGESVDEQAPSSSSSSK	CPs	76.227
AGELDGESVDEQAPSSSSSSK;EGEGGPDVPSR	CPs	0.603
EGEGGPDVPSR;DLKEHL	CPs	3286.005
DLKEHL;LPLWP	CPs	191.934
LPLWP;CWAHC	CPs	171.636
CWAHC;HGVAPPE	CPs	571.572
HGVAPPE;ARMPPPPPP	CPs	38.181
FVCAKCEKPF;VCAKCEKP	CPs	0.003
VCAKCEKP;LSELAARRAQPK	CPs	1.731

LSELAARRAQPK;RVHRLLR	CPs	527.76
RVHRLLR;AEMGSKGVTAGK	CPs	5.334
AEMGSKGVTAGK;ENFTERVQ	CPs	56.094
ENFTERVQ;FKSLGSNDLKF	CPs	622.233
FKSLGSNDLKF;SHDCLSEHLNGK	CPs	3015.642
SHDCLSEHLNGK;VEAVEQNTLQEFLK	CPs	633.809
VEAVEQNTLQEFLK;LTMDLLESCFPY	CPs	3977.281
LTMDLLESCFPY;VYKQSVTSSA	CPs	0.063
VYKQSVTSSA;GVAVREERFEALR	CPs	4570.368
GVAVREERFEALR;ELLKQTEGEETHR	CPs	79.203
ELLKQTEGEETHR;YEETQVEAKREVH	CPs	8.625
YEETQVEAKREVH;KEPAAPPK	CPs	4.362
KEPAAPPK;PEVPKKP	CPs	12.951
PEVPKKP;PEVPKKV	CPs	6.801
PEVPKKV;VPKVPPK	CPs	10.884
VPKVPPK;AAVPEKKV	CPs	2.703
AAVPEKKV;LDFAVP	CPs	16.254
LDFAVP;GKFETHL	CPs	64.103
GKFETHL;QVAQEALR	CPs	37.693
QVAQEALR;KKPLMH	CPs	55.226
KKPLMH;LLLPVR	CPs	2604.228
LLLPVR;HLFLTSKPMV	CPs	1633.624
HLFLTSKPMV;MTPEDFVQRY	CPs	2045.052
MTPEDFVQRY;QGGLGL	CPs	1079.754
QGGLGL;PRAKKPP	CPs	1029.395
PRAKKPP;MKLLSGGK	CPs	1208.047
MKLLSGGK;AAVRVYD	CPs	406.319
AAVRVYD;TGPKYPF	CPs	2184.603
TGPKYPF;KSFGDLSK	CPs	15.22
KSFGDLSK;FPPPPVA	CPs	1018.716
FPPPPVA;TVMLLLT	CPs	2881.601
TVMLLLT;LAPHALLR	CPs	5095.045
LAPHALLR;LPPFPR	CPs	4042.502
LPPFPR;HSELSELNVK	CPs	476.986
HSELSELNVK;YNDSPELR	CPs	396.498
YNDSPELR;VDTPEVVL	CPs	125.838
VDTPEVVL;VTPALLHVKYATK	CPs	58.743
VTPALLHVKYATK;FLDNLYP	CPs	11930.18
FLDNLYP;GLQCDSLR	CPs	4994.269
GLQCDSLR;KPSSSLR	CPs	90.165
KPSSSLR;KLPLEQV	CPs	495.906
KLPLEQV;GTGVVELVK	CPs	925.551
GTGVVELVK;LPGLF	CPs	1858.236
LPGLF;FFESF	CPs	6342.309
FFESF;MAAVLEPL	CPs	1381.392
MAAVLEPL;LTKELY	CPs	980.109
LTKELY;SKEDATKGKFAF	CPs	6.321
SKEDATKGKFAF;MDHEAESVLEVVAAVLK	CPs	6336.105
MDHEAESVLEVVAAVLK;YEEKLEASELFKDK	CPs	45.567
YEEKLEASELFKDK;SEVPLVDVTK	CPs	3.501
SEVPLVDVTK;AAGPLLKL	CPs	4947.516

AAGPLLKL;AGQAFRKFLPLF	CPs	1109.145
AGQAFRKFLPLF;VLQATVVAVGSGSKGK	CPs	0.117
VLQATVVAVGSGSKGK;AEGTAVLRR	CPs	14.394
AEGTAVLRR;KTPEAPAHPEKPR	CPs	2950.353
KTPEAPAHPEKPR;EAPAHPEKP	CPs	0.009
EAPAHPEKP;MEGGAELLF	CPs	78.585
MEGGAELLF;LTFVTSSRAHAK	CPs	32.274
LTFVTSSRAHAK;NNAGAFLDESLF	CPs	18.078
NNAGAFLDESLF;KGLAMVPL	CPs	2.964
KGLAMVPL;WGELAGY	CPs	2514.75
WGELAGY;GFGDLKSPAGLQVLNDY	CPs	2833.035
GFGDLKSPAGLQVLNDY;ALPTQPAEEAARK	CPs	1401.15
ALPTQPAEEAARK;LELFANVP	CPs	154.406
LELFANVP;KTPAAPK	CPs	560.913
KTPAAPK;NQPLPDLK	CPs	961.354
NQPLPDLK;EKAQAKVGSLDN	CPs	17.112
EKAQAKVGSLDN;EDGTKMKGYSF	CPs	793.545
EDGTKMKGYSF;SFGHPSSV	CPs	0.027
SFGHPSSV;SSVAGEVVF	CPs	0.015
SSVAGEVVF;AGEVVFNTGL	CPs	0.009
AGEVVFNTGL;PEALTDPAYK	CPs	6.201
PEALTDPAYK;EALTDPAYKG	CPs	0.003
EALTDPAYKG;LTDPAYK	CPs	0.006
LTDPAYK;DRKEPLF	CPs	14.529
DRKEPLF;TGLAAGAQVYK	CPs	0.045
TGLAAGAQVYK;AAGAQVYKMSMA	CPs	0.009
AAGAQVYKMSMA;AGAQVYKM	CPs	0.003
AGAQVYKM;YALDSTLPAGWKP	CPs	1.275
YALDSTLPAGWKP;ALDSTLPAGWKPL	CPs	0.003
ALDSTLPAGWKPL;LDSTLPAGWKP	CPs	0.003
LDSTLPAGWKP;DSTLPAGWKPLFVN	CPs	0.003
DSTLPAGWKPLFVN;STLPAGWKP	CPs	0.003
STLPAGWKP;LFVNVNDQTN	CPs	0.027
LFVNVNDQTN;FHPEVGPGPTD	CPs	0.675
FHPEVGPGPTD;PNNLAVPLYK	CPs	60.519
PNNLAVPLYK;NNTKFVHDN	CPs	34.278
NNTKFVHDN;KLFAEAVKKA	CPs	0.813
KLFAEAVKKA;LFAEAVKK	CPs	0.003
LFAEAVKK;RNVDSKSLFHYRQY	CPs	0.027
RNVDSKSLFHYRQY;VDSKSLFH	CPs	0.006
VDSKSLFH;RQYRAGKEL	CPs	0.027
RQYRAGKEL;VGVVEC	CPs	770.118
VGVVEC;VSDVDVP	CPs	318.261
VSDVDVP;TNLRLFHH	CPs	3793.926
TNLRLFHH;YTCSFCGK	CPs	1137.948
YTCSFCGK;LYDLENLPASK	CPs	1783.539
LYDLENLPASK;WLALGGKAPYASTK	CPs	165.762
WLALGGKAPYASTK;LLDVWDQV	CPs	0.834
LLDVWDQV;VGPQMHVHL	CPs	333.735
VGPQMHVHL;YPAPQVS	CPs	444.66
YPAPQVS;LDHKFDL	CPs	0.657

LDHKFDL;AYSSSQRVSSY	CPs	132.213
AYSSSQRVSSY;RRTFGGAPS	CPs	0.033
RRTFGGAPS;FGGAPSFPLGSPL	CPs	0.009
FGGAPSFPLGSPL;GAPSFPLG	CPs	0.006
GAPSFPLG;APSFPLGSPL	CPs	0.003
APSFPLGSPL;GSPLSSPVFPR	CPs	0.018
GSPLSSPVFPR;SSPVFPRAGFGTK	CPs	0.012
SSPVFPRAGFGTK;SPVFPRAGFGTKG	CPs	0.003
SPVFPRAGFGTKG;PVFPRAGFGTK	CPs	0.003
PVFPRAGFGTK;VFPRAGFG	CPs	0.003
VFPRAGFG;AGFGTKGSSSSVTSR	CPs	0.012
AGFGTKGSSSSVTSR;FGTKGSSSSVTSRVYQ	CPs	0.006
FGTKGSSSSVTSRVYQ;GSSSSVTSRVYQVS	CPs	0.012
GSSSSVTSRVYQVS;SSSSVTSRVYQ	CPs	0.003
SSSSVTSRVYQ;QVSRTSGGAGGLGALR	CPs	0.03
QVSRTSGGAGGLGALR;VSRTSGGAGGLGALRAS	CPs	0.003
VSRTSGGAGGLGALRAS;SRTSGGAGGLGALR	CPs	0.003
SRTSGGAGGLGALR;TSGGAGGLGALRAS	CPs	0.006
TSGGAGGLGALRAS;SGGAGGLGALRA	CPs	0.003
SGGAGGLGALRA;RASRLGSTRVP	CPs	0.03
RASRLGSTRVP;ASRLGSTRVPSS	CPs	0.003
ASRLGSTRVPSS;SRLGSTRVPS	CPs	0.003
SRLGSTRVPS;RLGSTRVPSS	CPs	0.003
RLGSTRVPSS;LGSTRVPS	CPs	0.003
LGSTRVPS;GSTRVPSSY	CPs	0.003
GSTRVPSSY;TRVPSSYGAGELLD	CPs	0.006
TRVPSSYGAGELLD;RVPSSYGAGELLDF	CPs	0.003
RVPSSYGAGELLDF;VPSSYGAGELLD	CPs	0.003
VPSSYGAGELLD;PSSYGAGELLDF	CPs	0.003
PSSYGAGELLDF;SSYGAGELLD	CPs	0.003
SSYGAGELLD;SYGAGELLDF	CPs	0.003
SYGAGELLDF;YGAGELLD	CPs	0.003
YGAGELLD;GAGELLDF	CPs	0.003
GAGELLDF;SLADAVNQEFL	CPs	0.024
SLADAVNQEFL;LADAVNQEF	CPs	0.003
LADAVNQEF;VELQELNDRFANY	CPs	0.051
VELQELNDRFANY;LKEEAENNLAA	CPs	1.254
LKEEAENNLAA;KEEAENNLAAF	CPs	0.003
KEEAENNLAAF;SEAEEWYKSK	CPs	0.507
SEAEEWYKSK;VSDLTQAANKNNDALRQAK	CPs	0.201
VSDLTQAANKNNDALRQAK;NDALRQAKQEMMEY	CPs	0.033
NDALRQAKQEMMEY;ETSPEQRGSEVH	CPs	3.591
ETSPEQRGSEVH;TSPEQRGSEVHTK	CPs	0.003
TSPEQRGSEVHTK;VVSEATQQQHE	CPs	0.291
VVSEATQQQHE;WTVTLK	CPs	136.29
WTVTLK;LLDRPLAL	CPs	2048.577
LLDRPLAL;SGPVPP	CPs	874.083
SGPVPP;VGRNLSEL	CPs	1375.556
VGRNLSEL;STPEVKPLK	CPs	3898.945
STPEVKPLK;KDFDTALK	CPs	158.937
KDFDTALK;DFDTALKHYDKAK	CPs	0.003

DFDTALKHYDKAK;AAALEAMKDYTK	CPs	0.585
AAALEAMKDYTK;HLGAFEESLNY	CPs	3033.21
HLGAFEESLNY;GVTAELV	CPs	127.335
GVTAELV;VDVVDPK	CPs	195.954
VDVVDPK;KDLKEKKEVVE	CPs	230.208
KDLKEKKEVVE;DLKEKKEVVEEAENGR	CPs	0.003
DLKEKKEVVEEAENGR;LKEKKEVVE	CPs	0.003
LKEKKEVVE;KEKKEVVEEAENGR	CPs	0.003
KEKKEVVEEAENGR;KEVVEEAENGREAPAN	CPs	0.009
KEVVEEAENGREAPAN;EVVEEAENGR	CPs	0.003
EVVEEAENGR;VVEEAENGREAPAN	CPs	0.003
VVEEAENGREAPAN;HTQTTPLVEYY	CPs	1257.621
HTQTTPLVEYY;KGPELPLVPVK	CPs	1434.441
KGPELPLVPVK;RDDGKEALKF	CPs	3531.609
RDDGKEALKF;YTDPSYFF	CPs	0.03
YTDPSYFF;QAAKLLPG	CPs	825.333
QAAKLLPG;ESVPEFPLSPPKKK	CPs	484.212
ESVPEFPLSPPKKK;AEKVLVTGGAGY	CPs	1954.452
AEKVLVTGGAGY;HVVDLAK	CPs	1.623
HVVDLAK;ALLERTGY	CPs	402.189
ALLERTGY;TLDVTTGQRK	CPs	0.024
TLDVTTGQRK;EDYYYHPPP	CPs	22.881
EDYYYHPPP;DYYGYDY	CPs	0.123
DYYGYDY;DDDMDM	CPs	231.597
DDDMDM;VTSPQGFQFR	CPs	1260.951
VTSPQGFQFR;FTRVPGGPY	CPs	7.593
FTRVPGGPY;GNFAHLGHESF	CPs	7.833
GNFAHLGHESF;AHLGHESFY	CPs	0.009
AHLGHESFY;YWQLPETY	CPs	0.024
YWQLPETY;AAPGPVPP	CPs	19.47
AAPGPVPP;VVPGQ	CPs	5.241
VVPGQ;FTQTPY	CPs	11.679
FTQTPY;SGGKSGPVEDF	CPs	3.483
SGGKSGPVEDF;VSLAMVGGHLEF	CPs	0.033
VSLAMVGGHLEF;DAPGQYGAY	CPs	4.602
DAPGQYGAY;CGKFAGY	CPs	443.585
CGKFAGY;ATDTSQGELVHPKALP	CPs	442.747
ATDTSQGELVHPKALP;QTPMASSPRPK	CPs	4.872
QTPMASSPRPK;SLELERR	CPs	3.114
SLELERR;ASGSFVVVQK	CPs	2.193
ASGSFVVVQK;VDPEPQVK	CPs	0.777
VDPEPQVK;TRLCEPKEAS	CPs	0.051
TRLCEPKEAS;TPVKSPGKAAAMKQR	CPs	18.549
TPVKSPGKAAAMKQR;KEVKLPGKGK	CPs	0.216
KEVKLPGKGK;EVKLPGKG	CPs	0.003
EVKLPGKG;GEFLYDNL	CPs	81.411
GEFLYDNL;LSDGKWVPF	CPs	12.162
LSDGKWVPF;DVSVVLD	CPs	519.801
DVSVVLD;KNDLVEALK	CPs	815.568
KNDLVEALK;NDLVEALKRKQ	CPs	0.003
NDLVEALKRKQ;GTSAPSMTSAALRWMY	CPs	29.397

GTSAPSMTSAALRWMY;DQAWRLVAHDCPNYFR	CPs	0.957
DQAWRLVAHDCPNYFR;DWRYPYMQ	CPs	423.966
DWRYPYMQ;VGAPVLF	CPs	413.1
VGAPVLF;LGGHAFQ	CPs	74.376
LGGHAFQ;NHAAGP	CPs	201.138
NHAAGP;QRVVSD	CPs	847.007
QRVVSD;MMATYKEKQASA	CPs	251.242
MMATYKEKQASA;MEKALSM	CPs	484.266
PVYEP;PGPVAPPPP	CPs	1153.401
PGPVAPPPP;VYEDKSGKQQY	CPs	284.895
VYEDKSGKQQY;LVVGSP	CPs	802.038
LVVGSP;RKVSSAEGAAKEEPKR	CPs	286.929
RKVSSAEGAAKEEPKR;KVSSAEGAAKEEPK	CPs	0.003
KVSSAEGAAKEEPK;VSSAEGAAKEEPKRR	CPs	0.003
VSSAEGAAKEEPKRR;SSAEGAAKEEPKR	CPs	0.003
SSAEGAAKEEPKR;SARLSAKPAPAKVETKPK	CPs	0.042
SARLSAKPAPAKVETKPK;PAPAKVETKPKKAAGKDK	CPs	0.021
PAPAKVETKPKKAAGKDK;AEVANQETKEDLPAE	CPs	0.105
AEVANQETKEDLPAE;RVQLAAK	CPs	1137.078
RVQLAAK;LTGPGM	CPs	1171.796
LTGPGM;LLVALLH	CPs	7524.466
LLVALLH;VVDNGSGMCKAGF	CPs	6277.839
VVDNGSGMCKAGF;VTNWDDMEK	CPs	0.201
VTNWDDMEK;YNELRVAP	CPs	0.045
YNELRVAP;DSGDGVTHTVP	CPs	0.189
DSGDGVTHTVP;AGRDLTDYL	CPs	0.081
AGRDLTDYL;GRDLTDY	CPs	0.003
GRDLTDY;LTDYLMK	CPs	0.009
LTDYLMK;ERFRCPEA	CPs	0.204
ERFRCPEA;DLYANTVL	CPs	0.117
DLYANTVL;ESVPEFPLSPPK	CPs	132.426
ESVPEFPLSPPK;VPEFPLSPP	CPs	0.006
VPEFPLSPP;EFPLSPPK	CPs	0.006
EFPLSPPK;EPAAYSP	CPs	1499.895
EPAAYSP;TSSQVHYP	CPs	5081.703
TSSQVHYP;SFEVEKELF	CPs	3045.266
SFEVEKELF;VGENAEFERQY	CPs	1147.381
VGENAEFERQY;PTGAPP	CPs	2355.198
PTGAPP;KPAKVSP	CPs	2062.005
KPAKVSP;CHFDKSK	CPs	469.001
CHFDKSK;AGLAPVPF	CPs	2195.833
AGLAPVPF;LSSFMWK	CPs	10.401
LSSFMWK;YFVVPAP	CPs	1605.18
YFVVPAP;VSSFDKSK	CPs	13234.41
VSSFDKSK;FGFKEDPF	CPs	11897.27
FGFKEDPF;GDLVEELK	CPs	400.692
GDLVEELK;PGRLPP	CPs	4413.465
PGRLPP;ADSRPKPANKTPPK	CPs	69.993
ADSRPKPANKTPPK;KTPPKSPGEPAKDKAAK	CPs	0.027
KTPPKSPGEPAKDKAAK;TDTSKFTGSHKE	CPs	5.379
TDTSKFTGSHKE;VDESGYVPGYK	CPs	0.087

VDESGYVPGYK;AGTYDQKVQGGK	CPs	0.036
LGKHEKFPEVLK;HEKFPEVL	CPs	0.009
HEKFPEVL;EKNPLPSKE	CPs	1759.954
EKNPLPSKE;KNPLPSK	CPs	0.003
KNPLPSK;AAPVLHL	CPs	4318.557
AAPVLHL;EFLDAGK	CPs	653.538
EFLDAGK;GTVPVGA	CPs	1822.929
GTVPVGA;VEVVQRLF	CPs	3556.128
VEVVQRLF;VVDGVLK	CPs	6961.11
VVDGVLK;RMEALMR	CPs	698.991
RMEALMR;LWLPLFL	CPs	6.033
LWLPLFL;GPRGHPPP	CPs	778.485
GPRGHPPP;QPPTVVPGGDLAK	CPs	2637.897
QPPTVVPGGDLAK;KAGASTPK	CPs	464.67
KAGASTPK;GPPVAPKP	CPs	1468.188
GPPVAPKP;PDPSLPA	CPs	70.122
PDPSLPA;GDYDLRK	CPs	251.888
GDYDLRK;VATSRPLV	CPs	276.661
VATSRPLV;LPPVPQ	CPs	1474.71
LPPVPQ;PNFAGTWKM	CPs	1596.084
PNFAGTWKM;TVGVP	CPs	853.304
TVGVP;KHYAMQEAKQQ	CPs	460.891
KHYAMQEAKQQ;NDTKVLR	CPs	1241.913
NDTKVLR;VEPTQPTP	CPs	394.815
VEPTQPTP;APLAAPR	CPs	852.885
APLAAPR;MFLDVS	CPs	110.318
MFLDVS;LEESSGMENSPAGARPK	CPs	6503.293
LEESSGMENSPAGARPK;TSSQVKTKP	CPs	1613.142
TSSQVKTKP;AHLVALAERD	CPs	2654.973
AHLVALAERD;KTCLDNLASKGK	CPs	2357.744
KTCLDNLASKGK;AVAVRTLQEQLEKAK	CPs	1256.812
AVAVRTLQEQLEKAK;GPPFPPPPPG	CPs	120.534
GPPFPPPPPG;VPEPTKP	CPs	5463.374
VPEPTKP;FLFDKPVSPL	CPs	596.332
FLFDKPVSPL;FDKPVSP	CPs	0.006
FDKPVSP;SDVLELTDDNFESR	CPs	44.808
SDVLELTDDNFESR;KSEEEFEKF	CPs	13.029
KSEEEFEKF;FKDLFSEAHSEFLK	CPs	1.554
FKDLFSEAHSEFLK;KDLFSEAH	CPs	0.003
KDLFSEAH;MTEDNKDLLQGK	CPs	3.39
MTEDNKDLLQGK;YDVDYEKNAK	CPs	0.054
YDVDYEKNAK;KFLDAGKK	CPs	0.561
KFLDAGKK;FLDAGKKLHF	CPs	0.003
FLDAGKKLHF;KLHFAVASRK	CPs	0.018
KLHFAVASRK;LHFAVASRKTF	CPs	0.003
LHFAVASRKTF;HFAVASRK	CPs	0.003
HFAVASRK;KTFSHELSDF	CPs	0.021
KTFSHELSDF;TFSHELSD	CPs	0.003
TFSHELSD;FSHELSDF	CPs	0.003
FSHELSDF;ALERFLEDY	CPs	0.963
ALERFLEDY;LEDYFDGNLK	CPs	0.015

LEDYFDGNLK;FDGNLKRYLK	CPs	0.012
FDGNLKRYLK;PSPYEVR	CPs	1.308
PSPYEVR;FSPANKKQNPKKY	CPs	0.387
FSPANKKQNPKKY;YEDELSEVLEKPNDQ	CPs	2181.282
YEDELSEVLEKPNDQ;EDELSEVLEKPNDQAEPK	CPs	0.003
EDELSEVLEKPNDQAEPK;DELSEVLEKPN	CPs	0.003
DELSEVLEKPN;SEVLEKPNDQAEPK	CPs	0.009
SEVLEKPNDQAEPK;SDEDSDGDRPQASPGLGPGPK	CPs	1.863
SDEDSDGDRPQASPGLGPGPK;EDSEGPSQGPASREK	CPs	0.168
EDSEGPSQGPASREK;MGAEEAKPPKGK	CPs	0.936
MGAEEAKPPKGK;SSEAFPTNF	CPs	1645.515
SSEAFPTNF;LGKVTNPSEA	CPs	5.34
LGKVTNPSEA;MTLGLETPY	CPs	23.097
MTLGLETPY;TSLPLHPLNVSF	CPs	3.609
TSLPLHPLNVSF;LPENVVVK	CPs	14.625
LPENVVVK;LSVGNAMF	CPs	0.312
LSVGNAMF;VQEELKLLDKF	CPs	0.024
VQEELKLLDKF;SSEAFPTNFRDPEAAK	CPs	0.057
SSEAFPTNFRDPEAAK;MTLDLETPY	CPs	2.082
MTLDLETPY;VSEELKLLDKFRK	CPs	27.516
VSEELKLLDKFRK;LGKVTNPKEA	CPs	5.547
LGKVTNPKEA;AATEPWP	CPs	64.958
AATEPWP;KAHVVL	CPs	1720.174
KAHVVL;ASDEFLKER	CPs	128.271
ASDEFLKER;AAAPPPPPP	CPs	2857.662
AAAPPPPPP;GVVAHVP	CPs	232.308
GVVAHVP;RSGDPR	CPs	784.893
RSGDPR;LLEDGGEAPAALEAALEEK	CPs	1648.701
LLEDGGEAPAALEAALEEK;WLNLERALY	CPs	10.68
WLNLERALY;VQRLAETVF	CPs	6.351
VQRLAETVF;SLLSDVFPGVQYH	CPs	17.058
SLLSDVFPGVQYH;DGDVDPEWVENLNSVLDDNK	CPs	1.344
DGDVDPEWVENLNSVLDDNK;FTSSGLVTK	CPs	2.337
FTSSGLVTK;TAAMVEFY	CPs	1.92
TAAMVEFY;VLFNEVLDH	CPs	4.293
VLFNEVLDH;LDDAFRK	CPs	7.809
LDDAFRK;PPPLRPPP	CPs	418.359
PPPLRPPP;AARGHVP	CPs	503.718
AARGHVP;AMGPLVLTEVL	CPs	175.641
AMGPLVLTEVL;SVPKVEAVKSDN	CPs	1.356
SVPKVEAVKSDN;LTFATKTPG	CPs	175.749
LTFATKTPG;ADLQTER	CPs	493.512
ADLQTER;FVPVEAG	CPs	197.127
FVPVEAG;KQHPPR	CPs	873.144
KQHPPR;ASKCPKCDKT	CPs	153.777
ASKCPKCDKT;HDGKPFCHKPC	CPs	3.075
HDGKPFCHKPC;FCHKPCYA	CPs	0.015
FCHKPCYA;YEKPSAEKPQ	CPs	0.339
YEKPSAEKPQ;TEERKASGPPKGPSR	CPs	0.063
TEERKASGPPKGPSR;TGEPNMCPRCNK	CPs	0.138
TGEPNMCPRCNK;AEKVTSLGK	CPs	0.123

AEKVTSLGK;HDGQPYCHKPC	CPs	0.6
HDGQPYCHKPC;YCHKPCY	CPs	0.015
YCHKPCY;YDKDPEGKAQP	CPs	0.147
YDKDPEGKAQP;DWHRPCLKCEKCGK	CPs	8.13
DWHRPCLKCEKCGK;HEGKPYCNHPC	CPs	0.159
HEGKPYCNHPC;YCNHPCYA	CPs	0.015
AVNLSRNGPALLEA;ARLLAAP	CPs	10.491
ARLLAAP;DEGWWRG	CPs	1.098
DEGWWRG;VGVGVP	CPs	3.482
VGVGVP;CDDDPQ	CPs	1770.247
CDDDPQ;AEPSAPESKHKSS	CPs	3883.758
AEPSAPESKHKSS;AELDVPW	CPs	297.399
AELDVPW;AAAPLKVC	CPs	254.934
AAAPLKVC;MEEPQRAR	CPs	121.332
MEEPQRAR;KPARQGP	CPs	433.718
KPARQGP;TSEVDLAKPLVK	CPs	876.937
TSEVDLAKPLVK;GPPLRHE	CPs	3926.357
GPPLRHE;RPGHPR	CPs	1323.312
RPGHPR;VFRRFVEVGRVA	CPs	0.014
VFRRFVEVGRVA;RRFVEVGRVAY	CPs	0.006
RRFVEVGRVAY;VSFGPHAGK	CPs	0.033
VSFGPHAGK;KCMQLTDF	CPs	0.654
KCMQLTDF;WAATRWAK	CPs	1.425
WAATRWAK;MTDFDRYK	CPs	0.147
MTDFDRYK;KLQKAALLKASPK	CPs	0.282
KLQKAALLKASPK;AAKGAAAVAAAAAKVPAKK	CPs	0.048
AAKGAAAVAAAAAKVPAKK;AVAAAAAKVPAK	CPs	0.018
AVAAAAAKVPAK;VAAAAAKVPAKK	CPs	0.003
VAAAAAKVPAKK;AAAAKVPAK	CPs	0.006
AAAAKVPAK;AAVMVHQLSK	CPs	534.156
AAVMVHQLSK;NAVRLHY	CPs	5.19
NAVRLHY;AAAVLFRMSEDKPQDYK	CPs	3.675
AAAVLFRMSEDKPQDYK;PVDGLPDL	CPs	2.118
PVDGLPDL;VDGLPDLG	CPs	0.003
VDGLPDLG;LPVKPAK	CPs	590.823
LPVKPAK;GVLKPGM	CPs	1872.15
GVLKPGM;VGFNVKN	CPs	0.114
VGFNVKN;PVLDCHT	CPs	0.153
PVLDCHT;DGPKFLKSGDA	CPs	0.09
DGPKFLKSGDA;QWALDDVEEELMAK	CPs	460.638
QWALDDVEEELMAK;DLHFEGMFKK	CPs	0.042
DLHFEGMFKK;LDLDGPK	CPs	6078.63
LDLDGPK;LNLQEYQSKKLMSDN	CPs	11073.83
LNLQEYQSKKLMSDN;KLMSDNGVKVQRF	CPs	0.027
KLMSDNGVKVQRF;FVADTANEALEAAK	CPs	0.039
FVADTANEALEAAK;QTPKEGVKVK	CPs	71.991
QTPKEGVKVK;KKLLPVA	CPs	2799.027
KKLLPVA;YGSSH	CPs	829.025
YGSSH;GLVPPPK	CPs	5061.475
GLVPPPK;MEGNPVHR	CPs	134.739
MEGNPVHR;LLMLSGVGNADDLRK	CPs	4317.225

LLMLSGVGNADDLRK;MEAYHKPDQQKLQ	CPs	568.701
MEAYHKPDQQKLQ;AYGQALAK	CPs	18.627
AYGQALAK;KKRPPVKEEDLK	CPs	330.324
KKRPPVKEEDLK;RPPVKEEDLKG	CPs	0.006
RPPVKEEDLKG;PPVKEEDLK	CPs	0.003
PPVKEEDLK;TGAETVFEKPKAGPAK	CPs	0.345
TGAETVFEKPKAGPAK;GAETVFEKPK	CPs	0.003
GAETVFEKPK;AETVFEKPKAGPAK	CPs	0.003
AETVFEKPKAGPAK;ETVFEKPKAGPA	CPs	0.003
ETVFEKPKAGPA;VFEKPKAGPAK	CPs	0.006
VFEKPKAGPAK;ELVYEELLARHLGAY	CPs	6.414
ELVYEELLARHLGAY;YQEVANPLMTSVAF	CPs	2.838
YQEVANPLMTSVAF;EKEKPVP	CPs	354.722
EKEKPVP;LYDEVHPKQHLAK	CPs	246.418
LYDEVHPKQHLAK;KMPTGGSR	CPs	269.907
KMPTGGSR;ASTKAKPPSAAAPAAK	CPs	0.129
ASTKAKPPSAAAPAAK;AKPPSAAAPAAKGAQAPTK	CPs	0.012
AKPPSAAAPAAKGAQAPTK;KPPSAAAPAAK	CPs	0.003
KPPSAAAPAAK;AAAPAAKGAQAPTKAPE	CPs	0.012
AAAPAAKGAQAPTKAPE;AAAGQPQR	CPs	19.797
AAAGQPQR;LVDVFV	CPs	1428.306
LVDVFV;LSPSVLR	CPs	88.875
LSPSVLR;VFGGAGVP	CPs	52.002
VFGGAGVP;FPPLSPLR	CPs	83.321
FPPLSPLR;REVLPTPSD	CPs	142.462
REVLPTPSD;VVASVPRAMDY	CPs	135.087
VVASVPRAMDY;AALDSLSLFT	CPs	23.496
AALDSLSLFT;PGVRNPPP	CPs	6.236
PGVRNPPP;CAHAPPK	CPs	79.126
CAHAPPK;MTNRLLQRK	CPs	121.02
MTNRLLQRK;TATYAQAL	CPs	155.916
TATYAQAL;STREHTAYY	CPs	3.036
STREHTAYY;NYLHATAF	CPs	0.366
NYLHATAF;LSRADLTEY	CPs	0.606
LSRADLTEY;PTPDTLP	CPs	165.78
PTPDTLP;TLEAEAFDDVVGETVGK	CPs	373.878
TLEAEAFDDVVGETVGK;DDVVGETVGKTD	CPs	0.021
DDVVGETVGKTD;DVVGETVGK	CPs	0.003
DVVGETVGK;VVGETVGKTD	CPs	0.003
VVGETVGKTD;VGETVGKTDY	CPs	0.003
VGETVGKTDY;WPENTNF	CPs	46.689
WPENTNF;CFEPEHM	CPs	0.021
CFEPEHM;EAEMVLAKDVVPATETGVALAK	CPs	5.829
EAEMVLAKDVVPATETGVALAK;DMESPTKPDEALVK	CPs	0.066
DMESPTKPDEALVK;KDVVLPVQTEETAVK	CPs	0.081
KDVVLPVQTEETAVK;ETEVALSR	CPs	0.333
ETEVALSR;LPPETEVT	CPs	0.246
LPPETEVT;SEAEVVPVPVK	CPs	0.096
SEAEVVPVPVK;KELPPSPEKK	CPs	23.829
KELPPSPEKK;TQPTSLPKQT	CPs	0.858
TQPTSLPKQT;SGSVPAAPPK	CPs	0.078

SGSVPAAPPK;PPGLP	CPs	0.045
PPGLP;RVSPSKPASAPAVKPGSK	CPs	3.081
RVSPSKPASAPAVKPGSK;AVPKAPAT	CPs	0.063
AVPKAPAT;ADLSRPKSTTTSSVK	CPs	0.78
ADLSRPKSTTTSSVK;TPPVDKKP	CPs	0.132
TPPVDKKP;TEAAAPARKPEPN	CPs	1.581
TEAAAPARKPEPN;VGSLDNVGHLPAGGAVK	CPs	7.047
VGSLDNVGHLPAGGAVK;KPPLE	CPs	235.475
KPPLE;LGGTFY	CPs	355.21
LGGTFY;TAEKALPLGN	CPs	2382.903
TAEKALPLGN;AAAMGDPGLSRLQFAPF	CPs	467.211
AAAMGDPGLSRLQFAPF;MLPSGFRK	CPs	1022.058
MLPSGFRK;FLVHNVKELEVLLMCNK	CPs	0.024
FLVHNVKELEVLLMCNK;LVHNVKELEV	CPs	0.003
LVHNVKELEV;KGQPHRP	CPs	13.749
KGQPHRP;PDPLPPPPP	CPs	1660.665
PDPLPPPPP;LGPVEKF	CPs	872.19
LGPVEKF;AKDERPQ	CPs	64.595
AKDERPQ;AQVQLR	CPs	172.912
AQVQLR;LNVLEQKQKALNAGY	CPs	6.042
LNVLEQKQKALNAGY;TLEPSEKPFDLK	CPs	0.453
TLEPSEKPFDLK;AGSGSSPRAASF	CPs	141.135
AGSGSSPRAASF;LLALEPELEARLAH	CPs	0.036
LLALEPELEARLAH;DYQRQPDSGVSSVRSF	CPs	7.092
DYQRQPDSGVSSVRSF;ALKMRLVDH	CPs	1.461
ALKMRLVDH;ANYYQVLGVQA	CPs	80.154
ANYYQVLGVQA;KGYTRP	CPs	50.243
MAFSTAL;VKLVT	CPs	92.376
VKLVT;KELANLVP	CPs	531.308
KELANLVP;LDSPVSRSY	CPs	1837.357
LDSPVSRSY;GEVKPEVT	CPs	729.248
GEVKPEVT;SPAAPGGP	CPs	1128.538
SPAAPGGP;AGVPPHRP	CPs	452.43
AGVPPHRP;SSQPLASKQEKDGAEK	CPs	505.128
SSQPLASKQEKDGAEK;KEPSEVPTPK	CPs	1.842
KEPSEVPTPK;CCCDC	CPs	1096.083
CCCDC;SLGNATVL	CPs	1.998
SLGNATVL;YEGGKGS	CPs	5632.311
YEGGKGS;PGLVP	CPs	664.277
PGLVP;EEELAVLAL	CPs	650.812
EEELAVLAL;ALEVVL	CPs	612.266
ALEVVL;PEEVHHGEEEVETFA	CPs	755.443
PEEVHHGEEEVETFA;KSLTNDWEDHLAVK	CPs	1.569
KSLTNDWEDHLAVK;CVQQLKEFDGK	CPs	1.431
CVQQLKEFDGK;LFETALLSSGF	CPs	0.66
LFETALLSSGF;LEDPQTHSNR	CPs	0.036
LEDPQTHSNR;FEAEPVSKPK	CPs	2898.561
FEAEPVSKPK;WLSKEP	CPs	985.335
WLSKEP;TLDAATAHPALF	CPs	5344.8
TLDAATAHPALF;TLDAATAHPAL	CPs	65.589
TLDAATAHPAL;TGLFTLH	CPs	426.084

TGLFTLH;LLLPEVGPEDQGTY	CPs	0.636
LLLPEVGPEDQGTY;SCVATHPSHGPQESR	CPs	0.042
SCVATHPSHGPQESR;LGEVTLEEAAPH	CPs	98.091
LGEVTLEEAAPH;YWKGFDFSVPF	CPs	20.409
YWKGFDFSVPF;KNPELLD	CPs	17.982
KNPELLD;TVKVPLKVQEADEAK	CPs	211.56
TVKVPLKVQEADEAK;PELQLVA	CPs	11.627
PELQLVA;AEEQPQVELF	CPs	19.039
AEEQPQVELF;ADCNLLPK	CPs	3.693
ADCNLLPK;AYEQVAKALK	CPs	1.431
AYEQVAKALK;PQAAVSPP	CPs	77.835
PQAAVSPP;SDSGLFGF	CPs	138.675
SDSGLFGF;DSGLFGFY	CPs	0.003
DSGLFGFY;SGNLGHTPF	CPs	0.327
SGNLGHTPF;GSHSLRYF	CPs	31.746
GSHSLRYF;VSLMVP	CPs	4.323
VSLMVP;DWRQSPGEAGDR	CPs	447.285
DWRQSPGEAGDR;EPPMVR	CPs	25.563
EPPMVR;PNPYLR	CPs	0.164
PNPYLR;TSVEDSLP	CPs	616.903
TSVEDSLP;MKASASLR	CPs	1359.168
MKASASLR;PAAANL	CPs	284.456
PAAANL;AGAPVYL	CPs	352.264
AGAPVYL;LAAVLEY	CPs	0.018
LAAVLEY;AAVLEYLTAE	CPs	0.003
AAVLEYLTAE;AVLEYLTA	CPs	0.003
AVLEYLTA;VLEYLTAE	CPs	0.003
VLEYLTAE;PAETAAPAPVEKSPAKKK	CPs	14.952
PAETAAPAPVEKSPAKKK;AAPAPVEKSPAKK	CPs	0.012
AAPAPVEKSPAKK;SLAALKKALAA	CPs	0.153
SLAALKKALAA;LAALKKALA	CPs	0.003
LAALKKALA;AALKKALAA	CPs	0.003
AALKKALAA;KGTLVQTKGTGASGSFK	CPs	0.09
KGTLVQTKGTGASGSFK;GTLVQTKGTGASGSFKLNKK	CPs	0.003
GTLVQTKGTGASGSFKLNKK;LVQTKGTGASGSFK	CPs	0.006
LVQTKGTGASGSFK;GTGASGSFKLNKK	CPs	0.015
GTGASGSFKLNKK;TGASGSFKL	CPs	0.003
TGASGSFKL;GASGSFKLNKK	CPs	0.003
GASGSFKLNKK;KAAKSPAKPKAVKPK	CPs	0.246
KAAKSPAKPKAVKPK;KSPAKPKAVKP	CPs	0.009
KSPAKPKAVKP;SPAKPKAVKPKAAKPK	CPs	0.003
SPAKPKAVKPKAAKPK;RLLLPGELAKHAVSEGTKAVTKY	CPs	1.566
RLLLPGELAKHAVSEGTKAVTKY;KGNYSERVG	CPs	10.818
KGNYSERVG;YLAAVLEYL	CPs	0.042
YLAAVLEYL;VLLPKKTESHHK	CPs	0.192
VLLPKKTESHHK;ALKRQGRTLYGFGG	CPs	8.961
ALKRQGRTLYGFGG;AGAPVYLA	CPs	13.176
AGAPVYLA;RLLLPGELAKHAVSEGTKAV	CPs	352.362
RLLLPGELAKHAVSEGTKAV;KHAVSEGTKAVTKYTSAK	CPs	0.027
KHAVSEGTKAVTKYTSAK;HAVSEGTKAVTKYTSA	CPs	0.003
HAVSEGTKAVTKYTSA;SEGTKAVTKYTSAK	CPs	0.009

SEGTKAVTKYTSAK;KVLKQVHPD	CPs	8.511
KVLKQVHPD;TYTEHAKRKTV	CPs	332.844
TYTEHAKRKTV;PVAPAAPAPAEK	CPs	32.535
PVAPAAPAPAEK;APAAPAPAEKTPVK	CPs	0.006
APAAPAPAEKTPVK;PAAPAPAEK	CPs	0.003
PAAPAPAEK;AAPAPAEKTPVKKK	CPs	0.003
AAPAPAEKTPVKKK;APAPAEKTPVKK	CPs	0.003
APAPAEKTPVKK;PAPAEKTPVK	CPs	0.003
PAPAEKTPVK;TPKKVKKPAAAAGT	CPs	0.429
TPKKVKKPAAAAGT;PEPSKSAPAPKK	CPs	154.161
PEPSKSAPAPKK;VLLPKKTESH	CPs	6.834
VLLPKKTESH;SAPATGGVKKPH	CPs	1.074
SAPATGGVKKPH;RYRPGTVAL	CPs	0.036
RYRPGTVAL;KTDLRFQSSAV	CPs	0.117
KTDLRFQSSAV;QSSAVMALQEACEA	CPs	0.018
QSSAVMALQEACEA;SSAVMALQEACEAY	CPs	0.003
SSAVMALQEACEAY;AGSESQLKK	CPs	56.322
AGSESQLKK;LPGELAK	CPs	203.982
LPGELAK;KHAVSEGTKAVTKYTSSK	CPs	0.018
KHAVSEGTKAVTKYTSSK;TMQVSSLQREPF	CPs	7274.268
TMQVSSLQREPF;KLKKPPTDEELKETVK	CPs	21.201
KLKKPPTDEELKETVK;TDEELKETVKK	CPs	0.018
TDEELKETVKK;AAAAELTLLEK	CPs	7983.246
AAAAELTLLEK;VDCTADSEVCSAQGVRGYPT	CPs	114.066
VDCTADSEVCSAQGVRGYPT;ALGLEHSETVK	CPs	14.925
ALGLEHSETVK;VLRQAKDEL	CPs	6.558
VLRQAKDEL;EPGALLEP	CPs	1166.871
EPGALLEP;ATGSYG	CPs	3.687
ATGSYG;AASSRAQVLDLYR	CPs	746.799
AASSRAQVLDLYR;GHYTEGAELVDSVLDVVRK	CPs	1093.404
GHYTEGAELVDSVLDVVRK;HYTEGAELVDSVLDVVR	CPs	0.003
HYTEGAELVDSVLDVVR;YTEGAELVD	CPs	0.003
YTEGAELVD;TEGAELVDSVLDVVRK	CPs	0.003
TEGAELVDSVLDVVRK;EGAELVDSVLDVVRKE	CPs	0.003
EGAELVDSVLDVVRKE;AELVDSVLDVVRK	CPs	0.006
AELVDSVLDVVRK;ESCDCLQGF	CPs	0.045
ESCDCLQGF;NATLSVHQ	CPs	0.177
NATLSVHQ;ATLSVHQL	CPs	0.003
ATLSVHQL;KLTTPTYGDLNHL	CPs	0.093
KLTTPTYGDLNHL;PGQLNADLR	CPs	0.081
PGQLNADLR;ADLRKLAVN	CPs	0.015
ADLRKLAVN;LAVNMVPFPRLHF	CPs	0.015
LAVNMVPFPRLHF;TVPELTQQMFDSK	CPs	0.096
TVPELTQQMFDSK;SMKEVDEQMLNVQNK	CPs	0.111
SMKEVDEQMLNVQNK;KEVDEQMLNVQN	CPs	0.006
KEVDEQMLNVQN;EVDEQMLNVQNK	CPs	0.003
EVDEQMLNVQNK;MLNVQNKNSS	CPs	0.015
MLNVQNKNSS;LHWYTGEGMD	CPs	0.195
LHWYTGEGMD;VDLEPGTMDSVRSGP	CPs	20.1
VDLEPGTMDSVRSGP;VDLEPGTMDS	CPs	13.302
VDLEPGTMDS;EPGTMDSVR	CPs	0.009

EPGTMDSVR;DSVRSGPFGQ	CPs	0.015
DSVRSGPFGQ;LVDSVLDVVRK	CPs	0.816
LVDSVLDVVRK;VDSVLDVVRKE	CPs	0.003
VDSVLDVVRKE;DSVLDVVRK	CPs	0.003
DSVLDVVRK;SVLDVVRKE	CPs	0.003
SVLDVVRKE;MPSPKVSDTVVEPY	CPs	0.165
MPSPKVSDTVVEPY;AQAAAHCPGACGAGDAETGKPRKVA	CPs	616.404
AQAAAHCPGACGAGDAETGKPRKVAL;WLMLQKDEPEDF	CPs	359.649
AAPGAVDAMAAQKRPSQ;KMLASLDVF	CPs	1632.984
KMLASLDVF;AEESSKAVK	CPs	214.245
AEESSKAVK;YKVYDLTKF	CPs	0.09
YKVYDLTKF;GDVPKKP	CPs	17316.44
GDVPKKP;AAVRGLAP	CPs	504.036
AAVRGLAP;FQSLNQYLHAGPPVYF	CPs	11210.66
FQSLNQYLHAGPPVYF;DLEDKKVNQQPK	CPs	2083.545
DLEDKKVNQQPK;LLQSVNQLL	CPs	1778.649
LLQSVNQLL;TMFLTMFGEKL	CPs	466.296
TMFLTMFGEKL;ASASGTMAKHEQ	CPs	4493.046
ASASGTMAKHEQ;SGPFGQLFRP	CPs	909.723
SGPFGQLFRP;GPFGQLFRPDN	CPs	0.003
GPFGQLFRPDN;ADLRKLA	CPs	5.367
ADLRKLA;LVFAFEKQQ	CPs	712.029
LVFAFEKQQ;VERVLK	CPs	1837.53
VERVLK;MENSSAASASSEAGSSR	CPs	2881.689
MENSSAASASSEAGSSR;LHTKEKGKPLM	CPs	2107.056
LHTKEKGKPLM;AFDLKCDSVCVNPY	CPs	130.431
AFDLKCDSVCVNPY;KGMPTVR	CPs	3687.627
KGMPTVR;LLALGDSGVGK	CPs	37.467
LLALGDSGVGK;LWDTAGQER	CPs	2.16
LWDTAGQER;APAPPPAPPPPPP	CPs	2442.858
APAPPPAPPPPPP;ARFPP	CPs	196.962
ARFPP;AAPPPPPP	CPs	1049.223
AAPPPPPP;RPREKP	CPs	1941.509
RPREKP;TQPDLNQ	CPs	688.512
TQPDLNQ;FGRAAGP	CPs	354.118
FGRAAGP;MEALRLANSAF	CPs	828.588
SLAAAPPP;AAPGPALCL	CPs	119.272
AAPGPALCL;DYVFPENGLVAY	CPs	11.49
DYVFPENGLVAY;FVEDLRKEFAGK	CPs	4.173
FVEDLRKEFAGK;LEKLVTNF	CPs	2264.057
LEKLVTNF;PGAAPSP	CPs	251.128
PGAAPSP;EKLAL	CPs	237.879
EKLAL;LDGPVVL	CPs	821.208
LDGPVVL;TPFPHVDF	CPs	2633.37
TPFPHVDF;VGPLH	CPs	866.883
VGPLH;LVTAP	CPs	2197.713
LVTAP;HDPEKLTQELQEHVKR	CPs	1326.078
HDPEKLTQELQEHVKR;KPKKPR	CPs	1186.799
KPKKPR;HTPFMDEL	CPs	368.329
HTPFMDEL;YVLDDAVFVLKPV	CPs	469.197
YVLDDAVFVLKPV;KPAGAP	CPs	929.733

KPAGAP;HDVKFPLDSTGSELKQ	CPs	152.739
HDVKFPLDSTGSELKQ;ATAHPPPA	CPs	57.41
ATAHPPPA;MELGELLYNK	CPs	6.595
MELGELLYNK;ADPAAGPPPSEGEESTVR	CPs	209.361
ADPAAGPPPSEGEESTVR;DWEKLERKE	CPs	346.881
DWEKLERKE;LEKAGYP	CPs	3758.327
LEKAGYP;AVEAKKTYVR	CPs	340.216
AVEAKKTYVR;LLPVESVY	CPs	1.821
LLPVESVY;LEELLTTQC	CPs	592.233
LEELLTTQC;FPPDVGGNVD	CPs	0.159
FPPDVGGNVD;VGGNVDYKN	CPs	0.012
VGGNVDYKN;EFGAG	CPs	29.958
EFGAG;TDRETGKLKGEAT	CPs	685.527
TDRETGKLKGEAT;KAPKPDGPGGGPGGSH	CPs	1.266
KAPKPDGPGGGPGGSH;PGLLHQELS	CPs	19.629
PGLLHQELS;VTAECGHSFCR	CPs	0.06
VTAECGHSFCR;GPEKMPP	CPs	107.604
GPEKMPP;LSDLETTTSHMPR	CPs	102.066
LSDLETTTSHMPR;AAVKTLNPKAEVAR	CPs	221.55
AAVKTLNPKAEVAR;EEFPDKVT	CPs	5014.946
EEFPDKVT;MAAVQSFLA	CPs	785.644
MAAVQSFLA;KWAPPK	CPs	159.336
KWAPPK;GAPARGGPP	CPs	122.118
GAPARGGPP;ASEPAEHEQLRK	CPs	395.172
ASEPAEHEQLRK;EAAHEHL	CPs	2.94
EAAHEHL;AKVAVLGA	CPs	43.905
AKVAVLGA;NSPLVSRLTLY	CPs	0.063
NSPLVSRLTLY;VSPFEEKM	CPs	8.811
VSPFEEKM;KGEEFVKNMK	CPs	0.063
KGEEFVKNMK;AERRVPFSLLRG	CPs	78.786
AERRVPFSLLRG;WDPFRDWYP	CPs	0.042
WDPFRDWYP;DPFRDWY	CPs	0.003
DPFRDWY;RLFDQAF	CPs	0.033
RLFDQAF;FGLPRLPEEW	CPs	0.018
FGLPRLPEEW;SHSGWPGYVRAL	CPs	0.042
SHSGWPGYVRAL;HSGWPGYVR	CPs	0.003
HSGWPGYVR;SGWPGYVRAL	CPs	0.003
SGWPGYVRAL;EGPAYNRAL	CPs	0.045
EGPAYNRAL;NRALSRQL	CPs	0.015
NRALSRQL;LDVNHFAPEELTVK	CPs	0.075
LDVNHFAPEELTVK;DVNHFAPEEL	CPs	0.003
DVNHFAPEEL;HFAPEELTVKT	CPs	0.009
HFAPEELTVKT;FAPEELTVK	CPs	0.003
FAPEELTVK;APEELTVKT	CPs	0.003
APEELTVKT;SLSPEGTL	CPs	0.978
SLSPEGTL;LSPEGTLTVEAPLPK	CPs	0.003
LSPEGTLTVEAPLPK;APLPKSATQ	CPs	0.03
APLPKSATQ;LPKSATQSA	CPs	0.006
LPKSATQSA;QARAQLGGPEAGKSEQPENK	CPs	0.051
QARAQLGGPEAGKSEQPENK;PLEAWR	CPs	1504.712
PLEAWR;HLLMDPR	CPs	21.726

HLLMDPR;GTEQK	CPs	1505.406
GTEQK;HTALKLLQ	CPs	676.96
HTALKLLQ;TNWDDMEK	CPs	763.374
TNWDDMEK;PTLLVW	CPs	120.071
PTLLVW;KEKPPP	CPs	19.869
KEKPPP;LEMTRL	CPs	615.418
LEMTRL;SPGVVKHR	CPs	12.867
SPGVVKHR;APEVL	CPs	626.313
APEVL;RVVPPPR	CPs	354.462
RVVPPPR;LGGVDVP	CPs	471.168
LGGVDVP;ALALEYL	CPs	714.678
ALALEYL;VLSAADKGNVKAA	CPs	877.353
VLSAADKGNVKAA;LSAADKGNVKAAWGK	CPs	0.003
LSAADKGNVKAAWGK;SAADKGNVKAA	CPs	0.003
SAADKGNVKAA;AADKGNVK	CPs	0.003
AADKGNVK;ADKGNVKAAWGK	CPs	0.003
ADKGNVKAAWGK;LLSHSLLVT	CPs	0.489
LLSHSLLVT;LSHSLLVTL	CPs	0.003
LSHSLLVTL;SHSLLVTLA	CPs	0.003
SHSLLVTLA;SLLVTLASHLPSDFTPA	CPs	0.006
SLLVTLASHLPSDFTPA;TLASHLPSDFTPAVHA	CPs	0.012
TLASHLPSDFTPAVHA;LASHLPSDFTPAVH	CPs	0.003
LASHLPSDFTPAVH;ASHLPSDF	CPs	0.003
ASHLPSDF;SHLPSDFTPAVHASLDKF	CPs	0.003
SHLPSDFTPAVHASLDKF;HLPSDFTPAVHASLDK	CPs	0.003
HLPSDFTPAVHASLDK;LPSDFTPAVHASL	CPs	0.003
LPSDFTPAVHASL;PSDFTPAVH	CPs	0.003
PSDFTPAVH;SDFTPAVHASLDKF	CPs	0.003
SDFTPAVHASLDKF;DFTPAVHASLDK	CPs	0.003
DFTPAVHASLDK;FTPAVHA	CPs	0.003
FTPAVHA;VHASLDKF	CPs	0.012
VHASLDKF;ASLDKFLAN	CPs	0.006
ASLDKFLAN;LDKFLANV	CPs	0.006
LDKFLANV;TVLTSKY	CPs	3.072
TVLTSKY;AMSLTEL	CPs	80.441
AMSLTEL;GAPQAGLRAKVL	CPs	93.466
GAPQAGLRAKVL;ASPLQRLGN	CPs	0.036
ASPLQRLGN;ASPLASFVTGAL	CPs	0.06
ASPLASFVTGAL;GPSLVPVP	CPs	380.463
GPSLVPVP;GAAPPTRP	CPs	93.219
GAAPPTRP;EVHGPSYF	CPs	128.67
EVHGPSYF;RVQLREQHL	CPs	0.024
RVQLREQHL;DESGPVQTRP	CPs	298.485
DESGPVQTRP;AKPASKDSGLK	CPs	298.902
AKPASKDSGLK;APLDRGV	CPs	120.537
APLDRGV;MCRALPP	CPs	0.63
MCRALPP;TAGPVEL	CPs	3.519
TAGPVEL;EPLGRDQ	CPs	43.629
EPLGRDQ;DTVGRVH	CPs	42.099
DTVGRVH;SAELETSEGLDEPEK	CPs	1818.654
GPPHQGPPM;VLEEEVLDPK	CPs	1854.231

VLEEEVLDPK;VFRKKK	CPs	2008.026
VFRKKK;PLEEAVLY	CPs	2342.961
PLEEAVLY;PPLPPPP	CPs	3449.297
PPLPPPP;PVTFRFG	CPs	49.458
PVTFRFG;DGFKHR	CPs	1258.945
DGFKHR;MLPPPPRQPPP	CPs	176.76
MLPPPPRQPPP;EVALPV	CPs	3022.799
EVALPV;MEHLGPH	CPs	90.172
MEHLGPH;LYLPSR	CPs	864.398
LYLPSR;ADAGLRRVVP	CPs	4.351
ADAGLRRVVP;PPPSVPPPK	CPs	6.27
PPPSVPPPK;EDVLLPH	CPs	217.536
EDVLLPH;MEADLSDF	CPs	244.713
MEADLSDF;AAMEESFPR	CPs	658.935
AAMEESFPR;QNSPEAYDYGL	CPs	849.339
QNSPEAYDYGL;SFEDDTVPLSPLK	CPs	5504.25
SFEDDTVPLSPLK;MQPPPPPPPP	CPs	498.294
MQPPPPPPPP;VVLPLDEMAFEK	CPs	247.326
VVLPLDEMAFEK;EHPVP	CPs	2596.313
EHPVP;SGMPPHV	CPs	182.809
SGMPPHV;PSFLKATK	CPs	65.388
PSFLKATK;KTVTY	CPs	3350.961
KTVTY;LLQNQYEPVQPH	CPs	2338.452
LLQNQYEPVQPH;KQLWMPF	CPs	0.063
KQLWMPF;AAVATSAKGQEESAQKAK	CPs	18.408
AAVATSAKGQEESAQKAK;TSSSFLYPVTSSPY	CPs	2460.291
TSSSFLYPVTSSPY;QGLQHQVAH	CPs	367.461
QGLQHQVAH;MATQLEAARLLTY	CPs	0.027
MATQLEAARLLTY;LDPSPEVQKK	CPs	104.517
LDPSPEVQKK;LLQHADK	CPs	1266.795
LLQHADK;ADSKEAGAETPPAGAAAR	CPs	1030.398
ADSKEAGAETPPAGAAAR;CSCATC	CPs	90.039
CSCATC;KGDPPP	CPs	2188.896
KGDPPP;EAEAVPEVSEKY	CPs	1882.221
EAEAVPEVSEKY;APPRAGPP	CPs	1696.815
APPRAGPP;RELELRRR	CPs	536.118
MDQRGPP;LPAAAPSPAH	CPs	157.035
LPAAAPSPAH;EDDLDWL	CPs	7092.426
EDDLDWL;MTAKTLVLL	CPs	2665.98
MTAKTLVLL;TAKTLVLLM	CPs	0.003
TAKTLVLLM;TGTVDCWRK	CPs	5132.766
TGTVDCWRK;DVSLPF	CPs	6323.021
DVSLPF;RPPPKAY	CPs	2988.208
RPPPKAY;SHGQRTFGENY	CPs	8300.862
SHGQRTFGENY;AAAGSGPGLGVSAGPGPA	CPs	241.326
AAAGSGPGLGVSAGPGPA;APVPKKLLM	CPs	2252.148
APVPKKLLM;VVDQVVGK	CPs	4586.616
VVDQVVGK;KESPKFF	CPs	9.071
KESPKFF;GLMSP	CPs	4868.215
LVVLLL;ALLYK	CPs	183.687
ALLYK;DPVLEL	CPs	4770.105

DPVLEL;LGGYCR	CPs	2507.688
LGGYCR;KYSPELLK	CPs	144.054
KYSPELLK;EGLLLWCQRK	CPs	499.194
EGLLLWCQRK;NTLQTKLR	CPs	12.963
NTLQTKLR;HEAFESDLAAH	CPs	4.497
HEAFESDLAAH;GSVPGALDY	CPs	19.014
GSVPGALDY;SSALYGESDL	CPs	0.036
SSALYGESDL;PVRQTAQR	CPs	4399.329
PVRQTAQR;PAASALP	CPs	5698.662
PAASALP;PVTVACF	CPs	4883.634
PVTVACF;DNQGGYGSVGR	CPs	313.434
DNQGGYGSVGR;TMCCFF	CPs	4.347
TMCCFF;AQFGGQKNPP	CPs	284.298
AQFGGQKNPP;PGKLLSDADLESDAAVAK	CPs	171.729
PGKLLSDADLESDAAVAK;VETSEVDVNSPK	CPs	2.1
VETSEVDVNSPK;NEVTDKK	CPs	2217.935
NEVTDKK;APPVPPFG	CPs	2230.384
APPVPPFG;RFPNKPHLS	CPs	39.168
RFPNKPHLS;VPPPRPP	CPs	298.818
VPPPRPP;DDYEPELL	CPs	3.345
DDYEPELL;KTGELLW	CPs	11.661
KTGELLW;AAAEEEPKPKK	CPs	18.705
AAAEEEPKPKK;QGFLVF	CPs	5391.995
QGFLVF;DSALSTHKP	CPs	5956.705
DSALSTHKP;SASPVYQA	CPs	5.052
SASPVYQA;RSSNLQSRSFR	CPs	0.075
RSSNLQSRSFR;ERFETERNSPRFAK	CPs	15.132
ERFETERNSPRFAK;ETERNSPRFA	CPs	0.009
ETERNSPRFA;TERNSPRF	CPs	0.003
TERNSPRF;RNSPRFAKL	CPs	0.006
RNSPRFAKL;NSPRFAKLR	CPs	0.003
NSPRFAKLR;RNWHHGLS	CPs	0.024
RNWHHGLS;VATSPAPAAH	CPs	9.942
VATSPAPAAH;TSPAPAAHTY	CPs	0.006
TSPAPAAHTY;SGGPAESASRPP	CPs	5.703
SGGPAESASRPP;FAPGKTTTTVSK	CPs	0.066
FAPGKTTTTVSK;ASKGLQDLKKQ	CPs	210.171
ASKGLQDLKKQ;FGCPCM	CPs	327.3
FGCPCM;PAAFTSSK	CPs	3771.444
PAAFTSSK;DRVYVHPF	CPs	132.843
PLLMP;GVTSVP	CPs	1044.996
GVTSVP;GHVDSGKSTTTGHL	CPs	212.341
GHVDSGKSTTTGHL;ELSELKPK	CPs	3072.6
ELSELKPK;GPAPY	CPs	2877.284
GPAPY;GELAKHAV	CPs	0.25
GELAKHAV;EVYDDFLHYRK	CPs	58.059
EVYDDFLHYRK;DPFNPFELTNHA	CPs	0.06
DPFNPFELTNHA;PFNPFELTNH	CPs	0.003
PFNPFELTNH;SQPGEKKLTGGSN	CPs	2184.099
SQPGEKKLTGGSN;YTVYK	CPs	2881.781
YTVYK;MMTGRQGRATFQ	CPs	5521.375

MMTGRQGRATFQ;LELVNP	CPs	46.361
LELVNP;FGGLTHQVEKKD	CPs	7925.038
FGGLTHQVEKKD;KYDPPPP	CPs	2414.597
KYDPPPP;FNYRTTRF	CPs	910.081
FNYRTTRF;ADDPLLALLQEAQRS	CPs	687.408
ADDPLLALLQEAQRS;VLELLDKY	CPs	2639.937
VLELLDKY;LKMKGDYFRY	CPs	0.063
LKMKGDYFRY;SHRDFVTPGQQKR	CPs	4941.357
SHRDFVTPGQQKR;MRYVASYLL	CPs	184.581
MRYVASYLL;YVASYLLA	CPs	0.006
YVASYLLA;MGPAL	CPs	2364.753
MGPAL;MNPVVVVH	CPs	1130.751
MNPVVVVH;VNLARLALF	CPs	17.292
VNLARLALF;LPLVD	CPs	127.004
LPLVD;KWPQPLE	CPs	216.594
KWPQPLE;EDFREAKPR	CPs	1139.329
EDFREAKPR;LSLQRTGGGGGGSGN	CPs	274.836
LSLQRTGGGGGGSGN;QLTGGHEAF	CPs	1.569
QLTGGHEAF;EKEPGNKVNLA	CPs	85.575
EKEPGNKVNLA;FVEQADALK	CPs	0.807
FVEQADALK;NDVFVTEEW	CPs	0.063
NDVFVTEEW;EAPRPVH	CPs	182.283
EAPRPVH;AQPLP	CPs	406.773
AQPLP;PGAPGLPPP	CPs	181.563
PGAPGLPPP;TGVSAQVQKQ	CPs	14.496
TGVSAQVQKQ;AQVQKQRAKELG	CPs	0.012
AQVQKQRAKELG;YGGRESGCSF	CPs	16.989
YGGRESGCSF;AAVVENVVKL	CPs	120.459
AAVVENVVKL;VDDDSLGEFPVTNSR	CPs	5.334
VDDDSLGEFPVTNSR;MDGPEDSTGQPGPADR	CPs	38.271
MDGPEDSTGQPGPADR;MWAGGGVG	CPs	236.562
MWAGGGVG;TVALLQ	CPs	251.214
TVALLQ;VVLGQFLVLKK	CPs	20.331
VVLGQFLVLKK;AAEHPEPPK	CPs	42.747
AAEHPEPPK;VGNKSDLTTK	CPs	192.135
VGNKSDLTTK;KSDLTTKKVVDN	CPs	0.009
KSDLTTKKVVDN;SDLTTKKVVDNTTAK	CPs	0.003
SDLTTKKVVDNTTAK;LETSAKNATNVEQAF	CPs	0.21
LETSAKNATNVEQAF;LEDLATY	CPs	137.376
LEDLATY;FEWLVASAEQLLK	CPs	72.114
FEWLVASAEQLLK;MMMVLQPEGLG	CPs	44.514
MMMVLQPEGLG;QGPLKASC	CPs	823.959
QGPLKASC;SFADYNLL	CPs	1.692
SFADYNLL;PLLSAYVA	CPs	0.075
PLLSAYVA;DQGQSWKEEVV	CPs	28.329
DQGQSWKEEVV;QSWLQGPLKA	CPs	0.039
QSWLQGPLKA;VNDGVEDLRCKY	CPs	0.825
VNDGVEDLRCKY;ALPQHLKPF	CPs	0.876
ALPQHLKPF;AYVARLN	CPs	0.336
AYVARLN;VARLNSRPKLKA	CPs	0.006
VARLNSRPKLKA;AFLASLEHMN	CPs	0.033

AFLASLEHMN;TLKEKELETGPK	CPs	2075.868
TLKEKELETGPK;VSEGVYESTEAPGH	CPs	16.014
VSEGVYESTEAPGH;YESTEAPGHY	CPs	0.015
YESTEAPGHY;PQPPEPG	CPs	1624.086
PQPPEPG;GYFLPAEF	CPs	219.938
GYFLPAEF;SPGGVTVQR	CPs	312.133
SPGGVTVQR;SPEDYALKV	CPs	2.559
SPEDYALKV;RVDAVLLL	CPs	342.054
RVDAVLLL;GQSVRLLAK	CPs	334.536
GQSVRLLAK;SGDTKWLPY	CPs	2.184
SGDTKWLPY;YGEGHVVTF	CPs	2.652
YGEGHVVTF;DGQRFVFDGNCEY	CPs	0.027
DGQRFVFDGNCEY;KFHATTQE	CPs	0.719
KFHATTQE;VNSWKESPL	CPs	0.202
VNSWKESPL;SAPVPTATQR	CPs	2.472
SAPVPTATQR;TTTPTATPLTK	CPs	2.277
TTTPTATPLTK;TMTPTATPLTK	CPs	1.323
TMTPTATPLTK;TTTSTATPLTK	CPs	3.252
TTTSTATPLTK;TTTPTATPRTK	CPs	0.171
TTTPTATPRTK;ATPLTETSTK	CPs	1.131
ATPLTETSTK;TTTETTTPTATPLTK	CPs	0.858
TTTETTTPTATPLTK;TTTETTTSTATPLTK	CPs	1.719
TTTETTTSTATPLTK;TTAPTVPPLTK	CPs	0.045
TTAPTVPPLTK;TAPTVPPL	CPs	0.003
TAPTVPPL;TTVLPTGTSF	CPs	0.282
TTVLPTGTSF;HLDNYKKSSAA	CPs	649.497
VGDPKHSSPKVQGGSADN;VQGGSADNLK	CPs	0.03
VQGGSADNLK;FDPEKGGKP	CPs	700.329
FDPEKGGKP;EGALLDF	CPs	349.721
EGALLDF;NVSPVELPF	CPs	1610.305
NVSPVELPF;RDLSGPK	CPs	5015.093
RDLSGPK;FQTVADYGKDLVEKAK	CPs	108.877
FQTVADYGKDLVEKAK;DYGKDLVEKA	CPs	0.015
DYGKDLVEKA;YGKDLVEK	CPs	0.003
YGKDLVEK;EELTPFFK	CPs	0.447
EELTPFFK;LPVERPK	CPs	24.659
LPVERPK;KPEHVNP	CPs	624.582
KPEHVNP;MLTKFETK	CPs	424.9
MLTKFETK;FETKSARVK	CPs	0.012
FETKSARVK;FGTTDAVVKH	CPs	15.504
FGTTDAVVKH;LDDKNCWEK	CPs	19.926
LDDKNCWEK;GDVSERVR	CPs	0.336
GDVSERVR;VEATEGLGDDALGK	CPs	2.949
VEATEGLGDDALGK;KPVLVL	CPs	67.475
KPVLVL;LAGGAVY	CPs	124.174
LAGGAVY;ANRGPAYGLS	CPs	184.596
ANRGPAYGLS;RGPAYGLSR	CPs	0.006
RGPAYGLSR;GSESVKDTPVK	CPs	964.284
GSESVKDTPVK;RQKEYKCGDLVF	CPs	3529.476
RQKEYKCGDLVF;STANKYQVFF	CPs	6.21
STANKYQVFF;EGERPLPVE	CPs	1.548

EGERPLPVE;MMGHRPVL	CPs	395.883
MMGHRPVL;AGEMLSVAEH	CPs	7.113
AGEMLSVAEH;ALTEKSKAMTGVEQWPY	CPs	5.994
ALTEKSKAMTGVEQWPY;HVVEVPQ	CPs	10.71
HVVEVPQ;AARAAPPP	CPs	97.737
AARAAPPP;EEGARLLASK	CPs	149.394
EEGARLLASK;KPCLPR	CPs	120.482
KPCLPR;PVKFLFPQ	CPs	279.597
PVKFLFPQ;YRDTF	CPs	400.323
YRDTF;DPLTGF	CPs	18.847
DPLTGF;MLSPLVKDWAPK	CPs	150.281
MLSPLVKDWAPK;ECVHKLGSSC	CPs	254.773
ECVHKLGSSC;KLENAVQGS	CPs	789.264
KLENAVQGS;ADSYTELEKAVV	CPs	7.512
ADSYTELEKAVV;MLQKELNH	CPs	0.117
MLQKELNH;AYPLEKALDVMVSTFH	CPs	49.572
AYPLEKALDVMVSTFH;YPLEKALDV	CPs	0.003
YPLEKALDV;NEFFEGFPDKQPR	CPs	1.254
NEFFEGFPDKQPR;MKENFPNF	CPs	114.009
MKENFPNF;VVLVSRVLK	CPs	173.916
VVLVSRVLK;CCAPP	CPs	1574.432
CCAPP;LLYKPF	CPs	52.203
LLYKPF;AKVSSPTETER	CPs	49.276
AKVSSPTETER;NTELGAFTK	CPs	0.126
NTELGAFTK;PGVLDRMMK	CPs	1.02
PGVLDRMMK;PSQMEHAME	CPs	36.135
PSQMEHAME;NDYFVVHMKQKGKK	CPs	3.591
NDYFVVHMKQKGKK;FVVHMKQKGK	CPs	0.009
FVVHMKQKGK;GFDDFF	CPs	115.205
GFDDFF;VGMLGK	CPs	184.135
VGMLGK;THFNKGPSYG	CPs	146.223
THFNKGPSYG;HLYDPKMQ	CPs	0.555
HLYDPKMQ;YDPKMQTDKP	CPs	0.006
YDPKMQTDKP;VYDPKYC	CPs	0.234
VYDPKYC;RHEKVQTL	CPs	237.183
RHEKVQTL;SLDGTRLYVTT	CPs	1.686
SLDGTRLYVTT;MEHHQPEHPAPGETR	CPs	317.586
MEHHQPEHPAPGETR;LHNLEHSMG	CPs	217.089
LHNLEHSMG;MHSYELG	CPs	0.027
MHSYELG;TTHPVTNTTEKQ	CPs	98.337
TTHPVTNTTEKQ;VELDPEVEGTK	CPs	0.216
VELDPEVEGTK;KNKQLPSAGPDKN	CPs	19.068
KNKQLPSAGPDKN;ANVELSSL	CPs	311.031
ANVELSSL;PPPLMPPHG	CPs	272.862
PPPLMPPHG;HPTGVRP	CPs	15.969
HPTGVRP;LLRKGNYAER	CPs	23.124
LLRKGNYAER;KGNYAERVG	CPs	0.009
KGNYAERVG;VLLPKKTESHKPGKN	CPs	0.234
VLLPKKTESHKPGKN;LLPKKTESHKPGK	CPs	0.003
LLPKKTESHKPGK;FPVGRVHRLL	CPs	33.984
FPVGRVHRLL;YMAAVLEYL	CPs	0.075

YMAAVLEYL;MAAVLEYLT	CPs	0.003
MAAVLEYLT;ADFENTCSR	CPs	701.952
ADFENTCSR;ADVLDLHEAGGEDF	CPs	61.098
ADVLDLHEAGGEDF;DYGYPFDT	CPs	1072.755
DYGYPFDT;PFLEDSVKVVK	CPs	3.654
PFLEDSVKVVK;LEDSVKVVKN	CPs	0.006
LEDSVKVVKN;APLTLGN	CPs	295.251
APLTLGN;LVLCEAAFPKKLAF	CPs	441.546
LVLCEAAFPKKLAF;VLCEAAFPK	CPs	0.003
VLCEAAFPK;LEDLHSEFDEQHGK	CPs	0.045
LEDLHSEFDEQHGK;KVPTVSRP	CPs	0.042
KVPTVSRP;EEVLQRGEAL	CPs	1.776
EEVLQRGEAL;FSTVPAVPLAK	CPs	460.914
FSTVPAVPLAK;TVPAVPLAKT	CPs	0.006
TVPAVPLAKT;KDVDKAFLK	CPs	9.387
KDVDKAFLK;GSGLAASF	CPs	1.428
GSGLAASF;LPGAAF	CPs	1545.887
LPGAAF;KLVFLPP	CPs	711.282
KLVFLPP;SLGEVRL	CPs	844.57
SLGEVRL;TTMSPYL	CPs	896.859
TTMSPYL;RDTEEFY	CPs	1948.272
RDTEEFY;LGPAWEVGNR	CPs	8.826
LGPAWEVGNR;MTMRKT	CPs	92.318
MTMRKT;TGNVCKK	CPs	1148.362
TGNVCKK;EPVPAVT	CPs	1124.985
EPVPAVT;FSHWTPF	CPs	1230.863
FSHWTPF;DGWRLPETS	CPs	551.977
DGWRLPETS;ASSKSMVL	CPs	5.64
ASSKSMVL;PGQLKQF	CPs	2.469
PGQLKQF;LDEFPNLKA	CPs	0.216
LDEFPNLKA;MQSDRFLKMPVNNK	CPs	0.348
MQSDRFLKMPVNNK;KWGFTLY	CPs	364.868
KWGFTLY;VDALDTASHTHKSG	CPs	78.517
VDALDTASHTHKSG;PHAADSR	CPs	38.859
PHAADSR;EGMGAFY	CPs	941.157
EGMGAFY;VVYENMKQTLGVTQK	CPs	17.76
VVYENMKQTLGVTQK;GDGRTFPKR	CPs	1343.643
GDGRTFPKR;RGQTCVVHY	CPs	0.024
RGQTCVVHY;TGMLEDGKK	CPs	0.027
TGMLEDGKK;MSVGQRAK	CPs	0.117
MSVGQRAK;SVGQRAKLT	CPs	0.003
SVGQRAKLT;LAPDYDALDVANK	CPs	6571.5
LAPDYDALDVANK;KLYYNLDDTAY	CPs	117.357
KLYYNLDDTAY;SYLENPAFMLLDLK	CPs	26.502
SYLENPAFMLLDLK;KSLQKPQK	CPs	122.052
KSLQKPQK;THFNKGP	CPs	5372.529
THFNKGP;KGPSYGL	CPs	0.012
KGPSYGL;YGLSAEVKNK	CPs	0.012
YGLSAEVKNK;HLASPEGK	CPs	1703.64
HLASPEGK;TLVFEQRK	CPs	0.024
TLVFEQRK;LNELRMQ	CPs	3169.455

LNELRMQ;KEKHTLVL	CPs	1268.241
KEKHTLVL;KFDTPVDKLPQL	CPs	177.432
KFDTPVDKLPQL;LLPKKL	CPs	2918.997
LLPKKL;ASLGHPATFGRATHVVVR	CPs	652.665
ASLGHPATFGRATHVVVR;AAEEKDPLSY	CPs	218.79
AAEEKDPLSY;LANTEF	CPs	10823.83
LANTEF;ATGQKLMR	CPs	337.932
ATGQKLMR;ALAADHTVY	CPs	5.481
ALAADHTVY;KLLHQPH	CPs	165.462
KLLHQPH;PGHLQEGFGCVVTNR	CPs	7727.487
PGHLQEGFGCVVTNR;NRFDQLFD	CPs	0.039
NRFDQLFD;DDESDPFEVLKAAENK	CPs	0.021
DDESDPFEVLKAAENK;DESDPFEVLK	CPs	0.003
DESDPFEVLK;KNPLPPSVGVVDKK	CPs	0.168
KNPLPPSVGVVDKK;KEETQPPVALK	CPs	0.039
KEETQPPVALK;EETQPPVALKK	CPs	0.003
EETQPPVALKK;TQPPVALK	CPs	0.006
TQPPVALK;FEKPLEEK	CPs	3.846
FEKPLEEK;ENEVEEVKEEGPK	CPs	4.149
ENEVEEVKEEGPK;DVDDPEAFPAL	CPs	5.712
DVDDPEAFPAL;PVGKVG	CPs	2916.246
PVGKVG;APMVTVK	CPs	1204.239
APMVTVK;LDDFLFR	CPs	9334.581
LDDFLFR;MESDFYLR	CPs	1726.563
MESDFYLR;PYRPPP	CPs	972.345
PYRPPP;QLEKTVP	CPs	14.933
QLEKTVP;LGLDLWQVK	CPs	764.317
LGLDLWQVK;APAASHPR	CPs	2767.194
APAASHPR;SSAVMALQEASEA	CPs	1101.08
SSAVMALQEASEA;GVVVFG	CPs	491.88
GVVVFG;KGGGPAAP	CPs	732.808
KGGGPAAP;LPPKPPKP	CPs	204.264
LPPKPPKP;SDRAEEFKK	CPs	531.996
SDRAEEFKK;RDKRPDL	CPs	2746.947
RDKRPDL;NKSVP	CPs	1280.403
NKSVP;GRELPLR	CPs	50.235
GRELPLR;MNAAASSYPMASLY	CPs	1841.307
MNAAASSYPMASLY;FTLGPPK	CPs	159.308
FTLGPPK;APARTAGSS	CPs	0.005
APARTAGSS;LVTQQK	CPs	59.34
LVTQQK;SGVLDTLTK	CPs	401.658
SGVLDTLTK;VEFEELLK	CPs	865.173
VEFEELLK;AAPLLTVP	CPs	183.51
AAPLLTVP;KNGGEPL	CPs	1324.071
KNGGEPL;APATSLK	CPs	54.156
APATSLK;FGLAPLPP	CPs	284.075
FGLAPLPP;RHCPR	CPs	205.8
RHCPR;STPGGHPKPPH	CPs	531.145
STPGGHPKPPH;VSNELLEEAF	CPs	1.464
VSNELLEEAF;FGQGGAGPVGGQGPR	CPs	5.052
FGQGGAGPVGGQGPR;EEYEGPNKKPR	CPs	0.087

EEYEGPNKKPR;APPPQALG	CPs	1126.121
APPPQALG;LSPLLHR	CPs	386.569
LSPLLHR;ASEELASKL	CPs	332.355
ASEELASKL;LLEVEVL	CPs	108.762
LLEVEVL;PVVGAPG	CPs	34.512
PVVGAPG;AESSFSFKKLLD	CPs	3996.684
AESSFSFKKLLD;PSKGPLQSVQVFGRKK	CPs	327.759
PSKGPLQSVQVFGRKK;TLQYKLLEPV	CPs	0.132
TLQYKLLEPV;KLLEPVLLLGKERF	CPs	0.012
KLLEPVLLLGKERF;VEERPEKDFTEK	CPs	325.008
VEERPEKDFTEK;KSPVPAELKDVQK	CPs	15.165
KSPVPAELKDVQK;DDDAAHA	CPs	1451.997
DDDAAHA;PAVELFP	CPs	922.071
PAVELFP;TAFAV	CPs	19.577
TAFAV;EDFKEQTRL	CPs	645.466
EDFKEQTRL;MQDLQEVTQDL	CPs	1.215
MQDLQEVTQDL;GDGDSGVHGQHV	CPs	1.812
TDAAVSFAKDF;CFVYPLDF	CPs	1.344
CFVYPLDF;NSGPVGR	CPs	15117.64
NSGPVGR;AEKFDCH	CPs	1696.452
AEKFDCH;FDCHYCRDNLQGK	CPs	0.009
FDCHYCRDNLQGK;DNLQGKKYVQK	CPs	0.021
DNLQGKKYVQK;DGHHCCLKCFDKFC	CPs	0.033
DGHHCCLKCFDKFC;HDTCFRCSKC	CPs	0.27
HDTCFRCSKC;RCSKCLQPL	CPs	0.015
RCSKCLQPL;ASETFVAKDN	CPs	0.027
ASETFVAKDN;PKCKGCLKP	CPs	0.069
PKCKGCLKP;KGTVWHKDCFTCSNCKQ	CPs	0.645
KGTVWHKDCFTCSNCKQ;VWHKDCFTCSN	CPs	0.009
VWHKDCFTCSN;WHKDCFTCSNCKQ	CPs	0.003
WHKDCFTCSNCKQ;GEDFYCVTCHEAKFAKHCVKCN	CPs	0.069
GEDFYCVTCHEAKFAKHCVKCN;EDFYCVTCHEAK	CPs	0.003
EDFYCVTCHEAK;YCVTCHEA	CPs	0.009
YCVTCHEA;HAECFVCVTCSK	CPs	0.522
HAECFVCVTCSK;VCVTCSKKLAGQ	CPs	0.015
VCVTCSKKLAGQ;KLAGQRFTAVEDQ	CPs	0.021
KLAGQRFTAVEDQ;TAVEDQYYCVDCYK	CPs	0.021
TAVEDQYYCVDCYK;YYCVDCYKNFVAKK	CPs	0.018
YYCVDCYKNFVAKK;YCVDCYKNFVAK	CPs	0.003
YCVDCYKNFVAK;EGQSWHDYCFHCKK	CPs	1.221
EGQSWHDYCFHCKK;SWHDYCFHCK	CPs	0.009
SWHDYCFHCK;WHDYCFHCKK	CPs	0.003
WHDYCFHCKK;HDYCFHCK	CPs	0.003
HDYCFHCK;RFVFHQEQ	CPs	0.051
RFVFHQEQ;VFHQEQVYCPDCAKK	CPs	0.006
VFHQEQVYCPDCAKK;FHQEQVYCPDCAKKL	CPs	0.003
FHQEQVYCPDCAKKL;HQEQVYCPDCAKK	CPs	0.003
HQEQVYCPDCAKK;QEQVYCPDCAKKL	CPs	0.003
QEQVYCPDCAKKL;EQVYCPDCAKK	CPs	0.003
EQVYCPDCAKK;QVYCPDCAK	CPs	0.003
QVYCPDCAK;VYCPDCAKKL	CPs	0.003

VYCPDCAKKL;YCPDCAKK	CPs	0.003
YCPDCAKK;EHNTEKPEPTEQKR	CPs	3182.241
EHNTEKPEPTEQKR;KDWYDVK	CPs	1441.548
KDWYDVK;MEDLVVEVR	CPs	6606.291
MEDLVVEVR;GASVKP	CPs	0.495
GASVKP;AGGAVPNLKEK	CPs	3681.462
AGGAVPNLKEK;DKEVPHPREP	CPs	1020.174
DKEVPHPREP;RAAAAAP	CPs	1521.606
RAAAAAP;TLKASEGEGGGGMR	CPs	441.6
TLKASEGEGGGGMR;DDFKGLQHL	CPs	569.838
DDFKGLQHL;PAGLPDLK	CPs	2.184
PAGLPDLK;LPLVDT	CPs	474.891
LPLVDT;PAPGMALPP	CPs	0.063
PAPGMALPP;VDSDMYDLPKKEDA	CPs	214.938
VDSDMYDLPKKEDA;DLPKKEDAL	CPs	0.018
DLPKKEDAL;SDADPFHR	CPs	0.324
SDADPFHR;MLLYTEVTRY	CPs	70.542
MLLYTEVTRY;LDFKVVEGSF	CPs	0.03
LDFKVVEGSF;MEQPGAAAASGVGGSGEEPGGGR	CPs	7799.691
MEQPGAAAASGVGGSGEEPGGGR;PELRQVSV	CPs	4900.203
PELRQVSV;LSLDF	CPs	6897.929
LSLDF;EPPSGPEL	CPs	1002.709
EPPSGPEL;LYTLVTYVPVTTFK	CPs	1008.468
LYTLVTYVPVTTFK;TLVTYVPVTT	CPs	0.006
TLVTYVPVTT;SAFLDKVVGLQK	CPs	3107.625
SAFLDKVVGLQK;GVVVFP	CPs	52.424
GVVVFP;LLQAQGVEVPSKD	CPs	15605.59
LLQAQGVEVPSKD;MSLAQKK	CPs	9847.461
MSLAQKK;APAAH	CPs	764.616
APAAH;AAVWQQVL	CPs	850.17
AAVWQQVL;LPAPGPSPSDKK	CPs	61.134
LPAPGPSPSDKK;TSTGQDSTTPKER	CPs	147.423
TSTGQDSTTPKER;EELAPYPK	CPs	102.42
EELAPYPK;DPLHLLPP	CPs	3008.307
DPLHLLPP;SSVQRVR	CPs	2082.987
SSVQRVR;LLFDP	CPs	27.654
LLFDP;DPGALVPL	CPs	962.151
DPGALVPL;LETALPV	CPs	1038.204
LETALPV;VPKSVTGP	CPs	1557.009
VPKSVTGP;DDHNWWQGK	CPs	7569.855
DDHNWWQGK;EEVVKLPAFKRK	CPs	17.625
EEVVKLPAFKRK;SAEFLLHMLK	CPs	668.778
SAEFLLHMLK;ALKRFS	CPs	6105.603
ALKRFS;VTSLFQVLPQ	CPs	1837.452
VTSLFQVLPQ;ESVGNLQ	CPs	907.386
ESVGNLQ;VTQPVVAKETAVSK	CPs	93.195
VTQPVVAKETAVSK;NLSTLEDLNTRWK	CPs	344.991
NLSTLEDLNTRWK;QDSPLSQPR	CPs	285
QDSPLSQPR;GPSPPPPPPP	CPs	417.999
GPSPPPPPPP;GVPRLP	CPs	226.719
GVPRLP;AGDPVANVRF	CPs	6724.671

AGDPVANVRF;FRPGAGQPPR	CPs	3585.27
FRPGAGQPPR;SELKYVPK	CPs	6.729
SELKYVPK;FDPEFTAKTPK	CPs	1467.591
FDPEFTAKTPK;CFDGVKRLC	CPs	28.023
CFDGVKRLC;RPKKPPPPSAP	CPs	481.908
RPKKPPPPSAP;EACLGGHPSCVK	CPs	5117.997
EACLGGHPSCVK;LLKLLGL	CPs	3388.575
LLKLLGL;VQQTLLEK	CPs	4663.944
VQQTLLEK;QGAMHTL	CPs	8833.221
QGAMHTL;MDLVKS	CPs	644.922
MDLVKS;PAASK	CPs	4404.048
LTTTKGF;KSVKEFH	CPs	7137.367
KSVKEFH;FTAYQ	CPs	4.097
FTAYQ;ETVHPPR	CPs	2916.201
ETVHPPR;PPGPPGPP	CPs	1334.449
PPGPPGPP;EFGFDGDFY	CPs	0.054
EFGFDGDFY;EDLLSENRK	CPs	105.036
EDLLSENRK;LLLPGELAKHAV	CPs	937.803
LLLPGELAKHAV;LPGELAKHAVS	CPs	0.006
LPGELAKHAVS;HYVDPTTM	CPs	2472.654
HYVDPTTM;LVYHLRPY	CPs	0.039
LVYHLRPY;KSPGNK	CPs	323.235
KSPGNK;LPPDPGY	CPs	734.885
LPPDPGY;KYEPPLL	CPs	4118.871
KYEPPLL;TLLQKLDFPTMKF	CPs	1494.043
TLLQKLDFPTMKF;FADKVPK	CPs	9610.77
FADKVPK;TGEKGFGYK	CPs	0.048
TGEKGFGYK;MDMSPLRPQNY	CPs	1653.537
MDMSPLRPQNY;KDPLRLK	CPs	8585.982
KDPLRLK;DYLHATAY	CPs	2741.385
DYLHATAY;TQVFP	CPs	867.905
TQVFP;KMFYLL	CPs	1277.796
KMFYLL;ALMMTAK	CPs	421.906
ALMMTAK;KDFSMNK	CPs	1353.272
KDFSMNK;MEELLRK	CPs	154.393
MEELLRK;TTKPSR	CPs	216.678
TTKPSR;AAVSAARA	CPs	2110.097
AAVSAARA;MDTRLPR	CPs	1702.597
MDTRLPR;PDDMGM	CPs	1390.038
PDDMGM;EDEEVKATFRP	CPs	797.349
EDEEVKATFRP;ADTFGLN	CPs	0.126
ADTFGLN;SVEKPLGPT	CPs	39.066
SVEKPLGPT;KDVKRH	CPs	1746.165
KDVKRH;FTPSKK	CPs	183.999
FTPSKK;VRPPQHP	CPs	4323.51
VRPPQHP;VDTPSGTNSGAGK	CPs	845.691
VDTPSGTNSGAGK;MGGSH	CPs	1042.584
MGGSH;MLNQDSAVHVK	CPs	1064.379
MLNQDSAVHVK;KPTHPLP	CPs	2114.022
KPTHPLP;DAVFAP	CPs	1285.677
DAVFAP;ASKTPVGF	CPs	1278.408

ASKTPVGF;MVGGVEEEF	CPs	81.762
MVGGVEEEF;VSGPPK	CPs	4285.725
VSGPPK;MEDGRELDL	CPs	2005.485
MEDGRELDL;DDEMPSHDLH	CPs	52.746
DDEMPSHDLH;SESPVGPKP	CPs	43.05
SESPVGPKP;ESPVGPKPT	CPs	0.003
ESPVGPKPT;AEHQWVETSPK	CPs	1.56
AEHQWVETSPK;EPPEALGPPSNQ	CPs	0.147
EPPEALGPPSNQ;PVRAAAP	CPs	1150.086
PVRAAAP;AAPEPLH	CPs	410.037
AAPEPLH;AERGYSF	CPs	1567.224
AERGYSF;KGTVMTFL	CPs	4438.653
KGTVMTFL;PGGLLLGDEAPNFEAN	CPs	1389.078
PGGLLLGDEAPNFEAN;KLAPEFAKR	CPs	0.162
KLAPEFAKR;DSVEDHLA	CPs	0.054
DSVEDHLA;SVEDHLAW	CPs	0.003
SVEDHLAW;KELPSGKKYLR	CPs	0.402
KELPSGKKYLR;ELPSGKKY	CPs	0.003
ELPSGKKY;LRYTPQP	CPs	0.024
LRYTPQP;LRAAL	CPs	2502.803
LRAAL;SSPVAFPR	CPs	1981.578
SSPVAFPR;MKTVP	CPs	3090.114
MKTVP;ASSAQSGGSSGGPAVPTVQRG	CPs	361.204
ASSAQSGGSSGGPAVPTVQRG;RLAECEEQAKASKK	CPs	44.292
RLAECEEQAKASKK;LPGLVQEGEPFSEEATLFTK	CPs	277.26
LPGLVQEGEPFSEEATLFTK;TAERSAYY	CPs	85.476
TAERSAYY;LSAEEAAKQKKEK	CPs	0.033
LSAEEAAKQKKEK;LLNVEHL	CPs	11.622
LLNVEHL;DYDHDAFLGAEEAK	CPs	435.306
DYDHDAFLGAEEAK;VTVDELK	CPs	4.92
VTVDELK;TAFLHPEEYDY	CPs	4.254
TAFLHPEEYDY;ADEPEWVKTEREQ	CPs	2.166
ADEPEWVKTEREQ;VGSQATDFGEALVRHDEF	CPs	1.863
VGSQATDFGEALVRHDEF;FGEALVR	CPs	0.021
FGEALVR;VRHDEF	CPs	0.015
VRHDEF;PGFGLGDEVD	CPs	55.452
PGFGLGDEVD;NRDWQDGK	CPs	5.1
NRDWQDGK;AKLKPGAPVRSK	CPs	1.869
AKLKPGAPVRSK;VTDNGDGTYRVQY	CPs	8.838
VTDNGDGTYRVQY;GDFLGRER	CPs	7.494
GDFLGRER;SVRFVPQEMGPH	CPs	0.327
SVRFVPQEMGPH;VVPVASLSDDAR	CPs	0.732
VVPVASLSDDAR;VLVETVTK	CPs	2.184
VLVETVTK;MGNRVYNVT	CPs	0.348
MGNRVYNVT;EESGKPGAHVT	CPs	364.701
EESGKPGAHVT;MDDFERRR	CPs	5427.897
MDDFERRR;KREEMRLEAER	CPs	0.039
KREEMRLEAER;TTNTQAEGDDEAALLERLA	CPs	69.66
TTNTQAEGDDEAALLERLA;AEGDDEAALLERL	CPs	0.015
AEGDDEAALLERL;EGDDEAALLERLA	CPs	0.003
EGDDEAALLERLA;RMQNDTTDNETAEKEGR	CPs	0.141

RMQNDTTDNETAEKEGR;SHQKNDWMEAEEKK	CPs	0.105
SHQKNDWMEAEEKK;VEVVVDEK	CPs	0.111
VEVVVDEK;KGEKVEEKTAGKGVNEK	CPs	0.504
KGEKVEEKTAGKGVNEK;KVQEDKPHTAVPK	CPs	0.051
KVQEDKPHTAVPK;SQEDKAAFKK	CPs	1.296
SQEDKAAFKK;FLDAKKGFTEVK	CPs	6.153
FLDAKKGFTEVK;VSEAKEAEGTSQVEAGK	CPs	4.659
VSEAKEAEGTSQVEAGK;VEAGKRLEELR	CPs	0.036
VEAGKRLEELR;RLEELRRR	CPs	0.015
RLEELRRR;RGETESEEFEKLKQK	CPs	0.024
RGETESEEFEKLKQK;ETESEEFEKLK	CPs	0.006
ETESEEFEKLK;TESEEFEKLKQK	CPs	0.003
TESEEFEKLKQK;QQEAALELEELKK	CPs	0.036
QQEAALELEELKK;LELEELKKK	CPs	0.015
LELEELKKK;VLEEEEQRKK	CPs	0.045
VLEEEEQRKK;RQKMPEDGLSEDKKPF	CPs	2.763
RQKMPEDGLSEDKKPF;EDGLSEDKKPFK	CPs	0.015
EDGLSEDKKPFK;KKPFKCFTPK	CPs	0.021
KKPFKCFTPK;SGVKSTHQAAVVSK	CPs	7.485
SGVKSTHQAAVVSK;DSRLEQY	CPs	0.045
DSRLEQY;NEWLTKTPEG	CPs	3.12
NEWLTKTPEG;TPEGSKSPAPKP	CPs	0.018
TPEGSKSPAPKP;DLRPGDVSGKR	CPs	6.243
DLRPGDVSGKR;LWEKQSVDK	CPs	0.036
LWEKQSVDK;WEKQSVDKVTSPTK	CPs	0.003
WEKQSVDKVTSPTK;EKQSVDKVTSPT	CPs	0.003
EKQSVDKVTSPT;VDKVTSPTKV	CPs	0.012
VDKVTSPTKV;DVDAKKKPPSC	CPs	799.479
DVDAKKKPPSC;TQTNLKR	CPs	749.138
TQTNLKR;EVDCCC	CPs	604.809
EVDCCC;EELLDL	CPs	557.874
EELLDL;VSSPPLLK	CPs	447.925
VSSPPLLK;HLQPPPP	CPs	964.311
HLQPPPP;APSAPAPK	CPs	1679.493
APSAPAPK;NLSRGP	CPs	260.124
NLSRGP;QPPAMLPHKAVYLR	CPs	1894.89
QPPAMLPHKAVYLR;AMLPHKAVY	CPs	0.009
AMLPHKAVY;PVVAPKP	CPs	127.524
PVVAPKP;APKPKVNPFRP	CPs	0.009
APKPKVNPFRP;LLDDMTKNDPF	CPs	1.008
LLDDMTKNDPF;DDMTKNDPFKA	CPs	0.006
DDMTKNDPFKA;VPPPVTTPF	CPs	0.168
VPPPVTTPF;TQPRGPLAPSPAPK	CPs	0.207
TQPRGPLAPSPAPK;KFSPVTPK	CPs	0.039
KFSPVTPK;FSPGAPGGPGSQPHQK	CPs	0.045
FSPGAPGGPGSQPHQK;APGPLTLKEVEELEQLTQK	CPs	4.35
APGPLTLKEVEELEQLTQK;PGPLTLKE	CPs	0.003
PGPLTLKE;VEELEQLTQK	CPs	0.024
VEELEQLTQK;SLEGAPY	CPs	0.372
SLEGAPY;LSKKGPK	CPs	208.329
LSKKGPK;LADTERFY	CPs	5007.24

LADTERFY;AATGQFP	CPs	347.997
AATGQFP;LSGSLP	CPs	234.42
LSGSLP;LAPLML	CPs	167.879
LAPLML;GSTRVPSS	CPs	590.332
GSTRVPSS;VPGPPPPPP	CPs	405.414
VPGPPPPPP;AQKKYLQ	CPs	152.268
AQKKYLQ;YLRAEPEDH	CPs	1224.603
YLRAEPEDH;LAATLTK	CPs	311.126
LAATLTK;LTCESFPHAVDH	CPs	0.052
LTCESFPHAVDH;KVVKAEK	CPs	1016.307
KVVKAEK;VEDELKAEKRK	CPs	1503.216
VEDELKAEKRK;FEGDFKTRPK	CPs	472.731
FEGDFKTRPK;HGGLL	CPs	182.418
HGGLL;VDYYEVL	CPs	0.003
QAKLPR;KKPEWGK	CPs	19.571
KKPEWGK;VPVAGF	CPs	659.506
VPVAGF;ETLFQP	CPs	5736.101
ETLFQP;SSGGGEYKTHF	CPs	3013.354
SSGGGEYKTHF;VVGKEDSLK	CPs	175.122
VVGKEDSLK;AFRKAVFVK	CPs	0.219
AFRKAVFVK;VASRLVQ	CPs	7000.58
VASRLVQ;YPLEHA	CPs	1531.819
YPLEHA;KPCDVSR	CPs	1439.774
KPCDVSR;LPGELAKHAVSEGTKAVTKYTSSK	CPs	1418.116
LPGELAKHAVSEGTKAVTKYTSSK;HAVSEGTKAVTKYTS	CPs	0.021
HAVSEGTKAVTKYTS;KAAQATR	CPs	2051.999
KAAQATR;LSGAPRK	CPs	388.306
LSGAPRK;ELTSKYR	CPs	151.07
ELTSKYR;MEQDHSPRK	CPs	645.712
MEQDHSPRK;KRVQSVVL	CPs	185.69
KRVQSVVL;CCDTQ	CPs	4.503
CCDTQ;PKPPPPPP	CPs	84.91
PKPPPPPP;LMAVPEV	CPs	862.014
LMAVPEV;DVPGGLK	CPs	97.427
DVPGGLK;LLNELERLGQDL	CPs	109.906
LLNELERLGQDL;VTQKSYKVSTSAPR	CPs	192.543
VTQKSYKVSTSAPR;TSAPRSFSSRS	CPs	0.027
TSAPRSFSSRS;SSAFSRVGN	CPs	0.063
SSAFSRVGN;YGGAPGL	CPs	0.078
YGGAPGL;YEELQTLAGK	CPs	5.022
YEELQTLAGK;LAELEAALRNAK	CPs	0.96
LAELEAALRNAK;SSYGTPGFN	CPs	0.813
SSYGTPGFN;TSSKAVVVKK	CPs	0.057
TSSKAVVVKK;SSKAVVVK	CPs	0.003
SSKAVVVK;DGKLVSESSDVLSK	CPs	0.039
DGKLVSESSDVLSK;TAAENEFVVLKK	CPs	54.672
TAAENEFVVLKK;MNKVELEAK	CPs	19.698
MNKVELEAK;LLEGEESR	CPs	109.611
LLEGEESR;ATAENEFVVLKK	CPs	377.766
ATAENEFVVLKK;LAGLEEAL	CPs	1.95
LAGLEEAL;ADSLPDSVRN	CPs	2141.049

ADSLPDSVRN;DEGFGHRPHKD	CPs	34.299
DEGFGHRPHKD;CQLVAWQ	CPs	56.514
CQLVAWQ;TAAPTMG	CPs	127.122
TAAPTMG;EEKSQMASLTKTGQN	CPs	132.173
EEKSQMASLTKTGQN;EEALPL	CPs	153.322
EEALPL;TGAGKHVPR	CPs	454.473
TGAGKHVPR;VDWCPTGF	CPs	1.377
VDWCPTGF;QPPTVVPGGDLAKVQR	CPs	0.042
QPPTVVPGGDLAKVQR;EDMAALEK	CPs	0.195
EDMAALEK;LMERLSVDYGKK	CPs	45.726
LMERLSVDYGKK;LSVDYGKKS	CPs	0.012
LSVDYGKKS;SVDYGKKSKLEF	CPs	0.003
SVDYGKKSKLEF;ARLDHKFDLMYA	CPs	0.693
ARLDHKFDLMYA;RAFVHW	CPs	0.039
RAFVHW;EGEFSEAREDMAALEK	CPs	0.039
EGEFSEAREDMAALEK;LFPAAY	CPs	85.773
LFPAAY;AGELADKKDR	CPs	150.843
AGELADKKDR;KNERF	CPs	1296.828
KNERF;YLLSQSSPAPL	CPs	4440.03
YLLSQSSPAPL;LYEEVHPKQHAHKQ	CPs	3.888
LYEEVHPKQHAHKQ;AEAYEVL	CPs	453.069
AEAYEVL;TSACPLLR	CPs	3476.31
TSACPLLR;YQPDHV	CPs	8328.735
YQPDHV;KAATSPTP	CPs	141.477
KAATSPTP;LVGAKLQ	CPs	247.629
LVGAKLQ;RAGPH	CPs	1472.103
RAGPH;KKSLP	CPs	5144.318
KKSLP;VEDSKDVNVNFEKS	CPs	951.844
VEDSKDVNVNFEKS;SCLGGSDNF	CPs	0.201
SCLGGSDNF;GGSDNFKHL	CPs	0.009
GGSDNFKHL;DVDLPEVD	CPs	3.681
DVDLPEVD;LPRPPGP	CPs	9.792
LPRPPGP;DGTEGLVRGQK	CPs	1.017
DGTEGLVRGQK;AEAPEFVEM	CPs	0.681
AEAPEFVEM;FRDQEGQDVLLF	CPs	1.122
FRDQEGQDVLLF;AHLDATTVL	CPs	0.954
AHLDATTVL;DVARGVQK	CPs	0.126
DVARGVQK;QVAEVFTGHLGK	CPs	0.531
QVAEVFTGHLGK;VAEVFTGHL	CPs	0.003
VAEVFTGHL;EYDHLPEQA	CPs	0.633
EYDHLPEQA;YDHLPEQ	CPs	0.003
YDHLPEQ;EAVAKADKLAEEHS	CPs	0.048
EAVAKADKLAEEHS;HRSKDH	CPs	32.693
HRSKDH;GPAGP	CPs	130.153
GPAGP;VEEAAKVTGK	CPs	189.66
VEEAAKVTGK;AAGPPTVH	CPs	3.033
AAGPPTVH;ATETVELHKLK	CPs	292.455
ATETVELHKLK;LLVTLPAALHY	CPs	112.335
LLVTLPAALHY;MGGGCC	CPs	10.623
MGGGCC;KGYLVSVDGY	CPs	2478.84
KGYLVSVDGY;AGGHRLLLE	CPs	38.364

AGGHRLLLE;QSAPGHRP	CPs	25.494
QSAPGHRP;PEFLEDPSVL	CPs	2403.318
PEFLEDPSVL;FLEDPSVLTK	CPs	0.006
FLEDPSVLTK;EDPSVLTKEKL	CPs	0.006
EDPSVLTKEKL;LQHLTARNRPPL	CPs	0.108
LQHLTARNRPPL;SNEDLLDQLVK	CPs	7.365
SNEDLLDQLVK;NTAQLQR	CPs	4.532
NTAQLQR;GRPLELSDF	CPs	11.941
GRPLELSDF;ANTLRDAAPK	CPs	39.45
ANTLRDAAPK;ELERLQKE	CPs	2264.994
ELERLQKE;FVALPGVGVSML	CPs	288.108
FVALPGVGVSML;DDEVDVDGTVEEDLGKSR	CPs	2295.132
DDEVDVDGTVEEDLGKSR;VDVDGTVEEDLGKS	CPs	0.009
VDVDGTVEEDLGKS;VDGTVEEDLGKSR	CPs	0.006
VDGTVEEDLGKSR;SLTDENALAGNEELTVK	CPs	1.725
SLTDENALAGNEELTVK;VTDTGVGMTREELVK	CPs	1.128
VTDTGVGMTREELVK;DTGVGMTREELV	CPs	0.006
DTGVGMTREELV;SGTSEFLNK	CPs	0.06
SGTSEFLNK;VLKEEASDY	CPs	5.25
VLKEEASDY;VEKTVWDWEL	CPs	1.818
VEKTVWDWEL;TAEGEVTFK	CPs	1.884
TAEGEVTFK;PRGLFDEY	CPs	0.054
PRGLFDEY;SDDLPLNV	CPs	0.351
SDDLPLNV;DEKYNDTFWK	CPs	0.537
DEKYNDTFWK;ESSPFVERLLKK	CPs	0.486
ESSPFVERLLKK;LTEPVDEY	CPs	0.054
LTEPVDEY;RFQNVAKEGVK	CPs	0.057
RFQNVAKEGVK;EAVEKEFEPL	CPs	0.066
EAVEKEFEPL;LTESPCAL	CPs	0.594
LTESPCAL;EDEDDKTVSDLAVV	CPs	1.83
EDEDDKTVSDLAVV;AVETLSPDWEFDRVD	CPs	1022.925
AVETLSPDWEFDRVD;VSLDV	CPs	1383.558
VSLDV;KVPLTGKK	CPs	234.609
KVPLTGKK;TQTLRPGVK	CPs	320.121
TQTLRPGVK;EVPLP	CPs	494.402
EVPLP;LVELSGR	CPs	522.916
LVELSGR;MDFLLGNPF	CPs	1979.871
MDFLLGNPF;DHDPLQAPELHR	CPs	80.721
DHDPLQAPELHR;APQDLR	CPs	130.008
APQDLR;TVFQVVVEH	CPs	526.389
TVFQVVVEH;ALKDKLEK	CPs	7732.934
ALKDKLEK;PLSEVRDLDF	CPs	1123.168
PLSEVRDLDF;VYTFAKK	CPs	289.49
VYTFAKK;TEYKLVVVGAGGVGKSA	CPs	1302.412
TEYKLVVVGAGGVGKSA;KLVVVGAGGVGK	CPs	0.009
KLVVVGAGGVGK;KDLASPP	CPs	199.395
KDLASPP;MEDVHSL	CPs	2788.158
MEDVHSL;KLKDEEVAQLKK	CPs	785.772
KLKDEEVAQLKK;TEKAPEPHVEEDDDELD	CPs	6421.665
TEKAPEPHVEEDDDELD;PTAPNVTVTRLT	CPs	1.008
PTAPNVTVTRLT;VLKEGVEY	CPs	2.04

VLKEGVEY;MVGSYGPR	CPs	4.422
MVGSYGPR;KPPGPPPGPPPP	CPs	147.105
KPPGPPPGPPPP;TSSSRLSFS	CPs	232.785
TSSSRLSFS;VHKSVMEEL	CPs	3623.196
VHKSVMEEL;STQFGGYAK	CPs	1236.438
STQFGGYAK;KELDLGP	CPs	24.66
KELDLGP;SLCALRAVDQ	CPs	565.446
SLCALRAVDQ;FVELTMPY	CPs	5.988
FVELTMPY;KDEFPF	CPs	17.109
KDEFPF;TPQPSGP	CPs	2030.724
TPQPSGP;KPPGPPP	CPs	446.997
KPPGPPP;AAPEVPK	CPs	393.57
AAPEVPK;VLSGVHELEQ	CPs	276.111
VLSGVHELEQ;KDYREPP	CPs	394.227
KDYREPP;LPPPVAP	CPs	580.071
LPPPVAP;NADEFENMVAEK	CPs	2101.209
NADEFENMVAEK;GVRVELA	CPs	298.866
GVRVELA;VDAGSGSPHSPPQ	CPs	32.178
VDAGSGSPHSPPQ;DSLVFF	CPs	21.399
DSLVFF;APALQTAQR	CPs	1309.005
APALQTAQR;AVPRASPTT	CPs	103.665
AVPRASPTT;SLRFDGA	CPs	257.247
SLRFDGA;PPTVVPGGDLA	CPs	4.377
PPTVVPGGDLA;VVPGGDLAK	CPs	0.009
VVPGGDLAK;ARLDHKFDLMYAK	CPs	0.081
ARLDHKFDLMYAK;DLMYAKRA	CPs	0.021
DLMYAKRA;VHWYVGEGME	CPs	0.027
VHWYVGEGME;YDSLVVGKL	CPs	36.033
YDSLVVGKL;LGPSLLTEL	CPs	1.533
LGPSLLTEL;AGQHDLVPH	CPs	72.504
AGQHDLVPH;EFKFPNRL	CPs	12.192
EFKFPNRL;LSAGGDFK	CPs	0.045
LSAGGDFK;QPGGGPRP	CPs	40.536
QPGGGPRP;TENSTSTPAAKPKRA	CPs	63.882
TENSTSTPAAKPKRA;ENSTSTPAAKPK	CPs	0.003
ENSTSTPAAKPK;KSTDHPKYSDM	CPs	0.054
KSTDHPKYSDM;RLVTTGVLKQTK	CPs	0.159
RLVTTGVLKQTK;TGVLKQTKGVG	CPs	0.012
TGVLKQTKGVG;ASGSFRLAKSDEPKR	CPs	0.033
ASGSFRLAKSDEPKR;SGSFRLAK	CPs	0.003
SGSFRLAK;SDEPKRSVAF	CPs	0.024
SDEPKRSVAF;APVMVEL	CPs	133.245
APVMVEL;QGCVPR	CPs	165.141
QGCVPR;AAAAAGPGAPLSADELLPK	CPs	395.355
AAAAAGPGAPLSADELLPK;GKDPFLD	CPs	218.502
GKDPFLD;AHGSVVKSEDY	CPs	6.75
AHGSVVKSEDY;GPPPPPPG	CPs	566.547
GPPPPPPG;KRAQSKGR	CPs	668.528
KRAQSKGR;AAAMDVDTPSG	CPs	393.772
AAAMDVDTPSG;VTPQPGK	CPs	154.197
VTPQPGK;HYLPNVD	CPs	224.679

HYLPNVD;NFLATAGAK	CPs	59.292
NFLATAGAK;AYPGVLL	CPs	3.51
AYPGVLL;FTPDLAH	CPs	3.915
FTPDLAH;RAGSALNRMKELQK	CPs	6.561
RAGSALNRMKELQK;QDHPVLL	CPs	291.093
QDHPVLL;TLRFYTK	CPs	126.863
TLRFYTK;EAPDDLK	CPs	441.73
EAPDDLK;VSLKPM	CPs	87.534
VSLKPM;LGGPTSVRGF	CPs	1299.546
LGGPTSVRGF;VAVSLQR	CPs	254.76
VAVSLQR;ATNNP	CPs	715.299
ATNNP;MEVAQPK	CPs	131.556
MEVAQPK;KAAAVHLLQ	CPs	786.192
KAAAVHLLQ;RGPPPPPR	CPs	191.601
RGPPPPPR;PAALLRGP	CPs	86.829
PAALLRGP;SLEVGVP	CPs	2249.067
SLEVGVP;VVPVP	CPs	210.759
VVPVP;GVVGVLR	CPs	635.637
GLPAGPRP;KVVKFPDEDEVLEK	CPs	1495.015
KVVKFPDEDEVLEK;SNQTGPAWAGSERDY	CPs	0.093
SNQTGPAWAGSERDY;VLSKGHAAPLL	CPs	328.911
VLSKGHAAPLL;GHAAPLLY	CPs	0.012
GHAAPLLY;AGELKWGTDEEKF	CPs	1433.532
AGELKWGTDEEKF;TGLGPLE	CPs	5013.369
TGLGPLE;QPVLLTEL	CPs	1621.074
QPVLLTEL;SDAPPLTL	CPs	445.506
SDAPPLTL;SVNSHFMKD	CPs	0.09
SVNSHFMKD;ASTTTCTRF	CPs	852.234
ASTTTCTRF;KGTLEAP	CPs	3819.195
KGTLEAP;KLHFSPKSK	CPs	52.005
KLHFSPKSK;HGPPA	CPs	1533.804
HGPPA;TDAETLPALK	CPs	299.454
TDAETLPALK;YEEVARK	CPs	1601.511
YEEVARK;ALATDQGSKL	CPs	5656.182
ALATDQGSKL;RRALPR	CPs	183.876
RRALPR;VTRLPVVLGH	CPs	309.24
VTRLPVVLGH;SVGEGVTTVRP	CPs	0.051
SVGEGVTTVRP;ESVPKLVTDFMAKK	CPs	12.444
ESVPKLVTDFMAKK;SVPKLVTD	CPs	0.003
SVPKLVTD;VPKLVTDF	CPs	0.003
VPKLVTDF;LVTDFMAK	CPs	0.009
LVTDFMAK;TDFMAKKF	CPs	0.006
TDFMAKKF;AKKFSLDQL	CPs	0.012
AKKFSLDQL;THVLPLEK	CPs	0.03
THVLPLEK;NEGFDLLRS	CPs	0.027
NEGFDLLRS;EEVEVAPPKDHEVR	CPs	183.528
EEVEVAPPKDHEVR;EVEVAPPK	CPs	0.003
EVEVAPPK;EVAPPKDHEVR	CPs	0.006
EVAPPKDHEVR;VDEFVTH	CPs	74.049
VDEFVTH;AAPARPPK	CPs	3397.335
AAPARPPK;KGLLA	CPs	7099.452

KGLLA;CPLSKKPLR	CPs	146.991
CPLSKKPLR;KFNLPPP	CPs	368.442
KFNLPPP;LFRFSQDSA	CPs	546.999
LFRFSQDSA;KESELLDF	CPs	237.075
KESELLDF;LQKEFLQ	CPs	3085.331
LQKEFLQ;HLLPRP	CPs	9480.393
HLLPRP;WAAPAPPK	CPs	598.192
WAAPAPPK;AMFEQMR	CPs	3772.191
AMFEQMR;LGDLLETCHF	CPs	0.309
LGDLLETCHF;DLDGFEF	CPs	1293.624
DLDGFEF;KLSTFP	CPs	1008.443
KLSTFP;VHTSVHGVGH	CPs	561.25
VHTSVHGVGH;SFVQSAFK	CPs	0.03
SFVQSAFK;ELDELVSAVEEHF	CPs	16.14
ELDELVSAVEEHF;KYNLQPKGE	CPs	0.051
KYNLQPKGE;VLAESDPGFKRTK	CPs	943.56
VLAESDPGFKRTK;YGMLFDEY	CPs	4.731
YGMLFDEY;ASMLTELL	CPs	12.924
ASMLTELL;LTAHTVK	CPs	415.503
LTAHTVK;RDKKSVP	CPs	1590.405
RDKKSVP;CEEEAAVQPHSR	CPs	371.475
CEEEAAVQPHSR;PRSGQARP	CPs	5169.774
PRSGQARP;KAANRLP	CPs	6124.746
KAANRLP;TKEVYELLDSPGK	CPs	110.931
TKEVYELLDSPGK;ELLDSPGKVL	CPs	0.015
ELLDSPGKVL;KFYPPKVEMF	CPs	6.945
KFYPPKVEMF;GVHLPF	CPs	101.174
GVHLPF;NVHLFL	CPs	11448.08
NVHLFL;PENDPNVVPK	CPs	2433.195
PENDPNVVPK;EDFEKPK	CPs	291.069
EDFEKPK;DVKSSLPR	CPs	972.546
DVKSSLPR;TFLLLVPF	CPs	1155.839
TFLLLVPF;RFKDLGEEHF	CPs	309.19
RFKDLGEEHF;VKLVNELTEFAK	CPs	0.942
VKLVNELTEFAK;VNELTEF	CPs	0.009
VNELTEF;AKTCVADESHAGCEK	CPs	0.021
AKTCVADESHAGCEK;LSHKDDSPDLPK	CPs	1.899
LSHKDDSPDLPK;DDSPDLPKL	CPs	0.012
DDSPDLPKL;KADEKKFWGK	CPs	0.06
KADEKKFWGK;AEFVEVTKL	CPs	3.786
AEFVEVTKL;LKECCDKPLLEK	CPs	1.071
LKECCDKPLLEK;LVEVSRSLGK	CPs	4.182
LVEVSRSLGK;VGTRCCTKPESER	CPs	0.03
VGTRCCTKPESER;AFDEKLF	CPs	0.798
AFDEKLF;FDEKLFTF	CPs	0.003
FDEKLFTF;LKHKPKATEEQLK	CPs	1.344
LKHKPKATEEQLK;ATEEQLKTVMENF	CPs	0.018
ATEEQLKTVMENF;LKTVMEN	CPs	0.015
LKTVMEN;EGGPLYK	CPs	62.306
EGGPLYK;DSPQEHP	CPs	393.568
DSPQEHP;HNSANVPF	CPs	1679.658

HNSANVPF;ERGLQKGQETPSK	CPs	592.749
ERGLQKGQETPSK;DVGLGDGLG	CPs	0.183
DVGLGDGLG;EFLGELQKKEEEMRQ	CPs	643.677
EFLGELQKKEEEMRQ;ELEEEVNNFQKK	CPs	2.145
ELEEEVNNFQKK;KKKAAAQLLQ	CPs	0.03
KKKAAAQLLQ;ASVLSLPYPF	CPs	736.362
ASVLSLPYPF;VTLAVPQ	CPs	987.567
VTLAVPQ;ELVLP	CPs	1372.209
ELVLP;SELYLPSST	CPs	2291.088
SELYLPSST;ATTKPR	CPs	2162.94
ATTKPR;KEALSRL	CPs	989.688
KEALSRL;APAPEVP	CPs	1629.987
APAPEVP;VGSKQPK	CPs	5.283
VGSKQPK;FNEKDGQVERK	CPs	318.027
FNEKDGQVERK;AATPLGLR	CPs	591.807
AATPLGLR;EDDLPVYL	CPs	390.723
EDDLPVYL;LGTDGTHY	CPs	51.609
LGTDGTHY;ESVKKVQVEK	CPs	1448.334
ESVKKVQVEK;KALLT	CPs	662.543
KALLT;SQPSGLQASKTGKK	CPs	5912.622
SQPSGLQASKTGKK;FAENPKGADEFVAK	CPs	976.873
FAENPKGADEFVAK;ANSTTRQDRF	CPs	0.225
ANSTTRQDRF;SGEGFYK	CPs	0.447
SGEGFYK;GEGFYKYK	CPs	0.003
GEGFYKYK;PGAPATHVP	CPs	1883.211
PGAPATHVP;AALPLLQ	CPs	115.284
AALPLLQ;PAARPRP	CPs	566.175
PAARPRP;ELVGLGA	CPs	244.911
ELVGLGA;LTAFHEL	CPs	851.208
LTAFHEL;PPPPPRP	CPs	845.559
PPPPPRP;ALDVHESEPF	CPs	179.808
ALDVHESEPF;APGGFGPK	CPs	255.972
LTELPVMVEL;VGPPTRP	CPs	285.156
VGPPTRP;PAPQVSTA	CPs	505.575
PAPQVSTA;MMLGTEGGEGFVVK	CPs	2.7
MMLGTEGGEGFVVK;VEMDWVLKHTGP	CPs	1.761
VEMDWVLKHTGP;SHGSHETDEEFDARWVTY	CPs	79.335
SHGSHETDEEFDARWVTY;VPAQKPP	CPs	407.267
VPAQKPP;GNYSERVGAGAPVYL	CPs	398.893
GNYSERVGAGAPVYL;SERVGAGAPV	CPs	0.009
SERVGAGAPV;YLAAVLE	CPs	0.03
YLAAVLE;AVLEYLT	CPs	0.009
AVLEYLT;TGGVKKPH	CPs	16.902
TGGVKKPH;KNYPP	CPs	313.073
KNYPP;PGPASGGSGEVDELFDVKNAFY	CPs	1400.068
PGPASGGSGEVDELFDVKNAFY;RASGLLR	CPs	53.862
RASGLLR;AAARVLLL	CPs	75.978
AAARVLLL;MEAATEF	CPs	315.432
MEAATEF;LDDEEYVVPGR	CPs	37.419
LDDEEYVVPGR;PMAWAR	CPs	163.32
PMAWAR;GPGPPLPS	CPs	6.549

GPGPPLPS;LPKAASK	CPs	36.732
LPKAASK;SGPKVAP	CPs	96.063
SGPKVAP;KEKPNSK	CPs	281.978
KEKPNSK;PPAAP	CPs	536.125
PPAAP;TLMRY	CPs	278.565
TLMRY;EDPFKSDPF	CPs	454.827
EDPFKSDPF;HRQGLLVV	CPs	254.684
HRQGLLVV;PSPPGLLP	CPs	4156.72
PSPPGLLP;KPGVK	CPs	172.608
KPGVK;LTLHGLQQY	CPs	184.761
LTLHGLQQY;HMETGNHY	CPs	248.274
HMETGNHY;KDVHFDSL	CPs	286.199
KDVHFDSL;VPALPP	CPs	588.088
VPALPP;LDSGVPFL	CPs	5.67
LDSGVPFL;ADGPVAEVLLR	CPs	146.451
ADGPVAEVLLR;LAFVLYNDQPPK	CPs	49.404
LAFVLYNDQPPK;ACVCKAHVG	CPs	53.634
ACVCKAHVG;VGKPAPEFQ	CPs	0.021
VGKPAPEFQ;FFYPLDF	CPs	0.195
FFYPLDF;LADVTRKLSSDY	CPs	0.768
LADVTRKLSSDY;VDDSKEYFSKHN	CPs	1.209
VDDSKEYFSKHN;SALVPAVGF	CPs	114.819
SALVPAVGF;LLLPVRQAF	CPs	1.242
LLLPVRQAF;GSLYVVH	CPs	415.641
GSLYVVH;VGKVVLV	CPs	1.725
VGKVVLV;ADEEKLPPGWEK	CPs	1188.885
ADEEKLPPGWEK;PPVDRPH	CPs	555.585
PPVDRPH;RKLLFF	CPs	27.083
RKLLFF;AAVLQQR	CPs	624.235
AAVLQQR;TGVSRAVP	CPs	18.93
TGVSRAVP;ATVVLAF	CPs	62.163
ATVVLAF;KEEAEKPEEAAKDQHR	CPs	99.888
KEEAEKPEEAAKDQHR;SSAHFNRGPAYGLSAEVK	CPs	67.251
SSAHFNRGPAYGLSAEVK;RGPAYGLSAEVKNK	CPs	0.018
RGPAYGLSAEVKNK;EQELREW	CPs	2.073
EQELREW;KLQPGSVKK	CPs	3.6
KLQPGSVKK;VNESTQNWH	CPs	0.027
VNESTQNWH;YGVKPHD	CPs	0.207
YGVKPHD;DLFENTNHTQ	CPs	0.036
DLFENTNHTQ;AKTKGNKVNVGVK	CPs	0.261
AKTKGNKVNVGVK;VGVKYAEKQE	CPs	0.027
VGVKYAEKQE;RFEPEKLREGRN	CPs	0.033
RFEPEKLREGRN;FEPEKLREGR	CPs	0.003
FEPEKLREGR;EPEKLREGRN	CPs	0.003
EPEKLREGRN;TRRHLYDPK	CPs	0.534
TRRHLYDPK;RRHLYDP	CPs	0.003
RRHLYDP;HLYDPKLG	CPs	0.006
HLYDPKLG;YDPKLGTDQPLDQ	CPs	0.006
YDPKLGTDQPLDQ;DPKLGTDQP	CPs	0.003
DPKLGTDQP;PKLGTDQPLDQAT	CPs	0.003
PKLGTDQPLDQAT;LGTDQPLDQ	CPs	0.006

LGTDQPLDQ;GTDQPLDQAT	CPs	0.003
GTDQPLDQAT;QAGMTAPGTK	CPs	0.159
QAGMTAPGTK;FEPGLGMEHCDT	CPs	0.039
FEPGLGMEHCDT;EPGLGMEHCD	CPs	0.003
EPGLGMEHCD;PGLGMEHCDT	CPs	0.003
PGLGMEHCDT;MGSNKGASQRGMT	CPs	0.048
MGSNKGASQRGMT;KGASQRGMTVY	CPs	0.012
KGASQRGMTVY;YGLPRQVYD	CPs	0.03
YGLPRQVYD;LPRQVYDPKYC	CPs	0.006
LPRQVYDPKYC;YDPKYCL	CPs	0.015
YDPKYCL;CLTPEYPELGEPAHN	CPs	0.015
CLTPEYPELGEPAHN;LTPEYPELGEPAH	CPs	0.003
LTPEYPELGEPAH;TPEYPELG	CPs	0.003
TPEYPELG;PEYPELGEPAHN	CPs	0.003
PEYPELGEPAHN;EYPELGEPAH	CPs	0.003
EYPELGEPAH;YPELGEPAHN	CPs	0.003
YPELGEPAHN;ELGEPAHNHHP	CPs	0.006
ELGEPAHNHHP;LGEPAHNHHPHN	CPs	0.003
LGEPAHNHHPHN;SSMAEVDAAMAARPH	CPs	74.379
SSMAEVDAAMAARPH;FVTFDDHDPVDKLVLQK	CPs	0.261
FVTFDDHDPVDKLVLQK;VTFDDHDP	CPs	0.003
VTFDDHDP;TFDDHDPVDKLVLQ	CPs	0.003
TFDDHDPVDKLVLQ;FDDHDPVDKLVLQK	CPs	0.003
FDDHDPVDKLVLQK;DDHDPVDKLVLQ	CPs	0.003
DDHDPVDKLVLQ;DHDPVDKLVLQK	CPs	0.003
DHDPVDKLVLQK;HDPVDKLVL	CPs	0.003
HDPVDKLVL;PVDKLVLQK	CPs	0.006
PVDKLVLQK;MDETDTLFPKLK	CPs	362.016
MDETDTLFPKLK;WLLDF	CPs	73.688
WLLDF;RGPPNCR	CPs	60.718
RGPPNCR;AASSLEQKL	CPs	202.401
AASSLEQKL;NAVLEYL	CPs	120.215
NAVLEYL;LAPLLVK	CPs	67.69
LAPLLVK;AAGVEAAAEVAATEPKM	CPs	208.584
AAGVEAAAEVAATEPKM;VEAAAEVAATEPK	CPs	0.009
VEAAAEVAATEPK;GNRFEPY	CPs	8.925
GNRFEPY;RFEPYANPTKRYR	CPs	0.006
RFEPYANPTKRYR;EGPFGGGME	CPs	18.831
EGPFGGGME;VPAGMGAGLER	CPs	9.168
VPAGMGAGLER;ESPEVAERACR	CPs	2.691
ESPEVAERACR;GGPGGGNF	CPs	178.515
GGPGGGNF;RVFTVDH	CPs	441.117
RVFTVDH;KLLPVGQTVF	CPs	0.021
KLLPVGQTVF;SAEFEVKEY	CPs	2.079
SAEFEVKEY;LYGEQVDGTAF	CPs	0.339
LYGEQVDGTAF;VTVMQDF	CPs	12.936
VTVMQDF;EEVPAADLSDQVPDTESETK	CPs	3.324
EEVPAADLSDQVPDTESETK;LDSTDQWEKF	CPs	0.912
LDSTDQWEKF;GYTQQLAFRQKSSAY	CPs	0.666
GYTQQLAFRQKSSAY;ALALLGKLEGDRLTKF	CPs	5.442
ALALLGKLEGDRLTKF;ALLALLARK	CPs	1.77

ALLALLARK;MVFQALAQYQKDVPDHK	CPs	1.422
MVFQALAQYQKDVPDHK;VHLQAGQCGN	CPs	176.004
VHLQAGQCGN;DPTGTYHGDSDLQLER	CPs	0.597
DPTGTYHGDSDLQLER;NALLEYL	CPs	63.581
NALLEYL;GPPPPPPPK	CPs	152.911
GPPPPPPPK;EPPPPKKPPPP	CPs	8.175
EPPPPKKPPPP;MGLTAALVKMGQLEGWQVFAEPKV	CPs	270.852
MGLTAALVKMGQLEGWQVFAEPKV;YLDVPVDLSK	CPs	124.32
YLDVPVDLSK;AAALAVER	CPs	5.235
AAALAVER;VSEPTTVAF	CPs	843.741
VSEPTTVAF;VQTGMDTTK	CPs	148.899
VQTGMDTTK;GMDTTKTVLTGTK	CPs	0.009
GMDTTKTVLTGTK;DTTKTVLTGTKDT	CPs	0.006
DTTKTVLTGTKDT;GVAKGAVQTGMDTTK	CPs	0.363
GVAKGAVQTGMDTTK;DTTKTVLTGAKDT	CPs	0.528
DTTKTVLTGAKDT;SYVEVLVPLPQ	CPs	112.677
SYVEVLVPLPQ;KFEVYEKNN	CPs	10.925
KFEVYEKNN;GGGSGARLL	CPs	71.347
GGGSGARLL;KLEELELDEQQKK	CPs	95.775
KLEELELDEQQKK;LAGGPPRP	CPs	66.762
LAGGPPRP;EPYTF	CPs	338.376
EPYTF;KPPLAGP	CPs	350.393
KPPLAGP;PGQPARP	CPs	27.889
PGQPARP;EKNPAKP	CPs	155.865
EKNPAKP;SSSGSSVSTAGRPG	CPs	293.478
SSSGSSVSTAGRPG;VEEPPGTGSSGLGSSSSTSSGGSFH	CPs	0.066
VEEPPGTGSSGLGSSSSTSSGGSFH;KFTPKYVLR	CPs	0.321
KFTPKYVLR;MDFQHRPG	CPs	246.744
MDFQHRPG;EAPAQPAPEKVK	CPs	1.626
EAPAQPAPEKVK;AGELNPVAPK	CPs	37.461
AGELNPVAPK;ADSEKLPVQK	CPs	2.643
ADSEKLPVQK;EKEDVPLVEK	CPs	0.06
EKEDVPLVEK;KEDVPLVEKK	CPs	0.003
KEDVPLVEKK;EDVPLVEK	CPs	0.003
EDVPLVEK;AATDLERF	CPs	127.173
AATDLERF;SLDLVTMKK	CPs	12.879
SLDLVTMKK;SLQETYEAK	CPs	2.46
SLQETYEAK;FGLLNGN	CPs	966.26
FGLLNGN;PFGMALP	CPs	4713.559
PFGMALP;DERVDLLSF	CPs	26.616
DERVDLLSF;TGSTQVGKQVAL	CPs	0.027
TGSTQVGKQVAL;MRYVASY	CPs	5012.097
MRYVASY;RKHSQTK	CPs	2053.514
RKHSQTK;METPGRVTP	CPs	3501.901
METPGRVTP;AAPEERDLTQEQTEK	CPs	61.254
AAPEERDLTQEQTEK;TVVGLDK	CPs	933.714
TVVGLDK;NEASLYL	CPs	18.09
NEASLYL;EGAPERP	CPs	118.238
EGAPERP;ELPMDPKSN	CPs	296.806
ELPMDPKSN;KEEEPVKKVLEKK	CPs	0.057
KEEEPVKKVLEKK;PSPNMRP	CPs	1490.372

PSPNMRP;VAPFDLP	CPs	1759.509
VAPFDLP;KSSGNPLNDKDKDKNVRQ	CPs	562.876
KSSGNPLNDKDKDKNVRQ;YTSVTPVLR	CPs	164.886
YTSVTPVLR;LDPLPPPPPP	CPs	6.432
LDPLPPPPPP;VGPDFQKGL	CPs	159.99
VGPDFQKGL;YDTKLGTDKCDN	CPs	2.646
YDTKLGTDKCDN;MEVADPQQL	CPs	113.769
MEVADPQQL;ASDEGKLFV	CPs	52.545
ASDEGKLFV;SGGYGGSRDYY	CPs	0.645
SGGYGGSRDYY;TPPGGFPPP	CPs	131.706
TPPGGFPPP;PQGYGAPP	CPs	0.036
PQGYGAPP;GPPPPPPDQ	CPs	0.651
GPPPPPPDQ;AEVEQKKKRTFR	CPs	5.295
AEVEQKKKRTFR;FTYRGVDLDQ	CPs	0.039
FTYRGVDLDQ;AQALDHL	CPs	24.504
AQALDHL;EALPGHRR	CPs	1.44
EALPGHRR;KGLVVT	CPs	251.952
KGLVVT;MDFDKK	CPs	131.427
MDFDKK;ANLLK	CPs	253.709
ANLLK;VDMGHSSY	CPs	121.771
VDMGHSSY;NHAAPFSK	CPs	13.518
NHAAPFSK;VLTFYRKEPF	CPs	0.024
VLTFYRKEPF;GPPVASHPVTPS	CPs	1535.559
GPPVASHPVTPS;VVTVVTTKK	CPs	81.57
VVTVVTTKK;GPGVHRK	CPs	982.413
GPGVHRK;RRSLLR	CPs	231.117
RRSLLR;EPGALSTSVL	CPs	224.942
EPGALSTSVL;MFNYERPKHF	CPs	1640.557
MFNYERPKHF;TEQRYSASSTVSSH	CPs	0.141
TEQRYSASSTVSSH;EGEFQRLAA	CPs	14.055
EGEFQRLAA;QGLPGLVS	CPs	346.109
QGLPGLVS;TLAVERLLEPL	CPs	398.401
TLAVERLLEPL;LKEELVAAVEDVRK	CPs	1.257
LKEELVAAVEDVRK;DDWEEKRHF	CPs	494.703
DDWEEKRHF;FDDRGPSL	CPs	0.039
FDDRGPSL;VLDYDHGSR	CPs	0.03
VLDYDHGSR;QLLQLVEPF	CPs	7.407
QLLQLVEPF;SGYSDSAVLK	CPs	1.263
SGYSDSAVLK;GDVASDGKKEPSDKAVKK	CPs	2.427
GDVASDGKKEPSDKAVKK;VTLDEVGDEEDSELQK	CPs	1.566
VTLDEVGDEEDSELQK;DEEDSELQKLRK	CPs	0.021
DEEDSELQKLRK;KEFVPGVK	CPs	27.654
KEFVPGVK;YCGGGPL	CPs	87.384
YCGGGPL;YMMCSP	CPs	876.389
YMMCSP;EDFRKLLM	CPs	172.366
EDFRKLLM;HGVYDKPK	CPs	612.669
HGVYDKPK;LLLEAQ	CPs	24.834
LLLEAQ;PAAYRK	CPs	67.347
PAAYRK;EAAGPLLH	CPs	515.769
EAAGPLLH;PSEPAAP	CPs	7.92
PSEPAAP;MADVVY	CPs	36.915

MADVVY;TGGPLLS	CPs	4246.959
TGGPLLS;KLKLPEK	CPs	405.912
KLKLPEK;KGSSAPR	CPs	2804.238
KGSSAPR;DGGEHVVSSEKAPR	CPs	1280.355
DGGEHVVSSEKAPR;SSQGAKEKPVGELGTPK	CPs	0.114
SSQGAKEKPVGELGTPK;MEGCSEEASLLRHLEK	CPs	279.642
MEGCSEEASLLRHLEK;AKPKPKPNQNLSEASGK	CPs	1.386
AKPKPKPNQNLSEASGK;TSGLELLR	CPs	24.93
TSGLELLR;LRPSFNR	CPs	0.024
LRPSFNR;ADDFGFF	CPs	640.118
ADDFGFF;SVANKFYVH	CPs	310.039
SVANKFYVH;VLEETAPEDVQK	CPs	2.385
VLEETAPEDVQK;VVFDDSEPVQK	CPs	3.234
VVFDDSEPVQK;GKPVYM	CPs	2295.938
GKPVYM;GAAPSVNP	CPs	635.479
GAAPSVNP;ATQVEPLLPGGAP	CPs	0.156
ATQVEPLLPGGAP;KLSHPKL	CPs	3064.275
KLSHPKL;SVYVYKVLK	CPs	3983.373
SVYVYKVLK;LDSNAVHH	CPs	2272.233
LDSNAVHH;AVGTVP	CPs	5162.397
AVGTVP;MDALVAH	CPs	167.745
MDALVAH;EEMLRK	CPs	27.993
EEMLRK;TGVDNPGHP	CPs	727.209
TGVDNPGHP;LYDLVMTH	CPs	5222.841
LYDLVMTH;ALEWPSLTAQ	CPs	0.024
ALEWPSLTAQ;WLVSPPPP	CPs	405.849
WLVSPPPP;KYYVPK	CPs	8229.89
KYYVPK;TQAGEKAPVPK	CPs	901.942
TQAGEKAPVPK;SEQPPSEKSTKPK	CPs	45.288
SEQPPSEKSTKPK;DDAVEALAGSLGK	CPs	26.451
DDAVEALAGSLGK;KTGNYGY	CPs	11710.38
KTGNYGY;QSLLTLL	CPs	2586.101
KGPPAVT;FEVKLKHY	CPs	340.71
FEVKLKHY;SPRPPAGHPHGR	CPs	6.765
SPRPPAGHPHGR;ALSPGLVESEDL	CPs	9.534
ALSPGLVESEDL;LSEVFPK	CPs	403.742
LSEVFPK;FYASRPESK	CPs	4160.491
FYASRPESK;AKGSPAG	CPs	2969.982
AKGSPAG;DPAAAPNP	CPs	7678.302
DPAAAPNP;QLAKSLGV	CPs	1032.447
QLAKSLGV;PGELAKHAV	CPs	821.256
PGELAKHAV;AAGTLYTYPEN	CPs	3756.564
AAGTLYTYPEN;APPHFHF	CPs	0.096
APPHFHF;LCEKMAQFDAK	CPs	0.525
LCEKMAQFDAK;MAQFDAKK	CPs	0.012
MAQFDAKK;LLPGELAKHAVSEGTKAVTKY	CPs	1475.235
LLPGELAKHAVSEGTKAVTKY;AVTKYTSSK	CPs	0.048
AVTKYTSSK;KDPPYK	CPs	649.419
KDPPYK;ASVAVEPQL	CPs	1165.509
ASVAVEPQL;TRDFKPGDL	CPs	3669.201
TRDFKPGDL;AQLKP	CPs	9253.272

AQLKP;RAYGGPR	CPs	112.209
RAYGGPR;VSGPVVL	CPs	2217.492
VSGPVVL;VPPVLLM	CPs	3669.396
VPPVLLM;ATTLLEAMK	CPs	389.403
ATTLLEAMK;DYEGLEPKTTYY	CPs	110.649
DYEGLEPKTTYY;AVGSHVYSVAK	CPs	0.093
AVGSHVYSVAK;VYSVAKTDSF	CPs	0.015
VYSVAKTDSF;EYVDPVDGTVTKK	CPs	0.03
EYVDPVDGTVTKK;ESEPPRPR	CPs	664.098
ESEPPRPR;RPPEPQT	CPs	188.418
RPPEPQT;AARMPPLA	CPs	2980.887
AARMPPLA;VGGKQVVPF	CPs	123.291
VGGKQVVPF;AMVSEFLKQA	CPs	845.421
AMVSEFLKQA;MVSEFLK	CPs	0.003
MVSEFLK;KHGQERF	CPs	2982.957
KHGQERF;LTCKRLP	CPs	226.935
LTCKRLP;EVVLVKK	CPs	761.46
EVVLVKK;MLEDSVSPK	CPs	574.508
MLEDSVSPK;LEDSVSPKK	CPs	0.003
LEDSVSPKK;MDSLMPVVN	CPs	330.853
MDSLMPVVN;KQELK	CPs	2035.152
KQELK;PVERPK	CPs	1537.722
PVERPK;ASKPQN	CPs	1828.629
ASKPQN;RPLPPPP	CPs	128.084
RPLPPPP;AELDPFG	CPs	693.187
AELDPFG;LDGGAPGSQPHG	CPs	0.144
LDGGAPGSQPHG;LCDFNPK	CPs	17.13
LCDFNPK;LKQAPLVH	CPs	0.072
LKQAPLVH;MSVGVDHL	CPs	1053.321
MSVGVDHL;KDPNAPKRPPS	CPs	630.939
KDPNAPKRPPS;TAEFVNLEFL	CPs	978.402
TAEFVNLEFL;AEKLPNLTHL	CPs	3.114
AEKLPNLTHL;LSFDTALK	CPs	9.767
LSFDTALK;SELEYKFNRK	CPs	18.313
SELEYKFNRK;ALERPYTSK	CPs	0.03
ALERPYTSK;SHAQYKELQK	CPs	0.075
SHAQYKELQK;LHELNVPFFK	CPs	7.428
LHELNVPFFK;HELNVPF	CPs	0.003
HELNVPF;VGVPLPSSVSF	CPs	814.335
VGVPLPSSVSF;YTEPSSQVSREAAAF	CPs	0.273
YTEPSSQVSREAAAF;PAAPEKVPK	CPs	529.193
PAAPEKVPK;KGSSGTKP	CPs	735.586
KGSSGTKP;AAPELPVPTSGPL	CPs	2123.355
AAPELPVPTSGPL;KDGLW	CPs	1793.126
KDGLW;KRWLLAGP	CPs	362.677
KRWLLAGP;MQAPPY	CPs	390.837
MQAPPY;ADGGSPFLGRR	CPs	12.945
ADGGSPFLGRR;EDSEVFKM	CPs	24.159
EDSEVFKM;PPKLHTCEKCN	CPs	2.121
PPKLHTCEKCN;WVGDELYCEKHAR	CPs	1.149
WVGDELYCEKHAR;VGDELYCEKH	CPs	0.003

VGDELYCEKH;GPPPGLLTPP	CPs	14.508
GPPPGLLTPP;KSPAPPLLHVA	CPs	1.074
KSPAPPLLHVA;FQTSHTLH	CPs	0.306
FQTSHTLH;QAVLEYL	CPs	1031.748
QAVLEYL;LPPVTLALL	CPs	188.715
LPPVTLALL;FAGEPK	CPs	25.142
FAGEPK;WSKNWAK	CPs	3413.158
WSKNWAK;TSPAKQQAPPVRN	CPs	9.807
TSPAKQQAPPVRN;APPVRNL	CPs	0.021
APPVRNL;NLHQSGF	CPs	0.015
NLHQSGF;RDVMLPK	CPs	703.524
RDVMLPK;AYLEKVFY	CPs	142.968
AYLEKVFY;VGPLLL	CPs	841.511
VGPLLL;DFSLEKK	CPs	109.531
DFSLEKK;KYFPVVKLV	CPs	3604.71
KYFPVVKLV;EDVKLR	CPs	3162.045
EDVKLR;SGVHLTF	CPs	242.243
SGVHLTF;QTKGKGR	CPs	1081.936
QTKGKGR;VPPYVPP	CPs	1108.173
VPPYVPP;KPPKPPKPPK	CPs	142.764
KPPKPPKPPK;YEETRGVLK	CPs	2142.048
YEETRGVLK;KMTYYHF	CPs	1633.83
KMTYYHF;LPPPVPP	CPs	1395.279
LPPPVPP;ESLLLDLKQ	CPs	184.053
ESLLLDLKQ;KSKPGAK	CPs	4831.668
KSKPGAK;DRHPPPPP	CPs	3593.667
DRHPPPPP;MDHPSPL	CPs	878.094
MDHPSPL;DGEVTLTTLKK	CPs	0.426
DGEVTLTTLKK;LFEDDESEKEQYR	CPs	0.084
LFEDDESEKEQYR;LDEEHLESHKK	CPs	0.183
LDEEHLESHKK;GQSTPRLF	CPs	0.255
GQSTPRLF;LTQEELDSGLDELSVR	CPs	0.366
LTQEELDSGLDELSVR;SVDDPLEY	CPs	0.285
SVDDPLEY;TQESDVMVGPFK	CPs	0.414
TQESDVMVGPFK;TNTLETPTK	CPs	2086.74
TNTLETPTK;LDEQPAMVTKK	CPs	16.932
LDEQPAMVTKK;NQMNSKL	CPs	17.877
NQMNSKL;KFEMEP	CPs	836.678
KFEMEP;LRARWP	CPs	3242.998
LRARWP;DVGVVV	CPs	4724.172
DVGVVV;VVEHPEFLK	CPs	98.382
VVEHPEFLK;EVVVQRLFQ	CPs	7.803
EVVVQRLFQ;AALKTASDF	CPs	7.401
AALKTASDF;ANVERVPF	CPs	0.339
ANVERVPF;DAATLHTSTAMAAQHG	CPs	0.024
DAATLHTSTAMAAQHG;VLKTPSAA	CPs	6.483
VLKTPSAA;PGELMQEDL	CPs	1.968
PGELMQEDL;ATDDVMLLDTWDQ	CPs	0.027
ATDDVMLLDTWDQ;LGWDDNYWSVDPLDRA	CPs	5.556
LGWDDNYWSVDPLDRA;DDNYWSVDPLDR	CPs	0.009
DDNYWSVDPLDR;WSVDPLDRALAELAA	CPs	0.012

WSVDPLDRALAELAA;SVDPLDRAL	CPs	0.003
SVDPLDRAL;PAARFKK	CPs	277.728
PAARFKK;EVGLTFQ	CPs	1310.603
KLYSSSGP;PPTRPTDKPL	CPs	7099.951
PPTRPTDKPL;TRPTDKPLR	CPs	0.006
TRPTDKPLR;FAPVNVTTEVK	CPs	0.123
FAPVNVTTEVK;APVNVTTEVKS	CPs	0.003
APVNVTTEVKS;VNVTTEVKSVEMH	CPs	0.006
VNVTTEVKSVEMH;VAGDSKNDPPMEAAGF	CPs	0.126
VAGDSKNDPPMEAAGF;PPMEAA	CPs	0.024
PPMEAA;SDYPPLG	CPs	0.249
SDYPPLG;AYRPPK	CPs	4737.6
AYRPPK;ELLARAVP	CPs	566.799
ELLARAVP;VSGVSLTK	CPs	154.407
VSGVSLTK;VSNFFVK	CPs	2227.836
VSNFFVK;MGHFDLLNY	CPs	3211.427
MGHFDLLNY;VNPTVFF	CPs	964.474
VNPTVFF;ELFADKVPK	CPs	0.063
ELFADKVPK;FADKVPKTAEN	CPs	0.006
FADKVPKTAEN;VAHFTFQPDPEPVEY	CPs	308.751
VAHFTFQPDPEPVEY;ANPPPPPPP	CPs	1668.993
ANPPPPPPP;VGGVPSNF	CPs	16832.41
VGGVPSNF;TQSRAASY	CPs	1.521
TQSRAASY;EDEFPEV	CPs	59.013
EDEFPEV;TALDPEV	CPs	785.798
TALDPEV;GSTVLSF	CPs	1316.809
GSTVLSF;AETVADTRRL	CPs	1397.496
AETVADTRRL;VVVPPLPGK	CPs	45.921
VVVPPLPGK;ALKGVRT	CPs	1511.259
ALKGVRT;DTVAFVP	CPs	2154.159
DTVAFVP;ALPGDNVGFNVK	CPs	0.348
ALPGDNVGFNVK;AVPTFPS	CPs	252.762
AVPTFPS;EATFQVP	CPs	278.457
EATFQVP;GPPVNVPQ	CPs	3800.943
GPPVNVPQ;FPGVESP	CPs	10449.54
FPGVESP;KDSNPLLF	CPs	222.059
KDSNPLLF;TPFHRPL	CPs	392.077
TPFHRPL;KTAFP	CPs	1346.442
KTAFP;GPPHMPPP	CPs	1533.261
GPPHMPPP;DVAPYFK	CPs	4081.494
DVAPYFK;DLGEEPLERAA	CPs	1885.212
DLGEEPLERAA;LVEALCAEHQ	CPs	1221.153
LVEALCAEHQ;LMFDVQ	CPs	3275.087
LMFDVQ;AAPGVHR	CPs	616.645
AAPGVHR;AEVLEPDPR	CPs	1301.7
AEVLEPDPR;MNVDHEVKL	CPs	573.789
MNVDHEVKL;SGELLLQGVHD	CPs	9.39
SGELLLQGVHD;TTGDPSK	CPs	3072.113
TTGDPSK;RAASKLR	CPs	283.702
RAASKLR;STPQQLEK	CPs	748.872
STPQQLEK;RLQHLL	CPs	547.68

RLQHLL;DAPKGSK	CPs	300.701
DAPKGSK;AVSEGTKAVTKYTSSK	CPs	543.829
AVSEGTKAVTKYTSSK;DKLQDGSVK	CPs	4524.255
DKLQDGSVK;TPTPLGEGKST	CPs	1121.025
TPTPLGEGKST;GTPVFVHAGPF	CPs	25.473
GTPVFVHAGPF;MPGLPTRP	CPs	99.264
MPGLPTRP;QPPEPQANDVGFKK	CPs	242.187
QPPEPQANDVGFKK;PPEPQANDVGFK	CPs	0.003
PPEPQANDVGFK;RLSESDKEDEADKVK	CPs	1.356
RLSESDKEDEADKVK;EPGPEAAAAGSQDH	CPs	0.291
EPGPEAAAAGSQDH;DAVEREKTEAQPAPK	CPs	0.393
DAVEREKTEAQPAPK;VDVDVSEELSK	CPs	0.072
VDVDVSEELSK;VEEAEEGSQALDLK	CPs	0.567
VEEAEEGSQALDLK;VQDTETETLTQEK	CPs	0.072
VQDTETETLTQEK;TEVVLEEDVTK	CPs	0.594
TEVVLEEDVTK;DVTKKVEF	CPs	0.021
DVTKKVEF;KDDASGK	CPs	15.678
KDDASGK;HFFNPVPL	CPs	4519.878
HFFNPVPL;FNPVPLMK	CPs	0.006
FNPVPLMK;GFAKPGME	CPs	1557.651
GFAKPGME;FDWLYE	CPs	2.358
FDWLYE;VSPKVP	CPs	467.01
VSPKVP;TLPEVKVRPP	CPs	1344.546
TLPEVKVRPP;DDSDEDLLHV	CPs	0.189
DDSDEDLLHV;AKEKGKFEDMA	CPs	2114.979
AKEKGKFEDMA;KKFKDPNAPK	CPs	0.099
KKFKDPNAPK;KFKDPNAPKRPP	CPs	0.003
KFKDPNAPKRPP;LFCSEYRPK	CPs	0.048
LFCSEYRPK;AATAAAVVAEEDTELR	CPs	3408.738
AATAAAVVAEEDTELR;LDSDRTPSTSSTAER	CPs	527.979
LDSDRTPSTSSTAER;YEPAGPP	CPs	42.648
YEPAGPP;LTELENELNTK	CPs	2.775
LTELENELNTK;SDGVLKV	CPs	295.335
RLASLDR;KDAATTK	NCP	201.769
KDAATTK;VLLHVLPV	NCP	55.889
VLLHVLPV;KAGALVLS	NCP	3.679
KAGALVLS;SGPLNFTV	NCP	104.429
SGPLNFTV;FAKLV	NCP	83.922
FAKLV;RNPPPR	NCP	3.828
RNPPPR;KTAEDGKK	NCP	153.013
KTAEDGKK;TRELLP	NCP	230.294
TRELLP;KEDPKQPP	NCP	2.568
KEDPKQPP;QRLLANYK	NCP	65.277
QRLLANYK;AAPVDK	NCP	27.381
AAPVDK;QATHNVP	NCP	90.814
QATHNVP;LSPAVLP	NCP	3.849
LSPAVLP;LSTLKY	NCP	98.805
LSTLKY;LVELPF	NCP	5.45
LVELPF;MMDFLM	NCP	1.48
MMDFLM;LLTVPF	NCP	71.195
LLTVPF;RGTFLR	NCP	6.036

RGTFLR;LLPGELAK	NCP	12.132
LLPGELAK;TVAPDPK	NCP	86.355
TVAPDPK;KYPPPPLP	NCP	16.642
KYPPPPLP;VTLMAELK	NCP	1.752
VTLMAELK;KAYFPK	NCP	67.046
KAYFPK;KFPSLVH	NCP	2.679
KFPSLVH;FPEFLQ	NCP	63.787
FPEFLQ;SPAKPEVQ	NCP	53.184
SPAKPEVQ;VAVTPSLP	NCP	61.778
VAVTPSLP;LVGGLL	NCP	23.973
LVGGLL;KPSVVT	NCP	206.04
KPSVVT;KKSHVVLS	NCP	20.701
KKSHVVLS;VALEYL	NCP	4.611
VALEYL;LPGVLP	NCP	46.875
LPGVLP;SHMDLR	NCP	93.12
SHMDLR;DDDPSEK	NCP	155.319
DDDPSEK;VGNSLL	NCP	3.813
VGNSLL;RPHLFL	NCP	36.05
RPHLFL;KEAPKR	NCP	2.11
KEAPKR;LLMGYHLL	NCP	38.232
LLMGYHLL;VSSLPVEVT	NCP	3.65
VSSLPVEVT;KLRSPK	NCP	3.904
KLRSPK;CSFRCAKC	NCP	20.195
CSFRCAKC;STVYVP	NCP	3.949
STVYVP;SCPSRVPT	NCP	63.41
SCPSRVPT;AAVSL	NCP	48.036
AAVSL;FVKHLM	NCP	9.768
FVKHLM;SVGSVLTK	NCP	130.053
SVGSVLTK;KPGKM	NCP	5.547
KPGKM;VPELQ	NCP	18.891
VPELQ;SFKPLR	NCP	223.072
SFKPLR;TGGGGVGK	NCP	3.051
TGGGGVGK;AGPKAFSP	NCP	70.715
AGPKAFSP;TLYGN	NCP	14.268
TLYGN;SMPDP	NCP	115.837
SMPDP;AELAFR	NCP	17.634
AELAFR;REQVVR	NCP	22.91
REQVVR;LPLGEL	NCP	71.824
LPLGEL;KRGPPPP	NCP	43.865
KRGPPPP;QPATPLM	NCP	145.12
QPATPLM;AETPKP	NCP	79.536
AETPKP;VGPPFR	NCP	147.239
VGPPFR;EPATEYL	NCP	0.514
EPATEYL;VLSAAD	NCP	78.962
VLSAAD;LHPTPAVH	NCP	38.529
LHPTPAVH;GVMKLLT	NCP	1.171
GVMKLLT;SLAVGLF	NCP	27.611
SLAVGLF;TGAPGLL	NCP	4.132
TGAPGLL;WHPEKPP	NCP	77.36
WHPEKPP;AAPEPPR	NCP	8.016
AAPEPPR;RGRPPPPPSR	NCP	141.129

RGRPPPPPSR;LERMVLSSL	NCP	109.171
LERMVLSSL;KDYVT	NCP	34.905
KDYVT;FYTVPGK	NCP	62.673
FYTVPGK;LPLSLLK	NCP	48.857
LPLSLLK;AYHVQPRP	NCP	31.638
AYHVQPRP;NDLLDF	NCP	32.287
NDLLDF;ELGVKHL	NCP	2.847
ELGVKHL;NGFSSFLK	NCP	7.67
NGFSSFLK;RGPMPSP	NCP	13.266
RGPMPSP;SLSAPAPAPG	NCP	259.809
SLSAPAPAPG;KSLFK	NCP	64.912
KSLFK;PAAALHL	NCP	67.844
PAAALHL;TFKFPVG	NCP	11.467
TFKFPVG;RANAK	NCP	7.29
RANAK;VDLNLK	NCP	109.461
VDLNLK;LPGVP	NCP	24.924
LPGVP;KKKKDAPGR	NCP	15.641
KKKKDAPGR;LSYVYK	NCP	225.226
LSYVYK;EPTAP	NCP	52.182
EPTAP;KLPGDVP	NCP	232.11
KLPGDVP;LHALFP	NCP	20.876
LHALFP;KGAQVAK	NCP	2.664
KGAQVAK;EAPGKKPR	NCP	134.344
EAPGKKPR;KGATK	NCP	51.257
KGATK;PGVGPP	NCP	33.021
PGVGPP;VYLTH	NCP	4.144
VYLTH;NPNNARPL	NCP	24.554
NPNNARPL;KPKKGPK	NCP	27.483
KPKKGPK;RLVLEPF	NCP	66.205
RLVLEPF;KDAAK	NCP	109.05
KDAAK;QTSLPDL	NCP	63.266
QTSLPDL;LNLPVLLE	NCP	6.697
LNLPVLLE;KKYKQ	NCP	47.697
KKYKQ;FPPPYMK	NCP	8.031
FPPPYMK;HVGLAQY	NCP	28.013
HVGLAQY;EVHRAYF	NCP	0.144
EVHRAYF;DLRLQDVTVL	NCP	4.686
DLRLQDVTVL;RLYTEPP	NCP	13.266
RLYTEPP;LLDNSRNV	NCP	48.099
LLDNSRNV;PGKSSR	NCP	80.248
PGKSSR;FDELKKVKK	NCP	76.277
FDELKKVKK;QMKKKL	NCP	30.294
QMKKKL;QQKHTK	NCP	71.805
QQKHTK;SGTLFDN	NCP	107.371
SGTLFDN;FVPGDNK	NCP	66.956
FVPGDNK;KGGGTAPK	NCP	71.413
KGGGTAPK;LPPPPLPPKY	NCP	8.351
LPPPPLPPKY;VAAPRSAPP	NCP	151.851
VAAPRSAPP;TTKHGPP	NCP	147.891
TTKHGPP;VPSLKK	NCP	70.755
VPSLKK;ATQVEPL	NCP	24.66

ATQVEPL;KESGQ	NCP	70.243
KESGQ;VPGLVLR	NCP	71.558
VPGLVLR;CNDDCP	NCP	51.631
CNDDCP;VPAAL	NCP	111.96
VPAAL;YNLQNYE	NCP	5.382
YNLQNYE;GDSSSC	NCP	16.175
GDSSSC;ATAPKF	NCP	148.152
ATAPKF;QWGKEGP	NCP	37.806
QWGKEGP;FWGLP	NCP	47.995
FWGLP;VSYVY	NCP	33.831
VSYVY;VPFPFT	NCP	176.816
VPFPFT;AKKTYDLLFK	NCP	65.019
AKKTYDLLFK;ANLTLF	NCP	47.236
ANLTLF;DSHGP	NCP	5.024
DSHGP;PWTKYQ	NCP	55.989
PWTKYQ;VAFVEL	NCP	22.122
VAFVEL;ALPLVL	NCP	4.659
ALPLVL;KPFNQNM	NCP	11.044
KPFNQNM;TASVGPLN	NCP	9.158
TASVGPLN;MDPSGVK	NCP	25.765
MDPSGVK;YGDGFF	NCP	103.869
YGDGFF;EGPPARP	NCP	59.546
EGPPARP;PAAGRHVGP	NCP	0.858
PAAGRHVGP;KKPPPK	NCP	89.19
KKPPPK;TVFQA	NCP	9.315
TVFQA;KTEPDK	NCP	25.422
KTEPDK;LEQAFKR	NCP	28.603
LEQAFKR;EKLFQPK	NCP	152.969
EKLFQPK;AEENK	NCP	59.796
AEENK;AARTLMPK	NCP	31.203
AARTLMPK;MHELPVQ	NCP	25.936
MHELPVQ;HPAACH	NCP	8.288
HPAACH;LEESHE	NCP	17.737
LEESHE;PSGKK	NCP	80.181
PSGKK;CSRPCL	NCP	65.601
CSRPCL;GKPPL	NCP	15.896
GKPPL;FEKPAK	NCP	11.338
FEKPAK;KQGALTF	NCP	109.98
KQGALTF;KGLALQ	NCP	1.386
KGLALQ;QKNSK	NCP	74.579
QKNSK;LDKEDQQK	NCP	69.897
LDKEDQQK;KGEVL	NCP	188.566
KGEVL;VPKSH	NCP	111.614
VPKSH;TDDLK	NCP	71.139
TDDLK;VTDAKQP	NCP	2.853
VTDAKQP;RAELLR	NCP	21.441
RAELLR;LQGAFPVG	NCP	21.603
LQGAFPVG;SVLPEC	NCP	33.207
SVLPEC;KRPKVPP	NCP	39.549
KRPKVPP;AAKVTPA	NCP	25.404
AAKVTPA;DDDGEY	NCP	71.307

DDDGEY;RKLLKTEL	NCP	17.865
RKLLKTEL;SDFRYS	NCP	14.233
SDFRYS;NLVEPPR	NCP	35.145
NLVEPPR;ADVFAP	NCP	7.763
ADVFAP;TVSLL	NCP	88.626
TVSLL;YPPMKLP	NCP	41.104
YPPMKLP;KPACMLR	NCP	34.116
KPACMLR;FPSSPP	NCP	10.298
FPSSPP;DGTQG	NCP	97.005
DGTQG;KKPPTL	NCP	7.257
KKPPTL;ATLGVVLP	NCP	123.916
ATLGVVLP;FRGWR	NCP	2.253
FRGWR;RAPKSSLA	NCP	88.193
RAPKSSLA;KPSQLAK	NCP	69.931
KPSQLAK;LDLECFGL	NCP	130.935
LDLECFGL;TNKVP	NCP	86.523
TNKVP;ESFVVEVK	NCP	81.993
ESFVVEVK;ALPPP	NCP	62.021
ALPPP;KVFYPP	NCP	133.884
KVFYPP;DVSER	NCP	117.609
DVSER;CCCEASP	NCP	1.206
CCCEASP;NKGDFPP	NCP	8.407
NKGDFPP;PSFLLK	NCP	67.302
PSFLLK;VGNLPF	NCP	73.013
VGNLPF;MDDDPP	NCP	14.712
MDDDPP;VHLMEPK	NCP	93.289
VHLMEPK;SKPAAP	NCP	97.25
SKPAAP;PLGVLAP	NCP	112.215
PLGVLAP;KGPDYSK	NCP	53.671
KGPDYSK;LPEKPF	NCP	99.828
LPEKPF;KEQEVVDKVME	NCP	85.193
KEQEVVDKVME;TTACHEFFEHE	NCP	0.099
TTACHEFFEHE;WLLLGT	NCP	36.321
WLLLGT;LEVMVPK	NCP	120.783
LEVMVPK;TVQHH	NCP	68.598
TVQHH;SAANPML	NCP	11.25
SAANPML;VGLVPLKA	NCP	177.165
VGLVPLKA;GFRHLDSA	NCP	192.522
GFRHLDSA;ALAACPK	NCP	78.612
ALAACPK;ALTVS	NCP	2.901
ALTVS;LPHGPPP	NCP	35.25
LPHGPPP;SLVRGPR	NCP	69.609
SLVRGPR;FSYGPN	NCP	267.402
FSYGPN;KRLGETR	NCP	147.621
KRLGETR;GLLPP	NCP	19.884
GLLPP;TVEEELLK	NCP	84.592
TVEEELLK;SGGHVL	NCP	57.048
SGGHVL;AGTALKRLM	NCP	237.104
AGTALKRLM;AEALTAW	NCP	57.789
AEALTAW;APLPVQ	NCP	33.834
APLPVQ;SALEFMR	NCP	69.786

SALEFMR;ALHVP	NCP	60.276
ALHVP;ACMKHH	NCP	44.187
ACMKHH;QPRFNSR	NCP	14.97
QPRFNSR;PFPVPTLR	NCP	38.553
PFPVPTLR;VSPVLGPP	NCP	31.194
VSPVLGPP;KKQWVR	NCP	28.035
KKQWVR;LPVCL	NCP	53.364
LPVCL;QNLEP	NCP	28.8
QNLEP;DKFSM	NCP	0.672
DKFSM;KGVRY	NCP	11.926
KGVRY;KKFPVFKALEF	NCP	335.189
KKFPVFKALEF;ALEFKSQLVAGK	NCP	0.021
ALEFKSQLVAGK;VDEDDFVH	NCP	0.48
VDEDDFVH;FESLPHEN	NCP	0.033
FESLPHEN;KPVALTSYQTNKGRHDELTY	NCP	0.024
KPVALTSYQTNKGRHDELTY;TRCLVH	NCP	2.451
TRCLVH;ESLPHEN	NCP	24.99
ESLPHEN;KPVALTSY	NCP	0.021
KPVALTSY;SYQTNKSRHDELTYF	NCP	0.018
SYQTNKSRHDELTYF;QTNKSRHDELTY	NCP	0.006
QTNKSRHDELTY;KLFGP	NCP	40.743
KLFGP;LHYLKELK	NCP	22.189
LHYLKELK;KLYVPP	NCP	4.254
KLYVPP;TGGPLPL	NCP	2.363
TGGPLPL;MHVPLR	NCP	15.978
MHVPLR;TSDKGNVK	NCP	10.296
TSDKGNVK;PALPK	NCP	401.607
PALPK;SLSAPLTVLPTVTCEH	NCP	97.512
SLSAPLTVLPTVTCEH;GVTLPL	NCP	44.766
GVTLPL;MHFVVPR	NCP	126.73
MHFVVPR;SAKTPVGF	NCP	1.236
SAKTPVGF;KQNPEYSN	NCP	65.975
KQNPEYSN;LALVLF	NCP	89.245
LALVLF;APVQPL	NCP	45.938
APVQPL;TCTYLP	NCP	169.237
TCTYLP;TAAVY	NCP	6.299
TAAVY;LPWPKL	NCP	8.205
LPWPKL;KFTEGH	NCP	76.924
KFTEGH;SLGVTPVQ	NCP	58.586
SLGVTPVQ;YNELARV	NCP	39.801
YNELARV;ATTLM	NCP	42.421
ATTLM;DLLGHPKT	NCP	21.762
DLLGHPKT;KTPGY	NCP	25.307
KTPGY;FPSGAV	NCP	15.807
FPSGAV;SGPLM	NCP	14.304
SGPLM;LSENDLPK	NCP	85.213
LSENDLPK;RPVFARVR	NCP	34.37
RPVFARVR;TVDLPL	NCP	27.378
TVDLPL;TVVSF	NCP	17.562
TVVSF;KGWFSLAP	NCP	0.912
KGWFSLAP;TVPLDVT	NCP	74.757

TVPLDVT;PKTEC	NCP	17.779
PKTEC;KDVLR	NCP	14.544
KDVLR;KDPEV	NCP	21.426
KDPEV;LGDVLRGL	NCP	9.119
LGDVLRGL;PGVAH	NCP	6.363
PGVAH;KGRPRPP	NCP	19.077
KGRPRPP;QEGLLLAR	NCP	0.345
QEGLLLAR;LKGLGH	NCP	226.162
LKGLGH;WAPEL	NCP	30.345
WAPEL;PVAAMRG	NCP	46.461
PVAAMRG;LGPKSNP	NCP	16.721
LGPKSNP;KVMLGW	NCP	15.723
KVMLGW;TLPSPYS	NCP	20.232
TLPSPYS;DPVDD	NCP	135.849
DPVDD;HNALVLY	NCP	8.611
HNALVLY;KDSPLQEL	NCP	146.405
KDSPLQEL;LAGSLLGK	NCP	241.06
LAGSLLGK;EHQSMLF	NCP	45.599
EHQSMLF;HVVLLH	NCP	5.506
HVVLLH;FPESL	NCP	68.049
FPESL;LYGSEKF	NCP	44.942
LYGSEKF;EKPFEPK	NCP	7.278
EKPFEPK;KDVVGP	NCP	35.556
KDVVGP;DLKSSKFKA	NCP	156.579
DLKSSKFKA;VPVPPKY	NCP	49.917
VPVPPKY;QGDVLDF	NCP	105.465
QGDVLDF;PVVCSHVL	NCP	88.257
PVVCSHVL;AAVPAV	NCP	194.94
AAVPAV;KPAALSYS	NCP	79.437
KPAALSYS;LSPKRP	NCP	70.713
LSPKRP;HARLAY	NCP	93.384
HARLAY;PVAGP	NCP	27.93
PVAGP;PLGAPF	NCP	49.041
PLGAPF;VFLPALQV	NCP	81.09
VFLPALQV;KELGSVPK	NCP	20.319
KELGSVPK;LLGVGH	NCP	63.438
LLGVGH;KYPFF	NCP	18.072
KYPFF;GKFSQK	NCP	0.219
GKFSQK;TREHVVP	NCP	1.695
TREHVVP;ELLMEQ	NCP	102.567
ELLMEQ;PAMYV	NCP	15.649
PAMYV;TEDPKVP	NCP	166.775
TEDPKVP;VLLVF	NCP	56.622
VLLVF;RPTGKF	NCP	101.817
RPTGKF;LLHLEL	NCP	101.58
LLHLEL;VVNPLLA	NCP	66.108
VVNPLLA;QSLPPGLAVKELK	NCP	161.859
QSLPPGLAVKELK;LSGGLL	NCP	58.152
LSGGLL;CMEPP	NCP	11.392
CMEPP;NVVVF	NCP	15.86
NVVVF;VGVLSA	NCP	55.95

VGVLSA;YGLGSY	NCP	222.877
YGLGSY;ATKLEN	NCP	42.36
ATKLEN;GKKGL	NCP	77.933
GKKGL;TAEHKPP	NCP	8.724
TAEHKPP;ENLDFL	NCP	20.002
ENLDFL;VDAYEVFL	NCP	138.717
VDAYEVFL;KETFP	NCP	248.264
KETFP;KKGAFYG	NCP	22.504
KKGAFYG;TSLPP	NCP	29.267
TSLPP;LGNMLAK	NCP	37.762
LGNMLAK;KSYKADK	NCP	168.921
KSYKADK;YTLPK	NCP	78.902
YTLPK;AAHWY	NCP	33.799
AAHWY;PDPVN	NCP	204.519
PDPVN;KAHLTPH	NCP	40.983
KAHLTPH;LKLLTELK	NCP	7.215
LKLLTELK;NCMDS	NCP	59.696
NCMDS;VELAGRTPK	NCP	8.053
VELAGRTPK;KSTPMGK	NCP	111.119
KSTPMGK;LPPGAP	NCP	37.815
LPPGAP;EGPDALK	NCP	9.756
EGPDALK;KTTNPL	NCP	141.622
KTTNPL;KNYVPL	NCP	30.621
KNYVPL;HTLWF	NCP	36.768
HTLWF;FEKLR	NCP	15.93
FEKLR;DYMMSH	NCP	9.282
DYMMSH;TTVVLT	NCP	23.463
TTVVLT;KGACAKK	NCP	28.976
KGACAKK;LPPLHK	NCP	134.811
LPPLHK;AAGVPAMAESVERLQ	NCP	8.769
AAGVPAMAESVERLQ;AESVERLQQ	NCP	0.021
AESVERLQQ;LPLAH	NCP	173.674
LPLAH;GPKFLDK	NCP	1.146
GPKFLDK;DPEGFYK	NCP	34.359
DPEGFYK;LAAKAAGY	NCP	44.249
LAAKAAGY;EESSCM	NCP	65.1
EESSCM;GVDDPTFGY	NCP	22.14
GVDDPTFGY;RDKNMNR	NCP	33.487
RDKNMNR;LYLPL	NCP	14.333
LYLPL;TGPKTLPP	NCP	16.104
TGPKTLPP;LSQPF	NCP	52.72
LSQPF;GVDLFTGK	NCP	28.887
GVDLFTGK;ARPPFS	NCP	6.477
ARPPFS;QENWLSK	NCP	126.215
QENWLSK;DAPVLSVK	NCP	23.268
DAPVLSVK;LSLQGEVL	NCP	157.98
LSLQGEVL;LGRPPR	NCP	162.763
LGRPPR;AETEERSLDNF	NCP	48.44
AETEERSLDNF;EDPSDNWEEGGGGGGGVEK	NCP	9.582
EDPSDNWEEGGGGGGGVEK;SGVYRPPG	NCP	0.669
SGVYRPPG;ATLMEVPP	NCP	45.183

ATLMEVPP;KLNLGLP	NCP	47.004
KLNLGLP;LFVKSK	NCP	86.721
LFVKSK;EGDPP	NCP	9.717
EGDPP;MLGMLNK	NCP	1.243
MLGMLNK;LCQLCAGK	NCP	14.678
LCQLCAGK;FGYSGAFK	NCP	0.063
FGYSGAFK;VPPELLL	NCP	26.22
VPPELLL;VGVFTK	NCP	30.79
VGVFTK;YAVAVVKKGTDF	NCP	5.804
YAVAVVKKGTDF;KLNELRGK	NCP	0.036
KLNELRGK;LAMVPSHAVVAR	NCP	2.559
LAMVPSHAVVAR;LGYEYVTAL	NCP	0.909
LGYEYVTAL;LLEACTFHKP	NCP	24.072
LLEACTFHKP;VSRPGDVS	NCP	216.303
VSRPGDVS;TPYPR	NCP	31.545
TPYPR;CSVKLH	NCP	78.529
CSVKLH;EGHPFLFL	NCP	7.434
EGHPFLFL;VPKLKLM	NCP	29.88
VPKLKLM;MNFGELK	NCP	66.561
MNFGELK;SARGP	NCP	97.515
SARGP;VQEGPPK	NCP	145.16
VQEGPPK;DDFKKAF	NCP	8.196
DDFKKAF;FSPLTPLT	NCP	342.519
FSPLTPLT;KYRLLLH	NCP	228.895
KYRLLLH;LLPGVK	NCP	109.091
LLPGVK;MELAAKY	NCP	51.325
MELAAKY;AVGSLLA	NCP	34.505
AVGSLLA;SGPKPF	NCP	150.73
SGPKPF;SGPVLL	NCP	22.139
SGPVLL;TGVVPG	NCP	10.032
TGVVPG;KPLGLKK	NCP	213.004
KPLGLKK;KGACFLSF	NCP	135.483
KGACFLSF;VVAQRVP	NCP	52.796
VVAQRVP;ADGGSFP	NCP	41.01
ADGGSFP;LDRSHL	NCP	247.564
LDRSHL;ELLAGP	NCP	19.385
ELLAGP;MHKKAGK	NCP	1.957
MHKKAGK;PLLTLFP	NCP	140.057
PLLTLFP;GHNLF	NCP	74.535
GHNLF;CFCDC	NCP	54.444
CFCDC;YSRVPPP	NCP	39.996
YSRVPPP;LLYAFAEATVPK	NCP	3.894
LLYAFAEATVPK;PDPLSR	NCP	59.884
PDPLSR;VHDSKLK	NCP	323.582
VHDSKLK;KENPKALH	NCP	169.89
KENPKALH;EDNLAAK	NCP	16.576
EDNLAAK;KDFLYPF	NCP	20.226
KDFLYPF;VDMATVV	NCP	36.314
VDMATVV;QELLALNR	NCP	2.481
QELLALNR;KPTRP	NCP	57.847
KPTRP;AEEVVVVAKF	NCP	2.801

AEEVVVVAKF;SGDVAKK	NCP	28.773
SGDVAKK;MDFNVKK	NCP	2.212
MDFNVKK;SLLKKKP	NCP	29.04
SLLKKKP;NRVLVLL	NCP	16.209
NRVLVLL;EALVEL	NCP	86.615
EALVEL;DDCMC	NCP	16.59
DDCMC;RSLKTGY	NCP	158.776
RSLKTGY;KNHPVEYF	NCP	54.599
KNHPVEYF;NVFLVA	NCP	4.302
NVFLVA;LASMPLN	NCP	49.609
LASMPLN;LSSWRP	NCP	55.898
LSSWRP;HGRLNPY	NCP	69.021
HGRLNPY;YTGLK	NCP	98.979
YTGLK;NVLYL	NCP	22.3
NVLYL;LSGKPKK	NCP	48.153
LSGKPKK;LFSLLGD	NCP	25.004
LFSLLGD;ALLPAGP	NCP	91.521
ALLPAGP;NSRHVLN	NCP	24.18
NSRHVLN;LAALK	NCP	0.202
LAALK;APWAVTK	NCP	16.874
APWAVTK;ELATLLLE	NCP	54.46
ELATLLLE;APFPGQLP	NCP	28.988
APFPGQLP;TLFDQ	NCP	42.694
TLFDQ;LALNYSVF	NCP	69.054
LALNYSVF;EGKGFGYK	NCP	35.993
EGKGFGYK;DNFGKY	NCP	16.102
DNFGKY;FPPLVGPT	NCP	87.014
FPPLVGPT;LDAELLTK	NCP	47.859
LDAELLTK;AASPP	NCP	137.025
AASPP;LVGRP	NCP	17.028
LVGRP;FSLGGF	NCP	4.683
FSLGGF;FGEGLL	NCP	224.679
FGEGLL;KPRMKAK	NCP	227.764
KPRMKAK;APKRSFF	NCP	188.238
APKRSFF;FMSAFVK	NCP	16.491
FMSAFVK;NDSRSP	NCP	16.128
NDSRSP;LLPLQR	NCP	136.865
LLPLQR;KAKLKKDF	NCP	118.258
KAKLKKDF;KGLLSHSL	NCP	126.791
KGLLSHSL;GATKVRP	NCP	83.799
GATKVRP;QKAAKPR	NCP	7.371
QKAAKPR;KEGFTVP	NCP	73.098
KEGFTVP;YDKAY	NCP	125.395
YDKAY;LAGLLLKA	NCP	50.603
LAGLLLKA;RLKLPF	NCP	76.096
RLKLPF;PQQHADK	NCP	63.171
PQQHADK;VEDLP	NCP	132.95
VEDLP;KTMLTK	NCP	20.644
KTMLTK;PLLAA	NCP	30.557
PLLAA;MFPLPQK	NCP	38.182
MFPLPQK;QFKFR	NCP	102.552

QFKFR;LPDRF	NCP	21.267
LPDRF;LVLELRPG	NCP	21.203
LVLELRPG;LMCCC	NCP	81.354
LMCCC;KKFPVF	NCP	57.186
KKFPVF;EDDLRK	NCP	62.479
EDDLRK;VNLEF	NCP	43.01
VNLEF;LTVEK	NCP	85.819
LTVEK;AQPKY	NCP	60.596
AQPKY;YHMVYM	NCP	20.622
YHMVYM;VTAAPLY	NCP	90.663
VTAAPLY;VPLLLDQH	NCP	228.406
VPLLLDQH;TYGAPR	NCP	89.117
TYGAPR;FPKQYVL	NCP	127.456
FPKQYVL;KLVPHSKP	NCP	11.492
KLVPHSKP;FALDGL	NCP	28.041
FALDGL;TVEELLY	NCP	104.55
TVEELLY;VPSLDL	NCP	22.332
VPSLDL;SLYVYKVL	NCP	45.811
SLYVYKVL;EVLNQPM	NCP	79.514
EVLNQPM;ATFKLLPF	NCP	9.988
ATFKLLPF;AAPYHRLP	NCP	29.01
AAPYHRLP;KENLPEAK	NCP	69.918
KENLPEAK;KHFGPSP	NCP	79.427
KHFGPSP;KSDGLGLK	NCP	21.633
KSDGLGLK;PPSLFTGL	NCP	266.082
PPSLFTGL;MHEKPPP	NCP	111.606
MHEKPPP;YQLVK	NCP	122.473
YQLVK;VGLVGK	NCP	68.259
VGLVGK;GVAHT	NCP	196.226
GVAHT;YPVTR	NCP	83.142
YPVTR;LDDSDVLP	NCP	9.435
LDDSDVLP;PFEVP	NCP	146.887
PFEVP;GVLRL	NCP	3.899
GVLRL;PFLEYT	NCP	44.424
PFLEYT;NSPLYK	NCP	230.961
NSPLYK;AVLKYCR	NCP	44.664
AVLKYCR;SYPAAA	NCP	85.498
SYPAAA;NPLKV	NCP	12.599
NPLKV;DDDLEE	NCP	79.407
DDDLEE;AQMDLWK	NCP	78.924
AQMDLWK;KFLVAW	NCP	413.209
KFLVAW;GCVLQKA	NCP	119.835
GCVLQKA;QDDKLV	NCP	70.205
QDDKLV;CVFLLQ	NCP	35.958
CVFLLQ;KELLKK	NCP	74.664
KELLKK;NYLLKWN	NCP	24.01
NYLLKWN;GPQDLPAP	NCP	73.608
GPQDLPAP;MPKLPR	NCP	23.838
MPKLPR;VSVTDKF	NCP	25.22
VSVTDKF;TPQVVAKK	NCP	9.418
TPQVVAKK;RLVPY	NCP	137.006

RLVPY;VVPNDLA	NCP	213.936
VVPNDLA;PLLKKL	NCP	209.283
PLLKKL;MDCGP	NCP	89.086
MDCGP;FPFTSGQ	NCP	47.672
FPFTSGQ;VKEFSPK	NCP	534.574
VKEFSPK;ASVWK	NCP	72.233
ASVWK;KEHPLME	NCP	43.704
KEHPLME;LPKGVMY	NCP	34.636
LPKGVMY;QVTFT	NCP	72.951
QVTFT;CMDAC	NCP	145.487
CMDAC;KFLLT	NCP	182.655
KFLLT;MSLKNK	NCP	68.49
MSLKNK;ELEEYKL	NCP	136.563
ELEEYKL;QSMVPR	NCP	132.207
QSMVPR;VPHPQ	NCP	3.289
VPHPQ;KNLPP	NCP	28.673
KNLPP;VDHLV	NCP	99.333
VDHLV;AKNLEGY	NCP	31.956
AKNLEGY;KPRREP	NCP	122.673
KPRREP;YLDLLL	NCP	51.45
YLDLLL;MYLTF	NCP	22.258
MYLTF;ADKASFLL	NCP	76.598
ADKASFLL;LMLKL	NCP	167.067
LMLKL;FLEPDVP	NCP	0.574
FLEPDVP;TMKEVAR	NCP	8.99
TMKEVAR;KWEKKR	NCP	213.103
KWEKKR;LPKAHLN	NCP	208.458
LPKAHLN;VSRTYKNV	NCP	178.583
VSRTYKNV;PLPPLPPPP	NCP	289.074
PLPPLPPPP;HKTLGAH	NCP	45.481
HKTLGAH;VLLLML	NCP	81.635
VLLLML;KSMVLF	NCP	1.201
KSMVLF;RTCTPLM	NCP	1.5
RTCTPLM;EPKGKSW	NCP	165.834
EPKGKSW;VGLMLK	NCP	18.207
VGLMLK;KQPHKAP	NCP	0.755
KQPHKAP;LHLVPPNP	NCP	25.723
LHLVPPNP;PQHLVLF	NCP	60.119
PQHLVLF;QNYSYPG	NCP	195.934
QNYSYPG;KGTALVLT	NCP	119.783
KGTALVLT;GLNKCR	NCP	92.425
GLNKCR;YEQSTGY	NCP	79.092
YEQSTGY;VGQPLTK	NCP	55.521
VGQPLTK;SAKPALPKPEPRPK	NCP	33.47
SAKPALPKPEPRPK;AKPALPKPEPRP	NCP	0.003
AKPALPKPEPRP;ALPKPEPRPK	NCP	0.009
ALPKPEPRPK;LPKPEPRP	NCP	0.003
LPKPEPRP;KKWDL	NCP	165.15
KKWDL;KECPTAP	NCP	156.792
KECPTAP;GCDLDLRK	NCP	171.771
GCDLDLRK;ERVGLEL	NCP	79.711

ERVGLEL;KNSLLETL	NCP	116.234
KNSLLETL;GVFLN	NCP	92.89
GVFLN;NQSFLVL	NCP	31.952
NQSFLVL;KLPPPPYK	NCP	68.736
KLPPPPYK;HLLRPPP	NCP	139.467
HLLRPPP;KPSPR	NCP	3.636
KPSPR;SKKVF	NCP	4.389
SKKVF;DPGEF	NCP	27.225
DPGEF;KQWLHSP	NCP	44.217
KQWLHSP;CAAEHME	NCP	190.353
CAAEHME;EMCCQ	NCP	33.081
EMCCQ;FGNLL	NCP	20.577
FGNLL;TVTLKY	NCP	47.488
TVTLKY;VAYVLK	NCP	140.115
VAYVLK;KDGMMKK	NCP	44.57
KDGMMKK;MHSLVHR	NCP	7.273
MHSLVHR;CTTLYKLL	NCP	60.302
CTTLYKLL;KENHVFQ	NCP	56.742
KENHVFQ;ALLTK	NCP	22.402
ALLTK;GSPMLQ	NCP	66.09
GSPMLQ;KFGLG	NCP	113.943
KFGLG;DSCCAC	NCP	107.849
DSCCAC;TVHVYVR	NCP	65.022
TVHVYVR;AHRLQL	NCP	39.384
AHRLQL;ATKKAQR	NCP	50.346
ATKKAQR;KVLVVHHSF	NCP	54.628
KVLVVHHSF;MKGKY	NCP	9.825
MKGKY;KTHVAPK	NCP	16.985
KTHVAPK;NGHSLLLP	NCP	234.555
NGHSLLLP;NPYKAR	NCP	52.668
NPYKAR;LLVCCLPV	NCP	183.744
LLVCCLPV;GLHETTF	NCP	10.743
GLHETTF;MDLEALKK	NCP	32.509
MDLEALKK;LVLAL	NCP	6.383
LVLAL;VTPRVQ	NCP	104.781
VTPRVQ;VSVLPF	NCP	334.327
VSVLPF;YELPNPT	NCP	24.572
YELPNPT;YVTLF	NCP	44.302
YVTLF;DMLLRY	NCP	46.115
DMLLRY;AEEVVVVA	NCP	31.422
AEEVVVVA;REEKLK	NCP	13.057
REEKLK;KRLGVFF	NCP	215.096
KRLGVFF;SVNLTKLL	NCP	95.184
SVNLTKLL;KPMSPVH	NCP	27.697
KPMSPVH;KDFKV	NCP	65.052
KDFKV;PLLLK	NCP	8.276
PLLLK;VVGLK	NCP	42.162
VVGLK;RKVLQF	NCP	352.785
RKVLQF;KSYLVF	NCP	146.523
KSYLVF;MLFLYP	NCP	470.689
MLFLYP;TLVRLR	NCP	2.345

TLVRLR;KKGHVPP	NCP	37.861
KKGHVPP;KMNGML	NCP	92.216
KMNGML;LPPLPKRP	NCP	47.262
LPPLPKRP;AEHLTSHKY	NCP	11.325
AEHLTSHKY;KKEESH	NCP	5.569
KKEESH;NSLAALKK	NCP	19.748
NSLAALKK;AAVSGRGV	NCP	114.922
AAVSGRGV;RAKYP	NCP	76.307
RAKYP;CMMCEM	NCP	9.027
CMMCEM;TVYGSPLC	NCP	25.261
TVYGSPLC;TVGAP	NCP	8.94
TVGAP;YTVAK	NCP	58.197
YTVAK;QTLMF	NCP	24.432
QTLMF;SDFGVSKL	NCP	119.949
SDFGVSKL;SGPKPVH	NCP	7.526
SGPKPVH;LKLLEH	NCP	87.388
LKLLEH;MYTMWHK	NCP	167.489
MYTMWHK;ALQVELLP	NCP	52.974
ALQVELLP;KPGPVVY	NCP	58.336
KPGPVVY;SPGTLY	NCP	99.086
SPGTLY;SSRMEK	NCP	76.203
SSRMEK;KKCYPPP	NCP	13.831
KKCYPPP;KPFLP	NCP	51.426
KPFLP;LLLLPPK	NCP	167.007
LLLLPPK;LLCSPLLM	NCP	118.395
LLCSPLLM;PSLQLVSVL	NCP	15.747
PSLQLVSVL;LPAPSPPQC	NCP	19.308
LPAPSPPQC;LSKLLR	NCP	46.211
LSKLLR;MVKVP	NCP	140.184
MVKVP;QERKK	NCP	81.649
QERKK;KYGHAF	NCP	137.918
KYGHAF;DVKNLLTK	NCP	51.651
DVKNLLTK;HGPAP	NCP	110.269
HGPAP;ALKWVK	NCP	62.243
ALKWVK;DLDLR	NCP	193.053
DLDLR;REPASPK	NCP	5.251
REPASPK;SLLEELTL	NCP	9.578
SLLEELTL;TVDGLPF	NCP	71.632
TVDGLPF;NPLLSK	NCP	18.221
NPLLSK;SNELLMF	NCP	119.844
SNELLMF;AGLLKKTTGL	NCP	32.107
AGLLKKTTGL;VCLDP	NCP	103.517
VCLDP;FLQRQS	NCP	119.1
FLQRQS;EVKVLR	NCP	37.14
EVKVLR;LDFLPF	NCP	94.569
LDFLPF;TVKQVP	NCP	212.524
TVKQVP;DDEPK	NCP	1.026
DDEPK;TQPGLK	NCP	319.901
TQPGLK;NQDLLR	NCP	16.122
NQDLLR;HGVLLF	NCP	36.685
HGVLLF;GKLLY	NCP	96.507

GKLLY;KELGY	NCP	21.842
KELGY;FGVSST	NCP	28.686
FGVSST;LLQQPEPK	NCP	36.399
LLQQPEPK;GPPRPSF	NCP	29.743
GPPRPSF;AGPLTV	NCP	70.626
AGPLTV;MNPKLEW	NCP	83.651
MNPKLEW;VLASRVAP	NCP	39.144
VLASRVAP;NLALPP	NCP	58.872
NLALPP;QTLSSTEV	NCP	19.15
QTLSSTEV;PTQFK	NCP	135.465
PTQFK;VGGVP	NCP	241.596
VGGVP;KDAAMK	NCP	227.228
KDAAMK;LAPFP	NCP	33.723
LAPFP;TLLLNTK	NCP	75.778
TLLLNTK;LSLSGGTT	NCP	99.569
LSLSGGTT;LPPPLATT	NCP	16.975
LPPPLATT;SLTLPSPLVT	NCP	48.111
SLTLPSPLVT;KQTKTPLTP	NCP	53.838
KQTKTPLTP;GVNPLL	NCP	72.071
GVNPLL;FGLDLK	NCP	301.822
FGLDLK;DLKAVDK	NCP	86.709
DLKAVDK;KLADNPP	NCP	9.563
KLADNPP;AAADYKK	NCP	11.089
AAADYKK;YPMRMT	NCP	27.627
YPMRMT;MGVVTLK	NCP	217.724
MGVVTLK;LSYLPL	NCP	14.644
LSYLPL;KCAQSKR	NCP	67.268
KCAQSKR;TALDSVL	NCP	46.872
TALDSVL;LDSYLK	NCP	108.006
LDSYLK;LAGATGPL	NCP	214.069
LAGATGPL;YVLPPK	NCP	114.156
YVLPPK;KMMLLQ	NCP	164.199
KMMLLQ;YEKPLE	NCP	49.169
YEKPLE;QRHTVSPP	NCP	25.017
QRHTVSPP;LTMTF	NCP	54.643
LTMTF;LSYVYKVL	NCP	13.748
LSYVYKVL;KNMGLP	NCP	49.9
KNMGLP;HSSLLSHV	NCP	82.256
HSSLLSHV;EAFNLLHL	NCP	9.945
EAFNLLHL;LASLKQSR	NCP	2.878
LASLKQSR;KLHAPGR	NCP	0.11
KLHAPGR;THCLLR	NCP	7.291
THCLLR;YSDVP	NCP	42.378
YSDVP;KRHLEK	NCP	158.676
KRHLEK;EVELE	NCP	150.293
EVELE;LGPLKPF	NCP	46.518
LGPLKPF;TDVQPLEK	NCP	3.852
TDVQPLEK;SSVESLGK	NCP	6.519
SSVESLGK;HLLELPF	NCP	103.437
HLLELPF;SELRF	NCP	70.713
SELRF;DSVDF	NCP	8.086

DSVDF;AVSCFA	NCP	69.683
AVSCFA;HTLGPN	NCP	2.358
HTLGPN;FMELQGY	NCP	19.215
FMELQGY;RRPKTK	NCP	140.422
RRPKTK;DHMLPHQ	NCP	61.37
DHMLPHQ;KTLTPR	NCP	25.597
KTLTPR;VTDVPQ	NCP	31.976
VTDVPQ;LDLAHC	NCP	14.485
LDLAHC;LPKYPPP	NCP	57.68
LPKYPPP;KDGEPLVT	NCP	64.18
KDGEPLVT;LFKYH	NCP	59.283
LFKYH;LPLPYRST	NCP	3.68
LPLPYRST;FGPGP	NCP	80.209
FGPGP;LDQATLSL	NCP	101.357
LDQATLSL;TCKPR	NCP	46.425
TCKPR;KYHSATTL	NCP	249.855
KYHSATTL;PKAAQ	NCP	111.169
PKAAQ;VHVLF	NCP	70.35
VHVLF;SPDVH	NCP	23.109
SPDVH;GPPSRY	NCP	16.718
GPPSRY;AAGPK	NCP	18.636
AAGPK;VNPLR	NCP	44.511
VNPLR;YQRMLVVLL	NCP	16.206
YQRMLVVLL;ALLLLCGGEDD	NCP	0.474
ALLLLCGGEDD;YVGLGY	NCP	112.383
YVGLGY;PSLPVLP	NCP	67.806
PSLPVLP;YTPQP	NCP	5.307
YTPQP;GPKRVLLF	NCP	202.314
GPKRVLLF;KNLKLE	NCP	40.641
KNLKLE;GNRYP	NCP	71.625
GNRYP;VLEKSPGK	NCP	166.758
VLEKSPGK;LNTRY	NCP	24.799
LNTRY;VTKHGPQ	NCP	21.086
VTKHGPQ;LNPVVP	NCP	55.933
LNPVVP;TTYLRK	NCP	98.483
TTYLRK;LGPTFSP	NCP	51.966
LGPTFSP;EALEFK	NCP	95.577
EALEFK;YDGLL	NCP	32.806
YDGLL;WLPPPP	NCP	203.061
WLPPPP;KLVDKEF	NCP	9.116
KLVDKEF;LDQVEVVK	NCP	147.859
LDQVEVVK;VGFSTK	NCP	58.431
VGFSTK;LEKNWALP	NCP	83.852
LEKNWALP;QFFKFGM	NCP	94.617
QFFKFGM;MGSLFDL	NCP	55.486
MGSLFDL;PATGLQF	NCP	216.764
PATGLQF;MEDHLSLH	NCP	36.25
MEDHLSLH;MMGLYT	NCP	189.392
MMGLYT;DFPELKP	NCP	7.821
DFPELKP;KSPGYGL	NCP	2.272
KSPGYGL;KLLNESGK	NCP	141.971

KLLNESGK;DMPDLRK	NCP	241.812
DMPDLRK;MTNYQP	NCP	54.049
MTNYQP;PWTAH	NCP	173.171
PWTAH;KPEPPGK	NCP	31.134
KPEPPGK;NFPFEL	NCP	82.693
NFPFEL;TLEVMPY	NCP	221.795
TLEVMPY;SSGVAQLVK	NCP	47.104
SSGVAQLVK;RHCDDD	NCP	16.19
RHCDDD;ESLRVAE	NCP	92.701
ESLRVAE;DTPELLH	NCP	42.731
DTPELLH;FLRYP	NCP	288.561
FLRYP;PDDGMM	NCP	96.691
PDDGMM;RMPVVVK	NCP	13.545
RMPVVVK;VAFEKHY	NCP	21.914
VAFEKHY;KSLDTF	NCP	31.285
KSLDTF;KAHFRYK	NCP	21.888
KAHFRYK;GLLTL	NCP	71.246
GLLTL;RNLLYK	NCP	147.976
RNLLYK;GPGPYK	NCP	0.89
GPGPYK;TAAKYL	NCP	39.289
TAAKYL;HEPTLL	NCP	345.9
HEPTLL;TVEYTKF	NCP	51.873
TVEYTKF;ERMFLSF	NCP	91.25
ERMFLSF;EAAVPL	NCP	33.921
EAAVPL;WLFALKK	NCP	2.289
WLFALKK;AAVVDC	NCP	278.853
AAVVDC;KFPWSEL	NCP	80.917
KFPWSEL;HLSNEPK	NCP	46.994
HLSNEPK;LNHSMK	NCP	6.769
LNHSMK;VGELLRKN	NCP	42.195
VGELLRKN;FEPEKL	NCP	48.381
FEPEKL;VEVLTF	NCP	57.192
VEVLTF;DHPLK	NCP	7.257
DHPLK;KNPGLLRP	NCP	56.322
KNPGLLRP;GKVLFP	NCP	81.081
GKVLFP;EAAPALSPK	NCP	39.554
EAAPALSPK;MQLTDF	NCP	78.997
MQLTDF;PGLVPLH	NCP	2.886
PGLVPLH;TGYGMPR	NCP	12.552
TGYGMPR;KHLLN	NCP	153.836
KHLLN;PAGLAF	NCP	100.105
PAGLAF;EVEKVFK	NCP	103.215
EVEKVFK;SCVGVFK	NCP	16.07
SCVGVFK;KYVLPKSK	NCP	25.836
KYVLPKSK;LGAAPKSL	NCP	41.973
LGAAPKSL;KSTSVFP	NCP	30.138
KSTSVFP;RGVVP	NCP	142.606
RGVVP;GTGPLF	NCP	167.759
GTGPLF;PPSLHK	NCP	18.904
PPSLHK;LVTLANNR	NCP	102.645
LVTLANNR;VELLV	NCP	3.966

VELLV;SVNLVAL	NCP	50.579
SVNLVAL;GVPSAF	NCP	23.107
GVPSAF;LPGLPP	NCP	125.49
LPGLPP;VSPTWL	NCP	5.684
VSPTWL;KDPSAVP	NCP	43.017
KDPSAVP;DFGPK	NCP	9.528
DFGPK;RTDLEKPF	NCP	31.948
RTDLEKPF;MTKLNGN	NCP	8.87
MTKLNGN;KATVEPR	NCP	21.732
KATVEPR;LGALQRPK	NCP	31.782
LGALQRPK;SSSKPP	NCP	123.997
SSSKPP;DGKAPQK	NCP	90.971
DGKAPQK;VLLVKSSM	NCP	197.392
VLLVKSSM;KDFGP	NCP	31.886
KDFGP;VGNPLL	NCP	20.034
VGNPLL;RLPLQDV	NCP	32.665
RLPLQDV;DGPKFLK	NCP	0.42
DGPKFLK;VKLLFA	NCP	62.844
VKLLFA;PPAYKR	NCP	10.634
PPAYKR;ALPNLL	NCP	14.124
ALPNLL;QGPDAPPK	NCP	5.359
QGPDAPPK;LSGLHFK	NCP	124.509
LSGLHFK;ELNARK	NCP	9.057
ELNARK;KEMTVETL	NCP	53.139
KEMTVETL;GSYDN	NCP	42.027
GSYDN;FLVHVDK	NCP	62.019
FLVHVDK;GVKYPEL	NCP	52.22
GVKYPEL;AFRLW	NCP	43.461
AFRLW;KELFFPPP	NCP	73.306
KELFFPPP;CPLSLFPK	NCP	163.467
CPLSLFPK;KECCH	NCP	207.519
KECCH;DTVVVEK	NCP	393.179
DTVVVEK;LLKLSY	NCP	122.086
LLKLSY;SGVVP	NCP	21.147
SGVVP;TGFPTNF	NCP	77.184
TGFPTNF;KKYCFP	NCP	25.845
KKYCFP;EVVPQ	NCP	22.587
EVVPQ;GVAMVA	NCP	35.838
GVAMVA;TTSAG	NCP	29.069
TTSAG;NSSGPPPPP	NCP	9.87
NSSGPPPPP;QALKVFN	NCP	12.484
QALKVFN;FPVGEVVP	NCP	90.009
FPVGEVVP;LNGPLLSKL	NCP	85.631
LNGPLLSKL;LLHQRSPL	NCP	47.026
LLHQRSPL;KVTLLL	NCP	58.74
KVTLLL;LKFDRP	NCP	17.304
LKFDRP;KAPAKPPP	NCP	1.046
KAPAKPPP;APAKPPPK	NCP	0.003
APAKPPPK;PPGPPYK	NCP	159.157
PPGPPYK;SPRMKG	NCP	101.663
SPRMKG;MGHRVDY	NCP	7.176

MGHRVDY;TTRESVVDY	NCP	1.104
TTRESVVDY;TTATMPK	NCP	143.337
TTATMPK;KFQKGT	NCP	13.237
KFQKGT;KMTFP	NCP	78.111
KMTFP;GMMADM	NCP	210.149
GMMADM;RNPSF	NCP	21.696
RNPSF;NGDLPF	NCP	17.622
NGDLPF;LGRTL	NCP	16.648
LGRTL;MGPPKY	NCP	25.851
MGPPKY;FSHVNPLQ	NCP	70.731
FSHVNPLQ;KPGCLPLT	NCP	66.476
KPGCLPLT;SPGGADAF	NCP	216.984
SPGGADAF;LLMAPPG	NCP	18.096
LLMAPPG;KDFKEL	NCP	73.249
KDFKEL;KTTVPF	NCP	29.777
KTTVPF;SSLRFP	NCP	92.133
SSLRFP;KTLSLPL	NCP	142.096
KTLSLPL;KTETQEKNPLPSK	NCP	8.714
KTETQEKNPLPSK;TETQEKNPLPSKE	NCP	0.003
TETQEKNPLPSKE;KNPLPSKET	NCP	0.015
KNPLPSKET;TKSTFQQ	NCP	72.435
TKSTFQQ;SLNDLE	NCP	143.191
SLNDLE;CECCC	NCP	159.74
CECCC;EVSLLP	NCP	305.677
EVSLLP;KSTAAPPPP	NCP	9.917
KSTAAPPPP;AVFGL	NCP	179.8
AVFGL;NEPLFTVS	NCP	16.419
NEPLFTVS;GPPQHTT	NCP	5.738
GPPQHTT;MDMDDD	NCP	138.231
MDMDDD;KGPGVF	NCP	23.961
KGPGVF;LSLLRVVSS	NCP	59.052
LSLLRVVSS;LKKDLEK	NCP	64.246
LKKDLEK;KKPLL	NCP	19.524
KKPLL;KAFSMLLR	NCP	28.466
KAFSMLLR;VSSEDTLLP	NCP	115.419
VSSEDTLLP;HLLPPT	NCP	30.96
HLLPPT;TSPCCNP	NCP	5.25
TSPCCNP;TTKPGLRK	NCP	73.195
TTKPGLRK;TGVVGTLK	NCP	29.31
TGVVGTLK;PSDTWF	NCP	61.808
PSDTWF;LAAVL	NCP	15.036
LAAVL;KEPFP	NCP	41.991
KEPFP;LYYHR	NCP	2.521
LYYHR;VNEKF	NCP	8.444
VNEKF;PNWNGKK	NCP	226.06
PNWNGKK;RYFKTPP	NCP	113.237
RYFKTPP;LVTHPA	NCP	69.322
LVTHPA;CPPLKVSF	NCP	113.211
CPPLKVSF;LLAFAN	NCP	22.955
LLAFAN;MFSWLK	NCP	118.761
MFSWLK;KCNTLK	NCP	20.982

KCNTLK;MGSPFPK	NCP	0.073
MGSPFPK;KPRSEMLV	NCP	5.568
KPRSEMLV;KLNLGTVP	NCP	32.124
KLNLGTVP;KNSTL	NCP	246.992
KNSTL;VGGPF	NCP	11.341
VGGPF;KVVYPK	NCP	97.191
KVVYPK;FFLPQ	NCP	173.457
FFLPQ;TGPPVGGK	NCP	31.611
TGPPVGGK;KPGRKTP	NCP	30.495
KPGRKTP;LVTKLPK	NCP	97.184
LVTKLPK;VVLDNP	NCP	29.016
VVLDNP;AYLKNRCL	NCP	338.299
AYLKNRCL;KPTLFK	NCP	30.846
KPTLFK;RKLEPEH	NCP	295.589
RKLEPEH;EPHQKAK	NCP	24.402
EPHQKAK;KLPEL	NCP	34.39
KLPEL;LSYDLP	NCP	50.573
LSYDLP;SVVRKHY	NCP	170.761
SVVRKHY;SAKKLVLK	NCP	131.79
SAKKLVLK;LKVGSLEK	NCP	34.845
LKVGSLEK;ANSAALFY	NCP	109.25
ANSAALFY;RAGLKLS	NCP	12.952
RAGLKLS;RVAPPK	NCP	104.529
RVAPPK;LGVPTVRF	NCP	86.393
LGVPTVRF;MNANLRP	NCP	36.027
MNANLRP;LLVLML	NCP	186.393
LLVLML;PGAGKSGK	NCP	14.007
PGAGKSGK;TQSLPK	NCP	36.453
TQSLPK;EQQVKGK	NCP	7.098
EQQVKGK;KNFKPQNF	NCP	208.591
KNFKPQNF;KFYLPNP	NCP	106.743
KFYLPNP;DGAVKPPP	NCP	269.49
DGAVKPPP;GLFGPP	NCP	40.281
GLFGPP;VAPPPVFP	NCP	23.592
VAPPPVFP;DADDC	NCP	202.893
DADDC;FLNPRLY	NCP	176.822
FLNPRLY;AALELSGR	NCP	19.663
AALELSGR;AGHPPVK	NCP	12.489
AGHPPVK;ACKYA	NCP	188.537
ACKYA;LTGTLQ	NCP	4.834
LTGTLQ;TLQAQ	NCP	21.498
TLQAQ;TQPPPR	NCP	217.062
TQPPPR;LGGPL	NCP	54.827
LGGPL;LAPPLL	NCP	41.578
LAPPLL;KPFLSPM	NCP	82.173
KPFLSPM;PEMAAP	NCP	41.123
PEMAAP;AVNLSR	NCP	95.512
AVNLSR;HLPGSPF	NCP	0.251
HLPGSPF;TVTFE	NCP	23.709
TVTFE;ATAKPF	NCP	38.856
ATAKPF;RVGLVK	NCP	225.229

RVGLVK;DELVFF	NCP	95.657
DELVFF;EHLSYL	NCP	69.6
EHLSYL;PAPKLEVP	NCP	27.214
PAPKLEVP;QTDSRKL	NCP	13.032
QTDSRKL;KNWPPYK	NCP	92.504
KNWPPYK;WAGKPLF	NCP	32.287
WAGKPLF;APVPPGTP	NCP	2.298
APVPPGTP;LSTLPVT	NCP	102.653
LSTLPVT;KPFFPYLK	NCP	18.501
KPFFPYLK;TLNELL	NCP	3.847
TLNELL;QPTLLR	NCP	2.055
QPTLLR;EHVVLK	NCP	3.317
EHVVLK;FLQSAPSK	NCP	24.265
FLQSAPSK;YESCLY	NCP	73.802
YESCLY;MLKVALD	NCP	4.144
MLKVALD;RGPYH	NCP	26.582
RGPYH;QFVHRK	NCP	65.422
QFVHRK;TPVGVP	NCP	30.204
TPVGVP;VPFGVPK	NCP	19.356
VPFGVPK;CLHMASK	NCP	6.32
CLHMASK;RSVGGPVR	NCP	313.056
RSVGGPVR;KAFKTSSK	NCP	0.886
KAFKTSSK;TSLTK	NCP	88.677
TSLTK;HVPKKKD	NCP	80.22
HVPKKKD;KSYAHPP	NCP	50.375
KSYAHPP;LGVLT	NCP	199.602
LGVLT;LNPHPR	NCP	11.314
LNPHPR;RGPPLFK	NCP	37.031
RGPPLFK;RLESPPP	NCP	61.746
RLESPPP;EVKYTPY	NCP	26.124
EVKYTPY;TNPALP	NCP	11.212
TNPALP;LRLLDH	NCP	46.998
LRLLDH;KVPDLAVG	NCP	110.474
KVPDLAVG;PGGFVP	NCP	146.341
PGGFVP;KPESVPP	NCP	41.859
KPESVPP;VGMKVVK	NCP	18.875
VGMKVVK;VSPRHP	NCP	18.258
VSPRHP;KGYLTGR	NCP	6.286
KGYLTGR;MPALL	NCP	39.393
MPALL;KPMGP	NCP	33.744
KPMGP;VSPDLRGP	NCP	235.062
VSPDLRGP;KPHGVPK	NCP	83.028
KPHGVPK;FAFLFC	NCP	36.368
FAFLFC;VLAFSM	NCP	19.903
VLAFSM;TLASVP	NCP	11.646
TLASVP;RQHKFK	NCP	134.192
RQHKFK;EVRVQP	NCP	17.542
EVRVQP;YVLTGR	NCP	70.082
YVLTGR;SWKETL	NCP	43.752
SWKETL;FYAPEL	NCP	137.181
FYAPEL;KKPMAAP	NCP	28.077

KKPMAAP;AELTVEVR	NCP	100.038
AELTVEVR;ENAHKDFKKAVG	NCP	45.204
ENAHKDFKKAVG;SLLADF	NCP	34.119
SLLADF;SPSPPHLK	NCP	9.874
SPSPPHLK;LPAHKL	NCP	47.847
LPAHKL;LLSLKY	NCP	11.291
LLSLKY;KLSEYGP	NCP	21.723
KLSEYGP;ATYLQY	NCP	39.859
ATYLQY;KDDLNL	NCP	39.606
KDDLNL;KLHRTP	NCP	37.953
KLHRTP;LSELP	NCP	25.056
LSELP;LEAGSGLP	NCP	62.72
LEAGSGLP;LGETLP	NCP	28.35
LGETLP;KEPPFER	NCP	71.932
KEPPFER;LYGGLFL	NCP	17.198
LYGGLFL;WGVLW	NCP	180.885
WGVLW;GPHEAPK	NCP	90.813
GPHEAPK;LDVKAPK	NCP	83.376
LDVKAPK;KDGGDPK	NCP	14.998
KDGGDPK;TAPVNFK	NCP	101.376
TAPVNFK;DRPLNP	NCP	42.638
DRPLNP;MEMLCAA	NCP	73.252
MEMLCAA;PKMTFLH	NCP	21.623
PKMTFLH;LDGLQFF	NCP	89.17
LDGLQFF;VGEVLVTK	NCP	31.212
VGEVLVTK;WVLTSKY	NCP	62.501
WVLTSKY;KPFHGPP	NCP	22.204
KPFHGPP;FGGWK	NCP	5.732
FGGWK;MSDFPL	NCP	134.662
MSDFPL;LPPPPPSS	NCP	199.239
LPPPPPSS;YYPRLH	NCP	110.231
YYPRLH;RDAHF	NCP	5.82
RDAHF;HDGSFLK	NCP	19.147
HDGSFLK;RYTVKP	NCP	214.911
RYTVKP;GPVHPAK	NCP	4.125
GPVHPAK;YGGKSKK	NCP	87.237
YGGKSKK;TAKRPP	NCP	16.553
TAKRPP;QKFDLR	NCP	12.18
QKFDLR;DTVRNKTK	NCP	1.737
DTVRNKTK;KKYCGK	NCP	154.123
KKYCGK;KAACHPK	NCP	203.769
KAACHPK;WGVKK	NCP	24.995
WGVKK;LSPEELQK	NCP	58.261
LSPEELQK;KPFQGK	NCP	37.461
KPFQGK;VVHRKCF	NCP	50.96
VVHRKCF;KTFLAQC	NCP	11.749
KTFLAQC;LYCLY	NCP	128.874
LYCLY;KVTPVTR	NCP	17.801
KVTPVTR;KYLPPP	NCP	48.394
KYLPPP;DLVLK	NCP	3.77
DLVLK;RAGKHKQ	NCP	61.683

RAGKHKQ;TSTAPGKQ	NCP	18.294
TSTAPGKQ;FTKVKM	NCP	6.699
FTKVKM;LPQLGVSLP	NCP	1.944
LPQLGVSLP;LEVAKPP	NCP	0.234
LEVAKPP;KSQEFFH	NCP	50.107
KSQEFFH;ADLGE	NCP	107.312
ADLGE;AATVVAPPGALAS	NCP	0.153
AATVVAPPGALAS;ESGKVSSG	NCP	9.127
ESGKVSSG;SSGSLALR	NCP	16.529
SSGSLALR;LKVFEPKLN	NCP	15.972
LKVFEPKLN;NHGLF	NCP	21.874
NHGLF;PLSFSY	NCP	8.651
PLSFSY;NMAHNAEHEACDL	NCP	54.618
NMAHNAEHEACDL;GPAAHVY	NCP	15.909
GPAAHVY;FGFGFL	NCP	1.866
FGFGFL;MLQDVLDLRK	NCP	4.929
MLQDVLDLRK;EMEERSG	NCP	42.783
EMEERSG;LHPAARP	NCP	65.67
LHPAARP;YPSLKVLL	NCP	62.791
YPSLKVLL;SLPFFQRT	NCP	37.539
SLPFFQRT;KMFGEKL	NCP	10.746
KMFGEKL;RAAPF	NCP	67.916
RAAPF;TGLRPTT	NCP	0.519
TGLRPTT;NCTPK	NCP	51.121
NCTPK;KYKAPTK	NCP	150.387
KYKAPTK;MKPLRNLL	NCP	34.92
MKPLRNLL;WRYSSWK	NCP	91.973
WRYSSWK;VPTVFVG	NCP	75.198
VPTVFVG;FLTVPF	NCP	50.772
FLTVPF;RYELQR	NCP	99.141
RYELQR;LTEWG	NCP	12.76
LTEWG;HLGPSFP	NCP	4.283
HLGPSFP;NVLEY	NCP	4.342
NVLEY;GVTRVY	NCP	37.706
GVTRVY;TMMDPP	NCP	236.487
TMMDPP;LTMPLTLL	NCP	10.744
LTMPLTLL;TKGPRK	NCP	17.948
TKGPRK;ANPLL	NCP	38.736
ANPLL;FYWLLR	NCP	305.364
FYWLLR;KPLENQP	NCP	7.03
KPLENQP;LPLPNC	NCP	33.995
LPLPNC;TLSLETGF	NCP	111.534
TLSLETGF;PTVKMY	NCP	25.125
PTVKMY;DPGGY	NCP	8.842
DPGGY;LGPPMLK	NCP	10.322
LGPPMLK;HSRDP	NCP	17.212
HSRDP;VNFDP	NCP	21.776
VNFDP;PLSQGATR	NCP	7.351
PLSQGATR;AEGAFVLL	NCP	1.22
AEGAFVLL;TGGPDN	NCP	4.209
TGGPDN;KPTLGQQ	NCP	27.616

KPTLGQQ;KAQVALD	NCP	87.696
KAQVALD;LGFLEL	NCP	0.638
LGFLEL;VGDALSVS	NCP	112.989
VGDALSVS;VAWNGKK	NCP	13.045
VAWNGKK;KVGTRVP	NCP	68.33
KVGTRVP;QAVVL	NCP	22.872
QAVVL;LRMCGAF	NCP	8.785
LRMCGAF;LLLLPPQ	NCP	23.298
LLLLPPQ;KDWVSL	NCP	19.551
KDWVSL;DLNFPK	NCP	127.742
DLNFPK;KQKVNGF	NCP	4.768
KQKVNGF;DFFEDTELYRK	NCP	42.218
DFFEDTELYRK;FFAYTLPK	NCP	4.032
FFAYTLPK;NSENKSGNQFVLY	NCP	6.492
NSENKSGNQFVLY;NTTVEEMPMN	NCP	61.29
NTTVEEMPMN;TVEEMPMNVAD	NCP	0.006
TVEEMPMNVAD;LKDVKVSLY	NCP	51.675
LKDVKVSLY;DDFLHFK	NCP	183.141
DDFLHFK;NGAPTVKF	NCP	11.208
NGAPTVKF;CLGPK	NCP	184.113
CLGPK;HLYLSR	NCP	8.391
HLYLSR;LLFTT	NCP	68.902
LLFTT;PEAGK	NCP	70.134
PEAGK;TFVPKF	NCP	89.702
TFVPKF;KETVQL	NCP	53.914
KETVQL;LTVDKK	NCP	25.923
LTVDKK;LGVSVPVL	NCP	4.485
LGVSVPVL;QGHDVLT	NCP	130.082
QGHDVLT;LELKVE	NCP	67.636
LELKVE;LGSVLR	NCP	128.189
LGSVLR;DPYMM	NCP	327.603
DPYMM;ETEYTVK	NCP	68.946
ETEYTVK;TFGPGV	NCP	16.542
TFGPGV;SHPSWLPPKS	NCP	31.305
SHPSWLPPKS;SVSTQQPPKK	NCP	0.099
SVSTQQPPKK;KKFAPVVAPKPK	NCP	0.024
KKFAPVVAPKPK;FAPVVAPKP	NCP	0.006
FAPVVAPKP;APVVAPKPK	NCP	0.003
APVVAPKPK;FPPPPPLDDN	NCP	72.81
FPPPPPLDDN;AATFLK	NCP	27.976
AATFLK;SPYKPRPP	NCP	16.031
SPYKPRPP;KAMGKPK	NCP	14.212
KAMGKPK;KVEVPK	NCP	1.974
KVEVPK;LDFPP	NCP	19.664
LDFPP;ASPPVSTPVTGHK	NCP	63.381
ASPPVSTPVTGHK;PPVSTPVTGHKR	NCP	0.006
PPVSTPVTGHKR;VSTPVTGHK	NCP	0.006
VSTPVTGHK;PVPMSGQGPPPSTR	NCP	0.309
PVPMSGQGPPPSTR;NQGRYYEPY	NCP	0.111
NQGRYYEPY;CSVGPGYGGK	NCP	0.027
CSVGPGYGGK;VGPGYGGKN	NCP	0.006

VGPGYGGKN;DSDPSYGQQGHPNTWK	NCP	0.027
DSDPSYGQQGHPNTWK;PAGMYPVAGPK	NCP	0.09
PAGMYPVAGPK;CCGPP	NCP	4.878
CCGPP;DVDVSLPK	NCP	36
DVDVSLPK;PNLKNRDF	NCP	12.634
PNLKNRDF;MNKLVFY	NCP	10.287
MNKLVFY;FRPEDELEHLTK	NCP	33.065
FRPEDELEHLTK;EDELEHLT	NCP	0.009
EDELEHLT;DELEHLTK	NCP	0.003
DELEHLTK;KGGSER	NCP	37.231
KGGSER;QEAYLLK	NCP	26.93
QEAYLLK;NAVFP	NCP	29.88
NAVFP;PPFKN	NCP	27.172
PPFKN;SLFTVLAT	NCP	75.158
SLFTVLAT;KLPLR	NCP	16.228
KLPLR;KNKEC	NCP	246.8
KNKEC;EAYLV	NCP	119.064
EAYLV;KEHAAPPN	NCP	54.418
KEHAAPPN;EKTKCPV	NCP	12.092
EKTKCPV;SVTLF	NCP	24.691
SVTLF;LPTVEC	NCP	11.673
LPTVEC;QLLLEYL	NCP	68.919
QLLLEYL;PLFGSPP	NCP	52.094
PLFGSPP;LLAKVPGQ	NCP	20.643
LLAKVPGQ;QQAKGGKP	NCP	73
QQAKGGKP;SDLPFEL	NCP	171.704
SDLPFEL;CCMVN	NCP	46.974
CCMVN;QDDAAPR	NCP	96.174
QDDAAPR;MLGPL	NCP	101.907
MLGPL;RAKPAVP	NCP	73.933
RAKPAVP;APDFA	NCP	24.02
APDFA;LEAFLF	NCP	74.478
LEAFLF;HKLLGH	NCP	77.719
HKLLGH;MDDGH	NCP	121.167
MDDGH;CDMMDF	NCP	15.803
CDMMDF;KELTTPK	NCP	35.803
KELTTPK;KGVVP	NCP	97.482
KGVVP;KNYKYC	NCP	233.001
KNYKYC;NSPLGF	NCP	48.855
NSPLGF;LEPLVPVK	NCP	35.967
LEPLVPVK;KHHRAWP	NCP	72.969
KHHRAWP;PPYVP	NCP	5.192
PPYVP;ALKERK	NCP	76.566
ALKERK;AKFAH	NCP	17.859
AKFAH;AAAALY	NCP	25.656
AAAALY;AAPPSLP	NCP	57.214
AAPPSLP;MLCLVCKK	NCP	7.602
MLCLVCKK;LPPVSGPG	NCP	99.444
LPPVSGPG;AMLKRQP	NCP	106.611
AMLKRQP;GSLSYDTLP	NCP	37.937
GSLSYDTLP;ELTALAPS	NCP	95.089

ELTALAPS;KPAAYK	NCP	39.866
KPAAYK;LLPGLF	NCP	213.142
LLPGLF;SDGVLK	NCP	10.248
SDGVLK;KYLVPPP	NCP	59.031
KYLVPPP;PPVPY	NCP	101.205
PPVPY;RLPPPPP	NCP	12.783
RLPPPPP;TLPGATRP	NCP	16.179
TLPGATRP;LPVPPPPP	NCP	44.421
LPVPPPPP;ESHLPGSPF	NCP	13.703
ESHLPGSPF;TPLSAP	NCP	58.177
TPLSAP;RTGSLVLR	NCP	27.309
RTGSLVLR;LFEDPY	NCP	24.881
LFEDPY;KPPAFTK	NCP	27.178
KPPAFTK;DPPPREPP	NCP	37.92
DPPPREPP;GPVGVP	NCP	104.124
GPVGVP;TSFKEMK	NCP	26.922
TSFKEMK;KMVGDLC	NCP	108.789
KMVGDLC;CLGKP	NCP	57.038
CLGKP;NSPRMLK	NCP	84.817
NSPRMLK;KPAAAK	NCP	33.873
KPAAAK;KGLKFLP	NCP	7.178
KGLKFLP;KMLYF	NCP	51.528
KMLYF;TVLKVHA	NCP	71.037
TVLKVHA;ANLVTR	NCP	37.446
ANLVTR;TGALDLK	NCP	109.9
TGALDLK;KDGLELLL	NCP	70.122
KDGLELLL;CTECCC	NCP	275.9
CTECCC;VTPPYLV	NCP	85.312
VTPPYLV;PPLPS	NCP	22.038
PPLPS;LSGNGKLL	NCP	37.578
LSGNGKLL;NPPTFLSQ	NCP	50.654
NPPTFLSQ;ALLME	NCP	58.774
ALLME;EAELGLFG	NCP	41.832
EAELGLFG;YNLTELK	NCP	5.144
YNLTELK;KLAEC	NCP	131.422
KLAEC;MDGLVP	NCP	60.95
MDGLVP;VFEPKL	NCP	10.228
VFEPKL;QPSGLPG	NCP	70.487
QPSGLPG;LSSGYG	NCP	32.914
LSSGYG;LPWGGGKK	NCP	45.878
LPWGGGKK;LPGGNHH	NCP	9.393
LPGGNHH;LQKPRLL	NCP	38.797
LQKPRLL;LLSHSL	NCP	99.399
LLSHSL;PAPPPPPPPP	NCP	35.354
PAPPPPPPPP;PRPPPSPPPPP	NCP	0.624
PRPPPSPPPPP;PPPPRSPP	NCP	0.483
PPPPRSPP;LPVGVRPS	NCP	125.526
LPVGVRPS;YETPK	NCP	64.695
YETPK;AAHWC	NCP	109.465
AAHWC;LPAGGL	NCP	3.224
LPAGGL;VLATAAQ	NCP	21.837

VLATAAQ;LSTGFKTH	NCP	70.503
LSTGFKTH;KDGNPLPS	NCP	20.943
KDGNPLPS;TCPGF	NCP	164.325
TCPGF;LPPGPPGP	NCP	43.518
LPPGPPGP;AASSLGPK	NCP	11.533
AASSLGPK;VPTGKHF	NCP	130.922
VPTGKHF;DGCEELKK	NCP	2.379
DGCEELKK;LNKGM	NCP	58.726
LNKGM;MLVCVDGL	NCP	10.191
MLVCVDGL;SPNVYN	NCP	29.484
SPNVYN;VTFLQYE	NCP	17.957
VTFLQYE;LALELGHK	NCP	18.624
LALELGHK;VPAHPP	NCP	14.824
VPAHPP;KGGGGAGP	NCP	28.142
KGGGGAGP;EFGTNLK	NCP	124.87
EFGTNLK;PGPPPPP	NCP	257.09
PGPPPPP;TWLLT	NCP	245.092
TWLLT;APKSRFF	NCP	7.056
APKSRFF;APVMVLE	NCP	177.332
APVMVLE;PALTLKSAP	NCP	71.523
PALTLKSAP;ALLKCGR	NCP	1.026
ALLKCGR;EPFSLR	NCP	75.342
EPFSLR;DTLQFTSF	NCP	9.471
DTLQFTSF;KNGLHPP	NCP	137.802
KNGLHPP;EKSNGLQ	NCP	30.454
EKSNGLQ;KAMSVDS	NCP	23.42
KAMSVDS;KSVKVPPKE	NCP	177.978
KSVKVPPKE;SLKAVQVP	NCP	14.293
SLKAVQVP;FLRSMF	NCP	75.345
FLRSMF;TMMCMP	NCP	98.234
TMMCMP;GVVAVGPP	NCP	81.255
GVVAVGPP;KEKGPGR	NCP	90.694
KEKGPGR;KELTPPK	NCP	8.103
KELTPPK;AAPLLP	NCP	49.451
AAPLLP;MHDLRL	NCP	39.175
MHDLRL;FKTEDF	NCP	196.682
FKTEDF;LLFVPR	NCP	2.791
LLFVPR;QALPGVR	NCP	8.426
QALPGVR;KELVTL	NCP	78.816
KELVTL;RPALPP	NCP	50.494
RPALPP;VVSGPV	NCP	66.671
VVSGPV;KEEWNF	NCP	14.532
KEEWNF;TAVKHEQEY	NCP	58.473
TAVKHEQEY;LTMHVTK	NCP	19.905
LTMHVTK;LFVLY	NCP	15.553
LFVLY;KPSCCMK	NCP	62.205
KPSCCMK;LLPFL	NCP	8.381
LLPFL;AALSYL	NCP	38.409
AALSYL;SLKRGFPF	NCP	89.398
SLKRGFPF;MLWGLRL	NCP	13.19
MLWGLRL;KSDGEGNS	NCP	91.537

KSDGEGNS;MPGVP	NCP	194.411
MPGVP;PGPGLP	NCP	55.293
PGPGLP;VFLRPF	NCP	17.361
VFLRPF;DEGLRLR	NCP	73.299
DEGLRLR;KELPL	NCP	15.088
KELPL;VDMLVPK	NCP	51.987
VDMLVPK;SDPLLSK	NCP	13.979
SDPLLSK;LTKSAWR	NCP	27.538
LTKSAWR;KHGEYGKK	NCP	58.878
KHGEYGKK;KQETALL	NCP	6.104
KQETALL;QYAVLKN	NCP	163.695
QYAVLKN;EWLVFH	NCP	2.976
EWLVFH;SFDKA	NCP	71.397
SFDKA;KWAPLR	NCP	77.376
KWAPLR;HDFDTPK	NCP	23.94
HDFDTPK;QGFLVL	NCP	109.743
QGFLVL;VEAGRP	NCP	53.535
VEAGRP;MDVLF	NCP	48.088
MDVLF;LPGLK	NCP	23.681
LPGLK;APAAWP	NCP	44.313
APAAWP;LRLLGP	NCP	28.428
LRLLGP;CCADF	NCP	176.575
CCADF;WDHHGLS	NCP	145.25
WDHHGLS;GDVKLAAQTHVSK	NCP	86.295
GDVKLAAQTHVSK;LTQKPM	NCP	75.288
LTQKPM;LGSVTP	NCP	6.174
LGSVTP;ESVPTPK	NCP	19.431
ESVPTPK;RQVKPKTLSEEERK	NCP	3.612
RQVKPKTLSEEERK;LSEEERKVHGPQQ	NCP	0.021
LSEEERKVHGPQQ;SEEERKVHGPQQVDF	NCP	0.003
SEEERKVHGPQQVDF;AAEGLKPVGNAQPSGF	NCP	0.204
AAEGLKPVGNAQPSGF;AEGLKPVGN	NCP	0.003
AEGLKPVGN;VGNAKLADTPKPLS	NCP	0.054
VGNAKLADTPKPLS;TPKPLSSTKPAETPK	NCP	0.024
TPKPLSSTKPAETPK;SSTKPAETPKPLGN	NCP	0.015
SSTKPAETPKPLGN;STKPAETPKPLG	NCP	0.003
STKPAETPKPLG;TKPAETPKPLGN	NCP	0.003
TKPAETPKPLGN;KPAETPKPLG	NCP	0.003
KPAETPKPLG;AETPKPLGN	NCP	0.006
AETPKPLGN;VKPAETPKPLG	NCP	0.027
VKPAETPKPLG;VKPAETPKPLGN	NCP	0.108
VKPAETPKPLGN;KPTETPKPLG	NCP	0.039
KPTETPKPLG;SNVKPAETPK	NCP	0.279
SNVKPAETPK;AKPAETSKPLDN	NCP	0.042
AKPAETSKPLDN;EAPKPLGN	NCP	0.048
EAPKPLGN;RTVTVNTEQK	NCP	55.461
RTVTVNTEQK;TGSPTSPLNAEK	NCP	26.349
TGSPTSPLNAEK;GSPTSPLN	NCP	0.003
GSPTSPLN;SPTSPLNAEK	NCP	0.003
SPTSPLNAEK;LEAVAEEKPHVKPYFSK	NCP	1.53
LEAVAEEKPHVKPYFSK;EAVAEEKPHVKPY	NCP	0.003

EAVAEEKPHVKPY;LKELVTNF	NCP	37.573
LKELVTNF;VFYFT	NCP	1.746
VFYFT;PVMKFK	NCP	6.518
PVMKFK;LVGTVFP	NCP	11.457
LVGTVFP;TVMSY	NCP	39.733
TVMSY;YMMSCP	NCP	5.786
YMMSCP;EDSDC	NCP	1.344
EDSDC;ATDQEKR	NCP	35.874
ATDQEKR;EGTLHFK	NCP	3.114
EGTLHFK;KRTPP	NCP	125.826
KRTPP;VPPGPLQP	NCP	5.706
VPPGPLQP;GPVLVPR	NCP	24.219
GPVLVPR;MGYPMQL	NCP	124.33
MGYPMQL;WAKPENL	NCP	1.731
WAKPENL;KLTFLPQ	NCP	3.926
KLTFLPQ;LTAVTK	NCP	38.908
LTAVTK;REHEPK	NCP	23.663
REHEPK;TKKFL	NCP	41.907
TKKFL;KKLKLR	NCP	22.578
KKLKLR;KLVVDYC	NCP	23.197
KLVVDYC;APGHPVK	NCP	115.556
APGHPVK;NAFRAPS	NCP	155.425
NAFRAPS;PLPPKY	NCP	73.523
PLPPKY;VTFEPFQ	NCP	79.941
VTFEPFQ;VAGGNLY	NCP	56.218
VAGGNLY;TTPGKENFRLK	NCP	9.944
TTPGKENFRLK;VTGLDLPK	NCP	75.621
VTGLDLPK;KSMNVP	NCP	31.407
KSMNVP;GFPDVP	NCP	82.605
GFPDVP;EDVLLK	NCP	28.575
EDVLLK;LPLPNF	NCP	131.895
LPLPNF;MMMCEC	NCP	219.168
MMMCEC;AGGLALF	NCP	66.096
AGGLALF;YRLTK	NCP	110.79
YRLTK;MWKSVVGHDV	NCP	27.126
MWKSVVGHDV;DYKGEVEKHTSQK	NCP	11.868
DYKGEVEKHTSQK;EEPAAPAPLPK	NCP	15.687
EEPAAPAPLPK;FGFVSF	NCP	6.339
FGFVSF;GVPLT	NCP	175.174
GVPLT;PSVYPSFP	NCP	399.71
PSVYPSFP;FSMMPKAK	NCP	23.938
FSMMPKAK;NPLFAPP	NCP	29.249
NPLFAPP;MNESQK	NCP	4.75
MNESQK;FPAAPQ	NCP	136.076
FPAAPQ;FVTPSVP	NCP	4.311
FVTPSVP;LEFPT	NCP	8.175
LEFPT;KCWEPLK	NCP	0.879
KCWEPLK;LSLNNAPK	NCP	7.192
LSLNNAPK;SSLRSY	NCP	20.295
SSLRSY;KSASVLV	NCP	188.786
KSASVLV;LLVDPL	NCP	114.319

LLVDPL;LLAGAKEK	NCP	128.226
LLAGAKEK;AYTNF	NCP	46.965
AYTNF;SSLKLEER	NCP	32.345
SSLKLEER;ETLPVAY	NCP	10.107
ETLPVAY;KEVLMPK	NCP	84.229
KEVLMPK;EDGRR	NCP	86.375
EDGRR;LLLLYDR	NCP	2.89
LLLLYDR;VLAPEL	NCP	97.658
VLAPEL;VGPVP	NCP	4.384
VGPVP;QMVQTK	NCP	224.576
QMVQTK;MTLPL	NCP	72.928
MTLPL;APLLGAH	NCP	114.752
APLLGAH;KQKNLLE	NCP	1.086
KQKNLLE;LMVGRTHNL	NCP	26.634
LMVGRTHNL;AFEFLA	NCP	34.215
AFEFLA;LDLKER	NCP	1.252
LDLKER;LGPKKST	NCP	37.817
LGPKKST;KFHKK	NCP	95.098
KFHKK;AAAFLDK	NCP	59.235
AAAFLDK;KGGDPLK	NCP	141.434
KGGDPLK;VGLPNGKS	NCP	24.856
VGLPNGKS;KYWALFQ	NCP	36.959
KYWALFQ;HVVTP	NCP	98.901
HVVTP;VTLSP	NCP	14.17
VTLSP;AAAASQGGRAGGGGGSSG	NCP	168.335
AAAASQGGRAGGGGGSSG;TVALTPLP	NCP	191.946
TVALTPLP;KGLVGL	NCP	7.587
KGLVGL;VTRSPYK	NCP	65.877
VTRSPYK;MGPEA	NCP	51.969
MGPEA;VMLLQHS	NCP	164.491
VMLLQHS;MEPTFP	NCP	137.435
MEPTFP;VEKEKKEV	NCP	18.696
VEKEKKEV;PPPPKSR	NCP	82.597
PPPPKSR;KNEDF	NCP	61.952
KNEDF;GPALDY	NCP	26.761
GPALDY;EVLNPFL	NCP	3.485
EVLNPFL;VGEYL	NCP	0.39
VGEYL;VHLAC	NCP	32.728
VHLAC;YLCCFGY	NCP	72.509
YLCCFGY;MTLFLEYL	NCP	268.173
MTLFLEYL;EVWEK	NCP	71.308
EVWEK;ATPPKGY	NCP	186.584
ATPPKGY;KQAAGLPR	NCP	4.845
KQAAGLPR;VAALF	NCP	16.51
VAALF;CKRWLVL	NCP	8.484
CKRWLVL;GPPLVG	NCP	40.479
GPPLVG;AVRPPYK	NCP	20.393
AVRPPYK;KAGRELL	NCP	0.147
KAGRELL;AAKRPTT	NCP	7.708
AAKRPTT;CFTPGF	NCP	72.632
CFTPGF;YELLLK	NCP	97.357

YELLLK;DFDLPK	NCP	92.721
DFDLPK;DRPEPL	NCP	91.871
DRPEPL;LTTGP	NCP	6.172
LTTGP;ATDFSLPR	NCP	69.35
ATDFSLPR;ASLLAA	NCP	69.402
ASLLAA;YLVEPF	NCP	105.541
YLVEPF;KQYLQPP	NCP	15.306
KQYLQPP;WGKVK	NCP	18.225
WGKVK;MQTLKC	NCP	38.714
MQTLKC;QAGLAYSL	NCP	8.718
QAGLAYSL;KSKSTVP	NCP	27.939
KSKSTVP;VGAVPF	NCP	43.857
VGAVPF;VALSLK	NCP	48.171
VALSLK;TPLNL	NCP	181.546
TPLNL;KDTALPPPP	NCP	58.046
KDTALPPPP;KTTPVGPR	NCP	159.708
KTTPVGPR;KGPPQHR	NCP	78.934
KGPPQHR;LSFLL	NCP	200.016
LSFLL;AAKSNMK	NCP	119.751
AAKSNMK;KPRLLK	NCP	3.413
KPRLLK;DYYLR	NCP	11.751
DYYLR;LDVKLF	NCP	204.61
LDVKLF;SLAEPDV	NCP	30.032
SLAEPDV;LVAKFCK	NCP	36.963
LVAKFCK;FGEHLL	NCP	63.178
FGEHLL;MVLVEALK	NCP	3.771
MVLVEALK;TEKFLT	NCP	292.83
TEKFLT;KTLSPLLK	NCP	26.744
KTLSPLLK;NQNLLL	NCP	139.479
NQNLLL;KNKDPPCL	NCP	43.075
KNKDPPCL;YELLTAK	NCP	93.249
YELLTAK;WDFDKSK	NCP	28.439
WDFDKSK;LERYCE	NCP	62.628
LERYCE;WSFSEAR	NCP	63.114
WSFSEAR;NYALGP	NCP	1.057
NYALGP;LTHLE	NCP	32.019
LTHLE;KKDFRWQ	NCP	31.025
KKDFRWQ;SLDLYK	NCP	39.774
SLDLYK;EQALLR	NCP	7.764
EQALLR;LGPPPPHPP	NCP	16.683
LGPPPPHPP;KDANFH	NCP	93.802
KDANFH;NYVNK	NCP	65.354
NYVNK;AFSLYK	NCP	9.01
AFSLYK;TGLSPL	NCP	117.2
TGLSPL;KPFLEPYK	NCP	210.124
KPFLEPYK;EVVAF	NCP	101.768
EVVAF;MDMKR	NCP	5.65
MDMKR;CEARW	NCP	45.53
CEARW;KMGLR	NCP	0.967
KMGLR;NFKLLS	NCP	119.823
NFKLLS;AYLFE	NCP	39.918

AYLFE;SEDLLKF	NCP	23.774
SEDLLKF;KHNTQ	NCP	21.46
KHNTQ;QVAVPP	NCP	254.196
QVAVPP;QDVCALLL	NCP	312.197
QDVCALLL;QPVREK	NCP	25.648
QPVREK;LLFGP	NCP	45.518
LLFGP;SNSPRF	NCP	78.525
SNSPRF;KEEELVEV	NCP	3.003
KEEELVEV;DYTRHFT	NCP	88.53
DYTRHFT;ESYKNPRR	NCP	145.113
ESYKNPRR;LCFTVPL	NCP	37.047
LCFTVPL;KAHFRP	NCP	14.866
KAHFRP;LLGLQF	NCP	154.986
LLGLQF;TTWGP	NCP	59.537
TTWGP;KPHLTSK	NCP	37.404
KPHLTSK;GLMKP	NCP	130.774
GLMKP;KLDLSNRP	NCP	41.106
KLDLSNRP;ARYLLF	NCP	2.459
ARYLLF;VCLVA	NCP	3.558
VCLVA;KPDVPSH	NCP	140.037
KPDVPSH;STPMFAK	NCP	201.805
STPMFAK;VLSVEL	NCP	48.546
VLSVEL;TPLTRLY	NCP	153.636
TPLTRLY;MCCMP	NCP	24.524
MCCMP;QSPPTFYQ	NCP	10.635
QSPPTFYQ;LDAGVSRL	NCP	67.665
LDAGVSRL;HLLLPVNF	NCP	104.74
HLLLPVNF;QVTPGKK	NCP	22.967
QVTPGKK;KYVDY	NCP	57.978
KYVDY;MDRRKK	NCP	11.278
MDRRKK;NPGHFP	NCP	288.095
NPGHFP;RVFLPQ	NCP	133.255
RVFLPQ;KTMLE	NCP	47.385
KTMLE;LPDMP	NCP	30.357
LPDMP;SLLYQE	NCP	246.984
SLLYQE;AELKVEGK	NCP	3.914
AELKVEGK;KGPVLLH	NCP	62.892
KGPVLLH;LLLKSLP	NCP	36.295
LLLKSLP;GKQDLLSF	NCP	34.718
GKQDLLSF;CSYEGP	NCP	45.849
CSYEGP;EFTLL	NCP	24.327
EFTLL;ENFRAL	NCP	56.614
ENFRAL;RNALNVQ	NCP	284.412
RNALNVQ;VYVYK	NCP	291.212
VYVYK;KPMLPALR	NCP	33.744
KPMLPALR;YVSVY	NCP	250.497
YVSVY;GLSGLL	NCP	85.821
GLSGLL;KQADLLH	NCP	38.433
KQADLLH;KSLEF	NCP	102.229
KSLEF;CLCAF	NCP	67.787
CLCAF;VPLPRVFW	NCP	60.99

VPLPRVFW;LVPVAEGY	NCP	106.293
LVPVAEGY;LSLKDL	NCP	54.286
LSLKDL;NPLEVRK	NCP	22.709
NPLEVRK;QKAQLSY	NCP	275.08
QKAQLSY;LEEAEVLPL	NCP	0.428
LEEAEVLPL;KVVSGPK	NCP	15.481
KVVSGPK;VNLPK	NCP	85.925
VNLPK;KPTTVAP	NCP	9.291
KPTTVAP;KESDLTKGL	NCP	79.222
KESDLTKGL;FRSLFL	NCP	158.03
FRSLFL;KLYPPPP	NCP	131.389
KLYPPPP;RLALGGYL	NCP	9.398
RLALGGYL;KKVPAAP	NCP	13.623
KKVPAAP;MTMKYP	NCP	309.945
MTMKYP;RLVSPQTT	NCP	146.428
RLVSPQTT;TKGGSS	NCP	27.45
TKGGSS;KYFFVP	NCP	104.597
KYFFVP;PGAKPGAK	NCP	1.68
PGAKPGAK;HLAKDSGFKVVAH	NCP	14.749
HLAKDSGFKVVAH;PLPPPPYK	NCP	71.082
PLPPPPYK;NTLAFVLL	NCP	115.769
NTLAFVLL;DGLLSL	NCP	20.194
DGLLSL;KVAAAAPK	NCP	25.647
KVAAAAPK;ELKKVF	NCP	21.678
ELKKVF;YEVPPF	NCP	34.755
YEVPPF;PPPSLAAP	NCP	15.192
PPPSLAAP;KSYSDKGF	NCP	139.845
KSYSDKGF;EGLLLLPK	NCP	84.315
EGLLLLPK;YEGKSLR	NCP	3.579
YEGKSLR;PNYKLP	NCP	14.145
PNYKLP;FDGVKLR	NCP	53.745
FDGVKLR;FGPLKYY	NCP	45.086
FGPLKYY;LLLRAF	NCP	85.579
LLLRAF;AVPPKGM	NCP	49.856
AVPPKGM;KGTTFLRY	NCP	81.393
KGTTFLRY;ADDLRFK	NCP	292.36
ADDLRFK;KNFMPK	NCP	103.607
KNFMPK;NLLEFK	NCP	100.504
NLLEFK;GVAVLLEN	NCP	113.703
GVAVLLEN;QSAKALVY	NCP	3.219
QSAKALVY;LNVLPF	NCP	85.733
LNVLPF;LLVAVAF	NCP	37.684
LLVAVAF;FVLAP	NCP	213.032
FVLAP;KMATGHF	NCP	53.856
KMATGHF;KSLQP	NCP	72.508
KSLQP;AERPVR	NCP	163.229
AERPVR;TLPMMLP	NCP	32.127
TLPMMLP;KQGVPPR	NCP	23.764
KQGVPPR;PLLMFPPP	NCP	77.741
PLLMFPPP;PCGGM	NCP	66.141
PCGGM;NSFLKLFW	NCP	9.345

NSFLKLFW;DFLSF	NCP	0.514
DFLSF;VTEPHHLK	NCP	27.318
VTEPHHLK;KGNQRFK	NCP	70.31
KGNQRFK;RFGLTAH	NCP	222.472
RFGLTAH;YGPMK	NCP	326.7
YGPMK;KYCLR	NCP	114.785
KYCLR;NGFSLP	NCP	183.204
NGFSLP;TPAVSKY	NCP	47.484
TPAVSKY;RVKRRLFG	NCP	41.131
RVKRRLFG;PPPVLLFLL	NCP	157.623
PPPVLLFLL;VVRTLK	NCP	21.47
VVRTLK;KLLRPL	NCP	54.885
KLLRPL;LLALKY	NCP	33.304
LLALKY;ASKVLPR	NCP	65.393
ASKVLPR;ERVWY	NCP	7.077
ERVWY;LLEGEERRM	NCP	150.85
LLEGEERRM;RLSEPYK	NCP	203.529
RLSEPYK;CGKSP	NCP	106.476
CGKSP;KELDKLP	NCP	134.259
KELDKLP;KPKTTATK	NCP	69.708
KPKTTATK;LDELPL	NCP	10.047
LDELPL;LQVPL	NCP	46.227
LQVPL;KEVTQKP	NCP	16.476
KEVTQKP;TQYFR	NCP	151.503
TQYFR;SAAYVY	NCP	6.731
SAAYVY;GVGVL	NCP	11.658
GVGVL;EVLLK	NCP	215.532
EVLLK;LKLADF	NCP	93.669
LKLADF;VGVLCGKP	NCP	241.758
VGVLCGKP;LAQALSPK	NCP	10.534
LAQALSPK;DLPVYF	NCP	65.189
DLPVYF;QNTPTEK	NCP	274.572
QNTPTEK;FFKPK	NCP	66.292
FFKPK;LLSVTR	NCP	108.588
LLSVTR;GVHVLT	NCP	175.152
GVHVLT;WGMKVL	NCP	172.095
WGMKVL;VFQFLNAK	NCP	30.464
VFQFLNAK;VKPVKVSAPR	NCP	4.653
VKPVKVSAPR;PQRVVL	NCP	61.005
PQRVVL;LVLRLQQ	NCP	50.961
LVLRLQQ;LKGVVT	NCP	33.591
LKGVVT;SLDGLLQ	NCP	22.257
SLDGLLQ;LLAARRL	NCP	0.144
LLAARRL;EEMMMAF	NCP	13.098
EEMMMAF;KPPCLDK	NCP	96.516
KPPCLDK;SAAHHGR	NCP	58.494
SAAHHGR;VLGYSCST	NCP	81.649
VLGYSCST;LNTEF	NCP	10.812
LNTEF;KATSLGLK	NCP	31.112
KATSLGLK;KPMFPPP	NCP	52.014
KPMFPPP;LFKCGK	NCP	2.091

LFKCGK;KLNVTDF	NCP	95.244
KLNVTDF;VLDLDLF	NCP	56.985
VLDLDLF;KANLYL	NCP	77.044
KANLYL;KFVKLA	NCP	567.282
KFVKLA;AASKPVEAA	NCP	64.451
AASKPVEAA;WKEVAQDCTK	NCP	14.373
WKEVAQDCTK;AVELNPK	NCP	0.03
AVELNPK;KKFVPP	NCP	11.562
KKFVPP;YSLCDAAHAQTEVAK	NCP	10.77
YSLCDAAHAQTEVAK;YGLKPPTL	NCP	0.048
YGLKPPTL;DKLGTKL	NCP	62.175
DKLGTKL;LALLEPL	NCP	54.198
LALLEPL;KMVYVLT	NCP	28.459
KMVYVLT;LDGLLEK	NCP	46.505
LDGLLEK;VGLMKL	NCP	44.332
VGLMKL;EVADFL	NCP	16.944
EVADFL;PDSVKKP	NCP	11.09
PDSVKKP;LLAPQ	NCP	1.2
LLAPQ;TLDYVGW	NCP	6.597
TLDYVGW;FFPYK	NCP	12.09
FFPYK;METPTNR	NCP	0.43
METPTNR;KDPTRE	NCP	157.004
KDPTRE;VSLQFK	NCP	60.564
VSLQFK;HPAKKQ	NCP	66.685
HPAKKQ;KDVEHKN	NCP	21.272
KDVEHKN;GPVKVKC	NCP	64.657
GPVKVKC;TVPNAER	NCP	104.348
TVPNAER;VVTATLLE	NCP	25.59
VVTATLLE;LPLPPPKY	NCP	6.493
LPLPPPKY;PPVLLHV	NCP	248.105
PPVLLHV;ALGHTAPP	NCP	14.683
ALGHTAPP;RKKSQF	NCP	20.466
RKKSQF;GLVAKLQ	NCP	287.295
GLVAKLQ;FGGFGQQ	NCP	58.271
FGGFGQQ;LSYPPR	NCP	43.684
LSYPPR;EVSLFP	NCP	44.36
EVSLFP;FSPPRF	NCP	91.56
FSPPRF;CMLAV	NCP	6.372
CMLAV;KRLEVVTK	NCP	35.983
KRLEVVTK;FFVASQF	NCP	52.046
FFVASQF;LPPATEM	NCP	108.867
LPPATEM;ESNHPK	NCP	4.983
ESNHPK;EAVEPVH	NCP	22.425
EAVEPVH;LLLEVG	NCP	44.316
LLLEVG;KLNLSH	NCP	110.38
KLNLSH;MEHYK	NCP	42.204
MEHYK;EAVRVYST	NCP	34.329
EAVRVYST;LGLEVL	NCP	26.232
LGLEVL;SKLQPK	NCP	370.808
SKLQPK;VGDASHFE	NCP	8.679
VGDASHFE;GHAAKPL	NCP	29.964

GHAAKPL;EPDYQPR	NCP	7.914
EPDYQPR;DGAALLKE	NCP	24.757
DGAALLKE;KSLVEYLQ	NCP	29.306
KSLVEYLQ;AGDDKN	NCP	8.191
AGDDKN;TSTVPDFK	NCP	12.254
TSTVPDFK;KVEGL	NCP	56.694
KVEGL;LQVGELK	NCP	205.437
LQVGELK;PGVPVH	NCP	118.411
PGVPVH;QTWEKQS	NCP	2.087
QTWEKQS;ELMSY	NCP	0.477
ELMSY;KNNLLC	NCP	428.805
KNNLLC;AHLEHT	NCP	47.251
AHLEHT;KTLLFVH	NCP	19.074
KTLLFVH;MMKPPPP	NCP	72.873
MMKPPPP;MEHGVGY	NCP	211.863
MEHGVGY;ERDSWLK	NCP	23.181
ERDSWLK;RNSHLV	NCP	109.641
RNSHLV;KNEEPG	NCP	18.327
KNEEPG;LGRVVL	NCP	134.246
LGRVVL;LSNHAY	NCP	33.156
LSNHAY;TNPPPP	NCP	48.159
TNPPPP;LVLVAK	NCP	96.729
LVLVAK;RDDLRK	NCP	102.72
RDDLRK;EAELRAPV	NCP	26.698
EAELRAPV;KCHGVLGK	NCP	86.613
KCHGVLGK;DFMDGLK	NCP	77.291
DFMDGLK;TAERVAP	NCP	108.148
TAERVAP;KGYRL	NCP	39.105
KGYRL;KSTAAGPP	NCP	64.974
KSTAAGPP;PPFPHWP	NCP	26.127
PPFPHWP;VTGGGLL	NCP	120.939
VTGGGLL;NPGSLAVP	NCP	20.067
NPGSLAVP;GLPPYPK	NCP	2.838
GLPPYPK;RGVVVL	NCP	33.222
RGVVVL;KGVAKLY	NCP	128.166
KGVAKLY;LLLFLPK	NCP	226.578
LLLFLPK;ATMMDF	NCP	12.798
ATMMDF;KASSSQK	NCP	179.213
KASSSQK;NSVSAAPK	NCP	163.902
NSVSAAPK;LVLAMQ	NCP	14.659
LVLAMQ;LYSSSML	NCP	87.57
LYSSSML;KNYSLR	NCP	17.19
KNYSLR;PTHVK	NCP	56.258
PTHVK;LLPTL	NCP	37.57
LLPTL;KNQEF	NCP	48.908
KNQEF;DWSFY	NCP	118.68
DWSFY;AVRSF	NCP	18.573
AVRSF;DSKPVNL	NCP	18.166
DSKPVNL;LLLSKKL	NCP	207.479
LLLSKKL;ETHLVH	NCP	30.615
ETHLVH;HSLKL	NCP	39.961

HSLKL;VVFLQ	NCP	9.464
VVFLQ;VLLKEY	NCP	54.232
VLLKEY;PVVHPP	NCP	1.389
PVVHPP;LVCNGPS	NCP	231.14
LVCNGPS;NNDTH	NCP	18.415
NNDTH;KYFQSK	NCP	56.838
KYFQSK;LSEVVVVK	NCP	52.959
LSEVVVVK;VEEKKC	NCP	17.735
VEEKKC;FQFKLR	NCP	8.052
FQFKLR;LTLLATPL	NCP	347.004
LTLLATPL;KEVAAHK	NCP	49.653
KEVAAHK;VNTLLRPDGEKK	NCP	36.085
VNTLLRPDGEKK;ARLLCEF	NCP	3.066
ARLLCEF;KAYAAPK	NCP	58.746
KAYAAPK;TLHTAPPK	NCP	139.809
TLHTAPPK;SVAVFCR	NCP	131.565
SVAVFCR;LPLSADNK	NCP	68.244
LPLSADNK;LTTKPSLK	NCP	59.237
LTTKPSLK;SELLVRR	NCP	31.617
SELLVRR;GHVNN	NCP	123.948
GHVNN;FTKALSS	NCP	219.397
FTKALSS;PLLMQ	NCP	59.826
PLLMQ;RMLALK	NCP	210.633
RMLALK;KKSSKHK	NCP	29.601
KKSSKHK;TEMHK	NCP	20.909
TEMHK;SLPEV	NCP	14.112
SLPEV;KGGGKPNL	NCP	31.146
KGGGKPNL;KPTTYC	NCP	97.845
KPTTYC;LRNLPLQ	NCP	18.606
LRNLPLQ;KETTPSEK	NCP	70.359
KETTPSEK;MEPLTF	NCP	15.112
MEPLTF;QKVESK	NCP	161.342
QKVESK;LVEWT	NCP	94.02
LVEWT;NAYKL	NCP	251.461
NAYKL;GFTPGLG	NCP	34.278
GFTPGLG;RSVGPGVR	NCP	72.902
RSVGPGVR;QLLAF	NCP	83.607
QLLAF;LVLLLPL	NCP	143.34
LVLLLPL;GKDEPQR	NCP	138.3
GKDEPQR;ATLRVLEP	NCP	22.408
ATLRVLEP;NLRSHL	NCP	15.764
NLRSHL;TQCFR	NCP	19.104
TQCFR;KPVALT	NCP	316.747
KPVALT;LPELNGKLT	NCP	1.746
LPELNGKLT;KSDSFGF	NCP	140.294
KSDSFGF;EEDRLK	NCP	20.097
EEDRLK;KTYLNF	NCP	88.764
KTYLNF;LFSYVLT	NCP	33.16
LFSYVLT;GVLGVGH	NCP	20.322
GVLGVGH;NHLMEPK	NCP	21.237
NHLMEPK;DKVHL	NCP	34.419

DKVHL;LGKFTEV	NCP	108.3
LGKFTEV;SAKKLLV	NCP	145.494
SAKKLLV;CENND	NCP	122.706
CENND;KTAAYP	NCP	26.295
KTAAYP;FLLPLEF	NCP	79.241
FLLPLEF;NAPLF	NCP	0.558
NAPLF;PPLPFD	NCP	5.389
PPLPFD;NPPLLQLLL	NCP	23.436
NPPLLQLLL;LLHQLPTH	NCP	21.717
LLHQLPTH;NPPVT	NCP	0.645
NPPVT;TVSFGR	NCP	62.201
TVSFGR;LAKAANK	NCP	323.557
LAKAANK;RVPLFE	NCP	41.417
RVPLFE;SSLAKVTL	NCP	17.521
SSLAKVTL;KTPAWVK	NCP	69.665
KTPAWVK;PFFSLPR	NCP	10.665
PFFSLPR;PFVVLL	NCP	35.679
PFVVLL;QCVKTK	NCP	48.903
QCVKTK;AVVAPCN	NCP	205.564
AVVAPCN;VDTLP	NCP	206.001
VDTLP;NVLKVTK	NCP	73.601
NVLKVTK;KNEYA	NCP	31.317
KNEYA;LSLSMEGV	NCP	93.694
LSLSMEGV;KAAEVT	NCP	13.056
KAAEVT;LLMARL	NCP	105.192
LLMARL;SFKVEKK	NCP	23.832
SFKVEKK;ESGKQEC	NCP	19.595
ESGKQEC;PGSQKTK	NCP	44.227
PGSQKTK;KDSVH	NCP	46.211
KDSVH;KAVFGQ	NCP	282.265
KAVFGQ;ERKSFLFL	NCP	4.437
ERKSFLFL;TKNPQ	NCP	41.682
TKNPQ;VLSST	NCP	37.95
VLSST;PPPATHT	NCP	269.915
PPPATHT;KPNDK	NCP	194.442
KPNDK;SVYVY	NCP	23.892
SVYVY;LLSDDLLFL	NCP	0.451
LLSDDLLFL;SCFGPK	NCP	10.998
SCFGPK;FPLSLQPLL	NCP	92.85
FPLSLQPLL;RGVFSAY	NCP	19.97
RGVFSAY;SGDLMF	NCP	5.313
SGDLMF;SLVSVVR	NCP	172.956
SLVSVVR;HVVPEVR	NCP	3.513
HVVPEVR;KVVGFER	NCP	16.756
KVVGFER;RAYLFL	NCP	11.541
RAYLFL;NSSLPQR	NCP	50.124
NSSLPQR;MMVTKK	NCP	6.816
MMVTKK;CVGKPNP	NCP	48.741
CVGKPNP;KYMSKK	NCP	17.945
KYMSKK;YGGGKGGY	NCP	2.499
YGGGKGGY;LTGDW	NCP	16.003

LTGDW;EKSLVR	NCP	19.467
EKSLVR;TAGFY	NCP	127.971
TAGFY;APQLR	NCP	43.878
APQLR;RVSSFKLE	NCP	36.717
RVSSFKLE;VCDLP	NCP	326.057
VCDLP;LVVLL	NCP	272.148
LVVLL;KQKPEP	NCP	289.971
KQKPEP;KDFGKK	NCP	41.481
KDFGKK;GDCPP	NCP	114.63
GDCPP;ALELD	NCP	26.419
ALELD;VTHEYTV	NCP	26.127
VTHEYTV;KRWKY	NCP	46.412
KRWKY;FSLDQLLHT	NCP	19.122
FSLDQLLHT;AVSVTP	NCP	93.475
AVSVTP;KDPQPPK	NCP	70.185
KDPQPPK;LLTVDL	NCP	77.627
LLTVDL;KLLPLK	NCP	27
KLLPLK;RTLVPAER	NCP	13
RTLVPAER;ERLVE	NCP	124.068
ERLVE;KMALP	NCP	80.394
KMALP;NAFLVL	NCP	149.1
NAFLVL;SLNSTF	NCP	134.078
SLNSTF;GHMPPDG	NCP	30.997
GHMPPDG;DVGVYK	NCP	7.466
DVGVYK;KDHYPP	NCP	1.308
KDHYPP;AAPENKP	NCP	107.196
AAPENKP;PAKYNPS	NCP	21.28
PAKYNPS;LTYSPP	NCP	152.952
LTYSPP;PVVGQ	NCP	74.106
PVVGQ;LASESLLK	NCP	20.147
LASESLLK;FVDVLPF	NCP	42.939
FVDVLPF;TDTMLEK	NCP	41.103
TDTMLEK;RLRLQK	NCP	53.506
RLRLQK;NPLPAWF	NCP	21.998
NPLPAWF;PMSPK	NCP	14.77
PMSPK;EPLKSQ	NCP	18.144
EPLKSQ;YDLPP	NCP	69.483
YDLPP;GPMDC	NCP	44.519
GPMDC;GSNPLR	NCP	58.668
GSNPLR;KGLSPF	NCP	100.915
KGLSPF;LYVTSP	NCP	110.058
LYVTSP;ERPVSK	NCP	262.899
ERPVSK;RMPFFKY	NCP	78.206
RMPFFKY;EVNVNR	NCP	53.203
EVNVNR;KELVMPK	NCP	148.128
KELVMPK;KQRLGQP	NCP	72.954
KQRLGQP;WNPGRP	NCP	161.459
WNPGRP;FVVGF	NCP	11.575
FVVGF;AERLRFP	NCP	174.185
AERLRFP;KYELP	NCP	3.262
KYELP;LEMRL	NCP	195.162

LEMRL;FLKHGF	NCP	140.274
FLKHGF;KRLCC	NCP	125.367
KRLCC;VSAKGPVN	NCP	187.35
VSAKGPVN;FVFLVLGW	NCP	73.61
FVFLVLGW;EGLLAL	NCP	62.652
EGLLAL;KGPSSR	NCP	9.601
KGPSSR;VVVDLVAM	NCP	59.376
VVVDLVAM;PVKVLFW	NCP	19.628
PVKVLFW;KLNLQNK	NCP	49.27
KLNLQNK;VVPLQY	NCP	86.936
VVPLQY;KQELPY	NCP	171.28
KQELPY;KLATTVP	NCP	3.546
KLATTVP;KRDLLR	NCP	241.614
KRDLLR;KPEARFQ	NCP	57.075
KPEARFQ;DQLLTH	NCP	0.996
DQLLTH;RQLLLLC	NCP	140.958
RQLLLLC;DKLTP	NCP	107.7
DKLTP;VFPSLVG	NCP	27.482
VFPSLVG;KTVKP	NCP	5.256
KTVKP;MQFLTM	NCP	71.148
MQFLTM;LFAKPP	NCP	248.677
LFAKPP;EVHHLLL	NCP	10.134
EVHHLLL;KVMVFGPS	NCP	28.032
KVMVFGPS;HELSELLC	NCP	24.177
HELSELLC;KEPPGFPK	NCP	34.703
KEPPGFPK;TNVVPVP	NCP	172.06
TNVVPVP;LESGLLT	NCP	81.366
LESGLLT;SPLKLR	NCP	99.239
SPLKLR;SFLYL	NCP	12.669
SFLYL;DVKSF	NCP	14.643
DVKSF;KEGLN	NCP	14.809
KEGLN;MGKLVFH	NCP	25.884
MGKLVFH;TVMQAAH	NCP	74.037
TVMQAAH;SSSFRNLG	NCP	163.863
SSSFRNLG;LRLWN	NCP	26.544
LRLWN;RKGGK	NCP	28.758
RKGGK;ALKLLKGS	NCP	1.638
ALKLLKGS;HQGVMV	NCP	33.657
HQGVMV;VGDLLAY	NCP	67.145
VGDLLAY;DPPFKP	NCP	49.291
DPPFKP;VSVPNP	NCP	67.287
VSVPNP;SCNPPRP	NCP	16.281
SCNPPRP;VKKGVTP	NCP	135.113
VKKGVTP;FELVAVR	NCP	10.621
FELVAVR;LQNPVL	NCP	30.407
LQNPVL;QPKSVPK	NCP	26.529
QPKSVPK;EYAALLL	NCP	61.764
EYAALLL;YKSPPNR	NCP	112.936
YKSPPNR;WAHPAAY	NCP	10.812
WAHPAAY;KGGYVRL	NCP	87.96
KGGYVRL;TVQAH	NCP	38.022

TVQAH;LDVGF	NCP	12.924
LDVGF;GVKLP	NCP	71.442
GVKLP;CCCFMAP	NCP	137.997
CCCFMAP;MWKEK	NCP	5.726
MWKEK;FVRKKK	NCP	16.977
FVRKKK;QQLEVLRA	NCP	13.492
QQLEVLRA;KWDDLPQ	NCP	47.624
KWDDLPQ;SDGLRPLM	NCP	31.426
SDGLRPLM;APVPPPP	NCP	44.508
APVPPPP;VEKAM	NCP	139.041
VEKAM;KADLMR	NCP	92.009
KADLMR;VGPVGN	NCP	54.312
VGPVGN;GVTTL	NCP	31.224
GVTTL;NFGMLTH	NCP	86.895
NFGMLTH;RFVKKK	NCP	77.632
RFVKKK;KKKYG	NCP	160.388
KKKYG;PPVPE	NCP	38.758
PPVPE;ETTHAKGK	NCP	20.316
ETTHAKGK;VDNPPAF	NCP	13.926
VDNPPAF;TVFMKSP	NCP	21.377
TVFMKSP;EDLRLSPF	NCP	16.39
EDLRLSPF;KNVHSLTN	NCP	35.264
KNVHSLTN;ALQDLL	NCP	50.853
ALQDLL;TVHSLLCY	NCP	52.984
TVHSLLCY;KMLVGW	NCP	56.3
KMLVGW;ENCCC	NCP	163.831
ENCCC;KSPTGTVRF	NCP	74.585
KSPTGTVRF;LLVGD	NCP	177.7
LLVGD;WELLD	NCP	0.929
WELLD;NKLLS	NCP	1.839
NKLLS;QKLLLQ	NCP	29.029
QKLLLQ;APELW	NCP	151.884
APELW;QELEGMK	NCP	22.092
QELEGMK;NSGSPPP	NCP	124.028
NSGSPPP;KNMKVGS	NCP	58.963
KNMKVGS;MEQLLRN	NCP	206.727
MEQLLRN;MYDLHY	NCP	11.849
MYDLHY;FGQRQGF	NCP	49.894
FGQRQGF;ASLTTFL	NCP	57.735
ASLTTFL;LQVTPP	NCP	114.663
LQVTPP;VKPPLPHP	NCP	146.136
VKPPLPHP;REVKQLYF	NCP	79.713
REVKQLYF;LSLKV	NCP	16.386
LSLKV;LPPKYKK	NCP	41.468
LPPKYKK;HLLFMVM	NCP	28.221
HLLFMVM;LDLSP	NCP	45.171
LDLSP;DAPFVF	NCP	17.265
DAPFVF;KNKDRF	NCP	27.027
KNKDRF;VDLFEGK	NCP	76.845
VDLFEGK;LVTFAL	NCP	147.258
LVTFAL;VDDLTEF	NCP	9.576

VDDLTEF;ATRFAF	NCP	105.96
ATRFAF;LFGGF	NCP	50.151
LFGGF;KVPTKHG	NCP	96.588
KVPTKHG;YPFLP	NCP	168.612
YPFLP;KDTPPNP	NCP	12.22
KDTPPNP;NHLTMRP	NCP	36.222
NHLTMRP;HALMRLD	NCP	109.239
HALMRLD;MFLRGER	NCP	57.588
MFLRGER;KNVGL	NCP	44.37
KNVGL;KRTVTAM	NCP	67.916
KRTVTAM;YGPTLL	NCP	152.299
YGPTLL;EDTLQK	NCP	74.319
EDTLQK;VLLLV	NCP	156.15
VLLLV;AVTTNK	NCP	44.306
AVTTNK;TKEQER	NCP	163.855
TKEQER;CKGPARP	NCP	156.492
CKGPARP;LATKAVE	NCP	9.818
LATKAVE;SLDSGGLR	NCP	65.658
SLDSGGLR;FLEKGK	NCP	129.168
FLEKGK;GGPTH	NCP	145.777
GGPTH;LGSLAEAY	NCP	74.633
LGSLAEAY;PPPPLP	NCP	86.389
PPPPLP;MGHLLVK	NCP	77.54
MGHLLVK;AFLEMK	NCP	28.657
AFLEMK;KSVKKE	NCP	38.406
KSVKKE;VKMLK	NCP	9.11
VKMLK;FLQSQR	NCP	18.48
FLQSQR;KKLAVVHMDL	NCP	18.469
KKLAVVHMDL;LGSPAKPV	NCP	57.231
LGSPAKPV;KAVYW	NCP	72.647
KAVYW;RGFVFP	NCP	18.859
RGFVFP;DPGAAL	NCP	2.367
DPGAAL;LDNLPGM	NCP	59.078
LDNLPGM;PPAPF	NCP	4.243
PPAPF;PDPLPY	NCP	466.571
PDPLPY;RLLFLPK	NCP	33.636
RLLFLPK;GPPPPAPLR	NCP	118.783
GPPPPAPLR;NGLGNER	NCP	131.12
NGLGNER;LLVYSV	NCP	137.259
LLVYSV;KELTDPF	NCP	5.619
KELTDPF;EVEREVP	NCP	46.348
EVEREVP;MERTF	NCP	134.114
MERTF;VTHFRPK	NCP	15.006
VTHFRPK;KSHVPP	NCP	47.053
KSHVPP;EPPVVKT	NCP	54.089
EPPVVKT;QEPPGRPQ	NCP	66.265
QEPPGRPQ;ALLTQ	NCP	8.538
ALLTQ;TPLPPR	NCP	2.775
TPLPPR;APVEALLPF	NCP	48.867
APVEALLPF;TTYSKER	NCP	4.898
TTYSKER;KHYGGCSK	NCP	42.564

KHYGGCSK;AVPPCVQ	NCP	42.49
AVPPCVQ;LPLAPAR	NCP	27.129
LPLAPAR;VTVKKWT	NCP	108.303
VTVKKWT;VMFLH	NCP	180.843
VMFLH;LSGAGLML	NCP	8.133
LSGAGLML;EARVAC	NCP	189.146
EARVAC;TALKFYP	NCP	78.507
TALKFYP;VADDD	NCP	3.238
VADDD;FFEPQPP	NCP	7.406
FFEPQPP;QSARLL	NCP	16.069
QSARLL;DSLMCK	NCP	7.349
DSLMCK;GLAPLH	NCP	126.061
GLAPLH;FKFVL	NCP	17.777
FKFVL;EAVHPPR	NCP	1.678
EAVHPPR;NRGHDLSF	NCP	36.899
NRGHDLSF;KVALLSVP	NCP	18.703
KVALLSVP;DWDDMEK	NCP	29.964
DWDDMEK;KPSRPPP	NCP	24.36
KPSRPPP;LSDHGPVP	NCP	90.78
LSDHGPVP;KDCAFPK	NCP	74.733
KDCAFPK;DLPLTVE	NCP	0.243
DLPLTVE;TVGPSLVH	NCP	102.372
TVGPSLVH;AGPHKTLLE	NCP	24.374
AGPHKTLLE;PEAPP	NCP	78.121
PEAPP;DSLRVAP	NCP	6.357
DSLRVAP;PLLKSYST	NCP	13.296
PLLKSYST;AAKVPTA	NCP	38.475
AAKVPTA;NAHLNP	NCP	50.769
NAHLNP;GVPPPAPV	NCP	4.536
GVPPPAPV;NGGELL	NCP	120.575
NGGELL;KHTCGK	NCP	23.298
KHTCGK;DLHPHL	NCP	27.532
DLHPHL;FLDKVR	NCP	23.873
FLDKVR;DSFTPA	NCP	29.329
DSFTPA;DLVEPTA	NCP	113.643
DLVEPTA;PVATAA	NCP	106.769
PVATAA;APTVE	NCP	6.105
APTVE;EVSLP	NCP	144.144
EVSLP;LMVMRRC	NCP	7.15
LMVMRRC;RRPWE	NCP	124.635
RRPWE;KAVSRPT	NCP	15.192
KAVSRPT;LPGPM	NCP	68.31
LPGPM;DVPPPP	NCP	27.63
DVPPPP;KHGFSPP	NCP	104.75
KHGFSPP;KEHYTL	NCP	100.647
KEHYTL;PMLLTLL	NCP	8.254
PMLLTLL;FLDVLR	NCP	78.552
FLDVLR;KRPEYNP	NCP	38.013
KRPEYNP;KSPVAF	NCP	7.653
KSPVAF;KVEGAPV	NCP	81.411
KVEGAPV;WGGLVNAG	NCP	49.914

WGGLVNAG;GHPRALPP	NCP	86.37
GHPRALPP;KDVHEEF	NCP	26.888
KDVHEEF;LTALPGFG	NCP	35.689
LTALPGFG;NFTKVWF	NCP	67.04
NFTKVWF;GPFAVP	NCP	190.963
GPFAVP;PVGGP	NCP	133.935
PVGGP;TAPWFN	NCP	159.795
TAPWFN;RSLLVK	NCP	3.911
RSLLVK;KNPKYK	NCP	37.162
KNPKYK;LSPPVLTR	NCP	32.625
LSPPVLTR;PASLPTPP	NCP	12.351
PASLPTPP;LNNPVAQ	NCP	41.504
LNNPVAQ;RFAVVK	NCP	93.096
RFAVVK;AQKLLR	NCP	97.363
AQKLLR;VGVAPGP	NCP	214.746
VGVAPGP;WGPPTSRP	NCP	21.222
WGPPTSRP;KPVHDAP	NCP	7.199
KPVHDAP;LVVGL	NCP	61.345
LVVGL;VFAALPPP	NCP	95.319
VFAALPPP;YQEKVGK	NCP	2.423
YQEKVGK;DWFMAGK	NCP	35.047
DWFMAGK;ATTSTTLP	NCP	180.921
ATTSTTLP;KVFKPK	NCP	170.462
KVFKPK;LTVEL	NCP	76.449
LTVEL;NVEAPSL	NCP	11.638
NVEAPSL;CDRDLR	NCP	54.699
CDRDLR;SLDVLTHF	NCP	81.599
SLDVLTHF;KGVMFHL	NCP	103.915
KGVMFHL;LPPPP	NCP	146.988
LPPPP;LTVALK	NCP	82.544
LTVALK;APPLF	NCP	61.359
APPLF;PYPGP	NCP	106.237
PYPGP;SFVELK	NCP	11.12
SFVELK;LTVDPGF	NCP	53.323
LTVDPGF;KTNRH	NCP	80.651
KTNRH;QSFKEAF	NCP	30.178
QSFKEAF;LLGQL	NCP	21.371
LLGQL;TDSPK	NCP	131.101
TDSPK;RNLGTGM	NCP	11.979
RNLGTGM;RVMQKL	NCP	115.802
RVMQKL;VVLELKK	NCP	68.329
VVLELKK;LAFLLHK	NCP	85.241
LAFLLHK;EVHNGG	NCP	14.802
EVHNGG;VLNVHS	NCP	73.78
VLNVHS;MTFLTFM	NCP	36.98
MTFLTFM;RATVTRPV	NCP	9.429
RATVTRPV;KDGAKTK	NCP	10.03
KDGAKTK;EAFTRLQ	NCP	3.326
EAFTRLQ;ELYKVP	NCP	33.516
ELYKVP;VPKGPAAF	NCP	3.418
VPKGPAAF;TATFFL	NCP	46.61

TATFFL;LGEKWDR	NCP	6.714
LGEKWDR;LEASYAY	NCP	29.526
LEASYAY;RYPHK	NCP	148.929
RYPHK;NVDVLP	NCP	109.911
NVDVLP;TMGPKVR	NCP	56.422
TMGPKVR;LSAPGGLLS	NCP	13.776
LSAPGGLLS;VAMNLPR	NCP	110.061
VAMNLPR;KPPVPPP	NCP	69.225
KPPVPPP;KAGHEYK	NCP	61.832
KAGHEYK;VLGLQ	NCP	16.855
VLGLQ;SLLRNSDD	NCP	54.987
SLLRNSDD;YNPLSR	NCP	10.977
YNPLSR;KAFASSPK	NCP	42.936
KAFASSPK;TCRLFDL	NCP	26.108
TCRLFDL;GPPGPPVT	NCP	5.376
GPPGPPVT;GSHKER	NCP	64.018
GSHKER;KGWPGAK	NCP	1.41
KGWPGAK;FFGNPYF	NCP	66.761
FFGNPYF;EEGRPPYP	NCP	4.534
EEGRPPYP;KMQLAK	NCP	32.396
KMQLAK;TVLLF	NCP	87.592
TVLLF;FFSLVHKR	NCP	33.252
FFSLVHKR;FVNVEF	NCP	19.958
FVNVEF;KCKAAQE	NCP	121.957
KCKAAQE;LDEVLK	NCP	32.942
LDEVLK;WNDDMEK	NCP	51.553
WNDDMEK;FLGPFF	NCP	53.646
FLGPFF;LGKELLD	NCP	0.494
LGKELLD;KAAPYGK	NCP	124.164
KAAPYGK;LSSALDL	NCP	67.765
LSSALDL;KFKGRP	NCP	46.67
KFKGRP;FPGVPVGR	NCP	97.24
FPGVPVGR;LYKKKKKKF	NCP	46.616
LYKKKKKKF;VYKNVR	NCP	59.316
VYKNVR;TVPLPNSF	NCP	34.696
TVPLPNSF;TSRTLY	NCP	20.796
TSRTLY;MEKGDQK	NCP	67.979
MEKGDQK;CVTKKL	NCP	47.274
CVTKKL;FKKVL	NCP	68.277
FKKVL;RLNPVEF	NCP	28.716
RLNPVEF;APKNAHTK	NCP	18.465
APKNAHTK;KDLFT	NCP	21.672
KDLFT;VGKLMGK	NCP	44.733
VGKLMGK;LTVVQ	NCP	105.112
LTVVQ;APRSPKAP	NCP	23.25
APRSPKAP;KQAAVLP	NCP	67.674
KQAAVLP;VTLAF	NCP	28.14
VTLAF;AESHSVK	NCP	51.153
AESHSVK;RGPKFL	NCP	1.389
RGPKFL;APELRPT	NCP	14.009
APELRPT;AMLLNY	NCP	158.347

AMLLNY;GYSDN	NCP	67.571
GYSDN;LPEELL	NCP	103.029
LPEELL;RAPPERK	NCP	17.727
RAPPERK;KLALGAGK	NCP	92.11
KLALGAGK;LTAFWTK	NCP	43.869
LTAFWTK;FLVLVVLH	NCP	89.157
FLVLVVLH;TVYGLPR	NCP	166.787
TVYGLPR;VAKKPK	NCP	30.783
VAKKPK;LFKGGKK	NCP	7.098
LFKGGKK;AQMKLF	NCP	55.989
AQMKLF;LFSRSL	NCP	47.844
LFSRSL;KFVGFGP	NCP	77.715
KFVGFGP;VEVLLL	NCP	211.758
VEVLLL;VHAKSWT	NCP	86.55
VHAKSWT;KGFMP	NCP	142.824
KGFMP;PLMHPLK	NCP	4.921
PLMHPLK;LLTGPELK	NCP	141.077
LLTGPELK;KSATVYD	NCP	112.407
KSATVYD;TGLPALV	NCP	62.833
TGLPALV;PPPKAHTK	NCP	97.707
PPPKAHTK;EHFFEKH	NCP	60.417
EHFFEKH;PACHNP	NCP	1.473
PACHNP;GLTAPGP	NCP	88.578
GLTAPGP;EPKPPALGP	NCP	39.2
EPKPPALGP;KYSRHLLE	NCP	35.08
KYSRHLLE;LDSRLE	NCP	0.08
LDSRLE;KTMAHR	NCP	1.978
KTMAHR;YLLVEF	NCP	34.904
YLLVEF;KALNP	NCP	98.287
KALNP;PPKPEPKP	NCP	187.967
PPKPEPKP;NGAVLKK	NCP	11.292
NGAVLKK;PPKPEPKPK	NCP	11.733
PPKPEPKPK;LPLNPF	NCP	22.108
LPLNPF;KNKPFK	NCP	118.104
KNKPFK;EVPAACQ	NCP	160.545
EVPAACQ;ACPTTYM	NCP	154.518
ACPTTYM;GGPVR	NCP	32.037
GGPVR;ELRRF	NCP	92.516
ELRRF;ALLLK	NCP	11.566
ALLLK;LTWPAAF	NCP	58.278
LTWPAAF;DTMSV	NCP	54.533
DTMSV;LTLPV	NCP	2.554
LTLPV;VLEDLGRQ	NCP	42.192
VLEDLGRQ;SFLRKA	NCP	29.972
SFLRKA;LSLFALAP	NCP	21.865
LSLFALAP;AAFVDVT	NCP	43.443
AAFVDVT;EVSVQYR	NCP	62.576
EVSVQYR;LMLVA	NCP	60.751
LMLVA;KHAGQKR	NCP	11.606
KHAGQKR;HRLVPPH	NCP	53.478
HRLVPPH;YGTGPFN	NCP	141.433

YGTGPFN;QEALEMR	NCP	67.259
QEALEMR;PPPWN	NCP	104.343
PPPWN;LPPPPQK	NCP	26.709
LPPPPQK;AAVPYH	NCP	62.104
AAVPYH;KPLTLSQPP	NCP	91.932
KPLTLSQPP;ATKAVCVLK	NCP	18.752
ATKAVCVLK;KDPLWS	NCP	35.47
KDPLWS;PEVYRK	NCP	180.896
PEVYRK;NFPLTS	NCP	42.522
NFPLTS;EMLYG	NCP	4.026
EMLYG;LLLLSCST	NCP	39.456
LLLLSCST;VEVLKLGK	NCP	63.103
VEVLKLGK;DPPPLFD	NCP	119.205
DPPPLFD;RPLKDL	NCP	134.411
RPLKDL;QAHLYR	NCP	36.855
QAHLYR;LKVPVP	NCP	26.35
LKVPVP;KSEHPPR	NCP	2.249
KSEHPPR;KSTLKV	NCP	62.454
KSTLKV;RWKLR	NCP	3.216
RWKLR;LSPPFR	NCP	54.822
LSPPFR;KKLVVAP	NCP	4.225
KKLVVAP;VSFYY	NCP	169.637
VSFYY;LEPKYKS	NCP	42.886
LEPKYKS;TVELMFP	NCP	14.618
TVELMFP;AAAAVTRP	NCP	58.125
AAAAVTRP;PAGMRK	NCP	62.392
PAGMRK;KAPPDPK	NCP	17.037
KAPPDPK;VGGDGVVV	NCP	44.313
VGGDGVVV;KKHGPR	NCP	29.114
KKHGPR;LSVKPR	NCP	85.839
LSVKPR;VLRNPLLA	NCP	34.176
VLRNPLLA;KSSSYAH	NCP	7.371
KSSSYAH;SVCGYLLL	NCP	5.973
SVCGYLLL;KAETFGP	NCP	149.95
KAETFGP;VARPPR	NCP	110.222
VARPPR;LLETSL	NCP	31.113
LLETSL;YSESFK	NCP	139.917
YSESFK;KTPPRH	NCP	173.104
KTPPRH;PYLNPPE	NCP	10.809
PYLNPPE;TLFMMF	NCP	2.13
TLFMMF;NLLYPPPPP	NCP	14.462
NLLYPPPPP;KEMTFL	NCP	84.39
KEMTFL;KRSLVPSTL	NCP	165.621
KRSLVPSTL;SVQTCPK	NCP	31.173
SVQTCPK;LQEPGP	NCP	52.233
LQEPGP;AAAAKLAL	NCP	5.374
AAAAKLAL;KTSYVYSL	NCP	15.164
KTSYVYSL;GVLSWY	NCP	189.487
GVLSWY;LAPPF	NCP	0.321
LAPPF;PRVEF	NCP	26.034
PRVEF;VTLEVT	NCP	39.488

VTLEVT;LYDASDL	NCP	67.155
LYDASDL;CCPCS	NCP	66.117
CCPCS;SRSLLVD	NCP	49.599
SRSLLVD;VKCPKC	NCP	56.226
VKCPKC;GVPPLKM	NCP	82.863
GVPPLKM;PGVPGLVA	NCP	2.935
PGVPGLVA;LFGKKQ	NCP	28.419
LFGKKQ;VTVEVW	NCP	37.625
VTVEVW;SEMGSSGE	NCP	29.16
PLFLG;FLSSSGY	NCP	25.346
FLSSSGY;LDTLY	NCP	15.736
LDTLY;ATLSVH	NCP	59.772
ATLSVH;LMVLVL	NCP	11.747
LMVLVL;ESTLATRK	NCP	52.102
ESTLATRK;GVLAAA	NCP	136.616
GVLAAA;DVDLRK	NCP	30.843
DVDLRK;LHAELLT	NCP	51.153
LHAELLT;QDPLFK	NCP	61.738
QDPLFK;HTGPPPP	NCP	3.371
HTGPPPP;KAGALVL	NCP	87.907
KAGALVL;VGLPR	NCP	91.436
VGLPR;SYPYKK	NCP	14.311
SYPYKK;TVFNK	NCP	48.612
TVFNK;PEFLKA	NCP	53.22
PEFLKA;PGARAGPGPP	NCP	30.74
PGARAGPGPP;LSGEVLR	NCP	63.568
LSGEVLR;LPKEPL	NCP	34.497
LPKEPL;LEYAALL	NCP	23.465
LEYAALL;ESARLLR	NCP	23.817
ESARLLR;TSGDLLSK	NCP	0.444
TSGDLLSK;RVVTAF	NCP	82.066
RVVTAF;LENHWQL	NCP	12.045
LENHWQL;RGVFHH	NCP	9.926
RGVFHH;QRVPKAP	NCP	1.836
QRVPKAP;STMPF	NCP	3.649
STMPF;GVDKAVFP	NCP	24.027
GVDKAVFP;TVTSAY	NCP	7.734
TVTSAY;PTVGWV	NCP	13.1
PTVGWV;LDLSPST	NCP	35.821
LDLSPST;LPVEP	NCP	22.986
LPVEP;PQTHHT	NCP	7.827
PQTHHT;FNDLSL	NCP	38.079
FNDLSL;PSFGAPL	NCP	27.468
PSFGAPL;KNRLPVAPP	NCP	55.89
KNRLPVAPP;MLTAGAQ	NCP	61.994
MLTAGAQ;QHDSVR	NCP	34.444
QHDSVR;KTPRGTP	NCP	55.541
KTPRGTP;KEGLSPAGP	NCP	36.546
KEGLSPAGP;PAAAASSK	NCP	0.207
PAAAASSK;YPSDWLM	NCP	6.127
YPSDWLM;TQVVLD	NCP	3.027

TQVVLD;AFLMPK	NCP	0.021
AFLMPK;ESLHELL	NCP	148.436
ESLHELL;QSFPKAK	NCP	27.111
QSFPKAK;LPSRSQPP	NCP	24.882
LPSRSQPP;EVFLL	NCP	66.682
EVFLL;QALVLSLY	NCP	23.165
QALVLSLY;NKPVERK	NCP	11.643
NKPVERK;SGTRVSPS	NCP	58.23
SGTRVSPS;EPAMDPK	NCP	63.15
EPAMDPK;VPYALL	NCP	25.575
VPYALL;PGMDC	NCP	18.904
PGMDC;QMLGY	NCP	65.765
QMLGY;PVGMQPR	NCP	138.483
PVGMQPR;EGGHG	NCP	21.735
EGGHG;GPLQYR	NCP	9.367
GPLQYR;NDGMVP	NCP	4.653
NDGMVP;FDDNVF	NCP	140.472
FDDNVF;VEVGLLD	NCP	70.86
VEVGLLD;GNNSL	NCP	0.276
GNNSL;LTEGL	NCP	7.187
LTEGL;HLAAP	NCP	59.187
HLAAP;NDMSDC	NCP	9.267
NDMSDC;WPPGFPP	NCP	52.339
WPPGFPP;KVGLPL	NCP	109.76
KVGLPL;KDVLLEF	NCP	53.385
KDVLLEF;LAPPKSR	NCP	77.824
LAPPKSR;EPTMVK	NCP	1.31
EPTMVK;RCHVLPK	NCP	25.579
RCHVLPK;RTTYSGL	NCP	14.7
RTTYSGL;VGGFHFK	NCP	52.313
VGGFHFK;SGFNLG	NCP	54.468
SGFNLG;VSKLSWKP	NCP	14.457
VSKLSWKP;SPYKHR	NCP	11.319
SPYKHR;MWTAY	NCP	29.379
MWTAY;SGRPPVV	NCP	7.194
SGRPPVV;MELPP	NCP	11.997
MELPP;LSGGAAPG	NCP	6.021
LSGGAAPG;GVAVYG	NCP	58.162
GVAVYG;VVAAP	NCP	202.398
VVAAP;VSKPTLN	NCP	45.44
VSKPTLN;KTSTDRK	NCP	18.22
KTSTDRK;KAGTSGLP	NCP	45.038
KAGTSGLP;VSMTY	NCP	18.607
VSMTY;VSVPGPQ	NCP	31.589
VSVPGPQ;KGTLSVH	NCP	16.219
KGTLSVH;PPPPRQPPP	NCP	49.487
PPPPRQPPP;RKKKYAR	NCP	26.143
RKKKYAR;EGPFHYK	NCP	6.785
EGPFHYK;LGKSEQLK	NCP	78.706
LGKSEQLK;EHALLA	NCP	14.391
EHALLA;SALVMD	NCP	26.474

SALVMD;QVGLGP	NCP	22.791
QVGLGP;NPVAGF	NCP	1.788
NPVAGF;VAHVYKP	NCP	33.055
VAHVYKP;GADSY	NCP	28.14
GADSY;LLLTGKFL	NCP	33.656
LLLTGKFL;VTEEALLP	NCP	17.763
VTEEALLP;LLMVEPK	NCP	32.334
LLMVEPK;FMGQAW	NCP	53.341
FMGQAW;KHAAPPP	NCP	29.876
KHAAPPP;AAQGEPQVQFK	NCP	25.021
AAQGEPQVQFK;EPFLDPL	NCP	130.532
EPFLDPL;RDSGKNP	NCP	20.829
RDSGKNP;MSLLLNTFY	NCP	99.244
MSLLLNTFY;TYLVHR	NCP	16.109
TYLVHR;SVYGLA	NCP	56.979
SVYGLA;QTYVY	NCP	116.232
QTYVY;ATEPSL	NCP	15.292
ATEPSL;LGKRCAY	NCP	80.262
LGKRCAY;MPGGK	NCP	0.048
MPGGK;HSLLVTAL	NCP	75.009
HSLLVTAL;SLTLKY	NCP	114.533
SLTLKY;HVSPSKE	NCP	6.87
HVSPSKE;ARPFP	NCP	8.485
ARPFP;PSVPPG	NCP	128.459
PSVPPG;TPAKMPK	NCP	182.73
TPAKMPK;VVFAPPK	NCP	4.294
VVFAPPK;KPLKVSH	NCP	7.578
KPLKVSH;SREGMLTP	NCP	2.172
SREGMLTP;DEGLRPL	NCP	17.237
DEGLRPL;NGCPPPYHTFEEPTYVNLK	NCP	31.375
NGCPPPYHTFEEPTYVNLK;KPFWNK	NCP	59.351
KPFWNK;YALPHALL	NCP	10.864
YALPHALL;LPHALLEVR	NCP	0.006
LPHALLEVR;PGPHELL	NCP	60.408
PGPHELL;DLLYRAL	NCP	73.406
DLLYRAL;CGEQHYR	NCP	21.666
CGEQHYR;HGPPPP	NCP	19.054
HGPPPP;GPPYHPP	NCP	9
GPPYHPP;LVLSKH	NCP	16.979
LVLSKH;EAPTSMP	NCP	49.576
EAPTSMP;NDFTGKK	NCP	14.195
NDFTGKK;LTYGFN	NCP	29.026
LTYGFN;VSMLFM	NCP	17.658
VSMLFM;YLLQDM	NCP	3.395
YLLQDM;AEVQPKGP	NCP	56.521
AEVQPKGP;YAAPPP	NCP	5.997
YAAPPP;LYEYTQ	NCP	9.792
LYEYTQ;VPLTFWT	NCP	0.27
VPLTFWT;AMNLRK	NCP	12.086
AMNLRK;LGEAPA	NCP	115.18
LGEAPA;KYKLPPPP	NCP	0.441

KYKLPPPP;KHSPAAYRK	NCP	65.802
KHSPAAYRK;LPVKRGPPP	NCP	28.443
LPVKRGPPP;GLSVVK	NCP	7.791
GLSVVK;KYDDLP	NCP	38.817
KYDDLP;RKLTLQY	NCP	29.013
RKLTLQY;LDVSAKP	NCP	88.356
LDVSAKP;KPEGMF	NCP	6.371
KPEGMF;HLDVKP	NCP	36.037
HLDVKP;KYAPPPP	NCP	70.613
KYAPPPP;EGSYKS	NCP	63.106
EGSYKS;VKALGPH	NCP	61.463
VKALGPH;LKVFKPK	NCP	5.4
LKVFKPK;HLLKAPH	NCP	25.078
HLLKAPH;LLLDRMM	NCP	20.214
LLLDRMM;ESQYPVEKL	NCP	0.735
ESQYPVEKL;DEDCCCC	NCP	86.135
DEDCCCC;TKGKLLK	NCP	65.157
TKGKLLK;EHYKLSA	NCP	0.12
EHYKLSA;MYLVMM	NCP	69.667
MYLVMM;LPLPHHK	NCP	5.544
LPLPHHK;KLGYPF	NCP	21.701
KLGYPF;ATELLEL	NCP	39.39
ATELLEL;LAAASFFR	NCP	3.271
LAAASFFR;KYLCEP	NCP	11.906
KYLCEP;VSGFPYL	NCP	37.387
VSGFPYL;ALDNLTSL	NCP	5.597
ALDNLTSL;YSTVYK	NCP	55.873
YSTVYK;KDLKPV	NCP	23.364
KDLKPV;DYHKPK	NCP	55.07
DYHKPK;KEHFEV	NCP	26.829
KEHFEV;KHGNP	NCP	184.254
KHGNP;VGTNKEK	NCP	34.92
VGTNKEK;LTNNKYVNE	NCP	2.206
LTNNKYVNE;RTPTYH	NCP	124.215
RTPTYH;DSLNFF	NCP	2.297
DSLNFF;PSFLVSFP	NCP	25.768
PSFLVSFP;LPNKENP	NCP	24.93
LPNKENP;YVKLGFP	NCP	27.914
YVKLGFP;LERLKDY	NCP	14.154
LERLKDY;VSCKNK	NCP	8.379
VSCKNK;FFPPPPP	NCP	69.07
FFPPPPP;KFLQLGK	NCP	21.212
KFLQLGK;PVSPP	NCP	7.402
PVSPP;YPLLLST	NCP	5.619
YPLLLST;WDGTF	NCP	70.968
WDGTF;PGVKLLV	NCP	56.571
PGVKLLV;VGDVSSF	NCP	86.264
VGDVSSF;SGPAKYR	NCP	15.555
SGPAKYR;LEPTF	NCP	20.263
LEPTF;CCSGGY	NCP	75.711
CCSGGY;ASEGSRGGRAPGGPP	NCP	54.231

ASEGSRGGRAPGGPP;AHHRPTT	NCP	12.141
AHHRPTT;KCFTKP	NCP	27.105
KCFTKP;QQTLLEK	NCP	0.246
QQTLLEK;VDPKGKP	NCP	0.977
VDPKGKP;RLLLF	NCP	29.763
RLLLF;AEEELKK	NCP	42.087
AEEELKK;LATVLDF	NCP	70.149
LATVLDF;HKKYTSY	NCP	32.01
HKKYTSY;PAAMRPK	NCP	60.343
PAAMRPK;EGFKP	NCP	26.751
EGFKP;KGFTKY	NCP	131.496
KGFTKY;YFNQLSK	NCP	144.557
YFNQLSK;KYCGSW	NCP	3.345
KYCGSW;PFLKEWL	NCP	16.072
PFLKEWL;RCLHY	NCP	98.51
RCLHY;KSPDPPKP	NCP	1.525
KSPDPPKP;MEKPY	NCP	3.381
MEKPY;KESLALFP	NCP	46.556
KESLALFP;SPLSTLTLL	NCP	7.573
SPLSTLTLL;SAVRLR	NCP	53.685
SAVRLR;TGATLAW	NCP	97.365
TGATLAW;LSAAVTAL	NCP	66.236
LSAAVTAL;DLRKLAV	NCP	27.918
DLRKLAV;VRELME	NCP	68.971
VRELME;EVELSAF	NCP	18.467
EVELSAF;QVCVQ	NCP	12.796
QVCVQ;KYPLTLY	NCP	37.701
KYPLTLY;MKETLL	NCP	51.035
MKETLL;AMVSNSG	NCP	0.1
AMVSNSG;LGERLF	NCP	2.112
LGERLF;NVALSYK	NCP	91.218
NVALSYK;AAALALGR	NCP	67.602
AAALALGR;KETAGGK	NCP	16.322
KETAGGK;RALSGPK	NCP	12.312
RALSGPK;AALLTEA	NCP	6.16
AALLTEA;KSLKNVH	NCP	58.761
KSLKNVH;QHAVPL	NCP	97.143
QHAVPL;SGDVQRF	NCP	17.855
SGDVQRF;TMLPLKD	NCP	72.108
TMLPLKD;STVLKKAP	NCP	7.671
STVLKKAP;YEELVKE	NCP	144.186
YEELVKE;TDMGVPK	NCP	27.009
TDMGVPK;KNDPPPP	NCP	73.693
KNDPPPP;TVYFPC	NCP	6.06
TVYFPC;KFEVPLL	NCP	72.938
KFEVPLL;KAHPSSK	NCP	29.344
KAHPSSK;YRALFQ	NCP	29.25
YRALFQ;CHRLEWN	NCP	116.24
CHRLEWN;KTDPPPF	NCP	1.231
KTDPPPF;QPTVLN	NCP	102.327
QPTVLN;MPKRLP	NCP	5.246

MPKRLP;GSEGKPAELK	NCP	12.16
GSEGKPAELK;EGTLTFG	NCP	71.172
EGTLTFG;TSTTPTPLT	NCP	22.52
TSTTPTPLT;YYAGAK	NCP	121.228
YYAGAK;GLGVQ	NCP	0.503
GLGVQ;KDELGYVF	NCP	16.302
KDELGYVF;KGHHLVP	NCP	22.473
KGHHLVP;NPLNPK	NCP	78.493
NPLNPK;DLALKYFQK	NCP	171.563
DLALKYFQK;KELAVQP	NCP	75.849
KELAVQP;KFWGKY	NCP	6.997
KFWGKY;KPSPTPPP	NCP	3.27
KPSPTPPP;QSNALLRH	NCP	6.765
QSNALLRH;EKALSRL	NCP	21.498
EKALSRL;QPVYM	NCP	33.591
QPVYM;KEYEPK	NCP	44.406
KEYEPK;NYCVCC	NCP	16.512
NYCVCC;APGGLPL	NCP	54.89
APGGLPL;LPNFC	NCP	216.328
LPNFC;LDPSGL	NCP	11.297
LDPSGL;VKQLLVG	NCP	12.19
VKQLLVG;VAVLLKE	NCP	23.708
VAVLLKE;LDGVTVTG	NCP	43.701
LDGVTVTG;APLPHHK	NCP	99.967
APLPHHK;MNPVVS	NCP	15.486
MNPVVS;SVSHAKPP	NCP	45.87
SVSHAKPP;MGGKHS	NCP	19.116
MGGKHS;VPGATTPP	NCP	1.086
VPGATTPP;QTLGPLE	NCP	97.187
QTLGPLE;KAAAVLH	NCP	2.293
KAAAVLH;KQPVHN	NCP	20.663
KQPVHN;QFVELCS	NCP	179.985
QFVELCS;QKMVPR	NCP	136.053
QKMVPR;QFMRDF	NCP	33.465
QFMRDF;VHTKLAQ	NCP	8.685
VHTKLAQ;MHSDLR	NCP	53.217
MHSDLR;PGLPP	NCP	190.843
PGLPP;VKKPPAA	NCP	17.337
VKKPPAA;DGYSLKY	NCP	6.179
DGYSLKY;KPWSHAL	NCP	1.767
KPWSHAL;SAPGKQER	NCP	14.976
SAPGKQER;KGVLTMM	NCP	82.071
KGVLTMM;RVHLFL	NCP	49.293
RVHLFL;DYLKEK	NCP	5.653
DYLKEK;QVGCKP	NCP	9.753
QVGCKP;DHKFLM	NCP	103.613
DHKFLM;LDRLLVK	NCP	1.731
LDRLLVK;RNVLSYR	NCP	3.414
RNVLSYR;KVYLPPP	NCP	28.036
KVYLPPP;KPNPPKP	NCP	65.066
KPNPPKP;NPHKPR	NCP	21.207

NPHKPR;FGEGLGKG	NCP	12.034
FGEGLGKG;KELFHEK	NCP	11.189
KELFHEK;GRHVEF	NCP	57.244
GRHVEF;AAAEVNR	NCP	96.516
AAAEVNR;LSDMPPK	NCP	8.78
LSDMPPK;TATAPF	NCP	1.083
TATAPF;TALSPKVR	NCP	26.347
TALSPKVR;AAQRLGK	NCP	30.525
AAQRLGK;KMVLDLSE	NCP	69.851
KMVLDLSE;PPDLDLR	NCP	99.669
PPDLDLR;APVEPPGK	NCP	6.364
APVEPPGK;TVREPF	NCP	132.851
TVREPF;KPVVYFAK	NCP	20.589
KPVVYFAK;KAGPYDPP	NCP	0.061
KAGPYDPP;NPPALPP	NCP	4.248
NPPALPP;LKVSLGK	NCP	12.309
LKVSLGK;AVVVTVRA	NCP	3.204
AVVVTVRA;KSCKPLR	NCP	35.909
KSCKPLR;HHLAQKEK	NCP	3.328
HHLAQKEK;KEAVGGK	NCP	33.189
KEAVGGK;AGGAVVP	NCP	18.069
AGGAVVP;KMKPKP	NCP	9.57
KMKPKP;LPKPAKP	NCP	12.566
LPKPAKP;CPPPKF	NCP	20.317
CPPPKF;RFTPD	NCP	44.826
RFTPD;GMVPGKP	NCP	44.252
GMVPGKP;AKLCKAP	NCP	25.918
AKLCKAP;CMMYCACK	NCP	5.058
CMMYCACK;DAKPPPR	NCP	32.31
DAKPPPR;AAPLK	NCP	10.289
AAPLK;RPPGYLL	NCP	6.517
RPPGYLL;KMEGVTV	NCP	49.464
KMEGVTV;LSNANAK	NCP	43.616
LSNANAK;LLSPEHK	NCP	8.343
LLSPEHK;KMVFLT	NCP	112.825
KMVFLT;WAVPPGP	NCP	22.89
WAVPPGP;GRPNLYP	NCP	1.008
GRPNLYP;SLSKGPFP	NCP	9.389
SLSKGPFP;HCTKK	NCP	15.745
HCTKK;KGKYVLTH	NCP	0.609
KGKYVLTH;MLKPY	NCP	34.826
MLKPY;ASDPPLTL	NCP	41.368
ASDPPLTL;RGPAAYR	NCP	4.932
RGPAAYR;MVEKPPP	NCP	35.954
MVEKPPP;EPMAHF	NCP	42.877
EPMAHF;KDGSPPP	NCP	6.417
KDGSPPP;KPDRMGP	NCP	1.856
KPDRMGP;VPVAPGT	NCP	5.134
VPVAPGT;KPAHVAV	NCP	2.223
KPAHVAV;APTLGLPP	NCP	16.182
APTLGLPP;SEPLAGF	NCP	23.607

SEPLAGF;TCNQLPLT	NCP	62.291
TCNQLPLT;KVVGVGN	NCP	131.524
KVVGVGN;MQPPGPPP	NCP	76.395
MQPPGPPP;ANPSFPLG	NCP	64.445
ANPSFPLG;EVGLP	NCP	112.795
EVGLP;KVSRALD	NCP	1.377
KVSRALD;DGDTP	NCP	16.314
DGDTP;PKRDLP	NCP	4.287
PKRDLP;VTAPH	NCP	8.789
VTAPH;KAKHGKG	NCP	8.412
KAKHGKG;MEAKLKK	NCP	116.607
MEAKLKK;LTLQDY	NCP	9.724
LTLQDY;TSLAKP	NCP	22.74
TSLAKP;WAAVVK	NCP	93.696
WAAVVK;VLVGDGGTGK	NCP	42.974
VLVGDGGTGK;LGVEVHPL	NCP	0.087
LGVEVHPL;GVEVHPLVFHT	NCP	0.003
GVEVHPLVFHT;CFSKLKK	NCP	13.258
CFSKLKK;LFPPHGS	NCP	3.953
LFPPHGS;ASRALLR	NCP	128.067
ASRALLR;PATKY	NCP	4.992
PATKY;PGDGY	NCP	61.338
PGDGY;FARSNK	NCP	3.036
FARSNK;AARAAPVV	NCP	84.148
AARAAPVV;RLELLT	NCP	41.334
RLELLT;SSPKEKL	NCP	2.981
SSPKEKL;NGAQSQK	NCP	12.259
NGAQSQK;KPPTHLR	NCP	3.419
KPPTHLR;VELLLK	NCP	6.328
VELLLK;VGGGSLLA	NCP	135.263
VGGGSLLA;KALLSNR	NCP	12.952
KALLSNR;KALVQSP	NCP	14.696
KALVQSP;PPPSRPP	NCP	0.927
PPPSRPP;SETTLYPK	NCP	21.453
SETTLYPK;VGFVPKC	NCP	34.155
VGFVPKC;KYAPSP	NCP	8.275
KYAPSP;VAVESEDL	NCP	47.99
VAVESEDL;SHLLVTLA	NCP	18.132
SHLLVTLA;DSPLLRW	NCP	109.497
DSPLLRW;KAVLGLGA	NCP	27.262
KAVLGLGA;LLDLVTPM	NCP	51.914
LLDLVTPM;SAAVGAP	NCP	3.045
SAAVGAP;KETKKQEK	NCP	8.386
KETKKQEK;GSPMQL	NCP	10.94
GSPMQL;PFVSLPL	NCP	0.327
PFVSLPL;LAVSKSSK	NCP	11.971
LAVSKSSK;GVVAPGP	NCP	12.063
GVVAPGP;HDGHLNY	NCP	0.155
HDGHLNY;MPGERGK	NCP	52.473
MPGERGK;LVPTLRVP	NCP	8.923
LVPTLRVP;LEALVLL	NCP	13.211

LEALVLL;SYVNAG	NCP	5.752
SYVNAG;PAARPSPP	NCP	10.683
PAARPSPP;LHEGFLE	NCP	23.046
LHEGFLE;ARGPAVS	NCP	7.557
ARGPAVS;FVEDPF	NCP	5.936
FVEDPF;MGMMHP	NCP	53.196
MGMMHP;PEAQPGK	NCP	17.41
PEAQPGK;KALTY	NCP	6.039
KALTY;LPVQP	NCP	9.849
LPVQP;LSLLHLPL	NCP	1.448
LSLLHLPL;DLPRPLS	NCP	9.072
DLPRPLS;LDTPGTY	NCP	22.755
LDTPGTY;GLHKPAA	NCP	55.209
GLHKPAA;KRKPPSP	NCP	9.76
KRKPPSP;DDTGQQ	NCP	24.018
DDTGQQ;DHLKFGLL	NCP	77.223
DHLKFGLL;CCQSGM	NCP	54.267
CCQSGM;VPPVTPP	NCP	18.548
VPPVTPP;KHRNPP	NCP	57.897
KHRNPP;PPPPNRT	NCP	54.466
PPPPNRT;FKVSEKK	NCP	26.907
FKVSEKK;GPTLVFL	NCP	22.671
GPTLVFL;DFRKPGEK	NCP	17.985
DFRKPGEK;VPPKAVP	NCP	8.655
VPPKAVP;KPYVPP	NCP	16.11
KPYVPP;ETGAKNLV	NCP	92.453
ETGAKNLV;KPACNAK	NCP	36.355
KPACNAK;KERDSLP	NCP	43.226
KERDSLP;EVAKER	NCP	15.336
EVAKER;ESLLLTKA	NCP	42.207
ESLLLTKA;SVTKKPP	NCP	92.857
SVTKKPP;PPKTPNK	NCP	0.636
PPKTPNK;LEFGRGF	NCP	8.054
LEFGRGF;VVLLLLGV	NCP	39.166
VVLLLLGV;DPCPRGP	NCP	9.413
DPCPRGP;YGQVSR	NCP	11.649
YGQVSR;TLPFDK	NCP	2.313
TLPFDK;SLPAKAK	NCP	7.647
SLPAKAK;LKLTELKL	NCP	18.546
LKLTELKL;PAAPTY	NCP	39.504
PAAPTY;PHAPFR	NCP	6.535
PHAPFR;LEADPVS	NCP	41.751
LEADPVS;KSPDLDL	NCP	81.791
KSPDLDL;TLQALVLK	NCP	9.4
TLQALVLK;ELGGLDHK	NCP	8.157
ELGGLDHK;KDPPCLP	NCP	50
KDPPCLP;LTGLPGGR	NCP	55.657
LTGLPGGR;DHWQGPP	NCP	26.199
DHWQGPP;QFMPAK	NCP	13.605
QFMPAK;DVGELL	NCP	15.639
DVGELL;PMDMF	NCP	44.942

PMDMF;YDSPPLG	NCP	13.054
YDSPPLG;AATKLF	NCP	73.67
AATKLF;KPDQVPQ	NCP	6.958
KPDQVPQ;GVLMVG	NCP	16.155
GVLMVG;TTSFTLAK	NCP	4.862
TTSFTLAK;NDFKLFH	NCP	12.072
NDFKLFH;KNKVLYP	NCP	13.107
KNKVLYP;ATSDLPK	NCP	4.989
ATSDLPK;EWALVR	NCP	55.495
EWALVR;LLQQQM	NCP	4.989
LLQQQM;ETLFQPS	NCP	81.198
ETLFQPS;VKVTLLH	NCP	6.707
VKVTLLH;ECFKSSG	NCP	92.832
ECFKSSG;KSGVPEK	NCP	26.139
KSGVPEK;KHVDARP	NCP	32.377
KHVDARP;LDELLP	NCP	50.171
LDELLP;VTPTNP	NCP	8.977
VTPTNP;KLDPTK	NCP	46.743
KLDPTK;PLGKTNP	NCP	37.647
PLGKTNP;LPDRKPP	NCP	7.415
LPDRKPP;PGCDLR	NCP	8.688
PGCDLR;PPPVVPR	NCP	18.123
PPPVVPR;LPLKPPP	NCP	14.685
LPLKPPP;PVREPK	NCP	15.7
PVREPK;TVLPLLQ	NCP	11.432
TVLPLLQ;KASEDPK	NCP	37.374
KASEDPK;GVVPNGL	NCP	11.934
GVVPNGL;DCHSCM	NCP	76.108
DCHSCM;LQKAALL	NCP	9.581
LQKAALL;VLEELLF	NCP	7.461
VLEELLF;WPEPEF	NCP	16.971
WPEPEF;AASRRYP	NCP	6.435
AASRRYP;TRQGQPP	NCP	53.932
TRQGQPP;ASLVVPG	NCP	0.407
ASLVVPG;ERSLAVP	NCP	41.688
ERSLAVP;KFLMPK	NCP	26.883
KFLMPK;LLLVVKP	NCP	17.019
LLLVVKP;RLPLGVCA	NCP	54.735
RLPLGVCA;TAELGSPP	NCP	42.208
TAELGSPP;GSSME	NCP	37.412
GSSME;AAHVSHK	NCP	110.448
AAHVSHK;KNYFAAP	NCP	50.109
KNYFAAP;GTWKPR	NCP	67.426
GTWKPR;AAPLGLHQ	NCP	0.746
AAPLGLHQ;KDVQKP	NCP	67.156
KDVQKP;LDPTEF	NCP	22.155
LDPTEF;VFGGPK	NCP	60.891
VFGGPK;ANQGY	NCP	14.867
ANQGY;GVPPVF	NCP	2.361
GVPPVF;VGPFML	NCP	9.165
VGPFML;ALAATVF	NCP	0.085

ALAATVF;QTGAKFV	NCP	0.21
QTGAKFV;CLAFKCL	NCP	67.335
CLAFKCL;EDLVKLQ	NCP	25.7
EDLVKLQ;WFPFFC	NCP	54.6
WFPFFC;YAHRYH	NCP	6.505
YAHRYH;LPVKHSR	NCP	0.483
LPVKHSR;KDKHEPP	NCP	11.604
KDKHEPP;ADDFEV	NCP	18.947
ADDFEV;HESFL	NCP	6.055
HESFL;PSRGQARP	NCP	32.345
PSRGQARP;GKFPA	NCP	33.778
GKFPA;ANEFLPR	NCP	26.043
ANEFLPR;KWGGGVT	NCP	28.341
KWGGGVT;TSPAE	NCP	111.17
TSPAE;PCLKGK	NCP	6.663
PCLKGK;KEVAFAF	NCP	22.888
KEVAFAF;MLALKLD	NCP	43.761
MLALKLD;KDTPPP	NCP	76.512
KDTPPP;FPASTELL	NCP	18.006
FPASTELL;ELLLNNL	NCP	10.779
ELLLNNL;KLPTPLT	NCP	21.206
KLPTPLT;LSGSK	NCP	1.98
LSGSK;LVVSY	NCP	58.771
LVVSY;LTTFDN	NCP	39.248
LTTFDN;LGNMALK	NCP	9.297
LGNMALK;QPGSLAAP	NCP	11.773
QPGSLAAP;AALLF	NCP	46.296
AALLF;WDFALYE	NCP	4.311
WDFALYE;VGDSLLP	NCP	16.082
VGDSLLP;EPVLQ	NCP	38.67
EPVLQ;QLLLPK	NCP	0.079
QLLLPK;NPGPPPPP	NCP	41.495
NPGPPPPP;KARTVH	NCP	63.73
KARTVH;KDAAKRH	NCP	11.759
KDAAKRH;ESDLPLNL	NCP	129.631
ESDLPLNL;PGPLGVT	NCP	1.643
PGPLGVT;APPPTPR	NCP	13.555
APPPTPR;VPLGP	NCP	57.03
VPLGP;GVGRLVK	NCP	22.583
GVGRLVK;MKLQNL	NCP	11.91
MKLQNL;SGFLT	NCP	51.708
SGFLT;FGVNK	NCP	30.451
FGVNK;LSVDEVR	NCP	0.879
LSVDEVR;TFDWRC	NCP	1.565
TFDWRC;YDPPGLR	NCP	105.115
YDPPGLR;GLRQAM	NCP	20.099
GLRQAM;PPRGPPPPPPPP	NCP	31.786
PPRGPPPPPPPP;LDKLRL	NCP	95.759
LDKLRL;FPDPEK	NCP	2.325
FPDPEK;PPDEE	NCP	94.618
PPDEE;VLPPAP	NCP	65.526

VLPPAP;LAVDLLLP	NCP	20.529
LAVDLLLP;LSNHVLGL	NCP	34.659
LSNHVLGL;RSDLRLL	NCP	2.657
RSDLRLL;FDDLNLK	NCP	47.143
FDDLNLK;AAAEAPVVV	NCP	5.633
AAAEAPVVV;KPMYPR	NCP	1.81
KPMYPR;KDMCSPK	NCP	38.486
KDMCSPK;AVALLSY	NCP	12.135
AVALLSY;KGRPHSK	NCP	31.078
KGRPHSK;VPGTCKK	NCP	26.217
VPGTCKK;KCPPQ	NCP	28.937
KCPPQ;KLYFAAP	NCP	59.689
KLYFAAP;KDLADPV	NCP	61.472
KDLADPV;PQMHTKK	NCP	117.729
PQMHTKK;MRKPEKQ	NCP	29.343
MRKPEKQ;AVPPLKF	NCP	90.084
AVPPLKF;EHLLTRE	NCP	13.821
EHLLTRE;TPFQYPP	NCP	73.87
TPFQYPP;LPGLELR	NCP	44.262
LPGLELR;LPPNPPLP	NCP	4.857
LPPNPPLP;KKPRMAK	NCP	44.522
KKPRMAK;VSLLAYY	NCP	0.106
VSLLAYY;SLRPESL	NCP	45.765
SLRPESL;SLPLFGPG	NCP	7.785
SLPLFGPG;VRTVGGL	NCP	3.06
VRTVGGL;LGPLA	NCP	68.82
LGPLA;TVHLLPL	NCP	20.363
TVHLLPL;VYPALKPR	NCP	22.221
VYPALKPR;KVLEPY	NCP	51.063
KVLEPY;VNLEYL	NCP	32.835
VNLEYL;KDPSYF	NCP	17.908
KDPSYF;STTATPL	NCP	13.041
STTATPL;EDVNWY	NCP	92.684
EDVNWY;LPLVPAAH	NCP	3.387
LPLVPAAH;PFTMVP	NCP	77.665
PFTMVP;LGPPFLQ	NCP	129.438
LGPPFLQ;SSSEFPK	NCP	15.408
SSSEFPK;SALQY	NCP	85.542
SALQY;LDLTGL	NCP	10.334
LDLTGL;EAKSRTLG	NCP	44.22
EAKSRTLG;KKTGMSK	NCP	2.527
KKTGMSK;KSTLGHE	NCP	119.214
KSTLGHE;LDFTVW	NCP	24.864
LDFTVW;KPQGKTT	NCP	20.576
KPQGKTT;APESPLR	NCP	31.396
APESPLR;NGHLFY	NCP	15.485
NGHLFY;GVPSVPK	NCP	50.553
GVPSVPK;VPEPSAL	NCP	4.662
VPEPSAL;RLAPPER	NCP	17.961
RLAPPER;EVPDPT	NCP	25.65
EVPDPT;GSLNTEFK	NCP	22.83

GSLNTEFK;NLKAKAL	NCP	0.535
NLKAKAL;RETKNH	NCP	2.033
RETKNH;VLTLGNER	NCP	104.005
VLTLGNER;KFTAGRP	NCP	2.255
KFTAGRP;ELGSLNK	NCP	9.72
ELGSLNK;AAPPERK	NCP	136.402
AAPPERK;ETPKAVP	NCP	5.672
ETPKAVP;ERMALL	NCP	28.722
ERMALL;KEEVTSALR	NCP	15.631
KEEVTSALR;KKPPKK	NCP	12.473
KKPPKK;KGFHRK	NCP	5.415
KGFHRK;EGTFVRF	NCP	70.21
EGTFVRF;LPSLPYPF	NCP	55.409
LPSLPYPF;LAMLR	NCP	22.767
LAMLR;PVYPTAA	NCP	10.638
PVYPTAA;VVALP	NCP	33.267
VVALP;WKALDF	NCP	52.875
WKALDF;CQTGEKL	NCP	18.282
CQTGEKL;FCHKPCY	NCP	14.991
FCHKPCY;MDHYDSQQTNDY	NCP	10.113
MDHYDSQQTNDY;LGTLPVDF	NCP	1.408
LGTLPVDF;VWQKPTR	NCP	21.311
VWQKPTR;NLSLPF	NCP	8.235
NLSLPF;KSHPRP	NCP	1.587
KSHPRP;ATAGSLK	NCP	10.446
ATAGSLK;DDPLTNLNTAFDVAEK	NCP	30.165
DDPLTNLNTAFDVAEK;VSSFYHAF	NCP	1.344
VSSFYHAF;AADQAAPQ	NCP	8.364
AADQAAPQ;LYLEYAKRAAPF	NCP	6.375
LYLEYAKRAAPF;HVNMAGTNPY	NCP	0.996
HVNMAGTNPY;RRELPPDQAEY	NCP	6.933
RRELPPDQAEY;TGPDAVPGALDY	NCP	0.057
TGPDAVPGALDY;MSFSTALYGESDL	NCP	0.036
MSFSTALYGESDL;HQPPPPQG	NCP	41.01
HQPPPPQG;FKAPPPPP	NCP	12.264
FKAPPPPP;HSGGPPPP	NCP	13.194
HSGGPPPP;HLLPPPP	NCP	0.237
HLLPPPP;KGFVGP	NCP	59.916
KGFVGP;KNTLML	NCP	87.925
KNTLML;KPLKTH	NCP	26.288
KPLKTH;SKLRSGLL	NCP	31.221
SKLRSGLL;RGPRQLLS	NCP	13.347
RGPRQLLS;SHLLVTL	NCP	1.168
SHLLVTL;SSAKPPK	NCP	32.411
SSAKPPK;KEYCMP	NCP	2.433
KEYCMP;QKDNLT	NCP	10.12
QKDNLT;KKAMKPQ	NCP	11.669
KKAMKPQ;TQGRMLP	NCP	12.672
TQGRMLP;LGLPGLGW	NCP	8.094
LGLPGLGW;EELRTTP	NCP	2.367
EELRTTP;YYMFMC	NCP	25.348

YYMFMC;FDVAEK	NCP	33.984
FDVAEK;KESTLGSK	NCP	3.525
KESTLGSK;TEDKSK	NCP	8.457
TEDKSK;VGYPHK	NCP	99.428
VGYPHK;DGDMM	NCP	7.204
DGDMM;VLESLQ	NCP	69.593
VLESLQ;TLDDSLP	NCP	42.163
TLDDSLP;LPYLQPN	NCP	38.7
LPYLQPN;KGYLHP	NCP	70.163
KGYLHP;NNYLLLRV	NCP	178.647
NNYLLLRV;ATETVP	NCP	35.658
ATETVP;QQLPSGK	NCP	16.048
QQLPSGK;KAYGPL	NCP	18.108
KAYGPL;KDLQPC	NCP	174.417
KDLQPC;PQLVTPR	NCP	60.236
PQLVTPR;LPQSDLR	NCP	23.901
LPQSDLR;PAAGLPPK	NCP	97.084
PAAGLPPK;RPKEQK	NCP	3.23
RPKEQK;LFTMKPLL	NCP	72.801
LFTMKPLL;KGKYGNK	NCP	83.748
KGKYGNK;PGAPRVR	NCP	11.943
PGAPRVR;MELLQ	NCP	34.818
MELLQ;FVAHKF	NCP	158.961
FVAHKF;PGFLVRVQ	NCP	7.081
PGFLVRVQ;LLSKKL	NCP	147.423
LLSKKL;QGLALF	NCP	23.48
QGLALF;KTPFQEK	NCP	25.638
KTPFQEK;KGTLRAGP	NCP	80.691
KGTLRAGP;TETNKLR	NCP	3.789
TETNKLR;LSAKPPK	NCP	9.957
LSAKPPK;TTKAGKK	NCP	3.963
TTKAGKK;NYLTELK	NCP	19.548
NYLTELK;TVLLPF	NCP	62.958
TVLLPF;KVKLPLQ	NCP	48.414
KVKLPLQ;LLMLK	NCP	103.176
LLMLK;HVRGFPL	NCP	29.667
HVRGFPL;FPGKDT	NCP	80.706
FPGKDT;FLDAGKK	NCP	10.515
FLDAGKK;QWCYVPK	NCP	20.692
QWCYVPK;VVSLA	NCP	116.022
VVSLA;KSNLEL	NCP	66.578
KSNLEL;EPRHHSKR	NCP	28.563
EPRHHSKR;KKYVLST	NCP	4.824
KKYVLST;KVLFPAP	NCP	12.909
KVLFPAP;MMTANR	NCP	26.124
MMTANR;QPDVGPK	NCP	82.146
QPDVGPK;LLCMNPM	NCP	117.522
LLCMNPM;NLLLSVSK	NCP	18.966
NLLLSVSK;SVMCVD	NCP	102.712
SVMCVD;DPLLQK	NCP	15.506
DPLLQK;EQPKLP	NCP	7.518

EQPKLP;MMGVY	NCP	160.27
MMGVY;YLLTAVT	NCP	2.807
YLLTAVT;LTGGLEL	NCP	4.489
LTGGLEL;AVSAVPQ	NCP	4.991
AVSAVPQ;LGSYNHR	NCP	11.34
LGSYNHR;YYGTY	NCP	19.201
YYGTY;GAVFLAP	NCP	1.224
GAVFLAP;KGKPAAR	NCP	153.596
KGKPAAR;EDLFTGL	NCP	12.133
EDLFTGL;GHVANTK	NCP	23.885
GHVANTK;PGADFPK	NCP	0.826
PGADFPK;KPYGAP	NCP	26.727
KPYGAP;KVPSLALP	NCP	6.08
KVPSLALP;LLLLLPS	NCP	39.148
LLLLLPS;KETAVLL	NCP	49.067
KETAVLL;ESLTVGP	NCP	6.45
ESLTVGP;YESKVY	NCP	6.858
YESKVY;SLLEETLL	NCP	57.289
SLLEETLL;VMGELLC	NCP	17.69
VMGELLC;FLLSDH	NCP	2.937
FLLSDH;KAFPPGP	NCP	25.327
KAFPPGP;KHFYAR	NCP	30.528
KHFYAR;TPAGVPP	NCP	24.395
TPAGVPP;PPKEPKPP	NCP	20.211
PPKEPKPP;SLGTPEK	NCP	6.774
SLGTPEK;KLAPARAL	NCP	15.898
KLAPARAL;AEEFLVL	NCP	21.852
AEEFLVL;EAAPGLR	NCP	20.741
EAAPGLR;LPTHLRC	NCP	8.61
LPTHLRC;PAAGPGRGP	NCP	14.394
PAAGPGRGP;GKPLASHVS	NCP	23.742
GKPLASHVS;KGVTFSLC	NCP	3.435
KGVTFSLC;LLNLEEE	NCP	29.713
LLNLEEE;PRFGMR	NCP	7.454
PRFGMR;TGPGLDL	NCP	135.253
TGPGLDL;KQHKPP	NCP	88.085
KQHKPP;AFKRSP	NCP	4.873
AFKRSP;LLYDRYN	NCP	78.71
LLYDRYN;ERFVQNL	NCP	50.235
ERFVQNL;AELLRA	NCP	13.8
AELLRA;WPDGAR	NCP	40.804
WPDGAR;EPVLNLRNT	NCP	9.549
EPVLNLRNT;KTYFLL	NCP	72.105
KTYFLL;QELRTPL	NCP	7.946
QELRTPL;LLQVKEL	NCP	9.867
LLQVKEL;QRSPPLGP	NCP	7.923
QRSPPLGP;NDKWP	NCP	25.536
NDKWP;KDMTKK	NCP	2.883
KDMTKK;HSPPARK	NCP	70.212
HSPPARK;KADVVRV	NCP	41.718
KADVVRV;LPVQVFN	NCP	247.314

LPVQVFN;KHSSAFK	NCP	3.195
KHSSAFK;KYPPPPAP	NCP	46.641
KYPPPPAP;GEVFP	NCP	97.339
GEVFP;KAYPPPP	NCP	16.427
KAYPPPP;LPASP	NCP	125.457
LPASP;MKMEAMSR	NCP	49.683
MKMEAMSR;KLGLLY	NCP	147.046
KLGLLY;YVLTGP	NCP	36.428
YVLTGP;LYEESTPK	NCP	24.696
LYEESTPK;KVHNNKK	NCP	41.698
KVHNNKK;APKPPL	NCP	81.57
APKPPL;KLVVTSK	NCP	49.56
KLVVTSK;LMKFFR	NCP	78.095
LMKFFR;STKPLYT	NCP	173.341
STKPLYT;KNRSPRP	NCP	4.052
KNRSPRP;LRAAPP	NCP	56.602
LRAAPP;QNAAPY	NCP	40.934
QNAAPY;DLVLVKK	NCP	16.953
DLVLVKK;EAPAPPKAEAKAK	NCP	102.354
EAPAPPKAEAKAK;DLRASKP	NCP	4.237
DLRASKP;PLLKMLQ	NCP	6.044
PLLKMLQ;QPTPK	NCP	86.242
QPTPK;HYLPPLQ	NCP	31.131
HYLPPLQ;DNELLP	NCP	5.706
DNELLP;FPRPKFS	NCP	3.6
FPRPKFS;KVLLMEC	NCP	1.827
KVLLMEC;LLESTML	NCP	43.457
LLESTML;HGCFSQ	NCP	18.931
HGCFSQ;KFTVP	NCP	0.719
KFTVP;LSLTSKY	NCP	86.548
LSLTSKY;VLLAF	NCP	7.833
VLLAF;RLHFLP	NCP	47.603
RLHFLP;LHVTFPK	NCP	25.251
LHVTFPK;SGLEMRK	NCP	1.371
SGLEMRK;PGAGLK	NCP	54.754
PGAGLK;DGLPKYK	NCP	6.362
DGLPKYK;AHRLQLLR	NCP	29.962
AHRLQLLR;LLKSLYR	NCP	23.324
LLKSLYR;KMYLSFG	NCP	10.78
KMYLSFG;YFMGPK	NCP	8.829
YFMGPK;GSPPFKF	NCP	0.752
GSPPFKF;TLGMKGY	NCP	1.231
TLGMKGY;LSKPLPP	NCP	67.983
LSKPLPP;LSKLWDV	NCP	52.854
LSKLWDV;ALPLHAL	NCP	14.483
ALPLHAL;QNCAKLK	NCP	112.923
QNCAKLK;DEAVPLP	NCP	9.643
DEAVPLP;QALTVH	NCP	2.615
QALTVH;VGVAPLT	NCP	18.114
VGVAPLT;ALTVLDF	NCP	29.737
ALTVLDF;LATKLE	NCP	5.576

LATKLE;RYVPQA	NCP	3.582
RYVPQA;RSGFKGK	NCP	10.455
RSGFKGK;PDVLSH	NCP	78.096
PDVLSH;KPQSKHR	NCP	35.566
KPQSKHR;THVELL	NCP	26.876
THVELL;LDLLLKKK	NCP	23.743
LDLLLKKK;MDDFER	NCP	0.372
MDDFER;KETPVP	NCP	6.524
KETPVP;PAKMQPF	NCP	20.793
PAKMQPF;NPMVFV	NCP	38.938
NPMVFV;CAQDLRK	NCP	71.036
CAQDLRK;RYLQNNF	NCP	3.234
RYLQNNF;LSPLVDTHS	NCP	7.491
LSPLVDTHS;AMKSKR	NCP	71.848
AMKSKR;KNLEVLN	NCP	12.822
KNLEVLN;PGAPVNL	NCP	57.429
PGAPVNL;AFANLR	NCP	13.596
AFANLR;TSAPRSFSS	NCP	58.157
TSAPRSFSS;KDVDEAYMNK	NCP	0.573
KDVDEAYMNK;MNKVELESRLEGLTDELNF	NCP	0.021
MNKVELESRLEGLTDELNF;KYEELQTLAGK	NCP	0.24
KYEELQTLAGK;LLEGEESRLESGMQNM	NCP	0.318
LLEGEESRLESGMQNM;MEVMNL	NCP	8.014
MEVMNL;RAAPPKP	NCP	67.214
RAAPPKP;RGKTLPL	NCP	4.005
RGKTLPL;KETQARP	NCP	125.986
KETQARP;LNLTNR	NCP	8.612
LNLTNR;YVLGAPL	NCP	21.96
YVLGAPL;MEEPQR	NCP	19.768
MEEPQR;RLNSLTK	NCP	1.616
RLNSLTK;LPLLLLP	NCP	2.188
LPLLLLP;SGVSAVP	NCP	29.991
SGVSAVP;KGSSSPR	NCP	117.938
KGSSSPR;MELLKT	NCP	65.656
MELLKT;LSPPDKP	NCP	33.234
LSPPDKP;KVGNSPF	NCP	9.083
KVGNSPF;YAASEY	NCP	0.805
YAASEY;KAPTDKK	NCP	25.284
KAPTDKK;DGKPF	NCP	128.039
DGKPF;VFVKSDR	NCP	36.631
VFVKSDR;LVPHYK	NCP	12.419
LVPHYK;PGGLL	NCP	7.974
PGGLL;SLAFF	NCP	71.683
SLAFF;KRVAVFF	NCP	28.364
KRVAVFF;TFLHLQH	NCP	64.641
TFLHLQH;GPVLVQTC	NCP	1.68
GPVLVQTC;APRPQLPP	NCP	2.547
APRPQLPP;KGAATALPK	NCP	78.225
KGAATALPK;KQPTYFAP	NCP	156.831
KQPTYFAP;ATDVSVP	NCP	8.971
ATDVSVP;KEFVPYPK	NCP	54.912

KEFVPYPK;LFDTPPP	NCP	50.805
LFDTPPP;GCDKLR	NCP	21.83
GCDKLR;VAWVGSH	NCP	13.005
VAWVGSH;LALGAER	NCP	7.878
LALGAER;WLPVKK	NCP	12.295
WLPVKK;VNMLP	NCP	20.03
VNMLP;MQVLNC	NCP	93.813
MQVLNC;NNPPPR	NCP	71.086
NNPPPR;KKDAGPK	NCP	46.215
KKDAGPK;FPPHRT	NCP	39.809
FPPHRT;MTEDDL	NCP	51.996
MTEDDL;MRQLVQL	NCP	56.1
MRQLVQL;KSLLF	NCP	100.573
KSLLF;KPPKGLH	NCP	22.062
KPPKGLH;VHSPPPK	NCP	87.654
VHSPPPK;ELGAFH	NCP	4.335
ELGAFH;PSTSPSR	NCP	177.717
PSTSPSR;LLLATKK	NCP	21.761
LLLATKK;ATCQAY	NCP	24.375
ATCQAY;YRLPTAA	NCP	4.768
YRLPTAA;EGPDAGR	NCP	0.276
EGPDAGR;PHYVSF	NCP	34.142
PHYVSF;SLTSPL	NCP	35.478
SLTSPL;KTKLLL	NCP	5.088
KTKLLL;EALTLKNQ	NCP	10.519
EALTLKNQ;PPLMPK	NCP	18.791
PPLMPK;ALLEYLT	NCP	31.891
ALLEYLT;LYRTTVL	NCP	42.941
LYRTTVL;KPMVRP	NCP	43.194
KPMVRP;EHLKPQ	NCP	22.776
EHLKPQ;LSVNPMLL	NCP	31.863
LSVNPMLL;PGLDGK	NCP	12.714
PGLDGK;RGPNSLLP	NCP	89.542
RGPNSLLP;WSVLGCG	NCP	8.97
WSVLGCG;RTVLLF	NCP	5.757
RTVLLF;RNVGVPR	NCP	26.736
RNVGVPR;LQLVL	NCP	25.524
LQLVL;FVDKLK	NCP	22.734
FVDKLK;KPKEKLQC	NCP	13.139
KPKEKLQC;EVPLLT	NCP	36.664
EVPLLT;TSPTGWK	NCP	41.319
TSPTGWK;LPPPPDLPPPPP	NCP	53.79
LPPPPDLPPPPP;LAVVEY	NCP	52.629
LAVVEY;LAKKTLNL	NCP	28.781
LAKKTLNL;FPKLTGV	NCP	67.092
FPKLTGV;QPPRMAK	NCP	35.958
QPPRMAK;ECCCNF	NCP	2.338
ECCCNF;KEYLNP	NCP	48.479
KEYLNP;SVVGTTN	NCP	16.039
SVVGTTN;KGGSLLR	NCP	114.188
KGGSLLR;KADLGGLL	NCP	115.45

KADLGGLL;KAVQFPP	NCP	230.357
KAVQFPP;YGVDAP	NCP	120.934
YGVDAP;LVTLDY	NCP	23
LVTLDY;KRMPPPP	NCP	35.968
KRMPPPP;AETFAAK	NCP	104.129
AETFAAK;LAPRPVH	NCP	13.464
LAPRPVH;LTGAVAF	NCP	34.264
LTGAVAF;TVPLEGK	NCP	18.384
TVPLEGK;KYSMKK	NCP	14.538
KYSMKK;KLGTKMQ	NCP	179.598
KLGTKMQ;KLSWT	NCP	37.568
KLSWT;CSHAAPK	NCP	64.705
CSHAAPK;VGGLDL	NCP	20.012
VGGLDL;DPVLRPP	NCP	12.075
DPVLRPP;REVLSEK	NCP	57.846
REVLSEK;LTAELLQLL	NCP	78.126
LTAELLQLL;KPGSGLR	NCP	23.161
KPGSGLR;SVARLAKLEK	NCP	8.652
SVARLAKLEK;LMMKKT	NCP	156.875
LMMKKT;PKKMPK	NCP	34.992
PKKMPK;PTVVGPP	NCP	43.279
PTVVGPP;EAKPPLH	NCP	69.048
EAKPPLH;VTLLDF	NCP	22.352
VTLLDF;RPTAYFP	NCP	13.062
RPTAYFP;YVRSLD	NCP	15.364
YVRSLD;METMNLP	NCP	26.819
METMNLP;TSPLPLKP	NCP	58.239
TSPLPLKP;KAAGGLPR	NCP	72.063
KAAGGLPR;FLLVVK	NCP	18.57
FLLVVK;SVDMRS	NCP	4.035
SVDMRS;DDFERR	NCP	7.227
DDFERR;AAPPKATG	NCP	24.459
AAPPKATG;KSAALPK	NCP	6.951
KSAALPK;HPKPSTP	NCP	32.694
HPKPSTP;SLAVTNK	NCP	2.602
SLAVTNK;KPSVDKK	NCP	90.824
KPSVDKK;VLPLLTQ	NCP	32.325
VLPLLTQ;HGPSK	NCP	1.738
HGPSK;NVTLLAS	NCP	54.908
NVTLLAS;LEKGLDG	NCP	26.148
LEKGLDG;MEALAK	NCP	47.647
MEALAK;QTPKVHP	NCP	11.199
QTPKVHP;YGLVDY	NCP	42.059
YGLVDY;NAGFVF	NCP	19.437
NAGFVF;APNPAK	NCP	15.87
APNPAK;PVGVP	NCP	109.366
PVGVP;SAAKPLE	NCP	7.634
SAAKPLE;KGPKF	NCP	4.456
KGPKF;SLMVHGK	NCP	59.697
SLMVHGK;KPPTVPP	NCP	112.026
KPPTVPP;KNLQAF	NCP	33.287

KNLQAF;QLELQK	NCP	54.474
QLELQK;KAAERR	NCP	24.183
KAAERR;LPPPPLFFP	NCP	0.658
LPPPPLFFP;PLSLLKPF	NCP	84.627
PLSLLKPF;SCLRSLGY	NCP	2.214
SCLRSLGY;KFLLVPP	NCP	86.922
KFLLVPP;NLRRFN	NCP	51.732
NLRRFN;KPVERT	NCP	39.666
KPVERT;NPEKPVK	NCP	8.247
NPEKPVK;LGLGGAV	NCP	3.075
LGLGGAV;GPEAPP	NCP	29.799
GPEAPP;VLYRLAV	NCP	14.631
VLYRLAV;HTPPP	NCP	8.498
HTPPP;LPPLVPK	NCP	19.657
LPPLVPK;LSDLSAPK	NCP	42.765
LSDLSAPK;LESVVPY	NCP	11.013
LESVVPY;KFVVKPP	NCP	13.962
KFVVKPP;KPFGTGR	NCP	0.794
KPFGTGR;KKLAAPG	NCP	10.632
KKLAAPG;NPAGPRP	NCP	0.153
NPAGPRP;VDKPPSVL	NCP	3.705
VDKPPSVL;VFECR	NCP	42.234
VFECR;EDFHNHP	NCP	7.2
EDFHNHP;VEFPGLKE	NCP	43.753
VEFPGLKE;SFPLG	NCP	78.842
SFPLG;KMTDRK	NCP	5.049
KMTDRK;QTLVWCLHKE	NCP	31.147
QTLVWCLHKE;ADLLKEPQE	NCP	7.266
ADLLKEPQE;KVGPRLL	NCP	57.162
KVGPRLL;RLAEFR	NCP	25.677
RLAEFR;RFAAPPPP	NCP	5.181
RFAAPPPP;TERWW	NCP	11.076
TERWW;MDALKK	NCP	3.788
MDALKK;NPARPPP	NCP	3.844
NPARPPP;GHSLEK	NCP	13.071
GHSLEK;VGKLSHSL	NCP	324.551
VGKLSHSL;KDYSGP	NCP	22.84
KDYSGP;VHVVYP	NCP	14.747
VHVVYP;FPVRGHL	NCP	32.776
FPVRGHL;VTLLEEL	NCP	16.649
VTLLEEL;AAVAVPL	NCP	10.816
AAVAVPL;WMHMG	NCP	20.936
WMHMG;KPPAAPQP	NCP	49
KPPAAPQP;KADFHH	NCP	13.583
KADFHH;SLLTQKL	NCP	16.12
SLLTQKL;VDLSNTT	NCP	195.474
VDLSNTT;VFCER	NCP	19.143
VFCER;ADVSKEP	NCP	35.484
ADVSKEP;DVGDSF	NCP	22.26
DVGDSF;AAKPLAL	NCP	23.699
AAKPLAL;KAADMPK	NCP	51.588

KAADMPK;LVELK	NCP	14.493
LVELK;PPNVKPP	NCP	82.909
PPNVKPP;KPTEWYK	NCP	6.2
KPTEWYK;YELRLSH	NCP	9.028
YELRLSH;QPTPTPLL	NCP	12.807
QPTPTPLL;EDVPTLL	NCP	0.276
EDVPTLL;KYLWVT	NCP	2.835
KYLWVT;KFVYPP	NCP	39.039
KFVYPP;APMTKP	NCP	15.99
APMTKP;APAEKPP	NCP	52.775
APAEKPP;MNYPGHG	NCP	78.144
MNYPGHG;LLVSFFV	NCP	22.627
LLVSFFV;GVPLK	NCP	25.056
GVPLK;VYPKPR	NCP	24.963
VYPKPR;FSPGTKK	NCP	10.29
FSPGTKK;KGYPPPP	NCP	50.943
KGYPPPP;KLTLKGYKQ	NCP	57.59
KLTLKGYKQ;YKLTSGWV	NCP	11.814
YKLTSGWV;PVCPLK	NCP	44.46
PVCPLK;VGMFLQG	NCP	2.523
VGMFLQG;GSVLLNR	NCP	38.98
GSVLLNR;KSALKLY	NCP	24.767
KSALKLY;VAVHVH	NCP	7.186
VAVHVH;AAAKPENLSL	NCP	37.181
AAAKPENLSL;QCCGPGP	NCP	16.384
QCCGPGP;LGNKVLVC	NCP	9.14
LGNKVLVC;SKSVNVKPLVT	NCP	8.361
SKSVNVKPLVT;ERYVPR	NCP	18.244
ERYVPR;LGAAVLSL	NCP	20.354
LGAAVLSL;AMLSGVT	NCP	17.254
AMLSGVT;EPKTPLSPP	NCP	1.896
EPKTPLSPP;PVNGP	NCP	4.784
PVNGP;KDEAAAK	NCP	4.54
KDEAAAK;LEKGDLQ	NCP	5.382
LEKGDLQ;LYPHKVVF	NCP	232.757
LYPHKVVF;LLKLSYR	NCP	7.099
LLKLSYR;VADGPV	NCP	46.229
VADGPV;HQGKGPK	NCP	7.95
HQGKGPK;VDLSGPK	NCP	60.625
VDLSGPK;PLLMFCK	NCP	5.612
PLLMFCK;GSLGPGAYK	NCP	21.994
GSLGPGAYK;SWEVY	NCP	12.282
SWEVY;LSEAYFRK	NCP	43.667
LSEAYFRK;SPPDVLK	NCP	12.819
SPPDVLK;VDVDKDTLQHK	NCP	0.051
VDVDKDTLQHK;EVYGPK	NCP	3.408
EVYGPK;FGERALK	NCP	22.311
FGERALK;AEYKKL	NCP	61.534
AEYKKL;HLLFVV	NCP	18.465
HLLFVV;NTGLLLAR	NCP	33.93
NTGLLLAR;ASDRLSL	NCP	94.77

ASDRLSL;VLLGHR	NCP	52.13
VLLGHR;KCYEPKL	NCP	27.67
KCYEPKL;ELVMPEKF	NCP	28.02
ELVMPEKF;MNPVVG	NCP	64.119
MNPVVG;VHDLTK	NCP	29.067
VHDLTK;ALMFL	NCP	38.718
ALMFL;KDTTSLC	NCP	5.886
KDTTSLC;KPANEC	NCP	22.275
KPANEC;VSPLTF	NCP	81.608
VSPLTF;KNTLTPV	NCP	6.576
KNTLTPV;LLLDVALP	NCP	19.155
LLLDVALP;DSRLTPV	NCP	103.186
DSRLTPV;PKVTVLGL	NCP	127.934
PKVTVLGL;DLLHPSPEEEKR	NCP	53.443
DLLHPSPEEEKR;RLVQSPNS	NCP	0.051
RLVQSPNS;VESSVEVP	NCP	30.761
VESSVEVP;ESPPK	NCP	104.322
ESPPK;KPPTKGK	NCP	55.999
KPPTKGK;HGQDNY	NCP	3.36
HGQDNY;ELGPP	NCP	87.783
ELGPP;YFANPK	NCP	165.962
YFANPK;LMTVGC	NCP	313.152
LMTVGC;LAETLYK	NCP	10.476
LAETLYK;KPTRLT	NCP	9.235
KPTRLT;EFVRPLF	NCP	269.094
EFVRPLF;KKQPTKK	NCP	8.273
KKQPTKK;NTDLSGPK	NCP	23.136
NTDLSGPK;QTLDVV	NCP	14.02
QTLDVV;EGLKLAY	NCP	43.745
EGLKLAY;THDTPLL	NCP	31.837
THDTPLL;KVMFLM	NCP	34.295
KVMFLM;YEHNSP	NCP	4.119
YEHNSP;GPAAAAAGK	NCP	5.884
GPAAAAAGK;QAKAPVP	NCP	33.425
QAKAPVP;KPDVPP	NCP	24.861
KPDVPP;LPHQKVGL	NCP	22.707
LPHQKVGL;KEYNGP	NCP	360.72
KEYNGP;LDQVKYF	NCP	89.797
LDQVKYF;GVSVQKR	NCP	6.254
GVSVQKR;FFKGSPR	NCP	6.531
FFKGSPR;KELGGSF	NCP	15.463
KELGGSF;KLEDKKTW	NCP	24.047
KLEDKKTW;YPLPQML	NCP	6.237
YPLPQML;ASSDLQVK	NCP	16.089
ASSDLQVK;MLFWY	NCP	83.296
MLFWY;LLKGGAK	NCP	30.06
LLKGGAK;AGVFLKK	NCP	4.725
AGVFLKK;GPATKE	NCP	67.619
GPATKE;QNTMDP	NCP	14.536
QNTMDP;PLTVLPP	NCP	37.284
PLTVLPP;RFFSK	NCP	96.867

RFFSK;LGRSPEDK	NCP	39.108
LGRSPEDK;QTRKLAV	NCP	41.244
QTRKLAV;KPDRTPP	NCP	213.356
KPDRTPP;KVVSSGK	NCP	3.25
KVVSSGK;NLHQVTK	NCP	25.959
NLHQVTK;LCVSP	NCP	21.885
LCVSP;TVYLLP	NCP	115.016
TVYLLP;DVDLR	NCP	84.52
DVDLR;NGPSFP	NCP	0.308
NGPSFP;QTVLLFP	NCP	38.404
QTVLLFP;APNSPVFL	NCP	21.594
APNSPVFL;SVVVL	NCP	26.705
SVVVL;EFLDF	NCP	21.877
EFLDF;KERAALE	NCP	27.885
KERAALE;ESGVVTEK	NCP	9.095
ESGVVTEK;ATEGPAVRR	NCP	4.887
ATEGPAVRR;MFLGKVK	NCP	76.365
MFLGKVK;MKLVFK	NCP	77.929
MKLVFK;EKVLWK	NCP	156.186
EKVLWK;LTRSLK	NCP	6.399
LTRSLK;NMKLHM	NCP	2.607
NMKLHM;DLEVRGLK	NCP	30.533
DLEVRGLK;GVPVWF	NCP	36.495
GVPVWF;TLEHSLK	NCP	232.975
TLEHSLK;QLVVSTQ	NCP	31.232
QLVVSTQ;KDSTFQQ	NCP	22.695
KDSTFQQ;KGTLLPGK	NCP	94.113
KGTLLPGK;ELPFYQK	NCP	70.762
ELPFYQK;LSVFHPPP	NCP	52.884
LSVFHPPP;YNEALRV	NCP	15.054
YNEALRV;PAAALPPP	NCP	195.993
PAAALPPP;PTLGNPK	NCP	31.61
PTLGNPK;LDEKDEL	NCP	8.122
LDEKDEL;YLGDVKK	NCP	13.607
YLGDVKK;QGYVLY	NCP	111.457
QGYVLY;LEDGK	NCP	32.553
LEDGK;SALQKLK	NCP	28.095
SALQKLK;KHDQPF	NCP	78.002
KHDQPF;VPSLDLP	NCP	21.534
VPSLDLP;ESPLYQ	NCP	55.234
ESPLYQ;VPDPAAP	NCP	96.042
VPDPAAP;QAFDSFY	NCP	27.35
QAFDSFY;NCHCCH	NCP	38.607
NCHCCH;VELPF	NCP	117.901
VELPF;AHVPEQ	NCP	21.603
AHVPEQ;LVGPRLL	NCP	91.613
LVGPRLL;SVGLSPF	NCP	9.993
SVGLSPF;TRSSWTR	NCP	64.948
TRSSWTR;LDVQPK	NCP	29.42
LDVQPK;APHQPVR	NCP	20.712
APHQPVR;RSLRSY	NCP	93.919

RSLRSY;KGDQKK	NCP	38.7
KGDQKK;DNSGGL	NCP	7.982
DNSGGL;QALLLKAL	NCP	39.886
QALLLKAL;FFKKP	NCP	66.846
FFKKP;MGNPLLR	NCP	3.305
MGNPLLR;NGEGPF	NCP	64.342
NGEGPF;VGAPP	NCP	127.922
VGAPP;KASPVFPR	NCP	19.375
KASPVFPR;GLPPKNF	NCP	17.247
GLPPKNF;SLLNKPK	NCP	54.938
SLLNKPK;SLLVQTK	NCP	28.684
SLLVQTK;ALETNTHVKYF	NCP	21.275
ALETNTHVKYF;KSCFRAC	NCP	47.008
KSCFRAC;VPPGLFY	NCP	12.615
VPPGLFY;KDHPHK	NCP	50.784
KDHPHK;KELDFKG	NCP	72.881
KELDFKG;ELPEEQ	NCP	28.567
ELPEEQ;RGYLL	NCP	11.064
RGYLL;TSKKPPPP	NCP	20.988
TSKKPPPP;QQELPLR	NCP	24.032
QQELPLR;PPHAVGK	NCP	11.685
PPHAVGK;VVPKWQ	NCP	7.545
VVPKWQ;DLGTRKP	NCP	17.095
DLGTRKP;NVGVLPF	NCP	46.391
NVGVLPF;KGVFK	NCP	24.619
KGVFK;PGPRPPPP	NCP	2.793
PGPRPPPP;KLKKKPPG	NCP	38.087
KLKKKPPG;LLLKTK	NCP	50.706
LLLKTK;TVAKL	NCP	8.745
TVAKL;KTGSLLST	NCP	44.974
KTGSLLST;TQYVYK	NCP	46.955
TQYVYK;KANMPT	NCP	25.351
KANMPT;MLSYK	NCP	108.705
MLSYK;YHNLVE	NCP	25.4
YHNLVE;STELLLR	NCP	47.875
STELLLR;EHNVTTF	NCP	26.676
EHNVTTF;KAFVGP	NCP	1.889
KAFVGP;KYVGSP	NCP	46.615
KYVGSP;PALPETK	NCP	27.282
PALPETK;GPRLLEY	NCP	11.066
GPRLLEY;KPTMHR	NCP	28
KPTMHR;SVDFVLY	NCP	28.46
SVDFVLY;SPGSPP	NCP	11.841
SPGSPP;FGTPGLG	NCP	102.1
FGTPGLG;KPTLFN	NCP	41.876
KPTLFN;ALDRLGF	NCP	62.406
ALDRLGF;AEKLLR	NCP	63.793
AEKLLR;GKVLQY	NCP	26.015
GKVLQY;PSPVVPP	NCP	33.319
PSPVVPP;LSALAAF	NCP	13.712
LSALAAF;SLCSSPLLT	NCP	5.874

SLCSSPLLT;RLSMLPH	NCP	56.397
RLSMLPH;KPATTVP	NCP	160.063
KPATTVP;MEAEPK	NCP	33.053
MEAEPK;KVFVVF	NCP	4.612
KVFVVF;GVDYPL	NCP	30.107
GVDYPL;LATALQK	NCP	12.297
LATALQK;LLLAAK	NCP	12.517
LLLAAK;CCKDYAK	NCP	15.965
CCKDYAK;LMLLLN	NCP	48.579
LMLLLN;VLPVEP	NCP	3.159
VLPVEP;RALVLLAK	NCP	19.387
RALVLLAK;NLAHRY	NCP	14.987
NLAHRY;CAVVED	NCP	28.146
CAVVED;LPPLRPKP	NCP	34.192
LPPLRPKP;KAALTAL	NCP	108.099
KAALTAL;FNGRMH	NCP	29.256
FNGRMH;LLEAPVE	NCP	68.412
LLEAPVE;YGDNYLR	NCP	129.161
YGDNYLR;LEAVDP	NCP	90.856
LEAVDP;KPHSPP	NCP	56.579
KPHSPP;ELYKKLR	NCP	5.518
ELYKKLR;KARKLR	NCP	116.858
KARKLR;VTAALMLL	NCP	15.52
VTAALMLL;KDKVFPGK	NCP	20.264
KDKVFPGK;QNSFLVL	NCP	5.001
QNSFLVL;AAVTKTH	NCP	1.069
AAVTKTH;KMLSDF	NCP	11.493
KMLSDF;EYVSQL	NCP	27.462
EYVSQL;VPPERK	NCP	5.514
VPPERK;KGLLGLL	NCP	1.908
KGLLGLL;LAGLDAR	NCP	20.934
LAGLDAR;YVVAGP	NCP	72.686
YVVAGP;PTVPPP	NCP	45.693
PTVPPP;KVGQP	NCP	18.139
KVGQP;QGKEYPGP	NCP	7.77
QGKEYPGP;EPPLLVD	NCP	25.124
EPPLLVD;VLNAVVT	NCP	9.943
VLNAVVT;VPPHVKP	NCP	38.364
VPPHVKP;PEPLKTP	NCP	37.55
PEPLKTP;RPPVFK	NCP	218.551
RPPVFK;YTFHPP	NCP	29.355
YTFHPP;LGDKVEF	NCP	17.234
LGDKVEF;ANPLPL	NCP	14.646
ANPLPL;FNNTKK	NCP	67.363
FNNTKK;PVHKLRP	NCP	43.145
PVHKLRP;ESAHVPR	NCP	60.151
ESAHVPR;NAVLTAH	NCP	11.244
NAVLTAH;TMFLTM	NCP	33.563
TMFLTM;APGVHPK	NCP	74.887
APGVHPK;EDAAVTK	NCP	1.188
EDAAVTK;NPLGKKP	NCP	7.076

NPLGKKP;LSELDSL	NCP	10.419
LSELDSL;KSWLVP	NCP	8.994
KSWLVP;CCCYCDP	NCP	30.993
CCCYCDP;KDLLYH	NCP	96.973
KDLLYH;NQMVFK	NCP	34.391
NQMVFK;DYGLL	NCP	28.564
DYGLL;LTGALLK	NCP	39.864
LTGALLK;GPAPKPDP	NCP	35.682
GPAPKPDP;AARDLKEK	NCP	37.892
AARDLKEK;TPVKFPP	NCP	2.698
TPVKFPP;NMSLEKK	NCP	23.894
NMSLEKK;KHGTSSK	NCP	6.934
KHGTSSK;PFVEDL	NCP	107.382
PFVEDL;VGGGGLLS	NCP	68.711
VGGGGLLS;SVGSPP	NCP	0.141
SVGSPP;LGPAFSK	NCP	5.887
LGPAFSK;FVGMPF	NCP	51.99
FVGMPF;SSVPTVK	NCP	46.547
SSVPTVK;SADRMHL	NCP	13.081
SADRMHL;EFLFD	NCP	17.336
EFLFD;AAQALAL	NCP	59.119
AAQALAL;DSEAV	NCP	30.651
DSEAV;MAVVKK	NCP	9.201
MAVVKK;KDRALTH	NCP	25.848
KDRALTH;KPSYCCH	NCP	15.741
KPSYCCH;TELLSLPY	NCP	8.826
TELLSLPY;GTVPSP	NCP	32.237
GTVPSP;KPPKPKL	NCP	88.534
KPPKPKL;KVEKEF	NCP	47.667
KVEKEF;SYCRVP	NCP	95.751
SYCRVP;DRTPLV	NCP	23.049
DRTPLV;AVRSDSVH	NCP	16.667
AVRSDSVH;KNVTPFE	NCP	3.6
KNVTPFE;KFVAPP	NCP	61.672
KFVAPP;DTPKPL	NCP	50.207
DTPKPL;VGPAREK	NCP	15.757
VGPAREK;SLDGNLH	NCP	88.65
SLDGNLH;EYPKAGK	NCP	124.133
EYPKAGK;KLQNKP	NCP	40.216
KLQNKP;FLDEER	NCP	29.147
FLDEER;ASVGGQPK	NCP	6.804
ASVGGQPK;LDVAGFK	NCP	21.592
LDVAGFK;LQAVLSL	NCP	52.326
LQAVLSL;PPGMGLPP	NCP	69.83
PPGMGLPP;RRVLLFK	NCP	38.85
RRVLLFK;LLFPLE	NCP	84.465
LLFPLE;NTTSAVTVK	NCP	9.691
NTTSAVTVK;RLKELKDQ	NCP	0.039
RLKELKDQ;TGLRLGE	NCP	20.088
TGLRLGE;TAYLVF	NCP	6.885
TAYLVF;RCRASLLR	NCP	3.71

RCRASLLR;LVAMGSL	NCP	66.691
LVAMGSL;KKHMPK	NCP	28.443
KKHMPK;EVYVYK	NCP	57.47
EVYVYK;KLVDEKF	NCP	18.678
KLVDEKF;APLDRVP	NCP	83.577
APLDRVP;MYVLK	NCP	14.521
MYVLK;NGPSWL	NCP	60.881
NGPSWL;HHALHY	NCP	5.535
HHALHY;SHSLTVM	NCP	15.819
SHSLTVM;PTVKVSR	NCP	9.972
PTVKVSR;VTLAKHF	NCP	11.058
VTLAKHF;KPVVMK	NCP	39.685
KPVVMK;ASAPNK	NCP	20.849
ASAPNK;TDLDLRK	NCP	73.35
TDLDLRK;KVHLMK	NCP	5.07
KVHLMK;KAERMMH	NCP	79.288
KAERMMH;LALPPR	NCP	15.194
LALPPR;TRVLELTTKKT	NCP	61.173
TRVLELTTKKT;FDQRVSSY	NCP	43.263
FDQRVSSY;EHFLTLL	NCP	21.991
EHFLTLL;KQEHPRP	NCP	118.748
KQEHPRP;VPKVVHG	NCP	47.209
VPKVVHG;LSGVGGEK	NCP	25.317
LSGVGGEK;AAEANLALR	NCP	32.904
AAEANLALR;AVFDT	NCP	37.929
AVFDT;YFPDSGF	NCP	62.199
YFPDSGF;QGPLSGR	NCP	43.929
QGPLSGR;LVEEELDRAQERL	NCP	133.821
LVEEELDRAQERL;LEEAEKAADESERGMK	NCP	0.057
LEEAEKAADESERGMK;RFSALQY	NCP	1.104
RFSALQY;MNCCLK	NCP	15.695
MNCCLK;GVMLKLT	NCP	21.511
GVMLKLT;VPHKMV	NCP	2.991
VPHKMV;LPTLLR	NCP	7.872
LPTLLR;FGVGFY	NCP	36.303
FGVGFY;KESDHK	NCP	17.678
KESDHK;LVVPA	NCP	4.344
LVVPA;TTMVKM	NCP	9.829
TTMVKM;WAAVP	NCP	8.141
WAAVP;RFEALGK	NCP	28.549
RFEALGK;CFFKPVL	NCP	5.499
CFFKPVL;MDRECC	NCP	5.657
MDRECC;ELSAELY	NCP	21.055
ELSAELY;MGGKSH	NCP	32.228
MGGKSH;NGSLPLH	NCP	54.054
NGSLPLH;SDMEDE	NCP	49.563
SDMEDE;VMLSLGPQ	NCP	26.031
VMLSLGPQ;TVLDELR	NCP	6.207
TVLDELR;PVGHKAK	NCP	9.261
PVGHKAK;LDCPSLL	NCP	16.987
LDCPSLL;FYLKKL	NCP	118.811

FYLKKL;VGAAVL	NCP	59.148
VGAAVL;LQPPVL	NCP	66.556
LQPPVL;EALSPL	NCP	18.891
EALSPL;AKYLPH	NCP	41.292
AKYLPH;GLLVDYK	NCP	28.077
GLLVDYK;KNQKTK	NCP	66.174
KNQKTK;KDVSR	NCP	126.063
KDVSR;LLQYE	NCP	65.904
LLQYE;HALLPAAR	NCP	0.104
HALLPAAR;KNQSLAR	NCP	86.71
KNQSLAR;WATLLAH	NCP	15.342
WATLLAH;KLKSPAK	NCP	8.069
KLKSPAK;CNYMPLL	NCP	31.917
CNYMPLL;MELVE	NCP	72.57
MELVE;LEVATY	NCP	45.39
LEVATY;QAACKKR	NCP	7.066
QAACKKR;EDELLVK	NCP	8.514
EDELLVK;YSTFGE	NCP	60.893
YSTFGE;QRAAPAK	NCP	146.017
QRAAPAK;NLATLKT	NCP	60.818
NLATLKT;MLDLL	NCP	19.552
MLDLL;RPHQEK	NCP	2.673
RPHQEK;MATVLLPR	NCP	6.944
MATVLLPR;RDLKEKL	NCP	10.245
RDLKEKL;QTWLGGS	NCP	25.981
QTWLGGS;KDPMHQ	NCP	31.74
KDPMHQ;VQLYY	NCP	4.224
VQLYY;FSPTTTK	NCP	16.047
FSPTTTK;WAGKVEA	NCP	17.549
WAGKVEA;KTPHGPK	NCP	36.316
KTPHGPK;KLEGLKFQ	NCP	3.858
KLEGLKFQ;KDWQTK	NCP	49.365
KDWQTK;GDDDL	NCP	29.499
GDDDL;TTAFPAST	NCP	74.573
TTAFPAST;KLQDKDK	NCP	5.88
KLQDKDK;YLDRSGF	NCP	80.737
YLDRSGF;NSASKPK	NCP	71.981
NSASKPK;LPGLSQ	NCP	127.24
LPGLSQ;RTAVQK	NCP	46.521
RTAVQK;KEVTLPK	NCP	45.179
KEVTLPK;PKLGLFY	NCP	39.981
PKLGLFY;LVAVY	NCP	83.016
LVAVY;LDDLKP	NCP	99.067
LDDLKP;KYLDLR	NCP	19.62
KYLDLR;KPFVEP	NCP	216.015
KPFVEP;KQGVLQF	NCP	4.5
KQGVLQF;HNEYGN	NCP	2.474
HNEYGN;DMYEMM	NCP	8.137
DMYEMM;KVGLEPV	NCP	74.919
KVGLEPV;WGQGY	NCP	25.166
WGQGY;CTQGELK	NCP	33.244

CTQGELK;VYLKV	NCP	9.389
VYLKV;PAAKNF	NCP	42.804
PAAKNF;NVGFRYK	NCP	15.309
NVGFRYK;KVDPLVSL	NCP	19.219
KVDPLVSL;VSLVPVKD	NCP	8.685
VSLVPVKD;KGVWAAR	NCP	32.564
KGVWAAR;LVFKELEK	NCP	58.552
LVFKELEK;ANELEF	NCP	169.91
ANELEF;HLLEAK	NCP	25.903
HLLEAK;LGPHHKQ	NCP	70.733
LGPHHKQ;VSTRLLT	NCP	2.736
VSTRLLT;ELGNYG	NCP	7.446
ELGNYG;KKNFPGP	NCP	5.91
KKNFPGP;KLFDF	NCP	32.191
KLFDF;WAAVVPK	NCP	27.311
WAAVVPK;VFKLAP	NCP	26.001
VFKLAP;LPRLLF	NCP	15.798
LPRLLF;EVPQHLNK	NCP	4.791
EVPQHLNK;KVGDYL	NCP	20.974
KVGDYL;EPEPTLL	NCP	91.913
EPEPTLL;PVVVPSRR	NCP	43.248
PVVVPSRR;HFVVK	NCP	9.28
HFVVK;KDGKVPK	NCP	23.094
KDGKVPK;KDYVYK	NCP	9.618
KDYVYK;TTTVHEK	NCP	29.399
TTTVHEK;TQPVEL	NCP	7.419
TQPVEL;VYKVNY	NCP	78.319
VYKVNY;MLPTLLVK	NCP	28.856
MLPTLLVK;KRHTSSK	NCP	147.651
KRHTSSK;QLAPVPF	NCP	80.554
QLAPVPF;QFPFPK	NCP	37.425
QFPFPK;TKFGGQR	NCP	134.97
TKFGGQR;MCKMFP	NCP	127.35
MCKMFP;MEMTVP	NCP	73.682
MEMTVP;RTPVLTL	NCP	12.763
RTPVLTL;DMALLR	NCP	0.71
DMALLR;KSVMLGP	NCP	33.861
KSVMLGP;VTHSLLV	NCP	34.809
VTHSLLV;LSPVARK	NCP	74.164
LSPVARK;GKNLPEN	NCP	114.977
GKNLPEN;TYGVSFF	NCP	17.214
TYGVSFF;KLVPRLLG	NCP	14.367
KLVPRLLG;VDEKTKEVLQ	NCP	0.048
VDEKTKEVLQ;RWAASPK	NCP	0.054
RWAASPK;EPELLGFN	NCP	4.713
EPELLGFN;KYPLT	NCP	62.352
KYPLT;YPHGV	NCP	8.401
YPHGV;SVVSPP	NCP	47.387
SVVSPP;RMELVH	NCP	40.162
RMELVH;KFTKTK	NCP	27.504
KFTKTK;NFALNVP	NCP	5.045

NFALNVP;ATTKKFK	NCP	4.435
ATTKKFK;LKTVETR	NCP	73.398
LKTVETR;LGGVLLF	NCP	8.139
LGGVLLF;KEDDLRK	NCP	44.493
KEDDLRK;KLKLVSHK	NCP	11.703
KLKLVSHK;RNCARL	NCP	10.136
RNCARL;MGVFLQQ	NCP	39.099
MGVFLQQ;KMQMLK	NCP	16.83
KMQMLK;KAEDSLLAADEAAAK	NCP	0.927
KAEDSLLAADEAAAK;CAELEEELK	NCP	18.297
CAELEEELK;EEELKTVTNNLK	NCP	0.012
EEELKTVTNNLK;AEFAERSVTK	NCP	0.888
AEFAERSVTK;HAKEENLSMH	NCP	8.574
HAKEENLSMH;AKEENLSMHQ	NCP	0.003
AKEENLSMHQ;MHQMLDQTLLELNNM	NCP	0.021
MHQMLDQTLLELNNM;AFVQRT	NCP	11.809
AFVQRT;TVGDLLK	NCP	76.292
TVGDLLK;FDLYPP	NCP	17.724
FDLYPP;QQAVYK	NCP	76.564
QQAVYK;FFANRP	NCP	19.67
FFANRP;NEGSPFL	NCP	31.12
NEGSPFL;PSPLLAGK	NCP	68.405
PSPLLAGK;KPPAFAK	NCP	135.648
KPPAFAK;SHALALF	NCP	45.945
SHALALF;VVAPK	NCP	59.736
VVAPK;MDDLFLP	NCP	120.757
MDDLFLP;HLKVPQ	NCP	48.519
HLKVPQ;PTVAHVP	NCP	79.31
PTVAHVP;NELATLL	NCP	5.269
NELATLL;ALGGF	NCP	55.517
ALGGF;KVWSHH	NCP	7.456
KVWSHH;TGPELKY	NCP	6.125
TGPELKY;TQWEK	NCP	4.854
TQWEK;KGFDGDR	NCP	36.414
KGFDGDR;MEDLNGK	NCP	0.93
MEDLNGK;KRFATLK	NCP	49.792
KRFATLK;ALDYSN	NCP	28.626
ALDYSN;VGAGSFL	NCP	8.706
VGAGSFL;KAFASPR	NCP	12.21
KAFASPR;EGGPKSH	NCP	46.331
EGGPKSH;LLSAAGT	NCP	132.853
LLSAAGT;PSFPWTK	NCP	10.317
PSFPWTK;TSSHRAAP	NCP	18.462
TSSHRAAP;LSAAPLVM	NCP	41.015
LSAAPLVM;LPAGGGKN	NCP	51.021
LPAGGGKN;MVPLLEL	NCP	80.859
MVPLLEL;KMKVLR	NCP	57.996
KMKVLR;EGLKKKMQ	NCP	4.384
EGLKKKMQ;SSFGWQ	NCP	1.743
SSFGWQ;KLNSGLK	NCP	35
KLNSGLK;RFRCTEP	NCP	7.587

RFRCTEP;KTVVP	NCP	117.735
KTVVP;HPKTQ	NCP	1.476
HPKTQ;PLSSSPVFP	NCP	1.872
PLSSSPVFP;KPAEGV	NCP	30.69
KPAEGV;QRVPPPP	NCP	0.673
QRVPPPP;EPTFYK	NCP	337.946
EPTFYK;VAAHEVR	NCP	33.186
VAAHEVR;GLPKAGK	NCP	11.488
GLPKAGK;SGPETESSPLDPHTKER	NCP	48.086
SGPETESSPLDPHTKER;LLVGHTPVHF	NCP	13.308
LLVGHTPVHF;TDVDLHAY	NCP	0.03
TDVDLHAY;ALEKPGWENGF	NCP	0.135
ALEKPGWENGF;LNVDRVFAL	NCP	3.117
LNVDRVFAL;SDEEKVLRF	NCP	0.03
SDEEKVLRF;VSFTVP	NCP	59.689
VSFTVP;EMHCEC	NCP	17.388
EMHCEC;VVLFPF	NCP	10.503
VVLFPF;SLHTLYVW	NCP	69.293
SLHTLYVW;LFNSLK	NCP	49.075
LFNSLK;PDFVKS	NCP	57.563
PDFVKS;KMQVLYR	NCP	54.015
KMQVLYR;QEKLTSK	NCP	33.504
QEKLTSK;DPSVKAK	NCP	50.166
DPSVKAK;MTGLKKK	NCP	150.882
MTGLKKK;LPVGPDP	NCP	87.024
LPVGPDP;AQKYLLR	NCP	7.047
AQKYLLR;LGLWYK	NCP	152.947
LGLWYK;EEEGCKC	NCP	21.339
EEEGCKC;QMDSQK	NCP	0.783
QMDSQK;VRNCML	NCP	134.15
VRNCML;TATTEMK	NCP	16.494
TATTEMK;YLLPKM	NCP	3.702
YLLPKM;GYKLL	NCP	74.284
GYKLL;TQAFK	NCP	1.332
TQAFK;AKPLTDQEKRRQ	NCP	43.568
AKPLTDQEKRRQ;PSPSVLK	NCP	27.531
PSPSVLK;SQFLEKEY	NCP	5.076
SQFLEKEY;SPKQPDL	NCP	4.338
SPKQPDL;VALAPPPR	NCP	60.405
VALAPPPR;KTAVPGR	NCP	10.071
KTAVPGR;TCQLLLH	NCP	40.747
TCQLLLH;ASLDKF	NCP	1.211
ASLDKF;KFLLPK	NCP	21.87
KFLLPK;TVPGWVP	NCP	0.963
TVPGWVP;PVALPPPL	NCP	10.867
PVALPPPL;GAVAVHPP	NCP	17.118
GAVAVHPP;KDRTLNK	NCP	3.699
KDRTLNK;KSEPLLH	NCP	77.51
KSEPLLH;ATSGYG	NCP	11.245
ATSGYG;KHNARP	NCP	4.781
KHNARP;PVFPQR	NCP	5.749

PVFPQR;RVALALT	NCP	30.485
RVALALT;GVLKAVD	NCP	40.435
GVLKAVD;TGGLLL	NCP	9.615
TGGLLL;FALLMFLK	NCP	22.694
FALLMFLK;KPAVVNK	NCP	49.147
KPAVVNK;NPNGF	NCP	1.326
NPNGF;CCPPPP	NCP	17.606
CCPPPP;CCCDYS	NCP	26.641
CCCDYS;TLDWVAF	NCP	26.282
TLDWVAF;KLLQF	NCP	36.928
KLLQF;LPSFY	NCP	9.686
LPSFY;LSKVVEF	NCP	17.745
LSKVVEF;GKFNLL	NCP	126.006
GKFNLL;KPHYGPK	NCP	114.597
KPHYGPK;MHVKHR	NCP	61.083
MHVKHR;VLLTKKP	NCP	15.004
VLLTKKP;LRVPPLTPQ	NCP	40.64
LRVPPLTPQ;HLVALLH	NCP	9.964
HLVALLH;LVEKVLR	NCP	33.029
LVEKVLR;LAKAQ	NCP	17.734
LAKAQ;AMRAQR	NCP	21.606
AMRAQR;GFKTAF	NCP	44.507
GFKTAF;LVRTLF	NCP	30.964
LVRTLF;KSGSKAP	NCP	16.664
KSGSKAP;VNVPKTR	NCP	14.58
VNVPKTR;LECVEPNCRSK	NCP	0.204
LECVEPNCRSK;KYVPPP	NCP	1.845
KYVPPP;KGELFR	NCP	10.155
KGELFR;LYEYSGQ	NCP	1.201
LYEYSGQ;SYCRPV	NCP	2.736
SYCRPV;AKALVK	NCP	101.532
AKALVK;AEELGLK	NCP	30.224
AEELGLK;KNYRPP	NCP	19.965
KNYRPP;KAKPL	NCP	35.386
KAKPL;MGVTTK	NCP	7.94
MGVTTK;KEYLP	NCP	8.814
KEYLP;TVYYLT	NCP	33.808
TVYYLT;VGPGY	NCP	0.95
VGPGY;LNDTEM	NCP	20.262
LNDTEM;PLLRLLK	NCP	33.06
PLLRLLK;SGDLFGF	NCP	3.654
SGDLFGF;KTNETSK	NCP	6.613
KTNETSK;KPEGEP	NCP	118.647
KPEGEP;MSVFHKK	NCP	34.569
MSVFHKK;LFLGGLS	NCP	3.009
LFLGGLS;VFLTAAH	NCP	148.892
VFLTAAH;SPTVKY	NCP	29.271
SPTVKY;YCMSH	NCP	47.991
YCMSH;ATNHLK	NCP	183.123
ATNHLK;KQLFMP	NCP	0.078
KQLFMP;AVPFLVVLL	NCP	24.813

AVPFLVVLL;PANSSKK	NCP	19.429
PANSSKK;LSGPPEK	NCP	101.25
LSGPPEK;LTVLEEL	NCP	40.281
LTVLEEL;NVLVVLV	NCP	276.123
NVLVVLV;LVYDVL	NCP	89.759
LVYDVL;FSHKLT	NCP	28.213
FSHKLT;FDAGLL	NCP	82.781
FDAGLL;WNAAPLL	NCP	60.033
WNAAPLL;LEVLSQK	NCP	12.319
LEVLSQK;GAPGFFL	NCP	142.911
GAPGFFL;DPYLVRT	NCP	19.583
DPYLVRT;HEEPTLL	NCP	61.387
HEEPTLL;VPPPLP	NCP	125.61
VPPPLP;ETPKHEQEK	NCP	42.606
ETPKHEQEK;KPLYPPP	NCP	75.81
KPLYPPP;EDVPSE	NCP	0.359
EDVPSE;ASVNKYK	NCP	93.625
ASVNKYK;KDVKFQ	NCP	63.921
KDVKFQ;VNSCDSF	NCP	14.048
VNSCDSF;DYSPLP	NCP	26.319
DYSPLP;GPKMHTR	NCP	12.042
GPKMHTR;WSLHSTM	NCP	70.875
WSLHSTM;KVLLAPD	NCP	13.696
KVLLAPD;VQEFKK	NCP	41.298
VQEFKK;DSFSTPF	NCP	15.083
DSFSTPF;TLQYYLT	NCP	7.368
TLQYYLT;KMTAMVP	NCP	14.911
KMTAMVP;RLNLVTEK	NCP	42.893
RLNLVTEK;LFRPDN	NCP	74.937
LFRPDN;KQDLDLR	NCP	24.538
KQDLDLR;KGMDFH	NCP	58.497
KGMDFH;KYLNPLP	NCP	40.254
KYLNPLP;QAVLSYL	NCP	64.202
QAVLSYL;KGDKLR	NCP	36.279
KGDKLR;MELPV	NCP	55.831
MELPV;WGFTKF	NCP	108.182
WGFTKF;VPYFS	NCP	18.999
VPYFS;DPPKF	NCP	20.374
DPPKF;PGGDLVV	NCP	88.146
PGGDLVV;NMDQLQ	NCP	53.223
NMDQLQ;KRCAPR	NCP	44.496
KRCAPR;KHLVDFK	NCP	54.987
KHLVDFK;TVTLNA	NCP	57.711
TVTLNA;KGAGL	NCP	6.935
KGAGL;LPCKKPP	NCP	19.689
LPCKKPP;LDPNPL	NCP	19.927
LDPNPL;KNLTAVT	NCP	23.136
KNLTAVT;TAKNLYL	NCP	7.722
TAKNLYL;LTRLHY	NCP	79.898
LTRLHY;WLPTSN	NCP	13.504
WLPTSN;PLYDAPK	NCP	80.822

PLYDAPK;EAASKPK	NCP	90.922
EAASKPK;AQFLVA	NCP	30.278
AQFLVA;CLRVPVP	NCP	56.169
CLRVPVP;SHLSLV	NCP	23.623
SHLSLV;KLVVFEP	NCP	20.37
KLVVFEP;SLERLLV	NCP	7.46
SLERLLV;LTVFPK	NCP	6.465
LTVFPK;VSKPGLT	NCP	5.527
VSKPGLT;LPYPQYP	NCP	69.057
LPYPQYP;HLLHKAT	NCP	19.635
HLLHKAT;KHWCSP	NCP	31.61
KHWCSP;KDYVPK	NCP	68.424
KDYVPK;FVMMVL	NCP	7.66
FVMMVL;ETGHF	NCP	1.745
ETGHF;TLFLSAN	NCP	93.625
TLFLSAN;EMQRPK	NCP	40.913
EMQRPK;PLLYM	NCP	19.791
PLLYM;MHQSY	NCP	4.585
MHQSY;LARLHS	NCP	28.524
LARLHS;GAPAPPPP	NCP	18.303
GAPAPPPP;RSAPGSGSKVPQKKVK	NCP	9.858
RSAPGSGSKVPQKKVK;DDDDDFDEEVEEKAPVKK	NCP	0.102
DDDDDFDEEVEEKAPVKK;NWAEGK	NCP	24.177
NWAEGK;LYANSYL	NCP	25.532
LYANSYL;PVSELCFR	NCP	1.708
PVSELCFR;VVGMDL	NCP	27.015
VVGMDL;VHEVLYR	NCP	73.112
VHEVLYR;KAQNQLGL	NCP	48.486
KAQNQLGL;NPLVLF	NCP	17.341
NPLVLF;PKHGP	NCP	46.941
PKHGP;GVFLQ	NCP	4.824
GVFLQ;HGVKGLP	NCP	70.878
HGVKGLP;CCCFPHRP	NCP	89.471
CCCFPHRP;RFTATKK	NCP	94.506
RFTATKK;PGNTRPP	NCP	15.052
PGNTRPP;AFSTVVP	NCP	40.739
AFSTVVP;KPYQPP	NCP	45.949
KPYQPP;KLLLPPA	NCP	18.281
KLLLPPA;KNKKGM	NCP	37.578
KNKKGM;LDLTNLL	NCP	12.418
LDLTNLL;SGDFVHRLPL	NCP	72.128
SGDFVHRLPL;DVAKL	NCP	50.118
DVAKL;KAPSPLR	NCP	145.596
KAPSPLR;PSVPEVL	NCP	9.649
PSVPEVL;KTESLGKP	NCP	27.518
KTESLGKP;VLPLEEAY	NCP	15.999
VLPLEEAY;VVDPKKPFRK	NCP	24.612
VVDPKKPFRK;AGDFPKVK	NCP	56.367
AGDFPKVK;GMKAPY	NCP	67.128
GMKAPY;KPAAPGPV	NCP	19.152
KPAAPGPV;KFFPNPP	NCP	44.199

KFFPNPP;NLAKKLA	NCP	79.363
NLAKKLA;PKVPHHK	NCP	2.154
PKVPHHK;QPAPPR	NCP	2.135
QPAPPR;NHRAKK	NCP	88.872
NHRAKK;PFVYPP	NCP	0.999
PFVYPP;KATLRLY	NCP	89.157
KATLRLY;WDLEY	NCP	47.085
WDLEY;PPAAAMH	NCP	16.483
PPAAAMH;PPFFPPP	NCP	4.485
PPFFPPP;LHTDRP	NCP	63.942
LHTDRP;PGKQAWT	NCP	24.198
PGKQAWT;TAKKPQH	NCP	0.959
TAKKPQH;EPTLKDF	NCP	28.474
EPTLKDF;DTVPTPK	NCP	53.006
DTVPTPK;EFLAGT	NCP	24.409
EFLAGT;GPVRPPTP	NCP	57.174
GPVRPPTP;PMAYQL	NCP	26.337
PMAYQL;QRYFPK	NCP	50.922
QRYFPK;LPGGVP	NCP	20.531
LPGGVP;LEKQPY	NCP	13.257
LEKQPY;KHDLNQK	NCP	6.715
KHDLNQK;MLPTAF	NCP	52.953
MLPTAF;LVMGGGKR	NCP	63.021
LVMGGGKR;VDVML	NCP	9.492
VDVML;RGGPLSF	NCP	29.858
RGGPLSF;HYASSPR	NCP	72.985
HYASSPR;KTTPVR	NCP	1.64
KTTPVR;KDFPPK	NCP	16.095
KDFPPK;FCCFGPF	NCP	70.426
FCCFGPF;CCQDDP	NCP	31.874
CCQDDP;EVADWL	NCP	14.131
EVADWL;KELDDG	NCP	7.475
KELDDG;EPAPPRP	NCP	51.543
EPAPPRP;PEHAP	NCP	1.794
PEHAP;HSRSH	NCP	66.855
HSRSH;VPGVPGSP	NCP	1.317
VPGVPGSP;ALGGFWL	NCP	43.242
ALGGFWL;KDLLEPF	NCP	73.567
KDLLEPF;ALAAKVS	NCP	17.585
ALAAKVS;PPPDPPPP	NCP	122.346
PPPDPPPP;YHLSPPP	NCP	39.643
YHLSPPP;KNFFPGGP	NCP	15.737
KNFFPGGP;KSGDVAK	NCP	60.798
KSGDVAK;CADPLR	NCP	18.444
CADPLR;KPPAGAPP	NCP	10.575
KPPAGAPP;LDKDRL	NCP	91.692
LDKDRL;QGDPLEY	NCP	52.447
QGDPLEY;ESKTKSK	NCP	3.992
ESKTKSK;DCDSGY	NCP	37.588
DCDSGY;LLLENY	NCP	111.972
LLLENY;MDMPCC	NCP	18.366

MDMPCC;KPDVLN	NCP	37.815
KPDVLN;MRLVQF	NCP	18.12
MRLVQF;KNSGLVR	NCP	51.045
KNSGLVR;EAGMAK	NCP	4.805
EAGMAK;HQVHRK	NCP	16.939
HQVHRK;HTGGKLLG	NCP	41.214
HTGGKLLG;VLLTTF	NCP	6.92
VLLTTF;LSPLMFP	NCP	44.235
LSPLMFP;NRSHVLN	NCP	18.376
NRSHVLN;GHQQLYW	NCP	15.516
GHQQLYW;LARALPF	NCP	43.449
LARALPF;KHMELR	NCP	21.633
KHMELR;KPAVP	NCP	7.671
KPAVP;VVLKEPK	NCP	16.502
VVLKEPK;VTLAED	NCP	37.234
VTLAED;FSPYLLR	NCP	175.244
FSPYLLR;KNKMVP	NCP	82.459
KNKMVP;KSHAK	NCP	24.434
KSHAK;EARLLR	NCP	47.475
EARLLR;EDAAMGK	NCP	90.246
EDAAMGK;TEDLRDK	NCP	48.592
TEDLRDK;YVLTKK	NCP	53.576
YVLTKK;QVTWL	NCP	28.425
QVTWL;KLTTSTK	NCP	20.649
KLTTSTK;APATLHVG	NCP	16.299
APATLHVG;EVFMYA	NCP	18.291
EVFMYA;SLAALKK	NCP	77.626
SLAALKK;TDPEATPK	NCP	70.767
TDPEATPK;PFSLL	NCP	0.56
PFSLL;GHAAHSN	NCP	15.33
GHAAHSN;PAEKD	NCP	105.849
PAEKD;ECCFQ	NCP	34.018
ECCFQ;TLEALRY	NCP	27.587
TLEALRY;KNTHTLLL	NCP	7.713
KNTHTLLL;LSLTAVPQ	NCP	47.79
LSLTAVPQ;TKFRPM	NCP	39.178
TKFRPM;KERLT	NCP	90.279
KERLT;KLSYL	NCP	84.756
KLSYL;YGVTGP	NCP	49.448
YGVTGP;TPSKAPK	NCP	7.113
TPSKAPK;DNPAP	NCP	43.951
DNPAP;VDLVL	NCP	105.816
VDLVL;PAKPSVK	NCP	7.407
PAKPSVK;LVQRVVL	NCP	21.135
LVQRVVL;PEVAADP	NCP	16.932
PEVAADP;KPEAPPN	NCP	114.066
KPEAPPN;LEGLLN	NCP	33.965
LEGLLN;PLVTKTK	NCP	344.953
PLVTKTK;LEEELKR	NCP	21.338
LEEELKR;LSSTFQQ	NCP	25.068
LSSTFQQ;LLEEKLK	NCP	41.971

LLEEKLK;KPKKYSK	NCP	16.037
KPKKYSK;KDPAPPP	NCP	53.038
KDPAPPP;VASKKAAP	NCP	39
VASKKAAP;KVGDLEK	NCP	21.98
KVGDLEK;KDGPLK	NCP	37.771
KDGPLK;DLKEHLH	NCP	17.375
DLKEHLH;LDKDNLR	NCP	11.535
LDKDNLR;RLLLSD	NCP	23.491
RLLLSD;KPGLAY	NCP	55.271
KPGLAY;SYLGKP	NCP	6.87
SYLGKP;EVLTGL	NCP	122.064
EVLTGL;MHGTLR	NCP	42.403
MHGTLR;QALLNKVV	NCP	12.918
QALLNKVV;QGPLGLVSL	NCP	1.734
QGPLGLVSL;LVQLGSLF	NCP	46.869
LVQLGSLF;TRSLFVN	NCP	29.619
TRSLFVN;VEKGPKV	NCP	17.135
VEKGPKV;PPNQEEC	NCP	17.742
PPNQEEC;GPVKVG	NCP	45.829
GPVKVG;TGKGKLLK	NCP	31.262
TGKGKLLK;RTSLFVN	NCP	23.389
RTSLFVN;LMLVGK	NCP	7.776
LMLVGK;LLMGM	NCP	39.647
LLMGM;KLAEDR	NCP	8.581
KLAEDR;TNFVL	NCP	118.236
TNFVL;KGMVVR	NCP	44.133
KGMVVR;KGLALVR	NCP	2.13
KGLALVR;KRYWSK	NCP	35.528
KRYWSK;VGALLRGP	NCP	17.335
VGALLRGP;LVAPPG	NCP	16.589
LVAPPG;YVKPEM	NCP	25.02
YVKPEM;PLFSPR	NCP	160.764
PLFSPR;TARMGK	NCP	65.353
TARMGK;KSRGLLTL	NCP	53.103
KSRGLLTL;NLEFVAR	NCP	65.382
NLEFVAR;ACLFLQ	NCP	12.54
ACLFLQ;EVFTGHL	NCP	15.506
EVFTGHL;TSPFLPR	NCP	28.692
TSPFLPR;GALGQRK	NCP	13.014
GALGQRK;ESTLVLK	NCP	21.861
ESTLVLK;GPLGHLKP	NCP	0.634
GPLGHLKP;AAQGEPQVQFKL	NCP	2.07
AAQGEPQVQFKL;DGFHFK	NCP	15.944
DGFHFK;PPLPP	NCP	281.298
PPLPP;HWRSPPP	NCP	189.123
HWRSPPP;KNLVFST	NCP	70.249
KNLVFST;LPTLAPD	NCP	7.638
LPTLAPD;AASLVFPR	NCP	2.289
AASLVFPR;TCKLVQT	NCP	112.763
TCKLVQT;ECMTAK	NCP	27.699
ECMTAK;LGPVG	NCP	128.227

LGPVG;YCADY	NCP	37.586
YCADY;PVEGVHK	NCP	173.088
PVEGVHK;FLVGVA	NCP	65.359
FLVGVA;TLENLKK	NCP	8.534
TLENLKK;AALYLTKP	NCP	8.038
AALYLTKP;DLDQLRK	NCP	5.945
DLDQLRK;DDEETTALVCDNGSGLVK	NCP	14.412
DDEETTALVCDNGSGLVK;WDDMEK	NCP	1.365
WDDMEK;QPLGKHL	NCP	0.579
QPLGKHL;DLAGRDL	NCP	0.492
DLAGRDL;RFRCPET	NCP	0.351
RFRCPET;VTMELLK	NCP	14.497
VTMELLK;LRLRYL	NCP	17.213
LRLRYL;AEEFPV	NCP	52.882
AEEFPV;KDLGK	NCP	16.826
KDLGK;LSPDSVPV	NCP	41.542
LSPDSVPV;LKEPLTV	NCP	97.076
LKEPLTV;KFLEPQ	NCP	30.982
KFLEPQ;TVLKFLLK	NCP	7.491
TVLKFLLK;HDLGHF	NCP	4.655
HDLGHF;HEPFLH	NCP	15.448
HEPFLH;TLHFRFL	NCP	12.749
TLHFRFL;AQLSLVY	NCP	50.848
AQLSLVY;LMLKPPP	NCP	40.596
LMLKPPP;MVSLAPFP	NCP	23.796
MVSLAPFP;LSVLF	NCP	34.449
LSVLF;KKMATK	NCP	10.815
KKMATK;LSYEPN	NCP	29.99
LSYEPN;RLPSHVTP	NCP	0.381
RLPSHVTP;VPLQVSH	NCP	0.439
VPLQVSH;KSFMVPR	NCP	20.021
KSFMVPR;RLLGPLF	NCP	4.489
RLLGPLF;QSPPAATL	NCP	17.69
QSPPAATL;KSKALLY	NCP	0.558
KSKALLY;LLPRSKV	NCP	20.488
LLPRSKV;ELVAEAL	NCP	8.127
ELVAEAL;APEYNK	NCP	86.247
APEYNK;MMYLLSH	NCP	3.042
MMYLLSH;NPPGVVL	NCP	55.139
NPPGVVL;KEVFVK	NCP	10.056
KEVFVK;TSFNTPA	NCP	40.741
TSFNTPA;DGNVGF	NCP	82.655
DGNVGF;NRLPPPP	NCP	34.234
NRLPPPP;LLNTFY	NCP	21.563
LLNTFY;KNSSL	NCP	73.15
KNSSL;KLLLPR	NCP	57.104
KLLLPR;AYSDF	NCP	2.385
AYSDF;FDRGPPL	NCP	64.137
FDRGPPL;KEFVTVVR	NCP	14.658
KEFVTVVR;LEFTPTN	NCP	93.121
LEFTPTN;CYPSLP	NCP	17.897

CYPSLP;LPPWL	NCP	14.439
LPPWL;GGLSFRK	NCP	5.757
GGLSFRK;SEEFYQK	NCP	40.776
SEEFYQK;KMKVPGK	NCP	168.049
KMKVPGK;MLSVAA	NCP	33.611
MLSVAA;LSLDVKN	NCP	44.355
LSLDVKN;GLPPSSVT	NCP	78.61
GLPPSSVT;MANVLFP	NCP	17.58
MANVLFP;VFELL	NCP	8.849
VFELL;KYREN	NCP	18.231
KYREN;ECCSSD	NCP	21.637
ECCSSD;LSAAAGLK	NCP	60.036
LSAAAGLK;RSMPSR	NCP	131.334
RSMPSR;EMLLQR	NCP	1.091
EMLLQR;ERLFSNF	NCP	60.091
ERLFSNF;QTDFFLR	NCP	18.441
QTDFFLR;KAPLKKP	NCP	43.247
KAPLKKP;PVEPTKP	NCP	15.817
PVEPTKP;ERGDFV	NCP	7.419
ERGDFV;DPVTYF	NCP	6.54
DPVTYF;YTLMVK	NCP	59.622
YTLMVK;QTLKPM	NCP	36.869
QTLKPM;TLAAAVK	NCP	54.543
TLAAAVK;KCGMLK	NCP	44.998
KCGMLK;NDPPV	NCP	16.625
NDPPV;LNLVRQ	NCP	6.943
LNLVRQ;ATPLLSK	NCP	27.018
ATPLLSK;PAKPVTT	NCP	108.729
PAKPVTT;KDWFPK	NCP	35.297
KDWFPK;MLLSMY	NCP	44.322
MLLSMY;SRLLQE	NCP	23.923
SRLLQE;MMHLVG	NCP	54.981
MMHLVG;SVGPVQN	NCP	0.042
SVGPVQN;VTFDPH	NCP	171.884
VTFDPH;FHLLF	NCP	0.28
FHLLF;KMPKLR	NCP	101.309
KMPKLR;HSLELST	NCP	0.343
HSLELST;TVKNLKEK	NCP	70.809
TVKNLKEK;FAYVYPFP	NCP	15.999
FAYVYPFP;GVVEL	NCP	95.448
GVVEL;NTLDATP	NCP	14.981
NTLDATP;QGDLKGPK	NCP	16.843
QGDLKGPK;LTLVFMT	NCP	16.269
LTLVFMT;KDRWPKK	NCP	84.683
KDRWPKK;DVGQLPLT	NCP	61.023
DVGQLPLT;PDCCAL	NCP	52.537
PDCCAL;QQLAELR	NCP	28.256
QQLAELR;LTFTPARP	NCP	4.678
LTFTPARP;QDSKLK	NCP	47.261
QDSKLK;LSTDNPK	NCP	24.225
LSTDNPK;MLTVPPPP	NCP	102.673

MLTVPPPP;YDDMPK	NCP	50.805
YDDMPK;PSRSLLL	NCP	92.06
PSRSLLL;WSAPSDKEGY	NCP	8.328
WSAPSDKEGY;LALKFLP	NCP	0.792
LALKFLP;KSEAKPR	NCP	62.224
KSEAKPR;GPGFTAAP	NCP	152.976
GPGFTAAP;KDGKPPP	NCP	46.962
KDGKPPP;GAAVLSL	NCP	10.887
GAAVLSL;SDGLVVK	NCP	47.739
SDGLVVK;LVSAF	NCP	2.67
LVSAF;KHALSPR	NCP	16.076
KHALSPR;LPPHRP	NCP	84.976
LPPHRP;DLFTGHL	NCP	4.758
DLFTGHL;MLLLAPP	NCP	43.616
MLLLAPP;KPPLYK	NCP	12.268
KPPLYK;HLVLKTQ	NCP	2.126
HLVLKTQ;KPAPVH	NCP	40.326
KPAPVH;PGARAVLP	NCP	3.127
PGARAVLP;FASLVHR	NCP	12.872
FASLVHR;LTVLLM	NCP	10.468
LTVLLM;LPPGLSSK	NCP	18.407
LPPGLSSK;MSSLVHR	NCP	26.133
MSSLVHR;GKVPPP	NCP	58.161
GKVPPP;KGWKQLK	NCP	41.238
KGWKQLK;DRPKVGL	NCP	23.956
DRPKVGL;LSAGLDF	NCP	15.186
LSAGLDF;EGQYRPP	NCP	1.277
EGQYRPP;QPSWWL	NCP	14.565
QPSWWL;RLVSVK	NCP	18.637
RLVSVK;VTGGKLR	NCP	34.31
VTGGKLR;KSSLEPMK	NCP	1.855
KSSLEPMK;MEFYPV	NCP	26.676
MEFYPV;QVPPPP	NCP	69.605
QVPPPP;MEKVPPP	NCP	43.83
MEKVPPP;MTHPPY	NCP	21.474
MTHPPY;LPLPGP	NCP	45.594
LPLPGP;QELLDF	NCP	45.876
QELLDF;HGLPPPP	NCP	76.603
HGLPPPP;VVGSVL	NCP	15.798
VVGSVL;ALPYAP	NCP	4.671
ALPYAP;AYAAPA	NCP	68.88
AYAAPA;RYYPK	NCP	23.186
RYYPK;LSQGKLL	NCP	111.855
LSQGKLL;VPAGFL	NCP	40.033
VPAGFL;LLLAGKSF	NCP	3.504
LLLAGKSF;AVKPFR	NCP	165.689
AVKPFR;LTTLWF	NCP	92.236
LTTLWF;AEGKPLH	NCP	13.881
AEGKPLH;FNFFSLP	NCP	143.231
FNFFSLP;HSTASPP	NCP	5.14
HSTASPP;VERVDK	NCP	0.065

VERVDK;LSNGEGP	NCP	17.121
LSNGEGP;FKMAPK	NCP	19.812
FKMAPK;VPNLSSAC	NCP	97.696
VPNLSSAC;LAPGKPN	NCP	8.909
LAPGKPN;YHDELF	NCP	53.158
YHDELF;KTLCYK	NCP	78.639
KTLCYK;PEVNKR	NCP	21.302
PEVNKR;QMWKSK	NCP	5.002
QMWKSK;VYHMYR	NCP	48.908
VYHMYR;LSWLKL	NCP	37.753
LSWLKL;KTPWYQ	NCP	50.426
KTPWYQ;KYTVQE	NCP	91.273
KYTVQE;LVSVTRPL	NCP	1.707
LVSVTRPL;EHKGPQ	NCP	178.403
EHKGPQ;KEFSSFLK	NCP	28.809
KEFSSFLK;PRNGPLQ	NCP	22.21
PRNGPLQ;LGERQSY	NCP	15.758
LGERQSY;PLAKDLL	NCP	10.723
PLAKDLL;KNDTPL	NCP	15.966
KNDTPL;GLMVWKQ	NCP	0.42
GLMVWKQ;LLVVPGP	NCP	40.275
LLVVPGP;LPLPVLE	NCP	131.064
LPLPVLE;RLSGP	NCP	60.989
RLSGP;KAAVLSL	NCP	61.132
KAAVLSL;KFFYHK	NCP	3.428
KFFYHK;LFGRLGR	NCP	36.163
LFGRLGR;TVFPL	NCP	30.036
TVFPL;KLRWYLH	NCP	90.17
KLRWYLH;YVYSVN	NCP	15.706
YVYSVN;KFVHVPP	NCP	54.474
KFVHVPP;RDGKVK	NCP	3.308
RDGKVK;LAPPPPPP	NCP	5.832
LAPPPPPP;AVGSLPF	NCP	68.391
AVGSLPF;QYLLPLL	NCP	68.89
QYLLPLL;GLVEPY	NCP	119.724
GLVEPY;QSGSFKL	NCP	36.588
QSGSFKL;SDLRLSFP	NCP	0.393
SDLRLSFP;LALDDD	NCP	23.997
LALDDD;ESLMALH	NCP	18.198
ESLMALH;VPVQPLC	NCP	25.136
VPVQPLC;SLDPDFK	NCP	28.492
SLDPDFK;ESALLVR	NCP	78.192
ESALLVR;KGDCLK	NCP	14.844
KGDCLK;KAKLLVP	NCP	40.434
KAKLLVP;FGSLPY	NCP	36.426
FGSLPY;GVTALLQ	NCP	8.961
GVTALLQ;LTVDLM	NCP	38.235
LTVDLM;LAPGLALAL	NCP	32.976
LAPGLALAL;SGGHFRP	NCP	56.265
SGGHFRP;DKVGNDK	NCP	1.166
DKVGNDK;EDVGLLP	NCP	49.228

EDVGLLP;LHAPPPPPP	NCP	3.669
LHAPPPPPP;GRPPKAAP	NCP	25.224
GRPPKAAP;RPAHKL	NCP	18.012
RPAHKL;FAPLRST	NCP	3.714
FAPLRST;LDLSAPK	NCP	11.61
LDLSAPK;THSLDLR	NCP	19.169
THSLDLR;LTPFQLTN	NCP	72.855
LTPFQLTN;YGVKP	NCP	16.092
YGVKP;RPGVLLRQ	NCP	2.085
RPGVLLRQ;VNSHEFE	NCP	31.657
VNSHEFE;RKPEPPR	NCP	30.107
RKPEPPR;TPGDCK	NCP	18.178
TPGDCK;EKLGDHT	NCP	41.75
EKLGDHT;SGFGDRK	NCP	15.129
SGFGDRK;WSPPHLK	NCP	17.064
WSPPHLK;HNFHKN	NCP	3.348
HNFHKN;KPLLKNP	NCP	36.609
KPLLKNP;MPLPVAP	NCP	54.64
MPLPVAP;PSGVPSPR	NCP	11.925
PSGVPSPR;GVLLNKVV	NCP	8.334
GVLLNKVV;LVLPVPQ	NCP	9.243
LVLPVPQ;GPVMKVH	NCP	7.358
GPVMKVH;FSCPPK	NCP	23.715
FSCPPK;MEVLAP	NCP	41.178
MEVLAP;KAWLVGP	NCP	25.09
KAWLVGP;KDLPF	NCP	36.899
KDLPF;KDEAAKK	NCP	14.886
KDEAAKK;LGKYGPAN	NCP	1.485
LGKYGPAN;DGLVWGSSK	NCP	0.273
DGLVWGSSK;PLASFVTQ	NCP	4.341
PLASFVTQ;PFHPPLP	NCP	35.806
PFHPPLP;PDRLVN	NCP	27.749
PDRLVN;GDLKFNP	NCP	24.85
GDLKFNP;LLEGEFPQ	NCP	36.602
LLEGEFPQ;HGVVPLAT	NCP	28.716
HGVVPLAT;GVVLH	NCP	11.95
GVVLH;PGCGFPPP	NCP	47.474
PGCGFPPP;LGLEFAQ	NCP	28.825
LGLEFAQ;LFKPVGN	NCP	67.163
LFKPVGN;YLRPPSFL	NCP	19.617
YLRPPSFL;VNEAPP	NCP	19.534
VNEAPP;KGPGLTTP	NCP	16.32
KGPGLTTP;TTLPELK	NCP	84.851
TTLPELK;LGCLKL	NCP	60.01
LGCLKL;FPVPYPP	NCP	27.864
FPVPYPP;LSGVYPL	NCP	9.602
LSGVYPL;GQMKVVYF	NCP	19.899
GQMKVVYF;EPRPPGL	NCP	26.905
EPRPPGL;DLGFLKDL	NCP	2.954
DLGFLKDL;SPCCLVT	NCP	11.664
SPCCLVT;MLYGAR	NCP	21.022

MLYGAR;VAGAGLV	NCP	6.902
VAGAGLV;LYKPSGK	NCP	16.362
LYKPSGK;LQYKKM	NCP	4.225
LQYKKM;TPATDVFP	NCP	15.303
TPATDVFP;VVARAGPV	NCP	8.624
VVARAGPV;GPAEPLN	NCP	52.819
GPAEPLN;NPALPLE	NCP	9.903
NPALPLE;VPGFP	NCP	91.83
VPGFP;VTLVLT	NCP	93.653
VTLVLT;KNGCLVH	NCP	43.968
KNGCLVH;ELSDEP	NCP	19.975
ELSDEP;QNSLDF	NCP	26.22
QNSLDF;KAADFQLH	NCP	12.545
KAADFQLH;GVPLNP	NCP	37.773
GVPLNP;ARRNPLE	NCP	21.942
ARRNPLE;ALLTKAVVPEMA	NCP	8.97
ALLTKAVVPEMA;LTKNLALEL	NCP	0.519
LTKNLALEL;AELNVRVNCL	NCP	0.027
AELNVRVNCL;ELNVRVN	NCP	0.003
ELNVRVN;AGGSLSHL	NCP	2.118
AGGSLSHL;AGRGQARP	NCP	1.737
AGRGQARP;RLVDHP	NCP	6.49
RLVDHP;LPSLPAAGP	NCP	8.981
LPSLPAAGP;NNNLGGGL	NCP	20.895
NNNLGGGL;YPELG	NCP	12.099
YPELG;ATLRVLPEAQAK	NCP	23.262
ATLRVLPEAQAK;VDVFREDLCTK	NCP	0.579
VDVFREDLCTK;TENLLGSYFPK	NCP	0.177
TENLLGSYFPK;FSERGDAVTK	NCP	1.185
FSERGDAVTK;GHAAPVAA	NCP	8.742
GHAAPVAA;LSALPF	NCP	14.802
LSALPF;GTLEFK	NCP	4.818
GTLEFK;LTEKPQK	NCP	1.534
LTEKPQK;PPPPRGPP	NCP	4.85
PPPPRGPP;MELMMD	NCP	14.541
MELMMD;AAAAVVEF	NCP	19.596
AAAAVVEF;TVPVP	NCP	40.128
TVPVP;EDHLERL	NCP	0.771
EDHLERL;YMPALPR	NCP	7.926
YMPALPR;YGPLTL	NCP	31.152
YGPLTL;LSPTGVP	NCP	87.9
LSPTGVP;TAAELYP	NCP	118.651
TAAELYP;KDREVAK	NCP	37.163
KDREVAK;MEGAEEKKK	NCP	49.456
MEGAEEKKK;KNNGDP	NCP	21.822
KNNGDP;QSPLSPR	NCP	8.006
QSPLSPR;KGVLVDP	NCP	53.418
KGVLVDP;RPGVMTK	NCP	39.109
RPGVMTK;MLGMLK	NCP	15.708
MLGMLK;KNSPR	NCP	21.363
KNSPR;MTKAEG	NCP	42.582

MTKAEG;GGAVLE	NCP	1.787
GGAVLE;RTPLAVR	NCP	18.801
RTPLAVR;KEEMY	NCP	126.543
KEEMY;GVPPPPPPP	NCP	63.511
GVPPPPPPP;PSVPKKP	NCP	6.131
PSVPKKP;RLLSVLNK	NCP	57.703
RLLSVLNK;STLFFE	NCP	37.172
STLFFE;TTPYPK	NCP	4.714
TTPYPK;PSLFER	NCP	35.268
PSLFER;KDVKP	NCP	5.612
KDVKP;KANGSLK	NCP	15.217
KANGSLK;LHLSFK	NCP	21.218
LHLSFK;QPWKYT	NCP	76.641
QPWKYT;VSGDLKP	NCP	36.306
VSGDLKP;EFGLP	NCP	4.446
EFGLP;FHDGAR	NCP	81.043
FHDGAR;SELLLTK	NCP	1.694
SELLLTK;MFLLQGA	NCP	11.197
MFLLQGA;KQLDPAL	NCP	11.609
KQLDPAL;KCPVK	NCP	108.13
KCPVK;AAFKGNK	NCP	46.362
AAFKGNK;RSKVALD	NCP	169.512
RSKVALD;AAVPV	NCP	57.915
AAVPV;QALHQPL	NCP	60.789
QALHQPL;TMPPPPPP	NCP	116.856
TMPPPPPP;MVGAP	NCP	16.047
MVGAP;MLAAQL	NCP	17.481
MLAAQL;SGMNVARLNF	NCP	19.673
SGMNVARLNF;VTEVENGGSLGSK	NCP	4.374
VTEVENGGSLGSK;SGETAKGDYPLEAVRMQ	NCP	2.451
SGETAKGDYPLEAVRMQ;LFEELARASSH	NCP	3.066
LFEELARASSH;GWRPGSGFT	NCP	3.636
GWRPGSGFT;SGFTNTMRVVPVP	NCP	0.015
SGFTNTMRVVPVP;SVTPEVR	NCP	19.951
SVTPEVR;PVGEAQL	NCP	7.46
PVGEAQL;GLSVELP	NCP	7.045
GLSVELP;TFKTLGS	NCP	30.269
TFKTLGS;LHWFY	NCP	11.13
LHWFY;VGLKTEL	NCP	38.599
VGLKTEL;RHDNP	NCP	25.928
RHDNP;TAAAFDN	NCP	87.391
TAAAFDN;LHPYKK	NCP	6.111
LHPYKK;NRTPKK	NCP	10.691
NRTPKK;LLPASGK	NCP	19.189
LLPASGK;KRHCDK	NCP	14.496
KRHCDK;AALSVP	NCP	75.755
AALSVP;SGVHKSR	NCP	42.228
SGVHKSR;PHALTKT	NCP	0.631
PHALTKT;LPQPAL	NCP	6.939
LPQPAL;TAVAGGFL	NCP	10.073
TAVAGGFL;AVSLFRA	NCP	3.843

AVSLFRA;PWGGSYG	NCP	29.808
PWGGSYG;KSLRTVL	NCP	134.23
KSLRTVL;YGPLKN	NCP	81.815
YGPLKN;QEWGWAK	NCP	47.001
QEWGWAK;TPKNKK	NCP	25.104
TPKNKK;SAPYYRQ	NCP	20.575
SAPYYRQ;KVVCKP	NCP	14.169
KVVCKP;KHVTPP	NCP	85.115
KHVTPP;GAAGPSR	NCP	23.221
GAAGPSR;TMAPKPK	NCP	29.765
TMAPKPK;TVVPPKF	NCP	29.647
TVVPPKF;AQTAKAP	NCP	28.535
AQTAKAP;AVLSRAF	NCP	27.819
AVLSRAF;KNNTVVEL	NCP	53.439
KNNTVVEL;FEMASF	NCP	18
FEMASF;TKFVLF	NCP	105.054
TKFVLF;KGVRCR	NCP	1.545
KGVRCR;LCLVCK	NCP	75.886
LCLVCK;NPPGRPP	NCP	88.44
NPPGRPP;SVNPLVP	NCP	85.776
SVNPLVP;PPPDKPPP	NCP	4.707
PPPDKPPP;KLGVLLAE	NCP	34.401
KLGVLLAE;ADLDLTKR	NCP	12.524
ADLDLTKR;AEELTFP	NCP	20.905
AEELTFP;PGEPKPP	NCP	148.983
PGEPKPP;RPFCGT	NCP	7.695
RPFCGT;LEYLG	NCP	4.515
LEYLG;EGVSPP	NCP	64.908
EGVSPP;TKTTHGK	NCP	1.761
TKTTHGK;GPSVMLK	NCP	7.349
GPSVMLK;AFLGYK	NCP	52.155
AFLGYK;EPKVHR	NCP	9.099
EPKVHR;KPPPKP	NCP	29.118
KPPPKP;YSCDDSL	NCP	16.773
YSCDDSL;KEALTLL	NCP	19.333
KEALTLL;TGAGPSFP	NCP	35.192
TGAGPSFP;APGAAAAGK	NCP	36.319
APGAAAAGK;RAPPPGWP	NCP	20.745
RAPPPGWP;VPGAKPR	NCP	37.592
VPGAKPR;FPSSNL	NCP	39.078
FPSSNL;LDFTGHL	NCP	38.316
LDFTGHL;AAPLTK	NCP	20.745
AAPLTK;LDVTLP	NCP	1.437
LDVTLP;KTHFGP	NCP	7.125
KTHFGP;LFAPMK	NCP	8.518
LFAPMK;VGLFLVK	NCP	72.387
VGLFLVK;PAPGAVF	NCP	13.448
PAPGAVF;KLLPKKTE	NCP	33.408
KLLPKKTE;TYSLGGTL	NCP	8.664
TYSLGGTL;KKNSFHR	NCP	73.233
KKNSFHR;MAVELH	NCP	12.522

MAVELH;AVLQPSHL	NCP	25.933
AVLQPSHL;NAPRVVK	NCP	4.745
NAPRVVK;LGPEFP	NCP	15.025
LGPEFP;LVMMPQ	NCP	1.19
LVMMPQ;KPFVVLP	NCP	39.48
KPFVVLP;KELCKPQ	NCP	38.43
KELCKPQ;VDLTVFP	NCP	51.553
VDLTVFP;ASLALLR	NCP	75.962
ASLALLR;RGTNAPG	NCP	3.939
RGTNAPG;ELAPF	NCP	11.142
ELAPF;LLGGLA	NCP	9.649
LLGGLA;MQSLK	NCP	29.18
MQSLK;KPFAQK	NCP	22.536
KPFAQK;YKLPPGP	NCP	9.72
YKLPPGP;TGTSVPR	NCP	102.858
TGTSVPR;SASFSRP	NCP	73.891
SASFSRP;PPPFLG	NCP	6.902
PPPFLG;RLGTLVL	NCP	71.239
RLGTLVL;ERPLDW	NCP	64.05
ERPLDW;KLMKKPP	NCP	15.272
KLMKKPP;AALKP	NCP	38.541
AALKP;MEDLLDLGEER	NCP	75.538
MEDLLDLGEER;HLLEHLE	NCP	19.229
HLLEHLE;SAQKNPP	NCP	16.153
SAQKNPP;PHQDDD	NCP	0.174
PHQDDD;FSLLY	NCP	8.448
FSLLY;APTKNAK	NCP	2.636
APTKNAK;LFFVPPP	NCP	10.678
LFFVPPP;SLDPAYP	NCP	45.477
SLDPAYP;LYSSWYL	NCP	35.378
LYSSWYL;FARAQR	NCP	24.001
FARAQR;TAAKQVK	NCP	73.718
TAAKQVK;WEVLR	NCP	6.673
WEVLR;PPETTAHP	NCP	34.749
PPETTAHP;KMTAPPR	NCP	51.732
KMTAPPR;SPPLVVP	NCP	17.765
SPPLVVP;KDYGVP	NCP	1.402
KDYGVP;TEQALSAF	NCP	42.608
TEQALSAF;AALVEC	NCP	13.632
AALVEC;SELQNLK	NCP	14.796
SELQNLK;LLFKAGP	NCP	17.944
LLFKAGP;KLDSDGLL	NCP	26.393
KLDSDGLL;KLERQPGL	NCP	5.101
KLERQPGL;ALPHALL	NCP	1.076
ALPHALL;LLARAEVV	NCP	15.288
LLARAEVV;KTALRSP	NCP	21.562
KTALRSP;KPGVPGKP	NCP	5.961
KPGVPGKP;KGVHPP	NCP	7.619
KGVHPP;NLPGSFP	NCP	5.908
NLPGSFP;LDDKLL	NCP	31.863
LDDKLL;GLYGPGD	NCP	10.026

GLYGPGD;PSLFPP	NCP	125.721
PSLFPP;LHVLLST	NCP	6.935
LHVLLST;WVLGGSL	NCP	76.386
WVLGGSL;MQPPP	NCP	3.007
MQPPP;SPTGSLP	NCP	60.306
SPTGSLP;TLHVSSQH	NCP	14.069
TLHVSSQH;GPPSLR	NCP	2.071
GPPSLR;VDKPLR	NCP	3.857
VDKPLR;RVTKLLT	NCP	72.153
RVTKLLT;VCVLGAGK	NCP	49.027
VCVLGAGK;FPGAFLLG	NCP	4.68
FPGAFLLG;TGQLKSH	NCP	7.083
TGQLKSH;QVEPP	NCP	25.821
QVEPP;PLQVPEPP	NCP	54.195
PLQVPEPP;LYFVVL	NCP	7.1
LYFVVL;PWVAEKQ	NCP	19.963
PWVAEKQ;LTVGLVPL	NCP	14.211
LTVGLVPL;LVSKASLV	NCP	9.563
LVSKASLV;VPTAASPL	NCP	17.986
VPTAASPL;KLDHPTP	NCP	24.54
KLDHPTP;GPPLLSF	NCP	6.591
GPPLLSF;LNYDAL	NCP	7.862
LNYDAL;KETSHPPP	NCP	35.823
KETSHPPP;RSPLQRF	NCP	18.324
RSPLQRF;AFKPPPPP	NCP	1.5
AFKPPPPP;CDMSQPP	NCP	22.987
CDMSQPP;EGPGDN	NCP	25.26
EGPGDN;KPDVPPP	NCP	7.965
KPDVPPP;KARLPLR	NCP	16.746
KARLPLR;HMALKKK	NCP	5.019
HMALKKK;RAGVLAH	NCP	96.654
RAGVLAH;KPHLVSH	NCP	24.635
KPHLVSH;TGVSLLF	NCP	50.332
TGVSLLF;NKYLGY	NCP	10.995
NKYLGY;VSVLL	NCP	39.612
VSVLL;KAATN	NCP	81.017
KAATN;YHTKSR	NCP	8.199
YHTKSR;EDSVLLR	NCP	82.048
EDSVLLR;KPTTTKAK	NCP	58.58
KPTTTKAK;LSCSAGPF	NCP	96.604
LSCSAGPF;KERAAPP	NCP	11.378
KERAAPP;TSGLELL	NCP	13.734
TSGLELL;GPAAVTWK	NCP	15.369
GPAAVTWK;STGSEFL	NCP	3.64
STGSEFL;TVVMP	NCP	8.337
TVVMP;QPGLGGLT	NCP	23.351
QPGLGGLT;KAATKPK	NCP	17.586
KAATKPK;AAPKRPP	NCP	5.28
AAPKRPP;KRRLDP	NCP	23.943
KRRLDP;TGLRAGP	NCP	23.605
TGLRAGP;LGQALGR	NCP	8.015

LGQALGR;LSVAVP	NCP	11.476
LSVAVP;SLDAFVP	NCP	3.591
SLDAFVP;LSLTEFL	NCP	59.223
LSLTEFL;NTPMQL	NCP	5.964
NTPMQL;AALLECT	NCP	16.952
AALLECT;MELQKAK	NCP	110.355
MELQKAK;DNCMSH	NCP	28.669
DNCMSH;AVPPLLY	NCP	10.983
AVPPLLY;KYTLACK	NCP	23.292
KYTLACK;KAPPLP	NCP	18.272
KAPPLP;NGELRHV	NCP	54.766
NGELRHV;ANGGLGL	NCP	4.178
ANGGLGL;KSSYLMK	NCP	13.815
KSSYLMK;PLVGGP	NCP	1.149
PLVGGP;ALVEF	NCP	7.99
ALVEF;LPPPPLFR	NCP	15.171
LPPPPLFR;KNHEPTR	NCP	59.801
KNHEPTR;RVVVGY	NCP	44.107
RVVVGY;KPQFRPP	NCP	19.247
KPQFRPP;VCLLHKE	NCP	13.843
VCLLHKE;EAVLLLKK	NCP	7.469
EAVLLLKK;KLNPYAK	NCP	1.431
KLNPYAK;SDQKGVQGKKPVVGNKEKKAVGDK	NCP	0.189
SDQKGVQGKKPVVGNKEKKAVGDK;KEKKAVGDKKLKKPV	NCP	0.045
KEKKAVGDKKLKKPVVG;GMEVVLL	NCP	15.006
GMEVVLL;VPGHNLP	NCP	6.75
VPGHNLP;MQFLT	NCP	83.445
MQFLT;NGSSLDF	NCP	38.983
NGSSLDF;KRHPPPPG	NCP	50.717
KRHPPPPG;LAGGAP	NCP	80.019
LAGGAP;KKVGLEPL	NCP	7.47
KKVGLEPL;KLTTDK	NCP	1.519
KLTTDK;LSGPSPQA	NCP	22.673
LSGPSPQA;PVPPPPPP	NCP	38.949
PVPPPPPP;ESNPPLR	NCP	34.575
ESNPPLR;AAPSLSGK	NCP	31.74
AAPSLSGK;GVFYQ	NCP	4.452
GVFYQ;VTEADLP	NCP	81.072
VTEADLP;PKVPPPPPP	NCP	3.6
PKVPPPPPP;MAMLQF	NCP	31.971
MAMLQF;VPAFLGF	NCP	3.916
VPAFLGF;PLVLPPPR	NCP	26.525
PLVLPPPR;EAPQGG	NCP	80.317
EAPQGG;FFPPPPG	NCP	35.982
FFPPPPG;TLRDQGF	NCP	25.233
TLRDQGF;KVGPRLK	NCP	11.228
KVGPRLK;MLYVVQFL	NCP	1.212
MLYVVQFL;FSNRPK	NCP	37.297
FSNRPK;YAVKPY	NCP	44.997
YAVKPY;KSPGGGP	NCP	136.451
KSPGGGP;KMWHLK	NCP	34.824

KMWHLK;MNNLLR	NCP	28.054
MNNLLR;MKLAVL	NCP	55.164
MKLAVL;HTYMR	NCP	25.287
HTYMR;KRLVPPP	NCP	14.726
KRLVPPP;RLGPTYPP	NCP	36.381
RLGPTYPP;SLHSYPF	NCP	4.525
SLHSYPF;TLKPPLAL	NCP	23.904
TLKPPLAL;SPVYAGA	NCP	88.844
SPVYAGA;VPPPPLP	NCP	27.717
VPPPPLP;EPPGHKP	NCP	86.694
EPPGHKP;VVDLVR	NCP	17.415
VVDLVR;PLYTPVM	NCP	61.765
PLYTPVM;FGGFVDF	NCP	9.581
FGGFVDF;LSSNPLLF	NCP	89.94
LSSNPLLF;KAVAVLG	NCP	12.355
KAVAVLG;NPQVKK	NCP	100.65
NPQVKK;AYHLQK	NCP	67.335
AYHLQK;QFSKGGP	NCP	164.343
QFSKGGP;MNQLAK	NCP	29.847
MNQLAK;FKLLDN	NCP	50.514
FKLLDN;CCLVHR	NCP	6.131
CCLVHR;NSKLLAR	NCP	17.382
NSKLLAR;VWFKLSS	NCP	121.054
VWFKLSS;VSVNSLKK	NCP	131.208
VSVNSLKK;VGQLY	NCP	78.542
VGQLY;KGWAGPK	NCP	5.205
KGWAGPK;SGPMQL	NCP	3.246
SGPMQL;NPSFPLG	NCP	10.668
NPSFPLG;KSSVTSP	NCP	47.329
KSSVTSP;LAAKFLL	NCP	7.863
LAAKFLL;GDVKNF	NCP	1.437
GDVKNF;FDPAWK	NCP	23.591
FDPAWK;MRFSLLR	NCP	22.635
MRFSLLR;TAAVSPF	NCP	23.199
TAAVSPF;LVGEAPPL	NCP	5.022
LVGEAPPL;KPAGLDY	NCP	31.927
KPAGLDY;FETTPF	NCP	18.999
FETTPF;PLKKHP	NCP	22.382
PLKKHP;KLLTDSK	NCP	31.75
KLLTDSK;TDTATLPV	NCP	3.699
TDTATLPV;PPPPKRS	NCP	17.744
PPPPKRS;PLLKVFW	NCP	32.52
PLLKVFW;DLTVNH	NCP	74.917
DLTVNH;KHLPPK	NCP	6.428
KHLPPK;KFETLLK	NCP	116.469
KFETLLK;KDGPP	NCP	3.171
KDGPP;HALLKQDG	NCP	59.715
HALLKQDG;KTPWAP	NCP	35.665
KTPWAP;HLLYR	NCP	54.047
HLLYR;LLNPFL	NCP	49.651
LLNPFL;QDLLEF	NCP	26.303

QDLLEF;YELDLRK	NCP	8.683
YELDLRK;SADEYG	NCP	89.703
SADEYG;SLADAVN	NCP	55.212
SLADAVN;FVGAAP	NCP	5.565
FVGAAP;VGDVLLP	NCP	0.596
VGDVLLP;LLLSRGK	NCP	34.231
LLLSRGK;DKPLTPT	NCP	23.357
DKPLTPT;AARPRAAP	NCP	58.032
AARPRAAP;GPAAYVP	NCP	1.675
GPAAYVP;LSYCPPPPP	NCP	87.785
LSYCPPPPP;KPFAAVP	NCP	35.238
KPFAAVP;DLKLCSF	NCP	50.784
DLKLCSF;KPCFQE	NCP	1.291
KPCFQE;TGDCVKK	NCP	109.536
TGDCVKK;KQVPSGK	NCP	10.589
KQVPSGK;KSRPLH	NCP	1.431
KSRPLH;TAPAVRLK	NCP	2.473
TAPAVRLK;FTPVASK	NCP	65.771
FTPVASK;VSKATTK	NCP	3.334
VSKATTK;EAALPC	NCP	74.651
EAALPC;LSDLATY	NCP	39.808
LSDLATY;NRMFLT	NCP	18.384
NRMFLT;VAGTVKR	NCP	0.599
VAGTVKR;APGLP	NCP	70.099
APGLP;GVLVLLGT	NCP	176.039
GVLVLLGT;KKLTEVR	NCP	46.693
KKLTEVR;VSNGPQ	NCP	47.708
VSNGPQ;KGGWPF	NCP	3.501
KGGWPF;KDNLLL	NCP	16.119
KDNLLL;FVPGKTHVLLH	NCP	3.081
FVPGKTHVLLH;ALLDRML	NCP	22.326
ALLDRML;LNLVYKYL	NCP	11.418
LNLVYKYL;KPVFAAP	NCP	87.273
KPVFAAP;RSLFSNF	NCP	18.232
RSLFSNF;TVLVTHK	NCP	1.248
TVLVTHK;YVLRAS	NCP	40.46
YVLRAS;MMGLK	NCP	6.612
MMGLK;LTHWERK	NCP	1.048
LTHWERK;APPPPPPP	NCP	2.492
APPPPPPP;QTLLHPR	NCP	1.089
QTLLHPR;LYFAAGP	NCP	14.106
LYFAAGP;ADPRVRQ	NCP	44.703
ADPRVRQ;SHRLSAF	NCP	13.716
SHRLSAF;DLSMCHP	NCP	62.128
DLSMCHP;KPLERVK	NCP	36.819
KPLERVK;LNLVVLGH	NCP	95.433
LNLVVLGH;MNFKFV	NCP	61.874
MNFKFV;KMSLP	NCP	27.81
KMSLP;AESVHVPPP	NCP	6.237
AESVHVPPP;RQLFQEK	NCP	94.431
RQLFQEK;LLLEMEQ	NCP	44.263

LLLEMEQ;KTSSYKP	NCP	24.329
KTSSYKP;GTGLSPPK	NCP	55.716
GTGLSPPK;RYVASY	NCP	101.829
RYVASY;NMDKKK	NCP	28.288
NMDKKK;TGRELPP	NCP	9.809
TGRELPP;REMFL	NCP	54.339
REMFL;DCDCPP	NCP	30.577
DCDCPP;PSVEF	NCP	61.7
PSVEF;NSPLQFR	NCP	9.268
NSPLQFR;AYVKAGP	NCP	72.014
AYVKAGP;NVVLLR	NCP	29.58
NVVLLR;HLAAPPR	NCP	30.093
HLAAPPR;TVYTLLR	NCP	18.243
TVYTLLR;KYEFRV	NCP	60.867
KYEFRV;PYFGPLP	NCP	86.575
PYFGPLP;YMAAV	NCP	42.872
YMAAV;LSVPHRGP	NCP	22.968
LSVPHRGP;AYLRDF	NCP	16.069
AYLRDF;NELGGLK	NCP	123.746
NELGGLK;MRLSPP	NCP	82.359
MRLSPP;ERLPHF	NCP	83.361
ERLPHF;ANLRMAP	NCP	38.304
ANLRMAP;SFEVLGR	NCP	19.765
SFEVLGR;KGGPMPK	NCP	16.52
KGGPMPK;SYNLVLY	NCP	137.251
SYNLVLY;QPVPPGP	NCP	218.112
QPVPPGP;VNVRTKP	NCP	0.323
VNVRTKP;FTKEKT	NCP	8.536
FTKEKT;VNFKEYK	NCP	6.396
VNFKEYK;SLFRLH	NCP	153.732
SLFRLH;PTPPP	NCP	33.756
PTPPP;PDAGDE	NCP	45.309
PDAGDE;LGKMHMK	NCP	23.265
LGKMHMK;KDLKEF	NCP	0.219
KDLKEF;APVLEYL	NCP	11.613
APVLEYL;KRFSLRP	NCP	39.324
KRFSLRP;KDANLLY	NCP	28.793
KDANLLY;LSTREVL	NCP	61.234
LSTREVL;HTAPY	NCP	6.476
HTAPY;RRKLLK	NCP	14.671
RRKLLK;WGAKPLF	NCP	27.749
WGAKPLF;VVVAPGP	NCP	2.7
VVVAPGP;KKAPSPK	NCP	37.753
KKAPSPK;KGEHYGP	NCP	1.082
KGEHYGP;ERQFSFP	NCP	95.617
ERQFSFP;PPVAAPN	NCP	29.201
PPVAAPN;SALGLEL	NCP	94.767
SALGLEL;KVRLGGGN	NCP	30.66
KVRLGGGN;KPPTVPN	NCP	116.77
KPPTVPN;QVKNKPK	NCP	33.594
QVKNKPK;LKLLAPEPR	NCP	51.236

LKLLAPEPR;NPVVLEPA	NCP	29.406
NPVVLEPA;TNFAP	NCP	36.079
TNFAP;PTGYCLK	NCP	165.783
PTGYCLK;MFVLLQ	NCP	25.127
MFVLLQ;AEEGDGN	NCP	46.426
AEEGDGN;NAPSFPL	NCP	0.311
NAPSFPL;NEGFDLL	NCP	12.484
NEGFDLL;RHLMPTR	NCP	30.696
RHLMPTR;EKPPDP	NCP	40.715
EKPPDP;CTYCCC	NCP	15.18
CTYCCC;AMVSEFL	NCP	69.639
AMVSEFL;GDDGP	NCP	10.383
GDDGP;VAPVT	NCP	79.327
VAPVT;NSSFTLH	NCP	6.357
NSSFTLH;HVVVLHP	NCP	18.884
HVVVLHP;PVVVMLC	NCP	24.288
PVVVMLC;KNKKLT	NCP	5.632
KNKKLT;PDLPLPD	NCP	87.968
PDLPLPD;KDELPY	NCP	31.711
KDELPY;PDVTLER	NCP	4.802
PDVTLER;KQPAEPP	NCP	2.025
KQPAEPP;LEKGLDL	NCP	18.429
LEKGLDL;RLKKKP	NCP	50.596
RLKKKP;YDHME	NCP	134.481
YDHME;AVLELY	NCP	15.437
AVLELY;KLHQELS	NCP	78.769
KLHQELS;AAATPDLG	NCP	100.533
AAATPDLG;AYLSLEL	NCP	99.018
AYLSLEL;KYPQSR	NCP	84.752
KYPQSR;KSVYVLK	NCP	1.257
KSVYVLK;KPPRKGQ	NCP	21.807
KPPRKGQ;NPGLLLG	NCP	18.016
NPGLLLG;KPGSLDL	NCP	82.257
KPGSLDL;SRLLQRF	NCP	0.149
SRLLQRF;SGPKPHVL	NCP	5.899
SGPKPHVL;KSKYPHR	NCP	5.394
KSKYPHR;STFTHKTVY	NCP	69.833
STFTHKTVY;KGGDSLK	NCP	35.878
KGGDSLK;MDPCHLSVPWGK	NCP	0.317
MDPCHLSVPWGK;DEGAKRLT	NCP	0.298
DEGAKRLT;KKTKCGK	NCP	84.78
KKTKCGK;VMPGHRP	NCP	0.293
VMPGHRP;KSKAFPK	NCP	46.252
KSKAFPK;KGVSLFPK	NCP	8.813
KGVSLFPK;KFVEAF	NCP	46.587
KFVEAF;TLGYTPF	NCP	26.284
TLGYTPF;QLNMH	NCP	7.457
QLNMH;APALHAGP	NCP	63.754
APALHAGP;DFLRA	NCP	72.882
DFLRA;FRQANF	NCP	30.045
FRQANF;ATLFGPK	NCP	15.185

ATLFGPK;LGPPPPPPL	NCP	20.52
LGPPPPPPL;AALAFGV	NCP	46.236
AALAFGV;LPPFE	NCP	22.794
LPPFE;KGVLSHSL	NCP	16.962
KGVLSHSL;MAPKPK	NCP	130.654
MAPKPK;FGVPPPP	NCP	5.723
FGVPPPP;AAVVD	NCP	16.704
AAVVD;SAGPLGGP	NCP	11.059
SAGPLGGP;SALLVPR	NCP	16.91
SALLVPR;PPPYVPP	NCP	48.504
PPPYVPP;PPYVPPP	NCP	0.003
PPYVPPP;KPGNEEK	NCP	52.762
KPGNEEK;ALVAVLG	NCP	110.762
ALVAVLG;NPYKLR	NCP	49.606
NPYKLR;GPVAAMM	NCP	16.5
GPVAAMM;NVDGSHM	NCP	258.374
NVDGSHM;EQKQPQ	NCP	3.127
EQKQPQ;PVKLSHK	NCP	30.2
PVKLSHK;SEPVCSK	NCP	41.842
SEPVCSK;RMVTLK	NCP	22.196
RMVTLK;RRSLF	NCP	50.281
RRSLF;VLQDVKL	NCP	39.581
VLQDVKL;SRELQGK	NCP	3.565
SRELQGK;QTTERGY	NCP	26.454
QTTERGY;PVLST	NCP	23.654
PVLST;TQLLLGP	NCP	63.201
TQLLLGP;KAAWAGAP	NCP	7.606
KAAWAGAP;WKAFR	NCP	33.741
WKAFR;KDLTLRPK	NCP	51.072
KDLTLRPK;ADKLTR	NCP	107.906
ADKLTR;RTVYLSE	NCP	21.622
RTVYLSE;VDFPR	NCP	13.112
VDFPR;PQGAKP	NCP	65.035
PQGAKP;ALGNPLR	NCP	58.425
ALGNPLR;DPPPYK	NCP	73.839
DPPPYK;SSVASPNPR	NCP	71.688
SSVASPNPR;QRFPPPP	NCP	14.627
QRFPPPP;LQDPPK	NCP	2.436
LQDPPK;VGGGLVT	NCP	8.545
VGGGLVT;KPPGAAPR	NCP	16.032
KPPGAAPR;RDLVRKL	NCP	103.55
RDLVRKL;VTRLLSK	NCP	23.682
VTRLLSK;KMPPDAK	NCP	2.095
KMPPDAK;RVSGLSPK	NCP	5.325
RVSGLSPK;QLTGSPK	NCP	11.714
QLTGSPK;EVDVNLP	NCP	3.747
EVDVNLP;KSHGVPP	NCP	28.477
KSHGVPP;KSLEFK	NCP	3.575
KSLEFK;GPRAPAPP	NCP	14.521
GPRAPAPP;VYYTVPL	NCP	29.372
VYYTVPL;MSFSTAL	NCP	115.153

MSFSTAL;LPGVGVP	NCP	29.673
LPGVGVP;VQLHYV	NCP	8
VQLHYV;VEVTY	NCP	32.278
VEVTY;SSFHL	NCP	9.266
SSFHL;KFQHPP	NCP	58.767
KFQHPP;LLRPSQEL	NCP	69.283
LLRPSQEL;LDAAKGVG	NCP	8.892
LDAAKGVG;KTGKPNW	NCP	91.989
KTGKPNW;TLPPRHP	NCP	14.055
TLPPRHP;NHGEAGK	NCP	120.545
NHGEAGK;SLGLENR	NCP	0.577
SLGLENR;EEELAAL	NCP	12.066
EEELAAL;FMYSAK	NCP	71.246
FMYSAK;LQGAQML	NCP	4.402
LQGAQML;LLEPF	NCP	63.296
LLEPF;GNVQLSR	NCP	78.859
GNVQLSR;VDKDLLEPGCSV	NCP	15.537
VDKDLLEPGCSV;DGPKLVRE	NCP	3.492
DGPKLVRE;VYAGAF	NCP	31.28
VYAGAF;PPAAEVP	NCP	8.278
PPAAEVP;CCCCCDL	NCP	43.428
CCCCCDL;SKTPPPP	NCP	44.156
SKTPPPP;ELKRTP	NCP	50.065
ELKRTP;KEVSFQVD	NCP	87.359
KEVSFQVD;PPPPPRGP	NCP	1.624
PPPPPRGP;PATTPP	NCP	0.33
PATTPP;CGFFTP	NCP	5.676
CGFFTP;VPLRGSY	NCP	27.077
VPLRGSY;AHRLQLR	NCP	45.288
AHRLQLR;VGELALK	NCP	20.499
VGELALK;QLKKLLH	NCP	15.216
QLKKLLH;MLVARLT	NCP	20.787
MLVARLT;PGLGHVGP	NCP	18.093
PGLGHVGP;GHLLKFK	NCP	13.128
GHLLKFK;PVPLPPPP	NCP	80.824
PVPLPPPP;DPKAVPP	NCP	2.055
DPKAVPP;HLPLPL	NCP	16.019
HLPLPL;GPKGYLK	NCP	121.426
GPKGYLK;NDPLEP	NCP	186.666
NDPLEP;LLLGPLY	NCP	120.996
LLLGPLY;EEAPGHRP	NCP	45.983
EEAPGHRP;CDVDLR	NCP	56.553
CDVDLR;GVKYPLE	NCP	22.086
GVKYPLE;MGPDLLR	NCP	86.53
MGPDLLR;EGSPPLTL	NCP	14.475
EGSPPLTL;LSGAVP	NCP	14.967
LSGAVP;SLENFQ	NCP	1.689
SLENFQ;KRLSSH	NCP	160.647
KRLSSH;GYDEEF	NCP	10.653
VGAPVP;ATLAPAF	NCP	75.099
ATLAPAF;KTPVP	NCP	108.765

KTPVP;YVVED	NCP	37.761
YVVED;NQAGY	NCP	129.796
NQAGY;RLPDVN	NCP	53.441
RLPDVN;LSRQL	NCP	3.354
LSRQL;KARLGLP	NCP	77.916
KARLGLP;PLGFAF	NCP	20.79
PLGFAF;QTARLLR	NCP	50.43
QTARLLR;PGAGNVP	NCP	58.443
PGAGNVP;AALDNP	NCP	3.705
AALDNP;FLPRAAV	NCP	29.388
FLPRAAV;PVPLGPK	NCP	5.31
PVPLGPK;GVETLPV	NCP	0.984
GVETLPV;CRTPGPP	NCP	87.036
CRTPGPP;AGLGRPGPAPR	NCP	31.95
AGLGRPGPAPR;PVVAVTK	NCP	17.196
PVVAVTK;MVGHFGVQEDR	NCP	5.361
MVGHFGVQEDR;PLAGPAPP	NCP	7.917
PLAGPAPP;KPSGVP	NCP	154.885
KPSGVP;GPPRPPP	NCP	10.367
GPPRPPP;SVVPSPK	NCP	10.149
SVVPSPK;KGPPRTP	NCP	9.885
KGPPRTP;AESDWDTVTVLRKK	NCP	53.04
AESDWDTVTVLRKK;PPLRFPGP	NCP	16.938
PPLRFPGP;LLTLCDF	NCP	74.331
LLTLCDF;DVEPLY	NCP	1.005
DVEPLY;TGPDVLL	NCP	44.631
TGPDVLL;PGFVLLR	NCP	56.202
PGFVLLR;KEPRTH	NCP	8.379
KEPRTH;KQEAGP	NCP	12.411
KQEAGP;KASGGAVPK	NCP	25.212
KASGGAVPK;PAPWS	NCP	112.965
PAPWS;KGGHVLP	NCP	46.098
KGGHVLP;KLFGGAH	NCP	11.367
KLFGGAH;KDPLVTD	NCP	102.579
KDPLVTD;QAAPGPP	NCP	24.399
QAAPGPP;LPAEPV	NCP	4.056
LPAEPV;EHVPDL	NCP	30.958
EHVPDL;FPGEGP	NCP	20.567
FPGEGP;KPEPAAP	NCP	75.531
KPEPAAP;HLELCLK	NCP	101.875
HLELCLK;YPAAHLL	NCP	23.487
YPAAHLL;RKNAAHP	NCP	38.466
RKNAAHP;LSAVTNK	NCP	2.927
LSAVTNK;PLPMPGAP	NCP	14.097
PLPMPGAP;PFHAAF	NCP	6.295
PFHAAF;LPPRPPP	NCP	2.021
LPPRPPP;VPPAPPGP	NCP	14.752
VPPAPPGP;TTGAAAP	NCP	7.898
TTGAAAP;DPPPFR	NCP	25.386
DPPPFR;GSAPAPPK	NCP	2.616
GSAPAPPK;QAGLAARP	NCP	31.725

QAGLAARP;LTALLAK	NCP	24.19
LTALLAK;GVWHVP	NCP	20.735
GVWHVP;ASPLK	NCP	17.595
ASPLK;ESPCAL	NCP	24.361
ESPCAL;HGARPPP	NCP	12.557
HGARPPP;VLLPPGV	NCP	33.366
VLLPPGV;KHAAPGA	NCP	2.385
KHAAPGA;AVSAASRL	NCP	44.007
AVSAASRL;KECLSLK	NCP	69.604
KECLSLK;PRLTPP	NCP	42.113
PRLTPP;LTTLRAL	NCP	10.173
LTTLRAL;EGAAPGG	NCP	15.168
EGAAPGG;LEGAGYR	NCP	18.033
LEGAGYR;VRVPVLL	NCP	23.247
VRVPVLL;GDGLSVSPP	NCP	4.396
GDGLSVSPP;KPAAPRP	NCP	9.905
KPAAPRP;YESSVGY	NCP	2.785
YESSVGY;RPAEC	NCP	5.981
RPAEC;KKRWRP	NCP	9.939
KKRWRP;KSPEMEQK	NCP	45.88
KSPEMEQK;EPKTNR	NCP	83.901
EPKTNR;ATPGMP	NCP	19.151
ATPGMP;RLRACPP	NCP	34.854
RLRACPP;MAKLLLPT	NCP	28.47
MAKLLLPT;ADALPA	NCP	43.257
ADALPA;QGGLQLR	NCP	2.244
QGGLQLR;KFAAAP	NCP	7.851
KFAAAP;KYTPAAP	NCP	24.484
KYTPAAP;KAHGQEK	NCP	16.355
KAHGQEK;LAGRTPK	NCP	14.733
LAGRTPK;PPAVP	NCP	5.757
PPAVP;KHTPPGP	NCP	30.381
KHTPPGP;DALPVQ	NCP	89.185
DALPVQ;KELPDK	NCP	4.136
KELPDK;GTLPALV	NCP	0.666
GTLPALV;PRKMSK	NCP	5.914
PRKMSK;EGTLP	NCP	48.332
EGTLP;QSAGP	NCP	18.462
QSAGP;FALLDF	NCP	33.748
FALLDF;KGSSGVP	NCP	36.165
KGSSGVP;TPPTRAAP	NCP	15.635
TPPTRAAP;LLLALVGQ	NCP	17.808
LLLALVGQ;HGAGLPQ	NCP	1.257
HGAGLPQ;GPPLVGT	NCP	28.521
GPPLVGT;SPVLLHQ	NCP	36.234
SPVLLHQ;VDVLNPGGPQ	NCP	5.976
VDVLNPGGPQ;KSLKGLP	NCP	9.37
KSLKGLP;KAPQAAAPP	NCP	22.593
KAPQAAAPP;VTGPAARP	NCP	7.676
VTGPAARP;SAAPPPP	NCP	66.774
SAAPPPP;KATAMPK	NCP	18.838

KATAMPK;VLPHQLAH	NCP	32.327
VLPHQLAH;VSVRDF	NCP	28.273
VSVRDF;LPPPPGP	NCP	13.085
LPPPPGP;VPLKGRP	NCP	6.486
VPLKGRP;KHEARP	NCP	2.787
KHEARP;KERLVF	NCP	5.695
KERLVF;FPDRSP	NCP	10.418
FPDRSP;QANPKR	NCP	28.311
QANPKR;KMGAAP	NCP	26.46
KMGAAP;WAGCLHGL	NCP	23.992
WAGCLHGL;PPSPVVPV	NCP	34.391
PPSPVVPV;PQGPNPR	NCP	35.943
PQGPNPR;MSRPPA	NCP	84.529
MSRPPA;LQLEYPV	NCP	14.214
LQLEYPV;SPGRTLVL	NCP	3.504
SPGRTLVL;PGVPGP	NCP	2.282
PGVPGP;PPPLPP	NCP	29.157
PPPLPP;KEPRPP	NCP	14.607
KEPRPP;KDLVAF	NCP	7.987
KDLVAF;PGMTDLR	NCP	35.432
PGMTDLR;LGATAASG	NCP	2.517
LGATAASG;PLLYLFLP	NCP	47.662
PLLYLFLP;RLGLTLK	NCP	12.009
RLGLTLK;TVSRRPLT	NCP	24.051
TVSRRPLT;TKAAGVLP	NCP	2.993
TKAAGVLP;FTKGAP	NCP	21.271
FTKGAP;KPTAGP	NCP	45.245
KPTAGP;KSLLLGSR	NCP	45.09
KSLLLGSR;KAGREQR	NCP	8.043
KAGREQR;MTRTRVLL	NCP	158.095
MTRTRVLL;ELLEAPV	NCP	24.332
ELLEAPV;VTLVTLAS	NCP	0.12
VTLVTLAS;TDHPTLLT	NCP	3.25
TDHPTLLT;QPYLPY	NCP	7.427
QPYLPY;ARPNPP	NCP	11.748
ARPNPP;KELDTF	NCP	2.098
KELDTF;RGMRVP	NCP	53.07
RGMRVP;PPPPKLE	NCP	41.753
PPPPKLE;LVEVP	NCP	12.006
LVEVP;KVPPPPPPP	NCP	1.572
KVPPPPPPP;PGGGVPPGP	NCP	0.496
PGGGVPPGP;LGRPWYP	NCP	16.811
LGRPWYP;AAVSGVLPQVPEPR	NCP	53.67
AAVSGVLPQVPEPR;GPPSRPGP	NCP	5.41
GPPSRPGP;KPVSFC	NCP	7.749
KPVSFC;LVPRQPP	NCP	22.142
LVPRQPP;VHSSEETVSGAKK	NCP	12.249
VHSSEETVSGAKK;VADEKLR	NCP	13.108
VADEKLR;DPPPP	NCP	27.548
DPPPP;ALPKAVR	NCP	34.596
ALPKAVR;LPPPHPEN	NCP	73.474

LPPPHPEN;KGPPALPH	NCP	7.308
KGPPALPH;GMGHDP	NCP	1.817
GMGHDP;RPFGP	NCP	33.67
RPFGP;APPVTRD	NCP	0.66
APPVTRD;LSGEGLPF	NCP	25.862
LSGEGLPF;VGGLP	NCP	5.19
VGGLP;RPVEC	NCP	87.235
RPVEC;RLGRFGY	NCP	84.158
RLGRFGY;VVRQLH	NCP	9.231
VVRQLH;KGAMVPR	NCP	41.494
KGAMVPR;EPAALYQ	NCP	1.121
EPAALYQ;NAFNLK	NCP	52.291
NAFNLK;LSVAGVTK	NCP	70.146
LSVAGVTK;QSQTN	NCP	24.663
QSQTN;RFLKRQ	NCP	1.7
RFLKRQ;QELNPVP	NCP	31.153
QELNPVP;LALSLRFVP	NCP	56.268
LALSLRFVP;PSRKFF	NCP	1.227
PSRKFF;LELPVP	NCP	7.07
LELPVP;VAPPSPR	NCP	95.112
VAPPSPR;APGGKKTK	NCP	73.683
APGGKKTK;CGLVSP	NCP	19.785
CGLVSP;QKQLTQ	NCP	91.627
QKQLTQ;PPKPMYG	NCP	17.732
PPKPMYG;GTAGPPPP	NCP	17.199
GTAGPPPP;DPGHPYP	NCP	14.055
DPGHPYP;AVRPALY	NCP	8.478
AVRPALY;LGLAGA	NCP	9.285
LGLAGA;RVGTLR	NCP	59.119
RVGTLR;SSEEMC	NCP	0.774
SSEEMC;LGPAVPAVT	NCP	8.774
LGPAVPAVT;QAHVLAT	NCP	4.578
QAHVLAT;QGLVQFK	NCP	14.932
QGLVQFK;PVETLPR	NCP	58.175
PVETLPR;SVACHDF	NCP	49.734
SVACHDF;GPMLLK	NCP	0.427
GPMLLK;ELGSFLVPN	NCP	15.164
ELGSFLVPN;TKLVTVK	NCP	15.3
TKLVTVK;LPPPLPPKP	NCP	9.339
LPPPLPPKP;SGACKMP	NCP	3.915
SGACKMP;KPSTPGSGP	NCP	14.083
KPSTPGSGP;TEALGSPP	NCP	1.431
TEALGSPP;PRGSAPPP	NCP	3.315
PRGSAPPP;EGEAFW	NCP	39.267
EGEAFW;TYLVK	NCP	19.472
TYLVK;LHNLVSV	NCP	42.594
LHNLVSV;SGDQPVVP	NCP	49.048
SGDQPVVP;DKPPAPP	NCP	15.131
DKPPAPP;WGSPGY	NCP	13.539
WGSPGY;KSRHAPK	NCP	25.794
KSRHAPK;AAPEPRP	NCP	7.974

AAPEPRP;PEAPPER	NCP	21.51
PEAPPER;SGRHGVP	NCP	12.813
SGRHGVP;FVEKTP	NCP	59.836
FVEKTP;QAPFLRP	NCP	127.445
QAPFLRP;APPLPPPPP	NCP	65.157
APPLPPPPP;LSPSVPK	NCP	67.429
LSPSVPK;ATNKWLP	NCP	6.596
ATNKWLP;PPAGAR	NCP	28.329
PPAGAR;PPGRPPP	NCP	30.537
PPGRPPPP;LLLLKNVT	NCP	73.972
LLLLKNVT;VYSPTFL	NCP	81.251
VYSPTFL;PPANP	NCP	10.725
PPANP;QTALLLF	NCP	44.32
QTALLLF;RELDLH	NCP	2.174
RELDLH;PAAEFFLP	NCP	17.811
PAAEFFLP;HESPAPPP	NCP	28.614
HESPAPPP;TVAAF	NCP	6.408
TVAAF;AVEPNK	NCP	26.037
AVEPNK;KFPGTVP	NCP	0.846
KFPGTVP;LLFTEV	NCP	40.542
LLFTEV;KPSPTYK	NCP	20.245
KPSPTYK;ERMLRL	NCP	4.638
ERMLRL;DGDDP	NCP	25.16
DGDDP;VSALTVVYF	NCP	56.34
VSALTVVYF;WVPSPK	NCP	27.061
WVPSPK;ADMMFP	NCP	10.674
ADMMFP;TGGFAL	NCP	33.929
TGGFAL;KPFGGSK	NCP	37.374
KPFGGSK;QAAGP	NCP	6.225
QAAGP;LVTELR	NCP	22.779
LVTELR;CYAEQ	NCP	17.728
CYAEQ;GYPGAK	NCP	22.781
GYPGAK;KFHTVPP	NCP	77.427
KFHTVPP;RAAGPLR	NCP	4.56
RAAGPLR;QHPELLK	NCP	1.192
QHPELLK;EVGVLR	NCP	2.447
EVGVLR;LPPPMPP	NCP	23.242
LPPPMPP;GPASFHSP	NCP	26.504
GPASFHSP;AATRWAK	NCP	87.072
AATRWAK;PPPPAVGL	NCP	7.488
PPPPAVGL;RRLPQ	NCP	41.359
RRLPQ;AVSRVY	NCP	43.353
AVSRVY;NGAPGGL	NCP	59.213
NGAPGGL;PASARGAK	NCP	39.406
PASARGAK;RPRHVLL	NCP	22.721
RPRHVLL;LNADKG	NCP	5.247
LNADKG;PGVHKPP	NCP	23.103
PGVHKPP;LPDFSVT	NCP	50.841
LPDFSVT;QLEVPRP	NCP	2.322
QLEVPRP;DEMDC	NCP	23.302
DEMDC;APRTVYA	NCP	9.693

APRTVYA;KDPLTK	NCP	21.237
KDPLTK;KEPVPTP	NCP	23.657
KEPVPTP;PAARPKE	NCP	4.417
PAARPKE;VGDMVVP	NCP	116.174
VGDMVVP;PKLPP	NCP	31.198
PKLPP;ALLSSLDL	NCP	75.327
ALLSSLDL;KATPSK	NCP	21.653
KATPSK;SGPSLVH	NCP	16.315
SGPSLVH;KERALDP	NCP	47.18
KERALDP;FPPPVAP	NCP	65.496
FPPPVAP;AGGHRLLN	NCP	3.306
AGGHRLLN;YCPSLP	NCP	37.65
YCPSLP;FAFFAE	NCP	41.241
FAFFAE;EDKPAW	NCP	7.056
EDKPAW;LAATPGGK	NCP	0.898
LAATPGGK;RLLAVPF	NCP	35.297
RLLAVPF;FETKTPP	NCP	51.165
FETKTPP;LPPPKAPPP	NCP	0.784
LPPPKAPPP;FAFVQT	NCP	97.341
FAFVQT;RDPGSLR	NCP	8.885
RDPGSLR;KAPLPP	NCP	6.168
KAPLPP;GDGAP	NCP	23.211
GDGAP;SSGSHPAG	NCP	44.343
SSGSHPAG;ATLPKY	NCP	36.039
ATLPKY;PPVVLRL	NCP	63.817
PPVVLRL;LDFHALPY	NCP	12.335
LDFHALPY;RLSKLKK	NCP	1.981
RLSKLKK;GPPAAE	NCP	11.298
GPPAAE;ATLPK	NCP	60.635
ATLPK;PDGMLRL	NCP	30.217
PDGMLRL;DDLDGLHL	NCP	11.175
DDLDGLHL;NVPRFM	NCP	41.4
NVPRFM;KVGPP	NCP	140.592
KVGPP;GPAAVP	NCP	3.74
GPAAVP;LLGKLAL	NCP	11.433
LLGKLAL;RGKGGPPK	NCP	1.518
RGKGGPPK;PKGGLGSL	NCP	20.608
PKGGLGSL;TTGLVLD	NCP	27.662
TTGLVLD;KESAFLH	NCP	16.326
KESAFLH;TVEALER	NCP	29.118
TVEALER;KHLQCF	NCP	85.068
KHLQCF;EHARAG	NCP	17.025
EHARAG;ELVPVA	NCP	34.603
ELVPVA;EGAGLSLF	NCP	40.721
EGAGLSLF;AHTRFSK	NCP	46.906
AHTRFSK;LVVLY	NCP	8.142
LVVLY;KSVTPP	NCP	62.273
KSVTPP;DKKKMR	NCP	30.597
DKKKMR;SNGLLTLL	NCP	73.441
SNGLLTLL;SPPGPT	NCP	87.863
SPPGPT;KAVFAPK	NCP	53.284

KAVFAPK;VDDLNL	NCP	118.697
VDDLNL;TPKPAAP	NCP	5.229
TPKPAAP;LVVVLV	NCP	17.512
LVVVLV;FDCHYC	NCP	9.273
FDCHYC;HMASKE	NCP	26.738
HMASKE;VLTYC	NCP	25.036
VLTYC;VEVGLFT	NCP	41.297
VEVGLFT;YPPDRP	NCP	49.131
YPPDRP;VYRSAF	NCP	42.096
VYRSAF;ARPLAFC	NCP	117.189
ARPLAFC;APEPRP	NCP	6.513
APEPRP;LLLLTVVV	NCP	5.158
LLLLTVVV;KYVPPPP	NCP	2.717
KYVPPPP;AVAPPV	NCP	50.988
AVAPPV;GQKGPLQ	NCP	7.83
GQKGPLQ;MEVPGSL	NCP	53.886
MEVPGSL;KMTFPP	NCP	33.243
KMTFPP;HPALGGLL	NCP	8.913
HPALGGLL;GPGGM	NCP	27.393
GPGGM;DCDVD	NCP	53.236
DCDVD;LTVTFM	NCP	9.458
LTVTFM;KPASAKK	NCP	0.513
KPASAKK;MTKPMR	NCP	1.582
MTKPMR;VEDKLR	NCP	21.944
VEDKLR;VFGPPF	NCP	63.336
VFGPPF;MPCRQK	NCP	29.722
MPCRQK;KETANLG	NCP	105.987
KETANLG;PRRCAC	NCP	11.024
PRRCAC;GVLTK	NCP	61.791
GVLTK;NATKLSK	NCP	4.345
NATKLSK;PGPTPP	NCP	1.511
PGPTPP;GVEFK	NCP	67.015
GVEFK;FPKLLT	NCP	23.556
FPKLLT;KAAFLQR	NCP	7.151
KAAFLQR;KLNVSTK	NCP	13.869
KLNVSTK;SHNGF	NCP	5.923
SHNGF;LSMGML	NCP	91.601
LSMGML;LKKKVS	NCP	5.628
LKKKVS;LPPPPRPNP	NCP	10.078
LPPPPRPNP;HLSAAGR	NCP	31.575
HLSAAGR;MENFV	NCP	44.999
MENFV;VSPPPT	NCP	9.265
VSPPPT;APVLDF	NCP	33.282
APVLDF;HLGPPPPPP	NCP	1.845
HLGPPPPPP;PSTTTPK	NCP	15.285
PSTTTPK;ALAFSPTQ	NCP	80.924
ALAFSPTQ;PGAPRYK	NCP	14.949
PGAPRYK;SPGHRTP	NCP	25.429
SPGHRTP;MLSRLLK	NCP	25.686
MLSRLLK;VYKEKL	NCP	95.978
VYKEKL;CSQDDAV	NCP	43.954

CSQDDAV;VFFGPHQ	NCP	20.364
VFFGPHQ;SSVTSVR	NCP	2.552
SSVTSVR;NQLTSNPENTVFDAK	NCP	0.447
NQLTSNPENTVFDAK;DLGGGTFDV	NCP	1.221
DLGGGTFDV;NGDTHLGGEDFDQRVMEHF	NCP	0.072
NGDTHLGGEDFDQRVMEHF;YEGEDFSETLTRAK	NCP	0.195
YEGEDFSETLTRAK;EGEDFSETLTRA	NCP	0.003
EGEDFSETLTRA;SETLTRAK	NCP	0.015
SETLTRAK;VLEDSDLKK	NCP	0.201
VLEDSDLKK;VLLDVCPL	NCP	0.519
VLLDVCPL;LYGSAGPPPTNEEEAADKDEL	NCP	1.047
LYGSAGPPPTNEEEAADKDEL;FWKAPK	NCP	15.222
FWKAPK;KYLLVEF	NCP	48.625
KYLLVEF;RTRDKSK	NCP	21.582
RTRDKSK;ALRQSLSK	NCP	4.743
ALRQSLSK;TGPLAVP	NCP	37.919
TGPLAVP;PKEYVK	NCP	26.223
PKEYVK;KVELSQLRVAKVTGGAASK	NCP	108.549
KVELSQLRVAKVTGGAASK;KYKPLDLRPK	NCP	3.426
KYKPLDLRPK;QPTYP	NCP	2.235
QPTYP;MGWPVQ	NCP	30.054
MGWPVQ;TVEGGAP	NCP	0.508
TVEGGAP;RNGRPK	NCP	86.772
RNGRPK;RQAGRFF	NCP	13.752
RQAGRFF;TALNVP	NCP	8.282
TALNVP;ACARPLLS	NCP	121.017
ACARPLLS;VSAPEYR	NCP	51.031
VSAPEYR;TADFLAT	NCP	2.172
TADFLAT;DPQGEKQ	NCP	17.898
DPQGEKQ;ATQKRL	NCP	15.713
ATQKRL;PGAPPNP	NCP	11.056
PGAPPNP;AVSGAGP	NCP	26.999
AVSGAGP;SSNLELHSL	NCP	6.51
SSNLELHSL;KWGFTK	NCP	9.031
KWGFTK;SPAGLRGP	NCP	8.174
SPAGLRGP;VYKVLKQVHPD	NCP	70.411
VYKVLKQVHPD;LDDLEL	NCP	9.75
LDDLEL;KPTPP	NCP	10.634
KPTPP;TPPGPLQ	NCP	3.72
TPPGPLQ;KLTTVPP	NCP	17.212
KLTTVPP;PLGPFAV	NCP	23.907
PLGPFAV;HATSR	NCP	49.496
HATSR;LDGLQPC	NCP	16.053
LDGLQPC;APKQT	NCP	77.259
APKQT;KKLLKK	NCP	87.355
KKLLKK;VKNPPPP	NCP	17.642
VKNPPPP;LLKKPP	NCP	14.191
LLKKPP;VVPSPKV	NCP	36.137
VVPSPKV;LAPDPSR	NCP	93.976
LAPDPSR;FDPAL	NCP	140.897
FDPAL;KDLVT	NCP	23.91

KDLVT;HGTPH	NCP	9.025
HGTPH;TLHGSFR	NCP	40.842
TLHGSFR;NPPLAPP	NCP	54.78
NPPLAPP;MRGDKYR	NCP	110.736
MRGDKYR;AAPLHVP	NCP	21.746
AAPLHVP;LTAPEPP	NCP	8.469
LTAPEPP;VLHTPH	NCP	22.485
VLHTPH;VTGLPDLR	NCP	4.233
VTGLPDLR;TQAGLK	NCP	136.878
TQAGLK;VLEMDPLPSSKPFQ	NCP	27.939
VLEMDPLPSSKPFQ;KKHYGR	NCP	292.599
KKHYGR;MQTATLP	NCP	13.561
MQTATLP;TTLDDKLLGEKL	NCP	22.01
TTLDDKLLGEKL;TVRMNTF	NCP	33.981
TVRMNTF;QFPKALG	NCP	9.055
QFPKALG;SLPLVDT	NCP	87.563
SLPLVDT;PVVPPPF	NCP	59.632
PVVPPPF;DPVSPP	NCP	0.84
DPVSPP;CVPKK	NCP	80.403
CVPKK;AGKRASPP	NCP	38.87
AGKRASPP;WPKVPK	NCP	5.368
WPKVPK;LSGSFPF	NCP	124.398
LSGSFPF;KTALYT	NCP	14.471
KTALYT;KEVLCSF	NCP	48.358
KEVLCSF;DLQTLPK	NCP	18.426
DLQTLPK;QGQFE	NCP	83.915
QGQFE;LEEVALT	NCP	75.184
LEEVALT;LEMYRDL	NCP	36.012
LEMYRDL;VGKLWN	NCP	82.46
VGKLWN;DPKGKGK	NCP	1.297
DPKGKGK;ASKQLFR	NCP	2.516
ASKQLFR;HALPPT	NCP	5.889
HALPPT;KPFYHKGPP	NCP	10.851
KPFYHKGPP;APGALR	NCP	2.595
APGALR;LPDTQQK	NCP	8.61
LPDTQQK;YSAFF	NCP	32.751
YSAFF;KELRVPF	NCP	9.801
KELRVPF;KNDLLL	NCP	22.552
KNDLLL;LGFSPP	NCP	31.539
LGFSPP;KVSSSVLKA	NCP	67.172
KVSSSVLKA;VTAGL	NCP	15.876
VTAGL;LLAPERP	NCP	34.114
LLAPERP;EFSAQR	NCP	1.095
EFSAQR;MGAVVVR	NCP	66.299
MGAVVVR;LNGKAPK	NCP	2.635
LNGKAPK;EPMEEP	NCP	16.136
EPMEEP;ESPQLLH	NCP	7.017
ESPQLLH;ESRPVFL	NCP	70.236
ESRPVFL;EGPFG	NCP	126.994
EGPFG;PPPVAP	NCP	11.756
PPPVAP;KEEVTLP	NCP	1.927

KEEVTLP;TTEEDLK	NCP	64.869
TTEEDLK;RLPKKT	NCP	8.73
RLPKKT;PGNCCC	NCP	18.353
PGNCCC;RLSTHDP	NCP	3.586
RLSTHDP;YAPSLGP	NCP	23.31
YAPSLGP;LGGEAGRP	NCP	57.465
LGGEAGRP;TKSPVP	NCP	15.522
TKSPVP;SERPLP	NCP	13.148
SERPLP;EKALLAP	NCP	9.102
EKALLAP;NHRPPK	NCP	53.109
NHRPPK;RPPGSLGR	NCP	9.73
RPPGSLGR;LPPPPPLFTAY	NCP	6.558
LPPPPPLFTAY;WRKKGP	NCP	23.756
WRKKGP;GPDDKLR	NCP	0.012
GPDDKLR;RGFVLEEV	NCP	7.965
RGFVLEEV;ESGPPP	NCP	17.26
ESGPPP;ENHPVLL	NCP	8.784
ENHPVLL;LQVPPP	NCP	11.535
LQVPPP;KRLNAK	NCP	30.021
KRLNAK;LYVFF	NCP	6.282
LYVFF;LDDLSAPK	NCP	59.103
LDDLSAPK;KVVRVH	NCP	85.047
KVVRVH;PPPFAGPPP	NCP	18.114
PPPFAGPPP;MLPRAE	NCP	71.495
MLPRAE;PLSLLLLF	NCP	15.828
PLSLLLLF;GLSTLRL	NCP	24.457
GLSTLRL;KPKPPK	NCP	43.389
KPKPPK;KNVVAPK	NCP	53.436
KNVVAPK;QEVVPVP	NCP	56.372
QEVVPVP;KAVKELK	NCP	20.821
KAVKELK;LSAVMD	NCP	12.065
LSAVMD;NFLGSKAK	NCP	13.09
NFLGSKAK;VKVGV	NCP	86.466
VKVGV;VLTGKSVQ	NCP	7.092
VLTGKSVQ;APVLGF	NCP	15.023
APVLGF;VPAKLP	NCP	11.761
VPAKLP;LWTRSLY	NCP	24.905
LWTRSLY;KLLYPV	NCP	82.764
KLLYPV;KVLLLVP	NCP	25.201
KVLLLVP;CLGPCAP	NCP	69.32
CLGPCAP;QELDPK	NCP	2.847
QELDPK;HVSPLH	NCP	15.102
HVSPLH;QRMSLLL	NCP	2.076
QRMSLLL;HVLPLEK	NCP	11.823
HVLPLEK;KLGYTQ	NCP	64.794
KLGYTQ;SLDGALLT	NCP	1.017
SLDGALLT;GVPGV	NCP	76.489
GVPGV;GLPAYK	NCP	23.649
GLPAYK;PLLSVC	NCP	30.414
PLLSVC;KNKMKPF	NCP	24.218
KNKMKPF;MCCLEKK	NCP	33.903

MCCLEKK;QLPGKAKL	NCP	8.302
QLPGKAKL;VLNVHVT	NCP	40.311
VLNVHVT;QHTVEVL	NCP	49.538
QHTVEVL;LPPPL	NCP	16.773
LPPPL;LEALLY	NCP	4.185
LEALLY;PLMFPLLM	NCP	60.004
PLMFPLLM;LTVGEC	NCP	18.434
LTVGEC;KSAYFFH	NCP	7.483
KSAYFFH;LYMSSSL	NCP	4.776
LYMSSSL;QQPFDLK	NCP	1.31
QQPFDLK;DDDEPP	NCP	61.086
DDDEPP;FEMHPF	NCP	54.072
FEMHPF;HCKPK	NCP	7.723
HCKPK;KAGEKTH	NCP	74.649
KAGEKTH;LPAGAHC	NCP	43.61
LPAGAHC;APVLPPLP	NCP	15.333
APVLPPLP;AMWYM	NCP	13.989
AMWYM;KSFSPLPP	NCP	53.016
KSFSPLPP;KFSVTH	NCP	5.004
KFSVTH;KHLADGF	NCP	16.95
KHLADGF;TLRDLKY	NCP	19.393
TLRDLKY;ALGSDVLL	NCP	30.204
ALGSDVLL;GEPLR	NCP	38.042
GEPLR;ELSEP	NCP	1.774
ELSEP;YSVPVEF	NCP	106.181
YSVPVEF;LPPPPLK	NCP	84.283
LPPPPLK;LPDLPPPP	NCP	154.733
LPDLPPPP;DNAPVLK	NCP	72.052
DNAPVLK;NSLLRPR	NCP	31.878
NSLLRPR;APELLSK	NCP	35.306
APELLSK;RAGGPRPP	NCP	20.757
RAGGPRPP;KPFYSP	NCP	30.604
KPFYSP;MLSLAKF	NCP	6.873
MLSLAKF;VAGPPHL	NCP	82.802
VAGPPHL;DSVGPK	NCP	40.437
DSVGPK;VATVSPLR	NCP	70.795
VATVSPLR;HSLLVT	NCP	31.632
HSLLVT;EAFKENE	NCP	110.663
EAFKENE;DGKLP	NCP	31.174
DGKLP;MLDWYT	NCP	64.146
MLDWYT;KDSTVP	NCP	48.53
KDSTVP;NALQFR	NCP	2.536
NALQFR;EVAAP	NCP	174.431
EVAAP;KQVTAF	NCP	33.561
KQVTAF;RLVALLH	NCP	180.937
RLVALLH;FAPLTS	NCP	103.517
FAPLTS;LLQAELL	NCP	186.007
LLQAELL;CAASLPPPP	NCP	174.956
CAASLPPPP;AGHSKSQ	NCP	18.615
AGHSKSQ;NEHAPQ	NCP	24.471
NEHAPQ;PPPPVHQK	NCP	29.902

PPPPVHQK;KRPLLNF	NCP	12.57
KRPLLNF;QAYGRGP	NCP	44.837
QAYGRGP;KNSLLTTH	NCP	71.074
KNSLLTTH;VMTYL	NCP	12.225
VMTYL;LDDPPR	NCP	6.402
LDDPPR;DGDGP	NCP	4.056
DGDGP;KPSYPYK	NCP	79.314
KPSYPYK;PDTASRP	NCP	11.231
PDTASRP;VALPYLK	NCP	7.411
VALPYLK;GFETSVLQK	NCP	7.748
GFETSVLQK;MVQQPR	NCP	56.257
MVQQPR;MLSLQLK	NCP	104.675
MLSLQLK;ELGPAY	NCP	60.435
ELGPAY;LPAKLAK	NCP	12.21
LPAKLAK;KSLAFAF	NCP	17.592
KSLAFAF;KTTKAVGP	NCP	3.303
KTTKAVGP;KMSFTP	NCP	40.681
KMSFTP;KPDKLGK	NCP	1.61
KPDKLGK;KYLLPP	NCP	42.895
KYLLPP;LSVAPSPP	NCP	31.031
LSVAPSPP;TVREVPT	NCP	55.815
TVREVPT;KLFLASNL	NCP	5.598
KLFLASNL;LDLKEF	NCP	31.486
LDLKEF;NKVAVLG	NCP	48.722
NKVAVLG;KGKLY	NCP	10.183
KGKLY;AASSYG	NCP	72.755
AASSYG;AGLLLPK	NCP	7.746
AGLLLPK;ADKPDLG	NCP	43.977
ADKPDLG;LVCEFK	NCP	33.21
LVCEFK;LSLPVFR	NCP	14.547
LSLPVFR;DEPWAF	NCP	64.983
DEPWAF;GPGPAPP	NCP	54.543
GPGPAPP;LLGGPLWG	NCP	28.642
LLGGPLWG;PPGPPPGP	NCP	4.424
PPGPPPGP;CDSSD	NCP	103.627
CDSSD;SPFKQP	NCP	43.691
SPFKQP;VGPALRP	NCP	239.934
VGPALRP;LYLDAGK	NCP	42.997
LYLDAGK;VLAAC	NCP	11.339
VLAAC;MGPGMGP	NCP	52.279
MGPGMGP;KPKFPHTF	NCP	21.98
KPKFPHTF;LSVVTPGP	NCP	14.178
LSVVTPGP;FRGGPLAK	NCP	69.598
FRGGPLAK;KAPEC	NCP	1.152
KAPEC;KDAYVRK	NCP	15.177
KDAYVRK;PPSVTFL	NCP	68.054
PPSVTFL;KNRFR	NCP	40.984
KNRFR;KPEVPGP	NCP	15.5
KPEVPGP;DPRVAPR	NCP	28.243
DPRVAPR;KTDGP	NCP	88.215
KTDGP;AAGPLAQR	NCP	4.158

AAGPLAQR;EAARLKP	NCP	13.653
EAARLKP;EETPG	NCP	14.777
EETPG;DVFLKSH	NCP	13.444
DVFLKSH;MDLEKV	NCP	37.566
MDLEKV;KDKLGDLL	NCP	3.339
KDKLGDLL;KMMMVK	NCP	212.214
KMMMVK;EPVGL	NCP	0.51
EPVGL;KGSYF	NCP	3.648
KGSYF;WKGPSH	NCP	13.274
WKGPSH;WTFFK	NCP	8.781
WTFFK;LAATFPF	NCP	140.343
LAATFPF;PAGKPAAP	NCP	4.098
PAGKPAAP;PVEEK	NCP	27.651
PVEEK;KMSLVLF	NCP	13.384
KMSLVLF;DPLVP	NCP	2.204
DPLVP;GVVGGGPP	NCP	13.986
GVVGGGPP;ARFTKK	NCP	76.129
ARFTKK;KDVASY	NCP	27.786
KDVASY;KPVTEPK	NCP	6.447
KPVTEPK;RLHGALP	NCP	141.327
RLHGALP;DWHRPC	NCP	47.039
DWHRPC;NWRAGGP	NCP	0.069
NWRAGGP;LRHVRP	NCP	5.497
LRHVRP;NLTVLDF	NCP	8.783
NLTVLDF;DQAVYF	NCP	26.445
DQAVYF;VLGVP	NCP	132.826
VLGVP;SDPRLYP	NCP	44.418
SDPRLYP;HSSLRR	NCP	27.743
HSSLRR;KYLPPPP	NCP	126.574
KYLPPPP;LFMQPK	NCP	168.825
LFMQPK;RGMKWPVD	NCP	64.181
RGMKWPVD;SPAAPGPV	NCP	7.818
SPAAPGPV;MVHFVK	NCP	100.708
MVHFVK;AAMAKKK	NCP	3.129
AAMAKKK;DFNRVAL	NCP	37.298
DFNRVAL;QMFLLQ	NCP	94.015
QMFLLQ;KSTLLE	NCP	90.566
KSTLLE;FTHRLLA	NCP	1.395
FTHRLLA;LAKPGAK	NCP	40.891
LAKPGAK;MAELVK	NCP	18.561
MAELVK;KCWPK	NCP	12.471
KCWPK;LSETFK	NCP	5.06
LSETFK;KEPPLYR	NCP	11.389
KEPPLYR;TMLQRPQ	NCP	69.447
TMLQRPQ;GFDLL	NCP	11.273
GFDLL;RKAPVP	NCP	52.24
RKAPVP;LASPKK	NCP	67.916
LASPKK;APEVK	NCP	35.419
APEVK;LGPHKPY	NCP	4.971
LGPHKPY;KNAQLLK	NCP	32.765
KNAQLLK;VVPGSPS	NCP	1.464

VVPGSPS;VGEVP	NCP	46.968
VGEVP;VASRFK	NCP	10.609
VASRFK;AKLSHK	NCP	133.173
AKLSHK;KSGSKPK	NCP	40.886
KSGSKPK;HMPKKRKK	NCP	19.549
HMPKKRKK;TGKLNLLK	NCP	39.068
TGKLNLLK;PVLPLTPK	NCP	27.481
PVLPLTPK;TATLPQH	NCP	11.99
TATLPQH;QFLKDK	NCP	56.911
QFLKDK;PAKNDSP	NCP	38.621
PAKNDSP;QHPLYR	NCP	134.877
QHPLYR;KEAPAPPKAEAK	NCP	6.892
KEAPAPPKAEAK;DYDALDVANK	NCP	0.408
DYDALDVANK;ASPFAVP	NCP	6.434
ASPFAVP;PQSWK	NCP	77.883
PQSWK;SCLDFSLPL	NCP	39.352
SCLDFSLPL;FEEVLK	NCP	38.033
FEEVLK;KEVTVYK	NCP	69.816
KEVTVYK;GVFLQQ	NCP	69.313
GVFLQQ;AFELLH	NCP	15.009
AFELLH;LFGVVA	NCP	64.163
LFGVVA;RPEERF	NCP	17.313
RPEERF;KGVHYR	NCP	3.067
KGVHYR;MEEFPK	NCP	33.698
MEEFPK;VGEEKLK	NCP	11.08
VGEEKLK;KTPHFPPR	NCP	31.538
KTPHFPPR;KASVVL	NCP	6.28
KASVVL;ALRYKT	NCP	0.069
ALRYKT;VGLLANF	NCP	12.342
VGLLANF;RQAFGVQSL	NCP	0.783
RQAFGVQSL;GSSSVKL	NCP	7.466
GSSSVKL;KMKLATP	NCP	4.231
KMKLATP;KVPGWVE	NCP	12.942
KVPGWVE;GDLLDF	NCP	29.393
GDLLDF;SGLGSKFR	NCP	79.633
SGLGSKFR;ENFMVNL	NCP	73.443
ENFMVNL;LEVATAL	NCP	20.876
LEVATAL;QPPNMPR	NCP	33.039
QPPNMPR;SFDSLP	NCP	15.49
SFDSLP;RVTLLTF	NCP	14.013
RVTLLTF;HLLEQLP	NCP	5.727
HLLEQLP;LHGSFLF	NCP	1.322
LHGSFLF;SHLEKTL	NCP	32.016
SHLEKTL;KAFFSPP	NCP	21.532
KAFFSPP;VSFLTPK	NCP	20.604
VSFLTPK;KNYKMPK	NCP	9.417
KNYKMPK;KNAGEF	NCP	1.014
KNAGEF;TVLSLA	NCP	8.78
TVLSLA;LPVVRPG	NCP	1.83
LPVVRPG;GDEEC	NCP	102.793
GDEEC;AFFLM	NCP	13.442

AFFLM;KFFEV	NCP	12.555
KFFEV;RTVPLAN	NCP	29.488
RTVPLAN;KEGMPH	NCP	60.063
KEGMPH;KAQAVHLQ	NCP	0.882
KAQAVHLQ;VVVPVNPK	NCP	43.838
VVVPVNPK;LHLGYLPNQLFR	NCP	9.171
LHLGYLPNQLFR;LQYVPLL	NCP	2.035
LQYVPLL;QDLTQKL	NCP	9.006
QDLTQKL;LADTPPK	NCP	44.246
LADTPPK;EPAASPGGP	NCP	0.03
EPAASPGGP;KVPMGLK	NCP	7.738
KVPMGLK;ASPKAPPP	NCP	0.453
ASPKAPPP;GSPLKKL	NCP	48.651
GSPLKKL;EGPEGKL	NCP	6.365
EGPEGKL;KQKWVR	NCP	63.222
KQKWVR;KPSFFYQ	NCP	38.46
KPSFFYQ;LNLTGL	NCP	7.627
LNLTGL;LNSFDAK	NCP	25.619
LNSFDAK;RAVKEKY	NCP	25.249
RAVKEKY;KYKAPTE	NCP	42.764
KYKAPTE;MDGPAAL	NCP	12.891
MDGPAAL;GFLLDGN	NCP	1.599
GFLLDGN;GDGRLFR	NCP	92.739
GDGRLFR;KGGLLSH	NCP	23.614
KGGLLSH;KAMVP	NCP	60.113
KAMVP;APFSRK	NCP	27.018
APFSRK;KEELYPK	NCP	134.685
KEELYPK;MLYLPRS	NCP	69.978
MLYLPRS;KYPRPP	NCP	15.072
KYPRPP;EEELTVK	NCP	18.415
EEELTVK;PYVTR	NCP	29.862
PYVTR;RALLNYL	NCP	12.242
RALLNYL;LGAWCPR	NCP	37.29
LGAWCPR;TVHLFGK	NCP	134.443
TVHLFGK;KVYLGFP	NCP	26.994
KVYLGFP;SKHTRPP	NCP	45.456
SKHTRPP;AVPLPPLP	NCP	27.879
AVPLPPLP;LDTLTPK	NCP	6.764
LDTLTPK;LPPGHLP	NCP	35.892
LPPGHLP;KGMTHVP	NCP	13.729
KGMTHVP;MGEGDC	NCP	40.643
MGEGDC;KDLEHGP	NCP	42.18
KDLEHGP;YSQALLR	NCP	22.408
YSQALLR;GSAMPK	NCP	6.665
GSAMPK;HLYDPK	NCP	14.908
HLYDPK;KAVSYPK	NCP	26.46
KAVSYPK;KFYKRH	NCP	16.046
KFYKRH;NSLVLVSP	NCP	10.944
NSLVLVSP;FSQQHP	NCP	78.468
FSQQHP;KTTPVVR	NCP	95.256
KTTPVVR;KQSTQRQ	NCP	39.96

KQSTQRQ;VSLEKNR	NCP	1.881
VSLEKNR;KYYKVG	NCP	32.874
KYYKVG;PLTNTHSKR	NCP	23.476
PLTNTHSKR;VGGRELVL	NCP	0.885
VGGRELVL;KFQPPPP	NCP	2.79
KFQPPPP;YAMPGF	NCP	1.914
YAMPGF;QPLPKPP	NCP	8.384
QPLPKPP;PVFVL	NCP	21.021
PVFVL;AGKLHPR	NCP	17.871
AGKLHPR;AALVD	NCP	160.417
AALVD;AGGLP	NCP	82.812
AGGLP;LNRMAF	NCP	38.463
LNRMAF;ELEKFDK	NCP	46.739
ELEKFDK;LVGAPLQ	NCP	7.353
LVGAPLQ;KEAAQR	NCP	77.035
KEAAQR;VSKDLTK	NCP	41.919
VSKDLTK;AGALVRK	NCP	96.264
AGALVRK;FEKLRF	NCP	4.041
FEKLRF;KERYVL	NCP	29.177
KERYVL;TAYKAR	NCP	56.859
TAYKAR;KTSFGK	NCP	221.112
KTSFGK;RLGSPVTR	NCP	12.319
RLGSPVTR;AELRALV	NCP	28.338
AELRALV;LPPPPLPPP	NCP	19.992
LPPPPLPPP;MPKLDR	NCP	26.208
MPKLDR;RGPPLLY	NCP	2.931
RGPPLLY;AMGLM	NCP	77.613
AMGLM;GPLTPRP	NCP	66.444
GPLTPRP;PDPPP	NCP	2.087
PDPPP;ERYWPF	NCP	3.297
ERYWPF;APRTVY	NCP	33.294
APRTVY;KPPDLLA	NCP	12.334
KPPDLLA;QKAAPVP	NCP	27.851
QKAAPVP;TYVTPR	NCP	79.516
TYVTPR;MFSGLP	NCP	106.76
MFSGLP;PLNPKGSP	NCP	141.255
PLNPKGSP;FGVGMGK	NCP	17.227
FGVGMGK;FPGGGSL	NCP	81.651
FPGGGSL;KGLSSGK	NCP	10.577
KGLSSGK;PAAPP	NCP	8.043
PAAPP;NPKRPP	NCP	12.711
NPKRPP;GHERQY	NCP	5.074
GHERQY;RVLVP	NCP	22.164
RVLVP;FDSPKF	NCP	74.489
FDSPKF;KNLFPSN	NCP	29.304
KNLFPSN;YGGSSSSSSYGSGR	NCP	23.266
YGGSSSSSSYGSGR;KLNLKYVC	NCP	9.788
KLNLKYVC;VTDLPF	NCP	46.306
VTDLPF;ALTALTY	NCP	44.597
ALTALTY;CMDDQ	NCP	29.974
CMDDQ;YNFPDPP	NCP	29.54

YNFPDPP;ATTRWLL	NCP	7.003
ATTRWLL;KLFPPPPP	NCP	13.623
KLFPPPPP;FVGPLK	NCP	264.075
FVGPLK;VGPVPLT	NCP	86.126
VGPVPLT;KKMEPF	NCP	56.646
KKMEPF;SRPLT	NCP	11.127
SRPLT;KEYTH	NCP	91.905
KEYTH;KHQNPPP	NCP	45.303
KHQNPPP;STVPP	NCP	68.013
STVPP;AELRADP	NCP	2.604
AELRADP;SLLEEVN	NCP	10.015
SLLEEVN;PVVPLDP	NCP	5.853
PVVPLDP;DTAGQEDYDRLRP	NCP	27.371
DTAGQEDYDRLRP;SLGVLPK	NCP	14.229
SLGVLPK;SKLTGYQP	NCP	9.54
SKLTGYQP;KGHVLGL	NCP	29.005
KGHVLGL;LRSDLRLL	NCP	0.512
LRSDLRLL;KPPPTAP	NCP	42.705
KPPPTAP;KPTRNV	NCP	25.51
KPTRNV;KRWAAFY	NCP	40.401
KRWAAFY;YALPPPPP	NCP	1.089
YALPPPPP;LEDNP	NCP	18.342
LEDNP;MELLKLG	NCP	14.31
MELLKLG;LARSLKT	NCP	29.421
LARSLKT;KVYTAF	NCP	24.794
KVYTAF;AAVYQA	NCP	1.734
AAVYQA;LQKQPY	NCP	1.045
LQKQPY;VSCNP	NCP	1.107
VSCNP;AAGRGWK	NCP	21.561
AAGRGWK;LLVLDL	NCP	2.873
LLVLDL;TLFGNER	NCP	83.614
TLFGNER;VGFPP	NCP	4.61
VGFPP;QERLHLV	NCP	23.898
QERLHLV;CPCDAL	NCP	23.035
CPCDAL;REPLTRHN	NCP	62.024
REPLTRHN;GTLLEL	NCP	3.867
GTLLEL;PPLLEAP	NCP	32.188
PPLLEAP;KLLFVPP	NCP	0.618
KLLFVPP;MDFEDDYTHS	NCP	127.719
MDFEDDYTHS;EHGLSNY	NCP	5.588
EHGLSNY;ANPSFPL	NCP	63.211
ANPSFPL;KPFKFVLGK	NCP	2.409
KPFKFVLGK;FDVELLKLE	NCP	0.165
FDVELLKLE;APTHKPK	NCP	26.306
APTHKPK;PERPGPGSAG	NCP	42.22
PERPGPGSAG;QLYVPR	NCP	3.449
QLYVPR;LYLRPP	NCP	72.921
LYLRPP;KPHVPPPPP	NCP	6.603
KPHVPPPPP;VKDEQPR	NCP	7.651
VKDEQPR;KCVPP	NCP	0.57
KCVPP;NQLLNVSK	NCP	49.748

NQLLNVSK;GDNVGF	NCP	48.196
GDNVGF;LTYVMVLV	NCP	18.644
LTYVMVLV;AGPSLV	NCP	16.272
AGPSLV;ADELAK	NCP	30.46
ADELAK;MFKFVSP	NCP	1.049
MFKFVSP;SLAMTKH	NCP	16.539
SLAMTKH;LAPSPAPK	NCP	17.404
LAPSPAPK;RDPSFN	NCP	29.18
RDPSFN;EMVHAK	NCP	37.798
EMVHAK;MMKAH	NCP	28.328
MMKAH;MELLQDT	NCP	15.913
MELLQDT;KSHVEQG	NCP	2.237
KSHVEQG;RNCLLLH	NCP	55.242
RNCLLLH;QGELEF	NCP	13.47
QGELEF;ESLLTY	NCP	29.292
ESLLTY;ENMLEK	NCP	0.267
ENMLEK;AAPSVTGK	NCP	7.653
AAPSVTGK;NTLPEQ	NCP	58.054
NTLPEQ;MLLLKLVQ	NCP	26.747
MLLLKLVQ;SAHLQW	NCP	42.541
SAHLQW;EPELT	NCP	6.063
EPELT;KSYSLPP	NCP	79.694
KSYSLPP;PASELPR	NCP	26.863
PASELPR;KSTPLQTP	NCP	6.912
KSTPLQTP;KSTSVGK	NCP	25.584
KSTSVGK;YFPPVR	NCP	1.656
YFPPVR;LPLEGKG	NCP	49.629
LPLEGKG;ALPVLPPP	NCP	8.913
ALPVLPPP;KNDTLP	NCP	13.509
KNDTLP;KFFPF	NCP	43.61
KFFPF;APSCYPQ	NCP	3.766
APSCYPQ;YQGKSLR	NCP	5.208
YQGKSLR;LLKPKP	NCP	64.631
LLKPKP;DLDVKGSP	NCP	132.544
DLDVKGSP;ALTTYG	NCP	2.034
ALTTYG;WWLLLVE	NCP	6.132
WWLLLVE;TVHAKPP	NCP	14.438
TVHAKPP;HAKPPPP	NCP	0.006
HAKPPPP;GKPPAVK	NCP	16.128
GKPPAVK;FGRSFSP	NCP	26.614
FGRSFSP;NVNANY	NCP	16.787
NVNANY;MTDTFAP	NCP	17.079
MTDTFAP;VDCVTPK	NCP	34.701
VDCVTPK;CDMDMC	NCP	30.741
CDMDMC;KEPLGGP	NCP	2.601
KEPLGGP;DLVAPPK	NCP	1.074
DLVAPPK;KDEDP	NCP	11.029
KDEDP;KPSSKGPP	NCP	5.046
KPSSKGPP;HMGGPL	NCP	10.896
HMGGPL;LKLLSYR	NCP	48.875
LKLLSYR;LAPLLKDV	NCP	67.218

LAPLLKDV;VPGGALG	NCP	4.768
VPGGALG;SQGLLALR	NCP	67.815
SQGLLALR;PCCDAL	NCP	2.67
PCCDAL;CGDDPK	NCP	5.372
CGDDPK;TVALPVDF	NCP	6.487
TVALPVDF;TKGVHLR	NCP	24.93
TKGVHLR;CPGVLAK	NCP	10.646
CPGVLAK;PPRKLLK	NCP	63.388
PPRKLLK;KDVEEN	NCP	53.607
KDVEEN;NPTRQPP	NCP	198.564
NPTRQPP;MDARKK	NCP	56.724
MDARKK;FSVLP	NCP	7.401
FSVLP;GLVLEAP	NCP	22.188
GLVLEAP;AAEVLKR	NCP	5.949
AAEVLKR;MAVYPP	NCP	32.459
MAVYPP;CEHAAA	NCP	39.796
CEHAAA;KRGELKT	NCP	24.852
KRGELKT;DFDLPKG	NCP	1.217
DFDLPKG;DPLSP	NCP	2.884
DPLSP;LGKAVLGA	NCP	54.452
LGKAVLGA;LGSHESP	NCP	5.506
LGSHESP;GPLDLLF	NCP	61.304
GPLDLLF;NSARLLR	NCP	4.752
NSARLLR;KQNLKE	NCP	12.142
KQNLKE;QDRGQL	NCP	57.893
QDRGQL;KQKFC	NCP	16.516
KQKFC;LLSGTSY	NCP	45.077
LLSGTSY;PGPVVYLR	NCP	45.841
PGPVVYLR;PALGT	NCP	3.189
PALGT;KDDLGKF	NCP	61.832
KDDLGKF;FVKSPF	NCP	14.124
FVKSPF;GPEGAP	NCP	227.637
GPEGAP;PLSGRAAP	NCP	16.615
PLSGRAAP;KGTFY	NCP	1.556
KGTFY;KPEAFR	NCP	3.861
KPEAFR;TVGDLF	NCP	5.701
TVGDLF;AEVRELQ	NCP	0.854
AEVRELQ;SPQGVPFL	NCP	5.346
SPQGVPFL;LMPNVLL	NCP	15.763
LMPNVLL;KSMAPR	NCP	59.697
KSMAPR;QLLWH	NCP	40.2
QLLWH;EPSLVHRK	NCP	9.45
EPSLVHRK;KESSVLS	NCP	31.05
KESSVLS;GPPSPPPPPP	NCP	7.479
GPPSPPPPPP;VTLGTFP	NCP	4.419
VTLGTFP;VFEVELL	NCP	66.681
VFEVELL;RMLKSSH	NCP	1.185
RMLKSSH;LPEQKVL	NCP	30.797
LPEQKVL;YRVFYM	NCP	46.098
YRVFYM;LGALLLLK	NCP	69.714
LGALLLLK;TFHRK	NCP	8.622

TFHRK;KLGKSVE	NCP	16.674
KLGKSVE;KDVLEL	NCP	152.677
KDVLEL;LAKVAVP	NCP	24.107
LAKVAVP;NLPGHGP	NCP	2.874
NLPGHGP;KVLLCQF	NCP	3.016
KVLLCQF;KMKPPR	NCP	9.695
KMKPPR;LDVGNF	NCP	9.268
LDVGNF;HSLLK	NCP	91.238
HSLLK;RPPVVP	NCP	61.167
RPPVVP;FESAENK	NCP	24.399
FESAENK;LFPLTQV	NCP	38.125
LFPLTQV;QKKMPK	NCP	3.017
QKKMPK;ARLPKKK	NCP	37.32
ARLPKKK;MSFQLYW	NCP	88.111
MSFQLYW;LSQRPR	NCP	71.456
LSQRPR;KDSGTLLK	NCP	0.339
KDSGTLLK;KAPAHYK	NCP	36.538
KAPAHYK;LSYNFPAL	NCP	100.112
LSYNFPAL;KNLEVTVK	NCP	105.645
KNLEVTVK;PGMLEY	NCP	53.098
PGMLEY;KFLRNP	NCP	32.547
KFLRNP;AAALTVR	NCP	39.128
AAALTVR;DPLENHPP	NCP	32.211
DPLENHPP;HLDRAP	NCP	72.973
HLDRAP;RLGVGAPV	NCP	1.505
RLGVGAPV;VGAGMF	NCP	38.1
VGAGMF;HSDFPK	NCP	75.163
HSDFPK;FTLKSK	NCP	60.176
FTLKSK;MMLDLNKAK	NCP	38.526
MMLDLNKAK;STSETMGHSADRLAAAF	NCP	0.441
STSETMGHSADRLAAAF;KAQDEGLLSDVVPF	NCP	0.108
KAQDEGLLSDVVPF;VGLPPLEKF	NCP	5.604
VGLPPLEKF;FGATGCRLVM	NCP	0.063
FGATGCRLVM;CALKFFC	NCP	15.457
CALKFFC;NAPKSEK	NCP	73.128
NAPKSEK;KQKPSSH	NCP	2.285
KQKPSSH;AFFPPRP	NCP	51.679
AFFPPRP;LLHKKM	NCP	24.914
LLHKKM;PPSGGPL	NCP	5.934
PPSGGPL;KYFLLPP	NCP	1.672
KYFLLPP;GVARVQ	NCP	82.883
GVARVQ;EEHDMD	NCP	18.646
EEHDMD;VTVPGGDL	NCP	29.447
VTVPGGDL;QLNGAHRSY	NCP	34.924
QLNGAHRSY;PSARLPSP	NCP	8.582
PSARLPSP;QEFTPR	NCP	7.105
QEFTPR;ARASHTQ	NCP	19.175
ARASHTQ;WAPRML	NCP	22.554
WAPRML;VPGRVLL	NCP	20.289
VPGRVLL;TKNLP	NCP	15.4
TKNLP;YGPGAP	NCP	7.859

YGPGAP;KTVEVF	NCP	50.608
KTVEVF;KACYLSP	NCP	42.518
KACYLSP;KLTVNKT	NCP	0.813
KLTVNKT;SGTSQNEG	NCP	21.939
SGTSQNEG;NPPSPSSPF	NCP	11.202
NPPSPSSPF;VPPPAVEPKKP	NCP	60.051
VPPPAVEPKKP;YPAPVFH	NCP	4.573
YPAPVFH;VPPLSNSV	NCP	4.946
VPPLSNSV;KGYKNF	NCP	27.739
KGYKNF;AEEFTLP	NCP	26.148
AEEFTLP;LNEVVTR	NCP	11.034
LNEVVTR;VPLGGP	NCP	1.887
VPLGGP;RKLVETL	NCP	48.264
RKLVETL;AALVLEDGSVLRGQPF	NCP	63.818
AALVLEDGSVLRGQPF;GPALPDGRF	NCP	17.805
GPALPDGRF;EETPAFF	NCP	17.668
EETPAFF;KPSSERP	NCP	50.561
KPSSERP;KGSPRAGP	NCP	41.412
KGSPRAGP;ETFFKVS	NCP	28.239
ETFFKVS;MAPSRK	NCP	11.457
MAPSRK;KVRAVF	NCP	4.305
KVRAVF;PSTGKGGK	NCP	5.967
PSTGKGGK;KVMVFK	NCP	101.994
KVMVFK;STSSVFV	NCP	10.593
STSSVFV;ATPVKVT	NCP	13.779
ATPVKVT;QEWYEL	NCP	51.973
QEWYEL;LVHLYP	NCP	5.127
LVHLYP;MDYHGS	NCP	81.155
MDYHGS;KLLEQEKQ	NCP	50.446
KLLEQEKQ;TERLLAL	NCP	94.631
TERLLAL;VEVEL	NCP	10.845
VEVEL;ASSTLLD	NCP	23.475
ASSTLLD;NKPSVPK	NCP	74.985
NKPSVPK;AVSLWGF	NCP	87.583
AVSLWGF;EGLDFY	NCP	43.455
EGLDFY;KANLLGK	NCP	28.052
KANLLGK;EHAAVTK	NCP	45.969
EHAAVTK;SGNFTPK	NCP	0.181
SGNFTPK;KGHVGPK	NCP	13.151
KGHVGPK;EALTVLD	NCP	16.677
EALTVLD;VEFPASP	NCP	5.061
VEFPASP;YLAAVL	NCP	19.659
YLAAVL;YEELVK	NCP	7.438
YEELVK;KPAAHAR	NCP	0.26
KPAAHAR;VPLAALL	NCP	41.055
VPLAALL;QLLEC	NCP	13.543
QLLEC;QKDPQQ	NCP	73.638
QKDPQQ;THLTLP	NCP	112.923
THLTLP;FLDELVS	NCP	7.268
FLDELVS;TRYFPL	NCP	45.358
TRYFPL;MLLEEAR	NCP	16.575

MLLEEAR;KGFKTPK	NCP	8.207
KGFKTPK;VGMFLGA	NCP	27.033
VGMFLGA;NVASGF	NCP	1.842
NVASGF;ASCLLL	NCP	78.627
ASCLLL;YTDLLRLFEY	NCP	30.117
YTDLLRLFEY;TFGADVVSK	NCP	8.457
TFGADVVSK;KKWLPLT	NCP	61.281
KKWLPLT;KDRLNAG	NCP	13.272
KDRLNAG;HAAVKPP	NCP	28.263
HAAVKPP;YGAADL	NCP	15.606
YGAADL;HSPMPQ	NCP	105.861
HSPMPQ;KEVDQGK	NCP	17.856
KEVDQGK;VFLPALT	NCP	43.413
VFLPALT;PDAPPRP	NCP	66.238
PDAPPRP;RPSPGE	NCP	33.527
RPSPGE;AKVLGLG	NCP	14.055
AKVLGLG;LAEAW	NCP	17.811
LAEAW;ELKEELKK	NCP	140.068
ELKEELKK;VGLLETL	NCP	39.227
VGLLETL;KMKVPPP	NCP	35.952
KMKVPPP;LSCGSSSTSL	NCP	92.235
LSCGSSSTSL;DCLQGF	NCP	47.725
DCLQGF;DLVTPF	NCP	30.792
DLVTPF;MKLFVK	NCP	28.214
MKLFVK;DYDMC	NCP	3.457
DYDMC;YVDYK	NCP	46.883
YVDYK;ETTTPLP	NCP	113.284
ETTTPLP;GPDRLVP	NCP	60.596
GPDRLVP;VGSFKV	NCP	42.594
VGSFKV;LSFPTTK	NCP	44.628
LSFPTTK;FQAKKF	NCP	31.9
FQAKKF;LVFVQ	NCP	13.278
LVFVQ;GGLSAPGA	NCP	7.125
GGLSAPGA;KSMLPEK	NCP	82.1
KSMLPEK;KTKHPP	NCP	1.906
KTKHPP;VGPLELK	NCP	10.959
VGPLELK;RVLPL	NCP	22.959
RVLPL;KGGVLQR	NCP	2.742
KGGVLQR;WPPEEF	NCP	50.987
WPPEEF;AAGTLY	NCP	19.713
AAGTLY;NYPELK	NCP	51.808
NYPELK;FPQTEC	NCP	36.776
FPQTEC;HLAWEKK	NCP	16.227
HLAWEKK;GKGVSHR	NCP	11.112
GKGVSHR;TQTHNVP	NCP	31.678
TQTHNVP;PGVFMLR	NCP	138.008
PGVFMLR;NSSVGKKP	NCP	5.809
NSSVGKKP;SLVVHSR	NCP	10.403
SLVVHSR;SLDRPF	NCP	11.506
SLDRPF;KAFLLSQL	NCP	22.004
KAFLLSQL;LAMPV	NCP	70.125

LAMPV;KPRGGLH	NCP	2.619
KPRGGLH;KPASLPGP	NCP	15.219
KPASLPGP;AAYLVK	NCP	6.265
AAYLVK;VGCVFV	NCP	5.135
VGCVFV;LEVNSHLV	NCP	97.404
LEVNSHLV;AEVKRF	NCP	28.363
AEVKRF;TALPDT	NCP	17.993
TALPDT;KPTPNPH	NCP	65.757
KPTPNPH;EVGVLFT	NCP	4.41
EVGVLFT;LSARPST	NCP	1.471
LSARPST;KPSQQPK	NCP	31.895
KPSQQPK;QFHPDR	NCP	10.329
QFHPDR;YGGPAP	NCP	90.376
YGGPAP;LAAQFCC	NCP	0.099
LAAQFCC;PVLMH	NCP	46.86
PVLMH;LAPDVEL	NCP	1.451
LAPDVEL;PFGQYPP	NCP	29.517
PFGQYPP;KPALPTPP	NCP	22.236
KPALPTPP;KAYPPK	NCP	46.648
KAYPPK;RFVLPQ	NCP	20.1
RFVLPQ;FPAVPEV	NCP	28.263
FPAVPEV;VTVLYT	NCP	7.383
VTVLYT;KLEDVKF	NCP	28.091
KLEDVKF;KELAVTK	NCP	29.758
KELAVTK;KGRLLLK	NCP	7.419
KGRLLLK;EKLVA	NCP	8.066
EKLVA;LAKYTSSK	NCP	25.402
LAKYTSSK;NNMHAA	NCP	6.419
NNMHAA;PAPPVDR	NCP	32.902
PAPPVDR;AAAVRRP	NCP	11.763
AAAVRRP;LTVLPF	NCP	15.959
LTVLPF;NAKLGLL	NCP	23.016
NAKLGLL;CFTKFYL	NCP	12.247
CFTKFYL;DPPPPQPP	NCP	43.394
DPPPPQPP;AEAPGHGP	NCP	2.043
AEAPGHGP;WGMLPPPPP	NCP	72.907
WGMLPPPPP;LEGLGAL	NCP	26.693
LEGLGAL;KFNHFFK	NCP	55.821
KFNHFFK;KGVYPR	NCP	160.936
KGVYPR;YRLLNGN	NCP	45.453
YRLLNGN;KVETFSGVYKK	NCP	3.938
KVETFSGVYKK;ERMLYR	NCP	11.577
ERMLYR;LTVSLVLP	NCP	8.107
LTVSLVLP;VVLAC	NCP	21.119
VVLAC;KGMPPR	NCP	7.609
KGMPPR;VNATPQ	NCP	7.892
VNATPQ;DAFVRSP	NCP	57.778
DAFVRSP;APLPL	NCP	14.168
APLPL;LKLSQKNS	NCP	0.123
LKLSQKNS;MYDNRESQDTSF	NCP	134.001
MYDNRESQDTSF;VDSTTCLFPVEEK	NCP	6.273

VDSTTCLFPVEEK;TSQFVSLVMF	NCP	19.515
TSQFVSLVMF;VVTYKP	NCP	23.593
VVTYKP;LEEVEPTP	NCP	16.406
LEEVEPTP;KQLEDGRTL	NCP	23.209
KQLEDGRTL;PVLHLLFP	NCP	1.892
PVLHLLFP;DMCWK	NCP	3.043
DMCWK;NEHYAK	NCP	4.206
NEHYAK;LTAAVAL	NCP	21.389
LTAAVAL;EVDALV	NCP	13.419
EVDALV;EVLPLLAK	NCP	20.235
EVLPLLAK;YLLKLK	NCP	10.834
YLLKLK;LDFAFGL	NCP	17.531
LDFAFGL;DLDKLLK	NCP	75.381
DLDKLLK;FEPELK	NCP	15.579
FEPELK;DLLKGPK	NCP	1.341
DLLKGPK;KPAAETPK	NCP	10.467
KPAAETPK;LSSSALSH	NCP	11.559
LSSSALSH;LDVLGVTK	NCP	3.957
LDVLGVTK;LTSDTGEL	NCP	6.604
LTSDTGEL;DSVSEFLK	NCP	38.816
DSVSEFLK;FTQLYKP	NCP	12.114
FTQLYKP;GPLGDLR	NCP	17.151
GPLGDLR;MYMDPP	NCP	88.87
MYMDPP;NHTKKKK	NCP	95.784
NHTKKKK;VAKTAVT	NCP	52.229
VAKTAVT;PPTLGPP	NCP	17.91
PPTLGPP;EKLSYDK	NCP	79.206
EKLSYDK;LTPLVT	NCP	71.118
LTPLVT;VTASNVLK	NCP	36.348
VTASNVLK;FNVDPF	NCP	2.104
FNVDPF;QDCHK	NCP	6.476
QDCHK;KTLLQPL	NCP	14.275
KTLLQPL;KLHFDP	NCP	77.402
KLHFDP;VYQFPK	NCP	92.523
VYQFPK;GDLPGLVRL	NCP	4.326
GDLPGLVRL;GKELDSL	NCP	7.629
GKELDSL;KYAKPP	NCP	38.146
KYAKPP;LDDALVT	NCP	50.448
LDDALVT;RELTQAR	NCP	205.467
RELTQAR;LDTEFR	NCP	9.981
LDTEFR;VPDDFP	NCP	51.918
VPDDFP;EAEVSVP	NCP	6.108
EAEVSVP;QNGKLFK	NCP	65.79
QNGKLFK;LNLFEML	NCP	7.46
LNLFEML;PTSTPKC	NCP	33.142
PTSTPKC;VFFNTLR	NCP	35.492
VFFNTLR;PKQMPAP	NCP	38.103
PKQMPAP;VAALYLE	NCP	128.523
VAALYLE;VHLSHAK	NCP	201.025
VHLSHAK;LSVPPFK	NCP	121.82
LSVPPFK;GCKAKGK	NCP	36.204

GCKAKGK;ELRYF	NCP	98.13
ELRYF;LLVGG	NCP	59.851
LLVGG;EEVRLPP	NCP	101.318
EEVRLPP;FPPLSLLK	NCP	53.074
FPPLSLLK;KGLLLGL	NCP	40.046
KGLLLGL;KSQDTPK	NCP	29.353
KSQDTPK;DLETPY	NCP	18.08
DLETPY;KSYLSSH	NCP	54.559
KSYLSSH;ARPLM	NCP	19.208
ARPLM;FAFVQTC	NCP	59.704
FAFVQTC;RFPLMR	NCP	17.027
RFPLMR;MCGSC	NCP	30.271
MCGSC;GFSTAPF	NCP	36.687
GFSTAPF;MDFSCW	NCP	1.125
MDFSCW;EMKPFP	NCP	277.437
EMKPFP;SHPLGPK	NCP	12.788
SHPLGPK;ALGNLMNK	NCP	8.866
ALGNLMNK;GVPPLK	NCP	8.861
GVPPLK;TPVKGPQ	NCP	105.747
TPVKGPQ;HDTLLLL	NCP	47.092
HDTLLLL;VATGLCK	NCP	26.925
VATGLCK;KGGGLTLH	NCP	0.897
KGGGLTLH;NSSVAPY	NCP	85.28
NSSVAPY;PRPLNL	NCP	5.443
PRPLNL;DDLLLK	NCP	9.839
DDLLLK;HDLKNPP	NCP	35.854
HDLKNPP;GDEWQL	NCP	73.035
GDEWQL;RLGFTLT	NCP	28.557
RLGFTLT;MYLPK	NCP	11.655
MYLPK;VTLVSVL	NCP	106.151
VTLVSVL;EVFGK	NCP	24.802
EVFGK;VVLLPL	NCP	161.22
VVLLPL;HSLLVTN	NCP	13.997
HSLLVTN;AAGPVYL	NCP	13.264
AAGPVYL;LVPVPQK	NCP	21.611
LVPVPQK;KLHRPT	NCP	35.803
KLHRPT;KLGYGP	NCP	82.047
KLGYGP;AYPGHGP	NCP	80.924
AYPGHGP;KGHGAPPP	NCP	73.026
KGHGAPPP;KDWPVR	NCP	9.877
KDWPVR;ADSYLN	NCP	8.034
ADSYLN;QGCCC	NCP	30.18
QGCCC;KCPYLFQ	NCP	3.245
KCPYLFQ;RSLGLAH	NCP	2.734
RSLGLAH;KAYSTAL	NCP	10.116
KAYSTAL;CDKPLR	NCP	24.164
CDKPLR;TAPLYE	NCP	66.75
TAPLYE;KYPPKF	NCP	34.992
KYPPKF;GSLEKNF	NCP	54.249
GSLEKNF;LLVGNKCDLTTKK	NCP	12.468
LLVGNKCDLTTKK;LVGNKCDLTTK	NCP	0.003

LVGNKCDLTTK;VGNKCDLTTKK	NCP	0.003
VGNKCDLTTKK;LETSAKNATNVEQSF	NCP	0.42
LETSAKNATNVEQSF;KRMGPGATAGGAEK	NCP	0.069
KRMGPGATAGGAEK;MGPGATAGGAEKSNVK	NCP	0.006
MGPGATAGGAEKSNVK;KCEPRF	NCP	45.679
KCEPRF;KPEFSR	NCP	15.548
KPEFSR;HHLLEKL	NCP	137.89
HHLLEKL;KFTVMK	NCP	101.63
KFTVMK;VDYSPR	NCP	6.354
VDYSPR;CSSPLLLH	NCP	89.7
CSSPLLLH;VYGSLQ	NCP	56.025
VYGSLQ;QGPWEGK	NCP	3.738
QGPWEGK;PLPPPP	NCP	0.72
PLPPPP;KGFLVGP	NCP	6.978
KGFLVGP;LGMSRK	NCP	33.823
LGMSRK;KPPGVAY	NCP	167.781
KPPGVAY;METGWPK	NCP	48.953
METGWPK;PSRPVVK	NCP	43.866
PSRPVVK;LAQLQEA	NCP	40.123
LAQLQEA;HGSTK	NCP	4.919
HGSTK;KHATAA	NCP	30.69
KHATAA;NYMLAK	NCP	11.611
NYMLAK;VGLFSPR	NCP	124.115
VGLFSPR;PVLLWFL	NCP	83.037
PVLLWFL;DLPLTL	NCP	26.266
DLPLTL;EVFPGLK	NCP	22.049
EVFPGLK;KDLPPPP	NCP	17.179
KDLPPPP;FLYPP	NCP	19.034
FLYPP;NRAAF	NCP	38.317
NRAAF;TVGAYVPL	NCP	9.678
TVGAYVPL;LCPGDLK	NCP	3.363
LCPGDLK;CKDLKEK	NCP	6.056
CKDLKEK;TVLDL	NCP	223.5
TVLDL;RLQTVSF	NCP	109.317
RLQTVSF;GPVSMAH	NCP	18.811
GPVSMAH;DSVRLF	NCP	0.746
DSVRLF;NWKGVT	NCP	3.178
NWKGVT;ADYSLN	NCP	3.362
ADYSLN;VGHGVSH	NCP	149.424
VGHGVSH;FDHLRK	NCP	47.593
FDHLRK;VVKKLAL	NCP	58.068
VVKKLAL;MMMGHP	NCP	18.669
MMMGHP;LSGGFLK	NCP	39.435
LSGGFLK;ANCGL	NCP	53.016
ANCGL;DLHYMVY	NCP	16.014
DLHYMVY;VESTGVFT	NCP	0.171
VESTGVFT;PELNGKLTGM	NCP	0.378
PELNGKLTGM;TCRLEKPAK	NCP	0.072
TCRLEKPAK;KAVLLKK	NCP	55.907
KAVLLKK;KPFPVHT	NCP	57.892
KPFPVHT;PAPGKPPK	NCP	5.037

PAPGKPPK;KEALWL	NCP	30.708
KEALWL;ERPLPAH	NCP	35.367
ERPLPAH;VPVPP	NCP	8.786
VPVPP;NLSSVFVQ	NCP	32.281
NLSSVFVQ;MQLFVK	NCP	5.583
MQLFVK;KSPGLKL	NCP	12.018
KSPGLKL;LPTAAQPP	NCP	125.039
LPTAAQPP;LEQLLEQL	NCP	33.522
LEQLLEQL;NCKPLR	NCP	30.682
NCKPLR;FYLKPP	NCP	0.294
FYLKPP;RGPMPP	NCP	55.856
RGPMPP;GTFLK	NCP	98.106
GTFLK;KSMCSP	NCP	4.011
KSMCSP;EGLVLP	NCP	13.575
EGLVLP;PGVYWK	NCP	58.324
PGVYWK;TTGLTTF	NCP	1.152
TTGLTTF;AQPLVH	NCP	23.675
AQPLVH;QQAKNR	NCP	28.012
QQAKNR;KDPKVTK	NCP	32.168
KDPKVTK;ASGKPQ	NCP	17.037
ASGKPQ;QPSVHR	NCP	25.453
QPSVHR;MTLSVQK	NCP	44.963
MTLSVQK;PFLFTEV	NCP	11.425
PFLFTEV;QFAEFCF	NCP	9.227
QFAEFCF;KVLFCYF	NCP	93.66
KVLFCYF;ATLTVFPK	NCP	37.993
ATLTVFPK;LPLKDLT	NCP	61.662
LPLKDLT;DSEDC	NCP	9.063
DSEDC;VSGAALK	NCP	2.175
VSGAALK;VGLNVKS	NCP	19.478
VGLNVKS;ALRLTEY	NCP	9.117
ALRLTEY;SRTLSGMESY	NCP	0.021
SRTLSGMESY;LEELVVQPL	NCP	2.028
LEELVVQPL;TNGGVGK	NCP	32.929
TNGGVGK;DKPKTY	NCP	6.264
DKPKTY;SLRPLQF	NCP	19.812
SLRPLQF;EKLGSR	NCP	28.521
EKLGSR;LTTKLSPK	NCP	121.281
LTTKLSPK;CMDHSGK	NCP	31.238
CMDHSGK;TMLADHAARQLLDF	NCP	50.58
TMLADHAARQLLDF;FFPPPKY	NCP	37.5
FFPPPKY;FLGFL	NCP	101.874
FLGFL;MHAVPY	NCP	27.384
MHAVPY;APAEPPR	NCP	1.744
APAEPPR;MLSSFLR	NCP	0.128
MLSSFLR;MVPPWR	NCP	2.158
MVPPWR;ALVFVPVL	NCP	63.618
ALVFVPVL;VGEPPRG	NCP	38.556
VGEPPRG;SAAGPPPP	NCP	2.163
SAAGPPPP;PPMLK	NCP	0.993
PPMLK;RALPHM	NCP	79.818

RALPHM;PALLTNRL	NCP	28.95
PALLTNRL;KAAHLLY	NCP	41.202
KAAHLLY;LSEGKPL	NCP	16.242
LSEGKPL;VPGVR	NCP	39.045
VPGVR;FARPPD	NCP	109.307
FARPPD;LGPELTP	NCP	10.392
LGPELTP;LGLDKV	NCP	3.046
LGLDKV;KDYEEVGVD	NCP	64.812
KDYEEVGVD;NLTLTLR	NCP	118.202
NLTLTLR;LDDVVKL	NCP	29.445
LDDVVKL;MLLLLE	NCP	60.285
MLLLLE;PYSSFLR	NCP	5.425
PYSSFLR;SQLPFLQ	NCP	88.647
SQLPFLQ;EKNTEF	NCP	158.612
EKNTEF;CSPAK	NCP	4.362
CSPAK;LDAPGHR	NCP	88.026
LDAPGHR;SGAELLEL	NCP	18.739
SGAELLEL;ALPPYVP	NCP	35.679
ALPPYVP;GVHVLFL	NCP	31.848
GVHVLFL;EEHALLL	NCP	7.059
EEHALLL;KFTLEQEK	NCP	34.842
KFTLEQEK;PAPPVLPPP	NCP	28.773
PAPPVLPPP;PAAAMP	NCP	44.847
PAAAMP;ALPKPEP	NCP	15.201
ALPKPEP;KNVLGAR	NCP	13.881
KNVLGAR;LQKGAPY	NCP	36.114
LQKGAPY;LTAAPEL	NCP	3.732
LTAAPEL;KNTKPNL	NCP	18.566
KNTKPNL;LMTVMP	NCP	22.18
LMTVMP;KQDAGPLP	NCP	188.019
KQDAGPLP;YAAYLF	NCP	5.378
YAAYLF;LQALYTVS	NCP	12.418
LQALYTVS;LLSVLTKH	NCP	18.314
LLSVLTKH;LDNLKGPT	NCP	62.062
LDNLKGPT;LLQTQPR	NCP	28.122
LLQTQPR;LSPQEEPGK	NCP	35.724
LSPQEEPGK;DSFAY	NCP	29.165
DSFAY;KPMLKP	NCP	254.002
KPMLKP;DHTSLPL	NCP	17.759
DHTSLPL;FLTLKGS	NCP	18.592
FLTLKGS;QTPKYH	NCP	10.091
QTPKYH;ATMSPMK	NCP	47.785
ATMSPMK;AGGPR	NCP	14.646
AGGPR;TYTLLK	NCP	22.017
TYTLLK;TPPPAPK	NCP	62.097
TPPPAPK;YLEAVLAT	NCP	16.214
YLEAVLAT;CMWEVGK	NCP	15.652
CMWEVGK;GPPPNRPP	NCP	12.231
GPPPNRPP;KGSSSRP	NCP	41.16
KGSSSRP;APELEAE	NCP	0.345
APELEAE;KELPLAGK	NCP	19.26

KELPLAGK;AALPLKK	NCP	9.33
AALPLKK;PLATGLP	NCP	1.497
PLATGLP;ESNRGPP	NCP	136.485
ESNRGPP;LTTVGHF	NCP	22.016
LTTVGHF;KDMENF	NCP	23.358
KDMENF;DPFLDL	NCP	120.277
DPFLDL;KTVPGAR	NCP	110.31
KTVPGAR;FSNPPGP	NCP	57.267
FSNPPGP;EKVLL	NCP	14.505
EKVLL;LAFLTKK	NCP	4.2
LAFLTKK;PAAEPP	NCP	32.505
PAAEPP;ETMKFER	NCP	4.199
ETMKFER;PAAWHAK	NCP	63.457
PAAWHAK;MLRSGK	NCP	69.489
MLRSGK;QALKLLKT	NCP	14.808
QALKLLKT;KPWTQ	NCP	27.462
KPWTQ;MVLDAP	NCP	6.954
MVLDAP;VVGSGSPK	NCP	7.611
VVGSGSPK;LSYSEFR	NCP	10.659
LSYSEFR;KHPREVK	NCP	70.137
KHPREVK;ENLLTLL	NCP	3.726
ENLLTLL;VLEEDLSK	NCP	31.523
VLEEDLSK;NGSLPF	NCP	29.083
NGSLPF;TQGPLCP	NCP	40.932
TQGPLCP;PGVTVKDVN	NCP	21.531
PGVTVKDVN;TKVNLP	NCP	25.394
TKVNLP;PGRLTPP	NCP	3.112
PGRLTPP;KAEVVAP	NCP	40.131
KAEVVAP;LEPGSYK	NCP	2.169
LEPGSYK;VLLAAAV	NCP	49.725
VLLAAAV;KNGSLPF	NCP	7.623
KNGSLPF;KLLKPEK	NCP	59.996
KLLKPEK;VGFDLRLPY	NCP	4.194
VGFDLRLPY;KGMGDLK	NCP	21.763
KGMGDLK;PDDPPR	NCP	46.68
PDDPPR;TVNPPF	NCP	68.75
TVNPPF;LFDRDL	NCP	0.247
LFDRDL;PPLHKPK	NCP	12.804
PPLHKPK;LPPELP	NCP	109.059
LPPELP;KEMHVW	NCP	12.303
KEMHVW;PAGASN	NCP	56.454
PAGASN;KTFGVT	NCP	44.186
KTFGVT;DDDSG	NCP	47.374
DDDSG;AAVSSVR	NCP	5.622
AAVSSVR;PHALMRL	NCP	105.156
PHALMRL;VFQAAVPK	NCP	6.824
VFQAAVPK;VERLLEPL	NCP	22.002
VERLLEPL;KYRLLPG	NCP	49.888
KYRLLPG;AAVPWN	NCP	29.103
AAVPWN;LELLTDR	NCP	34.403
LELLTDR;NPPSSKP	NCP	45.837

NPPSSKP;ESSHAPK	NCP	27.279
ESSHAPK;HWMLDK	NCP	39.76
HWMLDK;PLPPHMT	NCP	104.685
PLPPHMT;AAVLGDA	NCP	15.695
AAVLGDA;LPRAGTP	NCP	8.901
LPRAGTP;DSLTSKY	NCP	9.534
DSLTSKY;LPPAAGPL	NCP	35.044
LPPAAGPL;LSLPFVR	NCP	75.158
LSLPFVR;SETFVAK	NCP	4.74
SETFVAK;SRYKKM	NCP	61.254
SRYKKM;KAAPPPR	NCP	4.549
KAAPPPR;AVCAAY	NCP	2.934
AVCAAY;LLWHQK	NCP	34.589
LLWHQK;TPKMPP	NCP	45.637
TPKMPP;DFLNNP	NCP	14.651
DFLNNP;EELFKRV	NCP	58.438
EELFKRV;WLPVVR	NCP	24.33
WLPVVR;SPFVLGV	NCP	82.329
SPFVLGV;TDLRKEE	NCP	16.523
TDLRKEE;AVLPMTR	NCP	36.045
AVLPMTR;LTVRPP	NCP	52.789
LTVRPP;ALEFVA	NCP	3.303
ALEFVA;MLPQNA	NCP	27.363
MLPQNA;GTFGLA	NCP	12.134
GTFGLA;LHAGLAAP	NCP	37.806
LHAGLAAP;KMSRAP	NCP	60.609
KMSRAP;FPEPLK	NCP	24.882
FPEPLK;RLHEEGP	NCP	20.655
RLHEEGP;VLPLLYK	NCP	77.2
VLPLLYK;KPQMPPP	NCP	143.724
KPQMPPP;SCLLLH	NCP	72.462
SCLLLH;NPVSTFHV	NCP	9.266
NPVSTFHV;NYALLRL	NCP	40.638
NYALLRL;KPFNPPP	NCP	8.127
KPFNPPP;KHVWMK	NCP	45.088
KHVWMK;DAYVRP	NCP	22.662
DAYVRP;VTLNV	NCP	9.315
VTLNV;KAQLYYP	NCP	28.17
KAQLYYP;EFCRPL	NCP	74.891
EFCRPL;APDLPKL	NCP	3.516
APDLPKL;VSDSLLP	NCP	8.218
VSDSLLP;TSMMSVH	NCP	40.65
TSMMSVH;AYLFD	NCP	12.092
AYLFD;QTYVYK	NCP	72.174
QTYVYK;KVPQLELV	NCP	109.216
KVPQLELV;LLGVETL	NCP	75.039
LLGVETL;KPQHDAK	NCP	143.865
KPQHDAK;VTPLK	NCP	78.809
VTPLK;ALGVGL	NCP	15.934
ALGVGL;MVFHHK	NCP	242.015
MVFHHK;NPSKYR	NCP	82.594

NPSKYR;LKDYL	NCP	47.157
LKDYL;MNPVV	NCP	27.237
MNPVV;DSLQLRR	NCP	125.331
DSLQLRR;FLVHNVK	NCP	114.882
FLVHNVK;APPPPP	NCP	96.764
APPPPP;KLLLLLKLK	NCP	184.269
KLLLLLKLK;PAATTS	NCP	14.532
PAATTS;KHLVYP	NCP	15.547
KHLVYP;MDMKRR	NCP	147.596
MDMKRR;NWLHRP	NCP	46.152
NWLHRP;DLFAP	NCP	42.307
DLFAP;GVVAPPG	NCP	11.877
GVVAPPG;TVFGLSPK	NCP	73.556
TVFGLSPK;KEDMLKK	NCP	11.049
KEDMLKK;AAAVHC	NCP	49.081
AAAVHC;VAFLPM	NCP	57.171
VAFLPM;REGSVAM	NCP	82.503
REGSVAM;MVYGTF	NCP	0.233
MVYGTF;KSGGL	NCP	52.932
KSGGL;LLLDFTAL	NCP	18.939
LLLDFTAL;NCTHMD	NCP	129.277
NCTHMD;HALRTSSK	NCP	51.549
HALRTSSK;KPFHVGP	NCP	48.408
KPFHVGP;FRTVGQ	NCP	46.44
FRTVGQ;CDFKDC	NCP	35.561
CDFKDC;KKESVPK	NCP	19.809
KKESVPK;APPKLPN	NCP	215.083
APPKLPN;KSKKLSL	NCP	7.41
KSKKLSL;LEYLNSKK	NCP	40.946
LEYLNSKK;VELARC	NCP	5.481
VELARC;MLPGVR	NCP	152.895
MLPGVR;KGVLVPN	NCP	19.063
KGVLVPN;MGMVKK	NCP	21.806
MGMVKK;AVTWKP	NCP	32.238
AVTWKP;HNTALPK	NCP	59.583
HNTALPK;KERYVLE	NCP	30.31
KERYVLE;LPSKETLEQ	NCP	233.174
LPSKETLEQ;KAGKVGL	NCP	137.58
KAGKVGL;GKSHVLE	NCP	42.705
GKSHVLE;CYEPKR	NCP	43.06
CYEPKR;VSLDFP	NCP	87.026
VSLDFP;TPLVTK	NCP	58.162
TPLVTK;EVPGGGW	NCP	63.209
EVPGGGW;LPAKSVPK	NCP	42.381
LPAKSVPK;VTVEPY	NCP	56.74
VTVEPY;YFAPKGP	NCP	4.496
YFAPKGP;DVDLLR	NCP	71.079
DVDLLR;HQLFY	NCP	57.798
HQLFY;KKLGMQ	NCP	13.779
KKLGMQ;KPWTVR	NCP	23.227
KPWTVR;PPDNP	NCP	20.319

PPDNP;ESLLTAK	NCP	75.855
ESLLTAK;EDHMSE	NCP	20.841
EDHMSE;GVLDAL	NCP	10.995
GVLDAL;LEKFDKS	NCP	51.134
LEKFDKS;APGSSP	NCP	30.808
APGSSP;PPPLDPP	NCP	60.282
PPPLDPP;VGAPSWL	NCP	45.173
VGAPSWL;ADKPDLGE	NCP	16.317
ADKPDLGE;SFDKAKLKKTETQEKN	NCP	0.03
SFDKAKLKKTETQEKN;FDKAKLKKTETQEK	NCP	0.003
FDKAKLKKTETQEK;KTETQEKNTLPTK	NCP	0.021
KTETQEKNTLPTK;EQEKQAK	NCP	0.327
EQEKQAK;SVPSPPAR	NCP	0.147
SVPSPPAR;AATANFP	NCP	48.34
AATANFP;LRLVKL	NCP	4.769
LRLVKL;MEGELKK	NCP	22.413
MEGELKK;AAHRKFC	NCP	10.24
AAHRKFC;KDCRLL	NCP	19.373
KDCRLL;LNWKPK	NCP	122.968
LNWKPK;KYPPRPP	NCP	26.057
KYPPRPP;KDHFLT	NCP	0.664
KDHFLT;SVTPSLAH	NCP	43.761
SVTPSLAH;LVRASKE	NCP	33.101
LVRASKE;VTLPF	NCP	3.429
VTLPF;LSRLPY	NCP	21.441
LSRLPY;VVPNDL	NCP	98.512
VVPNDL;SLLALYK	NCP	21.879
SLLALYK;TTYAFSS	NCP	32.079
TTYAFSS;QQRQPSPP	NCP	114.167
QQRQPSPP;PLLVVLK	NCP	1.05
PLLVVLK;RDSFPWEVPKK	NCP	5.325
RDSFPWEVPKK;DSFPWEVPK	NCP	0.003
DSFPWEVPK;LLELAK	NCP	29.925
LLELAK;GPAMLLK	NCP	110.517
GPAMLLK;PFMDLK	NCP	16.918
PFMDLK;QDKTY	NCP	97.14
QDKTY;LLVLDPF	NCP	14.846
LLVLDPF;LLAGMPK	NCP	28.042
LLAGMPK;VPFHLRP	NCP	5.489
VPFHLRP;CRHMRVC	NCP	1.494
CRHMRVC;GPDAASQP	NCP	53.688
GPDAASQP;VGSPVK	NCP	15.472
VGSPVK;VTLVT	NCP	1.952
VTLVT;KAVSPVT	NCP	45.657
KAVSPVT;SVEDLPL	NCP	23.601
SVEDLPL;KKYFGP	NCP	12.853
KKYFGP;TGPLNP	NCP	46.841
TGPLNP;LGKTVLK	NCP	1.045
LGKTVLK;LDDLNPR	NCP	44.124
LDDLNPR;PDWGKNK	NCP	13.253
PDWGKNK;GVWEPR	NCP	18.783

GVWEPR;KEKKPKK	NCP	58.554
KEKKPKK;KEKTAPP	NCP	38.172
KEKTAPP;FNFPEAF	NCP	37.719
FNFPEAF;VASVTP	NCP	17.56
VASVTP;FSYPGN	NCP	20.927
FSYPGN;VLYDRK	NCP	8.185
VLYDRK;ASYKQHV	NCP	123.306
ASYKQHV;MTMSRVP	NCP	13.091
MTMSRVP;NCCCCGY	NCP	11.607
NCCCCGY;FDDTSF	NCP	7.326
FDDTSF;KDMLSKE	NCP	64.632
KDMLSKE;QLVKDLH	NCP	18.324
QLVKDLH;TPPLKNYL	NCP	59.106
TPPLKNYL;KPGSPRP	NCP	7.177
KPGSPRP;VFGLK	NCP	25.272
VFGLK;LGPGF	NCP	123.659
LGPGF;PFVELN	NCP	50.25
PFVELN;KSADLHL	NCP	19.659
KSADLHL;RLYQLQR	NCP	61.161
RLYQLQR;EKNGAPK	NCP	23.494
EKNGAPK;LKQAQQVK	NCP	89.184
LKQAQQVK;KAMVQPP	NCP	126.524
KAMVQPP;LGRVMKF	NCP	10.284
LGRVMKF;SVELLFP	NCP	89.472
SVELLFP;VAPLVPL	NCP	133.416
VAPLVPL;APLQP	NCP	29.76
APLQP;KLYVPK	NCP	35.176
KLYVPK;VMPAAPPK	NCP	115.562
VMPAAPPK;ATFKP	NCP	33.354
ATFKP;TKYGFN	NCP	16.177
TKYGFN;GMQDGSVNMREKPF	NCP	26.729
GMQDGSVNMREKPF;TAPVNKF	NCP	102.711
TAPVNKF;KDAVQPK	NCP	198.312
KDAVQPK;PSKVPGK	NCP	4.455
PSKVPGK;YSALAL	NCP	1.26
YSALAL;LPPPPLRLT	NCP	105.105
LPPPPLRLT;KWGAPK	NCP	5.214
KWGAPK;PRVEC	NCP	28.833
PRVEC;HQVPEPR	NCP	4.413
HQVPEPR;VAVVDTGR	NCP	17.76
VAVVDTGR;LGQQLAF	NCP	21.636
LGQQLAF;DMGVGF	NCP	52.932
DMGVGF;RLGPQKP	NCP	13.731
RLGPQKP;TWVHGLR	NCP	18.567
TWVHGLR;RCRVSEP	NCP	1.428
RCRVSEP;FYPPVR	NCP	58.662
FYPPVR;RLSDVAHY	NCP	53.959
RLSDVAHY;PTKTGP	NCP	137.432
PTKTGP;LPSTWQV	NCP	26.011
LPSTWQV;TFGGLSY	NCP	6.02
TFGGLSY;LDSLVVPQ	NCP	15.159

LDSLVVPQ;KLSLLC	NCP	16.237
KLSLLC;PKSGLPQ	NCP	42.545
PKSGLPQ;MGLMSN	NCP	1.396
MGLMSN;KPERRAP	NCP	24.578
KPERRAP;DSLQLF	NCP	8.272
DSLQLF;TQKEVLK	NCP	9.291
TQKEVLK;MSKRPT	NCP	37.877
MSKRPT;PVWGNN	NCP	37.378
PVWGNN;LAVTF	NCP	17.493
LAVTF;KPEFYP	NCP	71.103
KPEFYP;QSPMQL	NCP	72.999
QSPMQL;LPAVDLLK	NCP	53.171
LPAVDLLK;KGPPHPQ	NCP	33.897
KGPPHPQ;RPGGFKL	NCP	6.615
RPGGFKL;GAASQSF	NCP	101.899
GAASQSF;HEEPVLL	NCP	31.847
HEEPVLL;GVVKPC	NCP	17.325
GVVKPC;DPAAVPK	NCP	0.09
DPAAVPK;EAKQLR	NCP	4.638
EAKQLR;KCLTVSL	NCP	12.208
KCLTVSL;KVKLSYR	NCP	230.091
KVKLSYR;KVEQP	NCP	5.381
KVEQP;KEFNRF	NCP	67.566
KEFNRF;RNQPTKP	NCP	3.826
RNQPTKP;KDVHLQ	NCP	47.604
KDVHLQ;PVFKPH	NCP	5.126
PVFKPH;VPLSFVG	NCP	47.482
VPLSFVG;AAPVHVT	NCP	119.039
AAPVHVT;EALYG	NCP	91.302
EALYG;MAGLMN	NCP	111.187
MAGLMN;FAAPD	NCP	1.328
FAAPD;KKHSLYDP	NCP	85.45
KKHSLYDP;KSNPLMK	NCP	49.311
KSNPLMK;VPPVP	NCP	20.097
VPPVP;YEMQAY	NCP	0.383
YEMQAY;MEALEFD	NCP	172.407
MEALEFD;FDSVSRLAVD	NCP	0.015
FDSVSRLAVD;AMASPRMLK	NCP	4.218
AMASPRMLK;ASPRMLKT	NCP	0.006
ASPRMLKT;LPPSFLEEN	NCP	0.042
LPPSFLEEN;GVVGDWKNHFT	NCP	3.435
GVVGDWKNHFT;ADTTLTL	NCP	0.078
ADTTLTL;LYSAFT	NCP	27.832
LYSAFT;DLQFPSR	NCP	28.112
DLQFPSR;DMPPGT	NCP	15.343
DMPPGT;LAETLGRK	NCP	57.662
LAETLGRK;TGPGLLSMA	NCP	68.625
TGPGLLSMA;KVTKSPP	NCP	30.924
KVTKSPP;VGCLHY	NCP	41.446
VGCLHY;RDVLKY	NCP	52.287
RDVLKY;QEPPPPPP	NCP	11.537

QEPPPPPP;LVGSKHPP	NCP	18.841
LVGSKHPP;FDLGAAR	NCP	0.251
FDLGAAR;MDMPF	NCP	13.141
MDMPF;VECLAR	NCP	44.138
VECLAR;CDVDKLR	NCP	4.578
CDVDKLR;GPDYCL	NCP	7.711
GPDYCL;LPAPW	NCP	17.3
LPAPW;PAATRPP	NCP	60.315
PAATRPP;PAADKAH	NCP	58.332
PAADKAH;AMRPPR	NCP	12.183
AMRPPR;EFAYL	NCP	194.437
EFAYL;SLVKL	NCP	94.094
SLVKL;LRGEC	NCP	10.585
LRGEC;ALETVPK	NCP	59.444
ALETVPK;KSGLLLAK	NCP	11.133
KSGLLLAK;WFLLMPL	NCP	19.828
WFLLMPL;TQLLPR	NCP	1.398
TQLLPR;WLYYK	NCP	44.969
WLYYK;LEAAQPT	NCP	0.838
LEAAQPT;QTESHHK	NCP	55.529
QTESHHK;AGLVKELS	NCP	38.431
AGLVKELS;KNPFLR	NCP	1.739
KNPFLR;FEQVCK	NCP	37.869
FEQVCK;GPMEHHQ	NCP	61.876
GPMEHHQ;WVKLENTK	NCP	61.827
WVKLENTK;ESLVR	NCP	50.813
ESLVR;ELTSSTTF	NCP	15.607
ELTSSTTF;GKQGL	NCP	32.643
GKQGL;HDLKLP	NCP	24.705
HDLKLP;ENGRPP	NCP	34.634
ENGRPP;LVAPKPH	NCP	86.781
LVAPKPH;KGVVLF	NCP	20.179
KGVVLF;ASAYVY	NCP	85.274
ASAYVY;KPHNFR	NCP	28.579
KPHNFR;AAPPQ	NCP	1.718
AAPPQ;LSLLNVGL	NCP	61.81
LSLLNVGL;WLEEKLL	NCP	27.206
WLEEKLL;KAFYPK	NCP	4.252
KAFYPK;DAAEVLK	NCP	11.471
DAAEVLK;SPLKEK	NCP	15.879
SPLKEK;HLLPPP	NCP	4.515
HLLPPP;LEAEALSL	NCP	103.519
LEAEALSL;MNFGLS	NCP	13.635
MNFGLS;PPPPTLPR	NCP	1.137
PPPPTLPR;GSTRVSP	NCP	38.826
GSTRVSP;KHYPP	NCP	20.648
KHYPP;PFASHLL	NCP	1.309
PFASHLL;VTVLP	NCP	37.049
VTVLP;LLGLGP	NCP	36.456
LLGLGP;TMLNPPP	NCP	25.51
TMLNPPP;FALPVD	NCP	50.826

FALPVD;PLLPPPP	NCP	129.39
PLLPPPP;YKLLPP	NCP	5.003
YKLLPP;ESPERPF	NCP	2.869
ESPERPF;RGKALPP	NCP	17.826
RGKALPP;KTDTSLPK	NCP	73.485
KTDTSLPK;GVGASL	NCP	40.494
GVGASL;PLGVPHP	NCP	77.679
PLGVPHP;FPLGLLP	NCP	3.039
FPLGLLP;SDAMSN	NCP	23.021
SDAMSN;TAPGLPY	NCP	10.012
TAPGLPY;ALRQLLL	NCP	11.975
ALRQLLL;PVGAPSS	NCP	7.93
PVGAPSS;QPNAPGF	NCP	74.715
QPNAPGF;LLLFLEPQ	NCP	81.315
LLLFLEPQ;MNADDC	NCP	26.637
MNADDC;NDEFGY	NCP	44.721
NDEFGY;RPLAKAH	NCP	95.144
RPLAKAH;VSLNPFL	NCP	18.81
VSLNPFL;CKRPK	NCP	2.286
CKRPK;QPGGPLP	NCP	31.053
QPGGPLP;KELSY	NCP	39.492
KELSY;QSHHKF	NCP	58.593
QSHHKF;KLGVGGGK	NCP	46.731
KLGVGGGK;VPPPSKRH	NCP	11.643
VPPPSKRH;KAARVDN	NCP	15.199
KAARVDN;LYKQKPLE	NCP	1.097
LYKQKPLE;EEEVYK	NCP	14.188
EEEVYK;HFENFK	NCP	18.614
HFENFK;QLLLF	NCP	23.544
QLLLF;PRHFPK	NCP	42.486
PRHFPK;GLSKGY	NCP	65.547
GLSKGY;LPGLQR	NCP	83.586
LPGLQR;KNSFKKAF	NCP	1.374
KNSFKKAF;SSGKFPP	NCP	60.424
SSGKFPP;FTMALQ	NCP	35.273
FTMALQ;PLLTNM	NCP	23.134
PLLTNM;EETAAPL	NCP	145.358
EETAAPL;RVYELGK	NCP	20.91
RVYELGK;PDPVE	NCP	86.362
PDPVE;EKPPWLPA	NCP	3.183
EKPPWLPA;LLKSRQP	NCP	8.693
LLKSRQP;AAWLGER	NCP	54.342
AAWLGER;SLHKRTF	NCP	67.978
SLHKRTF;PAGSR	NCP	264.624
PAGSR;NGMCD	NCP	27.117
NGMCD;ALLKT	NCP	5.954
ALLKT;LEKPEDL	NCP	18.919
LEKPEDL;KTSMPK	NCP	2.585
KTSMPK;KVSLKKP	NCP	104.92
KVSLKKP;EVLLL	NCP	84.668
EVLLL;GLSDEK	NCP	24.273

GLSDEK;VDVPY	NCP	147.597
VDVPY;PLAVKTH	NCP	1.689
PLAVKTH;KSMLEP	NCP	17.337
KSMLEP;NGMLEL	NCP	10.263
NGMLEL;FAFPGPL	NCP	12.702
FAFPGPL;VVLFP	NCP	10.866
VVLFP;NMPVVG	NCP	53.554
NMPVVG;KAQVEELK	NCP	3.428
KAQVEELK;LEELEK	NCP	30.015
LEELEK;VSLMGCK	NCP	59.569
VSLMGCK;SLLVTLA	NCP	41.789
SLLVTLA;NPALKKE	NCP	142.954
NPALKKE;LVNMTL	NCP	35.099
LVNMTL;THLWF	NCP	0.295
THLWF;NSFVVT	NCP	46.263
NSFVVT;LSDLLP	NCP	47.601
LSDLLP;KPAGP	NCP	95.147
KPAGP;LNWELGK	NCP	61.684
LNWELGK;MEKGPK	NCP	84.459
MEKGPK;SEVEL	NCP	36.122
SEVEL;VFGFC	NCP	101.412
VFGFC;DRTKLF	NCP	85.078
DRTKLF;PVVSK	NCP	14.049
PVVSK;FGGLNSR	NCP	8.355
FGGLNSR;SPGSLVHR	NCP	83.93
SPGSLVHR;SLLAA	NCP	13.327
SLLAA;LPKRKPE	NCP	1.32
LPKRKPE;LGSLAVTK	NCP	24.686
LGSLAVTK;KRLYFPH	NCP	11.664
KRLYFPH;PYELKK	NCP	54.616
PYELKK;LSRAAVP	NCP	98.103
LSRAAVP;TVAAGVP	NCP	59.786
TVAAGVP;SHNGKHR	NCP	38.634
SHNGKHR;RYMPPP	NCP	0.222
RYMPPP;AMQKLF	NCP	0.181
AMQKLF;KLAELKK	NCP	6.978
KLAELKK;GVPLE	NCP	21.417
GVPLE;LAFPLLK	NCP	25.427
LAFPLLK;RDGKAGM	NCP	39.82
RDGKAGM;SHLLFWP	NCP	45.222
SHLLFWP;PYQSL	NCP	13.923
PYQSL;FSLPL	NCP	89.235
FSLPL;LDLPK	NCP	15.312
LDLPK;KFYPPAGP	NCP	92.949
KFYPPAGP;ALMCGL	NCP	38.481
ALMCGL;LGNKLTC	NCP	20.748
LGNKLTC;DGHVCAC	NCP	14.366
DGHVCAC;LLEDHGK	NCP	28.29
LLEDHGK;LDLESF	NCP	74.836
LDLESF;VSSKPQK	NCP	10.914
VSSKPQK;NLATLTK	NCP	0.515

NLATLTK;SLPPSLY	NCP	15.903
SLPPSLY;PVKPY	NCP	69.454
PVKPY;GGVNSF	NCP	49.472
GGVNSF;MDSDC	NCP	22.489
MDSDC;LNLGSGL	NCP	40.001
LNLGSGL;KPLFHVP	NCP	18.198
KPLFHVP;LDSLFP	NCP	30.973
LDSLFP;EHTLEP	NCP	33.969
EHTLEP;ASPVVPP	NCP	22.173
ASPVVPP;KTHVPP	NCP	114.081
KTHVPP;LSHAPMYY	NCP	20.091
LSHAPMYY;THGDLR	NCP	99.797
THGDLR;KDWNYK	NCP	14.77
KDWNYK;MEDMDQSPLVSSSSDSPPRPQPAF	NCP	90.458
MEDMDQSPLVSSSSDSPPRPQPAF;VSSSSDSPPRPQPA	NCP	0.027
VSSSSDSPPRPQPA;SSSDSPPRPQP	NCP	0.006
SSSDSPPRPQP;VLERKPAAA	NCP	0.132
VLERKPAAA;LVHRSM	NCP	62.239
LVHRSM;LGLANKNVKDAMAK	NCP	11.15
LGLANKNVKDAMAK;MKVTKK	NCP	15.814
MKVTKK;VKNTLLK	NCP	11.117
VKNTLLK;GSSGPK	NCP	11.904
GSSGPK;KYNPP	NCP	4.242
KYNPP;KELLLK	NCP	5.989
KELLLK;DVDRLK	NCP	0.914
DVDRLK;LDVPKL	NCP	5.368
LDVPKL;DLKPML	NCP	22.854
DLKPML;RNVLLAE	NCP	22.878
RNVLLAE;AKYLNA	NCP	0.291
AKYLNA;DLVVL	NCP	18.932
DLVVL;AAAFKKK	NCP	170.7
AAAFKKK;KGALLVQ	NCP	48.069
KGALLVQ;LYTLAE	NCP	7.929
LYTLAE;RLGNTHPK	NCP	12.51
RLGNTHPK;MQVPGGP	NCP	5.052
MQVPGGP;NVERSAGK	NCP	26.436
NVERSAGK;VGRGSNR	NCP	0.153
VGRGSNR;AALGQK	NCP	10.309
AALGQK;TVPLLPP	NCP	21.462
TVPLLPP;NWSGPK	NCP	15.48
NWSGPK;APPDM	NCP	19.569
APPDM;LPYAGP	NCP	30.443
LPYAGP;DPFKLR	NCP	23.214
DPFKLR;MMAEEM	NCP	35.539
MMAEEM;SSSEKPF	NCP	7.662
SSSEKPF;LYMSAQ	NCP	12.305
LYMSAQ;KTYPEF	NCP	62.497
KTYPEF;KTFEPLL	NCP	68.009
KTFEPLL;RLYVW	NCP	8.622
RLYVW;KVELC	NCP	7.729
KVELC;DFTLPN	NCP	49.911

DFTLPN;FPSALGL	NCP	6.168
FPSALGL;LQKAAPQ	NCP	6.231
LQKAAPQ;KDVHGP	NCP	16.539
KDVHGP;TVTLLNK	NCP	1.157
TVTLLNK;KTSKSQ	NCP	1.762
KTSKSQ;PTTPDLP	NCP	29.634
PTTPDLP;KPEAFAAK	NCP	10.125
KPEAFAAK;NKQLKE	NCP	4.376
NKQLKE;FTPKYP	NCP	55.302
FTPKYP;LDRFVSF	NCP	6.421
LDRFVSF;VLPYLY	NCP	20.045
VLPYLY;FGYWY	NCP	37.398
FGYWY;PQDLGAR	NCP	0.01
PQDLGAR;LSGKSKK	NCP	13.23
LSGKSKK;KLPKMVP	NCP	6.302
KLPKMVP;GHALGYP	NCP	31.327
GHALGYP;PPWLKK	NCP	3.371
PPWLKK;CHKYLM	NCP	37.965
CHKYLM;LPPPFFK	NCP	16.095
LPPPFFK;VLKAQAQ	NCP	9.568
VLKAQAQ;MPLLKR	NCP	10.559
MPLLKR;VSLELALR	NCP	80.596
VSLELALR;LGPAEVY	NCP	70.685
LGPAEVY;LGLDF	NCP	26.605
LGLDF;HDALK	NCP	159.107
HDALK;AFLKME	NCP	56.476
AFLKME;AERLAY	NCP	2.226
AERLAY;MAATT	NCP	35.967
MAATT;HTLVSF	NCP	25.22
HTLVSF;VTEVVR	NCP	80.416
VTEVVR;KSTTSPSK	NCP	117.465
KSTTSPSK;PGVRLKL	NCP	4.439
PGVRLKL;LELSQAAL	NCP	2.203
LELSQAAL;NVVGVLK	NCP	19.073
NVVGVLK;KVVGPD	NCP	16.768
KVVGPD;RKLVHA	NCP	10.974
RKLVHA;KPPQPPR	NCP	31.98
KPPQPPR;LLSKGH	NCP	4.145
LLSKGH;KGLTVDLK	NCP	21.27
KGLTVDLK;VCDLPPR	NCP	26.319
VCDLPPR;RLLLFSPF	NCP	12.367
RLLLFSPF;AAVWGNK	NCP	29.351
AAVWGNK;LPPLPKPR	NCP	81.123
LPPLPKPR;GPAAVR	NCP	29.218
GPAAVR;DPFATAK	NCP	23.946
DPFATAK;FENLCKL	NCP	7.458
FENLCKL;QSLCK	NCP	8.783
QSLCK;MPAGAP	NCP	29.503
MPAGAP;FQLPPPPP	NCP	130.197
FQLPPPPP;SCCFRAK	NCP	164.856
SCCFRAK;SDLRLL	NCP	22.943

SDLRLL;GNLRLLDH	NCP	3.889
GNLRLLDH;LGNKVDSY	NCP	49.775
LGNKVDSY;KEVALPK	NCP	19.141
KEVALPK;PLPVQ	NCP	16.094
PLPVQ;TPMQL	NCP	29.893
TPMQL;KTLLFTL	NCP	9.201
KTLLFTL;KVVLLCF	NCP	12.29
KVVLLCF;LYPLPH	NCP	66.688
LYPLPH;KSWELC	NCP	140.259
KSWELC;PNPGKHT	NCP	79.97
PNPGKHT;QLAPCQ	NCP	1.369
QLAPCQ;EEPKKL	NCP	10.563
EEPKKL;YCKCK	NCP	23.606
YCKCK;LDVTGPK	NCP	1.927
LDVTGPK;KVDPPP	NCP	10.673
KVDPPP;TGSDLRK	NCP	45.622
TGSDLRK;LGMDPK	NCP	24.323
LGMDPK;MEYFPV	NCP	7.962
MEYFPV;KASQPL	NCP	23.638
KASQPL;SAHVPPR	NCP	58.613
SAHVPPR;KNWGF	NCP	25.528
KNWGF;EHLSEKP	NCP	32.322
EHLSEKP;RVGVVLA	NCP	52.629
RVGVVLA;KLVKLKLP	NCP	24.272
KLVKLKLP;KMTTKAV	NCP	32.749
KMTTKAV;KDSQLDK	NCP	10.517
KDSQLDK;VSLDNP	NCP	36.073
VSLDNP;EVLRGWEE	NCP	9.177
EVLRGWEE;ELPPSLKK	NCP	16.619
ELPPSLKK;TPSVHKT	NCP	7.204
TPSVHKT;KELPASP	NCP	9.576
KELPASP;KGSNGP	NCP	66.42
KGSNGP;KEFLE	NCP	18.533
KEFLE;LGPGPPGP	NCP	100.462
LGPGPPGP;LHGSFFL	NCP	135.357
LHGSFFL;LEVPVAR	NCP	5.484
LEVPVAR;KTQPK	NCP	65.535
KTQPK;KPFDLP	NCP	76.014
KPFDLP;LLDVALP	NCP	33.999
LLDVALP;TGDSKKK	NCP	41.846
TGDSKKK;SLLKNC	NCP	35.838
SLLKNC;PLLQKLVS	NCP	26.503
PLLQKLVS;HQVSFLSF	NCP	8.802
HQVSFLSF;KENNFKK	NCP	66.972
KENNFKK;KLRRDV	NCP	46.025
KLRRDV;MAAGPK	NCP	45.037
MAAGPK;QFVWL	NCP	14.727
QFVWL;LDAAGYR	NCP	144.989
LDAAGYR;LPEVPPP	NCP	42.07
LPEVPPP;TSLLTRF	NCP	12.17
TSLLTRF;MPGAEFR	NCP	22.557

MPGAEFR;ELKLPHE	NCP	24.838
ELKLPHE;AVSVGVY	NCP	38.004
AVSVGVY;AEAGVVPPR	NCP	20.013
AEAGVVPPR;YLYYTLTS	NCP	21.581
YLYYTLTS;ALGPP	NCP	5.56
ALGPP;ELVTYLA	NCP	116.181
ELVTYLA;DPTVL	NCP	8.442
DPTVL;HKKLDLR	NCP	191.333
HKKLDLR;PRFRMG	NCP	21.339
PRFRMG;KPCVLR	NCP	0.9
KPCVLR;KPPRVL	NCP	20.011
KPPRVL;VHVLC	NCP	44.295
VHVLC;AMTLHT	NCP	46.34
AMTLHT;VGPPLD	NCP	59.884
VGPPLD;DRELPPP	NCP	16.056
DRELPPP;NKRRGAP	NCP	11.07
NKRRGAP;QAAGLPR	NCP	2.826
QAAGLPR;LPPPAPR	NCP	15.102
LPPPAPR;ATVVPLH	NCP	78.347
ATVVPLH;AVPYLTL	NCP	53.871
AVPYLTL;PDVKPPP	NCP	17.617
PDVKPPP;VGPPPRT	NCP	13.467
VGPPPRT;QAVMPR	NCP	11.819
QAVMPR;ENFTLRK	NCP	33
ENFTLRK;TVPLLGH	NCP	17.059
TVPLLGH;YNSPPLK	NCP	78.864
YNSPPLK;LSLSH	NCP	3.378
LSLSH;EAFLPK	NCP	53.679
EAFLPK;PSVPFYK	NCP	70.304
PSVPFYK;KYNPPPPPP	NCP	88.3
KYNPPPPPP;LEMKDDF	NCP	126.68
LEMKDDF;NCCTNF	NCP	77.896
NCCTNF;PFLDLH	NCP	19.541
PFLDLH;FTNLLLY	NCP	41.628
FTNLLLY;PDPPMRK	NCP	2.637
PDPPMRK;LWVSVPP	NCP	12.465
LWVSVPP;SFSKL	NCP	29.646
SFSKL;KALKKPP	NCP	6.999
KALKKPP;QLLVCKGP	NCP	40.035
QLLVCKGP;TFEPQR	NCP	84.202
TFEPQR;RLKPGMV	NCP	112.461
RLKPGMV;PKSALP	NCP	16.785
PKSALP;VGPFQP	NCP	42.957
VGPFQP;PNPPPPRPP	NCP	29.403
PNPPPPRPP;DLVAPGPG	NCP	6.978
DLVAPGPG;KESVVPK	NCP	20.256
KESVVPK;VPFLPRH	NCP	19.869
VPFLPRH;KQATLKT	NCP	23.52
KQATLKT;TAWCP	NCP	52.004
TAWCP;KDFRR	NCP	29.853
KDFRR;MLRPKA	NCP	28.723

MLRPKA;DGSAAPK	NCP	85.748
DGSAAPK;VSVPSP	NCP	6.927
VSVPSP;KPYVPK	NCP	0.666
KPYVPK;KGKANRP	NCP	38.184
KGKANRP;YDTKCP	NCP	40.23
YDTKCP;LTAGPSSP	NCP	1.165
LTAGPSSP;LTAEAQR	NCP	2.325
LTAEAQR;MLSCMNPK	NCP	56.033
MLSCMNPK;EATLLEL	NCP	31.944
EATLLEL;NKELKTDF	NCP	10.096
NKELKTDF;MERLDK	NCP	7.608
MERLDK;EELLLP	NCP	28.863
EELLLP;KEHYLT	NCP	44.249
KEHYLT;MDDCA	NCP	6.741
MDDCA;QSLVRQP	NCP	16.222
QSLVRQP;DSLPNPL	NCP	50.762
DSLPNPL;FGVLL	NCP	3.582
FGVLL;KSYSLTPL	NCP	34.044
KSYSLTPL;TVKNLP	NCP	20.388
TVKNLP;KTYVLLSC	NCP	0.457
KTYVLLSC;KNMHLP	NCP	0.216
KNMHLP;LDEKVELY	NCP	74.477
LDEKVELY;LLSGHK	NCP	21.541
LLSGHK;DAHLFL	NCP	33.078
DAHLFL;VAAGLLSL	NCP	94.695
VAAGLLSL;KGYEGPP	NCP	2.385
KGYEGPP;PQLEKVLV	NCP	39.432
PQLEKVLV;LTGGELL	NCP	49.086
LTGGELL;LTGVTVY	NCP	45.207
LTGVTVY;KNLAFLTH	NCP	30.267
KNLAFLTH;KGEVEPK	NCP	3.365
KGEVEPK;VLNEC	NCP	37.651
VLNEC;DLYFEP	NCP	16.655
DLYFEP;QAPLLQN	NCP	106.093
QAPLLQN;AGPPVSR	NCP	32.214
AGPPVSR;HGGVLLK	NCP	35.154
HGGVLLK;LAFLG	NCP	40.613
LAFLG;AVVVF	NCP	6.813
AVVVF;SVFLRP	NCP	4.176
SVFLRP;LAEPKPR	NCP	7.546
LAEPKPR;PTEDAPR	NCP	106.416
PTEDAPR;GVHPCR	NCP	3.65
GVHPCR;PPPPGPK	NCP	51.91
PPPPGPK;KGVWLHA	NCP	93.402
KGVWLHA;KRSLWP	NCP	42.437
KRSLWP;FGLCFTVL	NCP	17.695
FGLCFTVL;LSGVPQN	NCP	10.7
LSGVPQN;GHRGPPPP	NCP	121.098
GHRGPPPP;SMSYE	NCP	54.405
SMSYE;LTELK	NCP	27.174
LTELK;KMVPSR	NCP	24.658

KMVPSR;CLPPGAYR	NCP	17.774
CLPPGAYR;YYFPR	NCP	59.73
YYFPR;VQMMTAK	NCP	17.49
VQMMTAK;LADKVLK	NCP	6.126
LADKVLK;KSTWAR	NCP	1.26
KSTWAR;PLAKPP	NCP	20.988
PLAKPP;PTTLAKR	NCP	68.982
PTTLAKR;TAPPYK	NCP	7.05
TAPPYK;EVTGAPP	NCP	4.287
EVTGAPP;KPKLEMP	NCP	10.104
KPKLEMP;CGSPK	NCP	1.965
CGSPK;PLMLLL	NCP	44.961
PLMLLL;CEPALA	NCP	6.595
CEPALA;RGGVARP	NCP	5.579
RGGVARP;LFKFKAR	NCP	18.783
LFKFKAR;KYVPEM	NCP	2.484
KYVPEM;AEFAKSLQ	NCP	22.617
AEFAKSLQ;GVVDVP	NCP	32.17
GVVDVP;HMLSAVY	NCP	28.568
HMLSAVY;DPEASEK	NCP	135.577
DPEASEK;RMYPK	NCP	0.51
RMYPK;QNRHEYF	NCP	43.059
QNRHEYF;NHLLVSV	NCP	58.614
NHLLVSV;KYFAPR	NCP	13.812
KYFAPR;YPPFP	NCP	8.049
YPPFP;PFSLLGR	NCP	10.821
PFSLLGR;LGFTLF	NCP	63.948
LGFTLF;TMAERK	NCP	76.583
TMAERK;GVGPTVF	NCP	89.397
GVGPTVF;GSENTLMK	NCP	30.415
GSENTLMK;KPMFLQ	NCP	63.668
KPMFLQ;WPFLFW	NCP	10.536
WPFLFW;HNLSKM	NCP	84.52
HNLSKM;YVTKGYP	NCP	4.44
YVTKGYP;DPMPDE	NCP	15.389
DPMPDE;CCCAP	NCP	9.364
CCCAP;VLWELN	NCP	16.566
VLWELN;QTKDF	NCP	43.467
QTKDF;PAAWPGPK	NCP	78.651
PAAWPGPK;PVRPPVP	NCP	88.08
PVRPPVP;KALNVKS	NCP	87.267
KALNVKS;KNYVLVK	NCP	7.188
KNYVLVK;KSALVSV	NCP	9.776
KSALVSV;PDEQFH	NCP	28.473
PDEQFH;LDVLTF	NCP	24.66
LDVLTF;SPPVSP	NCP	7.795
SPPVSP;KYYPPP	NCP	0.059
KYYPPP;RKVVENL	NCP	43.777
RKVVENL;APPTLHP	NCP	3.305
APPTLHP;YGAAGK	NCP	29.446
YGAAGK;FTSTTLF	NCP	35.45

FTSTTLF;TLFKFH	NCP	77.437
TLFKFH;NGLGGGL	NCP	79.854
NGLGGGL;VTPVLEA	NCP	4.73
VTPVLEA;LCPVML	NCP	14.605
LCPVML;KLSPVLP	NCP	179.808
KLSPVLP;KFYQTP	NCP	24.78
KFYQTP;DFENEM	NCP	36.479
DFENEM;GVYMTF	NCP	6.868
GVYMTF;KVAFAE	NCP	41.799
KVAFAE;TGAYVY	NCP	22.794
TGAYVY;GPAMPLK	NCP	19.932
GPAMPLK;GKKAA	NCP	52.482
GKKAA;LSYTLLR	NCP	22.818
LSYTLLR;VSPLSGR	NCP	27.644
VSPLSGR;GVFDF	NCP	61.746
GVFDF;LTPTKET	NCP	23.955
LTPTKET;LSVLE	NCP	9.583
LSVLE;SVNLPPV	NCP	5.078
SVNLPPV;TANFLP	NCP	10.164
TANFLP;CLTML	NCP	39.375
CLTML;HGGGLF	NCP	1.414
HGGGLF;AEVRKF	NCP	41.582
AEVRKF;AAAAAAALYPL	NCP	16.86
AAAAAAALYPL;QEYAP	NCP	72.019
QEYAP;FSLKTQD	NCP	63.878
FSLKTQD;SLVSKASL	NCP	22.701
SLVSKASL;FTLNEK	NCP	89.131
FTLNEK;MKLDLC	NCP	15.303
MKLDLC;EVPGLAR	NCP	48.167
EVPGLAR;KQHMML	NCP	17.194
KQHMML;SYSVAPR	NCP	41.9
SYSVAPR;DVELNLK	NCP	61.762
DVELNLK;LLRTEPF	NCP	5.937
LLRTEPF;KKGGPPK	NCP	24.611
KKGGPPK;KPMMRP	NCP	80.646
KPMMRP;QPVAAWRL	NCP	11.412
QPVAAWRL;QKLERVP	NCP	199.335
QKLERVP;EAPPKLQ	NCP	2.205
EAPPKLQ;KMQPPPP	NCP	12.471
KMQPPPP;LLLLSLVV	NCP	53.023
LLLLSLVV;KLNAYPQ	NCP	26.841
KLNAYPQ;TSENPN	NCP	9.03
TSENPN;KNLATLEL	NCP	2.39
KNLATLEL;KMGNVKK	NCP	47.11
KMGNVKK;VSMPSPK	NCP	38.562
VSMPSPK;LNPPLPP	NCP	44.945
LNPPLPP;GPVGF	NCP	69.157
GPVGF;KMGPPVP	NCP	60.369
KMGPPVP;SRKEKLDR	NCP	10.829
SRKEKLDR;VGYAGL	NCP	26.265
VGYAGL;LPPPPPPPP	NCP	24.756

LPPPPPPPP;QELQKQK	NCP	25.438
QELQKQK;LPPPPVAP	NCP	6.603
LPPPPVAP;HLLPPYK	NCP	60.876
HLLPPYK;KKPNKP	NCP	1.091
KKPNKP;ASFDLEK	NCP	0.011
ASFDLEK;AMQKLFA	NCP	94.691
AMQKLFA;VQVSY	NCP	6.48
VQVSY;MSESPVF	NCP	82.711
MSESPVF;RLDVLSH	NCP	32.774
RLDVLSH;LVRFL	NCP	35.469
LVRFL;QELLSKH	NCP	23.332
QELLSKH;SVAFTPK	NCP	53.747
SVAFTPK;EVFTGL	NCP	37.183
EVFTGL;APAPEVR	NCP	41.504
APAPEVR;YLSLENY	NCP	53.427
YLSLENY;KESGP	NCP	16.582
KESGP;LSPGMSGK	NCP	2.42
LSPGMSGK;KPTTRVR	NCP	11.487
KPTTRVR;NLGGGLG	NCP	69.897
NLGGGLG;DPARPP	NCP	3.048
DPARPP;RGGPGPLQ	NCP	0.117
RGGPGPLQ;ATPSSLFP	NCP	10.545
ATPSSLFP;MKLVFR	NCP	29.751
MKLVFR;GSKYQEC	NCP	8.644
GSKYQEC;LLEPSLY	NCP	17.274
LLEPSLY;MERPVRVK	NCP	0.113
MERPVRVK;EGDMLLRVL	NCP	7.62
EGDMLLRVL;EGSSVVTMEGQRL	NCP	6.039
EGSSVVTMEGQRL;LSVTQDPACKKS	NCP	0.213
LSVTQDPACKKS;EVTSYK	NCP	52.599
EVTSYK;KKGKSSK	NCP	47.365
KKGKSSK;LTRNFK	NCP	31.934
LTRNFK;VVPAFH	NCP	20.7
VVPAFH;LFEVL	NCP	30.741
LFEVL;LVKYPPP	NCP	4.104
LVKYPPP;KGATMLM	NCP	9.753
KGATMLM;EHVLPD	NCP	6.424
EHVLPD;SLHSYPK	NCP	9.732
SLHSYPK;KGALTLPL	NCP	6.377
KGALTLPL;QMAAPPK	NCP	41.722
QMAAPPK;HEGFLL	NCP	20.12
HEGFLL;KAGLNET	NCP	50.949
KAGLNET;YLPKRP	NCP	12.58
YLPKRP;KGVTDGWK	NCP	74.301
KGVTDGWK;DFEKPK	NCP	106.749
DFEKPK;PHLALLR	NCP	25.671
PHLALLR;HPPTPMPP	NCP	7.002
HPPTPMPP;KYPNQK	NCP	13.025
KYPNQK;KHLFDP	NCP	8.583
KHLFDP;LKEEVKR	NCP	2.973
LKEEVKR;TAGDW	NCP	8.199

TAGDW;APVHSD	NCP	27.07
APVHSD;SLLLF	NCP	57.305
SLLLF;VGTGLGLR	NCP	16.62
VGTGLGLR;EVGGGPL	NCP	37.137
EVGGGPL;AYEEF	NCP	5.589
AYEEF;KGLVLLAF	NCP	10.878
KGLVLLAF;LLATEY	NCP	5.541
LLATEY;VYALHNK	NCP	37.078
VYALHNK;LTGLLL	NCP	127.13
LTGLLL;TVGLKGK	NCP	172.128
TVGLKGK;KSSNPK	NCP	25.792
KSSNPK;KTATLSK	NCP	74.049
KTATLSK;EGRLWSK	NCP	15.185
EGRLWSK;FAAPK	NCP	56.869
FAAPK;KMKQQGP	NCP	88.496
KMKQQGP;VGAPVY	NCP	64.582
VGAPVY;LVLLAF	NCP	64.424
LVLLAF;KPPSAQP	NCP	11.206
KPPSAQP;VSGPTRP	NCP	16.704
VSGPTRP;NVHTAPK	NCP	8.273
NVHTAPK;EGMMTQ	NCP	36.442
EGMMTQ;APAAAPAPG	NCP	31.623
APAAAPAPG;LSASTFT	NCP	160.163
LSASTFT;EEHPVLL	NCP	70.882
EEHPVLL;DSLLLP	NCP	44.391
DSLLLP;VGEAVGRV	NCP	56.346
VGEAVGRV;KGLPLQK	NCP	20.568
KGLPLQK;LLVTLA	NCP	24.02
LLVTLA;TLDEF	NCP	37.882
TLDEF;NRLNLSSP	NCP	6.065
NRLNLSSP;SLVGRPR	NCP	42.547
SLVGRPR;LSVYF	NCP	23.408
LSVYF;RWKKTPPP	NCP	3.943
RWKKTPPP;KCVRVPQ	NCP	110.298
KCVRVPQ;LVSAFVG	NCP	13.215
LVSAFVG;LGAAR	NCP	158.865
LGAAR;PAALVKP	NCP	0.6
PAALVKP;RDDVLKN	NCP	8.738
RDDVLKN;HVVLWG	NCP	90.171
HVVLWG;PPNLYLK	NCP	16.758
PPNLYLK;KEAFSLEK	NCP	76.068
KEAFSLEK;RNPLDK	NCP	8.398
RNPLDK;KVDKVLQ	NCP	48.537
KVDKVLQ;HLPNK	NCP	0.063
HLPNK;VYGHRPV	NCP	17.94
VYGHRPV;TMLLAPV	NCP	10.883
TMLLAPV;CDYEYK	NCP	36.025
CDYEYK;SSAVRLR	NCP	5.159
SSAVRLR;APDTVLHC	NCP	24.102
APDTVLHC;ESPHLPL	NCP	66.447
ESPHLPL;QRMHTK	NCP	9.918

QRMHTK;LRPNGGK	NCP	35.052
LRPNGGK;AELRAPKG	NCP	53.391
AELRAPKG;LFGELL	NCP	57.289
LFGELL;KKFASGAP	NCP	42.827
KKFASGAP;CGMMGK	NCP	0.132
CGMMGK;KSSLE	NCP	12.336
KSSLE;ADTLSV	NCP	86.86
ADTLSV;KPRYLK	NCP	66.804
KPRYLK;LYPSRL	NCP	111.491
LYPSRL;LSPLSGH	NCP	116.98
LSPLSGH;TGSSHLK	NCP	66.438
TGSSHLK;CGPMKK	NCP	37.187
CGPMKK;LVTVKLP	NCP	61.95
LVTVKLP;LGPPPEFP	NCP	72.735
LGPPPEFP;LSPRK	NCP	242.295
LSPRK;KMLAHSTF	NCP	55.078
KMLAHSTF;MRDLVT	NCP	124.232
MRDLVT;KNGDYE	NCP	48.624
KNGDYE;LLELQN	NCP	124.525
LLELQN;CPTLLPML	NCP	4.625
CPTLLPML;SSNVRLR	NCP	49.546
SSNVRLR;LPGELAKH	NCP	14.213
LPGELAKH;SEGTKAVTKYTSSK	NCP	0.03
SEGTKAVTKYTSSK;SNAPYLR	NCP	2.196
SNAPYLR;KNLKVHA	NCP	78.261
KNLKVHA;AEGDEVTRSQEPHQGL	NCP	29.838
AEGDEVTRSQEPHQGL;GWPGSPPA	NCP	0.219
GWPGSPPA;VSFSDL	NCP	63.855
VSFSDL;GHNWF	NCP	85.086
GHNWF;NRYFVK	NCP	23.851
NRYFVK;KNPFYQK	NCP	113.427
KNPFYQK;WDPDPR	NCP	36.167
WDPDPR;LTVDVFP	NCP	113.64
LTVDVFP;LGGLK	NCP	77.085
LGGLK;VFAPEA	NCP	10.684
VFAPEA;LPVLEF	NCP	38.469
LPVLEF;LLSSSPM	NCP	55.421
LLSSSPM;ESFGPGK	NCP	26.355
ESFGPGK;EHTPP	NCP	41.1
EHTPP;RLEER	NCP	6.088
RLEER;EFCGVP	NCP	3.776
EFCGVP;LKEKLDR	NCP	4.866
LKEKLDR;VGKDPK	NCP	8.146
VGKDPK;ESVPTVK	NCP	74.277
ESVPTVK;KGFKPK	NCP	29.478
KGFKPK;LLPKKMDEY	NCP	63.636
LLPKKMDEY;FLKAAPK	NCP	39.893
FLKAAPK;TLEVMYP	NCP	47.832
TLEVMYP;PPWPP	NCP	27.505
PPWPP;KDGVTRP	NCP	39.965
KDGVTRP;KVFLSR	NCP	5.284

KVFLSR;AAAEKPLEF	NCP	36.689
AAAEKPLEF;SWVCRK	NCP	4.513
SWVCRK;KPYSGP	NCP	22.41
KPYSGP;TLLRT	NCP	85.751
TLLRT;KDREPGH	NCP	55.816
KDREPGH;KGVLTVK	NCP	37.244
KGVLTVK;YPGSDLY	NCP	48.075
YPGSDLY;MAFLLFK	NCP	10.593
MAFLLFK;MESLYR	NCP	8.02
MESLYR;VEKPSPR	NCP	11.483
VEKPSPR;KYVTTYQ	NCP	91.32
KYVTTYQ;YKPPPR	NCP	102.568
YKPPPR;ELPRVPV	NCP	2.769
ELPRVPV;VSALVK	NCP	20.31
VSALVK;QAVKFQ	NCP	25.986
QAVKFQ;KAWLGVP	NCP	29.229
KAWLGVP;LALVGQ	NCP	13.16
LALVGQ;GRTGPVVE	NCP	61.654
GRTGPVVE;MMDDTN	NCP	3.296
MMDDTN;TGPGLLP	NCP	40.539
TGPGLLP;PGPPHDAAP	NCP	0.075
PGPPHDAAP;LSVFMPK	NCP	12.448
LSVFMPK;PFSALAV	NCP	49.626
PFSALAV;LSVSSPAP	NCP	40.676
LSVSSPAP;AVSRGVPA	NCP	20.323
AVSRGVPA;AAGPFLPR	NCP	45.329
AAGPFLPR;LFVPTGT	NCP	31.126
LFVPTGT;KPGSVHR	NCP	19.38
KPGSVHR;GVLTL	NCP	36.194
GVLTL;TYGVSF	NCP	2.398
TYGVSF;SLFPPPK	NCP	3.873
SLFPPPK;VAPGSTQ	NCP	61.422
VAPGSTQ;RALLEVY	NCP	52.452
RALLEVY;DLLGHPK	NCP	20.451
DLLGHPK;TSTVLGLK	NCP	7.235
TSTVLGLK;RQAGVSLY	NCP	6.243
RQAGVSLY;NGKWLLR	NCP	35.014
NGKWLLR;LDNRLP	NCP	15.332
LDNRLP;KLLLGFK	NCP	7.684
KLLLGFK;LTSTHSC	NCP	1.094
LTSTHSC;KEHYPPP	NCP	16.6
KEHYPPP;PRSPLNF	NCP	101.904
PRSPLNF;LDLKVLR	NCP	12.191
LDLKVLR;AELVLDT	NCP	10.648
AELVLDT;YSNRLY	NCP	81.27
YSNRLY;GVGSTVLT	NCP	56.657
GVGSTVLT;WMGKK	NCP	12.96
WMGKK;LPPSSLE	NCP	5.848
LPPSSLE;PEVPPKAP	NCP	19.908
PEVPPKAP;PPLPPPPPPG	NCP	7.881
PPLPPPPPPG;GYVAV	NCP	16.13

GYVAV;VPPVKLY	NCP	11.164
VPPVKLY;YEESH	NCP	68.006
YEESH;KAALQY	NCP	60.129
KAALQY;GCCEKR	NCP	20.755
GCCEKR;THQSPPK	NCP	1.26
THQSPPK;EPLPRHP	NCP	90.162
EPLPRHP;KPKAAPP	NCP	20.409
KPKAAPP;RGVGSTR	NCP	3.927
RGVGSTR;EGTPLTWKP	NCP	21.975
EGTPLTWKP;GDDLLPAGTEDY	NCP	58.692
GDDLLPAGTEDY;PAGALR	NCP	58.575
PAGALR;MKLPLL	NCP	22.455
MKLPLL;QGGVH	NCP	13.08
QGGVH;GPKPVVF	NCP	30.558
GPKPVVF;EVPVL	NCP	17.258
EVPVL;PEMEEP	NCP	21.858
PEMEEP;LTAEVL	NCP	10.102
LTAEVL;KVRALTL	NCP	14.342
KVRALTL;SDRWPR	NCP	104.548
SDRWPR;KGFWYK	NCP	3.857
KGFWYK;EALLQ	NCP	5.115
EALLQ;DPVDSF	NCP	32.854
DPVDSF;RKLNLRLK	NCP	33.023
RKLNLRLK;SGPKAPR	NCP	11.191
SGPKAPR;EGPDF	NCP	57.879
EGPDF;PMDMVY	NCP	3.047
PMDMVY;KYLGPP	NCP	54.735
KYLGPP;GPRPPYK	NCP	1.735
GPRPPYK;AAGYH	NCP	3.192
AAGYH;VPPSHLLK	NCP	28.389
VPPSHLLK;LDRLLDH	NCP	30.228
LDRLLDH;KPACFFK	NCP	38.022
KPACFFK;HMLEY	NCP	91.334
HMLEY;LHPYFR	NCP	67.965
LHPYFR;GKGNVL	NCP	37.372
GKGNVL;AALVAY	NCP	4.389
AALVAY;NRMYGY	NCP	36.335
NRMYGY;SSAQSQK	NCP	115.846
SSAQSQK;ELKDRF	NCP	1.545
ELKDRF;PPGPEKPS	NCP	104.988
PPGPEKPS;PYELKKV	NCP	11.144
PYELKKV;KGPPPYK	NCP	44.274
KGPPPYK;LGLKVF	NCP	22.641
LGLKVF;PVNFKLL	NCP	43.413
PVNFKLL;KDPLTPT	NCP	11.479
KDPLTPT;AAPGFFK	NCP	55.524
AAPGFFK;VSKAPVT	NCP	72.702
VSKAPVT;LMLGMPK	NCP	21.66
LMLGMPK;WFPQKQ	NCP	126.689
WFPQKQ;YPHEGPP	NCP	10.855
YPHEGPP;HYTMR	NCP	9.348

HYTMR;EFAKAG	NCP	13.751
EFAKAG;KDAGVPR	NCP	20.775
KDAGVPR;HLDLVELL	NCP	54.561
HLDLVELL;CASFKAAP	NCP	5.173
CASFKAAP;ANVLYAR	NCP	22.596
ANVLYAR;TVGWLPP	NCP	28.146
TVGWLPP;LLAARQDLSK	NCP	36.992
LLAARQDLSK;ELVKRL	NCP	141.537
ELVKRL;KDPGVLLR	NCP	1.942
KDPGVLLR;LPVSLLP	NCP	30.821
LPVSLLP;VSLAEC	NCP	73.714
VSLAEC;AAEVLK	NCP	3.528
AAEVLK;AFLADPSA	NCP	31.172
AFLADPSA;NKGGGLG	NCP	56.737
NKGGGLG;RVHLLF	NCP	4.767
RVHLLF;VLHFLL	NCP	26.549
VLHFLL;KRDQPR	NCP	3.346
KRDQPR;LGVQFF	NCP	58.698
LGVQFF;KAFLLY	NCP	21.168
KAFLLY;LSGKVALK	NCP	64.467
LSGKVALK;LSRTVVKL	NCP	22.181
LSRTVVKL;LSGALLGK	NCP	2.775
LSGALLGK;RHLELEE	NCP	30.637
RHLELEE;VTGPPQ	NCP	3.239
VTGPPQ;PHALLLVG	NCP	56.497
PHALLLVG;KEMLDLK	NCP	75.962
KEMLDLK;EVGWQL	NCP	86.151
EVGWQL;AVDLDLH	NCP	39.316
AVDLDLH;KLQKCVP	NCP	9.038
KLQKCVP;LFAGPARL	NCP	9.685
LFAGPARL;KEMTVPK	NCP	55.481
KEMTVPK;FYQKHR	NCP	93.276
FYQKHR;TVLTLP	NCP	98.116
TVLTLP;KDPENLP	NCP	1.572
KDPENLP;PAATFAK	NCP	7.562
PAATFAK;AMTLAC	NCP	12.733
AMTLAC;SVKAPVTK	NCP	17.214
SVKAPVTK;MFFLPLT	NCP	67.851
MFFLPLT;NQLLVLSH	NCP	10.815
NQLLVLSH;YAVELK	NCP	44.508
YAVELK;FGFPFVL	NCP	54.135
FGFPFVL;PVAAEPF	NCP	43.596
PVAAEPF;CEMCGP	NCP	39.339
CEMCGP;DSSVRLK	NCP	5.334
DSSVRLK;KENGKLLK	NCP	8.316
KENGKLLK;LASYLLKNKAQFEDRN	NCP	59.819
LASYLLKNKAQFEDRN;VRAFRK	NCP	77.346
VRAFRK;MGNAP	NCP	16.294
MGNAP;YVMHVR	NCP	12.416
YVMHVR;AAGALTVH	NCP	14.487
AAGALTVH;DAVAPF	NCP	100.263

DAVAPF;KADLPF	NCP	16.381
KADLPF;VHQVKF	NCP	83.634
VHQVKF;TADELVFF	NCP	86.423
TADELVFF;VAPPKYP	NCP	26.026
VAPPKYP;LEGLPV	NCP	38.759
LEGLPV;SDFTAP	NCP	7.542
SDFTAP;VRHLLSG	NCP	23.52
VRHLLSG;LPEPL	NCP	37.828
LPEPL;DNAQPK	NCP	72.138
DNAQPK;ETHKNH	NCP	9.513
ETHKNH;GPLVPG	NCP	68.196
GPLVPG;VYEFEP	NCP	4.523
VYEFEP;QADRVLDR	NCP	10.218
QADRVLDR;TWLEDY	NCP	54.709
TWLEDY;TVTAL	NCP	9.923
TVTAL;LTSEPLK	NCP	23.505
LTSEPLK;EGFDNL	NCP	32.214
EGFDNL;KELPSGPK	NCP	19.159
KELPSGPK;CNTHTTL	NCP	55.388
CNTHTTL;FNSNATL	NCP	44.494
FNSNATL;LDDPLEQ	NCP	94.13
LDDPLEQ;AAAVPPPR	NCP	19.521
AAAVPPPR;DNMYDLR	NCP	19.462
DNMYDLR;MGVLLTG	NCP	6.639
MGVLLTG;YRVASYL	NCP	13.196
YRVASYL;VHTLTSAL	NCP	31.038
VHTLTSAL;AVPAPGPE	NCP	33.741
AVPAPGPE;APASARAR	NCP	5.817
APASARAR;KPAPAP	NCP	32.304
KPAPAP;RDKKDL	NCP	6.78
RDKKDL;LSPAAPP	NCP	40.984
LSPAAPP;VKQAMF	NCP	32.492
VKQAMF;LAVAY	NCP	18.675
LAVAY;EHAARLK	NCP	10.348
EHAARLK;QPAVKPP	NCP	28.365
QPAVKPP;RFGKQK	NCP	0.213
RFGKQK;NGPAVP	NCP	27.117
NGPAVP;ADEGLPK	NCP	26.874
ADEGLPK;HAVAAL	NCP	35.888
HAVAAL;VVKVPK	NCP	90.132
VVKVPK;KPDDRLK	NCP	0.592
KPDDRLK;RKESHH	NCP	24.861
RKESHH;MLSCQLVR	NCP	25.064
MLSCQLVR;TKKKVEC	NCP	2.758
TKKKVEC;KKGHPRP	NCP	41.345
KKGHPRP;ALALKPF	NCP	31.236
ALALKPF;VPPASLP	NCP	28.657
VPPASLP;SGCALLVR	NCP	3.132
SGCALLVR;AVPPVLF	NCP	15.569
AVPPVLF;EVAVLPL	NCP	3.955
EVAVLPL;AFLGAPP	NCP	50.892

AFLGAPP;PGGPGPPLS	NCP	0.165
PGGPGPPLS;VPARPP	NCP	2.916
VPARPP;VPQAKN	NCP	15.621
VPQAKN;GVLMNK	NCP	7.824
GVLMNK;MFLPYL	NCP	68.438
MFLPYL;RELSF	NCP	101.322
RELSF;LTRRQ	NCP	32.188
LTRRQ;GPAHRQTP	NCP	6.266
GPAHRQTP;KEMPPPP	NCP	0.663
KEMPPPP;ANVELSLS	NCP	19.029
ANVELSLS;NVHTTKSK	NCP	50.799
NVHTTKSK;SMQNK	NCP	2.637
SMQNK;KMSGMC	NCP	43.371
KMSGMC;TGKLQTP	NCP	22.041
TGKLQTP;NFQSL	NCP	10.072
NFQSL;YSVPLPV	NCP	79.487
YSVPLPV;MPLSP	NCP	65.116
MPLSP;SVTQVVR	NCP	10.313
SVTQVVR;KPPAVLQ	NCP	2.914
KPPAVLQ;KETTLDNP	NCP	31.242
KETTLDNP;KPHPPPPP	NCP	10.863
KPHPPPPP;ATLPLT	NCP	1.146
ATLPLT;KPTALPFP	NCP	0.153
KPTALPFP;PQSPGNLK	NCP	54.774
PQSPGNLK;FNNKGM	NCP	0.773
FNNKGM;ELGVLPY	NCP	13
ELGVLPY;LGQAYA	NCP	19.592
LGQAYA;KTFCKGP	NCP	26.589
KTFCKGP;KRADLLK	NCP	139.68
KRADLLK;LFMGV	NCP	36.831
LFMGV;GFAHM	NCP	1.69
GFAHM;MLHLQ	NCP	67.956
MLHLQ;TTPTVP	NCP	56.267
TTPTVP;FLQRTN	NCP	36.265
FLQRTN;KGYLSDH	NCP	11.069
KGYLSDH;AALLEMN	NCP	61.002
AALLEMN;KGDPETPR	NCP	52.171
KGDPETPR;ETYTPMN	NCP	3.213
ETYTPMN;CMMDQ	NCP	0.906
CMMDQ;HLGPPPP	NCP	65.822
HLGPPPP;TFVPPPPPP	NCP	82.546
TFVPPPPPP;KLKPPR	NCP	38.732
KLKPPR;WLDVSLL	NCP	29.473
WLDVSLL;RHKKKE	NCP	20.591
RHKKKE;LPLNPP	NCP	7.738
LPLNPP;EEAGRLLA	NCP	7.226
EEAGRLLA;FMLMTP	NCP	131.704
FMLMTP;LPSGVVPK	NCP	23.381
LPSGVVPK;WPTYSGH	NCP	9.162
WPTYSGH;KHELYT	NCP	40.561
KHELYT;AEQVTKSVLF	NCP	27.902

AEQVTKSVLF;RGAAPAAP	NCP	57.771
RGAAPAAP;LLDVVHPAAK	NCP	32.055
LLDVVHPAAK;LDDLLQLK	NCP	2.91
LDDLLQLK;LTVLSLA	NCP	145.869
LTVLSLA;SAPGLDL	NCP	34.129
SAPGLDL;KSGSFKL	NCP	64.649
KSGSFKL;VTTGLAR	NCP	53.485
VTTGLAR;LLRHHLAK	NCP	23.463
LLRHHLAK;YNSGEC	NCP	30.821
YNSGEC;DRGQVVF	NCP	91.833
DRGQVVF;EEETTALVCDNGSGLCKAGFAGDDAPR	NCP	7.815
EEETTALVCDNGSGLCKAGFAGDDAPR;TTALVCDNGSGL	NCP	0.009
TTALVCDNGSGLCK;TALVCDNGSGLCKAGF	NCP	0.003
TALVCDNGSGLCKAGF;ALVCDNGSGLCKAG	NCP	0.003
ALVCDNGSGLCKAG;LVCDNGSGLCKAGF	NCP	0.003
LVCDNGSGLCKAGF;VCDNGSGLCKAGFAGDDAPR	NCP	0.003
VCDNGSGLCKAGFAGDDAPR;DNGSGLCKAGFAGDD	NCP	0.006
DNGSGLCKAGFAGDD;GSGLCKAGFAGDDAPR	NCP	0.006
GSGLCKAGFAGDDAPR;VGMGQKDSY	NCP	0.486
VGMGQKDSY;WHHSFYNELR	NCP	6.48
WHHSFYNELR;YNELRVAPEE	NCP	0.015
YNELRVAPEE;EEHPTLL	NCP	0.024
EEHPTLL;APLNPK	NCP	0.027
APLNPK;RDLTDYL	NCP	2.145
RDLTDYL;SSSLEKSY	NCP	0.912
SSSLEKSY;DLYANNVL	NCP	1.824
DLYANNVL;ANNVLSGGTTM	NCP	0.009
ANNVLSGGTTM;LAPSTMK	NCP	0.075
LAPSTMK;DFLQVK	NCP	1.945
DFLQVK;PNVRPP	NCP	69.059
PNVRPP;LGRFVPF	NCP	19.414
LGRFVPF;AAAHDYR	NCP	50.817
AAAHDYR;AVCVAVL	NCP	20.909
AVCVAVL;FNFLQ	NCP	51.432
FNFLQ;NSSLLNKE	NCP	3.63
NSSLLNKE;VEGVLLG	NCP	17.871
VEGVLLG;DSSDDC	NCP	17.809
DSSDDC;RVLKTK	NCP	6.519
RVLKTK;QVTHKK	NCP	9.057
QVTHKK;TAARLEL	NCP	23.585
TAARLEL;PVPGGVGP	NCP	21.882
PVPGGVGP;ETVSLPR	NCP	29.044
ETVSLPR;KYAPVM	NCP	18.68
KYAPVM;KLSAPKSP	NCP	2.299
KLSAPKSP;MASSL	NCP	6.146
MASSL;NFRSKP	NCP	116.983
NFRSKP;QGEEPK	NCP	4.349
QGEEPK;SPDVP	NCP	5.517
SPDVP;VPPGLPF	NCP	52.845
VPPGLPF;YLSPPPPPP	NCP	18.576
YLSPPPPPP;KGPGLAHA	NCP	32.791

KGPGLAHA;YGDMDP	NCP	11.231
YGDMDP;AAPAPETR	NCP	16.293
AAPAPETR;NGDCM	NCP	24.706
NGDCM;PPPAYPP	NCP	8.328
PPPAYPP;YAAKPK	NCP	2.292
YAAKPK;PGLAGVP	NCP	15.381
PGLAGVP;TVDLL	NCP	1.857
TVDLL;LSFAAPR	NCP	60.609
LSFAAPR;KTEGHTK	NCP	30.159
KTEGHTK;PPVSFSPR	NCP	108.912
PPVSFSPR;KFGWKP	NCP	0.072
KFGWKP;QPNVLPLP	NCP	24.315
QPNVLPLP;RKCML	NCP	87.93
RKCML;YLLTLGT	NCP	8.96
YLLTLGT;LEESLLTL	NCP	25.032
LEESLLTL;LSGGLLDL	NCP	21.888
LSGGLLDL;KDGAWR	NCP	18.993
KDGAWR;KNLLVAP	NCP	9.531
KNLLVAP;WPGTRTV	NCP	8.539
WPGTRTV;KQCPPR	NCP	19.038
KQCPPR;FHVHQT	NCP	85.107
FHVHQT;KPWKWP	NCP	4.7
KPWKWP;CNCGQP	NCP	69.73
CNCGQP;RPSPPHT	NCP	39.381
RPSPPHT;KYLFGPK	NCP	6.801
KYLFGPK;KSSSKAP	NCP	109.47
KSSSKAP;LPPEPAPL	NCP	22.841
LPPEPAPL;KARFPPP	NCP	36.123
KARFPPP;KGEAKLTK	NCP	11.227
KGEAKLTK;EMAHMS	NCP	36.303
EMAHMS;TPTLRPR	NCP	14.243
TPTLRPR;GPLTP	NCP	13.29
GPLTP;VLELSGR	NCP	13.797
VLELSGR;EAALK	NCP	13.66
EAALK;TGPGLPL	NCP	80.412
TGPGLPL;SLLKGSLF	NCP	21.018
SLLKGSLF;VDLALPA	NCP	25.782
VDLALPA;KPGPPPK	NCP	28.043
KPGPPPK;LTMFKP	NCP	115.81
LTMFKP;AAYFMC	NCP	24.906
AAYFMC;TASLPLP	NCP	115.478
TASLPLP;LGNMKF	NCP	33.597
LGNMKF;QANPLAK	NCP	33.94
QANPLAK;DLFTGL	NCP	46.268
DLFTGL;MDKYP	NCP	51.984
MDKYP;AAGWGKR	NCP	50.521
AAGWGKR;KNVKPPP	NCP	0.51
KNVKPPP;GDCSMW	NCP	6.915
GDCSMW;KAMADLR	NCP	16.116
KAMADLR;PSKLF	NCP	33.561
PSKLF;THPQSVK	NCP	30.161

THPQSVK;KKGDGAP	NCP	21.402
KKGDGAP;VPVSRTPT	NCP	0.084
VPVSRTPT;FRAPTR	NCP	43.416
FRAPTR;AAPHKKT	NCP	89.025
AAPHKKT;LGLSL	NCP	8.572
LGLSL;KPSSLLKK	NCP	22.71
KPSSLLKK;PAGAAK	NCP	17.796
PAGAAK;KFCVPK	NCP	228.182
KFCVPK;PMMKKP	NCP	10.347
PMMKKP;LPPLSPLPP	NCP	4.447
LPPLSPLPP;PTSFRCP	NCP	11.7
PTSFRCP;LPEKF	NCP	69.465
LPEKF;WCSLGK	NCP	41.789
WCSLGK;KNMFPK	NCP	35.289
KNMFPK;PLPPPSP	NCP	29.433
PLPPPSP;LLLDFP	NCP	23.76
LLLDFP;LPPCFFR	NCP	23.11
LPPCFFR;TYVLRR	NCP	10.106
TYVLRR;AHTVYK	NCP	13.597
AHTVYK;SPLGEQ	NCP	9.162
SPLGEQ;GKVWGNK	NCP	26.948
GKVWGNK;VVTKLLM	NCP	13.974
VVTKLLM;KLVPSSK	NCP	4.561
KLVPSSK;WSPTQK	NCP	31.439
WSPTQK;GCKPP	NCP	52.102
GCKPP;SNALLEY	NCP	28.587
SNALLEY;HLVTPLLE	NCP	8.403
HLVTPLLE;VLLMKEPT	NCP	89.184
VLLMKEPT;LDPPPP	NCP	3.348
LDPPPP;ADFGLSA	NCP	0.6
ADFGLSA;LWHHTF	NCP	30.632
LWHHTF;KGPGLWL	NCP	39.876
KGPGLWL;LPNSPGGP	NCP	5.11
LPNSPGGP;TQRETNL	NCP	1.965
TQRETNL;RKPVEK	NCP	8.931
RKPVEK;PSLFGPP	NCP	14.055
PSLFGPP;KAFAHR	NCP	43.016
KAFAHR;YVPAPLR	NCP	24.52
YVPAPLR;QPAVSPPP	NCP	0.108
QPAVSPPP;WLAGAVA	NCP	18.716
WLAGAVA;PFVELDT	NCP	3.048
PFVELDT;LPRGLLVD	NCP	24.636
LPRGLLVD;WRQAGLS	NCP	26.512
WRQAGLS;KTCTAPK	NCP	19.379
KTCTAPK;SLHKPPPR	NCP	4.111
SLHKPPPR;FPAGRFL	NCP	153.431
FPAGRFL;APGFGTVP	NCP	131.968
APGFGTVP;VLKAVDK	NCP	5.687
VLKAVDK;KPMPRGP	NCP	18.648
KPMPRGP;KGVAPCK	NCP	15.652
KGVAPCK;EGPLP	NCP	14.619

EGPLP;VQYFKP	NCP	42.443
VQYFKP;AELTAL	NCP	1.744
AELTAL;MVHKKT	NCP	1.461
MVHKKT;RQKSP	NCP	32.952
RQKSP;GVPGLPP	NCP	76.602
GVPGLPP;QDSDLR	NCP	80.357
QDSDLR;KLAGAV	NCP	48.898
KLAGAV;QLRKKKRK	NCP	14.864
QLRKKKRK;KPLAADP	NCP	6.964
KPLAADP;KSLGNVF	NCP	4.937
KSLGNVF;LLELGAP	NCP	36.001
LLELGAP;LALELEA	NCP	13.917
LALELEA;PGLEGSAK	NCP	33.018
PGLEGSAK;TPLPKPR	NCP	17.601
TPLPKPR;APPLVYY	NCP	68.331
APPLVYY;WGGLVF	NCP	40.941
WGGLVF;FVPHRPS	NCP	21.167
FVPHRPS;VTGWHK	NCP	45.976
VTGWHK;QATPAAKP	NCP	29.213
QATPAAKP;PVHTVK	NCP	25.371
PVHTVK;TVLTGTK	NCP	37.339
TVLTGTK;SLYPAGAP	NCP	34.347
SLYPAGAP;LRYTVKP	NCP	46.358
LRYTVKP;CGSRPLGP	NCP	29.305
CGSRPLGP;PRNPFF	NCP	50.835
PRNPFF;SVGGAFP	NCP	53.784
SVGGAFP;STAHH	NCP	15.437
STAHH;APMVKLR	NCP	0.021
APMVKLR;KKGQKVGEF	NCP	30.429
KKGQKVGEF;LNVTDF	NCP	9.708
LNVTDF;RAPTPP	NCP	22.225
RAPTPP;LPSLVPP	NCP	31.875
LPSLVPP;KGTPYVP	NCP	18.149
KGTPYVP;EVTTWKP	NCP	45.967
EVTTWKP;DSSLVF	NCP	52.74
DSSLVF;KLRALAK	NCP	14.906
KLRALAK;LPAEGKP	NCP	51.39
LPAEGKP;SLDLPDL	NCP	30.234
SLDLPDL;EKLFDPK	NCP	26.625
EKLFDPK;ESPLVGAL	NCP	9.049
ESPLVGAL;VSGKKPK	NCP	18.708
VSGKKPK;KGTYAPP	NCP	19.961
KGTYAPP;AKLLHLLR	NCP	7.701
AKLLHLLR;KASLEPH	NCP	14.749
KASLEPH;VSGNLPK	NCP	7.019
VSGNLPK;AAAARGVAL	NCP	11.206
AAAARGVAL;ALSFALL	NCP	37.559
ALSFALL;KHAFPR	NCP	7.201
KHAFPR;VAPGRVL	NCP	25.532
VAPGRVL;KEKGRGP	NCP	0.436
KEKGRGP;RVMVLDG	NCP	7.91

RVMVLDG;KHGPP	NCP	36.312
KHGPP;LSPLMLM	NCP	18.672
LSPLMLM;LLVSTVPP	NCP	10.755
LLVSTVPP;LTGTEHL	NCP	15.618
LTGTEHL;QGVGALF	NCP	13.053
QGVGALF;MKKGQPK	NCP	22.813
MKKGQPK;ALKELR	NCP	5.891
ALKELR;LVLLGELL	NCP	0.087
LVLLGELL;VLMVPKL	NCP	23.982
VLMVPKL;RPPSLRPP	NCP	31.644
RPPSLRPP;LSLPTGTS	NCP	0.655
LSLPTGTS;MYTLPAP	NCP	21.341
MYTLPAP;LLTVDLLP	NCP	33.205
LLTVDLLP;LGPVF	NCP	49.601
LGPVF;VHFLAD	NCP	63.925
VHFLAD;APAASSY	NCP	1.664
APAASSY;GVFAPP	NCP	79.653
GVFAPP;KYELVSF	NCP	12.421
KYELVSF;TQTHTVP	NCP	65.237
TQTHTVP;VPAAVSLP	NCP	61.87
VPAAVSLP;EPPAL	NCP	4.899
EPPAL;NGGELLD	NCP	0.269
NGGELLD;PVLPVPLP	NCP	46.477
PVLPVPLP;NDGNTN	NCP	75.249
NDGNTN;KCFSLSP	NCP	36.651
KCFSLSP;AAAGALAL	NCP	17.694
AAAGALAL;KYSVPPP	NCP	14.969
KYSVPPP;PPDPPPPP	NCP	21.784
PPDPPPPP;NPNLPLE	NCP	5.411
NPNLPLE;WMFVLFPP	NCP	0.265
WMFVLFPP;KPHMARP	NCP	17.208
KPHMARP;PVGPA	NCP	82.482
PVGPA;LPESRHSR	NCP	43.4
LPESRHSR;KPLLLAK	NCP	60.169
KPLLLAK;PPRTPP	NCP	5.127
PPRTPP;APPARPV	NCP	21.804
APPARPV;SGGLPALV	NCP	1.959
SGGLPALV;AEQVALSR	NCP	63.707
AEQVALSR;DMAYSG	NCP	3.288
DMAYSG;QEGKVTL	NCP	2.857
QEGKVTL;LTAVSPK	NCP	4.742
LTAVSPK;LETLSF	NCP	18.909
LETLSF;LNPYAK	NCP	3.093
LNPYAK;DGWSPR	NCP	26.769
DGWSPR;MAQSQK	NCP	53.424
MAQSQK;EPPHSPK	NCP	45.007
EPPHSPK;HELAAQ	NCP	8.513
HELAAQ;RKSLVSK	NCP	80.827
RKSLVSK;PSGDPER	NCP	31.613
PSGDPER;TSVPGHRP	NCP	10.425
TSVPGHRP;AQVLHVS	NCP	2.506

AQVLHVS;PASLKDK	NCP	51.855
PASLKDK;GPQSPVFL	NCP	12.818
GPQSPVFL;GKGLL	NCP	38.506
GKGLL;KQQTLLK	NCP	40.221
KQQTLLK;LVEELR	NCP	43.908
LVEELR;PRYYEDKP	NCP	25.838
PRYYEDKP;ASPGRLF	NCP	102.133
ASPGRLF;KPAASH	NCP	4.352
KPAASH;LPPPAQN	NCP	9.69
LPPPAQN;LAGAGALL	NCP	16.471
LAGAGALL;QWTPLP	NCP	10.286
QWTPLP;PVVSKTGP	NCP	13.474
PVVSKTGP;VTADLLP	NCP	13.11
VTADLLP;YDPLP	NCP	2.868
YDPLP;EELRLTP	NCP	34.658
EELRLTP;MLTSVWK	NCP	1.906
MLTSVWK;PSLLPAPY	NCP	4.961
PSLLPAPY;GKVLCTE	NCP	26.557
GKVLCTE;DTVHPHL	NCP	9.729
DTVHPHL;PSACEPP	NCP	1.548
PSACEPP;GVGPAM	NCP	7.626
GVGPAM;LAHPASR	NCP	52.49
LAHPASR;VGSKPF	NCP	8.563
VGSKPF;KLPLVVL	NCP	24.956
KLPLVVL;NSQFLVL	NCP	46.305
NSQFLVL;KGSAVLA	NCP	75.808
KGSAVLA;ALNTEFKN	NCP	1.985
ALNTEFKN;YMLVLQ	NCP	60.859
YMLVLQ;NYALLLR	NCP	7.262
NYALLLR;RSQVPRP	NCP	15.933
RSQVPRP;TANPFP	NCP	24.094
TANPFP;GPKAGG	NCP	15.801
GPKAGG;LGSARLTP	NCP	28.026
LGSARLTP;KQASLLR	NCP	2.114
KQASLLR;KEDLYP	NCP	76.861
KEDLYP;SSWSFPK	NCP	25.592
SSWSFPK;KLQPLPLT	NCP	13.813
KLQPLPLT;QPSFVR	NCP	9.77
QPSFVR;YEQTY	NCP	25.006
YEQTY;ADLLPY	NCP	6.452
ADLLPY;KPMPGSP	NCP	9.238
KPMPGSP;DCPALVR	NCP	41.733
DCPALVR;VYSKDQLQTF	NCP	19.694
VYSKDQLQTF;VLDSGDGVTH	NCP	0.954
VLDSGDGVTH;KFTKPP	NCP	9.706
KFTKPP;PPALKLYP	NCP	10.53
PPALKLYP;KLVPHQLAH	NCP	0.383
KLVPHQLAH;ARAAPPPP	NCP	28.981
ARAAPPPP;VSYVYVK	NCP	1.971
VSYVYVK;KHGGPPP	NCP	13.877
KHGGPPP;NDLFER	NCP	17.941

NDLFER;THVVPET	NCP	1.25
THVVPET;VGVAPPG	NCP	65.901
VGVAPPG;AAPQTLP	NCP	5.103
AAPQTLP;PELTWR	NCP	16.275
PELTWR;KRTAFPP	NCP	19.234
KRTAFPP;GAAVH	NCP	4.47
GAAVH;KLQAKALL	NCP	9.33
KLQAKALL;TGRGLVP	NCP	28.938
TGRGLVP;KCEPTR	NCP	4.968
KCEPTR;VRAPP	NCP	23.51
VRAPP;RNFCATL	NCP	0.412
RNFCATL;PGGLVAL	NCP	7.487
PGGLVAL;LPDTTRPP	NCP	49.498
LPDTTRPP;VVHLMV	NCP	37.425
VVHLMV;KVTPGK	NCP	43.28
KVTPGK;THKPPR	NCP	30.154
THKPPR;LGPLE	NCP	0.867
LGPLE;PEVVRK	NCP	15.197
PEVVRK;KQAALLQ	NCP	18.969
KQAALLQ;EMDGV	NCP	2.899
EMDGV;KPNPN	NCP	16.746
KPNPN;RLKGGAK	NCP	28.263
RLKGGAK;KPAAQGAP	NCP	101.703
KPAAQGAP;VDGPTHK	NCP	22.737
VDGPTHK;NRVLTL	NCP	1.374
NRVLTL;MSPQDLR	NCP	29.358
MSPQDLR;QASLKDL	NCP	9.012
QASLKDL;AGPRKASP	NCP	8.603
AGPRKASP;GPPAEP	NCP	11.25
GPPAEP;QVVHGRP	NCP	8.626
QVVHGRP;GRVPPLL	NCP	9.797
GRVPPLL;RATPPP	NCP	43.159
RATPPP;CDCCP	NCP	37.203
CDCCP;PGPGPPPP	NCP	87.588
PGPGPPPP;KLGPVGV	NCP	28.967
KLGPVGV;RSLTFE	NCP	38.187
RSLTFE;LGFQP	NCP	32.364
LGFQP;KLVEPAP	NCP	32.302
KLVEPAP;EPAALVN	NCP	4.014
EPAALVN;HLAPPP	NCP	1.857
HLAPPP;AVPPPPRC	NCP	61.182
AVPPPPRC;LGLAK	NCP	68.964
LGLAK;VVPVGPA	NCP	27.65
VVPVGPA;PPRAPLPP	NCP	123.817
PPRAPLPP;SEVHPHL	NCP	12.284
SEVHPHL;DLTARALPF	NCP	2.806
DLTARALPF;ERPRPPGP	NCP	1.518
ERPRPPGP;ASRPRLP	NCP	20.463
ASRPRLP;KSALRGP	NCP	17.046
KSALRGP;ALEGAKH	NCP	3.186
ALEGAKH;NTHVTYR	NCP	37.998

NTHVTYR;LHGPPLP	NCP	32.319
LHGPPLP;KVMLPH	NCP	4.976
KVMLPH;MCMLGPPP	NCP	9.892
MCMLGPPP;AYLASA	NCP	8.445
AYLASA;CPGDLRK	NCP	39.783
CPGDLRK;SQPGGGHA	NCP	87.903
SQPGGGHA;FCPRP	NCP	13.527
FCPRP;PPPLRLP	NCP	13.713
PPPLRLP;RPSPFD	NCP	11.754
RPSPFD;SAHALAL	NCP	2.103
SAHALAL;RLFVVK	NCP	31.124
RLFVVK;AAPPGRPP	NCP	17.014
AAPPGRPP;KPGQPPP	NCP	0.807
KPGQPPP;SLDPAGP	NCP	12.315
SLDPAGP;GVTGPP	NCP	26.877
GVTGPP;PSPAGPAPR	NCP	36.108
PSPAGPAPR;RKSGAP	NCP	3.137
RKSGAP;HPRGPPP	NCP	4.672
HPRGPPP;EHLSSPPQ	NCP	25.719
EHLSSPPQ;KGRHAH	NCP	84.536
KGRHAH;ASGPFGR	NCP	28.633
ASGPFGR;GVGLSA	NCP	19.638
GVGLSA;KDEPCP	NCP	2.49
KDEPCP;LSPPFPK	NCP	9.362
LSPPFPK;TVPESAR	NCP	27.462
TVPESAR;ASRPTVP	NCP	52.839
ASRPTVP;APGKPQ	NCP	16.684
APGKPQ;EPLLRGP	NCP	20.219
EPLLRGP;RSAPGPK	NCP	15.105
RSAPGPK;KGPWAPK	NCP	65.26
KGPWAPK;GRWAF	NCP	2.166
GRWAF;VLGVSHAP	NCP	26.934
VLGVSHAP;MADMEPN	NCP	32.585
MADMEPN;TPDRSHG	NCP	6.673
TPDRSHG;KAPAHPK	NCP	2.372
KAPAHPK;KPLSPGPR	NCP	61.564
KPLSPGPR;THKNPAL	NCP	19.008
THKNPAL;GMKGP	NCP	28.565
GMKGP;PPTPMPP	NCP	0.643
PPTPMPP;VGSNPVPP	NCP	1.929
VGSNPVPP;PWKFVF	NCP	1.869
PWKFVF;DGPGPAR	NCP	10.365
DGPGPAR;KPQGKVP	NCP	14.603
KPQGKVP;PVDLFF	NCP	86.98
PVDLFF;FPPPPLPD	NCP	27.236
FPPPPLPD;KAPAPPKP	NCP	0.436
KAPAPPKP;VDVLPVQ	NCP	15.089
VDVLPVQ;VGVELVKR	NCP	27.465
VGVELVKR;EVPMAP	NCP	21.354
EVPMAP;NPPKFL	NCP	32.457
NPPKFL;LSTAEPK	NCP	19.251

LSTAEPK;LVETW	NCP	60.895
LVETW;PLVFGK	NCP	6.278
PLVFGK;VEDDF	NCP	18.192
VEDDF;KHRWPR	NCP	5.796
KHRWPR;LPVPGVR	NCP	21.99
LPVPGVR;KVPVEK	NCP	3.937
KVPVEK;FACPPPP	NCP	19.983
FACPPPP;PGAFVPRP	NCP	0.477
PGAFVPRP;SVNPMLL	NCP	3.572
SVNPMLL;VFNDMK	NCP	27.738
VFNDMK;AAFPGSPK	NCP	42.001
AAFPGSPK;YECFTLY	NCP	67.769
YECFTLY;LGGFPPK	NCP	21.286
LGGFPPK;LSPPFVE	NCP	11.4
LSPPFVE;FGGPK	NCP	25.212
FGGPK;DGPVPLPT	NCP	25.227
DGPVPLPT;VTWGLPL	NCP	43.722
VTWGLPL;GPPVSER	NCP	14.142
GPPVSER;LELLLP	NCP	84.42
LELLLP;QPPEPPG	NCP	36.156
QPPEPPG;QGPRAAQP	NCP	14.247
QGPRAAQP;ASPAHPPGP	NCP	102.537
ASPAHPPGP;DSSAPPPPP	NCP	44.652
DSSAPPPPP;RAAPPRP	NCP	19.575
RAAPPRP;KLGAAV	NCP	77.382
KLGAAV;PQNLQR	NCP	23.286
PQNLQR;VTLDPL	NCP	53.673
VTLDPL;PPLSPPVP	NCP	8.349
PPLSPPVP;YSLPAY	NCP	13.407
YSLPAY;RDGPSAPP	NCP	34.104
RDGPSAPP;MVSPQLR	NCP	36.609
MVSPQLR;RPPPP	NCP	82.191
RPPPP;ARPLLAPP	NCP	34.344
ARPLLAPP;VGDHGPGP	NCP	5.097
VGDHGPGP;WPPPGVP	NCP	52.935
WPPPGVP;TPYRP	NCP	2.211
TPYRP;APFRPK	NCP	22.692
APFRPK;CSAPGLCP	NCP	39.483
CSAPGLCP;PKTRPP	NCP	38.247
PKTRPP;VVPGKLA	NCP	23.37
VVPGKLA;SPTMAGF	NCP	23.739
SPTMAGF;GPAAL	NCP	70.116
GPAAL;KAPGSAVP	NCP	76.362
KAPGSAVP;SAQPGVR	NCP	0.21
SAQPGVR;RAPVSPR	NCP	44.64
RAPVSPR;CCSCP	NCP	14.04
CCSCP;PVFPRQ	NCP	2.331
PVFPRQ;LPPAPKP	NCP	107.43
LPPAPKP;VTRMKP	NCP	17.01
VTRMKP;RGSGLLR	NCP	39.26
RGSGLLR;ELRVGR	NCP	6.526

ELRVGR;VPPGPAL	NCP	45.753
VPPGPAL;EHSATK	NCP	27.564
EHSATK;KQMSP	NCP	12.263
KQMSP;PPDRKP	NCP	192.34
KVFKPP;TVEPR	NCP	22.23
TVEPR;AAPVPPP	NCP	60.281
AAPVPPP;NRRPPP	NCP	46.539
NRRPPP;LLEAVPL	NCP	9.936
LLEAVPL;KPFALR	NCP	23.325
KPFALR;LVLDTPP	NCP	3.582
LVLDTPP;YSVWLN	NCP	19.041
YSVWLN;MDKDGSATHR	NCP	43.278
MDKDGSATHR;TPPVKNP	NCP	3.615
TPPVKNP;APAVLQGK	NCP	43.848
APAVLQGK;APATLRPPP	NCP	18.825
APATLRPPP;RLSLESF	NCP	13.911
RLSLESF;DAPLLTR	NCP	28.761
DAPLLTR;PSGRSVPP	NCP	14.814
PSGRSVPP;ATAGPK	NCP	7.17
ATAGPK;RGPPPLHM	NCP	26.322
RGPPPLHM;AERPSVP	NCP	38.982
AERPSVP;APGDK	NCP	3.027
APGDK;VGDALGFGHAA	NCP	36.705
VGDALGFGHAA;EPTAG	NCP	3.777
EPTAG;PERGPSGP	NCP	6.345
PERGPSGP;EVKPSPR	NCP	3.315
EVKPSPR;VGPAQHP	NCP	4.93
VGPAQHP;WGPGYVR	NCP	41.201
WGPGYVR;RRHAK	NCP	28.107
RRHAK;SSAVRAPP	NCP	47.169
SSAVRAPP;GPVDPPR	NCP	0.192
GPVDPPR;EVVGLLD	NCP	12.696
EVVGLLD;APPGAPVLP	NCP	12.612
APPGAPVLP;PVGTPRP	NCP	7.545
PVGTPRP;DHLSPPP	NCP	4.188
DHLSPPP;AGEKLTFVDF	NCP	10.171
AGEKLTFVDF;PATSAPR	NCP	4.538
PATSAPR;FANAPK	NCP	66.987
FANAPK;EDTQLH	NCP	25.506
EDTQLH;LPPRVPT	NCP	18.075
LPPRVPT;AAGLHY	NCP	4.176
AAGLHY;LPAAERK	NCP	19.653
LPAAERK;FKLTR	NCP	30.625
FKLTR;KQCCLSVH	NCP	127.833
KQCCLSVH;MFLLFLNK	NCP	109.254
MFLLFLNK;LDGLTAL	NCP	10.646
LDGLTAL;ASGRGGAP	NCP	36.921
ASGRGGAP;GWFTLR	NCP	26.428
GWFTLR;LSLKKKP	NCP	58.295
LSLKKKP;NYEELAK	NCP	76.195
NYEELAK;TVGGGLL	NCP	56.252

TVGGGLL;CHKPVK	NCP	16.221
CHKPVK;EGVFP	NCP	17.707
EGVFP;SGQPVLL	NCP	0.857
SGQPVLL;VGAPSW	NCP	0.33
VGAPSW;EGPSRPRP	NCP	1.338
EGPSRPRP;KAARLR	NCP	11.292
KAARLR;EKPSVPA	NCP	13.764
EKPSVPA;ALVPMP	NCP	70.231
ALVPMP;VGALSQL	NCP	2.867
VGALSQL;EAALDLK	NCP	44.802
EAALDLK;TAVQLL	NCP	64.836
TAVQLL;GVAAAL	NCP	69.733
GVAAAL;TALRTPT	NCP	38.966
TALRTPT;KKDGTPK	NCP	30.057
KKDGTPK;LDVEAP	NCP	20.211
LDVEAP;EGVPGVR	NCP	63.453
EGVPGVR;WDVCTP	NCP	35.61
WDVCTP;ECSAK	NCP	45.727
ECSAK;APAPPAAP	NCP	16.175
APAPPAAP;KDFSRLL	NCP	23.436
KDFSRLL;QALLPLR	NCP	8.082
QALLPLR;GVTAEVL	NCP	16.581
GVTAEVL;DKFLSAL	NCP	16.888
DKFLSAL;VAPPPFPK	NCP	4.242
VAPPPFPK;KPMMPQ	NCP	17.787
KPMMPQ;VTTSTRYT	NCP	22.608
VTTSTRYT;MGAVL	NCP	34.346
MGAVL;VGPPLKG	NCP	44.392
VGPPLKG;EFLKTLSK	NCP	6.621
EFLKTLSK;GPAFLVK	NCP	4.568
GPAFLVK;TGMDPP	NCP	59.706
TGMDPP;YEAFVK	NCP	30.604
YEAFVK;KSELYKK	NCP	4.353
KSELYKK;HLVPPRP	NCP	5.813
HLVPPRP;EVVMFL	NCP	3.457
EVVMFL;APRHPK	NCP	27.602
APRHPK;PGVGLPGLK	NCP	11.181
PGVGLPGLK;PGPPP	NCP	46.002
PGPPP;RVNLTLK	NCP	34.23
RVNLTLK;KLLDPK	NCP	6.616
KLLDPK;NLVAYY	NCP	21.735
NLVAYY;KKLPGSM	NCP	77.066
KKLPGSM;GVSVLE	NCP	56.481
GVSVLE;ELGLY	NCP	18.391
ELGLY;KVHELR	NCP	62.804
KVHELR;KSNELFL	NCP	35.302
KSNELFL;AAHRKCF	NCP	8.711
AAHRKCF;YFEDRFSR	NCP	4.623
YFEDRFSR;NPPRQPP	NCP	58.273
NPPRQPP;KGNKRAP	NCP	18.839
KGNKRAP;KMNSF	NCP	5.76

KMNSF;RNVCML	NCP	26.269
RNVCML;LALLY	NCP	1.752
LALLY;PHRVFP	NCP	39.953
PHRVFP;KPTAAKP	NCP	5.718
KPTAAKP;MFALPKL	NCP	4.842
MFALPKL;STGRGKK	NCP	17.775
STGRGKK;RGVGLTL	NCP	89.458
RGVGLTL;KTPVWLP	NCP	115.604
KTPVWLP;EVLTAW	NCP	4.017
EVLTAW;LSADAVN	NCP	56.025
LSADAVN;KDSFKVSL	NCP	19.32
KDSFKVSL;NRLLHNFG	NCP	64.258
NRLLHNFG;TVLGLLT	NCP	50.972
TVLGLLT;TPEVVL	NCP	62.317
TPEVVL;LDVSGLRP	NCP	37.436
LDVSGLRP;PLAAAPL	NCP	36.021
PLAAAPL;VLVSY	NCP	27.448
VLVSY;QAPPVLTR	NCP	40.403
QAPPVLTR;LKDFDN	NCP	22.12
LKDFDN;SLPLFP	NCP	1.79
SLPLFP;LSPRPTTQ	NCP	22.941
LSPRPTTQ;LEVLSGVVK	NCP	32.214
LEVLSGVVK;PAFFPWK	NCP	1.422
PAFFPWK;LLETLDQ	NCP	2.857
LLETLDQ;DKDNK	NCP	117.12
DKDNK;LLLNTYF	NCP	38.036
LLLNTYF;TKHPAK	NCP	57.538
TKHPAK;VPPPCPLP	NCP	27.689
VPPPCPLP;TLDWVA	NCP	35.761
TLDWVA;YSHVPR	NCP	62.31
YSHVPR;EPRSLPLQ	NCP	131.54
EPRSLPLQ;LTAAPQQ	NCP	40.447
LTAAPQQ;LDELLRK	NCP	8.006
LDELLRK;ESLLSPLK	NCP	52.879
ESLLSPLK;LGVNQE	NCP	50.108
LGVNQE;VPEMT	NCP	28.893
VPEMT;VAVELLA	NCP	188.482
VAVELLA;KGTMVYH	NCP	4.683
KGTMVYH;THSLAF	NCP	5.726
THSLAF;VTKLVDL	NCP	89.802
VTKLVDL;KNHWLK	NCP	5.968
KNHWLK;KYQPPP	NCP	63.017
KYQPPP;VVPWKQ	NCP	27.102
VVPWKQ;FDGELL	NCP	3.459
FDGELL;QGAQLVH	NCP	43.098
QGAQLVH;TQYVY	NCP	28.954
TQYVY;EPFLPYK	NCP	79.907
EPFLPYK;EPFLSLR	NCP	203.574
EPFLSLR;KGGHP	NCP	21.366
KGGHP;LLLLLLKLLLPP	NCP	88.342
LLLLLLKLLLPP;VVPFPLQPE	NCP	13.619

VVPFPLQPE;APRSFK	NCP	53.53
APRSFK;PSSPM	NCP	183.98
PSSPM;KQLEAFP	NCP	10.383
KQLEAFP;NGCVLVA	NCP	62.205
NGCVLVA;KNMPGN	NCP	33.666
KNMPGN;DDGGSC	NCP	16.767
DDGGSC;EMSDLRK	NCP	0.615
EMSDLRK;KWAVQR	NCP	8.865
KWAVQR;EVPLLPA	NCP	11.362
EVPLLPA;QRVPGLTLL	NCP	34.53
QRVPGLTLL;LNLKGPK	NCP	24.276
LNLKGPK;LKVPAAL	NCP	20.367
LKVPAAL;VGFPSKA	NCP	46.505
VGFPSKA;LLVNYR	NCP	56.082
LLVNYR;LLSHSLL	NCP	117.829
LLSHSLL;LSFPL	NCP	33.03
LSFPL;KNLKVE	NCP	26.364
KNLKVE;FFDHVR	NCP	52.905
FFDHVR;KHDFMP	NCP	41.348
KHDFMP;KHHYGY	NCP	70.747
KHHYGY;PTGNQ	NCP	30.578
PTGNQ;KLFNG	NCP	1.657
KLFNG;DYLHLR	NCP	225.215
DYLHLR;HGPMKPP	NCP	3.021
HGPMKPP;GQPLLE	NCP	45.037
GQPLLE;SLRLYP	NCP	45.029
SLRLYP;KGAELYK	NCP	54.681
KGAELYK;KYMLM	NCP	1.734
KYMLM;PLDWSK	NCP	4.353
PLDWSK;LTHWKNLY	NCP	16.498
LTHWKNLY;KLTVKYTC	NCP	63.245
KLTVKYTC;VAFVLQ	NCP	43.641
VAFVLQ;KDAPVYP	NCP	46.695
KDAPVYP;TDPEPK	NCP	32.184
TDPEPK;ETWVVSL	NCP	3.651
ETWVVSL;AKPLT	NCP	35.064
AKPLT;YGESDL	NCP	22.11
YGESDL;KEDEPYR	NCP	34.344
KEDEPYR;MDRLKAR	NCP	36.627
MDRLKAR;LDNLFRF	NCP	56.772
LDNLFRF;VGTVTPL	NCP	42.948
VGTVTPL;KSPVPVK	NCP	175.678
KSPVPVK;AEENDN	NCP	15.627
AEENDN;PVDASVH	NCP	145.931
PVDASVH;KMAKGPK	NCP	81.724
KMAKGPK;PNWGREK	NCP	3.062
PNWGREK;FAFLF	NCP	46.734
FAFLF;ASYEEFR	NCP	136.887
ASYEEFR;LKMYGDK	NCP	12.304
LKMYGDK;LLESVNH	NCP	8.607
LLESVNH;KPAGHAK	NCP	114.368

KPAGHAK;EVLSRTP	NCP	57.151
EVLSRTP;HNRSTTLC	NCP	33.467
HNRSTTLC;LPPPHPMP	NCP	27.136
LPPPHPMP;KYLPPKY	NCP	169.878
KYLPPKY;PVVPP	NCP	99.573
PVVPP;LGELHQK	NCP	33.593
LGELHQK;PADLRSP	NCP	29.058
PADLRSP;QMAAPK	NCP	46.197
QMAAPK;RFKPPP	NCP	99.534
RFKPPP;GVLEQPL	NCP	2.244
GVLEQPL;LPAEHKK	NCP	12.808
LPAEHKK;PSRRKPP	NCP	164.678
PSRRKPP;GVKDTEFK	NCP	21.654
GVKDTEFK;PRWGGGKK	NCP	1.516
PRWGGGKK;EGGDPRVP	NCP	5.21
EGGDPRVP;FDLVVSG	NCP	6.432
FDLVVSG;PPSRPPP	NCP	92.592
PPSRPPP;LSRTPPM	NCP	54.292
LSRTPPM;RKTLAAP	NCP	10.794
RKTLAAP;QTLHFK	NCP	109.881
QTLHFK;LKSHGVL	NCP	85.58
LKSHGVL;NTLQQPR	NCP	93.085
NTLQQPR;NLQHEGK	NCP	9.987
NLQHEGK;LLLLLLGY	NCP	46.806
LLLLLLGY;GAPTLRE	NCP	58.853
GAPTLRE;TPELKPR	NCP	4.155
TPELKPR;KWVDR	NCP	67.695
KWVDR;QPPVKMK	NCP	46.83
QPPVKMK;NPSRKFF	NCP	5.944
NPSRKFF;ANLNLEK	NCP	46.74
ANLNLEK;ASLVCKK	NCP	2.087
ASLVCKK;TLYKKT	NCP	71.895
TLYKKT;NLATLKTK	NCP	2.239
NLATLKTK;KTYFAP	NCP	16.692
KTYFAP;NRLAEAL	NCP	8.009
NRLAEAL;LAKLLKK	NCP	55.015
LAKLLKK;LAPGLP	NCP	40.154
LAPGLP;PPPPWP	NCP	16.434
PPPPWP;CEDSTT	NCP	39.525
CEDSTT;AMRELL	NCP	76.854
AMRELL;KALLLAP	NCP	25.822
KALLLAP;LSLFPPYK	NCP	73.694
LSLFPPYK;WEVPKK	NCP	22.809
WEVPKK;VAAKGA	NCP	29.128
VAAKGA;GFSTLYP	NCP	26.45
GFSTLYP;GDPNLPR	NCP	36.375
GDPNLPR;LELDSL	NCP	118.506
LELDSL;LAETLKY	NCP	118.729
LAETLKY;GHGEGTRL	NCP	82.16
GHGEGTRL;PALVVM	NCP	15.177
PALVVM;TTMLP	NCP	2.209

TTMLP;LGDGLAR	NCP	65.84
LGDGLAR;RETLKK	NCP	18.484
RETLKK;RNPPLLV	NCP	22.283
RNPPLLV;SLDDLPL	NCP	50.668
SLDDLPL;LFRKGV	NCP	17.669
LFRKGV;PYLLLS	NCP	11.097
PYLLLS;CTLAKPV	NCP	1.185
CTLAKPV;LSAPLKR	NCP	49.392
LSAPLKR;LPTAMFK	NCP	2.545
LPTAMFK;KHAVKK	NCP	25.014
KHAVKK;NVPPLEF	NCP	21.227
NVPPLEF;KESTYSLG	NCP	40.048
KESTYSLG;NAELLL	NCP	49.728
NAELLL;ELDVPSL	NCP	122.267
ELDVPSL;TEHPFK	NCP	106.882
TEHPFK;KPPHKL	NCP	15.216
KPPHKL;TTLRALY	NCP	32.565
TTLRALY;KPETLSR	NCP	54.309
KPETLSR;LRTGSSRL	NCP	46.566
LRTGSSRL;MPLWERT	NCP	4.905
MPLWERT;FFLVASRT	NCP	31.97
FFLVASRT;TPEDAF	NCP	19.09
TPEDAF;SGKLDAF	NCP	2.03
SGKLDAF;VSPTVKL	NCP	40.42
VSPTVKL;LTKKTPP	NCP	142.931
LTKKTPP;PAPPPPPPK	NCP	134.905
PAPPPPPPK;KTVHC	NCP	44.34
KTVHC;NKTPPD	NCP	27.923
NKTPPD;MGVKPLL	NCP	29.409
MGVKPLL;SCVGVFQ	NCP	2.727
SCVGVFQ;KDKPHK	NCP	6.63
KDKPHK;VYLVAPA	NCP	111.984
VYLVAPA;DRASFK	NCP	6.072
DRASFK;KAPAPPPP	NCP	2.487
KAPAPPPP;ELLDVSP	NCP	27.105
ELLDVSP;NKKLLVK	NCP	7.405
NKKLLVK;KTVHEELTK	NCP	49.307
KTVHEELTK;CCFCEC	NCP	41.971
CCFCEC;KFKVL	NCP	0.234
KFKVL;VGFLQ	NCP	40.355
VGFLQ;RGLLTLK	NCP	103.807
RGLLTLK;TGLLFNT	NCP	2.991
TGLLFNT;HVGVGRPR	NCP	2.13
HVGVGRPR;RGPPPPPPPP	NCP	7.428
RGPPPPPPPP;LVHSP	NCP	76.76
LVHSP;LGQVGAF	NCP	13.878
LGQVGAF;FPSLVHR	NCP	153.726
FPSLVHR;ACGPVNL	NCP	85.248
ACGPVNL;VPNPYD	NCP	55.32
VPNPYD;LLGLLYP	NCP	36.697
LLGLLYP;APSFPLG	NCP	47.961

APSFPLG;SPPPLKEK	NCP	51.657
SPPPLKEK;KPGVVLP	NCP	23.015
KPGVVLP;LLMVGY	NCP	47.749
LLMVGY;TVTPVAK	NCP	0.774
TVTPVAK;VGLTTGPP	NCP	84.677
VGLTTGPP;HSSLLAE	NCP	7.644
HSSLLAE;QLHKFK	NCP	9.901
QLHKFK;PQSDN	NCP	29.055
PQSDN;RGVGLTLK	NCP	16.524
RGVGLTLK;DGPVL	NCP	62.465
DGPVL;GVVVTAP	NCP	94.218
GVVVTAP;WEPVLLR	NCP	0.519
WEPVLLR;DGVRVPQ	NCP	20.977
DGVRVPQ;HLNFLVSV	NCP	41.72
HLNFLVSV;YLLNAPQ	NCP	42.826
YLLNAPQ;LTKALPR	NCP	31.74
LTKALPR;KVTALLP	NCP	24.216
KVTALLP;KPLSP	NCP	7.461
KPLSP;DNLFKFPP	NCP	49.647
DNLFKFPP;FAFPGLP	NCP	44.102
FAFPGLP;AEYKKQ	NCP	8.871
AEYKKQ;ESPASPHL	NCP	48.633
ESPASPHL;TGPLFTP	NCP	3.06
TGPLFTP;QSLGRNPLL	NCP	7.144
QSLGRNPLL;LVLLLPK	NCP	5.501
LVLLLPK;GAGELL	NCP	25.548
GAGELL;LLKLLE	NCP	3.544
LLKLLE;QHALEPK	NCP	43.388
QHALEPK;SDLFYNTSL	NCP	54.672
SDLFYNTSL;GDLEKF	NCP	46.869
GDLEKF;ESVGRVH	NCP	12.151
ESVGRVH;FYKGPK	NCP	26.624
FYKGPK;RGSRAAPP	NCP	22.834
RGSRAAPP;QGMHVPG	NCP	74.315
QGMHVPG;KEPSGEKK	NCP	71.433
KEPSGEKK;KAAMVAV	NCP	9.093
KAAMVAV;AHKAKGK	NCP	67.381
AHKAKGK;KDVGVP	NCP	54.83
KDVGVP;VGVEKKL	NCP	11.112
VGVEKKL;QRNLFQKR	NCP	221.019
QRNLFQKR;LSKPEY	NCP	22.512
LSKPEY;LVKAALLV	NCP	61.101
LVKAALLV;MTLMDLK	NCP	106.344
MTLMDLK;SQNFLVL	NCP	13.344
SQNFLVL;SPNAAP	NCP	43.81
SPNAAP;LGFPML	NCP	0.966
LGFPML;VVAPPK	NCP	29.618
VVAPPK;KVYVSW	NCP	32.548
KVYVSW;NVLELY	NCP	3.545
NVLELY;AFVRQT	NCP	64.174
AFVRQT;TFKEPK	NCP	92.066

TFKEPK;LLAGPVA	NCP	6.798
LLAGPVA;MFLLQG	NCP	9.655
MFLLQG;KGYVP	NCP	19.947
KGYVP;EVPSSLKE	NCP	40.299
EVPSSLKE;WSEEVP	NCP	5.895
WSEEVP;TLRFAK	NCP	10.566
TLRFAK;GVPVLP	NCP	0.597
GVPVLP;TVPELT	NCP	52.979
TVPELT;LLLQYA	NCP	6.556
LLLQYA;LPLTTK	NCP	22.074
LPLTTK;CHMLR	NCP	51.183
CHMLR;AAKLHT	NCP	13.742
AAKLHT;KAASMPK	NCP	20.661
KAASMPK;LAEALLK	NCP	2.26
LAEALLK;KTMSPLR	NCP	5.307
KTMSPLR;DPVKPPP	NCP	25.727
DPVKPPP;KGNHVPR	NCP	13.83
KGNHVPR;KGMHTK	NCP	3.868
KGMHTK;KDLLGPV	NCP	10.008
KDLLGPV;ELAMLP	NCP	15.929
ELAMLP;GPGHRTP	NCP	14.746
GPGHRTP;VCVKGAP	NCP	90.101
VCVKGAP;AAPDGKSK	NCP	11.175
AAPDGKSK;KFKPPPP	NCP	1.612
KFKPPPP;LEMFST	NCP	1.014
LEMFST;KNMGLPP	NCP	14.409
KNMGLPP;SGHGFLV	NCP	35.471
SGHGFLV;TPLAAPP	NCP	18.844
TPLAAPP;HTPLYK	NCP	15.902
HTPLYK;KPLPPPAGP	NCP	9.067
KPLPPPAGP;MDWKKK	NCP	79.605
MDWKKK;RGSRTLY	NCP	28.385
RGSRTLY;LGPPKYP	NCP	19.702
LGPPKYP;LDLFP	NCP	26.804
LDLFP;EAEVGLF	NCP	19.278
EAEVGLF;VVPVDQ	NCP	63.456
VVPVDQ;ATVYALK	NCP	5.569
ATVYALK;RFPLRL	NCP	38.804
RFPLRL;GDVPF	NCP	38.799
GDVPF;PVATPPR	NCP	42.367
PVATPPR;ALKKLKVQ	NCP	1.077
ALKKLKVQ;AAARLVL	NCP	14.031
AAARLVL;SSLNELHSL	NCP	67.674
SSLNELHSL;KGLLEY	NCP	13.365
KGLLEY;FGGASP	NCP	28.55
FGGASP;QAQKSQ	NCP	141.937
QAQKSQ;SALPALV	NCP	138.117
SALPALV;VAGEK	NCP	13.5
VAGEK;WAPECALP	NCP	16.206
WAPECALP;KDLPVTD	NCP	39.996
KDLPVTD;LVWVPSEK	NCP	15.197

LVWVPSEK;DSGVLK	NCP	14.884
DSGVLK;SVNPPLV	NCP	21.069
SVNPPLV;VTEYLK	NCP	6.806
VTEYLK;DFVLK	NCP	5.653
DFVLK;GDDMC	NCP	70.287
GDDMC;AQSGLLR	NCP	57.272
AQSGLLR;LGKKTPK	NCP	98.794
LGKKTPK;KKYHHK	NCP	2.31
KKYHHK;EVPKEVL	NCP	11.586
EVPKEVL;AVQPEGLK	NCP	15.297
AVQPEGLK;LRPPYK	NCP	55.52
LRPPYK;LEDLGFK	NCP	239.466
LEDLGFK;TVPSTAF	NCP	80.554
TVPSTAF;PLYPQFL	NCP	4.653
PLYPQFL;KTLAANK	NCP	38.192
KTLAANK;AALATLKT	NCP	69.541
AALATLKT;KLSGVSHP	NCP	5.241
KLSGVSHP;GQFQLTH	NCP	20.484
GQFQLTH;TGKGVP	NCP	77.517
TGKGVP;LTLKCYN	NCP	19.518
LTLKCYN;REVDLL	NCP	80.004
REVDLL;AGTALKR	NCP	11.606
AGTALKR;VLQGQF	NCP	17.178
VLQGQF;KGMAKR	NCP	73.032
KGMAKR;KDTVP	NCP	66.214
KDTVP;TVTALAP	NCP	79.841
TVTALAP;GFVVTGY	NCP	16.552
GFVVTGY;KELYLD	NCP	64.589
KELYLD;KLKKGPK	NCP	32.164
KLKKGPK;ESVHPLH	NCP	32.006
ESVHPLH;VASNL	NCP	24.585
VASNL;YVFLQLPV	NCP	25.165
YVFLQLPV;ELDSLVT	NCP	91.34
ELDSLVT;TGGAGY	NCP	34.768
TGGAGY;GPLGHAPFQ	NCP	15.876
GPLGHAPFQ;SLYVYK	NCP	9.513
SLYVYK;MLSVPF	NCP	14.472
MLSVPF;QHNKPP	NCP	132.119
QHNKPP;NLVGL	NCP	16.38
NLVGL;AYGGLLK	NCP	67.972
AYGGLLK;AARAVRP	NCP	165.431
AARAVRP;KMEASCV	NCP	29.154
KMEASCV;DLVPQ	NCP	62.629
DLVPQ;LTGNYGT	NCP	32.376
LTGNYGT;YDHYME	NCP	77.739
YDHYME;KLTLDKLDVKGKR	NCP	9.807
KLTLDKLDVKGKR;LDKLDVK	NCP	0.009
LDKLDVK;LWLARK	NCP	62.172
LWLARK;LLQLPQ	NCP	121.214
LLQLPQ;KPFTP	NCP	118.674
KPFTP;ARVLLR	NCP	64.527

ARVLLR;ASFEKRQ	NCP	82.621
ASFEKRQ;VPPPPPLA	NCP	0.648
VPPPPPLA;LEAVP	NCP	71.343
LEAVP;KHLLPPTP	NCP	0.405
KHLLPPTP;CYLLEVL	NCP	148.539
CYLLEVL;QKPAAPP	NCP	13.319
QKPAAPP;GHCPPAA	NCP	24.156
GHCPPAA;KCSDL	NCP	89.938
KCSDL;PKELSH	NCP	64.523
PKELSH;LDSLVFK	NCP	17.772
LDSLVFK;ACARPLL	NCP	18.561
ACARPLL;YSAPPK	NCP	4.881
YSAPPK;LLHLLLLKK	NCP	182.79
LLHLLLLKK;KEVVP	NCP	10.084
KEVVP;KNYPPPP	NCP	44.969
KNYPPPP;LEGAGYL	NCP	26.554
LEGAGYL;PVNFKLLS	NCP	4.218
PVNFKLLS;THVLYLR	NCP	9.609
THVLYLR;RYLKPD	NCP	34.182
RYLKPD;VEVEALK	NCP	13.443
VEVEALK;FVGAG	NCP	16.28
FVGAG;AAALQAV	NCP	99.13
AAALQAV;KNSLAPE	NCP	148.013
KNSLAPE;KHNPP	NCP	152.917
KHNPP;RLSWY	NCP	73.436
RLSWY;KPFYVP	NCP	32.617
KPFYVP;HGDPG	NCP	58.541
HGDPG;KPPDWK	NCP	57.747
KPPDWK;QHNPPDR	NCP	83.152
QHNPPDR;VQLSVPH	NCP	16.802
VQLSVPH;MGTSAF	NCP	28.963
MGTSAF;PGLPELKL	NCP	21.657
PGLPELKL;ERVWYF	NCP	24.363
ERVWYF;KLTLKGY	NCP	1.695
KLTLKGY;SLLQYE	NCP	14.594
SLLQYE;VSGSTGPK	NCP	44.626
VSGSTGPK;VLLKERY	NCP	65.223
VLLKERY;YGAEAL	NCP	32.529
YGAEAL;TMDDLR	NCP	34.44
TMDDLR;PPPPKKD	NCP	40.628
PPPPKKD;MFLCLT	NCP	118.734
MFLCLT;MALLNY	NCP	37.462
MALLNY;HVPPP	NCP	107.321
HVPPP;LSGLKSGR	NCP	4.855
LSGLKSGR;LTHTAPK	NCP	9.084
LTHTAPK;EVDALFK	NCP	18.398
EVDALFK;DKNQRP	NCP	21.1
DKNQRP;AALLHGR	NCP	17.207
AALLHGR;KVRPSK	NCP	62.425
KVRPSK;STAKVPR	NCP	128.082
STAKVPR;KPFLSHM	NCP	25.224

KPFLSHM;LDFLRML	NCP	20.622
LDFLRML;PLHSLAK	NCP	29.589
PLHSLAK;GVFLVA	NCP	21.674
GVFLVA;QKPNVR	NCP	76.482
QKPNVR;KYWMPP	NCP	41.815
KYWMPP;LYTLGSF	NCP	38.478
LYTLGSF;KNHPP	NCP	54.63
KNHPP;AHLGTALK	NCP	4.217
AHLGTALK;TATPVLT	NCP	24.373
TATPVLT;CHMYCGSP	NCP	22.644
CHMYCGSP;LSVLNY	NCP	113.642
LSVLNY;TVFPSL	NCP	118.998
TVFPSL;AVPPEKP	NCP	63.403
AVPPEKP;TVTSKEGP	NCP	30.147
TVTSKEGP;EQHKLR	NCP	16.04
EQHKLR;KEPLLGP	NCP	24.745
KEPLLGP;PGAALAHL	NCP	0.756
PGAALAHL;LSSPQGF	NCP	18.222
LSSPQGF;KFTAPR	NCP	4.461
KFTAPR;PTVGVP	NCP	40.233
PTVGVP;YAPLL	NCP	40.308
YAPLL;PALLPLLK	NCP	18.057
PALLPLLK;TSSPVSR	NCP	20.547
TSSPVSR;MHSDLRK	NCP	10.757
MHSDLRK;KMTPPLDL	NCP	8.773
KMTPPLDL;KDLPEL	NCP	2.231
KDLPEL;KDPCPSK	NCP	22.825
KDPCPSK;PGFHKKK	NCP	52.815
PGFHKKK;DPLPPR	NCP	125.43
DPLPPR;PGLATT	NCP	1.694
PGLATT;SGLTPKET	NCP	43.923
SGLTPKET;KPLLRSP	NCP	153.168
KPLLRSP;VSHSLLDH	NCP	137.101
VSHSLLDH;VTSALG	NCP	0.056
VTSALG;AAVLM	NCP	9.01
AAVLM;RLVYP	NCP	6.002
RLVYP;VALGGGL	NCP	9.549
VALGGGL;CRHLPVQ	NCP	68.794
CRHLPVQ;CVFLLFPPL	NCP	2.106
CVFLLFPPL;YLSAPR	NCP	62.255
YLSAPR;DLPFP	NCP	41.991
DLPFP;YLQNNF	NCP	43.495
YLQNNF;KCSTKK	NCP	38.52
KCSTKK;QRKKPR	NCP	5.36
QRKKPR;DQAFGL	NCP	2.119
DQAFGL;VSPAAPPK	NCP	33.23
VSPAAPPK;LLASLVK	NCP	72.349
LLASLVK;DPFRDW	NCP	11.979
DPFRDW;VLLKP	NCP	5.733
VLLKP;LPPGFVT	NCP	43.59
LPPGFVT;KDEPWP	NCP	8.582

KDEPWP;KWSVKR	NCP	0.28
KWSVKR;MTKKTK	NCP	60.855
MTKKTK;TTVAVP	NCP	8.232
TTVAVP;NTLGL	NCP	103.292
NTLGL;VLHNLLY	NCP	55.102
VLHNLLY;KVLLESR	NCP	9.054
KVLLESR;EVPAGPR	NCP	113.766
EVPAGPR;EHKLAR	NCP	112.593
EHKLAR;QCVLPG	NCP	18.489
QCVLPG;NYHSSSK	NCP	9.141
NYHSSSK;KTKLDHK	NCP	51.504
KTKLDHK;KDPVSPL	NCP	101.478
KDPVSPL;KGNARPK	NCP	15.351
KGNARPK;PPLKTSSK	NCP	102.477
PPLKTSSK;NSVTLLMF	NCP	85.536
NSVTLLMF;RRAFFL	NCP	0.44
RRAFFL;YDLRSP	NCP	42.739
YDLRSP;VSAEPQ	NCP	111.591
VSAEPQ;KYLDLPK	NCP	15.599
KYLDLPK;ELEAMSR	NCP	129.354
ELEAMSR;NVELPK	NCP	14.854
NVELPK;KLHDLP	NCP	29.739
KLHDLP;KKTTLLK	NCP	45.452
KKTTLLK;KEELKKY	NCP	9.054
KEELKKY;KFLAEDGR	NCP	102.042
KFLAEDGR;YGTLVLH	NCP	7.504
YGTLVLH;DHCPQ	NCP	91.401
DHCPQ;KPASVAK	NCP	22.875
KPASVAK;TTVEKR	NCP	40.845
TTVEKR;FVGKSEL	NCP	27.543
FVGKSEL;LVFGGEK	NCP	96.504
LVFGGEK;LPESFFL	NCP	115.46
LPESFFL;LSPLVLRK	NCP	21.208
LSPLVLRK;GSLPELQ	NCP	15.702
GSLPELQ;QGPEATPK	NCP	5.346
QGPEATPK;KGKLLGPP	NCP	58.022
KGKLLGPP;VELQTPK	NCP	72.027
VELQTPK;DLLLKEL	NCP	40.528
DLLLKEL;STLLDPN	NCP	3.155
STLLDPN;EGPFVF	NCP	96.079
EGPFVF;SGVVFC	NCP	68.414
SGVVFC;PVLKLF	NCP	20.731
PVLKLF;PQRPP	NCP	146.586
PQRPP;PYVPFP	NCP	65.802
PYVPFP;VNHPQVSALLGEEDEEALHY	NCP	14.205
VNHPQVSALLGEEDEEALHY;LTRVEVTEF	NCP	0.06
LTRVEVTEF;EKCCCKE	NCP	31.868
EKCCCKE;TGSPPVFR	NCP	12.171
TGSPPVFR;KTKGNAP	NCP	33.645
KTKGNAP;VGNQAPK	NCP	8.331
VGNQAPK;MHKAKSK	NCP	20.461

MHKAKSK;WGVRLE	NCP	16.359
WGVRLE;HAPGHRP	NCP	11.096
HAPGHRP;VGFPPPP	NCP	16.539
VGFPPPP;MMKELR	NCP	70.741
MMKELR;ESLTDPSKLDSGKELH	NCP	68.093
ESLTDPSKLDSGKELH;SAYLVAEK	NCP	0.234
SAYLVAEK;KSLTNDWEDHLAVKH	NCP	0.522
KSLTNDWEDHLAVKH;SLTNDWEDHLAVKHF	NCP	0.003
SLTNDWEDHLAVKHF;SVEGQLEF	NCP	0.045
SVEGQLEF;RALLFVPRRAPF	NCP	0.024
RALLFVPRRAPF;KKFYEQF	NCP	0.291
KKFYEQF;FYEQFSK	NCP	0.006
FYEQFSK;CVQQLKEFEGK	NCP	0.276
CVQQLKEFEGK;ALLSSGFSLEDPQTHA	NCP	0.423
ALLSSGFSLEDPQTHA;PLHKP	NCP	29.898
PLHKP;VLKAQAQSQKAK	NCP	129.211
VLKAQAQSQKAK;DPEAPTVP	NCP	5.274
DPEAPTVP;SLGRFKK	NCP	14.558
SLGRFKK;CVFGR	NCP	16.86
CVFGR;DVKKKAA	NCP	12.285
DVKKKAA;SLHVNAF	NCP	23.836
SLHVNAF;AALLVEN	NCP	9.273
AALLVEN;KMPKFS	NCP	9.345
KMPKFS;PVSWESF	NCP	50.715
PVSWESF;LETAVNL	NCP	73.26
LETAVNL;KDPVPR	NCP	112.521
KDPVPR;ELVAAGM	NCP	51.537
ELVAAGM;ALFPMK	NCP	12.644
ALFPMK;KAGYSRR	NCP	73.465
KAGYSRR;NLEVRVLL	NCP	2.429
NLEVRVLL;TAGAVLL	NCP	30.384
TAGAVLL;VSVLP	NCP	64.317
VSVLP;ASDLLLH	NCP	1.132
ASDLLLH;AFWTDF	NCP	39.923
AFWTDF;KHFAVP	NCP	7.212
KHFAVP;WRGAGPP	NCP	28.887
WRGAGPP;KVTVSRF	NCP	82.929
KVTVSRF;CRVKCR	NCP	75.123
CRVKCR;KETVQYP	NCP	51.57
KETVQYP;DPDLKLK	NCP	50.851
DPDLKLK;DFGKEF	NCP	28.103
DFGKEF;KNYSEKQ	NCP	67.285
KNYSEKQ;KMSRAT	NCP	36.497
KMSRAT;KYPLSHG	NCP	48.49
KYPLSHG;LLDNMK	NCP	107.325
LLDNMK;PPLTPPK	NCP	206.612
PPLTPPK;ASLHF	NCP	47.394
ASLHF;KGGVKLQ	NCP	16.885
KGGVKLQ;LPGSWT	NCP	54.863
LPGSWT;GSFSKLST	NCP	88.333
GSFSKLST;PAKSYR	NCP	71.28

PAKSYR;EEHDDM	NCP	135.852
EEHDDM;KDTLFK	NCP	46.964
KDTLFK;PAGFLTS	NCP	18.76
PAGFLTS;KTTKPSL	NCP	109.215
KTTKPSL;EVATTVL	NCP	11.34
EVATTVL;WLVVPR	NCP	86.817
WLVVPR;KVYGVG	NCP	11.237
KVYGVG;LLLLL	NCP	149.998
LLLLL;APLVVM	NCP	275.442
APLVVM;LLPSRLAV	NCP	33.042
LLPSRLAV;KPGPVREP	NCP	65.597
KPGPVREP;KDFMQPK	NCP	7.072
KDFMQPK;LRSPY	NCP	1.926
LRSPY;NPPYK	NCP	58.271
NPPYK;VLPVVY	NCP	158.727
VLPVVY;SLLKGAP	NCP	12.453
SLLKGAP;KVLCY	NCP	16.362
KVLCY;SPPVGVVLVL	NCP	51.448
SPPVGVVLVL;EEKTKK	NCP	2.859
EEKTKK;VSHKYP	NCP	26.111
VSHKYP;LAQKAMAP	NCP	42.258
LAQKAMAP;NALVQF	NCP	20.442
NALVQF;CLQLNR	NCP	107.703
CLQLNR;QPSGKKP	NCP	78.589
QPSGKKP;KKKKEYQT	NCP	5.139
KKKKEYQT;EGFDL	NCP	38.819
EGFDL;RSFSPPP	NCP	36.577
RSFSPPP;VTFPT	NCP	80.445
VTFPT;LSNAFKPP	NCP	66.872
LSNAFKPP;KERVYS	NCP	62.032
KERVYS;TPPVVP	NCP	2.481
TPPVVP;RPYRKPP	NCP	53.319
RPYRKPP;NYLSLMF	NCP	97.143
NYLSLMF;YGYLMP	NCP	72.594
YGYLMP;KPLRVAP	NCP	55.202
KPLRVAP;LSVHKR	NCP	7.426
LSVHKR;KHPDDD	NCP	64.112
KHPDDD;MMMVLL	NCP	50.064
MMMVLL;LALLLVPL	NCP	46.999
LALLLVPL;VQPNPP	NCP	1.496
VQPNPP;SSPGGVY	NCP	16.252
SSPGGVY;MWAGGGR	NCP	75.579
MWAGGGR;LLVYLM	NCP	11.352
LLVYLM;ESLTPL	NCP	25.296
ESLTPL;TYVMKK	NCP	8.934
TYVMKK;YSPLS	NCP	40.166
YSPLS;LVAVP	NCP	15.766
LVAVP;AQNVLK	NCP	5.403
AQNVLK;FVSSGPF	NCP	16.02
FVSSGPF;KEAEGPR	NCP	42.743
KEAEGPR;HGVEPTF	NCP	14.094

HGVEPTF;ELHFG	NCP	50.917
ELHFG;EVGPP	NCP	63.996
EVGPP;ESSNKP	NCP	14.382
ESSNKP;LDEVFL	NCP	20.732
LDEVFL;LYRLPLT	NCP	216.555
LYRLPLT;VVPPLTPP	NCP	13.828
VVPPLTPP;SGHSLRYF	NCP	101.52
SGHSLRYF;LPASVLF	NCP	22.004
LPASVLF;PLPFD	NCP	49.419
PLPFD;ESAYLV	NCP	180.3
ESAYLV;QTLLELY	NCP	37.12
QTLLELY;KGHKVF	NCP	9.677
KGHKVF;WKKCS	NCP	47.073
WKKCS;NPAVGPR	NCP	67.149
NPAVGPR;DGLGYSR	NCP	148.963
DGLGYSR;VGFEQL	NCP	7.665
VGFEQL;EMVVFK	NCP	64.613
EMVVFK;NNLLLTG	NCP	73.368
NNLLLTG;PAAKDPK	NCP	12.037
PAAKDPK;ALDKHK	NCP	3.462
ALDKHK;YSVLML	NCP	7.454
YSVLML;DTPELQR	NCP	0.459
DTPELQR;VKAVPVK	NCP	147.321
VKAVPVK;FCMPCY	NCP	52.873
FCMPCY;TALPKY	NCP	71.162
TALPKY;AFLPQ	NCP	4.525
AFLPQ;QMGGP	NCP	7.341
QMGGP;GPAPERPP	NCP	11.676
GPAPERPP;DKYVPR	NCP	29.96
DKYVPR;RHQLKSY	NCP	23.845
RHQLKSY;SGDVQFR	NCP	52.242
SGDVQFR;LLLGGGKK	NCP	31.545
LLLGGGKK;ETVHTYL	NCP	37.44
ETVHTYL;KSDVEF	NCP	97.409
KSDVEF;ATLGLAF	NCP	28.06
ATLGLAF;DSQEEM	NCP	41.454
DSQEEM;KEALGRLT	NCP	14
KEALGRLT;KLELDLPLV	NCP	17.001
KLELDLPLV;LPGNHKP	NCP	40.938
LPGNHKP;YAAVK	NCP	0.081
YAAVK;NYDKAKL	NCP	11.791
NYDKAKL;KSMPNFK	NCP	11.147
KSMPNFK;EKYVLE	NCP	11.734
EKYVLE;VSPTVLK	NCP	1.823
VSPTVLK;LAGKPHV	NCP	44.011
LAGKPHV;KKKKTGPK	NCP	21.825
KKKKTGPK;LAKHRW	NCP	1.059
LAKHRW;PGFVPA	NCP	47.508
PGFVPA;QRTYVY	NCP	74.316
QRTYVY;VSWLGGS	NCP	1.178
VSWLGGS;VPSLKLPL	NCP	87.103

VPSLKLPL;LLSPQKEP	NCP	13.659
LLSPQKEP;STPKVVH	NCP	1.212
STPKVVH;NGACRLL	NCP	27.956
NGACRLL;KAAAAVL	NCP	31.998
KAAAAVL;GLCVAVL	NCP	32.194
GLCVAVL;DPLLQDR	NCP	10.635
DPLLQDR;VAPSYR	NCP	29.501
VAPSYR;PAAPKPP	NCP	76.843
PAAPKPP;SGYVTPR	NCP	76.436
SGYVTPR;LNVVAQ	NCP	14.179
LNVVAQ;RQPPAVP	NCP	36.126
RQPPAVP;RPPRPP	NCP	0.285
RPPRPP;LAWGLL	NCP	107.331
LAWGLL;MPKFLK	NCP	22.073
MPKFLK;LPPGKFG	NCP	0.622
LPPGKFG;QSLNFPK	NCP	15.269
QSLNFPK;TGPLGGP	NCP	23.179
TGPLGGP;DNVMLK	NCP	35.531
DNVMLK;ENLLKLAR	NCP	0.016
ENLLKLAR;MMGQK	NCP	14.996
MMGQK;QGPAAG	NCP	19.762
QGPAAG;LTPPDPL	NCP	0.261
LTPPDPL;EELRFQ	NCP	4.829
EELRFQ;QLQERL	NCP	83.56
QLQERL;HGWTPK	NCP	4.983
HGWTPK;LPQSVER	NCP	0.939
LPQSVER;DPPTTLL	NCP	29.478
DPPTTLL;LSALVGLP	NCP	151.118
LSALVGLP;SGGKRKP	NCP	15.64
SGGKRKP;QPRAHGP	NCP	8.607
QPRAHGP;KGAVVLG	NCP	53.418
KGAVVLG;FPTAQQ	NCP	84.026
FPTAQQ;VPGVRLL	NCP	114.628
VPGVRLL;GPPPLP	NCP	14.232
GPPPLP;FEPFPK	NCP	32.151
FEPFPK;HLLFPLE	NCP	130.691
HLLFPLE;KCKFEKV	NCP	265.329
KCKFEKV;KPPRAY	NCP	46.402
KPPRAY;VGLEPPP	NCP	63.159
VGLEPPP;NVLYTSKY	NCP	20.153
NVLYTSKY;MQVKSKT	NCP	41.316
MQVKSKT;AAGFPGSR	NCP	8.74
AAGFPGSR;YVLMYG	NCP	7.085
YVLMYG;KPFPVAF	NCP	0.426
KPFPVAF;GVMFLQ	NCP	43.189
GVMFLQ;FDMADF	NCP	35.178
FDMADF;VLTELK	NCP	6.812
VLTELK;DYYAQR	NCP	23.065
DYYAQR;GTRNLR	NCP	149.793
GTRNLR;LPDRPP	NCP	14.547
LPDRPP;PPPLVPPPPP	NCP	32.043

PPPLVPPPPP;PDPAAP	NCP	50.769
PDPAAP;KGAQY	NCP	48.051
KGAQY;GYGPY	NCP	14.972
GYGPY;KTVYMFVP	NCP	17.301
KTVYMFVP;ENTHHPK	NCP	11.674
ENTHHPK;LDVPDV	NCP	36.581
LDVPDV;WHQLYW	NCP	25.03
WHQLYW;EVVDLVR	NCP	52.35
EVVDLVR;EQRYHKK	NCP	2.294
EQRYHKK;QPRYGLS	NCP	7.705
QPRYGLS;KTLSSSS	NCP	144.006
KTLSSSS;VGGNSLLR	NCP	29.373
VGGNSLLR;KPSLALD	NCP	20.112
KPSLALD;TLLVKLYLF	NCP	52.98
TLLVKLYLF;AYLFS	NCP	33.026
AYLFS;SLYPAPGA	NCP	44.749
SLYPAPGA;ALLEYL	NCP	47.741
ALLEYL;LLYTLGT	NCP	10.539
LLYTLGT;KDNYKLK	NCP	19.026
KDNYKLK;QRTPVVT	NCP	50.194
QRTPVVT;VGFYQ	NCP	78.252
VGFYQ;HLLPLP	NCP	8.906
HLLPLP;RSLRVSP	NCP	16.222
RSLRVSP;HELNVFP	NCP	3.14
HELNVFP;AVKLLKK	NCP	55.636
AVKLLKK;THLDALK	NCP	150.305
THLDALK;ALGAPVFP	NCP	8.875
ALGAPVFP;KNLNVKS	NCP	84.45
KNLNVKS;YQTNKR	NCP	29.565
YQTNKR;SVDLDE	NCP	28.661
SVDLDE;LHPPKSGP	NCP	15.663
LHPPKSGP;VGPPLAP	NCP	31.033
VGPPLAP;TSVLDAK	NCP	5.807
TSVLDAK;KQEVQLK	NCP	89.664
KQEVQLK;EVAGKLK	NCP	59.787
EVAGKLK;KDGAVPF	NCP	19.717
KDGAVPF;EGDPLLR	NCP	7.137
EGDPLLR;HRAGLR	NCP	16.338
HRAGLR;DMCDC	NCP	28.067
DMCDC;DMSGVLR	NCP	10.138
DMSGVLR;NLAGVPG	NCP	16.587
NLAGVPG;KEETDR	NCP	23.219
KEETDR;VPGFML	NCP	1.908
VPGFML;GVLLY	NCP	62.454
GVLLY;EDTGP	NCP	7.579
EDTGP;AAAKLHK	NCP	24.098
AAAKLHK;KSYDLP	NCP	17.857
KSYDLP;EPPGAP	NCP	86.235
EPPGAP;VGGPTV	NCP	9.288
VGGPTV;QVPHALL	NCP	6.726
QVPHALL;VMFLNPR	NCP	17.591

VMFLNPR;KQFSVAK	NCP	22.084
KQFSVAK;LAVAYY	NCP	2.925
LAVAYY;VEPLP	NCP	10.467
VEPLP;VLKLGHQ	NCP	18.468
VLKLGHQ;PVGMLFKK	NCP	16.907
PVGMLFKK;STPLPPRK	NCP	11.392
STPLPPRK;YRFQR	NCP	25.826
YRFQR;LVEKLVR	NCP	21.084
LVEKLVR;VDWCTP	NCP	28.633
VDWCTP;AYLFRN	NCP	46.532
AYLFRN;PAAALEGAP	NCP	10.504
PAAALEGAP;DRPAPHF	NCP	23.411
DRPAPHF;KSPETLEDQ	NCP	33.517
KSPETLEDQ;SPGVLPPP	NCP	22.161
SPGVLPPP;KQTMSF	NCP	42.819
KQTMSF;YTRKCF	NCP	53.597
YTRKCF;KVGPAPPVGL	NCP	31
KVGPAPPVGL;CMYTSSK	NCP	18.656
CMYTSSK;MLSREMT	NCP	26.884
MLSREMT;LKQWDL	NCP	61.401
LKQWDL;DSSLELHSL	NCP	76.118
DSSLELHSL;DWRHPK	NCP	42.814
DWRHPK;LDLDPFL	NCP	39.885
LDLDPFL;ASVQKR	NCP	50.229
ASVQKR;TAVNLP	NCP	55.442
TAVNLP;RQTVVE	NCP	0.901
RQTVVE;KSPPARY	NCP	9.753
KSPPARY;DPLLFNVK	NCP	45.525
DPLLFNVK;LSKDTPK	NCP	120.033
LSKDTPK;ALLAP	NCP	206.376
ALLAP;PFRPQR	NCP	34.787
PFRPQR;VLFVGGV	NCP	12.706
VLFVGGV;GDGPD	NCP	64.929
GDGPD;QDLLLEK	NCP	49.466
QDLLLEK;MLDEVKR	NCP	122.715
MLDEVKR;LAPTTSP	NCP	86.592
LAPTTSP;KDDDPK	NCP	49.534
KDDDPK;HPPMPPHP	NCP	158.58
HPPMPPHP;YLAGGP	NCP	90.545
YLAGGP;FSLYMNF	NCP	144.916
FSLYMNF;LQPMVKP	NCP	27.89
LQPMVKP;KVFKAGP	NCP	55.438
KVFKAGP;VLPLEK	NCP	5.544
VLPLEK;CSLDSF	NCP	54.372
CSLDSF;LLDLAYY	NCP	121.898
LLDLAYY;FCLKLK	NCP	23.719
FCLKLK;PVMPPPF	NCP	134.402
PVMPPPF;CPAKEFF	NCP	75.438
CPAKEFF;KSYPPPPPP	NCP	24.658
KSYPPPPPP;GVYVP	NCP	17.996
GVYVP;MASLGRL	NCP	97.98

MASLGRL;ALKMVK	NCP	30.183
ALKMVK;QAEFYK	NCP	49.114
QAEFYK;AAQVHS	NCP	53.12
AAQVHS;YDCMQKF	NCP	63.174
YDCMQKF;KQEPQP	NCP	116.983
KQEPQP;LKKLVR	NCP	60.608
LKKLVR;GKLHVLH	NCP	127.77
GKLHVLH;KGGVYVP	NCP	77.008
KGGVYVP;VSLLLK	NCP	33.518
VSLLLK;LTLQQPR	NCP	95.34
LTLQQPR;PPPPVKAH	NCP	18.889
PPPPVKAH;FAKSEC	NCP	76.571
FAKSEC;VRGFYV	NCP	6.226
VRGFYV;LYKMFLP	NCP	41.471
LYKMFLP;VGWHHSFY	NCP	124.573
VGWHHSFY;NSSSSKK	NCP	35.382
NSSSSKK;QATQR	NCP	24.432
QATQR;LSLQMGPK	NCP	106.133
LSLQMGPK;HSTLPDF	NCP	5.452
HSTLPDF;QSSYAP	NCP	12.128
QSSYAP;LLRKKKKL	NCP	19.516
LLRKKKKL;VRDPGPP	NCP	60.662
VRDPGPP;PLSPLFP	NCP	69.406
PLSPLFP;LPLTGNSS	NCP	54.452
LPLTGNSS;PEPAKSAPAPK	NCP	24.669
PEPAKSAPAPK;KQGLPKT	NCP	89.289
KQGLPKT;MNVFK	NCP	145.585
MNVFK;PGREPGSP	NCP	74.249
PGREPGSP;VAKKER	NCP	82.656
VAKKER;KYGLSP	NCP	9.498
KYGLSP;KGENFH	NCP	132.592
KGENFH;HTHKPPPPP	NCP	1.728
HTHKPPPPP;QSLFP	NCP	138.807
QSLFP;LQVTDF	NCP	17.364
LQVTDF;LGPPVFK	NCP	52.307
LGPPVFK;KTHLRSE	NCP	56.334
KTHLRSE;TAVVEYP	NCP	14.416
TAVVEYP;GHALMAV	NCP	131.231
GHALMAV;LSNMGMF	NCP	108.483
LSNMGMF;DDCFYP	NCP	25.143
DDCFYP;KPVLMGF	NCP	19.209
KPVLMGF;HMKLPR	NCP	15.991
HMKLPR;KLVSGQK	NCP	63.065
KLVSGQK;PVLTSSFL	NCP	4.237
PVLTSSFL;KDTFWK	NCP	0.363
KDTFWK;KGLRQLP	NCP	265.841
KGLRQLP;RFSMK	NCP	247.563
RFSMK;RFAAMK	NCP	18.318
RFAAMK;DWLEY	NCP	13.378
DWLEY;TYVKPK	NCP	46.098
TYVKPK;LDKEDLHD	NCP	19.044

LDKEDLHD;ATPLPKL	NCP	7.961
ATPLPKL;NLGAQLL	NCP	54.519
NLGAQLL;VKVHDV	NCP	17.341
VKVHDV;SSTKPGAK	NCP	66.338
SSTKPGAK;KPGFCCP	NCP	35.202
KPGFCCP;KNLAAVP	NCP	28.38
KNLAAVP;NSRSPFN	NCP	33.118
NSRSPFN;KEEHPK	NCP	70.674
KEEHPK;KVHVFPP	NCP	5.843
KVHVFPP;KGLYPK	NCP	74.86
KGLYPK;EMVTSK	NCP	14.547
EMVTSK;NAFVAEL	NCP	105.689
NAFVAEL;TATPLTK	NCP	299.532
TATPLTK;KGETPF	NCP	8.098
KGETPF;QTTDLY	NCP	51.887
QTTDLY;ALADSTRGLPN	NCP	7.056
ALADSTRGLPN;FFWGLF	NCP	139.321
FFWGLF;KLAGAP	NCP	12.578
KLAGAP;VSDLYPF	NCP	42.147
VSDLYPF;AMGLY	NCP	3.139
AMGLY;ASLLKLQ	NCP	54.995
ASLLKLQ;KNSHLSTF	NCP	41.85
KNSHLSTF;VVPLN	NCP	43.473
VVPLN;VPFYS	NCP	0.093
VPFYS;TPPVP	NCP	16.633
TPPVP;GNTVRLGL	NCP	8.874
GNTVRLGL;VLLPRCM	NCP	3.173
VLLPRCM;CCECC	NCP	28.986
CCECC;VQLTGY	NCP	10.851
VQLTGY;KYGAGT	NCP	114.778
KYGAGT;KFLMK	NCP	15.456
KFLMK;RLLLGPEL	NCP	70.181
RLLLGPEL;CMCCCE	NCP	83.17
CMCCCE;DYGVLA	NCP	7.116
DYGVLA;TTAPSK	NCP	20.91
TTAPSK;MQGALLPRF	NCP	54.116
MQGALLPRF;EGQLEF	NCP	77.646
EGQLEF;YDNRVEL	NCP	218.119
YDNRVEL;VTYPPPP	NCP	75.444
VTYPPPP;SPYKNP	NCP	65.969
SPYKNP;MMPKPR	NCP	14.673
MMPKPR;MLTEAK	NCP	4.189
MLTEAK;KNPTVV	NCP	56.136
KNPTVV;LTEKPLDK	NCP	101.582
LTEKPLDK;VGVNGF	NCP	39.322
VGVNGF;RPVAPTL	NCP	43.484
RPVAPTL;ESLAMPK	NCP	92.413
ESLAMPK;MMDLD	NCP	42.11
MMDLD;EDHPQH	NCP	4.989
EDHPQH;KDNVGKK	NCP	29.716
KDNVGKK;LPMNVKD	NCP	44.223

LPMNVKD;LNKRDL	NCP	37.916
LNKRDL;MCCCCFP	NCP	120.904
MCCCCFP;LPSNTN	NCP	208.319
LPSNTN;MAQLRK	NCP	0.361
MAQLRK;SGMFVP	NCP	207.206
SGMFVP;PLLKLGK	NCP	15.252
PLLKLGK;TLLESYK	NCP	0.984
TLLESYK;KGAMKKK	NCP	22.455
KGAMKKK;SGLPFDK	NCP	43.093
SGLPFDK;LMKLLTQ	NCP	13.536
LMKLLTQ;KFNGF	NCP	43.172
KFNGF;KFAASPL	NCP	148.974
KFAASPL;ALERYP	NCP	42.456
ALERYP;KMLKRDF	NCP	15.735
KMLKRDF;QFSTLYE	NCP	17.407
QFSTLYE;LPPYA	NCP	74.318
LPPYA;KMKKPP	NCP	45.184
KMKKPP;VMKSTPP	NCP	47.589
VMKSTPP;TSSHLQPP	NCP	0.309
TSSHLQPP;CLLPPS	NCP	16.112
CLLPPS;QDSLLTPL	NCP	4.566
QDSLLTPL;CCDDDP	NCP	6.631
CCDDDP;KFLNAQSP	NCP	56.3
KFLNAQSP;GSPVVPP	NCP	52.543
GSPVVPP;AGLSASFF	NCP	100.724
AGLSASFF;KVLLA	NCP	4.977
KVLLA;ALLKYVW	NCP	49.095
ALLKYVW;KSSGGRK	NCP	49.183
KSSGGRK;LLLTKFF	NCP	1.842
LLLTKFF;DLKLPVG	NCP	10.926
DLKLPVG;ADRLNL	NCP	48.152
ADRLNL;LPLEPP	NCP	98.622
LPLEPP;NSANVPF	NCP	65.748
NSANVPF;KPYLYR	NCP	13.2
KPYLYR;AQPKGLLK	NCP	21.423
AQPKGLLK;RLVFPLE	NCP	30.738
RLVFPLE;PTTKTYF	NCP	30.007
PTTKTYF;KDLKLGF	NCP	9.374
KDLKLGF;PSGSYRP	NCP	123.051
PSGSYRP;LTWMMLL	NCP	34.029
LTWMMLL;KMLTSYL	NCP	9.48
KMLTSYL;DVPLNF	NCP	14.037
DVPLNF;QQPRVPL	NCP	20.754
QQPRVPL;TVYENK	NCP	9.424
TVYENK;GVLSLK	NCP	64.511
GVLSLK;LGEFAK	NCP	12.615
LGEFAK;KMLTDP	NCP	7.803
KMLTDP;KEHKSY	NCP	4.534
KEHKSY;SPERFP	NCP	37.095
SPERFP;NPFVLEK	NCP	64.547
NPFVLEK;ELFDSV	NCP	48.814

ELFDSV;GPELALLK	NCP	43.512
GPELALLK;VKLKLFL	NCP	1.238
VKLKLFL;HLSKQHS	NCP	6.48
HLSKQHS;EHFDFK	NCP	66.634
EHFDFK;LRYTPEP	NCP	64.517
LRYTPEP;GKSFSVF	NCP	13.153
GKSFSVF;QTLFKNN	NCP	13.758
QTLFKNN;MTFLTM	NCP	37.305
MTFLTM;PVSAAKLA	NCP	0.201
PVSAAKLA;KEAKQQP	NCP	27.926
KEAKQQP;VLPEL	NCP	90.774
VLPEL;FLLPKVS	NCP	20.236
FLLPKVS;LLSKGEL	NCP	66.669
LLSKGEL;QAPVYL	NCP	115.427
QAPVYL;EWGKVK	NCP	103.606
EWGKVK;FDNLSSNF	NCP	41.961
FDNLSSNF;DPPTFPK	NCP	49.221
DPPTFPK;TAKGSPK	NCP	26.592
TAKGSPK;GVLELNNL	NCP	91.976
GVLELNNL;VELHSEDL	NCP	47.911
VELHSEDL;WLLLDH	NCP	46.445
WLLLDH;KPFCKK	NCP	37.399
KPFCKK;EVDDF	NCP	44.219
EVDDF;LGDQAQN	NCP	100.101
LGDQAQN;GSLKPNL	NCP	36.853
GSLKPNL;REVTH	NCP	12.296
REVTH;TELDMM	NCP	3.201
TELDMM;KANAAK	NCP	5.41
KANAAK;LPLLQQ	NCP	180.153
LPLLQQ;FEKRGGK	NCP	15.878
FEKRGGK;RVGDWK	NCP	141.499
RVGDWK;PLLPVVLE	NCP	41.867
PLLPVVLE;LLDKKVEK	NCP	40.954
LLDKKVEK;KNVGEEL	NCP	30.522
KNVGEEL;NADLVRY	NCP	70.226
NADLVRY;VAVLEVP	NCP	47.863
VAVLEVP;KTLFKP	NCP	36.323
KTLFKP;PMLSVPP	NCP	223.333
PMLSVPP;EKLNEGF	NCP	3.993
EKLNEGF;LPPPPEK	NCP	111.675
LPPPPEK;RAPPFST	NCP	76.701
RAPPFST;LHKKLLY	NCP	18
LHKKLLY;AMVEAY	NCP	44.298
AMVEAY;FLGTTKPV	NCP	110.1
FLGTTKPV;KSPAATE	NCP	6.66
KSPAATE;KVFVNK	NCP	32.123
KVFVNK;VLLLLMQ	NCP	16.78
VLLLLMQ;QHAFVL	NCP	67.62
QHAFVL;GPATPPPP	NCP	22.572
GPATPPPP;KSLSLTKP	NCP	30.867
KSLSLTKP;KFFPAVH	NCP	93.588

KFFPAVH;KASVSKY	NCP	5.604
KASVSKY;KRELLEPL	NCP	137.954
KRELLEPL;NLPHALLR	NCP	47.457
NLPHALLR;KSNMKK	NCP	4.173
KSNMKK;PPVKLHH	NCP	90.801
PPVKLHH;VHVLFNY	NCP	17.037
VHVLFNY;GLYVSPR	NCP	1.843
GLYVSPR;KPLEENP	NCP	16.176
KPLEENP;PLQTPP	NCP	138.378
PLQTPP;KSEMNK	NCP	57.963
KSEMNK;LEPTPPF	NCP	21.915
LEPTPPF;VYEFL	NCP	35.813
VYEFL;TKKLGLK	NCP	49.593
TKKLGLK;NTSLWH	NCP	20.377
NTSLWH;ADKGPSY	NCP	3.308
ADKGPSY;VNFTAFF	NCP	4.107
VNFTAFF;LTPHSPK	NCP	37.669
LTPHSPK;LFLLKC	NCP	84.974
LFLLKC;RVGLLVK	NCP	7.45
RVGLLVK;KTHEGK	NCP	1.401
KTHEGK;KKLPPK	NCP	27.17
KKLPPK;KDLLK	NCP	161.178
KDLLK;KGSQRVK	NCP	180.166
KGSQRVK;KSLYL	NCP	190.215
KSLYL;KRFLLPPG	NCP	159.939
KRFLLPPG;VLAFF	NCP	11.042
VLAFF;SMCFC	NCP	23.67
SMCFC;FGGMAL	NCP	108.921
FGGMAL;TELYKHK	NCP	352.245
TELYKHK;VSPLPPPPPP	NCP	17.038
VSPLPPPPPP;LSGLVAF	NCP	49.67
LSGLVAF;DGFGAQR	NCP	50.83
DGFGAQR;TAVPY	NCP	30.371
TAVPY;LAPPVRP	NCP	8.791
LAPPVRP;GPLNFT	NCP	20.192
GPLNFT;LEGHLDY	NCP	13.983
LEGHLDY;QCDLDLR	NCP	9.603
QCDLDLR;MVGMFLR	NCP	2.136
MVGMFLR;RFHLGKS	NCP	54.25
RFHLGKS;LRVPFLE	NCP	42.744
LRVPFLE;YVKGLPL	NCP	94.947
YVKGLPL;LKTFSLC	NCP	5.22
LKTFSLC;MEALFLL	NCP	43.865
MEALFLL;GVPKRPP	NCP	17.809
GVPKRPP;KPPTPLP	NCP	11.814
KPPTPLP;KPLGP	NCP	42.24
KPLGP;LVALELL	NCP	32.283
LVALELL;TVTVYK	NCP	4.821
TVTVYK;YTAPP	NCP	33.312
YTAPP;LLEFF	NCP	7.268
LLEFF;NLHKKM	NCP	119.829

NLHKKM;EMRKVQK	NCP	25.29
EMRKVQK;MQVPKD	NCP	3.79
MQVPKD;VARPQV	NCP	53.646
VARPQV;DRVHEEP	NCP	19.049
DRVHEEP;KFFQPP	NCP	16.303
KFFQPP;FKLLMGP	NCP	45.756
FKLLMGP;MKLGP	NCP	25.929
MKLGP;LGLRVNP	NCP	61.422
LGLRVNP;VFEKPK	NCP	220.32
VFEKPK;ESPLGAF	NCP	14.667
ESPLGAF;KLEYLTA	NCP	9.761
KLEYLTA;LLVAN	NCP	67.449
LLVAN;KKPPK	NCP	8.241
KKPPK;AAKPPVH	NCP	42.22
AAKPPVH;CKVSPPPP	NCP	69.177
CKVSPPPP;QTQTLPK	NCP	31.386
QTQTLPK;KDLPGVR	NCP	5.352
KDLPGVR;KAEVP	NCP	32.682
KAEVP;GSLDLTK	NCP	132.453
GSLDLTK;LNEASY	NCP	97.968
LNEASY;EGKVLRL	NCP	119.445
EGKVLRL;SLTTDLR	NCP	16.577
SLTTDLR;AQPVYL	NCP	33.975
AQPVYL;VSLLEGF	NCP	16.161
VSLLEGF;LAATNKK	NCP	4.255
LAATNKK;VTVNVSL	NCP	38.78
VTVNVSL;KTMLHA	NCP	53.956
KTMLHA;FRRSVLLL	NCP	83.567
FRRSVLLL;QAFLDF	NCP	29.292
QAFLDF;QAPAPRP	NCP	55.012
QAPAPRP;MLTRY	NCP	9.92
MLTRY;KLDLFQ	NCP	56.212
KLDLFQ;RFGPP	NCP	4.611
RFGPP;LVYMNP	NCP	295.614
LVYMNP;QQPEPKPK	NCP	5.733
QQPEPKPK;KTMTKP	NCP	40.121
KTMTKP;EVAVKLVL	NCP	1.71
EVAVKLVL;LLVLPY	NCP	166.603
LLVLPY;TKTVD	NCP	44.339
TKTVD;KHSSPHF	NCP	8.037
KHSSPHF;KEGFG	NCP	79.932
KEGFG;LLNKFDK	NCP	27.412
LLNKFDK;VALSLY	NCP	27.74
VALSLY;KTWTSPK	NCP	132.736
KTWTSPK;GPRSAKP	NCP	9.063
GPRSAKP;NLLSSFG	NCP	69.314
NLLSSFG;AALLTML	NCP	9.684
AALLTML;NAVPRAF	NCP	68.676
NAVPRAF;MELLKGL	NCP	40.503
MELLKGL;FSVRALT	NCP	27.037
FSVRALT;RQRHFH	NCP	47.949

RQRHFH;PVMGAP	NCP	14.931
PVMGAP;FPVTAVT	NCP	26.835
FPVTAVT;LSVGYPK	NCP	23.361
LSVGYPK;AAPPQEV	NCP	20.985
AAPPQEV;VGGVSML	NCP	45.207
VGGVSML;EDFPEV	NCP	45.873
EDFPEV;EPFAP	NCP	2.952
EPFAP;CRVLSQM	NCP	81.381
CRVLSQM;LPEVPYN	NCP	54.48
LPEVPYN;KKCPVEF	NCP	18.689
KKCPVEF;VPVTGK	NCP	0.145
VPVTGK;PVELLTKF	NCP	32.409
PVELLTKF;GSCCC	NCP	25.062
GSCCC;KPSMKSK	NCP	6.311
KPSMKSK;LSLEFY	NCP	10.842
LSLEFY;QSRPVNF	NCP	41.365
QSRPVNF;YSDMLLQ	NCP	58.248
YSDMLLQ;WGSLLGKK	NCP	22.524
WGSLLGKK;LPPHPKPP	NCP	21.212
LPPHPKPP;PPSKPPPR	NCP	9.517
PPSKPPPR;ARVLLGV	NCP	17.562
ARVLLGV;ESGKF	NCP	124.296
ESGKF;AVRGADP	NCP	37.797
AVRGADP;PLSHFLR	NCP	4.229
PLSHFLR;NSCCCCH	NCP	98.998
NSCCCCH;YPPPVPP	NCP	87.737
YPPPVPP;KQTVAVL	NCP	7.9
KQTVAVL;APGNTFK	NCP	18.87
APGNTFK;KDVPF	NCP	194.118
KDVPF;KGFAKKK	NCP	78.207
KGFAKKK;RGVKLPF	NCP	22.398
RGVKLPF;YVGSFV	NCP	60.156
YVGSFV;KGFVLRGP	NCP	33.123
KGFVLRGP;TPLLEAH	NCP	64.851
TPLLEAH;LDEELK	NCP	30.825
LDEELK;HSGPK	NCP	68.096
HSGPK;KAAPPTL	NCP	43.269
KAAPPTL;KFSLKKT	NCP	0.865
KFSLKKT;NLSAVLA	NCP	19.545
NLSAVLA;NSDVP	NCP	14.658
NSDVP;LLDKTLT	NCP	42.099
LLDKTLT;KAPVLQK	NCP	26.264
KAPVLQK;KLTEVR	NCP	224.086
KLTEVR;ERELSPK	NCP	62.384
ERELSPK;FPGNKD	NCP	28.47
FPGNKD;VELSLPK	NCP	66.457
VELSLPK;AYVLEF	NCP	27.555
AYVLEF;QPTDKK	NCP	34.86
QPTDKK;LYDSGYL	NCP	24.38
LYDSGYL;LYAGL	NCP	19.21
LYAGL;SLANLLYY	NCP	116.223

SLANLLYY;SVFVP	NCP	20.441
SVFVP;KKKKKLVKY	NCP	14.953
KKKKKLVKY;KEPVRDFL	NCP	31.022
KEPVRDFL;NLAEVK	NCP	50.143
NLAEVK;KLQVQ	NCP	157.329
KLQVQ;TMAMSY	NCP	7.556
TMAMSY;KTAPEVL	NCP	8.533
KTAPEVL;KNFADKV	NCP	140.717
KNFADKV;KVDLVVL	NCP	241.632
KVDLVVL;KFNFQPK	NCP	31.276
KFNFQPK;KWVPEAK	NCP	39.039
KWVPEAK;LTPVK	NCP	20.007
LTPVK;KNNAP	NCP	55.623
KNNAP;PLAGY	NCP	126.506
PLAGY;LSNFRP	NCP	10.141
LSNFRP;ESGHSKLK	NCP	23.994
ESGHSKLK;TSCKMP	NCP	42.867
TSCKMP;HKKFTK	NCP	64.988
HKKFTK;DPFVLK	NCP	22.228
DPFVLK;AATRLEF	NCP	51.158
AATRLEF;GCQPCL	NCP	22.104
GCQPCL;LGPPPPP	NCP	16.668
LGPPPPP;LRELQLK	NCP	97.188
LRELQLK;DGPDFL	NCP	79.993
DGPDFL;PHALGHF	NCP	52.745
PHALGHF;PFDLLR	NCP	53.572
PFDLLR;KYTMPP	NCP	42.698
KYTMPP;GVLLANF	NCP	8.709
GVLLANF;FADLRK	NCP	61.498
FADLRK;KCVCR	NCP	22.788
KCVCR;KTEKSSK	NCP	47.576
KTEKSSK;PGRPPRP	NCP	101.352
PGRPPRP;MLKHC	NCP	28.066
MLKHC;VVLTPAQ	NCP	41.891
VVLTPAQ;LDPGMTL	NCP	58.212
LDPGMTL;VPSRGPR	NCP	32.343
VPSRGPR;LGLMFGP	NCP	131.997
LGLMFGP;KSYAPQ	NCP	1.372
KSYAPQ;AAAELPY	NCP	8.519
AAAELPY;AAGQEPQ	NCP	21.27
AAGQEPQ;APAPPSP	NCP	53.562
APAPPSP;KTQECT	NCP	6.781
KTQECT;AGAELRA	NCP	7.14
AGAELRA;KLVYSPK	NCP	5.273
KLVYSPK;TQPVPM	NCP	28.887
TQPVPM;RLDYH	NCP	16.756
RLDYH;KGRSPWK	NCP	8.429
KGRSPWK;FVFGLVT	NCP	30.225
FVFGLVT;APRSHPLP	NCP	58.812
APRSHPLP;KERQPP	NCP	3.024
KERQPP;QQTSLLNY	NCP	83.671

QQTSLLNY;NNCKGVTK	NCP	5.37
NNCKGVTK;KLLVFPY	NCP	0.791
KLLVFPY;LTAAHF	NCP	120.607
LTAAHF;NAVLELREQ	NCP	157.914
NAVLELREQ;GGPVVYR	NCP	21.713
GGPVVYR;RPQDLPQR	NCP	22.71
RPQDLPQR;LPLLFWP	NCP	20.889
LPLLFWP;RPEPERP	NCP	0.147
RPEPERP;TLGGVPR	NCP	5.976
TLGGVPR;GKPAEF	NCP	9.505
GKPAEF;VTVASQ	NCP	1.496
VTVASQ;FLVNEM	NCP	30.967
FLVNEM;YPGAEPP	NCP	22.367
YPGAEPP;KGALPGPP	NCP	59.961
KGALPGPP;LPGAH	NCP	12.456
LPGAH;KAHDPPR	NCP	2.448
KAHDPPR;GSLLR	NCP	41.907
GSLLR;LGDFLEH	NCP	40.131
LGDFLEH;KNFDPPP	NCP	85.999
KNFDPPP;APVSLNLA	NCP	87.422
APVSLNLA;LKAVGP	NCP	23.556
LKAVGP;ACCFPK	NCP	29.601
ACCFPK;MSLVWKP	NCP	100.197
MSLVWKP;DKLLF	NCP	1.93
DKLLF;VLHLRP	NCP	36.095
VLHLRP;LKAGPVDSF	NCP	18.243
LKAGPVDSF;LATGGDLTR	NCP	28.896
LATGGDLTR;LLLKFNT	NCP	79.567
LLLKFNT;VGVVQY	NCP	71.957
VGVVQY;KPAVPPK	NCP	17.859
KPAVPPK;KAAHLYL	NCP	10.597
KAAHLYL;NSLTAELL	NCP	12.414
NSLTAELL;PTFVEP	NCP	49.092
PTFVEP;TAEAP	NCP	36.845
TAEAP;FQKEPK	NCP	88.041
FQKEPK;DVKTHK	NCP	68.641
DVKTHK;KKSVSLPK	NCP	98.637
KKSVSLPK;ATLADKE	NCP	40.616
ATLADKE;KYGPP	NCP	7.686
KYGPP;VVTQP	NCP	8.205
VVTQP;VGPSSLPL	NCP	32.118
VGPSSLPL;KTWVAV	NCP	4.84
KTWVAV;ELVHLQA	NCP	85.799
ELVHLQA;AAVPELV	NCP	7.879
AAVPELV;TAELLER	NCP	6.074
TAELLER;YGAGEL	NCP	13.188
YGAGEL;KADGF	NCP	77.802
KADGF;LRKFT	NCP	4.689
LRKFT;DYDPSR	NCP	54.885
DYDPSR;ARPALFC	NCP	180.6
ARPALFC;KQKHETFP	NCP	39.864

KQKHETFP;SFLPVYFL	NCP	88.231
SFLPVYFL;PLGLRVP	NCP	52.241
PLGLRVP;VPSLR	NCP	14.598
VPSLR;FLKLWN	NCP	72.261
FLKLWN;VGGLLLS	NCP	8.263
VGGLLLS;GLREGVK	NCP	20.156
GLREGVK;ALSAVAL	NCP	2.776
ALSAVAL;KVPAETPK	NCP	80.853
KVPAETPK;KYPPKY	NCP	16.176
KYPPKY;FDALKKK	NCP	26.72
FDALKKK;GPGPRLDP	NCP	39.948
GPGPRLDP;KSTEASK	NCP	77.142
KSTEASK;RLALPCLK	NCP	40.48
RLALPCLK;FDLWKEK	NCP	5.267
FDLWKEK;KRLAERS	NCP	2.103
KRLAERS;TAAASPVPL	NCP	20.781
TAAASPVPL;KENVVGK	NCP	96.207
KENVVGK;QSLALKQ	NCP	18.661
QSLALKQ;KRETALYF	NCP	22.839
KRETALYF;ETTFKGP	NCP	20.432
ETTFKGP;LLRVGDH	NCP	6.657
LLRVGDH;PTYLPVY	NCP	23.077
PTYLPVY;EFYRP	NCP	43.392
EFYRP;TNVVEPY	NCP	23.837
TNVVEPY;KVLVTAK	NCP	33.345
KVLVTAK;APLLYDY	NCP	22.819
APLLYDY;VTLSLP	NCP	50.234
VTLSLP;KHPKGGK	NCP	99.516
KHPKGGK;PLTNF	NCP	57.834
PLTNF;QKRAAGL	NCP	4.254
QKRAAGL;VTPELT	NCP	24.222
VTPELT;RKPKFN	NCP	32.676
RKPKFN;FSLGLER	NCP	14.268
FSLGLER;GSSDL	NCP	72.198
GSSDL;RHVSSK	NCP	10.791
RHVSSK;KYFAGKS	NCP	16.255
KYFAGKS;VGLLAAR	NCP	70.127
VGLLAAR;LQELPK	NCP	19.815
LQELPK;APPLLHP	NCP	45.816
APPLLHP;GSAQVVLR	NCP	51.354
GSAQVVLR;SCLLLLPQ	NCP	67.077
SCLLLLPQ;QDLQKF	NCP	4.509
QDLQKF;RVQLREQ	NCP	93.72
RVQLREQ;KNQTGLR	NCP	76.255
KNQTGLR;TSNLPTLK	NCP	46.598
TSNLPTLK;LQAFYP	NCP	19.579
LQAFYP;KAFKNK	NCP	18.402
KAFKNK;ATAKALTH	NCP	52.904
ATAKALTH;VDLGKKP	NCP	17.889
VDLGKKP;EPPPPPPPP	NCP	5.277
EPPPPPPPP;KVSTTFL	NCP	9.529

KVSTTFL;KVGTGLK	NCP	7.979
KVGTGLK;KASHKTPP	NCP	54.963
KASHKTPP;NSLTRNK	NCP	20.406
NSLTRNK;TVKGGLP	NCP	89.518
TVKGGLP;NDGLLW	NCP	14.913
NDGLLW;EVGGLPY	NCP	45.957
EVGGLPY;KGDYFPK	NCP	14.271
KGDYFPK;ERQDFK	NCP	38.483
ERQDFK;QLDLDLR	NCP	38.112
QLDLDLR;LDVMVH	NCP	18.751
LDVMVH;KCTSLEK	NCP	8.256
KCTSLEK;YAFQPL	NCP	3.959
YAFQPL;FDKKSLK	NCP	27.015
FDKKSLK;PVDLVNP	NCP	66.273
PVDLVNP;LAPTSPKQ	NCP	42.405
LAPTSPKQ;KGCMFLK	NCP	42.832
KGCMFLK;NQHGLP	NCP	10.361
NQHGLP;KQATYAH	NCP	114.96
KQATYAH;PPAAGPPR	NCP	6.408
PPAAGPPR;LPNTRPP	NCP	58.071
LPNTRPP;GDEGW	NCP	10.732
GDEGW;MDRSLYP	NCP	41.45
MDRSLYP;GSPPVP	NCP	120.165
GSPPVP;AEKYLDLPK	NCP	101.197
AEKYLDLPK;APACLRL	NCP	38.444
APACLRL;NLYSL	NCP	4.591
NLYSL;KGRGKEK	NCP	97.409
KGRGKEK;DAVMLK	NCP	12.645
DAVMLK;SPPTYAL	NCP	0.876
SPPTYAL;KKMQKK	NCP	77.656
KKMQKK;LHSLLVT	NCP	9.05
LHSLLVT;ENAVLKQ	NCP	9.684
ENAVLKQ;TDPDLLL	NCP	7.06
TDPDLLL;KCSGVGK	NCP	13.773
KCSGVGK;FPLLEL	NCP	32.475
FPLLEL;VVPGSP	NCP	158.307
VVPGSP;TSEWPK	NCP	43.065
TSEWPK;KDVYPK	NCP	156.839
KDVYPK;GKTPAEV	NCP	84.873
GKTPAEV;VGGPVT	NCP	6.7
VGGPVT;HTLPPPPP	NCP	25.046
HTLPPPPP;DFLDF	NCP	17.428
DFLDF;LYVEVH	NCP	2.535
LYVEVH;KYPPSW	NCP	88.844
KYPPSW;NNKSVLD	NCP	91.705
NNKSVLD;DDMVGP	NCP	25.355
DDMVGP;LPTRAAP	NCP	8.781
LPTRAAP;ADLKRL	NCP	11.919
ADLKRL;CDVTGR	NCP	47.322
CDVTGR;KVLVTVK	NCP	9.835
KVLVTVK;KTAADLR	NCP	17.189

KTAADLR;KYATAR	NCP	9.33
KYATAR;KMVLGW	NCP	119.632
KMVLGW;LVPVLNY	NCP	29.351
LVPVLNY;KPKGLPPPP	NCP	3.654
KPKGLPPPP;GVTFLEN	NCP	22.792
GVTFLEN;ALAAVSK	NCP	1.575
ALAAVSK;KKDQAR	NCP	61.572
KKDQAR;KPAHPR	NCP	53.781
KPAHPR;LAAHPN	NCP	25.937
LAAHPN;VLEVKNR	NCP	75.019
VLEVKNR;KDFLSAL	NCP	109.832
KDFLSAL;QQLPTHGP	NCP	73.086
QQLPTHGP;ESPLQY	NCP	20.869
ESPLQY;KELLGR	NCP	15.546
KELLGR;KGLVLLA	NCP	71.649
KGLVLLA;EKGGK	NCP	41.643
EKGGK;EGLLFGK	NCP	10.271
EGLLFGK;SFKLSGF	NCP	8.809
SFKLSGF;GVGVVSY	NCP	26.298
GVGVVSY;MVFLPR	NCP	21.825
MVFLPR;TSDKPVL	NCP	37.805
TSDKPVL;KYVPSR	NCP	24.927
KYVPSR;KLGQGNK	NCP	52.614
KLGQGNK;EQAGTK	NCP	16.215
EQAGTK;AALKAVDK	NCP	19.87
AALKAVDK;YNHTKK	NCP	56.919
YNHTKK;TAPMVN	NCP	46.788
TAPMVN;TVPKEKK	NCP	8.204
TVPKEKK;ESPKDPVLPAR	NCP	110.364
ESPKDPVLPAR;VTDSEEKLGMDDLSK	NCP	9.84
VTDSEEKLGMDDLSK;RPPKPLGGGTPK	NCP	0.21
RPPKPLGGGTPK;KDGAVTKN	NCP	25.708
KDGAVTKN;EGSLGLR	NCP	40.757
EGSLGLR;LLLKKKKTT	NCP	73.249
LLLKKKKTT;SYLMMC	NCP	30.711
SYLMMC;SPFYL	NCP	10.758
SPFYL;TTYGPK	NCP	26.216
TTYGPK;AAHFSPK	NCP	47.007
AAHFSPK;SLHSGP	NCP	172.842
SLHSGP;KQFPPPP	NCP	61.012
KQFPPPP;KGTPVKH	NCP	50.3
KGTPVKH;LLLLFMR	NCP	58.137
LLLLFMR;GFAFKKF	NCP	37.539
GFAFKKF;VLYPALVK	NCP	227.497
VLYPALVK;VGVLGLK	NCP	35.033
VGVLGLK;PTKVSK	NCP	59.95
PTKVSK;LTSMNTSL	NCP	87.704
LTSMNTSL;KFLKPLL	NCP	29.466
KFLKPLL;KGASSLVP	NCP	109.593
KGASSLVP;KTRLSAP	NCP	47.752
KTRLSAP;EHKQPP	NCP	58.031

EHKQPP;KFYEYK	NCP	30.604
KFYEYK;FVLLPK	NCP	16.265
FVLLPK;GVPKLR	NCP	4.698
GVPKLR;GLFKGR	NCP	8.331
GLFKGR;KGEAGKK	NCP	3.489
KGEAGKK;DGEPP	NCP	118.932
DGEPP;KDTVPVK	NCP	12.301
KDTVPVK;KPVPEKP	NCP	36.245
KPVPEKP;NMMDLR	NCP	18.406
NMMDLR;PPPHLAK	NCP	179.91
PPPHLAK;LTNVGK	NCP	35.201
LTNVGK;LPGDLLF	NCP	4.968
LPGDLLF;KFETVW	NCP	10.306
KFETVW;ESFGL	NCP	156.041
ESFGL;SKFVLVK	NCP	113.305
SKFVLVK;HVLTRM	NCP	49.14
HVLTRM;TPGHVLR	NCP	30.711
TPGHVLR;RAAYTLR	NCP	108.462
RAAYTLR;VGLPKRP	NCP	40.08
VGLPKRP;MEHLER	NCP	126.983
MEHLER;EDDDC	NCP	41.277
EDDDC;SPNPRPP	NCP	87.877
SPNPRPP;KSPPVME	NCP	10.545
KSPPVME;EADHPYKK	NCP	8.346
EADHPYKK;KLALAK	NCP	70.727
KLALAK;LGATVKK	NCP	23.497
LGATVKK;KELNYKP	NCP	4.665
KELNYKP;EMGGLRK	NCP	12.23
EMGGLRK;VHGPKSGP	NCP	23.668
VHGPKSGP;KGDWHRP	NCP	34.823
KGDWHRP;KDLQPQ	NCP	45.402
KDLQPQ;KVPSPLLL	NCP	27.471
KVPSPLLL;PPPPPVH	NCP	90.124
PPPPPVH;KDPGVR	NCP	105.372
KDPGVR;ANYLDVK	NCP	26.241
ANYLDVK;GFPPPFP	NCP	47.099
GFPPPFP;VDLTY	NCP	9.556
VDLTY;HVVPFGLP	NCP	44.031
HVVPFGLP;EFAVP	NCP	52.232
EFAVP;LSRVRPT	NCP	90.307
LSRVRPT;DKSPSVW	NCP	200.781
DKSPSVW;NVHFLL	NCP	31.422
NVHFLL;LQGKVMK	NCP	4.626
LQGKVMK;KGHVLC	NCP	6.998
KGHVLC;GVLRPAP	NCP	1.095
GVLRPAP;GPTVSAAF	NCP	1.521
GPTVSAAF;DVGLK	NCP	5.464
DVGLK;KETLLDPK	NCP	92.009
KETLLDPK;KSELP	NCP	55.156
KSELP;YQQTLM	NCP	45.828
YQQTLM;AALLKTP	NCP	11.808

AALLKTP;KEAHGMPK	NCP	14.091
KEAHGMPK;PVAALQL	NCP	42.339
PVAALQL;LVPVGY	NCP	47.852
LVPVGY;PASTSL	NCP	6.03
PASTSL;NVLLAERS	NCP	0.454
NVLLAERS;AKKVAVLG	NCP	11.265
AKKVAVLG;KEGLSLE	NCP	0.327
KEGLSLE;KTEAP	NCP	9.626
KTEAP;APGGDLH	NCP	94.492
APGGDLH;LYKKSV	NCP	10.43
LYKKSV;KGCRPK	NCP	1.879
KGCRPK;KEPLLLSH	NCP	179.483
KEPLLLSH;KEFVRK	NCP	128.496
KEFVRK;EGAKSFLL	NCP	14.724
EGAKSFLL;KRSEHGK	NCP	37.491
KRSEHGK;LFVLSGPK	NCP	47.436
LFVLSGPK;KNVYLVFF	NCP	73.534
KNVYLVFF;EPPLL	NCP	48.975
EPPLL;LLRKPVV	NCP	45.536
LLRKPVV;LPVASRVP	NCP	26.919
LPVASRVP;ASYEYL	NCP	1.888
ASYEYL;KFPNPGK	NCP	48.135
KFPNPGK;RKLLPPQ	NCP	90.426
RKLLPPQ;GFVKVVKNKAY	NCP	54.189
GFVKVVKNKAY;LAFDVKK	NCP	3.878
LAFDVKK;LGHEVR	NCP	61.069
LGHEVR;LSKLNR	NCP	24.047
LSKLNR;LEFAQ	NCP	52.864
LEFAQ;LTVDPY	NCP	40.334
LTVDPY;LLALKAF	NCP	78.405
LLALKAF;PGAVVPP	NCP	36.709
PGAVVPP;GPDLCF	NCP	2.282
GPDLCF;KVDLHF	NCP	7.947
KVDLHF;VEDHLVSL	NCP	47.88
VEDHLVSL;LPGVAC	NCP	3.553
LPGVAC;WGAKWF	NCP	17.175
WGAKWF;EAHVPPR	NCP	9.62
EAHVPPR;WLDLR	NCP	15.139
WLDLR;KPPPPGK	NCP	230.441
KPPPPGK;FKLATPY	NCP	28.597
FKLATPY;KQRCLSH	NCP	19.31
KQRCLSH;AEGSGEVVVVPATG	NCP	128.028
AEGSGEVVVVPATG;FFFMEM	NCP	63.94
FFFMEM;PVVVKK	NCP	16.551
PVVVKK;DVLRSH	NCP	23.198
DVLRSH;NAKPKPP	NCP	2.445
NAKPKPP;VSKPTGLR	NCP	31.056
VSKPTGLR;MCMMAA	NCP	28.623
MCMMAA;DDPKPLG	NCP	14.899
DDPKPLG;DKYVVP	NCP	33.113
DKYVVP;WMSFVPK	NCP	29.673

WMSFVPK;RGLEVSK	NCP	11.623
RGLEVSK;KSGTVSK	NCP	6.765
KSGTVSK;YPDKLR	NCP	6.492
YPDKLR;KEKLPLH	NCP	104.687
KEKLPLH;KATNAF	NCP	26.256
KATNAF;QPHPVK	NCP	39.03
QPHPVK;NDGAKVLR	NCP	114.118
NDGAKVLR;VSVVSKPP	NCP	129.125
VSVVSKPP;DDPPPPR	NCP	75.267
DDPPPPR;HVVTKH	NCP	45.328
HVVTKH;AAPPT	NCP	4.037
AAPPT;KPCDMSK	NCP	11.628
KPCDMSK;VVGAP	NCP	34.773
VVGAP;YGPEC	NCP	109.711
YGPEC;PVGFDPK	NCP	52.562
PVGFDPK;NLVVPQ	NCP	5.814
NLVVPQ;HHTFY	NCP	7.015
HHTFY;NVLTYC	NCP	109.623
NVLTYC;MPLTHK	NCP	19.554
MPLTHK;AAPMK	NCP	20.195
AAPMK;EDLVGP	NCP	12.196
EDLVGP;SVVFP	NCP	143.576
SVVFP;LDPGPTY	NCP	56.002
LDPGPTY;APVDPPP	NCP	69.707
APVDPPP;KLVPHQ	NCP	5.436
KLVPHQ;VDLSLPK	NCP	11.463
VDLSLPK;HFKLEAH	NCP	35.064
HFKLEAH;RGPPLYL	NCP	50.694
RGPPLYL;TDLRQF	NCP	91.879
TDLRQF;GNDKLQ	NCP	14.183
GNDKLQ;VGAPLNF	NCP	53.581
VGAPLNF;LERLPGF	NCP	11.72
LERLPGF;KGGYVPR	NCP	14.046
KGGYVPR;KSVYAPG	NCP	36.382
KSVYAPG;DGVNLSK	NCP	4.361
DGVNLSK;QVYSRPP	NCP	15.294
QVYSRPP;RLSTPVLR	NCP	105.228
RLSTPVLR;NTVLKYPK	NCP	4.291
NTVLKYPK;LTSPGHK	NCP	59.094
LTSPGHK;QPPVLPP	NCP	22.205
QPPVLPP;KSPNKENP	NCP	57.525
KSPNKENP;LPRPTYG	NCP	47.43
LPRPTYG;KPPMSLP	NCP	92.793
KPPMSLP;SPAKKPAV	NCP	27.366
SPAKKPAV;CWADF	NCP	0.669
CWADF;DDDLRF	NCP	27.177
DDDLRF;KVFSRS	NCP	27.759
KVFSRS;ELDPRHP	NCP	6.463
ELDPRHP;ARQAK	NCP	28.676
ARQAK;LETVP	NCP	3.043
LETVP;KNFRPP	NCP	12.602

KNFRPP;STLQKLAL	NCP	21.099
STLQKLAL;LPGDFE	NCP	17.826
LPGDFE;MCDWPP	NCP	39.129
MCDWPP;KESTLVP	NCP	29.317
KESTLVP;VGMAGK	NCP	8.301
VGMAGK;GPWNSY	NCP	15.125
GPWNSY;KLFNSLK	NCP	15.952
KLFNSLK;EASAVYK	NCP	31.424
EASAVYK;DAKSFLL	NCP	27.747
DAKSFLL;LEKGVLK	NCP	7.875
LEKGVLK;NSLLTTH	NCP	89.688
NSLLTTH;VSSLLP	NCP	6.591
VSSLLP;YPALR	NCP	191.298
YPALR;KAKAAPK	NCP	9.991
KAKAAPK;TTMKAGP	NCP	212.117
TTMKAGP;DWLYE	NCP	136.377
DWLYE;AENPGLENHR	NCP	164.265
AENPGLENHR;KHLGQLP	NCP	72.424
KHLGQLP;GPVRSLK	NCP	143.603
GPVRSLK;MMELEF	NCP	52.792
MMELEF;KWDLLLL	NCP	2.517
KWDLLLL;KRELPF	NCP	2.849
KRELPF;PTLVVQSP	NCP	49.414
PTLVVQSP;YRDDLNY	NCP	11.627
YRDDLNY;LPKDEL	NCP	71.539
LPKDEL;QNKVLKV	NCP	6.434
QNKVLKV;ASTSYVA	NCP	15.55
ASTSYVA;PVAYRK	NCP	189.797
PVAYRK;MSPGPELS	NCP	65.68
MSPGPELS;QAQSQK	NCP	47.724
QAQSQK;LPPLKHG	NCP	10.373
LPPLKHG;DLVLVK	NCP	25.186
DLVLVK;HGKHNTF	NCP	12.566
HGKHNTF;LVLDL	NCP	91.081
LVLDL;GKGGPVA	NCP	89.727
GKGGPVA;DVPLSGF	NCP	160.043
DVPLSGF;RNSKKLL	NCP	89.293
RNSKKLL;MEPMTL	NCP	20.789
MEPMTL;RFKKRTEL	NCP	13.518
RFKKRTEL;NPVEHPF	NCP	0.411
NPVEHPF;LAVLK	NCP	44.437
LAVLK;AASTNLK	NCP	16.325
AASTNLK;KVVLTSK	NCP	11.343
KVVLTSK;LAEVGSLL	NCP	38.242
LAEVGSLL;QSLPFLQ	NCP	31.181
QSLPFLQ;YGKGTVP	NCP	7.404
YGKGTVP;WTLYR	NCP	52.131
WTLYR;HEYLSMEL	NCP	51.456
HEYLSMEL;KTNGQKTSK	NCP	120.672
KTNGQKTSK;GSGEWEM	NCP	27.717
GSGEWEM;FLLVFP	NCP	48.669

FLLVFP;KGGDNPK	NCP	96.705
KGGDNPK;EPVAP	NCP	8.316
EPVAP;TPSKPSR	NCP	100.461
TPSKPSR;MTSDLP	NCP	1.42
MTSDLP;EVPPPLQ	NCP	76.727
EVPPPLQ;FAKVK	NCP	32.731
FAKVK;EVELF	NCP	41.111
EVELF;LFGPEVP	NCP	172.797
LFGPEVP;KPDLRLK	NCP	18.106
KPDLRLK;KHLKPV	NCP	37.202
KHLKPV;NLQMLQ	NCP	16.326
NLQMLQ;GLPPRSS	NCP	107.58
GLPPRSS;EHAFLK	NCP	22.557
EHAFLK;TSFTEK	NCP	1.2
TSFTEK;LTLPGY	NCP	3.388
LTLPGY;KSFYQKKR	NCP	23.075
KSFYQKKR;LAPFQMT	NCP	22.977
LAPFQMT;APLSVSPP	NCP	4.603
APLSVSPP;LDRNLQ	NCP	35.268
LDRNLQ;ALKQVSSSKCFGGLQ	NCP	77.393
ALKQVSSSKCFGGLQ;LTEKMK	NCP	4.653
LTEKMK;YVDATEDLWKTNY	NCP	0.099
YVDATEDLWKTNY;ALKNPGKYK	NCP	1.5
ALKNPGKYK;DATYLVK	NCP	2.781
DATYLVK;LVVGVSP	NCP	31.716
LVVGVSP;ALKRDQK	NCP	13.986
ALKRDQK;ERFPPR	NCP	16.662
ERFPPR;VGNLELK	NCP	15.213
VGNLELK;KTPNQFL	NCP	70.014
KTPNQFL;PGVRLP	NCP	37.335
PGVRLP;VRPHPFQ	NCP	105.03
VRPHPFQ;KAAHGASLR	NCP	28.671
KAAHGASLR;YWKSNKK	NCP	149.761
YWKSNKK;DLPPYK	NCP	6.54
DLPPYK;HLLEQVAPK	NCP	17.174
HLLEQVAPK;AVELGKNQAKF	NCP	10.671
AVELGKNQAKF;KPATFPP	NCP	21.927
KPATFPP;TGLPVEF	NCP	49.707
TGLPVEF;LYDGLVS	NCP	32.641
LYDGLVS;KTPLPPP	NCP	14.369
KTPLPPP;VTRLAV	NCP	61.557
VTRLAV;TGCLR	NCP	87.978
TGCLR;GSPLAAPP	NCP	83.751
GSPLAAPP;GVYNVP	NCP	54.846
GVYNVP;KPFEPE	NCP	46.108
KPFEPE;GLPKVTPL	NCP	84.584
GLPKVTPL;PGKPTALP	NCP	63.297
PGKPTALP;NHMDDPR	NCP	16.494
NHMDDPR;KAALPGKP	NCP	44.28
KAALPGKP;GVSSSSK	NCP	10.06
GVSSSSK;LTMSVPR	NCP	83.327

LTMSVPR;REDGVTPY	NCP	2.976
REDGVTPY;EFLSF	NCP	7.389
EFLSF;VCSGKKK	NCP	7.167
VCSGKKK;LGTALKANPF	NCP	25.003
LGTALKANPF;ADPAGP	NCP	86.193
ADPAGP;KSDKPLR	NCP	85.764
KSDKPLR;TFGGLML	NCP	7.187
TFGGLML;LSKVVH	NCP	10.809
LSKVVH;ALSHFFP	NCP	1.78
ALSHFFP;EPGKLHHP	NCP	14.163
EPGKLHHP;SLEKFDK	NCP	8.564
SLEKFDK;VEVLLALP	NCP	36.666
VEVLLALP;QTLSAPK	NCP	119.299
QTLSAPK;KMVELC	NCP	12.797
KMVELC;LPQPP	NCP	0.451
LPQPP;KPRLPEE	NCP	1.02
KPRLPEE;MELESV	NCP	20.721
MELESV;NRPMHK	NCP	37.752
NRPMHK;KPMFTAK	NCP	2.249
KPMFTAK;TVLTQKL	NCP	2.092
TVLTQKL;DLEVPFPR	NCP	14.025
DLEVPFPR;VPVPAAR	NCP	20.915
VPVPAAR;ALGSLA	NCP	38.301
ALGSLA;MVETRK	NCP	137.979
MVETRK;KLSGLDLR	NCP	17.29
KLSGLDLR;PGLPLPPPP	NCP	19.229
PGLPLPPPP;KLSSLGVL	NCP	84.336
KLSSLGVL;VTVLPEK	NCP	21.108
VTVLPEK;QKEVP	NCP	19.846
QKEVP;VLLVGHP	NCP	56.192
VLLVGHP;VGLKVFN	NCP	117.694
VGLKVFN;VVPPK	NCP	35.564
VVPPK;LLLLEL	NCP	115.057
LLLLEL;KNHVVY	NCP	10.34
KNHVVY;SPVAALS	NCP	23.775
SPVAALS;KSSGYHP	NCP	89.38
KSSGYHP;KLELLGV	NCP	53.541
KLELLGV;VSAFKAE	NCP	10.851
VSAFKAE;VTYLG	NCP	15.644
VTYLG;LHYLSMF	NCP	16.021
LHYLSMF;KEYMKK	NCP	49.788
KEYMKK;KEGVTLK	NCP	19.722
KEGVTLK;AVGVPPR	NCP	48.737
AVGVPPR;VGKGSFY	NCP	16.716
VGKGSFY;PLPTRYS	NCP	51.867
PLPTRYS;PVFDLN	NCP	76.576
PVFDLN;PVVVPKD	NCP	30.204
PVVVPKD;NSECMDC	NCP	4.086
NSECMDC;TMPSGSEK	NCP	42.33
TMPSGSEK;LYLMVPP	NCP	116.163
LYLMVPP;LYAQVK	NCP	8.921

LYAQVK;NVLEYL	NCP	0.169
NVLEYL;NCMDY	NCP	4.056
NCMDY;KEELLKT	NCP	9.908
KEELLKT;GRLLNHP	NCP	4.812
GRLLNHP;CCCQTP	NCP	110.529
CCCQTP;DHKKVV	NCP	13.615
DHKKVV;EPRLLTLQ	NCP	42.663
EPRLLTLQ;YGRLVK	NCP	22.07
YGRLVK;NVPPVG	NCP	53.31
NVPPVG;DSLERL	NCP	2.331
DSLERL;LLGLL	NCP	32.95
LLGLL;HCYLLSE	NCP	0.665
HCYLLSE;KDAALKK	NCP	8.877
KDAALKK;KGSHVY	NCP	18.558
KGSHVY;MSFYEP	NCP	7.081
MSFYEP;DDFFLR	NCP	52.37
DDFFLR;PAATMRH	NCP	5.208
PAATMRH;QSPFLP	NCP	13.965
QSPFLP;VVVHLFR	NCP	23.074
VVVHLFR;LEVPVA	NCP	49.161
LEVPVA;MELPVP	NCP	9.617
MELPVP;VTEVPR	NCP	32.319
VTEVPR;KERMFQ	NCP	31.614
KERMFQ;AALGL	NCP	38.382
AALGL;LNLKTVR	NCP	47.436
LNLKTVR;ESGVSLEK	NCP	49.723
ESGVSLEK;QTNLGL	NCP	10.172
QTNLGL;KYVKPP	NCP	75.471
KYVKPP;KNYGAFF	NCP	34.854
KNYGAFF;KPVEY	NCP	57.234
KPVEY;SGEPLPTL	NCP	59.053
SGEPLPTL;LTVSGLK	NCP	11.184
LTVSGLK;AYTVP	NCP	17.655
AYTVP;SPEKLER	NCP	103.98
SPEKLER;MEGSVGK	NCP	80.664
MEGSVGK;LPVPT	NCP	36.671
LPVPT;MLEPAP	NCP	50.23
MLEPAP;LAVGPGV	NCP	51.363
LAVGPGV;KFSLDQL	NCP	54.816
KFSLDQL;AHQEHALT	NCP	28.958
AHQEHALT;LSGHLFF	NCP	137.577
LSGHLFF;EHLDALK	NCP	7.122
EHLDALK;KTGFVAH	NCP	2.463
KTGFVAH;LKGVVPPGS	NCP	3.199
LKGVVPPGS;RLEKF	NCP	33.726
RLEKF;QSLLFY	NCP	171.021
QSLLFY;KSTDPQ	NCP	42.915
KSTDPQ;LVPLLLVL	NCP	16.815
LVPLLLVL;GVYLPSR	NCP	14.802
GVYLPSR;MNLLVK	NCP	30.477
MNLLVK;ATLLVAL	NCP	20.084

ATLLVAL;VVFLLAK	NCP	0.898
VVFLLAK;DPEPLPPR	NCP	44.598
DPEPLPPR;NHSVPPP	NCP	37.281
NHSVPPP;DPLVHRT	NCP	67.316
DPLVHRT;AESLLLR	NCP	38.836
AESLLLR;KQLCVHL	NCP	21.008
KQLCVHL;KCLSVELE	NCP	135.151
KCLSVELE;DFKGPP	NCP	6.246
DFKGPP;QPVDKRH	NCP	46.355
QPVDKRH;TVLVALLP	NCP	20.19
TVLVALLP;KVESLGP	NCP	30.955
KVESLGP;VSAFPPPP	NCP	17.445
VSAFPPPP;KGKCTLK	NCP	14.022
KGKCTLK;RPLKLAK	NCP	113.937
RPLKLAK;STNWSDVGK	NCP	109.178
STNWSDVGK;DDMEWKKY	NCP	0.051
DDMEWKKY;LLPVLFK	NCP	7.47
LLPVLFK;LSHFTDYL	NCP	13.375
LSHFTDYL;PLGLVLPL	NCP	6.722
PLGLVLPL;RRPAGLLS	NCP	22.395
RRPAGLLS;GHVSAPR	NCP	12.679
GHVSAPR;LPRALDLL	NCP	22.364
LPRALDLL;SPFPPNP	NCP	13.23
SPFPPNP;VASAVVR	NCP	192.888
VASAVVR;WLLLTPP	NCP	9.267
WLLLTPP;LDLLLK	NCP	16.258
LDLLLK;AAKPPP	NCP	1.535
AAKPPP;NPHALLR	NCP	9.375
NPHALLR;PPPHPPVE	NCP	18.604
PPPHPPVE;VQVLPF	NCP	64.067
VQVLPF;LEDFLVS	NCP	16.854
LEDFLVS;LRVYMLRLK	NCP	3.888
LRVYMLRLK;LLVANY	NCP	24.88
LLVANY;AGGHLLR	NCP	4.773
AGGHLLR;KLSLGAAP	NCP	13.019
KLSLGAAP;FAARPR	NCP	51.982
FAARPR;PTPLGPGP	NCP	33.66
PTPLGPGP;ASKRALVL	NCP	34.932
ASKRALVL;KAAPK	NCP	25.53
KAAPK;LVPPPAPPP	NCP	12.992
LVPPPAPPP;VPGKPMC	NCP	0.582
VPGKPMC;KQKEDGT	NCP	64.437
KQKEDGT;QPVYGK	NCP	78.204
QPVYGK;NMDLRK	NCP	41.688
NMDLRK;KGWFRPP	NCP	59.382
KGWFRPP;RGMYGK	NCP	24.384
RGMYGK;KTMQY	NCP	1.273
KTMQY;NVLTSPR	NCP	66.986
NVLTSPR;MTDSFF	NCP	3.645
MTDSFF;LDTLSPAK	NCP	65.236
LDTLSPAK;KSNPYP	NCP	21.306

KSNPYP;VTFMVP	NCP	26.37
VTFMVP;VSGVVLE	NCP	18.435
VSGVVLE;PAAPHLK	NCP	22.476
PAAPHLK;NAVLE	NCP	96.131
NAVLE;KPDCAM	NCP	10.11
KPDCAM;ALFLK	NCP	19.36
ALFLK;GDLLRVLL	NCP	178.883
GDLLRVLL;FDLHLK	NCP	30.664
FDLHLK;EAAPKPK	NCP	82.572
EAAPKPK;MLPPYK	NCP	153.713
MLPPYK;EDGPLLR	NCP	41.116
EDGPLLR;FGGGPTA	NCP	50.019
FGGGPTA;LLFGPKG	NCP	111.569
LLFGPKG;DPPFPK	NCP	9.1
DPPFPK;ASLNQLKL	NCP	44.439
ASLNQLKL;GASDY	NCP	129.488
GASDY;TLLGVPD	NCP	34.792
TLLGVPD;DLRGGLGK	NCP	63.165
DLRGGLGK;TTLPEKL	NCP	9.515
TTLPEKL;VPTLNPK	NCP	59.431
VPTLNPK;VSDFVLY	NCP	63.936
VSDFVLY;DASPNP	NCP	8.132
DASPNP;KELKAPL	NCP	93.076
KELKAPL;MQPASAK	NCP	5.637
MQPASAK;PASTHEQ	NCP	5.451
PASTHEQ;MPLSGP	NCP	101.049
MPLSGP;STELAFK	NCP	22.721
STELAFK;LQVEPF	NCP	9.535
LQVEPF;CNCWC	NCP	14.66
CNCWC;PMPLYK	NCP	81.925
PMPLYK;FNDLSLA	NCP	33.266
FNDLSLA;TFEKPVK	NCP	15.205
TFEKPVK;YLNFVK	NCP	58.451
YLNFVK;HKEPPR	NCP	121.056
HKEPPR;AVSAVEP	NCP	63.028
AVSAVEP;WEQKNPK	NCP	135.422
WEQKNPK;TMEAYRG	NCP	25.141
TMEAYRG;GRAKLAL	NCP	7.08
GRAKLAL;LPFYK	NCP	155.523
LPFYK;KPGKFK	NCP	68.283
KPGKFK;DVGTNLK	NCP	23
DVGTNLK;LVVFM	NCP	8.599
LVVFM;LNNFAFF	NCP	204.333
LNNFAFF;VPSYF	NCP	38.91
VPSYF;RTQMLK	NCP	75.399
RTQMLK;DLERTMR	NCP	24.032
DLERTMR;KEWAKKM	NCP	71.545
KEWAKKM;QYEVFH	NCP	3.254
QYEVFH;KPFEC	NCP	141.607
KPFEC;PNPFGK	NCP	114.723
PNPFGK;SFLGLSL	NCP	44.438

SFLGLSL;KGGELQK	NCP	30.82
KGGELQK;VGGELY	NCP	72.911
VGGELY;GVSPK	NCP	0.304
GVSPK;PGLEVL	NCP	11.523
PGLEVL;KHVPPR	NCP	6.005
KHVPPR;PPPRHP	NCP	22.03
PPPRHP;KMDKLEK	NCP	15.366
KMDKLEK;QPPMLPLL	NCP	0.746
QPPMLPLL;KPGAGKT	NCP	3.724
KPGAGKT;KPLVWSK	NCP	44
KPLVWSK;KDELYP	NCP	80.707
KDELYP;HDMPLP	NCP	86.271
HDMPLP;YGEDDC	NCP	8.481
YGEDDC;KVTPGKK	NCP	17.877
KVTPGKK;RNLLSQL	NCP	170.931
RNLLSQL;FTLGNST	NCP	24.192
FTLGNST;KSAADF	NCP	31.449
KSAADF;KKKTHK	NCP	89.379
KKKTHK;GVHLPFLE	NCP	2.951
GVHLPFLE;LLGKNKF	NCP	23.688
LLGKNKF;KEQVKQK	NCP	55.671
KEQVKQK;VVGLLWW	NCP	55.89
VVGLLWW;AAMKTKK	NCP	10.555
AAMKTKK;TAACRLL	NCP	68.52
TAACRLL;SLDVK	NCP	120.347
SLDVK;LTVVVKP	NCP	30.187
LTVVVKP;ERLFYLP	NCP	47.217
ERLFYLP;KKPTFK	NCP	65.283
KKPTFK;LSAPLEP	NCP	50.222
LSAPLEP;KMYLPPP	NCP	0.205
KMYLPPP;LSDGLPF	NCP	22.269
LSDGLPF;LSLEQL	NCP	5.952
LSLEQL;PPPAAPP	NCP	4.71
PPPAAPP;KPRSGAP	NCP	39.666
KPRSGAP;ELTYHV	NCP	37.134
ELTYHV;TPPAAGKQ	NCP	44.349
TPPAAGKQ;RPPFGK	NCP	60.981
RPPFGK;PTVEEVL	NCP	1.35
PTVEEVL;TSARFSP	NCP	42.822
TSARFSP;SDVDF	NCP	4.44
SDVDF;TLGMRDE	NCP	65.895
TLGMRDE;KNKKST	NCP	8.049
KNKKST;LPTLDPA	NCP	25.638
LPTLDPA;EVGPAAW	NCP	8.934
EVGPAAW;RRGVTPP	NCP	100.463
RRGVTPP;VTAPNLL	NCP	83.968
VTAPNLL;KEPSPTPK	NCP	78.69
KEPSPTPK;LHHLGNLK	NCP	29.162
LHHLGNLK;LPLNYHRP	NCP	11.709
LPLNYHRP;TTKKKPK	NCP	6.001
TTKKKPK;LPSHSLK	NCP	101.948

LPSHSLK;APPRGAPP	NCP	9.46
APPRGAPP;QWNAY	NCP	56.127
QWNAY;KGPFQFP	NCP	78.948
KGPFQFP;MQKDPSV	NCP	16.559
MQKDPSV;SLRKSSE	NCP	107.983
SLRKSSE;KVLEGVP	NCP	52.523
KVLEGVP;YASQGGK	NCP	6.309
YASQGGK;FVPLVK	NCP	0.439
FVPLVK;CRSRP	NCP	5.877
CRSRP;PVNFKLTP	NCP	66.432
PVNFKLTP;NGDAK	NCP	68.267
NGDAK;FGTYLLSH	NCP	64.156
FGTYLLSH;DLSVNP	NCP	32.391
DLSVNP;ELGPF	NCP	311.273
ELGPF;LTLELNNL	NCP	55.926
LTLELNNL;VAHTLLTN	NCP	3.06
VAHTLLTN;KFPHMF	NCP	55.011
KFPHMF;KMTAP	NCP	38.427
KMTAP;KEVLQGK	NCP	116.842
KEVLQGK;QFGDPR	NCP	93.27
QFGDPR;YPEPEL	NCP	70.853
YPEPEL;KDYPPPP	NCP	66.91
KDYPPPP;FLSGGFD	NCP	27.552
FLSGGFD;KRGLDK	NCP	3.728
KRGLDK;KQHVFPP	NCP	10.305
KQHVFPP;YELVL	NCP	1.518
YELVL;KETAYP	NCP	23.94
KETAYP;KAHPKY	NCP	18.271
KAHPKY;FVAQEF	NCP	13.482
FVAQEF;LPDFSSL	NCP	5.304
LPDFSSL;KNFSWP	NCP	68.919
KNFSWP;MCMSPL	NCP	6.452
MCMSPL;WLEAPW	NCP	6.396
WLEAPW;HLKLWH	NCP	4.3
HLKLWH;PYPKFS	NCP	30.191
PYPKFS;KKNVQTK	NCP	6.937
KKNVQTK;LLVSK	NCP	146
LLVSK;FFPPP	NCP	7.323
FFPPP;EHVTPR	NCP	17.908
EHVTPR;EHSSG	NCP	17.189
EHSSG;AEQVLA	NCP	6.1
AEQVLA;STSSMS	NCP	143.73
STSSMS;GDVLK	NCP	13.665
GDVLK;LLTGLA	NCP	17.138
LLTGLA;DRFRTF	NCP	58.761
DRFRTF;SSTLVPR	NCP	11.133
SSTLVPR;AVLGEY	NCP	4.935
AVLGEY;FQGLKH	NCP	25.078
FQGLKH;FLSFKTTP	NCP	3.315
FLSFKTTP;KLDVKP	NCP	12.546
KLDVKP;EPAYFLL	NCP	13.86

EPAYFLL;EFLGKKT	NCP	1.833
EFLGKKT;VLKLGY	NCP	64.8
VLKLGY;FNDRPP	NCP	45.069
FNDRPP;RVTVEP	NCP	46.778
RVTVEP;KAMEWT	NCP	67.077
KAMEWT;MAAPVLL	NCP	16.75
MAAPVLL;SGMSVTR	NCP	17.249
SGMSVTR;NGPLSPF	NCP	3.663
NGPLSPF;KPHFPK	NCP	18.564
KPHFPK;LADCGKVK	NCP	18.591
LADCGKVK;YWAGY	NCP	36.648
YWAGY;KTYAGP	NCP	6.007
KTYAGP;EVDLRKN	NCP	53.487
EVDLRKN;HQTSVLQK	NCP	29.957
HQTSVLQK;RPPEKPP	NCP	0.892
RPPEKPP;KEVYNK	NCP	55.586
KEVYNK;MLARKC	NCP	48.789
MLARKC;KAYFWP	NCP	18.513
KAYFWP;VAGDKLK	NCP	7.275
VAGDKLK;VTPGTM	NCP	28.681
VTPGTM;RQGLQAV	NCP	36.791
RQGLQAV;VGLLRLP	NCP	22.501
VGLLRLP;YALAHA	NCP	66.968
YALAHA;VYPTFR	NCP	37.159
VYPTFR;VTLVYK	NCP	43.631
VTLVYK;GFGFGQ	NCP	1.189
GFGFGQ;GPLSPRPP	NCP	5.574
GPLSPRPP;VGTGLRAV	NCP	19.697
VGTGLRAV;ELSQRL	NCP	50.482
ELSQRL;GPAGVSPRPPP	NCP	13.335
GPAGVSPRPPP;FPLKTKLLL	NCP	14.235
FPLKTKLLL;TLSVSVH	NCP	16.019
TLSVSVH;KPPKYP	NCP	11.071
KPPKYP;PPPAVYK	NCP	3.458
PPPAVYK;CDECC	NCP	4.009
CDECC;HLPGKGK	NCP	38.829
HLPGKGK;KGPLSKP	NCP	0.558
KGPLSKP;AAPRTPGK	NCP	56.301
AAPRTPGK;AAPRPP	NCP	21.075
AAPRPP;DPPPRAVP	NCP	4.731
DPPPRAVP;KERLVP	NCP	20.046
KERLVP;KAFNPK	NCP	36.26
KAFNPK;YLKNPR	NCP	57.345
YLKNPR;EPATHE	NCP	49.201
EPATHE;LSRPPPL	NCP	16.104
LSRPPPL;KTGFLGPK	NCP	51.029
KTGFLGPK;RAGPPGVP	NCP	24.493
RAGPPGVP;RPNPPGP	NCP	43.695
RPNPPGP;VGDDLLK	NCP	0.11
VGDDLLK;QPLMLLR	NCP	40.914
QPLMLLR;MSAARPP	NCP	41.626

MSAARPP;EAALRRK	NCP	0.87
EAALRRK;RVGVGPR	NCP	64.119
RVGVGPR;EPSFP	NCP	175.83
EPSFP;MEVKR	NCP	59.184
MEVKR;LAPKHAAP	NCP	24.465
LAPKHAAP;PEAPGP	NCP	23.349
PEAPGP;TRVFRST	NCP	5.811
TRVFRST;YETRGY	NCP	2.594
YETRGY;AVPPRP	NCP	53.689
AVPPRP;KDVTCHQ	NCP	8.847
KDVTCHQ;NNSRLKL	NCP	54.057
NNSRLKL;TDVTVCK	NCP	15.444
TDVTVCK;PPTLHRP	NCP	23.694
PPTLHRP;TDLVR	NCP	16.131
TDLVR;TTVRDLP	NCP	9.008
TTVRDLP;PHLGKVP	NCP	43.24
PHLGKVP;AAALQLR	NCP	23.658
AAALQLR;VPAGCAGP	NCP	18.3
VPAGCAGP;RKGGPAAP	NCP	7.389
RKGGPAAP;EELDRDP	NCP	13.404
EELDRDP;LPFPEDE	NCP	3.339
LPFPEDE;NGQTREHALLA	NCP	0.209
NGQTREHALLA;GQTREHALL	NCP	0.003
GQTREHALL;EHALLAYTLG	NCP	0.012
EHALLAYTLG;FAPVNVTTEVKSVEMHHEA	NCP	0.435
FAPVNVTTEVKSVEMHHEA;APVNVTTEVKSVEMHH	NCP	0.003
APVNVTTEVKSVEMHH;EDGPKFL	NCP	0.321
EDGPKFL;TPLAPPR	NCP	25.393
TPLAPPR;DRPLAAR	NCP	26.433
DRPLAAR;KQTNAGP	NCP	32.928
KQTNAGP;YAALFPR	NCP	30.39
YAALFPR;SLKLQTLK	NCP	11.345
SLKLQTLK;APPRGPPPP	NCP	43.102
APPRGPPPP;DDPPP	NCP	12.951
DDPPP;AYTLPK	NCP	40.349
AYTLPK;KDVSAPP	NCP	38.629
KDVSAPP;RFWAAP	NCP	12.279
RFWAAP;GPREALL	NCP	10.089
GPREALL;EMADLR	NCP	54.861
EMADLR;LVPHQLA	NCP	22.104
LVPHQLA;TVPGGAVR	NCP	7.932
TVPGGAVR;PSLVFG	NCP	12.875
PSLVFG;KLGVLLA	NCP	49.567
KLGVLLA;TRSPWSY	NCP	67.089
TRSPWSY;PDDGT	NCP	8.694
PDDGT;LLFVD	NCP	47.126
LLFVD;YKYFLSF	NCP	3.765
YKYFLSF;RYLKYLTK	NCP	5.05
RYLKYLTK;PAAADK	NCP	51.882
QHGGEC;SSGPGVY	NCP	37.194
SSGPGVY;LQSTPVK	NCP	19.335

LQSTPVK;LEEALLP	NCP	17.997
LEEALLP;NMKRSP	NCP	13.07
NMKRSP;ASLFTLH	NCP	1.501
ASLFTLH;EQLALF	NCP	20.393
EQLALF;AALLP	NCP	23.518
AALLP;TLGKSVV	NCP	95.207
TLGKSVV;TVWGPV	NCP	34.158
TVWGPV;YEVKSY	NCP	34.404
YEVKSY;PPPPHPP	NCP	6.064
PPPPHPP;AAPTLPKL	NCP	57.698
AAPTLPKL;AEVKQN	NCP	97.591
AEVKQN;DCHPGLK	NCP	60.086
DCHPGLK;QTQEGKH	NCP	7.695
QTQEGKH;QKPALPP	NCP	27.42
QKPALPP;CCESSST	NCP	17.4
CCESSST;KLKEKL	NCP	34.761
KLKEKL;FVPGVT	NCP	171.279
FVPGVT;VKGTVR	NCP	39.91
VKGTVR;RDGVLTSP	NCP	14.972
RDGVLTSP;PPYQLQ	NCP	5.698
PPYQLQ;LWHHSF	NCP	40.314
LWHHSF;RLSRPA	NCP	16.694
RLSRPA;KDLFL	NCP	44.371
KDLFL;RMLLT	NCP	2.417
RMLLT;PGVHGSLS	NCP	3.583
PGVHGSLS;GVASPF	NCP	12.083
GVASPF;KAVLEY	NCP	40.671
KAVLEY;MKTST	NCP	15.357
MKTST;LLLEN	NCP	0.676
LLLEN;AAGRAPGGP	NCP	7.823
AAGRAPGGP;LDMFPS	NCP	66.574
LDMFPS;DKGAVAL	NCP	120.348
DKGAVAL;ELPRSNFK	NCP	2.45
ELPRSNFK;AGSGK	NCP	58.29
AGSGK;APVHRVAL	NCP	23.962
APVHRVAL;LEAMLP	NCP	26.189
LEAMLP;KTVMLRN	NCP	86.745
KTVMLRN;KLCFMGK	NCP	4.713
KLCFMGK;RKKLTP	NCP	7.879
RKKLTP;KLFHSKK	NCP	76.22
KLFHSKK;PEVAAQPP	NCP	56.88
PEVAAQPP;LPTAPSP	NCP	25.08
LPTAPSP;WGLLLP	NCP	1.554
WGLLLP;VPASW	NCP	25.468
VPASW;DDDDDDDC	NCP	43.971
DDDDDDDC;LPYEPPP	NCP	31.308
LPYEPPP;EPSPPLP	NCP	11.075
EPSPPLP;KQKSHPK	NCP	37.656
KQKSHPK;LSDLVFK	NCP	142.755
LSDLVFK;LSRGSPP	NCP	49.797
LSRGSPP;SPSSKRP	NCP	9.502

SPSSKRP;ASPLRLR	NCP	70.676
ASPLRLR;CSEKPKA	NCP	10.131
CSEKPKA;KGAPRPP	NCP	36.16
KGAPRPP;APGPLPKL	NCP	26.022
APGPLPKL;LDPPRLP	NCP	34.181
LDPPRLP;FGELKKQ	NCP	144.396
FGELKKQ;PSSVAPSP	NCP	1.422
PSSVAPSP;AAPELF	NCP	101.679
AAPELF;AAPGLLH	NCP	38.299
AAPGLLH;KGPFMPRP	NCP	17.706
KGPFMPRP;KYPPPPPPPP	NCP	132.26
KYPPPPPPPP;GVDLGL	NCP	17.692
GVDLGL;PPPPGPKP	NCP	0.567
PPPPGPKP;PEGLRASP	NCP	8.085
PEGLRASP;TVDLLGK	NCP	42.212
TVDLLGK;SVRTLCTT	NCP	74.596
SVRTLCTT;KDRLSVSLK	NCP	118.289
KDRLSVSLK;DCCDQ	NCP	5.751
DCCDQ;TSSGK	NCP	18.132
TSSGK;KSMGLPK	NCP	15.028
KSMGLPK;RKELKRN	NCP	24.569
RKELKRN;VADLLL	NCP	27.465
VADLLL;VNLNFPK	NCP	85.377
VNLNFPK;RVHLMK	NCP	14.712
RVHLMK;GDVKNFLY	NCP	124.441
GDVKNFLY;EPPVAAG	NCP	46.694
EPPVAAG;YHLLPQR	NCP	0.562
YHLLPQR;STGLQPK	NCP	64.307
STGLQPK;PDEAKAK	NCP	91.821
PDEAKAK;TTAAPHK	NCP	107.844
TTAAPHK;DTLGKLK	NCP	22.951
DTLGKLK;KYPLVKK	NCP	28.662
KYPLVKK;DHGVRLK	NCP	3.294
DHGVRLK;TPAHAK	NCP	29.112
TPAHAK;TRDGQVL	NCP	3.443
TRDGQVL;TLVTCSK	NCP	3.793
TLVTCSK;KYGPQR	NCP	35.319
KYGPQR;NHAKLVR	NCP	118.614
NHAKLVR;DPGHFP	NCP	18.72
DPGHFP;LHGVFR	NCP	20.64
LHGVFR;RNGLLGL	NCP	74.123
RNGLLGL;NSLLTTHT	NCP	9.631
NSLLTTHT;GPPPSWP	NCP	38.717
GPPPSWP;KACKPQ	NCP	36.064
KACKPQ;AAPFPQ	NCP	85.079
AAPFPQ;TPPTKPPP	NCP	33.601
TPPTKPPP;YMLLVQ	NCP	37.551
YMLLVQ;PVAGV	NCP	42.149
PVAGV;HDHVPR	NCP	27.222
HDHVPR;MAVAFVV	NCP	24.057
MAVAFVV;GTKGKLLK	NCP	74.562

GTKGKLLK;GVLELAK	NCP	8.443
GVLELAK;KKMTPPP	NCP	28.679
KKMTPPP;FMAPPPP	NCP	9.15
FMAPPPP;MLTAEK	NCP	34.348
MLTAEK;LTLPL	NCP	45.827
LTLPL;VEPVPK	NCP	17.848
VEPVPK;RDAPPVR	NCP	25.949
RDAPPVR;PAGCRKP	NCP	30.564
PAGCRKP;YFYGN	NCP	12.115
YFYGN;GVWHHS	NCP	65.171
GVWHHS;LEEVLK	NCP	2.505
LEEVLK;TLDAKVH	NCP	6.586
TLDAKVH;FSLLQYE	NCP	0.378
FSLLQYE;LKETLK	NCP	6.297
LKETLK;FGDCMQ	NCP	48.465
FGDCMQ;DSLTLKKL	NCP	21.816
DSLTLKKL;AALPH	NCP	11.93
AALPH;FPPDVN	NCP	27.301
FPPDVN;KTEDGAP	NCP	24.008
KTEDGAP;KMFAFW	NCP	63.694
KMFAFW;KEAPTLPP	NCP	5.594
KEAPTLPP;MGQANFR	NCP	43.585
MGQANFR;PPPPGGTR	NCP	2.81
PPPPGGTR;VPPVFRL	NCP	8.451
VPPVFRL;NVPPELF	NCP	24.54
NVPPELF;PLRAAPGP	NCP	92.667
PLRAAPGP;NWDLR	NCP	5.862
NWDLR;MSLHYP	NCP	9.019
MSLHYP;APVTP	NCP	40.931
APVTP;LQHAFE	NCP	63.88
LQHAFE;VGVTGVP	NCP	51.797
VGVTGVP;EERVGP	NCP	65.946
EERVGP;LEAVPDS	NCP	12.942
LEAVPDS;CCKDT	NCP	4.81
CCKDT;SALLVTTV	NCP	20.15
SALLVTTV;PDVGLPY	NCP	11.008
PDVGLPY;AGVGGPR	NCP	95.948
AGVGGPR;DSAMSN	NCP	62.274
DSAMSN;APALVKVP	NCP	92.509
APALVKVP;LEAAML	NCP	119.634
LEAAML;GVKVLLG	NCP	21.663
GVKVLLG;NPGDWF	NCP	3.135
NPGDWF;KVRRELSQ	NCP	80.828
KVRRELSQ;RGPVTRPP	NCP	0.663
RGPVTRPP;MPLELAF	NCP	30.352
MPLELAF;SLAVGL	NCP	97.796
SLAVGL;GHQKPR	NCP	55.308
GHQKPR;THLTQPK	NCP	19.477
THLTQPK;APEVKGK	NCP	79.28
APEVKGK;SLGAGAAPK	NCP	92.35
SLGAGAAPK;DVSNPLR	NCP	13.895

DVSNPLR;RSGGLLR	NCP	7.515
RSGGLLR;GHSLDDM	NCP	52.951
GHSLDDM;VLSLY	NCP	16.133
VLSLY;RFEPEK	NCP	71.658
RFEPEK;LQPKSR	NCP	3.115
LQPKSR;LRTTSPRT	NCP	28.889
LRTTSPRT;MEDRRPCPEVGPR	NCP	94.059
MEDRRPCPEVGPR;CSCKYP	NCP	93.81
CSCKYP;MYKPVDPY	NCP	7.131
MYKPVDPY;RASGNLLR	NCP	17.014
RASGNLLR;APQTPN	NCP	29.448
APQTPN;LSSSCVP	NCP	54.467
LSSSCVP;VGAKVP	NCP	46.521
VGAKVP;RAPVYM	NCP	1.155
RAPVYM;RHPPN	NCP	7.623
RHPPN;KPPGRTP	NCP	93.987
KPPGRTP;GESDL	NCP	13.003
GESDL;TWRLTHN	NCP	38.171
TWRLTHN;GSHAAPA	NCP	42.069
GSHAAPA;KLWVGPR	NCP	1.621
KLWVGPR;ALGAGLER	NCP	8.177
ALGAGLER;VTGLPLP	NCP	4.293
VTGLPLP;AALAPP	NCP	5.973
AALAPP;LVGGVVV	NCP	24.067
LVGGVVV;TFKATDL	NCP	1.446
TFKATDL;APPHVSR	NCP	11.787
APPHVSR;PVNEQC	NCP	23.345
PVNEQC;RLGVFY	NCP	20.121
RLGVFY;TTPSLPPR	NCP	14.461
TTPSLPPR;LTARAAC	NCP	34.133
LTARAAC;RALLNL	NCP	39.768
RALLNL;VKSVY	NCP	12.082
VKSVY;AAPPAPPK	NCP	32.36
AAPPAPPK;HFFKGP	NCP	65.593
HFFKGP;RPLPERAF	NCP	40.211
RPLPERAF;EQAALW	NCP	29.596
EQAALW;LLSELVP	NCP	11.871
LLSELVP;AAKLGKL	NCP	116.81
AAKLGKL;LGAERALR	NCP	10.905
LGAERALR;SVFRASP	NCP	26.28
SVFRASP;SLRKTY	NCP	42.412
SLRKTY;PRPTYP	NCP	49.055
PRPTYP;AMLSGLS	NCP	21.757
AMLSGLS;CDTKPPPPP	NCP	37.799
CDTKPPPPP;LSRPPGK	NCP	1.585
LSRPPGK;FKLHERK	NCP	33.263
FKLHERK;RHGGKLF	NCP	36.585
RHGGKLF;KDSKKK	NCP	26.019
KDSKKK;LAPGMLL	NCP	2.124
LAPGMLL;KTSQMPK	NCP	14.787
KTSQMPK;EKPRQAK	NCP	6.744

EKPRQAK;VTGVLPGK	NCP	11.886
VTGVLPGK;HSPSAPPP	NCP	79.332
HSPSAPPP;NVLVP	NCP	2.946
NVLVP;TTSSKTK	NCP	30.703
TTSSKTK;KPGAHAAP	NCP	124.556
KPGAHAAP;LPVVDY	NCP	18.349
LPVVDY;KVAGME	NCP	45.714
KVAGME;KSFHPGPL	NCP	86.138
KSFHPGPL;ADGMSSL	NCP	5.697
ADGMSSL;GEEAWF	NCP	9.372
GEEAWF;GGGPRRPP	NCP	85.707
GGGPRRPP;KTFHVPA	NCP	171.852
KTFHVPA;LLEENSLLL	NCP	14.209
LLEENSLLL;FSYLLLPC	NCP	1.403
FSYLLLPC;NAPKGSF	NCP	26.934
NAPKGSF;PSLGY	NCP	41.164
PSLGY;EGHRAPC	NCP	0.257
EGHRAPC;DPRKAAP	NCP	8.46
DPRKAAP;AAPDHKR	NCP	27.816
AAPDHKR;MRTPPPPP	NCP	5.127
MRTPPPPP;DKSLF	NCP	89.188
DKSLF;CPSCAPW	NCP	2.894
CPSCAPW;LTAVDF	NCP	36.907
LTAVDF;ASLGAKHGP	NCP	40.802
ASLGAKHGP;KLKVDEF	NCP	24.376
KLKVDEF;TSYMTT	NCP	48.003
TSYMTT;AALGLAGR	NCP	19.124
AALGLAGR;LGMGGSF	NCP	35.631
LGMGGSF;KPPGFSK	NCP	11.98
KPPGFSK;MVSHHPV	NCP	15.8
MVSHHPV;LSFGPPP	NCP	68.337
LSFGPPP;GSPNPLL	NCP	73.279
GSPNPLL;VAPVNMK	NCP	46.133
VAPVNMK;NFYTLPK	NCP	15.256
NFYTLPK;RPGVLEAP	NCP	3.659
RPGVLEAP;APPNPPPL	NCP	21.793
APPNPPPL;DSARLLR	NCP	37.16
DSARLLR;TVYGSSH	NCP	63.579
TVYGSSH;ELGLVL	NCP	6.276
ELGLVL;RQEKGVH	NCP	8.23
RQEKGVH;KKLDDY	NCP	31.931
KKLDDY;LGQTPTKEELD	NCP	29.991
LGQTPTKEELD;KKSHKK	NCP	57.234
KKSHKK;NLRNLGK	NCP	4.648
NLRNLGK;MEDPNKP	NCP	8.511
MEDPNKP;TKKPEL	NCP	2.829
TKKPEL;DGLSKGPK	NCP	15.749
DGLSKGPK;KDGGVP	NCP	18.784
KDGGVP;EFLAHA	NCP	2.832
EFLAHA;LLPPRVF	NCP	24.542
LLPPRVF;QFPPPP	NCP	3.324

QFPPPP;AVAPLH	NCP	80.893
AVAPLH;KTGLEPA	NCP	25.299
KTGLEPA;LARPSAPK	NCP	33.083
LARPSAPK;ESGVTK	NCP	37.125
ESGVTK;ASNELR	NCP	31.696
ASNELR;QHELSCP	NCP	21.057
QHELSCP;QALSSLR	NCP	35.324
QALSSLR;KDAVLFL	NCP	3.477
KDAVLFL;APGGHVGGP	NCP	4.426
APGGHVGGP;TVMDLR	NCP	78.912
TVMDLR;EDLMK	NCP	69.98
EDLMK;SGPFVP	NCP	30.654
SGPFVP;TALQP	NCP	57.109
TALQP;VGVLSAS	NCP	24.048
VGVLSAS;GPAMLGK	NCP	28.244
GPAMLGK;LSATVPK	NCP	10.351
LSATVPK;YELMMD	NCP	19.574
YELMMD;VYVLF	NCP	47.164
VYVLF;KWSPAP	NCP	87.161
KWSPAP;NYVPVK	NCP	7.125
NYVPVK;VGTPLLSN	NCP	41.958
VGTPLLSN;KLKESQSPR	NCP	23.193
KLKESQSPR;AAADVLA	NCP	13.705
AAADVLA;PPPPHPGP	NCP	152.36
PPPPHPGP;TTSTVQK	NCP	1.438
TTSTVQK;RSLLQE	NCP	2.108
RSLLQE;PPRDYP	NCP	42.087
PPRDYP;KNFPLKDL	NCP	27.523
KNFPLKDL;SKMRGP	NCP	15.074
SKMRGP;ALRYMP	NCP	2.172
ALRYMP;FRCPET	NCP	0.985
FRCPET;LTRLPNN	NCP	20.871
LTRLPNN;SDTSKGLK	NCP	53.946
SDTSKGLK;KHKSLAGK	NCP	14.363
KHKSLAGK;EVFKRLP	NCP	0.498
EVFKRLP;AAVEVLK	NCP	31.837
AAVEVLK;DSPGKVL	NCP	0.072
DSPGKVL;LSATLQF	NCP	5.481
LSATLQF;VAGKWVL	NCP	18.737
VAGKWVL;QATLKGY	NCP	35.374
QATLKGY;VTDVEPY	NCP	26.289
VTDVEPY;KVVVKVK	NCP	10.212
KVVVKVK;FPLGPEV	NCP	71.459
FPLGPEV;YAMFD	NCP	6.22
YAMFD;RDVVDL	NCP	34.935
RDVVDL;GYVFL	NCP	71.559
GYVFL;LSPVFG	NCP	15.725
LSPVFG;RLPFK	NCP	0.391
RLPFK;EFVEMKAK	NCP	33.428
EFVEMKAK;NRLRQENM	NCP	3.42
NRLRQENM;PEWAP	NCP	20.184

PEWAP;DLLEFK	NCP	25.098
DLLEFK;LESYK	NCP	18.328
LESYK;VRLLHYK	NCP	69.458
VRLLHYK;CRQHMSL	NCP	121.479
CRQHMSL;AETYEL	NCP	29.77
AETYEL;EVMELK	NCP	18.923
EVMELK;FEAYK	NCP	9.042
FEAYK;TPTPPRPV	NCP	49.203
TPTPPRPV;LSTHLAK	NCP	15.081
LSTHLAK;KNTTN	NCP	234.393
KNTTN;GSDLVLP	NCP	2.968
GSDLVLP;SSSSAK	NCP	15.965
SSSSAK;ELAGPCQ	NCP	1.408
ELAGPCQ;FKTCVV	NCP	31.829
FKTCVV;NPGGGGGF	NCP	36.285
NPGGGGGF;KPATSLR	NCP	4.539
KPATSLR;LADRMQK	NCP	14.532
LADRMQK;QAPLLVLG	NCP	25.401
QAPLLVLG;KYMPPPPP	NCP	15.537
KYMPPPPP;LAARPLQ	NCP	12.891
LAARPLQ;KPVSPL	NCP	14.113
KPVSPL;LTGDWK	NCP	58.416
LTGDWK;LPPLAPPP	NCP	76.346
LPPLAPPP;KNNSLPL	NCP	1.974
KNNSLPL;NLKLLK	NCP	66.841
NLKLLK;VTLEKFDK	NCP	46.257
VTLEKFDK;SLESFRP	NCP	12.371
SLESFRP;PSKATLPP	NCP	24.241
PSKATLPP;KEAVGSLP	NCP	30.893
KEAVGSLP;SARLPNP	NCP	6.381
SARLPNP;CMMGW	NCP	15.738
CMMGW;QTPKVPK	NCP	31.105
QTPKVPK;ALGRALPP	NCP	15.578
ALGRALPP;GLPVEY	NCP	0.183
GLPVEY;VTYTPF	NCP	13.567
VTYTPF;AALYTPK	NCP	21.405
AALYTPK;KERMGPK	NCP	108.36
KERMGPK;SPAAPAVS	NCP	12.275
SPAAPAVS;ERNSPRF	NCP	29.722
ERNSPRF;LGTALLK	NCP	10.367
LGTALLK;ADMGSSL	NCP	47.284
ADMGSSL;MAAVEF	NCP	22.407
MAAVEF;LNDAHLAK	NCP	43.1
LNDAHLAK;GVCYGL	NCP	29.4
GVCYGL;RFLKGDP	NCP	41.31
RFLKGDP;QDPGSPPK	NCP	0.579
QDPGSPPK;GVNAERK	NCP	30.861
GVNAERK;EGWGGPK	NCP	7.579
EGWGGPK;VAELLLP	NCP	13.013
VAELLLP;NPEPKPR	NCP	11.19
NPEPKPR;KDVVPAP	NCP	7.24

KDVVPAP;FNKRP	NCP	0.036
FNKRP;FARNDK	NCP	12.195
FARNDK;LSGVVVK	NCP	35.405
LSGVVVK;ESDDMM	NCP	84.775
ESDDMM;RLVSLTK	NCP	122.01
RLVSLTK;RLLFGP	NCP	32.79
RLLFGP;HTRSELP	NCP	3.302
HTRSELP;QHKGAPR	NCP	35.622
QHKGAPR;DDDLKGPK	NCP	0.261
DDDLKGPK;TSRVPSSY	NCP	7.402
TSRVPSSY;LHSSVHPV	NCP	39.36
LHSSVHPV;KPPRLQ	NCP	29.94
KPPRLQ;LALDMY	NCP	1.716
LALDMY;KLLLLPT	NCP	4.101
KLLLLPT;VGSYGRP	NCP	30.678
VGSYGRP;SLLFDKAK	NCP	21.861
SLLFDKAK;AEPEPKVR	NCP	4.623
AEPEPKVR;QSLLYEK	NCP	8.228
QSLLYEK;LSWMSW	NCP	11.878
LSWMSW;GGPNFQPA	NCP	24.723
GGPNFQPA;HLNLSF	NCP	55.802
HLNLSF;KAGNLKE	NCP	21.012
KAGNLKE;YVASYLL	NCP	13.248
YVASYLL;LLWHKQ	NCP	102.934
LLWHKQ;VTKQPLLR	NCP	3.887
VTKQPLLR;APKSKHV	NCP	18.066
APKSKHV;RRPLK	NCP	13.882
RRPLK;ALGKNNP	NCP	2.561
ALGKNNP;LPLAAHLP	NCP	113.817
LPLAAHLP;MGTKMLR	NCP	23.266
MGTKMLR;KNGVQGQ	NCP	87.627
KNGVQGQ;LGTAARAAP	NCP	34.539
LGTAARAAP;KHAGPVP	NCP	37.682
KHAGPVP;QPHLKY	NCP	112.645
QPHLKY;RAVPSK	NCP	61.716
RAVPSK;KSTTDVP	NCP	46.677
KSTTDVP;LYSWNK	NCP	202.608
LYSWNK;TADFLVL	NCP	5.49
TADFLVL;LPSVETLG	NCP	9.084
LPSVETLG;KYQPPR	NCP	34.931
KYQPPR;RPGGPAL	NCP	80.874
RPGGPAL;TKVPAVG	NCP	7.755
TKVPAVG;YMGPCPP	NCP	1.023
YMGPCPP;KTGSQP	NCP	3.351
KTGSQP;KTEEMMK	NCP	83.463
KTEEMMK;LVTNSN	NCP	38.035
LVTNSN;RPPWLK	NCP	41.345
RPPWLK;VELGAP	NCP	58.861
VELGAP;LSSNLPLF	NCP	3.051
LSSNLPLF;SFRFPSGN	NCP	5.951
SFRFPSGN;KPMTT	NCP	12.799

KPMTT;PVGPLYPP	NCP	56.831
PVGPLYPP;AGPMVR	NCP	4.758
AGPMVR;AFEGCVP	NCP	11.418
AFEGCVP;KSGSGK	NCP	38.319
KSGSGK;SPLSKTH	NCP	70.311
SPLSKTH;ALNFLL	NCP	39.205
ALNFLL;DSLDELVR	NCP	43.76
DSLDELVR;STEEDM	NCP	10.089
STEEDM;FGGFK	NCP	96.622
FGGFK;WLLPFLL	NCP	30.909
WLLPFLL;KMGPKR	NCP	3.887
KMGPKR;LVVVSRVL	NCP	16.419
LVVVSRVL;LTWVVSP	NCP	41.155
LTWVVSP;AVHVV	NCP	27.707
AVHVV;LAHLWSF	NCP	1.146
LAHLWSF;DDQLLR	NCP	7.438
DDQLLR;MSGFLT	NCP	0.959
MSGFLT;QQLQKEKK	NCP	40.827
QQLQKEKK;QLLPPAL	NCP	36.18
QLLPPAL;VGENPK	NCP	58.366
VGENPK;AAGDFVK	NCP	69.842
AAGDFVK;EVLPKPP	NCP	11.347
EVLPKPP;ELSKKLT	NCP	27.99
ELSKKLT;GVTAAH	NCP	29.583
GVTAAH;LPPRNVP	NCP	53.825
LPPRNVP;KEFPKP	NCP	33.358
KEFPKP;LSRVTVLK	NCP	47.717
LSRVTVLK;ELLKVHA	NCP	43.456
ELLKVHA;LELYY	NCP	11.511
LELYY;CGHPWH	NCP	8.603
CGHPWH;KAENTLK	NCP	85.926
KAENTLK;NVGVLT	NCP	16.339
NVGVLT;LPETLAK	NCP	43.581
LPETLAK;ALLKFPL	NCP	52.314
ALLKFPL;PYSQKAK	NCP	39.585
PYSQKAK;KTTCYK	NCP	132.042
KTTCYK;NFAPEKK	NCP	107.102
NFAPEKK;NPVVYR	NCP	53.997
NPVVYR;MPDGPP	NCP	52.636
MPDGPP;EPPAGP	NCP	1.512
EPPAGP;STAPFLQL	NCP	18.624
STAPFLQL;RGPPYNL	NCP	18.357
RGPPYNL;GSPQALPK	NCP	43.127
GSPQALPK;TFAVSM	NCP	2.827
TFAVSM;PAAAPPAL	NCP	12.393
PAAAPPAL;HALVHHQ	NCP	38.676
HALVHHQ;DSAPPLTL	NCP	8.955
DSAPPLTL;KGEYYVK	NCP	17.297
KGEYYVK;LPRPPVE	NCP	50.911
LPRPPVE;ASLPF	NCP	23.405
ASLPF;KVVGTH	NCP	6.726

KVVGTH;QVYPLR	NCP	63.906
QVYPLR;NAVLEYLT	NCP	72.678
NAVLEYLT;GSSLPLKD	NCP	35.143
GSSLPLKD;VPLREQ	NCP	2.787
VPLREQ;NAAVNP	NCP	35.024
NAAVNP;KYVGPSP	NCP	15.804
KYVGPSP;TQSNRSP	NCP	8.541
TQSNRSP;GVQPLQY	NCP	22
GVQPLQY;LDDGLLP	NCP	122.901
LDDGLLP;PGLLGPV	NCP	194.105
PGLLGPV;ERVFAPF	NCP	12.459
ERVFAPF;KELNAVQ	NCP	47.688
KELNAVQ;YEPDN	NCP	29.092
YEPDN;THGPRPP	NCP	0.423
THGPRPP;VGMVFLVL	NCP	98.699
VGMVFLVL;KSAVKSP	NCP	53.983
KSAVKSP;LSADKELL	NCP	4.07
LSADKELL;THVKLPP	NCP	14.289
THVKLPP;VAPPAPR	NCP	12.72
VAPPAPR;TQPKLVTD	NCP	66.09
TQPKLVTD;FGEHLLE	NCP	0.001
FGEHLLE;LSVKQR	NCP	28.193
LSVKQR;PAGGAPPR	NCP	9.429
PAGGAPPR;WSDRLSM	NCP	10.342
WSDRLSM;TPHDALK	NCP	6.381
TPHDALK;PDEEALR	NCP	71.735
PDEEALR;RVPPLR	NCP	0.642
RVPPLR;AAVSAL	NCP	42.18
AAVSAL;PGKFGV	NCP	36.781
PGKFGV;FLCLVCK	NCP	59.418
FLCLVCK;LLETGRP	NCP	0.24
LLETGRP;KSPETM	NCP	68.367
KSPETM;AAMVPR	NCP	38.15
AAMVPR;LTLGGVR	NCP	13.672
LTLGGVR;PMGDLR	NCP	1.415
PMGDLR;APSGMSQ	NCP	52.381
APSGMSQ;KMLPPPPPP	NCP	3.684
KMLPPPPPP;ALDQGRK	NCP	12.9
ALDQGRK;RGLVLPGE	NCP	0.056
RGLVLPGE;CHTKPR	NCP	4.411
CHTKPR;PLLLA	NCP	4.536
PLLLA;WVETSPK	NCP	35.249
WVETSPK;GPVPLY	NCP	3.993
GPVPLY;RLDPHK	NCP	62.659
RLDPHK;NGLVP	NCP	30.819
NGLVP;HVGSLVT	NCP	8.78
HVGSLVT;EMALRP	NCP	16.53
EMALRP;KTTEAH	NCP	13.074
KTTEAH;YNRKPVL	NCP	82.575
YNRKPVL;PAAPDFR	NCP	44.907
PAAPDFR;KDLYPM	NCP	28.981

KDLYPM;ASTRLFQ	NCP	64.002
ASTRLFQ;EPSTTAPK	NCP	79.101
EPSTTAPK;ASLEPDV	NCP	40.08
ASLEPDV;YCDCCC	NCP	5.666
YCDCCC;KVPLPPR	NCP	19.042
KVPLPPR;KSKKNL	NCP	0.024
KSKKNL;DRPLNLQ	NCP	11.897
DRPLNLQ;YCCYGY	NCP	7.27
YCCYGY;EDVYPG	NCP	26.09
EDVYPG;VLPFK	NCP	34.467
VLPFK;KYFLQGMGY	NCP	13.944
KYFLQGMGY;ELNYFAK	NCP	18.124
ELNYFAK;YVRLR	NCP	6.17
YVRLR;GKQLQPG	NCP	45.171
GKQLQPG;RTAPVAM	NCP	43.476
RTAPVAM;KNRGAPR	NCP	61.551
KNRGAPR;KPHSVPP	NCP	8.478
KPHSVPP;APAKPPR	NCP	10.692
APAKPPR;VHQVEF	NCP	5.247
VHQVEF;MLKFFVG	NCP	213.826
MLKFFVG;QHVGPK	NCP	30.957
QHVGPK;CAMVGK	NCP	7.647
CAMVGK;ELEPGVP	NCP	41.291
ELEPGVP;ALPLLVG	NCP	0.168
ALPLLVG;HEHLR	NCP	100.44
HEHLR;QTPKVGKP	NCP	22.498
QTPKVGKP;SLGGAKAL	NCP	66.987
SLGGAKAL;EPLLQSK	NCP	1.614
EPLLQSK;FTHRLL	NCP	18.174
FTHRLL;CEDDF	NCP	118.706
CEDDF;VEFVDVSSVPL	NCP	0.051
VEFVDVSSVPL;LPTRAAPP	NCP	91.176
LPTRAAPP;KVLSLLF	NCP	21.48
KVLSLLF;KTPAWP	NCP	1.588
KTPAWP;LPAAPPK	NCP	13.931
LPAAPPK;LGLHLHPP	NCP	6.159
LGLHLHPP;LGLPLEQV	NCP	4.044
LGLPLEQV;VPPRVLLY	NCP	0.522
VPPRVLLY;EYLLTAK	NCP	96.058
EYLLTAK;DLLKF	NCP	11.208
DLLKF;NSALLEF	NCP	11.937
NSALLEF;ATLKEKL	NCP	4.805
ATLKEKL;GYSLVYF	NCP	4.927
GYSLVYF;LAGPGL	NCP	0.5
LAGPGL;KEFQYRR	NCP	12.489
KEFQYRR;PSDRVR	NCP	42.748
PSDRVR;LLLLLCCLL	NCP	28.266
LLLLLCCLL;TPTLRLY	NCP	66.971
TPTLRLY;VPESLHW	NCP	4.812
VPESLHW;GSNLPF	NCP	118.72
GSNLPF;EGPFEL	NCP	0.809

EGPFEL;MGVVDM	NCP	31.38
MGVVDM;TALSELP	NCP	40.602
TALSELP;KEPAP	NCP	2.131
KEPAP;QHLETTY	NCP	26.528
QHLETTY;LLLPPVVSC	NCP	108.37
LLLPPVVSC;SLALLDT	NCP	19.838
SLALLDT;KPFEQP	NCP	7.699
KPFEQP;KHLAYVK	NCP	168.228
KHLAYVK;KVPMFK	NCP	99.972
KVPMFK;EYAPLR	NCP	25.218
EYAPLR;RLEVLAR	NCP	38.045
RLEVLAR;ELRQKYNVR	NCP	6.162
ELRQKYNVR;DDEVQVVRGHY	NCP	0.045
DDEVQVVRGHY;LDKSFF	NCP	27.124
LDKSFF;KVKTFHE	NCP	40.194
KVKTFHE;PSKETLP	NCP	14.583
PSKETLP;TLYFKDEPL	NCP	24.242
TLYFKDEPL;AEGRLVNLG	NCP	3.177
AEGRLVNLG;LTRLTEKQAQY	NCP	3.207
LTRLTEKQAQY;LGMSREGPFKPDHY	NCP	0.033
LGMSREGPFKPDHY;MSREGPFKPDHYRY	NCP	0.006
MSREGPFKPDHYRY;EGPFKPDH	NCP	0.009
EGPFKPDH;GVEYLT	NCP	39.967
GVEYLT;LSKKHPSLV	NCP	1.472
LSKKHPSLV;GDAVVKK	NCP	12.112
GDAVVKK;PLFKSLP	NCP	3.389
PLFKSLP;AADPPK	NCP	39.913
AADPPK;TSSAVLFH	NCP	2.676
TSSAVLFH;KCKQNAK	NCP	17.532
KCKQNAK;YSPYKK	NCP	37.863
YSPYKK;DVPNGM	NCP	22.332
DVPNGM;SLPLPNFS	NCP	9.413
SLPLPNFS;DASDDLDDLNFFNQK	NCP	8.826
DASDDLDDLNFFNQK;PVAPP	NCP	22.644
PVAPP;HAGLQSY	NCP	3.15
HAGLQSY;RTYLLPP	NCP	39.148
RTYLLPP;NFLYN	NCP	33.543
NFLYN;PAAPL	NCP	8.211
PAAPL;PCQLVR	NCP	33.618
PCQLVR;GKEHLLP	NCP	10.664
GKEHLLP;LPVFLPLD	NCP	6.882
LPVFLPLD;LSSSMYL	NCP	18.724
LSSSMYL;HLDPLLK	NCP	1.727
HLDPLLK;KRQAEDGH	NCP	15.201
KRQAEDGH;EKPLF	NCP	2.655
EKPLF;ADSFK	NCP	109.648
ADSFK;FPVHGLR	NCP	8.468
FPVHGLR;RDLTDY	NCP	148.795
RDLTDY;KKPAPPP	NCP	230.603
KKPAPPP;LVPSP	NCP	33.255
LVPSP;GPETFGY	NCP	9.45

GPETFGY;LHVPPPPP	NCP	23.193
LHVPPPPP;EVFGKLFG	NCP	41.028
EVFGKLFG;HALELVT	NCP	137.014
HALELVT;APPKAYT	NCP	16.757
APPKAYT;PPNRSPPPP	NCP	123.012
PPNRSPPPP;PPPPPPPSRPP	NCP	0.018
PPPPPPPSRPP;LVFMT	NCP	21.697
LVFMT;VGMLL	NCP	64.766
VGMLL;PVVVRP	NCP	59.823
PVVVRP;LDRETNL	NCP	17.806
LDRETNL;KKPGPTK	NCP	12.884
KKPGPTK;DPSKPPR	NCP	51.897
DPSKPPR;AAWLGRE	NCP	9.777
AAWLGRE;FVCFNK	NCP	97.314
FVCFNK;LTAALFY	NCP	76.959
LTAALFY;KDAPG	NCP	86.352
KDAPG;TSHKAK	NCP	52.905
TSHKAK;KPGACAK	NCP	20.992
KPGACAK;KEALGLRT	NCP	76.733
KEALGLRT;LVPHLLPPPP	NCP	4.644
LVPHLLPPPP;LEYLT	NCP	11.527
LEYLT;KPDNMK	NCP	1.23
KPDNMK;GEVHKR	NCP	82.209
GEVHKR;GVGQRVK	NCP	92.639
GVGQRVK;AFLVYL	NCP	6.051
AFLVYL;VVHKVM	NCP	110.326
VVHKVM;LDGWQL	NCP	44.804
LDGWQL;FLLKKT	NCP	73.308
FLLKKT;SPLLHPR	NCP	84.864
SPLLHPR;LSYCNSL	NCP	134.551
LSYCNSL;KHLPNVK	NCP	33.806
KHLPNVK;LLPSL	NCP	2.29
LLPSL;MQALGC	NCP	7.007
MQALGC;KSNSPWR	NCP	27.789
KSNSPWR;MTFAPR	NCP	57.567
MTFAPR;ANFSGLR	NCP	42.343
ANFSGLR;LANALAH	NCP	42.21
LANALAH;LFGHAPK	NCP	21.777
LFGHAPK;TDLTPF	NCP	90.768
TDLTPF;TDRGLDR	NCP	0.053
TDRGLDR;EPKTEEL	NCP	17.914
EPKTEEL;PAELPLK	NCP	15.669
PAELPLK;EHLSENL	NCP	23.111
EHLSENL;KPLTVSF	NCP	9.771
KPLTVSF;LPEKPL	NCP	41.005
LPEKPL;TCVQCRKP	NCP	3.812
TCVQCRKP;LQPGQGR	NCP	30.771
LQPGQGR;ADPAP	NCP	5.868
ADPAP;QLPLEQV	NCP	7.917
QLPLEQV;PGLRPP	NCP	2.359
PGLRPP;QKNLLKELK	NCP	78.525

QKNLLKELK;WFEPK	NCP	16.481
WFEPK;VPPPLPP	NCP	35.127
VPPPLPP;LLLSQSPF	NCP	113.659
LLLSQSPF;KDFRGAP	NCP	50.501
KDFRGAP;GLPFFSL	NCP	16.005
GLPFFSL;VVADTEL	NCP	70.096
VVADTEL;EAFVRHLL	NCP	5.688
EAFVRHLL;LALEEVD	NCP	4.79
LALEEVD;MPGVLLR	NCP	23.982
MPGVLLR;GPAMKK	NCP	49.672
GPAMKK;AVRPPPAL	NCP	6.65
AVRPPPAL;GFLSL	NCP	24.577
GFLSL;GLEYKP	NCP	19.931
GLEYKP;AGLEVLFA	NCP	72.201
AGLEVLFA;PFEVPRP	NCP	29.352
PFEVPRP;LPPGAVPP	NCP	7.938
LPPGAVPP;PVVPV	NCP	186.099
PVVPV;AAAGLAQLP	NCP	3.34
AAAGLAQLP;DHHKAVA	NCP	13.691
DHHKAVA;GLRPPR	NCP	3.993
GLRPPR;LAELK	NCP	4.644
LAELK;KNNKPF	NCP	104.95
KNNKPF;ELSTPP	NCP	16.655
ELSTPP;ATKLLR	NCP	0.954
ATKLLR;GPAEPKRP	NCP	67.918
GPAEPKRP;LSAALKK	NCP	9.611
LSAALKK;VDRRVFP	NCP	99.645
VDRRVFP;LFVVTVT	NCP	76.834
LFVVTVT;VYALDF	NCP	15.903
VYALDF;LKPVLLGLK	NCP	15.422
LKPVLLGLK;LAKKAAL	NCP	12.04
LAKKAAL;LVMSPK	NCP	10.872
LVMSPK;LAATL	NCP	21.833
LAATL;LLDLVALP	NCP	46.788
LLDLVALP;KLNLQP	NCP	68.065
KLNLQP;LDEFQK	NCP	73.091
LDEFQK;YHPRST	NCP	17.778
YHPRST;VDARALT	NCP	17.292
VDARALT;LPPLPPPPP	NCP	3.715
LPPLPPPPP;VGVAAH	NCP	9.555
VGVAAH;MALSEEK	NCP	18.315
MALSEEK;KQGGSPK	NCP	23.22
KQGGSPK;PNLVLF	NCP	3.609
PNLVLF;SLHQPLQ	NCP	30.285
SLHQPLQ;VREPVLS	NCP	12.404
VREPVLS;AVPEDAP	NCP	58.194
AVPEDAP;EVFSPKGPR	NCP	28.192
EVFSPKGPR;QTKPDL	NCP	51.614
QTKPDL;PVVVGL	NCP	2.532
PVVVGL;SVGAALK	NCP	61.78
SVGAALK;QPVGLLSK	NCP	10.46

QPVGLLSK;EGVLLVK	NCP	12.343
EGVLLVK;KPVYKP	NCP	93.803
KPVYKP;RNKFVV	NCP	25.549
RNKFVV;TGYGMRP	NCP	58.271
TGYGMRP;YGGLF	NCP	5.017
YGGLF;EPQFKP	NCP	44.951
EPQFKP;AAPLRGK	NCP	1.107
AAPLRGK;ANKGPSYG	NCP	0.394
ANKGPSYG;KGMGVQ	NCP	57.012
KGMGVQ;RSLPGAAP	NCP	79.373
RSLPGAAP;PAHVPAR	NCP	46.417
PAHVPAR;KGLEVTP	NCP	12.743
KGLEVTP;PMGRPP	NCP	29.121
PMGRPP;ACGLVASN	NCP	9.234
ACGLVASN;LNTALAT	NCP	59.961
LNTALAT;RALGTQGP	NCP	35.638
RALGTQGP;KELDP	NCP	4.11
KELDP;MELMSPV	NCP	27.372
MELMSPV;NEPLYK	NCP	55.82
NEPLYK;ESRLLTK	NCP	10.185
ESRLLTK;FVLFY	NCP	102.909
FVLFY;HNVKVF	NCP	19.776
HNVKVF;AKVEQVLSLEPQHELK	NCP	3.768
AKVEQVLSLEPQHELK;VLSLEPQHELKF	NCP	0.015
VLSLEPQHELKF;FMGLPK	NCP	0.217
FMGLPK;CEREQ	NCP	27.888
CEREQ;PAELHRP	NCP	6.029
PAELHRP;MHCNK	NCP	5.472
MHCNK;LDDTEVKK	NCP	3.621
LDDTEVKK;KRWQVP	NCP	12.271
KRWQVP;SLGASPPK	NCP	22.415
SLGASPPK;PSLTPPPQ	NCP	27.864
PSLTPPPQ;LQELPTK	NCP	31.761
LQELPTK;HVVPPK	NCP	17.253
HVVPPK;EGGLL	NCP	37.99
EGGLL;DDRLPR	NCP	1.962
DDRLPR;VSVGLVE	NCP	77.786
VSVGLVE;KLLKTE	NCP	3.316
KLLKTE;KDSGAVP	NCP	38.934
KDSGAVP;ELPYKPSP	NCP	33.707
ELPYKPSP;ERGLFGA	NCP	16.254
ERGLFGA;PPALPPPPPP	NCP	11.877
PPALPPPPPP;VFPLHSF	NCP	13.66
VFPLHSF;DGPPL	NCP	42.479
DGPPL;WFHLTK	NCP	38.086
WFHLTK;GPPPPPPD	NCP	15.497
GPPPPPPD;ATHRLLL	NCP	1.041
ATHRLLL;QDLLAEKVL	NCP	17.292
QDLLAEKVL;VGGPTVE	NCP	13.234
VGGPTVE;PQHGLRP	NCP	65.534
PQHGLRP;DLEPGTM	NCP	21.717

DLEPGTM;NAVLALHQLVL	NCP	0.489
NAVLALHQLVL;MVPFPRLHF	NCP	0.219
MVPFPRLHF;FMPGFAPL	NCP	0.027
FMPGFAPL;MPGFAPLT	NCP	0.003
MPGFAPLT;DVLGGSY	NCP	22.453
DVLGGSY;VSAGLLA	NCP	63.5
VSAGLLA;KYKAP	NCP	113.223
KYKAP;SVLRGEAF	NCP	86.455
SVLRGEAF;ARYAPP	NCP	41.841
ARYAPP;DLKLP	NCP	6.638
DLKLP;GVLPK	NCP	12.309
GVLPK;GVFTTFL	NCP	24.795
GVFTTFL;NFTDLE	NCP	47.05
NFTDLE;RPVVHV	NCP	6.224
RPVVHV;PPRPSHA	NCP	6.744
PPRPSHA;EFDDLPF	NCP	136.696
EFDDLPF;WPDARG	NCP	20.058
WPDARG;QVLLA	NCP	2.702
QVLLA;YKNPSK	NCP	10.789
YKNPSK;KTMVSR	NCP	30.195
KTMVSR;QLLSQGLR	NCP	11.171
QLLSQGLR;KYLDKPV	NCP	94.306
KYLDKPV;LLVPPPR	NCP	14.798
LLVPPPR;LTLELNQV	NCP	88.738
LTLELNQV;KPWGSR	NCP	6.695
KPWGSR;YNVEAL	NCP	52.12
YNVEAL;FPSLL	NCP	41.604
FPSLL;GFPASGP	NCP	25.569
GFPASGP;MSPKLL	NCP	32.048
MSPKLL;VDPLLLR	NCP	23.887
VDPLLLR;FLEWLLP	NCP	4.664
FLEWLLP;PLLSA	NCP	1.006
PLLSA;KEDFGF	NCP	50.552
KEDFGF;VGPVPGSP	NCP	12.819
VGPVPGSP;KEPLSH	NCP	68.262
KEPLSH;CDGSDC	NCP	20.896
CDGSDC;TAFPALK	NCP	71.868
TAFPALK;ESGCCC	NCP	45.146
ESGCCC;RGLAGP	NCP	79.14
RGLAGP;HERTTPK	NCP	27.937
HERTTPK;TVSDSLP	NCP	66.554
TVSDSLP;KQNLPF	NCP	20.875
KQNLPF;ASLSKLRK	NCP	36.855
ASLSKLRK;GKPLEM	NCP	7.499
GKPLEM;KPTAVPP	NCP	7.872
KPTAVPP;LGKKELP	NCP	34.048
LGKKELP;RTPPGPK	NCP	12.194
RTPPGPK;KPHKKM	NCP	34.429
KPHKKM;KYKKAP	NCP	28.245
KYKKAP;PPVPAP	NCP	62.861
PPVPAP;NHHVPR	NCP	26.748

NHHVPR;KDQHVP	NCP	3.784
KDQHVP;APGLLPR	NCP	4.634
APGLLPR;KGLAARP	NCP	0.072
KGLAARP;SAEKELNL	NCP	4.974
SAEKELNL;VSYLL	NCP	58.578
VSYLL;ANFLDC	NCP	98.425
ANFLDC;NVPMLLL	NCP	0.041
NVPMLLL;VPLLWFL	NCP	11.403
VPLLWFL;CTTTVAF	NCP	97.824
CTTTVAF;ATPRVM	NCP	38.922
ATPRVM;SQAPLLR	NCP	82.488
SQAPLLR;LSLNV	NCP	29.647
LSLNV;PPMVH	NCP	21.162
PPMVH;TTLLL	NCP	0.144
TTLLL;VPACL	NCP	16.667
VPACL;LHGPQPP	NCP	25.831
LHGPQPP;FSLAPP	NCP	86.943
FSLAPP;DLVKTLK	NCP	18.84
DLVKTLK;KHQEPPR	NCP	4.805
KHQEPPR;AVGPPLH	NCP	1.693
AVGPPLH;MEVSEFL	NCP	12.822
MEVSEFL;MNGEEEFF	NCP	38.81
MNGEEEFF;DPDLTPF	NCP	21.069
DPDLTPF;MQNDAGEFVDL	NCP	82.119
MQNDAGEFVDL;VPLVEP	NCP	19.74
VPLVEP;AAFNLR	NCP	32.769
AAFNLR;WRTPAP	NCP	26.682
WRTPAP;ALATLKT	NCP	19.02
ALATLKT;VEAPPTR	NCP	16.899
VEAPPTR;KTGLELW	NCP	21.139
KTGLELW;ADAWPH	NCP	8
ADAWPH;GPGHPPGP	NCP	10.527
GPGHPPGP;KEFTMGP	NCP	14.611
KEFTMGP;RAAPFSLL	NCP	33.044
RAAPFSLL;WGDDFVG	NCP	29.118
WGDDFVG;ASAFGAPR	NCP	33.222
ASAFGAPR;APLVTP	NCP	53.358
APLVTP;PPHMPPP	NCP	2.661
PPHMPPP;LLDNMKK	NCP	14.58
LLDNMKK;YVETKPK	NCP	45.009
YVETKPK;TVGRKPPG	NCP	6.828
TVGRKPPG;PAAMPAK	NCP	4.342
PAAMPAK;ELRVTH	NCP	26.138
ELRVTH;CRRHFLV	NCP	9.912
CRRHFLV;AGWLGH	NCP	11.805
AGWLGH;KCPWPP	NCP	4.981
KCPWPP;GDSPLVL	NCP	3.305
GDSPLVL;AWSLP	NCP	22.272
AWSLP;LPSPP	NCP	30.508
LPSPP;LHGPSFP	NCP	9.516
LHGPSFP;FGPPNTK	NCP	41.288

FGPPNTK;VGGLPLCL	NCP	25.227
VGGLPLCL;GPHRGGP	NCP	10.098
GPHRGGP;SVGPAPCN	NCP	6.946
SVGPAPCN;VPAVLPTSP	NCP	17.952
VPAVLPTSP;TAEVA	NCP	41.843
TAEVA;NTAPLLR	NCP	10.212
NTAPLLR;AVTAQQ	NCP	27.073
AVTAQQ;LVHELR	NCP	51.233
LVHELR;NSRQAPR	NCP	47.586
NSRQAPR;LEAAYV	NCP	6.972
LEAAYV;SGPVRQP	NCP	38.706
SGPVRQP;KTAHRK	NCP	33.409
KTAHRK;FPSVLG	NCP	12.569
FPSVLG;KKTQETLSQAGQK	NCP	38.538
KKTQETLSQAGQK;PDMRPP	NCP	10.266
PDMRPP;HLYPGAP	NCP	17.412
HLYPGAP;FNCCC	NCP	6.783
FNCCC;GQLVTSK	NCP	5.374
GQLVTSK;RVTEPV	NCP	3.636
RVTEPV;AALTSKY	NCP	11.391
AALTSKY;RSPPELP	NCP	79.52
RSPPELP;HTFKTH	NCP	9.862
HTFKTH;GPPVVH	NCP	29.378
GPPVVH;FTKQKT	NCP	27.412
FTKQKT;VTYVYKV	NCP	4.722
VTYVYKV;QKAQK	NCP	4.869
QKAQK;AHAWPA	NCP	20.972
AHAWPA;VPAPPAAP	NCP	12.414
VPAPPAAP;AGKNLE	NCP	23.521
AGKNLE;MSGPPPPPP	NCP	11.798
MSGPPPPPP;PVAMLGVP	NCP	92.97
PVAMLGVP;QPWLPSP	NCP	85.138
QPWLPSP;KDKLEVVK	NCP	51.255
KDKLEVVK;KPRGGPP	NCP	25.479
KPRGGPP;LPVGSYL	NCP	73.337
LPVGSYL;ETPSTATK	NCP	42.271
ETPSTATK;AHLGEPP	NCP	33.497
AHLGEPP;WPGGK	NCP	0.463
WPGGK;DCFKAG	NCP	19.781
DCFKAG;MDRRPKY	NCP	11.362
MDRRPKY;LGKRTDL	NCP	13.574
LGKRTDL;KPGGKPP	NCP	25.258
KPGGKPP;ASVQVQRK	NCP	3.704
ASVQVQRK;RLALYL	NCP	103.851
RLALYL;ETGAP	NCP	9.486
ETGAP;AEEGRGF	NCP	147.198
AEEGRGF;LFNEKNF	NCP	14.161
LFNEKNF;EHMVLPK	NCP	21.552
EHMVLPK;NLAGALV	NCP	1.532
NLAGALV;VEEGPLP	NCP	0.324
VEEGPLP;TPKTLPK	NCP	114.631

TPKTLPK;EPSGAPR	NCP	49.574
EPSGAPR;LERML	NCP	93.87
LERML;VLLHVLFEHAVGYALLALK	NCP	125.484
VLLHVLFEHAVGYALLALK;VLLGVGDPK	NCP	1.236
VLLGVGDPK;GTGSLPK	NCP	3.504
GTGSLPK;DTSPGAP	NCP	134.418
DTSPGAP;ADFLP	NCP	121.582
ADFLP;LSGKPPR	NCP	12.812
LSGKPPR;PKELKPK	NCP	99.504
PKELKPK;VMTPTP	NCP	0.486
VMTPTP;NEEFTVK	NCP	17.467
NEEFTVK;RYYDPK	NCP	4.859
RYYDPK;PAATGSPP	NCP	9.876
PAATGSPP;LPRSPWP	NCP	12.42
LPRSPWP;LPGPVDP	NCP	2.115
LPGPVDP;ASAFPGR	NCP	4.917
ASAFPGR;LSLVYAW	NCP	7.573
LSLVYAW;LAVGLSH	NCP	17.789
LAVGLSH;CDNPSNF	NCP	32.818
CDNPSNF;STCVSLPK	NCP	7.286
STCVSLPK;PLVPPPPP	NCP	17.859
PLVPPPPP;TPVLLL	NCP	53.983
TPVLLL;VSDDML	NCP	5.304
VSDDML;LWTYKK	NCP	28.269
LWTYKK;APGAP	NCP	7.611
APGAP;LSDPNPL	NCP	12.065
LSDPNPL;LNVDVP	NCP	53.953
LNVDVP;KDCEGK	NCP	77.391
KDCEGK;LRLLGPF	NCP	42.305
LRLLGPF;VYQVSR	NCP	90.261
VYQVSR;YWELLD	NCP	14.998
YWELLD;DVRGPR	NCP	30.333
DVRGPR;LHQELGL	NCP	58.013
LHQELGL;QPNLQR	NCP	53.467
QPNLQR;MLAVPEMD	NCP	26.132
MLAVPEMD;LSRTASP	NCP	30.972
LSRTASP;VLLSEKK	NCP	41.395
VLLSEKK;YPPRDP	NCP	40.425
YPPRDP;PPPSSAPPR	NCP	34.082
PPPSSAPPR;VYGAVSR	NCP	5.808
VYGAVSR;KLYPLE	NCP	4.176
KLYPLE;PDLLQ	NCP	0.457
PDLLQ;TVSVGY	NCP	25.877
TVSVGY;ADGAPQPR	NCP	107.466
ADGAPQPR;NLPVLL	NCP	70.545
NLPVLL;CRHLLLT	NCP	4.197
CRHLLLT;ALAAGY	NCP	7.944
ALAAGY;PPPPNPPPPP	NCP	143.718
PPPPNPPPPP;LWLLLTDL	NCP	102.733
LWLLLTDL;ARPKMT	NCP	60.273
ARPKMT;APLLSHPP	NCP	34.089

APLLSHPP;FHGQWP	NCP	13.541
FHGQWP;SSMLLQQ	NCP	38.98
SSMLLQQ;VESAVLR	NCP	18.434
VESAVLR;DLAFSPKEN	NCP	1.803
DLAFSPKEN;KRRCAP	NCP	24.145
KRRCAP;KKCRNQ	NCP	50.181
KKCRNQ;PQAYK	NCP	31.085
PQAYK;VPRVTSL	NCP	14.862
VPRVTSL;YDPEA	NCP	17.755
YDPEA;PKPGHFK	NCP	110.28
PKPGHFK;TVLPLKD	NCP	44.729
TVLPLKD;VGAAAK	NCP	39.934
VGAAAK;LAEHAWYLL	NCP	67.224
LAEHAWYLL;EMFGELK	NCP	21.837
EMFGELK;HGCPWH	NCP	37.803
HGCPWH;CCCCSF	NCP	38.154
CCCCSF;DWGTF	NCP	7.32
DWGTF;QWPLK	NCP	5.769
QWPLK;AGEELLK	NCP	130.095
AGEELLK;KDNGRLK	NCP	24.057
KDNGRLK;KSPVPLL	NCP	27.975
KSPVPLL;PAAKVH	NCP	21.221
PAAKVH;APLGKV	NCP	123.792
APLGKV;NDSRLKL	NCP	46.77
NDSRLKL;PAPERK	NCP	91.228
PAPERK;VPSWESF	NCP	56.282
VPSWESF;FPLVPG	NCP	136.159
FPLVPG;AAAYKPR	NCP	4.103
AAAYKPR;QARLKV	NCP	1.2
QARLKV;KGYEFK	NCP	155.275
KGYEFK;KPVPEFK	NCP	66.26
KPVPEFK;KRLTPF	NCP	40.063
KRLTPF;LGSKYR	NCP	125.621
LGSKYR;EAWGC	NCP	79.227
EAWGC;LVYRTH	NCP	1.672
LVYRTH;GVHRYR	NCP	136.16
GVHRYR;DHYWY	NCP	53.95
DHYWY;KMGKPQ	NCP	55.202
KMGKPQ;SEPFMF	NCP	49.255
SEPFMF;KPGEPLP	NCP	2.019
KPGEPLP;MSAGPK	NCP	2.741
MSAGPK;AAGVNK	NCP	28.366
AAGVNK;EVRPC	NCP	10.545
EVRPC;KSESPQ	NCP	35.328
KSESPQ;ALKQV	NCP	46.746
ALKQV;AALRVFL	NCP	1.875
AALRVFL;QKDPKE	NCP	24.539
QKDPKE;NGHLTVK	NCP	68.821
NGHLTVK;GPLKYGP	NCP	77.312
GPLKYGP;EADLDLR	NCP	27.004
EADLDLR;SLLHY	NCP	3.819

SLLHY;DDPGGK	NCP	20.844
DDPGGK;DLDLSK	NCP	32.459
DLDLSK;DNTLSVH	NCP	7.486
DNTLSVH;ALVPPLPP	NCP	4.637
ALVPPLPP;LPNAPLE	NCP	130.963
LPNAPLE;QLGSVLP	NCP	49.694
QLGSVLP;AADFLAR	NCP	22.117
AADFLAR;EALTTLF	NCP	21.176
EALTTLF;CVVKPPCD	NCP	55.101
CVVKPPCD;KPPVVDR	NCP	3.951
KPPVVDR;LPRTTPP	NCP	18.24
LPRTTPP;KPFYG	NCP	18.768
KPFYG;ATFKLCK	NCP	15.366
ATFKLCK;PSLADPP	NCP	106.515
PSLADPP;NNQPC	NCP	16.942
NNQPC;VGPGSTP	NCP	6.633
VGPGSTP;KMKGAP	NCP	4.256
KMKGAP;KKCAAQQ	NCP	26.695
KKCAAQQ;SLQVVL	NCP	65.427
SLQVVL;AQPRPL	NCP	24.339
AQPRPL;VSSFY	NCP	18.602
VSSFY;VLPSL	NCP	55.708
VLPSL;LLYYAA	NCP	31.766
LLYYAA;VEGFLVR	NCP	18.192
VEGFLVR;KPKAME	NCP	10.629
KPKAME;KNPSVPK	NCP	76.666
KNPSVPK;SSSLMDSF	NCP	48.941
SSSLMDSF;ALRYLNF	NCP	83.271
ALRYLNF;ATPLSPTVT	NCP	0.03
ATPLSPTVT;VTTPSPK	NCP	34.345
VTTPSPK;GVLTTL	NCP	11.24
GVLTTL;KGPKHYR	NCP	24.658
KGPKHYR;ADLKLAY	NCP	90.45
ADLKLAY;KAGFLEN	NCP	34.262
KAGFLEN;KQLPGPP	NCP	166.314
KQLPGPP;SLDFLK	NCP	19.009
SLDFLK;HLPPPPP	NCP	4.929
HLPPPPP;SVAEK	NCP	56.909
SVAEK;LPKGPN	NCP	55.077
LPKGPN;VTGFLSW	NCP	63.895
VTGFLSW;LVELELF	NCP	20.228
LVELELF;DDDESAC	NCP	79.333
DDDESAC;LNHDKPK	NCP	36.894
LNHDKPK;KYPLPPPPPL	NCP	34.473
KYPLPPPPPL;KPPAAGGK	NCP	15.452
KPPAAGGK;PWAEAE	NCP	95.55
PWAEAE;KGFMLR	NCP	13.561
KGFMLR;PGPVAPPPAP	NCP	8.144
PGPVAPPPAP;NKNLLPP	NCP	0.186
NKNLLPP;SRTRGPP	NCP	6.897
SRTRGPP;LRAAVP	NCP	12.63

LRAAVP;PGRVEAP	NCP	58.576
PGRVEAP;AALCPAA	NCP	17.393
AALCPAA;LSFVPPK	NCP	15.124
LSFVPPK;SSSQSGFK	NCP	19.052
SSSQSGFK;LPNPPPPPP	NCP	127.902
LPNPPPPPP;VPVRVLM	NCP	17.673
VPVRVLM;KDPLLPP	NCP	6.909
KDPLLPP;KEPTHP	NCP	10.072
KEPTHP;DVLAADAL	NCP	15.806
DVLAADAL;DPPKPR	NCP	38.152
DPPKPR;TVRLEPT	NCP	27.273
TVRLEPT;KNTTEY	NCP	11.196
KNTTEY;RPAAVYR	NCP	13.908
RPAAVYR;VVGHFLL	NCP	14.673
VVGHFLL;WGLEPL	NCP	102.635
WGLEPL;ALGELLEA	NCP	63.583
ALGELLEA;VLAAGAVL	NCP	13.365
VLAAGAVL;GVPSLKR	NCP	27.675
GVPSLKR;SLDPENPTK	NCP	155.291
SLDPENPTK;ATKYLKDVTLKK	NCP	0.117
ATKYLKDVTLKK;SSVNCFP	NCP	12.912
SSVNCFP;AGLAFPP	NCP	87.493
AGLAFPP;KLLYPP	NCP	53.058
KLLYPP;YSGSMSL	NCP	14.769
YSGSMSL;HSSQPPK	NCP	9.953
HSSQPPK;VGPQP	NCP	8.628
VGPQP;EMMPHW	NCP	29.677
EMMPHW;LPLAVP	NCP	8.564
LPLAVP;RGAAPLR	NCP	157.015
RGAAPLR;RAPAAAAK	NCP	84.008
RAPAAAAK;ASWLP	NCP	50.536
ASWLP;KPLSYDK	NCP	15.075
KPLSYDK;SLLPLP	NCP	39.969
SLLPLP;MQALCK	NCP	20.601
MQALCK;LPVDPVP	NCP	24.006
LPVDPVP;VTFPGGV	NCP	10.679
VTFPGGV;FGPPNR	NCP	2.799
FGPPNR;MDGHDSPQTPKGS	NCP	0.6
MDGHDSPQTPKGS;PDGPVK	NCP	30.019
PDGPVK;PSELPPS	NCP	22.074
PSELPPS;VPAALLL	NCP	0.635
VPAALLL;TLLFQPVAELKK	NCP	0.282
TLLFQPVAELKK;ALPHALRM	NCP	30.274
ALPHALRM;APTFLER	NCP	38.079
APTFLER;CNKDLVP	NCP	27.594
CNKDLVP;HTLPHV	NCP	75.44
HTLPHV;LTTLMDM	NCP	21.21
LTTLMDM;PVRLLPP	NCP	18.676
PVRLLPP;KKVHGGP	NCP	9.182
KKVHGGP;APAFPQ	NCP	50.08
APAFPQ;GPAHVFV	NCP	10.352

GPAHVFV;SGYVPR	NCP	92.413
SGYVPR;PVKADP	NCP	13.641
PVKADP;EQWYEL	NCP	10.703
EQWYEL;DTVHLFK	NCP	8.269
DTVHLFK;LGYAQP	NCP	49.325
LGYAQP;AGSELLEL	NCP	45.525
AGSELLEL;PLGVCA	NCP	12.142
PLGVCA;LGKERK	NCP	32.891
LGKERK;LLKEPLL	NCP	42.454
LLKEPLL;LGPPFLT	NCP	53.265
LGPPFLT;LAHELLTN	NCP	45.24
LAHELLTN;LFPVP	NCP	0.656
LFPVP;DLPVDT	NCP	78.732
DLPVDT;PPPPPGRP	NCP	37
PPPPPGRP;HALMLR	NCP	1.467
HALMLR;AFVPLSG	NCP	1.326
AFVPLSG;VEHLAF	NCP	0.932
VEHLAF;VTTKPF	NCP	12.069
VTTKPF;PLPGLP	NCP	81.109
PLPGLP;KVLQF	NCP	48.296
KVLQF;PVLALALR	NCP	24.798
PVLALALR;ELLDNVP	NCP	7.039
ELLDNVP;PHKVPPP	NCP	43.227
PHKVPPP;LLGAVL	NCP	188.186
LLGAVL;RLSPTLK	NCP	53.268
RLSPTLK;YPPPPPPPPP	NCP	15.298
YPPPPPPPPP;KGDMPVP	NCP	82.392
KGDMPVP;ECHPYLNQS	NCP	17.432
ECHPYLNQS;AALGAQLLSEW	NCP	0.075
AALGAQLLSEW;RPNFRVR	NCP	28.819
RPNFRVR;KGAVNF	NCP	37.968
KGAVNF;KDNFPPPPP	NCP	13.934
KDNFPPPPP;EVFGKLF	NCP	47.146
EVFGKLF;SKAGGPL	NCP	66.413
SKAGGPL;KALYE	NCP	85.957
KALYE;LLVDTAPL	NCP	40.484
LLVDTAPL;ESHGPAGP	NCP	25.72
ESHGPAGP;MEFDVT	NCP	68.331
MEFDVT;LWVKKPP	NCP	20.586
LWVKKPP;ARLGLTV	NCP	5.25
ARLGLTV;DPAVGPR	NCP	24.89
DPAVGPR;YPPLLL	NCP	37.407
YPPLLL;AAGPVHR	NCP	19.768
AAGPVHR;ERGPTVP	NCP	10.718
ERGPTVP;LLKPLM	NCP	61.594
LLKPLM;TPGHYPR	NCP	8.984
TPGHYPR;KPAGVRK	NCP	4.342
KPAGVRK;PGRPLPP	NCP	16.295
PGRPLPP;KEEVLKP	NCP	44.929
KEEVLKP;SPATLLAH	NCP	13.811
SPATLLAH;QEYDFFK	NCP	87.093

QEYDFFK;DLWEKQS	NCP	18.997
DLWEKQS;QKAPTLR	NCP	25.574
QKAPTLR;LGPSR	NCP	32.212
LGPSR;VPGLAHLK	NCP	25.703
VPGLAHLK;KVPMTPP	NCP	2.962
KVPMTPP;AWELNKFP	NCP	39.879
AWELNKFP;PGAQPPR	NCP	6.062
PGAQPPR;QVVKKPG	NCP	4.306
QVVKKPG;PSFLSVQ	NCP	46.035
PSFLSVQ;RSFRLL	NCP	8.141
RSFRLL;KSGPVPP	NCP	10.402
KSGPVPP;LLNVSK	NCP	3.483
LLNVSK;KPALPKP	NCP	4.785
KPALPKP;AAFLLLP	NCP	76.805
AAFLLLP;VEEWVK	NCP	10.155
VEEWVK;KDDVGDVDVVVK	NCP	10.422
KDDVGDVDVVVK;LLACLTPK	NCP	108.696
LLACLTPK;GPVSLHLSP	NCP	24.489
GPVSLHLSP;MTKGPS	NCP	46.882
MTKGPS;HLGDHLP	NCP	8.271
HLGDHLP;LDLFTGKK	NCP	7.962
LDLFTGKK;GPATFLQ	NCP	26.613
GPATFLQ;EPEPAL	NCP	20.78
EPEPAL;ELGQGLR	NCP	1.038
ELGQGLR;TRFNPLP	NCP	9.912
TRFNPLP;LLLKVV	NCP	41.373
LLLKVV;GVEDLPP	NCP	27.228
GVEDLPP;QTHHLP	NCP	35.364
QTHHLP;LCGLL	NCP	76.248
LCGLL;LQSHESPK	NCP	7.095
LQSHESPK;VAGVPPR	NCP	16.329
VAGVPPR;VGWKEVR	NCP	31.992
VGWKEVR;LEPLK	NCP	12.639
LEPLK;VAALEYL	NCP	38.085
VAALEYL;KLGPPPSP	NCP	31.839
KLGPPPSP;SAKDYFFK	NCP	8.019
SAKDYFFK;KPFGKVT	NCP	14.643
KPFGKVT;EGLDPLW	NCP	22.503
EGLDPLW;PVASW	NCP	18.612
PVASW;RMLPLDT	NCP	14.361
RMLPLDT;VEKVGPK	NCP	40.767
VEKVGPK;NPSFAPV	NCP	0.984
NPSFAPV;KKVKPGK	NCP	12.274
KKVKPGK;WSVCKK	NCP	17.259
WSVCKK;QMDVPL	NCP	4.656
QMDVPL;RGHPTAV	NCP	57.707
RGHPTAV;KNLSSLK	NCP	30.963
KNLSSLK;HMPVNPK	NCP	25.479
HMPVNPK;VTLMPK	NCP	55.977
VTLMPK;SAPTTRL	NCP	12.456
SAPTTRL;VLSKDNW	NCP	0.27

VLSKDNW;KKPLLLSW	NCP	36.184
KKPLLLSW;LPPLDL	NCP	10.577
LPPLDL;KAHAAPR	NCP	6.937
KAHAAPR;KGDLAEHP	NCP	6.077
KGDLAEHP;PVGPGLPP	NCP	58.012
PVGPGLPP;SSGLFPF	NCP	193.794
SSGLFPF;EPRFVLLW	NCP	167.474
EPRFVLLW;KTPLAPHV	NCP	2.758
KTPLAPHV;PSDPKHR	NCP	7.266
PSDPKHR;KNLLPAA	NCP	17.741
KNLLPAA;RKLTQEN	NCP	0.655
RKLTQEN;LANGKMK	NCP	9.461
LANGKMK;KRGPEPP	NCP	35.074
KRGPEPP;EEQRLLA	NCP	22.53
EEQRLLA;TATFPA	NCP	31.475
TATFPA;KEDPVEK	NCP	15.426
KEDPVEK;KHKGK	NCP	20.17
KHKGK;VLGLLAF	NCP	8.01
VLGLLAF;TSLKPAAP	NCP	25.953
TSLKPAAP;ANKGPLQ	NCP	5.651
ANKGPLQ;VKRTNPK	NCP	29.779
VKRTNPK;CTVLWLR	NCP	15.548
CTVLWLR;QPRPPPAPP	NCP	4.558
QPRPPPAPP;QAAKPRK	NCP	3.686
QAAKPRK;GPDGKTR	NCP	49.137
GPDGKTR;LSSVLAK	NCP	0.556
LSSVLAK;NCETF	NCP	69.725
NCETF;LKLSHPVV	NCP	2.155
LKLSHPVV;ASDRLSLQ	NCP	33.821
ASDRLSLQ;RKVVQY	NCP	3.276
RKVVQY;LSRTHLPP	NCP	42.559
LSRTHLPP;KFYLPP	NCP	8.315
KFYLPP;YGPGY	NCP	4.387
YGPGY;FGHPSF	NCP	34.935
FGHPSF;TGGKVP	NCP	4.335
TGGKVP;CHSSLMT	NCP	1.838
CHSSLMT;GPPGPTVP	NCP	22.507
GPPGPTVP;KVVPKK	NCP	29.378
KVVPKK;RHQSSNPQ	NCP	5.02
RHQSSNPQ;LPWGVH	NCP	40.068
LPWGVH;GGAGGLGAL	NCP	31.191
GGAGGLGAL;PVGKLMP	NCP	9.21
PVGKLMP;TQGTLAK	NCP	8.082
TQGTLAK;SGPKFP	NCP	42.831
SGPKFP;AARSVLGP	NCP	1.173
AARSVLGP;GPPPKY	NCP	1.089
GPPPKY;KSTTPP	NCP	16.064
KSTTPP;KSNYA	NCP	29.914
KSNYA;HELTGAP	NCP	0.249
HELTGAP;EVAVF	NCP	0.11
EVAVF;AAGPVP	NCP	11.983

AAGPVP;MLDFAY	NCP	18.72
MLDFAY;VPGRTPP	NCP	24.921
VPGRTPP;RDGPPLR	NCP	7.539
RDGPPLR;HLLMNLE	NCP	65.168
HLLMNLE;AVPKFY	NCP	2.988
AVPKFY;KTGPFP	NCP	82.243
KTGPFP;TVLLPL	NCP	0.648
TVLLPL;PDEELAR	NCP	10.107
PDEELAR;KRELKPNEEVTWK	NCP	5.688
KRELKPNEEVTWK;LPPGVA	NCP	88.184
LPPGVA;APPKLNP	NCP	8.728
APPKLNP;PPRDKP	NCP	33.36
PPRDKP;ATMLYR	NCP	18.35
ATMLYR;VVQVYRK	NCP	5.226
VVQVYRK;PQVPPGP	NCP	12.444
PQVPPGP;VTVPPFK	NCP	22.111
VTVPPFK;LATGPPEVP	NCP	31.892
LATGPPEVP;LYGSQYL	NCP	11.826
LYGSQYL;FDYWYT	NCP	0.88
FDYWYT;GRPVTPP	NCP	18.078
GRPVTPP;LLKVLRK	NCP	56.333
LLKVLRK;YKGAP	NCP	13.909
YKGAP;KTLAHHQ	NCP	11.439
KTLAHHQ;YSALGSQRVK	NCP	8.13
YSALGSQRVK;VLLEDPVL	NCP	1.095
VLLEDPVL;MDPKSQRVKLN	NCP	15.846
MDPKSQRVKLN;SNFNHKQLE	NCP	6.288
SNFNHKQLE;VLLEDPVLS	NCP	1.854
VLLEDPVLS;MDPKGQKVKLN	NCP	17.007
MDPKGQKVKLN;DALEFTPF	NCP	3.624
DALEFTPF;RLLVENL	NCP	39.722
RLLVENL;LRDDVVK	NCP	1.813
LRDDVVK;GHVVPLA	NCP	4.557
GHVVPLA;SDVLARK	NCP	12.548
SDVLARK;PEYPFSEEY	NCP	2.803
PEYPFSEEY;VPKLSLP	NCP	0.305
VPKLSLP;KSPLL	NCP	14.521
KSPLL;SENPPLR	NCP	8.328
SENPPLR;LSYKNDL	NCP	48.758
LSYKNDL;GVYLSPR	NCP	11.344
GVYLSPR;QRGVVVL	NCP	17.154
QRGVVVL;LQLDYV	NCP	16.545
LQLDYV;FGGAPS	NCP	29.442
FGGAPS;SVDLCRTWE	NCP	0.954
SVDLCRTWE;KQTPALVVL	NCP	2.691
KQTPALVVL;MDPKGQRVKLN	NCP	18.9
MDPKGQRVKLN;ALEVTKF	NCP	2.334
ALEVTKF;KNLHLDYV	NCP	0.498
KNLHLDYV;ETGKFC	NCP	0.303
ETGKFC;GVSNFNHKQLE	NCP	1.536
GVSNFNHKQLE;YGALGSQRVK	NCP	0.984

YGALGSQRVK;LNLPVLLED	NCP	0.912
LNLPVLLED;NNLQLDYV	NCP	34.41
NNLQLDYV;ECHPYFNQS	NCP	2.937
ECHPYFNQS;NSNMRYNEL	NCP	1.368
NSNMRYNEL;VGHPEYPFVEEY	NCP	2.229
VGHPEYPFVEEY;RPDLLDH	NCP	56.639
RPDLLDH;VPVVGVN	NCP	4.506
VPVVGVN;RNSALLR	NCP	3.786
RNSALLR;YCHAELK	NCP	74.965
YCHAELK;HVNMSR	NCP	16.691
HVNMSR;LPQKTPQ	NCP	53.238
LPQKTPQ;KKKYTT	NCP	40.5
KKKYTT;QPPTML	NCP	89.251
QPPTML;ELVTTLTP	NCP	17.126
ELVTTLTP;HPRGKY	NCP	28.62
HPRGKY;KPEPALQ	NCP	64.461
KPEPALQ;GAVKEW	NCP	24.456
GAVKEW;PVPGSPF	NCP	65.385
PVPGSPF;ERNSRPF	NCP	4.89
ERNSRPF;RDAHTK	NCP	36.051
RDAHTK;KTPPVEGP	NCP	2.344
KTPPVEGP;KEGRTTP	NCP	32.381
KEGRTTP;SHSLLVTL	NCP	33.22
SHSLLVTL;KDKEEVP	NCP	51.699
KDKEEVP;GMPKSH	NCP	25.665
GMPKSH;LMDFVPLL	NCP	25.205
LMDFVPLL;RDQLLTH	NCP	3.849
RDQLLTH;QLLLTTK	NCP	9.319
QLLLTTK;LEKSVGK	NCP	27.963
LEKSVGK;TPPKGPQ	NCP	60.978
TPPKGPQ;LATPRSLA	NCP	55.553
LATPRSLA;LDPNLP	NCP	8.595
LDPNLP;PAAGHWR	NCP	11.655
PAAGHWR;VELLPF	NCP	21.741
VELLPF;RLAVTAK	NCP	7.831
RLAVTAK;PEELTVH	NCP	92.259
PEELTVH;SLSLL	NCP	15.611
SLSLL;APAVM	NCP	36.033
APAVM;EHGGVG	NCP	2.238
EHGGVG;PPNAQPP	NCP	61.053
PPNAQPP;GVLDVTLP	NCP	20.397
GVLDVTLP;TVMYSPP	NCP	11.613
TVMYSPP;LTSKVGN	NCP	18.81
LTSKVGN;GLSYPPTP	NCP	18.361
GLSYPPTP;KAQGHRP	NCP	26.109
KAQGHRP;AAVPEVLQQQEEDRSK	NCP	82.118
AAVPEVLQQQEEDRSK;KSADEVLF	NCP	14.82
KSADEVLF;FLLTKK	NCP	38.14
FLLTKK;PSLAPTF	NCP	41.15
PSLAPTF;TELTP	NCP	31.08
TELTP;AGSKPQ	NCP	1.042

AGSKPQ;KTYTSSK	NCP	10.364
KTYTSSK;SPAGPDVPV	NCP	19.447
SPAGPDVPV;KLSPPER	NCP	51.243
KLSPPER;AMPQEKLASSPKD	NCP	22.16
AMPQEKLASSPKD;VAKVEPAVSSVVN	NCP	0.06
VAKVEPAVSSVVN;EVPMVV	NCP	0.09
EVPMVV;VVVSPVGAKGSAPATSSTQGK	NCP	0.012
VVVSPVGAKGSAPATSSTQGK;VSPVGAKGSAPATSSTQG	NCP	0.006
VSPVGAKGSAPATSSTQGKK;EGAQNQGKKVEGAQNQGK	NCP	0.423
EGAQNQGKKVEGAQNQGK;VDASANQGKKAESAPTQGK	NCP	1.257
VDASANQGKKAESAPTQGK;ADMVQSQEAPKQE	NCP	0.06
ADMVQSQEAPKQE;VQSQEAPKQEAPAK	NCP	0.009
VQSQEAPKQEAPAK;DDTATN	NCP	7.84
DDTATN;VLSEGEAQRL	NCP	1.262
VLSEGEAQRL;VQDTWHK	NCP	0.06
VQDTWHK;LLATEQEDAAVAK	NCP	1.122
LLATEQEDAAVAK;ALEAKAAAVEKQ	NCP	2.418
ALEAKAAAVEKQ;LEEVVEKL	NCP	8.268
LEEVVEKL;TTQEQLAKEKDTVQK	NCP	2.955
TTQEQLAKEKDTVQK;NHKPLPP	NCP	9.744
NHKPLPP;KNRLLEK	NCP	45.996
KNRLLEK;KGCGP	NCP	80.2
KGCGP;EPPLRPP	NCP	93.227
EPPLRPP;LSFPTTTK	NCP	93.535
LSFPTTTK;ESAHFN	NCP	61.52
ESAHFN;HMSDLR	NCP	19.275
HMSDLR;PYACLLPK	NCP	15.715
PYACLLPK;KLPEEFF	NCP	53.73
KLPEEFF;MENLRK	NCP	91.782
MENLRK;KTEMASK	NCP	3.885
KTEMASK;YLVKNN	NCP	29.901
YLVKNN;YLDSTGKP	NCP	37.553
YLDSTGKP;KGPTKET	NCP	36.252
KGPTKET;KGAGSLLR	NCP	0.357
KGAGSLLR;FDAVYT	NCP	42.124
FDAVYT;KYDFPPK	NCP	58.5
KYDFPPK;KGGLTA	NCP	3.651
KGGLTA;KGAGP	NCP	16.776
KGAGP;MDEDVLTTLK	NCP	161.33
MDEDVLTTLK;KETPLYK	NCP	13.696
KETPLYK;YMTLSGF	NCP	40.164
YMTLSGF;KNLTAPP	NCP	7.458
KNLTAPP;KEGLTLE	NCP	58.724
KEGLTLE;LPVLGPP	NCP	27.921
LPVLGPP;SVDVPK	NCP	23.148
SVDVPK;PLGGPPK	NCP	2.146
PLGGPPK;AQFVRN	NCP	58.637
AQFVRN;LDGAVRY	NCP	4.917
LDGAVRY;SLTFK	NCP	32.727
SLTFK;MGCFMLH	NCP	2.134
MGCFMLH;AETASLR	NCP	17.88

AETASLR;SLLVVFF	NCP	11.892
SLLVVFF;GPKPPL	NCP	23.021
GPKPPL;AAARVLL	NCP	19.191
AAARVLL;MKFNPF	NCP	4.158
MKFNPF;VGATWL	NCP	21.628
VGATWL;HCGAP	NCP	197.163
HCGAP;PPVPFRPP	NCP	62.037
PPVPFRPP;HSPTFP	NCP	85.181
HSPTFP;ATLADKL	NCP	10.816
ATLADKL;KTYQP	NCP	17.013
KTYQP;KTPCAGP	NCP	27.396
KTPCAGP;KSTEVAF	NCP	74.166
KSTEVAF;LYVMNP	NCP	20.811
LYVMNP;LMLPQPY	NCP	26.349
LMLPQPY;SGGAPVLL	NCP	86.102
SGGAPVLL;KAKSLGV	NCP	44.016
KAKSLGV;KGVFLPP	NCP	180.309
KGVFLPP;KNYHHL	NCP	2.377
KNYHHL;NRPFFP	NCP	176.654
NRPFFP;LGPNYAK	NCP	30.705
LGPNYAK;LRTEC	NCP	32.431
LRTEC;LDSRLEQ	NCP	32.268
LDSRLEQ;TVPPNPGK	NCP	62.489
TVPPNPGK;SGRTSTR	NCP	159.729
SGRTSTR;SLDESLPL	NCP	49.933
SLDESLPL;LALGGAF	NCP	85.733
LALGGAF;AALPRGK	NCP	139.221
AALPRGK;AALGAGK	NCP	27.484
AALGAGK;LTAELL	NCP	14.157
LTAELL;LKLVAKS	NCP	35.822
LKLVAKS;AGFFKFK	NCP	6.544
AGFFKFK;FRLLVTT	NCP	16.62
FRLLVTT;YYRKP	NCP	71.921
YYRKP;KPLLNVN	NCP	5.158
KPLLNVN;ASTLEL	NCP	14.178
ASTLEL;AAVPVA	NCP	34.422
AAVPVA;QGKHVPR	NCP	120.821
QGKHVPR;LLPRHLQ	NCP	6.333
LLPRHLQ;MGHVKK	NCP	17.259
MGHVKK;PDDSFGATEQK	NCP	40.863
PDDSFGATEQK;SNNSAVPYKK	NCP	37.314
SNNSAVPYKK;KAASLQGK	NCP	9.34
KAASLQGK;EEDLR	NCP	13.001
EEDLR;SGELLLKT	NCP	5.889
SGELLLKT;KDAWNDLKK	NCP	21.124
KDAWNDLKK;QGVLALH	NCP	21.201
QGVLALH;KKMAGP	NCP	41.204
KKMAGP;LMFPLVQ	NCP	11.679
LMFPLVQ;KDEFP	NCP	33.028
KDEFP;KRSDMEE	NCP	43.073
KRSDMEE;KEVGGP	NCP	9.831

KEVGGP;SVFALTN	NCP	193.065
SVFALTN;MGPALK	NCP	81.688
MGPALK;KDYNSPK	NCP	162.009
KDYNSPK;HELSSPP	NCP	21.659
HELSSPP;RGPQTAL	NCP	109.737
RGPQTAL;PPRLLTPP	NCP	20.022
PPRLLTPP;LDLSLPK	NCP	14.103
LDLSLPK;DFVEHL	NCP	65.056
DFVEHL;LPEKPKP	NCP	1.155
LPEKPKP;EPAYEKN	NCP	24.156
EPAYEKN;KVGVL	NCP	8.906
KVGVL;KPYSKP	NCP	89.593
KPYSKP;VTSLDSL	NCP	14.639
VTSLDSL;PTLGAPP	NCP	18.546
PTLGAPP;KLLEVPLL	NCP	13.759
KLLEVPLL;FTMELAK	NCP	41.862
FTMELAK;KAFDSKEK	NCP	52.604
KAFDSKEK;QKMLVR	NCP	0.312
QKMLVR;KATLKC	NCP	92.724
KATLKC;RVPAALSK	NCP	87.147
RVPAALSK;SLVPVGN	NCP	107.392
SLVPVGN;TMQPRF	NCP	10.266
TMQPRF;LTGKVQT	NCP	12.857
LTGKVQT;KNFGAA	NCP	8.958
KNFGAA;RGFMK	NCP	64.635
RGFMK;MKGVNK	NCP	183.03
MKGVNK;VGAPVSP	NCP	11.256
VGAPVSP;TELMYG	NCP	2.671
TELMYG;NDKLLK	NCP	109.904
NDKLLK;VPGDMK	NCP	12.186
VPGDMK;VVVCPK	NCP	47.739
VVVCPK;QHTPPDR	NCP	187.314
QHTPPDR;KVEAGAP	NCP	41.625
KVEAGAP;LPLTNF	NCP	8.335
LPLTNF;NELLLE	NCP	53.663
NELLLE;TAAEPPGP	NCP	21.126
TAAEPPGP;TMYNY	NCP	25.959
TMYNY;KFTLSGH	NCP	14.203
KFTLSGH;KGVTGLK	NCP	67.941
KGVTGLK;PVTVGPP	NCP	104.235
PVTVGPP;PPLPMPPH	NCP	7.752
PPLPMPPH;KGQEQLY	NCP	0.098
KGQEQLY;KYLLGPP	NCP	47.494
KYLLGPP;YNDPLKK	NCP	36.434
YNDPLKK;WTLSWVA	NCP	61.446
WTLSWVA;KELACR	NCP	16.297
KELACR;GKVLL	NCP	75.795
GKVLL;KPTEF	NCP	73.671
KPTEF;GVLNKP	NCP	32.508
GVLNKP;PGAPY	NCP	69.093
PGAPY;LSGSPMKF	NCP	49.767

LSGSPMKF;WGSRAAF	NCP	6.321
WGSRAAF;PGGSPPP	NCP	7.485
PGGSPPP;QGYKEDR	NCP	11.241
QGYKEDR;AFKVKPF	NCP	13.317
AFKVKPF;KTVGATF	NCP	10.398
KTVGATF;HTSSAPR	NCP	13.062
HTSSAPR;EPGMHVK	NCP	19.26
EPGMHVK;KPYNVR	NCP	93.278
KPYNVR;LPPKSPF	NCP	43.579
LPPKSPF;KPDSLLR	NCP	79.944
KPDSLLR;ADGFSLN	NCP	37.848
ADGFSLN;KFHKHE	NCP	35.867
KFHKHE;KWGLDLR	NCP	25.735
KWGLDLR;KNLEVLNF	NCP	91.952
KNLEVLNF;LFVGAVA	NCP	19.15
LFVGAVA;NALPL	NCP	15.876
NALPL;EGRFC	NCP	18.68
EGRFC;AKYGP	NCP	104.691
AKYGP;SAQNKPR	NCP	4.884
SAQNKPR;TFAPPPPP	NCP	5.674
TFAPPPPP;VGSKQKP	NCP	17.435
VGSKQKP;GSVNQEF	NCP	206.616
GSVNQEF;AVNPF	NCP	121.84
AVNPF;PPVVFVK	NCP	41.804
PPVVFVK;SDFLLY	NCP	59.194
SDFLLY;LTSGYG	NCP	85.196
LTSGYG;KESPFFK	NCP	80.544
KESPFFK;LLAESSSF	NCP	13.713
LLAESSSF;EGFDLL	NCP	4.581
EGFDLL;SHVVGF	NCP	43.705
SHVVGF;DVLLEF	NCP	126.117
DVLLEF;FNLDVDKLTVY	NCP	8.723
FNLDVDKLTVY;VGPAEF	NCP	103.006
VGPAEF;TCNQLPTL	NCP	32.147
TCNQLPTL;MMLHGF	NCP	13.372
MMLHGF;LTQLEY	NCP	63.636
LTQLEY;VGGHVPR	NCP	55.296
VGGHVPR;LPTPVTP	NCP	16.247
LPTPVTP;METVQRL	NCP	8.359
METVQRL;VDLKGKP	NCP	11.99
VDLKGKP;SEHPLDF	NCP	30.859
SEHPLDF;CCDDDND	NCP	42.872
CCDDDND;TLHPHV	NCP	75.183
TLHPHV;AVKYKK	NCP	78.709
AVKYKK;WDTQPVPK	NCP	5.936
WDTQPVPK;PAMKKK	NCP	4.29
PAMKKK;SPLFLHV	NCP	2.652
SPLFLHV;KAYFAPK	NCP	5.805
KAYFAPK;MGVPP	NCP	30.367
MGVPP;KVEPER	NCP	36.248
KVEPER;PLLPPGP	NCP	107.395

PLLPPGP;KNKEPP	NCP	3.669
KNKEPP;PPAPKY	NCP	22.367
PPAPKY;KPVYTQK	NCP	14.698
KPVYTQK;TLADLY	NCP	5.946
TLADLY;LGPPTYA	NCP	1.452
LGPPTYA;KGLAAKR	NCP	20.985
KGLAAKR;KLSYPLVY	NCP	17.189
KLSYPLVY;PMEAAP	NCP	8.674
PMEAAP;LLLLMTW	NCP	13.109
LLLLMTW;EVPLLTD	NCP	3.445
EVPLLTD;PGSPK	NCP	11.172
PGSPK;GVGPHAP	NCP	9.534
GVGPHAP;VLPPPP	NCP	32.373
VLPPPP;KGNQVPK	NCP	15.446
KGNQVPK;MRLPPGP	NCP	18.907
MRLPPGP;STRSPAL	NCP	0.48
STRSPAL;KVLWQP	NCP	4.965
KVLWQP;LSGATPK	NCP	13.602
LSGATPK;LLNVLSA	NCP	15.374
LLNVLSA;EQGSLLR	NCP	67.465
EQGSLLR;KLMAPPP	NCP	4.911
KLMAPPP;HPVPPRP	NCP	3.729
HPVPPRP;GPSPVGPP	NCP	18.99
GPSPVGPP;LPPPRF	NCP	6.753
LPPPRF;NDPVYK	NCP	38.096
NDPVYK;NGDCLRL	NCP	15.124
NGDCLRL;KYQCKL	NCP	22.11
KYQCKL;KGVLVK	NCP	60.137
KGVLVK;VASVTPL	NCP	0.534
VASVTPL;KGHSTLPP	NCP	35.19
KGHSTLPP;KDLPK	NCP	2.244
KDLPK;TLAAPGGH	NCP	6.793
TLAAPGGH;KPEVHR	NCP	33.62
KPEVHR;SSPVFRP	NCP	28.986
SSPVFRP;GPAPGLPPP	NCP	16.372
GPAPGLPPP;LSGGTPG	NCP	26.583
LSGGTPG;AALRAAR	NCP	29.282
AALRAAR;SAPLTLKP	NCP	0.145
SAPLTLKP;KPGGPRP	NCP	9.276
KPGGPRP;KGPSVPK	NCP	28.566
KGPSVPK;AAGAQLLL	NCP	33.009
AAGAQLLL;PLSLP	NCP	4.827
PLSLP;SLGAVVPH	NCP	5.658
SLGAVVPH;LVVLPAP	NCP	8.175
LVVLPAP;GASEVPR	NCP	0.323
GASEVPR;APMHVP	NCP	31.11
APMHVP;DNELPL	NCP	97.369
DNELPL;GLKVHR	NCP	18.147
GLKVHR;ASPLDPGP	NCP	39.63
ASPLDPGP;VLLAAPK	NCP	10.718
VLLAAPK;LSPKVVPP	NCP	2.212

LSPKVVPP;PPVPAPK	NCP	7.203
PPVPAPK;GPAPGPPQP	NCP	36.147
GPAPGPPQP;SLESVP	NCP	3.149
SLESVP;PKATLPK	NCP	7.506
PKATLPK;PVLYYLL	NCP	1.983
PVLYYLL;VGGLVYPF	NCP	30.549
VGGLVYPF;SPVLSRP	NCP	39.157
SPVLSRP;GTTGAAGP	NCP	59.103
GTTGAAGP;PLSAPP	NCP	15.813
PLSAPP;KGHLLPPP	NCP	4.899
KGHLLPPP;HNRPPPP	NCP	2.495
HNRPPPP;VEAPLPK	NCP	26.383
VEAPLPK;AAHLMAP	NCP	58.341
AAHLMAP;PPPRYKL	NCP	14.742
PPPRYKL;SLALGQR	NCP	17.838
SLALGQR;GFGGAP	NCP	14.76
GFGGAP;EVSTPLPK	NCP	16.044
EVSTPLPK;TSSKPQK	NCP	33.678
TSSKPQK;LSELTKP	NCP	0.687
LSELTKP;HSSGPLQ	NCP	3.717
HSSGPLQ;HLVSPPKH	NCP	2.307
HLVSPPKH;SLQDPNL	NCP	26.849
SLQDPNL;GVARSLK	NCP	54.619
GVARSLK;KPVDAPPR	NCP	33.702
KPVDAPPR;AAAPPPP	NCP	11.736
AAAPPPP;ALVSSVR	NCP	3.372
ALVSSVR;EPGPAAPK	NCP	31.692
EPGPAAPK;NEGPAF	NCP	54.564
NEGPAF;KTAPDE	NCP	9.959
KTAPDE;PPKKAAP	NCP	48.814
PPKKAAP;LGPPLGAF	NCP	8.595
LGPPLGAF;SLLVKGPR	NCP	17.075
SLLVKGPR;AVFTR	NCP	32.457
AVFTR;PLYEGY	NCP	15.528
PLYEGY;KLENAVQ	NCP	4.366
KLENAVQ;LTSRPGP	NCP	43.871
LTSRPGP;MDTVLSR	NCP	5.944
MDTVLSR;PALGERK	NCP	38.202
PALGERK;LAAGAVL	NCP	12.557
LAAGAVL;LAEVKTR	NCP	85.717
LAEVKTR;TEQALSF	NCP	66.987
TEQALSF;TAGGVLP	NCP	2.319
TAGGVLP;GVSVAPT	NCP	22.944
GVSVAPT;RTFLRDLR	NCP	28.107
RTFLRDLR;GPALSKPN	NCP	8.091
GPALSKPN;WCAGGP	NCP	4.581
WCAGGP;EGMLKPP	NCP	18.794
EGMLKPP;KDLLPGK	NCP	30.654
KDLLPGK;KSSLEKP	NCP	95.874
KSSLEKP;LATELK	NCP	3.795
LATELK;KLVPRLL	NCP	60.916

KLVPRLL;KLHGPPP	NCP	4.851
KLHGPPP;QTPALLA	NCP	62.319
QTPALLA;NPLPGL	NCP	56.472
NPLPGL;QVVLAPV	NCP	52.824
QVVLAPV;AAVHKPP	NCP	65.447
AAVHKPP;DEFMK	NCP	20.632
DEFMK;LLYMQP	NCP	34.875
LLYMQP;LPDFA	NCP	10.124
LPDFA;TVFTPNT	NCP	32.838
TVFTPNT;DQPLYK	NCP	63.702
DQPLYK;KGVTSVP	NCP	2.974
KGVTSVP;YVQLK	NCP	3.474
YVQLK;WDFWKAL	NCP	145.166
WDFWKAL;KPSDVRP	NCP	14.179
KPSDVRP;VDVAHC	NCP	23.807
VDVAHC;TTAHGK	NCP	1.663
TTAHGK;EKLSFGN	NCP	1.8
EKLSFGN;AVPLM	NCP	46.632
AVPLM;LALPHPP	NCP	2.505
LALPHPP;VAFKRPP	NCP	17.252
VAFKRPP;MSSYKK	NCP	2.94
MSSYKK;PLGLPP	NCP	1.471
PLGLPP;RQDHRLLR	NCP	10.548
RQDHRLLR;TSPGLLP	NCP	62.85
TSPGLLP;DGVTKLF	NCP	46.322
DGVTKLF;FEGKNF	NCP	54.189
FEGKNF;VELKMC	NCP	7.192
VELKMC;VEVVRK	NCP	53.576
VEVVRK;PVPPAKAT	NCP	23.128
PVPPAKAT;VPGAPLPP	NCP	14.523
VPGAPLPP;KNKSFVN	NCP	7.64
KNKSFVN;MRELDK	NCP	26.965
MRELDK;CDMSN	NCP	13.473
CDMSN;YYTHTS	NCP	18.333
YYTHTS;EPAAYK	NCP	3.657
EPAAYK;KCGLTPK	NCP	70.559
KCGLTPK;VLLKFPF	NCP	61.836
VLLKFPF;LLSPKPQ	NCP	76.939
LLSPKPQ;KGRPVPP	NCP	10.047
KGRPVPP;TGHSTFP	NCP	189.5
TGHSTFP;PPGDHLL	NCP	12.241
PPGDHLL;HKPLYP	NCP	9.701
HKPLYP;LQFPPPPP	NCP	19.345
LQFPPPPP;LRFDELL	NCP	152.667
LRFDELL;TTTTTFK	NCP	35.772
TTTTTFK;PLLHP	NCP	3.501
PLLHP;EPVTKE	NCP	15.377
EPVTKE;AASVVLLP	NCP	59.653
AASVVLLP;GGPKLSY	NCP	9.258
GGPKLSY;KPKKKLY	NCP	17.297
KPKKKLY;NRSLVAP	NCP	3.964

NRSLVAP;FPGVASKK	NCP	5.772
FPGVASKK;YVTVPQ	NCP	32.787
YVTVPQ;KPFEPV	NCP	14.775
KPFEPV;LPPVAHK	NCP	3.069
LPPVAHK;KEPTAALR	NCP	34.632
KEPTAALR;KAFEMPV	NCP	3.221
KAFEMPV;PGADVPF	NCP	0.666
PGADVPF;SLLGLY	NCP	12.051
SLLGLY;KKAAAPK	NCP	10.882
KKAAAPK;KHAALYL	NCP	25.229
KHAALYL;QKNDLT	NCP	165.571
QKNDLT;GPKVPP	NCP	4.895
GPKVPP;KLANKNVK	NCP	16.488
KLANKNVK;SVTTQNK	NCP	4.155
SVTTQNK;WNDAKKL	NCP	52.339
WNDAKKL;KPTDTQPP	NCP	131.999
KPTDTQPP;PMFVVN	NCP	2.059
PMFVVN;SQTSSK	NCP	44.12
SQTSSK;LLNQDL	NCP	27.388
LLNQDL;RLKLSF	NCP	8.262
RLKLSF;KENPLPSK	NCP	18.524
KENPLPSK;ELGGGVP	NCP	14.25
ELGGGVP;TVPLVDT	NCP	64.894
TVPLVDT;LAFEGPK	NCP	55.707
LAFEGPK;KGDSKADK	NCP	75.552
KGDSKADK;ELVVPLP	NCP	1.175
ELVVPLP;KLPSKEPQ	NCP	64.417
KLPSKEPQ;EPVPLGRP	NCP	31.161
EPVPLGRP;KMELPLSE	NCP	32.849
KMELPLSE;PPPGPHPPP	NCP	11.362
PPPGPHPPP;KNPSFN	NCP	6.962
KNPSFN;KEMKPPR	NCP	20.959
KEMKPPR;DGADLRL	NCP	10.137
DGADLRL;PAGRPPLP	NCP	36.318
PAGRPPLP;NTLRKEH	NCP	109.566
NTLRKEH;APAYKR	NCP	17.171
APAYKR;VSPLSPLP	NCP	27.9
VSPLSPLP;NCPDLRK	NCP	18.172
NCPDLRK;RELSPPPP	NCP	133.47
RELSPPPP;LGESLLH	NCP	49.328
LGESLLH;YSDNLLL	NCP	66.091
YSDNLLL;PLLGVLR	NCP	33.59
PLLGVLR;THASLPGL	NCP	22.327
THASLPGL;KEGGPV	NCP	19.785
KEGGPV;YRPFTF	NCP	11.816
YRPFTF;DGFKKF	NCP	10.137
DGFKKF;VSELKEELK	NCP	12.774
VSELKEELK;HVNRSPK	NCP	38.896
HVNRSPK;DKDAA	NCP	162.656
DKDAA;VELALPM	NCP	24.3
VELALPM;LDVFEDY	NCP	6.193

LDVFEDY;NFGLPLL	NCP	7.352
NFGLPLL;SAGVTLK	NCP	84.18
SAGVTLK;ERELVRDLK	NCP	49.956
ERELVRDLK;VPSPL	NCP	3.73
VPSPL;KSVHPAR	NCP	10.257
KSVHPAR;VGLVLPP	NCP	63.393
VGLVLPP;MDLVSK	NCP	108.521
MDLVSK;KQFFHT	NCP	30.981
KQFFHT;KELRGGK	NCP	14.971
KELRGGK;LSDLP	NCP	5.505
LSDLP;KMKAAPQ	NCP	32.594
KMKAAPQ;WFKPPPPP	NCP	27.226
WFKPPPPP;NGPRPSK	NCP	49.997
NGPRPSK;KLLPKKT	NCP	12.936
KLLPKKT;PGTHLGFG	NCP	12.199
PGTHLGFG;LEPAHML	NCP	29.066
LEPAHML;GLTDTTVPR	NCP	21.141
GLTDTTVPR;KVTKPGK	NCP	18.282
KVTKPGK;RDHVPY	NCP	4.602
RDHVPY;KDAAKHR	NCP	37.173
KDAAKHR;GLPASC	NCP	36.256
GLPASC;LSLVAVPK	NCP	23.117
LSLVAVPK;TGSPPPHP	NCP	38.304
TGSPPPHP;VRLLLPGELAKHAV	NCP	29.869
VRLLLPGELAKHAV;RLLLPGELAK	NCP	0.003
RLLLPGELAK;QTPKL	NCP	79.044
QTPKL;KPMEPK	NCP	26.291
KPMEPK;WTLKLVSN	NCP	18.172
WTLKLVSN;SLERPPPP	NCP	72.636
SLERPPPP;PLCPALL	NCP	60.687
PLCPALL;KDFAER	NCP	36.693
KDFAER;KVPAGPK	NCP	9.632
KVPAGPK;DRLPLGR	NCP	10.831
DRLPLGR;GSPLK	NCP	7.026
GSPLK;GTLGLEL	NCP	113.603
GTLGLEL;KGYEKKPYCN	NCP	68.982
KGYEKKPYCN;LALNWEL	NCP	4.357
LALNWEL;MKATEKR	NCP	3.027
MKATEKR;LGDPPA	NCP	22.475
LGDPPA;RHGVPH	NCP	51.382
RHGVPH;RGLESFK	NCP	16.782
RGLESFK;SLAALK	NCP	30.815
SLAALK;TTVADTPENLRLK	NCP	10.245
TTVADTPENLRLK;TVADTPENLR	NCP	0.003
TVADTPENLR;VADTPENLRLK	NCP	0.003
VADTPENLRLK;ADTPENLR	NCP	0.003
ADTPENLR;DTPENLRLKQQSE	NCP	0.003
DTPENLRLKQQSE;TALKLLGL	NCP	12.102
TALKLLGL;FPLLKSLP	NCP	28.462
FPLLKSLP;TDTPELQR	NCP	22.541
TDTPELQR;QLLFPR	NCP	52.794

QLLFPR;TFQPCLSH	NCP	47.425
TFQPCLSH;SAWPK	NCP	5.34
SAWPK;LPSQK	NCP	30.272
LPSQK;VRLSKPP	NCP	24.888
VRLSKPP;DGAVVKK	NCP	5.8
DGAVVKK;AASLGAGK	NCP	42.137
AASLGAGK;TELLLTK	NCP	16.431
TELLLTK;ATGLLALR	NCP	1.561
ATGLLALR;DPLTTTPK	NCP	48.312
DPLTTTPK;GRPNPAK	NCP	15.786
GRPNPAK;AALLENF	NCP	61.152
AALLENF;PNWLWK	NCP	12.522
PNWLWK;KEVPPTL	NCP	101.022
KEVPPTL;KCDLRPP	NCP	2.528
KCDLRPP;YAVTSF	NCP	4.558
YAVTSF;SLRFKAP	NCP	49.382
SLRFKAP;KTMARP	NCP	29.758
KTMARP;QPFKGPQ	NCP	31.563
QPFKGPQ;PPPPGSRSP	NCP	22.938
PPPPGSRSP;YYPPRP	NCP	0.705
YYPPRP;RPLLF	NCP	95.124
RPLLF;EGSLLVEK	NCP	18.314
EGSLLVEK;ASPLAPV	NCP	62.629
ASPLAPV;LMVEGLK	NCP	24.036
LMVEGLK;DSLKLK	NCP	25.718
DSLKLK;QQHNKSP	NCP	9.853
QQHNKSP;MGEVPKK	NCP	150.396
MGEVPKK;PTLARWP	NCP	24.585
PTLARWP;NTEEHM	NCP	16.295
NTEEHM;THTVGVP	NCP	59.374
THTVGVP;TDEVLR	NCP	14.133
TDEVLR;AARPPR	NCP	15.312
AARPPR;GPARPQPP	NCP	2.388
GPARPQPP;QTPTPK	NCP	3.792
QTPTPK;NLRLTPP	NCP	30.284
NLRLTPP;VTGPLLP	NCP	10.921
VTGPLLP;VPELT	NCP	31.131
VPELT;VARHGPHT	NCP	5.991
VARHGPHT;KTVFVQP	NCP	1.556
KTVFVQP;LVLPKYK	NCP	7.108
LVLPKYK;HRLEPVG	NCP	3.434
HRLEPVG;PPPEPPR	NCP	29.998
PPPEPPR;GLGTAPSP	NCP	11.955
GLGTAPSP;ALPGLELR	NCP	97.545
ALPGLELR;NLVGPPP	NCP	1.83
NLVGPPP;CTFGEKL	NCP	58.788
CTFGEKL;PGVNGK	NCP	103.466
PGVNGK;PTSYEL	NCP	44.536
PTSYEL;KLSTGAP	NCP	7.68
KLSTGAP;NGGVSLPR	NCP	9.602
NGGVSLPR;AAKVFPQ	NCP	55.143

AAKVFPQ;LVVSYVN	NCP	43.657
LVVSYVN;KGWTQK	NCP	60.432
KGWTQK;HLLGP	NCP	34.133
HLLGP;PGAATNF	NCP	31.972
PGAATNF;RDPGTLR	NCP	51.468
RDPGTLR;KDFEPK	NCP	67.715
KDFEPK;LAKEPPK	NCP	4.654
LAKEPPK;CFFVPKL	NCP	17.42
CFFVPKL;ESLQNLK	NCP	23.415
ESLQNLK;LDAAVFL	NCP	0.193
LDAAVFL;VKVGPP	NCP	7.83
VKVGPP;APGDKQL	NCP	0.363
APGDKQL;DKYPHK	NCP	3.296
DKYPHK;EHLGGPR	NCP	6.951
EHLGGPR;CHVLKH	NCP	126.631
CHVLKH;EHPVLL	NCP	55.439
EHPVLL;NKYADF	NCP	22.95
NKYADF;KGVVTTAM	NCP	2.371
KGVVTTAM;PSDPAGK	NCP	16.527
PSDPAGK;NVPFPPP	NCP	15.641
NVPFPPP;TAPLPGR	NCP	111.135
TAPLPGR;MGPFAPL	NCP	2.274
MGPFAPL;LGPTVKTK	NCP	21.792
LGPTVKTK;ALEVGF	NCP	44.734
ALEVGF;LDVGAVLP	NCP	7.089
LDVGAVLP;SGKKLPY	NCP	0.888
SGKKLPY;SAAGPRPL	NCP	6.684
SAAGPRPL;LSMEY	NCP	5.622
LSMEY;SLKEPY	NCP	22.136
SLKEPY;LPPSHSV	NCP	5.001
LPPSHSV;KPADP	NCP	39.405
KPADP;QKSGLLR	NCP	59.722
QKSGLLR;KELLSK	NCP	27.974
KELLSK;ESDNLAF	NCP	22.017
ESDNLAF;KASFLKGL	NCP	11.548
KASFLKGL;GMKGVNK	NCP	3.323
GMKGVNK;SGPKKNK	NCP	7.278
SGPKKNK;VGSLLEEL	NCP	55.737
VGSLLEEL;DPGLLPSK	NCP	81.871
DPGLLPSK;KHSPFVSP	NCP	2.544
KHSPFVSP;ALTSEAK	NCP	4.862
ALTSEAK;PAKPLR	NCP	9.151
PAKPLR;SVRFYKR	NCP	23.234
SVRFYKR;LALFK	NCP	24.156
LALFK;LTTVDPF	NCP	32.317
LTTVDPF;KNLLQAE	NCP	7.884
KNLLQAE;LPERHP	NCP	7.391
LPERHP;YFSGKR	NCP	10.59
YFSGKR;VGGSKYR	NCP	41.08
VGGSKYR;VSSSADR	NCP	139.61
VSSSADR;ALLLTT	NCP	74.671

ALLLTT;VVDPFSK	NCP	35.637
VVDPFSK;KFHVPK	NCP	38.681
KFHVPK;DLASLLVP	NCP	78.202
DLASLLVP;DLLFSCLF	NCP	112.056
DLLFSCLF;EGCEAG	NCP	11.756
EGCEAG;KMTVP	NCP	15.861
KMTVP;SAHPPKK	NCP	37.411
SAHPPKK;SYMDLRK	NCP	3.149
SYMDLRK;TKFMLGV	NCP	137.098
TKFMLGV;PEPREC	NCP	21.867
PEPREC;AAKGSPK	NCP	4.535
AAKGSPK;KARPSF	NCP	27.192
KARPSF;MDGLTT	NCP	34.206
MDGLTT;KEHFTK	NCP	44.157
KEHFTK;KVALVGNQ	NCP	38.425
KVALVGNQ;LAPPHHK	NCP	36.296
LAPPHHK;LAVLLAK	NCP	11.439
LAVLLAK;LTLFT	NCP	21.183
LTLFT;KLGQWY	NCP	0.351
KLGQWY;LCEKPLR	NCP	14.098
LCEKPLR;SVPMPKP	NCP	16.65
SVPMPKP;LTGKALQ	NCP	28.413
LTGKALQ;SAPAAVK	NCP	61.578
SAPAAVK;NYLKNK	NCP	43.656
NYLKNK;VVPGAKL	NCP	12.828
VVPGAKL;DLDLNLK	NCP	4.008
DLDLNLK;GVRLLPG	NCP	34.668
GVRLLPG;LPGVDALS	NCP	4.253
LPGVDALS;AACKVVK	NCP	45
AACKVVK;LYVMGVLP	NCP	46.203
LYVMGVLP;MEFNPSDHPR	NCP	19.977
MEFNPSDHPR;MMYCCC	NCP	73.953
MMYCCC;PLPPPPPGP	NCP	29.736
PLPPPPPGP;EDLDPV	NCP	20.415
EDLDPV;PGPPGGGGGEQDF	NCP	11.937
PGPPGGGGGEQDF;VPELSH	NCP	1.492
VPELSH;LQAPVFP	NCP	19.2
LQAPVFP;TRVAGGKP	NCP	29.261
TRVAGGKP;SCFRAC	NCP	35.511
SCFRAC;PQVPGPP	NCP	6.466
PQVPGPP;KDLSCR	NCP	7.734
KDLSCR;NFYHQP	NCP	9.567
NFYHQP;GAEGPGK	NCP	3.573
GAEGPGK;APSTSQP	NCP	50.633
APSTSQP;GLPGAGKT	NCP	16.741
GLPGAGKT;PTVEVS	NCP	19.386
PTVEVS;VDELLK	NCP	31.358
VDELLK;KDTEPK	NCP	112.863
KDTEPK;RLVAPGP	NCP	73.837
RLVAPGP;VAEGALPY	NCP	10.364
VAEGALPY;PLTGPLQ	NCP	61.351

PLTGPLQ;AEKDQLK	NCP	5.238
AEKDQLK;GHVPPRP	NCP	27.326
GHVPPRP;KAPAYW	NCP	22.189
KAPAYW;DSCYEP	NCP	6.107
DSCYEP;KAGGP	NCP	18.571
KAGGP;AELDPGF	NCP	37.164
AELDPGF;YTAFH	NCP	34.217
YTAFH;FEVLK	NCP	25.386
FEVLK;AKEELRK	NCP	17.707
AKEELRK;KKVGAPR	NCP	6.74
KKVGAPR;GLGGLTA	NCP	0.682
GLGGLTA;YRFQLLN	NCP	11.286
YRFQLLN;NMLRLR	NCP	97.931
NMLRLR;MKFKKM	NCP	74.388
MKFKKM;KEYVPP	NCP	115.216
KEYVPP;MAVDVP	NCP	0.096
MAVDVP;KTPPSPLSPP	NCP	18.038
KTPPSPLSPP;FTFLPAP	NCP	33.718
FTFLPAP;GPRGH	NCP	26.447
GPRGH;RLEPVTR	NCP	32.629
RLEPVTR;PSPPKPP	NCP	21.09
PSPPKPP;YAPFKR	NCP	2.19
YAPFKR;KEAMVV	NCP	22.616
KEAMVV;PVPNPPPP	NCP	37.734
PVPNPPPP;AMAGVPK	NCP	28.225
AMAGVPK;FLYVKPP	NCP	8.831
FLYVKPP;PALYR	NCP	1.588
PALYR;SLTPP	NCP	40.119
SLTPP;PPGSGRLP	NCP	35.801
PPGSGRLP;ELAAVY	NCP	3.712
ELAAVY;VDPAMY	NCP	5.687
VDPAMY;KEDMK	NCP	114.496
KEDMK;ELTFE	NCP	6.749
ELTFE;VNDLFER	NCP	7.605
VNDLFER;RLLLPGELAKHAV	NCP	0.099
RLLLPGELAKHAV;HAVSEGTKAVT	NCP	0.03
HAVSEGTKAVT;DVPKKFP	NCP	15.009
DVPKKFP;LLRTKLLL	NCP	5.242
LLRTKLLL;SLTVPKV	NCP	20.412
SLTVPKV;SEDPLLL	NCP	54.347
SEDPLLL;NLAGAVL	NCP	27.406
NLAGAVL;SLEETLH	NCP	1.064
SLEETLH;EKMLLEK	NCP	11.457
EKMLLEK;ANKLGLL	NCP	6.171
ANKLGLL;TTLLLPP	NCP	1.519
TTLLLPP;LAALA	NCP	63.854
LAALA;KAFLLTH	NCP	11.559
KAFLLTH;GPARSPY	NCP	24.042
GPARSPY;SLAGKTPSL	NCP	10.317
SLAGKTPSL;EKVEPQ	NCP	37.372
EKVEPQ;LQEKLLY	NCP	16.283

LQEKLLY;LLRVGSGD	NCP	8.388
LLRVGSGD;KMVLGE	NCP	12.588
KMVLGE;PPPKLH	NCP	21.49
PPPKLH;CPVVQKM	NCP	15.971
CPVVQKM;GTGTPKR	NCP	0.898
GTGTPKR;GNTDGLR	NCP	4.871
GNTDGLR;VLEKELK	NCP	70.32
VLEKELK;QCPKEK	NCP	69.981
QCPKEK;MCCDV	NCP	6.598
MCCDV;TCDSSKP	NCP	74.846
TCDSSKP;KMFGAN	NCP	5.089
KMFGAN;KGAELRP	NCP	58.706
KGAELRP;KSRSPFVP	NCP	25.458
KSRSPFVP;LPPELLSL	NCP	0.462
LPPELLSL;DAVVDLR	NCP	54.46
DAVVDLR;AVLLPKK	NCP	24.108
AVLLPKK;PSPAVASAPAPK	NCP	43.058
PSPAVASAPAPK;MGSPPPPP	NCP	2.859
MGSPPPPP;RLVGVNK	NCP	63.598
RLVGVNK;ARMLPRP	NCP	83.934
ARMLPRP;LNPVVFP	NCP	11.031
LNPVVFP;GAAPLR	NCP	22.371
GAAPLR;RPPQHP	NCP	93.911
RPPQHP;SMKMVAAAK	NCP	7.539
SMKMVAAAK;SEAANLAAAGKEVK	NCP	3.234
SEAANLAAAGKEVK;AAQHLPP	NCP	8.781
AAQHLPP;EAFGG	NCP	76.458
EAFGG;APAFAVK	NCP	20.313
APAFAVK;GPPDKN	NCP	8.128
GPPDKN;CKRGPPPPP	NCP	41.447
CKRGPPPPP;SLNVRGF	NCP	78.852
SLNVRGF;LLADATAK	NCP	61.339
LLADATAK;YCTNKMP	NCP	83.261
YCTNKMP;PPKTGQAR	NCP	89.19
PPKTGQAR;ASGTTEVK	NCP	20.277
ASGTTEVK;VPLAVY	NCP	53.287
VPLAVY;RYSSKLL	NCP	20.768
RYSSKLL;LSQADLR	NCP	53.163
LSQADLR;SVGHRTP	NCP	7.168
SVGHRTP;MFSQLRD	NCP	17.186
MFSQLRD;RTKPGVR	NCP	49.668
RTKPGVR;DPELLRH	NCP	5.899
DPELLRH;KSNGLVV	NCP	12.599
KSNGLVV;PAGFKEP	NCP	29.883
PAGFKEP;KAVEKGP	NCP	52.966
KAVEKGP;KPATVPP	NCP	4.479
KPATVPP;LASFVRT	NCP	4.053
LASFVRT;AAVPPLL	NCP	6.918
AAVPPLL;PAKSAPAPK	NCP	174.686
PAKSAPAPK;KYDLPR	NCP	2.371
KYDLPR;KLEEWVK	NCP	17.471

KLEEWVK;KSTTLVK	NCP	13.579
KSTTLVK;KHPPKRP	NCP	16.388
KHPPKRP;LGPTVVP	NCP	83.644
LGPTVVP;KDVTAAL	NCP	69.645
KDVTAAL;MLPLSKLL	NCP	30.192
MLPLSKLL;KDLKAQGK	NCP	18.972
KDLKAQGK;SLGDQLR	NCP	124.802
SLGDQLR;QLMPPK	NCP	13.291
QLMPPK;LEGLYL	NCP	2.705
LEGLYL;LDKPPLP	NCP	30.742
LDKPPLP;GKGHPPR	NCP	2.352
GKGHPPR;SKAFFPLG	NCP	5
SKAFFPLG;QVLDLLL	NCP	21.78
QVLDLLL;QLTGTEHL	NCP	80.113
QLTGTEHL;QVAVLY	NCP	18.347
QVAVLY;PADLPKL	NCP	32.361
PADLPKL;GFNKQ	NCP	42.856
GFNKQ;QELRHVAL	NCP	36.525
QELRHVAL;MPTFY	NCP	9.429
MPTFY;KLQEAK	NCP	92.661
KLQEAK;LPEPPAPL	NCP	39.468
LPEPPAPL;HVSLTSL	NCP	30.852
HVSLTSL;LECLTGL	NCP	21.179
LECLTGL;VGCMM	NCP	64.377
VGCMM;VPAKSPQ	NCP	9.768
VPAKSPQ;KDRGPEP	NCP	3.661
KDRGPEP;KPWAC	NCP	41.382
KPWAC;AFDDDH	NCP	41.802
AFDDDH;KQFGPVP	NCP	52.083
KQFGPVP;CVHHTKS	NCP	73.799
CVHHTKS;LALAGP	NCP	4.183
LALAGP;KMVGAVL	NCP	50.79
KMVGAVL;PPPEARL	NCP	23.37
PPPEARL;MDPGAAL	NCP	39.507
MDPGAAL;PPKTVGHL	NCP	0.521
PPKTVGHL;LSPALPV	NCP	37.702
LSPALPV;SGPALLQR	NCP	33.965
SGPALLQR;FNFVFT	NCP	63.156
FNFVFT;LEVRVPQ	NCP	19.213
LEVRVPQ;TVSTFVAK	NCP	24.144
TVSTFVAK;QLGVVGA	NCP	13.619
QLGVVGA;GKPLLVG	NCP	31.093
GKPLLVG;KPHLAGK	NCP	23.642
KPHLAGK;PTTPPNP	NCP	76.566
PTTPPNP;APSRKPTP	NCP	57.327
APSRKPTP;PVYQLPF	NCP	9.21
PVYQLPF;GAAAAP	NCP	17.941
GAAAAP;KYSSLPAA	NCP	5.03
KYSSLPAA;VPEPKPP	NCP	30.558
VPEPKPP;LYELC	NCP	27.447
LYELC;WVLLGPL	NCP	195.877

WVLLGPL;CDNPPPP	NCP	7.628
CDNPPPP;KLEELKK	NCP	39.619
KLEELKK;KSSEPY	NCP	32.498
KSSEPY;KGAQPYK	NCP	30.591
KGAQPYK;RGVVEPK	NCP	32.76
RGVVEPK;EKHVPR	NCP	7.149
EKHVPR;ESGPRVWF	NCP	61.462
ESGPRVWF;YARNFLP	NCP	1.326
YARNFLP;VGPLE	NCP	13.55
VGPLE;KLDLMPL	NCP	23.095
KLDLMPL;KEHTVLP	NCP	9.915
KEHTVLP;RPCLCR	NCP	44.682
RPCLCR;LLHLNVSK	NCP	1.695
LLHLNVSK;KEAAPP	NCP	11.039
KEAAPP;APVPP	NCP	0.558
APVPP;KQPVQPP	NCP	2.518
KQPVQPP;GPCSK	NCP	3.639
GPCSK;GKLALQ	NCP	4.319
GKLALQ;FLFNTP	NCP	2.89
FLFNTP;LSQGPQPY	NCP	48.56
LSQGPQPY;KQQAALR	NCP	106.42
KQQAALR;EGGKYV	NCP	23.067
EGGKYV;ATEGLLL	NCP	40.5
ATEGLLL;KCFSVYCP	NCP	158.153
KCFSVYCP;TPVTLKQ	NCP	11.91
TPVTLKQ;VFGDEKR	NCP	57.591
VFGDEKR;EGRNLLG	NCP	6.423
EGRNLLG;AELCPL	NCP	6.766
AELCPL;MMFLLQ	NCP	39.108
MMFLLQ;NGEHLQK	NCP	4.101
NGEHLQK;RSLQPPGP	NCP	46.131
RSLQPPGP;YCDSSPP	NCP	41.9
YCDSSPP;KPSAVDCP	NCP	15.192
KPSAVDCP;QAFLVAA	NCP	18.33
QAFLVAA;GPAGPPQPP	NCP	18.609
GPAGPPQPP;KSSELRP	NCP	10.129
KSSELRP;KATPDE	NCP	14.31
KATPDE;DYEVSVK	NCP	1.07
DYEVSVK;AVPFLPK	NCP	147.906
AVPFLPK;AMRFLT	NCP	48.019
AMRFLT;VLDAGPR	NCP	130.515
VLDAGPR;GLKTTKP	NCP	49.646
GLKTTKP;KTYCLLR	NCP	5.505
KTYCLLR;DKYGPPV	NCP	19.756
DKYGPPV;MEVKLV	NCP	54.422
MEVKLV;AVPPPPGH	NCP	71.136
AVPPPPGH;KLMPQK	NCP	6.337
KLMPQK;QMPEPK	NCP	53.241
QMPEPK;SQSSLRPF	NCP	23.967
SQSSLRPF;KVVGHT	NCP	15.789
KVVGHT;PGVGEPQR	NCP	45.953

PGVGEPQR;AVVLLRLG	NCP	43.921
AVVLLRLG;EHTLAG	NCP	33.128
EHTLAG;PVSPEVL	NCP	15.319
PVSPEVL;AVNVYGM	NCP	30.912
AVNVYGM;FKPQAPK	NCP	21.69
FKPQAPK;TQPVRVP	NCP	111.597
TQPVRVP;LVQLEKF	NCP	93.006
LVQLEKF;RAPVSPKP	NCP	50.349
RAPVSPKP;PAAAAAKN	NCP	40.086
PAAAAAKN;CGLPP	NCP	100.923
CGLPP;NLKPAVP	NCP	92.547
NLKPAVP;PSLAPPP	NCP	0.611
PSLAPPP;GPQLVAH	NCP	10.408
GPQLVAH;HRPGAALG	NCP	131.994
HRPGAALG;LPLAAPR	NCP	4.565
LPLAAPR;LLEPRSL	NCP	11.041
LLEPRSL;YTKVMSV	NCP	12.392
YTKVMSV;EVLVTSK	NCP	144.448
EVLVTSK;KADHLSF	NCP	2.768
KADHLSF;CPPPSYF	NCP	36.802
CPPPSYF;APGYLYP	NCP	89.816
APGYLYP;TTLLPPQ	NCP	16.231
TTLLPPQ;GVFLYK	NCP	49.877
GVFLYK;PHALANL	NCP	66.102
PHALANL;LPPPPCK	NCP	29.563
LPPPPCK;VGGFLKSH	NCP	79.187
VGGFLKSH;GVPGPAP	NCP	9.496
GVPGPAP;KDFFVK	NCP	25.538
KDFFVK;TLGARQR	NCP	1.393
TLGARQR;PGMSK	NCP	47.064
PGMSK;LRKVTEL	NCP	11.891
LRKVTEL;KVGLEVP	NCP	9.117
KVGLEVP;KNKAE	NCP	21.558
KNKAE;QCYQGK	NCP	27.487
QCYQGK;NCALLRC	NCP	36.087
NCALLRC;YSLFDT	NCP	18.666
YSLFDT;QNEVPR	NCP	16.859
QNEVPR;VEQLLG	NCP	67.225
VEQLLG;DRGRPLP	NCP	17.085
DRGRPLP;GMKKTK	NCP	26.306
GMKKTK;DDLEHC	NCP	55.198
DDLEHC;DTLQNLK	NCP	42.729
DTLQNLK;FAYVFP	NCP	38.88
FAYVFP;TLLMQLLR	NCP	3.639
TLLMQLLR;KPFEDL	NCP	131.681
KPFEDL;ATVYDLR	NCP	41.709
ATVYDLR;MMDKVEF	NCP	4.704
MMDKVEF;PAFPRSPP	NCP	35.524
PAFPRSPP;HLPYKK	NCP	12.428
HLPYKK;HLGKLSR	NCP	139.401
HLGKLSR;GPRKVLLF	NCP	8.992

GPRKVLLF;DPPKAAA	NCP	7.716
DPPKAAA;PAKGKKP	NCP	32.321
PAKGKKP;HGWVVK	NCP	45.538
HGWVVK;VSDHGPK	NCP	39.585
VSDHGPK;MQEGKH	NCP	42.777
MQEGKH;PAAASSY	NCP	33.368
PAAASSY;LNDPHVRK	NCP	5.605
LNDPHVRK;LPLPDFS	NCP	0.333
LPLPDFS;SRFLNRQPY	NCP	1.821
SRFLNRQPY;ADGGAPP	NCP	10.785
ADGGAPP;TALPHQP	NCP	27.921
TALPHQP;LAPKPEP	NCP	2.115
LAPKPEP;LVPYLY	NCP	8.064
LVPYLY;VGGFKKK	NCP	33.792
VGGFKKK;PLATL	NCP	2.4
PLATL;PHALLAL	NCP	28.374
PHALLAL;KPNPKPP	NCP	38.763
KPNPKPP;LTATHPK	NCP	21.549
LTATHPK;PPPPPPYK	NCP	10.212
PPPPPPYK;KNYVAPR	NCP	10.551
KNYVAPR;GDRGTFP	NCP	2.595
GDRGTFP;KGPAAGN	NCP	4.476
KGPAAGN;PPVPP	NCP	29.165
PPVPP;APPTLPGPGP	NCP	24.022
APPTLPGPGP;PRPPVPP	NCP	23.835
PRPPVPP;DFHCKMD	NCP	1.943
DFHCKMD;KDTWK	NCP	37.161
KDTWK;KETPLM	NCP	33.294
KETPLM;SEVVRTPR	NCP	22.153
SEVVRTPR;PFPPFPP	NCP	15.129
PFPPFPP;RAGLP	NCP	0.755
RAGLP;DDCPPAA	NCP	83.808
DDCPPAA;WVVTLPP	NCP	150.25
WVVTLPP;SAATR	NCP	27.407
SAATR;KDCHMT	NCP	2.701
KDCHMT;LLNFSSAPK	NCP	1.068
LLNFSSAPK;VPVWQP	NCP	18.947
VPVWQP;SLRYGGP	NCP	20.271
SLRYGGP;EDVCRLF	NCP	8.877
EDVCRLF;ANTVGKL	NCP	27.579
ANTVGKL;DPTVFF	NCP	10.354
DPTVFF;TRSTLY	NCP	56.883
TRSTLY;LPGPAA	NCP	67.049
LPGPAA;HPTLL	NCP	84.793
HPTLL;EYTELLKKK	NCP	81.374
EYTELLKKK;VCCCCCCF	NCP	32.367
VCCCCCCF;FSLRLWN	NCP	16.489
FSLRLWN;PAAPEK	NCP	5.607
PAAPEK;EGSARLLR	NCP	4.334
EGSARLLR;VALEYLT	NCP	19.603
VALEYLT;SAALTHPK	NCP	19.773

SAALTHPK;EVFPAST	NCP	10.479
EVFPAST;KVMPER	NCP	3.125
KVMPER;LDVNKL	NCP	72.276
LDVNKL;GEHPK	NCP	134.007
GEHPK;DPHLPPP	NCP	34.9
DPHLPPP;PPAPPPPPP	NCP	11.448
PPAPPPPPP;KPAEKR	NCP	21.539
KPAEKR;AVPKGAK	NCP	32.206
AVPKGAK;VSGLSPF	NCP	38.43
VSGLSPF;FFAPPGP	NCP	2.1
FFAPPGP;EGVMLK	NCP	3.95
EGVMLK;ATAQLQR	NCP	30.427
ATAQLQR;YVEKQGK	NCP	25.662
YVEKQGK;QAFYSK	NCP	10.976
QAFYSK;TVVPQNT	NCP	10.303
TVVPQNT;HKAAPR	NCP	12.104
HKAAPR;KKHTLPPPP	NCP	16.498
KKHTLPPPP;KHGTPPPPPP	NCP	97.893
KHGTPPPPPP;FASSVPR	NCP	4.919
FASSVPR;NHQVGPSP	NCP	35.184
NHQVGPSP;LGPPKGM	NCP	14.191
LGPPKGM;WSSQLPPPP	NCP	11.124
WSSQLPPPP;EAESLKL	NCP	3.074
EAESLKL;AAEKLNK	NCP	18.601
AAEKLNK;TVLAEGAK	NCP	128.784
TVLAEGAK;PALSLP	NCP	29.547
PALSLP;QPLATTP	NCP	0.461
QPLATTP;VVPGKTF	NCP	11.25
VVPGKTF;SLEETLHT	NCP	37.189
SLEETLHT;GSMAPLR	NCP	51.351
GSMAPLR;RPAFMK	NCP	24.264
RPAFMK;LVVTGGK	NCP	33.458
LVVTGGK;FMMKPP	NCP	30.174
FMMKPP;TALGETPL	NCP	117.469
TALGETPL;HEEGGGGK	NCP	17.135
HEEGGGGK;DAVYPK	NCP	11.923
DAVYPK;QVLPGLP	NCP	31.161
QVLPGLP;NPLLAGK	NCP	23.756
NPLLAGK;CCCDGCE	NCP	27.364
CCCDGCE;DLTAAPG	NCP	19.914
DLTAAPG;LDLLEGF	NCP	28.107
LDLLEGF;VNVGPLP	NCP	88.317
VNVGPLP;FGFGQQ	NCP	31.032
FGFGQQ;KGFCDF	NCP	24.489
KGFCDF;DKCKKK	NCP	0.641
DKCKKK;GSVPVK	NCP	169.753
GSVPVK;QLPVPPK	NCP	149.133
QLPVPPK;LHSPPPPP	NCP	66.144
LHSPPPPP;FYHCQQ	NCP	29.541
FYHCQQ;YVTDK	NCP	2.886
YVTDK;YEGSQY	NCP	30.803

YEGSQY;VPLLLLPG	NCP	12.663
VPLLLLPG;MAYPYK	NCP	36.447
MAYPYK;PFPPL	NCP	3.877
PFPPL;GLDLPHK	NCP	9.489
GLDLPHK;KGCCPQ	NCP	12.812
KGCCPQ;KMYPWH	NCP	3.691
KMYPWH;KEPFGP	NCP	68.768
KEPFGP;CTPLKFF	NCP	37.15
CTPLKFF;KTCDPK	NCP	34.175
KTCDPK;KVTFLTALLR	NCP	59.817
KVTFLTALLR;KLLFLLLLK	NCP	43.041
KLLFLLLLK;GSLVRLR	NCP	26.088
GSLVRLR;KTTLPVP	NCP	57.331
KTTLPVP;KGAEAGP	NCP	32.949
KGAEAGP;KMVPPAH	NCP	9.881
KMVPPAH;GPVLP	NCP	13.873
GPVLP;SPVPEVL	NCP	13.148
SPVPEVL;PALFKAL	NCP	6.849
PALFKAL;GPMGRPP	NCP	42.864
GPMGRPP;AAPLLPK	NCP	3.075
AAPLLPK;KPLALEM	NCP	22.278
KPLALEM;APALR	NCP	40.369
APALR;DKPVSLP	NCP	50.859
DKPVSLP;TVLALPR	NCP	13.86
TVLALPR;WEPLAL	NCP	5.061
WEPLAL;LTSKGLP	NCP	7.259
LTSKGLP;ATPSTLR	NCP	23.634
ATPSTLR;APLPDL	NCP	2.284
APLPDL;GPQLPPR	NCP	9.066
GPQLPPR;ATGLEHK	NCP	32.526
ATGLEHK;KCLPPSH	NCP	130.863
KCLPPSH;APLEPF	NCP	49.434
APLEPF;AASKVEAP	NCP	12.861
AASKVEAP;EKGMVP	NCP	16.169
EKGMVP;FFPGRPQ	NCP	55.27
FFPGRPQ;MAKLLR	NCP	30.212
MAKLLR;WGNAY	NCP	3.184
WGNAY;TKKLLL	NCP	23.792
TKKLLL;LRVNGF	NCP	9.67
LRVNGF;QSYGF	NCP	25.593
QSYGF;KVGKKQ	NCP	95.691
KVGKKQ;RETVPPPP	NCP	151.326
RETVPPPP;FGQPSFPL	NCP	61.587
FGQPSFPL;DFAQPD	NCP	33.356
DFAQPD;KSPQAAPSP	NCP	161.308
KSPQAAPSP;VSAEKPK	NCP	8.559
VSAEKPK;ARVVTAR	NCP	18.816
ARVVTAR;LTTKGK	NCP	21.206
LTTKGK;ESQAPHLK	NCP	4.231
ESQAPHLK;EVPAGPP	NCP	21.537
EVPAGPP;RRAKHT	NCP	82.716

RRAKHT;SCDPF	NCP	17.135
SCDPF;TTKLMLSK	NCP	4.098
TTKLMLSK;PMPEAQ	NCP	0.807
PMPEAQ;LSALEPK	NCP	2.004
LSALEPK;VKQDKGP	NCP	23.383
VKQDKGP;LVPPHQ	NCP	42.489
LVPPHQ;LQSTVPK	NCP	8.103
LQSTVPK;KEPTVP	NCP	0.54
KEPTVP;KAHALLY	NCP	7.778
KAHALLY;KDQAASK	NCP	26.554
KDQAASK;AAGPVYM	NCP	38.783
AAGPVYM;VEDFLP	NCP	43.407
VEDFLP;RPPQLQ	NCP	15.948
RPPQLQ;PSFGAPLG	NCP	35.634
PSFGAPLG;ALLAKKH	NCP	11.488
ALLAKKH;EAAVRRPP	NCP	4.184
EAAVRRPP;ASHHGPK	NCP	5.749
ASHHGPK;LHPGKPP	NCP	14.15
LHPGKPP;FRWEL	NCP	78.826
FRWEL;GASSK	NCP	240.9
GASSK;KKLVKGP	NCP	23.984
KKLVKGP;VTSLDFHK	NCP	77.535
VTSLDFHK;TALREGL	NCP	34.026
TALREGL;VGEVHPL	NCP	50.958
VGEVHPL;VSLLEGC	NCP	17.472
VSLLEGC;PTSSLKL	NCP	28.698
PTSSLKL;ADSLLLLKR	NCP	71.028
ADSLLLLKR;VSPKVAK	NCP	40.875
VSPKVAK;RLLVPYL	NCP	27.157
RLLVPYL;CCSPAP	NCP	7.814
CCSPAP;LEHVDP	NCP	57.586
LEHVDP;TVEGKPL	NCP	12.362
TVEGKPL;PVSVPP	NCP	143.089
PVSVPP;EDEVKLP	NCP	27.837
EDEVKLP;TVHELLKT	NCP	1.358
TVHELLKT;DKFGDL	NCP	22.816
DKFGDL;LNQPQY	NCP	14.717
LNQPQY;EVPPLLL	NCP	3.52
EVPPLLL;DTFSVVP	NCP	42.125
DTFSVVP;LNAVFQ	NCP	10.857
LNAVFQ;KTQNPK	NCP	2.853
KTQNPK;LLFTQPT	NCP	102.898
LLFTQPT;LELVQK	NCP	195.663
LELVQK;TLAETLF	NCP	5.694
TLAETLF;CRRPPLL	NCP	4.374
CRRPPLL;AHGDRAP	NCP	78.609
AHGDRAP;AATKL	NCP	20.966
AATKL;MHLFEK	NCP	10.339
MHLFEK;GDLVKLR	NCP	3.563
GDLVKLR;FLNYVK	NCP	46.023
FLNYVK;CPERLLT	NCP	0.805

CPERLLT;VPSLKLP	NCP	104.991
VPSLKLP;LPDYLF	NCP	109.44
LPDYLF;SPGGAQKR	NCP	24.522
SPGGAQKR;AAHQVLY	NCP	3.482
AAHQVLY;YSCCFE	NCP	69.031
YSCCFE;KAHTAPR	NCP	20.604
KAHTAPR;LLAPP	NCP	66.933
LLAPP;EPLPTPR	NCP	13.032
EPLPTPR;PGPPAPPPP	NCP	68.096
PGPPAPPPP;KPPFSVP	NCP	27.123
KPPFSVP;SGALPPAL	NCP	2.812
SGALPPAL;VWLGGS	NCP	30.086
VWLGGS;TVPPP	NCP	26.461
TVPPP;TPVNVFF	NCP	12.545
TPVNVFF;AVYSNR	NCP	3.888
AVYSNR;LPPVLLPDL	NCP	54.543
LPPVLLPDL;VPVVG	NCP	145.821
VPVVG;LLEKVRK	NCP	14.439
LLEKVRK;EPRLLTL	NCP	18.249
EPRLLTL;KPHAPR	NCP	25.173
KPHAPR;LPRHPSP	NCP	15.673
LPRHPSP;GYLLK	NCP	5.246
GYLLK;KMMSMK	NCP	12.541
KMMSMK;FANLNLL	NCP	38.867
FANLNLL;LMGPK	NCP	1.354
LMGPK;EPPLPAHPR	NCP	15.222
EPPLPAHPR;KTTPVLH	NCP	15.948
KTTPVLH;AGLEVLF	NCP	85.034
AGLEVLF;PADLLSR	NCP	5.662
PADLLSR;KDLLRPE	NCP	12.24
KDLLRPE;GHPGPVLL	NCP	4.806
GHPGPVLL;AAPKPPR	NCP	24.867
AAPKPPR;WSHMASEK	NCP	5.235
WSHMASEK;PCCPK	NCP	8.898
PCCPK;LFNLHED	NCP	3.374
LFNLHED;KQTVPH	NCP	0.919
KQTVPH;EVHLTKL	NCP	60.074
EVHLTKL;VYLLPLFL	NCP	57.724
VYLLPLFL;PSHVSLP	NCP	49.031
PSHVSLP;VAFLMP	NCP	2.461
VAFLMP;AVVALPR	NCP	1.38
AVVALPR;YEKYTQ	NCP	41.291
YEKYTQ;KPALSED	NCP	13.813
KPALSED;VQLYHV	NCP	29.187
VQLYHV;KYDGRP	NCP	105.291
KYDGRP;LSLLDVK	NCP	56.504
LSLLDVK;KGPPTLP	NCP	112.486
KGPPTLP;DMHMMS	NCP	87.458
DMHMMS;ESLLYL	NCP	29.878
ESLLYL;LGPPKGF	NCP	23.628
LGPPKGF;SAKHQQP	NCP	255.774

SAKHQQP;EVPFP	NCP	3.728
EVPFP;RKKATNE	NCP	22.234
RKKATNE;LELYK	NCP	20.396
LELYK;APNGQRK	NCP	105.052
APNGQRK;LDQTLPK	NCP	161.426
LDQTLPK;KLSVY	NCP	6.025
KLSVY;KHAPLF	NCP	15.513
KHAPLF;MMEDC	NCP	4.604
MMEDC;LPVLVLK	NCP	28.702
LPVLVLK;PVGHHGK	NCP	38.613
PVGHHGK;AKLRYH	NCP	20.823
DKELY;NWRQGP	NCP	67.437
NWRQGP;KHYCSP	NCP	267.288
KHYCSP;GPAFFPK	NCP	51.175
GPAFFPK;HYRPQLT	NCP	38.414
HYRPQLT;MREATPY	NCP	51.384
MREATPY;VAPPLLY	NCP	30.939
VAPPLLY;EEEFFG	NCP	38.004
EEEFFG;PLENQ	NCP	14.824
PLENQ;QVGKNGP	NCP	24.537
QVGKNGP;AALEKELK	NCP	5.606
AALEKELK;PEHLRQ	NCP	28.005
PEHLRQ;LSHSLL	NCP	0.235
LSHSLL;LAPFFSKVL	NCP	4.352
LAPFFSKVL;TLEPLLPR	NCP	4.182
TLEPLLPR;KFPLP	NCP	7.621
KFPLP;MAHKKP	NCP	16.473
MAHKKP;QLKHGLK	NCP	177.866
QLKHGLK;KEDEPK	NCP	11.665
KEDEPK;KAGLELR	NCP	217.254
KAGLELR;PPPDPLK	NCP	8.231
PPPDPLK;LARGL	NCP	0.187
LARGL;CGAPPPK	NCP	26.468
CGAPPPK;LDEKQMP	NCP	56.628
LDEKQMP;KGPLLVG	NCP	143.569
KGPLLVG;SDGGVFF	NCP	17.633
SDGGVFF;GMGQDLR	NCP	122.371
GMGQDLR;LSPLPF	NCP	51.8
LSPLPF;GPRRVLK	NCP	41.631
GPRRVLK;LEVNLF	NCP	41.62
LEVNLF;AARPPAPR	NCP	64.592
AARPPAPR;HVHGVEF	NCP	4.218
HVHGVEF;GSYNEC	NCP	10.158
GSYNEC;DVYLEF	NCP	1.623
DVYLEF;ETTAG	NCP	77.971
ETTAG;VRPTLHY	NCP	27.683
VRPTLHY;PPDPKPPP	NCP	34.708
PPDPKPPP;YPNDRK	NCP	6.836
YPNDRK;SLAFSPGG	NCP	4.983
SLAFSPGG;KVHFAK	NCP	34.293
KVHFAK;KEAYHEQL	NCP	202.533

KEAYHEQL;SGWPGY	NCP	13.902
SGWPGY;PDPKPPP	NCP	66.427
PDPKPPP;GHPYK	NCP	56.508
GHPYK;KPDLR	NCP	38.136
KPDLR;LFLVAGL	NCP	11.043
LFLVAGL;FLLLLLGY	NCP	46.28
FLLLLLGY;DSLLDQLVK	NCP	19.173
DSLLDQLVK;PAAAPAGR	NCP	57.601
PAAAPAGR;TVLRFEK	NCP	1.844
TVLRFEK;DLGLSP	NCP	96.397
DLGLSP;CEKPVK	NCP	21.233
CEKPVK;SHSLLRP	NCP	34.591
SHSLLRP;MCMCCC	NCP	85.334
MCMCCC;LHRGFAK	NCP	109.365
LHRGFAK;EVVLFLP	NCP	115.447
EVVLFLP;PAAQHPK	NCP	54.621
PAAQHPK;KAKPTAP	NCP	30.656
KAKPTAP;PPYPP	NCP	8.8
PPYPP;DSGLLLDA	NCP	23.705
DSGLLLDA;MKVVHF	NCP	0.403
MKVVHF;PAAAALLH	NCP	1.071
PAAAALLH;VLPYPR	NCP	4.413
VLPYPR;QVLLLSRN	NCP	35.996
QVLLLSRN;PGGVVTPPP	NCP	4.96
PGGVVTPPP;HPPAHPPP	NCP	16.611
HPPAHPPP;LTAGLRP	NCP	3.006
LTAGLRP;GPPPPLPAR	NCP	90.027
GPPPPLPAR;RYKPPPP	NCP	20.769
RYKPPPP;PTPPVVP	NCP	13.386
PTPPVVP;GPVGRVE	NCP	37.194
GPVGRVE;LPPGLG	NCP	67.59
LPPGLG;KNPRVP	NCP	23.469
KNPRVP;EPPRAPP	NCP	8.604
EPPRAPP;DGKLW	NCP	1.427
DGKLW;GVAGGGLG	NCP	25.306
GVAGGGLG;QPPARPG	NCP	15.183
QPPARPG;VLLPTVCPV	NCP	26.445
VLLPTVCPV;LPPGVDP	NCP	53.226
LPPGVDP;MGVAPA	NCP	5.376
MGVAPA;LRPVGSY	NCP	86.475
LRPVGSY;CRSLQ	NCP	8.655
CRSLQ;KGAARQPP	NCP	3.966
KGAARQPP;REQLLAEF	NCP	12.234
REQLLAEF;ETLRDLAQGTK	NCP	0.498
ETLRDLAQGTK;DLPLP	NCP	17.4
DLPLP;TAVHPGK	NCP	16.32
TAVHPGK;NHLPVGR	NCP	35.24
NHLPVGR;RAPPPVR	NCP	30.661
RAPPPVR;TAAGL	NCP	68.478
TAAGL;AAAAEEFGWRRVGF	NCP	0.207
AAAAEEFGWRRVGF;LPPPPLLLLP	NCP	9.99

LPPPPLLLLP;AVVPPKE	NCP	37.035
AVVPPKE;WSLLACK	NCP	28.95
WSLLACK;EGATLRL	NCP	19.143
EGATLRL;FLPALAA	NCP	49.416
FLPALAA;PVGVSPPGP	NCP	9.033
PVGVSPPGP;GPLEEAR	NCP	3.701
GPLEEAR;GPVRVPP	NCP	6.208
GPVRVPP;KQPGSPR	NCP	55.242
KQPGSPR;ACGLVAS	NCP	29.22
ACGLVAS;PAAEKLH	NCP	19.074
PAAEKLH;ELSVGV	NCP	0.96
ELSVGV;KPLPDFR	NCP	1.398
KPLPDFR;LGGRVEVS	NCP	0.519
LGGRVEVS;ALPAHPV	NCP	43.587
ALPAHPV;LTGTPAY	NCP	6.272
LTGTPAY;LPKGRPL	NCP	24.22
LPKGRPL;NVERVFP	NCP	3.14
NVERVFP;KPVVLLL	NCP	22.074
KPVVLLL;KAPELGP	NCP	53.338
KAPELGP;MLPPSP	NCP	58.278
MLPPSP;GPPALCSPL	NCP	36.597
GPPALCSPL;AGPKR	NCP	15.018
AGPKR;VPPFTF	NCP	22.697
VPPFTF;AGVLAPPL	NCP	45.265
AGVLAPPL;APPLPLPP	NCP	3.819
APPLPLPP;GLVLPPQ	NCP	19.107
GLVLPPQ;PVELLF	NCP	17.267
PVELLF;EALPSRP	NCP	13.639
EALPSRP;GVDVGNF	NCP	35.477
GVDVGNF;LKGAL	NCP	1.093
LKGAL;DPVFKP	NCP	11.841
DPVFKP;LALGL	NCP	32.868
LALGL;QAGPPPPR	NCP	0.039
QAGPPPPR;AAAVAV	NCP	38.468
AAAVAV;KESLKKP	NCP	1.152
KESLKKP;PSAAPGVP	NCP	92.527
PSAAPGVP;LPGGLLG	NCP	21.249
LPGGLLG;KTWRGSP	NCP	208.859
KTWRGSP;EDDDGD	NCP	41.377
EDDDGD;TVPRVPV	NCP	27.918
TVPRVPV;KLAQALK	NCP	14.837
KLAQALK;PSGGL	NCP	13.579
PSGGL;PGAGQS	NCP	25.827
PGAGQS;KEVVTPQ	NCP	0.938
KEVVTPQ;TLDRME	NCP	40.657
TLDRME;NSVVSCDF	NCP	62.36
NSVVSCDF;LPTELPTL	NCP	65.712
LPTELPTL;PLVHPPP	NCP	2.029
PLVHPPP;KDSTPPPPP	NCP	30.744
KDSTPPPPP;PQPSQMP	NCP	7.863
PQPSQMP;PALPGL	NCP	16.029

PALPGL;APPKERPP	NCP	3.333
APPKERPP;VLLVGPS	NCP	15.183
VLLVGPS;GVCRPP	NCP	2.757
GVCRPP;TEGTCLR	NCP	22.065
TEGTCLR;VVTPW	NCP	3.777
VVTPW;PAAPGF	NCP	2.412
PAAPGF;LEPLNEKR	NCP	0.209
LEPLNEKR;NAPGVP	NCP	29.436
NAPGVP;GPVLPPPP	NCP	28.153
GPVLPPPP;AAPVDKARP	NCP	37.89
AAPVDKARP;AKEWGYADHN	NCP	8.294
AKEWGYADHN;EPPVPLVR	NCP	9.012
EPPVPLVR;HVSPNR	NCP	7.165
HVSPNR;PAGRALSP	NCP	10.253
PAGRALSP;VGDANPALQK	NCP	13.419
VGDANPALQK;DPDGAP	NCP	15.997
DPDGAP;VSGQVPR	NCP	14.844
VSGQVPR;LDSLP	NCP	18.696
LDSLP;NAPVNLH	NCP	78.303
NAPVNLH;FLLKWN	NCP	4.425
FLLKWN;PPSPVV	NCP	35.532
PPSPVV;GNVTKLF	NCP	11.691
GNVTKLF;KTGDWSK	NCP	20.387
KTGDWSK;RWTPAP	NCP	15.067
RWTPAP;SVGPAVLP	NCP	34.755
SVGPAVLP;ESLSVPR	NCP	51.516
ESLSVPR;FMVGLGA	NCP	26.028
FMVGLGA;LSLFVPT	NCP	3.06
LSLFVPT;VAPPPPPPP	NCP	12
VAPPPPPPP;PAATKF	NCP	18.875
PAATKF;AKTVAY	NCP	50.07
AKTVAY;SPQFPHS	NCP	43.047
SPQFPHS;LVPAP	NCP	6.19
LVPAP;VAAVP	NCP	42.981
VAAVP;SLRSPY	NCP	6.341
SLRSPY;VEGPVP	NCP	41.388
VEGPVP;VLPAGPV	NCP	4.6
VLPAGPV;VLAGLHSV	NCP	2.304
VLAGLHSV;EHMTPK	NCP	0.335
EHMTPK;VTGDLLK	NCP	76.482
VTGDLLK;LPTKW	NCP	32.638
LPTKW;HDGLHLP	NCP	28.311
HDGLHLP;GPVFPSLA	NCP	13.346
GPVFPSLA;NGVADP	NCP	14.563
NGVADP;AGKLERP	NCP	7.848
AGKLERP;DPGCRRV	NCP	9.09
DPGCRRV;VAASRGS	NCP	18.629
VAASRGS;KCWHLLT	NCP	42.268
KCWHLLT;PAAPGWPR	NCP	29.729
PAAPGWPR;RALATLV	NCP	20.707
RALATLV;FNTYKLLPPKNPATK	NCP	15.449

FNTYKLLPPKNPATK;GPPKRPP	NCP	11.545
GPPKRPP;LQGVPG	NCP	3.477
LQGVPG;PPALEPR	NCP	20.67
PPALEPR;VGVTLTPL	NCP	0.816
VGVTLTPL;PEDVLFPP	NCP	173.025
PEDVLFPP;REPCVH	NCP	34.914
REPCVH;KNVSPPGR	NCP	16.505
KNVSPPGR;VQEPKVG	NCP	64.705
VQEPKVG;LSQAFPP	NCP	0.111
LSQAFPP;LALVV	NCP	15.515
LALVV;TSGPRLT	NCP	5.074
TSGPRLT;KMMAA	NCP	119.139
KMMAA;CAMPPQ	NCP	59.871
CAMPPQ;TPYHRLP	NCP	90.429
TPYHRLP;NVLRDAV	NCP	13.083
NVLRDAV;GPGVPP	NCP	24.879
GPGVPP;LQFEA	NCP	21.651
LQFEA;TGFYK	NCP	41.444
TGFYK;KGGAAPPR	NCP	13.618
KGGAAPPR;WVPSEK	NCP	5.946
WVPSEK;KERGPN	NCP	59.85
KERGPN;TAAAVF	NCP	56.466
TAAAVF;QVPPP	NCP	79.743
QVPPP;MEECW	NCP	56.829
MEECW;KNVDLL	NCP	24.023
KNVDLL;APLNPPK	NCP	29.482
APLNPPK;AAVTAAPN	NCP	19.671
AAVTAAPN;PKAPWVP	NCP	0.552
PKAPWVP;KDALTF	NCP	26.36
KDALTF;NVQELRF	NCP	1.441
NVQELRF;QTAGFP	NCP	45.377
QTAGFP;KSKHLAGK	NCP	78.012
KSKHLAGK;YNLPL	NCP	0.412
YNLPL;DFTPVK	NCP	130.452
DFTPVK;LVKPGVP	NCP	107.993
LVKPGVP;RPKHTK	NCP	13.228
RPKHTK;ARDKHT	NCP	33.795
ARDKHT;APHFWCS	NCP	25.368
APHFWCS;PGALYPK	NCP	11.979
PGALYPK;TTSNFPP	NCP	16.907
TTSNFPP;NPLSQF	NCP	33.571
NPLSQF;DPELSPP	NCP	27.903
DPELSPP;KVYMKP	NCP	36.953
KVYMKP;YMQVP	NCP	40.171
YMQVP;KQPSPPP	NCP	0.842
KQPSPPP;MAGDC	NCP	49.375
MAGDC;EDVYKLN	NCP	19.113
EDVYKLN;KDGSAL	NCP	11.697
KDGSAL;QVRGNPP	NCP	84.582
QVRGNPP;PTAKTDPP	NCP	8.724
PTAKTDPP;LSYAPR	NCP	224.051

LSYAPR;EVARVY	NCP	93.666
EVARVY;LFGGFPLY	NCP	25.356
LFGGFPLY;VEVLL	NCP	72.894
VEVLL;KMVLLKFCF	NCP	130.69
KMVLLKFCF;KPVYQ	NCP	25.611
KPVYQ;SHSLVHRK	NCP	112.665
SHSLVHRK;GCYSGP	NCP	12.627
GCYSGP;RPPPPPP	NCP	3.575
RPPPPPP;QPNLPSK	NCP	8.295
QPNLPSK;LFNKENF	NCP	1.545
LFNKENF;TTAFVHR	NCP	20.166
TTAFVHR;FAVTL	NCP	3.546
FAVTL;SGPAALQP	NCP	44.263
SGPAALQP;EHLQLPP	NCP	46.839
EHLQLPP;APAKPHV	NCP	3.006
APAKPHV;MLKAC	NCP	17.223
MLKAC;KPSGPRP	NCP	85.289
KPSGPRP;GYMDLRK	NCP	21.621
GYMDLRK;MFAEC	NCP	27.153
MFAEC;TVAPNLL	NCP	2.677
TVAPNLL;CDDYH	NCP	32.25
CDDYH;NLKKALA	NCP	17.211
NLKKALA;GKSVRPL	NCP	21.276
GKSVRPL;NHQPPK	NCP	139.112
NHQPPK;PEVLF	NCP	40.882
PEVLF;LMKANLL	NCP	9.741
LMKANLL;KPPEFLL	NCP	107.45
KPPEFLL;AREVAAF	NCP	27.571
AREVAAF;FTLLLF	NCP	11.204
FTLLLF;NYGGPK	NCP	47.374
NYGGPK;VVFKPP	NCP	43.625
VVFKPP;DYAPPPR	NCP	64.114
DYAPPPR;KKPHLLFP	NCP	140.643
KKPHLLFP;KAFPPPPP	NCP	24.959
KAFPPPPP;EGLSSG	NCP	7.282
EGLSSG;LSVPPAN	NCP	61.361
LSVPPAN;EKEKR	NCP	2.907
EKEKR;KKPKPPP	NCP	0.894
KKPKPPP;FPTWTPP	NCP	36.219
FPTWTPP;DSTLVKV	NCP	36.442
DSTLVKV;KMKPEAKK	NCP	4.923
KMKPEAKK;SVEKLLK	NCP	73.434
SVEKLLK;KAFLLLCS	NCP	64.401
KAFLLLCS;GPDDF	NCP	6.593
GPDDF;LTPVLSAK	NCP	128.281
LTPVLSAK;KDLGPP	NCP	77.61
KDLGPP;LKLLKTEL	NCP	83.256
LKLLKTEL;FPPPPHP	NCP	93.488
FPPPPHP;VGTHTVP	NCP	17.482
VGTHTVP;QPPLSSFH	NCP	54.803
QPPLSSFH;HAPKPR	NCP	128.212

HAPKPR;PVSPK	NCP	9.542
PVSPK;MVPFPLR	NCP	61.584
MVPFPLR;YWMEP	NCP	123.754
YWMEP;PTPVMY	NCP	5.384
PTPVMY;LQSLK	NCP	98.395
LQSLK;DPFKP	NCP	46.88
DPFKP;MELLEN	NCP	17.251
MELLEN;KQATVR	NCP	6.486
KQATVR;VEDLPFL	NCP	55.163
VEDLPFL;WAVLDKL	NCP	11.551
WAVLDKL;LNGRVVL	NCP	36.135
LNGRVVL;QTRPVR	NCP	17.145
QTRPVR;AVPFFS	NCP	153.404
AVPFFS;SPGGVY	NCP	19.104
SPGGVY;KGVWN	NCP	15.685
KGVWN;KYPPEL	NCP	62.187
KYPPEL;LACYDGH	NCP	29.469
LACYDGH;LLFPEL	NCP	25.092
LLFPEL;SHSLLVVP	NCP	25.604
SHSLLVVP;CEVCFP	NCP	79.191
CEVCFP;FAFLHP	NCP	72.79
FAFLHP;EHAAQG	NCP	12.561
EHAAQG;NHLMTP	NCP	37.518
NHLMTP;LLLLESR	NCP	100.535
LLLLESR;KEGKMVK	NCP	54.919
KEGKMVK;LLGLLPY	NCP	30.122
LLGLLPY;MGGPVV	NCP	22.495
MGGPVV;VSGLDLK	NCP	23.757
VSGLDLK;LEEKLK	NCP	2.97
LEEKLK;DPLSVL	NCP	44.301
DPLSVL;PSVPKPP	NCP	49.07
PSVPKPP;KVVWE	NCP	0.217
KVVWE;YPGVPA	NCP	85.26
YPGVPA;KDLGSNK	NCP	0.036
KDLGSNK;VVFHVK	NCP	10.223
VVFHVK;TVAPVLLR	NCP	11.094
TVAPVLLR;VLKTLR	NCP	4.515
VLKTLR;MLSHFFR	NCP	53.392
MLSHFFR;NLLEY	NCP	11.784
NLLEY;DDMEAE	NCP	0.585
DDMEAE;VLLLDPF	NCP	1.817
VLLLDPF;VPGHKKK	NCP	68.575
VPGHKKK;RSAPGSGSKVPQK	NCP	11.628
RSAPGSGSKVPQK;SAPGSGSKVPQKK	NCP	0.003
SAPGSGSKVPQKK;PGSGSKVPQK	NCP	0.006
PGSGSKVPQK;QGSPEKK	NCP	0.012
QGSPEKK;PAPRPAP	NCP	32.103
PAPRPAP;TSALWR	NCP	21.065
TSALWR;YVVAPR	NCP	51.343
YVVAPR;PSLVFGV	NCP	60.881
PSLVFGV;KPPTLAPQ	NCP	5.635

KPPTLAPQ;ELEVME	NCP	51.927
ELEVME;QEEVLLL	NCP	8.852
QEEVLLL;NLRTPT	NCP	42.922
NLRTPT;ENPLYK	NCP	35.676
ENPLYK;PPRFAL	NCP	62.279
PPRFAL;TTALVC	NCP	1.059
TTALVC;PLVASGGRT	NCP	84.409
PLVASGGRT;ESKKGSK	NCP	20.592
ESKKGSK;GVTLL	NCP	138.555
GVTLL;LDPGLDK	NCP	12.929
LDPGLDK;KSTLAAH	NCP	38.499
KSTLAAH;LPGAVLT	NCP	21.184
LPGAVLT;VEGVSLTG	NCP	6.978
VEGVSLTG;LDVEPA	NCP	0.264
LDVEPA;RSLAGGPK	NCP	11.891
RSLAGGPK;LPDGNLL	NCP	5.811
LPDGNLL;TVDGPW	NCP	83.412
TVDGPW;KSMATVP	NCP	6.225
KSMATVP;PPAVVGF	NCP	36.732
PPAVVGF;GPPGSLPP	NCP	9.847
GPPGSLPP;FVTLQYE	NCP	5.189
FVTLQYE;DSPGLF	NCP	73.074
DSPGLF;HAALGLQ	NCP	9.297
HAALGLQ;GPPVP	NCP	2.196
GPPVP;SLKHTPQ	NCP	42.628
SLKHTPQ;MVQPK	NCP	15.744
MVQPK;YTVPPPP	NCP	39.525
YTVPPPP;LMYPPLP	NCP	21.399
LMYPPLP;LPVTRAGP	NCP	51.57
LPVTRAGP;GVKLA	NCP	65.237
GVKLA;LSLVLT	NCP	2.719
LSLVLT;WKAETRT	NCP	102.936
WKAETRT;KLLSHSL	NCP	15.941
KLLSHSL;LAPLKGR	NCP	14.028
LAPLKGR;ESPLAM	NCP	61.257
ESPLAM;KEDFP	NCP	13.023
KEDFP;PSPMPPK	NCP	14.166
PSPMPPK;NHKKTEP	NCP	23.61
NHKKTEP;MDHPSPF	NCP	40.605
MDHPSPF;KEAPVDF	NCP	53.901
KEAPVDF;PVVVVLV	NCP	19.219
PVVVVLV;QGSFHR	NCP	10.881
QGSFHR;HSLLVNT	NCP	32.619
HSLLVNT;ASALLEY	NCP	92.994
ASALLEY;WTLDF	NCP	8.475
WTLDF;LELNPK	NCP	6.704
LELNPK;FPELLHK	NCP	89.872
FPELLHK;GRGPQ	NCP	11.997
GRGPQ;LFKHR	NCP	3.855
LFKHR;LELMGVQL	NCP	15.624
LELMGVQL;DNQLRL	NCP	3.54

DNQLRL;LTDLPAE	NCP	99.857
LTDLPAE;PNAAPP	NCP	7.791
PNAAPP;MSFARP	NCP	0.132
MSFARP;TAPVNF	NCP	18.031
TAPVNF;GYEPTLP	NCP	3.429
GYEPTLP;EGGDLPL	NCP	26.082
EGGDLPL;TAGHPGPP	NCP	6.155
TAGHPGPP;KAVPLPP	NCP	5.499
KAVPLPP;NKDVKF	NCP	2.94
NKDVKF;SLGVQLP	NCP	81.048
SLGVQLP;DQTPRD	NCP	15.274
DQTPRD;TGSSGAAGE	NCP	25.619
TGSSGAAGE;KQCLRP	NCP	9.567
KQCLRP;AEPKYK	NCP	22.416
AEPKYK;LDGPNVG	NCP	5.581
LDGPNVG;SLHWPK	NCP	90.129
SLHWPK;MRWFWK	NCP	5.019
MRWFWK;LVFNWRL	NCP	135.402
LVFNWRL;EEETSY	NCP	14.894
EEETSY;ALGPLELR	NCP	42.573
ALGPLELR;DKPDLG	NCP	63.033
DKPDLG;AKSSHG	NCP	5.964
AKSSHG;LAKLEKL	NCP	7.717
LAKLEKL;TYFPHDF	NCP	32.339
TYFPHDF;KSDRLDL	NCP	72.085
KSDRLDL;KAHAPPPP	NCP	189.518
KAHAPPPP;SSFQLLY	NCP	12.09
SSFQLLY;QPLFGK	NCP	50.545
QPLFGK;VPSPKVS	NCP	32.045
VPSPKVS;HSLSLLV	NCP	1.036
HSLSLLV;KEGHAF	NCP	12.021
KEGHAF;KEDHAF	NCP	22.697
KEDHAF;LPRTSY	NCP	21.25
LPRTSY;LEGPAY	NCP	0.954
LEGPAY;KPMKRSF	NCP	42.098
KPMKRSF;DLPKTPP	NCP	103.326
DLPKTPP;KSLVQGK	NCP	11.503
KSLVQGK;LLGSPVG	NCP	61.361
LLGSPVG;TLPFAP	NCP	21.279
TLPFAP;LEKGEELL	NCP	32.553
LEKGEELL;VSKKKK	NCP	8.494
VSKKKK;KVAPEPR	NCP	43.769
KVAPEPR;APGFLMP	NCP	25.858
APGFLMP;SGPTLVG	NCP	10.241
SGPTLVG;EEMSSTL	NCP	2.925
EEMSSTL;WLRMK	NCP	48.96
WLRMK;EDRWPR	NCP	39.96
EDRWPR;KPTRPC	NCP	24.547
KPTRPC;REEAAAAAPAPAADDQGVLK	NCP	59.687
REEAAAAAPAPAADDQGVLK;EVAEEEVVRTPR	NCP	15.348
EVAEEEVVRTPR;TEQVTTASFPVGSKK	NCP	14.523

TEQVTTASFPVGSKK;GEGALPTGK	NCP	17.19
GEGALPTGK;PGLLLLY	NCP	39.262
PGLLLLY;SHAALAL	NCP	47.037
SHAALAL;RMPPSPK	NCP	72.626
RMPPSPK;KSALWPP	NCP	17.878
KSALWPP;PRDPPR	NCP	26.849
PRDPPR;QQDLDLR	NCP	0.249
QQDLDLR;SPVLYK	NCP	3.493
SPVLYK;LLFLP	NCP	34.817
LLFLP;KGGLLTK	NCP	13.908
KGGLLTK;PPPLTPK	NCP	148.825
PPPLTPK;KLVFEVK	NCP	3.723
KLVFEVK;AVPPLHT	NCP	21.254
AVPPLHT;VYCNSP	NCP	48.369
VYCNSP;APTSLPNP	NCP	8.419
APTSLPNP;QYCWHK	NCP	82.656
QYCWHK;KMVYPP	NCP	6.132
KMVYPP;AAELK	NCP	42.534
AAELK;TVPFYQ	NCP	195.114
TVPFYQ;DLVQNLKN	NCP	71.186
DLVQNLKN;GLSAAAF	NCP	32.398
GLSAAAF;LLQNVYLF	NCP	126.461
LLQNVYLF;KNLMPPP	NCP	3.351
KNLMPPP;PLFSGPP	NCP	59.718
PLFSGPP;MTPLDF	NCP	2.17
MTPLDF;KDGHTVPL	NCP	5.747
KDGHTVPL;WSFHSH	NCP	10.515
WSFHSH;KPGSDPR	NCP	56.76
KPGSDPR;AALEY	NCP	4.744
AALEY;LLTPL	NCP	71.085
LLTPL;YNGPGY	NCP	2.103
YNGPGY;NQSVLL	NCP	12.579
NQSVLL;KRLLASK	NCP	61.214
KRLLASK;LKELAK	NCP	30.511
LKELAK;KSVKDAK	NCP	51.723
KSVKDAK;LELDTLK	NCP	29.58
LELDTLK;PVTPVSP	NCP	7.398
PVTPVSP;KMGKP	NCP	11.031
KMGKP;EGAVRQL	NCP	62.168
EGAVRQL;FVRPSL	NCP	110.793
FVRPSL;NPDPFF	NCP	17.584
NPDPFF;WQKLR	NCP	18.152
WQKLR;GYLDF	NCP	45.657
GYLDF;DLHVK	NCP	24.397
DLHVK;TVPSRRK	NCP	12.117
TVPSRRK;FKLLS	NCP	34.746
FKLLS;MPSLKT	NCP	81.8
MPSLKT;YLEGEKL	NCP	0.099
YLEGEKL;VLVVEQR	NCP	51.46
VLVVEQR;VSGLFSH	NCP	22.577
VSGLFSH;VLKEKKPL	NCP	6.117

VLKEKKPL;NLHNFG	NCP	33.225
NLHNFG;YSVLY	NCP	12.874
YSVLY;FLLFCTLQ	NCP	11.37
FLLFCTLQ;QFKPQK	NCP	14.549
QFKPQK;KERTPHW	NCP	25.536
KERTPHW;EVSLRGF	NCP	12.621
EVSLRGF;KHMTYL	NCP	173.592
KHMTYL;TVKAQGK	NCP	61.362
TVKAQGK;EPKKSQP	NCP	8.928
EPKKSQP;LSLHLFK	NCP	41.31
LSLHLFK;HMHERVG	NCP	8.632
HMHERVG;TEYLF	NCP	12.366
TEYLF;DPGQVTN	NCP	64.854
DPGQVTN;DSPLFMY	NCP	66.935
DSPLFMY;PLLMQAW	NCP	4.072
PLLMQAW;CATYDF	NCP	5.037
CATYDF;VTAPET	NCP	134.409
VTAPET;LYPGENL	NCP	17.022
LYPGENL;YSSPPHL	NCP	7.431
YSSPPHL;AEKVLVT	NCP	45.083
AEKVLVT;MLAPGGQ	NCP	2.295
MLAPGGQ;KAYADF	NCP	12.414
KAYADF;KGFMKPG	NCP	1.938
KGFMKPG;GVFGPP	NCP	73.927
GVFGPP;AMCDLRK	NCP	1.554
AMCDLRK;LSAAQPDK	NCP	110.196
LSAAQPDK;MTVFL	NCP	66.633
MTVFL;GPHCMSF	NCP	4.424
GPHCMSF;SLKLEER	NCP	8.82
SLKLEER;QLGRAAK	NCP	31.567
QLGRAAK;EPSELNK	NCP	5.136
EPSELNK;MSEGL	NCP	248.24
MSEGL;GAVADLK	NCP	1.524
GAVADLK;KGNKKLKT	NCP	15.595
KGNKKLKT;LHLPKN	NCP	17.364
LHLPKN;LDNYLPL	NCP	23.979
LDNYLPL;MSVNKKL	NCP	1.737
MSVNKKL;FEEVKAR	NCP	66.243
FEEVKAR;TLVHSPK	NCP	1.212
TLVHSPK;AQLLFLRP	NCP	146.852
AQLLFLRP;DFSLPR	NCP	58.992
DFSLPR;QTVPSPK	NCP	8.796
QTVPSPK;WDPFDR	NCP	18.672
WDPFDR;EALPSPR	NCP	4.615
EALPSPR;KFLLLPW	NCP	3.377
KFLLLPW;PGVKLVH	NCP	79.702
PGVKLVH;AVSRVYS	NCP	23.13
AVSRVYS;VLGSLSPF	NCP	180.341
VLGSLSPF;FPAQVLH	NCP	26.779
FPAQVLH;RNASLLR	NCP	17.954
RNASLLR;NNFPFFC	NCP	135.015

NNFPFFC;LSRSKSE	NCP	0.466
LSRSKSE;NPAAPP	NCP	25.307
NPAAPP;KTLQELKP	NCP	20.262
KTLQELKP;TLSVEAP	NCP	16.803
TLSVEAP;NALTKAV	NCP	6.267
NALTKAV;KTQLDHY	NCP	46.746
KTQLDHY;MTPPPPPP	NCP	57.52
MTPPPPPP;GVGLVV	NCP	51.374
GVGLVV;KDLVQTK	NCP	13.942
KDLVQTK;KSMQLQ	NCP	3.795
KSMQLQ;KGGKPRP	NCP	19.29
KGGKPRP;LPPVTPK	NCP	63.359
LPPVTPK;LPDFRM	NCP	55.09
LPDFRM;AAAAAGRL	NCP	0.071
AAAAAGRL;CAMCPP	NCP	49.671
CAMCPP;TVYRPAP	NCP	49.723
TVYRPAP;RKPAHPP	NCP	36.291
RKPAHPP;KSHDLVL	NCP	90.386
KSHDLVL;AGSPFLP	NCP	13.386
AGSPFLP;KGMHAHLK	NCP	33.246
KGMHAHLK;KARLQPVP	NCP	48.9
KARLQPVP;VGFLY	NCP	82.875
VGFLY;PLYAHKT	NCP	28.77
PLYAHKT;KWRLPR	NCP	20.091
KWRLPR;KPNPLPP	NCP	11.826
KPNPLPP;KFPSPPPP	NCP	0.811
KFPSPPPP;LLPPTPR	NCP	0.269
LLPPTPR;KNGGLDHK	NCP	3.45
KNGGLDHK;KTGMFRP	NCP	23.308
KTGMFRP;LKEGFLA	NCP	8.642
LKEGFLA;NFQLSGK	NCP	39.429
NFQLSGK;ELPVAER	NCP	42.426
ELPVAER;MELRH	NCP	15.937
MELRH;GPAVLRQP	NCP	59.063
GPAVLRQP;HLLLLGGY	NCP	2.203
HLLLLGGY;HPEPKV	NCP	65.027
HPEPKV;EVTKLF	NCP	24.507
EVTKLF;LQKCHC	NCP	49.638
LQKCHC;PLLKGLK	NCP	27.024
PLLKGLK;KAPKPLQK	NCP	3.937
KAPKPLQK;FLWFKPP	NCP	116.6
FLWFKPP;DGFVVLK	NCP	34.327
DGFVVLK;VREEKAR	NCP	2.897
VREEKAR;TAKTLVL	NCP	19.881
TAKTLVL;LELRVLL	NCP	112.675
LELRVLL;KFALL	NCP	29.798
KFALL;LLKLVTP	NCP	71.775
LLKLVTP;HMRVLNG	NCP	12.012
HMRVLNG;GSHLPLA	NCP	63.724
GSHLPLA;LPQKLLY	NCP	21.926
LPQKLLY;SFLSK	NCP	18.081

SFLSK;FDSTPA	NCP	15.43
FDSTPA;KLLVFEC	NCP	30.842
KLLVFEC;KHDLQGP	NCP	97.722
KHDLQGP;KKSHLAGK	NCP	97.576
KKSHLAGK;MKFLFQ	NCP	0.503
MKFLFQ;HSLLVDV	NCP	4.294
HSLLVDV;NVLLPKK	NCP	3.012
NVLLPKK;VPYRVFT	NCP	90.066
VPYRVFT;MGTSPK	NCP	60.092
MGTSPK;LAFYAPP	NCP	5.422
LAFYAPP;DLKKKPP	NCP	58.884
DLKKKPP;REFLR	NCP	10.409
REFLR;AAPPTFL	NCP	6.139
AAPPTFL;FLDGVLT	NCP	48.335
FLDGVLT;LASLPFVR	NCP	9.76
LASLPFVR;PLDKPTP	NCP	13.737
PLDKPTP;VLEWT	NCP	32.736
VLEWT;YQLTLLK	NCP	37.142
YQLTLLK;HLLLK	NCP	6.868
HLLLK;NHLMRTP	NCP	39.899
NHLMRTP;ATPVTAR	NCP	87.025
ATPVTAR;MCSFHR	NCP	110.877
MCSFHR;GPPPLPR	NCP	170.486
GPPPLPR;KFHVPPP	NCP	20.827
KFHVPPP;RSDSVSPK	NCP	7.2
RSDSVSPK;NDVAKRSL	NCP	92.325
NDVAKRSL;AKLLL	NCP	6.131
AKLLL;KVRKLPF	NCP	24.111
KVRKLPF;RYGYPK	NCP	29.304
RYGYPK;VDLRVGL	NCP	46.411
VDLRVGL;ANALFK	NCP	76.452
ANALFK;SLDAALK	NCP	94.443
SLDAALK;EPTLLR	NCP	73.157
EPTLLR;ASSCKVC	NCP	11.743
ASSCKVC;DRKLVETK	NCP	1.656
DRKLVETK;GPPPFTGPP	NCP	30.216
GPPPFTGPP;EVTRLPP	NCP	34.521
EVTRLPP;LKQTLSPR	NCP	7.818
LKQTLSPR;VDRAP	NCP	45.027
VDRAP;AVGSPDL	NCP	16.659
AVGSPDL;GREVKNR	NCP	6.594
GREVKNR;HALEVLK	NCP	26.858
HALEVLK;NLWELLNK	NCP	22.191
NLWELLNK;VSVVDL	NCP	5.431
VSVVDL;LLGFFPD	NCP	71.603
LLGFFPD;EGSPETG	NCP	3.12
EGSPETG;KWLVAP	NCP	20.227
KWLVAP;TVGLELLK	NCP	29.349
TVGLELLK;EYVTAL	NCP	2.642
EYVTAL;KMAGP	NCP	29.815
KMAGP;MRGQPK	NCP	68.313

MRGQPK;PSPRFQSP	NCP	184.856
PSPRFQSP;NVLRGPV	NCP	51.915
NVLRGPV;YVAPP	NCP	6.552
YVAPP;AVVLMGK	NCP	49.069
AVVLMGK;HFMPVMF	NCP	5.144
HFMPVMF;APHTKTLH	NCP	21.271
APHTKTLH;KWVGNPK	NCP	74.844
KWVGNPK;PNRNLLV	NCP	94.647
PNRNLLV;KHATHL	NCP	14.931
KHATHL;TNGSVKK	NCP	70.313
TNGSVKK;KFLLTNK	NCP	21.015
KFLLTNK;RGKTFN	NCP	136.881
RGKTFN;VSDGVLK	NCP	28.495
VSDGVLK;KRPGSGK	NCP	129.825
KRPGSGK;KAAMDLR	NCP	24.135
KAAMDLR;ALSGDSK	NCP	61.041
ALSGDSK;QVNHQF	NCP	129.945
QVNHQF;TVVAV	NCP	15.651
TVVAV;PLSVGPP	NCP	41.795
PLSVGPP;QSLLPL	NCP	9.046
QSLLPL;SVALPTA	NCP	43.781
SVALPTA;DLLNFRK	NCP	24.441
DLLNFRK;RVYSTK	NCP	44.167
RVYSTK;LDGNLLR	NCP	24.041
LDGNLLR;PFQSAGK	NCP	10.459
PFQSAGK;KTPLLF	NCP	25.683
KTPLLF;VPSEVLK	NCP	41.28
VPSEVLK;HLGGK	NCP	53.822
HLGGK;DHMLLR	NCP	5.863
DHMLLR;LGWPPSG	NCP	11.793
LGWPPSG;PAVPKVT	NCP	75.684
PAVPKVT;KDLYK	NCP	86.921
KDLYK;PAAPPTRLR	NCP	49.852
PAAPPTRLR;KLTYLE	NCP	28.367
KLTYLE;SLYLKVK	NCP	30.144
SLYLKVK;MDGSYP	NCP	119.13
MDGSYP;TPVNKQ	NCP	205.539
TPVNKQ;KDLKGPV	NCP	57.279
KDLKGPV;LSGELQK	NCP	97.405
LSGELQK;KDTLSKSH	NCP	19.842
KDTLSKSH;YHEEETK	NCP	19.217
YHEEETK;LPMDP	NCP	107.245
LPMDP;DPFKMH	NCP	88.544
DPFKMH;LTTPLHSP	NCP	0.349
LTTPLHSP;SAEFLLH	NCP	95.849
SAEFLLH;RQAVSLR	NCP	41.124
RQAVSLR;FTEVLK	NCP	21.78
FTEVLK;EEHPVL	NCP	17.257
EEHPVL;LAGKPSR	NCP	107.475
LAGKPSR;TVSSMPK	NCP	35.189
TVSSMPK;AAAPKE	NCP	22.489

AAAPKE;LGFHG	NCP	12.771
LGFHG;GVKELLGR	NCP	82.518
GVKELLGR;VGFFPR	NCP	143.43
VGFFPR;AGVALPLP	NCP	29.073
AGVALPLP;RLEGLLR	NCP	16.971
RLEGLLR;GVAPLNF	NCP	8.399
GVAPLNF;KNVFVEF	NCP	44.011
KNVFVEF;AAPPEPK	NCP	17.246
AAPPEPK;KFAYVPK	NCP	0.616
KFAYVPK;FAAPSWK	NCP	31.466
FAAPSWK;KRQSCH	NCP	17.47
KRQSCH;QLWVPSK	NCP	48.005
QLWVPSK;ANCCC	NCP	40.468
ANCCC;AVTLK	NCP	5.631
AVTLK;NQTPKKPK	NCP	5.532
NQTPKKPK;GHAGPRSP	NCP	87.399
GHAGPRSP;LGEMGPR	NCP	18.434
LGEMGPR;KSTTHRF	NCP	21.031
KSTTHRF;HSPFAGP	NCP	28.355
HSPFAGP;KAPFCPK	NCP	48.625
KAPFCPK;FPLKLT	NCP	58.413
FPLKLT;GCLHPLE	NCP	37.161
GCLHPLE;MNKDAQMR	NCP	67.607
MNKDAQMR;QPLLSFLK	NCP	82.945
QPLLSFLK;PPDTVHR	NCP	28.215
PPDTVHR;ALHVKP	NCP	51.578
ALHVKP;SPTFFKP	NCP	11.277
SPTFFKP;SEVLDVP	NCP	138.598
SEVLDVP;RLKNGPK	NCP	109.547
RLKNGPK;MEVSEF	NCP	86.334
MEVSEF;QFGLLAL	NCP	17.937
QFGLLAL;KLLLCA	NCP	72.825
KLLLCA;KYLPPQP	NCP	121.441
KYLPPQP;HQTNRPP	NCP	17.181
HQTNRPP;SSLKLKGK	NCP	29.402
SSLKLKGK;AKNFF	NCP	67.794
AKNFF;KDEVLKL	NCP	25.272
KDEVLKL;ASVSEL	NCP	166.924
ASVSEL;NSPEFHF	NCP	209.49
NSPEFHF;GDPLCPP	NCP	9.608
GDPLCPP;KVHLFK	NCP	13.29
KVHLFK;ALTDPKY	NCP	36.178
ALTDPKY;VLTSKYR	NCP	8.204
VLTSKYR;TVEEWPR	NCP	65.125
TVEEWPR;NRFVHW	NCP	1.041
NRFVHW;HGRKLP	NCP	1.389
HGRKLP;KVFGGP	NCP	16.476
KVFGGP;GTPRVLT	NCP	106.43
GTPRVLT;KSFMWPK	NCP	6.691
KSFMWPK;HDPFLFL	NCP	15.876
HDPFLFL;EVLGGF	NCP	9.41

EVLGGF;LDDLKGT	NCP	6.052
LDDLKGT;MFLVGG	NCP	67.629
MFLVGG;VSKVALR	NCP	46.164
VSKVALR;KMPDAH	NCP	9.41
KMPDAH;SYLGPK	NCP	49.698
SYLGPK;SLMTE	NCP	39.853
SLMTE;WVKYLH	NCP	5.45
WVKYLH;GKGNL	NCP	119.881
GKGNL;PRENRK	NCP	109.809
PRENRK;AHLLPF	NCP	68.235
AHLLPF;FDKALK	NCP	65.507
FDKALK;EEVEVLQ	NCP	93.964
EEVEVLQ;KLVGV	NCP	74.487
KLVGV;KPYPGA	NCP	23.555
KPYPGA;LLADFK	NCP	127.719
LLADFK;SDPVVSY	NCP	6.04
SDPVVSY;KLGRSHVL	NCP	52.742
KLGRSHVL;TSLMYLK	NCP	79.753
TSLMYLK;PLAVFR	NCP	0.876
PLAVFR;TAMVP	NCP	24.195
TAMVP;LGTRLL	NCP	3.093
LGTRLL;VGVPFLP	NCP	3.696
VGVPFLP;AASLSGY	NCP	27.545
AASLSGY;AKTLLPW	NCP	16.624
AKTLLPW;FSVLLPR	NCP	22.023
FSVLLPR;KNFPPPPP	NCP	67.914
KNFPPPPP;WALSML	NCP	26.514
WALSML;HTAELGAP	NCP	23.571
HTAELGAP;LPACLPLT	NCP	23.865
LPACLPLT;ESSCDC	NCP	24.972
ESSCDC;KDDGLP	NCP	45.858
KDDGLP;KSSAAPR	NCP	2.139
KSSAAPR;VGFLVAA	NCP	29.964
VGFLVAA;HEEPTL	NCP	122.445
HEEPTL;VLPAVGLSY	NCP	222.897
VLPAVGLSY;LKFLLPPP	NCP	102.704
LKFLLPPP;VELKEF	NCP	50.469
VELKEF;KPGMLPK	NCP	94.446
KPGMLPK;LGTDQPL	NCP	7.996
LGTDQPL;KSRKHPV	NCP	35.609
KSRKHPV;MLKGVQL	NCP	43.824
MLKGVQL;VHLLQKD	NCP	17.638
VHLLQKD;ELNPRHP	NCP	56.796
ELNPRHP;GPPPPRSP	NCP	62.868
GPPPPRSP;AAPPVYL	NCP	13.089
AAPPVYL;GFTYL	NCP	117.21
GFTYL;KQRNYK	NCP	318.164
KQRNYK;KNGVEEL	NCP	24.709
KNGVEEL;QLFLLG	NCP	59.19
QLFLLG;VTLATLK	NCP	86.921
VTLATLK;YLLPKF	NCP	41.431

YLLPKF;PRTVAP	NCP	3.636
PRTVAP;KCDSLFK	NCP	30.129
KCDSLFK;AAKVELR	NCP	79.938
AAKVELR;VVTEDLR	NCP	79.901
VVTEDLR;TDDYLAK	NCP	41.134
TDDYLAK;LDVTGVLK	NCP	41.105
LDVTGVLK;KKFVPFK	NCP	111.777
KKFVPFK;MQTSLPGA	NCP	52.05
MQTSLPGA;LALLNRSL	NCP	60.822
LALLNRSL;KPNKKSC	NCP	70.407
KPNKKSC;LGVLSK	NCP	12.483
LGVLSK;PVNMLYF	NCP	51.036
PVNMLYF;KSGKKCS	NCP	28.035
KSGKKCS;VGLNVLAL	NCP	184.273
VGLNVLAL;CFCCSK	NCP	1.518
CFCCSK;KSHKPPP	NCP	10.956
KSHKPPP;VTSNF	NCP	73.346
VTSNF;KTGLVLG	NCP	39.282
KTGLVLG;VAYPH	NCP	117.753
VAYPH;GTYLRVP	NCP	61.945
GTYLRVP;TVGPLTP	NCP	6.936
TVGPLTP;MQVLN	NCP	39.144
MQVLN;KQALEHR	NCP	73.092
KQALEHR;KSSKKFH	NCP	11.018
KSSKKFH;LSEKMR	NCP	19.048
LSEKMR;KGKLKPVLK	NCP	27.632
KGKLKPVLK;YVMLALY	NCP	139.321
YVMLALY;HCCCCCSK	NCP	11.223
HCCCCCSK;KDRGLK	NCP	10.686
KDRGLK;TATLLLT	NCP	61.583
TATLLLT;AVTGLP	NCP	4.984
AVTGLP;QDEFTVK	NCP	36.782
QDEFTVK;LDEVLKE	NCP	15.078
LDEVLKE;LTEVAAK	NCP	124.404
LTEVAAK;KSKKGGL	NCP	72.204
KSKKGGL;KFLVPR	NCP	38.721
KFLVPR;KPPRALQ	NCP	102.318
KPPRALQ;SPGALK	NCP	15.454
SPGALK;ESLLTKA	NCP	3.401
ESLLTKA;TSKTYFR	NCP	35.143
TSKTYFR;EVLLLH	NCP	9.941
EVLLLH;TLNLHRK	NCP	8.28
TLNLHRK;GLKAKSK	NCP	57.042
GLKAKSK;GVVRDLKEK	NCP	3.153
GVVRDLKEK;LATDLTK	NCP	22.138
LATDLTK;EPDFTPPP	NCP	9.081
EPDFTPPP;KAMFLY	NCP	26.684
KAMFLY;PKTHC	NCP	74.739
PKTHC;DLKEKLC	NCP	7.992
DLKEKLC;KDLLLYP	NCP	22.806
KDLLLYP;YALKRQGRTL	NCP	24.108

YALKRQGRTL;ALKRQGRTLYG	NCP	0.003
ALKRQGRTLYG;VLAVP	NCP	21.721
VLAVP;RVFSSS	NCP	7.689
RVFSSS;KVLDSF	NCP	11.082
KVLDSF;QVWWH	NCP	11.034
QVWWH;LPKAPKP	NCP	54.273
LPKAPKP;TAEAPAA	NCP	28.719
TAEAPAA;RLALGPH	NCP	56.904
RLALGPH;KAGSLTAL	NCP	78.362
KAGSLTAL;ALRTDY	NCP	20.299
ALRTDY;NDQSPR	NCP	111.656
NDQSPR;RDHKFDL	NCP	29.862
RDHKFDL;NHGVVNR	NCP	33.402
NHGVVNR;EPPKYVH	NCP	36.187
EPPKYVH;KVLEPQR	NCP	36.986
KVLEPQR;SSPVYVGR	NCP	63.577
SSPVYVGR;FGGWKGVT	NCP	95.877
FGGWKGVT;TLSTAFR	NCP	5.405
TLSTAFR;KVVDYSQFQESDDADEDYGR	NCP	9.991
KVVDYSQFQESDDADEDYGR;ADEDYGRDSGPPAKK	NCP	0.039
ADEDYGRDSGPPAKK;DEDYGRDSGPPAK	NCP	0.003
DEDYGRDSGPPAK;EEEDEAPFQEK	NCP	0.285
EEEDEAPFQEK;SGSDEDFLVE	NCP	0.036
SGSDEDFLVE;VTPSPVKGK	NCP	0.144
VTPSPVKGK;NTPPTPPP	NCP	0.161
NTPPTPPP;ASSGRPPP	NCP	7.578
ASSGRPPP;KGDVVVLS	NCP	2.893
KGDVVVLS;RHLPPPP	NCP	23.921
RHLPPPP;QLVRFV	NCP	5.013
QLVRFV;KETPTKE	NCP	23.094
KETPTKE;LYANLGK	NCP	23.091
LYANLGK;LLNPPSLLF	NCP	12.751
LLNPPSLLF;RPKKGLP	NCP	45.029
RPKKGLP;KDSLTSYK	NCP	25.36
KDSLTSYK;ALELCKP	NCP	18.287
ALELCKP;EPLTFDK	NCP	12.073
EPLTFDK;KVYWEK	NCP	2.511
KVYWEK;ETKKAGP	NCP	63.759
ETKKAGP;LPHDPNL	NCP	21.552
LPHDPNL;EVEST	NCP	8.274
EVEST;LLSSNELL	NCP	43.082
LLSSNELL;DDPLSG	NCP	58.552
DDPLSG;QTNPML	NCP	60.743
QTNPML;PLLVEF	NCP	29.101
PLLVEF;LPDLSPLP	NCP	0.071
LPDLSPLP;KSPFSVG	NCP	54.138
KSPFSVG;LYKKAKSL	NCP	58.833
LYKKAKSL;VVLLSAAF	NCP	16.131
VVLLSAAF;RVVFQ	NCP	1.987
RVVFQ;KVHLLFL	NCP	64.418
KVHLLFL;LSYQSSL	NCP	0.525

LSYQSSL;ATDGLVL	NCP	12.483
ATDGLVL;QYLLFDP	NCP	15.933
QYLLFDP;KRCAARP	NCP	67.432
KRCAARP;VAALP	NCP	201.651
VAALP;SLAAQP	NCP	5.028
SLAAQP;LMQLHHSK	NCP	38.127
LMQLHHSK;EHFALK	NCP	2.601
EHFALK;KKKKTSP	NCP	20.715
KKKKTSP;DFAPQD	NCP	66.21
DFAPQD;FCKPR	NCP	8.808
FCKPR;QGNADKLR	NCP	18.912
QGNADKLR;KSAHFFL	NCP	69.432
KSAHFFL;SYMLGPP	NCP	0.161
SYMLGPP;LSLVTLA	NCP	39.46
LSLVTLA;KNQQVVK	NCP	30.506
KNQQVVK;TATFVP	NCP	40.416
TATFVP;KQPDDM	NCP	30.732
KQPDDM;NSSLLLMF	NCP	47.439
NSSLLLMF;VKTEC	NCP	6.12
VKTEC;VPDLF	NCP	23.785
VPDLF;GEPTLQPK	NCP	51.578
GEPTLQPK;EFLKLCV	NCP	16.774
EFLKLCV;KQATMGP	NCP	12.647
KQATMGP;VPPPPHPP	NCP	20.821
VPPPPHPP;GHLMRSS	NCP	31.707
GHLMRSS;VSHFYR	NCP	0.38
VSHFYR;DPEASVVP	NCP	36.699
DPEASVVP;VLQPVP	NCP	10.044
VLQPVP;LPHVFPP	NCP	257.82
LPHVFPP;KTYNLF	NCP	133.545
KTYNLF;VELSLPKK	NCP	3.822
VELSLPKK;MTFSKTP	NCP	11.73
MTFSKTP;EEVALP	NCP	65.235
EEVALP;VRGVGLGR	NCP	153.889
VRGVGLGR;DLLSL	NCP	16.58
DLLSL;LLHKGLLY	NCP	13.162
LLHKGLLY;KALEC	NCP	17.961
KALEC;MWVTLPK	NCP	55.464
MWVTLPK;LSTKKAAP	NCP	4.05
LSTKKAAP;KNLQPGN	NCP	51.678
KNLQPGN;TEKMLEP	NCP	37.658
TEKMLEP;TVHHYHR	NCP	116.385
TVHHYHR;SGPSLHVR	NCP	30.184
SGPSLHVR;SHSLLLV	NCP	8.594
SHSLLLV;KAAVLEY	NCP	166.713
KAAVLEY;LDLRLPY	NCP	54.39
LDLRLPY;LPGGSVLPA	NCP	34.093
LPGGSVLPA;FFVPQCR	NCP	117.791
FFVPQCR;KDLFNPK	NCP	28.414
KDLFNPK;KAGPKKK	NCP	8.781
KAGPKKK;VKPEK	NCP	12.941

VKPEK;LRNLPY	NCP	0.006
LRNLPY;KYFKHR	NCP	5.506
KYFKHR;PHTFGH	NCP	13.607
PHTFGH;ESSVPGP	NCP	29.356
ESSVPGP;KPADN	NCP	22.806
KPADN;AVGVLK	NCP	191.214
AVGVLK;KPPAGMK	NCP	39.545
KPPAGMK;ALQAGLR	NCP	5.782
ALQAGLR;KSVPPPP	NCP	7.671
KSVPPPP;MPNVLLL	NCP	4.722
MPNVLLL;APASKTPP	NCP	112.676
APASKTPP;HRPDLR	NCP	39.696
HRPDLR;KASLLAGP	NCP	38.778
KASLLAGP;ASKGPAG	NCP	21.24
ASKGPAG;VGPPH	NCP	13.23
VGPPH;KQPLSVK	NCP	22.851
KQPLSVK;RLEVLR	NCP	125.314
RLEVLR;NTPPALP	NCP	12.575
NTPPALP;LDSTVPK	NCP	117.3
LDSTVPK;KSLEAPVP	NCP	11.943
KSLEAPVP;KPMNKP	NCP	34.225
KPMNKP;NLPHALM	NCP	47.817
NLPHALM;DVEFDLK	NCP	8.486
DVEFDLK;YGVLVSV	NCP	18.961
YGVLVSV;LEYLTA	NCP	16.95
LEYLTA;SLYTLLR	NCP	46.604
SLYTLLR;PPPPPGAPAR	NCP	34.41
PPPPPGAPAR;MTTPYP	NCP	56.512
MTTPYP;TPGLLNR	NCP	43.944
TPGLLNR;KPHSGDH	NCP	16.043
KPHSGDH;TTTVKSK	NCP	15.3
TTTVKSK;SDDCK	NCP	121.665
SDDCK;MVSEPQVK	NCP	25.467
MVSEPQVK;TGLELLK	NCP	15.616
TGLELLK;KKTSETLSQAGQK	NCP	124.313
KKTSETLSQAGQK;KSFEEKVENLK	NCP	13.353
KSFEEKVENLK;KLMEPK	NCP	110.815
KLMEPK;VGLDAAGKTT	NCP	1.431
VGLDAAGKTT;MLQEDELRDAVLL	NCP	0.261
MLQEDELRDAVLL;MRMGTL	NCP	132.483
MRMGTL;FNFSSGK	NCP	10.439
FNFSSGK;PCAGPP	NCP	4.6
PCAGPP;PPFFKY	NCP	5.534
PPFFKY;HLDARK	NCP	25.197
HLDARK;NTLERVH	NCP	8.409
NTLERVH;CMDLRK	NCP	93.144
CMDLRK;FGGFVT	NCP	76.417
FGGFVT;RGYMPR	NCP	18.732
RGYMPR;ENFGYSNR	NCP	10.292
ENFGYSNR;KFCCD	NCP	304.902
KFCCD;GSELLLTK	NCP	60.079

GSELLLTK;AFRVLP	NCP	48.927
AFRVLP;VVSPVA	NCP	61.044
VVSPVA;KYSKVP	NCP	65.91
KYSKVP;NPLSKYK	NCP	1.382
NPLSKYK;EWDGKVK	NCP	4.324
EWDGKVK;MLPLSLLK	NCP	14.954
MLPLSLLK;KKNEYPL	NCP	35.353
KKNEYPL;KDALLPTP	NCP	3.426
KDALLPTP;LELPRLQ	NCP	36.056
LELPRLQ;KELTPA	NCP	85.381
KELTPA;FLLQG	NCP	6.303
FLLQG;KEDPGAK	NCP	15.837
KEDPGAK;RGPNAPK	NCP	43.569
RGPNAPK;KPEAFMK	NCP	76.631
KPEAFMK;EHTGALR	NCP	51.247
EHTGALR;AQYQL	NCP	58.164
AQYQL;KPRNVSN	NCP	23.528
KPRNVSN;CVCCCFN	NCP	37.008
CVCCCFN;QFVDQR	NCP	15.049
QFVDQR;VPQVKMF	NCP	51.935
VPQVKMF;PLTLTTF	NCP	111.666
PLTLTTF;VTTYLGF	NCP	20.191
VTTYLGF;NLVPFK	NCP	4.389
NLVPFK;PGNGK	NCP	19.265
PGNGK;LLALDFF	NCP	76.06
LLALDFF;KGLLSHH	NCP	99.993
KGLLSHH;DYPLGP	NCP	76.217
DYPLGP;NQRWPP	NCP	25.059
NQRWPP;RDHDFK	NCP	21.705
RDHDFK;KFNPP	NCP	16.932
KFNPP;FEVHAAK	NCP	2.995
FEVHAAK;QSDLSLP	NCP	10.433
QSDLSLP;RVKLNF	NCP	151.56
RVKLNF;LVRLSTLY	NCP	1.894
LVRLSTLY;GLLEPPLE	NCP	39.432
GLLEPPLE;KEMTPK	NCP	14.099
KEMTPK;LSLVEGK	NCP	77.13
LSLVEGK;YLLKDK	NCP	123.987
YLLKDK;GVTLLFP	NCP	82.551
GVTLLFP;PVSPLLLA	NCP	100.036
PVSPLLLA;RLYQLQ	NCP	19.493
RLYQLQ;KVLLPVP	NCP	86.211
KVLLPVP;PEAPAYR	NCP	38.452
PEAPAYR;QGSALAF	NCP	16.637
QGSALAF;KPLAPP	NCP	31.243
KPLAPP;TVPPSL	NCP	26.834
TVPPSL;NKFPPPPP	NCP	8.83
NKFPPPPP;TYRKCF	NCP	46.572
TYRKCF;KEHLPAP	NCP	19.964
KEHLPAP;LLAAEYL	NCP	43.063
LLAAEYL;VGSHCDL	NCP	44.604

VGSHCDL;LDNPVV	NCP	48.279
LDNPVV;KSHDLV	NCP	1.083
KSHDLV;SVNLLPR	NCP	19.515
SVNLLPR;AAPGTRAR	NCP	41.73
AAPGTRAR;FCNSCR	NCP	55.689
FCNSCR;KLLPRL	NCP	16.056
KLLPRL;FVLGK	NCP	0.929
FVLGK;YGKLLLP	NCP	1.74
YGKLLLP;LGVPN	NCP	78.592
LGVPN;KNLDYLPP	NCP	34.865
KNLDYLPP;VEPTKTP	NCP	15.292
VEPTKTP;APFRSK	NCP	47.367
APFRSK;LPSRPLE	NCP	18.767
LPSRPLE;ALLKKP	NCP	131.589
ALLKKP;KPPMSLH	NCP	23.68
KPPMSLH;LLTKKVL	NCP	13.542
LLTKKVL;FLTPLEF	NCP	1.199
FLTPLEF;NDSMDC	NCP	91.269
NDSMDC;RDLPNP	NCP	109.503
RDLPNP;SFPLGSPL	NCP	1.48
SFPLGSPL;PMLRLY	NCP	117.986
PMLRLY;LPFDP	NCP	116.616
LPFDP;SYELDP	NCP	18.267
SYELDP;VSPLL	NCP	106.171
VSPLL;LGHVKYK	NCP	21.272
LGHVKYK;MHSYTVP	NCP	28.012
MHSYTVP;TWQSL	NCP	35.654
TWQSL;RTLVGER	NCP	61.443
RTLVGER;LKLEEF	NCP	9.541
LKLEEF;RVHKRFC	NCP	2.582
RVHKRFC;HDTKPK	NCP	89.496
HDTKPK;FPPLAF	NCP	8.953
FPPLAF;TTDKPK	NCP	13.668
TTDKPK;LPPLYK	NCP	24.929
LPPLYK;PPAMK	NCP	43.914
PPAMK;FGTEVK	NCP	65.827
FGTEVK;FPASP	NCP	7.089
FPASP;PGKPVTC	NCP	26.031
PGKPVTC;ELTPAVY	NCP	1.811
ELTPAVY;NGKLRF	NCP	99.549
NGKLRF;VRALPLAL	NCP	88.024
VRALPLAL;CTFEYL	NCP	48.954
CTFEYL;GQYPK	NCP	18.707
GQYPK;KADLLPL	NCP	102.262
KADLLPL;LLFGPK	NCP	114.426
LLFGPK;SKFEVD	NCP	75.194
SKFEVD;NGLDVP	NCP	75.208
NGLDVP;GLGSQRVK	NCP	2.016
GLGSQRVK;ELFGLLY	NCP	385.925
ELFGLLY;AERRVFP	NCP	72.292
AERRVFP;LKGNF	NCP	7.416

LKGNF;AAVNKPLT	NCP	37.583
AAVNKPLT;CPSRPVT	NCP	83.355
CPSRPVT;TNEKLQQL	NCP	55.896
TNEKLQQL;RCKGPK	NCP	19.05
RCKGPK;MEEKLKK	NCP	13.479
MEEKLKK;SVALVSLK	NCP	34.329
SVALVSLK;EFPEPLK	NCP	80.544
EFPEPLK;ALELLMA	NCP	25.491
ALELLMA;REKLSPK	NCP	41.803
REKLSPK;KDMQYK	NCP	45.258
KDMQYK;VTSVLP	NCP	84.387
VTSVLP;KTYGFGL	NCP	43.727
KTYGFGL;LSALEL	NCP	30.393
LSALEL;KTLHRSE	NCP	13.632
KTLHRSE;KPGNPPP	NCP	23.713
KPGNPPP;DKGVVSV	NCP	40.014
DKGVVSV;LYNEP	NCP	81.882
LYNEP;NTVNLP	NCP	10.031
NTVNLP;AGPSFPLG	NCP	29.343
AGPSFPLG;SLEGKPL	NCP	0.516
SLEGKPL;LAVAPVGF	NCP	43.371
LAVAPVGF;DPKAAPR	NCP	87.897
DPKAAPR;NMAVLEY	NCP	39.75
NMAVLEY;VGKNWL	NCP	59.868
VGKNWL;KPPSVHR	NCP	32.031
KPPSVHR;NGVLLSK	NCP	28.735
NGVLLSK;QGSKLP	NCP	44.202
QGSKLP;TGAGPLL	NCP	7.866
TGAGPLL;MHGKKK	NCP	13.602
MHGKKK;KLRAALK	NCP	5.507
KLRAALK;MDYQK	NCP	76.831
MDYQK;KELKMEK	NCP	26.393
KELKMEK;SSLLDPR	NCP	25.165
SSLLDPR;VELKDVK	NCP	45.158
VELKDVK;FTDSDF	NCP	167.632
FTDSDF;VAPPMLK	NCP	86.88
VAPPMLK;KSLAAGY	NCP	57.941
KSLAAGY;NPPGVFP	NCP	10.726
NPPGVFP;NFLAGYSP	NCP	44.964
NFLAGYSP;SVLTMR	NCP	27.621
SVLTMR;LSAALEL	NCP	42.672
LSAALEL;KPCKPC	NCP	6.3
KPCKPC;LFAGFEA	NCP	60.302
LFAGFEA;TGALLQ	NCP	9.87
TGALLQ;LKEFDGK	NCP	0.283
LKEFDGK;LVVPSK	NCP	99.987
LVVPSK;KDPLQLP	NCP	2.898
KDPLQLP;LPELNGK	NCP	35.445
LPELNGK;ELPDVPR	NCP	41.523
ELPDVPR;TVGGPAP	NCP	57.443
TVGGPAP;HPEVKP	NCP	15.364

HPEVKP;LPLENK	NCP	22.071
LPLENK;KKVGLVK	NCP	42.822
KKVGLVK;MLALLSR	NCP	9.665
MLALLSR;TPARVGH	NCP	39.679
TPARVGH;QRGDLH	NCP	21.186
QRGDLH;KVLAPRSD	NCP	15.009
KVLAPRSD;VLFPP	NCP	50.03
VLFPP;ALLELEA	NCP	14.736
ALLELEA;GVMFLQG	NCP	85.401
GVMFLQG;LSFPTTKT	NCP	39.667
LSFPTTKT;NGLTTFK	NCP	35.664
NGLTTFK;CAPLDT	NCP	41.064
CAPLDT;LLDCPPP	NCP	26.838
LLDCPPP;KYLSVP	NCP	5.055
KYLSVP;VGYGPK	NCP	55.635
VGYGPK;LSNGKKP	NCP	16.305
LSNGKKP;SKYEPLL	NCP	64.508
SKYEPLL;KLVTDF	NCP	57.978
KLVTDF;FTKYTSSK	NCP	6.631
FTKYTSSK;VVSYT	NCP	45.009
VVSYT;KVPVPDPP	NCP	18.995
KVPVPDPP;NKKEFDK	NCP	130.663
NKKEFDK;QDKEFR	NCP	4.68
QDKEFR;PLLPHHK	NCP	6.006
PLLPHHK;KGYLFS	NCP	18.263
KGYLFS;QANSRPF	NCP	72.609
QANSRPF;VTTKPQK	NCP	12.168
VTTKPQK;VLLDTPP	NCP	34.219
VLLDTPP;KSDSENR	NCP	72.164
KSDSENR;GVRDAP	NCP	75.081
GVRDAP;KGSYVGL	NCP	12.729
KGSYVGL;KMQWA	NCP	9.859
KMQWA;WLYCGCK	NCP	83.772
WLYCGCK;LTVLSNLSY	NCP	30.207
LTVLSNLSY;KDSVP	NCP	64.253
KDSVP;KDALLGP	NCP	18.403
KDALLGP;WSSKAPK	NCP	67.85
WSSKAPK;NVGGLTA	NCP	59.484
NVGGLTA;PDDSL	NCP	8.1
PDDSL;NASVPK	NCP	58.984
NASVPK;PDLPS	NCP	63.074
PDLPS;ASPLGGPK	NCP	28.002
ASPLGGPK;KPPGGKP	NCP	39.687
KPPGGKP;VRHKCK	NCP	3.966
VRHKCK;EGQMKK	NCP	31.83
EGQMKK;MDPDLRK	NCP	8.859
MDPDLRK;NELLKPHL	NCP	7.983
NELLKPHL;EEERYF	NCP	12.754
EEERYF;DGPKFL	NCP	2.502
DGPKFL;EPPVPY	NCP	19.953
EPPVPY;VDHETLH	NCP	52.746

VDHETLH;LGVADLQ	NCP	44.342
LGVADLQ;NVHQPSW	NCP	29.88
NVHQPSW;FLAGPHL	NCP	56.715
FLAGPHL;CANPVKL	NCP	13.878
CANPVKL;KTTAPPK	NCP	140.776
KTTAPPK;DLENVKLL	NCP	15.15
DLENVKLL;KMLAHVP	NCP	25.559
KMLAHVP;MQFGEKL	NCP	34.684
MQFGEKL;TADGVSLP	NCP	1.218
TADGVSLP;SSSVPTVK	NCP	3.209
SSSVPTVK;KTYLLF	NCP	132.963
KTYLLF;EAALGEPL	NCP	23.241
EAALGEPL;LTTVPK	NCP	20.526
LTTVPK;ELPLGGP	NCP	14.791
ELPLGGP;LHFRFL	NCP	21.27
LHFRFL;KPLFT	NCP	27.186
KPLFT;RLLGPFM	NCP	145.04
RLLGPFM;LEDLDQVL	NCP	40.869
LEDLDQVL;LLPKKWM	NCP	182.473
LLPKKWM;VTDLATY	NCP	33.065
VTDLATY;LGSGKELP	NCP	6.67
LGSGKELP;YTKYTSSK	NCP	8.886
YTKYTSSK;VLTSPPP	NCP	44.916
VLTSPPP;VEELDPV	NCP	30.105
VEELDPV;FPPPAPV	NCP	9.497
FPPPAPV;KKTKKGP	NCP	75.295
KKTKKGP;CEDDP	NCP	78.221
CEDDP;YSSYLAK	NCP	29.935
YSSYLAK;KQFLLPPP	NCP	57.554
KQFLLPPP;KTKLAY	NCP	37.869
KTKLAY;DDSKPK	NCP	0.727
DDSKPK;LEVNELKE	NCP	112.05
LEVNELKE;MDMVDF	NCP	14.589
MDMVDF;AVQLSKK	NCP	13.758
AVQLSKK;LSEELYL	NCP	3.582
LSEELYL;VSVGTAPL	NCP	40.943
VSVGTAPL;TQLLEYL	NCP	36.934
TQLLEYL;LLAVTP	NCP	140.415
LLAVTP;VDKVLK	NCP	12.639
VDKVLK;VPEVKPK	NCP	53.253
VPEVKPK;SLAKY	NCP	33.942
SLAKY;MKHLER	NCP	2.639
MKHLER;RLPEPKQ	NCP	21.504
RLPEPKQ;CNMDV	NCP	54.931
CNMDV;LVQTVAY	NCP	61.746
LVQTVAY;ASPGLPLR	NCP	5.115
ASPGLPLR;KEPLF	NCP	16.059
KEPLF;VGPLL	NCP	19.398
VGPLL;KERKTAP	NCP	14.256
KERKTAP;RASLVPA	NCP	9.816
RASLVPA;SADPPLTL	NCP	24.279

SADPPLTL;GPSQKTK	NCP	57.221
GPSQKTK;AKPTPP	NCP	216.742
AKPTPP;KEETAH	NCP	2.601
KEETAH;QAPVP	NCP	43.106
QAPVP;AASGSGMAQK	NCP	38.706
AASGSGMAQK;SLLTLV	NCP	9.34
SLLTLV;VVVCKP	NCP	88.463
VVVCKP;ANLQTER	NCP	1.137
ANLQTER;AAVWG	NCP	52.99
AAVWG;KYSLE	NCP	150.146
KYSLE;HTSDPPR	NCP	127.512
HTSDPPR;TAGLLKK	NCP	8.886
TAGLLKK;QWKPLF	NCP	14.127
QWKPLF;VAPYPQ	NCP	39.864
VAPYPQ;LSAAKSPK	NCP	34.482
LSAAKSPK;TAAAVLFL	NCP	53.284
TAAAVLFL;EALLELA	NCP	67.146
EALLELA;FKAGFF	NCP	35.265
FKAGFF;LDKLPVG	NCP	7.935
LDKLPVG;AAVKLKAL	NCP	73.172
AAVKLKAL;METRSPQ	NCP	0.499
METRSPQ;GLEEFL	NCP	61.044
GLEEFL;PGRFHPK	NCP	18.254
PGRFHPK;AEQLLLP	NCP	32.226
AEQLLLP;VTRQPK	NCP	23.023
VTRQPK;RPTGHAPK	NCP	1.032
RPTGHAPK;WQPLAL	NCP	16.878
WQPLAL;PLLVGLR	NCP	116.832
PLLVGLR;AAKNVVSQF	NCP	39.248
AAKNVVSQF;TCVYSPPKP	NCP	159.939
TCVYSPPKP;SCDDPG	NCP	20.956
SCDDPG;MESLKQ	NCP	12.245
MESLKQ;SVLSSTEV	NCP	67.909
SVLSSTEV;ASLSTF	NCP	11.304
ASLSTF;YGFQLLR	NCP	23.069
YGFQLLR;LPSRSLL	NCP	0.592
LPSRSLL;QFSSLLY	NCP	75.384
QFSSLLY;TGADLAPR	NCP	46.467
TGADLAPR;WGGLRKPS	NCP	43.868
WGGLRKPS;KLPLTLK	NCP	106.684
KLPLTLK;VDLRKTP	NCP	6.866
VDLRKTP;KELVTTF	NCP	142.612
KELVTTF;KRTSQPE	NCP	25.118
KRTSQPE;YDPPL	NCP	34.243
YDPPL;KVPSKH	NCP	66.291
KVPSKH;EVDPQ	NCP	29.381
EVDPQ;SDGVLVK	NCP	13.177
SDGVLVK;APASGHGP	NCP	136.646
APASGHGP;FFVYQ	NCP	137.722
FFVYQ;KTFTVPP	NCP	50.003
KTFTVPP;YDDAFLG	NCP	23.518

YDDAFLG;GFVGTLK	NCP	31.433
GFVGTLK;KGLPEKEN	NCP	11.439
KGLPEKEN;QHPKY	NCP	37.543
QHPKY;KPVPAH	NCP	12.347
KPVPAH;KWGPKP	NCP	144.453
KWGPKP;LPGSFPNL	NCP	186.697
LPGSFPNL;RWYALH	NCP	44.343
RWYALH;KDDPSR	NCP	43.067
KDDPSR;NDKGHSS	NCP	68.773
NDKGHSS;NAPFPR	NCP	24.071
NAPFPR;VRQNPP	NCP	67.923
VRQNPP;PGPPYK	NCP	4.155
PGPPYK;ACMHHK	NCP	21.582
ACMHHK;DYLKEML	NCP	68.305
DYLKEML;RHFKMY	NCP	34.425
RHFKMY;AEARPGPAGLQ	NCP	4.049
AEARPGPAGLQ;LSPAVS	NCP	2.508
LSPAVS;DWHHAV	NCP	124.875
DWHHAV;FLSTPWF	NCP	15.57
FLSTPWF;RHLQSFVPK	NCP	71.425
RHLQSFVPK;FLLALEVW	NCP	18.839
FLLALEVW;LTVPEHK	NCP	33.154
LTVPEHK;EGPLLTR	NCP	186.548
EGPLLTR;NFEKPFL	NCP	102.046
NFEKPFL;KASHVTL	NCP	32.765
KASHVTL;VSEASKPP	NCP	96.084
VSEASKPP;RGTKPR	NCP	137.079
RGTKPR;VLMAKPKC	NCP	96.193
VLMAKPKC;YLLMAK	NCP	46.989
YLLMAK;LPGGVLF	NCP	15.23
LPGGVLF;RPCLCRK	NCP	23.4
RPCLCRK;FTLSKK	NCP	183.169
FTLSKK;CEMKK	NCP	53.844
CEMKK;LSDALAK	NCP	30.743
LSDALAK;LERRAGP	NCP	43.188
LERRAGP;LHLEQLP	NCP	154.893
LHLEQLP;QSCCFKPP	NCP	15.282
QSCCFKPP;MMDPF	NCP	11.185
MMDPF;SLAGLL	NCP	1.1
SLAGLL;GPKPPPPP	NCP	8.229
GPKPPPPP;DPPKLQ	NCP	55.11
DPPKLQ;LLFLLGLQL	NCP	41.449
LLFLLGLQL;HLQLALR	NCP	195.224
HLQLALR;VERVPF	NCP	1.042
VERVPF;LSDLDLSL	NCP	53.255
LSDLDLSL;NDMPQ	NCP	82.59
NDMPQ;LTGGGLK	NCP	13.897
LTGGGLK;PPAAAPPPP	NCP	1.31
PPAAAPPPP;LPPVSER	NCP	5.634
LPPVSER;TLDEAE	NCP	12.034
TLDEAE;CDSSDC	NCP	69.546

CDSSDC;FDLMAY	NCP	64.881
FDLMAY;FYHDEL	NCP	189.029
FYHDEL;QMKVR	NCP	73.611
QMKVR;VGDLLVA	NCP	109.429
VGDLLVA;VEMHEP	NCP	132.377
VEMHEP;NELLTLL	NCP	17.49
NELLTLL;LMKFFVG	NCP	1.87
LMKFFVG;SLYVY	NCP	36.636
SLYVY;VHLPGAK	NCP	66.453
VHLPGAK;VVANLPR	NCP	52.941
VVANLPR;KPPMYSK	NCP	17.727
KPPMYSK;VLLMTQLF	NCP	7.271
VLLMTQLF;GLTLTL	NCP	212.793
GLTLTL;CLSSASW	NCP	7.059
CLSSASW;EDLRNLP	NCP	27.43
EDLRNLP;ENSYKYP	NCP	93.666
ENSYKYP;QPSLKSK	NCP	64.569
QPSLKSK;KYNGL	NCP	4.205
KYNGL;KGSLVAF	NCP	3.261
KGSLVAF;EVAEPKPVK	NCP	28.728
EVAEPKPVK;TNGLLK	NCP	76.369
TNGLLK;KLVPHQLA	NCP	54.995
KLVPHQLA;KGYAMK	NCP	24.552
KGYAMK;ADLVFL	NCP	139.308
ADLVFL;LLALADL	NCP	55.89
LLALADL;WETHTKK	NCP	55.221
WETHTKK;VPPKY	NCP	19.177
VPPKY;LDVLVKK	NCP	39.239
LDVLVKK;VLLNL	NCP	3.384
VLLNL;QGSLGLHR	NCP	4.756
QGSLGLHR;VYKLRAK	NCP	53.85
VYKLRAK;KERVGFVK	NCP	4.235
KERVGFVK;QTLNFRK	NCP	67.996
QTLNFRK;VPPFFT	NCP	12.887
VPPFFT;MDDKKK	NCP	81.66
MDDKKK;MTSLLLP	NCP	13.312
MTSLLLP;AAHTVP	NCP	23.069
AAHTVP;AAVTYYL	NCP	2.337
AAVTYYL;YTVVY	NCP	22.821
YTVVY;SGKYVGL	NCP	27.09
SGKYVGL;LVAPSK	NCP	89.467
LVAPSK;VQHVLY	NCP	18.18
VQHVLY;LSKKKEC	NCP	12.213
LSKKKEC;PPPAPPP	NCP	12.66
PPPAPPP;KVHFALH	NCP	1.461
KVHFALH;KEAVFPP	NCP	53.676
KEAVFPP;CYMVH	NCP	55.044
CYMVH;EPGKTLK	NCP	32.519
EPGKTLK;TLRAELK	NCP	4.569
TLRAELK;LPHPVLPP	NCP	46.195
LPHPVLPP;NRELSDF	NCP	52.691

NRELSDF;NSSSLEK	NCP	10.654
NSSSLEK;LTVVTLA	NCP	42.378
LTVVTLA;LPGELLA	NCP	25.022
LPGELLA;HKDVRV	NCP	33.396
HKDVRV;WLFKLD	NCP	32.154
WLFKLD;WELLLP	NCP	5.599
WELLLP;PWAPSPP	NCP	13.418
PWAPSPP;TWLKDY	NCP	2.425
TWLKDY;KATLVLL	NCP	17.004
KATLVLL;YCCSPP	NCP	44.825
YCCSPP;VVNELK	NCP	49.639
VVNELK;HGQQLPR	NCP	0.896
HGQQLPR;KQAGVLT	NCP	45.528
KQAGVLT;KSFPPPPP	NCP	12.249
KSFPPPPP;KKEPNR	NCP	45.502
KKEPNR;FVKPGFP	NCP	31.13
FVKPGFP;LVSGLSPF	NCP	9.013
LVSGLSPF;EFQTK	NCP	3.104
EFQTK;LSEPGKA	NCP	16.956
LSEPGKA;RVLTLKR	NCP	17.959
RVLTLKR;EPKEEPP	NCP	19.176
EPKEEPP;LVMDPK	NCP	28.643
LVMDPK;LHLKLSV	NCP	17.902
LHLKLSV;AYFSF	NCP	3.486
AYFSF;LSAKTPK	NCP	22.979
LSAKTPK;EVHVPPP	NCP	15.084
EVHVPPP;KDARPPP	NCP	34.957
KDARPPP;KHCKKGA	NCP	63.314
KHCKKGA;LSLLNTFY	NCP	41.916
LSLLNTFY;LDLDF	NCP	51.834
LDLDF;GVFPTP	NCP	13.581
GVFPTP;KACAPR	NCP	74.26
KACAPR;TLNPFRQ	NCP	79.128
TLNPFRQ;LSLDPNL	NCP	33
LSLDPNL;SWLHY	NCP	87.992
SWLHY;AVLSL	NCP	35.8
AVLSL;MGHAAGK	NCP	12.065
MGHAAGK;TLPVVE	NCP	57.519
TLPVVE;PSWLDRL	NCP	15.673
PSWLDRL;AYAFPF	NCP	2.489
AYAFPF;KLSGMVP	NCP	28.065
KLSGMVP;VTFQA	NCP	89.041
VTFQA;LDLETPY	NCP	80.955
LDLETPY;DKAQVLH	NCP	2.22
DKAQVLH;RPLKKGP	NCP	5.939
RPLKKGP;WPSFLPG	NCP	89.691
WPSFLPG;ALNQPSP	NCP	123.756
ALNQPSP;EPWQEH	NCP	1.609
EPWQEH;MRSLPF	NCP	5.084
MRSLPF;TALTTQQKLAGVTAL	NCP	115.956
TALTTQQKLAGVTAL;VFGDAGK	NCP	44.112

VFGDAGK;NSLRPTT	NCP	46.276
NSLRPTT;QRKFFV	NCP	11.495
QRKFFV;KSPLVL	NCP	98.874
KSPLVL;LGEPGAV	NCP	27.141
LGEPGAV;LAPGLVHR	NCP	125.098
LAPGLVHR;GVATPK	NCP	88.901
GVATPK;RLPKTK	NCP	5.028
RLPKTK;CCCMV	NCP	45.969
CCCMV;LTDPSHP	NCP	13.852
LTDPSHP;DDEPP	NCP	16.613
DDEPP;LQFSQAAK	NCP	4.318
LQFSQAAK;ARLEKK	NCP	35.982
ARLEKK;SGSEEEAS	NCP	97.302
SGSEEEAS;ASKYLVP	NCP	18.309
ASKYLVP;VVLGDGP	NCP	11.699
VVLGDGP;EKKLAPQ	NCP	54.777
EKKLAPQ;VAAKQ	NCP	1.348
VAAKQ;LFVYFLPF	NCP	8.331
LFVYFLPF;KLDLLAGF	NCP	55.542
KLDLLAGF;VWVGLSSP	NCP	1.601
VWVGLSSP;MFLLFLKN	NCP	97.483
MFLLFLKN;ELNLKAPK	NCP	35.421
ELNLKAPK;SVLFPF	NCP	6.776
SVLFPF;KGGNSPN	NCP	2.131
KGGNSPN;AVGSLL	NCP	60.368
AVGSLL;CSTVLKK	NCP	105.217
CSTVLKK;DKLLNVSK	NCP	14.427
DKLLNVSK;PYVLGAGK	NCP	65.226
PYVLGAGK;KSDLTER	NCP	77.327
KSDLTER;DAEHPPLQ	NCP	396.51
DAEHPPLQ;LTSALSR	NCP	37.528
LTSALSR;GPSHTLPP	NCP	29.243
GPSHTLPP;YGVKPH	NCP	47.38
YGVKPH;HKDFAW	NCP	7.637
HKDFAW;LLHMLK	NCP	6.613
LLHMLK;KDWHY	NCP	46.421
KDWHY;TALNLL	NCP	120.528
TALNLL;QQCFRP	NCP	57.075
QQCFRP;LNEPPK	NCP	50.706
LNEPPK;SPVHKPP	NCP	46.264
SPVHKPP;RAAHLLGS	NCP	102.83
RAAHLLGS;KVTNWD	NCP	12.912
KVTNWD;GPPDKLP	NCP	10.29
GPPDKLP;APVVEL	NCP	48.514
APVVEL;KAEVSAY	NCP	64.079
KAEVSAY;KTVSVAV	NCP	16.114
KTVSVAV;LNMFYL	NCP	34.211
LNMFYL;LASRSPLL	NCP	3.441
LASRSPLL;VGMLY	NCP	7.818
VGMLY;YFTQHQ	NCP	14.055
YFTQHQ;LDLFKNN	NCP	38.325

LDLFKNN;GSAKSFLL	NCP	16.027
GSAKSFLL;KQPDKK	NCP	65.48
KQPDKK;TVSTAF	NCP	52.608
TVSTAF;FVTATQR	NCP	87.966
FVTATQR;LPPGLPPP	NCP	12.366
LPPGLPPP;EDVPLV	NCP	22.818
EDVPLV;TLPPTPP	NCP	21.934
TLPPTPP;KLLSK	NCP	22.193
KLLSK;VKVWKR	NCP	42.51
VKVWKR;HKPPER	NCP	15.15
HKPPER;TDFPKM	NCP	11.911
TDFPKM;HRTSLLVY	NCP	12.851
HRTSLLVY;EPGPSE	NCP	71.922
EPGPSE;KLLLPSV	NCP	31.588
KLLLPSV;KCGALVH	NCP	12.284
KCGALVH;PQGTEKQ	NCP	30.556
PQGTEKQ;EPSVGTY	NCP	72.485
EPSVGTY;QAVGF	NCP	19.825
QAVGF;LHNLS	NCP	0.704
LHNLS;KKTVP	NCP	19.753
KKTVP;KPGGFAK	NCP	25.248
KPGGFAK;KKSRPK	NCP	23.844
KKSRPK;QDMKYR	NCP	119.946
QDMKYR;VPGVSPK	NCP	6.785
VPGVSPK;QSLLVL	NCP	12.714
QSLLVL;KPAAYGQ	NCP	45.798
KPAAYGQ;VQLKLR	NCP	119.985
VQLKLR;VPLYEG	NCP	44.341
VPLYEG;YSSGE	NCP	58.163
YSSGE;DFVPVPR	NCP	35.758
DFVPVPR;NVLLFN	NCP	27.834
NVLLFN;DDAAAK	NCP	27.588
DDAAAK;KFKAYP	NCP	12.747
KFKAYP;VAAPPPK	NCP	59.618
VAAPPPK;PLDKN	NCP	112.552
PLDKN;KEVLLL	NCP	3.717
KEVLLL;KNEEMLR	NCP	93.306
KNEEMLR;LKLEAAA	NCP	33.855
LKLEAAA;VRDLWF	NCP	22.191
VRDLWF;DKLLVRF	NCP	11.567
DKLLVRF;HVKMVP	NCP	29.313
HVKMVP;KCVLFPP	NCP	261.757
KCVLFPP;LKVCAL	NCP	48.788
LKVCAL;KNFKEQTRL	NCP	33.666
KNFKEQTRL;QADETLR	NCP	50.265
QADETLR;KEFKYP	NCP	55.845
KEFKYP;PAAQNY	NCP	18.781
PAAQNY;WLEKEAR	NCP	20.643
WLEKEAR;KELPFL	NCP	3.113
KELPFL;KSPPVQ	NCP	10.087
KSPPVQ;THKTVYF	NCP	121.344

THKTVYF;VKVQGPK	NCP	40.056
VKVQGPK;LLDCYQ	NCP	50.925
LLDCYQ;VLEPFN	NCP	39.33
VLEPFN;KWGPQ	NCP	24.059
KWGPQ;SGAPLQL	NCP	23.137
SGAPLQL;SGPLVTK	NCP	71.265
SGPLVTK;KGGHPPR	NCP	116.588
KGGHPPR;AAAPRPK	NCP	8.559
AAAPRPK;LTYKK	NCP	242.539
LTYKK;PPKTVH	NCP	107.577
PPKTVH;ELGVLLA	NCP	3.065
ELGVLLA;VTVNGPL	NCP	6.903
VTVNGPL;KVYFFN	NCP	13.446
KVYFFN;GVLLTEA	NCP	11.752
GVLLTEA;RGLDPVH	NCP	17.378
RGLDPVH;AALCPGVR	NCP	75.547
AALCPGVR;KDAAVP	NCP	116.543
KDAAVP;LPSHEGV	NCP	10.551
LPSHEGV;VAPPPPFT	NCP	23.617
VAPPPPFT;DGSSLPVV	NCP	5.435
DGSSLPVV;GVPLSAY	NCP	18.309
GVPLSAY;YSPLLTH	NCP	98.061
YSPLLTH;NEVKKAP	NCP	4.572
NEVKKAP;KPPGCHP	NCP	25.326
KPPGCHP;PAAGLPTR	NCP	0.505
PAAGLPTR;GASLTLLL	NCP	5.802
GASLTLLL;CGDNNPT	NCP	9.051
CGDNNPT;DGTELL	NCP	4.502
DGTELL;HGPPRSP	NCP	4.137
HGPPRSP;TPPVPLT	NCP	64.03
TPPVPLT;SSYDAAL	NCP	33.663
SSYDAAL;HLLTGTK	NCP	62.898
HLLTGTK;ATVVEPY	NCP	34.535
ATVVEPY;PGEFGLF	NCP	11.889
PGEFGLF;LGASSNV	NCP	76.732
LGASSNV;LDKLVPG	NCP	15.699
LDKLVPG;GTTELFP	NCP	1.467
GTTELFP;LPVPRAK	NCP	21.278
LPVPRAK;LHPRPP	NCP	13.474
LHPRPP;VTVPNDL	NCP	22.502
VTVPNDL;QGELLDF	NCP	31.912
QGELLDF;VGAVLPF	NCP	9.639
VGAVLPF;TGAGPAAR	NCP	28.089
TGAGPAAR;KSLDCLP	NCP	67.061
KSLDCLP;FTEHKK	NCP	87.492
FTEHKK;AFLSSGAYLTHQQK	NCP	2.106
AFLSSGAYLTHQQK;HGQHPQPY	NCP	3.24
HGQHPQPY;AAVPSLK	NCP	8.515
AAVPSLK;VPALPPAP	NCP	0.714
VPALPPAP;VLTYSK	NCP	128.013
VLTYSK;KNGFL	NCP	4.665

KNGFL;KGNDWLP	NCP	9.437
KGNDWLP;PPKHPPP	NCP	13.539
PPKHPPP;CYVALL	NCP	33.558
CYVALL;LRDDREL	NCP	17.809
LRDDREL;TDMEEE	NCP	9.617
TDMEEE;MQRAPLQ	NCP	21.972
MQRAPLQ;SPGSLPC	NCP	16.183
SPGSLPC;HCKPVK	NCP	32.003
HCKPVK;SDDNNL	NCP	91.716
SDDNNL;KAETRP	NCP	69.99
KAETRP;MDLKVV	NCP	13.05
MDLKVV;FRAEGM	NCP	7.138
FRAEGM;LGAYGPL	NCP	102.069
LGAYGPL;KYTLGK	NCP	14.805
KYTLGK;PGGGK	NCP	1.757
PGGGK;GATSR	NCP	41.62
GATSR;QEFVL	NCP	37.151
QEFVL;LPAAALE	NCP	27.369
LPAAALE;DPLKPS	NCP	135.3
DPLKPS;RVTVGKP	NCP	5.619
RVTVGKP;MPVLVVL	NCP	35.184
MPVLVVL;DGSLFGF	NCP	52.074
DGSLFGF;QRAPVSFP	NCP	22.183
QRAPVSFP;QVLPVL	NCP	16.469
QVLPVL;SSGSYK	NCP	10.11
SSGSYK;VHFSP	NCP	87.478
VHFSP;DLHNHDL	NCP	55.586
DLHNHDL;LDVLP	NCP	47.844
LDVLP;ERFPPPP	NCP	19.33
ERFPPPP;KDFVPEK	NCP	30.594
KDFVPEK;ELAGLQR	NCP	11.49
ELAGLQR;MLALLN	NCP	7.632
MLALLN;KKAAEC	NCP	18.632
KKAAEC;PESKGM	NCP	78.237
PESKGM;DPDWF	NCP	49.246
DPDWF;TANLLL	NCP	8.807
TANLLL;LSYKVVAGF	NCP	33.25
LSYKVVAGF;KGPTYVH	NCP	51.788
KGPTYVH;VTREVPF	NCP	73.024
VTREVPF;FSLWHMT	NCP	127.487
FSLWHMT;KGFPPPPPP	NCP	25.95
KGFPPPPPP;SAKKLLVK	NCP	124.776
SAKKLLVK;CMCDTK	NCP	91.69
CMCDTK;VLYKKT	NCP	13.587
VLYKKT;VDPLY	NCP	47.633
VDPLY;TSVFH	NCP	24.165
TSVFH;NLTKLF	NCP	2.311
NLTKLF;KPTLGFG	NCP	56.016
KPTLGFG;KAMSC	NCP	3.228
KAMSC;GSHSLSY	NCP	47.829
GSHSLSY;GFGLK	NCP	61.247

GFGLK;VPGMPLR	NCP	73.27
VPGMPLR;EFKLLCV	NCP	86.382
EFKLLCV;LPHTGNK	NCP	39.363
LPHTGNK;MKVMLC	NCP	49.401
MKVMLC;KADETF	NCP	176.241
KADETF;VKKKKF	NCP	1.971
VKKKKF;RAPALR	NCP	4.689
RAPALR;AMTRGE	NCP	26.861
AMTRGE;VLKLQGH	NCP	0.193
VLKLQGH;PKNHKP	NCP	19.148
PKNHKP;LPLSRTP	NCP	148.078
LPLSRTP;VPGVEVL	NCP	33.414
VPGVEVL;VGFFC	NCP	49.166
VGFFC;SSGPSPP	NCP	45.877
SSGPSPP;VYLFPL	NCP	2.061
VYLFPL;EHDLLLQ	NCP	19.629
EHDLLLQ;NLECWRR	NCP	80.061
NLECWRR;LPAAHTVP	NCP	142.821
LPAAHTVP;ELVAYLT	NCP	3.285
ELVAYLT;RRPATL	NCP	0.039
RRPATL;KLEDVH	NCP	177.393
KLEDVH;TPGTTLP	NCP	56.591
TPGTTLP;ARQVN	NCP	12.964
ARQVN;AVPPNSGK	NCP	27.669
AVPPNSGK;WLNSLL	NCP	80.687
WLNSLL;LPRDTSSST	NCP	10.219
LPRDTSSST;KPMLLK	NCP	66.456
KPMLLK;ELLAHLL	NCP	7.691
ELLAHLL;NQERRQP	NCP	2.646
NQERRQP;CGFAMK	NCP	11.164
CGFAMK;WTRVQF	NCP	18.582
WTRVQF;FPAFEL	NCP	35.079
FPAFEL;TTPVAPK	NCP	13.094
TTPVAPK;QLELYL	NCP	6.922
QLELYL;CQPPR	NCP	18.623
CQPPR;DSLLTTH	NCP	33.301
DSLLTTH;VMTLY	NCP	9.708
VMTLY;PNKGLPP	NCP	41.069
PNKGLPP;QRALHL	NCP	19.236
QRALHL;GVTLVAF	NCP	12.478
GVTLVAF;TSAEVPR	NCP	67.47
TSAEVPR;DYPLPGR	NCP	49.655
DYPLPGR;EAAPVGP	NCP	45.588
EAAPVGP;TVMVK	NCP	6.357
TVMVK;MLSGLSGR	NCP	10.029
MLSGLSGR;FVTTPGP	NCP	43.89
FVTTPGP;LAGAPFP	NCP	24.753
LAGAPFP;PGVTPAPP	NCP	39.345
PGVTPAPP;DPYLRVT	NCP	104.74
DPYLRVT;PVGVLEPK	NCP	48.14
PVGVLEPK;DLSSPEF	NCP	14.295

DLSSPEF;LQLEC	NCP	121.323
LQLEC;ANVKLFK	NCP	70.248
ANVKLFK;AVKGKGK	NCP	36.718
AVKGKGK;VLAPKR	NCP	73.667
VLAPKR;KPGWGP	NCP	12.751
KPGWGP;TVTVQPR	NCP	12.495
TVTVQPR;SVAAAQSP	NCP	2.637
SVAAAQSP;LPPPAPV	NCP	45.876
LPPPAPV;TAELLNK	NCP	0.911
TAELLNK;VTPKFKF	NCP	19.44
VTPKFKF;TVGTSPK	NCP	14.533
TVGTSPK;ELDLSFK	NCP	40.385
ELDLSFK;AHLKVHR	NCP	54.871
AHLKVHR;LGTKGAR	NCP	19.431
LGTKGAR;PVFGPL	NCP	3.15
PVFGPL;VAVQCYPK	NCP	6.803
VAVQCYPK;KLEVVR	NCP	68.527
KLEVVR;VPPPPLK	NCP	10.052
VPPPPLK;LPPPPKY	NCP	14.35
LPPPPKY;KGSSDPK	NCP	0.065
KGSSDPK;KDCQRPP	NCP	6.384
KDCQRPP;KCYVYP	NCP	36.429
KCYVYP;KPVTPFLF	NCP	46.639
KPVTPFLF;RGVCQK	NCP	5.625
RGVCQK;LGQFLK	NCP	184.397
LGQFLK;EPPAATPK	NCP	29.401
EPPAATPK;KGKPSPR	NCP	83.669
KGKPSPR;AGKREK	NCP	59.658
AGKREK;SDNLALDL	NCP	9.127
SDNLALDL;KGFTEVK	NCP	44.97
KGFTEVK;KDKLEF	NCP	26.265
KDKLEF;ESVTLPY	NCP	34.356
ESVTLPY;EMEFHS	NCP	34.271
EMEFHS;AELSL	NCP	0.381
AELSL;AAPLLSE	NCP	6.652
AAPLLSE;VNKSLVSK	NCP	25.368
VNKSLVSK;QVTRVLPQ	NCP	154.334
QVTRVLPQ;KSTTGKSK	NCP	103.086
KSTTGKSK;PAAKPP	NCP	113.961
PAAKPP;FAKPAVP	NCP	1.563
FAKPAVP;KLTELR	NCP	40.386
KLTELR;RAGSK	NCP	11.524
RAGSK;FYSPRP	NCP	82.115
FYSPRP;EKWWM	NCP	9.982
EKWWM;RKTAAT	NCP	1.322
RKTAAT;GDVRVPQ	NCP	104.227
GDVRVPQ;MLPPVPP	NCP	2.456
MLPPVPP;PAAGTPSR	NCP	26.281
PAAGTPSR;MDEMSS	NCP	45.717
MDEMSS;KTQTSPK	NCP	56.417
KTQTSPK;TVRLQKK	NCP	16.477

TVRLQKK;PAYDPK	NCP	10.599
PAYDPK;VVEVPN	NCP	221.523
VVEVPN;LVAMDV	NCP	139.505
LVAMDV;KFFLPPP	NCP	10.015
KFFLPPP;ELLLK	NCP	39.821
ELLLK;CCCCEEK	NCP	42.004
CCCCEEK;LHKSKAR	NCP	39.068
LHKSKAR;GRSSLRFP	NCP	29.254
GRSSLRFP;VPVVAGP	NCP	28.139
VPVVAGP;LYKEEPK	NCP	29.565
LYKEEPK;ERPLTDRR	NCP	38.521
ERPLTDRR;LTAKSDGP	NCP	4.314
LTAKSDGP;VESLLEF	NCP	83.217
VESLLEF;LFRTQLH	NCP	56.792
LFRTQLH;KPMMDLR	NCP	77.244
KPMMDLR;APEWPR	NCP	4.332
APEWPR;DDDGP	NCP	8.052
DDDGP;KLMPEK	NCP	124.518
KLMPEK;APSTSL	NCP	9.864
APSTSL;PPPPSPR	NCP	1.143
PPPPSPR;FNLSY	NCP	11.808
FNLSY;ERQFSSR	NCP	52.776
ERQFSSR;WDLYE	NCP	91.806
WDLYE;TSPGGVP	NCP	46.143
TSPGGVP;NDLLMK	NCP	15.943
NDLLMK;EPMVKF	NCP	14.576
EPMVKF;LVKKSTPK	NCP	4.071
LVKKSTPK;RSLLPGK	NCP	31.74
RSLLPGK;KDKPYP	NCP	48.528
KDKPYP;TLAVPTLP	NCP	11.487
TLAVPTLP;PGPMP	NCP	9.232
PGPMP;LDDKAFR	NCP	10.017
LDDKAFR;VLPLK	NCP	1.344
VLPLK;TKSTF	NCP	0.762
TKSTF;QESGAYFK	NCP	17.136
QESGAYFK;YLSGP	NCP	10.428
YLSGP;DLDSSSM	NCP	0.71
DLDSSSM;LSVPLDLR	NCP	22.131
LSVPLDLR;TGLELLKK	NCP	60.115
TGLELLKK;KLVRDVK	NCP	61.413
KLVRDVK;DDDLVR	NCP	36.041
DDDLVR;LKFDPR	NCP	71.806
LKFDPR;QPTAAKP	NCP	25.298
QPTAAKP;LNLVGL	NCP	6.291
LNLVGL;SVCLNPP	NCP	8.544
SVCLNPP;EVGPLLS	NCP	17.055
EVGPLLS;KSPNFFL	NCP	75.712
KSPNFFL;KTSCAPR	NCP	29.844
KTSCAPR;MGALRTTP	NCP	27.113
MGALRTTP;PFEMPK	NCP	7.954
PFEMPK;YKPGLYT	NCP	82.829

YKPGLYT;PGKEVFP	NCP	59.676
PGKEVFP;GPLFLASP	NCP	24.708
GPLFLASP;RERVVLAK	NCP	8.703
RERVVLAK;MGPNLLR	NCP	9.367
MGPNLLR;MMVMMP	NCP	21.926
MMVMMP;EDAADPK	NCP	23.574
EDAADPK;MENFYQ	NCP	45.682
MENFYQ;KSGGPP	NCP	1.604
KSGGPP;NDLSRPK	NCP	0.852
NDLSRPK;EVFSHSQ	NCP	55.138
EVFSHSQ;LGSKEELL	NCP	9.828
LGSKEELL;HYRLPPP	NCP	68.67
HYRLPPP;KCAYPP	NCP	50.544
KCAYPP;VAMAVGGK	NCP	1.326
VAMAVGGK;DPRRLSP	NCP	33.48
DPRRLSP;KSGAGP	NCP	42.743
KSGAGP;GPVFSLAP	NCP	14.386
GPVFSLAP;PGKYFK	NCP	90.68
PGKYFK;RGGPLEK	NCP	33.318
RGGPLEK;PLPFP	NCP	98.578
PLPFP;SPSVPDK	NCP	12.041
SPSVPDK;RPNNPK	NCP	24.958
RPNNPK;KLEATL	NCP	9.437
KLEATL;PDDGVT	NCP	79.021
PDDGVT;GVEYL	NCP	20.618
GVEYL;KYPFVF	NCP	35.269
KYPFVF;MPLAPN	NCP	65.223
MPLAPN;LRPVTF	NCP	29.421
LRPVTF;KPVSFY	NCP	8.358
KPVSFY;ESVHPHL	NCP	10.176
ESVHPHL;TLLKEF	NCP	18.438
TLLKEF;GSSLKLK	NCP	80.601
GSSLKLK;GVPEAH	NCP	6.047
GVPEAH;KYVEMAP	NCP	42.591
KYVEMAP;LEYAVLT	NCP	8.739
LEYAVLT;LDAVFL	NCP	68.719
LDAVFL;VLLGPVH	NCP	92.697
VLLGPVH;HALALNF	NCP	3.645
HALALNF;SLANLKK	NCP	21.965
SLANLKK;HAGPAGP	NCP	58.677
HAGPAGP;KQDVAR	NCP	51.508
KQDVAR;LLMKLFAR	NCP	24.153
LLMKLFAR;FLVLTK	NCP	13.991
FLVLTK;VSELTKP	NCP	157.83
VSELTKP;APLNAKP	NCP	42.858
APLNAKP;EADMSS	NCP	46.978
EADMSS;HSLLQPF	NCP	9.198
HSLLQPF;PPPLPVE	NCP	8.228
PPPLPVE;GKDPL	NCP	112.243
GKDPL;PPVLKLF	NCP	47.166
PPVLKLF;TNQLVR	NCP	54.291

TNQLVR;SVGGLVQ	NCP	21.071
SVGGLVQ;SADYFKP	NCP	142.924
SADYFKP;SPVFLGFP	NCP	9.597
SPVFLGFP;AHRPPPP	NCP	113.16
AHRPPPP;ESPLLR	NCP	24.395
ESPLLR;LSGVARL	NCP	116.449
LSGVARL;QRNWPP	NCP	49.148
QRNWPP;NSLPFLTV	NCP	21.867
NSLPFLTV;NDEYF	NCP	42.384
NDEYF;KETVYPK	NCP	126.675
KETVYPK;DLELLR	NCP	61.465
DLELLR;KPKAPKG	NCP	39.984
KPKAPKG;TVGVLPK	NCP	40.058
TVGVLPK;KAGSD	NCP	106.938
KAGSD;LDLDVLF	NCP	126.997
LDLDVLF;QNKPVR	NCP	44.141
QNKPVR;SLKAAGGK	NCP	69.594
SLKAAGGK;VQQELM	NCP	31.515
VQQELM;KKFYAR	NCP	10.549
KKFYAR;KHNHTPK	NCP	49.251
KHNHTPK;EGSVKSSG	NCP	58.425
EGSVKSSG;KSNDLHL	NCP	0.773
KSNDLHL;NSAPVLL	NCP	9.333
NSAPVLL;PTPTSPLK	NCP	77.107
PTPTSPLK;GSLGEKMK	NCP	70.595
GSLGEKMK;LCYVALD	NCP	124.299
LCYVALD;ASAKKLL	NCP	13.144
ASAKKLL;KAFDKPQ	NCP	95.673
KAFDKPQ;KRGSPPK	NCP	167.301
KRGSPPK;TERLFTSY	NCP	21.768
TERLFTSY;LADTARY	NCP	14.642
LADTARY;VLLMAF	NCP	113.488
VLLMAF;MMLVSKK	NCP	2.301
MMLVSKK;CPKLTVP	NCP	5.057
CPKLTVP;VDLTEF	NCP	20.647
VDLTEF;HLAGPVLF	NCP	22.113
HLAGPVLF;DYAPPRP	NCP	51.95
DYAPPRP;AAALVVV	NCP	5.646
AAALVVV;KTYAP	NCP	2.295
KTYAP;KTYDQLK	NCP	11.095
KTYDQLK;KPVPGPP	NCP	2.216
KPVPGPP;PGGMK	NCP	7.329
PGGMK;KGTLAGTP	NCP	18.192
KGTLAGTP;PRPLMSN	NCP	52.335
PRPLMSN;KPRVVL	NCP	27.213
KPRVVL;DSDDD	NCP	41.184
DSDDD;EHLPAPK	NCP	73.452
EHLPAPK;YETGAY	NCP	24.384
YETGAY;SLDAVLT	NCP	41.389
SLDAVLT;PVPAPP	NCP	6.959
PVPAPP;LTEKLL	NCP	29.188

LTEKLL;RPPGTHL	NCP	31.922
RPPGTHL;LPPPVHK	NCP	16.746
LPPPVHK;DLRKLA	NCP	19.338
DLRKLA;MPPAQR	NCP	111.726
MPPAQR;MDALRLN	NCP	24.927
MDALRLN;PSPLVHM	NCP	129.315
PSPLVHM;VGDVLAR	NCP	2.139
VGDVLAR;VPPEQ	NCP	0.87
VPPEQ;LARFP	NCP	38.83
LARFP;GVGPVP	NCP	30.806
GVGPVP;GFLSRAGT	NCP	11.106
GFLSRAGT;KSDSMYF	NCP	34.684
KSDSMYF;VPFLLR	NCP	54.174
VPFLLR;DALRYH	NCP	16.617
DALRYH;PVTAGPP	NCP	57.824
PVTAGPP;LPPVTDR	NCP	5.799
LPPVTDR;RCEPPK	NCP	11.536
RCEPPK;PAAAKLHT	NCP	86.874
PAAAKLHT;ASCLLLH	NCP	9.6
ASCLLLH;ADMVVGKDK	NCP	23.927
ADMVVGKDK;YPHSPPP	NCP	43.428
YPHSPPP;TPTYLPY	NCP	15.229
TPTYLPY;TTVFVH	NCP	34.868
TTVFVH;YSAPPPPPP	NCP	11.818
YSAPPPPPP;GMSLYV	NCP	0.515
GMSLYV;QAELAQL	NCP	4.323
QAELAQL;APSVDKP	NCP	5.007
APSVDKP;KERDEFLK	NCP	0.567
KERDEFLK;VAQAK	NCP	69.223
VAQAK;RQKVLAK	NCP	26.432
RQKVLAK;GPGALP	NCP	67.455
GPGALP;ELAVKAQ	NCP	37.686
ELAVKAQ;CKLTPPPPP	NCP	7.5
CKLTPPPPP;TVGGLAP	NCP	16.294
TVGGLAP;KDVGSSLR	NCP	62.391
KDVGSSLR;ATVLEL	NCP	9.161
ATVLEL;VLVAVSR	NCP	21.22
VLVAVSR;KYAATM	NCP	76.947
KYAATM;FSADLK	NCP	13.62
FSADLK;LPTAPGLP	NCP	4.148
LPTAPGLP;GPLGTLGP	NCP	24.366
GPLGTLGP;HGPSVRP	NCP	31.335
HGPSVRP;LLVPGPV	NCP	5.673
LLVPGPV;FELNDKK	NCP	7.021
FELNDKK;KYGPPGP	NCP	36.479
KYGPPGP;KWTELR	NCP	4.621
KWTELR;KNNPP	NCP	9.056
KNNPP;AVRPGSQ	NCP	28.722
AVRPGSQ;NQRLLL	NCP	102.69
NQRLLL;LPPRHP	NCP	53.802
LPPRHP;KPGCPAK	NCP	13.392

KPGCPAK;KTPSPKR	NCP	45.777
KTPSPKR;APPRQPP	NCP	32.52
APPRQPP;TPVTTLLP	NCP	30.192
TPVTTLLP;LSAKPER	NCP	79.78
LSAKPER;QQPLTPP	NCP	25.826
QQPLTPP;EETVVCTSR	NCP	26.361
EETVVCTSR;ALTWPT	NCP	78.549
ALTWPT;SLHALAT	NCP	18.106
SLHALAT;KLVYAMP	NCP	69.837
KLVYAMP;GPARPGPL	NCP	74.237
GPARPGPL;PGEAG	NCP	31.8
PGEAG;TTDNFLSK	NCP	1.024
TTDNFLSK;QPHNPQ	NCP	17.348
QPHNPQ;QGGLGAL	NCP	108.667
QGGLGAL;KPNSTSSK	NCP	6.984
KPNSTSSK;KDGLPLH	NCP	46.475
KDGLPLH;PPPPTGSRPP	NCP	158.985
PPPPTGSRPP;GVGGGVGL	NCP	41.409
GVGGGVGL;KLADVR	NCP	115.815
KLADVR;FHGGGAK	NCP	37.645
FHGGGAK;AAAAPPLPP	NCP	15.086
AAAAPPLPP;HGVGVSH	NCP	11.175
HGVGVSH;KHWFER	NCP	19.339
KHWFER;ASAFEKVVR	NCP	19.325
ASAFEKVVR;GVAAVLE	NCP	8.058
GVAAVLE;LGQLHPR	NCP	6.717
LGQLHPR;KVVPKCL	NCP	23.59
KVVPKCL;EPANP	NCP	15.608
EPANP;EHEPVL	NCP	20.574
EHEPVL;DQKRPDN	NCP	1.527
DQKRPDN;ASKSGK	NCP	21.913
ASKSGK;VATRWY	NCP	3.026
VATRWY;SPAAGVRP	NCP	8.064
SPAAGVRP;APPTGV	NCP	4.434
APPTGV;AAALPPPR	NCP	1.275
AAALPPPR;VSVTPL	NCP	2.322
VSVTPL;SPAGGPPR	NCP	34.836
SPAGGPPR;CRRWLR	NCP	1.758
CRRWLR;EPYELP	NCP	23.847
EPYELP;FFKCLPQ	NCP	14.203
FFKCLPQ;SARLLY	NCP	30.858
SARLLY;CAPGPVPRP	NCP	1.745
CAPGPVPRP;RPPEPHF	NCP	34.38
RPPEPHF;PAAAGPPR	NCP	7.485
PAAAGPPR;GEAADLL	NCP	10.257
GEAADLL;FLRLPPPP	NCP	196.764
FLRLPPPP;EGLAL	NCP	72.06
EGLAL;PPAAAPL	NCP	16.761
PPAAAPL;GRPAAGPP	NCP	7.041
GRPAAGPP;TCRLVH	NCP	44.689
TCRLVH;DPCCAL	NCP	23.901

DPCCAL;PGDLCPP	NCP	47.909
PGDLCPP;LEKFD	NCP	42.514
LEKFD;SLCALL	NCP	96.683
SLCALL;VVFRDPYRFKK	NCP	13.269
VVFRDPYRFKK;KHVVFV	NCP	7.755
KHVVFV;VELNF	NCP	3.214
VELNF;TPAEKP	NCP	28.838
TPAEKP;MFSWVSKD	NCP	54.153
MFSWVSKD;EFTSK	NCP	88.738
EFTSK;KGTSGK	NCP	48.968
KGTSGK;PLSLAY	NCP	56.527
PLSLAY;KEQGTGPK	NCP	5.672
KEQGTGPK;KGPKEPK	NCP	13.861
KGPKEPK;KFQFPN	NCP	40.662
KFQFPN;TVQFK	NCP	43.344
TVQFK;QESHLGK	NCP	19.383
QESHLGK;CDGDDC	NCP	25.617
CDGDDC;LPFEL	NCP	53.222
LPFEL;MCVVAG	NCP	89.779
MCVVAG;VSLVT	NCP	27.785
VSLVT;GQRAAP	NCP	127.122
GQRAAP;FEPDN	NCP	74.632
FEPDN;LALRL	NCP	35.63
LALRL;RPVTPPR	NCP	60.667
RPVTPPR;PPDPKPP	NCP	35.072
PPDPKPP;PVLSTPR	NCP	53.533
PVLSTPR;WPDRQ	NCP	23.345
WPDRQ;TLNPF	NCP	9.121
TLNPF;FSKTWLF	NCP	47.079
FSKTWLF;KPNVGPP	NCP	66.498
KPNVGPP;TLVTSF	NCP	44.556
TLVTSF;SSQPRRPP	NCP	9.093
SSQPRRPP;AAVSLLR	NCP	28.104
AAVSLLR;KQGPPVP	NCP	176.574
KQGPPVP;HFKVPK	NCP	70.386
HFKVPK;TGKVLP	NCP	133.646
TGKVLP;LHPPERK	NCP	6.304
LHPPERK;PSLQHLR	NCP	6.203
PSLQHLR;TVNVPP	NCP	62.167
TVNVPP;NVPPYLL	NCP	0.006
NVPPYLL;PRGPVPP	NCP	24.485
PRGPVPP;KDALVPFG	NCP	40.234
KDALVPFG;LSARTSP	NCP	49.341
LSARTSP;SGARLVH	NCP	8.808
SGARLVH;PAAPHKP	NCP	48.915
PAAPHKP;TQLVLGL	NCP	46.652
TQLVLGL;VGWDAA	NCP	136.585
VGWDAA;APHGVKP	NCP	37.134
APHGVKP;QAPHPVR	NCP	208.932
QAPHPVR;CGPFPK	NCP	10.905
CGPFPK;KSMKAGF	NCP	20.529

KSMKAGF;QLAAAAK	NCP	1.395
QLAAAAK;KVLHPPP	NCP	33.816
KVLHPPP;GETQK	NCP	63.842
GETQK;LSPLVDT	NCP	11.853
LSPLVDT;PPALPPK	NCP	54.307
PPALPPK;FAERAL	NCP	109.415
FAERAL;KSPVP	NCP	27.235
KSPVP;VDLDSVL	NCP	20.151
VDLDSVL;VTAALK	NCP	235.139
VGPLPD;RTNLETSL	NCP	20.053
RTNLETSL;GELLDF	NCP	21.815
GELLDF;RDSRKL	NCP	19.425
RDSRKL;PRPPPPAP	NCP	4.93
PRPPPPAP;LMVPLR	NCP	66.834
LMVPLR;LSDGPW	NCP	101.39
LSDGPW;AVPSL	NCP	56.157
AVPSL;VPLQER	NCP	30.73
VPLQER;PAAREPP	NCP	17.156
PAAREPP;GLTPPGVP	NCP	27.264
GLTPPGVP;TMLLAVP	NCP	35.374
TMLLAVP;RDPAPRK	NCP	23.949
RDPAPRK;PAQTPN	NCP	21.924
PAQTPN;GVKKPDL	NCP	77.426
GVKKPDL;SSPFKV	NCP	80.359
SSPFKV;WNVGLKK	NCP	1.496
WNVGLKK;KDAAPQ	NCP	160.287
KDAAPQ;PVHKMV	NCP	4.828
PVHKMV;SHSLLV	NCP	47.76
SHSLLV;AAKDEPQR	NCP	116.495
AAKDEPQR;KPRASPTT	NCP	126.162
KPRASPTT;SVLNRDM	NCP	66.999
SVLNRDM;KKVRAAK	NCP	52.509
KKVRAAK;NTENAK	NCP	205.935
NTENAK;VTPNAAF	NCP	66.916
VTPNAAF;LLPRLTK	NCP	86.933
LLPRLTK;GSAAMPK	NCP	65.826
GSAAMPK;KDNAGK	NCP	34.201
KDNAGK;FGAQPVA	NCP	57.761
FGAQPVA;HQPPAPRP	NCP	3.534
HQPPAPRP;ELLAYL	NCP	12.754
ELLAYL;KCFCNPK	NCP	49.05
KCFCNPK;WQQLMTF	NCP	87.759
WQQLMTF;LYGKPPPPP	NCP	16.344
LYGKPPPPP;MFLLQGAQMLQ	NCP	36.362
MFLLQGAQMLQ;LQGAQMLQMLE	NCP	0.009
LQGAQMLQMLE;KELTPSLLDVK	NCP	8.808
KELTPSLLDVK;SSADPSHAAVVNRF	NCP	0.513
SSADPSHAAVVNRF;RGFPVYSHVDPKN	NCP	0.258
RGFPVYSHVDPKN;FYELLK	NCP	24.276
FYELLK;MFHLQGPQLLQ	NCP	0.279
MFHLQGPQLLQ;KFFLTGP	NCP	22.678

KFFLTGP;LLQGAQM	NCP	10.184
LLQGAQM;DLDHYTN	NCP	6.501
DLDHYTN;PEMFKL	NCP	2.817
PEMFKL;LTQVAF	NCP	18.912
LTQVAF;QTPVLKP	NCP	12.432
QTPVLKP;FDSKYP	NCP	25.396
FDSKYP;SVTTPPR	NCP	64.541
SVTTPPR;SDLQLR	NCP	111
SDLQLR;HLLEQPL	NCP	10.992
HLLEQPL;VFMKR	NCP	20.425
VFMKR;SFPTKLVL	NCP	23.066
SFPTKLVL;VPLKVGP	NCP	59.73
VPLKVGP;KMKAAP	NCP	78.055
KMKAAP;GAVTPP	NCP	90.726
GAVTPP;KGMEPR	NCP	40.974
KGMEPR;GPTVYNK	NCP	74.852
GPTVYNK;SGLQKRPYR	NCP	19.725
SGLQKRPYR;TCDEC	NCP	9.933
TCDEC;PVAGAYL	NCP	2.44
PVAGAYL;VGLLAARR	NCP	2.096
VGLLAARR;MDAKK	NCP	30.012
MDAKK;EDKGVVP	NCP	163.429
EDKGVVP;LLFPSPK	NCP	53.292
LLFPSPK;KKQGNPP	NCP	16.392
KKQGNPP;KLAPEPR	NCP	6.593
KLAPEPR;KPSCCC	NCP	11.539
KPSCCC;EVHFKE	NCP	15.408
EVHFKE;ATFDALSK	NCP	2.495
ATFDALSK;PKCLRP	NCP	33.816
PKCLRP;PGKPPPPP	NCP	120.243
PGKPPPPP;PGPAPPP	NCP	0.045
PGPAPPP;PPHPPPP	NCP	84.51
PPHPPPP;PVGPSFP	NCP	13.581
PVGPSFP;QHAKPR	NCP	159.001
QHAKPR;DDTLP	NCP	41.522
DDTLP;TVLRVLL	NCP	62.094
TVLRVLL;KPWTPPP	NCP	79.206
KPWTPPP;DRKEPNF	NCP	14.836
DRKEPNF;DDFVEKNVL	NCP	19.926
DDFVEKNVL;QLLAFH	NCP	25.77
QLLAFH;FATVSTPP	NCP	19.865
FATVSTPP;DPLTAPR	NCP	34.812
DPLTAPR;PDARLCL	NCP	80.184
PDARLCL;GKGFGSP	NCP	16.566
GKGFGSP;TALPP	NCP	32.691
TALPP;RTCVFKP	NCP	45.915
RTCVFKP;EVVLV	NCP	112.036
EVVLV;TMKNP	NCP	157.059
TMKNP;LYEVELL	NCP	27.591
LYEVELL;HFLRF	NCP	143.942
HFLRF;RLQHADK	NCP	94.978

RLQHADK;HVQVHYK	NCP	122.562
HVQVHYK;KLRPEELK	NCP	55.641
KLRPEELK;DLLKMP	NCP	17.562
DLLKMP;KAHALFP	NCP	53.936
KAHALFP;YLKNRP	NCP	22.312
YLKNRP;KVLPKSFK	NCP	34.332
KVLPKSFK;PEMMAKP	NCP	31.136
PEMMAKP;LTATLPF	NCP	128.614
LTATLPF;MGTTVKW	NCP	24.74
MGTTVKW;NYPSVGL	NCP	11.565
NYPSVGL;DDLLSPK	NCP	32.923
DDLLSPK;RGLLTL	NCP	73.445
RGLLTL;GKTFT	NCP	61.198
GKTFT;KGNCPK	NCP	0.705
KGNCPK;KAFLPGAP	NCP	15.015
KAFLPGAP;WVLNSLL	NCP	25.394
WVLNSLL;ETRELAK	NCP	22.45
ETRELAK;SGLYGGQ	NCP	36.434
SGLYGGQ;SSGPRKP	NCP	19.639
SSGPRKP;KYHPSPK	NCP	26.82
KYHPSPK;MVKALR	NCP	50.184
MVKALR;NSLTSSS	NCP	120.765
NSLTSSS;EGKSFLL	NCP	34.088
EGKSFLL;KVMNLL	NCP	87.492
KVMNLL;LGNKTK	NCP	32.751
LGNKTK;KHAVPR	NCP	17.052
KHAVPR;KDDAVL	NCP	70.957
KDDAVL;PACWLSK	NCP	17.36
PACWLSK;PEFELYL	NCP	175.863
PEFELYL;ELRLLT	NCP	15.997
ELRLLT;NVLNH	NCP	81.884
NVLNH;KFNQDK	NCP	12.486
KFNQDK;VVLLHKC	NCP	179.68
VVLLHKC;KEYHGAH	NCP	176.588
KEYHGAH;FMGVLQ	NCP	54.276
FMGVLQ;DPFDLL	NCP	8.028
DPFDLL;KYSTP	NCP	1.944
KYSTP;TDERYF	NCP	123.678
TDERYF;KGLHSGPF	NCP	61.438
KGLHSGPF;QPPKEPFP	NCP	208.235
QPPKEPFP;AGPAAVTK	NCP	1.488
AGPAAVTK;QALLPLTP	NCP	27.613
QALLPLTP;PAAAPAPAPA	NCP	7.397
PAAAPAPAPA;VGPDKRL	NCP	70.338
VGPDKRL;AAAGAVER	NCP	47.109
AAAGAVER;EPAELTKL	NCP	21.319
EPAELTKL;AAKLVTVK	NCP	18.224
AAKLVTVK;PQLVTSK	NCP	12.528
PQLVTSK;ELVASQ	NCP	5.616
ELVASQ;SGVDLLK	NCP	6.489
SGVDLLK;LTFLYN	NCP	25.98

LTFLYN;EPAPSVK	NCP	27.729
EPAPSVK;ADAASQVLLG	NCP	22.959
ADAASQVLLG;AFGFT	NCP	19.362
AFGFT;RSLKVH	NCP	9.345
RSLKVH;RYHFLRD	NCP	8.622
RYHFLRD;EEAMPTC	NCP	3.004
EEAMPTC;KDNVPPP	NCP	1.874
KDNVPPP;EEGPPAA	NCP	18.798
EEGPPAA;LPTLYNQNL	NCP	72.279
LPTLYNQNL;KMAWP	NCP	2.952
KMAWP;GPPGTGKTL	NCP	39.447
GPPGTGKTL;LGSVDKLR	NCP	9.57
LGSVDKLR;VGGSAAAL	NCP	15.642
VGGSAAAL;KPCLPVG	NCP	13.983
KPCLPVG;KSQHTPK	NCP	27.241
KSQHTPK;KLSALRL	NCP	5.016
KLSALRL;EQPPGRP	NCP	1.589
EQPPGRP;DGFLASA	NCP	38.826
DGFLASA;LDLFDDVGTFASGPPK	NCP	74.883
LDLFDDVGTFASGPPK;VGVLPGVR	NCP	2.979
VGVLPGVR;LASGLAVT	NCP	2.733
LASGLAVT;SRDALRGP	NCP	2.511
SRDALRGP;KDSDC	NCP	126.808
KDSDC;VTELAGP	NCP	11.006
VTELAGP;PLKREEN	NCP	64.3
PLKREEN;MDPLSELQD	NCP	216.053
MDPLSELQD;DDPSQALNQLK	NCP	0.039
DDPSQALNQLK;SDEMPDFPK	NCP	0.063
SDEMPDFPK;LSELSEHVK	NCP	11.874
LSELSEHVK;VLRETVAY	NCP	0.933
VLRETVAY;LLDHQGELFPSPDAKGL	NCP	1.323
LLDHQGELFPSPDAKGL;REKFDK	NCP	23.626
REKFDK;QTEKLLQY	NCP	11.531
QTEKLLQY;AYKSSTVL	NCP	2.446
AYKSSTVL;YLTLPVYL	NCP	27.257
YLTLPVYL;AAPPPPGGP	NCP	36.846
AAPPPPGGP;GVFSVAR	NCP	17.946
GVFSVAR;GGLGSLA	NCP	27.664
GGLGSLA;PVDKLVL	NCP	17.652
PVDKLVL;VYHLRPY	NCP	71.003
VYHLRPY;PPVLPPPP	NCP	8.97
PPVLPPPP;LPGEPPPP	NCP	17.316
LPGEPPPP;SGPGSRVT	NCP	3.54
SGPGSRVT;VGRAGP	NCP	42.081
VGRAGP;EGVTLHR	NCP	7.257
EGVTLHR;WGLLLLPK	NCP	17.331
WGLLLLPK;HPPRPGAP	NCP	19.54
HPPRPGAP;GPSSLR	NCP	104.246
GPSSLR;KYSVNF	NCP	9.334
KYSVNF;ALQKKR	NCP	7.875
ALQKKR;VPLEMYK	NCP	4.035

VPLEMYK;WSAPGVPR	NCP	13.866
WSAPGVPR;MMCLEP	NCP	45.185
MMCLEP;EVKLPVE	NCP	44.886
EVKLPVE;NATEGRL	NCP	81.469
NATEGRL;LPSRSL	NCP	21.74
LPSRSL;AELFRA	NCP	10.197
AELFRA;SDLRQLR	NCP	0.897
SDLRQLR;TAEYTEL	NCP	8.364
TAEYTEL;GPATQK	NCP	65.601
GPATQK;VGNLPVLL	NCP	124.054
VGNLPVLL;VGVAPP	NCP	10.676
VGVAPP;ETPLRLGTVA	NCP	0.738
ETPLRLGTVA;SGTSALSR	NCP	17.43
SGTSALSR;EMAAF	NCP	7.606
EMAAF;QRVPPGD	NCP	4.614
QRVPPGD;LSTLKGP	NCP	3.329
LSTLKGP;LQKAALLK	NCP	14.085
LQKAALLK;DRDRLK	NCP	34.825
DRDRLK;CCSLEF	NCP	32.652
CCSLEF;AQYVLR	NCP	55.053
AQYVLR;KPKKRH	NCP	23.943
KPKKRH;PAAKKKE	NCP	31.301
PAAKKKE;LSGLGVPK	NCP	63.177
LSGLGVPK;KALLGAGK	NCP	50.415
KALLGAGK;AAFRP	NCP	19.234
AAFRP;LSVAPY	NCP	72.89
LSVAPY;LPVLP	NCP	26.232
LPVLP;APSPREY	NCP	122.983
APSPREY;ALGALQEA	NCP	134.477
ALGALQEA;AAKQPPPLMK	NCP	4.956
AAKQPPPLMK;KKTHFPS	NCP	1.321
KKTHFPS;GNACLRL	NCP	187.94
GNACLRL;KDALMLK	NCP	23.325
KDALMLK;VAVLLRL	NCP	1.416
VAVLLRL;KHPNPLP	NCP	69.828
KHPNPLP;TADLSLP	NCP	16.057
TADLSLP;GSLPFDK	NCP	20.531
GSLPFDK;ARGPGRPP	NCP	7.971
ARGPGRPP;EALEFMR	NCP	20.893
EALEFMR;EGKPVGR	NCP	11.825
EGKPVGR;TDVKAPAP	NCP	42.798
TDVKAPAP;GPLPELLK	NCP	11.364
GPLPELLK;RLVEPY	NCP	2.307
RLVEPY;LPSCHLP	NCP	81.219
LPSCHLP;ALPFP	NCP	3.333
ALPFP;VGTDAPK	NCP	118.92
VGTDAPK;APPRPPAF	NCP	8.472
APPRPPAF;PNLQAVLV	NCP	6.717
PNLQAVLV;KDTVPKV	NCP	80.845
KDTVPKV;ADLPP	NCP	76.301
ADLPP;AAPPPPRYK	NCP	31.893

AAPPPPRYK;KHSGSPPP	NCP	38.997
KHSGSPPP;PVADPKP	NCP	27.402
PVADPKP;KKVNAKK	NCP	45.676
KKVNAKK;EPKRQAK	NCP	22.433
EPKRQAK;ASSLLSFSN	NCP	30.187
ASSLLSFSN;LRLVEPY	NCP	251.576
LRLVEPY;MFRRFN	NCP	26.052
MFRRFN;AALVPL	NCP	90.667
AALVPL;YVLGGP	NCP	15.149
YVLGGP;GPKKKKDF	NCP	12.276
GPKKKKDF;LVVLH	NCP	11.278
LVVLH;TTLKVH	NCP	14.427
TTLKVH;PGALDY	NCP	7.674
PGALDY;TKTPP	NCP	57.606
TKTPP;NGKVDY	NCP	65.969
NGKVDY;KGDGLLL	NCP	17.458
KGDGLLL;RDGVVST	NCP	5.568
RDGVVST;QPAQPP	NCP	11.942
QPAQPP;RPVCE	NCP	28.165
RPVCE;EVPAY	NCP	23.483
EVPAY;TLRQVSF	NCP	20.412
TLRQVSF;WTPLSLLK	NCP	85.497
WTPLSLLK;YFTNPPK	NCP	6.205
YFTNPPK;KDNAGEF	NCP	177.209
KDNAGEF;KPFFKVGL	NCP	81.055
KPFFKVGL;LVYCV	NCP	5.718
LVYCV;GPVSLTR	NCP	182.928
GPVSLTR;KLFRDPN	NCP	11.246
KLFRDPN;SDFGKPPP	NCP	46.299
SDFGKPPP;TVTLTPV	NCP	37.462
TVTLTPV;VPTLVVP	NCP	13.047
VPTLVVP;KARALLLA	NCP	13.659
KARALLLA;PPATKF	NCP	75.59
PPATKF;LDVTEF	NCP	52.452
LDVTEF;LDKEKL	NCP	5.089
LDKEKL;MLLEAKVK	NCP	100.004
MLLEAKVK;QTSPVF	NCP	5.154
QTSPVF;AATLYY	NCP	62.389
AATLYY;GSLLDF	NCP	83.996
GSLLDF;AVTWVGK	NCP	10.93
AVTWVGK;LSPGCKR	NCP	68.781
LSPGCKR;NVVTGPK	NCP	0.698
NVVTGPK;HLYDPLK	NCP	19.021
HLYDPLK;VEPLFP	NCP	25.418
VEPLFP;CPPERK	NCP	11.779
CPPERK;VAMGFKF	NCP	41.013
VAMGFKF;NLKVV	NCP	1.116
NLKVV;KQNLEK	NCP	13.292
KQNLEK;LTPYTAA	NCP	257.788
LTPYTAA;KNALP	NCP	27.95
KNALP;RKFVPP	NCP	94.357

RKFVPP;EAPASLM	NCP	151.298
EAPASLM;KNVHEVR	NCP	71.1
KNVHEVR;NLVERDK	NCP	155.505
NLVERDK;KSPGPGK	NCP	150.93
KSPGPGK;VASVYT	NCP	6.321
VASVYT;TSSPVAH	NCP	31.953
TSSPVAH;MKGVP	NCP	73.744
MKGVP;KVEGKNVK	NCP	46.764
KVEGKNVK;PGAFVL	NCP	6.791
PGAFVL;NSTFL	NCP	33.186
NSTFL;LSKAALQ	NCP	40.972
LSKAALQ;TDFYVYYT	NCP	65.06
TDFYVYYT;KKFKKK	NCP	68.211
KKFKKK;GFLKLD	NCP	0.483
GFLKLD;KQVLSH	NCP	14.166
KQVLSH;KSAMMLR	NCP	13.882
KSAMMLR;CVAFE	NCP	28.487
CVAFE;EPHVVF	NCP	8.025
EPHVVF;KGDGMDL	NCP	32.136
KGDGMDL;ALAVRVF	NCP	52.645
ALAVRVF;CVLSQLR	NCP	24.995
CVLSQLR;FKSLFDP	NCP	9.036
FKSLFDP;QTKEKLC	NCP	37.548
QTKEKLC;NCSDD	NCP	101.812
NCSDD;GFRHLDC	NCP	30.855
GFRHLDC;PGVLEEVL	NCP	331.616
PGVLEEVL;KVLPTKGK	NCP	17.173
KVLPTKGK;VVPAVTK	NCP	102.554
VVPAVTK;PGTVMPLK	NCP	6.931
PGTVMPLK;KYNNAY	NCP	62.249
KYNNAY;LGPVEL	NCP	21.424
LGPVEL;METPS	NCP	30.639
METPS;LLVVLPK	NCP	58.959
LLVVLPK;RPPMQP	NCP	57.966
RPPMQP;VSLLLY	NCP	11.571
VSLLLY;KPLRLGP	NCP	18.81
KPLRLGP;YTLEAE	NCP	57.519
YTLEAE;LAVKELK	NCP	183.911
LAVKELK;CNYVCC	NCP	65.686
CNYVCC;HPTKE	NCP	19.578
HPTKE;EKVSF	NCP	1.799
EKVSF;MFSRML	NCP	37.006
MFSRML;LTMFGEK	NCP	30.533
LTMFGEK;KPFDPR	NCP	36.037
KPFDPR;LDLPNFE	NCP	77.289
LDLPNFE;EGLDF	NCP	14.475
EGLDF;KETTPKSK	NCP	16.91
KETTPKSK;MDASLEKK	NCP	50.379
MDASLEKK;PTLAEMGK	NCP	0.033
PTLAEMGK;VVDGFNVLYNRKPVLY	NCP	0.453
VVDGFNVLYNRKPVLY;VDGFNV	NCP	0.003

VDGFNV;NVLYNRKPVLY	NCP	0.012
NVLYNRKPVLY;VSSSVLAATK	NCP	0.345
VSSSVLAATK;FQELPGSDPGLN	NCP	0.339
FQELPGSDPGLN;KLNELESDLTFK	NCP	0.468
KLNELESDLTFK;ANEERLLEEGVLR	NCP	0.228
ANEERLLEEGVLR;DDRPQVEEPESLR	NCP	0.441
DDRPQVEEPESLR;LTAESF	NCP	25.869
LTAESF;KTSGKLF	NCP	70.116
KTSGKLF;SLKDPSL	NCP	106.801
SLKDPSL;NKEEPGL	NCP	27.144
NKEEPGL;NQVAVPP	NCP	106.743
NQVAVPP;KGVAY	NCP	55.127
KGVAY;KDLHY	NCP	2.485
KDLHY;FPHMVP	NCP	52.227
FPHMVP;REHDRP	NCP	337.931
REHDRP;RKKAEFL	NCP	26.073
RKKAEFL;KCYKLSH	NCP	264.277
KCYKLSH;KTLPPGN	NCP	54.873
KTLPPGN;KMPLLL	NCP	37.619
KMPLLL;LVELAGR	NCP	25.597
LVELAGR;ATVLPVQ	NCP	1.731
ATVLPVQ;TKAKNP	NCP	77.045
TKAKNP;LDYVVP	NCP	11.58
LDYVVP;KELKGK	NCP	16.806
KELKGK;LLTPNAP	NCP	161.923
LLTPNAP;PEADD	NCP	65.615
PEADD;MKENLEK	NCP	18.045
MKENLEK;EDPLRWT	NCP	109.075
EDPLRWT;CTLRLQKR	NCP	9.839
CTLRLQKR;APEMKC	NCP	41.217
APEMKC;VTLLMEL	NCP	106.582
VTLLMEL;VPDLVNP	NCP	40.257
VPDLVNP;VRELMEEK	NCP	9.116
VRELMEEK;QGVLTL	NCP	14.182
QGVLTL;RLLLPHSR	NCP	46.235
RLLLPHSR;GMSLF	NCP	8.362
GMSLF;LSPRLK	NCP	3.029
LSPRLK;KHAAYEP	NCP	59.479
KHAAYEP;LEVLKAH	NCP	14.577
LEVLKAH;DPYLLRS	NCP	35.916
DPYLLRS;KGHLTPP	NCP	29.258
KGHLTPP;KCQKDKKK	NCP	7.858
KCQKDKKK;KAFANK	NCP	46.026
KAFANK;YDVEF	NCP	105.788
YDVEF;PLLERVL	NCP	49.629
PLLERVL;KDPAAQ	NCP	13.671
KDPAAQ;SGFGKKL	NCP	9.424
SGFGKKL;YPSHKP	NCP	61.34
YPSHKP;KAPEKTEF	NCP	63.939
KAPEKTEF;LVRNLAGY	NCP	22.69
LVRNLAGY;VTGLL	NCP	8.042

VTGLL;KKLLGPL	NCP	26.025
KKLLGPL;AAVGVVQ	NCP	9.045
AAVGVVQ;LHLLK	NCP	21.031
LHLLK;TPGPDHGP	NCP	44.339
TPGPDHGP;NELLMF	NCP	71.068
NELLMF;VLLENTA	NCP	118.214
VLLENTA;AHVGYF	NCP	61.414
AHVGYF;KDLTSYK	NCP	59.682
KDLTSYK;PPFPQVN	NCP	16.799
PPFPQVN;MLLVTLPK	NCP	4.963
MLLVTLPK;DMMMPF	NCP	47.375
DMMMPF;ALTLPLGL	NCP	75.129
ALTLPLGL;LPPLSFL	NCP	16.66
LPPLSFL;AASHKPP	NCP	41.232
AASHKPP;GPPLVATPP	NCP	94.208
GPPLVATPP;PAGTPRP	NCP	79.908
PAGTPRP;LELEL	NCP	40.293
LELEL;KNSLLR	NCP	13.308
KNSLLR;MDVMF	NCP	62.056
MDVMF;QPPTSSK	NCP	14.033
QPPTSSK;KAFAFNH	NCP	67.687
KAFAFNH;KPVSPLL	NCP	6.332
KPVSPLL;PHALLR	NCP	19.335
PHALLR;VALEYLTA	NCP	26.193
VALEYLTA;DEFFP	NCP	118.987
DEFFP;RFGTFH	NCP	9.995
RFGTFH;VLLLKLT	NCP	64.843
VLLLKLT;KCGPVLVL	NCP	12.912
KCGPVLVL;CDCNPLP	NCP	29.109
CDCNPLP;LGAFEPK	NCP	44.127
LGAFEPK;HGTFPTE	NCP	22.154
HGTFPTE;EEFRPF	NCP	13.884
EEFRPF;TAEKDK	NCP	25.11
TAEKDK;KNFLPP	NCP	10.395
KNFLPP;ALLKLPF	NCP	117.453
ALLKLPF;LTLSF	NCP	40.303
LTLSF;KSSLRLP	NCP	1.433
KSSLRLP;VAKAQAK	NCP	82.074
VAKAQAK;ESGHKKF	NCP	27.723
ESGHKKF;NEEVALSR	NCP	73.899
NEEVALSR;LSGGLVA	NCP	22.942
LSGGLVA;VGLEQLP	NCP	63.143
VGLEQLP;LLQLAGP	NCP	2.799
LLQLAGP;HLLFLKKD	NCP	20.35
HLLFLKKD;DERLAVPA	NCP	9.704
DERLAVPA;LGLASSY	NCP	46.885
LGLASSY;LLEEGKEL	NCP	4.337
LLEEGKEL;KGQGKPH	NCP	16.593
KGQGKPH;KPKYPK	NCP	4.695
KPKYPK;AFLDDM	NCP	34.707
AFLDDM;LQVLPEKF	NCP	5.395

LQVLPEKF;HRGLEL	NCP	10.256
HRGLEL;CCKAKGK	NCP	32.877
CCKAKGK;GSAAPSPK	NCP	0.327
GSAAPSPK;ATTLVPF	NCP	41.508
ATTLVPF;LTFLTK	NCP	8.67
LTFLTK;LVHLML	NCP	31.882
LVHLML;GPVLAL	NCP	20.459
GPVLAL;KFSNFVR	NCP	17.076
KFSNFVR;VGPLLF	NCP	54.795
VGPLLF;LPPPPRPP	NCP	80.556
LPPPPRPP;ALDVP	NCP	108.985
ALDVP;RTEAFH	NCP	11.556
RTEAFH;PLWGGKGK	NCP	34.383
PLWGGKGK;KPKGNLGP	NCP	62.444
KPKGNLGP;LPPPPEY	NCP	2.992
LPPPPEY;LRQELAV	NCP	57.026
LRQELAV;FSQGGQFLR	NCP	44.656
FSQGGQFLR;VGSVLFL	NCP	88.725
VGSVLFL;AVRSKLV	NCP	28.047
AVRSKLV;SGGVPGVR	NCP	14.939
SGGVPGVR;EELAF	NCP	37.822
EELAF;NFQVTKL	NCP	69.804
NFQVTKL;FHKPKFF	NCP	62.831
FHKPKFF;PAEGFK	NCP	31.461
PAEGFK;FSLLGAY	NCP	69.513
FSLLGAY;TPSLVSK	NCP	66.82
TPSLVSK;QTSSSSYR	NCP	21.497
QTSSSSYR;GPGTLL	NCP	1.791
GPGTLL;AAHGNLRP	NCP	3.786
AAHGNLRP;GVVMKLK	NCP	38.385
GVVMKLK;ASLVKSVQ	NCP	33.819
ASLVKSVQ;MPGAT	NCP	5.563
MPGAT;RYLLTN	NCP	17.943
RYLLTN;MHLPAPP	NCP	7.112
MHLPAPP;AVSYLGPK	NCP	6.072
AVSYLGPK;KGDLLK	NCP	100.095
KGDLLK;KGSNRLK	NCP	119.868
KGSNRLK;AAEFVAR	NCP	27.81
AAEFVAR;QHVTAGK	NCP	12.091
QHVTAGK;HQPGPVS	NCP	128.931
HQPGPVS;RLFGPEL	NCP	51.872
RLFGPEL;HVLQGW	NCP	99.648
HVLQGW;AALMLK	NCP	153.337
AALMLK;KSTVAPK	NCP	38.517
KSTVAPK;VLSVLAL	NCP	30.36
VLSVLAL;CPCCC	NCP	29.184
CPCCC;VSNPG	NCP	1.295
VSNPG;KAVEPF	NCP	49.266
KAVEPF;LSGKKPK	NCP	18.3
LSGKKPK;KFEPPPP	NCP	132.775
KFEPPPP;DGRFDR	NCP	15.968

DGRFDR;NSVFSKY	NCP	22.093
NSVFSKY;KAFYKSF	NCP	0.446
KAFYKSF;TTAPNPL	NCP	8.737
TTAPNPL;GRPAGRW	NCP	42.195
GRPAGRW;TSSPVGR	NCP	20.352
TSSPVGR;SGVMLK	NCP	38.654
SGVMLK;KGRLTDL	NCP	14.725
KGRLTDL;PSVPPKP	NCP	76.62
PSVPPKP;TQVLPF	NCP	9.63
TQVLPF;QHGVMR	NCP	19.743
QHGVMR;LLSGEC	NCP	34.236
LLSGEC;TTVLFPPP	NCP	134.271
TTVLFPPP;RNPVLR	NCP	36.213
RNPVLR;LGMTLHLV	NCP	32.813
LGMTLHLV;KGPPQ	NCP	21.505
KGPPQ;LLDLDKRL	NCP	27.918
LLDLDKRL;KYLVTSR	NCP	10.22
KYLVTSR;VTDLLLP	NCP	13.531
VTDLLLP;KDDNLRP	NCP	25.131
KDDNLRP;KGPLE	NCP	23.79
KGPLE;KRGDLVL	NCP	133.196
KRGDLVL;SLMQPSH	NCP	14.418
SLMQPSH;VALAP	NCP	104.101
VALAP;FAAGK	NCP	211.701
FAAGK;LPPPKYR	NCP	5.313
LPPPKYR;YNLWPP	NCP	10.562
YNLWPP;VHSFTPLK	NCP	74.364
VHSFTPLK;WHLVLGS	NCP	3.438
WHLVLGS;VSAALL	NCP	10.927
VSAALL;KEGLL	NCP	4.781
KEGLL;KFFKPP	NCP	43.162
KFFKPP;TTMVHRK	NCP	4.032
TTMVHRK;KFSPLVG	NCP	26.414
KFSPLVG;VGVVLA	NCP	49.386
VGVVLA;KGTVPF	NCP	35.806
KGTVPF;KSNFGPK	NCP	39.281
KSNFGPK;ETGRAGP	NCP	3.156
ETGRAGP;VERLLKK	NCP	5.616
VERLLKK;LDPTNM	NCP	33.433
LDPTNM;TTDVLML	NCP	52.925
TTDVLML;GHSLLQ	NCP	64.996
GHSLLQ;GHAKYVF	NCP	118.613
GHAKYVF;VTPGGVF	NCP	68.449
VTPGGVF;EVGDRPQ	NCP	56.448
EVGDRPQ;LMVTR	NCP	22.787
LMVTR;APPARPPF	NCP	88.936
APPARPPF;KRGKLK	NCP	106.131
KRGKLK;MAELEKF	NCP	26.522
MAELEKF;RYTPQP	NCP	66.76
RYTPQP;KDKFMVP	NCP	0.309
KDKFMVP;GEELLVY	NCP	1.427

GEELLVY;NYFSKVVF	NCP	22.855
NYFSKVVF;AAEVKLR	NCP	14.465
AAEVKLR;LSHQLVE	NCP	55.426
LSHQLVE;KEQNAF	NCP	35.27
KEQNAF;CTQVLYR	NCP	18.03
CTQVLYR;VGLGRPP	NCP	7.885
VGLGRPP;KYYTTFK	NCP	62.498
KYYTTFK;YAGLP	NCP	83.071
YAGLP;NSVRLR	NCP	96.227
NSVRLR;FVKWPR	NCP	5.38
FVKWPR;LLDVLTSLP	NCP	6.428
LLDVLTSLP;GVSKP	NCP	61.381
GVSKP;MPKKSK	NCP	8.253
MPKKSK;LSPLTTTP	NCP	4.305
LSPLTTTP;MPLFVL	NCP	11.054
MPLFVL;KECLEK	NCP	15.331
KECLEK;RLPLQR	NCP	100.524
RLPLQR;VSGLGLLP	NCP	90.701
VSGLGLLP;KSYTLF	NCP	70.845
KSYTLF;KNFGTVP	NCP	19.629
KNFGTVP;CPTQKML	NCP	104.286
CPTQKML;AQLPP	NCP	24.001
AQLPP;LPPNPLPP	NCP	5.339
LPPNPLPP;KVYSMKK	NCP	30.868
KVYSMKK;KNEFAP	NCP	133.97
KNEFAP;ALQERQEPDV	NCP	54.361
ALQERQEPDV;LSLLMEN	NCP	67.98
LSLLMEN;KALTVLL	NCP	47.414
KALTVLL;KEVSP	NCP	147.015
KEVSP;FGKTVPK	NCP	1.83
FGKTVPK;VEMDGFV	NCP	52.821
VEMDGFV;TLDDVAP	NCP	30.637
TLDDVAP;LETAFPK	NCP	127.382
LETAFPK;KGGYTLQ	NCP	13.407
KGGYTLQ;PGGVFLK	NCP	39.322
PGGVFLK;RLVTASPQ	NCP	62.831
RLVTASPQ;AGAEARL	NCP	14.749
AGAEARL;LGDVP	NCP	51.935
LGDVP;GTPPKDPK	NCP	26.815
GTPPKDPK;AAPGLLVR	NCP	42.729
AAPGLLVR;VSADKMR	NCP	95.081
VSADKMR;PPGMP	NCP	56.439
PPGMP;ASLTFLH	NCP	24.612
ASLTFLH;LKYFTNK	NCP	4.483
LKYFTNK;LDQDLY	NCP	34.673
LDQDLY;VEVDL	NCP	2.344
VEVDL;VTLAAF	NCP	50.796
VTLAAF;RTCLVH	NCP	59.129
RTCLVH;RKDPPPL	NCP	30.868
RKDPPPL;CDDPK	NCP	6.578
CDDPK;LPPHSVS	NCP	2.821

LPPHSVS;LPPKY	NCP	21.924
LPPKY;VSAGLTPK	NCP	26.688
VSAGLTPK;RVLLEKY	NCP	72.491
RVLLEKY;CYNLPPR	NCP	12.69
CYNLPPR;NGSPFPLG	NCP	4.428
NGSPFPLG;LSLTEVR	NCP	49.218
LSLTEVR;KDAKTLL	NCP	38.244
KDAKTLL;APYVARSP	NCP	37.752
APYVARSP;KGHDHHF	NCP	16.744
KGHDHHF;WDLHQY	NCP	47.453
WDLHQY;AAFPSLVG	NCP	11.019
AAFPSLVG;VGSGHLFF	NCP	5.656
VGSGHLFF;RYSSPP	NCP	27.378
RYSSPP;EMGPPKP	NCP	40.82
EMGPPKP;EKFPPSF	NCP	23.326
EKFPPSF;PVLEPSLC	NCP	24.891
PVLEPSLC;DGLGLRK	NCP	12.659
DGLGLRK;MMGWAGP	NCP	21.394
MMGWAGP;STKYEC	NCP	72.528
STKYEC;VGSRAAPL	NCP	6.608
VGSRAAPL;VVSSLLRP	NCP	102.69
VVSSLLRP;KASFLR	NCP	37.198
KASFLR;KEPAPPH	NCP	11.754
KEPAPPH;SLYLLTR	NCP	43.11
SLYLLTR;LTAAPGLA	NCP	129.084
LTAAPGLA;RPPKAAK	NCP	8.316
RPPKAAK;LDAEVR	NCP	13.974
LDAEVR;VAVGVFL	NCP	51.116
VAVGVFL;KEKSKPP	NCP	9.19
KEKSKPP;RVGLVQY	NCP	44.484
RVGLVQY;LGDDPLKK	NCP	20.523
LGDDPLKK;TDVRLK	NCP	70.875
TDVRLK;KPTGELK	NCP	9.539
KPTGELK;NDFQWK	NCP	94.137
NDFQWK;AAAAPK	NCP	92.811
AAAAPK;LEVSAPK	NCP	55.657
LEVSAPK;KSVFCPK	NCP	21.632
KSVFCPK;GPSALGEGP	NCP	20.938
GPSALGEGP;SLMFKP	NCP	13.148
SLMFKP;LGPRS	NCP	21.073
LGPRS;KGKYRSS	NCP	2.538
KGKYRSS;EPLGNLLP	NCP	8.43
EPLGNLLP;AAVTQAR	NCP	42.132
AAVTQAR;PAGGNT	NCP	2.886
PAGGNT;KMELA	NCP	44.024
KMELA;MRLMAK	NCP	16.119
MRLMAK;GCMPPPP	NCP	40.021
GCMPPPP;LQVTVR	NCP	60.41
LQVTVR;SPPKYSPP	NCP	31.684
SPPKYSPP;REAVVPK	NCP	19.274
REAVVPK;RLVAPNP	NCP	49.389

RLVAPNP;ALGETLP	NCP	31.372
ALGETLP;YDDMSPR	NCP	83.051
YDDMSPR;LLQNSVKQ	NCP	0.486
LLQNSVKQ;LPTKLFQ	NCP	8.956
LPTKLFQ;MLTAAAN	NCP	93.345
MLTAAAN;LYAAV	NCP	0.759
LYAAV;AESKKLT	NCP	19.037
AESKKLT;DPMDEP	NCP	22.083
DPMDEP;HQLQR	NCP	7.653
HQLQR;SVDVTKLP	NCP	66.418
SVDVTKLP;GPLPPPGP	NCP	16.97
GPLPPPGP;MLCSFP	NCP	41.611
MLCSFP;VYKPPR	NCP	52.554
VYKPPR;VELALMP	NCP	42.395
VELALMP;VGPVVPP	NCP	3.607
VGPVVPP;AEEDLLFR	NCP	29.241
AEEDLLFR;RASLLR	NCP	9.434
RASLLR;KMATLKL	NCP	7.83
KMATLKL;TSPLPLPK	NCP	40.297
TSPLPLPK;VLRNPLAL	NCP	21.486
VLRNPLAL;KMTATVP	NCP	18.596
KMTATVP;LSVDPY	NCP	13.842
LSVDPY;DDEFTLH	NCP	10.191
DDEFTLH;KWAGFLP	NCP	5.331
KWAGFLP;YSLTSNP	NCP	147.418
YSLTSNP;RGSADPK	NCP	18.759
RGSADPK;MPGPVGM	NCP	37.196
MPGPVGM;GPKVYSL	NCP	86.257
GPKVYSL;LVAPKYF	NCP	28.229
LVAPKYF;SGLVRLR	NCP	48.91
SGLVRLR;RPDRPP	NCP	176.916
RPDRPP;APPME	NCP	23.553
APPME;KPPAAHLK	NCP	63.344
KPPAAHLK;NPSRKF	NCP	1.65
NPSRKF;LAAAWPQ	NCP	18.621
LAAAWPQ;LRPVGSYL	NCP	13.243
LRPVGSYL;DDDLDP	NCP	37.161
DDDLDP;KDFLALN	NCP	25.229
KDFLALN;KHVLFGVGV	NCP	42.925
KHVLFGVGV;KPPGKLH	NCP	32.352
KPPGKLH;KGRSLLSP	NCP	45.113
KGRSLLSP;PPEKPAR	NCP	177.271
PPEKPAR;EGSRLGH	NCP	7.053
EGSRLGH;LEFDSV	NCP	33.651
LEFDSV;ESGKGYG	NCP	52.286
ESGKGYG;KKLDAWN	NCP	9.661
KKLDAWN;GTVLEL	NCP	39.77
GTVLEL;VGGSVFL	NCP	3.534
VGGSVFL;TAPGRVL	NCP	17.997
TAPGRVL;TVPPTPK	NCP	49.548
TVPPTPK;YDLGLVS	NCP	7.849

YDLGLVS;KPPHLK	NCP	2.27
KPPHLK;VYVPSQR	NCP	22.671
VYVPSQR;TLGPPF	NCP	19.728
TLGPPF;MLKFLAK	NCP	12.504
MLKFLAK;ALDGKLKHEDTN	NCP	22.26
ALDGKLKHEDTN;VAPPRP	NCP	4.128
VAPPRP;PDKPY	NCP	34.542
PDKPY;ATPVSLK	NCP	14.871
ATPVSLK;ASVQLLR	NCP	23.604
ASVQLLR;TASPALK	NCP	50.64
TASPALK;MSLLEAK	NCP	17.989
MSLLEAK;CGEMCP	NCP	71.57
CGEMCP;KMAAM	NCP	28.293
KMAAM;GKELLAM	NCP	8.916
GKELLAM;LPPGKN	NCP	83.598
LPPGKN;QPVLRN	NCP	39.015
QPVLRN;VLLDGRP	NCP	5.536
VLLDGRP;SLLTGTK	NCP	7.916
SLLTGTK;AVPPLYL	NCP	7.723
AVPPLYL;LGVAKGGK	NCP	14.736
LGVAKGGK;EPGAV	NCP	0.678
EPGAV;SLALF	NCP	35.543
SLALF;AASKGLP	NCP	2.809
AASKGLP;AARPAK	NCP	14.54
AARPAK;KALSYAF	NCP	48.507
KALSYAF;DDALWL	NCP	27.825
DDALWL;KFPGNSP	NCP	13.263
KFPGNSP;KFVSPL	NCP	140.497
KFVSPL;LGPVPMY	NCP	31.478
LGPVPMY;KGVLSH	NCP	88.117
KGVLSH;VKYLLF	NCP	0.824
VKYLLF;TADLHLK	NCP	16.899
TADLHLK;ATVVVE	NCP	97.156
ATVVVE;RNYDSMKDFEEMRK	NCP	7.007
RNYDSMKDFEEMRK;DDDVP	NCP	12.882
DDDVP;SSSRPLGP	NCP	5.473
SSSRPLGP;LGVLNL	NCP	44.646
LGVLNL;RVCLPW	NCP	49.736
RVCLPW;LLQLEEEP	NCP	3.85
LLQLEEEP;WGGLGAL	NCP	24.276
WGGLGAL;EAKGYG	NCP	1.101
EAKGYG;VEAYK	NCP	2.933
VEAYK;LSPVLQLV	NCP	21.397
LSPVLQLV;KEPFAK	NCP	37.791
KEPFAK;VTMPPY	NCP	87.326
VTMPPY;SKPVGAR	NCP	0.658
SKPVGAR;LVPAAYR	NCP	11.595
LVPAAYR;KSLVYK	NCP	77.412
KSLVYK;QCSKSAA	NCP	10.151
QCSKSAA;CMCCC	NCP	25.099
CMCCC;KVRSSPQ	NCP	138.869

KVRSSPQ;AAVVPAL	NCP	12.37
AAVVPAL;LFDRQGT	NCP	39.476
LFDRQGT;VALPLEQV	NCP	77.706
VALPLEQV;HGPSPP	NCP	5.187
HGPSPP;TPRAGP	NCP	0.339
TPRAGP;SVERSPP	NCP	33.148
SVERSPP;METTPGGR	NCP	44.157
METTPGGR;KPAKSPV	NCP	74.156
KPAKSPV;VPCCMVV	NCP	23.077
VPCCMVV;TPTDPK	NCP	16.433
TPTDPK;DYTVKGH	NCP	52.725
DYTVKGH;LDLLAY	NCP	70.362
LDLLAY;TVPLSR	NCP	1.396
TVPLSR;RLGSTRVSP	NCP	40.29
RLGSTRVSP;KTVQP	NCP	65.24
KTVQP;KNPSLR	NCP	63.919
KNPSLR;FGRAANF	NCP	10.73
FGRAANF;ASASAAASTLSEPPR	NCP	48.9
ASASAAASTLSEPPR;GLGLPLK	NCP	27.955
GLGLPLK;APLKV	NCP	83.4
APLKV;LTYVTPF	NCP	105.875
LTYVTPF;VEVLP	NCP	84.184
VEVLP;YLLDLL	NCP	47.436
YLLDLL;LDQLHK	NCP	6.558
LDQLHK;VGTRVY	NCP	35.457
VGTRVY;KPKAGAVP	NCP	12.821
KPKAGAVP;MKQWAK	NCP	65.929
MKQWAK;APGGP	NCP	11.441
APGGP;GLADFLR	NCP	3.772
GLADFLR;LLHAKFLL	NCP	84.846
LLHAKFLL;QVLASEPVPF	NCP	25.127
QVLASEPVPF;KDNVEMP	NCP	33.082
KDNVEMP;KWALLLE	NCP	5.037
KWALLLE;EELSPPSHQ	NCP	34.115
EELSPPSHQ;DLLGL	NCP	17.934
DLLGL;KDGMEVP	NCP	66.274
KDGMEVP;HVPVV	NCP	10.55
HVPVV;LALAAQY	NCP	52.753
LALAAQY;VVLKRGCP	NCP	56.744
VVLKRGCP;EKALLRS	NCP	27.519
EKALLRS;FSPKYTK	NCP	11.94
FSPKYTK;KGTVAQ	NCP	11.715
KGTVAQ;ETVPQGLK	NCP	47.706
ETVPQGLK;LDLKLR	NCP	40.258
LDLKLR;KHTEYK	NCP	4.302
KHTEYK;KGAFFH	NCP	15.698
KGAFFH;LPNKAGP	NCP	2.688
LPNKAGP;KEQGGPLP	NCP	21.091
KEQGGPLP;EWLLLDH	NCP	22.971
EWLLLDH;DCEEPK	NCP	40.019
DCEEPK;AAEVVRK	NCP	9.322

AAEVVRK;KDWNFGK	NCP	7.607
KDWNFGK;KLANLL	NCP	13.876
KLANLL;QQVALPL	NCP	35.387
QQVALPL;LPLNGPAA	NCP	20.152
LPLNGPAA;VEKLPDSPAL	NCP	17.438
VEKLPDSPAL;SPPDPDTPSPAGDSR	NCP	0.153
SPPDPDTPSPAGDSR;NDLFYG	NCP	14.851
NDLFYG;LAPHAKM	NCP	58.434
LAPHAKM;LPKLFLH	NCP	12.32
LPKLFLH;LDDLLKL	NCP	17.058
LDDLLKL;GLLSGVAF	NCP	4.126
GLLSGVAF;LSPVQ	NCP	48.285
LSPVQ;TLWLLR	NCP	46.745
TLWLLR;SGVSLF	NCP	20.391
SGVSLF;QLYRAP	NCP	3.802
QLYRAP;EGGALLVAK	NCP	0.417
EGGALLVAK;MHWAK	NCP	47.711
MHWAK;TFMYDP	NCP	80.856
TFMYDP;VFSPLVG	NCP	78.343
VFSPLVG;KQRAVEP	NCP	15.605
KQRAVEP;GMPLGL	NCP	99.079
GMPLGL;KYSVAGK	NCP	43.698
KYSVAGK;CPPLLVT	NCP	64.529
CPPLLVT;KPEVFGP	NCP	16.291
KPEVFGP;LDLLEH	NCP	9.194
LDLLEH;AHVPLPGH	NCP	37.293
AHVPLPGH;LSTLRFN	NCP	75.684
LSTLRFN;KEALLSR	NCP	135.748
KEALLSR;MLQFLL	NCP	74.034
MLQFLL;MLNVNK	NCP	29.522
MLNVNK;LSSKP	NCP	8.208
LSSKP;PLSSSVQLL	NCP	85.498
PLSSSVQLL;MKLAC	NCP	35.738
MKLAC;GPKLPAGP	NCP	38.815
GPKLPAGP;KGVLP	NCP	10.635
KGVLP;PSVPAPK	NCP	8.643
PSVPAPK;LPPPPPPPG	NCP	108.294
LPPPPPPPG;TALRPASLP	NCP	15.606
TALRPASLP;LSVHGNLQ	NCP	64.47
LSVHGNLQ;LPRLPEE	NCP	14.637
LPRLPEE;KQFWAK	NCP	27.33
KQFWAK;APLVPPPL	NCP	91.469
APLVPPPL;LSEVP	NCP	34.798
LSEVP;PVDGLPY	NCP	49.509
PVDGLPY;RHRYLKE	NCP	54.198
RHRYLKE;VGEPLT	NCP	63.666
VGEPLT;KGGAA	NCP	6.486
KGGAA;KSYVPR	NCP	4.562
KSYVPR;QRNAARP	NCP	43.009
QRNAARP;RSTLTK	NCP	16.29
RSTLTK;RHGAGPP	NCP	24.006

RHGAGPP;TSRLLR	NCP	20.286
TSRLLR;LDGYY	NCP	99.605
LDGYY;LQPEV	NCP	135.361
LQPEV;SLDDLLKL	NCP	81.989
SLDDLLKL;DVQDPL	NCP	23.626
DVQDPL;MGGCGC	NCP	12.324
MGGCGC;LSHLTPK	NCP	91.665
LSHLTPK;PKLHN	NCP	73.116
PKLHN;DRVVDL	NCP	2.082
DRVVDL;KGVDYL	NCP	1.964
KGVDYL;PHNLK	NCP	39.189
PHNLK;PAPPKRGP	NCP	24.961
PAPPKRGP;MDSFCC	NCP	28.613
MDSFCC;VDEVGGEAL	NCP	25.431
VDEVGGEAL;LAELELA	NCP	109.048
LAELELA;DYFEYK	NCP	42.087
DYFEYK;RGSPKKL	NCP	2.598
RGSPKKL;DTPRFM	NCP	30.204
DTPRFM;PHTPP	NCP	2.577
PHTPP;KAGVNLK	NCP	95.868
KAGVNLK;PVVAYGY	NCP	25.272
PVVAYGY;EEVLLR	NCP	78.704
EEVLLR;LSLLDNP	NCP	13.927
LSLLDNP;CPLLNVKS	NCP	137.579
CPLLNVKS;HDPLPPP	NCP	123.709
HDPLPPP;MYKRPP	NCP	66.569
MYKRPP;KKQGTLH	NCP	38.655
KKQGTLH;PAAQYN	NCP	52.579
PAAQYN;ATVLLE	NCP	2.525
ATVLLE;TVPAEKQ	NCP	62.596
TVPAEKQ;CCDDDD	NCP	59.058
CCDDDD;PSSLPH	NCP	31.208
PSSLPH;HHYNY	NCP	15.073
HHYNY;AHQFAP	NCP	101.25
AHQFAP;EKSPAQ	NCP	237.252
EKSPAQ;YALFD	NCP	56.921
YALFD;KACRLL	NCP	19.636
KACRLL;TLLEKVLDEF	NCP	252.317
TLLEKVLDEF;TPKKLTP	NCP	0.123
TPKKLTP;TSALELL	NCP	0.336
TSALELL;LTLELVDQ	NCP	4.609
LTLELVDQ;ESQSDLSFSK	NCP	38.511
ESQSDLSFSK;LRLLSTPC	NCP	0.804
LRLLSTPC;PLDTSSY	NCP	30.387
PLDTSSY;KPPLLAQR	NCP	0.539
KPPLLAQR;VSLGLLAG	NCP	34.081
VSLGLLAG;KYGGP	NCP	40.676
KYGGP;KEDARAP	NCP	4.951
KEDARAP;PGGLVLF	NCP	8.196
PGGLVLF;FPGPG	NCP	4.553
FPGPG;LHLKGGP	NCP	0.16

LHLKGGP;RVTTKRR	NCP	24.282
RVTTKRR;EPAYTLR	NCP	22.34
EPAYTLR;TASLGHPK	NCP	22.284
TASLGHPK;GVVRGPR	NCP	14.83
GVVRGPR;FPMQPQ	NCP	46.397
FPMQPQ;GPPPPPGP	NCP	60.189
GPPPPPGP;KTKEAR	NCP	10.78
KTKEAR;DRPDLR	NCP	10.385
DRPDLR;YPVAP	NCP	35.997
YPVAP;FVVDQSQAVKF	NCP	38.478
FVVDQSQAVKF;VVDQSQAVK	NCP	0.003
VVDQSQAVK;VDQSQAVKF	NCP	0.003
VDQSQAVKF;ALDMARGMAF	NCP	0.027
ALDMARGMAF;LDMARGMA	NCP	0.003
LDMARGMA;DMARGMAF	NCP	0.003
DMARGMAF;LWELVTREVPF	NCP	0.447
LWELVTREVPF;MNEDPAKRPK	NCP	0.321
MNEDPAKRPK;EDDGC	NCP	54.33
EDDGC;VGLAQK	NCP	29.56
VGLAQK;AFFNL	NCP	3.575
AFFNL;GSFPCGFF	NCP	134.368
GSFPCGFF;HTPKPK	NCP	46.29
HTPKPK;KDLVLPVQ	NCP	20.709
KDLVLPVQ;AHQMTDLR	NCP	3.177
AHQMTDLR;VHVLFN	NCP	120.764
VHVLFN;PKAPPPPPPP	NCP	81.427
PKAPPPPPPP;QELDLR	NCP	45.296
QELDLR;KVKLDP	NCP	29.49
KVKLDP;MTPVASK	NCP	50.296
MTPVASK;SPLVFR	NCP	18.776
SPLVFR;VGVPLEK	NCP	135.322
VGVPLEK;LRELSF	NCP	3.366
LRELSF;LPVELQP	NCP	66.485
LPVELQP;KDVYFPP	NCP	59.851
KDVYFPP;MLDLFCF	NCP	31.604
MLDLFCF;VEADRPGKLF	NCP	20.22
VEADRPGKLF;RDGYEGPPR	NCP	0.555
RDGYEGPPR;SYRDPFE	NCP	0.282
SYRDPFE;HDFPLFL	NCP	42.429
HDFPLFL;VSKLPKK	NCP	102.027
VSKLPKK;LTLLAF	NCP	27.889
LTLLAF;FSQGVHP	NCP	77.258
FSQGVHP;MLDKLLK	NCP	28.03
MLDKLLK;AAPLLT	NCP	13.794
AAPLLT;ERLVRP	NCP	50.867
ERLVRP;PQLVER	NCP	12.651
PQLVER;RPAHKLS	NCP	13.753
RPAHKLS;EMPFPK	NCP	15.359
EMPFPK;CLPQVGK	NCP	12.721
CLPQVGK;PMAKER	NCP	40.538
PMAKER;PPVVGPV	NCP	47.788

PPVVGPV;SSELLKVR	NCP	63.176
SSELLKVR;LDHVGAP	NCP	21.462
LDHVGAP;TTKLFP	NCP	4.483
TTKLFP;AVPSKGK	NCP	15.425
AVPSKGK;KLAVGWQ	NCP	155.509
KLAVGWQ;SLPVV	NCP	22.385
SLPVV;SLPLVD	NCP	105.049
SLPLVD;YNAPGL	NCP	2.873
YNAPGL;FDLVHRK	NCP	28.056
FDLVHRK;KSCPYP	NCP	87.127
KSCPYP;EYLFDSF	NCP	8.643
EYLFDSF;TTGNLFLK	NCP	14.996
TTGNLFLK;STLSVRP	NCP	58.804
STLSVRP;LPPPHDQP	NCP	41.642
LPPPHDQP;KDPLPLK	NCP	28.852
KDPLPLK;KPAFLGP	NCP	14.409
KPAFLGP;VGLGLQ	NCP	41.432
VGLGLQ;QNEEVR	NCP	14.745
QNEEVR;LQTFKT	NCP	94.681
LQTFKT;LSPPLKT	NCP	33.972
LSPPLKT;FLLEAH	NCP	62.012
FLLEAH;PQHSLG	NCP	13.317
PQHSLG;PAAQEKPP	NCP	12.133
PAAQEKPP;KTVRVEF	NCP	25.821
KTVRVEF;VFFDAPK	NCP	25.323
VFFDAPK;LPPWVAA	NCP	10.994
LPPWVAA;KASSLGLL	NCP	14.541
KASSLGLL;SGVPWPK	NCP	62.224
SGVPWPK;VPPLYK	NCP	93.885
VPPLYK;KEAPP	NCP	111.275
KEAPP;SCCYAP	NCP	3.961
SCCYAP;LFFPLPRLP	NCP	23.12
LFFPLPRLP;QTLHVWC	NCP	48.024
QTLHVWC;TVEELEF	NCP	21.97
TVEELEF;YSVGV	NCP	39.899
YSVGV;AASLLRK	NCP	0.687
AASLLRK;ESAVVPR	NCP	45.48
ESAVVPR;LSKAE	NCP	63.193
LSKAE;PLSPP	NCP	14.438
PLSPP;TVFLPEEY	NCP	76.077
TVFLPEEY;AAPDPPR	NCP	6.382
AAPDPPR;HGDPK	NCP	28.329
HGDPK;AGKPVPGP	NCP	45.197
AGKPVPGP;PLTSTSE	NCP	2.59
PLTSTSE;GVHYLT	NCP	11.925
GVHYLT;PAAAKKR	NCP	103.893
PAAAKKR;LRKLPF	NCP	18.708
LRKLPF;KHALLPS	NCP	53.162
KHALLPS;MEMFKK	NCP	0.612
MEMFKK;LLEQEEY	NCP	9.165
LLEQEEY;AMMEED	NCP	12.652

AMMEED;GVHFPK	NCP	22.557
GVHFPK;LQCCRLP	NCP	2.144
LQCCRLP;FFPPPPK	NCP	43.416
FFPPPPK;EKFDKSKLK	NCP	36.336
EKFDKSKLK;FDKSKLKKTE	NCP	0.006
FDKSKLKKTE;LFEVAPG	NCP	28.248
LFEVAPG;LKEVDGVKK	NCP	59.695
LKEVDGVKK;GSLKDELLKG	NCP	65.906
GSLKDELLKG;CGCDDT	NCP	11.664
CGCDDT;ELGLYEV	NCP	22.734
ELGLYEV;TTLKFSLQ	NCP	31.371
TTLKFSLQ;QSAAKHQ	NCP	131.926
QSAAKHQ;LLLFK	NCP	8.96
LLLFK;KFPWAP	NCP	31.9
KFPWAP;GSPLSPTS	NCP	119.915
GSPLSPTS;AQLPTHH	NCP	11.929
AQLPTHH;ARLELTD	NCP	28.595
ARLELTD;KDSSSPP	NCP	8.478
KDSSSPP;PVRSSTT	NCP	105.922
PVRSSTT;PSKETLL	NCP	4.617
PSKETLL;KGVPWAP	NCP	34.044
KGVPWAP;GPAKLPP	NCP	42.66
GPAKLPP;GPPPPPR	NCP	23.801
GPPPPPR;AAARAPVV	NCP	140.179
AAARAPVV;AAVEPLV	NCP	86.343
AAVEPLV;RLQEFNLL	NCP	61.686
RLQEFNLL;RGFDPK	NCP	37.425
RGFDPK;KEHLQQP	NCP	54.764
KEHLQQP;GGPGGPGGPGGPMGR	NCP	64.83
GGPGGPGGPGGPMGR;GPPGPLM	NCP	0.444
GPPGPLM;FVDKLR	NCP	9.648
FVDKLR;LPQTTATF	NCP	15.049
LPQTTATF;AGLAAKLA	NCP	11.121
AGLAAKLA;KDQKELGK	NCP	64.668
KDQKELGK;KSLVHKK	NCP	83.954
KSLVHKK;LPGVL	NCP	32.152
LPGVL;FHEPVGP	NCP	140.406
FHEPVGP;GAAVHR	NCP	64.424
GAAVHR;LSHVPPQP	NCP	15.775
LSHVPPQP;VTGGLWV	NCP	13.625
VTGGLWV;YGFGY	NCP	2.554
YGFGY;KPGSKK	NCP	24.644
KPGSKK;APKVHGP	NCP	81.474
APKVHGP;EPLTTHLK	NCP	31.095
EPLTTHLK;WMKPLR	NCP	20.182
WMKPLR;GSLEPLL	NCP	9.011
GSLEPLL;GPWGMR	NCP	30.987
GPWGMR;ATFLGY	NCP	14.47
ATFLGY;PATALPP	NCP	8.598
PATALPP;SLGPPPPR	NCP	4.938
SLGPPPPR;AAMTYL	NCP	3.033

AAMTYL;KFQCPK	NCP	98.804
KFQCPK;NTPLSM	NCP	127.596
NTPLSM;PDPTTLL	NCP	91.666
PDPTTLL;LPPPPLPPPPP	NCP	6.603
LPPPPLPPPPP;KHPAVP	NCP	23.291
KHPAVP;NDLFELR	NCP	115.59
NDLFELR;EVNLPK	NCP	6.066
EVNLPK;MYFKAEPY	NCP	8.64
MYFKAEPY;LGPRLL	NCP	24.063
LGPRLL;KQKTHTFLQ	NCP	32.82
KQKTHTFLQ;HLTERPK	NCP	8.709
HLTERPK;PPSVPQP	NCP	3.441
PPSVPQP;KNAGPPR	NCP	29.484
KNAGPPR;TFPAPPK	NCP	79.062
TFPAPPK;NSNPFE	NCP	8.625
NSNPFE;SKLFF	NCP	14.508
SKLFF;FTVLQKEK	NCP	24.958
FTVLQKEK;VYVKSV	NCP	41.178
VYVKSV;VVPPF	NCP	4.883
VVPPF;EPVTVW	NCP	19.872
EPVTVW;AESVSVP	NCP	17.961
AESVSVP;VTASPV	NCP	18.21
VTASPV;AYVTPKQ	NCP	69.108
AYVTPKQ;ATSPQKSPSVPK	NCP	10.527
ATSPQKSPSVPK;SVPKSPTPK	NCP	0.024
SVPKSPTPK;SPTPKSPPSRK	NCP	0.012
SPTPKSPPSRK;KDDSFLGK	NCP	0.033
KDDSFLGK;DDSFLGKLGGTL	NCP	0.003
DDSFLGKLGGTL;QPRGLSF	NCP	12.703
QPRGLSF;LERRFTL	NCP	15.339
LERRFTL;PRNPPGP	NCP	58.917
PRNPPGP;AKHLFT	NCP	33.917
AKHLFT;LKGAVP	NCP	0.766
LKGAVP;FLTPDSFEQK	NCP	29.36
FLTPDSFEQK;KKYKK	NCP	57.663
KKYKK;VDVQLK	NCP	2.218
VDVQLK;NVVVK	NCP	86.144
NVVVK;SLESALK	NCP	31.132
SLESALK;HGGSHMVP	NCP	14.009
HGGSHMVP;LLDGRPPP	NCP	85.887
LLDGRPPP;LDVKQY	NCP	66.843
LDVKQY;KGEEVR	NCP	6.667
KGEEVR;KFMPPPP	NCP	9.594
KFMPPPP;MELEAM	NCP	32.115
MELEAM;LSHVPPR	NCP	27.155
LSHVPPR;VFKSL	NCP	7.836
VFKSL;FHVFAK	NCP	29.706
FHVFAK;EWTLAY	NCP	69.721
EWTLAY;KASTVPR	NCP	24.399
KASTVPR;NHQALLR	NCP	9.894
NHQALLR;LPYLQGGP	NCP	44.108

LPYLQGGP;LEEKLQNL	NCP	30.934
LEEKLQNL;KSFLMTLP	NCP	13.031
KSFLMTLP;VPPELVC	NCP	18.253
VPPELVC;MVLLHK	NCP	24.779
MVLLHK;CNLLLR	NCP	75.145
CNLLLR;MLSVLARL	NCP	40.622
MLSVLARL;PLSSKHT	NCP	9.388
PLSSKHT;EPLLPR	NCP	46.067
EPLLPR;VELKPAP	NCP	50.682
VELKPAP;LLERF	NCP	55.963
LLERF;EGLLDF	NCP	17.96
EGLLDF;SDLPNPL	NCP	24.087
SDLPNPL;TQVKLF	NCP	16.392
TQVKLF;VASYLLA	NCP	18.769
VASYLLA;VGVKGHVL	NCP	9.348
VGVKGHVL;KERLFVK	NCP	32.657
KERLFVK;VTTVSPK	NCP	86.746
VTTVSPK;FYTSAK	NCP	34.76
FYTSAK;PDVGRPP	NCP	27.505
PDVGRPP;NVRPPR	NCP	13.263
NVRPPR;KEYGFN	NCP	27.009
KEYGFN;MPAPVLL	NCP	15.021
MPAPVLL;AALTKAVG	NCP	91.379
AALTKAVG;RSKFF	NCP	42.84
RSKFF;FPGVPAG	NCP	56.518
FPGVPAG;VVPML	NCP	11.394
VVPML;NTLPDVN	NCP	29.024
NTLPDVN;RPPAATL	NCP	62.217
RPPAATL;KCESRP	NCP	35.937
KCESRP;PGPKVF	NCP	5.461
PGPKVF;KYLPPDPL	NCP	103.412
KYLPPDPL;KDEGKLP	NCP	1.212
KDEGKLP;KELKPLH	NCP	36.718
KELKPLH;HLLLFPK	NCP	50.553
HLLLFPK;FNPLSRT	NCP	13.286
FNPLSRT;RLFAPK	NCP	25.467
RLFAPK;AHQFLLH	NCP	3.573
AHQFLLH;KPPTTSK	NCP	14.451
KPPTTSK;WNDAK	NCP	14.962
WNDAK;ENAPTL	NCP	75.495
ENAPTL;KASKKPK	NCP	32.678
KASKKPK;GYDPL	NCP	77.86
GYDPL;KLPHHLK	NCP	3.371
KLPHHLK;NLPPF	NCP	81.37
NLPPF;LAPPVPR	NCP	3.06
LAPPVPR;PAPPPPRPP	NCP	198.015
PAPPPPRPP;QKALCK	NCP	19.362
QKALCK;MERCYH	NCP	28.457
MERCYH;LPPPPPPRP	NCP	46.722
LPPPPPPRP;KTFGYRP	NCP	71.524
KTFGYRP;TLFQRQ	NCP	5.789

TLFQRQ;TQDNPLY	NCP	16.321
TQDNPLY;LVLVGY	NCP	94.674
LVLVGY;PPAVYK	NCP	17.498
PPAVYK;CELPPPPP	NCP	18.187
CELPPPPP;SPFGQP	NCP	44.429
SPFGQP;EGSVE	NCP	28.554
EGSVE;YSPLSLT	NCP	33.031
YSPLSLT;PSLVHKR	NCP	43.646
PSLVHKR;LPPPPP	NCP	5.008
LPPPPP;SLPLSPVLG	NCP	8.576
SLPLSPVLG;PPLLHAPP	NCP	60.658
PPLLHAPP;TAAVKPK	NCP	77.828
TAAVKPK;QALPTHH	NCP	17.083
QALPTHH;PGKFR	NCP	23.222
PGKFR;VGVPGAP	NCP	10.072
VGVPGAP;LLLPL	NCP	21.048
LLLPL;KTKKLR	NCP	30.825
KTKKLR;LPFFVKN	NCP	21.551
LPFFVKN;PTLPW	NCP	43.219
PTLPW;MVGLYR	NCP	7.41
MVGLYR;PLFPAG	NCP	4.803
PLFPAG;MLALAT	NCP	22.787
MLALAT;ASSPAPSL	NCP	27.475
ASSPAPSL;GPLATPLP	NCP	57.779
GPLATPLP;PVLLLLPG	NCP	35.443
PVLLLLPG;AAEPPRPP	NCP	108.092
AAEPPRPP;PGPREGSP	NCP	74.85
PGPREGSP;LAFLTK	NCP	13.519
LAFLTK;TPGTAAP	NCP	17.622
TPGTAAP;VTLLK	NCP	18.044
VTLLK;PAASMLK	NCP	190.902
PAASMLK;LPHLPR	NCP	50.872
LPHLPR;TATFP	NCP	9.818
TATFP;PPVFRFP	NCP	2.229
PPVFRFP;PEVSLPLK	NCP	9.354
PEVSLPLK;VDLLKFF	NCP	40.548
VDLLKFF;KDYLLEPK	NCP	59.658
KDYLLEPK;FFPMPK	NCP	75.55
FFPMPK;APGHVPK	NCP	23.082
APGHVPK;WMVLKAK	NCP	32.777
WMVLKAK;VETLSKY	NCP	92.2
VETLSKY;KSLPKY	NCP	71.978
KSLPKY;VTGAPVA	NCP	37.159
VTGAPVA;KPKGPTK	NCP	223.719
KPKGPTK;QKVVAGVANALAHR	NCP	36.453
QKVVAGVANALAHR;KVVAGVANALAHRYH	NCP	0.003
KVVAGVANALAHRYH;VKVDEVGGEALGR	NCP	15.429
VKVDEVGGEALGR;KPCTVK	NCP	181.845
KPCTVK;EVFGPL	NCP	86.309
EVFGPL;MRLLT	NCP	4.593
MRLLT;LGPALP	NCP	64.828

LGPALP;CNATLLLF	NCP	75.653
CNATLLLF;KEVFDKK	NCP	146.671
KEVFDKK;SKELDLVSHHVRTK	NCP	10.034
SKELDLVSHHVRTK;DELKRQEVAR	NCP	0.045
DELKRQEVAR;EDLQRQHDQLEAQ	NCP	17.346
EDLQRQHDQLEAQ;PEVGPPR	NCP	84.012
PEVGPPR;VGVLAY	NCP	78.273
VGVLAY;LSLKLE	NCP	12.697
LSLKLE;GPPEAP	NCP	4.919
GPPEAP;VQYVPR	NCP	72.444
VQYVPR;LDKVAL	NCP	44.179
LDKVAL;TPKGLPR	NCP	44.708
TPKGLPR;TGPEFGE	NCP	18.684
TGPEFGE;DPGLWF	NCP	5.856
DPGLWF;RDNSRKL	NCP	9.319
RDNSRKL;ADGGSPF	NCP	33.353
ADGGSPF;GCALLVR	NCP	12.111
GCALLVR;KSSLLHF	NCP	19.918
KSSLLHF;ASEVAHR	NCP	5.294
ASEVAHR;TLNAPLTL	NCP	169.56
TLNAPLTL;STGQHTVP	NCP	32.658
STGQHTVP;LLDSTLL	NCP	0.339
LLDSTLL;KETLGFK	NCP	72.016
KETLGFK;NLVLLAK	NCP	23.754
NLVLLAK;PFVELD	NCP	0.197
PFVELD;RHFHR	NCP	3.811
RHFHR;KRAHSPK	NCP	11.555
KRAHSPK;VYVRHKD	NCP	77.182
VYVRHKD;KNVGVTK	NCP	84.632
KNVGVTK;RLLPQ	NCP	8.574
RLLPQ;RYYSPL	NCP	15.91
RYYSPL;KGAAGPGGP	NCP	37.817
KGAAGPGGP;LDVKLFD	NCP	141.805
LDVKLFD;MKLAY	NCP	61.182
MKLAY;GDFSPPPP	NCP	23.12
GDFSPPPP;KLHPHGR	NCP	110.583
KLHPHGR;APAVFDF	NCP	67.587
APAVFDF;LSGEFL	NCP	5.088
LSGEFL;DPEESKGP	NCP	41.235
DPEESKGP;MVVNDAGRPKVQVEY	NCP	48.324
MVVNDAGRPKVQVEY;KGETKSFYPEEVSSM	NCP	0.045
KGETKSFYPEEVSSM;AASMDRK	NCP	20.878
AASMDRK;MGGDMD	NCP	50.717
MGGDMD;VESLP	NCP	42.243
VESLP;KQTELP	NCP	1.681
KQTELP;GDDGM	NCP	23.844
GDDGM;FLDLYPPL	NCP	14.874
FLDLYPPL;CSVNKVH	NCP	38.991
CSVNKVH;PPPFPL	NCP	30.188
PPPFPL;MLNGV	NCP	57.004
MLNGV;VLPAAPK	NCP	5.786

VLPAAPK;MERPY	NCP	0.714
MERPY;RFAQNK	NCP	8.131
RFAQNK;NDSRLKN	NCP	69.123
NDSRLKN;RSLTAAH	NCP	71.568
RSLTAAH;NVGKPPP	NCP	24.678
NVGKPPP;CFMDML	NCP	54.656
CFMDML;LGVFQLR	NCP	9.138
LGVFQLR;VDDLSLP	NCP	79.965
VDDLSLP;KAVGLSH	NCP	27.87
KAVGLSH;FTTGPPM	NCP	38.949
FTTGPPM;GPASSAK	NCP	63.799
GPASSAK;VGPLGKL	NCP	1.5
VGPLGKL;TTLTMT	NCP	113.376
TTLTMT;MLLELLT	NCP	0.888
MLLELLT;KPSEVPK	NCP	25.281
KPSEVPK;SATFLGN	NCP	147.756
SATFLGN;SCLTF	NCP	15.794
SCLTF;TGPLEC	NCP	3.655
TGPLEC;EGPVK	NCP	0.543
EGPVK;PPGAGPQPP	NCP	31.035
PPGAGPQPP;KDGAPL	NCP	86.619
KDGAPL;MLRLFEP	NCP	55.622
MLRLFEP;TALRVLP	NCP	44.884
TALRVLP;LFGYGVR	NCP	114.516
LFGYGVR;AVPTTPL	NCP	20.597
AVPTTPL;HLVLMFW	NCP	128.38
HLVLMFW;MLQVTLK	NCP	21.99
MLQVTLK;LQKELQF	NCP	110.553
LQKELQF;LQFNWK	NCP	7.913
LQFNWK;WNPPGR	NCP	159.45
WNPPGR;KKKER	NCP	72.814
KKKER;AAPGV	NCP	14.034
AAPGV;TQLLAYY	NCP	93.788
TQLLAYY;PLLLAPPQ	NCP	1.816
PLLLAPPQ;GPPKVYK	NCP	128.226
GPPKVYK;LQLHLLR	NCP	42.297
LQLHLLR;ALPLGRK	NCP	27.249
ALPLGRK;KASLRAGP	NCP	97.236
KASLRAGP;PFSSMLGV	NCP	8.435
PFSSMLGV;QGFPLLK	NCP	32.892
QGFPLLK;AGELLDF	NCP	76.777
AGELLDF;VGPSLA	NCP	52.029
VGPSLA;KRLSHSL	NCP	0.821
KRLSHSL;LTVLPL	NCP	96.492
LTVLPL;SLLEEL	NCP	5.977
SLLEEL;KDKGLAEP	NCP	35.168
KDKGLAEP;EAVLLLK	NCP	1.768
EAVLLLK;RSLGPVK	NCP	25.337
RSLGPVK;TVPKYK	NCP	67.401
TVPKYK;DPVPTYK	NCP	22.483
DPVPTYK;NKLDPP	NCP	66.86

NKLDPP;LPAVTKYTSSK	NCP	60.502
LPAVTKYTSSK;KGFQGPK	NCP	8.277
KGFQGPK;KSPPTPPP	NCP	60.347
KSPPTPPP;KLVVGSLE	NCP	25.621
KLVVGSLE;WTKTLSNQ	NCP	30.992
WTKTLSNQ;CKLEA	NCP	6.646
CKLEA;PATPFSR	NCP	34.793
PATPFSR;SFLMS	NCP	45.832
SFLMS;FAKRGF	NCP	11.012
FAKRGF;EDPAAAK	NCP	90.367
EDPAAAK;GFAPL	NCP	32.714
GFAPL;VVFLAPL	NCP	21.846
VVFLAPL;VTYVPV	NCP	20.356
VTYVPV;KPRWAPK	NCP	37.115
KPRWAPK;KVWAPDL	NCP	50.832
KVWAPDL;KSHYFFK	NCP	37.018
KSHYFFK;KPFPEV	NCP	5.898
KPFPEV;QSLDAAH	NCP	6.644
QSLDAAH;AGKVDY	NCP	22.953
AGKVDY;QALPP	NCP	55.009
QALPP;WELPKEQ	NCP	44.6
WELPKEQ;VTDLFP	NCP	17.031
VTDLFP;TPQPKPP	NCP	130.413
TPQPKPP;DTVFK	NCP	20.62
DTVFK;DDSLAVP	NCP	21.942
DDSLAVP;LASELHC	NCP	81.963
LASELHC;EFVVEVK	NCP	33.464
EFVVEVK;LPTGTTLP	NCP	10.533
LPTGTTLP;FLEAVP	NCP	10.636
FLEAVP;LERVHL	NCP	15.962
LERVHL;WDPDRQ	NCP	7.579
WDPDRQ;ELLVVVH	NCP	10.092
ELLVVVH;VSAPPPRP	NCP	46.823
VSAPPPRP;PLLTPP	NCP	15.186
PLLTPP;FPVGRVH	NCP	96.201
FPVGRVH;GHYAERVGAGAPV	NCP	0.036
GHYAERVGAGAPV;LAAVLEYL	NCP	0.042
LAAVLEYL;LLPKKTSATVGPKAPAGGKK	NCP	0.192
LLPKKTSATVGPKAPAGGKK;LPKKTSATVGPKAPAGGK	NCP	0.003
LPKKTSATVGPKAPAGGK;KKTSATVGPKAPAGGKK	NCP	0.006
KKTSATVGPKAPAGGKK;KTSATVGPKAPAGGK	NCP	0.003
KTSATVGPKAPAGGK;TSATVGPKAPAGGKK	NCP	0.003
TSATVGPKAPAGGKK;ATVGPKAPAGGK	NCP	0.006
ATVGPKAPAGGK;TVGPKAPAGGKK	NCP	0.003
TVGPKAPAGGKK;VGPKAPAGGK	NCP	0.003
VGPKAPAGGK;APAGGKKATQASQEY	NCP	0.012
APAGGKKATQASQEY;DGEWQL	NCP	8.196
DGEWQL;KYRWGKG	NCP	11.419
KYRWGKG;VKPGVPME	NCP	14.198
VKPGVPME;VTRAEFEEL	NCP	3.312
VTRAEFEEL;VGGATRVPK	NCP	0.276

VGGATRVPK;VQEVLLK	NCP	0.027
VQEVLLK;MAALDREVQ	NCP	4.362
MAALDREVQ;AALDREVQY	NCP	0.003
AALDREVQY;MGEVVLL	NCP	22.303
MGEVVLL;AALQELLSK	NCP	21.824
AALQELLSK;VPAGAYLA	NCP	8.157
VPAGAYLA;GPPPPYH	NCP	82.446
GPPPPYH;LLVAPK	NCP	6.838
LLVAPK;NESLLTLP	NCP	56.757
NESLLTLP;DPVLVNP	NCP	20.346
DPVLVNP;GHSSLSYF	NCP	2.592
GHSSLSYF;MLRAAVK	NCP	9
MLRAAVK;RGPVKPSGGPGGGGTQ	NCP	15.209
RGPVKPSGGPGGGGTQ;SATFPLSVQKF	NCP	16.443
SATFPLSVQKF;MEELTLKGVTQY	NCP	0.507
MEELTLKGVTQY;LDWVA	NCP	40.626
LDWVA;GVPGGPAP	NCP	10.836
GVPGGPAP;EPKVPPP	NCP	61.287
EPKVPPP;ALGSQLR	NCP	15.847
ALGSQLR;EADPKL	NCP	49.613
EADPKL;PGNSDPRP	NCP	75.325
PGNSDPRP;RRGAPPK	NCP	19.935
RRGAPPK;PGSNMKP	NCP	1.065
PGSNMKP;LREGRLR	NCP	21.521
LREGRLR;EDPQPK	NCP	47.247
EDPQPK;LDLDSGP	NCP	9.279
LDLDSGP;LDLHY	NCP	12.106
LDLHY;LPGMLPVS	NCP	76.142
LPGMLPVS;PVTVPLP	NCP	33.502
PVTVPLP;TLSLPEVL	NCP	10.116
TLSLPEVL;LPGSALPP	NCP	47.975
LPGSALPP;KPMLEP	NCP	13.17
KPMLEP;NSCKKS	NCP	42.261
NSCKKS;PNDPPR	NCP	57.54
PNDPPR;KCLALK	NCP	14.782
KCLALK;QSDRGTP	NCP	14.082
QSDRGTP;DLAMGLK	NCP	42.311
DLAMGLK;PRVPPP	NCP	50.247
PRVPPP;RLPLQ	NCP	8.736
RLPLQ;RLAAPAH	NCP	2.355
RLAAPAH;KPGTLPK	NCP	28.525
KPGTLPK;LPFTDVLR	NCP	1.586
LPFTDVLR;VGVLKAVD	NCP	39.679
VGVLKAVD;KMGPPP	NCP	16.152
KMGPPP;RDGVRLP	NCP	38.612
RDGVRLP;TVEAPPQP	NCP	2.061
TVEAPPQP;PPCSSSP	NCP	24.622
PPCSSSP;VTLGNF	NCP	36.269
VTLGNF;PLVGSQL	NCP	48.562
PLVGSQL;NRLGTGM	NCP	107.715
NRLGTGM;WGYLYR	NCP	44.217

WGYLYR;SSGVHRPP	NCP	72.095
SSGVHRPP;NKKDKEPN	NCP	48.148
NKKDKEPN;PAAKGTSPK	NCP	49.712
PAAKGTSPK;CEDPPP	NCP	2.773
CEDPPP;KCGPVVK	NCP	59.622
KCGPVVK;AAAPKPR	NCP	9.378
AAAPKPR;TLPAT	NCP	22.638
TLPAT;HLGPPPPP	NCP	45.192
HLGPPPPP;SGPKKLP	NCP	0.977
SGPKKLP;PGPRPRPAP	NCP	20.832
PGPRPRPAP;PAGQPPR	NCP	1.392
PAGQPPR;KPPCPK	NCP	7.609
KPPCPK;PVYQLFP	NCP	60.077
PVYQLFP;VTPVGRVE	NCP	95.466
VTPVGRVE;KLEMNLK	NCP	26.328
KLEMNLK;KGEFPK	NCP	24.375
KGEFPK;LAAAPSPP	NCP	48.223
LAAAPSPP;PLSKVH	NCP	12.33
PLSKVH;DLLGAP	NCP	49.742
DLLGAP;GVHLLPF	NCP	43.597
GVHLLPF;MLAAQLL	NCP	6.509
MLAAQLL;DDPQSSWDRVKDF	NCP	7.44
DDPQSSWDRVKDF;LDNWDTLASTLSK	NCP	0.744
LDNWDTLASTLSK;DLEEVKQK	NCP	0.126
DLEEVKQK;VQELQDKLSPL	NCP	0.135
VQELQDKLSPL;ESLAMLR	NCP	16.666
ESLAMLR;EPFLKSP	NCP	0.587
EPFLKSP;FLGEAAP	NCP	5.26
FLGEAAP;GGKYRVP	NCP	0.399
GGKYRVP;GEVSTYTDDLQK	NCP	1.967
GEVSTYTDDLQK;VQQLDTLRQK	NCP	0.756
VQQLDTLRQK;FGLLFDP	NCP	63.189
FGLLFDP;EPAHVP	NCP	53.097
EPAHVP;PLSGYK	NCP	52.977
PLSGYK;PPLTRHP	NCP	44.142
PPLTRHP;GPPLAPPP	NCP	55.629
GPPLAPPP;KGGHVPPP	NCP	4.827
KGGHVPPP;LSLGFGR	NCP	119.445
LSLGFGR;KPNLGAGK	NCP	23.857
KPNLGAGK;GPLLPTK	NCP	65.564
GPLLPTK;TVGGLL	NCP	89.55
TVGGLL;KGYPPPPP	NCP	11.235
KGYPPPPP;LVKYPP	NCP	28.548
LVKYPP;KKHFGH	NCP	20.896
KKHFGH;CACEC	NCP	107.573
CACEC;TGAPYLR	NCP	26.542
TGAPYLR;LSFEKKP	NCP	11.045
LSFEKKP;QPTFEPK	NCP	21.651
QPTFEPK;APKLSPP	NCP	26.097
APKLSPP;EMMMMG	NCP	4.753
EMMMMG;AAPLSYL	NCP	2.066

AAPLSYL;SNLWVSLL	NCP	59.103
SNLWVSLL;LSGPKHL	NCP	2.01
LSGPKHL;PLYKKPQ	NCP	3.1
PLYKKPQ;TQLMF	NCP	8.946
TQLMF;KASDLLR	NCP	10.374
KASDLLR;HVTPSQ	NCP	45.908
HVTPSQ;KAYEPQR	NCP	19.015
KAYEPQR;TLGADSLP	NCP	132.998
TLGADSLP;VDESAF	NCP	15.751
VDESAF;TGPCAPK	NCP	7.227
TGPCAPK;LSANLLY	NCP	30.789
LSANLLY;APLSLP	NCP	30.389
APLSLP;CGTKQP	NCP	39.973
CGTKQP;LDYDPKY	NCP	4.284
LDYDPKY;LLQADWAAK	NCP	16.781
LLQADWAAK;LNGFSAL	NCP	9.445
LNGFSAL;VYLFLP	NCP	51.084
VYLFLP;PLAEDP	NCP	15.14
PLAEDP;LLGFVP	NCP	24.664
LLGFVP;PRLKPM	NCP	105.236
PRLKPM;RPPFNK	NCP	1.207
RPPFNK;KKTVVSK	NCP	4.734
KKTVVSK;QSPNPKL	NCP	110.501
QSPNPKL;GCDLRK	NCP	1.249
GCDLRK;KGPHVSPK	NCP	1.461
KGPHVSPK;RVKVKEK	NCP	7.905
RVKVKEK;QSLGLPY	NCP	47.823
QSLGLPY;VEEWLGR	NCP	21.701
VEEWLGR;KGHFPPP	NCP	63.096
KGHFPPP;PSTMKLK	NCP	44.257
PSTMKLK;PDMYG	NCP	33.782
PDMYG;VHLPYL	NCP	76.779
VHLPYL;GSTAEC	NCP	19.926
GSTAEC;APLDRVG	NCP	160.674
APLDRVG;LTVAVPK	NCP	80.116
LTVAVPK;RSLQEKGH	NCP	29.324
RSLQEKGH;PPPPGRPP	NCP	0.327
PPPPGRPP;MQMKGP	NCP	60.87
MQMKGP;KFPEQP	NCP	138.559
KFPEQP;KDSTQK	NCP	84.32
KDSTQK;APLGPL	NCP	145.224
APLGPL;CKTKVR	NCP	6.742
CKTKVR;LKRDSPP	NCP	8.501
LKRDSPP;KVGNWL	NCP	93.22
KVGNWL;AKLSTLP	NCP	56.351
AKLSTLP;MESMLP	NCP	66.21
MESMLP;QPYVLR	NCP	85.381
QPYVLR;MLLEGVP	NCP	1.793
MLLEGVP;VAPPKGF	NCP	46.389
VAPPKGF;PHDSSK	NCP	38.338
PHDSSK;QTTLSKY	NCP	58.704

QTTLSKY;HHHDC	NCP	4.442
HHHDC;VGFCPE	NCP	19.554
VGFCPE;QSLGLSH	NCP	12.373
QSLGLSH;ALEKNVLF	NCP	42.905
ALEKNVLF;MMSMSMM	NCP	5.494
MMSMSMM;ASFVTEVLAH	NCP	32.327
ASFVTEVLAH;ELQPL	NCP	53.005
ELQPL;AACLEY	NCP	53.249
AACLEY;PDVTK	NCP	5.77
PDVTK;MHSPRHP	NCP	1.326
MHSPRHP;KGSYVKL	NCP	14.13
KGSYVKL;KLHPKP	NCP	4.439
KLHPKP;LDDLF	NCP	47.605
LDDLF;ATEVSK	NCP	79.328
ATEVSK;PTPPLMQ	NCP	18.315
PTPPLMQ;LNLKMLGL	NCP	15.676
LNLKMLGL;ELATYRK	NCP	3.429
ELATYRK;ASALVPGR	NCP	53.639
ASALVPGR;GPPLPPPG	NCP	21.556
GPPLPPPG;AEEVP	NCP	57.581
AEEVP;PRPPQY	NCP	18.024
PRPPQY;ELTFFLAK	NCP	77.499
ELTFFLAK;KPGALFVP	NCP	73.156
KPGALFVP;KAPSLPR	NCP	36.611
KAPSLPR;KMEEKP	NCP	5.506
KMEEKP;KTFPVAV	NCP	37.619
KTFPVAV;CMTPKF	NCP	221.244
CMTPKF;SRPPVTL	NCP	185.748
SRPPVTL;ATLSLFPK	NCP	15.621
ATLSLFPK;PPPLGYK	NCP	45.387
PPPLGYK;ELWSM	NCP	1.99
ELWSM;APPFYLK	NCP	17.346
APPFYLK;EPPLYHH	NCP	6.579
EPPLYHH;DFDDLHR	NCP	54.111
DFDDLHR;KPPSFDK	NCP	20.696
KPPSFDK;AAPPF	NCP	13.27
AAPPF;MNSLF	NCP	11.01
MNSLF;SVDFNK	NCP	9.497
SVDFNK;KFYLLPP	NCP	36.793
KFYLLPP;KDYLLY	NCP	15.227
KDYLLY;GVGPVAH	NCP	6.162
GVGPVAH;QEEQPK	NCP	64.386
QEEQPK;KNKLSKP	NCP	6.106
KNKLSKP;DCCHR	NCP	12.029
DCCHR;SLYGYLK	NCP	21.411
SLYGYLK;SDYLLK	NCP	47.521
SDYLLK;PSKPHTP	NCP	5.564
PSKPHTP;DKAPSPVK	NCP	17.02
DKAPSPVK;LDKLLR	NCP	1.554
LDKLLR;NPRPGPP	NCP	68.046
NPRPGPP;EANLDLR	NCP	34.569

EANLDLR;YDPKCY	NCP	3.177
YDPKCY;AVVLD	NCP	3.128
AVVLD;KRWWHQ	NCP	1.665
KRWWHQ;KPSSLLR	NCP	13.404
KPSSLLR;EEYLRSLE	NCP	3.886
EEYLRSLE;GYKPVSP	NCP	16.73
GYKPVSP;KTGFNGK	NCP	84.178
KTGFNGK;VGPLLEK	NCP	27.342
VGPLLEK;KDLSSYG	NCP	29.862
KDLSSYG;DLESDHFGK	NCP	36.773
DLESDHFGK;QAGLPAAK	NCP	39.145
QAGLPAAK;KDCLAAL	NCP	56.502
KDCLAAL;KELYSPP	NCP	14.771
KELYSPP;LLRNPF	NCP	6.954
LLRNPF;QALLLTHL	NCP	12.024
QALLLTHL;QAFSPY	NCP	55.321
QAFSPY;LFDQFFGEH	NCP	51.584
LFDQFFGEH;ESDLFPASTSL	NCP	0.033
ESDLFPASTSL;SDLFPAST	NCP	0.003
SDLFPAST;SPFYLRPPS	NCP	0.03
SPFYLRPPS;FYLRPPSFL	NCP	0.006
FYLRPPSFL;SSDGVLTV	NCP	2.886
SSDGVLTV;REEKPAVTAAPKK	NCP	0.075
REEKPAVTAAPKK;AAHLKFP	NCP	4.491
AAHLKFP;KSWLTERF	NCP	64.021
KSWLTERF;YLKTSKY	NCP	16.383
YLKTSKY;CFPKQLDF	NCP	30.602
CFPKQLDF;NSLTRTP	NCP	48.006
NSLTRTP;KYAPPP	NCP	23.61
KYAPPP;PLVCL	NCP	96.331
PLVCL;MDLFL	NCP	3.126
MDLFL;HVFPEPR	NCP	26.85
HVFPEPR;FKDVDY	NCP	60.942
FKDVDY;KKGHLRY	NCP	27.509
KKGHLRY;NLLKFPL	NCP	37.177
NLLKFPL;LGGHVLY	NCP	16.074
LGGHVLY;PGLPGP	NCP	130.373
PGLPGP;ALGVSSLPK	NCP	4.872
ALGVSSLPK;VGPSSVLTY	NCP	10.395
VGPSSVLTY;CLLFAQ	NCP	28.389
CLLFAQ;LSGAVAL	NCP	13.059
LSGAVAL;AVPFPEV	NCP	23.146
AVPFPEV;EKAFYVPP	NCP	261.212
EKAFYVPP;KNALVV	NCP	12.957
KNALVV;RMPVLR	NCP	74.146
RMPVLR;QRLLLEK	NCP	28.592
QRLLLEK;ADWFCR	NCP	20.431
ADWFCR;PLEVLY	NCP	28.449
PLEVLY;SWDGLLF	NCP	94.557
SWDGLLF;GVTSL	NCP	88.239
GVTSL;VDPVFN	NCP	24.819

VDPVFN;VTLVKY	NCP	32.186
VTLVKY;LPPPPAAHK	NCP	15.528
LPPPPAAHK;LLHAPFLE	NCP	30.772
LLHAPFLE;AAAFLEN	NCP	57.87
AAAFLEN;LPTHN	NCP	154.82
LPTHN;VFEHLGK	NCP	28.458
VFEHLGK;ANLFP	NCP	141.711
ANLFP;GNLLVN	NCP	63.049
GNLLVN;LSMSSESL	NCP	51.312
LSMSSESL;LQKFDK	NCP	18.578
LQKFDK;AQLLKTQR	NCP	25.854
AQLLKTQR;AAQEDLEKTKEEL	NCP	39.804
AAQEDLEKTKEEL;PPDDC	NCP	68.008
PPDDC;KPAAL	NCP	39.777
KPAAL;DAGNAP	NCP	8.349
DAGNAP;ESLEF	NCP	36.053
ESLEF;GSAEPAP	NCP	13.273
GSAEPAP;PAAGGPPR	NCP	0.354
PAAGGPPR;LGLGL	NCP	97.8
LGLGL;KSVSWVP	NCP	92.607
KSVSWVP;ATLSVHQ	NCP	14.334
ATLSVHQ;KKSSKK	NCP	72.498
KKSSKK;YVLPFL	NCP	32.114
YVLPFL;KDSPGSF	NCP	11.109
KDSPGSF;VGPLD	NCP	0.616
VGPLD;QGPLVG	NCP	12.198
QGPLVG;KGGYGPK	NCP	11.987
KGGYGPK;KECKAPK	NCP	7.348
KECKAPK;NRPMPP	NCP	131.906
NRPMPP;KELFHVT	NCP	3.115
KELFHVT;LTGVKQL	NCP	17.135
LTGVKQL;TVLKPHLS	NCP	153.837
TVLKPHLS;KQLNYFAK	NCP	5.236
KQLNYFAK;PPPPEPPR	NCP	3.822
PPPPEPPR;ADLPLNL	NCP	46.644
ADLPLNL;VTGGLLA	NCP	22.245
VTGGLLA;LPADLPAP	NCP	31.329
LPADLPAP;KELTQGLR	NCP	3.23
KELTQGLR;FPASTSL	NCP	48.46
FPASTSL;TYYQKAK	NCP	73.046
TYYQKAK;MNQLKD	NCP	140.961
MNQLKD;VLLTML	NCP	16.336
VLLTML;VGSLPGVR	NCP	7.368
VGSLPGVR;FLAKSSPQ	NCP	21.341
FLAKSSPQ;SLDPLY	NCP	151.497
SLDPLY;GGMKKQR	NCP	61.012
GGMKKQR;PRHFPLG	NCP	16.887
PRHFPLG;KERTGTSF	NCP	16.851
KERTGTSF;SHSLLVGR	NCP	57.624
SHSLLVGR;DLLERKN	NCP	28.254
DLLERKN;TLAAMGR	NCP	81.695

TLAAMGR;QVLRGPV	NCP	35.943
QVLRGPV;KYVLFGP	NCP	72.718
KYVLFGP;SLTPHRP	NCP	55.431
SLTPHRP;ATAPYNY	NCP	0.23
ATAPYNY;KGKFLR	NCP	9.294
KGKFLR;DDDLTH	NCP	8.499
DDDLTH;KAAAKLH	NCP	2.016
KAAAKLH;LTHSGNK	NCP	7.914
LTHSGNK;KHCFPY	NCP	19.713
KHCFPY;KPAHPTPP	NCP	42.168
KPAHPTPP;KTVMHK	NCP	12.555
KTVMHK;PFQFSSR	NCP	88.302
PFQFSSR;WLLLT	NCP	16.644
WLLLT;RSPPVTLR	NCP	64.537
RSPPVTLR;KVMGTSLL	NCP	1.589
KVMGTSLL;KEREFTPF	NCP	22.285
KEREFTPF;GMGATK	NCP	17.082
GMGATK;LEMRVHL	NCP	0.756
LEMRVHL;LPNLP	NCP	121.704
LPNLP;LGRLTL	NCP	168.651
LGRLTL;PPPEHF	NCP	25.242
PPPEHF;FLSHVLP	NCP	49.946
FLSHVLP;TGPALT	NCP	12.439
TGPALT;KTGVPVG	NCP	75.54
KTGVPVG;EMYMST	NCP	23.006
EMYMST;FGVPGPG	NCP	0.688
FGVPGPG;LDHPREL	NCP	68.424
LDHPREL;AAASFLK	NCP	28.553
AAASFLK;CGSCDGK	NCP	17.469
CGSCDGK;HAYVWY	NCP	30.67
HAYVWY;CTEHLMR	NCP	75.156
CTEHLMR;CSFDKK	NCP	8.901
CSFDKK;MRSPPPPPP	NCP	41.727
MRSPPPPPP;SGPPVP	NCP	3.858
SGPPVP;DSDVP	NCP	2.613
DSDVP;KEALLQ	NCP	62.39
KEALLQ;HCKVPK	NCP	71.239
HCKVPK;KDPMPK	NCP	98.375
KDPMPK;VLAAPPP	NCP	35.736
VLAAPPP;PTMPPQK	NCP	20.037
PTMPPQK;SPWLAK	NCP	105.757
SPWLAK;ENATLSL	NCP	95.754
ENATLSL;SFLHLGK	NCP	20.237
SFLHLGK;GFSSPQ	NCP	14.965
GFSSPQ;TYPPAL	NCP	4.932
TYPPAL;MVKFPP	NCP	2.921
MVKFPP;RGPLVPK	NCP	92.317
RGPLVPK;MDDLDALL	NCP	61.662
MDDLDALL;VGGMKHH	NCP	23.474
VGGMKHH;KEWQALL	NCP	130.147
KEWQALL;AVSFQVF	NCP	75.279

AVSFQVF;KAAGW	NCP	260.888
KAAGW;RVQQLYK	NCP	164.682
RVQQLYK;LLFPD	NCP	270.49
LLFPD;LTGTEF	NCP	8.342
LTGTEF;ALLVG	NCP	38.506
ALLVG;GRLQP	NCP	4.745
GRLQP;LVGVLLR	NCP	33.31
LVGVLLR;KEVLSPF	NCP	70.484
KEVLSPF;RSFCRLP	NCP	77.943
RSFCRLP;TLVLPF	NCP	15.534
TLVLPF;FQLGKK	NCP	69.036
FQLGKK;RNTPLV	NCP	55.792
RNTPLV;VQLLG	NCP	14.28
VQLLG;MNELEP	NCP	25.457
MNELEP;VGMKLLT	NCP	21.268
VGMKLLT;PDGQVDD	NCP	184.16
PDGQVDD;LQAKKL	NCP	0.775
LQAKKL;HPFLLH	NCP	115.592
HPFLLH;VDGDVAK	NCP	11.791
VDGDVAK;EVDFNLK	NCP	19.709
EVDFNLK;EEELVR	NCP	145.656
EEELVR;KTYDVQK	NCP	82.563
KTYDVQK;EYLDP	NCP	31.936
EYLDP;ASDLDLLK	NCP	20.714
ASDLDLLK;LLLKKPL	NCP	32.484
LLLKKPL;KPPVAMP	NCP	156.42
KPPVAMP;PADLP	NCP	6.171
PADLP;QSLLFAQL	NCP	61.008
QSLLFAQL;KTVHHLK	NCP	2.964
KTVHHLK;FRCPE	NCP	29.484
FRCPE;GLREC	NCP	133.855
GLREC;RFEFYR	NCP	14.264
RFEFYR;NCHSDLR	NCP	35.451
NCHSDLR;SLTERGP	NCP	15.387
SLTERGP;FGGYLGT	NCP	5.458
FGGYLGT;KHYEVN	NCP	1.188
KHYEVN;LQELLEA	NCP	2.913
LQELLEA;LLGGPLVL	NCP	86.345
LLGGPLVL;DHVFGK	NCP	46.824
DHVFGK;YADDDY	NCP	3.958
YADDDY;DPACHPR	NCP	42.44
DPACHPR;KEKPAAK	NCP	49.522
KEKPAAK;YGVSF	NCP	11.595
YGVSF;WFVVGG	NCP	121.715
WFVVGG;RKMNVSYY	NCP	95.832
RKMNVSYY;QTSTELR	NCP	35.583
QTSTELR;LVHLLF	NCP	198.876
LVHLLF;TACKMP	NCP	117.592
TACKMP;VGTHVL	NCP	103.35
VGTHVL;RKLLSAK	NCP	95.886
RKLLSAK;FGADLR	NCP	36.357

FGADLR;YTSPK	NCP	77.949
YTSPK;VSFVLE	NCP	23.079
VSFVLE;TVVGF	NCP	4.695
TVVGF;KDLSSPH	NCP	53.139
KDLSSPH;GVMLLVQ	NCP	7.188
GVMLLVQ;CGRAKL	NCP	34.512
CGRAKL;SLGVSTNP	NCP	10.88
SLGVSTNP;KLFLPPP	NCP	26.065
KLFLPPP;AHPYK	NCP	13.044
AHPYK;EGPTSSK	NCP	97.899
EGPTSSK;CPNDLR	NCP	93.11
CPNDLR;VLNLP	NCP	22.648
VLNLP;LPPPPHP	NCP	102.651
LPPPPHP;MFGGPAS	NCP	105.704
MFGGPAS;RKLGYDV	NCP	35.79
RKLGYDV;KQQTLLSP	NCP	2.374
KQQTLLSP;VGGGLPF	NCP	68.675
VGGGLPF;LAAHLLF	NCP	77.371
LAAHLLF;KLSLPA	NCP	112.082
KLSLPA;KDSFSKL	NCP	14.769
KDSFSKL;NMVESAF	NCP	14.446
NMVESAF;DASKYR	NCP	52.029
DASKYR;PEVLQ	NCP	74.415
PEVLQ;DKPKTR	NCP	8.379
DKPKTR;NGVNNP	NCP	8.687
NGVNNP;RQAGFFR	NCP	55.722
RQAGFFR;GVVAMLQ	NCP	11.833
GVVAMLQ;VNEMV	NCP	85.799
VNEMV;KFRSLPK	NCP	19.872
KFRSLPK;HLVRALY	NCP	1.108
HLVRALY;HNNLLNY	NCP	87.383
HNNLLNY;NAKPWK	NCP	52.575
NAKPWK;LDLVVPL	NCP	63.843
LDLVVPL;KRLLGLK	NCP	15.582
KRLLGLK;KSLYFP	NCP	23.383
KSLYFP;FSYEFNK	NCP	46.5
FSYEFNK;PVLPPPPP	NCP	1.847
PVLPPPPP;KPPPPPYK	NCP	68.986
KPPPPPYK;SQVRVLEL	NCP	23.552
SQVRVLEL;AAVLGLTK	NCP	9.684
AAVLGLTK;VSALGFK	NCP	13.633
VSALGFK;HVVKAP	NCP	80.996
HVVKAP;FGTVT	NCP	54.196
FGTVT;KDELFPP	NCP	48.564
KDELFPP;VFEFLP	NCP	29.744
VFEFLP;MNGCC	NCP	91.774
MNGCC;KAASSSPK	NCP	31.47
KAASSSPK;YALFE	NCP	62.97
YALFE;NYYQRLQ	NCP	108.69
NYYQRLQ;TLALK	NCP	63.231
TLALK;KDSTLERK	NCP	199.994

KDSTLERK;HPPFLT	NCP	57.312
HPPFLT;GFVEF	NCP	33.418
GFVEF;PMRGPP	NCP	117.285
PMRGPP;LDVVHAP	NCP	53.121
LDVVHAP;EGWLLLR	NCP	6.363
EGWLLLR;TGAKSPK	NCP	23.942
TGAKSPK;KFNLTPY	NCP	172.804
KFNLTPY;EVPGWF	NCP	9.837
EVPGWF;VSLDVVR	NCP	61.584
VSLDVVR;DRHVYP	NCP	53.148
DRHVYP;KLTNPR	NCP	20.846
KLTNPR;RKGALQP	NCP	28.936
RKGALQP;AAAKPGELM	NCP	17.676
AAAKPGELM;AAAAAPVP	NCP	0.132
AAAAAPVP;REPLSGCKLR	NCP	0.168
REPLSGCKLR;ALLVTSK	NCP	21.254
ALLVTSK;GLALEC	NCP	10.693
GLALEC;VTGRNLY	NCP	17.855
VTGRNLY;KDTQPPK	NCP	16.981
KDTQPPK;GTDCLGP	NCP	7.89
GTDCLGP;AEFTRLE	NCP	11.613
AEFTRLE;KPQKGPP	NCP	12.303
KPQKGPP;TSALTQGL	NCP	16.553
TSALTQGL;RTHPERR	NCP	83.656
RTHPERR;EDRKAVLK	NCP	1.779
EDRKAVLK;KTVAP	NCP	95.739
KTVAP;KDFPPPPP	NCP	19.314
KDFPPPPP;EGDSWQL	NCP	18.552
EGDSWQL;VDLLLAPL	NCP	21.837
VDLLLAPL;DQLVP	NCP	75.57
DQLVP;ATVHYK	NCP	78.605
ATVHYK;VSLAPE	NCP	43.309
VSLAPE;TADDC	NCP	8.402
TADDC;KEPVP	NCP	18.775
KEPVP;MFPGRAA	NCP	32.442
MFPGRAA;AGSHARPP	NCP	51.873
AGSHARPP;EGLRVYS	NCP	68.615
EGLRVYS;LEASTLVP	NCP	17.812
LEASTLVP;VGPRLP	NCP	24.774
VGPRLP;SLMTLF	NCP	21.327
SLMTLF;CCASKLK	NCP	8.147
CCASKLK;KNFKEL	NCP	30.859
KNFKEL;LPPVCMP	NCP	13.616
LPPVCMP;QHGFWP	NCP	64.102
QHGFWP;VPCPGAGP	NCP	11.094
VPCPGAGP;LAPPFG	NCP	9.273
LAPPFG;SPVGRLAP	NCP	22.203
SPVGRLAP;LLPPPPP	NCP	19.734
LLPPPPP;QGALECC	NCP	54.866
QGALECC;AFPLTS	NCP	142.063
AFPLTS;PPVLHHL	NCP	5.205

PPVLHHL;SGHLALP	NCP	44.484
SGHLALP;NELLEL	NCP	36.764
NELLEL;KDLSSSL	NCP	16.915
KDLSSSL;AVSSLLVP	NCP	13.449
AVSSLLVP;PPPGKPQP	NCP	31.134
PPPGKPQP;SKDMRLF	NCP	36.741
SKDMRLF;KEPVAP	NCP	1.856
KEPVAP;QPLDPGR	NCP	10.107
QPLDPGR;GLFHHWR	NCP	33.666
GLFHHWR;KNVTDF	NCP	9.21
KNVTDF;AGEKAEKPDTKEKKPEAK	NCP	9.675
AGEKAEKPDTKEKKPEAK;AEKPDTKEKKPEA	NCP	0.012
AEKPDTKEKKPEA;VGVSNPR	NCP	4.468
VGVSNPR;TVHRKTF	NCP	179.276
TVHRKTF;AASDPR	NCP	26.575
AASDPR;DPSLFLLPL	NCP	9.018
DPSLFLLPL;FRSGLPT	NCP	11.703
FRSGLPT;FPEVP	NCP	1.425
FPEVP;NTKKQGR	NCP	10.92
NTKKQGR;PKASYPF	NCP	47.397
PKASYPF;LPPTRTP	NCP	143.13
LPPTRTP;AELREVQ	NCP	2.709
AELREVQ;KSMAGP	NCP	23.064
KSMAGP;PALYRAL	NCP	22.766
PALYRAL;LAAFDK	NCP	81.013
LAAFDK;ESNLLQ	NCP	10.01
ESNLLQ;FSLGTSYF	NCP	22.8
FSLGTSYF;KDFYPPT	NCP	78.538
KDFYPPT;PSHRK	NCP	32.705
PSHRK;ALERPY	NCP	70.267
ALERPY;TALLGLTK	NCP	26.67
TALLGLTK;TLAAGRM	NCP	119.7
TLAAGRM;KRPGHRP	NCP	17.007
KRPGHRP;KWHVMP	NCP	11.459
KWHVMP;ETDLLFL	NCP	13.774
ETDLLFL;QTKKAAP	NCP	61.593
QTKKAAP;LAELLMA	NCP	2.43
LAELLMA;PPLELLSL	NCP	9.914
PPLELLSL;LQLGVKK	NCP	69.67
LQLGVKK;KSDGVLK	NCP	7.926
KSDGVLK;GPYDMK	NCP	34.845
GPYDMK;PGAPVLPP	NCP	3.99
PGAPVLPP;PGLPELL	NCP	36.396
PGLPELL;LSPGPLHK	NCP	4.539
LSPGPLHK;EVTAVPK	NCP	35.426
EVTAVPK;SGNLLL	NCP	33.975
SGNLLL;GLLQLMT	NCP	10.845
GLLQLMT;ETLGFK	NCP	66.132
ETLGFK;TPALEPKR	NCP	15.064
TPALEPKR;WFRAPS	NCP	17.294
WFRAPS;LSAPPG	NCP	240.4

LSAPPG;MVLHAPQ	NCP	19.578
MVLHAPQ;KALLVPL	NCP	45.192
KALLVPL;VLRRLLA	NCP	8.502
VLRRLLA;TDLPPN	NCP	2.654
TDLPPN;QVLYRA	NCP	30.126
QVLYRA;RALAPAH	NCP	1.615
RALAPAH;TGTPKPL	NCP	0.099
TGTPKPL;YKGPP	NCP	18.174
YKGPP;LPPGLPVP	NCP	17.199
LPPGLPVP;PSPALRPK	NCP	31.251
PSPALRPK;LAGANL	NCP	103.896
LAGANL;NSAVTKN	NCP	40.724
NSAVTKN;EMWYG	NCP	1.761
EMWYG;LGSKKHG	NCP	25.752
LGSKKHG;MLEPAQ	NCP	28.425
MLEPAQ;VKYPPPPP	NCP	135.667
VKYPPPPP;LSAAPASPK	NCP	102.969
LSAAPASPK;HVLSGVT	NCP	26.067
HVLSGVT;SAAWK	NCP	31.203
SAAWK;LSRGLAF	NCP	31.293
LSRGLAF;VEEFLC	NCP	6.468
VEEFLC;GPATPP	NCP	63.333
GPATPP;VTDVDLP	NCP	17.571
VTDVDLP;LGLPGAGK	NCP	4.428
LGLPGAGK;LREHLE	NCP	65.348
LREHLE;TFDPPPP	NCP	38.287
TFDPPPP;KCCDSK	NCP	30.651
KCCDSK;YGDPMD	NCP	65.924
YGDPMD;QTFTYP	NCP	43.401
QTFTYP;VSPKPP	NCP	31.114
VSPKPP;KVKVAGK	NCP	7.185
KVKVAGK;HDQSLVV	NCP	84.27
HDQSLVV;NYLPL	NCP	9.861
NYLPL;KPLRKP	NCP	3.818
KPLRKP;YKLEAGK	NCP	11.263
YKLEAGK;MTLHSK	NCP	19.043
MTLHSK;RLEVLK	NCP	75.442
RLEVLK;KSLEC	NCP	4.661
KSLEC;MEQKVGK	NCP	35.571
MEQKVGK;PLPPPKY	NCP	8.809
PLPPPKY;EFEEKK	NCP	84.992
EFEEKK;MHFPRQ	NCP	7.573
MHFPRQ;LALLNRVT	NCP	11.004
LALLNRVT;ALARRML	NCP	19.479
ALARRML;VGGVLLPP	NCP	184.628
VGGVLLPP;PVVTVAM	NCP	72.186
PVVTVAM;LRLESF	NCP	50.422
LRLESF;CSDCLQGF	NCP	8.144
CSDCLQGF;SGGFL	NCP	103.504
SGGFL;KNTEVVP	NCP	73.676
KNTEVVP;AGSYAF	NCP	45.774

AGSYAF;FDKSLKK	NCP	26.508
FDKSLKK;DSVYKLN	NCP	20.913
DSVYKLN;DSFPLHNL	NCP	22.527
DSFPLHNL;EHFLMP	NCP	86.718
EHFLMP;LTPEKLDF	NCP	73.441
LTPEKLDF;LGTPVQAT	NCP	94.664
LGTPVQAT;PSVPAVH	NCP	2.175
PSVPAVH;TMERPVT	NCP	13.569
TMERPVT;EHPKVVP	NCP	86.767
EHPKVVP;VPLFK	NCP	74.517
VPLFK;TWAPKPW	NCP	62.082
TWAPKPW;HSLKFVML	NCP	3.108
HSLKFVML;APAGDWPK	NCP	117.356
APAGDWPK;VPVVT	NCP	139.998
VPVVT;QHLTVK	NCP	25.291
QHLTVK;FATGM	NCP	25.932
FATGM;LTAEPN	NCP	42.626
LTAEPN;GHDSKLK	NCP	264.877
GHDSKLK;GPLLP	NCP	123.201
GPLLP;LELLRL	NCP	17.822
LELLRL;ENKGKLLK	NCP	65.485
ENKGKLLK;GTVPVGR	NCP	90.363
GTVPVGR;YSLTAELL	NCP	45.923
YSLTAELL;KYNLLPK	NCP	21.238
KYNLLPK;REPLSPP	NCP	204.843
REPLSPP;AAVPSLYK	NCP	54.932
AAVPSLYK;TGAPGLLH	NCP	20.353
TGAPGLLH;SLQPGDPA	NCP	46.503
SLQPGDPA;FYDLL	NCP	127.847
FYDLL;QPSGPPR	NCP	51.769
QPSGPPR;KYFQHPK	NCP	75.648
KYFQHPK;EFLDPY	NCP	287.82
EFLDPY;QQTLSK	NCP	22.392
QQTLSK;KSSCPPK	NCP	13.074
KSSCPPK;PGFGAGLF	NCP	75.411
PGFGAGLF;FLGTLLDR	NCP	41.943
FLGTLLDR;DSEMDS	NCP	86.553
DSEMDS;DLSLPL	NCP	31.662
DLSLPL;MFQRVVL	NCP	338.847
MFQRVVL;FSMLEK	NCP	11.306
FSMLEK;ALFKAGP	NCP	108.081
ALFKAGP;VLLLF	NCP	13.089
VLLLF;DVLPEVD	NCP	27.607
DVLPEVD;SSRVNMK	NCP	19.586
SSRVNMK;ETYSKLK	NCP	53.235
ETYSKLK;APALEC	NCP	140.728
APALEC;QAQCLLK	NCP	119.54
QAQCLLK;QHRKCF	NCP	0.753
QHRKCF;ESVPELK	NCP	9.807
ESVPELK;KSFTPGR	NCP	44.62
KSFTPGR;EHLQTTY	NCP	56.664

EHLQTTY;ECGLL	NCP	232.082
ECGLL;MKAAMRR	NCP	203.797
MKAAMRR;PVPGSK	NCP	47.48
PVPGSK;QLGMPQ	NCP	2.116
QLGMPQ;KTGAAP	NCP	2.853
KTGAAP;LFSHVPL	NCP	40.347
LFSHVPL;KNYQFF	NCP	6.939
KNYQFF;KELLDQ	NCP	75.108
KELLDQ;TAEPKLP	NCP	256.67
TAEPKLP;PLLPPK	NCP	156.493
PLLPPK;LEPEPLAP	NCP	8.796
LEPEPLAP;KELKLP	NCP	10.083
KELKLP;LPTMLVY	NCP	202.026
LPTMLVY;LASVKAR	NCP	108.408
LASVKAR;RAALVP	NCP	208.308
RAALVP;RHPCR	NCP	16.79
RHPCR;LTKVPEFQ	NCP	71.437
LTKVPEFQ;EEWVPLPR	NCP	3.105
EEWVPLPR;HVVPP	NCP	45.68
HVVPP;KGESSLL	NCP	54.225
EMVKHR;GKALPL	NCP	32.985
GKALPL;QPQAGLR	NCP	112.86
QPQAGLR;DARLNL	NCP	10.149
DARLNL;SPPAGRPAGP	NCP	268.887
SPPAGRPAGP;MNKLHM	NCP	17.493
MNKLHM;PHKPVPP	NCP	39.591
PHKPVPP;GFGAGP	NCP	92.22
GFGAGP;GPGTWPK	NCP	47.67
GPGTWPK;ALGVPP	NCP	65.778
ALGVPP;RPAAGAPP	NCP	2.826
RPAAGAPP;QKRLPW	NCP	50.292
QKRLPW;VAPLPPPPPP	NCP	66.042
VAPLPPPPPP;LEKHHF	NCP	18.219
LEKHHF;LGQGAHV	NCP	32.43
LGQGAHV;VLELAGR	NCP	20.616
VLELAGR;PLGVCAG	NCP	63.111
PLGVCAG;YPQDPKAGP	NCP	10.632
YPQDPKAGP;GATELRL	NCP	56.352
GATELRL;LHSNVAHY	NCP	13.533
LHSNVAHY;APGQGP	NCP	0.099
APGQGP;ATGFN	NCP	18.342
ATGFN;EVEGPLP	NCP	33.033
EVEGPLP;AGPHVP	NCP	14.989
AGPHVP;MGVPGKP	NCP	7.532
MGVPGKP;GPPPPLVP	NCP	1.716
GPPPPLVP;AAPGK	NCP	35.427
AAPGK;RALPGVGR	NCP	5.736
RALPGVGR;AVSTTLP	NCP	9.84
AVSTTLP;AAPPPPR	NCP	136.065
AAPPPPR;RVSLDVP	NCP	57.63
RVSLDVP;LYESALTENQKLKTK	NCP	36.252

LYESALTENQKLKTK;DPEPLPRP	NCP	33.789
DPEPLPRP;ETCLLF	NCP	38.242
ETCLLF;LAAGPPP	NCP	4.793
LAAGPPP;PQAAPRP	NCP	10.836
PQAAPRP;GPNPPHL	NCP	1.023
GPNPPHL;AARLVP	NCP	6.157
AARLVP;HLPFHLL	NCP	96.321
HLPFHLL;EGAPARPP	NCP	22.49
EGAPARPP;NAPAQAP	NCP	14.865
NAPAQAP;FVPAP	NCP	0.118
FVPAP;TSAGPLLP	NCP	13.355
TSAGPLLP;RVTGLAH	NCP	2.725
RVTGLAH;WDPGTR	NCP	103.025
WDPGTR;RPLRPGP	NCP	3.933
RPLRPGP;PFHLPR	NCP	4.59
PFHLPR;GKNPVAQ	NCP	9.457
GKNPVAQ;HVLPAAR	NCP	53.549
HVLPAAR;SAPGFN	NCP	12.834
SAPGFN;EDFPQSPSTHP	NCP	102.807
EDFPQSPSTHP;VLGARVVA	NCP	34.935
VLGARVVA;PTPAKVPP	NCP	13.452
PTPAKVPP;KDHYVK	NCP	3.669
KDHYVK;ELTAL	NCP	2.343
ELTAL;LPPLAPR	NCP	24.009
LPPLAPR;GPPGPPPP	NCP	13.725
GPPGPPPP;TSAPGLLP	NCP	205.65
TSAPGLLP;AVRFNL	NCP	106.797
AVRFNL;MSMCPL	NCP	24.846
MSMCPL;RLVTRF	NCP	14.859
RLVTRF;ALSMLP	NCP	176.532
ALSMLP;QQARPK	NCP	5.257
QQARPK;AAGFM	NCP	19.557
AAGFM;AFLGKY	NCP	26.465
AFLGKY;PGGLQAV	NCP	125.196
PGGLQAV;GCKRSP	NCP	14.676
GCKRSP;KLWQAVQ	NCP	9.909
KLWQAVQ;VVPSTGP	NCP	50.865
VVPSTGP;FSDKGMP	NCP	21.894
FSDKGMP;LSGHDQ	NCP	47.884
LSGHDQ;NLPRHLQ	NCP	61.394
NLPRHLQ;GVPLR	NCP	60.429
GVPLR;GSPTWLPP	NCP	7.179
GSPTWLPP;KLCKSAP	NCP	125.421
KLCKSAP;KSTYGLF	NCP	29.811
KSTYGLF;LGLGLLY	NCP	47.976
LGLGLLY;NGKELTAL	NCP	265.233
NGKELTAL;LPFSP	NCP	51.453
LPFSP;TSRVAPGK	NCP	12.772
TSRVAPGK;RGLDPHV	NCP	40.224
RGLDPHV;VVPGKYN	NCP	13.575
VVPGKYN;AQLLNKV	NCP	34.808

AQLLNKV;DPWVK	NCP	15.852
DPWVK;KPSPCAC	NCP	11.781
KPSPCAC;PAGKEVPR	NCP	6.646
PAGKEVPR;RAPPP	NCP	44.52
RAPPP;KTARPSP	NCP	3.114
KTARPSP;LMVEDR	NCP	47.568
LMVEDR;FNLDKL	NCP	32.228
FNLDKL;LNERLTN	NCP	55.36
LNERLTN;MRYVAS	NCP	51.372
MRYVAS;MDVPNLK	NCP	36.794
MDVPNLK;AKVAVL	NCP	106.459
AKVAVL;LPPVQP	NCP	14.04
LPPVQP;LLLTQEK	NCP	9.663
LLLTQEK;KYTVAGF	NCP	13.335
KYTVAGF;VVEVMVH	NCP	46.011
VVEVMVH;TGATDYL	NCP	25.701
TGATDYL;NNPVFNK	NCP	3.662
NNPVFNK;EVHNLK	NCP	0.214
EVHNLK;YVARLN	NCP	21.605
YVARLN;VLPGWP	NCP	19.801
VLPGWP;FDKNK	NCP	4.046
FDKNK;VPGKLVLE	NCP	170.665
VPGKLVLE;LTASVEK	NCP	6.419
LTASVEK;PPVSPAPP	NCP	9.535
PPVSPAPP;SLPYAGK	NCP	49.835
SLPYAGK;LAHPALL	NCP	37.693
LAHPALL;SPLPDP	NCP	12.609
SPLPDP;KADLQPP	NCP	19.209
KADLQPP;KSFSTAL	NCP	30.789
KSFSTAL;PPTVV	NCP	70.76
PPTVV;PRAPP	NCP	30.213
PRAPP;KGFVPPL	NCP	22.179
KGFVPPL;KLQSAGK	NCP	85.473
KLQSAGK;TLMQY	NCP	76.921
TLMQY;KRVLPR	NCP	9.807
KRVLPR;TLLMY	NCP	18.44
TLLMY;RHVVLAF	NCP	13.05
RHVVLAF;MLGMLKN	NCP	21.447
MLGMLKN;APRFPK	NCP	15.064
APRFPK;PAPVGP	NCP	32.399
PAPVGP;EVKDLR	NCP	255.492
EVKDLR;KGFGPPP	NCP	41.536
KGFGPPP;PGLPT	NCP	18.564
PGLPT;LKVGKNL	NCP	19.262
LKVGKNL;PPFLKDK	NCP	39.664
PPFLKDK;AVRVPAP	NCP	16.065
AVRVPAP;APGGNAGP	NCP	47.199
APGGNAGP;KKPAMTK	NCP	26.556
KKPAMTK;PERAGPQ	NCP	100.44
PERAGPQ;LGGVLPF	NCP	28.872
LGGVLPF;DLGPLL	NCP	115.256

DLGPLL;QPMEPRP	NCP	8.236
QPMEPRP;FGGAP	NCP	39.645
FGGAP;PPNLAAPR	NCP	1.194
PPNLAAPR;ALKATF	NCP	23.813
ALKATF;LAHPARS	NCP	0.454
LAHPARS;NHALVLY	NCP	12.102
NHALVLY;MGKEVP	NCP	39.942
MGKEVP;KPACVSGK	NCP	93.822
KPACVSGK;SNPLGF	NCP	57.315
SNPLGF;TTYQDKAATATE	NCP	15.131
TTYQDKAATATE;LGSRVEH	NCP	30.447
LGSRVEH;PGAAEGLR	NCP	36.904
PGAAEGLR;CDGVV	NCP	14.199
CDGVV;PVGGGLLG	NCP	3.356
PVGGGLLG;LPLQAKA	NCP	31.803
LPLQAKA;REETALGK	NCP	10.528
REETALGK;SSPALLLK	NCP	43.295
SSPALLLK;EPMQGF	NCP	20.482
EPMQGF;PATLHPK	NCP	4.575
PATLHPK;KPSPYTK	NCP	57.72
KPSPYTK;LPPKRD	NCP	28.094
LPPKRD;NSVAPSK	NCP	32.334
NSVAPSK;PPQGHVK	NCP	6.543
PPQGHVK;NRGPPR	NCP	49.444
NRGPPR;ECAKP	NCP	26.964
ECAKP;MDLKPPP	NCP	1.047
MDLKPPP;KFVFVPH	NCP	22.113
KFVFVPH;NLPGPM	NCP	50.856
NLPGPM;TVAGGLP	NCP	60.813
TVAGGLP;KTYFP	NCP	8.097
KTYFP;GSWGLVR	NCP	54.644
GSWGLVR;AQSSK	NCP	27.196
AQSSK;DGLVHLQ	NCP	4.151
DGLVHLQ;VTERYL	NCP	11.211
VTERYL;VDHCLPK	NCP	2.785
VDHCLPK;KELTVDP	NCP	1.452
KELTVDP;VTWLAQ	NCP	2.127
VTWLAQ;NKTKQCE	NCP	8.159
NKTKQCE;VTTLLF	NCP	21.184
VTTLLF;RPHLPPPPP	NCP	52.581
RPHLPPPPP;TGKRAAAP	NCP	35.382
TGKRAAAP;WVDRPK	NCP	0.191
WVDRPK;KKLSSQP	NCP	56.146
KKLSSQP;HTMKHT	NCP	202.44
HTMKHT;MGTTL	NCP	158.339
MGTTL;VLLEP	NCP	42.517
VLLEP;FDDSEF	NCP	267.126
FDDSEF;LNKLSFPA	NCP	3.101
LNKLSFPA;MMTCCP	NCP	6.15
MMTCCP;GAGLPGLVS	NCP	74.44
GAGLPGLVS;PVTSPP	NCP	17.094

PVTSPP;QELPLQK	NCP	47.037
QELPLQK;RMKAPS	NCP	6.518
RMKAPS;QRAFLEEV	NCP	139.531
QRAFLEEV;MTLSGF	NCP	47.861
MTLSGF;KGSDVRP	NCP	18.003
KGSDVRP;FLVPPF	NCP	5.976
FLVPPF;VPGANGPR	NCP	19.464
VPGANGPR;AAFQPL	NCP	6.781
AAFQPL;MNGPSPK	NCP	3.984
MNGPSPK;EGPCPRGP	NCP	8.057
EGPCPRGP;KLNFRK	NCP	48.672
KLNFRK;EESCSPK	NCP	16.74
EESCSPK;PKGNPPP	NCP	43.581
PKGNPPP;LPFLE	NCP	23.379
LPFLE;RPPVGPP	NCP	6.822
RPPVGPP;VLRAPP	NCP	64.194
VLRAPP;TSVLVKVP	NCP	19.458
TSVLVKVP;MLVML	NCP	46.507
MLVML;GWNAY	NCP	15.48
GWNAY;LTPLPDA	NCP	13.118
LTPLPDA;KSGFPPPPP	NCP	23.113
KSGFPPPPP;YCCDC	NCP	197.664
YCCDC;LPKKTK	NCP	8.418
LPKKTK;QPGAPLGN	NCP	245.552
QPGAPLGN;EDHRWNPP	NCP	121.543
EDHRWNPP;SLQREFP	NCP	19.691
SLQREFP;NVLVV	NCP	68.343
NVLVV;PGPVVT	NCP	12.886
PGPVVT;KPPSR	NCP	13.881
KPPSR;SAAFPGR	NCP	19.667
SAAFPGR;PVHYPP	NCP	7.125
PVHYPP;KCGPPP	NCP	17.565
KCGPPP;LPVSKLLP	NCP	27.372
LPVSKLLP;KMAFLQ	NCP	63.886
KMAFLQ;LVPGAKKA	NCP	7.634
LVPGAKKA;RSVPKTTL	NCP	23.646
RSVPKTTL;KAYSMTSL	NCP	67.531
KAYSMTSL;MDSSSMP	NCP	31.292
MDSSSMP;AFNKL	NCP	55.461
AFNKL;LPEQP	NCP	54.28
LPEQP;DMERLMK	NCP	16.916
DMERLMK;TLTSKRY	NCP	22.789
TLTSKRY;AATVGRP	NCP	2.795
AATVGRP;VERVFP	NCP	20.514
VERVFP;KGWEPK	NCP	112.872
KGWEPK;LTGTSLFY	NCP	51.795
LTGTSLFY;KPHVKK	NCP	202.38
KPHVKK;HQVLYR	NCP	44.316
HQVLYR;FSLVHR	NCP	29.799
FSLVHR;AATYVY	NCP	17.749
AATYVY;LGERVSK	NCP	54.794

LGERVSK;LPGKPVY	NCP	14.101
LPGKPVY;CMTPK	NCP	1.202
CMTPK;LSHKLSTF	NCP	143.146
LSHKLSTF;VLYKPGP	NCP	2.234
VLYKPGP;DNEKQAK	NCP	41.496
DNEKQAK;KAMLL	NCP	29.253
KAMLL;TMVNL	NCP	26.931
TMVNL;KSDFGFY	NCP	32.055
KSDFGFY;RFLPFK	NCP	8.89
RFLPFK;PGSLTKHR	NCP	48.51
PGSLTKHR;LVLAP	NCP	8.883
LVLAP;GFSRVGR	NCP	56.312
GFSRVGR;KTSLRFQ	NCP	64.366
KTSLRFQ;HPPRGPAP	NCP	6.245
HPPRGPAP;KEAPAPPKA	NCP	24.291
KEAPAPPKA;FGDSLV	NCP	84.207
FGDSLV;PGCPAC	NCP	18.975
PGCPAC;KRLPRL	NCP	135.27
KRLPRL;KGLAEPRP	NCP	4.083
KGLAEPRP;GSVLGQETK	NCP	1.762
GSVLGQETK;PAPTPRP	NCP	45.983
PAPTPRP;KLREELH	NCP	62.877
KLREELH;HPSSAK	NCP	12.39
HPSSAK;AAWTVP	NCP	15.681
AAWTVP;GPVQPEK	NCP	10.476
GPVQPEK;PLSTGPAP	NCP	80.952
PLSTGPAP;YGTWK	NCP	34.996
YGTWK;LEEELRK	NCP	55.098
LEEELRK;AEAVKALVKPK	NCP	2.225
AEAVKALVKPK;PSLMVPSS	NCP	9.648
PSLMVPSS;VAEAPA	NCP	17.796
VAEAPA;NELPPE	NCP	31.978
NELPPE;PSGFLDN	NCP	41.285
PSGFLDN;FTRAVEDQ	NCP	23.808
FTRAVEDQ;FHKMEK	NCP	16.504
FHKMEK;LSAEP	NCP	26.291
LSAEP;SPPAPPP	NCP	125.634
SPPAPPP;ASVVFPK	NCP	60.51
ASVVFPK;KVGLVP	NCP	20.934
KVGLVP;MRLLTHD	NCP	4.326
MRLLTHD;KKNGAPR	NCP	34.615
KKNGAPR;GFGQSL	NCP	42.402
GFGQSL;PFVTLK	NCP	56.294
PFVTLK;SALLL	NCP	14.232
SALLL;QDVGLAL	NCP	12.921
QDVGLAL;KSSGPPLP	NCP	34.356
KSSGPPLP;YHKYSL	NCP	21.454
YHKYSL;LGAGLER	NCP	8.439
LGAGLER;NFTPLAK	NCP	80.015
NFTPLAK;ERFRCP	NCP	36.784
ERFRCP;KTVPDK	NCP	31.716

KTVPDK;KPAHNGK	NCP	129.183
KPAHNGK;LELVSH	NCP	0.629
LELVSH;MFYPK	NCP	0.291
MFYPK;ANLVF	NCP	6.927
ANLVF;PNRKTH	NCP	19.266
PNRKTH;MLQGKMG	NCP	15.316
MLQGKMG;PLLMF	NCP	75.528
PLLMF;LPLLF	NCP	197.556
LPLLF;ALGELP	NCP	7.626
ALGELP;VTLLT	NCP	16.341
VTLLT;YGDGP	NCP	9.542
YGDGP;AAGNLPF	NCP	35.01
AAGNLPF;VYKVLK	NCP	90.798
VYKVLK;RLHVLFPL	NCP	19.641
RLHVLFPL;VNVRVQVR	NCP	72.465
VNVRVQVR;PTLHSVPQ	NCP	16.632
PTLHSVPQ;MGGAF	NCP	10.009
MGGAF;RKNYLAD	NCP	91.79
RKNYLAD;DDLAQN	NCP	15.777
DDLAQN;VSLVC	NCP	37.935
VSLVC;KNEKFDK	NCP	213.522
KNEKFDK;KMLNVTD	NCP	50.115
KMLNVTD;NMCCC	NCP	88.896
NMCCC;APDLP	NCP	61.413
APDLP;ATFPEFQ	NCP	27.808
ATFPEFQ;ALMGM	NCP	11.975
ALMGM;CSPLYE	NCP	13.354
CSPLYE;KTKKLLLD	NCP	56.186
KTKKLLLD;DPLLL	NCP	151.204
DPLLL;ASRVLNY	NCP	205.118
ASRVLNY;LPGSAPLP	NCP	7.674
LPGSAPLP;NVFLKSH	NCP	75.765
NVFLKSH;PQVGKTK	NCP	6.9
PQVGKTK;KTHTFSSK	NCP	12.282
KTHTFSSK;AAKVFE	NCP	165.478
AAKVFE;AHLVVRK	NCP	141.668
AHLVVRK;LKMQPK	NCP	118.735
LKMQPK;GPDMEQL	NCP	79.488
GPDMEQL;PGRPYVQ	NCP	9.302
PGRPYVQ;MELPEKK	NCP	22.606
MELPEKK;KEVVEK	NCP	4.088
KEVVEK;EVEHQLQ	NCP	340.272
EVEHQLQ;MLKPGAK	NCP	56.499
MLKPGAK;QMLKSK	NCP	25.302
QMLKSK;KFNSTH	NCP	56.013
KFNSTH;EHYMYT	NCP	281.617
EHYMYT;KLSALR	NCP	19.199
KLSALR;LSPLLMP	NCP	20.026
LSPLLMP;KTKFSLCK	NCP	22.802
KTKFSLCK;NRGLLGL	NCP	8.604
NRGLLGL;GRAPKGGK	NCP	43.35

GRAPKGGK;SAEFL	NCP	57.334
SAEFL;ASTSVPFK	NCP	24.491
ASTSVPFK;KHETTF	NCP	8.263
KHETTF;PAWTSDK	NCP	50.144
PAWTSDK;FLDAKGK	NCP	55.302
FLDAKGK;SQALP	NCP	31.482
SQALP;RPGVLRL	NCP	8.397
RPGVLRL;LYLKTAA	NCP	27.811
LYLKTAA;HMPQSVL	NCP	75.659
HMPQSVL;VLLLGELLK	NCP	69.577
VLLLGELLK;NAFNKL	NCP	44.654
NAFNKL;VTSPTVK	NCP	10.077
VTSPTVK;KTLKLLVK	NCP	132.738
KTLKLLVK;PVVVPKP	NCP	79.423
PVVVPKP;EKENMQPF	NCP	69.197
EKENMQPF;VVLPKEFPVYL	NCP	0.024
VVLPKEFPVYL;AFLEAVQFF	NCP	56.112
AFLEAVQFF;LPSESVSSLTDLK	NCP	27.24
LPSESVSSLTDLK;TDLKTPAG	NCP	0.027
TDLKTPAG;KQPEVPSP	NCP	0.126
KQPEVPSP;KVTATSH	NCP	3.805
KVTATSH;LHYALPP	NCP	6.465
LHYALPP;KSSPPEKK	NCP	179.993
KSSPPEKK;HSFTPLK	NCP	81.945
HSFTPLK;PAHHEK	NCP	8.242
PAHHEK;NQRVTHFH	NCP	12.581
NQRVTHFH;VTVSKASL	NCP	12.994
VTVSKASL;MRYVA	NCP	5.036
MRYVA;WSARSP	NCP	11.706
WSARSP;ESFSTP	NCP	26.653
ESFSTP;KADPVYP	NCP	8.762
KADPVYP;QPSLV	NCP	20.754
QPSLV;STEPVP	NCP	8.253
STEPVP;NADFLRA	NCP	33.972
NADFLRA;QPDCVR	NCP	41.905
QPDCVR;LDLLLKK	NCP	11.864
LDLLLKK;LLVPF	NCP	69.576
LLVPF;KPTRFPC	NCP	41.401
KPTRFPC;GQARAP	NCP	84.515
GQARAP;FGGQVSR	NCP	71.328
FGGQVSR;MSHFPK	NCP	11.008
MSHFPK;SSPFKVSLP	NCP	15.923
SSPFKVSLP;VVLFFVT	NCP	12.052
VVLFFVT;KTLVELPF	NCP	151.455
KTLVELPF;PHVKAGP	NCP	10.656
PHVKAGP;KMSLKK	NCP	35.604
KMSLKK;MDMMEP	NCP	0.938
MDMMEP;ERQVVR	NCP	3.037
ERQVVR;GVPPGSSK	NCP	22.398
GVPPGSSK;ALRGLTV	NCP	9.899
ALRGLTV;VLTPELY	NCP	31.683

VLTPELY;PVELL	NCP	33.664
PVELL;LYSDLK	NCP	5.015
LYSDLK;AGELLD	NCP	98.808
AGELLD;CERMLSL	NCP	59.32
CERMLSL;TQVPDK	NCP	3.632
TQVPDK;KLWSVPK	NCP	0.943
KLWSVPK;KEMDPLE	NCP	32.811
KEMDPLE;VAPGKLP	NCP	51.725
VAPGKLP;TVLLAE	NCP	74.724
TVLLAE;ALLKFLP	NCP	12.84
ALLKFLP;SHGSRYK	NCP	2.331
SHGSRYK;TVGAGMG	NCP	22.542
TVGAGMG;KDLSPPP	NCP	147.859
KDLSPPP;RLEELR	NCP	37.833
RLEELR;VVFMSKFP	NCP	2.285
VVFMSKFP;ALPLLVK	NCP	102.228
ALPLLVK;VVKPP	NCP	43.194
VVKPP;ASGSRLPEDA	NCP	55.146
ASGSRLPEDA;VASPRPVKG	NCP	0.141
VASPRPVKG;SPRPVKGTAGREAA	NCP	0.006
SPRPVKGTAGREAA;KYKVQLH	NCP	26.731
KYKVQLH;KASVEVR	NCP	50.906
KASVEVR;RNGPPGK	NCP	24.657
RNGPPGK;LVVACLLP	NCP	64.992
LVVACLLP;TGTLDSRL	NCP	9.913
TGTLDSRL;KDLLQ	NCP	23.126
KDLLQ;VAPFPE	NCP	91.185
VAPFPE;GVHVVPV	NCP	93.529
GVHVVPV;KDLQNEKP	NCP	46.556
KDLQNEKP;EACKLTK	NCP	26.094
EACKLTK;PASFLP	NCP	15.972
PASFLP;LLNETGDEPFQYKN	NCP	34.656
LLNETGDEPFQYKN;NETGDEPFQYK	NCP	0.006
NETGDEPFQYK;KLYGLPA	NCP	144.328
KLYGLPA;FARKAVF	NCP	11.265
FARKAVF;HPSDDD	NCP	48.899
HPSDDD;TAAPEF	NCP	50.062
TAAPEF;AQLLSKN	NCP	41.612
AQLLSKN;LPQRPP	NCP	54.498
LPQRPP;KYLPPVP	NCP	8.346
KYLPPVP;MALTSKP	NCP	57.565
MALTSKP;NYAGP	NCP	15.435
NYAGP;QGAPVYL	NCP	107.193
QGAPVYL;LSVEVR	NCP	7.256
LSVEVR;MLSVAAR	NCP	17.257
MLSVAAR;GVLVKT	NCP	80.543
GVLVKT;KEGGLVT	NCP	88.11
KEGGLVT;LPNPL	NCP	53.658
LPNPL;VGGSLWVP	NCP	42.075
VGGSLWVP;MGHPSE	NCP	16.086
MGHPSE;FTSHFKAV	NCP	34.027

FTSHFKAV;LQELQLK	NCP	19.952
LQELQLK;KYPELQ	NCP	10.671
KYPELQ;PPSPGP	NCP	6.657
PPSPGP;RLKTTAF	NCP	174.366
RLKTTAF;EPTFPAA	NCP	42.58
EPTFPAA;KKKGFK	NCP	51.257
KKKGFK;KHPGNQ	NCP	0.987
KHPGNQ;KPPFGR	NCP	24.3
KPPFGR;KPPEPPR	NCP	29.604
KPPEPPR;RNPPP	NCP	8.598
RNPPP;GVPGKQD	NCP	24.784
GVPGKQD;LPPKKFG	NCP	89.264
LPPKKFG;TPHLGSNR	NCP	15.348
TPHLGSNR;PSPALLTH	NCP	20.028
PSPALLTH;MKAVP	NCP	9.339
MKAVP;KELTELTP	NCP	50.065
KELTELTP;LAGPVG	NCP	61.07
LAGPVG;EDGFDLL	NCP	70.366
EDGFDLL;YNQSL	NCP	58.322
YNQSL;KNRVVGP	NCP	39.606
KNRVVGP;EVHFTR	NCP	2.796
EVHFTR;KATKTLHT	NCP	290.184
KATKTLHT;GLGEF	NCP	17.061
GLGEF;SGPTSLP	NCP	17.352
SGPTSLP;ATPVSLLLR	NCP	19.29
ATPVSLLLR;VGPGYG	NCP	11.568
VGPGYG;WPGGP	NCP	34.242
WPGGP;KWLLL	NCP	90.613
KWLLL;RLVLTLH	NCP	116.684
RLVLTLH;YDELKK	NCP	108.214
YDELKK;TDRGLRD	NCP	5.018
TDRGLRD;FGSVKSR	NCP	9.006
FGSVKSR;YDSPLGP	NCP	92.233
YDSPLGP;KGFKPKHR	NCP	37.295
KGFKPKHR;RVVYTSAK	NCP	98.803
RVVYTSAK;KDSSYPK	NCP	20.598
KDSSYPK;LPSVLGP	NCP	17.732
LPSVLGP;TGPVNELR	NCP	29.329
TGPVNELR;RLAMLK	NCP	52.653
RLAMLK;PLPKLSH	NCP	86.984
PLPKLSH;GVRASLTK	NCP	5.94
GVRASLTK;LSKYAQP	NCP	75.736
LSKYAQP;KGVASKE	NCP	18.236
KGVASKE;SRYVPR	NCP	12.387
SRYVPR;YETFKEY	NCP	36.259
YETFKEY;AARLLLR	NCP	14.24
AARLLLR;APVAP	NCP	6.228
APVAP;GPVVGN	NCP	19.782
GPVVGN;KGGPKYK	NCP	54.4
KGGPKYK;LTVLCEK	NCP	95.891
LTVLCEK;MDLSPH	NCP	11.853

MDLSPH;LYSAAYL	NCP	47.796
LYSAAYL;VDVSVPK	NCP	8.325
VDVSVPK;VLWGKNKC	NCP	8.185
VLWGKNKC;PLSPLPF	NCP	23.958
PLSPLPF;QPMPPC	NCP	19.959
QPMPPC;KGVLSSH	NCP	0.483
KGVLSSH;AFAAVEL	NCP	6.114
AFAAVEL;RYVHR	NCP	32.751
RYVHR;KDVGLRK	NCP	35.253
KDVGLRK;PLDVTHS	NCP	22.601
PLDVTHS;RAPCE	NCP	12.384
RAPCE;MDLKDKK	NCP	229.902
MDLKDKK;ELVAVP	NCP	23.133
ELVAVP;HAVGSK	NCP	35.145
HAVGSK;LEELKLKK	NCP	67.462
LEELKLKK;KEVTWMR	NCP	253.4
KEVTWMR;EYWQLE	NCP	8.233
EYWQLE;LLLKNVT	NCP	14.633
LLLKNVT;LGRFGY	NCP	38.808
LGRFGY;VTKLQTLK	NCP	55.827
VTKLQTLK;YVVARP	NCP	80.694
YVVARP;DLPFL	NCP	131.458
DLPFL;MLKPLR	NCP	32.97
MLKPLR;LQKVLQ	NCP	15.159
LQKVLQ;VFLLNLV	NCP	291.567
VFLLNLV;KKYLKDY	NCP	152.583
KKYLKDY;LDTDLVLLQ	NCP	4.466
LDTDLVLLQ;TARLQAL	NCP	11.908
TARLQAL;NQVRPP	NCP	22.106
NQVRPP;KPYHLVP	NCP	45.12
KPYHLVP;KGPPVP	NCP	114.981
KGPPVP;KPPGPPPP	NCP	21.711
KPPGPPPP;GVDPF	NCP	52.923
GVDPF;CLPQN	NCP	26.497
CLPQN;EEETYS	NCP	49.199
EEETYS;KGEVLPL	NCP	9.661
KGEVLPL;VYCLAL	NCP	94.236
VYCLAL;LPVKKGC	NCP	28.769
LPVKKGC;PPFPAPSP	NCP	32.059
PPFPAPSP;LARAAGF	NCP	0.318
LARAAGF;AALSAAN	NCP	11.552
AALSAAN;ELVHLQ	NCP	12.412
ELVHLQ;TLDVVRK	NCP	23.268
TLDVVRK;VSTPML	NCP	124.073
VSTPML;KGSLLA	NCP	38.103
KGSLLA;KWLPQN	NCP	8.268
KWLPQN;LNYVNVP	NCP	137.785
LNYVNVP;KLRKVSGP	NCP	28.754
KLRKVSGP;EQYSCH	NCP	23.151
EQYSCH;KPYGVR	NCP	60.565
KPYGVR;ECDGP	NCP	52.649

ECDGP;DHLMTRP	NCP	86.143
DHLMTRP;TVLLSR	NCP	41.645
TVLLSR;WFTKK	NCP	139.573
WFTKK;VLEERK	NCP	43.506
VLEERK;TAAKP	NCP	91.17
TAAKP;EQLLTGK	NCP	28.307
EQLLTGK;LNPPLP	NCP	121.947
LNPPLP;LSAYLGGK	NCP	73.41
LSAYLGGK;FLEKKPR	NCP	50.478
FLEKKPR;RLLLPG	NCP	61.884
RLLLPG;LSPPLR	NCP	74.346
LSPPLR;KDPAP	NCP	13.803
KDPAP;RAHVFLT	NCP	16.795
RAHVFLT;ARDLNLK	NCP	0.755
ARDLNLK;ATDLSLP	NCP	1.144
ATDLSLP;DDFFSRT	NCP	17.282
DDFFSRT;MGRLKAT	NCP	9.132
MGRLKAT;RAPPPP	NCP	3.429
RAPPPP;NAVPLEY	NCP	23.851
NAVPLEY;SLKTSNPK	NCP	32.504
SLKTSNPK;KPDPPR	NCP	9.067
KPDPPR;AASLDVV	NCP	1.91
AASLDVV;LEEVVR	NCP	70.921
LEEVVR;FVLLSN	NCP	117.156
FVLLSN;TKAPLVM	NCP	19.805
TKAPLVM;MGPQKPR	NCP	114.192
MGPQKPR;VGTVRLQ	NCP	9.547
VGTVRLQ;TPPLPP	NCP	6.914
TPPLPP;YPLPG	NCP	8.14
YPLPG;YDAVVK	NCP	175.398
YDAVVK;LDYLLHF	NCP	3.66
LDYLLHF;SSLVRNL	NCP	84.198
SSLVRNL;FPKVM	NCP	105.911
FPKVM;NSVLAPL	NCP	91.08
NSVLAPL;NRAPPKK	NCP	438.033
NRAPPKK;NEGMP	NCP	129.154
NEGMP;VEDVCTAKPR	NCP	10.895
VEDVCTAKPR;FTKTSAV	NCP	18.573
FTKTSAV;KAHVLLLY	NCP	51.898
KAHVLLLY;DDDSDM	NCP	18.726
DDDSDM;LDDNLRP	NCP	59.364
LDDNLRP;LNPLG	NCP	55.818
LNPLG;DGSLMF	NCP	31.752
DGSLMF;KEYYPPP	NCP	36.032
KEYYPPP;ETLKL	NCP	69.631
ETLKL;KCSSN	NCP	20.088
KCSSN;WTTTK	NCP	24.869
WTTTK;YWYSS	NCP	108.468
YWYSS;LMTTQA	NCP	121.971
LMTTQA;KASLQAP	NCP	28.623
KASLQAP;SVGPKV	NCP	4.795

SVGPKV;SGPGPAFK	NCP	62.762
SGPGPAFK;FPPLLV	NCP	49.302
FPPLLV;YTAVLP	NCP	94.134
YTAVLP;YLSSVAS	NCP	64.567
YLSSVAS;EALVLE	NCP	2.993
EALVLE;DCMMP	NCP	39.723
DCMMP;VDGLLKK	NCP	87.247
VDGLLKK;PGPGNP	NCP	185.342
PGPGNP;KWVHPP	NCP	42.007
KWVHPP;VTGKKVP	NCP	17.753
VTGKKVP;KDHSL	NCP	42.876
KDHSL;FGKMYPP	NCP	111.315
FGKMYPP;SGSLPLK	NCP	10.506
SGSLPLK;AVPGV	NCP	9.588
AVPGV;NLEALNYL	NCP	1.116
NLEALNYL;LPWKWP	NCP	81.03
LPWKWP;VNGALPP	NCP	111.499
VNGALPP;LDHKYLP	NCP	55.728
LDHKYLP;LDNLP	NCP	41.651
LDNLP;LNVLVK	NCP	25.881
LNVLVK;VTALGL	NCP	21.231
VTALGL;KNGLGFHG	NCP	47.976
KNGLGFHG;SGKLAARP	NCP	2.898
SGKLAARP;NFPLT	NCP	111.625
NFPLT;TVESAKP	NCP	47.61
TVESAKP;GDFKLK	NCP	12.992
GDFKLK;KDFLSKK	NCP	53.364
KDFLSKK;VASGGLLS	NCP	12.567
VASGGLLS;KHSYQ	NCP	17.802
KHSYQ;KFLHPPPP	NCP	125.811
KFLHPPPP;RQGDLH	NCP	46.707
RQGDLH;KGSSARP	NCP	0.802
KGSSARP;GPLLSKKV	NCP	74.898
GPLLSKKV;KPFFKVL	NCP	25.473
KPFFKVL;VSKPPP	NCP	67.919
VSKPPP;VPAGYLAA	NCP	26.049
VPAGYLAA;KEFLDLRK	NCP	38.173
KEFLDLRK;KEGVPPP	NCP	56.27
KEGVPPP;KMSPLTLL	NCP	39.082
KMSPLTLL;VGGAAVL	NCP	21.506
VGGAAVL;KKPDFH	NCP	46.177
KKPDFH;MNLLQ	NCP	18.341
MNLLQ;KGATFAL	NCP	182.08
KGATFAL;KWGPPK	NCP	12.587
KWGPPK;LWVANKLP	NCP	50.515
LWVANKLP;NPAAPVKP	NCP	46.097
NPAAPVKP;KPPGCKE	NCP	3.756
KPPGCKE;PDKNVPP	NCP	104.412
PDKNVPP;KHHVPP	NCP	28.092
KHHVPP;LTLLK	NCP	20.161
LTLLK;CRKKLP	NCP	66.564

CRKKLP;LSGHPKD	NCP	3.164
LSGHPKD;VLHSPP	NCP	26.439
VLHSPP;EALERM	NCP	38.436
EALERM;VDLRV	NCP	12.226
VDLRV;VPGFFP	NCP	4.889
VPGFFP;YPGEAPP	NCP	6.376
YPGEAPP;ATDELASKLSRR	NCP	1.745
ATDELASKLSRR;QQPRPVL	NCP	8.796
QQPRPVL;GLAEVLF	NCP	9.03
GLAEVLF;TQHKAF	NCP	94.893
TQHKAF;HAVSEGTKAVTKYTSS	NCP	22.858
HAVSEGTKAVTKYTSS;GPMLEY	NCP	9.387
GPMLEY;VAKTVPF	NCP	13.773
VAKTVPF;YAANK	NCP	107.912
YAANK;VAAMG	NCP	31.954
VAAMG;VLTSKY	NCP	0.123
VLTSKY;GNDKLLK	NCP	42.207
GNDKLLK;NVGVNAK	NCP	40.17
NVGVNAK;NFLEAH	NCP	20.672
NFLEAH;KVVQRWK	NCP	30.42
KVVQRWK;DARVLLK	NCP	0.018
DARVLLK;KMRKPP	NCP	3.246
KMRKPP;KVSHCVRK	NCP	19.782
KVSHCVRK;QHWPPPP	NCP	93.801
QHWPPPP;VSLVPLPQ	NCP	9.639
VSLVPLPQ;EPRMHP	NCP	0.811
EPRMHP;VSGPKFQ	NCP	30.471
VSGPKFQ;VGGLPLR	NCP	9.782
VGGLPLR;HPPFL	NCP	12.906
HPPFL;PPVPWW	NCP	14.001
PPVPWW;LEAVDPS	NCP	10.567
LEAVDPS;EALTAL	NCP	34.703
EALTAL;RNSLGR	NCP	4.821
RNSLGR;YTPGAP	NCP	78.21
YTPGAP;VGHGGLSFL	NCP	3.174
VGHGGLSFL;GPVAEY	NCP	2.142
GPVAEY;PGHPLLVP	NCP	21.453
PGHPLLVP;ASTAHPK	NCP	13.728
ASTAHPK;VVGGGGL	NCP	16.182
VVGGGGL;ERLVEL	NCP	30.744
ERLVEL;PPAQGPLPA	NCP	48.045
PPAQGPLPA;YDPEAA	NCP	4.521
YDPEAA;AATRPPR	NCP	0.993
AATRPPR;EWGLPVP	NCP	63.504
EWGLPVP;FPLLTG	NCP	11.416
FPLLTG;NKPAAGPP	NCP	6.245
NKPAAGPP;AAPLSL	NCP	12.024
AAPLSL;AADRLVG	NCP	8.241
AADRLVG;RDVSGPK	NCP	9.313
RDVSGPK;PAKSSLKQ	NCP	25.94
PAKSSLKQ;GFVAPL	NCP	12.384

GFVAPL;LSPFELRT	NCP	11.937
LSPFELRT;ASVSELAC	NCP	26.049
ASVSELAC;RQSLAPR	NCP	65.268
RQSLAPR;VPLHM	NCP	55.273
VPLHM;KPGRSGQ	NCP	87.111
KPGRSGQ;TTPHAPP	NCP	6.428
TTPHAPP;VFRGAPS	NCP	63.885
VFRGAPS;PTVHAVP	NCP	9.927
PTVHAVP;KLCWVP	NCP	2.488
KLCWVP;SGLAPPAL	NCP	29.622
SGLAPPAL;TPMLDK	NCP	76.593
TPMLDK;ADVLTHR	NCP	0.684
ADVLTHR;GPPAGP	NCP	12.941
GPPAGP;ELEPAL	NCP	53.506
ELEPAL;GKHPALL	NCP	35.999
GKHPALL;KGAYGCK	NCP	3.981
KGAYGCK;PVVDLPL	NCP	31.455
PVVDLPL;KLAGPLGA	NCP	18.3
KLAGPLGA;NLRASRGL	NCP	3.453
NLRASRGL;LERYTP	NCP	32.62
LERYTP;PLLAFDL	NCP	0.875
PLLAFDL;KGPQRSPP	NCP	18.784
KGPQRSPP;GAPPAE	NCP	72.275
GAPPAE;ATPPHWK	NCP	41.794
ATPPHWK;WPPAGGP	NCP	62.546
WPPAGGP;TGKTSAK	NCP	88.876
TGKTSAK;YEGLDF	NCP	31.884
YEGLDF;GSGLCKAGL	NCP	51.689
GSGLCKAGL;KEKLCYVA	NCP	0.6
KEKLCYVA;VTGLDPK	NCP	81.408
VTGLDPK;MTMKHP	NCP	10.648
MTMKHP;GGPVGPV	NCP	8.814
GGPVGPV;LPGGTL	NCP	50.684
LPGGTL;TTHPPR	NCP	4.926
TTHPPR;PGPFPPPPP	NCP	8.02
PGPFPPPPP;TAGKVLK	NCP	53.343
TAGKVLK;RYKALSM	NCP	15.03
RYKALSM;MGAPSP	NCP	10.751
MGAPSP;GAFLAAP	NCP	42.124
GAFLAAP;TTHPSR	NCP	4.322
TTHPSR;QPNVTSK	NCP	43.41
QPNVTSK;QLWGNK	NCP	2.289
QLWGNK;LGPALLG	NCP	7.281
LGPALLG;NPKRPSP	NCP	14.541
NPKRPSP;FVEPAPR	NCP	4.371
FVEPAPR;QVGLPP	NCP	62.4
QVGLPP;KDMAPK	NCP	39.907
KDMAPK;GPPPQEPK	NCP	33.632
GPPPQEPK;ETLQGGL	NCP	11.223
ETLQGGL;DFEWL	NCP	15.271
DFEWL;KDAKPFMR	NCP	13.749

KDAKPFMR;PSPVGPP	NCP	49.91
PSPVGPP;LSHSLLV	NCP	72.775
LSHSLLV;HGPFSLLR	NCP	7.148
HGPFSLLR;PTADKQR	NCP	67.785
PTADKQR;AAAAGK	NCP	98.638
AAAAGK;YSMSLL	NCP	41.289
YSMSLL;DSPLGL	NCP	4.769
DSPLGL;VSLAVLK	NCP	9.189
VSLAVLK;YVHSPPK	NCP	2.071
YVHSPPK;VVPGAVGK	NCP	160.863
VVPGAVGK;KGPTNY	NCP	41.238
KGPTNY;VKPAAF	NCP	76.238
VKPAAF;LPLLS	NCP	15.051
LPLLS;SALNAAPK	NCP	29.296
SALNAAPK;TVLLAPR	NCP	37.238
TVLLAPR;PSLRMAP	NCP	21.498
PSLRMAP;DGLLLPGK	NCP	18.565
DGLLLPGK;VPFLHPR	NCP	56.538
VPFLHPR;KMHYLT	NCP	16.889
KMHYLT;AAYSEYL	NCP	19.534
AAYSEYL;CAPVET	NCP	63.359
CAPVET;APKHQKK	NCP	43.573
APKHQKK;KAMVRP	NCP	18.281
KAMVRP;LPAAVKP	NCP	2.602
LPAAVKP;VSEAPL	NCP	17.481
VSEAPL;LSPKV	NCP	2.573
LSPKV;EDPWAF	NCP	6.996
EDPWAF;LHKLAAF	NCP	24.064
LHKLAAF;SKKLPR	NCP	40.545
SKKLPR;VPLNP	NCP	55.11
VPLNP;QSLLLV	NCP	25.134
QSLLLV;PTFGF	NCP	134.882
PTFGF;RTTVFVN	NCP	34.669
RTTVFVN;LLGAL	NCP	55.352
LLGAL;WPVSAK	NCP	21.448
WPVSAK;AGKQAVR	NCP	20.52
AGKQAVR;LVRGP	NCP	32.516
LVRGP;VENGAP	NCP	24.948
VENGAP;EFFPEL	NCP	92.191
EFFPEL;HSPVLL	NCP	32.247
HSPVLL;KPRSGLPG	NCP	25.736
KPRSGLPG;VLKLGQH	NCP	37.887
VLKLGQH;PGHLPPPP	NCP	26.148
PGHLPPPP;SLLGPV	NCP	16.636
SLLGPV;QPGRHWQ	NCP	3.08
QPGRHWQ;QGEPGK	NCP	15.597
QGEPGK;DALVKPK	NCP	2.821
DALVKPK;EAAKYQP	NCP	15.581
EAAKYQP;ERLAVPAK	NCP	7.362
ERLAVPAK;WFVVN	NCP	54.046
WFVVN;TKSPKKN	NCP	7.073

TKSPKKN;QDLLPR	NCP	5.077
QDLLPR;AVNNLP	NCP	35.889
AVNNLP;APKLTVP	NCP	12.381
APKLTVP;LDGVGKR	NCP	28.073
LDGVGKR;GVSLLC	NCP	9.178
GVSLLC;LTPGLT	NCP	51.768
LTPGLT;PHYTTF	NCP	64.995
PHYTTF;LQTSVPK	NCP	12.404
LQTSVPK;LAQSPTK	NCP	3.202
LAQSPTK;KHHPPK	NCP	114.567
KHHPPK;VGKGLVV	NCP	21.794
VGKGLVV;PVTHWR	NCP	10.239
PVTHWR;THPSARPLR	NCP	64.542
THPSARPLR;PPVPLPPP	NCP	23.832
PPVPLPPP;KPPAPL	NCP	34.392
KPPAPL;KPQTKAPP	NCP	14.706
KPQTKAPP;DLEKSLK	NCP	13.975
DLEKSLK;SGVLPGVR	NCP	10.484
SGVLPGVR;TGAGPPPP	NCP	32.181
TGAGPPPP;LSKQLFR	NCP	57.07
LSKQLFR;ASTPKPGL	NCP	16.925
ASTPKPGL;ALEQAMQAAR	NCP	81.336
ALEQAMQAAR;FGGFGQG	NCP	23.145
FGGFGQG;VTKLF	NCP	1.972
VTKLF;KAMPPK	NCP	104.996
KAMPPK;LPYPQP	NCP	28.843
LPYPQP;KLAFGG	NCP	122.999
KLAFGG;GPARPPPP	NCP	49.644
GPARPPPP;LLDRVVRP	NCP	45.444
LLDRVVRP;GSVFPK	NCP	33.091
GSVFPK;ETGNAK	NCP	53.028
ETGNAK;AAGALFPP	NCP	10.367
AAGALFPP;EKKHTK	NCP	44.236
EKKHTK;LPWQSK	NCP	145.959
LPWQSK;EGAGLEF	NCP	56.268
EGAGLEF;LPRPGPP	NCP	38.897
LPRPGPP;LTMVRP	NCP	225.525
LTMVRP;KNSLPF	NCP	22.303
KNSLPF;MEGPLS	NCP	17.61
MEGPLS;RGPFKP	NCP	29.843
RGPFKP;KQWTVPK	NCP	18.349
KQWTVPK;QVKEGN	NCP	45.548
QVKEGN;LPTRPC	NCP	111.384
LPTRPC;ACDLDLR	NCP	11.565
ACDLDLR;LFAFYKGL	NCP	54.525
LFAFYKGL;GPARF	NCP	31.596
GPARF;TTNAPAGSSAAAGSSR	NCP	1.959
TTNAPAGSSAAAGSSR;VLERDQKL	NCP	3.891
VLERDQKL;TAAPEVH	NCP	6.688
TAAPEVH;APAAAPPP	NCP	13.869
APAAAPPP;KMKAGQP	NCP	53.916

KMKAGQP;VTPLEC	NCP	25.973
VTPLEC;KDMLTQ	NCP	77.406
KDMLTQ;KTPWKGP	NCP	3.373
KTPWKGP;GSKVTTHPLAK	NCP	19.988
GSKVTTHPLAK;VEVLVGKEVADQVK	NCP	6.06
VEVLVGKEVADQVK;KEVADQVKAPLVLKD	NCP	0.018
KEVADQVKAPLVLKD;LPWTPV	NCP	35.173
LPWTPV;KELPLLGV	NCP	7.323
KELPLLGV;ELDVAPDV	NCP	30.011
ELDVAPDV;TGYHKAK	NCP	65.547
TGYHKAK;KTDLTK	NCP	35.508
KTDLTK;AAPELL	NCP	32.164
AAPELL;DPRGLYG	NCP	11.465
DPRGLYG;FFSKAK	NCP	43.219
FFSKAK;VSCSPLVF	NCP	54.348
VSCSPLVF;MAPSPLPR	NCP	119.027
MAPSPLPR;LGPPPYK	NCP	38.097
LGPPPYK;AMLRLK	NCP	5.107
AMLRLK;ELTTKDLK	NCP	32.105
ELTTKDLK;KSYPPR	NCP	39.612
KSYPPR;KGSNVLR	NCP	33.054
KGSNVLR;ASLKYTVPP	NCP	46.68
ASLKYTVPP;LDNLLK	NCP	42.433
LDNLLK;AGAPVY	NCP	89.253
AGAPVY;EHTPTK	NCP	33.848
EHTPTK;DGPFLP	NCP	45.705
DGPFLP;TDFHPVR	NCP	13.597
TDFHPVR;GPPHLLTH	NCP	20.226
GPPHLLTH;GPMKK	NCP	0.735
GPMKK;APSFPL	NCP	1.493
APSFPL;VSPQVYP	NCP	39.723
VSPQVYP;VHQVL	NCP	3.12
VHQVL;VSEYDP	NCP	37.356
VSEYDP;GCEAELR	NCP	8.722
GCEAELR;LPLDADTE	NCP	94.158
LPLDADTE;TQVPSPK	NCP	40.106
TQVPSPK;MACCSD	NCP	80.799
MACCSD;LLQHVRVPH	NCP	18.444
LLQHVRVPH;MDYDAFLG	NCP	0.432
MDYDAFLG;HPAVPPP	NCP	3.576
HPAVPPP;GGAPGL	NCP	33.207
GGAPGL;YKAVSPP	NCP	23.809
YKAVSPP;LPTDTPP	NCP	22.151
LPTDTPP;NELPGPR	NCP	62.652
NELPGPR;VTGRLLEQ	NCP	61.602
VTGRLLEQ;VNFTEAP	NCP	13.131
VNFTEAP;VNFLGP	NCP	124.32
VNFLGP;VNPMLLL	NCP	21.346
VNPMLLL;KPAWKPP	NCP	61.293
KPAWKPP;FEAAPVP	NCP	0.018
FEAAPVP;PGPLTFLF	NCP	15.248

PGPLTFLF;RQGYGGGGGP	NCP	47.763
RQGYGGGGGP;MNLYAMT	NCP	50.449
MNLYAMT;RGPTVFF	NCP	7.14
RGPTVFF;KAGHHTP	NCP	10.064
KAGHHTP;EVSLLPF	NCP	44.709
EVSLLPF;SDLDLSR	NCP	94.641
SDLDLSR;YGDEDC	NCP	60.48
YGDEDC;EVVLNLP	NCP	47.889
EVVLNLP;CDCPP	NCP	23.574
CDCPP;AYPLEK	NCP	20.149
AYPLEK;KNNSFDK	NCP	40.887
KNNSFDK;KHKRDP	NCP	101.912
KHKRDP;NRTVSKVDDF	NCP	45.549
NRTVSKVDDF;VDDFLANEAKGTK	NCP	0.018
VDDFLANEAKGTK;SFTLYLGP	NCP	39.261
SFTLYLGP;SLAARAGF	NCP	15.225
SLAARAGF;DAPEPF	NCP	139.807
DAPEPF;TGKPY	NCP	9.213
TGKPY;KEGDFR	NCP	29.238
KEGDFR;EAVAGAP	NCP	52.307
EAVAGAP;ETVGFFF	NCP	6.015
ETVGFFF;TLMPMLP	NCP	8.628
TLMPMLP;QPLGNVG	NCP	8.041
QPLGNVG;AAHPDAP	NCP	21.344
AAHPDAP;TPPDPRPP	NCP	36.561
TPPDPRPP;QGQKTLK	NCP	29.953
QGQKTLK;AGKELER	NCP	31.667
AGKELER;QAHSLHV	NCP	2.329
QAHSLHV;MEVPPNSR	NCP	24.797
MEVPPNSR;LGPALALAL	NCP	5.169
LGPALALAL;GSGHGGGK	NCP	38.565
GSGHGGGK;LPPVGDP	NCP	13.575
LPPVGDP;ESCSC	NCP	4.431
ESCSC;PHEHAH	NCP	81.826
PHEHAH;KTPAELT	NCP	121.085
KTPAELT;AEALP	NCP	101.832
AEALP;LVPGVL	NCP	44.031
LVPGVL;LSHSTPLH	NCP	27.118
LSHSTPLH;PSSSLLR	NCP	21.239
PSSSLLR;KVHTPP	NCP	0.055
KVHTPP;AVTPLL	NCP	3.875
AVTPLL;NEDPLLP	NCP	96.348
NEDPLLP;KMAAPQ	NCP	100.584
KMAAPQ;DPVLQPL	NCP	11.766
DPVLQPL;KPPAAGLH	NCP	0.054
KPPAAGLH;VPLTVPVP	NCP	57.207
VPLTVPVP;LSVPSPK	NCP	8.553
LSVPSPK;EVPADPESKAHVTR	NCP	50.175
EVPADPESKAHVTR;PLTTVP	NCP	35.847
PLTTVP;LTGKPPR	NCP	0.558
LTGKPPR;LPPCKP	NCP	69.057

LPPCKP;EKCLAPV	NCP	25.524
EKCLAPV;VARVAY	NCP	59.817
VARVAY;QWELVTK	NCP	2.031
QWELVTK;ALSLPF	NCP	9.885
ALSLPF;KDSSMPP	NCP	29.194
KDSSMPP;PVGREVT	NCP	8.18
PVGREVT;NGALKVR	NCP	49.572
NGALKVR;PSLPAGK	NCP	3.726
PSLPAGK;GPSLKVRH	NCP	3.028
GPSLKVRH;KAAPGKAGPP	NCP	0.794
KAAPGKAGPP;PVLSPSPPPP	NCP	66.06
PVLSPSPPPP;KLSYMP	NCP	10.915
KLSYMP;LKAAAPP	NCP	152.594
LKAAAPP;KAAHQNM	NCP	14.952
KAAHQNM;QVTMLK	NCP	60.316
QVTMLK;LPPLPLPLP	NCP	27.446
LPPLPLPLP;TGDLLL	NCP	57.966
TGDLLL;AAPFLPR	NCP	167.352
AAPFLPR;HHSVTAK	NCP	23.38
HHSVTAK;VGFVNVN	NCP	26.913
VGFVNVN;AAKHAYT	NCP	73.703
AAKHAYT;TVDASVP	NCP	52.929
TVDASVP;KEVPPP	NCP	68.019
KEVPPP;DALLHVL	NCP	18.394
DALLHVL;ELTLPVTF	NCP	43.449
ELTLPVTF;WGKVWR	NCP	28.271
WGKVWR;TTPSRRP	NCP	41.998
TTPSRRP;CSSEC	NCP	17.735
CSSEC;APLSPPVPR	NCP	13.671
APLSPPVPR;EQWRL	NCP	6.085
EQWRL;ANHLVSPN	NCP	34.053
ANHLVSPN;LSHSLLCY	NCP	55.344
LSHSLLCY;PATMP	NCP	87.159
PATMP;PAVLEY	NCP	39.168
PAVLEY;LSPVLPL	NCP	31.839
LSPVLPL;RRTLLNP	NCP	38.771
RRTLLNP;TEVLGKNK	NCP	44.356
TEVLGKNK;DPVTVLK	NCP	30.291
DPVTVLK;MEDSMDMDMSPLRPQNY	NCP	33.224
MEDSMDMDMSPLRPQNY;APVYL	NCP	244.299
APVYL;PHQPPR	NCP	8.703
PHQPPR;HSNQTVVL	NCP	11.134
HSNQTVVL;KPEGSPP	NCP	30.782
KPEGSPP;KSTSFTH	NCP	45.19
KSTSFTH;KALLYP	NCP	127.29
KALLYP;PTTTLLR	NCP	54.003
PTTTLLR;MPSSKNK	NCP	11.48
MPSSKNK;MLLLEV	NCP	16.602
MLLLEV;YQFSKPK	NCP	32.773
YQFSKPK;KKMMAK	NCP	85.173
KKMMAK;TPATTPL	NCP	68.598

TPATTPL;EGLFELK	NCP	6.476
EGLFELK;KAPWPK	NCP	11.614
KAPWPK;TQAVKPK	NCP	17.285
TQAVKPK;LSKKVF	NCP	18.169
LSKKVF;AFLLSF	NCP	7.601
AFLLSF;CMMMTC	NCP	40.671
CMMMTC;LSGFTER	NCP	59.746
LSGFTER;AMALLVR	NCP	152.6
AMALLVR;VRLEGGLK	NCP	60.573
VRLEGGLK;VVDKSH	NCP	6.645
VVDKSH;LDLSSKQ	NCP	1.516
LDLSSKQ;KGVSSMH	NCP	42.179
KGVSSMH;SAAKAP	NCP	47.043
SAAKAP;KYEPMPP	NCP	23.415
KYEPMPP;QSGLVGL	NCP	17.784
QSGLVGL;KPLLRLF	NCP	72.739
KPLLRLF;SPSLVHR	NCP	11.76
SPSLVHR;PSDTASK	NCP	7.875
PSDTASK;KLLLSH	NCP	1.361
KLLLSH;WFEPKLN	NCP	6.601
WFEPKLN;VSLYLH	NCP	233.552
VSLYLH;RPAPY	NCP	15.43
RPAPY;MEALKKK	NCP	39.609
MEALKKK;KGALLYY	NCP	44.733
KGALLYY;ALPDELP	NCP	70.562
ALPDELP;KHERPP	NCP	203.029
KHERPP;EVERPDL	NCP	20.523
EVERPDL;PAAWFR	NCP	129.708
PAAWFR;PAAGAK	NCP	104.28
PAAGAK;PGMMVY	NCP	19.494
PGMMVY;SHGSLKY	NCP	54.068
SHGSLKY;YMDDC	NCP	98.122
YMDDC;GDFLAAS	NCP	12.789
GDFLAAS;RPELLTL	NCP	24.299
RPELLTL;AAYLK	NCP	88.176
AAYLK;KMVTH	NCP	61.696
KMVTH;HQLPELLK	NCP	209.493
HQLPELLK;KELGKK	NCP	34.785
KELGKK;LPPSPEGF	NCP	4.898
LPPSPEGF;AFNLNLL	NCP	2.626
AFNLNLL;ARGLPAC	NCP	53.517
ARGLPAC;AVALLSK	NCP	50.873
AVALLSK;KGEDLLF	NCP	59.058
KGEDLLF;TPGYLR	NCP	8.205
TPGYLR;HVTVP	NCP	146.431
HVTVP;KNYPQ	NCP	71.807
KNYPQ;KAYFLKER	NCP	58.462
KAYFLKER;KFSLHP	NCP	1.485
KFSLHP;NTTFLPF	NCP	111.474
NTTFLPF;NKSDRAK	NCP	6.029
NKSDRAK;LLMSSLGK	NCP	53.079

LLMSSLGK;KKSDCR	NCP	4.989
KKSDCR;YESLGPK	NCP	37.027
YESLGPK;TACEHH	NCP	77.976
TACEHH;KDYTKK	NCP	4.218
KDYTKK;EELVRM	NCP	86.454
EELVRM;VVEPY	NCP	4.548
VVEPY;CECCHK	NCP	50.403
CECCHK;KGLLYSK	NCP	62.898
KGLLYSK;AASFSKL	NCP	10.722
AASFSKL;RLGFSKP	NCP	116.132
RLGFSKP;KYTLYP	NCP	47.139
KYTLYP;ESCCCCCFF	NCP	178.39
ESCCCCCFF;MRGATTTF	NCP	12.338
MRGATTTF;LPSDFTP	NCP	67.447
LPSDFTP;PPPVDHK	NCP	95.586
PPPVDHK;CSHNVPP	NCP	45.71
CSHNVPP;VSDLCPK	NCP	27.285
VSDLCPK;LYGNF	NCP	59.181
LYGNF;EWGNLP	NCP	24.352
EWGNLP;LLEDPLH	NCP	1.257
LLEDPLH;TYLER	NCP	48.443
TYLER;HPKHAAAP	NCP	13.569
HPKHAAAP;KAWAAKP	NCP	3.849
KAWAAKP;MQVAM	NCP	3.774
MQVAM;LAHEVLK	NCP	71.827
LAHEVLK;AVLFKAA	NCP	128.447
AVLFKAA;GKMMT	NCP	19.837
GKMMT;NKSKLK	NCP	96.185
NKSKLK;QQLPPL	NCP	16.648
QQLPPL;LSGLEC	NCP	108.528
LSGLEC;VAAKAG	NCP	22.11
VAAKAG;NLSELTASP	NCP	4.248
NLSELTASP;PAAVSARGP	NCP	53.279
PAAVSARGP;DLPPTAHP	NCP	39.271
DLPPTAHP;MATLPQ	NCP	1.892
MATLPQ;KVMFSM	NCP	36.372
KVMFSM;ADSPPP	NCP	56.092
ADSPPP;KLDLMPK	NCP	26.945
KLDLMPK;PLAVRF	NCP	4.873
PLAVRF;FPPPLGP	NCP	49.467
FPPPLGP;KVGTFS	NCP	11.915
KVGTFS;LADRLGF	NCP	34.455
LADRLGF;ELEAPL	NCP	30.859
ELEAPL;LDLDP	NCP	11.559
LDLDP;KGVHGFAK	NCP	7.565
KGVHGFAK;ATGRGLVP	NCP	3.706
ATGRGLVP;KPASRPQP	NCP	26.619
KPASRPQP;VHVSLP	NCP	4.007
VHVSLP;KSLVLDN	NCP	25.069
KSLVLDN;HSLLVPV	NCP	0.216
HSLLVPV;MFLVD	NCP	5.307

MFLVD;AAAPPP	NCP	13.637
AAAPPP;YKLSTL	NCP	8.392
YKLSTL;DMHEN	NCP	38.94
DMHEN;RVLPKKY	NCP	33.771
RVLPKKY;AAVPYSLQ	NCP	102.684
AAVPYSLQ;KLRLEV	NCP	14.873
KLRLEV;LKLLTK	NCP	4.92
LKLLTK;PGMLEK	NCP	9.919
PGMLEK;SRNGPLP	NCP	10.566
SRNGPLP;LLSHS	NCP	50.046
LLSHS;VSKDTAK	NCP	14.282
VSKDTAK;KVGTF	NCP	35.577
KVGTF;GTDFVPL	NCP	24.172
GTDFVPL;NHTMF	NCP	1.991
NHTMF;LVNRPFLF	NCP	90.883
LVNRPFLF;ERLAAPK	NCP	173.603
ERLAAPK;HGDVRLK	NCP	39.73
HGDVRLK;WRLLDH	NCP	83.667
WRLLDH;SLHRKTF	NCP	40.125
SLHRKTF;YVTGYPK	NCP	28.184
YVTGYPK;VSSLDF	NCP	134.334
VSSLDF;RVTLMPK	NCP	9.993
RVTLMPK;VENLP	NCP	14.38
VENLP;ESKSLPL	NCP	45.051
ESKSLPL;ESGSKFC	NCP	13.761
ESGSKFC;VYFNGLK	NCP	39.774
VYFNGLK;KEPLKEPK	NCP	30.723
KEPLKEPK;APGSRKY	NCP	70.188
APGSRKY;LELSSW	NCP	67.914
LELSSW;RGPAY	NCP	1.988
RGPAY;AVAVVK	NCP	101.245
AVAVVK;LHVYPL	NCP	3.584
LHVYPL;AEAGPLL	NCP	21.682
AEAGPLL;EHGFKKV	NCP	6.05
EHGFKKV;QAVLHV	NCP	110.082
QAVLHV;LSLAEVK	NCP	42.994
LSLAEVK;ALPHASR	NCP	6.129
ALPHASR;KGNMVR	NCP	67.829
KGNMVR;VSPTKTP	NCP	17.383
VSPTKTP;LETSLLPY	NCP	30.743
LETSLLPY;DDSFLGK	NCP	60.169
DDSFLGK;KGYPVPQ	NCP	13.283
KGYPVPQ;YPGPGP	NCP	8.179
YPGPGP;LSGAVPVG	NCP	40.862
LSGAVPVG;SLVPLDLR	NCP	6.105
SLVPLDLR;VVTSPPG	NCP	15.564
VVTSPPG;VSVPGSP	NCP	50.638
VSVPGSP;KDAARGR	NCP	175.401
KDAARGR;DPAKKER	NCP	29.378
DPAKKER;KTHALTP	NCP	32.712
KTHALTP;YEKAK	NCP	8.328

YEKAK;ESLVGV	NCP	26.829
ESLVGV;GDHGLPPP	NCP	18.841
GDHGLPPP;KDYVY	NCP	26.897
KDYVY;SPGGHRK	NCP	71.758
SPGGHRK;AAVFRP	NCP	67.323
AAVFRP;VYKEDK	NCP	25.619
VYKEDK;VGLSYYK	NCP	171.646
VGLSYYK;GLRSPR	NCP	39.773
GLRSPR;NQGQQPK	NCP	9.277
NQGQQPK;FHPEVGP	NCP	16.832
FHPEVGP;LVTLSNL	NCP	51.393
LVTLSNL;VWFRKM	NCP	45.501
VWFRKM;RFYKSF	NCP	8.53
RFYKSF;KVVFKPP	NCP	43.608
KVVFKPP;APPLP	NCP	20.471
APPLP;VGSPLQ	NCP	10.126
VGSPLQ;MMDYEV	NCP	174.354
MMDYEV;LVRVF	NCP	56.687
LVRVF;EWEVY	NCP	19.527
EWEVY;KEVWAGP	NCP	75.312
KEVWAGP;VKFPKLSP	NCP	30.873
VKFPKLSP;SGLRTAPH	NCP	20.37
SGLRTAPH;APGTVVSPP	NCP	128.386
APGTVVSPP;GVDVLL	NCP	164.439
GVDVLL;LEVTLGK	NCP	31.958
LEVTLGK;KYPCLP	NCP	44.937
KYPCLP;TVALPF	NCP	94.953
TVALPF;KNSLDF	NCP	39.591
KNSLDF;NGNPPLR	NCP	3.93
NGNPPLR;GGLLAY	NCP	1.698
GGLLAY;SSEDPN	NCP	8.688
SSEDPN;MPEPLSF	NCP	18.432
MPEPLSF;QQLAL	NCP	55.486
QQLAL;TVQAEVP	NCP	25.143
TVQAEVP;FESAEQL	NCP	46.004
FESAEQL;NPKAHQP	NCP	18.028
NPKAHQP;EMMMGG	NCP	4.575
EMMMGG;VLLTM	NCP	26.696
VLLTM;GNCAHF	NCP	51.592
GNCAHF;DDCGP	NCP	27.306
DDCGP;QPVENLH	NCP	13.782
QPVENLH;KTLHPP	NCP	27.143
KTLHPP;VRAAP	NCP	13.759
VRAAP;DDDTM	NCP	31.34
DDDTM;PLLVN	NCP	19.41
PLLVN;KLSDEFK	NCP	59.184
KLSDEFK;PLPRSYP	NCP	108.529
PLPRSYP;VTESKVTL	NCP	107.861
VTESKVTL;VSPPPP	NCP	12.913
VSPPPP;KGGSGYVP	NCP	52.962
KGGSGYVP;EVCPLSQ	NCP	24.411

EVCPLSQ;ATQQRPFHLVVF	NCP	17.528
ATQQRPFHLVVF;VGPPPPPPP	NCP	27.595
VGPPPPPPP;SPGALVH	NCP	25.745
SPGALVH;KAAPSP	NCP	8.892
KAAPSP;RPLVDTH	NCP	43.156
RPLVDTH;LRGAADL	NCP	18.681
LRGAADL;YDKPPP	NCP	29.136
YDKPPP;KSDPTLP	NCP	70.629
KSDPTLP;KNSYN	NCP	60.563
KNSYN;RPAAGLGLR	NCP	46.789
RPAAGLGLR;GAFGK	NCP	169.866
GAFGK;QLPEVL	NCP	48.236
QLPEVL;KPSFF	NCP	10.935
KPSFF;PTPSLHAP	NCP	123.813
PTPSLHAP;VYKVKR	NCP	5.106
VYKVKR;LSPEVGK	NCP	1.123
LSPEVGK;KSQLAK	NCP	0.681
KSQLAK;KEDSQPL	NCP	24.011
KEDSQPL;VSGVGLVC	NCP	22.795
VSGVGLVC;NELRVAP	NCP	12.807
NELRVAP;KFNVVP	NCP	36.134
KFNVVP;AAVPNDL	NCP	5.899
AAVPNDL;LAPALQ	NCP	73.554
LAPALQ;RSLEKM	NCP	43.001
RSLEKM;VGALLAH	NCP	20.368
VGALLAH;LDVVLGH	NCP	6.777
LDVVLGH;VTADLLVP	NCP	9.51
VTADLLVP;MCGGD	NCP	19.271
MCGGD;VPPAMPP	NCP	24.274
VPPAMPP;VSFREK	NCP	39.852
VSFREK;EHNAEFR	NCP	53.463
EHNAEFR;KSEGLL	NCP	55.188
KSEGLL;DWHRPLC	NCP	34.47
DWHRPLC;PAAQKEK	NCP	15.941
PAAQKEK;AESSDKLYR	NCP	22.188
AESSDKLYR;SVLTAEDQETLKK	NCP	14.373
SVLTAEDQETLKK;LVFKGDAY	NCP	4.218
LVFKGDAY;VEPVEAVAPK	NCP	5.643
VEPVEAVAPK;AGYSLGPL	NCP	52.702
AGYSLGPL;KVPPL	NCP	60.45
KVPPL;GPGPPPPPP	NCP	36.815
GPGPPPPPP;KDFQDSK	NCP	14.208
KDFQDSK;AAVLSAERLEVS	NCP	21.627
AAVLSAERLEVS;MDRPKP	NCP	29.775
MDRPKP;CLAALGLN	NCP	24.486
CLAALGLN;FLLKVK	NCP	2.041
FLLKVK;KVGLNPK	NCP	1.77
KVGLNPK;KPSLERP	NCP	36.137
KPSLERP;LLNSLDA	NCP	24.399
LLNSLDA;ALLRGVFL	NCP	107.296
ALLRGVFL;LESPTVTTLTPTSSGK	NCP	26.384

LESPTVTTLTPTSSGK;PEHPPPP	NCP	66.787
PEHPPPP;KSGPVRP	NCP	6.15
KSGPVRP;CYAVPG	NCP	62.127
CYAVPG;LGPPPP	NCP	2.157
LGPPPP;PADLLYPR	NCP	63.812
PADLLYPR;APAPKPPQ	NCP	81.244
APAPKPPQ;DLLLRL	NCP	62.31
DLLLRL;ALGRPPAL	NCP	119.766
ALGRPPAL;YVAVPR	NCP	3.245
YVAVPR;EKVYPP	NCP	107.956
EKVYPP;SNSLFDNF	NCP	24.167
SNSLFDNF;KLKKTMM	NCP	3.783
KLKKTMM;LRLVGLEK	NCP	79.053
LRLVGLEK;VEVKGKP	NCP	6.031
VEVKGKP;VDLPR	NCP	13.163
VDLPR;LERLGLP	NCP	65.086
LERLGLP;NMGCMGP	NCP	35.198
NMGCMGP;KSDPQGK	NCP	38.158
KSDPQGK;KVVGVGGG	NCP	12.027
KVVGVGGG;PAAPTLHL	NCP	56.778
PAAPTLHL;VGQVETL	NCP	35.531
VGQVETL;NCHVRTK	NCP	0.702
NCHVRTK;AGVLSF	NCP	80.107
AGVLSF;APAHSPK	NCP	60.848
APAHSPK;KQGPAAP	NCP	41.59
KQGPAAP;WLRPPLM	NCP	18.627
WLRPPLM;DGVFL	NCP	3.873
DGVFL;DAAHR	NCP	18.327
DAAHR;KPLHFHD	NCP	18.917
KPLHFHD;DQNLSNRP	NCP	118.345
DQNLSNRP;AAPQPLH	NCP	19.488
AAPQPLH;APKRASPP	NCP	29.613
APKRASPP;RPPPQY	NCP	4.64
RPPPQY;PPPELRPP	NCP	15.511
PPPELRPP;ARAPPP	NCP	0.051
ARAPPP;PGGLLL	NCP	6.006
PGGLLL;STETGRP	NCP	4.826
STETGRP;FNKTKSK	NCP	8.754
FNKTKSK;KTQEAST	NCP	0.921
KTQEAST;EALLSHPK	NCP	1.996
EALLSHPK;GSGPVDK	NCP	24.405
GSGPVDK;RLCRP	NCP	2.865
RLCRP;LGPAAPLP	NCP	33.591
LGPAAPLP;PLNSPF	NCP	37.22
PLNSPF;KDTDPDSKPF	NCP	27.565
KDTDPDSKPF;PDGDT	NCP	40.881
PDGDT;KGVAGGAK	NCP	22.905
KGVAGGAK;KYPLTL	NCP	14.657
KYPLTL;KPHQAAP	NCP	20.323
KPHQAAP;KGAHPPR	NCP	165.231
KGAHPPR;RPPKVL	NCP	29.639

RPPKVL;SSHVSPR	NCP	10.786
SSHVSPR;KQNLE	NCP	15.209
KQNLE;SGSRCPE	NCP	13.627
SGSRCPE;KCGAAPPP	NCP	11.583
KCGAAPPP;AQRPRPP	NCP	30.75
AQRPRPP;AGALKVR	NCP	1.302
AGALKVR;KLVKGHAY	NCP	23.279
KLVKGHAY;LLPAAPG	NCP	2.443
LLPAAPG;TCLPPGP	NCP	6.708
TCLPPGP;AKEWG	NCP	36.714
AKEWG;GAPPK	NCP	5.918
GAPPK;VPLLPDLK	NCP	5.856
VPLLPDLK;KPAARPP	NCP	9.601
KPAARPP;VPPPHLK	NCP	14.525
VPPPHLK;SGTTAAL	NCP	66.196
SGTTAAL;SRPPVTLR	NCP	14.378
SRPPVTLR;MEAKPPP	NCP	2.479
MEAKPPP;THVHPK	NCP	13.356
THVHPK;RRHLME	NCP	17.415
RRHLME;TLVLLMGK	NCP	15.516
TLVLLMGK;TVPDRK	NCP	36.474
TVPDRK;KYHPVPP	NCP	21.027
KYHPVPP;GVPVMLP	NCP	50.312
GVPVMLP;KFLGSVP	NCP	17.356
KFLGSVP;KALMTW	NCP	34.8
KALMTW;TVPAPVP	NCP	10.095
TVPAPVP;MAAHM	NCP	98.402
MAAHM;KDPLGY	NCP	5.82
KDPLGY;KPAKPSVK	NCP	12.867
KPAKPSVK;KTKYFEPK	NCP	39.733
KTKYFEPK;TEAVLPKLHL	NCP	1.023
TEAVLPKLHL;KVGGAP	NCP	10.272
KVGGAP;SAATLPR	NCP	87.102
SAATLPR;KGRVALLK	NCP	13.202
KGRVALLK;GPPVKFYK	NCP	72.01
GPPVKFYK;HSGRVGP	NCP	31.143
HSGRVGP;PTPGGPAP	NCP	0.105
PTPGGPAP;LPPDKEF	NCP	10.007
LPPDKEF;LPALPPP	NCP	26.944
LPALPPP;VTLLLLP	NCP	39.399
VTLLLLP;DDRPVR	NCP	8.472
DDRPVR;LHLHQTLHKE	NCP	10.856
LHLHQTLHKE;KEAPAYK	NCP	3.945
KEAPAYK;KNSLTDGP	NCP	8.056
KNSLTDGP;FMTGYFF	NCP	12.356
FMTGYFF;VGPLVTK	NCP	3.948
VGPLVTK;TAAKFR	NCP	27.61
TAAKFR;NPAVGRP	NCP	16.407
NPAVGRP;GTVLMLK	NCP	35.33
GTVLMLK;PPPLLRPP	NCP	1.765
PPPLLRPP;APEPGPRP	NCP	3.486

APEPGPRP;RVGEELR	NCP	93.753
RVGEELR;ERLDPKF	NCP	11.642
ERLDPKF;SHPFFP	NCP	10.167
SHPFFP;KFHPSLP	NCP	53.019
KFHPSLP;PAALGL	NCP	46.933
PAALGL;KEMYH	NCP	83.697
KEMYH;MFCRPL	NCP	30.092
MFCRPL;LCDLYAK	NCP	23.835
LCDLYAK;SGPKKPL	NCP	8.088
SGPKKPL;AVPYPQ	NCP	2.41
AVPYPQ;HLLEQP	NCP	17.871
HLLEQP;EEETKFK	NCP	7.326
EEETKFK;QVVDKST	NCP	3.263
QVVDKST;QGSHPRP	NCP	47.221
QGSHPRP;LPPTPP	NCP	9.674
LPPTPP;RLDPVHSH	NCP	121.356
RLDPVHSH;AGVLLSK	NCP	51.304
AGVLLSK;ERGDVPL	NCP	11.919
ERGDVPL;GVFLLQV	NCP	27.482
GVFLLQV;PATVPRP	NCP	5.347
PATVPRP;KSMPPPPP	NCP	62.61
KSMPPPPP;KSTKLDLK	NCP	40.886
KSTKLDLK;MDPAT	NCP	0.573
MDPAT;DSYLLK	NCP	43.915
DSYLLK;PVDLK	NCP	13.046
PVDLK;DFSTVP	NCP	6.321
DFSTVP;VGSSGH	NCP	17.814
VGSSGH;TDEVNSP	NCP	37.938
TDEVNSP;KQEPKAK	NCP	157.533
KQEPKAK;APPKWKP	NCP	12.091
APPKWKP;VKVGVGGG	NCP	31.995
VKVGVGGG;LGRPLL	NCP	47.807
LGRPLL;SELGAP	NCP	3.178
SELGAP;GLYRALVS	NCP	19.599
GLYRALVS;PACKGSK	NCP	1.248
PACKGSK;NPGLTLH	NCP	83.84
NPGLTLH;LNPALHK	NCP	17.923
LNPALHK;KDGALLT	NCP	13.257
KDGALLT;LPKLPKL	NCP	32.507
LPKLPKL;KKVGAP	NCP	31.299
KKVGAP;SVKKTPKSK	NCP	39.423
SVKKTPKSK;LVLGAPV	NCP	5.743
LVLGAPV;WLPNNVK	NCP	22.506
WLPNNVK;NSALLELY	NCP	27.53
NSALLELY;VGWLGY	NCP	5.313
VGWLGY;KDLGSGK	NCP	42.697
KDLGSGK;KPPLY	NCP	43.944
KPPLY;ERPPRL	NCP	49.449
ERPPRL;QGQHNPK	NCP	69.278
QGQHNPK;KAGLPL	NCP	4.056
KAGLPL;APLLPLLK	NCP	40.656

APLLPLLK;AASGLRQA	NCP	64.165
AASGLRQA;HLVKHT	NCP	153.468
HLVKHT;FLVPML	NCP	39.815
FLVPML;PGPAHTK	NCP	35.571
PGPAHTK;VLYRTT	NCP	122.902
VLYRTT;GTVSPF	NCP	297.306
GTVSPF;DKVLVTSK	NCP	13.944
DKVLVTSK;SYMDLR	NCP	12.582
SYMDLR;AKLHVT	NCP	18.014
AKLHVT;ASSPTVLMRLVA	NCP	24.24
ASSPTVLMRLVA;AMKGAP	NCP	40.342
AMKGAP;AALLNQHY	NCP	13.619
AALLNQHY;PYPPVPP	NCP	28.015
PYPPVPP;YTLFGRSY	NCP	21.155
YTLFGRSY;QMPAK	NCP	61.536
QMPAK;RGPLKPP	NCP	83.731
RGPLKPP;DAPPRPK	NCP	94.722
DAPPRPK;KYPPLD	NCP	11.007
KYPPLD;LPELKAM	NCP	82.403
LPELKAM;KSLTSPVQ	NCP	110.422
KSLTSPVQ;SGNPLR	NCP	84.011
SGNPLR;PSFKPP	NCP	21.697
PSFKPP;SLGSFPF	NCP	12.444
SLGSFPF;LYFGVP	NCP	108.473
LYFGVP;KGDLGVK	NCP	8.562
KGDLGVK;FGGKLV	NCP	6.787
FGGKLV;TSAVKPK	NCP	12.152
TSAVKPK;KLVSWKK	NCP	26.481
KLVSWKK;EHYKK	NCP	18.333
EHYKK;ELFTPSQ	NCP	207.282
ELFTPSQ;LTGRTWGK	NCP	18.037
LTGRTWGK;KYKPEH	NCP	33.335
KYKPEH;TGSPLDF	NCP	2.346
TGSPLDF;YSAKLVR	NCP	89.935
YSAKLVR;KPAFKTP	NCP	81.027
KPAFKTP;EPTLSYQ	NCP	1.388
EPTLSYQ;EAPLVH	NCP	13.212
EAPLVH;QTSNRP	NCP	16.684
QTSNRP;VGLLNTSPF	NCP	32.76
VGLLNTSPF;YDLCR	NCP	68.477
YDLCR;RVHYLP	NCP	6.123
RVHYLP;HTMFDM	NCP	46.261
HTMFDM;PGAPPPSR	NCP	2.634
PGAPPPSR;AGRKFVF	NCP	173.271
AGRKFVF;YHAAPLL	NCP	9.803
YHAAPLL;KANAPEV	NCP	126.645
KANAPEV;VTPNVE	NCP	5.797
VTPNVE;ASPGPLLR	NCP	46.229
ASPGPLLR;KTLLLSVKQ	NCP	0.312
KTLLLSVKQ;AAVMAF	NCP	4.098
AAVMAF;LFLNYFR	NCP	51.981

LFLNYFR;GSLMVP	NCP	44.287
GSLMVP;MMPTPVL	NCP	22.335
MMPTPVL;NSVLPVVV	NCP	4.494
NSVLPVVV;VPNNEL	NCP	59.601
VPNNEL;FFVSLLLVL	NCP	3.054
FFVSLLLVL;FGLLGDN	NCP	62.18
FGLLGDN;MCTPP	NCP	14.283
MCTPP;VGLVK	NCP	74.685
VGLVK;AGKPPRP	NCP	0.879
AGKPPRP;QAMTK	NCP	9.057
QAMTK;GPAASTK	NCP	171.276
GPAASTK;LPGYLVPL	NCP	256.078
LPGYLVPL;VGFSPF	NCP	162.86
VGFSPF;RNFVHW	NCP	7.272
RNFVHW;DLLKNRF	NCP	36.157
DLLKNRF;KEGALPP	NCP	37.901
KEGALPP;KTLGATPK	NCP	17.665
KTLGATPK;YPGLP	NCP	72.344
YPGLP;HSLLSKSK	NCP	51.66
HSLLSKSK;GGAVLEY	NCP	4.044
GGAVLEY;HVLSSAK	NCP	11.929
HVLSSAK;ALHRVRKQ	NCP	140.394
ALHRVRKQ;AEFLLH	NCP	17.298
AEFLLH;KMPWSLK	NCP	115.655
KMPWSLK;MTVGL	NCP	27.651
MTVGL;GPQEPPLQ	NCP	77.944
GPQEPPLQ;KLNSGKK	NCP	33.912
KLNSGKK;LLRPNLY	NCP	34.311
LLRPNLY;SSLKPPR	NCP	63.267
SSLKPPR;TLPDLK	NCP	9.831
TLPDLK;ECYAPK	NCP	13.412
ECYAPK;LFCRP	NCP	16.572
LFCRP;YADFKRQ	NCP	69.249
YADFKRQ;CHMDF	NCP	56.722
CHMDF;AVVLLLR	NCP	70.478
AVVLLLR;VDFLYT	NCP	4.147
VDFLYT;LLVKKTL	NCP	185.072
LLVKKTL;KDLVTL	NCP	81.927
KDLVTL;VVVLLLKK	NCP	15.616
VVVLLLKK;KPGMETK	NCP	15.669
KPGMETK;CPPGVK	NCP	53.013
CPPGVK;KDDPYPK	NCP	94.761
KDDPYPK;LTPCWPK	NCP	90.432
LTPCWPK;AALGFHG	NCP	32.136
AALGFHG;FPLGVP	NCP	26.514
FPLGVP;CFDKFC	NCP	13.967
CFDKFC;KTLVAVP	NCP	10.128
KTLVAVP;MLECME	NCP	45.573
MLECME;KPLSPTEK	NCP	106.948
KPLSPTEK;EVASFR	NCP	18.087
EVASFR;PHLKLLSE	NCP	45.063

PHLKLLSE;GYDPKLG	NCP	77.829
GYDPKLG;YDPKL	NCP	0.003
YDPKL;VSKTLEK	NCP	55.922
VSKTLEK;VGLKP	NCP	18.691
VGLKP;VSKYDF	NCP	19.892
VSKYDF;AELMLP	NCP	89.436
AELMLP;KKLSL	NCP	32.143
KKLSL;KSAFLTPL	NCP	35.577
KSAFLTPL;ATVHMASK	NCP	9.713
ATVHMASK;KTELRK	NCP	0.811
KTELRK;PSQFKPR	NCP	182.913
PSQFKPR;MEFLGL	NCP	113.306
MEFLGL;TVEYL	NCP	18.846
TVEYL;YPVVKE	NCP	8.637
YPVVKE;PPVNLYL	NCP	58.975
PPVNLYL;MGVLYR	NCP	31.41
MGVLYR;KSLPLSM	NCP	5.934
KSLPLSM;SAAPLMP	NCP	50.106
SAAPLMP;YTLPL	NCP	51.885
YTLPL;KPYCLP	NCP	8.088
KPYCLP;NTDDPP	NCP	107.457
NTDDPP;KTGPKDR	NCP	4.077
KTGPKDR;ADVMLK	NCP	118.946
ADVMLK;HLKQLQ	NCP	105.87
HLKQLQ;LEYLGY	NCP	73.537
LEYLGY;LQEVLK	NCP	47.09
LQEVLK;LSLEGKPL	NCP	32.548
LSLEGKPL;VSLHYVR	NCP	15.635
VSLHYVR;NAVLEY	NCP	7.825
NAVLEY;VAKFN	NCP	8.769
VAKFN;PPAPP	NCP	14.159
PPAPP;KDSLPLT	NCP	52.081
KDSLPLT;MTEGP	NCP	72.348
MTEGP;KWCSVR	NCP	59.205
KWCSVR;DVSPHV	NCP	1.632
DVSPHV;SNVVFL	NCP	31.266
SNVVFL;KPLQYMR	NCP	107.147
KPLQYMR;DLELATY	NCP	22.972
DLELATY;KNEEP	NCP	62.9
KNEEP;PPLHLF	NCP	37.233
PPLHLF;GLYLFA	NCP	82.419
GLYLFA;KLTAPPK	NCP	7.863
KLTAPPK;KDCLKTVK	NCP	62.686
KDCLKTVK;AEAGLLLP	NCP	48.167
AEAGLLLP;LFVLL	NCP	20.227
LFVLL;HLLLSF	NCP	3.914
HLLLSF;SLLRKY	NCP	26.05
SLLRKY;ESSLPLL	NCP	104.405
ESSLPLL;CCCCDP	NCP	6.754
CCCCDP;TVEKFDK	NCP	114.749
TVEKFDK;LSSSQTK	NCP	24.457

LSSSQTK;KNTSFLGP	NCP	94.815
KNTSFLGP;AARHDEF	NCP	27.279
AARHDEF;MYVLMLK	NCP	268.191
MYVLMLK;PRPPYK	NCP	56.376
PRPPYK;EGFALL	NCP	4.55
EGFALL;VSLRNV	NCP	63.253
VSLRNV;PTPVPVP	NCP	0.77
PTPVPVP;MFAKAT	NCP	69.454
MFAKAT;KGEKAPR	NCP	46.542
KGEKAPR;PAEGKRP	NCP	58.005
PAEGKRP;LPVNPLPP	NCP	10.917
LPVNPLPP;HLKKLLY	NCP	12.161
HLKKLLY;LNVKVK	NCP	23.749
LNVKVK;TLLSF	NCP	23.573
TLLSF;VPSEELR	NCP	5.869
VPSEELR;VRPPKP	NCP	1.251
VRPPKP;TTFGN	NCP	177.764
TTFGN;KCSYAP	NCP	114.031
KCSYAP;QDVAVTK	NCP	17.712
QDVAVTK;MLYPVRT	NCP	54.834
MLYPVRT;TDMPNC	NCP	25.502
TDMPNC;KSKPNR	NCP	166.173
KSKPNR;QWKLPFL	NCP	25.705
QWKLPFL;LPPLHAPP	NCP	30.222
LPPLHAPP;APLNPAK	NCP	97.608
APLNPAK;TASASSHL	NCP	45.594
TASASSHL;KPSLK	NCP	159.639
KPSLK;KLVHPPP	NCP	94.029
KLVHPPP;VAAPGSLK	NCP	41.376
VAAPGSLK;VSGSSLVF	NCP	26.394
VSGSSLVF;KFVKVPP	NCP	45.143
KFVKVPP;KGPAPAVP	NCP	21.424
KGPAPAVP;LMSSPL	NCP	37.299
LMSSPL;KVMNLKP	NCP	8.42
KVMNLKP;KPFCPP	NCP	24.498
KPFCPP;PPAKM	NCP	11.434
PPAKM;HEMKHR	NCP	32.859
HEMKHR;PLLGSLP	NCP	6.594
PLLGSLP;YGLEY	NCP	18.027
YGLEY;KLHPKPH	NCP	9.132
KLHPKPH;VRALAAF	NCP	16.497
VRALAAF;RLSLSTSK	NCP	42.696
RLSLSTSK;LSTTDLR	NCP	5.988
LSTTDLR;APGKKPP	NCP	94.149
APGKKPP;VGVGF	NCP	10.575
VGVGF;LSSQLHS	NCP	16.89
LSSQLHS;FGAPLTS	NCP	46.187
FGAPLTS;KDGSPP	NCP	132.652
KDGSPP;TVSKLVLGL	NCP	8.046
TVSKLVLGL;KTPKYR	NCP	61.98
KTPKYR;KGNWNKP	NCP	25.595

KGNWNKP;PEALSEL	NCP	4.102
PEALSEL;PQDGVL	NCP	50.661
PQDGVL;HTMLRP	NCP	22.575
HTMLRP;QGLLKA	NCP	32.838
QGLLKA;AASGLYR	NCP	57.627
AASGLYR;LPSLVEC	NCP	13.227
LPSLVEC;AATEFK	NCP	66.371
AATEFK;HQFFPK	NCP	4.306
HQFFPK;TKPALSC	NCP	102.087
TKPALSC;LRNLPGP	NCP	29.562
LRNLPGP;EVWEKQS	NCP	3.849
EVWEKQS;LNNLTSSR	NCP	23.447
LNNLTSSR;HLPPPR	NCP	20.083
HLPPPR;PPALCAGK	NCP	2.304
PPALCAGK;GFRKKQ	NCP	4.118
GFRKKQ;AGPPLAY	NCP	19.177
AGPPLAY;CDSLTAH	NCP	47.475
CDSLTAH;KQLQHSPP	NCP	5.67
KQLQHSPP;WVHFKK	NCP	36.386
WVHFKK;VENLTEF	NCP	9.048
VENLTEF;EFKHLH	NCP	34.411
EFKHLH;WVEVAF	NCP	58.65
WVEVAF;YGDDEC	NCP	94.466
YGDDEC;KMKGQP	NCP	18.414
KMKGQP;LNRSKPP	NCP	71.812
LNRSKPP;SAVFKENK	NCP	0.606
SAVFKENK;EVYKLPK	NCP	83.589
EVYKLPK;KEVPRDF	NCP	34.959
KEVPRDF;KVMEAR	NCP	84.036
KVMEAR;LPGRLP	NCP	19.944
LPGRLP;VGTSSLLP	NCP	103.215
VGTSSLLP;KDHWLPP	NCP	19.094
KDHWLPP;RTEDTK	NCP	36.421
RTEDTK;KSAVPVGR	NCP	52.731
KSAVPVGR;VPFTPF	NCP	57.5
VPFTPF;LTPFK	NCP	19.536
LTPFK;LEPSLVG	NCP	24.238
LEPSLVG;LSPKGGDAK	NCP	9.039
LSPKGGDAK;LAGPLFG	NCP	15.894
LAGPLFG;QPSGPLG	NCP	7.703
QPSGPLG;AQYKVLG	NCP	75.663
AQYKVLG;ELLFTEL	NCP	101.176
ELLFTEL;PSGVLR	NCP	29.18
PSGVLR;PLFSLAK	NCP	54.012
PLFSLAK;LDGPLLS	NCP	94.467
LDGPLLS;VSVVLGH	NCP	64.945
VSVVLGH;FTPDVH	NCP	4.947
FTPDVH;MEESVNQMQPLNEK	NCP	119.252
MEESVNQMQPLNEK;PPLPPPPPP	NCP	27.91
PPLPPPPPP;ADVLSVLRQY	NCP	37.418
ADVLSVLRQY;KELVA	NCP	22.158

KELVA;DKVPSLP	NCP	29.047
DKVPSLP;AAYTVVP	NCP	17.06
AAYTVVP;KAAPPPPPP	NCP	33.72
KAAPPPPPP;AGMQAK	NCP	16.168
AGMQAK;PVSLKLP	NCP	18.849
PVSLKLP;PTGPAK	NCP	14.292
PTGPAK;KDLSKPK	NCP	13.109
KDLSKPK;MCVPP	NCP	106.123
MCVPP;KSGHFLM	NCP	119.502
KSGHFLM;VLELPF	NCP	11.972
VLELPF;PVPGPD	NCP	117.336
PVPGPD;LLMGSWLL	NCP	82.998
LLMGSWLL;GMPLLAPP	NCP	53.074
GMPLLAPP;VVLMRW	NCP	48.297
VVLMRW;KDLGGFP	NCP	83.274
KDLGGFP;KMVWLK	NCP	64.899
KMVWLK;KKKTAPP	NCP	153.938
KKKTAPP;PALLLFT	NCP	7.833
PALLLFT;CVGVCK	NCP	63.229
CVGVCK;MMKSTY	NCP	74.351
MMKSTY;CMQDC	NCP	30.4
CMQDC;EPRNLK	NCP	1.082
EPRNLK;KHNLPVK	NCP	3.145
KHNLPVK;KHHEVR	NCP	4.313
KHHEVR;ALVWGKN	NCP	3.108
ALVWGKN;EVPKPLP	NCP	8.62
EVPKPLP;KLHKPP	NCP	9.792
KLHKPP;DTFWK	NCP	2.231
DTFWK;MVMPK	NCP	1.776
MVMPK;AVPPACR	NCP	13.657
AVPPACR;NSGAEVP	NCP	2.715
NSGAEVP;VLVGL	NCP	25.607
VLVGL;TYLVVVP	NCP	21.13
TYLVVVP;RKRPPVE	NCP	91.185
RKRPPVE;GLSVYF	NCP	60.549
GLSVYF;YYPPPRP	NCP	15.657
YYPPPRP;KEVSSVP	NCP	43.38
KEVSSVP;VDKSKL	NCP	72.981
VDKSKL;FFVQKAK	NCP	2.358
FFVQKAK;KCKMGVK	NCP	98.373
KCKMGVK;LLGESFK	NCP	195.636
LLGESFK;PLFGLPW	NCP	100.709
PLFGLPW;QPTALL	NCP	4.435
QPTALL;KEPDVA	NCP	9.95
KEPDVA;WSYMF	NCP	141.769
WSYMF;YKVLG	NCP	89.507
YKVLG;LDKLPVE	NCP	19.158
LDKLPVE;AYLLPPP	NCP	33.58
AYLLPPP;FEKQF	NCP	83.655
FEKQF;FRLLPGTGY	NCP	113.712
FRLLPGTGY;LLLVKP	NCP	54.285

LLLVKP;EVRKLQ	NCP	77.553
EVRKLQ;LTYAQPP	NCP	6.396
LTYAQPP;FHAGY	NCP	32.522
FHAGY;KTTTYFKM	NCP	187.107
KTTTYFKM;SSVQF	NCP	152.572
SSVQF;HLQVDK	NCP	28.641
HLQVDK;TPALLSK	NCP	129.558
TPALLSK;VPNTPVS	NCP	169.386
VPNTPVS;LTMPK	NCP	7.814
LTMPK;MFLEAT	NCP	73.635
MFLEAT;VGVSLEK	NCP	79.177
VGVSLEK;PPSLVPQ	NCP	7.664
PPSLVPQ;AEKAALT	NCP	7.582
AEKAALT;KTYSTSK	NCP	21.419
KTYSTSK;KSYVGAK	NCP	60.04
KSYVGAK;KNSLLET	NCP	1.767
KNSLLET;KSTVKPY	NCP	26.805
KSTVKPY;LGLLK	NCP	39.648
LGLLK;KETPAQK	NCP	35.765
KETPAQK;EPPLLPKL	NCP	71.848
EPPLLPKL;GSPGVTSR	NCP	43.176
GSPGVTSR;EDHLLLQ	NCP	187.143
EDHLLLQ;LLSCPYPV	NCP	4.667
LLSCPYPV;VEEPN	NCP	44.164
VEEPN;AHNGEC	NCP	20.537
AHNGEC;KSLMLK	NCP	0.459
KSLMLK;SSLPF	NCP	78.582
SSLPF;LVGTPK	NCP	31.947
LVGTPK;KTLPLDL	NCP	11.412
KTLPLDL;TAWVPK	NCP	59.047
TAWVPK;RGPMPPS	NCP	115.977
RGPMPPS;RPKKRH	NCP	11.343
RPKKRH;PSTPPPP	NCP	10.08
PSTPPPP;KFDPAP	NCP	8.988
KFDPAP;KYGQLLT	NCP	6.828
KYGQLLT;KTSSYG	NCP	7.023
KTSSYG;MSSPKLG	NCP	66.887
MSSPKLG;PQLDLHR	NCP	76.008
PQLDLHR;GASARLL	NCP	35.937
GASARLL;LPLLLLKP	NCP	25.774
LPLLLLKP;GPKKWSE	NCP	16.313
GPKKWSE;AAHQPPPP	NCP	74.494
AAHQPPPP;GPRPPEY	NCP	18.936
GPRPPEY;DSYPPLG	NCP	36.35
DSYPPLG;DPRNHPTQ	NCP	46.578
DPRNHPTQ;LSSSGF	NCP	5.328
LSSSGF;FFPPQ	NCP	47.619
FFPPQ;HTLLLLLPS	NCP	24.519
HTLLLLLPS;KYLYK	NCP	14.952
KYLYK;KVLELQR	NCP	3.478
KVLELQR;MPYVPK	NCP	69.882

MPYVPK;GVPLL	NCP	88.133
GVPLL;QAVPF	NCP	5.152
QAVPF;RLPVVLG	NCP	5.187
RLPVVLG;KLSLK	NCP	70.341
KLSLK;ELEFTF	NCP	26.829
ELEFTF;KPLLK	NCP	120.741
KPLLK;YVGAMH	NCP	142.233
YVGAMH;TTLDLRK	NCP	48.002
TTLDLRK;VSPKSPTPK	NCP	7.579
VSPKSPTPK;PTEKFK	NCP	80.244
PTEKFK;NKVLVP	NCP	28.056
NKVLVP;KDGARGP	NCP	39.063
KDGARGP;SHEAPEL	NCP	2.982
SHEAPEL;KGTTVKNQ	NCP	46.988
KGTTVKNQ;ENAPAG	NCP	2.032
ENAPAG;TVSALP	NCP	62.139
TVSALP;KTLMVGP	NCP	61.107
KTLMVGP;VLETW	NCP	67.829
VLETW;KTHVPPR	NCP	22.528
KTHVPPR;AAWLAAPM	NCP	63.696
AAWLAAPM;YDLTNGK	NCP	69.294
YDLTNGK;KLYEVP	NCP	0.402
KLYEVP;LPHAPFR	NCP	14.028
LPHAPFR;HALFVV	NCP	283.278
HALFVV;KSLENW	NCP	26.724
KSLENW;KFFAFL	NCP	4.622
KFFAFL;TAEVNLP	NCP	35.83
TAEVNLP;TPPGAPA	NCP	131.94
TPPGAPA;TKYTSSK	NCP	47.856
TKYTSSK;VPSLTPL	NCP	64.521
VPSLTPL;LPGLERL	NCP	62.115
LPGLERL;LYVAVST	NCP	55.862
LYVAVST;RFLGPQGSQ	NCP	13.309
RFLGPQGSQ;KTHRLSE	NCP	34.839
KTHRLSE;SLDALLR	NCP	25.038
SLDALLR;KSSLKNK	NCP	69.207
KSSLKNK;KKTGF	NCP	12.885
KKTGF;QGKFCKGP	NCP	27.948
QGKFCKGP;LVSLENYK	NCP	10.43
LVSLENYK;VATVVTPR	NCP	37.096
VATVVTPR;FNLDF	NCP	160.683
FNLDF;AGKKCF	NCP	7.821
AGKKCF;TTLSEVKK	NCP	5.073
TTLSEVKK;AAHLNPK	NCP	14.829
AAHLNPK;KPSSFP	NCP	18.546
KPSSFP;ELSVP	NCP	97.365
ELSVP;PLYQPP	NCP	37.311
PLYQPP;AYAAPR	NCP	75.765
AYAAPR;DMMPF	NCP	157.008
DMMPF;LLYPP	NCP	22.029
LLYPP;LGAGPHR	NCP	48.872

LGAGPHR;YLVFLP	NCP	72.615
YLVFLP;LGWTHC	NCP	28.543
LGWTHC;KTLFLCSF	NCP	19.812
KTLFLCSF;GSRPKKK	NCP	2.382
GSRPKKK;HPPPNTPV	NCP	9.669
HPPPNTPV;TTYKC	NCP	3.29
TTYKC;PGGLVEF	NCP	72.882
PGGLVEF;DSPPLYG	NCP	2.737
DSPPLYG;VAVFV	NCP	9.986
VAVFV;ELYKKL	NCP	89.196
ELYKKL;LSAYTPF	NCP	36.877
LSAYTPF;NLVGY	NCP	2.916
NLVGY;KMPSFQQ	NCP	76.044
KMPSFQQ;KSCNLTK	NCP	80.282
KSCNLTK;STGEPAPPRPLP	NCP	66.159
STGEPAPPRPLP;SSSLEYKS	NCP	10.327
SSSLEYKS;FVSVPK	NCP	47.174
FVSVPK;KGEVTFLH	NCP	6.862
KGEVTFLH;QGHLVLY	NCP	1.527
QGHLVLY;RLSVAYT	NCP	11.109
RLSVAYT;LDPSFFEK	NCP	41.282
LDPSFFEK;LVSTLVPL	NCP	0.483
LVSTLVPL;ESTLTRAK	NCP	6.306
ESTLTRAK;KLHFST	NCP	21.442
KLHFST;LTLSPT	NCP	51.633
LTLSPT;NLGTGMS	NCP	65.48
NLGTGMS;VGNLLK	NCP	38.016
VGNLLK;KGVEPTR	NCP	5.134
KGVEPTR;VFLQY	NCP	2.871
VFLQY;DKPEF	NCP	19.989
DKPEF;PKMAP	NCP	8.816
PKMAP;KEGVQTK	NCP	18
KEGVQTK;PSPKAPR	NCP	2.325
PSPKAPR;KGFSNPK	NCP	14.661
KGFSNPK;GVTMLP	NCP	16.65
GVTMLP;TPPFNPP	NCP	179.142
TPPFNPP;PPYLSNR	NCP	58.857
PPYLSNR;VAHAPPPL	NCP	62.731
VAHAPPPL;KPGRAPP	NCP	14.091
KPGRAPP;MHPAAEPK	NCP	67.961
MHPAAEPK;VGRPP	NCP	26.469
VGRPP;MDKNELVQKAK	NCP	76.944
MDKNELVQKAK;KFGLFPP	NCP	28.717
KFGLFPP;KGFCSLGK	NCP	152.66
KGFCSLGK;AATPGPVG	NCP	16.854
AATPGPVG;PQCLDLR	NCP	22.995
PQCLDLR;FRCPEA	NCP	4.938
FRCPEA;KGPLTLP	NCP	85.068
KGPLTLP;EPPASAKP	NCP	0.36
EPPASAKP;GTLERA	NCP	32.058
GTLERA;CCCYP	NCP	5.394

CCCYP;TPSPKEEDEEPESPLEK	NCP	141.186
TPSPKEEDEEPESPLEK;KEEDEEPESPLEKK	NCP	0.012
KEEDEEPESPLEKK;EEDEEPESPLEK	NCP	0.003
EEDEEPESPLEK;NGVGAPL	NCP	1.696
NGVGAPL;TTTDAA	NCP	94.035
TTTDAA;PAPAPPPPP	NCP	26.156
PAPAPPPPP;APPPPPRP	NCP	0.009
APPPPPRP;KDGPPP	NCP	15.481
KDGPPP;LSVSKGTL	NCP	26.766
LSVSKGTL;LPGYLKA	NCP	75.005
LPGYLKA;AFFPGLP	NCP	23.304
AFFPGLP;EALLEL	NCP	45.244
EALLEL;PVEKRER	NCP	20.588
PVEKRER;KPAAHPPP	NCP	13.423
KPAAHPPP;SRLAALLE	NCP	82.628
SRLAALLE;PPLPPFF	NCP	66.838
PPLPPFF;LPGVLAP	NCP	19.802
LPGVLAP;EGLPGSST	NCP	16.435
EGLPGSST;MPLSGLP	NCP	3.05
MPLSGLP;KQAAFLR	NCP	39.858
KQAAFLR;VEPGFLP	NCP	26.205
VEPGFLP;KPFHLS	NCP	11.124
KPFHLS;AAVKKLET	NCP	0.792
AAVKKLET;RLTKAH	NCP	10.162
RLTKAH;LTRAAPL	NCP	25.947
LTRAAPL;VRLPEGS	NCP	25.43
VRLPEGS;VKGGGV	NCP	18.265
VKGGGV;KPLLGSGR	NCP	4.248
KPLLGSGR;NSLMVQKL	NCP	37.569
NSLMVQKL;GKPPPYK	NCP	47.52
GKPPPYK;LLMAK	NCP	22.532
LLMAK;KNPGLP	NCP	68.392
KNPGLP;KNLDLR	NCP	153.095
KNLDLR;EGPAAK	NCP	94.386
EGPAAK;KVLLTKLP	NCP	25.245
KVLLTKLP;FGVTLE	NCP	8.956
FGVTLE;ANSLEML	NCP	2.81
ANSLEML;TVYKQPP	NCP	1.677
TVYKQPP;NLRYPMAV	NCP	11.724
NLRYPMAV;SLPELT	NCP	66.162
SLPELT;AALTFAL	NCP	0.511
AALTFAL;RAVGAGPAG	NCP	11.663
RAVGAGPAG;PKAPWPK	NCP	5.445
PKAPWPK;EMMMTG	NCP	62.958
EMMMTG;AEEVGLFF	NCP	66.654
AEEVGLFF;VSNDKLR	NCP	0.999
VSNDKLR;PGDKKGPP	NCP	43.014
PGDKKGPP;VELEL	NCP	54.001
VELEL;HGHVPR	NCP	132.578
HGHVPR;KNVFYPLP	NCP	104.545
KNVFYPLP;QFAVPR	NCP	11.373

QFAVPR;DAGLAGP	NCP	15.126
DAGLAGP;KPRRHKPDQ	NCP	68.114
KPRRHKPDQ;LVQHLPR	NCP	52.972
LVQHLPR;KTPENLPP	NCP	1.661
KTPENLPP;QNLVHRK	NCP	161.481
QNLVHRK;LSSKKLL	NCP	36.18
LSSKKLL;KGPTYQ	NCP	25.819
KGPTYQ;AARGNNP	NCP	11.766
AARGNNP;THSLFYR	NCP	12.716
THSLFYR;LSAPNLL	NCP	8.856
LSAPNLL;KTGPARP	NCP	50.593
KTGPARP;PAWPGAP	NCP	19.157
PAWPGAP;KQAHAPP	NCP	119.299
KQAHAPP;KMYFAW	NCP	18.009
KMYFAW;VTPLLEP	NCP	75.485
VTPLLEP;TLKMDLP	NCP	3.148
TLKMDLP;HVELLPDY	NCP	51.71
HVELLPDY;GPGFYSPP	NCP	78.526
GPGFYSPP;PEKNNKP	NCP	114.518
PEKNNKP;AAFDY	NCP	39.576
AAFDY;KRVGAVP	NCP	67.957
KRVGAVP;KTKGLVR	NCP	25.158
KTKGLVR;FVTKLF	NCP	3.138
FVTKLF;KNVSGKH	NCP	159.032
KNVSGKH;KPAACNR	NCP	1.555
KPAACNR;LPVSL	NCP	35.376
LPVSL;MAPTP	NCP	104.982
MAPTP;EFEVVTK	NCP	0.831
EFEVVTK;TQAAPLH	NCP	10.955
TQAAPLH;KALLFL	NCP	21.75
KALLFL;MQMLK	NCP	9.07
MQMLK;EARGV	NCP	24.566
EARGV;TGLLL	NCP	8.188
TGLLL;QKGPQVP	NCP	6.608
QKGPQVP;HVAPQ	NCP	6.909
HVAPQ;TNPHVVP	NCP	70.161
TNPHVVP;CALCLR	NCP	35.851
CALCLR;GPAAH	NCP	97.995
GPAAH;LALLLLK	NCP	20.583
LALLLLK;EPGAMNK	NCP	19.44
EPGAMNK;MMVKSK	NCP	158.019
MMVKSK;AAEKVLQ	NCP	32.211
AAEKVLQ;LTLRGNGP	NCP	32.085
LTLRGNGP;LPSTFT	NCP	19.485
LPSTFT;KPTLYKQ	NCP	35.019
KPTLYKQ;RLFKPLR	NCP	8.153
RLFKPLR;LLHVPK	NCP	253.831
LLHVPK;SPAGGFP	NCP	107.67
SPAGGFP;KPERGPP	NCP	5.157
KPERGPP;LSAKQGL	NCP	53.694
LSAKQGL;VLAELLL	NCP	15.552

VLAELLL;FGGSRHQ	NCP	116.028
FGGSRHQ;DMLGPK	NCP	25.647
DMLGPK;KPHFGPP	NCP	22.695
KPHFGPP;PAGEHQA	NCP	31.682
PAGEHQA;AVTTAP	NCP	0.001
AVTTAP;PFPGVR	NCP	49.407
PFPGVR;TAPNLPR	NCP	33.54
TAPNLPR;DPAPGPP	NCP	67.104
DPAPGPP;KVDDFTF	NCP	93.99
KVDDFTF;VGLSW	NCP	75.647
VGLSW;YVLEAK	NCP	10.378
YVLEAK;LDLGTASR	NCP	2.121
LDLGTASR;DPCRGPP	NCP	2.748
DPCRGPP;ELELAP	NCP	55.128
ELELAP;LAGPT	NCP	54.195
LAGPT;LSLTSPPP	NCP	42.666
LSLTSPPP;LGVSHPR	NCP	3.627
LGVSHPR;LDAPGKGT	NCP	6.048
LDAPGKGT;QMGKPR	NCP	33.528
QMGKPR;GPVPAPPPP	NCP	11.043
GPVPAPPPP;SAWLTK	NCP	26.322
SAWLTK;GVPTL	NCP	2.517
GVPTL;SGAKAGYGDGL	NCP	21.495
SGAKAGYGDGL;ACLLP	NCP	105.596
ACLLP;LGARALPP	NCP	0.274
LGARALPP;CKDDDD	NCP	47.283
CKDDDD;SVFYY	NCP	184.475
SVFYY;AALGAQL	NCP	62.758
AALGAQL;KPGKKEK	NCP	220.704
KPGKKEK;GPPPSRPP	NCP	12.66
GPPPSRPP;AGAMCPR	NCP	14.325
AGAMCPR;DDEQNP	NCP	123.927
DDEQNP;KAPGLLPR	NCP	55.956
KAPGLLPR;QALVRGP	NCP	22.071
QALVRGP;ETSSPKPP	NCP	23.004
ETSSPKPP;TLRLLM	NCP	31.581
TLRLLM;PPPGPPSP	NCP	45.108
PPPGPPSP;PQGPADPP	NCP	15.726
PQGPADPP;GGHTLLL	NCP	21.657
GGHTLLL;CDKALSK	NCP	15.798
CDKALSK;VGPPPAVP	NCP	108.342
VGPPPAVP;PVTRPTL	NCP	144.531
PVTRPTL;TRPPGA	NCP	13.737
TRPPGA;PAPPPPPPP	NCP	86.649
PAPPPPPPP;TGACVRP	NCP	5.772
TGACVRP;PVPPLPPP	NCP	250.158
PVPPLPPP;PWGLGPP	NCP	29.139
PWGLGPP;TVVAVP	NCP	77.478
TVVAVP;ESGPPSP	NCP	9.414
ESGPPSP;LCSRSWP	NCP	6.456
LCSRSWP;SPATLLL	NCP	71.958

SPATLLL;GSYLVYF	NCP	45.747
VGVTATPLP;APSHTRPP	NCP	9.501
APSHTRPP;PNPPKK	NCP	74.074
PNPPKK;ACGLV	NCP	61.538
ACGLV;LPPGRSP	NCP	8.34
LPPGRSP;RPGPPPPP	NCP	0.093
RPGPPPPP;RGAPGHRP	NCP	29.673
RGAPGHRP;LAGGLL	NCP	49.095
LAGGLL;MHRDSCPLDCK	NCP	21.697
MHRDSCPLDCK;KQLRAVV	NCP	0.176
KQLRAVV;ARPHPFQ	NCP	27.679
ARPHPFQ;GPTSLKMP	NCP	122.483
GPTSLKMP;KCLEGTL	NCP	29.007
KCLEGTL;SLSVPL	NCP	64.323
SLSVPL;KPVPPPPP	NCP	8.148
KPVPPPPP;RSGTLPP	NCP	3.924
RSGTLPP;KGPEGLPR	NCP	22.674
KGPEGLPR;RLFPNW	NCP	1.516
RLFPNW;LDAALVV	NCP	99.284
LDAALVV;GSAAGPG	NCP	0.477
GSAAGPG;APPPAPPRP	NCP	3.186
APPPAPPRP;LPFPEED	NCP	50.028
LPFPEED;STAVVEPY	NCP	40.644
STAVVEPY;VQRAVCM	NCP	2.385
VQRAVCM;LTGSTLSF	NCP	10.807
LTGSTLSF;KPAPPP	NCP	11.849
KPAPPP;AASFFNLH	NCP	3.478
AASFFNLH;AADVNLP	NCP	71.351
AADVNLP;RGGGPPR	NCP	3.696
RGGGPPR;STNFW	NCP	35.872
STNFW;ETPALP	NCP	1.544
ETPALP;YPLYR	NCP	38.596
YPLYR;WQHVPY	NCP	3.86
WQHVPY;AASRRLM	NCP	8.094
AASRRLM;YGEKRPVD	NCP	23.43
YGEKRPVD;AGVRPPL	NCP	88.53
AGVRPPL;VSGASF	NCP	25.486
VSGASF;QLMLGLK	NCP	16.65
QLMLGLK;DLRNLGK	NCP	24.641
DLRNLGK;KDEPPAPP	NCP	35.185
KDEPPAPP;PAASLMP	NCP	22.847
PAASLMP;KYVYVSVL	NCP	19.768
KYVYVSVL;WPAPPRP	NCP	34.763
WPAPPRP;PLSGW	NCP	13.077
PLSGW;LASEGPLK	NCP	62.391
LASEGPLK;LEEFL	NCP	5.907
LEEFL;TPGMAALQ	NCP	64.917
TPGMAALQ;ALEKLR	NCP	78.741
ALEKLR;LVNGLVV	NCP	39.646
LVNGLVV;SRGRLLNP	NCP	24.689
SRGRLLNP;LVDLTPAL	NCP	94.143

LVDLTPAL;GPVGGRPP	NCP	13.314
GPVGGRPP;KKTVHNP	NCP	69.298
KKTVHNP;GPPLHPP	NCP	0.224
GPPLHPP;NLLSYQ	NCP	24.586
NLLSYQ;SFNRTP	NCP	100.706
SFNRTP;RPRPVPP	NCP	2.991
RPRPVPP;VGLENPGR	NCP	5.347
VGLENPGR;VVPSSLVT	NCP	5.738
VVPSSLVT;PPGRAAPP	NCP	31.671
PPGRAAPP;YGEQLL	NCP	22.329
YGEQLL;APEGKRP	NCP	34.341
APEGKRP;FARAGAR	NCP	38.223
FARAGAR;FGEKLKG	NCP	2.833
FGEKLKG;LAPERPP	NCP	69.029
LAPERPP;TGLEEP	NCP	150.486
TGLEEP;TGGFAP	NCP	0.993
TGGFAP;VGPVSLPR	NCP	70.725
VGPVSLPR;QQLLY	NCP	21.282
QQLLY;AFGCLN	NCP	194.628
AFGCLN;TAPGNPK	NCP	6.123
TAPGNPK;KLQPLLPT	NCP	75.457
KLQPLLPT;KLHCVPP	NCP	14.96
KLHCVPP;KPHGTLR	NCP	42.027
KPHGTLR;PMFAMK	NCP	15.354
PMFAMK;GPPLRPLP	NCP	2.166
GPPLRPLP;KSPELY	NCP	74.389
KSPELY;KETTVFPK	NCP	12.944
KETTVFPK;ATLGLAA	NCP	13.018
ATLGLAA;RTVLEFP	NCP	11.621
RTVLEFP;VPPEPKP	NCP	38.892
VPPEPKP;VNRGLPTP	NCP	5.877
VNRGLPTP;PNRLSR	NCP	20.433
PNRLSR;ENTCMKK	NCP	11.478
ENTCMKK;ELPWPLPR	NCP	56.494
ELPWPLPR;FAAPR	NCP	80.54
FAAPR;KAAALLAP	NCP	79.845
KAAALLAP;PSCPGTF	NCP	23.088
PSCPGTF;RLDGSLDFKK	NCP	58.843
RLDGSLDFKK;GHLSPTGNTEF	NCP	0.057
GHLSPTGNTEF;ASFKVTGENDKYRLTY	NCP	1.59
ASFKVTGENDKYRLTY;KVTGENDKY	NCP	0.009
KVTGENDKY;DFGDDSSDKFFTSH	NCP	0.084
DFGDDSSDKFFTSH;GPPAWC	NCP	29.534
GPPAWC;TYSGLP	NCP	0.198
TYSGLP;RFFEY	NCP	90.453
RFFEY;APLVRGPG	NCP	91.44
APLVRGPG;FGSPK	NCP	14.101
FGSPK;AAVSLA	NCP	25.823
AAVSLA;MRAMAG	NCP	32.298
MRAMAG;PPAGHKL	NCP	72.526
PPAGHKL;KTVSEAP	NCP	14.978

KTVSEAP;RGVGAPA	NCP	17.199
RGVGAPA;FSLKENT	NCP	4.162
FSLKENT;ATGVPSPK	NCP	8.948
ATGVPSPK;YRDMPP	NCP	4.797
YRDMPP;TLEHPF	NCP	9.223
TLEHPF;AVTPHPR	NCP	5.126
AVTPHPR;PKSGPLQ	NCP	5.043
PKSGPLQ;QAAASEAPR	NCP	32.415
QAAASEAPR;LVAAVL	NCP	30.231
LVAAVL;GRTPDL	NCP	17.781
GRTPDL;KNQSQK	NCP	23.356
KNQSQK;ELSMGML	NCP	27.389
ELSMGML;LSSAGL	NCP	72.585
LSSAGL;KAPGFYVL	NCP	66.559
KAPGFYVL;LRHQKPP	NCP	25.052
LRHQKPP;PGKVVGP	NCP	7.636
PGKVVGP;AAAARKPV	NCP	66.638
AAAARKPV;VSALKSVQ	NCP	73.378
VSALKSVQ;LLQVTR	NCP	9.975
LLQVTR;YGACGP	NCP	74.714
YGACGP;DWAFLVL	NCP	91.413
DWAFLVL;KPVSVP	NCP	50.871
KPVSVP;EGRALGK	NCP	28.875
EGRALGK;QYSPPK	NCP	8.616
QYSPPK;QARFVF	NCP	12.121
QARFVF;SKDNEGSWFR	NCP	20.027
SKDNEGSWFR;KEPSLTPK	NCP	63.363
KEPSLTPK;FTKTAG	NCP	22.365
FTKTAG;TPSGKVPK	NCP	13.816
TPSGKVPK;TDSKYFTTTKK	NCP	12.842
TDSKYFTTTKK;VLLTFPAR	NCP	5.538
VLLTFPAR;KLAPPLVTLL	NCP	3.63
KLAPPLVTLL;DLGPPVSGPPL	NCP	10.305
DLGPPVSGPPL;GTGGLGL	NCP	1.134
GTGGLGL;LNGLTGL	NCP	3.415
LNGLTGL;AWGAP	NCP	9.599
AWGAP;MFLVNS	NCP	27.94
MFLVNS;KPTVNGP	NCP	32.151
KPTVNGP;QLMRPTVP	NCP	27.716
QLMRPTVP;KPHDLFL	NCP	18.933
KPHDLFL;LEQGAAPL	NCP	74.496
LEQGAAPL;ALLRGVF	NCP	24.492
ALLRGVF;PSTTTKP	NCP	17.067
PSTTTKP;KPGKF	NCP	8.74
KPGKF;KVRSPK	NCP	12.437
KVRSPK;KRTAPP	NCP	15.009
KRTAPP;MREALHT	NCP	18.456
MREALHT;RLAGVDP	NCP	7.908
RLAGVDP;KDLKKKP	NCP	19.269
KDLKKKP;PVAWP	NCP	17.395
PVAWP;KTYALVP	NCP	17.519

KTYALVP;ARGGPPRP	NCP	4.576
ARGGPPRP;PAAFGRR	NCP	36.155
PAAFGRR;KESKPYAP	NCP	56.26
KESKPYAP;KFFYLK	NCP	4.667
KFFYLK;LLLLSFVT	NCP	12.303
LLLLSFVT;AATLVTL	NCP	98.377
AATLVTL;LEFVAR	NCP	8.934
LEFVAR;AVTKGRP	NCP	8.865
AVTKGRP;VGVVAPT	NCP	67.511
VGVVAPT;DLSFAPST	NCP	13.93
DLSFAPST;GTRGP	NCP	196.449
GTRGP;RLAKFLY	NCP	36.116
RLAKFLY;GLPVG	NCP	124.929
GLPVG;GVEPGPK	NCP	25.269
GVEPGPK;VWEAVRRQ	NCP	120.516
VWEAVRRQ;KLKLAVP	NCP	25.209
KLKLAVP;FNGTKR	NCP	15.406
FNGTKR;RAPKAHP	NCP	22.652
RAPKAHP;PVLEDFSL	NCP	112.644
PVLEDFSL;EKYGPLY	NCP	27.903
EKYGPLY;YKVFPLG	NCP	9.289
YKVFPLG;ARSLVAP	NCP	63.494
ARSLVAP;VDLEKLK	NCP	25.555
VDLEKLK;RPGRHV	NCP	24.21
RPGRHV;QEALDPR	NCP	48.257
QEALDPR;KYAHLGPP	NCP	9.111
KYAHLGPP;VELDHL	NCP	31.617
VELDHL;SKLWS	NCP	77.644
SKLWS;KGRGAGPR	NCP	21.899
KGRGAGPR;MMPTP	NCP	74.55
MMPTP;AMQAAKAR	NCP	24.933
AMQAAKAR;TQVPF	NCP	14.091
TQVPF;KSGPHAP	NCP	24.205
KSGPHAP;VGVTTLPL	NCP	127.542
VGVTTLPL;ALFKAGK	NCP	5.189
ALFKAGK;NDDMSK	NCP	21.072
NDDMSK;PANPPPPP	NCP	51.553
PANPPPPP;KNMAGP	NCP	21.485
KNMAGP;GGREPNLK	NCP	4.08
GGREPNLK;HGRDAVYL	NCP	61.461
HGRDAVYL;GETPLM	NCP	45.411
GETPLM;RPGAPAVP	NCP	11.745
RPGAPAVP;NYLTVR	NCP	4.051
NYLTVR;MPERK	NCP	11.912
MPERK;LSATEF	NCP	11.314
LSATEF;VKKLALF	NCP	20.28
VKKLALF;YDLCRR	NCP	4.278
YDLCRR;MGGGG	NCP	37.802
MGGGG;LLARQAP	NCP	3.186
LLARQAP;GPGPRYPK	NCP	3.999
GPGPRYPK;LLTERGY	NCP	43.867

LLTERGY;FLVSPPPP	NCP	4.328
FLVSPPPP;LGPLSET	NCP	49.749
LGPLSET;LGSFTKPR	NCP	63.273
LGSFTKPR;YGVAV	NCP	19.81
YGVAV;MPYNPR	NCP	10.875
MPYNPR;FFSLKVHR	NCP	9.641
FFSLKVHR;SGRGPP	NCP	100.063
SGRGPP;KKRKAPP	NCP	17.298
KKRKAPP;VARLGPG	NCP	121.817
VARLGPG;QAVLH	NCP	25.2
QAVLH;AAEPL	NCP	44.223
AAEPL;LLEGFK	NCP	19.23
LLEGFK;VMPPPPFQ	NCP	23.157
VMPPPPFQ;PGSLEFNK	NCP	4.275
PGSLEFNK;VVGPEC	NCP	18.144
VVGPEC;VEDVLDF	NCP	101.734
VEDVLDF;AAKLKAL	NCP	168.5
AAKLKAL;ALPGLA	NCP	5.55
ALPGLA;LDVELAT	NCP	19.605
LDVELAT;DDDLQQ	NCP	5.874
DDDLQQ;PGPAEP	NCP	47.982
PGPAEP;QEFTRY	NCP	1.894
QEFTRY;CGMDP	NCP	59.628
CGMDP;KPVLRPP	NCP	5.789
KPVLRPP;LHAHNK	NCP	7.086
LHAHNK;PAPGLFPP	NCP	2.592
PAPGLFPP;GSAAPN	NCP	72.495
GSAAPN;SWKLSM	NCP	31.102
SWKLSM;MGTTVP	NCP	33.599
MGTTVP;KVHGNPP	NCP	9.45
KVHGNPP;LLTHVLPL	NCP	24.648
LLTHVLPL;QPPPPR	NCP	3.744
QPPPPR;LLVNR	NCP	103.479
LLVNR;GNPSFLP	NCP	1.158
GNPSFLP;PAYQR	NCP	80.061
PAYQR;AGTALKLR	NCP	12.561
AGTALKLR;KPGPPRGP	NCP	8.269
KPGPPRGP;ASLLLAAA	NCP	62.654
ASLLLAAA;EHVPVH	NCP	31.479
EHVPVH;NRYLMDF	NCP	57.661
NRYLMDF;CGCMPP	NCP	18.479
CGCMPP;LTFAK	NCP	5.683
LTFAK;TVVAEGPA	NCP	18.053
TVVAEGPA;VLGLQF	NCP	82.779
VLGLQF;HDLETLY	NCP	21.658
HDLETLY;LLPLFET	NCP	51.155
LLPLFET;RPPPLGV	NCP	35.616
RPPPLGV;FNLLW	NCP	102.412
FNLLW;QWPSF	NCP	3.596
QWPSF;SSDLKKE	NCP	32.667
SSDLKKE;TTTAG	NCP	131.367

TTTAG;VGTTVK	NCP	17.718
VGTTVK;MLKPDL	NCP	44.466
MLKPDL;PAHETKP	NCP	73.464
PAHETKP;LPQPDFR	NCP	184.2
LPQPDFR;QPLSMVP	NCP	8.166
QPLSMVP;QGLKPF	NCP	7.479
QGLKPF;QNGPPE	NCP	78.601
QNGPPE;EGAEVERL	NCP	83.672
EGAEVERL;KQCPLAF	NCP	4.588
KQCPLAF;SNLPALV	NCP	17.813
SNLPALV;PAALSPC	NCP	24.813
PAALSPC;VTLLPRE	NCP	41.475
VTLLPRE;ELGLPAAP	NCP	6.768
ELGLPAAP;VPPVLFN	NCP	15.462
VPPVLFN;LGLPP	NCP	49.14
LGLPP;LSLAGLT	NCP	76.194
LSLAGLT;MAAAP	NCP	1.785
MAAAP;TRPAGP	NCP	3.693
TRPAGP;TAPPGCD	NCP	36.744
TAPPGCD;CVDPAPG	NCP	14.763
CVDPAPG;APRSKAPP	NCP	28.044
APRSKAPP;LTAEVLELAGNA	NCP	48.492
LTAEVLELAGNA;AAPRMR	NCP	30.102
AAPRMR;EHAPTYF	NCP	26.154
EHAPTYF;LSTVP	NCP	48.945
LSTVP;LETLPVT	NCP	44.778
LETLPVT;SVTVPQR	NCP	55.567
SVTVPQR;GSRLLTF	NCP	4.626
GSRLLTF;PGALFVPP	NCP	11.22
PGALFVPP;AATPVNQ	NCP	86.847
AATPVNQ;LPEEVPF	NCP	29.259
LPEEVPF;DRPRVK	NCP	39.875
DRPRVK;FAVKASR	NCP	7.66
FAVKASR;KGGKVPK	NCP	77.372
KGGKVPK;SQEKEATETHF	NCP	14.925
SQEKEATETHF;LPLVAA	NCP	45.166
LPLVAA;PFKAE	NCP	12.848
PFKAE;FVKDPALR	NCP	3.543
FVKDPALR;WLWHHS	NCP	3.477
WLWHHS;HVSLVEL	NCP	7.744
HVSLVEL;KNLTKAV	NCP	70.077
KNLTKAV;KFPVVRGT	NCP	55.92
KFPVVRGT;ALAHPKQQ	NCP	15.999
ALAHPKQQ;LAGLGGF	NCP	1.289
LAGLGGF;PVFLSEETPY	NCP	20.055
PVFLSEETPY;VPPVPPPPSSE	NCP	0.069
VPPVPPPPSSE;PPKSPPRP	NCP	4.614
PPKSPPRP;VTDTGEVTEAGK	NCP	37.911
VTDTGEVTEAGK;LTEVLTDSHVKVQK	NCP	13.617
LTEVLTDSHVKVQK;ALCDPLEEVREAAAK	NCP	9.609
ALCDPLEEVREAAAK;TFEAAP	NCP	33.972

TFEAAP;LSSVPLFK	NCP	2.4
LSSVPLFK;KPLATEP	NCP	8.793
KPLATEP;KEMHLAL	NCP	69.526
KEMHLAL;KPHADGT	NCP	34.548
KPHADGT;KDLKL	NCP	11.994
KDLKL;AAVFGGP	NCP	15.851
AAVFGGP;MMTVCC	NCP	12.279
MMTVCC;LVDLDMVLK	NCP	7.263
LVDLDMVLK;VAEQTPLTALY	NCP	2.967
VAEQTPLTALY;TKDLDKANY	NCP	7.77
TKDLDKANY;VTVRVPQKNS	NCP	1.689
VTVRVPQKNS;YVAPQ	NCP	26.835
YVAPQ;KESPSPP	NCP	20.571
KESPSPP;METPQSR	NCP	28.248
METPQSR;ACDDDC	NCP	19.8
ACDDDC;YYRPP	NCP	23.911
YYRPP;LFQDGDFENPVLY	NCP	52.01
LFQDGDFENPVLY;AFQKKGGEKEEL	NCP	0.39
AFQKKGGEKEEL;KPQFVPK	NCP	21.661
KPQFVPK;LQGRSRLQ	NCP	3.839
LQGRSRLQ;ALELLKK	NCP	39.693
ALELLKK;CCEDP	NCP	93.412
CCEDP;MEEKYVPP	NCP	174.614
MEEKYVPP;DHTRVVLHDGDPN	NCP	48.723
DHTRVVLHDGDPN;FTGAKKT	NCP	65.148
FTGAKKT;CCCDDDD	NCP	7.725
CCCDDDD;CSGCC	NCP	138.772
CSGCC;GKLPVLL	NCP	39.11
GKLPVLL;NASCLLLH	NCP	5.436
NASCLLLH;LMQMLK	NCP	62.146
LMQMLK;QFYPMAY	NCP	103.904
QFYPMAY;TVGDQVGK	NCP	82.984
TVGDQVGK;SPGRLPK	NCP	9.485
SPGRLPK;QLEELKK	NCP	33.909
QLEELKK;EDMSDT	NCP	31.813
EDMSDT;ATAAAASASEAEAEPK	NCP	24.867
ATAAAASASEAEAEPK;LSQSAGP	NCP	33.377
LSQSAGP;KLLTWAL	NCP	3.489
KLLTWAL;AALSAGE	NCP	3.582
AALSAGE;AQAFVSN	NCP	33.72
AQAFVSN;ALLAY	NCP	17.14
ALLAY;PPPAYK	NCP	2.88
PPPAYK;LLSPP	NCP	3.264
LLSPP;LADVTRK	NCP	18.135
LADVTRK;MKSPLEECAEGEPF	NCP	20.186
MKSPLEECAEGEPF;KKGAAPR	NCP	6.061
KKGAAPR;MFSGPL	NCP	25.988
MFSGPL;EPAAPPR	NCP	7.296
EPAAPPR;CAPWLSK	NCP	35.914
CAPWLSK;KAPQAPPP	NCP	195.863
KAPQAPPP;KKWLPTL	NCP	34.819

KKWLPTL;AKSGAP	NCP	144.8
AKSGAP;LDPTASYHG	NCP	50.937
LDPTASYHG;KHYWPK	NCP	115.15
KHYWPK;NLAHPLK	NCP	47.697
NLAHPLK;KSTKPPK	NCP	31.079
KSTKPPK;NPKKYSH	NCP	64.801
NPKKYSH;PPPWAH	NCP	0.227
PPPWAH;QFHYYR	NCP	4.254
QFHYYR;RQPLGPP	NCP	87.802
RQPLGPP;ENLKL	NCP	40.977
ENLKL;LGSVFGLP	NCP	0.933
LGSVFGLP;LLAPERPK	NCP	17.927
LLAPERPK;KSGLVVT	NCP	20.619
KSGLVVT;LPPKLSH	NCP	4.468
LPPKLSH;LVDALPL	NCP	0.378
LVDALPL;KGTVVAP	NCP	26.268
KGTVVAP;YRSLG	NCP	11.03
YRSLG;GGGGLGGGL	NCP	1.8
GGGGLGGGL;EAFGLA	NCP	12.418
EAFGLA;CPPLLVVL	NCP	18.966
CPPLLVVL;KGKPEDK	NCP	46.056
KGKPEDK;LCALARP	NCP	113.49
LCALARP;SVGPAPPP	NCP	0.105
SVGPAPPP;KAFTPGAP	NCP	81.429
KAFTPGAP;DVPPP	NCP	63.212
DVPPP;GSLGVK	NCP	0.844
GSLGVK;PPPLF	NCP	72.956
PPPLF;KNKKLA	NCP	48.493
KNKKLA;SVGKPSPR	NCP	19.629
SVGKPSPR;DPMRPP	NCP	196.472
DPMRPP;GPLTAY	NCP	16.135
GPLTAY;KALLAK	NCP	21.138
KALLAK;KVYPSR	NCP	113.51
KVYPSR;LPHALLRL	NCP	56.536
LPHALLRL;KAAFQPK	NCP	40.718
KAAFQPK;PAALGF	NCP	104.466
PAALGF;GVTFGGPA	NCP	69.564
GVTFGGPA;LSPVP	NCP	17.781
LSPVP;RWKVVLL	NCP	51.445
RWKVVLL;KLVGPGV	NCP	8.99
KLVGPGV;ASQPLMR	NCP	40.765
ASQPLMR;KGLLVHR	NCP	33.554
KGLLVHR;YVLPLP	NCP	75.321
YVLPLP;LRVLSM	NCP	23.821
LRVLSM;APGNCAKK	NCP	4.196
APGNCAKK;VDLAEGRK	NCP	14.829
VDLAEGRK;GPPLPPGP	NCP	106.353
GPPLPPGP;VTLLF	NCP	6.288
VTLLF;HALLK	NCP	56.905
HALLK;LDLSGPK	NCP	130.235
LDLSGPK;YGAPP	NCP	9.039

YGAPP;VLLNLV	NCP	18.412
VLLNLV;KLLVGY	NCP	1.341
KLLVGY;HTVPLYE	NCP	62.628
HTVPLYE;ASLLERSPR	NCP	54.387
ASLLERSPR;LRYQLQ	NCP	3.446
LRYQLQ;HMMQP	NCP	32.844
HMMQP;KSVLRPYL	NCP	13.138
KSVLRPYL;HLLGKR	NCP	27.165
HLLGKR;APVPLQ	NCP	34.547
APVPLQ;YVYRQ	NCP	1.864
YVYRQ;VSLSLPK	NCP	23.706
VSLSLPK;VSVPSPVK	NCP	5.526
VSVPSPVK;SALRTTP	NCP	23.142
SALRTTP;ATRPVPP	NCP	7.764
ATRPVPP;CPRRAM	NCP	61.034
CPRRAM;KLTNW	NCP	28.618
KLTNW;VSTAPPPP	NCP	1.718
VSTAPPPP;KTYAPAP	NCP	62.745
KTYAPAP;EVFKKDP	NCP	84.013
EVFKKDP;LTSSFLPFQ	NCP	5.93
LTSSFLPFQ;KRFTLPTK	NCP	124.432
KRFTLPTK;MDPGF	NCP	41.262
MDPGF;KDTAPF	NCP	33.459
KDTAPF;RPLKGKP	NCP	93.911
RPLKGKP;VLPHQLA	NCP	195.24
VLPHQLA;KPGNPR	NCP	24.442
KPGNPR;KGPKELK	NCP	147.095
KGPKELK;PVDLSKWSGPLS	NCP	112.338
PVDLSKWSGPLS;DPGKLYTL	NCP	1.311
DPGKLYTL;QAEWDDYVPKLYEQLSGK	NCP	2.322
QAEWDDYVPKLYEQLSGK;LYVKL	NCP	0.742
LYVKL;DVPRRP	NCP	34.835
DVPRRP;KACPL	NCP	22.051
KACPL;HDKLVTL	NCP	9.392
HDKLVTL;FNALLFH	NCP	74.458
FNALLFH;GKGMLN	NCP	32.663
GKGMLN;ASSAEGGP	NCP	33.237
ASSAEGGP;WHHPPR	NCP	150.127
WHHPPR;KNALPKT	NCP	47.466
KNALPKT;LYFLAY	NCP	106.119
LYFLAY;KQRLAL	NCP	23.952
KQRLAL;LRNFGLR	NCP	6.794
LRNFGLR;SSLKVRLL	NCP	20.047
SSLKVRLL;KGDPML	NCP	2.082
KGDPML;ALSSGFL	NCP	72.891
ALSSGFL;KDLDPLP	NCP	11.891
KDLDPLP;MFPVRNP	NCP	20.214
MFPVRNP;EELLL	NCP	2.499
EELLL;ATTEPK	NCP	40.777
ATTEPK;KAFGEKK	NCP	47.15
KAFGEKK;KPLRLAPP	NCP	7.444

KPLRLAPP;TTGGLLL	NCP	19.667
TTGGLLL;LPKTTL	NCP	35.997
LPKTTL;KFPLLADD	NCP	77.08
KFPLLADD;DPFRDSP	NCP	1.025
DPFRDSP;ALRTPTS	NCP	49.605
ALRTPTS;VAVPVYK	NCP	16.27
VAVPVYK;PTPPEKK	NCP	13.28
PTPPEKK;PGAHAGP	NCP	115.947
PGAHAGP;GAKPP	NCP	38.607
GAKPP;QELPGNE	NCP	123.907
QELPGNE;SHSLPVV	NCP	1.133
SHSLPVV;KDLRGGAG	NCP	19.884
KDLRGGAG;LPKGGLK	NCP	9.091
LPKGGLK;CHAHKLR	NCP	39.755
CHAHKLR;MNFSGK	NCP	22.057
MNFSGK;AAKYVLK	NCP	18.464
AAKYVLK;VTHNPRE	NCP	5.299
VTHNPRE;KVLLSGAP	NCP	40.208
KVLLSGAP;PLHFGPK	NCP	188.32
PLHFGPK;DHGAAP	NCP	28.805
DHGAAP;VTTLL	NCP	1.596
VTTLL;LEVSDVP	NCP	99.45
LEVSDVP;KGCPVN	NCP	43.563
KGCPVN;TPAPRLLK	NCP	14.577
TPAPRLLK;KHALPR	NCP	8.278
KHALPR;VPELSKT	NCP	46.917
VPELSKT;VFLPALC	NCP	60.674
VFLPALC;NLSRSPR	NCP	72.228
NLSRSPR;GPVDLGF	NCP	86.746
GPVDLGF;KVSELLR	NCP	4.044
KVSELLR;FGPGPG	NCP	13.247
FGPGPG;ALRGPPAL	NCP	22.12
ALRGPPAL;VGLGWLR	NCP	54.569
VGLGWLR;TLEALYR	NCP	53.127
TLEALYR;RPNPAL	NCP	33.339
RPNPAL;GQEERLK	NCP	10.224
GQEERLK;DDMACK	NCP	62.853
DDMACK;SSVDSAPL	NCP	0.972
SSVDSAPL;KTYAKP	NCP	16.324
KTYAKP;LQMFGEKL	NCP	24.809
LQMFGEKL;DPEGKGVLK	NCP	0.642
DPEGKGVLK;TLLLLPV	NCP	6.961
TLLLLPV;ALGTAF	NCP	37.583
ALGTAF;EDHKPH	NCP	69.258
EDHKPH;DLLRLFEY	NCP	19.476
DLLRLFEY;KPAAGYR	NCP	244.776
KPAAGYR;GPAHKKP	NCP	34.641
GPAHKKP;VSRAPVTK	NCP	67.498
VSRAPVTK;SFFVKPP	NCP	35.381
SFFVKPP;KPSPP	NCP	41.791
KPSPP;RRATKHV	NCP	7.808

RRATKHV;ALLHFLR	NCP	134.364
ALLHFLR;TAPFKPR	NCP	21.504
TAPFKPR;PPVRP	NCP	7.66
PPVRP;LLLDV	NCP	24.627
LLLDV;VEPKM	NCP	13.284
VEPKM;EGSSG	NCP	39.891
EGSSG;VVETRLR	NCP	64.581
VVETRLR;LVTLDF	NCP	14.255
LVTLDF;NPSLWL	NCP	116.928
NPSLWL;HGAVKPK	NCP	8.478
HGAVKPK;LAGEALF	NCP	10.447
LAGEALF;GVFHLYR	NCP	0.546
GVFHLYR;CCDNPLP	NCP	21.591
CCDNPLP;KDLVTLA	NCP	7.862
KDLVTLA;PVLPT	NCP	109.093
PVLPT;LFVPLEF	NCP	19.205
LFVPLEF;VPPPPLPA	NCP	38.934
VPPPPLPA;AVGVVFL	NCP	1.494
AVGVVFL;KVTPKGK	NCP	66.118
KVTPKGK;KFVAAGN	NCP	15.408
KFVAAGN;LGVRPSY	NCP	128.376
LGVRPSY;KFAEAGK	NCP	42.782
KFAEAGK;ALTRPPR	NCP	3.525
ALTRPPR;KPPNGFSK	NCP	62.38
KPPNGFSK;LVGVSP	NCP	11.841
LVGVSP;KKVQGKK	NCP	116.912
KKVQGKK;SSLNPLLF	NCP	14.272
SSLNPLLF;GPKLPPPPPP	NCP	28.346
GPKLPPPPPP;KLPCLK	NCP	87.901
KLPCLK;LVGVN	NCP	13.569
LVGVN;KYFALPV	NCP	41.891
KYFALPV;AEAVRPP	NCP	92.844
AEAVRPP;LDADYPLRVLY	NCP	0.897
LDADYPLRVLY;GLSVKV	NCP	55.344
GLSVKV;KEVFP	NCP	1.236
KEVFP;ERGSVPH	NCP	13.632
ERGSVPH;SVKPQHLT	NCP	13.675
SVKPQHLT;NVPPHRP	NCP	6.875
NVPPHRP;LGVPRPH	NCP	120.597
LGVPRPH;RPTPATL	NCP	4.14
RPTPATL;KGMELR	NCP	49.15
KGMELR;YLGLP	NCP	5.24
YLGLP;AAPGLLHQ	NCP	76.959
AAPGLLHQ;DAEPTN	NCP	164.677
DAEPTN;VGGPTVFF	NCP	40.775
VGGPTVFF;CFGHEAP	NCP	42.234
CFGHEAP;VDDPLELV	NCP	52.56
VDDPLELV;PAALK	NCP	24.366
PAALK;PNWKGNK	NCP	3.073
PNWKGNK;VELLK	NCP	51.74
VELLK;VLLLNHP	NCP	2.295

VLLLNHP;AARTKLE	NCP	36.217
AARTKLE;LYLTTLG	NCP	72.104
LYLTTLG;LLNTF	NCP	59.962
LLNTF;PPMPEYP	NCP	4.65
PPMPEYP;KPAHNDP	NCP	33.261
KPAHNDP;YGPLEHA	NCP	12.576
YGPLEHA;LSAARLL	NCP	66.183
LSAARLL;TDAKPK	NCP	15.738
TDAKPK;KLRVSLKLK	NCP	24.45
KLRVSLKLK;TKEPDV	NCP	9.692
TKEPDV;KTKKLPF	NCP	132.711
KTKKLPF;KPTTWKN	NCP	1.272
KPTTWKN;LTEVGAVG	NCP	55.425
LTEVGAVG;CSDDF	NCP	16.639
CSDDF;YYLSMP	NCP	35.529
YYLSMP;LGDASAW	NCP	25.034
LGDASAW;PAAKPPTP	NCP	67.026
PAAKPPTP;TVAAL	NCP	2.544
TVAAL;KTPQARPP	NCP	101.946
KTPQARPP;VYHMVP	NCP	14.419
VYHMVP;RAPGKGK	NCP	13.827
RAPGKGK;SAALLEY	NCP	185.969
SAALLEY;TVAVAGP	NCP	96.804
TVAVAGP;ATKPLDY	NCP	22.401
ATKPLDY;KKGGHAK	NCP	85.767
KKGGHAK;FTLSKV	NCP	72.724
FTLSKV;LSPPLN	NCP	8.153
LSPPLN;VPTKVLP	NCP	33.393
VPTKVLP;DDDSD	NCP	44.61
DDDSD;KGDWPK	NCP	83.634
KGDWPK;GQGRRTPEP	NCP	16.878
GQGRRTPEP;MPGMFP	NCP	71.139
MPGMFP;PAGPSFP	NCP	7.227
PAGPSFP;GNASHK	NCP	9.738
GNASHK;GVTMR	NCP	91.768
GVTMR;HYNHPK	NCP	106.902
HYNHPK;LKLLDF	NCP	33.174
LKLLDF;MKPHTAP	NCP	114.264
MKPHTAP;VLYKET	NCP	29.439
VLYKET;ARPKHL	NCP	9.453
ARPKHL;APGSLVH	NCP	29.297
APGSLVH;KSPAAM	NCP	32.998
KSPAAM;VAALWE	NCP	134.729
VAALWE;DPVHLPL	NCP	3.093
DPVHLPL;RLMLQH	NCP	249.364
RLMLQH;AKLKHS	NCP	12.023
AKLKHS;LSLHY	NCP	99.144
LSLHY;KHKKDY	NCP	25.699
KHKKDY;LKVLNDL	NCP	95.172
LKVLNDL;KLELLC	NCP	31.893
KLELLC;KDPLEK	NCP	6.183

KDPLEK;VLLDL	NCP	49.299
VLLDL;SLVCGLR	NCP	7.394
SLVCGLR;RYFLSTF	NCP	41.223
RYFLSTF;CTPQFK	NCP	225.829
CTPQFK;VTARKSL	NCP	10.634
VTARKSL;PGSSMK	NCP	31.108
PGSSMK;RMGALP	NCP	23.627
RMGALP;QPSQK	NCP	53.835
QPSQK;LSVPK	NCP	154.227
LSVPK;KTYRVP	NCP	37.72
KTYRVP;GPKEVR	NCP	76.413
GPKEVR;AAMTLY	NCP	13.202
AAMTLY;DLPPHRP	NCP	131.562
DLPPHRP;MNFLYP	NCP	33.226
MNFLYP;LSMAK	NCP	71.354
LSMAK;PSKVDSTP	NCP	122.286
PSKVDSTP;KQPDFLP	NCP	4.62
KQPDFLP;TYSGVTR	NCP	2.256
TYSGVTR;SVYVYK	NCP	11.353
SVYVYK;RKNLYP	NCP	16.737
RKNLYP;FPPVT	NCP	3.363
FPPVT;KTDLRMK	NCP	35.075
KTDLRMK;ERSPPK	NCP	89.424
ERSPPK;PPPLSGNY	NCP	21.807
PPPLSGNY;FSVTSF	NCP	188.673
FSVTSF;KNNPDHP	NCP	84.124
KNNPDHP;PFVTKKDL	NCP	55.053
PFVTKKDL;VPEPVVF	NCP	9.975
VPEPVVF;NQTDGLR	NCP	193.853
NQTDGLR;VGSHSLV	NCP	180.207
VGSHSLV;KERFYVN	NCP	45.478
KERFYVN;KPSVP	NCP	2.702
KPSVP;KPPAPQR	NCP	96.486
KPPAPQR;LDRAQL	NCP	256.941
LDRAQL;EDVGPK	NCP	2.049
EDVGPK;FQKKPP	NCP	86.884
FQKKPP;KSPGPFAK	NCP	2.942
KSPGPFAK;PPVGVH	NCP	25.72
PPVGVH;VHPPG	NCP	45.965
VHPPG;MGNGFTP	NCP	55.918
MGNGFTP;DFHDLGVNTR	NCP	44.333
DFHDLGVNTR;ARLLGGQ	NCP	92.367
ARLLGGQ;LPPAKPP	NCP	19.074
LPPAKPP;APGVKLV	NCP	5.865
APGVKLV;FYPLAW	NCP	4.389
FYPLAW;CRDVTHT	NCP	20.662
CRDVTHT;LLGLAGF	NCP	22.805
LLGLAGF;KGPPDV	NCP	0.888
KGPPDV;TQFLPP	NCP	0.921
TQFLPP;EHLLLQH	NCP	51.609
EHLLLQH;KDDAGK	NCP	49.702

KDDAGK;KVAPGHR	NCP	125.936
KVAPGHR;YPVVS	NCP	50.466
YPVVS;LGVEVHPLVF	NCP	22.719
LGVEVHPLVF;AAQYEHDLEV	NCP	1.773
AAQYEHDLEV;LLSPDLPL	NCP	183.153
LLSPDLPL;TVPLLPR	NCP	53.367
TVPLLPR;LVLPEKF	NCP	115.771
LVLPEKF;KNAELLR	NCP	89.435
KNAELLR;VYALKRQGRTLYG	NCP	7.305
VYALKRQGRTLYG;FVFSKPK	NCP	0.162
FVFSKPK;KTTPFSL	NCP	11.056
KTTPFSL;TARRAF	NCP	172.535
TARRAF;PHLTGQP	NCP	24.738
PHLTGQP;QRTPK	NCP	6.873
QRTPK;KLLAGF	NCP	19.872
KLLAGF;KDVKHR	NCP	89.34
KDVKHR;TVTVPKV	NCP	10.947
TVTVPKV;FAKFC	NCP	17.463
FAKFC;RGPGLQ	NCP	18.069
RGPGLQ;PGGPKGK	NCP	59.727
PGGPKGK;ATNLDLH	NCP	5.173
ATNLDLH;VDNVNPK	NCP	30.072
VDNVNPK;KSAGLML	NCP	15.63
KSAGLML;DFGAPAD	NCP	1.406
DFGAPAD;GPGRPGPP	NCP	50.247
GPGRPGPP;LLLYLVLLL	NCP	56.004
LLLYLVLLL;VSVPGEHA	NCP	1.911
VSVPGEHA;KYLEGAK	NCP	46.783
KYLEGAK;LSVGPVP	NCP	68.096
LSVGPVP;DCCAAP	NCP	62.61
DCCAAP;KPGSTPPP	NCP	120.939
KPGSTPPP;CYRPGLP	NCP	89.325
CYRPGLP;CCCCP	NCP	90.699
CCCCP;GEAVPP	NCP	28.386
GEAVPP;RPALLTH	NCP	41.13
RPALLTH;EQPGPPK	NCP	11.457
EQPGPPK;EHEPALP	NCP	30.939
EHEPALP;LLALGAPL	NCP	8.088
LLALGAPL;KEEVLGFF	NCP	43.69
KEEVLGFF;DPLPPY	NCP	30.087
DPLPPY;ASKALLR	NCP	72.911
ASKALLR;LYRGPF	NCP	137.955
LYRGPF;KVPPFK	NCP	4.995
KVPPFK;GVSLLCEF	NCP	129.339
GVSLLCEF;LTESLTGF	NCP	58.92
LTESLTGF;APLYL	NCP	53.979
APLYL;SDMGL	NCP	5.929
SDMGL;AAPRAK	NCP	46.191
AAPRAK;DKLLP	NCP	21.666
DKLLP;YGCSDC	NCP	62.48
YGCSDC;TVPPGGR	NCP	9.315

TVPPGGR;KDLAEVK	NCP	108.783
KDLAEVK;MLSGKER	NCP	27.084
MLSGKER;CDCVAL	NCP	131.838
CDCVAL;CCMCDC	NCP	10.413
CCMCDC;KNGLLDH	NCP	62.38
KNGLLDH;KEYLPQ	NCP	109.407
KEYLPQ;QEKNAPK	NCP	106.866
QEKNAPK;LESLMT	NCP	13.535
LESLMT;VLYFPL	NCP	20.734
VLYFPL;KHYVSPQ	NCP	86.931
KHYVSPQ;QACYGY	NCP	15.864
QACYGY;DNPPLHK	NCP	14.391
DNPPLHK;AGVYRPQ	NCP	26.51
AGVYRPQ;LDGLL	NCP	53.083
LDGLL;YVTHF	NCP	28.824
YVTHF;KYPPPSP	NCP	109.857
KYPPPSP;LLLPTEF	NCP	7.592
LLLPTEF;LLPLTEF	NCP	82.383
LLPLTEF;SDDTLLP	NCP	53.62
SDDTLLP;TGNLSGNK	NCP	18.959
TGNLSGNK;LDMMPF	NCP	41.73
LDMMPF;SPPELRPP	NCP	124.104
SPPELRPP;KMHLQK	NCP	106.783
KMHLQK;QLVLY	NCP	29.172
QLVLY;GCGTPK	NCP	0.641
GCGTPK;KLGLLKVS	NCP	11.596
KLGLLKVS;HFRRVVL	NCP	51.911
HFRRVVL;ETVLHG	NCP	82.401
ETVLHG;DHLKL	NCP	34.309
DHLKL;PLFVLLP	NCP	48.116
PLFVLLP;HLLVLSL	NCP	82.771
HLLVLSL;CFQFLR	NCP	196.008
CFQFLR;GVVYK	NCP	134.669
GVVYK;PPPYPPPPP	NCP	101.619
PPPYPPPPP;SSLLAAQ	NCP	186.285
SSLLAAQ;KLWYK	NCP	191.332
KLWYK;NGLLL	NCP	133.413
NGLLL;LDLVVP	NCP	44.55
LDLVVP;PPGAW	NCP	44.987
PPGAW;LAKGVVK	NCP	96.33
LAKGVVK;CFNEC	NCP	62.227
CFNEC;LYKDKGK	NCP	35.073
LYKDKGK;KAAFLER	NCP	197.216
KAAFLER;KLLLCCE	NCP	69.477
KLLLCCE;ALKYHVK	NCP	4.51
ALKYHVK;TVREDK	NCP	11.732
TVREDK;YCLPQVH	NCP	0.67
YCLPQVH;LPRLHK	NCP	0.746
LPRLHK;FCMCAG	NCP	11.331
FCMCAG;GELPVQ	NCP	18.643
GELPVQ;PVVDFFP	NCP	48.692

PVVDFFP;GVLPWLS	NCP	90.46
GVLPWLS;KTMVKK	NCP	20.282
KTMVKK;ERPSSKK	NCP	24.201
ERPSSKK;VLDERLS	NCP	12.493
VLDERLS;AQVVAVK	NCP	27.203
AQVVAVK;DRKYRPP	NCP	78.126
DRKYRPP;FGYSNR	NCP	103.026
FGYSNR;PGLPVGT	NCP	12.936
PGLPVGT;GSVDGGP	NCP	40.041
GSVDGGP;KEDSLVHL	NCP	28.062
KEDSLVHL;TADVSVP	NCP	2.19
TADVSVP;KDELDVT	NCP	48.01
KDELDVT;PPPPPAL	NCP	133.251
PPPPPAL;FLLVAGL	NCP	30.017
FLLVAGL;ENGPYF	NCP	9.174
ENGPYF;PTRVAP	NCP	22.641
PTRVAP;KDQKNL	NCP	59.851
KDQKNL;KLEPLTV	NCP	0.86
KLEPLTV;LEEELPL	NCP	128.328
LEEELPL;PLLNV	NCP	8.17
PLLNV;KPERPK	NCP	54.612
KPERPK;AAFNKL	NCP	187.274
AAFNKL;VGDQLSF	NCP	39.222
VGDQLSF;MLPPLLHG	NCP	46.177
MLPPLLHG;VVELTGK	NCP	227.181
VVELTGK;EVKGF	NCP	49.989
EVKGF;EQVTY	NCP	94.649
EQVTY;FELGKKT	NCP	105.367
FELGKKT;KDFKHR	NCP	162.3
KDFKHR;KPLGFF	NCP	90.804
KPLGFF;EYTMY	NCP	261.252
EYTMY;KDKPEK	NCP	71.886
KDKPEK;QTPKQLR	NCP	61.569
QTPKQLR;DLSPLVD	NCP	38.748
DLSPLVD;LCGLVGRL	NCP	19.113
LCGLVGRL;EAKYC	NCP	34.611
EAKYC;PLKLLAVA	NCP	68.064
PLKLLAVA;TAQVLL	NCP	67.103
TAQVLL;KYVPPKY	NCP	18.568
KYVPPKY;TVYHF	NCP	16.538
TVYHF;SLKHC	NCP	71.224
SLKHC;YGVSFF	NCP	14
YGVSFF;KYPKMPT	NCP	11.83
KYPKMPT;FDSVPTVK	NCP	76.38
FDSVPTVK;FFKPLPP	NCP	18.39
FFKPLPP;SLGLL	NCP	63.222
SLGLL;QLLTANR	NCP	270.945
QLLTANR;HEVKK	NCP	53.396
HEVKK;KMLFLQLF	NCP	80.505
KMLFLQLF;KSSFK	NCP	0.267
KSSFK;WDMQWH	NCP	7.453

WDMQWH;DHWHEL	NCP	33.906
DHWHEL;LTPPFDK	NCP	108.663
LTPPFDK;NGQAFRK	NCP	11.327
NGQAFRK;PAFLLL	NCP	57.97
PAFLLL;MHSSVLK	NCP	6.035
MHSSVLK;VKVTL	NCP	101.323
VKVTL;EHTLLL	NCP	141.761
EHTLLL;VGDLGL	NCP	15.057
VGDLGL;EPAFYLL	NCP	35.728
EPAFYLL;VGDPTLV	NCP	11.987
VGDPTLV;KTAEVK	NCP	19.651
KTAEVK;RLMKL	NCP	89.549
RLMKL;AKLLAQDL	NCP	14.203
AKLLAQDL;AVPGPPS	NCP	98.25
AVPGPPS;KFALRP	NCP	19.376
KFALRP;DVTFMAP	NCP	185.826
DVTFMAP;VYYKVL	NCP	28.08
VYYKVL;KLRLFR	NCP	65.073
KLRLFR;GEVGL	NCP	99.436
GEVGL;QVYLK	NCP	40.779
QVYLK;RSLFQK	NCP	38.561
RSLFQK;KDSGKVAY	NCP	30.849
KDSGKVAY;EEELA	NCP	26.959
EEELA;AAHKKLF	NCP	76.589
AAHKKLF;KAVVSQK	NCP	0.219
KAVVSQK;LTLGLR	NCP	116.581
LTLGLR;NFPLL	NCP	95.288
NFPLL;LSEETLH	NCP	8.157
LSEETLH;KYLAPCQVP	NCP	101.742
KYLAPCQVP;APASPLLL	NCP	30.258
APASPLLL;RGALSPPP	NCP	35.904
RGALSPPP;LHVLML	NCP	76.782
LHVLML;RVFNY	NCP	24.927
RVFNY;KKYQNKP	NCP	98.542
KKYQNKP;GVYPLVY	NCP	42.665
GVYPLVY;KETLKGF	NCP	165.931
KETLKGF;KDALLA	NCP	11.648
KDALLA;FNTYSPK	NCP	24.13
FNTYSPK;MQSLLL	NCP	72.294
MQSLLL;VVQPELK	NCP	122.96
VVQPELK;YCCMYR	NCP	71.227
YCCMYR;VSKKTPT	NCP	51.341
VSKKTPT;NPVDRGPP	NCP	70.599
NPVDRGPP;PFYVPP	NCP	31.545
PFYVPP;SLTAVFVVY	NCP	144.078
SLTAVFVVY;LPTVKDF	NCP	20.706
LPTVKDF;SSVPTVLK	NCP	1.197
SSVPTVLK;SFLEGK	NCP	7.287
SFLEGK;KKTTAQK	NCP	15.832
KKTTAQK;LSPVFR	NCP	252.852
LSPVFR;PPTRLYR	NCP	250.34

PPTRLYR;VHVVATK	NCP	150.147
VHVVATK;VALQA	NCP	131.007
VALQA;KGLQVVA	NCP	13.923
KGLQVVA;DGLLDF	NCP	12.898
DGLLDF;PPLPLP	NCP	20.634
PPLPLP;YECQY	NCP	5.369
YECQY;EHGFKVE	NCP	1.644
EHGFKVE;DGSLMFQ	NCP	164.737
DGSLMFQ;AGPKYTL	NCP	40.226
AGPKYTL;ANQLLL	NCP	177.544
ANQLLL;KKLAMY	NCP	18.641
KKLAMY;LEQLP	NCP	9.204
LEQLP;TTPPKKK	NCP	25.366
TTPPKKK;VGDKVSPK	NCP	21.615
VGDKVSPK;TLYPFR	NCP	60.5
TLYPFR;KKYPPR	NCP	197.404
KKYPPR;KAPMGPK	NCP	22.964
KAPMGPK;KQMHFLK	NCP	246.928
KQMHFLK;LAGPKNP	NCP	25.965
LAGPKNP;FPAGKAH	NCP	35.589
FPAGKAH;VFLKLSSC	NCP	46.934
VFLKLSSC;LTKKMPF	NCP	40.363
LTKKMPF;KGLQVV	NCP	1.166
KGLQVV;LLLVKLLLLLL	NCP	79.339
LLLVKLLLLLL;YSSMSK	NCP	27.744
YSSMSK;KNARAGK	NCP	143.862
KNARAGK;YSAAAPK	NCP	7.677
YSAAAPK;LSECVTP	NCP	11.765
LSECVTP;RLTLNAP	NCP	136.447
RLTLNAP;KGMGSKP	NCP	1.736
KGMGSKP;AVTMGSK	NCP	47.248
AVTMGSK;EQLTPL	NCP	88.308
EQLTPL;TPGAP	NCP	49.029
TPGAP;VSVQP	NCP	15.927
VSVQP;VYHPSF	NCP	74.432
VYHPSF;THKNAPL	NCP	4.02
THKNAPL;VGLTTL	NCP	87.987
VGLTTL;KLLKT	NCP	67.422
KLLKT;DLFKLSKL	NCP	77.556
DLFKLSKL;DMAPP	NCP	18.162
DMAPP;KMGYLPP	NCP	11.47
KMGYLPP;LLSSYELP	NCP	13.611
LLSSYELP;KGCEPVK	NCP	39.387
KGCEPVK;LAVMSV	NCP	19.098
LAVMSV;TTPVVSGK	NCP	11.577
TTPVVSGK;VVFRQ	NCP	95.468
VVFRQ;VLSLYA	NCP	4.65
VLSLYA;PFPFG	NCP	120.061
PFPFG;KMRSLPP	NCP	6.623
KMRSLPP;KCTVAAP	NCP	8.085
KCTVAAP;SLDHFTW	NCP	194.287

SLDHFTW;AKLLPQ	NCP	69.116
AKLLPQ;HYAPKK	NCP	38.775
HYAPKK;VLGRWEE	NCP	11.958
VLGRWEE;PTVSVLH	NCP	36.07
PTVSVLH;VGFVGF	NCP	70.724
VGFVGF;VLLDNL	NCP	33.841
VLLDNL;EELMMM	NCP	63.801
EELMMM;NYLEF	NCP	29.634
NYLEF;MTVTLKLV	NCP	6.987
MTVTLKLV;VLWLPK	NCP	26.985
VLWLPK;VPAAK	NCP	32.93
VPAAK;QEGFNVK	NCP	58.741
QEGFNVK;KHVFRP	NCP	164.523
KHVFRP;PHKKPFQ	NCP	35.85
PHKKPFQ;TAPAMY	NCP	316.392
TAPAMY;DPHHK	NCP	52.196
DPHHK;KLYPPT	NCP	4.449
KLYPPT;NHEPPRP	NCP	16.404
NHEPPRP;NRTLLQ	NCP	33.049
NRTLLQ;PGATPVRP	NCP	38.043
PGATPVRP;KRGVTSK	NCP	23.717
KRGVTSK;FVLLNL	NCP	148.297
FVLLNL;AFKDKYKQLF	NCP	106.115
AFKDKYKQLF;KDEDLAQ	NCP	59.944
KDEDLAQ;LYRVR	NCP	12.266
LYRVR;VPWGPVV	NCP	35.656
VPWGPVV;NKKTLK	NCP	177.317
NKKTLK;LWSPMT	NCP	8.032
LWSPMT;QMLRLQK	NCP	59.633
QMLRLQK;VDSSLPR	NCP	122.248
VDSSLPR;LERLY	NCP	60.159
LERLY;KAWVKP	NCP	9.57
KAWVKP;AAPRKHV	NCP	40.043
AAPRKHV;LPHLLALP	NCP	159.678
LPHLLALP;FPHKTVP	NCP	31.447
FPHKTVP;FDAMRF	NCP	356.571
FDAMRF;VPPGVH	NCP	69.996
VPPGVH;VLAELY	NCP	46.319
VLAELY;KFTPAVR	NCP	12.001
KFTPAVR;MAAKPK	NCP	26.787
MAAKPK;LPPLPHL	NCP	71.469
LPPLPHL;WGAPK	NCP	81.629
WGAPK;AGRLPACVVDCGTGYTK	NCP	39.678
AGRLPACVVDCGTGYTK;YFLLTEPPLNTPENREY	NCP	0.315
YFLLTEPPLNTPENREY;DVGYERF	NCP	0.48
DVGYERF;LSPLDL	NCP	8.368
LSPLDL;PPPPDLK	NCP	13.065
PPPPDLK;MNVKLPQ	NCP	11.702
MNVKLPQ;PLCCGY	NCP	6.424
PLCCGY;VLLPK	NCP	188.153
VLLPK;KMVTSAP	NCP	8.484

KMVTSAP;PKWPALK	NCP	17.712
PKWPALK;GNKMTPQ	NCP	32.401
GNKMTPQ;FDLSEGK	NCP	99.399
FDLSEGK;APAEKY	NCP	89.991
APAEKY;KPRPTPP	NCP	223.617
KPRPTPP;SLFLLFAFLL	NCP	13.917
SLFLLFAFLL;TGPAYQR	NCP	6.48
TGPAYQR;EPDRLGAP	NCP	13.353
EPDRLGAP;LVAQTLLT	NCP	29.703
LVAQTLLT;YADHLKK	NCP	29.558
YADHLKK;VGLLGPLY	NCP	32.944
VGLLGPLY;LLSAW	NCP	10.59
LLSAW;VGLEQPL	NCP	10.152
VGLEQPL;CDDDSG	NCP	16.554
CDDDSG;TDALPL	NCP	34.622
TDALPL;VGLTGLA	NCP	4.9
VGLTGLA;GPPFN	NCP	1.539
GPPFN;APPTKAK	NCP	66.843
APPTKAK;TLGTVP	NCP	35.103
TLGTVP;KLGLRP	NCP	31.962
KLGLRP;LPPGVPD	NCP	8.931
LPPGVPD;CPAGPP	NCP	20.576
CPAGPP;LGPYPP	NCP	89.757
LGPYPP;EVLDSKP	NCP	38.653
EVLDSKP;VAAGLLS	NCP	12.464
VAAGLLS;PVFQNKP	NCP	160.042
PVFQNKP;LPNDN	NCP	65.531
LPNDN;MTHARP	NCP	11.509
MTHARP;AHVTTLT	NCP	17.403
AHVTTLT;AVWRQP	NCP	35.94
AVWRQP;QPPTVVP	NCP	78.057
QPPTVVP;TLDVVR	NCP	21.858
TLDVVR;FSKLLETN	NCP	9.132
FSKLLETN;AAKGVVD	NCP	52.95
AAKGVVD;EVALRTK	NCP	25.808
EVALRTK;QLTGLK	NCP	21.999
QLTGLK;LDYEVK	NCP	51.259
LDYEVK;TPVVTSK	NCP	57.708
TPVVTSK;TVHVP	NCP	6.209
TVHVP;QPGCPVGR	NCP	3.178
QPGCPVGR;LTLNAF	NCP	18.189
LTLNAF;KMAEVPP	NCP	1.865
KMAEVPP;APPGRPP	NCP	39.538
APPGRPP;EMSKAP	NCP	153.882
EMSKAP;GTLLAGP	NCP	12.708
GTLLAGP;TGAPVL	NCP	0.822
TGAPVL;ANLVFQ	NCP	85.838
ANLVFQ;PPPPHVP	NCP	11.251
PPPPHVP;PTVLVEE	NCP	0.129
PTVLVEE;VSPLLL	NCP	10.671
VSPLLL;DQAPFD	NCP	77.222

DQAPFD;TVGWPQ	NCP	213.814
TVGWPQ;NYKPAK	NCP	9.032
NYKPAK;LSAFASPK	NCP	170.439
LSAFASPK;TTVFANK	NCP	16.873
TTVFANK;VGGELKP	NCP	45.537
VGGELKP;LPQRFSP	NCP	69.642
LPQRFSP;AALRVF	NCP	48.402
AALRVF;DSLPFEL	NCP	134.898
DSLPFEL;APPKFAK	NCP	15.609
APPKFAK;LLMAPAEK	NCP	8.574
LLMAPAEK;TSLQKLAL	NCP	9.192
TSLQKLAL;PLRVTH	NCP	27.711
PLRVTH;KGPPLTF	NCP	4.947
KGPPLTF;PFLDL	NCP	31.262
PFLDL;NSVPH	NCP	22.446
NSVPH;RNSPNLK	NCP	42.301
RNSPNLK;VYSRPK	NCP	81.156
VYSRPK;TKHFEL	NCP	35.217
TKHFEL;SDFDVQK	NCP	16.842
SDFDVQK;LPPFP	NCP	140.087
LPPFP;RVADVLLL	NCP	9.97
RVADVLLL;DDKLLK	NCP	11.286
DDKLLK;VPPLPEK	NCP	8.166
VPPLPEK;VSLDPFL	NCP	28.604
VSLDPFL;ARVSGPK	NCP	5.184
ARVSGPK;LSVFSLDQL	NCP	66.19
LSVFSLDQL;VAQVPQ	NCP	36.083
VAQVPQ;VPKVPGK	NCP	6.295
VPKVPGK;KHLARP	NCP	198.294
KHLARP;SGLTVTRR	NCP	79.604
SGLTVTRR;ALSELHC	NCP	356.842
ALSELHC;KDNQMPH	NCP	58.464
KDNQMPH;LPLHPK	NCP	37.313
LPLHPK;VAAPP	NCP	154.665
VAAPP;DLRKL	NCP	20.073
DLRKL;RLDLAGR	NCP	32.404
RLDLAGR;FTKLTSG	NCP	45.218
FTKLTSG;KPCVAK	NCP	10.581
KPCVAK;VDVDSLP	NCP	4.885
VDVDSLP;AAPRYQ	NCP	4.046
AAPRYQ;MKEPSLPL	NCP	83.965
MKEPSLPL;KKLLAVP	NCP	29.168
KKLLAVP;SFYPHK	NCP	56.388
SFYPHK;KGRDVGF	NCP	108.463
KGRDVGF;LTGDWKS	NCP	6.135
LTGDWKS;LYPSLR	NCP	50.195
LYPSLR;ENVPKRH	NCP	268.002
ENVPKRH;VKLGPLY	NCP	91.564
VKLGPLY;GHLAPPP	NCP	170.811
GHLAPPP;TAGKVMLK	NCP	2.126
TAGKVMLK;EPVSGPK	NCP	169.959

EPVSGPK;FPKSDF	NCP	18.859
FPKSDF;VPLLLLPT	NCP	139.565
VPLLLLPT;PLLPNF	NCP	79.971
PLLPNF;SKPAADR	NCP	113.196
SKPAADR;DVSKPP	NCP	184.687
DVSKPP;NPGHPF	NCP	95.055
NPGHPF;YLAAVKLY	NCP	31.676
YLAAVKLY;TNYVKD	NCP	19.321
TNYVKD;MAVAVP	NCP	132.036
MAVAVP;MLGAVP	NCP	4.491
MLGAVP;DVPLDFP	NCP	64.365
DVPLDFP;VTVGVPH	NCP	49.065
VTVGVPH;SVPSGVLP	NCP	23.211
SVPSGVLP;KAFWPFQ	NCP	118.806
KAFWPFQ;LSPSVKL	NCP	202.499
LSPSVKL;DSWGLLF	NCP	60.766
DSWGLLF;KESYPQR	NCP	69.959
KESYPQR;LLQQPKEP	NCP	11.101
LLQQPKEP;VDFKVDH	NCP	4.437
VDFKVDH;GSLPQEL	NCP	66.138
GSLPQEL;KEFGP	NCP	52.214
KEFGP;SALRLGE	NCP	59.494
SALRLGE;NPPNPLPP	NCP	100.452
NPPNPLPP;NHMKLGK	NCP	38.507
NHMKLGK;ESLPVGR	NCP	21.85
ESLPVGR;WALKPTW	NCP	0.321
WALKPTW;PVTLSASVAP	NCP	30.603
PVTLSASVAP;LVALANQ	NCP	10.094
LVALANQ;FPGGV	NCP	0.478
FPGGV;KVRAVFF	NCP	19.664
KVRAVFF;PSPGLP	NCP	45.58
PSPGLP;LLSGTSYK	NCP	27.086
LLSGTSYK;KNHTPPT	NCP	71.158
KNHTPPT;FGPGPGS	NCP	19.542
FGPGPGS;VGNVLPF	NCP	9.576
VGNVLPF;PVSSSPF	NCP	51.732
PVSSSPF;QTNLYLP	NCP	29.313
QTNLYLP;PLPRANY	NCP	5.255
PLPRANY;AAPLLTH	NCP	59.467
AAPLLTH;FLFAFC	NCP	12.734
FLFAFC;VTHVPPP	NCP	13.039
VTHVPPP;TNDMLRF	NCP	84.678
TNDMLRF;SDQEKRKQ	NCP	13.302
SDQEKRKQ;LLAYALL	NCP	47.555
LLAYALL;KDQPPS	NCP	108.199
KDQPPS;KRVKLGGK	NCP	60.281
KRVKLGGK;LLRLTK	NCP	18.637
LLRLTK;TVGFYPK	NCP	6.933
TVGFYPK;LVRDLK	NCP	39.093
LVRDLK;ALPPPK	NCP	17.592
ALPPPK;MVPRLSP	NCP	36.573

MVPRLSP;YNYLT	NCP	4.259
YNYLT;QEHVTK	NCP	51.009
QEHVTK;LSLEELTL	NCP	36.22
LSLEELTL;LGVHVVP	NCP	146.939
LGVHVVP;PPTLPMK	NCP	1.489
PPTLPMK;PPPPPAPAGR	NCP	4.797
PPPPPAPAGR;CSSVTPP	NCP	31.221
CSSVTPP;AGGGVLPH	NCP	49.632
AGGGVLPH;QFPAPQP	NCP	2.931
QFPAPQP;PDVKNTR	NCP	5.172
PDVKNTR;KSQHAVP	NCP	32.13
KSQHAVP;GDCVAL	NCP	13.731
GDCVAL;QPLPPAP	NCP	21.924
QPLPPAP;LDVARA	NCP	64.668
LDVARA;AGGVSEARF	NCP	21
AGGVSEARF;DGPKLVER	NCP	7.875
DGPKLVER;VGVSPTK	NCP	19.761
VGVSPTK;VPPDKPP	NCP	22.512
VPPDKPP;GVEPLK	NCP	51.237
GVEPLK;MCSSMPP	NCP	22.44
MCSSMPP;VDETLFK	NCP	34.536
VDETLFK;VAPPCK	NCP	7.497
VAPPCK;TVEALL	NCP	39.737
TVEALL;DGVVPKD	NCP	221.845
DGVVPKD;MEPTML	NCP	11.7
MEPTML;TVPLYE	NCP	161.409
TVPLYE;PPLRPLPL	NCP	33.114
PPLRPLPL;KCYPPAAP	NCP	3.444
KCYPPAAP;KQRTAE	NCP	3.192
KQRTAE;LPGGQPP	NCP	6.93
LPGGQPP;APGLLTLP	NCP	45.171
APGLLTLP;VGRLLDH	NCP	18.147
VGRLLDH;ELSDKPLR	NCP	17.759
ELSDKPLR;LNVPF	NCP	90.633
LNVPF;YTPVAPK	NCP	40.861
YTPVAPK;KYLPGF	NCP	15.593
KYLPGF;YHYHRP	NCP	3.907
YHYHRP;FSLLEVT	NCP	45.284
FSLLEVT;DPAAAM	NCP	5.245
DPAAAM;RGGDCCT	NCP	1.089
RGGDCCT;LAGVNLF	NCP	54.039
LAGVNLF;LLKDSFK	NCP	58.269
LLKDSFK;APRPKFS	NCP	115.76
APRPKFS;VTVTLL	NCP	47.751
VTVTLL;KDLDEVR	NCP	10.956
KDLDEVR;DPTSLSPK	NCP	19.662
DPTSLSPK;KDRPGAK	NCP	62.971
KDRPGAK;NTKPQPY	NCP	70.757
NTKPQPY;DVTLP	NCP	47.502
DVTLP;HLLEDYEMAAK	NCP	39.315
HLLEDYEMAAK;EEHVPLL	NCP	107.362

EEHVPLL;EFSPK	NCP	22.964
EFSPK;KTCVTKLF	NCP	29.691
KTCVTKLF;MEYYLH	NCP	37.233
MEYYLH;ERGFDLL	NCP	39.484
ERGFDLL;CGLKKAP	NCP	6.923
CGLKKAP;RQGPLEK	NCP	95.079
RQGPLEK;NLELLMA	NCP	21.91
NLELLMA;TLVVPGK	NCP	0.182
TLVVPGK;AAKSHVH	NCP	18.441
AAKSHVH;TLFMTM	NCP	82.233
TLFMTM;KSELTL	NCP	47.4
KSELTL;EPKGTPK	NCP	122.343
EPKGTPK;KELKC	NCP	9.711
KELKC;KPQRLDS	NCP	13.15
KPQRLDS;WLFPKPP	NCP	9.582
WLFPKPP;KFFLPP	NCP	13.188
KFFLPP;PKETPDKP	NCP	17.643
PKETPDKP;LPATLPGP	NCP	18.087
LPATLPGP;TPPNFPPP	NCP	8.957
TPPNFPPP;QALVTRF	NCP	16.827
QALVTRF;MFSWLKR	NCP	114.403
MFSWLKR;VPPPPAPL	NCP	51.555
VPPPPAPL;APVTTTPK	NCP	33.224
APVTTTPK;TKHTCCE	NCP	7.77
TKHTCCE;KLVPEDK	NCP	6.108
KLVPEDK;PPFTP	NCP	9.733
PPFTP;AVTTVAKY	NCP	37.029
AVTTVAKY;LPSRPLP	NCP	56.958
LPSRPLP;VGHLPPPP	NCP	4.722
VGHLPPPP;GNLGGGLG	NCP	95.334
GNLGGGLG;LGKSLDVP	NCP	33.674
LGKSLDVP;KDLSFGV	NCP	8.463
KDLSFGV;AETPKLP	NCP	65.049
AETPKLP;SPFLVAP	NCP	71.062
SPFLVAP;KSLWE	NCP	176.021
KSLWE;LKLSLK	NCP	76.279
LKLSLK;AQLNVKS	NCP	36.386
AQLNVKS;LSTPELPP	NCP	22.281
LSTPELPP;HPLPAGP	NCP	6.003
HPLPAGP;EVADLLL	NCP	84.373
EVADLLL;LLAKTYSK	NCP	40.19
LLAKTYSK;KPAHCKK	NCP	1.38
KPAHCKK;KLKYL	NCP	108.145
KLKYL;NFAELL	NCP	32.279
NFAELL;LSLNTKPK	NCP	20.782
LSLNTKPK;PNLPSKET	NCP	65.948
PNLPSKET;MMPPLR	NCP	61.368
MMPPLR;PAGHGPP	NCP	14.464
PAGHGPP;VGTGLAA	NCP	45.807
VGTGLAA;MAVESFLK	NCP	15.789
MAVESFLK;KALHLPK	NCP	62.946

KALHLPK;VCLLGCG	NCP	26.064
VCLLGCG;LSAKPL	NCP	39.131
LSAKPL;PEMKPN	NCP	139.42
PEMKPN;DLEMTK	NCP	20.661
DLEMTK;RLDDLLK	NCP	25.632
RLDDLLK;VTGKSPY	NCP	20.088
VTGKSPY;KPVYLPP	NCP	26.918
KPVYLPP;PVTPLK	NCP	21.52
PVTPLK;VLLEQPL	NCP	26.172
VLLEQPL;LSPPNYT	NCP	10.257
LSPPNYT;MFFPGAP	NCP	16.643
MFFPGAP;LELNQVK	NCP	13.497
LELNQVK;HHHCD	NCP	87.349
HHHCD;MLQSLLK	NCP	84.471
MLQSLLK;PANHK	NCP	143.993
PANHK;GPAKSRP	NCP	56.389
GPAKSRP;SKLHYNK	NCP	0.374
SKLHYNK;KHFSVVL	NCP	7.267
KHFSVVL;TATLRMLL	NCP	36.206
TATLRMLL;KLPPTP	NCP	16.665
KLPPTP;WAVLDLK	NCP	54.355
WAVLDLK;QGVVLVT	NCP	41.702
QGVVLVT;KPGAEV	NCP	31.227
KPGAEV;KVPLT	NCP	62.113
KVPLT;LPHAPYR	NCP	0.831
LPHAPYR;EPPLPQPP	NCP	10.38
EPPLPQPP;CNVPKK	NCP	31.28
CNVPKK;PPPPGKGQ	NCP	3.967
PPPPGKGQ;KLLAFC	NCP	172.181
KLLAFC;YKPVPP	NCP	61.558
YKPVPP;QDSSGY	NCP	26.315
QDSSGY;KLTDYVPKA	NCP	21.075
KLTDYVPKA;SALSTF	NCP	24.75
SALSTF;EVLDL	NCP	24.22
EVLDL;DATLSVH	NCP	29.946
DATLSVH;FPLDV	NCP	6.414
FPLDV;VGPALPLT	NCP	38.373
VGPALPLT;LGYGPGD	NCP	13.794
LGYGPGD;PAFHVLP	NCP	34.977
PAFHVLP;HSSLLVTL	NCP	0.41
HSSLLVTL;KPTCK	NCP	216.052
KPTCK;KFFVGV	NCP	2.084
KFFVGV;TGAPVPG	NCP	133.365
TGAPVPG;DLLKP	NCP	32.392
DLLKP;DNAKSLFL	NCP	1.019
DNAKSLFL;MAELEFK	NCP	11.611
MAELEFK;LPAGY	NCP	131.007
LPAGY;CCQPF	NCP	2.117
CCQPF;RGGVGYSR	NCP	21.543
RGGVGYSR;KPGPLAAQP	NCP	88.993
KPGPLAAQP;PAAPTTF	NCP	4.83

PAAPTTF;APPPPPKY	NCP	69.126
APPPPPKY;YPLEAH	NCP	121.155
YPLEAH;LSAGKCL	NCP	59.511
LSAGKCL;QPPAAKK	NCP	33.176
QPPAAKK;KDPKESF	NCP	40.563
KDPKESF;LDLFPVL	NCP	86.311
LDLFPVL;LVGSVQFY	NCP	9.432
LVGSVQFY;VTLFA	NCP	65.448
VTLFA;KAAAAPGK	NCP	71.574
KAAAAPGK;DVRCR	NCP	1.914
DVRCR;PPPKKPQ	NCP	16.128
PPPKKPQ;RPRQLLS	NCP	3.048
RPRQLLS;KHHDLR	NCP	67.232
KHHDLR;LTTTAAP	NCP	21.286
LTTTAAP;VEKPSLP	NCP	56.375
VEKPSLP;KETVSGP	NCP	17.58
KETVSGP;TYSVTVP	NCP	65.449
TYSVTVP;KSPAAP	NCP	61.041
KSPAAP;TALHLT	NCP	38.255
TALHLT;KEMYVP	NCP	69.999
KEMYVP;SSVHGVPP	NCP	26.14
SSVHGVPP;AFSSRF	NCP	67.634
AFSSRF;AAFGQP	NCP	17.772
AAFGQP;ALSGALKK	NCP	0.306
ALSGALKK;GPPRLLK	NCP	9.439
GPPRLLK;VSNSHF	NCP	52.68
VSNSHF;EPQALGR	NCP	64.983
EPQALGR;LAALKA	NCP	16.893
LAALKA;ALVKKLK	NCP	236.702
ALVKKLK;KTALEL	NCP	3.979
KTALEL;VLVAGPP	NCP	21.656
VLVAGPP;TGKMAPPP	NCP	43.953
TGKMAPPP;LEEVALSR	NCP	47.803
LEEVALSR;LSGPPGAGK	NCP	8.702
LSGPPGAGK;APALLLF	NCP	6.166
APALLLF;MKVEL	NCP	25.415
MKVEL;QFGLVF	NCP	1.317
QFGLVF;SPVFPQR	NCP	7.438
SPVFPQR;KSTMPP	NCP	65.18
KSTMPP;NFKRSP	NCP	29.145
NFKRSP;QPGAP	NCP	22.816
QPGAP;FKDEPL	NCP	8.253
FKDEPL;LVAPMT	NCP	22.698
LVAPMT;SCDQQAS	NCP	37.391
SCDQQAS;VPNGFQ	NCP	31.594
VPNGFQ;PVNGKDF	NCP	8.636
PVNGKDF;GPPVFNL	NCP	19.98
GPPVFNL;PVPVFLR	NCP	29.344
PVPVFLR;QPAPPPP	NCP	51.747
QPAPPPP;KQMCKP	NCP	204.554
KQMCKP;RCLVCKK	NCP	35.068

RCLVCKK;NRQGPP	NCP	87.585
NRQGPP;SGHLPLA	NCP	55.698
SGHLPLA;ANVDPK	NCP	100.98
ANVDPK;ALPPPP	NCP	0.324
ALPPPP;PLFLLL	NCP	27.234
PLFLLL;LPAAPLDF	NCP	23.847
LPAAPLDF;VLFQGL	NCP	32.199
VLFQGL;DSPPP	NCP	21.882
DSPPP;GVPPRRK	NCP	9.789
GVPPRRK;FALKK	NCP	3.452
FALKK;LPPTRSEP	NCP	4.327
LPPTRSEP;SRGPP	NCP	0.684
SRGPP;HQKPPPP	NCP	19.073
HQKPPPP;LPPLD	NCP	24.736
LPPLD;PLGPLPGF	NCP	131.424
PLGPLPGF;TSAPLNP	NCP	84.765
TSAPLNP;KGSPLPLK	NCP	77.766
KGSPLPLK;ESPLVF	NCP	36.386
ESPLVF;LKLLSTVK	NCP	2.364
LKLLSTVK;HHKEVR	NCP	6.591
HHKEVR;CPLRDEK	NCP	12.999
CPLRDEK;SVVHPPR	NCP	10.332
SVVHPPR;PTVGF	NCP	115.614
PTVGF;YKGPPPP	NCP	5.776
YKGPPPP;NADDKLR	NCP	19.338
NADDKLR;KQKTMT	NCP	106.508
KQKTMT;SGSFDSLP	NCP	46.587
SGSFDSLP;TVFPPPPP	NCP	25.632
TVFPPPPP;MTSST	NCP	31.563
MTSST;TSMLPTP	NCP	22.444
TSMLPTP;GVGLLPT	NCP	14.346
GVGLLPT;LPTEKPP	NCP	0.012
LPTEKPP;MNTEL	NCP	27.995
MNTEL;KDTAELSPL	NCP	59.475
KDTAELSPL;LLKPFK	NCP	106.032
LLKPFK;GVLTERG	NCP	42.511
GVLTERG;AFASPPPPP	NCP	48.795
AFASPPPPP;LPGGL	NCP	58.085
LPGGL;SFPTNR	NCP	27.678
SFPTNR;CMGGH	NCP	73.395
CMGGH;CMAELEK	NCP	89.481
CMAELEK;GCENNM	NCP	148.392
GCENNM;VSRTTNTY	NCP	57.579
VSRTTNTY;PRHPPPDP	NCP	1.549
PRHPPPDP;LAPGPALPH	NCP	5.577
LAPGPALPH;LTLELNN	NCP	37.173
LTLELNN;KAHAPQR	NCP	0.618
KAHAPQR;RGRPPPR	NCP	7.47
RGRPPPR;GKPTKET	NCP	84.765
GKPTKET;PLKRAGY	NCP	6.15
PLKRAGY;KAHRGGFE	NCP	2.1

KAHRGGFE;LSDLL	NCP	58.493
LSDLL;QAPPVT	NCP	36.61
QAPPVT;MELLLGK	NCP	26.283
MELLLGK;VNLLY	NCP	127.868
VNLLY;ASRALNR	NCP	18.394
ASRALNR;VGLFF	NCP	13.038
VGLFF;EVPASYK	NCP	74.561
EVPASYK;RETSGEKK	NCP	33.604
RETSGEKK;DYLPY	NCP	21.414
DYLPY;KTGLELLK	NCP	283.215
KTGLELLK;GKPPPP	NCP	28.041
GKPPPP;KKHGGVP	NCP	15.033
KKHGGVP;PAVSRPHT	NCP	31.656
PAVSRPHT;DANRNKQ	NCP	33.255
DANRNKQ;SLPAGW	NCP	67.059
SLPAGW;TSVGP	NCP	8.946
TSVGP;DNSPP	NCP	43.437
DNSPP;APHFWFT	NCP	40.799
APHFWFT;FGGFVTF	NCP	96.667
FGGFVTF;EDLGFKG	NCP	108.564
EDLGFKG;KTGTAGP	NCP	7.5
KTGTAGP;AHSRHSVDG	NCP	8.652
AHSRHSVDG;MRELVH	NCP	54.534
MRELVH;NMLLQ	NCP	60.963
NMLLQ;NAHLPEK	NCP	14.999
NAHLPEK;ARVST	NCP	0.154
ARVST;VPRDHS	NCP	17.121
VPRDHS;LSGKSLLF	NCP	44.799
LSGKSLLF;DRTPLP	NCP	87.935
DRTPLP;GFFPFVL	NCP	54.454
GFFPFVL;LDWAPPP	NCP	16.017
LDWAPPP;ACCEC	NCP	13.844
ACCEC;SNAPVLL	NCP	34.278
SNAPVLL;DRVVNL	NCP	26.029
DRVVNL;HKAPPPPPP	NCP	104.694
HKAPPPPPP;LGDGGVL	NCP	43.134
LGDGGVL;EKAVSR	NCP	50.358
EKAVSR;VSLGL	NCP	13.535
VSLGL;LSSHKMF	NCP	6.705
LSSHKMF;KPAKHP	NCP	22.422
KPAKHP;LPSPSLWY	NCP	14.13
LPSPSLWY;TVVLLVK	NCP	2.304
TVVLLVK;SLWWEELD	NCP	53.407
SLWWEELD;TAGKDKK	NCP	9.941
TAGKDKK;LPPLAFAL	NCP	7.399
LPPLAFAL;PPPAPL	NCP	14.685
PPPAPL;YLGWTKF	NCP	52.124
YLGWTKF;LSVVGSR	NCP	27.118
LSVVGSR;LSTHRAPP	NCP	25.569
LSTHRAPP;FHDLLR	NCP	130.437
FHDLLR;CMAPSC	NCP	28.796

CMAPSC;PGQLK	NCP	68.316
PGQLK;SHVGKPP	NCP	42.286
SHVGKPP;DGNFAVVK	NCP	29.552
DGNFAVVK;TTGPTLP	NCP	56.829
TTGPTLP;KLSGPTLP	NCP	7.627
KLSGPTLP;VGAGLLQTK	NCP	18.756
VGAGLLQTK;NAVVLLS	NCP	30.675
NAVVLLS;SLLWG	NCP	6.399
SLLWG;MRPPPHP	NCP	5.535
MRPPPHP;KKGAPAP	NCP	6.288
KKGAPAP;CPRMMT	NCP	29.208
CPRMMT;LPPPKPP	NCP	19.365
LPPPKPP;LPVLPLP	NCP	39.651
LPVLPLP;LPKPRPP	NCP	81.087
LPKPRPP;KSAPAVR	NCP	23.222
KSAPAVR;SLLRVVSS	NCP	97.54
SLLRVVSS;GGPLVLF	NCP	16.824
GGPLVLF;KHKSKLK	NCP	15.14
KHKSKLK;FNRTPLP	NCP	9.198
FNRTPLP;GASGLTPF	NCP	13.438
GASGLTPF;LPVDGSF	NCP	51.225
LPVDGSF;KGFLDLF	NCP	14.187
KGFLDLF;AAHEKR	NCP	1.484
AAHEKR;AKGDAPP	NCP	27.259
AKGDAPP;LGEFVQ	NCP	7.869
LGEFVQ;PPPKGQK	NCP	69.342
PPPKGQK;LPMAFAL	NCP	17.727
LPMAFAL;ARLLNYL	NCP	7.322
ARLLNYL;ALLTWDL	NCP	14.841
ALLTWDL;GTLEKF	NCP	10.876
GTLEKF;KHRGCLK	NCP	64.56
KHRGCLK;KMMLQQ	NCP	114.626
KMMLQQ;VDVKTTDGY	NCP	8.766
VDVKTTDGY;LMELHGEGSSSGKATGDETGAK	NCP	1.038
LMELHGEGSSSGKATGDETGAK;KSLGRDP	NCP	40.39
KSLGRDP;NGFLNGN	NCP	26.354
NGFLNGN;SLTACH	NCP	13.869
SLTACH;VRFPSL	NCP	17.134
VRFPSL;VQPAMGT	NCP	140.612
VQPAMGT;GVPAP	NCP	10.351
GVPAP;AGRALPR	NCP	52.109
AGRALPR;LLTVMMM	NCP	0.915
LLTVMMM;VGPVTL	NCP	93.982
VGPVTL;DLGEYF	NCP	66.441
DLGEYF;QVPYTKL	NCP	29.756
QVPYTKL;NTVSLH	NCP	14.215
NTVSLH;EEDMDH	NCP	20.61
EEDMDH;LSVLDNP	NCP	51.753
LSVLDNP;SHLLV	NCP	6.383
SHLLV;PVGLAF	NCP	49.534
PVGLAF;MVMPLAP	NCP	99.588

MVMPLAP;VTDTPEL	NCP	45.552
VTDTPEL;FEMSAF	NCP	115.299
FEMSAF;KDYLPK	NCP	1.823
KDYLPK;HPAVRGP	NCP	46.41
HPAVRGP;MPAVKK	NCP	12.274
MPAVKK;NLHFRY	NCP	23.307
NLHFRY;KLRVDVP	NCP	23.238
KLRVDVP;GFSLT	NCP	24.666
GFSLT;KSHLGLQ	NCP	34.935
KSHLGLQ;RMYHKAGP	NCP	118.134
RMYHKAGP;KPPAPRVP	NCP	70.959
KPPAPRVP;LSRPTLW	NCP	22.2
LSRPTLW;TVRTDLP	NCP	68.859
TVRTDLP;VYALK	NCP	84.968
VYALK;EVEGPPL	NCP	45.706
EVEGPPL;KPGGGRPP	NCP	55.62
KPGGGRPP;FRPGTK	NCP	22.088
FRPGTK;LSKLSNY	NCP	75.067
LSKLSNY;LQSLVAL	NCP	103.395
LQSLVAL;LTVPSR	NCP	24.639
LTVPSR;LQGLK	NCP	62.034
LQGLK;QPGVAGPR	NCP	49.001
QPGVAGPR;LGDAFADLSLK	NCP	59.988
LGDAFADLSLK;GYNADTQKLLAK	NCP	6.243
GYNADTQKLLAK;FAGNQKQLEQTLK	NCP	42.372
FAGNQKQLEQTLK;KSVSTNP	NCP	73.896
KSVSTNP;KYQLSGK	NCP	43.594
KYQLSGK;LEGSVAPG	NCP	63.804
LEGSVAPG;LPSGPAPP	NCP	16.593
LPSGPAPP;YMALRLSF	NCP	6.398
YMALRLSF;LPPLSPR	NCP	21.678
LPPLSPR;VGLPL	NCP	0.772
VGLPL;DGAPGHRP	NCP	29.661
DGAPGHRP;PGVGPVS	NCP	110.633
PGVGPVS;VMVGAGP	NCP	10.345
VMVGAGP;FGGYPVLP	NCP	38.337
FGGYPVLP;LPADLDA	NCP	84.185
LPADLDA;PAAPAP	NCP	35.515
PAAPAP;KPHGSVR	NCP	79.083
KPHGSVR;RFLLPK	NCP	41.576
RFLLPK;KDPTPK	NCP	29.686
KDPTPK;VGGFKGR	NCP	31.953
VGGFKGR;RLARPPP	NCP	45.675
RLARPPP;KDLAL	NCP	66.891
KDLAL;KFAAGGR	NCP	5.976
KFAAGGR;TVLGK	NCP	54.092
TVLGK;GPLGPP	NCP	26.224
GPLGPP;SGHLPFH	NCP	6.041
SGHLPFH;STPFPPK	NCP	4.782
STPFPPK;AAVLEYL	NCP	45.882
AAVLEYL;ALERLK	NCP	27.603

ALERLK;NFLLQVLK	NCP	65.775
NFLLQVLK;KGDLEVP	NCP	20.062
KGDLEVP;GPPHAWK	NCP	7.26
GPPHAWK;PTLSP	NCP	51.501
PTLSP;KSYARP	NCP	45.171
KSYARP;PGAKPPP	NCP	23.892
PGAKPPP;LPELPP	NCP	22.668
LPELPP;LRGVFLVA	NCP	0.201
LRGVFLVA;KATSVPK	NCP	46.967
KATSVPK;EPLSDVPP	NCP	109.065
EPLSDVPP;LGMPSPPP	NCP	16.978
LGMPSPPP;WMMVQL	NCP	10.982
WMMVQL;FARSLQ	NCP	100.125
FARSLQ;GLALW	NCP	119.934
GLALW;PAAPPPPP	NCP	34.42
PAAPPPPP;CSKSC	NCP	21.828
CSKSC;RYHGH	NCP	0.093
RYHGH;EECCC	NCP	42.371
EECCC;LPFCFPGL	NCP	52.128
LPFCFPGL;LTMEKPK	NCP	13.939
LTMEKPK;KEEAPSLRP	NCP	29.951
KEEAPSLRP;ARPATATVGQK	NCP	0.06
ARPATATVGQK;GRKWDPY	NCP	2.925
GRKWDPY;TNMGPTKL	NCP	0.33
TNMGPTKL;EGFTVQNEANKY	NCP	0.072
EGFTVQNEANKY;TVQNEANKYQLSVSKY	NCP	0.009
TVQNEANKYQLSVSKY;WGGAYTWDMAK	NCP	0.864
WGGAYTWDMAK;EMGQRAK	NCP	40.446
EMGQRAK;KSGPF	NCP	4.861
KSGPF;CSPALA	NCP	116.058
CSPALA;SPGGGWTQ	NCP	40.905
SPGGGWTQ;VPEAAPF	NCP	17.4
VPEAAPF;HTLNGS	NCP	41.493
HTLNGS;PSTTVP	NCP	1.941
PSTTVP;RLLTYY	NCP	18.285
RLLTYY;GPPDPLL	NCP	0.678
GPPDPLL;KFGGPPP	NCP	28.31
KFGGPPP;CSGAYSG	NCP	15.243
CSGAYSG;LSLAAMLL	NCP	34.675
LSLAAMLL;LVSKGTL	NCP	120.45
LVSKGTL;LAPKSPR	NCP	59.432
LAPKSPR;EGPAFN	NCP	1.456
EGPAFN;VGKVELR	NCP	9.885
VGKVELR;GPPQPLPP	NCP	15.954
GPPQPLPP;RVPHPFQ	NCP	66.941
RVPHPFQ;LHKAGPV	NCP	19.917
LHKAGPV;KSYSGVGK	NCP	58.759
KSYSGVGK;MPLLGPL	NCP	2.253
MPLLGPL;LPGGLVV	NCP	1.191
LPGGLVV;MLMNKGK	NCP	8.468
MLMNKGK;NKTSDVK	NCP	8.905

NKTSDVK;KYAPPPPPAP	NCP	49.025
KYAPPPPPAP;FSKTVLSF	NCP	18.858
FSKTVLSF;EGDSFY	NCP	36.949
EGDSFY;KTKHPGP	NCP	22.127
KTKHPGP;KELVLAL	NCP	55.198
KELVLAL;NPAVW	NCP	52.239
NPAVW;TEDKRP	NCP	98.775
TEDKRP;NPPLGP	NCP	39.948
NPPLGP;VLDEREK	NCP	95.504
VLDEREK;MDLAKLL	NCP	39.628
MDLAKLL;SPVFPR	NCP	58.434
SPVFPR;KFYRGQPR	NCP	4.553
KFYRGQPR;TVGPQP	NCP	76.207
TVGPQP;PPPKGPSR	NCP	12.582
PPPKGPSR;PPNAGFR	NCP	20.682
PPNAGFR;RKLTLKY	NCP	17.156
RKLTLKY;LSRRVPP	NCP	2.398
LSRRVPP;LHLVPPP	NCP	0.189
LHLVPPP;DALRVPR	NCP	1.938
DALRVPR;QGSPTRLN	NCP	2.501
QGSPTRLN;LTLTSM	NCP	44.508
LTLTSM;PGAGNT	NCP	1.756
PGAGNT;GPVTPAAP	NCP	2.127
GPVTPAAP;PAGPSDVP	NCP	68.895
PAGPSDVP;LLAPRQP	NCP	1.431
LLAPRQP;NEGSFP	NCP	47.07
NEGSFP;SAALLQ	NCP	1.523
SAALLQ;LPVLAPQT	NCP	7.357
LPVLAPQT;KLGPAARPP	NCP	34.365
KLGPAARPP;KGAQVPR	NCP	33.528
KGAQVPR;AAPRTVY	NCP	116.133
AAPRTVY;REVSAR	NCP	2.958
REVSAR;KERGGGPK	NCP	111.503
KERGGGPK;PGAAPPYK	NCP	19.294
PGAAPPYK;TSGPA	NCP	48.507
TSGPA;PLTLRD	NCP	12.677
PLTLRD;AVTRVLTP	NCP	42.763
AVTRVLTP;AVACLK	NCP	109.8
AVACLK;GPPVSGPP	NCP	11.025
GPPVSGPP;CWCPP	NCP	55.857
CWCPP;QPTYAG	NCP	34.077
QPTYAG;GRFLPP	NCP	0.174
GRFLPP;PPPGRKPP	NCP	0.522
PPPGRKPP;KALAGPPP	NCP	22.176
KALAGPPP;GSTLFDN	NCP	5.255
GSTLFDN;LGPSALP	NCP	5.509
LGPSALP;LSDYVPK	NCP	9.557
LSDYVPK;KTYLGAP	NCP	3.816
KTYLGAP;KTLNP	NCP	21.982
KTLNP;VVQAPSP	NCP	26.586
VVQAPSP;FGFLLGLQ	NCP	61.31

FGFLLGLQ;VGLQMKGP	NCP	3.361
VGLQMKGP;NMLLKK	NCP	14.358
NMLLKK;TGAPVT	NCP	85.458
TGAPVT;LHEEKAK	NCP	31.658
LHEEKAK;KFKCLP	NCP	6.769
KFKCLP;ASTPRPK	NCP	35.889
ASTPRPK;QAGHAPRP	NCP	77.598
QAGHAPRP;PSRPPP	NCP	2.787
PSRPPP;TGPAATP	NCP	19.388
TGPAATP;KPLGKAPK	NCP	29.208
KPLGKAPK;KNFVQ	NCP	12.144
KNFVQ;NLPHGGP	NCP	25.906
NLPHGGP;RFPTTKT	NCP	19.038
RFPTTKT;PVAFLA	NCP	7.947
PVAFLA;PLQELHD	NCP	38.262
PLQELHD;PRGAAATL	NCP	10.095
PRGAAATL;NTGSVKK	NCP	8.675
NTGSVKK;PGPPPPDP	NCP	7.231
PGPPPPDP;VAGLTA	NCP	29.979
VAGLTA;LLAPPER	NCP	40.217
LLAPPER;KPGAVVR	NCP	30.073
KPGAVVR;HHPFPK	NCP	4.38
HHPFPK;MVGAPP	NCP	8.985
MVGAPP;LLESLP	NCP	2.517
LLESLP;RGADVP	NCP	2.154
RGADVP;LGPEGP	NCP	32.355
LGPEGP;MGFAV	NCP	32.165
MGFAV;VGAVP	NCP	94.036
VGAVP;PGMDPLR	NCP	6.342
PGMDPLR;AVGEAGPR	NCP	3.321
AVGEAGPR;AGPVRSP	NCP	10.215
AGPVRSP;MLAQPR	NCP	1.104
MLAQPR;KVHSPQ	NCP	20.697
LVDADADLEERLK;VDADADLEERLKN	NCP	0.003
VDADADLEERLKN;ADADLEERLK	NCP	0.006
ADADLEERLK;DTAGEPG	NCP	69.519
DTAGEPG;LLFDRT	NCP	44.311
LLFDRT;LLRLEGEDQ	NCP	18.545
LLRLEGEDQ;MCADLR	NCP	59.616
MCADLR;LAVGEQVL	NCP	2.856
LAVGEQVL;WGVNRLP	NCP	74.895
WGVNRLP;DMFGEKL	NCP	9.955
DMFGEKL;NNPPK	NCP	14.324
NNPPK;LRFGPGAL	NCP	46.338
LRFGPGAL;KTLFGGH	NCP	44.071
KTLFGGH;KECKALP	NCP	0.509
KECKALP;DDMGFGLFD	NCP	52.794
DDMGFGLFD;APERPLT	NCP	17.217
APERPLT;AEEFPTL	NCP	49.152
AEEFPTL;ELELPQ	NCP	24.1
ELELPQ;LGAAVL	NCP	120.164

LGAAVL;DGRVVKP	NCP	55.977
DGRVVKP;GCMYPK	NCP	94.986
GCMYPK;SAEAG	NCP	40.788
SAEAG;DGFSAQR	NCP	27.88
DGFSAQR;SVMLFM	NCP	0.531
SVMLFM;LEALQ	NCP	65.54
LEALQ;NSLTSVK	NCP	65.166
NSLTSVK;LPTRCP	NCP	82.683
LPTRCP;VYVTHLAQ	NCP	9.961
VYVTHLAQ;KACLLSL	NCP	2.322
KACLLSL;VPPGPD	NCP	84.249
VPPGPD;VTLTDPF	NCP	78.812
VTLTDPF;LMPPPP	NCP	13.441
LMPPPP;AAGSVHR	NCP	64.629
AAGSVHR;KNERNSP	NCP	49.485
KNERNSP;EGCPFPQ	NCP	14.435
EGCPFPQ;LAAPV	NCP	58.396
LAAPV;QAPGVLR	NCP	24.207
QAPGVLR;ESAAG	NCP	35.874
ESAAG;VPTMRRP	NCP	101.837
VPTMRRP;QVTLL	NCP	10.123
QVTLL;LSPGASYR	NCP	10.887
LSPGASYR;NPRPPR	NCP	42.876
NPRPPR;KQFVVPP	NCP	64.776
KQFVVPP;GVLGAPP	NCP	3.215
GVLGAPP;MVGTTK	NCP	97.377
MVGTTK;PLSGFP	NCP	18.103
PLSGFP;AAEPEFK	NCP	47.166
AAEPEFK;APPSEPK	NCP	222.231
APPSEPK;PDRFDS	NCP	0.62
PDRFDS;SDHFPK	NCP	39.712
SDHFPK;FAAAG	NCP	1.455
FAAAG;MVPFRLP	NCP	2.034
MVPFRLP;NSFDKAKLK	NCP	10.583
NSFDKAKLK;SFDKAKLKK	NCP	0.003
SFDKAKLKK;GVPFGP	NCP	20.157
GVPFGP;GTLLELA	NCP	20.248
GTLLELA;VGPTVP	NCP	53.597
VGPTVP;VSPVY	NCP	54.235
VSPVY;FPPLPEK	NCP	3.725
FPPLPEK;ESKGLPL	NCP	5.64
ESKGLPL;GMMVPV	NCP	75.942
GMMVPV;LPPLVEKP	NCP	14.413
LPPLVEKP;DPSLLGPQ	NCP	45.609
DPSLLGPQ;NHHALNV	NCP	52.887
NHHALNV;SWQPGTK	NCP	0.807
SWQPGTK;EAFNLL	NCP	23.225
EAFNLL;VTTRFVD	NCP	33.567
VTTRFVD;NLPMHK	NCP	23.38
NLPMHK;EDFLDP	NCP	19.139
EDFLDP;KLTEFH	NCP	0.372

KLTEFH;ELEVLLMCNK	NCP	83.89
ELEVLLMCNK;EVQLLT	NCP	9.846
EVQLLT;PVARSWVCR	NCP	11.588
PVARSWVCR;GLAKDDQLK	NCP	92.518
GLAKDDQLK;DDQLKVHGF	NCP	0.012
DDQLKVHGF;YVREPAK	NCP	79.965
YVREPAK;APLGGVPR	NCP	38.435
APLGGVPR;VTTVP	NCP	27.913
VTTVP;AAPGVR	NCP	94.518
AAPGVR;HPTGPVAP	NCP	1.4
HPTGPVAP;GVCLK	NCP	101.464
GVCLK;TGLEMMK	NCP	55.113
TGLEMMK;LSVKRQ	NCP	257.757
LSVKRQ;GDNLRLK	NCP	20.616
GDNLRLK;KVHVGNK	NCP	135.669
KVHVGNK;LPRQTP	NCP	108.293
LPRQTP;KTEAKC	NCP	150.123
KTEAKC;SSLLQKF	NCP	101.638
SSLLQKF;PGVATWPP	NCP	9.113
PGVATWPP;LVLYAN	NCP	156.477
LVLYAN;NVVTLY	NCP	95.454
NVVTLY;SLAQP	NCP	106.113
SLAQP;VGPVVFSR	NCP	57.228
VGPVVFSR;LSTKGPP	NCP	17.899
LSTKGPP;PAAPKLAP	NCP	201.573
PAAPKLAP;QRDLTDY	NCP	215.663
QRDLTDY;MFLNDVW	NCP	53.877
MFLNDVW;ERRKFF	NCP	20.835
ERRKFF;APFLSN	NCP	74.611
APFLSN;ALGVERTR	NCP	55.385
ALGVERTR;VGGPEYVHC	NCP	0.885
VGGPEYVHC;VLELDNVK	NCP	4.134
VLELDNVK;ELVSDANQHVK	NCP	2.76
ELVSDANQHVK;KEHPPK	NCP	47.185
KEHPPK;VPTNGPLK	NCP	32.376
VPTNGPLK;EFTLVP	NCP	26.43
EFTLVP;RAVLNTR	NCP	116.111
RAVLNTR;YVVAAPL	NCP	16.12
YVVAAPL;PKWQAP	NCP	9.191
PKWQAP;LDSPGL	NCP	28.195
LDSPGL;PPNPEEK	NCP	11.178
PPNPEEK;PVVVPPK	NCP	17.013
PVVVPPK;RKLVFY	NCP	7.433
RKLVFY;KLGTAPK	NCP	76.942
KLGTAPK;EYEVAK	NCP	25.236
EYEVAK;KGVSWK	NCP	36.282
KGVSWK;KQSRPGP	NCP	5.555
KQSRPGP;PLSCVV	NCP	59.386
PLSCVV;HATGLSF	NCP	26.979
HATGLSF;FLQPPPPPP	NCP	114.594
FLQPPPPPP;SLAAPQ	NCP	90.099

SLAAPQ;LVRVY	NCP	70.773
LVRVY;EVPPAAG	NCP	27.713
EVPPAAG;KFTAAP	NCP	18.646
KFTAAP;AAGQDAF	NCP	5.655
AAGQDAF;LTGLPLVQ	NCP	40.581
LTGLPLVQ;KDPNGF	NCP	4.182
KDPNGF;TVPPASLF	NCP	3.606
TVPPASLF;LAPSTM	NCP	54.945
LAPSTM;VGVNLPK	NCP	11.55
VGVNLPK;KQGLLEF	NCP	25.253
KQGLLEF;VATVSRLP	NCP	47.872
VATVSRLP;PAPPQRPP	NCP	4.098
PAPPQRPP;PPYPVPP	NCP	4.434
PPYPVPP;QAQPATK	NCP	22.001
QAQPATK;AGGHRQPL	NCP	9.988
AGGHRQPL;RMLPAQK	NCP	22.925
RMLPAQK;MEYLGL	NCP	33.298
MEYLGL;PSHEKPK	NCP	5.357
PSHEKPK;TTTPTVT	NCP	24.346
TTTPTVT;GVVGRPR	NCP	5.327
GVVGRPR;NSLMKC	NCP	68.262
NSLMKC;SLAAP	NCP	59.239
SLAAP;KRAGP	NCP	92.484
KRAGP;ERLQNQ	NCP	45.396
ERLQNQ;DPLTFGKN	NCP	2.055
DPLTFGKN;KVKLGLERL	NCP	56.276
KVKLGLERL;GPGFGPSN	NCP	8.659
GPGFGPSN;PPPGKKKR	NCP	17.544
PPPGKKKR;PAPPKEPP	NCP	19.473
PAPPKEPP;PQPPHL	NCP	104.036
PQPPHL;APGSPRHL	NCP	27.877
APGSPRHL;KAGFEVP	NCP	10.857
KAGFEVP;LKAKFPP	NCP	77.003
LKAKFPP;AASFTPY	NCP	122.287
AASFTPY;VGSSTNPK	NCP	10.611
VGSSTNPK;VQARVCML	NCP	82.358
VQARVCML;HPHYF	NCP	17.89
HPHYF;DKHLPP	NCP	80.553
DKHLPP;LLLNTF	NCP	20.483
LLLNTF;MDQRPS	NCP	169.675
MDQRPS;AAYKVL	NCP	13.509
AAYKVL;ATPLYE	NCP	32.088
ATPLYE;AAGFLPSWL	NCP	14.286
AAGFLPSWL;NSHVTPR	NCP	15.279
NSHVTPR;DDLPKPP	NCP	14.987
DDLPKPP;KFTLYPLP	NCP	11.986
KFTLYPLP;TSCFLL	NCP	9.792
TSCFLL;VYGGP	NCP	61.008
VYGGP;NHGFKTR	NCP	63.201
NHGFKTR;TTLAPQ	NCP	18.738
TTLAPQ;PPQATGK	NCP	12.471

PPQATGK;VAPAGLQ	NCP	18.501
VAPAGLQ;FPVTP	NCP	38.72
FPVTP;FFPPAAK	NCP	8.992
FFPPAAK;QGGLGALR	NCP	69.3
QGGLGALR;LPPLGP	NCP	2.429
LPPLGP;ASKFPPT	NCP	48.493
ASKFPPT;EAAPGP	NCP	97.151
EAAPGP;HAAPGVAK	NCP	65.887
HAAPGVAK;TFGPARP	NCP	41.397
TFGPARP;PWPKL	NCP	3.245
PWPKL;EEDNP	NCP	16.017
EEDNP;KYSPPPPP	NCP	24.999
KYSPPPPP;RLLPGLF	NCP	65.737
RLLPGLF;MVVSGWR	NCP	47.451
MVVSGWR;LRELPLR	NCP	12.15
LRELPLR;MDRKMR	NCP	11.133
MDRKMR;VPGGHAVP	NCP	18.984
VPGGHAVP;AAGFSPY	NCP	3.749
AAGFSPY;GAPSFP	NCP	5.05
GAPSFP;TTLVLLGL	NCP	120.426
TTLVLLGL;FGEKLVR	NCP	29.477
FGEKLVR;PPPLFD	NCP	6.937
PPPLFD;CVFEKPK	NCP	39.884
CVFEKPK;SNAGFLVL	NCP	12.55
SNAGFLVL;RGDPVLP	NCP	25.2
RGDPVLP;DYPPLG	NCP	31.631
DYPPLG;DGKAPYP	NCP	23.742
DGKAPYP;MAGECK	NCP	36.342
MAGECK;AVFPFP	NCP	6.454
AVFPFP;PVTVTRTT	NCP	9.275
PVTVTRTT;TVTRTTVTT	NCP	0.006
TVTRTTVTT;LPPPPPPV	NCP	5.553
LPPPPPPV;LDPFL	NCP	15.84
LDPFL;EDVLKEAK	NCP	57.021
EDVLKEAK;FPEFQL	NCP	2.367
FPEFQL;STTPVPR	NCP	6.318
STTPVPR;GAPAL	NCP	53.721
GAPAL;KGEPARP	NCP	22.96
KGEPARP;AAPPVP	NCP	148.886
AAPPVP;ESLLLP	NCP	5.052
ESLLLP;PAAAPLVP	NCP	0.012
PAAAPLVP;APPRLPP	NCP	35.139
APPRLPP;KTHPQR	NCP	19.188
KTHPQR;GDDPF	NCP	39.063
GDDPF;AAPFPLRH	NCP	44.691
AAPFPLRH;LPTEVPPP	NCP	10.779
LPTEVPPP;TVGLK	NCP	7.044
TVGLK;LFQDQNPLFR	NCP	9.192
LFQDQNPLFR;LPDPLPD	NCP	29.232
LPDPLPD;VAPGPRPP	NCP	10.35
VAPGPRPP;DPVLVPN	NCP	29.295

DPVLVPN;VYFKEQF	NCP	12.289
VYFKEQF;MDARKEGLLR	NCP	90.035
MDARKEGLLR;LSVPGGDL	NCP	98.169
LSVPGGDL;FLVMPP	NCP	6.142
FLVMPP;GPDVDRQ	NCP	4.037
GPDVDRQ;RKVFYP	NCP	126.571
RKVFYP;LGTTRSGP	NCP	68.147
LGTTRSGP;KTFEKER	NCP	34.258
KTFEKER;VPYVPPP	NCP	29.664
VPYVPPP;KPVVFNQ	NCP	81.327
KPVVFNQ;GQSTPLR	NCP	36.417
GQSTPLR;LSGGPGY	NCP	7.728
LSGGPGY;VPAPAALH	NCP	101.763
VPAPAALH;LTPPPEA	NCP	9.111
LTPPPEA;VGTVLEVK	NCP	85.464
VGTVLEVK;PAAETP	NCP	0.543
PAAETP;ELAAAP	NCP	5.693
ELAAAP;LANLGAK	NCP	4.855
LANLGAK;EGTFLVL	NCP	15.207
EGTFLVL;KTTGFQP	NCP	97.391
KTTGFQP;SPVKPP	NCP	3.12
SPVKPP;KFGAEVR	NCP	18.87
KFGAEVR;EVGPPN	NCP	14.1
EVGPPN;AAALLRQ	NCP	19.39
AAALLRQ;PRHLPPPPP	NCP	38.903
PRHLPPPPP;KYVNPPPPP	NCP	35.64
KYVNPPPPP;LVFKEELK	NCP	19.282
LVFKEELK;KPTNPPLP	NCP	37.71
KPTNPPLP;RGPPHHR	NCP	4.361
RGPPHHR;VGLKLLG	NCP	8.5
VGLKLLG;SGPPAQPTR	NCP	11.273
SGPPAQPTR;PGCSVPV	NCP	0.054
PGCSVPV;GTVTPSR	NCP	44.824
GTVTPSR;YPVGP	NCP	12.005
YPVGP;VEGLGFK	NCP	2.398
VEGLGFK;VPLVPQ	NCP	3.311
VPLVPQ;YPLGP	NCP	11.808
YPLGP;ALDRVPRKVG	NCP	40.086
ALDRVPRKVG;AAPVQP	NCP	37.543
AAPVQP;SAALRLP	NCP	6.501
SAALRLP;PEVAAPF	NCP	21.303
PEVAAPF;GTGPASVR	NCP	17.339
GTGPASVR;VRPWSV	NCP	33.64
VRPWSV;FGGFVFT	NCP	8.484
FGGFVFT;GAQVGLARARPR	NCP	5.603
GAQVGLARARPR;APEPHRP	NCP	0.994
APEPHRP;GPGKPP	NCP	20.261
GPGKPP;GSPPYFK	NCP	35.788
GSPPYFK;LPVYGTPP	NCP	3.024
LPVYGTPP;RHKVNPP	NCP	20.84
RHKVNPP;DKPHEGK	NCP	5.734

DKPHEGK;VPPDLW	NCP	37.488
VPPDLW;KAPAVLH	NCP	28.77
KAPAVLH;SRSGKLV	NCP	17.516
SRSGKLV;HDKPAPP	NCP	41.224
HDKPAPP;PPPLFQ	NCP	3.837
PPPLFQ;VGGLLP	NCP	2.324
VGGLLP;CKLLHV	NCP	14.475
CKLLHV;APGKPAAP	NCP	2.662
APGKPAAP;AARPPVPP	NCP	55.211
AARPPVPP;PVLVVGLR	NCP	25.333
PVLVVGLR;SDLDLNLK	NCP	15.17
SDLDLNLK;NRAMLTP	NCP	22.566
NRAMLTP;PPAGVESR	NCP	25.737
PPAGVESR;VPAGQAF	NCP	41.493
VPAGQAF;KLVVVGGGGVGK	NCP	44.538
KLVVVGGGGVGK;VKDRDDFPV	NCP	2.292
VKDRDDFPV;ASHHVAY	NCP	0.858
ASHHVAY;VDEAFEQLVR	NCP	0.051
VDEAFEQLVR;LQSLQPL	NCP	42.088
LQSLQPL;AALLRPL	NCP	13.539
AALLRPL;RQLVTLF	NCP	63.096
RQLVTLF;QEPGRPP	NCP	5.169
QEPGRPP;AAPAPVP	NCP	4.698
AAPAPVP;KPTTRPQ	NCP	74.055
KPTTRPQ;SGGGLLL	NCP	5.984
SGGGLLL;LLTLP	NCP	34.038
LLTLP;EDFPDL	NCP	39.541
EDFPDL;AGKPRPP	NCP	5.973
AGKPRPP;PEGQLVK	NCP	13.983
PEGQLVK;LPNNVKT	NCP	89.403
LPNNVKT;CDEYTP	NCP	0.033
CDEYTP;VEPPTAW	NCP	1.671
VEPPTAW;VPLARP	NCP	25.773
VPLARP;ERLLPR	NCP	22.341
ERLLPR;LDSKVRVQ	NCP	30.156
LDSKVRVQ;VPPPGQL	NCP	14.609
VPPPGQL;TVRTVDL	NCP	28.177
TVRTVDL;PPGPPSPP	NCP	22.392
PPGPPSPP;APPLPPPPPPP	NCP	39.084
APPLPPPPPPP;GVVPEC	NCP	43.295
GVVPEC;AHEWMLPV	NCP	10.287
AHEWMLPV;LAGVPTHRP	NCP	0.688
LAGVPTHRP;AMLSGERK	NCP	39.185
AMLSGERK;PPPPPHVEL	NCP	0.756
PPPPPHVEL;TAGGFLP	NCP	21.313
TAGGFLP;GLPVT	NCP	28.785
GLPVT;LKRVLLGP	NCP	27.608
LKRVLLGP;KPWAARP	NCP	0.691
KPWAARP;MNQLE	NCP	22.695
MNQLE;RHFGKAPGTPH	NCP	9.698
RHFGKAPGTPH;RPAEGKP	NCP	12.909

RPAEGKP;NSLRLR	NCP	60.7
NSLRLR;KTVPSGAP	NCP	3.506
KTVPSGAP;APVLSAEK	NCP	39.846
APVLSAEK;RCDPVP	NCP	21.487
RCDPVP;WLEPA	NCP	2.754
WLEPA;LSRPPLP	NCP	14.868
LSRPPLP;KEMPTTK	NCP	10.419
KEMPTTK;EGPVEPK	NCP	34.118
EGPVEPK;DVLFLK	NCP	3.849
DVLFLK;TTGLPY	NCP	36.699
TTGLPY;DDAWYK	NCP	53.278
DDAWYK;KPYSAH	NCP	3.351
KPYSAH;KKLRQGK	NCP	56.993
KKLRQGK;VPEYKPR	NCP	45.277
VPEYKPR;APLLFRD	NCP	13.511
APLLFRD;WPAGP	NCP	102.115
WPAGP;AGSAQQGRP	NCP	0.135
AGSAQQGRP;PAAAAAP	NCP	121.616
PAAAAAP;KTHPGPP	NCP	2.751
KTHPGPP;VEGPL	NCP	0.42
VEGPL;PGHAPKP	NCP	31.474
PGHAPKP;ATVMSPR	NCP	47.745
ATVMSPR;AVCYAA	NCP	19.311
AVCYAA;LSYPAPQ	NCP	33.596
LSYPAPQ;QHTPPR	NCP	21.354
QHTPPR;VLGHQKP	NCP	15.339
VLGHQKP;AVTRALAA	NCP	10.572
AVTRALAA;GFRGLGR	NCP	22.342
GFRGLGR;KGGVTDF	NCP	37.193
KGGVTDF;LVVGPS	NCP	5.427
LVVGPS;ESLVVP	NCP	17.268
ESLVVP;HETLL	NCP	0.22
HETLL;LPTEM	NCP	25.031
LPTEM;ASRVGPN	NCP	32.868
ASRVGPN;QHLLSK	NCP	0.462
QHLLSK;LTQQGF	NCP	18.999
LTQQGF;EVLRLPY	NCP	3.864
EVLRLPY;LPPNPPPL	NCP	28.071
LPPNPPPL;KPGKTF	NCP	66.214
KPGKTF;VAGESQR	NCP	64.526
VAGESQR;AALVLP	NCP	7.372
AALVLP;LLVPATE	NCP	24.432
LLVPATE;LQVWVRQ	NCP	22.679
LQVWVRQ;PGVPPPPP	NCP	11.241
PGVPPPPP;WLLLTG	NCP	18.765
WLLLTG;GSAGLRPP	NCP	9.471
GSAGLRPP;DEMCPP	NCP	33.919
DEMCPP;LPTPPVVP	NCP	29.135
LPTPPVVP;RPGGRHP	NCP	5.446
RPGGRHP;FGLPLR	NCP	6.342
FGLPLR;VPPYQPP	NCP	2.987

VPPYQPP;ETAVGKLK	NCP	27.831
ETAVGKLK;LLMLQSLE	NCP	22.836
LLMLQSLE;LTDFVEL	NCP	12.822
LTDFVEL;CCQFGPK	NCP	13.09
CCQFGPK;VASVVKGP	NCP	37.407
VASVVKGP;NPPSL	NCP	0.951
NPPSL;GVRGPPPP	NCP	28.359
GVRGPPPP;AQLRPAT	NCP	18.258
AQLRPAT;KGPVVYL	NCP	18.875
KGPVVYL;ATVRVPA	NCP	3.004
ATVRVPA;PGVRPTPP	NCP	73.887
PGVRPTPP;DLRFY	NCP	4.674
DLRFY;MLVLAHT	NCP	159.032
MLVLAHT;DAPAPPPR	NCP	41.895
DAPAPPPR;ASAWLT	NCP	12.525
ASAWLT;TGTTTLR	NCP	4.639
TGTTTLR;QPRSPY	NCP	91.875
QPRSPY;AASSGDSVTRR	NCP	23.882
AASSGDSVTRR;LPRTGAP	NCP	43.849
LPRTGAP;VTPLVD	NCP	4.49
VTPLVD;PVGHRPP	NCP	15.448
PVGHRPP;CVNDWP	NCP	18.539
CVNDWP;QGPLPKQP	NCP	1.971
QGPLPKQP;AEASKPK	NCP	51.37
AEASKPK;PGASPP	NCP	17.382
PGASPP;SVLQWRVV	NCP	19.487
SVLQWRVV;VVGAFPMAKL	NCP	22.56
VVGAFPMAKL;VAAAVSGPKAK	NCP	97.305
VAAAVSGPKAK;APPLSGDT	NCP	62.581
APPLSGDT;GVDPVGP	NCP	41.391
GVDPVGP;LGLNVTDF	NCP	1.515
LGLNVTDF;ADRPVR	NCP	0.987
ADRPVR;PFGNTHNKHKLN	NCP	18.152
PFGNTHNKHKLN;VAGDEESYTVFKDL	NCP	1.257
VAGDEESYTVFKDL;GDEESYTVFKD	NCP	0.006
GDEESYTVFKD;VVDGVKL	NCP	5.346
VVDGVKL;PDLGVAPP	NCP	1.594
PDLGVAPP;KPDLLPP	NCP	27.149
KPDLLPP;DLGRTKP	NCP	8.241
DLGRTKP;RLALPR	NCP	16.744
RLALPR;EPMELPP	NCP	9.167
EPMELPP;HGGPRPPP	NCP	11.281
HGGPRPPP;CALLASPPR	NCP	42.084
CALLASPPR;AAGVPPLP	NCP	23.552
AAGVPPLP;GPDRPTLP	NCP	48.84
GPDRPTLP;KEQSLVL	NCP	8.392
KEQSLVL;VGLVTVRA	NCP	2.441
VGLVTVRA;KMGGPL	NCP	21.234
KMGGPL;FAGVPPR	NCP	20.041
FAGVPPR;PGLPTFP	NCP	4.23
PGLPTFP;KGSPLAAP	NCP	43.886

KGSPLAAP;PAPVGPP	NCP	1.702
PAPVGPP;SPAARPPQK	NCP	27.983
SPAARPPQK;KDEKSLR	NCP	8.845
KDEKSLR;HGRGPPPP	NCP	33.32
HGRGPPPP;PAQLGW	NCP	12.79
PAQLGW;NVPAMY	NCP	1.818
NVPAMY;PTAALPP	NCP	7.262
PTAALPP;LPLSS	NCP	59.22
LPLSS;VDPVNF	NCP	0.945
VDPVNF;VTKECP	NCP	6.786
VTKECP;VAKKEK	NCP	15.639
VAKKEK;TASQAMP	NCP	115.617
TASQAMP;MLMPKKNR	NCP	57.996
MLMPKKNR;NVSAPGR	NCP	56.487
NVSAPGR;KNQVLGH	NCP	57.769
KNQVLGH;ASAKGEP	NCP	29.394
ASAKGEP;YTSQKAK	NCP	70.556
YTSQKAK;VLLMFYK	NCP	34.515
VLLMFYK;TKAVTKYTSSK	NCP	175.29
TKAVTKYTSSK;CCYCCW	NCP	32.205
CCYCCW;AGVRHALL	NCP	0.463
AGVRHALL;GVGVF	NCP	23.106
GVGVF;VSVLAL	NCP	21.83
VSVLAL;NVGVLK	NCP	61.053
NVGVLK;KPNPPAH	NCP	2.028
KPNPPAH;TSEPKP	NCP	8.713
TSEPKP;KDPTMK	NCP	3.122
KDPTMK;YGVVPQA	NCP	13.302
YGVVPQA;VTFVQAYT	NCP	13.33
VTFVQAYT;SQHTGSGAPVLLR	NCP	2.171
SQHTGSGAPVLLR;VLNDHSMAF	NCP	14.49
VLNDHSMAF;RFKKPT	NCP	98.581
RFKKPT;PKSLGPQ	NCP	69.545
PKSLGPQ;THSLVHR	NCP	62.484
THSLVHR;YSTLGL	NCP	28.153
YSTLGL;KGAHLNF	NCP	7.281
KGAHLNF;RAALNVQ	NCP	68.924
RAALNVQ;GCMPKK	NCP	1.168
GCMPKK;KTLLAAP	NCP	27.119
KTLLAAP;EVSYY	NCP	55.987
EVSYY;KAPDP	NCP	16.584
KAPDP;MEAEDVAR	NCP	124.583
MEAEDVAR;KPPAQPP	NCP	7.926
KPPAQPP;PVPSEGRL	NCP	1.722
PVPSEGRL;QPPTPR	NCP	48.381
QPPTPR;SLAAYL	NCP	126.571
SLAAYL;CGVQAPR	NCP	105.377
CGVQAPR;APAHGSD	NCP	15.831
APAHGSD;LAPGPGP	NCP	9.327
LAPGPGP;KAMGPPPP	NCP	35.769
KAMGPPPP;APGSRGPPP	NCP	4.887

APGSRGPPP;LPLPDFMR	NCP	45.576
LPLPDFMR;REPPAHPR	NCP	26.482
REPPAHPR;DLEKGAAP	NCP	50.667
DLEKGAAP;APPKKAH	NCP	0.974
APPKKAH;KVGTVAK	NCP	23.637
KVGTVAK;HRLLVQD	NCP	25.78
HRLLVQD;LLHFVV	NCP	17.631
LLHFVV;ASMCCNK	NCP	53.828
ASMCCNK;KNLEKPGM	NCP	62.943
KNLEKPGM;YLRAALH	NCP	3.907
YLRAALH;KPKWDPK	NCP	96.494
KPKWDPK;RQKFDEY	NCP	31.801
RQKFDEY;LPSVPLLPP	NCP	139.187
LPSVPLLPP;AAPRPPPKPM	NCP	56.259
AAPRPPPKPM;LSSALG	NCP	27.748
LSSALG;CMSSAPF	NCP	6.485
CMSSAPF;LLGCGF	NCP	60.622
LLGCGF;ATPPV	NCP	64.628
ATPPV;DPEAHKK	NCP	43.503
DPEAHKK;FDYEDKEFL	NCP	7.053
FDYEDKEFL;LTPNPLFF	NCP	107.913
LTPNPLFF;SAAAKKK	NCP	16.72
SAAAKKK;EGPPLAG	NCP	24.803
EGPPLAG;EVPTPTLP	NCP	15.381
EVPTPTLP;GPFPK	NCP	41.607
GPFPK;QELRPTL	NCP	1.836
QELRPTL;KAVVAGV	NCP	9.36
KAVVAGV;LQELPKT	NCP	41.644
LQELPKT;LQTTSVP	NCP	8.306
LQTTSVP;AQLAL	NCP	50.115
AQLAL;AHGVDKVVAC	NCP	41.298
AHGVDKVVAC;QSGLKYVA	NCP	1.365
QSGLKYVA;TTDEYDGHTTYPSHVYE	NCP	4.335
TTDEYDGHTTYPSHVYE;DGHTTYP	NCP	0.015
DGHTTYP;TTYPSHVYE	NCP	0.009
TTYPSHVYE;VEGPVPAAPTRR	NCP	36.093
VEGPVPAAPTRR;TEAGELVPGAAELEGK	NCP	2.232
TEAGELVPGAAELEGK;SPVEQEK	NCP	45.99
SPVEQEK;LANDLTSL	NCP	4.362
LANDLTSL;KVDALF	NCP	50.766
KVDALF;LNTLVL	NCP	49.995
LNTLVL;LLAPLAL	NCP	134.488
LLAPLAL;VGLVTVRAY	NCP	15.245
VGLVTVRAY;NHAVSLARGEVGF	NCP	0.027
NHAVSLARGEVGF;EEETGVL	NCP	4.015
EEETGVL;LALSLPVRF	NCP	0.404
LALSLPVRF;FPLGSP	NCP	15.414
FPLGSP;VSLDSPGH	NCP	7.824
VSLDSPGH;KGEFVWK	NCP	85.245
KGEFVWK;GSLPLNL	NCP	17.461
GSLPLNL;PAGGHAAP	NCP	26.52

PAGGHAAP;VGLQHLL	NCP	68.475
VGLQHLL;KGGARGPR	NCP	22.854
KGGARGPR;VELSGPK	NCP	2.234
VELSGPK;ASLWVG	NCP	66.543
ASLWVG;QFRYPPP	NCP	9.348
QFRYPPP;PRAVVP	NCP	66.417
PRAVVP;TTDDLTEAWLKEK	NCP	4.773
TTDDLTEAWLKEK;AALFY	NCP	59.029
AALFY;LRGGPTL	NCP	82.649
LRGGPTL;LLYPFQ	NCP	87.96
LLYPFQ;PMELLR	NCP	72.555
PMELLR;PGPPPPPPP	NCP	31.159
PGPPPPPPP;AGKWAK	NCP	16.787
AGKWAK;PSPGHKP	NCP	7.647
PSPGHKP;VSSPRVQ	NCP	5.652
VSSPRVQ;PPPPTKEEPR	NCP	79.092
PPPPTKEEPR;KPPFHDP	NCP	15.483
KPPFHDP;AAAGGGGGGGRY	NCP	9.957
AAAGGGGGGGRY;GHPAFVNY	NCP	2.703
GHPAFVNY;HDEGYGPPPPHYEG	NCP	5.139
HDEGYGPPPPHYEG;DEGYGPPPPHYEGR	NCP	0.003
DEGYGPPPPHYEGR;GPPPPHYEG	NCP	0.012
GPPPPHYEG;NYLPSGV	NCP	2.344
NYLPSGV;SDALETLGF	NCP	0.242
SDALETLGF;TLKLCFSTAQH	NCP	0.63
TLKLCFSTAQH;LCFSTAQHAS	NCP	0.009
LCFSTAQHAS;NAAKGDK	NCP	0.094
NAAKGDK;VMLAPPP	NCP	111.063
VMLAPPP;APPPPLAW	NCP	40.419
APPPPLAW;ATLSVVR	NCP	0.875
ATLSVVR;GVEPK	NCP	2.845
GVEPK;SFGGKLVTF	NCP	4.475
SFGGKLVTF;RYRPPG	NCP	29.43
RYRPPG;DLLYRAVL	NCP	43.761
DLLYRAVL;APAPTAKG	NCP	77.367
APAPTAKG;PAATPPKK	NCP	22.092
PAATPPKK;RGQPERP	NCP	20.877
RGQPERP;KEVVGGY	NCP	230.938
KEVVGGY;PAAHC	NCP	67.71
PAAHC;GSLGTSSK	NCP	12.108
GSLGTSSK;KVGSPLL	NCP	3.513
KVGSPLL;PGPPPP	NCP	19.122
PGPPPP;PGPGELR	NCP	134.028
PGPGELR;AAGRVTPR	NCP	19.224
AAGRVTPR;VEAGLLG	NCP	36.453
VEAGLLG;VETRPYK	NCP	29.232
VETRPYK;KMLLVPP	NCP	10.389
KMLLVPP;NHLTSSS	NCP	121.43
NHLTSSS;YEKSYR	NCP	107.958
YEKSYR;MLSRKPP	NCP	81.837
MLSRKPP;KDRLLSKL	NCP	123.315

KDRLLSKL;DPFLMK	NCP	43.755
DPFLMK;GVSGAPPK	NCP	9.036
GVSGAPPK;PVHKHPPL	NCP	22.2
PVHKHPPL;FHLQTAP	NCP	1.198
FHLQTAP;MGQKSSF	NCP	23.163
MGQKSSF;NGYVRP	NCP	28.625
NGYVRP;MKVELLPALTDNY	NCP	65.422
MKVELLPALTDNY;KGHEPAK	NCP	42.225
KGHEPAK;QPAAF	NCP	0.29
QPAAF;DSSLP	NCP	58.072
DSSLP;LSAEQ	NCP	76.793
LSAEQ;KDVGGEVP	NCP	31.155
KDVGGEVP;LNKGVPH	NCP	13.728
LNKGVPH;QHSLLLP	NCP	38.182
QHSLLLP;SVWGL	NCP	82.49
SVWGL;TLALGG	NCP	44.262
TLALGG;RLYTSSK	NCP	18.754
RLYTSSK;VNPFP	NCP	43.331
VNPFP;KKMDTVPL	NCP	3.259
KKMDTVPL;VPPLPPPP	NCP	9.815
VPPLPPPP;KVYFKP	NCP	37.692
KVYFKP;RSLRVSPH	NCP	20.22
RSLRVSPH;PVPVQPSW	NCP	39.795
PVPVQPSW;VPVQPSWLRR	NCP	0.003
VPVQPSWLRR;APGRLFDQ	NCP	0.057
APGRLFDQ;FGEGLLEA	NCP	0.027
FGEGLLEA;YLRAPSVA	NCP	0.063
YLRAPSVA;VSTDPGHF	NCP	1.047
VSTDPGHF;VVGDHVEVH	NCP	0.075
VVGDHVEVH;GSLPSPTS	NCP	4.731
GSLPSPTS;LYRFLPQ	NCP	4.68
LYRFLPQ;RFGPPP	NCP	2.682
RFGPPP;LTKHMGK	NCP	54.603
LTKHMGK;LLQTEKYF	NCP	14.02
LLQTEKYF;LSDLVVPQ	NCP	17.393
LSDLVVPQ;KAFAGNK	NCP	142.537
KAFAGNK;CNCPP	NCP	67.211
CNCPP;QARLPR	NCP	112.536
QARLPR;GPPPPLKHP	NCP	63.552
GPPPPLKHP;KFYLLH	NCP	98.355
KFYLLH;RVPFLE	NCP	58.35
RVPFLE;SLRSYP	NCP	82.65
SLRSYP;LHEPPPPP	NCP	12.907
LHEPPPPP;SPGVVKR	NCP	5.124
SPGVVKR;LPRAPESD	NCP	3.618
LPRAPESD;AEFKEAF	NCP	12.821
AEFKEAF;EEFVQMMTAK	NCP	0.387
EEFVQMMTAK;HMNCCT	NCP	77.185
HMNCCT;RSGVLGK	NCP	26.927
RSGVLGK;VLSLYRNT	NCP	23.518
VLSLYRNT;LLTANRK	NCP	92.942

LLTANRK;HLTEAPK	NCP	38.178
HLTEAPK;TDEWRL	NCP	17.02
TDEWRL;LELLDMVK	NCP	98.777
LELLDMVK;YEGHFLK	NCP	152.778
YEGHFLK;QHTTKSK	NCP	114.633
QHTTKSK;KDSKWPR	NCP	13.782
KDSKWPR;KNLTGL	NCP	17.772
KNLTGL;YELAPL	NCP	11.988
YELAPL;CTLNNEC	NCP	71.446
CTLNNEC;AGSLLK	NCP	51.678
AGSLLK;PLSCLKL	NCP	56.57
PLSCLKL;LPESTRPP	NCP	17.667
LPESTRPP;VTPSEKFP	NCP	56.809
VTPSEKFP;KHSMLGFM	NCP	148.278
KHSMLGFM;LRMQP	NCP	95.049
LRMQP;HSLLV	NCP	10.896
HSLLV;PTLLVHG	NCP	15.138
PTLLVHG;KTDPSP	NCP	42.606
KTDPSP;SNYDNY	NCP	74.037
SNYDNY;WEGLPVP	NCP	53.549
WEGLPVP;ESGKGGP	NCP	56.82
ESGKGGP;SPLVP	NCP	13.941
SPLVP;AAQPLLR	NCP	30.868
AAQPLLR;TAPPP	NCP	32.789
TAPPP;GVPPPPP	NCP	170.502
GVPPPPP;WSGDGLL	NCP	0.183
WSGDGLL;NYGPPPP	NCP	17.377
NYGPPPP;SPEAPL	NCP	54.756
SPEAPL;MEALKK	NCP	66.222
MEALKK;KQLRSPK	NCP	15.825
KQLRSPK;LSSLFGGK	NCP	13.08
LSSLFGGK;QPGEGRP	NCP	77.66
QPGEGRP;KGEAAQ	NCP	1.627
KGEAAQ;DGQVLTL	NCP	26.421
DGQVLTL;ALGYE	NCP	15.843
ALGYE;VTAEVVLAH	NCP	88.733
VTAEVVLAH;LGGHL	NCP	67.981
LGGHL;THCDPK	NCP	3.57
THCDPK;FASSCR	NCP	141.59
FASSCR;GFAQPVA	NCP	78.649
GFAQPVA;VPFAKPPLGSLR	NCP	0.033
VPFAKPPLGSLR;WNELLSREVAK	NCP	22.623
WNELLSREVAK;SREVAKKAPHL	NCP	0.015
SREVAKKAPHL;FDLEAF	NCP	34.956
FDLEAF;PVTAPRP	NCP	10.416
PVTAPRP;VSYAPAK	NCP	44.262
VSYAPAK;LSVNPSR	NCP	66.239
LSVNPSR;SAEAAVP	NCP	1.719
SAEAAVP;KSADKPK	NCP	40.078
KSADKPK;TDDYDF	NCP	7.413
TDDYDF;FGPPVLSP	NCP	113.298

FGPPVLSP;LPGGEVAV	NCP	6.456
LPGGEVAV;AAPEKKV	NCP	73.257
AAPEKKV;KNQKNPTN	NCP	53.102
KNQKNPTN;LLQLLLSH	NCP	50.521
LLQLLLSH;VLLLPRE	NCP	19.031
VLLLPRE;KLLLE	NCP	38.379
KLLLE;DWHHGL	NCP	14.626
DWHHGL;KETPY	NCP	11.757
KETPY;AFPPPPPPPP	NCP	138.623
AFPPPPPPPP;WNELLSQEATK	NCP	70.966
WNELLSQEATK;QQHVRP	NCP	36.551
QQHVRP;GKTAL	NCP	93.178
GKTAL;PVPVTPP	NCP	18.069
PVPVTPP;DCDPK	NCP	0.914
DCDPK;QPGKPPR	NCP	30.072
QPGKPPR;AAVLF	NCP	80.884
AAVLF;TAPAPP	NCP	102.761
TAPAPP;YGYAAPK	NCP	102.25
YGYAAPK;LDGPSPK	NCP	26.207
LDGPSPK;AVPSGY	NCP	24.135
AVPSGY;EHLVDLK	NCP	44.046
EHLVDLK;EDLGKF	NCP	11.71
EDLGKF;KPDLP	NCP	44.631
KPDLP;LPVTRPP	NCP	2.525
LPVTRPP;VNSTVLTS	NCP	72.516
VNSTVLTS;TTPSAAKV	NCP	8.836
TTPSAAKV;EPEFPPP	NCP	31.251
EPEFPPP;ALRAMGR	NCP	11.733
ALRAMGR;RLPTHH	NCP	1.106
RLPTHH;NNVPRP	NCP	35.028
NNVPRP;AALPKL	NCP	35.512
AALPKL;DSSSFPR	NCP	7.044
DSSSFPR;KMLGVLLT	NCP	32.769
KMLGVLLT;KHMSRP	NCP	33.149
KHMSRP;GLYRVP	NCP	41.143
GLYRVP;KPLKQQK	NCP	6.662
KPLKQQK;GEPWPL	NCP	159.262
GEPWPL;KYPPPPPSP	NCP	99.783
KYPPPPPSP;VQATLTK	NCP	47.444
VQATLTK;KEGWPK	NCP	25.125
KEGWPK;LKEPQGTL	NCP	79.087
LKEPQGTL;ESHPLDF	NCP	30.473
ESHPLDF;ALSPPHLK	NCP	91.371
ALSPPHLK;NAPSFPGL	NCP	53.296
NAPSFPGL;QPPVCMP	NCP	8.225
QPPVCMP;LDAVVFT	NCP	3.25
LDAVVFT;KMSFPK	NCP	110.786
KMSFPK;WGPRFHPP	NCP	23.986
WGPRFHPP;ETKPPPA	NCP	17.099
ETKPPPA;VLLPEKF	NCP	2.07
VLLPEKF;QEKAKGLG	NCP	26.422

QEKAKGLG;CCVLLKK	NCP	2.016
CCVLLKK;HEGPFLFL	NCP	141.345
HEGPFLFL;YVTFH	NCP	26.328
YVTFH;KWFPK	NCP	99.053
KWFPK;PGLKSPE	NCP	9.031
PGLKSPE;LPPGL	NCP	101.933
LPPGL;KVSLGV	NCP	68.151
KVSLGV;LSSSMLP	NCP	34.806
LSSSMLP;WDDLRDK	NCP	37.027
WDDLRDK;SVYSDF	NCP	16.914
SVYSDF;KGEQLLR	NCP	9.842
KGEQLLR;LLPGG	NCP	25.279
LLPGG;QEALERM	NCP	120.999
QEALERM;KTFTTPT	NCP	94.235
KTFTTPT;RPAAKLT	NCP	9.232
RPAAKLT;RGPLPVK	NCP	53.729
RGPLPVK;TWLSWVA	NCP	19.86
TWLSWVA;QGSNQLK	NCP	57.291
QGSNQLK;NKYVPR	NCP	8.383
NKYVPR;SPPAPRLLS	NCP	43.755
SPPAPRLLS;KLKLP	NCP	14.208
KLKLP;MPAVLLL	NCP	17.834
MPAVLLL;RPPKLQ	NCP	140.65
RPPKLQ;KTKMPPK	NCP	7.991
KTKMPPK;LSQELQLK	NCP	22.588
LSQELQLK;VSGPLPLK	NCP	43.448
VSGPLPLK;TVPFVP	NCP	3.582
TVPFVP;CMDEC	NCP	42.567
CMDEC;KNSSRP	NCP	54.744
KNSSRP;KPGSGNKK	NCP	84.774
KPGSGNKK;ESVPKHK	NCP	85.207
ESVPKHK;QQQVPR	NCP	10.88
QQQVPR;APRSKPTP	NCP	8.673
APRSKPTP;EHEPLAP	NCP	0.354
EHEPLAP;RVMNVL	NCP	72.129
RVMNVL;AKFYTQL	NCP	19.809
AKFYTQL;WPSPKN	NCP	49.137
WPSPKN;RKLTLCE	NCP	30.523
RKLTLCE;TTAKNLYLG	NCP	34.832
TTAKNLYLG;SPPAARAGP	NCP	45.837
SPPAARAGP;QDKSPKKK	NCP	7.468
QDKSPKKK;DLGVLR	NCP	142.806
DLGVLR;TTFTTH	NCP	54.27
TTFTTH;DWMKDK	NCP	166.362
DWMKDK;LELLNNL	NCP	0.113
LELLNNL;HSSVD	NCP	84.265
HSSVD;KLLDL	NCP	13.391
KLLDL;PDAVHVK	NCP	45.136
PDAVHVK;HLLLPPP	NCP	59.111
HLLLPPP;TLAPQAV	NCP	27.855
TLAPQAV;ERYLRPP	NCP	16.071

ERYLRPP;SLLVTLH	NCP	9.327
SLLVTLH;AVPTLLK	NCP	10.374
AVPTLLK;RGSKSLFH	NCP	67.287
RGSKSLFH;EANGSG	NCP	9.588
EANGSG;LTVDHPK	NCP	67.608
LTVDHPK;PTAGKE	NCP	18.906
PTAGKE;ADAAPPT	NCP	1.054
ADAAPPT;AQVFRK	NCP	86.159
AQVFRK;RVSKSPK	NCP	47.319
RVSKSPK;ALHVPK	NCP	5.503
ALHVPK;VSRSHL	NCP	29.093
VSRSHL;VCTTF	NCP	14.137
VCTTF;SLTTGSH	NCP	27.063
SLTTGSH;LGPLYLK	NCP	111.738
LGPLYLK;KTTLSSLH	NCP	0.905
KTTLSSLH;VAVLSLLA	NCP	20.092
VAVLSLLA;KSSSKTGK	NCP	138.722
KSSSKTGK;RLNRVF	NCP	43.759
RLNRVF;SVLRLLVK	NCP	67.644
SVLRLLVK;LPLTPRKF	NCP	63.303
LPLTPRKF;MDRKKGP	NCP	8.735
MDRKKGP;PLYMQPL	NCP	74.104
PLYMQPL;MTGFC	NCP	14.417
MTGFC;ERTVFV	NCP	5.367
ERTVFV;DNLFYP	NCP	37.216
DNLFYP;TPQQVR	NCP	155.399
TPQQVR;EVLKPM	NCP	102.958
EVLKPM;PPKHQP	NCP	2.385
PPKHQP;DLLDL	NCP	53.747
DLLDL;LLKVLRKK	NCP	88.669
LLKVLRKK;MLELAVQKVVVHPL	NCP	22.301
MLELAVQKVVVHPL;QQLRVP	NCP	97.342
QQLRVP;VLAPPG	NCP	18.828
VLAPPG;HNKPPPP	NCP	100.16
HNKPPPP;SMPLRK	NCP	25.66
SMPLRK;KPDQLSP	NCP	107.216
KPDQLSP;KRLLDLR	NCP	32.829
KRLLDLR;KAGHFTK	NCP	38.46
KAGHFTK;LSYNLR	NCP	12.397
LSYNLR;KVPPHL	NCP	18.254
KVPPHL;EEVTVLTK	NCP	56.055
EEVTVLTK;SLGPVR	NCP	50.004
SLGPVR;PLFFLRD	NCP	57.04
PLFFLRD;LTDSHNK	NCP	24.173
LTDSHNK;KPDVAEP	NCP	0.6
KPDVAEP;EAPTVDLR	NCP	13.741
EAPTVDLR;KGAVGP	NCP	32.282
KGAVGP;MWYAKGK	NCP	12.001
MWYAKGK;HVLPPD	NCP	54.125
HVLPPD;QVTVP	NCP	34.413
QVTVP;VNELPK	NCP	18.415

VNELPK;AATKST	NCP	4.31
AATKST;KELEC	NCP	8.178
KELEC;NPPRGLED	NCP	130.05
NPPRGLED;AFLDF	NCP	2.656
AFLDF;LLALVGQL	NCP	147.524
LLALVGQL;KSLTFPK	NCP	14.497
KSLTFPK;PLLKWT	NCP	28.76
PLLKWT;LSTEEF	NCP	40.629
LSTEEF;EDFPLD	NCP	34.723
EDFPLD;LAGTL	NCP	61.706
LAGTL;TAESAF	NCP	22.828
TAESAF;KTSHRASP	NCP	21.461
KTSHRASP;AAPAEPLLK	NCP	11.34
AAPAEPLLK;RVPPPLSP	NCP	20.634
RVPPPLSP;EVTNLK	NCP	12.946
EVTNLK;PVAADPPL	NCP	107.579
PVAADPPL;QNNRLN	NCP	1.36
QNNRLN;EFVVQE	NCP	16.056
EFVVQE;KGYLLGP	NCP	5.031
KGYLLGP;VAGHVLY	NCP	22.071
VAGHVLY;NDSLLL	NCP	22.284
NDSLLL;GPEPAP	NCP	25.331
GPEPAP;TLELVEETVQAMD	NCP	7.293
TLELVEETVQAMD;VEETVQAMDVE	NCP	0.012
VEETVQAMDVE;KPVSLP	NCP	6.058
KPVSLP;MECDKT	NCP	12.033
MECDKT;YNKPPPP	NCP	1.964
YNKPPPP;EAAPGVP	NCP	19.701
EAAPGVP;SLLGTTSPK	NCP	41.787
SLLGTTSPK;EVGLPPVP	NCP	14.325
EVGLPPVP;LEDGPKF	NCP	16.095
LEDGPKF;AALDEVR	NCP	98.178
AALDEVR;ATPPDMR	NCP	4.626
ATPPDMR;APLDAPLP	NCP	6.558
APLDAPLP;GPVWLP	NCP	10.068
GPVWLP;GVLLP	NCP	1.185
GVLLP;SGVAVVGLK	NCP	76.125
SGVAVVGLK;FPGLP	NCP	20.238
FPGLP;NFEPV	NCP	20.506
NFEPV;AASTDVAGLEESFRK	NCP	66.422
AASTDVAGLEESFRK;TGGAVERLTDTSK	NCP	0.579
TGGAVERLTDTSK;AVERLTDTSKYTGSHKER	NCP	0.009
AVERLTDTSKYTGSHKER;YKNAGTYDAKVKK	NCP	0.129
YKNAGTYDAKVKK;NFGLKRE	NCP	33.898
NFGLKRE;GDLPY	NCP	0.122
GDLPY;KYMSSK	NCP	68.698
KYMSSK;FPGENK	NCP	49.313
FPGENK;MSNRRR	NCP	22.569
MSNRRR;LLSEAQLL	NCP	21.406
LLSEAQLL;LPLPHKH	NCP	1.467
LPLPHKH;SPPEEKPPPP	NCP	11.081

SPPEEKPPPP;PSSVLP	NCP	59.307
PSSVLP;AQPGKLLK	NCP	199.758
AQPGKLLK;GSSVAQLVK	NCP	47.47
GSSVAQLVK;VNLLP	NCP	5.762
VNLLP;KTGSPGSPGAGGVQSTSK	NCP	28.257
KTGSPGSPGAGGVQSTSK;LEVRWH	NCP	76.554
LEVRWH;KPTEVVH	NCP	22.164
KPTEVVH;TVAFVSPL	NCP	43.339
TVAFVSPL;VGGPLSP	NCP	11.18
VGGPLSP;GVTGKHF	NCP	34.587
GVTGKHF;LRPVVVV	NCP	7.483
LRPVVVV;PAPPPVPP	NCP	30.122
PAPPPVPP;VDTVRGP	NCP	212.004
VDTVRGP;CGPPK	NCP	156.369
CGPPK;SCKYHPH	NCP	18.514
SCKYHPH;KRLELGSSG	NCP	151.842
KRLELGSSG;MEDLMGK	NCP	3.587
MEDLMGK;LALSSGF	NCP	38.535
LALSSGF;TSDGP	NCP	100.422
TSDGP;PDLPGFP	NCP	2.748
PDLPGFP;NAAPQPR	NCP	224.773
NAAPQPR;TAVRVPA	NCP	85.637
TAVRVPA;TATLRPY	NCP	68.499
TATLRPY;LPPTPGL	NCP	18.937
LPPTPGL;DRPAY	NCP	18.659
DRPAY;MPFGAPL	NCP	10.189
MPFGAPL;HDRGP	NCP	48.267
HDRGP;VDLKGPK	NCP	102.515
VDLKGPK;QESAVPL	NCP	22.185
QESAVPL;KTGNPRP	NCP	5.95
KTGNPRP;ALEAHK	NCP	4.691
ALEAHK;SPMEL	NCP	8.206
SPMEL;APARGPPP	NCP	82.73
APARGPPP;LAASAPLRPP	NCP	0.978
LAASAPLRPP;GPPVLA	NCP	9.753
GPPVLA;VGPGTSLK	NCP	48.283
VGPGTSLK;LPAGVPK	NCP	69.957
LPAGVPK;LRHFPL	NCP	43.125
LRHFPL;VRVNPP	NCP	17.073
VRVNPP;APVKKLVA	NCP	92.409
APVKKLVA;KPCQAAP	NCP	1.232
KPCQAAP;VSVDLVR	NCP	24.856
VSVDLVR;KQAQKAP	NCP	30.747
KQAQKAP;AGGAVLNP	NCP	43.283
AGGAVLNP;ASSAH	NCP	102.691
ASSAH;LYEEEE	NCP	9.954
LYEEEE;ELGVPL	NCP	7.779
ELGVPL;ESLAF	NCP	31.922
ESLAF;NPPVTME	NCP	55.137
NPPVTME;TDLRFQ	NCP	2.5
TDLRFQ;GPLDGK	NCP	53.211

GPLDGK;LGAHVFN	NCP	70.536
LGAHVFN;TFSLLC	NCP	61.302
TFSLLC;HPRPGPP	NCP	10.641
HPRPGPP;VHQVLYR	NCP	71.81
VHQVLYR;KHETTY	NCP	6.003
KHETTY;QFLLPK	NCP	24.841
QFLLPK;LVHRKCF	NCP	49.223
LVHRKCF;FGHPGAK	NCP	46.173
FGHPGAK;DDDDDY	NCP	12.378
DDDDDY;KDGGF	NCP	2.548
KDGGF;DPERYF	NCP	60.56
DPERYF;LQGLEL	NCP	43.936
LQGLEL;DKALFMR	NCP	19.104
DKALFMR;AQAFV	NCP	69.336
AQAFV;DLGTLA	NCP	36.542
DLGTLA;YSTFW	NCP	47.353
YSTFW;KKMGP	NCP	109.755
KKMGP;LEAPDVS	NCP	28.937
LEAPDVS;QVVPF	NCP	7.21
QVVPF;PAAVVE	NCP	42.906
PAAVVE;VRELTF	NCP	12.671
VRELTF;VGRPPPPPP	NCP	25.822
VGRPPPPPP;VLCVRF	NCP	137.246
VLCVRF;AQLKVFN	NCP	8.851
AQLKVFN;VPDLDVK	NCP	9.323
VPDLDVK;LGRLPH	NCP	111.265
LGRLPH;KNMAPQ	NCP	102.953
KNMAPQ;ALLVLF	NCP	2.758
ALLVLF;NPSTMKLK	NCP	27.569
NPSTMKLK;AKSLVAL	NCP	17.815
AKSLVAL;KYFAPPGP	NCP	9.234
KYFAPPGP;DNQLLR	NCP	22.392
DNQLLR;ENLLTHV	NCP	1.658
ENLLTHV;LNLSQL	NCP	56.134
LNLSQL;LLPLPEVT	NCP	2.619
LLPLPEVT;NYYHR	NCP	100.917
NYYHR;LVLGMGT	NCP	11.568
LVLGMGT;DLERTP	NCP	75.387
DLERTP;NNNEC	NCP	16.484
NNNEC;KVVGVPQ	NCP	57.085
KVVGVPQ;CKLMHN	NCP	117.578
CKLMHN;KAGELQVP	NCP	64.543
KAGELQVP;CFFKG	NCP	81.032
CFFKG;PFFFP	NCP	53.613
PFFFP;ASARLLAR	NCP	10.654
ASARLLAR;LALNL	NCP	72.341
LALNL;DFLFM	NCP	125.286
DFLFM;LPLEGK	NCP	13.152
LPLEGK;GYKSC	NCP	13.626
GYKSC;KYLPPFF	NCP	13.312
KYLPPFF;MELSFY	NCP	110.091

MELSFY;GDKLGP	NCP	20.75
GDKLGP;LPGKSLL	NCP	49.459
LPGKSLL;AAPKKPP	NCP	8.505
AAPKKPP;MARKAAL	NCP	9.668
MARKAAL;SFLVLKK	NCP	32.518
SFLVLKK;LDDLSGP	NCP	62.393
LDDLSGP;TEEYGY	NCP	42.178
TEEYGY;SGSFVK	NCP	16.593
SGSFVK;TKCVF	NCP	173.892
TKCVF;TVLSL	NCP	5.339
TVLSL;NPMLKNQ	NCP	0.36
NPMLKNQ;GVTHVL	NCP	11.034
GVTHVL;GWLEPL	NCP	44.269
GWLEPL;SKFFEE	NCP	124.397
SKFFEE;KANPPDK	NCP	91.336
KANPPDK;SRPLNPK	NCP	21.162
SRPLNPK;VGGAKPR	NCP	35.691
VGGAKPR;PRFQP	NCP	60.47
PRFQP;LSMGPF	NCP	76.359
LSMGPF;PGAVAK	NCP	20.424
PGAVAK;YEDFMP	NCP	182.934
YEDFMP;FPVELHT	NCP	270.207
FPVELHT;QVELACR	NCP	10.558
QVELACR;WAKVDVP	NCP	0.987
WAKVDVP;LAKKANA	NCP	174.804
LAKKANA;YGVGF	NCP	98.723
YGVGF;LVLLPAT	NCP	28.056
LVLLPAT;GKDLAPL	NCP	10.105
GKDLAPL;ETVAAH	NCP	177.42
ETVAAH;FSATRYP	NCP	43.17
FSATRYP;HKLKFK	NCP	121.076
HKLKFK;LGTGDLRK	NCP	7.29
LGTGDLRK;LALALL	NCP	182.097
LALALL;YESAMGY	NCP	5.224
YESAMGY;AALLTVK	NCP	74.36
AALLTVK;KVTKKGP	NCP	5.878
KVTKKGP;AKLGVLG	NCP	20.733
AKLGVLG;KGATRYK	NCP	25.344
KGATRYK;RKYLVVC	NCP	0.702
RKYLVVC;QPFESPR	NCP	26.325
QPFESPR;PGELTVHAV	NCP	63.573
PGELTVHAV;VVDKLEK	NCP	3.339
VVDKLEK;DLDSMSS	NCP	35.889
DLDSMSS;TLLLYK	NCP	42.422
TLLLYK;KKKPPAT	NCP	72.432
KKKPPAT;LFVLPFE	NCP	63.93
LFVLPFE;QAAFVSN	NCP	5.308
QAAFVSN;TVAVFHK	NCP	65.184
TVAVFHK;TVFPDLL	NCP	12.072
TVFPDLL;QQCMFF	NCP	146.724
QQCMFF;APPEVPK	NCP	62.025

APPEVPK;LGPTETM	NCP	1.265
LGPTETM;YGVVMP	NCP	13.567
YGVVMP;MGESSGK	NCP	63.017
MGESSGK;LPLPPPAPP	NCP	43.513
LPLPPPAPP;YGYVPGL	NCP	76.644
YGYVPGL;GVLPPE	NCP	146.196
GVLPPE;LAGLSV	NCP	63.323
LAGLSV;SWLHSTMR	NCP	64.833
SWLHSTMR;KPVSRHL	NCP	11.53
KPVSRHL;SSWWAHVE	NCP	16.487
SSWWAHVE;MKPLLLQ	NCP	16.249
MKPLLLQ;LGPPFQL	NCP	3.953
LGPPFQL;GPPGPPLS	NCP	36.424
GPPGPPLS;TMEVP	NCP	32.87
TMEVP;NVELTKP	NCP	58.74
NVELTKP;YSAALLEY	NCP	20.044
YSAALLEY;WGSGNLG	NCP	22.527
WGSGNLG;YDLKKVG	NCP	10.647
YDLKKVG;GPYTSWP	NCP	28.052
GPYTSWP;PSGVHG	NCP	27.526
PSGVHG;FKFKPAP	NCP	102.644
FKFKPAP;RHEELK	NCP	15.12
RHEELK;PLFLVKP	NCP	13.311
PLFLVKP;PAAAMPSP	NCP	7.969
PAAAMPSP;CQPNPR	NCP	5.942
CQPNPR;HSMDLRK	NCP	17.404
HSMDLRK;VSRKHD	NCP	2.75
VSRKHD;KDFSPPK	NCP	11.349
KDFSPPK;GPGPPVP	NCP	7.144
GPGPPVP;VGAAQHV	NCP	17.294
VGAAQHV;GCHGHPR	NCP	29.004
GCHGHPR;ETSKPL	NCP	10.338
ETSKPL;ALPHA	NCP	1.776
ALPHA;LCLAA	NCP	35.472
LCLAA;EAAAE	NCP	19.255
EAAAE;TEELV	NCP	33.941
TEELV;RPPGPRPP	NCP	38.127
RPPGPRPP;KSGHFFP	NCP	78.301
KSGHFFP;KHFKGPK	NCP	85.302
KHFKGPK;DLYPEF	NCP	12.761
DLYPEF;KPPAQGHF	NCP	44.376
KPPAQGHF;NLSLK	NCP	5.491
NLSLK;RGEDVLP	NCP	0.71
RGEDVLP;CCCCCCEK	NCP	67.062
CCCCCCEK;LEVERK	NCP	20.955
LEVERK;KPRSLGR	NCP	10.968
KPRSLGR;LSGPK	NCP	87.52
LSGPK;RTDPVGP	NCP	19.881
RTDPVGP;LTSSNGSL	NCP	32.79
LTSSNGSL;AEFPSKVNT	NCP	74.258
AEFPSKVNT;VGPVLSVK	NCP	20.205

VGPVLSVK;TATLP	NCP	18.093
TATLP;SYFQV	NCP	6.339
SYFQV;NRLPVVL	NCP	49.195
NRLPVVL;SPPLF	NCP	25.604
SPPLF;NGCMD	NCP	0.378
NGCMD;KPPERPP	NCP	30.822
KPPERPP;LPPPYK	NCP	1.216
LPPPYK;RHGAPK	NCP	28.449
RHGAPK;RVSSRSPL	NCP	12.953
RVSSRSPL;VPGMNPP	NCP	38.997
VPGMNPP;MCCYSF	NCP	56.121
MCCYSF;NSKLLR	NCP	50.209
NSKLLR;FTTLKF	NCP	8.955
FTTLKF;TVYFK	NCP	45.605
TVYFK;KDDDLR	NCP	42.751
KDDDLR;VTGGLWR	NCP	0.569
VTGGLWR;KFNAANN	NCP	7.284
KFNAANN;YLSAML	NCP	78.222
YLSAML;KYPSPK	NCP	192.166
KYPSPK;TSPVDLR	NCP	3.944
TSPVDLR;KVLLLLL	NCP	12.994
KVLLLLL;KPGGCLR	NCP	8.009
KPGGCLR;MGPWF	NCP	10.38
MGPWF;EMSSGDM	NCP	3.63
EMSSGDM;PEKTEL	NCP	17.658
PEKTEL;CNPAPKK	NCP	105.987
CNPAPKK;FLLVKK	NCP	5.247
FLLVKK;SGEDVKQYKPVV	NCP	68.034
SGEDVKQYKPVV;KASKPPK	NCP	9.409
KASKPPK;VSSSLTK	NCP	56.643
VSSSLTK;LGGVPSP	NCP	6.104
LGGVPSP;KEKGLK	NCP	84.597
KEKGLK;KDLPAGL	NCP	61.702
KDLPAGL;RSPLQFR	NCP	29.187
RSPLQFR;CPVGCAKCAQGCVCKGASDK	NCP	48.758
CPVGCAKCAQGCVCKGASDK;CVCKGASDKCSCCA	NCP	0.033
CVCKGASDKCSCCA;PPPFSRDV	NCP	6.261
PPPFSRDV;SCCPVGCAK	NCP	6.756
SCCPVGCAK;LSFGSFL	NCP	14.832
LSFGSFL;TPATVPLPT	NCP	66.588
TPATVPLPT;KEALSRAL	NCP	39.055
KEALSRAL;GQVLEY	NCP	11.831
GQVLEY;FCLRVF	NCP	74.116
FCLRVF;PLSLPPLPP	NCP	33.117
PLSLPPLPP;RPPLEQK	NCP	28.74
RPPLEQK;KHKLP	NCP	22.59
KHKLP;DPASVAKK	NCP	18.995
DPASVAKK;LTGKPEK	NCP	51.49
LTGKPEK;KGACPPK	NCP	19.856
KGACPPK;SLDLPVE	NCP	41.247
SLDLPVE;NAVLPDP	NCP	26.692

NAVLPDP;RPAVTKY	NCP	41.613
RPAVTKY;PGAEYSK	NCP	13.683
PGAEYSK;HDTPLLL	NCP	3.728
HDTPLLL;KEWQPLGK	NCP	8.967
KEWQPLGK;ERSLQTAV	NCP	48.237
ERSLQTAV;SEVGGPQ	NCP	13.606
SEVGGPQ;SASLDPRN	NCP	11.565
SASLDPRN;LLGGGP	NCP	116.877
LLGGGP;ESLKTPA	NCP	1.203
ESLKTPA;SGAKALR	NCP	0.867
SGAKALR;EETPPA	NCP	57.258
EETPPA;TPRVHYR	NCP	6.08
TPRVHYR;GPAGKPTPP	NCP	18.628
GPAGKPTPP;LAPAH	NCP	5.681
LAPAH;MEKPSFP	NCP	102.289
MEKPSFP;MKPLGL	NCP	8.558
MKPLGL;RFHGQP	NCP	76.983
RFHGQP;KMGVP	NCP	4.134
KMGVP;RELGGSL	NCP	10.095
RELGGSL;ESATVPR	NCP	7.114
ESATVPR;QALVK	NCP	284.262
QALVK;LRKSPP	NCP	62.735
LRKSPP;ALELLKT	NCP	185.896
ALELLKT;LVTTSAK	NCP	94.11
LVTTSAK;LTLAL	NCP	14.721
LTLAL;YEAATF	NCP	4.109
YEAATF;MVKKPP	NCP	62.487
MVKKPP;KLVHAVP	NCP	60.778
KLVHAVP;KAQDLTLP	NCP	82.247
KAQDLTLP;TMVQPK	NCP	46.344
TMVQPK;RTGDFQLH	NCP	11.535
RTGDFQLH;LSALVDGKS	NCP	0.255
LSALVDGKS;NNNLSK	NCP	14.166
NNNLSK;VLERVGLC	NCP	107.512
VLERVGLC;KGFVLAP	NCP	12.494
KGFVLAP;KDLSGPK	NCP	0.129
KDLSGPK;LSVTTAGP	NCP	47.982
LSVTTAGP;PDKKSVP	NCP	12.69
PDKKSVP;ESKKKHE	NCP	91.137
ESKKKHE;KFVHLYK	NCP	106.428
KFVHLYK;SAETTK	NCP	77.814
SAETTK;LSGGLV	NCP	57.265
LSGGLV;DLAVAP	NCP	59.442
DLAVAP;HKAHLVP	NCP	1.131
HKAHLVP;VGPKPLN	NCP	54.306
VGPKPLN;PLLALPG	NCP	22.488
PLLALPG;EPKPAW	NCP	22.326
EPKPAW;NAVVLVT	NCP	7.896
NAVVLVT;TTKAP	NCP	42.702
TTKAP;CMFNPLL	NCP	13.865
CMFNPLL;AVQAAPR	NCP	35.773

AVQAAPR;KGKPTHH	NCP	60.264
KGKPTHH;TLMLTY	NCP	7.473
TLMLTY;LNPTHPL	NCP	31.485
LNPTHPL;TPPLAAPQ	NCP	90.528
TPPLAAPQ;VTGPPL	NCP	52.614
VTGPPL;TTKDPP	NCP	60.501
TTKDPP;LSLNNM	NCP	30.065
LSLNNM;KEHEYK	NCP	12.291
KEHEYK;KSMGPP	NCP	12.343
KSMGPP;MAMLQ	NCP	69.921
MAMLQ;VGDLGVK	NCP	57.183
VGDLGVK;LNFMP	NCP	58.779
LNFMP;QFLHQQKG	NCP	15.879
QFLHQQKG;ELLEL	NCP	0.786
ELLEL;LRTGAVF	NCP	28.847
LRTGAVF;EPLVL	NCP	53.173
EPLVL;ASRPLLR	NCP	4.008
ASRPLLR;LDADHSPK	NCP	17.316
LDADHSPK;LHPPKVHQ	NCP	39.633
LHPPKVHQ;KPTREPS	NCP	87.618
KPTREPS;THYKSLP	NCP	18.407
THYKSLP;FEPFQL	NCP	33.643
FEPFQL;VYQLVK	NCP	33.372
VYQLVK;EPLELGFN	NCP	22.713
EPLELGFN;SVLMGCK	NCP	131.235
SVLMGCK;LLFGFDP	NCP	12.866
LLFGFDP;LSRVHL	NCP	28.923
LSRVHL;KVSATSPR	NCP	56.799
KVSATSPR;LAAGASF	NCP	7.582
LAAGASF;LPPNVQ	NCP	160.403
LPPNVQ;HLKGSQYK	NCP	13.783
HLKGSQYK;YTMVKRH	NCP	215.447
YTMVKRH;SVATQPK	NCP	128.697
SVATQPK;KASYSAK	NCP	56.226
KASYSAK;KCMPR	NCP	30.436
KCMPR;VPRAASQT	NCP	22.523
VPRAASQT;KALQPVP	NCP	5.659
KALQPVP;SFYNEL	NCP	169.664
SFYNEL;KCLSAGP	NCP	27.577
KCLSAGP;RGLLTLL	NCP	13.755
RGLLTLL;FPGAFMP	NCP	3.422
FPGAFMP;APGVHKP	NCP	71.506
APGVHKP;RAPNYK	NCP	40.175
RAPNYK;AATPGLLR	NCP	60.853
AATPGLLR;LKLLQL	NCP	16.634
LKLLQL;PAAKQPR	NCP	39.117
PAAKQPR;EAPPSCE	NCP	17.713
EAPPSCE;PGPHMPPP	NCP	153.579
PGPHMPPP;GKGGPEVP	NCP	80.156
GKGGPEVP;LKTPEVPP	NCP	11.499
LKTPEVPP;HDFLVGL	NCP	7.33

HDFLVGL;QPGDPTK	NCP	15.707
QPGDPTK;KQHYVL	NCP	13.662
KQHYVL;RPVGKSL	NCP	5.052
RPVGKSL;LPLKD	NCP	13.365
LPLKD;LELVPD	NCP	32.275
LELVPD;LPALATK	NCP	63.284
LPALATK;GVVTL	NCP	27.663
GVVTL;QGLLELY	NCP	51.514
QGLLELY;LKYHWP	NCP	5.891
LKYHWP;YPFPGLP	NCP	57.16
YPFPGLP;KVKTGPP	NCP	35.987
KVKTGPP;WHFRSYG	NCP	3.262
WHFRSYG;RLLLPGE	NCP	80.256
RLLLPGE;CMDDC	NCP	23.708
CMDDC;PPPHVLLCPK	NCP	39.847
PPPHVLLCPK;GPPDLLPP	NCP	145.878
GPPDLLPP;DHPLPPP	NCP	43.055
DHPLPPP;AARPRPP	NCP	58.299
AARPRPP;TLPPRPH	NCP	51.94
TLPPRPH;SPLKSSFR	NCP	57.908
SPLKSSFR;LSLYF	NCP	55.999
LSLYF;VVAPLP	NCP	38.489
VVAPLP;RVTLMC	NCP	16.549
RVTLMC;EDVESP	NCP	9.47
EDVESP;APARYK	NCP	83.131
APARYK;DGLKKAR	NCP	36.242
DGLKKAR;MLNVQNK	NCP	36.118
MLNVQNK;KNPVGPP	NCP	95.099
KNPVGPP;KRPPLQ	NCP	20.767
KRPPLQ;RDYPQ	NCP	35.958
RDYPQ;SLVGL	NCP	24.84
SLVGL;ESPFKH	NCP	57.803
ESPFKH;MTVHRK	NCP	120.195
MTVHRK;PPPLVQPP	NCP	119.356
PPPLVQPP;KVYLR	NCP	24.276
KVYLR;PAKVETH	NCP	18.854
PAKVETH;GDLPPP	NCP	2.167
GDLPPP;NSLMKCDV	NCP	56.985
NSLMKCDV;LKHRFV	NCP	144.443
LKHRFV;ASLSL	NCP	141.408
ASLSL;PSVGVN	NCP	113.349
PSVGVN;PVSPNKK	NCP	15.663
PVSPNKK;PAAMKQR	NCP	29.676
PAAMKQR;KDAAPPPPP	NCP	23.871
KDAAPPPPP;VCAGGLVA	NCP	41.88
VCAGGLVA;PPGPALP	NCP	50.118
PPGPALP;LAPKNPT	NCP	12.585
LAPKNPT;RLVWKVSN	NCP	0.345
RLVWKVSN;KVRKLLSH	NCP	21.544
KVRKLLSH;PVGTGLR	NCP	14.255
PVGTGLR;KRLLKTEL	NCP	80.613

KRLLKTEL;TANLHRK	NCP	16.53
TANLHRK;LPPPPPV	NCP	21.769
LPPPPPV;VGAASELL	NCP	5.888
VGAASELL;FKELEKK	NCP	9.486
FKELEKK;EGDDS	NCP	40.684
EGDDS;GLVPPL	NCP	5.451
GLVPPL;VPPVGP	NCP	1.2
VPPVGP;KMLAAPG	NCP	75.929
KMLAAPG;PPTPGLHA	NCP	9.01
PPTPGLHA;LSGGLLA	NCP	52.02
LSGGLLA;TQCDLR	NCP	0.287
TQCDLR;MAPEV	NCP	4.213
MAPEV;KDHLTP	NCP	43.763
KDHLTP;QAPGVR	NCP	43.825
QAPGVR;PKLPAPP	NCP	17.802
PKLPAPP;AVSHAPGP	NCP	12.18
AVSHAPGP;SLPMGP	NCP	8.802
SLPMGP;KFHPPVT	NCP	8.685
KFHPPVT;LTLPVT	NCP	64.509
LTLPVT;TKPGM	NCP	25.869
TKPGM;NGPPPVR	NCP	17.406
NGPPPVR;RKGRLP	NCP	15.054
RKGRLP;TPFPPNP	NCP	111.477
TPFPPNP;KFKLD	NCP	26.555
KFKLD;EEHPTL	NCP	60.28
EEHPTL;LERLYLSK	NCP	8.855
LERLYLSK;PKAPRPP	NCP	36.631
PKAPRPP;VADTLTTPL	NCP	54.453
VADTLTTPL;PHALALL	NCP	0.216
PHALALL;KPELTPP	NCP	6.198
KPELTPP;LYKVSSK	NCP	293.984
LYKVSSK;KPGSVAP	NCP	12.951
KPGSVAP;PEGPRGPSA	NCP	3.853
PEGPRGPSA;KDDPMK	NCP	78.674
KDDPMK;AEPPPPR	NCP	40.081
AEPPPPR;DDHGG	NCP	35.786
DDHGG;AAPEFW	NCP	5.646
AAPEFW;CGPLLL	NCP	48.949
CGPLLL;DDMECGCE	NCP	81.648
DDMECGCE;TAEVSLP	NCP	7.307
TAEVSLP;TTYLEF	NCP	7.003
TTYLEF;MMDTTK	NCP	135.81
MMDTTK;QHGPTLR	NCP	75.632
QHGPTLR;GPPPPHLNK	NCP	20.811
GPPPPHLNK;LSPWSYTTYRGS	NCP	0.354
LSPWSYTTYRGS;KGVAFTK	NCP	26.397
KGVAFTK;VAFTKP	NCP	0.006
VAFTKP;HQDAPPP	NCP	11.587
HQDAPPP;AAAALP	NCP	4.304
AAAALP;KGPAAVPR	NCP	15.313
KGPAAVPR;KWEAGP	NCP	45.239

KWEAGP;PPPPPHQ	NCP	22.309
PPPPPHQ;KPSPVHR	NCP	59.544
KPSPVHR;ARLHKP	NCP	11.439
ARLHKP;ESAAGPK	NCP	55.872
ESAAGPK;GRLPPR	NCP	4.934
GRLPPR;RAGPPAAP	NCP	41.47
RAGPPAAP;APYRAK	NCP	15.675
APYRAK;LHFRGAK	NCP	24.417
LHFRGAK;FTGAAG	NCP	30.987
FTGAAG;SAAPELGLR	NCP	56.612
SAAPELGLR;WQPAPHF	NCP	10.185
WQPAPHF;WTGPGP	NCP	31.492
WTGPGP;VAAPPK	NCP	24.848
VAAPPK;ELLVK	NCP	15.042
ELLVK;AGAKHPP	NCP	43.56
AGAKHPP;CSKKAAP	NCP	28.99
CSKKAAP;WAALLR	NCP	22.739
WAALLR;AGFLSQRT	NCP	7.378
AGFLSQRT;PGAAPTRP	NCP	51.033
PGAAPTRP;DRWRSPP	NCP	36.209
DRWRSPP;CTLPAGWK	NCP	22.975
CTLPAGWK;ADPELQLVAR	NCP	57.041
ADPELQLVAR;RGFLFGPSL	NCP	1.644
RGFLFGPSL;EQASPVGP	NCP	1.8
EQASPVGP;KGVVSPK	NCP	5.035
KGVVSPK;MVEMTPV	NCP	5.726
MVEMTPV;AQWNQL	NCP	17.523
AQWNQL;GTEFL	NCP	22.903
GTEFL;LPPPPSPSLP	NCP	3.365
LPPPPSPSLP;ALPAVP	NCP	6.153
ALPAVP;PGPAPP	NCP	2.778
PGPAPP;LGFLKDL	NCP	0.976
LGFLKDL;PLGLVP	NCP	3.189
PLGLVP;LKSQP	NCP	14.298
LKSQP;APEPGPPR	NCP	8.255
APEPGPPR;KLEERGFDKAY	NCP	30.16
KLEERGFDKAY;APLPEKT	NCP	18.605
APLPEKT;QGAWPPP	NCP	10.305
QGAWPPP;VHLQK	NCP	23.352
VHLQK;RNKDP	NCP	13.507
RNKDP;APGAHALL	NCP	48.737
APGAHALL;RKGPLL	NCP	40.387
RKGPLL;HGRPPPP	NCP	47.385
HGRPPPP;PGARPP	NCP	21.315
PGARPP;HSSGVLVT	NCP	5.924
HSSGVLVT;PVGPPPPP	NCP	41.272
PVGPPPPP;DVEVA	NCP	14.639
DVEVA;LPPVVGE	NCP	2.398
LPPVVGE;LTPLL	NCP	34.56
LTPLL;TALKHALRQ	NCP	6.266
TALKHALRQ;LGSAADF	NCP	17.658

LGSAADF;RSAPSLLR	NCP	9.165
RSAPSLLR;PANMLPR	NCP	8.937
PANMLPR;KEAGAGP	NCP	70.03
KEAGAGP;KNPLTGGV	NCP	102.401
KNPLTGGV;RPVPARP	NCP	30.006
RPVPARP;KPGTVAVP	NCP	2.872
KPGTVAVP;TAEFKEAF	NCP	4.026
TAEFKEAF;QLSGVLP	NCP	16.496
QLSGVLP;VSPAVEK	NCP	53.131
VSPAVEK;KVGPTVL	NCP	30.012
KVGPTVL;VGDSF	NCP	23.21
VGDSF;KGYHLVV	NCP	18.373
KGYHLVV;LLLTPLPD	NCP	8.862
LLLTPLPD;QGPPKPP	NCP	10.532
QGPPKPP;RGYPPPRD	NCP	5.635
RGYPPPRD;PKMVRPP	NCP	19.809
PKMVRPP;ALPGLP	NCP	7.664
ALPGLP;DKPPCPGP	NCP	158.158
DKPPCPGP;CWLLVLH	NCP	24.725
CWLLVLH;QPSWLR	NCP	25.036
QPSWLR;AAPVGR	NCP	54.797
AAPVGR;EDADGLPF	NCP	38.859
EDADGLPF;LTHSGPP	NCP	6.021
LTHSGPP;KPRDDR	NCP	37.779
KPRDDR;PLSVLPAP	NCP	53.55
PLSVLPAP;PHVVLAA	NCP	27.379
PHVVLAA;RGPPQRP	NCP	44.997
RGPPQRP;PGPRAAPPPP	NCP	0.495
PGPRAAPPPP;ERPPPQT	NCP	26.666
ERPPPQT;KVRPTGP	NCP	12.331
KVRPTGP;LGERAGSY	NCP	31.467
LGERAGSY;ELAAAPG	NCP	45.347
ELAAAPG;KSGVGDVP	NCP	5.406
KSGVGDVP;ASAPSLGV	NCP	24.349
ASAPSLGV;SPLRKL	NCP	0.179
SPLRKL;LPTVG	NCP	42.388
LPTVG;RKPAGEK	NCP	82.038
RKPAGEK;PAAPEVP	NCP	103.109
PAAPEVP;FVGAPL	NCP	16.395
FVGAPL;APAPADRP	NCP	5.391
APAPADRP;EDMVHRK	NCP	8.677
EDMVHRK;PPPPRY	NCP	18.426
PPPPRY;KTVRSLP	NCP	120.815
KTVRSLP;QEGTLSL	NCP	86.422
QEGTLSL;PQKLPAP	NCP	26.375
PQKLPAP;RLSFLKK	NCP	29.82
RLSFLKK;KELGGVP	NCP	29.602
KELGGVP;VAVGVLAK	NCP	4.442
VAVGVLAK;LEKGYEKK	NCP	45.111
LEKGYEKK;PRPWPK	NCP	9.421
PRPWPK;SLTPDRQ	NCP	104.189

SLTPDRQ;CRRLLLD	NCP	19.506
CRRLLLD;SGLPELL	NCP	38.874
SGLPELL;RVPEAKGP	NCP	70.926
RVPEAKGP;EGVPAGAGK	NCP	8.355
EGVPAGAGK;EHSPAPPP	NCP	32.253
EHSPAPPP;TGPGK	NCP	0.339
TGPGK;VSGKTLVQ	NCP	0.604
VSGKTLVQ;KGYNGPK	NCP	29.553
KGYNGPK;PAAYGGP	NCP	28.916
PAAYGGP;EHGLQ	NCP	6.148
EHGLQ;VLVLK	NCP	24.113
VLVLK;APAAPPPK	NCP	70.803
APAAPPPK;PEPLLRP	NCP	23.418
PEPLLRP;VSALTVVR	NCP	9.867
VSALTVVR;KPVPRK	NCP	80.796
KPVPRK;LLPYLS	NCP	43.351
LLPYLS;KDEDPK	NCP	58.107
KDEDPK;SDLRLVT	NCP	70.374
SDLRLVT;APAAGPPR	NCP	25.164
APAAGPPR;CKLYKC	NCP	27.164
CKLYKC;EPPLPRP	NCP	6.366
EPPLPRP;GPLRPLPP	NCP	23.505
GPLRPLPP;PVVLLYR	NCP	30.871
PVVLLYR;RPPKHL	NCP	4.152
RPPKHL;ENSGMDF	NCP	43.08
ENSGMDF;RLVHQP	NCP	0.407
RLVHQP;LLALGPH	NCP	13.908
LLALGPH;KAGSPY	NCP	3.834
KAGSPY;GVSYVP	NCP	30.151
GVSYVP;VPGVRL	NCP	83.351
VPGVRL;VPLREQGN	NCP	17.121
VPLREQGN;LGPAPPVK	NCP	18.481
LGPAPPVK;KCVGAP	NCP	129.206
KCVGAP;PALGQPK	NCP	28.599
PALGQPK;LPVAAREF	NCP	38.607
LPVAAREF;EGSSEMG	NCP	96.691
EGSSEMG;PGGPPAPPP	NCP	9.462
PGGPPAPPP;TQPVP	NCP	1.307
TQPVP;TVVHLQ	NCP	17.218
TVVHLQ;LPAPAAGP	NCP	28.439
LPAPAAGP;GLLPLTH	NCP	98.785
GLLPLTH;LPDTVPR	NCP	80.313
LPDTVPR;EAVGSLPK	NCP	69.177
EAVGSLPK;KDFLYP	NCP	51.959
KDFLYP;VPLLE	NCP	9.219
VPLLE;MMSVNK	NCP	53.647
MMSVNK;LAELL	NCP	8.363
LAELL;PVAAPPPP	NCP	42.892
PVAAPPPP;KKLPVAL	NCP	0.824
KKLPVAL;QAFLRVW	NCP	65.959
QAFLRVW;LDDVLKN	NCP	25.565

LDDVLKN;EPVKLPK	NCP	87.004
EPVKLPK;LYGFN	NCP	3.929
LYGFN;PPPGRPP	NCP	48.534
PPPGRPP;EFHPLSR	NCP	11.514
EFHPLSR;KPAAVVTK	NCP	104.244
KPAAVVTK;PLNGYPK	NCP	94.77
PLNGYPK;KKMRHGP	NCP	52.867
KKMRHGP;LDNLPY	NCP	11.036
LDNLPY;VGSDPDK	NCP	4.752
VGSDPDK;VSPEVLP	NCP	1.245
VSPEVLP;THDAKAA	NCP	6.763
THDAKAA;ALAVLEY	NCP	104.912
ALAVLEY;YMVNKR	NCP	74.538
YMVNKR;LLGALFL	NCP	42.421
LLGALFL;VGPTSQPP	NCP	4.287
VGPTSQPP;AAAAATAR	NCP	6
AAAAATAR;KHKTKSK	NCP	7.157
KHKTKSK;KDRSAPPP	NCP	54.853
KDRSAPPP;LKNHG	NCP	4.376
LKNHG;AAPSAF	NCP	13.042
AAPSAF;PGPRLPP	NCP	88.269
PGPRLPP;LPGVHVL	NCP	4.674
LPGVHVL;AHVVPPPP	NCP	18.3
AHVVPPPP;EGELSFK	NCP	77.237
EGELSFK;TQQDS	NCP	38.038
TQQDS;KGGDTPL	NCP	44.187
KGGDTPL;DDENQP	NCP	63.324
DDENQP;VPNHTPPP	NCP	10.632
VPNHTPPP;DPGGGWF	NCP	70.545
DPGGGWF;KEFPSFP	NCP	142.575
KEFPSFP;CSHMLR	NCP	140.88
CSHMLR;KTENLP	NCP	75.995
KTENLP;KELRLR	NCP	97.195
KELRLR;LSPLPNFS	NCP	9.069
LSPLPNFS;KMVLMAQ	NCP	7.833
KMVLMAQ;KDPPPP	NCP	10.895
KDPPPP;VASGLL	NCP	13.507
VASGLL;KGGGRFT	NCP	4.469
KGGGRFT;AELAEVP	NCP	24.618
AELAEVP;HARGYVSK	NCP	58.662
HARGYVSK;VTGVP	NCP	18.817
VTGVP;AADGFLPR	NCP	28.772
AADGFLPR;AVPPKYP	NCP	82.95
AVPPKYP;KPRLLL	NCP	3.06
KPRLLL;TAKVPTPK	NCP	66.612
TAKVPTPK;VPHRGPF	NCP	21.357
VPHRGPF;DGPLAQL	NCP	94.074
DGPLAQL;AAPSTVPR	NCP	31.149
AAPSTVPR;FDARAM	NCP	76.633
FDARAM;QTGGLGL	NCP	14.892
QTGGLGL;KGPVVQL	NCP	120.518

KGPVVQL;KEFCPK	NCP	31.693
KEFCPK;LMPLSGW	NCP	81.335
LMPLSGW;KWPAAKP	NCP	131.457
KWPAAKP;KPFPE	NCP	74.698
KPFPE;MMMEEM	NCP	22.569
MMMEEM;CFSSSLC	NCP	357.068
CFSSSLC;SSTFYQKGK	NCP	57.741
SSTFYQKGK;KNHPPPP	NCP	9.438
KNHPPPP;GYEVLY	NCP	67.491
GYEVLY;MLGASL	NCP	18.829
MLGASL;QTKQLQ	NCP	18.057
QTKQLQ;CSSCC	NCP	32.72
CSSCC;YVVPPGRE	NCP	22.515
YVVPPGRE;GTMLTEL	NCP	61.905
GTMLTEL;LLEALMA	NCP	103.306
LLEALMA;TGPAPLR	NCP	41.046
TGPAPLR;SPAAALR	NCP	1.289
SPAAALR;KHNAPR	NCP	90.93
KHNAPR;LPKTPR	NCP	7.914
LPKTPR;KDWKK	NCP	22.585
KDWKK;LPNAHKL	NCP	11.999
LPNAHKL;RELVRNL	NCP	12.811
RELVRNL;AGPVYLAA	NCP	210.969
AGPVYLAA;VEGPTLM	NCP	28.469
VEGPTLM;KSALAFH	NCP	59.335
KSALAFH;AFSRP	NCP	68.465
AFSRP;GHSPGYK	NCP	3.342
GHSPGYK;LFRGAP	NCP	33.816
LFRGAP;ATVPEC	NCP	91.641
ATVPEC;RTEHALL	NCP	65.512
RTEHALL;ASQRVTP	NCP	25.433
ASQRVTP;NLGLSV	NCP	39.01
NLGLSV;FVTTAQR	NCP	1.289
FVTTAQR;MCMYN	NCP	60.486
MCMYN;AGAEEPRQ	NCP	46.389
AGAEEPRQ;KQLLGAP	NCP	12.463
KQLLGAP;FPAVAH	NCP	105.753
FPAVAH;RMEKQ	NCP	42.08
RMEKQ;GTRLL	NCP	26.929
GTRLL;LGGEAGPR	NCP	35.943
LGGEAGPR;LGEMGRP	NCP	2.366
LGEMGRP;PDAVMK	NCP	9.036
PDAVMK;LMGQVP	NCP	65.928
LMGQVP;VTHLPR	NCP	29.667
VTHLPR;KKTLAY	NCP	112.429
KKTLAY;KQQTLLEK	NCP	12.128
KQQTLLEK;ADGHSPP	NCP	30.052
ADGHSPP;PGPLT	NCP	64.161
PGPLT;WDWSPK	NCP	45.927
WDWSPK;VVKKLALF	NCP	95.75
VVKKLALF;KYAELFK	NCP	67.878

KYAELFK;KHLTPLSY	NCP	110.028
KHLTPLSY;CAAPL	NCP	18.325
CAAPL;GTSLTPV	NCP	10.713
GTSLTPV;LSTLEY	NCP	24.179
LSTLEY;GPPPPGPP	NCP	14.655
GPPPPGPP;GPVLLA	NCP	72.585
GPVLLA;CYVLTH	NCP	84.13
CYVLTH;LLQPQ	NCP	2.348
LLQPQ;KEKAAPGT	NCP	35.631
KEKAAPGT;VSKDAPF	NCP	4.44
VSKDAPF;PTALAPP	NCP	117.262
PTALAPP;LPELPRP	NCP	11.613
LPELPRP;NLLHVLH	NCP	24.756
NLLHVLH;LLDFLK	NCP	27.947
LLDFLK;VVPLAT	NCP	251.983
VVPLAT;ERRNPF	NCP	25.085
ERRNPF;TAELAT	NCP	32.061
TAELAT;KAVTLLPV	NCP	41.635
KAVTLLPV;LQWLN	NCP	22.1
LQWLN;QYPWSP	NCP	92.332
QYPWSP;TGTPDVY	NCP	2.214
TGTPDVY;LSVVLEGKT	NCP	1.827
LSVVLEGKT;VVAAERE	NCP	153.099
VVAAERE;LGTTVL	NCP	36.186
LGTTVL;TLLLP	NCP	126.272
TLLLP;VPAKTVP	NCP	47.928
VPAKTVP;LEVRDL	NCP	32.047
LEVRDL;LTHAKLLQ	NCP	77.186
LTHAKLLQ;GPLDD	NCP	32.943
GPLDD;NTLRAH	NCP	47.614
NTLRAH;KSVFNLQ	NCP	44.304
KSVFNLQ;ALELRDN	NCP	14.685
ALELRDN;AEALGPLE	NCP	95.18
AEALGPLE;TAWCTP	NCP	63.046
TAWCTP;QLDAER	NCP	14.114
QLDAER;PPHVVAK	NCP	26.445
PPHVVAK;DAWKGAGP	NCP	36.421
DAWKGAGP;RVGPP	NCP	11.853
RVGPP;ADVQGAEVK	NCP	55.583
ADVQGAEVK;PVGGAP	NCP	68.233
PVGGAP;GKPLP	NCP	58.844
GKPLP;SEHVGTH	NCP	31.183
SEHVGTH;ETSQEAKSVFKQTEKG	NCP	3.77
ETSQEAKSVFKQTEKG;MEEFDSK	NCP	20.097
MEEFDSK;EETEETSSSNLVK	NCP	9.159
EETEETSSSNLVK;VKVEELEKPK	NCP	0.033
VKVEELEKPK;KVEELEKPKKAEEVK	NCP	0.003
KVEELEKPKKAEEVK;VEELEKPK	NCP	0.003
VEELEKPK;ELEKPKKAEEVK	NCP	0.006
ELEKPKKAEEVK;GHGLEVF	NCP	4.83
GHGLEVF;VGVGPA	NCP	5.797

VGVGPA;APGLLPPPPL	NCP	34.323
APGLLPPPPL;LYTKKVGKAPK	NCP	7.271
LYTKKVGKAPK;QAPALRP	NCP	31.081
QAPALRP;PAALPL	NCP	72.332
PAALPL;KSAGLA	NCP	84.33
KSAGLA;SVWLGGS	NCP	0.091
SVWLGGS;SQVGDVL	NCP	20.61
SQVGDVL;MVMVVQP	NCP	121.496
MVMVVQP;KAHGLDF	NCP	34.144
KAHGLDF;LLLAV	NCP	25.964
LLLAV;GVLEPLSR	NCP	16.597
GVLEPLSR;AEKFPSSN	NCP	52.008
AEKFPSSN;MKSGPVS	NCP	44.424
MKSGPVS;SLPAVS	NCP	73.995
SLPAVS;EGPDAKL	NCP	27.602
EGPDAKL;ETHRLV	NCP	5.032
ETHRLV;KSSVP	NCP	20.826
KSSVP;MPGLMF	NCP	2.369
MPGLMF;KDVMKK	NCP	41.779
KDVMKK;ESAQPK	NCP	9.854
ESAQPK;RFCPEA	NCP	66.798
RFCPEA;KGYPVR	NCP	16.641
KGYPVR;HVMCR	NCP	48.528
HVMCR;ARAVLVGL	NCP	41.418
ARAVLVGL;RSGVPGVR	NCP	1.11
RSGVPGVR;NLPVPV	NCP	68.083
NLPVPV;TTGPAP	NCP	57.657
TTGPAP;PKGLDAR	NCP	0.285
PKGLDAR;QMTNF	NCP	20.607
QMTNF;HFAKGPP	NCP	35.475
HFAKGPP;QTAALVV	NCP	22.895
QTAALVV;KANTLVYH	NCP	17.154
KANTLVYH;ELDLGLNHVVRK	NCP	0.726
ELDLGLNHVVRK;AQLGLEN	NCP	88.219
AQLGLEN;HALGSYP	NCP	13.596
HALGSYP;LPVVP	NCP	33.075
LPVVP;KDAGLLR	NCP	28.856
KDAGLLR;SMAGMVD	NCP	185.748
SMAGMVD;EKVQVTK	NCP	11.374
EKVQVTK;LALNVAA	NCP	4.613
LALNVAA;APDLLVK	NCP	116.011
APDLLVK;EDKPDM	NCP	31.755
EDKPDM;MVMGPK	NCP	43.212
MVMGPK;VGGFGVGK	NCP	26.597
VGGFGVGK;SASADVLAMSK	NCP	29.457
SASADVLAMSK;KGVHKDK	NCP	24.856
KGVHKDK;TLKTSR	NCP	7.337
TLKTSR;HSTTT	NCP	0.477
HSTTT;SETLPEK	NCP	107.202
SETLPEK;GYALLRL	NCP	210.201
GYALLRL;PGLLHCNP	NCP	86.913

PGLLHCNP;VDLHSR	NCP	173.011
VDLHSR;SFGTKPFL	NCP	106.406
SFGTKPFL;HLKFST	NCP	15.498
HLKFST;QRGELAF	NCP	157.081
QRGELAF;FRAPP	NCP	5.559
FRAPP;QAPPPPPPP	NCP	65.883
THMPPPPPP;KPHAAPR	NCP	66.772
KPHAAPR;TAKHKPP	NCP	14.541
TAKHKPP;VSEGPLP	NCP	36.23
VSEGPLP;KNLMKPK	NCP	102.705
KNLMKPK;WNQALYE	NCP	34.447
WNQALYE;LLMLLN	NCP	24.479
LLMLLN;VGLLGP	NCP	98.032
VGLLGP;PKVPPPP	NCP	132.005
PKVPPPP;TLFGFGT	NCP	60.12
TLFGFGT;AAPTF	NCP	26.172
AAPTF;GSPPPLP	NCP	31.641
GSPPPLP;LVGQPPW	NCP	71.097
LVGQPPW;ANGVFF	NCP	4.422
ANGVFF;KANLPKT	NCP	1.926
KANLPKT;VGVHLQK	NCP	4.641
VGVHLQK;PRVMPPP	NCP	36.738
PRVMPPP;SYMGKPP	NCP	57.052
SYMGKPP;VLDLRAF	NCP	13.667
VLDLRAF;GMQSAGK	NCP	34.098
GMQSAGK;TLLTGAK	NCP	56.37
TLLTGAK;TDASDVKPC	NCP	4.122
TDASDVKPC;TPAACRPP	NCP	65.805
TPAACRPP;KLLLNSLYK	NCP	16.345
KLLLNSLYK;VVPSP	NCP	152.774
VVPSP;LLVPPSK	NCP	14.53
LLVPPSK;MEPKPK	NCP	55.221
MEPKPK;SPVSRLGP	NCP	33.617
SPVSRLGP;RKPPVK	NCP	161.746
RKPPVK;GPLRPPLP	NCP	22.676
GPLRPPLP;EQGLLLAR	NCP	87.114
EQGLLLAR;PAALLSE	NCP	5.22
PAALLSE;GPPDF	NCP	13.038
GPPDF;LPDGQPP	NCP	10.038
LPDGQPP;RHVFLL	NCP	156.69
RHVFLL;ALEPVQF	NCP	107.496
ALEPVQF;GGGPSLPF	NCP	213.186
GGGPSLPF;TKGPKQ	NCP	22.678
TKGPKQ;KARAALD	NCP	144.458
KARAALD;LTPFDK	NCP	26.677
LTPFDK;PPGYK	NCP	82.088
PPGYK;AAPFCKA	NCP	157.482
AAPFCKA;DLSAVLT	NCP	37.569
DLSAVLT;KNSAPGR	NCP	9.477
KNSAPGR;FPPPPP	NCP	32.851
FPPPPP;PKEKLGR	NCP	5.631

PKEKLGR;CCCPD	NCP	86.273
CCCPD;QAPPKLQ	NCP	86.617
QAPPKLQ;KRPLLL	NCP	134.553
KRPLLL;GPFMC	NCP	5.312
GPFMC;PVFPQPY	NCP	162.478
PVFPQPY;PVGFYGY	NCP	48.653
PVGFYGY;VTRSVYQ	NCP	8.859
VTRSVYQ;DMDDSS	NCP	3.348
DMDDSS;MFPPP	NCP	47.484
MFPPP;RSGLPEK	NCP	0.676
RSGLPEK;KPHSWNP	NCP	234.401
KPHSWNP;VGVTEN	NCP	93.535
VGVTEN;VTTHKYE	NCP	180.11
VTTHKYE;LKNYKL	NCP	6.217
LKNYKL;GPVAFSN	NCP	35.024
GPVAFSN;STPLPKRP	NCP	179.115
STPLPKRP;ESLKYVP	NCP	103.044
ESLKYVP;EVSGPVK	NCP	62.283
EVSGPVK;TKNPQP	NCP	50.62
TKNPQP;TVDDLPL	NCP	33.047
TVDDLPL;LDPFP	NCP	29.181
LDPFP;PKEFSVK	NCP	222.835
PKEFSVK;FEFPLE	NCP	48.857
FEFPLE;VSLAD	NCP	152.094
VSLAD;SAGYVPH	NCP	15.648
SAGYVPH;KFLPAK	NCP	80.68
KFLPAK;REEYMK	NCP	20.367
REEYMK;MMMDGP	NCP	22.533
MMMDGP;MDGLVT	NCP	35.822
MDGLVT;GLQNK	NCP	146.878
GLQNK;ESTKAFSK	NCP	17.49
ESTKAFSK;QQSGLNVK	NCP	20.219
QQSGLNVK;TTPPM	NCP	28.725
TTPPM;GQEAQSK	NCP	28.536
GQEAQSK;TVDQLLT	NCP	8.121
TVDQLLT;FGDLVSGL	NCP	31.086
FGDLVSGL;SLLTSF	NCP	73.617
SLLTSF;QLPACQ	NCP	69.762
QLPACQ;FGVGP	NCP	31.896
FGVGP;KKSSTVP	NCP	9.181
KKSSTVP;MNGSK	NCP	45.903
MNGSK;CGMPPPP	NCP	54.257
CGMPPPP;DGLLK	NCP	29.047
DGLLK;PNGPAP	NCP	22.253
PNGPAP;AANPKL	NCP	25.339
AANPKL;GVPTVG	NCP	29.972
GVPTVG;AAAAASSALK	NCP	31.629
AAAAASSALK;QVGTLAK	NCP	27.822
QVGTLAK;LGSPVP	NCP	22.638
LGSPVP;TQKGTVPK	NCP	25.605
TQKGTVPK;RALLLEP	NCP	48.397

RALLLEP;KNLHYP	NCP	62.298
KNLHYP;KFWLAA	NCP	5.706
KFWLAA;LHRYSP	NCP	191.444
LHRYSP;EAALAW	NCP	57.909
EAALAW;KSGTVP	NCP	43.32
KSGTVP;VSDSTDVLS	NCP	39.712
VSDSTDVLS;AETLPPL	NCP	14.783
AETLPPL;APGTQKFY	NCP	19.66
APGTQKFY;EPAARPP	NCP	18.608
EPAARPP;GPVPP	NCP	11.544
GPVPP;SLVGGGGR	NCP	83.697
SLVGGGGR;PSPLPFPAP	NCP	55.767
PSPLPFPAP;AALGAGALL	NCP	14.523
AALGAGALL;GVGLLFN	NCP	10.149
GVGLLFN;KGYSGGH	NCP	31.069
KGYSGGH;EEKSKLK	NCP	136.788
EEKSKLK;HSSNQK	NCP	2.169
HSSNQK;KGSADPR	NCP	21.116
KGSADPR;LDKLLLR	NCP	18.223
LDKLLLR;GPDPHGR	NCP	16.862
GPDPHGR;ERPLGFF	NCP	6.556
ERPLGFF;EPQDN	NCP	0.501
EPQDN;MGTVEKR	NCP	22.862
MGTVEKR;EVEGLVA	NCP	77.148
EVEGLVA;LNVNKMP	NCP	2.814
LNVNKMP;RDALVSP	NCP	39.447
RDALVSP;LGPPPLTPP	NCP	1.395
LGPPPLTPP;FFPPPGP	NCP	10.807
FFPPPGP;QHSGPGSR	NCP	12.161
QHSGPGSR;VGPFEP	NCP	1.707
VGPFEP;MSQDDN	NCP	0.753
MSQDDN;EVPALLL	NCP	117.054
EVPALLL;LPGLSA	NCP	28.393
LPGLSA;EHKPT	NCP	22.868
EHKPT;GPGPPPP	NCP	11.769
GPGPPPP;KDAWSK	NCP	26.097
KDAWSK;EYNVAG	NCP	26.617
EYNVAG;LLGDSGVGK	NCP	24.629
LLGDSGVGK;EHLAPR	NCP	25.656
EHLAPR;MLARPAV	NCP	29.295
MLARPAV;LAGLTFPP	NCP	28.587
LAGLTFPP;KPCRQGP	NCP	27.519
KPCRQGP;QADRGPP	NCP	53.634
QADRGPP;ATPPMAK	NCP	17.745
ATPPMAK;KPPHLHL	NCP	8.488
KPPHLHL;TRVGLKH	NCP	149.432
TRVGLKH;VLLDY	NCP	69.433
VLLDY;KENAGPK	NCP	36.62
KENAGPK;LHKAVPG	NCP	63.954
LHKAVPG;FGLLAL	NCP	54.648
FGLLAL;SGLGKAPR	NCP	47.574

SGLGKAPR;AVRPPPLA	NCP	8.124
AVRPPPLA;LEFGAA	NCP	28.494
LEFGAA;KGPRTPPP	NCP	37.752
KGPRTPPP;AAMMY	NCP	3.237
AAMMY;NKSSLW	NCP	4.134
NKSSLW;PASTVT	NCP	107.631
PASTVT;QGVFPRQ	NCP	15.576
QGVFPRQ;AAPTPGKK	NCP	17.847
AAPTPGKK;KAQAFYP	NCP	39.822
KAQAFYP;NRWASKPP	NCP	58.647
NRWASKPP;KGVEKPP	NCP	11.439
KGVEKPP;LNLVQR	NCP	5.497
LNLVQR;LVLLDL	NCP	91.64
LVLLDL;QTPFPR	NCP	13.036
QTPFPR;MAELEK	NCP	25.647
MAELEK;GNPPPP	NCP	4.251
GNPPPP;QAPSVNP	NCP	28.61
QAPSVNP;VGPMG	NCP	32.583
VGPMG;LPGGPAR	NCP	65.856
LPGGPAR;PDGSF	NCP	196.602
PDGSF;KGHHPK	NCP	71.86
KGHHPK;CCCYDC	NCP	0.138
CCCYDC;HGPPAP	NCP	0.315
HGPPAP;AVMLKV	NCP	85.773
AVMLKV;LAVRL	NCP	31.878
LAVRL;KMGEGTLH	NCP	9.597
KMGEGTLH;GNVDTSH	NCP	31.826
GNVDTSH;TSAASSHL	NCP	39.309
TSAASSHL;GPPPPPPK	NCP	1.423
GPPPPPPK;KDNGLLPL	NCP	0.752
KDNGLLPL;GHVAVPQ	NCP	3.271
GHVAVPQ;RGPNPFE	NCP	16.248
RGPNPFE;DLLGTNT	NCP	41.699
DLLGTNT;GSTYLVK	NCP	36.36
GSTYLVK;DCCSFQP	NCP	26.142
DCCSFQP;PGPPPPGP	NCP	4.971
PGPPPPGP;HESFRSH	NCP	8.221
HESFRSH;PAAEVLN	NCP	0.521
PAAEVLN;VDLLK	NCP	4.318
VDLLK;KHTPGPP	NCP	40.458
KHTPGPP;VYVYKVL	NCP	78.636
VYVYKVL;RTPKGPP	NCP	39.32
RTPKGPP;VGMTH	NCP	10.285
VGMTH;EVEEVETPNSTPPR	NCP	89.879
EVEEVETPNSTPPR;SGPKAEPVSR	NCP	0.192
SGPKAEPVSR;AEALGHKTPEPAPR	NCP	0.114
AEALGHKTPEPAPR;ADTPRDAALKQPPAPR	NCP	67.629
ADTPRDAALKQPPAPR;VPPEPPK	NCP	71.049
VPPEPPK;LPPLK	NCP	6.823
LPPLK;PPPRALPP	NCP	60.611
PPPRALPP;MYKLPL	NCP	24.34

MYKLPL;KPAACH	NCP	0.782
KPAACH;PPSRGPAPP	NCP	11.947
PPSRGPAPP;FPGLGK	NCP	18.741
FPGLGK;PTRPGKQ	NCP	46.044
PTRPGKQ;DDELH	NCP	13.932
DDELH;PPKSPRPP	NCP	4.049
PPKSPRPP;PAEPHRP	NCP	13.986
PAEPHRP;LSFEVPP	NCP	17.401
LSFEVPP;KAGTPLR	NCP	19.52
KAGTPLR;VLERPK	NCP	12.103
VLERPK;TLDAGALSL	NCP	17.196
TLDAGALSL;PPPPPGPR	NCP	108.302
PPPPPGPR;AGMHPPR	NCP	53.478
AGMHPPR;MESGAYGAPR	NCP	14.502
MESGAYGAPR;VDPTPDPST	NCP	2.826
VDPTPDPST;RLVAPER	NCP	171.903
RLVAPER;KETPAGP	NCP	60.21
KETPAGP;LPPLPP	NCP	9.525
LPPLPP;AAPCCPK	NCP	53.553
AAPCCPK;EAPGAVPG	NCP	10.248
EAPGAVPG;TSCCC	NCP	10.749
TSCCC;TAGVLL	NCP	38.64
TAGVLL;FEYRQSP	NCP	19.888
FEYRQSP;PSPKPPAAP	NCP	4.4
PSPKPPAAP;PSAVVPP	NCP	68.064
PSAVVPP;YDVAK	NCP	47.14
YDVAK;VLLKEF	NCP	7.823
VLLKEF;ASGSFLAR	NCP	84.03
ASGSFLAR;HGAFPFH	NCP	65.034
HGAFPFH;DNPRPP	NCP	8.362
DNPRPP;KPFPKGW	NCP	14.483
KPFPKGW;WHPGPPP	NCP	24.681
WHPGPPP;KQTAP	NCP	28.162
KQTAP;KPTPDFK	NCP	7.853
KPTPDFK;QNVKGLA	NCP	17.379
QNVKGLA;LPPPPPDPL	NCP	17.259
LPPPPPDPL;ALEHAGFR	NCP	0.472
ALEHAGFR;AFTLLY	NCP	21.617
AFTLLY;ELTVRAP	NCP	21.364
ELTVRAP;SHPAAKGK	NCP	52.049
SHPAAKGK;THKLFR	NCP	50.17
THKLFR;KAMVPR	NCP	38.892
KAMVPR;GPGRPPPPPP	NCP	2.748
GPGRPPPPPP;THVLQGK	NCP	0.378
THVLQGK;VKTTSY	NCP	52.053
VKTTSY;KEHAELP	NCP	47.679
KEHAELP;APFFPK	NCP	12.719
APFFPK;SLGDVAKK	NCP	132.747
SLGDVAKK;KNMSPPPP	NCP	42.729
KNMSPPPP;EGPLLAEP	NCP	40.806
EGPLLAEP;QPGEPALP	NCP	217.992

QPGEPALP;AYLKKATNE	NCP	7.846
AYLKKATNE;LMLLLLK	NCP	59.862
LMLLLLK;KLSELLRY	NCP	5.411
KLSELLRY;KCVLVR	NCP	47.956
KCVLVR;PSKET	NCP	90.44
PSKET;KQNPVNF	NCP	2.026
KQNPVNF;RPPTGT	NCP	31.145
RPPTGT;CCCSV	NCP	6.475
CCCSV;LPCEDEPP	NCP	10.818
LPCEDEPP;HPAAPK	NCP	89.493
HPAAPK;SSATRGGK	NCP	0.363
SSATRGGK;LFEPGLG	NCP	16.938
LFEPGLG;GPHQPGPP	NCP	25.055
GPHQPGPP;KGPCKL	NCP	7.728
KGPCKL;PGESAGP	NCP	47.611
PGESAGP;KELLVT	NCP	120.674
KELLVT;LGGQGRPP	NCP	92.701
LGGQGRPP;ATDLTPKP	NCP	0.365
ATDLTPKP;KPFDLK	NCP	26.562
KPFDLK;NGLSH	NCP	7.062
NGLSH;MCSDF	NCP	50.644
MCSDF;RMGRW	NCP	42.299
RMGRW;KEFQPPPP	NCP	2.719
KEFQPPPP;APNKKTK	NCP	29.339
APNKKTK;GRGSK	NCP	5.571
GRGSK;KAPLQFR	NCP	148.332
KAPLQFR;KDLEPLV	NCP	0.877
KDLEPLV;KPPVQPP	NCP	26.792
KPPVQPP;KLAWDQ	NCP	201.798
KLAWDQ;ALPAPA	NCP	23.346
ALPAPA;EGPKLSPP	NCP	47.496
EGPKLSPP;VNPVHV	NCP	23.982
VNPVHV;LGPLPPLP	NCP	58.989
LGPLPPLP;ADEARVSR	NCP	17.43
ADEARVSR;GPPLPFPN	NCP	55.524
GPPLPFPN;EPSHWLPP	NCP	65.847
EPSHWLPP;VMVDGKPVNL	NCP	115.206
VMVDGKPVNL;PSAVPPP	NCP	1.537
PSAVPPP;KPSDPR	NCP	48.893
KPSDPR;LMKPPR	NCP	4.098
LMKPPR;VSADMRK	NCP	6.741
VSADMRK;GPGGKGH	NCP	28.119
GPGGKGH;HVEHSLP	NCP	66.595
HVEHSLP;LYKYD	NCP	1.659
LYKYD;YALKLWN	NCP	94.293
YALKLWN;CFPLDLRK	NCP	17.441
CFPLDLRK;DPNWF	NCP	29.053
DPNWF;LSPAVP	NCP	106.268
LSPAVP;GRAAP	NCP	121.803
GRAAP;LGPPFAK	NCP	19.141
LGPPFAK;PSTGELK	NCP	16.719

PSTGELK;QPPVRPP	NCP	65.099
QPPVRPP;TPAALLLP	NCP	6.225
TPAALLLP;KLYHTF	NCP	60.766
KLYHTF;FEGSEEK	NCP	16.659
FEGSEEK;QALGY	NCP	106.452
QALGY;AAPRNPPGP	NCP	11.136
AAPRNPPGP;MDVPEQ	NCP	20.045
MDVPEQ;KWDPLR	NCP	58.039
KWDPLR;RDELVARPF	NCP	22.859
RDELVARPF;FVGAGL	NCP	20.439
FVGAGL;GPPMGQL	NCP	44.472
GPPMGQL;PHVKGR	NCP	40.02
PHVKGR;QQQMQLF	NCP	35.871
QQQMQLF;LGSALP	NCP	5.013
LGSALP;TVLPL	NCP	91.699
TVLPL;LPSPPP	NCP	89.753
LPSPPP;PDLTRPS	NCP	11.074
PDLTRPS;SSAAYG	NCP	104.079
SSAAYG;APSFLP	NCP	0.543
APSFLP;GPPPPPRAAP	NCP	7.128
GPPPPPRAAP;TEVGWW	NCP	28.827
TEVGWW;LAAFMGM	NCP	25.647
LAAFMGM;QAKKSGK	NCP	2.307
QAKKSGK;LFEEPF	NCP	135.813
LFEEPF;VVTRR	NCP	12.891
VVTRR;LSSLAGPR	NCP	42.348
LSSLAGPR;LLRTPNN	NCP	22.214
LLRTPNN;SYVPGGG	NCP	36.394
SYVPGGG;ETRSPAL	NCP	22.595
ETRSPAL;LAAVLE	NCP	23.302
LAAVLE;CSCCE	NCP	42.917
CSCCE;LPVVPPTP	NCP	7.999
LPVVPPTP;ADKLEPV	NCP	7.958
ADKLEPV;LSLQY	NCP	9.363
LSLQY;CSGPGF	NCP	40.633
CSGPGF;KAVAF	NCP	6.567
KAVAF;KFGGDGK	NCP	174.245
KFGGDGK;VLPVPGAK	NCP	18.81
VLPVPGAK;LPGELTP	NCP	26.065
LPGELTP;TVFAVSLSV	NCP	1.613
TVFAVSLSV;NLVSL	NCP	41.625
NLVSL;AGPLSGKKFGNPGEKL	NCP	50.487
AGPLSGKKFGNPGEKL;MNFYN	NCP	39.511
MNFYN;LVLLGPG	NCP	85.856
LVLLGPG;VGGLDGLLGV	NCP	73.012
VGGLDGLLGV;LPTPP	NCP	11.532
LPTPP;APRGAPPP	NCP	11.688
APRGAPPP;EVEPKESPHPPR	NCP	28.154
EVEPKESPHPPR;LEDLLP	NCP	28.398
LEDLLP;ALLARSLGK	NCP	9.933
ALLARSLGK;KPVAP	NCP	151.692

KPVAP;APHSGDA	NCP	30.208
APHSGDA;KDQPP	NCP	156.54
KDQPP;KDFNPP	NCP	39.375
KDFNPP;TPPPLHLEP	NCP	4.625
TPPPLHLEP;DMEALLPLMNM	NCP	24.309
DMEALLPLMNM;DPGHPPE	NCP	39.841
DPGHPPE;WPAKPK	NCP	43.947
WPAKPK;RSPPS	NCP	54.216
RSPPS;KGLPLQ	NCP	16.97
KGLPLQ;TVAGPL	NCP	12.606
TVAGPL;KDVASPP	NCP	4.3
KDVASPP;PRAVFPS	NCP	69.635
PRAVFPS;SELLVRK	NCP	48.267
SELLVRK;MEPWLP	NCP	295.156
MEPWLP;LSYFTP	NCP	99.288
LSYFTP;AGMSSLKG	NCP	0.168
AGMSSLKG;TVTPLTK	NCP	45.17
TVTPLTK;KTPVLPLEK	NCP	60.673
KTPVLPLEK;PASSVP	NCP	121.034
PASSVP;CCECD	NCP	19.894
CCECD;FRLLQR	NCP	64.826
FRLLQR;LTALEAM	NCP	16.285
LTALEAM;VGTVPPPF	NCP	16.536
VGTVPPPF;LVELKVLR	NCP	154.073
LVELKVLR;VVRKPL	NCP	36.811
VVRKPL;LSAVPVG	NCP	21.318
LSAVPVG;GLDASH	NCP	0.852
GLDASH;MGAGGP	NCP	11.555
MGAGGP;ELHMC	NCP	29.226
ELHMC;MEMKKKP	NCP	105.468
MEMKKKP;LKKGYL	NCP	39.999
LKKGYL;TRVSPSY	NCP	127.51
TRVSPSY;HKPKSK	NCP	16.1
HKPKSK;FGLSSGAP	NCP	15.058
FGLSSGAP;PEPAKSAPAP	NCP	6.812
PEPAKSAPAP;NALLELY	NCP	0.613
NALLELY;LHSDLVK	NCP	4.2
LHSDLVK;AVPLLFL	NCP	54.803
AVPLLFL;VNGYVFF	NCP	152.059
VNGYVFF;HMCCC	NCP	90.393
HMCCC;KMESGLK	NCP	27.267
KMESGLK;KEWRRGY	NCP	3.206
KEWRRGY;LSLGPPG	NCP	34.426
LSLGPPG;ALRLLR	NCP	46.364
ALRLLR;LPLPPATHT	NCP	70.432
LPLPPATHT;VPGFPS	NCP	27.243
VPGFPS;LGTLTLK	NCP	3.216
LGTLTLK;VGPVPVP	NCP	93.092
VGPVPVP;ESPLLL	NCP	15.84
ESPLLL;PPLLSPLPP	NCP	27.864
PPLLSPLPP;KGEQQLY	NCP	0.591

KGEQQLY;TFFFVM	NCP	15.886
TFFFVM;WPGTLR	NCP	107.858
WPGTLR;LSLHTFR	NCP	63.301
LSLHTFR;SKPPP	NCP	42.771
SKPPP;VLEDGELLVQQTK	NCP	66.677
VLEDGELLVQQTK;LLNPQGK	NCP	3.901
LLNPQGK;LPDLGETE	NCP	19.269
LPDLGETE;PDCDAR	NCP	118.701
PDCDAR;GGSLMKC	NCP	24.233
GGSLMKC;SPAAVVEF	NCP	24.381
SPAAVVEF;KEPPHK	NCP	5.202
KEPPHK;FDGYPF	NCP	59.253
FDGYPF;DEVLNLK	NCP	8.79
DEVLNLK;KVFEPKL	NCP	6.87
KVFEPKL;KGMNSPP	NCP	60.72
KGMNSPP;QAPRPTP	NCP	15.441
QAPRPTP;LPMLAFP	NCP	0.039
LPMLAFP;RQTVLR	NCP	13.864
RQTVLR;KPHSLK	NCP	19.284
KPHSLK;CCCVC	NCP	56.553
CCCVC;DGMLLVRL	NCP	48.516
DGMLLVRL;HTPVRN	NCP	62.313
HTPVRN;KALTTALY	NCP	46.469
KALTTALY;GMSLSKK	NCP	40.612
GMSLSKK;WLVTKFL	NCP	22.026
WLVTKFL;TPPPPER	NCP	5.286
TPPPPER;VGPAARVPR	NCP	44.597
VGPAARVPR;PLLPPGLP	NCP	28.443
PLLPPGLP;LDVAPL	NCP	15.708
LDVAPL;TVAPPGPP	NCP	168.3
TVAPPGPP;KSTFHP	NCP	2.284
KSTFHP;GHALSTP	NCP	38.126
GHALSTP;VFARP	NCP	19.749
VFARP;AAPSAVAAATSSERD	NCP	66.045
AAPSAVAAATSSERD;LLARPPP	NCP	25.455
LLARPPP;AVATL	NCP	74.203
AVATL;FDVNFK	NCP	67.616
FDVNFK;KPCPPP	NCP	41.452
KPCPPP;LGPGAMPP	NCP	42.104
LGPGAMPP;APLYPFSP	NCP	63.771
APLYPFSP;MKNGLFP	NCP	27.1
MKNGLFP;WSLLELQKAFAK	NCP	18.302
WSLLELQKAFAK;NSLSHL	NCP	72.915
NSLSHL;RAQGGGGE	NCP	11.946
RAQGGGGE;KYVVR	NCP	2.694
KYVVR;WQMPPPPP	NCP	17.163
WQMPPPPP;GVLSAAGT	NCP	37.911
GVLSAAGT;AAPPGEYF	NCP	27.309
AAPPGEYF;KKRHG	NCP	41.712
KKRHG;KKPPDKK	NCP	19.42
KKPPDKK;RHYKFY	NCP	12.285

RHYKFY;AFKNVPF	NCP	38.232
AFKNVPF;LPQKPS	NCP	98.217
LPQKPS;EVLRK	NCP	42.867
EVLRK;PQDPKHR	NCP	49.662
PQDPKHR;PSLAYLY	NCP	94.796
PSLAYLY;DLPPK	NCP	98.794
DLPPK;WKDVDR	NCP	40.605
WKDVDR;GVNLGGAG	NCP	17.276
GVNLGGAG;GLFCETG	NCP	5.548
GLFCETG;THKPEDR	NCP	23.757
THKPEDR;ACPSLHV	NCP	2.877
ACPSLHV;GGHLE	NCP	36.09
GGHLE;SSPPLPL	NCP	37.34
SSPPLPL;CLVPTLK	NCP	22.386
CLVPTLK;TMEVLA	NCP	30.513
TMEVLA;VEDAAPF	NCP	106.3
VEDAAPF;KPQPPHPK	NCP	19.547
KPQPPHPK;PLGGSDTAK	NCP	11.337
PLGGSDTAK;NLLKPMGK	NCP	0.033
NLLKPMGK;GMANLLTGPK	NCP	0.573
GMANLLTGPK;KVLLVPGPEKEN	NCP	0.45
KVLLVPGPEKEN;VLLVPGPEK	NCP	0.003
VLLVPGPEK;LVPGPEKEN	NCP	0.006
LVPGPEKEN;PLLEDGTK	NCP	96.852
PLLEDGTK;KFLKPM	NCP	7.432
KFLKPM;SEMTDKLGL	NCP	19.047
SEMTDKLGL;EGLDWLSNELSKR	NCP	0.093
EGLDWLSNELSKR;WGSGGGLG	NCP	1.644
WGSGGGLG;VLLLN	NCP	17.277
VLLLN;VPPKVP	NCP	33.479
VPPKVP;NLKLL	NCP	25.174
NLKLL;ATPGPGDPVDAK	NCP	40.778
ATPGPGDPVDAK;GPGDPVDAKSGKAP	NCP	0.009
GPGDPVDAKSGKAP;SLKVDVK	NCP	11.878
SLKVDVK;NPPSGSFK	NCP	30.401
NPPSGSFK;LLGLAGG	NCP	79.809
LLGLAGG;KGPPQHP	NCP	15.865
KGPPQHP;PPPPKVP	NCP	66.438
PPPPKVP;MHVTPLP	NCP	42.171
MHVTPLP;KNMLK	NCP	6.896
KNMLK;FYALGPP	NCP	26.383
FYALGPP;AAPLAPFP	NCP	6.93
AAPLAPFP;SDQVNGVLVL	NCP	19.247
SDQVNGVLVL;LGKAHATTSNTVSK	NCP	0.15
LGKAHATTSNTVSK;LEKVRKVSVNVK	NCP	0.045
LEKVRKVSVNVK;KLEVNEAELLRRR	NCP	0.078
KLEVNEAELLRRR;RDGLP	NCP	1.486
RDGLP;FGPKTNP	NCP	8.381
FGPKTNP;SLKESETLPEK	NCP	1.224
SLKESETLPEK;RVDDFKKAFSKEK	NCP	0.171
RVDDFKKAFSKEK;VDDFKKAFSK	NCP	0.003

VDDFKKAFSK;DDFKKAFSKEK	NCP	0.003
DDFKKAFSKEK;AFSKEKMEKTKVR	NCP	0.015
AFSKEKMEKTKVR;TKVRTRENLER	NCP	0.027
TKVRTRENLER;TRLKTKENLEKTR	NCP	0.033
TRLKTKENLEKTR;RLKTKENLEK	NCP	0.003
RLKTKENLEK;KTKENLEKTRHTLEK	NCP	0.006
KTKENLEKTRHTLEK;ENLEKTRHTLEKR	NCP	0.009
ENLEKTRHTLEKR;RMNKLGTRLVPAER	NCP	0.036
RMNKLGTRLVPAER;KSFTPDHV	NCP	0.078
KSFTPDHV;SFTPDHVVYA	NCP	0.003
SFTPDHVVYA;FTPDHVVY	NCP	0.003
FTPDHVVY;TAVYKVPPF	NCP	0.036
TAVYKVPPF;VPPFTFHVKK	NCP	0.015
VPPFTFHVKK;EGQVEVLK	NCP	0.036
EGQVEVLK;ATEMVEVGAEEEEGGAER	NCP	0.024
ATEMVEVGAEEEEGGAER;TEESDAVLVDK	NCP	0.114
TEESDAVLVDK;TGVFAPR	NCP	51.603
TGVFAPR;LEELQQKVSY	NCP	24.318
LEELQQKVSY;RCRFLR	NCP	86.428
RCRFLR;YAKVSPP	NCP	6.831
YAKVSPP;TMPDLR	NCP	24.974
TMPDLR;QPPGPYP	NCP	18.24
QPPGPYP;PLLTPLQ	NCP	7.855
PLLTPLQ;LSTLAL	NCP	2.949
LSTLAL;HSLAPMYY	NCP	26.045
HSLAPMYY;KGPTLPH	NCP	11.058
KGPTLPH;RGLPPR	NCP	25.62
RGLPPR;WSPLVLEK	NCP	20.863
WSPLVLEK;RPSAAPK	NCP	17.604
RPSAAPK;AVEAAK	NCP	2.918
AVEAAK;GPKFR	NCP	11.745
GPKFR;PSSVPLK	NCP	29.07
PSSVPLK;AEGELSK	NCP	72.901
AEGELSK;KAHALYL	NCP	24.161
KAHALYL;APSYRQL	NCP	4.912
APSYRQL;SQTPEV	NCP	28.128
SQTPEV;MKVENC	NCP	3.194
MKVENC;KASELFK	NCP	21.126
KASELFK;TCVPSVSSK	NCP	16.263
TCVPSVSSK;APFPLL	NCP	9.754
APFPLL;LWLPF	NCP	16.721
LWLPF;LAPPERK	NCP	25.104
LAPPERK;KPEAAPP	NCP	17.601
KPEAAPP;TPGNPPR	NCP	43.107
TPGNPPR;SHRSLLSH	NCP	37.975
SHRSLLSH;VPFFPT	NCP	8.402
VPFFPT;TLSVALGK	NCP	35.304
TLSVALGK;PQNAS	NCP	38.751
PQNAS;TSYSYRQSSSTSS	NCP	7.38
TSYSYRQSSSTSS;SSSTSSFGGMGGGSMR	NCP	0.021
SSSTSSFGGMGGGSMR;FGGMGGGSM	NCP	0.018

FGGMGGGSM;FGAGGAFRAPS	NCP	0.03
FGAGGAFRAPS;TEELNREVAGHTE	NCP	3.06
TEELNREVAGHTE;RNLLEGQDA	NCP	0.45
RNLLEGQDA;YFNDLSLAK	NCP	0.027
YFNDLSLAK;FNDLSLAKAL	NCP	0.003
FNDLSLAKAL;PGAGP	NCP	1.281
PGAGP;FPRPC	NCP	49.041
FPRPC;NRGAP	NCP	1.575
NRGAP;GSQVKDR	NCP	14.59
GSQVKDR;KDNDM	NCP	10.544
KDNDM;FNPLRST	NCP	108.609
FNPLRST;SPTSLF	NCP	117.064
SPTSLF;VVPAAA	NCP	107.45
VVPAAA;LNPDLY	NCP	106.756
LNPDLY;KHGTPP	NCP	2.811
KHGTPP;KVSVPKK	NCP	70.001
KVSVPKK;YDAMAQENLQK	NCP	87.072
YDAMAQENLQK;KVVSSEVKEVEE	NCP	3.915
KVVSSEVKEVEE;VVSSEVKEVEESV	NCP	0.003
VVSSEVKEVEESV;LPPALSPY	NCP	45.799
LPPALSPY;KRDKFF	NCP	33.437
KRDKFF;KNGKMPP	NCP	13.879
KNGKMPP;LPSNLSPP	NCP	30.396
LPSNLSPP;KEPLKK	NCP	5.723
KEPLKK;KLGTGDW	NCP	18.984
KLGTGDW;VGGGYVFF	NCP	41.194
VGGGYVFF;LVEEMSA	NCP	52.871
LVEEMSA;LPGYLVP	NCP	8.385
LPGYLVP;KPLPP	NCP	97.333
KPLPP;VDPLVVE	NCP	29.064
VDPLVVE;PRGLFD	NCP	6.474
PRGLFD;LFGVEVAVA	NCP	1.793
LFGVEVAVA;MQVSKTK	NCP	29.106
MQVSKTK;KPVLPAL	NCP	25.797
KPVLPAL;LPKAPPPP	NCP	156.678
LPKAPPPP;YAEPFF	NCP	133.761
YAEPFF;FAPPGPP	NCP	14.46
FAPPGPP;KPFVWP	NCP	9.343
KPFVWP;VHGGPP	NCP	47.148
VHGGPP;LSGGPQ	NCP	85.974
LSGGPQ;PLLSFL	NCP	1.932
PLLSFL;VGVKL	NCP	25.805
VGVKL;VEGPLL	NCP	54.15
VEGPLL;WVPRHWP	NCP	117.285
WVPRHWP;SFKLSR	NCP	34.921
SFKLSR;APGVAP	NCP	16.845
APGVAP;FVNVN	NCP	30.089
FVNVN;LMPKATLL	NCP	45.13
LMPKATLL;VDPNLQA	NCP	5.465
VDPNLQA;MELPVVP	NCP	48.886
MELPVVP;KFREQTAQR	NCP	11.808

KFREQTAQR;ADEPF	NCP	17.342
ADEPF;KHPGYR	NCP	35.707
KHPGYR;LKVAY	NCP	45.158
LKVAY;SPAPVSNL	NCP	13.176
SPAPVSNL;AELKLE	NCP	21.793
AELKLE;PAPPPPYK	NCP	192.231
PAPPPPYK;GHRAGAH	NCP	17.568
GHRAGAH;GPLALP	NCP	123.77
GPLALP;PPVTSRP	NCP	104.877
PPVTSRP;APLSP	NCP	30.199
APLSP;GPVDKVH	NCP	19.466
GPVDKVH;ESGPSLHV	NCP	1.468
ESGPSLHV;PMAPPGHR	NCP	4.748
PMAPPGHR;AKGPTL	NCP	14.521
AKGPTL;TRLLDVV	NCP	20.516
TRLLDVV;AALSW	NCP	31.59
AALSW;PMMPP	NCP	7.092
PMMPP;PLYGAP	NCP	6.19
PLYGAP;DDDLAA	NCP	107.087
DDDLAA;ERPLMF	NCP	102.991
ERPLMF;LPMLLR	NCP	27.875
LPMLLR;TAPKVYP	NCP	2.523
TAPKVYP;LLQRKASPPT	NCP	16.671
LLQRKASPPT;YAKGALQY	NCP	40.572
YAKGALQY;LNELREGCLEA	NCP	8.769
LNELREGCLEA;NMELLL	NCP	45.928
NMELLL;MKVPPR	NCP	114.23
MKVPPR;TCMRPP	NCP	17.778
TCMRPP;ELSAG	NCP	86.826
ELSAG;PAWLK	NCP	3.027
PAWLK;KKPAAAVA	NCP	5.049
KKPAAAVA;LAEATAW	NCP	39.465
LAEATAW;MDGSMC	NCP	49.342
MDGSMC;PFLDLHY	NCP	0.234
PFLDLHY;ALGSAL	NCP	35.861
ALGSAL;ESLLVGRL	NCP	7.23
ESLLVGRL;KNLLAGN	NCP	41.682
KNLLAGN;GVVLSAS	NCP	68.436
GVVLSAS;KNYGSLVN	NCP	0.261
KNYGSLVN;LDGRFYR	NCP	11.1
LDGRFYR;YMKLDR	NCP	180.582
YMKLDR;QTLLPPDP	NCP	7.735
QTLLPPDP;MTPRPGP	NCP	94.694
MTPRPGP;ALAHHYR	NCP	208.695
ALAHHYR;QGKGQPK	NCP	5.347
QGKGQPK;APKFSR	NCP	15.048
APKFSR;SLGAAGK	NCP	10.395
SLGAAGK;KGSHMFL	NCP	22.404
KGSHMFL;PGVFAAQ	NCP	34.727
PGVFAAQ;LYKAGQP	NCP	53.187
LYKAGQP;AESPTEEAATATAGAGAAGPG	NCP	2.811

AESPTEEAATATAGAGAAGPG;EDAGPSLV	NCP	26.091
EDAGPSLV;DYPLP	NCP	49.984
DYPLP;LPAAAK	NCP	0.09
LPAAAK;PRTLAPP	NCP	128.703
PRTLAPP;NPSPPPR	NCP	46.934
NPSPPPR;MKLYCL	NCP	1.861
MKLYCL;APLMVFF	NCP	49.746
APLMVFF;PGPLVG	NCP	35.706
PGPLVG;GFAAKPK	NCP	39.438
GFAAKPK;AAGLEL	NCP	30.39
AAGLEL;RGPPKLQ	NCP	73.08
RGPPKLQ;GPLLVLRLP	NCP	24.413
GPLLVLRLP;PLFFLR	NCP	1.558
PLFFLR;CGEAHC	NCP	69.353
CGEAHC;HLHPVDQ	NCP	4.797
HLHPVDQ;PTDKLEK	NCP	10.105
PTDKLEK;LQLLVEPF	NCP	114.635
LQLLVEPF;CFTKSKLK	NCP	45.099
CFTKSKLK;PPNGAPR	NCP	18.433
PPNGAPR;KWTPPK	NCP	28.001
KWTPPK;SSAPLQYL	NCP	15.682
SSAPLQYL;TVLNKK	NCP	3.249
TVLNKK;PLMGPLP	NCP	38.351
PLMGPLP;LEGLYLD	NCP	3.195
LEGLYLD;LDVWEH	NCP	56.914
LDVWEH;GPPLVE	NCP	2.12
GPPLVE;AAPDTT	NCP	30.463
AAPDTT;KQMVELP	NCP	35.697
KQMVELP;VPNSEAQ	NCP	11.304
VPNSEAQ;LSEAPLPK	NCP	1.352
LSEAPLPK;VDGDLSLP	NCP	32.403
VDGDLSLP;QPPAELSP	NCP	38.979
QPPAELSP;AHSPP	NCP	27.447
AHSPP;KSGPLHH	NCP	50.958
KSGPLHH;SGLLTF	NCP	69.343
SGLLTF;LSATRTSF	NCP	49.436
LSATRTSF;AHVLVRK	NCP	17.749
AHVLVRK;GLLLK	NCP	43.548
GLLLK;DYEELP	NCP	27.263
DYEELP;DHTVPLPVG	NCP	1.983
DHTVPLPVG;FGFWLF	NCP	4.948
FGFWLF;GTTENSPVTF	NCP	4.814
GTTENSPVTF;AAPPPLP	NCP	4.764
AAPPPLP;KCLGTFPP	NCP	47.524
KCLGTFPP;PDGGY	NCP	103.013
PDGGY;KGGRHPPP	NCP	5.778
KGGRHPPP;VRKPAAP	NCP	150.082
VRKPAAP;PLEAP	NCP	263.049
PLEAP;NLVVGAP	NCP	26.043
NLVVGAP;KEVRLR	NCP	8.162
KEVRLR;RFEQKGFRLVAMK	NCP	34.806

RFEQKGFRLVAMK;MHSGPVVAM	NCP	2.463
MHSGPVVAM;WFRPEELVNFK	NCP	1.704
WFRPEELVNFK;AHAERTF	NCP	6.354
AHAERTF;MNSGPVVAM	NCP	1.095
MNSGPVVAM;NSGPVVAMV	NCP	0.003
NSGPVVAMV;VWEGLNVVK	NCP	1.599
VWEGLNVVK;WFKPEEL	NCP	0.939
WFKPEEL;LREVTMK	NCP	21.505
LREVTMK;EHGLFL	NCP	2.675
EHGLFL;VNKHELQ	NCP	13.518
VNKHELQ;GLGLLPG	NCP	22.687
GLGLLPG;ADDLDF	NCP	21.789
ADDLDF;TGDAGASATFPMQCSALRK	NCP	0.021
TGDAGASATFPMQCSALRK;LTGALEY	NCP	87.878
LTGALEY;PPEHAPP	NCP	1.15
PPEHAPP;PLRPPPP	NCP	68.375
PLRPPPP;RSTVLR	NCP	31.216
RSTVLR;PAGLHRY	NCP	64.169
PAGLHRY;PDGPPVR	NCP	12.82
PDGPPVR;VGEVRAV	NCP	22.341
VGEVRAV;LPPPALMLP	NCP	17.822
LPPPALMLP;KTHPPPPPP	NCP	57.042
KTHPPPPPP;RGPPPLAW	NCP	20.56
RGPPPLAW;ALKKVGV	NCP	14.802
ALKKVGV;ARAAP	NCP	29.594
ARAAP;PPPPGGAGK	NCP	21.966
PPPPGGAGK;SAAKENPCRK	NCP	12.294
SAAKENPCRK;PFLLSR	NCP	45.034
PFLLSR;ARLAPLP	NCP	1.235
ARLAPLP;AELRAL	NCP	0.591
AELRAL;LPSPYVL	NCP	17.809
LPSPYVL;PGGGPPHL	NCP	13.769
PGGGPPHL;ARNGPVL	NCP	3.774
ARNGPVL;LPGLSET	NCP	9.64
LPGLSET;KENPPP	NCP	0.357
KENPPP;APPLPPLPR	NCP	24.074
APPLPPLPR;ADDDLP	NCP	24.961
ADDDLP;LLLTG	NCP	58.599
LLLTG;APGHDSA	NCP	25.052
APGHDSA;LPVSLPAP	NCP	5.05
LPVSLPAP;RAAKRH	NCP	29.084
RAAKRH;PRDLHPH	NCP	63.405
PRDLHPH;NPTRAQP	NCP	1.29
NPTRAQP;VLEGELK	NCP	18.118
VLEGELK;RPLGVQP	NCP	83.927
RPLGVQP;WDGTRP	NCP	41.377
WDGTRP;EYHLGLLK	NCP	70.325
EYHLGLLK;APVGDT	NCP	17.046
APVGDT;HLAGP	NCP	12.594
HLAGP;LSVPLP	NCP	12.027
LSVPLP;KSGPLAAP	NCP	31.806

KSGPLAAP;KHHWK	NCP	58.81
KHHWK;ATVVAPEH	NCP	22.373
ATVVAPEH;PGMLDF	NCP	115.926
PGMLDF;LSPGVKR	NCP	70.515
LSPGVKR;ELAGN	NCP	86.812
ELAGN;VEPGSFP	NCP	13.989
VEPGSFP;KGPMPR	NCP	0.138
KGPMPR;NCCYP	NCP	37.428
NCCYP;NVVSKPR	NCP	120.66
NVVSKPR;KDSAPPP	NCP	37.994
KDSAPPP;FPPPGTPP	NCP	20.788
FPPPGTPP;LPPLHP	NCP	5.522
LPPLHP;LLCRM	NCP	23.445
LLCRM;KAASLQP	NCP	32.271
KAASLQP;KSTTELR	NCP	14.431
KSTTELR;VVVSPVG	NCP	93.47
VVVSPVG;LPTVP	NCP	4.959
LPTVP;RGAAVSPP	NCP	3.981
RGAAVSPP;LRVPGSFM	NCP	12.705
LRVPGSFM;KPGLV	NCP	52.576
KPGLV;RAGGQN	NCP	56.196
RAGGQN;YEVSQL	NCP	0.738
YEVSQL;KHGEPF	NCP	1.49
KHGEPF;GVVLL	NCP	47.379
GVVLL;LPPSHL	NCP	27.225
LPPSHL;EPEFFLQ	NCP	21.268
EPEFFLQ;LLVAGAGK	NCP	101.174
LLVAGAGK;KDELPYK	NCP	7.969
KDELPYK;PGLPVG	NCP	7.778
PGLPVG;AGPVKP	NCP	58.615
AGPVKP;TPRYSPPP	NCP	50.804
TPRYSPPP;FSDSGSLP	NCP	56.541
FSDSGSLP;KNVGVLK	NCP	175.677
KNVGVLK;CDCCCP	NCP	50.07
CDCCCP;LGPLTRF	NCP	39.106
LGPLTRF;LLGHCCPL	NCP	57.053
LLGHCCPL;LPLGSLP	NCP	3.558
LPLGSLP;DTGDVPR	NCP	7.858
DTGDVPR;QVFKLKSL	NCP	12.777
QVFKLKSL;GPPYP	NCP	36.641
GPPYP;KVPDLA	NCP	85.378
KVPDLA;KEFQA	NCP	18.995
KEFQA;KHFPK	NCP	38.203
KHFPK;SQLAF	NCP	48.966
SQLAF;KHKPLKF	NCP	26.162
KHKPLKF;LSQAPSR	NCP	159.216
LSQAPSR;DMHPPTK	NCP	85.425
DMHPPTK;AELRLAV	NCP	126.264
AELRLAV;VLLLHNPL	NCP	8.7
VLLLHNPL;LENRVVL	NCP	30.256
LENRVVL;GFHRD	NCP	30.398

GFHRD;PVNFKLSL	NCP	56.107
PVNFKLSL;LSLLLGM	NCP	72.347
LSLLLGM;LREAVLK	NCP	142.785
LREAVLK;KYLAFPH	NCP	34.453
KYLAFPH;KGKPPSQR	NCP	23.576
KGKPPSQR;NSSLLTLP	NCP	33.798
NSSLLTLP;VASFRK	NCP	7.158
VASFRK;RETSQH	NCP	355.287
RETSQH;FAAPVT	NCP	24.315
FAAPVT;DNTPTSTR	NCP	6.198
DNTPTSTR;NEEDLLFR	NCP	68.887
NEEDLLFR;ENSAPF	NCP	39.324
ENSAPF;HHTVSK	NCP	13.215
HHTVSK;LDVPK	NCP	16.047
LDVPK;ASLLQTDR	NCP	87.768
ASLLQTDR;RSGPKPF	NCP	142.61
RSGPKPF;KKNQVLLY	NCP	9.319
KKNQVLLY;AHELGF	NCP	1.089
AHELGF;KEQLKVV	NCP	4.379
KEQLKVV;PAATV	NCP	24.507
PAATV;RLLAEAK	NCP	49.512
RLLAEAK;HAPPPPPP	NCP	19.14
HAPPPPPP;DDDQLQ	NCP	38.154
DDDQLQ;KSTTEVH	NCP	60.928
KSTTEVH;MDPVLAP	NCP	14.493
MDPVLAP;KLTAGP	NCP	34.583
KLTAGP;TPPPPP	NCP	5.536
TPPPPP;RDLKEKLC	NCP	53.901
RDLKEKLC;LYTCGAA	NCP	17.091
LYTCGAA;AAVDGVLK	NCP	13.391
AAVDGVLK;CMMNP	NCP	29.865
CMMNP;KPYSKGP	NCP	64.42
KPYSKGP;PAAADPP	NCP	19.79
PAAADPP;KMFHGPP	NCP	27.217
KMFHGPP;KSLDPKV	NCP	29.202
KSLDPKV;ELMMCL	NCP	39.446
ELMMCL;GPLSVHR	NCP	24.184
GPLSVHR;KMSTAGP	NCP	78.147
KMSTAGP;LGEGFL	NCP	1.55
LGEGFL;NGSPVEL	NCP	0.723
NGSPVEL;SSFDVAKQ	NCP	8.977
SSFDVAKQ;AGEML	NCP	58.008
AGEML;LLGMNQ	NCP	40.049
LLGMNQ;QCPPR	NCP	60.813
QCPPR;TRTHLDY	NCP	28.359
TRTHLDY;VPVEC	NCP	40.299
VPVEC;LLGVN	NCP	20.419
LLGVN;MLTTAF	NCP	7.164
MLTTAF;KAEDGAP	NCP	141.171
KAEDGAP;YGPFL	NCP	4.584
YGPFL;AGGSFKK	NCP	45.098

AGGSFKK;EANVFPR	NCP	10.249
EANVFPR;KSRGLGL	NCP	5.145
KSRGLGL;KNYLSM	NCP	20.715
KNYLSM;RPCLRCK	NCP	24.228
RPCLRCK;DLTDY	NCP	47.207
DLTDY;LAGRTPY	NCP	5.226
LAGRTPY;VHVLYH	NCP	41.31
VHVLYH;LVGLFEDT	NCP	63.516
LVGLFEDT;KCSMDPK	NCP	31.864
KCSMDPK;PTAVSAG	NCP	17.255
PTAVSAG;EAAPYLR	NCP	43.674
EAAPYLR;YQKSALKTL	NCP	8.469
YQKSALKTL;VADPKESFVK	NCP	21.12
VADPKESFVK;VGALAKAV	NCP	4.572
VGALAKAV;YQKSSVKTL	NCP	2.427
YQKSSVKTL;MDLERAK	NCP	5.049
MDLERAK;KMPNLLSH	NCP	11.673
KMPNLLSH;LLEAFGNAK	NCP	10.686
LLEAFGNAK;EFLEF	NCP	49.219
EFLEF;STHPHFVR	NCP	3.146
STHPHFVR;RFSLFL	NCP	10.938
RFSLFL;FGEAAPYLR	NCP	32.169
FGEAAPYLR;GEAAPYLRK	NCP	0.003
GEAAPYLRK;EAAPYLRKS	NCP	0.003
EAAPYLRKS;AAPYLRK	NCP	0.003
AAPYLRK;ANPLLEAFGNA	NCP	5.115
ANPLLEAFGNA;DEAPT	NCP	19.261
DEAPT;EWRPPPPP	NCP	13.866
EWRPPPPP;KPAAPPGP	NCP	50.121
KPAAPPGP;KDPKGR	NCP	81.444
KDPKGR;ELRVNP	NCP	51.447
ELRVNP;RVTPP	NCP	6.641
RVTPP;RHSAPPP	NCP	0.42
RHSAPPP;FFGWLF	NCP	88.735
FFGWLF;QARPVQV	NCP	0.659
QARPVQV;TLGLLL	NCP	12.447
TLGLLL;KAQPAPPP	NCP	22.807
KAQPAPPP;VGQTRVP	NCP	11.868
VGQTRVP;DKQRPD	NCP	140.637
DKQRPD;LLEMLGK	NCP	46.367
LLEMLGK;LVPTHH	NCP	7.084
LVPTHH;QPPHHA	NCP	11.226
QPPHHA;KDALAAF	NCP	33.734
KDALAAF;PPLDP	NCP	44.823
PPLDP;QALKP	NCP	47.289
QALKP;LPPPQVP	NCP	29.793
LPPPQVP;GRPTRPP	NCP	3.81
GRPTRPP;PPLPLMQ	NCP	24.741
PPLPLMQ;GVPLD	NCP	27.223
GVPLD;PAYLFPP	NCP	3.842
PAYLFPP;MVPPP	NCP	61.861

MVPPP;SGPLPRPK	NCP	78.332
SGPLPRPK;ALPSPAPK	NCP	1.467
ALPSPAPK;NGFYLGGSHL	NCP	10.087
NGFYLGGSHL;KPDVQ	NCP	31.064
KPDVQ;KFSHAH	NCP	9.942
KFSHAH;PTVEAQ	NCP	58.246
PTVEAQ;VKYLYN	NCP	43.53
VKYLYN;LGRLSSP	NCP	5.436
LGRLSSP;DVPRPGPP	NCP	32.337
DVPRPGPP;KPEPP	NCP	16.446
KPEPP;AAPVNKAPR	NCP	59.088
AAPVNKAPR;AQRTGLEDPERY	NCP	6.521
AQRTGLEDPERY;NAEVFK	NCP	0.771
NAEVFK;RHEMPPH	NCP	13.377
RHEMPPH;KHLLLAY	NCP	7.98
KHLLLAY;KVLLY	NCP	10.458
KVLLY;QHTEEYE	NCP	6.933
QHTEEYE;LVVLG	NCP	13.041
LVVLG;TNEKLQQLFNH	NCP	17.709
TNEKLQQLFNH;LFNHTMF	NCP	0.021
LFNHTMF;LDEECWFPKATDK	NCP	2.643
LDEECWFPKATDK;SLKDFSK	NCP	11.899
SLKDFSK;KGMFRTVGQLY	NCP	0.197
KGMFRTVGQLY;GMFRTVGQ	NCP	0.003
GMFRTVGQ;TVGQLYK	NCP	0.012
TVGQLYK;ALELDPNLY	NCP	2.178
ALELDPNLY;LARALEEALEA	NCP	11.592
LARALEEALEA;HQGVMVG	NCP	3.24
HQGVMVG;LGPVPA	NCP	45.706
LGPVPA;VSVGPGP	NCP	7.476
VSVGPGP;MAPRPK	NCP	49.179
MAPRPK;PSVTRPEVH	NCP	7.767
PSVTRPEVH;SGALRTPTS	NCP	8.439
SGALRTPTS;ALRTPTSRT	NCP	0.006
ALRTPTSRT;PVPGAFF	NCP	41.184
PVPGAFF;EVHVPR	NCP	43.425
EVHVPR;DPLDRRP	NCP	46.503
DPLDRRP;PGGVSELP	NCP	56.555
PGGVSELP;PETPFP	NCP	6.562
PETPFP;AAKKK	NCP	5.768
AAKKK;VSKGPLQ	NCP	17.875
VSKGPLQ;AVKPPPK	NCP	56.328
AVKPPPK;ALEGPAY	NCP	6.177
ALEGPAY;ADSDL	NCP	5.39
ADSDL;AAYVLK	NCP	12.174
AAYVLK;LLLLFRM	NCP	26.398
LLLLFRM;DAGKYA	NCP	8.325
DAGKYA;PVPYK	NCP	11.138
PVPYK;LDGELPQ	NCP	104.026
LDGELPQ;GVLVDLP	NCP	51.203
GVLVDLP;GPSLAMR	NCP	21.336

GPSLAMR;LLGGVLSA	NCP	5.983
LLGGVLSA;KYPAPQP	NCP	62.6
KYPAPQP;AETKAAAADGERPGPGP	NCP	32.232
AETKAAAADGERPGPGP;GDSVSKLPK	NCP	12.678
GDSVSKLPK;LLPRPPGD	NCP	16.638
LLPRPPGD;NPKRASPP	NCP	0.381
NPKRASPP;STELLLKR	NCP	14.026
STELLLKR;VDLNPR	NCP	9.245
VDLNPR;VGKAPLR	NCP	29.25
VGKAPLR;GVKGALLR	NCP	22.866
GVKGALLR;KPERAPP	NCP	100.785
KPERAPP;SHELSDF	NCP	1.716
SHELSDF;GEGLAEL	NCP	23.86
GEGLAEL;VGATMQK	NCP	9.749
VGATMQK;VGPPLLG	NCP	10.053
VGPPLLG;GLEVGKAP	NCP	9.672
GLEVGKAP;LLEQPGTP	NCP	1.083
LLEQPGTP;LPGKTAGP	NCP	100.494
LPGKTAGP;NPPPPPPPP	NCP	20.316
NPPPPPPPP;NAHTDPLG	NCP	41.619
NAHTDPLG;TKEYQDLQHLDNEEN	NCP	10.845
TKEYQDLQHLDNEEN;LPKESR	NCP	34.075
LPKESR;FLYLAY	NCP	32.924
FLYLAY;EPLLAVP	NCP	106.186
EPLLAVP;KPSCVGTK	NCP	74.513
KPSCVGTK;GKRDFVDHL	NCP	12.111
GKRDFVDHL;CLFSTAQ	NCP	2.229
CLFSTAQ;LGPQEV	NCP	26.457
LGPQEV;SNLRTPST	NCP	10.758
SNLRTPST;KSTLPLP	NCP	157.783
KSTLPLP;AEGTAVLR	NCP	2.639
AEGTAVLR;KAHVPPP	NCP	69.366
KAHVPPP;VSPSPL	NCP	122.811
VSPSPL;FSLKKLG	NCP	71.1
FSLKKLG;RKVSPHL	NCP	24.082
RKVSPHL;RLEAERLA	NCP	19.268
RLEAERLA;MGPPEVGP	NCP	55.873
MGPPEVGP;MQKDTSLPPP	NCP	39.633
MQKDTSLPPP;PPPSGPLASN	NCP	0.021
PPPSGPLASN;TPVLGEP	NCP	30.834
TPVLGEP;SATPDRQ	NCP	117.864
SATPDRQ;ADMLMHL	NCP	53.888
ADMLMHL;SPGKRFA	NCP	60.469
SPGKRFA;VGGGLGGQ	NCP	10.842
VGGGLGGQ;LGARDL	NCP	27.513
LGARDL;VTVTPALT	NCP	57.215
VTVTPALT;VGATESSK	NCP	16.521
VGATESSK;ALKKRE	NCP	10.282
ALKKRE;MLGPRST	NCP	15.981
MLGPRST;LPAGKLLY	NCP	55.346
LPAGKLLY;WSAGEKL	NCP	34.57

WSAGEKL;MELLDQ	NCP	12.465
MELLDQ;TQTPWKT	NCP	142.383
TQTPWKT;CHSDEKK	NCP	31.829
CHSDEKK;KPLLSPR	NCP	33.468
KPLLSPR;KVPGEAK	NCP	16.074
KVPGEAK;ASYEEVSV	NCP	61.932
ASYEEVSV;AYFSGSKDAEGK	NCP	0.081
AYFSGSKDAEGK;NLKLTAVPTLLK	NCP	1.161
NLKLTAVPTLLK;ANLVEMLFSED	NCP	0.711
ANLVEMLFSED;VVVPP	NCP	42.069
VVVPP;FPGVQP	NCP	12.877
FPGVQP;LATALTK	NCP	56.676
LATALTK;DLPPLPPPPP	NCP	16.274
DLPPLPPPPP;GVGGGL	NCP	52.824
GVGGGL;LPLPNFS	NCP	43.225
LPLPNFS;KPPLAPP	NCP	84.471
KPPLAPP;PGPEAP	NCP	4.866
PGPEAP;TMPQPR	NCP	29.988
TMPQPR;TELQSALLLR	NCP	9.407
TELQSALLLR;VGVFAL	NCP	22.128
VGVFAL;KYLLP	NCP	78.675
KYLLP;DPWGGGK	NCP	7.444
DPWGGGK;LLAAQAKK	NCP	1.802
LLAAQAKK;LCAVPR	NCP	44.58
LCAVPR;FSGNPPPP	NCP	57.864
FSGNPPPP;AFSPRLK	NCP	72.613
AFSPRLK;LLGPLPPP	NCP	5.244
LLGPLPPP;TGDGVNDAPALKK	NCP	30.515
TGDGVNDAPALKK;GDGVNDAPALK	NCP	0.003
GDGVNDAPALK;RGAVP	NCP	16.785
RGAVP;VGPVLS	NCP	30.444
VGPVLS;LFMNYL	NCP	71.845
LFMNYL;ASLDRVK	NCP	20.684
ASLDRVK;SVKRVR	NCP	28.974
SVKRVR;LTVLSNL	NCP	12.879
LTVLSNL;ALSFFGY	NCP	49.398
ALSFFGY;PVFGWVP	NCP	26.685
PVFGWVP;HVKPPPP	NCP	12.027
HVKPPPP;SGLRLLM	NCP	24.072
SGLRLLM;DDDLHT	NCP	91.986
DDDLHT;SPPVAP	NCP	23.4
SPPVAP;VHLGKAAP	NCP	47.865
VHLGKAAP;PYPLSLLK	NCP	10.762
PYPLSLLK;AAASP	NCP	19.16
AAASP;KTHPPGP	NCP	130.684
KTHPPGP;QGVELSLS	NCP	46.047
QGVELSLS;TVLLLP	NCP	33.83
TVLLLP;GPLPP	NCP	38.31
GPLPP;LPSGPLL	NCP	62.355
LPSGPLL;SAGPP	NCP	11.203
SAGPP;KENVPPP	NCP	11.463

KENVPPP;VPGVK	NCP	43.724
VPGVK;KSAHTAK	NCP	32.179
KSAHTAK;KLGLTPK	NCP	15.876
KLGLTPK;YYRPK	NCP	45.942
YYRPK;DCPESLKKEEGPAEPPREN	NCP	20.315
DCPESLKKEEGPAEPPREN;KGLLG	NCP	37.882
KGLLG;EPLLHRP	NCP	76.928
EPLLHRP;LGSALCGK	NCP	23.008
LGSALCGK;TGPKAFK	NCP	26.006
TGPKAFK;EVVLVK	NCP	2.439
EVVLVK;ALLATLRP	NCP	65.971
ALLATLRP;PLLVAPA	NCP	3.476
PLLVAPA;LPGAAPP	NCP	20.227
LPGAAPP;VTPVP	NCP	12.401
VTPVP;GVSKPSP	NCP	3.189
GVSKPSP;GKGSNFK	NCP	3.976
GKGSNFK;PPPLMPHP	NCP	8.961
PPPLMPHP;KGHGKPR	NCP	0.035
KGHGKPR;LVLSAL	NCP	16.137
LVLSAL;EDSHKPR	NCP	43.282
EDSHKPR;LVAPGSF	NCP	58.742
LVAPGSF;QAFVR	NCP	48.075
QAFVR;FLMLLF	NCP	36.619
FLMLLF;ARFKVK	NCP	2.358
ARFKVK;PPHPPPPP	NCP	39.146
PPHPPPPP;KGDSAPY	NCP	71.749
KGDSAPY;PSLVGRP	NCP	64.752
PSLVGRP;TVGLLL	NCP	7.368
TVGLLL;RSVPAAK	NCP	38.325
RSVPAAK;LGMESQ	NCP	39.3
LGMESQ;TMAPPPPP	NCP	41.771
TMAPPPPP;TGPAVLSM	NCP	58.836
TGPAVLSM;PSAPPL	NCP	129.648
PSAPPL;MESALTARDR	NCP	7.845
MESALTARDR;LGYKPEEY	NCP	11.115
LGYKPEEY;FVDHVRT	NCP	1.089
FVDHVRT;VDHVRTSF	NCP	0.003
VDHVRTSF;KGKKDNYPQSVPR	NCP	1.683
KGKKDNYPQSVPR;GPPLLPVP	NCP	1.848
GPPLLPVP;KDPLPPL	NCP	19.801
KDPLPPL;RGGPVHR	NCP	11.18
RGGPVHR;MDDREDLVYQAK	NCP	17.154
MDDREDLVYQAK;EVVVL	NCP	6.585
EVVVL;FWLSR	NCP	37.945
FWLSR;WPSFP	NCP	12.984
WPSFP;HSFVPL	NCP	64.311
HSFVPL;LSFFNSYL	NCP	8.517
LSFFNSYL;PVGDPP	NCP	3.123
PVGDPP;LASLRPVT	NCP	20.708
LASLRPVT;LAARPPTV	NCP	38.382
LAARPPTV;KAAAKYP	NCP	33.843

KAAAKYP;ERVVPY	NCP	45.532
ERVVPY;MEKNALNK	NCP	43.952
MEKNALNK;VVVSVPG	NCP	15.514
VVVSVPG;DMPPR	NCP	28.626
DMPPR;KPMGKGK	NCP	33.615
KPMGKGK;TGGPLLP	NCP	198.336
TGGPLLP;KPRAAGGP	NCP	53.036
KPRAAGGP;FGMAV	NCP	54.67
FGMAV;HPGASKR	NCP	23.64
HPGASKR;PTSGLP	NCP	23.418
PTSGLP;APLNKAP	NCP	36.89
APLNKAP;PTLGPPR	NCP	56.766
PTLGPPR;RTSAAPP	NCP	10.714
RTSAAPP;TAFPEFQ	NCP	31.937
TAFPEFQ;VSFSKL	NCP	9.586
VSFSKL;KEQPQ	NCP	54.501
KEQPQ;ELRLSQ	NCP	123.62
ELRLSQ;NPAPPPPPP	NCP	62.265
NPAPPPPPP;GVLGGSLDA	NCP	87.48
GVLGGSLDA;AAWGKVG	NCP	27.117
AAWGKVG;QRKFVF	NCP	20.628
QRKFVF;AAAPLA	NCP	155.67
AAAPLA;HVDDNM	NCP	107.469
HVDDNM;THRKCF	NCP	1.405
THRKCF;TLSEGAP	NCP	68.627
TLSEGAP;VSVPGSPS	NCP	52.368
VSVPGSPS;GHQLVLY	NCP	19.858
GHQLVLY;GRVALPLQ	NCP	0.458
GRVALPLQ;VVRQSKF	NCP	1.551
VVRQSKF;MVAPERP	NCP	41.862
MVAPERP;EEFHPVR	NCP	36.376
EEFHPVR;GNTGLSSK	NCP	28.473
GNTGLSSK;APALLPL	NCP	10.131
APALLPL;LLLPHE	NCP	7.721
LLLPHE;FPGVLQAL	NCP	31.645
FPGVLQAL;PLDLP	NCP	28.791
PLDLP;KDQVPF	NCP	8.733
KDQVPF;APERLPT	NCP	3.642
APERLPT;PLLTLPVP	NCP	11.121
PLLTLPVP;AAERRQP	NCP	0.465
AAERRQP;KDMGSLER	NCP	17.793
KDMGSLER;GNHLYF	NCP	26.763
GNHLYF;SVPLLL	NCP	1.994
SVPLLL;SLQDKLLP	NCP	27.582
SLQDKLLP;MADMDC	NCP	9.24
MADMDC;KWAETEM	NCP	47.812
KWAETEM;ANFPLLK	NCP	171.486
ANFPLLK;AYTPLK	NCP	50.847
AYTPLK;PGCHRP	NCP	4.44
PGCHRP;EGPAPPRP	NCP	20.684
EGPAPPRP;VSAGVEVEP	NCP	1.08

VSAGVEVEP;LPLAKAQ	NCP	17.865
LPLAKAQ;NMCPPPPP	NCP	54.087
NMCPPPPP;TVTPY	NCP	6.457
TVTPY;PALTLGN	NCP	87.975
PALTLGN;GPPPPPDP	NCP	17.508
GPPPPPDP;ALVSC	NCP	2.334
ALVSC;QECEQ	NCP	45.291
QECEQ;ELFTPT	NCP	11.912
ELFTPT;VLAAVYKALSDH	NCP	8.022
VLAAVYKALSDH;FSYGRALQ	NCP	0.405
FSYGRALQ;LRDSLRLL	NCP	8.821
LRDSLRLL;KPVQPMGP	NCP	12.293
KPVQPMGP;GVDML	NCP	75.745
GVDML;LHANT	NCP	1.232
LHANT;KNELLP	NCP	16.252
KNELLP;NRKLVAP	NCP	15.321
NRKLVAP;RGAGPSF	NCP	79.392
RGAGPSF;KEGRPGK	NCP	30.978
KEGRPGK;AAALTLGK	NCP	12.003
AAALTLGK;PQASNVP	NCP	129.45
PQASNVP;KKGVNAK	NCP	50.318
KKGVNAK;TMMVRF	NCP	129.687
TMMVRF;KFDLNK	NCP	10.69
KFDLNK;PVLHL	NCP	44.03
PVLHL;PFAAGP	NCP	13.542
PFAAGP;VSLLPR	NCP	16.047
VSLLPR;VPQNLQ	NCP	62.934
VPQNLQ;VYGAGL	NCP	93.157
VYGAGL;EVLLV	NCP	28.427
EVLLV;KKKKKQFGY	NCP	18.013
KKKKKQFGY;SLPDRSSL	NCP	41.849
SLPDRSSL;MLFFK	NCP	78.829
MLFFK;DYGGF	NCP	199.137
DYGGF;RPPLKHG	NCP	41.907
RPPLKHG;LVEKPSLP	NCP	32.85
LVEKPSLP;KVVSLPK	NCP	25.901
KVVSLPK;RVTPGGP	NCP	124.099
RVTPGGP;LPPPPLPPPLPP	NCP	52.581
LPPPPLPPPLPP;RLHPPPP	NCP	31.817
RLHPPPP;NPSRKGP	NCP	7.294
NPSRKGP;KAARGGLP	NCP	1.175
KAARGGLP;LLGLAN	NCP	4.912
LLGLAN;TVDAAPR	NCP	1.754
TVDAAPR;HGPKHVQ	NCP	4.284
HGPKHVQ;KPTERVK	NCP	1.462
KPTERVK;AVEKPK	NCP	93.599
AVEKPK;EPPGPLLP	NCP	17.005
EPPGPLLP;PPRLQRQ	NCP	58.974
PPRLQRQ;LSGGTGLK	NCP	41.64
LSGGTGLK;GVPPLS	NCP	15.797
GVPPLS;GCKLERL	NCP	68.304

GCKLERL;PPPPPPSR	NCP	36.454
PPPPPPSR;LANKTLEL	NCP	3.857
LANKTLEL;LEPLLPR	NCP	16.942
LEPLLPR;VVPRAFVD	NCP	145.122
VVPRAFVD;NGYLF	NCP	70.692
NGYLF;LASLLQ	NCP	0.924
LASLLQ;PAAFLGPR	NCP	33.495
PAAFLGPR;QFLPDP	NCP	14.637
QFLPDP;CCMECC	NCP	54.35
CCMECC;AATYFLK	NCP	25.275
AATYFLK;GVESKAVP	NCP	101.533
GVESKAVP;AAAAVVEFQRAQSLL	NCP	7.052
AAAAVVEFQRAQSLL;QGTFSLPR	NCP	13.327
QGTFSLPR;FALKV	NCP	121.305
FALKV;DGETEEL	NCP	65.892
DGETEEL;VGMGDLR	NCP	33.287
VGMGDLR;ASAAFK	NCP	70.543
ASAAFK;DHDAAC	NCP	6.878
DHDAAC;VLSKPR	NCP	5.85
VLSKPR;FPLVFK	NCP	38.148
FPLVFK;VLLSEKLK	NCP	38.409
VLLSEKLK;PAAARPPPPP	NCP	84.175
PAAARPPPPP;LTGGELP	NCP	8.276
LTGGELP;KQEDPLP	NCP	39.94
KQEDPLP;AAFPREVP	NCP	11.696
AAFPREVP;LSFPTT	NCP	46.15
LSFPTT;KAHAQPQR	NCP	26.813
KAHAQPQR;DTSSPP	NCP	201.076
DTSSPP;GPVTVHV	NCP	11.658
GPVTVHV;KGGGL	NCP	78.036
KGGGL;FLSGVQ	NCP	11.156
FLSGVQ;LAVKEYK	NCP	77.94
LAVKEYK;VSFNLP	NCP	59.473
VSFNLP;KGPVFAK	NCP	82.17
KGPVFAK;KDGVSVK	NCP	6.617
KDGVSVK;SGRLP	NCP	128.532
SGRLP;APFPEV	NCP	33.867
APFPEV;NELLTHVL	NCP	29.194
NELLTHVL;ATKSL	NCP	37.539
ATKSL;VSLQVVH	NCP	17.094
VSLQVVH;LPPSLSE	NCP	52.994
LPPSLSE;QKRLSPP	NCP	0.109
QKRLSPP;ERFVKDL	NCP	92.189
ERFVKDL;WTGYP	NCP	34.588
WTGYP;VPPGKC	NCP	109.085
VPPGKC;VNLVGPS	NCP	93.933
VNLVGPS;VTAEVLP	NCP	7.047
VTAEVLP;KAPNF	NCP	24.543
KAPNF;TKTLPVP	NCP	109.806
TKTLPVP;LQAAEYR	NCP	40.104
LQAAEYR;KCEFYG	NCP	113.398

KCEFYG;TDKLLER	NCP	11.994
TDKLLER;AVSDPWP	NCP	53.691
AVSDPWP;EATPKLP	NCP	3.998
EATPKLP;DPVDPPR	NCP	54.247
DPVDPPR;TRTSLYT	NCP	227.168
TRTSLYT;KLVSP	NCP	24.61
KLVSP;DPMDKLR	NCP	5.868
DPMDKLR;CCCNNYL	NCP	36.6
CCCNNYL;YMGVPP	NCP	24.698
YMGVPP;RLENAP	NCP	16.125
RLENAP;KTESLLTK	NCP	53.083
KTESLLTK;VPSPKP	NCP	51.59
VPSPKP;EQFWDQ	NCP	42.21
EQFWDQ;PPPPALW	NCP	75.873
PPPPALW;ASETFYKVGDGEE	NCP	5.754
ASETFYKVGDGEE;LPPDM	NCP	110.04
LPPDM;RKVGQK	NCP	27.093
RKVGQK;LLPFEL	NCP	49.396
LLPFEL;LPAPHHK	NCP	56.925
LPAPHHK;KMRPPPPP	NCP	86.447
KMRPPPPP;ELGVLLP	NCP	25.228
ELGVLLP;FRCPETL	NCP	64.146
FRCPETL;CMEDC	NCP	7.284
CMEDC;MLFFLL	NCP	111.068
MLFFLL;CCCYG	NCP	16.156
CCCYG;VAGPGLR	NCP	73.938
VAGPGLR;MGVPY	NCP	100.011
MGVPY;NSHPVLL	NCP	4.838
NSHPVLL;PAADF	NCP	40.023
PAADF;KHTPRP	NCP	26.854
KHTPRP;KGVELF	NCP	6.087
KGVELF;PTCPGGK	NCP	55.07
PTCPGGK;ADYVGF	NCP	11.286
ADYVGF;KDFQAL	NCP	93.76
KDFQAL;AEAGE	NCP	89.031
AEAGE;ATWNK	NCP	27.617
ATWNK;KDVRGPR	NCP	23.443
KDVRGPR;KVLFH	NCP	30.626
KVLFH;APPSHAK	NCP	50.04
APPSHAK;PGDVVYV	NCP	170.211
PGDVVYV;EDDDDP	NCP	7.624
EDDDDP;KLHTADT	NCP	24.869
KLHTADT;EKLVTL	NCP	25.368
EKLVTL;HFDARG	NCP	54.925
HFDARG;CATKSYSK	NCP	108.225
CATKSYSK;KVPVHE	NCP	43.901
KVPVHE;PVDLTLP	NCP	130.38
PVDLTLP;GTVVLK	NCP	110.424
GTVVLK;DSQALLR	NCP	74.587
DSQALLR;KGPKPYP	NCP	8.222
KGPKPYP;CSCYPP	NCP	39.396

CSCYPP;VWVKVFG	NCP	81.222
VWVKVFG;AKLQP	NCP	47.728
AKLQP;AALHQLR	NCP	0.329
AALHQLR;QKKGTPPK	NCP	15.216
QKKGTPPK;VSRGPTPP	NCP	33.774
VSRGPTPP;MDFRHP	NCP	14.155
MDFRHP;RDGLAAP	NCP	77.643
RDGLAAP;GKTHHKT	NCP	48.591
GKTHHKT;LGAPKSR	NCP	42.281
LGAPKSR;TSTATPLTK	NCP	3.336
TSTATPLTK;KGRAALH	NCP	15.328
KGRAALH;TSFNVP	NCP	29.457
TSFNVP;KHLLGH	NCP	0.939
KHLLGH;LGPPAP	NCP	0.384
LGPPAP;TADRLPP	NCP	140.442
TADRLPP;LAQKNYR	NCP	17.6
LAQKNYR;KVDAGP	NCP	62.308
KVDAGP;VPASSGPP	NCP	8.052
VPASSGPP;PATTVLVT	NCP	40.737
PATTVLVT;HLMHEL	NCP	25.082
HLMHEL;PVLLG	NCP	157.078
PVLLG;PSAGLQPGK	NCP	56.973
PSAGLQPGK;LDSPVL	NCP	2.544
LDSPVL;MFVPP	NCP	9.8
MFVPP;KLGAPLPL	NCP	79.639
KLGAPLPL;KTTFDP	NCP	11.633
KTTFDP;TLVHTV	NCP	11.113
TLVHTV;GFLSSGAP	NCP	90.27
GFLSSGAP;NYEVVF	NCP	3.32
NYEVVF;NDCPEW	NCP	8.856
NDCPEW;MDCDC	NCP	15.433
MDCDC;CCMGAK	NCP	17.793
CCMGAK;TVAEHFP	NCP	40.347
TVAEHFP;LVLAQM	NCP	36.983
LVLAQM;GVGPL	NCP	107.862
GVGPL;EGLALSR	NCP	19.519
EGLALSR;KGLAWK	NCP	21.602
KGLAWK;VSDKPP	NCP	44.248
VSDKPP;KGYFFVP	NCP	29.444
KGYFFVP;LAERPY	NCP	16.186
LAERPY;HNAAPPP	NCP	28.785
HNAAPPP;MGAPAGGK	NCP	49.104
MGAPAGGK;KAMVPL	NCP	42.678
KAMVPL;EPGPLP	NCP	8.529
EPGPLP;HHTLKK	NCP	2.765
HHTLKK;VNPTVF	NCP	28.077
VNPTVF;RTSNAGGL	NCP	8.842
RTSNAGGL;GVVGDW	NCP	78.66
GVVGDW;EVGVLYT	NCP	36.459
EVGVLYT;QSGSFLK	NCP	8.34
QSGSFLK;YPMVKP	NCP	104.166

YPMVKP;RYAGGPR	NCP	12.24
RYAGGPR;KFRGPVY	NCP	45.548
KFRGPVY;PGGGDVLK	NCP	0.981
PGGGDVLK;VGYPSRL	NCP	90.402
VGYPSRL;ECCCP	NCP	5.11
ECCCP;GDKGLLY	NCP	27.743
GDKGLLY;PPGGP	NCP	24.01
PPGGP;VPPQPAPL	NCP	19.053
VPPQPAPL;KDTTLC	NCP	16.94
KDTTLC;PFLVGK	NCP	119.341
PFLVGK;PGGWSMQ	NCP	16.136
PGGWSMQ;LPVTAGPR	NCP	5.014
LPVTAGPR;RKQEPK	NCP	4.79
RKQEPK;FHKSCF	NCP	37.695
FHKSCF;TLVLRT	NCP	34.909
TLVLRT;DLWRK	NCP	22.098
DLWRK;KPQQEH	NCP	40.86
KPQQEH;VLVPPAA	NCP	23.7
VLVPPAA;PLKVHGF	NCP	79.643
PLKVHGF;LCLLPR	NCP	85.951
LCLLPR;TATESLP	NCP	1.695
TATESLP;DEFSTFP	NCP	46.088
DEFSTFP;QGSHHPR	NCP	14.82
QGSHHPR;EEETTAL	NCP	62.022
EEETTAL;PAPRALPE	NCP	32.359
PAPRALPE;LLFGRHL	NCP	30.357
LLFGRHL;AFAPLVK	NCP	188.741
AFAPLVK;APALL	NCP	19.768
APALL;VPVVAPG	NCP	30.909
VPVVAPG;SALNF	NCP	26.25
SALNF;KVKLSH	NCP	14.57
KVKLSH;GKEGGPL	NCP	77.917
GKEGGPL;LSPLQFR	NCP	29.78
LSPLQFR;LPPPKVPP	NCP	28.96
LPPPKVPP;REPLPSKE	NCP	30.06
REPLPSKE;PAWGKER	NCP	11.238
PAWGKER;ESVLVVRV	NCP	96.488
ESVLVVRV;APPVVLM	NCP	100.671
APPVVLM;RPGAAPPP	NCP	72.258
RPGAAPPP;VPMSQLR	NCP	0.565
VPMSQLR;DDKEYVGF	NCP	5.168
DDKEYVGF;KVPWAAP	NCP	75.175
KVPWAAP;AALATL	NCP	3.945
AALATL;ALRAMR	NCP	58.979
ALRAMR;SEGALRL	NCP	7.035
SEGALRL;TTPTVTPL	NCP	3.192
TTPTVTPL;KEAGPV	NCP	63.79
KEAGPV;AASLGL	NCP	13.152
AASLGL;PPGQEPTR	NCP	0.633
PPGQEPTR;PSSAVAK	NCP	43.89
PSSAVAK;TGGCETQ	NCP	37.977

TGGCETQ;PRPETLK	NCP	5.037
PRPETLK;GAPDPPK	NCP	20.391
GAPDPPK;GPARLTPR	NCP	5.939
GPARLTPR;PKTAGPR	NCP	77.461
PKTAGPR;LRLLPGE	NCP	19.404
LRLLPGE;YLANP	NCP	10.742
YLANP;KPALADP	NCP	23.929
KPALADP;NTAVLL	NCP	25.559
NTAVLL;SVGPSL	NCP	4.156
SVGPSL;HLAADPK	NCP	77.313
HLAADPK;YGLVK	NCP	30.984
YGLVK;ERPFWK	NCP	29.583
ERPFWK;EDMTYAVRK	NCP	55.928
EDMTYAVRK;GPAAAVTK	NCP	20.599
GPAAAVTK;RPPLSNPGSQ	NCP	31.962
RPPLSNPGSQ;CSDDAR	NCP	4.713
CSDDAR;VTAAAH	NCP	30.099
VTAAAH;WMWYGP	NCP	15.698
WMWYGP;LSPGVV	NCP	23.397
LSPGVV;NVLPPR	NCP	72.562
NVLPPR;QLPTLGPPP	NCP	23.919
QLPTLGPPP;PRGPWPP	NCP	59.016
PRGPWPP;TVGGPDV	NCP	38.474
TVGGPDV;KPVTKAAP	NCP	24.553
KPVTKAAP;LGSALRTP	NCP	4.914
LGSALRTP;KLRVDP	NCP	4.433
KLRVDP;KTVGPVR	NCP	0.03
KTVGPVR;DLAALVVD	NCP	64.632
DLAALVVD;LPLLP	NCP	99.828
LPLLP;GPSALLR	NCP	33.999
GPSALLR;VPRLPGP	NCP	141.238
VPRLPGP;KTAPGP	NCP	18.734
KTAPGP;KGQVP	NCP	48.765
KGQVP;TVLWGKK	NCP	1.111
TVLWGKK;CNPDLRK	NCP	26.297
CNPDLRK;KVSPQ	NCP	5.803
KVSPQ;AKESQKTK	NCP	0.188
AKESQKTK;GSPPGHMT	NCP	26.341
GSPPGHMT;DYLGLSV	NCP	63.899
DYLGLSV;EPVAPSPP	NCP	17.209
EPVAPSPP;EAGGLF	NCP	0.582
EAGGLF;HGPPP	NCP	42.897
HGPPP;LSPGLGCG	NCP	35.742
LSPGLGCG;AMKEPGK	NCP	57.983
AMKEPGK;KHAVTDK	NCP	26.62
KHAVTDK;AELCPLAEEL	NCP	3.605
AELCPLAEEL;GSSEHLVNPK	NCP	17.268
GSSEHLVNPK;VGATLYQ	NCP	2.308
VGATLYQ;PLVFALLF	NCP	1.197
PLVFALLF;VAAALTK	NCP	26.072
VAAALTK;GPAYP	NCP	34.207

GPAYP;APQLEP	NCP	24.834
APQLEP;LAGFPCL	NCP	76.818
LAGFPCL;GGTPPV	NCP	63.794
GGTPPV;QQLPGM	NCP	20.031
QQLPGM;MPAPPKP	NCP	6.024
MPAPPKP;AARLGGGM	NCP	13.882
AARLGGGM;VPGAGTAF	NCP	59.066
VPGAGTAF;PPPLPPGP	NCP	59.367
PPPLPPGP;LPSPPCCGP	NCP	8.532
LPSPPCCGP;KEQYLTK	NCP	8.646
KEQYLTK;CPPGAP	NCP	84.679
CPPGAP;KGGPG	NCP	232.772
KGGPG;LPPERV	NCP	21.526
LPPERV;QPVYL	NCP	21.441
QPVYL;RKGPLPLP	NCP	9.408
RKGPLPLP;VPSPPPPP	NCP	55.338
VPSPPPPP;TTSPHPPKP	NCP	2.187
TTSPHPPKP;MDWVMK	NCP	6.854
MDWVMK;SLLVLT	NCP	42.097
SLLVLT;KPSKERKK	NCP	7.595
KPSKERKK;KGETVAHF	NCP	15.786
KGETVAHF;KKPSELKDQ	NCP	18.394
KKPSELKDQ;GPSLQGHD	NCP	66.549
GPSLQGHD;AAAAFVSEK	NCP	39.893
AAAAFVSEK;LADATLP	NCP	21.759
LADATLP;KGNPGNKK	NCP	51.564
KGNPGNKK;RGPPVPPP	NCP	39.604
RGPPVPPP;PPPARLPP	NCP	14.229
PPPARLPP;GPGMLLL	NCP	53.496
GPGMLLL;HRPAPPLPP	NCP	21.588
HRPAPPLPP;CRPKF	NCP	1.601
CRPKF;TPNAPAP	NCP	11.803
TPNAPAP;QEDDP	NCP	17.348
QEDDP;KNFCVP	NCP	121.513
KNFCVP;RNSLL	NCP	2.294
RNSLL;KGSHLLSE	NCP	5.167
KGSHLLSE;DSMMVHR	NCP	19.278
DSMMVHR;CAAFNKL	NCP	22.964
CAAFNKL;GPPLPPP	NCP	9.802
GPPLPPP;VPDPP	NCP	191.727
VPDPP;SPGGRAF	NCP	111.165
SPGGRAF;MKPLNR	NCP	50.73
MKPLNR;TVGMSGKK	NCP	28.478
TVGMSGKK;DSTHKHR	NCP	101.622
DSTHKHR;YRGPP	NCP	90.007
YRGPP;EAVDKPV	NCP	3.467
EAVDKPV;PSSDVSVP	NCP	33.016
PSSDVSVP;KEAGP	NCP	71.841
KEAGP;RPMVVDL	NCP	10.353
RPMVVDL;KNWGAGP	NCP	30.699
KNWGAGP;LPHVLPPP	NCP	92.07

LPHVLPPP;TVPMRPR	NCP	6.056
TVPMRPR;WDFLAR	NCP	73.429
WDFLAR;PAGWGPK	NCP	14.319
PAGWGPK;TPHEELL	NCP	24.45
TPHEELL;GPQHKPP	NCP	33.873
GPQHKPP;LSGAAEKK	NCP	91.086
LSGAAEKK;VCHLPLP	NCP	22.539
VCHLPLP;KAHGTFK	NCP	22.652
KAHGTFK;RPQQVH	NCP	31.146
RPQQVH;FLGASQR	NCP	29.488
FLGASQR;LSGLLEEL	NCP	36.012
LSGLLEEL;YAVAK	NCP	31.704
YAVAK;SGLPEFRP	NCP	62.097
SGLPEFRP;KTSAP	NCP	17.229
KTSAP;PAANK	NCP	106.988
PAANK;KYVRSK	NCP	3.33
KYVRSK;MLEHLSSLPTQMDY	NCP	3.207
MLEHLSSLPTQMDY;PLAPPP	NCP	72.25
PLAPPP;APGVLSVP	NCP	67.983
APGVLSVP;TGGPPTVR	NCP	5.082
TGGPPTVR;RPPGVPPP	NCP	66.102
RPPGVPPP;VGELPT	NCP	138.497
VGELPT;APAPPLPSG	NCP	1.9
APAPPLPSG;KGGGLWSP	NCP	4.014
KGGGLWSP;LDSVLLGK	NCP	51.558
LDSVLLGK;SPVSFPK	NCP	76.632
SPVSFPK;LMRTPPPP	NCP	44.28
LMRTPPPP;QQGPPPPP	NCP	28.014
QQGPPPPP;KKFTPK	NCP	68.417
KKFTPK;TPHSPP	NCP	79.033
TPHSPP;KSPVVGP	NCP	20.835
KSPVVGP;LNTEFK	NCP	119.922
LNTEFK;AGGLRLSG	NCP	22.602
AGGLRLSG;AEPEKPLL	NCP	24.414
AEPEKPLL;VNNWKTK	NCP	79.517
VNNWKTK;SRGLLTF	NCP	60.205
SRGLLTF;VGLLSK	NCP	46.926
VGLLSK;LDHYMVL	NCP	24.584
LDHYMVL;KKKVPP	NCP	13.224
KKKVPP;PFQAP	NCP	30.594
PFQAP;SGPKPLG	NCP	125.608
SGPKPLG;DRPPLK	NCP	27.057
DRPPLK;MGGLDPQ	NCP	15.821
MGGLDPQ;TGATLLT	NCP	113.353
TGATLLT;LNSLFEL	NCP	179.306
LNSLFEL;DYLPP	NCP	25.167
DYLPP;GHAPPPPPP	NCP	8.719
GHAPPPPPP;GLAPHL	NCP	146.669
GLAPHL;LNFSVF	NCP	100.629
LNFSVF;LFTPPVVQ	NCP	102.358
LFTPPVVQ;NPVSGVGF	NCP	34.371

NPVSGVGF;KLVEL	NCP	61.766
KLVEL;KAAHASF	NCP	6.961
KAAHASF;VLTLLM	NCP	23.387
VLTLLM;TLDEAGP	NCP	0.46
TLDEAGP;LDEAGPK	NCP	0.003
LDEAGPK;GVFAAP	NCP	86.877
GVFAAP;HESTLVF	NCP	69.141
HESTLVF;DLSLP	NCP	52.902
DLSLP;FYGPRP	NCP	14.277
FYGPRP;NVEAGAP	NCP	46.052
NVEAGAP;DALSL	NCP	56.566
DALSL;GQEHVKR	NCP	11.378
GQEHVKR;LSKFAHHR	NCP	54.325
LSKFAHHR;NLGGTM	NCP	1.985
NLGGTM;LPVPQ	NCP	6.414
LPVPQ;GPKDL	NCP	125.011
GPKDL;KTAPGGQ	NCP	44.501
KTAPGGQ;STMKL	NCP	16.521
STMKL;FKRLP	NCP	123.217
FKRLP;ELQTAVR	NCP	47.973
ELQTAVR;APGRPAPP	NCP	27.498
APGRPAPP;VPTTF	NCP	11.945
VPTTF;EVSVGSR	NCP	39.634
EVSVGSR;RFVDAP	NCP	48.183
RFVDAP;VGSSHLVT	NCP	39.888
VGSSHLVT;VEFENPDEAEKALK	NCP	35.72
VEFENPDEAEKALK;LPTHATD	NCP	0.586
LPTHATD;EDCCCCH	NCP	29.1
EDCCCCH;KETLP	NCP	10.184
KETLP;LAPHAVP	NCP	9.49
LAPHAVP;FVMYVR	NCP	80.139
FVMYVR;KNVPANP	NCP	97.749
KNVPANP;KNVVVEK	NCP	8.444
KNVVVEK;PQHPPPP	NCP	74.293
PQHPPPP;TRVSAVH	NCP	13.626
TRVSAVH;KQFEKK	NCP	52.325
KQFEKK;KKMVPR	NCP	6.267
KKMVPR;SAPEPKKAEENPASK	NCP	0.606
SAPEPKKAEENPASK;KVGVLAK	NCP	100.648
KVGVLAK;GPVNPRP	NCP	65.397
GPVNPRP;HGPAPP	NCP	34.254
HGPAPP;STTGK	NCP	35.499
STTGK;KPSVTVP	NCP	73.053
KPSVTVP;KSDGVPK	NCP	2.357
KSDGVPK;KAVYTVP	NCP	52.498
KAVYTVP;AAPSKKPP	NCP	70.494
AAPSKKPP;VGSELEMR	NCP	45.978
VGSELEMR;KDNPP	NCP	22.73
KDNPP;NYPAP	NCP	37.488
NYPAP;KKTRPR	NCP	12.994
KKTRPR;PNWGLYK	NCP	10.31

PNWGLYK;KAPGY	NCP	39.823
KAPGY;FPPSVR	NCP	39.987
FPPSVR;NNSLVHRK	NCP	9.006
NNSLVHRK;AASTPP	NCP	141.549
AASTPP;LVTAF	NCP	7.77
LVTAF;QGPKEPK	NCP	7.434
QGPKEPK;AHGVP	NCP	10.584
AHGVP;TLMTTPF	NCP	43.127
TLMTTPF;LENSLLMF	NCP	22.761
LENSLLMF;ALVAGP	NCP	19.732
ALVAGP;KDVPVRP	NCP	64.569
KDVPVRP;KEGSFLL	NCP	39.054
KEGSFLL;WVTPP	NCP	59.768
WVTPP;VGANPF	NCP	15.537
VGANPF;GPPQPPHP	NCP	10.792
GPPQPPHP;PAHFKGPP	NCP	12.885
PAHFKGPP;TVPKV	NCP	46.43
TVPKV;LDTDYL	NCP	59.304
LDTDYL;RSAALLLR	NCP	42.094
RSAALLLR;KHGSPMY	NCP	20.327
KHGSPMY;TTPAFTP	NCP	24.445
TTPAFTP;KGGKKGK	NCP	23.085
KGGKKGK;APGTPRP	NCP	172.398
APGTPRP;LPPVVVSP	NCP	107.607
LPPVVVSP;PNSVAKYR	NCP	20.087
PNSVAKYR;KDAGVVR	NCP	3.463
KDAGVVR;WTPPG	NCP	11.15
WTPPG;KEVVTG	NCP	31.53
KEVVTG;ALKQVS	NCP	121.042
ALKQVS;TSLPAK	NCP	3.936
TSLPAK;PVALTPQ	NCP	5.913
PVALTPQ;EVTTPP	NCP	43.509
EVTTPP;GKSRSPLL	NCP	29.07
GKSRSPLL;DGFKLK	NCP	217.131
DGFKLK;AELSELR	NCP	113.358
AELSELR;PPVGPPPP	NCP	53.802
PPVGPPPP;VRPPYK	NCP	28.184
VRPPYK;THGGGP	NCP	12.73
THGGGP;VVLSRAGP	NCP	39.885
VVLSRAGP;SLHVGPK	NCP	30.522
LTAELLE;MLPEF	NCP	40.404
MLPEF;FLLQQ	NCP	7.343
FLLQQ;PGAGSQ	NCP	33.168
PGAGSQ;NPRVSK	NCP	33.183
NPRVSK;AELRHLV	NCP	142.65
AELRHLV;TDLKTQP	NCP	15.6
TDLKTQP;QSVQPK	NCP	26.661
QSVQPK;PAGGLRASP	NCP	23.94
PAGGLRASP;LPSMVLR	NCP	11.703
LPSMVLR;VPASTVLP	NCP	16.998
VPASTVLP;QALKV	NCP	18.204

QALKV;KPAEMR	NCP	172.624
KPAEMR;YGKKKSLE	NCP	24.197
YGKKKSLE;TLKYSFQK	NCP	15.171
TLKYSFQK;TGLLKPHL	NCP	41.272
TGLLKPHL;ESVLCVKPDVH	NCP	73.4
ESVLCVKPDVH;GPLLVP	NCP	11.55
GPLLVP;VPDSLRP	NCP	9.781
VPDSLRP;AKTVLR	NCP	43.155
AKTVLR;ATNFML	NCP	25.793
ATNFML;LSFLP	NCP	30.916
LSFLP;TVGPKKGK	NCP	5.178
TVGPKKGK;SATSAKK	NCP	27.459
SATSAKK;LLSAQT	NCP	4.437
LLSAQT;SVGSKSSK	NCP	0.306
SVGSKSSK;KGYSSPK	NCP	115.863
KGYSSPK;VPVEKKP	NCP	130.176
VPVEKKP;ELVDLGKK	NCP	13.074
ELVDLGKK;APGED	NCP	42.869
APGED;LKARML	NCP	8.317
LKARML;QTNLLVL	NCP	12.699
QTNLLVL;KGLLDSLR	NCP	89.309
KGLLDSLR;AGPSVPR	NCP	101.028
AGPSVPR;LPLPPP	NCP	20.643
LPLPPP;LPAAPL	NCP	46.734
LPAAPL;LDLRKDL	NCP	9.759
LDLRKDL;VEFPPGT	NCP	9.976
VEFPPGT;RGPSTLVL	NCP	108.938
RGPSTLVL;GDLYLGY	NCP	17.764
GDLYLGY;GTGLTHA	NCP	28.475
GTGLTHA;EDFYRPP	NCP	134.181
EDFYRPP;SAPDPGAK	NCP	77.448
SAPDPGAK;CDGAF	NCP	83.106
CDGAF;RAKGQPGK	NCP	38.883
RAKGQPGK;GSALRLGE	NCP	22.432
GSALRLGE;KPEAMNK	NCP	41.333
KPEAMNK;LPVQPP	NCP	32.554
LPVQPP;KEVWPP	NCP	53.738
KEVWPP;MVERGTKE	NCP	92.053
MVERGTKE;PPLRLPPL	NCP	93.749
PPLRLPPL;AAARPAGL	NCP	32.58
AAARPAGL;VEDLNPKK	NCP	35.232
VEDLNPKK;EAPRAF	NCP	19.359
EAPRAF;AAPRPPKPP	NCP	11.985
AAPRPPKPP;FPPPPPLD	NCP	53.469
FPPPPPLD;SLMKAFFL	NCP	0.772
SLMKAFFL;PQELQ	NCP	156.149
PQELQ;NLATL	NCP	18.312
NLATL;LDNQLYF	NCP	76.611
LDNQLYF;GPKLLSH	NCP	58.566
GPKLLSH;APQVHPP	NCP	15.918
APQVHPP;PPPKNF	NCP	57.22

PPPKNF;TLGTLQ	NCP	16.055
TLGTLQ;RLTQPY	NCP	14.586
RLTQPY;RPPEPL	NCP	0.021
RPPEPL;FLLNQL	NCP	7.144
FLLNQL;PTAHRYH	NCP	13.763
PTAHRYH;LERVLH	NCP	109.032
LERVLH;AQPQSYPR	NCP	13.524
AQPQSYPR;VEKALQA	NCP	6.027
VEKALQA;PPPPPNPR	NCP	20.488
PPPPPNPR;FLSLLF	NCP	29.499
FLSLLF;PFFLVLPP	NCP	2.715
PFFLVLPP;SAVTPP	NCP	57.314
SAVTPP;HLLPF	NCP	53.574
HLLPF;TAALRGAP	NCP	174.525
TAALRGAP;LLNLFK	NCP	61.699
LLNLFK;EEEKYV	NCP	16.443
EEEKYV;VPEPKR	NCP	5.064
VPEPKR;HLAQP	NCP	32.435
HLAQP;KTPPKFP	NCP	104.646
KTPPKFP;LTCPDGLK	NCP	8.755
LTCPDGLK;PDAPPPR	NCP	25.562
PDAPPPR;GRPFSHV	NCP	8.1
GRPFSHV;AAHNMPK	NCP	89.287
AAHNMPK;TREFR	NCP	62.567
TREFR;EPKGPSY	NCP	67.822
EPKGPSY;MPAHPL	NCP	5.013
MPAHPL;KYTAFVP	NCP	272.264
KYTAFVP;PEPELEPPAL	NCP	34.735
PEPELEPPAL;LPPVQ	NCP	9.813
LPPVQ;EDPLGK	NCP	67.031
EDPLGK;LAHPLAM	NCP	35.637
LAHPLAM;KATNLYL	NCP	0.058
KATNLYL;KPSSPTHP	NCP	2.99
KPSSPTHP;PAPPAPR	NCP	46.746
PAPPAPR;QPAAVHL	NCP	158.379
QPAAVHL;MDLRAL	NCP	59.031
MDLRAL;VTLEEL	NCP	68.37
VTLEEL;APPPAPR	NCP	99.984
APPPAPR;NTLKLVSN	NCP	11.889
NTLKLVSN;KTAHHWP	NCP	13.89
KTAHHWP;AAPAL	NCP	84.598
AAPAL;NVVLLLR	NCP	17.145
NVVLLLR;TALTNL	NCP	42.881
TALTNL;VPVGRVE	NCP	25.561
VPVGRVE;EVADF	NCP	80.432
EVADF;LGGVGL	NCP	26.869
LGGVGL;TAPPSPR	NCP	56.622
TAPPSPR;PGAAFP	NCP	8.024
PGAAFP;ETVGWW	NCP	33.642
ETVGWW;EPLLARHP	NCP	120.231
EPLLARHP;LEATLELL	NCP	43.236

LEATLELL;VSPPRLP	NCP	32.607
VSPPRLP;AGEKAEKPDTKEKKPE	NCP	52.6
AGEKAEKPDTKEKKPE;MYVTKK	NCP	6.389
MYVTKK;LPPPPRPPP	NCP	51.294
LPPPPRPPP;ELEAPPK	NCP	42.655
ELEAPPK;VGNVHKR	NCP	23.613
VGNVHKR;TRLSSF	NCP	5.751
TRLSSF;RATLDCF	NCP	51.602
RATLDCF;ALEPPEPK	NCP	126.927
ALEPPEPK;FARKGF	NCP	11.266
FARKGF;YGPKM	NCP	16.536
YGPKM;TWNSPRF	NCP	21.794
TWNSPRF;KPGAGV	NCP	1.02
KPGAGV;LAARVL	NCP	0.441
LAARVL;PLHVPFW	NCP	10.296
PLHVPFW;KAGVLSH	NCP	36.735
KAGVLSH;TATPAP	NCP	16.224
TATPAP;GRKASVR	NCP	39.123
GRKASVR;LDDPTLL	NCP	6.04
LDDPTLL;HLRPPP	NCP	45.713
HLRPPP;TVTLL	NCP	81.865
TVTLL;GVWPK	NCP	41.153
GVWPK;TVPLQER	NCP	115.236
TVPLQER;LVGPPRP	NCP	211.2
LVGPPRP;MLFPDTEA	NCP	20.526
MLFPDTEA;DFKLTLH	NCP	15.906
DFKLTLH;SLAVGVLK	NCP	106.509
SLAVGVLK;EKSEVVF	NCP	99.877
EKSEVVF;PPHGP	NCP	113.133
PPHGP;KNVLQR	NCP	11.555
KNVLQR;KGFATRQ	NCP	34.621
KGFATRQ;NRLLEATSY	NCP	0.272
NRLLEATSY;GVLLT	NCP	41.218
GVLLT;AAHGT	NCP	8.643
AAHGT;PHPYN	NCP	71.168
PHPYN;AVALK	NCP	2.976
AVALK;VVLSL	NCP	40.317
VVLSL;LVKLGDK	NCP	112.624
LVKLGDK;GFGDRAK	NCP	73.097
GFGDRAK;EVLLGALEE	NCP	127.089
EVLLGALEE;GVFTAL	NCP	18.22
GVFTAL;EPCRFTL	NCP	27.956
EPCRFTL;ASRPVTGP	NCP	16.908
ASRPVTGP;APVALRP	NCP	6.438
APVALRP;AAESALQVVEKLQ	NCP	91.05
AAESALQVVEKLQ;VTHLMK	NCP	34.384
VTHLMK;APGFPP	NCP	10.631
APGFPP;PLKEKLC	NCP	10.66
PLKEKLC;TPKVYQP	NCP	29.814
TPKVYQP;VGHLG	NCP	137.672
VGHLG;CKFNAK	NCP	135.163

CKFNAK;VEKLP	NCP	12.399
VEKLP;VAPPSDW	NCP	25.994
VAPPSDW;PPPPQQPEL	NCP	37.176
PPPPQQPEL;APGLLSLPR	NCP	131.169
APGLLSLPR;LSSMQN	NCP	9.574
LSSMQN;EPPKVR	NCP	58.682
EPPKVR;LPFFPPP	NCP	207.279
LPFFPPP;MDAGFFR	NCP	58.32
MDAGFFR;VLLSC	NCP	4.581
VLLSC;ESPSPAPKPR	NCP	12.258
ESPSPAPKPR;RQAFLT	NCP	14.16
RQAFLT;MTQQPRPVL	NCP	56.661
MTQQPRPVL;LEEVLCPPK	NCP	57.357
LEEVLCPPK;LSPKHPESN	NCP	7.38
LSPKHPESN;TLQKLDEY	NCP	15.273
TLQKLDEY;RDEFTNTCPSDKE	NCP	1.419
RDEFTNTCPSDKE;SDVAKRLTK	NCP	0.054
SDVAKRLTK;RPPPPRPP	NCP	91.08
RPPPPRPP;LPSLTSTLL	NCP	153.681
LPSLTSTLL;KYAQPP	NCP	15.69
KYAQPP;EVVNTP	NCP	23.017
EVVNTP;DLKKLPYQ	NCP	41.105
DLKKLPYQ;SYKAAG	NCP	8.277
SYKAAG;GAAPP	NCP	71.958
GAAPP;TLVEQPK	NCP	206.991
TLVEQPK;GKSAVLA	NCP	91.176
GKSAVLA;LMWKLQ	NCP	1.654
LMWKLQ;KLYSNGY	NCP	40.377
KLYSNGY;KLLEPV	NCP	78.929
KLLEPV;LPLKPCP	NCP	36.195
LPLKPCP;KVKSSQK	NCP	61.297
KVKSSQK;LTEVVVLL	NCP	5.69
LTEVVVLL;KLGAEKVV	NCP	138.559
KLGAEKVV;MTFLT	NCP	10.443
MTFLT;HVLAV	NCP	6.624
HVLAV;FEGLP	NCP	30.623
FEGLP;PSHVAK	NCP	40.732
PSHVAK;REVKFLLQ	NCP	13.799
REVKFLLQ;GLVEF	NCP	3.168
GLVEF;VAPYQP	NCP	100.512
VAPYQP;ELFVEAVEQNTLQEFLKLA	NCP	0.597
ELFVEAVEQNTLQEFLKLA;KPAPPKPEPKPKK	NCP	175.539
KPAPPKPEPKPKK;PAPPKPEPKPK	NCP	0.003
PAPPKPEPKPK;APPKPEPKPKKAPA	NCP	0.003
APPKPEPKPKKAPA;KSVVHPP	NCP	3.621
KSVVHPP;AANLPK	NCP	1.333
AANLPK;PVPVP	NCP	64.928
PVPVP;LPQSPDY	NCP	48.916
LPQSPDY;MCKAC	NCP	88.512
MCKAC;VSFLNQ	NCP	49.092
VSFLNQ;HLPGPPP	NCP	56.489

HLPGPPP;TPLRGLTGL	NCP	81.793
TPLRGLTGL;AAAAGVRAL	NCP	39.35
AAAAGVRAL;KYLPSQR	NCP	24.325
KYLPSQR;ELLFKEGVMVAKK	NCP	60.14
ELLFKEGVMVAKK;GLEGERPAR	NCP	0.279
GLEGERPAR;KAAHPPPP	NCP	3.669
KAAHPPPP;HRPYLFPL	NCP	37.704
HRPYLFPL;NASPRHP	NCP	120.471
NASPRHP;PLFPTPPP	NCP	33.744
PLFPTPPP;RTDAPPPP	NCP	5.055
RTDAPPPP;LELAEVP	NCP	53.131
LELAEVP;PPAGP	NCP	92.12
PPAGP;PPPLLLAP	NCP	10.686
PPPLLLAP;KRTGDSR	NCP	63.312
KRTGDSR;AASKHDP	NCP	22.047
AASKHDP;KPWHKQ	NCP	20.497
KPWHKQ;AQNWLEY	NCP	20.873
AQNWLEY;LDFDM	NCP	85.992
LDFDM;RSRRVFP	NCP	85.446
RSRRVFP;EPPLLPKLR	NCP	20.43
EPPLLPKLR;RNSPSFNR	NCP	58.641
RNSPSFNR;KGAEVP	NCP	21.3
KGAEVP;DDKLLR	NCP	43.635
DDKLLR;HDVFVR	NCP	170.202
HDVFVR;GDNVRSSAGAVR	NCP	36.687
GDNVRSSAGAVR;SAGAVRDAGGAFGK	NCP	0.018
SAGAVRDAGGAFGK;EQHLGLEA	NCP	25.767
EQHLGLEA;EAGAFVLL	NCP	2.166
EAGAFVLL;KETSPR	NCP	32.44
KETSPR;VVHKPF	NCP	8.873
VVHKPF;KHVVMLLS	NCP	97.923
KHVVMLLS;FKPSRL	NCP	5.553
FKPSRL;AAYLRR	NCP	49.638
AAYLRR;EPYVPEK	NCP	58.609
EPYVPEK;SLRNFVLK	NCP	1.841
SLRNFVLK;TLVVY	NCP	118.228
TLVVY;GFALPPPP	NCP	7.103
GFALPPPP;ALPHAL	NCP	108.027
ALPHAL;AEVPK	NCP	33.498
AEVPK;DRPAPHFER	NCP	107.535
DRPAPHFER;RPAPHFE	NCP	0.003
RPAPHFE;TATLFF	NCP	27.084
TATLFF;FLLLALLTA	NCP	49.171
FLLLALLTA;DLPEVH	NCP	24.32
DLPEVH;APVLSA	NCP	46.719
APVLSA;KSLYT	NCP	91.36
KSLYT;KPLKSR	NCP	22.377
KPLKSR;KLEFGAA	NCP	191.144
KLEFGAA;WPVPS	NCP	10.632
WPVPS;WSLMLK	NCP	72.948
WSLMLK;DTTEKPF	NCP	15.919

DTTEKPF;PKLWHH	NCP	74.079
PKLWHH;VHKAKSK	NCP	6.558
VHKAKSK;LLGVG	NCP	14.684
LLGVG;FGRAM	NCP	71.476
FGRAM;TLYCKL	NCP	23.223
TLYCKL;KPKAAPPP	NCP	24.803
KPKAAPPP;NKFASPY	NCP	0.706
NKFASPY;TTEKKV	NCP	77.285
TTEKKV;VPHVSGRN	NCP	8.088
VPHVSGRN;LYLFTEK	NCP	76.567
LYLFTEK;ALLSAAL	NCP	71.069
ALLSAAL;LKGKRSP	NCP	40.3
LKGKRSP;MFDDPK	NCP	53.741
MFDDPK;TGLPSPR	NCP	0.498
TGLPSPR;KELAVT	NCP	23.449
KELAVT;QLFGGFK	NCP	99.377
QLFGGFK;LEFQA	NCP	7.408
LEFQA;MLLVEPQ	NCP	24.431
MLLVEPQ;LSAPPP	NCP	3.126
LSAPPP;GDPQGAGK	NCP	9.36
GDPQGAGK;KSWAPK	NCP	4.003
KSWAPK;KHQPPPP	NCP	21.854
KHQPPPP;GALGRAGK	NCP	19.467
GALGRAGK;KSSAFALL	NCP	104.358
KSSAFALL;LSPLLRPH	NCP	42.945
LSPLLRPH;KPWKKT	NCP	82.971
KPWKKT;LDLPPL	NCP	36.438
LDLPPL;PEKPPPK	NCP	26.19
PEKPPPK;PGEAPP	NCP	19.545
PGEAPP;KNCKSGL	NCP	4.809
KNCKSGL;APPPYK	NCP	26.418
APPPYK;EKALSR	NCP	50.415
EKALSR;PLDGPPR	NCP	0.75
PLDGPPR;VFATPK	NCP	45.643
VFATPK;LHNPTPFP	NCP	13.409
LHNPTPFP;NLPLSK	NCP	16.867
NLPLSK;KTSSRK	NCP	1.083
KTSSRK;NVVRDL	NCP	36.342
NVVRDL;PSETLGRK	NCP	163.878
PSETLGRK;LPGGAREK	NCP	28.556
LPGGAREK;KGWEAH	NCP	67.584
KGWEAH;FENDP	NCP	14.29
FENDP;VVPGKFY	NCP	53.141
VVPGKFY;LDMPGY	NCP	55.716
LDMPGY;KPVVKY	NCP	102.21
KPVVKY;RLGTVRPSSF	NCP	40.59
RLGTVRPSSF;MRRRYM	NCP	18.346
MRRRYM;VEKLKKGP	NCP	28.649
VEKLKKGP;PVDLEA	NCP	30.861
PVDLEA;MTKQE	NCP	5.257
MTKQE;PPPPALR	NCP	3.152

PPPPALR;DELFVVT	NCP	11.725
DELFVVT;YVPFPGLP	NCP	29.925
YVPFPGLP;ASEHVAR	NCP	21.881
ASEHVAR;NPPHLK	NCP	28.575
NPPHLK;LSVLY	NCP	94.828
LSVLY;KVGLAPK	NCP	144.873
KVGLAPK;KPVSYF	NCP	7.887
KPVSYF;ALMAHAMEEVKK	NCP	22.4
ALMAHAMEEVKK;LNKNKKLVKKLAK	NCP	72.42
LNKNKKLVKKLAK;APMEGFQL	NCP	33.288
APMEGFQL;SVNGERLGTY	NCP	46.668
SVNGERLGTY;STDKQMGYQC	NCP	1.599
STDKQMGYQC;PFVGK	NCP	28.723
PFVGK;NKHNP	NCP	150.083
NKHNP;LGYEY	NCP	105.814
LGYEY;LGKPE	NCP	30.225
LGKPE;KDGVLPLF	NCP	84.395
KDGVLPLF;KLKRPPPPPR	NCP	20.454
KLKRPPPPPR;AADALK	NCP	8.535
AADALK;GFEKLSK	NCP	11.541
GFEKLSK;LPAAR	NCP	16.794
LPAAR;MRMNSF	NCP	0.406
MRMNSF;KTHVPLAP	NCP	79.241
KTHVPLAP;NCVSMY	NCP	1.701
NCVSMY;KDGPKKP	NCP	3.331
KDGPKKP;KVVQY	NCP	159.513
KVVQY;GLPPPPRC	NCP	6.641
GLPPPPRC;GPVTLLPR	NCP	180.804
GPVTLLPR;KHLQPAH	NCP	2.2
KHLQPAH;CMSMAA	NCP	13.277
CMSMAA;KDPPHPAP	NCP	3.723
KDPPHPAP;NCVPEK	NCP	90.897
NCVPEK;RALLYK	NCP	3.661
RALLYK;GLELYL	NCP	103.296
GLELYL;KQKKAC	NCP	18.624
KQKKAC;KAMPLLLG	NCP	149.397
KAMPLLLG;ESSVKELK	NCP	170.49
ESSVKELK;KQKAFSK	NCP	78.005
KQKAFSK;KAKFYYM	NCP	68.919
KAKFYYM;MSCKPK	NCP	22.92
MSCKPK;KYNFYVQ	NCP	78.105
KYNFYVQ;KREFKK	NCP	9.934
KREFKK;LYARRK	NCP	354.984
LYARRK;ATTPAKKMVVSPTK	NCP	19.55
ATTPAKKMVVSPTK;TTPAKKMVVSPTKK	NCP	0.003
TTPAKKMVVSPTKK;VAVATPAKKAVVTPGKK	NCP	0.042
VAVATPAKKAVVTPGKK;VATPAKKAVVTPGK	NCP	0.006
VATPAKKAVVTPGK;ATPAKKAVVTPGKK	NCP	0.003
ATPAKKAVVTPGKK;TPAKKAVVTPGK	NCP	0.003
TPAKKAVVTPGK;AMPAKKTVTPVK	NCP	0.045
AMPAKKTVTPVK;KTVTPVKAVVTPGKK	NCP	0.015

KTVTPVKAVVTPGKK;TVTPVKAVVTPGK	NCP	0.003
TVTPVKAVVTPGK;VTPVKAVVTPGKK	NCP	0.003
VTPVKAVVTPGKK;TPVKAVVTPGKKGATPGK	NCP	0.003
TPVKAVVTPGKKGATPGK;ATPGKAVVATSGK	NCP	0.039
ATPGKAVVATSGK;AVVATSGKKGAATPGK	NCP	0.015
AVVATSGKKGAATPGK;SATEETLQEVFEK	NCP	4.431
SATEETLQEVFEK;PGELAK	NCP	33.469
PGELAK;RPTPYP	NCP	14.339
RPTPYP;KPVPPLPP	NCP	4.575
KPVPPLPP;SGFKLRM	NCP	239.922
SGFKLRM;KYELLPP	NCP	39.057
KYELLPP;KPTVHF	NCP	30.091
KPTVHF;TGGPLLMA	NCP	13.505
TGGPLLMA;VAAPKHP	NCP	169.225
VAAPKHP;KGGGAP	NCP	16.49
KGGGAP;PAAFPQ	NCP	1.101
PAAFPQ;MGKSFPP	NCP	22.186
MGKSFPP;SSGMKL	NCP	19.662
SSGMKL;KFLDQ	NCP	202.19
KFLDQ;KNMEVP	NCP	5.181
KNMEVP;FHPEQL	NCP	96.574
FHPEQL;LTSLT	NCP	32.733
LTSLT;SHSKLKTL	NCP	118.562
SHSKLKTL;GYGYP	NCP	21.628
GYGYP;LPTQKFF	NCP	1.788
LPTQKFF;TVQAKLK	NCP	59.343
TVQAKLK;QRKSQ	NCP	83.673
QRKSQ;PAPEPKPK	NCP	77.394
PAPEPKPK;MMTAL	NCP	22.71
MMTAL;KDFVDK	NCP	17.903
KDFVDK;KLFALSQL	NCP	41.29
KLFALSQL;GVRVSNH	NCP	167.693
GVRVSNH;FAARRF	NCP	168.87
FAARRF;GPTMKALL	NCP	111.78
GPTMKALL;AAAAAPVYL	NCP	161.349
AAAAAPVYL;YQPVVQQ	NCP	34.098
YQPVVQQ;ALYLL	NCP	173.889
ALYLL;YSDSLF	NCP	147.402
YSDSLF;WFPLLK	NCP	86.454
WFPLLK;KTLVSQQK	NCP	42.555
KTLVSQQK;VGGLEFL	NCP	74.673
VGGLEFL;QGLGY	NCP	28.003
QGLGY;AAAAVTDF	NCP	219.597
AAAAVTDF;HGYFPP	NCP	25.352
HGYFPP;DLDLEF	NCP	34.759
DLDLEF;AGAGK	NCP	42.723
AGAGK;DQLLLEK	NCP	48.831
DQLLLEK;DYALRP	NCP	6.593
DYALRP;LSFAAGTK	NCP	83.256
LSFAAGTK;KELWHH	NCP	52.267
KELWHH;QPTGLPR	NCP	0.764

QPTGLPR;VPKGHLQK	NCP	44.755
VPKGHLQK;VAKFAAK	NCP	15.804
VAKFAAK;LAAATL	NCP	9.192
LAAATL;LGLTL	NCP	18.39
LGLTL;LQPSRL	NCP	34.232
LQPSRL;WPKNKGP	NCP	21.807
WPKNKGP;VPPRKFR	NCP	84.831
VPPRKFR;KSYRGF	NCP	35.563
KSYRGF;LPNNNPPL	NCP	23.15
LPNNNPPL;SVGALAAF	NCP	35.448
SVGALAAF;KPALCKQR	NCP	38.538
KPALCKQR;KTDFLR	NCP	66.571
KTDFLR;MSGGK	NCP	44.466
MSGGK;NAAAVTK	NCP	75.666
NAAAVTK;ETAGKLLL	NCP	73.487
ETAGKLLL;VPSLKKLP	NCP	72.192
VPSLKKLP;CGTVRFN	NCP	38.098
CGTVRFN;VGNPL	NCP	83.598
VGNPL;EAPLKGS	NCP	42.203
EAPLKGS;EQLKTVK	NCP	55.263
EQLKTVK;VTGLYL	NCP	65.886
VTGLYL;DYTRHTF	NCP	81.285
DYTRHTF;AVGGL	NCP	16.056
AVGGL;GPPLFD	NCP	22.789
GPPLFD;VVLLHV	NCP	6.435
VVLLHV;KYSPPPP	NCP	38.771
KYSPPPP;TVKSDRK	NCP	140.121
TVKSDRK;NATMYFM	NCP	138.316
NATMYFM;GFGFG	NCP	76.595
GFGFG;VARDAPK	NCP	5.989
VARDAPK;ALRYPM	NCP	23.697
ALRYPM;TVDLLDH	NCP	6.744
TVDLLDH;LPYLKN	NCP	158.096
LPYLKN;FAPDG	NCP	9.937
FAPDG;PWKWP	NCP	84.131
PWKWP;RVFKHLF	NCP	18.456
RVFKHLF;RELSVGR	NCP	83.511
RELSVGR;GSSAWL	NCP	57.63
GSSAWL;GTAALYHL	NCP	10.819
GTAALYHL;PYLELEMS	NCP	82.155
PYLELEMS;PHAALL	NCP	97.745
PHAALL;LAAAAGY	NCP	98.086
LAAAAGY;KPFRNK	NCP	10.061
KPFRNK;VSLLAY	NCP	17.643
VSLLAY;HELGF	NCP	42.624
HELGF;LNLQEY	NCP	25.599
LNLQEY;QGAPAL	NCP	150.471
QGAPAL;ASVVPLKEKK	NCP	87.922
ASVVPLKEKK;KTLSLAP	NCP	44.351
KTLSLAP;FSLLFDSK	NCP	28.837
FSLLFDSK;VTDDLP	NCP	14.274

VTDDLP;LQLKPR	NCP	22.095
LQLKPR;SLLLPLLP	NCP	44.882
SLLLPLLP;RPGGPPR	NCP	5.578
RPGGPPR;PAAPEKPVK	NCP	28.854
PAAPEKPVK;RLVGKLFP	NCP	2.391
RLVGKLFP;MLTAQQ	NCP	32.819
MLTAQQ;MLKGV	NCP	132.828
MLKGV;LLPTVF	NCP	8.824
LLPTVF;FSPKPPD	NCP	7.506
FSPKPPD;EPVGAF	NCP	35.574
EPVGAF;ARERVP	NCP	6.114
ARERVP;VLKDLR	NCP	17.121
VLKDLR;GCYQKRH	NCP	17.727
GCYQKRH;PLLGYK	NCP	108.176
PLLGYK;EVEVLK	NCP	4.357
EVEVLK;TSGDFK	NCP	20.343
TSGDFK;ECVKQLC	NCP	31.203
ECVKQLC;PLYPQPY	NCP	57.846
PLYPQPY;SHVPL	NCP	55.883
SHVPL;KDYDGK	NCP	66.591
KDYDGK;VPYQPK	NCP	34.686
VPYQPK;RGLRSY	NCP	104.808
RGLRSY;LNLNSRSPEYL	NCP	15.537
LNLNSRSPEYL;KGLKVSTP	NCP	103.059
KGLKVSTP;KDHCPK	NCP	77.812
KDHCPK;KSVSPTAL	NCP	92.367
KSVSPTAL;VLAAF	NCP	104.706
VLAAF;PAPAGP	NCP	115.079
PAPAGP;KGSETLK	NCP	115.041
KGSETLK;PGGSLPRC	NCP	34.23
PGGSLPRC;AAVLENLM	NCP	135.535
AAVLENLM;LPVETPPP	NCP	47.672
LPVETPPP;GPPFP	NCP	15.286
GPPFP;KGLLGH	NCP	34.658
KGLLGH;NTAPLL	NCP	12.394
NTAPLL;LSGGLPK	NCP	38.187
LSGGLPK;RAVGLSLK	NCP	37.356
RAVGLSLK;LSDNPHLL	NCP	99.612
LSDNPHLL;LSLTTLLP	NCP	33.542
LSLTTLLP;VPLLNLL	NCP	20.244
VPLLNLL;TGKHPAN	NCP	21.978
TGKHPAN;TPDHVVY	NCP	6.402
TPDHVVY;QAVKAEK	NCP	46.642
QAVKAEK;DFLLPLGK	NCP	9.654
DFLLPLGK;KDLGMH	NCP	81.462
KDLGMH;SARLLR	NCP	59.846
SARLLR;LKKGYNP	NCP	15.066
LKKGYNP;MASLPVP	NCP	63.562
MASLPVP;SSNPLLR	NCP	50.807
SSNPLLR;KLLTPQ	NCP	16.027
KLLTPQ;ALSMLA	NCP	0.65

ALSMLA;EGAGLL	NCP	35.331
EGAGLL;DALKPNL	NCP	135.253
DALKPNL;VPVVGM	NCP	104.48
VPVVGM;TVPFPL	NCP	7.828
TVPFPL;YLLFPLG	NCP	54.573
YLLFPLG;LDEFLRPR	NCP	39.276
LDEFLRPR;KYHQGPK	NCP	155.342
KYHQGPK;CKKKPP	NCP	6.765
CKKKPP;GFAGF	NCP	66.628
GFAGF;ALNTKAV	NCP	59.57
ALNTKAV;KGWTLHT	NCP	169.147
KGWTLHT;KTGDWKS	NCP	46.79
KTGDWKS;VVNLAAR	NCP	81.102
VVNLAAR;PLLPPQFRL	NCP	54.301
PLLPPQFRL;KPAAHYK	NCP	63.681
KPAAHYK;QECAK	NCP	12.363
QECAK;EPPGKPAP	NCP	118.47
EPPGKPAP;ESYAKK	NCP	22.82
ESYAKK;KFACCPP	NCP	125.724
KFACCPP;ATMANKR	NCP	7.021
ATMANKR;LESLFLKP	NCP	91.023
LESLFLKP;FLTQHKSH	NCP	32.403
FLTQHKSH;FRPGQRQ	NCP	46.035
FRPGQRQ;KMKVR	NCP	70.886
KMKVR;GSVPAVLP	NCP	60.732
GSVPAVLP;LLCASLK	NCP	59.767
LLCASLK;MDEFM	NCP	208.641
MDEFM;SLKPRF	NCP	12.299
SLKPRF;QTKVAPTK	NCP	35.584
QTKVAPTK;HSNTPPR	NCP	200.097
HSNTPPR;KPWGKP	NCP	11.751
KPWGKP;RGGLYSR	NCP	95.934
RGGLYSR;HDLPPPPPP	NCP	56.259
HDLPPPPPP;RSLLGDR	NCP	83.991
RSLLGDR;GRPANKP	NCP	153.645
GRPANKP;KDRPSPY	NCP	9.966
KDRPSPY;KSLAP	NCP	3.899
KSLAP;KDFDLKN	NCP	18.748
KDFDLKN;RLSNTTLH	NCP	39.387
RLSNTTLH;LQEVLKL	NCP	83.099
LQEVLKL;VRKVLQ	NCP	151.542
VRKVLQ;FFDAR	NCP	2.356
FFDAR;NGVVTVAR	NCP	87.098
NGVVTVAR;RGPVAVVL	NCP	40.201
RGPVAVVL;RVGGLT	NCP	10.076
RVGGLT;RFYQRKRM	NCP	14.448
RFYQRKRM;PTGTY	NCP	35.79
PTGTY;GSLYGFN	NCP	25.147
GSLYGFN;LGFLKLD	NCP	57.947
LGFLKLD;FPGYPP	NCP	53.971
FPGYPP;LGRVPHN	NCP	80.333

LGRVPHN;LLMKQLLL	NCP	288.931
LLMKQLLL;VTPLLGH	NCP	77.123
VTPLLGH;VAGPLHP	NCP	6.789
VAGPLHP;MFKPPPP	NCP	91.99
MFKPPPP;VGAAGALPL	NCP	4.664
VGAAGALPL;HPAVPPPP	NCP	15.507
HPAVPPPP;GFVFC	NCP	6.603
GFVFC;VVESTGVFTTMEKAGAHL	NCP	91.476
VVESTGVFTTMEKAGAHL;TMEKAGAHLKGGAK	NCP	0.027
TMEKAGAHLKGGAK;DQVVSCDF	NCP	0.522
DQVVSCDF;SNRVVDLMVHMASKE	NCP	0.126
SNRVVDLMVHMASKE;VDLMVH	NCP	0.012
VDLMVH;MHLEKYH	NCP	6.52
MHLEKYH;ADHTT	NCP	16.77
ADHTT;PKFWVGP	NCP	25.077
PKFWVGP;LVFHSFGGGTGSGF	NCP	10.307
LVFHSFGGGTGSGF;YPAPQVSTAVVEPY	NCP	0.108
YPAPQVSTAVVEPY;TTHTTLEHSDCAF	NCP	0.054
TTHTTLEHSDCAF;SLPPPPP	NCP	0.004
SLPPPPP;PPTVVPGGDL	NCP	0.494
PPTVVPGGDL;VVPGGDLA	NCP	0.009
VVPGGDLA;LDHKFDLMYA	NCP	0.087
LDHKFDLMYA;KFDLMYAK	NCP	0.009
KFDLMYAK;RAFVHWY	NCP	0.024
RAFVHWY;YVGEGMEEGEFSEARE	NCP	0.018
YVGEGMEEGEFSEARE;VGEGMEEGEFSEAR	NCP	0.003
VGEGMEEGEFSEAR;GMEEGEFS	NCP	0.009
GMEEGEFS;EEGEFSEAR	NCP	0.006
EEGEFSEAR;TGAAVR	NCP	39.069
TGAAVR;TYGLLDM	NCP	148.846
TYGLLDM;GPSSKGQP	NCP	59.999
GPSSKGQP;PLTTPLPSPP	NCP	28.074
PLTTPLPSPP;LPGKYMQ	NCP	34.009
LPGKYMQ;YVASKGK	NCP	64.671
YVASKGK;HEAFNK	NCP	6.036
HEAFNK;LGSGLEQ	NCP	121.37
LGSGLEQ;VTVLK	NCP	119.844
VTVLK;VGTSL	NCP	10.252
VGTSL;PAANTAF	NCP	2.057
PAANTAF;WFGVGK	NCP	248.484
WFGVGK;AAPFH	NCP	0.177
AAPFH;KLYSPLVY	NCP	37.116
KLYSPLVY;LSGFKTYY	NCP	31.209
LSGFKTYY;PQLPPR	NCP	3.259
PQLPPR;VAAELYL	NCP	63.45
VAAELYL;LDDLGP	NCP	108.65
LDDLGP;PLHVPR	NCP	5.725
PLHVPR;KSRGPGV	NCP	6.92
KSRGPGV;KYRARL	NCP	5.913
KYRARL;RSNLREVAQ	NCP	52.131
RSNLREVAQ;TLSVSSDSGNSTASTK	NCP	47.322

TLSVSSDSGNSTASTK;AEPPMSGLPEFPR	NCP	0.879
AEPPMSGLPEFPR;LDLEPATVGAPLHK	NCP	0.093
LDLEPATVGAPLHK;SGSSCQSHPLTQSR	NCP	0.099
SGSSCQSHPLTQSR;SGHSLGTPEPAPR	NCP	0.057
SGHSLGTPEPAPR;TPEPAPRAP	NCP	0.018
TPEPAPRAP;ESVPTGRP	NCP	0.03
ESVPTGRP;DYQPCPTGPN	NCP	0.036
DYQPCPTGPN;QEPEPKSF	NCP	28.014
QEPEPKSF;PAAQAYGHETP	NCP	0.03
PAAQAYGHETP;YGHETPL	NCP	0.015
YGHETPL;SVAGVHTVPGSPQ	NCP	3.663
SVAGVHTVPGSPQ;DRHVPYG	NCP	0.45
DRHVPYG;RQGSPTPALPEKR	NCP	0.18
RQGSPTPALPEKR;PGAPP	NCP	12.81
PGAPP;VSKVMLSAGQKR	NCP	0.162
VSKVMLSAGQKR;VMLSAGQK	NCP	0.009
VMLSAGQK;PGVPP	NCP	8.43
PGVPP;KLMHAK	NCP	61.261
KLMHAK;ADYKP	NCP	20.838
ADYKP;LTSLETGF	NCP	12.521
LTSLETGF;QASLVQR	NCP	52.215
QASLVQR;DPAMLLR	NCP	148.932
DPAMLLR;VHMRPP	NCP	69.811
VHMRPP;EGVEVLK	NCP	42.485
EGVEVLK;QTMLKLH	NCP	24.013
QTMLKLH;PPGRKFN	NCP	35.276
PPGRKFN;YGLLFGP	NCP	17.397
YGLLFGP;KETSSLY	NCP	64.962
KETSSLY;AENLLKTE	NCP	13.564
AENLLKTE;LSTGPPPP	NCP	115.637
LSTGPPPP;FPPPLP	NCP	39.669
FPPPLP;SYKTEC	NCP	35.803
SYKTEC;EHKLGAP	NCP	0.303
EHKLGAP;DLSYLTFP	NCP	74.429
DLSYLTFP;KSGKAHP	NCP	2.689
KSGKAHP;KLAPTKY	NCP	161.451
KLAPTKY;LEKLLK	NCP	52.848
LEKLLK;VPREC	NCP	18.075
VPREC;EGNGPAA	NCP	46.865
EGNGPAA;ASPPKLLL	NCP	0.676
ASPPKLLL;REAKMP	NCP	144.05
REAKMP;LALMGL	NCP	4.858
LALMGL;PPPQPGPK	NCP	15.95
PPPQPGPK;LSGKEVP	NCP	19.462
LSGKEVP;KFEDTNL	NCP	104.057
KFEDTNL;RSHPPAP	NCP	29.805
RSHPPAP;KEGLTPLP	NCP	3.306
KEGLTPLP;ESGSRKYR	NCP	37.608
ESGSRKYR;LSQSKL	NCP	151.014
LSQSKL;EPKAHTK	NCP	28.009
EPKAHTK;VGPYE	NCP	3.122

VGPYE;KPSGRPVG	NCP	20.373
KPSGRPVG;PTAPGKAD	NCP	6.171
PTAPGKAD;KPVEEK	NCP	70.917
KPVEEK;GKELLMA	NCP	5.97
GKELLMA;AVNSWVKGK	NCP	22.665
AVNSWVKGK;SWVKGKSCLA	NCP	0.009
SWVKGKSCLA;YDYLTKDLLN	NCP	28.404
YDYLTKDLLN;VKVGVN	NCP	15.142
VKVGVN;LLSPAASFVTGQ	NCP	0.659
LLSPAASFVTGQ;MKLQRF	NCP	12.777
MKLQRF;CCCCPC	NCP	20.568
CCCCPC;LLYPLR	NCP	49.279
LLYPLR;KMSSLGK	NCP	185.685
KMSSLGK;KNLLQ	NCP	86.316
KNLLQ;SGFHFK	NCP	47.931
SGFHFK;VQTLQNF	NCP	11.543
VQTLQNF;KSARVMP	NCP	92.212
KSARVMP;LVLKTLNL	NCP	23.868
LVLKTLNL;KTDLLK	NCP	40.821
KTDLLK;PLFLRPVT	NCP	40.841
PLFLRPVT;EKDLLLK	NCP	20.104
EKDLLLK;KNGPGP	NCP	58.271
KNGPGP;KTVGPGK	NCP	0.027
KTVGPGK;LLLKSCL	NCP	140.068
LLLKSCL;GPGQLSKK	NCP	74.298
GPGQLSKK;VPHEEL	NCP	30.044
VPHEEL;KSLSLAW	NCP	20.787
KSLSLAW;FVLFHLK	NCP	19.419
FVLFHLK;QSLDLRK	NCP	155.466
QSLDLRK;PGTLHWLPF	NCP	74.025
PGTLHWLPF;LDLFTGK	NCP	37.47
LDLFTGK;EYLRMK	NCP	2.716
EYLRMK;TVWAKSK	NCP	105.386
TVWAKSK;KMVVLSL	NCP	239.287
KMVVLSL;VEDTKVLR	NCP	18.246
VEDTKVLR;LSAVGL	NCP	36.831
LSAVGL;SKPFFG	NCP	155.001
SKPFFG;PTLLEH	NCP	77.778
PTLLEH;DLHQKPK	NCP	152.688
DLHQKPK;CFTPK	NCP	21.642
CFTPK;VSWMRK	NCP	343.055
VSWMRK;QQMKKK	NCP	5.524
QQMKKK;REKFDKS	NCP	50.226
REKFDKS;GKKGLGK	NCP	93.221
GKKGLGK;AHDLKK	NCP	305.172
AHDLKK;KPWFVF	NCP	38.098
KPWFVF;TVPLLM	NCP	162.231
TVPLLM;KESPF	NCP	26.541
KESPF;VAKAYR	NCP	14.507
VAKAYR;EFFTL	NCP	14.494
EFFTL;KYLPPYK	NCP	8.33

KYLPPYK;PLLSTALQ	NCP	14.2
PLLSTALQ;KVLDPF	NCP	29.445
KVLDPF;KSRTLPFP	NCP	18.408
KSRTLPFP;FPPPP	NCP	59.021
FPPPP;GYVYLPQ	NCP	117.561
GYVYLPQ;PLLAKSK	NCP	48.055
PLLAKSK;LLVPFHS	NCP	235.883
LLVPFHS;YFTLKTKR	NCP	69.369
YFTLKTKR;VAKTLLK	NCP	60.954
VAKTLLK;LSWGKDLAF	NCP	107.322
LSWGKDLAF;PDVSKKP	NCP	38.824
PDVSKKP;TVKVVY	NCP	22.692
TVKVVY;APAGFKK	NCP	227.981
APAGFKK;KYVLY	NCP	5.757
KYVLY;GSKYV	NCP	42.616
GSKYV;LKFAPP	NCP	153.693
LKFAPP;LTTPPDK	NCP	75.341
LTTPPDK;LKETLKGF	NCP	54.46
LKETLKGF;SLEKTPLK	NCP	1.691
SLEKTPLK;QHYCC	NCP	37.339
QHYCC;LQPSHLFY	NCP	18.878
LQPSHLFY;VGGLLLF	NCP	22.438
VGGLLLF;VGGLLK	NCP	54.381
VGGLLK;AMGAKLF	NCP	16.761
AMGAKLF;KNPFLLPF	NCP	103.22
KNPFLLPF;RLPPYM	NCP	61.128
RLPPYM;DPPANK	NCP	87.735
DPPANK;SNFKSPVK	NCP	60.06
SNFKSPVK;EGAWLR	NCP	218.203
EGAWLR;YPLHARP	NCP	82.554
YPLHARP;YELHFSPL	NCP	22.887
YELHFSPL;SYTFS	NCP	58.364
SYTFS;LLKGQK	NCP	91.165
LLKGQK;YQSLFKK	NCP	83.756
YQSLFKK;PADLAK	NCP	84.424
PADLAK;KCTPQ	NCP	159.936
KCTPQ;PLTSLGLP	NCP	49.026
PLTSLGLP;KKVHGLQ	NCP	15.107
KKVHGLQ;LAEFKEAF	NCP	147.828
LAEFKEAF;AEVKKVLGN	NCP	0.111
AEVKKVLGN;RVFDKEGNGTVMGAELRH	NCP	3.378
RVFDKEGNGTVMGAELRH;GEKMKEEEVEALM	NCP	0.525
GEKMKEEEVEALM;VEPGPL	NCP	9.529
VEPGPL;NGLVL	NCP	0.92
NGLVL;KVAPLLK	NCP	2.314
KVAPLLK;LPHNRP	NCP	5.676
LPHNRP;KVYPFPLL	NCP	4.059
KVYPFPLL;VPASLP	NCP	45.635
VPASLP;QSLGLEGK	NCP	17.07
QSLGLEGK;LSVPPKF	NCP	8.335
LSVPPKF;DHVERK	NCP	31.905

DHVERK;LDGKF	NCP	1.005
LDGKF;KEYVLE	NCP	2.313
KEYVLE;KGPTYPK	NCP	57.246
KGPTYPK;QALLPQ	NCP	17.259
QALLPQ;MLKVVR	NCP	23.774
MLKVVR;LERMFL	NCP	20.235
LERMFL;KANLGLGK	NCP	76.624
KANLGLGK;VLAAQLN	NCP	54.797
VLAAQLN;NNVAVP	NCP	54.844
NNVAVP;PLPFPR	NCP	73.892
PLPFPR;ESYGGGKK	NCP	5.139
ESYGGGKK;PPQPHRP	NCP	9.402
PPQPHRP;PPMLPPP	NCP	30.928
PPMLPPP;AASLP	NCP	46.281
AASLP;GKLLVP	NCP	5.219
GKLLVP;FDKSKL	NCP	52.344
FDKSKL;TLFKSK	NCP	124.116
TLFKSK;RHLASFRK	NCP	48.043
RHLASFRK;KSDLKLLL	NCP	180.885
KSDLKLLL;KYGKLWN	NCP	21.759
KYGKLWN;THLPFH	NCP	80.892
THLPFH;KVTKKSAP	NCP	176.277
KVTKKSAP;LEVLS	NCP	7.095
LEVLS;MNRTLLL	NCP	17.507
MNRTLLL;QMNLRK	NCP	2.593
QMNLRK;EWMAC	NCP	189.354
EWMAC;ALSKPP	NCP	3.141
ALSKPP;AHSSFQMALSK	NCP	96.383
AHSSFQMALSK;LNTFEFMDKLGENLQLKLAQAK	NCP	7.023
LNTFEFMDKLGENLQLKLAQAK;LGENLQLK	NCP	0.027
LGENLQLK;RPPGF	NCP	91.587
RPPGF;EQELLK	NCP	72.675
EQELLK;KFRGKK	NCP	23.895
KFRGKK;PVGRPP	NCP	446.805
PVGRPP;AYENF	NCP	47.394
AYENF;YSSVLGPV	NCP	101.818
YSSVLGPV;FPAAP	NCP	99.227
FPAAP;QGTPSL	NCP	196.654
QGTPSL;YGTPGFN	NCP	21.407
YGTPGFN;LKRTRPP	NCP	282.555
LKRTRPP;NPLPPPPPP	NCP	53.352
NPLPPPPPP;KRLLKK	NCP	90.04
KRLLKK;HLSSVLGH	NCP	169.043
HLSSVLGH;REATSN	NCP	72.681
REATSN;VAAYY	NCP	7.707
VAAYY;SELLRY	NCP	155.748
SELLRY;AMLLSRY	NCP	229.116
AMLLSRY;SPFLSVQ	NCP	31.833
SPFLSVQ;AGLASTLR	NCP	72.192
AGLASTLR;KAWATGK	NCP	17.317
KAWATGK;KNFLPPPPP	NCP	77.6

KNFLPPPPP;PPPPPP	NCP	0.012
PPPPPP;QGTLQP	NCP	21.487
QGTLQP;SLGPK	NCP	13.991
SLGPK;NRLNPR	NCP	6.424
NRLNPR;MCYTSSK	NCP	164.582
MCYTSSK;KKPLLL	NCP	23.179
KKPLLL;SSPVNPVV	NCP	75.092
SSPVNPVV;NMKLLTER	NCP	7.842
NMKLLTER;GPPPPPVAP	NCP	17.239
GPPPPPVAP;NNKLGLL	NCP	93.518
NNKLGLL;CAKFDKSK	NCP	66.735
CAKFDKSK;NKTGLSPK	NCP	39.141
NKTGLSPK;RLAHLFL	NCP	119.872
RLAHLFL;KLLAPP	NCP	38.975
KLLAPP;LSGKKLC	NCP	93.19
LSGKKLC;LGPEL	NCP	74.033
LGPEL;AEYDNK	NCP	116.193
AEYDNK;REAGLAAGQ	NCP	15.153
REAGLAAGQ;EDEDFKV	NCP	89.562
EDEDFKV;SVLKKNLN	NCP	70.942
SVLKKNLN;KGELAALP	NCP	2.006
KGELAALP;KDVQPP	NCP	34.522
KDVQPP;LPDYKQT	NCP	4.157
LPDYKQT;KPFYPMP	NCP	135.3
KPFYPMP;LSVKL	NCP	58.236
LSVKL;APNLPLE	NCP	14.121
APNLPLE;RVSKSH	NCP	99.162
RVSKSH;ESYPHVK	NCP	40.309
ESYPHVK;NVPPNFK	NCP	17.526
NVPPNFK;WLDVSLLL	NCP	0.912
WLDVSLLL;KGKVFVY	NCP	69.806
KGKVFVY;NHDCAC	NCP	54.634
NHDCAC;APFQMT	NCP	179.826
APFQMT;TMPPLAR	NCP	32.22
TMPPLAR;KTYVH	NCP	128.957
KTYVH;PAGVSSPR	NCP	122.589
PAGVSSPR;HQSPPK	NCP	52.308
HQSPPK;RQLLTTK	NCP	20.73
RQLLTTK;CDLLSLPK	NCP	16.162
CDLLSLPK;TLVVTPF	NCP	113.696
TLVVTPF;HVELTLR	NCP	19.093
HVELTLR;PPAASPP	NCP	38.867
PPAASPP;NDSTK	NCP	44.995
NDSTK;HLEASVPK	NCP	70.268
HLEASVPK;QGAKHKT	NCP	25.116
QGAKHKT;KPGFFSPK	NCP	123.034
KPGFFSPK;GLPTP	NCP	18.104
GLPTP;SLQLTSL	NCP	38.772
SLQLTSL;GDDAC	NCP	20.931
GDDAC;TQWLSN	NCP	65.817
TQWLSN;LHELYT	NCP	97.365

LHELYT;TSTPLGKP	NCP	39.526
TSTPLGKP;RYSPTTSH	NCP	0.269
RYSPTTSH;VRFPLEF	NCP	42.61
VRFPLEF;NFFVQTC	NCP	38.229
NFFVQTC;YAAEVK	NCP	2.492
YAAEVK;ETYLFD	NCP	3.03
ETYLFD;VPAWPK	NCP	35.739
VPAWPK;VYKVL	NCP	9.687
VYKVL;KEKAPPPP	NCP	34.179
KEKAPPPP;KECYVH	NCP	111.849
KECYVH;MNTVVRT	NCP	59.524
MNTVVRT;KVTTYSF	NCP	16.682
KVTTYSF;RKLTMDF	NCP	7.975
RKLTMDF;PVPLVVVP	NCP	14.163
PVPLVVVP;VRSVTFK	NCP	9.617
VRSVTFK;KPMKGMP	NCP	16.932
KPMKGMP;LFNHEDL	NCP	166.447
LFNHEDL;ELNKAR	NCP	66.302
ELNKAR;VPPSK	NCP	56.835
VPPSK;QSSKVSGP	NCP	76.3
QSSKVSGP;KGHNHLK	NCP	37.712
KGHNHLK;YTFYKDP	NCP	134.136
YTFYKDP;DTLEF	NCP	23.913
DTLEF;ASAVPL	NCP	205.849
ASAVPL;KLPKMP	NCP	91.796
KLPKMP;NPYPSWQ	NCP	43.785
NPYPSWQ;KDTVTAK	NCP	5.124
KDTVTAK;LSEKLQ	NCP	20.482
LSEKLQ;KDGNKP	NCP	182.448
KDGNKP;KLTKPGK	NCP	127.265
KLTKPGK;LLPFLL	NCP	8.799
LLPFLL;NTASSF	NCP	34.642
NTASSF;VDAPLTK	NCP	59.979
VDAPLTK;PALTMAK	NCP	162.032
PALTMAK;KSNDPEN	NCP	116.701
KSNDPEN;GVKLWN	NCP	30.731
GVKLWN;TVNPDL	NCP	22.941
TVNPDL;FFLLWYR	NCP	60.963
FFLLWYR;DYVEF	NCP	72.312
DYVEF;GYHLRK	NCP	16.809
GYHLRK;KDVKLVK	NCP	21.571
KDVKLVK;LPSSRPL	NCP	78.494
LPSSRPL;AAKPSVPK	NCP	5.896
AAKPSVPK;KLEFNPK	NCP	54.308
KLEFNPK;KDSHPRF	NCP	44.622
KDSHPRF;KFPSL	NCP	15.876
KFPSL;EVYLL	NCP	26.442
EVYLL;NNPSFLP	NCP	86.737
NNPSFLP;ELLPK	NCP	13.569
ELLPK;EFFYRE	NCP	76.106
EFFYRE;HFGVLF	NCP	44.376

HFGVLF;HSPPPPPP	NCP	11.289
HSPPPPPP;PTVTSP	NCP	19
PTVTSP;DGLKM	NCP	193.248
DGLKM;LHVEVKD	NCP	50.471
LHVEVKD;KPYCPP	NCP	0.531
KPYCPP;AAEDDEDDDVDTKKQK	NCP	10.006
AAEDDEDDDVDTKKQK;AEDDEDDDVDTK	NCP	0.003
AEDDEDDDVDTK;VPPDKPK	NCP	25.46
VPPDKPK;FVGHSLR	NCP	116.461
FVGHSLR;GVLHY	NCP	0.522
GVLHY;VSYVPR	NCP	156.021
VSYVPR;PKKGYSY	NCP	3.722
PKKGYSY;LPLLLFSSD	NCP	5.601
LPLLLFSSD;KMSPPSH	NCP	100.855
KMSPPSH;DSRLLL	NCP	37.8
DSRLLL;GSPKKPLT	NCP	52.899
GSPKKPLT;AAVFL	NCP	31.199
AAVFL;SALRDHLV	NCP	0.064
SALRDHLV;MLTAEEK	NCP	42.719
MLTAEEK;PLRPLST	NCP	107.364
PLRPLST;KLYPLQ	NCP	47.481
KLYPLQ;LTKYD	NCP	45.427
LTKYD;LTLYKLP	NCP	125.384
LTLYKLP;YDPLPR	NCP	70.731
YDPLPR;EHPSAPPP	NCP	39.544
EHPSAPPP;QNGNSRK	NCP	41.636
QNGNSRK;KSSLPR	NCP	3.247
KSSLPR;PLFLP	NCP	55.415
PLFLP;YNMYL	NCP	137.542
YNMYL;KPHKYAP	NCP	22.121
KPHKYAP;TLSLEY	NCP	54.592
TLSLEY;LCYMF	NCP	135.114
LCYMF;LLFKAPG	NCP	25.056
LLFKAPG;SLSTF	NCP	140.09
SLSTF;LFDSLY	NCP	86.607
LFDSLY;LLAPLDLR	NCP	8.472
LLAPLDLR;FMEVLK	NCP	38.797
FMEVLK;KPPLAAFK	NCP	51.746
KPPLAAFK;KLHVKAY	NCP	73.896
KLHVKAY;QWRFR	NCP	71.866
QWRFR;LLGHRKT	NCP	203.256
LLGHRKT;AKDVKFGADARALML	NCP	27.458
AKDVKFGADARALML;GVDLLADAVAV	NCP	0.048
GVDLLADAVAV;VDLLADAVAVTMGPK	NCP	0.003
VDLLADAVAVTMGPK;GSPKVTKDGVTVAK	NCP	1.032
GSPKVTKDGVTVAK;VGLQVVAVK	NCP	5.484
VGLQVVAVK;LQVVAVKAPGFGDN	NCP	0.006
LQVVAVKAPGFGDN;LNLEDVQPHDLGK	NCP	0.669
LNLEDVQPHDLGK;LEDVQPHDLG	NCP	0.006
LEDVQPHDLG;EDVQPHDLGK	NCP	0.003
EDVQPHDLGK;DDAMLLK	NCP	0.054

DDAMLLK;TSEYEKEKLNERLA	NCP	0.087
TSEYEKEKLNERLA;LSDGVAVLK	NCP	0.045
LSDGVAVLK;VGGTSDVEVNEKK	NCP	0.147
VGGTSDVEVNEKK;EKKDRVTDALN	NCP	0.03
EKKDRVTDALN;DRVTDALNATR	NCP	0.009
DRVTDALNATR;TALLDAAGVASL	NCP	0.696
TALLDAAGVASL;ELPLTK	NCP	63.154
ELPLTK;KKFFAP	NCP	7.442
KKFFAP;MEELGLL	NCP	42.321
MEELGLL;KGFPVTP	NCP	315.951
KGFPVTP;KSMKVVKN	NCP	66.478
KSMKVVKN;FEKATHLK	NCP	16.644
FEKATHLK;KDHEKTL	NCP	19.935
KDHEKTL;LDNLEVKKK	NCP	0.422
LDNLEVKKK;AAPMEL	NCP	24.721
AAPMEL;MKLPLFH	NCP	39.612
MKLPLFH;TLMGRAA	NCP	36.59
TLMGRAA;KEDLLD	NCP	68.302
KEDLLD;CWVVLYH	NCP	29.028
CWVVLYH;LNDLLK	NCP	395.822
LNDLLK;LSLVGSKP	NCP	56.88
LSLVGSKP;KSRHYSGP	NCP	48.844
KSRHYSGP;QLSTCF	NCP	0.476
QLSTCF;KFVLGPP	NCP	97.696
KFVLGPP;KEDVVPK	NCP	10.905
KEDVVPK;EYSLLKK	NCP	41.538
EYSLLKK;MNFEY	NCP	8.949
MNFEY;KFDYLP	NCP	192.036
KFDYLP;VFSLGK	NCP	91.779
VFSLGK;TELYHY	NCP	8.096
TELYHY;EPKFLLM	NCP	120.34
EPKFLLM;VSFALT	NCP	34.559
VSFALT;ELLCSLSN	NCP	65.667
ELLCSLSN;SHLELST	NCP	55.522
SHLELST;MLVTLKTLQ	NCP	49.715
MLVTLKTLQ;NFPLLK	NCP	17.382
NFPLLK;VVSGKGPK	NCP	28.486
VVSGKGPK;MLPLTVLK	NCP	48.323
MLPLTVLK;CHYKK	NCP	29.638
CHYKK;KTYYLNE	NCP	51.822
KTYYLNE;VAKFYLY	NCP	64.22
VAKFYLY;SPVEVLK	NCP	93.817
SPVEVLK;KSRNLLY	NCP	21.419
KSRNLLY;AGKPY	NCP	85.06
AGKPY;KNCLGLAK	NCP	16.272
KNCLGLAK;ESDMTEAP	NCP	84.17
ESDMTEAP;QMCPTGF	NCP	147.789
QMCPTGF;PPHLSVQ	NCP	7.933
PPHLSVQ;NDAVRAP	NCP	121.544
NDAVRAP;QGFLVFH	NCP	90.153
QGFLVFH;KVSEMVA	NCP	16.593

KVSEMVA;APPKGVH	NCP	40.084
APPKGVH;AVFLV	NCP	281.87
AVFLV;PALLR	NCP	9.499
PALLR;KLEGDLK	NCP	8.466
KLEGDLK;KMCEGK	NCP	17.604
KMCEGK;RYRPGP	NCP	6.611
RYRPGP;KELVMVP	NCP	18.73
KELVMVP;NGFSWLK	NCP	36.837
NGFSWLK;EDLTLKK	NCP	23.421
EDLTLKK;SHSLLVSK	NCP	5.261
SHSLLVSK;GVLMLK	NCP	172.404
GVLMLK;KTVAKP	NCP	77.86
KTVAKP;LKALTK	NCP	47.376
LKALTK;QTHLKAPK	NCP	35.502
QTHLKAPK;KNTCPPPPP	NCP	203.759
KNTCPPPPP;KSGPK	NCP	98.529
KSGPK;RVMVP	NCP	8.593
RVMVP;KSVPP	NCP	111.143
KSVPP;KLLPNC	NCP	3.519
KLLPNC;LLQFEDLK	NCP	94.765
LLQFEDLK;LPGGLAGK	NCP	85.667
LPGGLAGK;KNMPP	NCP	15.103
KNMPP;ELTHPKEN	NCP	351.282
ELTHPKEN;QNMNSLK	NCP	15.183
QNMNSLK;EMMCCC	NCP	32.879
EMMCCC;LTVKALL	NCP	24.777
LTVKALL;TKNSLPK	NCP	105.798
TKNSLPK;VSLKKPK	NCP	94.695
VSLKKPK;TVHHVN	NCP	49.09
TVHHVN;VLLSAL	NCP	14.103
VLLSAL;KLTQP	NCP	2.979
KLTQP;KPGKTFV	NCP	4.827
KPGKTFV;MEKLELR	NCP	80.543
MEKLELR;VRLLLEGP	NCP	89.187
VRLLLEGP;VKYLYP	NCP	125.112
VKYLYP;GPQLEMT	NCP	161.454
GPQLEMT;GEDAVQAEKFQHPGPN	NCP	52.191
GEDAVQAEKFQHPGPN;LSKYQASTSNTVSK	NCP	0.294
LSKYQASTSNTVSK;KEPVSSPGEEKEGEHADENK	NCP	10.245
KEPVSSPGEEKEGEHADENK;SLEETLHTVDL	NCP	0.06
SLEETLHTVDL;SSLRKVDSLKK	NCP	0.126
SSLRKVDSLKK;VDSLKKAFSR	NCP	0.015
VDSLKKAFSR;SSPFKVSPL	NCP	0.144
SSPFKVSPL;KVSPLTFGRKK	NCP	0.012
KVSPLTFGRKK;VSPLTFGRK	NCP	0.003
VSPLTFGRK;AEGLSEASLTSALVEGK	NCP	0.123
AEGLSEASLTSALVEGK;TSALVEGKSMEGDAGTATSR	NCP	0.027
TSALVEGKSMEGDAGTATSR;SEEEPVQPAVL	NCP	0.198
SEEEPVQPAVL;EEEPVQPA	NCP	0.003
EEEPVQPA;EEPVQPAVL	NCP	0.003
EEPVQPAVL;VLGMN	NCP	39.843

VLGMN;MDVDPK	NCP	20.739
MDVDPK;KCDEM	NCP	12.012
KCDEM;VDLLKDR	NCP	41.188
VDLLKDR;GPSLQ	NCP	56.996
GPSLQ;LVKAAYK	NCP	83.835
LVKAAYK;DRVPPR	NCP	2.811
DRVPPR;SVPKVPGK	NCP	49.206
SVPKVPGK;MDALDRVVK	NCP	6.672
MDALDRVVK;PEGTKP	NCP	0.405
PEGTKP;LSSSG	NCP	69.756
LSSSG;LSRLPET	NCP	12.984
LSRLPET;CFGYDSF	NCP	15.967
CFGYDSF;KNELNVP	NCP	168.455
KNELNVP;FLASLLTK	NCP	17.973
FLASLLTK;ATSNSKLK	NCP	14.016
ATSNSKLK;LSDRTLH	NCP	10.797
LSDRTLH;LPLKAF	NCP	220.762
LPLKAF;SYLTDS	NCP	60.585
SYLTDS;FHAVEPY	NCP	55.595
FHAVEPY;AAPNTAF	NCP	24.006
AAPNTAF;KEFTFH	NCP	37.911
KEFTFH;QHSVLDPK	NCP	147.636
QHSVLDPK;PTLPWLS	NCP	54.762
PTLPWLS;LHSRGKTL	NCP	19.602
LHSRGKTL;HGPFLM	NCP	24.48
HGPFLM;TQKLPVE	NCP	7.107
TQKLPVE;KPDYKRL	NCP	244.608
KPDYKRL;LGKSPLC	NCP	5.151
LGKSPLC;KNFFLLEY	NCP	42.301
KNFFLLEY;YNVDLRK	NCP	42.756
YNVDLRK;RDLLGLDQ	NCP	20.435
RDLLGLDQ;SLKMPK	NCP	1.311
SLKMPK;YSGLF	NCP	18.685
YSGLF;YEAFVKH	NCP	21.165
YEAFVKH;KKKPNVG	NCP	70.923
KKKPNVG;YTLPVGF	NCP	28.883
YTLPVGF;KFFLYK	NCP	56.805
KFFLYK;FPGDLP	NCP	211.635
FPGDLP;YLFLAY	NCP	199.036
YLFLAY;VGVLK	NCP	52.979
VGVLK;LRSGSLR	NCP	349.756
LRSGSLR;LSSSGNK	NCP	1.722
LSSSGNK;LSKGP	NCP	3.645
LSKGP;VTDREVSPL	NCP	44.595
VTDREVSPL;KHGHLK	NCP	30.941
KHGHLK;CTTLLQ	NCP	17.091
CTTLLQ;KTLHYVSL	NCP	64.252
KTLHYVSL;AANPML	NCP	36.081
AANPML;TQPLKFLK	NCP	85.454
TQPLKFLK;CACCE	NCP	44.955
CACCE;AELVQ	NCP	46.699

AELVQ;LSQKPEW	NCP	120.377
LSQKPEW;KGLTDL	NCP	68.397
KGLTDL;LSFDHP	NCP	373.341
LSFDHP;SGGHALAL	NCP	93.165
SGGHALAL;FAPVN	NCP	25.807
FAPVN;FGLVEK	NCP	57.035
FGLVEK;SGPPGHTM	NCP	22.966
SGPPGHTM;LGGPVTN	NCP	102.227
LGGPVTN;PDSLFLLPL	NCP	21.613
PDSLFLLPL;KPLTPAPR	NCP	103.505
KPLTPAPR;NMGLMSN	NCP	11.493
NMGLMSN;QHYVYF	NCP	53.694
QHYVYF;KELSFFYK	NCP	144.517
KELSFFYK;HLVVP	NCP	46.916
HLVVP;KLEYL	NCP	91.051
KLEYL;KKAWKK	NCP	98.021
KKAWKK;GLLQDGY	NCP	25.522
GLLQDGY;VTLKV	NCP	88.244
VTLKV;KQLESYK	NCP	64.329
KQLESYK;LRLMDPY	NCP	62.622
LRLMDPY;EGSSKFC	NCP	54.088
EGSSKFC;NVVAPS	NCP	79.689
NVVAPS;EHRKFC	NCP	5.024
EHRKFC;KLVKPVK	NCP	107.817
KLVKPVK;ELFQA	NCP	35.881
ELFQA;LVVKYEPK	NCP	9.138
LVVKYEPK;LTESRLQT	NCP	42.918
LTESRLQT;LFLLDHVP	NCP	50.616
LFLLDHVP;NSYSSGLP	NCP	25.341
NSYSSGLP;ESTTQQK	NCP	17.286
ESTTQQK;LDTFLVH	NCP	41.543
LDTFLVH;KPQTLSVH	NCP	45.411
KPQTLSVH;VLMLK	NCP	33.927
VLMLK;FKLHDK	NCP	28.069
FKLHDK;YCYCGK	NCP	57.234
YCYCGK;TSMPF	NCP	38.777
TSMPF;GPSLRVH	NCP	161.689
GPSLRVH;TAVDKK	NCP	11.168
TAVDKK;PPPGKLQ	NCP	51.633
PPPGKLQ;PDETRD	NCP	64.528
PDETRD;TAVTVP	NCP	38.645
TAVTVP;MKQVSL	NCP	185.035
MKQVSL;PKGVEPK	NCP	27.56
PKGVEPK;KKLEEL	NCP	8.035
KKLEEL;TSRPSR	NCP	47.112
TSRPSR;EMDEEDKAFKQKQKEEQKK	NCP	51.186
EMDEEDKAFKQKQKEEQKK;APKGHVP	NCP	29.321
APKGHVP;KDLEEL	NCP	104.442
KDLEEL;LGKEKR	NCP	167.334
LGKEKR;LLLLPKP	NCP	130.881
LLLLPKP;PVPVRAAP	NCP	28.165

PVPVRAAP;RTGAHSPK	NCP	111.83
RTGAHSPK;PSEKPDP	NCP	69.612
PSEKPDP;GVLLVK	NCP	16.453
GVLLVK;ELLKPEK	NCP	114.984
ELLKPEK;KTETQEKNPLPSKET	NCP	48.767
KTETQEKNPLPSKET;ETQEKNPLPSKE	NCP	0.006
ETQEKNPLPSKE;TQEKNPLPSKET	NCP	0.003
TQEKNPLPSKET;KPTHQPR	NCP	87.637
KPTHQPR;VSDHLFK	NCP	23.64
VSDHLFK;MELVPP	NCP	67.529
MELVPP;NELRAVEP	NCP	13.179
NELRAVEP;AAFGGPAKPLLS	NCP	52.62
AAFGGPAKPLLS;TAVKEPK	NCP	49.641
TAVKEPK;KTQLLL	NCP	12.459
KTQLLL;EAGASGNS	NCP	108.303
EAGASGNS;LSDDLPL	NCP	19.842
LSDDLPL;HSVPL	NCP	1.671
HSVPL;TVGALL	NCP	24.417
TVGALL;LSKTEVR	NCP	13.734
LSKTEVR;TTALLFP	NCP	206.89
TTALLFP;SPSRHPL	NCP	142.781
SPSRHPL;SVANNPK	NCP	13.14
SVANNPK;LLQHEGK	NCP	80.619
LLQHEGK;GKVTVYF	NCP	42.336
GKVTVYF;VEVPGPGR	NCP	3.201
VEVPGPGR;TPALP	NCP	12.696
TPALP;QATVPKE	NCP	38.715
QATVPKE;QAPFVS	NCP	81.852
QAPFVS;AAAPFTK	NCP	27.139
AAAPFTK;LMPLLLQ	NCP	45.579
LMPLLLQ;LYREGPQ	NCP	13.346
LYREGPQ;PLLYGK	NCP	34.828
PLLYGK;FFHMPPP	NCP	53.765
FFHMPPP;ENKLLC	NCP	10.41
ENKLLC;FPGEMPP	NCP	46.2
FPGEMPP;KFTYR	NCP	55.242
KFTYR;PPVSSK	NCP	115.222
PPVSSK;KSSSPTP	NCP	1.575
KSSSPTP;HAAPNPK	NCP	3.251
HAAPNPK;APSRKF	NCP	9.888
APSRKF;AAFEKR	NCP	32.391
AAFEKR;KGTLSPK	NCP	16.194
KGTLSPK;KSRGLLT	NCP	108.387
KSRGLLT;RYHLPAP	NCP	23.97
RYHLPAP;AQLLPLR	NCP	91.828
AQLLPLR;KVGGPLLF	NCP	26.033
KVGGPLLF;GGVRN	NCP	141.268
GGVRN;DLGFK	NCP	67.248
DLGFK;PEPLRFK	NCP	364.518
PEPLRFK;ATLLL	NCP	10.898
ATLLL;LVSLYA	NCP	17.472

LVSLYA;GNGVSF	NCP	38.904
GNGVSF;KAMCP	NCP	44.146
KAMCP;KTPALT	NCP	113.486
KTPALT;KWLWKN	NCP	12.684
KWLWKN;KLHDMAY	NCP	205.89
KLHDMAY;VADKVLV	NCP	25.65
VADKVLV;PLLGVMLQ	NCP	20.446
PLLGVMLQ;SLLVYK	NCP	5.006
SLLVYK;AAPKKKA	NCP	27.637
AAPKKKA;TKAAPML	NCP	45.3
TKAAPML;KPEVLQPP	NCP	27.893
KPEVLQPP;CCCAN	NCP	0.963
CCCAN;KEQPF	NCP	14.467
KEQPF;GVVKK	NCP	38.964
GVVKK;KSPALGF	NCP	90.443
KSPALGF;KSGHMEM	NCP	265.512
KSGHMEM;KTSSGNSK	NCP	97.642
KTSSGNSK;HVEFFSK	NCP	1.733
HVEFFSK;YFTGEAL	NCP	89.049
YFTGEAL;KTTLARAL	NCP	44.41
KTTLARAL;LGHVDSGK	NCP	3.893
LGHVDSGK;KVLDLAGF	NCP	1.539
KVLDLAGF;TTKKLVL	NCP	99.763
TTKKLVL;NAFSPLP	NCP	63.356
NAFSPLP;PTNSPLD	NCP	71.115
PTNSPLD;FLLKLQL	NCP	0.331
FLLKLQL;LYTNY	NCP	54.902
LYTNY;KDSGPG	NCP	34.209
KDSGPG;LALMFDLS	NCP	25
LALMFDLS;TVKFPR	NCP	57.24
TVKFPR;SRFWY	NCP	97.844
SRFWY;KGHHVK	NCP	7.018
KGHHVK;TDWYK	NCP	221.111
TDWYK;TNDGL	NCP	47.955
TNDGL;QFPLYNQL	NCP	179.176
QFPLYNQL;EALVK	NCP	61.368
EALVK;MYVLDF	NCP	75.281
MYVLDF;KSPTLR	NCP	124.791
KSPTLR;NSALEF	NCP	120.576
NSALEF;PSVKPR	NCP	2.889
PSVKPR;FSLLYQ	NCP	70.381
FSLLYQ;AQPVYLA	NCP	91.244
AQPVYLA;KLHNAWR	NCP	73.594
KLHNAWR;NTFAL	NCP	97.464
NTFAL;ATSASSHLNK	NCP	65.022
ATSASSHLNK;KKNYAP	NCP	5.861
KKNYAP;KQARLL	NCP	0.534
KQARLL;KMDSF	NCP	86.257
KMDSF;KTVFPR	NCP	171.026
KTVFPR;ELHWSQV	NCP	29.059
ELHWSQV;RLGVEKK	NCP	60.365

RLGVEKK;KEQFL	NCP	14.604
KEQFL;KKLPYL	NCP	42.463
KKLPYL;FFPGYK	NCP	129.863
FFPGYK;KYTLGGF	NCP	64.429
KYTLGGF;KELVLT	NCP	19.973
KELVLT;FDDGFF	NCP	174.046
FDDGFF;LLCKKP	NCP	35.402
LLCKKP;CMHECH	NCP	4.711
CMHECH;ELYLGY	NCP	26.57
ELYLGY;LSVNNN	NCP	143.583
LSVNNN;VKKTMFC	NCP	21.843
VKKTMFC;THKPLSE	NCP	12.021
THKPLSE;QHGVFV	NCP	6.108
QHGVFV;KKAKPAKP	NCP	85.308
KKAKPAKP;VKKAMMF	NCP	4.438
VKKAMMF;EGYEC	NCP	21.06
EGYEC;RVDTHSK	NCP	27.189
RVDTHSK;CGLFAY	NCP	27.492
CGLFAY;EVSEVYP	NCP	115.601
EVSEVYP;KESLEEY	NCP	50.698
KESLEEY;KKPAAFK	NCP	177.027
KKPAAFK;RTASK	NCP	111.426
RTASK;RLPPPP	NCP	5.547
RLPPPP;REGLEW	NCP	15.092
REGLEW;KKYGEK	NCP	115.77
KKYGEK;QAGPVTPP	NCP	313.132
QAGPVTPP;LRSLAR	NCP	16.575
LRSLAR;FSSLQ	NCP	73.736
FSSLQ;FDLLPLGK	NCP	79.41
FDLLPLGK;KAKVVK	NCP	50.083
KAKVVK;LPAAAL	NCP	78.6
LPAAAL;DDGWF	NCP	3.75
DDGWF;AGFVNRQ	NCP	28.029
AGFVNRQ;KMGLKHQ	NCP	9.822
KMGLKHQ;KNVGSPPP	NCP	30.783
KNVGSPPP;LSAAATPK	NCP	6.822
LSAAATPK;KPKWRPP	NCP	93.267
KPKWRPP;KLACRY	NCP	22.29
KLACRY;MPPSLP	NCP	44.514
MPPSLP;AGEFLAR	NCP	1.919
AGEFLAR;VGPNLLG	NCP	10.548
VGPNLLG;LRFSLAQ	NCP	68.086
LRFSLAQ;LAGLT	NCP	23.619
LAGLT;AVTPAGPA	NCP	48.048
AVTPAGPA;CKFWGK	NCP	98.802
CKFWGK;YGLHPPPP	NCP	111.951
YGLHPPPP;ATLFGKP	NCP	46.334
ATLFGKP;KDLMQPK	NCP	233.785
KDLMQPK;VTPVSP	NCP	67.794
VTPVSP;KVVPRSK	NCP	0.732
KVVPRSK;KNPLNPK	NCP	111.563

KNPLNPK;ALLRLDL	NCP	1.66
ALLRLDL;KSKSWH	NCP	14.024
KSKSWH;ARGFPP	NCP	9.274
ARGFPP;ASAYEVL	NCP	26.384
ASAYEVL;NTSTSSSSSS	NCP	174.271
NTSTSSSSSS;HHEVLYL	NCP	49.803
HHEVLYL;DGFAL	NCP	181.67
DGFAL;RTSVPYE	NCP	69.156
RTSVPYE;GEKPDL	NCP	11.065
GEKPDL;MCSDPK	NCP	187.934
MCSDPK;SHSGVGKY	NCP	1.269
SHSGVGKY;PAAELAK	NCP	75.798
PAAELAK;FVRGKRF	NCP	33.118
FVRGKRF;KLWALLE	NCP	88.424
KLWALLE;EGPVLD	NCP	18.457
EGPVLD;QAGPGPKP	NCP	23.412
QAGPGPKP;LPTKLPP	NCP	138.749
LPTKLPP;EGYVGLER	NCP	88.495
EGYVGLER;KGGLTHK	NCP	5.091
KGGLTHK;VPTLP	NCP	25.032
VPTLP;REVPKK	NCP	83.841
REVPKK;LTAGRSSP	NCP	15.804
LTAGRSSP;GPPPPPP	NCP	6.525
GPPPPPP;NSKALLR	NCP	26.378
NSKALLR;KDRLAVP	NCP	17.412
KDRLAVP;RPVNR	NCP	67.732
RPVNR;RGLYFLSH	NCP	40.559
RGLYFLSH;KPGGVK	NCP	49.95
KPGGVK;LPPLPGP	NCP	9.616
LPPLPGP;VGFKPPP	NCP	10.188
VGFKPPP;YQRKEK	NCP	231.447
YQRKEK;VGTLAL	NCP	1.092
VGTLAL;AGAQY	NCP	79.74
AGAQY;KPGKAQ	NCP	55.056
KPGKAQ;KGFAVPPP	NCP	89.343
KGFAVPPP;QMLHERK	NCP	7.718
QMLHERK;RPKKPER	NCP	58.242
RPKKPER;VNPFQ	NCP	15.493
VNPFQ;PTKGKK	NCP	4.782
PTKGKK;LAAKFR	NCP	89.315
LAAKFR;PGGFPK	NCP	49.284
PGGFPK;GVGPV	NCP	46.437
GVGPV;ALPTLGN	NCP	194.892
ALPTLGN;LEKFDSK	NCP	66.648
LEKFDSK;VQVLA	NCP	6.393
VQVLA;YLVQESTDKEAK	NCP	44.397
YLVQESTDKEAK;KGEVTY	NCP	37.236
KGEVTY;DDDFLKT	NCP	83.109
DDDFLKT;GTSKKNLP	NCP	105.232
GTSKKNLP;KHSPF	NCP	80.244
KHSPF;KKNGAEL	NCP	5.246

KKNGAEL;RYVEER	NCP	89.637
RYVEER;ATLLP	NCP	100.462
ATLLP;GPANKPP	NCP	10.017
GPANKPP;EHYYAT	NCP	25.136
EHYYAT;SAAVLM	NCP	12.603
SAAVLM;PAGTHNPK	NCP	70.653
PAGTHNPK;KGTTNVR	NCP	57.609
KGTTNVR;KTTVLGD	NCP	55.896
KTTVLGD;KDFFLVCF	NCP	6.628
KDFFLVCF;MLFLVR	NCP	0.263
MLFLVR;GSMFLT	NCP	66.06
GSMFLT;EVGSLLGK	NCP	180.516
EVGSLLGK;GVQTLVRL	NCP	70.063
GVQTLVRL;VGFMKN	NCP	47.973
VGFMKN;LPPHLK	NCP	1.83
LPPHLK;KLLDLAGF	NCP	22.949
KLLDLAGF;LLMVGL	NCP	0.597
LLMVGL;HDLVVGKY	NCP	83.332
HDLVVGKY;LFGVSLPK	NCP	7.943
LFGVSLPK;FLRLNF	NCP	68.509
FLRLNF;VPPGKAV	NCP	62.036
VPPGKAV;EGLTMM	NCP	3.742
EGLTMM;PPSLH	NCP	109.05
PPSLH;RVGLKVF	NCP	115.179
RVGLKVF;TLMAT	NCP	54.825
TLMAT;QYPVPQQ	NCP	6.434
QYPVPQQ;MDVLVP	NCP	0.553
MDVLVP;SGPVVPP	NCP	122.705
SGPVVPP;GKKSTN	NCP	4.962
GKKSTN;KGATTTH	NCP	14.608
KGATTTH;VSLLV	NCP	69.527
VSLLV;RPPPKY	NCP	114.318
RPPPKY;KLGHPQPP	NCP	168.685
KLGHPQPP;KASLHVPK	NCP	34.26
KASLHVPK;KDVAP	NCP	44.867
KDVAP;VCWHKPK	NCP	42.532
VCWHKPK;VLPKCVK	NCP	104.417
VLPKCVK;KDLEQLR	NCP	71.941
KDLEQLR;KATMPR	NCP	137.342
KATMPR;KYLPLPPP	NCP	16.395
KYLPLPPP;QVFKLSKL	NCP	95.346
QVFKLSKL;MLNTDL	NCP	47.814
MLNTDL;DTLKDFP	NCP	181.53
DTLKDFP;AASYVYK	NCP	139.114
AASYVYK;ELSYLYR	NCP	116.817
ELSYLYR;KKEVR	NCP	22.085
KKEVR;KLLRCS	NCP	45.66
KLLRCS;PVVKEHM	NCP	9.244
PVVKEHM;KYLPVPPP	NCP	2.444
KYLPVPPP;KLATRP	NCP	57.324
KLATRP;PPGKHTR	NCP	63.751

PPGKHTR;ANSPVLL	NCP	271.968
ANSPVLL;KFAEF	NCP	21.129
KFAEF;RQKLYLSK	NCP	23.37
RQKLYLSK;RTSAN	NCP	88.4
RTSAN;PGRST	NCP	35.191
PGRST;LSHSGV	NCP	78.375
LSHSGV;VPAQPP	NCP	6.09
VPAQPP;KLFFPLG	NCP	181.586
KLFFPLG;YAPTTTK	NCP	60.646
YAPTTTK;GLMNLLL	NCP	19.088
GLMNLLL;GVLSY	NCP	47.538
GVLSY;GNKTKSPK	NCP	127.771
GNKTKSPK;LSLVTLTGH	NCP	20.997
LSLVTLTGH;RKRLGFM	NCP	258.542
RKRLGFM;LPLLGPS	NCP	50.365
LPLLGPS;TAGDLSLP	NCP	24.116
TAGDLSLP;AVKKNK	NCP	75.162
AVKKNK;NCALLCR	NCP	148.675
NCALLCR;PLLLYKK	NCP	62.318
PLLLYKK;KYLPPT	NCP	35.418
KYLPPT;QPAYNLR	NCP	25.666
QPAYNLR;RPRVY	NCP	62.226
RPRVY;VGSVP	NCP	43.616
VGSVP;LDKSPKL	NCP	159.901
LDKSPKL;AAVPGRPP	NCP	230.397
AAVPGRPP;LDKFVR	NCP	20.325
LDKFVR;LHKVMSN	NCP	41.67
LHKVMSN;KPPPPSP	NCP	188.778
KPPPPSP;GQYVPR	NCP	42.375
GQYVPR;LVVDLL	NCP	64.088
LVVDLL;KFSVA	NCP	36.01
KFSVA;KYVEAR	NCP	66.71
KYVEAR;LMDLL	NCP	80.91
LMDLL;LLLFLVSPK	NCP	22.618
LLLFLVSPK;NALLVRY	NCP	58.728
NALLVRY;EKLRF	NCP	34.439
EKLRF;MVPVLL	NCP	50.862
MVPVLL;TAAADFN	NCP	1.497
TAAADFN;LPFNPK	NCP	9.874
LPFNPK;LAVGSLLK	NCP	1.791
LAVGSLLK;LSGLYT	NCP	58.422
LSGLYT;AGPTLTAL	NCP	68.886
AGPTLTAL;QPQAAVR	NCP	107.261
QPQAAVR;LTFKSK	NCP	14.455
LTFKSK;DSLAARAGF	NCP	62.285
DSLAARAGF;KERFKK	NCP	59.047
KERFKK;LVPGVR	NCP	176.208
LVPGVR;DLQAP	NCP	29.439
DLQAP;GAAVL	NCP	161.301
GAAVL;VPGTPLLQ	NCP	8.993
VPGTPLLQ;YLHFFT	NCP	19.434

YLHFFT;PTVEFK	NCP	53.707
PTVEFK;VMTLVL	NCP	61.526
VMTLVL;LSEKLER	NCP	112.374
LSEKLER;ALSFRK	NCP	173.836
ALSFRK;KDLYFLKN	NCP	33.168
KDLYFLKN;KMFGK	NCP	42.354
KMFGK;CHKAKGK	NCP	33.77
CHKAKGK;KPASLLH	NCP	50.55
KPASLLH;KELQPK	NCP	43.227
KELQPK;RLLVLML	NCP	1.206
RLLVLML;KDKHYVK	NCP	125.401
KDKHYVK;PSKGPLQ	NCP	91.296
PSKGPLQ;TSKKLVN	NCP	86.768
TSKKLVN;FGGAK	NCP	199.018
FGGAK;KSQEKFY	NCP	237.036
KSQEKFY;LTAGLPLF	NCP	110.991
LTAGLPLF;DYKAKGK	NCP	59.855
DYKAKGK;KPDKRFF	NCP	47.07
KPDKRFF;LSSKSHE	NCP	26.529
LSSKSHE;AYPGHPG	NCP	70.218
AYPGHPG;KPTAMLK	NCP	18.264
KPTAMLK;VYTPLEK	NCP	102.874
VYTPLEK;KAYYSK	NCP	29.994
KAYYSK;EYLLTA	NCP	134.372
EYLLTA;VLLEYK	NCP	0.552
VLLEYK;LDLSGKP	NCP	36.342
LDLSGKP;FEMFPH	NCP	15.777
FEMFPH;GLKVYER	NCP	109.041
GLKVYER;TTVVKPV	NCP	27.948
TTVVKPV;LDWKVVLY	NCP	93.376
LDWKVVLY;FFMGP	NCP	90.66
FFMGP;MPLLLR	NCP	84.56
MPLLLR;YPPLG	NCP	86.043
YPPLG;VDEKLK	NCP	140.194
VDEKLK;ECWVTKK	NCP	48.074
ECWVTKK;KTDAQP	NCP	10.129
KTDAQP;PSSGGKK	NCP	22.998
PSSGGKK;KQSTFGP	NCP	96.059
KQSTFGP;LDKRPDL	NCP	31.677
LDKRPDL;EHMVRK	NCP	249.396
EHMVRK;DRPSFNR	NCP	77.538
DRPSFNR;TKFVTPQ	NCP	107.496
TKFVTPQ;KASQNPH	NCP	70.174
KASQNPH;MKLHSK	NCP	15.48
MKLHSK;VEGSTK	NCP	23.637
VEGSTK;DPLLENR	NCP	4.065
DPLLENR;LTPSYK	NCP	100.35
LTPSYK;FPGAVP	NCP	151.937
FPGAVP;NPSKFR	NCP	2.011
NPSKFR;KVLDMHP	NCP	62.003
KVLDMHP;ATPVNF	NCP	311.587

ATPVNF;TSAEPLKK	NCP	1.607
TSAEPLKK;KLTTVGLR	NCP	122.632
KLTTVGLR;EEMCD	NCP	18.414
EEMCD;HPETLN	NCP	195.563
HPETLN;PLVPA	NCP	18.684
PLVPA;SKVPEHK	NCP	115.537
SKVPEHK;SDTPLL	NCP	69.819
SDTPLL;KGNKLR	NCP	156.107
KGNKLR;AKVMPSR	NCP	40.537
AKVMPSR;YNGGELL	NCP	12.543
YNGGELL;VEGAVFN	NCP	52.254
VEGAVFN;TTNSLR	NCP	39.597
TTNSLR;EFKAPK	NCP	39.254
EFKAPK;WYLKEK	NCP	111.273
WYLKEK;KFLPGVGV	NCP	43.495
KFLPGVGV;AAGQVETL	NCP	96.402
AAGQVETL;SLPVRF	NCP	23.685
SLPVRF;VSGSGH	NCP	22.218
VSGSGH;PVGPTPVP	NCP	112.377
PVGPTPVP;AAKLVSLK	NCP	92.775
AAKLVSLK;ASPLQFR	NCP	2.631
ASPLQFR;DLRKDLY	NCP	27.128
DLRKDLY;RLHLEGY	NCP	10.632
RLHLEGY;NDLFLPY	NCP	96.076
NDLFLPY;MFEKK	NCP	33.519
MFEKK;VYKGPK	NCP	38.001
VYKGPK;RLKAVD	NCP	141.356
RLKAVD;RGFAAPP	NCP	75.519
RGFAAPP;NVEALSP	NCP	66.424
NVEALSP;LPQKGRLR	NCP	5.564
LPQKGRLR;PAKLLCY	NCP	27.801
PAKLLCY;CCCCCSF	NCP	92.628
CCCCCSF;KEGFGYK	NCP	19.336
KEGFGYK;ETPKLP	NCP	51.317
ETPKLP;GLLSEKK	NCP	193.53
GLLSEKK;MEEVVLT	NCP	166.5
MEEVVLT;YTPSVPP	NCP	38.085
YTPSVPP;NKKCE	NCP	6.787
NKKCE;FLDPKK	NCP	53.523
FLDPKK;QPVKPYN	NCP	110.117
QPVKPYN;AYFMKK	NCP	12.496
AYFMKK;KNGSSLP	NCP	47.516
KNGSSLP;LLDMLL	NCP	46.231
LLDMLL;ENFLEK	NCP	60.296
ENFLEK;PPPPSRPP	NCP	14.358
PPPPSRPP;TTGLVMD	NCP	112.447
TTGLVMD;MLLYR	NCP	0.548
MLLYR;FASPR	NCP	18.702
FASPR;EGMMFP	NCP	29.455
EGMMFP;QSGRVLGL	NCP	126.636
QSGRVLGL;TVFTPTN	NCP	8.724

TVFTPTN;HHKLFLLL	NCP	24.009
HHKLFLLL;KLQLEK	NCP	0.746
KLQLEK;EGQSNRK	NCP	53.361
EGQSNRK;LTGMAF	NCP	17.472
LTGMAF;EPLFLVLQ	NCP	8.073
EPLFLVLQ;MALNVLF	NCP	1.498
MALNVLF;TQDDN	NCP	180.464
TQDDN;LFDSFVS	NCP	53.107
LFDSFVS;NHDSLPQL	NCP	89.943
NHDSLPQL;LCGVGLLR	NCP	10.154
LCGVGLLR;KSVTFH	NCP	38.622
KSVTFH;RLLLLM	NCP	99.364
RLLLLM;STYLGEC	NCP	102.876
STYLGEC;CSDSHMM	NCP	158.547
CSDSHMM;NMRAVEF	NCP	3.248
NMRAVEF;LDLNTAY	NCP	72.067
LDLNTAY;PPTPSPP	NCP	17.181
PPTPSPP;RAVCML	NCP	2.907
RAVCML;EVPLEM	NCP	88.302
EVPLEM;MYKPR	NCP	127.691
MYKPR;FKEQF	NCP	21.363
FKEQF;PPSLGGTK	NCP	27.87
PPSLGGTK;MMCCQ	NCP	27.3
MMCCQ;AAKCK	NCP	94.962
AAKCK;KLTGRGP	NCP	112.081
KLTGRGP;KNQLPK	NCP	30.311
KNQLPK;KPYMQH	NCP	12.835
KPYMQH;LPFSYST	NCP	53.741
LPFSYST;VVFFAM	NCP	85.644
VVFFAM;LSFLTR	NCP	7.036
LSFLTR;VLDLGGGLR	NCP	19.281
VLDLGGGLR;SLGLVK	NCP	46.368
SLGLVK;LATLADKL	NCP	51.177
LATLADKL;SVGPSLATDKS	NCP	24.219
SVGPSLATDKS;GLPFE	NCP	34.166
GLPFE;KPFFSL	NCP	95.5
KPFFSL;FTTAPAK	NCP	49.427
FTTAPAK;TDMEC	NCP	104.901
TDMEC;YSVPP	NCP	13.743
YSVPP;RGYKLPK	NCP	52.444
RGYKLPK;KFLKDL	NCP	22.808
KFLKDL;LAPGVGV	NCP	149.095
LAPGVGV;LAPLTK	NCP	141.791
LAPLTK;FTKRTT	NCP	64.387
FTKRTT;VELSATY	NCP	86.882
VELSATY;HERMR	NCP	141.52
HERMR;DLLAHC	NCP	356.154
DLLAHC;MEVKSLPK	NCP	6.437
MEVKSLPK;PSFKKWK	NCP	136.551
PSFKKWK;YGYEDF	NCP	7.74
YGYEDF;KELMH	NCP	73.366

KELMH;KSGVYL	NCP	26.072
KSGVYL;ATELGSPP	NCP	2.139
ATELGSPP;VLVLHK	NCP	34.545
VLVLHK;AELNVR	NCP	120.915
AELNVR;LTLYKPL	NCP	13.066
LTLYKPL;APNDVVP	NCP	40.494
APNDVVP;ESTKVP	NCP	16.196
ESTKVP;VLRPELLCK	NCP	250.849
VLRPELLCK;KVNPLDK	NCP	250.055
KVNPLDK;TNEFDLK	NCP	1.863
TNEFDLK;KPMAARP	NCP	38.064
KPMAARP;VGAGFEA	NCP	98.944
VGAGFEA;LSLETLLK	NCP	121.718
LSLETLLK;ADVVF	NCP	55.333
ADVVF;QSAKL	NCP	15.15
QSAKL;FHVAVPP	NCP	148.238
FHVAVPP;TAPEVVPK	NCP	64.116
TAPEVVPK;MVKELC	NCP	37.338
MVKELC;LDVSAPK	NCP	104.823
LDVSAPK;KSDTPR	NCP	236.125
KSDTPR;WEHVKR	NCP	121.944
WEHVKR;ASPGRLLDL	NCP	192.296
ASPGRLLDL;SDMVL	NCP	268.006
SDMVL;YAFPELY	NCP	29.906
YAFPELY;DDGFFF	NCP	40.84
DDGFFF;HGLHAR	NCP	178.871
HGLHAR;TVRGNQL	NCP	173.685
TVRGNQL;DLPFVVRP	NCP	28.837
DLPFVVRP;KFVPYP	NCP	13.905
KFVPYP;EMFLKPP	NCP	0.338
EMFLKPP;LQHLSAQR	NCP	20.611
LQHLSAQR;VLAAGFMM	NCP	81.623
VLAAGFMM;AMPLLL	NCP	39.829
AMPLLL;KDTTVLLK	NCP	10.622
KDTTVLLK;AHLHGAK	NCP	3.057
AHLHGAK;EPPHPGAK	NCP	40.666
EPPHPGAK;SLLVD	NCP	110.813
SLLVD;PLLFVT	NCP	65.743
PLLFVT;KLGVNPLK	NCP	29.46
KLGVNPLK;KGLDDFF	NCP	22.937
KGLDDFF;LGDVLLR	NCP	157.354
LGDVLLR;AAPVLL	NCP	46.464
AAPVLL;YLLQKD	NCP	28.809
YLLQKD;LDGSLLGK	NCP	54.84
LDGSLLGK;AFPTLVYF	NCP	52.293
AFPTLVYF;EPPAYLT	NCP	76.187
EPPAYLT;AMGRSLLL	NCP	84.663
AMGRSLLL;DEVLR	NCP	23.725
DEVLR;VSVALLLP	NCP	45.503
VSVALLLP;KKCAAK	NCP	9.129
KKCAAK;DLRETNL	NCP	63.514

DLRETNL;DKLRL	NCP	0.012
DKLRL;MAPVP	NCP	47.519
MAPVP;LYEAVLT	NCP	64.836
LYEAVLT;VLLLFA	NCP	83.931
VLLLFA;SMSPLK	NCP	73.344
SMSPLK;NCLDLRK	NCP	45.423
NCLDLRK;PLVNVH	NCP	64.012
PLVNVH;TAKFDK	NCP	57.257
TAKFDK;KLTAPY	NCP	69.604
KLTAPY;TAELL	NCP	35.636
TAELL;CDCLY	NCP	91.546
CDCLY;KGTHLPPK	NCP	2.298
KGTHLPPK;EALRAL	NCP	127.992
EALRAL;PPYLLL	NCP	162.237
PPYLLL;KRPPSPG	NCP	8.739
KRPPSPG;QQPLSPPR	NCP	81.498
QQPLSPPR;QTPLSR	NCP	11.48
QTPLSR;APALEAKH	NCP	18.555
APALEAKH;VPLNL	NCP	119.515
VPLNL;SCCELF	NCP	77.792
SCCELF;AGAAFV	NCP	91.528
AGAAFV;LRLVQDY	NCP	22.128
LRLVQDY;FLGVYLT	NCP	55.383
FLGVYLT;KQHMDVK	NCP	6.569
KQHMDVK;FKTGRP	NCP	23.826
FKTGRP;GPPFL	NCP	7.447
GPPFL;NSSSN	NCP	54.428
NSSSN;DLLNTF	NCP	2.055
DLLNTF;AALVLH	NCP	5.683
AALVLH;KSVVGM	NCP	238.581
KSVVGM;KDPVLL	NCP	30.234
KDPVLL;YSSLHSAH	NCP	196.374
YSSLHSAH;WAYQL	NCP	16.748
WAYQL;ATKDLR	NCP	29.241
ATKDLR;KSHCSKSL	NCP	1.017
KSHCSKSL;MTKAPW	NCP	2.565
MTKAPW;YSAALLKT	NCP	68.512
YSAALLKT;ECGRVFL	NCP	280.557
ECGRVFL;TLKYPLEAH	NCP	22.86
TLKYPLEAH;VLEKHV	NCP	42.159
VLEKHV;ARPLAM	NCP	7.082
ARPLAM;PGAGNK	NCP	537.159
PGAGNK;NVPPTQAR	NCP	47.67
NVPPTQAR;ASATRF	NCP	18.168
ASATRF;VLPKPGLVA	NCP	213.31
VLPKPGLVA;VGKLKTEL	NCP	24.852
VGKLKTEL;GVAEQLHNEGFK	NCP	65.49
GVAEQLHNEGFK;CKKVSML	NCP	76.859
CKKVSML;GWKQVDL	NCP	11.061
GWKQVDL;QVTKGPK	NCP	80.742
QVTKGPK;ELGRVYST	NCP	42.555

ELGRVYST;VSGLTSPK	NCP	134.287
VSGLTSPK;KLLFHS	NCP	16.56
KLLFHS;VTPLELT	NCP	44.12
VTPLELT;MNLSPPPPP	NCP	220.731
MNLSPPPPP;LKDSKL	NCP	1.618
LKDSKL;KTVYLK	NCP	144.171
KTVYLK;FPGPAP	NCP	21.054
FPGPAP;NVPLP	NCP	13.677
NVPLP;KDYELL	NCP	29.427
KDYELL;TTRLKLLL	NCP	106.128
TTRLKLLL;LFVTPK	NCP	14.517
LFVTPK;YPVEPF	NCP	186.428
YPVEPF;LHGLSPF	NCP	18.316
LHGLSPF;DVSVPK	NCP	132.888
DVSVPK;VLSNTTM	NCP	28.394
VLSNTTM;TKLTTK	NCP	321.459
TKLTTK;KRSLL	NCP	18.618
KRSLL;KYTLFLLN	NCP	26.376
KYTLFLLN;MDKLKK	NCP	47.244
MDKLKK;TFLNEK	NCP	55.936
TFLNEK;KQSALKTL	NCP	4.835
KQSALKTL;ASLVKF	NCP	22.096
ASLVKF;KHFLFF	NCP	74.118
KHFLFF;PGMVP	NCP	108.986
PGMVP;NDGPK	NCP	4.159
NDGPK;KNMMLK	NCP	109.398
KNMMLK;GAALF	NCP	271.008
GAALF;VVTAYPPK	NCP	0.501
VVTAYPPK;LPYSSGHS	NCP	10.44
LPYSSGHS;KTVPVEK	NCP	5.967
KTVPVEK;KQLEVDGK	NCP	22.011
KQLEVDGK;EVDHYTN	NCP	3.102
EVDHYTN;YLFPELY	NCP	102.303
YLFPELY;RNGPRP	NCP	4.664
RNGPRP;LTSLALA	NCP	97.3
LTSLALA;LAAMLDTVVFK	NCP	71
LAAMLDTVVFK;VGTVYP	NCP	49.588
VGTVYP;VAGLEF	NCP	91.178
VAGLEF;PVLFTLTF	NCP	65.434
PVLFTLTF;KDSLAALK	NCP	96.243
KDSLAALK;QRLLRL	NCP	13.203
QRLLRL;QSMKKF	NCP	0.686
QSMKKF;LDVPF	NCP	15.333
LDVPF;WGSLN	NCP	33.61
WGSLN;VNESHFE	NCP	3.563
VNESHFE;RLPPMF	NCP	47.262
RLPPMF;APGEQPK	NCP	56.187
APGEQPK;ESAKGSK	NCP	110.791
ESAKGSK;SGLPTKET	NCP	5.651
SGLPTKET;AARTRVQLR	NCP	2.673
AARTRVQLR;VAHSL	NCP	16.156

VAHSL;EHEGLGK	NCP	16.113
EHEGLGK;MALPLK	NCP	15.872
MALPLK;LSLEEL	NCP	46.987
LSLEEL;SVLLDKLK	NCP	12.302
SVLLDKLK;KDAVTK	NCP	2.698
KDAVTK;KVELCSF	NCP	69.78
KVELCSF;RVYYEK	NCP	119.433
RVYYEK;MLAHVAF	NCP	22.095
MLAHVAF;KTAPALLSH	NCP	15.927
KTAPALLSH;LVGAFVP	NCP	37.566
LVGAFVP;KGPDQ	NCP	2.145
KGPDQ;NKALQRL	NCP	25.16
NKALQRL;VFLLNL	NCP	135.754
VFLLNL;NTPPV	NCP	13.914
NTPPV;PVLNLLL	NCP	31.131
PVLNLLL;QNVSNQLK	NCP	1.938
QNVSNQLK;LAVSY	NCP	203.369
LAVSY;MHLLAMM	NCP	37.894
MHLLAMM;VTQKLK	NCP	189.159
VTQKLK;LLFAHF	NCP	72.651
LLFAHF;KHGGPP	NCP	281.007
KHGGPP;KRGYSR	NCP	16.356
KRGYSR;KETFEK	NCP	106.826
KETFEK;LAATVQRVP	NCP	2.233
LAATVQRVP;DRHLPDL	NCP	14.381
DRHLPDL;LTGKNHM	NCP	33.444
LTGKNHM;HSSLLLLV	NCP	178.18
HSSLLLLV;TMSGNF	NCP	16.401
TMSGNF;PDLLTK	NCP	17.277
PDLLTK;PMSGL	NCP	278.664
PMSGL;AAAKPDALM	NCP	24.03
AAAKPDALM;TNKLYRGQ	NCP	24.024
TNKLYRGQ;TTHAVKK	NCP	55.181
TTHAVKK;LLTTL	NCP	47.704
LLTTL;KGYALP	NCP	150.99
KGYALP;ALPHALM	NCP	142.398
ALPHALM;GPATMFLP	NCP	10.899
GPATMFLP;DPAPLPR	NCP	55.176
DPAPLPR;GLYEY	NCP	89.808
GLYEY;AMGLMNS	NCP	70.254
AMGLMNS;KQLEVPY	NCP	17.016
KQLEVPY;KEHLLMP	NCP	25.728
KEHLLMP;KEKKLC	NCP	81.152
KEKKLC;AVVAPGLP	NCP	3.409
AVVAPGLP;FDGNY	NCP	12.923
FDGNY;HLPLLP	NCP	20.779
HLPLLP;LVPNAPK	NCP	31.451
LVPNAPK;KPPRGAAP	NCP	14.026
KPPRGAAP;KNVAVRK	NCP	2.76
KNVAVRK;KTFFPP	NCP	63.807
KTFFPP;TGSFGLN	NCP	127.883

TGSFGLN;AQVATLR	NCP	4.192
AQVATLR;KNMLQ	NCP	63.566
KNMLQ;RNDPK	NCP	81.037
RNDPK;FSQSVLH	NCP	13.766
FSQSVLH;KVVHVR	NCP	65.878
KVVHVR;QWQSY	NCP	7.47
QWQSY;LDVALP	NCP	18.089
LDVALP;KKGGLNL	NCP	20.425
KKGGLNL;SPAPY	NCP	18.843
SPAPY;AVPEELP	NCP	2.3
AVPEELP;YPLLL	NCP	268.749
YPLLL;MHSYEAV	NCP	81.292
MHSYEAV;LSLQYE	NCP	16.509
LSLQYE;TLLVENK	NCP	33.161
TLLVENK;KSGVMLK	NCP	18.727
KSGVMLK;MGKPEPPR	NCP	6.362
MGKPEPPR;VTAERAAV	NCP	12.412
VTAERAAV;ETHVRL	NCP	61.773
ETHVRL;PTPPFP	NCP	11.274
PTPPFP;LPYPQPY	NCP	23.504
LPYPQPY;TVGPP	NCP	8.061
TVGPP;KLCSPVVR	NCP	83.695
KLCSPVVR;LAGAGLL	NCP	135.843
LAGAGLL;MLPLLSLK	NCP	76.424
MLPLLSLK;LGGAPL	NCP	18.139
LGGAPL;WDPLL	NCP	3.98
WDPLL;QVVEC	NCP	2.512
QVVEC;PPPPLAP	NCP	16.833
PPPPLAP;VAPRASPAP	NCP	4.482
VAPRASPAP;AYGVALK	NCP	54.102
AYGVALK;LLGEVL	NCP	144.854
LLGEVL;ADFDGVLY	NCP	21.967
ADFDGVLY;DSYPPL	NCP	13.347
DSYPPL;LTMLVL	NCP	196.401
LTMLVL;TPKALK	NCP	1.26
TPKALK;LKQNKELK	NCP	23.004
LKQNKELK;LLRFALSN	NCP	79.151
LLRFALSN;THLGLVTN	NCP	8.896
THLGLVTN;KMPPPP	NCP	192.447
KMPPPP;TTVLP	NCP	103.193
TTVLP;GKPPPPPPP	NCP	13.546
GKPPPPPPP;ADVFAPA	NCP	1.863
ADVFAPA;YRMDP	NCP	14.58
YRMDP;YFGKPP	NCP	18.296
YFGKPP;TSLRLLTSL	NCP	6.28
TSLRLLTSL;FGGVH	NCP	21.197
FGGVH;LRLSEAK	NCP	80.767
LRLSEAK;MAAPP	NCP	148.644
MAAPP;FPWLN	NCP	56.474
FPWLN;LLEYL	NCP	47.704
LLEYL;KPAALHV	NCP	50.364

KPAALHV;GSRGPP	NCP	171.332
GSRGPP;GLGGLT	NCP	19.858
GLGGLT;LLMPLNPA	NCP	123.758
LLMPLNPA;PPKVSPGP	NCP	18.964
PPKVSPGP;YGYGLM	NCP	22.238
YGYGLM;TMFLTFM	NCP	11.007
TMFLTFM;RLFQVK	NCP	37.083
RLFQVK;MDLEAK	NCP	69.543
MDLEAK;CVPQGLQL	NCP	58.657
CVPQGLQL;APLVVL	NCP	40.295
APLVVL;SVEEFL	NCP	5.64
SVEEFL;KTSLKAK	NCP	102.417
KTSLKAK;ADALLLLPK	NCP	6.927
ADALLLLPK;KGPCLPLT	NCP	20.835
KGPCLPLT;SALRTPTS	NCP	42.25
SALRTPTS;PLLFSL	NCP	67.074
PLLFSL;FVMGVNHE	NCP	10.182
FVMGVNHE;ADGAGALL	NCP	63.539
ADGAGALL;APHSSTTY	NCP	13.15
APHSSTTY;EAPGPLNF	NCP	165.434
EAPGPLNF;ELGLKR	NCP	70.71
ELGLKR;EPSKTLNP	NCP	47.052
EPSKTLNP;LKGVL	NCP	17.316
LKGVL;KNGPP	NCP	108.171
KNGPP;HALPFR	NCP	14.508
HALPFR;VGLFE	NCP	70.318
VGLFE;ELFLQ	NCP	74.939
ELFLQ;DTEYLF	NCP	94.629
DTEYLF;RTSLSF	NCP	11.614
RTSLSF;GHPPPP	NCP	167.981
GHPPPP;KDFVAH	NCP	178.923
KDFVAH;VRQELF	NCP	17.571
VRQELF;KMVMPK	NCP	19.438
KMVMPK;KVSKLDSR	NCP	101.327
KVSKLDSR;AAALDMK	NCP	2.412
AAALDMK;PFQSL	NCP	31.651
PFQSL;KGDSSKK	NCP	40.829
KGDSSKK;VKGTC	NCP	69.574
VKGTC;GPDSF	NCP	35.447
GPDSF;FTRPPPP	NCP	16.662
FTRPPPP;NSLGVRGGF	NCP	19.08
NSLGVRGGF;SPSAHSLR	NCP	290.874
SPSAHSLR;QPQVYR	NCP	105.273
QPQVYR;LDGFGVY	NCP	122.512
LDGFGVY;TTKAT	NCP	34.184
TTKAT;PLFAFL	NCP	38.451
PLFAFL;KYNPLLT	NCP	9.651
KYNPLLT;MGTGYGRP	NCP	32.91
MGTGYGRP;AGLLKKT	NCP	3.934
AGLLKKT;LLYSRK	NCP	3.168
LLYSRK;DPVGLPY	NCP	4.122

DPVGLPY;KFFVVELY	NCP	10.266
KFFVVELY;LLELAGN	NCP	269.561
LLELAGN;KQYFERR	NCP	49.695
KQYFERR;ANVEKPVK	NCP	14.601
ANVEKPVK;DGANENVWH	NCP	0.135
DGANENVWH;EAKPLKLY	NCP	1.707
EAKPLKLY;KPPAPPGHR	NCP	10.608
KPPAPPGHR;TPPVVPGVS	NCP	2.445
TPPVVPGVS;KPVTYGP	NCP	2.038
KPVTYGP;KMGSGPVP	NCP	35.013
KMGSGPVP;AKYGLP	NCP	60.458
AKYGLP;RGGVLR	NCP	437.497
RGGVLR;HPDGFR	NCP	23.118
HPDGFR;KSGVSESK	NCP	22.164
KSGVSESK;PVPPPP	NCP	138.276
PVPPPP;KPPEYLR	NCP	58.094
KPPEYLR;DKEGKLP	NCP	20.538
DKEGKLP;KQSLGLQ	NCP	19.105
KQSLGLQ;KAPGLLRP	NCP	73.934
KAPGLLRP;AVEKKLAEY	NCP	34.839
AVEKKLAEY;SFALK	NCP	26.989
SFALK;KGPLFK	NCP	118.163
KGPLFK;LVNLTN	NCP	26.242
LVNLTN;LAPGLELR	NCP	57.542
LAPGLELR;ALAEAW	NCP	15.096
ALAEAW;AAHGPPK	NCP	19.815
AAHGPPK;KVDLRFQ	NCP	15.442
KVDLRFQ;VGPAV	NCP	57.563
VGPAV;TRKVLLT	NCP	55.912
TRKVLLT;DMMDAP	NCP	70.769
DMMDAP;SVGLQFF	NCP	27.729
SVGLQFF;LLEPVLL	NCP	7.122
LLEPVLL;KWAETYP	NCP	22.515
KWAETYP;LDLLLKKKK	NCP	75.427
LDLLLKKKK;KPFQAR	NCP	22.589
KPFQAR;SGKHVVF	NCP	115.623
SGKHVVF;LGAPA	NCP	59.427
LGAPA;EEEAG	NCP	183.066
EEEAG;SLRQKY	NCP	19.275
SLRQKY;EVVASPK	NCP	9.211
EVVASPK;ADLLL	NCP	49.49
ADLLL;LDLLVLG	NCP	4.863
LDLLVLG;PVATAGEK	NCP	32.238
PVATAGEK;TVLLP	NCP	58.975
TVLLP;RFHVEPP	NCP	199.403
RFHVEPP;SLEGKLP	NCP	10.765
SLEGKLP;KLGVVAAP	NCP	25.071
KLGVVAAP;LSLNVSKP	NCP	30.513
LSLNVSKP;EHLKTTL	NCP	16.662
EHLKTTL;VHGGALF	NCP	1.187
VHGGALF;KPPTVPKS	NCP	49.821

KPPTVPKS;SPPKVTSP	NCP	197.641
SPPKVTSP;MPNLLL	NCP	161.603
MPNLLL;GTVAL	NCP	108.555
GTVAL;KTVMENF	NCP	59.599
KTVMENF;LSKFF	NCP	36.524
LSKFF;ALQHADK	NCP	67.669
ALQHADK;KTLGELLK	NCP	90.237
KTLGELLK;VDLLRPK	NCP	107.658
VDLLRPK;VPLTTEY	NCP	23.7
VPLTTEY;KCCLLLH	NCP	7.68
KCCLLLH;PNAASPNP	NCP	161.456
PNAASPNP;ESTMLEK	NCP	79.206
ESTMLEK;PNMLLR	NCP	1.68
PNMLLR;KKTFYK	NCP	84.058
KKTFYK;RPQYGLS	NCP	93.906
RPQYGLS;PFLYR	NCP	64.194
PFLYR;KPQTVPK	NCP	51.74
KPQTVPK;QSTFPAST	NCP	7.665
QSTFPAST;KYFQPPP	NCP	75.867
KYFQPPP;KPKPPKL	NCP	106.497
KPKPPKL;MKPLLL	NCP	4.644
MKPLLL;KDEFFP	NCP	142.084
KDEFFP;DSGVTPY	NCP	2.571
DSGVTPY;KYTSRY	NCP	40.428
KYTSRY;STAHTYG	NCP	32.835
STAHTYG;LERPTY	NCP	79.604
LERPTY;VLDVVKR	NCP	115.602
VLDVVKR;LYFVGHL	NCP	29.427
LYFVGHL;CCMGK	NCP	40.108
CCMGK;HSPTLL	NCP	14.205
HSPTLL;HESFRK	NCP	54.317
HESFRK;KECAPK	NCP	41.034
KECAPK;GNDEVR	NCP	10.852
GNDEVR;FLFGTLPP	NCP	15.717
FLFGTLPP;ENTGKPSQ	NCP	20.646
ENTGKPSQ;DPPLYP	NCP	25.47
DPPLYP;GHAARAAP	NCP	58.472
GHAARAAP;PARGPP	NCP	0.021
PARGPP;SPAAAYQ	NCP	23.136
SPAAAYQ;TALKLR	NCP	109.857
TALKLR;QTKAPK	NCP	193.09
QTKAPK;KPGVP	NCP	45.705
KPGVP;KDAAP	NCP	57.341
KDAAP;VSGPLR	NCP	11.206
VSGPLR;EDAASNK	NCP	49.491
EDAASNK;MEQVNELK	NCP	5.817
MEQVNELK;KGDYGQCRELCEK	NCP	0.813
KGDYGQCRELCEK;DYGQCRELCEKA	NCP	0.006
DYGQCRELCEKA;LLYFAPG	NCP	53.009
LLYFAPG;ELLFK	NCP	11.233
ELLFK;TVFKVT	NCP	37.241

TVFKVT;LRQMKLH	NCP	37.872
LRQMKLH;FYEAFSK	NCP	126.364
FYEAFSK;MYQFLF	NCP	68.591
MYQFLF;KSAGVVPG	NCP	84.462
KSAGVVPG;ATVQLSR	NCP	5.889
ATVQLSR;PPPRYK	NCP	82.743
PPPRYK;CSELGAM	NCP	25.632
CSELGAM;GFHLSLR	NCP	77.158
GFHLSLR;SCVEEVDAAMCARPHKVD	NCP	46.379
SCVEEVDAAMCARPHKVD;SVKPGAHLTVKK	NCP	0.177
SVKPGAHLTVKK;DALLVRY	NCP	106.731
DALLVRY;LVELFP	NCP	77.562
LVELFP;KNCVHTKK	NCP	24.481
KNCVHTKK;QASVPLLK	NCP	169.349
QASVPLLK;KVNHKAK	NCP	17.613
KVNHKAK;LGPEF	NCP	5.344
LGPEF;KAHALKF	NCP	8.756
KAHALKF;MLYVY	NCP	95.058
MLYVY;QTPKVPKG	NCP	47.934
QTPKVPKG;LFLKLLQ	NCP	22.135
LFLKLLQ;NKNHRPP	NCP	116.127
NKNHRPP;LDDLRKDL	NCP	5.124
LDDLRKDL;FEDLKSM	NCP	82.212
FEDLKSM;KNRVVL	NCP	23.049
KNRVVL;LSAALKAK	NCP	16.745
LSAALKAK;NSLTLL	NCP	40.96
NSLTLL;VSDVVPD	NCP	452.003
VSDVVPD;MHVMGP	NCP	41.913
MHVMGP;WKFPYPQ	NCP	74.445
WKFPYPQ;MPKCGP	NCP	49.692
MPKCGP;TLDLKN	NCP	46.746
TLDLKN;AALLEY	NCP	4.725
AALLEY;CDDMC	NCP	46.212
CDDMC;LSVGGPM	NCP	67.689
LSVGGPM;FKVSPPPPP	NCP	7.051
FKVSPPPPP;FSLVKHR	NCP	72.239
FSLVKHR;GPVVHVK	NCP	51.847
GPVVHVK;KERSQPK	NCP	11.88
KERSQPK;VYFNGKL	NCP	11.411
VYFNGKL;ACGLLG	NCP	95.005
ACGLLG;HLLLTTKK	NCP	45.134
HLLLTTKK;LFDNFAL	NCP	0.232
LFDNFAL;DGTELLK	NCP	127.433
DGTELLK;VPVELL	NCP	118.902
VPVELL;RLTLNM	NCP	41.976
RLTLNM;PPPVAKPP	NCP	27.052
PPPVAKPP;KRTKPHY	NCP	4.538
KRTKPHY;KPLAK	NCP	7.938
KPLAK;NGSFF	NCP	57.265
NGSFF;LVLKPMT	NCP	111.536
LVLKPMT;KTGPSLTKK	NCP	48.099

KTGPSLTKK;KQRVAPK	NCP	25.45
KQRVAPK;ALEFVAR	NCP	47.193
ALEFVAR;RKLPLK	NCP	36.389
RKLPLK;STLFF	NCP	37.435
STLFF;WQNAY	NCP	20.069
WQNAY;KELRELR	NCP	72.552
KELRELR;MQKPEAT	NCP	13.74
MQKPEAT;GKTFY	NCP	1.203
GKTFY;KTYLGAK	NCP	67.285
KTYLGAK;VPPLPR	NCP	5.322
VPPLPR;VDPVLS	NCP	66.026
VDPVLS;LLSYVSK	NCP	11.025
LLSYVSK;VPNKLSHK	NCP	93.694
VPNKLSHK;GVFHG	NCP	15.06
GVFHG;DPGKPNL	NCP	81.819
DPGKPNL;LLAPEPR	NCP	67.059
LLAPEPR;FVLLN	NCP	54.468
FVLLN;KPLVT	NCP	25.863
KPLVT;EVWLHQ	NCP	8.601
EVWLHQ;CCCPP	NCP	51
CCCPP;TKRSHL	NCP	10.895
TKRSHL;KTTTYPF	NCP	103.084
KTTTYPF;APGSRPKP	NCP	27.248
APGSRPKP;WGHTPR	NCP	172.315
WGHTPR;MGMAPRPP	NCP	38.574
MGMAPRPP;QKPDL	NCP	18.147
QKPDL;KHCDPPP	NCP	10.992
KHCDPPP;MEGHDPKEPEQLRK	NCP	68.123
MEGHDPKEPEQLRK;HDPKEPEQLR	NCP	0.009
HDPKEPEQLR;PKEPEQLRK	NCP	0.006
PKEPEQLRK;PKFFNP	NCP	3.87
PKFFNP;LPPTVPP	NCP	135.859
LPPTVPP;APAYGP	NCP	84.282
APAYGP;AVLFDT	NCP	50.246
AVLFDT;GPSVVKSR	NCP	8.929
GPSVVKSR;LKLELGR	NCP	64.785
LKLELGR;GPPPAGRPPP	NCP	10.182
GPPPAGRPPP;KSTELLLR	NCP	19.068
KSTELLLR;KLKTVLLK	NCP	25.028
KLKTVLLK;QGKGVKPK	NCP	54.997
QGKGVKPK;ATKKAFA	NCP	19.797
ATKKAFA;KHLVDF	NCP	55.629
KHLVDF;VPSLP	NCP	25.704
VPSLP;KAAHATK	NCP	23.763
KAAHATK;TEPGLLH	NCP	12.911
TEPGLLH;VQPPPP	NCP	115.774
VQPPPP;SSAKGYK	NCP	197.823
SSAKGYK;TPVNQF	NCP	5.183
TPVNQF;ASPPP	NCP	0.859
ASPPP;TTKLLTK	NCP	54.32
TTKLLTK;DDGGH	NCP	37.693

DDGGH;TFSMKLP	NCP	33.441
TFSMKLP;YDSAPR	NCP	85.38
YDSAPR;GFLSQRT	NCP	8.586
GFLSQRT;SLGDKLNL	NCP	165.536
SLGDKLNL;EVLLA	NCP	62.395
EVLLA;KGFFKGP	NCP	24.701
KGFFKGP;VFGVNVN	NCP	13.098
VFGVNVN;KAMFKK	NCP	20.221
KAMFKK;LPFASKPP	NCP	8.649
LPFASKPP;QTHVGQQ	NCP	5.267
QTHVGQQ;RHVAPR	NCP	90.442
RHVAPR;TQFLP	NCP	16.011
TQFLP;RVLLGEMK	NCP	10.926
RVLLGEMK;LDSLLPP	NCP	0.507
LDSLLPP;PAATTPP	NCP	41.346
PAATTPP;CGQPVKL	NCP	17.715
CGQPVKL;AASCLLL	NCP	22.379
AASCLLL;KLLTTGM	NCP	17.31
KLLTTGM;PGLPV	NCP	16.738
PGLPV;KESVYSK	NCP	73.709
KESVYSK;AAPPAKPP	NCP	29.211
AAPPAKPP;VGVAPGVP	NCP	177.94
VGVAPGVP;RKVFKPK	NCP	138.527
RKVFKPK;VPYFSLR	NCP	86.278
VPYFSLR;AELARL	NCP	63.123
AELARL;KLLPKKET	NCP	3.362
KLLPKKET;NEKVLQGK	NCP	7.008
NEKVLQGK;VSLPLVPQ	NCP	2.178
VSLPLVPQ;LEKLQNHEK	NCP	3.261
LEKLQNHEK;HADSPLEK	NCP	87.849
HADSPLEK;LLLTLL	NCP	132.759
LLLTLL;KPFGP	NCP	46.405
KPFGP;SFLEHPLGSG	NCP	64.179
SFLEHPLGSG;TPTTK	NCP	35.765
TPTTK;LSREVDY	NCP	20.782
LSREVDY;ALNPKTC	NCP	12.146
ALNPKTC;LLLGK	NCP	63.951
LLLGK;SRALAEL	NCP	39.106
SRALAEL;MNMMLK	NCP	101.051
MNMMLK;KPPEPVP	NCP	11.941
KPPEPVP;EYRDSVK	NCP	150.755
EYRDSVK;GPPALMGP	NCP	16.261
GPPALMGP;MGMADM	NCP	113.271
MGMADM;TVLMG	NCP	10.935
TVLMG;FMGPK	NCP	7.062
FMGPK;KMSKTVKR	NCP	6.609
KMSKTVKR;TDFGLLN	NCP	140.138
TDFGLLN;ASVFEYK	NCP	10.233
ASVFEYK;NGHGPGPP	NCP	98.824
NGHGPGPP;APAPPFPK	NCP	42.693
APAPPFPK;LQTQGP	NCP	52.806

LQTQGP;PPTTL	NCP	2.997
PPTTL;DCKPCM	NCP	9.533
DCKPCM;LPAARTY	NCP	15.463
LPAARTY;RALKELW	NCP	23.973
RALKELW;PFPPPPR	NCP	55.974
PFPPPPR;TTMKPPP	NCP	4.329
TTMKPPP;KDALP	NCP	44.463
KDALP;VTVGSTPP	NCP	63.54
VTVGSTPP;AALLV	NCP	101.103
AALLV;SGTFPF	NCP	1.478
SGTFPF;PAPWELR	NCP	23.551
PAPWELR;LPPPDKYK	NCP	20.58
LPPPDKYK;GPPSPVGT	NCP	71.517
GPPSPVGT;KTGLAF	NCP	7.586
KTGLAF;PPPPLA	NCP	15.573
PPPPLA;KGKGHSR	NCP	10.017
KGKGHSR;SGPLNFT	NCP	51.291
SGPLNFT;MRDFC	NCP	92.248
MRDFC;EYLFDAY	NCP	63.155
EYLFDAY;LPDVMKP	NCP	134.989
LPDVMKP;KMTAPR	NCP	83.585
KMTAPR;DFFGGQ	NCP	8.653
DFFGGQ;NQLEP	NCP	7.868
NQLEP;MKASGTLREY	NCP	154.933
MKASGTLREY;KHTPPSK	NCP	12.344
KHTPPSK;QDLMH	NCP	18.525
QDLMH;GRGVLR	NCP	99.109
GRGVLR;EMDGMC	NCP	31.439
EMDGMC;KMVPSAK	NCP	67.414
KMVPSAK;THMPVPV	NCP	32.177
THMPVPV;DGMPLKK	NCP	27.532
DGMPLKK;KAHKYKQ	NCP	48.329
KAHKYKQ;TVSLLT	NCP	166.915
TVSLLT;ERTGNTKK	NCP	12.804
ERTGNTKK;KLQRMGP	NCP	44.742
KLQRMGP;LVYPTEK	NCP	44.078
LVYPTEK;PPQRGTPP	NCP	79.513
PPQRGTPP;TTSAK	NCP	12.642
TTSAK;KNLTRPP	NCP	13.635
KNLTRPP;KSMFRP	NCP	2.715
KSMFRP;AGPVP	NCP	3.525
AGPVP;KPAYPK	NCP	44.886
KPAYPK;ALPKVSAP	NCP	105.024
ALPKVSAP;ADYLRQQ	NCP	9.023
ADYLRQQ;KYFGPR	NCP	51.045
KYFGPR;LNNWLHE	NCP	154.356
LNNWLHE;QSKRSLL	NCP	89.692
QSKRSLL;LLDVLALP	NCP	13.776
LLDVLALP;TGPPAPR	NCP	6.807
TGPPAPR;RAGPGPP	NCP	13.194
RAGPGPP;PGPRPRP	NCP	37.626

PGPRPRP;PEAAYPS	NCP	112.38
PEAAYPS;TVRTTLH	NCP	11.625
TVRTTLH;RVDAERP	NCP	198.123
RVDAERP;FTSSHK	NCP	0.933
FTSSHK;NVAASKER	NCP	104.358
NVAASKER;RTVPSSY	NCP	29.645
RTVPSSY;PAGPGP	NCP	45.004
PAGPGP;KGSWVEGR	NCP	57.051
KGSWVEGR;VGGRPFH	NCP	59.34
VGGRPFH;FGYSKLK	NCP	34.832
FGYSKLK;TSRKKVL	NCP	57.73
TSRKKVL;AHQRFYH	NCP	13.449
AHQRFYH;NLLVLF	NCP	123.918
NLLVLF;RVYTLR	NCP	2.613
RVYTLR;VFTTVGGK	NCP	132.15
VFTTVGGK;LEVKLQ	NCP	153.531
LEVKLQ;KYTVH	NCP	26.129
KYTVH;VELPKPR	NCP	8.083
VELPKPR;KGRVGAAT	NCP	34.59
KGRVGAAT;SLSAPGAK	NCP	0.255
SLSAPGAK;PLLALR	NCP	10.125
PLLALR;TLGGAL	NCP	53.202
TLGGAL;AAVPDK	NCP	82.182
AAVPDK;LPRLEP	NCP	30.756
LPRLEP;MEYLAV	NCP	88.331
MEYLAV;KATTMR	NCP	67.056
KATTMR;YNPEPLK	NCP	22.267
YNPEPLK;NVVVSY	NCP	14.789
NVVVSY;PGASGQ	NCP	240.028
PGASGQ;PLEKPP	NCP	0.113
PLEKPP;SGVPVPP	NCP	91.39
SGVPVPP;SAAWLG	NCP	20.001
SAAWLG;VVTQFFR	NCP	12.572
VVTQFFR;KTEAAGRP	NCP	41.637
KTEAAGRP;TGPFQEK	NCP	95.619
TGPFQEK;KGPTAMK	NCP	5.209
KGPTAMK;RVAPP	NCP	8.328
RVAPP;PTDLSPPK	NCP	71.886
PTDLSPPK;MQLARK	NCP	22.137
MQLARK;LSVGVP	NCP	119.736
LSVGVP;MDRGAPR	NCP	47.295
MDRGAPR;GLGMEHC	NCP	134.361
GLGMEHC;PAATPP	NCP	101.982
PAATPP;NAVATTN	NCP	35.571
NAVATTN;RMMGGS	NCP	146.883
RMMGGS;TLVLRR	NCP	49.875
TLVLRR;LNQEVKY	NCP	3.71
LNQEVKY;KDPLDVTH	NCP	1.359
KDPLDVTH;KGSDSP	NCP	11.47
KGSDSP;KRDKLLVQ	NCP	36.945
KRDKLLVQ;KEVGPGK	NCP	12.177

KEVGPGK;AGGFAPYYG	NCP	50.954
AGGFAPYYG;TNTTFRH	NCP	13.12
TNTTFRH;ERGLGKGQK	NCP	3.875
ERGLGKGQK;RDAPGGLK	NCP	64.843
RDAPGGLK;KRMMVPP	NCP	37.755
KRMMVPP;AVDVP	NCP	0.327
AVDVP;LPPLP	NCP	15.264
LPPLP;DLDLRDK	NCP	170.252
DLDLRDK;LSFALR	NCP	13.189
LSFALR;NPVSL	NCP	4.133
NPVSL;PTSGSLP	NCP	4.486
PTSGSLP;KLELGPRR	NCP	129.102
KLELGPRR;YKLNQ	NCP	28.931
YKLNQ;SSLFP	NCP	44.767
SSLFP;GDACRLL	NCP	52.317
GDACRLL;PLTQPP	NCP	129.132
PLTQPP;MPPPVVV	NCP	35.63
MPPPVVV;SPRPPTR	NCP	76.783
SPRPPTR;VRHLEK	NCP	67.112
VRHLEK;KGPAVSPK	NCP	80.689
KGPAVSPK;MEFDL	NCP	115.283
MEFDL;NKVEP	NCP	66.655
NKVEP;ANAAFR	NCP	11.565
ANAAFR;ASALLE	NCP	22.08
ASALLE;SLTLAL	NCP	100.482
SLTLAL;PHALALM	NCP	70.631
PHALALM;KTVLMK	NCP	14.529
KTVLMK;LDYLLHS	NCP	37.47
LDYLLHS;KAAVFHG	NCP	6.1
KAAVFHG;NPPLKK	NCP	48.792
NPPLKK;YVLASR	NCP	21.554
YVLASR;EVWLK	NCP	90.693
EVWLK;SGNPYP	NCP	4.609
SGNPYP;VGGTVP	NCP	37.983
VGGTVP;VGLRF	NCP	2.108
VGLRF;EGSMSE	NCP	14.464
EGSMSE;PAAAAPAPAP	NCP	50.843
PAAAAPAPAP;ATLAGNM	NCP	32.689
ATLAGNM;EGDTSSK	NCP	60.999
EGDTSSK;LSLGNVVK	NCP	18.33
LSLGNVVK;VPAAWAP	NCP	12.27
VPAAWAP;KVHVAC	NCP	0.731
KVHVAC;LSGSAPLR	NCP	85.737
LSGSAPLR;PGPLP	NCP	32.946
PGPLP;AALAKTR	NCP	23.857
AALAKTR;NPVLAFM	NCP	6.813
NPVLAFM;ACKPQR	NCP	90.872
ACKPQR;GPAEALY	NCP	9.379
GPAEALY;MGMTVPR	NCP	39.495
MGMTVPR;AEFMVL	NCP	131.157
AEFMVL;KQMSTAVP	NCP	7.244

KQMSTAVP;TPSGSLP	NCP	14.535
TPSGSLP;MLRPPGP	NCP	46.498
MLRPPGP;PPNPPPPP	NCP	20.843
PPNPPPPP;RRLLEP	NCP	24.733
RRLLEP;CGLLLLGL	NCP	11.58
CGLLLLGL;LMCQPP	NCP	72.188
LMCQPP;SSMLLE	NCP	0.828
SSMLLE;TLPPMDK	NCP	2.053
TLPPMDK;ESPAGLHT	NCP	6.017
ESPAGLHT;MGKALPP	NCP	34.515
MGKALPP;GAPSAPT	NCP	32.949
GAPSAPT;SSGPKSSG	NCP	81.849
SSGPKSSG;MTKKLPFL	NCP	45.795
MTKKLPFL;AAVPEVPK	NCP	33.091
AAVPEVPK;RQKLAL	NCP	6.233
RQKLAL;PGSPGGAPR	NCP	165.94
PGSPGGAPR;GATVKGPK	NCP	78.528
GATVKGPK;GPPKPFF	NCP	6.32
GPPKPFF;KETPFP	NCP	24.664
KETPFP;STEYKAKDDTQVK	NCP	30.005
STEYKAKDDTQVK;KGPQSP	NCP	4.231
KGPQSP;NYVAQEVDDYHAF	NCP	15.134
NYVAQEVDDYHAF;SLFKEVFESSKMDF	NCP	34.242
SLFKEVFESSKMDF;MVESVRTPY	NCP	8.304
MVESVRTPY;MLAPL	NCP	83.89
MLAPL;AALLPPR	NCP	49.304
AALLPPR;VGPLVP	NCP	53.662
VGPLVP;PTARPP	NCP	35.322
PTARPP;QVAFP	NCP	63.188
QVAFP;LVVLP	NCP	77.305
LVVLP;KKPPLL	NCP	9.474
KKPPLL;DCEGP	NCP	32.003
DCEGP;QGQGP	NCP	5.457
QGQGP;AEQLSPGK	NCP	8.038
AEQLSPGK;VDKHTK	NCP	22.098
VDKHTK;LGSKNKL	NCP	31.962
LGSKNKL;PDVGNMT	NCP	12.623
PDVGNMT;PAKGLSR	NCP	137.464
PAKGLSR;KPRGAVY	NCP	89.541
KPRGAVY;LSLERL	NCP	57.179
LSLERL;KFGGP	NCP	35.479
KFGGP;MLAPNTL	NCP	11.768
MLAPNTL;FTECSL	NCP	9.211
FTECSL;EGPPSLR	NCP	15.657
EGPPSLR;KAGETLRK	NCP	74.721
KAGETLRK;VSTAK	NCP	87.39
VSTAK;TTEHPGVP	NCP	13.902
TTEHPGVP;VPYTPPPP	NCP	19.578
VPYTPPPP;PRPPEY	NCP	24.945
PRPPEY;QGPALLRP	NCP	81.084
QGPALLRP;VTALYL	NCP	29.441

VTALYL;DSKPAAP	NCP	1.21
DSKPAAP;PGVFP	NCP	24.309
PGVFP;NVLPTL	NCP	33.708
NVLPTL;PGPVL	NCP	6.755
PGPVL;LRPPLL	NCP	85.984
LRPPLL;EDHAMC	NCP	27.059
EDHAMC;KDMDLK	NCP	0.889
KDMDLK;RLFGPGAL	NCP	1.23
RLFGPGAL;AFVTVGP	NCP	102.845
AFVTVGP;LDVLV	NCP	78.97
LDVLV;SVTPVLR	NCP	93.585
SVTPVLR;RAADPVP	NCP	24.069
RAADPVP;NFFFSLP	NCP	61.547
NFFFSLP;DDSLLLF	NCP	77.692
DDSLLLF;PGKMRGP	NCP	6.546
PGKMRGP;LACFP	NCP	43.203
LACFP;PLLHR	NCP	14.712
PLLHR;LERLVDY	NCP	39.474
LERLVDY;GVVHL	NCP	5.367
GVVHL;KDGLWHH	NCP	239.034
KDGLWHH;FPTRPPP	NCP	37.992
FPTRPPP;GFWKAP	NCP	30.27
GFWKAP;REGAPLL	NCP	25.866
REGAPLL;KGVGVLLA	NCP	13.818
KGVGVLLA;KYGEC	NCP	0.443
KYGEC;LGPTSAPK	NCP	47.526
LGPTSAPK;ASSAVYK	NCP	31.147
ASSAVYK;QEKEPK	NCP	85.571
QEKEPK;GPPVSGPPL	NCP	9.25
GPPVSGPPL;KQAGTAPP	NCP	94.287
KQAGTAPP;RPVVPR	NCP	12.219
RPVVPR;GDTKPLSK	NCP	10.902
GDTKPLSK;TPHKPP	NCP	40.46
TPHKPP;AGPSFPSH	NCP	12.696
AGPSFPSH;YGPLP	NCP	27.523
YGPLP;TVFTVGGP	NCP	21.651
TVFTVGGP;RQTLRVL	NCP	81.84
RQTLRVL;KSMPQKK	NCP	34.257
KSMPQKK;EATPKLSP	NCP	22.701
EATPKLSP;QAPLSP	NCP	8.598
QAPLSP;GPPSAP	NCP	16.004
GPPSAP;PATKPQQ	NCP	14.685
PATKPQQ;PGVKLLA	NCP	6.483
PGVKLLA;ESPASLQR	NCP	41.332
ESPASLQR;ESGPSPP	NCP	30.669
ESGPSPP;LERVCLL	NCP	20.849
LERVCLL;AEAGPLLH	NCP	1.579
AEAGPLLH;GVPPEC	NCP	220.611
GVPPEC;DGGPLL	NCP	12.573
DGGPLL;VERPQL	NCP	161.577
VERPQL;SLEVPF	NCP	68.897

SLEVPF;LLDGGMK	NCP	23.509
LLDGGMK;KNGVVTK	NCP	76.788
KNGVVTK;LGCLL	NCP	37.454
LGCLL;KGARTPPP	NCP	27.58
KGARTPPP;LSLPAVFF	NCP	13.098
LSLPAVFF;KEGNKK	NCP	23.387
KEGNKK;FPVHSKP	NCP	40.413
FPVHSKP;KTYMGLVF	NCP	24.135
KTYMGLVF;KPAWHHT	NCP	170.827
KPAWHHT;MSLLRTER	NCP	127.241
MSLLRTER;LEYAAVL	NCP	32.88
LEYAAVL;SLMLPN	NCP	1.528
SLMLPN;RSKFFV	NCP	118.667
RSKFFV;VAPPARP	NCP	40.035
VAPPARP;KERPYEDL	NCP	81.429
LELEAPAA;DLASRDVVSR	NCP	28.503
DLASRDVVSR;PDPPLP	NCP	67.012
PDPPLP;DASRALLR	NCP	33.411
DASRALLR;KDLEPL	NCP	34.071
KDLEPL;FKVSLP	NCP	23.604
FKVSLP;MFTGDC	NCP	0.524
MFTGDC;LEERLR	NCP	10.032
LEERLR;KGAPGKT	NCP	24.405
KGAPGKT;GVPLTASP	NCP	0.042
GVPLTASP;EQAGPG	NCP	5.7
EQAGPG;PVTSRPPP	NCP	37.392
PVTSRPPP;WVSGGLLL	NCP	67.167
WVSGGLLL;PPPLRYK	NCP	18.306
PPPLRYK;LHVQS	NCP	22.144
LHVQS;RLLFQEK	NCP	18.74
RLLFQEK;EHLPFHPL	NCP	12.472
EHLPFHPL;WTPGP	NCP	17.153
WTPGP;KPRVGAK	NCP	3.096
KPRVGAK;MDEAVGDLK	NCP	26.415
MDEAVGDLK;LPYPQLLKY	NCP	43.321
LPYPQLLKY;GLSRDPLL	NCP	63.896
GLSRDPLL;TAAPVTR	NCP	30.939
TAAPVTR;HTPAVP	NCP	58.785
HTPAVP;WRAPGWP	NCP	5.991
WRAPGWP;AAEVLL	NCP	50.139
AAEVLL;TQRPAFK	NCP	15.345
TQRPAFK;ELAAPQ	NCP	37.78
ELAAPQ;VVVVHK	NCP	26.115
VVVVHK;QQGSKQGP	NCP	29.37
QQGSKQGP;EPGPAL	NCP	19.133
EPGPAL;SVGVLP	NCP	49.959
SVGVLP;KLLEARS	NCP	62.134
KLLEARS;RAPVTAAP	NCP	3.041
RAPVTAAP;KDHSLVTL	NCP	10.261
KDHSLVTL;NSDDC	NCP	48.206
NSDDC;QAPLH	NCP	147

QAPLH;DPLGLAP	NCP	103.995
DPLGLAP;DLLGP	NCP	61.318
DLLGP;WTLSVH	NCP	1.229
WTLSVH;VPAAPP	NCP	93.732
VPAAPP;LRAAVLAK	NCP	29.991
LRAAVLAK;KPGPL	NCP	2.982
KPGPL;ALHPPP	NCP	26.817
ALHPPP;ERLVHLQ	NCP	19.29
ERLVHLQ;PTQTLPSR	NCP	46.696
PTQTLPSR;KPYTGPP	NCP	20.928
KPYTGPP;LLSWY	NCP	30.618
LLSWY;LSLLLRNL	NCP	7.014
LSLLLRNL;FGGMPAP	NCP	63.66
FGGMPAP;EAVHRPP	NCP	12.617
EAVHRPP;PNLPP	NCP	43.723
PNLPP;WPSFVAP	NCP	57.842
WPSFVAP;RVHGGVR	NCP	67.519
RVHGGVR;KEAQTLK	NCP	97.139
KEAQTLK;HSGLDFK	NCP	41.815
HSGLDFK;AYLDF	NCP	1.602
AYLDF;SLTPK	NCP	11.963
SLTPK;APKGGPA	NCP	6.487
APKGGPA;PYPVVPP	NCP	45.498
PYPVVPP;DEAPP	NCP	4.218
DEAPP;LENPHAK	NCP	81.66
LENPHAK;RVDRAEP	NCP	55.73
RVDRAEP;SAALKKPS	NCP	37.821
SAALKKPS;VGGPSSSF	NCP	18.507
VGGPSSSF;VVLLL	NCP	23.079
VVLLL;AQVRVY	NCP	9.46
AQVRVY;KPRDLP	NCP	4.175
KPRDLP;KKLHPP	NCP	15.516
KKLHPP;RGLDAQGHA	NCP	81.975
RGLDAQGHA;LGSSYG	NCP	70.666
LGSSYG;RQVWVSPEK	NCP	9.51
RQVWVSPEK;HANLLP	NCP	3.977
HANLLP;QPSFP	NCP	38.877
QPSFP;DLLLK	NCP	5.578
DLLLK;ELSVDPF	NCP	24.077
ELSVDPF;TVTMVHQ	NCP	84.405
TVTMVHQ;KPFAAGH	NCP	22.368
KPFAAGH;CCDQT	NCP	179.422
CCDQT;AVALLT	NCP	44.183
AVALLT;EEGLPK	NCP	46.152
EEGLPK;MRLPKAL	NCP	75.606
MRLPKAL;LVEAPSLR	NCP	11.218
LVEAPSLR;QEPGPPK	NCP	58.592
QEPGPPK;MHQSF	NCP	88.644
MHQSF;AFQVH	NCP	26.191
AFQVH;LDLGSLNF	NCP	121.392
LDLGSLNF;AALAFRK	NCP	188.612

AALAFRK;KHNNPR	NCP	20.926
KHNNPR;PAELPTLK	NCP	158.592
PAELPTLK;LAPPFS	NCP	37.527
LAPPFS;GPPVSEL	NCP	13.56
GPPVSEL;GPPVSELRT	NCP	0.096
GPPVSELRT;LHLVL	NCP	34.266
LHLVL;LPVTLPP	NCP	35.211
LPVTLPP;GRHLYP	NCP	29.739
GRHLYP;AAAQPPPP	NCP	48.831
AAAQPPPP;AHFGKPP	NCP	33.756
AHFGKPP;QAGKRPL	NCP	86.223
QAGKRPL;SVMMPL	NCP	18.678
SVMMPL;LLKPMP	NCP	42.794
LLKPMP;TTSLLAAP	NCP	8.05
TTSLLAAP;KGANAPR	NCP	158.364
KGANAPR;TVGGLLA	NCP	10.776
TVGGLLA;RGELVK	NCP	129.99
RGELVK;HPERLTP	NCP	22.788
HPERLTP;KRGHPPLGP	NCP	20.256
KRGHPPLGP;HGLAKLY	NCP	31.869
HGLAKLY;GKLLG	NCP	9.942
GKLLG;APLKVG	NCP	38.73
APLKVG;GGPVSLPR	NCP	3.591
GGPVSLPR;RPGGLEK	NCP	16.212
RPGGLEK;VPGNEP	NCP	19.593
VPGNEP;CPHSSML	NCP	49.863
CPHSSML;GVSYLK	NCP	124.391
GVSYLK;LLKSPR	NCP	111.49
LLKSPR;AAEHRVP	NCP	10.029
AAEHRVP;TYPRP	NCP	44.957
TYPRP;PNPPPPP	NCP	109.591
PNPPPPP;GVPLDLY	NCP	58.476
GVPLDLY;VPLSSPE	NCP	14.984
VPLSSPE;YSFTWKP	NCP	30.432
YSFTWKP;KPCGPAK	NCP	103.446
KPCGPAK;KGLLGGLA	NCP	12.733
KGLLGGLA;KDPLCFK	NCP	9.14
KDPLCFK;LKTDLPPP	NCP	51.534
LKTDLPPP;SRGPK	NCP	54.504
SRGPK;SLKPF	NCP	17.349
SLKPF;PGLLGVP	NCP	124.386
PGLLGVP;SSLLNPR	NCP	11.796
SSLLNPR;DHGSPVLL	NCP	23.943
DHGSPVLL;VPPLFWP	NCP	17.827
VPPLFWP;QGPRVVK	NCP	49.607
QGPRVVK;KTTAFHV	NCP	24.834
KTTAFHV;GYPEV	NCP	75.444
GYPEV;KWLSGTL	NCP	3.586
KWLSGTL;DHKPMC	NCP	25.079
DHKPMC;LSKKHYDP	NCP	93.043
LSKKHYDP;LPKTLPP	NCP	79.683

LPKTLPP;RPTAVAM	NCP	140.318
RPTAVAM;AVVTKPGK	NCP	68.812
AVVTKPGK;EGFKDK	NCP	25.407
EGFKDK;KSEPFF	NCP	41.753
KSEPFF;QEVPHK	NCP	84.529
QEVPHK;KFGPSRP	NCP	37.92
KFGPSRP;VPPFT	NCP	51.758
VPPFT;LSSTF	NCP	36.64
LSSTF;LLYPTHY	NCP	23.352
LLYPTHY;EPLNKDS	NCP	37.119
EPLNKDS;YLRWGFP	NCP	269.943
YLRWGFP;PAVLGF	NCP	69.131
PAVLGF;VVADLRP	NCP	34.348
VVADLRP;MLALKKK	NCP	98.666
MLALKKK;HFPEVGP	NCP	18.432
HFPEVGP;KSQVPR	NCP	159.525
KSQVPR;KPVSEEY	NCP	5.641
KPVSEEY;KGPVLYF	NCP	46.248
KGPVLYF;YDLNKL	NCP	2.069
YDLNKL;CNGAVP	NCP	27.739
CNGAVP;TVLLK	NCP	27.74
TVLLK;LHKTMAK	NCP	3.376
LHKTMAK;KPPGEKR	NCP	55.725
KPPGEKR;KDVLPVLQ	NCP	35.501
KDVLPVLQ;LSDHFQVSK	NCP	73.429
LSDHFQVSK;LKPVER	NCP	14.783
LKPVER;TVMVMPR	NCP	165.127
TVMVMPR;CAPGLCP	NCP	6.294
CAPGLCP;NYGHRK	NCP	7.809
NYGHRK;HTKTRK	NCP	60.39
HTKTRK;MSPPGA	NCP	40.443
MSPPGA;KPVSEHK	NCP	36.038
KPVSEHK;PPPVAPKP	NCP	105.442
PPPVAPKP;VGELGLK	NCP	27.605
VGELGLK;FPPLE	NCP	34.042
FPPLE;TRRYNDF	NCP	49.838
TRRYNDF;NHLLFLPE	NCP	65.121
NHLLFLPE;KPLLNV	NCP	51.39
KPLLNV;CGYKVLL	NCP	47.671
CGYKVLL;GYGALGP	NCP	97.365
GYGALGP;QTGSLLGKK	NCP	1.709
QTGSLLGKK;EENPLR	NCP	0.769
EENPLR;GAPLHPR	NCP	54.797
GAPLHPR;YKPPPPP	NCP	5.443
YKPPPPP;FYPRVP	NCP	181.095
FYPRVP;PALVK	NCP	129.924
PALVK;TQDLSGPK	NCP	3.876
TQDLSGPK;HHLLGK	NCP	98.27
HHLLGK;CFVYK	NCP	144.589
CFVYK;HPVDTAK	NCP	30.821
HPVDTAK;VKSMC	NCP	34.663

VKSMC;PALPDL	NCP	1.457
PALPDL;ASVGTLAFDE	NCP	50.49
ASVGTLAFDE;NSALLR	NCP	111.67
NSALLR;HPKKLL	NCP	20.4
HPKKLL;KSHKFTEK	NCP	145.698
KSHKFTEK;LPASKDLG	NCP	34.524
LPASKDLG;SPKSLPYL	NCP	5.253
SPKSLPYL;KSTLSK	NCP	3.141
KSTLSK;KNEQLR	NCP	22.764
KNEQLR;RPDSLPQL	NCP	26.654
RPDSLPQL;RMRQPK	NCP	18.78
RMRQPK;KCAAP	NCP	343.911
KCAAP;WRFEPY	NCP	64.999
WRFEPY;EPNDN	NCP	19.595
EPNDN;ASGSFFPK	NCP	95.244
ASGSFFPK;YDSGC	NCP	10.098
YDSGC;VLLPKTK	NCP	28.468
VLLPKTK;EPLMSK	NCP	4.235
EPLMSK;PSCCC	NCP	6.636
PSCCC;NACCC	NCP	83.199
NACCC;NGSVHF	NCP	9.661
NGSVHF;SPPDLP	NCP	4.367
SPPDLP;DSTPY	NCP	98.281
DSTPY;TNYTVP	NCP	18.185
TNYTVP;MHAAPP	NCP	17.281
MHAAPP;PLPVA	NCP	79.59
PLPVA;VLLKK	NCP	15.173
VLLKK;TAGLK	NCP	231.558
TAGLK;QAGKFVH	NCP	53.95
QAGKFVH;NFHFTR	NCP	99.266
NFHFTR;KNLMPLY	NCP	41.743
KNLMPLY;VTPLDKT	NCP	130.59
VTPLDKT;PLAMGPLP	NCP	48.998
PLAMGPLP;LGDKPPP	NCP	20.863
LGDKPPP;ALALAF	NCP	206.726
ALALAF;YCCCCEC	NCP	122.337
YCCCCEC;NGPAPSPR	NCP	6.016
NGPAPSPR;AATPSELP	NCP	6.087
AATPSELP;FYPPV	NCP	80.159
FYPPV;TPTATPLT	NCP	11.992
TPTATPLT;KSGPARP	NCP	13.236
KSGPARP;QEYSGKR	NCP	4.809
QEYSGKR;GNAVPF	NCP	294.137
GNAVPF;NSPPLPK	NCP	121.183
NSPPLPK;KNRLAL	NCP	6.839
KNRLAL;LSYVY	NCP	297.567
LSYVY;KPSQPPPL	NCP	180.31
KPSQPPPL;NPGGGNF	NCP	187.986
NPGGGNF;TVLTRCNK	NCP	35.615
TVLTRCNK;LPMLQA	NCP	69.343
LPMLQA;EETPGAGN	NCP	39.734

EETPGAGN;TLFNAP	NCP	61.053
TLFNAP;HRDPPVP	NCP	57.786
HRDPPVP;GLVDL	NCP	27.163
GLVDL;VLAAFH	NCP	68.378
VLAAFH;KPPKYVP	NCP	65.551
KPPKYVP;LASLRLP	NCP	9.317
LASLRLP;VPEAAPK	NCP	68.994
VPEAAPK;VSLSV	NCP	54.427
VSLSV;KPYAFPP	NCP	53.507
KPYAFPP;KVALLPD	NCP	84.249
KVALLPD;LGGHSMK	NCP	148.644
LGGHSMK;KVMAM	NCP	0.435
KVMAM;KWRTGALF	NCP	67.726
KWRTGALF;KSPRFM	NCP	38.453
KSPRFM;AALGAY	NCP	145.497
AALGAY;KDMAVAP	NCP	30.537
KDMAVAP;MPDDLR	NCP	31.465
MPDDLR;PLAGDLK	NCP	64.382
PLAGDLK;HLLSSRP	NCP	8.886
HLLSSRP;QAPNRLT	NCP	46.681
QAPNRLT;MVKGY	NCP	99.72
MVKGY;FSLAGT	NCP	2.145
FSLAGT;VRGGSAPP	NCP	147.317
VRGGSAPP;GKVLCC	NCP	15.3
GKVLCC;LPLCLVLLLH	NCP	76.725
LPLCLVLLLH;LPWKW	NCP	133.611
LPWKW;FRWKL	NCP	95.932
FRWKL;KPGAQEKQ	NCP	32.46
KPGAQEKQ;KYFEK	NCP	17.726
KYFEK;GEHLLF	NCP	93.49
GEHLLF;KELWHHS	NCP	46.202
KELWHHS;VYVPTVK	NCP	25.203
VYVPTVK;TDDLPLNL	NCP	63.618
TDDLPLNL;TDKSYL	NCP	44.389
TDKSYL;LGAFSSLY	NCP	41.642
LGAFSSLY;NSPFK	NCP	73.369
NSPFK;MLRKAPL	NCP	91.122
MLRKAPL;MEPGPNL	NCP	155.483
MEPGPNL;LLTDFR	NCP	199.624
LLTDFR;KGELSRP	NCP	73.292
KGELSRP;YSVGGKGKK	NCP	40.471
YSVGGKGKK;QGKDKK	NCP	23.118
QGKDKK;GMYMP	NCP	431.205
GMYMP;LLMNY	NCP	5.039
LLMNY;KFVVPPP	NCP	109.326
KFVVPPP;EVPNLP	NCP	129.987
EVPNLP;ENEAQAAAETPEVK	NCP	49.44
ENEAQAAAETPEVK;ASEAEAAEPAAPTKDDKSK	NCP	0.258
ASEAEAAEPAAPTKDDKSK;SDGAPASDSKPSSTEAAPSS	NCP	0.117
SDGAPASDSKPSSTEAAPSSK;KNTAHDV	NCP	30.526
KNTAHDV;KLVPLRLG	NCP	21.42

KLVPLRLG;VAVFF	NCP	10.836
VAVFF;DLKLGL	NCP	49.091
DLKLGL;LLPFLE	NCP	52.9
LLPFLE;LSLPEAL	NCP	127.511
LSLPEAL;KGFLV	NCP	0.513
KGFLV;EHLTLPPE	NCP	78.234
EHLTLPPE;MKVKKT	NCP	9.838
MKVKKT;KLGGALL	NCP	9.725
KLGGALL;VEYTLPK	NCP	155.2
VEYTLPK;SVDVVETV	NCP	51.9
SVDVVETV;KSGKPAK	NCP	71.285
KSGKPAK;GVSPLK	NCP	47.299
GVSPLK;LPGYP	NCP	37.05
LPGYP;STTVVPR	NCP	156.783
STTVVPR;VSLVK	NCP	5.435
VSLVK;RVMKKE	NCP	29.229
RVMKKE;KKVSDH	NCP	14.578
KKVSDH;KKPKP	NCP	37.761
KKPKP;PFQSQK	NCP	50.136
PFQSQK;FETPV	NCP	26.481
FETPV;LTYSLGN	NCP	10.593
LTYSLGN;ALLLVAN	NCP	4.275
ALLLVAN;VAPFFK	NCP	65.055
VAPFFK;KPGAGK	NCP	43.533
KPGAGK;KELLLF	NCP	12.948
KELLLF;GTYGK	NCP	93.696
GTYGK;VAFLDQ	NCP	42.327
VAFLDQ;LLKFPH	NCP	46.448
LLKFPH;PFKVALD	NCP	58.93
PFKVALD;DFSGSLP	NCP	124.773
DFSGSLP;DLTNLK	NCP	35.658
DLTNLK;KVFQPK	NCP	203.822
KVFQPK;HSQPNEK	NCP	58.462
HSQPNEK;VHPEAR	NCP	34.239
VHPEAR;ASGVHVPF	NCP	76.002
ASGVHVPF;GDFSQAR	NCP	52.517
GDFSQAR;MLLQPPGP	NCP	18.93
MLLQPPGP;PVGPP	NCP	49.129
PVGPP;YTSLGSAL	NCP	116.156
YTSLGSAL;KVVSSAVH	NCP	59.409
KVVSSAVH;SKLLFK	NCP	146.238
SKLLFK;PDHRK	NCP	43.852
PDHRK;ESMDM	NCP	31.937
ESMDM;AAGRVGLH	NCP	11.289
AAGRVGLH;KVVAAPK	NCP	58.335
KVVAAPK;KLQFPVGR	NCP	125.227
KLQFPVGR;AAVLK	NCP	61.686
AAVLK;MSCYPPP	NCP	37.587
MSCYPPP;LSLLNTF	NCP	430.83
LSLLNTF;PLYSQEY	NCP	73.878
PLYSQEY;KGFLSRLK	NCP	631.85

KGFLSRLK;KEMGVTV	NCP	15.601
KEMGVTV;DLLAL	NCP	113.016
DLLAL;PPMYALKR	NCP	9.699
PPMYALKR;KEVDGAP	NCP	185.109
KEVDGAP;WQQLVH	NCP	33.878
WQQLVH;LDLTGKN	NCP	122.313
LDLTGKN;ANQVLK	NCP	24.895
ANQVLK;TELKY	NCP	32.664
TELKY;FSKGKPP	NCP	33.164
FSKGKPP;LVPVEP	NCP	100.573
LVPVEP;ETFSGELSK	NCP	2.781
ETFSGELSK;MPLRLQ	NCP	4.515
MPLRLQ;TMEAYVN	NCP	87.485
TMEAYVN;PSTKYPP	NCP	196.138
PSTKYPP;KYLHFGT	NCP	202.653
KYLHFGT;HYGLLAFFLE	NCP	217.523
HYGLLAFFLE;LVLTML	NCP	208.963
LVLTML;MLAHVL	NCP	17.075
MLAHVL;VPLSLGP	NCP	15.508
VPLSLGP;TNDLSLPK	NCP	54.267
TNDLSLPK;LAHTC	NCP	155.262
LAHTC;LQKRSP	NCP	9.428
LQKRSP;EVEVVR	NCP	77.682
EVEVVR;ASSVLSF	NCP	61.392
ASSVLSF;VTPLFSE	NCP	4.174
VTPLFSE;VGFPLK	NCP	118.884
VGFPLK;FALNSNSG	NCP	55.296
FALNSNSG;VPDPNK	NCP	247.791
VPDPNK;MENEELL	NCP	92.133
MENEELL;VTPSVKP	NCP	14.202
VTPSVKP;ERVVYP	NCP	209.313
ERVVYP;MMDDGS	NCP	489.099
MMDDGS;TEGTEPSK	NCP	22.899
TEGTEPSK;VLLPEKL	NCP	325.391
VLLPEKL;LLKPLLL	NCP	173.815
LLKPLLL;MAMDC	NCP	48.699
MAMDC;TRDPLV	NCP	170.283
TRDPLV;SATAFSLK	NCP	132.455
SATAFSLK;FSPLGSPL	NCP	44.74
FSPLGSPL;KFFFAW	NCP	70.68
KFFFAW;KTPPPVP	NCP	27.144
KTPPPVP;RLLRCDM	NCP	72.329
RLLRCDM;KFAAPPPPPP	NCP	75.382
KFAAPPPPPP;VTLPLEP	NCP	204.021
VTLPLEP;YSNEALR	NCP	161.879
YSNEALR;LGVGVLKA	NCP	39.844
LGVGVLKA;LTAAELVT	NCP	32.412
LTAAELVT;GNNMLK	NCP	119.729
GNNMLK;HSFPTLK	NCP	8.905
HSFPTLK;KNSAAP	NCP	7.427
KNSAAP;LLTMLLTK	NCP	33.816

LLTMLLTK;GKNLLF	NCP	58.74
GKNLLF;LSSVLY	NCP	30.675
LSSVLY;KFGSL	NCP	33.48
KFGSL;GLTLTLK	NCP	72.669
GLTLTLK;VFKEPK	NCP	7.068
VFKEPK;AVLAELK	NCP	13.35
AVLAELK;KQCGFGH	NCP	41.389
KQCGFGH;PFLDLWN	NCP	20.025
PFLDLWN;DKVLR	NCP	31.841
DKVLR;FPDNLMP	NCP	252.831
FPDNLMP;LVQVDQ	NCP	84.195
LVQVDQ;LSDTLKAF	NCP	89.122
LSDTLKAF;KSSSLPK	NCP	25.136
KSSSLPK;QTLLP	NCP	82.107
QTLLP;PEVQF	NCP	43.81
PEVQF;FMWVLSF	NCP	42.131
FMWVLSF;CMEDLRK	NCP	109.138
CMEDLRK;FDKNPNY	NCP	14.411
FDKNPNY;QPPSK	NCP	100.113
QPPSK;EAFLL	NCP	235.341
EAFLL;VTAVTDK	NCP	9.907
VTAVTDK;LATYK	NCP	197.588
LATYK;KVPEGP	NCP	173.521
KVPEGP;KTPLPEE	NCP	1.098
KTPLPEE;RSLAPTF	NCP	44.061
RSLAPTF;KVLGWM	NCP	151.122
KVLGWM;LTVYF	NCP	16.815
LTVYF;KAFAPSSK	NCP	99.582
KAFAPSSK;DPCVEQ	NCP	92.496
DPCVEQ;QGPSFP	NCP	193.716
QGPSFP;ELQVLL	NCP	95.727
ELQVLL;RLRSLLK	NCP	18.293
RLRSLLK;KCLLGP	NCP	126.544
KCLLGP;SLAKSPK	NCP	83.544
SLAKSPK;KFVTP	NCP	94.451
KFVTP;RFLMLK	NCP	26.158
RFLMLK;KPPSFK	NCP	5.868
KPPSFK;KYQPFR	NCP	16.457
KYQPFR;MDLYQK	NCP	140.463
MDLYQK;WPGYVR	NCP	76.96
WPGYVR;AAKRTR	NCP	74.412
AAKRTR;QTVVLD	NCP	61.481
QTVVLD;PKAAHLK	NCP	35.965
PKAAHLK;KLEVQLQQ	NCP	9.816
KLEVQLQQ;PTNEGP	NCP	59.729
PTNEGP;ALGLVT	NCP	5.218
ALGLVT;PLYFYELP	NCP	99.324
PLYFYELP;RPLEPEW	NCP	337.809
RPLEPEW;GDVVFK	NCP	105.603
GDVVFK;DPKKVHT	NCP	325.226
DPKKVHT;KNLLGK	NCP	168.763

KNLLGK;VEPGGLP	NCP	17.931
VEPGGLP;FGGPGT	NCP	28.281
FGGPGT;DLQKKR	NCP	61.422
DLQKKR;CLPTSLPK	NCP	193.734
CLPTSLPK;EAAMGK	NCP	4.412
EAAMGK;TMSGAPK	NCP	423.982
TMSGAPK;GVKRAYY	NCP	95.826
GVKRAYY;QKPAC	NCP	185.069
QKPAC;MNSLKFR	NCP	12.319
MNSLKFR;KGPVPKP	NCP	32.468
KGPVPKP;KAATVP	NCP	68.514
KAATVP;KNPPKR	NCP	102.633
KNPPKR;VTPFFPQ	NCP	18.546
VTPFFPQ;YLPTAQ	NCP	144.148
YLPTAQ;MAALPDR	NCP	107.057
MAALPDR;KQVAPEKPVKKQ	NCP	5.988
KQVAPEKPVKKQ;QVAPEKPVK	NCP	0.003
QVAPEKPVK;VAPEKPVKKQ	NCP	0.003
VAPEKPVKKQ;PPGKYKP	NCP	17.638
PPGKYKP;RVGMGVL	NCP	24.542
RVGMGVL;NFLDKL	NCP	91.69
NFLDKL;VCVTCSK	NCP	10.265
VCVTCSK;ASAPGLLH	NCP	49.62
ASAPGLLH;KDVEVDK	NCP	160.999
KDVEVDK;AAVFHFPK	NCP	19.416
AAVFHFPK;PMGGGKAPR	NCP	27.653
PMGGGKAPR;VVVLQGK	NCP	130.626
VVVLQGK;ELHSL	NCP	13.231
ELHSL;KPSCSFR	NCP	22.212
KPSCSFR;QADQLH	NCP	11.63
QADQLH;TVLYL	NCP	28.167
TVLYL;VATPLP	NCP	1.78
VATPLP;SLFPDLL	NCP	8.814
SLFPDLL;KADAR	NCP	60.368
KADAR;NRNPVNY	NCP	11.481
NRNPVNY;LDDNLPR	NCP	61.222
LDDNLPR;RPGLVLR	NCP	7.601
RPGLVLR;KNSLEL	NCP	9.831
KNSLEL;KETLLEMF	NCP	6.081
KETLLEMF;VPFLPR	NCP	29.203
VPFLPR;LSLPLKK	NCP	9.065
LSLPLKK;EPPLLPK	NCP	25.048
EPPLLPK;TPMHTGKL	NCP	36.473
TPMHTGKL;ALTGDEVKK	NCP	33.622
ALTGDEVKK;KDEGLP	NCP	109.05
KDEGLP;LGLEL	NCP	19.952
LGLEL;QPERK	NCP	79.362
QPERK;MMSCD	NCP	20.988
MMSCD;KQQVPHY	NCP	68.619
KQQVPHY;LAALKK	NCP	0.412
LAALKK;GPNAVKK	NCP	237.035

GPNAVKK;LTGLA	NCP	80.611
LTGLA;KPEYKN	NCP	7.26
KPEYKN;TNYVKLFL	NCP	18.048
TNYVKLFL;LVLDDV	NCP	46.256
LVLDDV;CMCMHP	NCP	49.251
CMCMHP;KRLQPF	NCP	66.445
KRLQPF;KYSARP	NCP	132.092
KYSARP;EVERLPPL	NCP	14.73
EVERLPPL;FGMAK	NCP	25.338
FGMAK;MEENPPP	NCP	127.14
MEENPPP;YGLSF	NCP	101.628
YGLSF;DPLPSKET	NCP	12.046
DPLPSKET;HSPAHH	NCP	138.257
HSPAHH;SPVFRPQ	NCP	18.358
SPVFRPQ;KTLLPLNV	NCP	25.34
KTLLPLNV;TKPEC	NCP	24.741
TKPEC;LYVLR	NCP	58.524
LYVLR;MPPLFNTV	NCP	59.641
MPPLFNTV;LNYFAK	NCP	82.895
LNYFAK;NDGLK	NCP	91.239
NDGLK;KTSLAFK	NCP	53.145
KTSLAFK;FLLDFRT	NCP	12.465
FLLDFRT;RPGGLQ	NCP	38.473
RPGGLQ;PEAEAAR	NCP	63.362
PEAEAAR;ALHSP	NCP	26.254
ALHSP;GVFLNS	NCP	125.228
GVFLNS;KALNF	NCP	73.749
KALNF;NSPLGSLH	NCP	170.112
NSPLGSLH;KPCAPAK	NCP	42.835
KPCAPAK;KEVPEQ	NCP	37.835
KEVPEQ;DRASLLR	NCP	2.745
DRASLLR;KCFLHTYE	NCP	8.194
KCFLHTYE;NVRLL	NCP	43.857
NVRLL;GVVPLAT	NCP	34.442
GVVPLAT;AAMAK	NCP	13.983
AAMAK;TDHPVLL	NCP	54.229
TDHPVLL;KNKEVKDL	NCP	139.202
KNKEVKDL;KDTQLKGK	NCP	8.796
KDTQLKGK;AFGLSL	NCP	9.577
AFGLSL;LQFGGF	NCP	7.064
LQFGGF;ESMPVPR	NCP	25.185
ESMPVPR;LPLPPPYK	NCP	3.102
LPLPPPYK;PVDYGYN	NCP	25.887
PVDYGYN;HLTVSF	NCP	52.363
HLTVSF;MPPGV	NCP	340.146
MPPGV;DSDGLK	NCP	162.531
DSDGLK;KMVTHKK	NCP	55.058
KMVTHKK;LVGLF	NCP	21.777
LVGLF;VSCPLSQ	NCP	84.079
VSCPLSQ;RAFEPF	NCP	41.435
RAFEPF;KTSSMRP	NCP	39.727

KTSSMRP;SGGPR	NCP	44.913
SGGPR;MDLKSVM	NCP	60.072
MDLKSVM;KDLLPL	NCP	204.827
KDLLPL;DWHHTVL	NCP	14.406
DWHHTVL;KTTKAPQ	NCP	56.254
KTTKAPQ;KLWST	NCP	49.479
KLWST;TQNPMLL	NCP	59.424
TQNPMLL;LAVWGKN	NCP	38.733
LAVWGKN;AAGVLKA	NCP	65.289
AAGVLKA;TAGSLADP	NCP	103.169
TAGSLADP;KYPPAAF	NCP	84.075
KYPPAAF;RRYPGP	NCP	6.489
RRYPGP;KAHLTTPP	NCP	82.153
KAHLTTPP;ELEGGWK	NCP	3.836
ELEGGWK;ALLLFK	NCP	67.554
ALLLFK;HLGTTMK	NCP	122.53
HLGTTMK;CHRDPVQ	NCP	10.959
CHRDPVQ;KTKYSSTK	NCP	100.871
KTKYSSTK;PVVTGK	NCP	85.395
PVVTGK;APPPPPP	NCP	19.606
APPPPPP;QLVLVT	NCP	32.076
QLVLVT;VPAKVP	NCP	19.131
VPAKVP;LGPKLR	NCP	8.202
LGPKLR;EDLPF	NCP	159.878
EDLPF;KEMFKL	NCP	30.894
KEMFKL;RSDLLRL	NCP	92.68
RSDLLRL;KVKVAF	NCP	71.052
KVKVAF;SVVHMR	NCP	6.533
SVVHMR;LYEVSELKD	NCP	78.196
LYEVSELKD;DHSEQ	NCP	63.216
DHSEQ;EFPTLRL	NCP	60.702
EFPTLRL;KAPLLFK	NCP	77.355
KAPLLFK;AVAPKYF	NCP	191.267
AVAPKYF;RQFKKKA	NCP	8.608
RQFKKKA;VTAAVLY	NCP	6.69
VTAAVLY;DLAVY	NCP	275.976
DLAVY;QKALNDH	NCP	109.982
QKALNDH;KEGAK	NCP	4.54
KEGAK;MPQVLK	NCP	21.299
MPQVLK;DKAPNLL	NCP	171.109
DKAPNLL;FAARP	NCP	116.586
FAARP;PRPPPPP	NCP	22.346
PRPPPPP;LTDGCTSL	NCP	46.942
LTDGCTSL;QPNLSLPPL	NCP	96.092
QPNLSLPPL;VGLSGADF	NCP	108.201
VGLSGADF;HLPPPGP	NCP	4.513
HLPPPGP;LLFYERLL	NCP	60.755
LLFYERLL;LGEAVFA	NCP	40.465
LGEAVFA;PGLQK	NCP	96.728
PGLQK;ASFLMP	NCP	81.999
ASFLMP;KYLARGL	NCP	208.582

KYLARGL;DGVVKLF	NCP	103.115
DGVVKLF;LALAVHDK	NCP	44.088
LALAVHDK;KGDLWAH	NCP	30.135
KGDLWAH;HLQPNVP	NCP	95.421
HLQPNVP;SCLLTVP	NCP	7.648
SCLLTVP;GNNSGPK	NCP	29.834
GNNSGPK;MLLLLTF	NCP	64.246
MLLLLTF;AAASSSVVR	NCP	0.599
AAASSSVVR;KHDYLK	NCP	88.591
KHDYLK;RKYLPPY	NCP	16.542
RKYLPPY;EAWHLKK	NCP	50.823
EAWHLKK;KPKELEK	NCP	48.176
KPKELEK;TPPKY	NCP	0.061
TPPKY;AEEESK	NCP	33.138
AEEESK;VLLRPP	NCP	92.601
VLLRPP;KGKPT	NCP	23.555
KGKPT;RVPLNC	NCP	35.643
RVPLNC;AAKTVY	NCP	5.575
AAKTVY;GHVQPLR	NCP	14.891
GHVQPLR;QTTLVQ	NCP	182.19
QTTLVQ;PGLEPLL	NCP	74.532
PGLEPLL;FPLGLP	NCP	43.9
FPLGLP;MEEYHRPCDEVGF	NCP	61.422
MEEYHRPCDEVGF;KEPPVKS	NCP	165.476
KEPPVKS;LLEPVTR	NCP	14.767
LLEPVTR;KALEALRQ	NCP	79.221
KALEALRQ;QSLQFVK	NCP	66.725
QSLQFVK;KAGPKGP	NCP	31.183
KAGPKGP;LSESELR	NCP	6.986
LSESELR;APLLPD	NCP	30.93
APLLPD;LGTVPVGR	NCP	36.216
LGTVPVGR;GVPEV	NCP	50.992
GVPEV;DSEGHLY	NCP	10.988
DSEGHLY;KFLNPPP	NCP	41.751
KFLNPPP;LPLTFN	NCP	2.869
LPLTFN;VGVNK	NCP	45.996
VGVNK;STLLKLTTL	NCP	4.802
STLLKLTTL;GHWGSRK	NCP	35.382
GHWGSRK;LYLLPAGTFVGGY	NCP	47.232
LYLLPAGTFVGGY;VSDGVLVK	NCP	35.328
VSDGVLVK;EVRKLA	NCP	387.709
EVRKLA;SLDLFP	NCP	120.051
SLDLFP;KLEDVPK	NCP	67.466
KLEDVPK;KLLWN	NCP	66.55
KLLWN;MTQKPPP	NCP	228.06
MTQKPPP;GKERVR	NCP	18.633
GKERVR;KEELLKK	NCP	32.639
KEELLKK;GRMPLR	NCP	105.439
GRMPLR;AAPFPRLH	NCP	59.622
AAPFPRLH;PAASLHPK	NCP	74.136
PAASLHPK;RQDLLAK	NCP	19.983

RQDLLAK;VSLLPF	NCP	73.326
VSLLPF;ATKLRF	NCP	40.76
ATKLRF;CVMMVPP	NCP	86.692
CVMMVPP;KMRLHF	NCP	49.926
KMRLHF;DLGELNSF	NCP	35.07
DLGELNSF;SFTKLP	NCP	30.347
SFTKLP;RRLAAY	NCP	123.603
RRLAAY;WHFEL	NCP	99.46
WHFEL;EMMEC	NCP	143.445
EMMEC;LTLLNT	NCP	20.507
LTLLNT;KGGLT	NCP	16.056
KGGLT;DGLEFK	NCP	23.115
DGLEFK;EAASG	NCP	147.415
EAASG;KATTNLR	NCP	97.181
KATTNLR;EGGGNL	NCP	62.821
EGGGNL;TYRATPP	NCP	8.405
TYRATPP;KSKFADL	NCP	30.859
KSKFADL;KELPK	NCP	133.917
KELPK;YPVVKAD	NCP	63.89
YPVVKAD;MKTFPP	NCP	124.977
MKTFPP;FLQPPYK	NCP	171.159
FLQPPYK;MLLVRF	NCP	175.914
MLLVRF;FSTPK	NCP	66.007
FSTPK;KTNAP	NCP	53.279
KTNAP;LPLTTTL	NCP	55.098
LPLTTTL;NAPLALFN	NCP	43.783
NAPLALFN;KWALLR	NCP	119.345
KWALLR;KDTTFK	NCP	183.057
KDTTFK;LDLYLHW	NCP	26.584
LDLYLHW;GRVPGQP	NCP	31.581
GRVPGQP;ALSHHPR	NCP	46.692
ALSHHPR;LCLHY	NCP	59.523
LCLHY;MRPLVL	NCP	34.128
MRPLVL;KYTVKF	NCP	71.015
KYTVKF;WEGLPV	NCP	24.348
WEGLPV;LHTVYQ	NCP	84.259
LHTVYQ;PGKMK	NCP	165.269
PGKMK;RQPYGLS	NCP	6.192
RQPYGLS;SHFLTDY	NCP	34.021
SHFLTDY;RNLAELA	NCP	60.714
RNLAELA;RTFHGLK	NCP	117.359
RTFHGLK;KTNKCPQ	NCP	15.421
KTNKCPQ;YKVLPK	NCP	126.27
YKVLPK;GDLEFK	NCP	256.275
GDLEFK;WHYLK	NCP	50.907
WHYLK;APSYP	NCP	144.689
APSYP;VLSDAVVK	NCP	115.837
VLSDAVVK;SGPVEDMF	NCP	19.73
SGPVEDMF;SSTEEVLVAAKK	NCP	31.002
SSTEEVLVAAKK;CDLPRDFL	NCP	165.403
CDLPRDFL;NDSHVAA	NCP	80.99

NDSHVAA;KPNPPQPP	NCP	192.105
KPNPPQPP;SCYKPS	NCP	95.619
SCYKPS;PPPKY	NCP	44.188
PPPKY;KVVSY	NCP	39.602
KVVSY;KLEGLNR	NCP	6.931
KLEGLNR;VDFLTY	NCP	93.495
VDFLTY;PKKAPPP	NCP	7.224
PKKAPPP;PSLLFYP	NCP	18.809
PSLLFYP;STKEDAVAFAEK	NCP	21.42
STKEDAVAFAEK;MMCDP	NCP	31.465
MMCDP;ENCKN	NCP	183.396
ENCKN;PELSRVLHL	NCP	78.176
PELSRVLHL;GQAFRK	NCP	57.966
GQAFRK;ATDYWVP	NCP	19.195
ATDYWVP;ESFTVAK	NCP	67.529
ESFTVAK;NSSPRPK	NCP	27.727
NSSPRPK;VKTTLSF	NCP	36.678
VKTTLSF;KVELKP	NCP	0.84
KVELKP;TVEFL	NCP	66.092
TVEFL;ELTLSSPP	NCP	3.868
ELTLSSPP;HSKDLLSH	NCP	200.12
HSKDLLSH;LSSLQVA	NCP	69.738
LSSLQVA;VPRQAF	NCP	50.184
VPRQAF;ESGKAG	NCP	51.891
ESGKAG;AAPFHLSE	NCP	81.078
AAPFHLSE;KSFASSLK	NCP	233.394
KSFASSLK;DRVLDF	NCP	34.507
DRVLDF;PLRVRP	NCP	93.11
PLRVRP;KQRGVTH	NCP	81.645
KQRGVTH;ADAFGDEL	NCP	111.18
ADAFGDEL;HLLVL	NCP	11.382
HLLVL;KAVAELH	NCP	25.704
KAVAELH;YAPLPTLT	NCP	77.022
YAPLPTLT;KPKHLK	NCP	30.256
KPKHLK;YEKKYP	NCP	53.007
YEKKYP;KPMTRP	NCP	144.273
KPMTRP;APHKTTVL	NCP	41.946
APHKTTVL;KFLDAGK	NCP	37.014
KFLDAGK;KFAKRTSL	NCP	248.235
KFAKRTSL;KSVLVR	NCP	26.021
KSVLVR;LTTGYAA	NCP	50.004
LTTGYAA;LKKLLG	NCP	16.735
LKKLLG;RVTAPK	NCP	64.424
RVTAPK;PNLPLR	NCP	209.092
PNLPLR;LSNGKSVL	NCP	16.494
LSNGKSVL;LLRKLPM	NCP	9.504
LLRKLPM;LTQSSAPP	NCP	177.275
LTQSSAPP;KAVAP	NCP	19.054
KAVAP;EPAYSKP	NCP	19.787
EPAYSKP;PSEPAL	NCP	77.658
PSEPAL;VSGYTPK	NCP	14.037

VSGYTPK;VGAPPGHKK	NCP	101.662
VGAPPGHKK;DMVLY	NCP	19.67
DMVLY;KDASLTPK	NCP	34.369
KDASLTPK;SGLCKAGF	NCP	179.531
SGLCKAGF;YFPQKK	NCP	40.962
YFPQKK;KNFKRK	NCP	88.165
KNFKRK;KLLKPPP	NCP	1.404
KLLKPPP;VSLVGRP	NCP	98.21
VSLVGRP;LPFLLL	NCP	11.005
LPFLLL;KHLSHLK	NCP	152.924
KHLSHLK;EELLDVT	NCP	27.28
EELLDVT;YTGSLF	NCP	22.577
YTGSLF;LLTPSFAK	NCP	38.622
LLTPSFAK;LGVEL	NCP	245.934
LGVEL;KSTQRKKG	NCP	32.79
KSTQRKKG;HPVLL	NCP	37.621
HPVLL;APEAYSP	NCP	6.144
APEAYSP;KDLTKLPR	NCP	81.578
KDLTKLPR;GHGFGAL	NCP	69.492
GHGFGAL;KEVSALK	NCP	158.842
KEVSALK;MNKPAVK	NCP	11.619
MNKPAVK;PSKKHK	NCP	49.598
PSKKHK;EPTLFQ	NCP	51.381
EPTLFQ;DLKDLR	NCP	56.172
DLKDLR;LTTWY	NCP	9.324
LTTWY;REVFAPF	NCP	176.799
REVFAPF;FGSDSPF	NCP	58.596
FGSDSPF;KTMAAKK	NCP	62.187
KTMAAKK;VGGYK	NCP	78.246
VGGYK;RGGGLP	NCP	62.418
RGGGLP;VLVSKSVP	NCP	100.956
VLVSKSVP;SPTMTKF	NCP	2.115
SPTMTKF;ESLLTGLK	NCP	56.097
ESLLTGLK;GGLKKALA	NCP	271.462
GGLKKALA;VAFDPK	NCP	13.863
VAFDPK;KYPPPPSPP	NCP	1.217
KYPPPPSPP;ENRLSDF	NCP	11.923
ENRLSDF;VFLSPK	NCP	31.424
VFLSPK;TSKYR	NCP	48.81
TSKYR;LGLEF	NCP	70.341
LGLEF;VGLGL	NCP	4.029
VGLGL;KPLSAMT	NCP	131.616
KPLSAMT;KAPSKQ	NCP	0.676
KAPSKQ;KAMPKK	NCP	46.491
KAMPKK;PTPSLVHRK	NCP	15.978
PTPSLVHRK;LSVNP	NCP	20.289
LSVNP;NALQLRE	NCP	24.351
NALQLRE;ATSTKKK	NCP	6.72
ATSTKKK;CFFPKVL	NCP	28.406
CFFPKVL;EKYVPM	NCP	2.584
EKYVPM;KMVKPPP	NCP	4.469

KMVKPPP;SALLRRDS	NCP	76.578
SALLRRDS;DLVLPF	NCP	22.363
DLVLPF;KKGLF	NCP	18.231
KKGLF;LVSKTSLQ	NCP	62.165
LVSKTSLQ;VASKFR	NCP	79.672
VASKFR;TSEYYCK	NCP	168.204
TSEYYCK;KAMKVVK	NCP	72.248
KAMKVVK;QQPMLLL	NCP	43.594
QQPMLLL;MELEV	NCP	33.555
MELEV;AALLNL	NCP	60.416
AALLNL;YRPGTVA	NCP	77.217
YRPGTVA;GLHETTY	NCP	9.819
GLHETTY;AYPTTK	NCP	21.352
AYPTTK;TALFV	NCP	106.88
TALFV;LPEPDLF	NCP	72.562
LPEPDLF;LNPHRP	NCP	176.654
LNPHRP;SLLRAY	NCP	243.537
SLLRAY;RMKKLVH	NCP	61.288
RMKKLVH;LSLVF	NCP	4.991
LSLVF;KSRTPFPL	NCP	92.328
KSRTPFPL;AAASGLAR	NCP	18.114
AAASGLAR;PLGYQPF	NCP	111.951
PLGYQPF;KDLNPK	NCP	43.476
KDLNPK;FAGVHLLV	NCP	13.899
FAGVHLLV;PYPPP	NCP	44.305
PYPPP;PLSAPPP	NCP	96.695
PLSAPPP;KPVSPVKP	NCP	10.402
KPVSPVKP;LPPGSLL	NCP	73.115
LPPGSLL;KFELLC	NCP	9.133
KFELLC;KHNSPR	NCP	0.114
KHNSPR;GPAYGAP	NCP	12.281
GPAYGAP;KSHVGPP	NCP	136.102
KSHVGPP;PGLVED	NCP	111.596
PGLVED;DYCFHCK	NCP	12.439
DYCFHCK;NPPHSVK	NCP	38.258
NPPHSVK;NFPLYVW	NCP	93.865
NFPLYVW;WPCKMP	NCP	11.573
WPCKMP;EDRKPEE	NCP	27.574
EDRKPEE;LTEKNF	NCP	81.807
LTEKNF;KWLSM	NCP	15.777
KWLSM;MDFLGL	NCP	16.877
MDFLGL;TVLKV	NCP	13.459
TVLKV;TKTYF	NCP	35.435
TKTYF;NFGLLVP	NCP	139.477
NFGLLVP;EVFCC	NCP	50.481
EVFCC;KTSSLLC	NCP	56.376
KTSSLLC;KLAVLY	NCP	26.534
KLAVLY;KGSRDLR	NCP	76.809
KGSRDLR;KEPFELLK	NCP	120.553
KEPFELLK;KTNPLAH	NCP	58.887
KTNPLAH;VPLAPP	NCP	24.672

VPLAPP;FPSRGFW	NCP	244.887
FPSRGFW;LSLFK	NCP	40.736
LSLFK;MEMGP	NCP	77.442
MEMGP;LLEEL	NCP	169.731
LLEEL;KPNAY	NCP	75.456
KPNAY;KLQKPPP	NCP	10.465
KLQKPPP;KPSYTK	NCP	33.405
KPSYTK;AFLLRKD	NCP	77.745
AFLLRKD;LSPVVK	NCP	9.231
LSPVVK;MLFHHV	NCP	27.372
MLFHHV;LPLLKTSD	NCP	45.771
LPLLKTSD;TPKDPSP	NCP	89.709
TPKDPSP;LSEDLVY	NCP	98.556
LSEDLVY;HHVKAGP	NCP	8.366
HHVKAGP;GTDLLNF	NCP	59.133
GTDLLNF;AVLEY	NCP	118.444
AVLEY;AEFPTPF	NCP	82.131
AEFPTPF;KEHKAK	NCP	101.274
KEHKAK;YPPLLR	NCP	0.509
YPPLLR;VFEWLP	NCP	115.183
VFEWLP;LLQNSV	NCP	3.374
LLQNSV;PLVYAV	NCP	25.215
PLVYAV;KVVGSPK	NCP	36.406
KVVGSPK;KVVFVSNL	NCP	39.47
KVVFVSNL;AFLMKP	NCP	18.378
AFLMKP;KGGVAPV	NCP	10.06
KGGVAPV;EYLVLTA	NCP	82.937
EYLVLTA;KPDEAVP	NCP	61.045
KPDEAVP;TKLLEF	NCP	10.143
TKLLEF;VGMLK	NCP	18.666
VGMLK;DKELFL	NCP	10.238
DKELFL;MLLHKNL	NCP	14.413
MLLHKNL;AALLDQV	NCP	9.221
AALLDQV;ALLFLPR	NCP	324.649
ALLFLPR;PEELT	NCP	28.805
PEELT;VTFEKKP	NCP	30.34
VTFEKKP;KGLADPR	NCP	119.45
KGLADPR;KDRSP	NCP	138.849
KDRSP;KPEFPR	NCP	3.697
KPEFPR;LGKLNVK	NCP	21.593
LGKLNVK;PGKLPPPPPP	NCP	16.627
PGKLPPPPPP;LGALL	NCP	32.087
LGALL;GVLQHLL	NCP	90.006
GVLQHLL;MTQTEELKY	NCP	56.502
MTQTEELKY;KNGMK	NCP	58.707
KNGMK;LPGSSVLLP	NCP	11.832
LPGSSVLLP;KDAVLP	NCP	9.895
KDAVLP;THLLLFLPK	NCP	237.011
THLLLFLPK;QTQTLKP	NCP	67.681
QTQTLKP;RPKVPR	NCP	65.952
RPKVPR;NPVLLR	NCP	30.048

NPVLLR;YKDFM	NCP	182.468
YKDFM;FMLLQ	NCP	137.376
FMLLQ;KDFKVT	NCP	52.834
KDFKVT;VALSVSN	NCP	52.904
VALSVSN;GPRPLYL	NCP	12.087
GPRPLYL;VDLAAF	NCP	10.308
VDLAAF;TFLVGVT	NCP	6.177
TFLVGVT;LVGGP	NCP	31.231
LVGGP;FLSMSPGP	NCP	69.747
FLSMSPGP;RLGQSKLF	NCP	42.48
RLGQSKLF;VPEVEHE	NCP	13.541
VPEVEHE;FLTHASK	NCP	39.205
FLTHASK;ARPPFG	NCP	157.965
ARPPFG;SGLKMRP	NCP	2.64
SGLKMRP;HPATVPPP	NCP	56.099
HPATVPPP;VYTVY	NCP	95.641
VYTVY;VLPLE	NCP	7.818
VLPLE;KGELLKT	NCP	74.997
KGELLKT;LALRGVF	NCP	21.467
LALRGVF;TLFALPK	NCP	17.194
TLFALPK;KEFLVPPP	NCP	24.798
KEFLVPPP;HVVLGFE	NCP	5.645
HVVLGFE;YVGTTTF	NCP	75.912
YVGTTTF;VSGYVY	NCP	145.069
VSGYVY;MEDTVHV	NCP	21.633
MEDTVHV;EPVRSF	NCP	118.227
EPVRSF;AAQSLLL	NCP	238.547
AAQSLLL;DAVLDLH	NCP	76.449
DAVLDLH;ESPERFP	NCP	239.373
ESPERFP;KDFLY	NCP	34.489
KDFLY;VGVLLL	NCP	108.261
VGVLLL;PGGVYSN	NCP	15.026
PGGVYSN;KPLLVN	NCP	152.905
KPLLVN;KDLLKK	NCP	274.832
KDLLKK;HLDDLPG	NCP	37.218
HLDDLPG;CNDSHP	NCP	94.254
CNDSHP;KHFKKAT	NCP	12.675
KHFKKAT;PAKRGPK	NCP	13.032
PAKRGPK;VVGVL	NCP	14.776
VVGVL;DVVAH	NCP	1.916
DVVAH;KEGWVSLL	NCP	51.432
KEGWVSLL;KSGLRMK	NCP	55.498
KSGLRMK;LPFLKPGL	NCP	22.247
LPFLKPGL;NFGVWLK	NCP	44.307
NFGVWLK;QEYDEQR	NCP	29.496
QEYDEQR;NAFLKK	NCP	27.072
NAFLKK;KLGPAPP	NCP	11.886
KLGPAPP;NFFLQ	NCP	18.379
NFFLQ;PPPLSDYVER	NCP	79.505
PPPLSDYVER;VEDFRQPH	NCP	6.669
VEDFRQPH;LDVRDL	NCP	40.243

LDVRDL;DPVSGYC	NCP	14.865
DPVSGYC;KTLVAQP	NCP	63.371
KTLVAQP;QGGGLLQ	NCP	2.671
QGGGLLQ;AAPPTETR	NCP	20.156
AAPPTETR;APKPAE	NCP	6.561
APKPAE;MSMHLN	NCP	11.766
MSMHLN;GPGVP	NCP	10.642
GPGVP;NSYPPPP	NCP	32.604
NSYPPPP;VEAPGPK	NCP	156.794
VEAPGPK;WLEAP	NCP	16.444
WLEAP;TVWDPPPPP	NCP	10.173
TVWDPPPPP;ESSFYL	NCP	32.184
ESSFYL;LVTLGN	NCP	13.791
LVTLGN;PLTSVFL	NCP	21.312
PLTSVFL;EPELQ	NCP	20.696
EPELQ;EMHLKPP	NCP	169.044
EMHLKPP;DVVVF	NCP	12.543
DVVVF;DLPRPP	NCP	88.161
DLPRPP;VSFLER	NCP	29.254
VSFLER;ATHTAPK	NCP	22.689
ATHTAPK;MMDPEAL	NCP	23.585
MMDPEAL;TTTTPEVK	NCP	0.249
TTTTPEVK;KTYVAGF	NCP	19.195
KTYVAGF;PAHGVPK	NCP	7.491
PAHGVPK;EPPTK	NCP	3.399
EPPTK;QAPGDPKL	NCP	2.379
QAPGDPKL;KVLKSR	NCP	17.846
KVLKSR;LELDF	NCP	13.555
LELDF;EDGLLL	NCP	61.316
EDGLLL;KMLTDS	NCP	129.564
KMLTDS;SLYASGR	NCP	2.724
SLYASGR;SSTKEEFQQF	NCP	93.771
SSTKEEFQQF;WLPGA	NCP	33.618
WLPGA;LQSVVL	NCP	168.331
LQSVVL;VLGPHPPPPP	NCP	37.877
VLGPHPPPPP;LPPPELVF	NCP	29.971
LPPPELVF;AQLVLLEH	NCP	2.78
AQLVLLEH;LLPEGLA	NCP	219.615
LLPEGLA;KKELVR	NCP	22.339
KKELVR;NFGLPLC	NCP	103.481
NFGLPLC;YGKKP	NCP	89.061
YGKKP;QCMDGP	NCP	103.839
QCMDGP;LQMKAPR	NCP	3.655
LQMKAPR;LTAAGGPK	NCP	1.754
LTAAGGPK;TADEVLK	NCP	18.166
TADEVLK;VRGPPPPPP	NCP	50.21
VRGPPPPPP;TLLELDNL	NCP	21.954
TLLELDNL;ENLPLHF	NCP	31.974
ENLPLHF;SFPLGSP	NCP	31.83
SFPLGSP;KPHYVPK	NCP	59.74
KPHYVPK;NAPPPPPPP	NCP	114.284

NAPPPPPPP;LAHAP	NCP	35.317
LAHAP;PFSLAALK	NCP	287.921
PFSLAALK;KTLQETR	NCP	18.168
KTLQETR;GWPGYVR	NCP	9.471
GWPGYVR;SYFLN	NCP	20.539
SYFLN;KVYLLM	NCP	33.312
KVYLLM;LPPLKPRP	NCP	6.896
LPPLKPRP;KAGGVVP	NCP	23.767
KAGGVVP;VAVLGA	NCP	111.543
VAVLGA;KTGRARP	NCP	182.522
KTGRARP;QEFVPPP	NCP	37.506
QEFVPPP;FEPFLPLK	NCP	112.491
FEPFLPLK;LAAGVPL	NCP	6.879
LAAGVPL;TSPLVTK	NCP	91.308
TSPLVTK;NSNLLR	NCP	13.423
NSNLLR;KMAVH	NCP	47.073
KMAVH;EAPYG	NCP	164.154
EAPYG;KSDPTPL	NCP	7.317
KSDPTPL;KYDLPK	NCP	83.732
KYDLPK;KDDNK	NCP	3.856
KDDNK;KEPRPK	NCP	19.176
KEPRPK;WEPLK	NCP	38.769
WEPLK;KPVDPAK	NCP	45.185
KPVDPAK;MVVMMPV	NCP	71.283
MVVMMPV;LPPVVSVP	NCP	22.143
LPPVVSVP;LHVLFL	NCP	8.668
LHVLFL;LGDVHL	NCP	108.006
LGDVHL;VVFLPA	NCP	7.653
VVFLPA;MAGLM	NCP	10.986
MAGLM;NATPR	NCP	136.218
NATPR;KDVAAPR	NCP	68.085
KDVAAPR;KCSKPQ	NCP	45.531
KCSKPQ;GPVFP	NCP	141.342
GPVFP;VGSLAAT	NCP	38.384
VGSLAAT;MAPLLR	NCP	15.621
MAPLLR;KTALLPW	NCP	43.902
KTALLPW;EDVLKLQ	NCP	54.411
EDVLKLQ;LLSGGLA	NCP	39.325
LLSGGLA;KDSSLPSK	NCP	41.585
KDSSLPSK;KPYFLEA	NCP	12.246
KPYFLEA;KDSAVPW	NCP	46.68
KDSAVPW;KGDFY	NCP	68.361
KGDFY;TFELNPR	NCP	67.624
TFELNPR;VSGHL	NCP	56.738
VSGHL;KPNPEQR	NCP	11.353
KPNPEQR;KSCTTKT	NCP	39.722
KSCTTKT;FTFSKPK	NCP	12.642
FTFSKPK;QPKPPR	NCP	35.439
QPKPPR;KDGPVRVLP	NCP	54.043
KDGPVRVLP;PTAHKPK	NCP	36.408
PTAHKPK;YQWAPP	NCP	169.503

YQWAPP;QTGGGLGL	NCP	23.582
QTGGGLGL;LQGVGV	NCP	22.296
LQGVGV;VPAAEPF	NCP	11.079
VPAAEPF;KNLLLHR	NCP	108.592
KNLLLHR;KGLLF	NCP	6.227
KGLLF;APKRPSPA	NCP	0.246
APKRPSPA;LLLVYPV	NCP	38.496
LLLVYPV;APHTPPK	NCP	31.819
APHTPPK;VLEGKELEFY	NCP	48.284
VLEGKELEFY;KEFPNLP	NCP	23.455
KEFPNLP;YGRVALLC	NCP	10.238
YGRVALLC;ESNVTPP	NCP	10.497
ESNVTPP;LPAAPRSK	NCP	19.542
LPAAPRSK;AANPTVF	NCP	8.88
AANPTVF;VTGPPLD	NCP	21.228
VTGPPLD;GLVAV	NCP	6.667
GLVAV;KFHNR	NCP	5.051
KFHNR;MAVVP	NCP	154.935
MAVVP;RDDLKL	NCP	56.46
RDDLKL;CRDPVP	NCP	7.749
CRDPVP;VTTSVPK	NCP	12.732
VTTSVPK;GPPRLYL	NCP	22.378
GPPRLYL;PVSPY	NCP	21.155
PVSPY;PRGLPP	NCP	25.012
PRGLPP;SPDPF	NCP	13.202
SPDPF;AELRANV	NCP	68.311
AELRANV;KALLPAGP	NCP	105.293
KALLPAGP;KPGGLSH	NCP	52.635
KPGGLSH;FKSRWGP	NCP	30.256
FKSRWGP;TSHVLR	NCP	33.308
TSHVLR;PLLGVP	NCP	63.108
PLLGVP;FTPVPP	NCP	8.419
FTPVPP;EDCGGLP	NCP	3.098
EDCGGLP;AFSFYVC	NCP	65.533
AFSFYVC;AVPPPKE	NCP	1.56
AVPPPKE;QVKLL	NCP	60.774
QVKLL;CPPKWP	NCP	48.921
CPPKWP;SVFYYEL	NCP	25.079
SVFYYEL;LEVGF	NCP	45.468
LEVGF;CKDAPL	NCP	25.168
CKDAPL;LEKLLKK	NCP	52.833
LEKLLKK;PDLYKP	NCP	58.016
PDLYKP;CTCCE	NCP	49.273
CTCCE;LPLKP	NCP	61.176
LPLKP;FFSLLTPL	NCP	61.224
FFSLLTPL;KSAPKLSLK	NCP	7.179
KSAPKLSLK;HLKGKCK	NCP	122.237
HLKGKCK;VALLET	NCP	29.065
VALLET;TQVDKLR	NCP	14.594
TQVDKLR;PQEKPK	NCP	24.084
PQEKPK;KYLHGAK	NCP	91.158

KYLHGAK;DVVRPSDK	NCP	53.157
DVVRPSDK;GQDKLPR	NCP	2.458
GQDKLPR;HGMPPLT	NCP	52.313
HGMPPLT;LTFRKHSTF	NCP	41.865
LTFRKHSTF;KGPPDPK	NCP	133.432
KGPPDPK;LLPPPPER	NCP	4.47
LLPPPPER;VLGSPVK	NCP	6.26
VLGSPVK;FVLLF	NCP	22.759
FVLLF;DDVCKK	NCP	51.423
DDVCKK;AAPSFPR	NCP	32.457
AAPSFPR;APPGFQN	NCP	0.696
APPGFQN;KRPAPPP	NCP	0.039
KRPAPPP;FGSKH	NCP	0.23
FGSKH;GPLTPALP	NCP	4.192
GPLTPALP;LGSLGVPR	NCP	3.495
LGSLGVPR;PPTTLLE	NCP	40.983
PPTTLLE;FPGYP	NCP	25.097
FPGYP;PAMNVYV	NCP	9.763
PAMNVYV;VEGGLALP	NCP	188.364
VEGGLALP;VTDGTPK	NCP	50.481
VTDGTPK;MGTLVTPL	NCP	4.266
MGTLVTPL;APPPGVRG	NCP	9.807
APPPGVRG;GHASVLT	NCP	3.627
GHASVLT;KPMSP	NCP	32.319
KPMSP;TVVPANP	NCP	25.508
TVVPANP;GEFDL	NCP	5.22
GEFDL;PPPVY	NCP	64.453
PPPVY;LHLLEQPL	NCP	6.401
LHLLEQPL;GSPLPGKKPPVPR	NCP	87.717
GSPLPGKKPPVPR;VGPALLPT	NCP	0.543
VGPALLPT;APAGFLPP	NCP	0.796
APAGFLPP;LVGSPLP	NCP	27.279
LVGSPLP;LLDDHF	NCP	16.79
LLDDHF;KEADRAP	NCP	29.188
KEADRAP;QLFGGF	NCP	94.253
QLFGGF;QELMGM	NCP	14.136
QELMGM;KLSAFVP	NCP	39.187
KLSAFVP;KNLTMPP	NCP	5.1
KNLTMPP;LPPAKFK	NCP	9.495
LPPAKFK;QGEPLDR	NCP	2.781
QGEPLDR;YPAGP	NCP	36.714
YPAGP;AATLK	NCP	12.564
AATLK;WMMHG	NCP	13.701
WMMHG;VLTAED	NCP	129.125
VLTAED;LPGGLL	NCP	9.082
LPGGLL;LRVAPEQ	NCP	63.366
LRVAPEQ;PPHPVEL	NCP	54.291
PPHPVEL;QTVLVKK	NCP	34.472
QTVLVKK;EEELAA	NCP	260.935
EEELAA;KESRPVP	NCP	14.811
KESRPVP;WSVLKSSH	NCP	4.686

WSVLKSSH;LLMRPGSL	NCP	7.232
LLMRPGSL;EGDEKPAK	NCP	10.309
EGDEKPAK;TGLEKF	NCP	195.932
TGLEKF;PGPLQ	NCP	12.339
PGPLQ;LPPFLEP	NCP	0.645
LPPFLEP;APLVYSL	NCP	35.772
APLVYSL;QWAAP	NCP	12.763
QWAAP;PPDLRK	NCP	94.013
PPDLRK;RPQLPGP	NCP	47.881
RPQLPGP;ALVKAF	NCP	3.971
ALVKAF;PSPRPP	NCP	32.005
PSPRPP;AVSVTLPR	NCP	75.207
AVSVTLPR;LAPAHPV	NCP	13.701
LAPAHPV;CDDPP	NCP	6.303
CDDPP;FQVLL	NCP	1.754
FQVLL;GSCPGF	NCP	13.434
GSCPGF;EVGVFP	NCP	7.441
EVGVFP;RPQPEV	NCP	24.594
RPQPEV;AHVHPP	NCP	17.658
AHVHPP;QDPKPR	NCP	50.534
QDPKPR;TLAAVCL	NCP	58.008
TLAAVCL;AEVKPEVK	NCP	28.749
AEVKPEVK;VGLAEAAR	NCP	12.673
VGLAEAAR;AGPPLGFP	NCP	22.631
AGPPLGFP;PPRPLGAPA	NCP	20.236
PPRPLGAPA;PPAPGVPP	NCP	65.562
PPAPGVPP;DSPKVHR	NCP	19.419
DSPKVHR;HTLPAL	NCP	12.165
HTLPAL;SLRVGPR	NCP	40.742
SLRVGPR;LLHYF	NCP	21.058
LLHYF;LGVPG	NCP	156.315
LGVPG;TPLSM	NCP	68.82
AVPTHH;MNPLWTL	NCP	51.552
MNPLWTL;KGKRAH	NCP	76.053
KGKRAH;RPSPPPR	NCP	241.959
RPSPPPR;MKPPSVY	NCP	64.554
MKPPSVY;TCVVGHEALPH	NCP	0.258
TCVVGHEALPH;PPGVLMSS	NCP	8.775
PPGVLMSS;ALLQT	NCP	138.397
ALLQT;VYVLAPPQEELSK	NCP	6.395
VYVLAPPQEELSK;LYSKLRVDRNSWQ	NCP	0.18
LYSKLRVDRNSWQ;EALHNHYTQKSTSKSAG	NCP	0.072
EALHNHYTQKSTSKSAG;STTAPKVYPLSSCCGDK	NCP	23.061
STTAPKVYPLSSCCGDK;TCNVAHPASSTK	NCP	0.237
TCNVAHPASSTK;VGHDNPEVQF	NCP	0.654
VGHDNPEVQF;PPKEELSK	NCP	0.474
PPKEELSK;AERSPR	NCP	99.966
AERSPR;PLRPLPP	NCP	9.489
PLRPLPP;QDNSGKK	NCP	5.85
QDNSGKK;KSEEPR	NCP	139.272
KSEEPR;ELGAW	NCP	1.746

ELGAW;AAPAVAPL	NCP	61.116
AAPAVAPL;EVVKLTH	NCP	27.535
EVVKLTH;SAPSKAVEAAVDVSLPK	NCP	182.813
SAPSKAVEAAVDVSLPK;VELPAAELEVK	NCP	0.111
VELPAAELEVK;AAEVGVKLPEGHLPEAELK	NCP	0.033
AAEVGVKLPEGHLPEAELK;DARSSY	NCP	77.727
DARSSY;KQSTN	NCP	2.433
KQSTN;LEKVRL	NCP	87.22
LEKVRL;GVPPLMQ	NCP	1.547
GVPPLMQ;TLAARGM	NCP	15.306
TLAARGM;LTASVP	NCP	16.863
LTASVP;GPAPPVPP	NCP	84.654
GPAPPVPP;GPPGPPP	NCP	52.185
GPPGPPP;LVAGTVLQA	NCP	25.161
LVAGTVLQA;QRLLPPP	NCP	45.645
QRLLPPP;TSVWK	NCP	172.959
TSVWK;SVMFKHL	NCP	50.773
SVMFKHL;LGPGP	NCP	5.207
LGPGP;PGPAVPP	NCP	4.563
PGPAVPP;GRGPQMD	NCP	23.364
GRGPQMD;TPGEPPTP	NCP	15.756
TPGEPPTP;TPAPAP	NCP	9.054
TPAPAP;LRQDL	NCP	4.266
LRQDL;EVGALGRS	NCP	10.275
EVGALGRS;AALPPPP	NCP	6.225
AALPPPP;PRATAGPP	NCP	18
PRATAGPP;EKTFEC	NCP	25.399
EKTFEC;PVDLLPPP	NCP	107.471
PVDLLPPP;LSSTSVK	NCP	66.93
LSSTSVK;DEDAP	NCP	31.068
DEDAP;SFDKSGY	NCP	19.378
SFDKSGY;VVEVGLAP	NCP	203.249
VVEVGLAP;KVLDLF	NCP	40.357
KVLDLF;GLLAL	NCP	19.505
GLLAL;AAPLEYK	NCP	32.77
AAPLEYK;AAARPALG	NCP	47.279
AAARPALG;SGVSL	NCP	14.359
SGVSL;AAALPPPAR	NCP	5.141
AAALPPPAR;PFSNSHNTLKLR	NCP	5.448
PFSNSHNTLKLR;PAEDEFPDLSGHN	NCP	0.039
PAEDEFPDLSGHN;AEDEFPDLSGHNN	NCP	0.003
AEDEFPDLSGHNN;EDEFPDLSGHNNHMA	NCP	0.003
EDEFPDLSGHNNHMA;PDLSGHNNHMAK	NCP	0.012
PDLSGHNNHMAK;VLTPELYAELRAKSTPSGF	NCP	0.036
VLTPELYAELRAKSTPSGF;TPELYAELRAK	NCP	0.006
TPELYAELRAK;MTVGCVAGDEESYDVFK	NCP	0.291
MTVGCVAGDEESYDVFK;SSLDGDLAGRY	NCP	0.66
SSLDGDLAGRY;DGDLAGRYY	NCP	0.009
DGDLAGRYY;NYEFMWNPHLG	NCP	1.146
NYEFMWNPHLG;YEFMWNPHLGY	NCP	0.003
YEFMWNPHLGY;EFMWNPHLG	NCP	0.003

EFMWNPHLG;MWNPHLGY	NCP	0.006
MWNPHLGY;VQMVVDGVKL	NCP	0.318
VQMVVDGVKL;MVVDGVK	NCP	0.006
MVVDGVK;DDLMPAQK	NCP	0.066
DDLMPAQK;WKAAHK	NCP	112.98
WKAAHK;VGLLK	NCP	93.591
VGLLK;LAPAAAL	NCP	137.715
LAPAAAL;PAAAH	NCP	10.698
PAAAH;KTVEPY	NCP	66.421
KTVEPY;EPRRAH	NCP	17.837
EPRRAH;VPLLC	NCP	8.649
VPLLC;MWTAF	NCP	17.803
MWTAF;VGPPVH	NCP	19.553
VGPPVH;VDLLAPL	NCP	21.264
VDLLAPL;FVVSKP	NCP	15.99
FVVSKP;VLLGDC	NCP	72.082
VLLGDC;KTHLQP	NCP	42.104
KTHLQP;KETAK	NCP	91.329
KETAK;GHAAPQP	NCP	46.696
GHAAPQP;DRAPY	NCP	11.526
DRAPY;EHTRPP	NCP	43.191
EHTRPP;NDADLRDK	NCP	34.566
NDADLRDK;SPPLPGPAP	NCP	24.005
SPPLPGPAP;LFEVSGPP	NCP	78.672
LFEVSGPP;TAYLEF	NCP	94.051
TAYLEF;VAPPPPP	NCP	35.753
VAPPPPP;VGKPRLL	NCP	17.173
VGKPRLL;LEPLLK	NCP	45.666
LEPLLK;ESLTDPSKLDSGK	NCP	60.692
ESLTDPSKLDSGK;RDNSTMGYMAAKK	NCP	3.186
RDNSTMGYMAAKK;LYETALLSSGF	NCP	0.138
LYETALLSSGF;LERPTYT	NCP	13.707
LERPTYT;PGVRLLK	NCP	29.562
PGVRLLK;ACARLP	NCP	10.626
ACARLP;DPKPPPAAP	NCP	16.548
DPKPPPAAP;EVPAP	NCP	46.485
EVPAP;PPLGL	NCP	0.561
PPLGL;SNSDVLLY	NCP	48.387
SNSDVLLY;LVREDF	NCP	137.838
LVREDF;VNLML	NCP	43.465
VNLML;LADDML	NCP	29.565
LADDML;TKWGAPK	NCP	107.282
TKWGAPK;KSGGP	NCP	4.012
KSGGP;LDVVRK	NCP	85.424
LDVVRK;VEVLPLPQ	NCP	218.811
VEVLPLPQ;PELLTQ	NCP	26.451
PELLTQ;KATLVL	NCP	6.316
KATLVL;NLSRVQQV	NCP	5.337
NLSRVQQV;PKPPGGNPR	NCP	39.161
PKPPGGNPR;LGPPPYFR	NCP	56.1
LGPPPYFR;ATKKLQP	NCP	4.65

ATKKLQP;GPGCLLK	NCP	46.332
GPGCLLK;KGVVPLRL	NCP	11.775
KGVVPLRL;SLLRVVS	NCP	54.556
SLLRVVS;AALKVF	NCP	126.906
AALKVF;TVPGAP	NCP	90.632
TVPGAP;KGYLQ	NCP	67.719
KGYLQ;KWLLR	NCP	0.549
KWLLR;RLLDDF	NCP	74.949
RLLDDF;TGNRFGP	NCP	52.423
TGNRFGP;APVLS	NCP	11.1
APVLS;GSFRLK	NCP	26.483
GSFRLK;LPMLQ	NCP	41.871
LPMLQ;TKKKPR	NCP	81.042
TKKKPR;PAAMLPK	NCP	5.368
PAAMLPK;TNVARENK	NCP	13.113
TNVARENK;AAQEELVRDLK	NCP	48.851
AAQEELVRDLK;ADEDFVDPW	NCP	5.712
ADEDFVDPW;MTDDEKYLWK	NCP	8.349
MTDDEKYLWK;DLTLDQAYGY	NCP	0.03
DLTLDQAYGY;MTPRKLSYDFQ	NCP	7.161
MTPRKLSYDFQ;DYGGEY	NCP	16.795
DYGGEY;SLARVLTV	NCP	19.652
SLARVLTV;RLLPNR	NCP	39.322
RLLPNR;AEPSLR	NCP	10.109
AEPSLR;LPPLFPQ	NCP	41.056
LPPLFPQ;LLEPSL	NCP	45.263
LLEPSL;LERLYL	NCP	73.015
LERLYL;KRPLLK	NCP	3.519
KRPLLK;KVVKLK	NCP	121.446
KVVKLK;VSLGAL	NCP	48.663
VSLGAL;ARLLNY	NCP	45.833
ARLLNY;YVETQGK	NCP	14.146
YVETQGK;KGHPPFR	NCP	145.149
KGHPPFR;KQSVPR	NCP	10.904
KQSVPR;ADPFP	NCP	18.729
ADPFP;VGKKKVPVG	NCP	6.579
VGKKKVPVG;GPPVVLPY	NCP	10.17
GPPVVLPY;GPPFG	NCP	32.262
GPPFG;LPMPPPP	NCP	16.179
LPMPPPP;SVPTGPR	NCP	0.207
SVPTGPR;LGRLL	NCP	10.194
LGRLL;FGPFVL	NCP	20.917
FGPFVL;TSSLLLR	NCP	20.096
TSSLLLR;KAVSLGLK	NCP	31.242
KAVSLGLK;LVHLFL	NCP	0.208
LVHLFL;LVDPGVK	NCP	84.128
LVDPGVK;PAASRTK	NCP	56.326
PAASRTK;MGLMNS	NCP	28.268
MGLMNS;SFRDPPP	NCP	10.131
SFRDPPP;AAPSPDPR	NCP	0.225
AAPSPDPR;TLSLLF	NCP	45.67

TLSLLF;QKLASLR	NCP	6.495
QKLASLR;QRLRLQ	NCP	2.4
QRLRLQ;RVVPAATR	NCP	14.225
RVVPAATR;MLPPVT	NCP	90.511
MLPPVT;PAAELL	NCP	147.267
PAAELL;AYPGPH	NCP	67.019
AYPGPH;QPPPPP	NCP	105.151
QPPPPP;KDAHGLT	NCP	75.662
KDAHGLT;ERMGKNK	NCP	12.783
ERMGKNK;KTSAAPK	NCP	34.732
KTSAAPK;KPAPFMP	NCP	54.138
KPAPFMP;NTVLLL	NCP	71.175
NTVLLL;YYPPR	NCP	38.627
YYPPR;AEEFTW	NCP	54.19
AEEFTW;TGACVPR	NCP	21.477
TGACVPR;WVGKVK	NCP	39.921
WVGKVK;KFVPKSV	NCP	117.29
KFVPKSV;MTVNL	NCP	1.246
MTVNL;FLPYP	NCP	30.341
FLPYP;KDLHQPK	NCP	20.911
KDLHQPK;DKPGVLLR	NCP	77.427
DKPGVLLR;VYLKRAK	NCP	294.215
VYLKRAK;SPKYFPP	NCP	15.865
SPKYFPP;VGPAPPK	NCP	118.101
VGPAPPK;EVKLP	NCP	20.499
EVKLP;PAGKGAPP	NCP	109.653
PAGKGAPP;ALRASRGL	NCP	109.791
ALRASRGL;FYGKVYSQ	NCP	11.303
FYGKVYSQ;LCEKP	NCP	28.294
LCEKP;PAPEM	NCP	49.431
PAPEM;VKQKVA	NCP	0.921
VKQKVA;TGDTAKK	NCP	19.037
TGDTAKK;KDEGVL	NCP	32.641
KDEGVL;QSALRL	NCP	38.712
QSALRL;AKAVVL	NCP	41.09
AKAVVL;SGPPVPLT	NCP	17.983
SGPPVPLT;YEEHS	NCP	25.791
YEEHS;GRSLYGFN	NCP	19.686
GRSLYGFN;KMVMPP	NCP	75.644
KMVMPP;NDPKGSK	NCP	1.861
NDPKGSK;AAMLAA	NCP	36.068
AAMLAA;KMLTLGV	NCP	51.976
KMLTLGV;DPEGC	NCP	16.257
DPEGC;KLLMGK	NCP	73.953
KLLMGK;PKLPVGV	NCP	2.289
PKLPVGV;ERPGNPPR	NCP	47.604
ERPGNPPR;VAPLVL	NCP	23.229
VAPLVL;LLSDT	NCP	195.072
LLSDT;SFNNVPK	NCP	3.446
SFNNVPK;GVVLF	NCP	55.714
GVVLF;LPLPK	NCP	14.043

LPLPK;PVEAQSK	NCP	24.32
PVEAQSK;QTPTKP	NCP	18.897
QTPTKP;LAGTVRVAP	NCP	17.683
LAGTVRVAP;MQFLVP	NCP	8.165
MQFLVP;TAEGHVPR	NCP	51.1
TAEGHVPR;PPPTLPR	NCP	12.167
PPPTLPR;LPPPVPA	NCP	8.022
LPPPVPA;KKPSNGK	NCP	15.225
KKPSNGK;LSVPP	NCP	168.84
LSVPP;RTSKYR	NCP	17.956
RTSKYR;NHQCE	NCP	52.049
NHQCE;PPRNLVSF	NCP	19.5
PPRNLVSF;PAAFLSAP	NCP	34.059
PAAFLSAP;QTVPPNR	NCP	19.426
QTVPPNR;GKRSAFN	NCP	114.879
GKRSAFN;MGMDD	NCP	21.119
MGMDD;LVGGK	NCP	168.055
LVGGK;LRKLSY	NCP	210.549
LRKLSY;VGKLP	NCP	22.143
VGKLP;SFDKSLK	NCP	123.119
SFDKSLK;QPFLHQ	NCP	6.915
QPFLHQ;KDDDYAK	NCP	66.637
KDDDYAK;PMLLSVLLT	NCP	54.824
PMLLSVLLT;KSSLLGL	NCP	42.249
KSSLLGL;EGWSLK	NCP	196.041
EGWSLK;QTPVEL	NCP	5.805
QTPVEL;NSAPYLR	NCP	14.955
NSAPYLR;KGPPPKY	NCP	28.62
KGPPPKY;KRMTALQ	NCP	72.265
KRMTALQ;RPPTYP	NCP	22.499
RPPTYP;NFSPLRK	NCP	1.701
NFSPLRK;YPSLVHR	NCP	14.173
YPSLVHR;KFTLAGP	NCP	47.217
KFTLAGP;GNPRPSP	NCP	26.835
GNPRPSP;LGNPPRP	NCP	16.358
LGNPPRP;PPPVP	NCP	60.048
PPPVP;KELATKP	NCP	8.386
KELATKP;AAPGLAAPK	NCP	135.23
AAPGLAAPK;PHPPYP	NCP	22.047
PHPPYP;GKWLK	NCP	38.2
GKWLK;KNLRAT	NCP	27.84
KNLRAT;METPSRQ	NCP	5.168
METPSRQ;DLEKDEL	NCP	4.108
DLEKDEL;LTPLFE	NCP	4.908
LTPLFE;VLLSNR	NCP	37.418
VLLSNR;VTPLDEY	NCP	19.096
VTPLDEY;AAPGW	NCP	30.002
AAPGW;VVPVQP	NCP	73.095
VVPVQP;SLGLVTL	NCP	2.112
SLGLVTL;LLFVSTPA	NCP	4.383
LLFVSTPA;RNLPGLT	NCP	163.596

RNLPGLT;VLQLEYSG	NCP	24.759
VLQLEYSG;KETSWLK	NCP	55.203
KETSWLK;VKVLDPNTVF	NCP	21.102
VKVLDPNTVF;NLPHALRYP	NCP	8.395
NLPHALRYP;QLKDKETF	NCP	30.491
QLKDKETF;PLGPLP	NCP	35.98
PLGPLP;KAMSGPK	NCP	239.169
KAMSGPK;KPPKEPQP	NCP	98.519
KPPKEPQP;KHLNEL	NCP	23.532
KHLNEL;TTFEKPK	NCP	28.72
TTFEKPK;AALFH	NCP	56.532
AALFH;MDLLF	NCP	93.023
MDLLF;KMHPSPP	NCP	44.76
KMHPSPP;NGVTLKF	NCP	347.082
NGVTLKF;KEYPLPP	NCP	122.97
KEYPLPP;LPDHRP	NCP	78.288
LPDHRP;VLNLPF	NCP	14.17
VLNLPF;KFPPRP	NCP	29.814
KFPPRP;ASPHP	NCP	21.711
ASPHP;ALPKY	NCP	30.171
ALPKY;DPLKAAK	NCP	30.974
DPLKAAK;GGGRPRPP	NCP	45.033
GGGRPRPP;AARLAPGP	NCP	15.826
AARLAPGP;VTLVAQ	NCP	105.746
VTLVAQ;KNPPLE	NCP	4.609
KNPPLE;ASYERPS	NCP	55.782
ASYERPS;ATTAANPRESSY	NCP	44.138
ATTAANPRESSY;LMQFQGLKH	NCP	2.394
LMQFQGLKH;QFQGLKHQ	NCP	0.006
QFQGLKHQ;KVAVYPL	NCP	7.468
KVAVYPL;TPLSVPPTSP	NCP	73.127
TPLSVPPTSP;PGAVP	NCP	12.45
PGAVP;KDPTEK	NCP	3.208
KDPTEK;MELGP	NCP	23.196
MELGP;KHNAP	NCP	62.175
KHNAP;PVGYKP	NCP	24.602
PVGYKP;VKMPVW	NCP	9.144
VKMPVW;TEDLHLK	NCP	20.662
TEDLHLK;KFLFTQ	NCP	3.888
KFLFTQ;NATLSVH	NCP	11.489
NATLSVH;APPHLK	NCP	41.095
APPHLK;TWDDF	NCP	154.86
TWDDF;LPVVQ	NCP	40.736
LPVVQ;NMVGF	NCP	93.912
NMVGF;FGEKLR	NCP	46.452
FGEKLR;VSSDLEETAGTGSLPK	NCP	0.369
VSSDLEETAGTGSLPK;LPDFS	NCP	27.436
LPDFS;SVGPKTY	NCP	34.628
SVGPKTY;KAGKFYG	NCP	0.372
KAGKFYG;FGDGLFS	NCP	15.963
FGDGLFS;FFLAF	NCP	4.926

FFLAF;ASPTSKNK	NCP	35.308
ASPTSKNK;PLEPCPK	NCP	40.727
PLEPCPK;RDMDVVSGR	NCP	18.75
RDMDVVSGR;GSTYF	NCP	117.085
GSTYF;ATQLLPLK	NCP	76.41
ATQLLPLK;WVPHVAV	NCP	45.771
WVPHVAV;QEHKAGK	NCP	27.53
QEHKAGK;LLQHDAK	NCP	96.519
LLQHDAK;NGGCC	NCP	71.151
NGGCC;APRKPF	NCP	1.336
APRKPF;VPAVP	NCP	24.953
VPAVP;VLLPRP	NCP	21.787
VLLPRP;QQPGRPP	NCP	8.057
QQPGRPP;LLGAYA	NCP	17.376
LLGAYA;AGAQP	NCP	65.202
AGAQP;VPAAFMH	NCP	7.129
VPAAFMH;ESSAG	NCP	96.693
ESSAG;QSLNVP	NCP	58.133
QSLNVP;FLRTQLH	NCP	14.481
FLRTQLH;VPVPVE	NCP	28.227
VPVPVE;VGLSFVPK	NCP	67.599
VGLSFVPK;LVLLRP	NCP	58.909
LVLLRP;SVGLHY	NCP	23.351
SVGLHY;VGLMVG	NCP	20.881
VGLMVG;LFGAGP	NCP	19.523
LFGAGP;LNLLEKDYF	NCP	63.918
LNLLEKDYF;KNRFDN	NCP	22.266
KNRFDN;AALSA	NCP	3.198
AALSA;DPLKSP	NCP	28.644
DPLKSP;AAVLFP	NCP	14.761
AAVLFP;KEAPTP	NCP	10.151
KEAPTP;KPSPYVP	NCP	36.952
KPSPYVP;LPGGFGP	NCP	92.24
LPGGFGP;KSGSFPVF	NCP	160.45
KSGSFPVF;LFVKLY	NCP	75.149
LFVKLY;MATPP	NCP	45.142
MATPP;KGALP	NCP	15.372
KGALP;PVPDL	NCP	9.035
PVPDL;SCLLLRF	NCP	62.728
SCLLLRF;LPGAYK	NCP	21.791
LPGAYK;LLMNENE	NCP	43.212
LLMNENE;CKLLLPPP	NCP	40.105
CKLLLPPP;TMLPPPP	NCP	55.977
TMLPPPP;EAHGK	NCP	158.268
EAHGK;KGVALSH	NCP	21.543
KGVALSH;RGLRHF	NCP	6.771
RGLRHF;KVVAHTT	NCP	6.408
KVVAHTT;KNGAAPP	NCP	0.09
KNGAAPP;YLVPP	NCP	2.574
YLVPP;ALLMF	NCP	240.311
ALLMF;PVYLAQA	NCP	9.682

PVYLAQA;EVHLVEL	NCP	52.869
EVHLVEL;VEVPPNR	NCP	36.923
VEVPPNR;HLKVPQN	NCP	19.695
HLKVPQN;VGPFPF	NCP	223.149
VGPFPF;CHASLDPL	NCP	3.781
CHASLDPL;GLSSNPKK	NCP	184.191
GLSSNPKK;VYKAALK	NCP	57.866
VYKAALK;KGGVL	NCP	1.75
KGGVL;RDVLDF	NCP	2.334
RDVLDF;GVPLMQ	NCP	249.944
GVPLMQ;GPEYKVK	NCP	1.014
GPEYKVK;KDKLVVTS	NCP	116.538
KDKLVVTS;KMGEPLR	NCP	8.442
KMGEPLR;ATGARLK	NCP	79.351
ATGARLK;VNPTVE	NCP	13.476
VNPTVE;LVCSGNP	NCP	108.276
LVCSGNP;MHTPPR	NCP	38.679
MHTPPR;LLVVNQ	NCP	94.379
LLVVNQ;VFAPR	NCP	74.394
VFAPR;KDSPSVW	NCP	95.13
KDSPSVW;TPFVP	NCP	241.579
TPFVP;VLRVFL	NCP	98.321
VLRVFL;TGEQNLK	NCP	135.121
TGEQNLK;VLNLGWKQ	NCP	161.672
VLNLGWKQ;TMEVL	NCP	53.863
TMEVL;LSPVRHVPF	NCP	5.043
LSPVRHVPF;LLTGTQ	NCP	23.132
LLTGTQ;ALTLW	NCP	41.308
ALTLW;VTNLLF	NCP	21.581
VTNLLF;HAHVPR	NCP	64.246
HAHVPR;DSTPLL	NCP	152.442
DSTPLL;AAVFPR	NCP	261.608
AAVFPR;TAGLLLAR	NCP	59.56
TAGLLLAR;NTGVVLVT	NCP	40.199
NTGVVLVT;VGLKLVPT	NCP	250.122
VGLKLVPT;DLTNLKR	NCP	80.311
DLTNLKR;LGTGL	NCP	45.546
LGTGL;CSEKLWH	NCP	2.861
CSEKLWH;RMREPY	NCP	108.526
RMREPY;MESLLQL	NCP	8.084
MESLLQL;FTKSAAK	NCP	17.214
FTKSAAK;EAPLGGP	NCP	8.317
EAPLGGP;EDGFRK	NCP	38.831
EDGFRK;QGKGPLQ	NCP	75.628
QGKGPLQ;LYKLGQ	NCP	84.531
LYKLGQ;AAAWGPQ	NCP	20.622
AAAWGPQ;LMTAP	NCP	13.284
LMTAP;KEAKPR	NCP	3.027
KEAKPR;PPTGK	NCP	41.967
PPTGK;VTTPPPP	NCP	44.655
VTTPPPP;YKLHA	NCP	218.358

YKLHA;GKLQRGK	NCP	0.747
GKLQRGK;TVPTFH	NCP	261.693
TVPTFH;LSGVLKP	NCP	25.803
LSGVLKP;PVGLP	NCP	2.714
PVGLP;AMTLNPR	NCP	7.564
AMTLNPR;KDPPLLP	NCP	73.269
KDPPLLP;VELTLQ	NCP	104.405
VELTLQ;AHAQNYK	NCP	34.555
AHAQNYK;LTPRVM	NCP	20.178
LTPRVM;LSVLP	NCP	57.357
LSVLP;PQLPVEP	NCP	62.805
PQLPVEP;VGEELN	NCP	79.596
VGEELN;RPAPHF	NCP	3.084
RPAPHF;EETVKSLL	NCP	122.78
EETVKSLL;VPPKPLPP	NCP	63.007
VPPKPLPP;VNHKNGK	NCP	16.79
VNHKNGK;KGSDPGSK	NCP	4.96
KGSDPGSK;EPSFPLG	NCP	227.126
EPSFPLG;KQLSL	NCP	6.697
KQLSL;KNKAAP	NCP	1.539
KNKAAP;LSMVVKK	NCP	41.193
LSMVVKK;PPNARAP	NCP	90.939
PPNARAP;CTKLPPPP	NCP	121.275
CTKLPPPP;KLAVSSAA	NCP	16.097
KLAVSSAA;PPYPHT	NCP	50.325
PPYPHT;LPLPPPP	NCP	184.156
LPLPPPP;TPLWAKE	NCP	9.068
TPLWAKE;TVTLLK	NCP	9.081
TVTLLK;KDLVCVK	NCP	20.373
KDLVCVK;TNVQV	NCP	51.838
TNVQV;EASSG	NCP	50.622
EASSG;PAGVP	NCP	139.152
PAGVP;RRKAH	NCP	8.814
RRKAH;PPPPKNQ	NCP	19.074
PPPPKNQ;QGSPLWSP	NCP	32.256
QGSPLWSP;MVHVTF	NCP	54.128
MVHVTF;FPFPPPP	NCP	27.996
FPFPPPP;QAPVYM	NCP	2.172
QAPVYM;QGCDLRK	NCP	9.388
QGCDLRK;KPFTNPPP	NCP	41.748
KPFTNPPP;AAGGN	NCP	5.892
AAGGN;PRSSN	NCP	31.242
PRSSN;SASAYRP	NCP	32.241
SASAYRP;APPSYG	NCP	12.48
APPSYG;TKFQR	NCP	18.462
TKFQR;VPPPVP	NCP	11.955
VPPPVP;NSLRVHLGW	NCP	4.806
NSLRVHLGW;LPPSLL	NCP	31.92
LPPSLL;KLTLPEKF	NCP	121.895
KLTLPEKF;DDERD	NCP	51.574
DDERD;FLLPKSV	NCP	40.887

FLLPKSV;NTPMLQ	NCP	10.359
NTPMLQ;LVFTLK	NCP	59.73
LVFTLK;VLRFVLA	NCP	4.143
VLRFVLA;KKTLMH	NCP	17.562
KKTLMH;EPQLLKTE	NCP	27.054
EPQLLKTE;KKAVLQ	NCP	14.483
KKAVLQ;KMGVPLL	NCP	60.66
KMGVPLL;KSLKVLKK	NCP	144.852
KSLKVLKK;PPPGPP	NCP	2.737
PPPGPP;AMLSEK	NCP	5.844
AMLSEK;LLKYN	NCP	41.681
LLKYN;VFGLDR	NCP	32.671
VFGLDR;SGPKLT	NCP	8.954
SGPKLT;TLPPHRP	NCP	30.427
TLPPHRP;LSPSDLK	NCP	15.968
LSPSDLK;PALVKKFQ	NCP	16.132
PALVKKFQ;TENGPK	NCP	33.926
TENGPK;GAPSFPAAR	NCP	38.421
GAPSFPAAR;KVSLLLH	NCP	130.743
KVSLLLH;PVGDMF	NCP	6.439
PVGDMF;EAPGKEQ	NCP	20.514
EAPGKEQ;KGCHLEVR	NCP	216.899
KGCHLEVR;SVNPMVK	NCP	39.336
SVNPMVK;TGVFELNF	NCP	5.719
TGVFELNF;YQGELL	NCP	147.909
YQGELL;DNNKTFL	NCP	93.381
DNNKTFL;MSEEGP	NCP	3.959
MSEEGP;LEGKLP	NCP	118.906
LEGKLP;LGPFNPLL	NCP	143.363
LGPFNPLL;QAPVYLA	NCP	65.07
QAPVYLA;YLHATAF	NCP	52.02
YLHATAF;KGVAAP	NCP	12.34
KGVAAP;KGTSLVV	NCP	21.273
KGTSLVV;KNFVPP	NCP	56.505
KNFVPP;PALGARPP	NCP	21.789
PALGARPP;LDDLPQL	NCP	3.414
LDDLPQL;QSALLEY	NCP	25.451
QSALLEY;SVKPRPH	NCP	12.802
SVKPRPH;QMSLLLR	NCP	145.511
QMSLLLR;KPFTPR	NCP	41.784
KPFTPR;KGPVP	NCP	23.296
KGPVP;RLFTSSK	NCP	95.217
RLFTSSK;MGKVP	NCP	26.774
MGKVP;HVKKAH	NCP	33.103
HVKKAH;FSCCYTF	NCP	121.167
FSCCYTF;LVVDN	NCP	3.372
LVVDN;DKVLG	NCP	25.4
DKVLG;TVASKPK	NCP	51.121
TVASKPK;ELFGTST	NCP	35.685
ELFGTST;EYASSFSF	NCP	4.896
EYASSFSF;AAPKL	NCP	33.893

AAPKL;DALKKK	NCP	24.931
DALKKK;QMCCC	NCP	7.476
QMCCC;GLDASLF	NCP	5.046
GLDASLF;VVENFFK	NCP	226.53
VVENFFK;KNPLAQP	NCP	17.952
KNPLAQP;VTFRLK	NCP	19.82
VTFRLK;AVGVG	NCP	91.032
AVGVG;FLDLL	NCP	24.856
FLDLL;VSQPGY	NCP	3.002
VSQPGY;ASYKQVH	NCP	38.254
ASYKQVH;VFNDMKVRK	NCP	34.481
VFNDMKVRK;FNDMKVR	NCP	0.003
FNDMKVR;LLPLNDCR	NCP	0.177
LLPLNDCR;ALYDATYETKESKKEDL	NCP	0.027
ALYDATYETKESKKEDL;LYDATYETKESK	NCP	0.003
LYDATYETKESK;YDATYETKESKKEDL	NCP	0.003
YDATYETKESKKEDL;HSNFSHV	NCP	38.824
HSNFSHV;EGLEW	NCP	13.781
EGLEW;PFVSPLL	NCP	0.906
PFVSPLL;VLLPVPAG	NCP	145.584
VLLPVPAG;KNFGPPP	NCP	67.773
KNFGPPP;APAELL	NCP	24.892
APAELL;KNYKKM	NCP	163.254
KNYKKM;EGFDLLR	NCP	33.437
EGFDLLR;NSALL	NCP	149.85
NSALL;FMPLP	NCP	12.364
FMPLP;HSLLMTAL	NCP	117.093
HSLLMTAL;KDRWQAV	NCP	98.645
KDRWQAV;KPGENAP	NCP	33.708
KPGENAP;EMMEPP	NCP	14.449
EMMEPP;LGYPYPR	NCP	48.65
LGYPYPR;KAAVHPK	NCP	14.538
KAAVHPK;MKSLVMK	NCP	25.489
MKSLVMK;VSPRFAP	NCP	77.676
VSPRFAP;CTHVQKH	NCP	46.421
CTHVQKH;QHLENLGN	NCP	13.228
QHLENLGN;SSDDC	NCP	19.734
SSDDC;LGVVK	NCP	29.022
LGVVK;SCYCC	NCP	57.846
SCYCC;PQANS	NCP	131.615
PQANS;LAPLLK	NCP	6.352
LAPLLK;LVPNELR	NCP	216.782
LVPNELR;GVKGLL	NCP	19.477
GVKGLL;NSPYVCLL	NCP	92.331
NSPYVCLL;PLSGRP	NCP	7.124
PLSGRP;KFLEGG	NCP	9.795
KFLEGG;VPQLNL	NCP	14.353
VPQLNL;GDDAFK	NCP	59.322
GDDAFK;PVVNVKS	NCP	129.009
PVVNVKS;PVSKVP	NCP	128.88
PVSKVP;GPPHCPP	NCP	31.875

GPPHCPP;LRGLTL	NCP	27.681
LRGLTL;VPSYVH	NCP	26.276
VPSYVH;KTQKSPPP	NCP	4.029
KTQKSPPP;LLLLTMTT	NCP	118.708
LLLLTMTT;PLSHAAFK	NCP	11.603
PLSHAAFK;MNTLV	NCP	65.524
MNTLV;VGVVL	NCP	165.284
VGVVL;TPTGHP	NCP	150.276
TPTGHP;KSMVPPP	NCP	7.317
KSMVPPP;AAHVVPPP	NCP	12.634
AAHVVPPP;KACSSSAH	NCP	16.596
KACSSSAH;VPHGRVP	NCP	9.921
VPHGRVP;EPPDP	NCP	15.57
EPPDP;HPPNTRQ	NCP	4.533
HPPNTRQ;YPALTP	NCP	408.569
YPALTP;VGAVLK	NCP	46.615
VGAVLK;GGPPERP	NCP	15.849
GGPPERP;DALKKKLT	NCP	81.627
DALKKKLT;KKMMQLK	NCP	34.85
KKMMQLK;LVETLK	NCP	5.211
LVETLK;KMGRTLE	NCP	158.044
KMGRTLE;GPFEL	NCP	16.419
GPFEL;NPHAKGK	NCP	176.979
NPHAKGK;APVAGP	NCP	29.121
APVAGP;RLSTPLK	NCP	0.624
RLSTPLK;ASEPRLH	NCP	94.518
ASEPRLH;RWLPR	NCP	64.851
RWLPR;KGNPPVP	NCP	128.471
KGNPPVP;YSLVGL	NCP	66.144
YSLVGL;TAALLK	NCP	8.43
TAALLK;LDDLP	NCP	109.566
LDDLP;LVHQPP	NCP	30.84
LVHQPP;AYSSMFF	NCP	300.267
AYSSMFF;GVRFAVP	NCP	75.571
GVRFAVP;PPPPGKGE	NCP	37.53
PPPPGKGE;WFMKK	NCP	79.202
WFMKK;LKVLLP	NCP	86.457
LKVLLP;SPDYAVP	NCP	16.116
SPDYAVP;TKAGATK	NCP	44.109
TKAGATK;LLEKEVLDK	NCP	51.546
LLEKEVLDK;DTSLPEGLK	NCP	0.117
DTSLPEGLK;QVGSTFF	NCP	93.154
QVGSTFF;DPPNE	NCP	25.038
DPPNE;KKTSGGK	NCP	49.781
KKTSGGK;VGAPPER	NCP	25.51
VGAPPER;DMCHTK	NCP	37.47
DMCHTK;VLPSMT	NCP	74.13
VLPSMT;KTTPQP	NCP	129.185
KTTPQP;CGFPVKK	NCP	141.604
CGFPVKK;QPGFFL	NCP	27.75
QPGFFL;LGVVSVK	NCP	275.574

LGVVSVK;KACLRL	NCP	20.859
KACLRL;TTPGF	NCP	59.736
TTPGF;GLPPT	NCP	5.177
GLPPT;RFTLKL	NCP	35.134
RFTLKL;EGEFSEARE	NCP	12.149
EGEFSEARE;ESEQLK	NCP	44.97
ESEQLK;QSGFNVK	NCP	18.207
QSGFNVK;QEALYLK	NCP	81.511
QEALYLK;LSATPF	NCP	36.059
LSATPF;ALASGF	NCP	28.531
ALASGF;NGYAATF	NCP	81.951
NGYAATF;KKFKTGP	NCP	110.91
KKFKTGP;KQGLSKP	NCP	39.504
KQGLSKP;KEVLL	NCP	52.757
KEVLL;LSLLN	NCP	10.26
LSLLN;LEVSLGGNK	NCP	32.916
LEVSLGGNK;PPPSP	NCP	34.075
PPPSP;ELDGVTWK	NCP	27.465
ELDGVTWK;LPTLRLL	NCP	11.589
LPTLRLL;KVLTAF	NCP	33.678
KVLTAF;TTGKF	NCP	47.127
TTGKF;TTLGALVL	NCP	109.29
TTLGALVL;LSSPP	NCP	33.941
LSSPP;VYLKTQ	NCP	193.636
VYLKTQ;TLTVLPTLG	NCP	37.191
TLTVLPTLG;SLLVSVL	NCP	14.802
SLLVSVL;KPTVPFY	NCP	13.073
KPTVPFY;VGPFG	NCP	79.723
VGPFG;TTLLVK	NCP	0.101
TTLLVK;KPAANVH	NCP	30.877
KPAANVH;KTYLYPK	NCP	141.065
KTYLYPK;KDSLMAK	NCP	1.873
KDSLMAK;MGVVTF	NCP	94.233
MGVVTF;YLLLSHVT	NCP	33.791
YLLLSHVT;PGVFYNP	NCP	66.781
PGVFYNP;QFQRYK	NCP	105.057
QFQRYK;VPLAP	NCP	106.881
VPLAP;VGAVGL	NCP	15.269
VGAVGL;KSVGKPLK	NCP	29.217
KSVGKPLK;KDPALPP	NCP	175.258
KDPALPP;QHMAGK	NCP	1.617
QHMAGK;ALEVLRP	NCP	30.726
ALEVLRP;AVQLSK	NCP	28.688
AVQLSK;LGASPTP	NCP	51.373
LGASPTP;KGVELLP	NCP	5.676
KGVELLP;FPACAP	NCP	37.044
FPACAP;PPPPPKY	NCP	16.119
PPPPPKY;FEELGF	NCP	76.155
FEELGF;RDGPDPK	NCP	84.24
RDGPDPK;QMRGVEF	NCP	73.761
QMRGVEF;TVHKPQ	NCP	89.469

TVHKPQ;YLDLPK	NCP	116.985
YLDLPK;ECCYLR	NCP	28.19
ECCYLR;DKPFLNT	NCP	80.685
DKPFLNT;LCWLVLH	NCP	108.13
LCWLVLH;DDDLRPR	NCP	57.876
DDDLRPR;KPNGVPP	NCP	34.056
KPNGVPP;APSSD	NCP	27.912
APSSD;GHDSFLK	NCP	33.149
GHDSFLK;DHFEYM	NCP	12.843
DHFEYM;MLDLLR	NCP	76.668
MLDLLR;VEVPVLA	NCP	98.605
VEVPVLA;DSALLHV	NCP	95.127
DSALLHV;CHGPTSK	NCP	79.316
CHGPTSK;SSLLLPKK	NCP	27.832
SSLLLPKK;YRAGPP	NCP	24.861
YRAGPP;GPGPAL	NCP	2.784
GPGPAL;KRAFPPP	NCP	79.869
KRAFPPP;FPGAG	NCP	34.614
FPGAG;GDLLSL	NCP	9.915
GDLLSL;VPTKHAK	NCP	37.142
VPTKHAK;LHPDP	NCP	22.222
LHPDP;YEHVGLHD	NCP	174.009
YEHVGLHD;GLPNAKPP	NCP	1.014
GLPNAKPP;MAFLLFKT	NCP	11.202
MAFLLFKT;LLFKTSGK	NCP	0.009
LLFKTSGK;AEVKPGMM	NCP	0.969
AEVKPGMM;KDLKVH	NCP	102.798
KDLKVH;KLAPPP	NCP	9.06
KLAPPP;LLVHRR	NCP	132.03
LLVHRR;YMSSPLKK	NCP	85.293
YMSSPLKK;QDALLEF	NCP	3.468
QDALLEF;VPDDD	NCP	9.702
VPDDD;ESRVMNK	NCP	76.08
ESRVMNK;YMTLST	NCP	198.062
YMTLST;FVLTAAH	NCP	1.509
FVLTAAH;KALKLLKT	NCP	73.084
KALKLLKT;LSGCCPF	NCP	34.151
LSGCCPF;VDPAM	NCP	52.75
VDPAM;HLELSY	NCP	35.051
HLELSY;KVTEPNK	NCP	58.152
KVTEPNK;SGLAALKK	NCP	32.908
SGLAALKK;PLLPPPRPPP	NCP	54.839
PLLPPPRPPP;KVPGPP	NCP	8.428
KVPGPP;VPEALYV	NCP	211.598
VPEALYV;TPRANQP	NCP	18.807
TPRANQP;NSGFDLL	NCP	5.317
NSGFDLL;TVFLFHEC	NCP	85.281
TVFLFHEC;SYQALY	NCP	137.852
SYQALY;SSKMAP	NCP	85.393
SSKMAP;ERLVPR	NCP	87.602
ERLVPR;MEDSHVL	NCP	44.067

MEDSHVL;GDSPLVLY	NCP	51.147
GDSPLVLY;VPYLTK	NCP	160.719
VPYLTK;VVPSVPK	NCP	11.916
VVPSVPK;AAPFP	NCP	39.555
AAPFP;LHGVLPF	NCP	26.547
LHGVLPF;KAGRLGQP	NCP	31.689
KAGRLGQP;KTHVTK	NCP	17.37
KTHVTK;FPPPPGTP	NCP	169.233
FPPPPGTP;LVPVLMEF	NCP	34.017
LVPVLMEF;SHMLRR	NCP	86.827
SHMLRR;VPAKAPP	NCP	56.987
VPAKAPP;YCVAL	NCP	45.579
YCVAL;KSCDMKP	NCP	2.001
KSCDMKP;KVCVHRK	NCP	4.027
KVCVHRK;AMCCC	NCP	26.934
AMCCC;VEGLL	NCP	0.119
VEGLL;SYHVPR	NCP	78.453
SYHVPR;VGGMP	NCP	42.366
VGGMP;ESAGTLGF	NCP	34.281
ESAGTLGF;QGSPPKHL	NCP	59.853
QGSPPKHL;WQLLLDH	NCP	41.329
WQLLLDH;DSLFPAST	NCP	76.977
DSLFPAST;GSVPKV	NCP	135.492
GSVPKV;QKKCVL	NCP	278.922
QKKCVL;FGGAGAGAL	NCP	21.716
FGGAGAGAL;VRRPLQ	NCP	46.854
VRRPLQ;LTKYPLE	NCP	8.251
LTKYPLE;ALTVY	NCP	33.66
ALTVY;GYTVEAF	NCP	3.773
GYTVEAF;KDFVLT	NCP	275.026
KDFVLT;YSSFGR	NCP	53.549
YSSFGR;DVFLYT	NCP	29.65
DVFLYT;GVPGVRL	NCP	3.173
GVPGVRL;PPKVFNK	NCP	2.194
PPKVFNK;EAHPAKF	NCP	12.492
EAHPAKF;VLVAQHDAY	NCP	114.962
VLVAQHDAY;YDLADQLHA	NCP	6.801
YDLADQLHA;VVPEMTELLKKK	NCP	47.982
VVPEMTELLKKK;RPQLPD	NCP	25.488
RPQLPD;KYVPQ	NCP	3.597
KYVPQ;ETMKAK	NCP	55.063
ETMKAK;SPVVLVT	NCP	99.833
SPVVLVT;KMAEPPL	NCP	279.225
KMAEPPL;GPPVQP	NCP	6.723
GPPVQP;LTPLRLL	NCP	59.727
LTPLRLL;CQYDC	NCP	34.381
CQYDC;EKLAC	NCP	112.05
EKLAC;PVFRPQ	NCP	88.445
PVFRPQ;QQNALTK	NCP	130.441
QQNALTK;YAATVVP	NCP	183.734
YAATVVP;AAPVLLR	NCP	31.206

AAPVLLR;LSFPPPPP	NCP	4.26
LSFPPPPP;KPPQFHF	NCP	1.09
KPPQFHF;HPPGRPP	NCP	53.408
HPPGRPP;KMYFFPP	NCP	39.798
KMYFFPP;KLVALLPF	NCP	3.367
KLVALLPF;LDAKVFL	NCP	11.198
LDAKVFL;KYLSNLL	NCP	15.342
KYLSNLL;EGRNLLGL	NCP	106.657
EGRNLLGL;NSRLKL	NCP	25.979
NSRLKL;PDTLSGK	NCP	53.17
PDTLSGK;TSSLLVAK	NCP	67.587
TSSLLVAK;EPVYE	NCP	34.211
EPVYE;KYLANVL	NCP	96.043
KYLANVL;KHDFGGE	NCP	4.563
KHDFGGE;KTKSSPLQ	NCP	314.682
KTKSSPLQ;RVRGRP	NCP	48.287
RVRGRP;DCCFLR	NCP	103.788
DCCFLR;AFVTLK	NCP	3.814
AFVTLK;PKPAQPP	NCP	25.844
PKPAQPP;KALTF	NCP	21.444
KALTF;SDTALLSR	NCP	88.291
SDTALLSR;HYEKGP	NCP	9.095
HYEKGP;QAHELVK	NCP	103.617
QAHELVK;AVGELL	NCP	91.972
AVGELL;APGYKNK	NCP	46.154
APGYKNK;LSGGGLL	NCP	10.773
LSGGGLL;EHPSWLPP	NCP	10.548
EHPSWLPP;FEAVP	NCP	34.749
FEAVP;DKLETR	NCP	68.01
DKLETR;KREEPK	NCP	123.667
KREEPK;CLSELGQK	NCP	10.451
CLSELGQK;PVAPTPK	NCP	7.929
PVAPTPK;APPFL	NCP	200.215
APPFL;ERPLGAF	NCP	2.342
ERPLGAF;KPLLFER	NCP	55.612
KPLLFER;AVDRLNY	NCP	36.245
AVDRLNY;PAPSASAVNPHVD	NCP	4.341
PAPSASAVNPHVD;LEEPSPAPPSSDSPHADS	NCP	0.054
LEEPSPAPPSSDSPHADS;RAPGTEAAHVQLK	NCP	0.057
RAPGTEAAHVQLK;HEEPPAPAPGYDVHGR	NCP	1.107
HEEPPAPAPGYDVHGR;AGPKVPPEPKP	NCP	0.078
AGPKVPPEPKP;VPPEPKPY	NCP	0.012
VPPEPKPY;NETAHKPDLS	NCP	7.215
NETAHKPDLS;FGKSHQH	NCP	34.404
FGKSHQH;VDTRKF	NCP	51.577
VDTRKF;EKPNKK	NCP	47.004
EKPNKK;LVFPER	NCP	134.27
LVFPER;RKPLGR	NCP	98.079
RKPLGR;FVRLAGP	NCP	13.902
FVRLAGP;KDGLLEF	NCP	9.652
KDGLLEF;GLASAPR	NCP	60.503

GLASAPR;QTPVP	NCP	7.296
QTPVP;KLLVWPPP	NCP	24.996
KLLVWPPP;RLGVF	NCP	13.729
RLGVF;LGPAANVP	NCP	7.928
LGPAANVP;LVEYL	NCP	15.783
LVEYL;LFRPPFG	NCP	14.913
LFRPPFG;ASMLK	NCP	18.702
ASMLK;CCNPQPP	NCP	50.634
CCNPQPP;KPKLYFH	NCP	24.616
KPKLYFH;LPPVAL	NCP	54.656
LPPVAL;AFLGPPPP	NCP	6.09
AFLGPPPP;VFAARPPPP	NCP	91.617
VFAARPPPP;PLRSGP	NCP	41.388
PLRSGP;KGVAGG	NCP	48.967
KGVAGG;LTPEL	NCP	71.63
LTPEL;MFSLKPPRP	NCP	25.347
MFSLKPPRP;ALLNL	NCP	3.816
ALLNL;ASNFKK	NCP	64.164
ASNFKK;CMMSSY	NCP	64.374
CMMSSY;PAGGK	NCP	43.675
PAGGK;FYDTDLR	NCP	89.115
FYDTDLR;MMFDVD	NCP	30.618
MMFDVD;TFKAQ	NCP	140.634
TFKAQ;MSGKFPP	NCP	13.145
MSGKFPP;GDLEK	NCP	119.289
GDLEK;EGPAETPGEATPPPR	NCP	77.118
EGPAETPGEATPPPR;MLLYVLLL	NCP	42.769
MLLYVLLL;ETLRVT	NCP	17.652
ETLRVT;MCPPPRP	NCP	37.29
MCPPPRP;NHLHQVTK	NCP	123.719
NHLHQVTK;TPQLT	NCP	42.717
TPQLT;LEAYTL	NCP	94.309
LEAYTL;LETLPK	NCP	49.13
LETLPK;KGPSLT	NCP	25.656
KGPSLT;LTTAKF	NCP	54.075
LTTAKF;QEEAEPK	NCP	140.341
QEEAEPK;VELLAY	NCP	88.124
VELLAY;KSSALVV	NCP	48.135
KSSALVV;HLGAPPPPP	NCP	17.004
HLGAPPPPP;DTSLLYL	NCP	13.753
DTSLLYL;MADVLE	NCP	14.637
MADVLE;AAAVAVSGAKRS	NCP	45.857
AAAVAVSGAKRS;WGKPLF	NCP	15.607
WGKPLF;LQSQDVKPYTL	NCP	0.188
LQSQDVKPYTL;QKFPPPPP	NCP	62.401
QKFPPPPP;PLPPMPP	NCP	74.384
PLPPMPP;KPGGP	NCP	56.436
KPGGP;KETVP	NCP	67.501
KETVP;VLLGKLAL	NCP	115.034
VLLGKLAL;PLLPPPYK	NCP	16.419
PLLPPPYK;KELQAKGK	NCP	15.058

KELQAKGK;WAGVP	NCP	68.709
WAGVP;VGPGLP	NCP	22.133
VGPGLP;VLSATKPP	NCP	52.492
VLSATKPP;MMRKKK	NCP	35.264
MMRKKK;VGGPLLK	NCP	31.842
VGGPLLK;KKLEVWEK	NCP	48.99
KKLEVWEK;CDDAGPK	NCP	8.598
CDDAGPK;EEGSVK	NCP	40.822
EEGSVK;KLADWK	NCP	7.58
KLADWK;LPPPPPAP	NCP	2.667
LPPPPPAP;AFEVTADF	NCP	17.88
AFEVTADF;KDGSLELL	NCP	10.767
KDGSLELL;KPGLSPPP	NCP	11.476
KPGLSPPP;GDCCC	NCP	58.277
GDCCC;VVNPPPP	NCP	52.009
VVNPPPP;LPSPLLP	NCP	32.631
LPSPLLP;DMLVVHP	NCP	16.338
DMLVVHP;RVFPLE	NCP	44.457
RVFPLE;PKPPPPPPP	NCP	34.979
PKPPPPPPP;TEVLLPKK	NCP	55.821
TEVLLPKK;CDDEE	NCP	227.298
CDDEE;YTVPVN	NCP	29.023
YTVPVN;VGHSLV	NCP	15.375
VGHSLV;GSALYC	NCP	41.972
GSALYC;VGVVPPR	NCP	13.582
VGVVPPR;DLMAP	NCP	10.371
DLMAP;EPAVPGKP	NCP	52.823
EPAVPGKP;VDPGFM	NCP	50.938
VDPGFM;NLDPFLD	NCP	0.542
NLDPFLD;FASPPP	NCP	35.632
FASPPP;VLTTPQLV	NCP	44.706
VLTTPQLV;VDGVSSF	NCP	14.805
VDGVSSF;KHSSK	NCP	16.934
KHSSK;LHPLP	NCP	61.432
LHPLP;KLEFY	NCP	29.859
KLEFY;DLSFLPL	NCP	1.277
DLSFLPL;GYVNF	NCP	21.942
GYVNF;KAKAEC	NCP	7.093
KAKAEC;KNYPSK	NCP	17.159
KNYPSK;KFHYYR	NCP	37.252
KFHYYR;VLAHVDH	NCP	97.523
VLAHVDH;VFALKK	NCP	15.228
VFALKK;TTLPVKFL	NCP	112.123
TTLPVKFL;MEPPLP	NCP	35.303
MEPPLP;SVAPFVK	NCP	160.521
SVAPFVK;RYQLQGV	NCP	37.635
RYQLQGV;PSAAPFF	NCP	66.757
PSAAPFF;RVPTP	NCP	14.145
RVPTP;KRSSVDLL	NCP	57.669
KRSSVDLL;KNEEPSEEELGAPKPK	NCP	126.665
KNEEPSEEELGAPKPK;PVLVAYR	NCP	109.734

PVLVAYR;LDGVLFT	NCP	33.813
LDGVLFT;LQFYTQL	NCP	146.659
LQFYTQL;LDRLSE	NCP	17.013
LDRLSE;GLPTVP	NCP	14.816
GLPTVP;MTPVPP	NCP	128.505
MTPVPP;VTHLMKR	NCP	141.762
VTHLMKR;TGPAAMLR	NCP	21.526
TGPAAMLR;VKPLP	NCP	49.608
VKPLP;KDGLFP	NCP	109.505
KDGLFP;KMVKGGP	NCP	25.618
KMVKGGP;AVPTW	NCP	70.466
AVPTW;PAAAAVVK	NCP	91.2
PAAAAVVK;LLALLAGP	NCP	28.965
LLALLAGP;SDLPVP	NCP	29.98
SDLPVP;LFVLVHRE	NCP	82.718
LFVLVHRE;VLENAG	NCP	31.585
VLENAG;LVPELF	NCP	59.4
LVPELF;LDRKDL	NCP	28.752
LDRKDL;LGNFFSPK	NCP	35.177
LGNFFSPK;LFKLGLAPQ	NCP	10.989
LFKLGLAPQ;QSVPTVK	NCP	28.065
QSVPTVK;SDLVTLTKP	NCP	10.14
SDLVTLTKP;LGEGAGNLNDPNKEALAK	NCP	2.532
LGEGAGNLNDPNKEALAK;DPNKEALAKTEVSL	NCP	0.027
DPNKEALAKTEVSL;TNKFDVPGDEN	NCP	0.048
TNKFDVPGDEN;LTELGTVDPKN	NCP	2.718
LTELGTVDPKN;TELGTVDPK	NCP	0.003
TELGTVDPK;LQQTYAALNSK	NCP	0.855
LQQTYAALNSK;NSKATFYGEQVDYY	NCP	0.024
NSKATFYGEQVDYY;STKNTLLK	NCP	58.984
STKNTLLK;LLELNQV	NCP	196.995
LLELNQV;ESKLTSLP	NCP	2.997
ESKLTSLP;AQLTVK	NCP	91.122
AQLTVK;PLVPLLP	NCP	16.478
PLVPLLP;QLKAPVP	NCP	61.843
QLKAPVP;SGLAHRY	NCP	8.733
SGLAHRY;EPAAHAP	NCP	67.286
EPAAHAP;APAGAPP	NCP	14.607
APAGAPP;KKHEVM	NCP	33.24
KKHEVM;KHFPPGP	NCP	41.988
KHFPPGP;KVVFTPKDGSGPK	NCP	38.379
KVVFTPKDGSGPK;KEALAVT	NCP	7.08
KEALAVT;EPRFVHL	NCP	21.301
EPRFVHL;APCLPLQR	NCP	20.759
APCLPLQR;SPADS	NCP	182.22
SPADS;PAKWHPK	NCP	45.294
PAKWHPK;VVPVGV	NCP	1.596
VVPVGV;LSQEGPQR	NCP	33.07
LSQEGPQR;KSPTNR	NCP	82.691
KSPTNR;LVDLEK	NCP	140.199
LVDLEK;GLPKGM	NCP	56.247

GLPKGM;SSHYTKL	NCP	21.886
SSHYTKL;PSERLPPP	NCP	21.068
PSERLPPP;DGEVEPR	NCP	4.518
DGEVEPR;AANTPVF	NCP	8.403
AANTPVF;RMFELSP	NCP	44.185
RMFELSP;LVEPLF	NCP	24.098
LVEPLF;DMPDLR	NCP	22.665
DMPDLR;PLLLSVK	NCP	176.274
PLLLSVK;VGLVSKRP	NCP	147.823
VGLVSKRP;EVLLKKK	NCP	14.744
EVLLKKK;LDAPTSR	NCP	105.462
LDAPTSR;GAAYL	NCP	18.675
GAAYL;GFPLP	NCP	19.69
GFPLP;KAAPLQ	NCP	73.749
KAAPLQ;RKYLLFV	NCP	18.948
RKYLLFV;KGELFQA	NCP	39.708
KGELFQA;AYTLGVK	NCP	91.584
AYTLGVK;EYLRY	NCP	19.854
EYLRY;TGCGETQ	NCP	135.218
TGCGETQ;AAVELV	NCP	85.341
AAVELV;KGKVYVFF	NCP	224.836
KGKVYVFF;ADVFP	NCP	33.356
ADVFP;LGPPK	NCP	147.783
LGPPK;KVTKF	NCP	155.908
KVTKF;EVPGANY	NCP	83.655
EVPGANY;FTGTVVP	NCP	58.472
FTGTVVP;EAQFK	NCP	49.324
EAQFK;EVQEPLQK	NCP	28.244
EVQEPLQK;QLEVLK	NCP	88.86
QLEVLK;CHFLQPK	NCP	16.446
CHFLQPK;KAVVK	NCP	180.801
KAVVK;SESQFWN	NCP	6.543
SESQFWN;LWGAVG	NCP	204.162
LWGAVG;KPAPAVVK	NCP	246.04
KPAPAVVK;LPLVLTT	NCP	12.27
LPLVLTT;GYLAK	NCP	113.865
GYLAK;KGVSVPLP	NCP	41.016
KGVSVPLP;VTVLKPF	NCP	69.387
VTVLKPF;LSVLEY	NCP	9.399
LSVLEY;KASSKLVP	NCP	1.266
KASSKLVP;FEPEFQL	NCP	17.51
FEPEFQL;LLTERGYS	NCP	138.276
LLTERGYS;KYSQF	NCP	58.14
KYSQF;VGQYGPL	NCP	32.575
VGQYGPL;LLSHVTLA	NCP	42.179
LLSHVTLA;VLKRLLTE	NCP	135.694
VLKRLLTE;DSMPL	NCP	9.821
DSMPL;YRLKY	NCP	16.354
YRLKY;DEVWKTY	NCP	32.117
DEVWKTY;KRTFRKF	NCP	38.209
KRTFRKF;LAGGTEF	NCP	22.965

LAGGTEF;CRFGEKL	NCP	55.157
CRFGEKL;LLEQPL	NCP	26.488
LLEQPL;LLTMPVSK	NCP	7.523
LLTMPVSK;SSYMPR	NCP	235.878
SSYMPR;KVYFEP	NCP	9.574
KVYFEP;AACLYE	NCP	154.997
AACLYE;RYPVRT	NCP	16.321
RYPVRT;QGLPQYK	NCP	102.431
QGLPQYK;TVVAP	NCP	170.47
TVVAP;APLGGPVR	NCP	147.134
APLGGPVR;QQLAY	NCP	153.547
QQLAY;KEQNPLP	NCP	9.449
KEQNPLP;ALYLVR	NCP	6.361
ALYLVR;ELKEKL	NCP	33.824
ELKEKL;ALSMPL	NCP	57.132
ALSMPL;EDLFTVA	NCP	3.93
EDLFTVA;ALLHLFR	NCP	49.716
ALLHLFR;PMSPPLGN	NCP	19.173
PMSPPLGN;YFGPKP	NCP	54.808
YFGPKP;KTLGVL	NCP	21.818
KTLGVL;GVVVL	NCP	29.848
GVVVL;RPTAPTL	NCP	4.538
RPTAPTL;LFLPLEF	NCP	146.793
LFLPLEF;GKLPPR	NCP	58.671
GKLPPR;NTPLYPQ	NCP	106.354
NTPLYPQ;PPYGKPP	NCP	9.251
PPYGKPP;MPPKY	NCP	146.224
MPPKY;GVFHSTC	NCP	7.358
GVFHSTC;FNFHV	NCP	12.789
FNFHV;RHLLPR	NCP	91.012
RHLLPR;RSFWLQH	NCP	13.88
RSFWLQH;QEELREY	NCP	141.619
QEELREY;VSGPLK	NCP	145.476
VSGPLK;LSWAPSL	NCP	117.225
LSWAPSL;MMKAKGK	NCP	10.794
MMKAKGK;TAAHGNK	NCP	61.818
TAAHGNK;KMHFESR	NCP	79.239
KMHFESR;AFLPQP	NCP	9.875
AFLPQP;NYDEYR	NCP	5.892
NYDEYR;PFLGLP	NCP	71.472
PFLGLP;KPLGGAPP	NCP	180.325
KPLGGAPP;AHLVERK	NCP	96.644
AHLVERK;PPPPPRFLQ	NCP	38.565
PPPPPRFLQ;VPGLPPVQ	NCP	27.903
VPGLPPVQ;EDDMPP	NCP	77.131
EDDMPP;PGLTRPP	NCP	99.065
PGLTRPP;MEVPGVT	NCP	105.645
MEVPGVT;KEFESPK	NCP	14.665
KEFESPK;LSLGAYE	NCP	178.182
LSLGAYE;LGLQM	NCP	3.393
LGLQM;YGLYFVT	NCP	9.977

YGLYFVT;DLGLDL	NCP	25.843
DLGLDL;HVGGVSH	NCP	5.483
HVGGVSH;TGLLGDPP	NCP	10.266
TGLLGDPP;VAFLEN	NCP	125.313
VAFLEN;KGAKLAR	NCP	30.742
KGAKLAR;KSSMFHF	NCP	17.253
KSSMFHF;GPASSPPGP	NCP	52.496
GPASSPPGP;MSLFL	NCP	206.43
MSLFL;LDLVL	NCP	9.906
LDLVL;EAGLYSPK	NCP	10.071
EAGLYSPK;AVNTP	NCP	50.799
AVNTP;KDLGVP	NCP	55.407
KDLGVP;EVAMPVK	NCP	5.644
EVAMPVK;LFTKSK	NCP	12.566
LFTKSK;PGNFPPS	NCP	76.176
PGNFPPS;VTMSVLLK	NCP	68.308
VTMSVLLK;GVAGPN	NCP	120.713
GVAGPN;LSPGCLPK	NCP	51.597
LSPGCLPK;FHLVRK	NCP	70.515
FHLVRK;LAVPV	NCP	28.48
LAVPV;YGMFW	NCP	85.25
YGMFW;FVFAAPL	NCP	117.651
FVFAAPL;PVVSQPG	NCP	224.163
PVVSQPG;LSAYA	NCP	19.66
LSAYA;LLHVFV	NCP	88.854
LLHVFV;KKECVH	NCP	86.36
KKECVH;PPPYRP	NCP	8.125
PPPYRP;MGSLVFE	NCP	5.763
MGSLVFE;KGPRVLLF	NCP	14.634
KGPRVLLF;LGLTNHD	NCP	12.452
LGLTNHD;MEAVP	NCP	14.124
MEAVP;KGPSLCPP	NCP	75.771
KGPSLCPP;RHGKPR	NCP	18.277
RHGKPR;KYVLG	NCP	43.137
KYVLG;KGLTSLY	NCP	20.873
KGLTSLY;VPVTHVL	NCP	68.868
VPVTHVL;DLEETFF	NCP	189.795
DLEETFF;TPSLVHR	NCP	233.433
TPSLVHR;AGLSSALLR	NCP	63.034
AGLSSALLR;GSTLPSR	NCP	102.96
GSTLPSR;HGSNLE	NCP	100.796
HGSNLE;VPVGAP	NCP	15.823
VPVGAP;GLRYK	NCP	22.53
GLRYK;WLYVYAF	NCP	72.806
WLYVYAF;VPPLF	NCP	70.027
VPPLF;LELGF	NCP	18.252
LELGF;PRHGPAP	NCP	5.886
PRHGPAP;LPPFPP	NCP	7.638
LPPFPP;QFTKTK	NCP	5.36
QFTKTK;KEHQSCK	NCP	16.863
KEHQSCK;CCCCCCCAK	NCP	3.192

CCCCCCCAK;HYKLL	NCP	45.667
HYKLL;EVPPGLLK	NCP	85.518
EVPPGLLK;LSPFMR	NCP	40.215
LSPFMR;KGLLGY	NCP	24.996
KGLLGY;ASKLLRGL	NCP	19.451
ASKLLRGL;ALSKKPT	NCP	31.471
ALSKKPT;APPLL	NCP	29.312
APPLL;VSYLLHW	NCP	30.147
VSYLLHW;ELLLDVLK	NCP	107.295
ELLLDVLK;VTLEKPLPL	NCP	8.311
VTLEKPLPL;PANKTPPP	NCP	44.859
PANKTPPP;LSLEGKP	NCP	68.276
LSLEGKP;LQMRTP	NCP	6.27
LQMRTP;GVFYY	NCP	63.031
GVFYY;YGGLLTK	NCP	7.769
YGGLLTK;NPKRPASP	NCP	81.547
NPKRPASP;KPHLPSK	NCP	70.002
KPHLPSK;ELALLMA	NCP	61.017
ELALLMA;LHATGPK	NCP	39.672
LHATGPK;LEASVP	NCP	41.643
LEASVP;LDAKLVE	NCP	91.724
LDAKLVE;KAMSKQP	NCP	3.879
KAMSKQP;NVSLL	NCP	6.135
NVSLL;WVKPR	NCP	21.252
WVKPR;QTVFP	NCP	13.813
QTVFP;SPLQFR	NCP	45.254
SPLQFR;AANPLDK	NCP	98.271
AANPLDK;EAAPSNR	NCP	91.864
EAAPSNR;RGFLTK	NCP	73.148
RGFLTK;PPEHFP	NCP	27.235
PPEHFP;AEEGGDN	NCP	25.7
AEEGGDN;AQFVR	NCP	31.96
AQFVR;FVKKAPQ	NCP	3.17
FVKKAPQ;QFPDPPPP	NCP	43.066
QFPDPPPP;AALGFGH	NCP	63.213
AALGFGH;ELYLTA	NCP	47.295
ELYLTA;LAALEYL	NCP	53.072
LAALEYL;KNFLL	NCP	113.869
KNFLL;FESVTSR	NCP	25.244
FESVTSR;SCAKLDPA	NCP	44.23
SCAKLDPA;VGGSPRGP	NCP	151.269
VGGSPRGP;ALPVKYL	NCP	109.131
ALPVKYL;LHNLSF	NCP	36.704
LHNLSF;HALLRL	NCP	93.19
HALLRL;KDWEF	NCP	16.601
KDWEF;QLQFPVG	NCP	132.274
QLQFPVG;RQKCTDF	NCP	64.788
RQKCTDF;NLTKAVHG	NCP	0.084
NLTKAVHG;KEYGF	NCP	35.762
KEYGF;AAAFQQQ	NCP	95.737
AAAFQQQ;KSLLELT	NCP	24.039

KSLLELT;LELLEL	NCP	21.189
LELLEL;VYGPRGP	NCP	2.703
VYGPRGP;MDSVL	NCP	13.677
MDSVL;TELLSK	NCP	78.294
TELLSK;PNLLL	NCP	39.699
PNLLL;DKELKTDF	NCP	13.431
DKELKTDF;NLAVPP	NCP	4.824
NLAVPP;KGTDYLP	NCP	46.325
KGTDYLP;SGTRVSP	NCP	123.015
SGTRVSP;PKWTAGK	NCP	138.391
PKWTAGK;LPSVP	NCP	32.459
LPSVP;TFGGLG	NCP	24.111
TFGGLG;NKVAVLQ	NCP	3.208
NKVAVLQ;KKSAN	NCP	64.155
KKSAN;KPHTPLP	NCP	83.354
KPHTPLP;LSGFT	NCP	116.877
LSGFT;YLVGFPP	NCP	69.583
YLVGFPP;FFVVYLR	NCP	49.361
FFVVYLR;PLPPGHRP	NCP	64.35
PLPPGHRP;RALLEGESNPE	NCP	62.133
RALLEGESNPE;YTTSVLQK	NCP	2.289
YTTSVLQK;TTAWQENAYQK	NCP	0.159
TTAWQENAYQK;SPTPGLLRN	NCP	0.066
SPTPGLLRN;WGKKTEAKATKEQER	NCP	0.201
WGKKTEAKATKEQER;KREEKMFDSKEK	NCP	0.069
KREEKMFDSKEK;VAPQGLQTPLKEDAGD	NCP	0.216
VAPQGLQTPLKEDAGD;TLGASDTTASLK	NCP	0.087
TLGASDTTASLK;FTHSPEAEASAESFPDTK	NCP	0.093
FTHSPEAEASAESFPDTK;YVDRKEVPGDRK	NCP	0.06
YVDRKEVPGDRK;VDRKEVPGDR	NCP	0.003
VDRKEVPGDR;LLSKDAKEVELKGK	NCP	0.108
LLSKDAKEVELKGK;LSKDAKEVELK	NCP	0.003
LSKDAKEVELK;SKDAKEVELKGK	NCP	0.003
SKDAKEVELKGK;KDAKEVELK	NCP	0.003
KDAKEVELK;DAKEVELKGK	NCP	0.003
DAKEVELKGK;VSSEKEDEFSTPF	NCP	0.135
VSSEKEDEFSTPF;LEGDTASAPWKEQTR	NCP	0.432
LEGDTASAPWKEQTR;EGDTASAPWKEQ	NCP	0.003
EGDTASAPWKEQ;GDTASAPWKEQTR	NCP	0.003
GDTASAPWKEQTR;SGEFHAKP	NCP	0.039
SGEFHAKP;LEENLPERM	NCP	0.126
LEENLPERM;KEELSALTREGQGGP	NCP	0.027
KEELSALTREGQGGP;KVQSAGSRAVT	NCP	0.069
KVQSAGSRAVT;SQTVDADQLDLEELSK	NCP	0.054
SQTVDADQLDLEELSK;VDADQLDLEELSKD	NCP	0.009
VDADQLDLEELSKD;LDLEELSK	NCP	0.015
LDLEELSK;RRSPGPGSPEAEAGRG	NCP	0.084
RRSPGPGSPEAEAGRG;EVTSPTGFVHA	NCP	0.144
EVTSPTGFVHA;VLEDVSQSGGRVQ	NCP	0.036
VLEDVSQSGGRVQ;RTEQVSAEGR	NCP	0.06
RTEQVSAEGR;SSQAEESAGTQTSVK	NCP	0.339

SSQAEESAGTQTSVK;SDSESSTHREGSADETLATSSY	NCP	0.249
SDSESSTHREGSADETLATSSY;VDSPEVSEVADSGR	NCP	0.288
VDSPEVSEVADSGR;YLDSQEEENEGHW	NCP	0.387
YLDSQEEENEGHW;EEENEGHWF	NCP	0.015
EEENEGHWF;EFLASA	NCP	127.653
EFLASA;KSELNKPQ	NCP	157.156
KSELNKPQ;KPGLPH	NCP	34.602
KPGLPH;ARLLDY	NCP	30.668
ARLLDY;LLEKSAR	NCP	20.793
LLEKSAR;LTREELLF	NCP	1.069
LTREELLF;SSSMGCD	NCP	34.008
SSSMGCD;ERRTLEF	NCP	163.131
ERRTLEF;FAAPLVK	NCP	0.935
FAAPLVK;LSGRADLR	NCP	54.912
LSGRADLR;VSDPMLL	NCP	53.977
VSDPMLL;TMHVYLP	NCP	1.578
TMHVYLP;RVTLPTN	NCP	19.632
RVTLPTN;GLGVHG	NCP	32.07
GLGVHG;VPPAFVV	NCP	238.53
VPPAFVV;NGPAHTP	NCP	27.683
NGPAHTP;EAGWYEL	NCP	1.861
EAGWYEL;LAAKAR	NCP	134.463
LAAKAR;KTKKWPSP	NCP	30.246
KTKKWPSP;VEGML	NCP	49.542
VEGML;PGLNF	NCP	46.322
PGLNF;RPPVPLLF	NCP	25.441
RPPVPLLF;PSLVHR	NCP	42.417
PSLVHR;ETFNTAP	NCP	20.991
ETFNTAP;NGGKKKP	NCP	19.509
NGGKKKP;LAPLLVG	NCP	20.43
LAPLLVG;LPPKKPPP	NCP	17.787
LPPKKPPP;PDPLTPPAP	NCP	17.802
PDPLTPPAP;LPTGVEL	NCP	38.781
LPTGVEL;LGDNVL	NCP	34.709
LGDNVL;ATLPLGPP	NCP	68.494
ATLPLGPP;QPGPPPPP	NCP	9.6
QPGPPPPP;HELSDF	NCP	7.343
HELSDF;ATLLSHSL	NCP	29.815
ATLLSHSL;TLMGPQK	NCP	58.238
TLMGPQK;CMCCNK	NCP	25.057
CMCCNK;TDPTPVK	NCP	154.746
TDPTPVK;KATPVRV	NCP	14.961
KATPVRV;GHPPTGGK	NCP	67.05
GHPPTGGK;KPAVSPPP	NCP	32.324
KPAVSPPP;KGVHKPP	NCP	24.694
KGVHKPP;KAPLANV	NCP	94.967
KAPLANV;ASGTLGGF	NCP	264.163
ASGTLGGF;VPKYPK	NCP	29.297
VPKYPK;KVVPPP	NCP	190.699
KVVPPP;EFRASP	NCP	16.032
EFRASP;AAPAGP	NCP	23.355

AAPAGP;KGLLPMLL	NCP	6.918
KGLLPMLL;KAVPP	NCP	17.994
KAVPP;KLKKDLR	NCP	9.89
KLKKDLR;AARSLLLLR	NCP	184.621
AARSLLLLR;CPERLSK	NCP	21.462
CPERLSK;ATGMK	NCP	93.519
ATGMK;TVSVPSPR	NCP	48.096
TVSVPSPR;TGTPKPLGN	NCP	95.607
TGTPKPLGN;VKMLKKPKFELGK	NCP	71.42
VKMLKKPKFELGK;RPTAFKP	NCP	180.757
RPTAFKP;PTSSLF	NCP	19.898
PTSSLF;QPSHNPF	NCP	35.347
QPSHNPF;SVPRRP	NCP	211.238
SVPRRP;SPVFPRQ	NCP	16.557
SPVFPRQ;AAEPD	NCP	15.076
AAEPD;KDAGEGP	NCP	57.11
KDAGEGP;GVPGFP	NCP	3.088
GVPGFP;GGVFP	NCP	9.933
GGVFP;VFLAP	NCP	39.026
VFLAP;KHSPPGK	NCP	28.378
KHSPPGK;QKWYPK	NCP	25.749
QKWYPK;YNGTDS	NCP	14.862
YNGTDS;LSGPVR	NCP	103.404
LSGPVR;EVSKFAE	NCP	54.851
EVSKFAE;PVMLFVAR	NCP	201.712
PVMLFVAR;EEVMMS	NCP	20.004
EEVMMS;EAAALL	NCP	31.533
EAAALL;LALSPQP	NCP	103.065
LALSPQP;LPTTKN	NCP	24.588
LPTTKN;KLMPPPP	NCP	47.736
KLMPPPP;PLSGPPP	NCP	24.723
PLSGPPP;KDVLGLFR	NCP	96.615
KDVLGLFR;PPKLDRR	NCP	106.377
PPKLDRR;PFVLKYM	NCP	6.255
PFVLKYM;REPPNPV	NCP	31.764
REPPNPV;VDPTVFF	NCP	37.073
VDPTVFF;PPHLPSAAP	NCP	109.47
PPHLPSAAP;KGPVH	NCP	59.149
KGPVH;PGLVL	NCP	84.113
PGLVL;LAALFMD	NCP	14.679
LAALFMD;SLYVPK	NCP	16.935
SLYVPK;LPVAGV	NCP	12.706
LPVAGV;VGPAFL	NCP	61.032
VGPAFL;AVRAAPGP	NCP	36.618
AVRAAPGP;EQAGPPK	NCP	60.939
EQAGPPK;CPVATR	NCP	14.022
CPVATR;TTSSK	NCP	37.364
TTSSK;PPVPPP	NCP	16.66
PPVPPP;LLLCEF	NCP	114.335
LLLCEF;LTDCVVMRDPASK	NCP	54.331
LTDCVVMRDPASK;PPPPGAFL	NCP	79.95

PPPPGAFL;VFYLLSL	NCP	8.777
VFYLLSL;VSNVHVF	NCP	29.038
VSNVHVF;AKALQY	NCP	139.736
AKALQY;PAATHLF	NCP	5.911
PAATHLF;KHAAFNK	NCP	24.005
KHAAFNK;VGLEPSLR	NCP	16.492
VGLEPSLR;KLLHAE	NCP	42.896
KLLHAE;AGGLGLAR	NCP	0.127
AGGLGLAR;LPLKAAK	NCP	290.852
LPLKAAK;KGYGK	NCP	6.948
KGYGK;ARAAGPERRLL	NCP	37.333
ARAAGPERRLL;KEGGEPK	NCP	56.702
KEGGEPK;PPAGQRPP	NCP	58.846
PPAGQRPP;PAGQRPPGP	NCP	0.003
PAGQRPPGP;WTGNPW	NCP	23.859
WTGNPW;KTSVVAV	NCP	110.994
KTSVVAV;PSSVLWC	NCP	67.238
PSSVLWC;LQEFKEA	NCP	12.961
LQEFKEA;LSVKP	NCP	6.636
LSVKP;KGPPPPRPP	NCP	24.918
KGPPPPRPP;AELSEECH	NCP	8.75
AELSEECH;ASLQNL	NCP	25.108
ASLQNL;LPMVRGLE	NCP	49.577
LPMVRGLE;KDSGH	NCP	42.667
KDSGH;PPFPPPP	NCP	5.817
PPFPPPP;PAAMNK	NCP	19.926
PAAMNK;AAWSSK	NCP	9.161
AAWSSK;THPGTPF	NCP	124.438
THPGTPF;EDRFGDLL	NCP	33.308
EDRFGDLL;DCWGPPPP	NCP	41.176
DCWGPPPP;EEFDAVP	NCP	80.042
EEFDAVP;ARPPM	NCP	61.018
ARPPM;RPPLPR	NCP	28.557
RPPLPR;KKGNLEPK	NCP	13.092
KKGNLEPK;GSPVPP	NCP	98.88
GSPVPP;HLPSQPPTH	NCP	5.13
HLPSQPPTH;PRQPPLP	NCP	11.184
PRQPPLP;APASPPR	NCP	29.442
APASPPR;ATSLSGPK	NCP	33.617
ATSLSGPK;GPSLPW	NCP	31.213
GPSLPW;KAFYWW	NCP	21.557
KAFYWW;KMMPVK	NCP	1.812
KMMPVK;VLLTFV	NCP	49.545
VLLTFV;PKCPKCSKEV	NCP	58.12
PKCPKCSKEV;QDDAARP	NCP	47.805
QDDAARP;AFPSERP	NCP	75.807
AFPSERP;APGAPPVLP	NCP	41.823
APGAPPVLP;GSQGGLGAL	NCP	22.599
GSQGGLGAL;LTVKDL	NCP	16.793
LTVKDL;GVFVPE	NCP	66.694
GVFVPE;PVPVGP	NCP	26.502

PVPVGP;KNATKLG	NCP	48.641
KNATKLG;AGRLPAC	NCP	32.398
AGRLPAC;HPPGPRP	NCP	1.47
HPPGPRP;KCDSD	NCP	44.669
KCDSD;SDLRLLS	NCP	24.154
SDLRLLS;GNRPRP	NCP	67.745
GNRPRP;FGFLG	NCP	130.864
FGFLG;ETLEEDE	NCP	12.072
ETLEEDE;SKRGGVP	NCP	32.081
VTVGPH;LRAAPL	NCP	5.487
LRAAPL;TGDLLLQKE	NCP	125.823
TGDLLLQKE;KGVESGSK	NCP	34.375
KGVESGSK;AHEAVVY	NCP	10.902
AHEAVVY;GPGRPAAPP	NCP	8.048
GPGRPAAPP;TPPLTPP	NCP	41.724
TPPLTPP;EELMFF	NCP	108.145
EELMFF;WPGAP	NCP	95.225
WPGAP;PPASTPSR	NCP	1.095
PPASTPSR;PSPAAHLP	NCP	176.361
PSPAAHLP;AKELLR	NCP	32.11
AKELLR;VLPVPQ	NCP	4.923
VLPVPQ;PPLPPP	NCP	18.488
PPLPPP;EEPFFP	NCP	14.325
EEPFFP;LKQEPP	NCP	33.199
LKQEPP;ELLLLE	NCP	62.535
ELLLLE;RVEAAPK	NCP	5.567
RVEAAPK;LPPFEDE	NCP	13.596
LPPFEDE;LSAALPFL	NCP	0.954
LSAALPFL;KMAWLGP	NCP	0.567
KMAWLGP;LAGGGPLR	NCP	63.678
LAGGGPLR;MLAAGLP	NCP	143.952
MLAAGLP;SSSLLLD	NCP	17.571
SSSLLLD;CSSMAYP	NCP	110.347
CSSMAYP;KKAVGPR	NCP	1.53
KKAVGPR;YGRFSSP	NCP	2.145
YGRFSSP;VTTPGLP	NCP	105.935
VTTPGLP;TDLLPEGK	NCP	2.289
TDLLPEGK;GAAPRGGPP	NCP	52.818
GAAPRGGPP;APGAPTLH	NCP	17.13
APGAPTLH;TFLGL	NCP	116.589
TFLGL;MHKSLK	NCP	55.216
MHKSLK;TSRKKVLLK	NCP	14.36
TSRKKVLLK;MNQYVNKK	NCP	2.157
MNQYVNKK;AALLL	NCP	1.557
AALLL;SVELEEALPLTTAEGAAKKAAK	NCP	25.062
SVELEEALPLTTAEGAAKKAAK;PLTTAEGAAKK	NCP	0.024
PLTTAEGAAKK;LTTAEGAAKKAAK	NCP	0.003
LTTAEGAAKKAAK;AGGSAALSPSK	NCP	0.039
AGGSAALSPSK;LVQNDTLLQVK	NCP	0.216
LVQNDTLLQVK;RGAPAPASAPAPAAHKAK	NCP	0.099
RGAPAPASAPAPAAHKAK;PASAPAPAAHK	NCP	0.015

PASAPAPAAHK;ASAPAPAAHKAKKAAPGAAAPR	NCP	0.003
ASAPAPAAHKAKKAAPGAAAPR;PAPAAHKAK	NCP	0.009
PAPAAHKAK;KAAPGAAAPR	NCP	0.027
KAAPGAAAPR;KAAKPSVPKVPK	NCP	0.159
KAAKPSVPKVPK;KPSVPKVPKGR	NCP	0.009
KPSVPKVPKGR;PSVPKVPK	NCP	0.003
PSVPKVPK;NPRRPP	NCP	28.602
NPRRPP;KRVLAL	NCP	39.024
KRVLAL;ALHRLQLR	NCP	37.656
ALHRLQLR;VGWGGSLL	NCP	178.668
VGWGGSLL;KQAATKKP	NCP	19.453
KQAATKKP;APKRSF	NCP	2.774
APKRSF;SLPAEC	NCP	43.065
SLPAEC;FHPLVGP	NCP	32.823
FHPLVGP;PASPHVAR	NCP	36.099
PASPHVAR;APAKPEPR	NCP	22.998
APAKPEPR;HAAPGGSR	NCP	21.726
HAAPGGSR;ANFSPP	NCP	44.209
ANFSPP;KPGGPPGV	NCP	36.89
KPGGPPGV;RHGRTPP	NCP	14.769
RHGRTPP;LQLLVLP	NCP	13.071
LQLLVLP;GSDYPPP	NCP	23.076
GSDYPPP;CSYGKK	NCP	70.005
CSYGKK;MESLLLQ	NCP	19.704
MESLLLQ;QVFSNR	NCP	0.976
QVFSNR;FPGLSA	NCP	62.324
FPGLSA;YVYQR	NCP	45.397
YVYQR;LSDLPLVD	NCP	133.667
LSDLPLVD;PGASAN	NCP	50.454
PGASAN;EGPLLTP	NCP	29.754
EGPLLTP;DLEGPLPD	NCP	15.375
DLEGPLPD;RPSFFV	NCP	55.866
RPSFFV;ATPVAPQ	NCP	66.862
ATPVAPQ;PPWKPPGP	NCP	36.479
PPWKPPGP;LPPYKPR	NCP	60.309
LPPYKPR;RTLDAQP	NCP	7.825
RTLDAQP;DAPSLR	NCP	86.894
DAPSLR;RLLNPR	NCP	51.652
RLLNPR;DDSPPA	NCP	45.494
DDSPPA;VDAAP	NCP	8.676
VDAAP;PLTSKALGV	NCP	6.786
PLTSKALGV;LYFGET	NCP	16.672
LYFGET;ETGEHLVH	NCP	45.342
ETGEHLVH;CRPSF	NCP	60.575
CRPSF;QKEELK	NCP	12.159
QKEELK;VASEATALF	NCP	53.436
VASEATALF;VLSLEGKPL	NCP	43.125
VLSLEGKPL;QQYYRVP	NCP	95.845
QQYYRVP;RAGGPLEK	NCP	39.488
RAGGPLEK;LDLSSVAL	NCP	74.2
LDLSSVAL;AALRPLVKPK	NCP	32.177

AALRPLVKPK;LELGPAAP	NCP	71.509
LELGPAAP;RYWFK	NCP	98.936
RYWFK;AVRGSLALR	NCP	67.576
AVRGSLALR;LLLLPPA	NCP	10.583
LLLLPPA;AVPKY	NCP	1.525
AVPKY;VDGVLK	NCP	79.758
VDGVLK;FPPVYPP	NCP	9.105
FPPVYPP;DGFPALK	NCP	10.866
DGFPALK;KVLDGSDG	NCP	87.95
KVLDGSDG;VGFSK	NCP	26.187
VGFSK;TVCCLPPPP	NCP	116.415
TVCCLPPPP;FSHSEPY	NCP	87.588
FSHSEPY;LPAKEFL	NCP	32.809
LPAKEFL;VPPEKPP	NCP	4.358
VPPEKPP;GPFHHGL	NCP	38.749
GPFHHGL;SMDSKLK	NCP	4.14
SMDSKLK;HPPTTTK	NCP	75.518
HPPTTTK;SPGRLLEH	NCP	15.573
SPGRLLEH;AELRALVA	NCP	118.014
AELRALVA;PAEPP	NCP	17.508
PAEPP;WAKLFS	NCP	188.724
WAKLFS;ALKSFR	NCP	55.185
ALKSFR;LVLVAY	NCP	5.068
LVLVAY;KRHTSSKSK	NCP	65.519
KRHTSSKSK;QAVLR	NCP	72.507
QAVLR;WNLLL	NCP	22.492
WNLLL;FPKNR	NCP	33.062
FPKNR;PLFEFQ	NCP	27.184
PLFEFQ;KPRLL	NCP	64.064
KPRLL;DSFENRLLL	NCP	45.889
DSFENRLLL;VDPVGGF	NCP	46.631
VDPVGGF;GRPPPALGP	NCP	16.872
GRPPPALGP;MLRKPAL	NCP	17.374
MLRKPAL;KAAHYLL	NCP	145.064
KAAHYLL;PEPAKSAPAPKKGS	NCP	21.822
PEPAKSAPAPKKGS;RLPPNR	NCP	59.941
RLPPNR;GASARLLR	NCP	62.592
GASARLLR;DPTKVVR	NCP	31.53
DPTKVVR;PAPTLNY	NCP	99.96
PAPTLNY;PTKPRP	NCP	15.788
PTKPRP;AYEQVAK	NCP	29.797
AYEQVAK;CMDGW	NCP	32.04
CMDGW;HTAGPKP	NCP	73.622
HTAGPKP;TLAPPER	NCP	54.037
TLAPPER;EPLLAHRP	NCP	12.125
EPLLAHRP;DSPVVSY	NCP	82.419
DSPVVSY;PALPG	NCP	134.616
PALPG;AAALPPR	NCP	2.403
AAALPPR;LVHNPG	NCP	27.267
LVHNPG;SVTEPTK	NCP	79.581
SVTEPTK;VGVLL	NCP	2.178

VGVLL;KHGSVNC	NCP	50.859
KHGSVNC;SPPATRPP	NCP	143.268
SPPATRPP;KVYTFNSVRK	NCP	50.148
KVYTFNSVRK;LPPPPPPLGP	NCP	30.646
LPPPPPPLGP;LPNGMLP	NCP	16.734
LPNGMLP;LEAAKAT	NCP	92.66
LEAAKAT;KNRHPEM	NCP	60.678
KNRHPEM;HPSGKR	NCP	35.284
HPSGKR;KSVPAK	NCP	105.461
KSVPAK;APGTPP	NCP	38.887
APGTPP;KYAALKQ	NCP	44.181
KYAALKQ;GPFDP	NCP	45.687
GPFDP;KGFFKQ	NCP	7.383
KGFFKQ;PSLELF	NCP	67.986
PSLELF;GPPRMGP	NCP	21.222
GPPRMGP;AVTRLC	NCP	29.213
AVTRLC;KPHVMLK	NCP	57.198
KPHVMLK;TTVPTTLL	NCP	45.289
TTVPTTLL;MDKYDDLGLEASK	NCP	3.176
MDKYDDLGLEASK;AVGEVKK	NCP	32.925
AVGEVKK;KMRVGAP	NCP	33.393
KMRVGAP;AATTYERLK	NCP	15.507
AATTYERLK;GPAFPGAK	NCP	26.356
GPAFPGAK;ENPFLK	NCP	22.157
ENPFLK;VPGKPWMG	NCP	35.757
VPGKPWMG;KMGGGAPR	NCP	123.631
KMGGGAPR;KLHTYP	NCP	32.394
KLHTYP;KVAVK	NCP	26.13
KVAVK;TLNLLP	NCP	19.223
TLNLLP;WRPLNPP	NCP	20.307
WRPLNPP;RAANSPLP	NCP	106.156
RAANSPLP;VAPLLQ	NCP	56.4
VAPLLQ;VGVPLK	NCP	101.741
VGVPLK;GCDGF	NCP	28.462
GCDGF;VAAGALVL	NCP	41.873
VAAGALVL;LALAY	NCP	33.568
LALAY;RRGVFF	NCP	86.852
RRGVFF;LPPVSHK	NCP	9.118
LPPVSHK;VGVVALK	NCP	66.239
VGVVALK;RVTAAAPP	NCP	26.791
RVTAAAPP;LGPLF	NCP	10.8
LGPLF;EHGLFSR	NCP	4.487
EHGLFSR;PLLPPPPRPP	NCP	132.375
PLLPPPPRPP;LPSPF	NCP	19.434
LPSPF;RYGFN	NCP	17.725
RYGFN;KFVGL	NCP	94.296
KFVGL;TGPVFP	NCP	251.331
TGPVFP;LVVVVL	NCP	130.656
LVVVVL;GFAGK	NCP	12.156
GFAGK;ADELKKVL	NCP	69.684
ADELKKVL;TGNLLEAT	NCP	3.686

TGNLLEAT;ARPSPAL	NCP	70.716
ARPSPAL;SGVPGVLR	NCP	20.103
SGVPGVLR;KPPMC	NCP	39.004
KPPMC;VSVEALK	NCP	12.246
VSVEALK;MEVLV	NCP	10.701
MEVLV;KLPDRYL	NCP	57.578
KLPDRYL;LVEVLK	NCP	132.13
LVEVLK;NLDMRCV	NCP	19.491
NLDMRCV;LPWKWPW	NCP	67.853
LPWKWPW;EDLGTRKP	NCP	5.79
EDLGTRKP;MNGAP	NCP	3.568
MNGAP;ALSYREAVLR	NCP	9.689
ALSYREAVLR;PSSVTSR	NCP	6.816
PSSVTSR;HLTPPQ	NCP	25.266
HLTPPQ;PAPLLPPP	NCP	116.478
PAPLLPPP;KEVFAGP	NCP	5.586
KEVFAGP;KGSRSPLL	NCP	33.702
KGSRSPLL;QFLPPD	NCP	29.301
QFLPPD;EGWKVQE	NCP	19.732
EGWKVQE;KEAVP	NCP	72.008
KEAVP;ERVGL	NCP	11.574
ERVGL;DGLLP	NCP	18.102
DGLLP;GPVPVH	NCP	7.491
GPVPVH;GNVLYRAL	NCP	116.262
GNVLYRAL;DPASAVPQ	NCP	126.981
DPASAVPQ;TPASTLSPL	NCP	4.068
TPASTLSPL;KLYPLLVE	NCP	2.617
KLYPLLVE;SGRFGPP	NCP	104.135
SGRFGPP;KHPPVTH	NCP	3.378
KHPPVTH;ADNGLRL	NCP	23.329
ADNGLRL;KQPGVGAP	NCP	64.911
KQPGVGAP;GKTYFGV	NCP	144.848
GKTYFGV;TPYRSPPP	NCP	37.434
TPYRSPPP;PVVTGPC	NCP	1.986
PVVTGPC;KAAAKPK	NCP	59.979
KAAAKPK;QPTGKPPRQ	NCP	90.282
QPTGKPPRQ;KDHSLGVP	NCP	12.637
KDHSLGVP;AQGVVP	NCP	62.102
AQGVVP;LSAHPPVP	NCP	3.999
LSAHPPVP;VDLFF	NCP	82.035
VDLFF;TTMGL	NCP	74.196
TTMGL;RTGRDLPK	NCP	11.872
RTGRDLPK;AALRL	NCP	87.929
AALRL;GPSKTLPPP	NCP	18.423
GPSKTLPPP;PPPPKKN	NCP	133.549
PPPPKKN;LEVPPAN	NCP	56.571
LEVPPAN;ESSGG	NCP	16.782
ESSGG;PAAATRPP	NCP	57.872
PAAATRPP;GGPGAP	NCP	20.334
GGPGAP;KSHPAAK	NCP	49.195
KSHPAAK;FTLLDY	NCP	13.223

FTLLDY;KGLPDVPR	NCP	53.632
KGLPDVPR;AGAPLLSGP	NCP	3.341
AGAPLLSGP;KVNALQ	NCP	140.956
KVNALQ;ERGNLLGL	NCP	57.135
ERGNLLGL;QHLAVR	NCP	51.939
QHLAVR;ALASLMT	NCP	1.65
ALASLMT;KPSSRGAP	NCP	40.461
KPSSRGAP;LDLPP	NCP	26.594
LDLPP;AGRKLALK	NCP	2.23
AGRKLALK;KLRVKELKK	NCP	8.366
KLRVKELKK;LDDWGETCKGCAEK	NCP	0.03
LDDWGETCKGCAEK;VGAMELR	NCP	70.828
VGAMELR;KTHNK	NCP	90.852
KTHNK;PGPRPRAPP	NCP	53.553
PGPRPRAPP;ASPVRPAP	NCP	45.321
ASPVRPAP;FGEKL	NCP	52.695
FGEKL;LPEPEAPL	NCP	51.429
LPEPEAPL;SVPVLPSES	NCP	65.417
SVPVLPSES;DPRPPR	NCP	16.755
DPRPPR;FDVELL	NCP	18.615
FDVELL;QPHLLR	NCP	36.682
QPHLLR;QCPELQR	NCP	98.036
QCPELQR;QDLLTHVL	NCP	12.312
QDLLTHVL;TAVSVP	NCP	91.78
TAVSVP;GLNPEMK	NCP	14.105
GLNPEMK;QHLQSY	NCP	41.334
QHLQSY;TLVDLAK	NCP	15.535
TLVDLAK;VPPLSNGP	NCP	33.482
VPPLSNGP;PNLVLAK	NCP	16.575
PNLVLAK;PYSGPLR	NCP	38.685
PYSGPLR;QPPHRP	NCP	34.153
QPPHRP;APGLFLPK	NCP	58.319
APGLFLPK;LARAVL	NCP	58.242
LARAVL;VPGYRPP	NCP	118.984
VPGYRPP;LLLPPPP	NCP	87.861
LLLPPPP;QAVNRFS	NCP	5.484
QAVNRFS;VELPKTAF	NCP	51.725
VELPKTAF;PEARKK	NCP	60.759
PEARKK;DLPPAAN	NCP	11.227
DLPPAAN;KPPKHL	NCP	12.737
KPPKHL;KLEVP	NCP	86.508
KLEVP;LPAVP	NCP	132.585
LPAVP;YRMPPP	NCP	51.306
YRMPPP;KPASKTK	NCP	31.722
KPASKTK;GPVETTK	NCP	39.142
GPVETTK;LPSTKRP	NCP	10.049
LPSTKRP;GESLL	NCP	26.113
GESLL;YKYTSSK	NCP	27.69
YKYTSSK;LTVDPF	NCP	3.603
LTVDPF;KQKATPP	NCP	75.116
KQKATPP;VGLPS	NCP	32.253

VGLPS;KGTTLVY	NCP	1.365
KGTTLVY;VGPLG	NCP	151.905
VGPLG;PSKKKH	NCP	49.765
PSKKKH;VGLKV	NCP	34.125
VGLKV;ETVGW	NCP	85.467
ETVGW;DPLPKGPP	NCP	27.26
DPLPKGPP;GEPKTPK	NCP	59.262
GEPKTPK;QNLGAL	NCP	136.026
QNLGAL;KTYFPH	NCP	15.361
KTYFPH;KPAAPEKL	NCP	148.416
KPAAPEKL;TVENLVT	NCP	23.097
TVENLVT;TSAPLGGP	NCP	84.699
TSAPLGGP;ALAVFPP	NCP	117.32
ALAVFPP;KAVQP	NCP	13.471
KAVQP;LVAVEL	NCP	37.298
LVAVEL;KDTVVPK	NCP	14.209
KDTVVPK;KNKRDF	NCP	31.031
KNKRDF;KPMGRPPP	NCP	10.888
KPMGRPPP;AAKHLSM	NCP	186.678
AAKHLSM;APLAPTMV	NCP	41.394
APLAPTMV;LNPVLF	NCP	169.535
LNPVLF;KDLFRK	NCP	37.5
KDLFRK;TALGL	NCP	100.186
TALGL;CSDDN	NCP	94.151
CSDDN;PSFGGAP	NCP	0.166
PSFGGAP;ERPAFDR	NCP	61.409
ERPAFDR;LTMYK	NCP	32.653
LTMYK;HGGPLAPP	NCP	108.146
HGGPLAPP;PPPNAQR	NCP	55.201
PPPNAQR;NLGPHFK	NCP	16.658
NLGPHFK;NGAAVPPP	NCP	1.671
NGAAVPPP;LYKSL	NCP	39.669
LYKSL;VGMQSL	NCP	24.522
VGMQSL;KVKEPLL	NCP	6.273
KVKEPLL;MELGPDAF	NCP	36.418
MELGPDAF;MHYYR	NCP	35.198
MHYYR;DPPVHRP	NCP	10.152
DPPVHRP;EQLVLAEH	NCP	18.388
EQLVLAEH;TLREC	NCP	113.259
TLREC;AMVEP	NCP	9.312
AMVEP;VAAASL	NCP	37.428
VAAASL;KAPLRF	NCP	36.372
KAPLRF;ALMRVHL	NCP	23.123
ALMRVHL;SGLKDH	NCP	12.949
SGLKDH;LAAAPP	NCP	166.212
LAAAPP;PLYKPP	NCP	0.24
PLYKPP;LTELPVTF	NCP	365.049
LTELPVTF;KAAPYRL	NCP	23.472
KAAPYRL;LQSLLR	NCP	29.205
LQSLLR;VLEKSLKGP	NCP	4.967
VLEKSLKGP;KGYTLGP	NCP	16.717

KGYTLGP;KQPGTPPP	NCP	12.197
KQPGTPPP;HGQEVR	NCP	22.816
HGQEVR;NTALLRR	NCP	77.114
NTALLRR;GFAPLT	NCP	0.072
GFAPLT;RGAPAPK	NCP	0.238
RGAPAPK;TVVLL	NCP	48.464
TVVLL;PSLVRGPR	NCP	29.521
PSLVRGPR;YDCFHCK	NCP	2.945
YDCFHCK;LPPSGPP	NCP	11.646
LPPSGPP;KVRPDR	NCP	43.96
KVRPDR;AAFVLGPK	NCP	12.014
AAFVLGPK;APVRPAK	NCP	37.303
APVRPAK;APAVLQPQ	NCP	53.613
APAVLQPQ;SLLTPRLK	NCP	137.363
SLLTPRLK;VFFRQYLK	NCP	59.832
VFFRQYLK;FATTALF	NCP	16.872
FATTALF;TPGPKFT	NCP	8.16
TPGPKFT;NRGVLQC	NCP	82.89
NRGVLQC;KMGSENLNDQLEEF	NCP	30.102
KMGSENLNDQLEEF;LGGFRYG	NCP	60.792
LGGFRYG;LQVFDF	NCP	8.2
LQVFDF;HVPLLLGM	NCP	26.58
HVPLLLGM;EALTTL	NCP	4.026
EALTTL;LTALPT	NCP	49.688
LTALPT;LGAAP	NCP	36.106
LGAAP;KEYSFLC	NCP	26.096
KEYSFLC;QALLLP	NCP	71.11
QALLLP;LGADLSLT	NCP	36.042
LGADLSLT;KGVVHPP	NCP	57.603
KGVVHPP;DEPAGPP	NCP	4.419
DEPAGPP;CGALLVR	NCP	65.24
CGALLVR;ESTKNVP	NCP	18.222
ESTKNVP;MDDQDLNEPLAK	NCP	55.53
MDDQDLNEPLAK;QHALLK	NCP	27.142
QHALLK;DLAEDAPWKK	NCP	45.203
DLAEDAPWKK;WCNEHLKC	NCP	0.054
WCNEHLKC;KKQLGVGK	NCP	27.769
KKQLGVGK;CNPTMM	NCP	8.511
CNPTMM;KGGFTDK	NCP	25.065
KGGFTDK;LSQFPKAK	NCP	3.059
LSQFPKAK;MLGVP	NCP	18.942
MLGVP;KGAGTGGLGL	NCP	10.221
KGAGTGGLGL;DDVPVPNSPF	NCP	2.868
DDVPVPNSPF;PASLPVEF	NCP	7.971
PASLPVEF;KHVLRK	NCP	19.846
KHVLRK;EFLPF	NCP	26.495
EFLPF;LPPLEDG	NCP	61.78
LPPLEDG;DDLDF	NCP	151.466
DDLDF;KGGELGPA	NCP	55.239
KGGELGPA;VGSLK	NCP	145.791
VGSLK;FGFGQ	NCP	81.631

FGFGQ;VSAHTGPK	NCP	1.566
VSAHTGPK;NGLVEPF	NCP	46.377
NGLVEPF;KEPGVMK	NCP	95.945
KEPGVMK;VSFGPH	NCP	66.13
VSFGPH;LEDALP	NCP	441.098
LEDALP;VTAPK	NCP	11.937
VTAPK;KLVELPF	NCP	65.412
KLVELPF;HFALR	NCP	224.133
HFALR;PGSPF	NCP	81.742
PGSPF;LALLQPL	NCP	26.856
LALLQPL;MDDDS	NCP	59.697
MDDDS;LDGFSM	NCP	12.954
LDGFSM;VPAGLNL	NCP	2.283
VPAGLNL;LMTTLR	NCP	1.305
LMTTLR;VPTMWKP	NCP	49.914
VPTMWKP;PPLPPSP	NCP	66.462
PPLPPSP;DPTEAFFP	NCP	119.496
DPTEAFFP;RYPHY	NCP	209.517
RYPHY;GPFPD	NCP	71.9
GPFPD;VQPKLGP	NCP	38.017
VQPKLGP;WGEPF	NCP	184.149
WGEPF;LKLWLY	NCP	35.709
LKLWLY;LTGVSGVR	NCP	182.523
LTGVSGVR;KSKWPK	NCP	75.374
KSKWPK;KKEVLR	NCP	24.994
KKEVLR;KAAQPPP	NCP	156.458
KAAQPPP;EKPSVPKQ	NCP	96.183
EKPSVPKQ;VNPPLVL	NCP	84.238
VNPPLVL;TVAPY	NCP	101.474
TVAPY;NTALSF	NCP	66.868
NTALSF;AVVNY	NCP	90.104
AVVNY;KYHSPP	NCP	306.129
KYHSPP;KGGFGK	NCP	7.005
KGGFGK;SSDAM	NCP	159.442
SSDAM;ELNPKL	NCP	50.502
ELNPKL;TAQGHQEV	NCP	24.372
TAQGHQEV;LLPLLSQ	NCP	7.137
LLPLLSQ;GPGLP	NCP	1.761
GPGLP;LMKSKL	NCP	222.233
LMKSKL;NALRPP	NCP	19.555
NALRPP;KGHKASK	NCP	21.042
KGHKASK;HTTNK	NCP	17.567
HTTNK;SLRVAAY	NCP	90.402
SLRVAAY;LPGPP	NCP	33.021
LPGPP;SSGHVRPPS	NCP	36.697
SSGHVRPPS;DLAGCLH	NCP	10.746
DLAGCLH;PVLLT	NCP	25.055
PVLLT;MTLGT	NCP	17.986
MTLGT;LPVTAHGK	NCP	30.068
LPVTAHGK;TQGTHGGVP	NCP	14.181
TQGTHGGVP;KTPGLN	NCP	118.047

KTPGLN;MTQLMF	NCP	130.512
MTQLMF;PEYFAK	NCP	24.91
PEYFAK;VSELLT	NCP	6.966
VSELLT;ASDLLNF	NCP	4.125
ASDLLNF;KGGPVKK	NCP	1.808
KGGPVKK;PVLHKAW	NCP	1.029
PVLHKAW;TQFPYPP	NCP	93.355
TQFPYPP;MCMGW	NCP	142.277
MCMGW;ATAALPK	NCP	54.583
ATAALPK;HGFPPQ	NCP	77.235
HGFPPQ;LFVGVA	NCP	75.83
LFVGVA;KYLFA	NCP	16.956
KYLFA;KSTPY	NCP	6.384
KSTPY;MLLKPPP	NCP	47.11
MLLKPPP;YVNLK	NCP	21.635
YVNLK;EHALVWF	NCP	14.929
EHALVWF;KLLDF	NCP	63.885
KLLDF;DRPPTSEK	NCP	136.98
DRPPTSEK;LVTTLRNP	NCP	21.638
LVTTLRNP;KMGGSF	NCP	12.189
KMGGSF;MLVDVAK	NCP	12.069
MLVDVAK;MKSPPK	NCP	142.503
MKSPPK;KGMGP	NCP	58.384
KGMGP;GPPVSELPV	NCP	10.449
GPPVSELPV;RSELPCLGP	NCP	99.141
RSELPCLGP;ESTAKPP	NCP	43.032
ESTAKPP;LDSPGFK	NCP	75.074
LDSPGFK;ESPLPKN	NCP	78.114
ESPLPKN;KNGATPP	NCP	9.864
KNGATPP;PLTDTK	NCP	16.345
PLTDTK;VSFSKSTL	NCP	149.711
VSFSKSTL;MLVLQHS	NCP	133.762
MLVLQHS;SYPPVQ	NCP	26.826
SYPPVQ;LGLESFK	NCP	9.662
LGLESFK;KGVTFS	NCP	104.215
KGVTFS;YGKEVL	NCP	169.799
YGKEVL;KPDFN	NCP	24.534
KPDFN;KPVVKPP	NCP	3.033
KPVVKPP;KTVPQR	NCP	17.956
KTVPQR;FEEPL	NCP	0.659
FEEPL;LWFPKPP	NCP	16.935
LWFPKPP;LENHNPK	NCP	58.329
LENHNPK;PPNRKRY	NCP	35.262
PPNRKRY;FDKEKSQL	NCP	134.265
FDKEKSQL;PTLLSN	NCP	42.921
PTLLSN;RDLSLPK	NCP	12.828
RDLSLPK;LYLVLPLF	NCP	35.619
LYLVLPLF;RATPGAPAL	NCP	1.077
RATPGAPAL;PNGAPEL	NCP	145.788
PNGAPEL;FAEFLA	NCP	34.918
FAEFLA;LFVNVN	NCP	4.313

LFVNVN;KAAKP	NCP	8.506
KAAKP;SFPTTTK	NCP	251.015
SFPTTTK;KLSTVRP	NCP	1.011
KLSTVRP;KDLLYPP	NCP	51.96
KDLLYPP;CWDLDLR	NCP	30.366
CWDLDLR;KKNEHQ	NCP	19.768
KKNEHQ;KTLPP	NCP	78.578
KTLPP;NDHFVLK	NCP	0.099
NDHFVLK;HLYDP	NCP	108.804
HLYDP;KGAHLVY	NCP	30.249
KGAHLVY;MAGPK	NCP	158.719
MAGPK;LVVVDF	NCP	36.836
LVVVDF;RHKLPPP	NCP	42.397
RHKLPPP;VTVLF	NCP	13.49
VTVLF;HDPLVPP	NCP	16.644
HDPLVPP;PTVAPC	NCP	66.541
PTVAPC;KRDLEF	NCP	38.598
KRDLEF;QLEVLKLG	NCP	24.764
QLEVLKLG;DLPMHK	NCP	32.931
DLPMHK;AMAGKLF	NCP	185.503
AMAGKLF;RVVPP	NCP	2.132
RVVPP;KGGSVPR	NCP	21.766
KGGSVPR;NFGMLHT	NCP	5.964
NFGMLHT;EHPRQVP	NCP	10.682
EHPRQVP;YLVFPL	NCP	4.503
YLVFPL;VLEMGLLK	NCP	110.2
VLEMGLLK;DCVMPLR	NCP	47.079
DCVMPLR;NPLVAL	NCP	249.584
NPLVAL;NELALK	NCP	113.184
NELALK;REETELVTK	NCP	4.359
REETELVTK;KDSAKVP	NCP	48.285
KDSAKVP;EYKPP	NCP	56.823
EYKPP;MTMPPPP	NCP	13.887
MTMPPPP;MKVELCS	NCP	35.662
MKVELCS;KYVPQSP	NCP	146.267
KYVPQSP;LNKRQGRTL	NCP	47.556
LNKRQGRTL;APLLY	NCP	26.356
APLLY;RKVVLS	NCP	95.919
RKVVLS;KKKTTK	NCP	137.997
KKKTTK;KYPLPP	NCP	64.373
KYPLPP;GPLLT	NCP	2.85
GPLLT;QFVVR	NCP	64.669
QFVVR;QAPRPLPQ	NCP	56.649
QAPRPLPQ;CSMSPHP	NCP	11.412
CSMSPHP;GKLYY	NCP	72.479
GKLYY;ERTVPP	NCP	56.176
ERTVPP;KCCFPK	NCP	77.48
KCCFPK;GPVPVT	NCP	19.093
GPVPVT;KPAYMPR	NCP	73.131
KPAYMPR;KNEKHLSC	NCP	83.076
KNEKHLSC;RYNGKEL	NCP	127.743

RYNGKEL;TPLLVR	NCP	98.723
TPLLVR;GVASPALT	NCP	113.895
GVASPALT;SLRVTQ	NCP	9.978
SLRVTQ;VGPGLK	NCP	24.342
VGPGLK;ESLTEKPP	NCP	82.773
ESLTEKPP;PPNQAAPK	NCP	101.422
PPNQAAPK;LAVSAQAL	NCP	95.184
LAVSAQAL;DMMCCP	NCP	234.45
DMMCCP;VRKQSVVL	NCP	33.642
VRKQSVVL;AETPKLGP	NCP	3.218
AETPKLGP;GPPLFAK	NCP	63.355
GPPLFAK;DLHLVEK	NCP	117.212
DLHLVEK;VPELTLTF	NCP	162.159
VPELTLTF;NGKKMMM	NCP	102.208
NGKKMMM;EAAVLP	NCP	102.08
EAAVLP;KKQGFHG	NCP	126.133
KKQGFHG;GPAVFVK	NCP	28.674
GPAVFVK;YVAFLDQ	NCP	24.021
YVAFLDQ;MRLLTH	NCP	150.236
MRLLTH;FGVVPPK	NCP	350.608
FGVVPPK;KGALGW	NCP	55.077
KGALGW;QAPWPP	NCP	54.132
QAPWPP;WALVKM	NCP	48.869
WALVKM;GSAAPYLR	NCP	88.035
GSAAPYLR;KPTDCGAP	NCP	137.311
KPTDCGAP;KPCFLAV	NCP	97.154
KPCFLAV;KEGPVVL	NCP	0.201
KEGPVVL;TFLEP	NCP	41.311
TFLEP;ALLEQR	NCP	38.078
ALLEQR;LSGFAPK	NCP	28.591
LSGFAPK;SVVLL	NCP	4.472
SVVLL;EDAKLSPK	NCP	1.558
EDAKLSPK;HDPVTVY	NCP	29.213
HDPVTVY;AVASTNK	NCP	8.232
AVASTNK;KVSPK	NCP	64.807
KVSPK;LPPKLHTP	NCP	10.595
LPPKLHTP;EEELAAVL	NCP	4.941
EEELAAVL;DLEFPR	NCP	19.471
DLEFPR;PNQQHNK	NCP	42.995
PNQQHNK;EVFGTKK	NCP	9.591
EVFGTKK;FVKDWGNF	NCP	46.033
FVKDWGNF;LTGLPG	NCP	25.731
LTGLPG;TVNKSSY	NCP	78.75
TVNKSSY;RNPPPPK	NCP	74.006
RNPPPPK;MHPVTQF	NCP	35.746
MHPVTQF;GVLTVDK	NCP	66.284
GVLTVDK;MTQLM	NCP	29.673
MTQLM;DVGLGL	NCP	21.768
DVGLGL;EHMPPHL	NCP	52.272
EHMPPHL;THSPF	NCP	38.161
THSPF;FRLPFK	NCP	27.035

FRLPFK;PVDDP	NCP	73.3
PVDDP;PDAHGK	NCP	199.007
PDAHGK;AFVLDL	NCP	126.588
AFVLDL;MTLGSAG	NCP	51.66
MTLGSAG;PNFPK	NCP	63.741
PNFPK;KLPPWPH	NCP	1.401
KLPPWPH;EAEEY	NCP	4.969
EAEEY;VPALEY	NCP	150.927
VPALEY;EALRSL	NCP	10.928
EALRSL;TTLVAVVK	NCP	123.271
TTLVAVVK;HFDLKLK	NCP	33.54
HFDLKLK;TTRKPEGK	NCP	5.331
TTRKPEGK;KYPPQAP	NCP	133.053
KYPPQAP;SFLKAR	NCP	80.111
SFLKAR;KAFPTTK	NCP	0.133
KAFPTTK;VSTLPY	NCP	46.788
VSTLPY;ALKLLK	NCP	18.063
ALKLLK;EDHVAAK	NCP	292.397
EDHVAAK;SNLPAVL	NCP	17.151
SNLPAVL;LHKDYPKK	NCP	44.296
LHKDYPKK;PPPSHSVP	NCP	81.977
PPPSHSVP;GVVHLFN	NCP	25.038
GVVHLFN;YVTPVYV	NCP	74.965
YVTPVYV;SPSCLLLGH	NCP	172.683
SPSCLLLGH;LPVRGSYL	NCP	0.143
LPVRGSYL;PVAHC	NCP	11.856
PVAHC;TTLDHY	NCP	41.625
TTLDHY;TVDLLKG	NCP	63.193
TVDLLKG;NVLVGPS	NCP	24.702
NVLVGPS;LLLSGALK	NCP	88.752
LLLSGALK;DDSLVRQ	NCP	25.586
DDSLVRQ;KAHEPK	NCP	5.032
KAHEPK;LLLLFTW	NCP	1.829
LLLLFTW;TTAPAGKD	NCP	94.155
TTAPAGKD;AAFPTTKT	NCP	140.622
AAFPTTKT;PGSVLGVK	NCP	43.129
PGSVLGVK;RLPVVGL	NCP	29.412
RLPVVGL;KMAMPLK	NCP	8.316
KMAMPLK;VKLLSWY	NCP	65.414
VKLLSWY;VLPVG	NCP	5.301
VLPVG;LVLVF	NCP	15.097
LVLVF;AHLKKKY	NCP	14.841
AHLKKKY;MLKDLLK	NCP	25.335
MLKDLLK;MAKTKSK	NCP	119.375
MAKTKSK;AHPLSVTK	NCP	529.636
AHPLSVTK;KGVTF	NCP	12.527
KGVTF;RPAVVLR	NCP	33.22
RPAVVLR;GMPPPFP	NCP	0.437
GMPPPFP;QYFLCF	NCP	212.736
QYFLCF;SSMAG	NCP	6.408
SSMAG;KVPHPDP	NCP	75.223

KVPHPDP;SVASHPK	NCP	101.907
SVASHPK;KATYA	NCP	75.056
KATYA;GHPKY	NCP	20.073
GHPKY;KAVVKK	NCP	18.909
KAVVKK;KGQTFLSH	NCP	4.071
KGQTFLSH;LLTHRKFC	NCP	81.027
LLTHRKFC;KGLEFQA	NCP	7.752
KGLEFQA;GLSRGPYK	NCP	29.361
GLSRGPYK;FDCPPPAP	NCP	45.253
FDCPPPAP;KAENAPR	NCP	49.529
KAENAPR;KFFAVPP	NCP	71.754
KFFAVPP;AAPAVHQ	NCP	30.423
AAPAVHQ;QLLLKE	NCP	3.094
QLLLKE;PDTVLHC	NCP	19.394
PDTVLHC;KHPVGAL	NCP	17.005
KHPVGAL;FNGLL	NCP	64.757
FNGLL;KGPSP	NCP	219.265
KGPSP;FALLL	NCP	107.804
FALLL;EVAAVKK	NCP	16.576
EVAAVKK;LVGKLLAFS	NCP	39.464
LVGKLLAFS;VNPML	NCP	45.882
VNPML;ELLLSC	NCP	18.421
ELLLSC;MNLVVP	NCP	11.271
MNLVVP;DVDLSLP	NCP	50.19
DVDLSLP;QLLGSQR	NCP	6.116
QLLGSQR;AKARLK	NCP	102.624
AKARLK;KLFKPN	NCP	23.604
KLFKPN;FEVSSK	NCP	95.067
FEVSSK;KNGPKFRLL	NCP	25.447
KNGPKFRLL;KPRVTPP	NCP	67.811
KPRVTPP;VGWKAPQ	NCP	132.88
VGWKAPQ;KWFEPF	NCP	13.241
KWFEPF;FSPGAVAP	NCP	3.925
FSPGAVAP;YPLAHQ	NCP	59.216
YPLAHQ;HEKETPK	NCP	336.283
HEKETPK;THLGLLTN	NCP	54.087
THLGLLTN;KGLVRDK	NCP	23.103
KGLVRDK;MCPPP	NCP	1.014
MCPPP;PHALTVM	NCP	16.235
PHALTVM;LGHLVDY	NCP	11.139
LGHLVDY;EDAAG	NCP	12.363
EDAAG;KGPNP	NCP	120.085
KGPNP;TVPFY	NCP	146.48
TVPFY;KPRALY	NCP	66.567
KPRALY;NRPGKK	NCP	22.246
NRPGKK;RWAVGPSP	NCP	40.527
RWAVGPSP;APVHGPK	NCP	64.19
APVHGPK;KTQVRAP	NCP	67.78
KTQVRAP;RFPVM	NCP	63.87
RFPVM;CAALLL	NCP	23.051
CAALLL;LLVHNV	NCP	243.346

LLVHNV;WEEALLP	NCP	65.637
WEEALLP;LLKFWPR	NCP	52.73
LLKFWPR;SVADLPF	NCP	23.361
SVADLPF;VSLVLTPM	NCP	37.23
VSLVLTPM;TEALLTPG	NCP	30.13
TEALLTPG;TLDMLK	NCP	44.723
TLDMLK;LQLNV	NCP	93.739
LQLNV;GSMAKLK	NCP	52.176
GSMAKLK;LAYLL	NCP	87.077
LAYLL;LLMPNNP	NCP	0.994
LLMPNNP;PSRLPP	NCP	27.047
PSRLPP;VTVKRTGT	NCP	3.739
VTVKRTGT;VGPAHKL	NCP	147.168
VGPAHKL;LGLAY	NCP	199.224
LGLAY;PEPLL	NCP	47.262
PEPLL;TPATFP	NCP	51.519
TPATFP;HRRAL	NCP	9.707
HRRAL;FTKFPL	NCP	6.775
FTKFPL;REELVREE	NCP	236.703
REELVREE;NFETTVVY	NCP	4.484
NFETTVVY;LPPAKKGT	NCP	34.245
LPPAKKGT;QRKLL	NCP	67.153
QRKLL;KSREYVLE	NCP	25.157
KSREYVLE;EGKKTS	NCP	40.762
EGKKTS;PGVFPATR	NCP	91.221
PGVFPATR;KPPALHAK	NCP	37.76
KPPALHAK;KDPPRP	NCP	48.699
KDPPRP;LHPAMPP	NCP	17.722
LHPAMPP;RLPPLL	NCP	86.3
RLPPLL;LTPQKGP	NCP	1.36
LTPQKGP;KEKPPSR	NCP	38.868
KEKPPSR;EVENLPR	NCP	13.896
EVENLPR;YCCFFVL	NCP	15.341
YCCFFVL;LFKLLQL	NCP	87.624
LFKLLQL;GVLSH	NCP	14.04
GVLSH;LDVAP	NCP	119.355
LDVAP;VGLALVGQ	NCP	15.54
VGLALVGQ;LELSL	NCP	111.724
LELSL;LMTTAF	NCP	0.35
LMTTAF;ATDLSLPK	NCP	28.867
ATDLSLPK;GMPYK	NCP	45.071
GMPYK;KFYNPPPPP	NCP	132.96
KFYNPPPPP;KPDMTQP	NCP	130.878
KPDMTQP;KGPPP	NCP	189.394
KGPPP;LPGLSE	NCP	80.4
LPGLSE;KPNGETF	NCP	18.419
KPNGETF;GKKTEPK	NCP	40.224
GKKTEPK;FDSGSLP	NCP	9.028
FDSGSLP;KHASPP	NCP	164.511
KHASPP;PMLLL	NCP	1.689
PMLLL;DETKKK	NCP	16.068

DETKKK;AAVLEYLT	NCP	115.427
AAVLEYLT;VLEYLTA	NCP	0.006
VLEYLTA;TDEAQSK	NCP	21.037
TDEAQSK;LSKHPV	NCP	205.308
LSKHPV;SVGFLSAL	NCP	21.687
SVGFLSAL;TSPVTLTLL	NCP	9.305
TSPVTLTLL;MPPLLG	NCP	41.545
MPPLLG;NHLKGYH	NCP	7.515
NHLKGYH;KPGYVKK	NCP	26.984
KPGYVKK;VYLVPV	NCP	126.496
VYLVPV;LPEFRP	NCP	27.935
LPEFRP;PAPTHPSK	NCP	10.137
PAPTHPSK;KTSDLNLK	NCP	176.508
KTSDLNLK;SSMLLQ	NCP	52.024
SSMLLQ;KLTAASR	NCP	32.709
KLTAASR;ETHRVLM	NCP	391.014
ETHRVLM;LGKALVAK	NCP	63.617
LGKALVAK;LQRSKP	NCP	138.975
LQRSKP;NGRPRPP	NCP	34.447
NGRPRPP;TVVNPMLL	NCP	67.112
TVVNPMLL;KDALLTP	NCP	68.823
KDALLTP;GMEGL	NCP	47.506
GMEGL;LPGCLSLP	NCP	50.538
LPGCLSLP;LPPVFKK	NCP	37.944
LPPVFKK;KVYFPK	NCP	45.893
KVYFPK;YNPGL	NCP	130.137
YNPGL;KLDDNR	NCP	70.599
KLDDNR;NRPLSF	NCP	24.337
NRPLSF;MVLTK	NCP	2.709
MVLTK;EPDMRKK	NCP	108.338
EPDMRKK;VGSYL	NCP	2.301
VGSYL;CGAATPK	NCP	19.063
CGAATPK;MMAEEHTDLE	NCP	138.998
MMAEEHTDLE;TPSHVAL	NCP	78.201
TPSHVAL;RMELL	NCP	13.267
RMELL;KGLVGTVA	NCP	59.991
KGLVGTVA;VSVRNPP	NCP	18.069
VSVRNPP;SHLLMLST	NCP	3.966
SHLLMLST;RVHAPK	NCP	64.328
RVHAPK;PRPPGHV	NCP	161.719
PRPPGHV;VPTAGPLK	NCP	33.575
VPTAGPLK;TEPTSLPK	NCP	70.306
TEPTSLPK;LPGLYVLP	NCP	63.251
LPGLYVLP;MHSFPK	NCP	79.915
MHSFPK;GSPLHAK	NCP	18.185
GSPLHAK;HGVVPLATY	NCP	26.614
HGVVPLATY;TGRVYNVTQH	NCP	0.117
TGRVYNVTQH;SLYGNG	NCP	44.91
SLYGNG;KVHMLP	NCP	10.5
KVHMLP;GPKREPPP	NCP	73.172
GPKREPPP;AARYTLR	NCP	10.348

AARYTLR;TATLRPYLS	NCP	54.761
TATLRPYLS;AALCLENF	NCP	0.048
AALCLENF;HTEQMYKHKLVDF	NCP	3.768
HTEQMYKHKLVDF;ASRAGPRAAGTD	NCP	57.027
ASRAGPRAAGTD;EHHVLYL	NCP	128.692
EHHVLYL;VPSSVKP	NCP	60.455
VPSSVKP;LSPAAW	NCP	18.615
LSPAAW;AAVLLFLA	NCP	107.242
AAVLLFLA;LELVCR	NCP	11.397
LELVCR;VLELGGF	NCP	3.801
VLELGGF;KARAQ	NCP	44.706
KARAQ;GLLPPPPPP	NCP	310.542
GLLPPPPPP;VSLVAL	NCP	109.662
VSLVAL;KEFKGK	NCP	52.176
KEFKGK;VGHYLT	NCP	0.627
VGHYLT;DGDEKLR	NCP	41.481
DGDEKLR;QPPTTPK	NCP	84.032
QPPTTPK;TAFLGY	NCP	40.582
TAFLGY;SNSRPF	NCP	145.356
SNSRPF;WGGLP	NCP	64.703
WGGLP;CQPLKK	NCP	30.174
CQPLKK;EPDAAPA	NCP	23.982
EPDAAPA;TSLGDLKS	NCP	72.319
TSLGDLKS;QEPLYPQ	NCP	36.891
QEPLYPQ;PGPYPHPP	NCP	8.633
PGPYPHPP;APLTLF	NCP	67.567
APLTLF;VPVVDPL	NCP	14.304
VPVVDPL;KTVWG	NCP	9.627
KTVWG;MMDDDDSS	NCP	32.067
MMDDDDSS;WMVLEY	NCP	14.595
WMVLEY;VLPNKK	NCP	135.021
VLPNKK;KGATRSP	NCP	25.365
KGATRSP;QRVVEL	NCP	14.421
QRVVEL;LPHVKSK	NCP	54.342
LPHVKSK;TAVHYVK	NCP	43.67
TAVHYVK;CPLNL	NCP	49.609
CPLNL;RVGVPGSYL	NCP	27.887
RVGVPGSYL;KPVLLAGP	NCP	10.249
KPVLLAGP;GDFDAVL	NCP	176.186
GDFDAVL;FAYKSF	NCP	13.482
FAYKSF;KYKPPPPP	NCP	21.865
KYKPPPPP;YKPPPPPP	NCP	0.003
YKPPPPPP;KPNPARP	NCP	8.27
KPNPARP;DPKKK	NCP	8.083
DPKKK;KNSSVP	NCP	129.21
KNSSVP;QSLAAKR	NCP	5.189
QSLAAKR;LFAENETK	NCP	124.251
LFAENETK;AALTL	NCP	37.306
AALTL;LLEHAK	NCP	62.873
LLEHAK;TVSGKF	NCP	17.112
TVSGKF;YKELQK	NCP	120.141

YKELQK;EFLKQ	NCP	82.989
EFLKQ;TFRCSF	NCP	49.257
TFRCSF;TSMGK	NCP	120.285
TSMGK;KSMAKKP	NCP	7.81
KSMAKKP;LVHPAGP	NCP	1.995
LVHPAGP;DHLMRTP	NCP	11.273
DHLMRTP;RLSPLQF	NCP	13.362
RLSPLQF;LGPAATK	NCP	22.077
LGPAATK;GVFLGL	NCP	14.023
GVFLGL;FPGQLN	NCP	253.55
FPGQLN;LSFVPK	NCP	70.177
LSFVPK;EVGGMV	NCP	227.609
EVGGMV;KDKPGPP	NCP	156.616
KDKPGPP;KNVTEVP	NCP	18.158
KNVTEVP;EHLGDPR	NCP	35.061
EHLGDPR;KLPPP	NCP	57.801
KLPPP;GTAGEK	NCP	4.11
GTAGEK;TSVHRSTN	NCP	72.268
TSVHRSTN;AARPPPR	NCP	40.815
AARPPPR;FVNSP	NCP	10.119
FVNSP;KLVSPKK	NCP	113.879
KLVSPKK;KRVLRP	NCP	38.307
KRVLRP;KRLLLPFQ	NCP	44.857
KRLLLPFQ;EELRPT	NCP	32.016
EELRPT;RVNCYP	NCP	28.035
RVNCYP;VQVVFL	NCP	39.126
VQVVFL;GLFLEN	NCP	7.34
GLFLEN;LGLVSQR	NCP	26.683
LGLVSQR;LPMML	NCP	22.958
LPMML;MEFKAPP	NCP	7.845
MEFKAPP;CQDKPR	NCP	60.084
CQDKPR;PTTGPPR	NCP	62.467
PTTGPPR;GVLFPDSP	NCP	22.101
GVLFPDSP;FAAGP	NCP	41.583
FAAGP;KGRFP	NCP	3.489
KGRFP;PLDKPP	NCP	101.694
PLDKPP;RVPKTEP	NCP	173.861
RVPKTEP;KMAAF	NCP	48.999
KMAAF;GPLSRPP	NCP	28.9
GPLSRPP;ELNDLRK	NCP	80.601
ELNDLRK;VSAFTPK	NCP	64.872
VSAFTPK;SGHMPPP	NCP	32.48
SGHMPPP;ETGLSP	NCP	25.905
ETGLSP;NSRPPR	NCP	22.647
NSRPPR;VMPALLL	NCP	34.864
VMPALLL;PFGVHKP	NCP	27.132
PFGVHKP;SAEDVVVVH	NCP	142.952
SAEDVVVVH;TKTKKK	NCP	0.22
TKTKKK;LSPTLRP	NCP	13.263
LSPTLRP;PLEKVNPLGGAV	NCP	9.905
PLEKVNPLGGAV;AAAVFEYPGN	NCP	0.402

AAAVFEYPGN;EGKDLQK	NCP	21.808
EGKDLQK;MCAPPPPP	NCP	6.45
MCAPPPPP;LGLAGAVL	NCP	14.007
LGLAGAVL;GPYPP	NCP	28.296
GPYPP;KGMLLR	NCP	6.242
KGMLLR;GDVRGPR	NCP	24.273
GDVRGPR;VKVALQD	NCP	22.545
VKVALQD;FPFWY	NCP	29.736
FPFWY;PRDPRP	NCP	72.034
PRDPRP;TLLMGM	NCP	67.934
TLLMGM;RETPKNQ	NCP	48.904
RETPKNQ;TNTLRYPL	NCP	95.555
TNTLRYPL;YPGAP	NCP	7.722
YPGAP;KPANP	NCP	22.983
KPANP;AAHDLPK	NCP	6.531
AAHDLPK;LGLEFQA	NCP	4.54
LGLEFQA;TVYPVT	NCP	98.081
TVYPVT;KDSGSGPK	NCP	4.245
KDSGSGPK;EHLPPR	NCP	32.004
EHLPPR;PALQPV	NCP	39.729
PALQPV;VAEEGP	NCP	23.892
VAEEGP;KPRLEEN	NCP	7.218
KPRLEEN;KEQSPLPP	NCP	311.124
KEQSPLPP;VGAPFPF	NCP	111.486
VGAPFPF;PLECPPK	NCP	11.734
PLECPPK;HTTLGPSPP	NCP	8
HTTLGPSPP;AAPKVPKG	NCP	21.757
AAPKVPKG;MQLLLK	NCP	74.234
MQLLLK;GVALDL	NCP	18.826
GVALDL;KHRGPHL	NCP	15.878
KHRGPHL;KPHPSLRP	NCP	68.332
KPHPSLRP;KNLDTTP	NCP	48.614
KNLDTTP;KLSLVSGK	NCP	40.665
KLSLVSGK;LFGNST	NCP	18.757
LFGNST;PGVMLLR	NCP	63.804
PGVMLLR;HGFLV	NCP	11.415
HGFLV;ALLHAKK	NCP	44.312
ALLHAKK;SLVVP	NCP	85.986
SLVVP;VSGNKLLV	NCP	0.195
VSGNKLLV;PAMGLVP	NCP	3.727
PAMGLVP;KPPAHFH	NCP	22.668
KPPAHFH;PLGAAKPP	NCP	20.409
PLGAAKPP;PVREC	NCP	35.726
PVREC;KDQVP	NCP	14.703
KDQVP;LPPAW	NCP	6.22
LPPAW;KRGDLK	NCP	29.598
KRGDLK;DGVREVFE	NCP	97.344
DGVREVFE;AAQRSAAALA	NCP	2.337
AAQRSAAALA;AAAAPRTVYAFSA	NCP	0.027
AAAAPRTVYAFSA;AAAPRTVYA	NCP	0.003
AAAPRTVYA;AAPRTVYAFS	NCP	0.003

AAPRTVYAFS;RDYTQMNDL	NCP	0.129
RDYTQMNDL;APAGAPPGP	NCP	0.141
APAGAPPGP;AGHRLVL	NCP	6.437
AGHRLVL;LPLMLLVG	NCP	25.936
LPLMLLVG;ALADTAY	NCP	16.062
ALADTAY;MGYPM	NCP	9.719
MGYPM;TSGAYPP	NCP	24.598
TSGAYPP;QLSLR	NCP	18.041
QLSLR;KPELSH	NCP	123.409
KPELSH;KRHTHAT	NCP	5.067
KRHTHAT;RFEEPK	NCP	39.945
RFEEPK;NMFGEKL	NCP	34.865
NMFGEKL;TNATHNVP	NCP	6.366
TNATHNVP;PAARSRLP	NCP	22.702
PAARSRLP;KRHMKK	NCP	3.68
KRHMKK;LLGGPP	NCP	163.618
LLGGPP;PPDLPY	NCP	64.653
PPDLPY;NAPYAP	NCP	43.319
NAPYAP;PPAPDKPS	NCP	48.376
PPAPDKPS;KMYVAH	NCP	87.356
KMYVAH;FPQSQK	NCP	150.354
FPQSQK;VGGAGP	NCP	85.723
VGGAGP;VELGLQM	NCP	40.914
VELGLQM;VHMASEK	NCP	0.143
VHMASEK;LLPPYK	NCP	185.938
LLPPYK;KPVEC	NCP	93.869
KPVEC;GPCGDCL	NCP	102.087
GPCGDCL;ESVPCKLR	NCP	3.556
ESVPCKLR;KPFPTHGP	NCP	21.953
KPFPTHGP;YEKGLSR	NCP	34.215
YEKGLSR;ALPYK	NCP	5.22
ALPYK;DPLPL	NCP	60.688
DPLPL;FARAPGP	NCP	245.565
FARAPGP;ASGVAV	NCP	38.979
ASGVAV;RMVVWGK	NCP	12.777
RMVVWGK;LPPQFLR	NCP	22.158
LPPQFLR;LLHMLKNAESNAELK	NCP	61.682
LLHMLKNAESNAELK;RQDLDLR	NCP	117.787
RQDLDLR;MAALRR	NCP	10.662
MAALRR;FPLPPEK	NCP	89.775
FPLPPEK;KFFSPP	NCP	59.904
KFFSPP;KAASLAVP	NCP	35.951
KAASLAVP;KDLLCEF	NCP	21.568
KDLLCEF;GPELCTVEFVDKPVFR	NCP	94.871
GPELCTVEFVDKPVFR;ELCTVEFVDKPVF	NCP	0.006
ELCTVEFVDKPVF;VDKPVFR	NCP	0.021
VDKPVFR;AALGVGK	NCP	60.493
AALGVGK;NGTKAVTK	NCP	104.193
NGTKAVTK;MENGQVYR	NCP	228.29
MENGQVYR;GPWPPKP	NCP	73.735
GPWPPKP;YLTAPAG	NCP	149.892

YLTAPAG;QMFLT	NCP	161.057
QMFLT;QPVALPK	NCP	11.545
QPVALPK;DGLVRL	NCP	69.986
DGLVRL;TKKSPGK	NCP	41.56
TKKSPGK;NGLWLPP	NCP	20.307
NGLWLPP;EPGLAF	NCP	37.062
EPGLAF;QAGPSLVH	NCP	68.376
QAGPSLVH;KKKAPAVL	NCP	77.304
KKKAPAVL;AAADHPRPP	NCP	76.034
AAADHPRPP;PPMGP	NCP	175.102
PPMGP;VALPVT	NCP	90.857
VALPVT;KPAPKPP	NCP	139.365
KPAPKPP;RDPTGLR	NCP	63.805
RDPTGLR;RLGFGR	NCP	42.243
RLGFGR;PAAGTPPK	NCP	54.219
PAAGTPPK;KGGVPTPK	NCP	44.232
KGGVPTPK;LSRLFLL	NCP	11.354
LSRLFLL;ALPVLPPH	NCP	31.581
ALPVLPPH;VELAF	NCP	13.537
VELAF;LSSPVLP	NCP	42.225
LSSPVLP;PAPGVSPP	NCP	2.754
PAPGVSPP;GVPPVT	NCP	31.115
GVPPVT;AAAKTAPP	NCP	5.758
AAAKTAPP;PRVAC	NCP	35.903
PRVAC;KEVGVVP	NCP	38.794
KEVGVVP;KPKTPRP	NCP	22.898
KPKTPRP;PTRHGKK	NCP	15.925
PTRHGKK;VERAFPPP	NCP	74.556
VERAFPPP;FVMLN	NCP	52.469
FVMLN;EEKASPP	NCP	4.192
EEKASPP;DCDGDAQ	NCP	6.728
DCDGDAQ;MLSVA	NCP	17.191
MLSVA;EHHLYDR	NCP	32.873
EHHLYDR;WLPLGL	NCP	22.384
WLPLGL;FGLKLD	NCP	64.578
FGLKLD;LDQLLTH	NCP	196.539
LDQLLTH;GVVSLYA	NCP	135.905
GVVSLYA;KAGYHPK	NCP	81.721
KAGYHPK;KPACGHKDP	NCP	7.484
KPACGHKDP;AWVLQGK	NCP	210.172
AWVLQGK;YLRLR	NCP	97.14
YLRLR;KARGEVP	NCP	44.325
KARGEVP;LSELK	NCP	4.838
LSELK;EFKPGAK	NCP	27.994
EFKPGAK;KYEAAPR	NCP	20.336
KYEAAPR;STELLLYP	NCP	23.718
STELLLYP;KGPPPPSVP	NCP	0.889
KGPPPPSVP;TSPVDVR	NCP	8.523
TSPVDVR;LLLLHAEP	NCP	11.409
LLLLHAEP;TGFGK	NCP	61.575
TGFGK;GVPLSLVG	NCP	151.176

GVPLSLVG;GPLFQNVP	NCP	110.147
GPLFQNVP;MLPLSLL	NCP	377.983
MLPLSLL;KDGCCC	NCP	87.866
KDGCCC;KLGLDL	NCP	37.83
KLGLDL;APPVD	NCP	126.462
APPVD;SLERLL	NCP	36.744
SLERLL;ESGPTTQ	NCP	10.129
ESGPTTQ;RAYMEP	NCP	82.295
RAYMEP;KSVHPPR	NCP	84.607
KSVHPPR;TPVSAPPT	NCP	9.894
TPVSAPPT;KSGKELP	NCP	7.791
KSGKELP;VGGPTVF	NCP	0.933
VGGPTVF;RSVGKLG	NCP	117.567
RSVGKLG;SVLRLL	NCP	172.653
SVLRLL;PSLSPKHRP	NCP	68.814
PSLSPKHRP;KTQPQR	NCP	66.396
KTQPQR;HAAPGHR	NCP	48.639
HAAPGHR;APGHSPVP	NCP	19.938
APGHSPVP;RKPAEPP	NCP	14.265
RKPAEPP;SPAGLAQLP	NCP	108.435
SPAGLAQLP;MFSRPF	NCP	3.233
MFSRPF;LHPNK	NCP	53.122
LHPNK;ELSTTPP	NCP	28.809
ELSTTPP;TFAGPK	NCP	1.242
TFAGPK;WPHRYLL	NCP	11.484
WPHRYLL;EEELV	NCP	7.356
EEELV;LPPPPPRPP	NCP	0.333
LPPPPPRPP;RPPLQPP	NCP	0.018
RPPLQPP;KSSSMSH	NCP	18.224
KSSSMSH;LLMFNKPP	NCP	45.048
LLMFNKPP;AASTGLK	NCP	17.677
AASTGLK;DGPPVPR	NCP	127.734
DGPPVPR;ERWLLT	NCP	54.714
ERWLLT;GVSGTSLP	NCP	32.994
GVSGTSLP;KSFLMT	NCP	0.402
KSFLMT;MMMHR	NCP	1.349
MMMHR;LTPLETAA	NCP	29.824
LTPLETAA;KPPVPR	NCP	217.709
KPPVPR;SAAFPQR	NCP	41.43
SAAFPQR;GLGTVPVGR	NCP	32.995
GLGTVPVGR;LELNNL	NCP	8.561
LELNNL;LPEPLP	NCP	16.849
LPEPLP;GHDWM	NCP	20.012
GHDWM;LGPLGAP	NCP	37.315
LGPLGAP;VSLPPPPG	NCP	0.699
VSLPPPPG;KNFLQPP	NCP	24.948
KNFLQPP;EQLLPEQ	NCP	57.03
EQLLPEQ;SRAHVAH	NCP	56.646
SRAHVAH;PDCMD	NCP	45.123
PDCMD;KSVLGAGK	NCP	83.106
KSVLGAGK;FRWKPP	NCP	29.958

FRWKPP;APAPPHSAPG	NCP	21.447
APAPPHSAPG;LPLPPPPPP	NCP	47.778
LPLPPPPPP;QSACDLRK	NCP	17.208
QSACDLRK;VEELCHK	NCP	55.535
VEELCHK;AWAAVPG	NCP	43.747
AWAAVPG;VPALPEGP	NCP	97.641
VPALPEGP;HVLRAAP	NCP	22.404
HVLRAAP;GGGVLK	NCP	89.546
GGGVLK;TMNLLQ	NCP	42.619
TMNLLQ;RVPEC	NCP	132.284
RVPEC;ALGGAP	NCP	6.805
ALGGAP;APPLPKRP	NCP	113.949
APPLPKRP;ALLTPQ	NCP	11.349
ALLTPQ;PGPALPPP	NCP	40.694
PGPALPPP;LLLLMTTT	NCP	25.341
LLLLMTTT;LGDEETVRK	NCP	92.85
LGDEETVRK;AMEAVAAQGKAK	NCP	0.027
AMEAVAAQGKAK;SPQRPLSPP	NCP	51.775
SPQRPLSPP;PPPSMQ	NCP	31.701
PPPSMQ;AAVGAP	NCP	27.474
AAVGAP;RLLPHN	NCP	34.995
RLLPHN;ALAPR	NCP	95.259
ALAPR;ECGQC	NCP	41.838
ECGQC;NQPVL	NCP	97.854
NQPVL;YPLPLEQP	NCP	5.115
YPLPLEQP;DGPVPRP	NCP	29.04
DGPVPRP;GPPHYPP	NCP	0.843
GPPHYPP;KSRGS	NCP	50.823
KSRGS;AASPRAPAM	NCP	50.136
AASPRAPAM;GQAGRDL	NCP	30.141
GQAGRDL;GVTAL	NCP	125.415
GVTAL;SNLPF	NCP	86.636
LLRSPQQL;LDFGENLL	NCP	20.472
LDFGENLL;VTVVLL	NCP	119.253
VTVVLL;ACGPLPTP	NCP	13.32
ACGPLPTP;CVLGY	NCP	48.03
CVLGY;RLRGLVT	NCP	160.041
RLRGLVT;RAQHKEP	NCP	37.281
RAQHKEP;HSSLLVT	NCP	48.379
HSSLLVT;LGASLK	NCP	78.908
LGASLK;HSFYNEL	NCP	33.468
HSFYNEL;KVHHE	NCP	58.495
KVHHE;KLLSHSSHV	NCP	15.876
KLLSHSSHV;HCAPPRPP	NCP	50.891
HCAPPRPP;FGTPPP	NCP	39.685
FGTPPP;PPPVMPR	NCP	47.465
PPPVMPR;PAGRGLP	NCP	68.934
PAGRGLP;LTSNKF	NCP	344.751
LTSNKF;PPLPSPVLP	NCP	182.055
PPLPSPVLP;KACSSPQ	NCP	26.358
KACSSPQ;QDPLLR	NCP	24.414

QDPLLR;HFWSG	NCP	25.162
HFWSG;KQRGN	NCP	20.561
KQRGN;TGAPP	NCP	50.187
TGAPP;ECPALA	NCP	25.224
ECPALA;FQKPQK	NCP	258.993
FQKPQK;SRPHPPP	NCP	87.105
SRPHPPP;QGPVL	NCP	2.964
QGPVL;PPPPPPKY	NCP	45.78
PPPPPPKY;FAGFPP	NCP	29.859
FAGFPP;LTVEQPK	NCP	13.194
LTVEQPK;PAGPAPVPL	NCP	4.293
PAGPAPVPL;CRVSGKR	NCP	1.95
CRVSGKR;VVVLKKE	NCP	169.399
VVVLKKE;PPPLREP	NCP	29.15
PPPLREP;PPPTLRP	NCP	93.033
PPPTLRP;AAAEAPSLR	NCP	36.537
AAAEAPSLR;NPLVAP	NCP	57.876
NPLVAP;KSLEPY	NCP	19.755
KSLEPY;QPAGP	NCP	61.62
QPAGP;AAPKSNK	NCP	45.735
AAPKSNK;KNPELQR	NCP	253.536
KNPELQR;FPVSYQ	NCP	89.017
FPVSYQ;KRFNSLK	NCP	2.571
KRFNSLK;AKLLS	NCP	72.873
AKLLS;YVVAP	NCP	0.324
YVVAP;TQKEHLH	NCP	17.927
TQKEHLH;DPGRGP	NCP	39.667
DPGRGP;GPAFWAP	NCP	137.216
GPAFWAP;QGEPSPP	NCP	73.897
QGEPSPP;AQLLP	NCP	4.343
AQLLP;EAPAAVK	NCP	36.924
EAPAAVK;GLAEVL	NCP	29.862
GLAEVL;VTATPP	NCP	117.03
VTATPP;KGLLY	NCP	9.543
KGLLY;TAPLQRVL	NCP	150.213
TAPLQRVL;MTTKPF	NCP	33.195
MTTKPF;KSSPH	NCP	188.283
KSSPH;KDEMF	NCP	174.412
KDEMF;RKLVTR	NCP	24.395
RKLVTR;MGSLDFL	NCP	29.958
MGSLDFL;FGEESEK	NCP	53.937
FGEESEK;KDLHAPR	NCP	46.983
KDLHAPR;LPSVETGL	NCP	128.767
LPSVETGL;QKAAVTQ	NCP	115.944
QKAAVTQ;KKVLPAP	NCP	39.902
KKVLPAP;PPLKPKP	NCP	14.763
PPLKPKP;VKGGLLKT	NCP	6
VKGGLLKT;RKGLDDF	NCP	82.915
RKGLDDF;EPGAAPPP	NCP	99.959
EPGAAPPP;LLGFF	NCP	45.51
LLGFF;TGLLLLSVQKR	NCP	5.403

TGLLLLSVQKR;KLPYPPPPP	NCP	2.487
KLPYPPPPP;DLRTRP	NCP	44.736
DLRTRP;PAAEPPK	NCP	60.741
PAAEPPK;FPGLSPL	NCP	54.141
FPGLSPL;VPPGTP	NCP	111.768
VPPGTP;EPLYSQQK	NCP	4.183
EPLYSQQK;APVPKGH	NCP	43.307
APVPKGH;VTPGVELL	NCP	93.621
VTPGVELL;NVEVPHK	NCP	210.748
NVEVPHK;VPSLG	NCP	72.885
VPSLG;AEDLQTKL	NCP	29.009
AEDLQTKL;DQLLLT	NCP	35.01
DQLLLT;QVMPGLR	NCP	18.88
QVMPGLR;VEEMLVA	NCP	36.914
VEEMLVA;AKVAVLG	NCP	95.767
AKVAVLG;EKFMHLK	NCP	67.815
EKFMHLK;WTSLVG	NCP	83.033
WTSLVG;KFAGHRP	NCP	401.503
KFAGHRP;TVVAY	NCP	34.526
TVVAY;PVGGF	NCP	45.841
PVGGF;MNTVLSR	NCP	52.73
MNTVLSR;YEKQY	NCP	59.901
YEKQY;ESLLAF	NCP	10.048
ESLLAF;ALKKSC	NCP	1.487
ALKKSC;MESLQK	NCP	80.889
MESLQK;VPAWP	NCP	75.522
VPAWP;GLDQFM	NCP	18.684
GLDQFM;TKPTP	NCP	85.374
TKPTP;KSPKGGPP	NCP	289.299
KSPKGGPP;CTLEDPLE	NCP	3.061
CTLEDPLE;PGVLQP	NCP	8.495
PGVLQP;EPGLLTP	NCP	102.657
EPGLLTP;VPEVDQ	NCP	50.295
VPEVDQ;KQRLLAP	NCP	38.19
KQRLLAP;KALDN	NCP	44.335
KALDN;QATAHLVL	NCP	68.459
QATAHLVL;VFLMVFLE	NCP	142.632
VFLMVFLE;KRELPFG	NCP	44.889
KRELPFG;GLPPQC	NCP	26.367
GLPPQC;VKFQHL	NCP	13.987
VKFQHL;SYAFF	NCP	26.61
SYAFF;DSPLPY	NCP	148.626
DSPLPY;KYPPQPK	NCP	16.677
KYPPQPK;LGVSLRL	NCP	88.76
LGVSLRL;DLTEF	NCP	115.83
DLTEF;SLSTFQ	NCP	51.186
SLSTFQ;VSNFPK	NCP	21.279
VSNFPK;ELSGFT	NCP	46.128
ELSGFT;QRPDFLSF	NCP	28.816
QRPDFLSF;TLGAP	NCP	82.865
TLGAP;RGAQPAPK	NCP	2.34

RGAQPAPK;KPGPPPAP	NCP	114.331
KPGPPPAP;ALALK	NCP	23.366
ALALK;MPAVGQR	NCP	182.241
MPAVGQR;HSFWEGH	NCP	43.971
HSFWEGH;PLLGPL	NCP	120.874
PLLGPL;FLLSD	NCP	67.355
FLLSD;APLNPKA	NCP	29.746
APLNPKA;TNVPLK	NCP	11.292
TNVPLK;NYCPKSK	NCP	4.436
NYCPKSK;KEHALKSP	NCP	18.564
KEHALKSP;AVSEGTKAVTK	NCP	38.515
AVSEGTKAVTK;KTMPPGP	NCP	33.312
KTMPPGP;SSLVKR	NCP	66.273
SSLVKR;QKVTLGN	NCP	57.423
QKVTLGN;PPFPVP	NCP	112.884
PPFPVP;KLSVPVP	NCP	66.365
KLSVPVP;KKPSHAK	NCP	130.749
KKPSHAK;KGDCAYL	NCP	8.844
KGDCAYL;PPPLD	NCP	41.167
PPPLD;KTVSVHH	NCP	40.767
KTVSVHH;HMSLRAP	NCP	19.589
HMSLRAP;ARLPEVF	NCP	7.765
ARLPEVF;ALLPK	NCP	23.592
ALLPK;RGHLLF	NCP	60.393
RGHLLF;GEAADLLR	NCP	94.019
GEAADLLR;NVVKKAP	NCP	129.697
NVVKKAP;PLTEFLL	NCP	20.979
PLTEFLL;HSSLTPLH	NCP	23.406
HSSLTPLH;KPLSHPPP	NCP	52.284
KPLSHPPP;HGGLMTP	NCP	13.785
HGGLMTP;TVLLPPP	NCP	18.933
TVLLPPP;FEPEKLR	NCP	7.007
FEPEKLR;YGDRLER	NCP	116.367
YGDRLER;VMLSGPT	NCP	122.179
VMLSGPT;VPPLLP	NCP	55.956
VPPLLP;TVFSDVP	NCP	34.916
TVFSDVP;AAPRPPP	NCP	15.304
AAPRPPP;LGGPPPPPPP	NCP	166.74
LGGPPPPPPP;HLMLSY	NCP	18.029
HLMLSY;GPGAPAP	NCP	48.178
GPGAPAP;RKKKKTF	NCP	57.392
RKKKKTF;KNGVTPF	NCP	0.838
KNGVTPF;VSLLA	NCP	148.071
VSLLA;VPAELHE	NCP	23.498
VPAELHE;LDRPVVR	NCP	111.009
LDRPVVR;DGRPPLR	NCP	9.091
DGRPPLR;GVPPPP	NCP	93.572
GVPPPP;DVPLAKK	NCP	65.056
DVPLAKK;AATEEGD	NCP	17.814
AATEEGD;KVLMPSR	NCP	25.647
KVLMPSR;STMGKPQRLY	NCP	53.778

STMGKPQRLY;LAPPDFR	NCP	0.728
LAPPDFR;PGKGH	NCP	45.529
PGKGH;APPVPPGF	NCP	1.037
APPVPPGF;HTPKPR	NCP	0.153
HTPKPR;LHAQQPL	NCP	122.688
LHAQQPL;VTLYK	NCP	0.481
VTLYK;HFMCVPL	NCP	65.339
HFMCVPL;TAPKKFP	NCP	4.569
TAPKKFP;LSPPTPH	NCP	10.213
LSPPTPH;NGDMC	NCP	102.092
NGDMC;LDKEFR	NCP	100.723
LDKEFR;GVKGTKL	NCP	94.101
GVKGTKL;LATAKRLD	NCP	147.14
LATAKRLD;KGARDPQ	NCP	50.892
KGARDPQ;VGGAALTK	NCP	85.195
VGGAALTK;KELLPP	NCP	134.349
KELLPP;RPGSLRP	NCP	5.006
RPGSLRP;HAAVLT	NCP	46.189
HAAVLT;EDFHVK	NCP	106.98
EDFHVK;VGPSVVGA	NCP	95.367
VGPSVVGA;LKSDKL	NCP	17.654
LKSDKL;KGFSL	NCP	48.522
KGFSL;LLTFT	NCP	19.794
LLTFT;PLDGP	NCP	53.023
PLDGP;PLASL	NCP	38.118
PLASL;FAKFPEPPP	NCP	25.935
FAKFPEPPP;WLQNKLPQ	NCP	6.666
WLQNKLPQ;VLLLPY	NCP	40.416
VLLLPY;RPHPPPDP	NCP	2.49
RPHPPPDP;KSPLGM	NCP	33.803
KSPLGM;YTLPQV	NCP	33.051
YTLPQV;VLVLKK	NCP	65.602
VLVLKK;QMVETQQL	NCP	183.177
QMVETQQL;EKQAG	NCP	50.631
EKQAG;PVVAKAH	NCP	19.433
PVVAKAH;AQVLR	NCP	110.554
AQVLR;PLGSASLR	NCP	44.712
PLGSASLR;LASEPGVL	NCP	2.601
LASEPGVL;TSVLLT	NCP	31.437
TSVLLT;DSVFHRKK	NCP	298.797
DSVFHRKK;LSPLQ	NCP	27.992
LSPLQ;EPGLP	NCP	17.655
EPGLP;VTFKV	NCP	31.036
VTFKV;KVEPAAAK	NCP	52.049
KVEPAAAK;RVYLFTLC	NCP	83.776
RVYLFTLC;VSSLML	NCP	3.225
VSSLML;LSLALMLP	NCP	22.263
LSLALMLP;GDGALF	NCP	206.456
GDGALF;KSTVPP	NCP	152.277
KSTVPP;YGATNKR	NCP	98.988
YGATNKR;HVLLP	NCP	297.481

HVLLP;TVTMKVK	NCP	7.199
TVTMKVK;GVPPYK	NCP	47.218
GVPPYK;RRSKRRC	NCP	106.22
RRSKRRC;ALAAVEARR	NCP	50.947
ALAAVEARR;AALKRGP	NCP	29.024
AALKRGP;GSPVLLL	NCP	60.331
GSPVLLL;RFGPFSP	NCP	8
RFGPFSP;LDLTGGLL	NCP	4.297
LDLTGGLL;ETHVLR	NCP	69.096
ETHVLR;KPHSAPPP	NCP	91.58
KPHSAPPP;TLQLQ	NCP	439.45
TLQLQ;LSHGYK	NCP	31.848
LSHGYK;DLLDRFPK	NCP	12.161
DLLDRFPK;LMFWKCF	NCP	9.045
LMFWKCF;KEAWGVK	NCP	113.169
KEAWGVK;GPALYK	NCP	220.186
GPALYK;AKLHLLR	NCP	15.86
AKLHLLR;KELFDKK	NCP	45.399
KELFDKK;AWFRAQP	NCP	58.723
AWFRAQP;AMVHLLLK	NCP	59.177
AMVHLLLK;SRLFPPPPP	NCP	22.344
SRLFPPPPP;AAALTKAV	NCP	118.608
AAALTKAV;VFEDVVK	NCP	75.399
VFEDVVK;YQKGRSL	NCP	25.074
YQKGRSL;LTVMMK	NCP	68.634
LTVMMK;EVGTLAK	NCP	156.919
EVGTLAK;TAQPLHK	NCP	154.785
TAQPLHK;HGSLFF	NCP	104.987
HGSLFF;VSAGWGV	NCP	260.089
VSAGWGV;PGFLEEVP	NCP	90.102
PGFLEEVP;GLHETAPP	NCP	1.722
GLHETAPP;TDLALHMA	NCP	51.617
TDLALHMA;NKFFSPK	NCP	101.166
NKFFSPK;PGFPL	NCP	23.988
PGFPL;PVFGPPP	NCP	20.731
PVFGPPP;LSGKVYGY	NCP	76.14
LSGKVYGY;VTALLD	NCP	25.137
VTALLD;TGKVLGAGK	NCP	128.439
TGKVLGAGK;KSGVAAVK	NCP	10.968
KSGVAAVK;VDSAMELPF	NCP	11.591
VDSAMELPF;EHLTKTL	NCP	138.502
EHLTKTL;MKNVP	NCP	56.571
MKNVP;KPGEAV	NCP	61.629
KPGEAV;WLLLALPN	NCP	27.2
WLLLALPN;KDASFLL	NCP	14.005
KDASFLL;PLSVPLLPP	NCP	7.449
PLSVPLLPP;VMASPP	NCP	4.8
VMASPP;KTVLLK	NCP	28.104
KTVLLK;ALGPTVF	NCP	70.5
ALGPTVF;NSLHMH	NCP	29.819
NSLHMH;AHKERH	NCP	30.445

AHKERH;SVELP	NCP	334.382
SVELP;VDSVYRTR	NCP	7.648
VDSVYRTR;RNLLFLPR	NCP	84.698
RNLLFLPR;VELLKEL	NCP	98.472
VELLKEL;LSYTLAAP	NCP	28.411
LSYTLAAP;EVAPP	NCP	48.105
EVAPP;VRAGQLY	NCP	79.965
VRAGQLY;GPPAPP	NCP	141.933
GPPAPP;VLLAL	NCP	31.079
VLLAL;LLEAAPK	NCP	74.935
LLEAAPK;KYPPGVP	NCP	128.061
KYPPGVP;HNSLLLP	NCP	20.093
HNSLLLP;CHCCCCSH	NCP	5.599
CHCCCCSH;PATLQPL	NCP	3.345
PATLQPL;LLNPL	NCP	36.147
LLNPL;LPPPGAQR	NCP	1.77
LPPPGAQR;RGPKEPHL	NCP	35.97
RGPKEPHL;TAVDLSPA	NCP	42.195
TAVDLSPA;VLARCVP	NCP	177.425
VLARCVP;VPPPPPL	NCP	0.61
VPPPPPL;TVGVPL	NCP	58.665
TVGVPL;AAAPVPF	NCP	6.612
AAAPVPF;MEVLRPPAGPGGFSP	NCP	25.092
MEVLRPPAGPGGFSP;SANFLEWF	NCP	144.599
SANFLEWF;LELKYVCFGGL	NCP	15.096
LELKYVCFGGL;DPPKVYSL	NCP	95.497
DPPKVYSL;HSSLLEPLSK	NCP	2.969
HSSLLEPLSK;ASVDLMNK	NCP	16.221
ASVDLMNK;MRVGLTH	NCP	46.464
MRVGLTH;YQAAAAP	NCP	99.243
YQAAAAP;VELLLDFP	NCP	24.466
VELLLDFP;KKAGAP	NCP	17.789
KKAGAP;CWVLTV	NCP	36.802
CWVLTV;AVGVGKTW	NCP	28.808
AVGVGKTW;SLGLVVLP	NCP	189.76
SLGLVVLP;MGAVP	NCP	5.967
MGAVP;FVEPK	NCP	153.3
FVEPK;AKVSVL	NCP	37.755
AKVSVL;VGKACAP	NCP	149.586
VGKACAP;VLHDHGF	NCP	84.888
VLHDHGF;LTAKLH	NCP	24.852
LTAKLH;VSEGTKAVTK	NCP	10.427
VSEGTKAVTK;CFTEYL	NCP	83.14
CFTEYL;VGPGTF	NCP	5.211
VGPGTF;ELGLL	NCP	46.541
ELGLL;LLTTLPDK	NCP	88.882
LLTTLPDK;KDVPSLP	NCP	76.563
KDVPSLP;RKGNYAERVGAGAPV	NCP	26.996
RKGNYAERVGAGAPV;KGNYAERVGAGAPVYLAA	NCP	0.003
KGNYAERVGAGAPVYLAA;GNYAERVGAGAPV	NCP	0.003
GNYAERVGAGAPV;APAAPAAPAPAEK	NCP	32.1

APAAPAAPAPAEK;PAAPAAPAPAEKTPVKKKAR	NCP	0.003
PAAPAAPAPAEKTPVKKKAR;AAPAAPAPAEK	NCP	0.003
AAPAAPAPAEK;SGPPVSEL	NCP	0.087
SGPPVSEL;KERSGVSL	NCP	0.048
KERSGVSL;SLVSKGTL	NCP	0.102
SLVSKGTL;AGAAKPKKPAGAA	NCP	0.111
AGAAKPKKPAGAA;KAPKSPAKAKAVKPK	NCP	0.18
KAPKSPAKAKAVKPK;PEPTKSAPAPKK	NCP	1.194
PEPTKSAPAPKK;TTLKKVKPATGAK	NCP	23.952
TTLKKVKPATGAK;VAKPKAVQPK	NCP	0.126
VAKPKAVQPK;KSAPAPK	NCP	4.962
KSAPAPK;VDLLSFPK	NCP	31.755
VDLLSFPK;KAVTKYTSSK	NCP	22.122
KAVTKYTSSK;RARAFF	NCP	13.725
RARAFF;VPAVLLP	NCP	27.603
VPAVLLP;NQEVPRP	NCP	55.636
NQEVPRP;RDASTVQSQKAEGTGDAK	NCP	48.212
RDASTVQSQKAEGTGDAK;LDGRVVEPK	NCP	25.293
LDGRVVEPK;FNDDLC	NCP	53.613
FNDDLC;HGPTRVP	NCP	18.832
HGPTRVP;KMVTKL	NCP	31.46
KMVTKL;KCPAALVL	NCP	209.454
KCPAALVL;RLDYAKK	NCP	91.531
RLDYAKK;KTPPGTK	NCP	117.462
KTPPGTK;AVLLPKKTESHHKTK	NCP	9.575
AVLLPKKTESHHKTK;LGGELL	NCP	2.631
LGGELL;RLLLPGEL	NCP	26.226
RLLLPGEL;HAVSEGTKAVTK	NCP	0.03
HAVSEGTKAVTK;EGTKAVTKYTS	NCP	0.012
EGTKAVTKYTS;HMLPANP	NCP	13.677
HMLPANP;LLPGELAKHAVSEGTK	NCP	14.208
LLPGELAKHAVSEGTK;KPVREK	NCP	12.837
KPVREK;PFFPYK	NCP	16.899
PFFPYK;VAKKGNR	NCP	40.416
VAKKGNR;KRPPSPH	NCP	59.59
KRPPSPH;KQLGW	NCP	130.946
KQLGW;KAPVTYN	NCP	83.947
KAPVTYN;PRGSLSP	NCP	147.776
PRGSLSP;MTEVL	NCP	149.935
MTEVL;PNRRPP	NCP	103.278
PNRRPP;RVDVGLK	NCP	69.105
RVDVGLK;KPEGSLPP	NCP	13.592
KPEGSLPP;VNVTTEVKS	NCP	15.561
VNVTTEVKS;SLAPEC	NCP	14.454
SLAPEC;WLLLPV	NCP	49.137
WLLLPV;KGLYKK	NCP	73.392
KGLYKK;KPTFRAP	NCP	99.45
KPTFRAP;KTLFDVS	NCP	3.481
KTLFDVS;LTFKFK	NCP	19.281
LTFKFK;WFKSSL	NCP	141.758
WFKSSL;ALELL	NCP	59.952

ALELL;KSSPLQ	NCP	3.753
KSSPLQ;VLLWY	NCP	92.724
VLLWY;LSKAEFL	NCP	281.662
LSKAEFL;VKPKELKPK	NCP	21.084
VKPKELKPK;KAKSHVP	NCP	35.198
KAKSHVP;LVLLF	NCP	166.551
LVLLF;FYKGPL	NCP	58.299
FYKGPL;KGAKKLR	NCP	15.568
KGAKKLR;QPMVGM	NCP	29.735
QPMVGM;WDLWH	NCP	104.436
WDLWH;PMAPGGLP	NCP	17.37
PMAPGGLP;DRPLAQR	NCP	98.244
DRPLAQR;HLLMEPE	NCP	10.098
HLLMEPE;GSHSMRYF	NCP	9.306
GSHSMRYF;VDDTQFVRF	NCP	0.081
VDDTQFVRF;DSAQGSDVSLTVPK	NCP	2.667
DSAQGSDVSLTVPK;KPLGPEP	NCP	24.618
KPLGPEP;KYVPPL	NCP	21.822
KYVPPL;PGNGEAA	NCP	84.471
PGNGEAA;LVLMAQ	NCP	0.889
LVLMAQ;TSVFVDH	NCP	133.008
TSVFVDH;MVAALTK	NCP	41.27
MVAALTK;LLGLLQPGL	NCP	1.393
LLGLLQPGL;QPMLLK	NCP	3.464
QPMLLK;LPPAPPLP	NCP	49.656
LPPAPPLP;DPTGTYHGDSDLQ	NCP	51.018
DPTGTYHGDSDLQ;PTGTYHGDSDL	NCP	0.003
PTGTYHGDSDL;HGDSDLQLDR	NCP	0.015
HGDSDLQLDR;SVLDVVRKEAESCD	NCP	0.771
SVLDVVRKEAESCD;DVVRKEAESCDCLQGF	NCP	0.009
DVVRKEAESCDCLQGF;TVPELTQQVFDAK	NCP	0.501
TVPELTQQVFDAK;EGKMGAK	NCP	205.773
EGKMGAK;KPLWVPP	NCP	57.573
KPLWVPP;KTTLGP	NCP	48.345
KTTLGP;AAALVGPGV	NCP	54.993
AAALVGPGV;PSFPL	NCP	10.978
PSFPL;LPGGSVVPP	NCP	62.159
LPGGSVVPP;LALPFP	NCP	52.669
LALPFP;QTTFLR	NCP	24.864
QTTFLR;VGKHLDAY	NCP	5.07
VGKHLDAY;ASLLRLP	NCP	25.244
ASLLRLP;TPPVDPKL	NCP	42.033
TPPVDPKL;RPPEERPP	NCP	2.676
RPPEERPP;APPLLPPP	NCP	12.753
APPLLPPP;QAASNFKSPVKT	NCP	19.404
QAASNFKSPVKT;ASNFKSPVK	NCP	0.006
ASNFKSPVK;SNFKSPVKT	NCP	0.003
SNFKSPVKT;RGLLT	NCP	37.281
RGLLT;KPPRAF	NCP	11.58
KPPRAF;FAALKGY	NCP	57.576
FAALKGY;NALESYAFNMK	NCP	2.457

NALESYAFNMK;QFFGTHK	NCP	58.456
QFFGTHK;MYDTPAEVAEAF	NCP	54.023
MYDTPAEVAEAF;SHVLQLTNH	NCP	42.018
SHVLQLTNH;VLSPTLHY	NCP	45.295
VLSPTLHY;ATGRLGVP	NCP	34.214
ATGRLGVP;LCSALL	NCP	11.979
LCSALL;CCCCCCCCPPP	NCP	53.742
CCCCCCCCPPP;CCCCCCPP	NCP	0.006
CCCCCCPP;TGQSVPK	NCP	42.106
TGQSVPK;PSEMGPK	NCP	44.255
PSEMGPK;FLQASEELLKQHY	NCP	22.293
FLQASEELLKQHY;SGPVVAM	NCP	0.09
SGPVVAM;SLYPQFPP	NCP	136.698
SLYPQFPP;ELGEFL	NCP	16.708
ELGEFL;PLLTPVLP	NCP	66.819
PLLTPVLP;NPKYAR	NCP	37.308
NPKYAR;SVKKRC	NCP	88.685
SVKKRC;PPTFP	NCP	36.772
PPTFP;FPVTLK	NCP	5.312
FPVTLK;GTKLLYH	NCP	37.455
GTKLLYH;PSLGMDK	NCP	18.493
PSLGMDK;LMKGPP	NCP	59.255
LMKGPP;PSRGAGAGP	NCP	105.948
PSRGAGAGP;TADQLFR	NCP	16.455
TADQLFR;VLAPPN	NCP	42.12
VLAPPN;KKSSSLR	NCP	248.286
KKSSSLR;RPPKGF	NCP	80.65
RPPKGF;KSFTPK	NCP	238.241
KSFTPK;ELLMNLEL	NCP	25.78
ELLMNLEL;PENNLF	NCP	145.455
PENNLF;EVMVY	NCP	0.231
EVMVY;VGSGHKPP	NCP	234.099
VGSGHKPP;HDVSH	NCP	34.343
HDVSH;KEAPFAK	NCP	231.087
KEAPFAK;AAAPGNPK	NCP	23.517
AAAPGNPK;LTPLTP	NCP	35.307
LTPLTP;KEEGK	NCP	0.511
KEEGK;KTGLLHA	NCP	0.845
KTGLLHA;TLAQMPY	NCP	98.328
TLAQMPY;VDLNK	NCP	15.807
VDLNK;HLLTMFE	NCP	136.999
HLLTMFE;AGKPKLH	NCP	297.761
AGKPKLH;LYYVEELDPSLLANFPLLK	NCP	13.488
LYYVEELDPSLLANFPLLK;YYVEELDPSLL	NCP	0.003
YYVEELDPSLL;YVEELDPSLLANFPLLK	NCP	0.003
YVEELDPSLLANFPLLK;EELDPSLL	NCP	0.006
EELDPSLL;PSLLANFPLLK	NCP	0.012
PSLLANFPLLK;VSNLPAVKK	NCP	0.663
VSNLPAVKK;AGKPTLHYFN	NCP	11.538
AGKPTLHYFN;LANFPLLK	NCP	6.87
LANFPLLK;VSGVFLP	NCP	19.438

VSGVFLP;HGQDYLVGNKL	NCP	1.016
HGQDYLVGNKL;AGKPKLHYFN	NCP	20.247
AGKPKLHYFN;TQNSVLLL	NCP	25.774
TQNSVLLL;WVVSGFR	NCP	44.708
WVVSGFR;GKPGDY	NCP	84.192
GKPGDY;LRSSSSKK	NCP	95.313
LRSSSSKK;GSHSLSYF	NCP	0.919
GSHSLSYF;AGTVVLDDVELR	NCP	120.167
AGTVVLDDVELR;VSAPKNVR	NCP	56.955
VSAPKNVR;TVVPPFK	NCP	91.344
TVVPPFK;PVSPLLL	NCP	1.248
PVSPLLL;EPAFPALA	NCP	122.268
EPAFPALA;LAVEPVK	NCP	58.248
LAVEPVK;SFGDL	NCP	58.761
SFGDL;LLYPL	NCP	44.191
LLYPL;RKPHATP	NCP	2.501
RKPHATP;KSPPK	NCP	25.045
KSPPK;ALFASTR	NCP	19.361
ALFASTR;QTVLL	NCP	39.655
QTVLL;PGDVNAP	NCP	26.517
PGDVNAP;EKPPPAH	NCP	48.735
EKPPPAH;DTWKLCS	NCP	45.041
DTWKLCS;QLGSQLR	NCP	15.759
QLGSQLR;HASPPPPF	NCP	27.502
HASPPPPF;LVELNF	NCP	171.083
LVELNF;LSPGSVHNK	NCP	14.113
LSPGSVHNK;GVLAPEPR	NCP	124.671
GVLAPEPR;GVPVGP	NCP	40.317
GVPVGP;YRDLTVPF	NCP	37.67
YRDLTVPF;TSTVGPK	NCP	54.114
TSTVGPK;VASPKL	NCP	18.351
VASPKL;SLKVAPKVK	NCP	47.326
SLKVAPKVK;LDQDALH	NCP	40.148
LDQDALH;MLLKVK	NCP	46.248
MLLKVK;GYRKK	NCP	37.912
GYRKK;DVPAMY	NCP	23.621
DVPAMY;PDLDPPR	NCP	18.43
PDLDPPR;EEVRLPPL	NCP	23.229
EEVRLPPL;GSHSLRYFY	NCP	25.173
GSHSLRYFY;VGLPLLSQ	NCP	70.995
VGLPLLSQ;PGPAAAGF	NCP	63.723
PGPAAAGF;LPPVFK	NCP	10.031
LPPVFK;LTAGLENL	NCP	1.39
LTAGLENL;KAYFK	NCP	95.609
KAYFK;PGKPRPP	NCP	149.149
PGKPRPP;GPPLVFT	NCP	38.655
GPPLVFT;AVGGHVK	NCP	46.781
AVGGHVK;LYREPGK	NCP	112.389
LYREPGK;HSLLVTLA	NCP	229.683
HSLLVTLA;VDFNVPMK	NCP	1.971
VDFNVPMK;DGVPMPDKY	NCP	0.135

DGVPMPDKY;VNDAFGTAHR	NCP	0.282
VNDAFGTAHR;VDMVPGKPM	NCP	48.888
VDMVPGKPM;KGPLPPPPPP	NCP	16.27
KGPLPPPPPP;PPLPDNR	NCP	18.735
PPLPDNR;LSPAEC	NCP	216.02
LSPAEC;KTTHQK	NCP	102.387
KTTHQK;SLTYDKRL	NCP	167.061
SLTYDKRL;GSHGY	NCP	52.149
GSHGY;PGGLQA	NCP	28.695
PGGLQA;DLTLCHK	NCP	321.928
DLTLCHK;KMLSNC	NCP	44.772
KMLSNC;KALSLPK	NCP	48.405
KALSLPK;TASVHVNK	NCP	35.121
TASVHVNK;FAYLAMM	NCP	90.681
FAYLAMM;ELQEHKLK	NCP	14.117
ELQEHKLK;LTLELNLN	NCP	86.781
LTLELNLN;PPNGMVT	NCP	19.465
PPNGMVT;LGLEFSL	NCP	56.115
LGLEFSL;MKCDLDLR	NCP	8.667
MKCDLDLR;KLEYN	NCP	6.198
KLEYN;TVVGL	NCP	126.872
TVVGL;RDKDLR	NCP	164.505
RDKDLR;AAVLPQ	NCP	3.987
AAVLPQ;VLADNGPR	NCP	16.371
VLADNGPR;YCVNAFL	NCP	71.314
YCVNAFL;LHAPPPP	NCP	14.069
LHAPPPP;KGARPVK	NCP	4.389
KGARPVK;RTLLPR	NCP	31.03
RTLLPR;LTGLAA	NCP	153.587
LTGLAA;LPQGTP	NCP	307.281
LPQGTP;DFGNY	NCP	76.251
DFGNY;RSKFFVG	NCP	54.963
RSKFFVG;EQLLFF	NCP	14.919
EQLLFF;YVYKVLK	NCP	68.059
YVYKVLK;VYKVLKQ	NCP	0.003
VYKVLKQ;NGSVCVWNHETQTLVK	NCP	71.471
NGSVCVWNHETQTLVK;AWAHDSSEY	NCP	1.077
AWAHDSSEY;FLEKQGFK	NCP	0.687
FLEKQGFK;VEAESEQKWK	NCP	0.114
VEAESEQKWK;GLPTGC	NCP	33.514
GLPTGC;DPSLFLLLP	NCP	163.536
DPSLFLLLP;LPSSSPVFP	NCP	3.408
LPSSSPVFP;VGAGAPVYL	NCP	72.492
VGAGAPVYL;AGAPVYLAAVLEY	NCP	0.006
AGAPVYLAAVLEY;GAPVYLAA	NCP	0.003
GAPVYLAA;LAAVLEYLTAE	NCP	0.015
LAAVLEYLTAE;VLLPKKTESHH	NCP	0.189
VLLPKKTESHH;MALQEACEAY	NCP	2.19
MALQEACEAY;NVFPSVLG	NCP	14.733
NVFPSVLG;GEYVLPDEPY	NCP	9.629
GEYVLPDEPY;PPPNLLPP	NCP	3.627

PPPNLLPP;LEYLTAE	NCP	33.631
LEYLTAE;AVLLPKKTESH	NCP	0.174
AVLLPKKTESH;AAGYDVEKN	NCP	78.216
AAGYDVEKN;AGYDVEK	NCP	0.003
AGYDVEK;KENNGPK	NCP	6.795
KENNGPK;LLLSSGK	NCP	16.314
LLLSSGK;APAAPAAAPPAEKTPVKKK	NCP	8.457
APAAPAAAPPAEKTPVKKK;PAAPAAAPPAEK	NCP	0.003
PAAPAAAPPAEK;AAPAAAPPAEKTPVKK	NCP	0.003
AAPAAAPPAEKTPVKK;APAAAPPAEK	NCP	0.003
APAAAPPAEK;AAPPAEKTPVKKK	NCP	0.009
AAPPAEKTPVKKK;APPAEKTPVKK	NCP	0.003
APPAEKTPVKK;AVAASKERSGV	NCP	0.105
AVAASKERSGV;AASKERSGVSLA	NCP	0.006
AASKERSGVSLA;ASKERSGVSL	NCP	0.003
ASKERSGVSL;ERSGVSLAALKK	NCP	0.009
ERSGVSLAALKK;SGVSLAALKKALA	NCP	0.006
SGVSLAALKKALA;GVSLAALK	NCP	0.003
GVSLAALK;VSLAALKKALA	NCP	0.003
VSLAALKKALA;SLAALKKALAAA	NCP	0.003
SLAALKKALAAA;AALKKALAAAGYDVEK	NCP	0.006
AALKKALAAAGYDVEK;ALKKALAA	NCP	0.003
ALKKALAA;KKALAAAGYDVEK	NCP	0.006
KKALAAAGYDVEK;KALAAAGYDVEKN	NCP	0.003
KALAAAGYDVEKN;ALAAAGYDVEK	NCP	0.003
ALAAAGYDVEK;LKSLVSKGTLVQTK	NCP	0.057
LKSLVSKGTLVQTK;VSKGTLVQTKGTGASGSFK	NCP	0.012
VSKGTLVQTKGTGASGSFK;KGTLVQTK	NCP	0.006
KGTLVQTK;GTGASGSFKLNKKAATGEAKPK	NCP	0.024
GTGASGSFKLNKKAATGEAKPK;TPKKAKKPAAAAVTK	NCP	0.168
TPKKAKKPAAAAVTK;KKPAAAAVTKKVAKSPK	NCP	0.015
KKPAAAAVTKKVAKSPK;KPAAAAVTKKVA	NCP	0.003
KPAAAAVTKKVA;AAAAVTKKVAKSPK	NCP	0.006
AAAAVTKKVAKSPK;SAAKAVKP	NCP	0.078
SAAKAVKP;AKPKVAKP	NCP	0.03
AKPKVAKP;HAVSEGTKAVTKY	NCP	8.199
HAVSEGTKAVTKY;VVGLNEF	NCP	7.082
VVGLNEF;ADVAKRLS	NCP	1.251
ADVAKRLS;LEPLKK	NCP	7.003
LEPLKK;VSLAALKKA	NCP	12.387
VSLAALKKA;KDLKPVLDSY	NCP	36.447
KDLKPVLDSY;VDFAE	NCP	121.823
VDFAE;KNHQPAP	NCP	17.148
KNHQPAP;LLLLFSSL	NCP	102.171
LLLLFSSL;KQSARYH	NCP	287.002
KQSARYH;RVHCCP	NCP	187.952
RVHCCP;KSNPP	NCP	72.964
KSNPP;RYLVR	NCP	95.843
RYLVR;GVNMF	NCP	9.79
GVNMF;LLPWNP	NCP	70.709
LLPWNP;KQGPVVP	NCP	212.896

KQGPVVP;TAHAEFK	NCP	55.239
TAHAEFK;PPKVNPP	NCP	90.332
PPKVNPP;RELRDPSP	NCP	32.986
RELRDPSP;DVDLNLK	NCP	25.517
DVDLNLK;GSVVKP	NCP	102.265
GSVVKP;AVTFLPYK	NCP	4.988
AVTFLPYK;PDWGKN	NCP	7.141
PDWGKN;TVRVLP	NCP	29.15
TVRVLP;PSAHKLPP	NCP	43.809
PSAHKLPP;FVEYF	NCP	11.668
FVEYF;GVPLLF	NCP	66.725
GVPLLF;PHKKKS	NCP	50.491
PHKKKS;LLPMHK	NCP	11.904
LLPMHK;LTKHYVSL	NCP	37.475
LTKHYVSL;TGRPLVLYH	NCP	201.195
TGRPLVLYH;LAELRDN	NCP	12.637
LAELRDN;YCPDCA	NCP	16.733
YCPDCA;LDPTKTP	NCP	1.356
LDPTKTP;EVPFSTVP	NCP	26.613
EVPFSTVP;WGFPSR	NCP	29.043
WGFPSR;LRGVFY	NCP	18.69
LRGVFY;KGAAAV	NCP	6.202
KGAAAV;RPAAAGP	NCP	112.451
RPAAAGP;TVPVK	NCP	150.631
TVPVK;KPAAPGVP	NCP	21.989
KPAAPGVP;KMVGELR	NCP	99.882
KMVGELR;KDFPT	NCP	20.569
KDFPT;QWTPNP	NCP	52.506
QWTPNP;VLCPPKY	NCP	4.478
VLCPPKY;MLVTLK	NCP	134.892
MLVTLK;LPVFASK	NCP	79.915
LPVFASK;PQLEY	NCP	234.267
PQLEY;VELDLH	NCP	38.226
VELDLH;PGEVPP	NCP	69.962
PGEVPP;VGFGSNT	NCP	20.784
VGFGSNT;AELEFK	NCP	151.692
AELEFK;PEDLPR	NCP	3.088
PEDLPR;SLEPGAVP	NCP	17.729
SLEPGAVP;STFLRP	NCP	28.683
STFLRP;CSCEFL	NCP	18.36
CSCEFL;DGAAPLYR	NCP	263.418
DGAAPLYR;LVAGP	NCP	111.243
LVAGP;EPPLLKPL	NCP	25.128
EPPLLKPL;RQPAPPREAH	NCP	11.178
RQPAPPREAH;QVDKVNF	NCP	276.406
QVDKVNF;LLLDDF	NCP	44.612
LLLDDF;RASHKPTYENLRK	NCP	47.01
RASHKPTYENLRK;APPGPQ	NCP	22.479
APPGPQ;LEAGPL	NCP	184.572
LEAGPL;DTALPL	NCP	167.94
DTALPL;NLPPEF	NCP	47.164

NLPPEF;DAGMSSL	NCP	71.45
DAGMSSL;AVVEGL	NCP	17.166
AVVEGL;LSLWG	NCP	68.581
LSLWG;TSLLELPS	NCP	11.666
TSLLELPS;AAGPYPP	NCP	75.411
AAGPYPP;EHKLVQ	NCP	82.224
EHKLVQ;LLALYK	NCP	21.685
LLALYK;DHALLVY	NCP	14.682
DHALLVY;KMFRPP	NCP	5.859
KMFRPP;TPKAAP	NCP	69.783
TPKAAP;LPMNSVLL	NCP	28.172
LPMNSVLL;ESLAAGPP	NCP	1.896
ESLAAGPP;SGPVLK	NCP	131.592
SGPVLK;KPPYK	NCP	19.384
KPPYK;VDLLKG	NCP	126.38
VDLLKG;YSDPLP	NCP	1.077
YSDPLP;TNVTTEVK	NCP	182.53
TNVTTEVK;NVFAQAE	NCP	25.613
NVFAQAE;NLKARVS	NCP	205.501
NLKARVS;VPGPVAAK	NCP	39.465
VPGPVAAK;KPRKCV	NCP	104.253
KPRKCV;VYPLLP	NCP	9.221
VYPLLP;LLSVTLA	NCP	26.059
LLSVTLA;LRKLLRDG	NCP	176.195
LRKLLRDG;TLLPDK	NCP	4.071
TLLPDK;LLDGRLA	NCP	116.149
LLDGRLA;ELELGAP	NCP	71.336
ELELGAP;TQLGPLE	NCP	58.621
TQLGPLE;KNSELLTL	NCP	69.705
KNSELLTL;ASPLASFVT	NCP	6.6
ASPLASFVT;LFALAK	NCP	42.395
LFALAK;GMGRAAGF	NCP	16.38
GMGRAAGF;NVLLPK	NCP	4.611
NVLLPK;VLVLKF	NCP	143.074
VLVLKF;PYFLP	NCP	2.9
PYFLP;KTLETSE	NCP	40.65
KTLETSE;LYESSF	NCP	51.187
LYESSF;MPSLT	NCP	30.261
MPSLT;NALFKK	NCP	22.121
NALFKK;KYFPLNP	NCP	51.289
KYFPLNP;SPVKFFF	NCP	12.228
SPVKFFF;VAPVPRSH	NCP	72.782
VAPVPRSH;VTPAVLP	NCP	167.346
VTPAVLP;SYGGK	NCP	114.856
SYGGK;TTLTAHR	NCP	25.781
TTLTAHR;GTYTK	NCP	73.741
GTYTK;ALKLL	NCP	24.797
ALKLL;MNPLLK	NCP	14.796
MNPLLK;KPPLSPP	NCP	120.51
KPPLSPP;TDKPVP	NCP	81.792
TDKPVP;TAKLPF	NCP	52.168

TAKLPF;APDSF	NCP	2.646
APDSF;VPLFSG	NCP	105.419
VPLFSG;DPVRLPP	NCP	98.493
DPVRLPP;NSSLLLTPL	NCP	88.116
NSSLLLTPL;VGRLVK	NCP	76.027
VGRLVK;PGCVK	NCP	268.679
PGCVK;TVPGGAF	NCP	25.569
TVPGGAF;QNLETY	NCP	63.468
QNLETY;PCPPGSW	NCP	102.441
PCPPGSW;LGTLPVQ	NCP	131.227
LGTLPVQ;NFLLL	NCP	105.777
NFLLL;GKAQEALR	NCP	23.387
GKAQEALR;KDDPTGK	NCP	8.487
KDDPTGK;QSPFP	NCP	29.175
QSPFP;QRFPPP	NCP	22.639
QRFPPP;FNSSGTH	NCP	106.401
FNSSGTH;TNWKSLP	NCP	47.034
TNWKSLP;KGKATHK	NCP	8.49
KGKATHK;KALQPG	NCP	20.435
KALQPG;PPGDLPP	NCP	49.062
PPGDLPP;EPVLLAP	NCP	16.611
EPVLLAP;TPPYK	NCP	182.719
TPPYK;VASKGY	NCP	390.333
VASKGY;AVSRP	NCP	97.725
AVSRP;KLWGGGKK	NCP	17.135
KLWGGGKK;KNLKVH	NCP	20.074
KNLKVH;APKGMNK	NCP	0.708
APKGMNK;SVGKQAK	NCP	99.546
SVGKQAK;KPAAAGPP	NCP	56.619
KPAAAGPP;PAGGGSSK	NCP	1.607
PAGGGSSK;KDSFPLK	NCP	7.566
KDSFPLK;PFSALGL	NCP	89.218
PFSALGL;KKTPVGP	NCP	46.902
KKTPVGP;TDVEENVPVKDPGD	NCP	49.601
TDVEENVPVKDPGD;KTQEYNF	NCP	80.547
KTQEYNF;LFRWHLQ	NCP	8.658
LFRWHLQ;KNLFPQ	NCP	70.702
KNLFPQ;FAPPKPR	NCP	0.534
FAPPKPR;ALEPLL	NCP	18.963
ALEPLL;VSYVPPP	NCP	57.333
VSYVPPP;MLSSKPR	NCP	79.985
MLSSKPR;LPEPEPAL	NCP	2.694
LPEPEPAL;LAGGLG	NCP	112.618
LAGGLG;WAGKVLK	NCP	88.83
WAGKVLK;VGSVYK	NCP	30.632
VGSVYK;KKAEAGAGSATEFQ	NCP	73.263
KKAEAGAGSATEFQ;APVSLALR	NCP	30.295
APVSLALR;GMQSQK	NCP	80.354
GMQSQK;NNTLPYK	NCP	27.813
NNTLPYK;LPPTRP	NCP	47.37
LPPTRP;EKVVVLTK	NCP	24.766

EKVVVLTK;PDEYL	NCP	29.199
PDEYL;NPNHKTPP	NCP	56.757
NPNHKTPP;LELEGP	NCP	71.666
LELEGP;VEKELDLK	NCP	51.982
VEKELDLK;SYAEAVL	NCP	17.402
SYAEAVL;AENLR	NCP	60.036
AENLR;PVPAG	NCP	42.871
PVPAG;LLVVSK	NCP	189.837
LLVVSK;TLSPTED	NCP	4.874
TLSPTED;DPPRRP	NCP	28.428
DPPRRP;PPPATPHK	NCP	49.024
PPPATPHK;RWTAPKK	NCP	69.747
RWTAPKK;KGVRPLK	NCP	8.042
KGVRPLK;TAAAGMP	NCP	23.412
TAAAGMP;LTGLAAF	NCP	58.033
LTGLAAF;KTVPSQP	NCP	36.699
KTVPSQP;KDTSHVP	NCP	39.984
KDTSHVP;DLVHVER	NCP	42.231
DLVHVER;YQPPP	NCP	34.829
YQPPP;HSNFPR	NCP	30.744
HSNFPR;AALGPP	NCP	44.844
AALGPP;KPGGPPR	NCP	38.481
KPGGPPR;ACSGSPVPR	NCP	61.212
ACSGSPVPR;SDLMHQYREASQ	NCP	12.966
SDLMHQYREASQ;RYMVGFQ	NCP	12.733
RYMVGFQ;DLKFL	NCP	59.292
DLKFL;KKQGHR	NCP	43.352
KKQGHR;PVEAVP	NCP	32.818
PVEAVP;MFGGPPK	NCP	5.399
MFGGPPK;VPEVP	NCP	93.876
VPEVP;GPVPPEPP	NCP	23.205
GPVPPEPP;MVAAPQ	NCP	55.69
MVAAPQ;KNKYQPP	NCP	13.373
KNKYQPP;KTSGDPK	NCP	36.502
KTSGDPK;TGTPKLP	NCP	83.777
TGTPKLP;KDAALL	NCP	9.588
KDAALL;MDANKKK	NCP	36.097
MDANKKK;PSVVPRK	NCP	38.925
PSVVPRK;KDGTNLR	NCP	6.774
KDGTNLR;NPGEKGH	NCP	68.844
NPGEKGH;EVWGLF	NCP	3.351
EVWGLF;MDFEKHGKGS	NCP	42.482
MDFEKHGKGS;EERLFEH	NCP	41.871
EERLFEH;RLDAVK	NCP	13.171
RLDAVK;VSLATKPP	NCP	49.031
VSLATKPP;LDDLNL	NCP	11.029
LDDLNL;EVPLL	NCP	6.578
EVPLL;LPTHSQLGDTSK	NCP	5.742
LPTHSQLGDTSK;EMVFC	NCP	42.672
EMVFC;VPAMY	NCP	138.09
VPAMY;TYLYVP	NCP	112.105

TYLYVP;MDFELQ	NCP	120.941
MDFELQ;LSLSASPK	NCP	2.692
LSLSASPK;DPPAVVK	NCP	30.06
DPPAVVK;LLLYAK	NCP	88.745
LLLYAK;AKEGPVGK	NCP	6.409
AKEGPVGK;PDTKAVGH	NCP	25.826
PDTKAVGH;SFLRLWN	NCP	69.933
SFLRLWN;LTLGNRE	NCP	235.776
LTLGNRE;RGEAPSLR	NCP	166.291
RGEAPSLR;KHALPVP	NCP	17.739
KHALPVP;ATPDLF	NCP	17.925
ATPDLF;ANHLVDY	NCP	4.118
ANHLVDY;DLLGK	NCP	130.144
DLLGK;KSFDGVP	NCP	105.519
KSFDGVP;TGLNALSK	NCP	58.83
TGLNALSK;LCKVAGY	NCP	42.6
LCKVAGY;KVDLKLP	NCP	46.053
KVDLKLP;FKQFFK	NCP	107.124
FKQFFK;KVTFPR	NCP	96.843
KVTFPR;HYLVF	NCP	128.144
HYLVF;KLHKFK	NCP	202.278
KLHKFK;DVKKGGK	NCP	122.056
DVKKGGK;FPPAPNYP	NCP	73.608
FPPAPNYP;PPAEK	NCP	27.986
PPAEK;KKGSGAPK	NCP	19.291
KKGSGAPK;WWLLK	NCP	211.274
WWLLK;LAAATLKT	NCP	26.937
LAAATLKT;KAASTKR	NCP	46.974
KAASTKR;KDAGVPF	NCP	46.033
KDAGVPF;QGPSAFYL	NCP	11.075
QGPSAFYL;PLSPLK	NCP	6.322
PLSPLK;LFVNV	NCP	2.598
LFVNV;TLHFNLLP	NCP	58.626
TLHFNLLP;KCHVTFK	NCP	160.44
KCHVTFK;MLAPAPK	NCP	2.381
MLAPAPK;VSGKNLQK	NCP	10.053
VSGKNLQK;KEDAP	NCP	6.411
KEDAP;AEKLVVT	NCP	334.48
AEKLVVT;ASDPLPTL	NCP	17.604
ASDPLPTL;LTVKPQE	NCP	42.765
LTVKPQE;VSYVYK	NCP	16.238
VSYVYK;TVKPLP	NCP	14.857
TVKPLP;LSSGADAR	NCP	2.88
LSSGADAR;RGSALRPT	NCP	26.939
RGSALRPT;KLFDKK	NCP	71.584
KLFDKK;VVATEAPP	NCP	73.503
VVATEAPP;NKSGLSKK	NCP	5.744
NKSGLSKK;QEWEK	NCP	39.946
QEWEK;AYAEAKK	NCP	53.787
AYAEAKK;ALPGV	NCP	23.481
ALPGV;NLKKALAA	NCP	48.764

NLKKALAA;YLLAA	NCP	87.927
YLLAA;AVSRQ	NCP	142.882
AVSRQ;KNNDLL	NCP	61.209
KNNDLL;KQTPP	NCP	18.603
KQTPP;MMMSNLH	NCP	54.693
MMMSNLH;MHGTPSPK	NCP	111.999
MHGTPSPK;CEMPP	NCP	44.388
CEMPP;KQLFGK	NCP	41.672
KQLFGK;TLDVDAP	NCP	3.636
TLDVDAP;GPPEAPPP	NCP	120.43
GPPEAPPP;NKLTDR	NCP	51.824
NKLTDR;VAPVNPR	NCP	83.971
VAPVNPR;LSPEVPQ	NCP	57.198
LSPEVPQ;LSDLAEVK	NCP	40.598
LSDLAEVK;PEHAVEVL	NCP	80.791
PEHAVEVL;VGPAP	NCP	89.634
VGPAP;KELYMPK	NCP	100.608
KELYMPK;YLRNK	NCP	41.501
YLRNK;LAVEAL	NCP	42.48
LAVEAL;RPAPQ	NCP	77.68
RPAPQ;VRMVLDG	NCP	4.694
VRMVLDG;LELLN	NCP	73.332
LELLN;AATDNLR	NCP	11.29
AATDNLR;VVHTEPT	NCP	151.874
VVHTEPT;GKNLNN	NCP	6.805
GKNLNN;LSPVPLE	NCP	162.249
LSPVPLE;SGLPRMK	NCP	6.458
SGLPRMK;KNCPPPP	NCP	66.163
KNCPPPP;LVRLSM	NCP	16.367
LVRLSM;TDVTGTLR	NCP	62.02
TDVTGTLR;KPGVAP	NCP	108.987
KPGVAP;MQTKPPP	NCP	3.135
MQTKPPP;KVGAL	NCP	15.492
KVGAL;KPTRPFC	NCP	16.634
KPTRPFC;ASLLQ	NCP	113.577
ASLLQ;LQLLFL	NCP	1.953
LQLLFL;PPAGFGA	NCP	5.37
PPAGFGA;YGVPPPP	NCP	20.905
YGVPPPP;LSVNYL	NCP	47.199
LSVNYL;AAPGELK	NCP	12.936
AAPGELK;DKPLQLP	NCP	4.323
DKPLQLP;EHPTLL	NCP	15.509
EHPTLL;KPNQLHGK	NCP	175.056
KPNQLHGK;KLTDYVP	NCP	14.314
KLTDYVP;LPALSK	NCP	31.142
LPALSK;EDHAA	NCP	23.208
EDHAA;LHAHASK	NCP	7.846
LHAHASK;EPPLLDV	NCP	19.013
EPPLLDV;MLASH	NCP	47.464
MLASH;RTVNPK	NCP	13.233
RTVNPK;LKDKLEK	NCP	63.407

LKDKLEK;WFLDC	NCP	0.741
WFLDC;HLGVPPPP	NCP	126.615
HLGVPPPP;AGAPLSP	NCP	34.261
AGAPLSP;KLKKLP	NCP	1.005
KLKKLP;LVGVK	NCP	9.323
LVGVK;GSPDAKL	NCP	9.682
GSPDAKL;LDLGCLLGR	NCP	111.399
LDLGCLLGR;GVPVLEF	NCP	61.638
GVPVLEF;PPHPVR	NCP	330.951
PPHPVR;GVTLP	NCP	11.754
GVTLP;MMMWNVK	NCP	127.232
MMMWNVK;DLGPLLS	NCP	214.657
DLGPLLS;SNSVPKGR	NCP	429.189
SNSVPKGR;EHAVTTF	NCP	2.714
EHAVTTF;SHVLP	NCP	8.188
SHVLP;LSPSLPLVG	NCP	26.004
LSPSLPLVG;DDFFLRN	NCP	9.834
DDFFLRN;QWRVR	NCP	99.699
NNPLKP;SYSLSPPGP	NCP	96.909
SYSLSPPGP;DHPFLFL	NCP	29.157
DHPFLFL;FLEVLKPF	NCP	208.587
FLEVLKPF;SLGVVP	NCP	94.66
SLGVVP;RPFLLR	NCP	6.627
RPFLLR;KFTTEGK	NCP	4.997
KFTTEGK;GESDLKA	NCP	49.267
GESDLKA;KDHLVTL	NCP	10.008
KDHLVTL;LDFENTN	NCP	110.429
LDFENTN;KPSEQK	NCP	32.88
KPSEQK;VELLS	NCP	46.047
VELLS;QGLAL	NCP	38.013
QGLAL;LDVSGKPV	NCP	30.156
LDVSGKPV;YHGPPR	NCP	6.133
YHGPPR;KTTHKRLK	NCP	16.934
KTTHKRLK;FLSAL	NCP	32.493
FLSAL;PHALSSPK	NCP	70.647
PHALSSPK;HKLMGAR	NCP	24.534
HKLMGAR;MFLSA	NCP	75.354
MFLSA;VPPNLEF	NCP	57.921
VPPNLEF;HSSLLVVP	NCP	6.442
HSSLLVVP;PGGHLTPP	NCP	23.312
PGGHLTPP;RLLLLGAGESGK	NCP	4.516
RLLLLGAGESGK;KLLKKP	NCP	93.152
KLLKKP;GVPLPN	NCP	3.943
GVPLPN;CCQDT	NCP	44.994
CCQDT;QAFVP	NCP	74.831
QAFVP;NLDLFY	NCP	91.635
NLDLFY;VTVGEPK	NCP	9.514
VTVGEPK;WPFADNK	NCP	25.302
WPFADNK;YSNRLAE	NCP	116.787
YSNRLAE;NLAGVGP	NCP	51.117
NLAGVGP;VPAASSST	NCP	49.629

VPAASSST;AALFGR	NCP	1.128
AALFGR;DALDF	NCP	10.44
DALDF;SFGVKEV	NCP	8.472
SFGVKEV;HVPPG	NCP	28.713
HVPPG;KPAANRP	NCP	28.914
KPAANRP;APGSSLLR	NCP	8.151
APGSSLLR;PAASFDK	NCP	16.611
PAASFDK;HEAPTYF	NCP	181.266
HEAPTYF;VPVTHVLP	NCP	9.726
VPVTHVLP;AAFLVSFP	NCP	32.618
AAFLVSFP;HLVPPPPP	NCP	18.976
HLVPPPPP;PAAHEPPP	NCP	134.538
PAAHEPPP;AALPLP	NCP	16.071
AALPLP;KHLDAEFK	NCP	30.279
KHLDAEFK;VTWPK	NCP	2.471
VTWPK;TPAPVER	NCP	61.623
TPAPVER;LLLLLLL	NCP	21.882
LLLLLLL;PGAVPP	NCP	7.308
PGAVPP;GRHLLF	NCP	33.711
GRHLLF;LAELRK	NCP	3.819
LAELRK;TDLVLRR	NCP	62.953
TDLVLRR;ELFTPAGS	NCP	98.25
ELFTPAGS;AGAPQ	NCP	29.915
AGAPQ;LERVCLGL	NCP	53.94
LERVCLGL;LTVLLVPP	NCP	40.651
LTVLLVPP;GKLSQL	NCP	20.493
GKLSQL;ATWLLR	NCP	8.319
ATWLLR;PAALT	NCP	107.795
PAALT;VHKPP	NCP	42.439
VHKPP;CHLTVK	NCP	5.763
CHLTVK;PPPETF	NCP	41.399
PPPETF;LKVVVPPE	NCP	29.332
LKVVVPPE;YDPAVP	NCP	9.119
YDPAVP;TRLHPVPP	NCP	20.481
TRLHPVPP;RAHVMK	NCP	1.116
RAHVMK;PPARHHK	NCP	71.751
PPARHHK;WTLVGK	NCP	2.545
WTLVGK;WYPKF	NCP	37.259
WYPKF;VVLSP	NCP	80.796
VVLSP;VELVT	NCP	116.19
VELVT;KFPPLAF	NCP	16.855
KFPPLAF;ESLKVR	NCP	46.457
ESLKVR;LSHPTRPP	NCP	43.119
LSHPTRPP;EHAAPPPP	NCP	17.311
EHAAPPPP;CGCMD	NCP	35.388
CGCMD;KGRLLFYQ	NCP	46.095
KGRLLFYQ;LTAELAPST	NCP	56.067
LTAELAPST;KAAVDLR	NCP	48.609
KAAVDLR;VPGLNVTDF	NCP	34.049
VPGLNVTDF;HSLFRK	NCP	25.737
HSLFRK;HRDLRAGP	NCP	43.45

HRDLRAGP;EEFQKDHPDLQ	NCP	195.683
EEFQKDHPDLQ;WNVMLPK	NCP	30.352
WNVMLPK;LKVDAVP	NCP	49.34
LKVDAVP;PDLLL	NCP	145.017
PDLLL;PVGPAP	NCP	29.973
PVGPAP;LTAELLEL	NCP	126.328
LTAELLEL;APVVPNH	NCP	45.273
APVVPNH;QSLMPT	NCP	63.765
QSLMPT;GAKPHPR	NCP	59.124
GAKPHPR;PPPPVPY	NCP	10.841
PPPPVPY;TNKAAPK	NCP	2.137
TNKAAPK;PAPAGGP	NCP	25.434
PAPAGGP;KTKLVGAP	NCP	72.818
KTKLVGAP;LTFGPKG	NCP	1.891
LTFGPKG;AAVLASSPR	NCP	68.864
AAVLASSPR;SGFKHVK	NCP	10.429
SGFKHVK;PVVKPP	NCP	22.871
PVVKPP;RRHPT	NCP	7.107
RRHPT;ETHRLVM	NCP	117.183
ETHRLVM;WEEALPL	NCP	84.219
WEEALPL;KETPP	NCP	69.303
KETPP;PPYKP	NCP	80.76
PPYKP;ALTALKT	NCP	1.953
ALTALKT;LFKTSGK	NCP	4.741
LFKTSGK;RPVPSLPP	NCP	4.791
RPVPSLPP;AGAAFVNS	NCP	124.731
AGAAFVNS;CCHCCC	NCP	14.786
CCHCCC;AAPGGH	NCP	37.441
AAPGGH;EDLFPGLK	NCP	104.412
EDLFPGLK;PVLPFH	NCP	6.975
PVLPFH;SVQVFPR	NCP	72.599
SVQVFPR;GQLPFR	NCP	17.415
GQLPFR;STVPGFFK	NCP	7.134
STVPGFFK;LLGGSLDA	NCP	34.435
LLGGSLDA;LAPGLFG	NCP	54.311
LAPGLFG;EPKALSM	NCP	183.774
EPKALSM;LPASFVTQ	NCP	57.636
LPASFVTQ;PLFELT	NCP	30.685
PLFELT;CASLVQ	NCP	20.915
CASLVQ;QPSLPRRGP	NCP	18.033
QPSLPRRGP;TLSAGH	NCP	56.026
TLSAGH;EALEKYLN	NCP	139.512
EALEKYLN;GSLDMLK	NCP	21.621
GSLDMLK;APYDPK	NCP	174.504
APYDPK;YDPLK	NCP	37.008
YDPLK;ELLKVH	NCP	91.616
ELLKVH;CGKFTQH	NCP	73.45
CGKFTQH;PVLGGP	NCP	41.082
PVLGGP;MYLLQG	NCP	76.436
MYLLQG;KSFSSSKP	NCP	29.176
KSFSSSKP;LLAAHQDK	NCP	63.009

LLAAHQDK;PPLSPPPVL	NCP	6.788
PPLSPPPVL;VLLHVL	NCP	130.885
VLLHVL;QAVVKK	NCP	247.533
QAVVKK;MEMVGKY	NCP	79.94
MEMVGKY;KAYFPP	NCP	51.243
KAYFPP;KDVTLHK	NCP	44.142
KDVTLHK;FRGLVKY	NCP	281.584
FRGLVKY;PPNEKAR	NCP	4.419
PPNEKAR;KPELKVKP	NCP	18.395
KPELKVKP;KKLDDDKK	NCP	108.891
KKLDDDKK;QMLREEF	NCP	122.193
QMLREEF;KRPKPR	NCP	7.675
KRPKPR;APKNPLA	NCP	32.846
APKNPLA;CPCCDP	NCP	458.713
CPCCDP;PPKYFPQ	NCP	8.367
PPKYFPQ;VSELVDEV	NCP	43.52
VSELVDEV;KNVEP	NCP	39.681
KNVEP;LNPVVPF	NCP	64.032
LNPVVPF;YELDP	NCP	87.81
YELDP;EKVSFK	NCP	396.889
EKVSFK;KQKLEK	NCP	77.645
KQKLEK;DPPLTSSK	NCP	34.995
DPPLTSSK;SLFLLF	NCP	54.366
SLFLLF;NSLPKSPF	NCP	3.157
NSLPKSPF;LSKGSNVL	NCP	97.728
LSKGSNVL;KGVNFSN	NCP	170.261
KGVNFSN;LLDHKAEM	NCP	66.028
LLDHKAEM;VSPGPK	NCP	89.796
VSPGPK;KYPSVPP	NCP	332.406
KYPSVPP;LPPKLH	NCP	27.215
LPPKLH;KGFTVP	NCP	55.684
KGFTVP;KNVYL	NCP	79.71
KNVYL;GVMLK	NCP	37.442
GVMLK;MFLLE	NCP	108.958
MFLLE;LVQLKEF	NCP	225.035
LVQLKEF;TMLPP	NCP	103.021
TMLPP;LQDQLLK	NCP	33.803
LQDQLLK;KDLSSSLK	NCP	5.055
KDLSSSLK;LWHHSFY	NCP	31.143
LWHHSFY;PPYLL	NCP	58.725
PPYLL;HVLLKPGM	NCP	0.871
HVLLKPGM;LLQEC	NCP	91.367
LLQEC;EVPLPSP	NCP	199.492
EVPLPSP;AAAPDLK	NCP	154.446
AAAPDLK;LLGGAAVL	NCP	123.909
LLGGAAVL;NAFPL	NCP	49.346
NAFPL;KDATVPK	NCP	133.266
KDATVPK;DLFMKGP	NCP	47.742
DLFMKGP;ELKKVLR	NCP	36.978
ELKKVLR;ELTKQENLK	NCP	1.203
ELTKQENLK;KDYSPPPPPP	NCP	295.792

KDYSPPPPPP;MPPGAAR	NCP	111.245
MPPGAAR;VEAPGR	NCP	44.284
VEAPGR;KQKQRSS	NCP	6.285
KQKQRSS;RDKPL	NCP	23.519
RDKPL;NSRRKP	NCP	37.09
NSRRKP;VLAMLQ	NCP	21.755
VLAMLQ;AEMFC	NCP	5.107
AEMFC;ELSFKK	NCP	83.346
ELSFKK;PLALLPKTL	NCP	123.461
PLALLPKTL;VFKLLSWY	NCP	40.173
VFKLLSWY;EHVLVL	NCP	165.078
EHVLVL;LSPRPLP	NCP	273.48
LSPRPLP;RGPLPPNP	NCP	56.874
RGPLPPNP;KDNVFHVP	NCP	55.543
KDNVFHVP;YGMFVLK	NCP	8.985
YGMFVLK;PPSHTLK	NCP	7.643
PPSHTLK;AKRTPFG	NCP	9.276
AKRTPFG;MAEFAARA	NCP	0.06
MAEFAARA;EDTLWTGLTDTH	NCP	14.31
EDTLWTGLTDTH;FDNEMAPVEVK	NCP	2.01
FDNEMAPVEVK;EMAPVEVKTRKG	NCP	0.009
EMAPVEVKTRKG;MAPVEVKTRK	NCP	0.003
MAPVEVKTRK;KGKQTMQVDEHPRPQT	NCP	0.027
KGKQTMQVDEHPRPQT;KQTMQVDEHPRP	NCP	0.006
KQTMQVDEHPRP;VDEHPRPQT	NCP	0.015
VDEHPRPQT;EHPRPQTTMEQ	NCP	0.006
EHPRPQTTMEQ;LPPVFKKEGT	NCP	0.042
LPPVFKKEGT;VFKKEGTVTAGN	NCP	0.009
VFKKEGTVTAGN;KHNFTPLAR	NCP	1.554
KHNFTPLAR;LDPSKTNVN	NCP	6.228
LDPSKTNVN;APLGGRVP	NCP	23.478
APLGGRVP;LPTLVS	NCP	21.621
LPTLVS;TGAFK	NCP	26.361
TGAFK;LLNLHR	NCP	107.412
LLNLHR;SKYLFT	NCP	227.178
SKYLFT;AVGVFF	NCP	274.62
AVGVFF;ESKPFDLK	NCP	1.918
ESKPFDLK;DATPL	NCP	23.894
DATPL;MKLGLLGQ	NCP	31.768
MKLGLLGQ;VGSHGPPP	NCP	86.462
VGSHGPPP;VDLEKKL	NCP	38.521
VDLEKKL;FPGASL	NCP	27.764
FPGASL;KEVENLQQ	NCP	47.076
KEVENLQQ;KVFFTVK	NCP	24.742
KVFFTVK;VGGASL	NCP	10.65
VGGASL;TKGGPQP	NCP	108.911
TKGGPQP;DDDSM	NCP	30.244
DDDSM;VTKLFNY	NCP	26.853
VTKLFNY;GVGLK	NCP	260.738
GVGLK;PPAPPPK	NCP	171.088
PPAPPPK;SDGLFGF	NCP	32.33

SDGLFGF;PTLPKGKP	NCP	11.331
PTLPKGKP;KVPARFN	NCP	25.26
KVPARFN;KLEGVLPT	NCP	50.67
KLEGVLPT;KDFPK	NCP	196.587
KDFPK;GPVLRGVF	NCP	61.266
GPVLRGVF;LDAAL	NCP	0.432
LDAAL;FESAEEL	NCP	57.385
FESAEEL;LPPPPPPG	NCP	26.291
LPPPPPPG;KNQALVP	NCP	0.615
KNQALVP;GVPLPP	NCP	10.315
GVPLPP;PGNSCKK	NCP	44.007
PGNSCKK;YLDLKP	NCP	15.939
YLDLKP;RRLESHK	NCP	175.682
RRLESHK;LPKPERPP	NCP	204.339
LPKPERPP;HVGSRLY	NCP	4.326
HVGSRLY;HSSSSTVPEK	NCP	84.669
HSSSSTVPEK;VERKLAV	NCP	24.702
VERKLAV;EPDFNLK	NCP	26.262
EPDFNLK;RFRWE	NCP	26.946
RFRWE;YEKPETK	NCP	162.601
YEKPETK;YLPLPH	NCP	47.615
YLPLPH;PHFKLLSH	NCP	22.606
PHFKLLSH;TVPRPYG	NCP	42.344
TVPRPYG;TGHGPSPGP	NCP	24.384
TGHGPSPGP;MVVSKVNL	NCP	100.306
MVVSKVNL;KAKPSR	NCP	73.062
KAKPSR;LVPAHGP	NCP	85.436
LVPAHGP;VPGVTF	NCP	17.683
VPGVTF;GVTFLLPV	NCP	0.006
GVTFLLPV;KLDTRR	NCP	80.723
KLDTRR;KDFLSL	NCP	147.16
KDFLSL;TVMVP	NCP	20.849
TVMVP;PNWRSGK	NCP	78.169
PNWRSGK;GPAHTPL	NCP	3.26
GPAHTPL;VGEELLGR	NCP	7.896
VGEELLGR;LHSRLLERAAK	NCP	1.338
LHSRLLERAAK;SVSRVGSAAQT	NCP	0.717
SVSRVGSAAQT;DNSRLKL	NCP	98.058
DNSRLKL;AEAVRPPR	NCP	33.651
AEAVRPPR;TVTGLP	NCP	56.796
TVTGLP;LSAAPGVL	NCP	2.952
LSAAPGVL;TKVYPK	NCP	53.101
TKVYPK;VNYGFVF	NCP	56.148
VNYGFVF;LTVLDF	NCP	12.182
LTVLDF;WDDLRK	NCP	126.028
WDDLRK;KQAALER	NCP	88.868
KQAALER;QALKAVDK	NCP	20.217
QALKAVDK;GYLSRVF	NCP	2.856
GYLSRVF;YMLMTP	NCP	47.247
YMLMTP;DNDKGVNK	NCP	28.977
DNDKGVNK;LPVDP	NCP	31.23

LPVDP;QAFPP	NCP	3.37
QAFPP;NCCCCPQ	NCP	70.196
NCCCCPQ;KVFPESP	NCP	283.893
KVFPESP;MTTKPY	NCP	8.214
MTTKPY;KSHTLLSY	NCP	28.42
KSHTLLSY;TAALLC	NCP	153.413
TAALLC;GVLVDPL	NCP	43.831
GVLVDPL;YPVAFHK	NCP	11.702
YPVAFHK;KWNPN	NCP	4.474
KWNPN;TDMFRP	NCP	19.853
TDMFRP;NTELLLH	NCP	161.952
NTELLLH;WNLGF	NCP	179.694
WNLGF;KMAEYKLL	NCP	466.497
KMAEYKLL;TKGLPLGN	NCP	219.39
TKGLPLGN;HNSVVF	NCP	14.853
HNSVVF;PNWNKGK	NCP	6.619
PNWNKGK;ASKLL	NCP	17.582
ASKLL;RLTTQC	NCP	9.147
RLTTQC;GLSVTRLP	NCP	64.53
GLSVTRLP;KTTGEAKPK	NCP	3.216
KTTGEAKPK;LGGHLS	NCP	7.735
LGGHLS;NAVLQF	NCP	2.12
NAVLQF;KSRGLKFQ	NCP	63.525
KSRGLKFQ;TLLLKYM	NCP	87.352
TLLLKYM;VKAFC	NCP	199.929
VKAFC;KDVPL	NCP	20.298
KDVPL;ENDDVKR	NCP	36.608
ENDDVKR;NDALKLR	NCP	1.036
NDALKLR;LHFAVWK	NCP	52.287
LHFAVWK;VPAPASLSK	NCP	53.675
VPAPASLSK;WAPERP	NCP	40.689
WAPERP;KFPSLVGR	NCP	76.168
KFPSLVGR;ENLRMQP	NCP	163.518
ENLRMQP;PAAQK	NCP	17.111
PAAQK;EPPAAGGPK	NCP	105.465
EPPAAGGPK;EVFSM	NCP	9.586
EVFSM;LSPTTLP	NCP	15.704
LSPTTLP;TPPSAPAPK	NCP	159.127
TPPSAPAPK;DPKTALR	NCP	32.939
DPKTALR;SPLPDNSR	NCP	16.371
SPLPDNSR;SFGKRSH	NCP	6.994
SFGKRSH;LPAAGP	NCP	56.627
LPAAGP;LEPNKT	NCP	67.818
LEPNKT;QKPKLHYF	NCP	76.275
QKPKLHYF;PPPSPPR	NCP	38.874
PPPSPPR;YQLSHF	NCP	0.696
YQLSHF;PVPPLGPPP	NCP	23.175
PVPPLGPPP;TARSLTPA	NCP	19.368
TARSLTPA;LSLYPK	NCP	21.949
LSLYPK;AYLAP	NCP	5.784
AYLAP;LAVDF	NCP	16.928

LAVDF;VVLLYRPR	NCP	40.642
VVLLYRPR;KQGPT	NCP	18.38
KQGPT;LPEEPPAL	NCP	15.357
LPEEPPAL;APKGVTRP	NCP	1.36
APKGVTRP;VGAPV	NCP	5.813
VGAPV;KWFTTLQ	NCP	61.54
KWFTTLQ;FFGRPPP	NCP	25.397
FFGRPPP;ATVAAAPR	NCP	17.958
ATVAAAPR;VAGPPPPK	NCP	48.636
VAGPPPPK;VGPDDGLP	NCP	29.41
VGPDDGLP;LEMTF	NCP	54.525
LEMTF;RLLLAEAY	NCP	3.831
RLLLAEAY;PEKPGPK	NCP	36.984
PEKPGPK;FLAGPTVF	NCP	57.87
FLAGPTVF;TAGLLALR	NCP	22.023
TAGLLALR;CRAHAGP	NCP	2.127
CRAHAGP;QGPSFLPG	NCP	15.036
QGPSFLPG;RLLPPL	NCP	65.139
RLLPPL;AAPTVRY	NCP	3.39
AAPTVRY;LGGPAVTK	NCP	45.909
LGGPAVTK;LSAAP	NCP	43.137
LSAAP;VVGVKFQ	NCP	39.359
VVGVKFQ;AAPAPKPR	NCP	47.691
AAPAPKPR;NKVAVL	NCP	206.722
NKVAVL;LKVLVGK	NCP	100.85
LKVLVGK;MDQLEP	NCP	293.763
MDQLEP;KTKLSH	NCP	12.442
KTKLSH;NPPLHVLN	NCP	24.581
NPPLHVLN;EMLRLQK	NCP	27.681
EMLRLQK;LMPLVL	NCP	73.5
LMPLVL;AASGPP	NCP	166.945
AASGPP;MNVVP	NCP	167.574
MNVVP;LSPSRVLSP	NCP	62.237
LSPSRVLSP;KELLYLGKE	NCP	11.566
KELLYLGKE;LPLPPPEY	NCP	69.924
LPLPPPEY;CNALLCR	NCP	77.699
CNALLCR;KDLMVAP	NCP	12.517
KDLMVAP;KNWAKGK	NCP	26.748
KNWAKGK;KLVQRLT	NCP	24.477
KLVQRLT;PSAYSKP	NCP	23.808
PSAYSKP;DYEAC	NCP	11.228
DYEAC;PPHQKSK	NCP	12.087
PPHQKSK;QPMSFK	NCP	281.434
QPMSFK;VVLSGAK	NCP	39.704
VVLSGAK;EVGPDPK	NCP	19.123
EVGPDPK;AKLHSK	NCP	5.226
AKLHSK;KLPRLL	NCP	10.154
KLPRLL;KSGGLL	NCP	114.375
KSGGLL;LSDGALY	NCP	148.545
LSDGALY;VNAKNPK	NCP	50.223
VNAKNPK;YLYGHLL	NCP	44.133

YLYGHLL;QPVPPPPPP	NCP	37.113
QPVPPPPPP;EEKNY	NCP	4.716
EEKNY;EVLYR	NCP	33.763
EVLYR;WDWPK	NCP	43.137
WDWPK;QPHQVQR	NCP	14.483
QPHQVQR;EWRFR	NCP	32.907
EWRFR;KDCCYH	NCP	90.838
KDCCYH;LTDSHVVT	NCP	36.209
LTDSHVVT;EDTKDGP	NCP	98.431
EDTKDGP;NAYLMHY	NCP	9.312
NAYLMHY;LAVLQN	NCP	83.355
LAVLQN;KTPLP	NCP	37.602
KTPLP;AAAVTPGK	NCP	22.554
AAAVTPGK;AGPCSL	NCP	15.567
AGPCSL;LSKFT	NCP	15.081
LSKFT;NGASR	NCP	31.844
NGASR;LAEFRKK	NCP	4.972
LAEFRKK;KAPQNLH	NCP	7.088
KAPQNLH;RHALLR	NCP	116.325
RHALLR;GPHAQKEL	NCP	8.053
GPHAQKEL;LDVDRPK	NCP	250.583
LDVDRPK;TDYMPSL	NCP	119.808
TDYMPSL;KFAPPPPP	NCP	49.101
KFAPPPPP;MLKFQ	NCP	182.296
MLKFQ;VNPTV	NCP	61.778
VNPTV;VAPQPRP	NCP	9.723
VAPQPRP;KLQPLG	NCP	77.289
KLQPLG;DGNTR	NCP	117.504
DGNTR;DRSYRNRDLS	NCP	86.871
DRSYRNRDLS;DETAP	NCP	184.734
DETAP;LVVPLK	NCP	7.113
LVVPLK;KSVVTL	NCP	9.109
KSVVTL;KNEGP	NCP	3.512
KNEGP;EKPRVDF	NCP	67.899
EKPRVDF;TGGHVRP	NCP	387.292
TGGHVRP;LYEASNY	NCP	35.883
LYEASNY;LYRSPL	NCP	54.912
LYRSPL;AAFLLY	NCP	1.764
AAFLLY;NLLEYL	NCP	18.869
NLLEYL;VGNLSQL	NCP	22.008
VGNLSQL;KASELHF	NCP	54.16
KASELHF;HVHNR	NCP	149.553
HVHNR;QTWLLK	NCP	272.046
QTWLLK;VEVPRNP	NCP	22.997
VEVPRNP;PVPGSFP	NCP	81.184
PVPGSFP;SPPVHKP	NCP	65.99
SPPVHKP;EGVFPA	NCP	25.797
EGVFPA;KPSELR	NCP	16.879
KPSELR;PPMEAAG	NCP	12.209
PPMEAAG;WRAPP	NCP	115.161
WRAPP;NSLLTHVL	NCP	36.639

NSLLTHVL;LASVPDAL	NCP	33.163
LASVPDAL;LPKETPLD	NCP	63.111
LPKETPLD;YDYEARTTEDL	NCP	34.631
YDYEARTTEDL;RSSPF	NCP	166.96
RSSPF;RLLVHK	NCP	130.787
RLLVHK;KPAEEFN	NCP	135.045
KPAEEFN;LVAFHDF	NCP	75.955
LVAFHDF;WHAHKL	NCP	42.141
WHAHKL;NYMPGF	NCP	59.154
NYMPGF;PTEPPR	NCP	12.534
PTEPPR;KETQPK	NCP	104.067
KETQPK;FPKAKVP	NCP	102.218
FPKAKVP;LNVAPNLP	NCP	2.442
LNVAPNLP;TAWMLR	NCP	114.105
TAWMLR;YVDLNLK	NCP	5.679
YVDLNLK;KGDRAAPP	NCP	120.171
KGDRAAPP;NKWGF	NCP	206.223
NKWGF;GDLDLLR	NCP	109.002
GDLDLLR;LDLLK	NCP	96.393
LDLLK;ESVVFK	NCP	89.674
ESVVFK;KDPAKPS	NCP	3.308
KDPAKPS;LLRRF	NCP	52.032
LLRRF;AFPPPPPPSPP	NCP	158.622
AFPPPPPPSPP;QAPHPR	NCP	12.337
QAPHPR;AVELK	NCP	64.16
AVELK;KSNYEKF	NCP	42.3
KSNYEKF;VDKLP	NCP	23.548
VDKLP;KWAQY	NCP	88.203
KWAQY;PNGTRPP	NCP	53.916
PNGTRPP;YKNFVSW	NCP	24.791
YKNFVSW;PPPPGP	NCP	43.518
PPPPGP;PPPELPPR	NCP	164.487
PPPELPPR;VAAAPPPR	NCP	278.214
VAAAPPPR;KSAEVKN	NCP	49.228
KSAEVKN;YSVKRHQ	NCP	4.377
YSVKRHQ;VSLLLDPF	NCP	20.993
VSLLLDPF;GLFVSR	NCP	4.854
GLFVSR;GVKPLQ	NCP	136.038
GVKPLQ;TLLAKR	NCP	148.741
TLLAKR;MMMVLQ	NCP	238.46
MMMVLQ;FDFKGHF	NCP	10.419
FDFKGHF;HEAFLK	NCP	14.979
HEAFLK;ATTTP	NCP	44.527
ATTTP;EVLPK	NCP	8.423
EVLPK;MGLVP	NCP	94.774
MGLVP;KLLSH	NCP	35.606
KLLSH;LSSGY	NCP	15.711
LSSGY;GVPLKGL	NCP	3.066
GVPLKGL;TSMKLK	NCP	181.404
TSMKLK;LCFQYL	NCP	95.757
LCFQYL;LFLGGL	NCP	57.855

LFLGGL;MTSLGF	NCP	33.043
MTSLGF;FFLLVSL	NCP	143.61
FFLLVSL;TAESLP	NCP	54.725
TAESLP;TKKLLR	NCP	329.493
TKKLLR;RLGRGF	NCP	299.493
RLGRGF;KLGLGH	NCP	125.184
KLGLGH;YPAVP	NCP	4.653
YPAVP;LPQAVP	NCP	60.339
LPQAVP;VGHGYLP	NCP	1.968
VGHGYLP;KCGHGAP	NCP	4.347
KCGHGAP;PPGDLPR	NCP	13.738
PPGDLPR;TSLGHVVL	NCP	69.98
TSLGHVVL;AAVSPTKK	NCP	55.878
AAVSPTKK;PPVPLP	NCP	48.432
PPVPLP;GSAALQN	NCP	15.241
GSAALQN;SLKFKFV	NCP	60.285
SLKFKFV;KDGSTLL	NCP	70.698
KDGSTLL;KALKSWK	NCP	42.336
KALKSWK;KKSSAGK	NCP	7.259
KKSSAGK;VPPDHY	NCP	4.465
VPPDHY;VPLSGW	NCP	47.759
VPLSGW;KQGVPVP	NCP	81.697
KQGVPVP;KNEAKEGK	NCP	35.478
KNEAKEGK;VLLAP	NCP	3.618
VLLAP;KFAAGTK	NCP	18.498
KFAAGTK;LHVLL	NCP	39.881
LHVLL;MPPLG	NCP	16.429
MPPLG;QVVVR	NCP	120.084
QVVVR;VLPELY	NCP	22.653
VLPELY;KSGELL	NCP	2.249
KSGELL;RHTLGR	NCP	65.733
RHTLGR;AAVLL	NCP	20.773
AAVLL;KERVY	NCP	13.757
KERVY;KFFPLPP	NCP	2.143
KFFPLPP;GTLAAW	NCP	75.41
GTLAAW;GVASSLFP	NCP	113.118
GVASSLFP;LLEHKVTF	NCP	113.368
LLEHKVTF;LKLCEF	NCP	44.519
LKLCEF;KGHFGFG	NCP	68.074
KGHFGFG;ESVNLTK	NCP	79.439
ESVNLTK;VPLLGF	NCP	51.553
VPLLGF;LSSVKLKSP	NCP	47.778
LSSVKLKSP;GTPKPLLL	NCP	35.7
GTPKPLLL;TAAVKDF	NCP	10.833
TAAVKDF;FGEGLLE	NCP	34.292
FGEGLLE;LSEKP	NCP	20.076
LSEKP;PLLALLGP	NCP	83.691
PLLALLGP;KAAAAAAKVAP	NCP	12.949
KAAAAAAKVAP;SKLEF	NCP	125.937
SKLEF;KRVPALY	NCP	45.738
KRVPALY;PQKKKR	NCP	18.858

PQKKKR;EPDLPR	NCP	87.615
EPDLPR;HSEFLAK	NCP	6.186
HSEFLAK;LVEKVST	NCP	37.565
LVEKVST;KVRGSR	NCP	9.978
KVRGSR;LSQASLR	NCP	146.091
LSQASLR;VGVKN	NCP	268.639
VGVKN;KPHFPP	NCP	31.953
KPHFPP;VKVLT	NCP	30.923
VKVLT;LQDELPK	NCP	27.376
LQDELPK;LPELPR	NCP	148.94
LPELPR;SLGMTNF	NCP	235.225
SLGMTNF;MAPPT	NCP	57.006
MAPPT;RSEQLF	NCP	128.298
RSEQLF;STPPYKN	NCP	84.665
STPPYKN;KDAAPPPK	NCP	48.042
KDAAPPPK;KDQLLR	NCP	33.912
KDQLLR;AVHLQPAH	NCP	102.705
AVHLQPAH;LSGGVDNF	NCP	210.853
LSGGVDNF;VGEKF	NCP	37.32
VGEKF;KLDALLK	NCP	5.54
KLDALLK;EVLALAVM	NCP	239.511
EVLALAVM;KYPGSP	NCP	10.242
KYPGSP;CDMAC	NCP	272.215
CDMAC;EPGSLSCK	NCP	10.853
EPGSLSCK;LPMPPP	NCP	10.968
LPMPPP;GSSMKL	NCP	15.73
GSSMKL;LSELPY	NCP	134.007
LSELPY;KRYFLP	NCP	51.468
KRYFLP;KTLYF	NCP	453.303
KTLYF;VTLPL	NCP	38.931
VTLPL;WGVFP	NCP	47.802
WGVFP;GLTSKFP	NCP	11.102
GLTSKFP;ALSLLK	NCP	149.592
ALSLLK;EGPAFQP	NCP	33.648
EGPAFQP;LLVAVGY	NCP	27.537
LLVAVGY;HVVTF	NCP	120.72
HVVTF;RNAVTFK	NCP	34.725
RNAVTFK;PVFELTL	NCP	9.478
PVFELTL;SPYSYSTTALVSSPK	NCP	9.665
SPYSYSTTALVSSPK;VLEYLT	NCP	63.327
VLEYLT;LLGPGSEK	NCP	16.194
LLGPGSEK;QGLFSC	NCP	63.621
QGLFSC;FEDTNL	NCP	115.434
FEDTNL;EEMFKL	NCP	162.738
EEMFKL;LPLKL	NCP	123.28
LPLKL;MKPLLA	NCP	28.067
MKPLLA;KLLGSNGK	NCP	32.008
KLLGSNGK;PGPSSPPPP	NCP	240.35
PGPSSPPPP;RGSSKK	NCP	66.606
RGSSKK;PTSVPK	NCP	61.249
PTSVPK;FLYKK	NCP	222.327

FLYKK;NMLVKYF	NCP	121.707
NMLVKYF;KESLM	NCP	28.82
KESLM;SARPPPP	NCP	20.827
SARPPPP;NFSHKAK	NCP	35.634
NFSHKAK;KQCACQTK	NCP	35.123
KQCACQTK;PGASGK	NCP	67.443
PGASGK;KKSLKKP	NCP	19.488
KKSLKKP;RATSNVF	NCP	340.813
RATSNVF;DKEDLHDML	NCP	0.099
DKEDLHDML;LTKFP	NCP	17.933
LTKFP;KPKFQPP	NCP	47.512
KPKFQPP;PVGPKPSK	NCP	58.649
PVGPKPSK;PTVPLNE	NCP	14.559
PTVPLNE;YGKVK	NCP	41.377
YGKVK;LPPPPPPP	NCP	46.247
LPPPPPPP;EYTAF	NCP	220.903
EYTAF;TTLVPATK	NCP	65.753
TTLVPATK;LYEFLT	NCP	230.259
LYEFLT;PGPGSRLPP	NCP	5.689
PGPGSRLPP;KVKQLLR	NCP	116.808
KVKQLLR;PPPPLVP	NCP	20.6
PPPPLVP;GLDKLTEK	NCP	40.413
GLDKLTEK;KTHSKPP	NCP	220.5
KTHSKPP;LRLLCHF	NCP	147.876
LRLLCHF;DALCLF	NCP	40.861
DALCLF;KTHEWYK	NCP	20.304
KTHEWYK;QGLAHYRH	NCP	2.576
QGLAHYRH;LSSVPFPR	NCP	13.572
LSSVPFPR;VPGVGN	NCP	21.922
VPGVGN;KGVPLTK	NCP	9.483
KGVPLTK;MDVVLKK	NCP	33.873
MDVVLKK;MLKLPPP	NCP	21.638
MLKLPPP;LDQVDRP	NCP	102.733
LDQVDRP;LNYGKDM	NCP	60.728
LNYGKDM;LHWEHR	NCP	67.45
LHWEHR;SSPASKPGSTPSRP	NCP	71.18
SSPASKPGSTPSRP;TAEEKN	NCP	74.058
TAEEKN;ELELPAG	NCP	18.994
ELELPAG;ELKLQT	NCP	39.878
ELKLQT;YGVTKDGGQ	NCP	45.205
YGVTKDGGQ;SSGHPHFVR	NCP	30.629
SSGHPHFVR;ALPGP	NCP	201.262
ALPGP;VHPVV	NCP	32.036
VHPVV;LTAAFLLQ	NCP	2.616
LTAAFLLQ;KSYLLVVSF	NCP	37.206
KSYLLVVSF;PGGKL	NCP	32.424
PGGKL;VPTFPF	NCP	41.989
VPTFPF;RWPKP	NCP	91.167
RWPKP;LPFLVLP	NCP	55.241
LPFLVLP;TALAL	NCP	38.689
TALAL;KLVSAPAK	NCP	11.693

KLVSAPAK;CGDDDC	NCP	157.596
CGDDDC;EDLGFL	NCP	32.827
EDLGFL;LTSKYR	NCP	90.705
LTSKYR;TGLVSH	NCP	23.495
TGLVSH;QPSWLW	NCP	184.891
QPSWLW;HPDGPTP	NCP	2.177
HPDGPTP;PLLHLP	NCP	31.639
PLLHLP;TSPKVDL	NCP	148.109
TSPKVDL;LPRTPP	NCP	21.441
LPRTPP;KMEAP	NCP	45.814
KMEAP;LMLVARLT	NCP	59.783
LMLVARLT;LEQPL	NCP	289.828
LEQPL;LASVGY	NCP	26.904
LASVGY;FLGLEF	NCP	35.06
FLGLEF;AAPGKP	NCP	101.071
AAPGKP;FPDLLR	NCP	57.341
FPDLLR;QAEPPRP	NCP	28.023
QAEPPRP;LLKYTSAK	NCP	114.568
LLKYTSAK;PLPPPKDC	NCP	5.597
PLPPPKDC;ELLTDR	NCP	9.169
ELLTDR;GTGPLLSM	NCP	10.694
GTGPLLSM;VGGGMF	NCP	22.962
VGGGMF;GVPGVR	NCP	40.455
GVPGVR;GESVGP	NCP	169.318
GESVGP;TEPKPR	NCP	6.009
TEPKPR;TDGLLGK	NCP	20.055
TDGLLGK;KCGSAGP	NCP	42.215
KCGSAGP;GVGALL	NCP	273.631
GVGALL;PPPWP	NCP	64.509
PPPWP;LRELLT	NCP	25.305
LRELLT;LLRGVF	NCP	378.18
LLRGVF;CSHKTT	NCP	50.316
CSHKTT;KTPGAVPP	NCP	76.26
KTPGAVPP;LTLGSAPP	NCP	86.286
LTLGSAPP;KLYVP	NCP	28.323
KLYVP;PRPCE	NCP	0.795
PRPCE;SMDATLK	NCP	15.708
SMDATLK;YVENK	NCP	21.086
YVENK;VMAAMPP	NCP	30.945
VMAAMPP;KNPLSP	NCP	275.199
KNPLSP;PLLMY	NCP	136.729
PLLMY;STDRAVLH	NCP	87.528
STDRAVLH;DTVTYVP	NCP	50.447
DTVTYVP;EMHMTM	NCP	130.761
EMHMTM;KYKGVVFF	NCP	2.139
KYKGVVFF;SSVKDLTK	NCP	41.701
SSVKDLTK;LNLVKK	NCP	204.95
LNLVKK;KAKSQR	NCP	13.578
KAKSQR;LDLLEC	NCP	19.828
LDLLEC;SPLLLR	NCP	10.221
SPLLLR;GVKLDF	NCP	17.06

GVKLDF;LTRDLEY	NCP	42.879
LTRDLEY;GKHVLY	NCP	158.662
GKHVLY;QNYKVLG	NCP	179.525
QNYKVLG;KPDKFQ	NCP	6.282
KPDKFQ;PVGPAPT	NCP	283.747
PVGPAPT;VVPRSK	NCP	49.812
VVPRSK;LDLVLYR	NCP	21.507
LDLVLYR;LAALP	NCP	120.129
LAALP;KLTERL	NCP	142.976
KLTERL;QDLVHY	NCP	0.259
QDLVHY;LVVTGAF	NCP	62.601
LVVTGAF;KGSHHTK	NCP	15.557
KGSHHTK;LFETKSK	NCP	94.275
LFETKSK;KPGVPVF	NCP	2.829
KPGVPVF;CHAQSRK	NCP	165.465
CHAQSRK;KHLEC	NCP	111.318
KHLEC;VDVMLPK	NCP	76.498
VDVMLPK;SELKLVSN	NCP	73.709
SELKLVSN;LGTLAK	NCP	22.78
LGTLAK;TVLALD	NCP	61.164
TVLALD;VEGGSQ	NCP	12.525
VEGGSQ;LHKGVFR	NCP	66.597
LHKGVFR;AAKNKLA	NCP	22.451
AAKNKLA;SYLDP	NCP	148.045
SYLDP;CCESQ	NCP	99.963
CCESQ;AEFQK	NCP	40.049
AEFQK;QHLFY	NCP	25.741
QHLFY;EPTAF	NCP	15.503
EPTAF;FSMFPKP	NCP	62.686
FSMFPKP;KCLQGRLF	NCP	18.279
KCLQGRLF;VPGGKDY	NCP	38.874
VPGGKDY;VHKFLFN	NCP	10.068
VHKFLFN;LLNAFKYN	NCP	1.275
LLNAFKYN;EPGAPLN	NCP	49.203
EPGAPLN;MHVPKPK	NCP	48.368
MHVPKPK;KVVLWVTK	NCP	30.513
KVVLWVTK;EGRPPVVL	NCP	7.126
EGRPPVVL;HHSDASKK	NCP	84.123
HHSDASKK;KNTVLVPF	NCP	72.218
KNTVLVPF;LHPPPP	NCP	0.921
LHPPPP;PESPF	NCP	165.205
PESPF;KWALVGP	NCP	29.858
KWALVGP;EPFELYL	NCP	72.405
EPFELYL;ADKMSLG	NCP	21.069
ADKMSLG;AAGPVT	NCP	6.607
AAGPVT;LDVAF	NCP	4.895
LDVAF;PKAHALK	NCP	27.495
PKAHALK;PDKDPK	NCP	141.01
PDKDPK;GPAKQMKP	NCP	30.197
GPAKQMKP;DGKLWH	NCP	32.62
DGKLWH;NESVPL	NCP	20.583

NESVPL;LPGVK	NCP	12.402
LPGVK;EEPLFPF	NCP	8.424
EEPLFPF;PPPPPRPP	NCP	22.374
PPPPPRPP;ELHRLEYL	NCP	27.119
ELHRLEYL;AFSRKP	NCP	89.101
AFSRKP;MGLPAQK	NCP	45.438
MGLPAQK;LSAAFTGK	NCP	174.678
LSAAFTGK;KAELFAFL	NCP	32.663
KAELFAFL;ASHPPF	NCP	106.285
ASHPPF;SNLSKKLR	NCP	150.09
SNLSKKLR;KHNYLK	NCP	107.439
KHNYLK;APPLPRP	NCP	30.348
APPLPRP;GPHFVR	NCP	31.646
GPHFVR;EPVSG	NCP	18.28
EPVSG;HARPPPP	NCP	27.483
HARPPPP;LTPAT	NCP	75.465
LTPAT;AMWSN	NCP	29.265
AMWSN;KQVVKPQ	NCP	77.588
KQVVKPQ;RDLEQVSR	NCP	0.724
RDLEQVSR;WMKVK	NCP	75.896
WMKVK;GVLEL	NCP	93.928
GVLEL;DLCFR	NCP	189.228
DLCFR;SHLTPR	NCP	0.789
SHLTPR;MFLLLF	NCP	26.733
MFLLLF;YQAWY	NCP	8.684
YQAWY;GHPSQPK	NCP	28.273
GHPSQPK;LSPRPPP	NCP	11.037
LSPRPPP;HSKLKK	NCP	34.79
HSKLKK;VGSVNPML	NCP	12.57
VGSVNPML;PMEPAAGF	NCP	3.724
PMEPAAGF;LTYMGTL	NCP	8.048
LTYMGTL;PPPPPPP	NCP	113.866
PPPPPPP;PHLNPR	NCP	13.026
PHLNPR;VLSGLSPF	NCP	11.58
VLSGLSPF;PPPPPQLP	NCP	1.089
PPPPPQLP;PLRPGPP	NCP	87.3
PLRPGPP;ARNGPLR	NCP	69.584
ARNGPLR;KENPLPS	NCP	52.309
KENPLPS;VNLYL	NCP	16.742
VNLYL;VPGGP	NCP	31.384
VPGGP;LTAKLL	NCP	158.942
LTAKLL;KEELVR	NCP	28.269
KEELVR;YPLLLS	NCP	37.591
YPLLLS;MKLLT	NCP	12.098
MKLLT;AMVMNS	NCP	25.036
AMVMNS;LEGLQL	NCP	14.622
LEGLQL;VPELTLT	NCP	44.129
VPELTLT;GVDLR	NCP	7.003
GVDLR;EGSKAG	NCP	82.817
EGSKAG;EDPAE	NCP	185.19
EDPAE;LDKAGPQ	NCP	33.55

LDKAGPQ;APGVGF	NCP	47.624
APGVGF;LHARFL	NCP	27.814
LHARFL;VSALRTTP	NCP	1.518
VSALRTTP;VKGSPLL	NCP	78.989
VKGSPLL;SGAKHTR	NCP	101.842
SGAKHTR;PSPTVLPP	NCP	138.969
PSPTVLPP;LLPKKT	NCP	31.652
LLPKKT;TSCLLF	NCP	95.614
TSCLLF;TLLSPP	NCP	134.468
TLLSPP;PPLRPAVP	NCP	43.878
PPLRPAVP;MKLLPLQ	NCP	20.8
MKLLPLQ;TGTGAAPG	NCP	45.948
TGTGAAPG;LGAGF	NCP	239.247
LGAGF;PVYSTPK	NCP	19.019
PVYSTPK;YLLAATK	NCP	0.277
YLLAATK;KVPGHFP	NCP	34.952
KVPGHFP;KAPAPP	NCP	116.743
KAPAPP;FDLLK	NCP	16.343
FDLLK;PMYMSS	NCP	73.956
PMYMSS;PLTPML	NCP	10.65
PLTPML;VAPPPARPC	NCP	104.962
VAPPPARPC;NPNLSMP	NCP	40.857
NPNLSMP;KETTQPY	NCP	22.316
KETTQPY;ALASFR	NCP	22.381
ALASFR;VPTQLPK	NCP	59.597
VPTQLPK;AGAPVLY	NCP	27.112
AGAPVLY;YHPTQFP	NCP	27.905
YHPTQFP;EDSLP	NCP	51
EDSLP;CSVAKSNR	NCP	21
CSVAKSNR;LDMSPQ	NCP	56.908
LDMSPQ;KFPVELR	NCP	300.98
KFPVELR;PAAAFL	NCP	2.623
PAAAFL;RLKEELER	NCP	88.38
RLKEELER;KGSGTPF	NCP	57.74
KGSGTPF;KGEMK	NCP	309
KGEMK;TAAFLLLP	NCP	13.519
TAAFLLLP;KWNPF	NCP	108.267
KWNPF;LPASGNPVP	NCP	25.517
LPASGNPVP;KLLAY	NCP	44.647
KLLAY;SLGHLEPP	NCP	15.842
SLGHLEPP;FGTDEEK	NCP	77.679
FGTDEEK;HVLGLQ	NCP	33.208
HVLGLQ;FPAGSL	NCP	119.142
FPAGSL;AEVPL	NCP	14.904
AEVPL;KYAAPR	NCP	183.54
KYAAPR;KLLMAT	NCP	41.943
KLLMAT;KYVPLE	NCP	15.254
KYVPLE;APDFERK	NCP	16.08
APDFERK;MEVKPPP	NCP	33.721
MEVKPPP;GCDEYD	NCP	288.446
GCDEYD;HTALKDLQ	NCP	5.427

HTALKDLQ;FRLLLTL	NCP	57.786
FRLLLTL;VAAGDSK	NCP	20.274
VAAGDSK;LDGDLL	NCP	94.821
LDGDLL;GDKTESH	NCP	1.219
GDKTESH;PHFLEY	NCP	146.622
PHFLEY;AAAAAPN	NCP	3.966
AAAAAPN;LKLRNWQ	NCP	361.233
LKLRNWQ;KPGSMLGV	NCP	94.811
KPGSMLGV;PQLDME	NCP	60.298
PQLDME;ETKPP	NCP	41.018
ETKPP;LSGTEGLR	NCP	10.617
LSGTEGLR;TGPPHFH	NCP	68.586
TGPPHFH;EAAPVPG	NCP	165.249
EAAPVPG;EAGVSVPK	NCP	79.468
EAGVSVPK;PVGFSPK	NCP	165.723
PVGFSPK;DHVMPK	NCP	91.548
DHVMPK;KDVPGKQ	NCP	66.273
KDVPGKQ;FNTRTFY	NCP	9.009
FNTRTFY;RKSLPMK	NCP	155.078
RKSLPMK;KDLQL	NCP	44.706
KDLQL;PLAQVP	NCP	132.084
PLAQVP;AQLSPF	NCP	104.272
AQLSPF;VLELY	NCP	118.298
VLELY;LGRVNF	NCP	321.64
LGRVNF;VGGMLPL	NCP	33.222
VGGMLPL;NSDRPTT	NCP	40.238
NSDRPTT;YYLLVK	NCP	11.301
YYLLVK;ENEQLL	NCP	101.623
ENEQLL;PVGKPTLY	NCP	7.166
PVGKPTLY;KMVGPP	NCP	102.301
KMVGPP;KPVAKASH	NCP	18.912
KPVAKASH;EALKLP	NCP	2.285
EALKLP;MVSVTP	NCP	27.273
MVSVTP;DRHVPY	NCP	32.596
DRHVPY;LDQLLL	NCP	113.307
LDQLLL;PAALKHK	NCP	56.261
PAALKHK;YGGFG	NCP	131.239
YGGFG;KGAAAAART	NCP	9.995
KGAAAAART;STHRVL	NCP	62.739
STHRVL;VARHELK	NCP	142.936
VARHELK;LPKGAKTPR	NCP	82.722
LPKGAKTPR;SLQHKK	NCP	82.271
SLQHKK;QDPTK	NCP	21.393
QDPTK;ALPSPSR	NCP	297.945
ALPSPSR;RLNWEK	NCP	6.538
RLNWEK;ELLPVP	NCP	8.091
ELLPVP;KEGLLA	NCP	32.775
KEGLLA;LDVVL	NCP	136.008
LDVVL;KTHLLQ	NCP	67.889
KTHLLQ;LPLDKLT	NCP	2.121
LPLDKLT;PGRPPR	NCP	0.204

PGRPPR;AEFLLF	NCP	54.06
AEFLLF;KEAGLAGH	NCP	45.778
KEAGLAGH;LSAAPFVL	NCP	44.349
LSAAPFVL;DAFFKDVDY	NCP	14.483
DAFFKDVDY;AAYLYEVSQLKD	NCP	4.536
AAYLYEVSQLKD;KFPSLV	NCP	62.752
KFPSLV;LLLCSK	NCP	19.185
LLLCSK;EVYSG	NCP	80.027
EVYSG;LTLLKLGK	NCP	69.078
LTLLKLGK;KGHALP	NCP	124.024
KGHALP;APPVLPPP	NCP	159.065
APPVLPPP;LPWPL	NCP	30.105
LPWPL;LLLLYNR	NCP	11.5
LLLLYNR;EKGSVMH	NCP	181.829
EKGSVMH;KEQLLAR	NCP	104.485
KEQLLAR;DSLKLDYL	NCP	13.086
DSLKLDYL;PTKGDGPP	NCP	130.851
PTKGDGPP;KGVAELPF	NCP	52.637
KGVAELPF;GPPPGYK	NCP	11.395
GPPPGYK;KPGVGAPR	NCP	5.292
KPGVGAPR;GPLNF	NCP	6.003
GPLNF;SEKVAHAK	NCP	11.028
SEKVAHAK;QRNLLM	NCP	2.792
QRNLLM;DSYPLP	NCP	2.233
DSYPLP;LYEFLNP	NCP	104.411
LYEFLNP;GRAPA	NCP	175.582
GRAPA;KGFKP	NCP	36.066
KGFKP;PGFVNRL	NCP	26.754
PGFVNRL;LPPVT	NCP	64.259
LPPVT;LDDLKL	NCP	102.583
LDDLKL;ARLLA	NCP	13.827
ARLLA;DRAPPHF	NCP	36.047
DRAPPHF;PMDDLR	NCP	98.41
PMDDLR;KACDGAP	NCP	308.582
KACDGAP;KPSSETAGK	NCP	13.359
KPSSETAGK;KAVVGGGK	NCP	52.569
KAVVGGGK;KSTKLM	NCP	51.997
KSTKLM;TTPKPVLN	NCP	40.494
TTPKPVLN;LLQLFL	NCP	126.281
LLQLFL;WKLLL	NCP	101.34
WKLLL;QGPNKPS	NCP	92.488
QGPNKPS;LNYKEPK	NCP	46.443
LNYKEPK;KNLTFP	NCP	38.838
KNLTFP;GPAVTPPK	NCP	46.448
GPAVTPPK;SQLMGELK	NCP	14.983
SQLMGELK;VGAAP	NCP	79.973
VGAAP;PPLPAGP	NCP	119.581
PPLPAGP;LGPPER	NCP	139.376
LGPPER;NSRVLSPY	NCP	31.672
NSRVLSPY;MGETP	NCP	9.975
MGETP;PGPWRP	NCP	13.922

PGPWRP;RGPPTAPP	NCP	37.047
RGPPTAPP;ASRGPF	NCP	72.106
ASRGPF;GVGASRPR	NCP	88.92
GVGASRPR;SSMESSAS	NCP	14.075
SSMESSAS;VDKLRP	NCP	18.529
VDKLRP;LLAAP	NCP	8.837
LLAAP;SEKKSFQP	NCP	10.909
SEKKSFQP;YSLKGPR	NCP	16.02
YSLKGPR;VPRPLAL	NCP	12.26
VPRPLAL;MQPAYR	NCP	67.668
MQPAYR;RPEEPRP	NCP	25.206
RPEEPRP;VSTGLTG	NCP	134.524
VSTGLTG;GLPVAGPR	NCP	42.918
GLPVAGPR;KQEPQ	NCP	139.047
KQEPQ;GPPRPNAP	NCP	89.436
GPPRPNAP;PSGTLY	NCP	15.72
PSGTLY;VGLLKF	NCP	47.31
VGLLKF;LPPDPPLP	NCP	17.301
LPPDPPLP;CMMLEP	NCP	34.127
CMMLEP;TAGKK	NCP	263.263
TAGKK;KLFPHQ	NCP	83.418
KLFPHQ;TGVGNKL	NCP	114.729
TGVGNKL;KGMMER	NCP	61.326
KGMMER;EPLTPRP	NCP	463.038
NPPKDLKKPR;LAFPP	NCP	36.37
LAFPP;SGLVTTLTP	NCP	6.476
SGLVTTLTP;VESPAPPK	NCP	2.77
VESPAPPK;GVELRYLS	NCP	24.356
GVELRYLS;APAHA	NCP	8.31
APAHA;PPLPPPPPG	NCP	70.281
PPLPPPPPG;VNGPPPH	NCP	0.363
VNGPPPH;RKLQYLAT	NCP	34.27
RKLQYLAT;AHASE	NCP	40.149
AHASE;CVLLL	NCP	62.87
CVLLL;ADKKLVVVFG	NCP	12.006
ADKKLVVVFG;DQPVL	NCP	46.936
DQPVL;VEAPP	NCP	15.206
VEAPP;LVDLEL	NCP	8.941
LVDLEL;LYELLPEAPF	NCP	26.447
LYELLPEAPF;GVPLAA	NCP	34.689
GVPLAA;CCAGPPPP	NCP	24.339
CCAGPPPP;PPSETHV	NCP	51.69
PPSETHV;PVTKHPP	NCP	203.775
PVTKHPP;TGVPRPLP	NCP	28.188
TGVPRPLP;AENLLDGPPNPK	NCP	0.924
AENLLDGPPNPK;EHVTKVK	NCP	69.61
EHVTKVK;YRLGP	NCP	40.892
YRLGP;THQKAAR	NCP	74.358
THQKAAR;NGRAAFC	NCP	357.019
NGRAAFC;MMDGH	NCP	37.802
MMDGH;AAAPR	NCP	44.848

AAAPR;PPGAPPP	NCP	191.642
PPGAPPP;RSSWPFPR	NCP	1.569
RSSWPFPR;KEVQVTKV	NCP	163.897
KEVQVTKV;TFLPPP	NCP	52.038
TFLPPP;TSMKPGAK	NCP	94.316
TSMKPGAK;PVPAAPK	NCP	59.037
PVPAAPK;MDLSGVKKK	NCP	174.234
MDLSGVKKK;KPHGNVP	NCP	8.98
KPHGNVP;AYLEKLRQR	NCP	10.094
AYLEKLRQR;FKLLKVKR	NCP	94.995
FKLLKVKR;DVGFLPP	NCP	3.039
DVGFLPP;PAATSPSPK	NCP	31.209
PAATSPSPK;VGGLLLSA	NCP	0.399
VGGLLLSA;FCEAAFF	NCP	10.84
FCEAAFF;FLGASLKDEVLK	NCP	105.737
FLGASLKDEVLK;THTRVSVQR	NCP	1.179
THTRVSVQR;PALKP	NCP	19.821
PALKP;AASKK	NCP	22.875
AASKK;VPVTVH	NCP	2.379
VPVTVH;KGTVP	NCP	46.144
KGTVP;LSAANYF	NCP	60.525
LSAANYF;FGRLVKY	NCP	17.27
FGRLVKY;NVKHELQ	NCP	98.878
NVKHELQ;PPPPLHLK	NCP	19.116
PPPPLHLK;RAPPPLP	NCP	16.949
RAPPPLP;VDWCSAP	NCP	14.202
VDWCSAP;SLGPPGQP	NCP	1.377
SLGPPGQP;SGLSPPR	NCP	180.045
SGLSPPR;FGTLTH	NCP	43.113
FGTLTH;ATLLLL	NCP	115.834
ATLLLL;GPADDE	NCP	27.392
GPADDE;RDPPPR	NCP	26.1
RDPPPR;RPPPVSER	NCP	51.435
RPPPVSER;GTRPSAGM	NCP	19.647
GTRPSAGM;LLAKTLHG	NCP	96.22
LLAKTLHG;QPTAPP	NCP	127.311
QPTAPP;GYCPQ	NCP	7.022
GYCPQ;LSVPEC	NCP	0.754
LSVPEC;PAWGP	NCP	0.956
PAWGP;PRPPPAPP	NCP	23.283
PRPPPAPP;TDLHPR	NCP	13.968
TDLHPR;LPGTPRP	NCP	14.596
LPGTPRP;PVLRAA	NCP	2.586
PVLRAA;MDSRLPY	NCP	100.56
MDSRLPY;LGRAGP	NCP	30.566
LGRAGP;PPLLP	NCP	7.719
PPLLP;LGTRTPK	NCP	25.557
LGTRTPK;KPHAVAV	NCP	74.697
KPHAVAV;QGLWR	NCP	10.327
QGLWR;HGPPLAPR	NCP	35.621
HGPPLAPR;MVSEF	NCP	76.878

MVSEF;KEDFGFY	NCP	113.334
KEDFGFY;KYCSPPGP	NCP	43.45
KYCSPPGP;KPPTGHR	NCP	186.435
KPPTGHR;AAGPF	NCP	16.313
AAGPF;LGNKCVP	NCP	100.312
LGNKCVP;PPPPKGGAP	NCP	11.852
PPPPKGGAP;DAPDNG	NCP	22.161
DAPDNG;CMLLKR	NCP	72.646
CMLLKR;APAPRGPPSY	NCP	2.225
APAPRGPPSY;VSTSWKP	NCP	8.508
VSTSWKP;VGVFL	NCP	75.75
VGVFL;KTAPLT	NCP	18.735
KTAPLT;AAAGKP	NCP	18.12
AAAGKP;TSPKPE	NCP	1.134
TSPKPE;GPAVHPK	NCP	1.185
GPAVHPK;KGACLVH	NCP	38.677
KGACLVH;YEVSHKK	NCP	13.656
YEVSHKK;DVRAHESL	NCP	28.446
DVRAHESL;HPAPL	NCP	9.659
HPAPL;AAAEAGKP	NCP	35.436
AAAEAGKP;LVPVPQ	NCP	40.809
LVPVPQ;HVVFLV	NCP	22.119
HVVFLV;GSPLPRPK	NCP	29.293
GSPLPRPK;PAVGHPK	NCP	62.81
PAVGHPK;APPQPVLP	NCP	16.167
APPQPVLP;SVNLNYPK	NCP	3.432
SVNLNYPK;AHTGRPP	NCP	16.218
AHTGRPP;VGPMPPPP	NCP	42.267
VGPMPPPP;MRRLQ	NCP	1.227
MRRLQ;QLVAKDL	NCP	60.034
QLVAKDL;ASLMALGP	NCP	46.629
ASLMALGP;GVHRSPPR	NCP	31.856
GVHRSPPR;TAEVLGGK	NCP	11.551
TAEVLGGK;FDVTVPR	NCP	33.8
FDVTVPR;DLRRGAAP	NCP	9.957
DLRRGAAP;VTLEAVGR	NCP	7.468
VTLEAVGR;DGFAQGR	NCP	8.928
DGFAQGR;KGDLPGM	NCP	36.248
KGDLPGM;DRHLLP	NCP	6.877
DRHLLP;ALVGVGGK	NCP	120.44
ALVGVGGK;HVAQP	NCP	7.581
HVAQP;SPVTVGPQ	NCP	14.223
SPVTVGPQ;PQNLTVPLP	NCP	24.111
PQNLTVPLP;AVWPLR	NCP	36.951
AVWPLR;SLLFP	NCP	0.256
SLLFP;CASRLYTL	NCP	1.385
CASRLYTL;EDDPK	NCP	2.817
EDDPK;VGPPPP	NCP	141.532
VGPPPP;SSTPKKAP	NCP	12.165
SSTPKKAP;KTGPVQP	NCP	2.475
KTGPVQP;ALLLY	NCP	68.345

ALLLY;KVLPVR	NCP	14.338
KVLPVR;RAGGGPLR	NCP	6.914
RAGGGPLR;GPPPPQGP	NCP	7.554
GPPPPQGP;VTLGVSK	NCP	66.205
VTLGVSK;SPQRKP	NCP	0.761
SPQRKP;SDDKGPPP	NCP	73.662
SDDKGPPP;GTVFPP	NCP	105.459
GTVFPP;EMLRLQ	NCP	8.199
EMLRLQ;TGVRLT	NCP	70.198
TGVRLT;QKEPQPY	NCP	22.443
QKEPQPY;LKTEL	NCP	69.978
LKTEL;NEEVVELK	NCP	153.359
NEEVVELK;SLPPHMPF	NCP	37.2
SLPPHMPF;HGSDP	NCP	29.794
HGSDP;TDVSLLQKQ	NCP	39.384
TDVSLLQKQ;PPPKGPQP	NCP	146.106
PPPKGPQP;PLLKGLAK	NCP	53.171
PLLKGLAK;LPPLG	NCP	98.169
LPPLG;KGTTATLH	NCP	119.514
KGTTATLH;AKLLPP	NCP	16.708
AKLLPP;ELSPPGAP	NCP	10.515
ELSPPGAP;VGRLSSPP	NCP	7.248
VGRLSSPP;LSSSLLD	NCP	63.669
LSSSLLD;VPHQGPP	NCP	40.88
VPHQGPP;KPSGGP	NCP	51.009
KPSGGP;REVKPPP	NCP	102.318
REVKPPP;GVLVTLLH	NCP	15.496
GVLVTLLH;LSLQLT	NCP	19.415
LSLQLT;AGEEVFR	NCP	22.68
AGEEVFR;QLHVETGP	NCP	9.15
QLHVETGP;THPGL	NCP	45.669
THPGL;ASLLL	NCP	35.301
ASLLL;VGTGALVR	NCP	4.947
VGTGALVR;DLTRF	NCP	148.113
DLTRF;FPGGPG	NCP	80.125
FPGGPG;RSLAMK	NCP	34.449
RSLAMK;LPALRP	NCP	39.171
LPALRP;KPLEHPF	NCP	156.578
KPLEHPF;TSKYG	NCP	88.74
TSKYG;GVPSPN	NCP	20.656
GVPSPN;HKPTGAP	NCP	52.728
HKPTGAP;MNSKMP	NCP	32.327
MNSKMP;PGFKPPP	NCP	33.87
PGFKPPP;PVEAL	NCP	22.171
PVEAL;TWHGSPK	NCP	173.789
TWHGSPK;VVLVSRVL	NCP	40.225
VVLVSRVL;KEAML	NCP	14.982
KEAML;GLGLP	NCP	92.678
GLGLP;LPPAAGLP	NCP	158.262
LPPAAGLP;FEYLLT	NCP	25.567
FEYLLT;CTRFLSF	NCP	99.245

CTRFLSF;LLALVPHN	NCP	47.614
LLALVPHN;DPVPEVR	NCP	19.475
DPVPEVR;PRHPPPP	NCP	15.135
PRHPPPP;KEALS	NCP	3.885
KEALS;GSPEELRP	NCP	2.739
GSPEELRP;PPPPKGAK	NCP	20.419
PPPPKGAK;QKETVLGK	NCP	5.207
QKETVLGK;VPVLKK	NCP	89.947
VPVLKK;VSVGKLET	NCP	24.624
VSVGKLET;LPLVVC	NCP	122.018
LPLVVC;LATPRPK	NCP	0.708
LATPRPK;VAQLPL	NCP	10.263
VAQLPL;EGVVF	NCP	6.903
EGVVF;ASPLPGL	NCP	48.541
ASPLPGL;HALLLR	NCP	25.7
HALLLR;LYEGY	NCP	19.75
LYEGY;NSTKPR	NCP	12.59
NSTKPR;ELGGTPL	NCP	27.186
ELGGTPL;PVVLKLPV	NCP	131.668
PVVLKLPV;TGLQKTGSK	NCP	80.226
TGLQKTGSK;KPPHVAV	NCP	11.037
KPPHVAV;VGPPPPP	NCP	60.84
VGPPPPP;VGPPSK	NCP	12.95
VGPPSK;LQGPVG	NCP	57.201
LQGPVG;KSFFPK	NCP	75.931
KSFFPK;NCDLDLR	NCP	49.74
NCDLDLR;TGVELKP	NCP	7.373
TGVELKP;PSYSS	NCP	84.708
PSYSS;LELLDT	NCP	85.641
LELLDT;KYWQPAPP	NCP	127.263
KYWQPAPP;QVLPP	NCP	72.778
QVLPP;VHDAKGK	NCP	44.399
VHDAKGK;APKGVHP	NCP	30.975
APKGVHP;PPLPPPPG	NCP	41.301
PPLPPPPG;KPMPQ	NCP	74.619
KPMPQ;GPAAFPPP	NCP	2.551
GPAAFPPP;TVEAPPK	NCP	9.09
TVEAPPK;VGPAPP	NCP	56.315
VGPAPP;KGPRLLPP	NCP	14.914
KGPRLLPP;AAPLL	NCP	28.053
AAPLL;DPVLSSPP	NCP	4.469
DPVLSSPP;KFQTPPPPP	NCP	15.67
KFQTPPPPP;NFCCC	NCP	83.471
NFCCC;KGPQTT	NCP	54.163
KGPQTT;PDLGL	NCP	4.941
PDLGL;RAHAPR	NCP	180.225
RAHAPR;VYWKAVM	NCP	6.647
VYWKAVM;VGPGLGDGER	NCP	84.006
VGPGLGDGER;LSRQPR	NCP	46.869
LSRQPR;RGLLPVT	NCP	16.797
RGLLPVT;KVGVPLRL	NCP	52.444

KVGVPLRL;SRMPPPPP	NCP	4.637
SRMPPPPP;PPNTKP	NCP	64.798
PPNTKP;AASLLLR	NCP	42.002
AASLLLR;MKPLR	NCP	59.466
MKPLR;KDYPPLL	NCP	213.985
KDYPPLL;KLRAEAAV	NCP	43.355
KLRAEAAV;LARPRP	NCP	13.272
LARPRP;AFVKGDLR	NCP	33.825
AFVKGDLR;MGKGP	NCP	163.843
MGKGP;LGAGALVSL	NCP	85.865
LGAGALVSL;QPPPRP	NCP	95.752
QPPPRP;SLLHPPVD	NCP	15.725
SLLHPPVD;EDLLLP	NCP	15.993
EDLLLP;KGPKPP	NCP	48.997
KGPKPP;KDCMVR	NCP	46.986
KDCMVR;RKTVWWW	NCP	13.016
RKTVWWW;KDAVPSPP	NCP	18.388
KDAVPSPP;LAVHY	NCP	32.355
LAVHY;PPPGPGGH	NCP	57.035
PPPGPGGH;LGSSFPF	NCP	39.249
LGSSFPF;PQNLVTPPL	NCP	9.474
PQNLVTPPL;LHSPPPP	NCP	1.932
LHSPPPP;VTQPQ	NCP	17.298
VTQPQ;WAAPKPK	NCP	38.716
WAAPKPK;VDREQLVQK	NCP	101.315
VDREQLVQK;RYLAEVATGEK	NCP	21.303
RYLAEVATGEK;ALNYSVFY	NCP	0.132
ALNYSVFY;APEQACHLAK	NCP	0.039
APEQACHLAK;KALAQAPQ	NCP	34.887
KALAQAPQ;GTPGF	NCP	23.323
GTPGF;LPGAAPGPQ	NCP	37.241
LPGAAPGPQ;VPFPLR	NCP	95.968
VPFPLR;LQGLL	NCP	127.65
LQGLL;KGSSLNR	NCP	18.041
KGSSLNR;KDLEH	NCP	119.229
KDLEH;VNVPKVK	NCP	10.33
VNVPKVK;PNLLSK	NCP	40.224
PNLLSK;LSVHLAL	NCP	7.085
LSVHLAL;DTPELLR	NCP	82.243
DTPELLR;KEVMK	NCP	62.862
KEVMK;ESTSASPR	NCP	166.121
ESTSASPR;QMAVVPS	NCP	115.638
QMAVVPS;QARPKGP	NCP	31.659
QARPKGP;LLLSPN	NCP	6.115
LLLSPN;ALHLPF	NCP	4.712
ALHLPF;SLGAP	NCP	135.247
SLGAP;ASPQAP	NCP	12.332
ASPQAP;LALSA	NCP	6.42
LALSA;ADFDTYDDRA	NCP	14.328
ADFDTYDDRA;EEVVHKEQE	NCP	19.296
EEVVHKEQE;LPRGPVPAP	NCP	8.547

LPRGPVPAP;QGLPSH	NCP	126.825
QGLPSH;LCPDGLK	NCP	173.523
LCPDGLK;LPKAHACP	NCP	21.793
LPKAHACP;HSTTEVTQPR	NCP	69.947
HSTTEVTQPR;VTVNPMLL	NCP	19.936
VTVNPMLL;AAEPKM	NCP	504.971
AAEPKM;KQMALK	NCP	15.993
KQMALK;KVGAP	NCP	46.08
KVGAP;LTPTP	NCP	14.607
LTPTP;VLDLPKTN	NCP	2.296
VLDLPKTN;AASSFY	NCP	57.089
AASSFY;FLGRLP	NCP	54.235
FLGRLP;PPMAK	NCP	15.639
PPMAK;YGELQ	NCP	51.852
YGELQ;TCFSPK	NCP	52.632
TCFSPK;EVACRER	NCP	20.72
EVACRER;KDGTEFK	NCP	10.515
KDGTEFK;LASVRGQA	NCP	29.494
LASVRGQA;SVQPHRP	NCP	109.025
SVQPHRP;GLPPKYP	NCP	46.668
GLPPKYP;LLLQLPPNP	NCP	2.913
LLLQLPPNP;QGLPALPPL	NCP	89.142
QGLPALPPL;RLEQR	NCP	49.227
RLEQR;PFSLLR	NCP	57.792
PFSLLR;MYMLVK	NCP	25.543
MYMLVK;LPPPPRKTP	NCP	24.054
LPPPPRKTP;LDLFKKGT	NCP	65.52
LDLFKKGT;PFASHL	NCP	194.337
PFASHL;ADDDC	NCP	26.823
ADDDC;KSTAPRP	NCP	51.869
KSTAPRP;NEELLK	NCP	61.306
NEELLK;AADFLQR	NCP	35.909
AADFLQR;VGGALY	NCP	236.215
VGGALY;ALSSNAR	NCP	10.713
ALSSNAR;AHVKWVP	NCP	74.192
AHVKWVP;PGSFVP	NCP	58.713
PGSFVP;DEPLLAH	NCP	99.382
DEPLLAH;QWLLLDH	NCP	52.325
QWLLLDH;AARAVLLL	NCP	6.615
AARAVLLL;DKNLSSK	NCP	187.837
DKNLSSK;NAPQALR	NCP	28.581
NAPQALR;FLSFPTKT	NCP	18.24
FLSFPTKT;VVETLRR	NCP	21.336
VVETLRR;YSMAL	NCP	27.582
YSMAL;PTLAKDLP	NCP	57.929
PTLAKDLP;LKYVVPSP	NCP	180.295
LKYVVPSP;VGLTVDK	NCP	37.563
VGLTVDK;LLRYCL	NCP	4.041
LLRYCL;LAPCKL	NCP	69.692
LAPCKL;LEPLDEY	NCP	8.467
LEPLDEY;DLKRLDP	NCP	69.821

DLKRLDP;KQGKPVP	NCP	45.78
KQGKPVP;KNLLPK	NCP	90.402
KNLLPK;APRSLLPA	NCP	64.324
APRSLLPA;RLPSPFL	NCP	49.754
RLPSPFL;EDVKLAP	NCP	48.322
EDVKLAP;MTLLLAA	NCP	100.635
MTLLLAA;VVVPPKF	NCP	134.009
VVVPPKF;SPEAASN	NCP	35.656
SPEAASN;GGPLV	NCP	13.91
GGPLV;PPREVGP	NCP	66.492
PPREVGP;LPPHVAK	NCP	78.399
LPPHVAK;AKRLAK	NCP	73.881
AKRLAK;CMMEGP	NCP	125.062
CMMEGP;GVDSF	NCP	55.1
GVDSF;VDLARSH	NCP	12.225
VDLARSH;LGPSH	NCP	163.213
LGPSH;LRGDPG	NCP	13.443
LRGDPG;SFVNSEFLKPEVKS	NCP	104.172
SFVNSEFLKPEVKS;MGFLPR	NCP	74.967
MGFLPR;LQPGTP	NCP	66.213
LQPGTP;EGPRPVVL	NCP	17.496
EGPRPVVL;LHATLKPP	NCP	9.986
LHATLKPP;KVVMHAY	NCP	133.762
KVVMHAY;SPPHPAP	NCP	9.506
SPPHPAP;HRVELLSK	NCP	54.672
HRVELLSK;RVLPLLKP	NCP	14.442
RVLPLLKP;FYHRFHQ	NCP	47.244
FYHRFHQ;LYRPGT	NCP	26.523
LYRPGT;DKFDCP	NCP	57.808
DKFDCP;RAQQTESPPHASPK	NCP	36.896
RAQQTESPPHASPK;LLLDEL	NCP	108.778
LLLDEL;VPVSL	NCP	36.678
VPVSL;TVWSM	NCP	6.477
TVWSM;SLAFTP	NCP	2.987
SLAFTP;MEVVLK	NCP	77.809
MEVVLK;VELLGALEE	NCP	53.408
VELLGALEE;PVVPPGR	NCP	66.94
PVVPPGR;CDLESGSVKVKEPSN	NCP	23.715
CDLESGSVKVKEPSN;LGFYVH	NCP	5.736
LGFYVH;YEAAPH	NCP	16.052
YEAAPH;LPTGYEKP	NCP	103.056
LPTGYEKP;SHGAAHK	NCP	1.8
SHGAAHK;AAPLRF	NCP	11.605
AAPLRF;LVRVFLA	NCP	67.106
LVRVFLA;EPAGVPGR	NCP	100.194
EPAGVPGR;GPPGSPPR	NCP	12.579
GPPGSPPR;KGDPL	NCP	26.626
KGDPL;KAWMYG	NCP	77.148
KAWMYG;KSGTAQR	NCP	84.128
KSGTAQR;PALPLS	NCP	70.012
PALPLS;PKLVHR	NCP	5.634

PKLVHR;VLLKVTQL	NCP	11.268
VLLKVTQL;SLEEKPY	NCP	110.732
SLEEKPY;LDAMP	NCP	16.695
LDAMP;APKRSPAP	NCP	0.054
APKRSPAP;KLKPSNE	NCP	38.107
KLKPSNE;CCLTV	NCP	35.43
CCLTV;NACLRL	NCP	72.396
NACLRL;ALVAL	NCP	150.413
ALVAL;HDCLL	NCP	9.184
HDCLL;QGVAP	NCP	38.03
QGVAP;LPNASAPLP	NCP	50.454
LPNASAPLP;VGPTLPP	NCP	30.591
VGPTLPP;RVPHNAAVQVY	NCP	54.456
RVPHNAAVQVY;ASSPRASK	NCP	130.989
ASSPRASK;AQRKPEGSSFH	NCP	62.007
AQRKPEGSSFH;PHQYPALTPEQKKEL	NCP	0.354
PHQYPALTPEQKKEL;KENLKAAQEEYVK	NCP	2.634
KENLKAAQEEYVK;KKHVPTPP	NCP	53.487
KKHVPTPP;SLQELDTSSGVLLPF	NCP	23.586
SLQELDTSSGVLLPF;SLVPVDQ	NCP	6.867
SLVPVDQ;YQEGDLEKCQRAP	NCP	3.531
YQEGDLEKCQRAP;LRVPFLEF	NCP	0.045
LRVPFLEF;LKDTPAPRLLK	NCP	0.144
LKDTPAPRLLK;KDTPAPRLLKT	NCP	0.003
KDTPAPRLLKT;KDVAVSYYHF	NCP	0.624
KDVAVSYYHF;TSFKEMKK	NCP	0.9
TSFKEMKK;ELFEAHYAEKMAGCKL	NCP	0.264
ELFEAHYAEKMAGCKL;TVYLT	NCP	70.639
TVYLT;SGEELVTKP	NCP	24.518
SGEELVTKP;MEAAHSKTTEE	NCP	82.26
MEAAHSKTTEE;AWDLPVLP	NCP	39.864
AWDLPVLP;CGKPPLLAL	NCP	11.671
CGKPPLLAL;SLVHRK	NCP	162.3
SLVHRK;LDRPAP	NCP	117.894
LDRPAP;DYPPQRP	NCP	37.383
DYPPQRP;PHGGSKR	NCP	25.461
PHGGSKR;KGAKSSK	NCP	75.714
KGAKSSK;SQVPPPP	NCP	28.797
SQVPPPP;KLGAKPP	NCP	1.149
KLGAKPP;NLFKAGP	NCP	67.952
NLFKAGP;FLLLR	NCP	99.372
FLLLR;KMRSL	NCP	201.097
KMRSL;SSFYAH	NCP	33.983
SSFYAH;PVSELLTK	NCP	94.237
PVSELLTK;VPPPPPPP	NCP	114.51
VPPPPPPP;PAGSGPRP	NCP	0.588
PAGSGPRP;ANPLVG	NCP	34.23
ANPLVG;KHHDEVK	NCP	48.792
KHHDEVK;AAAVFF	NCP	10.935
AAAVFF;EVMPPFL	NCP	67.532
EVMPPFL;KGVFAHLK	NCP	293.737

KGVFAHLK;ALEPLLPK	NCP	25.065
ALEPLLPK;VSATLKPP	NCP	41.951
VSATLKPP;TSRFDSP	NCP	34.108
TSRFDSP;AAAAAAQGGGGGGEPR	NCP	31.764
AAAAAAQGGGGGGEPR;KAQGLVP	NCP	6.564
KAQGLVP;KYCLHLLQ	NCP	5.43
KYCLHLLQ;KTPLFN	NCP	103.343
KTPLFN;SALLEL	NCP	9.09
SALLEL;RPPSRPP	NCP	135.274
RPPSRPP;KRGFYPLP	NCP	28.08
KRGFYPLP;LPEFPR	NCP	98.514
LPEFPR;EPAPF	NCP	134.772
EPAPF;GSLPTKP	NCP	49.032
GSLPTKP;KSACDTK	NCP	158.153
KSACDTK;VLAGGY	NCP	11.473
VLAGGY;VREPVL	NCP	34.025
VREPVL;RLGWSL	NCP	168.343
RLGWSL;SLLERL	NCP	17.432
SLLERL;LSGQVP	NCP	57.46
LSGQVP;PVPGK	NCP	381.797
PVPGK;KDPAVFF	NCP	38.509
KDPAVFF;PSSLK	NCP	27.257
PSSLK;ECQMHD	NCP	146.772
ECQMHD;FPTTAGA	NCP	7.878
FPTTAGA;ALFPP	NCP	22.884
ALFPP;PPPPPQRPPP	NCP	75.834
PPPPPQRPPP;DLEEPK	NCP	42.201
DLEEPK;ATLEAVW	NCP	10.284
ATLEAVW;DGPLWRT	NCP	19.72
DGPLWRT;PAASFRP	NCP	1.326
PAASFRP;VVHLLF	NCP	40.394
VVHLLF;VLLRVY	NCP	64.549
VLLRVY;EDDSH	NCP	0.959
EDDSH;RPPPAAP	NCP	93.925
RPPPAAP;LVAPGGPR	NCP	37.887
LVAPGGPR;SDVLEL	NCP	12.237
SDVLEL;LPPGAYL	NCP	26.46
LPPGAYL;PNVMNYK	NCP	166.799
PNVMNYK;VHLLTH	NCP	7.939
VHLLTH;KGPPPP	NCP	17.022
KGPPPP;EVGAPPLP	NCP	0.497
EVGAPPLP;MEKSPPLL	NCP	11.853
MEKSPPLL;PESHNDP	NCP	106.083
PESHNDP;MHEAAPR	NCP	63.748
MHEAAPR;MDACHAK	NCP	104.186
MDACHAK;VLELP	NCP	67.026
VLELP;NFEQEKQ	NCP	0.667
NFEQEKQ;KVGCLK	NCP	109.853
KVGCLK;KELGK	NCP	13.048
KELGK;KNDKLGDL	NCP	7.316
KNDKLGDL;PHVSRPP	NCP	122.754

PHVSRPP;KEVEVLK	NCP	3.082
KEVEVLK;LDTRLR	NCP	121.925
LDTRLR;LLAASGDY	NCP	69.954
LLAASGDY;KHLSPP	NCP	55.69
KHLSPP;LLEFYDATAHFAK	NCP	5.501
LLEFYDATAHFAK;DATLTGF	NCP	24.61
DATLTGF;VMAKPHE	NCP	13.667
VMAKPHE;PHQVLYR	NCP	17.815
PHQVLYR;TPPAPTPGP	NCP	92.012
TPPAPTPGP;KKTDKP	NCP	40.875
KKTDKP;LTDVVLM	NCP	261.09
LTDVVLM;KDRPGLN	NCP	88.68
KDRPGLN;AGAHLMV	NCP	89.608
AGAHLMV;MDALAAP	NCP	17.637
MDALAAP;DVRPPGT	NCP	78.528
DVRPPGT;ATLGGLL	NCP	44.804
ATLGGLL;KYESPPQ	NCP	2.725
KYESPPQ;APQQH	NCP	66.722
APQQH;TPGNSF	NCP	3.753
TPGNSF;DDDLAPGPA	NCP	67.458
DDDLAPGPA;KPFLF	NCP	109.48
KPFLF;KSEAPEK	NCP	129.729
KSEAPEK;LLVPSP	NCP	63.707
LLVPSP;LMVEL	NCP	8.464
LMVEL;NFNLDC	NCP	48.27
NFNLDC;HLLGLLL	NCP	32.726
HLLGLLL;KGACLVHL	NCP	0.124
KGACLVHL;DGAATVP	NCP	6.14
DGAATVP;LYKMPEM	NCP	14.131
LYKMPEM;LNLNK	NCP	16.715
LNLNK;GHVLRF	NCP	22.609
GHVLRF;HGEGGGGK	NCP	76.161
HGEGGGGK;TQSKQ	NCP	33.093
TQSKQ;KGNNLKEK	NCP	20.643
KGNNLKEK;KPGAFYK	NCP	33.027
KPGAFYK;GNSPLR	NCP	194.513
GNSPLR;KSEQNLLR	NCP	97.621
KSEQNLLR;EEVGLF	NCP	7.563
EEVGLF;FGVPN	NCP	30.534
FGVPN;MWHMG	NCP	70.608
MWHMG;KLVYDSWSSGK	NCP	97.994
KLVYDSWSSGK;RYHVSGK	NCP	147.78
RYHVSGK;KEELL	NCP	5.397
KEELL;KDVAFMPP	NCP	31.947
KDVAFMPP;QMNDYYC	NCP	176.391
QMNDYYC;DLPAEPE	NCP	47.977
DLPAEPE;AKLLDLPF	NCP	171.125
AKLLDLPF;DGNVLLVL	NCP	20.086
DGNVLLVL;KPNEAGP	NCP	144.767
KPNEAGP;KQPFPK	NCP	3.672
KQPFPK;KPSPQEK	NCP	16.47

KPSPQEK;LNKRPP	NCP	99.214
LNKRPP;YGVLARN	NCP	223.865
YGVLARN;ANFLSPP	NCP	68.67
ANFLSPP;NLKGM	NCP	30.735
NLKGM;PRQPHP	NCP	15.463
PRQPHP;LEMELL	NCP	82.269
LEMELL;AQKELR	NCP	2.703
AQKELR;QPVHRP	NCP	9.849
QPVHRP;NPVTTYK	NCP	18.791
NPVTTYK;KMYQFH	NCP	35.794
KMYQFH;SNHTH	NCP	29.66
SNHTH;FGVGGK	NCP	31.414
FGVGGK;PLLFGPG	NCP	17.664
PLLFGPG;ETQTKGV	NCP	37.542
ETQTKGV;VPPPP	NCP	106.475
VPPPP;GFGFGQQ	NCP	45.684
GFGFGQQ;LSLVKSAGP	NCP	78.778
LSLVKSAGP;KLRVDPV	NCP	56.628
KLRVDPV;PPPHPTDP	NCP	29.388
PPPHPTDP;PPPPLPA	NCP	13.896
PPPPLPA;KGTMARP	NCP	20.303
KGTMARP;TAQLP	NCP	156.793
TAQLP;CSPGPK	NCP	94.47
CSPGPK;ASTSLGVT	NCP	67.664
ASTSLGVT;SDCDN	NCP	1.002
SDCDN;KHPFQPP	NCP	3.363
KHPFQPP;KPPLPAP	NCP	112.276
KPPLPAP;FFQGPPR	NCP	2.235
FFQGPPR;EGKDPLGE	NCP	10.272
EGKDPLGE;ETFEC	NCP	39.612
ETFEC;VSYYTVLK	NCP	235.146
VSYYTVLK;KPGAP	NCP	3.087
KPGAP;GKGFLAQ	NCP	4.947
GKGFLAQ;QVGDVLR	NCP	61.266
QVGDVLR;LGVGAGGF	NCP	180.714
LGVGAGGF;AKPAAKPK	NCP	45.152
AKPAAKPK;SRKFFR	NCP	15.154
SRKFFR;YAAAL	NCP	4.149
YAAAL;TQTPAMK	NCP	5.589
TQTPAMK;SRGAP	NCP	21.804
SRGAP;LGPFAV	NCP	2.853
LGPFAV;ETPAG	NCP	23.028
ETPAG;LSLGK	NCP	74.322
LSLGK;VHEPPPP	NCP	56.568
VHEPPPP;PFLSLR	NCP	76.752
PFLSLR;DGGPHLK	NCP	29.828
DGGPHLK;DPAVNPR	NCP	73.585
DPAVNPR;KRLLAQ	NCP	22.797
KRLLAQ;QELHGVP	NCP	56.702
QELHGVP;LAVFQK	NCP	0.933
LAVFQK;LYEAAVLT	NCP	57.454

LYEAAVLT;HYLSVDF	NCP	43.242
HYLSVDF;SGPLNF	NCP	62.805
SGPLNF;LLVTEK	NCP	11.259
LLVTEK;LELPF	NCP	54.855
LELPF;LPSPKPP	NCP	24.321
LPSPKPP;LTPWKSRL	NCP	98.658
LTPWKSRL;QGGYKPR	NCP	17.702
QGGYKPR;CMDEN	NCP	153.864
CMDEN;VSGHFVK	NCP	11.134
VSGHFVK;LGAPEV	NCP	5.712
LGAPEV;KKLGF	NCP	6.422
KKLGF;SPTPLEK	NCP	16.245
SPTPLEK;EVLALQLK	NCP	180.256
EVLALQLK;SPRPPRT	NCP	6.051
SPRPPRT;PGEPAP	NCP	0.813
PGEPAP;KPTNGVP	NCP	2.894
KPTNGVP;LTMNPGY	NCP	11.782
LTMNPGY;SSNQKLR	NCP	20.291
SSNQKLR;NKPHF	NCP	25.401
NKPHF;LPNPKEP	NCP	17.646
LPNPKEP;VTPKV	NCP	31.186
VTPKV;VGALLEPL	NCP	7.398
VGALLEPL;PEVSGPK	NCP	36.984
PEVSGPK;SPKVMHK	NCP	14.939
SPKVMHK;MPPHM	NCP	113.715
MPPHM;PKRSP	NCP	119.875
PKRSP;EDAACK	NCP	17.195
EDAACK;YGPMKS	NCP	24.381
YGPMKS;KDGSLPP	NCP	2.562
KDGSLPP;GHPPP	NCP	93.61
GHPPP;PKPPPPP	NCP	60.573
PKPPPPP;KPFPEK	NCP	19.206
KPFPEK;PAPPPPPP	NCP	122.31
PAPPPPPP;AQKDQLSEDEKFLF	NCP	50.924
AQKDQLSEDEKFLF;NNPVAQADWAAK	NCP	0.06
NNPVAQADWAAK;PVAQADWAAKK	NCP	0.006
PVAQADWAAKK;VAQADWAAK	NCP	0.003
VAQADWAAK;KLVWVPSEKQGF	NCP	0.027
KLVWVPSEKQGF;VENGKKVTVGK	NCP	0.087
VENGKKVTVGK;FCVVVNPYK	NCP	9.636
FCVVVNPYK;GVPQPDL	NCP	15.328
GVPQPDL;KAAGKLLE	NCP	9.042
KAAGKLLE;LAMVASSHKGKKDT	NCP	8.441
LAMVASSHKGKKDT;MVASSHKGKK	NCP	0.006
MVASSHKGKK;GVPLLK	NCP	21.717
GVPLLK;VGRDVVQK	NCP	1.995
VGRDVVQK;ATDKSFVEKL	NCP	3.66
ATDKSFVEKL;VEKLCTEQGSHPK	NCP	0.018
VEKLCTEQGSHPK;CTEQGSHPKFQKPK	NCP	0.012
CTEQGSHPKFQKPK;QLKDKTEF	NCP	0.042
QLKDKTEF;NASAWLTKNMDPLNDNVTSLLNASSDKF	NCP	1.557

NASAWLTKNMDPLNDNVTSLLNASSDKF;ASAWLTK	NCP	0.003
ASAWLTK;NMDPLNDNVTSLLNASSDKF	NCP	0.021
NMDPLNDNVTSLLNASSDKF;MDPLNDNVTSLLNASSDK	NCP	0.003
MDPLNDNVTSLLNASSDK;DPLNDNVTSL	NCP	0.003
DPLNDNVTSL;PLNDNVTSLLNASSDKFM	NCP	0.003
PLNDNVTSLLNASSDKFM;DNVTSLLNASSDKF	NCP	0.009
DNVTSLLNASSDKF;LNASSDKFM	NCP	0.018
LNASSDKFM;VGLDQMAK	NCP	2.682
VGLDQMAK;MTESSLPSASK	NCP	0.024
MTESSLPSASK;TESSLPSASKTKK	NCP	0.003
TESSLPSASKTKK;ESSLPSASKTKKG	NCP	0.003
ESSLPSASKTKKG;SSLPSASK	NCP	0.003
SSLPSASK;FRTVGQLYKEQLGK	NCP	0.039
FRTVGQLYKEQLGK;TVGQLYKEQLGKLM	NCP	0.006
TVGQLYKEQLGKLM;LYKEQLGK	NCP	0.012
LYKEQLGK;LMTTLRNTTPN	NCP	0.024
LMTTLRNTTPN;TTLRNTTPNFVR	NCP	0.006
TTLRNTTPNFVR;QAMCRGYLAR	NCP	2.283
QAMCRGYLAR;LQVTRQEEEMQA	NCP	3.894
LQVTRQEEEMQA;EDELQKTKERQQK	NCP	0.039
EDELQKTKERQQK;REQEVTMLK	NCP	7.26
REQEVTMLK;KLEVQLQELQSK	NCP	3.807
KLEVQLQELQSK;ASTVELLEEGKKKF	NCP	2.238
ASTVELLEEGKKKF;AEEKN	NCP	2.178
AEEKN;AEMEDLVSSK	NCP	0.159
AEMEDLVSSK;KKLEGDLK	NCP	0.894
KKLEGDLK;EALLQVEDERK	NCP	3.93
EALLQVEDERK;EVGSPGAK	NCP	91.464
EVGSPGAK;VVVLVL	NCP	4.332
VVVLVL;PPAGFQ	NCP	111.441
PPAGFQ;GPFPG	NCP	114.946
GPFPG;PTLTP	NCP	133.61
PTLTP;FGVPG	NCP	8.61
FGVPG;SLYVP	NCP	40.333
SLYVP;QRVAP	NCP	13.835
QRVAP;KPLLNQK	NCP	189.18
KPLLNQK;LSVGLGPK	NCP	2.295
LSVGLGPK;VTQLDLDGPK	NCP	29.5
VTQLDLDGPK;PPGVP	NCP	3.51
PPGVP;VGPPP	NCP	3.111
VGPPP;KHEHAAP	NCP	6.219
KHEHAAP;PKPDDGQVP	NCP	15.318
PKPDDGQVP;AALPLEK	NCP	64.887
AALPLEK;LTPGTTPL	NCP	9.801
LTPGTTPL;SEGPYLT	NCP	52.44
SEGPYLT;FPLLEV	NCP	39.663
FPLLEV;SSPKKLFPP	NCP	26.597
SSPKKLFPP;VLVLLLLLL	NCP	69.117
VLVLLLLLL;KPPEPTR	NCP	116.947
KPPEPTR;YGDDCE	NCP	30.366
YGDDCE;TPSLQAGP	NCP	67.305

TPSLQAGP;LDVDAPA	NCP	16.292
LDVDAPA;DALKK	NCP	272.133
DALKK;DMMCAC	NCP	10.936
DMMCAC;EGLPPAG	NCP	64.029
EGLPPAG;KAHKLE	NCP	18.578
KAHKLE;DLHFF	NCP	58.404
DLHFF;VTTAL	NCP	48.616
VTTAL;SGGSLSGY	NCP	151.194
SGGSLSGY;VGKGG	NCP	24.203
VGKGG;QRSPVNF	NCP	31.896
QRSPVNF;KDKLEFL	NCP	14.26
KDKLEFL;NRQPLNR	NCP	30.897
NRQPLNR;LSPLP	NCP	193.004
LSPLP;PKGHVVK	NCP	1.119
PKGHVVK;VSGGLDY	NCP	76.63
VSGGLDY;KLETVVKK	NCP	70.4
KLETVVKK;CDEWLKK	NCP	2.313
CDEWLKK;AGRSAVTL	NCP	28.714
AGRSAVTL;LGPVSAY	NCP	42.986
LGPVSAY;LVPAPP	NCP	80.245
LVPAPP;KGPLY	NCP	45.488
KGPLY;RVKPVK	NCP	54.726
RVKPVK;KDLVK	NCP	24.337
KDLVK;AERRVP	NCP	113.414
AERRVP;PVGTRP	NCP	38.794
PVGTRP;APAAGPRP	NCP	100.749
APAAGPRP;ATGSKP	NCP	55.199
ATGSKP;RVLPPR	NCP	12.016
RVLPPR;MAPALDR	NCP	6.978
MAPALDR;CCCQDAC	NCP	15.171
CCCQDAC;VCDLRPPG	NCP	18.503
VCDLRPPG;VGLVPD	NCP	149.505
VGLVPD;KPFGK	NCP	54.72
KPFGK;KSRVFH	NCP	66.027
KSRVFH;VHAAVQR	NCP	48.915
VHAAVQR;VLVGY	NCP	78.595
VLVGY;RGTLLR	NCP	48.611
RGTLLR;LPYRKPP	NCP	81.825
LPYRKPP;LTDFGR	NCP	22.428
LTDFGR;DMSLLHN	NCP	29.896
DMSLLHN;VVAPPGAPK	NCP	17.207
VVAPPGAPK;VGPTGF	NCP	17.539
VGPTGF;SLDAFVPL	NCP	26.616
SLDAFVPL;GPRDPTLP	NCP	225.539
GPRDPTLP;HPQTKFT	NCP	58.501
HPQTKFT;KPPGGQPK	NCP	5.06
KPPGGQPK;CSCPPPP	NCP	17.679
CSCPPPP;LVAPPNR	NCP	15.378
LVAPPNR;ASGRVR	NCP	118.024
ASGRVR;TLLGRCPL	NCP	78.429
TLLGRCPL;LSKRRGVP	NCP	0.956

LSKRRGVP;HGCAKAA	NCP	45.603
HGCAKAA;ERVAPVP	NCP	23.758
ERVAPVP;SPPGQQGSGQQGETEHPDQAR	NCP	8.219
SPPGQQGSGQQGETEHPDQAR;MDTKTQSLPNTHAQPHSN	NCP	6.102
MDTKTQSLPNTHAQPHSN;ARGKPWPK	NCP	136.125
ARGKPWPK;GPRALGPP	NCP	53.149
GPRALGPP;NGVLY	NCP	23.525
NGVLY;GPPGPAP	NCP	96.414
GPPGPAP;QGVAFPR	NCP	87.853
QGVAFPR;PTAAGF	NCP	64.914
PTAAGF;NACRLL	NCP	18.285
NACRLL;KAPHPR	NCP	275.946
KAPHPR;AAPERPP	NCP	304.644
AAPERPP;APWALQ	NCP	13.056
APWALQ;MLGAPV	NCP	13.733
MLGAPV;TPKVQQPP	NCP	3.723
TPKVQQPP;KAASRQP	NCP	0.55
KAASRQP;LPTASP	NCP	8.682
LPTASP;ARSHVAT	NCP	23.342
ARSHVAT;LARAVP	NCP	92.884
LARAVP;PVGSLGP	NCP	16.824
PVGSLGP;FGSAPPPPP	NCP	2.865
FGSAPPPPP;SEPALPR	NCP	13.194
SEPALPR;GPHLVPAP	NCP	61.008
GPHLVPAP;GDPMGVAQ	NCP	49.175
GDPMGVAQ;VNFKLLSH	NCP	17.32
VNFKLLSH;SGVVLPGP	NCP	31.719
SGVVLPGP;EVRLLDT	NCP	124.347
EVRLLDT;LHLKPP	NCP	5.016
LHLKPP;PGATSTHR	NCP	98.076
PGATSTHR;KPMGRP	NCP	43.671
KPMGRP;NLFGPLGL	NCP	18.536
NLFGPLGL;KTFKQ	NCP	79.501
KTFKQ;WTPAP	NCP	16.703
WTPAP;KTTGGP	NCP	88.693
KTTGGP;LWAPKER	NCP	149.495
LWAPKER;LRVDPVNF	NCP	91.75
LRVDPVNF;RVDPVNFK	NCP	0.003
RVDPVNFK;RSSGPSLVH	NCP	1.85
RSSGPSLVH;LRVDPVN	NCP	12.142
LRVDPVN;RVDPVNF	NCP	0.003
RVDPVNF;VDPVNFK	NCP	0.003
VDPVNFK;VLSAADKGNVKAAWGKVGGHAAEY	NCP	3.351
VLSAADKGNVKAAWGKVGGHAAEY;AADKGNVKAAWGKVG	NCP	0.009
AADKGNVKAAWGKVG;AAWGKVGGHAAEY	NCP	0.024
AAWGKVGGHAAEY;AWGKVGGHA	NCP	0.003
AWGKVGGHA;VGGHAAEYGAEALER	NCP	0.012
VGGHAAEYGAEALER;TYFPHFDLSQGSAQVKGHGAK	NCP	0.171
TYFPHFDLSQGSAQVKGHGAK;FDLSQGSAQVK	NCP	0.015
FDLSQGSAQVK;SQGSAQVKGHGAKVA	NCP	0.009
SQGSAQVKGHGAKVA;KVAAALTKAVE	NCP	0.036

KVAAALTKAVE;VAAALTKAVEH	NCP	0.003
VAAALTKAVEH;AAALTKAVEHL	NCP	0.003
AAALTKAVEHL;AALTKAVEHLD	NCP	0.003
AALTKAVEHLD;ALTKAVEHL	NCP	0.003
ALTKAVEHL;VEHLDDLPGALSELSDLHAHKL	NCP	0.015
VEHLDDLPGALSELSDLHAHKL;EHLDDLPGALSELSDLH	NCP	0.003
EHLDDLPGALSELSDLHAHK;ASLDKFLANVST	NCP	0.261
ASLDKFLANVST;SLDKFLANVSTVLTSKY	NCP	0.003
SLDKFLANVSTVLTSKY;KFLANVSTVLTSKYR	NCP	0.009
KFLANVSTVLTSKYR;LANVSTVLTSK	NCP	0.006
LANVSTVLTSK;ANVSTVLTSKYR	NCP	0.003
ANVSTVLTSKYR;NVSTVLTSK	NCP	0.003
NVSTVLTSK;VSTVLTSKYR	NCP	0.003
VSTVLTSKYR;STVLTSKY	NCP	0.003
STVLTSKY;TVLTSKYR	NCP	0.003
TVLTSKYR;MFLSFPTTK	NCP	2.637
MFLSFPTTK;FLSFPTTKT	NCP	0.003
FLSFPTTKT;LSFPTTKTYFPHFDLSHGSAQVK	NCP	0.003
LSFPTTKTYFPHFDLSHGSAQVK;FPTTKTYFPH	NCP	0.006
FPTTKTYFPH;PTTKTYFPHFDLS	NCP	0.003
PTTKTYFPHFDLS;TTKTYFPH	NCP	0.003
TTKTYFPH;TYFPHFDLSHGSAQVK	NCP	0.009
TYFPHFDLSHGSAQVK;FDLSHGSAQVKGHGAK	NCP	0.015
FDLSHGSAQVKGHGAK;VKGHGAKVAAALTK	NCP	0.027
VKGHGAKVAAALTK;KGHGAKVAAALT	NCP	0.003
KGHGAKVAAALT;GHGAKVAAALTK	NCP	0.003
GHGAKVAAALTK;KVAAALTKAV	NCP	0.012
KVAAALTKAV;VAAALTKAVGH	NCP	0.003
VAAALTKAVGH;AAALTKAVG	NCP	0.003
AAALTKAVG;AALTKAVGHLDDLPGAL	NCP	0.003
AALTKAVGHLDDLPGAL;AVGHLDDLPGALSELSDLHAHK	NCP	0.015
AVGHLDDLPGALSELSDLHAHKL;VGHLDDLPGALSE	NCP	0.003
VGHLDDLPGALSE;GHLDDLPGALSELSDLHAHKL	NCP	0.003
GHLDDLPGALSELSDLHAHKL;HLDDLPGALSELSDLHAH	NCP	0.003
HLDDLPGALSELSDLHAHK;LDDLPGALSELSDLHAHKL	NCP	0.003
LDDLPGALSELSDLHAHKL;DDLPGALSELSDLHAHK	NCP	0.003
DDLPGALSELSDLHAHK;DLPGALSELSDLHAHKL	NCP	0.003
DLPGALSELSDLHAHKL;PGALSELSDLHAH	NCP	0.006
PGALSELSDLHAH;LSELSDLHAHKL	NCP	0.009
LSELSDLHAHKL;SELSDLHAH	NCP	0.003
SELSDLHAH;LSDLHAHKL	NCP	0.006
LSDLHAHKL;SDLHAHKLR	NCP	0.003
SDLHAHKLR;KLRVDPVNFK	NCP	0.018
KLRVDPVNFK;LPSFP	NCP	24.162
LPSFP;KGSLENF	NCP	12.852
KGSLENF;DPPKAHGK	NCP	2.849
DPPKAHGK;ELGSGVKQ	NCP	63.756
ELGSGVKQ;AAPGGRVP	NCP	20.587
AAPGGRVP;AAHLP	NCP	20.751
AAHLP;SSEPPPPSQPPTHQS	NCP	84.095
SSEPPPPSQPPTHQS;HETWSGHVV	NCP	3.099

HETWSGHVV;SCACPPAPP	NCP	5.391
SCACPPAPP;PPPVPPP	NCP	110.38
PPPVPPP;KPWPPPR	NCP	12.624
KPWPPPR;PDWGGGK	NCP	38.006
PDWGGGK;AGLRPPSV	NCP	105.142
AGLRPPSV;PGAQPP	NCP	3.807
PGAQPP;PARGNLGH	NCP	181.407
PARGNLGH;LCLGCG	NCP	31.494
LCLGCG;FRDDPP	NCP	3.705
FRDDPP;APGGSTPK	NCP	22.215
APGGSTPK;NATPKPK	NCP	151.482
NATPKPK;PLEPQ	NCP	74.673
PLEPQ;LGVDTPAP	NCP	37.971
LGVDTPAP;LLVLSQL	NCP	45.143
LLVLSQL;PAVGALVL	NCP	55.834
PAVGALVL;REPLPSK	NCP	84.704
REPLPSK;GPLPPLAT	NCP	148.555
GPLPPLAT;KNKATY	NCP	60.738
KNKATY;CGCYEP	NCP	90.506
CGCYEP;YRALVS	NCP	81.781
YRALVS;LLLVTE	NCP	52.554
LLLVTE;VVPGWF	NCP	19.775
VVPGWF;ALKLLQSEL	NCP	262.392
ALKLLQSEL;YPLLVT	NCP	33.385
YPLLVT;NEPLPPP	NCP	103.427
NEPLPPP;NAGPP	NCP	9.225
NAGPP;KEPLFG	NCP	424.179
KEPLFG;ATATGTK	NCP	15.514
ATATGTK;TVYVYK	NCP	79.782
TVYVYK;RGPAGGKK	NCP	6.455
RGPAGGKK;TEQAHLP	NCP	8.478
TEQAHLP;KTTLLLVK	NCP	27.465
KTTLLLVK;KQPGNLK	NCP	44.785
KQPGNLK;SSGKFG	NCP	168.308
SSGKFG;KDSPLTVP	NCP	40.696
KDSPLTVP;AAAPPPPVPPPPP	NCP	51.96
AAAPPPPVPPPPP;AVPPGKM	NCP	81.332
AVPPGKM;VGGFDP	NCP	19.006
VGGFDP;HLRAFH	NCP	63.75
HLRAFH;EGPPAFNPPVP	NCP	41.718
EGPPAFNPPVP;AQERLL	NCP	21.609
AQERLL;APPPGVGR	NCP	94.371
APPPGVGR;AAPAVALP	NCP	93.596
AAPAVALP;PGKVGEPPP	NCP	76.677
PGKVGEPPP;TKPRPP	NCP	44.221
TKPRPP;KGSQP	NCP	176.082
KGSQP;KACEVGAP	NCP	54.314
GKLVP;KGGGLPV	NCP	38.401
KGGGLPV;AAPVMLH	NCP	180.131
AAPVMLH;LNRPKALN	NCP	139.221
LNRPKALN;NRPKALNAL	NCP	0.003

NRPKALNAL;EMTLAEGVK	NCP	4.443
EMTLAEGVK;RKEGMAAFVEKRK	NCP	2.691
RKEGMAAFVEKRK;FVEKRKANFKDQ	NCP	0.021
FVEKRKANFKDQ;TAGHFSR	NCP	11.67
TAGHFSR;TAQVLLRF	NCP	55.002
TAQVLLRF;AAPAPLP	NCP	131.799
AAPAPLP;SDLKLDYL	NCP	165.517
SDLKLDYL;RALGK	NCP	170.132
RALGK;MEPPPRGP	NCP	24.396
MEPPPRGP;YPKPPPPPP	NCP	121.218
YPKPPPPPP;LLDPRHK	NCP	31.719
LLDPRHK;KSGKQPR	NCP	14.248
KSGKQPR;PPRMPPP	NCP	11.45
PPRMPPP;MRELLTL	NCP	85.342
MRELLTL;AAGKLLR	NCP	21.392
AAGKLLR;KLKFGF	NCP	31.588
KLKFGF;AHLALF	NCP	29.471
AHLALF;APGGLLSQL	NCP	6.046
APGGLLSQL;QALLQFR	NCP	133.224
QALLQFR;TACHKR	NCP	21.558
TACHKR;SNKELF	NCP	15.798
SNKELF;EKQLDF	NCP	6.902
EKQLDF;LPGSPLL	NCP	40.782
LPGSPLL;SLPWVP	NCP	6.507
SLPWVP;ESHNRRP	NCP	121.614
ESHNRRP;LMLNPK	NCP	104.479
LMLNPK;KSKPLR	NCP	35.172
KSKPLR;QTWLSN	NCP	8.997
QTWLSN;TSPGAAHR	NCP	156.602
TSPGAAHR;VVLSDF	NCP	1.827
VVLSDF;LPGPSLL	NCP	1.794
LPGPSLL;LSVGGP	NCP	112.674
LSVGGP;ALLPPGYT	NCP	36.055
ALLPPGYT;LGAPPK	NCP	9.306
LGAPPK;VDVSGPK	NCP	8.9
VDVSGPK;RELYRNL	NCP	12.42
RELYRNL;PRGSTLVL	NCP	63.411
PRGSTLVL;VLHLPR	NCP	82.215
VLHLPR;ASLAPE	NCP	27.654
ASLAPE;AASVGAPP	NCP	45.201
AASVGAPP;LPSLGP	NCP	283.779
LPSLGP;VTPELPRP	NCP	126.486
VTPELPRP;TAPLP	NCP	37.479
TAPLP;LPDPGKPP	NCP	18.616
LPDPGKPP;MKLSPVL	NCP	10.988
MKLSPVL;DGVLGLR	NCP	195.984
DGVLGLR;KNQYSELE	NCP	120.421
KNQYSELE;SPAKPTPP	NCP	19.616
SPAKPTPP;QWPLRQQ	NCP	104.736
QWPLRQQ;RPGPTTT	NCP	59.061
RPGPTTT;FPSVAAR	NCP	84.261

FPSVAAR;VPPVVP	NCP	80.679
VPPVVP;MKTTF	NCP	293.302
MKTTF;LSDHQPK	NCP	350.226
LSDHQPK;VPSVPGLP	NCP	77.975
VPSVPGLP;KPGNWPR	NCP	95.052
KPGNWPR;LLLPGFLDF	NCP	74.65
LLLPGFLDF;SLKFF	NCP	58.179
SLKFF;TDPFPF	NCP	18.528
TDPFPF;KTLKPAK	NCP	72.794
KTLKPAK;PPKSYRK	NCP	6.846
PPKSYRK;KLVPH	NCP	35.052
KLVPH;HKSQPGPG	NCP	4.335
HKSQPGPG;KYLTMGP	NCP	9.051
KYLTMGP;GVTPL	NCP	24.19
GVTPL;NLVYNR	NCP	124.119
NLVYNR;YETLAAK	NCP	47.091
YETLAAK;LPALDD	NCP	151.892
LPALDD;QELRHVL	NCP	66.64
QELRHVL;FPDLDL	NCP	19.997
FPDLDL;DMMPCC	NCP	520.344
DMMPCC;KTVPVGR	NCP	89.299
KTVPVGR;KGPLHV	NCP	264.041
KGPLHV;DLANKFR	NCP	52.546
DLANKFR;EDKATNY	NCP	83.835
EDKATNY;RPKKDL	NCP	58.307
RPKKDL;NPGSVF	NCP	37.371
NPGSVF;KPVHTYK	NCP	8.401
KPVHTYK;PEDVLR	NCP	45.182
PEDVLR;DNLLHVLH	NCP	89.92
DNLLHVLH;ADSLLP	NCP	120.056
ADSLLP;GGFLFKHN	NCP	11.145
GGFLFKHN;SSAVQFQ	NCP	65.884
SSAVQFQ;KGHQFER	NCP	4.982
KGHQFER;AASPLSR	NCP	15.03
AASPLSR;YLKPPP	NCP	12.123
YLKPPP;LKKAYV	NCP	58.212
LKKAYV;AHNRPH	NCP	72.357
AHNRPH;NQKLVR	NCP	122.158
NQKLVR;DVALLPA	NCP	139.79
DVALLPA;MELLDTQ	NCP	61.305
MELLDTQ;LDFEHFLLL	NCP	13.8
LDFEHFLLL;PPYYKPPP	NCP	46.228
PPYYKPPP;AFANL	NCP	131.765
AFANL;EELRTLP	NCP	124.45
EELRTLP;KVEPY	NCP	35.732
KVEPY;FNHVF	NCP	15.648
FNHVF;MLPPP	NCP	62.418
MLPPP;KAALP	NCP	28.071
KAALP;TPSLRVTP	NCP	135.42
TPSLRVTP;SLPAVP	NCP	23.88
SLPAVP;GVSVLFL	NCP	21.708

GVSVLFL;FPPTF	NCP	171.162
FPPTF;AFSPLKR	NCP	26.001
AFSPLKR;EPKKEL	NCP	45.759
EPKKEL;KSCRPR	NCP	17.977
KSCRPR;KDFVSPE	NCP	21.797
KDFVSPE;LVLLD	NCP	11.1
LVLLD;AVDKKAAGAGKVTK	NCP	41.983
AVDKKAAGAGKVTK;GPPGPPA	NCP	15.881
GPPGPPA;KVGLGVPG	NCP	2.214
KVGLGVPG;FPEAG	NCP	76.533
FPEAG;GVAVP	NCP	30.687
GVAVP;VAPGPHL	NCP	4.02
VAPGPHL;VGPLK	NCP	1.89
VGPLK;QPKTLENP	NCP	54.532
QPKTLENP;KGGPPPPR	NCP	21.617
KGGPPPPR;LEAGFP	NCP	93.195
LEAGFP;KDQPF	NCP	110.656
KDQPF;KPGSFP	NCP	2.135
KPGSFP;LPAAEC	NCP	33.846
LPAAEC;KGDYPF	NCP	0.154
KGDYPF;PGRSTLVL	NCP	36.602
PGRSTLVL;TVVEPL	NCP	34.194
TVVEPL;DLGFGK	NCP	78.384
DLGFGK;KKQKLVE	NCP	55.468
KKQKLVE;EEYCN	NCP	17.693
EEYCN;PAAMNPP	NCP	94.17
PAAMNPP;PPPLP	NCP	135.441
PPPLP;AKLQPGK	NCP	157.059
AKLQPGK;KMLEVPP	NCP	36.562
KMLEVPP;KTLPAKR	NCP	225.483
KTLPAKR;RPDAKLR	NCP	11.16
RPDAKLR;TLETAP	NCP	49.208
TLETAP;DVKFA	NCP	45.981
DVKFA;SPGLFSP	NCP	332.491
SPGLFSP;PVSSLP	NCP	29.483
PVSSLP;PAYLR	NCP	40.086
PAYLR;KDAPP	NCP	11.577
KDAPP;VGGPL	NCP	4.908
VGGPL;TSKLNQP	NCP	11.91
TSKLNQP;ALLSL	NCP	155.02
ALLSL;PTYLRP	NCP	12.335
PTYLRP;LPMNLT	NCP	4.498
LPMNLT;RDLSLKP	NCP	56.847
RDLSLKP;SSQVPLLF	NCP	26.409
SSQVPLLF;MEMLP	NCP	36.914
MEMLP;PGFADPQ	NCP	22.518
PGFADPQ;QAFLKVLT	NCP	38.824
QAFLKVLT;NVDLH	NCP	196.742
NVDLH;PPLMFPP	NCP	12.279
PPLMFPP;STQLK	NCP	145.605
STQLK;SLLAGL	NCP	208.747

SLLAGL;RDDPLGP	NCP	153.933
RDDPLGP;YTTLL	NCP	19.619
YTTLL;EVSLDAF	NCP	54.291
EVSLDAF;AFVLFDP	NCP	299.275
AFVLFDP;GVDVLPF	NCP	5.135
GVDVLPF;FSLLQY	NCP	39.367
FSLLQY;DKPLRLP	NCP	399.404
DKPLRLP;LSHTPRY	NCP	221.263
LSHTPRY;APGHTPK	NCP	130.19
APGHTPK;KPPSFR	NCP	368.58
KPPSFR;LPPYLL	NCP	10.785
LPPYLL;FLLNS	NCP	18.622
FLLNS;VGPVGYR	NCP	41.201
VGPVGYR;NEYLL	NCP	136.564
NEYLL;KLLSYR	NCP	93.986
KLLSYR;DLCPLSQ	NCP	40.039
DLCPLSQ;RTAGLAPR	NCP	63.344
RTAGLAPR;GPPPVTLR	NCP	19.249
GPPPVTLR;PAVTQHAPYFK	NCP	69.185
PAVTQHAPYFK;KEAKPGK	NCP	6.472
KEAKPGK;QVWLK	NCP	5.552
QVWLK;FQRPENALKR	NCP	49.806
FQRPENALKR;ADDDRPSWR	NCP	25.308
ADDDRPSWR;DDRGPRRGLDDDR	NCP	0.276
DDRGPRRGLDDDR;DDDRGPWRN	NCP	0.057
DDDRGPWRN;GDDSRPGPWRP	NCP	0.171
GDDSRPGPWRP;FVRVMP	NCP	13.467
FVRVMP;THTSKYR	NCP	14.977
THTSKYR;TAVLR	NCP	5.85
TAVLR;HVDFVNP	NCP	6.963
HVDFVNP;ETAPK	NCP	214.233
ETAPK;LGVVD	NCP	313.127
LGVVD;NPPNN	NCP	55.3
NPPNN;KPAMVKL	NCP	108.44
KPAMVKL;AEEVVVGL	NCP	27.372
AEEVVVGL;KPVVSP	NCP	20.703
KPVVSP;LSGKP	NCP	318.802
LSGKP;DLFKL	NCP	54.3
DLFKL;FDAPPRP	NCP	113.349
FDAPPRP;LSPGFP	NCP	8.411
LSPGFP;KFTLK	NCP	40.054
KFTLK;AMNVL	NCP	5.375
AMNVL;MRYPPP	NCP	15.13
MRYPPP;ATNFL	NCP	34.725
ATNFL;ERPTLQQ	NCP	78.911
ERPTLQQ;TLALPWP	NCP	36.586
TLALPWP;VPPYK	NCP	190.472
VPPYK;WLGPVDK	NCP	91.971
WLGPVDK;LSLLK	NCP	221.671
LSLLK;YLHPTK	NCP	67.413
YLHPTK;QVELL	NCP	21.71

QVELL;TGREFF	NCP	3.637
TGREFF;LLEAEVP	NCP	19.956
LLEAEVP;EVPLV	NCP	0.012
EVPLV;KPVVK	NCP	12.132
KPVVK;PKGPVVF	NCP	111.191
PKGPVVF;LQLLS	NCP	9.673
LQLLS;VPHKLPP	NCP	17.294
VPHKLPP;VYLKTK	NCP	147.597
VYLKTK;EKREK	NCP	18.292
EKREK;GKAFSY	NCP	17.667
GKAFSY;TGKLLR	NCP	17.46
TGKLLR;DSMEH	NCP	27.779
DSMEH;NKSFPVNF	NCP	36.441
NKSFPVNF;CTCEC	NCP	16.881
CTCEC;KSSHEEV	NCP	28.705
KSSHEEV;QHVEVLK	NCP	80.897
QHVEVLK;VTVPGVK	NCP	61.203
VTVPGVK;SLFRAMLR	NCP	21.54
SLFRAMLR;LEHAQ	NCP	140.4
LEHAQ;LGFKKKYM	NCP	11.347
LGFKKKYM;KVYGP	NCP	234.012
KVYGP;DDSPP	NCP	70.52
DDSPP;KVSKER	NCP	510.481
KVSKER;RYKMLLN	NCP	46.166
RYKMLLN;EFRAPS	NCP	6.124
EFRAPS;DPGKK	NCP	33.632
DPGKK;RLTSFK	NCP	119.151
RLTSFK;VYFNKGL	NCP	166.348
VYFNKGL;NFLSY	NCP	35.258
NFLSY;HLLAKGK	NCP	42.868
HLLAKGK;PLLTMFK	NCP	7.526
PLLTMFK;PAGPK	NCP	66.06
PAGPK;PPSKPRPP	NCP	138.15
PPSKPRPP;VLFVGKVLH	NCP	69.471
VLFVGKVLH;LPDEPTP	NCP	24.594
LPDEPTP;KDRSPK	NCP	44.028
KDRSPK;KSSLRGAP	NCP	4.3
KSSLRGAP;WEVPPPPP	NCP	3.513
WEVPPPPP;TSLLAQY	NCP	292.424
TSLLAQY;ANNPFSK	NCP	45.078
ANNPFSK;FVGDAGK	NCP	30.609
FVGDAGK;KNSGTVYH	NCP	133.879
KNSGTVYH;KPPFY	NCP	176.495
KPPFY;CMTTSTR	NCP	114.699
CMTTSTR;WVAVP	NCP	5.343
WVAVP;RAHLFQ	NCP	72.436
RAHLFQ;KNDLR	NCP	54.017
KNDLR;GTPGAP	NCP	25.219
GTPGAP;LPLLA	NCP	33.932
LPLLA;AEFGAL	NCP	17.839
AEFGAL;LSNFKK	NCP	21.095

LSNFKK;PAAAPAPEK	NCP	15.298
PAAAPAPEK;KAQFNLTK	NCP	0.839
KAQFNLTK;PALPEAK	NCP	249.153
PALPEAK;ATAFKYP	NCP	88.488
ATAFKYP;GVGVFYP	NCP	189.502
GVGVFYP;MDFQQL	NCP	60.233
MDFQQL;KSSKPGD	NCP	129.28
KSSKPGD;NDVKCR	NCP	113.873
NDVKCR;YSKLVH	NCP	31.305
YSKLVH;FYAFKPP	NCP	47.439
FYAFKPP;DTAHTH	NCP	61.066
DTAHTH;KVLAFK	NCP	64.242
KVLAFK;LATYFV	NCP	11.262
LATYFV;PLEFLY	NCP	98.762
PLEFLY;RKKLEFQP	NCP	59.074
RKKLEFQP;LTVKSF	NCP	67.274
LTVKSF;LSPLK	NCP	79.377
LSPLK;ASLGHLKP	NCP	96.571
ASLGHLKP;EVAALEDK	NCP	126.72
EVAALEDK;KGLVVFY	NCP	64.662
KGLVVFY;KAAVWTPP	NCP	14.25
KAAVWTPP;PRMPP	NCP	189.141
PRMPP;GVPPFC	NCP	118.071
GVPPFC;LPHMLK	NCP	0.486
LPHMLK;LGPYLP	NCP	1.299
LGPYLP;QEKVP	NCP	18.68
QEKVP;LSRLLK	NCP	126.903
LSRLLK;DVEMM	NCP	143.397
DVEMM;LVPACR	NCP	6.192
LVPACR;SLEVLERK	NCP	61.942
SLEVLERK;SLPPLK	NCP	164.768
SLPPLK;KPLPPPPPP	NCP	71.445
KPLPPPPPP;VPGGLL	NCP	86.67
VPGGLL;TGCFATP	NCP	33.552
TGCFATP;GPPLP	NCP	3.432
GPPLP;YEVADF	NCP	92.17
YEVADF;DFKALKKK	NCP	113.975
DFKALKKK;MSEPK	NCP	32.457
MSEPK;GCPEK	NCP	9.37
GCPEK;SGLLEC	NCP	17.756
SGLLEC;KLGSKVE	NCP	76.5
KLGSKVE;LDLVKLR	NCP	42.097
LDLVKLR;VDHAKGK	NCP	55.661
VDHAKGK;KAAVLLPK	NCP	28.465
KAAVLLPK;KFTLN	NCP	65.106
KFTLN;LDKVFR	NCP	31.368
LDKVFR;LLSPEPKQ	NCP	33.551
LLSPEPKQ;SSAPK	NCP	231.831
SSAPK;EGNNL	NCP	28.903
EGNNL;LSGSY	NCP	19.881
LSGSY;RMLVK	NCP	3.561

RMLVK;KNPNP	NCP	135.057
KNPNP;YLLQNN	NCP	5.436
YLLQNN;KHRHHK	NCP	111.33
KHRHHK;LGLTV	NCP	2.811
LGLTV;KPVLVLG	NCP	98.675
KPVLVLG;AAPDR	NCP	201.1
AAPDR;LSFHKQLL	NCP	7.551
LSFHKQLL;EPVYTVF	NCP	18.936
EPVYTVF;SSHHPF	NCP	142.892
SSHHPF;KALFPP	NCP	88.672
KALFPP;QGYLDKV	NCP	208.889
QGYLDKV;KHLLPTPP	NCP	145.765
KHLLPTPP;PAASRY	NCP	20.108
PAASRY;PTLGF	NCP	218.968
PTLGF;VSKVYL	NCP	101.607
VSKVYL;EVLLRL	NCP	49.269
EVLLRL;KACPSR	NCP	3.08
KACPSR;SLGCK	NCP	8.959
SLGCK;TADVTVP	NCP	92.418
TADVTVP;LLVVL	NCP	39.405
LLVVL;AQPPRL	NCP	84.183
AQPPRL;SVFALT	NCP	1.107
SVFALT;VLGPR	NCP	10.878
VLGPR;DATFGLN	NCP	56.561
DATFGLN;ALPLL	NCP	10.333
ALPLL;VSFGGAGK	NCP	88.878
VSFGGAGK;PLKNLH	NCP	96.509
PLKNLH;KVFQPPP	NCP	289.843
KVFQPPP;KGCAFLSF	NCP	63.239
KGCAFLSF;KTLNLF	NCP	22.356
KTLNLF;NPGKPVK	NCP	3.856
NPGKPVK;LQNVPG	NCP	35.697
LQNVPG;KFHLPSP	NCP	28.134
KFHLPSP;DPCDLRK	NCP	37.827
DPCDLRK;VTPLTK	NCP	43.886
VTPLTK;LFVLSGKP	NCP	97.627
LFVLSGKP;CGSLR	NCP	158.007
CGSLR;KDLTRYK	NCP	294.992
KDLTRYK;FCKGLK	NCP	17.764
FCKGLK;VEVVLGH	NCP	72.429
VEVVLGH;KPDVTLGK	NCP	30.63
KPDVTLGK;KSVPN	NCP	66.317
KSVPN;KTKTYSSK	NCP	92.994
KTKTYSSK;ELDLSF	NCP	134.713
ELDLSF;PPDNRPP	NCP	6.455
PPDNRPP;AMLKGRK	NCP	121.507
AMLKGRK;KLVAPLPF	NCP	39.005
KLVAPLPF;KNDPF	NCP	24.39
KNDPF;TAFLKMT	NCP	43.366
TAFLKMT;PAMTLER	NCP	31.185
PAMTLER;PERGCDF	NCP	95.945

PERGCDF;KPLLKY	NCP	201.031
KPLLKY;TVEVVR	NCP	76.329
TVEVVR;KNGAQ	NCP	56.072
KNGAQ;LKLPL	NCP	166.812
LKLPL;LSAAGL	NCP	70.791
LSAAGL;LTLVGH	NCP	139.638
LTLVGH;AALVT	NCP	73.848
AALVT;TLKLRAK	NCP	27.594
TLKLRAK;LKLMGK	NCP	5.73
LKLMGK;RKHATGPK	NCP	197.962
RKHATGPK;GPPGPPGP	NCP	239.882
GPPGPPGP;KLLEVP	NCP	24.202
KLLEVP;HAFKGPP	NCP	61.932
HAFKGPP;NSSPPVF	NCP	72.909
NSSPPVF;AFLEF	NCP	83.247
AFLEF;VPEVPK	NCP	20.252
VPEVPK;SLMTFL	NCP	2.695
SLMTFL;DLDLSAPK	NCP	37.892
DLDLSAPK;KKMEPEF	NCP	22.912
KKMEPEF;HLPPSTTK	NCP	75.527
HLPPSTTK;QRGGNLAL	NCP	25.315
QRGGNLAL;KPTDVPK	NCP	83.618
KPTDVPK;LRSVFAL	NCP	3.159
LRSVFAL;VLLSSGK	NCP	65.154
VLLSSGK;LPLPPGH	NCP	14.289
LPLPPGH;KNAVCVHL	NCP	13.144
KNAVCVHL;KPPTSYSLK	NCP	116.45
KPPTSYSLK;TLAPL	NCP	52.795
TLAPL;APLKSRP	NCP	0.006
APLKSRP;LPKNKQTP	NCP	117.566
LPKNKQTP;QAYTSGHK	NCP	12.282
QAYTSGHK;CSCKFN	NCP	184.486
CSCKFN;MAPDRKK	NCP	46.104
MAPDRKK;PTSVDPK	NCP	12.626
PTSVDPK;FSFKNEDPALK	NCP	35.679
FSFKNEDPALK;KGYRVP	NCP	12.112
KGYRVP;PTNDN	NCP	8.57
PTNDN;NHPHN	NCP	60.136
NHPHN;DFDTALK	NCP	33.369
DFDTALK;GPGPP	NCP	16.118
GPGPP;VVPQKVG	NCP	3.25
VVPQKVG;THAGPPK	NCP	49.07
THAGPPK;KPSLGKGK	NCP	10.326
KPSLGKGK;TGGLDLK	NCP	125.508
TGGLDLK;LQVAKDLN	NCP	51.355
LQVAKDLN;LTLVSF	NCP	53.799
LTLVSF;KYLSTAK	NCP	200.039
KYLSTAK;VRYTLR	NCP	58.239
VRYTLR;LYASNYL	NCP	10.95
LYASNYL;AVLFN	NCP	4.341
AVLFN;EFLFE	NCP	16.525

EFLFE;KAKAF	NCP	10.304
KAKAF;VGHLEAK	NCP	14.44
VGHLEAK;RFVAQP	NCP	24.149
RFVAQP;AVDTHSKR	NCP	95.857
AVDTHSKR;SASPRGPK	NCP	4.43
SASPRGPK;KAFVVPP	NCP	74.127
KAFVVPP;FPLGLLPY	NCP	6.387
FPLGLLPY;ESLGTSF	NCP	44.682
ESLGTSF;DLALVP	NCP	26.01
DLALVP;YPPPPERP	NCP	40.587
YPPPPERP;LERKLDY	NCP	42.889
LERKLDY;RPLKLD	NCP	58.542
RPLKLD;VFPSQK	NCP	22.46
VFPSQK;KESLYFK	NCP	52.435
KESLYFK;RDGLPP	NCP	12.245
RDGLPP;LPPPAPLP	NCP	30.054
LPPPAPLP;SGLPRHLQR	NCP	28.834
SGLPRHLQR;PVLNP	NCP	37.524
PVLNP;PPPHPSEP	NCP	16.448
PPPHPSEP;NYVAVVK	NCP	5.697
NYVAVVK;GRPPKLGA	NCP	20.358
GRPPKLGA;PFVELTD	NCP	17.056
PFVELTD;VLDPL	NCP	35.184
VLDPL;KTASVELR	NCP	174.411
KTASVELR;PGAVTPVP	NCP	137.234
PGAVTPVP;AGKPPPR	NCP	42.657
AGKPPPR;LLPCLK	NCP	184.992
LLPCLK;KGQGPTGK	NCP	30.966
KGQGPTGK;KSLHYLVQ	NCP	94.32
KSLHYLVQ;KLDAYSK	NCP	110.865
KLDAYSK;KMVPP	NCP	15.016
KMVPP;KSCALAK	NCP	93.693
KSCALAK;KNTAT	NCP	63.5
KNTAT;LPGGVG	NCP	112.204
LPGGVG;LPECL	NCP	230.346
LPECL;PTLMSKPL	NCP	0.408
PTLMSKPL;AALEYL	NCP	63.467
AALEYL;LNFSV	NCP	49.441
LNFSV;NFLDF	NCP	17.34
NFLDF;AASMWAK	NCP	12.204
AASMWAK;PSAYPRF	NCP	18.434
PSAYPRF;DSGKWVPF	NCP	116.067
DSGKWVPF;APLLM	NCP	21.978
APLLM;SGPGFKAP	NCP	1.419
SGPGFKAP;RAAQLR	NCP	15.966
RAAQLR;LLYVHSLL	NCP	48.619
LLYVHSLL;KDVFP	NCP	26.208
KDVFP;SVLYF	NCP	180.305
SVLYF;ALLGRVF	NCP	177.069
ALLGRVF;QLGKNEVK	NCP	291.945
QLGKNEVK;LPGLS	NCP	25.612

LPGLS;KSPQGLSPQ	NCP	4.839
KSPQGLSPQ;PSLLLPTP	NCP	60.005
PSLLLPTP;QPSYK	NCP	60.196
QPSYK;LDPSAL	NCP	125.271
LDPSAL;SPPVCLPK	NCP	5.918
SPPVCLPK;MNGVP	NCP	51.354
MNGVP;LPLRTPLPP	NCP	391.233
LPLRTPLPP;TVHTRAP	NCP	2.622
TVHTRAP;VLEDGLVK	NCP	37.537
VLEDGLVK;PVGPRPF	NCP	66.858
PVGPRPF;LDPVL	NCP	20.394
LDPVL;KTHATVL	NCP	57.954
KTHATVL;AAAAALEAVAPL	NCP	19.082
AAAAALEAVAPL;APAEGLVY	NCP	17.53
APAEGLVY;PGPVP	NCP	64.913
PGPVP;EGLRVY	NCP	10.282
EGLRVY;PKKGNP	NCP	106.08
PKKGNP;ALLPVPN	NCP	93.435
ALLPVPN;DGNPQGYS	NCP	129.801
DGNPQGYS;RVHLLGS	NCP	63.825
RVHLLGS;KMMVLSF	NCP	29.264
KMMVLSF;QLDPNA	NCP	42.648
QLDPNA;AAPARLPLSV	NCP	189.876
AAPARLPLSV;KGQVGQK	NCP	44.07
KGQVGQK;PQGTN	NCP	44.464
PQGTN;APGLKPK	NCP	55.314
APGLKPK;RLKTLPPP	NCP	92.124
RLKTLPPP;KEGFVPP	NCP	248.493
KEGFVPP;YELLR	NCP	177.086
YELLR;TVPLSRTHGK	NCP	178.659
TVPLSRTHGK;LTFSPSEVK	NCP	25.146
LTFSPSEVK;ADDAGGP	NCP	17.967
ADDAGGP;LAEPPPLK	NCP	34.468
LAEPPPLK;LLKTEL	NCP	48.507
LLKTEL;VSPQETPKQ	NCP	64.28
VSPQETPKQ;RSVHPSLPSSKPQ	NCP	10.428
RSVHPSLPSSKPQ;SVHPSLPSSKP	NCP	0.003
SVHPSLPSSKP;VPPVPEGL	NCP	6.144
VPPVPEGL;DDDSDLYSPR	NCP	29.664
DDDSDLYSPR;TDWEPPDKK	NCP	2.694
TDWEPPDKK;YEYQPGK	NCP	0.066
YEYQPGK;VAPPPYK	NCP	16.134
VAPPPYK;ERFGDLLN	NCP	12.366
ERFGDLLN;FDFKAQTLK	NCP	4.281
FDFKAQTLK;KGNDLVR	NCP	59.466
KGNDLVR;KQELNEPPKQ	NCP	36.807
KQELNEPPKQ;GDPNKPSGF	NCP	5.286
GDPNKPSGF;VCTDCGTNLKQ	NCP	0.6
VCTDCGTNLKQ;MLVRNL	NCP	123.489
MLVRNL;PSLTTAEL	NCP	252.186
PSLTTAEL;PKVNLVLL	NCP	109.533

PKVNLVLL;DLFVP	NCP	0.76
DLFVP;ATGGAKLM	NCP	108.099
ATGGAKLM;LPSSAKYG	NCP	101.373
LPSSAKYG;TAEVLLEL	NCP	55.848
TAEVLLEL;FVVSEFLK	NCP	63.438
FVVSEFLK;GPGGPSLF	NCP	194.843
GPGGPSLF;AELEK	NCP	42.564
AELEK;EGGKSHGK	NCP	40.893
EGGKSHGK;MLKSSMLLK	NCP	6.787
MLKSSMLLK;YAPTT	NCP	165.357
YAPTT;MEPLLYT	NCP	97.776
MEPLLYT;NVKPE	NCP	109.808
NVKPE;YVPGSLY	NCP	92.743
YVPGSLY;KNLDF	NCP	11.411
KNLDF;RKMTALQ	NCP	16.954
RKMTALQ;VTEELLEK	NCP	183.414
VTEELLEK;DRFLR	NCP	38.958
DRFLR;LDQARL	NCP	118.005
LDQARL;SDPGFK	NCP	159.428
SDPGFK;LETAL	NCP	35.949
LETAL;KDLVQK	NCP	234.549
KDLVQK;ESTELLLR	NCP	37.186
ESTELLLR;MAGPVVD	NCP	235.394
MAGPVVD;VSGPVV	NCP	18.795
VSGPVV;MEMLSVP	NCP	15.774
MEMLSVP;FTKKKT	NCP	75.795
FTKKKT;VVGSPP	NCP	19.975
VVGSPP;VTLLRK	NCP	10.8
VTLLRK;MKYPP	NCP	52.163
MKYPP;GNLVTGGK	NCP	99.652
GNLVTGGK;PDDEVTR	NCP	36.857
PDDEVTR;DLSFPAST	NCP	21.25
DLSFPAST;LEARSN	NCP	19.548
LEARSN;DMPWFK	NCP	49.569
DMPWFK;KLHLPTPP	NCP	126.081
KLHLPTPP;KYPPLP	NCP	99.363
KYPPLP;RQALPR	NCP	110.55
RQALPR;AVPVK	NCP	12.287
AVPVK;LTPML	NCP	95.599
LTPML;PLATVMK	NCP	23.832
PLATVMK;TTSPK	NCP	8.988
TTSPK;SVGYF	NCP	195.959
SVGYF;VPPLT	NCP	31.135
VPPLT;VSTKEL	NCP	38.877
VSTKEL;PLEMSLPLE	NCP	16.538
PLEMSLPLE;ERLEVDR	NCP	102.987
ERLEVDR;VWDCGTP	NCP	62.413
VWDCGTP;QDGPGKK	NCP	78.176
QDGPGKK;FHVYTLR	NCP	31.374
FHVYTLR;KLRKAEL	NCP	41.247
KLRKAEL;TTDVGW	NCP	12.258

TTDVGW;VPSLPPPPPRP	NCP	3.624
VPSLPPPPPRP;AAGVAQK	NCP	50.599
AAGVAQK;FELGP	NCP	23.006
FELGP;FDPMQK	NCP	43.918
FDPMQK;NGMDC	NCP	4.757
NGMDC;KGVFKDR	NCP	61.066
KGVFKDR;LTVVSVLE	NCP	30.089
LTVVSVLE;KGTVMKL	NCP	0.904
KGTVMKL;GPGVEPAP	NCP	137.118
GPGVEPAP;QKAAPPVS	NCP	12.873
QKAAPPVS;ESLVRK	NCP	65.867
ESLVRK;DSALLNF	NCP	6.79
DSALLNF;LGGSLL	NCP	31.797
LGGSLL;AAESLLKT	NCP	59.129
AAESLLKT;RYAPR	NCP	51.165
RYAPR;FFKGPSR	NCP	117.37
FFKGPSR;TAARDRK	NCP	55.494
TAARDRK;GGHDP	NCP	15.11
GGHDP;LPPSF	NCP	160.093
LPPSF;GKSLRTVL	NCP	21.485
GKSLRTVL;LKGDLR	NCP	3.178
LKGDLR;LMKLKY	NCP	68.402
LMKLKY;HNTKSK	NCP	6.885
HNTKSK;TAAGVLL	NCP	25.414
TAAGVLL;PEGYK	NCP	11.673
PEGYK;KTTVLE	NCP	175.727
KTTVLE;AAQPK	NCP	81.529
AAQPK;DFLKK	NCP	80.028
DFLKK;KPVVFLQ	NCP	15.626
KPVVFLQ;AAVLEY	NCP	29.004
AAVLEY;RPAPP	NCP	151.675
RPAPP;EYKDSEVF	NCP	37.167
EYKDSEVF;DYAEC	NCP	35.226
DYAEC;CEEEDSTALVCDNGSGLCKA	NCP	76.721
CEEEDSTALVCDNGSGLCKA;EEEDSTAL	NCP	0.003
EEEDSTAL;STALVCDNGSGLCK	NCP	0.012
STALVCDNGSGLCK;YNELRVAPEEHPTLL	NCP	4.542
YNELRVAPEEHPTLL;NELRVAPEEHPTL	NCP	0.003
NELRVAPEEHPTL;ELRVAPEEHPTLL	NCP	0.003
ELRVAPEEHPTLL;LRVAPEEHPTL	NCP	0.003
LRVAPEEHPTL;RVAPEEHPTLL	NCP	0.003
RVAPEEHPTLL;VAPEEHPTLLTEAPLNPK	NCP	0.003
VAPEEHPTLLTEAPLNPK;APEEHPTLL	NCP	0.003
APEEHPTLL;EEHPTLLTEAPLNPK	NCP	0.006
EEHPTLLTEAPLNPK;ETFNVPAM	NCP	2.37
ETFNVPAM;TFNVPAMY	NCP	0.003
TFNVPAMY;NVPAMYV	NCP	0.006
NVPAMYV;VLDSGDGVTHN	NCP	0.447
VLDSGDGVTHN;DSGDGVTHNVP	NCP	0.006
DSGDGVTHNVP;MRLDLAGRDLTDY	NCP	0.066
MRLDLAGRDLTDY;LCYVALDFENEMATAASSSSLEK	NCP	1.911

LCYVALDFENEMATAASSSSLEK;VALDFENEMATAASSS	NCP	0.009
VALDFENEMATAASSSSLEKSY;ALDFENEMATAASSSSL	NCP	0.003
ALDFENEMATAASSSSLEK;LDFENEM	NCP	0.003
LDFENEM;DFENEMATAASSSSLEK	NCP	0.003
DFENEMATAASSSSLEK;ENEMATAASSSSLEKSY	NCP	0.006
ENEMATAASSSSLEKSY;GNERFRCPETL	NCP	0.081
GNERFRCPETL;ERFRCPET	NCP	0.006
ERFRCPET;CPETLFQPSF	NCP	0.012
CPETLFQPSF;LPFVP	NCP	42.735
LPFVP;VVLVT	NCP	5.277
VVLVT;KTTFYTVP	NCP	181.716
KTTFYTVP;KEFPF	NCP	80.457
KEFPF;YHPGFPN	NCP	21.993
YHPGFPN;YPEPEAV	NCP	27.667
YPEPEAV;ALNTEF	NCP	28.905
ALNTEF;NLPATY	NCP	108.8
NLPATY;ALLVV	NCP	30.598
ALLVV;AARGF	NCP	73.341
AARGF;KYKPATM	NCP	84.557
KYKPATM;AAVSLR	NCP	15.204
AAVSLR;NNLLSPP	NCP	145.512
NNLLSPP;QVGAP	NCP	32.337
QVGAP;ACSRFPPP	NCP	22.054
ACSRFPPP;RPSLPL	NCP	132.252
RPSLPL;KEHLASSK	NCP	61.976
KEHLASSK;VKHLMSN	NCP	33.79
VKHLMSN;KGVADLLK	NCP	73.766
KGVADLLK;PAADY	NCP	172.651
PAADY;GPADVKK	NCP	16.739
GPADVKK;KFERSLT	NCP	53.922
KFERSLT;LSVAVGLK	NCP	34.561
LSVAVGLK;SKVLR	NCP	3.845
SKVLR;DPAPF	NCP	35.673
DPAPF;LAVRNVP	NCP	39.984
LAVRNVP;RTVAGPK	NCP	29.82
RTVAGPK;VGPSL	NCP	47.251
VGPSL;VKAPVTK	NCP	26.907
VKAPVTK;RVKRC	NCP	39.957
RVKRC;PGRRLQPPP	NCP	85.944
PGRRLQPPP;GSHSLRY	NCP	56.291
GSHSLRY;QTKPH	NCP	31.98
QTKPH;GAALKP	NCP	4.813
GAALKP;TSGKSLP	NCP	50.022
TSGKSLP;SMQTQR	NCP	30.579
SMQTQR;GPAAPP	NCP	49.689
GPAAPP;LHEALER	NCP	78.125
LHEALER;VAGGGK	NCP	156.781
VAGGGK;ALQSPQ	NCP	110.28
ALQSPQ;AAFLK	NCP	49.586
AAFLK;KTAASSKK	NCP	83.25
KTAASSKK;VLVGPS	NCP	63.853

VLVGPS;AAVSGGRV	NCP	12.183
AAVSGGRV;FSATPPL	NCP	171.114
FSATPPL;AALSLSF	NCP	35.115
AALSLSF;TQARLAK	NCP	141.131
TQARLAK;SLEPTTK	NCP	72.555
SLEPTTK;HAAAGP	NCP	63.781
HAAAGP;HLSPPPP	NCP	45.599
HLSPPPP;RAPSAKPP	NCP	0.613
RAPSAKPP;SLYPRP	NCP	125.198
SLYPRP;ALVSY	NCP	68.778
ALVSY;PAEVPGKP	NCP	17.794
PAEVPGKP;KDPKP	NCP	55.064
KDPKP;RPPVTL	NCP	73.519
RPPVTL;KLVGKK	NCP	189.744
KLVGKK;QAVRPVS	NCP	12.615
QAVRPVS;KPGSYR	NCP	53.847
KPGSYR;LATYF	NCP	34.751
LATYF;AAAVGVKL	NCP	109.014
AAAVGVKL;YFLFPGN	NCP	75.255
YFLFPGN;TSLTP	NCP	9.015
TSLTP;FSLFKDS	NCP	102.06
FSLFKDS;YTLTFPM	NCP	12.073
YTLTFPM;LFTSK	NCP	546.882
LFTSK;KAEESDVKLK	NCP	27.545
KAEESDVKLK;QNLLQ	NCP	90.339
QNLLQ;PGNWHLK	NCP	110.827
PGNWHLK;AAVPVP	NCP	70.47
AAVPVP;RSACHNK	NCP	48.797
RSACHNK;FYGLDL	NCP	10.188
FYGLDL;LTVLGAGK	NCP	79.698
LTVLGAGK;KPFEP	NCP	131.259
KPFEP;RQEGKHV	NCP	13.771
RQEGKHV;TGLLQEAK	NCP	35.295
TGLLQEAK;EPLPGGGK	NCP	98.136
EPLPGGGK;LPLVPN	NCP	5.462
LPLVPN;ETLAY	NCP	4.459
ETLAY;PVMLE	NCP	10.911
PVMLE;CSGLR	NCP	38.646
CSGLR;AAPLGGAH	NCP	43.983
AAPLGGAH;MSAVKPK	NCP	146.91
MSAVKPK;VGSNLYP	NCP	173.217
VGSNLYP;GHRGP	NCP	15.63
GHRGP;VGVLGFGH	NCP	73.131
VGVLGFGH;GKPSWL	NCP	44.877
GKPSWL;ENVLRDAV	NCP	109.701
ENVLRDAV;KSSHGQ	NCP	131.336
KSSHGQ;LEEAY	NCP	3.105
LEEAY;GWLGSL	NCP	88.885
GWLGSL;KGHVGVGL	NCP	46.869
KGHVGVGL;APSTMKQK	NCP	35.31
APSTMKQK;GPACAPLQ	NCP	26.64

GPACAPLQ;VTTVPKV	NCP	50.472
VTTVPKV;VPKVEPAP	NCP	13.917
VPKVEPAP;TVLVQPLK	NCP	119.622
TVLVQPLK;SLDLLVSM	NCP	107.291
SLDLLVSM;PPKKKK	NCP	1.137
PPKKKK;KDRERK	NCP	17.851
KDRERK;AGVMFDF	NCP	8.265
AGVMFDF;PLVEYQK	NCP	3.702
PLVEYQK;GSDLGVK	NCP	51.489
GSDLGVK;QTFALPP	NCP	48.111
QTFALPP;GLLPGGYK	NCP	0.321
GLLPGGYK;GVWLPEK	NCP	2.213
GVWLPEK;LLSAAG	NCP	87.142
LLSAAG;DGDDAPW	NCP	0.243
DGDDAPW;ELVPL	NCP	45.242
ELVPL;RNLWEK	NCP	73.917
RNLWEK;AVSFCA	NCP	13.399
AVSFCA;EVTEF	NCP	18.744
EVTEF;VTAELL	NCP	3.071
VTAELL;KYLSFNLP	NCP	273.69
KYLSFNLP;KTTGGAAR	NCP	211.801
KTTGGAAR;KCCRALLE	NCP	42.441
KCCRALLE;DKAEGP	NCP	18.459
DKAEGP;SVHPSLP	NCP	47.784
SVHPSLP;PEQLQ	NCP	59.388
PEQLQ;KAPGVAGK	NCP	24.018
KAPGVAGK;LLMKPPP	NCP	99.824
LLMKPPP;VSPPELR	NCP	14.503
VSPPELR;NRSLLL	NCP	84.036
NRSLLL;EASAG	NCP	73.038
EASAG;ELFNPPPPP	NCP	21.197
ELFNPPPPP;LLKLKSL	NCP	150.984
LLKLKSL;GDLAGK	NCP	8.214
GDLAGK;KPTASPVP	NCP	12.919
KPTASPVP;SGKRVGVP	NCP	63.06
SGKRVGVP;ELCMML	NCP	10.866
ELCMML;TASVGKSTK	NCP	240.453
TASVGKSTK;LLLLPKK	NCP	19.08
LLLLPKK;MTNYY	NCP	10.649
MTNYY;KVPGKPP	NCP	71.608
KVPGKPP;GPPPPP	NCP	9.375
GPPPPP;FVAVPHFL	NCP	16.691
FVAVPHFL;RSPVGME	NCP	49.219
RSPVGME;NPLPPPPPPL	NCP	34.467
NPLPPPPPPL;VGVNPL	NCP	3.369
VGVNPL;DPDDCCC	NCP	16.383
DPDDCCC;LLSAL	NCP	15.81
LLSAL;APMPK	NCP	59.169
APMPK;RCPSLVGRP	NCP	50.262
RCPSLVGRP;PPLKPW	NCP	88.55
PPLKPW;DLVEGTL	NCP	46.236

DLVEGTL;KAFYKS	NCP	5.349
KAFYKS;SLDLP	NCP	35.059
SLDLP;KGAPAP	NCP	151.473
KGAPAP;WGVGGSLL	NCP	60.995
WGVGGSLL;TVPPSA	NCP	94.024
TVPPSA;FDSLPR	NCP	53.408
FDSLPR;SGPLP	NCP	31.399
SGPLP;KAKLSPAAP	NCP	111.747
KAKLSPAAP;KDFYPLL	NCP	25.406
KDFYPLL;PGAPVP	NCP	15.544
PGAPVP;EVELTPY	NCP	57.653
EVELTPY;KVVLL	NCP	7.378
KVVLL;EPPQHPP	NCP	46.743
EPPQHPP;LTVLSP	NCP	20.847
LTVLSP;VATLL	NCP	5.381
VATLL;KLWPPAGP	NCP	80.23
KLWPPAGP;SSSSVPTVK	NCP	83.21
SSSSVPTVK;SPGVRTPP	NCP	7.066
SPGVRTPP;APLPGL	NCP	10.158
APLPGL;FYMMGLL	NCP	111.032
FYMMGLL;LGYQPEAK	NCP	40.975
LGYQPEAK;ASPLVGLR	NCP	42.072
ASPLVGLR;VGVLKA	NCP	70.26
VGVLKA;AAAALRGVR	NCP	80.997
AAAALRGVR;QAPPAPAP	NCP	10.896
QAPPAPAP;RPEPERPP	NCP	5.928
RPEPERPP;AAAPML	NCP	0.018
AAAPML;PGSPTPLT	NCP	35.823
PGSPTPLT;CGLAYP	NCP	43.892
CGLAYP;PAPPPPPPPP	NCP	49.471
PAPPPPPPPP;LPAGPVEP	NCP	118.005
LPAGPVEP;LSTYYKGP	NCP	112.038
LSTYYKGP;KPVLLL	NCP	40.415
KPVLLL;LMELSGK	NCP	32.484
LMELSGK;KDGMENP	NCP	54.169
SLPLK;FNPPTPPP	NCP	10.032
FNPPTPPP;MCGAC	NCP	51.316
MCGAC;RDSPTPPP	NCP	0.834
RDSPTPPP;KQQALVF	NCP	57.698
KQQALVF;LSQFPK	NCP	191.317
LSQFPK;LSKMPK	NCP	25.922
LSKMPK;GPARYGL	NCP	77.593
GPARYGL;ADGTFKK	NCP	18.473
ADGTFKK;YGPVVV	NCP	93.658
YGPVVV;LPPVF	NCP	67.383
LPPVF;LAGGP	NCP	23.915
LAGGP;PKKGGPP	NCP	299.599
PKKGGPP;KAAAAP	NCP	147.51
KAAAAP;KPPEMGP	NCP	65.685
KPPEMGP;NVALL	NCP	440.24
NVALL;KYMLQYVK	NCP	17.067

KYMLQYVK;PPPYK	NCP	3.645
PPPYK;PQRFP	NCP	13.68
PQRFP;LTHLGY	NCP	14.097
LTHLGY;EEPPQLK	NCP	13.788
EEPPQLK;PAGVH	NCP	11.526
PAGVH;PSLLL	NCP	3.987
PSLLL;VSEKP	NCP	98.496
VSEKP;LSADLSPAVF	NCP	60.645
LSADLSPAVF;ALSPVYAP	NCP	71.482
ALSPVYAP;AALHF	NCP	121.497
AALHF;EMCYLK	NCP	45.549
EMCYLK;TPKGLRP	NCP	177.626
TPKGLRP;SKKAHAG	NCP	116.332
SKKAHAG;QSVESLPK	NCP	100.85
QSVESLPK;HKKKKFTK	NCP	127.09
HKKKKFTK;LPGLKRK	NCP	71.441
LPGLKRK;VLELQR	NCP	15.274
VLELQR;WKVLR	NCP	184.83
WKVLR;LLNVKS	NCP	255.992
LLNVKS;KAAATVLP	NCP	24.427
KAAATVLP;LFGKEK	NCP	11.304
LFGKEK;VSPRTAVLL	NCP	35.234
VSPRTAVLL;PLSGSTLLP	NCP	14.889
PLSGSTLLP;WTDDF	NCP	56.572
WTDDF;VHFLEG	NCP	25.025
VHFLEG;KDPHREPP	NCP	169.983
KDPHREPP;KKMKSH	NCP	47.725
KKMKSH;DGSDLQLER	NCP	23.51
DGSDLQLER;SYWVGEDSTYK	NCP	119.943
SYWVGEDSTYK;RNTLQLHRYR	NCP	0.231
RNTLQLHRYR;LPGLSAP	NCP	151.993
LPGLSAP;TQVFF	NCP	405.932
TQVFF;VGPVQ	NCP	135.669
VGPVQ;PGLWP	NCP	39.541
PGLWP;LHVNAF	NCP	17.363
LHVNAF;PFLAVR	NCP	62.409
PFLAVR;KSDGEW	NCP	49.005
KSDGEW;LYAGK	NCP	11.761
LYAGK;LMVYPHR	NCP	177.03
LMVYPHR;TSSLDKLR	NCP	11.129
TSSLDKLR;THLPAL	NCP	91.578
THLPAL;MSVAKPK	NCP	18.144
MSVAKPK;KPPAPP	NCP	249.229
KPPAPP;GKSGGGFP	NCP	28.893
GKSGGGFP;KTDLRF	NCP	41.03
KTDLRF;KLTLK	NCP	46.011
KLTLK;RNGTLLR	NCP	170.325
RNGTLLR;GDDELYC	NCP	35.302
GDDELYC;AAVTQF	NCP	5.172
AAVTQF;SVLLPR	NCP	17.153
SVLLPR;LGPPAVT	NCP	53.028

LGPPAVT;DPAAVSH	NCP	25.698
DPAAVSH;NGLLHR	NCP	41.778
NGLLHR;VSTALG	NCP	86.842
VSTALG;ESLHQRK	NCP	190.161
ESLHQRK;LQLLPQ	NCP	6.753
LQLLPQ;RWTEY	NCP	43.334
RWTEY;KRMLP	NCP	63.472
KRMLP;KPLELLSH	NCP	117.141
KPLELLSH;PPPLPLR	NCP	31.749
PPPLPLR;GLPPGKM	NCP	50.01
GLPPGKM;EHAPK	NCP	5.825
EHAPK;PQGFQGK	NCP	39.364
PQGFQGK;CNDDL	NCP	109.682
CNDDL;NDHVPK	NCP	12.966
NDHVPK;ASKGLQDL	NCP	32.961
ASKGLQDL;NGALTVH	NCP	65.698
NGALTVH;DMCFN	NCP	93.792
DMCFN;LELRYL	NCP	18.144
LELRYL;PAYGVAK	NCP	52.352
PAYGVAK;PPPFVP	NCP	101.137
PPPFVP;DSGTP	NCP	179.843
DSGTP;AATSVYP	NCP	3.174
AATSVYP;MDRLLR	NCP	36.844
MDRLLR;LLPGEL	NCP	3.83
LLPGEL;KKGGFYEK	NCP	53.064
KKGGFYEK;DGLLY	NCP	6.076
DGLLY;KCGGLVT	NCP	57.066
KCGGLVT;CNLEKTR	NCP	1.704
CNLEKTR;ASAKLPK	NCP	52.707
ASAKLPK;LDVSPQ	NCP	19.589
LDVSPQ;TAAGLVF	NCP	161.155
TAAGLVF;KAANLRP	NCP	67.338
KAANLRP;ETVFEKP	NCP	40.146
ETVFEKP;KLLTK	NCP	61.466
KLLTK;DPSPF	NCP	8.158
DPSPF;LLDEPQR	NCP	49.619
LLDEPQR;TVHALLTN	NCP	178.77
TVHALLTN;KGGGSGRP	NCP	27.529
KGGGSGRP;GSHYSLR	NCP	108.786
GSHYSLR;FLAVV	NCP	15.893
FLAVV;AVRLDHP	NCP	121.984
AVRLDHP;KAQHGY	NCP	15.554
KAQHGY;VTLSLA	NCP	6.882
VTLSLA;NSLVPKK	NCP	2.508
NSLVPKK;GVSKLDSR	NCP	107.877
GVSKLDSR;RPAVFPS	NCP	15.097
RPAVFPS;PAVRPP	NCP	84.729
PAVRPP;RYQLQ	NCP	19.545
RYQLQ;LGPPDLK	NCP	10.746
LGPPDLK;RSGVPGVLR	NCP	59.778
RSGVPGVLR;GMTTL	NCP	58.55

GMTTL;ATLGL	NCP	38.695
ATLGL;LGAPPV	NCP	52.772
LGAPPV;ELRVTTD	NCP	14.868
ELRVTTD;SVPKL	NCP	54.309
SVPKL;PGGPR	NCP	94.68
PGGPR;VDVVLPP	NCP	3.823
VDVVLPP;LPRGPLPP	NCP	80.585
LPRGPLPP;TFKPVGF	NCP	139.68
TFKPVGF;KSYVLGP	NCP	145.477
KSYVLGP;KAEAGP	NCP	129.422
KAEAGP;KVSKPP	NCP	43.233
KVSKPP;EEAPLGPHL	NCP	29.037
EEAPLGPHL;MKGLPR	NCP	104.013
MKGLPR;KKAVP	NCP	155.119
KKAVP;MEDKHF	NCP	45.501
MEDKHF;SCRGT	NCP	2.295
SCRGT;PPVVPPPP	NCP	59.324
PPVVPPPP;WSEHPK	NCP	39.772
WSEHPK;RHVTQMYPK	NCP	62.333
RHVTQMYPK;PGSFP	NCP	262.893
PGSFP;WGLLPL	NCP	167.817
WGLLPL;KPWQAP	NCP	6.648
KPWQAP;PAALSKSP	NCP	222.585
PAALSKSP;RYATGTL	NCP	89.412
RYATGTL;PVVLSSVT	NCP	132.544
PVVLSSVT;EYASTRP	NCP	177.587
EYASTRP;ENLRLK	NCP	199.219
ENLRLK;ELVLK	NCP	108.431
ELVLK;VLLFAM	NCP	2.872
VLLFAM;KTQAAKK	NCP	13.356
KTQAAKK;LKDLY	NCP	96.497
LKDLY;LSLDLPF	NCP	16.002
LSLDLPF;PKRAALQP	NCP	185.637
PKRAALQP;NLPLRY	NCP	313.446
NLPLRY;VPVGY	NCP	120.27
VPVGY;GPALPPP	NCP	14.829
GPALPPP;KDTQSVGPP	NCP	22.329
KDTQSVGPP;SGLAKHL	NCP	108.975
SGLAKHL;AELDVWP	NCP	7.389
AELDVWP;KFPSLVG	NCP	25.467
KFPSLVG;FLSEPELLPL	NCP	24.345
FLSEPELLPL;TVSDSLKK	NCP	174.477
TVSDSLKK;LSCLTK	NCP	9.616
LSCLTK;NGGFD	NCP	2.201
NGGFD;KYPLEHA	NCP	28.23
KYPLEHA;KLKTEL	NCP	113.827
KLKTEL;DRVSATLG	NCP	102.971
DRVSATLG;APNDQ	NCP	18.648
APNDQ;LSFPT	NCP	45.381
LSFPT;KPAPK	NCP	11.502
KPAPK;FTLKKK	NCP	61.698

FTLKKK;EQARLL	NCP	34.237
EQARLL;LPPKPKPP	NCP	22.097
LPPKPKPP;VVVMVVDL	NCP	5.686
VVVMVVDL;AMEPV	NCP	56.138
AMEPV;WDVCPTG	NCP	72.594
WDVCPTG;KEPGSGVP	NCP	12.885
KEPGSGVP;YEVGGVF	NCP	16.413
YEVGGVF;APPPFLLQ	NCP	151.15
APPPFLLQ;FTPLAR	NCP	16.805
FTPLAR;YRKSK	NCP	21.231
YRKSK;PSSKK	NCP	14.713
PSSKK;EPGVKVC	NCP	84.314
EPGVKVC;EVLPF	NCP	2.019
EVLPF;VTRLAEDGTR	NCP	13.698
VTRLAEDGTR;GDFKLKC	NCP	75.616
GDFKLKC;QDDLLSPK	NCP	30.555
QDDLLSPK;TPVGV	NCP	48.551
TPVGV;HPVLK	NCP	17.688
HPVLK;AHLGPSFP	NCP	130.062
AHLGPSFP;AFFYP	NCP	197.968
AFFYP;KDYRLEK	NCP	156.413
KDYRLEK;KLSGVGK	NCP	66.654
KLSGVGK;PRGGGL	NCP	28.542
PRGGGL;TVMLLK	NCP	174.439
TVMLLK;RVEATHP	NCP	53.756
RVEATHP;LSEKGELL	NCP	7.651
LSEKGELL;DLQKHL	NCP	2.291
DLQKHL;PSAPKVR	NCP	86.692
PSAPKVR;VEVTLK	NCP	51.108
VEVTLK;SDDEFVK	NCP	58.236
SDDEFVK;LPAPPP	NCP	6.975
LPAPPP;EHVTAPR	NCP	44.748
EHVTAPR;LELLKK	NCP	122.87
LELLKK;KYKPF	NCP	168.534
KYKPF;TLLVPF	NCP	96.286
TLLVPF;FVVDK	NCP	18.384
FVVDK;TNLLEKKKC	NCP	268.482
TNLLEKKKC;KHRKYL	NCP	19.431
KHRKYL;KPPAAP	NCP	16.269
KPPAAP;TLSVPALT	NCP	190.026
TLSVPALT;LERVCL	NCP	223.128
LERVCL;KTERSP	NCP	54.098
KTERSP;PNQVP	NCP	98.367
PNQVP;RTKHTK	NCP	162.351
RTKHTK;KDRVLP	NCP	49.471
KDRVLP;SLLPF	NCP	31.019
SLLPF;EVLFL	NCP	295.162
EVLFL;TRGAETMK	NCP	84.876
TRGAETMK;QATSPLK	NCP	16.79
QATSPLK;FLDFM	NCP	79.624
FLDFM;RPPGFK	NCP	7.445

RPPGFK;KTSTDGGP	NCP	87.865
KTSTDGGP;AVPPKLM	NCP	89.918
AVPPKLM;PAAPV	NCP	105.657
PAAPV;LQVQHASKQ	NCP	12.628
LQVQHASKQ;GAVPPKVK	NCP	63.29
GAVPPKVK;GGVLL	NCP	95.409
GGVLL;KGTDYGY	NCP	110.865
KGTDYGY;KERPPP	NCP	91.494
KERPPP;KADVFK	NCP	16.18
KADVFK;LVKGFK	NCP	272.735
LVKGFK;PPLKMP	NCP	142.368
PPLKMP;ECKKYFER	NCP	40.804
ECKKYFER;QKPHRP	NCP	55.169
QKPHRP;SVGVLPLY	NCP	217.504
SVGVLPLY;KQEDYAGP	NCP	53.535
KQEDYAGP;KLGVVT	NCP	71.391
KLGVVT;LTEQSKTK	NCP	80.342
LTEQSKTK;EGHHLLSH	NCP	41.937
EGHHLLSH;KPPVQLRK	NCP	36.603
KPPVQLRK;YETQLY	NCP	47.788
YETQLY;KTKHK	NCP	167.853
KTKHK;LDTQLVNN	NCP	66.663
LDTQLVNN;TSVPR	NCP	82.335
TSVPR;NMLLLS	NCP	60.005
NMLLLS;YVTGKYP	NCP	1.386
YVTGKYP;PLDPK	NCP	59.322
PLDPK;TAKASH	NCP	182.862
TAKASH;LLSPLQFR	NCP	19.452
LLSPLQFR;YCFHCKK	NCP	4.642
YCFHCKK;HGEFWK	NCP	68.97
HGEFWK;MDDKAFTK	NCP	39.158
MDDKAFTK;TDLFDYLPL	NCP	14.238
TDLFDYLPL;VGAPL	NCP	6.001
VGAPL;ESVKFHK	NCP	79.184
ESVKFHK;NSRVVDN	NCP	63.727
NSRVVDN;PGVFELY	NCP	21.14
PGVFELY;KGMMGP	NCP	31.516
KGMMGP;VPVLPQ	NCP	5.325
VPVLPQ;LFGKQK	NCP	0.147
LFGKQK;KRAVSTK	NCP	174.725
KRAVSTK;VVTVSLRP	NCP	224.203
VVTVSLRP;SGPGFWTGL	NCP	48.584
SGPGFWTGL;AEVPF	NCP	172.909
AEVPF;KAQPLDK	NCP	137.852
KAQPLDK;KGRVAPP	NCP	32.607
KGRVAPP;KKLSVPLP	NCP	36.643
KKLSVPLP;CKAPEPVL	NCP	143.079
CKAPEPVL;KGMHTVK	NCP	36.396
KGMHTVK;LMKVALD	NCP	132.266
LMKVALD;VEVAL	NCP	30.417
VEVAL;HPPKKR	NCP	90.817

HPPKKR;LDETPY	NCP	125.303
LDETPY;KVEGTAP	NCP	15.924
KVEGTAP;AKAAK	NCP	68.272
AKAAK;LLLVV	NCP	47.588
LLLVV;VAPTVQEKKVKK	NCP	6.471
VAPTVQEKKVKK;SAGAK	NCP	55.854
SAGAK;RLPSNR	NCP	41.649
RLPSNR;KRLASH	NCP	128.398
KRLASH;KEGTPP	NCP	31.881
KEGTPP;PGPLAPPP	NCP	101.712
PGPLAPPP;PLVPVLPP	NCP	121.575
PLVPVLPP;LSDTSVVL	NCP	151.245
LSDTSVVL;ATLRVL	NCP	30.618
ATLRVL;GDLLEL	NCP	0.623
GDLLEL;FDLGMY	NCP	33.175
FDLGMY;KYKPPYK	NCP	244.022
KYKPPYK;GGDFLLA	NCP	35.07
GGDFLLA;KSVHVMPK	NCP	62.151
KSVHVMPK;QKLKPK	NCP	57.385
QKLKPK;VSSGPP	NCP	3.878
VSSGPP;MGVLSPP	NCP	11.389
MGVLSPP;AVSEGTKAVTKY	NCP	135.917
AVSEGTKAVTKY;FSLTSGYF	NCP	8.268
FSLTSGYF;MVKEKR	NCP	93.337
MVKEKR;PFDLP	NCP	0.299
PFDLP;AAGLLLP	NCP	56.016
AAGLLLP;KDSLVQ	NCP	93.195
KDSLVQ;GDLLK	NCP	218.484
GDLLK;NMFLLFKT	NCP	120.802
NMFLLFKT;TDDDAGN	NCP	36.488
TDDDAGN;EGPEKPTK	NCP	57.192
EGPEKPTK;PVSLPN	NCP	466.99
PVSLPN;MESLLQLVR	NCP	345.087
MESLLQLVR;VSRKPGAK	NCP	66.291
VSRKPGAK;KTGGKPK	NCP	46.076
KTGGKPK;TSNKEFLR	NCP	92.644
TSNKEFLR;LDVGYERF	NCP	117.131
LDVGYERF;TLLLK	NCP	174.874
TLLLK;REKMGK	NCP	107.712
REKMGK;EKVSFKLT	NCP	31.65
EKVSFKLT;PAPASPP	NCP	52.307
PAPASPP;NVRLT	NCP	104.944
NVRLT;NVMLQ	NCP	27.761
NVMLQ;KVTKAPK	NCP	123.418
KVTKAPK;EAVHG	NCP	191.157
EAVHG;EKNHPEQ	NCP	106.671
EKNHPEQ;NSGPARPP	NCP	93.972
NSGPARPP;LDKPTLH	NCP	10.175
LDKPTLH;NSCCCHY	NCP	7.885
NSCCCHY;VGTDKMR	NCP	147.266
VGTDKMR;KGSRKVQ	NCP	10.557

KGSRKVQ;AKLTMMT	NCP	48.396
AKLTMMT;NGELLDF	NCP	7.908
NGELLDF;RGGMALY	NCP	26.211
RGGMALY;AAMPGP	NCP	37.033
AAMPGP;THDLPAP	NCP	101.979
THDLPAP;LEEKF	NCP	9.309
LEEKF;KAAVLS	NCP	87.711
KAAVLS;VDLPEVD	NCP	38.111
VDLPEVD;KGMSYPR	NCP	102.801
KGMSYPR;EQVLDP	NCP	209.865
EQVLDP;PVFAPPP	NCP	8.062
PVFAPPP;TWVTLK	NCP	30.416
TWVTLK;SQAKL	NCP	75.6
SQAKL;KELNGVP	NCP	5.776
KELNGVP;FGKSKK	NCP	485.58
FGKSKK;SPSELKN	NCP	8.691
SPSELKN;RLSPF	NCP	193.547
RLSPF;TADLKGPK	NCP	56.101
TADLKGPK;ALCLPK	NCP	6.593
ALCLPK;DLGFLT	NCP	0.154
DLGFLT;LDLFT	NCP	12.299
LDLFT;CRRTESH	NCP	0.613
CRRTESH;DPVDPTTVTK	NCP	138.647
DPVDPTTVTK;LLYKDK	NCP	48.52
LLYKDK;AFKPPLA	NCP	128.73
AFKPPLA;YEEALKY	NCP	2.339
YEEALKY;KDLQYE	NCP	0.13
KDLQYE;KGELWLA	NCP	51.276
KGELWLA;LLKGMVLS	NCP	196.656
LLKGMVLS;GVDDLKL	NCP	156.917
GVDDLKL;KATLPWL	NCP	128.224
KATLPWL;VTGVGTLK	NCP	16.178
VTGVGTLK;LRGNVF	NCP	16.785
LRGNVF;YVLMELTNH	NCP	334.35
YVLMELTNH;QSVVEPY	NCP	73.987
QSVVEPY;MEHLDTQ	NCP	22.05
MEHLDTQ;ETSSG	NCP	70.232
ETSSG;MPLLLTG	NCP	59.032
MPLLLTG;NPRLLYD	NCP	15.029
NPRLLYD;LAEALKL	NCP	21.054
LAEALKL;MLFAFPSL	NCP	26.566
MLFAFPSL;KRTLDVH	NCP	19.583
KRTLDVH;NVLPK	NCP	153.852
NVLPK;LPRLAY	NCP	31.023
LPRLAY;KGVPTDLA	NCP	82.519
KGVPTDLA;KGYAQF	NCP	361.073
KGYAQF;VLDVVREK	NCP	62.844
VLDVVREK;VVPWN	NCP	2.112
VVPWN;FNFLL	NCP	44.242
FNFLL;GPAELSR	NCP	20.475
GPAELSR;VDLGL	NCP	218.928

VDLGL;KNLEGKVLK	NCP	73.269
KNLEGKVLK;FFLPPPP	NCP	34.191
FFLPPPP;AAHEVLY	NCP	3.326
AAHEVLY;RPGHVR	NCP	3.513
RPGHVR;LVKPSLVA	NCP	123.861
LVKPSLVA;WLNPVLF	NCP	119.185
WLNPVLF;AELDDGC	NCP	100.295
AELDDGC;YKPPSRP	NCP	37.6
YKPPSRP;LEELQKK	NCP	4.176
LEELQKK;KGEGNK	NCP	6.405
KGEGNK;KRKKMFQ	NCP	102.144
KRKKMFQ;YRTGAPP	NCP	15.245
YRTGAPP;MDVPLKF	NCP	23.01
MDVPLKF;RELEDFR	NCP	13.912
RELEDFR;KNFEKVFQ	NCP	38.327
KNFEKVFQ;MMMSCC	NCP	231.61
MMMSCC;LQTVDRPK	NCP	149.306
LQTVDRPK;VDRPKDW	NCP	0.009
VDRPKDW;DRPKDWYK	NCP	0.003
DRPKDWYK;HDWDPPDRK	NCP	5.826
HDWDPPDRK;DWDPPDR	NCP	0.003
DWDPPDR;FEYEPGK	NCP	0.063
FEYEPGK;VEVVRKHTT	NCP	37.125
VEVVRKHTT;TFPGNYVKRL	NCP	22.875
TFPGNYVKRL;NPVVKMD	NCP	19.347
NPVVKMD;RPLHEPSQ	NCP	16.932
RPLHEPSQ;TLVAR	NCP	5.926
TLVAR;KTVHPPR	NCP	12.909
KTVHPPR;VTEDGKAHPF	NCP	3.323
VTEDGKAHPF;TPSGVDGGSGRSTP	NCP	13.407
TPSGVDGGSGRSTP;QGQVSTALG	NCP	0.21
QGQVSTALG;HDNRNEPTQPRQSGS	NCP	1.596
HDNRNEPTQPRQSGS;GSFRVLQE	NCP	0.039
GSFRVLQE;VRAPVTKV	NCP	2.271
VRAPVTKV;VHGGAGGTQKMPF	NCP	0.021
VHGGAGGTQKMPF;VCADCNLNLKQK	NCP	1.524
VCADCNLNLKQK;VEGELYCETHARA	NCP	0.048
VEGELYCETHARA;DTVTLYPK	NCP	0.063
DTVTLYPK;KVKPLLQ	NCP	16.365
KVKPLLQ;AAPLEVLR	NCP	39.189
AAPLEVLR;GSYGF	NCP	74.055
GSYGF;RKKPPK	NCP	1.371
RKKPPK;PPLGPPP	NCP	93.096
PPLGPPP;KKVPFP	NCP	15.714
KKVPFP;SGPFLTR	NCP	83.064
SGPFLTR;MQAHELFQ	NCP	168.054
MQAHELFQ;ALEGALF	NCP	62.076
ALEGALF;LSKELR	NCP	62.22
LSKELR;MLTKELYF	NCP	54.102
MLTKELYF;MLASHL	NCP	14.386
MLASHL;PPLLL	NCP	15.143

PPLLL;VYVKSK	NCP	35.133
VYVKSK;LFAAGL	NCP	0.534
LFAAGL;LTQVGK	NCP	178.812
LTQVGK;LVLVY	NCP	55.519
LVLVY;KEKSLNEV	NCP	75.222
KEKSLNEV;VLPLL	NCP	60.06
VLPLL;HLAVFV	NCP	11.9
HLAVFV;MFETF	NCP	51.409
MFETF;LLGSRGAT	NCP	38.045
LLGSRGAT;TWFKF	NCP	39.022
TWFKF;VKALAGN	NCP	2.696
VKALAGN;GPALPPR	NCP	101.907
GPALPPR;REGDLKK	NCP	39.804
REGDLKK;KPFDWT	NCP	16.434
KPFDWT;KPGMK	NCP	108.1
KPGMK;GSVDLL	NCP	10.514
GSVDLL;MTQPPAA	NCP	48.877
MTQPPAA;PSQPAPAEF	NCP	40.311
PSQPAPAEF;PELVAVPP	NCP	34.763
PELVAVPP;PVDGGN	NCP	51.78
PVDGGN;PPAAFA	NCP	74.769
PPAAFA;KVLELQ	NCP	280.597
KVLELQ;EDLGFGK	NCP	94.799
EDLGFGK;KSVPLTF	NCP	148.336
KSVPLTF;TSSHETPR	NCP	67.8
TSSHETPR;RLGEC	NCP	139.878
RLGEC;LQVFD	NCP	10.265
LQVFD;KVKGLEP	NCP	91.117
KVKGLEP;DNSLLR	NCP	170.1
DNSLLR;VGDRPLAL	NCP	63.867
VGDRPLAL;SGSYLKKL	NCP	7.905
SGSYLKKL;KNYLTA	NCP	54.591
KNYLTA;FNDGKY	NCP	236.19
FNDGKY;RTLKLT	NCP	2.529
RTLKLT;KYMALPP	NCP	109.302
KYMALPP;CDHGM	NCP	8.534
CDHGM;RGLFVAR	NCP	4.668
RGLFVAR;DNFSAQR	NCP	114.418
DNFSAQR;VAVLG	NCP	372.654
VAVLG;SMVHGKP	NCP	194.153
SMVHGKP;DLEKPQ	NCP	9.054
DLEKPQ;RYEKK	NCP	36.178
RYEKK;DNHFVK	NCP	33.473
DNHFVK;ANLALF	NCP	27.898
ANLALF;KYVTLPP	NCP	28.391
KYVTLPP;HPAPHPPSP	NCP	46.822
HPAPHPPSP;VLSPT	NCP	248.67
VLSPT;VKSKKL	NCP	8.208
VKSKKL;CDDGP	NCP	72.591
CDDGP;STMKLKLL	NCP	176.442
STMKLKLL;KELPLH	NCP	77.276

KELPLH;KKHSEK	NCP	35.022
KKHSEK;MWVPMK	NCP	4.506
MWVPMK;QVSPDLG	NCP	38.311
QVSPDLG;DAAKSFLL	NCP	11.496
DAAKSFLL;PGKQDPP	NCP	114.561
PGKQDPP;PVSSADLH	NCP	144.258
PVSSADLH;LPKDRPP	NCP	29.838
LPKDRPP;YGFMYP	NCP	123.57
YGFMYP;LALEYL	NCP	4.074
LALEYL;KLSTVRLL	NCP	20.718
KLSTVRLL;SSMLMT	NCP	141.369
SSMLMT;LEYKKL	NCP	85.542
LEYKKL;KSLQKAPK	NCP	42.989
KSLQKAPK;LSMMTEL	NCP	268.9
LSMMTEL;KYDPK	NCP	228.422
KYDPK;KGLELY	NCP	55.225
KGLELY;VGLLDVTPL	NCP	113.315
VGLLDVTPL;KPGPKPH	NCP	99.223
KPGPKPH;KDPGYVP	NCP	18.309
KDPGYVP;KLVLPR	NCP	189.744
KLVLPR;MGPWFK	NCP	249.816
MGPWFK;SCDDN	NCP	34.446
SCDDN;GFWHQ	NCP	47.975
GFWHQ;TVPLQK	NCP	78.625
TVPLQK;VGPLP	NCP	211.113
VGPLP;KVGVLAWR	NCP	103.883
KVGVLAWR;QAVLSL	NCP	20.373
QAVLSL;KDGVLP	NCP	37.32
KDGVLP;DRLRLP	NCP	16.449
DRLRLP;QRYLQ	NCP	139.365
QRYLQ;YFNTLR	NCP	54.354
YFNTLR;FLMRSF	NCP	96.982
FLMRSF;QMYVP	NCP	30.479
QMYVP;LHDPPPP	NCP	16.831
LHDPPPP;PPPPLHGK	NCP	21.483
PPPPLHGK;AAVSLGNS	NCP	12.855
AAVSLGNS;WVDKLRP	NCP	81.564
WVDKLRP;LVKSYMT	NCP	16.961
LVKSYMT;LSVMFHK	NCP	53.574
LSVMFHK;KAMSQ	NCP	65.542
KAMSQ;TRFGLAA	NCP	40.044
TRFGLAA;VGPALH	NCP	37.301
VGPALH;KERVYST	NCP	104.911
KERVYST;LTVFKP	NCP	29.426
LTVFKP;NLKREFRK	NCP	24.217
NLKREFRK;FYERLL	NCP	146.637
FYERLL;LGKALL	NCP	46.238
LGKALL;LSASKPK	NCP	31.81
LSASKPK;TAMDVVV	NCP	384.723
TAMDVVV;NAAVPNL	NCP	236.316
NAAVPNL;EGFAG	NCP	29.244

EGFAG;VSFVPP	NCP	9.486
VSFVPP;LDLAVGPG	NCP	202.997
LDLAVGPG;PPVVG	NCP	29.884
PPVVG;DRGPPP	NCP	207.968
DRGPPP;HLGPPAPK	NCP	42.969
HLGPPAPK;KPSAPQ	NCP	34.315
KPSAPQ;NVLGNER	NCP	5.861
NVLGNER;LPLALR	NCP	26.448
LPLALR;VTLGK	NCP	187.069
VTLGK;DLPAEE	NCP	19.079
DLPAEE;KESGPASK	NCP	18.289
KESGPASK;FLGNS	NCP	108.279
FLGNS;EVSKVRVQ	NCP	51.863
EVSKVRVQ;PSLYFK	NCP	18.514
PSLYFK;VSFSKLST	NCP	13.965
VSFSKLST;EAPGAP	NCP	66.452
EAPGAP;PMARAW	NCP	91.66
PMARAW;ELELLGAPET	NCP	127.842
ELELLGAPET;VGLQY	NCP	215.927
VGLQY;CCMGR	NCP	34.687
CCMGR;LEKTTTPQ	NCP	6.566
LEKTTTPQ;VEWVA	NCP	8.511
VEWVA;WDGKP	NCP	117.481
WDGKP;KDPVL	NCP	9.437
KDPVL;EDAPP	NCP	179.491
EDAPP;ELHQF	NCP	86.808
ELHQF;LLVTLLH	NCP	101.199
LLVTLLH;EVNLPGM	NCP	4.917
EVNLPGM;KNGGAPV	NCP	24.188
KNGGAPV;KVGLCC	NCP	53.062
KVGLCC;PATEF	NCP	62.082
PATEF;KSVPLP	NCP	34.245
KSVPLP;VGFVHNVK	NCP	41.307
VGFVHNVK;SGVLTGTK	NCP	65.547
SGVLTGTK;SPPLLG	NCP	45.198
SPPLLG;PLGSSPP	NCP	35.024
PLGSSPP;KWLWRPP	NCP	292.248
KWLWRPP;FLVFPP	NCP	213.445
FLVFPP;LSDVDLP	NCP	10.914
LSDVDLP;EVDSLLP	NCP	50.837
EVDSLLP;PPMDVLLL	NCP	61.078
PPMDVLLL;VPAALH	NCP	27.714
VPAALH;MTELLK	NCP	14.726
MTELLK;DHLHPKA	NCP	2.497
DHLHPKA;VDGFAA	NCP	328.058
VDGFAA;AAVFP	NCP	4.965
AAVFP;LLLEGPAL	NCP	175.939
LLLEGPAL;ALGLL	NCP	123.042
ALGLL;KVVGGVN	NCP	153.044
KVVGGVN;PHALMLR	NCP	120.919
PHALMLR;DLERPTY	NCP	32.795

DLERPTY;KTLTDKAP	NCP	1.546
KTLTDKAP;AREGYSF	NCP	9.707
AREGYSF;TVVPP	NCP	102.067
TVVPP;GNVPKTR	NCP	5.465
GNVPKTR;LEPFR	NCP	12.433
LEPFR;KHKAGPP	NCP	36.498
KHKAGPP;PGTAPK	NCP	46.533
PGTAPK;LGELYL	NCP	9.965
LGELYL;TTFVT	NCP	88.779
TTFVT;RAKALR	NCP	44.326
RAKALR;KEGEGLPY	NCP	21.636
KEGEGLPY;QLEKPR	NCP	24.087
QLEKPR;EARAAAGY	NCP	57.446
EARAAAGY;TLPSPGR	NCP	133.131
TLPSPGR;LNFFT	NCP	8.01
LNFFT;EAPAYSP	NCP	12.091
EAPAYSP;KGAAGRLP	NCP	161.952
KGAAGRLP;LRKAEL	NCP	34.809
LRKAEL;VLLVAY	NCP	333.603
VLLVAY;LSAAY	NCP	27.227
LSAAY;KNMSPVP	NCP	261.915
KNMSPVP;NPVQY	NCP	0.177
NPVQY;LDVVLG	NCP	182.662
LDVVLG;KALVLKGE	NCP	94.794
KALVLKGE;KQWLKK	NCP	26.178
KQWLKK;KNLAQKY	NCP	35.157
KNLAQKY;KAEELVH	NCP	271.098
KAEELVH;PVVLR	NCP	16.086
PVVLR;ESSPVNVK	NCP	44.387
ESSPVNVK;VVRVLQ	NCP	18.583
VVRVLQ;YYSNR	NCP	7.887
YYSNR;HGPTLGDQ	NCP	95.444
HGPTLGDQ;KYPPTK	NCP	109.717
KYPPTK;SFADTF	NCP	5.541
SFADTF;APECAPLK	NCP	233.519
APECAPLK;SANTPVR	NCP	40.485
SANTPVR;PPAYGNP	NCP	15.702
PPAYGNP;KGPGEHK	NCP	118.434
KGPGEHK;DMCGPP	NCP	70.848
DMCGPP;NSGPYP	NCP	123.471
NSGPYP;NQGKLFK	NCP	218.536
NQGKLFK;GVPLDLP	NCP	192.563
GVPLDLP;PLMLL	NCP	0.036
PLMLL;YGVDPW	NCP	60.961
YGVDPW;AAPAPPGM	NCP	108.896
AAPAPPGM;ALGLSV	NCP	9.513
ALGLSV;PPLLPDP	NCP	2.242
PPLLPDP;QHKALPH	NCP	0.224
QHKALPH;HGVYTL	NCP	34.578
HGVYTL;KGGAP	NCP	17.371
KGGAP;ADLSAPK	NCP	22.311

ADLSAPK;LVVHVV	NCP	12.146
LVVHVV;MYPLP	NCP	95.761
TVVLK;LSPVEC	NCP	201.378
LSPVEC;DPGAK	NCP	80.733
DPGAK;LSSGAP	NCP	194.064
LSSGAP;LPPGSPR	NCP	39.297
LPPGSPR;WGLQDPPPP	NCP	84.804
WGLQDPPPP;LSVLYLF	NCP	113.362
LSVLYLF;TFKFLP	NCP	10.062
TFKFLP;SGFGKKLG	NCP	3.156
SGFGKKLG;SSSLSGYPKKPMT	NCP	219.965
SSSLSGYPKKPMT;TLLDAF	NCP	4.126
TLLDAF;LSGPVAF	NCP	32.138
LSGPVAF;PVLVR	NCP	52.552
PVLVR;TALRSH	NCP	17.918
TALRSH;MRVLPL	NCP	177.054
MRVLPL;LLLVWQ	NCP	74.319
LLLVWQ;KPHSTHLLR	NCP	17.803
KPHSTHLLR;KAMAASK	NCP	119.504
KAMAASK;KEETTK	NCP	15.699
KEETTK;PGAAP	NCP	47.095
PGAAP;TAAVVKK	NCP	75.338
TAAVVKK;AMLPP	NCP	94.338
AMLPP;PPPLPA	NCP	138.277
PPPLPA;VPSLDPLQ	NCP	42.584
VPSLDPLQ;GCMDC	NCP	119.592
GCMDC;KVRLRP	NCP	25.995
KVRLRP;VTLLGH	NCP	107.088
VTLLGH;LDEKPYKC	NCP	11.229
LDEKPYKC;LQLQPGAK	NCP	228.705
LQLQPGAK;EPPELPAL	NCP	161.035
EPPELPAL;DRPSFRN	NCP	120.506
DRPSFRN;GVLSV	NCP	2.311
GVLSV;LSPSLP	NCP	28.175
LSPSLP;VSVKLF	NCP	32.194
VSVKLF;QGLPFR	NCP	87.032
QGLPFR;KLTVY	NCP	112.96
KLTVY;YETFK	NCP	62.112
YETFK;TALPAHR	NCP	151.298
TALPAHR;EELTPF	NCP	13.339
EELTPF;FRLPLK	NCP	4.088
FRLPLK;KGLLPAAH	NCP	38.958
KGLLPAAH;KGLVLPQQ	NCP	102.421
KGLVLPQQ;LDLEDSPK	NCP	33.572
LDLEDSPK;TRFFLVDF	NCP	92.763
TRFFLVDF;SNFNHL	NCP	22.23
SNFNHL;KAKPPPP	NCP	58.023
KAKPPPP;PALDLSRR	NCP	33.52
PALDLSRR;PAPLPPPPP	NCP	60.21
PAPLPPPPP;APLPPPPPPPP	NCP	0.003
APLPPPPPPPP;GPAADSP	NCP	10.964

GPAADSP;PAPFVRP	NCP	6.84
PAPFVRP;QWAAVPP	NCP	145.677
QWAAVPP;TAPATP	NCP	15.547
TAPATP;GSPFVP	NCP	7.883
GSPFVP;AVLLET	NCP	3.222
AVLLET;PASQLPGP	NCP	99.828
PASQLPGP;NFLPT	NCP	91.513
NFLPT;EAPSSKLK	NCP	6.093
EAPSSKLK;KLLCSCSF	NCP	3.336
KLLCSCSF;KPHPSKPK	NCP	16.049
KPHPSKPK;AAMAVAR	NCP	218.544
AAMAVAR;EEEPLK	NCP	108.225
EEEPLK;KASHLR	NCP	36.499
KASHLR;VEPLLT	NCP	13.014
VEPLLT;PPYTL	NCP	42.519
PPYTL;VVQHNP	NCP	47.847
VVQHNP;FCWTK	NCP	10.574
FCWTK;SNVVF	NCP	2.524
SNVVF;KDALLAS	NCP	57.338
KDALLAS;VPDLFF	NCP	14.581
VPDLFF;KTPLPALK	NCP	150.459
KTPLPALK;FSPQP	NCP	20.537
FSPQP;YFSLVRHK	NCP	22.588
YFSLVRHK;TLTLLRL	NCP	230.94
TLTLLRL;STDVSVDEVKALASLMT	NCP	104.342
STDVSVDEVKALASLMT;ALASLMTYK	NCP	0.03
ALASLMTYK;TMELAKKGF	NCP	1.536
TMELAKKGF;LHRFGAK	NCP	12.795
LHRFGAK;NAGGVTVSY	NCP	2.772
NAGGVTVSY;FEWLKNLNHVSY	NCP	0.027
FEWLKNLNHVSY;LGLDLRTAAY	NCP	6.18
LGLDLRTAAY;NEAGVTFT	NCP	0.063
NEAGVTFT;LVTVHK	NCP	7.312
LVTVHK;QPKQGLSGP	NCP	33.242
QPKQGLSGP;SPVKP	NCP	7.446
SPVKP;ASKLLLRG	NCP	1.746
ASKLLLRG;KLKPRP	NCP	102.877
KLKPRP;LPVPGDP	NCP	136.865
LPVPGDP;LPANLPTPPP	NCP	2.226
LPANLPTPPP;GVAGFEA	NCP	32.11
GVAGFEA;KSWLPSGKQ	NCP	59.253
KSWLPSGKQ;KYRPPPPP	NCP	47.21
KYRPPPPP;KPRPPPPAP	NCP	95.22
KPRPPPPAP;ARLPPPP	NCP	1.836
ARLPPPP;VGTVLLTR	NCP	7.863
VGTVLLTR;LTALWPP	NCP	11.661
LTALWPP;GMYPM	NCP	139.656
GMYPM;QQAFR	NCP	94.741
QQAFR;VFRPVVVL	NCP	245.019
VFRPVVVL;LPGLVLP	NCP	22.58
LPGLVLP;LAMVA	NCP	48.339

LAMVA;PYLLL	NCP	32.352
PYLLL;PGGAF	NCP	41.059
PGGAF;LLGLQM	NCP	47.421
LLGLQM;KPPSPPP	NCP	40.98
KPPSPPP;KEVPN	NCP	130.79
KEVPN;GLFASPK	NCP	32.484
GLFASPK;RLFQR	NCP	7.321
RLFQR;LFMLSK	NCP	20.624
LFMLSK;TPLADT	NCP	10.549
TPLADT;LLVPY	NCP	30.828
LLVPY;ANLLYY	NCP	114.873
ANLLYY;RLGRPP	NCP	262.488
RLGRPP;KAPSVPPK	NCP	29.475
KAPSVPPK;EVAVGF	NCP	147.594
EVAVGF;LLEAPQ	NCP	23.681
LLEAPQ;LSDTSVV	NCP	1.965
LSDTSVV;ALVMQ	NCP	27.597
ALVMQ;TMLMDLK	NCP	18.997
TMLMDLK;EANERVP	NCP	252.186
EANERVP;KGLVRAL	NCP	24.351
KGLVRAL;NAGELLDF	NCP	68.897
NAGELLDF;LGVSALFK	NCP	14.769
LGVSALFK;FLEVY	NCP	117.903
FLEVY;LSVHRKCF	NCP	4.662
LSVHRKCF;LEPVP	NCP	72.964
LEPVP;QAEWDDYVPK	NCP	40.157
QAEWDDYVPK;VGKMHFR	NCP	209.392
VGKMHFR;HHVCKK	NCP	191.129
HHVCKK;KWAETLY	NCP	3.336
KWAETLY;KDSTF	NCP	7.35
KDSTF;KSVEC	NCP	48.403
KSVEC;SVGGLVAL	NCP	27.164
SVGGLVAL;KKSLLPDH	NCP	5.152
KKSLLPDH;VPSPGH	NCP	93.825
VPSPGH;VTLPEE	NCP	166.097
VTLPEE;FLRLQ	NCP	62.758
FLRLQ;EMLRLP	NCP	24.17
EMLRLP;YKAAPK	NCP	36.369
YKAAPK;VLSAC	NCP	134.578
VLSAC;TLATL	NCP	112.58
TLATL;KQYGTMP	NCP	84.294
KQYGTMP;RGHLFL	NCP	71.844
RGHLFL;AWRHF	NCP	219.312
AWRHF;ETGTFVV	NCP	94.104
ETGTFVV;RSPSTLGH	NCP	304.791
RSPSTLGH;KPPGFLT	NCP	104.638
KPPGFLT;VVGPSSP	NCP	61.754
VVGPSSP;TVGAMN	NCP	131.811
TVGAMN;GVPPSP	NCP	25.75
GVPPSP;KSLYLK	NCP	111.032
KSLYLK;FGLLDGN	NCP	180.462

FGLLDGN;MCCPR	NCP	180.967
MCCPR;CDESP	NCP	6.035
CDESP;TGGLHTET	NCP	1.174
TGGLHTET;KGASLL	NCP	92.169
KGASLL;LEFLPPKY	NCP	33.462
LEFLPPKY;LAKLLR	NCP	37.794
LAKLLR;MGGFQKP	NCP	35.654
MGGFQKP;MDFTSGP	NCP	13.218
MDFTSGP;FALAYPP	NCP	124.887
FALAYPP;LSELKALL	NCP	242.157
LSELKALL;ELVESLK	NCP	32.914
ELVESLK;AANVTPPFP	NCP	77.336
AANVTPPFP;QWLPGK	NCP	123.361
QWLPGK;NRLAEL	NCP	9.671
NRLAEL;LVVAY	NCP	17.901
LVVAY;AFSLVHR	NCP	11.628
AFSLVHR;AAAMGKAR	NCP	133.878
AAAMGKAR;LNGPP	NCP	8.904
LNGPP;GAPHCQP	NCP	361.71
GAPHCQP;PPPLPPKF	NCP	80.688
PPPLPPKF;PSKEQLF	NCP	0.562
PSKEQLF;QVPADPK	NCP	77.267
QVPADPK;LPPWLK	NCP	127.77
LPPWLK;KSLTM	NCP	11.523
KSLTM;MEEFAPV	NCP	10.863
MEEFAPV;LLLLPPGPQ	NCP	71.391
LLLLPPGPQ;SDPRLPY	NCP	142.011
SDPRLPY;YWGNAPP	NCP	49.482
YWGNAPP;HDQMAK	NCP	73.08
HDQMAK;FSLPAKK	NCP	43.635
FSLPAKK;TVGLPV	NCP	38.254
TVGLPV;NSVKLNEHF	NCP	55.701
NSVKLNEHF;KQPGGP	NCP	123.824
KQPGGP;GVGVFF	NCP	135.165
GVGVFF;TSYCLT	NCP	28.968
TSYCLT;KLTAKGPK	NCP	7.41
KLTAKGPK;KALDVR	NCP	131.13
KALDVR;WGELHC	NCP	11.646
WGELHC;EGAGLLD	NCP	43.557
EGAGLLD;TVGPEL	NCP	37.624
TVGPEL;GPPHKAAP	NCP	248.738
GPPHKAAP;CMAFQ	NCP	20.809
CMAFQ;GPALPFH	NCP	56.498
GPALPFH;PKSVGK	NCP	87.756
PKSVGK;QPPGSSFH	NCP	5.458
QPPGSSFH;KYGVPPPPP	NCP	74.216
KYGVPPPPP;AHAVF	NCP	206.976
AHAVF;EGAGNP	NCP	189.102
EGAGNP;AVVLLR	NCP	53.748
AVVLLR;PPPPFPT	NCP	47.844
PPPPFPT;PPPEPRT	NCP	84.778

PPPEPRT;TMMELP	NCP	19.224
TMMELP;RWMKK	NCP	9.462
RWMKK;MLPPRPP	NCP	30.855
MLPPRPP;AAFFC	NCP	25.382
AAFFC;ADTDALLK	NCP	252.034
ADTDALLK;TVKKLALF	NCP	215.651
TVKKLALF;FQVTKL	NCP	132.618
FQVTKL;QRVVVPGN	NCP	33.507
QRVVVPGN;AHRGAP	NCP	77.158
AHRGAP;DPTLSKY	NCP	40.782
DPTLSKY;ATLFTKSH	NCP	10.341
ATLFTKSH;TTLLYK	NCP	27.42
TTLLYK;TPVHVQP	NCP	6.008
TPVHVQP;GVKLD	NCP	112.965
GVKLD;VTPGALFF	NCP	45.981
VTPGALFF;MDMLYV	NCP	20.946
MDMLYV;TQPKVPK	NCP	75.28
TQPKVPK;ALGVMLR	NCP	10.251
ALGVMLR;TVRHLEYL	NCP	4.976
TVRHLEYL;KGVPP	NCP	1.662
KGVPP;YDPKLG	NCP	85.864
YDPKLG;KAQGLREN	NCP	19.778
KAQGLREN;KEPHH	NCP	29.544
KEPHH;VGVEGL	NCP	22.95
VGVEGL;KRRAAPQ	NCP	40.219
KRRAAPQ;KELSTQ	NCP	219.876
KELSTQ;TSGESKTGLKN	NCP	75.011
TSGESKTGLKN;FLPDV	NCP	17.782
FLPDV;AKVKAVNG	NCP	1.352
AKVKAVNG;TLLRDEK	NCP	27.427
TLLRDEK;SHLNVPSPP	NCP	4.593
SHLNVPSPP;FPLDL	NCP	0.483
FPLDL;GKNKPR	NCP	99.647
GKNKPR;LPKERP	NCP	162.745
LPKERP;YPDQP	NCP	15.284
YPDQP;LDKKDL	NCP	246.211
LDKKDL;AKPERK	NCP	28.463
AKPERK;TYVLR	NCP	275.109
TYVLR;AKELYL	NCP	4.461
AKELYL;TGKKLHC	NCP	100.575
TGKKLHC;ATTATCTRFTDDY	NCP	97.749
ATTATCTRFTDDY;TVTLLF	NCP	33.282
TVTLLF;VPGGWGNK	NCP	39.558
VPGGWGNK;PPVAPKP	NCP	60.48
PPVAPKP;PLSGTPAPNKKR	NCP	6.174
PLSGTPAPNKKR;TAGQGFSYSK	NCP	3.102
TAGQGFSYSK;DLGPLLSE	NCP	2.751
DLGPLLSE;YNQNLSNRPS	NCP	0.036
YNQNLSNRPS;NQNLSNRP	NCP	0.003
NQNLSNRP;QNLSNRPSF	NCP	0.003
QNLSNRPSF;SNRPSFNR	NCP	0.009

SNRPSFNR;SSGEPVDY	NCP	0.042
SSGEPVDY;NRHAA	NCP	12.984
NRHAA;KVELK	NCP	42.901
KVELK;YPPTGYPP	NCP	154.133
YPPTGYPP;FPPPGQYP	NCP	2.07
FPPPGQYP;QLAQEDAQRLYQ	NCP	12.213
QLAQEDAQRLYQ;CTSFNSKLK	NCP	162.618
CTSFNSKLK;GVRLL	NCP	140.931
GVRLL;LVLLM	NCP	21.616
LVLLM;KPRRPP	NCP	56.391
KPRRPP;LDPVP	NCP	36.597
LDPVP;QLRVFVY	NCP	212.714
QLRVFVY;KGPATR	NCP	140.919
KGPATR;SLGELL	NCP	50.644
SLGELL;KLTAMQK	NCP	134.642
KLTAMQK;AGPGVKVR	NCP	81.303
AGPGVKVR;PLKKK	NCP	40.749
PLKKK;ETAKKYK	NCP	66.388
ETAKKYK;KLELGF	NCP	7.944
KLELGF;GPMGP	NCP	103.592
GPMGP;PSFPLG	NCP	131.58
PSFPLG;VGAPE	NCP	53.134
VGAPE;PGPYLP	NCP	40.136
PGPYLP;SLPGAELR	NCP	52.164
SLPGAELR;AAPPASKF	NCP	117.847
AAPPASKF;STKVL	NCP	13.914
STKVL;KTAEPTGR	NCP	17.624
KTAEPTGR;AAELVLK	NCP	12.144
AAELVLK;AETLEF	NCP	57.919
AETLEF;TVLTPKQTH	NCP	49.052
TVLTPKQTH;GSANSK	NCP	29.58
GSANSK;DSGLVF	NCP	140.143
DSGLVF;GVPGLTTK	NCP	7.742
GVPGLTTK;KDGLYYT	NCP	112.773
KDGLYYT;KVLLAF	NCP	8.112
KVLLAF;QAPVY	NCP	67.378
QAPVY;VLRPPR	NCP	11.7
VLRPPR;PGGRLGVVT	NCP	78.359
PGGRLGVVT;AQLLPLRF	NCP	34.846
AQLLPLRF;KLTFS	NCP	177.03
KLTFS;AVLPA	NCP	23.582
AVLPA;APGLPPP	NCP	27.726
APGLPPP;RLQYQALL	NCP	55.75
RLQYQALL;ARGQVPR	NCP	66.608
ARGQVPR;TQDLVR	NCP	37.461
TQDLVR;QALMP	NCP	2.535
QALMP;PFAPP	NCP	16.567
PFAPP;KENYMDKLR	NCP	7.722
KENYMDKLR;KARGAPR	NCP	61.697
KARGAPR;EQYDVLH	NCP	96.694
EQYDVLH;VPAYY	NCP	71.567

VPAYY;PELGPSPP	NCP	2.248
PELGPSPP;SEERAAPF	NCP	18.749
SEERAAPF;PVGKVYT	NCP	2.808
PVGKVYT;VPQTGWAK	NCP	39.948
VPQTGWAK;AALEF	NCP	128.964
AALEF;AAVEVL	NCP	18.88
AAVEVL;VLLPGPP	NCP	14.279
VLLPGPP;SATAAEVR	NCP	77.505
SATAAEVR;KSHPPPPR	NCP	54.705
KSHPPPPR;TASGGAP	NCP	9.628
TASGGAP;MNQLEP	NCP	10.401
MNQLEP;HTVLM	NCP	96.762
HTVLM;TSPNPHKPP	NCP	49.838
TSPNPHKPP;ASHSLL	NCP	30.363
ASHSLL;VNNKSHLV	NCP	12.706
VNNKSHLV;PEPLF	NCP	23.129
PEPLF;VGEVLVT	NCP	7.125
VGEVLVT;GVMLP	NCP	382.059
GVMLP;KLDLDSYL	NCP	56.191
KLDLDSYL;MDFKQ	NCP	56.676
MDFKQ;NPGLLL	NCP	134.235
NPGLLL;LLTKKV	NCP	11.549
LLTKKV;HESFLK	NCP	16.411
HESFLK;QAHLKL	NCP	73.451
QAHLKL;VGDVLLAP	NCP	294.24
VGDVLLAP;TVVLY	NCP	13.206
TVVLY;ASGSAFVK	NCP	126.315
ASGSAFVK;LERPPK	NCP	208.555
LERPPK;RVLLLPGEL	NCP	137.879
RVLLLPGEL;NAPGL	NCP	35.142
NAPGL;PAAYPPK	NCP	13.539
PAAYPPK;KTPPKHV	NCP	67.218
KTPPKHV;ATFPR	NCP	95.776
ATFPR;QTCLLL	NCP	235.232
QTCLLL;LDLRPK	NCP	28.495
LDLRPK;VEVLPF	NCP	25.586
VEVLPF;KAAFLDH	NCP	25.012
KAAFLDH;ELLYL	NCP	94.445
ELLYL;PLFALKF	NCP	259.777
PLFALKF;KEALL	NCP	67.917
KEALL;CCNCA	NCP	49.367
CCNCA;ADLLKQH	NCP	131.94
ADLLKQH;FSKVPF	NCP	92.689
FSKVPF;RFDTQLH	NCP	45.692
RFDTQLH;SALRPTT	NCP	49.027
SALRPTT;VASSYPK	NCP	15.638
VASSYPK;LAALKAK	NCP	86.421
LAALKAK;KDGALL	NCP	145.699
KDGALL;NGGEVKK	NCP	19.941
NGGEVKK;KDLTPLRK	NCP	18.623
KDLTPLRK;RVHRKFC	NCP	63.292

RVHRKFC;PSLLCDLY	NCP	41.334
PSLLCDLY;RHTQVLEY	NCP	44.742
RHTQVLEY;DPAVLLL	NCP	249.803
DPAVLLL;EVKATHK	NCP	133.99
EVKATHK;YKLEQK	NCP	37.466
YKLEQK;LPWPLK	NCP	0.396
LPWPLK;HEGLFL	NCP	110.229
HEGLFL;KLAAY	NCP	107.766
KLAAY;LFQPTVF	NCP	0.927
LFQPTVF;RAELLE	NCP	46.954
RAELLE;VSGTLL	NCP	209.871
VSGTLL;ASFPTNF	NCP	75.678
ASFPTNF;VSPLLLA	NCP	317.186
VSPLLLA;VQVSPLLK	NCP	95.319
VQVSPLLK;DGLLFK	NCP	110.467
DGLLFK;VLGGVVVL	NCP	64.668
VLGGVVVL;MVGGLPL	NCP	53.733
MVGGLPL;VHLSKFSL	NCP	13.629
VHLSKFSL;AQLLNVKS	NCP	303.762
AQLLNVKS;SPGPLSHS	NCP	17.649
SPGPLSHS;LSFLAAHK	NCP	102.081
LSFLAAHK;EHRMV	NCP	40.754
EHRMV;KMGVAVL	NCP	81.34
KMGVAVL;LAKLYK	NCP	89.387
LAKLYK;LLKLGY	NCP	8.7
LLKLGY;KEDPTYP	NCP	56.404
KEDPTYP;EGPGKGK	NCP	9.698
EGPGKGK;VGLKVT	NCP	82.072
VGLKVT;AQCLLR	NCP	82.68
AQCLLR;LFAWAP	NCP	7.379
LFAWAP;AVPLKCL	NCP	5.724
AVPLKCL;PSADVP	NCP	76.248
PSADVP;RPDSR	NCP	200.287
RPDSR;KPFPQK	NCP	82.296
KPFPQK;VHLSNK	NCP	66.935
VHLSNK;NPLTTAAP	NCP	1.417
NPLTTAAP;LSGAAALLP	NCP	75.255
LSGAAALLP;AVTLLE	NCP	7.173
AVTLLE;TSAFSRP	NCP	65.406
TSAFSRP;KAKNAVPR	NCP	4.737
KAKNAVPR;TGPGGF	NCP	3.105
TGPGGF;VDRLVG	NCP	70.818
VDRLVG;TVQEVR	NCP	169.577
TVQEVR;KMGTTKK	NCP	14.214
KMGTTKK;NSSPTVK	NCP	35.841
NSSPTVK;FSGDL	NCP	6.745
FSGDL;QELSQH	NCP	108.837
QELSQH;ESSLYR	NCP	43.181
ESSLYR;AARDFN	NCP	50.769
AARDFN;KNLEPTLR	NCP	19.413
KNLEPTLR;LTKPHPTA	NCP	12.883

LTKPHPTA;KGGPKTR	NCP	26.208
KGGPKTR;QKMWK	NCP	12.905
QKMWK;TYSLG	NCP	108.354
TYSLG;LPKLLLLP	NCP	10.546
LPKLLLLP;ADKPVE	NCP	37.044
ADKPVE;PRELLT	NCP	91.013
PRELLT;LTGPALV	NCP	29.068
LTGPALV;PVPSTLGAL	NCP	53.15
PVPSTLGAL;KEGGPLPS	NCP	116.152
KEGGPLPS;KNDGPRK	NCP	25.262
KNDGPRK;KPPVPAVLG	NCP	66.946
KPPVPAVLG;KAHVLLF	NCP	2.277
KAHVLLF;MSCCW	NCP	6.234
MSCCW;PSHPKV	NCP	1.857
PSHPKV;GYFKAPP	NCP	18.264
GYFKAPP;PGSVNK	NCP	3.569
PGSVNK;HLVFLL	NCP	61.522
HLVFLL;QHVREDEK	NCP	86.115
QHVREDEK;DGGSPP	NCP	37.68
DGGSPP;NLPERK	NCP	69.741
NLPERK;HLGHPP	NCP	57.624
HLGHPP;NGSLAF	NCP	43.514
NGSLAF;KPTNP	NCP	13.41
KPTNP;TEDGP	NCP	78.975
TEDGP;APSFAVP	NCP	5.853
APSFAVP;KPTLL	NCP	15.502
KPTLL;TPLLH	NCP	39.27
TPLLH;KSPLLPR	NCP	100.878
KSPLLPR;KAHGQRP	NCP	111.354
KAHGQRP;VVPVG	NCP	108.023
VVPVG;HPLALMR	NCP	75.252
HPLALMR;LDTLSPK	NCP	44.044
LDTLSPK;SYAYK	NCP	49.265
SYAYK;NPLSFF	NCP	69.264
NPLSFF;NFPPK	NCP	32.019
NFPPK;LDNLEGPK	NCP	42.145
LDNLEGPK;KETDLL	NCP	168.401
KETDLL;KETYVPK	NCP	34.303
KETYVPK;TKGVP	NCP	79.616
TKGVP;KESEVP	NCP	99.72
KESEVP;PLDVKVSL	NCP	26.785
PLDVKVSL;CTFTKTP	NCP	99.341
CTFTKTP;TLFPP	NCP	81.654
TLFPP;TPRKAP	NCP	13.345
TPRKAP;PAPARPSGP	NCP	175.74
PAPARPSGP;SSFAE	NCP	352.664
SSFAE;LSLWCLL	NCP	105.166
LSLWCLL;HPPPT	NCP	76.34
HPPPT;KKGPK	NCP	234.21
KKGPK;KHNLTF	NCP	9.94
KHNLTF;TFSSY	NCP	48.444

TFSSY;KDFRTPV	NCP	67.431
KDFRTPV;GVPVLWW	NCP	61.898
GVPVLWW;PTGKAPEP	NCP	44.844
PTGKAPEP;KTHHRTK	NCP	36.108
KTHHRTK;APLPMK	NCP	7.875
APLPMK;KVTAGEAAP	NCP	135.553
KVTAGEAAP;ASVGTAL	NCP	136.149
ASVGTAL;KAFATPP	NCP	26.16
KAFATPP;MSSLK	NCP	31.767
MSSLK;FVPLSGW	NCP	58.413
FVPLSGW;WPSFLGP	NCP	216.372
WPSFLGP;SLLKVAH	NCP	126.867
SLLKVAH;YNLLQYE	NCP	58.809
YNLLQYE;LTVSVPK	NCP	187.391
LTVSVPK;DLGLQFF	NCP	7.918
DLGLQFF;KVLTKQLP	NCP	2.978
KVLTKQLP;KEYAGVP	NCP	60.894
KEYAGVP;PYGPP	NCP	120.936
PYGPP;VGPFK	NCP	1.972
VGPFK;KAPPNLF	NCP	145.67
KAPPNLF;DVGALVK	NCP	48.493
DVGALVK;FVDSLTK	NCP	115.44
FVDSLTK;MLGPEGGEGFVVKLR	NCP	69.827
MLGPEGGEGFVVKLR;DTANDGFVRLR	NCP	0.312
DTANDGFVRLR;VPGTFVM	NCP	34.344
VPGTFVM;GRVLDPK	NCP	38.772
GRVLDPK;KGGYTVP	NCP	7.623
KGGYTVP;KTPRGPPP	NCP	82.431
KTPRGPPP;RSHFLT	NCP	43.972
RSHFLT;LKSLK	NCP	66.36
LKSLK;MAAHVPPR	NCP	60.602
MAAHVPPR;QNFDTALK	NCP	109.342
QNFDTALK;ELAPP	NCP	59.483
ELAPP;KELTSLSK	NCP	56.83
KELTSLSK;DGLKPPP	NCP	24.584
DGLKPPP;GTAKLPK	NCP	67.573
GTAKLPK;KMPREVP	NCP	97.044
KMPREVP;KCALKK	NCP	1.766
KCALKK;LSQLK	NCP	2.562
LSQLK;LCNSPAD	NCP	5.554
LCNSPAD;DSRLKL	NCP	140.28
DSRLKL;TAPAPK	NCP	11.201
TAPAPK;DVFGAQR	NCP	33.658
DVFGAQR;HFPKGK	NCP	37.884
HFPKGK;PLLSMN	NCP	7.083
PLLSMN;ETPKSPP	NCP	44.397
ETPKSPP;CVSLLL	NCP	7.368
CVSLLL;VSLLL	NCP	0.095
VSLLL;TATLPPV	NCP	85.846
TATLPPV;PHYLTL	NCP	93.306
PHYLTL;AGPLLP	NCP	13.281

AGPLLP;LPEKLAP	NCP	3.504
LPEKLAP;MENFQY	NCP	28.121
MENFQY;EHPTL	NCP	5.548
EHPTL;GVSVNP	NCP	34.664
GVSVNP;KAGGGVPK	NCP	90.277
KAGGGVPK;ESQPPRVGP	NCP	7.644
ESQPPRVGP;GPGLLLG	NCP	18.689
GPGLLLG;EGHLVL	NCP	62.398
EGHLVL;FVPVT	NCP	111.944
FVPVT;QLDSGK	NCP	64.899
QLDSGK;LDLEL	NCP	2.049
LDLEL;NQLTAAP	NCP	151.804
NQLTAAP;AVPATLM	NCP	128.439
AVPATLM;LSAASKPK	NCP	80.349
LSAASKPK;PAELSKL	NCP	11.702
PAELSKL;MRPKK	NCP	46.587
MRPKK;APTAL	NCP	63.874
APTAL;SSYLEQTR	NCP	29.505
SSYLEQTR;KPHTPP	NCP	46.386
KPHTPP;SSYKAG	NCP	86.247
SSYKAG;REPGKAKL	NCP	57.596
REPGKAKL;FALLF	NCP	64.773
FALLF;KADRMQ	NCP	53.545
KADRMQ;LDHFDK	NCP	33.23
LDHFDK;VKAKVLE	NCP	23.07
VKAKVLE;KPSCARP	NCP	39.568
KPSCARP;FPLLLL	NCP	73.679
FPLLLL;MDVPQC	NCP	25.846
MDVPQC;AAALAGY	NCP	25.266
AAALAGY;SELPRVLVT	NCP	140.759
SELPRVLVT;KFKLE	NCP	6.28
KFKLE;KPTHVSQ	NCP	241.785
KPTHVSQ;KNSADLLK	NCP	74.282
KNSADLLK;KVGLAK	NCP	9.885
KVGLAK;TVPNHVP	NCP	9.225
TVPNHVP;LEVLSQKK	NCP	45.976
LEVLSQKK;YTSGLF	NCP	72.737
YTSGLF;VLCPPK	NCP	105.241
VLCPPK;KENMNPK	NCP	38.597
KENMNPK;LSLMLQLPL	NCP	81.904
LSLMLQLPL;DDNLPR	NCP	45.501
DDNLPR;HAVLEAH	NCP	50.829
HAVLEAH;QKWRL	NCP	38.925
QKWRL;TAYAAF	NCP	543.681
TAYAAF;AGLKMGM	NCP	90.837
AGLKMGM;GQDEH	NCP	20.84
GQDEH;LDSFP	NCP	2.923
LDSFP;KLNWL	NCP	214.592
KLNWL;KTLETK	NCP	10.981
KTLETK;LLMPPK	NCP	136.673
LLMPPK;QGNGAP	NCP	120.924

QGNGAP;NSPLNQ	NCP	40.941
NSPLNQ;LTAHTHTK	NCP	60.591
LTAHTHTK;LADLDTTDGLGK	NCP	141.348
LADLDTTDGLGK;ALLLF	NCP	11.809
ALLLF;SVDSVFVL	NCP	7.496
SVDSVFVL;KVVPFHS	NCP	8.89
KVVPFHS;KPLPVSQ	NCP	17.394
KPLPVSQ;AFDCVDKMVY	NCP	7.919
AFDCVDKMVY;VLEGLQYPFAVTSFGK	NCP	7.806
VLEGLQYPFAVTSFGK;KVEYDVP	NCP	3.973
KVEYDVP;LDNLYP	NCP	73.016
LDNLYP;KFALPPPP	NCP	36.166
KFALPPPP;MFLHTPP	NCP	87.831
MFLHTPP;VGRKLPF	NCP	169.611
VGRKLPF;MMLYP	NCP	15.645
MMLYP;TVPPLK	NCP	130.994
TVPPLK;LSFPK	NCP	127.581
LSFPK;RLVFAL	NCP	51.067
RLVFAL;QPQHADK	NCP	29.285
QPQHADK;DCCDF	NCP	29.305
DCCDF;YETQY	NCP	83.904
YETQY;MNAPAP	NCP	19.646
MNAPAP;VSLSSTEV	NCP	47.778
VSLSSTEV;QMAPPP	NCP	8.451
QMAPPP;ACGLGL	NCP	44.361
ACGLGL;MLTPF	NCP	58.506
MLTPF;WLLPF	NCP	107.199
WLLPF;KQTGDLR	NCP	71.712
KQTGDLR;GLPGF	NCP	43.249
GLPGF;LHLPLLAL	NCP	1.947
LHLPLLAL;KLFEGK	NCP	59.975
KLFEGK;TASPLLP	NCP	65.646
TASPLLP;FFRYDVP	NCP	75.931
FFRYDVP;PLKGY	NCP	80.393
PLKGY;AKRKSP	NCP	101.092
AKRKSP;EPVAPAG	NCP	1.559
EPVAPAG;EMYCE	NCP	44.301
EMYCE;LLERPH	NCP	8.056
LLERPH;RTPGKPPP	NCP	9.887
RTPGKPPP;PELPF	NCP	69.802
PELPF;MEFVTGF	NCP	216.264
MEFVTGF;KGVPVLSVK	NCP	7.383
KGVPVLSVK;SVNMV	NCP	51.117
SVNMV;VPAYMP	NCP	21.621
VPAYMP;KYHPPPPP	NCP	21.561
KYHPPPPP;RAPLNF	NCP	112.697
RAPLNF;MFLPLY	NCP	10.903
MFLPLY;NLPFP	NCP	20.498
NLPFP;VPPGSAW	NCP	79.26
VPPGSAW;FPVGRLH	NCP	66.276
FPVGRLH;VGPKV	NCP	15.871

VGPKV;EAKVHNLF	NCP	10.947
EAKVHNLF;KLGWPK	NCP	70.068
KLGWPK;PLLLF	NCP	73.343
PLLLF;KQPSE	NCP	12.66
KQPSE;KMFVFLT	NCP	150.831
KMFVFLT;KAMFAPR	NCP	78.478
KAMFAPR;RFSTYTK	NCP	93.335
RFSTYTK;ALLMGL	NCP	76.716
ALLMGL;GHVLAHK	NCP	144.901
GHVLAHK;KGELTF	NCP	1.623
KGELTF;PLRVC	NCP	13.479
PLRVC;KPMDLL	NCP	17.307
KPMDLL;TSVHELLC	NCP	73.86
TSVHELLC;SLLAL	NCP	68.357
SLLAL;MGRWGS	NCP	77.037
MGRWGS;KAAPPP	NCP	3.036
KAAPPP;WGPLTH	NCP	101.619
WGPLTH;QTAEK	NCP	50.089
QTAEK;KDPRK	NCP	19.424
KDPRK;AACPQ	NCP	110.664
AACPQ;SSYVLGPV	NCP	96.18
SSYVLGPV;DGLKKVF	NCP	13.284
DGLKKVF;HVAKAME	NCP	56.676
HVAKAME;KEDYALAY	NCP	39.522
KEDYALAY;SVVLPGLL	NCP	142.006
SVVLPGLL;LLGGPVN	NCP	101.981
LLGGPVN;ASFGGLG	NCP	49.116
ASFGGLG;KRSGLLSP	NCP	52.062
KRSGLLSP;LPVLM	NCP	19.602
LPVLM;VSLVPLP	NCP	181.029
VSLVPLP;YNEATGGKYVPR	NCP	41.898
YNEATGGKYVPR;DPEVKLR	NCP	38.506
DPEVKLR;KGDDGH	NCP	46.839
KGDDGH;HTLPQ	NCP	19.254
HTLPQ;DLWEK	NCP	146.414
DLWEK;KSHVDTP	NCP	25.327
KSHVDTP;PAPRLLSVF	NCP	0.054
PAPRLLSVF;EELRVH	NCP	7.157
EELRVH;GPDSVFY	NCP	6.412
GPDSVFY;CYCCCCP	NCP	47.907
CYCCCCP;KGAMKPP	NCP	114.548
KGAMKPP;SEGTKAVTKY	NCP	19.468
SEGTKAVTKY;KNPRASPR	NCP	23.631
KNPRASPR;LPPVVP	NCP	42.944
LPPVVP;KAPLLMF	NCP	42.276
KAPLLMF;PLHVPLPP	NCP	7.128
PLHVPLPP;MTALQK	NCP	23.551
MTALQK;YGRGALNV	NCP	59.13
YGRGALNV;LRKNH	NCP	187.95
LRKNH;NTLLAGPL	NCP	40.148
NTLLAGPL;KTKKRP	NCP	46.255

KTKKRP;AELGL	NCP	26.429
AELGL;LEGVAL	NCP	66.84
LEGVAL;TGHFDKSK	NCP	14.529
TGHFDKSK;PVLSPLLLQ	NCP	53.014
PVLSPLLLQ;LGAAFL	NCP	39.408
LGAAFL;ARPPPP	NCP	180.707
ARPPPP;GEYVLPK	NCP	92.818
GEYVLPK;VTAGY	NCP	61.017
VTAGY;KEVSFK	NCP	84.938
KEVSFK;QLQPL	NCP	189.505
QLQPL;PPMGPGPH	NCP	35.43
PPMGPGPH;AQGEQ	NCP	253.374
AQGEQ;GQFLVL	NCP	70.395
GQFLVL;LAPLQH	NCP	137.117
LAPLQH;KFLAGF	NCP	47.644
KFLAGF;GKGAAAPP	NCP	13.892
GKGAAAPP;KYAEP	NCP	134.182
KYAEP;NGVPL	NCP	21.689
NGVPL;PLLFC	NCP	16.119
PLLFC;KGDHLLL	NCP	150.112
KGDHLLL;ECYFE	NCP	209.597
ECYFE;KFPSQK	NCP	4.926
KFPSQK;AVAPNPR	NCP	26.817
AVAPNPR;VGPGSTPR	NCP	65.973
VGPGSTPR;LLPNTKQ	NCP	30.85
LLPNTKQ;TVEASSSK	NCP	30.491
TVEASSSK;DEDETTALVCDNGSGLVK	NCP	1.905
DEDETTALVCDNGSGLVK;LRVAPEE	NCP	0.384
LRVAPEE;WQPALVR	NCP	20.892
WQPALVR;NGVLP	NCP	162.517
NGVLP;RASLVAP	NCP	61.94
RASLVAP;PKQPSPP	NCP	167.458
PKQPSPP;RFPTLTH	NCP	25.416
RFPTLTH;LSKVP	NCP	41.931
LSKVP;TPVKLP	NCP	1.386
TPVKLP;SPRMLK	NCP	221.796
SPRMLK;GPPYPHP	NCP	294.791
GPPYPHP;AHSSLLV	NCP	110.509
AHSSLLV;RLPSFN	NCP	298.161
RLPSFN;LSLLGTK	NCP	20.063
LSLLGTK;LLVKQ	NCP	31.582
LLVKQ;AGALRVK	NCP	205.086
AGALRVK;ALELRK	NCP	0.351
ALELRK;KDLLAGF	NCP	73.506
KDLLAGF;PGGCV	NCP	5.292
PGGCV;VPGVSVF	NCP	0.6
VPGVSVF;AVAYPLR	NCP	63.051
AVAYPLR;KGLGPAP	NCP	63.042
NLLSAL;TVGTAK	NCP	13.58
TVGTAK;YGTVH	NCP	46.054
YGTVH;RPPPPPAP	NCP	89.888

RPPPPPAP;FAGPG	NCP	63.495
FAGPG;PPYTYGP	NCP	93.714
PPYTYGP;TAEPH	NCP	13.882
TAEPH;KSAAAVVK	NCP	50.318
KSAAAVVK;KENPLSP	NCP	26.994
KENPLSP;RAQPARPFR	NCP	20.226
RAQPARPFR;MENALDQLKQF	NCP	16.086
MENALDQLKQF;LLGELLKDHSK	NCP	6.522
LLGELLKDHSK;QQQLPR	NCP	17.295
QQQLPR;PHYVTFDGLYY	NCP	237.906
PHYVTFDGLYY;QLQLL	NCP	68.838
QLQLL;RHTLVQ	NCP	21.883
RHTLVQ;PLRGPP	NCP	44.225
PLRGPP;VLSVC	NCP	62.547
VLSVC;LLEPVT	NCP	27.67
LLEPVT;GPPAPPP	NCP	47.231
GPPAPPP;ALLGSRPP	NCP	68.217
ALLGSRPP;KPYAAAR	NCP	41.578
KPYAAAR;KYLLPL	NCP	56.666
KYLLPL;QKSELL	NCP	175.852
QKSELL;TAVQVP	NCP	24.323
TAVQVP;LLSAAAS	NCP	30.363
LLSAAAS;EPLQPF	NCP	21.211
EPLQPF;ERDLRDVG	NCP	5.741
ERDLRDVG;RDVGDWRKN	NCP	0.012
RDVGDWRKN;KKMFETES	NCP	0.057
KKMFETES;DPVVTGAPP	NCP	7.944
DPVVTGAPP;AEAPGHPGSEERRE	NCP	1.062
AEAPGHPGSEERRE;SWTLKK	NCP	9.846
SWTLKK;VLEGTEVGSPPLSPSTK	NCP	19.647
VLEGTEVGSPPLSPSTK;WEQKGGPK	NCP	2.727
WEQKGGPK;APPPPAEVPEVHEE	NCP	13.896
APPPPAEVPEVHEE;EVPEVHE	NCP	0.018
EVPEVHE;VPEVHEE	NCP	0.003
VPEVHEE;VSELLL	NCP	8.029
VSELLL;TAPKGKVGGRWK	NCP	2.474
TAPKGKVGGRWK;LSLFSRT	NCP	79.752
LSLFSRT;LLESGKEL	NCP	13.798
LLESGKEL;QPAAQP	NCP	82.697
QPAAQP;WLSPPG	NCP	135.612
WLSPPG;TGPGSHQP	NCP	99.186
TGPGSHQP;AALVP	NCP	113.49
AALVP;LGDAPP	NCP	17.217
LGDAPP;PLGSSLTP	NCP	77.64
PLGSSLTP;EAAVMGT	NCP	13.296
EAAVMGT;FTGLR	NCP	37.384
FTGLR;QPPLPDS	NCP	9.866
QPPLPDS;TTLKRLLL	NCP	58.744
TTLKRLLL;QLSVVL	NCP	95.016
QLSVVL;KSWALLL	NCP	36.704
KSWALLL;TTAKKGAP	NCP	17.757

TTAKKGAP;KGFSP	NCP	52.389
KGFSP;GHPVPPGP	NCP	168.294
GHPVPPGP;AAKSQPSAPK	NCP	168.684
AAKSQPSAPK;SVNPFP	NCP	89.823
SVNPFP;WASVRGLT	NCP	160.983
WASVRGLT;KMTLP	NCP	66.705
KMTLP;RGPCPF	NCP	64.324
RGPCPF;KDSDDKAK	NCP	132.185
KDSDDKAK;NGGAA	NCP	37.909
NGGAA;SGLRNLSF	NCP	38.885
SGLRNLSF;DPKRSAF	NCP	29.508
DPKRSAF;KEPAALLP	NCP	38.28
KEPAALLP;LAPHALM	NCP	17.863
LAPHALM;DLALPV	NCP	55.187
DLALPV;NPPGPGPP	NCP	32.208
NPPGPGPP;KGPRTAPP	NCP	26.907
KGPRTAPP;GVKPY	NCP	178.855
GVKPY;PRPEANR	NCP	114.185
PRPEANR;PVPRDP	NCP	23.832
PVPRDP;QAAVLAPGG	NCP	35.883
QAAVLAPGG;SPPDSLP	NCP	163.41
SPPDSLP;LGQKVAL	NCP	50.754
LGQKVAL;WSGPPA	NCP	18.843
WSGPPA;LLHKPSQ	NCP	36.072
LLHKPSQ;CPVARK	NCP	104.638
CPVARK;KLPVAG	NCP	183.716
KLPVAG;TALLGPLL	NCP	43.059
TALLGPLL;KELAQPK	NCP	2.931
KELAQPK;KTPLAK	NCP	6.564
KTPLAK;TWLDF	NCP	54.586
TWLDF;KLMHGC	NCP	151.289
KLMHGC;LTEHAAP	NCP	6.171
LTEHAAP;KPPEKK	NCP	34.014
KPPEKK;HVGDVLT	NCP	0.642
HVGDVLT;APASYWFH	NCP	70.536
APASYWFH;QMSLLL	NCP	74.883
QMSLLL;PKSPPPP	NCP	50.061
PKSPPPP;EHGKPQ	NCP	20.244
EHGKPQ;TVMLK	NCP	77.845
TVMLK;EPAPKGPP	NCP	60.41
EPAPKGPP;METSP	NCP	9.813
METSP;PCDPPRGP	NCP	40.503
PCDPPRGP;LPNGK	NCP	292.171
LPNGK;LVGAAA	NCP	5.336
LVGAAA;SPLVGHR	NCP	53.346
SPLVGHR;LAQAKL	NCP	5.157
LAQAKL;PLSVYE	NCP	30.507
PLSVYE;LPPPGPLS	NCP	24.654
LPPPGPLS;LADGKVFTTGF	NCP	126.102
LADGKVFTTGF;SDSRPAAAPGTSRPGPS	NCP	2.364
SDSRPAAAPGTSRPGPS;DSRPAAAPGTSRPGP	NCP	0.003

DSRPAAAPGTSRPGP;SRPAAAPGTSRPGPS	NCP	0.003
SRPAAAPGTSRPGPS;AAPGTSRPGP	NCP	0.012
AAPGTSRPGP;VPVAGK	NCP	27.72
VPVAGK;YSSPPPP	NCP	45.828
YSSPPPP;MEYLLG	NCP	17.145
MEYLLG;LPDVA	NCP	9.927
LPDVA;LPHSCLP	NCP	36.747
LPHSCLP;SCPKP	NCP	17.529
SCPKP;TLSPK	NCP	34.201
TLSPK;AMPEV	NCP	108.599
AMPEV;KPAQVHR	NCP	15.843
KPAQVHR;KPPPLW	NCP	55.539
KPPPLW;ALQPR	NCP	45.693
ALQPR;NPLAGGLL	NCP	0.444
NPLAGGLL;FPVHGRL	NCP	18.019
FPVHGRL;GSTDKLK	NCP	42.152
GSTDKLK;YLWEPAM	NCP	1.491
YLWEPAM;KEYAPK	NCP	56.787
KEYAPK;SSDLVF	NCP	61.945
SSDLVF;RPKEQKGP	NCP	1.427
RPKEQKGP;PPVHQLGK	NCP	96.333
PPVHQLGK;WHHSF	NCP	167.056
WHHSF;LSLDPVL	NCP	6.707
LSLDPVL;LLLPQM	NCP	48.162
LLLPQM;NPMLLL	NCP	5.938
NPMLLL;PDPPPLFD	NCP	65.312
PDPPPLFD;TAYVAKKL	NCP	0.399
TAYVAKKL;RTQENEPK	NCP	85.068
RTQENEPK;AETLK	NCP	8.047
AETLK;AEKLNSL	NCP	11.948
AEKLNSL;PPPLL	NCP	16.53
PPPLL;KKEAPAPPKAEAKA	NCP	45.402
KKEAPAPPKAEAKA;KEAPAPPKAEAKAK	NCP	0.003
KEAPAPPKAEAKAK;KAGVGHLY	NCP	62.673
KAGVGHLY;TLRAQAAGP	NCP	34.464
TLRAQAAGP;ASGVAVSD	NCP	102.615
ASGVAVSD;SSTPEEVKKRKKAVL	NCP	0.063
SSTPEEVKKRKKAVL;STPEEVKK	NCP	0.003
STPEEVKK;FCLSEDKK	NCP	0.042
FCLSEDKK;LVGDVGQTVDDPYATFVK	NCP	0.054
LVGDVGQTVDDPYATFVK;VDDPYATF	NCP	0.024
VDDPYATF;ATFVKMLPDKDCRY	NCP	0.015
ATFVKMLPDKDCRY;KMLPDKDCRYA	NCP	0.012
KMLPDKDCRYA;MLPDKDCR	NCP	0.003
MLPDKDCR;APECAPLKSKM	NCP	0.357
APECAPLKSKM;PECAPLKSK	NCP	0.003
PECAPLKSK;CYEEVKDRCT	NCP	0.303
CYEEVKDRCT;MYMCN	NCP	95.928
MYMCN;AAPVVP	NCP	54.138
AAPVVP;KGVLYQ	NCP	20.808
KGVLYQ;PPPGPKY	NCP	3.258

PPPGPKY;VPAAGQP	NCP	14.889
VPAAGQP;LSPETYQ	NCP	94.042
LSPETYQ;GVLQY	NCP	69.876
GVLQY;KPVGV	NCP	62.954
KPVGV;DGVLLSK	NCP	0.453
DGVLLSK;FEVKLK	NCP	13.393
FEVKLK;LSGNTKEK	NCP	15.12
LSGNTKEK;KMLPPKY	NCP	95.82
KMLPPKY;KKTPQR	NCP	3.95
KKTPQR;FKFAAPR	NCP	1.903
FKFAAPR;VLTSYK	NCP	99.098
VLTSYK;ARALVAAP	NCP	28.272
ARALVAAP;TMPALP	NCP	62.82
TMPALP;KQSAGH	NCP	133.125
KQSAGH;YKLKKK	NCP	111.625
YKLKKK;EGSAPSPSPTHTHHR	NCP	17.741
EGSAPSPSPTHTHHR;KPYFPK	NCP	59.508
KPYFPK;YPQPY	NCP	5.368
YPQPY;QAPAPPAPK	NCP	8.882
QAPAPPAPK;AEQVAL	NCP	11.397
AEQVAL;QTDSLLP	NCP	5.226
QTDSLLP;KPYEMVT	NCP	7.375
KPYEMVT;PPPPQNK	NCP	0.149
PPPPQNK;EFLGGF	NCP	163.923
EFLGGF;AARAPVR	NCP	115.728
AARAPVR;GPAANPPPP	NCP	28.221
GPAANPPPP;NFELKK	NCP	64.185
NFELKK;VGALDL	NCP	89.973
VGALDL;GAAPK	NCP	17.346
GAAPK;ATTLPQ	NCP	6.237
ATTLPQ;KLGSDLK	NCP	1.978
KLGSDLK;LAELAA	NCP	1.053
LAELAA;GPCPLK	NCP	80.252
GPCPLK;WMVEKSL	NCP	299.418
WMVEKSL;PQARHQAP	NCP	5.997
PQARHQAP;PVLRPP	NCP	5.118
PVLRPP;KPKEVKE	NCP	138.523
KPKEVKE;MTLGF	NCP	0.212
MTLGF;ERLLLVL	NCP	18.426
ERLLLVL;RSLGTRVP	NCP	6.144
RSLGTRVP;LLPLF	NCP	32.269
LLPLF;PGALP	NCP	56.511
PGALP;KLVGP	NCP	31.692
KLVGP;KLEYLT	NCP	77.258
KLEYLT;PKCREC	NCP	2.071
PKCREC;YVYSSK	NCP	26.829
YVYSSK;YVDNLQ	NCP	11.463
YVDNLQ;KTKTVDLL	NCP	211.358
KTKTVDLL;KPDKP	NCP	44.613
KPDKP;NPLLLG	NCP	11.22
NPLLLG;SPLVPLP	NCP	27.96

SPLVPLP;KNTEFKQ	NCP	163.684
KNTEFKQ;AADFLRQ	NCP	32.927
AADFLRQ;MEVKRLKVTELR	NCP	49.164
MEVKRLKVTELR;SYGFDGRGLKAENGQFEEF	NCP	3.795
SYGFDGRGLKAENGQFEEF;VVVVVPNEEDWKK	NCP	1.956
VVVVVPNEEDWKK;MVNDPPVP	NCP	12.876
MVNDPPVP;YEMNY	NCP	45.979
YEMNY;RLYNLDVF	NCP	4.256
RLYNLDVF;LMLGPSVFDVFRQY	NCP	0.819
LMLGPSVFDVFRQY;SGAWLR	NCP	2.623
SGAWLR;SLVFDPMQK	NCP	26.474
SLVFDPMQK;KDAYHLKK	NCP	6.109
KDAYHLKK;VSELLTK	NCP	17.438
VSELLTK;EHGAG	NCP	22.683
EHGAG;MEKEEETTRELL	NCP	0.198
MEKEEETTRELL;VDVTSREGAKD	NCP	1.821
VDVTSREGAKD;VQVTVPGVK	NCP	0.249
VQVTVPGVK;TGPSVEGAL	NCP	0.396
TGPSVEGAL;LEGTVTGPK	NCP	0.516
LEGTVTGPK;ADVDLNVAAPK	NCP	0.072
ADVDLNVAAPK;STPGVKGEGPDVDVSLPK	NCP	0.195
STPGVKGEGPDVDVSLPK;EGPDVDVSLP	NCP	0.021
EGPDVDVSLP;KGPDVQLPEVSVKTPK	NCP	0.111
KGPDVQLPEVSVKTPK;DVQLPEVSVK	NCP	0.009
DVQLPEVSVK;DVDFHVQGPK	NCP	0.051
DVDFHVQGPK;YDVTAPK	NCP	0.042
YDVTAPK;DVPDVDVQGPDWHLK	NCP	0.057
DVPDVDVQGPDWHLK;DVDVQGPDWH	NCP	0.009
DVDVQGPDWH;EGPEGKLKGPKF	NCP	0.174
EGPEGKLKGPKF;GPKFKMPEM	NCP	0.024
GPKFKMPEM;DVDLHLK	NCP	0.057
DVDLHLK;KGPSVKGEYDVTVPK	NCP	0.018
KGPSVKGEYDVTVPK;DAPDVDVHGPDWH	NCP	0.102
DAPDVDVHGPDWH;DVDVHGPDWHLK	NCP	0.009
DVDVHGPDWHLK;VDVHGPDWH	NCP	0.003
VDVHGPDWH;DVHGPDWHLK	NCP	0.003
DVHGPDWHLK;PGFKGEGPEVD	NCP	0.063
PGFKGEGPEVD;GFKGEGPEVDVNLPK	NCP	0.003
GFKGEGPEVDVNLPK;KGEGPEVDVN	NCP	0.006
KGEGPEVDVN;EGPEVDVNLPK	NCP	0.006
EGPEVDVNLPK;VDVEGPDVN	NCP	0.06
VDVEGPDVN;DVDLHVKGPK	NCP	0.111
DVDLHVKGPK;VPKLEGDLK	NCP	0.054
VPKLEGDLK;LEGDLKGPK	NCP	0.009
LEGDLKGPK;ELDMNLPK	NCP	0.138
ELDMNLPK;SLEGPEGKLKGPK	NCP	0.075
SLEGPEGKLKGPK;LEGPEGKLKGPKF	NCP	0.003
LEGPEGKLKGPKF;TLPDVDMDLKGPKMK	NCP	0.075
TLPDVDMDLKGPKMK;DVDMDLKGPK	NCP	0.009
DVDMDLKGPK;VDMDLKGPKMK	NCP	0.003
VDMDLKGPKMK;DMDLKGPK	NCP	0.003

DMDLKGPK;DLKGPKMK	NCP	0.006
DLKGPKMK;GSLDVSAPK	NCP	0.024
GSLDVSAPK;PSFKGQGPDVDVNLPK	NCP	0.162
PSFKGQGPDVDVNLPK;EMHFKGPK	NCP	0.15
EMHFKGPK;SMPDVDLNLKGPKVK	NCP	0.027
SMPDVDLNLKGPKVK;MPDVDLNLK	NCP	0.003
MPDVDLNLK;PDVDLNLKGPK	NCP	0.003
PDVDLNLKGPK;DVDLNLKGPKVK	NCP	0.003
DVDLNLKGPKVK;VDLNLKGPK	NCP	0.003
VDLNLKGPK;DLNLKGPKVK	NCP	0.003
DLNLKGPKVK;KGPKVKGDVDVSVPK	NCP	0.012
KGPKVKGDVDVSVPK;GPKVKGDVDVS	NCP	0.003
GPKVKGDVDVS;VKGDVDVSVPK	NCP	0.009
VKGDVDVSVPK;VDVDVPDVDVKGPDWHLK	NCP	0.081
VDVDVPDVDVKGPDWHLK;VDVPDVDVK	NCP	0.006
VDVPDVDVK;DVKGPDWH	NCP	0.018
DVKGPDWH;FKGEGPEVDVSLPK	NCP	0.837
FKGEGPEVDVSLPK;VKGDVDVSLPK	NCP	0.978
VKGDVDVSLPK;VEVPDVN	NCP	0.249
VEVPDVN;EGPDAKVKGPK	NCP	0.024
EGPDAKVKGPK;DVSLHLKGPK	NCP	0.081
DVSLHLKGPK;GDYDVTVPK	NCP	0.036
GDYDVTVPK;TDVDVSLPK	NCP	0.384
TDVDVSLPK;DLHLKGPK	NCP	0.798
DLHLKGPK;GDVDVSVPKL	NCP	0.03
GDVDVSVPKL;DFDLHLKGPK	NCP	1.317
DFDLHLKGPK;AEGPDMDVNLPK	NCP	0.21
AEGPDMDVNLPK;GDVDVTCPK	NCP	0.21
GDVDVTCPK;EGPDAKLKGPKF	NCP	1.035
EGPDAKLKGPKF;PDLDLNLK	NCP	0.078
PDLDLNLK;DLDLNLKGPK	NCP	0.003
DLDLNLKGPK;DMHVNMPK	NCP	0.186
DMHVNMPK;VQTPEVDVK	NCP	0.237
VQTPEVDVK;QTPEVDVKVK	NCP	0.003
QTPEVDVKVK;DVKVKKPDLDVTGPK	NCP	0.015
DVKVKKPDLDVTGPK;NAPDVEVQGK	NCP	0.054
NAPDVEVQGK;DVEVQGKVKGPK	NCP	0.009
DVEVQGKVKGPK;DVELNLKGPK	NCP	0.084
DVELNLKGPK;VELNLKGPKVK	NCP	0.003
VELNLKGPKVK;FSVPGLKAEGPDMAVDLPK	NCP	0.186
FSVPGLKAEGPDMAVDLPK;EGPEVKVEGL	NCP	0.093
EGPEVKVEGL;PDLDFNLK	NCP	0.705
PDLDFNLK;DLDFNLKGPK	NCP	0.003
DLDFNLKGPK;DLHLKAPK	NCP	0.954
DLHLKAPK;EVDLNLKGPK	NCP	0.036
EVDLNLKGPK;DFDLNLK	NCP	0.99
DFDLNLK;SLGKGDFDVSVPK	NCP	1.194
SLGKGDFDVSVPK;KGPSLDVEGPDAKL	NCP	0.075
KGPSLDVEGPDAKL;DLHLKTPK	NCP	0.108
DLHLKTPK;LEGGELDLKGPK	NCP	0.045
LEGGELDLKGPK;AEASLPSPK	NCP	0.507

AEASLPSPK;KLEGPDVSLKGPK	NCP	0.498
KLEGPDVSLKGPK;LEGPDVSLK	NCP	0.003
LEGPDVSLK;EGPDVSLKGPK	NCP	0.003
EGPDVSLKGPK;ESDFGVSLK	NCP	0.864
ESDFGVSLK;SGPKVEGGVKGGQVGL	NCP	0.198
SGPKVEGGVKGGQVGL;VQSGAGEGEWEESDVKLK	NCP	0.213
VQSGAGEGEWEESDVKLK;GVAGSPEASVSGSK	NCP	0.108
GVAGSPEASVSGSK;GSPEASVSGSKGDLKSSK	NCP	0.009
GSPEASVSGSKGDLKSSK;SLGSLEGEAEVDTSSPKG	NCP	0.057
SLGSLEGEAEVDTSSPKG;LGSLEGEAEVDTSSPK	NCP	0.003
LGSLEGEAEVDTSSPK;SLEGEAEVDTSSPKGK	NCP	0.006
SLEGEAEVDTSSPKGK;LEGEAEVDTSSPK	NCP	0.003
LEGEAEVDTSSPK;FEGGEVALEGGKVK	NCP	0.144
FEGGEVALEGGKVK;EGGEVALEGGKVKGK	NCP	0.003
EGGEVALEGGKVKGK;GEVALEGGKVK	NCP	0.006
GEVALEGGKVK;GTFGGLGSKSK	NCP	0.057
GTFGGLGSKSK;GHYEVTGSDDEAGKLQGSGVSSSSKK	NCP	0.033
GHYEVTGSDDEAGKLQGSGVSSSSKK;HYEVTGSDDEAGK	NCP	0.003
HYEVTGSDDEAGKLQGSGVSSSSK;YEVTGSDDEAGKLQG	NCP	0.003
YEVTGSDDEAGKLQGSGVSSSSKK;VTGSDDEAGKLQGSG	NCP	0.006
VTGSDDEAGKLQGSGVSSSSK;GSDDEAGKLQGSGVSSSS	NCP	0.006
GSDDEAGKLQGSGVSSSSKK;SDDEAGKLQGSGVSSSSK	NCP	0.003
SDDEAGKLQGSGVSSSSK;DDEAGKLQGSGVSSSSKK	NCP	0.003
DDEAGKLQGSGVSSSSKK;EVELTVSTKKE	NCP	0.114
EVELTVSTKKE;KPHTSR	NCP	132.886
KPHTSR;LLKFAC	NCP	73.772
LLKFAC;LFDKHTLGHSES	NCP	201.084
LFDKHTLGHSES;VLCSQK	NCP	182.826
VLCSQK;QDPPK	NCP	45.529
QDPPK;GPGLQA	NCP	26.96
GPGLQA;NPSLW	NCP	65.316
NPSLW;EHPKGSPP	NCP	18.705
EHPKGSPP;EFKSSG	NCP	13.392
EFKSSG;LTDANLR	NCP	80.358
LTDANLR;VVYHKAK	NCP	3.496
VVYHKAK;ASSASARPAP	NCP	1.358
ASSASARPAP;MGFVDDQQTFF	NCP	211.035
MGFVDDQQTFF;LAPHLRLLA	NCP	33.108
LAPHLRLLA;FGFFFW	NCP	7.903
FGFFFW;KETFPP	NCP	93.524
KETFPP;ATGQKVPR	NCP	72.507
ATGQKVPR;MDEKVFTK	NCP	7.86
MDEKVFTK;MEQLSA	NCP	0.039
MEQLSA;TVTLPV	NCP	116.593
TVTLPV;LTQLAF	NCP	237.537
LTQLAF;YLEGLY	NCP	9.503
YLEGLY;GFAPLTS	NCP	70.012
GFAPLTS;EHVTLL	NCP	74.796
EHVTLL;EVPSPRK	NCP	241.185
EVPSPRK;AASPKVPK	NCP	46.368
AASPKVPK;TPPTT	NCP	117.614

TPPTT;LRLYSH	NCP	57.901
LRLYSH;LRDNLT	NCP	80.933
LRDNLT;MNGLYE	NCP	15.385
MNGLYE;KGGGP	NCP	38.684
KGGGP;ATLSRSL	NCP	17.89
ATLSRSL;LGEPFP	NCP	63.692
LGEPFP;MLMMGPL	NCP	25.407
MLMMGPL;PGSGLLPP	NCP	5.589
PGSGLLPP;TLMVL	NCP	29.736
TLMVL;VAGPVP	NCP	50.103
VAGPVP;KPACLK	NCP	21.426
KPACLK;FFGTQF	NCP	57.64
FFGTQF;LSTAPDK	NCP	42.957
LSTAPDK;LLSGGL	NCP	20.16
LLSGGL;GLTVLLR	NCP	181.736
GLTVLLR;GKAAP	NCP	132.786
GKAAP;CGYPMC	NCP	12.418
CGYPMC;KTDAGP	NCP	30.377
KTDAGP;KDLVP	NCP	49.578
KDLVP;PPKGY	NCP	24.54
PPKGY;CLAML	NCP	126.579
CLAML;MEKQSPR	NCP	71.14
MEKQSPR;TLDARMK	NCP	109.083
TLDARMK;KESLTK	NCP	134.229
KESLTK;ERTLTK	NCP	54.555
ERTLTK;PVVVPQR	NCP	31.181
PVVVPQR;LPLVF	NCP	49.348
LPLVF;HFELNDKK	NCP	0.894
HFELNDKK;PRLLGPEF	NCP	36.965
PRLLGPEF;KGGLGAAPP	NCP	7.143
KGGLGAAPP;VAPPQL	NCP	138.222
VAPPQL;LSDLFP	NCP	384.678
LSDLFP;KPFEPY	NCP	56.379
KPFEPY;WFYPP	NCP	110.49
WFYPP;PVVNV	NCP	5.554
PVVNV;FLLDS	NCP	122.46
FLLDS;QRKGGPK	NCP	0.77
QRKGGPK;PVPGDP	NCP	457.347
PVPGDP;MFRML	NCP	101.442
MFRML;LVEFY	NCP	215.125
LVEFY;VTHVP	NCP	132.791
VTHVP;NAHLVL	NCP	565.26
NAHLVL;STYKEC	NCP	66.597
STYKEC;RLLLVK	NCP	51.046
RLLLVK;TAVLY	NCP	158.979
TAVLY;GVPGVK	NCP	170.216
GVPGVK;KFVSTHH	NCP	80.56
KFVSTHH;DLFTPK	NCP	19.25
DLFTPK;MDVME	NCP	81.102
MDVME;TDLSK	NCP	14.328
TDLSK;AAPLLLP	NCP	28.329

AAPLLLP;AALNHPQ	NCP	25.026
AALNHPQ;TAQVLLFR	NCP	3.105
TAQVLLFR;QHAAFTP	NCP	37.23
QHAAFTP;LHLGK	NCP	229.465
LHLGK;ETPLVPPP	NCP	123.701
ETPLVPPP;EVNLTGPK	NCP	24.46
EVNLTGPK;VPGAVSKL	NCP	4.494
VPGAVSKL;AALPL	NCP	1.961
AALPL;FYQTNRLD	NCP	143.658
FYQTNRLD;KPPAACPP	NCP	26.061
KPPAACPP;LGHLLT	NCP	110.151
LGHLLT;KGREGKH	NCP	16.182
KGREGKH;QAPSF	NCP	7.069
QAPSF;TAAVLAF	NCP	17.63
TAAVLAF;GKHYFP	NCP	4.125
GKHYFP;LSVRDK	NCP	35.661
LSVRDK;GTLLF	NCP	31.932
GTLLF;QGGLAGALR	NCP	351.64
QGGLAGALR;LPLLHP	NCP	103.452
LPLLHP;PLLSLPP	NCP	5.273
PLLSLPP;PAAMARP	NCP	8.874
PAAMARP;KAAALPH	NCP	88.2
KAAALPH;TAELLTAA	NCP	18.489
TAELLTAA;RGSAAK	NCP	15.772
RGSAAK;LGGGVGL	NCP	15.654
LGGGVGL;RAPGNLR	NCP	49.662
RAPGNLR;LEEFVAY	NCP	7.311
LEEFVAY;TVPTKL	NCP	241.338
TVPTKL;VLGVSP	NCP	168.843
VLGVSP;EYMKK	NCP	151.676
EYMKK;FTAVAVL	NCP	155.668
FTAVAVL;PVMFKHPP	NCP	51.185
PVMFKHPP;KETPL	NCP	4.014
KETPL;LDAVAP	NCP	34.776
LDAVAP;ANGVVGR	NCP	20.124
ANGVVGR;KEVLFP	NCP	69.016
KEVLFP;DFKRP	NCP	7.877
DFKRP;KNKYGK	NCP	48.007
KNKYGK;PVLFK	NCP	21.26
PVLFK;LLHLLLGGPP	NCP	50.31
LLHLLLGGPP;KLTVP	NCP	21.234
KLTVP;LSPLD	NCP	120.852
LSPLD;LPFEPR	NCP	16.591
LPFEPR;FSLEFK	NCP	129.5
FSLEFK;LYTQYL	NCP	10.024
LYTQYL;GPSLVHKR	NCP	13.652
GPSLVHKR;GVEFL	NCP	56.239
GVEFL;FTMLT	NCP	183.858
FTMLT;LDAGRDL	NCP	148.008
LDAGRDL;RSLHPP	NCP	67.733
RSLHPP;RLLWN	NCP	206.526

RLLWN;EVLNF	NCP	5.856
EVLNF;CCGDSS	NCP	44.838
CCGDSS;MVTYLP	NCP	17.137
MVTYLP;SGPSLVHRK	NCP	121.022
SGPSLVHRK;DSLVPSGR	NCP	67.191
DSLVPSGR;KDFFPK	NCP	75.85
KDFFPK;APKPPP	NCP	87.465
APKPPP;LLLMPNPA	NCP	61.949
LLLMPNPA;KTMHAPK	NCP	52.36
KTMHAPK;LYLQNN	NCP	23.703
LYLQNN;EKVAKY	NCP	4.775
EKVAKY;KTYLQK	NCP	147.369
KTYLQK;APPTGHVP	NCP	273.687
APPTGHVP;PTPVV	NCP	5.061
PTPVV;PGMPPVP	NCP	15.918
PGMPPVP;PPPPPHV	NCP	88.245
PPPPPHV;GVVLTK	NCP	15.448
GVVLTK;KTLTFF	NCP	66.18
KTLTFF;FEDSEKAKKPVR	NCP	64.313
FEDSEKAKKPVR;ATLVL	NCP	18.435
ATLVL;DSVQP	NCP	45.297
DSVQP;MPGLKEPP	NCP	7.065
MPGLKEPP;KARPPRP	NCP	13.185
KARPPRP;HLGSLPF	NCP	219.192
HLGSLPF;VKVGVNGFG	NCP	97.999
VKVGVNGFG;TVLVQ	NCP	35.355
TVLVQ;LKEKDLRR	NCP	79.023
LKEKDLRR;KSWHLAP	NCP	27.455
KSWHLAP;HDSLRP	NCP	92.587
HDSLRP;EPFPVAV	NCP	24.344
EPFPVAV;TSEFLNK	NCP	43.861
TSEFLNK;DDAGKVLK	NCP	31.55
DDAGKVLK;PSFGL	NCP	19.038
PSFGL;FPSLVGRP	NCP	5.289
FPSLVGRP;VNELDVR	NCP	109.579
VNELDVR;MFNAV	NCP	188.352
MFNAV;DLPPNP	NCP	56.705
DLPPNP;LLKPPK	NCP	101.632
LLKPPK;VLLPA	NCP	102.074
VLLPA;AMAGPKF	NCP	133.779
AMAGPKF;RTFGP	NCP	14.95
RTFGP;EKSPHVP	NCP	85.235
EKSPHVP;EVAGQK	NCP	27.004
EVAGQK;QFYTKGK	NCP	86.174
QFYTKGK;MSHAAPR	NCP	30.304
MSHAAPR;LGGPVVFP	NCP	16.404
LGGPVVFP;FEATRAK	NCP	74.259
FEATRAK;AVEPK	NCP	2.924
AVEPK;LLSSMRD	NCP	127.54
LLSSMRD;KLLFPPPPP	NCP	20.858
KLLFPPPPP;NMAVLEYL	NCP	171.262

NMAVLEYL;EKKGEAG	NCP	388.803
EKKGEAG;KERFLTP	NCP	98.358
KERFLTP;LPLSAY	NCP	20.612
LPLSAY;QLMAF	NCP	69.216
QLMAF;FGEKLK	NCP	97.183
FGEKLK;TTATKY	NCP	43.871
TTATKY;TGAPL	NCP	68.653
TGAPL;NPPQLPP	NCP	243.929
NPPQLPP;ELERLQ	NCP	23.109
ELERLQ;NHALVLYT	NCP	73.789
NHALVLYT;DPEQFH	NCP	136.346
DPEQFH;VPLVD	NCP	27.094
VPLVD;KELTYPPP	NCP	50.004
KELTYPPP;NVQKDLKF	NCP	0.59
NVQKDLKF;KQAAVTRV	NCP	94.332
KQAAVTRV;PEEVGQPR	NCP	20.907
PEEVGQPR;LVLDY	NCP	21.597
LVLDY;LLMRMK	NCP	248.104
LLMRMK;WSLYM	NCP	101.169
WSLYM;MLSRGK	NCP	120.665
MLSRGK;LVFSHL	NCP	77.061
LVFSHL;VSGPVK	NCP	11.674
VSGPVK;LPKKAVL	NCP	44.283
LPKKAVL;PPPVL	NCP	114.158
PPPVL;LLAGPKSAF	NCP	20.709
LLAGPKSAF;GPPVSELL	NCP	55.089
GPPVSELL;HELFK	NCP	50.029
HELFK;APPPPPPPPP	NCP	421.943
APPPPPPPPP;KFGDDRAP	NCP	43.839
KFGDDRAP;QSPFPL	NCP	47.787
QSPFPL;LYLKSR	NCP	28.605
LYLKSR;LQEVL	NCP	10
LQEVL;VEVTLP	NCP	32.13
VEVTLP;AETEALPKLR	NCP	110.759
AETEALPKLR;FMGVLGA	NCP	65.734
FMGVLGA;ATFQPKP	NCP	49.662
ATFQPKP;SGVFL	NCP	110.546
SGVFL;SARVKGL	NCP	31.897
SARVKGL;KSLRELNK	NCP	85.998
KSLRELNK;AALSL	NCP	263.781
AALSL;TGTFK	NCP	26.843
TGTFK;MPPPP	NCP	2.707
MPPPP;NSLMK	NCP	82.538
NSLMK;VVPLNP	NCP	34.363
VVPLNP;LGAHPPLP	NCP	47.055
LGAHPPLP;DVAVP	NCP	175.734
DVAVP;AAAVLG	NCP	31.404
AAAVLG;QPAEYSF	NCP	119.394
QPAEYSF;LPAPP	NCP	193.833
LPAPP;KNDKNLL	NCP	63.548
KNDKNLL;QLEVL	NCP	46.47

QLEVL;LLVVTK	NCP	111.163
LLVVTK;WVLLVK	NCP	140.468
WVLLVK;FDVSVPK	NCP	52.549
FDVSVPK;KDDDDDV	NCP	65.39
KDDDDDV;QWFAVP	NCP	17.869
QWFAVP;PFHLRP	NCP	206.018
PFHLRP;YKGQLL	NCP	20.482
YKGQLL;KRESVF	NCP	3.359
KRESVF;LAPPRVF	NCP	293.419
LAPPRVF;KPYTTKK	NCP	304.185
KPYTTKK;EGPLQR	NCP	55.086
EGPLQR;KAHEPAK	NCP	102.506
KAHEPAK;PAAPQ	NCP	107.044
PAAPQ;WGGGLGGGL	NCP	13.176
WGGGLGGGL;KDFLSPR	NCP	238.518
KDFLSPR;TTTST	NCP	8.265
TTTST;SVAAGP	NCP	28.68
SVAAGP;LSPKFC	NCP	88.368
LSPKFC;LALFKK	NCP	75.327
LALFKK;GSELLTK	NCP	111.788
GSELLTK;ERPLFR	NCP	57.028
ERPLFR;LEVTNF	NCP	145.206
LEVTNF;LPDSPGLK	NCP	28.332
LPDSPGLK;ELNAF	NCP	124.454
ELNAF;EALEKF	NCP	55.263
EALEKF;LAGQN	NCP	0.771
LAGQN;KKLRLP	NCP	10.725
KKLRLP;TVSKP	NCP	4.303
TVSKP;QSAPLL	NCP	44.373
QSAPLL;LTTMF	NCP	122.201
LTTMF;TTVVLLR	NCP	39.169
TTVVLLR;APPMPSPP	NCP	18.903
APPMPSPP;RCLTVHL	NCP	4.226
RCLTVHL;EGPSLRPR	NCP	23.326
EGPSLRPR;RLGRLVT	NCP	9.126
RLGRLVT;KNGNSLLA	NCP	134.295
KNGNSLLA;LGVDLT	NCP	8.264
LGVDLT;LLVAGK	NCP	28.525
LLVAGK;KGPWAPEGP	NCP	4.923
KGPWAPEGP;VTEVLEL	NCP	15.213
VTEVLEL;VALSL	NCP	155.055
VALSL;EAERLAY	NCP	15.972
EAERLAY;QSNLPK	NCP	14.736
QSNLPK;FLPLGAGK	NCP	67.056
FLPLGAGK;VEVKLR	NCP	86.436
VEVKLR;KSEHTKK	NCP	75.924
KSEHTKK;GSVSL	NCP	58.437
GSVSL;NLAHPL	NCP	2.607
NLAHPL;LEKELK	NCP	18.849
LEKELK;KNYMK	NCP	17.528
KNYMK;KNHLTF	NCP	10.836

KNHLTF;PGHGK	NCP	2.856
PGHGK;PPCMHWC	NCP	11.638
PPCMHWC;TPGFPSPVP	NCP	55.256
TPGFPSPVP;EVLGGKA	NCP	8.104
EVLGGKA;PPLPLA	NCP	65.283
PPLPLA;TVPELRPP	NCP	12.603
TVPELRPP;MMETQ	NCP	4.997
MMETQ;FQLSSG	NCP	4.339
FQLSSG;VGAAGALV	NCP	78.72
VGAAGALV;QKKPGQK	NCP	52.905
QKKPGQK;RLNLPR	NCP	63.374
RLNLPR;KTPLLDL	NCP	513.054
KTPLLDL;ERPPQTH	NCP	251.019
ERPPQTH;NSLSTF	NCP	33.624
NSLSTF;PTPVQRPP	NCP	35.313
PTPVQRPP;MAVSELF	NCP	21.331
MAVSELF;VSFVEL	NCP	105.174
VSFVEL;ELRVT	NCP	65.624
ELRVT;LDFEGKD	NCP	33.963
LDFEGKD;MGGKAQVL	NCP	8.418
MGGKAQVL;LSHDK	NCP	17.431
LSHDK;WRLSSLL	NCP	33.863
WRLSSLL;PSVHNPF	NCP	32.359
PSVHNPF;LVLDQV	NCP	79.347
LVLDQV;KGVKKPK	NCP	9.74
KGVKKPK;LPLTP	NCP	14.479
LPLTP;AYRPR	NCP	25.823
AYRPR;LQVDF	NCP	187.629
LQVDF;KHEENPP	NCP	107.062
KHEENPP;VLDVVRK	NCP	76.818
VLDVVRK;EVDLNL	NCP	73.755
EVDLNL;DDHFVKL	NCP	145.655
DDHFVKL;DLFMP	NCP	85.306
DLFMP;EVFVLP	NCP	236.567
EVFVLP;MKQAREAK	NCP	142.413
MKQAREAK;KFNSASVP	NCP	153.526
KFNSASVP;VWLNSL	NCP	272.73
VWLNSL;TLDMDPL	NCP	19.85
TLDMDPL;KDNPKENP	NCP	78.987
KDNPKENP;VVPSPVK	NCP	68.34
VVPSPVK;SGHLFF	NCP	32.109
SGHLFF;PCPSLVHR	NCP	79.987
PCPSLVHR;LSNLLSPL	NCP	212.627
LSNLLSPL;KVLVTSK	NCP	137.691
KVLVTSK;KTPRVP	NCP	157.822
KTPRVP;KPTPRC	NCP	55.94
KPTPRC;GSVFQFVFL	NCP	8.877
GSVFQFVFL;YACDY	NCP	134.268
YACDY;FDDLEPF	NCP	1.344
FDDLEPF;LSALGW	NCP	21.651
LSALGW;HVDAVTK	NCP	33.277

HVDAVTK;TAELPYLK	NCP	188.99
TAELPYLK;PFVETLD	NCP	34.828
PFVETLD;KVPSHP	NCP	2.258
KVPSHP;LEVLNF	NCP	76.983
LEVLNF;KNSQP	NCP	24.738
KNSQP;SNCDD	NCP	108.951
SNCDD;GYEVL	NCP	82.77
GYEVL;LHVAPAP	NCP	86.827
LHVAPAP;LPPLGG	NCP	46.587
LPPLGG;KPPMGAP	NCP	9.048
KPPMGAP;YDVTVPK	NCP	8.838
YDVTVPK;QLWVPL	NCP	202.161
QLWVPL;PLPPGPP	NCP	85.995
PLPPGPP;KAAMELR	NCP	21.033
KAAMELR;QDATLSL	NCP	185.805
QDATLSL;QPFVAQ	NCP	106.154
QPFVAQ;PQLTHGR	NCP	3.601
PQLTHGR;TTMTH	NCP	301.619
TTMTH;HLLFMTE	NCP	124.633
HLLFMTE;VVEYP	NCP	19.617
VVEYP;PVASQLR	NCP	166.938
PVASQLR;HLRFF	NCP	25.472
HLRFF;FNPLLR	NCP	7.672
FNPLLR;MHLKYTK	NCP	27.669
MHLKYTK;PTPLPSKY	NCP	10.173
PTPLPSKY;KPLVPPP	NCP	125.28
KPLVPPP;GPPGPLPS	NCP	76.137
GPPGPLPS;AAPLAPPF	NCP	65.31
AAPLAPPF;YGPEVTK	NCP	30.906
YGPEVTK;SPSVLP	NCP	63.644
SPSVLP;TMHPPY	NCP	13.306
TMHPPY;FVYSRF	NCP	16.919
FVYSRF;KKEATPP	NCP	17.164
KKEATPP;KGAPVSPK	NCP	48.909
KGAPVSPK;KTTEPPR	NCP	173.93
KTTEPPR;KPFLSY	NCP	30.84
KPFLSY;FKVKPPP	NCP	181.83
FKVKPPP;RGDVAKK	NCP	13.308
RGDVAKK;SLTVGYPF	NCP	42.247
SLTVGYPF;RGVLPHN	NCP	55.164
RGVLPHN;MPGGPRPP	NCP	10.467
MPGGPRPP;PLLALGP	NCP	128.169
PLLALGP;LLLLLLLL	NCP	1.488
LLLLLLLL;KNLGP	NCP	18.062
KNLGP;PGPVLK	NCP	30.142
PGPVLK;RPPPPAPP	NCP	0.216
RPPPPAPP;PDAPKGP	NCP	65.175
PDAPKGP;MCDFDK	NCP	31.611
MCDFDK;APKAAFT	NCP	4.781
APKAAFT;PATKT	NCP	80.587
PATKT;TSYLFD	NCP	22.355

TSYLFD;RYPSRL	NCP	74.92
RYPSRL;SLDPVP	NCP	100.62
SLDPVP;KDPPPL	NCP	119.922
KDPPPL;MLKDL	NCP	37.269
MLKDL;SLTALLA	NCP	168.312
SLTALLA;LLGDAF	NCP	5.352
LLGDAF;GHVLPAL	NCP	55.317
GHVLPAL;EVVPP	NCP	36.879
EVVPP;TTLLYKLK	NCP	111.741
TTLLYKLK;GETYDF	NCP	7.514
GETYDF;MVLHLF	NCP	74.383
MVLHLF;KSMQYR	NCP	37.412
KSMQYR;LGVDF	NCP	66.991
LGVDF;VKNQE	NCP	54.707
VKNQE;LPLLTAV	NCP	111.741
LPLLTAV;KQPPETRS	NCP	56.041
KQPPETRS;KPSRFQK	NCP	15.639
KPSRFQK;LLAEVK	NCP	83.198
LLAEVK;KKKMAKKV	NCP	94.662
KKKMAKKV;ESPLFK	NCP	43.734
ESPLFK;FGVLQ	NCP	14.313
FGVLQ;LPPDAP	NCP	50.769
LPPDAP;KAAVSH	NCP	64.098
KAAVSH;MDNALHH	NCP	177.63
MDNALHH;KTLYLYKF	NCP	214.614
KTLYLYKF;KPRKGK	NCP	438.427
KPRKGK;TTEKVK	NCP	51.588
TTEKVK;TTKVP	NCP	24.36
TTKVP;KFYPPP	NCP	43.718
KFYPPP;MEKPQGK	NCP	3.49
MEKPQGK;KEGGGP	NCP	115.83
KEGGGP;EVVLPF	NCP	7.716
EVVLPF;LKATVGDP	NCP	11.39
LKATVGDP;LVAVE	NCP	4.261
LVAVE;RALAELA	NCP	1.95
RALAELA;PQNTG	NCP	23.391
PQNTG;ALNLG	NCP	43.992
ALNLG;ATDRLGV	NCP	11.34
ATDRLGV;ARSLLH	NCP	53.376
ARSLLH;WPGEDPP	NCP	43.632
WPGEDPP;KCLVGP	NCP	53.909
KCLVGP;GSKYY	NCP	36.438
GSKYY;AGQVLYK	NCP	12.336
AGQVLYK;MMLDVAK	NCP	119.889
MMLDVAK;ANAVP	NCP	196.813
ANAVP;AHQEAP	NCP	143.061
AHQEAP;WQLHCLGF	NCP	19.769
WQLHCLGF;KNDVFK	NCP	145.902
KNDVFK;NHQEC	NCP	55.381
NHQEC;TSEAPHKP	NCP	51.365
TSEAPHKP;MTGEKLK	NCP	17.883

MTGEKLK;KYPLLT	NCP	56.478
KYPLLT;LSCKLR	NCP	48.985
LSCKLR;LPPPPPRP	NCP	6.905
LPPPPPRP;KNWPGAP	NCP	109.755
KNWPGAP;LPPPGLPS	NCP	25.612
LPPPGLPS;RYHYR	NCP	193.352
RYHYR;RQRSSKK	NCP	265.626
RQRSSKK;VGKSLK	NCP	59.403
VGKSLK;ALVGY	NCP	24.28
ALVGY;TEQALFS	NCP	185.934
TEQALFS;PAATPKRH	NCP	196.893
PAATPKRH;GVVLAKK	NCP	15.506
GVVLAKK;PAPHMVSK	NCP	80.503
PAPHMVSK;VVNVY	NCP	3.434
VVNVY;RGGSLL	NCP	11.248
RGGSLL;QGVALGLPL	NCP	8.391
QGVALGLPL;KGTGPKLK	NCP	59.502
KGTGPKLK;APALSL	NCP	105.963
APALSL;LSSVPPP	NCP	26.628
LSSVPPP;GVLQLNL	NCP	238.892
GVLQLNL;DLQPHGLQ	NCP	154.807
DLQPHGLQ;KPAHYGP	NCP	4.073
KPAHYGP;AARVMSK	NCP	266.314
AARVMSK;EVGPL	NCP	54.579
EVGPL;ECCMMSK	NCP	58.757
ECCMMSK;MYKKPP	NCP	142.15
MYKKPP;MELAH	NCP	141.588
MELAH;EYKEKL	NCP	91.586
EYKEKL;SVWLG	NCP	0.763
SVWLG;KEPKPK	NCP	63.389
KEPKPK;LALAP	NCP	132.208
LALAP;DPPLSR	NCP	7.128
DPPLSR;KTSTFT	NCP	58.524
KTSTFT;SLQRGPPPP	NCP	12.882
SLQRGPPPP;GPPRPRP	NCP	6.6
GPPRPRP;SLGSPF	NCP	81.657
SLGSPF;KVMGTVP	NCP	24.003
KVMGTVP;PALGPPR	NCP	58.161
PALGPPR;VEKGPVK	NCP	32.033
VEKGPVK;VEPVP	NCP	119.398
VEPVP;PPLLPK	NCP	1.943
PPLLPK;GGVGSLM	NCP	69.033
GGVGSLM;NAAPREP	NCP	111.94
NAAPREP;SPPSVKP	NCP	2.586
SPPSVKP;FLLVPF	NCP	17.303
FLLVPF;GVSEPK	NCP	0.729
GVSEPK;DLVLPL	NCP	39.496
DLVLPL;GVKFLT	NCP	216.62
GVKFLT;NPKRSPP	NCP	93.826
NPKRSPP;KDSLC	NCP	7.562
KDSLC;KPPCPL	NCP	12.3

KPPCPL;RDPGSF	NCP	14.253
RDPGSF;NPGALKP	NCP	180.318
NPGALKP;VPLLP	NCP	33.721
VPLLP;GAERAGAGP	NCP	78.948
GAERAGAGP;LPGALPLT	NCP	120.3
LPGALPLT;RSGGP	NCP	33.636
RSGGP;TVAGLPSR	NCP	45.129
TVAGLPSR;PLNFRTSP	NCP	0.894
PLNFRTSP;KELQRVNP	NCP	72.356
KELQRVNP;LVAPGP	NCP	37.597
LVAPGP;LWKLPP	NCP	30.549
LWKLPP;DGASKTK	NCP	202.827
DGASKTK;VEVPL	NCP	51.677
VEVPL;KVVHAAGP	NCP	68.857
KVVHAAGP;ELVPP	NCP	177.852
ELVPP;AALLPNPP	NCP	183.978
AALLPNPP;DGPFL	NCP	167.564
DGPFL;PAEGY	NCP	71.086
PAEGY;LEDLPL	NCP	9.033
LEDLPL;AAAAAGPQPL	NCP	3.9
AAAAAGPQPL;AARPPPP	NCP	31.296
AARPPPP;HNVPVY	NCP	16.376
HNVPVY;LTADVNR	NCP	81.93
LTADVNR;VSLLS	NCP	6.141
VSLLS;APALFC	NCP	113.014
APALFC;PAAGRPPP	NCP	35.028
PAAGRPPP;LGAGPPPK	NCP	40.596
LGAGPPPK;PGHLEPP	NCP	12.243
PGHLEPP;CHPDY	NCP	41.858
CHPDY;LLMPNLAP	NCP	48.13
LLMPNLAP;GCQGP	NCP	73.209
GCQGP;NVANWPP	NCP	85.542
NVANWPP;PPTLLPPPVL	NCP	34.245
PPTLLPPPVL;EAGRGVPK	NCP	130.704
EAGRGVPK;PPPPGTH	NCP	78.081
PPPPGTH;TPTGF	NCP	212.369
TPTGF;DEVGLPL	NCP	102.364
DEVGLPL;KPAAFAK	NCP	56.43
KPAAFAK;KYAVVF	NCP	36.834
KYAVVF;GVPPP	NCP	50.091
GVPPP;KPAKP	NCP	174.606
KPAKP;NLSELHC	NCP	88.634
NLSELHC;PPVPAKPP	NCP	24.466
PPVPAKPP;LAAFYR	NCP	3.521
LAAFYR;VPVVGVNK	NCP	32.427
VPVVGVNK;PHELYP	NCP	162.631
PHELYP;MEEGL	NCP	161.925
MEEGL;YEASAY	NCP	99.552
YEASAY;LAKEPKP	NCP	57.456
LAKEPKP;EELRPLT	NCP	19.856
EELRPLT;GPPVAPPK	NCP	8.203

GPPVAPPK;KYAGPP	NCP	96.69
KYAGPP;LGPVP	NCP	33.027
LGPVP;GEAPP	NCP	92.7
GEAPP;MMSPDLR	NCP	15.285
MMSPDLR;LEVLPEFK	NCP	13.661
LEVLPEFK;AAGVVKAL	NCP	25.93
AAGVVKAL;AEPHRGGP	NCP	148.632
AEPHRGGP;VPQLPP	NCP	52.136
VPQLPP;PPSGEPL	NCP	22.747
PPSGEPL;AALSVKM	NCP	71.405
AALSVKM;RAAFY	NCP	63.013
RAAFY;PGRMPLGPP	NCP	26.22
PGRMPLGPP;PGPSP	NCP	94.464
PGPSP;HGSPPPP	NCP	37.368
HGSPPPP;PPEPPPP	NCP	7.8
PPEPPPP;GVQGPSF	NCP	45.519
GVQGPSF;RHPPPPPP	NCP	19.32
RHPPPPPP;KNFKER	NCP	85.704
KNFKER;FGAGP	NCP	130.74
FGAGP;LGPVQR	NCP	12.417
LGPVQR;LLPVKTSN	NCP	3.878
LLPVKTSN;PGLTPRP	NCP	0.136
PGLTPRP;KDRGLAH	NCP	63.384
KDRGLAH;LTNLPF	NCP	24.638
LTNLPF;LEKGPVLQ	NCP	50.433
LEKGPVLQ;MFGNMFL	NCP	60.013
MFGNMFL;VGHSSLV	NCP	57.674
VGHSSLV;LPLSTPR	NCP	94.801
LPLSTPR;VTHPAKP	NCP	6.519
VTHPAKP;LETTGPKP	NCP	23.201
LETTGPKP;LPAAPGLP	NCP	103.672
LPAAPGLP;KPPPRP	NCP	68.904
KPPPRP;TARGAGGPP	NCP	48.636
TARGAGGPP;APPPAPE	NCP	1.296
APPPAPE;TTPTATPL	NCP	90.396
TTPTATPL;TETTTHTATPL	NCP	0.987
TETTTHTATPL;TPTPTPLTK	NCP	0.288
TPTPTPLTK;TTTPTVTLLTK	NCP	1.041
TTTPTVTLLTK;TTPTVTQL	NCP	0.624
TTPTVTQL;TTTPTVTPLTK	NCP	0.147
TTTPTVTPLTK;TTTPTGTPLTK	NCP	0.21
TTTPTGTPLTK;TTTETTTPTPLTK	NCP	0.249
TTTETTTPTPLTK;TTETTTPTPL	NCP	0.003
TTETTTPTPL;TETTTPTPLTK	NCP	0.003
TETTTPTPLTK;ETTTPTPL	NCP	0.003
ETTTPTPL;TTTQTTTVTATPLTK	NCP	2.49
TTTQTTTVTATPLTK;TTTVTPLTK	NCP	0.057
TTTVTPLTK;TTTETTTATPLTK	NCP	1.005
TTTETTTATPLTK;TTTETTTPLTK	NCP	0.225
TTTETTTPLTK;TMTTTETTTPTPLTK	NCP	0.102
TMTTTETTTPTPLTK;TTTEPTTAPPLTK	NCP	1.68

TTTEPTTAPPLTK;TTDTTTATPLTK	NCP	0.042
TTDTTTATPLTK;TTTTAETTTPR	NCP	0.063
TTTTAETTTPR;ATPLTETTTPTVTPLTK	NCP	0.033
ATPLTETTTPTVTPLTK;NLRGAP	NCP	10.673
NLRGAP;RYLSPR	NCP	74.271
RYLSPR;KFFPPLP	NCP	140.794
KFFPPLP;GAVSRKPP	NCP	58.755
GAVSRKPP;LGGPLL	NCP	72.303
LGGPLL;LDDCGLTEVRCKD	NCP	35.109
LDDCGLTEVRCKD;DDCGLTEVRCK	NCP	0.003
DDCGLTEVRCK;NELGDGGVL	NCP	0.09
NELGDGGVL;GDGGVLLVL	NCP	0.009
GDGGVLLVL;KEALKELSL	NCP	1.305
KEALKELSL;LQASTMAF	NCP	2.262
LQASTMAF;NLPHALLRL	NCP	18.432
NLPHALLRL;LLPLFE	NCP	90.899
LLPLFE;GVGVHRP	NCP	18.541
GVGVHRP;ELMCML	NCP	52.5
ELMCML;PPPPKPP	NCP	41.082
PPPPKPP;LPNYV	NCP	127.667
GHKVPLPP;RHVGGALP	NCP	82.581
RHVGGALP;GGPPLHK	NCP	8.136
GGPPLHK;PASAKPEAP	NCP	57.579
PASAKPEAP;LQKEPKY	NCP	50.919
LQKEPKY;MSSSKHF	NCP	10.222
MSSSKHF;KAAKRDPL	NCP	37.298
KAAKRDPL;SEPLSLK	NCP	33.064
SEPLSLK;TGEKEQLAQAVARVK	NCP	86.414
TGEKEQLAQAVARVK;LADVVDSEALQ	NCP	15.096
LADVVDSEALQ;TALPEVD	NCP	0.697
TALPEVD;PPVGAPPPAP	NCP	4.46
PPVGAPPPAP;QGGTVPLP	NCP	30.774
QGGTVPLP;LCALAPR	NCP	36.822
LCALAPR;AAQALLALR	NCP	19.644
AAQALLALR;YGSGAF	NCP	85.966
YGSGAF;CCRGM	NCP	6.324
CCRGM;VRFPLE	NCP	30.732
VRFPLE;GPPPPEQR	NCP	21.101
GPPPPEQR;KACRPP	NCP	9.237
KACRPP;VAYWRQAGLS	NCP	49.029
VAYWRQAGLS;AVRDALKTEF	NCP	0.06
AVRDALKTEF;TGPSLLGP	NCP	26.52
TGPSLLGP;AAPLC	NCP	11.199
AAPLC;LGDATVL	NCP	1.225
LGDATVL;AVSLHPR	NCP	30.038
AVSLHPR;KKPAAGGP	NCP	17.676
KKPAAGGP;GYVSAPR	NCP	170.622
GYVSAPR;EHLSSPP	NCP	89.44
EHLSSPP;TAAHKLY	NCP	28.266
TAAHKLY;DASLGLR	NCP	63.938
DASLGLR;GVLPFK	NCP	87.285

GVLPFK;LMSLAQVL	NCP	47.442
LMSLAQVL;HVLNVSQ	NCP	21.15
HVLNVSQ;LSAAVESTSKKPQGAGK	NCP	177.295
LSAAVESTSKKPQGAGK;HGLGDTGHGWAEAF	NCP	7.202
HGLGDTGHGWAEAF;VSYST	NCP	24.144
VSYST;LNLTAVT	NCP	29.628
LNLTAVT;TTLLTTH	NCP	48.03
TTLLTTH;LQYGPLA	NCP	81.99
LQYGPLA;QYGPLAY	NCP	0.003
QYGPLAY;VDSTTGDGKPLPVQ	NCP	0.885
VDSTTGDGKPLPVQ;RSGKFR	NCP	16.314
RSGKFR;AARHLEK	NCP	27.315
AARHLEK;GHGVGP	NCP	6.816
GHGVGP;KLQAV	NCP	22.422
KLQAV;KHLKVP	NCP	2.509
KHLKVP;PGTKVAVP	NCP	60.002
PGTKVAVP;PPEHSPK	NCP	84.495
PPEHSPK;KQSPPVH	NCP	14.055
KQSPPVH;THGLVHR	NCP	24.594
THGLVHR;PAAQP	NCP	23.928
PAAQP;LGPAVP	NCP	19.965
LGPAVP;FTTLDH	NCP	3.874
FTTLDH;HDVVL	NCP	42.758
HDVVL;PPKGPHK	NCP	10.944
PPKGPHK;SVGPNYK	NCP	28.974
SVGPNYK;NDKPDL	NCP	5.092
NDKPDL;GPLTF	NCP	12.081
GPLTF;HGGPLLPP	NCP	11.144
HGGPLLPP;KTWPLLQ	NCP	80.233
KTWPLLQ;NSLHMKLR	NCP	52.623
NSLHMKLR;AAEARSLP	NCP	16.367
AAEARSLP;VGVPVVA	NCP	24.396
VGVPVVA;KNYTVLEF	NCP	15.88
KNYTVLEF;ATPPPP	NCP	28.451
ATPPPP;SGTVRLK	NCP	32.337
SGTVRLK;VAVDVPF	NCP	5.985
VAVDVPF;TGVLKAVP	NCP	21.819
TGVLKAVP;APAHQQP	NCP	1.98
APAHQQP;NSDVAKR	NCP	29.298
NSDVAKR;SLHGAPLQ	NCP	2.739
SLHGAPLQ;LPAAKFG	NCP	15.091
LPAAKFG;AGEAFRK	NCP	32.426
AGEAFRK;KAAPQPPP	NCP	1.926
KAAPQPPP;HGAAPVAA	NCP	51.939
HGAAPVAA;LAKRSSVL	NCP	6.261
LAKRSSVL;ERVLAPGP	NCP	13.827
ERVLAPGP;SLGVAGP	NCP	82.833
SLGVAGP;SVEMHHEAL	NCP	68.983
SVEMHHEAL;MKLGFP	NCP	11.163
MKLGFP;PAALVKPV	NCP	5.003
PAALVKPV;FPPFPKPP	NCP	24.309

FPPFPKPP;MAPKKDF	NCP	35.496
MAPKKDF;LETLVP	NCP	112.306
LETLVP;EMMSRY	NCP	23.777
EMMSRY;CMMKGP	NCP	4.576
CMMKGP;VTRLLSQ	NCP	38.105
VTRLLSQ;KPHKGLP	NCP	42.348
KPHKGLP;EEELALGL	NCP	29.446
EEELALGL;PGAATK	NCP	64.172
PGAATK;GLGGGP	NCP	45.312
GLGGGP;KDVPLKD	NCP	69.48
KDVPLKD;PPLFP	NCP	6.921
PPLFP;TPARPK	NCP	81.783
TPARPK;APKTHPK	NCP	63.558
APKTHPK;KMERPK	NCP	21.739
KMERPK;LADKKR	NCP	102.953
LADKKR;RQSADRPP	NCP	2.49
RQSADRPP;TVPTAHR	NCP	2.622
TVPTAHR;PSMAKDT	NCP	14.835
PSMAKDT;EVFPP	NCP	127.44
EVFPP;LPSPLSE	NCP	38.265
LPSPLSE;LSFGLSW	NCP	93.612
LSFGLSW;STKAQVLDAVK	NCP	9.075
STKAQVLDAVK;YADTVRPEFY	NCP	4.65
YADTVRPEFY;ADTVRPEF	NCP	0.003
ADTVRPEF;DTVRPEFY	NCP	0.003
DTVRPEFY;FNTYPSK	NCP	0.024
FNTYPSK;MKALDGSAL	NCP	0.048
MKALDGSAL;TGSALDFVRN	NCP	0.03
TGSALDFVRN;LLVLVTGGKSLDAVSQPAQELK	NCP	0.081
LLVLVTGGKSLDAVSQPAQELK;LVLVTGGK	NCP	0.003
LVLVTGGK;SLDAVSQPAQELK	NCP	0.024
SLDAVSQPAQELK;DSSLVFTA	NCP	0.111
DSSLVFTA;LFDGSANLMGQFTAARDF	NCP	4.095
LFDGSANLMGQFTAARDF;TGKALNLGY	NCP	0.207
TGKALNLGY;ALDYAQRY	NCP	0.027
ALDYAQRY;LVLLVAGK	NCP	0.075
LVLLVAGK;KQSGVVPF	NCP	0.06
KQSGVVPF;SDRTRPEF	NCP	2.397
SDRTRPEF;DRTRPEFY	NCP	0.003
DRTRPEFY;SDDPRVEFL	NCP	3.135
SDDPRVEFL;DDPRVEF	NCP	0.003
DDPRVEF;LLNVHSSKDEVQN	NCP	0.021
LLNVHSSKDEVQN;NVHSSKDEVQNAVR	NCP	0.006
NVHSSKDEVQNAVR;VNVGGALEY	NCP	0.069
VNVGGALEY;KQFGVAPL	NCP	0.15
KQFGVAPL;VDQEELVK	NCP	0.039
VDQEELVK;SNDVFPEFQLK	NCP	2.106
SNDVFPEFQLK;LKTYKSQAS	NCP	0.027
LKTYKSQAS;KGGSPLNTGK	NCP	0.057
KGGSPLNTGK;ALEFVARNYF	NCP	0.03
ALEFVARNYF;EFVARNY	NCP	0.006

EFVARNY;VARNYFVK	NCP	0.006
VARNYFVK;RRDSFQEVLRF	NCP	2.655
RRDSFQEVLRF;RDSFQEVLR	NCP	0.003
RDSFQEVLR;DSFQEVLRF	NCP	0.003
DSFQEVLRF;NSDPTDEFF	NCP	0.093
NSDPTDEFF;RVGNVQELSELSEQ	NCP	0.342
RVGNVQELSELSEQ;DFAEYQPELFEKFQNMRAQHPY	NCP	0.939
DFAEYQPELFEKFQNMRAQHPY;VLTADTLKLY	NCP	0.066
VLTADTLKLY;LMQLEFGRGF	NCP	2.655
LMQLEFGRGF;FEGCVP	NCP	10.782
FEGCVP;PVFPTELAF	NCP	6.924
PVFPTELAF;ALDTSEGVTQDRFSQ	NCP	0.027
ALDTSEGVTQDRFSQ;NLQVSLTSK	NCP	0.207
NLQVSLTSK;TEAAKPVVTK	NCP	8.394
TEAAKPVVTK;AEAAKPTATKTEATK	NCP	0.03
AEAAKPTATKTEATK;NWQLLE	NCP	76.882
NWQLLE;NATLPP	NCP	33.837
NATLPP;DGVEL	NCP	20.942
DGVEL;KDPAEP	NCP	1.327
KDPAEP;LTEALPVT	NCP	3.579
LTEALPVT;MEPRPK	NCP	12.021
MEPRPK;VHAKPPP	NCP	14.957
VHAKPPP;ESPPARPP	NCP	21.742
ESPPARPP;FLNYLPR	NCP	105.33
FLNYLPR;KLSCPP	NCP	3.753
KLSCPP;PSVPK	NCP	9.894
PSVPK;VKNVGND	NCP	0.153
VKNVGND;QLNRVFP	NCP	86.429
QLNRVFP;LPGVLWFY	NCP	84.235
LPGVLWFY;KRPKFN	NCP	44.321
KRPKFN;VGGPSTSY	NCP	62.758
VGGPSTSY;ALLLVGQ	NCP	47.568
ALLLVGQ;KLAELK	NCP	59.978
KLAELK;KSELGPP	NCP	57.232
KSELGPP;EVANFKR	NCP	20.736
EVANFKR;VTTAERLQ	NCP	6.99
VTTAERLQ;AVGWVF	NCP	44.766
AVGWVF;KNVAVP	NCP	7.674
KNVAVP;LDVSGPK	NCP	38.417
LDVSGPK;KFQSAK	NCP	99.456
KFQSAK;VPAALLH	NCP	12.846
VPAALLH;RDFKGPK	NCP	43.62
RDFKGPK;PELTHAT	NCP	34.594
PELTHAT;KGGLLS	NCP	17.136
KGGLLS;YKEQLGK	NCP	96.614
YKEQLGK;FSYQRLE	NCP	22.636
FSYQRLE;DYGPVLK	NCP	0.149
DYGPVLK;QNALL	NCP	74.442
QNALL;VPAVPLA	NCP	8.512
VPAVPLA;KEEGRP	NCP	59.837
KEEGRP;KTEYK	NCP	31.671

KTEYK;LLKKTLV	NCP	55.645
LLKKTLV;TETHLTK	NCP	11.399
TETHLTK;KDDMGP	NCP	22.654
KDDMGP;LMLPLLQ	NCP	1.077
LMLPLLQ;KSSHVAVST	NCP	17.895
KSSHVAVST;KDHGGPP	NCP	129.191
KDHGGPP;GVTTTAAP	NCP	2.67
GVTTTAAP;WGALP	NCP	6.006
WGALP;SNKHEP	NCP	26.526
SNKHEP;TSPHPP	NCP	42.804
TSPHPP;KAGDHPK	NCP	28.452
KAGDHPK;HLVDGPPP	NCP	19.365
HLVDGPPP;PPPLRPP	NCP	28.419
PPPLRPP;KDDGAH	NCP	16.072
KDDGAH;GQQLPMG	NCP	35.525
GQQLPMG;VPVSY	NCP	17.761
VPVSY;LAVATPPRP	NCP	20.222
LAVATPPRP;FPPFLP	NCP	49.545
FPPFLP;LGLAAGVL	NCP	52.236
LGLAAGVL;ERLQTAV	NCP	13.737
ERLQTAV;VGMLP	NCP	14.845
VGMLP;QTDLRF	NCP	8.837
QTDLRF;AAGERLL	NCP	34.909
AAGERLL;ANPSFP	NCP	9.615
ANPSFP;KVGVN	NCP	49.836
KVGVN;AAAGGDAF	NCP	98.696
AAAGGDAF;VADVPL	NCP	0.645
VADVPL;REQFSSR	NCP	6.591
REQFSSR;AAPQPGPP	NCP	12.798
AAPQPGPP;AAVFLQ	NCP	149.424
AAVFLQ;NYLNPR	NCP	22.24
NYLNPR;DNFSQAR	NCP	10.899
DNFSQAR;CSGGPPRP	NCP	24.881
CSGGPPRP;LLLLSADC	NCP	3.045
LLLLSADC;KVLSSP	NCP	340.284
KVLSSP;KAFATLPS	NCP	26.83
KAFATLPS;RQSLEVGK	NCP	14.052
RQSLEVGK;LPVVVGPP	NCP	17.862
LPVVVGPP;HLSSSS	NCP	124.311
HLSSSS;CLFCCC	NCP	18.444
CLFCCC;KVMKPPP	NCP	90.773
KVMKPPP;PPPLC	NCP	60.147
PPPLC;TGAFPQR	NCP	191.754
TGAFPQR;PGDAPPP	NCP	47.409
PGDAPPP;ASKLLAVR	NCP	33.267
ASKLLAVR;VSYVRH	NCP	2.872
VSYVRH;AQAYEVL	NCP	3.653
AQAYEVL;SGLSHQY	NCP	2.703
SGLSHQY;KDEMAH	NCP	48.796
KDEMAH;LPAAHDK	NCP	58.412
LPAAHDK;LCTVWLR	NCP	41.085

LCTVWLR;DRPLPDH	NCP	69.177
DRPLPDH;TVTGPPP	NCP	7.806
TVTGPPP;AAALPK	NCP	3.006
AAALPK;KTVMLTK	NCP	52.863
KTVMLTK;KEVVK	NCP	57.831
KEVVK;FGYARGQ	NCP	55.87
FGYARGQ;KGPTPEK	NCP	32.94
KGPTPEK;KFSPPYK	NCP	40.499
KFSPPYK;THSGPG	NCP	16.078
THSGPG;ETTGKVLR	NCP	17.114
ETTGKVLR;PGAAH	NCP	2.67
PGAAH;EAPKSVH	NCP	94.38
EAPKSVH;LMLVEPQ	NCP	2.763
LMLVEPQ;EGCYKK	NCP	1.953
EGCYKK;GLPPEF	NCP	20.988
GLPPEF;VPPEPLK	NCP	5.176
VPPEPLK;KAPLRLR	NCP	47.354
KAPLRLR;APPPKY	NCP	83.598
APPPKY;PPNVPPPP	NCP	30.696
PPNVPPPP;VSELVPK	NCP	30.757
VSELVPK;KPFHVPP	NCP	13.448
KPFHVPP;SGNLPF	NCP	52.993
SGNLPF;PTVNVE	NCP	3.23
PTVNVE;FYTLGLL	NCP	0.291
FYTLGLL;KLPVVLL	NCP	170.586
KLPVVLL;PDPAKSAPAPKKGSKK	NCP	20.199
PDPAKSAPAPKKGSKK;RLLLPGELAKH	NCP	0.294
RLLLPGELAKH;AAGPLLLK	NCP	27.435
AAGPLLLK;LTTVLP	NCP	5.968
LTTVLP;QDDGAQK	NCP	13.392
QDDGAQK;PPAAGTTPP	NCP	92.135
PPAAGTTPP;PAGTPKGK	NCP	65.259
PAGTPKGK;AVAYTPK	NCP	5.409
AVAYTPK;AARFNM	NCP	15.339
AARFNM;HAAPA	NCP	13.984
HAAPA;ENKHPE	NCP	57.752
ENKHPE;LTMELH	NCP	1.666
LTMELH;KVETLR	NCP	44.831
KVETLR;KPNTPQP	NCP	67.063
KPNTPQP;FWKTLT	NCP	191.774
FWKTLT;PPHKPK	NCP	71.766
PPHKPK;YSLPPPPP	NCP	2.778
YSLPPPPP;LPPEEE	NCP	9.078
LPPEEE;EEALLP	NCP	90.781
EEALLP;TVPFELRT	NCP	16.847
TVPFELRT;VAELK	NCP	119.794
VAELK;PLRVGSYL	NCP	91.149
PLRVGSYL;KVLDREK	NCP	4.944
KVLDREK;PHYVTF	NCP	69.183
PHYVTF;PPPPPGKPQ	NCP	9.963
PPPPPGKPQ;TTGPVLP	NCP	71.796

TTGPVLP;KPTLSGL	NCP	17.417
KPTLSGL;ATQQRFP	NCP	62.211
ATQQRFP;KHKAPPG	NCP	15.516
KHKAPPG;LPDLP	NCP	138.112
LPDLP;KMLEAH	NCP	62.642
KMLEAH;AGSSPGL	NCP	25.194
AGSSPGL;VDNLPK	NCP	14.215
VDNLPK;KLLTGMT	NCP	4.319
KLLTGMT;GFGRLGRL	NCP	58.863
GFGRLGRL;VAVATPAK	NCP	19.3
VAVATPAK;KAVVTPGK	NCP	0.093
KAVVTPGK;AVPVKAKSTAED	NCP	0.357
AVPVKAKSTAED;ASFEDAKEALNSCNK	NCP	1.08
ASFEDAKEALNSCNK;ASRLL	NCP	97.65
ASRLL;NPTVF	NCP	46.437
NPTVF;VPSEVL	NCP	135.276
VPSEVL;LRSVFALTNG	NCP	21.206
LRSVFALTNG;GVSLMPF	NCP	12.094
GVSLMPF;ALLFPPPPP	NCP	0.117
ALLFPPPPP;LLVPLLR	NCP	174.561
LLVPLLR;KPDTARVP	NCP	36.56
KPDTARVP;GPPVVT	NCP	29.814
GPPVVT;MATSV	NCP	56.734
MATSV;YSSSKPF	NCP	9.912
YSSSKPF;KDPGRRM	NCP	128.81
KDPGRRM;KEHSPPPQ	NCP	29.74
KEHSPPPQ;TSTSVP	NCP	43.568
TSTSVP;KLSVT	NCP	166.371
KLSVT;PKAAVGK	NCP	15.787
PKAAVGK;VGGGLGGAG	NCP	2.625
VGGGLGGAG;KQFVGTK	NCP	42.099
KQFVGTK;KPVGPAPP	NCP	6.942
KPVGPAPP;AALDSLSLF	NCP	107.585
AALDSLSLF;SDAGLGDTKMK	NCP	19.23
SDAGLGDTKMK;FDDEPKFM	NCP	0.201
FDDEPKFM;EEEKSGKWEGL	NCP	0.057
EEEKSGKWEGL;EELEEESFSER	NCP	0.072
EELEEESFSER;DDPVDLR	NCP	5.643
DDPVDLR;EEEWDPEYTPKSK	NCP	3.174
EEEWDPEYTPKSK;EEWDPEYTPK	NCP	0.003
EEWDPEYTPK;WDPEYTPKSK	NCP	0.006
WDPEYTPKSK;KAEAAPK	NCP	17.517
KAEAAPK;LPAARGLL	NCP	43.491
LPAARGLL;PGPLVGT	NCP	0.621
PGPLVGT;LDLRDK	NCP	2.055
LDLRDK;VGSLDPY	NCP	3.85
VGSLDPY;GPTAKNP	NCP	10.419
GPTAKNP;AGEVVF	NCP	29.811
AGEVVF;EDCDP	NCP	64.427
EDCDP;NFAVHFK	NCP	0.586
NFAVHFK;ARASLLR	NCP	5.789

ARASLLR;LALPP	NCP	15.985
LALPP;YGYHNN	NCP	104.193
YGYHNN;YTMASKE	NCP	15.597
YTMASKE;LYKVHR	NCP	69.137
LYKVHR;LGSTRVSP	NCP	58.908
LGSTRVSP;MDAPL	NCP	0.291
MDAPL;PLLEAAP	NCP	7.95
PLLEAAP;VEDLPNK	NCP	0.133
VEDLPNK;LLEQMLR	NCP	15.353
LLEQMLR;CQFGEKL	NCP	13.438
CQFGEKL;VLLALLH	NCP	0.594
VLLALLH;KGSGAPP	NCP	11.705
KGSGAPP;YECKLC	NCP	65.46
YECKLC;PVSEHF	NCP	16.953
PVSEHF;GLCAFTD	NCP	11.971
GLCAFTD;MFLVN	NCP	49.326
MFLVN;APKPERPP	NCP	62.972
APKPERPP;LMREVHL	NCP	21.454
LMREVHL;AVPAL	NCP	22.94
AVPAL;LHDGALT	NCP	40.23
LHDGALT;NSAMGK	NCP	21.505
NSAMGK;DLALF	NCP	17.672
DLALF;KDVHRK	NCP	61.803
KDVHRK;MDPWF	NCP	15.573
MDPWF;LAKERNK	NCP	92.599
LAKERNK;PSLTPRQPP	NCP	155.444
PSLTPRQPP;VRHLF	NCP	53.328
VRHLF;EGDLVKR	NCP	21.897
EGDLVKR;LPSSAEQ	NCP	55.303
LPSSAEQ;VGGLEF	NCP	28.901
VGGLEF;TELFRP	NCP	39.783
TELFRP;AEKAPPTL	NCP	60.033
AEKAPPTL;KDTGTLK	NCP	7.695
KDTGTLK;GTSATLL	NCP	68.745
GTSATLL;PPLPPL	NCP	9.861
PPLPPL;VLSTGKNK	NCP	27.952
VLSTGKNK;KSRVAH	NCP	26.073
KSRVAH;LDTSLPK	NCP	79.737
LDTSLPK;TTASLP	NCP	53.169
TTASLP;LLVGKY	NCP	9.02
LLVGKY;FSAAAG	NCP	28.854
FSAAAG;VLPAATK	NCP	26.853
VLPAATK;GVGLTL	NCP	13.393
GVGLTL;DLKLLKT	NCP	24.555
DLKLLKT;TYHQE	NCP	1.167
TYHQE;AGTALKRYP	NCP	19.851
AGTALKRYP;TFYPPR	NCP	31.847
TFYPPR;KLRTSF	NCP	6.25
KLRTSF;LMTGVP	NCP	13.28
LMTGVP;LPRFPLK	NCP	7.297
LPRFPLK;VLSRAFF	NCP	6.522

VLSRAFF;LGVPRAQ	NCP	16.005
LGVPRAQ;PGSKKDP	NCP	66.119
PGSKKDP;KFTEY	NCP	328.444
KFTEY;ESGFK	NCP	13.167
ESGFK;WLLGY	NCP	5.361
WLLGY;LAKLTYK	NCP	43.376
LAKLTYK;MFLGRS	NCP	18.756
MFLGRS;DMMCCA	NCP	11.794
DMMCCA;KQKTPLP	NCP	33.203
KQKTPLP;KKCDLK	NCP	19.725
KKCDLK;KYLHPK	NCP	150.526
KYLHPK;PFFDVQK	NCP	9.929
PFFDVQK;EGVFVT	NCP	4.71
EGVFVT;SVPPPPP	NCP	40.839
SVPPPPP;KYLDLPP	NCP	96.33
KYLDLPP;KLLPK	NCP	3.972
KLLPK;VVGETEDSSSQMTPK	NCP	117.54
VVGETEDSSSQMTPK;LDVLELAEKF	NCP	81.564
LDVLELAEKF;KKVLGY	NCP	23.31
KKVLGY;AHSLAEK	NCP	66.996
AHSLAEK;LREPLAN	NCP	12.607
LREPLAN;VDWLTEK	NCP	68.838
VDWLTEK;ERPGPP	NCP	36.714
ERPGPP;LSHSLLLVT	NCP	19.691
LSHSLLLVT;MNGWEP	NCP	32.908
MNGWEP;LFEVY	NCP	286.736
LFEVY;PEGLKPF	NCP	16.227
PEGLKPF;ADMRNLVERLE	NCP	12.633
ADMRNLVERLE;ASDTHCGYGDSAAK	NCP	0.066
ASDTHCGYGDSAAK;DSLLAGPVAEY	NCP	0.657
DSLLAGPVAEY;VKEMNDAAMF	NCP	3.774
VKEMNDAAMF;KHVSDDMKTHKNPA	NCP	6.036
KHVSDDMKTHKNPA;HVSDDMKTHKNPALK	NCP	0.003
HVSDDMKTHKNPALK;LPLDG	NCP	77.07
LPLDG;LSATAPPK	NCP	164.766
LSATAPPK;PLPPPPP	NCP	20.575
PLPPPPP;KPPCDGK	NCP	32.043
KPPCDGK;MEKVPDT	NCP	18.99
MEKVPDT;KPALMP	NCP	7.964
KPALMP;ALGPDY	NCP	18.346
ALGPDY;VTTVPVK	NCP	0.53
VTTVPVK;LEVAGP	NCP	1.057
LEVAGP;MMGHVPR	NCP	20.691
MMGHVPR;PQHVPP	NCP	19.08
PQHVPP;ADLLSLGVKE	NCP	31.311
ADLLSLGVKE;AAPPPP	NCP	5.112
AAPPPP;APGWP	NCP	10.566
APGWP;MVLPSCSK	NCP	82.623
MVLPSCSK;LPPAKKF	NCP	36.554
LPPAKKF;NVLENF	NCP	3.021
NVLENF;KCLKDSFK	NCP	25.44

KCLKDSFK;RANSLGLK	NCP	33.988
RANSLGLK;GPRNNPPP	NCP	28.355
GPRNNPPP;TLLGAP	NCP	33.582
TLLGAP;VPAGRVVL	NCP	68.65
VPAGRVVL;AAPQAAGK	NCP	32.645
AAPQAAGK;KVMPPP	NCP	6.58
KVMPPP;LGVRL	NCP	18.935
LGVRL;QTPAAPPK	NCP	8.116
QTPAAPPK;DGDMC	NCP	27.036
DGDMC;GYEPV	NCP	10.545
GYEPV;KTVVVKPP	NCP	1.947
KTVVVKPP;LSVVRELPK	NCP	0.582
LSVVRELPK;GVRGLTL	NCP	1.197
GVRGLTL;KLHSRAY	NCP	36.89
KLHSRAY;ATMADR	NCP	36.411
ATMADR;KPPAAGVK	NCP	24.033
KPPAAGVK;LPLLD	NCP	56.911
LPLLD;QHLKY	NCP	13.037
QHLKY;AAMKK	NCP	8.916
AAMKK;MDGPP	NCP	25.516
MDGPP;TPPGEPVP	NCP	22.377
TPPGEPVP;VTLYR	NCP	87.848
VTLYR;CPLLLGL	NCP	5.212
CPLLLGL;QPGPTPP	NCP	8.826
QPGPTPP;LYENGYT	NCP	38.334
LYENGYT;SSEGPRVW	NCP	44.727
SSEGPRVW;DLDGGPK	NCP	4.401
DLDGGPK;RGPPKLE	NCP	57.792
RGPPKLE;KDFPQ	NCP	15.959
KDFPQ;RKPVVF	NCP	5.431
RKPVVF;FFLVG	NCP	123.852
FFLVG;PSPPPQ	NCP	18.866
PSPPPQ;AMLLLA	NCP	41.808
AMLLLA;YNALGP	NCP	42.232
YNALGP;AASKDLPFP	NCP	30.866
AASKDLPFP;CMETAPP	NCP	20.517
CMETAPP;MLWFQ	NCP	0.499
MLWFQ;VSLTGGLL	NCP	85.656
VSLTGGLL;VSATLFPK	NCP	44.604
VSATLFPK;GKYPLR	NCP	68.925
GKYPLR;KQHPPP	NCP	97.281
KQHPPP;QLPCTF	NCP	30.797
QLPCTF;KEKGDLGP	NCP	5.857
KEKGDLGP;YGALK	NCP	26.016
YGALK;NAPLVG	NCP	21.33
NAPLVG;VPGGKKQ	NCP	6.243
VPGGKKQ;RKLTLSY	NCP	52.421
RKLTLSY;LTTKSC	NCP	30.082
LTTKSC;GDPGGWF	NCP	10.101
GDPGGWF;APPPPPYK	NCP	45.774
APPPPPYK;PPPPGSAR	NCP	53.73

PPPPGSAR;HPKWVP	NCP	8.919
HPKWVP;VGPGVK	NCP	26.015
VGPGVK;LAPGFP	NCP	47.304
LAPGFP;AVPPSCSCS	NCP	20.061
AVPPSCSCS;LPTFK	NCP	63.144
LPTFK;GPFPVT	NCP	22.582
GPFPVT;PSLVAFVG	NCP	30.612
PSLVAFVG;SSPVNPVVFF	NCP	73.652
SSPVNPVVFF;TGPGGVPVQGSK	NCP	39.657
TGPGGVPVQGSK;AALLAH	NCP	32.958
AALLAH;LTVTVLA	NCP	2.085
LTVTVLA;VGHSSLLV	NCP	13.32
VGHSSLLV;GFFTPR	NCP	9.832
GFFTPR;WGLGK	NCP	140.609
WGLGK;AEMDLRK	NCP	75.853
AEMDLRK;VGPFPS	NCP	19.469
VGPFPS;YEELVEK	NCP	25.462
YEELVEK;SGFNTPK	NCP	7.079
SGFNTPK;PKTFLK	NCP	42.844
PKTFLK;VTLDPY	NCP	191.03
VTLDPY;LVVVL	NCP	39.919
LVVVL;GVALYL	NCP	66.513
GVALYL;KGETLSR	NCP	51.213
KGETLSR;KRSNPQDQ	NCP	15.42
KRSNPQDQ;TRVPSSY	NCP	19.212
TRVPSSY;DDELNW	NCP	23.46
DDELNW;PPKKKKMG	NCP	140.649
PPKKKKMG;LPRGPVPP	NCP	7.928
LPRGPVPP;LGTARLK	NCP	23.7
LGTARLK;KPTAAPR	NCP	5.718
KPTAAPR;ERPGPLP	NCP	34.3
ERPGPLP;KEANVLG	NCP	30.509
KEANVLG;RGFSP	NCP	53.952
RGFSP;LLSWEVP	NCP	19.629
LLSWEVP;HVGGK	NCP	122.451
HVGGK;LDTPF	NCP	52.845
LDTPF;LKAAKGL	NCP	12.072
LKAAKGL;TEPKSLPK	NCP	7.776
TEPKSLPK;VCVLLR	NCP	161.754
VCVLLR;FPHYAK	NCP	19.057
FPHYAK;VLPVGY	NCP	3.893
VLPVGY;ACCCYG	NCP	52.287
ACCCYG;KDDPFP	NCP	16.221
KDDPFP;EPASLPR	NCP	45.513
EPASLPR;KALHFFH	NCP	14.88
KALHFFH;KDRNKSE	NCP	23.944
KDRNKSE;ANVPAPK	NCP	77.339
ANVPAPK;KDAGPPK	NCP	17.079
KDAGPPK;KTKHSSK	NCP	44.875
KTKHSSK;KPGQKPP	NCP	14.166
KPGQKPP;SYTLMF	NCP	148.542

SYTLMF;ELPLT	NCP	95.715
ELPLT;LDARGNL	NCP	12.11
LDARGNL;SNDRLFF	NCP	209.971
SNDRLFF;KDMLK	NCP	33.053
KDMLK;NASNNELVRTK	NCP	14.22
NASNNELVRTK;RQWYESH	NCP	0.078
RQWYESH;ALPLGRKK	NCP	0.024
ALPLGRKK;LEEQFQQGK	NCP	0.348
LEEQFQQGK;TKYNLF	NCP	33.108
TKYNLF;FALADRL	NCP	21.612
FALADRL;RLKLVRK	NCP	14.856
RLKLVRK;KWKFRP	NCP	4.672
KWKFRP;EAVRYAL	NCP	42.633
EAVRYAL;YLTEKKGH	NCP	32.55
YLTEKKGH;PMKKMP	NCP	42.486
PMKKMP;MVEGGGSSTMSQAK	NCP	5.144
MVEGGGSSTMSQAK;GDTELL	NCP	6.718
GDTELL;KPDPHRP	NCP	50.363
KPDPHRP;KPVSKKH	NCP	52.435
KPVSKKH;QLHDC	NCP	109.629
QLHDC;SGMFLT	NCP	35.939
SGMFLT;HPLPP	NCP	199.21
HPLPP;EHLSEVK	NCP	6.27
EHLSEVK;RWQVQRKVS	NCP	34.682
RWQVQRKVS;AFSPEEMKQLDALNK	NCP	0.228
AFSPEEMKQLDALNK;SLLAAF	NCP	18.366
SLLAAF;MQALKC	NCP	79.365
MQALKC;DDFGFF	NCP	162.633
DDFGFF;LSHEYAK	NCP	1.851
LSHEYAK;VTFEKPK	NCP	1.416
VTFEKPK;AVWLALV	NCP	27.399
AVWLALV;YKPPPGH	NCP	0.831
YKPPPGH;SCCRFAK	NCP	70.26
SCCRFAK;LPNCEMP	NCP	111.505
LPNCEMP;PDWNGKK	NCP	5.903
PDWNGKK;FELHAA	NCP	88.404
FELHAA;QAPGLFPP	NCP	48.43
QAPGLFPP;CTLLAPDW	NCP	28.832
CTLLAPDW;YHKAKGK	NCP	12.072
YHKAKGK;GSVVKTH	NCP	22.815
GSVVKTH;QPGGSTW	NCP	53.047
QPGGSTW;EASQLSLK	NCP	6.999
EASQLSLK;AEHTPL	NCP	18.548
AEHTPL;GVAPPPP	NCP	0.192
GVAPPPP;VPVLAK	NCP	23.653
VPVLAK;APLPLN	NCP	5.573
APLPLN;KKAAFLRK	NCP	8.658
KKAAFLRK;PGEPSE	NCP	5.511
PGEPSE;AAANFPL	NCP	27.772
AAANFPL;TYPPVG	NCP	66.389
TYPPVG;KRKPG	NCP	37.909

KRKPG;MVPPPFP	NCP	14.619
MVPPPFP;AEPVRRGP	NCP	7.29
AEPVRRGP;ELLKPV	NCP	50.253
ELLKPV;KPTGY	NCP	24.905
KPTGY;VGGELPK	NCP	22.482
VGGELPK;PVVRQ	NCP	111.904
PVVRQ;VSSFKLE	NCP	10.586
VSSFKLE;LHLKPLGPGSEK	NCP	3.003
LHLKPLGPGSEK;GHHAAVEF	NCP	73.558
GHHAAVEF;EAVKLLK	NCP	1.499
EAVKLLK;VVRKLP	NCP	41.292
VVRKLP;LDPPQ	NCP	2.283
LDPPQ;VDKGVVS	NCP	37.219
VDKGVVS;SSEELVTLR	NCP	85.382
SSEELVTLR;KAAPPPPR	NCP	68.295
KAAPPPPR;PLLKLMQ	NCP	14.305
PLLKLMQ;PSRMPALP	NCP	2.153
PSRMPALP;KDASEED	NCP	5.014
KDASEED;VFDEVVK	NCP	119.231
VFDEVVK;FDLKPPP	NCP	38.148
FDLKPPP;LDLGRL	NCP	7.092
LDLGRL;LDKVELN	NCP	3.367
LDKVELN;EQHPPQ	NCP	6.8
EQHPPQ;AAGVLK	NCP	5.751
AAGVLK;SPQLN	NCP	41.11
SPQLN;KVLKQVHPDTG	NCP	159.735
KVLKQVHPDTG;TAVRLLLPGELAK	NCP	0.159
TAVRLLLPGELAK;LLPGELA	NCP	0.015
LLPGELA;SEGTKAVTKYTSS	NCP	0.033
SEGTKAVTKYTSS;LNPRHP	NCP	20.994
LNPRHP;AVRLLLPGELAK	NCP	1.323
AVRLLLPGELAK;RLLLPGELAKHAVS	NCP	0.006
RLLLPGELAKHAVS;KNLKFYLK	NCP	5.211
KNLKFYLK;SVYVYKVLKQ	NCP	26.16
SVYVYKVLKQ;LLPGELAKHAVSEGTKAVTKYTSSK	NCP	0.189
LLPGELAKHAVSEGTKAVTKYTSSK;ESVFCPK	NCP	5.955
ESVFCPK;MKLVPSL	NCP	34.41
MKLVPSL;CPPVCCD	NCP	6.434
CPPVCCD;LLMLG	NCP	16.221
LLMLG;AAPDPSR	NCP	50.224
AAPDPSR;RSVSLGGF	NCP	13.22
RSVSLGGF;TVTLNY	NCP	4.083
TVTLNY;ETAAPKSP	NCP	296.197
ETAAPKSP;LFGFL	NCP	57.092
LFGFL;APETPKP	NCP	1.714
APETPKP;GSPLGTLQ	NCP	60.023
GSPLGTLQ;KLKPVVK	NCP	91.299
KLKPVVK;HLPPP	NCP	33.841
HLPPP;WLPLLFL	NCP	75.336
WLPLLFL;KDGKLR	NCP	28.889
KDGKLR;LLDGSPL	NCP	9.645

LLDGSPL;PPPAGLHW	NCP	11.782
PPPAGLHW;LLEGLLR	NCP	5.684
LLEGLLR;KNEELGP	NCP	47.634
KNEELGP;PPLPSPP	NCP	67.977
PPLPSPP;DPLHPR	NCP	32.434
DPLHPR;KPGVREP	NCP	28.31
KPGVREP;AATAAGVGR	NCP	89.181
AATAAGVGR;NTDLAPY	NCP	13.048
NTDLAPY;VDPLDF	NCP	28.323
VDPLDF;TETLLLLPL	NCP	3.06
TETLLLLPL;KPPPSGSR	NCP	88.56
KPPPSGSR;PDCCC	NCP	32.648
PDCCC;YGSSCDK	NCP	42.571
YGSSCDK;SKADLHL	NCP	10.272
SKADLHL;KPLYSK	NCP	26.451
KPLYSK;KTHETKK	NCP	15.867
KTHETKK;CHYCPK	NCP	20.091
CHYCPK;KGVATLL	NCP	82.961
KGVATLL;GVSYY	NCP	3.465
GVSYY;LPLLMYLVL	NCP	56.416
LPLLMYLVL;LEHALLT	NCP	50.675
LEHALLT;PFCCCED	NCP	22.485
PFCCCED;AQLVK	NCP	62.668
AQLVK;MAAVLEYLTAE	NCP	173.364
MAAVLEYLTAE;AVLLPKKTESHHKAK	NCP	0.186
AVLLPKKTESHHKAK;VLLPKKTESHHKAKGK	NCP	0.003
VLLPKKTESHHKAKGK;LLPKKTESHHKAK	NCP	0.003
LLPKKTESHHKAK;LPKKTESHHKAKGK	NCP	0.003
LPKKTESHHKAKGK;DDKSPK	NCP	7.19
DDKSPK;VSRNLDF	NCP	13.785
VSRNLDF;KALAP	NCP	8.409
KALAP;KYPQLQ	NCP	38.859
KYPQLQ;CCCCCAF	NCP	166.758
CCCCCAF;DQHMD	NCP	77.725
DQHMD;SPSTVDLP	NCP	156.398
SPSTVDLP;LELLP	NCP	16.075
LELLP;KKGLKEK	NCP	7.05
KKGLKEK;VTANLLY	NCP	24.297
VTANLLY;GKPFGP	NCP	36.578
GKPFGP;CLLHY	NCP	4.165
CLLHY;VLLLA	NCP	18.896
VLLLA;VVSHLPY	NCP	0.637
VVSHLPY;VGFLDNGL	NCP	27.269
VGFLDNGL;VMVGM	NCP	16.566
VMVGM;ELLKVEGK	NCP	51.786
ELLKVEGK;PPPMKPPP	NCP	4.141
PPPMKPPP;KAGMRP	NCP	43.409
KAGMRP;MSPVLY	NCP	27.115
MSPVLY;FDLAP	NCP	30.126
FDLAP;QWHVLPK	NCP	26.003
QWHVLPK;AACLAPK	NCP	17.676

AACLAPK;VGPAPNR	NCP	40.602
VGPAPNR;DCDEFLK	NCP	111.387
DCDEFLK;MMCQP	NCP	42.964
MMCQP;GNDLLW	NCP	161.754
GNDLLW;KDEVKK	NCP	46.13
KDEVKK;VTAQPRCP	NCP	24.637
VTAQPRCP;KGATNPK	NCP	158.432
KGATNPK;LSRYVVF	NCP	80.86
LSRYVVF;PLPLPPP	NCP	128.633
PLPLPPP;YVELLE	NCP	1.965
YVELLE;KYPSGF	NCP	61.339
KYPSGF;HVLGPPPP	NCP	105.632
HVLGPPPP;LGPAPK	NCP	12.904
LGPAPK;KGAVLEP	NCP	16.88
KGAVLEP;KQTNPK	NCP	119.655
KQTNPK;MNCPPP	NCP	8.656
MNCPPP;VPAAKK	NCP	12.479
VPAAKK;HLDDLGK	NCP	245.382
HLDDLGK;EEMSSG	NCP	214.246
EEMSSG;SLPDL	NCP	9.983
SLPDL;LGEQLDF	NCP	25.873
LGEQLDF;TATADDKKLQSSLKK	NCP	60.944
TATADDKKLQSSLKK;CKDAQGLK	NCP	69.333
CKDAQGLK;CELARF	NCP	7.039
CELARF;VTGTLLGF	NCP	86.202
VTGTLLGF;KHVTTFY	NCP	66.981
KHVTTFY;TVERVTEF	NCP	68.819
TVERVTEF;LHKRVF	NCP	49.722
LHKRVF;LDVQEL	NCP	98.269
LDVQEL;LVLSVC	NCP	123.135
LVLSVC;DSTEKVVEKLEAL	NCP	296.049
DSTEKVVEKLEAL;VVGVGMTK	NCP	17.894
VVGVGMTK;KTTESK	NCP	0.924
KTTESK;FTKPGVEN	NCP	4.35
FTKPGVEN;VLALGFEKMEKGPVA	NCP	7.563
VLALGFEKMEKGPVA;ALGFEKMEKGPV	NCP	0.006
ALGFEKMEKGPV;LGFEKMEK	NCP	0.003
LGFEKMEK;GFEKMEKGPVA	NCP	0.003
GFEKMEKGPVA;KYGLSPSPVAP	NCP	15.315
KYGLSPSPVAP;GLSPSPVAPQMF	NCP	0.006
GLSPSPVAPQMF;VAPQMFGN	NCP	0.018
VAPQMFGN;LVVLGH	NCP	18.994
LVVLGH;MEDPTPK	NCP	24.987
MEDPTPK;SSSVDGFKAN	NCP	19.466
SSSVDGFKAN;DGPGGKEATWVVDVKN	NCP	16.407
DGPGGKEATWVVDVKN;EATWVVDVK	NCP	0.018
EATWVVDVK;LAMKLQNLQLQPGKA	NCP	9.435
LAMKLQNLQLQPGKA;MKLQNLQLQPGKAKL	NCP	0.006
MKLQNLQLQPGKAKL;KLQNLQLQPGKA	NCP	0.003
KLQNLQLQPGKA;LQNLQLQPGKAK	NCP	0.003
LQNLQLQPGKAK;QNLQLQPGKA	NCP	0.003

QNLQLQPGKA;NLQLQPGKAKL	NCP	0.003
NLQLQPGKAKL;LQLQPGKAK	NCP	0.003
LQLQPGKAK;QLQPGKAKL	NCP	0.003
QLQPGKAKL;MGFPEAAR	NCP	45.876
MGFPEAAR;QQAAWPP	NCP	32.49
QQAAWPP;KLLLKLLH	NCP	20.263
KLLLKLLH;ATPLP	NCP	29.777
ATPLP;LTASPLPT	NCP	14.928
LTASPLPT;KPWAEPP	NCP	28.428
KPWAEPP;KGDSVFDK	NCP	72.376
KGDSVFDK;TGAPALPR	NCP	38.286
TGAPALPR;NQKGLFK	NCP	47.334
NQKGLFK;KGATLKPK	NCP	24.767
KGATLKPK;KFLLPPP	NCP	55.246
KFLLPPP;KWGAPR	NCP	1.788
KWGAPR;ELVAVRK	NCP	42.782
ELVAVRK;TWLLLN	NCP	27.888
TWLLLN;KEPAAPP	NCP	54.708
KEPAAPP;VPPPPP	NCP	38.271
VPPPPP;KLHLY	NCP	3.772
KLHLY;VGKTEL	NCP	45.266
VGKTEL;KPAFCDK	NCP	7.77
KPAFCDK;ERQKAGP	NCP	4.857
ERQKAGP;SSLPTKET	NCP	120.198
SSLPTKET;TAASGK	NCP	1.935
TAASGK;LTHTHLVL	NCP	1.393
LTHTHLVL;PPPLPAR	NCP	2.837
PPPLPAR;CCCDADC	NCP	21.295
CCCDADC;GKELAML	NCP	115.988
GKELAML;FRCEP	NCP	0.841
FRCEP;PLTAGMC	NCP	1.454
PLTAGMC;HALELQR	NCP	16.516
HALELQR;KEALLT	NCP	22.494
KEALLT;LADVF	NCP	29.415
LADVF;VDMAVY	NCP	64.695
VDMAVY;QTCHMS	NCP	41.04
QTCHMS;GLPTH	NCP	67.814
GLPTH;KAEDKPK	NCP	57.229
KAEDKPK;KAALAFF	NCP	5.195
KAALAFF;QVDSLK	NCP	14.25
QVDSLK;SPKLWKDS	NCP	29.659
SPKLWKDS;LSGGKELP	NCP	35.139
LSGGKELP;HTKPGPP	NCP	14.031
HTKPGPP;KPGANSR	NCP	21.069
KPGANSR;PTAPEYP	NCP	1.418
PTAPEYP;YREPQR	NCP	31.071
YREPQR;SEFPR	NCP	18.273
SEFPR;LYALRQ	NCP	53.658
LYALRQ;PLMFFNK	NCP	1.201
PLMFFNK;PPPPLAR	NCP	7.418
PPPPLAR;TMALQK	NCP	1.603

TMALQK;PVARSW	NCP	46.616
PVARSW;GPKEKVER	NCP	116.406
GPKEKVER;GTTYGP	NCP	49.176
GTTYGP;LSMMVK	NCP	33.781
LSMMVK;PFAKQKA	NCP	5.4
PFAKQKA;VGPMVLTL	NCP	9.554
VGPMVLTL;QAAGGGW	NCP	105.651
QAAGGGW;PLEAGSK	NCP	26.322
PLEAGSK;GFLLFDP	NCP	0.423
GFLLFDP;KGMYSRP	NCP	10.083
KGMYSRP;KYAASSK	NCP	21.694
KYAASSK;LGQKMPSK	NCP	37.706
LGQKMPSK;LAEVAAK	NCP	2.352
LAEVAAK;LAVEVPK	NCP	65.778
LAVEVPK;NGLHPPPL	NCP	11.292
NGLHPPPL;GVVPNTN	NCP	121.095
GVVPNTN;SYQVHKR	NCP	27.196
SYQVHKR;LTGPLG	NCP	63.968
LTGPLG;AAKHLMS	NCP	77.107
AAKHLMS;LDLSGPKV	NCP	86.748
LDLSGPKV;LHVLYP	NCP	34.589
LHVLYP;PGVLPRP	NCP	13.66
PGVLPRP;RKALTK	NCP	96
RKALTK;KELTVSP	NCP	25.856
KELTVSP;SALSTRFT	NCP	148.591
SALSTRFT;KAAATNH	NCP	32.571
KAAATNH;GCKLELR	NCP	131.105
GCKLELR;VTFEFQP	NCP	13.467
VTFEFQP;YMAVPSH	NCP	25.951
YMAVPSH;CAPEEMY	NCP	256.154
CAPEEMY;NPPASF	NCP	73.065
NPPASF;TPKFVTK	NCP	26.646
TPKFVTK;KPDYEPR	NCP	60.175
KPDYEPR;LGALEYL	NCP	64.044
LGALEYL;QYSCGP	NCP	19.322
QYSCGP;ASGVMLSF	NCP	75.772
ASGVMLSF;YDLCFR	NCP	15.089
YDLCFR;HSLLPVV	NCP	83.094
HSLLPVV;PQVETK	NCP	35.722
PQVETK;MLALSLLP	NCP	32.381
MLALSLLP;PPGPPPPPP	NCP	4.588
PPGPPPPPP;RCNAR	NCP	67.925
RCNAR;LGPTSDELK	NCP	22.582
LGPTSDELK;LPLVPEL	NCP	59.147
LPLVPEL;RFLKSFP	NCP	33.145
RFLKSFP;MEPFPK	NCP	69.539
MEPFPK;ANKPDL	NCP	61.225
ANKPDL;KTAPLTS	NCP	132.899
KTAPLTS;KSTKAH	NCP	42.349
KSTKAH;TPVPK	NCP	98.291
TPVPK;EALRALV	NCP	2.343

EALRALV;NNAPP	NCP	82.251
NNAPP;LGQTPP	NCP	65.787
LGQTPP;TVPSFK	NCP	33.694
TVPSFK;KPYNPPP	NCP	6.734
KPYNPPP;FSLKLWN	NCP	71.397
FSLKLWN;GVGPLC	NCP	1.567
GVGPLC;DGKKNK	NCP	73.793
DGKKNK;LSGSPTLL	NCP	63.772
LSGSPTLL;ASLWVGKR	NCP	30.264
ASLWVGKR;EPGPK	NCP	73.551
EPGPK;GPAAAPP	NCP	4.248
GPAAAPP;TVGSK	NCP	0.591
TVGSK;NMTEVP	NCP	75.035
NMTEVP;FALLSF	NCP	84.814
FALLSF;YPLSP	NCP	102.663
YPLSP;TGLKVRH	NCP	22.107
TGLKVRH;LEELYL	NCP	35.208
LEELYL;VGSYLK	NCP	98.525
VGSYLK;AQKPLRLL	NCP	12.985
AQKPLRLL;LLLLMQC	NCP	5.543
LLLLMQC;PVYAGAM	NCP	92.064
PVYAGAM;KLEPME	NCP	73.647
KLEPME;FKLLSH	NCP	38.821
FKLLSH;SNVPGVR	NCP	68.873
SNVPGVR;KEVQMMP	NCP	20.659
KEVQMMP;KLTAAPVQ	NCP	6.917
KLTAAPVQ;KGAASPPP	NCP	34.327
KGAASPPP;LPLPPPPAP	NCP	98.316
LPLPPPPAP;LLLFP	NCP	45.822
LLLFP;TGHLQWM	NCP	11.933
TGHLQWM;LEYLQAPP	NCP	13.782
LEYLQAPP;LLPPPPPPP	NCP	36.724
LLPPPPPPP;MLKYC	NCP	61.244
MLKYC;KRAEY	NCP	130.086
KRAEY;LLEMEQ	NCP	10.795
LLEMEQ;LKGTVSLK	NCP	99.168
LKGTVSLK;YLVLAQM	NCP	212.961
YLVLAQM;FGKVDQK	NCP	80.279
FGKVDQK;GCDEY	NCP	98.601
GCDEY;TPGAPPR	NCP	1.708
TPGAPPR;VTRSVDY	NCP	0.326
VTRSVDY;KDFRDPN	NCP	4.441
KDFRDPN;QLPPKER	NCP	62.189
QLPPKER;TLHKK	NCP	77.433
TLHKK;LELAQ	NCP	34.636
LELAQ;KYLFAAP	NCP	17.717
KYLFAAP;GLHGFKL	NCP	64.713
GLHGFKL;AAVFK	NCP	46.558
AAVFK;MPVSEKLF	NCP	136.34
MPVSEKLF;KAKCAK	NCP	43.921
KAKCAK;YGEKPFL	NCP	59.033

YGEKPFL;VGMFLQ	NCP	35.251
VGMFLQ;VSVSAYPT	NCP	15.227
VSVSAYPT;ELGVEP	NCP	24.831
ELGVEP;LHAFPK	NCP	16.075
LHAFPK;HLDKPAH	NCP	62.513
HLDKPAH;LPEAPVP	NCP	21.346
LPEAPVP;QAPYAVP	NCP	9.888
QAPYAVP;PTGVSPP	NCP	20.001
PTGVSPP;KDGPTGR	NCP	5.592
KDGPTGR;LVGSLVST	NCP	65.565
LVGSLVST;APLRFPS	NCP	74.871
APLRFPS;PPPPKY	NCP	48.71
PPPPKY;APPLHLP	NCP	63.589
APPLHLP;PAKPGFP	NCP	55.697
PAKPGFP;QGELL	NCP	34.773
QGELL;FVLAT	NCP	78.942
FVLAT;KDKVTAQK	NCP	38.602
KDKVTAQK;KVLRGLSN	NCP	80.261
KVLRGLSN;RLLLQSK	NCP	0.658
RLLLQSK;AERRVVW	NCP	2.919
AERRVVW;VTATPAY	NCP	6.129
VTATPAY;LPPLSLPPP	NCP	113.379
LPPLSLPPP;EHVFKAK	NCP	2.865
EHVFKAK;KPHTAAH	NCP	106.233
KPHTAAH;LTTKAAP	NCP	75.579
LTTKAAP;RATNVP	NCP	87.471
RATNVP;QVLGTDLR	NCP	0.456
QVLGTDLR;GPGYFPP	NCP	38.355
GPGYFPP;TGLNVPVG	NCP	86.658
TGLNVPVG;HHKRK	NCP	40.187
HHKRK;ENPKVPP	NCP	32.704
ENPKVPP;NSLLTHV	NCP	13.878
NSLLTHV;YPPLM	NCP	160.392
YPPLM;NSMLFL	NCP	19.281
NSMLFL;YRAALC	NCP	7.344
YRAALC;KLLGNVL	NCP	76.332
KLLGNVL;KMVSGGR	NCP	4.229
KMVSGGR;KYAPGTVL	NCP	99.382
KYAPGTVL;KGAEGAPA	NCP	58.109
KGAEGAPA;LAPPER	NCP	68.998
LAPPER;VGLLTQ	NCP	20.054
VGLLTQ;ALEKL	NCP	25.764
ALEKL;CCCCVP	NCP	19.567
CCCCVP;KVDLGVP	NCP	121.787
KVDLGVP;YEALCK	NCP	63.567
YEALCK;GLRVDVL	NCP	19.542
GLRVDVL;FDPLF	NCP	5.694
FDPLF;KWAGRVG	NCP	101.646
KWAGRVG;KQVFKKVN	NCP	12.222
KQVFKKVN;EVVSGP	NCP	48.682
EVVSGP;VSYDQLK	NCP	22.962

VSYDQLK;KFVGGAK	NCP	49.736
KFVGGAK;PARPPGP	NCP	8.862
PARPPGP;KLGDY	NCP	5.455
KLGDY;LGRCML	NCP	78.636
LGRCML;VTVVP	NCP	56.678
VTVVP;LQPKYKS	NCP	119.031
LQPKYKS;KCGPAP	NCP	53.602
KCGPAP;ALQSMYA	NCP	76.419
ALQSMYA;KSKHTK	NCP	165.872
KSKHTK;GFPPL	NCP	97.9
GFPPL;KDVLFL	NCP	22.145
KDVLFL;PDTLRYP	NCP	40.516
PDTLRYP;KDNHPLN	NCP	126.072
KDNHPLN;PPDLPP	NCP	27.876
PPDLPP;VSVPP	NCP	101.217
VSVPP;LSLPK	NCP	46.082
LSLPK;KGVLDF	NCP	21.633
KGVLDF;LVVAV	NCP	13.45
LVVAV;VVPSK	NCP	100.4
VVPSK;ESLLQKY	NCP	7.867
ESLLQKY;MEATGTDDVDKLK	NCP	61.955
MEATGTDDVDKLK;TNYAP	NCP	33.724
TNYAP;TKSGAL	NCP	29.213
TKSGAL;VLLENF	NCP	43.444
VLLENF;PPAPPP	NCP	6.221
PPAPPP;VGLKPGM	NCP	4.972
VGLKPGM;TKKLAY	NCP	95.823
TKKLAY;YKLLEPV	NCP	76.17
YKLLEPV;AALQY	NCP	296.726
AALQY;RVVYKNEK	NCP	16.021
RVVYKNEK;LGVRKVFL	NCP	21.221
LGVRKVFL;HPSNAY	NCP	25.033
HPSNAY;DDDDC	NCP	29.612
DDDDC;NYNHLMPTRY	NCP	109.066
NYNHLMPTRY;VLVFNT	NCP	26.193
VLVFNT;RVVSAPK	NCP	29.624
RVVSAPK;PVASFP	NCP	6.018
PVASFP;PHLFPKN	NCP	8.836
PHLFPKN;LCQKVHL	NCP	0.269
LCQKVHL;DLENKFKPF	NCP	29.907
DLENKFKPF;DPLYKP	NCP	4.66
DPLYKP;DKLLR	NCP	68.978
DKLLR;TSSSVAPR	NCP	25.77
TSSSVAPR;KGSYPSK	NCP	39.948
KGSYPSK;NPKVPGK	NCP	17.952
NPKVPGK;VDSLTVVKP	NCP	138.685
VDSLTVVKP;EAHLT	NCP	22.851
EAHLT;GVFLV	NCP	22.253
GVFLV;KVYYPP	NCP	59.479
KVYYPP;TLYVPP	NCP	33.813
TLYVPP;ESLPVLPK	NCP	12.638

ESLPVLPK;NPVPELLK	NCP	245.833
NPVPELLK;QCPLKK	NCP	33.986
QCPLKK;LSMNPAH	NCP	29.005
LSMNPAH;QFALKPK	NCP	6.843
QFALKPK;KCNPW	NCP	5.744
KCNPW;LGPKMFL	NCP	74.797
LGPKMFL;KPCTLH	NCP	63.93
KPCTLH;LPPPPPYK	NCP	19.406
LPPPPPYK;VYLVK	NCP	139.642
VYLVK;ALTALTK	NCP	20.726
ALTALTK;AAVSKLD	NCP	121.647
AAVSKLD;VSGYGPK	NCP	128.823
VSGYGPK;LPVPF	NCP	33.447
LPVPF;LLEQPLS	NCP	4.305
LLEQPLS;LQKLLPRL	NCP	134.664
LQKLLPRL;PTLLT	NCP	137.628
PTLLT;KDDFK	NCP	6.085
KDDFK;KDQLL	NCP	14.892
KDQLL;GPPGSGKGTVCQ	NCP	29.276
GPPGSGKGTVCQ;MHAPPKP	NCP	34.864
MHAPPKP;DGHMW	NCP	2.999
DGHMW;KVTESKQP	NCP	152.053
KVTESKQP;SGFKRK	NCP	3.674
SGFKRK;WFVNP	NCP	62.976
WFVNP;KDRGVDL	NCP	22.854
KDRGVDL;KFATL	NCP	10.809
KFATL;ETSLTEL	NCP	31.008
ETSLTEL;KELGKKGP	NCP	165.924
KELGKKGP;FVLAV	NCP	171.738
FVLAV;MKDNP	NCP	200.76
MKDNP;NVELLSK	NCP	23.323
NVELLSK;RNEFSKK	NCP	252.776
RNEFSKK;DASAWL	NCP	36.291
DASAWL;FPFPF	NCP	63.81
FPFPF;ALKLLLSH	NCP	110.649
ALKLLLSH;VLLKTVQL	NCP	52.288
VLLKTVQL;SFPTTK	NCP	113.684
SFPTTK;EVPPF	NCP	19.818
EVPPF;PPLMPV	NCP	40.011
PPLMPV;KCAVP	NCP	46.767
KCAVP;ETMLQLQ	NCP	32.035
ETMLQLQ;YPVKVF	NCP	36.24
YPVKVF;KKVEPDA	NCP	27.242
KKVEPDA;KRHSSVK	NCP	22.731
KRHSSVK;FLVGFTNKGTEDF	NCP	95.376
FLVGFTNKGTEDF;SFRYPQDY	NCP	0.06
SFRYPQDY;KGKTSGPK	NCP	8.608
KGKTSGPK;NSLAVLP	NCP	2.369
NSLAVLP;RMKLLT	NCP	137.322
RMKLLT;PVAVELK	NCP	70.446
PVAVELK;KESTTL	NCP	72.355

KESTTL;TRLLPR	NCP	66.248
TRLLPR;NCMGPP	NCP	129.519
NCMGPP;KWAAFPPP	NCP	2.163
KWAAFPPP;NYLKSR	NCP	1.974
NYLKSR;YFPLDF	NCP	35.835
YFPLDF;MGTVVKQ	NCP	181.771
MGTVVKQ;TSSVPTGT	NCP	49.278
TSSVPTGT;APKPPTA	NCP	73.802
APKPPTA;CRSVMVL	NCP	19.762
CRSVMVL;SLAGLPSR	NCP	9.947
SLAGLPSR;YGKSPPR	NCP	6.991
YGKSPPR;LSRNPK	NCP	82.587
LSRNPK;TQLLKK	NCP	28.412
TQLLKK;MMMDDLR	NCP	19.215
MMMDDLR;NDLSLAK	NCP	72.268
NDLSLAK;KYPGP	NCP	51.492
KYPGP;HNLSF	NCP	22.785
HNLSF;LSAFDNF	NCP	28.571
LSAFDNF;QQLPMG	NCP	25.911
QQLPMG;EQPNLK	NCP	0.136
EQPNLK;LAGLYT	NCP	14.631
LAGLYT;VSPKVPK	NCP	67.232
VSPKVPK;KGSHSVH	NCP	9.24
KGSHSVH;ASGSFLK	NCP	64.519
ASGSFLK;MCEDLRK	NCP	59.727
MCEDLRK;LYVPAGAAA	NCP	13.439
LYVPAGAAA;KVKDTR	NCP	95.095
KVKDTR;VGGFLN	NCP	74.756
VGGFLN;GFVGFY	NCP	18.036
GFVGFY;YNSGKR	NCP	65.176
YNSGKR;ENERNLF	NCP	36.099
ENERNLF;LPPFLQ	NCP	137.427
LPPFLQ;FDEKPF	NCP	3.977
FDEKPF;VHTKSR	NCP	73.567
VHTKSR;GCADC	NCP	10.91
GCADC;TTVLEY	NCP	41.83
TTVLEY;KGMYPPP	NCP	84.99
KGMYPPP;HFELNDK	NCP	156.489
HFELNDK;VGPFELY	NCP	38.748
VGPFELY;APLPNL	NCP	77.022
APLPNL;YQSEEK	NCP	77.373
YQSEEK;LVKLTMP	NCP	149.816
LVKLTMP;ANPWEGK	NCP	48.99
ANPWEGK;KFAAFPR	NCP	14.794
KFAAFPR;KVNFPR	NCP	39.059
KVNFPR;MLSGL	NCP	23.127
MLSGL;NSLDLF	NCP	90.145
NSLDLF;TAGLPK	NCP	4.931
TAGLPK;QEKLHL	NCP	61.17
QEKLHL;LYGPVP	NCP	7.278
LYGPVP;KVRSLDN	NCP	119.431

KVRSLDN;VPGGDN	NCP	12.909
VPGGDN;VPYASPK	NCP	37.278
VPYASPK;LPPTK	NCP	115.382
LPPTK;LYKEQLAK	NCP	200.509
LYKEQLAK;KEGRHLAK	NCP	4.671
KEGRHLAK;LYKEQLG	NCP	116.81
LYKEQLG;SLEELYL	NCP	0.027
SLEELYL;LYPQYP	NCP	317.095
LYPQYP;TGKSKEAP	NCP	5.816
TGKSKEAP;EGVNQEF	NCP	13.227
EGVNQEF;FDRSPPP	NCP	76.657
FDRSPPP;MLPSPV	NCP	54.644
MLPSPV;NLDLRK	NCP	172.645
NLDLRK;YMPEEM	NCP	63.332
YMPEEM;LFKLLPV	NCP	6.978
LFKLLPV;EAMDC	NCP	0.346
EAMDC;KEGRSK	NCP	38.322
KEGRSK;GVPMLT	NCP	37.292
GVPMLT;QAMVYN	NCP	80.758
QAMVYN;NALCRL	NCP	150.57
NALCRL;KSLLNRLP	NCP	111.485
KSLLNRLP;TVYVY	NCP	1.804
TVYVY;VGQLYE	NCP	31.101
VGQLYE;LSKTLKP	NCP	74.094
LSKTLKP;KSKFLR	NCP	105.471
KSKFLR;DGPNWFM	NCP	54.255
DGPNWFM;NVSFCAK	NCP	32.882
NVSFCAK;KDRLLSK	NCP	53.656
KDRLLSK;VKVAYSK	NCP	35.753
VKVAYSK;ESFFNPK	NCP	71.976
ESFFNPK;KTHGRPK	NCP	7.236
KTHGRPK;TNTAMK	NCP	1.86
TNTAMK;ECSSC	NCP	112.647
ECSSC;CRFEGKL	NCP	76.962
CRFEGKL;RLSFP	NCP	7.395
RLSFP;NSRGKF	NCP	86.497
NSRGKF;MKDVQY	NCP	57.813
MKDVQY;KFVMPK	NCP	17.544
KFVMPK;VSLGCLLG	NCP	138.885
VSLGCLLG;ELLYY	NCP	450.012
ELLYY;YAVAVVK	NCP	64.392
YAVAVVK;LTALAPST	NCP	100.868
LTALAPST;EVPFLT	NCP	11.665
EVPFLT;TTPLAPL	NCP	33.708
TTPLAPL;NSFNHL	NCP	10.278
NSFNHL;SLRLLSK	NCP	123.804
SLRLLSK;ENLTALL	NCP	34.713
ENLTALL;YFFWLF	NCP	35.129
YFFWLF;RFKKPK	NCP	19.863
RFKKPK;QQTSLFPF	NCP	24.478
QQTSLFPF;AFLLF	NCP	71.409

AFLLF;KSLLYFEK	NCP	107.087
KSLLYFEK;LHLSKF	NCP	102.124
LHLSKF;KALEY	NCP	6.719
KALEY;PQFNG	NCP	61.749
PQFNG;SLTYETK	NCP	57.088
SLTYETK;LSGKKGGP	NCP	43.551
LSGKKGGP;KPEPPPP	NCP	2.631
KPEPPPP;KVLGPSPP	NCP	5.19
KVLGPSPP;RVHKKC	NCP	262.254
RVHKKC;VYNFHF	NCP	37.017
VYNFHF;WGMRVP	NCP	43.919
WGMRVP;QAAEFK	NCP	63.193
QAAEFK;QVLKPR	NCP	199.176
QVLKPR;KFLYLP	NCP	21.443
KFLYLP;KGPSFT	NCP	17.107
KGPSFT;LQTYYL	NCP	130.446
LQTYYL;TFLLTK	NCP	38.732
TFLLTK;SAAPLL	NCP	37.798
SAAPLL;TYFSEN	NCP	75.171
TYFSEN;NPGGLP	NCP	11.142
NPGGLP;RHLYDPK	NCP	28.017
RHLYDPK;RQVYDPK	NCP	1.761
RQVYDPK;HGEYQDDYPR	NCP	0.132
HGEYQDDYPR;KNHLFT	NCP	25.91
KNHLFT;AVVNP	NCP	29.892
AVVNP;PDTVVHF	NCP	184.596
PDTVVHF;LMETF	NCP	48.666
LMETF;KVSSPASK	NCP	118.774
KVSSPASK;VPKSYN	NCP	7.779
VPKSYN;DLNKHKSK	NCP	152.946
DLNKHKSK;LSVVLFLL	NCP	58.248
LSVVLFLL;LKSLVSK	NCP	47.936
LKSLVSK;NTPFPF	NCP	95.8
NTPFPF;VVLFPNDL	NCP	178.773
VVLFPNDL;EAGMKPP	NCP	54.03
EAGMKPP;EPKLFGGK	NCP	75.702
EPKLFGGK;PAAFHPP	NCP	3.332
PAAFHPP;KPDVPPGP	NCP	95.002
KPDVPPGP;PEERLRPP	NCP	2.544
PEERLRPP;YSVWLNS	NCP	0.915
YSVWLNS;PPLNGDP	NCP	13.182
PPLNGDP;KYPTTAP	NCP	56.961
KYPTTAP;WPKLED	NCP	28.295
WPKLED;LSLNLR	NCP	32.25
LSLNLR;AAATLF	NCP	84.198
AAATLF;LSTMPVFA	NCP	6.133
LSTMPVFA;PPKVPPPP	NCP	17.931
PPKVPPPP;LLAEMAMK	NCP	0.209
LLAEMAMK;YEDVKK	NCP	2.509
YEDVKK;EELRVT	NCP	33.947
EELRVT;ARALHLL	NCP	81.45

ARALHLL;VLLDYLY	NCP	13.062
VLLDYLY;DAAKNAF	NCP	31.039
DAAKNAF;TLRAVLP	NCP	57.977
TLRAVLP;TAAPYLG	NCP	41.79
TAAPYLG;KALSF	NCP	103.432
KALSF;YQLLLLP	NCP	46.682
YQLLLLP;CMCCCK	NCP	32.625
CMCCCK;TLLEFTDL	NCP	3.318
TLLEFTDL;KVNSGPQ	NCP	9.682
KVNSGPQ;HVLLSAC	NCP	68.273
HVLLSAC;ENLTVVK	NCP	33.741
ENLTVVK;RLHQVL	NCP	16.515
RLHQVL;PVGVEPF	NCP	134.719
PVGVEPF;FVHWY	NCP	28.398
FVHWY;AVPSAC	NCP	16.623
AVPSAC;YRGAP	NCP	147.435
YRGAP;MLSVPPK	NCP	25.38
MLSVPPK;PATKLF	NCP	3.314
PATKLF;LCARPLWP	NCP	22.72
LCARPLWP;KDSTKGLK	NCP	327.305
KDSTKGLK;EPKFPPP	NCP	1.345
EPKFPPP;MERVPVR	NCP	1.016
MERVPVR;KHAPPP	NCP	155.335
KHAPPP;VPLEY	NCP	182.468
VPLEY;KAPKEKLP	NCP	20.854
KAPKEKLP;KGLLSVSK	NCP	34.734
KGLLSVSK;KLPYLPEF	NCP	20.267
KLPYLPEF;RGPQEEV	NCP	21.061
RGPQEEV;LELFTEL	NCP	5.408
LELFTEL;PVLKF	NCP	40.798
PVLKF;KLRHTP	NCP	44.877
KLRHTP;KSLGPVG	NCP	0.267
KSLGPVG;KPRPNGH	NCP	94.442
KPRPNGH;HEPKVVP	NCP	23.442
HEPKVVP;KVGMGPR	NCP	29.122
KVGMGPR;PDHVVPPS	NCP	23.78
PDHVVPPS;RESFFK	NCP	241.425
RESFFK;VLLDLTMP	NCP	18.186
VLLDLTMP;KFLTPP	NCP	4.011
KFLTPP;AAVKKAL	NCP	46.078
AAVKKAL;DDEEEVSSKVEK	NCP	34.463
DDEEEVSSKVEK;VVDDDTPEMYK	NCP	17.628
VVDDDTPEMYK;KGLLNLA	NCP	9.129
KGLLNLA;FTHLKLVF	NCP	15.802
FTHLKLVF;AEFTRLQ	NCP	19.889
AEFTRLQ;ELEVVAR	NCP	41.271
ELEVVAR;LSEELYFL	NCP	35.766
LSEELYFL;YPLPQPY	NCP	19.96
YPLPQPY;VKKPWK	NCP	29.394
VKKPWK;YTVTNNLK	NCP	179.993
YTVTNNLK;LPPLS	NCP	16.284

LPPLS;VTLLLN	NCP	47.629
VTLLLN;MDLEALK	NCP	84.405
MDLEALK;KDRYGPP	NCP	89.541
KDRYGPP;KSSDAGK	NCP	113.072
KSSDAGK;MLAGSL	NCP	31.326
MLAGSL;KPSVGQK	NCP	41.34
KPSVGQK;GHFSK	NCP	4.23
GHFSK;YSLDP	NCP	43.69
YSLDP;KKDSNR	NCP	86.606
KKDSNR;EVFEGDK	NCP	169.566
EVFEGDK;SVWLN	NCP	54.535
SVWLN;AAAAAAAAAGSSL	NCP	16.061
AAAAAAAAAGSSL;ANFQAR	NCP	100.618
ANFQAR;VGAGAKY	NCP	50.166
VGAGAKY;CYSFRLL	NCP	79.859
CYSFRLL;QYPEAL	NCP	31.597
QYPEAL;PVVVPQ	NCP	50.6
PVVVPQ;HCPPR	NCP	62.568
HCPPR;ERFGDLL	NCP	44.871
ERFGDLL;LKYFNTK	NCP	9.379
LKYFNTK;VEMLF	NCP	21.003
VEMLF;VEPAP	NCP	30.377
VEPAP;LDEPK	NCP	51.975
LDEPK;PAPPYK	NCP	5.736
PAPPYK;KKLAA	NCP	73.239
KKLAA;YGDTF	NCP	4.15
YGDTF;LAKLL	NCP	9.548
LAKLL;NSDSNLK	NCP	109.326
NSDSNLK;MLMLATF	NCP	45.886
MLMLATF;VFVWVGK	NCP	89.645
VFVWVGK;EPPHLK	NCP	15.202
EPPHLK;FHLFTGK	NCP	12.039
FHLFTGK;VLEDLK	NCP	166.725
VLEDLK;KEMGP	NCP	72.564
KEMGP;LDEVLEK	NCP	77.147
LDEVLEK;TRSHALL	NCP	132.133
TRSHALL;SGKGLVLG	NCP	0.549
SGKGLVLG;LVLPL	NCP	2.229
LVLPL;FGSSPQ	NCP	2.873
FGSSPQ;TSALSL	NCP	63.25
TSALSL;KYMPPPP	NCP	4.004
KYMPPPP;YLLPSR	NCP	6.669
YLLPSR;PPPPLARP	NCP	105.559
PPPPLARP;KEVLRR	NCP	101.604
KEVLRR;GKSNGQY	NCP	18.462
GKSNGQY;KVVLLGT	NCP	11.748
KVVLLGT;PAPCKKP	NCP	14.174
PAPCKKP;EPEEAF	NCP	25.644
EPEEAF;KDPKPK	NCP	87.93
KDPKPK;MKVSTKK	NCP	6.985
MKVSTKK;KKELDGK	NCP	77.903

KKELDGK;LALYKGK	NCP	14.194
LALYKGK;GMRSPP	NCP	9.458
GMRSPP;MPTLP	NCP	233.652
MPTLP;ALHNFG	NCP	92.397
ALHNFG;FVDFNK	NCP	51.637
FVDFNK;KGFGSPR	NCP	54.945
KGFGSPR;DLLFLPML	NCP	53.327
DLLFLPML;FSPLGSP	NCP	27.166
FSPLGSP;RTENKAP	NCP	104.532
RTENKAP;KGPLHHF	NCP	12.56
KGPLHHF;LPKNGP	NCP	43.66
LPKNGP;EFVLMP	NCP	126.48
EFVLMP;LPQPDLT	NCP	22.1
LPQPDLT;SVGSLF	NCP	44.599
SVGSLF;DHVLQGK	NCP	5.334
DHVLQGK;WASLDASTQTTH	NCP	21.215
WASLDASTQTTH;VTQPRNP	NCP	35.007
VTQPRNP;DPMGLSPK	NCP	46.965
DPMGLSPK;QAFLDFM	NCP	39.939
QAFLDFM;VKLLL	NCP	167.269
VKLLL;GHASPPVH	NCP	47.235
GHASPPVH;KKVVLR	NCP	60.281
KKVVLR;ALLFK	NCP	10.759
ALLFK;NVSRGP	NCP	12.663
NVSRGP;AVRCTPLT	NCP	166.683
AVRCTPLT;FLTPL	NCP	66.819
FLTPL;KTVGVPR	NCP	156.687
KTVGVPR;KPVCPRVHP	NCP	52.527
KPVCPRVHP;DKVYLYK	NCP	77.6
DKVYLYK;KVDLEK	NCP	32.859
KVDLEK;TVLALYK	NCP	14.976
TVLALYK;SLFAV	NCP	100.644
SLFAV;NTDLDLR	NCP	56.824
NTDLDLR;PATTPPYP	NCP	205.518
PATTPPYP;VTLDLEL	NCP	9.633
VTLDLEL;PYELKVK	NCP	29.367
PYELKVK;AVELYL	NCP	26.631
AVELYL;ESFRK	NCP	106.344
ESFRK;LDLCSF	NCP	14.235
LDLCSF;AVLEYL	NCP	33.314
AVLEYL;FGAPL	NCP	124.611
FGAPL;LTKVLFLLY	NCP	117.465
LTKVLFLLY;VAAMLEY	NCP	66.045
VAAMLEY;AADTPKR	NCP	4.96
AADTPKR;EPVSPVLQ	NCP	79.824
EPVSPVLQ;HLDFSSPK	NCP	142.373
HLDFSSPK;LEVLGGF	NCP	44.103
LEVLGGF;KYPLE	NCP	28.765
KYPLE;KWGLYL	NCP	24.186
KWGLYL;KFLEN	NCP	110.46
KFLEN;EMFADLK	NCP	18.206

EMFADLK;PTLLRD	NCP	7.089
PTLLRD;HAYFVK	NCP	118.873
HAYFVK;SLKKKEC	NCP	125.951
SLKKKEC;GHFVRL	NCP	47.061
GHFVRL;HKGPLV	NCP	149.029
HKGPLV;LELDGAFF	NCP	22.845
LELDGAFF;SGHSLRFY	NCP	4.766
SGHSLRFY;PKLHAFPP	NCP	18.334
PKLHAFPP;AAYREVPCN	NCP	11.636
AAYREVPCN;LAKAKEDFLKK	NCP	35.172
LAKAKEDFLKK;KAKEDFLK	NCP	0.006
KAKEDFLK;QTVEVLK	NCP	63.18
QTVEVLK;VSLLLVD	NCP	5.38
VSLLLVD;AADLRF	NCP	70.127
AADLRF;VNLVVVL	NCP	135.753
VNLVVVL;KDSETLH	NCP	149.748
KDSETLH;TENKLK	NCP	5.65
TENKLK;LDMPECLK	NCP	49.61
LDMPECLK;LFAMLF	NCP	83.419
LFAMLF;NVLSLY	NCP	136.202
NVLSLY;YDLLVLY	NCP	14.521
YDLLVLY;EAGAFVL	NCP	13.604
EAGAFVL;YKPWPT	NCP	87.435
YKPWPT;LSKEELL	NCP	5.368
LSKEELL;KQNATDY	NCP	61.49
KQNATDY;KFVLFK	NCP	96.406
KFVLFK;SSPFVK	NCP	3.794
SSPFVK;KQTLKP	NCP	52.629
KQTLKP;AAHDVLT	NCP	7.561
AAHDVLT;GVHNLSF	NCP	47.999
GVHNLSF;KDLTGTK	NCP	1.15
KDLTGTK;DKKLDL	NCP	80.682
DKKLDL;QCPATL	NCP	17.715
QCPATL;VSAKMVQ	NCP	20.189
VSAKMVQ;LTGHLYL	NCP	26.632
LTGHLYL;WEVVKEW	NCP	40.268
WEVVKEW;GPSLVHR	NCP	77.103
GPSLVHR;TYRHHK	NCP	269.862
TYRHHK;KVYDLR	NCP	15.172
KVYDLR;EDSPSF	NCP	105.765
EDSPSF;KLLWEF	NCP	53.594
KLLWEF;ALAAPG	NCP	8.131
ALAAPG;PPTWKNF	NCP	53.967
PPTWKNF;SGSKVFSP	NCP	57.621
SGSKVFSP;YGRPALH	NCP	27.171
YGRPALH;ASTTDFHK	NCP	69.542
ASTTDFHK;YGKEFC	NCP	18.984
YGKEFC;PTLFAP	NCP	19.837
PTLFAP;QPSFGPL	NCP	52.061
QPSFGPL;AALLK	NCP	106.612
AALLK;LFSPF	NCP	11.688

LFSPF;KKTAAAVL	NCP	212.172
KKTAAAVL;PSHSVQL	NCP	266.751
PSHSVQL;KFGNP	NCP	8.745
KFGNP;GRVVEPK	NCP	33.221
GRVVEPK;ESPKKR	NCP	88.348
ESPKKR;ALKKVR	NCP	26.316
ALKKVR;HELQLPP	NCP	77.102
HELQLPP;VPTPPPP	NCP	86.07
VPTPPPP;CHSEHVTK	NCP	6.756
CHSEHVTK;KLPLE	NCP	12.358
KLPLE;KTLLP	NCP	75.38
KTLLP;KDPFE	NCP	14.647
KDPFE;LELDQFF	NCP	29.874
LELDQFF;LCPTVK	NCP	79.272
LCPTVK;LKKAVY	NCP	167.264
LKKAVY;NDLVGPR	NCP	1.083
NDLVGPR;AELTTLF	NCP	106.057
AELTTLF;KSTLVPPA	NCP	29.691
KSTLVPPA;GLASLR	NCP	35.192
GLASLR;FLLGAGA	NCP	44.709
FLLGAGA;ELLRTQ	NCP	56.106
ELLRTQ;SLFKVT	NCP	154.978
SLFKVT;YVRLY	NCP	2.897
YVRLY;ENAAVLR	NCP	26.547
ENAAVLR;TLEKNF	NCP	175.09
TLEKNF;GGKVAVLQ	NCP	3.41
GGKVAVLQ;VDKLVLQ	NCP	19.543
VDKLVLQ;PPDCD	NCP	9.06
PPDCD;GHQVLTK	NCP	68.504
GHQVLTK;LQLKK	NCP	30.21
LQLKK;ACPPMYP	NCP	15.645
ACPPMYP;AELPP	NCP	15.211
AELPP;FSPLG	NCP	55.269
FSPLG;CNLQK	NCP	13.37
CNLQK;MNQSMC	NCP	0.744
MNQSMC;FMTLEY	NCP	134.539
FMTLEY;PVEAPV	NCP	231.771
PVEAPV;SSKRN	NCP	42.854
SSKRN;KKGVTLY	NCP	35.373
KKGVTLY;FEPQFLE	NCP	6.681
FEPQFLE;LLLMLN	NCP	138.157
LLLMLN;QDLPVKK	NCP	60.755
QDLPVKK;TVTNNLK	NCP	76.242
TVTNNLK;CCWNW	NCP	37.297
CCWNW;YGCFTKP	NCP	3.816
YGCFTKP;SHSGPPR	NCP	177.383
SHSGPPR;GVYPHK	NCP	29.77
GVYPHK;LSPLAY	NCP	313.968
LSPLAY;QVFEAR	NCP	54.2
QVFEAR;KSCMSPP	NCP	159.427
KSCMSPP;KTHTVPP	NCP	238.22

KTHTVPP;VPAWS	NCP	73.5
VPAWS;LTVLL	NCP	44.776
LTVLL;PSRKP	NCP	9.834
PSRKP;LSLNLSPL	NCP	88.865
LSLNLSPL;FVVEF	NCP	81.726
FVVEF;LPADP	NCP	47.1
LPADP;RSAEVKNK	NCP	12.699
RSAEVKNK;TVYFKEQ	NCP	6.067
TVYFKEQ;RTALTY	NCP	12.228
RTALTY;KKPKLAKPVEK	NCP	161.909
KKPKLAKPVEK;KDDLVK	NCP	20.781
KDDLVK;FKHLENL	NCP	33.289
FKHLENL;FFQKSLGL	NCP	13.289
FFQKSLGL;KEHQPK	NCP	19.693
KEHQPK;VQPSWL	NCP	71.055
VQPSWL;KEFEPSM	NCP	63.215
KEFEPSM;KAPALLH	NCP	103.56
KAPALLH;LAGLEY	NCP	8.197
LAGLEY;QTYAPK	NCP	25.017
QTYAPK;QVYTAM	NCP	63.803
QVYTAM;MKVELC	NCP	5.761
MKVELC;KGGLVDR	NCP	45.743
KGGLVDR;EMESPVF	NCP	17.157
EMESPVF;KMNVHTK	NCP	1.137
KMNVHTK;YVRKSR	NCP	14.113
YVRKSR;KPSYMCK	NCP	129.944
KPSYMCK;FFPPPPPP	NCP	77.034
FFPPPPPP;MFVAR	NCP	33.898
MFVAR;YPVLFKNN	NCP	47.042
YPVLFKNN;VGLLVK	NCP	4.807
VGLLVK;LSLVK	NCP	186.201
LSLVK;VLKLT	NCP	5.006
VLKLT;VLDRV	NCP	40.075
VLDRV;VLTNAGY	NCP	130.2
VLTNAGY;DKYELL	NCP	57.656
DKYELL;AVVVPPS	NCP	64.657
AVVVPPS;GFGAPS	NCP	0.216
GFGAPS;KEWHAFR	NCP	31.616
KEWHAFR;VSTDYL	NCP	22.639
VSTDYL;KGVELEK	NCP	20.799
KGVELEK;VQWLK	NCP	51.981
VQWLK;VTGGLP	NCP	51.125
VTGGLP;ETEGRK	NCP	41.5
ETEGRK;VRKLPGEL	NCP	34.332
VRKLPGEL;KDCSTVP	NCP	28.5
KDCSTVP;KFGSSAP	NCP	162.284
KFGSSAP;LGNERF	NCP	62.038
LGNERF;ESMMSK	NCP	56.144
ESMMSK;KHGPDFHL	NCP	85.162
KHGPDFHL;GKQFLAV	NCP	41.867
GKQFLAV;GMPVVLVL	NCP	0.922

GMPVVLVL;GPGAKGSK	NCP	35.171
GPGAKGSK;DLRKDL	NCP	39.934
DLRKDL;PGPPAPPK	NCP	6.734
PGPPAPPK;TTSSP	NCP	15.522
TTSSP;TELPAPL	NCP	77.755
TELPAPL;KPLGY	NCP	6.039
KPLGY;MSSDDD	NCP	95.298
MSSDDD;LMEAVLR	NCP	67.683
LMEAVLR;VARVNY	NCP	32.931
VARVNY;MWVKSSH	NCP	15.528
MWVKSSH;PPFMM	NCP	99.192
PPFMM;KDAMLK	NCP	1.367
KDAMLK;ERPRGP	NCP	67.174
ERPRGP;CLAKGK	NCP	57
CLAKGK;LDDLGTK	NCP	19.382
LDDLGTK;VNGLPVLL	NCP	5.182
VNGLPVLL;MSLAQKKDRVAQK	NCP	5.741
MSLAQKKDRVAQK;PQGLK	NCP	7.477
PQGLK;DMVTDHL	NCP	31.253
DMVTDHL;FDLAEF	NCP	49.563
FDLAEF;CYVAL	NCP	9.039
CYVAL;NPLVFG	NCP	21.051
NPLVFG;KEEVPK	NCP	23.365
KEEVPK;KLDNNPK	NCP	36.713
KLDNNPK;KTCGL	NCP	29.92
KTCGL;YGLRDF	NCP	19.973
YGLRDF;TLFLRAN	NCP	14.466
TLFLRAN;CCECCC	NCP	2.326
CCECCC;KDATVL	NCP	36.869
KDATVL;QVLLNR	NCP	9.649
QVLLNR;AFVTL	NCP	53.999
AFVTL;DASCCC	NCP	91.566
DASCCC;LVEPF	NCP	93.468
LVEPF;KYLPPPPPP	NCP	4.287
KYLPPPPPP;DFGMDL	NCP	125.709
DFGMDL;VAPYN	NCP	244.416
VAPYN;KKLECCC	NCP	121.897
KKLECCC;PPGKPPPP	NCP	12.816
PPGKPPPP;DLLPF	NCP	50.76
DLLPF;SLFLL	NCP	5.66
SLFLL;SCYSVVKSR	NCP	106.891
SCYSVVKSR;SVGFDPY	NCP	23.361
SVGFDPY;VAAVVDQ	NCP	91.143
VAAVVDQ;RGVTTPK	NCP	163.922
RGVTTPK;MFLVLQ	NCP	15.337
MFLVLQ;NLFMP	NCP	0.252
NLFMP;AATLKY	NCP	1.035
AATLKY;VSSGSLVF	NCP	85.424
VSSGSLVF;KTTSFRP	NCP	30.459
KTTSFRP;KTLMVPE	NCP	46.098
KTLMVPE;CLTSKYR	NCP	57.21

CLTSKYR;RGPLFK	NCP	25.852
RGPLFK;FGFGQQG	NCP	29.627
FGFGQQG;LHNLSFL	NCP	12.541
LHNLSFL;ASSKLPW	NCP	17.012
ASSKLPW;LLLNTFY	NCP	16.117
LLLNTFY;FKGTVP	NCP	49.541
FKGTVP;GLPPPYK	NCP	10.837
GLPPPYK;VGASLVA	NCP	28.589
VGASLVA;LSNHGAP	NCP	1.689
LSNHGAP;VHVPV	NCP	68.676
VHVPV;KLGFTVP	NCP	15.759
KLGFTVP;ALNTGLK	NCP	27.594
ALNTGLK;VPLRSPFK	NCP	6.037
VPLRSPFK;VLFDDVL	NCP	98.033
VLFDDVL;ALKKVVG	NCP	13.924
ALKKVVG;EHNGNK	NCP	65.118
EHNGNK;KFFVVG	NCP	35.795
KFFVVG;LPAPGL	NCP	5.142
LPAPGL;VLGELLA	NCP	19.383
VLGELLA;KTPVFL	NCP	66.666
KTPVFL;FPKMPGW	NCP	77.995
FPKMPGW;KSKERAP	NCP	172.466
KSKERAP;PFKKY	NCP	26.665
PFKKY;KSSFFFH	NCP	36.716
KSSFFFH;KEHWYL	NCP	72.357
KEHWYL;RLGRFG	NCP	134.881
RLGRFG;PVSLK	NCP	173.969
PVSLK;NLVALK	NCP	4.552
NLVALK;EMTKTE	NCP	46.427
EMTKTE;GLAFP	NCP	10.089
GLAFP;MSKDDN	NCP	336.418
MSKDDN;STTFLFR	NCP	26.292
STTFLFR;SMLRKALRLQ	NCP	5.351
SMLRKALRLQ;KMLLTPP	NCP	62.772
KMLLTPP;FSLQNYE	NCP	84.238
FSLQNYE;TGPLNPK	NCP	14.801
TGPLNPK;KSGHVLE	NCP	88.473
KSGHVLE;VLMLGF	NCP	3.441
VLMLGF;STALSL	NCP	2.973
STALSL;LTAEVAF	NCP	17.529
LTAEVAF;TTYFEL	NCP	56.217
TTYFEL;HVKKQLH	NCP	34.746
HVKKQLH;FGGPPGF	NCP	21.748
FGGPPGF;YDYLTK	NCP	15.636
YDYLTK;VPTKGPD	NCP	172.106
VPTKGPD;LVFKEC	NCP	81.411
LVFKEC;DTAPL	NCP	94.528
DTAPL;HAAWYL	NCP	88.995
HAAWYL;SLGQDGKK	NCP	7.605
SLGQDGKK;SVGLAMKK	NCP	43.961
SVGLAMKK;MNMRLP	NCP	7.992

MNMRLP;TPVTLPL	NCP	273.624
TPVTLPL;MPAAKKH	NCP	2.598
MPAAKKH;NRLAPPL	NCP	47.461
NRLAPPL;TATVLAP	NCP	4.247
TATVLAP;KPPQQL	NCP	9.852
KPPQQL;VSNPMLLL	NCP	4.312
VSNPMLLL;LPPGGFS	NCP	96.308
LPPGGFS;NVPPMLE	NCP	192.141
NVPPMLE;LKDKTEF	NCP	9.925
LKDKTEF;QHSVLTL	NCP	14.846
QHSVLTL;TDLLYLHL	NCP	9.921
TDLLYLHL;KDMMPK	NCP	40.347
KDMMPK;MVAMNSF	NCP	28.344
MVAMNSF;LPPKYQ	NCP	21.157
LPPKYQ;KDTTVKLL	NCP	146.303
KDTTVKLL;FDAMDF	NCP	31.878
FDAMDF;LSNDNF	NCP	141.736
LSNDNF;EEALERM	NCP	33.513
EEALERM;FTSPP	NCP	0.803
FTSPP;KTRHVYLQ	NCP	63.406
KTRHVYLQ;VGSPPRDF	NCP	118.428
VGSPPRDF;KDNMGKK	NCP	25.04
KDNMGKK;KLYVPPP	NCP	52.723
KLYVPPP;LSYFKAFL	NCP	35.193
LSYFKAFL;KAELLR	NCP	151.359
KAELLR;ALALVL	NCP	51.947
ALALVL;VTGLTPK	NCP	116.421
VTGLTPK;NGPSFPL	NCP	29.676
NGPSFPL;ALKKER	NCP	52.83
ALKKER;VSPPK	NCP	7.87
VSPPK;TLLVDL	NCP	66.752
TLLVDL;DNSRLKS	NCP	43.591
DNSRLKS;EEVLPF	NCP	101.589
EEVLPF;EHLARK	NCP	0.272
EHLARK;KDTVKVP	NCP	13.906
KDTVKVP;KAKKAF	NCP	84.356
KAKKAF;VGLSPP	NCP	43.272
VGLSPP;ELERVV	NCP	22.294
ELERVV;LALEPLSV	NCP	13.232
LALEPLSV;HKPPLEEV	NCP	66.073
HKPPLEEV;LGAAGLL	NCP	18.809
LGAAGLL;VETLKGV	NCP	7.422
VETLKGV;KNLPMDN	NCP	8.746
KNLPMDN;FVQAEF	NCP	29.129
FVQAEF;PTRHAF	NCP	5.245
PTRHAF;QNHKASY	NCP	2.502
QNHKASY;NKNDLALR	NCP	48.98
NKNDLALR;KGRKPK	NCP	48.804
KGRKPK;NFKLLSHSL	NCP	88.815
NFKLLSHSL;FSQPHLM	NCP	311.289
FSQPHLM;KDKHFR	NCP	37.069

KDKHFR;PVFDNL	NCP	38.532
PVFDNL;PGGPSNF	NCP	99.005
PGGPSNF;QTAKPQR	NCP	215.302
QTAKPQR;VAGVLPF	NCP	2.75
VAGVLPF;ADKARYEREMK	NCP	35.217
ADKARYEREMK;WNNTAADDKQPYEKK	NCP	0.198
WNNTAADDKQPYEKK;EKLWHHS	NCP	29.218
EKLWHHS;LHDHFG	NCP	0.773
LHDHFG;CCDCS	NCP	87.532
CCDCS;LQLAF	NCP	38.468
LQLAF;SPNVY	NCP	68.679
SPNVY;KEAMK	NCP	73.288
KEAMK;SLVEAQRTKV	NCP	93.854
SLVEAQRTKV;VGLVGG	NCP	230.692
VGLVGG;RLLTQY	NCP	53.124
RLLTQY;KRRPNCL	NCP	0.096
KRRPNCL;YSASFDK	NCP	19.035
YSASFDK;KPDLPP	NCP	35.933
KPDLPP;KPRFYG	NCP	79.942
KPRFYG;HVGGP	NCP	71.157
HVGGP;LSESVVYP	NCP	53.238
LSESVVYP;YTLVYF	NCP	1.091
YTLVYF;AQVLSARV	NCP	97.557
AQVLSARV;GFLSVVFP	NCP	25.284
GFLSVVFP;PSKFYSP	NCP	43.486
PSKFYSP;QEEYLTK	NCP	86.201
QEEYLTK;NLLVFL	NCP	0.261
NLLVFL;KPPGGFR	NCP	167.881
KPPGGFR;KNSTAF	NCP	17.855
KNSTAF;TDKPFDQ	NCP	46.482
TDKPFDQ;EGSAWL	NCP	64.425
EGSAWL;RPGVAVVL	NCP	9.261
RPGVAVVL;VVFLAP	NCP	9.991
VVFLAP;KAAMNPK	NCP	24.861
KAAMNPK;RSMTLER	NCP	44.357
RSMTLER;LLVPYL	NCP	84.801
LLVPYL;NDDAK	NCP	44.208
NDDAK;ASSGAFVK	NCP	8.613
ASSGAFVK;SGLPLKD	NCP	166.732
SGLPLKD;MLLSFD	NCP	2.669
MLLSFD;YALLSGF	NCP	108.553
YALLSGF;EAKLLR	NCP	10.1
EAKLLR;LNASSDKF	NCP	31.57
LNASSDKF;VKLLQ	NCP	73.671
VKLLQ;EKTPVSN	NCP	127.545
EKTPVSN;PWLPL	NCP	58.259
PWLPL;TTTLAF	NCP	66.415
TTTLAF;FSAAPGN	NCP	41.967
FSAAPGN;KNHVDF	NCP	36.279
KNHVDF;VGSPKV	NCP	1.193
VGSPKV;VPSLEVP	NCP	30.96

VPSLEVP;SAAYPF	NCP	40.048
SAAYPF;KEVVPVK	NCP	36.126
KEVVPVK;KPYATT	NCP	96.852
KPYATT;FDLPP	NCP	24.545
FDLPP;YLEENYK	NCP	11.529
YLEENYK;KAALLKASPKK	NCP	188.694
KAALLKASPKK;AALLKASPK	NCP	0.003
AALLKASPK;AAGQKAAPPPKTQ	NCP	0.144
AAGQKAAPPPKTQ;KAPSQKAPAPK	NCP	0.048
KAPSQKAPAPK;LSNRSPF	NCP	28.422
LSNRSPF;LSHVPV	NCP	101.341
LSHVPV;KLNSATYK	NCP	67.794
KLNSATYK;TNFKKK	NCP	24.338
TNFKKK;KLVLLRPG	NCP	5.841
KLVLLRPG;KLFLLQLK	NCP	139.153
KLFLLQLK;KNHFTL	NCP	42.017
KNHFTL;QDPLVH	NCP	32.623
QDPLVH;LWHHGL	NCP	44.753
LWHHGL;ELVNY	NCP	55.462
ELVNY;VGSLEEL	NCP	104.774
VGSLEEL;CLVCKK	NCP	41.521
CLVCKK;LGKELLDL	NCP	53.381
LGKELLDL;LLVKESPF	NCP	98.236
LLVKESPF;KAKPP	NCP	18.585
KAKPP;GPERVPP	NCP	22.031
GPERVPP;LFHLGKS	NCP	47.98
LFHLGKS;KEDPVAV	NCP	8.856
KEDPVAV;FDRLDY	NCP	76.379
FDRLDY;MCEPLK	NCP	21.244
MCEPLK;AGSYR	NCP	50.816
AGSYR;VLPSVRK	NCP	109.882
VLPSVRK;MSLPQRK	NCP	38.21
MSLPQRK;VDYLEF	NCP	76.989
VDYLEF;YVPGAAM	NCP	62.227
YVPGAAM;KAWSL	NCP	146.934
KAWSL;KPFNPR	NCP	24.893
KPFNPR;LNFKLSKL	NCP	9.621
LNFKLSKL;KDPGWTK	NCP	95.296
KDPGWTK;GYEDDC	NCP	16.755
GYEDDC;LYMLVPP	NCP	109.784
LYMLVPP;TVGLFPM	NCP	59.422
TVGLFPM;DLPVYG	NCP	38.151
DLPVYG;KNYGLLDL	NCP	13.88
KNYGLLDL;KNPFY	NCP	69.807
KNPFY;SLRLLSKF	NCP	7.204
SLRLLSKF;KGWEKPP	NCP	7.38
KGWEKPP;KDLVLN	NCP	35.415
KDLVLN;LSVGL	NCP	24.753
LSVGL;SEFLKQ	NCP	9.089
SEFLKQ;KDLLFPY	NCP	370.876
KDLLFPY;PYSVP	NCP	30.393

PYSVP;LDELSAF	NCP	87.422
LDELSAF;VSVKLFD	NCP	41.877
VSVKLFD;LPGFLVAL	NCP	21.621
LPGFLVAL;KDHDK	NCP	25.651
KDHDK;GHLFF	NCP	179.756
GHLFF;NNWLVE	NCP	116.287
NNWLVE;AAAKHLK	NCP	38.918
AAAKHLK;AFLVP	NCP	8.674
AFLVP;SVTFL	NCP	159.761
SVTFL;DMGGMQR	NCP	65.338
DMGGMQR;FATRY	NCP	125.852
FATRY;KETFPPP	NCP	97.114
KETFPPP;KNKGGLH	NCP	16.29
KNKGGLH;FSKSPF	NCP	77.337
FSKSPF;AAAPPPTPR	NCP	117.101
AAAPPPTPR;LPAAKVDY	NCP	72.825
LPAAKVDY;AKMAPER	NCP	48.709
AKMAPER;YMSDLRK	NCP	7.601
YMSDLRK;LSHLFSF	NCP	61.198
LSHLFSF;YTQSGLY	NCP	11.265
YTQSGLY;KSLTFEY	NCP	3.035
KSLTFEY;EGSWFR	NCP	37.237
EGSWFR;LVGGVVVL	NCP	276.981
LVGGVVVL;ETLRGPK	NCP	4.923
ETLRGPK;DKLLC	NCP	118.131
DKLLC;TPALL	NCP	61.739
TPALL;FKPSDF	NCP	90.294
FKPSDF;LELYKK	NCP	4.273
LELYKK;AYFPEL	NCP	9.153
AYFPEL;TPFLTR	NCP	19.253
TPFLTR;QYTSLL	NCP	122.568
QYTSLL;PPMMGY	NCP	39.94
PPMMGY;HVLAAPR	NCP	19.064
HVLAAPR;KMVNVP	NCP	9.415
KMVNVP;KFLPSR	NCP	35.871
KFLPSR;VEELE	NCP	65.351
VEELE;YTLGLNEK	NCP	9.25
YTLGLNEK;KDPLN	NCP	87.726
KDPLN;KQAHVLSF	NCP	8.628
KQAHVLSF;VDGRVVEP	NCP	34.5
VDGRVVEP;SPPVEGR	NCP	50.502
SPPVEGR;QKPTDKK	NCP	1.686
QKPTDKK;LQVSFL	NCP	102.369
LQVSFL;EGLSPPKP	NCP	201.671
EGLSPPKP;VPSFP	NCP	3.964
VPSFP;LSGVLVY	NCP	6.489
LSGVLVY;GNFKT	NCP	83.156
GNFKT;PPCVDQ	NCP	10.792
PPCVDQ;ALLEY	NCP	58.887
ALLEY;PFGMSVR	NCP	43.725
PFGMSVR;FYPLDF	NCP	38.726

FYPLDF;KNPDK	NCP	12.751
KNPDK;EEHHLRDY	NCP	56.078
EEHHLRDY;KMMLH	NCP	7.483
KMMLH;KGGFHFK	NCP	57.468
KGGFHFK;SLANPLL	NCP	31.182
SLANPLL;FPLLKN	NCP	38.45
FPLLKN;RLNKAK	NCP	82.111
RLNKAK;TLDMLKK	NCP	29.366
TLDMLKK;LSLMKQL	NCP	46.159
LSLMKQL;MSSSYLHS	NCP	16.434
MSSSYLHS;KKHYRY	NCP	54.845
KKHYRY;LRAKVL	NCP	80.56
LRAKVL;LHTKSK	NCP	11.804
LHTKSK;LSKQTPLS	NCP	39.345
LSKQTPLS;GKPPALPS	NCP	10.71
GKPPALPS;KDLLLE	NCP	11.167
KDLLLE;RLVQAF	NCP	25.505
RLVQAF;KDALK	NCP	17.208
KDALK;LMFPGLP	NCP	47.2
LMFPGLP;VWEKMR	NCP	1.707
VWEKMR;KGFLGW	NCP	78.005
KGFLGW;KGGFKGK	NCP	92.371
KGGFKGK;TVLKNAA	NCP	9.311
TVLKNAA;MAVWGNK	NCP	99.618
MAVWGNK;TFPVSTLAP	NCP	6.562
TFPVSTLAP;TFFCRAK	NCP	28.359
TFFCRAK;KLAVWGNK	NCP	2.072
KLAVWGNK;LAVWGNK	NCP	12.594
LAVWGNK;VGVNVEF	NCP	59.446
VGVNVEF;KHGTAPG	NCP	25.757
KHGTAPG;LLSDLDLR	NCP	0.231
LLSDLDLR;PLPGVGEELK	NCP	178.833
PLPGVGEELK;ATVTKAPK	NCP	0.072
ATVTKAPK;SLLGF	NCP	32.55
SLLGF;LPFLP	NCP	2.403
LPFLP;VASLHLPSFDAH	NCP	2.994
VASLHLPSFDAH;KHDMSK	NCP	11.4
KHDMSK;DLWLEREVF	NCP	20.043
DLWLEREVF;MKPATFP	NCP	5.589
MKPATFP;QTPAHHP	NCP	29.379
QTPAHHP;LKKASGV	NCP	81.024
LKKASGV;KEGAF	NCP	179.124
KEGAF;KVLAKK	NCP	41.031
KVLAKK;SVMDEL	NCP	68.643
SVMDEL;EAALLSK	NCP	45.406
EAALLSK;QLKLAQAK	NCP	4.17
QLKLAQAK;KLAQAKL	NCP	0.006
KLAQAKL;DPEMLCP	NCP	31.481
DPEMLCP;RLVFAK	NCP	16.981
RLVFAK;MMVLVF	NCP	5.189
MMVLVF;TVGKPL	NCP	25.645

TVGKPL;DPHNNPK	NCP	109.854
DPHNNPK;KDRLMYF	NCP	4.109
KDRLMYF;KPLGVLAP	NCP	5.136
KPLGVLAP;LMLPTVLK	NCP	22.741
LMLPTVLK;VKPLVT	NCP	52.985
VKPLVT;FLDMPK	NCP	19.029
FLDMPK;LNVPP	NCP	27.174
LNVPP;MSKVVFH	NCP	41.143
MSKVVFH;VGLLHF	NCP	4.263
VGLLHF;RNKPRP	NCP	48.105
RNKPRP;RPPPHVR	NCP	69.683
RPPPHVR;AGALVKRA	NCP	54.016
AGALVKRA;LFAGPPPPP	NCP	152.607
LFAGPPPPP;LSGRDLKY	NCP	4.862
LSGRDLKY;APRSVAAP	NCP	13.275
APRSVAAP;STYLKKL	NCP	8.181
STYLKKL;PSRLLY	NCP	5.106
PSRLLY;MHVPPR	NCP	36.918
MHVPPR;KTFGKK	NCP	2.085
KTFGKK;LSFVKP	NCP	55.882
LSFVKP;NSTHKFK	NCP	49.338
NSTHKFK;NPALALFN	NCP	36.6
NPALALFN;VLRVY	NCP	94.793
VLRVY;ALVQKRHY	NCP	60.282
ALVQKRHY;KKAQAQPVM	NCP	6.138
KKAQAQPVM;KKRMGN	NCP	6.088
KKRMGN;PGLHMPP	NCP	13.877
PGLHMPP;DMDDPP	NCP	8.199
DMDDPP;PKKGSDP	NCP	43.293
PKKGSDP;ALLLLF	NCP	17.793
ALLLLF;KPSAGP	NCP	31.486
KPSAGP;NEPFPK	NCP	20.4
NEPFPK;YRSKK	NCP	63.713
YRSKK;KLVVLGSGGVGK	NCP	28.083
KLVVLGSGGVGK;TAMRDLYMK	NCP	6.666
TAMRDLYMK;MRDLYMKNGQGF	NCP	0.006
MRDLYMKNGQGF;STFNDLQDLREQ	NCP	0.063
STFNDLQDLREQ;AVSVTPLR	NCP	87.294
AVSVTPLR;VGHLPF	NCP	23.542
VGHLPF;NYALLR	NCP	35.589
NYALLR;PPAQGLVP	NCP	11.993
PPAQGLVP;YDTCMVF	NCP	86.937
YDTCMVF;ASAKLVF	NCP	2.439
ASAKLVF;RHPSLLPL	NCP	11.85
RHPSLLPL;ESGSHPAG	NCP	24.498
ESGSHPAG;KSGAAPQ	NCP	4.299
KSGAAPQ;LMTATVR	NCP	43.057
LMTATVR;SGLALL	NCP	61.37
SGLALL;GSPTLY	NCP	6.631
GSPTLY;KLPKFN	NCP	2.295
KLPKFN;KPLDPLP	NCP	27.239

KPLDPLP;KLLFLEL	NCP	40.252
KLLFLEL;SLAHFPF	NCP	1.166
SLAHFPF;AYPGELN	NCP	9.349
AYPGELN;LSGGLAL	NCP	21.926
LSGGLAL;PCGDLR	NCP	27.457
PCGDLR;AAKSLN	NCP	62.139
AAKSLN;CSCSC	NCP	103.122
CSCSC;RVSTLLT	NCP	78.251
RVSTLLT;QTMSDTTFK	NCP	183.48
QTMSDTTFK;KEAGLPT	NCP	37.788
KEAGLPT;DANPYK	NCP	47.377
DANPYK;APPGVPPPP	NCP	84.602
APPGVPPPP;KSWALTL	NCP	24.928
KSWALTL;CCYSSPPP	NCP	53.622
CCYSSPPP;KFGKLPM	NCP	36.051
KFGKLPM;KKQAVVGK	NCP	183.995
KKQAVVGK;PGVEAHR	NCP	14.229
PGVEAHR;LFLLLLGY	NCP	57.124
LFLLLLGY;MLPPPP	NCP	59.363
MLPPPP;KPYSPPPPP	NCP	27.871
KPYSPPPPP;FPASTVT	NCP	59.996
FPASTVT;GEDLLP	NCP	68.203
GEDLLP;KETLL	NCP	56.228
KETLL;EFPELPKL	NCP	25.143
EFPELPKL;DEMCAK	NCP	92.191
DEMCAK;KDPGVRL	NCP	0.2
KDPGVRL;KDVLNPLL	NCP	11.256
KDVLNPLL;EYAGLDR	NCP	9.783
EYAGLDR;KMFPEPP	NCP	64.467
KMFPEPP;NAGQFP	NCP	55.768
NAGQFP;LTGGLTLL	NCP	38.66
LTGGLTLL;KSSDFGF	NCP	116.581
KSSDFGF;LLYKGM	NCP	7.431
LLYKGM;LLDLKK	NCP	14.163
LLDLKK;LDGAPP	NCP	14.57
LDGAPP;EDFDEF	NCP	277.347
EDFDEF;ELADK	NCP	80.437
ELADK;LSPVLPLLQ	NCP	11.055
LSPVLPLLQ;DGLHALT	NCP	10.544
DGLHALT;CMPAPRPP	NCP	17.412
CMPAPRPP;TPRFKHG	NCP	62.742
TPRFKHG;EKLSHE	NCP	7.926
EKLSHE;FMWKGP	NCP	28.449
FMWKGP;KLYLDWK	NCP	30.325
KLYLDWK;KYLQVV	NCP	5.519
KYLQVV;KVLGA	NCP	40.933
KVLGA;LSLANLYY	NCP	29.388
LSLANLYY;SFCRLP	NCP	105.309
SFCRLP;TSVELVH	NCP	41.499
TSVELVH;QVNGRVP	NCP	19.193
QVNGRVP;DDCPK	NCP	24.001

DDCPK;LLGAVP	NCP	12.414
LLGAVP;EVATLL	NCP	36.165
EVATLL;AQFVRNLA	NCP	45.209
AQFVRNLA;LGYPLTL	NCP	28.873
LGYPLTL;KEGSTPR	NCP	38.241
KEGSTPR;FTAKRE	NCP	97.401
FTAKRE;PDHKQ	NCP	11.805
PDHKQ;MTHQTHAY	NCP	72.573
MTHQTHAY;EVPFFF	NCP	19.746
EVPFFF;LSDLPVD	NCP	31.479
LSDLPVD;ENVYAG	NCP	10.908
ENVYAG;VHVVPR	NCP	11.574
VHVVPR;RSLAPSP	NCP	7.088
RSLAPSP;VVPGNLK	NCP	28.815
VVPGNLK;KHEKRPP	NCP	5.239
KHEKRPP;DSGVWPK	NCP	102.714
DSGVWPK;GQVPDFP	NCP	37.701
GQVPDFP;DKLVTL	NCP	28.965
DKLVTL;RGASYQR	NCP	59.936
RGASYQR;KELMRSP	NCP	5.037
KELMRSP;VASVTLP	NCP	28.314
VASVTLP;FRDQEGQDVLL	NCP	29.727
FRDQEGQDVLL;LPGVEL	NCP	29.848
LPGVEL;LEFRRSGL	NCP	19.118
LEFRRSGL;KPWQPPP	NCP	27.178
KPWQPPP;TTVSNVP	NCP	117.78
TTVSNVP;PSPGQF	NCP	10.44
PSPGQF;LLGAYL	NCP	4.301
LLGAYL;KHFTMVP	NCP	1.062
KHFTMVP;VFMFAAL	NCP	0.03
VFMFAAL;KEVLELAF	NCP	31.734
KEVLELAF;KLLEASVP	NCP	4.188
KLLEASVP;VPAFPEV	NCP	5.712
VPAFPEV;PVGLAA	NCP	6.844
PVGLAA;VGLAAA	NCP	0.003
VGLAAA;EGSWCHW	NCP	0.872
EGSWCHW;LSSYK	NCP	14.502
LSSYK;VSEPLE	NCP	0.489
VSEPLE;VVVAGY	NCP	45.769
VVVAGY;SVDGK	NCP	38.366
SVDGK;FEPGL	NCP	9.238
FEPGL;PGCDP	NCP	16.871
PGCDP;ASSKKGK	NCP	55.138
ASSKKGK;PYKTEAF	NCP	87.482
PYKTEAF;KVKAAPP	NCP	13.243
KVKAAPP;KLSPVLPL	NCP	38.345
KLSPVLPL;FLDGL	NCP	21.015
FLDGL;HLLYEK	NCP	136.164
HLLYEK;SAAWLGGSL	NCP	11.785
SAAWLGGSL;PAAPVP	NCP	22.56
PAAPVP;KLSGLPTP	NCP	8.486

KLSGLPTP;KFAASLP	NCP	50.968
KFAASLP;KGGAGEF	NCP	39.674
KGGAGEF;PSRGGAARP	NCP	2.457
PSRGGAARP;SGPVPSEL	NCP	16.773
SGPVPSEL;KNMVPQ	NCP	64.662
KNMVPQ;KAAFLGK	NCP	12.222
KAAFLGK;VDLLLL	NCP	10.225
VDLLLL;EPGGLVPL	NCP	11.903
EPGGLVPL;LPDVP	NCP	40.246
LPDVP;ESKPVP	NCP	52.206
ESKPVP;ADKKDEKR	NCP	76.116
ADKKDEKR;LGPPYKP	NCP	13.091
LGPPYKP;KDGPLP	NCP	93.186
KDGPLP;GAQAKAT	NCP	7.728
GAQAKAT;PGVSGKGK	NCP	8.101
PGVSGKGK;LHSKND	NCP	0.866
LHSKND;KEQSLLGT	NCP	17.181
KEQSLLGT;LGLEPPV	NCP	12.403
LGLEPPV;KLLDLGK	NCP	14.553
KLLDLGK;SPPSPLEK	NCP	0.876
SPPSPLEK;MGGPPP	NCP	37.646
MGGPPP;LGSSDYKL	NCP	59.314
LGSSDYKL;THLPL	NCP	42.129
THLPL;ERRVPF	NCP	43.487
ERRVPF;VPALPPLP	NCP	21.378
VPALPPLP;VGLEHLR	NCP	26.166
VGLEHLR;RAAKGDP	NCP	51.654
RAAKGDP;KVLELAGR	NCP	16.012
KVLELAGR;PPLPDF	NCP	72.471
PPLPDF;KHRSSRP	NCP	13.488
KHRSSRP;LPEKP	NCP	34.673
LPEKP;GVPLPPPP	NCP	45.942
GVPLPPPP;RAPVNQP	NCP	73.782
RAPVNQP;SPTVP	NCP	11.107
SPTVP;LDGPAAW	NCP	36.567
LDGPAAW;RVGFHH	NCP	10.869
RVGFHH;RTVPDPK	NCP	4.53
RTVPDPK;VEEQPF	NCP	10.725
VEEQPF;LGKEQVL	NCP	0.72
LGKEQVL;LAGPP	NCP	2.007
LAGPP;YETGNY	NCP	1.376
YETGNY;LEADPL	NCP	0.163
LEADPL;GPVLKL	NCP	29.436
GPVLKL;DPGNVAPR	NCP	382.167
DPGNVAPR;EKLSDPK	NCP	12.105
EKLSDPK;VTMVALK	NCP	109.985
VTMVALK;LSAFVTPV	NCP	0.3
LSAFVTPV;QFLLLF	NCP	137.728
QFLLLF;MDLRAQ	NCP	17.735
MDLRAQ;QALLNVKS	NCP	47.101
QALLNVKS;FGAGGVVP	NCP	79.854

FGAGGVVP;KNLSYK	NCP	75.963
KNLSYK;LKDVTLKK	NCP	7.169
LKDVTLKK;ATPKAGPGVVRK	NCP	252.279
ATPKAGPGVVRK;KGVSMLP	NCP	31.168
KGVSMLP;TSMPDM	NCP	45.078
TSMPDM;DGRHQQK	NCP	11.976
DGRHQQK;KSSLGALR	NCP	16.926
KSSLGALR;LTFVFP	NCP	43.757
LTFVFP;KYAALPK	NCP	10.038
KYAALPK;EKRPEET	NCP	119.644
EKRPEET;TQLWEFF	NCP	37.43
TQLWEFF;KGMNVKK	NCP	74.358
KGMNVKK;VLEAAYL	NCP	91.983
VLEAAYL;ELAYG	NCP	78.439
ELAYG;RPFEP	NCP	52.416
RPFEP;VMRVLNG	NCP	172.367
VMRVLNG;FGRHLDC	NCP	6.675
FGRHLDC;AAVKPP	NCP	148.551
AAVKPP;VPHKKT	NCP	125.598
VPHKKT;AFANLRKVL	NCP	113.697
AFANLRKVL;AAFYD	NCP	18.213
AAFYD;LAEAGVQLLSY	NCP	12.573
LAEAGVQLLSY;KFRTQLH	NCP	9.94
KFRTQLH;LAPKRGY	NCP	10.514
LAPKRGY;KTWLFH	NCP	37.701
KTWLFH;FDVQLK	NCP	36.136
FDVQLK;GVPNLL	NCP	79.568
GVPNLL;VSEEFL	NCP	8.338
VSEEFL;LLEKVR	NCP	99.402
LLEKVR;LVHQK	NCP	24.401
LVHQK;KCLSYPLP	NCP	79.72
KCLSYPLP;FVKLLSWY	NCP	7.332
FVKLLSWY;NALAL	NCP	26.021
NALAL;GLPPYKP	NCP	88.731
GLPPYKP;AMYVMLK	NCP	15.156
AMYVMLK;FRDAGKK	NCP	94.771
FRDAGKK;KEYLE	NCP	39.737
KEYLE;GPPDLP	NCP	18.477
GPPDLP;EDLGPPP	NCP	77.22
EDLGPPP;SEGPV	NCP	22.495
SEGPV;SARFEPF	NCP	106.73
SARFEPF;DSVARLK	NCP	27.039
DSVARLK;AGEEVRF	NCP	95.73
AGEEVRF;LAMRLDL	NCP	33.037
LAMRLDL;KGFPKWP	NCP	111.641
KGFPKWP;GPPTANQ	NCP	6.901
GPPTANQ;YPFNQY	NCP	20.984
YPFNQY;SSADLQVY	NCP	38.613
SSADLQVY;APHLSAK	NCP	37.596
APHLSAK;LAPEPF	NCP	78.039
LAPEPF;VLEPP	NCP	129.868

VLEPP;KYVGLP	NCP	106.397
KYVGLP;KANHAPK	NCP	100.054
KANHAPK;FASAL	NCP	14.814
FASAL;PSSLDSKP	NCP	68.016
PSSLDSKP;PPSSTK	NCP	2.031
PPSSTK;PMKESLK	NCP	160.779
PMKESLK;KDNGPLP	NCP	98.046
KDNGPLP;SLVTLLR	NCP	22.13
SLVTLLR;RGKLEVR	NCP	66.189
RGKLEVR;GLAAKEL	NCP	12.825
GLAAKEL;QPSFPL	NCP	38.434
QPSFPL;EQRLSH	NCP	40.182
EQRLSH;KLEPVTR	NCP	47.588
KLEPVTR;GVRTYSK	NCP	53.017
GVRTYSK;QNWLLLL	NCP	52.227
QNWLLLL;TRPKAP	NCP	126.335
TRPKAP;KKLNRDY	NCP	182.196
KKLNRDY;NGDLGAR	NCP	76.866
NGDLGAR;PASGDFL	NCP	17.164
PASGDFL;ELHVPLGPP	NCP	4.479
ELHVPLGPP;LPFLRP	NCP	4.368
LPFLRP;KGAAVLP	NCP	10.265
KGAAVLP;KVHLDR	NCP	260.527
KVHLDR;EGTKAVTKYTSSK	NCP	36.77
EGTKAVTKYTSSK;TGGPSLHV	NCP	24.187
TGGPSLHV;SSRAGLQFPVGR	NCP	8.477
SSRAGLQFPVGR;KGNYAERVGAGAPVYMAA	NCP	0.054
KGNYAERVGAGAPVYMAA;GNYAERVGAGAPVYMAAV	NCP	0.003
GNYAERVGAGAPVYMAAV;VGAGAPVYM	NCP	0.018
VGAGAPVYM;AGAPVYMAA	NCP	0.006
AGAPVYMAA;APVYMAAV	NCP	0.006
APVYMAAV;VLLPKKTESHKAKSK	NCP	0.201
VLLPKKTESHKAKSK;LLPKKTESHKA	NCP	0.003
LLPKKTESHKA;EPAPPKP	NCP	3.244
EPAPPKP;LTSSLPF	NCP	85.94
LTSSLPF;LTPLV	NCP	2.748
LTPLV;MMPTPVLL	NCP	12.421
MMPTPVLL;LEVSPL	NCP	30.459
LEVSPL;WGPKF	NCP	11.907
WGPKF;QVYPK	NCP	11.811
QVYPK;ERPAPVP	NCP	52.647
ERPAPVP;PFSAL	NCP	33.939
PFSAL;HHLSPGK	NCP	12.098
HHLSPGK;KQETLSVR	NCP	85.135
KQETLSVR;YPTPGYPP	NCP	14.831
YPTPGYPP;VVFFLT	NCP	29.571
VVFFLT;VELKDQTKPTP	NCP	16.593
VELKDQTKPTP;LGHVDP	NCP	1.477
LGHVDP;ALAPEPR	NCP	19.56
ALAPEPR;KEAPSLK	NCP	21.774
KEAPSLK;KVMGLKK	NCP	6.903

KVMGLKK;RKKERL	NCP	44.328
RKKERL;LETVSPK	NCP	0.818
LETVSPK;FPTAEELR	NCP	28.983
FPTAEELR;MTSLYR	NCP	166.827
MTSLYR;TALKFPY	NCP	41.716
TALKFPY;QVRPP	NCP	97.344
QVRPP;KYFNPPL	NCP	30.716
KYFNPPL;GHGTE	NCP	26.458
GHGTE;PPFPFPP	NCP	3.047
PPFPFPP;TLAVKLLF	NCP	45.264
TLAVKLLF;SEPTTASLSKK	NCP	41.295
SEPTTASLSKK;GVPGKL	NCP	2.899
GVPGKL;KLLLP	NCP	2.01
KLLLP;TDVLLL	NCP	32.849
TDVLLL;NNENLR	NCP	21.819
NNENLR;QPVYLAA	NCP	8.778
QPVYLAA;KMTPF	NCP	118.344
KMTPF;DVMQDPEFL	NCP	3.507
DVMQDPEFL;TGKFL	NCP	24.897
TGKFL;EKEELK	NCP	45.453
EKEELK;YAFMGM	NCP	43.845
YAFMGM;PLLCW	NCP	62.005
PLLCW;SGDYADFQYL	NCP	17.192
SGDYADFQYL;VDMLGVAY	NCP	0.633
VDMLGVAY;EAPSLATGY	NCP	0.024
EAPSLATGY;VGEPLK	NCP	44.092
VGEPLK;KGHVEEGL	NCP	3.903
KGHVEEGL;ALMPP	NCP	31.922
ALMPP;KDDALGLQ	NCP	3.825
KDDALGLQ;SLMGPF	NCP	4.15
SLMGPF;PLTASRPS	NCP	9.147
PLTASRPS;MEQASTMAEPR	NCP	4.505
MEQASTMAEPR;GPVYP	NCP	52.401
GPVYP;VVVLK	NCP	165.492
VVVLK;VPSLLPWLP	NCP	98.484
VPSLLPWLP;AHPEPPVR	NCP	90.739
AHPEPPVR;QTDLPNK	NCP	52.109
QTDLPNK;KYHVRK	NCP	4.177
KYHVRK;EVELLPL	NCP	12.521
EVELLPL;DPPVLPE	NCP	26.097
DPPVLPE;RSYVSGKR	NCP	299.766
RSYVSGKR;VKVGVGG	NCP	10.09
VKVGVGG;SGPALPAL	NCP	161.822
SGPALPAL;VFGPGK	NCP	32.584
VFGPGK;KVVKKLE	NCP	150.578
KVVKKLE;TPKRPP	NCP	14.718
TPKRPP;KTPPVHK	NCP	359.737
KTPPVHK;LTFPLYY	NCP	22.934
LTFPLYY;LPSLPDLPF	NCP	82.477
LPSLPDLPF;RLKAVDK	NCP	2.222
RLKAVDK;QTQVTAF	NCP	10.036

QTQVTAF;KMMPPP	NCP	55.077
KMMPPP;ASTPKLP	NCP	24.429
ASTPKLP;LHMAYK	NCP	83.951
LHMAYK;ETMSP	NCP	39.654
ETMSP;ALNTEFK	NCP	72.825
ALNTEFK;RNTPNPK	NCP	80.412
RNTPNPK;LTVFKNV	NCP	187.305
LTVFKNV;NPPEPKP	NCP	51.289
NPPEPKP;KHGAGPR	NCP	12.885
KHGAGPR;KLLPL	NCP	33.273
KLLPL;KPHAARP	NCP	47.37
KPHAARP;PLTSMLR	NCP	0.849
PLTSMLR;ESAHF	NCP	54.407
ESAHF;GPYHGKP	NCP	23.274
GPYHGKP;KLLLEH	NCP	76.003
KLLLEH;MELEAMSR	NCP	38.298
MELEAMSR;VGPLLK	NCP	26.561
VGPLLK;AAQSAPKVVLKSTTK	NCP	20.487
AAQSAPKVVLKSTTK;CPSQKKR	NCP	100.204
CPSQKKR;VDRSRP	NCP	16.746
VDRSRP;PAAVHPK	NCP	31.737
PAAVHPK;ATPPFSL	NCP	3.477
ATPPFSL;NSSVSLY	NCP	18.524
NSSVSLY;APNSPKEY	NCP	241.065
APNSPKEY;PRLLLCD	NCP	17.551
PRLLLCD;LVDYVL	NCP	72.212
LVDYVL;KDGVVEL	NCP	70.959
KDGVVEL;SPEKLPNP	NCP	41.497
SPEKLPNP;LPTTKGP	NCP	71.844
LPTTKGP;SGAFAL	NCP	4.286
SGAFAL;SLGGDGLR	NCP	53.158
SLGGDGLR;RQTFT	NCP	8.88
RQTFT;TSFYPAF	NCP	36.671
TSFYPAF;FLAPASPR	NCP	46.587
FLAPASPR;LPPMAP	NCP	3.624
LPPMAP;HTLPYLK	NCP	49.423
HTLPYLK;GEVMLK	NCP	13.316
GEVMLK;TKLGV	NCP	9.163
TKLGV;RLEPSLR	NCP	1.979
RLEPSLR;AVGSHVY	NCP	4.986
AVGSHVY;GFGFK	NCP	41.407
GFGFK;VHSRPSPP	NCP	59.633
VHSRPSPP;KPVPPPPPP	NCP	4.647
KPVPPPPPP;KGSPSNR	NCP	23.88
KGSPSNR;GFEEVLK	NCP	22.764
GFEEVLK;RLSSLR	NCP	8.343
RLSSLR;KGQRAAPP	NCP	82.89
KGQRAAPP;WGKVCW	NCP	6.435
WGKVCW;EVPTVPTHP	NCP	1.404
EVPTVPTHP;APPHYPPP	NCP	1.086
APPHYPPP;PNKPSGF	NCP	4.677

PNKPSGF;WTLPL	NCP	35.308
WTLPL;GVGVP	NCP	7.163
GVGVP;AAPMELF	NCP	119.604
AAPMELF;RPWDLPP	NCP	45.225
RPWDLPP;TGEQLSLF	NCP	3.55
TGEQLSLF;SFYLPVAAAMY	NCP	51.35
SFYLPVAAAMY;PPPVPPR	NCP	4.215
PPPVPPR;LSLLPLPL	NCP	65.815
LSLLPLPL;KFQPKC	NCP	26.669
KFQPKC;VSAKVGHG	NCP	76.744
VSAKVGHG;KPPPPPP	NCP	22.061
KPPPPPP;KPSVQHE	NCP	84.276
KPSVQHE;LPGSAVSF	NCP	13.29
LPGSAVSF;STVPKAL	NCP	72.091
STVPKAL;VLAPGP	NCP	33.924
VLAPGP;GNVNKHV	NCP	24.14
GNVNKHV;LLLMPH	NCP	73.798
LLLMPH;EGRNLGL	NCP	16.805
EGRNLGL;PLSFVLPP	NCP	17.077
PLSFVLPP;NCSSF	NCP	53.291
NCSSF;VSLQFR	NCP	13.486
VSLQFR;MLRPKALT	NCP	5.672
MLRPKALT;LEAALGEAKK	NCP	63.882
LEAALGEAKK;KKDLEKTYSAK	NCP	0.546
KKDLEKTYSAK;VLCGTCGQPADK	NCP	3.738
VLCGTCGQPADK;SSGSSASSVTVTR	NCP	0.078
SSGSSASSVTVTR;SVGGSGGGSFGDSLVTR	NCP	0.048
SVGGSGGGSFGDSLVTR;TLVGPGALLS	NCP	24.609
TLVGPGALLS;LGPALPFK	NCP	28.14
LGPALPFK;MSTPL	NCP	69.604
MSTPL;KKCPLC	NCP	29.06
KKCPLC;LAEVAL	NCP	49.986
LAEVAL;KAALPPGL	NCP	106.038
KAALPPGL;RLPPP	NCP	5.679
RLPPP;KLPPPPP	NCP	16.32
KLPPPPP;GVSLVPV	NCP	1.32
GVSLVPV;NQLLPK	NCP	1.177
NQLLPK;RNSLLR	NCP	1.341
RNSLLR;EEGPPAV	NCP	22.616
EEGPPAV;LGGRFVPPALEKKY	NCP	46.107
LGGRFVPPALEKKY;HTLHSLR	NCP	6.694
HTLHSLR;LYHRYL	NCP	52.131
LYHRYL;LSMAHC	NCP	72.008
LSMAHC;VGGLLLMQ	NCP	11.103
VGGLLLMQ;AQLLQRF	NCP	79.974
AQLLQRF;LPMALLSP	NCP	11.905
LPMALLSP;EVGDLP	NCP	55.784
EVGDLP;VGSHVY	NCP	3.265
VGSHVY;MAKPEPRP	NCP	4.683
MAKPEPRP;MLSLLQK	NCP	7.773
MLSLLQK;LGPVAVL	NCP	42.677

LGPVAVL;APNLYTL	NCP	18.388
APNLYTL;ALGMF	NCP	20.802
ALGMF;WSLHTPK	NCP	98.457
WSLHTPK;PAVSSPP	NCP	43.328
PAVSSPP;KGVLNVP	NCP	23.746
KGVLNVP;MGVQVP	NCP	57.299
MGVQVP;AAKHLM	NCP	44.392
AAKHLM;PAGHPKK	NCP	154.629
PAGHPKK;MYDVCKK	NCP	91.988
MYDVCKK;MPHVAGP	NCP	9.955
MPHVAGP;NPAFFL	NCP	0.879
NPAFFL;KQHTGPP	NCP	45.021
KQHTGPP;KMVALGN	NCP	31.646
KMVALGN;GKFTLPPP	NCP	36.778
GKFTLPPP;PPLEEFH	NCP	32.517
PPLEEFH;LSGKKMVP	NCP	9.467
LSGKKMVP;KKLSPVLP	NCP	83.608
KKLSPVLP;EGAVVEK	NCP	51.84
EGAVVEK;PVGTPPP	NCP	14.19
PVGTPPP;LAASELYL	NCP	32.259
LAASELYL;KGVSNPPP	NCP	40.787
KGVSNPPP;LYTVVQP	NCP	53.784
LYTVVQP;FLTTVK	NCP	80.896
FLTTVK;TLAALF	NCP	1.17
TLAALF;AENHLAA	NCP	15.758
AENHLAA;ANQVLKC	NCP	64.833
ANQVLKC;KDVPPP	NCP	41.355
KDVPPP;ASGCDC	NCP	35.425
ASGCDC;TNDMRLF	NCP	63.191
TNDMRLF;KKGDEVR	NCP	34.2
KKGDEVR;EYKSGGG	NCP	54.997
EYKSGGG;GPPLDF	NCP	25.451
GPPLDF;GEVAPPK	NCP	37.819
GEVAPPK;NLQLQP	NCP	67.04
NLQLQP;VPVEF	NCP	49.84
VPVEF;KGEHGPR	NCP	9.51
KGEHGPR;LGQNPAPK	NCP	33.32
LGQNPAPK;ALRPVAL	NCP	136.878
ALRPVAL;KGTLPF	NCP	2.616
KGTLPF;GCFVVF	NCP	21.529
GCFVVF;DGALRMPQ	NCP	2.078
DGALRMPQ;TTSLEK	NCP	23.989
TTSLEK;KNRVVLAK	NCP	18.177
KNRVVLAK;VTDCEK	NCP	4.23
VTDCEK;ESVKGQVH	NCP	0.807
ESVKGQVH;KPTRPCF	NCP	2.769
KPTRPCF;LQVFVP	NCP	267.657
LQVFVP;KMCEW	NCP	77.202
KMCEW;PSPPNPK	NCP	173.208
PSPPNPK;SPAAKTRK	NCP	35.307
SPAAKTRK;TNSLPK	NCP	39.387

TNSLPK;KNQVVVPF	NCP	42.513
KNQVVVPF;GVVGP	NCP	6.126
GVVGP;KLLDLAG	NCP	244.086
KLLDLAG;AFFLF	NCP	10.61
AFFLF;VLPLFE	NCP	46.299
VLPLFE;KPGPPP	NCP	37.579
KPGPPP;KLGRSEVL	NCP	25.493
KLGRSEVL;RPGVLLR	NCP	0.505
RPGVLLR;VLAFMVP	NCP	4.025
VLAFMVP;AVDFAP	NCP	9.736
AVDFAP;RVVEC	NCP	55.085
RVVEC;VLLFP	NCP	34.309
VLLFP;MWCLHC	NCP	46.589
MWCLHC;CEFLNK	NCP	14.607
CEFLNK;NPLNLEP	NCP	39.27
NPLNLEP;LMSSFLR	NCP	88.579
LMSSFLR;LVTSKY	NCP	13.962
LVTSKY;AHKSLLGK	NCP	14.922
AHKSLLGK;QSTAVHR	NCP	28.199
QSTAVHR;SLTGRGK	NCP	25.099
SLTGRGK;LAQALK	NCP	49.004
LAQALK;LVAPNPR	NCP	42.091
LVAPNPR;KPPMT	NCP	2.186
KPPMT;FAAAL	NCP	45.513
FAAAL;LLALLVGQ	NCP	16.213
LLALLVGQ;PKYPP	NCP	24.437
PKYPP;SRAEATLP	NCP	5.905
SRAEATLP;GKLTGL	NCP	19.674
GKLTGL;VGAAPKLH	NCP	87.228
VGAAPKLH;QLSKKGGP	NCP	4.352
QLSKKGGP;MEKELR	NCP	33.334
MEKELR;FAPAG	NCP	32.6
FAPAG;FGGLG	NCP	20.58
FGGLG;GYVVVKPKL	NCP	17.274
GYVVVKPKL;KRHAGPK	NCP	97.582
KRHAGPK;TVLEVR	NCP	6.417
TVLEVR;AGKVTRP	NCP	5.385
AGKVTRP;KSLVLT	NCP	212.819
KSLVLT;KPPGPP	NCP	39.421
KPPGPP;KLRTGAF	NCP	3.423
KLRTGAF;QLMEYKK	NCP	36.611
QLMEYKK;KKRRF	NCP	0.787
KKRRF;TMLPPPPP	NCP	129.39
TMLPPPPP;APTDLF	NCP	13.443
APTDLF;KMEPK	NCP	50.405
KMEPK;GKAGMPK	NCP	34.857
GKAGMPK;PRQGRPP	NCP	3.651
PRQGRPP;KPVSVE	NCP	117.925
KPVSVE;MDEMCR	NCP	37.172
MDEMCR;NTQSGPK	NCP	59.848
NTQSGPK;ALLRVE	NCP	13.958

ALLRVE;NAYLELGGLGERVLG	NCP	65.223
NAYLELGGLGERVLG;ELLKLE	NCP	10.75
ELLKLE;AEYGTLL	NCP	63.341
AEYGTLL;LDAKGPH	NCP	58.224
LDAKGPH;GPDATVP	NCP	4.929
GPDATVP;KTCCDRP	NCP	2.541
KTCCDRP;TGGVVRSP	NCP	49.126
TGGVVRSP;RGPGPRP	NCP	23.168
RGPGPRP;HDFRSPPP	NCP	279.73
HDFRSPPP;KLYPV	NCP	149.922
KLYPV;NYKKPQ	NCP	47.114
NYKKPQ;SPGVVP	NCP	62.802
SPGVVP;LVSASLK	NCP	11.826
LVSASLK;EPLQLVA	NCP	5.838
EPLQLVA;KYKLWH	NCP	51.828
KYKLWH;LPKLLAVA	NCP	61.218
LPKLLAVA;MCGGH	NCP	9.18
MCGGH;LHLHGAK	NCP	93.204
LHLHGAK;KDTYTLLY	NCP	12.939
KDTYTLLY;KLHFGR	NCP	15.963
KLHFGR;VLLKPP	NCP	117.421
VLLKPP;CECMHK	NCP	38.322
CECMHK;RVSLPF	NCP	25.536
RVSLPF;LGGERVLK	NCP	2.301
LGGERVLK;SPRVLVGL	NCP	1.122
SPRVLVGL;ADEATRRVVS	NCP	4.19
ADEATRRVVS;RNVPKFGLAH	NCP	3.006
RNVPKFGLAH;TYETLKHEKPPQ	NCP	51.657
TYETLKHEKPPQ;ETLKHEKPP	NCP	0.006
ETLKHEKPP;RHFKKLP	NCP	40.065
RHFKKLP;TSPATNQAPGPEEK	NCP	13.746
TSPATNQAPGPEEK;SRAGMVP	NCP	12.241
SRAGMVP;VGHPLSC	NCP	27.006
VGHPLSC;TKNVP	NCP	35.507
TKNVP;LGPVSPR	NCP	63.138
LGPVSPR;VGPPPHP	NCP	54.916
VGPPPHP;LLLDVAPL	NCP	87.548
LLLDVAPL;KFETTSP	NCP	10.486
KFETTSP;KQTKNPTK	NCP	54.746
KQTKNPTK;TLGSFK	NCP	26.842
TLGSFK;FGVVRPP	NCP	18.996
FGVVRPP;AKRYSYL	NCP	35.688
AKRYSYL;VAGVKGGK	NCP	13.1
VAGVKGGK;LGLQD	NCP	68.037
LGLQD;KYSPLVLL	NCP	37.272
KYSPLVLL;ALTEYGR	NCP	31.561
ALTEYGR;LYPYPQ	NCP	37.31
LYPYPQ;DLGPVP	NCP	94.716
DLGPVP;MGHAYC	NCP	90.114
MGHAYC;APYSRPK	NCP	10.417
APYSRPK;KTQNH	NCP	97.326

KTQNH;QRPHPK	NCP	7.988
QRPHPK;VGLEY	NCP	5.04
VGLEY;KNVSLPLH	NCP	34.491
KNVSLPLH;KPLLQ	NCP	29.688
KPLLQ;EAAGLAQL	NCP	9.247
EAAGLAQL;EAQVVG	NCP	8.28
EAQVVG;CAGPPR	NCP	24.185
CAGPPR;KSHLPPK	NCP	55.725
KSHLPPK;YRAGKEL	NCP	48.078
YRAGKEL;FGRPP	NCP	89.644
FGRPP;KEVKAL	NCP	144.276
KEVKAL;WNELEEAQQVEL	NCP	16.184
WNELEEAQQVEL;GVHELVK	NCP	26.361
GVHELVK;AALAVFGV	NCP	40.42
AALAVFGV;QKALN	NCP	211.089
QKALN;RPVSVAPR	NCP	153.021
RPVSVAPR;SKFKMPK	NCP	13.353
SKFKMPK;RAVKEMT	NCP	43.656
RAVKEMT;LQVSF	NCP	176.412
LQVSF;TDVKRL	NCP	8.283
TDVKRL;EEATEP	NCP	14.481
EEATEP;KFSAPR	NCP	67.82
KFSAPR;VLPLLAT	NCP	18.156
VLPLLAT;NVRLLAEF	NCP	7.74
NVRLLAEF;AGSYVP	NCP	112.171
AGSYVP;LGHGKF	NCP	197.883
LGHGKF;LNLLF	NCP	58.883
LNLLF;KSDVVLK	NCP	96.162
KSDVVLK;LPYKPPP	NCP	26.613
LPYKPPP;KVKKELLP	NCP	20.287
KVKKELLP;QAGQLAY	NCP	61.032
QAGQLAY;KFPFVKL	NCP	22.556
KFPFVKL;EFKFPN	NCP	80.664
EFKFPN;LYLDLL	NCP	45.978
LYLDLL;MARALR	NCP	16.722
MARALR;PVVLVSA	NCP	29.767
PVVLVSA;QHFLPNP	NCP	12.965
QHFLPNP;MDCMH	NCP	122.137
MDCMH;PLRPGQD	NCP	48.699
PLRPGQD;VPVSKTGP	NCP	50.694
VPVSKTGP;PGVLLP	NCP	34.935
PGVLLP;TTLPVKLF	NCP	16.001
TTLPVKLF;PSMKRP	NCP	25.411
PSMKRP;TPGLLRN	NCP	48.195
TPGLLRN;ALSTPVK	NCP	15.344
ALSTPVK;TPVHLLF	NCP	128.214
TPVHLLF;LPHAYPR	NCP	85.618
LPHAYPR;FLFSPPNPL	NCP	5.544
FLFSPPNPL;HAKAPPPP	NCP	29.696
HAKAPPPP;APTTDR	NCP	61.975
APTTDR;PPKLYK	NCP	2.649

PPKLYK;NHTSKLK	NCP	0.902
NHTSKLK;FGSSSTAT	NCP	79.333
FGSSSTAT;TGALVAK	NCP	76.394
TGALVAK;EAGAGALE	NCP	20.77
EAGAGALE;KYPPPP	NCP	52.982
KYPPPP;MLLGPPPPP	NCP	139.465
MLLGPPPPP;KPANPPP	NCP	61.472
KPANPPP;TLPSVF	NCP	17.358
TLPSVF;KDTVPR	NCP	39.346
KDTVPR;SLCGVSLP	NCP	69.681
SLCGVSLP;ELLVT	NCP	22.598
ELLVT;AAAFL	NCP	70.756
AAAFL;VGPWLP	NCP	145.965
VGPWLP;KGVLAK	NCP	14.814
KGVLAK;AALLQGR	NCP	74.852
AALLQGR;EAALRLR	NCP	42.324
EAALRLR;HESPWLPP	NCP	7.728
HESPWLPP;TPVVKK	NCP	28.326
TPVVKK;KCLEDRL	NCP	119.793
KCLEDRL;RVNVFF	NCP	22.773
RVNVFF;TQPTKP	NCP	7.899
TQPTKP;APRMKFY	NCP	9.016
APRMKFY;SMWEK	NCP	30.224
SMWEK;SLQPL	NCP	4.851
SLQPL;LTKPF	NCP	3.42
LTKPF;CPLVTTHL	NCP	19.119
CPLVTTHL;VVPAPGK	NCP	22.332
VVPAPGK;KEHSDPK	NCP	36.36
KEHSDPK;DDDPLN	NCP	2.419
DDDPLN;LSSTAF	NCP	91.016
LSSTAF;SLAVDL	NCP	32.377
SLAVDL;GVPKGPSR	NCP	74.814
GVPKGPSR;KTSLVHR	NCP	35.711
KTSLVHR;ERLPVG	NCP	19.372
ERLPVG;ASASKPK	NCP	14.114
ASASKPK;GLLTEGR	NCP	182.599
GLLTEGR;DALNTEF	NCP	17.313
DALNTEF;RPPPPGH	NCP	21.369
RPPPPGH;KVVPMSP	NCP	181.077
KVVPMSP;KYALPPPPPP	NCP	12.197
KYALPPPPPP;VFKTDLRFQ	NCP	9.949
VFKTDLRFQ;PHALL	NCP	6.125
PHALL;AVQPT	NCP	16.935
AVQPT;REVGAPK	NCP	11.074
REVGAPK;LLQDHRLLR	NCP	35.421
LLQDHRLLR;TDTTLPP	NCP	23.217
TDTTLPP;LEVMKK	NCP	154.029
LEVMKK;VCVHM	NCP	37.497
VCVHM;FYCPQKN	NCP	35.19
FYCPQKN;AAPLVP	NCP	15.296
AAPLVP;PPSVPVT	NCP	15.811

PPSVPVT;YWAPAVH	NCP	31.647
YWAPAVH;KLLLLAE	NCP	1.958
KLLLLAE;KDYSTK	NCP	32.487
KDYSTK;HAAAPGP	NCP	45.199
HAAAPGP;LPPPPWKT	NCP	51.975
LPPPPWKT;VGGPATT	NCP	15.765
VGGPATT;ALRTPLP	NCP	14.751
ALRTPLP;PVLPVQ	NCP	17.199
PVLPVQ;DLPPPY	NCP	6.015
DLPPPY;PLKPAPP	NCP	12.963
PLKPAPP;RMAAPR	NCP	100.613
RMAAPR;KMTLH	NCP	18.484
KMTLH;FLGLYKK	NCP	16.281
FLGLYKK;DGPRPP	NCP	41.388
DGPRPP;KPNRGRPP	NCP	137.58
KPNRGRPP;RMHHSSP	NCP	11.651
RMHHSSP;KGEYV	NCP	142.72
KGEYV;EYVKPK	NCP	128.315
EYVKPK;SVFYELY	NCP	0.882
SVFYELY;VASAPRPAP	NCP	49.414
VASAPRPAP;FLYLVP	NCP	30.456
FLYLVP;KDKVQ	NCP	3.671
KDKVQ;GPAEQAR	NCP	41.785
GPAEQAR;LEDFTAM	NCP	70.088
LEDFTAM;LPPDKR	NCP	34.792
LPPDKR;KDNPPGK	NCP	21.612
KDNPPGK;DVSQWPK	NCP	85.818
DVSQWPK;VGVPDPK	NCP	16.418
VGVPDPK;KVESP	NCP	48.159
KVESP;VLADHKL	NCP	96.804
VLADHKL;LVLEQL	NCP	23.142
LVLEQL;WDMKDK	NCP	0.051
WDMKDK;KYYPPPPPP	NCP	12.846
KYYPPPPPP;AARPHVK	NCP	116.046
AARPHVK;NSRFEPF	NCP	9.976
NSRFEPF;PVKQPH	NCP	10.574
PVKQPH;APEPGRPP	NCP	47.236
APEPGRPP;LTPLVTK	NCP	60.255
LTPLVTK;LVSLLVK	NCP	45.347
LVSLLVK;FSMPGPP	NCP	78.046
FSMPGPP;GVPLH	NCP	6.686
GVPLH;CGMDCA	NCP	90.63
CGMDCA;FPTLSKQP	NCP	11.121
FPTLSKQP;LYVKVH	NCP	3.997
LYVKVH;QPAPP	NCP	51.792
QPAPP;VPKVEP	NCP	150.129
VPKVEP;DGFDP	NCP	56.729
DGFDP;DPPAPLPL	NCP	65.425
DPPAPLPL;PPTRTDP	NCP	25.692
PPTRTDP;MRELL	NCP	57.485
MRELL;WTAGSP	NCP	95.539

WTAGSP;LRMPLYN	NCP	1.407
LRMPLYN;YCPKPK	NCP	24.449
YCPKPK;RAAFPGP	NCP	74.488
RAAFPGP;PGPRHPP	NCP	101.421
PGPRHPP;LLEPSLR	NCP	11.798
LLEPSLR;LPRPPPP	NCP	6.895
LPRPPPP;ASHSLLVAPPAALSKP	NCP	59.091
ASHSLLVAPPAALSKP;APPAALSKPLF	NCP	0.021
APPAALSKPLF;SSLLPRK	NCP	11.427
SSLLPRK;EVKAAPK	NCP	84.6
EVKAAPK;HLKLPTPP	NCP	151.685
HLKLPTPP;PTVTVDP	NCP	9.178
PTVTVDP;KDGSAPY	NCP	26.679
KDGSAPY;KYVPVGP	NCP	47.463
KYVPVGP;VPGKLLA	NCP	87.257
VPGKLLA;KNQATDYL	NCP	9.075
KNQATDYL;PVGAPAEP	NCP	10.231
PVGAPAEP;AVGHGQLR	NCP	23.508
AVGHGQLR;PVTPVLP	NCP	72.276
PVTPVLP;PGGSLVSPM	NCP	166.317
PGGSLVSPM;LLLPPGY	NCP	42.009
LLLPPGY;SSPVYM	NCP	8.184
SSPVYM;KSFAAAK	NCP	174.693
KSFAAAK;NPNVSYGP	NCP	33.138
NPNVSYGP;TTGLVL	NCP	68.877
LPPLPPP;KKHESPK	NCP	39.54
KKHESPK;FPVDHVL	NCP	191.07
FPVDHVL;YVTPLER	NCP	57.381
YVTPLER;AGPSLVH	NCP	103.85
AGPSLVH;KRRRER	NCP	48.669
KRRRER;GMVTGHK	NCP	151.351
GMVTGHK;TAVSLP	NCP	58.976
TAVSLP;LVDLLVCPL	NCP	153.628
LVDLLVCPL;DKKWS	NCP	418.331
DKKWS;CCEDC	NCP	74.296
CCEDC;YHKSSFR	NCP	34.577
YHKSSFR;EMDEC	NCP	15.541
EMDEC;LPGVCA	NCP	49.868
LPGVCA;KCGVGLH	NCP	206.434
KCGVGLH;TTNTLEWL	NCP	3.314
TTNTLEWL;HNLFLVSV	NCP	119.281
HNLFLVSV;PQGNYEK	NCP	13.293
PQGNYEK;RNVLST	NCP	214.82
RNVLST;KPDRWGK	NCP	79.024
KPDRWGK;PPPFL	NCP	44.46
PPPFL;LGLPRL	NCP	192.759
LGLPRL;KANLSLGV	NCP	119.7
KANLSLGV;KSSVPKN	NCP	55.464
KSSVPKN;GTFDLEK	NCP	81.224
GTFDLEK;NKVPLV	NCP	202.813
NKVPLV;LSGSAL	NCP	173.319

LSGSAL;RDHAP	NCP	49.02
RDHAP;EPPGLP	NCP	89.919
EPPGLP;KMDVLY	NCP	36.128
KMDVLY;KLMPPVLF	NCP	2.671
KLMPPVLF;MTAEK	NCP	75.614
MTAEK;RTPLPP	NCP	17.554
RTPLPP;TSVALP	NCP	12.506
TSVALP;HTLRPP	NCP	69.495
HTLRPP;LDHKLDL	NCP	59.068
LDHKLDL;PARLLQ	NCP	108.87
PARLLQ;PPPFPPP	NCP	194.345
PPPFPPP;DDPMLR	NCP	69.066
DDPMLR;VSDLGP	NCP	173.085
VSDLGP;LGSVKLEK	NCP	65.893
LGSVKLEK;KVRVLQ	NCP	401.159
KVRVLQ;PPPLLPP	NCP	31.023
PPPLLPP;ATAVLL	NCP	175.917
ATAVLL;KTPERYK	NCP	68.926
KTPERYK;PVYAAPR	NCP	27.474
PVYAAPR;EGTLSVHQ	NCP	108.543
EGTLSVHQ;AAAFSHC	NCP	200.172
AAAFSHC;KHVQPP	NCP	92.939
KHVQPP;LALFVPR	NCP	85.186
LALFVPR;NPYLR	NCP	55.782
NPYLR;LAFYSHPR	NCP	103.767
LAFYSHPR;KNLSF	NCP	8.513
KNLSF;LHPVGGK	NCP	89.983
LHPVGGK;AEFAERSVAK	NCP	268.028
AEFAERSVAK;EKQPQ	NCP	10.777
EKQPQ;LKKKHG	NCP	31.98
LKKKHG;MVTYTSSK	NCP	197.796
MVTYTSSK;KGWLLR	NCP	171.291
KGWLLR;DCMTF	NCP	119.078
DCMTF;TFFLPPL	NCP	356.274
TFFLPPL;MVPHY	NCP	94.746
MVPHY;GVFLGA	NCP	20.979
GVFLGA;MGMKK	NCP	75.105
MGMKK;PGGLP	NCP	25.746
PGGLP;LGVFK	NCP	165.954
LGVFK;LALPPP	NCP	43.203
LALPPP;MTKLK	NCP	71.982
MTKLK;KSYVNF	NCP	56.715
KSYVNF;KVGVP	NCP	193.021
KVGVP;RKVLRK	NCP	464.25
RKVLRK;KTHLY	NCP	242.199
KTHLY;SGLYECRT	NCP	135.578
SGLYECRT;MRTALTH	NCP	11.097
MRTALTH;KLVEQLP	NCP	80.01
KLVEQLP;KLYEEK	NCP	87.268
KLYEEK;ALATLK	NCP	96.956
ALATLK;NPLVLV	NCP	73.965

NPLVLV;PWKFFV	NCP	248.326
PWKFFV;VTVKP	NCP	182.801
VTVKP;CMCCSQP	NCP	16.98
CMCCSQP;KAVAVL	NCP	40.885
KAVAVL;ELLNKP	NCP	11.712
ELLNKP;PPHTKK	NCP	257.877
PPHTKK;VLSHMNR	NCP	76.887
VLSHMNR;NADEARSLKA	NCP	164.642
NADEARSLKA;TAPTPLLA	NCP	165.853
TAPTPLLA;KRKKKH	NCP	324.213
KRKKKH;ALLGL	NCP	115.004
ALLGL;GFKLL	NCP	89.878
GFKLL;KGPLF	NCP	37.232
KGPLF;GVQMF	NCP	167.625
GVQMF;RPLLKR	NCP	240.696
RPLLKR;LENPV	NCP	15.541
LENPV;ATLLK	NCP	2.444
ATLLK;SVVCC	NCP	140.116
SVVCC;VSAAPP	NCP	259.44
VSAAPP;LSGQRL	NCP	42.351
LSGQRL;VQLSQL	NCP	10.17
VQLSQL;SLSLSPM	NCP	30.782
SLSLSPM;HLLLPP	NCP	60.393
HLLLPP;KYVVYLL	NCP	121.117
KYVVYLL;KMLPLL	NCP	46.482
KMLPLL;CCMCGLK	NCP	34.28
CCMCGLK;KAAAFP	NCP	14.512
KAAAFP;HLLVKK	NCP	15.327
HLLVKK;VPLPT	NCP	145.19
VPLPT;PPPFFPP	NCP	47.508
PPPFFPP;PPKLP	NCP	11.754
PPKLP;GVAHHYR	NCP	5.575
GVAHHYR;KTAPT	NCP	74.261
KTAPT;PLPDHGK	NCP	361.362
PLPDHGK;KSAEVKNK	NCP	49.234
KSAEVKNK;YGPQC	NCP	156.12
YGPQC;LFDLANY	NCP	83.556
LFDLANY;ESVGK	NCP	171.446
ESVGK;HDYYD	NCP	71.416
HDYYD;DSAKF	NCP	70.7
DSAKF;PTLEH	NCP	40.365
PTLEH;LLDGP	NCP	53.443
LLDGP;LPTLPDA	NCP	213.713
LPTLPDA;KKHLLL	NCP	184.416
KKHLLL;VPMLT	NCP	41.508
VPMLT;LNFAF	NCP	79.371
LNFAF;KLGLQR	NCP	123.871
KLGLQR;PGTTTPRP	NCP	133.455
PGTTTPRP;ETKKLT	NCP	137.603
ETKKLT;AMDKAK	NCP	16.179
AMDKAK;TVEDH	NCP	201.712

TVEDH;VPVQPS	NCP	70.305
VPVQPS;KEKPSAGP	NCP	199.014
KEKPSAGP;GVVKP	NCP	1.395
GVVKP;VGLGQRK	NCP	45.798
VGLGQRK;PRWHHF	NCP	30.162
PRWHHF;YPEFFG	NCP	72.093
YPEFFG;VLVNKQK	NCP	386.271
VLVNKQK;SLDSD	NCP	12.261
SLDSD;SPPVVKHL	NCP	144.188
SPPVVKHL;EVAAL	NCP	454.05
EVAAL;NVSLPMLLL	NCP	79.594
NVSLPMLLL;FRFEY	NCP	75.294
FRFEY;QSPDRP	NCP	16.874
QSPDRP;LPDAVL	NCP	14.413
LPDAVL;RMDAA	NCP	82.025
RMDAA;VHVTF	NCP	56.041
VHVTF;YVLAP	NCP	56.525
YVLAP;VLTPEL	NCP	94.411
VLTPEL;QPTLLK	NCP	29.804
QPTLLK;KPLAKKP	NCP	72.921
KPLAKKP;RETLLR	NCP	30.705
RETLLR;LTLGLKLF	NCP	250.278
LTLGLKLF;LLLVMY	NCP	153.826
LLLVMY;RSPVVFP	NCP	34.554
RSPVVFP;KDMPPK	NCP	106.68
KDMPPK;KDRLVK	NCP	116.799
KDRLVK;PLLFSN	NCP	120.197
PLLFSN;VAVDMVL	NCP	234.558
VAVDMVL;KLHYPT	NCP	0.649
KLHYPT;LPHTYT	NCP	14.276
LPHTYT;MEEKQLLC	NCP	15.856
MEEKQLLC;MGPPLSY	NCP	51.528
MGPPLSY;QPSWL	NCP	36.254
QPSWL;KPPLP	NCP	78.733
KPPLP;ASLTFGK	NCP	44.642
ASLTFGK;DFKTDFLR	NCP	148.416
DFKTDFLR;PLKAVDK	NCP	145.84
PLKAVDK;DYLFF	NCP	109.508
DYLFF;AVKVSDP	NCP	37.696
AVKVSDP;DFEKPF	NCP	4.328
DFEKPF;KPCPPGP	NCP	6.559
KPCPPGP;YTDPAPP	NCP	421.694
YTDPAPP;APRCE	NCP	2.448
APRCE;NMKLVQT	NCP	19.218
NMKLVQT;KAERPPPP	NCP	23.814
KAERPPPP;LLFARN	NCP	32.01
LLFARN;EPEPLL	NCP	23.622
EPEPLL;YGSLLC	NCP	8.488
YGSLLC;AALLA	NCP	18.347
AALLA;ESDLSPP	NCP	36.078
ESDLSPP;KSSPDPK	NCP	4.684

KSSPDPK;LTPLPK	NCP	3.107
LTPLPK;KPPLHK	NCP	94.89
KPPLHK;TTLSPP	NCP	91.824
TTLSPP;KEQWPK	NCP	3.517
KEQWPK;KLLVVH	NCP	0.089
KLLVVH;PLGPL	NCP	24.507
PLGPL;RFPTD	NCP	5.634
RFPTD;CDDSN	NCP	83.866
CDDSN;VPDLGP	NCP	2.223
VPDLGP;KGLGVLLAF	NCP	3.357
KGLGVLLAF;QSLGL	NCP	106.791
QSLGL;LLETPV	NCP	141.396
LLETPV;KTFVTAAP	NCP	74.759
KTFVTAAP;VTLPLLKP	NCP	6.912
VTLPLLKP;KNDVPVK	NCP	38.095
KNDVPVK;VGYAMH	NCP	23.043
VGYAMH;MNGLEY	NCP	21.362
MNGLEY;VLLVGY	NCP	17.997
VLLVGY;VTPLPF	NCP	2.436
VTPLPF;KEVFDPK	NCP	26.007
KEVFDPK;DVGLLT	NCP	83.295
DVGLLT;VNLERLYL	NCP	78.549
VNLERLYL;TLLARDLGL	NCP	58.09
TLLARDLGL;TLALKR	NCP	96.735
TLALKR;GVPQP	NCP	43.911
GVPQP;VKRHY	NCP	129.08
VKRHY;MLSAD	NCP	26.727
MLSAD;KHFVP	NCP	204.799
KHFVP;TKKPGVK	NCP	87.972
TKKPGVK;PDRLL	NCP	46.538
PDRLL;VGHVPLPG	NCP	29.805
VGHVPLPG;SAALL	NCP	24.82
SAALL;KVPAAF	NCP	12.389
KVPAAF;KEQVP	NCP	10.713
KEQVP;FPLLLN	NCP	160.503
FPLLLN;DPCCC	NCP	32.139
DPCCC;LPGGVVFP	NCP	44.419
LPGGVVFP;AEEHLRPP	NCP	63.401
AEEHLRPP;AAPVPLPRPVTH	NCP	95.274
AAPVPLPRPVTH;RLKVFN	NCP	3.664
RLKVFN;NTTKDLKEK	NCP	1.884
NTTKDLKEK;EVSLPY	NCP	37.446
EVSLPY;NRMAL	NCP	91.037
NRMAL;LRLQPP	NCP	10.698
LRLQPP;GLPPVP	NCP	21.765
GLPPVP;PLTVA	NCP	83.272
PLTVA;KGFMKGP	NCP	23.532
KGFMKGP;RLLLQ	NCP	17.102
RLLLQ;AGANAAFR	NCP	12.075
AGANAAFR;DSLRLR	NCP	23.266
DSLRLR;KNVPF	NCP	36.951

KNVPF;TQTLLT	NCP	22.182
TQTLLT;VPTFKHG	NCP	23.7
VPTFKHG;DDFLGAAR	NCP	115.997
DDFLGAAR;AEEEL	NCP	8.598
AEEEL;LTLPDPA	NCP	91.254
LTLPDPA;RPLVPAAFW	NCP	120.19
RPLVPAAFW;TNAPFP	NCP	28.421
TNAPFP;VGPTLLS	NCP	202.093
VGPTLLS;KGYPP	NCP	102.437
KGYPP;ASPGLL	NCP	37.374
ASPGLL;TPKSKQP	NCP	5.922
TPKSKQP;KDPLEKG	NCP	38.007
KDPLEKG;GDMAVTR	NCP	71.35
GDMAVTR;TATFPAST	NCP	35.105
TATFPAST;VLLAVAF	NCP	23.005
VLLAVAF;EKGLSR	NCP	93.167
EKGLSR;VQVLP	NCP	20.236
VQVLP;EGKGSVVL	NCP	16.512
EGKGSVVL;FRGASLK	NCP	129.036
FRGASLK;LLTTQC	NCP	54.213
LLTTQC;EVYPE	NCP	17.145
EVYPE;PGAPKPP	NCP	64.488
PGAPKPP;PAFSGL	NCP	219.074
PAFSGL;KGMKP	NCP	78.381
KGMKP;NLKKAAL	NCP	159.267
NLKKAAL;LKKRPP	NCP	71.401
LKKRPP;FVTLK	NCP	15.654
FVTLK;LVRVFL	NCP	243.578
LVRVFL;NFSVFY	NCP	8.343
NFSVFY;PVVRGPP	NCP	7.533
PVVRGPP;KSHDW	NCP	89.976
KSHDW;KALGV	NCP	101.758
KALGV;AGRKPPL	NCP	87.212
AGRKPPL;KLGNTVK	NCP	356.758
KLGNTVK;GSLDHK	NCP	219.569
GSLDHK;NAAPKAP	NCP	168.751
NAAPKAP;TSLRTDPK	NCP	166.818
TSLRTDPK;ALPAH	NCP	88.718
ALPAH;CDEDP	NCP	153.507
CDEDP;KGLEF	NCP	17.721
KGLEF;CVPPP	NCP	90.264
CVPPP;KGCDPF	NCP	104.439
KGCDPF;PRVTPP	NCP	83.19
PRVTPP;RPHPPPP	NCP	20.157
RPHPPPP;KSPEDPVL	NCP	190.314
KSPEDPVL;GPSLGTPPPPR	NCP	45.663
GPSLGTPPPPR;KHLAPR	NCP	1.057
KHLAPR;GRFTLK	NCP	22.374
GRFTLK;AVRPPR	NCP	37.556
AVRPPR;KTTGPPPPP	NCP	63.387
KTTGPPPPP;TKSLT	NCP	72.721

TKSLT;LAVHGPPP	NCP	7.193
LAVHGPPP;LEVQLRW	NCP	180.655
LEVQLRW;FPLEP	NCP	49.088
FPLEP;EALWVGK	NCP	15.582
EALWVGK;HCMLR	NCP	74.371
HCMLR;LDELATY	NCP	74.903
LDELATY;GVEHAYF	NCP	150.112
GVEHAYF;DPCDN	NCP	15.558
DPCDN;VTDLL	NCP	63.096
VTDLL;HLAFVV	NCP	175.331
HLAFVV;LGPTP	NCP	37.788
LGPTP;NGMSLLL	NCP	2.671
NGMSLLL;YVFLLLMF	NCP	88.194
YVFLLLMF;QFSPP	NCP	68.567
QFSPP;LQTLRVL	NCP	26.614
LQTLRVL;SGLQQTKR	NCP	268.302
SGLQQTKR;FKGVNFK	NCP	291.588
FKGVNFK;VLENQ	NCP	26.403
VLENQ;LAEKLR	NCP	70.422
LAEKLR;LRKDEL	NCP	49.962
LRKDEL;YGKPPPPPP	NCP	1021.367
YGKPPPPPP;LLQVQH	NCP	121.095
LLQVQH;KGLYL	NCP	3.958
KGLYL;PVRFTTH	NCP	200.046
PVRFTTH;EDEDC	NCP	123.998
EDEDC;SYLLA	NCP	40.456
SYLLA;ALLEM	NCP	128.529
ALLEM;RKCYP	NCP	13.136
RKCYP;NVYNP	NCP	124.764
NVYNP;VLGLQM	NCP	47.655
VLGLQM;KVFVEK	NCP	55.321
KVFVEK;LLLGHC	NCP	9.164
LLLGHC;QAFELLLK	NCP	20.944
QAFELLLK;LVPKEK	NCP	155.108
LVPKEK;TPQVLPK	NCP	262.879
TPQVLPK;CMNEC	NCP	211.184
CMNEC;LLLGP	NCP	32.059
LLLGP;MTMVCC	NCP	227.727
MTMVCC;EDLVFF	NCP	96.707
EDLVFF;KNPALPP	NCP	324.042
KNPALPP;CKVVPSSY	NCP	2.742
CKVVPSSY;AGPFP	NCP	15.172
AGPFP;GRVVAPPG	NCP	276.327
GRVVAPPG;NFVVLG	NCP	526.958
NFVVLG;KDGAKTGK	NCP	33.253
KDGAKTGK;KWDLDLR	NCP	95.831
KWDLDLR;ALFLTK	NCP	127.251
ALFLTK;WGMKPP	NCP	392.322
WGMKPP;ELGAVP	NCP	52.531
ELGAVP;RKLGLK	NCP	7.761
RKLGLK;LHLPNK	NCP	15.977

LHLPNK;PSTLPSTDK	NCP	64.113
PSTLPSTDK;LLYRVD	NCP	161.526
LLYRVD;KPSTP	NCP	294.423
KPSTP;VTDLVGH	NCP	93.024
VTDLVGH;VDWGNKK	NCP	73.789
VDWGNKK;PMAMV	NCP	71.106
PMAMV;KEELSMPP	NCP	109.089
KEELSMPP;DLWKEK	NCP	95.169
DLWKEK;GPVKV	NCP	38.85
GPVKV;AAKETLFQ	NCP	197.936
AAKETLFQ;STRRVP	NCP	14.986
STRRVP;KYLPPS	NCP	2.063
KYLPPS;VGHTVYQ	NCP	130.78
VGHTVYQ;CPVAQPK	NCP	45.63
CPVAQPK;WVPLN	NCP	10.611
WVPLN;LDAPGGP	NCP	108.06
LDAPGGP;KPDSPP	NCP	23.79
KPDSPP;RLVAGPGK	NCP	42.284
RLVAGPGK;VGALLQPL	NCP	74.223
VGALLQPL;LPVSSGMS	NCP	41.896
LPVSSGMS;TWLLE	NCP	171.854
TWLLE;AGPGMPK	NCP	24.559
AGPGMPK;WPTAAPK	NCP	66.903
WPTAAPK;KTLPKK	NCP	36.83
KTLPKK;PTVFF	NCP	43.774
PTVFF;PLLPE	NCP	38.27
PLLPE;VAAFP	NCP	12.21
VAAFP;KGYNHP	NCP	66.93
KGYNHP;KNLEVLD	NCP	51.991
KNLEVLD;REVWY	NCP	15.225
REVWY;KDMHNAPP	NCP	5.448
KDMHNAPP;TLPFK	NCP	121.848
TLPFK;AFAHW	NCP	41.259
AFAHW;ADAKSFLL	NCP	56.054
ADAKSFLL;AADPL	NCP	207.211
AADPL;QKGAAPR	NCP	18.843
QKGAAPR;LPSSAPAP	NCP	4.244
LPSSAPAP;KPESLVDP	NCP	80.916
KPESLVDP;KLTPPPFQ	NCP	130.101
KLTPPPFQ;PADHSPP	NCP	9.322
PADHSPP;KSTGFW	NCP	61.842
KSTGFW;DVERVPF	NCP	35.07
DVERVPF;FPQSP	NCP	22.878
FPQSP;LDPTGL	NCP	2.048
LDPTGL;KAADRKK	NCP	49.806
KAADRKK;QAGLAL	NCP	19.788
QAGLAL;SDRPSF	NCP	156.51
SDRPSF;ATVVAVAA	NCP	303.202
ATVVAVAA;LTDGKLEF	NCP	119.642
LTDGKLEF;VPCFF	NCP	64.053
VPCFF;SGGLRTPT	NCP	32.016

SGGLRTPT;KELQLFPF	NCP	39.511
KELQLFPF;VPLYE	NCP	113.706
VPLYE;CQVPLTL	NCP	160.775
CQVPLTL;KTTLQF	NCP	51.175
KTTLQF;CCMCEC	NCP	17.907
CCMCEC;WGPLPKL	NCP	21.441
WGPLPKL;ASVPVG	NCP	42.54
ASVPVG;ALEVL	NCP	56.444
ALEVL;APALCCG	NCP	89.479
APALCCG;SLGATFPK	NCP	57.369
SLGATFPK;KERAKR	NCP	40.836
KERAKR;LLGGPL	NCP	138.693
LLGGPL;SVLPL	NCP	64.073
SVLPL;KCAADLGLKK	NCP	88.051
KCAADLGLKK;LDGFGFS	NCP	224.132
LDGFGFS;DPPFEVR	NCP	7.545
DPPFEVR;NAVPNLK	NCP	2.157
NAVPNLK;HSPVP	NCP	155.614
HSPVP;KGAHLVAR	NCP	94.347
KGAHLVAR;AALSGGTT	NCP	44.316
AALSGGTT;PVGSPK	NCP	377.619
PVGSPK;DTSSPK	NCP	298.032
DTSSPK;QEGLLD	NCP	375.875
QEGLLD;YLLVSW	NCP	74.328
YLLVSW;LKAQAQKL	NCP	25.779
LKAQAQKL;FDMAKKF	NCP	45.915
FDMAKKF;EPKELR	NCP	120.235
EPKELR;VGEFK	NCP	9.86
VGEFK;DSYPGPP	NCP	337.087
DSYPGPP;NDPGLLHQ	NCP	220.038
NDPGLLHQ;GTVKKVL	NCP	52.86
GTVKKVL;PRAPHF	NCP	97.353
PRAPHF;PVLQVLLE	NCP	120.245
PVLQVLLE;DPHAKGK	NCP	97.117
DPHAKGK;ALLRG	NCP	182.987
ALLRG;PVLACR	NCP	130.215
PVLACR;KPPPPPPP	NCP	38.866
KPPPPPPP;NPSVTRLP	NCP	95.555
NPSVTRLP;EKSALNTEF	NCP	0.877
EKSALNTEF;AGTYEVRF	NCP	4.326
AGTYEVRF;MSQLLQ	NCP	89.385
MSQLLQ;NALGFAN	NCP	82.115
NALGFAN;AKSGF	NCP	48.703
AKSGF;EPLWR	NCP	47.655
EPLWR;PDRLP	NCP	120.606
PDRLP;ASPPLAGGK	NCP	180.852
ASPPLAGGK;YHMVY	NCP	252.687
YHMVY;PPRNGALY	NCP	36.056
PPRNGALY;VAPSGP	NCP	241.285
VAPSGP;LVPEPGK	NCP	86.922
LVPEPGK;AAFELL	NCP	365.948

AAFELL;KNLLSH	NCP	566.676
KNLLSH;FTASM	NCP	116.713
FTASM;LFLRLP	NCP	77.184
LFLRLP;KHLLMKKK	NCP	30.924
KHLLMKKK;LGGPVVPF	NCP	119.976
LGGPVVPF;PGFAPMR	NCP	62.889
PGFAPMR;SAQLGTLK	NCP	13.11
SAQLGTLK;KYEPPLLL	NCP	176.085
KYEPPLLL;KGTAL	NCP	57.465
KGTAL;PLLSAY	NCP	245.703
PLLSAY;AASNRVLN	NCP	197.472
AASNRVLN;VGPVF	NCP	149.754
VGPVF;VKSFPQ	NCP	29.075
VKSFPQ;HGSFM	NCP	181.671
HGSFM;YAKPPPPP	NCP	142.17
YAKPPPPP;PGKPVVF	NCP	32.392
PGKPVVF;KGGGVL	NCP	32.264
KGGGVL;ANCCYK	NCP	79.473
ANCCYK;LSMKV	NCP	249.817
LSMKV;GLQSARHD	NCP	90.096
GLQSARHD;LTFQLH	NCP	167.913
LTFQLH;TYMAF	NCP	125.012
TYMAF;KFFVL	NCP	16.906
KFFVL;KGVDSVVP	NCP	117.621
KGVDSVVP;VSLDKTK	NCP	10.634
VSLDKTK;KEAGKLLK	NCP	17.703
KEAGKLLK;MELEVS	NCP	16.786
MELEVS;LFYPP	NCP	5.936
LFYPP;LARTTP	NCP	125.772
LARTTP;LLKKSLL	NCP	56.07
LLKKSLL;LLLAFK	NCP	168.924
LLLAFK;MEEQRLLQ	NCP	43.005
MEEQRLLQ;MYKPLPP	NCP	135.055
MYKPLPP;VTLLFF	NCP	336.05
VTLLFF;KVLLLLLL	NCP	130.462
KVLLLLLL;NSDTMVP	NCP	1.581
NSDTMVP;MADAV	NCP	69.266
MADAV;KHHKPPP	NCP	626.698
KHHKPPP;LTTHAKP	NCP	196.166
LTTHAKP;KPPLAP	NCP	21.976
KPPLAP;KDSEPLPK	NCP	293.769
KDSEPLPK;KSMSP	NCP	27.524
KSMSP;KLTTVF	NCP	101.506
KLTTVF;TGNRLL	NCP	152.807
TGNRLL;KGFLFLPY	NCP	72.489
KGFLFLPY;KMLPLLQ	NCP	102.459
KMLPLLQ;KHFSVLV	NCP	92.913
KHFSVLV;PVQLNL	NCP	53.584
PVQLNL;LMLLLST	NCP	137.838
LMLLLST;KAPKLLP	NCP	135.728
KAPKLLP;DNEFY	NCP	99.813

DNEFY;KMLLHR	NCP	47.067
KMLLHR;TSCVPR	NCP	52.447
TSCVPR;AELLAT	NCP	1.176
AELLAT;FFPPPR	NCP	92.477
FFPPPR;LPTNTL	NCP	179.203
LPTNTL;GQCCC	NCP	4.826
GQCCC;KDVAFPK	NCP	52.926
KDVAFPK;LSLVT	NCP	29.254
LSLVT;LTEVM	NCP	19.322
LTEVM;TSALVT	NCP	4.215
TSALVT;LQVSLR	NCP	155.28
LQVSLR;LGKEPK	NCP	120.738
LGKEPK;FLLAKR	NCP	68.193
FLLAKR;ELPVP	NCP	10.65
ELPVP;NGKLFR	NCP	191.142
NGKLFR;MPVLLGL	NCP	18.262
MPVLLGL;KDLKLE	NCP	28.649
KDLKLE;PVEAFS	NCP	4.063
PVEAFS;YSLFTD	NCP	43.34
YSLFTD;WLRKP	NCP	199.401
WLRKP;YSRKLKY	NCP	53.4
YSRKLKY;AAFLLN	NCP	11.242
AAFLLN;FVEKP	NCP	5.028
FVEKP;PGMLYE	NCP	11.829
PGMLYE;LYAAVL	NCP	54.213
LYAAVL;PELPSF	NCP	47.381
PELPSF;KVLTEK	NCP	27.801
KVLTEK;TLATPL	NCP	58.519
TLATPL;ASAKKAVLGPLVG	NCP	56.144
ASAKKAVLGPLVG;FVEEVLL	NCP	31.6
FVEEVLL;LLLPAME	NCP	113.036
LLLPAME;KPRDLL	NCP	147.378
KPRDLL;NHANL	NCP	10.035
NHANL;GPEKSQGK	NCP	225.648
GPEKSQGK;KAAALD	NCP	199.753
KAAALD;QKKGPK	NCP	187.817
QKKGPK;KTFGGDM	NCP	7.093
KTFGGDM;KGELP	NCP	164.6
KGELP;LLELR	NCP	49.827
LLELR;VVAVLL	NCP	38.511
VVAVLL;GMEDC	NCP	68.659
GMEDC;KVKKNY	NCP	119.469
KVKKNY;KGDAM	NCP	221.135
KGDAM;ALYHG	NCP	10.881
ALYHG;KNLEVK	NCP	81.571
KNLEVK;KEYLVP	NCP	68.279
KEYLVP;YVPLLP	NCP	189.337
YVPLLP;LLLLFAGP	NCP	71.879
LLLLFAGP;HRGLLF	NCP	28.275
HRGLLF;HGVAP	NCP	45.889
HGVAP;RQGRTLY	NCP	16.947

RQGRTLY;PNLPLE	NCP	223.584
PNLPLE;VLDAVRTK	NCP	79.359
VLDAVRTK;VYLKK	NCP	394.047
VYLKK;SMMSEF	NCP	86.084
SMMSEF;FSVTT	NCP	22.57
FSVTT;AAEVLP	NCP	121.455
AAEVLP;PLSKKDL	NCP	57.744
PLSKKDL;PFYEGNK	NCP	28.538
PFYEGNK;KLEPHLLF	NCP	95.137
KLEPHLLF;PTKEC	NCP	85.295
PTKEC;KGLTPLK	NCP	191.622
KGLTPLK;NSKLLLGRL	NCP	50.082
NSKLLLGRL;RSLLMK	NCP	121.108
RSLLMK;VNGFRNL	NCP	54.06
VNGFRNL;ASFKKFH	NCP	22.499
ASFKKFH;PVMCPK	NCP	273.967
PVMCPK;KRNVYP	NCP	62.234
KRNVYP;KESTVSK	NCP	118.771
KESTVSK;EPPKKEKK	NCP	121.809
EPPKKEKK;DFPAL	NCP	12.335
DFPAL;NMLGER	NCP	147.669
NMLGER;KAKDF	NCP	5.706
KAKDF;AAQVRMK	NCP	50.482
AAQVRMK;KLLLPRVD	NCP	18.911
KLLLPRVD;TAALP	NCP	32.883
TAALP;SMKELR	NCP	23.518
SMKELR;TTMPP	NCP	41.138
TTMPP;LPAVV	NCP	42.738
LPAVV;VLYSPK	NCP	23.16
VLYSPK;ELLLP	NCP	30.874
ELLLP;LLALLLPA	NCP	81.813
LLALLLPA;KTLDLF	NCP	58.286
KTLDLF;RSMLLF	NCP	65.7
RSMLLF;LEHSL	NCP	125.098
LEHSL;GKLPV	NCP	61.398
GKLPV;NSALLK	NCP	47.642
NSALLK;KPKGGPK	NCP	10.03
KPKGGPK;QVMMTAK	NCP	48.65
QVMMTAK;VGVNVFF	NCP	271.786
VGVNVFF;LLPRSPK	NCP	18.87
LLPRSPK;MFLLQQ	NCP	9.497
MFLLQQ;KGTTVPR	NCP	6.709
KGTTVPR;SLERLQ	NCP	6.399
SLERLQ;APAPAKPP	NCP	67.263
APAPAKPP;TARLLR	NCP	0.09
TARLLR;KSSQYP	NCP	37.959
KSSQYP;QRERKK	NCP	236.015
QRERKK;CEMMAK	NCP	149.482
CEMMAK;WEGNLP	NCP	2.346
WEGNLP;HTMYM	NCP	125.271
HTMYM;AFELLHL	NCP	103.397

AFELLHL;SPFPL	NCP	222.033
SPFPL;MSGPQYK	NCP	39.042
MSGPQYK;LLVVVD	NCP	205.753
LLVVVD;GVALL	NCP	1.287
GVALL;EAFDL	NCP	153.651
EAFDL;QCCCMVY	NCP	147.305
QCCCMVY;KPMSQP	NCP	14.133
KPMSQP;TTTLHL	NCP	58.399
TTTLHL;KGLPL	NCP	65.4
KGLPL;KGFYLDY	NCP	35.231
KGFYLDY;KPDSLP	NCP	72.669
KPDSLP;PELYSK	NCP	141.373
PELYSK;MPEAALK	NCP	273.122
MPEAALK;QPLYPPP	NCP	21.18
QPLYPPP;KEEVLK	NCP	26.017
KEEVLK;SPFLP	NCP	65.304
SPFLP;AVFKLLLK	NCP	17.802
AVFKLLLK;YEKWY	NCP	56.376
YEKWY;RTHEQEKQ	NCP	94.113
RTHEQEKQ;TVNKP	NCP	10.955
TVNKP;LLEYLT	NCP	21.895
LLEYLT;HALKKK	NCP	86.196
HALKKK;SLLTDYSPQLQKPKF	NCP	1.466
SLLTDYSPQLQKPKF;PHFDLTHL	NCP	52.587
PHFDLTHL;LLGGGV	NCP	29.493
LLGGGV;PLVLEF	NCP	4.2
PLVLEF;TAVADVH	NCP	3.466
TAVADVH;QSGPVLL	NCP	113.741
QSGPVLL;LEGTVP	NCP	37.84
LEGTVP;RMAMGGS	NCP	12.596
RMAMGGS;DAGAK	NCP	69.451
DAGAK;ATALLK	NCP	6.408
ATALLK;KGFPPLR	NCP	138.764
KGFPPLR;GVGGSLL	NCP	71.14
GVGGSLL;EPVYP	NCP	85.031
EPVYP;LLLTPLF	NCP	47.682
LLLTPLF;LGPLPPPSF	NCP	0.024
LGPLPPPSF;LEGLL	NCP	243.621
LEGLL;TARLVHA	NCP	85.941
TARLVHA;AARPT	NCP	77.4
AARPT;AMLKLVA	NCP	250.764
AMLKLVA;YKPLPP	NCP	115.167
YKPLPP;LRGGGVPRA	NCP	39.153
LRGGGVPRA;DPPPPFP	NCP	12.288
DPPPPFP;KLQKDPKK	NCP	87.999
KLQKDPKK;LPNKP	NCP	62.656
LPNKP;PLLMSL	NCP	14.856
PLLMSL;DLKKLAPK	NCP	17.54
DLKKLAPK;AVPQAAA	NCP	31.755
AVPQAAA;TVAALK	NCP	128.034
TVAALK;FNTSKK	NCP	116.059

FNTSKK;KDFGQAK	NCP	39.71
KDFGQAK;VSLKVQL	NCP	40.674
VSLKVQL;EPFTKLLV	NCP	18.019
EPFTKLLV;PPKDH	NCP	177.141
PPKDH;KMTDFAN	NCP	81.053
KMTDFAN;QWLEW	NCP	260.751
QWLEW;NSQLPSR	NCP	25.303
NSQLPSR;KCLQPKLLE	NCP	51.692
KCLQPKLLE;AESLLLVR	NCP	79.564
AESLLLVR;KPSLCH	NCP	134.655
KPSLCH;VPGDVLGF	NCP	36.956
VPGDVLGF;RPPEPLT	NCP	256.731
RPPEPLT;NNFLDC	NCP	74.902
NNFLDC;RELEEL	NCP	125.354
RELEEL;PFVEL	NCP	37.581
PFVEL;NALLP	NCP	16.383
NALLP;VKLQY	NCP	22.02
VKLQY;YSPPMLK	NCP	7.828
YSPPMLK;VLGGVSK	NCP	1.373
VLGGVSK;RPSPVVV	NCP	71.143
RPSPVVV;HQTQLVE	NCP	25.428
HQTQLVE;KAKLVTK	NCP	40.716
KAKLVTK;KHHSLKK	NCP	1.283
KHHSLKK;TVSPP	NCP	517.06
TVSPP;PPQVPLPP	NCP	102.915
PPQVPLPP;KGKSY	NCP	167.463
KGKSY;AENQLLLK	NCP	148.02
AENQLLLK;KLYPLEAH	NCP	57.224
KLYPLEAH;KQTFPP	NCP	10.882
KQTFPP;GPDDSLK	NCP	32.688
GPDDSLK;KFLLTA	NCP	204.803
KFLLTA;CCGRM	NCP	78.6
CCGRM;VGGELLY	NCP	74.695
VGGELLY;EVAGLK	NCP	42.306
EVAGLK;SLDTTSF	NCP	1.178
SLDTTSF;KPLFQP	NCP	46.483
KPLFQP;NTALP	NCP	77.385
NTALP;VTVFDRVP	NCP	58.65
VTVFDRVP;LSPGAL	NCP	139.67
LSPGAL;LAPAPLD	NCP	9.324
LAPAPLD;VGPLKL	NCP	35.164
VGPLKL;KHHYGP	NCP	31.721
KHHYGP;KPAGLQ	NCP	53.643
KPAGLQ;QMGDLLRS	NCP	73.198
QMGDLLRS;PLVPP	NCP	30.032
PLVPP;YTLPPK	NCP	143.446
YTLPPK;GKVLP	NCP	133.457
GKVLP;FVHWYVGEGME	NCP	52.137
FVHWYVGEGME;VGEGMEEGEF	NCP	0.015
VGEGMEEGEF;ATVALAF	NCP	62.785
ATVALAF;YNPAP	NCP	208.763

YNPAP;AALTV	NCP	411.892
AALTV;DLALSR	NCP	90.149
DLALSR;KLLKTKP	NCP	54.798
KLLKTKP;LDLDAGR	NCP	62.278
LDLDAGR;FGGAGVGK	NCP	23.997
FGGAGVGK;LTVTSF	NCP	103.098
LTVTSF;KEYWEPK	NCP	102.816
KEYWEPK;LVVMLPKPR	NCP	9.263
LVVMLPKPR;MAKAAVQ	NCP	99.922
MAKAAVQ;TGLQNL	NCP	23.441
TGLQNL;ESNEYG	NCP	159.043
ESNEYG;RVAVVDK	NCP	49.424
RVAVVDK;FYYVLV	NCP	0.202
FYYVLV;KPSVK	NCP	69.387
KPSVK;LEVPN	NCP	128.439
LEVPN;MKTELL	NCP	1.866
MKTELL;HSSLL	NCP	52.199
HSSLL;PLYPQ	NCP	9.949
PLYPQ;VPDLVP	NCP	219.218
VPDLVP;LMKLLTE	NCP	16.512
LMKLLTE;YRALP	NCP	91.947
YRALP;PGVMP	NCP	65.953
PGVMP;LTKPEDK	NCP	2.099
LTKPEDK;QKQYS	NCP	26.373
QKQYS;LCKAGF	NCP	60.16
LCKAGF;FGGGL	NCP	43.449
FGGGL;YPVKPPGP	NCP	14.67
YPVKPPGP;TVTPL	NCP	21.585
TVTPL;LDLAAAR	NCP	43.211
LDLAAAR;LTHGP	NCP	238.665
LTHGP;LELTKAV	NCP	285.681
LELTKAV;EPASLAHAK	NCP	13.257
EPASLAHAK;PELGF	NCP	141.772
PELGF;PALVVL	NCP	9.45
PALVVL;VHQVLY	NCP	196.812
VHQVLY;LFLQQ	NCP	70.005
LFLQQ;VAVGY	NCP	27.621
VAVGY;HVGYDPK	NCP	40.166
HVGYDPK;EHEAVHSK	NCP	94.69
EHEAVHSK;RMNVK	NCP	44.6
RMNVK;GVSHLVT	NCP	159.588
GVSHLVT;SVFYLEY	NCP	523.195
SVFYLEY;FGLQYQDTKGF	NCP	109.781
FGLQYQDTKGF;ESPLLFKFRAK	NCP	1.425
ESPLLFKFRAK;YGDFNKEVHKSGY	NCP	0.183
YGDFNKEVHKSGY;ALELEQERK	NCP	8.508
ALELEQERK;EALLQASQDQKK	NCP	0.087
EALLQASQDQKK;ESEAVEWQQK	NCP	1.296
ESEAVEWQQK;VQEDLEKTRAELK	NCP	0.384
VQEDLEKTRAELK;STPHVAEPAENEQD	NCP	0.048
STPHVAEPAENEQD;ALTSELANARDESKK	NCP	0.762

ALTSELANARDESKK;LEHVL	NCP	39.799
LEHVL;AEVLEL	NCP	305.339
AEVLEL;PAVFE	NCP	128.929
PAVFE;SLLTLF	NCP	6.65
SLLTLF;CDYVTKH	NCP	108.493
CDYVTKH;VSLPEC	NCP	55.541
VSLPEC;HGRGP	NCP	133.891
HGRGP;EDLFLSV	NCP	314.921
EDLFLSV;KGEHYP	NCP	16.701
KGEHYP;ACDDNPK	NCP	113.596
ACDDNPK;EKAAETPQ	NCP	117.312
EKAAETPQ;GVDVFP	NCP	19.602
GVDVFP;QSSLPK	NCP	16.683
QSSLPK;KLAEVK	NCP	100.58
KLAEVK;QNMQPK	NCP	28.107
QNMQPK;VTPSVLK	NCP	27.039
VTPSVLK;KKLASHS	NCP	33.504
KKLASHS;WNLEGPK	NCP	50.127
WNLEGPK;REGELK	NCP	106.177
REGELK;ERGVLEL	NCP	28.77
ERGVLEL;DDDCCQ	NCP	43.893
DDDCCQ;GPKLLFP	NCP	78.792
GPKLLFP;KADRMQK	NCP	60.369
KADRMQK;FAPLTSR	NCP	355.05
FAPLTSR;MEMHLR	NCP	87.561
MEMHLR;TALSHR	NCP	32.441
TALSHR;LSVKKK	NCP	13.731
LSVKKK;DKPDL	NCP	23.497
DKPDL;KSNVVVK	NCP	16.355
KSNVVVK;QPKHAK	NCP	32.505
QPKHAK;WTLLK	NCP	162.358
WTLLK;EGGLLK	NCP	96.287
EGGLLK;KNNWPP	NCP	33.429
KNNWPP;PAKHSPK	NCP	67.425
PAKHSPK;VGPMLLR	NCP	180.483
VGPMLLR;RGKFVL	NCP	47.08
RGKFVL;PAKHLLH	NCP	27.251
PAKHLLH;CCVKLCK	NCP	238.288
CCVKLCK;LSTDYK	NCP	279.549
LSTDYK;FGGQPY	NCP	87.686
FGGQPY;AAACRSVR	NCP	157.299
AAACRSVR;LATAERLVG	NCP	0.414
LATAERLVG;TPLLTTLPDK	NCP	1.563
TPLLTTLPDK;EPPRELP	NCP	29.673
EPPRELP;YAVPK	NCP	29.982
YAVPK;LSSELL	NCP	140.502
LSSELL;LEQLPK	NCP	253.987
LEQLPK;DDMPF	NCP	44.307
DDMPF;QRFTKK	NCP	297.576
QRFTKK;YLLKRS	NCP	51.579
YLLKRS;MTLLPK	NCP	103.352

MTLLPK;KTQQYP	NCP	263.235
KTQQYP;QKARE	NCP	69.295
QKARE;DGPDVREE	NCP	14.693
DGPDVREE;VTAALKAK	NCP	74.937
VTAALKAK;GVAVMH	NCP	109.746
GVAVMH;MCCPPDP	NCP	541.102
MCCPPDP;VLPKLYV	NCP	128.765
VLPKLYV;APLLR	NCP	19.732
APLLR;TEEDVKFY	NCP	112.695
TEEDVKFY;LPPKHL	NCP	120.902
LPPKHL;VGPMSL	NCP	27.729
VGPMSL;SPVLG	NCP	20.227
SPVLG;YEYTQ	NCP	68.88
YEYTQ;MNCVLAK	NCP	169.706
MNCVLAK;LDAPGHK	NCP	33.876
LDAPGHK;VLKLVPEKD	NCP	1.002
VLKLVPEKD;AKVAVLQ	NCP	276.34
AKVAVLQ;EFKFRNP	NCP	63.222
EFKFRNP;MVPPMAK	NCP	318.425
MVPPMAK;ALLEVPP	NCP	64.537
ALLEVPP;QLEVKL	NCP	347.166
QLEVKL;LDRPLQ	NCP	52.526
LDRPLQ;QAGFKGHT	NCP	39.799
QAGFKGHT;LGNVVPL	NCP	27.984
LGNVVPL;KLDQL	NCP	71.913
KLDQL;HSLEK	NCP	67.07
HSLEK;TVESAPN	NCP	22.647
TVESAPN;EVGALLT	NCP	88.291
EVGALLT;YKWPK	NCP	138.47
YKWPK;KGKMPPK	NCP	21.087
KGKMPPK;STDQAL	NCP	7.54
STDQAL;LPGVPDP	NCP	90.764
LPGVPDP;LLPLFL	NCP	134.128
LLPLFL;MAPSP	NCP	48.738
MAPSP;KNLAGQEK	NCP	39.798
KNLAGQEK;APLPPPPP	NCP	20.846
APLPPPPP;HWLFPLE	NCP	17.265
HWLFPLE;DLFCC	NCP	44.142
DLFCC;LPGDLY	NCP	83.77
LPGDLY;GPLPAAPP	NCP	107.501
GPLPAAPP;KRFFPLG	NCP	8.703
KRFFPLG;KATNSVPQ	NCP	215.044
KATNSVPQ;TVLRMDP	NCP	155.342
TVLRMDP;SHAQTVVL	NCP	16.497
SHAQTVVL;ETTFG	NCP	39.802
ETTFG;FTSLS	NCP	190.811
FTSLS;KEMWSP	NCP	176.779
KEMWSP;TKVGGLP	NCP	268.94
TKVGGLP;HLLPFEL	NCP	144.874
HLLPFEL;FLGNST	NCP	31.184
FLGNST;LGVGPK	NCP	24.99

LGVGPK;LSELAKLL	NCP	31.426
LSELAKLL;LSGAVVPH	NCP	6.513
LSGAVVPH;VGSGPPKGTGLHRYVW	NCP	23.051
VGSGPPKGTGLHRYVW;LDYAAAL	NCP	122.932
LDYAAAL;FADLLTLT	NCP	56.466
FADLLTLT;LNVEFL	NCP	5.927
LNVEFL;PQETSKYR	NCP	24.57
PQETSKYR;LPPHLR	NCP	60.705
LPPHLR;VGGPLVP	NCP	1.269
VGGPLVP;KYVCGP	NCP	293.628
KYVCGP;LAAMGFM	NCP	188.938
LAAMGFM;KLVLRP	NCP	385.181
KLVLRP;KAWVP	NCP	137.463
KAWVP;DDDLCCN	NCP	33.192
DDDLCCN;LPTVNQAV	NCP	19.936
LPTVNQAV;EEPCLLK	NCP	10.046
EEPCLLK;SLLVK	NCP	129.888
SLLVK;LPPPPLLPPPPP	NCP	113.329
LPPPPLLPPPPP;VKPLAL	NCP	31.697
VKPLAL;LQLFE	NCP	38.38
LQLFE;EDKLPY	NCP	103.331
EDKLPY;ATVVL	NCP	124.893
ATVVL;VGPVGP	NCP	48.123
VGPVGP;VAPVFAK	NCP	14.134
VAPVFAK;KPQMYH	NCP	214.577
KPQMYH;HLKVPPP	NCP	160.495
HLKVPPP;FNPFEL	NCP	211.533
FNPFEL;KMFPPPP	NCP	72.203
KMFPPPP;KSLEKF	NCP	431.134
KSLEKF;FNPVPL	NCP	47.595
FNPVPL;ELSKVEL	NCP	203.171
ELSKVEL;LVLEVPF	NCP	15.039
LVLEVPF;TKTVKK	NCP	48.255
TKTVKK;VSGGDLLL	NCP	8.775
VSGGDLLL;GVPKEQK	NCP	60.999
GVPKEQK;YMNEYL	NCP	501.849
YMNEYL;AEAVESPGDPGTTTPRP	NCP	60.867
AEAVESPGDPGTTTPRP;AEEGF	NCP	125.599
AEEGF;MPMDHK	NCP	2.799
MPMDHK;LPPMPPPPPP	NCP	19.13
LPPMPPPPPP;AALDSSLLF	NCP	21.555
AALDSSLLF;EDPVKK	NCP	45.694
EDPVKK;MFLTR	NCP	33.462
MFLTR;RPPPPPG	NCP	13.608
RPPPPPG;KTYNRP	NCP	7.638
KTYNRP;FNRLPL	NCP	120.066
FNRLPL;VELFK	NCP	25.013
VELFK;PTGVP	NCP	27.465
PTGVP;SLLVTH	NCP	36.418
SLLVTH;LSPSLPR	NCP	43.343
LSPSLPR;VSMLWAP	NCP	24.909

VSMLWAP;KPFEVM	NCP	62.746
KPFEVM;KGYEVLYL	NCP	296.484
KGYEVLYL;MALKLCPP	NCP	7.499
MALKLCPP;SSNPVY	NCP	574.033
SSNPVY;KTHGHVP	NCP	52.416
KTHGHVP;LAHSKK	NCP	31.986
LAHSKK;LAFGGF	NCP	20.823
LAFGGF;LQELYL	NCP	175.365
LQELYL;KTPAALPP	NCP	11.445
KTPAALPP;KTFHAK	NCP	34.713
KTFHAK;KFCVTAF	NCP	87.117
KFCVTAF;LPGVG	NCP	74.748
LPGVG;PVLPLQ	NCP	65.133
PVLPLQ;LELFPPPPP	NCP	71.379
LELFPPPPP;KNHGPQ	NCP	104.537
KNHGPQ;GLKPGSPK	NCP	95.098
GLKPGSPK;APVGHPK	NCP	9.792
APVGHPK;SPVLP	NCP	92.859
SPVLP;HTPEAPAP	NCP	6.251
HTPEAPAP;VTEVL	NCP	71.1
VTEVL;RSPGTGAGLAEKSDR	NCP	5.058
RSPGTGAGLAEKSDR;EAPLNPK	NCP	271.492
EAPLNPK;KSLVVPP	NCP	19.302
KSLVVPP;NVLVVVL	NCP	1.391
NVLVVVL;LTLFVVPC	NCP	24.171
LTLFVVPC;KERAP	NCP	58.381
KERAP;AKPLGA	NCP	20.223
AKPLGA;CCTPHDP	NCP	79.608
CCTPHDP;GKVLPF	NCP	26.228
GKVLPF;LALPCLK	NCP	64.234
LALPCLK;FDVKSK	NCP	49.101
FDVKSK;PAFSF	NCP	89.265
PAFSF;CENDN	NCP	13.295
CENDN;KPLHPK	NCP	129.802
KPLHPK;KVNAP	NCP	40.427
KVNAP;PPVTPTML	NCP	331.803
PPVTPTML;KFTVEHF	NCP	10.752
KFTVEHF;ESPEVVL	NCP	306.298
ESPEVVL;RPLSNR	NCP	66.165
RPLSNR;KMPCHG	NCP	61.086
KMPCHG;LVSSRVL	NCP	64.118
LVSSRVL;FGELAL	NCP	144.631
FGELAL;VPSGL	NCP	75.75
VPSGL;LSLEEAVP	NCP	89.519
LSLEEAVP;SGHVL	NCP	223.636
SGHVL;VSALPV	NCP	72.284
VSALPV;LTKMP	NCP	261.744
LTKMP;APNLP	NCP	185.421
APNLP;LKLWN	NCP	72.307
LKLWN;PVYLHGK	NCP	376.502
PVYLHGK;EVGFF	NCP	89.988

EVGFF;DHKLSLGK	NCP	110.089
DHKLSLGK;KRWGGGKK	NCP	278.622
KRWGGGKK;KDHGLQ	NCP	66.956
KDHGLQ;VEVVLL	NCP	3.966
VEVVLL;FPLDFFC	NCP	256.594
FPLDFFC;EVGVLVT	NCP	91.412
EVGVLVT;SKPLF	NCP	10.153
SKPLF;LSYSVP	NCP	164.391
LSYSVP;LSVSDRP	NCP	277.734
LSVSDRP;KQFAEKF	NCP	1.763
KQFAEKF;NMGCC	NCP	62.044
NMGCC;KGLKLF	NCP	14.553
KGLKLF;LSMVHKY	NCP	83.685
LSMVHKY;LQVEP	NCP	172.683
LQVEP;SGNGLK	NCP	166.848
SGNGLK;ACARPL	NCP	178.307
ACARPL;CLDSGAKSVVLMSH	NCP	4.542
CLDSGAKSVVLMSH;SLQPVAVELK	NCP	0.081
SLQPVAVELK;ENLRFHVEEEGKGKDA	NCP	0.24
ENLRFHVEEEGKGKDA;KAGGFLMKK	NCP	4.131
KAGGFLMKK;KELNYFAK	NCP	0.024
KELNYFAK;AKALESPERPF	NCP	0.018
AKALESPERPF;YAEAVARAK	NCP	7.638
YAEAVARAK;KVLPGVDALSSV	NCP	0.897
KVLPGVDALSSV;LRGVL	NCP	42.444
LRGVL;GQQNSVKQ	NCP	15.259
GQQNSVKQ;QTVPSPKV	NCP	35.378
QTVPSPKV;LVLTM	NCP	226.77
LVLTM;SPGGPHWEK	NCP	103.726
SPGGPHWEK;ETEEVKK	NCP	32.756
ETEEVKK;MHSYEGL	NCP	325.201
MHSYEGL;TKMWYP	NCP	54.422
TKMWYP;RWKWP	NCP	32.757
RWKWP;AAPDCAPK	NCP	81.424
AAPDCAPK;TGPLVEDFLSDFQ	NCP	406.968
TGPLVEDFLSDFQ;LDPLP	NCP	160.784
LDPLP;RVLLEY	NCP	21.778
RVLLEY;LTHAGALY	NCP	106.398
LTHAGALY;KLFVK	NCP	11.67
KLFVK;KPGMYGK	NCP	58.71
KPGMYGK;VGYPKH	NCP	6.507
VGYPKH;SLGLLL	NCP	11.361
SLGLLL;CTPGF	NCP	141.003
CTPGF;EMNLRK	NCP	240.345
EMNLRK;VTLSL	NCP	178.167
VTLSL;TEGAM	NCP	20.94
TEGAM;DGALNVDL	NCP	83.261
DGALNVDL;SSPNVY	NCP	71.968
SSPNVY;AEAVRRPP	NCP	148.017
AEAVRRPP;CLTLTLEV	NCP	187.836
CLTLTLEV;QPVPPPK	NCP	45.087

QPVPPPK;FLGWYT	NCP	77.108
FLGWYT;GPVTVP	NCP	303.208
GPVTVP;WHTKKT	NCP	11.604
WHTKKT;GKFSVF	NCP	23.388
GKFSVF;LLMKL	NCP	90.384
LLMKL;EKTYG	NCP	84.596
EKTYG;APAVPP	NCP	62.766
APAVPP;FLDPKKQP	NCP	37.054
FLDPKKQP;QTYNRP	NCP	429.119
QTYNRP;YVDKY	NCP	28.276
YVDKY;SPVFL	NCP	74.157
SPVFL;VLPLAF	NCP	184.026
VLPLAF;LLAVP	NCP	105.383
LLAVP;VPVQP	NCP	85.276
VPVQP;LVPLAF	NCP	47.795
LVPLAF;QTVMLK	NCP	19.488
QTVMLK;KKLLPR	NCP	84.631
KKLLPR;EAFVKHL	NCP	40.866
EAFVKHL;TFGPR	NCP	3.713
TFGPR;KATYMPK	NCP	2.254
KATYMPK;VGLKHV	NCP	75.33
VGLKHV;SLYTFP	NCP	28.368
SLYTFP;LKLYKK	NCP	46.188
LKLYKK;LVTLLM	NCP	315.939
LVTLLM;NGFCF	NCP	19.568
NGFCF;KWRHPP	NCP	56.364
KWRHPP;RSLEMK	NCP	121.363
RSLEMK;LAEVVL	NCP	91.785
LAEVVL;VPPKEHK	NCP	43.929
VPPKEHK;HTLSY	NCP	159.711
HTLSY;GEKLPR	NCP	51.453
GEKLPR;AALSLHP	NCP	164.937
AALSLHP;SLPFE	NCP	47.592
SLPFE;LDSRLK	NCP	6.968
LDSRLK;TLKMPR	NCP	35.287
TLKMPR;NDCCQ	NCP	123.38
NDCCQ;ASLPQ	NCP	85.396
ASLPQ;PNGKVTH	NCP	9.3
PNGKVTH;PKDLPTP	NCP	37.22
PKDLPTP;KAYNWKF	NCP	90.432
KAYNWKF;TNPLYPQ	NCP	150.298
TNPLYPQ;QRVFC	NCP	66.392
QRVFC;VTLPREL	NCP	68.134
VTLPREL;TAVLL	NCP	85.061
TAVLL;FTVPF	NCP	120.588
FTVPF;ADETETPAETTHNLK	NCP	292.572
ADETETPAETTHNLK;WVYPR	NCP	76.884
WVYPR;KGGKAKL	NCP	130.879
KGGKAKL;TSTAK	NCP	26.666
TSTAK;LVHLPY	NCP	89.395
LVHLPY;ASKLLR	NCP	101.631

ASKLLR;PPTPEP	NCP	11.102
PPTPEP;MVGHK	NCP	57.945
MVGHK;KEESSKLK	NCP	582.928
KEESSKLK;TYSGF	NCP	293.348
TYSGF;FDLGKEK	NCP	19.08
FDLGKEK;KRTHHAT	NCP	211.915
KRTHHAT;KVLLMSSK	NCP	135.762
KVLLMSSK;YMDVR	NCP	1.962
YMDVR;VPGVEAVL	NCP	20.841
VPGVEAVL;TMMLR	NCP	50.826
TMMLR;PPVKAH	NCP	73.131
PPVKAH;KLSPRK	NCP	45.3
KLSPRK;DEPEKR	NCP	9.464
DEPEKR;LEFVN	NCP	12.535
LEFVN;HTLSALK	NCP	225.281
HTLSALK;KDDTVKK	NCP	108.708
KDDTVKK;LPVTP	NCP	102.487
LPVTP;RVLKDF	NCP	23.868
RVLKDF;SNDDC	NCP	35.051
SNDDC;PVGAP	NCP	112.518
PVGAP;VGPAGNPR	NCP	39.999
VGPAGNPR;YNPERLK	NCP	255.87
YNPERLK;EKYVFR	NCP	64.792
EKYVFR;ELPVKPP	NCP	510.432
ELPVKPP;KASDYGY	NCP	6.338
KASDYGY;YQRVTGK	NCP	50.295
YQRVTGK;LNFAK	NCP	430.266
LNFAK;NGSVDM	NCP	94.732
NGSVDM;SGRPLP	NCP	106.346
SGRPLP;TVENLF	NCP	107.353
TVENLF;KEYMVP	NCP	149.157
KEYMVP;LKYVP	NCP	173.724
LKYVP;DTLDPSLL	NCP	243.495
DTLDPSLL;RVVPPLLY	NCP	24.717
RVVPPLLY;LGETPL	NCP	183.708
LGETPL;QPYSGPK	NCP	124.088
QPYSGPK;SLFPPY	NCP	636.759
SLFPPY;LALHVLH	NCP	234.477
LALHVLH;YPQLP	NCP	93.267
YPQLP;EVPKLPKL	NCP	0.516
EVPKLPKL;SSQPK	NCP	40.15
SSQPK;PHWYHAF	NCP	11.151
PHWYHAF;YYECCC	NCP	96.446
YYECCC;EHMPHP	NCP	1.251
EHMPHP;QSGGMPK	NCP	58.053
QSGGMPK;VKLWHHT	NCP	39.699
VKLWHHT;LYSGER	NCP	86.335
LYSGER;TRSAN	NCP	129.053
TRSAN;RAVVVQY	NCP	15.325
RAVVVQY;MDALKRV	NCP	12.23
MDALKRV;APRKPFS	NCP	271.578

APRKPFS;LVYEL	NCP	14.098
LVYEL;LGLPLNVN	NCP	52.415
LGLPLNVN;KPYQP	NCP	88.05
KPYQP;KGGGLGN	NCP	302.427
KGGGLGN;NFNKL	NCP	8.151
NFNKL;VSGPLL	NCP	75.493
VSGPLL;LVPDGL	NCP	19.092
LVPDGL;PKWAKPP	NCP	197.186
PKWAKPP;REATLLH	NCP	154.96
REATLLH;YKPPVLAK	NCP	68.336
YKPPVLAK;HGQYRNR	NCP	117.444
HGQYRNR;LPQPDL	NCP	220.311
LPQPDL;TLDALP	NCP	9.223
TLDALP;KSTPV	NCP	27.902
KSTPV;KHKGLYR	NCP	5.563
KHKGLYR;PPVLTASPP	NCP	174.974
PPVLTASPP;CMVQKAK	NCP	23.125
CMVQKAK;KTPFPK	NCP	21.155
KTPFPK;VKLPAL	NCP	29.622
VKLPAL;SGSFKL	NCP	86.656
SGSFKL;KNKPLAR	NCP	64.602
KNKPLAR;KPFTK	NCP	40.524
KPFTK;GLGFPYR	NCP	92.136
GLGFPYR;QPKLPAP	NCP	21.77
QPKLPAP;FLKLLQL	NCP	66.709
FLKLLQL;PAGKGLTK	NCP	84.899
PAGKGLTK;TKGLELW	NCP	39.015
TKGLELW;FCLGK	NCP	140.526
FCLGK;LLVLF	NCP	70.779
LLVLF;VLEFY	NCP	278.289
VLEFY;LEGDLKAPK	NCP	30.13
LEGDLKAPK;VTLPN	NCP	157.997
VTLPN;KVVPLLR	NCP	270.952
KVVPLLR;KVPGK	NCP	43.118
KVPGK;LAVMGS	NCP	36.598
LAVMGS;LSPVMLTP	NCP	60.315
LSPVMLTP;KTTTPK	NCP	71.592
KTTTPK;EDCCC	NCP	39.764
EDCCC;TDPFKH	NCP	141.718
TDPFKH;LRPVVL	NCP	57.647
LRPVVL;LAPTP	NCP	113.424
LAPTP;TTATSL	NCP	23.988
TTATSL;VPLSL	NCP	46.245
VPLSL;DLEEFM	NCP	33.262
DLEEFM;PPPPYK	NCP	38.361
PPPPYK;LSLLNNL	NCP	40.722
LSLLNNL;CNPAPK	NCP	14.369
CNPAPK;KGYVRP	NCP	110.142
KGYVRP;WLNFVLL	NCP	69.657
WLNFVLL;LPAMVDL	NCP	28.774
LPAMVDL;PNLLLG	NCP	0.419

PNLLLG;QANLGGLP	NCP	31.683
QANLGGLP;ANLAL	NCP	104.8
ANLAL;YPPGL	NCP	102.386
YPPGL;TLELRVAY	NCP	179.619
TLELRVAY;YLVGL	NCP	279.249
YLVGL;VACPP	NCP	60.804
VACPP;AYLLF	NCP	25.348
AYLLF;YTTVP	NCP	69.624
YTTVP;FPVVLL	NCP	72.863
FPVVLL;WWYGPPK	NCP	33.36
WWYGPPK;TLASPEQLEKF	NCP	8.628
TLASPEQLEKF;YPPLGR	NCP	83.059
YPPLGR;LLGHKVF	NCP	48.683
LLGHKVF;PPLPA	NCP	72.289
PPLPA;QALEELR	NCP	46.346
QALEELR;APKRPASP	NCP	8.763
APKRPASP;KTGKSGK	NCP	13.125
KTGKSGK;KGATMPK	NCP	123.204
KGATMPK;EVYLLHW	NCP	134.074
EVYLLHW;YCCEPP	NCP	24.81
YCCEPP;RMTTF	NCP	115.175
RMTTF;DPGKYA	NCP	74.262
DPGKYA;FPPAR	NCP	26.562
FPPAR;ADDSYF	NCP	64.677
ADDSYF;KTLLGAAP	NCP	35.325
KTLLGAAP;YPAPV	NCP	29.361
YPAPV;FKPPLH	NCP	11.34
FKPPLH;VSWLLMLE	NCP	137.995
VSWLLMLE;GVFKLLLQ	NCP	21.023
GVFKLLLQ;KGAGPRPR	NCP	31.717
KGAGPRPR;VRTTLNK	NCP	148.973
VRTTLNK;LSLPF	NCP	66.082
LSLPF;LRLTQY	NCP	81.023
LRLTQY;KMHALLP	NCP	134.416
KMHALLP;PPPFPV	NCP	45.093
PPPFPV;GWGLGNL	NCP	33.417
GWGLGNL;PCGPGVPR	NCP	115.455
PCGPGVPR;AAPTPP	NCP	97.415
AAPTPP;KHVGFGLQ	NCP	18.646
KHVGFGLQ;AESGLLEL	NCP	58.241
AESGLLEL;GLTSVTK	NCP	67.704
GLTSVTK;VPETL	NCP	84.964
VPETL;RSKLTPPP	NCP	14.946
RSKLTPPP;KGYEPF	NCP	55.056
KGYEPF;TAATPVPP	NCP	88.107
TAATPVPP;KPQEV	NCP	122.187
KPQEV;EKAVP	NCP	80.733
EKAVP;VEVSPGV	NCP	144.26
VEVSPGV;GKLEY	NCP	85.077
GKLEY;HVQPGGPR	NCP	21.394
HVQPGGPR;AALEYLT	NCP	101.343

AALEYLT;VDLDLK	NCP	21.555
VDLDLK;KKCLTH	NCP	81.981
KKCLTH;KLFKPK	NCP	72.915
KLFKPK;TLTVSRG	NCP	8.315
TLTVSRG;AALVKVGK	NCP	14.026
AALVKVGK;LTMKPLL	NCP	16.187
LTMKPLL;LSGKLN	NCP	441.228
LSGKLN;STKLR	NCP	216.1
STKLR;PVSVHSVSY	NCP	183.938
PVSVHSVSY;KVCRLL	NCP	18.984
KVCRLL;TELRQR	NCP	51.043
TELRQR;EVLGL	NCP	209.999
EVLGL;LTSNALT	NCP	131.254
LTSNALT;KHFTCNP	NCP	212.705
KHFTCNP;TLSGF	NCP	22.243
TLSGF;KLTPSC	NCP	71.499
KLTPSC;KFAPPPP	NCP	23.283
KFAPPPP;LNELHSL	NCP	147.414
LNELHSL;MLNPFP	NCP	151.725
MLNPFP;KQKELQF	NCP	561.213
KQKELQF;TNSCFKR	NCP	44.691
TNSCFKR;TEKPPGR	NCP	15.161
TEKPPGR;MESDAHGGGASR	NCP	102.534
MESDAHGGGASR;KGLEDR	NCP	123.307
KGLEDR;EKPTPGK	NCP	1.503
EKPTPGK;AAPVSK	NCP	65.226
AAPVSK;KPGYLR	NCP	21.474
KPGYLR;MDKHTR	NCP	103.556
MDKHTR;LDHNHDL	NCP	39.39
LDHNHDL;LHGGLS	NCP	230.851
LHGGLS;VRVEC	NCP	48.875
VRVEC;NFFFL	NCP	35.064
NFFFL;VPGKV	NCP	41.605
VPGKV;DLEFPPPPP	NCP	50.687
DLEFPPPPP;LEFFCPL	NCP	80.755
LEFFCPL;LPEVP	NCP	68.357
LPEVP;VLTVFPK	NCP	69.909
VLTVFPK;DSLGFL	NCP	15.975
DSLGFL;FLKLYKKK	NCP	111.096
FLKLYKKK;DRFGRDL	NCP	106.626
DRFGRDL;KPLTARPP	NCP	177.69
KPLTARPP;KFVLLYPQ	NCP	20.026
KFVLLYPQ;VSYSLGPV	NCP	49.518
VSYSLGPV;VSLRKN	NCP	50.81
VSLRKN;CCENT	NCP	113.683
CCENT;APGGLPFK	NCP	15.393
APGGLPFK;SVALVGVP	NCP	33.005
SVALVGVP;LCYVA	NCP	66.498
LCYVA;NSELLMF	NCP	36.982
NSELLMF;CLVELYL	NCP	144.531
CLVELYL;KSLDPK	NCP	4.826

KSLDPK;ASYTY	NCP	1.438
ASYTY;VPKHS	NCP	9.773
VPKHS;FFTKLEP	NCP	6.636
FFTKLEP;DASVP	NCP	430.536
DASVP;KEVPR	NCP	9.802
KEVPR;KMPHPK	NCP	221.708
KMPHPK;FMKPPPP	NCP	146.523
FMKPPPP;KFMVLGVF	NCP	116.662
KFMVLGVF;KAATVAF	NCP	10.956
KAATVAF;MVPGLPR	NCP	85.118
MVPGLPR;HHGLKFL	NCP	83.638
HHGLKFL;HNPVAGP	NCP	1.233
HNPVAGP;VSTLETK	NCP	160.154
VSTLETK;EHEGGGKK	NCP	24.622
EHEGGGKK;EEVSLF	NCP	46.19
EEVSLF;LLLPGELAK	NCP	106.714
LLLPGELAK;PLQPPPPR	NCP	136.971
PLQPPPPR;ELDPVVK	NCP	105.975
ELDPVVK;CDDDDP	NCP	21.822
CDDDDP;VPGGF	NCP	85.673
VPGGF;TPKPLG	NCP	331.821
TPKPLG;KQCLVH	NCP	99.958
KQCLVH;EHSFLSD	NCP	55.665
EHSFLSD;KDWLTY	NCP	66.231
KDWLTY;RSPLLTL	NCP	259.556
RSPLLTL;RVRGSESK	NCP	329.191
RVRGSESK;VYDPKY	NCP	75.255
VYDPKY;FSLLQ	NCP	62.256
FSLLQ;GMHAK	NCP	21.668
GMHAK;SHSVLSGT	NCP	28.111
SHSVLSGT;WSLHY	NCP	94.104
WSLHY;LHVLF	NCP	46.173
LHVLF;MSLRLQ	NCP	13.368
MSLRLQ;EVSSG	NCP	103.44
EVSSG;EVNNG	NCP	149.103
EVNNG;LSTKSYC	NCP	170.312
LSTKSYC;LKLVLP	NCP	12.174
LKLVLP;MFQLT	NCP	72.565
MFQLT;NPLTLR	NCP	117.989
NPLTLR;KVQNF	NCP	40.999
KVQNF;QFLEYAK	NCP	57.858
QFLEYAK;MAKCPET	NCP	50.249
MAKCPET;DMLLLVVL	NCP	3.001
DMLLLVVL;APKYG	NCP	1.794
APKYG;ELVKLQ	NCP	92.223
ELVKLQ;ELLDGAFF	NCP	6.713
ELLDGAFF;SLPGEPPP	NCP	353.89
SLPGEPPP;RFTLSW	NCP	50.193
RFTLSW;DAQNP	NCP	62.481
DAQNP;KNSGTLK	NCP	151.031
KNSGTLK;KSAGAGP	NCP	78.217

KSAGAGP;LLELTAN	NCP	40.536
LLELTAN;MACFALP	NCP	8.571
MACFALP;VPVVRPSR	NCP	73.206
VPVVRPSR;LEVAV	NCP	14.487
LEVAV;GKLLNH	NCP	85.872
GKLLNH;RKRSAKL	NCP	29.663
RKRSAKL;FLTPAEAR	NCP	51.637
FLTPAEAR;WYLEAPH	NCP	15.276
WYLEAPH;VGHSLVTL	NCP	89.567
VGHSLVTL;LLSSCCC	NCP	194.437
LLSSCCC;AAPLKGR	NCP	25.407
AAPLKGR;KHLLPMKT	NCP	157.464
KHLLPMKT;KNKVK	NCP	30.452
KNKVK;HVLLLF	NCP	25.59
HVLLLF;ALALA	NCP	46.698
ALALA;NSGMAL	NCP	22.72
NSGMAL;TSPVKTP	NCP	67.416
TSPVKTP;LMLPF	NCP	255.836
LMLPF;GKVAY	NCP	97.089
GKVAY;NSFVSL	NCP	95.532
NSFVSL;PLKMNL	NCP	7.71
PLKMNL;VKLSGVS	NCP	343.062
VKLSGVS;CYCECCC	NCP	23.826
CYCECCC;TNKNLLTK	NCP	309.672
TNKNLLTK;ATTFHVLN	NCP	88.788
ATTFHVLN;KDPLYN	NCP	138.829
KDPLYN;QLEVLF	NCP	54.956
QLEVLF;HSLEPK	NCP	0.369
HSLEPK;TPFAHAL	NCP	201.859
TPFAHAL;SGLLDTF	NCP	58.902
SGLLDTF;FSPLL	NCP	111.207
FSPLL;TLGEKMKE	NCP	86.136
TLGEKMKE;VVTYY	NCP	109.887
VVTYY;VGAGSL	NCP	87.6
VGAGSL;LGPPKPY	NCP	108.255
LGPPKPY;EFLRR	NCP	283.805
EFLRR;KDHLSFDK	NCP	41.242
KDHLSFDK;KTHLK	NCP	146.553
KTHLK;KSFSLV	NCP	207.872
KSFSLV;KKFLSN	NCP	249.094
KKFLSN;QQPPP	NCP	167.078
QQPPP;EDCDF	NCP	140.568
EDCDF;CGCSVAKK	NCP	40.345
CGCSVAKK;KCLSSAH	NCP	3.507
KCLSSAH;ARGKLAL	NCP	35.445
ARGKLAL;MADPLNK	NCP	59.738
MADPLNK;ADGDC	NCP	44.764
ADGDC;TLKPF	NCP	34.701
TLKPF;EDDPQ	NCP	54.261
EDDPQ;PLMPVV	NCP	4.014
PLMPVV;NPGPAP	NCP	5.123

NPGPAP;FKPER	NCP	27.834
FKPER;NVFLETPY	NCP	47.02
NVFLETPY;TKVEC	NCP	4.512
TKVEC;TEVRL	NCP	22.32
TEVRL;DHALVLY	NCP	112.155
DHALVLY;KGYNGPP	NCP	0.834
KGYNGPP;EGWDLRK	NCP	228.624
EGWDLRK;GSHLLQ	NCP	5.873
GSHLLQ;KGGAVK	NCP	8.25
KGGAVK;KATSKGK	NCP	53.389
KATSKGK;QPSSAKPP	NCP	218.856
QPSSAKPP;LLKTVETR	NCP	43.301
LLKTVETR;MVGLK	NCP	148.905
MVGLK;TVVLPEKF	NCP	32.044
TVVLPEKF;SLNVPPP	NCP	287.72
SLNVPPP;VEELDVP	NCP	35.638
VEELDVP;NSPFL	NCP	22.622
NSPFL;TAALTC	NCP	86.848
TAALTC;GVTSPF	NCP	96.308
GVTSPF;NLNVHSSK	NCP	44.677
NLNVHSSK;KLPVL	NCP	188.331
KLPVL;EFMSQAF	NCP	52.871
EFMSQAF;KPLSVA	NCP	10.188
KPLSVA;KTVMLK	NCP	4.185
KTVMLK;LHVTTSTR	NCP	192.184
LHVTTSTR;KVLFTGK	NCP	207.62
KVLFTGK;VLGFHG	NCP	9.448
VLGFHG;TVGTTGR	NCP	87.756
TVGTTGR;LTSASLP	NCP	207.939
LTSASLP;FGHAMKR	NCP	19.931
FGHAMKR;QEKNH	NCP	263.448
QEKNH;KVYLPP	NCP	31.987
KVYLPP;HCPLVL	NCP	222.803
HCPLVL;GSPPLPP	NCP	190.452
GSPPLPP;DAEMPATEK	NCP	31.188
DAEMPATEK;DSWDASKP	NCP	3.705
DSWDASKP;RAEFTVETR	NCP	0.825
RAEFTVETR;GVRVKETADF	NCP	1.353
GVRVKETADF;SAEFTVDAK	NCP	0.972
SAEFTVDAK;VYGPGVAK	NCP	1.23
VYGPGVAK;GPGVAKTGLK	NCP	0.006
GPGVAKTGLK;TGLKAHEPT	NCP	0.018
TGLKAHEPT;AHEPTYF	NCP	0.012
AHEPTYF;AAGKGKLDVQF	NCP	0.609
AAGKGKLDVQF;SVGVSPSLDLSK	NCP	0.432
SVGVSPSLDLSK;DQEFTVK	NCP	0.168
DQEFTVK;VPCKVEPGLGADN	NCP	0.084
VPCKVEPGLGADN;GVPVPGSPF	NCP	0.099
GVPVPGSPF;GPGLQGGSAGSPAR	NCP	0.291
GPGLQGGSAGSPAR;SVDARALTQTGGPHVK	NCP	0.702
SVDARALTQTGGPHVK;DGSPVPSSPF	NCP	0.258

DGSPVPSSPF;KPNKFTVETR	NCP	0.108
KPNKFTVETR;LAVEGPSEAK	NCP	0.129
LAVEGPSEAK;MSCMDNKDGSCSVEYVPY	NCP	0.03
MSCMDNKDGSCSVEYVPY;SCMDNKDGSCSVEYVP	NCP	0.003
SCMDNKDGSCSVEYVP;SVEYVPY	NCP	0.03
SVEYVPY;KVPVHDVTDASK	NCP	0.174
KVPVHDVTDASK;SFQVDTSK	NCP	0.093
SFQVDTSK;MAPLQVKVQGPK	NCP	0.03
MAPLQVKVQGPK;VDNADGTQTVNYVPSREGPY	NCP	0.435
VDNADGTQTVNYVPSREGPY;VLYGDEEVPR	NCP	0.069
VLYGDEEVPR;RVRAVPTGDASK	NCP	1.026
RVRAVPTGDASK;TAPQPGKY	NCP	2.613
TAPQPGKY;APERPLVG	NCP	0.465
APERPLVG;VGVNGLDVSSLRPF	NCP	0.018
VGVNGLDVSSLRPF;VDYVNCGHVTAY	NCP	0.441
VDYVNCGHVTAY;ETGEHLVHVKK	NCP	0.834
ETGEHLVHVKK;TGEHLVHVK	NCP	0.003
TGEHLVHVK;GEHLVHVKK	NCP	0.003
GEHLVHVKK;APSVANVGSHCDL	NCP	0.591
APSVANVGSHCDL;KGQHVPGSPF	NCP	0.321
KGQHVPGSPF;EDRKDGSCGVAY	NCP	0.339
EDRKDGSCGVAY;GDYEVSVK	NCP	0.216
GDYEVSVK;VVPVASPSGDAR	NCP	0.057
VVPVASPSGDAR;RLVSNHSLH	NCP	2.097
RLVSNHSLH;SLHETSSVFVDSLTK	NCP	0.018
SLHETSSVFVDSLTK;HSAPGPGPTDASK	NCP	0.063
HSAPGPGPTDASK;SAPGPGPTDASKVLAKGVGLSK	NCP	0.003
SAPGPGPTDASKVLAKGVGLSK;APGPGPTDASKVLAK	NCP	0.003
APGPGPTDASKVLAK;TDASKVLAKGVGLSKAY	NCP	0.018
TDASKVLAKGVGLSKAY;VLAKGVGLSK	NCP	0.015
VLAKGVGLSK;FTVDCSK	NCP	0.054
FTVDCSK;HVGSRLYSVS	NCP	0.417
HVGSRLYSVS;VGSRLYSV	NCP	0.003
VGSRLYSV;LLKDKGEY	NCP	0.03
LLKDKGEY;LKDKGEYTLVVK	NCP	0.003
LKDKGEYTLVVK;MRSLLD	NCP	45.985
MRSLLD;PAAAHLQ	NCP	30.53
PAAAHLQ;DPGWTPK	NCP	9.888
DPGWTPK;PVTRPP	NCP	30.363
PVTRPP;SEEQDLQGLKDKPLK	NCP	49.176
SEEQDLQGLKDKPLK;FEKVGDTSLDPNDF	NCP	1.464
FEKVGDTSLDPNDF;ASEGVQVKRVLEK	NCP	0.183
ASEGVQVKRVLEK;VVAAATAEAK	NCP	0.216
VVAAATAEAK;HGAPVP	NCP	7.443
HGAPVP;MAVWGKN	NCP	94.771
MAVWGKN;TVEALP	NCP	19.065
TVEALP;KEMAPEK	NCP	28.469
KEMAPEK;AAVKTL	NCP	123.958
AAVKTL;YMEENDLLKK	NCP	27.986
YMEENDLLKK;DLKRLKDE	NCP	0.96
DLKRLKDE;KDELAVNKQ	NCP	0.015

KDELAVNKQ;TKEYDRLLEEHT	NCP	0.879
TKEYDRLLEEHT;EYDRLLEEHTKLQ	NCP	0.006
EYDRLLEEHTKLQ;YDRLLEEHTK	NCP	0.003
YDRLLEEHTK;DRLLEEHTKLQ	NCP	0.003
DRLLEEHTKLQ;LQAAVDGPSDKKEE	NCP	0.894
LQAAVDGPSDKKEE;WLRANRP	NCP	35.319
WLRANRP;APAAGRH	NCP	23.208
APAAGRH;QHGPGGPK	NCP	92.505
QHGPGGPK;PQRAPALGP	NCP	63.087
PQRAPALGP;SLPFEL	NCP	53.343
SLPFEL;GPFLP	NCP	2.715
GPFLP;PPGVYPK	NCP	22.041
PPGVYPK;GHDGK	NCP	67.266
GHDGK;AHELPFPQ	NCP	56.862
AHELPFPQ;HRVLFL	NCP	18.361
HRVLFL;DVLYL	NCP	4.739
DVLYL;TVPAELF	NCP	38.728
TVPAELF;LQAVAVL	NCP	12.651
LQAVAVL;LTLGNER	NCP	92.517
LTLGNER;TEAALLRL	NCP	0.801
TEAALLRL;RKSLPEE	NCP	130.295
RKSLPEE;KGGLSF	NCP	3.093
KGGLSF;ATPVVAEVVKTPK	NCP	6.072
ATPVVAEVVKTPK;MELLVKA	NCP	109.602
MELLVKA;METSTQRTGSQL	NCP	89.877
METSTQRTGSQL;RGGVF	NCP	26.226
RGGVF;KYPLPPG	NCP	19.459
KYPLPPG;QVPPF	NCP	24.149
QVPPF;AASKDGCGVGEVAAGN	NCP	133.233
AASKDGCGVGEVAAGN;HFVLVGP	NCP	149.827
HFVLVGP;MELVYP	NCP	170.375
MELVYP;LPCRALP	NCP	19.765
LPCRALP;PAMPK	NCP	64.691
PAMPK;SGPAPRLLK	NCP	244.561
SGPAPRLLK;GVFLP	NCP	115.179
GVFLP;DSLGFK	NCP	60.711
DSLGFK;KAFKLQ	NCP	85.088
KAFKLQ;KGEEPR	NCP	123.157
KGEEPR;SFLLEKL	NCP	70.077
SFLLEKL;GVEGL	NCP	0.996
GVEGL;KVVKHSSP	NCP	26.954
KVVKHSSP;FDRKCF	NCP	59.217
FDRKCF;TPVAL	NCP	17.934
TPVAL;GVAFLG	NCP	32.379
GVAFLG;MSSSG	NCP	133.549
MSSSG;PPADY	NCP	56.567
PPADY;GKVVFL	NCP	55.597
GKVVFL;SPVLGDA	NCP	56.151
SPVLGDA;KGDLRVP	NCP	48.599
KGDLRVP;NLPHAL	NCP	10.138
NLPHAL;KGHGP	NCP	5.318

KGHGP;AASGLR	NCP	28.341
AASGLR;PMLLG	NCP	34.678
PMLLG;KLEVLR	NCP	152.667
KLEVLR;MDPWFK	NCP	10.643
MDPWFK;LDGLVK	NCP	287.659
LDGLVK;NVPGLVL	NCP	258.518
NVPGLVL;WGDVFV	NCP	68.799
WGDVFV;PPPKYGR	NCP	63.673
PPPKYGR;RGVKALLK	NCP	13.103
RGVKALLK;NLLVP	NCP	142.546
NLLVP;KDEPDM	NCP	42.539
KDEPDM;KNLQTVNVDEN	NCP	61.291
KNLQTVNVDEN;KSLPPPP	NCP	236.301
KSLPPPP;CLKHSSK	NCP	160.193
CLKHSSK;GPVSKDK	NCP	78.108
GPVSKDK;KVPSPTPP	NCP	22.059
KVPSPTPP;TGPKKLT	NCP	8.236
TGPKKLT;VNEVTEY	NCP	81.78
VNEVTEY;LETVPFKL	NCP	0.252
LETVPFKL;RVALAERD	NCP	26.373
RVALAERD;MELSKLPS	NCP	96.315
MELSKLPS;KVCLVR	NCP	94.347
KVCLVR;CRLAEAW	NCP	136.532
CRLAEAW;NFTLP	NCP	20.598
NFTLP;TLGPGLP	NCP	40.924
TLGPGLP;LKSLF	NCP	10.659
LKSLF;APPVP	NCP	21.443
APPVP;KPPEPDR	NCP	256.057
KPPEPDR;KATVLRY	NCP	99.23
KATVLRY;NFLTKR	NCP	47.698
NFLTKR;ATAGVELL	NCP	61.923
ATAGVELL;LLESGLALRP	NCP	85.709
LLESGLALRP;SMLRLQ	NCP	50.401
SMLRLQ;LGAAVTR	NCP	104.036
LGAAVTR;RVHNYP	NCP	10.564
RVHNYP;MALGPLVL	NCP	40.361
MALGPLVL;QEHVPKK	NCP	2.041
QEHVPKK;GVKQLLVG	NCP	27.441
GVKQLLVG;MEDLFR	NCP	11.91
MEDLFR;QNSLSHK	NCP	19.29
QNSLSHK;LLPKTK	NCP	139.937
LLPKTK;ASPGAP	NCP	53.304
ASPGAP;KQVVSLTL	NCP	75.217
KQVVSLTL;LKHLF	NCP	64.619
LKHLF;FLWGKMPK	NCP	145.749
FLWGKMPK;LSQMLSEK	NCP	68.121
LSQMLSEK;KLSYDFQ	NCP	30.856
KLSYDFQ;PVRGPP	NCP	111.858
PVRGPP;MKPAV	NCP	186.336
MKPAV;PLAAFSC	NCP	65.595
PLAAFSC;QPTEKLR	NCP	39.527

QPTEKLR;KSLMSSP	NCP	57.885
KSLMSSP;KQHKRFC	NCP	21.199
KQHKRFC;SGPLSM	NCP	23.594
SGPLSM;ELPPGAG	NCP	56.916
ELPPGAG;VGNYF	NCP	392.251
VGNYF;TLAGRLY	NCP	4.409
TLAGRLY;YVVPMLK	NCP	114.901
YVVPMLK;KTTPP	NCP	184.746
KTTPP;KWLRY	NCP	103.44
KWLRY;TVDLLPF	NCP	31.73
TVDLLPF;AFHGY	NCP	23.254
AFHGY;TPGLL	NCP	83.003
TPGLL;SLLVT	NCP	168.694
SLLVT;PAQPP	NCP	101.843
PAQPP;KACSSGLQLF	NCP	122.467
KACSSGLQLF;LDHLAVRF	NCP	237.771
LDHLAVRF;LPKKLGL	NCP	85.283
LPKKLGL;LSSFK	NCP	208.116
LSSFK;MDNLKKK	NCP	139.279
MDNLKKK;KLAVVFF	NCP	17.145
KLAVVFF;PLPRSPY	NCP	1.848
PLPRSPY;CMCGAP	NCP	200.594
CMCGAP;VASLVP	NCP	17.742
VASLVP;MVYMF	NCP	83.773
MVYMF;LTCKV	NCP	16.923
LTCKV;PTDHRPP	NCP	93.807
PTDHRPP;LPVVLFK	NCP	0.861
LPVVLFK;RGPGAK	NCP	42.428
RGPGAK;NVDGLVR	NCP	287.937
NVDGLVR;AYGLP	NCP	259.765
AYGLP;MDSGC	NCP	74.607
MDSGC;LEALEL	NCP	4.547
LEALEL;PPAPSPP	NCP	58.251
PPAPSPP;NAGSVLL	NCP	72.225
NAGSVLL;LPPVTRD	NCP	175.198
LPPVTRD;VGLAELVT	NCP	54.485
VGLAELVT;KGMEPK	NCP	28.29
KGMEPK;NVPLLK	NCP	93.309
NVPLLK;VGVPL	NCP	181.444
VGVPL;KPKVPKN	NCP	9.572
KPKVPKN;QELLP	NCP	42.156
QELLP;KGMKSPLQ	NCP	722.578
KGMKSPLQ;LTSLGKDP	NCP	35.868
LTSLGKDP;HDVLEQGL	NCP	22.179
HDVLEQGL;VGLPW	NCP	6.639
VGLPW;RENVTELK	NCP	0.864
RENVTELK;VPLLYQL	NCP	0.509
VPLLYQL;NHVVLLSC	NCP	5.314
NHVVLLSC;QPLAK	NCP	15.092
QPLAK;LLTGAL	NCP	2.743
LLTGAL;VLSPL	NCP	6.33

VLSPL;LEAHLLT	NCP	34.173
LEAHLLT;NFSPLQ	NCP	210.512
NFSPLQ;CHVKPK	NCP	19.188
CHVKPK;PPPDLPK	NCP	86.365
PPPDLPK;NLSWLLC	NCP	25.796
NLSWLLC;LVENQ	NCP	42.118
LVENQ;KPNLYPPP	NCP	37.658
KPNLYPPP;CTAPA	NCP	51.586
CTAPA;GWFAY	NCP	28.863
GWFAY;NGCCC	NCP	124.676
NGCCC;PVVGLQD	NCP	180.016
PVVGLQD;PLESVF	NCP	15.968
PLESVF;AAPPYK	NCP	26.884
AAPPYK;KTLTAAP	NCP	102.341
KTLTAAP;KKLDRRTP	NCP	190.413
KKLDRRTP;QLFPPPPP	NCP	117.469
QLFPPPPP;KAAMKLR	NCP	22.893
KAAMKLR;RLVTLLH	NCP	57.89
RLVTLLH;ESLLT	NCP	45.139
ESLLT;KEGLKK	NCP	28.95
KEGLKK;PTDALLFG	NCP	60.392
PTDALLFG;NSPSAL	NCP	10.689
NSPSAL;LTLLFFK	NCP	181.123
LTLLFFK;HDKFSYR	NCP	82.307
HDKFSYR;CVSAL	NCP	35.229
CVSAL;SLQEVNLR	NCP	11.2
SLQEVNLR;RTPLGSH	NCP	54.669
RTPLGSH;WPSQT	NCP	121.445
WPSQT;QGLHPEK	NCP	75.19
QGLHPEK;GSVFF	NCP	22.326
GSVFF;RNAVLLEK	NCP	13.02
RNAVLLEK;KGVLK	NCP	109.17
KGVLK;ALPHALLR	NCP	199.389
ALPHALLR;HLLPPPPP	NCP	13.107
HLLPPPPP;RTAGMYG	NCP	27.503
RTAGMYG;RELGGVT	NCP	152.751
RELGGVT;KDGGLLL	NCP	33.114
KDGGLLL;LAMGMPL	NCP	8.239
LAMGMPL;NPRKAAH	NCP	243.942
NPRKAAH;RPLRPP	NCP	98.529
RPLRPP;LTAELYTL	NCP	95.868
LTAELYTL;FHSTDAPP	NCP	14.181
FHSTDAPP;LPPRPPPP	NCP	189.918
LPPRPPPP;GPFKKPC	NCP	270
GPFKKPC;TALLK	NCP	38.739
TALLK;AHLLFP	NCP	205.683
AHLLFP;HRPFPLG	NCP	25.152
HRPFPLG;FMKLK	NCP	126.788
FMKLK;LVPFAT	NCP	10.819
LVPFAT;VAVVLL	NCP	98.634
VAVVLL;KDYVFPP	NCP	132.014

KDYVFPP;KSHPPGGK	NCP	15.049
KSHPPGGK;APGACAPR	NCP	61.281
APGACAPR;TGSEVPRP	NCP	19.356
TGSEVPRP;LEAVPL	NCP	51.798
LEAVPL;KKKQHT	NCP	377.448
KKKQHT;EGVRLK	NCP	81.402
EGVRLK;PPSLPPP	NCP	1.016
PPSLPPP;QVLEY	NCP	53.634
QVLEY;AYYGT	NCP	47.152
AYYGT;KSHKKK	NCP	60.371
KSHKKK;ATVVEC	NCP	51.364
ATVVEC;KLVLKR	NCP	15.641
KLVLKR;LLDPVL	NCP	120.019
LLDPVL;LSVAGVF	NCP	28.668
LSVAGVF;KQQLLK	NCP	15.816
KQQLLK;QEEKLQ	NCP	99.579
QEEKLQ;FLQGASR	NCP	94.571
FLQGASR;VPAQADW	NCP	38.418
VPAQADW;FGDSLVRT	NCP	12.331
FGDSLVRT;EPTPPR	NCP	189.261
EPTPPR;CECCCCPP	NCP	29.714
CECCCCPP;FFPPPP	NCP	134.397
FFPPPP;AVKDNH	NCP	391.975
AVKDNH;NALAHRGE	NCP	114.86
NALAHRGE;PSAAHR	NCP	33.495
PSAAHR;VVKTK	NCP	95.11
VVKTK;AFLEKVR	NCP	9.689
AFLEKVR;NTTYG	NCP	5.02
NTTYG;TWRLLDN	NCP	3.063
TWRLLDN;LPGGKPP	NCP	0.782
LPGGKPP;RGPLPVKRGPPPR	NCP	27.942
RGPLPVKRGPPPR;GPPPRSGGPPP	NCP	0.024
GPPPRSGGPPP;RDSYGGPPR	NCP	0.384
RDSYGGPPR;YGGPPRR	NCP	0.009
YGGPPRR;APPTRGPPP	NCP	1.134
APPTRGPPP;LSVEY	NCP	67.014
LSVEY;DVLSW	NCP	100.842
DVLSW;LPGKQER	NCP	157.428
LPGKQER;AGPPP	NCP	34.849
AGPPP;RPPPPKQK	NCP	8.183
RPPPPKQK;RSTGALLL	NCP	183.175
RSTGALLL;GMYGL	NCP	2.033
GMYGL;LLVHVN	NCP	69.972
LLVHVN;APSGK	NCP	6.846
APSGK;LALGVVLP	NCP	10.146
LALGVVLP;RVSLHR	NCP	7.963
RVSLHR;THELHRK	NCP	197.645
THELHRK;ARGGGLLM	NCP	149.755
ARGGGLLM;LAGGF	NCP	120.828
LAGGF;LPTTTMKP	NCP	171.252
LPTTTMKP;NHAGPLLL	NCP	7.7

NHAGPLLL;SDDSD	NCP	82.485
SDDSD;LATLKTK	NCP	38.941
LATLKTK;ASLLK	NCP	75.801
ASLLK;LVDPL	NCP	38.66
LVDPL;PLLAFK	NCP	36.01
PLLAFK;LPPPPPP	NCP	59.933
LPPPPPP;LEELKK	NCP	108.258
LEELKK;WLRPP	NCP	11.631
WLRPP;VTPVK	NCP	133.618
VTPVK;PPVAVFR	NCP	137.628
PPVAVFR;AASVGVP	NCP	11.598
AASVGVP;AAPGVY	NCP	22.091
AAPGVY;HSSLGFTT	NCP	59.605
HSSLGFTT;QFLPPPPPS	NCP	75.669
QFLPPPPPS;DLDLLF	NCP	9.986
DLDLLF;LELLELLL	NCP	5.457
LELLELLL;CNDTP	NCP	22.27
CNDTP;AVSKAFR	NCP	12.648
AVSKAFR;LLTCLY	NCP	12.143
LLTCLY;ANLLP	NCP	86.205
ANLLP;KNEPYP	NCP	79.671
KNEPYP;SSRLLR	NCP	71.136
SSRLLR;KPHGER	NCP	59.23
KPHGER;FSTGLDF	NCP	95.783
FSTGLDF;KDTNNLK	NCP	25.707
KDTNNLK;LERPAAL	NCP	135.586
LERPAAL;GPPVTRSP	NCP	31.476
GPPVTRSP;KQLSYH	NCP	49.005
KQLSYH;MKVFGN	NCP	210.642
MKVFGN;KYHTPAAL	NCP	44.148
KYHTPAAL;TVLSDLR	NCP	127.461
TVLSDLR;SGPPP	NCP	62.649
SGPPP;YADLPK	NCP	141.164
YADLPK;KLETQEK	NCP	141.793
KLETQEK;LELYL	NCP	153.696
LELYL;YGHKKPR	NCP	62.093
YGHKKPR;ALNDLTLS	NCP	64.564
ALNDLTLS;QQLGTGL	NCP	41.163
QQLGTGL;KGYAAPR	NCP	8.787
KGYAAPR;LLNGVVV	NCP	55.304
LLNGVVV;LLLPGELAKHA	NCP	1.048
LLLPGELAKHA;LPVTF	NCP	1.76
LPVTF;KDKDR	NCP	35.484
KDKDR;KMCDLDL	NCP	153.957
KMCDLDL;KADGGK	NCP	60.43
KADGGK;LVHPGPA	NCP	9.183
LVHPGPA;PMVPKAPP	NCP	2.607
PMVPKAPP;LSLAAF	NCP	16.115
LSLAAF;AAKGLPL	NCP	358.993
AAKGLPL;YAHHCV	NCP	4.515
YAHHCV;QVKYP	NCP	5.751

QVKYP;SEVPLVDT	NCP	41.381
SEVPLVDT;VGEHEYL	NCP	9.804
VGEHEYL;KLGLGY	NCP	407.416
KLGLGY;KDMLAQE	NCP	30.885
KDMLAQE;VGHVLH	NCP	85.908
VGHVLH;CALHFL	NCP	198.3
CALHFL;GLPVVR	NCP	61.122
GLPVVR;TMSLLK	NCP	103.127
TMSLLK;TAMVFP	NCP	78.568
TAMVFP;TGHYPK	NCP	27.948
TGHYPK;WDHEVH	NCP	272.294
WDHEVH;GSVPPP	NCP	171.438
GSVPPP;NAKLLNTK	NCP	203.377
NAKLLNTK;KLLRFQ	NCP	112.632
KLLRFQ;KTKAM	NCP	16.529
KTKAM;DLLLLK	NCP	4.74
DLLLLK;KVMLPAA	NCP	9.492
KVMLPAA;PSSVPNYFR	NCP	43.554
PSSVPNYFR;CECAP	NCP	92.893
CECAP;KEHGVTL	NCP	33.572
KEHGVTL;CNLLPLRQ	NCP	53.124
CNLLPLRQ;EKSSVPPK	NCP	28.371
EKSSVPPK;SVATP	NCP	184.701
SVATP;TTTASL	NCP	35.776
TTTASL;KAAFFPR	NCP	15.708
KAAFFPR;GSKSVG	NCP	140.06
GSKSVG;KKRKKKAPR	NCP	89.973
KKRKKKAPR;LLLLPAP	NCP	19.597
LLLLPAP;LPPSK	NCP	114.053
LPPSK;SVLAD	NCP	66.681
SVLAD;DMESD	NCP	68.508
DMESD;PPYLLR	NCP	8.532
PPYLLR;DRYPQ	NCP	97.59
DRYPQ;KDFKELSF	NCP	99.516
KDFKELSF;FSRGSLK	NCP	6.904
FSRGSLK;LHQFR	NCP	29.712
LHQFR;TLVFERQK	NCP	20.414
TLVFERQK;PALSL	NCP	164.464
PALSL;LVGVGG	NCP	134.165
LVGVGG;LSLAGL	NCP	54.348
LSLAGL;KTMPML	NCP	3.643
KTMPML;EHSLF	NCP	67.712
EHSLF;MEVSA	NCP	159.97
MEVSA;HPFYSL	NCP	205.881
HPFYSL;VKVGK	NCP	78.239
VKVGK;VEFPP	NCP	2.613
VEFPP;LLRPDR	NCP	45.403
LLRPDR;FFEEAL	NCP	52.287
FFEEAL;TGALGPLE	NCP	14.868
TGALGPLE;YVPLDF	NCP	95.082
YVPLDF;DDMEKLW	NCP	32.9

DDMEKLW;PALHFRP	NCP	61.786
PALHFRP;SSPVFP	NCP	42.546
SSPVFP;PLVVLLY	NCP	18.984
PLVVLLY;LCYVAL	NCP	64.61
LCYVAL;VELGV	NCP	15.451
VELGV;ALVTV	NCP	168.969
ALVTV;SMHDLRK	NCP	196.058
SMHDLRK;VMPPWR	NCP	145.054
VMPPWR;KDLPLK	NCP	53.313
KDLPLK;KEAFT	NCP	191.139
KEAFT;LPPEPKP	NCP	145.037
LPPEPKP;HVKRH	NCP	4.303
HVKRH;AGLEVLAF	NCP	81.315
AGLEVLAF;RPFAGSP	NCP	95.528
RPFAGSP;KLLCEF	NCP	2.35
KLLCEF;QYLVPLL	NCP	21.41
QYLVPLL;ALELQR	NCP	89.445
ALELQR;APAPNP	NCP	50.617
APAPNP;LKLSH	NCP	93.353
LKLSH;KTKQEYQ	NCP	13.432
KTKQEYQ;HSLSEKY	NCP	106.395
HSLSEKY;FEAKHSK	NCP	35.393
FEAKHSK;HTPHLK	NCP	54.352
HTPHLK;DTYLHL	NCP	60.78
DTYLHL;LFGQVL	NCP	8.292
LFGQVL;KVGLP	NCP	2.966
KVGLP;FSALTH	NCP	189.703
FSALTH;MCGPP	NCP	108.744
MCGPP;KALKTSK	NCP	31.563
KALKTSK;LVDYM	NCP	15.686
LVDYM;AKLSLEPY	NCP	31.731
AKLSLEPY;KFVFPPPP	NCP	37.372
KFVFPPPP;TLKNLH	NCP	87.024
TLKNLH;KEESKK	NCP	30.212
KEESKK;KPKRNTP	NCP	196.167
KPKRNTP;VPLLAF	NCP	129.58
VPLLAF;PNLVLV	NCP	27.476
PNLVLV;LSPDTKP	NCP	21.975
LSPDTKP;KGRFLVPM	NCP	118.239
KGRFLVPM;SPLVPF	NCP	158.421
SPLVPF;WLGAF	NCP	69.618
WLGAF;HESFLKGT	NCP	128.61
HESFLKGT;MKAPWK	NCP	23.025
MKAPWK;HLTAGPLT	NCP	36.828
HLTAGPLT;LVPVQP	NCP	399.862
LVPVQP;FGAQRYG	NCP	179.603
FGAQRYG;GLSKVH	NCP	41.389
GLSKVH;KHVGAPP	NCP	11.025
KHVGAPP;EPCALA	NCP	50.802
EPCALA;LVPHSPK	NCP	42.767
LVPHSPK;TLPKNK	NCP	50.166

TLPKNK;KDTHTKK	NCP	97.249
KDTHTKK;AAATVVVPVEW	NCP	118.943
AAATVVVPVEW;KPLLAG	NCP	42.933
KPLLAG;KDVKQ	NCP	67.014
KDVKQ;FTAQY	NCP	242.295
FTAQY;GVEPV	NCP	0.267
GVEPV;KVLHEK	NCP	87.82
KVLHEK;KETLGAGK	NCP	191.855
KETLGAGK;TKEAVLL	NCP	89.133
TKEAVLL;AKPSVPK	NCP	58.567
AKPSVPK;RSELLQ	NCP	103.697
RSELLQ;QPSAKSK	NCP	38.14
QPSAKSK;VGGLVV	NCP	33.554
VGGLVV;KTPPP	NCP	75.951
KTPPP;DDDDY	NCP	290.187
DDDDY;STNPKKP	NCP	149.788
STNPKKP;LYVLK	NCP	19.235
LYVLK;AAYLPFR	NCP	151.887
AAYLPFR;LTAYFV	NCP	185.571
LTAYFV;ERSLGAK	NCP	46.929
ERSLGAK;LVRFPLEF	NCP	24.879
LVRFPLEF;EFLPPP	NCP	183.033
EFLPPP;YAPELMK	NCP	75.777
YAPELMK;PDTKP	NCP	209.328
PDTKP;LGPGVT	NCP	191.62
LGPGVT;KCQQL	NCP	28.995
KCQQL;LLQGGVK	NCP	78.188
LLQGGVK;EFCLP	NCP	25.44
EFCLP;LGKGFMY	NCP	4.227
LGKGFMY;MGPPK	NCP	113.674
MGPPK;DKPKLPEN	NCP	82.331
DKPKLPEN;VHEVVH	NCP	54.474
VHEVVH;RLAPVPGSGFL	NCP	36.105
RLAPVPGSGFL;VERLLK	NCP	56.118
VERLLK;PAAGL	NCP	113.545
PAAGL;ADLKPNL	NCP	221.327
ADLKPNL;EEAFG	NCP	29.493
EEAFG;PTKKGK	NCP	6.207
PTKKGK;FLVPP	NCP	193.923
FLVPP;VLERKAP	NCP	93.25
VLERKAP;QPPFTP	NCP	143.738
QPPFTP;ASLVFK	NCP	25.837
ASLVFK;MKEEKEHRPK	NCP	157.61
MKEEKEHRPK;KMRVAPGP	NCP	117.075
KMRVAPGP;ANPDQ	NCP	32.284
ANPDQ;KYVVL	NCP	135.206
KYVVL;EVDSLPL	NCP	197.641
EVDSLPL;LSTPSVPK	NCP	129.458
LSTPSVPK;LKEKQAGK	NCP	2.208
LKEKQAGK;LHVVPPP	NCP	17.412
LHVVPPP;ETLKEKPPR	NCP	83.268

ETLKEKPPR;LPQPVEP	NCP	7.302
LPQPVEP;KPKKAPP	NCP	196.932
KPKKAPP;SLTMP	NCP	15.873
SLTMP;MFPKPPP	NCP	1.272
MFPKPPP;LPLPHL	NCP	65.364
LPLPHL;STELLL	NCP	2.323
STELLL;PQKMP	NCP	25.071
PQKMP;NDFKLKKE	NCP	268.545
NDFKLKKE;KDNAQ	NCP	226.422
KDNAQ;VADLL	NCP	332.208
VADLL;KTLGVLPF	NCP	59.18
KTLGVLPF;AHLLFLR	NCP	28.428
AHLLFLR;KKQAAPK	NCP	10.83
KKQAAPK;VNLEFL	NCP	26.751
VNLEFL;YGAEPLK	NCP	168.291
YGAEPLK;AAAANL	NCP	168.192
AAAANL;KDPYTLK	NCP	25.669
KDPYTLK;HANTLPPP	NCP	73.652
HANTLPPP;WKDLR	NCP	70.026
WKDLR;LPFGMHK	NCP	63.922
LPFGMHK;LPVKVNPPP	NCP	146.723
LPVKVNPPP;KCPPLP	NCP	149.533
KCPPLP;LVLHAV	NCP	252.224
LVLHAV;SFCRPL	NCP	50.257
SFCRPL;DVSYSEP	NCP	114.477
DVSYSEP;EKKYG	NCP	106.139
EKKYG;GLSSCML	NCP	244.996
GLSSCML;VEAGLK	NCP	18.818
VEAGLK;NTPVP	NCP	198.376
NTPVP;ALATL	NCP	105.323
ALATL;YSSAPPPP	NCP	12.745
YSSAPPPP;KTHDAK	NCP	84.671
KTHDAK;ELFTEL	NCP	32.19
ELFTEL;LFDPK	NCP	97.938
PSLLAAPV;LYVGFN	NCP	39.48
LYVGFN;RGPPQF	NCP	48.051
RGPPQF;GVPDL	NCP	11.209
GVPDL;TPPMSK	NCP	26.22
TPPMSK;DKAALPC	NCP	14.615
DKAALPC;AASSK	NCP	8.631
AASSK;PPKAHH	NCP	10.933
PPKAHH;NVEAPKR	NCP	24.996
NVEAPKR;SLPGVP	NCP	14.861
SLPGVP;GPDLALQ	NCP	48.648
GPDLALQ;APSLLLQPL	NCP	13.278
APSLLLQPL;GKYLLAH	NCP	27.708
GKYLLAH;LTGSLRFQ	NCP	82.885
LTGSLRFQ;DLTEDGTRPHVV	NCP	126.203
DLTEDGTRPHVV;YLLTA	NCP	10.978
YLLTA;NGFQGLKH	NCP	5.76
NGFQGLKH;PGGKLQGH	NCP	207.68

PGGKLQGH;KGAAAAPK	NCP	9.705
KGAAAAPK;PPPPPPPPP	NCP	30.304
PPPPPPPPP;PLLVSPLPP	NCP	31.613
PLLVSPLPP;THNSSLEY	NCP	18.304
THNSSLEY;LSYLENY	NCP	26.712
LSYLENY;KENHYVK	NCP	23.961
KENHYVK;EVVTY	NCP	5.459
EVVTY;KTAVFGH	NCP	10.786
KTAVFGH;TTATVLKEK	NCP	43.022
TTATVLKEK;QPGVAPPP	NCP	8.136
QPGVAPPP;LLAWLGTH	NCP	33.828
LLAWLGTH;QANLLEV	NCP	205.564
QANLLEV;VLPALF	NCP	2.892
VLPALF;LSADPV	NCP	4.679
LSADPV;KNVVDPP	NCP	5.467
KNVVDPP;LSSLSPM	NCP	10.725
LSSLSPM;WAGAP	NCP	0.428
WAGAP;VDPLSF	NCP	36.838
VDPLSF;ENAVRPP	NCP	46.298
ENAVRPP;MDPSRP	NCP	13.203
MDPSRP;APAFH	NCP	13.407
APAFH;ANGPP	NCP	18.834
ANGPP;EWTLTPP	NCP	34.416
EWTLTPP;KPSGTRQ	NCP	0.333
KPSGTRQ;KAFLMRER	NCP	22.695
KAFLMRER;NDTPAHK	NCP	32.034
NDTPAHK;KDWQVL	NCP	43.57
KDWQVL;KAARPTT	NCP	10.337
KAARPTT;KNKKAVYLR	NCP	18.079
KNKKAVYLR;LPPTGTH	NCP	42.872
LPPTGTH;PPPPGGASP	NCP	35.067
PPPPGGASP;CPVRR	NCP	18.687
CPVRR;PLLLKRD	NCP	34.944
PLLLKRD;VHKKFL	NCP	10.261
VHKKFL;KAPAPPPK	NCP	86.631
KAPAPPPK;PVAPPPGP	NCP	107.837
PVAPPPGP;TFSHELYW	NCP	18.997
TFSHELYW;KCHLFFY	NCP	53.64
KCHLFFY;KKETH	NCP	2.526
KKETH;KLKPTGK	NCP	74.628
KLKPTGK;QAASLE	NCP	59.769
QAASLE;CPHGKGS	NCP	11.723
CPHGKGS;KEMVPPP	NCP	117.361
KEMVPPP;KDALTT	NCP	30.317
KDALTT;PNAASAPP	NCP	0.255
PNAASAPP;MALNVFL	NCP	24.697
MALNVFL;RGFTKL	NCP	36.782
RGFTKL;HAPAPPK	NCP	46.074
HAPAPPK;LEGGPL	NCP	42.37
LEGGPL;DPVVSY	NCP	20.337
DPVVSY;AHALGL	NCP	8.228

AHALGL;ASKLLAR	NCP	30.387
ASKLLAR;MFPGALP	NCP	4.848
MFPGALP;DVVSKL	NCP	15.228
DVVSKL;KGGERAP	NCP	14.868
KGGERAP;LLMPGGPA	NCP	38.358
LLMPGGPA;KPQTAVR	NCP	30.187
KPQTAVR;LLMLQPLL	NCP	23.219
LLMLQPLL;CNDCDP	NCP	99.438
CNDCDP;LPPPKY	NCP	81.444
LPPPKY;DGHHLQK	NCP	96.352
DGHHLQK;LPPGVFT	NCP	53.048
LPPGVFT;EPEAPGPQ	NCP	30.654
EPEAPGPQ;LSQWGPK	NCP	16.537
LSQWGPK;MDNLKK	NCP	82.101
MDNLKK;LPPEVR	NCP	5.957
LPPEVR;LGTAGCRK	NCP	6.528
LGTAGCRK;PSLTGPK	NCP	31.854
PSLTGPK;TLQKDLEY	NCP	12.691
TLQKDLEY;PPPPQKK	NCP	220.146
PPPPQKK;RELKDF	NCP	126.102
RELKDF;AKGSPQ	NCP	102.692
AKGSPQ;QRGGLPP	NCP	16.059
QRGGLPP;TSTGGPPP	NCP	17.977
TSTGGPPP;TTESQKTK	NCP	5.798
TTESQKTK;KSDFVW	NCP	0.011
KSDFVW;CESDSPP	NCP	48.734
CESDSPP;PPSLVHR	NCP	4.116
PPSLVHR;PSLVHRK	NCP	0.003
PSLVHRK;ECVLAV	NCP	46.101
ECVLAV;MGRPLFY	NCP	7.596
MGRPLFY;VSPPVP	NCP	0.303
VSPPVP;PNTQMFK	NCP	0.019
PNTQMFK;GAAFLVA	NCP	23.744
GAAFLVA;HVFLPR	NCP	37.005
HVFLPR;LELVPN	NCP	10.03
LELVPN;KQKLPVG	NCP	129.734
KQKLPVG;GLKLGQH	NCP	10.708
GLKLGQH;TLQKTTTL	NCP	8.058
TLQKTTTL;HLAPPPPL	NCP	20.066
HLAPPPPL;AVDLDL	NCP	30.729
AVDLDL;PPEKPRP	NCP	2.343
PPEKPRP;DSCDN	NCP	24.286
DSCDN;AAHFP	NCP	7.7
AAHFP;YWAKGK	NCP	33.567
YWAKGK;KSSTVQP	NCP	22.56
KSSTVQP;LSSSMNS	NCP	31.617
LSSSMNS;FAAHLFF	NCP	23.358
FAAHLFF;HDDFLMM	NCP	127.81
HDDFLMM;KFALPK	NCP	41.918
KFALPK;FSPTT	NCP	40.27
FSPTT;GPSLKVHR	NCP	67.499

GPSLKVHR;KGKLLTK	NCP	136.062
KGKLLTK;LALRNQLR	NCP	2.887
LALRNQLR;VHTAGMA	NCP	52.344
VHTAGMA;APVTVP	NCP	38.844
APVTVP;YGPFPP	NCP	36.23
YGPFPP;KQCDLK	NCP	37.2
KQCDLK;KPLVAGK	NCP	12.343
KPLVAGK;KGPEKPK	NCP	3.315
KGPEKPK;GTAVPL	NCP	50.922
GTAVPL;AAASGGLF	NCP	15.299
AAASGGLF;PVALRGVP	NCP	3.09
PVALRGVP;DRMALSK	NCP	42.853
DRMALSK;AVPVNPR	NCP	12.228
AVPVNPR;FLTYT	NCP	53.162
FLTYT;KTPADVH	NCP	64.146
KTPADVH;NFMTPAP	NCP	3.115
NFMTPAP;KLLFLPW	NCP	83.531
KLLFLPW;NLKDKLEK	NCP	16.435
NLKDKLEK;ECDRRPP	NCP	3.143
ECDRRPP;TPKVMFK	NCP	40.698
TPKVMFK;AGKKYDS	NCP	19.053
AGKKYDS;FRFVR	NCP	52.014
FRFVR;QGLTEL	NCP	27.715
QGLTEL;VLEKRAP	NCP	29.204
VLEKRAP;KGHLLLLKK	NCP	35.892
KGHLLLLKK;LLTPAP	NCP	86.349
LLTPAP;VGNKHLF	NCP	91.033
VGNKHLF;TPTGWKF	NCP	21.602
TPTGWKF;LPHDP	NCP	52.218
LPHDP;FLLVGVP	NCP	10.275
FLLVGVP;LNQVVTR	NCP	4.663
LNQVVTR;KDMTFL	NCP	8.64
KDMTFL;SSSGG	NCP	7.263
SSSGG;KPPMPKP	NCP	52.011
KPPMPKP;DEPSLSK	NCP	25.043
DEPSLSK;APSDS	NCP	45.261
APSDS;SRGHL	NCP	45.207
SRGHL;LLPSVHK	NCP	7.273
LLPSVHK;PLNPK	NCP	45.612
PLNPK;NDHFVKL	NCP	22.538
NDHFVKL;CCDDVP	NCP	20.311
CCDDVP;QALTVK	NCP	91.574
QALTVK;LVYELHF	NCP	44.157
LVYELHF;KPSLGRPP	NCP	82.26
KPSLGRPP;KGFYY	NCP	17.389
KGFYY;PPLSP	NCP	46.904
PPLSP;ASLTVKAY	NCP	8.268
ASLTVKAY;KWFPPPP	NCP	61.692
KWFPPPP;PGELAKHAVSEGTKAVTKY	NCP	13.14
PGELAKHAVSEGTKAVTKY;VSVPAGP	NCP	11.772
VSVPAGP;EPSSSVP	NCP	3.633

EPSSSVP;VSLPPP	NCP	12.18
VSLPPP;PGCDKRL	NCP	14.653
PGCDKRL;ECCDAQ	NCP	94.658
ECCDAQ;EKPQDKS	NCP	10.647
EKPQDKS;KEFGTNL	NCP	26.461
KEFGTNL;FPPDVGG	NCP	55.157
FPPDVGG;PPKAP	NCP	0.111
PPKAP;PAWVPPPAA	NCP	12.291
PAWVPPPAA;TFPPPR	NCP	5.361
TFPPPR;VGPVK	NCP	71.133
VGPVK;RHRYR	NCP	104.731
RHRYR;SLAVGMK	NCP	1.743
SLAVGMK;KLLVCDC	NCP	31.041
KLLVCDC;PPPPPLP	NCP	65.133
PPPPPLP;MDEMRC	NCP	74.948
MDEMRC;KRLDNAV	NCP	20.199
KRLDNAV;TDAAVSF	NCP	26.334
TDAAVSF;AVREGP	NCP	82.944
AVREGP;TPAAPP	NCP	35.919
TPAAPP;NGLQAQT	NCP	55.618
NGLQAQT;MGTLPN	NCP	28.773
MGTLPN;MALSF	NCP	63.213
MALSF;MARELL	NCP	16.77
MARELL;EAGPLPAP	NCP	28.076
EAGPLPAP;RQAPP	NCP	4.746
RQAPP;PPLPRGK	NCP	6.516
PPLPRGK;PLALRVG	NCP	1.095
PLALRVG;LLDVTLP	NCP	1.726
LLDVTLP;LTPPRHP	NCP	24.236
LTPPRHP;ELTTAQHRGLQ	NCP	35.172
ELTTAQHRGLQ;GVSVKHP	NCP	47.325
GVSVKHP;KTPPPP	NCP	47.811
KTPPPP;TPHNEGPP	NCP	22.27
TPHNEGPP;LVSTKSLQ	NCP	94.805
LVSTKSLQ;LMKWPK	NCP	54.789
LMKWPK;LLHGVPF	NCP	30.447
LLHGVPF;AGRKLAL	NCP	2.158
AGRKLAL;FDDDFK	NCP	4.746
FDDDFK;KVLDSGDG	NCP	40.292
KVLDSGDG;KGPPLYK	NCP	24.493
KGPPLYK;YQKVRGT	NCP	27.426
YQKVRGT;GPAVKK	NCP	30.434
GPAVKK;KGYVPR	NCP	33.531
KGYVPR;KGDFPR	NCP	16.177
KGDFPR;KALKWSK	NCP	1.232
KALKWSK;HTDALGP	NCP	21.316
HTDALGP;TTPVVG	NCP	26.582
TTPVVG;VSRRPP	NCP	4.803
VSRRPP;KMGTK	NCP	0.619
KMGTK;KFHHTF	NCP	17.213
KFHHTF;LVVMQPEKFQSK	NCP	0.06

LVVMQPEKFQSK;VLEVAKDFPEY	NCP	2.757
VLEVAKDFPEY;TFAVADEEDF	NCP	0.033
TFAVADEEDF;KGPVKVVVG	NCP	0.936
KGPVKVVVG;MDATANDVTSDRY	NCP	0.177
MDATANDVTSDRY;ATKLSRTKEEL	NCP	0.156
ATKLSRTKEEL;RERPPK	NCP	16.504
RERPPK;PTALPEK	NCP	10.952
PTALPEK;VTSRVYQ	NCP	16.569
VTSRVYQ;PVSLNY	NCP	9.349
PVSLNY;LTVNSN	NCP	4.634
LTVNSN;TDSLPLL	NCP	8.172
TDSLPLL;EGVPL	NCP	5.182
EGVPL;KPTHLY	NCP	26.874
KPTHLY;RGHSSPK	NCP	70.232
RGHSSPK;KAVEVFL	NCP	4.071
KAVEVFL;RLPPPPPPP	NCP	95.58
RLPPPPPPP;TELKGVGP	NCP	82.219
TELKGVGP;KAWSLLLN	NCP	84.386
KAWSLLLN;RTKHHAK	NCP	84.747
RTKHHAK;KKPTHKH	NCP	35.257
KKPTHKH;LDLSLK	NCP	67.971
LDLSLK;LLSQAAL	NCP	12.392
LLSQAAL;MLLLNL	NCP	0.621
MLLLNL;PVTAPLLA	NCP	65.853
PVTAPLLA;VPLGGDPVP	NCP	2.856
VPLGGDPVP;TLGAPEPP	NCP	285.097
TLGAPEPP;YQGPQPP	NCP	35.597
YQGPQPP;KLLLAF	NCP	4.621
KLLLAF;RGVFLVA	NCP	3.624
RGVFLVA;APGPP	NCP	42.894
APGPP;PPPPYR	NCP	11.876
PPPPYR;KEKPPYH	NCP	4.192
KEKPPYH;KEPVG	NCP	12.918
KEPVG;EGNMDLR	NCP	80.196
EGNMDLR;FLLPK	NCP	109.266
FLLPK;LGRFGYG	NCP	25.806
LGRFGYG;DLPLTMP	NCP	81.387
DLPLTMP;YFPNRVP	NCP	0.866
YFPNRVP;VVFMT	NCP	17.851
VVFMT;EVGPPT	NCP	14.21
EVGPPT;VVLLLPK	NCP	35.32
VVLLLPK;KDDEKVR	NCP	159.581
KDDEKVR;KLDAFKQ	NCP	235.885
KLDAFKQ;AVHTGNLN	NCP	214.371
AVHTGNLN;FAPVVVF	NCP	11.934
FAPVVVF;GVLLALVGQ	NCP	96.912
GVLLALVGQ;KHCYSF	NCP	16.836
KHCYSF;KQRKELVL	NCP	59.019
KQRKELVL;KPAFHPP	NCP	78.537
KPAFHPP;DTHST	NCP	7.883
DTHST;VLKKHT	NCP	111.636

VLKKHT;VTPSVPK	NCP	34.6
VTPSVPK;SVEVKSLK	NCP	17.166
SVEVKSLK;THKEERA	NCP	0.069
THKEERA;LPVNLE	NCP	2.187
LPVNLE;KTAASLR	NCP	67.32
KTAASLR;AFSCSR	NCP	67.073
AFSCSR;PHVAGPK	NCP	10.584
PHVAGPK;RPVALPQT	NCP	57.034
RPVALPQT;KSVVLGGLP	NCP	97.329
KSVVLGGLP;STMKDRF	NCP	264.3
STMKDRF;EVFDF	NCP	105.104
EVFDF;GVSSASLP	NCP	11.173
GVSSASLP;EVLSLPKK	NCP	101.229
EVLSLPKK;MKVRW	NCP	72.753
MKVRW;LTPVYMP	NCP	4.445
LTPVYMP;RRDRVR	NCP	78.069
RRDRVR;KPETLK	NCP	3.564
KPETLK;EFPLR	NCP	28.846
EFPLR;SPLDALLP	NCP	7.5
SPLDALLP;EDLPEKEKL	NCP	51.822
EDLPEKEKL;YEALK	NCP	81.462
YEALK;KKCGPQ	NCP	38.211
KKCGPQ;LDPVYG	NCP	2.951
LDPVYG;PQDPVGR	NCP	73.969
PQDPVGR;GMSKPDL	NCP	39.416
GMSKPDL;NSHVL	NCP	62.665
NSHVL;KDFMPK	NCP	21.318
KDFMPK;ASTLHSK	NCP	6.585
ASTLHSK;KGLKP	NCP	38.201
KGLKP;ASGKTLLR	NCP	95.818
ASGKTLLR;TPPEL	NCP	22.355
TPPEL;EVDLPNKK	NCP	17.008
EVDLPNKK;TPKYTTH	NCP	2.085
TPKYTTH;KHFAAK	NCP	31.316
KHFAAK;KGGHPPP	NCP	38.686
KGGHPPP;VVGPA	NCP	21.422
VVGPA;NSRLKLG	NCP	14.556
NSRLKLG;YGAFLEF	NCP	93.798
YGAFLEF;YELPD	NCP	183.537
YELPD;LSKPF	NCP	34.018
LSKPF;KDLSPF	NCP	35.25
KDLSPF;TVKLK	NCP	53.724
TVKLK;TALPLTK	NCP	40.961
TALPLTK;LFDDLK	NCP	20.515
LFDDLK;EFYTTLF	NCP	26.312
EFYTTLF;FDKDCP	NCP	16.159
FDKDCP;DLPVL	NCP	10.553
DLPVL;KLDFQ	NCP	57.186
KLDFQ;LQKPRLLL	NCP	16.402
LQKPRLLL;CVGVCKK	NCP	22.878
CVGVCKK;QTTYL	NCP	153.701

QTTYL;RPKLSPN	NCP	6.31
RPKLSPN;LLSHGK	NCP	86.339
LLSHGK;QFRASP	NCP	6.646
QFRASP;TSAGKVLK	NCP	128.778
TSAGKVLK;FEDPN	NCP	0.429
FEDPN;LVEAQLTPF	NCP	17.714
LVEAQLTPF;ATTPAAAFEAL	NCP	46.137
ATTPAAAFEAL;TAVQLSR	NCP	44.776
TAVQLSR;VPGKSGY	NCP	112.326
VPGKSGY;FGTGFPH	NCP	127.713
FGTGFPH;AVPPELF	NCP	2.097
AVPPELF;PPRGVPK	NCP	2.336
PPRGVPK;CGSAHC	NCP	18.057
CGSAHC;TAELLELL	NCP	70.018
TAELLELL;YKAPVPP	NCP	60.57
YKAPVPP;MFLTM	NCP	115.422
MFLTM;LSAALEY	NCP	42.669
LSAALEY;VGLAEVK	NCP	10.727
VGLAEVK;LLFASPVN	NCP	3.495
LLFASPVN;FGSLT	NCP	23.448
FGSLT;TKKVLQY	NCP	4.972
TKKVLQY;YGYSEY	NCP	8.252
YGYSEY;MKSLLRL	NCP	61.806
MKSLLRL;AATTPLK	NCP	42.154
AATTPLK;FELGGDKK	NCP	52.41
FELGGDKK;TSHVPQ	NCP	10.191
TSHVPQ;APAGPLAAP	NCP	21.362
APAGPLAAP;KPQRVFP	NCP	4.102
KPQRVFP;ASKLLRA	NCP	77.408
ASKLLRA;LQAVL	NCP	8.134
LQAVL;LSDDLLK	NCP	26.424
LSDDLLK;TLDGKGT	NCP	38.855
TLDGKGT;FDEVVK	NCP	14.662
FDEVVK;AELVP	NCP	131.3
AELVP;KGTVVVY	NCP	157.681
KGTVVVY;KSSQHEY	NCP	15.671
KSSQHEY;SSALRKQ	NCP	3.148
SSALRKQ;YTMLEY	NCP	11.319
YTMLEY;ELKLVLW	NCP	4.974
ELKLVLW;VLGFFPP	NCP	1.422
VLGFFPP;VGKSPML	NCP	57.839
VGKSPML;VTVVPA	NCP	22.827
VTVVPA;NMLHLKGKP	NCP	43.318
NMLHLKGKP;SEAAKVTTQV	NCP	4.07
SEAAKVTTQV;EDPGWG	NCP	16.995
EDPGWG;KDDEPK	NCP	4.069
KDDEPK;GHLRVLSE	NCP	57.701
GHLRVLSE;ANLLTGPK	NCP	72.109
ANLLTGPK;LDLTTPK	NCP	16.124
LDLTTPK;AALSLY	NCP	72.352
AALSLY;SYTLP	NCP	14.294

SYTLP;KATHVPP	NCP	74.79
KATHVPP;MYQVPK	NCP	85.245
MYQVPK;PSVKPPR	NCP	87.072
PSVKPPR;LPLLLTH	NCP	5.277
LPLLLTH;KKVVRP	NCP	20.475
KKVVRP;KPANSPPP	NCP	9.919
KPANSPPP;PSVPLD	NCP	61.946
PSVPLD;MEDTQLH	NCP	57.091
MEDTQLH;PGMNKLE	NCP	147.828
PGMNKLE;LSSPGSF	NCP	17.252
LSSPGSF;TLSHALT	NCP	15.546
TLSHALT;RSPPMEAK	NCP	11.256
RSPPMEAK;TAFKP	NCP	95.82
TAFKP;MMCSHP	NCP	37.384
MMCSHP;KYAALG	NCP	18.188
KYAALG;YADHTLP	NCP	11.323
YADHTLP;YLLKSR	NCP	11.504
YLLKSR;CALVHRK	NCP	32.841
CALVHRK;VLRPYY	NCP	20.218
VLRPYY;AALLEYL	NCP	39.285
AALLEYL;NPADQ	NCP	3.261
NPADQ;KVFKF	NCP	79.152
KVFKF;YGFYMP	NCP	154.269
YGFYMP;PFLLEV	NCP	9.797
PFLLEV;TKDLGVK	NCP	0.666
TKDLGVK;AATYVPR	NCP	141.244
AATYVPR;KEGWKNK	NCP	25.908
KEGWKNK;EDPPTKPF	NCP	31.205
EDPPTKPF;LTAKKGH	NCP	56.74
LTAKKGH;LLKVKLR	NCP	48.176
LLKVKLR;MGTKMKG	NCP	26.13
MGTKMKG;LLGLQ	NCP	11.415
LLGLQ;KPYPVLK	NCP	16.365
KPYPVLK;YTSFW	NCP	57.7
YTSFW;FHPPQ	NCP	21.539
FHPPQ;KTTAT	NCP	44.551
KTTAT;LVMVTQ	NCP	22.722
LVMVTQ;DPPAALEK	NCP	29.238
DPPAALEK;KLLTEMTK	NCP	91.623
KLLTEMTK;DWHCPR	NCP	44.534
DWHCPR;FGVPLK	NCP	14.281
FGVPLK;PPKPVA	NCP	9.377
PPKPVA;PKSPEQEK	NCP	22.528
PKSPEQEK;SKPPQ	NCP	47.873
SKPPQ;GVPVGN	NCP	184.596
GVPVGN;PGPVGP	NCP	25.08
PGPVGP;SPLPPTR	NCP	7.11
SPLPPTR;ELLLMEQ	NCP	26.241
ELLLMEQ;KPWVTPP	NCP	48.156
KPWVTPP;EEGHDPKEPEQLR	NCP	5.047
EEGHDPKEPEQLR;FETTDDSLREHFEK	NCP	0.063

FETTDDSLREHFEK;ETTDDSLREHFE	NCP	0.003
ETTDDSLREHFE;DDSLREHFEK	NCP	0.009
DDSLREHFEK;VVMRDPQTK	NCP	0.051
VVMRDPQTK;SVKPGAHLTVK	NCP	0.147
SVKPGAHLTVK;VSSKQLLT	NCP	52.649
VSSKQLLT;ESRLVC	NCP	67.444
ESRLVC;LPPPPGH	NCP	70.163
LPPPPGH;KGTSEPK	NCP	30.492
KGTSEPK;FQMDDF	NCP	46.978
FQMDDF;RSYSKLL	NCP	24.858
RSYSKLL;DLCRNP	NCP	19.566
DLCRNP;LRLLPG	NCP	63.254
LRLLPG;ADPGVCCF	NCP	0.918
ADPGVCCF;KYRCAP	NCP	1.197
KYRCAP;HDLVLFW	NCP	47.115
HDLVLFW;KEKMGK	NCP	17.52
KEKMGK;GPLLLF	NCP	27.586
GPLLLF;YLLHAY	NCP	16.587
YLLHAY;WMPPF	NCP	116.352
WMPPF;NFVSEF	NCP	161.04
NFVSEF;ADLLLKN	NCP	89.607
ADLLLKN;CCPSC	NCP	26.616
CCPSC;KVNVLFQ	NCP	73.065
KVNVLFQ;SLFKRH	NCP	5.781
SLFKRH;AAAASLL	NCP	147.798
AAAASLL;LLANFKNY	NCP	25.107
LLANFKNY;FDGVKRL	NCP	27.129
FDGVKRL;AKLNV	NCP	29.978
AKLNV;ALLLAM	NCP	107.142
ALLLAM;ENLKKSLE	NCP	58.296
ENLKKSLE;PSLRPP	NCP	1.431
PSLRPP;LSSTDLR	NCP	14.142
LSSTDLR;PRVYGL	NCP	164.433
PRVYGL;KPLLLR	NCP	20.551
KPLLLR;KGYLEPF	NCP	5.883
KGYLEPF;AHSLMKP	NCP	34.817
AHSLMKP;MSSKM	NCP	17.587
MSSKM;MEFVLL	NCP	63.26
MEFVLL;KDAVY	NCP	9.283
KDAVY;AATLPLKP	NCP	21.153
AATLPLKP;VYEPPPPP	NCP	7.302
VYEPPPPP;KSATVPR	NCP	16.34
KSATVPR;MEGSLP	NCP	6.784
MEGSLP;DAYVLPK	NCP	48.81
DAYVLPK;VTPAEC	NCP	97.005
VTPAEC;KPGTPVK	NCP	11.024
KPGTPVK;LTEPL	NCP	25.317
LTEPL;KYLKPP	NCP	53.671
KYLKPP;VGPRKGV	NCP	86.817
VGPRKGV;HYHKK	NCP	2.09
HYHKK;KMSVLLSH	NCP	35.191

KMSVLLSH;GHYNYG	NCP	2.573
GHYNYG;DALPQV	NCP	13.726
DALPQV;YSLGSAL	NCP	73.902
YSLGSAL;ERLLVK	NCP	42.384
ERLLVK;LPKSELL	NCP	18.239
LPKSELL;KDPSLPLK	NCP	57.616
KDPSLPLK;LEKGLLD	NCP	15.494
LEKGLLD;KAWPS	NCP	33.73
KAWPS;NEALLEF	NCP	31.58
NEALLEF;LARVFL	NCP	23.118
LARVFL;NSLFSNL	NCP	9.538
NSLFSNL;KCYVPY	NCP	2.676
KCYVPY;HSTPK	NCP	77.148
HSTPK;FPDGDLP	NCP	112.554
FPDGDLP;NDKSFLL	NCP	30.483
NDKSFLL;PLFKY	NCP	45.798
PLFKY;FGKAKVS	NCP	61.74
FGKAKVS;TSPAAGNK	NCP	27.906
TSPAAGNK;KEAAF	NCP	6.882
KEAAF;DCPSLVRH	NCP	77.394
DCPSLVRH;MKLQF	NCP	14.567
MKLQF;HNHVQLK	NCP	116.551
HNHVQLK;EELKHLKF	NCP	65.337
EELKHLKF;PSSNFR	NCP	36.569
PSSNFR;DPCCCCT	NCP	38.764
DPCCCCT;LLPHKLGP	NCP	41.783
LLPHKLGP;LTTRGFP	NCP	21
LTTRGFP;CDKEFMWALK	NCP	81.438
CDKEFMWALK;KTTTKDR	NCP	2.422
KTTTKDR;LDLLKLKKK	NCP	15.243
LDLLKLKKK;LSAVYEGHVSCVK	NCP	32.918
LSAVYEGHVSCVK;KSWFHV	NCP	39.27
KSWFHV;KAAGAP	NCP	41.154
KAAGAP;RYAEAKK	NCP	47.038
RYAEAKK;ATLPDWK	NCP	34.023
ATLPDWK;KEVSVPP	NCP	16.937
KEVSVPP;HLLPP	NCP	13.609
HLLPP;LTNLTLL	NCP	74.55
LTNLTLL;QTAAAAPK	NCP	45.854
QTAAAAPK;DLEERKLN	NCP	20.865
DLEERKLN;KEFEKK	NCP	3.616
KEFEKK;AAPTRVY	NCP	58.007
AAPTRVY;HDESRPK	NCP	22.758
HDESRPK;NLGTVR	NCP	9.099
NLGTVR;KLVMPAP	NCP	12.561
KLVMPAP;LGPVK	NCP	121.399
LGPVK;GPPPWVVP	NCP	47.975
GPPPWVVP;DLNLK	NCP	13.399
DLNLK;CAHVAPPR	NCP	6.312
CAHVAPPR;DGVRGPR	NCP	0.095
DGVRGPR;QSLTYK	NCP	42.937

QSLTYK;TCEHPNPL	NCP	48.887
TCEHPNPL;LLMYRPL	NCP	153.492
LLMYRPL;PKGVHVK	NCP	23.688
PKGVHVK;SVGPTPPS	NCP	4.383
SVGPTPPS;LGNFMEQ	NCP	53.979
LGNFMEQ;GKALLY	NCP	5.721
GKALLY;NGLLVEL	NCP	56.239
NGLLVEL;TTKFTLFP	NCP	22.187
TTKFTLFP;KLDYKT	NCP	41.164
KLDYKT;QVCLKK	NCP	23.319
QVCLKK;YETWR	NCP	50.724
YETWR;LPPPVQ	NCP	243.606
LPPPVQ;LAYHFYR	NCP	38.135
LAYHFYR;LSTKDVK	NCP	69.628
LSTKDVK;DLPHSVP	NCP	99.765
DLPHSVP;KLQEKVVKR	NCP	42.422
KLQEKVVKR;HPYLTQE	NCP	2.898
HPYLTQE;SHRDYPFHEEF	NCP	5.052
SHRDYPFHEEF;FAYNLLR	NCP	19.582
FAYNLLR;LPGFFQ	NCP	26.762
LPGFFQ;HGKGG	NCP	30.612
HGKGG;KLQLN	NCP	91.731
KLQLN;KVYMPK	NCP	86.842
KVYMPK;SPLTNKP	NCP	15.645
SPLTNKP;KPDTSLLK	NCP	26.906
KPDTSLLK;CHSGLP	NCP	31.822
CHSGLP;KDPTYP	NCP	32.156
KDPTYP;GHLLVPF	NCP	64.461
GHLLVPF;KSGLTTK	NCP	87.564
KSGLTTK;HGGFLQ	NCP	81.475
HGGFLQ;RHQHQ	NCP	83.705
RHQHQ;NSGGDFKLK	NCP	8.563
NSGGDFKLK;KFSLPPPPPP	NCP	118.487
KFSLPPPPPP;RGLSKAY	NCP	82.767
RGLSKAY;NDVFPR	NCP	172.027
NDVFPR;NVVLLA	NCP	19.182
NVVLLA;LTELAPVT	NCP	27.479
LTELAPVT;YLEAY	NCP	42.54
YLEAY;KVAHRYH	NCP	29.713
KVAHRYH;HDFELPVL	NCP	29.094
HDFELPVL;SSSFLPK	NCP	108.612
SSSFLPK;PLLFLGP	NCP	68.369
PLLFLGP;HYRLSG	NCP	44.191
HYRLSG;FDARVK	NCP	145.544
FDARVK;NVAAVLV	NCP	72.994
NVAAVLV;FFKLPR	NCP	88.868
FFKLPR;KFADP	NCP	277.689
KFADP;LKLTGMT	NCP	6.658
LKLTGMT;KGSHVYK	NCP	40.026
KGSHVYK;APPSRPPPP	NCP	201.821
APPSRPPPP;FPLPKRP	NCP	59.475

FPLPKRP;KYQDSLK	NCP	5.971
KYQDSLK;QFKRVP	NCP	117.728
QFKRVP;TVPKYKS	NCP	79.222
TVPKYKS;KGGGTLLH	NCP	29.171
KGGGTLLH;KHKTPK	NCP	128.142
KHKTPK;MELVP	NCP	4.609
MELVP;LAHELGF	NCP	102.089
LAHELGF;LDNLTKDDPEEEDTHL	NCP	82.944
LDNLTKDDPEEEDTHL;FSLKNKK	NCP	91.027
FSLKNKK;PGPHKGK	NCP	8.721
PGPHKGK;TLVGK	NCP	10.064
TLVGK;GVVRERN	NCP	117.555
GVVRERN;TSFKPK	NCP	46.645
TSFKPK;LDTDPLNL	NCP	4.205
LDTDPLNL;VKVALLH	NCP	24.357
VKVALLH;ELNSLELL	NCP	7.711
ELNSLELL;PLFELY	NCP	33.278
PLFELY;DELGKF	NCP	18.268
DELGKF;NQPKPK	NCP	8.501
NQPKPK;QLLLLAA	NCP	1.689
QLLLLAA;HDPEPPR	NCP	25.687
HDPEPPR;YAAPR	NCP	36.42
YAAPR;QLSSF	NCP	26.607
QLSSF;KNSSLW	NCP	27.656
KNSSLW;PHALPSL	NCP	42.126
PHALPSL;PLRSTGP	NCP	75.792
PLRSTGP;ALPAEC	NCP	43.434
ALPAEC;DKLVP	NCP	43.176
DKLVP;RFHSKGL	NCP	42.688
RFHSKGL;EVLVL	NCP	152.669
EVLVL;EPAKFR	NCP	109.966
EPAKFR;FSLGL	NCP	80.229
FSLGL;KAGQSEK	NCP	17.456
KAGQSEK;LLFWFV	NCP	33.192
LLFWFV;KQTALLSP	NCP	33.372
KQTALLSP;VPTELGGK	NCP	44.668
VPTELGGK;KHVKYK	NCP	129.989
KHVKYK;KHTLYQ	NCP	24.036
KHTLYQ;KPTSGPAK	NCP	0.51
KPTSGPAK;KMFLKY	NCP	27.967
KMFLKY;ASALL	NCP	2.349
ASALL;LLYWNL	NCP	1.614
LLYWNL;PPAPLPPPPPP	NCP	32.366
PPAPLPPPPPP;APLPPPPPPAAPP	NCP	0.006
APLPPPPPPAAPP;LDEPTVL	NCP	36.213
LDEPTVL;NAAVP	NCP	14.295
NAAVP;CLHMA	NCP	11.323
CLHMA;AMLLL	NCP	75.503
AMLLL;QQPKKR	NCP	8.596
QQPKKR;GPPLLP	NCP	7.496
GPPLLP;KLVGGGGK	NCP	33.784

KLVGGGGK;MDVQLK	NCP	5.951
MDVQLK;TGVGALP	NCP	10.548
TGVGALP;LGMAYGAK	NCP	42.714
LGMAYGAK;VPSLPEV	NCP	16.803
VPSLPEV;SPLLVPP	NCP	4.674
SPLLVPP;MDPMEL	NCP	17.704
MDPMEL;ENTYPP	NCP	119.541
ENTYPP;MFLDMK	NCP	57.983
MFLDMK;CLSRW	NCP	4.057
CLSRW;VPKPHHK	NCP	109.836
VPKPHHK;NFVPPR	NCP	98.433
NFVPPR;VGRGWK	NCP	32.67
VGRGWK;RNTLVP	NCP	56.844
RNTLVP;APGWVH	NCP	47.921
APGWVH;MEGAKPTLQ	NCP	60.414
MEGAKPTLQ;MTSMPY	NCP	41.121
MTSMPY;AFTGPK	NCP	49.725
AFTGPK;KEGPP	NCP	24.702
KEGPP;MLSRKG	NCP	22.537
MLSRKG;YVSPLEW	NCP	17.714
YVSPLEW;AQVRPVS	NCP	25.728
AQVRPVS;ACVLLK	NCP	36.09
ACVLLK;LVELVK	NCP	12.376
LVELVK;ASVHR	NCP	47.139
ASVHR;VPSLPFK	NCP	14.186
VPSLPFK;VMLYLPRS	NCP	32.103
VMLYLPRS;VPGFSP	NCP	6.648
VPGFSP;GKLTSGGP	NCP	7.542
GKLTSGGP;KVGVFL	NCP	2.026
KVGVFL;TLLEFVLL	NCP	60.426
TLLEFVLL;DLKGPKV	NCP	25.05
DLKGPKV;NPVTFF	NCP	43.793
NPVTFF;LQGYLR	NCP	17.587
LQGYLR;KVVEQFH	NCP	37.712
KVVEQFH;WGELPL	NCP	1.599
WGELPL;NKDDM	NCP	33.391
NKDDM;LSPLFTPAP	NCP	109.907
LSPLFTPAP;DPVVT	NCP	13.969
DPVVT;VPFSLL	NCP	15.165
VPFSLL;VEKPPSR	NCP	34.689
VEKPPSR;KTLYPTR	NCP	60.726
KTLYPTR;FVDNVF	NCP	71.4
FVDNVF;KQWGRTQ	NCP	9.965
KQWGRTQ;WGKAEELK	NCP	2.157
WGKAEELK;LDSFPAST	NCP	209.146
LDSFPAST;LSPASL	NCP	70.952
LSPASL;LPGGPPPLP	NCP	0.48
LPGGPPPLP;PLWGGGKK	NCP	7.767
PLWGGGKK;TQRDVKF	NCP	16.596
TQRDVKF;VCAPP	NCP	49.071
VCAPP;KGAVLSH	NCP	50.961

KGAVLSH;KKVYHTK	NCP	106.506
KKVYHTK;PPGPDCP	NCP	87.699
PPGPDCP;PVDVL	NCP	22.954
PVDVL;KCLEDLQQ	NCP	91.145
KCLEDLQQ;NSTATK	NCP	49.011
NSTATK;EAPGDN	NCP	2.52
EAPGDN;ALAVVYM	NCP	26.334
ALAVVYM;YLLPP	NCP	137.106
YLLPP;KDKPF	NCP	1.717
KDKPF;KVNLTLK	NCP	5.226
KVNLTLK;LLEKKVEK	NCP	2.808
LLEKKVEK;YLEVLT	NCP	60.285
YLEVLT;SLDLLP	NCP	21.699
SLDLLP;EPRYH	NCP	53.835
EPRYH;VTERLLV	NCP	28.155
VTERLLV;METPSQR	NCP	0.14
METPSQR;KDFLL	NCP	0.697
KDFLL;LALLLY	NCP	77.862
LALLLY;KNCGFLSF	NCP	48.329
KNCGFLSF;QRPVVL	NCP	10.189
QRPVVL;MYSVKPK	NCP	5.694
MYSVKPK;GPLTKLH	NCP	21.408
GPLTKLH;LPVPNLP	NCP	29.468
LPVPNLP;VQVLCTE	NCP	10.66
VQVLCTE;KDLHQPFP	NCP	246.62
KDLHQPFP;TLSDVPS	NCP	147.31
TLSDVPS;KGEQLLLQK	NCP	9.828
KGEQLLLQK;PAVVF	NCP	214.626
PAVVF;ESQYKLN	NCP	67.011
ESQYKLN;DYPRSL	NCP	32.318
DYPRSL;RLFDPK	NCP	31.363
RLFDPK;HQDPVLL	NCP	99.21
HQDPVLL;AVPEF	NCP	184.539
AVPEF;TVDLLV	NCP	7.604
TVDLLV;ASFSS	NCP	24.007
ASFSS;LNVEC	NCP	19.137
LNVEC;DFLNGK	NCP	15.836
DFLNGK;VVLFFP	NCP	16.911
VVLFFP;KMVLLLDQ	NCP	29.194
KMVLLLDQ;VGGHLEF	NCP	33.327
VGGHLEF;KDPPP	NCP	26.958
KDPPP;AQAPP	NCP	26.676
AQAPP;VSRMAHSH	NCP	93.201
VSRMAHSH;APEVTFQ	NCP	81.525
APEVTFQ;RYNLFLK	NCP	2.523
RYNLFLK;KSSTPHGR	NCP	20.036
KSSTPHGR;NGTALKR	NCP	34.666
NGTALKR;VTPFELRT	NCP	40.044
VTPFELRT;TLKEELP	NCP	29.019
TLKEELP;GSVYF	NCP	61.037
GSVYF;QTPKVPGK	NCP	17.869

QTPKVPGK;KGRWEKQS	NCP	112.455
KGRWEKQS;TPPVGPLP	NCP	32.124
TPPVGPLP;VSALGK	NCP	57.318
VSALGK;AAGLEFPP	NCP	99.867
AAGLEFPP;KHPGMVP	NCP	54.192
KHPGMVP;VPPVLLPK	NCP	369.447
VPPVLLPK;VDELLHK	NCP	109.392
VDELLHK;RAHVRP	NCP	13.557
RAHVRP;AEPLL	NCP	23.927
AEPLL;KGSLHG	NCP	12.727
KGSLHG;KAKKKLLL	NCP	93.732
KAKKKLLL;FHFYFLTF	NCP	35.855
FHFYFLTF;SGHKSHGK	NCP	36.372
SGHKSHGK;LSAFLLTK	NCP	21.007
LSAFLLTK;MVKLNL	NCP	14.948
MVKLNL;KEAYKTH	NCP	1.981
KEAYKTH;PPNHQAK	NCP	89.163
PPNHQAK;LAGLSRQ	NCP	106.698
LAGLSRQ;KSKPAQK	NCP	375.788
KSKPAQK;TREFLLP	NCP	18.247
TREFLLP;RVVVAF	NCP	39.375
RVVVAF;KGAPSTM	NCP	10.722
KGAPSTM;KELFPK	NCP	65.15
KELFPK;LTVLPQ	NCP	83.107
LTVLPQ;PPDHPPP	NCP	53.093
PPDHPPP;GLNVNQ	NCP	3.081
GLNVNQ;LAVLLEN	NCP	41.412
LAVLLEN;QNNTSKY	NCP	3.682
QNNTSKY;FDDSDT	NCP	71.574
FDDSDT;VSKPSALR	NCP	23.819
VSKPSALR;HKSSSC	NCP	72.105
HKSSSC;DVESAF	NCP	28.105
DVESAF;SLAGPP	NCP	35.468
SLAGPP;EHDDLLL	NCP	73.591
EHDDLLL;QTKLPLK	NCP	4.157
QTKLPLK;HSTHDVLE	NCP	130.941
HSTHDVLE;LTLGDP	NCP	25.143
LTLGDP;DQQALKAP	NCP	7.092
DQQALKAP;PSVVGPPP	NCP	29.124
PSVVGPPP;KGTTLEK	NCP	22.969
KGTTLEK;SDYPPL	NCP	34.752
SDYPPL;KPEHVP	NCP	22.929
KPEHVP;ASNKTTLQK	NCP	21.566
ASNKTTLQK;EERLTWH	NCP	0.504
EERLTWH;LDGPVVN	NCP	35.823
LDGPVVN;VSEPVLEY	NCP	43.932
VSEPVLEY;VLHPLVAK	NCP	5.769
VLHPLVAK;LMVML	NCP	37.356
LMVML;AVDYW	NCP	43.453
AVDYW;AALSK	NCP	157.571
AALSK;KSGPH	NCP	33.67

KSGPH;MVMLQ	NCP	13.041
MVMLQ;KMVHGM	NCP	33.54
KMVHGM;TTVHALT	NCP	35.123
TTVHALT;TTTKLR	NCP	9.129
TTTKLR;YAEAEVL	NCP	119.403
YAEAEVL;GVGFY	NCP	101.353
GVGFY;FDLSF	NCP	50.18
FDLSF;LDLELDSL	NCP	60.748
LDLELDSL;LYHRLY	NCP	138.758
LYHRLY;TVFTP	NCP	81.115
TVFTP;LSTTKKPK	NCP	47.639
LSTTKKPK;LSYSMTSL	NCP	17.191
LSYSMTSL;KDHTTSPK	NCP	48.258
KDHTTSPK;VVVDF	NCP	70.434
VVVDF;SYMSLL	NCP	25.058
SYMSLL;KTPVVGR	NCP	113.517
KTPVVGR;ATLGNER	NCP	31.575
ATLGNER;TPDYFL	NCP	2.448
TPDYFL;TLVYF	NCP	47.374
TLVYF;FCTPGF	NCP	38.205
FCTPGF;ELVLPEK	NCP	80.292
ELVLPEK;ENYVAG	NCP	5.759
ENYVAG;FAFHLP	NCP	17.35
FAFHLP;VSARDVY	NCP	106.859
VSARDVY;DFAARGN	NCP	42.634
DFAARGN;DTVLEESR	NCP	37.262
DTVLEESR;CWDVP	NCP	26.499
CWDVP;AELLELA	NCP	21.303
AELLELA;KVRLPR	NCP	94.245
KVRLPR;GVFRLK	NCP	3.624
GVFRLK;FSLMS	NCP	96.738
FSLMS;KPAPVAVK	NCP	115.717
KPAPVAVK;PVLPLLP	NCP	5.796
PVLPLLP;CHCLKP	NCP	3.483
CHCLKP;KGVPF	NCP	11.865
KGVPF;MEFADKL	NCP	24.327
MEFADKL;KTDSDC	NCP	12.003
KTDSDC;KEMEY	NCP	80.696
KEMEY;KVKAVR	NCP	16.572
KVKAVR;KVAGNY	NCP	93.766
KVAGNY;ATFRLK	NCP	7.118
ATFRLK;PVHEPQ	NCP	132.859
PVHEPQ;LAGAAVLS	NCP	107.663
LAGAAVLS;FYKRPP	NCP	78.559
FYKRPP;LEDLF	NCP	24.902
LEDLF;AKYADF	NCP	19.054
AKYADF;DSLRLL	NCP	139.74
DSLRLL;LKYQAKY	NCP	5.736
LKYQAKY;FPVAQTP	NCP	47.184
FPVAQTP;WDPHR	NCP	47.355
WDPHR;LASLPF	NCP	15.921

LASLPF;NSLGAP	NCP	296.672
NSLGAP;AQTLPKKK	NCP	119.982
AQTLPKKK;TQYFHK	NCP	29.824
TQYFHK;NALLVPP	NCP	75.623
NALLVPP;NSPLLK	NCP	234.732
NSPLLK;NGFEKPK	NCP	30.198
NGFEKPK;PPDLFM	NCP	39.294
PPDLFM;RLPYFEP	NCP	33.531
RLPYFEP;LFLVPF	NCP	67.879
LFLVPF;DFTVRQ	NCP	42.27
DFTVRQ;EHLLQPF	NCP	2.972
EHLLQPF;PPPVLPPP	NCP	76.717
PPPVLPPP;LAAHVLK	NCP	0.618
LAAHVLK;WNHAAK	NCP	1.23
WNHAAK;PPTVVP	NCP	102.287
PPTVVP;LQNFFG	NCP	65.452
LQNFFG;KGFFPLEY	NCP	33.092
KGFFPLEY;NVVPSKR	NCP	104.56
NVVPSKR;ERKGSL	NCP	0.491
ERKGSL;GNVLY	NCP	21.159
GNVLY;PFPFPKPP	NCP	4.629
PFPFPKPP;EYVKPF	NCP	111.373
EYVKPF;TVPDGLR	NCP	144.41
TVPDGLR;MSSSL	NCP	183.346
MSSSL;ALAELYL	NCP	66.743
ALAELYL;YHGVRK	NCP	5.07
YHGVRK;TSKETPF	NCP	42.3
TSKETPF;RYTAF	NCP	65.721
RYTAF;PPPPEVP	NCP	100.044
PPPPEVP;WSHTPR	NCP	65.355
WSHTPR;KTPNYK	NCP	99.585
KTPNYK;RPVLSVTK	NCP	151.539
RPVLSVTK;EHVTEKP	NCP	35.637
EHVTEKP;LYSNMT	NCP	81.937
LYSNMT;TALVLGVP	NCP	36.119
TALVLGVP;KVDLTY	NCP	82.807
KVDLTY;DLPQGPN	NCP	30.231
DLPQGPN;GKPPAYT	NCP	14.378
GKPPAYT;KTREAAP	NCP	5.694
KTREAAP;FYTAPHF	NCP	71.137
FYTAPHF;LSPLERKK	NCP	83.226
LSPLERKK;MNKAQVLH	NCP	53
MNKAQVLH;KVVRSPK	NCP	73.663
KVVRSPK;QMVVQ	NCP	47.457
QMVVQ;KSMCVPK	NCP	58.184
KSMCVPK;KEALQDVEDENQ	NCP	0.253
KEALQDVEDENQ;VGLNV	NCP	5.274
VGLNV;NVLSYL	NCP	4.466
NVLSYL;NNPKKP	NCP	162.301
NNPKKP;KQQPDPK	NCP	25.776
KQQPDPK;QWKFYR	NCP	106.881

QWKFYR;APGEPSEK	NCP	51.221
APGEPSEK;KQGVPPP	NCP	94.14
KQGVPPP;QPEPQ	NCP	43.677
QPEPQ;MNDCSP	NCP	133.114
MNDCSP;KDVWEH	NCP	129.551
KDVWEH;SAAMRPK	NCP	92.152
SAAMRPK;GTNPVLL	NCP	79.479
GTNPVLL;LPLAA	NCP	139.208
LPLAA;HSGHTKR	NCP	94.921
HSGHTKR;FGGSVF	NCP	1.221
FGGSVF;DKHMSK	NCP	30.608
DKHMSK;LVSVP	NCP	9.474
LVSVP;MLYSTSR	NCP	129.345
MLYSTSR;KELSYLE	NCP	1.323
KELSYLE;KDVSKNF	NCP	45.282
KDVSKNF;GSKKPVKP	NCP	18.964
GSKKPVKP;FLTVKF	NCP	73.13
FLTVKF;SLTSKPP	NCP	3.138
SLTSKPP;KDVHKR	NCP	30.897
KDVHKR;SGAKNLYL	NCP	13.767
SGAKNLYL;VYKVLAT	NCP	111.762
VYKVLAT;KENGLLSP	NCP	55.824
KENGLLSP;KNVAVL	NCP	37.758
KNVAVL;NPQLKP	NCP	62.506
NPQLKP;KQPAP	NCP	52.136
KQPAP;MTLPLK	NCP	165.514
MTLPLK;QYEVP	NCP	9.68
QYEVP;LYEQTYL	NCP	37.792
LYEQTYL;HPTPPLC	NCP	42.659
HPTPPLC;PSKVNY	NCP	2.548
PSKVNY;TARPLQ	NCP	87.557
TARPLQ;LLFVAGK	NCP	5.307
LLFVAGK;KPPGKFSL	NCP	60.439
KPPGKFSL;AAALQAVL	NCP	169.95
AAALQAVL;CAPGP	NCP	33.996
CAPGP;KGSFRK	NCP	5.826
KGSFRK;EFRRVLL	NCP	44.124
EFRRVLL;MLSWRLH	NCP	55.229
MLSWRLH;DTLQGNR	NCP	147.201
DTLQGNR;LSLTVL	NCP	31.08
LSLTVL;AALCL	NCP	5.013
AALCL;KLSGPAAQ	NCP	114.708
KLSGPAAQ;VEGTVGVL	NCP	31.875
VEGTVGVL;PKAPERP	NCP	7.411
PKAPERP;LVGRL	NCP	50.021
LVGRL;ADGFTKK	NCP	29.394
ADGFTKK;KYPVLC	NCP	8.626
KYPVLC;QPLEY	NCP	12.579
QPLEY;PCLPKP	NCP	0.02
PCLPKP;LHLPFDE	NCP	24.81
LHLPFDE;NCEEK	NCP	52.33

NCEEK;GPVLGYK	NCP	66.831
GPVLGYK;LSGSFDSP	NCP	15.668
LSGSFDSP;PLLGV	NCP	7.575
PLLGV;KPHSYAK	NCP	105.216
KPHSYAK;GFTEVK	NCP	47.722
GFTEVK;VAPTTPL	NCP	28.373
VAPTTPL;WSPFGLP	NCP	9.48
WSPFGLP;EEVVPVK	NCP	5.445
EEVVPVK;PEFLAK	NCP	67.828
PEFLAK;EKLDDLVSK	NCP	3.32
EKLDDLVSK;ARGGPPP	NCP	36.09
ARGGPPP;LTTTC	NCP	10.497
LTTTC;LGYLDVP	NCP	32.133
LGYLDVP;KSPVK	NCP	3.402
KSPVK;LPTHSKT	NCP	8.383
LPTHSKT;LLLFLPKSV	NCP	58.59
LLLFLPKSV;VNKTRVVL	NCP	57.045
VNKTRVVL;LLALQPL	NCP	14.9
LLALQPL;LDLAGNR	NCP	21.196
LDLAGNR;KHAWNPP	NCP	46.947
KHAWNPP;TAPVAAEPF	NCP	1.967
TAPVAAEPF;RDPAAAPATGNK	NCP	52.815
RDPAAAPATGNK;AFDLDAFKNFS	NCP	0.075
AFDLDAFKNFS;VDGEPLGR	NCP	46.359
VDGEPLGR;DGEPLGRVSF	NCP	0.003
DGEPLGRVSF;RALSTGEKGF	NCP	2.427
RALSTGEKGF;STGEKGFGYK	NCP	0.009
STGEKGFGYK;FPLLNL	NCP	95.079
FPLLNL;GFMEY	NCP	24.774
GFMEY;TVALPTLP	NCP	4.404
TVALPTLP;EALGCSM	NCP	36.021
EALGCSM;KPGDTLK	NCP	42.624
KPGDTLK;PNTKSK	NCP	51.037
PNTKSK;NPVLPMC	NCP	56.481
NPVLPMC;KKDTHSK	NCP	45.051
KKDTHSK;LAEGPAY	NCP	24.992
LAEGPAY;LTYLVF	NCP	16.609
LTYLVF;AGQAFRKF	NCP	5.942
AGQAFRKF;VVAVGSGSKGK	NCP	0.132
VVAVGSGSKGK;ARPGVHE	NCP	43.218
ARPGVHE;WPMAPG	NCP	12.276
WPMAPG;KPHSMRK	NCP	13.348
KPHSMRK;PQGFKPP	NCP	18.224
PQGFKPP;GVAGPPKP	NCP	93.09
GVAGPPKP;EPSSKKG	NCP	11.719
EPSSKKG;HSSLPAAP	NCP	24.14
HSSLPAAP;EESPMPK	NCP	15.45
EESPMPK;ELARQNK	NCP	2.133
ELARQNK;CPLLN	NCP	168.369
CPLLN;KPEFPPP	NCP	50.271
KPEFPPP;KLESLNK	NCP	7.743

KLESLNK;KGSVCKK	NCP	21.591
KGSVCKK;PAAGAPP	NCP	27.67
PAAGAPP;ATPPPHL	NCP	188.696
ATPPPHL;AAKPSP	NCP	14.025
AAKPSP;LPGPLSF	NCP	4.917
LPGPLSF;YLPQLM	NCP	8.221
YLPQLM;PVELTLFT	NCP	45.351
PVELTLFT;GVPGLK	NCP	26.882
GVPGLK;KGDTVYL	NCP	2.029
KGDTVYL;SLASTF	NCP	67.722
SLASTF;TEVRPSP	NCP	26.961
TEVRPSP;LSSLL	NCP	20.564
LSSLL;KTAVP	NCP	41.68
KTAVP;ETFHGK	NCP	3.558
ETFHGK;PAPQQH	NCP	25.941
PAPQQH;VPPPPLGT	NCP	27.36
VPPPPLGT;DPGQVDD	NCP	56.502
DPGQVDD;GSHYG	NCP	37.493
GSHYG;SFKLE	NCP	2.691
SFKLE;CYMEPP	NCP	55.665
CYMEPP;KDYGSPP	NCP	12.82
KDYGSPP;MKLMLQT	NCP	42.02
MKLMLQT;EHFLPM	NCP	54.639
EHFLPM;SLVEVGFLP	NCP	19.86
SLVEVGFLP;VPPAGVEF	NCP	15.963
VPPAGVEF;PDMMVY	NCP	33.154
PDMMVY;AVPPPTKTH	NCP	26.171
AVPPPTKTH;RLLGNLAK	NCP	5.721
RLLGNLAK;LPTVPTP	NCP	15.144
LPTVPTP;KHHLTFK	NCP	26.281
KHHLTFK;YPVNFK	NCP	4.19
YPVNFK;LLGGLP	NCP	1.893
LLGGLP;EPPALKPR	NCP	34.533
EPPALKPR;KEMTMGP	NCP	47.214
KEMTMGP;KMQAW	NCP	103.033
KMQAW;LSPVEPL	NCP	51.029
LSPVEPL;LRSMPPPPP	NCP	7.693
LRSMPPPPP;SLPPRKSK	NCP	13.775
SLPPRKSK;GYAPLS	NCP	9.715
GYAPLS;PSKYPF	NCP	0.159
PSKYPF;LLKLDF	NCP	1.47
LLKLDF;PVKFTAF	NCP	43.118
PVKFTAF;LHATAF	NCP	35.32
LHATAF;KFPTKK	NCP	87.554
KFPTKK;TLSGN	NCP	4.158
TLSGN;MDTDC	NCP	165.486
MDTDC;GTLSH	NCP	44.641
GTLSH;VSAQFPK	NCP	76.364
VSAQFPK;ADPFVF	NCP	32.695
ADPFVF;KFGAASK	NCP	6.246
KFGAASK;REPCFP	NCP	178.461

REPCFP;FGLVEKEF	NCP	2.771
FGLVEKEF;YNLKLWN	NCP	49.825
YNLKLWN;HDKFVK	NCP	12.39
HDKFVK;NYVAK	NCP	67.601
NYVAK;KQYVSSK	NCP	28.29
KQYVSSK;KVLGKLLL	NCP	14.997
KVLGKLLL;ALVAY	NCP	232.056
ALVAY;MGNLEP	NCP	69.46
MGNLEP;KMPVP	NCP	120.9
KMPVP;DFVKR	NCP	55.413
DFVKR;TADGYG	NCP	31.272
TADGYG;DLRGSVY	NCP	108.252
DLRGSVY;FVGVFR	NCP	107.99
FVGVFR;VFNGPA	NCP	9.864
VFNGPA;YNKGLSH	NCP	72.658
YNKGLSH;GRSLAF	NCP	42.384
GRSLAF;KPMRKAK	NCP	18.794
KPMRKAK;GVTAELLE	NCP	105.166
GVTAELLE;TSAGVLSP	NCP	92.363
TSAGVLSP;DSEGTKAVTKY	NCP	48.306
DSEGTKAVTKY;ALELLAM	NCP	79.657
ALELLAM;KESGQQK	NCP	20.258
KESGQQK;TTLPKKTQ	NCP	18.156
TTLPKKTQ;KATEEKR	NCP	45.861
KATEEKR;LYLSPR	NCP	24.973
LYLSPR;FGTHETAF	NCP	34.88
FGTHETAF;KTKHTEKK	NCP	21.091
KTKHTEKK;DLDTSLPL	NCP	12.219
DLDTSLPL;VLLTKPK	NCP	17.169
VLLTKPK;AYPGHPGAGGGY	NCP	56.018
AYPGHPGAGGGY;MHDLPP	NCP	3.984
MHDLPP;VSLLYT	NCP	11.097
VSLLYT;LFNKHGKY	NCP	21.144
LFNKHGKY;LLGKALL	NCP	33.793
LLGKALL;PGLPATLP	NCP	0.045
PGLPATLP;KDTTKF	NCP	108.915
KDTTKF;KPVVVNP	NCP	44.774
KPVVVNP;LLQNSVK	NCP	290.65
LLQNSVK;LVYKTSR	NCP	80.564
LVYKTSR;MPALSML	NCP	28.59
MPALSML;DDDCD	NCP	48.825
DDDCD;ENYQKAK	NCP	7.131
ENYQKAK;KSATRY	NCP	59.082
KSATRY;KADLMVH	NCP	6.073
KADLMVH;VLGLP	NCP	36.584
VLGLP;AAPLT	NCP	58.212
AAPLT;KLYTPFR	NCP	49.759
KLYTPFR;ATVTPSPVKGKGK	NCP	92.373
ATVTPSPVKGKGK;VTPSPVKGKGKVG	NCP	0.006
VTPSPVKGKGKVG;PSPVKGKGK	NCP	0.006
PSPVKGKGK;EDEEPESPLEK	NCP	0.081

EDEEPESPLEK;EGNPLYK	NCP	98.643
EGNPLYK;DKKKKKGY	NCP	11.6
DKKKKKGY;KQFCKP	NCP	24.949
KQFCKP;PPSCFYL	NCP	28.106
PPSCFYL;KLWPSPK	NCP	38.557
KLWPSPK;GPPPDRK	NCP	0.131
GPPPDRK;ALKLLQS	NCP	297.871
ALKLLQS;NAHPEYL	NCP	0.069
NAHPEYL;RKTVETR	NCP	0.702
RKTVETR;VYPLDF	NCP	2.87
VYPLDF;KLGLL	NCP	182.4
KLGLL;CCCSM	NCP	4.846
CCCSM;RYLTK	NCP	9.597
RYLTK;DHFVKL	NCP	31.799
DHFVKL;GKPVLL	NCP	56.103
GKPVLL;ALPAALVK	NCP	2.473
ALPAALVK;SYKSAG	NCP	48.51
SYKSAG;MMECCC	NCP	9.558
MMECCC;YENTTGY	NCP	72.519
YENTTGY;MAMTCQ	NCP	6.009
MAMTCQ;HPQTK	NCP	0.063
HPQTK;EVLTG	NCP	30.863
EVLTG;ATSKPDL	NCP	0.78
ATSKPDL;KAGDLK	NCP	106.324
KAGDLK;TSNQKKR	NCP	163.823
TSNQKKR;FTKVHR	NCP	30.975
FTKVHR;LLYRDV	NCP	26.743
LLYRDV;PVLVLVGR	NCP	20.733
PVLVLVGR;KEKSVP	NCP	42.029
KEKSVP;MVGPVGR	NCP	189.754
MVGPVGR;FPLYR	NCP	4.901
FPLYR;SLGDLPF	NCP	145.818
SLGDLPF;VSPPLLL	NCP	74.292
VSPPLLL;VRFPSLL	NCP	8.824
VRFPSLL;NYCRPL	NCP	115.568
NYCRPL;KKQNVTLEK	NCP	36.604
KKQNVTLEK;EGVRVHQ	NCP	3.36
EGVRVHQ;FPLEF	NCP	71.675
FPLEF;KAGRFFP	NCP	71.371
KAGRFFP;DVPLDF	NCP	65.915
DVPLDF;GPFKGK	NCP	116.949
GPFKGK;YLPVGR	NCP	30.049
YLPVGR;KYLLPPPP	NCP	1.347
KYLLPPPP;GVGPPAH	NCP	68.733
GVGPPAH;PPMCAPR	NCP	58.91
PPMCAPR;NTPPP	NCP	76.266
NTPPP;VGSSHG	NCP	1.653
VGSSHG;KDMVMF	NCP	32.817
KDMVMF;LLVPGPP	NCP	29.926
LLVPGPP;PGLVEL	NCP	13.157
PGLVEL;NMPLD	NCP	10.047

NMPLD;KGVLTER	NCP	71.148
KGVLTER;LSVNMP	NCP	65.098
LSVNMP;DGNLKST	NCP	55.436
DGNLKST;KPVPKQ	NCP	31.222
KPVPKQ;ALGTL	NCP	14.634
ALGTL;VVGVPAT	NCP	20.148
VVGVPAT;VHPRQA	NCP	89.019
VHPRQA;KSRSLVNL	NCP	24.717
KSRSLVNL;KSPVVR	NCP	8.636
KSPVVR;YLLWSP	NCP	57.831
YLLWSP;KDTQKQ	NCP	191.484
KDTQKQ;AVCML	NCP	6.25
AVCML;YVKHR	NCP	8.013
YVKHR;VGNLLSK	NCP	40.766
VGNLLSK;LDHPPPP	NCP	12.594
LDHPPPP;PEEVLTK	NCP	113.641
PEEVLTK;GPPVTW	NCP	24.573
GPPVTW;ALLRVGF	NCP	20.294
ALLRVGF;LGSDVALL	NCP	4.938
LGSDVALL;ERFVQ	NCP	22.41
ERFVQ;VAGKPPW	NCP	44.077
VAGKPPW;SDYLKLY	NCP	21.428
SDYLKLY;FVGDAA	NCP	54.334
FVGDAA;CVLLYPLP	NCP	98.645
CVLLYPLP;AAVLVMP	NCP	24.259
AAVLVMP;GVYMLQ	NCP	58.484
GVYMLQ;GNLPLC	NCP	7.327
GNLPLC;KGFGAPPK	NCP	6.015
KGFGAPPK;LTKYKKQ	NCP	11.549
LTKYKKQ;NGLGGGLG	NCP	9.903
NGLGGGLG;TPPDYRK	NCP	68.535
TPPDYRK;KTPALTS	NCP	18.078
KTPALTS;KPGLCFL	NCP	69.781
KPGLCFL;QPPRAFK	NCP	131.489
QPPRAFK;FEPGLG	NCP	39.678
FEPGLG;LASLK	NCP	17.586
LASLK;KMGDPK	NCP	15.921
KMGDPK;NTAPGVR	NCP	46.371
NTAPGVR;KRVYF	NCP	2.634
KRVYF;DSPPRPAGP	NCP	158.997
DSPPRPAGP;SKLEC	NCP	20.715
SKLEC;FPLAGPLL	NCP	67.44
FPLAGPLL;TDTEKFP	NCP	123.847
TDTEKFP;KKKLGKGQ	NCP	136.964
KKKLGKGQ;LTQMKKK	NCP	6.199
LTQMKKK;RSGTEGLR	NCP	38.51
RSGTEGLR;PPKSYKR	NCP	5.38
PPKSYKR;DYMAYE	NCP	3.276
DYMAYE;TTDALEK	NCP	38.339
TTDALEK;RQNALK	NCP	53.31
RQNALK;KEYAAPR	NCP	34.894

KEYAAPR;KLAADYK	NCP	17.84
KLAADYK;KVTKGPK	NCP	196.03
KVTKGPK;DADDDAF	NCP	32.666
DADDDAF;SEVPLLVY	NCP	8.092
SEVPLLVY;AVVTP	NCP	23.984
AVVTP;PLPPYK	NCP	14.89
PLPPYK;VCGVCK	NCP	43.718
VCGVCK;KEFPPPP	NCP	228.585
KEFPPPP;KGVPDF	NCP	53.091
KGVPDF;MDGAGAEEVLAPLR	NCP	56.259
MDGAGAEEVLAPLR;WADVEARYPVFEGQETGK	NCP	52.713
WADVEARYPVFEGQETGK;GVPEHA	NCP	15.924
GVPEHA;VGNLY	NCP	71.443
VGNLY;EDKLFTF	NCP	23.553
EDKLFTF;NPPVGFP	NCP	12.566
NPPVGFP;TAAKLAF	NCP	83.403
TAAKLAF;VDPLYE	NCP	31.735
VDPLYE;KNYAF	NCP	12.606
KNYAF;ARYLFL	NCP	99.056
ARYLFL;DTRGQVLN	NCP	38.496
DTRGQVLN;KGVDDK	NCP	28.861
KGVDDK;YDMCD	NCP	4.514
YDMCD;KTYANFFT	NCP	43.015
KTYANFFT;KSDFSLK	NCP	9.543
KSDFSLK;PHGGK	NCP	56.129
PHGGK;RVHDEF	NCP	24.187
RVHDEF;LNSSHL	NCP	92.676
LNSSHL;PLKLLVKP	NCP	49.652
PLKLLVKP;GKELLKK	NCP	226.27
GKELLKK;GFTALK	NCP	111.65
GFTALK;MLNPRHP	NCP	13.332
MLNPRHP;QSARLLR	NCP	4.635
QSARLLR;LLFKQP	NCP	69.523
LLFKQP;HLVPASLP	NCP	40.488
HLVPASLP;VLLPF	NCP	25.247
VLLPF;SGTVLRK	NCP	46.698
SGTVLRK;HPPEFQL	NCP	52.53
HPPEFQL;LTPTHY	NCP	49.402
LTPTHY;TPLHTP	NCP	23.568
TPLHTP;VLENLLK	NCP	40.452
VLENLLK;KPGPEQP	NCP	36.074
KPGPEQP;KASAAP	NCP	63.76
KASAAP;KREKFPK	NCP	11.538
KREKFPK;SYGKLSR	NCP	1.658
SYGKLSR;NDFKLK	NCP	7.768
NDFKLK;KFLSCLTL	NCP	61.103
KFLSCLTL;LYEKDY	NCP	54.846
LYEKDY;CCMEGK	NCP	20.074
CCMEGK;KTYPYL	NCP	2.64
KTYPYL;GSHHKTK	NCP	109.199
GSHHKTK;QERLHVLV	NCP	15.69

QERLHVLV;KGSLDHH	NCP	7.458
KGSLDHH;PAAPTPP	NCP	69.942
PAAPTPP;VTDLVEY	NCP	11.124
VTDLVEY;LAAVDMAK	NCP	125.431
LAAVDMAK;AAVFAVPK	NCP	38.798
AAVFAVPK;FGATP	NCP	110.983
FGATP;LEKAALLT	NCP	92.865
LEKAALLT;QAKRPK	NCP	2.858
QAKRPK;LYQTYL	NCP	19.704
LYQTYL;ALAPH	NCP	35.901
ALAPH;LPLPLH	NCP	15.718
LPLPLH;YWCDDNLF	NCP	9.767
YWCDDNLF;KDFYLP	NCP	21.283
KDFYLP;RSYPLKVR	NCP	26.585
RSYPLKVR;LAAEAGKY	NCP	40.377
LAAEAGKY;TPELY	NCP	79.287
TPELY;VAEKTLL	NCP	7.119
VAEKTLL;VDGGFL	NCP	31.972
VDGGFL;TPSLLC	NCP	54.791
TPSLLC;LTDDTKN	NCP	7.461
LTDDTKN;LSKPEYD	NCP	36.472
LSKPEYD;FVDGGVF	NCP	9.759
FVDGGVF;MCHSELK	NCP	65.244
MCHSELK;PTDALL	NCP	82.991
PTDALL;LEPSFGGF	NCP	30.858
LEPSFGGF;HMAWHC	NCP	21.048
HMAWHC;NTPKFGK	NCP	277.824
NTPKFGK;RLLLPEGLAKHAVSEGTKA	NCP	110.379
RLLLPEGLAKHAVSEGTKA;HAVSEGTKAVPKYTSSK	NCP	0.03
HAVSEGTKAVPKYTSSK;TVNVRVN	NCP	80.154
TVNVRVN;DDDTFT	NCP	46.186
DDDTFT;KTEYG	NCP	32.594
KTEYG;KEGERAPR	NCP	1.33
KEGERAPR;MLAFYLPS	NCP	44.99
MLAFYLPS;MLVNLRVL	NCP	45.477
MLVNLRVL;GSVLLVVP	NCP	7.222
GSVLLVVP;LPSPLL	NCP	29.588
LPSPLL;KSVKYFEK	NCP	40.747
KSVKYFEK;EYQPF	NCP	9.908
EYQPF;MELLWLVN	NCP	111.87
MELLWLVN;ELRLESF	NCP	32.931
ELRLESF;MELSH	NCP	20.46
MELSH;LTLTF	NCP	121.53
LTLTF;EHRKSY	NCP	38.622
EHRKSY;LSYKTTLY	NCP	48.229
LSYKTTLY;VYDPYK	NCP	25.665
VYDPYK;GDLTAL	NCP	8.808
GDLTAL;LLFFP	NCP	84.833
LLFFP;QFNVTKL	NCP	6.087
QFNVTKL;SHTVLV	NCP	60.456
SHTVLV;MKLFQ	NCP	38.185

MKLFQ;GHLAKLY	NCP	3.66
GHLAKLY;PHWKFK	NCP	7.983
PHWKFK;LATREPD	NCP	67.982
LATREPD;NNPLVGN	NCP	129.027
NNPLVGN;LEGYVGF	NCP	43.131
LEGYVGF;AWGVGPG	NCP	61.098
AWGVGPG;KTLSGPPK	NCP	9.943
KTLSGPPK;TKRKKLK	NCP	77.15
TKRKKLK;TQGPLLHQ	NCP	1.723
TQGPLLHQ;NALALG	NCP	13.352
NALALG;KWHVKS	NCP	46.228
KWHVKS;KTTGH	NCP	35.571
KTTGH;LPDGQVL	NCP	3.278
LPDGQVL;ERPLTSY	NCP	50.907
ERPLTSY;NGPLLP	NCP	290.52
NGPLLP;PVYEGY	NCP	10.729
PVYEGY;KLLEF	NCP	30.818
KLLEF;AVPYN	NCP	18.487
AVPYN;VQAAK	NCP	32.183
VQAAK;KECALWD	NCP	157.669
KECALWD;SLGSLTNK	NCP	5.549
SLGSLTNK;LPEKGGC	NCP	57.426
LPEKGGC;RDGPHLK	NCP	68.004
RDGPHLK;RPKLEEN	NCP	93.012
RPKLEEN;GPVPSER	NCP	157.176
GPVPSER;RPGPPPSY	NCP	8.524
RPGPPPSY;VPKFV	NCP	47.49
VPKFV;YGDTNMP	NCP	74.703
YGDTNMP;AATFAPK	NCP	122.496
AATFAPK;TEREF	NCP	38.19
TEREF;NLVMAQY	NCP	43.685
NLVMAQY;KNTHTLL	NCP	26.494
KNTHTLL;VGNACLRL	NCP	108.801
VGNACLRL;PTSLGTLK	NCP	25.608
PTSLGTLK;MDSVEK	NCP	32.568
MDSVEK;KPYEGK	NCP	79.605
KPYEGK;WRKDGN	NCP	57.701
WRKDGN;KLLLYSTP	NCP	113.494
KLLLYSTP;RSPHPPP	NCP	20.765
RSPHPPP;ESAPPVN	NCP	44.701
ESAPPVN;MELTV	NCP	2.706
MELTV;FTAVPP	NCP	56.027
FTAVPP;KEMRAGP	NCP	33.409
KEMRAGP;YMKVKEK	NCP	3.6
YMKVKEK;DLKEKKEV	NCP	13.832
DLKEKKEV;ESVLQFYPK	NCP	19.998
ESVLQFYPK;EVVGDAPEKVGPK	NCP	1.401
EVVGDAPEKVGPK;LTQLPVLQ	NCP	138.943
LTQLPVLQ;NGVLLSKL	NCP	12.462
NGVLLSKL;LEATLEL	NCP	20.589
LEATLEL;GFFNTLR	NCP	102.642

GFFNTLR;AAQKTPK	NCP	29.478
AAQKTPK;PVHKKT	NCP	113.891
PVHKKT;KWLPPPPP	NCP	58.648
KWLPPPPP;CNWMFC	NCP	3.266
CNWMFC;VTGGF	NCP	26.475
VTGGF;DACRLL	NCP	66.889
DACRLL;GVPAHKL	NCP	43.794
GVPAHKL;FAPLLR	NCP	30.503
FAPLLR;YVPAGAR	NCP	14.887
YVPAGAR;VEVPP	NCP	30.986
VEVPP;TDLMFF	NCP	100.167
TDLMFF;PPGPPPPP	NCP	40.981
PPGPPPPP;KQSEFLL	NCP	5.249
KQSEFLL;YVEELRK	NCP	12.579
YVEELRK;FALLVY	NCP	39.745
FALLVY;KKTKRGP	NCP	29.586
KKTKRGP;LMMLLRVL	NCP	106.775
LMMLLRVL;VDDVTVP	NCP	84.532
VDDVTVP;PPYTLVY	NCP	48.951
PPYTLVY;LHVADLVSL	NCP	18.647
LHVADLVSL;ALNFSV	NCP	109.006
ALNFSV;KDVMPK	NCP	14.436
KDVMPK;KLAPGHR	NCP	4.886
KLAPGHR;PAALSLY	NCP	30.732
PAALSLY;FPGEKN	NCP	148.198
FPGEKN;RYAPYAF	NCP	135.981
RYAPYAF;KPYSQR	NCP	103.263
KPYSQR;DELNVPR	NCP	79.896
DELNVPR;GLKGQCK	NCP	13.622
GLKGQCK;KHFPS	NCP	71.818
KHFPS;NFLSAR	NCP	56.658
NFLSAR;LAVWLW	NCP	8.108
LAVWLW;LTDSPVL	NCP	2.956
LTDSPVL;LLPGELAKH	NCP	69.369
LLPGELAKH;PLTELPTL	NCP	13.428
PLTELPTL;AANPMLL	NCP	122.955
AANPMLL;VHFRDH	NCP	28.397
VHFRDH;HHAPPTT	NCP	10.905
HHAPPTT;LFAAYAR	NCP	33.133
LFAAYAR;LPPGVTF	NCP	3.951
LPPGVTF;KTMDFR	NCP	7.115
KTMDFR;KNYVLP	NCP	17.448
KNYVLP;KVKFSH	NCP	41.473
KVKFSH;LDLREY	NCP	132.989
LDLREY;LTTDKLK	NCP	11.973
LTTDKLK;KGAGK	NCP	86.511
KGAGK;VEAWP	NCP	42.63
VEAWP;EHMDLRK	NCP	70.128
EHMDLRK;KQTTYME	NCP	20.313
KQTTYME;KDLGLAP	NCP	96.288
KDLGLAP;FYPKAP	NCP	153.601

FYPKAP;KYSSM	NCP	154.74
KYSSM;KYFNTK	NCP	129.048
KYFNTK;NYLHKFYP	NCP	3.891
NYLHKFYP;GYQCP	NCP	1.785
GYQCP;DGLLKF	NCP	11.34
DGLLKF;YESKDY	NCP	42.24
YESKDY;EHFRKF	NCP	36.086
EHFRKF;PPGPRPPP	NCP	74.026
PPGPRPPP;KLYPVFF	NCP	23.682
KLYPVFF;GLGVLPF	NCP	37.971
GLGVLPF;PGVDALLS	NCP	17.969
PGVDALLS;RYDLPK	NCP	15.39
RYDLPK;KVLLPTYK	NCP	44.829
KVLLPTYK;THLYDP	NCP	2.325
THLYDP;HTTLTM	NCP	17.34
HTTLTM;AYSLLL	NCP	2.5
AYSLLL;CLHMAS	NCP	62.295
CLHMAS;GTFAL	NCP	134.588
GTFAL;LLPFQSLE	NCP	49.264
LLPFQSLE;HLLPPL	NCP	72.918
HLLPPL;KPLYSDP	NCP	28.94
KPLYSDP;VLDLLTNN	NCP	29.956
VLDLLTNN;AAPLSV	NCP	37.236
AAPLSV;EYCQY	NCP	205.97
EYCQY;KGVVFL	NCP	7.248
KGVVFL;PLLMPF	NCP	27.91
PLLMPF;SGSPKPR	NCP	303.639
SGSPKPR;RGYDMP	NCP	45.693
RGYDMP;ETNLDSLPL	NCP	92.412
ETNLDSLPL;ESLLVRK	NCP	32.486
ESLLVRK;TLSLSVLP	NCP	25.072
TLSLSVLP;FKHVT	NCP	4.076
FKHVT;ASLPPPR	NCP	8.055
ASLPPPR;AQNWLYE	NCP	4.701
AQNWLYE;LSGVLK	NCP	0.115
LSGVLK;YGYGPY	NCP	8.565
YGYGPY;WTLEDY	NCP	9.627
WTLEDY;QAAPP	NCP	53.868
QAAPP;EVEGAPA	NCP	45.279
EVEGAPA;KGGGGVPR	NCP	62.439
KGGGGVPR;TYQPP	NCP	9.812
TYQPP;GVFVNVN	NCP	18.183
GVFVNVN;AAEGPLLK	NCP	76.483
AAEGPLLK;MPKASA	NCP	17.688
MPKASA;SLRLAEY	NCP	24.575
SLRLAEY;YVAFLEN	NCP	24.483
YVAFLEN;PEVPKVK	NCP	14.472
PEVPKVK;WGPTPLF	NCP	28.459
WGPTPLF;LAKGPPM	NCP	30.987
LAKGPPM;TMPLQ	NCP	29.721
TMPLQ;RALLFLPR	NCP	8.19

RALLFLPR;PPLPPPT	NCP	4.44
PPLPPPT;PTSLNQP	NCP	0.711
PTSLNQP;ERPLFGP	NCP	21.045
ERPLFGP;MDDTLF	NCP	51.648
MDDTLF;MLLDFLLL	NCP	1.188
MLLDFLLL;STPDKSKP	NCP	29.169
STPDKSKP;TGLPMHK	NCP	11.301
TGLPMHK;ALKLLKDP	NCP	15.224
ALKLLKDP;MAPAPRPP	NCP	67.674
MAPAPRPP;KPPAAL	NCP	5.929
KPPAAL;GVKLSPR	NCP	30.894
GVKLSPR;LGCGF	NCP	38.378
LGCGF;ESGTKVK	NCP	15.388
ESGTKVK;ALGHHKH	NCP	66.155
ALGHHKH;ESTLTARK	NCP	60.993
ESTLTARK;MSTYSPF	NCP	36.856
MSTYSPF;KESVVR	NCP	29.052
KESVVR;ESLTHFK	NCP	71.099
ESLTHFK;EDDLHY	NCP	62.617
EDDLHY;KKEHM	NCP	76.37
KKEHM;STPMTWKP	NCP	7.8
STPMTWKP;KGPQAFR	NCP	55.906
KGPQAFR;NPVLL	NCP	58.166
NPVLL;KPPTFPK	NCP	86.002
KPPTFPK;SLDLDLK	NCP	73.38
SLDLDLK;DDPEKLR	NCP	17.376
DDPEKLR;QYRLLQ	NCP	1.035
QYRLLQ;KGADRKK	NCP	3.878
KGADRKK;PAVGPA	NCP	81.306
PAVGPA;AYLFL	NCP	36.388
AYLFL;AAAME	NCP	42.996
AAAME;AESLVY	NCP	1.745
AESLVY;PVCPHAA	NCP	54.01
PVCPHAA;PHAAKCL	NCP	0.009
PHAAKCL;LGEAVF	NCP	15.21
LGEAVF;LPSVAPLP	NCP	2.724
LPSVAPLP;MLKFF	NCP	62.558
MLKFF;AQQLY	NCP	22.473
AQQLY;PFFDVQ	NCP	64.459
PFFDVQ;EPTTAK	NCP	28.1
EPTTAK;KSRFKL	NCP	15.901
KSRFKL;EAVLVK	NCP	90.555
EAVLVK;RAMRELL	NCP	79.971
RAMRELL;LEPYLQPF	NCP	41.937
LEPYLQPF;LNSVGALL	NCP	33.192
LNSVGALL;MDLEAKVKK	NCP	82.715
MDLEAKVKK;YRTLPL	NCP	23.356
YRTLPL;PVWSN	NCP	14.231
PVWSN;VGKLFEDTK	NCP	7.479
VGKLFEDTK;EWAPPVQ	NCP	0.147
EWAPPVQ;LPKEKQPVAGSEGAQ	NCP	1.035

LPKEKQPVAGSEGAQ;MLKRL	NCP	55.807
MLKRL;VGKMLQ	NCP	163.968
VGKMLQ;GVLLF	NCP	0.752
GVLLF;EPAAMW	NCP	19.791
EPAAMW;KPSGHPPP	NCP	7.093
KPSGHPPP;GLETEKADVQLF	NCP	7.742
GLETEKADVQLF;LETEKADVQL	NCP	0.003
LETEKADVQL;HSSVDYADPDK	NCP	0.057
HSSVDYADPDK;LDCTKPK	NCP	1.99
LDCTKPK;AEEMNEKQVYDAH	NCP	26.801
AEEMNEKQVYDAH;WLLFQDVP	NCP	11.419
WLLFQDVP;LLSYKSPF	NCP	140.598
LLSYKSPF;TTAFP	NCP	8.538
TTAFP;DHVVSF	NCP	9.761
DHVVSF;LMKVVK	NCP	75.952
LMKVVK;AVQLKSK	NCP	24.998
AVQLKSK;LEKGLDQ	NCP	63.681
LEKGLDQ;APLYR	NCP	18.438
APLYR;KNPAANP	NCP	40.623
KNPAANP;HALVFV	NCP	12.568
HALVFV;SPRDPQ	NCP	38.121
SPRDPQ;SPNPP	NCP	11.382
SPNPP;DGLFSM	NCP	1.637
DGLFSM;VLAYY	NCP	26.298
VLAYY;LSVAAKF	NCP	16.429
LSVAAKF;YGAGELL	NCP	13.58
YGAGELL;LGVGGKT	NCP	9.097
LGVGGKT;LETALTYL	NCP	77.208
LETALTYL;AVNPLEY	NCP	13.517
AVNPLEY;KGKLLKT	NCP	8.919
KGKLLKT;VPEVF	NCP	4.23
VPEVF;DDDVTN	NCP	8.275
DDDVTN;HALLPFLE	NCP	100.418
HALLPFLE;EPSLGK	NCP	17.035
EPSLGK;YLEAVLT	NCP	42.623
YLEAVLT;NNPMLR	NCP	18.393
NNPMLR;KTVHPP	NCP	19.375
KTVHPP;KVLNL	NCP	39.467
KVLNL;MMMCYT	NCP	44.95
MMMCYT;SGLTPY	NCP	53.514
SGLTPY;VELLKLE	NCP	42.213
VELLKLE;GSGPGPHR	NCP	39.369
GSGPGPHR;DSPWAFP	NCP	22.404
DSPWAFP;MVNDLAP	NCP	9.486
MVNDLAP;KSAVP	NCP	30.963
KSAVP;TPPKFPP	NCP	0.321
TPPKFPP;EGAAGVGLK	NCP	23.913
EGAAGVGLK;RNDPVVL	NCP	0.102
RNDPVVL;CCMCSC	NCP	75.989
CCMCSC;YNLYR	NCP	15.079
YNLYR;SLQLMK	NCP	190.656

SLQLMK;WLPPK	NCP	21.129
WLPPK;LSGSPF	NCP	33.15
LSGSPF;RLFTEL	NCP	124.538
RLFTEL;SDMLP	NCP	36.633
SDMLP;CNYYKQ	NCP	72.42
CNYYKQ;QAAKMMS	NCP	13.033
QAAKMMS;KGGKP	NCP	0.386
KGGKP;RAHFLK	NCP	7.249
RAHFLK;ASRLVPA	NCP	77.589
ASRLVPA;PQGLKQF	NCP	1.823
PQGLKQF;TVAAGTL	NCP	77.772
TVAAGTL;FLKLYKK	NCP	40.194
FLKLYKK;LFPLVKP	NCP	20.367
LFPLVKP;DRFPS	NCP	0.898
DRFPS;TLSALPA	NCP	0.258
TLSALPA;PVVVG	NCP	7.893
PVVVG;MCDVD	NCP	21.327
MCDVD;ETVPLER	NCP	11.322
ETVPLER;DYFEEYGK	NCP	0.846
DYFEEYGK;FDDHDPVDK	NCP	0.09
FDDHDPVDK;FGFGDSR	NCP	0.354
FGFGDSR;FGPGPGSNF	NCP	0.036
FGPGPGSNF;DGLPKTR	NCP	50.223
DGLPKTR;LPVQV	NCP	20.025
LPVQV;ESLQNLSK	NCP	42.234
ESLQNLSK;SELTF	NCP	47.987
SELTF;LLVEVAPL	NCP	162.634
LLVEVAPL;KGSVNPM	NCP	76.652
KGSVNPM;FTSCCYF	NCP	170.538
FTSCCYF;AHLVPK	NCP	43.632
AHLVPK;LLPPMRV	NCP	36.577
LLPPMRV;KESALKTL	NCP	7.182
KESALKTL;ASVSLE	NCP	71.279
ASVSLE;FFTFHPP	NCP	113.643
FFTFHPP;VLAVLNY	NCP	15.882
VLAVLNY;LAPPEKK	NCP	118.36
LAPPEKK;QGMKAR	NCP	12.357
QGMKAR;LPVRGPVT	NCP	16.578
LPVRGPVT;LPLAPD	NCP	13.788
LPLAPD;DPRHLP	NCP	21.453
DPRHLP;FTTLDHY	NCP	17.417
FTTLDHY;LRSSGLR	NCP	6.465
LRSSGLR;KNDVP	NCP	1.164
KNDVP;TVKQSAEDVKK	NCP	27.324
TVKQSAEDVKK;KPSLVH	NCP	70.455
KPSLVH;CCWMP	NCP	38.604
CCWMP;SLDPLVDT	NCP	91.191
SLDPLVDT;KVPKP	NCP	22.386
KVPKP;DLLDP	NCP	87.216
DLLDP;LRDKELK	NCP	69.432
LRDKELK;QAGDAK	NCP	15.432

QAGDAK;LNEDFDVK	NCP	30.795
LNEDFDVK;MDPPPP	NCP	9.337
MDPPPP;KPPPPR	NCP	14.208
KPPPPR;QSAVLFL	NCP	18.689
QSAVLFL;LHAELVT	NCP	13.288
LHAELVT;QGELWK	NCP	73.928
QGELWK;PPRLHPP	NCP	68.214
PPRLHPP;LPPVSHPP	NCP	37.074
LPPVSHPP;PGFPLS	NCP	3.877
PGFPLS;AKGLSMQ	NCP	12.263
AKGLSMQ;GPQKTK	NCP	33.565
GPQKTK;ELPLQK	NCP	13.406
ELPLQK;KPAASP	NCP	29.064
KPAASP;ESQPLKKP	NCP	164.848
ESQPLKKP;VPSSLPF	NCP	2.594
VPSSLPF;KGSAEFLL	NCP	68.848
KGSAEFLL;AMGFPN	NCP	101.19
AMGFPN;KGKEKQAK	NCP	45.132
KGKEKQAK;CLPWLLH	NCP	205.595
CLPWLLH;QTYVPPP	NCP	4.09
QTYVPPP;VAPPEVP	NCP	160.37
VAPPEVP;KPTHVP	NCP	33.726
KPTHVP;QLLYLFL	NCP	10.639
QLLYLFL;GYSPQP	NCP	18.519
GYSPQP;SLYVPPP	NCP	12.053
SLYVPPP;LDFEGK	NCP	68.028
LDFEGK;VFKDPVADPN	NCP	17.397
VFKDPVADPN;ESPFLP	NCP	59.92
ESPFLP;LPPEKPPP	NCP	53.021
LPPEKPPP;KQHVLY	NCP	82.492
KQHVLY;VSLPAGK	NCP	109.406
VSLPAGK;HVVGVLK	NCP	15.336
HVVGVLK;KAEDFLK	NCP	55.014
KAEDFLK;LSALF	NCP	43.498
LSALF;AVPTPYA	NCP	59.363
AVPTPYA;LDDVLEL	NCP	153.571
LDDVLEL;ANGFW	NCP	45.839
ANGFW;LEFVHK	NCP	21.769
LEFVHK;LMLLL	NCP	47.498
LMLLL;YGLDL	NCP	62.683
YGLDL;LEFLKK	NCP	19.583
LEFLKK;GFYSEVKK	NCP	9.849
GFYSEVKK;KEAPAPPK	NCP	86.352
KEAPAPPK;KESTLWSP	NCP	55.69
KESTLWSP;KSYMVH	NCP	0.653
KSYMVH;KVVNPLFEKRPK	NCP	1.14
KVVNPLFEKRPK;TQALDRQTATQLLK	NCP	0.177
TQALDRQTATQLLK;AKLEKAKAKELATK	NCP	0.498
AKLEKAKAKELATK;LEKAKAKELATKLG	NCP	0.006
LEKAKAKELATKLG;LLMELP	NCP	43.516
LLMELP;PGKPPEE	NCP	242.511

PGKPPEE;NWMVSKK	NCP	32.831
NWMVSKK;PFPLSLLK	NCP	40.38
PFPLSLLK;KHGKLSK	NCP	24.808
KHGKLSK;LSEGKLP	NCP	7.755
LSEGKLP;EGKPLK	NCP	2.15
EGKPLK;EEKLHL	NCP	52.21
EEKLHL;EPTLFAC	NCP	57.315
EPTLFAC;ATLFV	NCP	101.714
ATLFV;PSLPASQ	NCP	22.483
PSLPASQ;KPKLL	NCP	21.33
KPKLL;LDDLNLK	NCP	37.157
LDDLNLK;MSFLPR	NCP	42.909
MSFLPR;ATTSK	NCP	20.086
ATTSK;LSTTPMR	NCP	38.973
LSTTPMR;SRGTGVK	NCP	36.101
SRGTGVK;EDGPFK	NCP	23.58
EDGPFK;EDLVPGSR	NCP	70.021
EDLVPGSR;FHVAKR	NCP	91.971
FHVAKR;APMLPP	NCP	59.856
APMLPP;MLATLK	NCP	68.81
MLATLK;QKGGLE	NCP	45.235
QKGGLE;FLVLLSSGK	NCP	56.373
FLVLLSSGK;FVDLLH	NCP	82.668
FVDLLH;AAFLSVQ	NCP	128.382
AAFLSVQ;KDVAAAP	NCP	21.879
KDVAAAP;TAAQSQK	NCP	9.417
TAAQSQK;LDSYK	NCP	59.922
LDSYK;TLFSLAL	NCP	6.896
TLFSLAL;FAGPGEDR	NCP	51.058
FAGPGEDR;PLQLRVW	NCP	14.541
PLQLRVW;FDDKTSF	NCP	40.059
FDDKTSF;QTNPMLL	NCP	24.441
QTNPMLL;LDRTLR	NCP	46.563
LDRTLR;HQGLPQ	NCP	18.539
HQGLPQ;MEMDC	NCP	96.187
MEMDC;YHDSPK	NCP	66.357
YHDSPK;VWLGSGL	NCP	70.125
VWLGSGL;PTVLYNR	NCP	4.443
PTVLYNR;HLKCLK	NCP	70.023
HLKCLK;ENGTVC	NCP	130.61
ENGTVC;TPNVVEF	NCP	25.137
TPNVVEF;TLAVDF	NCP	144.699
TLAVDF;LVPEAQAK	NCP	67.866
LVPEAQAK;SPEPSK	NCP	118.659
SPEPSK;MVNPTVFF	NCP	55.614
MVNPTVFF;TANPP	NCP	31.636
TANPP;LADDLVT	NCP	32.841
LADDLVT;VPPPPALP	NCP	18.477
VPPPPALP;GAVVPP	NCP	71.403
GAVVPP;QPGFLQ	NCP	11.811
QPGFLQ;QDELNPR	NCP	33.212

QDELNPR;LSLVTL	NCP	191.205
LSLVTL;FPVSLPL	NCP	22.758
FPVSLPL;SLEDVEY	NCP	17.406
SLEDVEY;LLRGVFY	NCP	41.85
LLRGVFY;RHKKKPR	NCP	29.286
RHKKKPR;GKELLDL	NCP	7.446
GKELLDL;CDAAHC	NCP	12.1
CDAAHC;YSGAGELL	NCP	15.59
YSGAGELL;KPAAFYP	NCP	40.431
KPAAFYP;GRVGATAAV	NCP	25.57
GRVGATAAV;ATAAVYSAA	NCP	0.012
ATAAVYSAA;AGNASKDLKVK	NCP	4.389
AGNASKDLKVK;EAPAPPKAEAK	NCP	4.805
EAPAPPKAEAK;KGAAAP	NCP	8.428
KGAAAP;VPGGLP	NCP	145.23
VPGGLP;AALAPGVP	NCP	96.717
AALAPGVP;VNSTLKV	NCP	7.242
VNSTLKV;LPPAALAF	NCP	70.625
LPPAALAF;SLMGP	NCP	26.311
SLMGP;LKHVEL	NCP	74.402
LKHVEL;LGKQPR	NCP	86.193
LGKQPR;SHTPPPK	NCP	71.725
SHTPPPK;SPPSF	NCP	136.857
SPPSF;AKDFGPK	NCP	10.02
AKDFGPK;EHALLAYTLGVKQL	NCP	41.063
EHALLAYTLGVKQL;VETGVLKPGM	NCP	0.396
VETGVLKPGM;TGVLKPGMV	NCP	0.006
TGVLKPGMV;GAPVYL	NCP	88.819
GAPVYL;KEFPKAK	NCP	17.891
KEFPKAK;TAELLEL	NCP	25.449
TAELLEL;KEFKAW	NCP	11.289
KEFKAW;KFFEPLK	NCP	51.061
KFFEPLK;KDKLEER	NCP	19.461
KDKLEER;LETVPLER	NCP	20.643
LETVPLER;LAAGP	NCP	23.577
LAAGP;LVGTVLM	NCP	57.015
LVGTVLM;EHLTVK	NCP	68.403
EHLTVK;KDEPYK	NCP	102.797
KDEPYK;KEVLYFY	NCP	34.836
KEVLYFY;LSHSL	NCP	16.327
LSHSL;VNDSFF	NCP	32.142
VNDSFF;HATPKEK	NCP	24.26
HATPKEK;TSSSDK	NCP	27.355
TSSSDK;GPVALLR	NCP	28.193
GPVALLR;KAAMSPSK	NCP	61.12
KAAMSPSK;KSLKDDK	NCP	92.318
KSLKDDK;PPPSW	NCP	148.144
PPPSW;VSLLMDLL	NCP	47.729
VSLLMDLL;DLAYLPQ	NCP	12.952
DLAYLPQ;WAFKLK	NCP	71.409
WAFKLK;PMKLVPP	NCP	27.387

PMKLVPP;KTVFKT	NCP	26.388
KTVFKT;SVNPPVLL	NCP	10.539
SVNPPVLL;DLHFKEK	NCP	47.136
DLHFKEK;SSAFE	NCP	11.279
SSAFE;KDRPGAGN	NCP	130.086
KDRPGAGN;REDGEKAAREVK	NCP	8.796
REDGEKAAREVK;SAVKFKK	NCP	42.685
SAVKFKK;TFKDLHFK	NCP	41.969
TFKDLHFK;HPPPG	NCP	92.695
HPPPG;LMSSSYL	NCP	11.778
LMSSSYL;TMFLT	NCP	26.247
TMFLT;QRSLETSL	NCP	153.384
QRSLETSL;LGAGLM	NCP	135.021
LGAGLM;LLSVP	NCP	13.007
LLSVP;QVFLK	NCP	126.364
QVFLK;VYDVFPK	NCP	55.05
VYDVFPK;KHFSP	NCP	25.316
KHFSP;VSACMFF	NCP	56.766
VSACMFF;SVKSSAK	NCP	61.837
SVKSSAK;LEVLPEK	NCP	57.776
LEVLPEK;KGMTCKK	NCP	19.575
KGMTCKK;SLPVP	NCP	90.466
SLPVP;TSPKPLG	NCP	0.039
TSPKPLG;PFLEDSFF	NCP	7.116
PFLEDSFF;NSPMDW	NCP	19.254
NSPMDW;TAPLQL	NCP	5.445
TAPLQL;LRKLSN	NCP	2.763
LRKLSN;DGPLF	NCP	20.292
DGPLF;MDLNLR	NCP	11.808
MDLNLR;CYMSPF	NCP	80.519
CYMSPF;FLVPLEF	NCP	57.505
FLVPLEF;YAKAPPL	NCP	87.069
YAKAPPL;FGPLLRY	NCP	15.009
FGPLLRY;NTVVEPY	NCP	101.996
NTVVEPY;KTYPPK	NCP	77.365
KTYPPK;RLDHSK	NCP	9.215
RLDHSK;GPGVKVHL	NCP	11.926
GPGVKVHL;ATKPTLPK	NCP	84.813
ATKPTLPK;AHERVLK	NCP	72.612
AHERVLK;PWPAELK	NCP	6.903
PWPAELK;KEVNKGK	NCP	50.574
KEVNKGK;PTESF	NCP	59.436
PTESF;PLADFR	NCP	78.738
PLADFR;PVLKPPPP	NCP	6.725
PVLKPPPP;FMLLGAG	NCP	16.135
FMLLGAG;LSGGTLRK	NCP	2.346
LSGGTLRK;KPGKELK	NCP	1.871
KPGKELK;DQVPH	NCP	30.13
DQVPH;TMKGSP	NCP	83.783
TMKGSP;SCCSHST	NCP	21.057
SCCSHST;AKLEY	NCP	47.857

AKLEY;YRLVPYP	NCP	5.373
YRLVPYP;NFMLNQ	NCP	0.159
NFMLNQ;ALVEAGK	NCP	39.975
ALVEAGK;DGPLP	NCP	12.458
DGPLP;LLLNSLKY	NCP	89.125
LLLNSLKY;RKMTVVP	NCP	29.528
RKMTVVP;LSGGWVLL	NCP	17.17
LSGGWVLL;KMRSPP	NCP	100.137
KMRSPP;LGVLLAF	NCP	167.534
LGVLLAF;TLATLKTK	NCP	142.743
TLATLKTK;FLVLKK	NCP	9.571
FLVLKK;EVDLKKNP	NCP	22.031
EVDLKKNP;LPELLK	NCP	38.085
LPELLK;QDFFGEH	NCP	19.123
QDFFGEH;QKTFRAE	NCP	40.275
QKTFRAE;HPKPDR	NCP	30.927
HPKPDR;GHLAKKF	NCP	23.829
GHLAKKF;DLDLRKD	NCP	23.493
DLDLRKD;DLPAF	NCP	9.269
DLPAF;EEFLGP	NCP	19.003
EEFLGP;DCCGCCC	NCP	131.829
DCCGCCC;KPYELK	NCP	32.921
KPYELK;PVFHTR	NCP	22.41
PVFHTR;NEEAGW	NCP	46.017
NEEAGW;EKLKGY	NCP	14.064
EKLKGY;LLWFFV	NCP	217.795
LLWFFV;KLLPF	NCP	66.26
KLLPF;LQLLVPL	NCP	137.193
LQLLVPL;VTLVQTK	NCP	163.455
VTLVQTK;ERVAKT	NCP	6.22
ERVAKT;LDMNLLH	NCP	76.337
LDMNLLH;DEFPF	NCP	8.356
DEFPF;HLLLNK	NCP	66.021
HLLLNK;AADSLKK	NCP	243.675
AADSLKK;NASLLR	NCP	128.271
NASLLR;EWMYG	NCP	49.641
EWMYG;MLPVTEL	NCP	39.831
MLPVTEL;KDAMVP	NCP	212.693
KDAMVP;VAPGMK	NCP	45.282
VAPGMK;GYPLTL	NCP	115.704
GYPLTL;AVSVPLKEKK	NCP	246.42
AVSVPLKEKK;ALATLTK	NCP	7.998
ALATLTK;HSGGY	NCP	77.35
HSGGY;GRLFDQ	NCP	89.4
GRLFDQ;CYCKP	NCP	39.092
CYCKP;KAKKEVL	NCP	2.988
KAKKEVL;LHLPFLE	NCP	9.204
LHLPFLE;QMFLTM	NCP	17.913
QMFLTM;KHFYNGY	NCP	44.097
KHFYNGY;KYKKK	NCP	179.374
KYKKK;VELKGVT	NCP	103.584

VELKGVT;KGKVCLR	NCP	44.087
KGKVCLR;DGPVQF	NCP	47.106
DGPVQF;VPSLVHRK	NCP	1.882
VPSLVHRK;QCVLVA	NCP	72.417
QCVLVA;EMKLY	NCP	54.803
EMKLY;ENGVLRFQ	NCP	2.94
ENGVLRFQ;TELPT	NCP	46.327
TELPT;YDAPPF	NCP	58.919
YDAPPF;GVYKKL	NCP	84.256
GVYKKL;SSDLALHH	NCP	92.85
SSDLALHH;EGSNWR	NCP	85.449
EGSNWR;TTYYNLF	NCP	60.938
TTYYNLF;VYPLPL	NCP	48.327
VYPLPL;VPYPVPP	NCP	5.581
VPYPVPP;FNFNGLK	NCP	357.023
FNFNGLK;KHFHPP	NCP	91.806
KHFHPP;KGLDLLC	NCP	100.018
KGLDLLC;SLTRKFC	NCP	46.977
SLTRKFC;KDALHAK	NCP	50.841
KDALHAK;SCHWNPK	NCP	19.086
SCHWNPK;KVGVLK	NCP	15.966
KVGVLK;AANVP	NCP	25.446
AANVP;GRKVMSK	NCP	23.639
GRKVMSK;AVERLL	NCP	28.155
AVERLL;KCPTTK	NCP	22.737
KCPTTK;CMDMDC	NCP	0.781
CMDMDC;LTAYDY	NCP	79.73
LTAYDY;GSVHLTF	NCP	63.942
GSVHLTF;PPPAPPK	NCP	46.761
PPPAPPK;MGVQLSLK	NCP	93.195
MGVQLSLK;EVELLLP	NCP	105.675
EVELLLP;LPRLHP	NCP	98.583
LPRLHP;KVYFHPK	NCP	92.479
KVYFHPK;LGENSPRP	NCP	15.983
LGENSPRP;TNLLLYF	NCP	18.555
TNLLLYF;VFSPHSAPF	NCP	37.773
VFSPHSAPF;LERLYLS	NCP	69.259
LERLYLS;TVYLR	NCP	46.634
TVYLR;KMVECL	NCP	36.201
KMVECL;YLVKTK	NCP	288.085
YLVKTK;QTHSSP	NCP	86.955
QTHSSP;KDVMPF	NCP	25.265
KDVMPF;LSFAPPK	NCP	49.833
LSFAPPK;STFDK	NCP	109.455
STFDK;TKRCLEN	NCP	26.059
TKRCLEN;SSPVHSK	NCP	91.241
SSPVHSK;ECCMG	NCP	24.163
ECCMG;SPPPPPL	NCP	73.937
SPPPPPL;LEGSLLLK	NCP	36.036
LEGSLLLK;EPEKLER	NCP	64.284
EPEKLER;PSAGFPL	NCP	31.399

PSAGFPL;HLLKKM	NCP	75.302
HLLKKM;YHRLY	NCP	25.236
YHRLY;TLAAPL	NCP	22.027
TLAAPL;HFVGPVR	NCP	71.426
HFVGPVR;CMYFTGP	NCP	5.707
CMYFTGP;VVQVN	NCP	1.106
VVQVN;RALLFV	NCP	69.099
RALLFV;EVLSSVAL	NCP	52.101
EVLSSVAL;GKVPTC	NCP	40.053
GKVPTC;FGMPDM	NCP	77.749
FGMPDM;KPVSNKK	NCP	196.026
KPVSNKK;SVGGLLY	NCP	35.979
SVGGLLY;LFVGQDN	NCP	52.349
LFVGQDN;KYTLSP	NCP	14.622
KYTLSP;KTMGTKK	NCP	7.05
KTMGTKK;LGSPVDL	NCP	56.293
LGSPVDL;LKVGSELK	NCP	5.096
LKVGSELK;LPPQLFR	NCP	43.539
LPPQLFR;DVLPPGR	NCP	54.126
DVLPPGR;KQQAAKP	NCP	65.871
KQQAAKP;LYHVRK	NCP	38.541
LYHVRK;LSTGF	NCP	42.553
LSTGF;KMCTAK	NCP	9.902
KMCTAK;YAPPP	NCP	27.651
YAPPP;DKLVG	NCP	27.252
DKLVG;MEEKLK	NCP	24.897
MEEKLK;LSFMTVFL	NCP	103.065
LSFMTVFL;VVLDGGP	NCP	54.75
VVLDGGP;LAAVYL	NCP	66.648
LAAVYL;EVVQEL	NCP	2.301
EVVQEL;KQEVP	NCP	160.288
KQEVP;LAVFF	NCP	25.119
LAVFF;PFFFMM	NCP	12.401
PFFFMM;KKQLGVP	NCP	18.039
KKQLGVP;KMLDLRK	NCP	22.354
KMLDLRK;QSGPEKK	NCP	1.542
QSGPEKK;VGPAK	NCP	541.404
VGPAK;MDMGH	NCP	90.971
MDMGH;PPFPP	NCP	4.5
PPFPP;KPGFDEL	NCP	90.616
KPGFDEL;SVALK	NCP	42.57
SVALK;QVSGTFF	NCP	72.308
QVSGTFF;KLSLHMH	NCP	7.657
KLSLHMH;KVVGV	NCP	294.606
KVVGV;VTPLNPK	NCP	89.418
VTPLNPK;KNKPPP	NCP	60.197
KNKPPP;FVMTGSL	NCP	9.217
FVMTGSL;FSLKSNY	NCP	46.998
FSLKSNY;ATAKFPK	NCP	65.832
ATAKFPK;LLFPLSV	NCP	23.585
LLFPLSV;MNAVLEY	NCP	41.904

MNAVLEY;VSVPSPK	NCP	1.026
VSVPSPK;KLGKNPVQ	NCP	96.415
KLGKNPVQ;LTGYDF	NCP	17.612
LTGYDF;MKVLDFP	NCP	20.805
MKVLDFP;AAVFLAP	NCP	77.889
AAVFLAP;APMVL	NCP	18.622
APMVL;LPLKDL	NCP	20.982
LPLKDL;KFQMTRL	NCP	38.322
KFQMTRL;QKMLLC	NCP	11.082
QKMLLC;FDPKVK	NCP	15.495
FDPKVK;SALERLK	NCP	256.352
SALERLK;EKGDGKRK	NCP	6.855
EKGDGKRK;YSDNLLLY	NCP	44.47
YSDNLLLY;AAGCSVAPRPK	NCP	149.618
AAGCSVAPRPK;DLKCLSPLV	NCP	25.224
DLKCLSPLV;NAPVELR	NCP	163.071
NAPVELR;AEEVYVP	NCP	23.371
AEEVYVP;NTVAV	NCP	43.221
NTVAV;QDRPTF	NCP	13.686
QDRPTF;CENVLLT	NCP	37.479
CENVLLT;LPAEKHK	NCP	186.068
LPAEKHK;KCSAKH	NCP	1.555
KCSAKH;KLLEVDP	NCP	27.452
KLLEVDP;NYLLSF	NCP	25.086
NYLLSF;SKSTEQK	NCP	36.549
SKSTEQK;GPLLPAPP	NCP	50.086
GPLLPAPP;LSFAHVY	NCP	2.24
LSFAHVY;MVHPPGP	NCP	60.709
MVHPPGP;EMMCR	NCP	27.855
EMMCR;HLRPPPP	NCP	47.027
HLRPPPP;LAGSLRVAP	NCP	157.747
LAGSLRVAP;KVLFKKRP	NCP	128.871
KVLFKKRP;VTDVLLK	NCP	92.69
VTDVLLK;LGQFVL	NCP	8.763
LGQFVL;AARFAQE	NCP	97.2
AARFAQE;HSLLLYR	NCP	153.39
HSLLLYR;HSTTFLL	NCP	2.542
HSTTFLL;SLLVQK	NCP	42.51
SLLVQK;ALVLF	NCP	50.358
ALVLF;KPVGDYK	NCP	1.946
KPVGDYK;KSDVLNG	NCP	30.61
KSDVLNG;KEMMER	NCP	59.054
KEMMER;SACDK	NCP	102.139
SACDK;LRLVENL	NCP	26.648
LRLVENL;QPSFLPG	NCP	88.786
QPSFLPG;EHVTPQ	NCP	15.164
EHVTPQ;EPPAPLR	NCP	69.496
EPPAPLR;KSGRQPP	NCP	39.909
KSGRQPP;GSAAAL	NCP	43.218
GSAAAL;MASDLRK	NCP	73.967
MASDLRK;TLERY	NCP	23.395

TLERY;VEPKFPP	NCP	7.694
VEPKFPP;AVTLTLK	NCP	13.33
AVTLTLK;LADMGSS	NCP	57.135
LADMGSS;GMMAY	NCP	17.237
GMMAY;FLTPQ	NCP	90.42
FLTPQ;SWLKLK	NCP	4.057
SWLKLK;DSPVVLVS	NCP	20.937
DSPVVLVS;GKPLAPP	NCP	132.933
GKPLAPP;EMFGEKL	NCP	107.049
EMFGEKL;PNPPVH	NCP	2.42
PNPPVH;AVPLA	NCP	110.383
AVPLA;KMFAAVF	NCP	19.826
KMFAAVF;PLRTGAP	NCP	8.338
PLRTGAP;EVFAFGL	NCP	104.516
EVFAFGL;LLHAMK	NCP	16.791
LLHAMK;KQVKVA	NCP	229.275
KQVKVA;LSPDGQYVPR	NCP	32.346
LSPDGQYVPR;KPLLKYF	NCP	18.069
KPLLKYF;FAFNYL	NCP	14.787
FAFNYL;GVGTY	NCP	5.146
GVGTY;KDTTVKPGAKKDTKDSQLK	NCP	18.335
KDTTVKPGAKKDTKDSQLK;DSQLKLPQ	NCP	0.042
DSQLKLPQ;TYEEALYKSK	NCP	0.324
TYEEALYKSK;YEEALYK	NCP	0.003
YEEALYK;LAEQFVLL	NCP	1.452
LAEQFVLL;AEQFVLLNL	NCP	0.003
AEQFVLLNL;KHLSPDGQYVPR	NCP	2.682
KHLSPDGQYVPR;GRYSNRLY	NCP	3.423
GRYSNRLY;AYEPSDTAL	NCP	0.024
AYEPSDTAL;DNMKKALKLLKTEL	NCP	2.193
DNMKKALKLLKTEL;ALKLLKTE	NCP	0.015
ALKLLKTE;HVLVGPK	NCP	66.336
HVLVGPK;KGPAKWPP	NCP	31.53
KGPAKWPP;RFAKKN	NCP	4.198
RFAKKN;KPVSEPK	NCP	71.433
KPVSEPK;PPLMP	NCP	16.94
PPLMP;TAANLYP	NCP	68.937
TAANLYP;GLSCPSYG	NCP	7.744
GLSCPSYG;GCMMEE	NCP	21.911
GCMMEE;DGGLLDF	NCP	16.408
DGGLLDF;LVVDFV	NCP	93.39
LVVDFV;GVTERGY	NCP	22.022
GVTERGY;YCDVCYNK	NCP	52.702
YCDVCYNK;GEGALYK	NCP	173.832
GEGALYK;LVEAPP	NCP	65.964
LVEAPP;PQFPLPSK	NCP	9.783
PQFPLPSK;LPPCPK	NCP	45.311
LPPCPK;PFSLNMSL	NCP	0.168
PFSLNMSL;KARVVFF	NCP	65.074
KARVVFF;QGPLLE	NCP	47.073
QGPLLE;HVLPYL	NCP	3.497

HVLPYL;CPKKGK	NCP	9.586
CPKKGK;GPLDPP	NCP	342.786
GPLDPP;FPDPVN	NCP	13.104
FPDPVN;KAPSVPK	NCP	2.124
KAPSVPK;AAARVLPE	NCP	6.618
AAARVLPE;LLPTV	NCP	9.407
LLPTV;MFGGTGP	NCP	53.29
MFGGTGP;QRLLLEPF	NCP	56.25
QRLLLEPF;RATNKSY	NCP	35.45
RATNKSY;LDRNLAG	NCP	99.853
LDRNLAG;VPGRTPPP	NCP	14.379
VPGRTPPP;VEGPVPN	NCP	5.297
VEGPVPN;VGPLKD	NCP	1.444
VGPLKD;DWHRP	NCP	89.87
DWHRP;LSVAY	NCP	18.706
LSVAY;ETCCC	NCP	61.889
ETCCC;DAHLVLY	NCP	8.503
DAHLVLY;FPGVPQ	NCP	8.55
FPGVPQ;SPKHQE	NCP	75.342
SPKHQE;HPTMSK	NCP	19.824
HPTMSK;KQFKTGP	NCP	92.972
KQFKTGP;EVVKLF	NCP	4.678
EVVKLF;DLPQL	NCP	103.797
DLPQL;EAVLNR	NCP	3.935
EAVLNR;KVSAVAP	NCP	126.165
KVSAVAP;LTVHYK	NCP	8.92
LTVHYK;KMKRFET	NCP	12.711
KMKRFET;LLAGY	NCP	57.311
LLAGY;DTEPVVL	NCP	56.209
DTEPVVL;AAWLER	NCP	26.015
AAWLER;ACSLLL	NCP	4.941
ACSLLL;FPLNFFC	NCP	92.173
FPLNFFC;DLLFK	NCP	97.511
DLLFK;AAYSEY	NCP	25.86
AAYSEY;TVGGLALS	NCP	17.47
TVGGLALS;TAVALAA	NCP	130.892
TAVALAA;LAGAELF	NCP	8.02
LAGAELF;NSLPF	NCP	35.52
NSLPF;LENVYR	NCP	7.92
LENVYR;KGAYPP	NCP	15.662
KGAYPP;EVCFR	NCP	51.646
EVCFR;PFVPQPY	NCP	80.166
PFVPQPY;TVEHAK	NCP	4.967
TVEHAK;WGMSVR	NCP	6.81
WGMSVR;VDLKP	NCP	17.532
VDLKP;LTGTELH	NCP	71.02
LTGTELH;LADALNT	NCP	44.918
LADALNT;GVLGL	NCP	5.871
GVLGL;EVHLPPP	NCP	19.753
EVHLPPP;GVSTTSLF	NCP	46.383
GVSTTSLF;RYHRLY	NCP	62.192

RYHRLY;KADLTVH	NCP	60.919
KADLTVH;GPAPRHP	NCP	0.186
GPAPRHP;MKFKDSK	NCP	75.692
MKFKDSK;NYWDKLT	NCP	61.761
NYWDKLT;APPVKPPP	NCP	22.335
APPVKPPP;ASLNF	NCP	6.726
ASLNF;KDMHAGK	NCP	51.546
KDMHAGK;ERPLTAM	NCP	75.225
ERPLTAM;PNGPVAK	NCP	1.939
PNGPVAK;NHYPDR	NCP	8.345
NHYPDR;LELPAG	NCP	6.508
LELPAG;GSMFL	NCP	187.031
GSMFL;ALARPPL	NCP	63.595
ALARPPL;KMACP	NCP	71.601
KMACP;MRAAPPP	NCP	27.42
MRAAPPP;LPSHATSP	NCP	58.911
LPSHATSP;NKAPHLK	NCP	7.11
NKAPHLK;AEEMNEK	NCP	102.57
AEEMNEK;TMLPLK	NCP	3.75
TMLPLK;MDVKYFK	NCP	30.809
MDVKYFK;DLAALVV	NCP	40.492
DLAALVV;PLPPP	NCP	16.097
PLPPP;ELFTP	NCP	25.686
ELFTP;PALPLSK	NCP	7.209
PALPLSK;FDKGPK	NCP	65.227
FDKGPK;RGVVEPY	NCP	27.059
RGVVEPY;GLGPNFC	NCP	52.576
GLGPNFC;LFDTQLH	NCP	136.991
LFDTQLH;SLDLRPY	NCP	9.072
SLDLRPY;QDSTFRKP	NCP	48.163
QDSTFRKP;QLALFLM	NCP	46.238
QLALFLM;LSFFKAYL	NCP	168.373
LSFFKAYL;PLFKRD	NCP	21.665
PLFKRD;LNFPY	NCP	9.382
LNFPY;VGKRATP	NCP	77.066
VGKRATP;LFDQF	NCP	48.712
LFDQF;VGNLK	NCP	53.331
VGNLK;DYMCSC	NCP	51.867
DYMCSC;LSGFDNK	NCP	190.256
LSGFDNK;TFAQRLC	NCP	56.19
TFAQRLC;VEQDALH	NCP	46.411
VEQDALH;KPAAWR	NCP	49.647
KPAAWR;LEPLVVP	NCP	8.025
LEPLVVP;QSAKKLL	NCP	9.318
QSAKKLL;SLTAC	NCP	3.536
SLTAC;YSLLA	NCP	13.767
YSLLA;KDLAWYF	NCP	2.943
KDLAWYF;KVMNVRP	NCP	109.401
KVMNVRP;NAFPLLK	NCP	11.14
NAFPLLK;KGAELAK	NCP	23.714
KGAELAK;AAPVL	NCP	58.312

AAPVL;TRTSLY	NCP	6.786
TRTSLY;TMVPLSF	NCP	16.08
TMVPLSF;ESGLLTL	NCP	15.573
ESGLLTL;KTRGRPK	NCP	16.364
KTRGRPK;KNFQASK	NCP	199.375
KNFQASK;KFLTN	NCP	120.144
KFLTN;YWTVRCF	NCP	108.674
YWTVRCF;KPVAGP	NCP	47.671
KPVAGP;NKETDRK	NCP	29.222
NKETDRK;SLANLLY	NCP	74.476
SLANLLY;HMATTAK	NCP	8.372
HMATTAK;ERGVPR	NCP	33.229
ERGVPR;ELVTFNKF	NCP	10.943
ELVTFNKF;PPLPSRP	NCP	3.642
PPLPSRP;AVSTPKK	NCP	19.126
AVSTPKK;VDYSKLKKEGPDF	NCP	5.243
VDYSKLKKEGPDF;KVDLEDY	NCP	0.417
KVDLEDY;APAPEFTA	NCP	56.922
APAPEFTA;VAAPEFF	NCP	3.993
VAAPEFF;PVAKAVV	NCP	32.467
PVAKAVV;LTTPGTTPL	NCP	38.562
LTTPGTTPL;LLKKSL	NCP	23.927
LLKKSL;EDAGPLKL	NCP	10.528
EDAGPLKL;KPRLLLF	NCP	19.224
KPRLLLF;EPHVMP	NCP	9.912
EPHVMP;PAAPRPP	NCP	97.476
PAAPRPP;VGDSLPDL	NCP	22.646
VGDSLPDL;KSFAAPK	NCP	26.401
KSFAAPK;VLTVY	NCP	23.246
VLTVY;AHDGSPP	NCP	167.965
AHDGSPP;APVTTPK	NCP	5.901
APVTTPK;PLLVPDSF	NCP	106.491
PLLVPDSF;NKGELER	NCP	0.137
NKGELER;MFLLQ	NCP	2.473
MFLLQ;GPPVNVP	NCP	3.135
GPPVNVP;PGVPATVP	NCP	15.393
PGVPATVP;YVEWY	NCP	28.037
YVEWY;QPVFLL	NCP	18.831
QPVFLL;LPDML	NCP	27.883
LPDML;NNLGGGLG	NCP	70.469
NNLGGGLG;CGFMPTR	NCP	35.46
CGFMPTR;HGPVLPLK	NCP	107.113
HGPVLPLK;MHLSAVYH	NCP	11.13
MHLSAVYH;NSGSLDF	NCP	23.874
NSGSLDF;PAADQN	NCP	38.187
PAADQN;MCCYQ	NCP	32.792
MCCYQ;ANYLK	NCP	63.369
ANYLK;NGRLVY	NCP	98.308
NGRLVY;KLYDKLD	NCP	18.479
KLYDKLD;MNLLKLP	NCP	171.727
MNLLKLP;KMLVFEH	NCP	35.024

KMLVFEH;KDSLAAL	NCP	71.449
KDSLAAL;ELDALP	NCP	205.682
ELDALP;KTSNPPK	NCP	29.314
KTSNPPK;LLKLKS	NCP	6.632
LLKLKS;DDRKPVGL	NCP	4.096
DDRKPVGL;QASPPDK	NCP	9.606
QASPPDK;MDTLPRR	NCP	40.152
MDTLPRR;GFMKPLR	NCP	279.318
GFMKPLR;SHSLL	NCP	14.342
SHSLL;NYAPGL	NCP	64.758
NYAPGL;TEPCRF	NCP	5.479
TEPCRF;PPQAR	NCP	2.055
PPQAR;NTSVVDTL	NCP	10.238
NTSVVDTL;LPPPPYK	NCP	2.244
LPPPPYK;QAPLVH	NCP	1.737
QAPLVH;KRGKPPP	NCP	70.356
KRGKPPP;TTDTFDER	NCP	5.221
TTDTFDER;NVLVQP	NCP	25.385
NVLVQP;KDLKNFLN	NCP	31.282
KDLKNFLN;LTHAYK	NCP	6.888
LTHAYK;PGSTKPPP	NCP	1.374
PGSTKPPP;MEDMPL	NCP	2.829
MEDMPL;KYVMGAP	NCP	42.719
KYVMGAP;VGGFHG	NCP	66.847
VGGFHG;APGGLK	NCP	57.012
APGGLK;PACPL	NCP	136.439
PACPL;PEPLLVD	NCP	50.26
PEPLLVD;KKLLVFMS	NCP	26.283
KKLLVFMS;KDFPNVP	NCP	66.638
KDFPNVP;AAAVPQR	NCP	60.384
AAAVPQR;LPPEL	NCP	9.132
LPPEL;GHSSLSY	NCP	3.801
GHSSLSY;QPGLFQ	NCP	8.568
QPGLFQ;KNQDFKK	NCP	90.837
KNQDFKK;YRSLYNPQ	NCP	50.2
YRSLYNPQ;KLDPTSK	NCP	50.354
KLDPTSK;KREPLF	NCP	11.407
KREPLF;RLSGRVGP	NCP	24.911
RLSGRVGP;AYKLVLL	NCP	5.671
AYKLVLL;SATFL	NCP	39.116
SATFL;HLPVPLPP	NCP	28.804
HLPVPLPP;KQPMLAF	NCP	21.033
KQPMLAF;PVALRLF	NCP	7.107
PVALRLF;KGGQAKRGP	NCP	74.886
KGQAKRGP;KSMEPVP	NCP	25.718
KSMEPVP;KSLLDA	NCP	1.404
KSLLDA;LLAFAHH	NCP	91.186
LLAFAHH;LSFLSDH	NCP	32.769
LSFLSDH;LFKNVPP	NCP	35.927
LFKNVPP;DMTCPQ	NCP	1.662
DMTCPQ;LPPAPP	NCP	31.227

LPPAPP;TFSKPQ	NCP	88.144
TFSKPQ;KQGTSLVP	NCP	24.374
KQGTSLVP;PPVYP	NCP	30.804
PPVYP;TVSSFPK	NCP	34.693
TVSSFPK;LTDSLYP	NCP	32.087
LTDSLYP;LSPVLLP	NCP	29.611
LSPVLLP;FYSLPN	NCP	33.521
FYSLPN;KYNSPPPP	NCP	114.768
KYNSPPPP;TFANLHF	NCP	4.836
TFANLHF;GYSLPP	NCP	22.584
GYSLPP;LDHRFSSY	NCP	19.887
LDHRFSSY;TMHVNVP	NCP	0.612
TMHVNVP;EPRGPGL	NCP	60.022
EPRGPGL;SGMFLTM	NCP	15.932
SGMFLTM;SLRYFR	NCP	100.692
SLRYFR;TPPPAPKP	NCP	0.85
TPPPAPKP;MGMLKKK	NCP	13.94
MGMLKKK;PLSKLF	NCP	43.717
PLSKLF;LSDPLVDT	NCP	32.612
LSDPLVDT;TARTE	NCP	40.552
TARTE;KAAGK	NCP	37.358
KAAGK;AEAPL	NCP	35.698
AEAPL;DGLPASPK	NCP	190.064
DGLPASPK;NKVLHF	NCP	100.435
NKVLHF;PAVVYR	NCP	19.691
PAVVYR;KLGLGVVD	NCP	37.272
KLGLGVVD;NLGGLLRY	NCP	63.459
NLGGLLRY;LEWPF	NCP	1.515
LEWPF;QPTAVPK	NCP	58.731
QPTAVPK;ATNFSLPR	NCP	7.939
ATNFSLPR;LPPTRESP	NCP	12.483
LPPTRESP;LNVTY	NCP	117.599
LNVTY;KGVSM	NCP	33.345
KGVSM;KPVWCAP	NCP	12.343
KPVWCAP;PVDLGLLEEDD	NCP	12.074
PVDLGLLEEDD;RAELEKHGY	NCP	24.654
RAELEKHGY;KPSQAPK	NCP	52.69
KPSQAPK;KGLYY	NCP	32.781
KGLYY;FHSSPR	NCP	24.233
FHSSPR;LHLGSSK	NCP	17.493
LHLGSSK;AGWNAY	NCP	0.31
AGWNAY;WKTLKAF	NCP	94.4
WKTLKAF;KLHMGPP	NCP	32.056
KLHMGPP;QLVRYP	NCP	63.365
QLVRYP;GPTSPSLPR	NCP	13.399
GPTSPSLPR;ELELGPA	NCP	56.291
ELELGPA;EEDLRK	NCP	12.235
EEDLRK;KLASLLR	NCP	32.208
KLASLLR;LDASRLVY	NCP	188.742
LDASRLVY;LAPHA	NCP	7.935
LAPHA;LSGLKVQ	NCP	8.732

LSGLKVQ;LTGPDL	NCP	38.613
LTGPDL;MVLVPLN	NCP	8.035
MVLVPLN;NKELFL	NCP	49.74
NKELFL;KTAGALP	NCP	17.024
KTAGALP;TGLNYL	NCP	6.777
TGLNYL;DRQGYMK	NCP	45.729
DRQGYMK;KEQQPQ	NCP	12.951
KEQQPQ;KHHCLKP	NCP	18.571
KHHCLKP;LESLKLPF	NCP	4.115
LESLKLPF;KDLAAVP	NCP	103.237
KDLAAVP;HEGAGL	NCP	79.874
HEGAGL;PLGYEPP	NCP	61.084
PLGYEPP;DLSFPLL	NCP	56.157
DLSFPLL;KPAPRVP	NCP	46.527
KPAPRVP;ELVELLF	NCP	71.904
ELVELLF;TMKTHY	NCP	18.879
TMKTHY;MSHFDM	NCP	29.657
MSHFDM;APGKR	NCP	118.347
APGKR;TVFHKALYCEL	NCP	44.241
TVFHKALYCEL;NTVPKGC	NCP	7.89
NTVPKGC;LVLAMRLQ	NCP	16.296
LVLAMRLQ;TVFQKALYCEF	NCP	28.164
TVFQKALYCEF;KNTMPR	NCP	61.804
KNTMPR;RFLKRTVY	NCP	3.428
RFLKRTVY;NVLRTSP	NCP	55.108
NVLRTSP;KKASTGT	NCP	7.686
KKASTGT;DPDSL	NCP	50.132
DPDSL;TAADLLN	NCP	9.675
TAADLLN;FTTTAG	NCP	109.311
FTTTAG;AFLHWY	NCP	100.783
AFLHWY;SELVQKAK	NCP	8.13
SELVQKAK;KDSHLVTL	NCP	15.209
KDSHLVTL;RFEVLT	NCP	13.293
RFEVLT;LEKYNNK	NCP	22.012
LEKYNNK;CDMDC	NCP	3.251
CDMDC;HGTSEPT	NCP	24.199
HGTSEPT;LDTGRPL	NCP	1.491
LDTGRPL;KPHVPK	NCP	20.277
KPHVPK;YQRLQ	NCP	19.914
YQRLQ;FLLVQL	NCP	61.965
FLLVQL;FQDTQLH	NCP	202.811
FQDTQLH;DNQMTLR	NCP	66.867
DNQMTLR;RLPPSFL	NCP	22.101
RLPPSFL;SVMKPGN	NCP	94.114
SVMKPGN;VEDMGFV	NCP	86.181
VEDMGFV;NSLGVKP	NCP	40.373
NSLGVKP;HSLPFHLP	NCP	92.178
HSLPFHLP;VTGSKLK	NCP	86.94
VTGSKLK;LQFFMC	NCP	9.328
LQFFMC;VFVFF	NCP	129.6
VFVFF;GLENLLK	NCP	62.457

GLENLLK;EEGLVR	NCP	75.452
EEGLVR;KHVGVPQ	NCP	32.224
KHVGVPQ;KGNYGPP	NCP	104.24
KGNYGPP;KDAFPLK	NCP	24.022
KDAFPLK;VKFGNVK	NCP	6.695
VKFGNVK;ELLRVLL	NCP	123.288
ELLRVLL;LDDLLKLN	NCP	32.991
LDDLLKLN;EVDPLL	NCP	13.728
EVDPLL;AGRPAVS	NCP	1.87
AGRPAVS;GPLLLKY	NCP	62.801
GPLLLKY;KGKYCQK	NCP	2.542
KGKYCQK;FDDFQK	NCP	55.356
FDDFQK;VYFPPGLP	NCP	20.559
VYFPPGLP;LPLVEF	NCP	13.032
LPLVEF;PLEAHLLT	NCP	19.919
PLEAHLLT;KQHPSG	NCP	28.182
KQHPSG;SLREPSLH	NCP	100.104
SLREPSLH;KVSLLR	NCP	215.646
KVSLLR;GVPGLP	NCP	85.023
GVPGLP;QRLAFVP	NCP	19.911
QRLAFVP;KSLERQP	NCP	15.528
KSLERQP;KKHLPP	NCP	54.273
KKHLPP;QNVKLQ	NCP	26.458
QNVKLQ;NSPFLP	NCP	16.962
NSPFLP;SLPAETPKP	NCP	86.588
SLPAETPKP;PTVLEK	NCP	42.195
PTVLEK;AARGLSVF	NCP	37.017
AARGLSVF;MFGMTK	NCP	35.259
MFGMTK;KTKTLPR	NCP	42.156
KTKTLPR;MMFDL	NCP	51.151
MMFDL;KNFLPPP	NCP	3.548
KNFLPPP;LGVLRL	NCP	7.564
LGVLRL;VTPPH	NCP	62.726
VTPPH;DYNLDRL	NCP	40.156
DYNLDRL;VAELLY	NCP	34.034
VAELLY;KVSPKAP	NCP	127.896
KVSPKAP;CDDDSF	NCP	16.681
CDDDSF;ETPLER	NCP	36.879
ETPLER;ASRKLHA	NCP	88.928
ASRKLHA;LLHFNVL	NCP	10.102
LLHFNVL;LKARPT	NCP	103.424
LKARPT;PGPLGGF	NCP	14.508
PGPLGGF;KNEEPAV	NCP	39.36
KNEEPAV;CGFHF	NCP	106.558
CGFHF;EAVRYFK	NCP	18.786
EAVRYFK;NMKLLTC	NCP	81.363
NMKLLTC;LAPEKPK	NCP	45.476
LAPEKPK;NVVTPR	NCP	8.425
NVVTPR;PKAAPP	NCP	9.671
PKAAPP;NSMGAL	NCP	85.528
NSMGAL;GVSLP	NCP	7.026

GVSLP;EAPPKHR	NCP	0.659
EAPPKHR;YNQLN	NCP	28.669
YNQLN;LHGVPAK	NCP	33.956
LHGVPAK;KSLGTLK	NCP	28.153
KSLGTLK;KPAWETP	NCP	258.054
KPAWETP;ELDLF	NCP	43.926
ELDLF;DVPLDVK	NCP	68.535
DVPLDVK;LVELRVLK	NCP	2.717
LVELRVLK;HSGASKK	NCP	31.83
HSGASKK;LVPLFE	NCP	31.617
LVPLFE;GLSSFVP	NCP	81.988
GLSSFVP;LEATLLEL	NCP	53.261
LEATLLEL;KQPGAPR	NCP	137.8
KQPGAPR;KPSAGPPP	NCP	85.236
KPSAGPPP;RMQSVTR	NCP	51.353
RMQSVTR;KAVHVR	NCP	34.02
KAVHVR;VAPFPEV	NCP	28.744
VAPFPEV;LYLKMVT	NCP	23.193
LYLKMVT;MRELVHL	NCP	21.248
MRELVHL;FPSLVG	NCP	141.033
FPSLVG;AQPPVT	NCP	70.132
AQPPVT;KEVPSLFK	NCP	30.012
KEVPSLFK;VTPGMPK	NCP	117.212
VTPGMPK;YEGLSDL	NCP	7.023
YEGLSDL;PACLPLQR	NCP	28.06
PACLPLQR;APPLPPPPP	NCP	82.914
APPLPPPPP;KMGVPPPP	NCP	0.27
KMGVPPPP;RGPPYLL	NCP	3.767
RGPPYLL;TRVVLP	NCP	33.615
TRVVLP;HPALALL	NCP	32.028
HPALALL;SLAAKR	NCP	2.5
SLAAKR;VWVGK	NCP	28.617
VWVGK;CSSAAL	NCP	89.625
CSSAAL;MDCPPPPP	NCP	64.836
MDCPPPPP;FAAAAVTK	NCP	92.228
FAAAAVTK;KATWHSH	NCP	142.977
KATWHSH;KHLGPK	NCP	54.85
KHLGPK;FAELLH	NCP	105.114
FAELLH;YEPGAPP	NCP	3.585
YEPGAPP;TQHTKLSL	NCP	33.986
TQHTKLSL;KVFCAK	NCP	38.418
KVFCAK;EGSSVSR	NCP	9.616
EGSSVSR;AATRVNK	NCP	93.279
AATRVNK;MDRAPR	NCP	6.675
MDRAPR;APPLVP	NCP	4.887
APPLVP;PSLPLVPH	NCP	101.04
PSLPLVPH;AAPSHPQP	NCP	3.693
AAPSHPQP;TAPLQLR	NCP	13.568
TAPLQLR;AMVSELF	NCP	29.622
AMVSELF;KQQHVYP	NCP	25.065
KQQHVYP;AALKQHLL	NCP	36.523

AALKQHLL;VGDLLSK	NCP	6.798
VGDLLSK;FDSSK	NCP	100.232
FDSSK;GPPGLP	NCP	33.619
GPPGLP;LLLRKLPF	NCP	67.143
LLLRKLPF;THQRSS	NCP	29.078
THQRSS;RPDGK	NCP	14.638
RPDGK;KPKVPEP	NCP	44.555
KPKVPEP;PAEPQRK	NCP	5.923
PAEPQRK;EGCRPP	NCP	9.656
EGCRPP;YSAPAA	NCP	59.91
YSAPAA;VTLAGL	NCP	5.058
VTLAGL;AAKASFK	NCP	61.566
AAKASFK;VDLEGK	NCP	0.466
VDLEGK;QQLVVLV	NCP	10.865
QQLVVLV;CLQPRVPL	NCP	14.404
CLQPRVPL;KPPMPPP	NCP	59.801
KPPMPPP;LLAGL	NCP	37.753
LLAGL;LPFTKLLF	NCP	34.469
LPFTKLLF;VTAAANP	NCP	7.417
VTAAANP;MFTDFK	NCP	58.974
MFTDFK;PGAPPPP	NCP	13.871
PGAPPPP;TSAKASP	NCP	104.581
TSAKASP;KESTHPP	NCP	21.492
KESTHPP;MVPFLPR	NCP	27.824
MVPFLPR;AHRAP	NCP	8.341
AHRAP;RDRMPK	NCP	33.338
RDRMPK;PTGLQAPP	NCP	1.707
PTGLQAPP;LTWYVLP	NCP	0.753
LTWYVLP;RAPPLPSE	NCP	52.962
RAPPLPSE;CDLLPLQR	NCP	10.275
CDLLPLQR;VGHRYR	NCP	2.502
VGHRYR;AKGHVP	NCP	1.179
AKGHVP;LDDDSKP	NCP	16.068
LDDDSKP;AARVPPPR	NCP	62.071
AARVPPPR;DLPGVGRGP	NCP	91.904
DLPGVGRGP;QYGPEAK	NCP	97.992
QYGPEAK;KMMKN	NCP	40.465
KMMKN;GQPLAPLP	NCP	13.145
GQPLAPLP;KRVTAF	NCP	57.546
KRVTAF;AFRKTK	NCP	10.779
AFRKTK;KTEVSQH	NCP	9.969
KTEVSQH;LAMLLK	NCP	4.993
LAMLLK;EKAPSPK	NCP	32.535
EKAPSPK;VTAPSLF	NCP	20.793
VTAPSLF;PDLPNR	NCP	36.905
PDLPNR;KMTTSVF	NCP	126.801
KMTTSVF;TTLKNY	NCP	10.321
TTLKNY;MALLLPP	NCP	23.298
MALLLPP;AVVVCKP	NCP	37.346
AVVVCKP;KCDLDLRK	NCP	16.282
KCDLDLRK;KLKLPHF	NCP	17.1

KLKLPHF;KNGDALP	NCP	96.969
KNGDALP;KPTLPF	NCP	10.7
KPTLPF;MPVPPR	NCP	3.223
MPVPPR;KTEPTK	NCP	7.296
KTEPTK;YLESRPQ	NCP	4.637
YLESRPQ;KMPELPCL	NCP	13.795
KMPELPCL;DPLVL	NCP	26.87
DPLVL;DLGLGK	NCP	71.214
DLGLGK;KLENPKN	NCP	78.111
KLENPKN;KPAGVLH	NCP	11.368
KPAGVLH;ESGSKY	NCP	4.44
ESGSKY;EGNLLDF	NCP	30.035
EGNLLDF;NNLCVPSSL	NCP	32.484
NNLCVPSSL;NFKLLHS	NCP	0.777
NFKLLHS;KPSAGPPLP	NCP	20.197
KPSAGPPLP;KPTPPSP	NCP	7.767
KPTPPSP;VLLAH	NCP	25.853
VLLAH;EVSLFPK	NCP	154.113
EVSLFPK;VGTCMFF	NCP	24.373
VGTCMFF;LEELYLN	NCP	16.082
LEELYLN;KFAHKTK	NCP	27.529
KFAHKTK;SEGARLLA	NCP	50.898
SEGARLLA;LAARLP	NCP	64.659
LAARLP;RLTYGFN	NCP	2.934
RLTYGFN;ESDLDLR	NCP	68.444
ESDLDLR;VYKGSGL	NCP	13.461
VYKGSGL;TGGRWK	NCP	57.052
TGGRWK;QVSTSAPR	NCP	41.364
QVSTSAPR;TWLKVK	NCP	38.69
TWLKVK;YRKTL	NCP	34.458
YRKTL;PLLFAK	NCP	1.378
PLLFAK;TAWDQV	NCP	45.464
TAWDQV;LFANLFH	NCP	8.386
LFANLFH;YSTLFM	NCP	16.526
YSTLFM;RVVHAK	NCP	15.97
RVVHAK;AAESDSPF	NCP	11.256
AAESDSPF;AVGDLW	NCP	25.242
AVGDLW;GDDTTLC	NCP	42.558
GDDTTLC;DRTMLP	NCP	34.175
DRTMLP;QALGPVR	NCP	151.645
QALGPVR;KDVFLMR	NCP	30.195
KDVFLMR;KEPPPP	NCP	7.245
KEPPPP;GSPTSLP	NCP	190.608
GSPTSLP;RGPVTPPR	NCP	61.491
RGPVTPPR;PLFGLYY	NCP	1.223
PLFGLYY;KDNLSLGP	NCP	0.574
KDNLSLGP;LPPGYQ	NCP	7.184
LPPGYQ;WGSGL	NCP	5.329
WGSGL;VGLQERQ	NCP	7.481
VGLQERQ;KPGVLLH	NCP	66.025
KPGVLLH;WKGPPPR	NCP	26.853

WKGPPPR;KDLTSKY	NCP	6.276
KDLTSKY;PYKPPPP	NCP	4.785
PYKPPPP;HSSLLKPA	NCP	11.708
HSSLLKPA;VPRGTPP	NCP	5.848
VPRGTPP;CYDKVPP	NCP	15.999
CYDKVPP;YLCFCGY	NCP	46.725
YLCFCGY;AHAWAP	NCP	53.445
AHAWAP;VPPPPLPAR	NCP	18.117
VPPPPLPAR;KLDVPVK	NCP	103.674
KLDVPVK;SLLTRSPPP	NCP	184.419
SLLTRSPPP;PPHVPR	NCP	16.269
PPHVPR;RPPVPPP	NCP	138.459
RPPVPPP;VSKRVR	NCP	43.641
VSKRVR;KNQQLVL	NCP	13.367
KNQQLVL;QPPEDPR	NCP	251.404
QPPEDPR;TLLALAPL	NCP	0.029
TLLALAPL;KPVVMHH	NCP	95.823
KPVVMHH;LLTMRHF	NCP	62.229
LLTMRHF;SVAPSQGP	NCP	16.567
SVAPSQGP;AMLSGL	NCP	117.186
AMLSGL;DPGFL	NCP	6.63
DPGFL;KHGTVVP	NCP	15.216
KHGTVVP;DRHLERP	NCP	50.532
DRHLERP;GHAGKPR	NCP	71.247
GHAGKPR;GGPPAGP	NCP	18.816
GGPPAGP;PPSGPRP	NCP	31.266
PPSGPRP;VASTPVQ	NCP	6.006
VASTPVQ;ETHSKLK	NCP	19.193
ETHSKLK;MSMDSM	NCP	51.165
MSMDSM;KTARPR	NCP	29.137
KTARPR;KPTRGPPP	NCP	10.218
KPTRGPPP;PVLPNPL	NCP	10.395
PVLPNPL;STAVRVP	NCP	27.192
STAVRVP;APRGYGL	NCP	17.604
APRGYGL;VLLRENF	NCP	94.46
VLLRENF;HLVLF	NCP	146.569
HLVLF;LAGPLE	NCP	2.718
LAGPLE;KCAAK	NCP	24.176
KCAAK;LPTKVPLL	NCP	4.032
LPTKVPLL;EHLRMEL	NCP	11.47
EHLRMEL;RARMELL	NCP	2.415
RARMELL;STNVP	NCP	60.824
STNVP;PVPPPPPPP	NCP	43.353
PVPPPPPPP;LPLTQK	NCP	45.681
LPLTQK;TQPAAPPK	NCP	6.823
TQPAAPPK;LKYNFTK	NCP	23.57
LKYNFTK;VENVP	NCP	3.487
VENVP;MTFFPKTK	NCP	81.726
MTFFPKTK;KPAAPGPP	NCP	47.976
KPAAPGPP;VMSEF	NCP	29.183
VMSEF;KLGVLLAF	NCP	80.034

KLGVLLAF;LEEKHGVP	NCP	0.084
LEEKHGVP;LTHPVH	NCP	17.535
LTHPVH;ERALPTP	NCP	42.12
ERALPTP;PAACK	NCP	75.958
PAACK;EADSG	NCP	84.725
EADSG;SREVP	NCP	74.292
SREVP;KSMMGC	NCP	21.874
PGPHVAK;MEGSLVP	NCP	13.251
MEGSLVP;SCDLDLR	NCP	71.293
SCDLDLR;CDLDLRK	NCP	0.003
CDLDLRK;GPVEAH	NCP	12.254
GPVEAH;SLDLLPTLAAL	NCP	12.705
SLDLLPTLAAL;ARGGKLL	NCP	20.343
ARGGKLL;RNFLEKVR	NCP	7.035
RNFLEKVR;SLGGRA	NCP	20.58
SLGGRA;AEAHQAVAF	NCP	4.662
AEAHQAVAF;VAEPLPPR	NCP	2.517
VAEPLPPR;EPVPKPP	NCP	8.784
EPVPKPP;VVLVPPTP	NCP	22.32
VVLVPPTP;LEMYVR	NCP	38.928
LEMYVR;LPGKYFTL	NCP	22.221
LPGKYFTL;PGLLVPLLR	NCP	78.483
PGLLVPLLR;SGAPLQLR	NCP	61.332
SGAPLQLR;LPSVRPR	NCP	8.772
LPSVRPR;PAAAAGP	NCP	2.328
PAAAAGP;AAPRPPR	NCP	85.377
AAPRPPR;PVGPTRP	NCP	0.69
PVGPTRP;APRGHPK	NCP	1.263
APRGHPK;HLQKLQ	NCP	54.048
HLQKLQ;TRNMVTR	NCP	157.527
TRNMVTR;HVLPP	NCP	30.105
HVLPP;VGFLVA	NCP	10.095
VGFLVA;NLQATHDLLYH	NCP	4.512
NLQATHDLLYH;AARRP	NCP	64.518
AARRP;ATPVSKL	NCP	5.241
ATPVSKL;TGRKPPGP	NCP	8.091
TGRKPPGP;PVAPPKKP	NCP	18.337
PVAPPKKP;RGPPEYP	NCP	19.262
RGPPEYP;AKGTDLH	NCP	66.315
AKGTDLH;EHGFLLE	NCP	5.074
EHGFLLE;ALKLLSGK	NCP	13.248
ALKLLSGK;VFAAFAP	NCP	1.184
VFAAFAP;EDCCPH	NCP	21.39
EDCCPH;KPKPAPP	NCP	40.545
KPKPAPP;VNGSVSPR	NCP	5.046
VNGSVSPR;TPVAEAK	NCP	48.57
TPVAEAK;SFPLPTLT	NCP	6.066
SFPLPTLT;YGVVNR	NCP	21.256
YGVVNR;VSLLDVK	NCP	15.989
VSLLDVK;MEPTPF	NCP	67.746
MEPTPF;NPLYN	NCP	50.842

NPLYN;VDNFL	NCP	0.389
VDNFL;GPPAPN	NCP	85.974
GPPAPN;ENHTSKK	NCP	86.368
ENHTSKK;KEVLLP	NCP	3.576
KEVLLP;KAAVPPR	NCP	103.485
KAAVPPR;LPHRLWC	NCP	3.266
LPHRLWC;LLRLPGEL	NCP	51.882
LLRLPGEL;DPVLP	NCP	4.137
DPVLP;RLGVVLS	NCP	30.234
RLGVVLS;LPARPPP	NCP	61.926
LPARPPP;HSRKPP	NCP	64.065
HSRKPP;LDDLLK	NCP	19.657
LDDLLK;AAPMLE	NCP	4.859
AAPMLE;PPPFP	NCP	76.104
PPPFP;KSPNLEV	NCP	76.237
KSPNLEV;KNSKVVKL	NCP	24.399
KNSKVVKL;KGSAAPQ	NCP	148.385
KGSAAPQ;KGYRHPPP	NCP	43.114
KGYRHPPP;LPDPKP	NCP	37.958
LPDPKP;KQQMVP	NCP	146.245
KQQMVP;AEKGLPR	NCP	54.752
AEKGLPR;HDTVLPP	NCP	1.617
HDTVLPP;PSRPGHPP	NCP	11.895
PSRPGHPP;LDVSKGP	NCP	16.206
LDVSKGP;GMCDPP	NCP	49.009
GMCDPP;WGAPVPK	NCP	5.612
WGAPVPK;KELPVKE	NCP	0.81
KELPVKE;ACPGP	NCP	52.618
ACPGP;KGCLDKR	NCP	1.164
KGCLDKR;GVGLP	NCP	2.987
GVGLP;LTPSLTPL	NCP	2.4
LTPSLTPL;RMLVVLL	NCP	4.827
RMLVVLL;RPPFVK	NCP	48.453
RPPFVK;HKPHEVR	NCP	8.595
HKPHEVR;AQPLL	NCP	19.782
AQPLL;AGSVP	NCP	33.12
AGSVP;RLGSAGK	NCP	16.684
RLGSAGK;DPEAPAPS	NCP	4.259
DPEAPAPS;SPLGKHL	NCP	125.491
SPLGKHL;PPPVMRP	NCP	44.972
PPPVMRP;PRRLCPP	NCP	8.35
PRRLCPP;LSAPGGLL	NCP	13.247
LSAPGGLL;PSQEGQN	NCP	27.429
PSQEGQN;LHLTPQ	NCP	169.041
LHLTPQ;CDDTAPP	NCP	25.626
CDDTAPP;VGYGKP	NCP	13.806
VGYGKP;KNAHPPR	NCP	3.288
KNAHPPR;RMVLRPP	NCP	43.938
RMVLRPP;HLTLGPPP	NCP	31.323
HLTLGPPP;ATVKHAQ	NCP	5.859
ATVKHAQ;KQLGPAE	NCP	45.54

KQLGPAE;PLLEPP	NCP	18.801
PLLEPP;PPPPLPGP	NCP	16.485
PPPPLPGP;DPKLLAP	NCP	16.248
DPKLLAP;TEVPDPK	NCP	3.792
TEVPDPK;EPVGVKDR	NCP	11.566
EPVGVKDR;LPVLC	NCP	13.352
LPVLC;EVHTELL	NCP	18.19
EVHTELL;PGARPPNP	NCP	33.641
PGARPPNP;AQVVL	NCP	22.809
AQVVL;KLSLKVTF	NCP	35.671
KLSLKVTF;SHTARPP	NCP	1.607
SHTARPP;RVASLR	NCP	14.862
RVASLR;AARGLFQ	NCP	34.233
AARGLFQ;LQVVSSV	NCP	0.405
LQVVSSV;DPEPP	NCP	6.385
DPEPP;NASLLRP	NCP	54.596
NASLLRP;QVVPAAHG	NCP	86.079
QVVPAAHG;PSLTEGK	NCP	106.263
PSLTEGK;EHAGPPR	NCP	2.259
EHAGPPR;AVWLQAQ	NCP	1.89
AVWLQAQ;GLTPPM	NCP	41.04
GLTPPM;EDKMPK	NCP	22.704
EDKMPK;KLTTSPPPP	NCP	2.862
KLTTSPPPP;KGSVNPPPP	NCP	45.12
KGSVNPPPP;KRRGW	NCP	12.192
KRRGW;LTGLAAGA	NCP	160.542
LTGLAAGA;AAPPV	NCP	40.518
AAPPV;QGPAPLM	NCP	1.617
QGPAPLM;DDGYFSS	NCP	0.486
DDGYFSS;KGKVP	NCP	20.466
KGKVP;HHKPPH	NCP	0.193
HHKPPH;MEVKDTP	NCP	52.193
MEVKDTP;LPLPP	NCP	54.105
LPLPP;PGFVLNR	NCP	7.053
PGFVLNR;EVGVPF	NCP	83.4
EVGVPF;VSAGKTPK	NCP	92.484
VSAGKTPK;VETEF	NCP	39.544
VETEF;HAPPPPKP	NCP	30.848
HAPPPPKP;GFEDVKK	NCP	17.814
GFEDVKK;KYTPVVLL	NCP	7.24
KYTPVVLL;PPSRPPPP	NCP	5.645
PPSRPPPP;YLSPVGP	NCP	13.993
YLSPVGP;AELCLP	NCP	39.023
AELCLP;AGLKPKK	NCP	36.415
AGLKPKK;MVFGAPK	NCP	89.508
MVFGAPK;VAARVALV	NCP	17.879
VAARVALV;AAFGGAP	NCP	3.078
AAFGGAP;LPPLEGD	NCP	17.232
LPPLEGD;PVGPL	NCP	23.304
PVGPL;LGPGVP	NCP	35.763
LGPGVP;GPGGP	NCP	35.466

GPGGP;LPDPSPPLTL	NCP	71.52
LPDPSPPLTL;LPPPLGPP	NCP	14.079
LPPPLGPP;LGPDNVG	NCP	4.302
LGPDNVG;FEVLGR	NCP	45.772
FEVLGR;RAAPLN	NCP	118.01
RAAPLN;FRGPPGP	NCP	47.367
FRGPPGP;SNQFLVL	NCP	111.262
SNQFLVL;ARLPGVGR	NCP	47.708
ARLPGVGR;LFVKKK	NCP	75.696
LFVKKK;KAFPPPPSP	NCP	164.149
KAFPPPPSP;LHTLAVVP	NCP	231.935
LHTLAVVP;MEGNKKK	NCP	52.956
MEGNKKK;KYGDYP	NCP	14.938
KYGDYP;SGVPGVR	NCP	32.297
SGVPGVR;TPTTKSTT	NCP	66.651
TPTTKSTT;KPHGAPR	NCP	7.446
KPHGAPR;KAAPVPF	NCP	15.801
KAAPVPF;EKLWHH	NCP	35.653
EKLWHH;VPPTGLPPP	NCP	11.541
VPPTGLPPP;APPGSFP	NCP	47.954
APPGSFP;KTPSRPK	NCP	3.975
KTPSRPK;NGGGGLPR	NCP	40.179
NGGGGLPR;KGDGESKK	NCP	11.113
KGDGESKK;PRNARPP	NCP	30.27
PRNARPP;PVHHSGP	NCP	10.799
PVHHSGP;LPPKERPP	NCP	47.703
LPPKERPP;KPLPPPP	NCP	40.725
KPLPPPP;VTPPAC	NCP	2.772
VTPPAC;PVLGPG	NCP	40.5
PVLGPG;ALTERLK	NCP	32.628
ALTERLK;LELHLK	NCP	11.263
LELHLK;QHVPPPP	NCP	14.406
QHVPPPP;MQADPTPER	NCP	1.601
MQADPTPER;FYMPQQVA	NCP	7.176
FYMPQQVA;YDPEVVLK	NCP	5.751
YDPEVVLK;YGEQL	NCP	30.973
YGEQL;DRLPAAR	NCP	45.167
DRLPAAR;YNPFEDEDDTGSTVSEK	NCP	176.007
YNPFEDEDDTGSTVSEK;CPGLL	NCP	60.36
CPGLL;VELLA	NCP	183.16
VELLA;EGNLQLKL	NCP	10.644
EGNLQLKL;MELPPGPR	NCP	17.582
MELPPGPR;TLVFL	NCP	47.955
TLVFL;KPPAPMQ	NCP	27.354
KPPAPMQ;TAEHLDLP	NCP	6.099
TAEHLDLP;HVLGGSLP	NCP	10.419
HVLGGSLP;TSPTPP	NCP	75.636
TSPTPP;EGALLD	NCP	12.378
EGALLD;RATSL	NCP	17.872
RATSL;LPPNP	NCP	13.43
LPPNP;QELLAQR	NCP	3.831

QELLAQR;PPPPHLAK	NCP	12.294
PPPPHLAK;PANPVDF	NCP	6.336
PANPVDF;GLLPFK	NCP	32.457
GLLPFK;KPEASSH	NCP	56.691
KPEASSH;QLCAVPR	NCP	20.328
QLCAVPR;LVTPAPYQLCAVPR	NCP	22.359
LVTPAPYQLCAVPR;KCPKGDP	NCP	2.712
KCPKGDP;APYLR	NCP	2.635
APYLR;KNVAP	NCP	11.066
KNVAP;AASGLRQAA	NCP	4.974
AASGLRQAA;KFYVGHDP	NCP	10.878
KFYVGHDP;FYVGHD	NCP	0.003
FYVGHD;KGATRAAP	NCP	117.346
KGATRAAP;GFKKKT	NCP	1.823
GFKKKT;FHGLSVGSS	NCP	90.067
FHGLSVGSS;SGCAYSG	NCP	7.094
SGCAYSG;FVDSVLY	NCP	29.719
FVDSVLY;GVVTLA	NCP	20.109
GVVTLA;KYWFPP	NCP	9.302
KYWFPP;KDTPKVAP	NCP	6.918
KDTPKVAP;LSDLPLD	NCP	1.731
LSDLPLD;NSYLLQ	NCP	2.404
NSYLLQ;TEADVNPKAYPLADAHLTKK	NCP	24.662
TEADVNPKAYPLADAHLTKK;EGYTP	NCP	11.541
EGYTP;KKTTLSLK	NCP	5.652
KKTTLSLK;REVAAPK	NCP	15.445
REVAAPK;EGSPPP	NCP	88.079
EGSPPP;APAELQF	NCP	11.358
APAELQF;AKHHPDL	NCP	39.855
AKHHPDL;LELPNP	NCP	40.344
LELPNP;RLTGLLL	NCP	22.746
RLTGLLL;AAAAARVGL	NCP	41.934
AAAAARVGL;AVPPPRVL	NCP	27.024
AVPPPRVL;APGSWSLK	NCP	33.03
APGSWSLK;LFDAGAF	NCP	35.455
LFDAGAF;VPDSVLVS	NCP	20.348
VPDSVLVS;AVKNP	NCP	122.505
AVKNP;NLDPV	NCP	22.599
NLDPV;FAYLLH	NCP	80.713
FAYLLH;MDPRKVSELRAF	NCP	5.261
MDPRKVSELRAF;YVGLTR	NCP	129.478
YVGLTR;LGPVDP	NCP	1.524
LGPVDP;KLYHWP	NCP	34.941
KLYHWP;PHLAP	NCP	143.741
PHLAP;TMMLEP	NCP	15.003
TMMLEP;EEAPAPPKAEAKAK	NCP	33.612
EEAPAPPKAEAKAK;EAASLLR	NCP	22.893
EAASLLR;APPTPRP	NCP	8.43
APPTPRP;PAGPVWPP	NCP	87.507
PAGPVWPP;LNFPLY	NCP	88.869
LNFPLY;SAAKAVR	NCP	12.75

SAAKAVR;APLAPPFS	NCP	1.401
APLAPPFS;YFNGPK	NCP	31.764
YFNGPK;FDDDKF	NCP	47.143
FDDDKF;LPGPAAG	NCP	82.091
LPGPAAG;GFRQEGK	NCP	9.036
GFRQEGK;EPSLPK	NCP	9.762
EPSLPK;QTLELYLT	NCP	109.41
QTLELYLT;LKSGAP	NCP	22.083
LKSGAP;KFCEPRP	NCP	85.683
KFCEPRP;APRGQVH	NCP	8.478
APRGQVH;GPPALPPP	NCP	6.411
GPPALPPP;ASLQAVLP	NCP	11.205
ASLQAVLP;LSLQHE	NCP	14.014
LSLQHE;KAASMPPP	NCP	0.128
KAASMPPP;PVPGTPPK	NCP	15.69
PVPGTPPK;LFEDPF	NCP	17.01
LFEDPF;KAEFDPP	NCP	26.448
KAEFDPP;VGGPVPR	NCP	60.762
VGGPVPR;PGNPPVR	NCP	71.946
PGNPPVR;AYALY	NCP	16.906
AYALY;DLGLLP	NCP	38.369
DLGLLP;KDKPVPK	NCP	15.954
KDKPVPK;SHRKFSAPR	NCP	87.582
SHRKFSAPR;DDSSKPVHL	NCP	0.099
DDSSKPVHL;SVARAGQKGY	NCP	3.372
SVARAGQKGY;VHYGEVTNDF	NCP	1.041
VHYGEVTNDF;GEVTNDFVMLK	NCP	0.009
GEVTNDFVMLK;GCVVGTKKRVLTLRK	NCP	0.033
GCVVGTKKRVLTLRK;SLLVQTKRR	NCP	0.291
SLLVQTKRR;LAAPRL	NCP	32.55
LAAPRL;SGHLLQ	NCP	1.911
SGHLLQ;YVPRLLF	NCP	68.901
YVPRLLF;SNVRLR	NCP	3.532
SNVRLR;VGGLTL	NCP	39.536
VGGLTL;QQPVTPP	NCP	42.912
QQPVTPP;EEELAAT	NCP	29.824
EEELAAT;LLWHEK	NCP	19.263
LLWHEK;KLVEGF	NCP	20.751
KLVEGF;LPAFLAA	NCP	49.529
LPAFLAA;ARVCML	NCP	13.365
ARVCML;GDRLLLLK	NCP	29.956
GDRLLLLK;PAPQLGPSSDTHT	NCP	21.605
PAPQLGPSSDTHT;KPGAAPNP	NCP	9.219
KPGAAPNP;LPGGWP	NCP	25.098
LPGGWP;VDAPSPK	NCP	11.143
VDAPSPK;FCTEYLT	NCP	1.964
FCTEYLT;EAAALYHL	NCP	102.666
EAAALYHL;KEFALLF	NCP	34.876
KEFALLF;DFPPP	NCP	17.993
DFPPP;YPPPPPPPPPSRK	NCP	5.34
YPPPPPPPPPSRK;KTFYPP	NCP	38.062

KTFYPP;NPPDLP	NCP	102.732
NPPDLP;LSSPVFPR	NCP	58.739
LSSPVFPR;SSPVFPRQ	NCP	0.003
SSPVFPRQ;FPPYY	NCP	70.504
FPPYY;FYVHR	NCP	31.524
FYVHR;TLALP	NCP	2.747
TLALP;RTVHEK	NCP	68.017
RTVHEK;ATRLVLP	NCP	20.756
ATRLVLP;LSLYK	NCP	4.33
LSLYK;AALELYL	NCP	3.275
AALELYL;DGGAP	NCP	4.167
DGGAP;KNYPSL	NCP	30.094
KNYPSL;PKGTHKR	NCP	55.376
PKGTHKR;AFFMFALF	NCP	13.546
AFFMFALF;SLLGP	NCP	97.785
SLLGP;AAAVRNEL	NCP	53.297
AAAVRNEL;QGLPL	NCP	33.138
QGLPL;LGVQNFLVSSFKLE	NCP	43.608
LGVQNFLVSSFKLE;HTGPAA	NCP	57.645
HTGPAA;RLVAEPQ	NCP	47.775
RLVAEPQ;CMGSM	NCP	3.093
CMGSM;NGEGMAK	NCP	4.362
NGEGMAK;LGDVLR	NCP	20.095
LGDVLR;FFLLFTR	NCP	32.342
FFLLFTR;LSFCDLR	NCP	2.716
LSFCDLR;EVPPRP	NCP	26.351
EVPPRP;RSRLLR	NCP	3.865
RSRLLR;RMSEALLLH	NCP	9.284
RMSEALLLH;AAKVYSK	NCP	31.177
AAKVYSK;ALEVLV	NCP	6.11
ALEVLV;NPGSSLLR	NCP	14.386
NPGSSLLR;KTALHAP	NCP	10.139
KTALHAP;LEGALS	NCP	28.797
LEGALS;LGAELNVL	NCP	60.936
LGAELNVL;VSEGTKAVTKYTSSK	NCP	8.983
VSEGTKAVTKYTSSK;DYGLLEP	NCP	10.467
DYGLLEP;LQPKYSK	NCP	16.929
LQPKYSK;LPDSGFF	NCP	85.074
LPDSGFF;VVFLP	NCP	23.127
VVFLP;KSRPAGP	NCP	36.695
KSRPAGP;PLPARPP	NCP	26.421
PLPARPP;PPAPPPPPPP	NCP	0.015
PPAPPPPPPP;VEKRELLA	NCP	0.754
VEKRELLA;ERPLGVGH	NCP	73.328
ERPLGVGH;NALALF	NCP	153.858
NALALF;AAFLWGGP	NCP	8.616
AAFLWGGP;KRFLAK	NCP	5.218
KRFLAK;VYTAHH	NCP	9.65
VYTAHH;SPVRNVH	NCP	12.396
SPVRNVH;DRFSVLN	NCP	62.275
DRFSVLN;ALVKPR	NCP	14.273

ALVKPR;GSPLVTKH	NCP	20.605
GSPLVTKH;QTRAVLE	NCP	98.538
QTRAVLE;PVPPA	NCP	31.727
PVPPA;KFAACH	NCP	65.482
KFAACH;LPPQVDQ	NCP	43.625
LPPQVDQ;MVGVTLK	NCP	35.13
MVGVTLK;SGNLLTLL	NCP	0.465
SGNLLTLL;KHNLELL	NCP	28.534
KHNLELL;KAGLP	NCP	5.205
KAGLP;DFVTK	NCP	36.489
DFVTK;EVPEVH	NCP	32.573
EVPEVH;PPPPRPP	NCP	63.519
PPPPRPP;KYNFKPP	NCP	2.347
KYNFKPP;EMDEEDKAFKQK	NCP	9.327
EMDEEDKAFKQK;MDEEDKAFKQKQK	NCP	0.003
MDEEDKAFKQKQK;KEALAEP	NCP	31.598
KEALAEP;DGCCPT	NCP	1.372
DGCCPT;VPVLPE	NCP	20.451
VPVLPE;KGEETLP	NCP	83.447
KGEETLP;HLSRYSGLP	NCP	4.422
HLSRYSGLP;PGAYEPF	NCP	0.9
PGAYEPF;GVKLLGK	NCP	6.109
GVKLLGK;VLDEVWK	NCP	7.403
VLDEVWK;KAKTSPP	NCP	23.913
KAKTSPP;VDLTAK	NCP	9.582
VDLTAK;RWHHT	NCP	99.55
RWHHT;LCAVVGLR	NCP	15.182
LCAVVGLR;MHDKRP	NCP	32.548
MHDKRP;SVDFMFN	NCP	14.931
SVDFMFN;HSKAVTK	NCP	50.339
HSKAVTK;NLPTFT	NCP	21.661
NLPTFT;LLALY	NCP	60.489
LLALY;DTVYVPY	NCP	43.778
DTVYVPY;QTAGSLK	NCP	42.036
QTAGSLK;MELTVKF	NCP	6.088
MELTVKF;AARPGGPP	NCP	136.877
AARPGGPP;KDFVPSP	NCP	116.463
KDFVPSP;ELLAQ	NCP	24.454
ELLAQ;KTAGGTLK	NCP	22.448
KTAGGTLK;CGDVAL	NCP	0.204
CGDVAL;PRGGLVH	NCP	1.497
PRGGLVH;TTGGSLVD	NCP	43.834
TTGGSLVD;GEVFPAA	NCP	43.274
GEVFPAA;AAFKFRP	NCP	29.605
AAFKFRP;LDVRMGK	NCP	13.713
LDVRMGK;LVDKYEL	NCP	38.79
LVDKYEL;LSFCLF	NCP	95.955
LSFCLF;HSLQLPP	NCP	73.535
HSLQLPP;HKKLLL	NCP	42.925
HKKLLL;GLPLA	NCP	41.193
GLPLA;YTQPP	NCP	26.462

YTQPP;KDLVTPPV	NCP	12.904
KDLVTPPV;AVYSFCH	NCP	9.579
AVYSFCH;DSGFDLL	NCP	5.73
DSGFDLL;SHAAQG	NCP	3.015
SHAAQG;ADDTSSK	NCP	73.281
ADDTSSK;VVGGPALL	NCP	43.676
VVGGPALL;DFRSPPP	NCP	2.589
DFRSPPP;AAPVPPSHP	NCP	16.257
AAPVPPSHP;APVLPP	NCP	25.434
APVLPP;PGRWPPPP	NCP	2.325
PGRWPPPP;KLLLPP	NCP	44.782
KLLLPP;PDGGPV	NCP	12.536
PDGGPV;THWKQTVFY	NCP	149.199
THWKQTVFY;KQMSTVP	NCP	27.681
KQMSTVP;KSSVHMR	NCP	99.381
KSSVHMR;LSAALK	NCP	50.502
LSAALK;LERLGPF	NCP	35.238
LERLGPF;LLPGD	NCP	14.311
LLPGD;KVVGQVP	NCP	5.891
KVVGQVP;TPLPTPP	NCP	13.392
TPLPTPP;LAGPPK	NCP	39.066
LAGPPK;VGTPV	NCP	0.399
VGTPV;LVGGLY	NCP	5.242
LVGGLY;LAVSC	NCP	17.597
LAVSC;GLPRMVP	NCP	23.031
GLPRMVP;RLPVATP	NCP	44.112
RLPVATP;EVPNPL	NCP	10.966
EVPNPL;MTLSGAG	NCP	115.574
MTLSGAG;GVVPPPP	NCP	53.505
GVVPPPP;FLLLCPLP	NCP	1.5
FLLLCPLP;FPGMNSF	NCP	96.49
FPGMNSF;HTFEEPTYVNLK	NCP	80.781
HTFEEPTYVNLK;NFPLTA	NCP	152.045
NFPLTA;WSLYF	NCP	41.742
WSLYF;KTVPGGK	NCP	9.649
KTVPGGK;PVLRLALE	NCP	39.209
PVLRLALE;VLLHPR	NCP	64.074
VLLHPR;KPVSSYK	NCP	55.98
KPVSSYK;LSVVEVK	NCP	16.81
LSVVEVK;KQALEVR	NCP	10.091
KQALEVR;LSHSPNVP	NCP	72.621
LSHSPNVP;VGLLGT	NCP	11.916
VGLLGT;VSELATY	NCP	2.956
VSELATY;KLWLVTL	NCP	45.308
KLWLVTL;LTANELL	NCP	8.172
LTANELL;CCMAE	NCP	31.198
CCMAE;AARMSPTR	NCP	34.424
AARMSPTR;VMPVLL	NCP	23.01
VMPVLL;MTGGF	NCP	64.047
MTGGF;KGFVLL	NCP	17.805
KGFVLL;TYLHQLR	NCP	12.895

TYLHQLR;NSLLTTTH	NCP	5.079
NSLLTTTH;AAQRLT	NCP	35.168
AAQRLT;AVGVPKR	NCP	41.814
AVGVPKR;HGGVSNVP	NCP	23.145
HGGVSNVP;TADVSLPK	NCP	20.65
TADVSLPK;YGYADF	NCP	25.572
YGYADF;EVLVFFK	NCP	8.684
EVLVFFK;MLEKLSR	NCP	11.047
MLEKLSR;PLPPVPP	NCP	22.424
PLPPVPP;WQVLKCK	NCP	10.66
WQVLKCK;KFHLLLKT	NCP	14.646
KFHLLLKT;KAWAALK	NCP	7.058
KAWAALK;LTWKASP	NCP	35.526
LTWKASP;VSKALSPK	NCP	61.803
VSKALSPK;KDLVTLTG	NCP	7.662
KDLVTLTG;TPLTTTTPL	NCP	9.657
TPLTTTTPL;APPAVNF	NCP	82.06
APPAVNF;HFVAL	NCP	3.546
HFVAL;ARLGTAPL	NCP	12.893
ARLGTAPL;WKGKMPK	NCP	77.374
WKGKMPK;WHPGPPTP	NCP	30.2
WHPGPPTP;ELRVTQ	NCP	11.464
ELRVTQ;EEEPKR	NCP	66.386
EEEPKR;KSSLP	NCP	5.83
KSSLP;LDDEAPP	NCP	13.655
LDDEAPP;LGASAFLV	NCP	148.038
LGASAFLV;MCPALRP	NCP	27.489
MCPALRP;KVHVSNP	NCP	18.562
KVHVSNP;PHPVNFK	NCP	11.216
PHPVNFK;GRVHQVL	NCP	48.363
GRVHQVL;MFQYDSTHGKFNGTVK	NCP	111.366
MFQYDSTHGKFNGTVK;QYDSTHGKFN	NCP	0.006
QYDSTHGKFN;YDSTHGKFNGTVK	NCP	0.003
YDSTHGKFNGTVK;VVESTGVFT	NCP	0.255
VVESTGVFT;FVMGVNHEKY	NCP	0.213
FVMGVNHEKY;TATQKTVDGPSGK	NCP	0.426
TATQKTVDGPSGK;AFRVPTPN	NCP	0.234
AFRVPTPN;KVVKQASEGPLK	NCP	0.183
KVVKQASEGPLK;VVKQASEGPL	NCP	0.003
VVKQASEGPL;ALNDHFVK	NCP	0.207
ALNDHFVK;YDNEFGYSNR	NCP	0.132
YDNEFGYSNR;DNEFGYSNRVVDL	NCP	0.003
DNEFGYSNRVVDL;EFGYSNR	NCP	0.006
EFGYSNR;FGYSNRVVDL	NCP	0.003
FGYSNRVVDL;GYSNRVVD	NCP	0.003
GYSNRVVD;YSNRVVDL	NCP	0.003
YSNRVVDL;SNRVVDLMVH	NCP	0.003
SNRVVDLMVH;VDLMVHMASKE	NCP	0.012
VDLMVHMASKE;MVHMASK	NCP	0.009
MVHMASK;VQVSLPLK	NCP	36.115
VQVSLPLK;KPYPPPPP	NCP	42.464

KPYPPPPP;APLKGEP	NCP	2.827
APLKGEP;LVTPQPP	NCP	40.082
LVTPQPP;GTAHMPW	NCP	9.103
GTAHMPW;PAKKPPR	NCP	25.265
PAKKPPR;KVVDPFSK	NCP	3.708
KVVDPFSK;HDVFVRK	NCP	0.555
HDVFVRK;LMELHGEGSSSGK	NCP	0.06
LMELHGEGSSSGK;PPPDLP	NCP	18.267
PPPDLP;SEKSVEAAAELSAK	NCP	13.584
SEKSVEAAAELSAK;PGQPPP	NCP	1.752
PGQPPP;DLKEKKEKVEEKAGR	NCP	1.422
DLKEKKEKVEEKAGR;LPSSLRP	NCP	57.786
LPSSLRP;TSPKDVL	NCP	5.206
TSPKDVL;SAKLWDV	NCP	6.728
SAKLWDV;GPARLGPP	NCP	7.98
GPARLGPP;APSRKFF	NCP	14.283
APSRKFF;KPNPRPT	NCP	10.191
KPNPRPT;KLSGWMR	NCP	21.688
KLSGWMR;PTAPEPK	NCP	14.435
PTAPEPK;LGPVPHR	NCP	12.897
LGPVPHR;NLPAP	NCP	10.168
NLPAP;EDVYENVHSK	NCP	11.042
EDVYENVHSK;LSPLKKT	NCP	64.806
LSPLKKT;QTGLPK	NCP	56.002
QTGLPK;LEGVP	NCP	138.324
LEGVP;LALEY	NCP	54.219
LALEY;VGATELTPL	NCP	29.039
VGATELTPL;LMLPLL	NCP	14.257
LMLPLL;KLLKMTSP	NCP	80.088
KLLKMTSP;LELEVLF	NCP	338.333
LELEVLF;TPAWVPP	NCP	54.189
TPAWVPP;FRALKK	NCP	82.486
FRALKK;QNSLVHRK	NCP	20.477
QNSLVHRK;MGDSV	NCP	93.702
MGDSV;KDHLVT	NCP	61.57
KDHLVT;KLLFSST	NCP	124.061
KLLFSST;NVFPGTK	NCP	255.462
NVFPGTK;MDKEAMRS	NCP	62.227
MDKEAMRS;AVSVTPL	NCP	165.311
AVSVTPL;VLAPL	NCP	59.865
VLAPL;SVLDVV	NCP	98.31
SVLDVV;ASTLGVPQ	NCP	94.422
ASTLGVPQ;KYAHLPP	NCP	70.089
KYAHLPP;PFQLS	NCP	40.674
PFQLS;GGYGVLK	NCP	14.601
GGYGVLK;MSPSMLK	NCP	101.269
MSPSMLK;VPPTP	NCP	30.293
VPPTP;SSAGGDHR	NCP	36.849
SSAGGDHR;YGGPY	NCP	12.456
YGGPY;VVGVRLL	NCP	0.897
VVGVRLL;FALAQKK	NCP	71.836

FALAQKK;FGTEKDKVNC	NCP	39.281
FGTEKDKVNC;TTMMLM	NCP	42.147
TTMMLM;VRNVFF	NCP	85.479
VRNVFF;TALNQR	NCP	103.081
TALNQR;LKSGSK	NCP	97.092
LKSGSK;LPPVPP	NCP	57.293
LPPVPP;LVFTPP	NCP	53.893
LVFTPP;SLFAGFRE	NCP	83.817
SLFAGFRE;KDDAHGQ	NCP	274.208
KDDAHGQ;SLDQLTPL	NCP	72.273
SLDQLTPL;NKTAHAK	NCP	14.509
NKTAHAK;LDRPLAL	NCP	36.192
LDRPLAL;LLNELRH	NCP	40.311
LLNELRH;VSALLEY	NCP	26.541
VSALLEY;ASLMPSK	NCP	13.37
ASLMPSK;PMMLLR	NCP	96.264
PMMLLR;KTDGWK	NCP	20.971
KTDGWK;TGLSKLLR	NCP	18.861
TGLSKLLR;LALELMA	NCP	60.333
LALELMA;KNLSKGVP	NCP	40.278
KNLSKGVP;VPKFDP	NCP	34.41
VPKFDP;KSMFPN	NCP	97.811
KSMFPN;FATVHR	NCP	79.228
FATVHR;HSLLVTL	NCP	123.36
HSLLVTL;TAVSLPK	NCP	41.669
TAVSLPK;KLFGKPK	NCP	33.796
KLFGKPK;NNFLVL	NCP	99.009
NNFLVL;TQLMGCK	NCP	39.662
TQLMGCK;PLAPP	NCP	85.062
PLAPP;VDPLASTL	NCP	28.833
VDPLASTL;VDVLGTK	NCP	17.299
VDVLGTK;KDAAVGH	NCP	31.986
KDAAVGH;PPPPHYE	NCP	40.865
PPPPHYE;LLVKPVP	NCP	62.347
LLVKPVP;KVHDPLT	NCP	18.566
KVHDPLT;HLLFAK	NCP	0.508
HLLFAK;MQATAPP	NCP	44.961
MQATAPP;PQQRPP	NCP	138.836
PQQRPP;FVLLPM	NCP	33.918
FVLLPM;KSEGESLK	NCP	11.368
KSEGESLK;SLGVP	NCP	164.972
SLGVP;KPEDDLP	NCP	25.917
KPEDDLP;TDYGF	NCP	50.089
TDYGF;KTYPPPPP	NCP	37.268
KTYPPPPP;ALASPPPN	NCP	13.155
ALASPPPN;VPVVGL	NCP	121.662
VPVVGL;TSVVTLRP	NCP	102.357
TSVVTLRP;RFSLPPP	NCP	5.166
RFSLPPP;TSVVTSP	NCP	24.585
TSVVTSP;ATFRCK	NCP	2.746
ATFRCK;MEMMVP	NCP	8.915

MEMMVP;LAPLLKEK	NCP	8.164
LAPLLKEK;TFKLTW	NCP	23.166
TFKLTW;LAHVAPKGP	NCP	34.571
LAHVAPKGP;KTKLLLQN	NCP	36.726
KTKLLLQN;CYLKLYN	NCP	43.105
CYLKLYN;LDKFQ	NCP	59.067
LDKFQ;KEPPKR	NCP	4.227
KEPPKR;VGPLKGL	NCP	89.355
VGPLKGL;VSTTVPPK	NCP	128.172
VSTTVPPK;LLLKDMKTK	NCP	4.515
LLLKDMKTK;KTKPVSF	NCP	15.005
KTKPVSF;TRPAVR	NCP	244.551
TRPAVR;MVEQLSF	NCP	52.786
MVEQLSF;DGHLSVY	NCP	59.153
DGHLSVY;NVELYL	NCP	2.707
NVELYL;FAFVQ	NCP	58.326
FAFVQ;PGAEFKP	NCP	21.326
PGAEFKP;ATVQQLR	NCP	21.07
ATVQQLR;KPHVSFR	NCP	31.551
KPHVSFR;NHTGTLR	NCP	21.981
NHTGTLR;KQSYGVVNRAY	NCP	8.69
KQSYGVVNRAY;QSYGVVNR	NCP	0.003
QSYGVVNR;GVVNRAY	NCP	0.009
GVVNRAY;TRVSPYSDYEGRKDVS	NCP	4.272
TRVSPYSDYEGRKDVS;VNLTM	NCP	47.248
VNLTM;PLYEMK	NCP	53.663
PLYEMK;CGLVPS	NCP	52.059
CGLVPS;APAPVLQK	NCP	39.219
APAPVLQK;GDVGVLR	NCP	134.409
GDVGVLR;NKPFVP	NCP	10.669
NKPFVP;GFLLGDN	NCP	76.76
GFLLGDN;PPPPPNSSPP	NCP	17.326
PPPPPNSSPP;KNYYLLFK	NCP	5.694
KNYYLLFK;KYLPP	NCP	38.669
KYLPP;MLFNKPP	NCP	90.436
MLFNKPP;CDGSM	NCP	59.696
CDGSM;LSDPTVPTK	NCP	51.078
LSDPTVPTK;FDAGVGK	NCP	48.894
FDAGVGK;FPGPLGT	NCP	49.488
FPGPLGT;KVTVPVGR	NCP	15.312
KVTVPVGR;FEKAKGK	NCP	141.475
FEKAKGK;VSDDHK	NCP	118.106
VSDDHK;KPGHTRK	NCP	78.213
KPGHTRK;KPMPSLGK	NCP	16.299
KPMPSLGK;FLRKFC	NCP	101.616
FLRKFC;KLVPLR	NCP	52.824
KLVPLR;EVQTLPK	NCP	27.888
EVQTLPK;RPPVF	NCP	29.497
RPPVF;KNGLLLLRK	NCP	68.976
KNGLLLLRK;SPRQQMP	NCP	187.433
SPRQQMP;KYVVYVL	NCP	91.921

KYVVYVL;FFLVWTK	NCP	7.829
FFLVWTK;WGSPGYV	NCP	74.76
WGSPGYV;FEVLGLR	NCP	25.435
FEVLGLR;AERVGAGAPV	NCP	25.058
AERVGAGAPV;AVLLPKKTESQKTKSK	NCP	0.219
AVLLPKKTESQKTKSK;VLLPKKTESQKTK	NCP	0.003
VLLPKKTESQKTK;LLPKKTESQK	NCP	0.003
LLPKKTESQK;LPKKTESQKTKSK	NCP	0.003
LPKKTESQKTKSK;PKKTESQKTK	NCP	0.003
PKKTESQKTK;VPESMVP	NCP	177.532
VPESMVP;TPPVSPWF	NCP	68.48
TPPVSPWF;PRVNC	NCP	58.201
PRVNC;PGPKGAR	NCP	45.531
PGPKGAR;DSLLKDYL	NCP	95.214
DSLLKDYL;YNCEVR	NCP	6.351
YNCEVR;ALLPPR	NCP	35.279
ALLPPR;KTLDKR	NCP	7.87
KTLDKR;KDAPLL	NCP	51.15
KDAPLL;KPVFRSP	NCP	38.022
KPVFRSP;SGPLNFSL	NCP	148.958
SGPLNFSL;AQKGPPK	NCP	2.917
AQKGPPK;AADLPLN	NCP	38.561
AADLPLN;FNADFH	NCP	77.077
FNADFH;PGKAP	NCP	23.754
PGKAP;VGVPLAT	NCP	148.715
VGVPLAT;AMRQTPLT	NCP	74.562
AMRQTPLT;KKLGYNP	NCP	13.086
KKLGYNP;SSTPEVKA	NCP	6.531
SSTPEVKA;LGFTTVK	NCP	19.449
LGFTTVK;KMLVLLFR	NCP	6.928
KMLVLLFR;LQLEYL	NCP	277.563
LQLEYL;VPGGLYF	NCP	29.645
VPGGLYF;DSLPVKSP	NCP	11.563
DSLPVKSP;VPGKHRP	NCP	47.181
VPGKHRP;KLQAALK	NCP	65.327
KLQAALK;HSGEVFK	NCP	3.364
HSGEVFK;ANPEDCAGFGKGEN	NCP	49.565
ANPEDCAGFGKGEN;APAATPAPK	NCP	8.791
APAATPAPK;GTATRF	NCP	44.718
GTATRF;KTPHVSK	NCP	26.579
KTPHVSK;SPKSGPQ	NCP	75.334
SPKSGPQ;KKGSFHP	NCP	73.305
KKGSFHP;KPSYPQR	NCP	121.292
KPSYPQR;EPSVYM	NCP	36.922
EPSVYM;CNLLPLQR	NCP	9.273
CNLLPLQR;TKRLQVLL	NCP	41.49
TKRLQVLL;AAGCLLPPF	NCP	47.181
AAGCLLPPF;HPLAP	NCP	33.711
HPLAP;SPKAVTEP	NCP	16.419
SPKAVTEP;NLDAP	NCP	24.999
NLDAP;FGVRK	NCP	75.54

FGVRK;FPKYN	NCP	2.123
FPKYN;KLACVTK	NCP	123.637
KLACVTK;NSHDKVK	NCP	26.871
NSHDKVK;TFLGD	NCP	212.1
TFLGD;EAGSLLK	NCP	14.205
EAGSLLK;PEPLKFR	NCP	33.189
PEPLKFR;LLLATVK	NCP	247.611
LLLATVK;LKLSQSKN	NCP	19.607
LKLSQSKN;KTCQQFLKK	NCP	23.98
KTCQQFLKK;VMGGVEAP	NCP	9.804
VMGGVEAP;LEVLPR	NCP	14.616
LEVLPR;AFASPL	NCP	43.986
AFASPL;QPKQPDH	NCP	94.941
QPKQPDH;ADHLVLY	NCP	37.323
ADHLVLY;KFAAQNR	NCP	150.579
KFAAQNR;VFPPT	NCP	6.024
VFPPT;FGDLP	NCP	199.467
FGDLP;EKKTCGK	NCP	3.591
EKKTCGK;EAALE	NCP	13.416
EAALE;SPGQPVF	NCP	17.289
SPGQPVF;KYLPN	NCP	38.094
KYLPN;TLNNKFASF	NCP	5.997
TLNNKFASF;KVSRAGK	NCP	7.467
KVSRAGK;LVNQHKK	NCP	24.915
LVNQHKK;VELLL	NCP	29.256
VELLL;YQTKVGR	NCP	64.518
YQTKVGR;KTVAPGP	NCP	18.612
KTVAPGP;QLGSQRL	NCP	66.863
QLGSQRL;KLDNNK	NCP	11.866
KLDNNK;LYFPGN	NCP	2.504
LYFPGN;QPLLH	NCP	145.953
QPLLH;PPPPAPL	NCP	54.514
PPPPAPL;ATAAPRSPP	NCP	56.25
ATAAPRSPP;KRWKPK	NCP	16.371
KRWKPK;GHAAQPP	NCP	12.533
GHAAQPP;FHAPEEL	NCP	129.091
FHAPEEL;KVMLRL	NCP	76.265
KVMLRL;KSRVAAP	NCP	17.253
KSRVAAP;TWLDFM	NCP	19.581
TWLDFM;LDAPF	NCP	27.279
LDAPF;GVLKP	NCP	58.854
GVLKP;LPEKPKDPG	NCP	121.806
LPEKPKDPG;LLVDVEPK	NCP	7.41
LLVDVEPK;AGKPTL	NCP	3.112
AGKPTL;VSALLP	NCP	51.681
VSALLP;SNVFEHY	NCP	81.599
SNVFEHY;VNPAM	NCP	21.981
VNPAM;GPGAKDGK	NCP	15.736
GPGAKDGK;MGFPDM	NCP	56.571
MGFPDM;LTVYFSPK	NCP	50.54
LTVYFSPK;KQLEY	NCP	72.004

KQLEY;LLEVDNR	NCP	10.073
LLEVDNR;KMMLP	NCP	278.86
KMMLP;NTLAF	NCP	53.697
NTLAF;AAPELTAL	NCP	56.037
AAPELTAL;SLEVL	NCP	30.893
SLEVL;SMGLPK	NCP	35.8
SMGLPK;SPAMKP	NCP	2.685
SPAMKP;LKELEDD	NCP	114.747
LKELEDD;GLSTLVK	NCP	28.607
GLSTLVK;RKKLEMKP	NCP	1.48
RKKLEMKP;GVGASGGP	NCP	112.22
GVGASGGP;AAGVVL	NCP	0.591
AAGVVL;NVELVK	NCP	5.269
NVELVK;QNQLLDL	NCP	142.227
QNQLLDL;SSSLEK	NCP	56.085
SSSLEK;ATVPKPK	NCP	27.209
ATVPKPK;EADDQP	NCP	49.318
EADDQP;HKVPQ	NCP	76.725
HKVPQ;NGRGVVVL	NCP	47.7
NGRGVVVL;LHLLPLLL	NCP	109.254
LHLLPLLL;FGSVETWPWQVLSK	NCP	76.217
FGSVETWPWQVLSK;VGGSNDKGLGMG	NCP	0.168
VGGSNDKGLGMG;RTPPLP	NCP	49.435
RTPPLP;KAYFER	NCP	70.739
KAYFER;EHLDDLK	NCP	194.419
EHLDDLK;ALGTVLQ	NCP	22.985
ALGTVLQ;SLVEDP	NCP	113.136
SLVEDP;KKKKPK	NCP	41.869
KKKKPK;AQVLVTGR	NCP	8.913
AQVLVTGR;TLPSPAK	NCP	35.801
TLPSPAK;KPLHPSK	NCP	95.023
KPLHPSK;CWELYC	NCP	20.04
CWELYC;ETGAGKHVPRA	NCP	0.105
ETGAGKHVPRA;DEVRTGTYR	NCP	0.204
DEVRTGTYR;MTMLEP	NCP	9.918
MTMLEP;KPMVVR	NCP	27.744
KPMVVR;KVSVPQ	NCP	27.63
KVSVPQ;RRKKK	NCP	30.66
RRKKK;MDSVEKGAATS	NCP	3.566
MDSVEKGAATS;GRPPKLQ	NCP	0.063
GRPPKLQ;NDHFVQL	NCP	23.073
NDHFVQL;KDACEPK	NCP	17.365
KDACEPK;AEEFPLT	NCP	54.225
AEEFPLT;LPAVAL	NCP	40.484
LPAVAL;AHVVLRK	NCP	19.551
AHVVLRK;CLPVQGK	NCP	196.17
CLPVQGK;KQTTFYL	NCP	6.267
KQTTFYL;AMAVEF	NCP	11.493
AMAVEF;KYPPPPPP	NCP	272.199
KYPPPPPP;ALLDNM	NCP	137.691
ALLDNM;KQMFAEK	NCP	76.113

KQMFAEK;KSVESTAK	NCP	132.781
KSVESTAK;LLYPKF	NCP	117.675
LLYPKF;FLCDVDLR	NCP	3.551
FLCDVDLR;HNMKLMR	NCP	10.395
HNMKLMR;KFTVK	NCP	38.853
KFTVK;KPGQAPQP	NCP	22.692
KPGQAPQP;QGVSTALG	NCP	9.436
QGVSTALG;TLDQLHK	NCP	34.631
TLDQLHK;LVSVAAR	NCP	19.084
LVSVAAR;AGEFLRA	NCP	28.125
AGEFLRA;RDKEKL	NCP	67.191
RDKEKL;LGLFS	NCP	47.645
LGLFS;RPPPPPPP	NCP	117.133
RPPPPPPP;LPRVLLDL	NCP	5.682
LPRVLLDL;KHTQVSAS	NCP	62.547
KHTQVSAS;KVVCPK	NCP	58.949
KVVCPK;QTGVVPR	NCP	14.742
QTGVVPR;MKLLETR	NCP	0.33
MKLLETR;SLFQPK	NCP	95.925
SLFQPK;VSLLPGPW	NCP	16.981
VSLLPGPW;AKLLFL	NCP	28.032
AKLLFL;RPSAPAPK	NCP	79.238
RPSAPAPK;MDSGL	NCP	21.181
MDSGL;SVYVYVKL	NCP	13.536
SVYVYVKL;LSGDMLK	NCP	17.528
LSGDMLK;KEGAVP	NCP	32.121
KEGAVP;MGKYK	NCP	37.279
MGKYK;LAVMP	NCP	111.897
LAVMP;NDHLNLK	NCP	51.485
NDHLNLK;MDALKKQ	NCP	32.596
MDALKKQ;RLALNYL	NCP	130.508
RLALNYL;MGSSYKYG	NCP	9.748
MGSSYKYG;PFEVLK	NCP	9.923
PFEVLK;KPAAEVPK	NCP	2.536
KPAAEVPK;LPNFF	NCP	16.052
LPNFF;VHLGLQ	NCP	113.286
VHLGLQ;SAYLVRP	NCP	25.503
SAYLVRP;QQRHVP	NCP	6.519
QQRHVP;RYLPRS	NCP	6.769
RYLPRS;LPESVF	NCP	49.406
LPESVF;TGRLYTL	NCP	20.454
TGRLYTL;PVVAFH	NCP	31.836
PVVAFH;PPVTPTPF	NCP	16.209
PPVTPTPF;KAGPP	NCP	46.872
KAGPP;PKNRPP	NCP	22.032
PKNRPP;QLEPVL	NCP	1.233
QLEPVL;YNPEKLR	NCP	215.427
YNPEKLR;ENVYP	NCP	76.915
ENVYP;AGKPTLH	NCP	29.853
AGKPTLH;PVEDW	NCP	91.145
PVEDW;KKPPSSPK	NCP	10.573

KKPPSSPK;WLLHEY	NCP	78.075
WLLHEY;KELMRPK	NCP	11.886
KELMRPK;KALHLLR	NCP	5.654
KALHLLR;FDEVVPK	NCP	15.393
FDEVVPK;VLAPM	NCP	27.993
VLAPM;DDLQKKR	NCP	9.325
DDLQKKR;EPPYKEK	NCP	121.671
EPPYKEK;PLLMMC	NCP	35.481
PLLMMC;PAAVSLF	NCP	4.13
PAAVSLF;FMLLE	NCP	46.056
FMLLE;VLLFG	NCP	37.492
VLLFG;DPKVGPP	NCP	8.919
DPKVGPP;ALLSSGF	NCP	12.986
ALLSSGF;KEPGKGK	NCP	19.605
KEPGKGK;KRPALN	NCP	75.856
KRPALN;KHKETL	NCP	57.657
KHKETL;HKTTLAFT	NCP	96.362
HKTTLAFT;EDGRGN	NCP	98.254
EDGRGN;QGLFPR	NCP	105.471
QGLFPR;ALSSAGPK	NCP	16.583
ALSSAGPK;EDDYPP	NCP	66.231
EDDYPP;VVDEGLR	NCP	88.188
VVDEGLR;HGTFVTL	NCP	5.23
HGTFVTL;KPLLLLL	NCP	43.703
KPLLLLL;LKLEAVR	NCP	20.992
LKLEAVR;GVNLNQ	NCP	33.315
GVNLNQ;EAFNL	NCP	36.038
EAFNL;WGGGAY	NCP	59.023
WGGGAY;LTAELEL	NCP	103.544
LTAELEL;MCFPPR	NCP	10.398
MCFPPR;SGGPRVNK	NCP	11.881
SGGPRVNK;LEELKDF	NCP	93.045
LEELKDF;ALRLDYL	NCP	156.702
ALRLDYL;LVGAP	NCP	10.256
LVGAP;MELLQD	NCP	6.826
MELLQD;NLKLLKT	NCP	36.281
NLKLLKT;NSYRPPK	NCP	139.404
NSYRPPK;KLYPPYK	NCP	15.256
KLYPPYK;YPLDF	NCP	0.972
YPLDF;KPCLVR	NCP	64.848
KPCLVR;KAVWY	NCP	2.301
KAVWY;RVESHSK	NCP	26.109
RVESHSK;FTPVA	NCP	9.351
FTPVA;GFNLPLC	NCP	226.655
GFNLPLC;QDDKLR	NCP	98.986
QDDKLR;NYYTTF	NCP	1.878
NYYTTF;KYKPPPP	NCP	157.745
KYKPPPP;AAPPWAH	NCP	71.19
AAPPWAH;NLPHA	NCP	45.415
NLPHA;DSPRAF	NCP	34.683
DSPRAF;EVFAPST	NCP	46.743

EVFAPST;KMRDVP	NCP	3.633
KMRDVP;KVPVPP	NCP	81.423
KVPVPP;NDFLDKL	NCP	2.049
NDFLDKL;KYKPLHP	NCP	28.05
KYKPLHP;TVFGLLY	NCP	74.463
TVFGLLY;LAYLKK	NCP	41.535
LAYLKK;VGLLY	NCP	10.37
VGLLY;MKLQNLQ	NCP	92.527
MKLQNLQ;KYTFVKN	NCP	63.938
KYTFVKN;KPHSLKL	NCP	15.463
KPHSLKL;LPVELLLC	NCP	0.195
LPVELLLC;EVAAALK	NCP	34.118
EVAAALK;WLVTSK	NCP	4.336
WLVTSK;GTLGLVM	NCP	32.415
GTLGLVM;LGPPLQF	NCP	49.052
LGPPLQF;AMYELAVASFPKKQD	NCP	27.711
AMYELAVASFPKKQD;QQALLVK	NCP	7.902
QQALLVK;GKTGVLR	NCP	2.547
GKTGVLR;FHSLVHR	NCP	24.565
FHSLVHR;TSLADLR	NCP	42.029
TSLADLR;KPYWR	NCP	46.6
KPYWR;LDWVAF	NCP	36.38
LDWVAF;TKNVLP	NCP	16.579
TKNVLP;RWTHQ	NCP	133.773
RWTHQ;KDQVFLN	NCP	13.476
KDQVFLN;TVASLP	NCP	2.283
TVASLP;LVDTVEY	NCP	42.926
LVDTVEY;MERPR	NCP	84.601
MERPR;KQMWNK	NCP	51.273
KQMWNK;PPMLVVR	NCP	109.173
PPMLVVR;KNYVTAP	NCP	14.523
KNYVTAP;LPELLD	NCP	5.643
LPELLD;RGATTTF	NCP	52.065
RGATTTF;PDSKKVP	NCP	17.258
PDSKKVP;LKTKHAR	NCP	52.957
LKTKHAR;KEELYYR	NCP	10.254
KEELYYR;MFPLP	NCP	45.495
MFPLP;WTEVP	NCP	80.174
WTEVP;KGCALVH	NCP	63.361
KGCALVH;QVVVLFK	NCP	18.816
QVVVLFK;EDEVMRAYL	NCP	10.125
EDEVMRAYL;KFLKPK	NCP	110.22
KFLKPK;DLSGPVK	NCP	4.491
DLSGPVK;KPDDDK	NCP	93.836
KPDDDK;KPLLRPTT	NCP	186.504
KPLLRPTT;LDVFL	NCP	21.192
LDVFL;QKNLE	NCP	59.272
QKNLE;MDDLLP	NCP	87.968
MDDLLP;ATPLPEK	NCP	42.25
ATPLPEK;MTWSY	NCP	8.532
MTWSY;KNYHQ	NCP	96.827

KNYHQ;VTLYF	NCP	55.335
VTLYF;VLRVFLA	NCP	70.342
VLRVFLA;ALLFMC	NCP	54.609
ALLFMC;LVPLK	NCP	22.422
LVPLK;KYHEPK	NCP	3.287
KYHEPK;NAAAY	NCP	25.9
NAAAY;DFLVK	NCP	29.946
DFLVK;ALNYSVF	NCP	7.769
ALNYSVF;VFENY	NCP	165.579
VFENY;LTPTAV	NCP	4.371
LTPTAV;FMLTMF	NCP	24.498
FMLTMF;HLAPPK	NCP	239.182
HLAPPK;LDPAPN	NCP	14.657
LDPAPN;HSLPVLK	NCP	21.561
HSLPVLK;VGEVVAR	NCP	27.759
VGEVVAR;KEHVPQ	NCP	0.576
KEHVPQ;QNQSELLK	NCP	54.759
QNQSELLK;YEFFR	NCP	148.458
YEFFR;LKHKKKNF	NCP	50.844
LKHKKKNF;ATDGN	NCP	32.11
ATDGN;LGLVY	NCP	53.349
LGLVY;ELYLDLL	NCP	120.51
ELYLDLL;PSTEL	NCP	4.55
PSTEL;TELPALP	NCP	155.19
TELPALP;VDDCGLQ	NCP	11.709
VDDCGLQ;KKGEQK	NCP	41.026
KKGEQK;TYCLLSH	NCP	9.815
TYCLLSH;LLLLFWT	NCP	12.178
LLLLFWT;KDYVGL	NCP	182.624
KDYVGL;KLNGAVPK	NCP	14.178
KLNGAVPK;TEEHHLRDY	NCP	24.931
TEEHHLRDY;VYSDLMK	NCP	2.385
VYSDLMK;TMEEPP	NCP	124.217
TMEEPP;AASEGTKAVTKYTSSK	NCP	49.836
AASEGTKAVTKYTSSK;ELMYS	NCP	37.906
ELMYS;PLLHQAP	NCP	27.609
PLLHQAP;YVGKLPL	NCP	18.06
YVGKLPL;GSTPSLP	NCP	216.023
GSTPSLP;QKAPAPK	NCP	10.612
QKAPAPK;KSDSKEK	NCP	24.032
KSDSKEK;TNYYLLK	NCP	159.544
TNYYLLK;PHSVYEY	NCP	22.07
PHSVYEY;AAEEVLQTVDHY	NCP	13.938
AAEEVLQTVDHY;KGGPYGK	NCP	88.077
KGGPYGK;VFPPQPY	NCP	93
VFPPQPY;CLFLPR	NCP	12.193
CLFLPR;KFFGGPK	NCP	8.115
KFFGGPK;FVQSQK	NCP	1.385
FVQSQK;VVDVVR	NCP	44.359
VVDVVR;CWQSDR	NCP	102.258
CWQSDR;AYLGT	NCP	0.248

AYLGT;HALHKSY	NCP	51.541
HALHKSY;LELEQL	NCP	36.035
LELEQL;LEVVVP	NCP	104.604
LEVVVP;GKFLKSKL	NCP	5.556
GKFLKSKL;LVHVSPED	NCP	3.762
LVHVSPED;YESGQY	NCP	70.804
YESGQY;PTKPGVP	NCP	58.916
PTKPGVP;VGLLL	NCP	12.694
VGLLL;KPKKETK	NCP	62.406
KPKKETK;LEVLKPF	NCP	39.935
LEVLKPF;EVCGGPP	NCP	32.92
EVCGGPP;KTFKTK	NCP	101.399
KTFKTK;FFKEFQ	NCP	126.132
FFKEFQ;LKAGLT	NCP	61.143
LKAGLT;DDYLVY	NCP	105.553
DDYLVY;TLQQMTK	NCP	53.694
TLQQMTK;PNCDLH	NCP	74.261
PNCDLH;LEDVVVL	NCP	138.894
LEDVVVL;KTARPGK	NCP	0.933
KTARPGK;QKLAF	NCP	48.964
QKLAF;VSCGKKK	NCP	49.623
VSCGKKK;KEEPVKK	NCP	25.107
KEEPVKK;SLWFF	NCP	7.308
SLWFF;EVLRPF	NCP	54.456
EVLRPF;VSGTLSPK	NCP	28.772
VSGTLSPK;QSLVSAY	NCP	49.155
QSLVSAY;AMDELKK	NCP	45.787
AMDELKK;AHLTSSK	NCP	110.187
AHLTSSK;FGVTFAKK	NCP	13.484
FGVTFAKK;DPRHPLLR	NCP	7.917
DPRHPLLR;LTHDLAK	NCP	12.042
LTHDLAK;VDSKPVNL	NCP	111.354
VDSKPVNL;LETALAPS	NCP	51.549
LETALAPS;LLEMDHF	NCP	2.05
LLEMDHF;QSVADKD	NCP	42.231
QSVADKD;KREVALK	NCP	6.585
KREVALK;RAVVVLS	NCP	8.223
RAVVVLS;AAANFP	NCP	18.983
AAANFP;VHQVLYRAL	NCP	91.179
VHQVLYRAL;QVLYRALVS	NCP	0.006
QVLYRALVS;ATLNRSLLK	NCP	0.453
ATLNRSLLK;VHLSPPSS	NCP	8.721
VHLSPPSS;SPPRPQP	NCP	17.532
SPPRPQP;PSAALY	NCP	26.791
PSAALY;VDLGSGY	NCP	6.222
VDLGSGY;KEMQPK	NCP	83.102
KEMQPK;PDLAVG	NCP	48.823
PDLAVG;PFAVPEM	NCP	180.038
PFAVPEM;KLSLY	NCP	9.325
KLSLY;QRLVTLF	NCP	58.908
QRLVTLF;DYSSAK	NCP	12.075

DYSSAK;LQAVLLPK	NCP	12.318
LQAVLLPK;TGALLEY	NCP	10.641
TGALLEY;VADWTGATYQDK	NCP	28.487
VADWTGATYQDK;KSCMAPP	NCP	42.685
KSCMAPP;AAPGQSLK	NCP	8.255
AAPGQSLK;DHSALY	NCP	0.843
DHSALY;AQQAADKYL	NCP	109.35
AQQAADKYL;PLAQADWAAK	NCP	0.054
PLAQADWAAK;HEMPPH	NCP	4.167
HEMPPH;YLLSGAGEHLK	NCP	19.887
YLLSGAGEHLK;LLDEECWFPK	NCP	5.229
LLDEECWFPK;VDYKADEWL	NCP	1.968
VDYKADEWL;KADEWLMK	NCP	0.009
KADEWLMK;VGTGLA	NCP	1.651
VGTGLA;TVGQLYKEQLAK	NCP	0.938
TVGQLYKEQLAK;LYKEQLA	NCP	0.012
LYKEQLA;ATLRNTNPNFVR	NCP	0.03
ATLRNTNPNFVR;KVLQRNCAA	NCP	4.413
KVLQRNCAA;VLQRNCAAY	NCP	0.003
VLQRNCAAY;EEELVKVREKQ	NCP	1.131
EEELVKVREKQ;DLGEELEALK	NCP	3.261
DLGEELEALK;KLRLEVN	NCP	5.094
KLRLEVN;DLPFVVPR	NCP	6.081
DLPFVVPR;GKADGAEAKAAEAR	NCP	0.072
GKADGAEAKAAEAR;DGAEAKAAEARAAE	NCP	0.009
DGAEAKAAEARAAE;PLLAL	NCP	85.903
PLLAL;MAAHDSE	NCP	92.627
MAAHDSE;AATVSR	NCP	149.02
AATVSR;KQCYLSP	NCP	2.27
KQCYLSP;PKPLNVR	NCP	31.96
PKPLNVR;KFPRGL	NCP	16.77
KFPRGL;DPRSLGP	NCP	45.782
DPRSLGP;VTAVDL	NCP	13.017
VTAVDL;ESVPDW	NCP	45.097
ESVPDW;FENLCK	NCP	46.962
FENLCK;PAALPPP	NCP	28.313
PAALPPP;ANLLVR	NCP	25.203
ANLLVR;RPVVTQP	NCP	50.937
RPVVTQP;LLYFKHL	NCP	136.201
LLYFKHL;AELLQKK	NCP	37.368
AELLQKK;NVPFLP	NCP	10.799
NVPFLP;VPGGL	NCP	30.351
VPGGL;VEQLRL	NCP	24.964
VEQLRL;LCVVSM	NCP	4.409
LCVVSM;VGGPY	NCP	6.255
VGGPY;SDGEWQL	NCP	39.615
SDGEWQL;GKVEADVAGHGQE	NCP	0.036
GKVEADVAGHGQE;VAGHGQEVL	NCP	0.018
VAGHGQEVL;FTGHPETL	NCP	5.382
FTGHPETL;GHPETLEKFDK	NCP	0.006
GHPETLEKFDK;NDMAAQYKVLGFHG	NCP	4.524

NDMAAQYKVLGFHG;AAQYKVLG	NCP	0.009
AAQYKVLG;AQYKVLGFHG	NCP	0.003
AQYKVLGFHG;KPGVEVV	NCP	113.223
KPGVEVV;KDNAWPK	NCP	7.119
KDNAWPK;PMEAAGP	NCP	3.273
PMEAAGP;ALSLLQ	NCP	17.043
ALSLLQ;VPGEAH	NCP	6.795
VPGEAH;QVQVPG	NCP	7.12
QVQVPG;ELPPEPQ	NCP	21.404
ELPPEPQ;TPLLRHL	NCP	0.759
TPLLRHL;EPGAVATH	NCP	64.266
EPGAVATH;PPFESLK	NCP	36.424
PPFESLK;KEFHPP	NCP	26.163
KEFHPP;GPKYPR	NCP	19.481
GPKYPR;WLPLVA	NCP	94.054
WLPLVA;LLNERLH	NCP	102.446
LLNERLH;QELAALK	NCP	96.756
QELAALK;CFDLPVP	NCP	77.053
CFDLPVP;DKDSGLK	NCP	78.608
DKDSGLK;KPASSSGK	NCP	1.572
KPASSSGK;YGPLL	NCP	96.25
YGPLL;AADPVK	NCP	20.226
AADPVK;KVCLLGCG	NCP	56.37
KVCLLGCG;VPSLPLL	NCP	28.547
VPSLPLL;VGGKLRS	NCP	3.06
VGGKLRS;EAYKYT	NCP	2.098
EAYKYT;LLDRNVLL	NCP	6.594
LLDRNVLL;KTTLRFQ	NCP	4.929
KTTLRFQ;VLPLEKLN	NCP	31.161
VLPLEKLN;MGGNCPT	NCP	7.79
MGGNCPT;LSAAFL	NCP	55.092
LSAAFL;NLHRGVN	NCP	32.586
NLHRGVN;LLPPLELS	NCP	85.113
LLPPLELS;LASSEQVK	NCP	0.816
LASSEQVK;NDFGQAR	NCP	32.667
NDFGQAR;KESGQPK	NCP	7.296
KESGQPK;PYTFEC	NCP	117.148
PYTFEC;KSKLLDAY	NCP	68.307
KSKLLDAY;VEKPPVG	NCP	19.862
VEKPPVG;KTYLKVFN	NCP	22.506
KTYLKVFN;GKDPF	NCP	122.943
GKDPF;SGPALQRL	NCP	44.661
SGPALQRL;KGTNWPV	NCP	181.738
KGTNWPV;KVYPPPPP	NCP	26.709
KVYPPPPP;DLKLV	NCP	32.487
DLKLV;EFTRLQ	NCP	15.677
EFTRLQ;ENLVYL	NCP	6.685
ENLVYL;AVAVVKK	NCP	17.471
AVAVVKK;VSPAPN	NCP	5.119
VSPAPN;LSYASGR	NCP	195.525
LSYASGR;AATLKGNK	NCP	66.384

AATLKGNK;VAPLF	NCP	43.425
VAPLF;KSSNYVH	NCP	4.65
KSSNYVH;VPVPTNF	NCP	31.418
VPVPTNF;LDPKPLP	NCP	91.122
LDPKPLP;VVPTFGKKKGPNANS	NCP	26.005
VVPTFGKKKGPNANS;DDFLPGHL	NCP	0.98
DDFLPGHL;FPDPVSA	NCP	43.122
FPDPVSA;KPTFFK	NCP	2.319
KPTFFK;NLPKNGP	NCP	12.106
NLPKNGP;LKDKELK	NCP	21.288
LKDKELK;RTGKLK	NCP	13.092
RTGKLK;KEVEFYK	NCP	33.29
KEVEFYK;LGSGAAAR	NCP	15.097
LGSGAAAR;LHQYVPQ	NCP	40.067
LHQYVPQ;LSVKMK	NCP	168.657
LSVKMK;VYQAF	NCP	2.184
VYQAF;LDFQDVLDKL	NCP	71.88
LDFQDVLDKL;EAVGGP	NCP	56.955
EAVGGP;SHLLSTSR	NCP	20.439
SHLLSTSR;YGEVL	NCP	36.684
YGEVL;KLPAAKPP	NCP	36.409
KLPAAKPP;TMLVHRK	NCP	83.499
TMLVHRK;KLLVHT	NCP	17.169
KLLVHT;QGAPVY	NCP	36.761
QGAPVY;LALRG	NCP	10.434
LALRG;KPSHTEK	NCP	82.315
KPSHTEK;DLPLSPLP	NCP	1.97
DLPLSPLP;AELELPL	NCP	37.611
AELELPL;KELYL	NCP	68.838
KELYL;CERLLLLF	NCP	42.742
CERLLLLF;APPLPPLP	NCP	230.313
APPLPPLP;KSPPSRP	NCP	58.001
KSPPSRP;MELDL	NCP	21.04
MELDL;GPGLK	NCP	32.801
GPGLK;GKGDVLR	NCP	11.112
GKGDVLR;RGLKSFE	NCP	1.561
RGLKSFE;KGFGPQK	NCP	115.082
KGFGPQK;LSGGPVVK	NCP	60.339
LSGGPVVK;QSNMPK	NCP	13.731
QSNMPK;YVPAP	NCP	27.666
YVPAP;LAGTTHQ	NCP	35.626
LAGTTHQ;APELYFK	NCP	67.494
APELYFK;AAYYK	NCP	47.406
AAYYK;AAGFK	NCP	11.865
AAGFK;KHVYPK	NCP	50.63
KHVYPK;APSHDGA	NCP	113.007
APSHDGA;VSMKEKL	NCP	103.618
VSMKEKL;LYLEYAK	NCP	14.271
LYLEYAK;LTVLP	NCP	19.058
LTVLP;LPPKDGN	NCP	38.841
LPPKDGN;KPCRGLVE	NCP	67.891

KPCRGLVE;ETMMMG	NCP	29.279
ETMMMG;CDAKPK	NCP	25.669
CDAKPK;MGCHAFP	NCP	12.918
MGCHAFP;KPGATTH	NCP	65.568
KPGATTH;PGAGTN	NCP	27.261
PGAGTN;RRLLDH	NCP	13.364
RRLLDH;PNWPHSKC	NCP	15.411
PNWPHSKC;CFSKEPLK	NCP	88.222
CFSKEPLK;MGCCCN	NCP	68.757
MGCCCN;PLTKFLQ	NCP	1.956
PLTKFLQ;ADVSGPK	NCP	88.865
ADVSGPK;LLLLLLLLL	NCP	68.749
LLLLLLLLL;KDKLLKLR	NCP	6.455
KDKLLKLR;KPSSLPGN	NCP	14.838
KPSSLPGN;YYHLT	NCP	28.908
YYHLT;CSHLLLH	NCP	56.313
CSHLLLH;TPVFPSLA	NCP	20.859
TPVFPSLA;LPVVDP	NCP	117.099
LPVVDP;EVELVSH	NCP	38.274
EVELVSH;YVGAAV	NCP	29.79
YVGAAV;PSLVKHR	NCP	28.962
PSLVKHR;KVVRNVPP	NCP	172.275
KVVRNVPP;PQEFKP	NCP	30.87
PQEFKP;KEPFPPP	NCP	17.787
KEPFPPP;LPPGSPP	NCP	49.071
LPPGSPP;LGSVPN	NCP	29.524
LGSVPN;QHTPPGP	NCP	1.421
QHTPPGP;TVTHARP	NCP	1.453
TVTHARP;GPLVEL	NCP	6.098
GPLVEL;LSVMWPK	NCP	35.71
LSVMWPK;NVSLAPL	NCP	116.675
NVSLAPL;KDSHAAK	NCP	49.107
KDSHAAK;LGFAKPP	NCP	7.584
LGFAKPP;MLPPPPR	NCP	3.534
MLPPPPR;NKAAP	NCP	54.625
NKAAP;TMFPKGP	NCP	0.236
TMFPKGP;PSLTSKYR	NCP	11.044
PSLTSKYR;YLVHR	NCP	35.036
YLVHR;VLATEVT	NCP	9.478
VLATEVT;KRGLDDF	NCP	8.082
KRGLDDF;GPSNYK	NCP	111.501
GPSNYK;PDWGNKK	NCP	71.975
PDWGNKK;PSKLYTSP	NCP	6.708
PSKLYTSP;FLRPPPP	NCP	46.156
FLRPPPP;KGKPPP	NCP	14.66
KGKPPP;APGWQAK	NCP	106.222
APGWQAK;VLKDYE	NCP	17.955
VLKDYE;VDARFV	NCP	22.883
VDARFV;SVGGPML	NCP	64.206
SVGGPML;VGLAK	NCP	65.23
VGLAK;TPLPKRP	NCP	4.331

TPLPKRP;LQRLVDY	NCP	211.284
LQRLVDY;LTELPVT	NCP	1.494
LTELPVT;VSSYLGPV	NCP	2.724
VSSYLGPV;PDSVHTK	NCP	37.969
PDSVHTK;VHSLVEL	NCP	31.995
VHSLVEL;MKTPLER	NCP	74.579
MKTPLER;KTYKRSS	NCP	19.177
KTYKRSS;MLFHTPF	NCP	82.067
MLFHTPF;VTTAVP	NCP	3.664
VTTAVP;ELAAML	NCP	49.595
ELAAML;VTPSSDLP	NCP	23.731
VTPSSDLP;AAGQPGGK	NCP	44.853
AAGQPGGK;KGSLTVRL	NCP	30.252
KGSLTVRL;DKEDLFPK	NCP	75.323
DKEDLFPK;EFEPL	NCP	24.313
EFEPL;AATPYAL	NCP	222.473
AATPYAL;PVSELLSKV	NCP	14.173
PVSELLSKV;EAVTLP	NCP	191.555
EAVTLP;PSAPPPR	NCP	6.205
PSAPPPR;FVRHLEK	NCP	41.859
FVRHLEK;LMHWPY	NCP	2.957
LMHWPY;LVFLY	NCP	18.766
LVFLY;MFNKPF	NCP	14.412
MFNKPF;VPKHFVP	NCP	19.431
VPKHFVP;KEAALEK	NCP	43.752
KEAALEK;EVTTALK	NCP	4.247
EVTTALK;ERPLLTL	NCP	19.432
ERPLLTL;LQEYSPK	NCP	54.761
LQEYSPK;AYLEF	NCP	68.208
AYLEF;MDEDGLPL	NCP	17.421
MDEDGLPL;AVTHKAP	NCP	80.589
AVTHKAP;VPGSNAF	NCP	126.868
VPGSNAF;HNVSNVLP	NCP	54.473
HNVSNVLP;KGPFWL	NCP	25.941
KGPFWL;GLEPF	NCP	59.506
GLEPF;MELTAH	NCP	35.747
MELTAH;KEAGTPP	NCP	37.047
KEAGTPP;GKDLPGM	NCP	3.198
GKDLPGM;MDTMAPQ	NCP	10.389
MDTMAPQ;KDKGLPN	NCP	9.067
KDKGLPN;KWASRSP	NCP	31.865
KWASRSP;AQLKSLK	NCP	36.381
AQLKSLK;WDSFY	NCP	47.212
WDSFY;KVGLSH	NCP	10.365
KVGLSH;FGGFGQ	NCP	158.285
FGGFGQ;EPGTSKLK	NCP	116.41
EPGTSKLK;GVALLGF	NCP	18.827
GVALLGF;KEHGGVP	NCP	11.122
KEHGGVP;EVFPPGT	NCP	5.766
EVFPPGT;REEGSPR	NCP	15.698
REEGSPR;ASSSSK	NCP	25.278

ASSSSK;EQTFAG	NCP	25.047
EQTFAG;QAAANP	NCP	149.994
QAAANP;KGVHLGP	NCP	51.582
KGVHLGP;VTKLLFY	NCP	100.71
VTKLLFY;MHAFPK	NCP	19.656
MHAFPK;METRY	NCP	53.472
METRY;AKETHGP	NCP	62.623
AKETHGP;LPFAFL	NCP	209.834
LPFAFL;NSLQNP	NCP	3.886
NSLQNP;KKKHLLLY	NCP	44.139
KKKHLLLY;KPLTKK	NCP	23.249
KPLTKK;KLPGVGV	NCP	27.181
KLPGVGV;LDSLGPK	NCP	4.218
LDSLGPK;DNNLF	NCP	16.902
DNNLF;GSPLNFT	NCP	4.383
GSPLNFT;KPVSP	NCP	36.578
KPVSP;AVVHPYK	NCP	83.811
AVVHPYK;MTHLVR	NCP	0.816
MTHLVR;ELVAVAK	NCP	16.819
ELVAVAK;EVPRSPK	NCP	11.898
EVPRSPK;EKTPLM	NCP	188.388
EKTPLM;MGTDLRK	NCP	30.101
MGTDLRK;VGNLGQGSY	NCP	16.249
VGNLGQGSY;LLNVHSSK	NCP	5.571
LLNVHSSK;CYPVKK	NCP	5.574
CYPVKK;CEAPKPK	NCP	122.865
CEAPKPK;LPPNMCL	NCP	97.997
LPPNMCL;STVPKLA	NCP	52.36
STVPKLA;TVLLEEL	NCP	58.298
TVLLEEL;RNKGVSE	NCP	65.28
RNKGVSE;KRDSYPR	NCP	76.239
KRDSYPR;KTEPVR	NCP	53.548
KTEPVR;ETSSLRR	NCP	54.797
ETSSLRR;MLLRSLK	NCP	9.396
MLLRSLK;QEGYLL	NCP	102.708
QEGYLL;RDGVAKK	NCP	4.87
RDGVAKK;FAYTLPK	NCP	31.106
FAYTLPK;LGKSGY	NCP	10.314
LGKSGY;CHVKK	NCP	10.744
CHVKK;VPLEGK	NCP	55.074
VPLEGK;AAAKKGK	NCP	8.001
AAAKKGK;LYRPGP	NCP	89.864
LYRPGP;LPGREC	NCP	6.403
LPGREC;AASGPPPP	NCP	1.251
AASGPPPP;KTTKKK	NCP	14.681
KTTKKK;ASLGRNK	NCP	12.36
ASLGRNK;YVSAAYK	NCP	52.848
YVSAAYK;LPKRLGK	NCP	26.431
LPKRLGK;MSHMEY	NCP	15.914
MSHMEY;VPLCSFK	NCP	19.285
VPLCSFK;LMGPLRL	NCP	66.624

LMGPLRL;RQQVPK	NCP	17.103
RQQVPK;KEELLK	NCP	36.617
KEELLK;VEVVLG	NCP	126.652
VEVVLG;NVPTLLK	NCP	174.534
NVPTLLK;TAGLRLL	NCP	21.93
TAGLRLL;TWPTVK	NCP	19.859
TWPTVK;DDLPLKD	NCP	40.884
DDLPLKD;GVDLLLKK	NCP	10.281
GVDLLLKK;VSGRPVR	NCP	46.353
VSGRPVR;QNLGL	NCP	16.494
QNLGL;LHKAAFA	NCP	7.456
LHKAAFA;KNKDLKDL	NCP	63.048
KNKDLKDL;KEHLQK	NCP	19.064
KEHLQK;KVTEC	NCP	5.298
KVTEC;GDDSC	NCP	30.592
GDDSC;KEQAVWP	NCP	4.704
KEQAVWP;KPRAVGK	NCP	26.015
KPRAVGK;RPVLSVK	NCP	89.494
RPVLSVK;TQEHVKR	NCP	75.83
TQEHVKR;NSESTFARAY	NCP	49.138
NSESTFARAY;TSHLVGSALSDPY	NCP	6.648
TSHLVGSALSDPY;MKFVDSK	NCP	1.173
MKFVDSK;LLVGYK	NCP	24.056
LLVGYK;KYFGPPP	NCP	215.34
KYFGPPP;RSALLR	NCP	27.126
RSALLR;QRQLGLGP	NCP	25.722
QRQLGLGP;NPLVVL	NCP	8.328
NPLVVL;GGFLALK	NCP	49.338
GGFLALK;VLDPVPR	NCP	105.571
VLDPVPR;FPGSH	NCP	19.629
FPGSH;KPPNAPP	NCP	27.272
KPPNAPP;AQSFML	NCP	82.834
AQSFML;QLLHQGTK	NCP	84.252
QLLHQGTK;RHLVTFL	NCP	32.6
RHLVTFL;PAGVQPRP	NCP	56.236
PAGVQPRP;RFFLHP	NCP	84.836
RFFLHP;PVAPFTP	NCP	292.246
PVAPFTP;RNSHVL	NCP	68.703
RNSHVL;LPVLESY	NCP	11.405
LPVLESY;RRKSCRR	NCP	48.615
RRKSCRR;PVGPVQ	NCP	85.92
PVGPVQ;TPKRAP	NCP	46.785
TPKRAP;PEVVDTCSLASPASVCQTK	NCP	30.645
PEVVDTCSLASPASVCQTK;TNLAALKK	NCP	2.889
TNLAALKK;TLGERQSY	NCP	2.076
TLGERQSY;SSADLKDFSSHQVNEFLAQ	NCP	20.907
SSADLKDFSSHQVNEFLAQ;SSHQVNEF	NCP	0.024
SSHQVNEF;RDLAAF	NCP	4.236
RDLAAF;AMLVGKDLKVD	NCP	0.084
AMLVGKDLKVD;KTYFAGK	NCP	15.217
KTYFAGK;KQKPQSR	NCP	50.952

KQKPQSR;KVEGP	NCP	52.688
KVEGP;GCLCQ	NCP	18.387
GCLCQ;KGLQELAK	NCP	6.01
KGLQELAK;PPLPPDKK	NCP	13.862
PPLPPDKK;LDDSLLK	NCP	25.828
LDDSLLK;ALSCELL	NCP	10.949
ALSCELL;ARHVFLT	NCP	45.726
ARHVFLT;VSFSLK	NCP	16.017
VSFSLK;PRGNTLR	NCP	11.353
PRGNTLR;TLSWLT	NCP	79.938
TLSWLT;RLYCLEL	NCP	20.703
RLYCLEL;TAAFLLPL	NCP	43.85
TAAFLLPL;NPGGKGY	NCP	280.287
NPGGKGY;RERLLDH	NCP	12.399
RERLLDH;MRLAGGL	NCP	72.579
MRLAGGL;CSHYDS	NCP	147.198
CSHYDS;VVSLVAL	NCP	230.499
VVSLVAL;TVTVAGPR	NCP	1.623
TVTVAGPR;FLMVCF	NCP	19.038
FLMVCF;RKVKVLF	NCP	53.877
RKVKVLF;KELVVYL	NCP	322.728
KELVVYL;LLKQNKP	NCP	40.108
LLKQNKP;YCECPP	NCP	66.951
YCECPP;MVWVPR	NCP	1.881
MVWVPR;PLLKQLVS	NCP	181.877
PLLKQLVS;GPGGKAPR	NCP	133.687
GPGGKAPR;RSLGVGY	NCP	58.143
RSLGVGY;EKGLFY	NCP	2.807
EKGLFY;NPHLKCK	NCP	27.634
NPHLKCK;KSHAVY	NCP	327.842
KSHAVY;VTPPKSC	NCP	9.049
VTPPKSC;APLLVTP	NCP	7.356
APLLVTP;KDPTP	NCP	43.85
KDPTP;TALYMVP	NCP	82.614
TALYMVP;TDYYLT	NCP	26.676
TDYYLT;LPPPPVPA	NCP	11.355
LPPPPVPA;PGMFAYL	NCP	92.773
PGMFAYL;QDYVPR	NCP	20.853
QDYVPR;KPPNVNK	NCP	8.994
KPPNVNK;LYFTGEAL	NCP	34.653
LYFTGEAL;VGPKLL	NCP	40.739
VGPKLL;KVMPHT	NCP	3.325
KVMPHT;KLVLLGESAVGK	NCP	37.64
KLVLLGESAVGK;SLYVYVK	NCP	3.454
SLYVYVK;NNLGGGL	NCP	4.412
NNLGGGL;QRAPVF	NCP	17.683
QRAPVF;LSAALEYL	NCP	18.15
LSAALEYL;LHTVQPK	NCP	38.675
LHTVQPK;HELLQPF	NCP	36.885
HELLQPF;KSEMEVQDAELK	NCP	0.237
KSEMEVQDAELK;ELGEWNF	NCP	8.062

ELGEWNF;VLNPV	NCP	2.42
VLNPV;KSPPGFN	NCP	7.087
KSPPGFN;ALVRVGTQAF	NCP	1.496
ALVRVGTQAF;QLFDRTGDGK	NCP	26.034
QLFDRTGDGK;PTNAEVLKVLGNPK	NCP	0.078
PTNAEVLKVLGNPK;VLDFEHFLPMLQTVAK	NCP	0.261
VLDFEHFLPMLQTVAK;LDFEHFLPM	NCP	0.003
LDFEHFLPM;EHFLPMLQ	NCP	0.009
EHFLPMLQ;VRMVLNG	NCP	1.374
VRMVLNG;PTVPVEP	NCP	30.915
PTVPVEP;GPHPPPPP	NCP	24.288
GPHPPPPP;ALPTLF	NCP	86.106
ALPTLF;PPFFAK	NCP	35.719
PPFFAK;KLLDFC	NCP	78.695
KLLDFC;LHHVPR	NCP	30.324
LHHVPR;YPAVGPP	NCP	29.523
YPAVGPP;NAVKELK	NCP	8.161
NAVKELK;QAAQNTK	NCP	24.002
QAAQNTK;VALVL	NCP	5.797
VALVL;DLHDNK	NCP	63.294
DLHDNK;LFNHTM	NCP	78.153
LFNHTM;KERWAR	NCP	48.285
KERWAR;KQKSFGP	NCP	9.822
KQKSFGP;QSKPRPK	NCP	36.254
QSKPRPK;MDVPGPK	NCP	5.557
MDVPGPK;NSAVTNK	NCP	101.52
NSAVTNK;AFLEY	NCP	3.363
AFLEY;ELYTL	NCP	48.899
ELYTL;TQAALVV	NCP	69.928
TQAALVV;VLAMQ	NCP	7.416
VLAMQ;KVCVLVGQ	NCP	8.213
KVCVLVGQ;GVFVY	NCP	13.695
GVFVY;MTMEEE	NCP	10.047
MTMEEE;TTGYVPR	NCP	93.459
TTGYVPR;EVPGLLP	NCP	11.193
EVPGLLP;VQPYSGSPAN	NCP	20.991
VQPYSGSPAN;SGSPANLAA	NCP	0.012
SGSPANLAA;DSGLALLT	NCP	4.159
DSGLALLT;KHPYN	NCP	16.32
KHPYN;ETMVYP	NCP	150.032
ETMVYP;CGSHMEM	NCP	0.39
CGSHMEM;MDLALH	NCP	50.955
MDLALH;KQRKVVE	NCP	9.745
KQRKVVE;PLNLASVYLT	NCP	16.166
PLNLASVYLT;ALAVL	NCP	19.491
ALAVL;KLEAGPGV	NCP	17.326
KLEAGPGV;AAPGGNF	NCP	1.259
AAPGGNF;KYMPPLP	NCP	3.823
KYMPPLP;KGFSGH	NCP	6.78
KGFSGH;LSCLLP	NCP	128.58
LSCLLP;VGSLNLEELSEMRY	NCP	18.29

VGSLNLEELSEMRY;YFDLL	NCP	2.524
YFDLL;PMGLLVK	NCP	24.516
PMGLLVK;LKNWL	NCP	76.739
LKNWL;PALYRALV	NCP	25.125
PALYRALV;LDLDVL	NCP	20.949
LDLDVL;WGKPP	NCP	22.863
WGKPP;TLSRKFC	NCP	0.744
TLSRKFC;HVVFLAQ	NCP	31.836
HVVFLAQ;KNPFP	NCP	21.637
KNPFP;RGQGGGPLR	NCP	71.288
RGQGGGPLR;VPPVPPPP	NCP	26.871
VPPVPPPP;PCVKV	NCP	123
PCVKV;HGKDAC	NCP	0.583
HGKDAC;YMCSMP	NCP	22.415
YMCSMP;ETLTP	NCP	44.433
ETLTP;DGLVKLR	NCP	9.046
DGLVKLR;KEALPGLK	NCP	13.763
KEALPGLK;LVPLERN	NCP	7.225
LVPLERN;PPQHAAK	NCP	9.555
PPQHAAK;LSLHEYL	NCP	44.321
LSLHEYL;PNTLSPK	NCP	47.46
PNTLSPK;KWYLLFK	NCP	184.299
KWYLLFK;DDPLTPF	NCP	138.498
DDPLTPF;PYPASLTL	NCP	16.215
PYPASLTL;SLQYY	NCP	144.81
SLQYY;NSLGPYK	NCP	45.483
NSLGPYK;FGVVKVK	NCP	71.559
FGVVKVK;VGPLEE	NCP	13.227
VGPLEE;LEDLNLK	NCP	1.765
LEDLNLK;AQQLSPGK	NCP	15.828
AQQLSPGK;TYLVY	NCP	27.702
TYLVY;FGMEDF	NCP	18.585
FGMEDF;EYLLL	NCP	103.643
EYLLL;KGYSFDR	NCP	25.545
KGYSFDR;TSSTK	NCP	50.836
TSSTK;AELLF	NCP	5.343
AELLF;EPPVLP	NCP	168.453
EPPVLP;AAAAVVFE	NCP	95.301
AAAAVVFE;NLSAGLL	NCP	4.494
NLSAGLL;YNLSLF	NCP	75.186
YNLSLF;PDETDR	NCP	35.682
PDETDR;PHARYP	NCP	1.011
PHARYP;ANHFAK	NCP	24.498
ANHFAK;SSGLKGMSL	NCP	84.305
SSGLKGMSL;VVVFGNDKL	NCP	0.051
VVVFGNDKL;VDVPVGEELLGR	NCP	0.066
VDVPVGEELLGR;LVKRLTDADAMKY	NCP	0.363
LVKRLTDADAMKY;RLTDADAMK	NCP	0.009
RLTDADAMK;RQMSLLL	NCP	0.18
RQMSLLL;LELAQYREVAAF	NCP	0.339
LELAQYREVAAF;YLDKLEPSK	NCP	0.18

YLDKLEPSK;NFLAGFEA	NCP	0.153
NFLAGFEA;KDSVLNG	NCP	21.378
KDSVLNG;VVEKAQ	NCP	8.512
VVEKAQ;TQYVYKV	NCP	12.14
TQYVYKV;MHMKNNLG	NCP	43.644
MHMKNNLG;LQFVL	NCP	6.3
LQFVL;VGGFY	NCP	1.105
VGGFY;LCALKSL	NCP	14.208
LCALKSL;HNPHVNPLPTGYEDE	NCP	101.523
HNPHVNPLPTGYEDE;DLYVPR	NCP	8.306
DLYVPR;KANLPVQ	NCP	18.943
KANLPVQ;PFVHTK	NCP	28.02
PFVHTK;AATPLPKL	NCP	5.196
AATPLPKL;VAHVGKP	NCP	35.076
VAHVGKP;PNLPFL	NCP	62.238
PNLPFL;QSLDLFDF	NCP	21.594
QSLDLFDF;SLVVEL	NCP	73.964
SLVVEL;LSDPVP	NCP	30.732
LSDPVP;WMKEVF	NCP	24.136
WMKEVF;EVKDFLP	NCP	54.387
EVKDFLP;KAVSPPK	NCP	47.13
KAVSPPK;FVAEAKK	NCP	47.1
FVAEAKK;KLDLSRP	NCP	62.949
KLDLSRP;TLGLR	NCP	86.148
TLGLR;SLGVEKL	NCP	37.265
SLGVEKL;KSLEKLR	NCP	132.061
KSLEKLR;AEADLLK	NCP	84.786
AEADLLK;VELPL	NCP	39.606
VELPL;KHHFHVTPP	NCP	70.176
KHHFHVTPP;SMYDLR	NCP	28.641
SMYDLR;GRELDVR	NCP	37.227
GRELDVR;YVMEKC	NCP	6.027
YVMEKC;LGLQDGY	NCP	83.001
LGLQDGY;FMLLQG	NCP	1.256
FMLLQG;DQGAVLLA	NCP	84.798
DQGAVLLA;ESKALEPV	NCP	1.876
ESKALEPV;YASQPR	NCP	52.301
YASQPR;NGGSLLL	NCP	19.876
NGGSLLL;PSDYLPK	NCP	5.646
PSDYLPK;VHTVSY	NCP	3.663
VHTVSY;PTKEEGK	NCP	135.954
PTKEEGK;NAVLYE	NCP	85.811
NAVLYE;VYKAKGK	NCP	80.886
VYKAKGK;NGSLEP	NCP	22.903
NGSLEP;VSLAALK	NCP	24.458
VSLAALK;LSNKEVL	NCP	10.035
LSNKEVL;KLVNKKQ	NCP	19.587
KLVNKKQ;MDPVVLSY	NCP	29.661
MDPVVLSY;KGATLL	NCP	78.754
KGATLL;KFLEAVVP	NCP	32.864
KFLEAVVP;ADLPNLR	NCP	84.127

ADLPNLR;KPELVPGK	NCP	8.828
KPELVPGK;FWGKY	NCP	49.885
FWGKY;VLLSSSK	NCP	53.822
VLLSSSK;HRDMSR	NCP	49.311
HRDMSR;KGMVP	NCP	87.321
KGMVP;VLALLF	NCP	3.169
VLALLF;AALSKTH	NCP	95.417
AALSKTH;SMLNVFL	NCP	18.16
SMLNVFL;LLRKGVPF	NCP	9.426
LLRKGVPF;GNFPLR	NCP	62.898
GNFPLR;QPELFM	NCP	57.831
QPELFM;YVPTLLT	NCP	137.001
YVPTLLT;TAEFDLK	NCP	15.587
TAEFDLK;KPNVKK	NCP	70.618
KPNVKK;NFFVQ	NCP	7.203
NFFVQ;NSLKAKQ	NCP	0.926
NSLKAKQ;LYPQPY	NCP	58.437
LYPQPY;SAGKFPK	NCP	60.447
SAGKFPK;RPVPP	NCP	23.314
RPVPP;QALVPPP	NCP	169.497
QALVPPP;LAHLPF	NCP	8.072
LAHLPF;EPSLVH	NCP	10.888
EPSLVH;LAPVPLPP	NCP	8.298
LAPVPLPP;NHLLDE	NCP	1.998
NHLLDE;DDGPLGAGK	NCP	1.581
DDGPLGAGK;YHLKKVQ	NCP	81.027
YHLKKVQ;KVPVPPK	NCP	100.904
KVPVPPK;PGAYVPK	NCP	5.829
PGAYVPK;LEKDLKDL	NCP	359.143
LEKDLKDL;LSELLYR	NCP	151.404
LSELLYR;TLGFWLP	NCP	9.945
TLGFWLP;LLVPVLGL	NCP	70.341
LLVPVLGL;NLLSQL	NCP	7.953
NLLSQL;KRCWAAP	NCP	21.957
KRCWAAP;VTLDELR	NCP	129.474
VTLDELR;AAAVGL	NCP	149.073
AAAVGL;RLLLGPEAL	NCP	6.843
RLLLGPEAL;DVPNLK	NCP	32.549
DVPNLK;LLGLGPG	NCP	237.264
LLGLGPG;GPSLGP	NCP	7.075
GPSLGP;ARPHLK	NCP	137.472
ARPHLK;KSGSVRK	NCP	50.375
KSGSVRK;YAGPL	NCP	22.305
YAGPL;ATEGVLK	NCP	0.399
ATEGVLK;VLHPPPP	NCP	45.163
VLHPPPP;CLVKCK	NCP	4.113
CLVKCK;PKVPLA	NCP	44.123
PKVPLA;RAVSSKT	NCP	20.892
RAVSSKT;WDPFR	NCP	65.533
WDPFR;KLLGALL	NCP	26.36
KLLGALL;VALRGAGK	NCP	12.952

VALRGAGK;NLYKK	NCP	15.029
NLYKK;TDEELKVQK	NCP	112.438
TDEELKVQK;GQKEHLH	NCP	14.021
GQKEHLH;QTKTT	NCP	9.151
QTKTT;SKKALPQ	NCP	90.609
SKKALPQ;VYEPR	NCP	179.802
VYEPR;RCFLDQSL	NCP	97.118
RCFLDQSL;HLQHPAP	NCP	39.829
HLQHPAP;VGRLL	NCP	2.03
VGRLL;MMKVLSF	NCP	35.253
MMKVLSF;LSSLSPK	NCP	113.805
LSSLSPK;KGKNLAK	NCP	77.856
KGKNLAK;SPSKPRP	NCP	12.219
SPSKPRP;KPPWTY	NCP	22.851
KPPWTY;PPRGFGK	NCP	37.06
PPRGFGK;KQFAYP	NCP	2.483
KQFAYP;NAAVPQR	NCP	37.146
NAAVPQR;KAFGGF	NCP	43.321
KAFGGF;AVDLGVTK	NCP	11.063
AVDLGVTK;LQKPLLLR	NCP	22.104
LQKPLLLR;HGRGKF	NCP	8.374
HGRGKF;KQDKPK	NCP	40.965
KQDKPK;RALLLK	NCP	35.063
RALLLK;PAAFVP	NCP	18.334
PAAFVP;MTEAY	NCP	193.053
MTEAY;NDFKLKCV	NCP	42.203
NDFKLKCV;HDSALY	NCP	100.689
HDSALY;DPGLPK	NCP	21.262
DPGLPK;KKCELR	NCP	8.345
KKCELR;NVNMPW	NCP	11.584
NVNMPW;YAAREVP	NCP	4.688
YAAREVP;NHALVL	NCP	88.824
NHALVL;SSPLHTK	NCP	2.341
SSPLHTK;KAVLF	NCP	39.708
KAVLF;FEPLLR	NCP	26.721
FEPLLR;LEPVTR	NCP	47.319
LEPVTR;HGPPTGGK	NCP	40.851
HGPPTGGK;LLVEF	NCP	28.166
LLVEF;LPVRTPQ	NCP	67.195
LPVRTPQ;KSHEELK	NCP	72.545
KSHEELK;SEAKRSC	NCP	68.727
SEAKRSC;TTTKF	NCP	1.8
TTTKF;SFDAAPK	NCP	1.057
SFDAAPK;AVAKYC	NCP	54.458
AVAKYC;KHASDL	NCP	15.904
KHASDL;TAVHPAPL	NCP	67.83
TAVHPAPL;VDYVK	NCP	25.631
VDYVK;RSGVP	NCP	17.376
RSGVP;AALSKSH	NCP	36.499
AALSKSH;KYSHLTRQ	NCP	5.084
KYSHLTRQ;KPAAPP	NCP	4.275

KPAAPP;AQFSPY	NCP	55.039
AQFSPY;FPLEYT	NCP	47.423
FPLEYT;RPLFFL	NCP	10.917
RPLFFL;ADVFHAY	NCP	100.236
ADVFHAY;LYNETKVRK	NCP	7.104
LYNETKVRK;QPSFPLG	NCP	11.8
QPSFPLG;NGSPFP	NCP	1.76
NGSPFP;QSPQVLSP	NCP	67.647
QSPQVLSP;TSPGWLPP	NCP	81.297
TSPGWLPP;NSGSTVK	NCP	31.906
NSGSTVK;QASRSPF	NCP	1.361
QASRSPF;KGAMVRP	NCP	16.129
KGAMVRP;HPKGYR	NCP	88.655
HPKGYR;KDSLATP	NCP	51.175
KDSLATP;TVRPSPW	NCP	15.809
TVRPSPW;KVEASPP	NCP	125.434
KVEASPP;KKLEKQ	NCP	0.549
KKLEKQ;LSLPLDK	NCP	94.419
LSLPLDK;FGMSPR	NCP	1.922
FGMSPR;KTAEWPK	NCP	24.84
KTAEWPK;DFPLLK	NCP	16.89
DFPLLK;VDFPEFQ	NCP	18.568
VDFPEFQ;LKYPLE	NCP	13.587
LKYPLE;EALRGLE	NCP	50.078
EALRGLE;KNLEQVP	NCP	55.48
KNLEQVP;VPPPPLAP	NCP	101.196
VPPPPLAP;TAALDAY	NCP	4.995
TAALDAY;CLFKGKE	NCP	83.667
CLFKGKE;KSPYQEFTDHLVK	NCP	18.566
KSPYQEFTDHLVK;YGHLKP	NCP	25.635
YGHLKP;ESGCYSS	NCP	31.375
ESGCYSS;GCLFA	NCP	107.291
GCLFA;GDYYRH	NCP	82.411
GDYYRH;VTPSPVK	NCP	0.263
VTPSPVK;SVVDVPK	NCP	12.564
SVVDVPK;YGVAP	NCP	4.582
YGVAP;TMMEEE	NCP	60.294
TMMEEE;ERFEALR	NCP	6.786
ERFEALR;MSLTE	NCP	7.812
MSLTE;AARSLLL	NCP	0.303
AARSLLL;LKSVPP	NCP	9.161
LKSVPP;PSRSLL	NCP	14.722
PSRSLL;VRSWHLP	NCP	4.565
VRSWHLP;MREYKLVVL	NCP	3.411
MREYKLVVL;TAMRDLY	NCP	3.282
TAMRDLY;VDKYELL	NCP	7.954
VDKYELL;ESPVYM	NCP	51.336
ESPVYM;DPVLAKK	NCP	20.411
DPVLAKK;KSNFRP	NCP	42.043
KSNFRP;FPLLHKN	NCP	17.016
FPLLHKN;ESPLVLY	NCP	53.468

ESPLVLY;PVFPKSN	NCP	16.357
PVFPKSN;VAFMLP	NCP	52.124
VAFMLP;CEAGAK	NCP	44.077
CEAGAK;KSSKTPK	NCP	6.012
KSSKTPK;MAEVKK	NCP	109.013
MAEVKK;GAPGVVM	NCP	48.355
GAPGVVM;PLVWF	NCP	16.364
PLVWF;YNVMH	NCP	20.118
YNVMH;SAGPKYR	NCP	174.6
SAGPKYR;QVKKTSAP	NCP	2.422
QVKKTSAP;GDLLP	NCP	41.762
GDLLP;RVLLAEF	NCP	113.614
RVLLAEF;KSAGPP	NCP	147.687
KSAGPP;RLYSPH	NCP	35.718
RLYSPH;TPGGGFN	NCP	22.565
TPGGGFN;EYKELK	NCP	65.835
EYKELK;DHLTFCLK	NCP	46.707
DHLTFCLK;LYLEFP	NCP	323.185
LYLEFP;LEEEPLL	NCP	20.979
LEEEPLL;ELFADKV	NCP	31.575
ELFADKV;HLVGMGQK	NCP	63.885
HLVGMGQK;HLLVPPP	NCP	5.442
HLLVPPP;FDKGLPH	NCP	5.061
FDKGLPH;LGPSPAPR	NCP	115.503
LGPSPAPR;KVVAKK	NCP	2.375
KVVAKK;YPGPL	NCP	16.849
YPGPL;VSEAPQ	NCP	20.97
VSEAPQ;LLYKKD	NCP	1.374
LLYKKD;DLGFLKLD	NCP	44.801
DLGFLKLD;ESAPSFS	NCP	103.057
ESAPSFS;RAVCYP	NCP	4.512
RAVCYP;DGPKFPGDK	NCP	7.244
DGPKFPGDK;NTLLTLL	NCP	31.314
NTLLTLL;ERTFQK	NCP	3.031
ERTFQK;GPSPF	NCP	45.783
GPSPF;VSLPPFK	NCP	1.892
VSLPPFK;KFVPTVG	NCP	81.811
KFVPTVG;KSSPF	NCP	2.316
KSSPF;WELNPK	NCP	137.921
WELNPK;LPFPD	NCP	2.137
LPFPD;RVHKCK	NCP	30.467
RVHKCK;CMMDP	NCP	39.391
CMMDP;PGKKP	NCP	12.026
PGKKP;MDYLYD	NCP	123.775
MDYLYD;DKLVEL	NCP	27.56
DKLVEL;GVPRL	NCP	188.058
GVPRL;VVPFP	NCP	2.034
VVPFP;LTYLSGN	NCP	63.904
LTYLSGN;KVMLLL	NCP	11.568
KVMLLL;RSELLRY	NCP	117.234
RSELLRY;CCFAVTK	NCP	78.945

CCFAVTK;AEKFQ	NCP	35.187
AEKFQ;LVNQK	NCP	27.617
LVNQK;LSAYGKSK	NCP	2.31
LSAYGKSK;DLVPYPR	NCP	84.774
DLVPYPR;VTRAAFN	NCP	19.446
VTRAAFN;AFNSGKVD	NCP	0.012
AFNSGKVD;ASCTTNCLAPLAK	NCP	0.384
ASCTTNCLAPLAK;DNEFGY	NCP	0.495
DNEFGY;YSNRVVD	NCP	0.015
YSNRVVD;NRVVDL	NCP	0.006
NRVVDL;VHMASKE	NCP	0.021
VHMASKE;LNELMPK	NCP	67.071
LNELMPK;PHLKPPP	NCP	80.656
PHLKPPP;EVPHALL	NCP	90.161
EVPHALL;KVLLLG	NCP	149.44
KVLLLG;SYTKVK	NCP	49.445
SYTKVK;RLELHL	NCP	54.276
RLELHL;AAYPR	NCP	5.373
AAYPR;KGYNFGP	NCP	2.241
KGYNFGP;PPKNF	NCP	61.513
PPKNF;ESEHDLLK	NCP	60.081
ESEHDLLK;KAEHTK	NCP	25.796
KAEHTK;KSCGFLSF	NCP	49.903
KSCGFLSF;VNLRTSP	NCP	18.68
VNLRTSP;FPQSQFFP	NCP	11.115
FPQSQFFP;LLVGNPR	NCP	140.335
LLVGNPR;QKDLKEK	NCP	45.95
QKDLKEK;ERELVR	NCP	8.206
ERELVR;FGAKLFR	NCP	3.477
FGAKLFR;KHNCKGK	NCP	10.025
KHNCKGK;TSASVLLR	NCP	67.141
TSASVLLR;RLKTEL	NCP	31.773
RLKTEL;ASGPLNF	NCP	33.269
ASGPLNF;CSPDRK	NCP	24.646
CSPDRK;FAGSKPP	NCP	80.154
FAGSKPP;PKGVP	NCP	174.137
PKGVP;FKHLNEL	NCP	20.424
FKHLNEL;VTVSEP	NCP	28.48
VTVSEP;PTSLMTQK	NCP	55.65
PTSLMTQK;TPAANQP	NCP	282.096
TPAANQP;LPGKL	NCP	5.462
LPGKL;TVFEKPK	NCP	158.235
TVFEKPK;NLSLFP	NCP	112.234
NLSLFP;DLDLLDKT	NCP	14.477
DLDLLDKT;PLMLP	NCP	45.307
PLMLP;LPPTTRP	NCP	18.272
LPPTTRP;NLFLTK	NCP	45.688
NLFLTK;KPPPGRP	NCP	99.521
KPPPGRP;LLHAP	NCP	66.175
LLHAP;LPAPKN	NCP	244.362
LPAPKN;KPTRCP	NCP	133.266

KPTRCP;ATASLGF	NCP	10.466
ATASLGF;TVPLEKG	NCP	225.756
TVPLEKG;AFFVQ	NCP	53.634
AFFVQ;DRGTECKLF	NCP	100.686
DRGTECKLF;QDPKYK	NCP	116.682
QDPKYK;NATLVH	NCP	0.102
NATLVH;KQMGTNR	NCP	3.571
KQMGTNR;ATLLLVQ	NCP	25.812
ATLLLVQ;EAGAAPPPP	NCP	10.484
EAGAAPPPP;KMPGASPK	NCP	32.523
KMPGASPK;VGFVAL	NCP	72.382
VGFVAL;YFRPWK	NCP	18.042
YFRPWK;KGAKTYLK	NCP	33.173
KGAKTYLK;LNLEPGK	NCP	23.319
LNLEPGK;PVVKLE	NCP	12.025
PVVKLE;KGPLPPP	NCP	39.048
KGPLPPP;LYFTGE	NCP	12.116
LYFTGE;PAAGLR	NCP	158.656
PAAGLR;KHVTHLK	NCP	39.585
KHVTHLK;VVPKKPP	NCP	32.225
VVPKKPP;AQAGTWN	NCP	138.447
AQAGTWN;KPTAGPKP	NCP	16.131
KPTAGPKP;PVPLE	NCP	72.171
PVPLE;LGVHVSL	NCP	109.81
LGVHVSL;ERKAQAF	NCP	67.685
ERKAQAF;PLTVGQK	NCP	98.341
PLTVGQK;KGRRPP	NCP	44.582
KGRRPP;TAVAKPK	NCP	127.347
TAVAKPK;KYGPPLT	NCP	15.523
KYGPPLT;YGKLKNY	NCP	82.488
YGKLKNY;EKERK	NCP	26.31
EKERK;LTEVELLT	NCP	137.697
LTEVELLT;ALKERL	NCP	29.625
ALKERL;KPYKELY	NCP	30.896
KPYKELY;LWHHTFY	NCP	54.054
LWHHTFY;ETVSLEVK	NCP	3.901
ETVSLEVK;TSDTKSK	NCP	36.332
TSDTKSK;GSAYFGPK	NCP	20.991
GSAYFGPK;LSHVPKR	NCP	0.682
LSHVPKR;RKTTTSF	NCP	22.352
RKTTTSF;VPAGELH	NCP	7.72
VPAGELH;ASMPP	NCP	90.159
ASMPP;NPRLN	NCP	80.343
NPRLN;KVFLKKKQ	NCP	5.111
KVFLKKKQ;LLDVAPL	NCP	54.46
LLDVAPL;DKVKLVK	NCP	126.488
DKVKLVK;KALNFTWK	NCP	45.532
KALNFTWK;ENVLKLQ	NCP	17.477
ENVLKLQ;APMLP	NCP	39.802
APMLP;KNALGLL	NCP	20.877
KNALGLL;NFGLSPR	NCP	8.631

NFGLSPR;PKPPLTPP	NCP	13.23
PKPPLTPP;LERTPYT	NCP	7.719
LERTPYT;KLVLVGDGGTGK	NCP	4.266
KLVLVGDGGTGK;RHLTGEFEKK	NCP	0.051
RHLTGEFEKK;DVTSRVTY	NCP	0.186
DVTSRVTY;APPEVVMDPAL	NCP	0.273
APPEVVMDPAL;PALAAQYEHD	NCP	0.024
PALAAQYEHD;EHDLEVAQTTALPDEDDDL	NCP	0.021
EHDLEVAQTTALPDEDDDL;WNMLPFL	NCP	24.692
WNMLPFL;LPPALPPP	NCP	21.31
LPPALPPP;KEHLDLP	NCP	0.378
KEHLDLP;TTLENGD	NCP	50.24
TTLENGD;SPGLL	NCP	17.409
SPGLL;YGNAL	NCP	47.664
YGNAL;LWLPTE	NCP	116.977
LWLPTE;FDLFK	NCP	30.905
FDLFK;QTVAGFK	NCP	162.939
QTVAGFK;KMLPF	NCP	70.561
KMLPF;NDEEPQ	NCP	118.371
NDEEPQ;MTEDMP	NCP	85.041
MTEDMP;EKVVTK	NCP	61.346
EKVVTK;KPVVPPK	NCP	219.784
KPVVPPK;MTCDDC	NCP	5.712
MTCDDC;VYFPPPT	NCP	216.201
VYFPPPT;YNKPSK	NCP	41.772
YNKPSK;LGERTVEP	NCP	8.985
LGERTVEP;HYMVY	NCP	38.885
HYMVY;YATVLFR	NCP	16.267
YATVLFR;HGRAAV	NCP	7.503
HGRAAV;GVPSL	NCP	12.546
GVPSL;GVSLEEL	NCP	54.783
GVSLEEL;RTLYGFN	NCP	38.715
RTLYGFN;VGRMVQF	NCP	9.231
VGRMVQF;VLVYEDESRKETN	NCP	209.81
VLVYEDESRKETN;EHPSLYR	NCP	13.695
EHPSLYR;VVDNNLRPK	NCP	76.965
VVDNNLRPK;KSPFWQK	NCP	4.051
KSPFWQK;TDLFKKQ	NCP	73.106
TDLFKKQ;TLAHYLK	NCP	160.764
TLAHYLK;KDREDHR	NCP	68.004
KDREDHR;GVHLPFL	NCP	31.647
GVHLPFL;PAFHKPP	NCP	38.481
PAFHKPP;KPTLVK	NCP	69.541
KPTLVK;TAQCAY	NCP	28.184
TAQCAY;KNKKLAL	NCP	148.675
KNKKLAL;FYRAASN	NCP	205.289
FYRAASN;HTQLSKY	NCP	20.581
HTQLSKY;LGYLSGFK	NCP	61.685
LGYLSGFK;VASLL	NCP	20.064
VASLL;SAPLGPL	NCP	66.39
SAPLGPL;KDFMADL	NCP	7.167

KDFMADL;LGRRC	NCP	96.315
LGRRC;GVPTLSH	NCP	213.315
GVPTLSH;TMLLQTF	NCP	2.092
TMLLQTF;QDKLLK	NCP	79.199
QDKLLK;KVPALDK	NCP	0.874
KVPALDK;EAGKHKP	NCP	57.542
EAGKHKP;VGPPFE	NCP	15.418
VGPPFE;NMMKR	NCP	22.623
NMMKR;TLLLLVK	NCP	21.162
TLLLLVK;KRALVLA	NCP	59.576
KRALVLA;LSSLHG	NCP	26.007
LSSLHG;PGAEVPF	NCP	20.682
PGAEVPF;WVTDPR	NCP	32.928
WVTDPR;KTVYPK	NCP	7.036
KTVYPK;ALFMDL	NCP	4.388
ALFMDL;KGKVRK	NCP	19.744
KGKVRK;NLPLKGRK	NCP	81.486
NLPLKGRK;MEASA	NCP	20.553
MEASA;NELLTH	NCP	23.292
NELLTH;KSADW	NCP	70.547
KSADW;LTRPAPLH	NCP	88.816
LTRPAPLH;GVVLV	NCP	11.621
GVVLV;GKAFNLL	NCP	62.056
GKAFNLL;LQAVAVN	NCP	80.283
LQAVAVN;GVCYVA	NCP	6.818
GVCYVA;TAPVDY	NCP	3.448
TAPVDY;VPSEVPR	NCP	50.024
VPSEVPR;KPFFHY	NCP	66.808
KPFFHY;RLKKESN	NCP	12.842
RLKKESN;GPGKL	NCP	36.634
GPGKL;DPGFLER	NCP	51.359
DPGFLER;NAVLALH	NCP	20.925
NAVLALH;KSNLVQTK	NCP	2.686
KSNLVQTK;PALFF	NCP	119.774
PALFF;MMHLK	NCP	1.333
MMHLK;DHEEC	NCP	29.514
DHEEC;DPVLTLP	NCP	25.487
DPVLTLP;MPLVSLK	NCP	36.868
MPLVSLK;TTMNKGK	NCP	96.086
TTMNKGK;KYVLYFPL	NCP	27.537
KYVLYFPL;SGNQAVRLPL	NCP	13.317
SGNQAVRLPL;RALVFQPVTEL	NCP	8.064
RALVFQPVTEL;KPSMPK	NCP	55.383
KPSMPK;KSLKF	NCP	21.576
KSLKF;QGELLD	NCP	159.276
QGELLD;EPYFAK	NCP	181.134
EPYFAK;AMDVVY	NCP	5.608
AMDVVY;ELLNKPK	NCP	10.722
ELLNKPK;VLPVLAK	NCP	21.834
VLPVLAK;CCMGPP	NCP	19.544
CCMGPP;EPVVHE	NCP	129.303

EPVVHE;KLLLHK	NCP	9.613
KLLLHK;TAVHVTK	NCP	53.643
TAVHVTK;DFEPELF	NCP	46.329
DFEPELF;ENLASYK	NCP	33.141
ENLASYK;YPTFEC	NCP	90.386
YPTFEC;HESFLEH	NCP	95.889
HESFLEH;KNFPVP	NCP	183.087
KNFPVP;SLTVVPK	NCP	2.406
SLTVVPK;VWPFEK	NCP	81.021
VWPFEK;KYVPPR	NCP	23.364
KYVPPR;KGNVVLK	NCP	26.205
KGNVVLK;FPYVPPP	NCP	70.024
FPYVPPP;GVLNVKS	NCP	26.223
GVLNVKS;ASVNQPK	NCP	22.517
ASVNQPK;KWGVVR	NCP	12.271
KWGVVR;KGNLGFGG	NCP	5.822
KGNLGFGG;VGVTL	NCP	20.073
VGVTL;LLFTK	NCP	13.723
LLFTK;SGNVDM	NCP	41.646
SGNVDM;RTGLLR	NCP	7.841
RTGLLR;SAVYGFN	NCP	21.799
SAVYGFN;VSVNPY	NCP	12.651
VSVNPY;AAPPFT	NCP	94.064
AAPPFT;ACWELYCLEHG	NCP	4.287
ACWELYCLEHG;DDSFNTFFSETGAGKH	NCP	0.081
DDSFNTFFSETGAGKH;FFSETGAGKHVPR	NCP	0.018
FFSETGAGKHVPR;SETGAGKHVPRA	NCP	0.006
SETGAGKHVPRA;YRGDVV	NCP	3.018
YRGDVV;RGDVVPK	NCP	0.003
RGDVVPK;VDWCPT	NCP	12.429
VDWCPT;FDLMYA	NCP	0.153
FDLMYA;NLQEAEEWYKSK	NCP	19.635
NLQEAEEWYKSK;LQEAEEWYK	NCP	0.003
LQEAEEWYK;QEAEEWYKSK	NCP	0.003
QEAEEWYKSK;RKLLEGEESR	NCP	0.561
RKLLEGEESR;QAAYEVL	NCP	49.245
QAAYEVL;ESGVKSSG	NCP	13.506
ESGVKSSG;VLLEGGF	NCP	1.581
VLLEGGF;LSTVPGTK	NCP	145.651
LSTVPGTK;PGAASN	NCP	51.962
PGAASN;DKELKDF	NCP	35.721
DKELKDF;GVPGVLR	NCP	19.722
GVPGVLR;VPVVGLP	NCP	26.871
VPVVGLP;LDLSPGT	NCP	9.828
LDLSPGT;CYAPRV	NCP	29.085
CYAPRV;LGLVVVPN	NCP	5.994
LGLVVVPN;LHFLQEHL	NCP	16.083
LHFLQEHL;PPSFGPN	NCP	4.713
PPSFGPN;VSVLPLGL	NCP	21.841
VSVLPLGL;GRHEVR	NCP	87.8
GRHEVR;AAKNKTLL	NCP	70.597

AAKNKTLL;LAAARDK	NCP	11.549
LAAARDK;TMELTAH	NCP	12.618
TMELTAH;VPLPL	NCP	1.116
VPLPL;KPEAPAP	NCP	32.286
KPEAPAP;KMTVPYVP	NCP	0.831
KMTVPYVP;LNLAPK	NCP	15.493
LNLAPK;ASGAPQ	NCP	24.179
ASGAPQ;MDLLAFERK	NCP	27.273
MDLLAFERK;VPKQCV	NCP	175.762
VPKQCV;DPVEFK	NCP	37.331
DPVEFK;HMLLLG	NCP	90.816
HMLLLG;KQTLF	NCP	37.936
KQTLF;LATDTAY	NCP	2.538
LATDTAY;KKFQKSN	NCP	8.253
KKFQKSN;VGGFAPK	NCP	68.192
VGGFAPK;KVLAFPP	NCP	52.653
KVLAFPP;PVGRTPP	NCP	1.866
PVGRTPP;LNEGFDL	NCP	36.661
LNEGFDL;LPSLLGPK	NCP	69.122
LPSLLGPK;YYDPLKK	NCP	7.26
YYDPLKK;LMLVGKK	NCP	9.112
LMLVGKK;AMLKQNL	NCP	29.774
AMLKQNL;LRVVF	NCP	0.642
LRVVF;HKSLL	NCP	97.426
HKSLL;VLPLLQ	NCP	94.97
VLPLLQ;WSKGNPSP	NCP	65.437
WSKGNPSP;AVNGKLL	NCP	245.414
AVNGKLL;KLLSQSKN	NCP	17.724
KLLSQSKN;PLPPPPPP	NCP	14.77
PLPPPPPP;VALGQRK	NCP	49.601
VALGQRK;MLSGEKR	NCP	39.877
MLSGEKR;PGAPGPP	NCP	33.194
PGAPGPP;EHVTELL	NCP	15.826
EHVTELL;AAPLVVP	NCP	1.877
AAPLVVP;VMYAHEP	NCP	159.078
VMYAHEP;YLLKDQ	NCP	19.39
YLLKDQ;MFHLQ	NCP	57.918
MFHLQ;VSLPY	NCP	57.644
VSLPY;KALESLLF	NCP	231.139
KALESLLF;LGSTVRP	NCP	34.281
LGSTVRP;MARSHGM	NCP	21.737
MARSHGM;LLLEGPA	NCP	70.488
LLLEGPA;LAGSRPK	NCP	36.915
LAGSRPK;LAPPLEC	NCP	88.477
LAPPLEC;SVPKLA	NCP	18.366
SVPKLA;SSVRPGGFG	NCP	20.729
SSVRPGGFG;SLYGLGASRPR	NCP	0.036
SLYGLGASRPR;GLGASRPRV	NCP	0.009
GLGASRPRV;SSGGGPGTLKAY	NCP	13.692
SSGGGPGTLKAY;PALTSSK	NCP	0.259
PALTSSK;CMMFKM	NCP	96.231

CMMFKM;GKFGPAN	NCP	18.506
GKFGPAN;EDWPAFP	NCP	13.086
EDWPAFP;AAWLGPST	NCP	21.432
AAWLGPST;FELRR	NCP	2.037
FELRR;EVPLY	NCP	124.956
EVPLY;ESTTLGA	NCP	25.563
ESTTLGA;CKPLDQ	NCP	38.196
CKPLDQ;DLSGALY	NCP	29.379
DLSGALY;KNGEPF	NCP	7.539
KNGEPF;AVPTP	NCP	14.16
AVPTP;KTAAHG	NCP	8.445
KTAAHG;RVFVYVGY	NCP	14.989
RVFVYVGY;ALGPLE	NCP	24.008
ALGPLE;PPPPKRPP	NCP	39.549
PPPPKRPP;NNNGW	NCP	6.754
NNNGW;LVPPKP	NCP	92.087
LVPPKP;GTVPEVH	NCP	19.386
GTVPEVH;KEWFP	NCP	4.887
KEWFP;KLTAPV	NCP	3.462
KLTAPV;PKPEPKPP	NCP	11.691
PKPEPKPP;DTPEVVL	NCP	71.01
DTPEVVL;RHLTPF	NCP	1.473
RHLTPF;SFSTQSTFSNYR	NCP	43.68
SFSTQSTFSNYR;YRSLGSVQ	NCP	0.03
YRSLGSVQ;SVQSSGHRVRPVS	NCP	0.015
SVQSSGHRVRPVS;VRPVSSAASVYAGAGGSGSR	NCP	0.024
VRPVSSAASVYAGAGGSGSR;GWGSGNLG	NCP	0.093
GWGSGNLG;DLNDRLASY	NCP	0.087
DLNDRLASY;KGPQVRDWAH	NCP	0.099
KGPQVRDWAH;KEELLFMKK	NCP	1.32
KEELLFMKK;TVELDAPKPQDLSK	NCP	0.624
TVELDAPKPQDLSK;VELDAPKPQDL	NCP	0.003
VELDAPKPQDL;EALLNVKVK	NCP	0.675
EALLNVKVK;RRLLEDGEDF	NCP	0.051
RRLLEDGEDF;TTTLRLVDGKVVSETSDTK	NCP	0.375
TTTLRLVDGKVVSETSDTK;TLRLVDGKVVSETSD	NCP	0.006
TLRLVDGKVVSETSD;LRLVDGKVVSE	NCP	0.003
LRLVDGKVVSE;RLVDGKVVSETSDTK	NCP	0.003
RLVDGKVVSETSDTK;LVDGKVVSE	NCP	0.003
LVDGKVVSE;VDGKVVSETSD	NCP	0.003
VDGKVVSETSD;VVSETSDTKVLR	NCP	0.012
VVSETSDTKVLR;AAPVK	NCP	25.644
AAPVK;ASDDPLEK	NCP	12.274
ASDDPLEK;KSLLFVTP	NCP	13.875
KSLLFVTP;KEEDCQSPTSKPPKPEQPLK	NCP	19.22
KEEDCQSPTSKPPKPEQPLK;DENKVDGVSAPK	NCP	1.245
DENKVDGVSAPK;KPPVCAVCK	NCP	11.751
KPPVCAVCK;PLPNFS	NCP	26.325
PLPNFS;NPDPPK	NCP	83.601
NPDPPK;LPPPRPGPP	NCP	20.643
LPPPRPGPP;RPHHFVF	NCP	22.246

RPHHFVF;LPLDLLVLLPLP	NCP	24.887
LPLDLLVLLPLP;VNEGLR	NCP	36.592
VNEGLR;HRKAGQP	NCP	5.963
HRKAGQP;LYFTDA	NCP	61.909
LYFTDA;GTTLPLP	NCP	14.339
GTTLPLP;STAASRPPVTLR	NCP	61.77
STAASRPPVTLR;ASRPPVT	NCP	0.009
ASRPPVT;LDASAQTTSH	NCP	8.106
LDASAQTTSH;AAALP	NCP	47.034
AAALP;KLYFGKSK	NCP	111.751
KLYFGKSK;RLELSF	NCP	43.77
RLELSF;SVGWFD	NCP	161.268
SVGWFD;ELGFLEW	NCP	12.056
ELGFLEW;QSMKSR	NCP	8.536
QSMKSR;LTGEEFK	NCP	2.132
LTGEEFK;SSLNPLL	NCP	28.726
SSLNPLL;KGPPKLY	NCP	39.83
KGPPKLY;GSPVLKK	NCP	14.802
GSPVLKK;QNKPPK	NCP	61.35
QNKPPK;HGAAAAPV	NCP	10.689
HGAAAAPV;SFPGL	NCP	77.277
SFPGL;PDLYVT	NCP	63.633
PDLYVT;AQPVP	NCP	9.423
AQPVP;AVPGNP	NCP	29.409
AVPGNP;SLDDLLK	NCP	47.541
SLDDLLK;YGGGGPGY	NCP	0.462
YGGGGPGY;SSGPYGGGGQYFAKPR	NCP	0.804
SSGPYGGGGQYFAKPR;AAARHSTLDF	NCP	28.773
AAARHSTLDF;DGDAQGKEVDSLLNK	NCP	0.225
DGDAQGKEVDSLLNK;DLGSLF	NCP	16.497
DLGSLF;ERPLVG	NCP	48.606
ERPLVG;KTPSVCVL	NCP	130.9
KTPSVCVL;WDAPA	NCP	13.7
WDAPA;SSGHPGSSG	NCP	6.886
SSGHPGSSG;VGLDLFT	NCP	14.306
VGLDLFT;TVPLLRPH	NCP	160.675
TVPLLRPH;DAKPHPR	NCP	17.342
DAKPHPR;KNNNL	NCP	30.247
KNNNL;RYVKF	NCP	42.132
RYVKF;RPFDK	NCP	12.356
RPFDK;TTMPEM	NCP	56.611
TTMPEM;ALTTL	NCP	21.986
ALTTL;PRLVRP	NCP	26.265
PRLVRP;VLMAYVP	NCP	32.913
VLMAYVP;EGSMFLK	NCP	43.576
EGSMFLK;KDFLAL	NCP	107.901
KDFLAL;DDDRLPLL	NCP	64.931
DDDRLPLL;NAFGVGF	NCP	26.476
NAFGVGF;TPTPLTK	NCP	17.819
TPTPLTK;KADCVR	NCP	30.645
KADCVR;KHTPHLH	NCP	69.264

KHTPHLH;PLVDTHSK	NCP	6.87
PLVDTHSK;MELLQVLK	NCP	43.326
MELLQVLK;FDVAKK	NCP	1.678
FDVAKK;EVLTLGN	NCP	25.839
EVLTLGN;LGDVKLR	NCP	72.794
LGDVKLR;APRMWHR	NCP	52.597
APRMWHR;PGGPAPP	NCP	52.568
PGGPAPP;PPLLTSSK	NCP	4.246
PPLLTSSK;AELDPVW	NCP	39.344
AELDPVW;KFLNPR	NCP	10.51
KFLNPR;ESVPLVL	NCP	155.13
ESVPLVL;TAPESNQ	NCP	191.634
TAPESNQ;KGYLER	NCP	83.003
KGYLER;DPQVHR	NCP	10.02
DPQVHR;MSSAYVP	NCP	98.575
MSSAYVP;KELLDK	NCP	11.42
KELLDK;YTAGP	NCP	68.815
YTAGP;KPYPPPPP	NCP	51.528
KPYPPPPP;SGPLRKPP	NCP	46.086
SGPLRKPP;CGPDLRK	NCP	11.276
CGPDLRK;KPQPDLLK	NCP	16.53
KPQPDLLK;TGAGAP	NCP	55.305
TGAGAP;QLLVYDY	NCP	39.573
QLLVYDY;NLSLKK	NCP	15.439
NLSLKK;TNLTLLL	NCP	27.573
TNLTLLL;LAVFKK	NCP	145.32
LAVFKK;AAGLKP	NCP	72.332
AAGLKP;KMDSHK	NCP	20.481
KMDSHK;KYLPPPPP	NCP	5.86
KYLPPPPP;ENALLPVTK	NCP	8.48
ENALLPVTK;LGGAPAP	NCP	37.917
LGGAPAP;TQLLAY	NCP	40.792
TQLLAY;ELFLPEEY	NCP	3.269
ELFLPEEY;NLAEAW	NCP	221.484
NLAEAW;EAVYLLN	NCP	48.708
EAVYLLN;KVGKFPF	NCP	13.042
KVGKFPF;KEFSWL	NCP	11.007
KEFSWL;DVLDAP	NCP	44.349
DVLDAP;KFGVL	NCP	13.895
KFGVL;VNPKL	NCP	34.372
VNPKL;ATAEMSSF	NCP	16.496
ATAEMSSF;CSCEC	NCP	13.873
CSCEC;QASSLAALK	NCP	91.992
QASSLAALK;TKFFSPP	NCP	24.129
TKFFSPP;KLGGAVV	NCP	59.742
KLGGAVV;LSNSDLR	NCP	21.504
LSNSDLR;KPFFRP	NCP	48.927
KPFFRP;GQHLVL	NCP	23.189
GQHLVL;KSGTHLK	NCP	27.736
KSGTHLK;QAPLAQR	NCP	43.263
QAPLAQR;KGGTAELR	NCP	108.078

KGGTAELR;TDLLSKEK	NCP	54.507
TDLLSKEK;KGLDY	NCP	78.99
KGLDY;FFGPP	NCP	14.598
FFGPP;SLGQGP	NCP	62.721
SLGQGP;PFLEF	NCP	121.196
PFLEF;VPEVH	NCP	176.334
VPEVH;KYTPVVL	NCP	44.865
KYTPVVL;LLLCE	NCP	31.368
LLLCE;PAKKEGK	NCP	3.676
PAKKEGK;VPLLT	NCP	3.144
VPLLT;TPSLYRP	NCP	11.795
TPSLYRP;RFGPVY	NCP	6.718
RFGPVY;PQLAF	NCP	11.868
PQLAF;TVEGKLP	NCP	47.48
TVEGKLP;KGYQQM	NCP	2.721
KGYQQM;NPLSSAPP	NCP	44.796
NPLSSAPP;GVNQP	NCP	12.324
GVNQP;DEFSTPF	NCP	6.048
DEFSTPF;PLPPLKKKD	NCP	228.394
PLPPLKKKD;TNRLLQ	NCP	37.73
TNRLLQ;VLTGALR	NCP	10.789
VLTGALR;TLGQMLR	NCP	100.422
TLGQMLR;NQLKELPEKMPK	NCP	204.017
NQLKELPEKMPK;SVFNGLNQ	NCP	0.087
SVFNGLNQ;KSCGAVR	NCP	5.866
KSCGAVR;VDAASLKGLNNLAK	NCP	0.842
VDAASLKGLNNLAK;LRELHLNNNK	NCP	4.467
LRELHLNNNK;KASYSGVSL	NCP	4.389
KASYSGVSL;AAVQLGNY	NCP	0.09
AAVQLGNY;LNELL	NCP	17.785
LNELL;AAKQPPLP	NCP	8.751
AAKQPPLP;VFSKLKQLKK	NCP	13.592
VFSKLKQLKK;EDAVSAALKGLK	NCP	0.186
EDAVSAALKGLK;SLEYLDLSFNQMTKLPSGLPVSL	NCP	0.036
SLEYLDLSFNQMTKLPSGLPVSL;TLYLDNNK	NCP	0.072
TLYLDNNK;KRFSALQY	NCP	0.051
KRFSALQY;SAFLTQ	NCP	350.706
SAFLTQ;EGLALSRL	NCP	122.395
EGLALSRL;KLPLLK	NCP	76.122
KLPLLK;TPPEELL	NCP	113.948
TPPEELL;GYVVPQ	NCP	104.259
GYVVPQ;QHGLQP	NCP	24.888
QHGLQP;LVLEQLRC	NCP	56.322
LVLEQLRC;KWTHC	NCP	12.627
KWTHC;RSPLRFPQ	NCP	36.097
RSPLRFPQ;KSTQVPP	NCP	11.216
KSTQVPP;KQGNVVQK	NCP	41.277
KQGNVVQK;KHMPGR	NCP	50.215
KHMPGR;KWGKN	NCP	43.034
KWGKN;MMHEMY	NCP	85.845
MMHEMY;VFLQAR	NCP	48.853

VFLQAR;KGGSLL	NCP	31.007
KGGSLL;ALPMD	NCP	73.06
ALPMD;LQRSSFR	NCP	40.722
LQRSSFR;QGEFDHF	NCP	11.522
QGEFDHF;KSVLQGK	NCP	126.156
KSVLQGK;YAKAF	NCP	6.949
YAKAF;VGHVFPK	NCP	116.348
VGHVFPK;LLTLQ	NCP	7.813
LLTLQ;DEDYGY	NCP	132.402
DEDYGY;KTFGVP	NCP	8.801
KTFGVP;YGGMDL	NCP	8.899
YGGMDL;LLPGPV	NCP	109.725
LLPGPV;LLPLEEH	NCP	88.094
LLPLEEH;VTAVGVLK	NCP	89.77
VTAVGVLK;LSLAL	NCP	48.054
LSLAL;DPCKLKL	NCP	48.519
DPCKLKL;NAAFAWE	NCP	37.862
NAAFAWE;SGPVLKK	NCP	12.319
SGPVLKK;LAKLEK	NCP	15.044
LAKLEK;KNQTTPK	NCP	21.348
KNQTTPK;VSLFHK	NCP	276.838
VSLFHK;MDVLV	NCP	186.02
MDVLV;KPSLVGGK	NCP	42.372
KPSLVGGK;FLHGTVK	NCP	8.35
FLHGTVK;VTGPKPK	NCP	28.352
VTGPKPK;RVHGQVL	NCP	100.83
RVHGQVL;FSLYYTQ	NCP	10.906
FSLYYTQ;CCYARPP	NCP	56.877
CCYARPP;DRDFR	NCP	111.489
DRDFR;KDSPKEF	NCP	34.902
KDSPKEF;TLRHNLP	NCP	87.998
TLRHNLP;TSLVLTGK	NCP	49.45
TSLVLTGK;VGLCF	NCP	142.652
VGLCF;KATTFLK	NCP	31.408
KATTFLK;HMVHRK	NCP	21.803
HMVHRK;LTPPHRP	NCP	54.214
LTPPHRP;YEKDY	NCP	66.302
YEKDY;DHMQPR	NCP	33.573
DHMQPR;QNTSGK	NCP	198.838
QNTSGK;YYKPP	NCP	1.166
YYKPP;KGDFVTSF	NCP	83.982
KGDFVTSF;FSKEVR	NCP	17.238
FSKEVR;TQKPGKL	NCP	5.986
TQKPGKL;KNFAY	NCP	61.224
KNFAY;LDSAVP	NCP	105.099
LDSAVP;KSPSPP	NCP	74.672
KSPSPP;AATTSGPP	NCP	120.208
AATTSGPP;FRAAQR	NCP	151.569
FRAAQR;KDKKFPK	NCP	87.821
KDKKFPK;LEPSLR	NCP	5.461
LEPSLR;KFNLSGTPQ	NCP	75.252

KFNLSGTPQ;LQVAKDL	NCP	96.476
LQVAKDL;NADAVKVL	NCP	22.392
NADAVKVL;YAGRRR	NCP	56.608
YAGRRR;FNMKVF	NCP	50.33
FNMKVF;AAKVYN	NCP	88.992
AAKVYN;ADMSCL	NCP	10.855
ADMSCL;KLDKYLPP	NCP	39.437
KLDKYLPP;TGLLKPH	NCP	17.44
TGLLKPH;WVLTAAH	NCP	9.204
WVLTAAH;PLGVPLA	NCP	84.531
PLGVPLA;HEDDLLL	NCP	26.589
HEDDLLL;EGPVPSR	NCP	30.411
EGPVPSR;ASAAEGDMEAELTR	NCP	31.749
ASAAEGDMEAELTR;VTMKAY	NCP	62.291
VTMKAY;KASGLTAL	NCP	94.6
KASGLTAL;LRSLLGP	NCP	15.783
LRSLLGP;HTLHVP	NCP	179.145
HTLHVP;SGVLWGKK	NCP	171.894
SGVLWGKK;SDYLSKM	NCP	15.971
SDYLSKM;VGPEGF	NCP	29.95
VGPEGF;VPPPLYP	NCP	30.18
VPPPLYP;TRKPAP	NCP	50.733
TRKPAP;LSAMFLVP	NCP	48.729
LSAMFLVP;YGVFLYT	NCP	39.003
YGVFLYT;VGVHLGGF	NCP	6.582
VGVHLGGF;DMFNYL	NCP	41.882
DMFNYL;VYLKKGS	NCP	1.21
VYLKKGS;AHMKDRL	NCP	7.499
AHMKDRL;VKQVVGE	NCP	9.183
VKQVVGE;VKWPLW	NCP	22.551
VKWPLW;KWFYAPP	NCP	6.058
KWFYAPP;VVEGPLLL	NCP	91.355
VVEGPLLL;AGWNLR	NCP	62.488
AGWNLR;KSGYFP	NCP	40.82
KSGYFP;GLPPPSRS	NCP	13.302
GLPPPSRS;KVGALLRL	NCP	7.677
KVGALLRL;KLVLMVP	NCP	12.904
KLVLMVP;LKLLAPPE	NCP	2.646
LKLLAPPE;LSDYKEK	NCP	31.409
LSDYKEK;LSVPGHR	NCP	14.461
LSVPGHR;SVAGTLR	NCP	5.714
SVAGTLR;LGGAVFQ	NCP	27.141
LGGAVFQ;KVHHAF	NCP	45.778
KVHHAF;YPPVQPP	NCP	15.276
YPPVQPP;LLMLLL	NCP	13.083
LLMLLL;AAPGLLLK	NCP	43.494
AAPGLLLK;ATKKSYC	NCP	52.092
ATKKSYC;KPYPAG	NCP	30.303
KPYPAG;SSKPQ	NCP	2.838
SSKPQ;DLFPGLK	NCP	20.975
DLFPGLK;KMNAY	NCP	16.314

KMNAY;HHPKAK	NCP	46.306
HHPKAK;GPPNLSF	NCP	17.091
GPPNLSF;KELPP	NCP	37.986
KELPP;VPGGDL	NCP	37.323
VPGGDL;PPALLPPP	NCP	33.534
PPALLPPP;FSLLEEY	NCP	26.738
FSLLEEY;TRVVPV	NCP	92.401
TRVVPV;VPQEAWHTV	NCP	4.566
VPQEAWHTV;DMKPRP	NCP	41.769
DMKPRP;ARKYP	NCP	106.284
ARKYP;AARTVDK	NCP	81.597
AARTVDK;VDLLLLQ	NCP	163.842
VDLLLLQ;KNMPLY	NCP	2.426
KNMPLY;GPAAAP	NCP	4.009
GPAAAP;LPALDDA	NCP	65.907
LPALDDA;EAGKYG	NCP	153.696
EAGKYG;KDFTLFK	NCP	192.02
KDFTLFK;RKPGAFR	NCP	29.995
RKPGAFR;EYVRAP	NCP	26.507
EYVRAP;KMLPPPP	NCP	70.161
KMLPPPP;LEVTKLFP	NCP	24.19
LEVTKLFP;MRHELL	NCP	112.529
MRHELL;LPFFL	NCP	17.244
LPFFL;KGVAVRR	NCP	65.188
KGVAVRR;CCMDTK	NCP	105.989
CCMDTK;RGPPEPP	NCP	27.313
RGPPEPP;LSVRLL	NCP	71.78
LSVRLL;SLTPVPK	NCP	29.914
SLTPVPK;VDVLGAGK	NCP	0.405
VDVLGAGK;KPDAP	NCP	49.173
KPDAP;LANYSVF	NCP	13.139
LANYSVF;PARARPP	NCP	8.125
PARARPP;APGSRKPP	NCP	10.101
APGSRKPP;SYLKNAK	NCP	5.57
SYLKNAK;RDLRDK	NCP	15.82
RDLRDK;VPELGTP	NCP	59.346
VPELGTP;KSYCKGK	NCP	41.474
KSYCKGK;PPRAGPLGP	NCP	26.812
PPRAGPLGP;PDGMVGL	NCP	181.863
PDGMVGL;SKFDLD	NCP	26.543
SKFDLD;VGLHMLK	NCP	22.216
VGLHMLK;YYEDKSK	NCP	58.763
YYEDKSK;GPYKVPK	NCP	9.823
GPYKVPK;KEAPGLFG	NCP	26.135
KEAPGLFG;EELFKVR	NCP	128.034
EELFKVR;ALKAQVP	NCP	4.237
ALKAQVP;LGTSTFT	NCP	35.097
LGTSTFT;FGKVGMRHY	NCP	10.731
FGKVGMRHY;HAGPKQR	NCP	10.392
HAGPKQR;MQKDSPV	NCP	9.558
MQKDSPV;MEDHSVLH	NCP	89.085

MEDHSVLH;YFGYPK	NCP	14.138
YFGYPK;KPPPMPR	NCP	48.397
KPPPMPR;KDLKLFG	NCP	22.895
KDLKLFG;APGKHVP	NCP	30.268
APGKHVP;DRPSF	NCP	42.39
DRPSF;GSLLTF	NCP	34.533
GSLLTF;GSLLLK	NCP	63.941
GSLLLK;MEKFQ	NCP	141.043
MEKFQ;LVRDLKEK	NCP	7.763
LVRDLKEK;VTLSPL	NCP	10.551
VTLSPL;LDLPNKK	NCP	222.016
LDLPNKK;PVTPKNK	NCP	13.019
PVTPKNK;KDAALK	NCP	17.149
KDAALK;SLTQALDR	NCP	0.725
SLTQALDR;GPLLPAPLL	NCP	46.234
GPLLPAPLL;KKLLGPP	NCP	33.507
KKLLGPP;VGPPPPPRP	NCP	0.336
VGPPPPPRP;PPPPALPR	NCP	5.679
PPPPALPR;GDTPKPL	NCP	4.881
GDTPKPL;TLGLVGLP	NCP	14.664
TLGLVGLP;ANHLVLY	NCP	5.549
ANHLVLY;TADGVSVP	NCP	23.986
TADGVSVP;LSPFEL	NCP	4.049
LSPFEL;GSGPPK	NCP	1.042
GSGPPK;KDESKSK	NCP	59.459
KDESKSK;TPDKHAT	NCP	16.843
TPDKHAT;RPPVLAP	NCP	16.866
RPPVLAP;EMSDDD	NCP	251.162
EMSDDD;SLDDVLP	NCP	7.659
SLDDVLP;NCPGLCP	NCP	11.44
NCPGLCP;LLPTRPP	NCP	15.581
LLPTRPP;LTSYLK	NCP	66.718
LTSYLK;PAAAQQPPP	NCP	17.484
PAAAQQPPP;PTPPMPP	NCP	8.769
PTPPMPP;KPSPACC	NCP	13.77
KPSPACC;MSSFF	NCP	13.263
MSSFF;SPNVTPK	NCP	16.02
SPNVTPK;TAASVH	NCP	9.215
TAASVH;SLGSAL	NCP	111.166
SLGSAL;VPNALLG	NCP	16.686
VPNALLG;LDVLPF	NCP	59.976
LDVLPF;EGPVMVK	NCP	83.306
EGPVMVK;LLQDPK	NCP	19.921
LLQDPK;EPLVLY	NCP	122.132
EPLVLY;QSKTKDF	NCP	48.765
QSKTKDF;TSTLPVR	NCP	0.028
TSTLPVR;KNDGPKF	NCP	9.656
KNDGPKF;KPPSHVGP	NCP	23.419
KPPSHVGP;HGYQAPR	NCP	41.666
HGYQAPR;PPTTLL	NCP	16.855
PPTTLL;LYDAALA	NCP	10.8

LYDAALA;TKTHPH	NCP	0.258
TKTHPH;QSVSLPK	NCP	9.387
QSVSLPK;VDLRKDL	NCP	14.058
VDLRKDL;TGKLKLVK	NCP	76.034
TGKLKLVK;QAVERL	NCP	2.392
QAVERL;QVLSLA	NCP	52.608
QVLSLA;GSLTLLL	NCP	35.213
GSLTLLL;LPPPPVAS	NCP	33.793
LPPPPVAS;EVAPSQGP	NCP	6.207
EVAPSQGP;PVKKLAL	NCP	3.327
PVKKLAL;VDLGAATEEGEGPEAAGR	NCP	7.008
VDLGAATEEGEGPEAAGR;VTLPLPP	NCP	42.488
VTLPLPP;GVFVAL	NCP	86.755
GVFVAL;KGAVTY	NCP	7.518
KGAVTY;QPRPAPP	NCP	20.121
QPRPAPP;KDDLLL	NCP	27.198
KDDLLL;KPGDFR	NCP	20.733
KPGDFR;VFPPTF	NCP	20.288
VFPPTF;VGLNL	NCP	88.725
VGLNL;VCAGLVA	NCP	2.257
VCAGLVA;GSETDLEPPVVK	NCP	5.075
GSETDLEPPVVK;AVAVGRPS	NCP	3.889
AVAVGRPS;RSALAEL	NCP	38.751
RSALAEL;RFKKAKK	NCP	37.347
RFKKAKK;NSHAVTK	NCP	5.274
NSHAVTK;ELPKTR	NCP	19.098
ELPKTR;RWEDDLKK	NCP	20.258
RWEDDLKK;KDPTGLPR	NCP	22.414
KDPTGLPR;GQPLVG	NCP	12.963
GQPLVG;AYALPM	NCP	100.484
AYALPM;EDEFVAA	NCP	15.954
EDEFVAA;AAPTVTR	NCP	37.054
AAPTVTR;DLSLLP	NCP	107.744
DLSLLP;PSYKLV	NCP	12.115
PSYKLV;LNHLSFL	NCP	58.838
LNHLSFL;VPGNQFR	NCP	50.185
VPGNQFR;LEDEGHE	NCP	1.658
LEDEGHE;RTEKDPQ	NCP	128.377
RTEKDPQ;FTKCC	NCP	4.398
FTKCC;TLSTSFY	NCP	14.601
TLSTSFY;KKYLELH	NCP	12.374
KKYLELH;KFLLLPK	NCP	20.029
KFLLLPK;GPPTPGAK	NCP	27.468
GPPTPGAK;VAGSGKER	NCP	58.686
VAGSGKER;LEKGAPK	NCP	63.804
LEKGAPK;QGTSLKPK	NCP	16.101
QGTSLKPK;EKVALSPG	NCP	16.566
EKVALSPG;KGNKNHE	NCP	33.404
KGNKNHE;ETLPRQ	NCP	94.68
ETLPRQ;YVPLGGL	NCP	5.956
YVPLGGL;MVDFMP	NCP	20.301

MVDFMP;AASDVAPA	NCP	11.517
AASDVAPA;KGWGGPSP	NCP	0.81
KGWGGPSP;ATPLYEG	NCP	19.833
ATPLYEG;VPAAPPP	NCP	70.812
VPAAPPP;LPSVPP	NCP	29.036
LPSVPP;LPFLPR	NCP	116.578
LPFLPR;QGPLRPP	NCP	4.113
QGPLRPP;LPMKE	NCP	2.525
LPMKE;AVTLDHR	NCP	21.903
AVTLDHR;PLFNTFH	NCP	90.066
PLFNTFH;DLFMPK	NCP	4.371
DLFMPK;KLLSHSLL	NCP	52.392
KLLSHSLL;LPGREPP	NCP	9.652
LPGREPP;SAMMDKK	NCP	22.601
SAMMDKK;YSKSGGG	NCP	61.03
YSKSGGG;VGGLVFL	NCP	35.415
VGGLVFL;FELSDPV	NCP	20.123
FELSDPV;VGALSP	NCP	107.133
VGALSP;SHSLVVP	NCP	27.738
SHSLVVP;HSLMV	NCP	14.919
HSLMV;TEVHELL	NCP	220.933
TEVHELL;QESEE	NCP	40.353
QESEE;TAPAPPKP	NCP	93.279
TAPAPPKP;KLQQLK	NCP	3.545
KLQQLK;ELVSF	NCP	152.097
ELVSF;VAREC	NCP	60.862
VAREC;ATVYSSK	NCP	86.931
ATVYSSK;RGPVTPP	NCP	25.587
RGPVTPP;KYFSLPF	NCP	47.622
KYFSLPF;PVEPVAK	NCP	23.976
PVEPVAK;KLPGVVG	NCP	2.361
KLPGVVG;MDQLKD	NCP	0.549
MDQLKD;LPPPPHGP	NCP	41.247
LPPPPHGP;NHGGPK	NCP	64.916
NHGGPK;TGGPLL	NCP	0.685
TGGPLL;PNGPVPR	NCP	38.745
PNGPVPR;LVLPVPAF	NCP	16.226
LVLPVPAF;AQAPGV	NCP	3.385
AQAPGV;TGLFNGK	NCP	67.538
TGLFNGK;PLRLGVAC	NCP	33.328
PLRLGVAC;SATQAPY	NCP	33.477
SATQAPY;LDHLTR	NCP	70.482
LDHLTR;NSLLDV	NCP	6.699
NSLLDV;TPKGDDK	NCP	22.68
TPKGDDK;AFREMP	NCP	98.901
AFREMP;AAPGLL	NCP	54.894
AAPGLL;SLPESLVTAL	NCP	22.446
SLPESLVTAL;REAGVVR	NCP	86.933
REAGVVR;PVWGGGNKCGACGR	NCP	11.466
PVWGGGNKCGACGR;YGQGAGTL	NCP	2.889
YGQGAGTL;PNTSKFAQK	NCP	3.744

PNTSKFAQK;GCYAKNFGPK	NCP	1.458
GCYAKNFGPK;LCFSTAQ	NCP	24.424
LCFSTAQ;RAQEVPL	NCP	25.215
RAQEVPL;VGPFP	NCP	61.431
VGPFP;QGSPVLL	NCP	159.765
QGSPVLL;NDFKWK	NCP	78.485
NDFKWK;QLAGY	NCP	37.549
QLAGY;TADGWR	NCP	6.057
TADGWR;ALEGLL	NCP	58.893
ALEGLL;QALWE	NCP	86.058
QALWE;VRYSLDPENPTK	NCP	16.332
VRYSLDPENPTK;PHAPPPK	NCP	44.297
PHAPPPK;KNGDLL	NCP	31.05
KNGDLL;HAPPPPK	NCP	50.965
HAPPPPK;FYEEVHDLERK	NCP	0.278
FYEEVHDLERK;DRLHPQT	NCP	19.39
DRLHPQT;TACQAY	NCP	32.381
TACQAY;QVFPPP	NCP	3.024
QVFPPP;KVRVLVH	NCP	8.761
KVRVLVH;KNLFKNN	NCP	36.69
KNLFKNN;LLSVTVPP	NCP	107.922
LLSVTVPP;DLKKALLRL	NCP	62.445
DLKKALLRL;LAAAVVVL	NCP	89.394
LAAAVVVL;RRSLQQY	NCP	0.156
RRSLQQY;KSSKKAKP	NCP	74.358
KSSKKAKP;MFKGPK	NCP	4.422
MFKGPK;LVHLLM	NCP	39.114
LVHLLM;PGPLLP	NCP	14.367
PGPLLP;LGPAVPA	NCP	31.11
LGPAVPA;ASSKQNGPTTASGKSELR	NCP	29.231
ASSKQNGPTTASGKSELR;LVLLGPPDTL	NCP	12.423
LVLLGPPDTL;MKPVAAP	NCP	66.432
MKPVAAP;KPPEYR	NCP	57.54
KPPEYR;AMLQKN	NCP	51.81
AMLQKN;VVPGSSP	NCP	1.576
VVPGSSP;PPDLTRPPP	NCP	3.906
PPDLTRPPP;QCCCCCSK	NCP	23.228
QCCCCCSK;VAKTSLY	NCP	2.59
VAKTSLY;MLPKLLVT	NCP	55.265
MLPKLLVT;DSPAAKA	NCP	63.409
DSPAAKA;PPDPPLPL	NCP	40.247
PPDPPLPL;AGLLK	NCP	64.831
AGLLK;LAAVAPT	NCP	4.955
LAAVAPT;WPTKTP	NCP	6.592
WPTKTP;YLQQKR	NCP	37.628
YLQQKR;LLAAMVK	NCP	19.384
LLAAMVK;KLLATLL	NCP	23.181
KLLATLL;YPVFPP	NCP	76.728
YPVFPP;KDQSLK	NCP	24.828
KDQSLK;VGPPDVPG	NCP	2.733
VGPPDVPG;ECGGD	NCP	2.721

ECGGD;CDLLLPQR	NCP	87.476
CDLLLPQR;GHDMPPL	NCP	17.782
GHDMPPL;KMLPPPLP	NCP	31.872
KMLPPPLP;ALLAL	NCP	6.18
ALLAL;FDEKFP	NCP	4.784
FDEKFP;VPPTF	NCP	60.178
VPPTF;PAGPRHP	NCP	7.116
PAGPRHP;NVELLY	NCP	15.632
NVELLY;DHVPAPK	NCP	41.383
DHVPAPK;VNPSYN	NCP	116.949
VNPSYN;LHLFP	NCP	85.721
LHLFP;LTPLPP	NCP	18.496
LTPLPP;ALGGLL	NCP	14.672
ALGGLL;PAPPEQ	NCP	11.839
PAPPEQ;PPLVVVPP	NCP	6.606
PPLVVVPP;AAKVPPR	NCP	106.014
AAKVPPR;HTEHTH	NCP	6.692
HTEHTH;NGGLPP	NCP	41.728
NGGLPP;TPKPPR	NCP	89.142
TPKPPR;QDYPEDPK	NCP	37.1
QDYPEDPK;RTDPPK	NCP	100.225
RTDPPK;CTRFNLH	NCP	0.86
CTRFNLH;AACLFVK	NCP	175.279
AACLFVK;PAGFAL	NCP	49.548
PAGFAL;LDKELK	NCP	56.705
LDKELK;KVHVPP	NCP	80.379
KVHVPP;VLTGPVR	NCP	112.315
VLTGPVR;YWNFL	NCP	30.602
YWNFL;GPPLRPWC	NCP	3.523
GPPLRPWC;LVLMSQ	NCP	58.38
LVLMSQ;EQLQSSG	NCP	69.293
EQLQSSG;PPNPPPPPP	NCP	12.892
PPNPPPPPP;PVSKMAP	NCP	38.631
PVSKMAP;FSKEAG	NCP	34.857
FSKEAG;DGRLLPR	NCP	3.399
DGRLLPR;RTKQGKP	NCP	39.948
RTKQGKP;YPLHAQ	NCP	34.664
YPLHAQ;VSVLN	NCP	2.932
VSVLN;HGFLLQPP	NCP	41.451
HGFLLQPP;LLGKNYL	NCP	32.517
LLGKNYL;PVPPPAPP	NCP	29.319
PVPPPAPP;QHPPPPQG	NCP	9.966
QHPPPPQG;FNPLGR	NCP	5.817
FNPLGR;VGGDPFP	NCP	87.816
VGGDPFP;PGFFT	NCP	77.054
PGFFT;GVPGGLLG	NCP	82.711
GVPGGLLG;RALGPR	NCP	0.795
RALGPR;TSQTVATR	NCP	205.148
TSQTVATR;LDALVP	NCP	4.318
LDALVP;ASPLLGAR	NCP	17.775
ASPLLGAR;VGPAVP	NCP	3.6

VGPAVP;RLAKVLA	NCP	24.234
RLAKVLA;SPPSLR	NCP	71.79
SPPSLR;KLLLLGD	NCP	1.013
KLLLLGD;QFAGDD	NCP	3.721
QFAGDD;PVSGPPP	NCP	74.352
PVSGPPP;ALKQAAK	NCP	44.13
ALKQAAK;AQVKLSLP	NCP	41.834
AQVKLSLP;AAAGFLP	NCP	9.871
AAAGFLP;FTPDHV	NCP	16.716
FTPDHV;KDLLPV	NCP	61.764
KDLLPV;SLKAVPF	NCP	81.93
SLKAVPF;GPCGD	NCP	17.312
GPCGD;QVVVGH	NCP	45.763
QVVVGH;TGRYHY	NCP	29.723
TGRYHY;ASTLVRP	NCP	23.929
ASTLVRP;KDCKR	NCP	3.189
KDCKR;WPSFAVP	NCP	67.383
WPSFAVP;VTLDYP	NCP	10.049
VTLDYP;VLLLLPL	NCP	33.508
VLLLLPL;QLLLPPWL	NCP	93.222
QLLLPPWL;KPVVF	NCP	74.282
KPVVF;CSFDY	NCP	130.696
CSFDY;GQFLY	NCP	39.497
GQFLY;AAQLLLP	NCP	33.844
AAQLLLP;KWSRPQ	NCP	11.421
KWSRPQ;NASPHPR	NCP	0.024
NASPHPR;AKLEC	NCP	71.604
AKLEC;LGPPYPK	NCP	28.077
LGPPYPK;PPSPPQ	NCP	137.742
PPSPPQ;DLPPP	NCP	2.901
DLPPP;EVHVGPK	NCP	42.833
EVHVGPK;GPARAGPPGP	NCP	11.281
GPARAGPPGP;GRLEC	NCP	126.867
GRLEC;WMPAPN	NCP	41.538
WMPAPN;YPPAYR	NCP	8.523
YPPAYR;QAGRP	NCP	86.601
QAGRP;LSSGLPK	NCP	26.658
LSSGLPK;STPLP	NCP	0.158
STPLP;PVGSPH	NCP	74.812
PVGSPH;LSLHSLLVTL	NCP	21.812
LSLHSLLVTL;REPVAPR	NCP	3.157
REPVAPR;KAMPLLGL	NCP	119.154
KAMPLLGL;PSWLDLR	NCP	65.246
PSWLDLR;APTLVLLPGE	NCP	35.536
APTLVLLPGE;LRTHLC	NCP	9.528
LRTHLC;KLVSPQP	NCP	5.819
KLVSPQP;RKPSVLQV	NCP	15.583
RKPSVLQV;KSDSELR	NCP	64.644
KSDSELR;KCEPTAP	NCP	8.727
KCEPTAP;RATDKK	NCP	6.126
RATDKK;LPAAPARP	NCP	63.894

LPAAPARP;MTPGAP	NCP	41.928
MTPGAP;LSAPK	NCP	5.54
LSAPK;TARATLLP	NCP	2.503
TARATLLP;TLSVYAP	NCP	24.23
TLSVYAP;KGMGSPPP	NCP	111.442
KGMGSPPP;KDAAAVH	NCP	147.11
KDAAAVH;NFVTEE	NCP	4.084
NFVTEE;ETVHEL	NCP	11.726
ETVHEL;GAAHV	NCP	120.847
GAAHV;DPPRMWP	NCP	8.721
DPPRMWP;AAPPPPP	NCP	24.102
AAPPPPP;LSAGPL	NCP	226.953
LSAGPL;LKMKVR	NCP	9.867
LKMKVR;DHNTTKK	NCP	49.518
DPGTTRGP;KDQTLHT	NCP	30.126
KDQTLHT;KVDWAK	NCP	24.13
KVDWAK;TTLPPG	NCP	12.488
TTLPPG;EARLGAP	NCP	4.614
EARLGAP;RFVFH	NCP	16.159
RFVFH;GPGDRPK	NCP	46.691
GPGDRPK;LGAGAAL	NCP	4.611
LGAGAAL;LTVVY	NCP	56.851
LTVVY;KETPGPK	NCP	6.83
KETPGPK;VELEAPW	NCP	1.056
VELEAPW;PGANDVPP	NCP	4.744
PGANDVPP;FLGAGRGT	NCP	32.819
FLGAGRGT;YQKGLSR	NCP	4.132
YQKGLSR;GEEDLGV	NCP	14.114
GEEDLGV;GSGMK	NCP	43.795
GSGMK;AALPV	NCP	34.373
AALPV;EAPHLVRV	NCP	6.348
EAPHLVRV;KSPLAGPG	NCP	31.743
KSPLAGPG;AALPEC	NCP	6.081
AALPEC;KPQLQ	NCP	29.166
KPQLQ;KAGAK	NCP	47.856
KAGAK;LDPKPCTP	NCP	18.006
LDPKPCTP;GPPPPAAVPP	NCP	15.447
GPPPPAAVPP;LLRPATP	NCP	10.596
LLRPATP;AAPGLP	NCP	56.136
AAPGLP;TGERLPP	NCP	21.993
TGERLPP;VPGEWF	NCP	18.747
VPGEWF;VAAPRGGPP	NCP	4.173
VAAPRGGPP;LLFPF	NCP	65.836
LLFPF;AGLHTET	NCP	62.321
AGLHTET;VGPLEP	NCP	35.574
VGPLEP;PGRVVPP	NCP	32.709
PGRVVPP;KWAATPP	NCP	149.763
KWAATPP;EYKLPP	NCP	58.752
EYKLPP;TKDLHVNP	NCP	0.703
TKDLHVNP;PLLVAVR	NCP	58.736
PLLVAVR;PPLKTK	NCP	31.926

PPLKTK;AACCMM	NCP	23.538
AACCMM;HALALSPH	NCP	10.192
HALALSPH;LAPHAL	NCP	32.603
LAPHAL;HGLEVL	NCP	57.432
HGLEVL;APAYRK	NCP	91.662
APAYRK;KGPLTQ	NCP	46.86
KGPLTQ;LGLGPLWG	NCP	8.511
LGLGPLWG;KGVLA	NCP	1.128
KGVLA;KSTLTGP	NCP	12.607
KSTLTGP;LLAPRPE	NCP	104.198
LLAPRPE;VFGGNST	NCP	74.791
VFGGNST;LYTAPP	NCP	120.365
LYTAPP;TVPLPPR	NCP	40.863
TVPLPPR;TGNELLR	NCP	252.909
TGNELLR;SLYQPR	NCP	35.979
SLYQPR;EFGARVM	NCP	1.254
EFGARVM;LFGEAAP	NCP	45.612
LFGEAAP;LFTYT	NCP	55.006
LFTYT;PCPAARPPP	NCP	10.094
PCPAARPPP;GHAPSLG	NCP	32.382
GHAPSLG;VPGVDLW	NCP	30.543
VPGVDLW;VGRAP	NCP	17.13
VGRAP;SPQFPSH	NCP	38.476
SPQFPSH;LLLLLKLKK	NCP	20.316
LLLLLKLKK;APKRPPSA	NCP	28.478
APKRPPSA;RPPVTLR	NCP	14.871
RPPVTLR;LTPHPP	NCP	85.662
LTPHPP;KPQTLALK	NCP	35.974
KPQTLALK;AGTGPVLM	NCP	32.003
AGTGPVLM;NAGPAPY	NCP	21.451
NAGPAPY;KPPPDER	NCP	7.836
KPPPDER;KDAEEKK	NCP	36.684
KDAEEKK;LGLGGAVV	NCP	33.05
LGLGGAVV;MLTDGP	NCP	24.369
MLTDGP;HPSGAPR	NCP	27.585
HPSGAPR;ESDPLL	NCP	15.162
ESDPLL;LGGGLSA	NCP	81.159
LGGGLSA;LGVKK	NCP	34.95
LGVKK;PEKLREG	NCP	50.052
PEKLREG;KELFAGP	NCP	14.964
KELFAGP;DPSPPPR	NCP	55.383
DPSPPPR;VGKLSHSLLV	NCP	50.419
VGKLSHSLLV;PPTRPP	NCP	10.982
PPTRPP;VTHTGHG	NCP	17.256
VTHTGHG;KSWTRPKL	NCP	29.622
KSWTRPKL;APLQPTPP	NCP	27.603
APLQPTPP;KGGAVPQ	NCP	3.246
KGGAVPQ;SWKKKGVP	NCP	11.256
SWKKKGVP;KPSYNR	NCP	12.301
KPSYNR;LPDYP	NCP	19.832
LPDYP;PVAELLM	NCP	14.836

PVAELLM;LYLTLHLR	NCP	0.359
LYLTLHLR;QAPRPQP	NCP	49.083
QAPRPQP;RRDSSYL	NCP	11.173
RRDSSYL;KNTARER	NCP	54.515
KNTARER;RGPPGARP	NCP	1.992
RGPPGARP;LDAVTAL	NCP	29.115
LDAVTAL;LPPLFRY	NCP	21.88
LPPLFRY;KEGASPGAP	NCP	39.11
KEGASPGAP;KTTQFVK	NCP	7.837
KTTQFVK;PGGGLLLG	NCP	75.995
PGGGLLLG;FDEPLPPPP	NCP	8.739
FDEPLPPPP;VPVSKPG	NCP	7.497
VPVSKPG;RLLPGSPPP	NCP	36.216
RLLPGSPPP;AHTVP	NCP	58.326
AHTVP;LTVTTSH	NCP	81.625
LTVTTSH;NFTGKPP	NCP	35.927
NFTGKPP;GASPP	NCP	29.724
GASPP;VPGAVP	NCP	5.232
VPGAVP;KESDKKFP	NCP	68.044
KESDKKFP;KEGALLR	NCP	1.704
KEGALLR;RLNGTAAV	NCP	57.99
RLNGTAAV;GGNKYLR	NCP	46.206
GGNKYLR;PPPKANK	NCP	0.969
PPPKANK;DCCPRY	NCP	23.142
DCCPRY;VGPLAA	NCP	10.31
VGPLAA;LNEVP	NCP	1.11
LNEVP;FGGKAP	NCP	2.631
FGGKAP;EMVGPGPG	NCP	28.332
EMVGPGPG;WGCCTSF	NCP	20.094
WGCCTSF;PLHSVPK	NCP	16.368
PLHSVPK;DMMDPP	NCP	6.597
DMMDPP;HLAYEK	NCP	63.226
HLAYEK;LAFGPPPP	NCP	2.327
LAFGPPPP;PVPPPLP	NCP	30.237
PVPPPLP;KPRGEPP	NCP	142.551
KPRGEPP;DGPKF	NCP	48.996
DGPKF;LKELTPK	NCP	34.111
LKELTPK;LKKLKP	NCP	81.113
LKKLKP;MVLGPV	NCP	19.471
MVLGPV;GKDSLWF	NCP	61.628
GKDSLWF;QRVPLR	NCP	78.055
QRVPLR;LLKEPK	NCP	6.264
LLKEPK;HSVGLVT	NCP	40.58
HSVGLVT;QVKPTR	NCP	8.877
QVKPTR;SNPMLR	NCP	23.268
SNPMLR;SQLAHPP	NCP	138.712
SQLAHPP;SLKPDSL	NCP	11.814
SLKPDSL;DPPVTK	NCP	54.689
DPPVTK;VLPPRKF	NCP	2.403
VLPPRKF;KRKMPK	NCP	46.753
KRKMPK;AAAAGEARR	NCP	14.462

AAAAGEARR;QPGTKEK	NCP	1.131
QPGTKEK;AAVHQLCQSLAGK	NCP	14.397
AAVHQLCQSLAGK;SSWTPLEF	NCP	1.107
SSWTPLEF;TQLLRP	NCP	8.098
TQLLRP;RKRFL	NCP	58.023
RKRFL;VGPVLPK	NCP	7.28
VGPVLPK;VTLDGY	NCP	96.982
VTLDGY;GQNLGAL	NCP	3.65
GQNLGAL;CAAPGVR	NCP	17.667
CAAPGVR;ESDVVPS	NCP	6.246
ESDVVPS;KTKPPDK	NCP	183.268
KTKPPDK;TSPPYKN	NCP	214.701
TSPPYKN;MFLPLEK	NCP	48.138
MFLPLEK;RQTLVLR	NCP	48.452
RQTLVLR;LTNELL	NCP	3.579
LTNELL;KSVMVVE	NCP	25.533
KSVMVVE;MGLMN	NCP	2.691
MGLMN;VQRLPE	NCP	10.192
VQRLPE;LTAAKAF	NCP	71.87
LTAAKAF;KVLLCNC	NCP	38.994
KVLLCNC;PKVMHR	NCP	75.951
PKVMHR;LRDRDEL	NCP	82.272
LRDRDEL;ALEGDKLVGK	NCP	21.277
ALEGDKLVGK;DGLHSL	NCP	19.979
DGLHSL;ESFALFLK	NCP	5.541
ESFALFLK;REAGLGK	NCP	3.591
REAGLGK;EVAGHPK	NCP	25.548
EVAGHPK;RLLVEPY	NCP	28.317
RLLVEPY;LPPLDYLLQ	NCP	133.326
LPPLDYLLQ;PAAVP	NCP	50.496
PAAVP;KDAVPGP	NCP	99.957
KDAVPGP;WDDLR	NCP	59.098
WDDLR;STVHLYT	NCP	43.787
STVHLYT;LNEALP	NCP	36.934
LNEALP;VTRLPET	NCP	19.571
VTRLPET;EVEMLVA	NCP	64.534
EVEMLVA;ELLHNKP	NCP	31.421
ELLHNKP;KGSLL	NCP	0.439
KGSLL;VTLQNFVVR	NCP	39.365
VTLQNFVVR;GRDLTFK	NCP	27.622
GRDLTFK;LVTYGP	NCP	43.991
LVTYGP;SMGLLLKK	NCP	111.87
SMGLLLKK;MVKKYG	NCP	33.421
MVKKYG;VVFLPAL	NCP	27.14
VVFLPAL;KMTLVK	NCP	6.945
KMTLVK;VGLVTLLH	NCP	12.772
VGLVTLLH;APADTTK	NCP	29.666
APADTTK;KYDRPP	NCP	95.136
KYDRPP;KTFHKLKK	NCP	40.069
KTFHKLKK;NFVPPGP	NCP	8.093
NFVPPGP;NPARYFK	NCP	20.865

NPARYFK;EYPTTP	NCP	34.345
EYPTTP;KAMHMT	NCP	46.697
KAMHMT;FSLYL	NCP	58.534
FSLYL;HSSPLDF	NCP	7.896
HSSPLDF;GVGELPK	NCP	2.439
GVGELPK;VSVLPGH	NCP	94.119
VSVLPGH;MEKLHW	NCP	4.439
MEKLHW;CVPPLK	NCP	100.53
CVPPLK;KHYGKKN	NCP	61.12
KHYGKKN;HQVEVLK	NCP	89.286
HQVEVLK;HLLFYTP	NCP	88.413
HLLFYTP;QSLKGK	NCP	20.654
QSLKGK;APPDLR	NCP	178.705
APPDLR;QAMVVPS	NCP	2.687
QAMVVPS;VSKCNAK	NCP	188.539
VSKCNAK;RPTLSH	NCP	24.32
RPTLSH;KDEQK	NCP	265.926
KDEQK;KMPHKV	NCP	24.027
KMPHKV;ERAPATHQ	NCP	13.825
ERAPATHQ;LEHDVP	NCP	87.02
LEHDVP;PGVFPGVK	NCP	4.492
PGVFPGVK;RGGPDFK	NCP	18.252
RGGPDFK;AALRKGQ	NCP	21.363
AALRKGQ;LAFQDP	NCP	25.605
LAFQDP;HDSALFY	NCP	36.12
HDSALFY;FSCFTYE	NCP	37.686
FSCFTYE;WVRSLAP	NCP	34.37
WVRSLAP;LTGAAV	NCP	46.983
LTGAAV;APLVMRK	NCP	38.974
APLVMRK;MQVLCN	NCP	37.304
MQVLCN;AVESVVR	NCP	17.835
AVESVVR;KKTSPR	NCP	15.925
KKTSPR;DPNRPHF	NCP	3.599
DPNRPHF;TPLTVRP	NCP	7.329
TPLTVRP;WAHYTL	NCP	50.644
WAHYTL;VMKPGLM	NCP	72.897
VMKPGLM;LAGFEA	NCP	153.167
LAGFEA;EGLQTER	NCP	58.314
EGLQTER;PPLFSSPK	NCP	80.892
PPLFSSPK;TVFLNMF	NCP	66.4
TVFLNMF;PAPGSFP	NCP	44.789
PAPGSFP;QFVTKLF	NCP	3.849
QFVTKLF;ALMGCL	NCP	19.596
ALMGCL;AEAKSPK	NCP	62.919
AEAKSPK;GVPLP	NCP	11.304
GVPLP;TMFLTMF	NCP	203.928
TMFLTMF;FGRALPGF	NCP	181.206
FGRALPGF;KGKYPLP	NCP	129.765
KGKYPLP;NSDALKKK	NCP	4.498
NSDALKKK;PYLFVR	NCP	6.815
PYLFVR;VPFELTL	NCP	131.49

VPFELTL;LDSLPL	NCP	118.707
LDSLPL;LTLMSKLL	NCP	3.367
LTLMSKLL;LYKLVPN	NCP	14.568
LYKLVPN;VGLGK	NCP	22.893
VGLGK;YCLSPP	NCP	3.149
YCLSPP;TLEGPL	NCP	109.513
TLEGPL;KEDGSSK	NCP	39.476
KEDGSSK;LLSVFFV	NCP	144.114
LLSVFFV;KYGKLSR	NCP	12.882
KYGKLSR;PPEKPPR	NCP	3.982
PPEKPPR;VGHNLSF	NCP	34.277
VGHNLSF;LYPQLM	NCP	51.148
LYPQLM;VGLALGPH	NCP	96.881
VGLALGPH;CTECD	NCP	313.639
CTECD;RFVTPAP	NCP	18.095
RFVTPAP;KKENRP	NCP	17.883
KKENRP;PPFTPP	NCP	39.306
PPFTPP;QKHVPP	NCP	117.153
QKHVPP;FYELLLK	NCP	56.65
FYELLLK;LSSPKTT	NCP	20.049
LSSPKTT;EVPDP	NCP	17.022
EVPDP;SVFTGLH	NCP	8.901
SVFTGLH;SYRGALQ	NCP	32.282
SYRGALQ;KGAGALV	NCP	66.996
KGAGALV;MLFTMF	NCP	37.812
MLFTMF;LTGPKLR	NCP	16.641
LTGPKLR;SSVPFP	NCP	3.438
SSVPFP;LPPEHF	NCP	56.58
LPPEHF;FNLTTFF	NCP	69.99
FNLTTFF;KEVSPF	NCP	31.002
KEVSPF;HLFRF	NCP	33.07
HLFRF;YLAAV	NCP	15.003
YLAAV;PSLSLLYK	NCP	4.215
PSLSLLYK;WEKLEK	NCP	5.181
WEKLEK;QLRWCEAF	NCP	51.72
QLRWCEAF;LKLYH	NCP	17.988
LKLYH;CMECGP	NCP	101.78
CMECGP;EYARHPF	NCP	8.44
EYARHPF;SHALALFL	NCP	27.227
SHALALFL;KPSYPK	NCP	3.472
KPSYPK;AAVMPVK	NCP	76.155
AAVMPVK;VFGSKPP	NCP	39.293
VFGSKPP;EALNYL	NCP	112.57
EALNYL;QVRKLDLR	NCP	36.861
QVRKLDLR;QCCHMM	NCP	10.749
QCCHMM;KLGPALPLT	NCP	25.707
KLGPALPLT;PPGSRPK	NCP	14.501
PPGSRPK;PVHTKK	NCP	36.718
PVHTKK;NSTVPKV	NCP	0.467
NSTVPKV;LSLVD	NCP	14.533
LSLVD;AHQVVVAK	NCP	45.062

AHQVVVAK;FGDPL	NCP	4.167
FGDPL;WDSETGENDSFAGK	NCP	16.953
WDSETGENDSFAGK;TNLTLRDY	NCP	0.126
TNLTLRDY;SGQGVVKLDVQPK	NCP	1.122
SGQGVVKLDVQPK;LGTTLKDEGK	NCP	4.167
LGTTLKDEGK;LLEAKGPVTDLAF	NCP	0.03
LLEAKGPVTDLAF;LEAKGPVTDLA	NCP	0.003
LEAKGPVTDLA;KGPVTDLAF	NCP	0.009
KGPVTDLAF;LAVCDASKVVT	NCP	0.045
LAVCDASKVVT;LDEHTLVTTSHDASVK	NCP	3.06
LDEHTLVTTSHDASVK;QGRTLYG	NCP	1.602
QGRTLYG;ALFKGAP	NCP	25.977
ALFKGAP;KHFRFKF	NCP	44.13
KHFRFKF;KEAKGLQR	NCP	35.245
KEAKGLQR;TVMTFL	NCP	17.564
TVMTFL;KMPPKE	NCP	2.829
KMPPKE;TMEAY	NCP	4.519
TMEAY;KHAKKVP	NCP	3.9
KHAKKVP;FPLLGT	NCP	8.172
FPLLGT;LVELNKVLF	NCP	9.06
LVELNKVLF;TWGYPP	NCP	28.952
TWGYPP;LRMETLP	NCP	19.878
LRMETLP;LAAHLPP	NCP	42.102
LAAHLPP;TTLGALLV	NCP	34.488
TTLGALLV;DPLLAGK	NCP	34.986
DPLLAGK;ADLDLTK	NCP	5.701
ADLDLTK;SVLADPR	NCP	49.595
SVLADPR;KAMEPK	NCP	57.997
KAMEPK;LNEVKGGL	NCP	6.27
LNEVKGGL;HLMEPK	NCP	41.786
HLMEPK;MDALK	NCP	3.838
MDALK;RKAPPPP	NCP	19.962
RKAPPPP;LPVKMK	NCP	15.507
LPVKMK;LLGPGP	NCP	28.853
LLGPGP;LLAGV	NCP	182.626
LLAGV;PTLVVSQP	NCP	22.59
PTLVVSQP;KQKAPTP	NCP	61.775
KQKAPTP;FPSSVQ	NCP	14.953
FPSSVQ;SPVFP	NCP	1.289
SPVFP;DVTLRPY	NCP	26.739
DVTLRPY;VCEVP	NCP	86.638
VCEVP;DHPTTLK	NCP	43.838
DHPTTLK;EKAPVDF	NCP	29.53
EKAPVDF;VNEHSFE	NCP	136.209
VNEHSFE;LLYVLPFL	NCP	40.8
LLYVLPFL;PVAYLQ	NCP	22.712
PVAYLQ;PAQLDQ	NCP	88.389
PAQLDQ;HLGLKVF	NCP	99.82
HLGLKVF;TAVCDY	NCP	31.25
TAVCDY;VVTPP	NCP	54.766
VVTPP;GVTPAFK	NCP	22.142

GVTPAFK;MPKFD	NCP	155.154
MPKFD;LEEALPL	NCP	15.279
LEEALPL;VSGLSFP	NCP	42.424
VSGLSFP;VGLKPVL	NCP	7.376
VGLKPVL;CNADLRK	NCP	2.875
CNADLRK;LSNYPPP	NCP	28.376
LSNYPPP;KQAKPR	NCP	24.645
KQAKPR;ANEGAPG	NCP	108.064
ANEGAPG;VTGFLLY	NCP	22.722
VTGFLLY;MVKKGY	NCP	47.187
MVKKGY;RMTNLKP	NCP	6.266
RMTNLKP;KETHPK	NCP	15.445
KETHPK;EMHGGP	NCP	20.528
EMHGGP;PWTGPK	NCP	156.879
PWTGPK;KMVPLK	NCP	15.408
KMVPLK;NQPPPPPP	NCP	16.137
NQPPPPPP;ERTDRP	NCP	71.913
ERTDRP;NSLLTLL	NCP	35.797
NSLLTLL;KPKVVAK	NCP	27.56
KPKVVAK;TPDCL	NCP	134.434
TPDCL;EPGNPPP	NCP	26.663
EPGNPPP;VTDLLAPL	NCP	13.731
VTDLLAPL;EALQNL	NCP	9.865
EALQNL;TGALKGTP	NCP	1.404
TGALKGTP;FTPAVH	NCP	19.545
FTPAVH;ESSLGTFF	NCP	136.415
ESSLGTFF;LPPPPKPP	NCP	18.759
LPPPPKPP;KPAFNLV	NCP	17.065
KPAFNLV;FRKEY	NCP	15.009
FRKEY;PLGQP	NCP	6.95
PLGQP;DLKPP	NCP	10.41
DLKPP;LGPFLKY	NCP	0.294
LGPFLKY;LLHRFLLQ	NCP	36.594
LLHRFLLQ;MDLALHH	NCP	22.092
MDLALHH;PPLYK	NCP	8.749
PPLYK;LTPVTGLP	NCP	164.403
LTPVTGLP;PPRSGAPPP	NCP	14.646
PPRSGAPPP;CSMDLRK	NCP	37.724
CSMDLRK;VPNLVPF	NCP	18.538
VPNLVPF;KYPPTER	NCP	45.345
KYPPTER;HVPPD	NCP	19.634
HVPPD;SETLPWK	NCP	117.96
SETLPWK;KCPDGM	NCP	23.334
KCPDGM;GSKTELR	NCP	20.268
GSKTELR;VLLKSLQL	NCP	21.738
VLLKSLQL;KKGGVLQ	NCP	11.076
KKGGVLQ;KPQFHPP	NCP	34.536
KPQFHPP;KFNPPPP	NCP	39.264
KFNPPPP;APVLEY	NCP	33.616
APVLEY;KVARPLQ	NCP	25.952
KVARPLQ;AAPFLRP	NCP	29.656

AAPFLRP;PPPPARQPP	NCP	0.153
PPPPARQPP;PVCDFV	NCP	200.316
PVCDFV;MPPLT	NCP	8.948
MPPLT;SNKELFL	NCP	8.4
SNKELFL;PLRKPP	NCP	11.673
PLRKPP;PRQKHV	NCP	32.886
PRQKHV;LLHKTLY	NCP	20.472
LLHKTLY;KSFAKGK	NCP	34.495
KSFAKGK;EAFKEAF	NCP	86.226
EAFKEAF;LSWVGCG	NCP	71.88
LSWVGCG;KPPPNDTR	NCP	72.162
KPPPNDTR;LSSNF	NCP	15.467
LSSNF;LDRLLQP	NCP	9.724
LDRLLQP;PGLPNF	NCP	43.829
PGLPNF;EQEKQ	NCP	0.889
EQEKQ;VFLVA	NCP	6.893
VFLVA;SSFQL	NCP	0.331
SSFQL;KAKLLVK	NCP	115.563
KAKLLVK;GLSGWGGGK	NCP	16.959
GLSGWGGGK;LLAALSK	NCP	38.397
LLAALSK;NDPFLDL	NCP	129.341
NDPFLDL;SGLDLKF	NCP	4.174
SGLDLKF;GPVLL	NCP	20.279
GPVLL;PAGVHPK	NCP	38.427
PAGVHPK;GVVKGFK	NCP	77.907
GVVKGFK;KVPLKD	NCP	19.272
KVPLKD;KPGPAPPP	NCP	44.278
KPGPAPPP;KLSVWPK	NCP	4.514
KLSVWPK;NLPAH	NCP	16.707
NLPAH;HTLPPPP	NCP	23.551
HTLPPPP;LEKMQNK	NCP	67.535
LEKMQNK;EETAGTGSLPK	NCP	14.025
EETAGTGSLPK;NTDKPLR	NCP	75.619
NTDKPLR;KHDPAPP	NCP	61.005
KHDPAPP;ALAHYR	NCP	46.878
ALAHYR;MLFLPY	NCP	92.895
MLFLPY;LPPKDR	NCP	115.596
LPPKDR;TASVVDLT	NCP	251.72
TASVVDLT;DPVTTK	NCP	48.937
DPVTTK;GRPSAPPP	NCP	59.333
GRPSAPPP;WAKLTH	NCP	62.641
WAKLTH;LLGMM	NCP	13.928
LLGMM;RMYVASY	NCP	26.844
RMYVASY;EVSLGRF	NCP	29.122
EVSLGRF;KYVLQ	NCP	103.839
KYVLQ;GPPLAYK	NCP	45.437
GPPLAYK;FLFLLY	NCP	113.175
FLFLLY;PPGPGPPPP	NCP	24.585
PPGPGPPPP;KDAWLL	NCP	107.53
KDAWLL;KSCEQEK	NCP	13.968
KSCEQEK;KEPLDKL	NCP	155.213

KEPLDKL;SNCQEAS	NCP	18.715
SNCQEAS;EFVHPK	NCP	38.156
EFVHPK;HVAHLK	NCP	15.022
HVAHLK;TRNLP	NCP	115.307
TRNLP;QKGVLAK	NCP	77.071
QKGVLAK;MTEGKRP	NCP	90.221
MTEGKRP;LRVEYP	NCP	47.419
LRVEYP;SPTMKLR	NCP	19.704
SPTMKLR;APKRAPSP	NCP	47.421
APKRAPSP;AASLEVY	NCP	191.315
AASLEVY;AAKKLAL	NCP	77.485
AAKKLAL;KTSNKAGP	NCP	0.999
KTSNKAGP;PVGFP	NCP	207.003
PVGFP;KHLQL	NCP	30.474
KHLQL;PAVDPPP	NCP	56.582
PAVDPPP;KLVGNKV	NCP	25.932
KLVGNKV;LLSPL	NCP	66.451
LLSPL;MNPVVSQPG	NCP	54.179
MNPVVSQPG;AATSTF	NCP	86.203
AATSTF;FLAAEEE	NCP	72.6
FLAAEEE;VEDLLK	NCP	0.56
VEDLLK;TTLPEL	NCP	0.414
TTLPEL;MKLKEVDR	NCP	95.172
MKLKEVDR;LDLSDPSLDMK	NCP	0.807
LDLSDPSLDMK;ATPMTPGAK	NCP	3.981
ATPMTPGAK;KVNFEDDSRGK	NCP	12.807
KVNFEDDSRGK;VFCVEKL	NCP	10.188
VFCVEKL;EVLTLPRT	NCP	19.461
EVLTLPRT;SETSAFMPVLKVVLTQANKLGV	NCP	2.802
SETSAFMPVLKVVLTQANKLGV;EYMDLR	NCP	78.385
EYMDLR;LSPLVDHT	NCP	0.338
LSPLVDHT;PLSAPKP	NCP	220.638
PLSAPKP;ALFRK	NCP	7.483
ALFRK;YTDEEPVKK	NCP	135.002
YTDEEPVKK;TDEEPVKKLLE	NCP	0.003
TDEEPVKKLLE;SLDRVTK	NCP	38.004
SLDRVTK;KDLKDYFSK	NCP	25.341
KDLKDYFSK;FGEVVDC	NCP	0.027
FGEVVDC;VVDCTLK	NCP	0.009
VVDCTLK;VGGLSPDTPEEK	NCP	0.798
VGGLSPDTPEEK;TFKEEEPVKK	NCP	1.437
TFKEEEPVKK;YHNVGLSK	NCP	0.045
YHNVGLSK;GEPKKPK	NCP	6.259
GEPKKPK;FGPLLFK	NCP	10.163
FGPLLFK;KEGVDFR	NCP	270.807
KEGVDFR;VASMLK	NCP	71.914
VASMLK;ANVSVSR	NCP	84.81
ANVSVSR;KPSVFAK	NCP	11.439
KPSVFAK;KAPSVHP	NCP	56.433
KAPSVHP;PACLP	NCP	41.604
PACLP;EPPNMPR	NCP	110.001

EPPNMPR;SEYLLR	NCP	17.109
SEYLLR;YGSSK	NCP	23.274
YGSSK;TKKATHK	NCP	41.042
TKKATHK;VGRNLPL	NCP	5.376
VGRNLPL;PLTRDPT	NCP	43.327
PLTRDPT;KGLLVAR	NCP	18.653
KGLLVAR;KECAAKK	NCP	193.665
KECAAKK;KSQNYAVS	NCP	80.911
KSQNYAVS;TTGLVM	NCP	2.889
TTGLVM;TAEVLEL	NCP	27.309
TAEVLEL;KSMLVLF	NCP	36.503
KSMLVLF;SLRAPALT	NCP	11.119
SLRAPALT;PVVGEC	NCP	19.041
PVVGEC;FDLMYAK	NCP	36.227
FDLMYAK;LALNF	NCP	3.714
LALNF;NAHLVLY	NCP	25.969
NAHLVLY;KLLFASNL	NCP	34.217
KLLFASNL;VLLTLKNL	NCP	68.082
VLLTLKNL;NDSLLLTK	NCP	119.823
NDSLLLTK;MLAVPEV	NCP	53.182
MLAVPEV;LAFMRHLP	NCP	14.705
LAFMRHLP;EPPYSLGK	NCP	59.493
EPPYSLGK;PPTPLMQ	NCP	6.03
PPTPLMQ;HPGAK	NCP	56.857
HPGAK;NGPSFLP	NCP	19.403
NGPSFLP;KYDPEE	NCP	30.562
KYDPEE;VTAPYGK	NCP	8.621
VTAPYGK;TLSFFE	NCP	40.068
TLSFFE;NALKANQ	NCP	123.732
NALKANQ;KTRTVKP	NCP	122.887
KTRTVKP;ATYMAF	NCP	13.677
ATYMAF;QTYKAGK	NCP	2.73
QTYKAGK;LLTEEVTL	NCP	64.811
LLTEEVTL;LPPRDKP	NCP	35.272
LPPRDKP;YPGSSLQ	NCP	62.129
YPGSSLQ;ELDSLTV	NCP	31.813
ELDSLTV;NKGNVL	NCP	85.907
NKGNVL;TTLLKK	NCP	1.489
TTLLKK;TNFKTLR	NCP	124.281
TNFKTLR;FDKFC	NCP	9.389
FDKFC;AGGHRLLL	NCP	50.899
AGGHRLLL;RPSPFDS	NCP	8.576
RPSPFDS;DLPVP	NCP	173.676
DLPVP;MVVKPLR	NCP	97.342
MVVKPLR;KTALTSSK	NCP	70.217
KTALTSSK;GSDSSC	NCP	3.817
GSDSSC;TPKKPLT	NCP	40.962
TPKKPLT;NSHLQK	NCP	1.797
NSHLQK;LLSTKHT	NCP	10.235
LLSTKHT;LPAGGHLK	NCP	42.223
LPAGGHLK;KHLAGRK	NCP	46.823

KHLAGRK;LVKYN	NCP	47.448
LVKYN;TTGVMTW	NCP	10.828
TTGVMTW;NNTLSVH	NCP	11.001
NNTLSVH;KSVTQLR	NCP	26.177
KSVTQLR;HLLVFV	NCP	29.692
HLLVFV;PVAPK	NCP	140.241
PVAPK;FNSYYPF	NCP	15.506
FNSYYPF;FGPVTLP	NCP	27.181
FGPVTLP;LAKPEPR	NCP	46.071
LAKPEPR;PVGVGN	NCP	64.986
PVGVGN;NGCVLAV	NCP	73.859
NGCVLAV;APPPAAPP	NCP	140.481
APPPAAPP;QSLAPR	NCP	1.374
QSLAPR;WLQNLKYR	NCP	26.68
WLQNLKYR;KGEQKNPK	NCP	126.963
KGEQKNPK;NTAGY	NCP	48.971
NTAGY;YPGVAP	NCP	21.315
YPGVAP;EDPSPVGR	NCP	78.498
EDPSPVGR;ALDTLY	NCP	39.286
ALDTLY;VLDPFWL	NCP	4.908
VLDPFWL;PSLNFVK	NCP	43.023
PSLNFVK;PSKRP	NCP	68.324
PSKRP;VHGFPTPF	NCP	99.843
VHGFPTPF;KDKNLQK	NCP	4.027
KDKNLQK;FAPLT	NCP	11.678
FAPLT;VFLEF	NCP	18.681
VFLEF;LAELN	NCP	4.818
LAELN;TNMLSW	NCP	49.554
TNMLSW;LMTMVP	NCP	32.973
LMTMVP;EHTTKK	NCP	3.43
EHTTKK;DELRVAVK	NCP	35.295
DELRVAVK;KKDLEKYT	NCP	86.102
KKDLEKYT;FKRMSYL	NCP	33.045
FKRMSYL;VHLLLMP	NCP	62.391
VHLLLMP;LTSLPEVL	NCP	21.265
LTSLPEVL;VSLPLLAK	NCP	144.611
VSLPLLAK;AAVLWELNKPF	NCP	10.903
AAVLWELNKPF;VLWELNKP	NCP	0.006
VLWELNKP;WELNKPF	NCP	0.006
WELNKPF;EEVEVAPPKAHE	NCP	0.027
EEVEVAPPKAHE;EVEVAPPKAHEVR	NCP	0.003
EVEVAPPKAHEVR;VEVAPPKAHE	NCP	0.003
VEVAPPKAHE;EVAPPKAHEVR	NCP	0.003
EVAPPKAHEVR;KHPEANLCLK	NCP	3.6
KHPEANLCLK;LKEPRGTLN	NCP	0.132
LKEPRGTLN;TVVDEMSVAK	NCP	0.102
TVVDEMSVAK;FGLGGVGL	NCP	2.727
FGLGGVGL;SVNPMLLLT	NCP	6.006
SVNPMLLLT;VNPMLLLTG	NCP	0.003
VNPMLLLTG;LLLTGRTWK	NCP	0.012
LLLTGRTWK;MKFKKAP	NCP	14.982

MKFKKAP;FGNQSL	NCP	51.857
FGNQSL;KDLGRAP	NCP	19.624
KDLGRAP;KSEPLKD	NCP	30.165
KSEPLKD;KPMDK	NCP	1.761
KPMDK;TALCGTEMKMLKDKN	NCP	2.019
TALCGTEMKMLKDKN;DGTSRFTCKGKPVYH	NCP	3.015
DGTSRFTCKGKPVYH;VYASYLL	NCP	83.781
VYASYLL;VSAMELLM	NCP	0.54
VSAMELLM;WKGVTSVPKLAADYK	NCP	0.543
WKGVTSVPKLAADYK;KGVTSVPKLAAD	NCP	0.003
KGVTSVPKLAAD;GVTSVPKLAADYK	NCP	0.003
GVTSVPKLAADYK;SVPKLAAD	NCP	0.009
SVPKLAAD;PKLAADYKN	NCP	0.006
PKLAADYKN;DYKNKKLDLDAL	NCP	0.015
DYKNKKLDLDAL;KKLDLDALVTH	NCP	0.012
KKLDLDALVTH;KLDLDALVTHT	NCP	0.003
KLDLDALVTHT;DLDALVTH	NCP	0.006
DLDALVTH;LDALVTHTLPFDK	NCP	0.003
LDALVTHTLPFDK;KLTERLR	NCP	60.503
KLTERLR;LTPLTPML	NCP	29.967
LTPLTPML;VGLEL	NCP	19.495
VGLEL;LSFDLVK	NCP	58.635
LSFDLVK;KKKTCMC	NCP	4.949
KKKTCMC;VDLGVK	NCP	23.778
VDLGVK;ALNVHSSK	NCP	22.882
ALNVHSSK;KFLATYP	NCP	3.608
KFLATYP;APLLVVV	NCP	8.173
APLLVVV;GRELLPRL	NCP	5.312
GRELLPRL;RKRYEE	NCP	33.246
RKRYEE;KPATVYT	NCP	26.439
KPATVYT;EKSGVPW	NCP	3.094
EKSGVPW;MEVAKW	NCP	103.794
MEVAKW;LPFVAPGA	NCP	45.714
LPFVAPGA;PPPPVPPR	NCP	3.578
PPPPVPPR;LGSTRVP	NCP	17.992
LGSTRVP;ASCLMLH	NCP	70.796
ASCLMLH;KGVTFPPR	NCP	29.248
KGVTFPPR;ELLEAAPA	NCP	17.021
ELLEAAPA;PPPWL	NCP	59.236
PPPWL;TRKANQP	NCP	68.805
TRKANQP;LLEQLP	NCP	20.868
LLEQLP;EAASFGLL	NCP	9.263
EAASFGLL;AAGTPGP	NCP	17.871
AAGTPGP;RHKKVME	NCP	25.932
RHKKVME;LTDKGLL	NCP	22.023
LTDKGLL;DGLLEHF	NCP	50.823
DGLLEHF;KAAVCPK	NCP	0.022
KAAVCPK;PLRTPP	NCP	23.948
PLRTPP;EVTHELL	NCP	128.86
EVTHELL;STAREAPAP	NCP	65.66
STAREAPAP;RLHYF	NCP	118.965

RLHYF;NPPPNLK	NCP	107.466
NPPPNLK;EGVKGLR	NCP	64.824
EGVKGLR;VELGLAK	NCP	1.269
VELGLAK;NAFVRSP	NCP	3.135
NAFVRSP;TSSVPKK	NCP	3.901
TSSVPKK;LPNHPF	NCP	76.908
LPNHPF;LDGPALSEL	NCP	74.883
LDGPALSEL;MACVLAK	NCP	58.751
MACVLAK;GLREFLR	NCP	61.723
GLREFLR;SKKFPVFQ	NCP	10.829
SKKFPVFQ;TVYAFLR	NCP	6.556
TVYAFLR;KGLFVR	NCP	5.51
KGLFVR;LYKLAKK	NCP	14.589
LYKLAKK;MEKPSPLLVG	NCP	54.093
MEKPSPLLVG;TEELKPPVEEK	NCP	5.502
TEELKPPVEEK;VEAKPEVQSQPPR	NCP	5.55
VEAKPEVQSQPPR;RGHFGK	NCP	10.897
RGHFGK;VAMLLTL	NCP	19.736
VAMLLTL;HEPVKK	NCP	30.223
HEPVKK;KPLTTY	NCP	19.43
KPLTTY;FSGPSVT	NCP	146.955
FSGPSVT;LYNLFLK	NCP	9.555
LYNLFLK;KSTMLSK	NCP	45.195
KSTMLSK;MNSLLSR	NCP	127.087
MNSLLSR;MLGASAA	NCP	36.929
MLGASAA;CDAAAPQ	NCP	12.585
CDAAAPQ;PLGAPLR	NCP	26.814
PLGAPLR;VGGCDLL	NCP	131.76
VGGCDLL;GTLVQTK	NCP	2.728
GTLVQTK;TVAKPY	NCP	19.721
TVAKPY;MAKCEPT	NCP	37.944
MAKCEPT;KLHQTLD	NCP	9.34
KLHQTLD;GSSGK	NCP	27.935
GSSGK;LDLLKER	NCP	20.635
LDLLKER;ESPVGKPP	NCP	3.524
ESPVGKPP;QNKKTEL	NCP	50.664
QNKKTEL;FYKGPQP	NCP	64.917
FYKGPQP;EKRVY	NCP	1.587
EKRVY;ASVVVK	NCP	1.629
ASVVVK;SNMQDLRK	NCP	64.071
SNMQDLRK;VRVFKKLL	NCP	55.902
VRVFKKLL;GHMFKPP	NCP	91.273
GHMFKPP;QGPKPEP	NCP	46.355
QGPKPEP;DGVLK	NCP	32.784
DGVLK;EKSLC	NCP	83.133
EKSLC;NMRYLVP	NCP	32.058
NMRYLVP;LDVKELK	NCP	162.082
LDVKELK;NRTPLP	NCP	181.884
NRTPLP;PALWDKL	NCP	32.63
PALWDKL;CKVKHMN	NCP	12.541
CKVKHMN;ALEFDSV	NCP	20.271

ALEFDSV;TTCKSE	NCP	112.703
TTCKSE;SGMTPPR	NCP	24.277
SGMTPPR;SVGHTLR	NCP	159.105
SVGHTLR;SPPVL	NCP	45.455
SPPVL;FVDVLFP	NCP	5.202
FVDVLFP;KGELVFL	NCP	13.935
KGELVFL;WNLPLAL	NCP	24.418
WNLPLAL;PVRNFPS	NCP	0.858
PVRNFPS;KYVVAR	NCP	58.92
KYVVAR;EAQKLR	NCP	24.888
EAQKLR;KMDVAAP	NCP	36.666
KMDVAAP;AFFLFC	NCP	108.014
AFFLFC;ELLETLLK	NCP	43.831
ELLETLLK;MGPALER	NCP	62.3
MGPALER;SERASMH	NCP	57.159
SERASMH;HLEGLLLF	NCP	23.97
HLEGLLLF;KGPKNKP	NCP	13.372
KGPKNKP;KVSKKL	NCP	6.684
KVSKKL;ALERMF	NCP	114.822
ALERMF;KSVSRMK	NCP	409.293
KSVSRMK;TLKNFYK	NCP	135.93
TLKNFYK;CMMDCC	NCP	21.002
CMMDCC;GVAPGN	NCP	26.347
GVAPGN;KLTVVSK	NCP	7.898
KLTVVSK;FLLLLF	NCP	49.354
FLLLLF;VHPGTPECCTHEGLEKK	NCP	205.151
VHPGTPECCTHEGLEKK;PVRPVVV	NCP	174.573
PVRPVVV;PLLPHL	NCP	2.206
PLLPHL;KELWGER	NCP	42.57
KELWGER;RKDVTLKK	NCP	20.817
RKDVTLKK;KGYYPK	NCP	7.626
KGYYPK;YTLVY	NCP	100.064
YTLVY;ALSFKR	NCP	140.709
ALSFKR;KTYMVK	NCP	5.476
KTYMVK;LTRDLKY	NCP	144.054
LTRDLKY;KFPYPQ	NCP	139.044
KFPYPQ;EVLLHPR	NCP	43.49
EVLLHPR;FMLVPTQ	NCP	1.998
FMLVPTQ;FGVRDAA	NCP	14.977
FGVRDAA;VDTRFK	NCP	36.195
VDTRFK;AAFNAP	NCP	70.152
AAFNAP;FGEKLRT	NCP	2.357
FGEKLRT;KDYVVP	NCP	5.625
KDYVVP;KMNSVF	NCP	184.576
KMNSVF;SNYRKE	NCP	0.174
SNYRKE;SHAFSVH	NCP	84.534
SHAFSVH;VRPYALL	NCP	89.562
VRPYALL;APPKYLLT	NCP	4.815
APPKYLLT;TKVQNK	NCP	67.844
TKVQNK;VVPPFLQPEV	NCP	160.545
VVPPFLQPEV;HQPHQPLPPT	NCP	0.186

HQPHQPLPPT;VLPVPQK	NCP	0.075
VLPVPQK;MYPPP	NCP	57.102
MYPPP;TLPTE	NCP	18.066
TLPTE;LGVLPGVR	NCP	33.271
LGVLPGVR;CHPKQHL	NCP	99.462
CHPKQHL;SSLLPGVK	NCP	10.679
SSLLPGVK;TSEKPK	NCP	14.653
TSEKPK;TSPKDVLRKN	NCP	7.025
TSPKDVLRKN;PVKVLGV	NCP	39.435
PVKVLGV;LPKPEL	NCP	52.081
LPKPEL;TDGNTGPL	NCP	45.83
TDGNTGPL;EVAFSN	NCP	7.164
EVAFSN;LPMLL	NCP	24.225
LPMLL;PPTKPNPP	NCP	78.337
PPTKPNPP;RDPLP	NCP	232.221
RDPLP;AAVHLLQ	NCP	189.975
AAVHLLQ;STVHGVGH	NCP	25.895
STVHGVGH;VHGGYH	NCP	30.154
VHGGYH;GHPKTKAFVTHGGSN	NCP	51.482
GHPKTKAFVTHGGSN;PLVPVPLP	NCP	23.427
PLVPVPLP;YDGKKPDTLGPN	NCP	62.757
YDGKKPDTLGPN;NTDLSLP	NCP	95.355
NTDLSLP;HLPKAAM	NCP	1.311
HLPKAAM;HGVPMVGLPL	NCP	110.712
HGVPMVGLPL;WSAHL	NCP	162.655
WSAHL;TLLTF	NCP	147.737
TLLTF;GYVGF	NCP	39.345
GYVGF;LDLWQVK	NCP	37.287
LDLWQVK;WTPEVK	NCP	23.473
WTPEVK;TQMAPK	NCP	79.805
TQMAPK;EQLRTPL	NCP	6.196
EQLRTPL;KLSKECLKKY	NCP	14.658
KLSKECLKKY;LQKLLLPR	NCP	32.579
LQKLLLPR;NPPGKLF	NCP	31.627
NPPGKLF;KEQHVYLE	NCP	78.419
KEQHVYLE;YVEFPK	NCP	56.802
YVEFPK;LNQLLMK	NCP	29.661
LNQLLMK;AVFFY	NCP	7.699
AVFFY;TVAALLY	NCP	7.44
TVAALLY;TMFGELK	NCP	39.086
TMFGELK;AAVSGLV	NCP	60.24
AAVSGLV;KLVKPSAEK	NCP	4.983
KLVKPSAEK;KSSGGKR	NCP	78.393
KSSGGKR;SQGSVKK	NCP	73.335
SQGSVKK;HTLPLA	NCP	35.38
HTLPLA;LCLVCKK	NCP	9.336
LCLVCKK;QVVPVLS	NCP	21.992
QVVPVLS;SELLLLKPFP	NCP	25.309
SELLLLKPFP;ARAALTL	NCP	51.884
ARAALTL;KNRVVAL	NCP	28.5
KNRVVAL;KAAFGNK	NCP	108.519

KAAFGNK;DPPMLLK	NCP	8.239
DPPMLLK;CCMLT	NCP	9.06
CCMLT;TVGLY	NCP	99.528
TVGLY;VLGAFVP	NCP	32.829
VLGAFVP;GKDGDH	NCP	75.359
GKDGDH;LALLLC	NCP	68.616
LALLLC;GTTPKPL	NCP	5.35
GTTPKPL;KPGFR	NCP	6.273
KPGFR;KGSSKAK	NCP	121.685
KGSSKAK;YESVSGY	NCP	236.514
YESVSGY;FGFGQG	NCP	35.797
FGFGQG;DPLNVHRK	NCP	96.554
DPLNVHRK;KYCMLQ	NCP	27.178
KYCMLQ;YPPVVPP	NCP	15.051
YPPVVPP;MEKALS	NCP	76.637
MEKALS;LPFELY	NCP	60.621
LPFELY;LDFTPK	NCP	36.781
LDFTPK;MEAAHSKT	NCP	11.288
MEAAHSKT;PAELW	NCP	32.712
PAELW;LRDQASNL	NCP	10.056
LRDQASNL;ASLPFVT	NCP	20.637
ASLPFVT;MLVPQKM	NCP	56.175
MLVPQKM;SVPLK	NCP	26.883
SVPLK;RNLLNYL	NCP	55.102
RNLLNYL;QNYRPP	NCP	7.874
QNYRPP;KHSLF	NCP	4.413
KHSLF;KPTLLK	NCP	256.704
KPTLLK;KGDDRDK	NCP	5.917
KGDDRDK;LPVMAAN	NCP	39.351
LPVMAAN;QAHPHN	NCP	39.336
QAHPHN;NSSPVFPR	NCP	246.384
NSSPVFPR;MDCAC	NCP	36.03
MDCAC;KYERHF	NCP	175.61
KYERHF;SLHHKGK	NCP	7.779
SLHHKGK;GVLKT	NCP	29.61
GVLKT;LSHSLVSPL	NCP	84.699
LSHSLVSPL;KSLKVENK	NCP	40.935
KSLKVENK;PLPQHPPPP	NCP	15.67
PLPQHPPPP;AAPLGGMF	NCP	30.066
AAPLGGMF;QKLQPLDF	NCP	124.212
QKLQPLDF;LDKNF	NCP	8.381
LDKNF;QPEALP	NCP	117.703
QPEALP;KETAPY	NCP	36.806
KETAPY;AALVA	NCP	99.595
AALVA;KKWHYK	NCP	62.288
KKWHYK;KEKKEGD	NCP	101.907
KEKKEGD;KQGDRF	NCP	62.205
KQGDRF;LKLGY	NCP	135.535
LKLGY;DLRDYF	NCP	108.069
DLRDYF;SDAGLTPL	NCP	61.179
SDAGLTPL;ALPAT	NCP	37.665

ALPAT;KYPSPW	NCP	55.668
KYPSPW;LAVDLNK	NCP	48.3
LAVDLNK;EKFDKSKL	NCP	248.123
EKFDKSKL;FDKSKLK	NCP	0.006
FDKSKLK;EKNPLPSK	NCP	0.036
EKNPLPSK;KNPLPSKE	NCP	0.003
KNPLPSKE;LYNYM	NCP	38.343
LYNYM;MLEEPG	NCP	22.782
MLEEPG;MDLMM	NCP	12.595
MDLMM;YWPEM	NCP	160.628
YWPEM;PRVSKLK	NCP	56.662
PRVSKLK;LLDAL	NCP	40.917
LLDAL;KESVKPV	NCP	77.765
KESVKPV;YVEDRSP	NCP	15.67
YVEDRSP;DHEVEEK	NCP	133.041
DHEVEEK;MVPEHR	NCP	70.902
MVPEHR;KPGFDR	NCP	5.999
KPGFDR;SLSVLY	NCP	230.29
SLSVLY;HAWMTK	NCP	148.188
HAWMTK;VEGAVF	NCP	105.182
VEGAVF;KAVLLKVS	NCP	15.087
KAVLLKVS;LVLPKKY	NCP	5.793
LVLPKKY;EDVLSHR	NCP	76.435
EDVLSHR;VGANLET	NCP	46.592
VGANLET;PPMLYK	NCP	102.51
PPMLYK;VTGGLPF	NCP	5.607
VTGGLPF;ALTVLYR	NCP	10.788
ALTVLYR;LRAAVFL	NCP	15.679
LRAAVFL;TLLQLFPF	NCP	60.279
TLLQLFPF;KKYLP	NCP	138.392
KKYLP;RKKKDL	NCP	39.873
RKKKDL;KEAFQPK	NCP	172.372
KEAFQPK;KSLKCPK	NCP	7.152
KSLKCPK;FAARFR	NCP	29.546
FAARFR;VFNGVGK	NCP	56.742
VFNGVGK;KLVRERK	NCP	14.097
KLVRERK;MMPGLPR	NCP	123.207
MMPGLPR;GKLNLL	NCP	94.447
GKLNLL;ANHLVL	NCP	58.731
ANHLVL;MDLAL	NCP	75.599
MDLAL;NSLHPHRT	NCP	4.893
NSLHPHRT;LAYGEKK	NCP	37.311
LAYGEKK;ELSNKLT	NCP	48.105
ELSNKLT;AKAHAAK	NCP	44.584
AKAHAAK;KNMTTH	NCP	62.117
KNMTTH;NYVELKN	NCP	22.326
NYVELKN;KSGVCKK	NCP	11.545
KSGVCKK;TTKDWVK	NCP	164.768
TTKDWVK;HYLYE	NCP	134.326
HYLYE;KADLVFL	NCP	50.288
KADLVFL;KATLGM	NCP	98.514

KATLGM;LVDPLR	NCP	121.471
LVDPLR;TGGLLYFY	NCP	17.729
TGGLLYFY;KFYGNHF	NCP	3.492
KFYGNHF;AQAFVN	NCP	19.249
AQAFVN;LLQTFTK	NCP	72.234
LLQTFTK;FAPFAQR	NCP	32.309
FAPFAQR;FLPLTQV	NCP	30.469
FLPLTQV;MVLLKPG	NCP	108.323
MVLLKPG;APHVGKP	NCP	224.466
APHVGKP;FSLGMES	NCP	9.409
FSLGMES;ATLLLLLPT	NCP	9.666
ATLLLLLPT;TQFLPNPL	NCP	76.902
TQFLPNPL;KDHVALY	NCP	12.075
KDHVALY;KPSCKQP	NCP	81.098
KPSCKQP;MNEAYR	NCP	27.696
MNEAYR;NSRLLAR	NCP	17.161
NSRLLAR;FAALLN	NCP	7.448
FAALLN;KLEELQE	NCP	186.651
KLEELQE;KELNAPPP	NCP	6.208
KELNAPPP;RKGYTT	NCP	104.657
RKGYTT;LSPLYEG	NCP	0.862
LSPLYEG;FMYASK	NCP	5.408
FMYASK;QKTLKT	NCP	13.222
QKTLKT;VKVYNYNHLMPTRY	NCP	34.646
VKVYNYNHLMPTRY;NHLMPTR	NCP	0.018
NHLMPTR;SLFSK	NCP	125.464
SLFSK;KTLRRYHR	NCP	30.72
KTLRRYHR;FPGLKY	NCP	10.299
FPGLKY;NDLHKT	NCP	24.966
NDLHKT;CDEEM	NCP	108.777
CDEEM;AAHVLL	NCP	53.094
AAHVLL;AFHLF	NCP	79.785
AFHLF;LHSPML	NCP	8.765
LHSPML;MSNVL	NCP	144.556
MSNVL;LSHLSVL	NCP	55.559
LSHLSVL;KVLKPK	NCP	26.725
KVLKPK;GVHVL	NCP	29.96
GVHVL;NVFLK	NCP	94.824
NVFLK;ETLNLQ	NCP	42.115
ETLNLQ;KFFWAF	NCP	26.288
KFFWAF;LSFGMQQ	NCP	29.578
LSFGMQQ;TAGVPDA	NCP	37.98
TAGVPDA;PLLKL	NCP	137.187
PLLKL;TCKFQP	NCP	50.256
TCKFQP;MVVPYP	NCP	87.386
MVVPYP;LSPLYE	NCP	7.899
LSPLYE;KAQRPFC	NCP	12.168
KAQRPFC;DVPMHK	NCP	261.861
DVPMHK;RVGLLF	NCP	12
RVGLLF;KSGDLRF	NCP	40.338
KSGDLRF;LLKGSEL	NCP	0.774

LLKGSEL;VYNLF	NCP	45.024
VYNLF;EPFELQF	NCP	17.149
EPFELQF;RSFLHQ	NCP	32.244
RSFLHQ;PCQDPN	NCP	44.529
PCQDPN;ALPTSPKQ	NCP	125.82
ALPTSPKQ;TSFVK	NCP	89.232
TSFVK;KGADLK	NCP	117.078
KGADLK;RLPKMWK	NCP	25.941
RLPKMWK;MELLCDR	NCP	28.491
MELLCDR;VLAVYH	NCP	225.314
VLAVYH;ANHGEC	NCP	88.692
ANHGEC;LHPTKSK	NCP	45.831
LHPTKSK;KTFEVPP	NCP	60.421
KTFEVPP;EEGEEHF	NCP	3.72
EEGEEHF;KCGTKSK	NCP	166.032
KCGTKSK;YEKSALK	NCP	64.352
YEKSALK;ASHSLLV	NCP	74.203
ASHSLLV;CCFKM	NCP	6.642
CCFKM;KGKEHCK	NCP	12.164
KGKEHCK;EDRLNF	NCP	126.081
EDRLNF;PLMGAK	NCP	15.415
PLMGAK;KETPAAAAK	NCP	114.065
KETPAAAAK;EVPFKPP	NCP	12.277
EVPFKPP;KDEATM	NCP	26.7
KDEATM;KKLALVP	NCP	40.553
KKLALVP;VLVLH	NCP	16.429
VLVLH;KGGFMVP	NCP	39.747
KGGFMVP;VTLVEGRK	NCP	71.364
VTLVEGRK;AASHKAK	NCP	19.544
AASHKAK;NGRRPPP	NCP	66.843
NGRRPPP;YVKKKC	NCP	240.381
YVKKKC;DASDL	NCP	90.01
DASDL;VGGKLAL	NCP	3.438
VGGKLAL;MKLAVLG	NCP	69.018
MKLAVLG;LLGLMQ	NCP	14.255
LLGLMQ;VKVLLGT	NCP	82.33
VKVLLGT;GFLLNGN	NCP	24.648
GFLLNGN;AALPVPQ	NCP	53.228
AALPVPQ;CFAALP	NCP	6.996
CFAALP;TLHEC	NCP	13.437
TLHEC;EHLSELL	NCP	50.485
EHLSELL;FETKESRKEEL	NCP	13.16
FETKESRKEEL;AEKLGGSL	NCP	0.174
AEKLGGSL;AFEGCPV	NCP	0.03
AFEGCPV;KTVRGNTDN	NCP	26.182
KTVRGNTDN;LPGAKPP	NCP	18.693
LPGAKPP;MTKTPF	NCP	93.738
MTKTPF;FAAERK	NCP	11.544
FAAERK;SGPKARP	NCP	15.515
SGPKARP;LPVFF	NCP	109.023
LPVFF;KPEEFYR	NCP	18.378

KPEEFYR;LNRKPALN	NCP	3.4
LNRKPALN;FETGTLRK	NCP	49.436
FETGTLRK;LTFLGGY	NCP	117.801
LTFLGGY;AALSAAD	NCP	18.216
AALSAAD;GLDFAAP	NCP	82.351
GLDFAAP;SPLWTRE	NCP	38.507
SPLWTRE;YKDPDYVRK	NCP	6.672
YKDPDYVRK;VAPPKNM	NCP	60.867
VAPPKNM;AAKNLAK	NCP	2.574
AAKNLAK;AEEDLFL	NCP	59.172
AEEDLFL;KEFLLSM	NCP	1.002
KEFLLSM;VTDAFVP	NCP	25.788
VTDAFVP;PPLLLLPG	NCP	9.741
PPLLLLPG;LKMSVDL	NCP	12.121
LKMSVDL;RNVHAPK	NCP	10.422
RNVHAPK;PAAFLPF	NCP	20.285
PAAFLPF;PFCPCL	NCP	144.4
PFCPCL;DKYLDLL	NCP	198.56
DKYLDLL;SNRPSF	NCP	37.86
SNRPSF;KYAQYK	NCP	16.597
KYAQYK;FFQRPK	NCP	129.411
FFQRPK;LPVLQLV	NCP	95.135
LPVLQLV;DDFFTR	NCP	36.678
DDFFTR;EALTVPF	NCP	4.413
EALTVPF;FLVVGEP	NCP	9.478
FLVVGEP;AYAKLVL	NCP	16.107
AYAKLVL;GLAVKELK	NCP	77.568
GLAVKELK;KESVHQL	NCP	54.576
KESVHQL;VLAAEYL	NCP	42.419
VLAAEYL;KGPVEVV	NCP	50.221
KGPVEVV;SGVPVK	NCP	45.368
SGVPVK;MLSCLQVR	NCP	2.586
MLSCLQVR;TQLGHKP	NCP	156.684
TQLGHKP;HAGLKEV	NCP	51.481
HAGLKEV;KPLSLVKHR	NCP	2.441
KPLSLVKHR;KPGRDAL	NCP	6.156
KPGRDAL;NTCAAQP	NCP	228.57
NTCAAQP;METVQLRNPPR	NCP	23.523
METVQLRNPPR;LLAPVAPH	NCP	28.548
LLAPVAPH;VEEPTVL	NCP	62.644
VEEPTVL;AVALL	NCP	157.352
AVALL;LPAAFC	NCP	165.543
LPAAFC;DRLHPDL	NCP	9.801
DRLHPDL;DSTSGKP	NCP	17.893
DSTSGKP;LDPVGY	NCP	87.057
LDPVGY;FPKNNTK	NCP	7.622
FPKNNTK;TLADLP	NCP	105.159
TLADLP;KPVELPP	NCP	85.395
KPVELPP;EVDGLLR	NCP	12.546
EVDGLLR;YAAQSR	NCP	43.342
YAAQSR;LSDLPL	NCP	64.527

LSDLPL;SVLFLN	NCP	16.271
SVLFLN;KLYPPKP	NCP	20.82
KLYPPKP;KGKLF	NCP	116.35
KGKLF;LLVGVNK	NCP	80.672
LLVGVNK;LYMLPPP	NCP	92.953
LYMLPPP;KGLTSPK	NCP	25.319
KGLTSPK;TLAEPL	NCP	9.004
TLAEPL;MHVLDF	NCP	229.302
MHVLDF;GGKTAQQ	NCP	11.504
GGKTAQQ;KSGFKFK	NCP	8.037
KSGFKFK;KQKLGVP	NCP	30.922
KQKLGVP;FYESHTK	NCP	0.21
FYESHTK;NHESKPR	NCP	119.259
NHESKPR;ESKPFFG	NCP	19.31
ESKPFFG;ELLKV	NCP	25.485
ELLKV;KLSPL	NCP	25.011
KLSPL;LRLTEY	NCP	65.116
LRLTEY;FLNHTMF	NCP	168.363
FLNHTMF;KLVSNPP	NCP	31.685
KLVSNPP;KRAQKP	NCP	29.331
KRAQKP;VGQPGRK	NCP	9.942
VGQPGRK;KPVCPK	NCP	68.976
KPVCPK;GAFRMDF	NCP	41.967
GAFRMDF;LFAVP	NCP	36.84
LFAVP;SLFKDD	NCP	130.371
SLFKDD;ELHTMAK	NCP	70.725
ELHTMAK;VCLVDVAK	NCP	31.567
VCLVDVAK;NERPNLK	NCP	13.671
NERPNLK;KVYLLMPQ	NCP	180.087
KVYLLMPQ;GPKFPFL	NCP	7.283
GPKFPFL;LSVNPML	NCP	83.574
LSVNPML;QVGKCFK	NCP	27.531
QVGKCFK;QLPVK	NCP	1.869
QLPVK;PLATLPGP	NCP	76.317
PLATLPGP;SFVDVLY	NCP	47.509
SFVDVLY;HMVPQ	NCP	43.34
HMVPQ;LVLEGELE	NCP	51.801
LVLEGELE;APHYASPR	NCP	10.791
APHYASPR;TATPNPL	NCP	32.029
TATPNPL;LEGQLDF	NCP	212.204
LEGQLDF;GKFKLSLK	NCP	13.965
GKFKLSLK;QMFGEKL	NCP	141.852
QMFGEKL;KYPPPKY	NCP	263.503
KYPPPKY;TSPGDLR	NCP	148.233
TSPGDLR;LLTLV	NCP	125.919
LLTLV;AELEKF	NCP	24.786
AELEKF;WEYMWY	NCP	44.388
WEYMWY;LSAGFNK	NCP	16.184
LSAGFNK;AAQRVFLP	NCP	110.643
AAQRVFLP;KAAPNK	NCP	124.692
KAAPNK;KPMLGFK	NCP	99.273

KPMLGFK;EDDYGY	NCP	117.234
EDDYGY;QASKKKE	NCP	10.402
QASKKKE;LLTVKKL	NCP	6.861
LLTVKKL;LTFGYSK	NCP	0.273
LTFGYSK;MNFKVF	NCP	39.786
MNFKVF;LLVLLYFP	NCP	14.373
LLVLLYFP;KDVYVTF	NCP	165.26
KDVYVTF;YRLLEQPL	NCP	77.502
YRLLEQPL;SEEPVVL	NCP	81.625
SEEPVVL;RNLLNY	NCP	37.646
RNLLNY;KGVNVLK	NCP	205.48
KGVNVLK;AKFVL	NCP	124.002
AKFVL;LPRKLL	NCP	0.027
LPRKLL;AAKKLALF	NCP	71.535
AAKKLALF;YTKAAVGK	NCP	1.539
YTKAAVGK;TAVLVHT	NCP	5.72
TAVLVHT;VGLLTEA	NCP	20.275
VGLLTEA;KGTLFQK	NCP	14.4
KGTLFQK;DLKAPKL	NCP	63.135
DLKAPKL;VKKVALLG	NCP	109.932
VKKVALLG;KSVPAF	NCP	77.393
KSVPAF;LGNKLGK	NCP	82.008
LGNKLGK;GASGK	NCP	9.514
GASGK;RKNVKGD	NCP	2.298
RKNVKGD;KGLVLQR	NCP	207.486
KGLVLQR;VMNVV	NCP	14.942
VMNVV;KSYHYR	NCP	48.678
KSYHYR;EDSMSE	NCP	74.731
EDSMSE;GVLFL	NCP	7.988
GVLFL;MYDFVK	NCP	33.024
MYDFVK;LRKSLLVQ	NCP	35.235
LRKSLLVQ;LERMF	NCP	72.588
LERMF;AAKVPYY	NCP	51.804
AAKVPYY;DLGLPL	NCP	11.686
DLGLPL;SFRDNVN	NCP	8.133
SFRDNVN;KSMPPK	NCP	99.66
KSMPPK;KKTKTPG	NCP	56.309
KKTKTPG;TLLHA	NCP	10.389
TLLHA;VSKTLDP	NCP	64.077
VSKTLDP;FDLNNP	NCP	7.876
FDLNNP;ADLEH	NCP	241.737
ADLEH;KCHHAW	NCP	145.43
KCHHAW;HLYTL	NCP	45.028
HLYTL;KLVMEVK	NCP	254.064
KLVMEVK;LYAEAKK	NCP	59.226
LYAEAKK;RAECDD	NCP	99.324
RAECDD;DEEKLR	NCP	65.009
DEEKLR;LSELTPK	NCP	55.171
LSELTPK;LGPNDQYK	NCP	70.008
LGPNDQYK;VQSTPRL	NCP	42.83
VQSTPRL;LSPVELP	NCP	9.39

LSPVELP;GVTLAREK	NCP	10.36
GVTLAREK;GPQKAF	NCP	112.907
GPQKAF;FRANDK	NCP	77.137
FRANDK;HGPKRDP	NCP	6.537
HGPKRDP;NDFMKK	NCP	27.104
NDFMKK;AARGTAF	NCP	70.869
AARGTAF;QHLVTVR	NCP	26.272
QHLVTVR;DLLAFYY	NCP	7.577
DLLAFYY;LSGKNGR	NCP	32.415
LSGKNGR;RAFLSGAP	NCP	0.225
RAFLSGAP;KFQPLQP	NCP	33.807
KFQPLQP;KPQVHGK	NCP	74.491
KPQVHGK;GHSAGPR	NCP	16.022
GHSAGPR;VSPKSTPP	NCP	5.041
VSPKSTPP;HEVLYR	NCP	6.015
HEVLYR;KVSPLKS	NCP	71.403
KVSPLKS;RPAHRKCF	NCP	124.091
RPAHRKCF;KGYPYTY	NCP	36.459
KGYPYTY;FPLQPLL	NCP	37.911
FPLQPLL;ESRVHVH	NCP	13.254
ESRVHVH;RLGERGY	NCP	31.251
RLGERGY;KGRNQSLK	NCP	51.091
KGRNQSLK;VGRALAH	NCP	162.384
VGRALAH;KDTTCL	NCP	97.763
KDTTCL;DVTTEVK	NCP	43.413
DVTTEVK;GPPGP	NCP	2.751
GPPGP;KMAFLAT	NCP	27.019
KMAFLAT;KQLAK	NCP	19.458
KQLAK;VLEVAQH	NCP	1.905
VLEVAQH;LNFWK	NCP	33.704
LNFWK;CHVLPAP	NCP	15.847
CHVLPAP;QVSLK	NCP	33.866
QVSLK;LGSRGLAGP	NCP	144
LGSRGLAGP;LGVGR	NCP	122.919
LGVGR;PALTLF	NCP	30.732
PALTLF;ASSGTTDVTNR	NCP	31.854
ASSGTTDVTNR;CNDPPPP	NCP	0.834
CNDPPPP;RNPPFH	NCP	102.504
RNPPFH;AAGKGDVPTKRPPVLR	NCP	41.745
AAGKGDVPTKRPPVLR;VVFLPALCRK	NCP	0.135
VVFLPALCRK;TCTTVAFTQVNSEDKSALAK	NCP	0.093
TCTTVAFTQVNSEDKSALAK;TQVNSEDKSALA	NCP	0.021
TQVNSEDKSALA;NLFEVLY	NCP	50.077
NLFEVLY;KPVVYNK	NCP	94.365
KPVVYNK;GNNDV	NCP	26.54
GNNDV;RKDLDC	NCP	50.301
RKDLDC;KDVAYP	NCP	24.688
KDVAYP;YEKFT	NCP	88.841
YEKFT;RPGNDKR	NCP	63.676
RPGNDKR;FFVNHR	NCP	76.065
FFVNHR;KEFWAK	NCP	45.872

KEFWAK;KYHAAK	NCP	0.163
KYHAAK;YDPPLG	NCP	75.018
YDPPLG;GVSFSLMF	NCP	169.01
GVSFSLMF;HMNKK	NCP	24.426
HMNKK;VGQKLLVG	NCP	22.047
VGQKLLVG;LSLLPEYVVPY	NCP	42.393
LSLLPEYVVPY;ADESGPPAPSKPR	NCP	15.081
ADESGPPAPSKPR;KSLLFGY	NCP	106.483
KSLLFGY;VERLTF	NCP	22.584
VERLTF;VEKLVR	NCP	22.226
VEKLVR;AVSLVP	NCP	26.455
AVSLVP;HLSPKAGP	NCP	3.651
HLSPKAGP;HALQEK	NCP	8.075
HALQEK;PNWGTVGR	NCP	0.448
PNWGTVGR;TSGVGRLH	NCP	35.163
TSGVGRLH;KGVSQRK	NCP	6.552
KGVSQRK;TLEQQ	NCP	2.45
TLEQQ;DPPAPPVD	NCP	29.721
DPPAPPVD;TAPLPKL	NCP	81.276
TAPLPKL;VGVKPVK	NCP	14.641
VGVKPVK;EPASSLLP	NCP	12.234
EPASSLLP;VLVLP	NCP	12.542
VLVLP;VTKKGSK	NCP	63.126
VTKKGSK;KSLKPGK	NCP	3.187
KSLKPGK;GPVVLEF	NCP	78.506
GPVVLEF;QFVCKK	NCP	9.972
QFVCKK;NLAHHYR	NCP	89.152
NLAHHYR;KDAWAAF	NCP	88.89
KDAWAAF;RDDKPR	NCP	154.4
RDDKPR;FNKPPPP	NCP	35.523
FNKPPPP;SALEPAAPP	NCP	44.619
SALEPAAPP;KEAVLFG	NCP	22.896
KEAVLFG;MCDDV	NCP	23.4
MCDDV;AAAAALTK	NCP	29.584
AAAAALTK;PVKLHSK	NCP	52.4
PVKLHSK;AMAPMLF	NCP	9.172
AMAPMLF;DVGDWRK	NCP	83.517
DVGDWRK;TLGLLAF	NCP	62.126
TLGLLAF;HVLPL	NCP	39.912
HVLPL;VPPPKPP	NCP	11.733
VPPPKPP;FYNPKGP	NCP	32.581
FYNPKGP;LRLVENF	NCP	1.536
LRLVENF;VEFVELL	NCP	6.662
VEFVELL;LKVLASK	NCP	26.235
LKVLASK;QGTYLDF	NCP	72.265
QGTYLDF;SPGHVHR	NCP	19.658
SPGHVHR;TVNPRHP	NCP	32.373
TVNPRHP;QDMMC	NCP	40.434
QDMMC;VVAAAF	NCP	3.4
VVAAAF;SPPSPKVT	NCP	104.627
SPPSPKVT;HVCQGPK	NCP	119.835

HVCQGPK;GPVVK	NCP	9.931
GPVVK;KNVTW	NCP	38.301
KNVTW;QTEDK	NCP	0.885
QTEDK;LGGAVMK	NCP	123.233
LGGAVMK;CYCDC	NCP	165.022
CYCDC;KDLGLLC	NCP	6.642
KDLGLLC;PPGKLP	NCP	64.95
PPGKLP;KYVVHKR	NCP	77.567
KYVVHKR;GSSLKLEE	NCP	15.268
GSSLKLEE;SLLGK	NCP	26.51
SLLGK;VVDVGLK	NCP	51.132
VVDVGLK;PTMMCP	NCP	26.52
PTMMCP;QGVFPR	NCP	151.627
QGVFPR;EPYEPL	NCP	34.119
EPYEPL;LDVHVER	NCP	104.117
LDVHVER;QPGVYVT	NCP	32.413
QPGVYVT;MGMLKK	NCP	6.63
MGMLKK;MKLAVGL	NCP	35.412
MKLAVGL;KSPERPF	NCP	34.331
KSPERPF;KMVKAGP	NCP	33.541
KMVKAGP;KGGVDLL	NCP	57.752
KGGVDLL;LSHSTVTM	NCP	20.058
LSHSTVTM;VGAALK	NCP	14.53
VGAALK;LPGVDP	NCP	15.279
LPGVDP;FALEKYK	NCP	4.949
FALEKYK;RVKLVPV	NCP	6.6
RVKLVPV;LVSVER	NCP	47.889
LVSVER;TNAFVP	NCP	7.203
TNAFVP;RPHDLP	NCP	15.463
RPHDLP;LVLLQPAE	NCP	90.378
LVLLQPAE;KPSVCVP	NCP	0.522
KPSVCVP;KEEFEPL	NCP	18.365
KEEFEPL;LEAEALR	NCP	8.179
LEAEALR;EVHYFK	NCP	38.633
EVHYFK;ARNVP	NCP	23.343
ARNVP;DFRETSP	NCP	27.952
DFRETSP;RFAGTKK	NCP	30.513
RFAGTKK;TLPDPQY	NCP	1.538
TLPDPQY;PLVSYTL	NCP	32.295
PLVSYTL;EAALPELK	NCP	60.888
EAALPELK;EVFPPSA	NCP	2.922
EVFPPSA;SPGSFVT	NCP	62.347
SPGSFVT;LLSFQYE	NCP	90.197
LLSFQYE;LEADLP	NCP	43.975
LEADLP;KGCFDF	NCP	7.995
KGCFDF;KPQPFLK	NCP	90.563
KPQPFLK;VLADPR	NCP	68.151
VLADPR;KRVFR	NCP	28.92
KRVFR;GQLALF	NCP	51.994
GQLALF;KNLNQFVL	NCP	19.28
KNLNQFVL;NDVTKN	NCP	67.816

NDVTKN;ATYLEF	NCP	3.791
ATYLEF;PLRLLCD	NCP	66.571
PLRLLCD;KRTTLLR	NCP	4.992
KRTTLLR;VPTPFF	NCP	44.799
VPTPFF;AMELLK	NCP	87.53
AMELLK;ALPLML	NCP	135.315
ALPLML;VHAFLK	NCP	86.028
VHAFLK;DSEFLQ	NCP	19.272
DSEFLQ;GLGNLNN	NCP	40.515
GLGNLNN;VDLLGF	NCP	18.718
VDLLGF;KPWYVK	NCP	39.917
KPWYVK;TGDGKLL	NCP	276.138
TGDGKLL;YHLCTKH	NCP	62.382
YHLCTKH;KQELTVP	NCP	19.279
KQELTVP;KAFYSK	NCP	51.438
KAFYSK;KHSLQGK	NCP	55.2
KHSLQGK;LDPEVLN	NCP	163.31
LDPEVLN;LYASFK	NCP	32.581
LYASFK;MDFQLQ	NCP	251.931
MDFQLQ;LRMVQF	NCP	88.469
LRMVQF;WSKGCKL	NCP	52.231
WSKGCKL;LDLKEK	NCP	76.974
LDLKEK;TLGEEKF	NCP	40.388
TLGEEKF;TGNHPR	NCP	14.188
TGNHPR;ESTEKPF	NCP	121.767
ESTEKPF;FSVQTK	NCP	133.931
FSVQTK;NELLTHV	NCP	69.501
NELLTHV;RLSTRVPL	NCP	95.899
RLSTRVPL;HLSSVLHK	NCP	25.485
HLSSVLHK;TLPVK	NCP	1.878
TLPVK;KLTMAH	NCP	11.313
KLTMAH;KSPHLGY	NCP	44.039
KSPHLGY;VSELPTK	NCP	17.697
VSELPTK;ATVVTP	NCP	49.801
ATVVTP;KALKLLTK	NCP	10.562
KALKLLTK;APLGP	NCP	4.278
APLGP;LDSKGPK	NCP	110.898
LDSKGPK;DKLSYE	NCP	11.658
DKLSYE;KPYVPR	NCP	42.736
KPYVPR;QTKPTR	NCP	96.849
QTKPTR;QPGFVPH	NCP	53.787
QPGFVPH;VTTQVPR	NCP	81.695
VTTQVPR;DEKAKGK	NCP	10.002
DEKAKGK;TVLVVW	NCP	19.959
TVLVVW;KPEFP	NCP	165.754
KPEFP;LEFDKK	NCP	10.182
LEFDKK;KPVDVSR	NCP	107.477
KPVDVSR;KLTFSLC	NCP	21.405
KLTFSLC;LKLDLRK	NCP	131.334
LKLDLRK;KPYFFK	NCP	27.462
KPYFFK;LHGVGKVF	NCP	169.282

LHGVGKVF;ETHGKPK	NCP	31.698
ETHGKPK;ALFKVPT	NCP	19.347
ALFKVPT;VLALLEN	NCP	116.345
VLALLEN;VLLVY	NCP	24.819
VLLVY;KVYRTN	NCP	114.216
KVYRTN;KPEHAKK	NCP	25.192
KPEHAKK;LLLSWTLKK	NCP	143.76
LLLSWTLKK;KASSLNK	NCP	64.497
KASSLNK;PPETPF	NCP	50.067
PPETPF;VLGPPW	NCP	39.12
VLGPPW;LQKVGK	NCP	31.925
LQKVGK;LELKPHLS	NCP	67.165
LELKPHLS;LTGLAAQ	NCP	69.597
LTGLAAQ;KESRLSF	NCP	8.856
KESRLSF;KTGPAP	NCP	34.203
KTGPAP;ATGACSF	NCP	16.089
ATGACSF;VSTTALGK	NCP	26.703
VSTTALGK;MAKLELR	NCP	142.007
MAKLELR;PGYKGY	NCP	102.375
PGYKGY;KAVAVLQ	NCP	16.878
KAVAVLQ;CNCDAP	NCP	128.134
CNCDAP;HDSPPPR	NCP	46.737
HDSPPPR;KVFAYR	NCP	0.986
KVFAYR;LLAAPGP	NCP	113.446
LLAAPGP;RASHVLNV	NCP	37.566
RASHVLNV;FGVPAKK	NCP	80.258
FGVPAKK;MPLQTMLL	NCP	40.18
MPLQTMLL;ASKLMKF	NCP	12.461
ASKLMKF;LEGTYLDK	NCP	7
LEGTYLDK;VSLVEL	NCP	4.056
VSLVEL;KELPLEL	NCP	10.083
KELPLEL;NHLVYH	NCP	16.583
NHLVYH;KQLTLPL	NCP	12.039
KQLTLPL;VGTVTLPL	NCP	38.569
VGTVTLPL;LPVKCPK	NCP	22.965
LPVKCPK;FSLKTDQ	NCP	55.185
FSLKTDQ;KPTSCPPP	NCP	101.501
KPTSCPPP;KSSVAPK	NCP	44.896
KSSVAPK;MPAPKPP	NCP	36.441
MPAPKPP;TPGLLSQQ	NCP	56.937
TPGLLSQQ;TTKVKK	NCP	125.508
TTKVKK;KHGFYAK	NCP	30.771
KHGFYAK;ERYVLPL	NCP	21.264
ERYVLPL;LSWYDN	NCP	23.286
LSWYDN;FGPLPLH	NCP	121.605
FGPLPLH;SLVFKPQ	NCP	4.311
SLVFKPQ;ETLDV	NCP	13.743
ETLDV;LPDFMR	NCP	32.843
LPDFMR;PHLALTDL	NCP	36.967
PHLALTDL;KVDTVHSP	NCP	20.741
KVDTVHSP;VKLVTVK	NCP	30.24

VKLVTVK;EFTAL	NCP	57.669
EFTAL;PRGTHKK	NCP	20.413
PRGTHKK;KTSGT	NCP	105.438
KTSGT;QEDLSLP	NCP	22.71
QEDLSLP;CGMSM	NCP	181.185
CGMSM;CPAALKK	NCP	97.887
CPAALKK;LPPTLPL	NCP	176.54
LPPTLPL;QPCLKK	NCP	45.237
QPCLKK;SPKYTKL	NCP	87.615
SPKYTKL;KEGVPVL	NCP	26.653
KEGVPVL;VTAFKKL	NCP	12.569
VTAFKKL;RDYAPK	NCP	5.353
RDYAPK;LSHSLLVT	NCP	100.433
LSHSLLVT;LSSKKGP	NCP	53.448
LSSKKGP;KNPLP	NCP	5.223
KNPLP;MLVGPV	NCP	56.359
MLVGPV;KLSTGWV	NCP	135.863
KLSTGWV;YPCCC	NCP	74.847
YPCCC;RASGKKAA	NCP	7.372
RASGKKAA;GPVSVLN	NCP	20.427
GPVSVLN;KNYVPV	NCP	23.213
KNYVPV;EVYLLHF	NCP	44.352
EVYLLHF;NPTVFF	NCP	54.268
NPTVFF;KNFYK	NCP	81.053
KNFYK;FLEKPEDL	NCP	27.352
FLEKPEDL;NYHLLPF	NCP	108.476
NYHLLPF;GKDLALP	NCP	6.786
GKDLALP;LEKVYSLSK	NCP	195.45
LEKVYSLSK;VLALANQ	NCP	105.978
VLALANQ;MLRAGGL	NCP	23.707
MLRAGGL;VTNFFKK	NCP	8.913
VTNFFKK;DGSLNFR	NCP	151.356
DGSLNFR;SPKSKKARVP	NCP	68.36
SPKSKKARVP;RAMLRP	NCP	93.678
RAMLRP;LRTTA	NCP	24.304
LRTTA;LSGLLPK	NCP	117.431
LSGLLPK;LSVKMVP	NCP	4.09
LSVKMVP;PVLLH	NCP	46.374
PVLLH;PLAHKTP	NCP	57.905
PLAHKTP;ALMGF	NCP	41.796
ALMGF;PFAAVLYL	NCP	7.122
PFAAVLYL;KEVLALN	NCP	53.217
KEVLALN;AMCWVA	NCP	57.783
AMCWVA;NKVAPV	NCP	30.505
NKVAPV;ADTLFL	NCP	60.128
ADTLFL;LGKFTEVL	NCP	0.508
LGKFTEVL;AVELL	NCP	69.915
AVELL;SLQDLHR	NCP	66.818
SLQDLHR;LDLGHPK	NCP	87.303
LDLGHPK;HSLGLLNV	NCP	20.121
HSLGLLNV;KMFPL	NCP	36.301

KMFPL;TVPSLP	NCP	26.085
TVPSLP;ERFNLN	NCP	20.67
ERFNLN;AYWPFEL	NCP	14.567
AYWPFEL;FMGVLQG	NCP	43.656
FMGVLQG;KLLWLY	NCP	24.918
KLLWLY;EFCFSPK	NCP	2.98
EFCFSPK;NSNVLR	NCP	10.388
NSNVLR;FPPPPEVP	NCP	121.125
FPPPPEVP;FALTHKAK	NCP	5.343
FALTHKAK;GDLRYF	NCP	37.357
GDLRYF;KNGGPQ	NCP	7.82
KNGGPQ;RMVVAPA	NCP	66.354
RMVVAPA;QRHFGLQ	NCP	33.123
QRHFGLQ;ATLGSGY	NCP	5.988
ATLGSGY;KTVDPK	NCP	5.119
KTVDPK;PLGPELLK	NCP	49.385
PLGPELLK;DDLGFEF	NCP	32.103
DDLGFEF;PFGMLKN	NCP	57.045
PFGMLKN;PAPGSPF	NCP	139.584
PAPGSPF;SLPPCPP	NCP	48.247
SLPPCPP;VGPNRHP	NCP	32.127
VGPNRHP;AAPLLG	NCP	205.646
AAPLLG;EVNANLT	NCP	101.833
EVNANLT;LSGPL	NCP	1.772
LSGPL;KGGVLDR	NCP	20.35
KGGVLDR;MSFST	NCP	5.147
MSFST;QLLNVKS	NCP	49.768
QLLNVKS;TVAKSPK	NCP	23.469
TVAKSPK;ESALTAY	NCP	7.181
ESALTAY;LPPFHKT	NCP	38.905
LPPFHKT;LPAAYN	NCP	11.403
LPAAYN;RVVLLGV	NCP	38.733
RVVLLGV;RKQAAPAK	NCP	38.219
RKQAAPAK;PPLSYK	NCP	13.605
PPLSYK;LDGAPAW	NCP	7.933
LDGAPAW;KFFYKPK	NCP	116.073
KFFYKPK;KFRCRP	NCP	136.854
KFRCRP;LPFPPP	NCP	35.33
LPFPPP;KNKGPLTV	NCP	111.754
KNKGPLTV;FAPGEN	NCP	62.109
FAPGEN;KEVPHK	NCP	10.433
KEVPHK;KLVDLF	NCP	34.987
KLVDLF;ESYKAG	NCP	47.571
ESYKAG;KYLPFLP	NCP	27.344
KYLPFLP;LDLGEDYPSGKK	NCP	33.789
LDLGEDYPSGKK;LGEDYPSGK	NCP	0.006
LGEDYPSGK;VLVGETGSGK	NCP	7.278
VLVGETGSGK;TLDAGKF	NCP	21.189
TLDAGKF;CDDDGS	NCP	261.369
CDDDGS;KPKKKKC	NCP	2.193
KPKKKKC;LMKFF	NCP	11.403

LMKFF;DSVETKK	NCP	8.196
DSVETKK;PAGMGKK	NCP	17.211
PAGMGKK;ELPKYKS	NCP	9.003
ELPKYKS;LKLLLC	NCP	111.517
LKLLLC;TVVPPAN	NCP	74.517
TVVPPAN;LPPPYRK	NCP	10.355
LPPPYRK;KVFYVP	NCP	63.309
KVFYVP;DVAVY	NCP	15.451
DVAVY;NSLHCCN	NCP	38.189
NSLHCCN;LAVGY	NCP	218.512
LAVGY;QVSLP	NCP	11.885
QVSLP;GTMAKKY	NCP	61.002
GTMAKKY;GRPTMK	NCP	13.753
GRPTMK;HSEFLKA	NCP	152.658
HSEFLKA;LQDLELK	NCP	15.414
LQDLELK;LEPPKER	NCP	88.472
LEPPKER;VTPEEVL	NCP	7.902
VTPEEVL;KDSHLSL	NCP	92.583
KDSHLSL;LVLPK	NCP	39.471
LVLPK;VPEVHE	NCP	55.101
VPEVHE;ATTNF	NCP	41.508
ATTNF;TTLDDKL	NCP	0.754
TTLDDKL;QKLPKQ	NCP	53.078
QKLPKQ;SLFEKKP	NCP	102.604
SLFEKKP;MMMSYE	NCP	43.538
MMMSYE;KPPRFCP	NCP	62.98
KPPRFCP;KNSFMH	NCP	25.214
KNSFMH;KERVKLVQ	NCP	15.951
KERVKLVQ;KMLHPP	NCP	124.996
KMLHPP;KDYLKVK	NCP	24.597
KDYLKVK;TYFPLPQ	NCP	42.603
TYFPLPQ;TATTEVK	NCP	49.977
TATTEVK;LSCKYLPP	NCP	15.786
LSCKYLPP;PPNLSK	NCP	1.563
PPNLSK;RMRVVR	NCP	65.006
RMRVVR;EPRYGKQ	NCP	19.264
EPRYGKQ;ENETLK	NCP	42
ENETLK;KNYLVLEC	NCP	13.665
KNYLVLEC;MSAPLLR	NCP	21.095
MSAPLLR;VVLKGDP	NCP	40.234
VVLKGDP;LHLFLEP	NCP	91.847
LHLFLEP;KDYAGRS	NCP	26.104
KDYAGRS;TQRKLAV	NCP	0.09
TQRKLAV;YDGVLA	NCP	11.637
YDGVLA;GKDVVNT	NCP	4.748
GKDVVNT;RPVEF	NCP	47.515
RPVEF;SAAPFP	NCP	17.036
SAAPFP;ESSLRYR	NCP	6.592
ESSLRYR;PVTRSTP	NCP	7.77
PVTRSTP;FVRFR	NCP	5.922
FVRFR;FFYPLLF	NCP	97.283

FFYPLLF;LGRGFY	NCP	177.393
LGRGFY;KELNYFA	NCP	17.85
KELNYFA;GSRLW	NCP	96.993
GSRLW;LADFAA	NCP	130.653
LADFAA;TERLLK	NCP	81.205
TERLLK;TAANGF	NCP	48.653
TAANGF;MPPLCPK	NCP	99.757
MPPLCPK;LYKKSKGL	NCP	1.802
LYKKSKGL;CCPVKKQ	NCP	16.744
CCPVKKQ;EVPFL	NCP	27.8
EVPFL;TVGKLP	NCP	50.197
TVGKLP;FTSMKLP	NCP	54.224
FTSMKLP;ASVVVKT	NCP	438.421
ASVVVKT;TVMRAEP	NCP	99.896
TVMRAEP;PMTEGP	NCP	136.971
PMTEGP;LQKEQLF	NCP	262.809
LQKEQLF;NVKKWY	NCP	190.815
NVKKWY;LKLSYRL	NCP	63.012
LKLSYRL;LALLLLMP	NCP	44.464
LALLLLMP;NLPVDN	NCP	23.267
NLPVDN;HFLLPF	NCP	84.342
HFLLPF;YGYHF	NCP	137.043
YGYHF;VALDF	NCP	22.81
VALDF;HEEPTLLT	NCP	18.398
HEEPTLLT;NFVST	NCP	6.784
NFVST;KLLHVPP	NCP	5.79
KLLHVPP;VVDVPQ	NCP	25.628
VVDVPQ;TLTLDLPT	NCP	33.63
TLTLDLPT;YGVYFK	NCP	56.86
YGVYFK;DPGGPV	NCP	37.319
DPGGPV;MEEEVLR	NCP	53.574
MEEEVLR;LVRGKHL	NCP	110.487
LVRGKHL;EPKGVPG	NCP	39.48
EPKGVPG;KPGFK	NCP	292.995
KPGFK;VVNPYK	NCP	130.449
VVNPYK;LSLTELLK	NCP	113.37
LSLTELLK;MMHLLR	NCP	214.14
MMHLLR;MGLLFLH	NCP	34.05
MGLLFLH;HVTAEPK	NCP	15.39
HVTAEPK;LTLLYK	NCP	170.263
LTLLYK;KFYTSAK	NCP	34.452
KFYTSAK;NRHQPK	NCP	29.153
NRHQPK;PVVGVVA	NCP	57.349
PVVGVVA;NDLRMK	NCP	53.421
NDLRMK;AQTLLLF	NCP	21.606
AQTLLLF;KKDGEVR	NCP	32.393
KKDGEVR;LSWHSTL	NCP	191.724
LSWHSTL;LSESALDK	NCP	211.132
LSESALDK;KTLVSPV	NCP	34.314
KTLVSPV;LFEPGL	NCP	13.868
LFEPGL;KTKVPGK	NCP	19.689

KTKVPGK;PAASRVTPP	NCP	211.437
PAASRVTPP;KNTSLGEL	NCP	35.64
KNTSLGEL;LSGAGLPY	NCP	33.18
LSGAGLPY;KKPVFVP	NCP	86.296
KKPVFVP;NPPPHQK	NCP	64.763
NPPPHQK;SLPLLDF	NCP	5.926
SLPLLDF;EAPVLTPG	NCP	192.422
EAPVLTPG;MLKCGH	NCP	58.434
MLKCGH;MVLLFLLPP	NCP	19.051
MVLLFLLPP;AAWLK	NCP	173.63
AAWLK;GVLTTQC	NCP	14.299
GVLTTQC;KGVLELP	NCP	10.908
KGVLELP;NAKPLLC	NCP	5.766
NAKPLLC;AFFPQ	NCP	41.291
AFFPQ;AEPAYSP	NCP	166.821
AEPAYSP;YVTAL	NCP	12.702
YVTAL;TKDVLSVAF	NCP	25.084
TKDVLSVAF;EEVVAKL	NCP	61.695
EEVVAKL;VGVSF	NCP	86.222
VGVSF;EGLVF	NCP	64.801
EGLVF;KSPHLLK	NCP	44.847
KSPHLLK;SLDTLYP	NCP	18.614
SLDTLYP;FKQQR	NCP	169.27
FKQQR;WFPPM	NCP	29.544
WFPPM;LDLTPM	NCP	20.315
LDLTPM;VKMTNY	NCP	5.073
VKMTNY;LQGPLR	NCP	69.516
LQGPLR;CATLWNK	NCP	68.94
CATLWNK;VLSKY	NCP	74.692
VLSKY;MSLDPKY	NCP	31.121
MSLDPKY;AEYTEL	NCP	4.219
AEYTEL;KKLPSP	NCP	7.925
KKLPSP;KSGKYVL	NCP	199.084
KSGKYVL;KLSVGAP	NCP	76.253
KLSVGAP;WYYTSSK	NCP	63.742
WYYTSSK;HESFLSK	NCP	92.735
HESFLSK;VFEKPKA	NCP	78.319
VFEKPKA;GPPPAPPK	NCP	39.516
GPPPAPPK;ADVLDLH	NCP	24.983
ADVLDLH;VNPGLVL	NCP	157.945
VNPGLVL;KLLPRLF	NCP	4.233
KLLPRLF;QKHPVLQ	NCP	77.247
QKHPVLQ;VDLDF	NCP	5.642
VDLDF;HKVPPPP	NCP	57.574
HKVPPPP;LATATLY	NCP	103.926
LATATLY;KGAACPK	NCP	128.144
KGAACPK;TPPEAKQ	NCP	289.432
TPPEAKQ;KEELHL	NCP	73.404
KEELHL;VPLYFLP	NCP	20.331
VPLYFLP;KDCGF	NCP	40.166
KDCGF;GPVEELK	NCP	111.079

GPVEELK;VDKPVF	NCP	30.197
VDKPVF;AAPLSLY	NCP	31.299
AAPLSLY;TGLKADVK	NCP	74.722
TGLKADVK;DKSVSYC	NCP	4.499
DKSVSYC;KPFCVGP	NCP	19.114
KPFCVGP;LFVKGPP	NCP	117.095
LFVKGPP;PAKEC	NCP	79.024
PAKEC;HTAFLK	NCP	12.896
HTAFLK;LAPHALLTW	NCP	43.647
LAPHALLTW;TSLDDSLP	NCP	102.063
TSLDDSLP;RFENLEK	NCP	17.881
RFENLEK;VGSNLLL	NCP	136.292
VGSNLLL;MTFLPE	NCP	45.396
MTFLPE;RSRAAPK	NCP	67.227
RSRAAPK;KPKKFPL	NCP	21.456
KPKKFPL;LNVGCFE	NCP	140.332
LNVGCFE;KEVCSYK	NCP	24.458
KEVCSYK;LPWLS	NCP	21.375
LPWLS;SGVLPLKK	NCP	284.866
SGVLPLKK;NTMLLQ	NCP	72.921
NTMLLQ;KSLGYGK	NCP	191.105
KSLGYGK;ALAAF	NCP	223.9
ALAAF;MPSLP	NCP	128.237
MPSLP;KLLADQT	NCP	10.368
KLLADQT;LETYL	NCP	15.945
LETYL;ADDLKRF	NCP	32.301
ADDLKRF;ANELGKK	NCP	19.704
ANELGKK;KCHKMKLQ	NCP	7.321
KCHKMKLQ;KPLYGLP	NCP	49.605
KPLYGLP;FPTTK	NCP	48.261
FPTTK;STTHVPK	NCP	41.612
STTHVPK;ALTTVVPL	NCP	31.963
ALTTVVPL;EPLKPK	NCP	161.258
EPLKPK;FVTMGKP	NCP	18.004
FVTMGKP;LRVTLALGP	NCP	142.068
LRVTLALGP;VFPLVK	NCP	45.144
VFPLVK;EPNPFSK	NCP	45.27
EPNPFSK;KEMSVVP	NCP	0.03
KEMSVVP;KPGRHTK	NCP	0.834
KPGRHTK;FLNWGKN	NCP	64.029
FLNWGKN;NLCKTK	NCP	3.764
NLCKTK;FDKAKGK	NCP	19.908
FDKAKGK;HMCLR	NCP	49.375
HMCLR;DLSQVVH	NCP	14.394
DLSQVVH;KEGHPGL	NCP	108.93
KEGHPGL;GVHDHFG	NCP	39.807
GVHDHFG;AAHQVL	NCP	21.764
AAHQVL;LGGPP	NCP	1.374
LGGPP;SSLKEEEH	NCP	23.872
SSLKEEEH;RLAFKP	NCP	44.858
RLAFKP;KRVLFS	NCP	54.96

KRVLFS;KHVMLK	NCP	112.933
KHVMLK;NSLNQEFL	NCP	191.603
NSLNQEFL;KLLQY	NCP	21.895
KLLQY;KYYAF	NCP	54.311
KYYAF;TNLLTGK	NCP	88.467
TNLLTGK;ETPLMSP	NCP	32.2
ETPLMSP;TTFGRC	NCP	33.5
TTFGRC;VNHKDLP	NCP	9.391
VNHKDLP;VVTKK	NCP	0.063
VVTKK;LEPVPVQK	NCP	124.793
LEPVPVQK;SPPPVAAVTSPP	NCP	1.83
SPPPVAAVTSPP;EDTEDWRPR	NCP	10.809
EDTEDWRPR;DTEDWRP	NCP	0.003
DTEDWRP;TEDWRPR	NCP	0.003
TEDWRPR;HVFSPKYTKL	NCP	1.026
HVFSPKYTKL;DWHHEVSA	NCP	0.033
DWHHEVSA;HHEVSARALN	NCP	0.006
HHEVSARALN;ARALNVQ	NCP	0.015
ARALNVQ;ATAYY	NCP	65.35
ATAYY;RPGQFK	NCP	63.747
RPGQFK;KKSLVSK	NCP	59.393
KKSLVSK;PNDLDLR	NCP	53.34
PNDLDLR;MLDFPP	NCP	9.513
MLDFPP;SVVPMN	NCP	7.71
SVVPMN;KNFSPPP	NCP	110.596
KNFSPPP;NAPSFP	NCP	27.585
NAPSFP;LESLPFLK	NCP	18.845
LESLPFLK;MTMTSR	NCP	168.979
MTMTSR;MMGHT	NCP	13.677
MMGHT;LVPLEKL	NCP	37.373
LVPLEKL;TLELYKK	NCP	4.552
TLELYKK;FYAPELL	NCP	117.801
FYAPELL;YAPELLYY	NCP	0.003
YAPELLYY;LVVSTQTALA	NCP	11.373
LVVSTQTALA;EHLSTKK	NCP	58.358
EHLSTKK;SALNRLFY	NCP	32.905
SALNRLFY;KFVKPLR	NCP	22.125
KFVKPLR;PKGHRTK	NCP	22.632
PKGHRTK;TTQSTLLK	NCP	82.2
TTQSTLLK;ADHLVL	NCP	177.773
ADHLVL;RADAPTP	NCP	187.258
RADAPTP;ASTVCLLL	NCP	99.347
ASTVCLLL;ATVGLK	NCP	1.542
ATVGLK;QCVPLTL	NCP	21.868
QCVPLTL;MLKTNPK	NCP	118.908
MLKTNPK;YKLPPPP	NCP	45.066
YKLPPPP;TPTSKYR	NCP	3.906
TPTSKYR;KLHYVV	NCP	35.175
KLHYVV;PENVAPR	NCP	74.244
PENVAPR;LELSQH	NCP	32.6
LELSQH;LELLNNM	NCP	47.028

LELLNNM;LKAAEDY	NCP	25.192
LKAAEDY;TTCKLK	NCP	61.188
TTCKLK;MLPLCP	NCP	28.112
MLPLCP;PHLALMR	NCP	62.329
PHLALMR;FPPKY	NCP	2.765
FPPKY;KPLFDP	NCP	20.833
KPLFDP;APHVARP	NCP	14.249
APHVARP;RPPSGE	NCP	49.873
RPPSGE;KFEQK	NCP	8.57
KFEQK;KPPKENP	NCP	146.05
KPPKENP;MKPAVD	NCP	101.492
MKPAVD;QESDLRLT	NCP	135.586
QESDLRLT;TLAEDLM	NCP	39.831
TLAEDLM;PLSTSP	NCP	67.112
PLSTSP;YLLKAAT	NCP	182.587
YLLKAAT;MFPAAK	NCP	58.428
MFPAAK;NPSKRFF	NCP	19.214
NPSKRFF;LTASWLK	NCP	2.362
LTASWLK;LAFMRYL	NCP	10.857
LAFMRYL;LYVKTK	NCP	33.777
LYVKTK;LLLRHADP	NCP	36.813
LLLRHADP;LSHKSTAT	NCP	56.987
LSHKSTAT;QNGVGLK	NCP	15.706
QNGVGLK;NFDRPP	NCP	24.807
NFDRPP;VTGNSPL	NCP	124.199
VTGNSPL;VSGKHVF	NCP	15.823
VSGKHVF;LPGEMSP	NCP	14.499
LPGEMSP;KACCNTK	NCP	93.978
KACCNTK;PAQKNYQ	NCP	24.653
PAQKNYQ;PAKKPPP	NCP	7.447
PAKKPPP;YARALLH	NCP	99.149
YARALLH;EENDEVLVAGFGR	NCP	64.522
EENDEVLVAGFGR;PPPPPLA	NCP	14.604
PPPPPLA;WMPVK	NCP	86.499
WMPVK;AQHLGVK	NCP	10.215
AQHLGVK;QGLKLLSH	NCP	7.374
QGLKLLSH;LLLFELK	NCP	208.88
LLLFELK;TQDLRK	NCP	46.083
TQDLRK;SSMLLQG	NCP	26.014
SSMLLQG;LGGDPL	NCP	41.454
LGGDPL;MFLHERK	NCP	68.771
MFLHERK;TRTFSPP	NCP	2.566
TRTFSPP;MQEAPTC	NCP	43.487
MQEAPTC;KNVDLLSDMV	NCP	34.386
KNVDLLSDMV;VLEFHFEPNEYFTNEVLTK	NCP	0.111
VLEFHFEPNEYFTNEVLTK;VAPGKTF	NCP	217.006
VAPGKTF;EDFSTFP	NCP	0.821
EDFSTFP;AAVETRVCETDGCSSEAK	NCP	106.377
AAVETRVCETDGCSSEAK;TGKPDPPP	NCP	103.266
TGKPDPPP;KQKNMGP	NCP	50.356
KQKNMGP;LEVAL	NCP	2.055

LEVAL;LDGHTSSK	NCP	206.691
LDGHTSSK;PVLAALLR	NCP	11.034
PVLAALLR;DGLLHRK	NCP	82.328
DGLLHRK;QNPKPK	NCP	66.099
QNPKPK;HTTGLSLNNDRLYK	NCP	39.678
HTTGLSLNNDRLYK;LTVYGPK	NCP	6.342
LTVYGPK;LYEVSQL	NCP	51.619
LYEVSQL;AEGFLQ	NCP	116.306
AEGFLQ;LPHLRP	NCP	68.872
LPHLRP;LDVPP	NCP	60.675
LDVPP;AVSDALFP	NCP	51.528
AVSDALFP;GSSLKLE	NCP	13.553
GSSLKLE;VSATLSF	NCP	44.668
VSATLSF;LTPSLGP	NCP	63.747
LTPSLGP;TAGDFKLK	NCP	7.725
TAGDFKLK;LLSGGLVA	NCP	28.893
LLSGGLVA;TKGGVLP	NCP	9.783
TKGGVLP;DPFMGK	NCP	83.859
DPFMGK;ETKYG	NCP	12.57
ETKYG;NPNLP	NCP	21.944
NPNLP;MDMDDT	NCP	120.547
MDMDDT;KDPPTPP	NCP	27.813
KDPPTPP;EVLDPFL	NCP	8.741
EVLDPFL;FDTSYSF	NCP	17.238
FDTSYSF;YAPLG	NCP	37.519
YAPLG;SYTLGL	NCP	255.068
SYTLGL;RAVKSV	NCP	22.326
RAVKSV;DEKAKSK	NCP	63.097
DEKAKSK;EPFPLK	NCP	83.45
EPFPLK;CHTALLLL	NCP	219.406
CHTALLLL;LEMFWTL	NCP	105.99
LEMFWTL;AAAGLAGSPP	NCP	18.182
AAAGLAGSPP;FAMPPPPPP	NCP	1.86
FAMPPPPPP;LAGLLKF	NCP	60.625
LAGLLKF;HLEAPPP	NCP	132.308
HLEAPPP;VVLVRVSL	NCP	3.219
VVLVRVSL;EEYLAK	NCP	53.455
EEYLAK;KKMVAK	NCP	31.476
KKMVAK;DTMVVF	NCP	0.609
DTMVVF;NPEKVPK	NCP	31.425
NPEKVPK;EPGDDLR	NCP	9.177
EPGDDLR;TGRSLLKL	NCP	14.471
TGRSLLKL;VPPFTFH	NCP	66.655
VPPFTFH;KFMSPGF	NCP	2.556
KFMSPGF;RYSSKQP	NCP	36
RYSSKQP;TVMVAAR	NCP	1.412
TVMVAAR;SPLELL	NCP	75.514
SPLELL;TFLGNST	NCP	22.598
TFLGNST;NPLKPEGE	NCP	149.197
NPLKPEGE;WHAPVR	NCP	51.294
WHAPVR;KPQEDLK	NCP	31.941

KPQEDLK;ATFRLAL	NCP	48.93
ATFRLAL;MRKASP	NCP	48.822
MRKASP;KSVKKDP	NCP	20.798
KSVKKDP;CCSYEP	NCP	10.02
CCSYEP;SFDKAKL	NCP	32.007
SFDKAKL;EDFSTPF	NCP	52.053
EDFSTPF;HLAPPPPP	NCP	158.847
HLAPPPPP;CLSLFQP	NCP	1.654
CLSLFQP;THLALP	NCP	37.398
THLALP;SHLESLT	NCP	124.085
SHLESLT;LTAELAP	NCP	47.493
LTAELAP;VPTKGTLH	NCP	5.592
VPTKGTLH;VLESVAR	NCP	107.415
VLESVAR;CTSVLKQ	NCP	48.358
CTSVLKQ;KENPLP	NCP	31.035
KENPLP;TQHFKPP	NCP	5.663
TQHFKPP;KNYGPK	NCP	1.048
KNYGPK;LGLFDT	NCP	52.866
LGLFDT;KLAKAL	NCP	9.489
KLAKAL;TVFPNF	NCP	28.029
TVFPNF;TLPTPTPL	NCP	23.433
TLPTPTPL;SVDLAAF	NCP	43.416
SVDLAAF;MERPVR	NCP	10.037
MERPVR;ALLPLPAF	NCP	48.897
ALLPLPAF;CHTYEGL	NCP	55.317
CHTYEGL;AALPSRVK	NCP	172.689
AALPSRVK;DSLGFKG	NCP	50.547
DSLGFKG;YKMKGDYHR	NCP	6.429
YKMKGDYHR;KVLMSVSL	NCP	28.621
KVLMSVSL;MRSVLY	NCP	13.115
MRSVLY;NPRGPP	NCP	37.126
NPRGPP;QTWLKL	NCP	19.475
QTWLKL;ESFGDL	NCP	1.723
ESFGDL;YYLKLLK	NCP	19.193
YYLKLLK;HGAPKP	NCP	24.649
HGAPKP;KAHFVVL	NCP	16.193
KAHFVVL;KELRDEK	NCP	6.052
KELRDEK;NVVTPGK	NCP	24.684
NVVTPGK;KSKKPR	NCP	211.436
KSKKPR;RTLLETDL	NCP	13.719
RTLLETDL;LTLSLPW	NCP	85.027
LTLSLPW;LPPSRPP	NCP	114.578
LPPSRPP;LVALPQ	NCP	19.438
LVALPQ;KHTLPK	NCP	114.27
KHTLPK;GVSLK	NCP	1.352
GVSLK;LTTRSPQ	NCP	7.003
LTTRSPQ;PAAKLVL	NCP	41.259
PAAKLVL;KDMCSKP	NCP	4.407
KDMCSKP;KYLSKPP	NCP	28.272
KYLSKPP;KPGMRGK	NCP	72.558
KPGMRGK;DNHYGK	NCP	21.176

DNHYGK;VVFLLHFF	NCP	30.324
VVFLLHFF;CNLLNKPR	NCP	45.436
CNLLNKPR;PAAVRSPP	NCP	19.464
PAAVRSPP;AAAAAGPGAP	NCP	6.573
AAAAAGPGAP;GYGMDL	NCP	2.267
GYGMDL;KDEGKGK	NCP	61.747
KDEGKGK;KTDQPL	NCP	36.605
KTDQPL;DFLVLVD	NCP	9.115
DFLVLVD;AALVL	NCP	127.503
AALVL;KWMSDH	NCP	16.401
KWMSDH;LHLFPLE	NCP	10.484
LHLFPLE;VGLSRVVP	NCP	54.16
VGLSRVVP;EVDLLK	NCP	10.269
EVDLLK;KMVGPLL	NCP	11.735
KMVGPLL;KEYVPL	NCP	94.219
KEYVPL;VHVELPP	NCP	24.353
VHVELPP;VTLVER	NCP	17.14
VTLVER;LSDVHTK	NCP	52.109
LSDVHTK;SVPKVPK	NCP	55.203
SVPKVPK;LAAAAKAK	NCP	2.581
LAAAAKAK;KQPTKET	NCP	16.055
KQPTKET;KQAAHVL	NCP	36.142
KQAAHVL;LAKKAAN	NCP	21.132
LAKKAAN;ALEVLPR	NCP	2.223
ALEVLPR;LDKVPR	NCP	59.331
LDKVPR;LTAELLLE	NCP	4.901
LTAELLLE;VPGAYSP	NCP	173.757
VPGAYSP;TDMRVT	NCP	38.664
TDMRVT;PKNLYPP	NCP	16.78
PKNLYPP;CLGKWCL	NCP	103.781
CLGKWCL;FHSSRP	NCP	16.447
FHSSRP;VPDLGAPL	NCP	87.386
VPDLGAPL;KTDTHVQ	NCP	9.948
KTDTHVQ;KNLKPHL	NCP	140.331
KNLKPHL;RQKFVF	NCP	35.545
RQKFVF;KGELLDL	NCP	50.651
KGELLDL;CGGPDLR	NCP	12.714
CGGPDLR;LPPTKFR	NCP	52.57
LPPTKFR;ATGDAVLK	NCP	34.031
ATGDAVLK;EPSVPR	NCP	14.389
EPSVPR;KPPRLRC	NCP	119.621
KPPRLRC;PSLCAPPK	NCP	6.952
PSLCAPPK;NSPPLR	NCP	11.99
NSPPLR;LPYPQ	NCP	8.766
LPYPQ;LSGVDLRLL	NCP	31.707
LSGVDLRLL;GTFNLG	NCP	64.48
GTFNLG;EEELDDC	NCP	66.849
EEELDDC;LPERPGP	NCP	104.778
LPERPGP;LSMVFLSK	NCP	2.924
LSMVFLSK;ANDLRK	NCP	69.283
ANDLRK;FVGPPPP	NCP	89.589

FVGPPPP;EVVLL	NCP	50.459
EVVLL;KVGLVTK	NCP	10.975
KVGLVTK;YYPMGK	NCP	36.195
YYPMGK;LQLLF	NCP	4.44
LQLLF;RPNSAT	NCP	21.075
RPNSAT;VVRVLK	NCP	1.782
VVRVLK;LSLKPTP	NCP	10.68
LSLKPTP;KVLKTQLP	NCP	12.995
KVLKTQLP;EHFDNK	NCP	169.653
EHFDNK;DDTFWK	NCP	11.311
DDTFWK;APKAYR	NCP	71.318
APKAYR;ELVAV	NCP	104.314
ELVAV;FEKPLE	NCP	1.815
FEKPLE;LPSRKF	NCP	6.815
LPSRKF;KHFDLM	NCP	3.958
KHFDLM;AGELL	NCP	28.328
AGELL;EENDP	NCP	51.957
EENDP;VSVLMSH	NCP	67.762
VSVLMSH;SLLEELLT	NCP	41.292
SLLEELLT;TGVSGNK	NCP	105.852
TGVSGNK;NSLGRLE	NCP	44.069
NSLGRLE;NPYRAK	NCP	48.94
NPYRAK;KLLLCCCC	NCP	68.525
KLLLCCCC;LEKLEK	NCP	60.261
LEKLEK;AGLHFFNPVPL	NCP	16.809
AGLHFFNPVPL;VGLDTTKF	NCP	13.329
VGLDTTKF;LETSAFK	NCP	39.858
LETSAFK;AAVKPPR	NCP	3.657
AAVKPPR;DYVRAPP	NCP	14.562
DYVRAPP;LLGFFF	NCP	88.6
LLGFFF;ENVPHSK	NCP	76.752
ENVPHSK;KTDFNLK	NCP	37.482
KTDFNLK;PLGASAPGP	NCP	54.366
PLGASAPGP;HSGSLSYF	NCP	124.911
HSGSLSYF;KAEVLVT	NCP	1.359
KAEVLVT;RVNCML	NCP	9.752
RVNCML;KMNQLFH	NCP	38.229
KMNQLFH;LFGPALG	NCP	67.692
LFGPALG;MESLYRVPF	NCP	10.89
MESLYRVPF;RYHRY	NCP	4.264
RYHRY;ALLVNVF	NCP	9.683
ALLVNVF;PGVRL	NCP	71.46
PGVRL;LPPTSYR	NCP	31.654
LPPTSYR;LSGHLDLL	NCP	17.606
LSGHLDLL;VTDRL	NCP	6.6
VTDRL;MEVKPLC	NCP	89.433
MEVKPLC;AQQKNLEGY	NCP	13.549
AQQKNLEGY;LMVVGESGLGK	NCP	0.093
LMVVGESGLGK;RSRKLAAVT	NCP	0.771
RSRKLAAVT;AETPKPL	NCP	32.79
AETPKPL;FNLLGKLK	NCP	7.578

FNLLGKLK;NDLFQAAK	NCP	5.541
NDLFQAAK;VELGC	NCP	37.187
VELGC;HSLVLT	NCP	25.381
HSLVLT;KAFFMMT	NCP	36.959
KAFFMMT;PSAHAQDL	NCP	27.87
PSAHAQDL;ALPLK	NCP	9.81
ALPLK;HLMYAK	NCP	24.853
HLMYAK;RLKVLRK	NCP	10.358
RLKVLRK;KQKCDT	NCP	19.351
KQKCDT;DVPLVNP	NCP	60.051
DVPLVNP;FHVKQR	NCP	3.35
FHVKQR;KNLYTFFL	NCP	35.241
KNLYTFFL;APGPK	NCP	151.941
APGPK;FGLWYT	NCP	41.617
FGLWYT;AAAPAVPK	NCP	37.896
AAAPAVPK;FPDSGFF	NCP	55.392
FPDSGFF;RSVHYP	NCP	10.389
RSVHYP;MECAP	NCP	24.179
MECAP;LLELN	NCP	107.685
LLELN;KMEGTEV	NCP	111.912
KMEGTEV;GHWGGGKK	NCP	24.451
GHWGGGKK;YALSK	NCP	59.225
YALSK;MEKVVP	NCP	8.646
MEKVVP;YGMYGY	NCP	60.207
YGMYGY;EHTGTAF	NCP	12.637
EHTGTAF;WLLQVEP	NCP	223.935
WLLQVEP;KGVLF	NCP	20.961
KGVLF;YGKEC	NCP	40.806
YGKEC;NPLHVSEK	NCP	83.513
NPLHVSEK;VLDVMEK	NCP	20.272
VLDVMEK;GLPPKGM	NCP	4.415
GLPPKGM;SCCEFL	NCP	20.793
SCCEFL;YLKPPPPP	NCP	40.657
YLKPPPPP;KKGVRT	NCP	7.326
KKGVRT;LPLVA	NCP	10.796
LPLVA;VAQRPSGH	NCP	13.914
VAQRPSGH;KVFYPQR	NCP	19.47
KVFYPQR;PQQGS	NCP	120.423
PQQGS;ASAGTVPR	NCP	105.834
ASAGTVPR;MVAMLK	NCP	50.286
MVAMLK;LSREQHSL	NCP	39.378
LSREQHSL;GPAMPPP	NCP	199.318
GPAMPPP;KLDDLTK	NCP	199.169
KLDDLTK;GVCYVAL	NCP	15.106
GVCYVAL;KPTMLVP	NCP	96.722
KPTMLVP;NAVEFFT	NCP	109.969
NAVEFFT;TNMPGLK	NCP	30.02
TNMPGLK;CVLVA	NCP	21.631
CVLVA;VADPLQY	NCP	18.401
VADPLQY;VSEPGTM	NCP	120.514
VSEPGTM;LDKSKPL	NCP	66.539

LDKSKPL;LPMSGPPP	NCP	5.278
LPMSGPPP;LPGCLPTP	NCP	17.066
LPGCLPTP;LLLVHQ	NCP	33.379
LLLVHQ;SAGPDKK	NCP	6.914
SAGPDKK;GLEVPHK	NCP	115.258
GLEVPHK;VTDLVFK	NCP	38.378
VTDLVFK;VGLPHPPP	NCP	65.119
VGLPHPPP;EVDLNLK	NCP	104.226
EVDLNLK;DDDDDDDC	NCP	94.517
DDDDDDDC;VLVVVFF	NCP	60.343
VLVVVFF;MDGSCM	NCP	18.245
MDGSCM;LKQNSKKP	NCP	24.568
LKQNSKKP;REGPPK	NCP	32.769
REGPPK;LFVGVAA	NCP	5.406
LFVGVAA;TEVSSLPLVK	NCP	1.595
TEVSSLPLVK;QPGWPVK	NCP	27.865
QPGWPVK;KQTVTPL	NCP	177.813
KQTVTPL;QRTVPTL	NCP	12.302
QRTVPTL;HLMPAPP	NCP	32.58
HLMPAPP;KSRHQY	NCP	24.031
KSRHQY;TDTEKPF	NCP	60.38
TDTEKPF;PLTGTTPL	NCP	53.749
PLTGTTPL;KFAAP	NCP	204.323
KFAAP;YGAPSP	NCP	0.457
YGAPSP;FLDPK	NCP	53.543
FLDPK;SSSVVPPPPP	NCP	16.42
SSSVVPPPPP;KERLR	NCP	27.221
KERLR;DASLLNF	NCP	2.281
DASLLNF;DSVSPPR	NCP	17.166
DSVSPPR;VGPKMLY	NCP	2.46
VGPKMLY;PVLGMVVLL	NCP	49.956
PVLGMVVLL;LFQQTLN	NCP	29.333
LFQQTLN;AAVKKLAL	NCP	2.875
AAVKKLAL;YFENPLP	NCP	60.227
YFENPLP;KKQFALVS	NCP	60.123
KKQFALVS;GTPPW	NCP	1.438
GTPPW;KNFFELLY	NCP	19.463
KNFFELLY;EVDKAAP	NCP	5.487
EVDKAAP;VSGMTFL	NCP	70.686
VSGMTFL;GSNSLDF	NCP	39.862
GSNSLDF;PHSSAKGK	NCP	39.549
PHSSAKGK;KMKLTPP	NCP	81.846
KMKLTPP;TVGMPL	NCP	51.579
TVGMPL;KETLPFDK	NCP	127.115
KETLPFDK;KEVAAHT	NCP	7.252
KEVAAHT;RHGLER	NCP	89.246
RHGLER;KLLKP	NCP	16.929
KLLKP;KECFRP	NCP	69.3
KECFRP;KMARYH	NCP	265.488
KMARYH;VPLLG	NCP	21.337
VPLLG;NVDVYDKR	NCP	77.726

NVDVYDKR;YRHVASP	NCP	15.754
YRHVASP;KSLRVTLL	NCP	5.718
KSLRVTLL;ASSSSLEK	NCP	42.954
ASSSSLEK;LPPVVLLP	NCP	9.828
LPPVVLLP;KSLHCCK	NCP	39.389
KSLHCCK;LGEEWERL	NCP	76.425
LGEEWERL;TAVFPFR	NCP	28.656
TAVFPFR;YLSDPTK	NCP	97.225
YLSDPTK;PVHLLPFL	NCP	73.469
PVHLLPFL;NLSEKRA	NCP	15.138
NLSEKRA;KVEVPKL	NCP	19.471
KVEVPKL;LTDKTTLY	NCP	41.069
LTDKTTLY;AGARFVF	NCP	12.69
AGARFVF;KKAACK	NCP	5.592
KKAACK;FTGAL	NCP	18.708
FTGAL;APPFLL	NCP	159.592
APPFLL;KTTNF	NCP	59.915
KTTNF;VVPGGDL	NCP	7.041
VVPGGDL;RKENK	NCP	67.384
RKENK;TTLPLSLK	NCP	57.731
TTLPLSLK;KYLTFPP	NCP	20.43
KYLTFPP;WDAFLLV	NCP	77.139
WDAFLLV;VEMSFK	NCP	204.676
VEMSFK;QMFLTYM	NCP	3.687
QMFLTYM;CLLPATV	NCP	7.517
CLLPATV;TVGLL	NCP	68.41
TVGLL;AVFPEYL	NCP	40.02
AVFPEYL;AFLDLRK	NCP	87.519
AFLDLRK;TDAEAALL	NCP	56.784
TDAEAALL;ESDYDF	NCP	59.555
ESDYDF;CLLMLK	NCP	3.994
CLLMLK;KFRLPK	NCP	73.569
KFRLPK;PVVVGV	NCP	193.359
PVVVGV;KLLVCCD	NCP	237.375
KLLVCCD;LSNYP	NCP	0.402
LSNYP;KNFHKKT	NCP	0.383
KNFHKKT;LHKFVM	NCP	68.974
LHKFVM;PDVFPK	NCP	1.179
PDVFPK;HPYLTEQ	NCP	70.701
HPYLTEQ;EPDLGAR	NCP	13.812
EPDLGAR;PRAPPGP	NCP	8.238
PRAPPGP;KLVSANK	NCP	11.037
KLVSANK;GVEKFDK	NCP	128.397
GVEKFDK;MLSVPPML	NCP	103.893
MLSVPPML;EVYDKP	NCP	56.369
EVYDKP;LGSKETQP	NCP	31.302
LGSKETQP;DPPMLR	NCP	67.303
DPPMLR;RTLLTF	NCP	35.699
RTLLTF;CCNCPP	NCP	75.121
CCNCPP;AAGPSL	NCP	40.386
AAGPSL;ELPFYQ	NCP	73.781

ELPFYQ;RGPLRK	NCP	110.122
RGPLRK;ATEKPDLS	NCP	3.585
ATEKPDLS;PSYKL	NCP	71.604
PSYKL;QRGVLGL	NCP	3.572
QRGVLGL;KLRLGLK	NCP	58.71
KLRLGLK;HTDQPAT	NCP	69.334
HTDQPAT;KPEYK	NCP	22.094
KPEYK;GVKLGL	NCP	28.195
GVKLGL;KLVNFN	NCP	96.17
KLVNFN;VEESPLL	NCP	169.486
VEESPLL;LLLPTLF	NCP	22.479
LLLPTLF;YAHLLPF	NCP	14.768
YAHLLPF;QDCAALP	NCP	127.755
QDCAALP;VPGLPALS	NCP	9.99
VPGLPALS;LVVDFKP	NCP	33.828
LVVDFKP;GVALLQPL	NCP	7.954
GVALLQPL;LSVNAPP	NCP	42.75
LSVNAPP;HQVLY	NCP	54.557
HQVLY;KTHFVKL	NCP	70.782
KTHFVKL;VPAASKE	NCP	7.054
VPAASKE;NPAAWKP	NCP	9.771
NPAAWKP;QPGTPPP	NCP	7.46
QPGTPPP;KGMRCK	NCP	27.672
KGMRCK;GSTYLEC	NCP	120.651
GSTYLEC;LMSSLKG	NCP	6.093
LMSSLKG;APMVKK	NCP	47.698
APMVKK;AVNLHKP	NCP	46.799
AVNLHKP;RSPAAKP	NCP	18.394
RSPAAKP;CCLKVK	NCP	132.047
CCLKVK;QTGQLSLF	NCP	57.315
QTGQLSLF;KMASPY	NCP	28.954
KMASPY;KQDVWHQ	NCP	21.405
KQDVWHQ;GRVGWP	NCP	83.382
GRVGWP;KDLLA	NCP	2.357
KDLLA;GPVVTFK	NCP	70.555
GPVVTFK;AGWKP	NCP	19.163
AGWKP;NVSGELL	NCP	20.461
NVSGELL;LPLQT	NCP	12.252
LPLQT;DLNVNR	NCP	10.739
DLNVNR;VLMYGY	NCP	90.376
VLMYGY;AAEKVLR	NCP	30.6
AAEKVLR;KDYFK	NCP	44.186
KDYFK;SAAVVTLLGAEK	NCP	84.063
SAAVVTLLGAEK;SESLSEKQVKEAKSK	NCP	2.898
SESLSEKQVKEAKSK;ESLSEKQVKEAK	NCP	0.003
ESLSEKQVKEAK;LLTDAPNPNSK	NCP	0.06
LLTDAPNPNSK;MEMLPDTTGKGALM	NCP	0.3
MEMLPDTTGKGALM;EMLPDTTGK	NCP	0.003
EMLPDTTGK;REPDAAQTDGLRT	NCP	0.114
REPDAAQTDGLRT;RKEEESVRVQSS	NCP	0.054
RKEEESVRVQSS;EEESVRVQSSVSK	NCP	0.006

EEESVRVQSSVSK;TAKPFPGSGN	NCP	0.144
TAKPFPGSGN;ADFPAPPP	NCP	0.078
ADFPAPPP;FTFKEPK	NCP	0.249
FTFKEPK;TFKEPKVSPNPELL	NCP	0.003
TFKEPKVSPNPELL;FKEPKVSPNPE	NCP	0.003
FKEPKVSPNPE;KEPKVSPNPELL	NCP	0.003
KEPKVSPNPELL;SLLQNSEGK	NCP	0.036
SLLQNSEGK;TPPPVAPKPAVK	NCP	0.129
TPPPVAPKPAVK;AGPFKGPQ	NCP	0.21
AGPFKGPQ;YTPKPAAPTPTVNTAH	NCP	0.045
YTPKPAAPTPTVNTAH;TPKPAAPTPTVNT	NCP	0.003
TPKPAAPTPTVNT;VGPSNELPGMSGK	NCP	0.054
VGPSNELPGMSGK;VVDSDTVQAHAAR	NCP	0.084
VVDSDTVQAHAAR;VDSDTVQAH	NCP	0.003
VDSDTVQAH;TPSLPAGW	NCP	0.048
TPSLPAGW;KKGKKPLNALDVMK	NCP	0.147
KKGKKPLNALDVMK;TFQPPDAKDGLPAKPSVK	NCP	0.075
TFQPPDAKDGLPAKPSVK;DAKDGLPAKP	NCP	0.015
DAKDGLPAKP;KDGLPAKPSVK	NCP	0.006
KDGLPAKPSVK;FVAPRPK	NCP	0.246
FVAPRPK;VAPRPKFSAKK	NCP	0.003
VAPRPKFSAKK;VQVWKPSVVEE	NCP	0.045
VQVWKPSVVEE;CHFEACK	NCP	3.375
CHFEACK;KAFLPPPP	NCP	1.426
KAFLPPPP;LPGRPVPP	NCP	25.652
LPGRPVPP;TSKPTDGLEEAKKR	NCP	0.072
TSKPTDGLEEAKKR;SKPTDGLEEAKK	NCP	0.003
SKPTDGLEEAKK;LVDEAFRPR	NCP	0.078
LVDEAFRPR;VLPGQSEEWNARL	NCP	0.078
VLPGQSEEWNARL;YVPQTQK	NCP	0.042
YVPQTQK;KVFSAK	NCP	5.748
KVFSAK;LSAKSPK	NCP	4.792
LSAKSPK;LSGRLSP	NCP	42.666
LSGRLSP;KEVVKT	NCP	19.43
KEVVKT;MLSHNTMVK	NCP	24.693
MLSHNTMVK;KLKYLK	NCP	83.241
KLKYLK;KPVRVL	NCP	27.339
KPVRVL;FPKASY	NCP	42.708
FPKASY;CVEKGF	NCP	36.588
CVEKGF;ADPGFPL	NCP	33.217
ADPGFPL;SEPFHR	NCP	148.499
SEPFHR;DNDDD	NCP	38.674
DNDDD;APAGVTY	NCP	10.431
APAGVTY;KSVAPP	NCP	63.225
KSVAPP;GPSLPP	NCP	13.086
GPSLPP;LAFGVKK	NCP	61.305
LAFGVKK;YSVDF	NCP	82.463
YSVDF;EHTPRP	NCP	39.615
EHTPRP;GEHAYFP	NCP	13.384
GEHAYFP;QEGSKSK	NCP	52.458
QEGSKSK;FLVKEK	NCP	13.329

FLVKEK;GPSLL	NCP	12.158
GPSLL;PQLSVGK	NCP	139.791
PQLSVGK;REFVKLL	NCP	5.728
REFVKLL;MVAPK	NCP	50.973
MVAPK;VEADF	NCP	67.667
VEADF;SLGTPLK	NCP	30.772
SLGTPLK;QTLLSK	NCP	51.932
QTLLSK;LMKLLTER	NCP	0.832
LMKLLTER;YDLKEPY	NCP	49.886
YDLKEPY;KDSHRSPP	NCP	63.223
KDSHRSPP;VRTVAYK	NCP	5.859
VRTVAYK;DRKPVL	NCP	39.813
DRKPVL;NLEVWVK	NCP	146.672
NLEVWVK;VEEGF	NCP	15.631
VEEGF;VSHVPSP	NCP	9.975
VSHVPSP;PFKFDK	NCP	35.349
PFKFDK;NLRLMLK	NCP	64.185
NLRLMLK;TLEDLPY	NCP	73.077
TLEDLPY;PAPPAPPPPPP	NCP	45.315
PAPPAPPPPPP;GVPEAQL	NCP	58.439
GVPEAQL;CAPPPLAL	NCP	46.69
CAPPPLAL;PSLVGRPR	NCP	50.832
PSLVGRPR;TNRVLFL	NCP	1.644
TNRVLFL;LLLLGN	NCP	12.27
LLLLGN;KLNFKK	NCP	15.878
KLNFKK;KFFAAL	NCP	5.406
KFFAAL;KDGWELF	NCP	0.475
KDGWELF;RPPEC	NCP	75.009
RPPEC;KGGVGL	NCP	98.724
KGGVGL;LDLLS	NCP	81.587
LDLLS;LVHVEY	NCP	19.407
LVHVEY;LKAPP	NCP	41.977
LKAPP;VLGLALF	NCP	96.264
VLGLALF;NSGGFPPPPP	NCP	31.449
NSGGFPPPPP;DTPLAGF	NCP	57.057
DTPLAGF;RNVSLDLP	NCP	19.565
RNVSLDLP;ETSGKPPPPP	NCP	42.547
ETSGKPPPPP;PPSRPPPK	NCP	26.358
PPSRPPPK;VGFPKSA	NCP	64.683
VGFPKSA;KSLSLQGK	NCP	26.745
KSLSLQGK;KAKSLHH	NCP	12.927
KAKSLHH;TLYGFN	NCP	13.811
TLYGFN;KKPQGPK	NCP	51.51
KKPQGPK;KPTAHQL	NCP	80.454
KPTAHQL;TAPPAPPRP	NCP	2.329
TAPPAPPRP;APPPTKP	NCP	11.67
APPPTKP;PAASPK	NCP	27.993
PAASPK;CMCCEC	NCP	92.139
CMCCEC;YRAGKQL	NCP	15.252
YRAGKQL;VPAVEL	NCP	7.949
VPAVEL;TAELPC	NCP	25.777

TAELPC;WNFFHGK	NCP	23.243
WNFFHGK;LDLGGLK	NCP	91.308
LDLGGLK;GPPVFW	NCP	47.857
GPPVFW;VGSRRVQ	NCP	14.076
VGSRRVQ;ELLLHLPL	NCP	12.327
ELLLHLPL;VAWPLK	NCP	52.589
VAWPLK;NMPGF	NCP	8.091
NMPGF;HNESYR	NCP	29.904
HNESYR;WSLGCG	NCP	18.049
WSLGCG;PGGSLFR	NCP	4.589
PGGSLFR;KLLNKRT	NCP	33.438
KLLNKRT;ESLKKAL	NCP	6.045
ESLKKAL;SGLPRVYL	NCP	50.104
SGLPRVYL;STTTVPR	NCP	21.879
STTTVPR;VTPFE	NCP	6.813
VTPFE;FGEMDF	NCP	20.352
FGEMDF;VGPPERP	NCP	9.285
VGPPERP;APGDHSA	NCP	31.173
APGDHSA;GARGP	NCP	60.858
GARGP;SVCMVD	NCP	11.222
SVCMVD;LQLEY	NCP	5.709
LQLEY;PLDPP	NCP	4.873
PLDPP;QQLVVV	NCP	53.189
QQLVVV;LLPHPP	NCP	13.141
LLPHPP;KPAAGRH	NCP	5.415
KPAAGRH;ERPPKP	NCP	10.155
ERPPKP;PGGKR	NCP	6.465
PGGKR;KASLLDAP	NCP	29.93
KASLLDAP;VLHLFL	NCP	17.445
VLHLFL;KAQGPTAP	NCP	16.927
KAQGPTAP;AFRSKP	NCP	1.634
AFRSKP;PLGGP	NCP	52.213
PLGGP;QRETPPP	NCP	54.777
QRETPPP;AQVLH	NCP	5.135
AQVLH;MCEDLR	NCP	1.825
MCEDLR;AAVHGPPK	NCP	4.965
AAVHGPPK;AAHARPP	NCP	3.102
AAHARPP;DPSLPQAP	NCP	12.102
DPSLPQAP;PGVDPPR	NCP	60.663
PGVDPPR;TALSRH	NCP	60.032
TALSRH;NLKAALK	NCP	137.964
NLKAALK;RAPPKAN	NCP	70.008
RAPPKAN;SQFPKAK	NCP	2.572
SQFPKAK;PLLVF	NCP	16.782
PLLVF;ALETKF	NCP	2.334
ALETKF;DKSSQPL	NCP	35.643
DKSSQPL;ALSKVHF	NCP	24.927
ALSKVHF;KPAPAKVETKPK	NCP	59.162
KPAPAKVETKPK;PAPAKVETKP	NCP	0.003
PAPAKVETKP;APAKVETKPK	NCP	0.003
APAKVETKPK;ATVRVAP	NCP	22.669

ATVRVAP;LEVLEK	NCP	30.78
LEVLEK;GKLMLPK	NCP	90.174
GKLMLPK;EATAG	NCP	40.038
EATAG;PAATSKAK	NCP	161.175
PAATSKAK;AAFPR	NCP	37.344
AAFPR;RTPEFLR	NCP	25.524
RTPEFLR;GRAPNPR	NCP	12.105
GRAPNPR;KLGMWKP	NCP	53.232
KLGMWKP;GAVPPPK	NCP	7.368
GAVPPPK;AFALL	NCP	113.948
AFALL;GVLKVFN	NCP	20.301
GVLKVFN;PAARPPAL	NCP	68.269
PAARPPAL;PRAAPRP	NCP	17.763
PRAAPRP;FTCEYL	NCP	12.599
FTCEYL;AAPMLQL	NCP	90.076
AAPMLQL;VEATPL	NCP	48.078
VEATPL;AAPRYK	NCP	32.19
AAPRYK;LPFGGPL	NCP	3.897
LPFGGPL;VSPKVPP	NCP	26.321
VSPKVPP;ADPQP	NCP	0.049
ADPQP;SPGAAR	NCP	7.713
SPGAAR;ESPLVL	NCP	18.788
ESPLVL;NSSYCSW	NCP	17.632
NSSYCSW;CWLLVHL	NCP	57.345
CWLLVHL;MDMCCP	NCP	18.264
MDMCCP;QPPPRPT	NCP	24.306
QPPPRPT;PFLLSQLE	NCP	3.933
PFLLSQLE;FGPGGP	NCP	61.599
FGPGGP;CPGTLR	NCP	33.51
CPGTLR;RDPRPP	NCP	29.901
RDPRPP;PFVTP	NCP	21.602
PFVTP;PPPQLPPR	NCP	37.135
PPPQLPPR;QNTTELR	NCP	1.218
QNTTELR;KPTVPGK	NCP	115.793
KPTVPGK;KLLAERS	NCP	35.7
KLLAERS;YGQQKR	NCP	13.053
YGQQKR;AASKGTL	NCP	45.643
AASKGTL;VGHLLPF	NCP	27.489
VGHLLPF;PAAVVKK	NCP	16.262
PAAVVKK;KAAPPSH	NCP	98.749
KAAPPSH;KEMVL	NCP	4.037
KEMVL;AAERLPPP	NCP	7.99
EDRRPTL;SPARDFQ	NCP	35.719
SPARDFQ;AALQERL	NCP	47.046
AALQERL;WLGGSLL	NCP	27.048
WLGGSLL;TAPGGLLH	NCP	69.557
TAPGGLLH;LQSVNQGK	NCP	155.517
LQSVNQGK;DSGFF	NCP	135.778
DSGFF;EESCC	NCP	64.689
EESCC;QPVASLQR	NCP	72.848
QPVASLQR;EDFDLLL	NCP	56.769

EDFDLLL;LDLFSHK	NCP	65.454
LDLFSHK;VSPKRF	NCP	17.106
VSPKRF;ATGFLLL	NCP	28.809
ATGFLLL;AAVFLPA	NCP	19.06
AAVFLPA;ATPPSPPK	NCP	82.661
ATPPSPPK;LATAEAW	NCP	24.105
LATAEAW;KLELTML	NCP	5.673
KLELTML;MFGPK	NCP	18.996
MFGPK;KPYGWK	NCP	218.461
KPYGWK;LLPTEFL	NCP	26.222
LLPTEFL;RGVVESH	NCP	139.623
RGVVESH;STWLLLH	NCP	166.077
STWLLLH;PPLETP	NCP	6.258
PPLETP;AAQLP	NCP	72.754
AAQLP;KPVLLVG	NCP	109.253
KPVLLVG;CCCGSE	NCP	35.661
CCCGSE;VARLT	NCP	6.022
VARLT;AEAPGLR	NCP	76.907
AEAPGLR;EPAASR	NCP	139.129
EPAASR;FADEKLF	NCP	283.676
FADEKLF;LADLAL	NCP	4.066
LADLAL;SYTAY	NCP	4.614
SYTAY;ERMFLS	NCP	32.864
ERMFLS;ASPPARSPPP	NCP	89.613
ASPPARSPPP;PTMRPK	NCP	29.154
PTMRPK;AAVDL	NCP	66.381
AAVDL;RGHLER	NCP	200.677
RGHLER;AAELR	NCP	204.153
AAELR;AASLEDP	NCP	106.277
AASLEDP;PQTGN	NCP	32.481
PQTGN;NPVLS	NCP	24.484
NPVLS;PPVLDKP	NCP	174.59
PPVLDKP;DWDLRK	NCP	25.749
DWDLRK;EKKER	NCP	5.814
EKKER;ALPPF	NCP	7.665
ALPPF;LPKTLTP	NCP	83.274
LPKTLTP;GVVMDL	NCP	66.579
GVVMDL;GVVNVFF	NCP	48.489
GVVNVFF;LSALVDKGS	NCP	75.336
LSALVDKGS;GSLLEC	NCP	37.956
GSLLEC;LPPVP	NCP	186.297
LPPVP;QLHETTY	NCP	12.633
QLHETTY;VVVERVP	NCP	69.762
VVVERVP;APAKFN	NCP	44.913
APAKFN;PFTAQQ	NCP	22.662
PFTAQQ;TKELHC	NCP	4.776
TKELHC;CVFGEKL	NCP	4.608
CVFGEKL;TATPP	NCP	95.466
TATPP;LSTTKPPP	NCP	71.583
LSTTKPPP;KGVHGPK	NCP	2.514
KGVHGPK;EKGTTHL	NCP	163.08

EKGTTHL;NVEHLQK	NCP	259.728
NVEHLQK;VEKLHCV	NCP	34.485
VEKLHCV;RACKFH	NCP	109.941
RACKFH;SLRCTTF	NCP	106.548
SLRCTTF;SSMYLKK	NCP	24.984
SSMYLKK;PPPYP	NCP	160.83
PPPYP;LGAQL	NCP	47.922
LGAQL;LPPYK	NCP	124.332
LPPYK;VKVFNHL	NCP	200.976
VKVFNHL;ATPLQLR	NCP	220.599
ATPLQLR;KAHPVAV	NCP	84.216
KAHPVAV;ELHQKL	NCP	90.036
ELHQKL;DPRVEF	NCP	70.845
DPRVEF;AGQNKFR	NCP	32.094
AGQNKFR;KDAKKNK	NCP	490.806
KDAKKNK;KPPAAFY	NCP	25.818
KPPAAFY;KQWLEF	NCP	25.557
KQWLEF;RDYRLL	NCP	20.992
RDYRLL;LVGANLE	NCP	79.76
LVGANLE;TVHYLLR	NCP	623.715
TVHYLLR;TPPGK	NCP	116.211
TPPGK;RSFKQQ	NCP	74.727
RSFKQQ;YYSFNFF	NCP	26.298
YYSFNFF;PAQLR	NCP	186.34
PAQLR;RLLVKLY	NCP	60.545
RLLVKLY;MMVLTH	NCP	8.457
MMVLTH;AARKLAGP	NCP	20.344
AARKLAGP;RPKVTY	NCP	140.027
RPKVTY;CCMSGP	NCP	31.749
CCMSGP;MGMDC	NCP	45.57
MGMDC;VDPNLQ	NCP	146.358
VDPNLQ;LPPKKYK	NCP	34.539
LPPKKYK;AELNLGP	NCP	86.209
AELNLGP;AGRYGKP	NCP	84.635
AGRYGKP;KFGTLPL	NCP	21.774
KFGTLPL;KTYVQF	NCP	22.437
KTYVQF;LSMSPLEL	NCP	99.705
LSMSPLEL;HPPLFFT	NCP	81.036
HPPLFFT;KYKSRPP	NCP	68.544
KYKSRPP;GCVLQAK	NCP	46.2
GCVLQAK;SEVPTPK	NCP	108.27
SEVPTPK;KEPREKQ	NCP	62.466
KEPREKQ;LLLPDALA	NCP	154.818
LLLPDALA;AYMFD	NCP	86.637
AYMFD;LGACKAA	NCP	34.338
LGACKAA;GLVSLVLP	NCP	6.141
GLVSLVLP;RPLKLSPLQ	NCP	0.279
RPLKLSPLQ;VLPKLYVKL	NCP	30.153
VLPKLYVKL;KPNNP	NCP	238.359
KPNNP;KEVGFF	NCP	26.667
KEVGFF;VDMVPGKPMC	NCP	246.834

VDMVPGKPMC;EFLEKTR	NCP	88.684
EFLEKTR;KEALMLP	NCP	106.716
KEALMLP;DLGGAPL	NCP	4.226
DLGGAPL;DVLNFRK	NCP	281.41
DVLNFRK;LGEEFK	NCP	31.71
LGEEFK;PATVPGVK	NCP	108.534
PATVPGVK;NSMQPK	NCP	49.178
NSMQPK;EALPGRHR	NCP	26.008
EALPGRHR;VGGGAVL	NCP	23.238
VGGGAVL;AQTPKLGP	NCP	9.168
AQTPKLGP;KLTNWD	NCP	47.603
KLTNWD;FELVHG	NCP	13.459
FELVHG;LLKASPK	NCP	13.542
LLKASPK;QLPEVLAG	NCP	166.698
QLPEVLAG;MYMCCC	NCP	109.539
MYMCCC;KFLPCL	NCP	9.957
KFLPCL;MNKYN	NCP	58.586
MNKYN;VYVVFS	NCP	27.481
VYVVFS;TSLLK	NCP	76.977
TSLLK;TLRLLQT	NCP	19.277
TLRLLQT;GVPPH	NCP	14.956
GVPPH;QTLLDY	NCP	16.223
QTLLDY;AKVTPP	NCP	216.514
AKVTPP;NPGEFQ	NCP	35.166
NPGEFQ;KSNEPGK	NCP	9.219
KSNEPGK;KTTAYLH	NCP	18.404
KTTAYLH;KSGHPVPP	NCP	131.541
KSGHPVPP;SWVCKK	NCP	117.744
SWVCKK;RKHGVVPL	NCP	81.178
RKHGVVPL;KLHAYR	NCP	3.059
KLHAYR;LDPGPL	NCP	19.126
LDPGPL;KAAPTP	NCP	8.043
KAAPTP;KPVLDPPT	NCP	101.679
KPVLDPPT;LCSFNQT	NCP	87.026
LCSFNQT;ALKEKR	NCP	47.104
ALKEKR;KGGALVV	NCP	59.436
KGGALVV;KTRQN	NCP	24.062
KTRQN;LSDPLVD	NCP	14.599
LSDPLVD;VKVYNY	NCP	11.195
VKVYNY;SPVVRWK	NCP	28.656
SPVVRWK;NHDPK	NCP	92.935
NHDPK;KASFWAPK	NCP	15.68
KASFWAPK;TTAKGVLK	NCP	23.092
TTAKGVLK;KKTVSPP	NCP	6.876
KKTVSPP;TAHPEY	NCP	14.391
TAHPEY;SAEKAYHEQL	NCP	73.095
SAEKAYHEQL;AEKAYHEQLS	NCP	0.003
AEKAYHEQLS;EKAYHEQL	NCP	0.003
EKAYHEQL;KGLLSVGK	NCP	49.193
KGLLSVGK;DRLPPDH	NCP	2.307
DRLPPDH;NGVTKLF	NCP	79.563

NGVTKLF;SPVVVA	NCP	56.113
SPVVVA;YRSWLK	NCP	29.424
YRSWLK;DVGQLSF	NCP	11.465
DVGQLSF;LSGAVSYK	NCP	5.868
LSGAVSYK;LSVMKK	NCP	12.862
LSVMKK;VPLMAGP	NCP	89.952
VPLMAGP;MFLSHNL	NCP	7.632
MFLSHNL;PLTLGNER	NCP	119.327
PLTLGNER;MAQSKGR	NCP	65.844
MAQSKGR;KGVPCLK	NCP	5.839
KGVPCLK;LEQVVR	NCP	106.917
LEQVVR;VDEDYK	NCP	169.982
VDEDYK;LLHKPSE	NCP	81.918
LLHKPSE;KGGHVPL	NCP	30.07
KGGHVPL;AVPALK	NCP	15.57
AVPALK;AELLEL	NCP	20.981
AELLEL;LPPLE	NCP	24.243
LPPLE;PPVHVPK	NCP	69.805
PPVHVPK;MFSALKK	NCP	61.127
MFSALKK;QPSYLKN	NCP	93.81
QPSYLKN;KAATPYP	NCP	65.305
KAATPYP;RKTTHK	NCP	9.051
RKTTHK;AAGALPFP	NCP	16.404
AAGALPFP;KNGVL	NCP	41.987
KNGVL;QTLLEK	NCP	23.275
QTLLEK;KPYVRP	NCP	23.246
KPYVRP;TPLGLQP	NCP	71.584
TPLGLQP;KAAQRR	NCP	44.898
KAAQRR;LLPPS	NCP	71.067
LLPPS;DLRVQPF	NCP	29.079
DLRVQPF;LDDLVK	NCP	114.978
LDDLVK;KPMVG	NCP	24.59
KPMVG;LYQLDAS	NCP	28.041
LYQLDAS;EQDDP	NCP	57.859
EQDDP;EGRSVHP	NCP	102.426
EGRSVHP;KLENPNK	NCP	16.788
KLENPNK;LNVLSTH	NCP	34.365
LNVLSTH;APFVPE	NCP	39.222
APFVPE;TAPRLLLF	NCP	8.859
TAPRLLLF;AHSSLHL	NCP	89.157
AHSSLHL;KKPAVHR	NCP	41.688
KKPAVHR;VPWGLP	NCP	11.931
VPWGLP;PEVLVQPP	NCP	50.366
PEVLVQPP;PPPHLNK	NCP	1.441
PPPHLNK;VLPSRVK	NCP	3.108
VLPSRVK;RTASPSR	NCP	21.944
RTASPSR;NGLPLC	NCP	16.644
NGLPLC;PGSKMSK	NCP	5.785
PGSKMSK;HKEYGP	NCP	15
HKEYGP;MVHMA	NCP	72.711
MVHMA;ALPPVLM	NCP	51.801

ALPPVLM;KPCAKSK	NCP	63.111
KPCAKSK;QGKGLPQ	NCP	7.761
QGKGLPQ;GVPTVP	NCP	149.661
GVPTVP;KPTKRMP	NCP	0.194
KPTKRMP;LASEPLVVGLPY	NCP	22.416
LASEPLVVGLPY;GAGELLD	NCP	81.115
GAGELLD;PFPPPP	NCP	22.82
PFPPPP;LAPAHVP	NCP	28.27
LAPAHVP;LPAPGVPP	NCP	3.666
LPAPGVPP;TPPTRTAP	NCP	21.687
TPPTRTAP;VGVCAAP	NCP	53.85
VGVCAAP;KDNVPEM	NCP	6.341
KDNVPEM;LDLPF	NCP	17.092
LDLPF;AEFLTR	NCP	56.673
AEFLTR;DYLVHKR	NCP	18.005
DYLVHKR;FLEKELFY	NCP	19.491
FLEKELFY;WPSSSSSP	NCP	36.805
WPSSSSSP;ALPPVG	NCP	124.844
ALPPVG;SGKYVLG	NCP	27.607
SGKYVLG;ANNCPALRK	NCP	0.075
ANNCPALRK;KPTAAP	NCP	5.846
KPTAAP;KPGGGP	NCP	74.004
KPGGGP;KAELPF	NCP	92.875
KAELPF;LSLGPKF	NCP	88.679
LSLGPKF;GPAAEVP	NCP	9.963
GPAAEVP;VPKSLSPP	NCP	48.271
VPKSLSPP;KPGNAP	NCP	67.497
KPGNAP;KPGGRPP	NCP	9.702
KPGGRPP;KYSPLLV	NCP	2.894
KYSPLLV;TTKKKAV	NCP	128.5
TTKKKAV;TVAGGGFG	NCP	7.725
TVAGGGFG;AQAFVNS	NCP	2.786
AQAFVNS;KKGGLDV	NCP	5.505
KKGGLDV;PTELVLER	NCP	27.081
PTELVLER;KLLLPFCC	NCP	23.517
KLLLPFCC;LDFGPR	NCP	18.814
LDFGPR;KNSLNKT	NCP	3.483
KNSLNKT;LGMQPGK	NCP	68.502
LGMQPGK;SSAVDKK	NCP	11.618
SSAVDKK;TVDVDLP	NCP	24.238
TVDVDLP;PPPKGGPQ	NCP	1.332
PPPKGGPQ;NPRLLK	NCP	18.984
NPRLLK;NAAPKD	NCP	0.972
NAAPKD;YAGTEY	NCP	49.962
YAGTEY;AALGAQLL	NCP	40.356
AALGAQLL;KETLPPP	NCP	14.541
KETLPPP;KEVLLLSPF	NCP	91.644
KEVLLLSPF;VLMVPR	NCP	86.082
VLMVPR;KNFDPP	NCP	4.647
KNFDPP;ASQLLAR	NCP	40.482
ASQLLAR;PPVGFKG	NCP	8.808

PPVGFKG;RQELGLR	NCP	8.07
RQELGLR;WPHGPPP	NCP	33.12
WPHGPPP;YTLGL	NCP	88.404
YTLGL;EPELPMG	NCP	86.244
EPELPMG;RGRPAAPP	NCP	45.228
RGRPAAPP;KDRLGGQ	NCP	29.616
KDRLGGQ;KPEKPVKK	NCP	10.611
KPEKPVKK;YVALSR	NCP	61.203
YVALSR;KWKAPF	NCP	104.879
KWKAPF;MFLKGST	NCP	41.077
MFLKGST;TADLALL	NCP	4.311
TADLALL;FLDRQVP	NCP	14.208
FLDRQVP;CYAALTH	NCP	14.058
CYAALTH;VVVKPKL	NCP	27.636
VVVKPKL;WNPDRQ	NCP	83.654
WNPDRQ;STAPLQVF	NCP	53.596
STAPLQVF;TVELYL	NCP	126.5
TVELYL;VLRPVPGYP	NCP	73.828
VLRPVPGYP;QLEVLKL	NCP	27.323
QLEVLKL;LSPAPGHRP	NCP	7.014
LSPAPGHRP;STYGKLK	NCP	98.067
STYGKLK;LSSEYKL	NCP	27.33
LSSEYKL;VLGFR	NCP	22.813
VLGFR;YFGKSK	NCP	13.061
YFGKSK;LMPLPFLL	NCP	129.124
LMPLPFLL;PEWTN	NCP	70.497
PEWTN;VVLCNDR	NCP	5.624
VVLCNDR;RTLLDVV	NCP	60.738
RTLLDVV;EHSPLDF	NCP	1.81
EHSPLDF;PGLLH	NCP	99.692
PGLLH;VLVHVV	NCP	117.069
VLVHVV;VGLAGAK	NCP	17.482
VGLAGAK;EKALRVT	NCP	22.01
EKALRVT;SAPGVAW	NCP	73.878
SAPGVAW;KPYPP	NCP	41.746
KPYPP;MASKSKLK	NCP	0.593
MASKSKLK;QAAVFPR	NCP	46.687
QAAVFPR;QPGVGP	NCP	4.149
QPGVGP;VAAVSF	NCP	39.543
VAAVSF;TTQLWLLP	NCP	3.623
TTQLWLLP;GSLGAELR	NCP	4.819
GSLGAELR;LLGHLHPP	NCP	10.668
LLGHLHPP;LVVPKQP	NCP	33.629
LVVPKQP;MASFF	NCP	53.052
MASFF;VEDVSLP	NCP	7.494
VEDVSLP;DNQPQLP	NCP	78.325
DNQPQLP;VGLVTVAR	NCP	23.001
VGLVTVAR;WGLSLSH	NCP	43.812
WGLSLSH;KAAQTVK	NCP	29.019
KAAQTVK;EHKRKPP	NCP	53.459
EHKRKPP;PPPPPPR	NCP	1.585

PPPPPPR;VTPLREQ	NCP	55.175
VTPLREQ;SHTLPDF	NCP	143.766
SHTLPDF;KSKVSDLT	NCP	54.144
KSKVSDLT;LVRDLKE	NCP	96.799
LVRDLKE;LFPKKEN	NCP	159.149
LFPKKEN;KTFKPY	NCP	34.156
KTFKPY;ASLGLEL	NCP	22.812
ASLGLEL;KKLDVPL	NCP	111.113
KKLDVPL;KGVCVGDKL	NCP	26.976
KGVCVGDKL;LSYVTP	NCP	103.69
LSYVTP;NGGDLKK	NCP	18.104
NGGDLKK;PPFPVLM	NCP	43.158
PPFPVLM;KAMSP	NCP	22.974
KAMSP;TPLLLSK	NCP	53.322
TPLLLSK;AAVVL	NCP	47.347
AAVVL;TGLKHEL	NCP	116.066
TGLKHEL;KGAFLPP	NCP	66.396
KGAFLPP;NAMLDL	NCP	93.771
NAMLDL;FSQLQ	NCP	51.435
FSQLQ;TELVF	NCP	19.641
TELVF;VPLTP	NCP	172.275
VPLTP;WTVKGQK	NCP	4.152
WTVKGQK;VTPPSLY	NCP	147.643
VTPPSLY;AAADLTEF	NCP	18.62
AAADLTEF;NTWHPK	NCP	19.728
NTWHPK;EYLVHGK	NCP	68.7
EYLVHGK;LSSSPLGR	NCP	19.896
LSSSPLGR;CMMNQ	NCP	55.653
CMMNQ;QAKVLR	NCP	16.707
QAKVLR;KYPKNQ	NCP	5.767
KYPKNQ;GQAPVY	NCP	21.56
GQAPVY;TDARPK	NCP	92.349
TDARPK;TDSVFHK	NCP	49.087
TDSVFHK;VTVGVEL	NCP	35.121
VTVGVEL;MMSSMM	NCP	30.272
MMSSMM;APNLPL	NCP	63.459
APNLPL;LPAASPPR	NCP	78.601
LPAASPPR;YGRALQ	NCP	74.459
YGRALQ;KGYEVLY	NCP	75.141
KGYEVLY;VPPFVYP	NCP	103.375
VPPFVYP;VMVGPQ	NCP	2.907
VMVGPQ;FLRHG	NCP	17.211
FLRHG;AKPQPGK	NCP	30.09
AKPQPGK;ATTSG	NCP	81.369
ATTSG;KVKPLM	NCP	142.881
KVKPLM;VAKSFR	NCP	9.842
VAKSFR;YDSLLKN	NCP	34.455
YDSLLKN;KLAPETH	NCP	27.252
KLAPETH;VLALELL	NCP	84.207
VLALELL;FFPPNTSK	NCP	60.795
FFPPNTSK;PAPGFK	NCP	54.166

PAPGFK;FPPPGAHP	NCP	27.513
FPPPGAHP;EPPEPPAY	NCP	62.034
EPPEPPAY;SFNAK	NCP	24.033
SFNAK;SGNAAP	NCP	15.477
SGNAAP;GKNGLDRL	NCP	20.963
GKNGLDRL;CEDLSGPK	NCP	25.513
CEDLSGPK;VTVPL	NCP	43.458
VTVPL;TDPKLL	NCP	55.32
TDPKLL;YDRTQM	NCP	130.478
YDRTQM;ADPELQVL	NCP	8.409
ADPELQVL;AALGF	NCP	22.465
AALGF;PAGAP	NCP	11.307
PAGAP;KEAGNPK	NCP	64.443
KEAGNPK;KNKKLLT	NCP	10.974
KNKKLLT;LSLLKKL	NCP	12.171
LSLLKKL;QPKPAAP	NCP	4.83
QPKPAAP;FTEVLKTHGFL	NCP	28.338
FTEVLKTHGFL;EVLKTHGFLV	NCP	0.006
EVLKTHGFLV;PAAHA	NCP	49.068
PAAHA;TKGAATK	NCP	68.13
TKGAATK;KFLSSVP	NCP	11.304
KFLSSVP;LPRPELE	NCP	2.757
LPRPELE;KVGVPRLL	NCP	14.669
KVGVPRLL;EGFGYKY	NCP	13.063
EGFGYKY;LLMHLSYL	NCP	0.692
LLMHLSYL;KPWAGTK	NCP	7.507
KPWAGTK;ALSLK	NCP	154.241
ALSLK;VLLGKAVP	NCP	12.993
VLLGKAVP;VGFKLLQL	NCP	40.015
VGFKLLQL;TPPPLP	NCP	20.099
TPPPLP;TSRKAAP	NCP	3.244
TSRKAAP;GLPPPGM	NCP	27.782
GLPPPGM;MPPLGL	NCP	111.754
MPPLGL;SVFRPL	NCP	9.707
SVFRPL;QAVVLAK	NCP	60.222
QAVVLAK;AAPKSP	NCP	63.997
AAPKSP;LVAAQYL	NCP	17.613
LVAAQYL;CVLYF	NCP	42.65
CVLYF;KKACAK	NCP	21.421
KKACAK;AAAKHKT	NCP	66.663
AAAKHKT;LAPLPP	NCP	4.749
LAPLPP;LACMHALLR	NCP	17.714
LACMHALLR;NGYKVLG	NCP	3.367
NGYKVLG;LPPSGTAAP	NCP	66.213
LPPSGTAAP;LATKVPR	NCP	40.599
LATKVPR;VAGPPPPPPP	NCP	131.721
VAGPPPPPPP;KDVGL	NCP	55.374
KDVGL;FALMRP	NCP	20.664
FALMRP;LPVKKFP	NCP	23.832
LPVKKFP;SVTLFPP	NCP	42.368
SVTLFPP;LPSGLFG	NCP	7.476

LPSGLFG;DDDVKYV	NCP	45.42
DDDVKYV;KTMVVPR	NCP	52.258
KTMVVPR;KESYSVY	NCP	69.309
KESYSVY;KLATDF	NCP	8.549
KLATDF;AAAMKKK	NCP	30.325
AAAMKKK;YEFTK	NCP	23.111
YEFTK;KFAGP	NCP	9.702
KFAGP;EDSVKPGAHL	NCP	35.601
EDSVKPGAHL;HYMVYM	NCP	79.018
HYMVYM;NLFEVYL	NCP	83.631
NLFEVYL;YCGKCFS	NCP	97.862
YCGKCFS;VTVFHKRK	NCP	73.284
VTVFHKRK;LAGNQ	NCP	65.616
LAGNQ;VWLGGSLL	NCP	45.91
VWLGGSLL;VLRVF	NCP	152.51
VLRVF;LLVTL	NCP	106.405
LLVTL;LPKMLL	NCP	4.302
LPKMLL;KDFLPPV	NCP	3.287
KDFLPPV;DNVPP	NCP	80.592
DNVPP;FPPDVG	NCP	15.898
FPPDVG;RHGGVVAK	NCP	12.573
RHGGVVAK;LLLLSNSM	NCP	36.194
LLLLSNSM;PVTLLL	NCP	142.59
PVTLLL;LGPVPGHV	NCP	20.485
LGPVPGHV;TVPKYSK	NCP	6.918
TVPKYSK;LDPPLL	NCP	52.695
LDPPLL;SSSYL	NCP	102.6
SSSYL;DRVPLQ	NCP	49.758
DRVPLQ;KTSYVLTF	NCP	0.717
KTSYVLTF;ESLLWEK	NCP	11.769
ESLLWEK;PPAPPPPP	NCP	113.763
PPAPPPPP;GPPPP	NCP	50.28
GPPPP;EVASLNF	NCP	8.96
EVASLNF;SLSPGSF	NCP	40.723
SLSPGSF;NMCVLAK	NCP	19.949
NMCVLAK;LSLRLSP	NCP	1.258
LSLRLSP;LAALVL	NCP	33.564
LAALVL;SGALDVLQMKEEDVLK	NCP	27.947
SGALDVLQMKEEDVLK;MKEEDVLKF	NCP	0.024
MKEEDVLKF;GCMSPPR	NCP	74.026
GCMSPPR;AAGGPK	NCP	17.325
AAGGPK;DLRWEEL	NCP	0.581
DLRWEEL;APGHKVP	NCP	46.42
APGHKVP;AMCDLR	NCP	29.532
AMCDLR;SLARAAGF	NCP	51.534
SLARAAGF;ETAEG	NCP	33.42
ETAEG;KHLKAAP	NCP	8.613
KHLKAAP;EVPRPQ	NCP	8.409
EVPRPQ;SGGCEHT	NCP	3.408
SGGCEHT;GVSLGDLL	NCP	15.711
GVSLGDLL;YGVAAV	NCP	41.709

YGVAAV;PAAAADER	NCP	179.517
PAAAADER;PLARAPGP	NCP	123.579
PLARAPGP;MLFNERMLESY	NCP	33.471
MLFNERMLESY;KGSSLDL	NCP	2.996
KGSSLDL;LAYGESDL	NCP	66.586
LAYGESDL;MFSWKL	NCP	3.749
MFSWKL;AATLH	NCP	62.971
AATLH;LAAQEAK	NCP	28.794
LAAQEAK;VPGVP	NCP	1.08
VPGVP;QDSVDF	NCP	16.769
QDSVDF;RRTAVGAP	NCP	25.387
RRTAVGAP;LGSTPTLL	NCP	156.81
LGSTPTLL;GHHKSPP	NCP	9.921
GHHKSPP;AASPTVR	NCP	16.08
AASPTVR;RLPLF	NCP	3.153
RLPLF;KAQKLR	NCP	12.416
KAQKLR;GPPGAPP	NCP	83.761
GPPGAPP;KGPSPPPPPP	NCP	41.577
KGPSPPPPPP;NPNLEP	NCP	2.543
NPNLEP;LHPYKTL	NCP	66.084
LHPYKTL;CCHYME	NCP	34.06
CCHYME;VLCPPPPP	NCP	6.783
VLCPPPPP;DPSLHEK	NCP	46.868
DPSLHEK;KHDLVTAL	NCP	51.522
KHDLVTAL;MFPQAR	NCP	22.209
MFPQAR;FFALPQ	NCP	85.209
FFALPQ;ELHFGK	NCP	10.723
ELHFGK;TVVALQT	NCP	59.726
TVVALQT;LALTFGAP	NCP	20.179
LALTFGAP;MRGNTHN	NCP	19.671
MRGNTHN;LQLVEPF	NCP	4.98
LQLVEPF;EPSLLHT	NCP	134.162
EPSLLHT;LSQEERY	NCP	26.32
LSQEERY;EVDGLRVP	NCP	77.906
EVDGLRVP;LLKGGNL	NCP	55.903
LLKGGNL;HMPGAGK	NCP	9.921
HMPGAGK;AYPGHP	NCP	154.593
AYPGHP;RELVVRQ	NCP	2.969
RELVVRQ;PGKSSG	NCP	20.86
PGKSSG;KWMSLF	NCP	51.743
KWMSLF;LVRPPVL	NCP	82.929
LVRPPVL;QKYPLGSH	NCP	12.087
QKYPLGSH;PPWKSSP	NCP	8.614
PPWKSSP;ALQASGP	NCP	67.467
ALQASGP;RVPTPN	NCP	9.039
RVPTPN;VVGYGK	NCP	56.774
VVGYGK;PPPVVRP	NCP	75.142
PPPVVRP;NLPRQRN	NCP	7.532
NLPRQRN;QDGVLR	NCP	29.467
QDGVLR;HVVVP	NCP	0.753
HVVVP;DADTEEL	NCP	153.275

DADTEEL;AAPVFLLY	NCP	100.825
AAPVFLLY;GSVLPLKK	NCP	22.952
GSVLPLKK;ERVLLYQ	NCP	87.951
ERVLLYQ;EPTLHPK	NCP	125.173
EPTLHPK;LMNVFLK	NCP	42.228
LMNVFLK;NPPPP	NCP	2.94
NPPPP;TQPLL	NCP	38.39
TQPLL;AELTVLD	NCP	43.917
AELTVLD;QEVTKPM	NCP	9.994
QEVTKPM;SGYGVSF	NCP	23.694
SGYGVSF;LPTNLF	NCP	33.461
LPTNLF;KGDGLLR	NCP	23.329
KGDGLLR;NASAWLT	NCP	44.61
NASAWLT;RSLAVTAP	NCP	66.024
RSLAVTAP;KGPCAPE	NCP	76.716
KGPCAPE;KKDGLAL	NCP	1.574
KKDGLAL;LGVGRPP	NCP	11.128
LGVGRPP;SFPLL	NCP	11.289
SFPLL;KEGVLDP	NCP	25.979
KEGVLDP;KDAALVR	NCP	6.771
KDAALVR;TASVVDL	NCP	3.91
TASVVDL;SVDLPNK	NCP	5.246
SVDLPNK;TFMLTK	NCP	107.476
TFMLTK;NRMFPP	NCP	11.751
NRMFPP;PLPPLPP	NCP	14.574
PLPPLPP;CMGPPPP	NCP	2.592
CMGPPPP;VSLGVSLR	NCP	0.987
VSLGVSLR;SNDPKLR	NCP	101.057
SNDPKLR;KPVSPHF	NCP	197.104
KPVSPHF;EALERMFL	NCP	72.998
EALERMFL;KRLDPGGP	NCP	70.09
KRLDPGGP;VTLLQEK	NCP	150.672
VTLLQEK;PPPPDRV	NCP	5.886
PPPPDRV;SPHVAPR	NCP	18.363
SPHVAPR;KGGRFGK	NCP	41.643
KGGRFGK;LHAPPPPP	NCP	1.308
LHAPPPPP;KGEKEVELK	NCP	47.969
KGEKEVELK;PVGHLPPP	NCP	50.041
PVGHLPPP;PAAFH	NCP	9.984
PAAFH;VLRGAQY	NCP	52.902
VLRGAQY;AATEEDG	NCP	27.146
AATEEDG;TAAHPLPP	NCP	5.662
TAAHPLPP;DTLKTKP	NCP	7.452
DTLKTKP;WGGGW	NCP	48.248
WGGGW;RSHPHQ	NCP	0.907
RSHPHQ;KFLKVVH	NCP	4.724
KFLKVVH;SGTAG	NCP	8.026
SGTAG;LLKKAGN	NCP	11.438
LLKKAGN;KPEYPR	NCP	66.97
KPEYPR;CEGCGGP	NCP	6.075
CEGCGGP;LHRDPLK	NCP	88.095

LHRDPLK;CAMDLR	NCP	115.698
CAMDLR;LDLTGNK	NCP	14.739
LDLTGNK;RLPGELAK	NCP	27.876
RLPGELAK;VGLPNLQ	NCP	13.782
VGLPNLQ;GPDVHRK	NCP	2.508
GPDVHRK;QMPPP	NCP	89.712
QMPPP;VSGLVVVQ	NCP	0.639
VSGLVVVQ;LDHKYPL	NCP	12.054
LDHKYPL;KPAFAAK	NCP	3.806
KPAFAAK;KGPPPPPP	NCP	34.087
KGPPPPPP;TAEPLPGL	NCP	7.865
TAEPLPGL;KEAKVPL	NCP	136.848
KEAKVPL;VAGVLP	NCP	5.191
VAGVLP;VPFHLPR	NCP	17.895
VPFHLPR;KQGPVPPP	NCP	6.234
KQGPVPPP;GVKVRPGP	NCP	26.757
GVKVRPGP;VAPPFQL	NCP	3.819
VAPPFQL;LPAACGC	NCP	1.743
LPAACGC;ESLLKTL	NCP	19.046
ESLLKTL;STLTDSLVCK	NCP	16.672
STLTDSLVCK;DSPGHVDF	NCP	1.053
DSPGHVDF;EHAHNMRVMKF	NCP	3.159
EHAHNMRVMKF;SVSPVVR	NCP	0.033
SVSPVVR;VAVEAKNPADLPK	NCP	0.021
VAVEAKNPADLPK;PADLPKLVE	NCP	0.021
PADLPKLVE;LLLVWTP	NCP	30.405
LLLVWTP;TGAPLAVP	NCP	25.064
TGAPLAVP;AGHGLVL	NCP	44.892
AGHGLVL;VPPVKAP	NCP	40.248
VPPVKAP;DQEVKLAP	NCP	2.691
DQEVKLAP;RAGALLGA	NCP	18.769
RAGALLGA;FMCCAG	NCP	75.651
FMCCAG;DRPALRV	NCP	1.227
DRPALRV;LPLPN	NCP	40.758
LPLPN;SRGKRVR	NCP	41.126
SRGKRVR;RLGGVKS	NCP	37.398
RLGGVKS;AATPGLL	NCP	62.359
AATPGLL;EKGKPPP	NCP	55.953
EKGKPPP;DRTKPGW	NCP	60.485
DRTKPGW;KAAYK	NCP	24.099
KAAYK;LVGPKPT	NCP	85.15
LVGPKPT;ETEAGP	NCP	68.961
ETEAGP;KVLHTFLQ	NCP	32.994
KVLHTFLQ;MVGTVPPP	NCP	4.685
MVGTVPPP;PTKETLPC	NCP	68.59
PTKETLPC;GKVSLQVP	NCP	58.329
GKVSLQVP;FLTHLT	NCP	15.974
FLTHLT;QLGPVQT	NCP	19.582
QLGPVQT;PPQSKTK	NCP	99.9
PPQSKTK;YCLAVMP	NCP	60.557
YCLAVMP;FNDFLLA	NCP	12.522

FNDFLLA;TDMGPP	NCP	33.481
TDMGPP;VSAPQPR	NCP	62.871
VSAPQPR;FLYPPK	NCP	113.741
FLYPPK;KSLVDSVP	NCP	27.121
KSLVDSVP;LGAAVLS	NCP	25.947
LGAAVLS;PPPGGASPP	NCP	14.988
PPPGGASPP;AQVAKDL	NCP	91.293
AQVAKDL;YDLRCR	NCP	29.079
YDLRCR;TQVPSPKV	NCP	6.554
TQVPSPKV;EFGAQR	NCP	39.661
EFGAQR;HAGPLMV	NCP	5.003
HAGPLMV;SNSLK	NCP	105.258
SNSLK;KPPVTYK	NCP	2.793
KPPVTYK;DVRTPPP	NCP	37.861
DVRTPPP;HSAAVTK	NCP	23.949
HSAAVTK;CVRSLLL	NCP	2.342
CVRSLLL;ADRPVRQ	NCP	30.928
ADRPVRQ;TDLEWR	NCP	10.095
TDLEWR;LPPPKLHT	NCP	6.882
LPPPKLHT;PDDKKLR	NCP	189.191
PDDKKLR;MLLKVPA	NCP	0.808
MLLKVPA;KQTLVLP	NCP	62.135
KQTLVLP;TVGALLF	NCP	7.003
TVGALLF;ALKEVLN	NCP	144.893
ALKEVLN;FTDEPQR	NCP	67.071
FTDEPQR;TASVLP	NCP	58.008
TASVLP;LHKLLSV	NCP	26.91
LHKLLSV;LESVPLLK	NCP	2.457
LESVPLLK;EGRVLLT	NCP	84.012
EGRVLLT;FRVKKK	NCP	266.283
FRVKKK;EELKRDF	NCP	56.271
EELKRDF;ESDLLD	NCP	61.324
ESDLLD;KDDASPK	NCP	2.549
KDDASPK;FNEALLL	NCP	62.815
FNEALLL;KNFFSPK	NCP	60.482
KNFFSPK;FQVWLK	NCP	32.929
FQVWLK;ESPYLHS	NCP	10.14
ESPYLHS;KLDLVY	NCP	0.845
KLDLVY;KYPPKNP	NCP	6.058
KYPPKNP;YDGFG	NCP	156.413
YDGFG;RYPKNTP	NCP	43.867
RYPKNTP;TPDVPLP	NCP	0.318
TPDVPLP;KPSFHVP	NCP	33.393
KPSFHVP;WCLLLLT	NCP	16.076
WCLLLLT;GLYGPDG	NCP	26.692
GLYGPDG;LPDDRCK	NCP	7.889
LPDDRCK;KAPVFF	NCP	17.899
KAPVFF;KPPRPPT	NCP	57.468
KPPRPPT;LEKNF	NCP	56.768
LEKNF;KTVKLQY	NCP	37.749
KTVKLQY;MKVNNL	NCP	14.908

MKVNNL;KELELKY	NCP	83.381
KELELKY;NGAFLDF	NCP	17.562
NGAFLDF;CDAACH	NCP	55.164
CDAACH;VTPTH	NCP	18.618
VTPTH;MAGLMNS	NCP	194.077
MAGLMNS;RVFGFRH	NCP	15.002
RVFGFRH;WPKSGKK	NCP	38.272
WPKSGKK;SETLVLGTK	NCP	40.365
SETLVLGTK;VGPELT	NCP	24.153
VGPELT;HDWHEL	NCP	67.932
HDWHEL;LSDDLLP	NCP	21.506
LSDDLLP;NRALVMP	NCP	36.108
NRALVMP;KFFPP	NCP	0.039
KFFPP;KHALLSP	NCP	97
KHALLSP;SLGNVVK	NCP	5.87
SLGNVVK;MMAKPK	NCP	19.51
MMAKPK;KPSDLDLR	NCP	81.291
KPSDLDLR;LLPGNGSL	NCP	65.85
LLPGNGSL;KLLRAAK	NCP	122.144
KLLRAAK;TSTAG	NCP	17.16
TSTAG;HTPPVSSLK	NCP	106.14
HTPPVSSLK;VSDGLLR	NCP	36.417
VSDGLLR;YGLPPPGP	NCP	13.593
YGLPPPGP;KDGLFGPP	NCP	21.321
KDGLFGPP;EEQFNLK	NCP	16.333
EEQFNLK;SSGRAPY	NCP	16.194
SSGRAPY;LLYHVSLL	NCP	24.191
LLYHVSLL;EDGFVEATEGLGDDALGK	NCP	67.866
EDGFVEATEGLGDDALGK;KLHQEL	NCP	38.721
KLHQEL;NLVNFHL	NCP	110.812
NLVNFHL;MTPYKK	NCP	25.938
MTPYKK;LSPAVKK	NCP	90.623
LSPAVKK;KCWSPP	NCP	19.443
KCWSPP;GKALLYY	NCP	63.543
GKALLYY;AMLAKTGVHHY	NCP	5.298
AMLAKTGVHHY;LSHEKML	NCP	39.487
LSHEKML;LDMGGKK	NCP	14.663
LDMGGKK;KKATHKK	NCP	101.571
KKATHKK;KQTNLLK	NCP	65.553
KQTNLLK;DKAVTL	NCP	5.131
DKAVTL;AAPNLD	NCP	49.641
AAPNLD;LPLTTGW	NCP	40.766
LPLTTGW;MRFLAT	NCP	8.487
MRFLAT;LDKLPGN	NCP	14.868
LDKLPGN;MSSLKKC	NCP	91.431
MSSLKKC;LLPVESV	NCP	19.372
LLPVESV;EVVLK	NCP	111.243
EVVLK;RVQYSVR	NCP	150.581
RVQYSVR;KSATPPR	NCP	102.418
KSATPPR;PLPSKET	NCP	10.184
PLPSKET;FSLKNK	NCP	31.503

FSLKNK;LDGKWF	NCP	12.991
LDGKWF;FGAKFF	NCP	4.043
FGAKFF;TVDLNLKG	NCP	18.064
TVDLNLKG;VTGKLVR	NCP	13.367
VTGKLVR;TLADVP	NCP	51.006
TLADVP;LSVQLLDL	NCP	1.836
LSVQLLDL;DLLLKKKK	NCP	20.92
DLLLKKKK;TANPPP	NCP	95.312
TANPPP;LGDSLPGP	NCP	169.216
LGDSLPGP;KPAEWPP	NCP	0.039
KPAEWPP;LFPLK	NCP	5.193
LFPLK;LRVAMK	NCP	68.496
LRVAMK;LEELPL	NCP	47.385
LEELPL;KSPTFEK	NCP	33.929
KSPTFEK;LRTTGTSF	NCP	10.174
LRTTGTSF;YSVWLG	NCP	181.809
YSVWLG;MLLRWS	NCP	171.21
MLLRWS;KDFSLPR	NCP	23.252
KDFSLPR;KVFELN	NCP	20.352
KVFELN;AARPKVH	NCP	135.784
AARPKVH;LLDFPLFL	NCP	16.52
LLDFPLFL;EAVTK	NCP	39.882
EAVTK;KTLAMAP	NCP	96.34
KTLAMAP;KPYAPPPPP	NCP	33.018
KPYAPPPPP;EQFVLL	NCP	228.105
EQFVLL;GFFFW	NCP	16.691
GFFFW;RFSGKVT	NCP	5.362
RFSGKVT;LLETLLK	NCP	70.421
LLETLLK;KVMLDLRK	NCP	33.237
KVMLDLRK;KVHEPQR	NCP	45.675
KVHEPQR;ELEKF	NCP	108.523
ELEKF;KPAAKDC	NCP	95.49
KPAAKDC;EELPADVR	NCP	78.87
EELPADVR;SAVPKLL	NCP	25.998
SAVPKLL;LDPYK	NCP	1.328
LDPYK;NLEVLNF	NCP	31.761
NLEVLNF;TKYAPVT	NCP	56.244
TKYAPVT;KENHEQEK	NCP	41.427
KENHEQEK;RPRKTK	NCP	197.928
RPRKTK;PLPHVLPP	NCP	4.41
PLPHVLPP;TTLDYH	NCP	93.483
TTLDYH;QFQGLKH	NCP	38.991
QFQGLKH;KTAEPY	NCP	39.496
KTAEPY;KDPELLSH	NCP	0.918
KDPELLSH;KQELTAL	NCP	32.406
KQELTAL;RGGRLYK	NCP	7.968
RGGRLYK;NSLVLLF	NCP	24.419
NSLVLLF;QQAPGHRP	NCP	30.357
QQAPGHRP;CCCAM	NCP	7.131
CCCAM;KPLPAPP	NCP	60.918
KPLPAPP;KPSAQP	NCP	26.655

KPSAQP;MFRKVVR	NCP	26.28
MFRKVVR;HGVYDPK	NCP	0.037
HGVYDPK;VGSYHTK	NCP	65.091
VGSYHTK;TPPPEKK	NCP	45.317
TPPPEKK;KKLLLPT	NCP	1.077
KKLLLPT;KERLVAPP	NCP	48.588
KERLVAPP;ERLVAPPG	NCP	0.003
ERLVAPPG;TTLLY	NCP	36.798
TTLLY;RLLVSQ	NCP	16.14
RLLVSQ;KPPEPKP	NCP	13.239
KPPEPKP;VVVKKLE	NCP	3.993
VVVKKLE;KRMFHL	NCP	17.107
KRMFHL;SLAPNLL	NCP	183.609
SLAPNLL;KLLLT	NCP	86.477
KLLLT;NFLLW	NCP	55.105
NFLLW;EDWVGY	NCP	31.728
EDWVGY;FDLDF	NCP	58.881
FDLDF;ALGGFSVF	NCP	16.634
ALGGFSVF;VVTAA	NCP	199.137
VVTAA;KTHLAY	NCP	38.059
KTHLAY;KSSSFAGP	NCP	1.806
KSSSFAGP;AAVTAF	NCP	1.689
AAVTAF;LPPQVPP	NCP	30.036
LPPQVPP;LPPQVDS	NCP	34.302
LPPQVDS;VGSVNP	NCP	29.636
VGSVNP;ADLPLN	NCP	60.6
ADLPLN;SHLRRTLS	NCP	32.286
SHLRRTLS;YSLPPPP	NCP	43.909
YSLPPPP;LASLAAF	NCP	22.214
LASLAAF;VPDSDLFF	NCP	120.388
VPDSDLFF;QTGKAPR	NCP	26.829
QTGKAPR;MPFVV	NCP	41.121
MPFVV;KTYGVSF	NCP	13.844
KTYGVSF;LGPQYVP	NCP	1.236
LGPQYVP;KVKFVPP	NCP	21.093
KVKFVPP;LEGAFP	NCP	34.92
LEGAFP;SLLEELL	NCP	38.961
SLLEELL;KEGLSL	NCP	14.739
KEGLSL;AKEWGY	NCP	9.355
AKEWGY;RAGVF	NCP	11.957
RAGVF;LADMRAGK	NCP	1.05
LADMRAGK;DRYLVL	NCP	64.336
DRYLVL;KGKGVLGA	NCP	100.673
KGKGVLGA;LPVKHAQ	NCP	108.819
LPVKHAQ;KTGSSQK	NCP	26.827
KTGSSQK;TSTTPTPL	NCP	167.463
TSTTPTPL;FPPERP	NCP	116.949
FPPERP;NTPAMYV	NCP	1.149
NTPAMYV;VQVSWN	NCP	41.631
VQVSWN;NAPKGSK	NCP	59.331
NAPKGSK;LLGQQVH	NCP	251.784

LLGQQVH;CDHYVLP	NCP	21.306
CDHYVLP;LHVYGY	NCP	3.996
LHVYGY;TPGHVPR	NCP	72.159
TPGHVPR;LFGVSLA	NCP	67.157
LFGVSLA;SMAGK	NCP	36.904
SMAGK;PFLPP	NCP	28.601
PFLPP;RKLVAH	NCP	37.5
RKLVAH;YTVGKPP	NCP	75.075
YTVGKPP;KKTSKQPK	NCP	27.516
KKTSKQPK;RGELPLR	NCP	23.184
RGELPLR;HVLLGPP	NCP	12.138
HVLLGPP;TVGPDMV	NCP	29.317
TVGPDMV;DSYPAVP	NCP	13.607
DSYPAVP;VGCHSP	NCP	7.132
VGCHSP;KGSNGAP	NCP	16.289
KGSNGAP;LERRVFP	NCP	17.776
LERRVFP;LSEKPHP	NCP	55.049
LSEKPHP;CMCAGP	NCP	19.039
CMCAGP;DTTEKFP	NCP	73.17
DTTEKFP;VAVKY	NCP	95.729
VAVKY;KSYPPPPP	NCP	12.231
KSYPPPPP;MPVSEF	NCP	4.612
MPVSEF;YGEVLY	NCP	156.186
YGEVLY;HACKHQ	NCP	47.736
HACKHQ;SVLRLLV	NCP	3.501
SVLRLLV;WTGYPP	NCP	57.602
WTGYPP;LGTKGLN	NCP	14.202
LGTKGLN;LEKFDK	NCP	106.158
LEKFDK;VPVQF	NCP	32.539
VPVQF;QGVVLLS	NCP	4.337
QGVVLLS;FVLNLLV	NCP	85.509
FVLNLLV;TTTVKEK	NCP	58.078
TTTVKEK;PTVNKKP	NCP	8.28
PTVNKKP;LFHGLSK	NCP	120.693
LFHGLSK;RCPETL	NCP	1.5
RCPETL;DKMTFL	NCP	33.764
DKMTFL;KFLAL	NCP	19.926
KFLAL;LSEVVEY	NCP	12.021
LSEVVEY;ENGVLR	NCP	78.981
ENGVLR;ALEFL	NCP	60.132
ALEFL;NCTFHKP	NCP	37.312
NCTFHKP;SFLGTVQK	NCP	22.928
SFLGTVQK;RFSMKL	NCP	16.449
RFSMKL;VEDPVRK	NCP	134.209
VEDPVRK;YDVTKAP	NCP	156.323
YDVTKAP;SFDKALK	NCP	22.717
SFDKALK;REDSWLK	NCP	11.607
REDSWLK;FLRLL	NCP	38.114
FLRLL;FVVPPGP	NCP	50.823
FVVPPGP;KADVKF	NCP	69.082
KADVKF;LTTGLEK	NCP	15.107

LTTGLEK;VAPPKFL	NCP	151.441
VAPPKFL;LLMYRLP	NCP	45.999
LLMYRLP;LEKMLK	NCP	335.615
LEKMLK;FKSLP	NCP	54.786
FKSLP;MELGC	NCP	6.345
MELGC;GVLPLLSQ	NCP	90.624
GVLPLLSQ;DLELL	NCP	5.787
DLELL;LSLVLRC	NCP	12.739
LSLVLRC;DLKEVLK	NCP	25.896
DLKEVLK;NDEPQE	NCP	96.506
NDEPQE;RVETVH	NCP	60.325
RVETVH;CKQTFEV	NCP	10.008
CKQTFEV;EEEYTS	NCP	113.103
EEEYTS;YAPEFQL	NCP	19.526
YAPEFQL;YLHGPQ	NCP	64.513
YLHGPQ;VFEYPGN	NCP	59.936
VFEYPGN;RHKVPQN	NCP	4.158
RHKVPQN;YLLKATA	NCP	125.58
YLLKATA;FPTPF	NCP	2.577
FPTPF;PQHALVS	NCP	228.792
PQHALVS;APDGHSA	NCP	71.587
APDGHSA;YPGYGY	NCP	14.649
YPGYGY;MEVLRPP	NCP	6.308
MEVLRPP;CSLPPRW	NCP	15.729
CSLPPRW;MLLEF	NCP	47.302
MLLEF;EHLMP	NCP	70.01
EHLMP;SHSLLVT	NCP	36.285
SHSLLVT;LAHPADK	NCP	1.96
LAHPADK;TEHLKVR	NCP	12.156
TEHLKVR;AELALHE	NCP	14.831
AELALHE;NPVLEKFDK	NCP	174.333
NPVLEKFDK;SSCSMW	NCP	97.062
SSCSMW;MFFAFSK	NCP	13.648
MFFAFSK;MMMFPP	NCP	49.361
MMMFPP;LQTLLR	NCP	10.723
LQTLLR;KLALH	NCP	81.326
KLALH;CCTPF	NCP	17.662
CCTPF;NYHGPK	NCP	38.657
NYHGPK;YAFLTM	NCP	0.069
YAFLTM;KLLPKK	NCP	8.142
KLLPKK;SVGPSRK	NCP	69.766
SVGPSRK;VGPLLVQ	NCP	44.189
VGPLLVQ;NLPTFPK	NCP	7.308
NLPTFPK;CSMMAA	NCP	44.244
CSMMAA;CQYFMR	NCP	90.355
CQYFMR;EKRFYK	NCP	23.277
EKRFYK;KEVELK	NCP	16.281
KEVELK;KVYHRAP	NCP	49.881
KVYHRAP;PAAPVQK	NCP	117.384
PAAPVQK;PLLAYL	NCP	67.719
PLLAYL;KPSWVVK	NCP	143.132

KPSWVVK;KGMMGC	NCP	108.12
KGMMGC;RVKASFK	NCP	39.058
RVKASFK;LVSGGVLM	NCP	50.058
LVSGGVLM;EDFDVAGK	NCP	2.726
EDFDVAGK;KDLSFP	NCP	76.884
KDLSFP;FPVGLLT	NCP	98.986
FPVGLLT;VPLQSPK	NCP	20.205
VPLQSPK;KPDRAM	NCP	7.425
KPDRAM;LNNPVA	NCP	0.3
LNNPVA;KNLNHTL	NCP	210.566
KNLNHTL;YGGGPGY	NCP	5.635
YGGGPGY;NEFTRL	NCP	28.301
NEFTRL;RLLMFSSK	NCP	32.038
RLLMFSSK;FPLLQSLE	NCP	45.219
FPLLQSLE;LLRVENL	NCP	10.229
LLRVENL;KDALALS	NCP	32.229
KDALALS;DRNLTL	NCP	27.919
DRNLTL;ENEFK	NCP	42.345
ENEFK;LSLTNSLF	NCP	24.492
LSLTNSLF;PFKGLP	NCP	23.019
PFKGLP;LDGVLK	NCP	4.16
LDGVLK;HTDCLPPP	NCP	32.421
HTDCLPPP;LDDFRFL	NCP	30.291
LDDFRFL;LQLNVKS	NCP	8.425
LQLNVKS;KQKAAAR	NCP	13.565
KQKAAAR;CNCHSK	NCP	24.504
CNCHSK;LRMSSKP	NCP	52.683
LRMSSKP;SLTAVPK	NCP	57.12
SLTAVPK;RGVPTPP	NCP	43.849
RGVPTPP;KPSAAP	NCP	12.354
KPSAAP;SLVGSVQF	NCP	60.063
SLVGSVQF;KLFFNPL	NCP	11.915
KLFFNPL;QRPPP	NCP	15.705
QRPPP;SVDMTCEGCSNAVTRVLNK	NCP	13.473
SVDMTCEGCSNAVTRVLNK;VDMTCEGCSNAVTR	NCP	0.003
VDMTCEGCSNAVTR;SVDTLLETLGK	NCP	3.825
SVDTLLETLGK;VDTLLETLGKTGKA	NCP	0.003
VDTLLETLGKTGKA;KTGKAVSYLGPK	NCP	0.027
KTGKAVSYLGPK;TTTKPPR	NCP	13.353
TTTKPPR;EEMYQP	NCP	4.969
EEMYQP;GPFKGPQ	NCP	2.273
GPFKGPQ;ECEFAG	NCP	21.246
ECEFAG;DVMVPP	NCP	16.422
DVMVPP;LGAMVPL	NCP	55.533
LGAMVPL;GSNAEVP	NCP	31.083
GSNAEVP;FVVPP	NCP	63.468
FVVPP;LPLAAAER	NCP	46.894
LPLAAAER;PTHEKR	NCP	16.551
PTHEKR;KSQEVW	NCP	6.356
KSQEVW;KGLTEKAK	NCP	38.574
KGLTEKAK;VSANK	NCP	0.801

VSANK;QSLMLK	NCP	27.808
QSLMLK;ELGALDF	NCP	31.982
ELGALDF;GVVGF	NCP	21.627
GVVGF;TYQALYE	NCP	25.464
TYQALYE;ALKLLKT	NCP	62.388
ALKLLKT;ALEALKL	NCP	25.888
ALEALKL;PPAAVVR	NCP	3.939
PPAAVVR;ASLDKLF	NCP	33.32
ASLDKLF;GLFAPPK	NCP	15.912
GLFAPPK;LGGAGMER	NCP	44.385
LGGAGMER;ERPLPVE	NCP	57.621
ERPLPVE;LSLGPQ	NCP	10.939
LSLGPQ;PGWHVK	NCP	21.168
PGWHVK;LPQTPP	NCP	26.535
LPQTPP;VAPPKGM	NCP	72.638
VAPPKGM;LTTKGAR	NCP	3.756
LTTKGAR;ASLLQSERVL	NCP	39.603
ASLLQSERVL;HVELSLGPLLP	NCP	30.513
HVELSLGPLLP;DSYPLGP	NCP	5.946
DSYPLGP;CGVLGPK	NCP	32.808
CGVLGPK;PPGPPPPG	NCP	17.031
PPGPPPPG;GPKTAP	NCP	42.718
GPKTAP;KMFPLPP	NCP	127.008
KMFPLPP;LPRRPP	NCP	60.225
LPRRPP;AAPLLTPV	NCP	0.257
AAPLLTPV;PGPPHPR	NCP	2.596
PGPPHPR;LPLSKGP	NCP	6.57
LPLSKGP;EETAPAVP	NCP	5.142
EETAPAVP;NDTFVHVTDL	NCP	3.05
NDTFVHVTDL;AAMLAAQDVAQRCK	NCP	0.6
AAMLAAQDVAQRCK;ADPLKVYPQLK	NCP	25.71
ADPLKVYPQLK;APPAVVR	NCP	14.449
APPAVVR;AASELLD	NCP	17.874
AASELLD;SALAQLR	NCP	0.701
SALAQLR;RFALSR	NCP	48.472
RFALSR;CPERLTL	NCP	8.663
CPERLTL;VGNPLLG	NCP	32.557
VGNPLLG;PATAGLPP	NCP	6.705
PATAGLPP;EEMGSSEG	NCP	56.261
EEMGSSEG;PFLGTKL	NCP	35.193
PFLGTKL;VSELTLVRVK	NCP	40.566
VSELTLVRVK;HSSGY	NCP	6.954
HSSGY;KLKEPLP	NCP	3.625
KLKEPLP;QFFKME	NCP	91.799
QFFKME;KARLVLA	NCP	12.159
KARLVLA;LPPPLPP	NCP	19.408
LPPPLPP;YKLPPPPPP	NCP	29.501
YKLPPPPPP;RLGLLPL	NCP	20.944
RLGLLPL;LDEVTR	NCP	14.262
LDEVTR;CGKKAGK	NCP	43.743
CGKKAGK;PLGKSQP	NCP	39.899

PLGKSQP;RARYVLLL	NCP	9.207
RARYVLLL;KDTLLVR	NCP	2.257
KDTLLVR;LYCWYL	NCP	0.969
LYCWYL;LPNGHPK	NCP	21.413
LPNGHPK;KATTLVL	NCP	53.536
KATTLVL;EQRVGPP	NCP	6.527
EQRVGPP;FFSLHV	NCP	26.497
FFSLHV;LGLREVW	NCP	8.544
LGLREVW;SPPTSRPP	NCP	5.06
SPPTSRPP;KLPMPGP	NCP	47.224
KLPMPGP;AARVYK	NCP	3.48
AARVYK;KPWCPK	NCP	24.204
KPWCPK;YAAFML	NCP	14.621
YAAFML;KFEVR	NCP	51.165
KFEVR;QAGALSV	NCP	5.505
QAGALSV;ALLAERK	NCP	52.189
ALLAERK;REDKNLTGTAR	NCP	3.305
REDKNLTGTAR;EAMDLRK	NCP	61.165
EAMDLRK;AEGWPH	NCP	45.818
AEGWPH;KALVGAGK	NCP	16.275
KALVGAGK;PPLSPSTK	NCP	39.916
PPLSPSTK;EMKKY	NCP	18.315
EMKKY;LLVTRADN	NCP	95.544
LLVTRADN;RKAKYKR	NCP	73.155
RKAKYKR;EGAWRL	NCP	61.73
EGAWRL;EFDTNL	NCP	26.991
EFDTNL;VGPHLPK	NCP	10.698
VGPHLPK;KVSPPLL	NCP	56.782
KVSPPLL;LLCCCSS	NCP	37.469
LLCCCSS;FLEKQGF	NCP	6.291
FLEKQGF;PPPLKKPP	NCP	116.005
PPPLKKPP;RCKALKP	NCP	104.075
RCKALKP;PVVAF	NCP	38.775
PVVAF;LTVLDY	NCP	90.106
LTVLDY;EPASLVSS	NCP	3.171
EPASLVSS;YLRDSGF	NCP	42.654
YLRDSGF;VSTQDPL	NCP	69.689
VSTQDPL;LYEFATL	NCP	74.071
LYEFATL;RLKLLRD	NCP	9.164
RLKLLRD;GVVLPK	NCP	15.615
GVVLPK;QAFLVA	NCP	200.503
QAFLVA;RKPPGPL	NCP	4.998
RKPPGPL;KYLQVVY	NCP	0.497
KYLQVVY;TFAQY	NCP	5.055
TFAQY;PSFPTWK	NCP	0.871
PSFPTWK;KPEVDNR	NCP	5.369
KPEVDNR;PTTNKPK	NCP	12.207
PTTNKPK;YVVYKVL	NCP	34.648
YVVYKVL;NHASLY	NCP	57.017
NHASLY;VGPPPER	NCP	54.901
VGPPPER;AAAKPGEL	NCP	26.016

AAAKPGEL;GVSYL	NCP	7.745
GVSYL;LNNLNPA	NCP	113.637
LNNLNPA;KNGYSVP	NCP	146.002
KNGYSVP;QFLYVPLG	NCP	10.872
QFLYVPLG;KSLVPF	NCP	23.021
KSLVPF;LSVLKMSK	NCP	57.925
LSVLKMSK;KERNLSF	NCP	71.582
KERNLSF;VAHETTF	NCP	84.016
VAHETTF;SVPSLDF	NCP	22.313
SVPSLDF;PVGYVT	NCP	2.439
PVGYVT;SHEPVKK	NCP	70.011
SHEPVKK;KEKYKLK	NCP	51.741
KEKYKLK;HKKSEC	NCP	19.263
HKKSEC;KVNVP	NCP	29.926
KVNVP;KGMTTVDDFFQGTK	NCP	27.392
KGMTTVDDFFQGTK;KSGTLL	NCP	3.85
KSGTLL;MDENQFVAVTSTNAAK	NCP	19.619
MDENQFVAVTSTNAAK;YDGPVFDLTTTPK	NCP	5.379
YDGPVFDLTTTPK;SPTRPNPP	NCP	0.069
SPTRPNPP;ELPMLLLE	NCP	37.3
ELPMLLLE;EVADFY	NCP	44.07
EVADFY;CSRTPPPPP	NCP	2.306
CSRTPPPPP;RDPKFLR	NCP	18.067
RDPKFLR;DLLLKK	NCP	299.717
DLLLKK;KWDPGDR	NCP	61.473
KWDPGDR;EKPGP	NCP	35.388
EKPGP;LSPPPY	NCP	27.003
LSPPPY;KLKPSQD	NCP	20.038
KLKPSQD;LDLDL	NCP	37.463
LDLDL;SHEPEFDYLK	NCP	96.528
SHEPEFDYLK;LGPPKEK	NCP	14.887
LGPPKEK;YSLEAGGR	NCP	1.746
YSLEAGGR;MTQLK	NCP	86.879
MTQLK;VGVEL	NCP	49.366
VGVEL;LPGHKKM	NCP	29.291
LPGHKKM;DYLVAP	NCP	39.558
DYLVAP;RKKVPQ	NCP	21.732
RKKVPQ;LPPGDPPP	NCP	21.487
LPPGDPPP;VGAGVETL	NCP	4.289
VGAGVETL;KAGGGPP	NCP	62.299
KAGGGPP;TTGSPK	NCP	91.719
TTGSPK;VLLDSGD	NCP	13.044
VLLDSGD;RVEPVLS	NCP	2.28
RVEPVLS;CTRFGPP	NCP	115.397
CTRFGPP;DRFRGAP	NCP	1.189
DRFRGAP;MGKTFLYN	NCP	18.728
MGKTFLYN;MDSLFP	NCP	29.598
MDSLFP;GVPGGRPP	NCP	30.34
GVPGGRPP;EAGPSLV	NCP	20.088
EAGPSLV;NPKAPPP	NCP	12.117
NPKAPPP;LSLLEEL	NCP	11.444

LSLLEEL;PAGPAF	NCP	55.069
PAGPAF;TLGPPVP	NCP	21.948
TLGPPVP;RPVSLVLGD	NCP	14.421
RPVSLVLGD;GTVVP	NCP	9.909
GTVVP;LPSYPPPPP	NCP	45.903
LPSYPPPPP;GTQEK	NCP	38.724
GTQEK;VGGGSLL	NCP	63.72
VGGGSLL;VGSDLVK	NCP	12.402
VGSDLVK;PLAGP	NCP	60.543
PLAGP;GHLVFLGL	NCP	26.154
GHLVFLGL;TSSHG	NCP	27.572
TSSHG;NFYDPK	NCP	0.729
NFYDPK;PLVNHAAV	NCP	20.275
PLVNHAAV;HDYKKAF	NCP	1.979
HDYKKAF;GFNCEVR	NCP	20.256
GFNCEVR;PPPVKPPP	NCP	10.69
PPPVKPPP;PAVNVLR	NCP	4.491
PAVNVLR;TLSGPPPP	NCP	27.99
TLSGPPPP;KPTVGSP	NCP	94.686
KPTVGSP;FPVEVVP	NCP	30.348
FPVEVVP;KQVHPP	NCP	45.336
KQVHPP;VHSFPL	NCP	203.598
VHSFPL;LSGEVPK	NCP	32.22
LSGEVPK;AFTYVLM	NCP	6.383
AFTYVLM;GNKGTLLH	NCP	33.034
GNKGTLLH;YKTGAPR	NCP	5.19
YKTGAPR;LDDVLK	NCP	7.649
LDDVLK;LPLNVAVN	NCP	63.303
LPLNVAVN;FKEVR	NCP	67.978
FKEVR;TLNFELP	NCP	33.18
TLNFELP;PNARKPP	NCP	13.919
PNARKPP;RLLFH	NCP	64.536
RLLFH;ERCSD	NCP	54.849
ERCSD;PELKGR	NCP	74.265
PELKGR;DPVLKLR	NCP	53.619
DPVLKLR;VLDVRVKE	NCP	24.475
VLDVRVKE;RPEKKPP	NCP	10.175
RPEKKPP;LAPKEPR	NCP	53.791
LAPKEPR;VGLGKLD	NCP	7.461
VGLGKLD;SSLTSPQFP	NCP	4.164
SSLTSPQFP;LFPGLEK	NCP	59.352
LFPGLEK;LSNRPVSL	NCP	6.221
LSNRPVSL;LVAMFLK	NCP	1.065
LVAMFLK;LAVPT	NCP	20.842
LAVPT;TVTLPVT	NCP	1.791
TVTLPVT;ASARLLR	NCP	65.676
ASARLLR;PLSSPVFE	NCP	37.226
PLSSPVFE;KQKAGP	NCP	1.905
KQKAGP;KLLENER	NCP	84.652
KLLENER;LGHVLRK	NCP	12.68
LGHVLRK;DLSDRFPS	NCP	51.865

DLSDRFPS;LNCMW	NCP	2.493
LNCMW;KNMNLWP	NCP	33.752
KNMNLWP;WLDDKPR	NCP	12.657
WLDDKPR;AHRKLEFK	NCP	45.954
AHRKLEFK;KGTKAPK	NCP	43.857
KGTKAPK;KTGDTKK	NCP	2.676
KTGDTKK;LSVSAVTK	NCP	44.314
LSVSAVTK;NAAMYK	NCP	68.889
NAAMYK;VTVEYLL	NCP	0.032
VTVEYLL;TSVFPPP	NCP	26.134
TSVFPPP;VVAERAL	NCP	27.593
VVAERAL;PPLKAGAGP	NCP	0.925
PPLKAGAGP;KMLPPPPP	NCP	95.594
KMLPPPPP;LSFKVT	NCP	12.751
LSFKVT;KRLSHVT	NCP	79.815
KRLSHVT;MEQLAS	NCP	39.611
MEQLAS;DGVTHSLP	NCP	94.119
DGVTHSLP;MKDPC	NCP	113.617
MKDPC;ATVLL	NCP	47.466
ATVLL;KMAVLK	NCP	4.232
KMAVLK;LAAELYL	NCP	1.296
LAAELYL;DPLYGP	NCP	15.883
DPLYGP;DWPPP	NCP	21.852
DWPPP;RPKYSL	NCP	15.641
RPKYSL;KAQQTPK	NCP	3.573
KAQQTPK;FGGPLLK	NCP	7.939
FGGPLLK;EVSLAVF	NCP	0.432
EVSLAVF;LDRNLNY	NCP	47.517
LDRNLNY;AEFLLMF	NCP	9.72
AEFLLMF;DPRHLPLR	NCP	74.633
DPRHLPLR;ERELVRDL	NCP	15.037
ERELVRDL;GLPPPAFLK	NCP	10.727
GLPPPAFLK;TGAKAVKP	NCP	15.304
TGAKAVKP;GVGPLLK	NCP	34.188
GVGPLLK;PVLKHFT	NCP	45.404
PVLKHFT;TLQVLAL	NCP	25.362
TLQVLAL;MLPFELR	NCP	17.287
MLPFELR;SPAAAAN	NCP	61.323
SPAAAAN;VMPALEF	NCP	1.736
VMPALEF;LMGPPFP	NCP	17.884
LMGPPFP;LSDRPPP	NCP	54.116
LSDRPPP;THLMVPP	NCP	8.436
THLMVPP;VTEVLELF	NCP	8.361
VTEVLELF;AMGLMN	NCP	59.685
AMGLMN;VLKVGNK	NCP	87.786
VLKVGNK;MAEKLSM	NCP	69.595
MAEKLSM;VPGMKAT	NCP	8.342
VPGMKAT;SGVGCPT	NCP	14.479
SGVGCPT;SHLKSR	NCP	107.417
SHLKSR;APFPR	NCP	17.277
APFPR;KAYVVF	NCP	55.495

KAYVVF;LEGALDF	NCP	79.169
LEGALDF;VTSFSKL	NCP	19.525
VTSFSKL;RPFASGP	NCP	7.5
RPFASGP;LSKKKV	NCP	51.645
LSKKKV;LGNSKSVL	NCP	16.191
LGNSKSVL;CPSLYE	NCP	28.074
CPSLYE;DKLAAP	NCP	49.199
DKLAAP;AMLNVLF	NCP	19.266
AMLNVLF;GVTPVP	NCP	13.642
GVTPVP;GPSCDLR	NCP	13.329
GPSCDLR;LHDDLTGK	NCP	5.606
LHDDLTGK;KANFPGK	NCP	6.883
KANFPGK;LDSFFVSF	NCP	55.077
LDSFFVSF;QKFYVP	NCP	102.944
QKFYVP;CMSRRP	NCP	31.171
CMSRRP;PLLPLH	NCP	1.554
PLLPLH;SPTPGLLR	NCP	7.611
SPTPGLLR;PHAADN	NCP	65.321
PHAADN;NPLLL	NCP	3.381
NPLLL;KPGPRTP	NCP	96.786
KPGPRTP;KDGKACK	NCP	21.301
KDGKACK;LPQLFRE	NCP	26.622
LPQLFRE;LPPAPPP	NCP	28.458
LPPAPPP;MAVSEFL	NCP	8.373
MAVSEFL;LQKVFERY	NCP	5.96
LQKVFERY;LSHHHMTL	NCP	30.523
LSHHHMTL;LASGAVT	NCP	2.003
LASGAVT;APGCDLR	NCP	1.833
APGCDLR;KTSSQLT	NCP	2.395
KTSSQLT;KETKDLYAPKPSSK	NCP	6.494
KETKDLYAPKPSSK;ASLYAPK	NCP	16.6
ASLYAPK;KLVPPPP	NCP	44.36
KLVPPPP;MSKKKP	NCP	1.308
MSKKKP;KRGPLPLP	NCP	15.99
KRGPLPLP;LHGPEPP	NCP	25.089
LHGPEPP;RALVLLA	NCP	75.558
RALVLLA;KAGPPPPR	NCP	0.678
KAGPPPPR;GRSTLVP	NCP	11.415
GRSTLVP;VDDGF	NCP	15.771
VDDGF;MEPPGGGL	NCP	22.923
MEPPGGGL;PPGGGLGPGR	NCP	0.006
PPGGGLGPGR;GPGMLPPPP	NCP	45.045
GPGMLPPPP;AADARLL	NCP	7.027
AADARLL;KYAAL	NCP	63.671
KYAAL;EVMMPF	NCP	1.047
EVMMPF;LGHYEVR	NCP	6.837
LGHYEVR;QMVLNR	NCP	13.29
QMVLNR;LSKPDLLV	NCP	26.614
LSKPDLLV;PLNLAGK	NCP	7.668
PLNLAGK;RFLVVK	NCP	32.388
RFLVVK;RDKQHR	NCP	87.99

RDKQHR;LTGAGFF	NCP	26.796
LTGAGFF;KPPGHFK	NCP	60.261
KPPGHFK;GLQTQ	NCP	18.732
GLQTQ;RRPPPPP	NCP	24.125
RRPPPPP;DLHLVEL	NCP	28.275
DLHLVEL;LSPPMKT	NCP	4.453
LSPPMKT;VTLPLLQ	NCP	68.67
VTLPLLQ;GPPPYK	NCP	15.47
GPPPYK;AALVSP	NCP	17.598
AALVSP;VLAGP	NCP	124.957
VLAGP;KTFKPPYQ	NCP	21.015
KTFKPPYQ;QPAGVSML	NCP	8.451
QPAGVSML;PDKSGKP	NCP	15.231
PDKSGKP;MEHLQR	NCP	13.913
MEHLQR;EPLPF	NCP	40.234
EPLPF;AALHP	NCP	63.288
AALHP;KYGGPPP	NCP	70.937
KYGGPPP;EEALQLR	NCP	29.035
EEALQLR;REGCAGK	NCP	13.749
REGCAGK;GPPQPP	NCP	10.878
GPPQPP;AELYVKPG	NCP	18.995
AELYVKPG;KNYGAP	NCP	61.768
KNYGAP;NGLFKTT	NCP	8.534
NGLFKTT;RTFLAVT	NCP	45.039
RTFLAVT;ASAVKP	NCP	3.355
ASAVKP;KSLEEPK	NCP	84.801
KSLEEPK;DLEGPPL	NCP	10.952
DLEGPPL;FSTLNP	NCP	1.899
FSTLNP;AAPVDLELKK	NCP	3.834
AAPVDLELKK;QQARF	NCP	145.611
QQARF;TKRQPTDC	NCP	19.902
TKRQPTDC;KPSQTSK	NCP	42.472
KPSQTSK;FDDHLR	NCP	3.24
FDDHLR;KGVLFWR	NCP	15.545
KGVLFWR;VLTPP	NCP	105.204
VLTPP;LDLDSKP	NCP	21.438
LDLDSKP;LFMKFLLQ	NCP	33.523
LFMKFLLQ;PHALLRL	NCP	33.704
PHALLRL;PTEGLLR	NCP	86.734
PTEGLLR;SKRGLLTL	NCP	17.979
SKRGLLTL;LVQGQF	NCP	30.146
LVQGQF;HLKEPP	NCP	11.682
HLKEPP;KMVYVSL	NCP	8.95
KMVYVSL;EADAWF	NCP	17.219
EADAWF;PPKVFKK	NCP	88.096
PPKVFKK;LTAEVEL	NCP	42.429
LTAEVEL;GPDWHLK	NCP	17.492
GPDWHLK;ATPVPPR	NCP	4.902
ATPVPPR;LVLLN	NCP	43.503
LVLLN;PDLLVV	NCP	11.641
PDLLVV;FVDKNF	NCP	17.225

FVDKNF;DEASLTATAPELDDEEKF	NCP	25.809
DEASLTATAPELDDEEKF;QMWQHLTKAEAK	NCP	0.933
QMWQHLTKAEAK;EGWLAGY	NCP	95.68
EGWLAGY;FFSLP	NCP	99.696
FFSLP;APSGWLG	NCP	9.102
APSGWLG;LSLPEME	NCP	25.067
LSLPEME;ALTDL	NCP	41.229
ALTDL;RTVKTYG	NCP	16.41
RTVKTYG;MLPPAAPP	NCP	41.179
MLPPAAPP;LSATPLP	NCP	52.191
LSATPLP;KLGHPPP	NCP	1.173
KLGHPPP;WKQPPHT	NCP	4.41
WKQPPHT;KSELAGH	NCP	29.582
KSELAGH;KGSKGPLR	NCP	14.38
KGSKGPLR;GQAVPN	NCP	6.474
GQAVPN;LYAAVLE	NCP	82.878
LYAAVLE;KTHPTK	NCP	0.755
KTHPTK;YDNVLK	NCP	21.237
YDNVLK;LSHTVPF	NCP	36.471
LSHTVPF;KLQTAHK	NCP	139.518
KLQTAHK;KGSHKPP	NCP	8.766
KGSHKPP;PTAAALAY	NCP	23.826
PTAAALAY;LTMDASGPK	NCP	4.968
LTMDASGPK;KFTVDLPK	NCP	36.834
KFTVDLPK;TVLDEVR	NCP	39.991
TVLDEVR;GPKVLTH	NCP	14.388
GPKVLTH;CDRKLTAL	NCP	44.247
CDRKLTAL;SLFMAPP	NCP	16.41
SLFMAPP;MEDTQL	NCP	1.152
MEDTQL;MGPLWPP	NCP	52.56
MGPLWPP;TLPGEERL	NCP	29.25
TLPGEERL;KGCMEEK	NCP	13.227
KGCMEEK;EETTFGL	NCP	48.269
EETTFGL;GKSNVQT	NCP	9.339
GKSNVQT;KDHKRTF	NCP	9.696
KDHKRTF;TVDLLL	NCP	11.802
TVDLLL;PKVNVLL	NCP	74.049
PKVNVLL;APPPVLPPP	NCP	0.937
APPPVLPPP;NGFASLL	NCP	69.036
NGFASLL;VELKVR	NCP	5.79
VELKVR;KLMEAR	NCP	116.198
KLMEAR;LVNLLK	NCP	86.95
LVNLLK;TDCVVVVNPQTKR	NCP	6.563
TDCVVVVNPQTKR;SNVEEADAAMAASPHAVDGNTVELK	NCP	0.066
SNVEEADAAMAASPHAVDGNTVELK;SQFGTVEK	NCP	0.189
SQFGTVEK;FQNHDAADKAAVVK	NCP	0.081
FQNHDAADKAAVVK;KMALDV	NCP	20.859
KMALDV;HSFLVPL	NCP	43.185
HSFLVPL;KVESEDL	NCP	0.486
KVESEDL;ETTGLLLR	NCP	39.834
ETTGLLLR;KVEANFR	NCP	14.217

KVEANFR;DFEPLHK	NCP	26.677
DFEPLHK;GFSLY	NCP	3.549
GFSLY;KPSVTPR	NCP	20.81
KPSVTPR;KRGPSGK	NCP	57.073
KRGPSGK;LVGNTTGHL	NCP	9.101
LVGNTTGHL;NQSLVHR	NCP	46.296
NQSLVHR;EGPEFL	NCP	8.946
EGPEFL;PDGLLLYC	NCP	42.546
PDGLLLYC;MLLPDK	NCP	39.375
MLLPDK;KPQRLSD	NCP	126.42
KPQRLSD;NVPPKHL	NCP	21.004
NVPPKHL;KEFKSLEFK	NCP	2.441
KEFKSLEFK;HMQLLCV	NCP	70.167
HMQLLCV;RGPYAKP	NCP	46.008
RGPYAKP;GRGLWEK	NCP	44.316
GRGLWEK;DSRLKLG	NCP	5.964
DSRLKLG;KSCYPP	NCP	161.812
KSCYPP;KQQHTVDPQ	NCP	152.87
KQQHTVDPQ;LFFNPKYL	NCP	151.66
LFFNPKYL;KFQHFK	NCP	17.274
KFQHFK;MGSGKMP	NCP	5.036
MGSGKMP;LSLALA	NCP	71.329
LSLALA;PPFPPRP	NCP	16.617
PPFPPRP;EEQLGVEY	NCP	76.05
EEQLGVEY;ETRMWN	NCP	32.531
ETRMWN;GHRTTM	NCP	13.867
GHRTTM;PPDLGPP	NCP	36.587
PPDLGPP;GFSEVFH	NCP	6.135
GFSEVFH;LDPGAAW	NCP	244.236
LDPGAAW;LSMLGGK	NCP	52.845
LSMLGGK;PDTHTY	NCP	31.351
PDTHTY;VPYHPLR	NCP	169.971
VPYHPLR;EELYRV	NCP	70.172
EELYRV;GQEPGVK	NCP	23.976
GQEPGVK;VLREKAP	NCP	146.994
VLREKAP;RAKPGAK	NCP	6.177
RAKPGAK;LRPYFPEQ	NCP	8.769
LRPYFPEQ;KSTLKVS	NCP	14.109
KSTLKVS;YRAAQK	NCP	24.849
YRAAQK;GPPLRPP	NCP	5.46
GPPLRPP;NPKGFR	NCP	23.635
NPKGFR;APHPGVK	NCP	6.02
APHPGVK;DYHVPR	NCP	27.939
DYHVPR;VGAPVYMAA	NCP	65.289
VGAPVYMAA;VNGLVPK	NCP	27.976
VNGLVPK;VVTTLRP	NCP	174.989
VVTTLRP;EVSLRPY	NCP	13.048
EVSLRPY;VHSLPAAP	NCP	7.062
VHSLPAAP;TLAEEEK	NCP	20.681
TLAEEEK;LLGEPAPP	NCP	9.723
LLGEPAPP;KEMDPK	NCP	18.478

KEMDPK;KPASRSK	NCP	28.001
KPASRSK;GDPPQSVL	NCP	22.83
GDPPQSVL;VLGKKKP	NCP	51.889
VLGKKKP;PVPVLFR	NCP	9.836
PVPVLFR;LMKLLT	NCP	13.372
LMKLLT;CVLFASPR	NCP	5.523
CVLFASPR;KFLYRY	NCP	22.086
KFLYRY;NAMDLR	NCP	5.135
NAMDLR;LSTVALA	NCP	5.512
LSTVALA;MTLPF	NCP	19.142
MTLPF;SFAAL	NCP	17.557
SFAAL;TQDLHLK	NCP	18.323
TQDLHLK;KEAHVPH	NCP	19.738
KEAHVPH;GHQQLY	NCP	22.131
GHQQLY;RLELLR	NCP	20.093
RLELLR;VLNLT	NCP	142.267
VLNLT;KPRVEKR	NCP	9.149
KPRVEKR;KEEFP	NCP	11.181
KEEFP;LYKKTGKL	NCP	12.441
LYKKTGKL;THVWKP	NCP	29.532
THVWKP;LDGGAAPD	NCP	47.607
LDGGAAPD;EEELALA	NCP	2.436
EEELALA;KALKQPQ	NCP	33.295
KALKQPQ;DLPLTDL	NCP	20.963
DLPLTDL;KPLAASPC	NCP	1.059
KPLAASPC;PGPPLVGK	NCP	17.007
PGPPLVGK;KTVFPT	NCP	9.892
KTVFPT;KAVPSL	NCP	17.741
KAVPSL;LSAGFPK	NCP	81.355
LSAGFPK;MYLLQQ	NCP	5.868
MYLLQQ;LKVDQGTL	NCP	75.125
LKVDQGTL;LTKWTGR	NCP	53.148
LTKWTGR;RLVSDTK	NCP	60.273
RLVSDTK;PLPVP	NCP	1.651
PLPVP;YPKGGLH	NCP	1.962
YPKGGLH;VAAGP	NCP	3.026
VAAGP;AVPPTYA	NCP	9.816
AVPPTYA;GDSLARL	NCP	11.557
GDSLARL;LSALLDGKN	NCP	1.742
LSALLDGKN;PCCLP	NCP	16.992
PCCLP;KPTHLTH	NCP	42.858
KPTHLTH;KEVLAGGK	NCP	5.847
KEVLAGGK;LSLPL	NCP	0.017
LSLPL;LPLDPFS	NCP	3.174
LPLDPFS;KFNPPP	NCP	40.248
KFNPPP;LTNGVT	NCP	50.904
LTNGVT;AYVHRK	NCP	125.01
AYVHRK;CGSKFLSF	NCP	53.585
CGSKFLSF;LDNVTKPP	NCP	9.954
LDNVTKPP;YHFSSAF	NCP	142.905
YHFSSAF;PEADPF	NCP	16.396

PEADPF;LNGGKPK	NCP	20.127
LNGGKPK;RGELDVR	NCP	39.119
RGELDVR;RPEERPP	NCP	67.149
RPEERPP;AAPRV	NCP	6.069
AAPRV;GGYAGK	NCP	6.834
GGYAGK;PVSVKPP	NCP	23.191
PVSVKPP;KALEQEKQ	NCP	0.044
KALEQEKQ;GDLHWH	NCP	48.615
GDLHWH;RVEERK	NCP	16.323
RVEERK;ELLDPVL	NCP	7.254
ELLDPVL;CVKSHRY	NCP	32.596
CVKSHRY;VEVSAPK	NCP	59.099
VEVSAPK;MDDLPTT	NCP	144.009
MDDLPTT;QAGVTLR	NCP	127.15
QAGVTLR;ASPVFP	NCP	34.872
ASPVFP;QLSNLK	NCP	164.064
QLSNLK;KGEGSVVL	NCP	3.668
KGEGSVVL;AHEVLRK	NCP	5.911
AHEVLRK;KHGDYKK	NCP	0.018
KHGDYKK;KWSGPAPL	NCP	46.313
KWSGPAPL;VTAADLSQ	NCP	54.547
VTAADLSQ;RSPGLEPP	NCP	8.6
RSPGLEPP;PPCAPDP	NCP	28.38
PPCAPDP;AAPVTEP	NCP	7.266
AAPVTEP;DLGPPVVL	NCP	13.293
DLGPPVVL;KLSQPPPF	NCP	18.885
KLSQPPPF;KGSDPPR	NCP	21.786
KGSDPPR;VGPPPPPP	NCP	4.017
VGPPPPPP;RLGPVKP	NCP	42.22
RLGPVKP;RTTAPR	NCP	53.474
RTTAPR;KPFRQAK	NCP	93.598
KPFRQAK;LLPSPF	NCP	24.593
LLPSPF;QSLAF	NCP	4.099
QSLAF;RAAGLLL	NCP	21.674
RAAGLLL;SPGVLR	NCP	25.161
SPGVLR;AAPAAYDASDTHL	NCP	46.419
AAPAAYDASDTHL;ELVPPAN	NCP	2.631
ELVPPAN;TPLVTKH	NCP	0.051
TPLVTKH;GVMLLTG	NCP	23.109
GVMLLTG;FLPHLSR	NCP	19.912
FLPHLSR;LCTVATLR	NCP	33.338
LCTVATLR;GVPPPPR	NCP	30.957
GVPPPPR;SPPAGARPP	NCP	5.271
SPPAGARPP;TAGFVAP	NCP	13.689
TAGFVAP;QPAAGSP	NCP	26.958
QPAAGSP;VVGWVGK	NCP	59.368
VVGWVGK;TSATKPR	NCP	0.938
TSATKPR;LVPPGHA	NCP	51.576
LVPPGHA;GPCCC	NCP	40.347
GPCCC;GHLVTSK	NCP	14.92
GHLVTSK;RDPPSGP	NCP	13.547

RDPPSGP;RVPPKP	NCP	0.345
RVPPKP;YLPQPY	NCP	26.055
YLPQPY;YAGPF	NCP	2.377
YAGPF;LYEGLDF	NCP	11.325
LYEGLDF;KGELL	NCP	17.037
KGELL;LLFKKL	NCP	77.877
LLFKKL;EVGDLL	NCP	11.345
EVGDLL;KFLAPPPPP	NCP	23.292
KFLAPPPPP;APRKPDF	NCP	27.948
APRKPDF;QAALLFLPR	NCP	41.244
QAALLFLPR;ARHVFL	NCP	15.223
ARHVFL;QRAPGGPH	NCP	17.237
QRAPGGPH;MKLTDP	NCP	25.113
MKLTDP;TVGPLLP	NCP	8.352
TVGPLLP;QSAVPPQ	NCP	18.756
QSAVPPQ;KGLNSRP	NCP	3.52
KGLNSRP;APLAPLM	NCP	37.316
APLAPLM;KPKAPAT	NCP	45.399
KPKAPAT;PKAPGVFL	NCP	205.74
PKAPGVFL;ESLRPTT	NCP	47.278
ESLRPTT;LSVDLGPK	NCP	56.556
LSVDLGPK;DDDDFDEEVEEKAPVKK	NCP	46.445
DDDDFDEEVEEKAPVKK;DDDFDEEVEEKAPVK	NCP	0.003
DDDFDEEVEEKAPVK;FDEEVEEKAPVKK	NCP	0.009
FDEEVEEKAPVKK;PLGPP	NCP	2.43
PLGPP;VLLLPGCC	NCP	104.952
VLLLPGCC;PLGMPLP	NCP	13.759
PLGMPLP;NGVPLL	NCP	32.519
NGVPLL;ESDGLKK	NCP	1.084
ESDGLKK;LDYDSLP	NCP	14.772
LDYDSLP;EQRPTH	NCP	55.394
EQRPTH;KGDYFP	NCP	78.705
KGDYFP;MAAHPTPP	NCP	152.916
MAAHPTPP;DLGTLAK	NCP	18.345
DLGTLAK;KATAALK	NCP	5.025
KATAALK;EKVTVLTK	NCP	0.652
EKVTVLTK;MMMDC	NCP	81.47
MMMDC;GLCEYNQ	NCP	24.019
GLCEYNQ;TAAPVLL	NCP	108.561
TAAPVLL;KNKLLER	NCP	105.227
KNKLLER;LKSKVLNP	NCP	41.254
LKSKVLNP;KSYLHGTVK	NCP	111.698
KSYLHGTVK;PPYLVMK	NCP	217.789
PPYLVMK;KEYYTK	NCP	52.292
KEYYTK;MGLMNSF	NCP	43.39
MGLMNSF;YSYCYN	NCP	19.685
YSYCYN;KDVSFN	NCP	42.103
KDVSFN;GVWHHT	NCP	90.96
GVWHHT;GMNMLK	NCP	40.232
GMNMLK;LYKRTKK	NCP	110.04
LYKRTKK;TSLNQ	NCP	133.548

TSLNQ;HEEPVSK	NCP	60.592
HEEPVSK;VYLDTAK	NCP	9.236
VYLDTAK;RNGVYSR	NCP	3.321
RNGVYSR;LWFDK	NCP	10.251
LWFDK;KELQREK	NCP	15.417
KELQREK;KLFDT	NCP	1.666
KLFDT;HTVLGAGK	NCP	16.944
HTVLGAGK;CSCCVP	NCP	32.915
CSCCVP;GLDLY	NCP	10.735
GLDLY;KEGSYGY	NCP	105.758
KEGSYGY;MDPPR	NCP	20.862
MDPPR;TNDMFRL	NCP	17.883
TNDMFRL;VVPSPK	NCP	9.084
VVPSPK;TVDLF	NCP	126.265
TVDLF;KLTSGWV	NCP	14.838
KLTSGWV;TEQMTLRGTLK	NCP	47.762
TEQMTLRGTLK;AHTNMF	NCP	1.522
AHTNMF;WNTLGVCKY	NCP	0.59
WNTLGVCKY;NEGKHLYTL	NCP	1.329
NEGKHLYTL;VVLANAR	NCP	19.255
VVLANAR;VAFHYK	NCP	97.473
VAFHYK;GVPGVKL	NCP	15.102
GVPGVKL;THFGKSE	NCP	145.431
THFGKSE;EHNLKP	NCP	2.337
EHNLKP;EMDFPK	NCP	5.133
EMDFPK;WKNQSLR	NCP	2.016
WKNQSLR;QRAAPALP	NCP	3.48
QRAAPALP;LPAAPEH	NCP	37.568
LPAAPEH;RSLPGPDF	NCP	20.539
RSLPGPDF;EHLSKVK	NCP	134.933
EHLSKVK;VPGLLG	NCP	6.943
VPGLLG;VTTEVKSVEMHHEA	NCP	45.137
VTTEVKSVEMHHEA;ALPGDNVG	NCP	0.051
ALPGDNVG;SGKKLEDGPKF	NCP	0.243
SGKKLEDGPKF;KLEDGPKFLK	NCP	0.009
KLEDGPKFLK;LEDGPKFL	NCP	0.003
LEDGPKFL;PVPPP	NCP	51.487
PVPPP;PPMEAQ	NCP	167.802
PPMEAQ;HAAPGP	NCP	65.187
HAAPGP;KQRPTPP	NCP	23.954
KQRPTPP;PLLNSKSV	NCP	2.802
PLLNSKSV;ESGSYK	NCP	1.683
ESGSYK;WDPDQR	NCP	14.241
WDPDQR;CFVYLLPQ	NCP	9.961
CFVYLLPQ;VAVLY	NCP	19.989
VAVLY;HYLVVSG	NCP	53.031
HYLVVSG;QSRKPK	NCP	85.46
QSRKPK;KATGEVK	NCP	38.428
KATGEVK;VAPEKVP	NCP	19.419
VAPEKVP;LHLLEQLP	NCP	16.925
LHLLEQLP;KGPQPPTP	NCP	60.804

KGPQPPTP;PLPVAL	NCP	15.243
PLPVAL;DSGKSTTTGHL	NCP	99.336
DSGKSTTTGHL;FEKEAAEMGK	NCP	0.075
FEKEAAEMGK;LEPSANMPWFK	NCP	0.48
LEPSANMPWFK;PSANMPWF	NCP	0.006
PSANMPWF;SANMPWFK	NCP	0.003
SANMPWFK;DPPMEAAGFT	NCP	0.381
DPPMEAAGFT;TARPVLS	NCP	59.796
TARPVLS;MDLKALL	NCP	38.619
MDLKALL;MDSFKVVLE	NCP	17.559
MDSFKVVLE;KVVLEGPAPWGF	NCP	0.012
KVVLEGPAPWGF;AHLTGTEF	NCP	5.304
AHLTGTEF;QDPDEEHLKK	NCP	0.18
QDPDEEHLKK;QSPRYTRL	NCP	0.24
QSPRYTRL;HVLNVQS	NCP	0.051
HVLNVQS;VLEEGGFFEEK	NCP	2.64
VLEEGGFFEEK;YGDDMP	NCP	3.433
YGDDMP;SHAFSHV	NCP	6.962
SHAFSHV;PCAPDSF	NCP	4.353
PCAPDSF;GPPGPPPPP	NCP	45.447
GPPGPPPPP;EFSVGPK	NCP	46.926
EFSVGPK;LDPPVPE	NCP	10.218
LDPPVPE;RPLMSAL	NCP	69.334
RPLMSAL;THTSVVP	NCP	66.099
THTSVVP;EYGKLSR	NCP	0.218
EYGKLSR;LGLGAAVL	NCP	3.436
LGLGAAVL;AHLRQLLR	NCP	12.818
AHLRQLLR;NFGNVR	NCP	37.639
NFGNVR;DHKFDL	NCP	10.05
DHKFDL;ARPALPAP	NCP	102.453
ARPALPAP;GVLLGEP	NCP	60.837
GVLLGEP;LAGEFP	NCP	34.496
LAGEFP;LGHETTF	NCP	6.679
LGHETTF;KPSDTK	NCP	18.309
KPSDTK;DFAKAKVEQDN	NCP	40.118
DFAKAKVEQDN;AAYKL	NCP	141.723
AAYKL;GPGSVTRLP	NCP	3.282
GPGSVTRLP;KNEEGP	NCP	12.478
KNEEGP;MLPQQAV	NCP	3.162
MLPQQAV;DFGHRVP	NCP	7.653
DFGHRVP;KHAVTQSP	NCP	18.267
KHAVTQSP;KAWFLLQ	NCP	13.107
KAWFLLQ;MLNKPPP	NCP	2.465
MLNKPPP;VGAFLG	NCP	5.625
VGAFLG;RGVEVPPP	NCP	32.958
RGVEVPPP;LKMTSAL	NCP	9.766
LKMTSAL;DPKPLRPP	NCP	14.184
DPKPLRPP;VAGLSP	NCP	44.654
VAGLSP;HHSVLKP	NCP	37.281
HHSVLKP;VGGRVPP	NCP	71.056
VGGRVPP;VAPVDF	NCP	58.227

VAPVDF;TGEKLNF	NCP	22.98
TGEKLNF;KPCQPR	NCP	20.912
KPCQPR;KPLLKAP	NCP	13.8
KPLLKAP;VKESKEETPGTEWEK	NCP	14.541
VKESKEETPGTEWEK;VAQLCDFNPK	NCP	0.045
VAQLCDFNPK;MSLKQTPLSR	NCP	0.075
MSLKQTPLSR;DPGTKWSP	NCP	22.06
DPGTKWSP;LFTPLFE	NCP	4.967
LFTPLFE;PVFGLLT	NCP	7.206
PVFGLLT;MPVPLN	NCP	25.593
MPVPLN;TLYYDLK	NCP	76.494
TLYYDLK;TLLNAF	NCP	64.701
TLLNAF;VPPLFLK	NCP	23.541
VPPLFLK;RRTPH	NCP	3.304
RRTPH;ERKLDDR	NCP	1.118
ERKLDDR;SGGVVF	NCP	21.067
SGGVVF;KCKTLSP	NCP	19.122
KCKTLSP;TTLQLVLQ	NCP	6.471
TTLQLVLQ;AAVYTYL	NCP	27.788
AAVYTYL;TLLDVLPL	NCP	32.062
TLLDVLPL;LFCAGYL	NCP	5.721
LFCAGYL;TVMLKK	NCP	41.091
TVMLKK;AADFLRA	NCP	89.819
AADFLRA;KPSYHYR	NCP	119.259
KPSYHYR;NRALVPM	NCP	14.4
NRALVPM;YVLRGT	NCP	138.961
YVLRGT;KHLAPPP	NCP	114.146
KHLAPPP;LKLLEDL	NCP	24.247
LKLLEDL;VFEDPF	NCP	122.684
VFEDPF;LDLKPM	NCP	16.02
LDLKPM;FLGELL	NCP	16.89
FLGELL;PKLFL	NCP	48.843
PKLFL;KFLKLR	NCP	19.273
KFLKLR;KVWEGH	NCP	87.444
KVWEGH;NNLFFDL	NCP	247.653
NNLFFDL;KVLQY	NCP	54.641
KVLQY;MLLPNAHL	NCP	49.36
MLLPNAHL;MVGPPQR	NCP	16.83
MVGPPQR;MFLGR	NCP	18.056
MFLGR;MLLPLGL	NCP	23.143
MLLPLGL;LTKAGNK	NCP	51.93
LTKAGNK;NVAVVKK	NCP	25.239
NVAVVKK;MGLVCKK	NCP	110.606
MGLVCKK;KLLAGER	NCP	12.801
KLLAGER;GSSETLK	NCP	82.45
GSSETLK;GRASKPR	NCP	20.46
GRASKPR;VVYKKMM	NCP	91.758
VVYKKMM;SPHLEPK	NCP	7.304
SPHLEPK;PGATGN	NCP	80.506
PGATGN;GNLGDLR	NCP	17.838
GNLGDLR;FVTPF	NCP	18.521

FVTPF;EPVGFPK	NCP	0.232
EPVGFPK;RNKSLLY	NCP	23.454
RNKSLLY;YMLVMM	NCP	259.482
YMLVMM;KEFYP	NCP	66.149
KEFYP;MNVTHPPP	NCP	32.379
MNVTHPPP;WEERLFY	NCP	0.664
WEERLFY;LKVVPQK	NCP	92.651
LKVVPQK;RLKKVLR	NCP	30.091
RLKKVLR;DLPHPF	NCP	0.297
DLPHPF;LGKLPF	NCP	31.085
LGKLPF;FVGVY	NCP	13.51
FVGVY;KFSPP	NCP	12.84
KFSPP;VPVAFH	NCP	9.543
VPVAFH;LSYKNPP	NCP	89.951
LSYKNPP;YKYPPLLG	NCP	60.687
YKYPPLLG;LPHVP	NCP	43.225
LPHVP;DLRHDP	NCP	33.021
DLRHDP;EVATLLH	NCP	19.722
EVATLLH;KDFCFC	NCP	123.788
KDFCFC;SPDLEFK	NCP	25.458
SPDLEFK;VEVETLP	NCP	12.865
VEVETLP;MVVFHK	NCP	23.453
MVVFHK;WLLPALQN	NCP	31.636
WLLPALQN;KLYNLFK	NCP	18.998
KLYNLFK;CESSDC	NCP	9.762
CESSDC;LSVKAPK	NCP	11.608
LSVKAPK;YLVEFP	NCP	25.214
YLVEFP;KGLALQLK	NCP	26.022
KGLALQLK;KLYRMAP	NCP	9.345
KLYRMAP;ASLWVGK	NCP	0.154
ASLWVGK;KLLPVGP	NCP	11.115
KLLPVGP;MVPPPPF	NCP	12.449
MVPPPPF;ATDVFNSK	NCP	110.04
ATDVFNSK;NCCCGY	NCP	12.348
NCCCGY;ASTLRFN	NCP	31.983
ASTLRFN;VSELSAY	NCP	3.039
VSELSAY;DRYHLCR	NCP	57.822
DRYHLCR;HEELTVMP	NCP	31.08
HEELTVMP;MNHKAK	NCP	0.525
MNHKAK;AHDLVLY	NCP	31.537
AHDLVLY;VYVKPPF	NCP	36.389
VYVKPPF;CLLGCG	NCP	80.697
CLLGCG;AGHRLLV	NCP	34.72
AGHRLLV;KGGAVPV	NCP	2.403
KGGAVPV;ANAVPN	NCP	1.437
ANAVPN;GSVMLHR	NCP	2.148
GSVMLHR;ESLLSRSVP	NCP	13.203
ESLLSRSVP;KSELVEK	NCP	79.34
KSELVEK;VEDPTLL	NCP	12.301
VEDPTLL;NEYLNPK	NCP	109.076
NEYLNPK;LPVGLPQT	NCP	17.599

LPVGLPQT;KTKSLGV	NCP	3.222
KTKSLGV;APSYLRQ	NCP	232.994
APSYLRQ;VYPFPP	NCP	19.474
VYPFPP;KHSSAKT	NCP	46.19
KHSSAKT;KWSKKLR	NCP	77.407
KWSKKLR;PGKVAGSAP	NCP	7.737
PGKVAGSAP;TFVPPPPP	NCP	72.342
TFVPPPPP;GKSLL	NCP	41.891
GKSLL;QTKLLL	NCP	0.69
QTKLLL;HVRVLGF	NCP	29.686
HVRVLGF;VSLNTEFK	NCP	14.462
VSLNTEFK;VLDVRVK	NCP	29.902
VLDVRVK;NGDNMLEPSANMPWFK	NCP	58.286
NGDNMLEPSANMPWFK;TTLLEALDC	NCP	0.087
TTLLEALDC;TTEVKSVEMHHEA	NCP	0.18
TTEVKSVEMHHEA;DPPMEAAGFTAQ	NCP	0.138
DPPMEAAGFTAQ;PPMEAAGF	NCP	0.003
PPMEAAGF;VLDCHTAH	NCP	0.081
VLDCHTAH;SDYPPLGR	NCP	0.168
SDYPPLGR;QTDFNLK	NCP	34.126
QTDFNLK;MANVRVF	NCP	101.487
MANVRVF;HLLVHRLPL	NCP	16.88
HLLVHRLPL;LYKFKQD	NCP	10.677
LYKFKQD;FGFNTLR	NCP	105.772
FGFNTLR;SSPVFPR	NCP	64.4
SSPVFPR;GSLPGL	NCP	107.608
GSLPGL;LLSPPEKQ	NCP	59.97
LLSPPEKQ;EKPGSST	NCP	44.097
EKPGSST;VLDLEL	NCP	48.333
VLDLEL;EPAPK	NCP	69.498
EPAPK;HFKGLAL	NCP	1.545
HFKGLAL;ERSPVFL	NCP	24.165
ERSPVFL;PLLEKPD	NCP	12.648
PLLEKPD;TVVPLKY	NCP	26.943
TVVPLKY;LSANLL	NCP	10.835
LSANLL;KGQKPVP	NCP	34.519
KGQKPVP;LEPLLDF	NCP	68.855
LEPLLDF;HMTLKTK	NCP	92.635
HMTLKTK;QTVNPP	NCP	24.264
QTVNPP;KQGSPGK	NCP	4.928
KQGSPGK;NPLSL	NCP	119.391
NPLSL;GVVPK	NCP	1.899
GVVPK;SGLYGFN	NCP	66.607
SGLYGFN;YPKKAP	NCP	64.389
YPKKAP;DFLETHY	NCP	85.274
DFLETHY;KYLVYH	NCP	29.529
KYLVYH;TPHVYP	NCP	168.855
TPHVYP;KGKYVVL	NCP	41.892
KGKYVVL;EVAGLKK	NCP	182.367
EVAGLKK;KDDHAY	NCP	82.068
KDDHAY;PAELEEA	NCP	3.34

PAELEEA;EEYCMP	NCP	2.258
EEYCMP;CSMNYHK	NCP	50.97
CSMNYHK;PNWPVK	NCP	128.19
PNWPVK;QVVLPF	NCP	1.492
QVVLPF;NFKLLSH	NCP	88.883
NFKLLSH;TVYDTLP	NCP	29.335
TVYDTLP;LDEKDLK	NCP	59.259
LDEKDLK;AAEREPPPLGDGKPTDF	NCP	57.545
AAEREPPPLGDGKPTDF;VGKEDSSSTEFVEK	NCP	39.705
VGKEDSSSTEFVEK;ESDKL	NCP	113.121
ESDKL;KQQPVLS	NCP	15.13
KQQPVLS;AALPK	NCP	21.42
AALPK;THLPAF	NCP	41.294
THLPAF;LSLKPPTLL	NCP	5.911
LSLKPPTLL;DQAKKF	NCP	36.17
DQAKKF;EKEPGAPR	NCP	39.699
EKEPGAPR;WAAQME	NCP	70.254
WAAQME;FELNPR	NCP	117.924
FELNPR;VEEKLK	NCP	162.468
VEEKLK;KLSTGAF	NCP	25.857
KLSTGAF;KPAKFR	NCP	2.394
KPAKFR;GPGPPVH	NCP	11.898
GPGPPVH;RSPVPTLR	NCP	16.168
RSPVPTLR;YMPKKH	NCP	2.675
YMPKKH;CSKTLVL	NCP	24.463
CSKTLVL;GLTRVYLSF	NCP	13.313
GLTRVYLSF;NVLNFRK	NCP	17.541
NVLNFRK;KDPVSEPK	NCP	4.081
KDPVSEPK;KRSTVRL	NCP	14.279
KRSTVRL;GSECCE	NCP	37.828
GSECCE;GFVLL	NCP	30.675
GFVLL;FAFKRG	NCP	50.879
FAFKRG;KAAVLSLY	NCP	16.437
KAAVLSLY;QEEPVKK	NCP	48.703
QEEPVKK;KSLLVQ	NCP	47.003
KSLLVQ;KFFHF	NCP	31.863
KFFHF;PPVLAAC	NCP	13.965
PPVLAAC;TPKALRP	NCP	8.248
TPKALRP;GSAADLLR	NCP	34.481
GSAADLLR;KLRTLEL	NCP	94.41
KLRTLEL;SVYVYKV	NCP	8.193
SVYVYKV;KLEGPKP	NCP	74.986
KLEGPKP;ALLMY	NCP	15.377
ALLMY;AAGPLPPR	NCP	30.307
AAGPLPPR;KDELLM	NCP	68.253
KDELLM;KLRYFLKQ	NCP	134.033
KLRYFLKQ;AVVSLVLP	NCP	4.62
AVVSLVLP;LALVK	NCP	37.416
LALVK;KNASATK	NCP	20.655
KNASATK;PPPDKHK	NCP	54.375
PPPDKHK;NFGGGAF	NCP	42.87

NFGGGAF;CGDTAF	NCP	20.217
CGDTAF;KDRVYF	NCP	14.407
KDRVYF;VTGTPEK	NCP	40.662
VTGTPEK;VVQLP	NCP	89.913
VVQLP;NSLKPR	NCP	29.283
NSLKPR;LRLQSVVL	NCP	65.966
LRLQSVVL;EFSAEQL	NCP	16.66
EFSAEQL;VGLHY	NCP	34.011
VGLHY;YPKKMK	NCP	6.18
YPKKMK;WCPFPQ	NCP	32.624
WCPFPQ;YCCCCFF	NCP	34.993
YCCCCFF;KVSFQPP	NCP	6.624
KVSFQPP;LEGKVLK	NCP	25.065
LEGKVLK;KCFLH	NCP	10.064
KCFLH;KSAKQMK	NCP	16.177
KSAKQMK;FLPGFF	NCP	28.163
FLPGFF;AACHRDP	NCP	13.653
AACHRDP;KGSQKPR	NCP	77.134
KGSQKPR;LAPPLPK	NCP	43.265
LAPPLPK;TPTLPRR	NCP	6.424
TPTLPRR;KMPAGLH	NCP	37.632
KMPAGLH;VPEVPN	NCP	63.713
VPEVPN;KNLKKEP	NCP	5.037
KNLKKEP;YGTLEKK	NCP	55.506
YGTLEKK;MAAAK	NCP	33.237
MAAAK;TAVSF	NCP	9.961
TAVSF;RPLPNF	NCP	50.427
RPLPNF;RVHGYP	NCP	188.673
RVHGYP;TLAGNKF	NCP	120.998
TLAGNKF;SFGHLQK	NCP	173.586
SFGHLQK;SPVFPAR	NCP	66.327
SPVFPAR;KSCKVK	NCP	0.654
KSCKVK;LVAAEYL	NCP	55.12
LVAAEYL;FFLLYL	NCP	1.913
FFLLYL;KPGVLKE	NCP	4.545
KPGVLKE;LGVTKPK	NCP	297.406
LGVTKPK;TELQQDVDDTKSAK	NCP	146.228
TELQQDVDDTKSAK;KPATLFY	NCP	74.205
KPATLFY;ATATPVPPR	NCP	184.326
ATATPVPPR;GKLELY	NCP	10.608
GKLELY;EQVPDLG	NCP	10.11
EQVPDLG;KQLADETLLK	NCP	3.432
KQLADETLLK;YKEELEQTYHAK	NCP	4.788
YKEELEQTYHAK;KEDARP	NCP	49.743
KEDARP;PLGPLSAP	NCP	71.098
PLGPLSAP;KESSKQR	NCP	21.389
KESSKQR;SVFYLLRK	NCP	46.828
SVFYLLRK;MWNPPP	NCP	14.142
MWNPPP;QTHFKE	NCP	91.155
QTHFKE;VPAAAAAAAATA	NCP	122.432
VPAAAAAAAATA;SGLVLL	NCP	60.232

SGLVLL;LRMVSML	NCP	55.254
LRMVSML;KNTLQ	NCP	29.792
KNTLQ;GLDLFT	NCP	39.675
GLDLFT;VLTYSKR	NCP	94.147
VLTYSKR;LDLYKSK	NCP	26.606
LDLYKSK;LPAMFAL	NCP	12.445
LPAMFAL;KGDLDLK	NCP	40.742
KGDLDLK;VYLTKK	NCP	33.612
VYLTKK;KTTLSVH	NCP	46.459
KTTLSVH;GPLTEC	NCP	147.627
GPLTEC;TPLDPVK	NCP	31.676
TPLDPVK;KPPEKPRP	NCP	12.688
KPPEKPRP;YSVWLSN	NCP	14.967
YSVWLSN;EMHTMM	NCP	5.154
EMHTMM;ASYVFQ	NCP	41.904
ASYVFQ;SKKQPC	NCP	51.858
SKKQPC;NPYQGVK	NCP	16.233
NPYQGVK;KPAFLR	NCP	70.413
KPAFLR;KDSTLLMQ	NCP	15.59
KDSTLLMQ;PTKPLR	NCP	21.586
PTKPLR;SKFPPPPP	NCP	6.158
SKFPPPPP;EVFPVL	NCP	25.051
EVFPVL;VLRLNP	NCP	6.825
VLRLNP;HTYDFR	NCP	44.273
HTYDFR;LGLEYL	NCP	50.833
LGLEYL;AAEKVLGQ	NCP	4.65
AAEKVLGQ;FGLLN	NCP	6.191
FGLLN;QRGVHVL	NCP	10.521
QRGVHVL;EVMFC	NCP	41.236
EVMFC;AKKLLVK	NCP	149.586
AKKLLVK;LFHLGSK	NCP	13.803
LFHLGSK;EYPPEL	NCP	25.047
EYPPEL;LPKAA	NCP	73.329
LPKAA;SAMMYP	NCP	89.754
SAMMYP;FYNELR	NCP	96.318
FYNELR;FSPVTPK	NCP	5.054
FSPVTPK;VEYGLY	NCP	4.431
VEYGLY;NNCCQ	NCP	9.033
NNCCQ;KKFKGPK	NCP	69.694
KKFKGPK;VGLPASGK	NCP	3.557
VGLPASGK;VSLPEVLN	NCP	14.058
VSLPEVLN;AAAEPAVPALP	NCP	104.994
AAAEPAVPALP;RVPFSLL	NCP	1.375
RVPFSLL;EGLDKLR	NCP	45.744
EGLDKLR;CSTLSDH	NCP	250.512
CSTLSDH;VVPLRFQ	NCP	19.139
VVPLRFQ;NTLLELQL	NCP	39.21
NTLLELQL;VSGPVVN	NCP	45.823
VSGPVVN;KEFMPP	NCP	88.089
KEFMPP;SLAVEC	NCP	19.562
SLAVEC;KDYRPP	NCP	21.73

KDYRPP;CDYRFSQ	NCP	0.311
CDYRFSQ;NELFSLN	NCP	44.724
NELFSLN;ERHAAPR	NCP	5.311
ERHAAPR;KGSLN	NCP	6.974
KGSLN;NKKVHWK	NCP	216.498
NKKVHWK;LSYFKEQ	NCP	155.266
LSYFKEQ;KLQGFVP	NCP	5.184
KLQGFVP;QAALPKL	NCP	71.664
QAALPKL;MKRKFP	NCP	87.815
MKRKFP;FMLKGPK	NCP	23.016
FMLKGPK;GSDPLVL	NCP	9.369
GSDPLVL;DRPLL	NCP	133.386
DRPLL;TVAEALSL	NCP	23.899
TVAEALSL;VLEYL	NCP	0.744
VLEYL;KVPGAP	NCP	110.096
KVPGAP;KSDYLLK	NCP	26.643
KSDYLLK;KRVALTL	NCP	99.174
KRVALTL;TQTNLK	NCP	48.541
TQTNLK;YFPVH	NCP	20.718
YFPVH;ALADPV	NCP	26.253
ALADPV;KDLFF	NCP	98.162
KDLFF;MVWCC	NCP	52.329
MVWCC;NEQFVLL	NCP	28.359
NEQFVLL;HGLEVLY	NCP	61.267
HGLEVLY;PAGFEKP	NCP	17.174
PAGFEKP;QTDLRK	NCP	111.274
QTDLRK;NPLHPGK	NCP	108.072
NPLHPGK;PASMLR	NCP	75.318
PASMLR;NALKKAAL	NCP	7.376
NALKKAAL;KTKGQKK	NCP	7.572
KTKGQKK;DEPRPK	NCP	26.322
DEPRPK;DCRDLR	NCP	8.919
DCRDLR;VSKPDLT	NCP	42.642
VSKPDLT;PSLGTGEPK	NCP	5.208
PSLGTGEPK;AENWAA	NCP	8.98
AENWAA;LDEFPK	NCP	97.614
LDEFPK;KPTRPP	NCP	69.932
KPTRPP;PPEPT	NCP	57.495
PPEPT;VSLHVPK	NCP	5.976
VSLHVPK;VTQVLKC	NCP	62.883
VTQVLKC;KNGEGP	NCP	5.782
KNGEGP;CDSDP	NCP	1.673
CDSDP;KEYNLP	NCP	150
KEYNLP;KHKGPR	NCP	7.23
KHKGPR;NEGPP	NCP	67.896
NEGPP;SSEVKPPP	NCP	30.054
SSEVKPPP;TTVAGP	NCP	24.891
TTVAGP;VLVTLGEK	NCP	19.387
VLVTLGEK;DLKVKGPK	NCP	30.102
DLKVKGPK;WSLGLTM	NCP	100.559
WSLGLTM;TAELPLF	NCP	14.355

TAELPLF;WPSFLP	NCP	9.066
WPSFLP;LDEEFSM	NCP	32.338
LDEEFSM;KQDTLHT	NCP	41.64
KQDTLHT;STMKLKL	NCP	33.608
STMKLKL;KFLLHS	NCP	3.846
KFLLHS;KLHLP	NCP	8.644
KLHLP;YKVNGPT	NCP	41.144
YKVNGPT;GPVKLLA	NCP	11.844
GPVKLLA;KQEYRGP	NCP	3.384
KQEYRGP;QPPKTEK	NCP	16.113
QPPKTEK;KKPAATP	NCP	33.798
KKPAATP;LPVTMAVP	NCP	111.409
LPVTMAVP;AALYGGP	NCP	3.434
AALYGGP;VVLGRPP	NCP	9.367
VVLGRPP;LTVEEAP	NCP	11.541
LTVEEAP;VVLGSGGVGK	NCP	35.006
VVLGSGGVGK;KDPTGPR	NCP	44.517
KDPTGPR;FMEELTK	NCP	53.677
FMEELTK;DLSVLADP	NCP	29.174
DLSVLADP;KDYVYKVL	NCP	37.768
KDYVYKVL;GGAALYL	NCP	7.722
GGAALYL;LAVLDL	NCP	53.345
LAVLDL;KVRVPQ	NCP	78.897
KVRVPQ;APMLKF	NCP	3.802
APMLKF;VGSGLPP	NCP	35.279
VGSGLPP;RDPAGGLK	NCP	22.272
RDPAGGLK;NKGKMGK	NCP	39.709
NKGKMGK;ACRALPLS	NCP	44.147
ACRALPLS;PPPPSYK	NCP	10.749
PPPPSYK;DVYFPK	NCP	17.269
DVYFPK;LTREC	NCP	31.275
LTREC;DAGLTPL	NCP	6.965
DAGLTPL;KNKYPFQ	NCP	28.864
KNKYPFQ;FMTLV	NCP	33.383
FMTLV;TGLTTL	NCP	54.228
TGLTTL;VSELP	NCP	39.093
VSELP;PGLTDTTVPR	NCP	58.788
PGLTDTTVPR;EDDVRQYVVRKPLN	NCP	0.084
EDDVRQYVVRKPLN;KPSAVHQP	NCP	4.785
KPSAVHQP;PLPAKPR	NCP	59.403
PLPAKPR;VVPGG	NCP	4.956
VVPGG;ACCLSDEVKESK	NCP	19.215
ACCLSDEVKESK;TPVYPPPPP	NCP	91.764
TPVYPPPPP;YARPEP	NCP	25.704
YARPEP;PPTYVSP	NCP	12.324
PPTYVSP;RFPPPP	NCP	22.135
RFPPPP;TLSTGEL	NCP	20.435
TLSTGEL;AAARLPSP	NCP	22.746
AAARLPSP;QAPYVL	NCP	25.576
QAPYVL;KLHGAEL	NCP	10.481
KLHGAEL;SSPVKKTEMDK	NCP	23.808

SSPVKKTEMDK;LGKGGLGK	NCP	10.86
LGKGGLGK;TVLPQ	NCP	34.156
TVLPQ;VPLRMQAL	NCP	11.195
VPLRMQAL;AGPASPK	NCP	0.012
AGPASPK;TSALPGLP	NCP	46.443
TSALPGLP;MMSRRF	NCP	43.773
MMSRRF;EELKPK	NCP	64.24
EELKPK;ARGKGLL	NCP	36.65
ARGKGLL;LSLDESLR	NCP	0.145
LSLDESLR;VPGKAALPP	NCP	2.162
VPGKAALPP;KNFKPPP	NCP	76.638
KNFKPPP;DLVEALK	NCP	31.531
DLVEALK;VGPSTAVP	NCP	30.615
VGPSTAVP;TVPGLLL	NCP	17.057
TVPGLLL;KPWPSRH	NCP	17.736
KPWPSRH;LVGLFEDTNL	NCP	11.49
LVGLFEDTNL;KPSSSRT	NCP	3.601
KPSSSRT;APPPHLP	NCP	55.94
APPPHLP;ARMAYP	NCP	8.973
ARMAYP;REKMAP	NCP	27.148
REKMAP;AAPSVKP	NCP	15.567
AAPSVKP;PAAAR	NCP	68.039
PAAAR;RYKVLK	NCP	16.297
RYKVLK;RSGNK	NCP	4.068
RSGNK;FEPLSPP	NCP	33.09
FEPLSPP;PSVPELV	NCP	3.443
PSVPELV;VFVTVV	NCP	15.612
VFVTVV;KTATVP	NCP	62.752
KTATVP;QPAASFK	NCP	35.153
QPAASFK;CARHAGP	NCP	2.982
CARHAGP;KVSSLNR	NCP	22.585
KVSSLNR;KPPPAAAP	NCP	50.969
KPPPAAAP;AASGPGVR	NCP	37.515
AASGPGVR;CHWAAK	NCP	36.633
CHWAAK;PAKMPPP	NCP	59.46
PAKMPPP;TSPLTAKP	NCP	25.215
TSPLTAKP;KPCPVQP	NCP	15.79
KPCPVQP;ADSSKSK	NCP	10.598
ADSSKSK;PLMYAP	NCP	1.876
PLMYAP;LPGGP	NCP	18.158
LPGGP;AAPVTRAP	NCP	162.963
AAPVTRAP;APACPQR	NCP	9.033
APACPQR;ARATKPR	NCP	65.607
ARATKPR;GPPVPPP	NCP	9.264
GPPVPPP;GVLPDVP	NCP	21.817
GVLPDVP;KGRTLTLP	NCP	24.765
KGRTLTLP;GPLFL	NCP	9.608
GPLFL;TKVGVPR	NCP	4.887
TKVGVPR;SRLLPHPN	NCP	114.855
SRLLPHPN;HYLMK	NCP	2.2
HYLMK;KRDPRPP	NCP	25.25

KRDPRPP;ESLHRQK	NCP	83.892
ESLHRQK;DDDDTC	NCP	36.202
DDDDTC;PPLLAAPR	NCP	50.487
PPLLAAPR;VSPVEEL	NCP	15.276
VSPVEEL;KYNPGAGK	NCP	8.022
KYNPGAGK;APAKPRP	NCP	57.177
APAKPRP;AQETSSVGPDRK	NCP	6.749
AQETSSVGPDRK;VGPDRKL	NCP	0.018
VGPDRKL;AAHEPLQ	NCP	8.352
AAHEPLQ;RLGTFGG	NCP	17.496
RLGTFGG;DDVYWPEAK	NCP	11.493
DDVYWPEAK;SRPTDAHFTR	NCP	3.186
SRPTDAHFTR;ALRYPMA	NCP	11.784
ALRYPMA;SASSSLEK	NCP	71.709
SASSSLEK;TSPGPL	NCP	89.686
TSPGPL;ESARLL	NCP	7.475
ESARLL;TLDRLK	NCP	51.711
TLDRLK;KLGPHLK	NCP	50.151
KLGPHLK;APAPPPYR	NCP	145.533
APAPPPYR;MSSPLKG	NCP	34.326
MSSPLKG;KPTCAPR	NCP	43.857
KPTCAPR;KEASHK	NCP	19.641
KEASHK;DGVGFK	NCP	12.39
DGVGFK;QPGAPQ	NCP	12.357
QPGAPQ;GPGRTSF	NCP	6.453
GPGRTSF;CTGHRC	NCP	68.034
CTGHRC;PAPAPAAK	NCP	14.178
PAPAPAAK;PPLPLTAT	NCP	44.901
PPLPLTAT;ECCDPH	NCP	18.757
ECCDPH;ERSSTG	NCP	32.193
ERSSTG;TLWKMKE	NCP	65.061
TLWKMKE;MDFKLPP	NCP	26.099
MDFKLPP;QTGPLLH	NCP	14.316
QTGPLLH;LDVSGP	NCP	43.377
LDVSGP;LDPVNP	NCP	9.283
LDPVNP;KELGVAK	NCP	39.833
KELGVAK;PGLPTALP	NCP	3.441
PGLPTALP;KEPPAPR	NCP	30.574
KEPPAPR;LLLLSSKP	NCP	3.003
LLLLSSKP;LGYPLTLF	NCP	0.759
LGYPLTLF;SLQSKL	NCP	163.479
SLQSKL;NPANKPR	NCP	38.16
NPANKPR;KSSERP	NCP	20.39
KSSERP;VRLFN	NCP	63.076
VRLFN;KMPLGAP	NCP	6.143
KMPLGAP;TLKLPDG	NCP	48.954
TLKLPDG;VPPRTVP	NCP	85.206
VPPRTVP;VLQMGQLR	NCP	12.588
VLQMGQLR;APVLARP	NCP	36.681
APVLARP;SPPPPPPPP	NCP	21.843
SPPPPPPPP;KSSHLK	NCP	4.146

KSSHLK;THARPP	NCP	41.602
THARPP;LSVGRP	NCP	6.978
LSVGRP;PGPLV	NCP	14.354
PGPLV;ETTGKVL	NCP	26.176
ETTGKVL;YVEELERGPAA	NCP	24.962
YVEELERGPAA;KYVLPPP	NCP	42.327
KYVLPPP;AHLLFPLE	NCP	1.725
AHLLFPLE;VNSTVLT	NCP	31.083
VNSTVLT;EVEMLAV	NCP	56.934
EVEMLAV;MVLVEL	NCP	1.869
MVLVEL;GGPWVPSP	NCP	21.174
GGPWVPSP;KANSGKR	NCP	51.355
KANSGKR;SLGAVTNK	NCP	31.61
SLGAVTNK;ARFGPP	NCP	0.78
ARFGPP;FGGPVHH	NCP	17.865
FGGPVHH;KQRLEY	NCP	2.374
KQRLEY;SPPRATPP	NCP	36.431
SPPRATPP;KGPFAPP	NCP	11.586
KGPFAPP;QGVAELK	NCP	13.221
QGVAELK;PVKPCPQ	NCP	27.291
PVKPCPQ;KPTTSVR	NCP	5.22
KPTTSVR;EAQLDLR	NCP	2.529
EAQLDLR;KVMPSR	NCP	7.186
KVMPSR;AEMYEHKEPDDS	NCP	24.2
AEMYEHKEPDDS;RNMYGY	NCP	31.969
RNMYGY;YGPPPP	NCP	11.357
YGPPPP;SKSSH	NCP	33.76
SKSSH;SPRPTPR	NCP	63.107
SPRPTPR;AFVPAGP	NCP	65.37
AFVPAGP;CSFLATH	NCP	8.007
CSFLATH;GPVFPSAL	NCP	25.797
GPVFPSAL;GKAGALV	NCP	29.592
GKAGALV;LTAAEAW	NCP	22.416
LTAAEAW;LMPNVPK	NCP	82.893
LMPNVPK;PSLPYY	NCP	25.239
PSLPYY;VDLTLN	NCP	47.118
VDLTLN;RNFPTSR	NCP	37.51
RNFPTSR;YYCCH	NCP	145.064
YYCCH;GQPYAP	NCP	30.711
GQPYAP;TPAPMLR	NCP	17.754
TPAPMLR;AGLLLAPP	NCP	8.967
AGLLLAPP;AVLYP	NCP	24.15
AVLYP;TGSMYKK	NCP	9.579
TGSMYKK;KEVSLK	NCP	39.577
KEVSLK;RLVGEPP	NCP	7.265
RLVGEPP;VAAALT	NCP	9.37
VAAALT;NEVGLAL	NCP	28.352
NEVGLAL;LHFTNNK	NCP	38.697
LHFTNNK;PGPASPPAFPSKEPDVP	NCP	123.291
PGPASPPAFPSKEPDVP;SLRPPR	NCP	0.711
SLRPPR;QMVVSAV	NCP	87.688

QMVVSAV;GYGLRY	NCP	28.352
GYGLRY;VLPGVDA	NCP	11.652
VLPGVDA;VPEYPF	NCP	95.041
VPEYPF;AAADELAFH	NCP	33.596
AAADELAFH;TQMHHK	NCP	15.201
TQMHHK;LDPGNVG	NCP	26.061
LDPGNVG;PAPEF	NCP	14.877
PAPEF;QHGAP	NCP	7.14
QHGAP;NGRLP	NCP	22.434
NGRLP;ESFLK	NCP	7.531
ESFLK;KQHPKP	NCP	10.547
KQHPKP;LVLDSGD	NCP	8.499
LVLDSGD;SPGPTHR	NCP	13.737
SPGPTHR;KVTTAPR	NCP	89.34
KVTTAPR;LHNSSLTK	NCP	78.685
LHNSSLTK;LTTAHKP	NCP	0.476
LTTAHKP;TLKSKST	NCP	33.655
TLKSKST;LEELKAK	NCP	9.26
LEELKAK;VGMFETF	NCP	51.3
VGMFETF;MLYPSRL	NCP	43.888
MLYPSRL;HPAPHPPP	NCP	0.915
HPAPHPPP;VDTLSKDGFSK	NCP	4.679
VDTLSKDGFSK;SSEAKEGEEAGTGDPLLEATSK	NCP	3.873
SSEAKEGEEAGTGDPLLEATSK;EAELGLGF	NCP	26.388
EAELGLGF;LAKHWR	NCP	4.344
LAKHWR;LPPTGAN	NCP	47.892
LPPTGAN;GVSRQL	NCP	67.923
GVSRQL;RPGFP	NCP	22.443
RPGFP;AEKGKPPKP	NCP	37.083
AEKGKPPKP;AAQGFE	NCP	4.789
AAQGFE;PVRLPGSP	NCP	12.095
PVRLPGSP;ELTLTVP	NCP	0.645
ELTLTVP;LVAKALLV	NCP	6.889
LVAKALLV;QSYVAL	NCP	3.42
QSYVAL;KDTLGPMQK	NCP	20.843
KDTLGPMQK;LPKEHGP	NCP	45.681
LPKEHGP;AQVRDLK	NCP	58.641
AQVRDLK;RAKFQP	NCP	36.528
RAKFQP;PPPPERPP	NCP	0.177
PPPPERPP;FRDGPPL	NCP	20.757
FRDGPPL;DYAKL	NCP	56.64
DYAKL;LDRSLPR	NCP	32.604
LDRSLPR;GESLGLR	NCP	8.514
GESLGLR;EGTAH	NCP	111.981
EGTAH;GKLPLQR	NCP	12.264
GKLPLQR;AAGLSFP	NCP	13.801
AAGLSFP;DLASKSV	NCP	55.041
DLASKSV;MRLVLVE	NCP	55.26
MRLVLVE;LTHNTLR	NCP	7.196
LTHNTLR;VLFFVLYL	NCP	74.971
VLFFVLYL;GLSGPLK	NCP	24.243

GLSGPLK;LVGLLAF	NCP	114.006
LVGLLAF;EPGHVH	NCP	116.496
EPGHVH;SLATLLHA	NCP	97.64
SLATLLHA;KTVMVPR	NCP	113.661
KTVMVPR;KGTSCKK	NCP	78.763
KGTSCKK;NSANLSSP	NCP	7.031
NSANLSSP;FFPQERK	NCP	17.206
FFPQERK;ELLSL	NCP	100.8
ELLSL;DKPLEK	NCP	98.895
DKPLEK;DMHSMM	NCP	92.042
DMHSMM;KEGGVT	NCP	4.767
KEGGVT;MGFCAQ	NCP	42.348
MGFCAQ;DLEERLK	NCP	28.548
DLEERLK;AALDSLSL	NCP	18.234
AALDSLSL;LGPPVNQ	NCP	122.716
LGPPVNQ;LSVVGQK	NCP	31.649
LSVVGQK;VSKLNLLK	NCP	24.7
VSKLNLLK;PSSRRPSP	NCP	132.962
PSSRRPSP;KHHERV	NCP	29.931
KHHERV;DVLSTSK	NCP	49.077
DVLSTSK;APKFGE	NCP	14.832
APKFGE;MCAPLR	NCP	20.955
MCAPLR;PPPRLPPF	NCP	17.511
PPPRLPPF;LPPRHSP	NCP	0.129
LPPRHSP;DPTVYFKEQFL	NCP	86.655
DPTVYFKEQFL;TSQDARFY	NCP	0.678
TSQDARFY;YALSARFEPF	NCP	0.021
YALSARFEPF;SNKGQTLVVQF	NCP	0.03
SNKGQTLVVQF;DDEFTHL	NCP	0.774
DDEFTHL;ENFAVLGLDL	NCP	0.594
ENFAVLGLDL;AEEFGNETWGVTK	NCP	0.699
AEEFGNETWGVTK;AAAGQAKDEL	NCP	0.297
AAAGQAKDEL;FACEKNENLAANF	NCP	7.638
FACEKNENLAANF;YAPKEVGP	NCP	5.742
YAPKEVGP;AALHPY	NCP	42.162
AALHPY;QPPKGLH	NCP	7.293
QPPKGLH;KDCPPW	NCP	0.504
KDCPPW;KAPQSPK	NCP	64.134
KAPQSPK;LPPPPPVA	NCP	2.076
LPPPPPVA;PAAKQ	NCP	6.864
PAAKQ;VSFGAPF	NCP	48.831
VSFGAPF;AHKGAN	NCP	16.348
AHKGAN;PGLPHPP	NCP	23.117
PGLPHPP;EGTLSVH	NCP	23.473
EGTLSVH;KVLEERK	NCP	89.849
KVLEERK;RPPGVTK	NCP	2.59
RPPGVTK;LDADWL	NCP	12.941
LDADWL;KQSSSVLL	NCP	18.256
KQSSSVLL;KTPPHTKK	NCP	0.153
KTPPHTKK;KGTLSVGR	NCP	16.172
KGTLSVGR;LSGGFLGK	NCP	32.776

LSGGFLGK;KHFYTLN	NCP	54.03
KHFYTLN;VTLKKKP	NCP	65.889
VTLKKKP;QHGLKKT	NCP	26.417
QHGLKKT;KTPPFK	NCP	107.667
KTPPFK;LSGTCLK	NCP	7.528
LSGTCLK;KQRLPLA	NCP	17.342
KQRLPLA;RSKAVP	NCP	26.998
RSKAVP;LPPGAAPP	NCP	27.347
LPPGAAPP;GLEGAP	NCP	29.229
GLEGAP;ENGFDLL	NCP	8.016
ENGFDLL;VGVFNLK	NCP	47.617
VGVFNLK;GWLKGPP	NCP	69.644
GWLKGPP;TLSAPQH	NCP	4.827
TLSAPQH;EVAGRDL	NCP	8.224
EVAGRDL;LAAFYGV	NCP	28.256
LAAFYGV;VTPPLLSL	NCP	28.131
VTPPLLSL;PSSRSPRP	NCP	15.297
PSSRSPRP;SLTLTPR	NCP	9.465
SLTLTPR;TFGPPPPP	NCP	4.545
TFGPPPPP;LFGLELY	NCP	45.934
LFGLELY;PPPLAPP	NCP	21.29
PPPLAPP;LTRVLTF	NCP	48.12
LTRVLTF;YEAPPAP	NCP	12.417
YEAPPAP;GAPHCPQ	NCP	27.273
GAPHCPQ;KRAGPTVP	NCP	185.196
KRAGPTVP;LSVPPPPS	NCP	29.787
LSVPPPPS;DYPPL	NCP	7.89
DYPPL;KRTTHK	NCP	112.074
KRTTHK;RPGVRLL	NCP	26.628
RPGVRLL;KSTAKVK	NCP	16.801
KSTAKVK;DMDEDP	NCP	84.464
DMDEDP;KDHEKML	NCP	11.955
KDHEKML;LDPTGTY	NCP	121.468
LDPTGTY;ALRKSVVL	NCP	29.718
ALRKSVVL;PSRKDSG	NCP	35.634
PSRKDSG;VPTQPLK	NCP	3.6
VPTQPLK;KDSLASP	NCP	76.707
KDSLASP;LPPPRYK	NCP	60.777
LPPPRYK;LKTFSCL	NCP	7.598
LKTFSCL;KKKKKKKKRKLQ	NCP	122.859
KKKKKKKKRKLQ;PVVKQQK	NCP	38.355
PVVKQQK;GVHKMPK	NCP	74.02
GVHKMPK;VLPTLPK	NCP	38.958
VLPTLPK;QPGGGAW	NCP	49.142
QPGGGAW;VPPPPARPL	NCP	32.754
VPPPPARPL;KKDCALKK	NCP	29.641
KKDCALKK;AEFKQ	NCP	182.127
AEFKQ;KSGAASF	NCP	44.748
KSGAASF;LNGKKVAP	NCP	162.792
LNGKKVAP;VVGLL	NCP	9.332
VVGLL;VVVLA	NCP	106.066

VVVLA;KASHPKP	NCP	60.608
KASHPKP;EPAVH	NCP	4.248
EPAVH;ATVAFGF	NCP	23.685
ATVAFGF;LDMLKK	NCP	255.4
LDMLKK;KPSYFPK	NCP	56.004
KPSYFPK;KTYAFR	NCP	0.84
KTYAFR;VDLNFPK	NCP	37.2
VDLNFPK;VLTRSLGP	NCP	42.302
VLTRSLGP;TLYEYQT	NCP	121.477
TLYEYQT;PSKGLPQ	NCP	0.398
PSKGLPQ;TTEPSVPK	NCP	60.654
TTEPSVPK;GEGFSLN	NCP	39.636
GEGFSLN;CEDDGY	NCP	111.871
CEDDGY;STQGK	NCP	18.425
STQGK;SEAPGVQP	NCP	3.429
SEAPGVQP;AVTLMSF	NCP	115.819
AVTLMSF;VLGLGAM	NCP	83.832
VLGLGAM;KYRWFP	NCP	16.698
KYRWFP;PTPAVTK	NCP	34.989
PTPAVTK;ASSHTML	NCP	52.47
ASSHTML;AAPAAPLPP	NCP	70.856
AAPAAPLPP;KAMAAPK	NCP	95.044
KAMAAPK;LMLDLRK	NCP	35.321
LMLDLRK;EDSGP	NCP	32.461
EDSGP;HMKSKGK	NCP	60.599
HMKSKGK;YRGKCR	NCP	3.76
YRGKCR;KLGSTWV	NCP	84.488
KLGSTWV;VTMPGLK	NCP	22.723
VTMPGLK;EEESPALYN	NCP	9.575
EEESPALYN;KTKAPLR	NCP	9.403
KTKAPLR;KGSLTTK	NCP	7.055
KGSLTTK;KFLTKSK	NCP	20.542
KFLTKSK;LLQTF	NCP	22.259
LLQTF;PNHSTPK	NCP	23.43
PNHSTPK;MLGAP	NCP	11.958
MLGAP;LGGLEF	NCP	4.173
LGGLEF;PQTPYHM	NCP	43.408
PQTPYHM;HEKLPP	NCP	0.018
HEKLPP;SAPLNPK	NCP	10.347
SAPLNPK;GNLSEKK	NCP	8.634
GNLSEKK;LSTGSPR	NCP	3.488
LSTGSPR;DVNLPK	NCP	83.842
DVNLPK;MRGPL	NCP	26.24
MRGPL;KHVPPGP	NCP	49.53
KHVPPGP;TNRTTLP	NCP	0.09
TNRTTLP;KQHFTQ	NCP	16.137
KQHFTQ;MWNPN	NCP	12.981
MWNPN;EPRPC	NCP	48.567
EPRPC;LNSPPN	NCP	49.582
LNSPPN;SFLKLWN	NCP	23.775
SFLKLWN;SHSLLDPT	NCP	13.002

SHSLLDPT;LGGDLRP	NCP	12.003
LGGDLRP;KLPSGRT	NCP	60.788
KLPSGRT;DLGFKG	NCP	23.934
DLGFKG;TPMFYKLL	NCP	10.231
TPMFYKLL;TTAQF	NCP	48.672
TTAQF;RLEALR	NCP	37.478
RLEALR;KHVPPPP	NCP	16.62
KHVPPPP;MLELSLSR	NCP	44.781
MLELSLSR;LGKKKLY	NCP	69.436
LGKKKLY;VNFLP	NCP	120.024
VNFLP;KSLLP	NCP	81.111
KSLLP;LLQLLH	NCP	214.977
LLQLLH;VGTHTVPL	NCP	71.897
VGTHTVPL;AGLNSLEAVKRK	NCP	19.635
AGLNSLEAVKRK;VYLPFL	NCP	13.582
VYLPFL;AKTYDYLFK	NCP	8.387
AKTYDYLFK;LEPRPH	NCP	9.928
LEPRPH;KPYLER	NCP	2.055
KPYLER;PPRAQ	NCP	1.667
PPRAQ;QPHTLDP	NCP	33.819
QPHTLDP;EALTLLT	NCP	1.473
EALTLLT;FPPDVTG	NCP	24.618
FPPDVTG;EDDLLHV	NCP	26.338
EDDLLHV;GPRCSS	NCP	29.189
GPRCSS;AEQSLHF	NCP	20.791
AEQSLHF;LPQPLA	NCP	2.267
LPQPLA;YAPTVPR	NCP	32.262
YAPTVPR;DPALMM	NCP	1.806
DPALMM;TVCLDY	NCP	9.808
TVCLDY;AFPPPPPW	NCP	11.9
AFPPPPPW;DSFPPAK	NCP	53.088
DSFPPAK;KNRGKKPF	NCP	39.406
KNRGKKPF;APPSVHPAQ	NCP	86.606
APPSVHPAQ;TDEWLR	NCP	11.418
TDEWLR;KPEVP	NCP	20.842
KPEVP;KPTACK	NCP	29.793
KPTACK;ATLRPDP	NCP	66.563
ATLRPDP;YADDC	NCP	10.384
YADDC;RKGHPPP	NCP	19.662
RKGHPPP;KLFYFYK	NCP	80.769
KLFYFYK;YSSLK	NCP	16.659
YSSLK;AHAGGGGGVGPA	NCP	26.552
AHAGGGGGVGPA;TDKKKVL	NCP	12.333
TDKKKVL;PHALLLR	NCP	3.465
PHALLLR;AAPPPPPPP	NCP	6.255
AAPPPPPPP;KEVASPGAP	NCP	7.536
KEVASPGAP;KPSGHGAP	NCP	2.196
KPSGHGAP;KFLADSP	NCP	2.974
KFLADSP;RGPGPPR	NCP	14.234
RGPGPPR;TVPDLEY	NCP	28.117
TVPDLEY;EPGLD	NCP	43.454

EPGLD;TPVTSPR	NCP	3.306
TPVTSPR;STPGVTVP	NCP	13.104
STPGVTVP;KPALVTP	NCP	12.738
KPALVTP;ESKKSHQ	NCP	13.474
ESKKSHQ;THAAKLY	NCP	16.083
THAAKLY;SALRVAE	NCP	6.518
SALRVAE;LGSVTLPR	NCP	16.414
LGSVTLPR;VPPAKAH	NCP	109.019
VPPAKAH;KNGSSLQ	NCP	35.641
KNGSSLQ;KDFLYF	NCP	35.775
KDFLYF;VLHMLL	NCP	18.689
VLHMLL;KLLAPEP	NCP	8.388
KLLAPEP;MKLPLLQ	NCP	69.084
MKLPLLQ;EDPWVVK	NCP	34.866
EDPWVVK;YPGVP	NCP	46.887
YPGVP;LDEAAARLLTVPF	NCP	71.691
LDEAAARLLTVPF;KDLFTGKK	NCP	7.009
KDLFTGKK;KCAVPP	NCP	70.833
KCAVPP;FALAT	NCP	15.104
FALAT;LGPQQGY	NCP	6.868
LGPQQGY;GPWELLLL	NCP	42.753
GPWELLLL;KTVLSPT	NCP	28.895
KTVLSPT;LVNPPTRPP	NCP	75.432
LVNPPTRPP;LVGPK	NCP	27.835
LVGPK;KTCAPPPP	NCP	14.811
KTCAPPPP;EYKVVGR	NCP	19.406
EYKVVGR;PPTARP	NCP	39.108
PPTARP;VPGLMQ	NCP	1.101
VPGLMQ;KALGHRP	NCP	71.547
KALGHRP;MEKLRLLG	NCP	54.132
MEKLRLLG;VGPPDLPA	NCP	20.745
VGPPDLPA;KQQLKLM	NCP	43.63
KQQLKLM;HVELMPL	NCP	7.505
HVELMPL;WFPSVGL	NCP	13.693
WFPSVGL;ERVLSAG	NCP	20.669
ERVLSAG;KPWPTPP	NCP	34.569
KPWPTPP;MEQPRKAVVVT	NCP	124.575
MEQPRKAVVVT;VHEVTK	NCP	43.591
VHEVTK;PPSRS	NCP	30.188
PPSRS;HCLLHRP	NCP	8.358
HCLLHRP;SPPNPKPP	NCP	31.387
SPPNPKPP;GPARHPK	NCP	9.005
GPARHPK;MMAKRET	NCP	16.848
MMAKRET;HKRGPA	NCP	13.563
HKRGPA;CFVPPPP	NCP	0.408
CFVPPPP;LDGPGPL	NCP	16.233
LDGPGPL;ETAAPGAPR	NCP	21.087
ETAAPGAPR;KTAHVPP	NCP	8.25
KTAHVPP;KLYCPEK	NCP	37.491
KLYCPEK;TSWAAFR	NCP	23.121
TSWAAFR;EWSLYPP	NCP	24.792

EWSLYPP;LEYLNP	NCP	0.327
LEYLNP;KPSVYVM	NCP	1.12
KPSVYVM;PGGLLVV	NCP	4.58
PGGLLVV;KPRPPTP	NCP	8.677
KPRPPTP;PEKLTSGP	NCP	121.013
PEKLTSGP;PPPLW	NCP	11.73
PPPLW;EGMGVPR	NCP	38.7
EGMGVPR;LPYSSSGH	NCP	33.4
LPYSSSGH;VLVEGPLNNL	NCP	16.934
VLVEGPLNNL;EVVLAK	NCP	30.55
EVVLAK;TGAFVGK	NCP	13.469
TGAFVGK;KAVMQF	NCP	41.404
KAVMQF;ELGTLFLG	NCP	34.457
ELGTLFLG;ERFVQDL	NCP	23.623
ERFVQDL;APLPP	NCP	20.117
APLPP;KTVPPER	NCP	9.172
KTVPPER;PRLVEPPA	NCP	37.088
PRLVEPPA;PDLPVMA	NCP	91.29
PDLPVMA;RGMQLV	NCP	35.217
RGMQLV;KPALSAPP	NCP	27.144
KPALSAPP;NSFPEPLK	NCP	20.362
NSFPEPLK;AHLDME	NCP	8.078
AHLDME;MPGWPK	NCP	11.806
MPGWPK;PTKPPR	NCP	6.278
PTKPPR;KALTLL	NCP	38.998
KALTLL;LALFLAA	NCP	36.959
LALFLAA;VTGAGVEF	NCP	183.417
VTGAGVEF;LPPPENN	NCP	32.31
LPPPENN;RSFEYPP	NCP	52.69
RSFEYPP;ELEKLYN	NCP	218.034
ELEKLYN;MLLLLN	NCP	65.42
MLLLLN;LVDRPK	NCP	2.947
LVDRPK;LEGLAL	NCP	84.773
LEGLAL;VLLPPR	NCP	121.065
VLLPPR;GVLAA	NCP	7.014
GVLAA;PLTESKP	NCP	58.494
PLTESKP;MVGLDAAGK	NCP	33.288
MVGLDAAGK;MLAEDELR	NCP	0.426
MLAEDELR;DLFPGLEK	NCP	20.664
DLFPGLEK;LKRHWPP	NCP	9.168
LKRHWPP;KQEHLR	NCP	77.895
KQEHLR;AEEWYK	NCP	57.697
AEEWYK;KQNDFKK	NCP	55.521
KQNDFKK;TVVVVP	NCP	10.383
TVVVVP;ARGGGPAY	NCP	1.889
ARGGGPAY;TDLGAFVK	NCP	27.894
TDLGAFVK;KTLHTKK	NCP	32.953
KTLHTKK;EVGTEEFLKK	NCP	5.756
EVGTEEFLKK;AAPPRTH	NCP	75.258
AAPPRTH;PDPSKSAPAPK	NCP	57.294
PDPSKSAPAPK;VRLLLPGELAK	NCP	0.291

VRLLLPGELAK;CGPPLGAP	NCP	43.072
CGPPLGAP;APYLLSQ	NCP	7.152
APYLLSQ;HLVPPL	NCP	43.965
HLVPPL;LDGLGFK	NCP	11.843
LDGLGFK;AHLTKHQ	NCP	28.977
AHLTKHQ;LTSGY	NCP	52.638
LTSGY;LNRKLAL	NCP	38.896
LNRKLAL;HTGPPPPG	NCP	72.122
HTGPPPPG;KEDGAPK	NCP	6.823
KEDGAPK;GHMPPTL	NCP	46.319
GHMPPTL;KDANYK	NCP	18.313
KDANYK;FFPPKPP	NCP	47.244
FFPPKPP;LHLEQPL	NCP	27.084
LHLEQPL;KEGGLSL	NCP	54.89
KEGGLSL;NDSTPQ	NCP	13.555
NDSTPQ;KDKKAGQ	NCP	3.464
KDKKAGQ;LYLTSAL	NCP	11.05
LYLTSAL;KDLGHGK	NCP	25.866
KDLGHGK;KMVPAP	NCP	91.118
KMVPAP;YLELGLT	NCP	67.189
YLELGLT;PSTLDLH	NCP	1.584
PSTLDLH;EHFPMD	NCP	32.247
EHFPMD;KMPLLLS	NCP	9.207
KMPLLLS;VMKFLH	NCP	27.195
VMKFLH;MGAVNL	NCP	10.611
MGAVNL;FFGDDFSS	NCP	1.923
FFGDDFSS;MDDFDM	NCP	75.951
MDDFDM;MEMMYG	NCP	19.344
MEMMYG;PVPTGEVY	NCP	68.066
PVPTGEVY;WEVDEMK	NCP	1.029
WEVDEMK;VDEMKESKLPGDK	NCP	0.006
VDEMKESKLPGDK;TYFTDKKTHLY	NCP	2.199
TYFTDKKTHLY;FTDKKTHL	NCP	0.006
FTDKKTHL;TDKKTHLY	NCP	0.003
TDKKTHLY;VVNSGNLL	NCP	1.707
VVNSGNLL;EDLEPFKMTPF	NCP	4.494
EDLEPFKMTPF;KKTDAPQPDVK	NCP	2.103
KKTDAPQPDVK;SDVEEDGDTVSQEEDDRKPK	NCP	0.3
SDVEEDGDTVSQEEDDRKPK;LGVSHVF	NCP	0.703
LGVSHVF;SSYVGHR	NCP	36.311
SSYVGHR;AFMLT	NCP	55.029
AFMLT;EHLVTR	NCP	75.939
EHLVTR;CRPRVG	NCP	0.519
CRPRVG;WSDGLLF	NCP	4.866
WSDGLLF;VHKTLLSE	NCP	63.897
VHKTLLSE;TATPSP	NCP	45.336
TATPSP;EGPELGK	NCP	58.533
EGPELGK;ESSGP	NCP	18.894
ESSGP;STLLAL	NCP	192.522
STLLAL;KSRVALD	NCP	73.809
KSRVALD;GFEVGGP	NCP	65.448

GFEVGGP;GKFLRP	NCP	101.746
GKFLRP;PLTMLVY	NCP	143.418
PLTMLVY;TKAPLT	NCP	48.567
TKAPLT;KAVQVGL	NCP	101.13
KAVQVGL;GEDGD	NCP	19.931
GEDGD;HEFSLQR	NCP	46.735
HEFSLQR;KGQPQR	NCP	308.861
KGQPQR;EPVKM	NCP	60.439
EPVKM;GVDDLRK	NCP	86.145
GVDDLRK;AGSPK	NCP	61.914
AGSPK;KPTFCK	NCP	201.929
KPTFCK;KLDPR	NCP	81.342
KLDPR;KPSGPKL	NCP	25.078
KPSGPKL;GKLPVL	NCP	23.207
GKLPVL;RFTGPK	NCP	0.544
RFTGPK;LDLDLF	NCP	68.411
LDLDLF;TYSSNDF	NCP	67.594
TYSSNDF;MLQSL	NCP	18.039
MLQSL;KDMVPK	NCP	59.079
KDMVPK;QHFFWK	NCP	27.882
QHFFWK;RPPNGLQ	NCP	15.872
RPPNGLQ;RVTLSVPP	NCP	5.88
RVTLSVPP;DATPKFP	NCP	45.084
DATPKFP;PFDAAA	NCP	4.608
PFDAAA;KPVDKHR	NCP	1.996
KPVDKHR;FKSAGGLK	NCP	4.241
FKSAGGLK;KKHVVPP	NCP	49.686
KKHVVPP;GVVRDLK	NCP	97.255
GVVRDLK;CYMALLR	NCP	17.316
CYMALLR;KYPHVK	NCP	65.262
KYPHVK;KTTKQPK	NCP	26.655
KTTKQPK;KFANKK	NCP	221.058
KFANKK;FGSSLAL	NCP	4.323
FGSSLAL;VWFKRM	NCP	230.319
VWFKRM;SLSVPYFR	NCP	63.69
SLSVPYFR;NHVLFKK	NCP	36.123
NHVLFKK;LANGKPP	NCP	10.209
LANGKPP;SSLLER	NCP	43.728
SSLLER;VLGTVKWF	NCP	24.651
VLGTVKWF;LHVLDLY	NCP	112.791
LHVLDLY;PPGPM	NCP	99.954
PPGPM;YGSQRPP	NCP	45.831
YGSQRPP;VSLLEEPM	NCP	29.076
VSLLEEPM;SPVYQA	NCP	37.778
SPVYQA;DHLTVPL	NCP	188.248
DHLTVPL;ATLPVTK	NCP	10.218
ATLPVTK;LSHEYLL	NCP	161.976
LSHEYLL;PDNYFR	NCP	150.651
PDNYFR;KCELTSP	NCP	59.13
KCELTSP;TVYDLP	NCP	89.858
TVYDLP;PKFTLGT	NCP	37.954

PKFTLGT;KVLDPLK	NCP	110.991
KVLDPLK;YVPLSKC	NCP	45.345
YVPLSKC;VGYGFN	NCP	211.476
VGYGFN;KFGGLVL	NCP	359.589
KFGGLVL;KLLSHSHW	NCP	27.165
KLLSHSHW;VFDFL	NCP	5.157
VFDFL;AEGFLPA	NCP	6.779
AEGFLPA;PVGPSLM	NCP	125.596
PVGPSLM;NFELKVK	NCP	48.123
NFELKVK;YFGPPNP	NCP	201.747
YFGPPNP;DPELFLK	NCP	128.094
DPELFLK;TLGVKQL	NCP	186.276
TLGVKQL;DRPALVR	NCP	191.46
DRPALVR;LGGFMP	NCP	113.391
LGGFMP;DCEMLQ	NCP	263.481
DCEMLQ;KYGPLAK	NCP	16.278
KYGPLAK;EHVTVH	NCP	102.116
EHVTVH;DDMDED	NCP	52.675
DDMDED;SSKSSS	NCP	38.691
SSKSSS;MATLLRLP	NCP	36.078
MATLLRLP;TDSSKMR	NCP	6.855
TDSSKMR;CSNPPYK	NCP	333.471
CSNPPYK;LDDFL	NCP	97.769
LDDFL;SAPGLL	NCP	80.592
SAPGLL;KLSRQE	NCP	86.616
KLSRQE;ALEAKNAN	NCP	149.812
ALEAKNAN;DMLLKK	NCP	11.025
DMLLKK;VGFAGAPL	NCP	376.512
VGFAGAPL;RYHHVVP	NCP	43.431
RYHHVVP;KQKSYP	NCP	19.944
KQKSYP;KALLKLTK	NCP	58.875
KALLKLTK;APKRPPSAF	NCP	91.578
APKRPPSAF;KRPPSAFF	NCP	0.006
KRPPSAFF;FALGQSR	NCP	85.554
FALGQSR;EGVRLLT	NCP	9.858
EGVRLLT;LARMAYP	NCP	0.714
LARMAYP;KHTCY	NCP	12.87
KHTCY;TQKAPK	NCP	102.108
TQKAPK;WVLGGSLL	NCP	30.978
WVLGGSLL;PQNMKP	NCP	0.834
PQNMKP;AYLLY	NCP	81.327
AYLLY;VGPLKLGK	NCP	192.363
VGPLKLGK;DLEKTTEK	NCP	99.291
DLEKTTEK;DWHHSL	NCP	144.573
DWHHSL;NFGPLTS	NCP	141.45
NFGPLTS;KGLAY	NCP	6.489
KGLAY;KLEGFLA	NCP	46.416
KLEGFLA;PLAVSLK	NCP	5.292
PLAVSLK;KQFWLVL	NCP	75.648
KQFWLVL;YHASAR	NCP	202.157
YHASAR;MHDLPR	NCP	616.317

MHDLPR;MGSPK	NCP	196.873
MGSPK;HETLKGF	NCP	1.527
HETLKGF;EGVLDLH	NCP	123.771
EGVLDLH;QRAKN	NCP	161.787
QRAKN;RNVKMLAL	NCP	49.596
RNVKMLAL;TAVLLE	NCP	5.211
TAVLLE;KPNPTCR	NCP	10.836
KPNPTCR;KGPSVDH	NCP	125.244
KGPSVDH;APGDGAK	NCP	214.875
APGDGAK;PAAGK	NCP	17.514
PAAGK;ARFMP	NCP	91.835
ARFMP;KTTPGKLR	NCP	9.769
KTTPGKLR;ASDSLVK	NCP	59.709
ASDSLVK;EHGLFLE	NCP	9.596
EHGLFLE;LSYVYKV	NCP	33.103
LSYVYKV;AVTDQPL	NCP	101.609
AVTDQPL;VPQEP	NCP	100.33
VPQEP;QAPGVRL	NCP	35.55
QAPGVRL;LSEDDLK	NCP	45.819
LSEDDLK;QSHLSKFR	NCP	108.576
QSHLSKFR;KATGHKK	NCP	34.311
KATGHKK;TLARVLP	NCP	36.675
TLARVLP;GVLSGF	NCP	90.375
GVLSGF;ALPEKKP	NCP	10.719
ALPEKKP;LLDLVTPL	NCP	1.449
LLDLVTPL;MGPGMP	NCP	177.093
MGPGMP;KPYPQ	NCP	154.902
KPYPQ;MQSGVTT	NCP	32.529
MQSGVTT;RLLDMP	NCP	13.8
RLLDMP;PSYPELG	NCP	64.061
PSYPELG;VGPGAAPPP	NCP	63.124
VGPGAAPPP;AARSLL	NCP	3.852
AARSLL;ESAPVPR	NCP	29.61
ESAPVPR;VPGASSPP	NCP	24.993
TSANSLK;QPGQKPP	NCP	38.304
QPGQKPP;QVEALP	NCP	57.708
QVEALP;LLSGVLSPF	NCP	13.545
LLSGVLSPF;KSYVDF	NCP	1.035
KSYVDF;PPLPD	NCP	0.618
PPLPD;LMKCDV	NCP	64.19
LMKCDV;AAVLE	NCP	16.92
AAVLE;EVALTNK	NCP	26.404
EVALTNK;VQSLLF	NCP	33.222
VQSLLF;PTGEVLR	NCP	72.194
PTGEVLR;RAFLEKVR	NCP	37.272
RAFLEKVR;KWTRGSP	NCP	28.182
KWTRGSP;KMLTKSK	NCP	4.428
KMLTKSK;AGGAVP	NCP	146.226
AGGAVP;LTAAPPPL	NCP	12.67
LTAAPPPL;GYSLPSGH	NCP	44.211
GYSLPSGH;MEESPVF	NCP	0.843

MEESPVF;KLKPPVG	NCP	15.234
KLKPPVG;TGHALAL	NCP	12.089
TGHALAL;PAAEVVGK	NCP	11.82
PAAEVVGK;KAAALPK	NCP	12.262
KAAALPK;CAGVLPR	NCP	18.423
CAGVLPR;AVPPSS	NCP	8.478
AVPPSS;EMDFLP	NCP	36.026
EMDFLP;QVTKPGK	NCP	29.401
QVTKPGK;KAASQKPP	NCP	12.173
KAASQKPP;KPPPPYK	NCP	11.527
KPPPPYK;CPPRAPGP	NCP	5.513
CPPRAPGP;VLLVTNK	NCP	7.996
VLLVTNK;RPLPP	NCP	51.821
RPLPP;GVSVPP	NCP	94.122
GVSVPP;KLLTAF	NCP	48.039
KLLTAF;LPSKSVP	NCP	34.39
LPSKSVP;APSFFR	NCP	3.423
APSFFR;EGPARPPP	NCP	3.161
EGPARPPP;MFAHPP	NCP	148.108
MFAHPP;DPAALSPR	NCP	8.672
DPAALSPR;EVSLPDL	NCP	4.125
EVSLPDL;KVMELC	NCP	34.389
KVMELC;DHTEAF	NCP	4.89
DHTEAF;LQTLRVSL	NCP	73.311
LQTLRVSL;PTPVVPP	NCP	12.954
PTPVVPP;DLLSLK	NCP	28.363
DLLSLK;RTLGFN	NCP	12.894
RTLGFN;DPAMPVP	NCP	8.204
DPAMPVP;MLSDPK	NCP	30.216
MLSDPK;QSLPGRGH	NCP	6.279
QSLPGRGH;KFSPPPPP	NCP	3.285
KFSPPPPP;ESLQLNSK	NCP	1.128
ESLQLNSK;RVAPVTK	NCP	7.405
RVAPVTK;LQELLK	NCP	94.403
LQELLK;RVHRLL	NCP	70.485
RVHRLL;VMLARSP	NCP	4.575
VMLARSP;GPTWQP	NCP	21.429
GPTWQP;PAAHSPR	NCP	15.48
PAAHSPR;ASVYKKR	NCP	27.387
ASVYKKR;KPCKRP	NCP	15.537
KPCKRP;KGVLQY	NCP	3.775
KGVLQY;RPGPGAPP	NCP	26.637
RPGPGAPP;ATPKAAR	NCP	12.704
ATPKAAR;PLGPDPPP	NCP	29.148
PLGPDPPP;PPPPPPPPSPR	NCP	14.805
PPPPPPPPSPR;VFKELEK	NCP	2.698
VFKELEK;VLKLGEH	NCP	19.805
VLKLGEH;TPLLQPP	NCP	22.659
TPLLQPP;DLLKPM	NCP	45.844
DLLKPM;AAPGVKLH	NCP	96.03
AAPGVKLH;ALSMPLN	NCP	73.281

ALSMPLN;PPGMKRH	NCP	21.362
PPGMKRH;GPDAGP	NCP	74.874
GPDAGP;EFPEFQL	NCP	0.471
EFPEFQL;GVGVEPY	NCP	123.882
GVGVEPY;AATLSVQF	NCP	0.057
AATLSVQF;KTPLLNV	NCP	91.095
KTPLLNV;KLVSYKN	NCP	26.023
KLVSYKN;KSLPGLERA	NCP	28.733
KSLPGLERA;LPVLEFT	NCP	118.251
LPVLEFT;KTVEVLK	NCP	23.116
KTVEVLK;FGAPLT	NCP	6.183
FGAPLT;VLMLENVL	NCP	35.189
VLMLENVL;DSPLSQRP	NCP	7.915
DSPLSQRP;AVSVTLP	NCP	92.297
AVSVTLP;KMPGAP	NCP	79.422
KMPGAP;ASERRAGP	NCP	3.96
ASERRAGP;FATTGF	NCP	5.307
FATTGF;PAPATTF	NCP	114.855
PAPATTF;PAGVQPPR	NCP	0.312
PAGVQPPR;ADGMSSLG	NCP	2.571
ADGMSSLG;SGFLGNST	NCP	4.569
SGFLGNST;AAQALALLR	NCP	16.662
AAQALALLR;NSPGFVEY	NCP	20.4
NSPGFVEY;EGRLW	NCP	32.551
EGRLW;KFVHRP	NCP	26.613
KFVHRP;TVGLPL	NCP	61.448
TVGLPL;KPTSP	NCP	8.067
KPTSP;PAASPLLL	NCP	4.41
PAASPLLL;NLPPP	NCP	17.274
NLPPP;QFAYNP	NCP	71.317
QFAYNP;APGLF	NCP	14.756
APGLF;VGGLLLGT	NCP	2.077
VGGLLLGT;ACGLVA	NCP	21.89
ACGLVA;CSEEC	NCP	27.15
CSEEC;WPSFPLG	NCP	104.994
WPSFPLG;ESDLLELK	NCP	242.362
ESDLLELK;AMDPK	NCP	7.671
AMDPK;LDVPVPR	NCP	79.712
LDVPVPR;DHFKDL	NCP	0.78
DHFKDL;VTLVTL	NCP	28.498
VTLVTL;KDPLVDT	NCP	6.152
KDPLVDT;KPPQYRL	NCP	25.965
KPPQYRL;SVNDALR	NCP	72.906
SVNDALR;KSFKKYP	NCP	86.887
KSFKKYP;NNPLVG	NCP	8.114
NNPLVG;FYLFPGN	NCP	64.006
FYLFPGN;PAYMPK	NCP	40.361
PAYMPK;LTQTAELL	NCP	6.853
LTQTAELL;ETSSK	NCP	26.351
ETSSK;LPELNG	NCP	103.47
LPELNG;HHEKVR	NCP	36.828

HHEKVR;AAALSLK	NCP	11.502
AAALSLK;LSYAGRS	NCP	92.547
LSYAGRS;LKTYKPP	NCP	65.907
LKTYKPP;PAASGLK	NCP	39.723
PAASGLK;LPGLVLPL	NCP	7.833
LPGLVLPL;SAPVPRAP	NCP	3.861
SAPVPRAP;NLRHAK	NCP	38.614
NLRHAK;AVAVLPF	NCP	20.642
AVAVLPF;YKGGLR	NCP	202.567
YKGGLR;YRVASY	NCP	33.905
YRVASY;KAFEREK	NCP	0.963
KAFEREK;ESPELLH	NCP	4.212
ESPELLH;LPLFACLL	NCP	24.012
LPLFACLL;TFCATPL	NCP	30.931
TFCATPL;LEFAGA	NCP	36.044
LEFAGA;KMPQTNF	NCP	23.395
KMPQTNF;GDSLMF	NCP	3.933
GDSLMF;MTLAPY	NCP	45.014
MTLAPY;REFTLLK	NCP	5.169
REFTLLK;TTYFLE	NCP	3.126
TTYFLE;EKRLKH	NCP	3.565
EKRLKH;QSMKAVP	NCP	61.016
QSMKAVP;WELNKYV	NCP	12.508
WELNKYV;QAPGVRLL	NCP	38.391
QAPGVRLL;KYDPGAH	NCP	10.074
KYDPGAH;KYKNQPP	NCP	42.647
KYKNQPP;PVVGSPF	NCP	30.414
PVVGSPF;PNWFMK	NCP	24.196
PNWFMK;QLSNKL	NCP	0.386
QLSNKL;PAAHPPK	NCP	35.329
PAAHPPK;LSLLEELT	NCP	31.677
LSLLEELT;AMLSVA	NCP	11.802
AMLSVA;VVATQPK	NCP	139.892
VVATQPK;KEMHPK	NCP	26.838
KEMHPK;KLSEFLR	NCP	68.185
KLSEFLR;LVNELN	NCP	2.111
LVNELN;RSDLLL	NCP	38.703
RSDLLL;KTPLLS	NCP	42.279
KTPLLS;DPVQPY	NCP	67.294
DPVQPY;KDNEK	NCP	17.357
KDNEK;AGALHY	NCP	78.253
AGALHY;RSVAAPK	NCP	49.938
RSVAAPK;VTFEY	NCP	81.797
VTFEY;SLMLK	NCP	10.095
SLMLK;RLKDPK	NCP	2.982
RLKDPK;LGKLP	NCP	68.013
LGKLP;TPLRLGTLG	NCP	31.657
TPLRLGTLG;TALHPGK	NCP	0.423
TALHPGK;LQLMT	NCP	17.319
LQLMT;CLYALSF	NCP	11.58
CLYALSF;LDLGLQM	NCP	42.591

LDLGLQM;NSLVQTK	NCP	22.716
NSLVQTK;VTRLAEY	NCP	50.027
VTRLAEY;MSVLLPR	NCP	137.62
MSVLLPR;TLMRPP	NCP	23.343
TLMRPP;DLFPP	NCP	109.763
DLFPP;PGPGPPPPP	NCP	141.771
PGPGPPPPP;PKALP	NCP	28.197
PKALP;EVLGRDL	NCP	115.89
EVLGRDL;LTHVL	NCP	4.5
LTHVL;FEKLLCV	NCP	20.776
FEKLLCV;NVLVVV	NCP	11.382
NVLVVV;CPAGYG	NCP	0.902
CPAGYG;MYGMQT	NCP	44.583
MYGMQT;AHELVRK	NCP	134.586
AHELVRK;GVVLSA	NCP	15.963
GVVLSA;TVASGELR	NCP	17.826
TVASGELR;SEMDDD	NCP	49.851
SEMDDD;ELAPDVS	NCP	46.648
ELAPDVS;GDYVPR	NCP	35.349
GDYVPR;LNPPGLK	NCP	6.759
LNPPGLK;KPDLFPQ	NCP	5.45
KPDLFPQ;PNLQAVR	NCP	30.069
PNLQAVR;LLLFRMPL	NCP	90.039
LLLFRMPL;KMGAPP	NCP	9.888
KMGAPP;TLKYPLE	NCP	56.784
TLKYPLE;KLTVMENF	NCP	40.911
KLTVMENF;AQLKVHA	NCP	29.814
AQLKVHA;GVVLK	NCP	20.847
GVVLK;RLTERGY	NCP	37.392
RLTERGY;KVLGMW	NCP	116.226
KVLGMW;LDELSVR	NCP	35.122
LDELSVR;TSLRACP	NCP	3.608
TSLRACP;QSDDGP	NCP	58.447
QSDDGP;RAASRPLP	NCP	52.851
RAASRPLP;MLPVSLML	NCP	8.855
MLPVSLML;KPPNVTF	NCP	167.031
KPPNVTF;LFAAGLP	NCP	61.744
LFAAGLP;RWTYKK	NCP	0.863
RWTYKK;EGGPTK	NCP	40.116
EGGPTK;KPEHTVS	NCP	64.02
KPEHTVS;VELVPLP	NCP	41.448
VELVPLP;AQLLLP	NCP	32.607
AQLLLP;KPGEFR	NCP	29.457
KPGEFR;KGYLLSK	NCP	60.211
KGYLLSK;KERPLH	NCP	0.591
KERPLH;EGLVTSK	NCP	55.032
EGLVTSK;KYPLTLF	NCP	7.232
KYPLTLF;LVPLPAR	NCP	27.489
LVPLPAR;APKAPMP	NCP	4.531
APKAPMP;KDPLGRV	NCP	38.652
KDPLGRV;ATQALEKY	NCP	32.289

ATQALEKY;KVKVEAL	NCP	80.667
KVKVEAL;DKVLYHT	NCP	102.419
DKVLYHT;KQANVPK	NCP	109.506
KQANVPK;ELGEW	NCP	4.675
ELGEW;QLPLPT	NCP	181.317
QLPLPT;RKPFPS	NCP	12.813
RKPFPS;QTSLEERK	NCP	11.006
QTSLEERK;KSPSWPP	NCP	19.68
KSPSWPP;CGHPLK	NCP	82.573
CGHPLK;NNNHDY	NCP	27.254
NNNHDY;VLRGLEPGQEY	NCP	3.573
VLRGLEPGQEY;ALTNLEPATEY	NCP	37.287
ALTNLEPATEY;TDLSPSTYY	NCP	5.736
TDLSPSTYY;GQYELR	NCP	3.825
GQYELR;RLKVEGY	NCP	0.096
RLKVEGY;RVLYEVT	NCP	41.865
RVLYEVT;NHLYMEPK	NCP	44.307
NHLYMEPK;KLLNGVL	NCP	3.906
KLLNGVL;VTGRPPP	NCP	30.888
VTGRPPP;PDLAW	NCP	20.752
PDLAW;KPAQVFQ	NCP	0.623
KPAQVFQ;ETSEMAH	NCP	6.658
ETSEMAH;KHLFST	NCP	18.051
KHLFST;DHLWQK	NCP	17.066
DHLWQK;PGVLKVAH	NCP	44.241
PGVLKVAH;LSGFNDK	NCP	27.354
LSGFNDK;KGSLLV	NCP	41.926
KGSLLV;TGGSVNKGK	NCP	60.716
TGGSVNKGK;DPPLKL	NCP	31.338
DPPLKL;LPSSSSGP	NCP	13.429
LPSSSSGP;TVVGMPF	NCP	0.188
TVVGMPF;YQYTSSK	NCP	25.761
YQYTSSK;PGDPLKK	NCP	46.381
PGDPLKK;KWGNK	NCP	17.49
KWGNK;AADTRKF	NCP	12.443
AADTRKF;KTTTFPLG	NCP	26.713
KTTTFPLG;AAVPSPK	NCP	7.925
AAVPSPK;MLNSRNR	NCP	14.766
MLNSRNR;PVPLLP	NCP	184.759
PVPLLP;CDLDLR	NCP	28.784
CDLDLR;SGPLAPAL	NCP	6.309
SGPLAPAL;VVHTNYDEY	NCP	142.605
VVHTNYDEY;EVEQAGVP	NCP	26.587
EVEQAGVP;MGVSLLC	NCP	1.742
MGVSLLC;MEYGPK	NCP	2.691
MEYGPK;KTGVP	NCP	46.981
KTGVP;KGLHVPR	NCP	35.325
KGLHVPR;EVVGAW	NCP	0.551
EVVGAW;GEVLDLH	NCP	2.134
GEVLDLH;LGLLG	NCP	30.036
LGLLG;PGTSYNK	NCP	9.057

PGTSYNK;EFGGVP	NCP	17.66
EFGGVP;KEFLSW	NCP	5.845
KEFLSW;GVDQMAK	NCP	26.873
GVDQMAK;VGSPLKR	NCP	12.537
VGSPLKR;KPTYSLK	NCP	50.896
KPTYSLK;EMLRVAA	NCP	43.667
EMLRVAA;FSHVLPL	NCP	109.228
FSHVLPL;VSLPGP	NCP	67.22
VSLPGP;RTVSYP	NCP	26.172
RTVSYP;FPSATELL	NCP	19.051
FPSATELL;LPRSNR	NCP	64.868
LPRSNR;VGKVHL	NCP	4.158
VGKVHL;LSFGKPH	NCP	44.241
LSFGKPH;MHPQSVLHSGYFHPLLR	NCP	4.908
MHPQSVLHSGYFHPLLR;SGYFHPLL	NCP	0.024
SGYFHPLL;AHRLQLRLL	NCP	14.472
AHRLQLRLL;LSVPN	NCP	6.226
LSVPN;PPEKPKP	NCP	16.194
PPEKPKP;KAKETLK	NCP	25.179
KAKETLK;KAGTLGPK	NCP	81.996
KAGTLGPK;GVVEPF	NCP	2.315
GVVEPF;KDSKGPK	NCP	3.036
KDSKGPK;KVPSLP	NCP	157.974
KVPSLP;SPPERPP	NCP	66.169
SPPERPP;KDEMFQ	NCP	82.151
KDEMFQ;LHGDLPP	NCP	7.873
LHGDLPP;MEETNNFK	NCP	25.038
MEETNNFK;TPSLVPPP	NCP	23.192
TPSLVPPP;QTPKLA	NCP	90.619
QTPKLA;KPGLP	NCP	32.253
KPGLP;YKPLTLY	NCP	17.606
YKPLTLY;VGLPK	NCP	35.233
VGLPK;ELFHPEQ	NCP	6.245
ELFHPEQ;VLMRTVK	NCP	50.679
VLMRTVK;DDFKLK	NCP	9.202
DDFKLK;GVLAK	NCP	77.901
GVLAK;KLNGVLSK	NCP	71.124
KLNGVLSK;EGPVVSY	NCP	28.859
EGPVVSY;AAAEDELLLPR	NCP	25.104
AAAEDELLLPR;WDDWKDTHPR	NCP	0.957
WDDWKDTHPR;LELALMA	NCP	28.918
LELALMA;LVRPF	NCP	91.412
LVRPF;MDLRLQ	NCP	37.389
MDLRLQ;KGGSVLNP	NCP	52.275
KGGSVLNP;KEEFELP	NCP	12.468
KEEFELP;RLALYK	NCP	82.539
RLALYK;ETMCAQ	NCP	27.424
ETMCAQ;SSVPTVKL	NCP	7.562
SSVPTVKL;PAAFPQP	NCP	64.794
PAAFPQP;ETAGGKLR	NCP	80.515
ETAGGKLR;LSVDLAT	NCP	63.389

LSVDLAT;KTAVTVNQ	NCP	17.554
KTAVTVNQ;FDGVSLK	NCP	120.012
FDGVSLK;VHAKSWTLKKH	NCP	0.683
VHAKSWTLKKH;KHFVGYPTNSDFELK	NCP	0.027
KHFVGYPTNSDFELK;YLTVDPY	NCP	8.211
YLTVDPY;ELHLQGF	NCP	16.365
ELHLQGF;EHVTEGFENMPAAF	NCP	3.81
EHVTEGFENMPAAF;AANPPKLQ	NCP	16.164
AANPPKLQ;VGMRHY	NCP	4.005
VGMRHY;VPDLVPN	NCP	31.143
VPDLVPN;HGVVL	NCP	103.414
HGVVL;QYKVLG	NCP	116.384
QYKVLG;DLEPKPR	NCP	28.326
DLEPKPR;TLDVKR	NCP	35.413
TLDVKR;RMSFLSF	NCP	65.474
RMSFLSF;MNRAVEF	NCP	62.305
MNRAVEF;MDLAP	NCP	16.154
MDLAP;KDWLEW	NCP	72.678
KDWLEW;VFDFLYT	NCP	3.703
VFDFLYT;KHTEC	NCP	2.069
KHTEC;GFTKLR	NCP	14.428
GFTKLR;KERHWLP	NCP	88.014
KERHWLP;KDHPVGP	NCP	27.488
KDHPVGP;YEAPSLPR	NCP	2.107
YEAPSLPR;KPPDPRP	NCP	35.231
KPPDPRP;GHLNESP	NCP	18.549
GHLNESP;KAQLVK	NCP	217.669
KAQLVK;CDCCC	NCP	64.625
CDCCC;VHNLGPK	NCP	64.746
VHNLGPK;SKKPGAK	NCP	51.477
SKKPGAK;AFQEALNSAGEKLVVVDF	NCP	29.322
AFQEALNSAGEKLVVVDF;NSAGEKLVVVDFS	NCP	0.018
NSAGEKLVVVDFS;LLFYR	NCP	60.021
LLFYR;PLAVSKL	NCP	26.211
PLAVSKL;LVPLL	NCP	0.949
LVPLL;TALGGLL	NCP	16.286
TALGGLL;EPAVFP	NCP	5.772
EPAVFP;TTARKHF	NCP	18.498
TTARKHF;KAQELF	NCP	66.496
KAQELF;VQLPEM	NCP	38.268
VQLPEM;VASLLSPR	NCP	45.293
VASLLSPR;KNHLSRL	NCP	63.25
KNHLSRL;EKPAAPP	NCP	35.252
EKPAAPP;KPAAPPR	NCP	0.003
KPAAPPR;PPLALPPP	NCP	34.264
PPLALPPP;WYTGTLR	NCP	81.803
WYTGTLR;FEVVY	NCP	18.892
FEVVY;NKGELLP	NCP	0.426
NKGELLP;SGKDLAF	NCP	19.916
SGKDLAF;VLEPL	NCP	31.495
VLEPL;FTCKSPP	NCP	40.67

FTCKSPP;PLLSCV	NCP	10.909
PLLSCV;EPPAPPK	NCP	28.671
EPPAPPK;VEPLPPPPPP	NCP	83.714
VEPLPPPPPP;LDTVVFK	NCP	17.125
LDTVVFK;VGPGRPLL	NCP	130.553
VGPGRPLL;KDRVLSMP	NCP	15.786
KDRVLSMP;DSRKSPP	NCP	268.8
DSRKSPP;KGALYY	NCP	20.866
KGALYY;PVVYVF	NCP	68.195
PVVYVF;LNLVPLL	NCP	27.459
LNLVPLL;PNFAGTW	NCP	26.215
PNFAGTW;KNPLLPK	NCP	101.354
KNPLLPK;LLDVLAPL	NCP	1.434
LLDVLAPL;EDDRAR	NCP	32.619
EDDRAR;FLMGDSL	NCP	103.194
FLMGDSL;KYLQVVYL	NCP	51.607
KYLQVVYL;MELLARL	NCP	215.478
MELLARL;KDPVGGF	NCP	23.526
KDPVGGF;ELPGVR	NCP	1.019
ELPGVR;FFKLLDN	NCP	1.536
FFKLLDN;FQYLSK	NCP	8.835
FQYLSK;MTGPLPR	NCP	43.005
MTGPLPR;EHLFK	NCP	2.167
EHLFK;PPSFPP	NCP	3.032
PPSFPP;GVKSLVS	NCP	28.749
GVKSLVS;YVDLC	NCP	3.453
YVDLC;FLTMF	NCP	237.181
FLTMF;HGVAVPQ	NCP	51.17
HGVAVPQ;MHVDW	NCP	6.262
MHVDW;NLTKDLR	NCP	23.813
NLTKDLR;LGVRGGF	NCP	20.673
LGVRGGF;VLSLEGKLP	NCP	19.711
VLSLEGKLP;MHNDLR	NCP	80.256
MHNDLR;DSPLLWR	NCP	43.923
DSPLLWR;QPGEPTPP	NCP	34.127
QPGEPTPP;EVSGLPF	NCP	110.04
EVSGLPF;KMEYKY	NCP	63.087
KMEYKY;RVMMPH	NCP	17.458
RVMMPH;GTVGTFYK	NCP	3.797
GTVGTFYK;KFTYPP	NCP	18.082
KFTYPP;VAGLLA	NCP	22.517
VAGLLA;VCLLHEK	NCP	84.619
VCLLHEK;NAFSPPL	NCP	33.953
NAFSPPL;FGKTEC	NCP	70.566
FGKTEC;YLLKKN	NCP	34.242
YLLKKN;KNAHVDP	NCP	32.677
KNAHVDP;YAPLGSP	NCP	11.688
YAPLGSP;QLLTMF	NCP	17.9
QLLTMF;ELRVTQK	NCP	31.245
ELRVTQK;KPPVH	NCP	142.744
KPPVH;TLLPVTF	NCP	18.944

TLLPVTF;ALNPKCT	NCP	3.463
ALNPKCT;VVPKP	NCP	23.627
VVPKP;MMPFF	NCP	89.175
MMPFF;LGVQGGF	NCP	61.5
LGVQGGF;VAYPMK	NCP	3.411
VAYPMK;SMEGY	NCP	60.108
SMEGY;KKSKCHK	NCP	39.36
KKSKCHK;LTLKAY	NCP	5.362
LTLKAY;VKSLKK	NCP	3.792
VKSLKK;TFEVGW	NCP	26.565
TFEVGW;KVVPSPP	NCP	20.598
KVVPSPP;DLDMSSS	NCP	32.877
DLDMSSS;PPPFTP	NCP	44.489
PPPFTP;EEKRL	NCP	23.689
EEKRL;TPDTASPK	NCP	89.178
TPDTASPK;KQWLYG	NCP	16.496
KQWLYG;SLATQYSH	NCP	22.575
SLATQYSH;VPALEYL	NCP	44.278
VPALEYL;VTYLDLL	NCP	18.384
VTYLDLL;NRPGRSK	NCP	8.952
NRPGRSK;KPAAPAPP	NCP	9.053
KPAAPAPP;NSGAFLVL	NCP	3.811
NSGAFLVL;TTSALKK	NCP	23.76
TTSALKK;YELALLT	NCP	79.878
YELALLT;KSALL	NCP	129.587
KSALL;KWQGKP	NCP	0.699
KWQGKP;DSPTSPR	NCP	17.343
DSPTSPR;LQFLLG	NCP	137.053
LQFLLG;LTGHLPY	NCP	6.794
LTGHLPY;PPMKL	NCP	165.78
PPMKL;TVGDLPY	NCP	20.485
TVGDLPY;LVDLLLKFF	NCP	63.24
LVDLLLKFF;YATAEY	NCP	44.537
YATAEY;PLNKP	NCP	5.974
PLNKP;KMGAAPK	NCP	30.482
KMGAAPK;MEKLLRLG	NCP	1.597
MEKLLRLG;VTKAFSF	NCP	25.887
VTKAFSF;LYLAPGN	NCP	4.356
LYLAPGN;SLYPPR	NCP	44.784
SLYPPR;CTQGEKL	NCP	17.78
CTQGEKL;KETHLQH	NCP	0.648
KETHLQH;KRLPLDY	NCP	36.474
KRLPLDY;VGSLLCEF	NCP	77.977
VGSLLCEF;PLGVSTY	NCP	45.165
PLGVSTY;TVTVY	NCP	6.66
TVTVY;KEMEK	NCP	16.377
KEMEK;LGDRQEL	NCP	5.543
LGDRQEL;LPPVKHG	NCP	27.141
LPPVKHG;AAPGTSRP	NCP	49.05
AAPGTSRP;QDGPVP	NCP	50.079
QDGPVP;RFENQR	NCP	16.794

RFENQR;NGLLNVKS	NCP	18.814
NGLLNVKS;LYNKETVRK	NCP	66.902
LYNKETVRK;VGLVN	NCP	10.834
VGLVN;MDGVLKR	NCP	48.744
MDGVLKR;LSTKPLVF	NCP	30.417
LSTKPLVF;NDFLLA	NCP	22.458
NDFLLA;CKQCLSL	NCP	25.28
CKQCLSL;DDDHLKDLSL	NCP	30.504
DDDHLKDLSL;YFPKVP	NCP	31.89
YFPKVP;HALGL	NCP	25.86
HALGL;KMYAPR	NCP	157.743
KMYAPR;YGVVTP	NCP	1.308
YGVVTP;LPVARPK	NCP	9.25
LPVARPK;FLLLKK	NCP	15.036
FLLLKK;KTYTSPP	NCP	30.341
KTYTSPP;CCCLV	NCP	62.526
CCCLV;LDVEDVK	NCP	62.668
LDVEDVK;KLASKYR	NCP	7.551
KLASKYR;LQRLALY	NCP	8.928
LQRLALY;YAFPEL	NCP	146.409
YAFPEL;GPGKPEK	NCP	8.568
GPGKPEK;NYPFPK	NCP	33.035
NYPFPK;DPVPDAK	NCP	89.89
DPVPDAK;SLDDPPK	NCP	14.588
SLDDPPK;ASGVSKY	NCP	144.378
ASGVSKY;EELFKKQ	NCP	20.182
EELFKKQ;RWHHSF	NCP	79.835
RWHHSF;VNFVEF	NCP	182.676
VNFVEF;KSVLLFN	NCP	84.079
KSVLLFN;AAVGAGR	NCP	146.496
AAVGAGR;KDAMALK	NCP	108.627
KDAMALK;KPYLEVR	NCP	9.956
KPYLEVR;FGLQY	NCP	230.01
FGLQY;TLNTVPQ	NCP	27.487
TLNTVPQ;QAPGFGSR	NCP	107.559
QAPGFGSR;KPAAEYK	NCP	192.708
KPAAEYK;TVPLPF	NCP	173.067
TVPLPF;MGFVAY	NCP	68.117
MGFVAY;PMGPK	NCP	71.71
PMGPK;GFTPL	NCP	94.26
GFTPL;PSLGFQP	NCP	10.605
PSLGFQP;QKKVEK	NCP	236.63
QKKVEK;LSNKPSR	NCP	89.364
LSNKPSR;AGSKTFLL	NCP	196.593
AGSKTFLL;KLEDGLK	NCP	175.167
KLEDGLK;VSSSVPEY	NCP	57.339
VSSSVPEY;SVSLDF	NCP	16.488
SVSLDF;KSVPTLK	NCP	1.453
KSVPTLK;DLGTVPQ	NCP	103.388
DLGTVPQ;LAPSVLF	NCP	4.153
LAPSVLF;AHQFPAL	NCP	67.07

AHQFPAL;HETLYQK	NCP	1.08
HETLYQK;ETLYQKDGQGKLFR	NCP	0.003
ETLYQKDGQGKLFR;LSFSYGRALQ	NCP	6.21
LSFSYGRALQ;NSQAAKGQYVHMGSSGSAS	NCP	2.436
NSQAAKGQYVHMGSSGSAS;SQAAKGQYVHMGSSG	NCP	0.003
SQAAKGQYVHMGSSG;KEARKPK	NCP	18.058
KEARKPK;KLLLKK	NCP	34.056
KLLLKK;MERPQP	NCP	14.148
MERPQP;GALLSY	NCP	6.125
GALLSY;KALKGAQP	NCP	40.063
KALKGAQP;NERPNL	NCP	68.678
NERPNL;EVGLQFF	NCP	67.971
EVGLQFF;PGKRF	NCP	1.822
PGKRF;ALAGAVL	NCP	42.048
ALAGAVL;TSVALPK	NCP	33.171
TSVALPK;SNWLSK	NCP	1.767
SNWLSK;SLRDFQ	NCP	31.488
SLRDFQ;YGYQYGR	NCP	15.942
YGYQYGR;LGKARHP	NCP	114.863
LGKARHP;KSPRMF	NCP	19.393
KSPRMF;ERDREVGV	NCP	14.162
ERDREVGV;PKLFPH	NCP	151.153
PKLFPH;LPHVPLPP	NCP	57.972
LPHVPLPP;EVRVPQ	NCP	31.191
EVRVPQ;LMPLLTG	NCP	4.05
LMPLLTG;VKVGVNG	NCP	31.895
VKVGVNG;HVMPRP	NCP	21.183
HVMPRP;NAVVLSL	NCP	56.334
NAVVLSL;ELEGKLK	NCP	9.037
ELEGKLK;CPSVLLK	NCP	52.8
CPSVLLK;KERFPLE	NCP	47.382
KERFPLE;MGGMFTR	NCP	26.228
MGGMFTR;SAELTLP	NCP	7.666
SAELTLP;EVPLTGR	NCP	52.592
EVPLTGR;APALSLY	NCP	9.802
APALSLY;HHPPGAAP	NCP	31.458
HHPPGAAP;GPDLWF	NCP	8.466
GPDLWF;GHNLAAGQ	NCP	111.569
GHNLAAGQ;YQKGSLR	NCP	11.215
YQKGSLR;SPDFMVP	NCP	44.019
SPDFMVP;FGPPG	NCP	15.438
FGPPG;PGPATVF	NCP	131.916
PGPATVF;LELLDH	NCP	8.055
LELLDH;TAEFVH	NCP	78.758
TAEFVH;MMGHSF	NCP	71.515
MMGHSF;KLHKGKK	NCP	5.352
KLHKGKK;MKLGFHG	NCP	4.311
MKLGFHG;KPASTPK	NCP	107.886
KPASTPK;HSVLP	NCP	1.37
HSVLP;KRTPPNP	NCP	32.532
KRTPPNP;KTVKLLKK	NCP	21.793

KTVKLLKK;AALVV	NCP	170.57
AALVV;FDKPM	NCP	0.535
FDKPM;MRLLTHN	NCP	93.753
MRLLTHN;HNLLSSHVR	NCP	0.015
HNLLSSHVR;AALLEAADHL	NCP	0.138
AALLEAADHL;NPGRSPK	NCP	0.347
NPGRSPK;VGVFF	NCP	16.942
VGVFF;LAAARP	NCP	19.751
LAAARP;RGRKAPP	NCP	23.886
RGRKAPP;KMVARK	NCP	91.96
KMVARK;SGRGPF	NCP	22.734
SGRGPF;KCPGKLK	NCP	13.614
KCPGKLK;APLLLAL	NCP	23.178
APLLLAL;LGPVDALS	NCP	11.028
LGPVDALS;LLQSTGPK	NCP	37.856
LLQSTGPK;SALPALVM	NCP	6.276
SALPALVM;LAPGGHVGR	NCP	0.327
LAPGGHVGR;KAVGDKKLKKPVVGK	NCP	0.417
KAVGDKKLKKPVVGK;AVGDKKLKKPVVG	NCP	0.003
AVGDKKLKKPVVG;KPPVEC	NCP	38.571
KPPVEC;RSLAALKK	NCP	25.509
RSLAALKK;ASGPLL	NCP	12.991
ASGPLL;AQLLRPL	NCP	0.753
AQLLRPL;LHSLVTL	NCP	49.866
LHSLVTL;LLLPKK	NCP	15.167
LLLPKK;GVGPFP	NCP	44.934
GVGPFP;QYQTPY	NCP	20.971
QYQTPY;KSFSSPPK	NCP	4.634
KSFSSPPK;VRTQP	NCP	44.37
VRTQP;KESCFPV	NCP	7.476
KESCFPV;VVPGLQD	NCP	19.125
VVPGLQD;NGPVYL	NCP	6.058
NGPVYL;APVFFQ	NCP	3.023
APVFFQ;HLLLPLLL	NCP	15.573
HLLLPLLL;EAVPLTGP	NCP	5.388
EAVPLTGP;FPPVTF	NCP	26.772
FPPVTF;GVHTVQY	NCP	3.948
GVHTVQY;GVALNLL	NCP	21.682
GVALNLL;YNELRV	NCP	115.166
YNELRV;KATWTPK	NCP	47.832
KATWTPK;KPEFRP	NCP	12.987
KPEFRP;KAMLPLGL	NCP	25.512
KAMLPLGL;KNSDLHLV	NCP	138.876
KNSDLHLV;LPGMSPPP	NCP	73.389
LPGMSPPP;LTKFKK	NCP	6.735
LTKFKK;LGVYLR	NCP	14.311
LGVYLR;KDHAKP	NCP	67.53
KDHAKP;PLLPPP	NCP	112.008
PLLPPP;KMLGPSFP	NCP	19.649
KMLGPSFP;MPDDLRK	NCP	104.307
MPDDLRK;LSEFPPPP	NCP	18.664

LSEFPPPP;EKGVLFK	NCP	43.002
EKGVLFK;TYFLEWNF	NCP	57.084
TYFLEWNF;MEHPP	NCP	102.5
MEHPP;KLLAPPE	NCP	16.206
KLLAPPE;RPSLMDS	NCP	80.847
RPSLMDS;RVSLPK	NCP	33.781
RVSLPK;DLGVVLY	NCP	61.115
DLGVVLY;LHVEVEL	NCP	47.208
LHVEVEL;FPLQPLLY	NCP	16.033
FPLQPLLY;PLKGHLQ	NCP	17.484
PLKGHLQ;RHASPLK	NCP	140.498
RHASPLK;GDGANNLPFF	NCP	53.316
GDGANNLPFF;PPPLAP	NCP	50.19
PPPLAP;NGKVDYK	NCP	50.527
NGKVDYK;NAGLGAL	NCP	2.681
NAGLGAL;EVTDYL	NCP	87.654
EVTDYL;GMMHSF	NCP	38.448
GMMHSF;LAPAM	NCP	22.608
LAPAM;KGASLLN	NCP	9.622
KGASLLN;GVLLK	NCP	31.547
GVLLK;VSHEKR	NCP	142.632
VSHEKR;TMLNPPPP	NCP	16.119
TMLNPPPP;CDMMP	NCP	0.178
CDMMP;DEKLFTF	NCP	11.666
DEKLFTF;PPNLLLQ	NCP	63.568
PPNLLLQ;APEVP	NCP	15.395
APEVP;VVKKLET	NCP	21.129
VVKKLET;QLSLK	NCP	52.446
QLSLK;HPFLP	NCP	62.095
HPFLP;LPSQNVPP	NCP	23.679
LPSQNVPP;KNGKQPP	NCP	64.715
KNGKQPP;KTVEKNF	NCP	55.395
KTVEKNF;VTPFYQ	NCP	30.093
VTPFYQ;KDAVTNK	NCP	36.624
KDAVTNK;EAPMCMC	NCP	49.924
EAPMCMC;NLPVM	NCP	15.005
NLPVM;VTLPKTK	NCP	93.567
VTLPKTK;KESPSVPK	NCP	2.82
KESPSVPK;TTYKK	NCP	83.278
TTYKK;NVLSSL	NCP	10.245
NVLSSL;VEGNL	NCP	79.971
VEGNL;QTHKSR	NCP	36.432
QTHKSR;LFPYP	NCP	22.56
LFPYP;ASHNPK	NCP	82.959
ASHNPK;KFVVGR	NCP	92.036
KFVVGR;RTAVLLPRK	NCP	7.042
RTAVLLPRK;VVKRPP	NCP	33.195
VVKRPP;KPGMKGK	NCP	5.586
KPGMKGK;PDHAKGK	NCP	36.858
PDHAKGK;FDPLY	NCP	28.989
FDPLY;AGPEFKP	NCP	46.118

AGPEFKP;EQPDN	NCP	68.73
EQPDN;GDFKLCK	NCP	15.325
GDFKLCK;VEALLEY	NCP	49.766
VEALLEY;LLEAVY	NCP	43.339
LLEAVY;LWYAR	NCP	22.535
LWYAR;KNLVSGKP	NCP	50.026
KNLVSGKP;FSGGL	NCP	39.353
FSGGL;VGLLFK	NCP	88.12
VGLLFK;KHVYYK	NCP	4.706
KHVYYK;NPSRFK	NCP	8.89
NPSRFK;KHLVPPP	NCP	57.701
KHLVPPP;NVELTR	NCP	15.99
NVELTR;RKVGPVLL	NCP	4.785
RKVGPVLL;PQPNAPK	NCP	77.025
PQPNAPK;RQQHGPV	NCP	0.577
RQQHGPV;ELEVVR	NCP	35.64
ELEVVR;KQNRDFK	NCP	0.594
KQNRDFK;HLLQC	NCP	31.949
HLLQC;LHAMAGK	NCP	29.65
LHAMAGK;NEAVYF	NCP	20.226
NEAVYF;KPASPLR	NCP	0.999
KPASPLR;FYPKRP	NCP	81.686
FYPKRP;HVLAPAR	NCP	8.826
HVLAPAR;QKGKPK	NCP	29.254
QKGKPK;EAPLL	NCP	22.557
EAPLL;KEVFYSK	NCP	14.552
KEVFYSK;TTKVEK	NCP	2.526
TTKVEK;MMVKPPP	NCP	16.032
MMVKPPP;NLKLAKA	NCP	8.166
NLKLAKA;AALTH	NCP	12.723
AALTH;KPFASVP	NCP	73.455
KPFASVP;EWLKFRR	NCP	173.071
EWLKFRR;KKDEK	NCP	14.958
KKDEK;KETGSPP	NCP	13.445
KETGSPP;ALPAPHV	NCP	12.852
ALPAPHV;TVQLPSR	NCP	13.416
TVQLPSR;AADPFSK	NCP	30.061
AADPFSK;LATAKDLR	NCP	48.096
LATAKDLR;KLYDLDK	NCP	24.78
KLYDLDK;LQAQMK	NCP	23.436
LQAQMK;AFLTTKK	NCP	48.737
AFLTTKK;KGMNALH	NCP	0.141
KGMNALH;WEVQPATF	NCP	9.993
WEVQPATF;DYEDKYLDGK	NCP	34.797
DYEDKYLDGK;LYARFNK	NCP	8.325
LYARFNK;LSLLEELTLAENQLLKLPVLPPK	NCP	1.848
LSLLEELTLAENQLLKLPVLPPK;TLAENQLLKL	NCP	0.021
TLAENQLLKL;LLKLPVLPPKLTLF	NCP	0.018
LLKLPVLPPKLTLF;PVLPPKLTL	NCP	0.012
PVLPPKLTL;LHNLSFLYL	NCP	2.16
LHNLSFLYL;LDHNALESVPL	NCP	0.024

LDHNALESVPL;LYPLSR	NCP	2.334
LYPLSR;EMVLRPP	NCP	7.317
EMVLRPP;LFDRGV	NCP	173.967
LFDRGV;VSVLPTTADF	NCP	1.371
VSVLPTTADF;RCHYCR	NCP	20.101
RCHYCR;LLKLVPT	NCP	63.677
LLKLVPT;KGVWGNK	NCP	29.526
KGVWGNK;FFGQW	NCP	171.682
FFGQW;KELLK	NCP	23.037
KELLK;RCNFFC	NCP	190.859
RCNFFC;FLVPTSA	NCP	70.86
FLVPTSA;VVKVANVSL	NCP	40.341
VVKVANVSL;HCDFLLLL	NCP	128.653
HCDFLLLL;YALQPPP	NCP	24.789
YALQPPP;VGVPTKL	NCP	34.065
VGVPTKL;KNYAGF	NCP	159.861
KNYAGF;NPGVVLF	NCP	19.649
NPGVVLF;GMPRQLL	NCP	54.399
GMPRQLL;MVAPP	NCP	47.379
MVAPP;VEDVNLP	NCP	40.488
VEDVNLP;LPKYVPL	NCP	6.609
LPKYVPL;HDTLEY	NCP	247.345
HDTLEY;MSPFPK	NCP	14.354
MSPFPK;NVPTVFF	NCP	129.421
NVPTVFF;LPGAASLK	NCP	50.582
LPGAASLK;APGVYGK	NCP	49.633
APGVYGK;AAVGLKS	NCP	0.563
AAVGLKS;KTFPPY	NCP	71.28
KTFPPY;RSAGDLPK	NCP	18.192
RSAGDLPK;SLLVKPGR	NCP	22.45
SLLVKPGR;SSPPPPPR	NCP	49.919
SSPPPPPR;WLLFP	NCP	0.33
WLLFP;PELLAYY	NCP	22.975
PELLAYY;KAALERLQ	NCP	16.028
KAALERLQ;MFPFFLK	NCP	0.637
MFPFFLK;QPSRFFR	NCP	40.022
QPSRFFR;VTRNLTSF	NCP	22.182
VTRNLTSF;PAVVEVP	NCP	53.995
PAVVEVP;EVDLHLK	NCP	15.084
EVDLHLK;PVMAKSS	NCP	3.741
PVMAKSS;MLKPEQK	NCP	15.032
MLKPEQK;TSLPKRPP	NCP	178.96
TSLPKRPP;KVGLDFC	NCP	13.322
KVGLDFC;VQTPRG	NCP	25.084
VQTPRG;VPGDVNL	NCP	32.198
VPGDVNL;THFNKGPS	NCP	47.731
THFNKGPS;VPDKLY	NCP	20.997
VPDKLY;KYVPGLF	NCP	137.208
KYVPGLF;YCLFVKDN	NCP	56.478
YCLFVKDN;KEVWELP	NCP	10.395
KEVWELP;AFVLLVPF	NCP	21.507

AFVLLVPF;NTQVLR	NCP	70.112
NTQVLR;MNVWGNK	NCP	134.701
MNVWGNK;KVLVPVP	NCP	32.429
KVLVPVP;VTGDQVKKLDVLSN	NCP	12.402
VTGDQVKKLDVLSN;LDVLSNDLVVNVLK	NCP	0.024
LDVLSNDLVVNVLK;KPHGCLK	NCP	11.776
KPHGCLK;SAPYGARYVG	NCP	3.914
SAPYGARYVG;ADVHRTLVY	NCP	0.039
ADVHRTLVY;LTCVQKNQAGR	NCP	67.413
LTCVQKNQAGR;APFGLLFP	NCP	13.554
APFGLLFP;PAAFLMP	NCP	82.641
PAAFLMP;MELVKAL	NCP	31.309
MELVKAL;KPLTPR	NCP	57.65
KPLTPR;MKSVQGF	NCP	29.611
MKSVQGF;VDKLVL	NCP	71.406
VDKLVL;LPWKWWP	NCP	105.152
LPWKWWP;FPDGKQ	NCP	37.281
FPDGKQ;DPCLKTL	NCP	43.753
DPCLKTL;LGALGGF	NCP	30.899
LGALGGF;ELNAQ	NCP	69.063
ELNAQ;KVPVEP	NCP	32.196
KVPVEP;LAVKEKY	NCP	54.84
LAVKEKY;SPPKYMP	NCP	6.174
SPPKYMP;KLLDMD	NCP	214.848
KLLDMD;APGRQLP	NCP	96.018
APGRQLP;KMKKPSK	NCP	85.075
KMKKPSK;AAPRNPPP	NCP	0.125
AAPRNPPP;KTSLKPH	NCP	19.047
KTSLKPH;SQPMHVL	NCP	93.129
SQPMHVL;GNKLVVK	NCP	21.177
GNKLVVK;TRNHLYY	NCP	43.726
TRNHLYY;NDMKTN	NCP	14.576
NDMKTN;KDTEFYK	NCP	29.215
KDTEFYK;KLAGA	NCP	1.349
KLAGA;TVGLPLHR	NCP	0.876
TVGLPLHR;FYPVP	NCP	79
FYPVP;VGNGVPK	NCP	67.38
VGNGVPK;ALSLLGK	NCP	0.719
ALSLLGK;LLEEPM	NCP	75.858
LLEEPM;VLLLRNSN	NCP	21.297
VLLLRNSN;VSGSVDLP	NCP	49.683
VSGSVDLP;ECFTPK	NCP	75.685
ECFTPK;VGLVALLH	NCP	26.243
VGLVALLH;KEMQPV	NCP	45.723
KEMQPV;AKKLKAL	NCP	81.006
AKKLKAL;NQVMQNK	NCP	14.667
NQVMQNK;LAMHSLH	NCP	64.162
LAMHSLH;LPTVHSK	NCP	44.525
LPTVHSK;LPLNP	NCP	16.372
LPLNP;KAYPPPPP	NCP	165.455
KAYPPPPP;SNRPSFN	NCP	106.722

SNRPSFN;MPGKGF	NCP	26.698
MPGKGF;FEALLF	NCP	137.222
FEALLF;LPAKGPP	NCP	51.576
LPAKGPP;VLLLDL	NCP	2.718
VLLLDL;NTVQLSR	NCP	25.254
NTVQLSR;VCEWVK	NCP	45.787
VCEWVK;LEVLKML	NCP	35.439
LEVLKML;ARHVMK	NCP	0.435
ARHVMK;TGKVP	NCP	21.473
TGKVP;LQETLP	NCP	40.089
LQETLP;GPPLSP	NCP	15.882
GPPLSP;WGLGKPV	NCP	121.336
WGLGKPV;KSAPWQR	NCP	101.471
KSAPWQR;FYVPPPP	NCP	14.857
FYVPPPP;ALNVEFR	NCP	55.833
ALNVEFR;EGPEKVAK	NCP	17.273
EGPEKVAK;LLALVGQ	NCP	42.883
LLALVGQ;YDMDC	NCP	186.549
YDMDC;QTDLKEK	NCP	75.402
QTDLKEK;VGMLLLN	NCP	6.657
VGMLLLN;QLLFYYC	NCP	29.786
QLLFYYC;DPAAAPPP	NCP	21.603
DPAAAPPP;GVMKL	NCP	58.426
GVMKL;KHKLPPP	NCP	58.227
KHKLPPP;MKAPP	NCP	47.066
MKAPP;KQQKVVQ	NCP	45.069
KQQKVVQ;PLPPAPGK	NCP	81.45
PLPPAPGK;TSYLLYVN	NCP	0.595
TSYLLYVN;KMYAVP	NCP	54.42
KMYAVP;KRNVPY	NCP	25.736
KRNVPY;RLLVENF	NCP	31.15
RLLVENF;VLEARVP	NCP	18.17
VLEARVP;NTFLKKPK	NCP	12.186
NTFLKKPK;FDRMPPG	NCP	55.362
FDRMPPG;RDYDDMSPR	NCP	0.051
RDYDDMSPR;ALRLHL	NCP	13.53
ALRLHL;QYKAQGK	NCP	66.733
QYKAQGK;FDTTGDF	NCP	131.046
FDTTGDF;QHYKK	NCP	37.358
QHYKK;ENPSPTVGTTEPGHQQ	NCP	100.689
ENPSPTVGTTEPGHQQ;PSPTVGTTEPGHQQF	NCP	0.006
PSPTVGTTEPGHQQF;KSGSFPP	NCP	37.233
KSGSFPP;VPHDLPL	NCP	63.447
VPHDLPL;KNLRPLV	NCP	9.213
KNLRPLV;APPSVEV	NCP	135.981
APPSVEV;MKKMLT	NCP	114.637
MKKMLT;HLRPVLLL	NCP	58.737
HLRPVLLL;KEMVCK	NCP	108.428
KEMVCK;YRSALFL	NCP	11.724
YRSALFL;PPSPGKY	NCP	62.766
PPSPGKY;ARLAELA	NCP	0.654

ARLAELA;PLMPLK	NCP	2.437
PLMPLK;LTGMVTPL	NCP	99.081
LTGMVTPL;CDEGMGP	NCP	9.771
CDEGMGP;VTDLP	NCP	35.541
VTDLP;EGMLMHL	NCP	77.055
EGMLMHL;VTLSAL	NCP	6.881
VTLSAL;LPPKRDF	NCP	2.082
LPPKRDF;KEELYKK	NCP	3.805
KEELYKK;ASTATPPPPP	NCP	4.421
ASTATPPPPP;AAVTDVQRLQA	NCP	30.3
AAVTDVQRLQA;EPSGPP	NCP	22.74
EPSGPP;CTLPPPPPP	NCP	47.928
CTLPPPPPP;LTHPPP	NCP	0.72
LTHPPP;APLCKKQ	NCP	4.963
APLCKKQ;GAGVH	NCP	18.452
GAGVH;GFVKVVKNKAYFK	NCP	34.092
GFVKVVKNKAYFK;TDYYARK	NCP	0.078
TDYYARK;AHELPKYGVK	NCP	0.156
AHELPKYGVK;KASFLRAQERAAES	NCP	0.606
KASFLRAQERAAES;KFSSEGAP	NCP	15.847
KFSSEGAP;VSCLLSP	NCP	15.764
VSCLLSP;PGRARPP	NCP	2.655
PGRARPP;KLEEEET	NCP	25.683
KLEEEET;KHGRSF	NCP	68.517
KHGRSF;NPMLD	NCP	73.276
NPMLD;DCVGDLRK	NCP	126.299
DCVGDLRK;AAPEKVK	NCP	29.158
AAPEKVK;QTVLLKERK	NCP	50.859
QTVLLKERK;LGDDVLK	NCP	64.455
LGDDVLK;TTAPLVDT	NCP	80.091
TTAPLVDT;YTLVPLM	NCP	22.092
YTLVPLM;VAVEPY	NCP	107.672
VAVEPY;HLAGLFT	NCP	19.95
HLAGLFT;MQLTHQL	NCP	4.015
MQLTHQL;QYMSLC	NCP	51.345
QYMSLC;DTVEL	NCP	71.771
DTVEL;VGPPAPPPP	NCP	91.941
VGPPAPPPP;LPPPPVPPP	NCP	22.032
LPPPPVPPP;AGESMFNRAK	NCP	41.781
AGESMFNRAK;LDDNLRPR	NCP	36.67
LDDNLRPR;DDNLRPRT	NCP	0.003
DDNLRPRT;KSYGNLH	NCP	22.776
KSYGNLH;SEDGLLKLWKP	NCP	7.112
SEDGLLKLWKP;FKLFDP	NCP	86.881
FKLFDP;LLEKPK	NCP	250.905
LLEKPK;PPPPP	NCP	54.677
PPPPP;SFVLNF	NCP	55.108
SFVLNF;MELGTELS	NCP	38.594
MELGTELS;FVGLKY	NCP	93.49
FVGLKY;CMMFEM	NCP	32.156
CMMFEM;KGVDLPK	NCP	112.374

KGVDLPK;NLSAVAL	NCP	30.688
NLSAVAL;SARLDR	NCP	16.335
SARLDR;GNNLLVK	NCP	42.33
GNNLLVK;VHYSR	NCP	47.525
VHYSR;KESEVVF	NCP	18.234
KESEVVF;NQMLLLH	NCP	14.671
NQMLLLH;LLLHRP	NCP	67.566
LLLHRP;ESLKTPQ	NCP	3.467
ESLKTPQ;EVAYVF	NCP	2.503
EVAYVF;LCDLDLRK	NCP	38.157
LCDLDLRK;PPETE	NCP	42.425
PPETE;VPLSPFK	NCP	38.302
VPLSPFK;EHLRYY	NCP	23.994
EHLRYY;TSYVSLSP	NCP	9.566
TSYVSLSP;LGRYSAL	NCP	6.822
LGRYSAL;AVPKGM	NCP	10.845
AVPKGM;KSVFKK	NCP	22.293
KSVFKK;RYNALC	NCP	70.524
RYNALC;CSKEK	NCP	1.894
CSKEK;LGALYLFL	NCP	14.63
LGALYLFL;QAVLLTLV	NCP	29.322
QAVLLTLV;KDDSP	NCP	4.323
KDDSP;KDVRGRP	NCP	19.843
KDVRGRP;ENHKQF	NCP	50.526
ENHKQF;AERLALVA	NCP	5.913
AERLALVA;AATCAKKK	NCP	107.394
AATCAKKK;KPEVGVP	NCP	65.441
KPEVGVP;GPPPPPPPP	NCP	69.207
GPPPPPPPP;KDAAAPV	NCP	32.991
KDAAAPV;PPAKY	NCP	4.194
PPAKY;TNNTHNVP	NCP	38.59
TNNTHNVP;GASHSLLR	NCP	31.53
GASHSLLR;KDVPN	NCP	12.149
KDVPN;EFVKKT	NCP	107.866
EFVKKT;SVMETSML	NCP	265.334
SVMETSML;VVVAF	NCP	63.96
VVVAF;TACPKF	NCP	23.106
TACPKF;EFDKSLK	NCP	4.315
EFDKSLK;VGSMV	NCP	38.109
VGSMV;LDLHGNPK	NCP	5.132
LDLHGNPK;KYQPPPPP	NCP	90.57
KYQPPPPP;KHLSPPR	NCP	27.361
KHLSPPR;KDLWQV	NCP	50.505
KDLWQV;KTFVYP	NCP	32.745
KTFVYP;KKPNAPK	NCP	122.241
KKPNAPK;VVGHGD	NCP	2.984
VVGHGD;KSYPELLK	NCP	1.969
KSYPELLK;FMARDF	NCP	44.682
FMARDF;ATLAEAW	NCP	28.665
ATLAEAW;KLPVE	NCP	12.213
KLPVE;TLSGAMK	NCP	48.971

TLSGAMK;KVMSGLK	NCP	20.239
KVMSGLK;KSTLVK	NCP	14.609
KSTLVK;PVRFLLEF	NCP	15.823
PVRFLLEF;LVTHSLVT	NCP	39.795
LVTHSLVT;PVVTK	NCP	143.381
PVVTK;SVEKDLR	NCP	133.624
SVEKDLR;DLDNMKK	NCP	29.183
DLDNMKK;SGLAFAF	NCP	11.233
SGLAFAF;FELQAVP	NCP	19.752
FELQAVP;DHDFLL	NCP	37.262
DHDFLL;KPASGLVK	NCP	12.549
KPASGLVK;LLPKKMW	NCP	3.558
LLPKKMW;DSGVTRPEF	NCP	24.856
DSGVTRPEF;LLFFFAHF	NCP	13.826
LLFFFAHF;DLSHS	NCP	63.426
DLSHS;NKEDKLR	NCP	12.606
NKEDKLR;LLLDPF	NCP	2.646
LLLDPF;PLLTVPP	NCP	125.857
PLLTVPP;NMAKKN	NCP	26.732
NMAKKN;KGGFLMY	NCP	9.963
KGGFLMY;VVLRALL	NCP	22.987
VVLRALL;KLGSSPTE	NCP	66.524
KLGSSPTE;DVPPLAP	NCP	144.601
DVPPLAP;FLNAK	NCP	49.916
FLNAK;RFGGLGR	NCP	71.824
RFGGLGR;KPENSH	NCP	38.598
KPENSH;SQPMLQ	NCP	73.478
SQPMLQ;TVPETPPYT	NCP	54.634
TVPETPPYT;KQSWKAQ	NCP	6.519
KQSWKAQ;TPTLFQA	NCP	3.383
TPTLFQA;LDRLGGAG	NCP	8.031
LDRLGGAG;LQGRLF	NCP	13.468
LQGRLF;VHPDLK	NCP	16.643
VHPDLK;KAHAEPQR	NCP	69.339
KAHAEPQR;LETALGH	NCP	48.024
LETALGH;TLRLGTT	NCP	51.808
TLRLGTT;VDWGGGKK	NCP	7.38
VDWGGGKK;VPTPLTP	NCP	31.122
VPTPLTP;QAVLSLY	NCP	91.068
QAVLSLY;DAGPPVN	NCP	1.397
DAGPPVN;DDGGP	NCP	25.8
DDGGP;HPEASGK	NCP	2.604
HPEASGK;KSKTAGP	NCP	180.046
KSKTAGP;NVGRKN	NCP	1.803
NVGRKN;VPRCE	NCP	11.655
VPRCE;KGVATEEN	NCP	53.415
KGVATEEN;SHTPKPP	NCP	24.815
SHTPKPP;KNEEPGL	NCP	94.552
KNEEPGL;PSDLSGPK	NCP	14.904
PSDLSGPK;ELKVVYL	NCP	47.046
ELKVVYL;LWTYK	NCP	138.285

LWTYK;GSLVLPVV	NCP	81.278
GSLVLPVV;KYLVSGGK	NCP	19.135
KYLVSGGK;RPKNTH	NCP	1.182
RPKNTH;KPVVYK	NCP	39.699
KPVVYK;PPPPPPPAPP	NCP	26.918
PPPPPPPAPP;LYRALVS	NCP	18.933
LYRALVS;KGSLP	NCP	150.813
KGSLP;KGVDVSP	NCP	56.493
KGVDVSP;RTHPPR	NCP	73.372
RTHPPR;TAPPNMS	NCP	138.42
TAPPNMS;SGPTLRPR	NCP	134.252
SGPTLRPR;AEPDHF	NCP	38.916
AEPDHF;WRTVKL	NCP	36.579
WRTVKL;MDVHEK	NCP	83.277
MDVHEK;SGPVRPP	NCP	8.907
SGPVRPP;LSLDEVR	NCP	35.443
LSLDEVR;AASCLL	NCP	5.013
AASCLL;HLMPTRY	NCP	16.1
HLMPTRY;KGAWRL	NCP	62.997
KGAWRL;VYKVVGK	NCP	123.547
VYKVVGK;DPPRRPP	NCP	10.232
DPPRRPP;KPVVHEP	NCP	2.176
KPVVHEP;DLQQVLK	NCP	7.691
DLQQVLK;KREVSLP	NCP	70.207
KREVSLP;DGGTKFG	NCP	8.43
DGGTKFG;VTSSPP	NCP	42.839
VTSSPP;LRALDK	NCP	50.568
LRALDK;MEDVVVKQGF	NCP	58.965
MEDVVVKQGF;EAASNRP	NCP	25.816
EAASNRP;MEGVEL	NCP	21.398
MEGVEL;DLGRSVY	NCP	29.815
DLGRSVY;APGTK	NCP	19.115
APGTK;YDPPVAR	NCP	47.034
YDPPVAR;VHRLLR	NCP	0.396
VHRLLR;LFYERLL	NCP	3.295
LFYERLL;LAFLRK	NCP	19.104
LAFLRK;VGGSTRLP	NCP	5.345
VGGSTRLP;KACPPK	NCP	65.382
KACPPK;EKLHVLH	NCP	24.933
EKLHVLH;CFFLFKNC	NCP	56.773
CFFLFKNC;VVAEVGGP	NCP	28.128
VVAEVGGP;LYENGTY	NCP	42.627
LYENGTY;KLPGLFY	NCP	10.038
KLPGLFY;FHLKVR	NCP	34.326
FHLKVR;SRKFFVG	NCP	6.576
SRKFFVG;HLAHFLPFP	NCP	204.767
HLAHFLPFP;WEYPNLK	NCP	56.344
WEYPNLK;PLVPLLPD	NCP	35.18
PLVPLLPD;LPGGFVG	NCP	12.946
LPGGFVG;NVVKP	NCP	170.664
NVVKP;PAAFMVK	NCP	0.737

PAAFMVK;LENANAK	NCP	22.522
LENANAK;YFPPKAM	NCP	10.11
YFPPKAM;PTTTKP	NCP	63.228
PTTTKP;SGVKVHV	NCP	85.815
SGVKVHV;TGGFLAP	NCP	78.237
TGGFLAP;VLAVHY	NCP	4.694
VLAVHY;MNELPGP	NCP	47.757
MNELPGP;LVLGRGEEE	NCP	20.719
LVLGRGEEE;MFLQLQ	NCP	34.94
MFLQLQ;HSPVKKE	NCP	43.776
HSPVKKE;ARFEPF	NCP	15.415
ARFEPF;LSPSPVGK	NCP	209.994
LSPSPVGK;FLGASLK	NCP	45.617
FLGASLK;TYSYLTPDLWK	NCP	0.447
TYSYLTPDLWK;KPGLERP	NCP	21.7
KPGLERP;RLHPYK	NCP	23.183
RLHPYK;VSKTDVP	NCP	105.072
VSKTDVP;LPPLN	NCP	84.124
LPPLN;KEVGVP	NCP	13.77
KEVGVP;LVTNWLA	NCP	0.071
LVTNWLA;EGLNLDLK	NCP	14.908
EGLNLDLK;EPYFFT	NCP	3.074
EPYFFT;LSAALKKAL	NCP	24.063
LSAALKKAL;EAHKPAP	NCP	139.485
EAHKPAP;LVAESLPK	NCP	61.879
LVAESLPK;RLSLLSK	NCP	118.356
RLSLLSK;ADAKLLPS	NCP	38.349
ADAKLLPS;SNVLDY	NCP	2.87
SNVLDY;DDELDWL	NCP	32.428
DDELDWL;DPFKELR	NCP	59.417
DPFKELR;VGLGVGH	NCP	0.418
VGLGVGH;LLAQMTG	NCP	5.609
LLAQMTG;TVGKQLLY	NCP	19.555
TVGKQLLY;LLVPTREL	NCP	29.244
LLVPTREL;NLLSSGF	NCP	70.872
NLLSSGF;KVGPLLS	NCP	207.045
KVGPLLS;TSAMSYP	NCP	4.34
TSAMSYP;LVDKAF	NCP	67.333
LVDKAF;LPELN	NCP	160.667
LPELN;VPNQDY	NCP	30.385
VPNQDY;HLVPL	NCP	28.011
HLVPL;RNALVAR	NCP	33.023
RNALVAR;FRDQKF	NCP	1.794
FRDQKF;LEATKLG	NCP	15.144
LEATKLG;MDKLVV	NCP	131.541
MDKLVV;EVPFHLT	NCP	9.886
EVPFHLT;FHPVNNK	NCP	20.216
FHPVNNK;VPKVSRY	NCP	56.56
VPKVSRY;SDYPGLP	NCP	71.426
SDYPGLP;KSVVPPP	NCP	39.327
KSVVPPP;KPSPPHR	NCP	6.324

KPSPPHR;KFAPQKK	NCP	4.176
KFAPQKK;ESVQPLSK	NCP	14.776
ESVQPLSK;GVPLPPPPPP	NCP	44.651
GVPLPPPPPP;QYPPK	NCP	78.883
QYPPK;HPGVKPH	NCP	70.41
HPGVKPH;NAFRASP	NCP	0.338
NAFRASP;FGFGQGQ	NCP	51.651
FGFGQGQ;VNFATFF	NCP	14.716
VNFATFF;NGPLSPVP	NCP	26.667
NGPLSPVP;VNVPNFK	NCP	100.539
VNVPNFK;SVKEWT	NCP	23.103
SVKEWT;AVPPH	NCP	8.871
AVPPH;LVCGSNP	NCP	8.498
LVCGSNP;NSFLLAE	NCP	1.218
NSFLLAE;LSPNVEF	NCP	63.366
LSPNVEF;LHLGHAK	NCP	14.313
LHLGHAK;FLLRL	NCP	44.886
FLLRL;LPVGK	NCP	116.811
LPVGK;KVMGK	NCP	7.573
KVMGK;PLYMAF	NCP	84.534
PLYMAF;NPVPAAF	NCP	24.72
NPVPAAF;HELVGN	NCP	12.339
HELVGN;LALFQ	NCP	33.048
LALFQ;PPTVMDL	NCP	103.673
PPTVMDL;KAHLLHV	NCP	12.036
KAHLLHV;GLQKVYK	NCP	2.541
GLQKVYK;KFGLPPP	NCP	113.241
KFGLPPP;KANSLTK	NCP	6.627
KANSLTK;FEDKPF	NCP	4.411
FEDKPF;LSQNVYK	NCP	25.089
LSQNVYK;PPPPAL	NCP	11.973
PPPPAL;TTFRGC	NCP	0.344
TTFRGC;ESQPLDQ	NCP	3.889
ESQPLDQ;TVYLPL	NCP	68.909
TVYLPL;EVHLM	NCP	21.064
EVHLM;KLYREP	NCP	6.638
KLYREP;LLVFL	NCP	121.12
LLVFL;HAAKPQH	NCP	37.749
HAAKPQH;VVFEGNHY	NCP	32.45
VVFEGNHY;ALPRGGPA	NCP	92.832
ALPRGGPA;HADAYFP	NCP	62.211
HADAYFP;KDYCPP	NCP	31.065
KDYCPP;TDAFLVL	NCP	41.223
TDAFLVL;EPRLDPP	NCP	45.355
EPRLDPP;LDLHYM	NCP	31.181
LDLHYM;YLTQEY	NCP	9.757
YLTQEY;SADQVTRALENVLSSKA	NCP	101.744
SADQVTRALENVLSSKA;LGSSLQK	NCP	14.229
LGSSLQK;ESAFAAG	NCP	58.254
ESAFAAG;EGYGFQ	NCP	4.821
EGYGFQ;NTPAMY	NCP	12.357

NTPAMY;KPLTGPY	NCP	48.009
KPLTGPY;PFLKVR	NCP	3.201
PFLKVR;RSPKHFLQS	NCP	44.113
RSPKHFLQS;ADVLDL	NCP	60.317
ADVLDL;YGQPPAP	NCP	13.677
YGQPPAP;TVHLHDK	NCP	6.967
TVHLHDK;PSPPLML	NCP	47.645
PSPPLML;PLLYLPLF	NCP	80.959
PLLYLPLF;ATSASSHL	NCP	54.65
ATSASSHL;KPLSKVD	NCP	24.219
KPLSKVD;VEEFWGPF	NCP	21.315
VEEFWGPF;VTPLPKL	NCP	71.763
VTPLPKL;MTLFTM	NCP	85.746
MTLFTM;KEVAEMT	NCP	39.315
KEVAEMT;GPNSKGPK	NCP	59.193
GPNSKGPK;KDCCMK	NCP	5.511
KDCCMK;ERGLWSK	NCP	74.253
ERGLWSK;MDGLVPD	NCP	5.596
MDGLVPD;KLHFTS	NCP	10.29
KLHFTS;AMKTKSK	NCP	41.301
AMKTKSK;EGTSKVAL	NCP	27.749
EGTSKVAL;YTPGKGK	NCP	0.072
YTPGKGK;NSATLPR	NCP	51.219
NSATLPR;LPPYRPP	NCP	2.184
LPPYRPP;PGKSSVG	NCP	22.05
PGKSSVG;QPLSHVF	NCP	49.212
QPLSHVF;LPTTGQLP	NCP	0.133
LPTTGQLP;KTFLH	NCP	68.132
KTFLH;TSSKHYK	NCP	40.063
TSSKHYK;LQKGFAQ	NCP	184.811
LQKGFAQ;VPMNLK	NCP	61.683
VPMNLK;KVGDPSSGP	NCP	40.525
KVGDPSSGP;LYLQNNF	NCP	9.701
LYLQNNF;KKKKKKVPR	NCP	17.776
KKKKKKVPR;GPVRDLK	NCP	59.894
GPVRDLK;TSPLVPPPL	NCP	12.238
TSPLVPPPL;AVTHLAP	NCP	7.382
AVTHLAP;TMDMSGP	NCP	26.991
TMDMSGP;VGRLLQDS	NCP	103.722
VGRLLQDS;EHPSGLR	NCP	5.805
EHPSGLR;AVHLPTLQ	NCP	112.651
AVHLPTLQ;TFMLPLP	NCP	2.744
TFMLPLP;EVKMLC	NCP	15.255
EVKMLC;TLLKVTK	NCP	0.553
TLLKVTK;RYKLN	NCP	10.313
RYKLN;ALGVGKGHL	NCP	79.491
[truncated: 138,252 more chars]
